# Phylogeny of the plant receptor-like kinase (RLK) gene family and expression analysis of wheat RLK genes in response to biotic and abiotic stresses

**DOI:** 10.1186/s12864-023-09303-7

**Published:** 2023-05-01

**Authors:** Jun Yan, Peisen Su, Xianyong Meng, Pingzeng Liu

**Affiliations:** 1 Key Laboratory of Huang-Huai-Hai Smart Agricultural Technology of the Ministry of Agriculture and Rural Affairs, College of Information Science and Engineering, Shandong Agricultural University, Tai’an, Shandong, 271018 People’s Republic of China; 2 College of Agronomy, Liaocheng University, Liaocheng, 252059 People’s Republic of China

**Keywords:** Biotic and abiotic stresses, Conserved exon–intron structures, Collinearity events, Expression patterns in wheat, Evolution, Receptor-like kinase gene family

## Abstract

**Background:**

The receptor-like kinase (RLK) gene families in plants contains a large number of members. They are membrane proteins with an extracellular receptor domain and participate in biotic and abiotic stress responses.

**Results:**

In this study, we identified RLKs in 15 representative plant genomes, including wheat, and classified them into 64 subfamilies by using four types of phylogenetic trees and HMM models. Conserved exon‒intron structures with conserved exon phases in the kinase domain were found in many RLK subfamilies from *Physcomitrella patens* to *Triticum aestivum*. Domain distributions of RLKs were also diagrammed. Collinearity events and tandem gene clusters suggested that polyploidization and tandem duplication events contributed to the member expansions of *T. aestivum* RLKs. Global expression pattern analysis was performed by using public transcriptome data. These analyses were involved in *T. aestivum, Aegilops tauschii* and *Brachypodium distachyon* RLKs under biotic and abiotic stresses. We also selected 9 RLKs to validate the transcriptome prediction by using qRT‒PCR under drought treatment and with *Fusarium graminearum* infection. The expression trends of these 9 wheat RLKs from public transcriptome data were consistent with the results of qRT‒PCR, indicating that they might be stress response genes under drought or *F. graminearum* treatments.

**Conclusion:**

In this study, we identified, classified, evolved, and expressed RLKs in wheat and related plants. Thus, our results will provide insights into the evolutionary history and molecular mechanisms of wheat RLKs.

**Supplementary Information:**

The online version contains supplementary material available at 10.1186/s12864-023-09303-7.

## Introduction

Receptor-like kinases (RLKs) are membrane proteins with an extracellular receptor domain, such as leucine rich repeats (LRRs), lectin (Lec), lysine motif (LysM) or wall associated kinases (WAK) [1]. RLKs play important roles in resistance signalling pathways of various abiotic stresses, including drought, high temperature and low temperature. They can also defend against pathogen infection by activating immune signalling in plants. RLK gene families from various plants have been identified in a large number of articles. We summarized these findings in the following Introduction section.

(1) Whole RLKs: In 2003 and 2004, Shiu et al. identified more than 600 RLKs in *Arabidopsis thaliana* and more than 1200 RLKs in *Oryza sativa* [2, 3]. They play important roles in plant growth, development, and defence responses to stresses. More than 440 RLKs from *O. sativa* might have originated from domain fusion events after the split of rice and *Arabidopsis* in evolution. In 2009, Lehti-Shiu et al. found that the expansion of RLK members coincided with the establishment of land plants [4]. In 2018, Lin et al. identified 563 RLK genes in Jilin ginseng (*Panax ginseng* C.A. Meyer) and analysed their evolution, functional diversity and coexpression networks [5].

(2) LRR-RLKs (subfamily of RLK): In 2013, Zan et al. identified 379 LRR-type RLK genes in *Populus trichocarpa*. A total of 312 *Pt*LRR-RLK genes out of 379 are located in segmental duplication blocks. Genome-wide analysis of microarray data showed that some *Pt*LRR-RLKs responded to shoot organogenesis, low ammonium feeding, wounding, hypoxia and seasonal dormancy [6]. In 2017, Liu et al. studied the origin and diversity of LRR-RLKs in plants and found that most LRR-RLKs were established in early land plants [7]. In 2018, Sun et al. identified 1641 LRR-RLK genes in four *Gossypium* species (*Gossypium arboreum*, *Gossypium barbadense*, *Gossypium hirsutum*, and *Gossypium raimondii*). Tandem duplication played an important role in the expansion of the *Gossypium* LRR-RLK gene family. Expression pattern analysis showed that *Gossypium* LRR-RLKs were widely involved in various stress defences and diverse developmental processes [8]. In 2020, Meng et al. identified 329 LRR-RLK genes in *Medicago truncatula*. Analysis of classification, duplication events, exon/intron organization, and expression profiling were performed in *M. truncatula* LRR-RLKs [9]. In 2021, 437 LRR-RLK genes were identified in *Saccharum spontaneum* and categorized into 14 groups. Analysis of promoter sequences and expression profiles showed that *Ss*LRR-RLKs were strongly regulated by various environmental stimuli, transcription factors and phytohormonal factors, suggesting that they respond to various diverse biotic and abiotic stresses [10]. In 2022, Song et al. identified 444 *Bn*LRR-RLKs in the *Brassica napus* cultivar “Zhongshuang 11” and classified them into 22 subfamilies. Based on CRISPR/Cas9 technology, they obtained six partial knockouts of *BnBRI1* homologues to generate semidwarf lines without decreased yield compared with controls [11]. In 2022, 15 *Ta*RPK1 (receptor-like protein kinase 1, a calcium-independent serine-threonine kinase that belongs to the subfamily LRR-RLK) genes were identified in *Triticum aestivum*. Eighteen putative miRNA targeting and cis-regulatory elements (light-related, hormone responsiveness, and stress elements) were identified in *Ta*RPK1 genes. In silico expression analysis and qRT‒PCR validated that *Ta*RPK1 genes exhibited higher expression in the roots of drought-tolerant varieties than in drought-susceptible varieties [12].

(3) LecRLKs (subfamily of RLK): In 2020, 46 putative lectin receptor-like kinases were identified in cucumber (*Cucumis sativus* L.) genome and were classified into three groups, including 23 G-type, 22 L-type, and one C-type *Cs*LecRLK genes. Analysis of promoter regulatory elements and expression patterns revealed that some *Cs*LecRLKs were associated with phytohormones and stress responses [13]. In 2020, Singh et al. identified 73 putative *Vr*LecRLKs in mung bean (*Vigna radiata* L. Wilczek) and classified them into three families: G-type, L-type, and C-type *Vr*LecRLKs [14]. In 2021, 1311 *Ah*RLKs, including *Ah*LRR-RLKs and *Ah*Lec-RLKs, were identified from the peanut (*Arachis hypogaea*) genome. The results of mining transcriptome data showed that 14 of 90 Al-responsive *Ah*RLKs were expressed specifically in root tissue [15].

(4) LysM-RLKs (subfamily of RLK): In 2020, Yang et al. identified 493 RLKs (LysM-RLKs and LRR-RLKs) and 228 RLPs (LysM-RLPs and LRR-RLPs) in the genome of *Bra*s*sica juncea*. The majority of RLKs (90.17%) and RLPs (52.83%) of *B. juncea* are from duplication events, indicating that duplication events significantly contributed to the expansion of the RLK and RLP gene families [16]. In 2021, Abedi et al. identified 33 LysM-RLK genes (subfamily of RLK) in three *Brassica* species (17 in *Bra*s*sica napus*, 8 in *Brassica rapa* and 8 in *Brassica oleracea*). RNA-seq expression analysis revealed that *BnLYP6* exhibited high expression in response to various biotic stresses. Structural modelling and docking simulations revealed that several residues in the active sites of *BnLYP6* could directly contact chitin [17].

(5) CRKs (Cysteine-rich receptor-like kinases, subfamily of RLK): In 2019, Quezada et al. identified 46 CRKs in *Phaseolus vulgaris* and performed comprehensive analyses, including identification, chromosomal localization, gene structures, transcript expression profiles, and in silico promoter analysis [18]. In 2019, Shumayla et al. identified 43, 37, 36, 38 and 170 CRK genes in the genomes of *Brachypodium distachyon*, *Hordeum vulgare*, *O. sativa*, *Sorghum bicolor* and *T. aestivum*, respectively. These CRKs were tightly clustered into four phylogenetic groups and were variably conserved in exons/introns, domains and motifs. Tissue-specific expression analysis suggested that some CRK genes are involved in plant development [19].

(6) PERKs (proline-rich extensin-like receptor kinases, subfamily of RLK): In 2004, 15 *At*PERKs were predicted in *A. thaliana*, and some *At*PERK members were identified as tissue-specific genes [20]. In 2022, 37 *Ta*PERKs were identified in wheat (*T. aestivum* L.) and were classified into eight well-defined groups. Analysis of cis-acting regulatory elements and expression profiles revealed that *Ta*PERKs may respond to phytohormones and various biotic and abiotic stresses [21].

Some articles have reported that RLKs play important roles in the response to biotic and abiotic stresses, such as drought, heat, salinity and cold. Overexpression of *PdERECTA* (an LRR-type RLK from *Populus deltoides*) improves water use efficiency and enhances drought resistance in transgenic *Arabidopsis* plants [22]. Rice (*O. sativa*) *OsSIK1* (LRR RLK) improves tolerance to drought and salt stress. Transgenic rice plants overexpressing *OsSIK1* exhibit enhanced tolerance to salt and drought stresses, while knock-out and RNA interference plants exhibit sensitivity to drought and salt stresses [23]. *OsLecRLK* overexpression and downregulation (through artificial miRNA) transgenic lines showed that rice *OsLecRLK* enhances salinity tolerance through ion homeostasis [24]. The interaction network of 255 *Arabidopsis* LRR-RLKs (567 pairs of interaction relationships) was established by using a sensitized high-throughput interaction assay. Plants have evolved LRR-RLK networks to process extracellular signals into cells, functioning in plant growth and immunity [25]. MtDMI2 (a Leu-rich repeat-type receptor kinase) and MtPUB2 (a novel plant U-box (PUB)-type E3 ligase) interact to form a negative feedback loop, playing an important role in nodulation homeostasis [26]. The systemin receptor SYR1 (an LRR-RLK) of tomato is not decisive for local and systemic wound responses but is important for defence against insect herbivory [27]. The *A. thalian*a lectin RLK *AtLecRK-IX.2* can modify the pathogen effector AvrPtoB to dampen its virulence in *Arabidopsis* [28]. By using a map-based cloning strategy, Duriez et al. identified the sunflower protein HaOr7 (LRR-RLK) as an effector that confers resistance to *Orobanche cumana* [29]. Rao et al. constructed nine higher-order mutants of *A. thaliana* receptor-like cytoplasmic kinase (RLCK) subfamily VII, revealing that numerous RLCK VII members are involved in plant development and pattern-triggered immune signalling [30]. *A. thaliana AtRIPK* (RPM1-INDUCED PROTEIN KINASE), an RLCK VII subfamily member, contributes to ROS (reactive oxygen species) production in the plant immune system [31].

In recent years, the biological functions of wheat RLKs mediating the response to biotic stress have been reported. In 2016, Rajaraman et al. found that the barley LRR-type RLK gene *HvLEMK1* was a factor mediating nonhost resistance in barley and quantitative host resistance in wheat to the wheat powdery mildew fungus [32]. In 2018, Saintenac et al. discovered that the RLK gene *Stb6* (a conserved wall-associated receptor kinase (WAK)-like protein, subfamily of RLK) in wheat is a natural resistance gene to the fungal pathogen *Zymoseptoria tritici* [33]. In 2019, Wang et al. found that the LRR-type RLK gene *TaXa21* in wheat is highly homologous to the rice bacterial blight resistance gene *Xa21*. They also found that *TaXa21* is a positive regulator of wheat high-temperature seedling plant (HTSP) resistance to *Puccinia striiformis* f. sp. tritici. This process is mediated by H_2_O_2_ and ethylene (ET) signalling pathways and is associated with the transcription factors *TaWRKY76* and *TaWRKY62* [34]. In 2020, Gu et al. discovered a novel cysteine-rich RLK gene, *TaCRK2*, which positively regulates leaf rust resistance in wheat [35]. Using comparative genomics, mutagenesis and complementation, Saintenac et al. identified a wheat cysteine-rich RLK gene, *Stb16q*, which exhibited resistance against Septoria tritici blotch (Stb, pathogen *Zymoseptoria tritici*) and localized at the plasma membrane in the infection cycle [36]. In 2021, Guo et al. identified a novel CRK RLK gene, *TaCRK3*, that could defend against *Rhizoctonia cerealis* in wheat. The *Ta*CRK3 protein contains two DUF26 (DOMAIN OF UNKNOWN FUNCTION 26) domains that can inhibit the growth of *R. cerealis* mycelia [37]. In 2021, the wheat wall-associated receptor-like kinases (WAKs, subfamily of RLKs), *TaWAK-6D* and *TaWAK7D*, were identified to mediate broad resistance to fungal pathogens (*Fusarium pseudograminearum* and *R. cerealis*) [38, 39]. In 2022, *TaPsIPK1* (a wheat receptor-like cytoplasmic kinase gene) was identified as a susceptibility gene for stripe rust (caused by *P. striiformis* f. sp. tritici) effectors [40].

In this study, we performed genome-wide identification, classification, and evolutionary analysis of the RLK gene family in 15 representative plants, including four wheat and *Aegilops tauschii* species*.* Global expression analyses of stresses were performed in individual *T. aestivum* RLKs. qRT‒PCR procedures of 9 selected RLKs were performed to validate the prediction of the transcriptome under drought conditions and *Fusarium graminearum* infection. Our results provide insights into the evolutionary history and molecular mechanisms of wheat RLKs.

## Results

### Genome-wide identification and classification of the RLK gene family in wheat, *Ae. tauschii* and other plants

We identified RLK genes with typical kinase domains and corresponding HMM models (see Methods) in 15 representative plants, including four wheat and *Ae. tauschii* (Table 1, Table S1). Other PKs (protein kinases) with typical kinase domains were also identified and classified in 15 representative plants (Table S1). The results showed that most proportions of RLKs in PKs were 75–78% in 15 representative plants (Table 1).

**Table 1:** The numbers of PK gene superfamily and RLK gene family in 15 plants

Species	PK	RLK	RLK/PK
*Chlamydomonas reinhardtii*	497	4	0.80%
*Physcomitrella patens*	637	298	46.78%
*Selaginella moellendorffii*	975	532	54.56%
*Amborella trichopoda*	583	375	64.32%
*Vitis vinifera*	1141	864	75.72%
*Arabidopsis thaliana*	1004	615	61.25%
*Oryza sativa*	1134	810	71.43%
*Zea mays*	1292	787	60.91%
*Brachypodium distachyon*	1200	839	69.92%
*Aegilops tauschii*	1552	1183	76.22%
*Triticum urartu*	1223	958	78.33%
*Triticum dicoccoides*	2834	2153	75.97%
*Triticum turgidum*	3209	2510	78.22%
*Triticum spelta*	4660	3551	76.20%
*Triticum aestivum*	5015	3889	77.55%

**Fig. 1:**
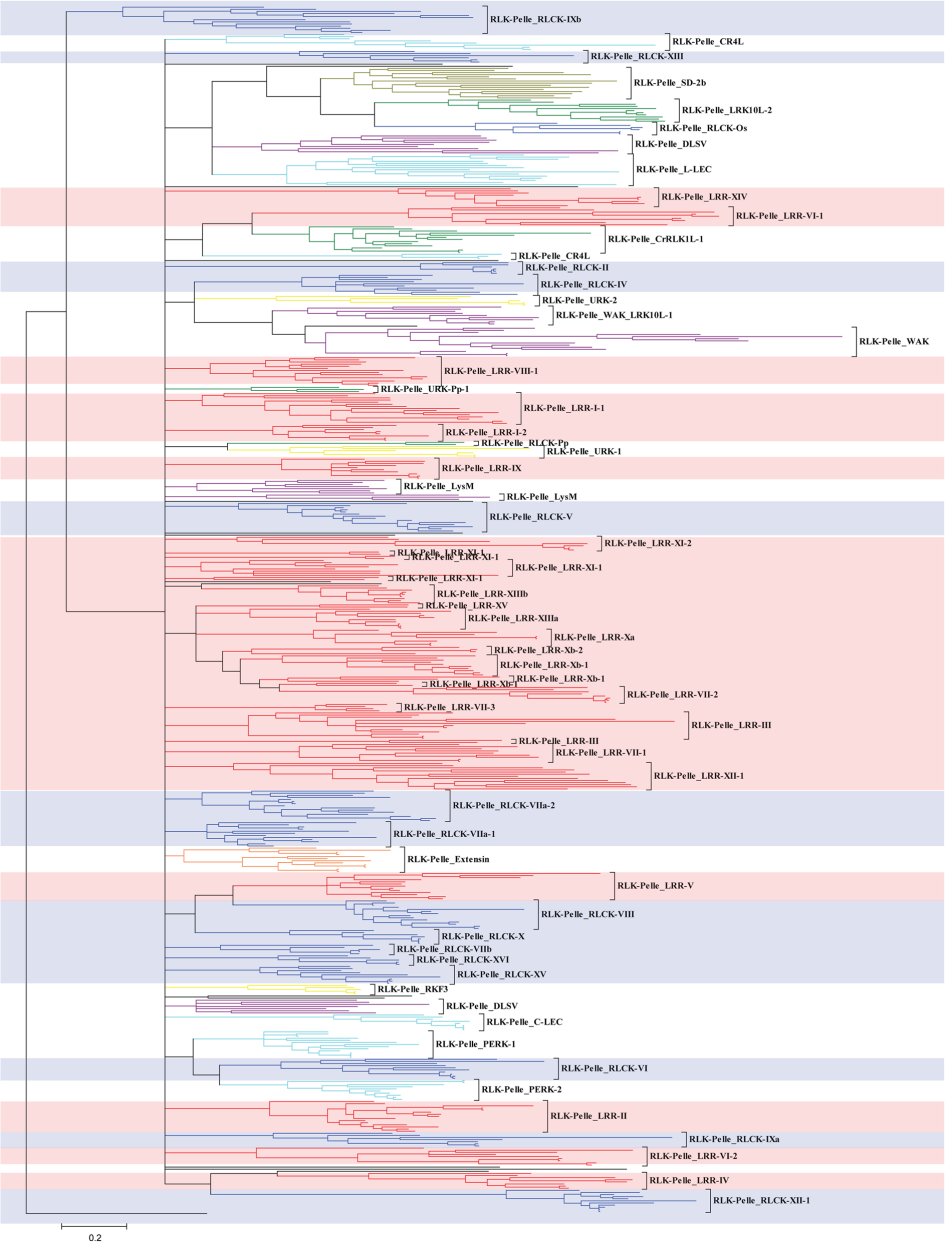
Classification and phylogenetic relationships of RLKs in wheat and 8 other representative plants. The Bayesian phylogenetic tree was built based on the kinase domain amino acid sequences from 9 representative plants (*C. reinhardtii, P. patens, S. moellendorffii*, *A. trichopoda*, *A. thaliana*, *B. distachyon*, *Ae. tauschii*, *T. urartu* and *T. aestivum*) by using MrBayes v3.2.7. Random representative samples of each subfamily were selected by the following criteria: members <  = 6, 1 RLK; 6 < members <  = 30, 2 RLKs; members > 30, 3 RLKs. Detailed information is provided in Fig. S1A

We classified the RLKs into 64 subfamilies by HMM models (Table S2). We only selected 1–3 members from every subfamily as the representative sequences to construct phylogenetic trees. To confirm the classification from HMM models, four types of phylogenetic trees (including Bayesian tree, maximum likelihood (ML) tree, neighbour-joining (NJ) tree with JTT model, and NJ tree with p-distance model) were constructed based on the truncated kinase domain sequences (Fig. 1, Fig. S1A-D and Table S3). The results showed that almost all the classifications of RLK subfamilies from HMM models and four phylogenetic trees were the same (Table S3). Among 64 RLK subfamilies, we noticed that all RLK-Pelle_RLCK-IXb clades from Bayesian and NJ phylogenetic trees contained a green algae sequence (PNW75571), suggesting that the RLK-Pelle_RLCK-IXb clade might be the ancestral subfamily of 64 RLK subfamilies. The HMM scan results showed that both green algae sequences (PNW75571 and PNW75214) belonged to the RLK-Pelle_RLCK-IXb clade.

### Evolution and conserved exon‒intron structures of RLK gene subfamilies

**Fig. 2:**
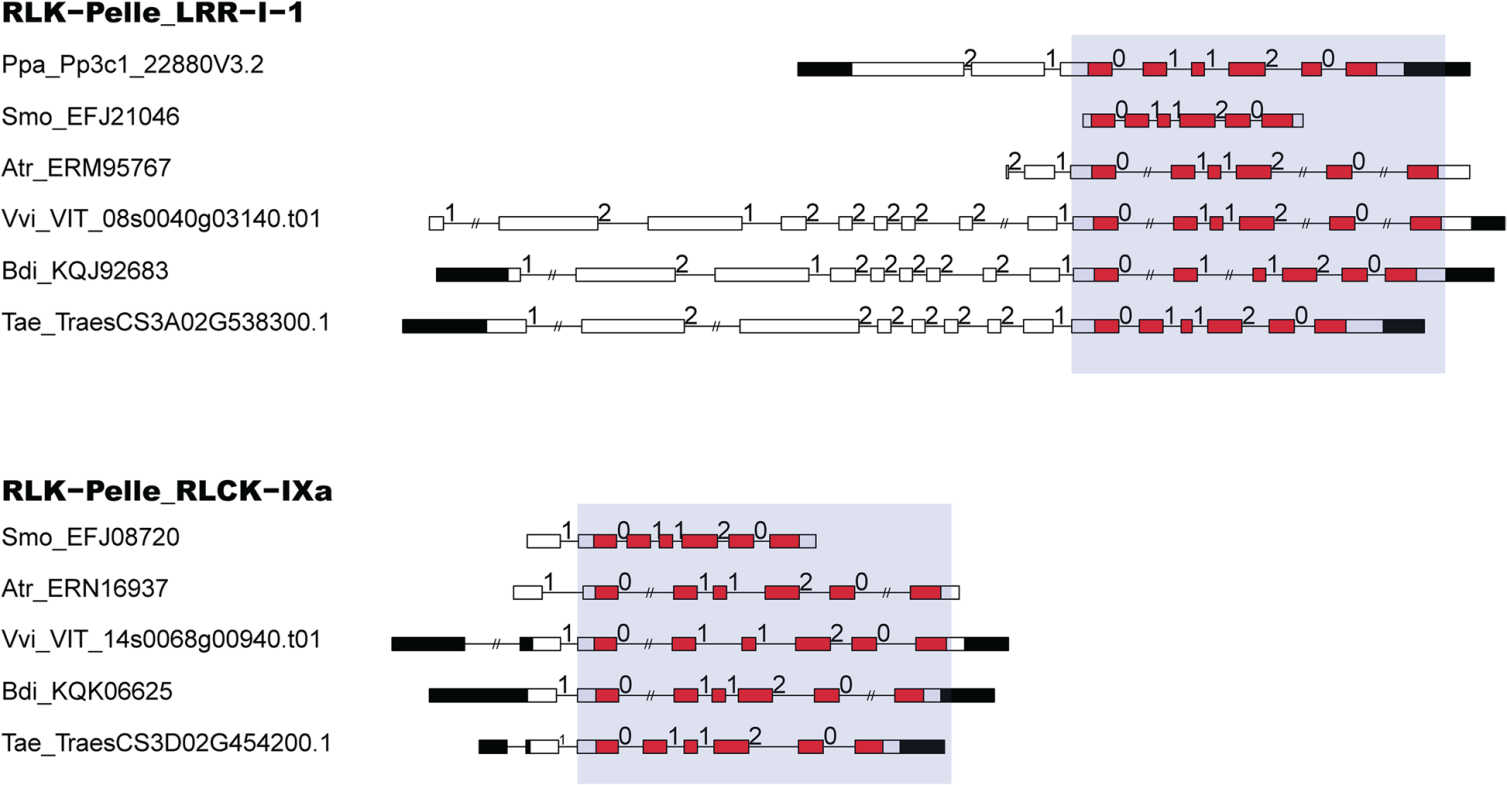
Two examples of conserved exon‒intron structures in RLKs. This diagram indicates that a conserved exon‒intron structure with conserved exon phases exists in the kinase domain. Filled boxes: red represents the kinase (PK_Tyr_Ser-Thr or Pkinase) domain; black boxes: untranslated regions (UTRs); white boxes: other exon regions; lines: introns. Numbers 0, 1, and 2: exon phases. The lengths of the boxes and lines are scaled based on the length of the genes. The long introns were shorted by “//”. (**A**) RLK-Pelle_LRR-I-1; **(B)** RLK-Pelle_RLCK-IXa

To obtain further insight into RLK evolution, we diagrammed the exon‒intron structures within the kinase domain in the 15 investigated plants (Fig. S2). The results showed that some conserved exon‒intron structures were present in the same RLK subfamilies across the investigated plants, especially in the kinase domain. We summarized these conserved exon‒intron structures in six representative plants, including *T. aestivum*, *B. distachyon*, *Vitis vinifera, Amborella trichopoda, Selaginella moellendorffii*, and *Physcomitrella patens* (Fig. S3). For instance, a conserved exon‒intron structure with exon phase “0112–0” existed in the RLK-Pelle_LRR-I-1 subfamily from *P. patens* to *T. aestivum* (Fig. 2). A similar example within the “0112–0” exon‒intron structure was also found in the RLK-Pelle_RLCK-IXa subfamily (Fig. 2).

To study the domain distributions, all RLK genes of 15 investigated plants were scanned against Pfam 34 in batches and diagrammed in our Perl and R scripts (Fig. S4). The results showed that some special domains existed across multiple RLK subfamilies. For example, LRR_8 Repeat domains (Pfam profile: PF13855) and the LRRNT_2 Family domain (Pfam profile: PF08263) existed in almost all RLK − Pelle_LRR (Leucine-rich repeat) subfamilies. Additionally, some RLK subfamilies contained only kinase domains. For example, some RLK − Pelle_DLSV members from *P. patens* (Pp3c22_10300V3.1), *S. moellendorffii* (EFJ35044), *A. trichopoda* (ERN15963), *B. distachyon* (PNT75271) and *T. aestivum* (TraesCS7B02G494200.1) contained only kinase domains.

### Chromosome location and duplication events of wheat RLKs

The chromosome locations of *T. aestivum* RLKs were mapped on 21 chromosomes (Fig. S5 and Table S4). The distribution of *T. aestivum* RLKs was among the A, B and D subgenomes.

**Fig. 3:**
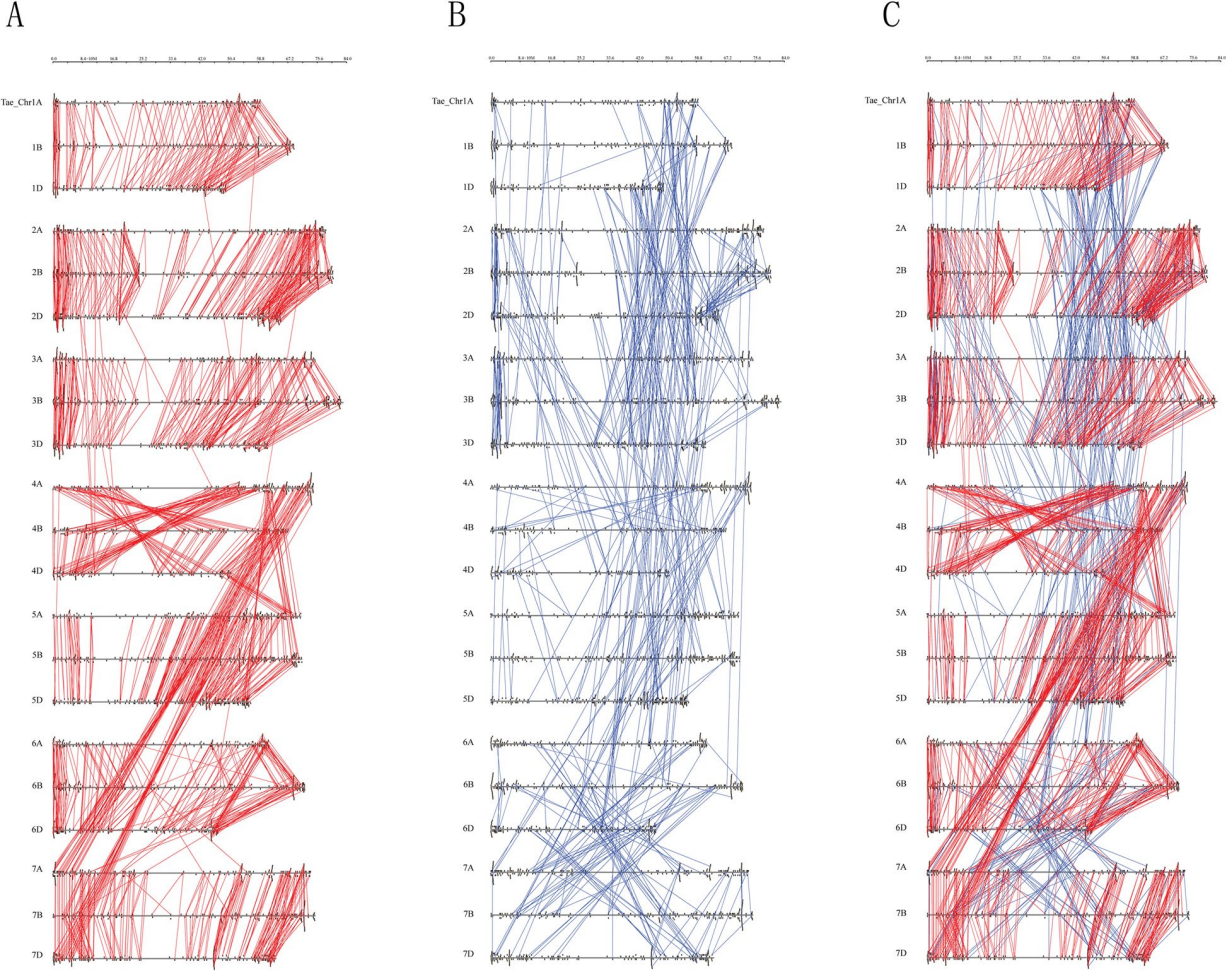
Collinearity events of *T. aestivum* RLK genes. (**A**) Collinearity events with *Ks* values of 0–0.35. (**B**) The other collinearity events. (**C**) All collinearity events. Red lines denote the collinearity events with *Ks* values of 0–0.35. Blue lines denote the other collinearity events

To study the whole genome duplication (WGD) events of wheat RLKs, we identified 2114 RLK gene pairs related to collinearity events by MCscanX (Table S5). Most *Ks* values of these wheat RLK collinearity events ranged from 0 to 0.35 and formed a peak of *Ks* at 0–0.15 (Fig. S6). These collinearity events of wheat RLKs were visualized on 21 chromosomes (Fig. 3A-C). The results showed that collinearity events within the *Ks* values of 0–0.35 were mainly located among the corresponding subgenomes, such as between 1A, 1B and 1D, suggesting that these collinearity events occurred along the polyploidization of *T. aestivum.* Moreover, collinearity events within *Ks* > 0.35 were mainly located across the corresponding subgenomes.

A total of 232 clusters of tandem duplications (TDs) were identified in *T. aestivum* RLKs (Fig. S7 and Table 2). Chromosomes 2D, 3B and 5D contained more than 15 clusters, which were 19, 17 and 18, respectively (Table S6). Some of these clusters belonged to the RLK-Pelle_DLSV subfamily. For example, a cluster on chromosome 5D within 4 genes (TraesCS5D02G357700.1, TraesCS5D02G357800.1, TraesCS5D02G358600.1 and TraesCS5D02G358700.1) belonged to the RLK-Pelle_DLSV subfamily. The largest cluster within 14 members was an RLK-Pelle_DLSV cluster, which was at the end of chromosome 2A. The second largest cluster within 13 members was also an RLK-Pelle_DLSV cluster, which was at the end of chromosome 7B.

**Table 2:** The tandem duplication clusters of RLKs in *T. aestivum* 21 chromosomes

Chromosome	Clusters of tandem duplication
1A	6
1B	9
1D	9
2A	13
2B	16
2D	19
3A	9
3B	17
3D	10
4A	9
4B	5
4D	4
5A	9
5B	15
5D	18
6A	10
6B	13
6D	10
7A	15
7B	8
7D	8

**Fig. 4:**
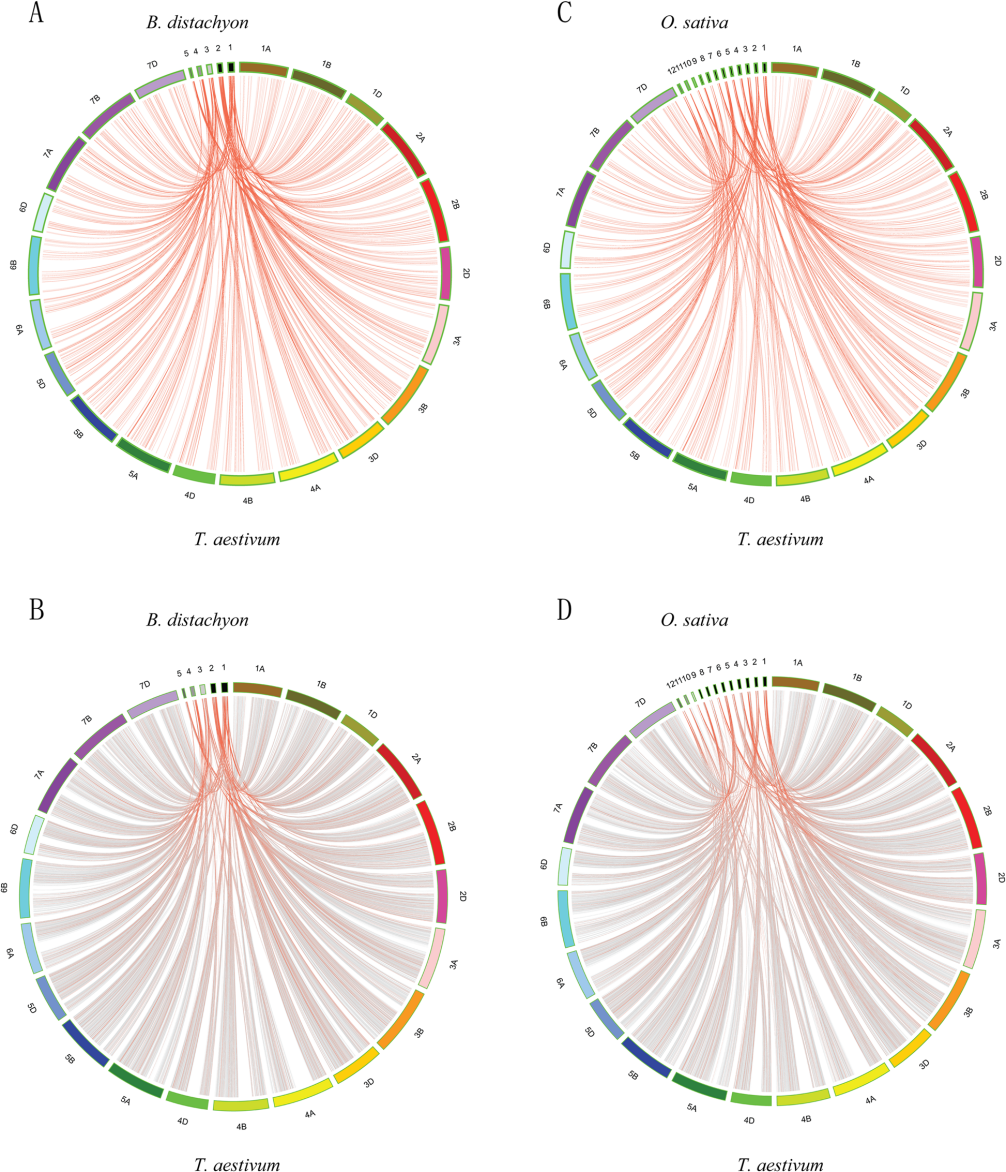
Synteny analysis of RLK genes. This graph displays syntenic maps among *T. aestivum*, *B. distachyon* and *O. sativa*. Red curves represent syntenic gene pairs between the RLKs, and grey curves represent other genes. (**A**) Synteny of RLKs between *T. aestivum* and *B. distachyon*; (**B**) Synteny of RLKs and other genes between *T. aestivum* and *B. distachyon*; (**C**) Synteny of RLKs between *T. aestivum* and *O. sativa*; (**D**) Synteny of RLKs and other genes between *T. aestivum* and *O. sativa*

To further study the mechanism of RLK duplication events, comparative syntenic maps of *T. aestivum, B. distachyon* and *O. sativa* RLKs were constructed (Fig. 4A-D). A total of 1081 RLK syntenic gene pairs were detected between *T. aestivum* and *B. distachyon.* Similarly, 961 RLK syntenic gene pairs were detected between *T. aestivum* and *O. sativa* (Table S7). Some RLK syntenic gene pairs shared the same *T. aestivum* RLK member associated with *B. distachyon* and *O. sativa,* suggesting that they might have descended from a single common ancestral sequence before the *Graminaceae* split in evolution. For instance, the *Tae*-*Bid* gene pair (TraesCS7D02G241500.1 and KQJ98873) and *Tae*-*Osa* gene pair (TraesCS7D02G241500.1 and Os08t0501200-00) shared the *T. aestivum* RLK gene (TraesCS7D02G241500.1), and all three of these genes belonged to the RLK-Pelle_WAK subfamily. We also calculated the *Ks* values between the *Tae*-*Bid* and *Tae*-*Osa* syntenic gene pairs (Table S7 and Fig. S8A-B). The results showed that the *Ks* values of *Tae*-*Bid* syntenic RLK gene pairs ranged from 0 to 0.95 and formed a peak within *Ks* values of 0.4–0.45. However, *Tae*-*Bid* syntenic gene pairs formed a peak within *Ks* values of 0.3–0.35. Similarly, the *Ks* values of *Tae*-*Osa* syntenic RLK gene pairs ranged from 0 to 1.35 and formed a peak within the *Ks* values of 0.5–0.55.

### Expression patterns of *T. aestivum*, *Ae. tauschii* and *B. distachyon* RLKs under drought stress

We studied the expression patterns of *T. aestivum, Ae. tauschii* and *B. distachyon* RLKs under biotic and abiotic stresses by using public transcriptome data at NCBI. According to the quality control performed by FastQC software, two transcriptome samples were excluded in the following analysis (Table S8). To search the expression pattern of wheat RLKs under drought stress, three public transcriptome datasets of wheat were selected for study (Fig. S9A-C and Table S9). (1) “TAM 111” and “TAM 112” (Bioproject: 659916): Wheat cultivars “TAM 111” and “TAM 112” (grown in southern America) have excellent drought tolerance. This RNA-seq analysis was conducted to compare gene expression differences in flag leaves of “TAM 111” and “TAM 112” under wet and dry conditions. We extracted the expression patterns of RLKs from this RNA-seq analysis. These results showed that some RLKs exhibited different expression patterns between “TAM 111” and “TAM 112”, suggesting that different drought-tolerance mechanisms existed in “TAM 111” and “TAM 112”. For example, TraesCS5B02G059000 (RLK-Pelle_DLSV) from the TAM112_Heading and TAM112_GrainFilling samples exhibited downregulation (log_2_ fold change, -1.71 and -2.07), while TraesCS5B02G059000 (RLK-Pelle_DLSV) from the TAM111_Heading and TAM111_GrainFilling samples exhibited upregulation (1.35 and 1.52). (2) “Svevo” and “IL20-2” (Bioproject: 686121): Two wheat genotypes, “Svevo” and “IL20-2”, were treated under well-watered and water-limited conditions. Then, the wheat seedling root tissues of “Svevo” and “IL20-2” underwent RNA extraction to perform transcriptome analysis. We studied the expression patterns of RLKs from this RNA-seq analysis. We noticed that some RLKs exhibited the same expression trends between “Svevo” and “IL20-2”. For instance, the expression trends of TraesCS2B02G008400 (RLK-Pelle_L-LEC) were downregulated with log_2_FC values of -1.57 and -1.99 in “IL20-2” and “Svevo”, respectively. (3) “L-82” and “Marvdasht” (Bioproject: 450487): Two wheat genotypes, “L-82” (drought-tolerant) and “Marvdasht” (drought-sensitive), were treated under well-watered and drought conditions. Then, RNA was extracted from the wheat root tissues of “L-82” and “Marvdasht” to perform transcriptome analysis. These results showed that some RLKs exhibited different expression patterns between “L-82” and “Marvdasht”, suggesting that different molecular mechanisms of drought tolerance existed in “L-82” (drought tolerant) and “Marvdasht” (drought sensitive). For instance, the expression trends of TraesCS5D02G374300 (RLK-Pelle_WAK) were down- and upregulated with log_2_FC values of -3.47 and 1.58 in “Marvdasht” and “L-82”, respectively.

**Fig. 5:**
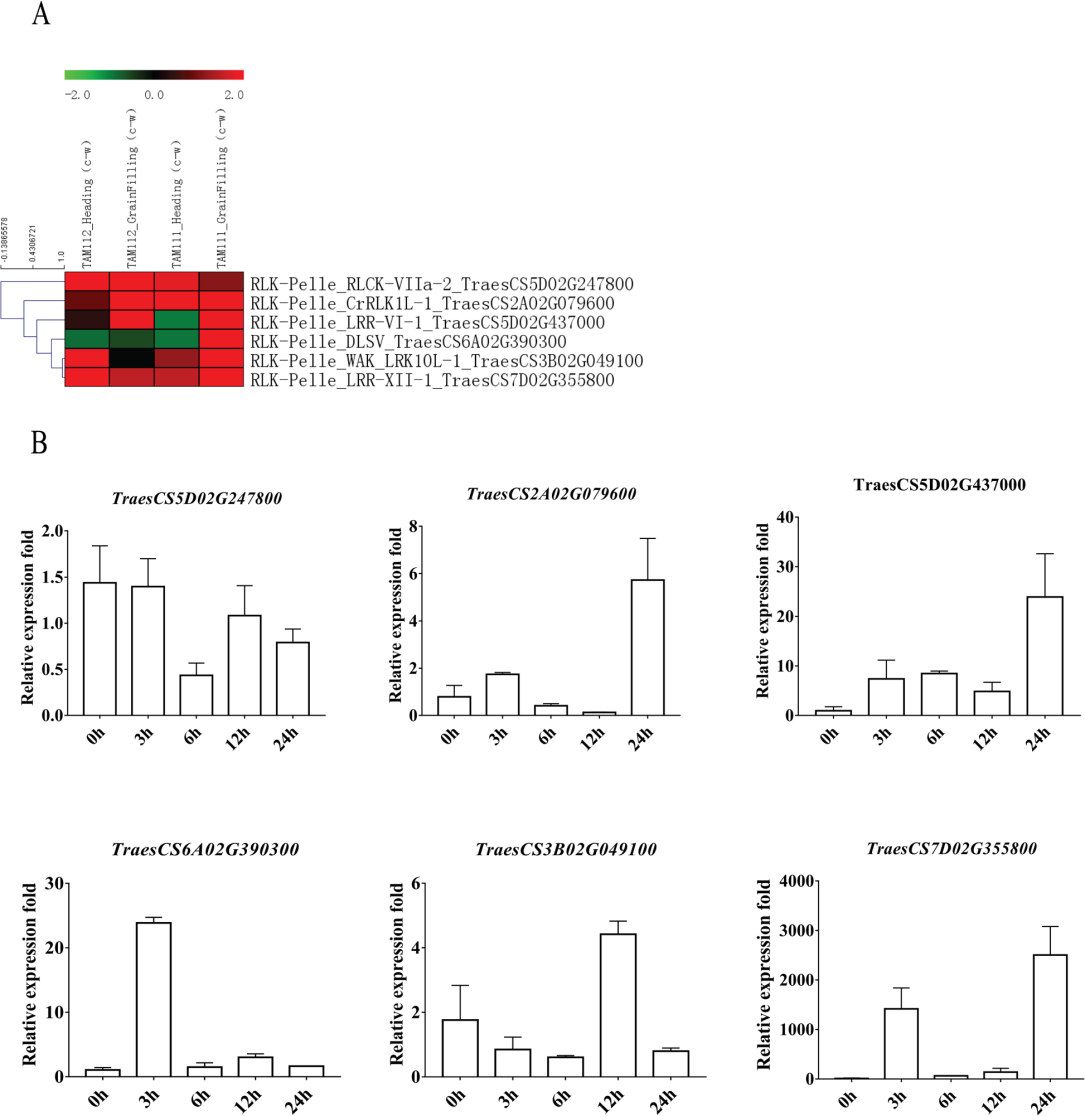
Heatmap of 6 selected wheat RLKs and their qRT‒PCR results under drought conditions. (**A**) Heatmap of the transcriptome; (**B**) qRT‒PCR under PEG-600 treatment

Drought is an abiotic stress that seriously affects wheat yield and quality. In our previous study, we found that some RLKs are involved in the drought response by bioinformatics analysis (Fig. 5 of article [41]). To further explore the expression patterns of RLK genes in the early stage of drought stress, we selected six wheat RLKs to examine their expression patterns under PEG (drought) treatment for 0, 3, 6, 12 and 24 h (hours) by using qRT‒PCR (Fig. 5A-B). We only compared the expression patterns between the public transcriptome (“TAM 111” and “TAM 112”, Bioproject: 659916) and qRT‒PCR because all of their sample tissues were from wheat leaves (the other two datasets were from root tissues). qRT‒PCR experiments also determined the expression patterns of RLKs from public transcriptome data under drought stress. The results showed that the expression trends were almost the same between the public transcriptome and qRT‒PCR. For example, the log_2_ values of TraesCS7D02G355800 (RLK-Pelle_LRR-XII-1) were 2.93, 1.50, 1.50 and 3.97 in the TAM112_Heading, TAM112_GrainFilling, TAM111_Heading and TAM111_GrainFilling samples, respectively. Our qRT‒PCR results validated that the expression trend of TraesCS7D02G355800 was the same as the transcriptome (Bioproject: 659916), exhibiting a peak within more than 1000-fold upregulation at 24 h.

The expression patterns of *Ae. tauschii* and *B. distachyon* RLKs under drought stress were also studied by using public transcriptome data at NCBI (Fig. S10A-B and Table S10). (1) *Ae. tauschii* cultivars “XJ98” and “XJ2” (Bioproject: 482066): Some *Ae. tauschii* RLKs exhibited similar expression trends between “XJ98” and “XJ2”. For example, the log_2_ values of AET5Gv20565600 (RLK-Pelle_SD-2b) were 2.30 and 3.33 in “XJ98” and “XJ2”, respectively. (2) *B. distachyon* cultivars “ABR4”, “ABR8” and “KOZ1” (Bioproject: 524106): Most *B. distachyon* RLKs exhibited different expression trends among “ABR4”, “ABR8” and “KOZ1”. For instance, the log_2_ values of BRADI_3g13827v3 (KQJ94884, RLK-Pelle_LRR-IV) were -1.05, 0.35, -2.0, -2.0, 2.15 and 2.09 in the “ABR4” t1, “ABR4” t2, “ABR8” t1, “ABR8” t2, “KOZ1” t1 and “KOZ1” t2 samples, respectively.

### Expression patterns of *T. aestivum* RLKs under other abiotic stresses

We also studied the expression patterns of *T. aestivum* RLKs under other abiotic stresses by using public transcriptome data at NCBI (Fig. S11A-C and Table S11). (1) Heat stress (Bioproject: 598150): The expression patterns of some *T. aestivum* RLKs exhibited a consecutive rise or decline at 10 days (d) and 14 d under heat stress treatment. For instance, the log_2_ values of TraesCS2D02G297100 (RLK-Pelle_SD-2b) were 2.66 and 5.55 at 10 d and 14 d, exhibiting the expression trend of a consecutive rise. Similarly, the log_2_ values of TraesCS7D02G386000 (RLK-Pelle_LRR-V) were -1.03 and -2.53 at 10 d and 14 d, exhibiting the expression trend of a consecutive decline. (2) Salinity stress (Bioproject: 573996): Under salinity stress, some *T. aestivum* RLKs exhibited different expression patterns between leaf and root tissue. For instance, the log_2_ values of TraesCS6B02G182900 (RLK-Pelle_LRR-III) were -4.06 in root tissue but 1.51 in leaf tissue. (3) Waterlogging stress (Bioproject: 604012): There were differences in waterlogging tolerance among different wheat varieties. Among the three investigated wheat varieties, the seeds of “Bainong 607” germinated earlier than those of “Bainong 207” and “Zhoumai 22” under waterlogging stress. Some *T. aestivum* RLKs exhibited upregulation in “Bainong 607” but downregulation in “Bainong 207” and “Zhoumai 22”, suggesting that different molecular mechanisms of waterlogging tolerance exist in different wheat varieties. For instance, the log_2_ values of TraesCS2D02G388000 (RLK-Pelle_RLCK-VIIa-2) were -1.47, -2.55 and 3.70 in “Zhoumai 22”, “Bainong 207” and “Bainong 607”, respectively.

### Expression patterns of *T. aestivum* RLKs under biotic stresses

We studied the expression patterns of *T. aestivum* RLKs under various abiotic stresses by using public transcriptome data at NCBI (Fig. S12A-D and Table S12). (1) *Fusarium graminearum* (EBI study accession:PRJEB12358): Near isogenic wheat lines (NILs) “NIL38” and “NIL51”, which were different in the presence of either or none of the FHB-resistance QTL Fhb1 and Qfhs.ifa-5A, were sequenced with *F. graminearum* treatments (3, 6, 12, 24, 36 and 48 h). Some *T. aestivum* RLKs exhibited similar expression patterns between “NIL38” and “NIL51”. For example, almost all the log_2_ values of TraesCS3A02G007200 (RLK-Pelle_LRR-XII-1) were upregulated between “NIL38” (4.23, 1.08, 3.45, 6.52, 4.00 and 4.56 at 3, 6, 12, 24, 36 and 48 h, respectively) and “NIL51” (5.66, 0.56, 3.22, 4.84, 2.56 and 5.89 at 3, 6, 12, 24, 36 and 48 h, respectively). However, other *T. aestivum* RLKs had expression patterns that were different between NIL38 and NIL51. For example, almost all the log_2_ values of TraesCS7A02G435000 (RLK-Pelle_LRR-Xb-1) were opposite (up- or downregulation) between “NIL38” (-0.02, -2.03, -0.32, -1.31, 0.30 and 0.45 at 3, 6, 12, 24, 36 and 48 h, respectively) and “NIL51” (-1.04, 0.38, 3.16, 1.49, -0.76 and 2.13 at 3, 6, 12, 24, 36 and 48 h, respectively). (2) Stripe rust (Bioproject: 613349): NILs “FLW29” (resistant) and cv. “PBW343” (susceptible) were subjected to transcriptome analysis in response to *P. striiformis* f. sp. *tritici* (*Pst*). Some *T. aestivum* RLKs exhibited upregulation in “FLW29” (resistant) after *Pst* treatment but downregulation in “PBW343” (susceptible), suggesting that these *T. aestivum* RLKs might participate in the signal pathways in response to *Pst*. For instance, the log_2_ values of TraesCS7D02G486900 (RLK-Pelle_DLSV) were opposite (up- or downregulation) between “FLW29” (2.63, 1.74 and 1.82 at 3, 6 and 12 h, respectively) and “PBW343” (-0.94, -1.84 and -1.73 at 3, 6 and 12 h, respectively). (3) *Xanthomonas translucens* with *Funneliformis mossae* (Bioproject: 474303): RNAseq of the wheat cultivar “Chinese Spring” was performed in root and leaf tissues during a long-term interaction with *F. mossae* (2 months) with or without pathogen infection by *X. translucens* CFBP 2054. Some *T. aestivum* RLKs exhibited opposite expression patterns between root and leaf tissues with Xanthomonas infection. For example, the log_2_ values of TraesCS5B02G208600 (RLK-Pelle_RLCK-VIIa-2) were -1.42 and 2.14 in roots and leaves, respectively. (4) *X. translucens* infection (Bioproject: 401247): The aim of this transcriptome was to detect the response of the wheat cultivar “Chinese Spring” to infection by the *X. translucens* pathogen. Some *T. aestivum* RLKs exhibited the opposite expression pattern between root and leaf tissues with Xanthomonas infection. For example, the log_2_ values of TraesCS3B02G043700 (RLK-Pelle_DLSV) were -1.96 and 4.37 in roots and leaves, respectively.

**Fig. 6:**
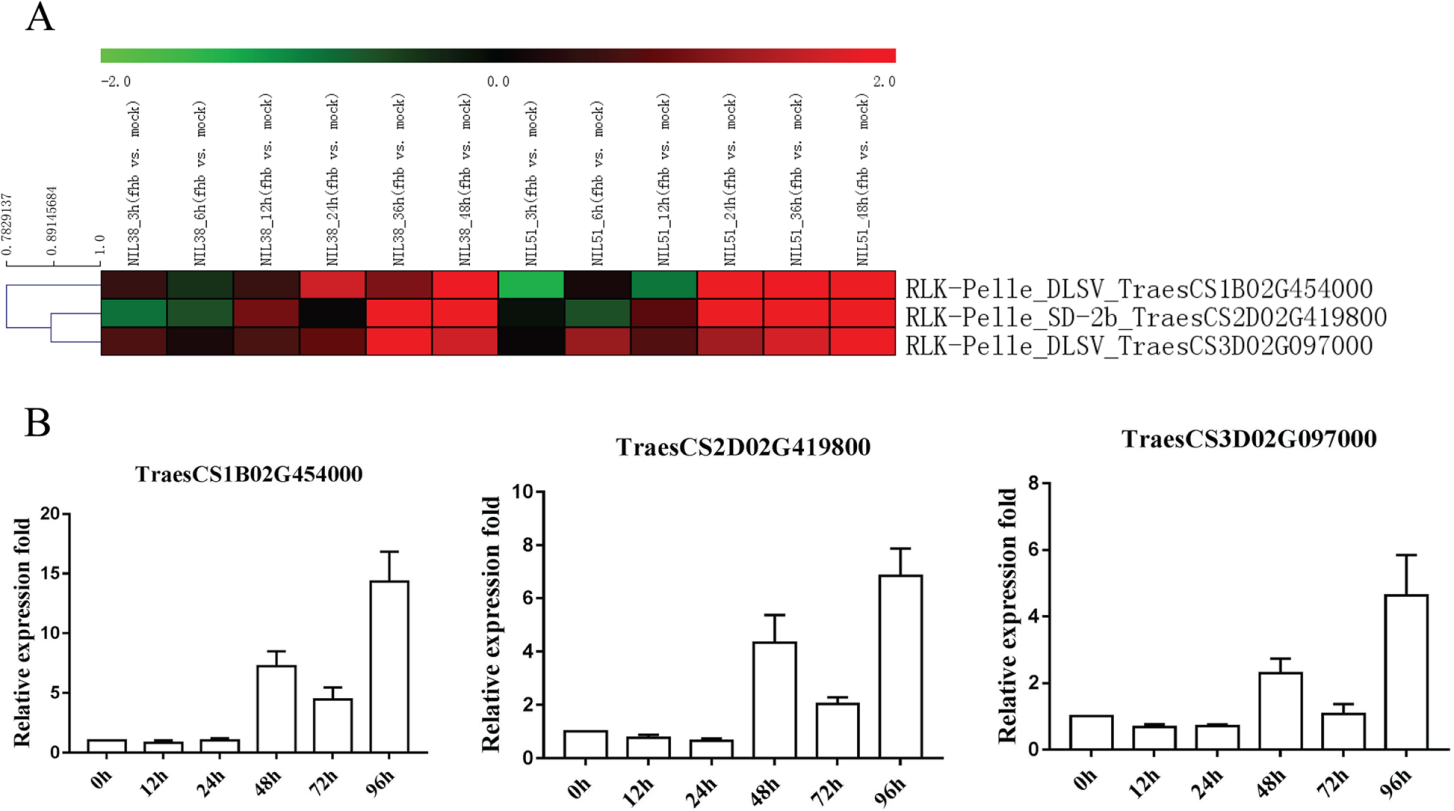
Heatmap of 3 selected wheat RLKs and their qRT‒PCR with *Fusarium graminearum* infection. (**A**) Heatmap of transcriptome; (**B**) qRT‒PCR with *F. graminearum* infection

To further determine the expression patterns of public transcriptome data under Fusarium head blight (FHB) stress, we selected three wheat RLKs to examine their expression patterns with *F. graminearum* treatments by using qRT‒PCR (Fig. 6A-B). The expression trends of three wheat RLKs from public transcriptome data were consistent with the results of qRT‒PCR. For instance, the log_2_ values of TraesCS1B02G454000 (RLK-Pelle_DLSV) in public transcriptome data were almost all upregulated to form a peak in NIL38 (0.48, -0.38, 0.50, 1.62, 1.00 and 1.89 at 3, 6, 12, 24, 36 and 48 h, respectively) and NIL51 (-1.36, 0.17, -0.92, 3.53, 1.89 and 4.28 at 3, 6, 12, 24, 36 and 48 h, respectively). Similarly, our qRT‒PCR results showed that TraesCS1B02G454000 (RLK-Pelle_DLSV) exhibited a peak of upregulation at 96 h after *F. graminearum* treatment.

## Discussion

### Evolution and duplication events of the RLK gene family in wheat and other plants

In this study, we identified RLKs in 15 representative plants, including green algae (*Chlamydomonas reinhardtii*) and moss (*P. patens*). Our results showed that there are only 4 RLKs (1 RLK-Pelle_C-LEC, 1 RLK-Pelle_L-LEC and 2 RLK-Pelle_RLCK-IXb) in *C. reinhardtii*, while the members have expanded into 298 RLKs (almost 64 RLK subfamilies) in *P. patens*. In 2021, Gong et al. also studied the early evolution and diversification of RLKs in 36 representative plants, including 5 rhodophytes, 1 glaucophyte, 1 prasinodermophyte, 18 chlorophytes, 6 charophytes, 2 bryophytes, and 3 vascular plants. Their results showed that RLKs have extensively diversified in charophytes, and charophyte RLKs mainly contribute to the diversity of land plant RLKs [42]. This was consistent with our results that moss RLKs had expanded into 298 members and almost 64 subfamilies. In 2021, rice *OsARK1* (ARBUSCULAR RECEPTOR-LIKE KINASE 1) was reported to have an ancient paralogue in spermatophytes, ARK2 [43]. Their results showed that *OsARK1* belongs to an unknown receptor kinase-2 (URK-2) subfamily, and a new domain, SPARK (Pfam ID: PF19160), was found in URK-2 orthologues. In our results, we also identified two RLK genes (Os07t0227300 − 00 and Os04t0465900 − 00) in the *O.sa* RLK − Pelle_URK − 2 subfamily. Interestingly, the rice sequence (Os07t0227300 − 00) also contained the SPARK domain (Fig. S4).

Among 64 RLK subfamilies, the members of some *T. aestivum* RLK subfamilies, such as RLK-Pelle_DLSV (829), RLK-Pelle_L-LEC (320), RLK-Pelle_LRR-XII-1 (337), RLK-Pelle_SD-2b (331), RLK-Pelle_WAK (385) and RLK-Pelle_LRR-XI-1 (237), were much larger than others, suggesting that certain RLK subfamilies experienced expansion during evolution (Table S2). This was consistent with the result of the 2009 article that RLKs had extensively expanding subfamilies, including DUF26, LRK10L-2, LRR-I, LRR-XII, SD1, SD-2b, and WAK [4]. The allohexaploid bread wheat (*T. aestivum*) genome contained three closely related subgenomes (A, B, and D). The A and B genomes diverged from a common ancestor approximately 7 million years ago, and the D genome diverged through homoploid hybrid speciation 5–6 million years ago. The bread wheat genome has experienced multiple rounds of hybrid speciation [44]. Our results showed that almost all RLKs were homologous sequences among the subgenome A, B and D chromosomes (Fig. 3). The *Ks* values of these RLK collinearity events were 0–0.35, suggesting that they were associated with polyploidization events among the wheat A, B and D subgenomes (Table S5 and Fig. S6). It has been reported that polyploidy, tandem duplications, segmental duplications and transposition events are the main mechanisms for the expansion of the wheat expansin gene family [45]. Consistent with their results, we also found some tandem RLK clusters on wheat chromosomes, suggesting that tandem duplication events also contributed to the expansion of RLK members during evolution (Fig. S7).

We also studied the exon‒intron structures among subfamilies of RLKs from 15 plants and found some conserved exon‒intron structures with conserved exon phases in the kinase domain of RLKs (Figure S3). Interestingly, a similar result was also reported that an unique intron phase pattern was also found in the plant cyclic nucleotide-gated ion channel (CNGC) gene family, which is involved in plant disease resistance [46]. For example, they found that group IVa CNGC genes had the unique phase pattern "0–0-0–0-0–0-2–2-0–1-2" (52%) for 11-intron genes and "0–0-0–0-0–2-2–0-1" (14%) for 10-intron genes.

There are several comprehensive reports about the genome-wide identification and classification of the RLK gene family in many plants [2–5]. However, our results were more comprehensive. We studied the exon‒intron structures of RLKs and found some conserved exon‒intron structures in evolution. In addition, our work included five different varieties of wheat (*T. aestivum, Triticum spelta, Triticum turgidum, Triticum dicoccoides, Triticum urartu*), which are diploid, tetraploid and hexaploid wheat, so our results will provide more detailed information on RLK evolution in wheat. Moreover, we studied the expression patterns of wheat RLKs under biotic and abiotic stresses by using public transcriptome data and qRT‒PCR. This study will provide candidate RLKs of drought and *F. graminearum* stresses for researchers.

### Expression pattern of wheat RLKs under drought and *F. graminearum* stresses

We studied the RLK expression patterns under drought stress by using public transcriptome data and qRT‒PCR. (1) DLSV-RLK: In 2021, *Arabidopsis* cysteine-rich receptor-like protein kinase *AtCRK33* was found to affect drought tolerance and stomatal density. CRKs contain the DUF26 (Domain of Unknown Function 26) domain [47]. The DUF26 domain, also known as the stress-antifungal domain (PF01657, Pfam domain name, Stress-antifungal Family) on the Pfam website [48], could be found in the RLK − Pelle_DLSV subfamily in our results (Fig. S4). We also detected the expression pattern of an RLK − Pelle_DLSV member (TraesCS6A02G390300) under drought stress by using public transcriptome data and qRT‒PCR (Fig. 5). The qRT‒PCR results showed that it exhibited a peak of upregulation at 3 h. The log_2_ value of TraesCS6A02G390300 in the TAM111_GrainFilling (c-w) sample was 4.29. (2) LRR-RLK: In 2011, it was reported that overexpression of *PdERECTA* (*Populus deltoides* LRR-RLK gene) in *Arabidopsis* enhances drought resistance [22]. In 2021, Li et al. discovered that overexpression of the *PdERECTA* gene in *poplar* improved water use efficiency and enhanced drought tolerance by reducing stomatal density and restricting water consumption [49]. A similar result was also reported in which overexpression of the *Sorghum bicolor* gene *SbERECTA* in *Arabidopsis* and maize enhanced their drought tolerance [50]. In our results, we also checked the expression pattern of two LRR-type RLKs (RLK-Pelle_LRR-VI-1 TraesCS5D02G437000 and RLK-Pelle_LRR-XII-1 TraesCS7D02G355800) under drought stress by using public transcriptome data and qRT‒PCR (Fig. 5). The qRT‒PCR results showed that they all exhibited a peak of upregulation at 24 h. Their log_2_ values in four samples were all upregulated. (3) SD-2b: In 2020, an S-domain RLK gene *OsESG1* (LOC_Os01g12410) in rice (*O. sativa*) was identified in early crown root development and drought response by controlling auxin response and distribution [51]. We obtained the protein sequence (LOC_Os01g12410, Os01t0223800-01) and converted the ID on the website (https://rapdb.dna.affrc.go.jp/). In our results, it belonged to the RLK subfamily RLK-Pelle_SD-2b (Table S1).

Some articles have studied the relationships between *F. graminearum* infection and RLKs in wheat and other plants. After *F. graminearum* infection, Manes et al. screened 227 RLKs and innate immune response genes in *Arabidopsis* and identified nine genes (including RLK7) that play roles in *F. graminearum* resistance [52]. An LRR-RLK gene (GRMZM2G132212) in maize was identified as a defence or recognition gene in the response to fungal pathogens (*Cochliobolus heterostrophus* and *F. graminearum*). However, *F. graminearum* might be able to exploit this LRR-RLK gene (GRMZM2G132212) function to increase its virulence [53]. We converted ID (GRMZM2G132212, Zm00001eb293660) in NCBI and Ensembl plant and found that it belonged to the RLK subfamily RLK-Pelle_LRR-XI-1 in our results (Table S1). Two LRR-RLK genes (*HvLRRK-6H* and *TaLRRK-6D*) were found to contribute to *Fusarium* resistance in cereals (*H. vulgare* and *T. aestivum*) [54]. In our results, we also checked the expression pattern of two DLSV-type RLKs (RLK-Pelle_DLSV TraesCS1B02G454000 and RLK-Pelle_DLSV TraesCS3D02G097000) with *F. graminearum* infection by using public transcriptome data and qRT‒PCR (Fig. 6). qRT‒PCR showed that they all exhibited a peak of upregulation at 48 and 96 h. The log_2_ values of these genes in some NIL38 and NIL51 samples (24, 36 and 48 h) were all upregulated. They might be new resistance genes to defend against *F. graminearum* infection*.* Indeed, it was reported that a novel CRK RLK (DLSV-RLK subfamily in our results) gene, *TaCRK3*, could defend against another fungal pathogen, *R. cerealis*, in wheat [37].

## Materials and methods

### Identification and classification of RLKs in plants

The genomes and proteomes of *T. aestivum*, *T. spelta*, *T. turgidum*, *T. dicoccoides*, *T. urartu*, *Ae. tauschii*, *B. distachyon*, *Zea mays*, *O. sativa*, *A. thaliana*, *V. vinifera*, *A. trichopoda*, *S. moellendorffii*, *P. patens* and *C. reinhardtii* were downloaded from Ensembl Plant release-51 (http://plants.ensembl.org/). To identify the PKs (protein kinases), all the proteomes of the fifteen plants were scanned by our local server HMMER3.1 (PK_Tyr_Ser-Thr.hmm pfam profile PF07714.19, Pkinase.hmm PF00069.27) and website pfam 34.0 (http://pfam.xfam.org/) in batch mode with an E value of 0.01. Atypical PKs with kinase (PK_Tyr_Ser-Thr or Pkinase) domains covering less than 50% alignment were excluded from the following analysis. Classifications of “typical” sequences of PK subfamilies were performed by HMMER 3.1 with HMM models developed by Legti-Shiu and Shui [55].

We selected 1–3 members as the representative sequences from every RLK subfamily to construct the phylogenetic trees. Each RLK subfamily was selected by the following criteria: members ≤ 6, 1 RLK; 6 < members ≤ 30, 2 RLKs; members > 30, 3 RLKs. The alignment of truncated RLK sequences in the kinase (PK_Tyr_Ser-Thr or Pkinase) domain was performed by ClustalW v2.0 [56]. A Bayesian phylogenetic tree was constructed using MrBayes v3.2.7 [57] with the mixed amino acid substitution model, and an MCMC chain with 10,000,000 generations was used. Markov chains were sampled every 100 generations, and the first 25% of the trees were discarded as burn-in. The results of MrBayes v3.2.7 were analysed by TreeGraph v2.14 [58] and our Perl scripts. The ML phylogenetic tree was constructed using PhyML v3.1 [59] with 100 bootstrap replicates. The appropriate model of the ML method, including model parameters, was calculated using the Akaike information criterion (AIC) with ProtTest v3.4 [60]. The NJ phylogenetic trees were constructed by Megacc 7.0 [61] with a model (p-distance or JTT) and 1000 bootstrap repetitions. The four types of phylogenetic trees were constructed by the above descriptions in our local server.

### Domain and intron–exon structure diagram of RLKs

The domain and intron–exon structures of RLK sequences in these fifteen plants were generated by our Perl and R scripts based on the corresponding GFF file information from Ensembl Plant release-51 (http://plants.ensembl.org/). The domain information of pfam-A models was downloaded from pfam 34.0 (http://pfam.xfam.org/) and then scanned in our local server.

## Chromosome locations, duplication events and synthetic analysis of wheat RLKs

Based on the extracted information in the GFF files from Ensembl Plants release-51 (http://plants.ensembl.org/), the chromosome locations of *T. aestivum* RLKs were diagrammed using GenomePixelizer software [62]. BLASTP was performed against RLKs of *T. aestivum*, *B. distachyon* and *O. sativa* with an E value of e-100. Based on the GFF files and BLAST results, tandem duplication and segmental duplication were searched using MCScanX [63]. The *Ka* and *Ks* values were calculated by “add_ka_and_ks_to_collinearity.pl” from MCScanX. Based on the GFF files and MCScanX results, synthetic diagrams among *T. aestivum*, *B. distachyon* and *O. sativa* were generated by using our Perl scripts and Circos software (http://circos.ca/). The chromosome locations of the tandem duplicate RLKs were mapped on each chromosome by using Mapchart v2.3 (http://www.wageningenur.nl/en/show/mapchart.htm).

### Bioinformatic analysis of public transcriptome expression data

Public wheat (*T. aestivum*), *Ae. tauschii* and *B. distachyon* transcriptome expression datasets were retrieved from the Sequence Read Archive (SRA) of NCBI. (1) Drought stress: Three drought RNA-seq datasets of wheat were about three groups of wheat cultivars, which were “TAM 111” and “TAM 112” (Bioproject: 659916), “Svevo” and “IL20-2” (Bioproject: 686121), and “L-82” and “Marvdasht” (Bioproject: 450487). Two drought RNA-seq datasets of *Ae. tauschii* and *B. distachyon* were *Ae. tauschii* cultivars “XJ98” and “XJ2” (Bioproject: 482066) and *B. distachyon* cultivars “ABR4”, “ABR8” and “KOZ1” (Bioproject: 524106), respectively. (2) Other abiotic stresses: Three wheat RNA-seq datasets of abiotic stresses included heat stress (Bioproject: 598150), salinity stress (Bioproject: 573996), and waterlogging stress (Bioproject: 604012). (3) Biotic stresses: Four wheat RNA-seq datasets of biotic stresses were about *F. graminearum* infection (EBI study accession: PRJEB12358), stripe rust (Bioproject: 613349), interactions with mycorhizal fungi (*F. mossae*) with and without pathogen attack by *X. translucens* (Bioproject: 474303), and *X. translucens* infection (Bioproject: 401247).

Quality control assessment of raw data was performed using FastQC v0.11.7 (https://www.bioinformatics.babraham.ac.uk/projects/fastqc/). High-quality RNA-seq reads were aligned to reference wheat (*T. aestivum*), *Ae. tauschii* and *B. distachyon* genomes of Ensembl Plants release-51 by Hisat2 v2.2 software [64]. The counts of expressed genes were performed using Samtools v1.10 [65] and HTseq v0.11.3 [66] software. The expression levels of the transcriptome (log_2_ value) were calculated by using R software and the R package DESeq2. Heatmaps of wheat RLK expression levels were generated using Mev4.9 [67].

### Plant materials and stress treatments

Wheat (*T. aestivum* L.) cultivar “SuMai 3” was used in this study. The wheat seedlings were planted into pots and grown at 22–25 °C with a photoperiods of 16 h of light and 8 h of darkness. The seedlings of wheat (*T. aestivum* L.) cultivar “SuMai 3” at the three-leaf and one-heart stage were drought stressed, which including 20% (m/V) PEG-6000 for 0, 3, 6, 12 and 24 h. The wheat leaves were harvested and immediately frozen in liquid nitrogen for expression analysis. At least 15 samples of each experimental replicate were analysed. The spikes of “SuMai 3” were inoculated with 10 μL of an *F. graminearum* (PH1-1) conidia suspension (5–10 × 10^4^ conidia mL^−1^). Then, the inoculated spikes were sealed in plastic bags to retain moisture for 72 h. The inoculated wheat spikes were collected after 0, 12, 24, 48, 72 and 96 h, and used to perform qRT‒PCR analysis.

### RNA Extraction and qRT‒PCR

The total RNA of each sample was extracted using the RNAprep Pure Plant Kit (Tiangen) and reverse transcribed into cDNA using the HiScript III 1st strand cDNA synthesis kit (Vazyme). The cDNA samples were used for qRT‒PCR analysis. qRT‒PCR analysis was performed using a Roche LightCycler® 480 (Roche Diagnostics GmbH, Mannheim, Germany). The wheat gene *β-Actin* was used as an endogenous control. Relative expression levels of genes were calculated using the Formula 2 ^−ΔΔCT^. Each experiment included three biological and technical replicates. All the qRT‒PCR primers in this study are provided in Table S13. The melting curves of qRT‒PCR samples are provided in Figure S13.

## Supplementary Information

**Additional file 1: Figure S1.** Phylogenetic classification of RLKs with 1-3 randomly chosen members in every subfamily from 9 representative plants (*C. reinhardtii, P. patens, S. moellendorffii, A. trichopoda, A. thaliana, B. distachyon, Ae. tauschii, T. urartu and T. aestivum*) by using the four methods. (A) Bayes; (B) ML (LG+I+G+F); (C) NJ (JTT); (D) NJ (p-distance).

**Additional file 2: Figure S2.** Exon−intron and domain diagrams of RLKs in 15 plants. The descriptions of the domain and exon phases are the same as those in Fig. 2. The lengths of the boxes and lines are scaled based on the lengths of the genes.

**Additional file 3: Figure S3.** Conserved exon−intron and domain diagrams of RLKs in *T. aestivum, B. distachyon, V. vinifera, A. trichopoda, S. moellendorffii and P. patens*. The descriptions of the domain and exon phases are the same as those in Fig. 2. The lengths of the boxes and lines are scaled based on the lengths of the genes.

**Additional file 4: Figure S4.** Domain diagrams of RLKs in 15 plants. Different domains are represented by boxes with different colours.

**Additional file 5: Figure S5.** Chromosome locations of RLKs in *T. aestivum*. Chromosomal locations of *T. aestivum* RLKs. Yellow boxes denote *T. aestivum* RLK genes.

**Additional file 6: Figure S6.** Collinearity (Ks values) of *T. aestivum* PK genes. Collinearity events of duplicated RLKs in the *T. aestivum* genome. The red bars denote the collinearity events contributed by polyploidizations (*Ks* values 0–0.35). The blue bars denote the other collinearity events. Information on the collinearity events is provided in Table S5.

**Additional file 7: Figure S7.** Chromosomal locations of the tandemly arrayed *T. aestivum* RLK genes. The tandemly arrayed T. aestivum RLK genes were grouped into 232 clusters distributed on the 21 chromosomes. Subfamilies and Gene IDs are labelled on the right of each chromosome, and the chromosomal location of each cluster is on the left of each chromosome. Genes in the same cluster are highlighted in the same colour. The information of chromosomal locations is shown in Table S6.

**Additional file 8: Figure S8.** Collinearity (Ks values) among *T. aestivum, B. distachyon* and *O. sativa*. (A) Collinearity events of duplicated RLKs and all genes between *T. aestivum, B. distachyon*. The green bars denote the collinearity events contributed by (A) polyploidizations of RLKs (*Ks* values 0.25–0.6) and all genes (*Ks* values of 0.3–0.45). The blue bars denote the other collinearity events. (B) Collinearity events of duplicated RLKs and all genes between *T. aestivum* and *O. sativa*. The pink bars denote the collinearity events contributed by polyploidizations of RLKs (*Ks* values of 0.3–0.8) and all genes (*Ks* values of 0.4–0.6). The blue bars denote the other collinearity events. Information on the collinearity events is provided in Table S7.

**Additional file 9: Figure S9.** Heatmap of the expression patterns of individual *T. aestivum* RLK genes under drought stress treatments. (A) In wheat cultivars “TAM 111” and “TAM 112”; (B) in two wheat genotypes “Svevo” and “IL20-2”; and (C) in two wheat genotypes “L-82” and “Marvdasht”.

**Additional file 10: Figure S10.** Heatmap of the expression patterns of individual *Ae. tauschii* and *B. distachyon* RLK genes under drought stress treatments. (A) In two Ae. tauschii cultivars “XJ98” and “XJ2” and (B) in three B. distachyon cultivars “ABR4”, “ABR8” and “KOZ1”.

**Additional file 11: Figure S11.** Heatmap of the expression patterns of individual *T. aestivum* RLK genes under other abiotic stress treatments. (A) Heat stress; (B) salinity stress; and (C) waterlogging stress.

**Additional file 12: Figure S12.** Heatmap of the expression patterns of individual *T. aestivum* RLK genes under biotic stress treatments. (A) *F. graminearum* infection; (B) stripe rust; (C) interactions with mycorhizal fungi (*F. mossae*) with and without pathogen attack by *X. translucens*; and (D) *X. translucens infection*.

**Additional file 13: Figure S13.** The melting curves of qRT‒PCR about 9 selected *T. aestivum* RLKs.

**Additional file 14: Table S1.** Classifications of protein kinases (including RLKs) in 15 plants. Each classification of PKs from 15 plants was in sheets 1-15.

**Additional file 15: Table S2.** Comparison of RLK subfamily sizes in 15 plants.

**Additional file 16: Table S3.** Classification of some representative members from RLK subfamilies in 9 representative plants.

**Additional file 17: Table S4.** Chromosome locations of *T. aestivum* RLKs.

**Additional file 18: Table S5.** Collinearity events and *Ka/Ks* values of *T. aestivum* RLKs.

**Additional file 19: Table S6.** Chromosome locations of *T. aestivum* tandem duplication RLKs.

**Additional file 20: Table S7.** Collinearity events and Ka/Ks values of RLKs among *T. aestivum, B. distachyon* and *O. sativa*. Sheets 1-2 show the *Ka/Ks* values of collinearity events from RLKs and all genes between *T. aestivum* and *B. distachyon*. Similarly, sheets 3-4 showed *T. aestivum* and *O. sativa*.

**Additional file 21: Table S8.** Public wheat, *Ae. tauschii* and *B. distachyon* RNA-seq expression data for use.

**Additional file 22: Table S9.** The expression of *T. aestivum* RLK genes under drought stress treatment. Sheet 1 was about the wheat (*Triticum aestivum L.*) cultivars "TAM 111" and "TAM 112" (US). Sheet 2 was about "Svevo" and "IL20-2". Sheet 3 was about “L-82” and “Marvdasht”.

**Additional file 23: Table S10.** The expression of *Ae. tauschii* and *B. distachyon* RLK genes under drought stress treatment. Sheet 1 was about Ae. tauschii. Sheet 2 was about *B. distachyon*.

**Additional file 24: Table S11.** Normalized gene expression log2-fold fold change (treatment vs. control) values of *T. aestivum* RLK genes from transcriptomes under abiotic stress treatments. Sheet 1 was about heat stress. Sheet 2 was about salinity stress. Sheet 3 was about waterlogging stress.

**Additional file 25: Table S12.** Normalized gene expression log2-fold fold change (treatment vs. control) values of *T. aestivum* RLK genes from transcriptomes under biotic stress treatments. Sheet 1 was about *F. graminearum* treatment. Sheet 2 was about stripe rust. Sheet 3 was about *Funneliformis* and *Xanthomonas* treatment. Sheet 4 was about *Xanthomonas* treatment.

**Additional file 26: Table S13.** Primers used in the qRT‒PCR analysis.

## Data Availability

The genomes, proteomes and GFF files of the investigated plants are available in Ensembl Plants 51-release (http://plants.ensembl.org/). The accession numbers of plants are *T. aestivum* (IWGSC), *T. spelta* (PGSBv2.0), *T. turgidum* (Svevo.v1), *T. dicoccoides* (WEWSeq_v.1.0), *T. urartu* (ASM34745v1), *Ae. tauschii* (Aet_v4.0), *B. distachyon* (v3.0), *Z. mays* (Zm-B73-REFERENCE-NAM-5.0), *O. sativa* (IRGSP-1.0), *A. thaliana* (TAIR10), *V. vinifera* (12X), *A. trichopoda* (AMTR1.0), *S. moellendorffii* (v1.0), *P. patens* (Phypa_V3) and *C. reinhardtii* (v5.5). Public wheat (*T. aestivum*), *Ae. tauschii* and *B. distachyon* transcriptome expression datasets were retrieved from the SRA database of NCBI and EBI. The SRA accession numbers of transcriptomes are 659916, 482066, 524106, 598150, 573996, 604012, 613349, 474303 and 401247. The EBI study accession of *F. graminearum* infection is PRJEB12358.
