# Supplementary figures and images for: Phylogeny of the plant receptor-like kinase (RLK) gene family and expression analysis of wheat RLK genes in response to biotic and abiotic stresses
Source: BMC Genomics. 2023 May 1;24:224. doi: 10.1186/s12864-023-09303-7 (PMC10152718; doi:10.1186/s12864-023-09303-7)

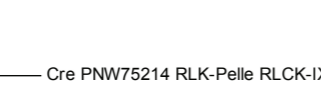





# D NJ(p-distance)

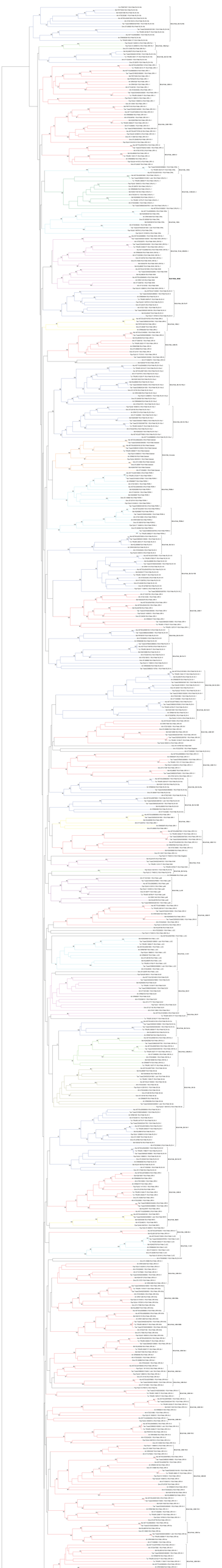

On PNT07511 RFLP-PuB\_LRR-VII

Supplement: Supplementary file 1 — Additional file 1: Figure S1. Phylogenetic classification of RLKs with 1-3 randomly chosen members in every subfamily from 9 representative plants (C. reinhardtii, P. patens, S. moellendorffii, A. trichopoda, A. thaliana, B. distachyon, Ae. tauschii, T. urartu and T. aestivum) by using the four methods. (A) Bayes; (B) ML (LG+I+G+F); (C) NJ (JTT); (D) NJ (p-distance). [file 12864_2023_9303_MOESM1_ESM.pdf]

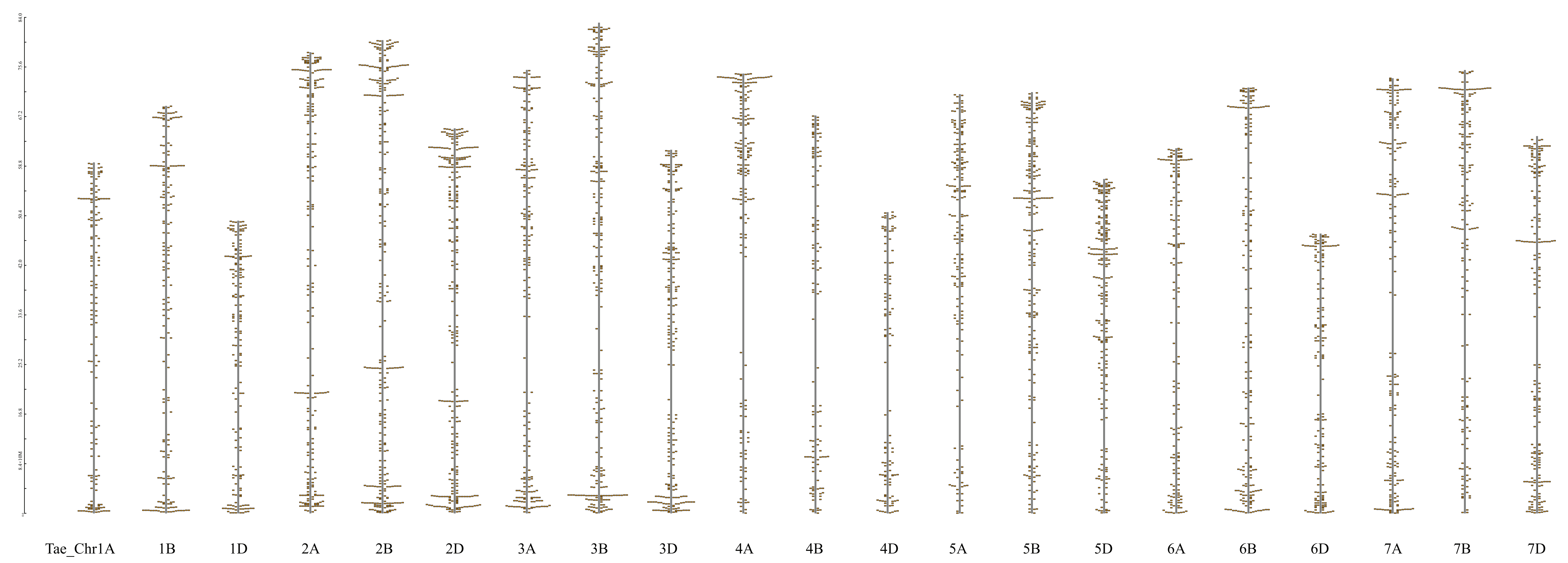

Supplement: Supplementary file 5 — Additional file 5: Figure S5. Chromosome locations of RLKs in T. aestivum. Chromosomal locations of T. aestivum RLKs. Yellow boxes denote T. aestivum RLK genes. [file 12864_2023_9303_MOESM5_ESM.pdf]

Frequency of duplication events

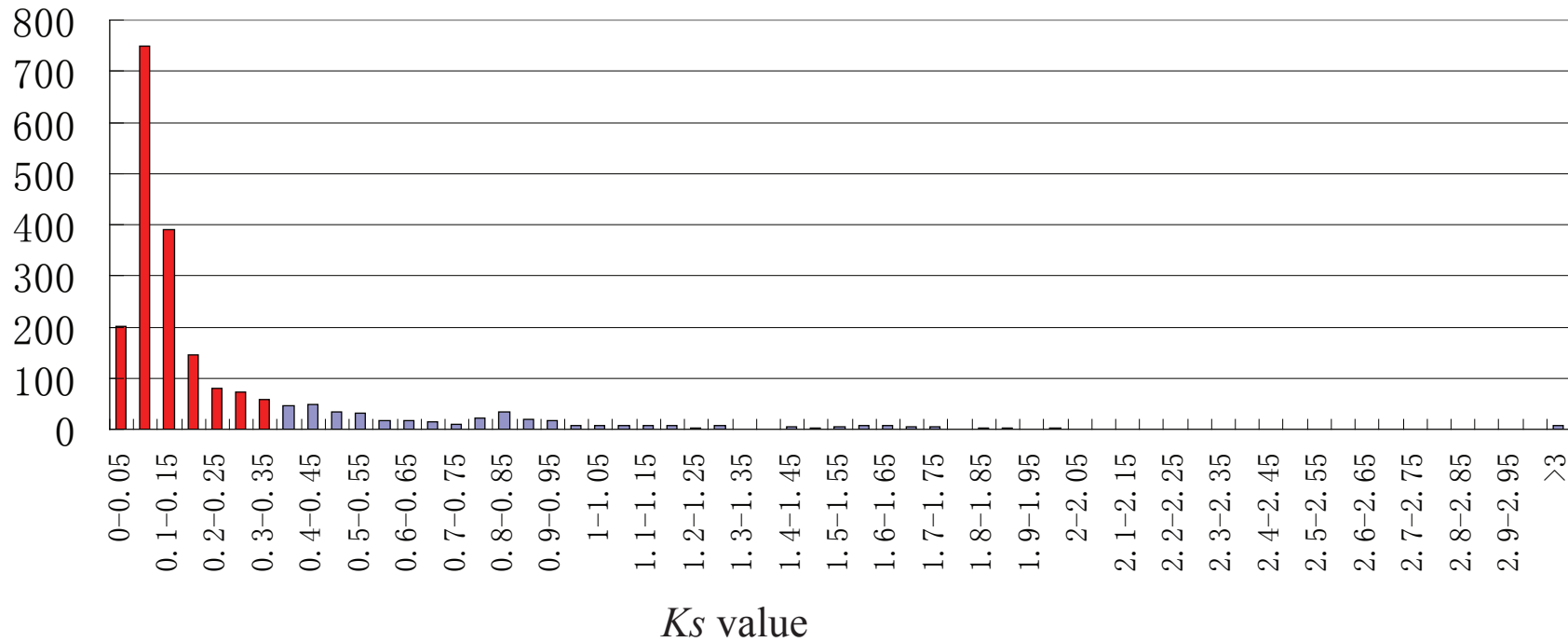

Supplement: Supplementary file 6 — Additional file 6: Figure S6. Collinearity (Ks values) of T. aestivum PK genes. Collinearity events of duplicated RLKs in the T. aestivum genome. The red bars denote the collinearity events contributed by polyploidizations (Ks values 0–0.35). The blue bars denote the other collinearity events. Information on the collinearity events is provided in Table S5. [file 12864_2023_9303_MOESM6_ESM.pdf]

Tae1A

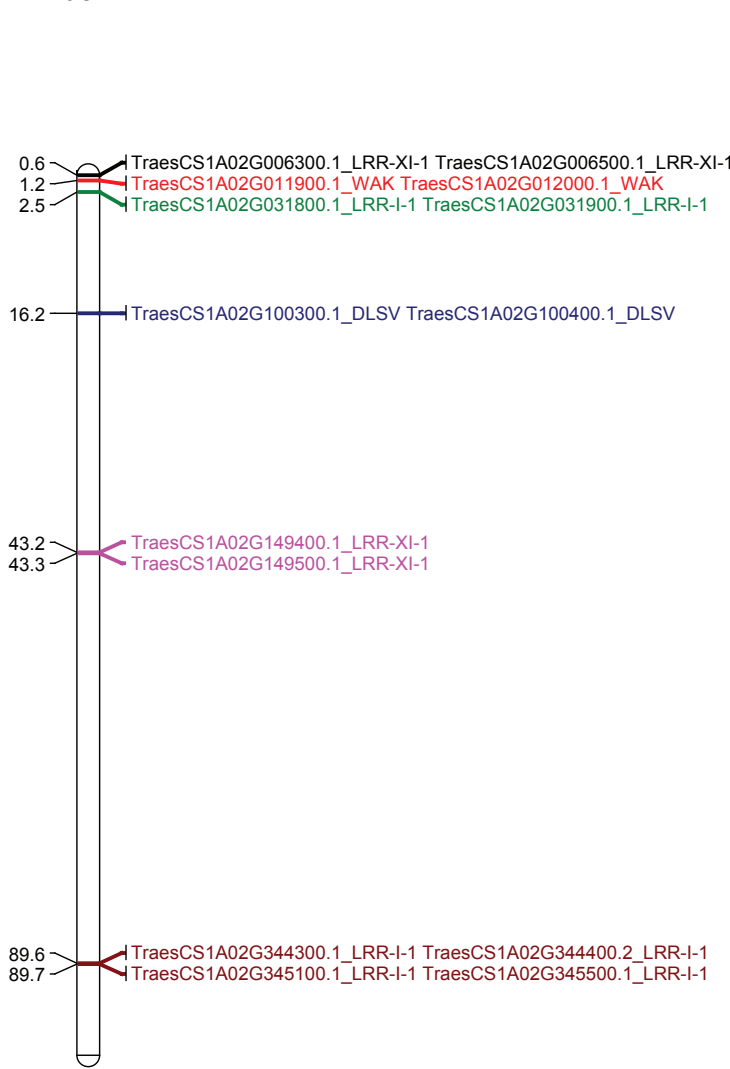

Tae1B

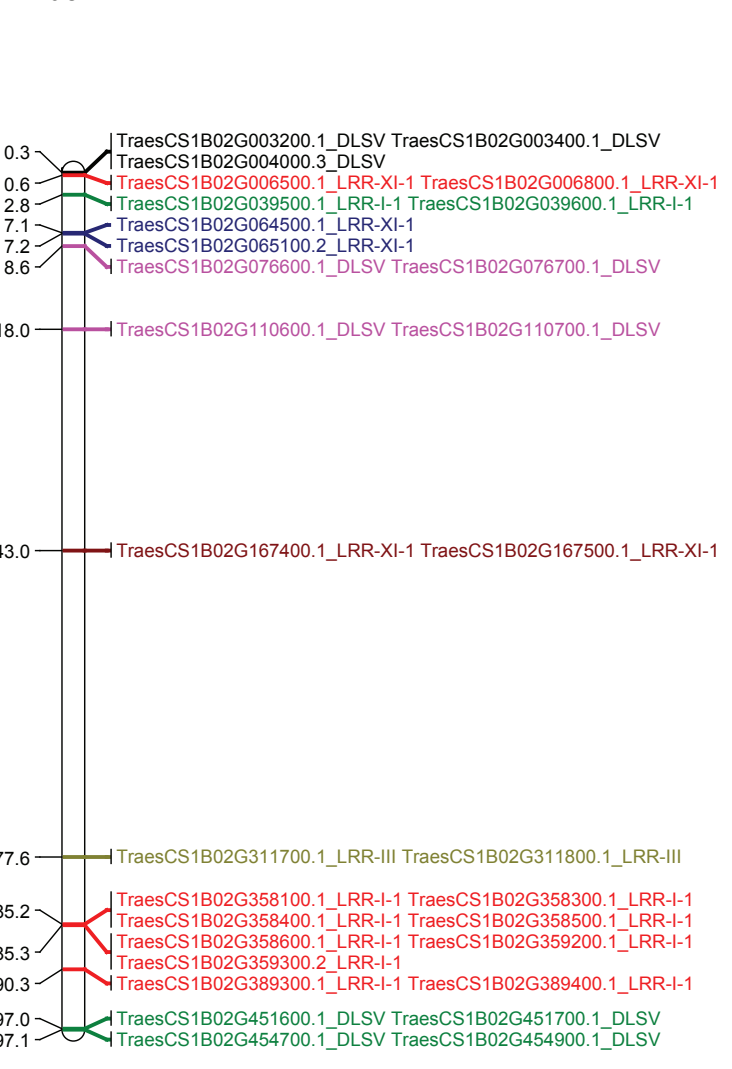

Tae1D

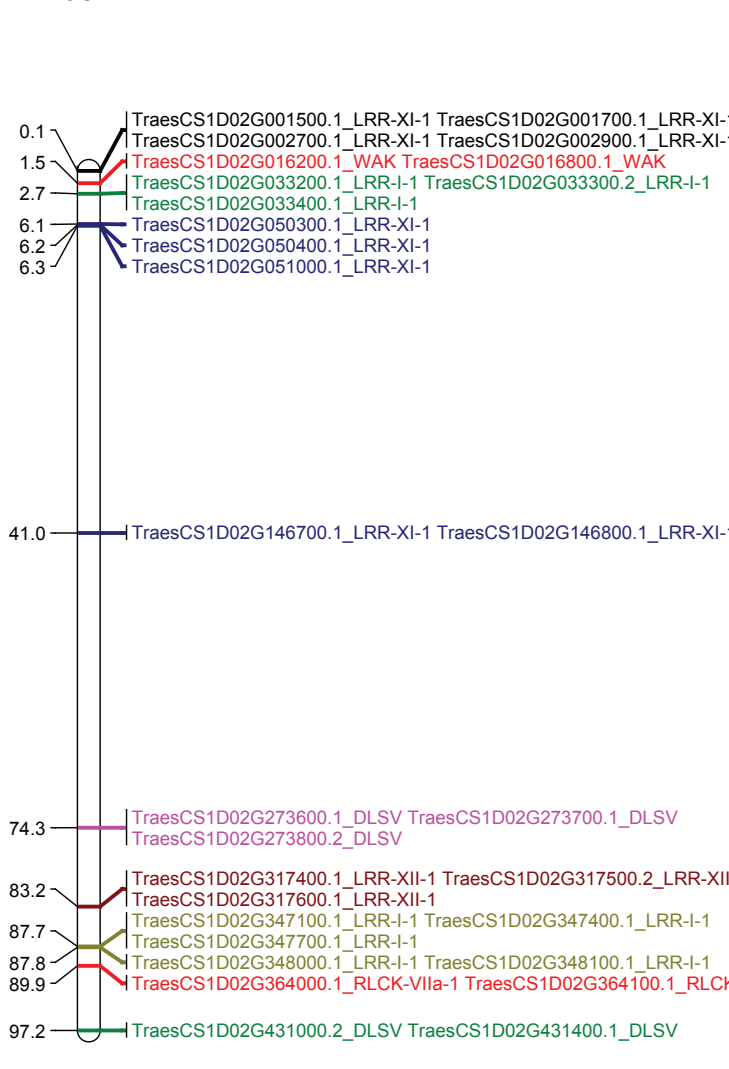

Tae2A

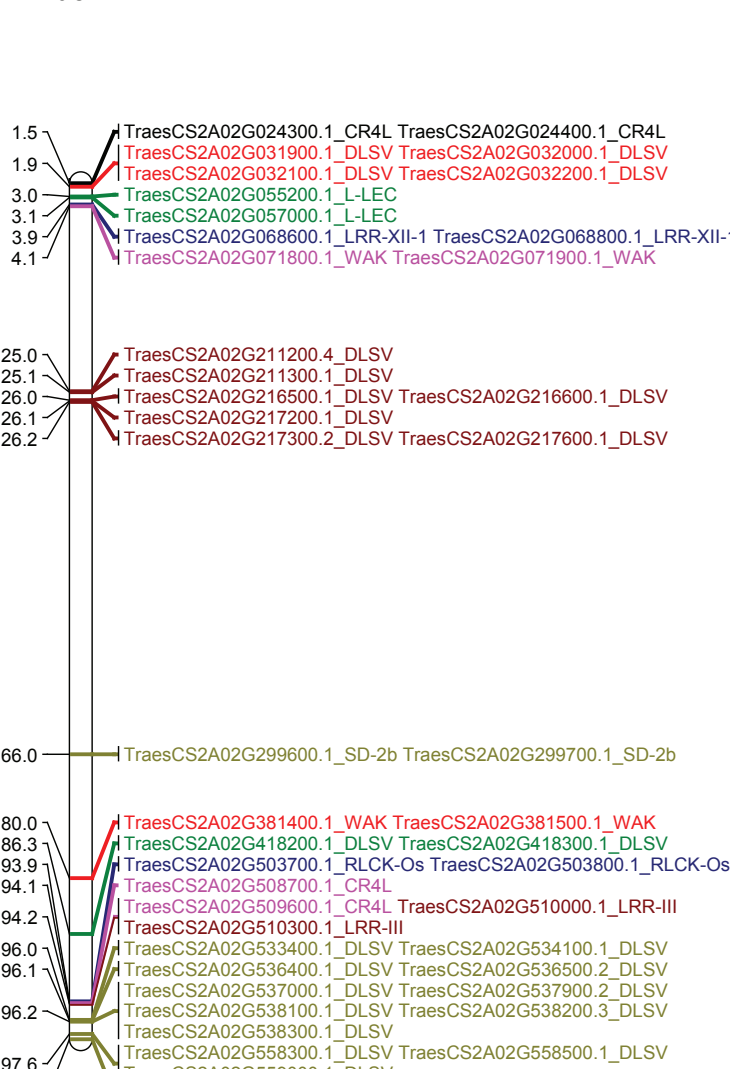

Tae2B

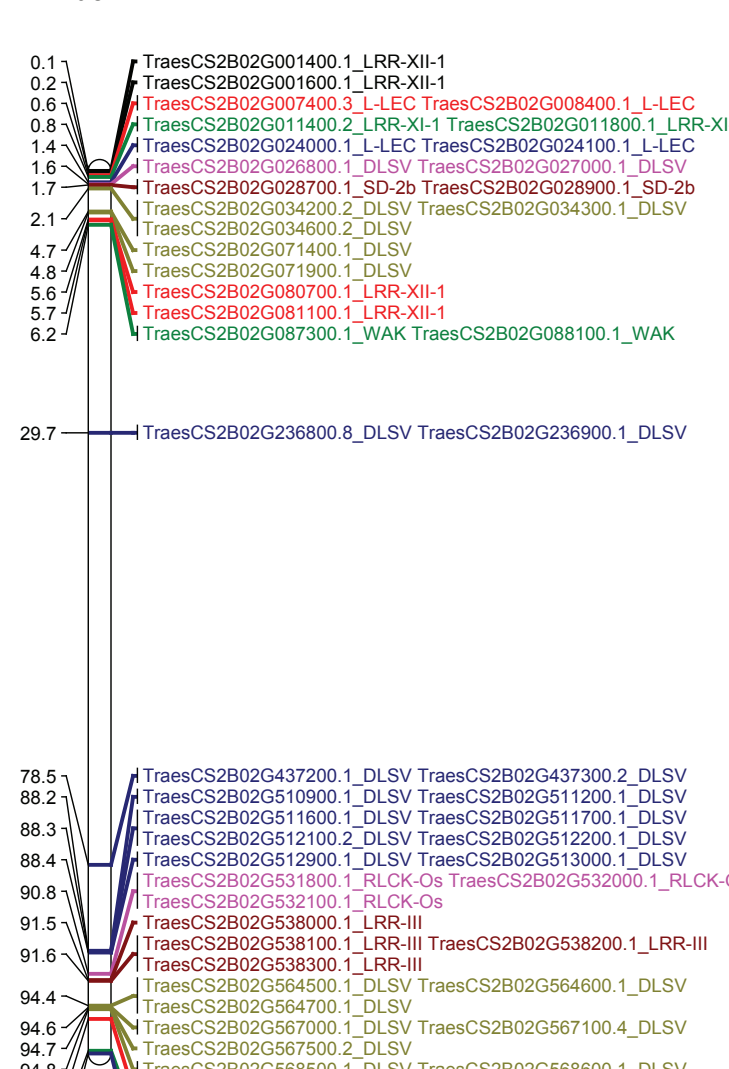

Tae2D

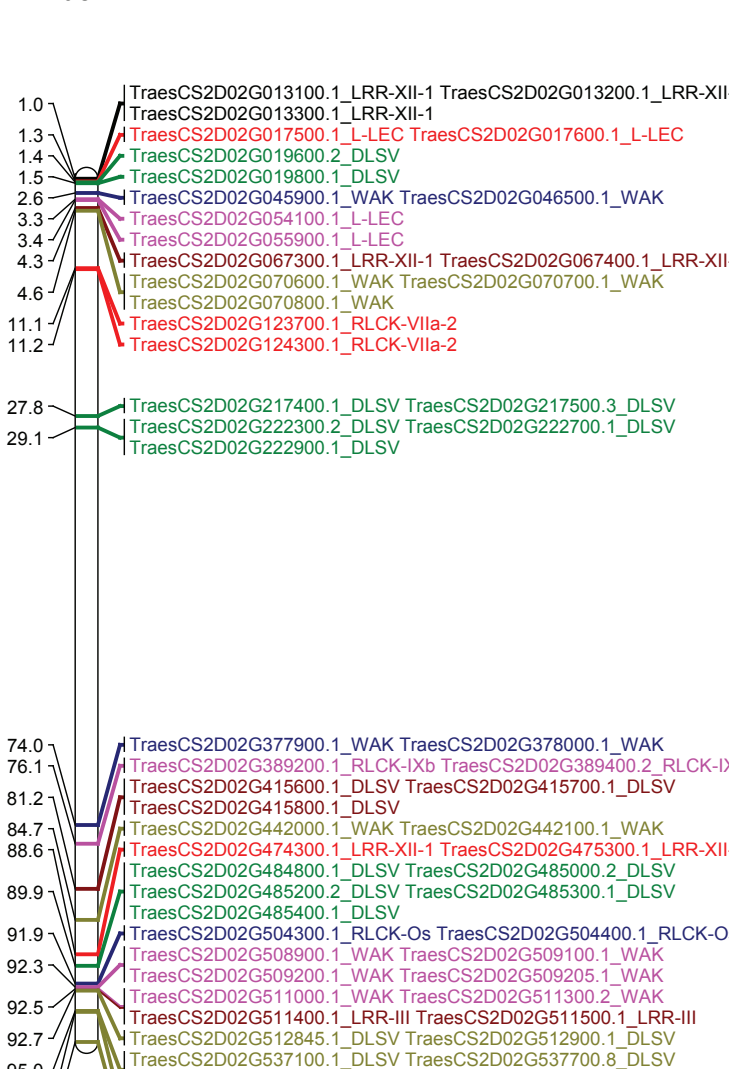

Tae3A

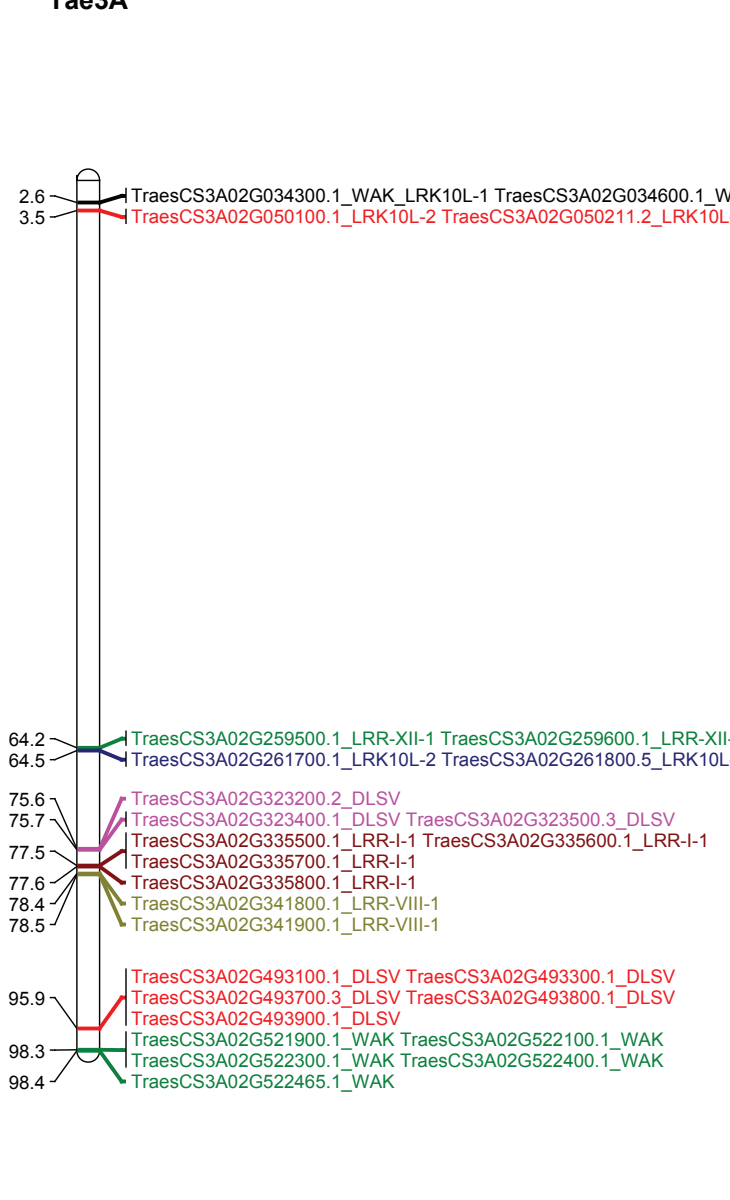

Tae3B

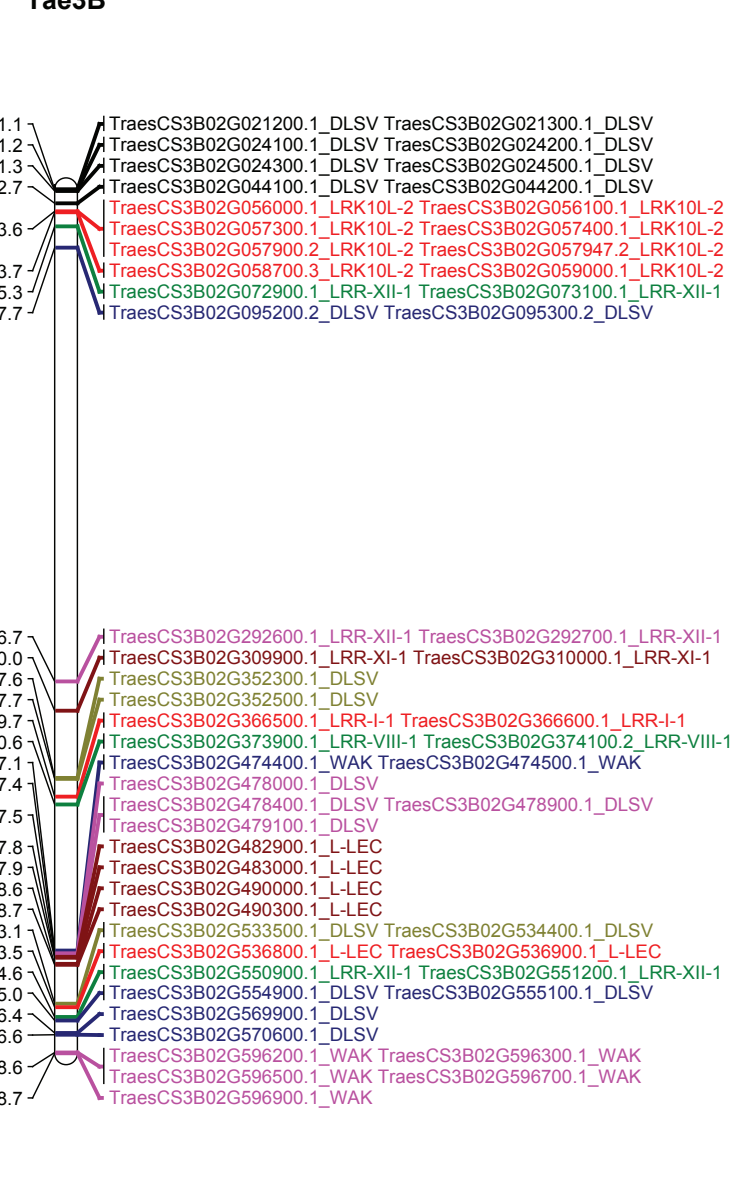

Tae3D

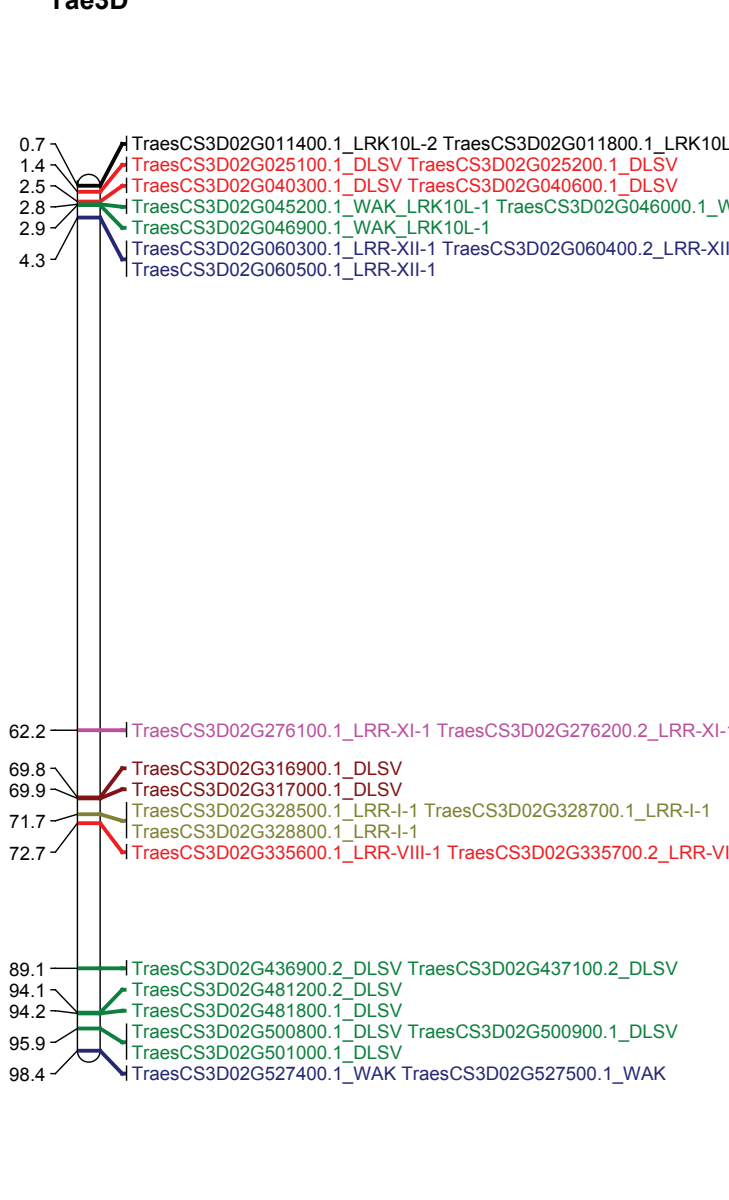

Tae4A

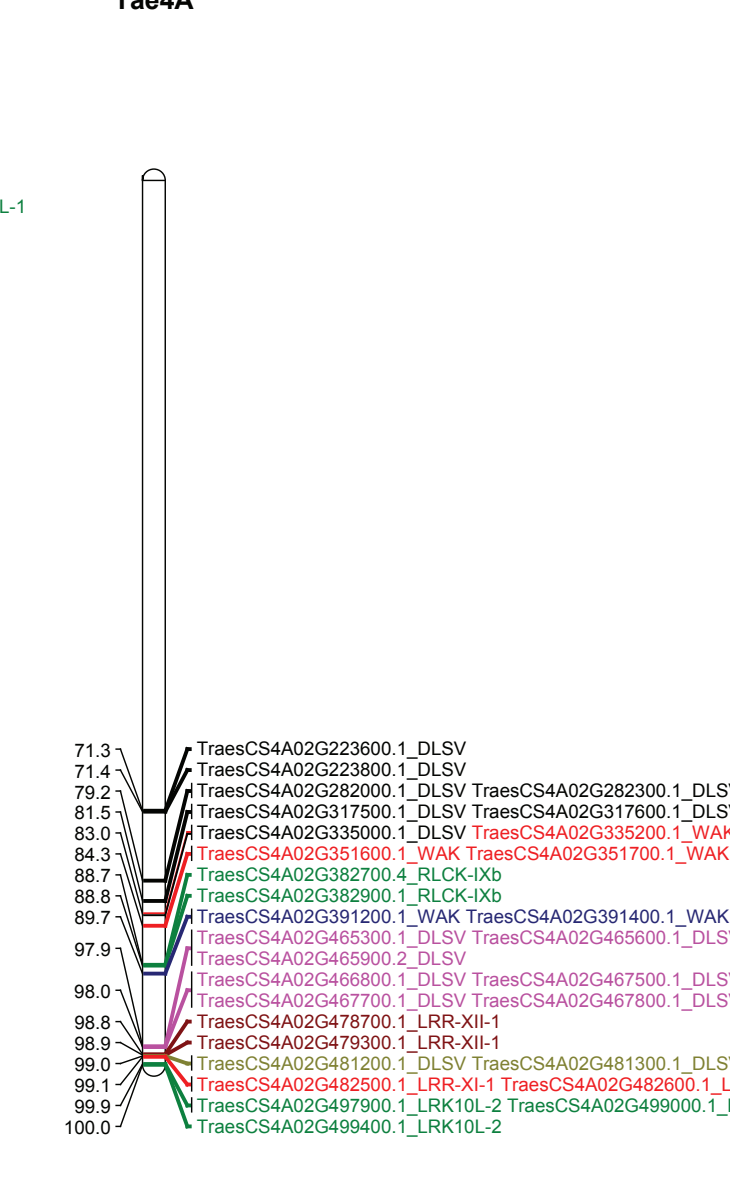

Tae4B

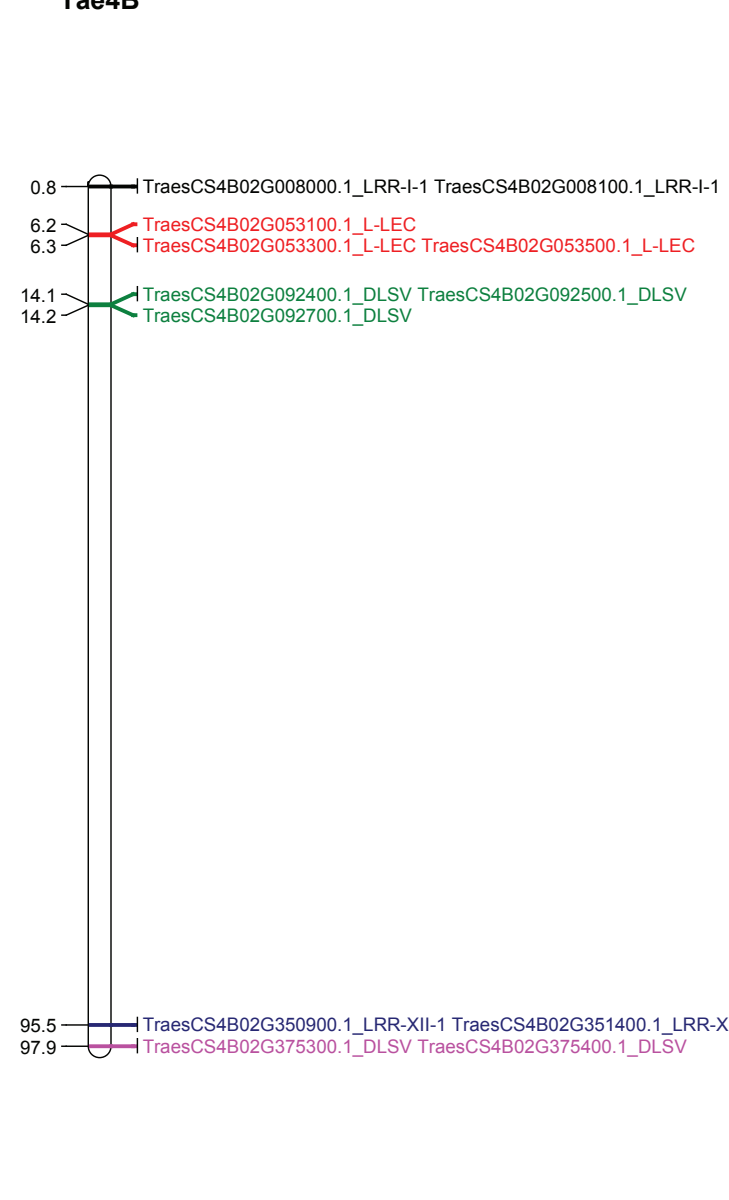

Tae4D

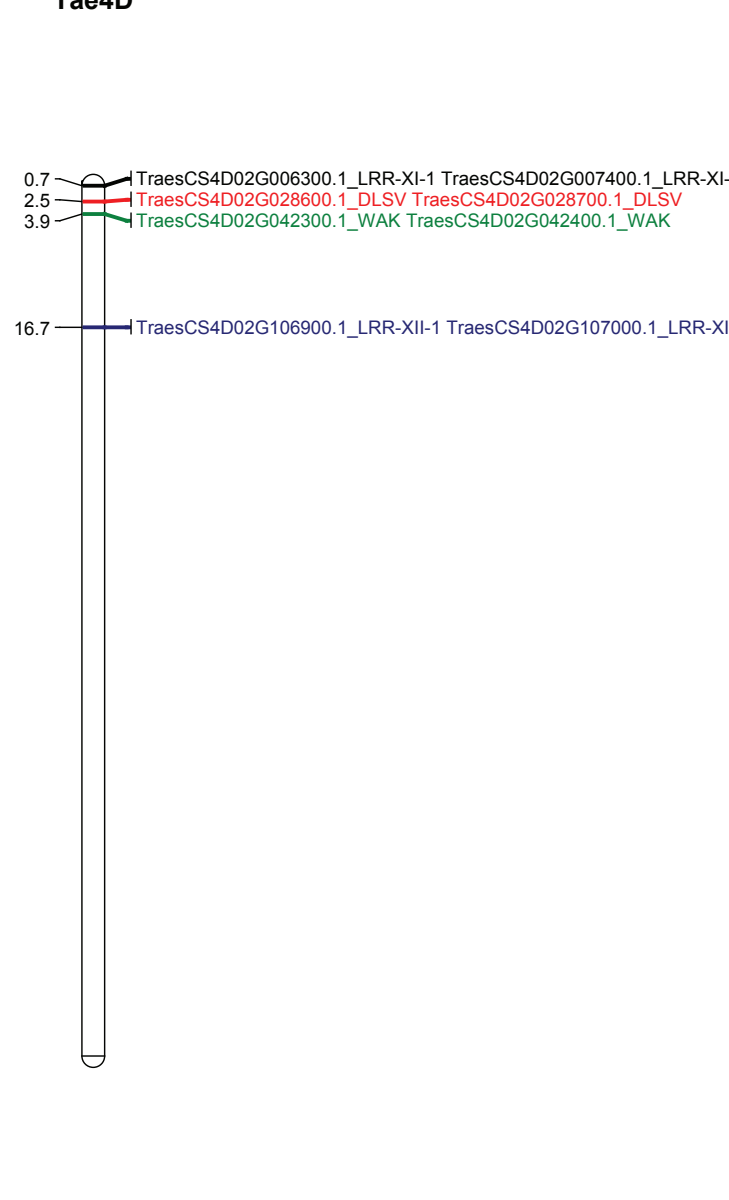

Tae5A

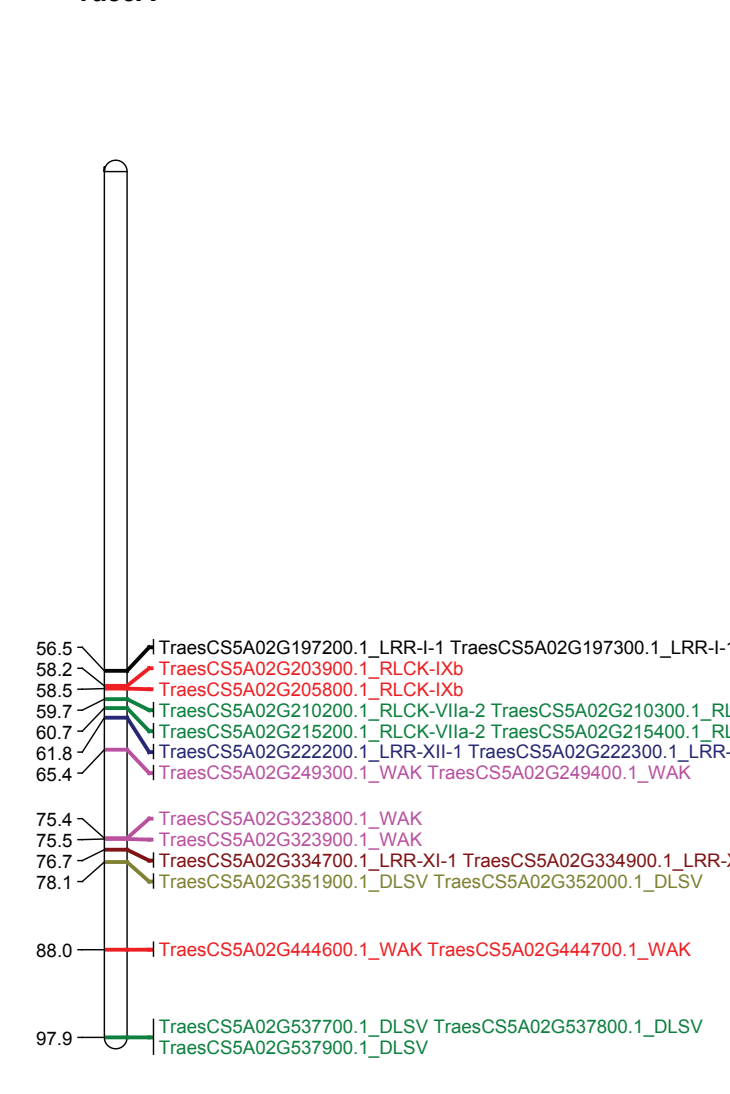

Tae5B

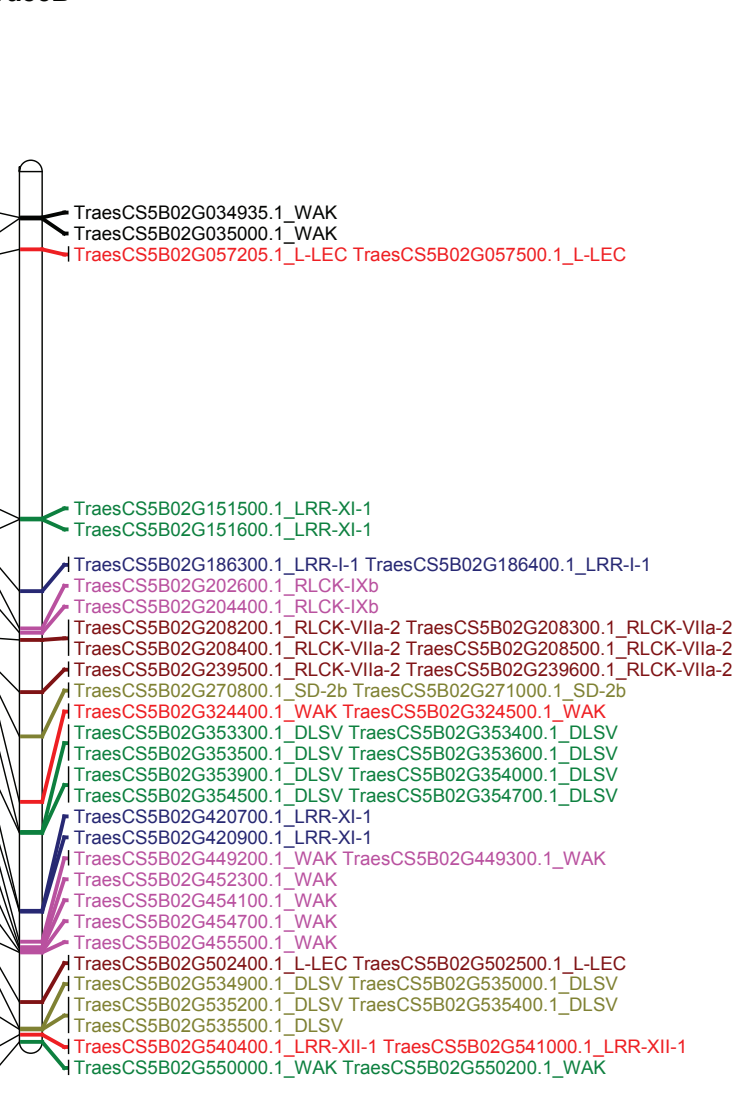

Supplement: Supplementary file 7 — Additional file 7: Figure S7. Chromosomal locations of the tandemly arrayed T. aestivum RLK genes. The tandemly arrayed T. aestivum RLK genes were grouped into 232 clusters distributed on the 21 chromosomes. Subfamilies and Gene IDs are labelled on the right of each chromosome, and the chromosomal location of each cluster is on the left of each chromosome. Genes in the same cluster are highlighted in the same colour. The information of chromosomal locations is shown in Table S6. [file 12864_2023_9303_MOESM7_ESM.pdf]
