# Supplementary material for: Phylogeny of the plant receptor-like kinase (RLK) gene family and expression analysis of wheat RLK genes in response to biotic and abiotic stresses
Source: BMC Genomics. 2023 May 1;24:224. doi: 10.1186/s12864-023-09303-7 (PMC10152718; doi:10.1186/s12864-023-09303-7)
Supplement: Supplementary file 3 — Additional file 3: Figure S3. Conserved exon−intron and domain diagrams of RLKs in T. aestivum, B. distachyon, V. vinifera, A. trichopoda, S. moellendorffii and P. patens. The descriptions of the domain and exon phases are the same as those in Fig. 2. The lengths of the boxes and lines are scaled based on the lengths of the genes. [file 12864_2023_9303_MOESM3_ESM.pdf]

RLK−Pelle subfamilies exon−intron and kinase domain diagram (part 1)

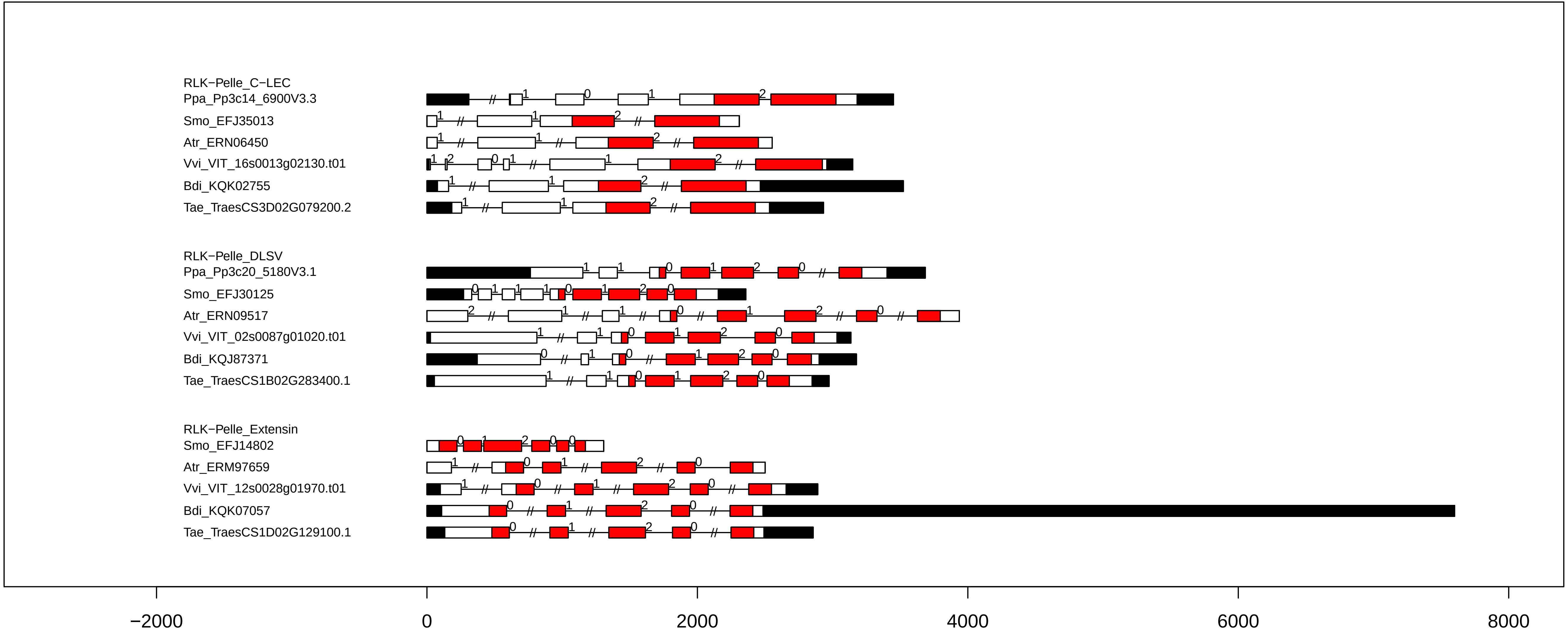

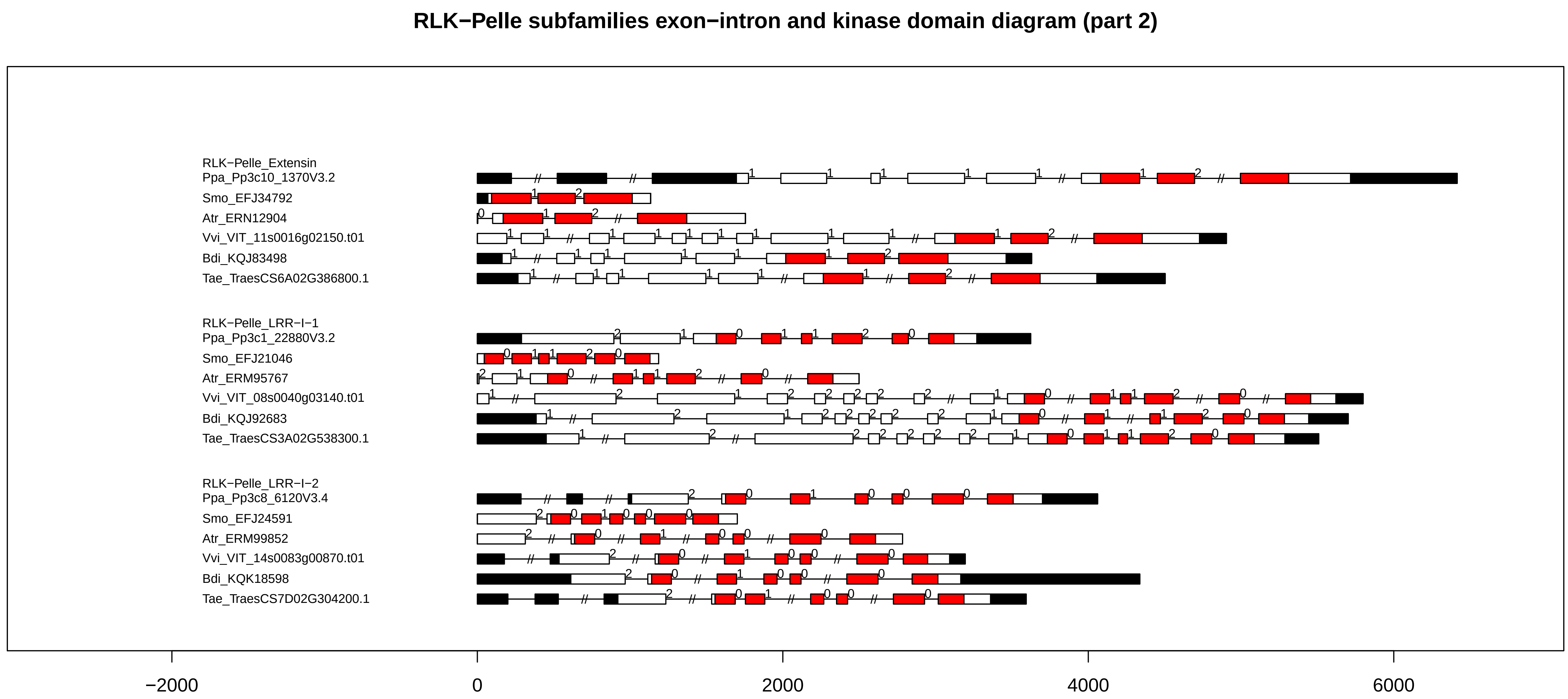
RLK−Pelle subfamilies exon−intron and kinase domain diagram (part 2)
RLK−Pelle_Extensin
Ppa_Pp3c10_1370V3.2
Smo_EFJ34792
Atr_ERN12904
Vvi_VIT_11s0016g02150.t01
Bdi_KQJ83498
Tae_TraesCS6A02G386800.1
RLK−Pelle_LRR−I−1
Ppa_Pp3c1_22880V3.2
Smo_EFJ21046
Atr_ERM95767
Vvi_VIT_08s0040g03140.t01
Bdi_KQJ92683
Tae_TraesCS3A02G538300.1
RLK−Pelle_LRR−I−2
Ppa_Pp3c8_6120V3.4
Smo_EFJ24591
Atr_ERM99852
Vvi_VIT_14s0083g00870.t01
Bdi_KQK18598
Tae_TraesCS7D02G304200.1
−2000
0
2000
4000
6000

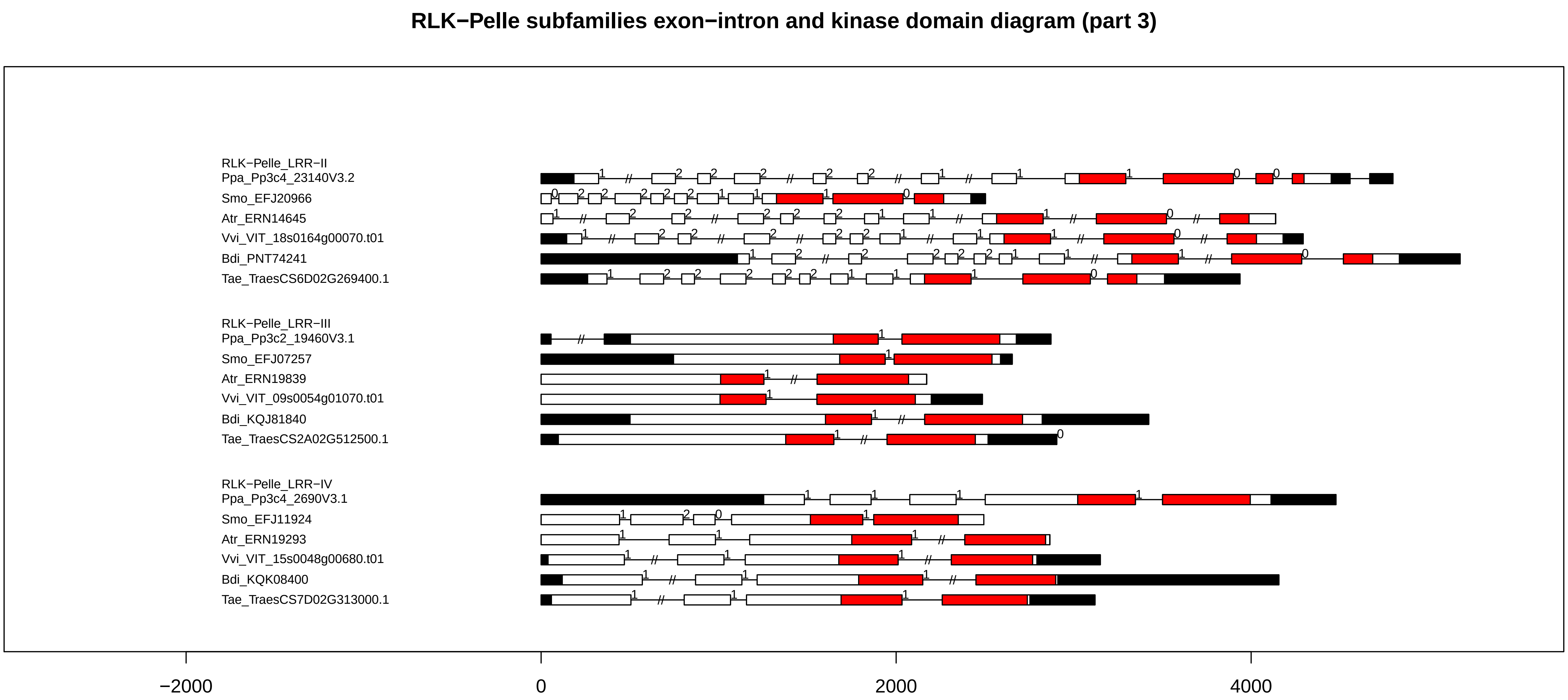

RLK−Pelle subfamilies exon−intron and kinase domain diagram (part 3)
RLK−Pelle_LRR−II
Ppa_Pp3c4_23140V3.2
Smo_EFJ20966
Atr_ERN14645
Vvi_VIT_18s0164g00070.t01
Bdi_PNT74241
Tae_TraesCS6D02G269400.1
RLK−Pelle_LRR−III
Ppa_Pp3c2_19460V3.1
Smo_EFJ07257
Atr_ERN19839
Vvi_VIT_09s0054g01070.t01
Bdi_KQJ81840
Tae_TraesCS2A02G512500.1
RLK−Pelle_LRR−IV
Ppa_Pp3c4_2690V3.1
Smo_EFJ11924
Atr_ERN19293
Vvi_VIT_15s0048g00680.t01
Bdi_KQK08400
Tae_TraesCS7D02G313000.1
−2000
0
2000
4000

RLK−Pelle subfamilies exon−intron and kinase domain diagram (part 4)

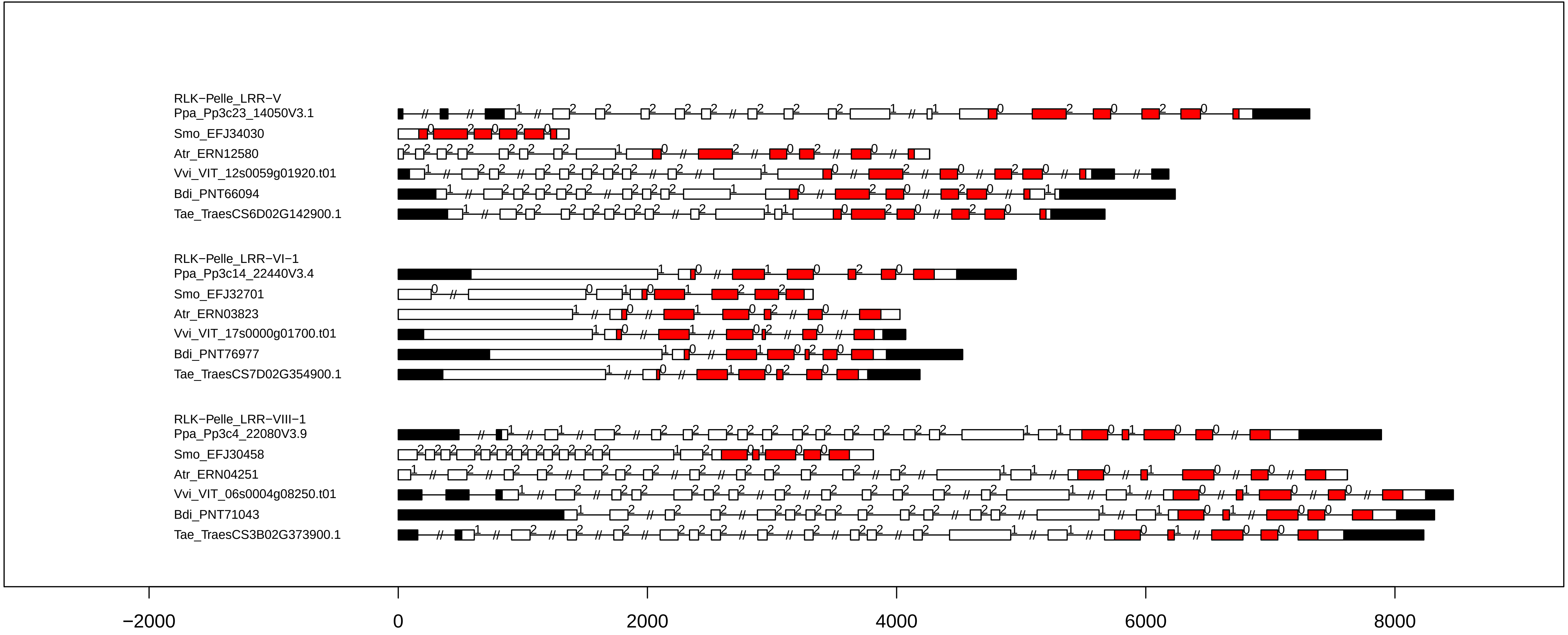

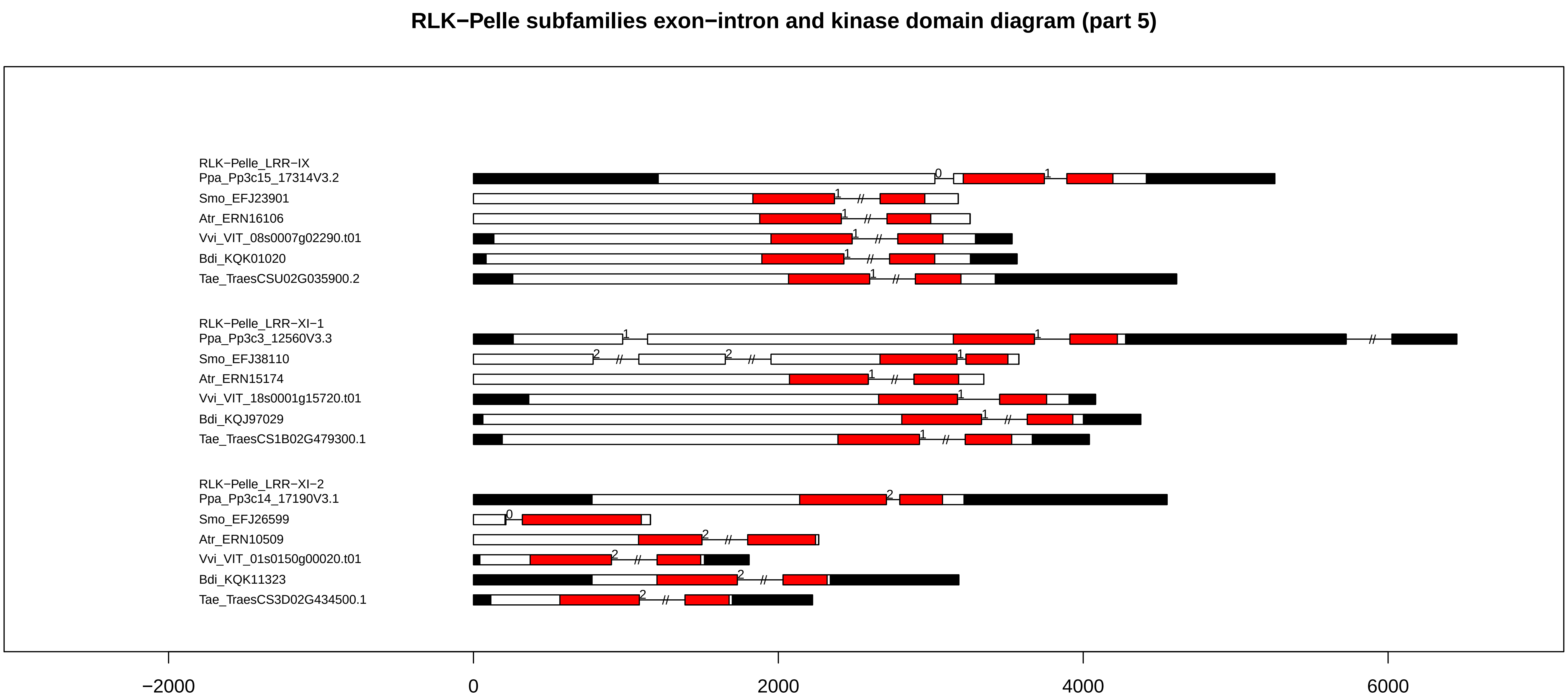
RLK−Pelle subfamilies exon−intron and kinase domain diagram (part 5)
RLK−Pelle_LRR−IX
Ppa_Pp3c15_17314V3.2
Smo_EFJ23901
Atr_ERN16106
Vvi_VIT_08s0007g02290.t01
Bdi_KQK01020
Tae_TraesCSU02G035900.2
RLK−Pelle_LRR−XI−1
Ppa_Pp3c3_12560V3.3
Smo_EFJ38110
Atr_ERN15174
Vvi_VIT_18s0001g15720.t01
Bdi_KQJ97029
Tae_TraesCS1B02G479300.1
RLK−Pelle_LRR−XI−2
Ppa_Pp3c14_17190V3.1
Smo_EFJ26599
Atr_ERN10509
Vvi_VIT_01s0150g00020.t01
Bdi_KQK11323
Tae_TraesCS3D02G434500.1
−2000
0
2000
4000
6000

RLK−Pelle subfamilies exon−intron and kinase domain diagram (part 6)

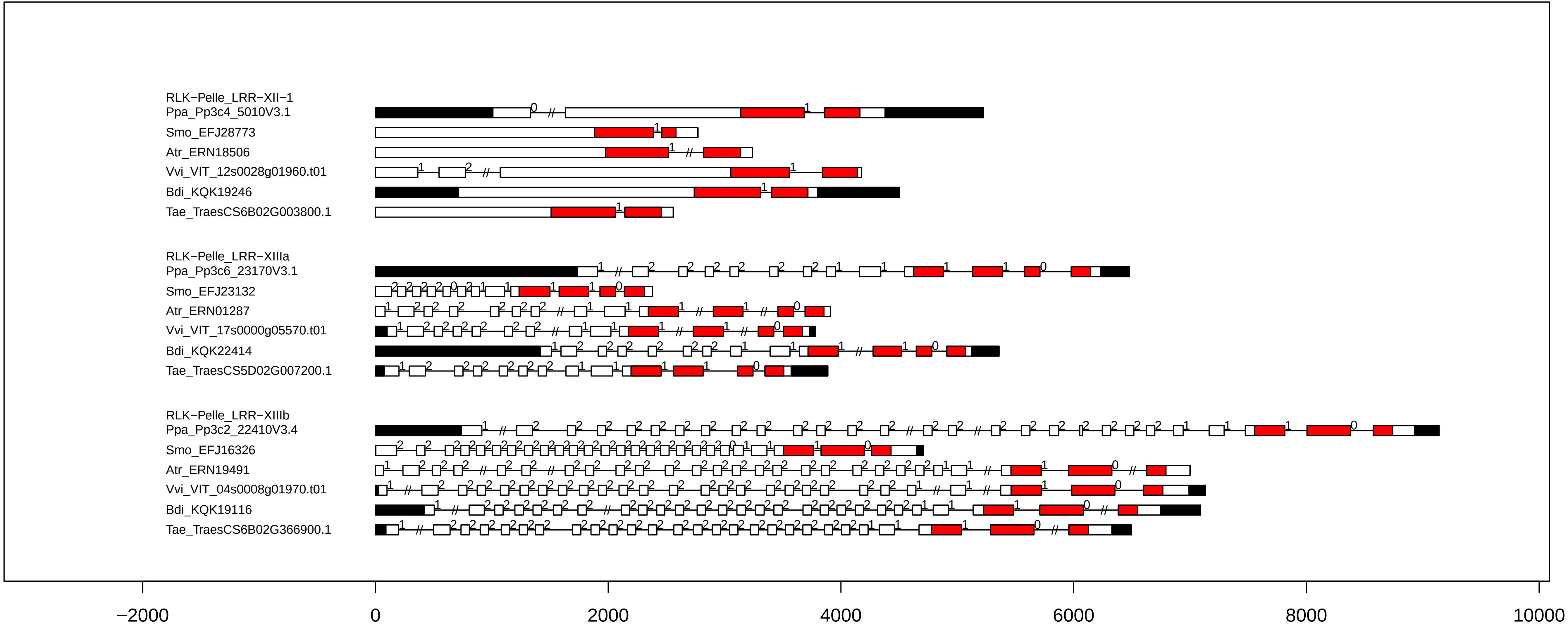

RLK−Pelle subfamilies exon−intron and kinase domain diagram (part 7)

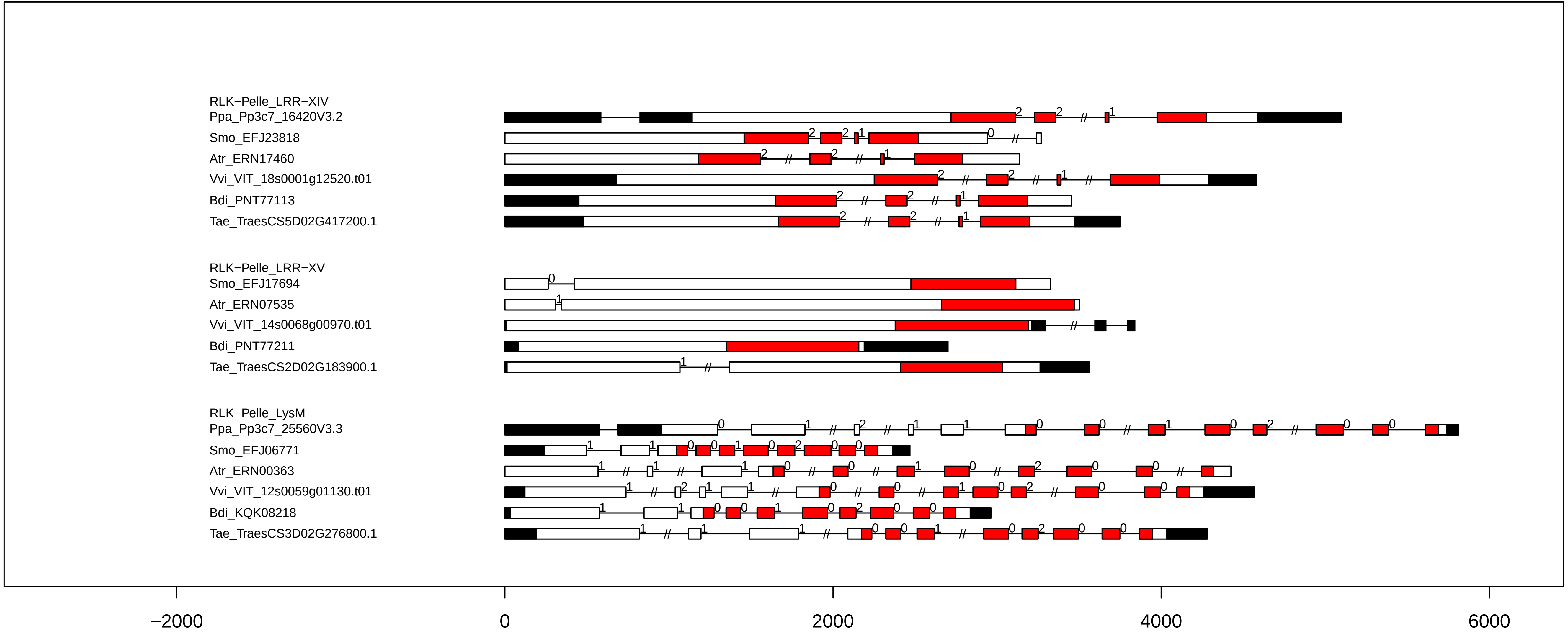

RLK−Pelle subfamilies exon−intron and kinase domain diagram (part 8)

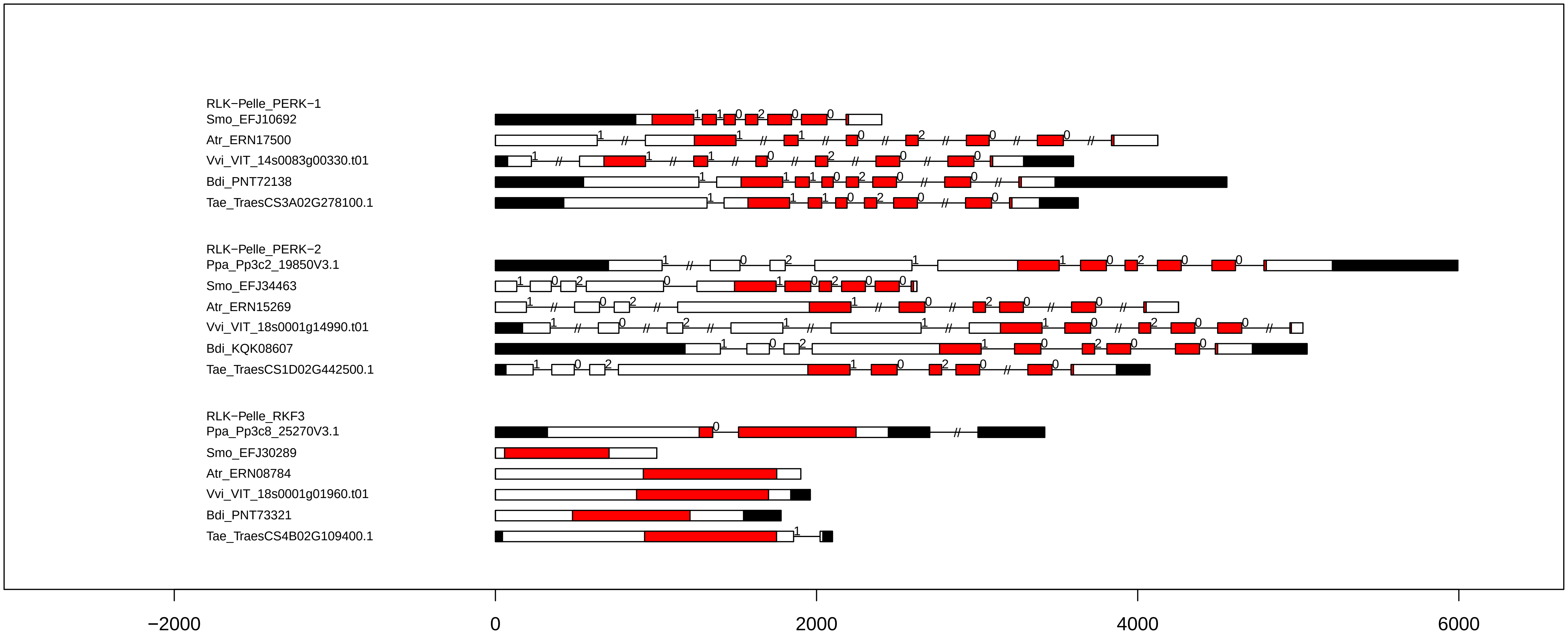

RLK−Pelle subfamilies exon−intron and kinase domain diagram (part 9)

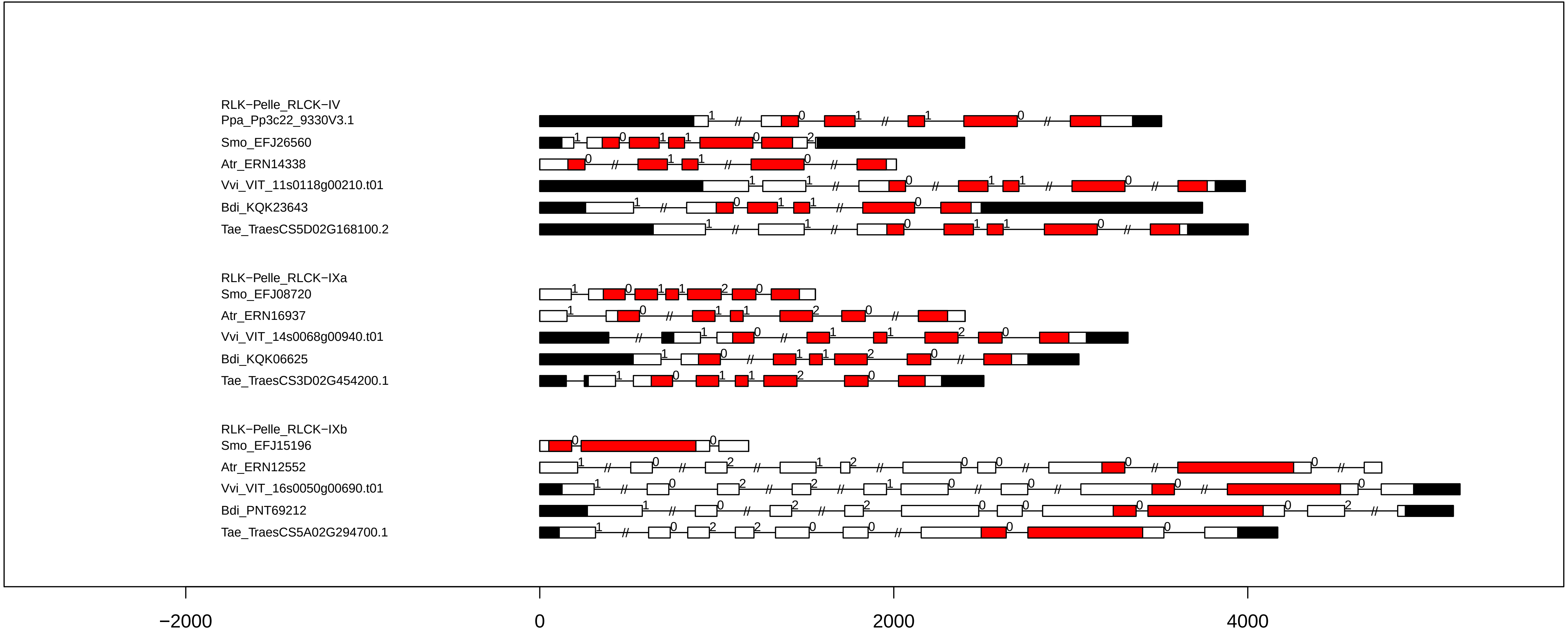

RLK−Pelle subfamilies exon−intron and kinase domain diagram (part 10)

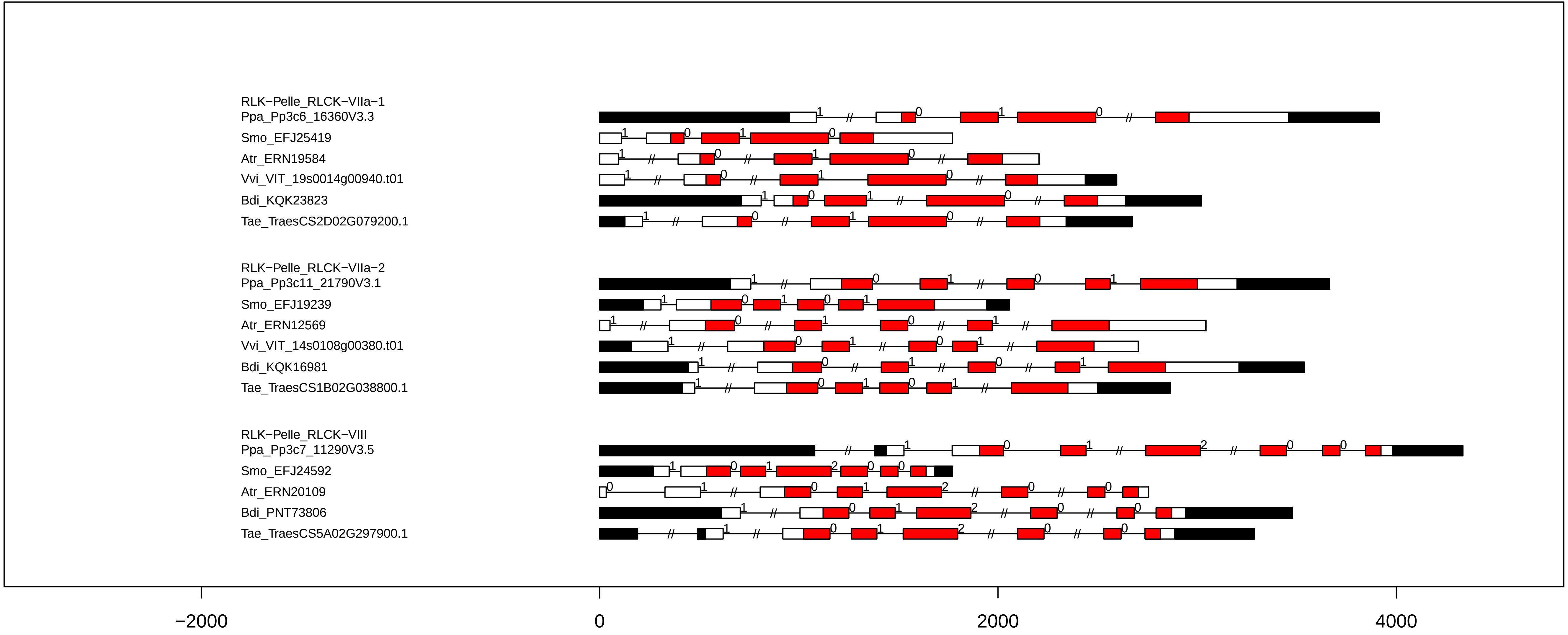

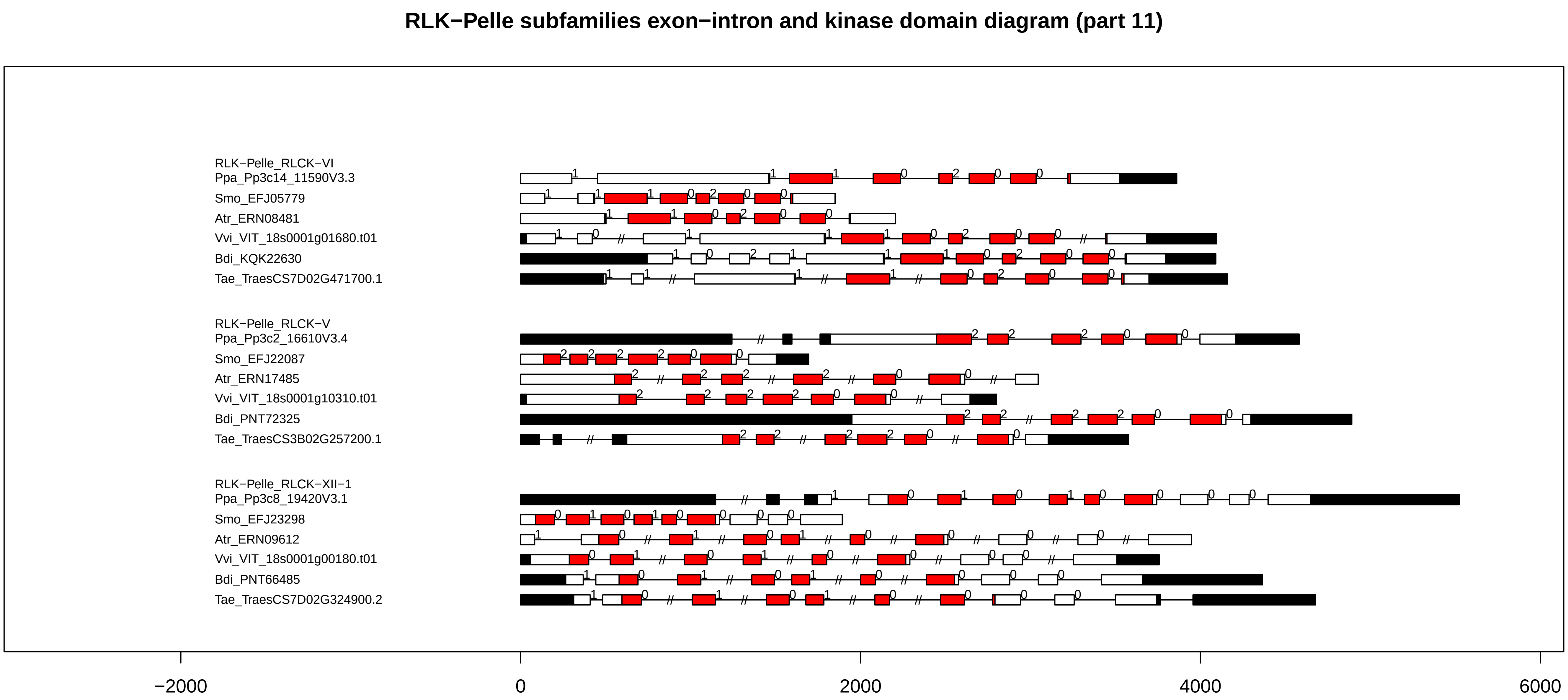

RLK−Pelle subfamilies exon−intron and kinase domain diagram (part 11)
RLK−Pelle_RLCK−VI
Ppa_Pp3c14_11590V3.3
Smo_EFJ05779
Atr_ERN08481
Vvi_VIT_18s0001g01680.t01
Bdi_KQK22630
Tae_TraesCS7D02G471700.1
RLK−Pelle_RLCK−V
Ppa_Pp3c2_16610V3.4
Smo_EFJ22087
Atr_ERN17485
Vvi_VIT_18s0001g10310.t01
Bdi_PNT72325
Tae_TraesCS3B02G257200.1
RLK−Pelle_RLCK−XII−1
Ppa_Pp3c8_19420V3.1
Smo_EFJ23298
Atr_ERN09612
Vvi_VIT_18s0001g00180.t01
Bdi_PNT66485
Tae_TraesCS7D02G324900.2
−2000
0
2000
4000
6000

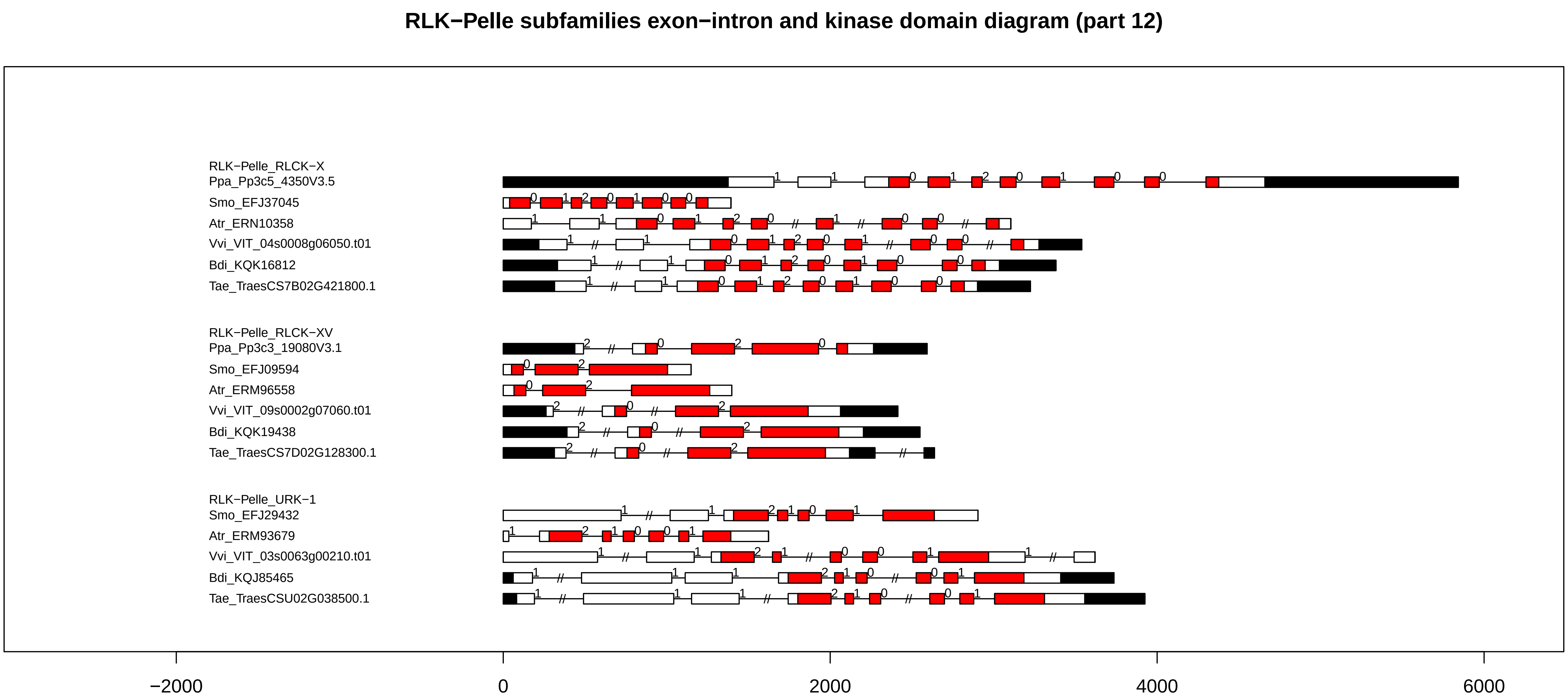
RLK−Pelle subfamilies exon−intron and kinase domain diagram (part 12)
RLK−Pelle_RLCK−X
Ppa_Pp3c5_4350V3.5
Smo_EFJ37045
Atr_ERN10358
Vvi_VIT_04s0008g06050.t01
Bdi_KQK16812
Tae_TraesCS7B02G421800.1
RLK−Pelle_RLCK−XV
Ppa_Pp3c3_19080V3.1
Smo_EFJ09594
Atr_ERM96558
Vvi_VIT_09s0002g07060.t01
Bdi_KQK19438
Tae_TraesCS7D02G128300.1
RLK−Pelle_URK−1
Smo_EFJ29432
Atr_ERM93679
Vvi_VIT_03s0063g00210.t01
Bdi_KQJ85465
Tae_TraesCSU02G038500.1
−2000
0
2000
4000
6000
