# Supplementary material for: Phylogeny of the plant receptor-like kinase (RLK) gene family and expression analysis of wheat RLK genes in response to biotic and abiotic stresses
Source: BMC Genomics. 2023 May 1;24:224. doi: 10.1186/s12864-023-09303-7 (PMC10152718; doi:10.1186/s12864-023-09303-7)
Supplement: Supplementary file 8 — Additional file 8: Figure S8. Collinearity (Ks values) among T. aestivum, B. distachyon and O. sativa. (A) Collinearity events of duplicated RLKs and all genes between T. aestivum, B. distachyon. The green bars denote the collinearity events contributed by (A) polyploidizations of RLKs (Ks values 0.25–0.6) and all genes (Ks values of 0.3–0.45). The blue bars denote the other collinearity events. (B) Collinearity events of duplicated RLKs and all genes between T. aestivum and O. sativa. The pink bars denote the collinearity events contributed by polyploidizations of RLKs (Ks values of 0.3–0.8) and all genes (Ks values of 0.4–0.6). The blue bars denote the other collinearity events. Information on the collinearity events is provided in Table S7. [file 12864_2023_9303_MOESM8_ESM.pdf]

A

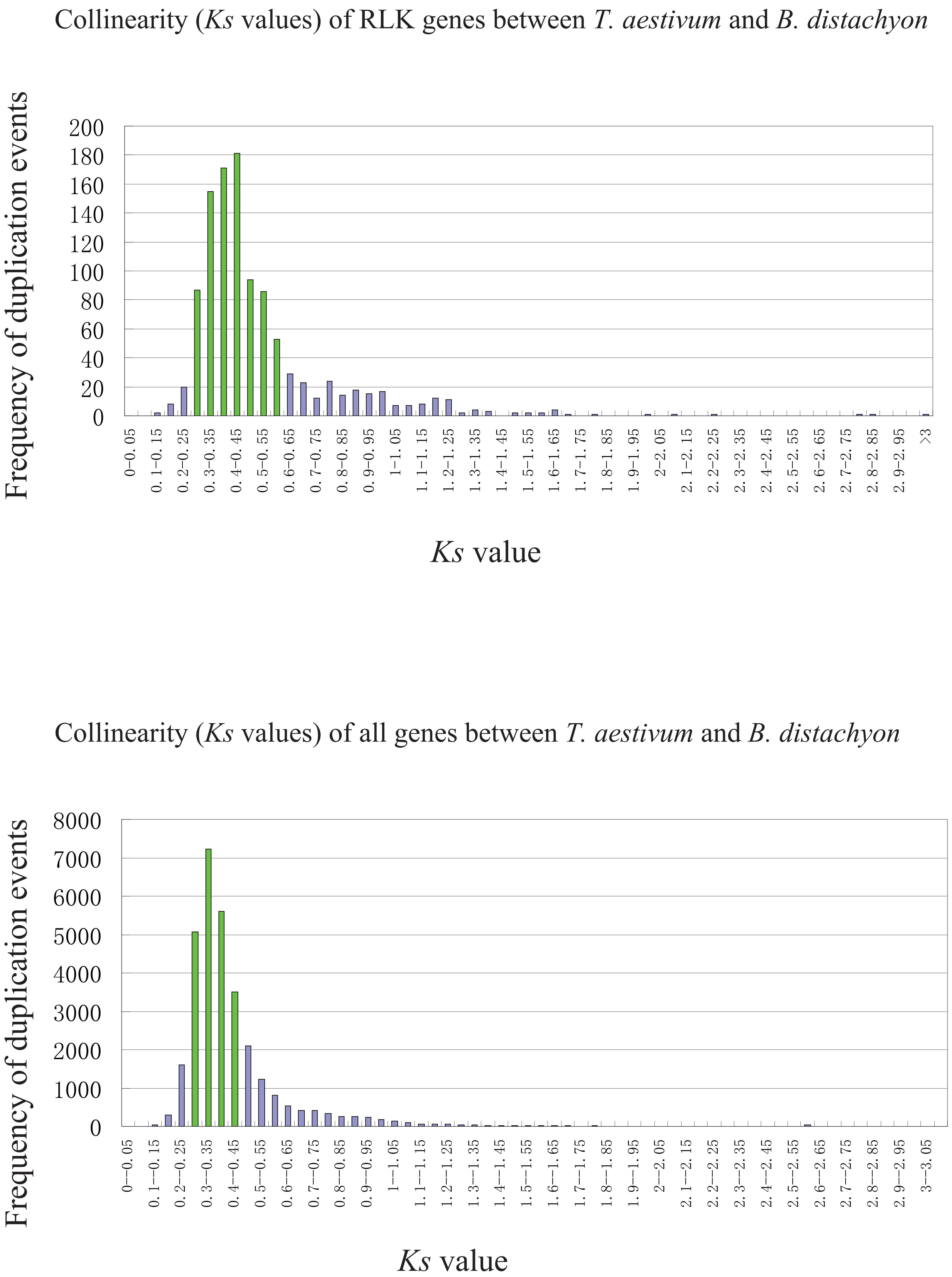

Collinearity (*Ks* values) of RLK genes between *T. aestivum* and *B. distachyon*
Frequency of duplication events
200
180
160
140
120
100
80
60
40
20
0
0−0. 05
0. 1−0. 15
0. 2−0. 25
0. 3−0. 35
0. 4−0. 45
0. 5−0. 55
0. 6−0. 65
0. 7−0. 75
0. 8−0. 85
0. 9−0. 95
1−1. 05
1. 1−1. 15
1. 2−1. 25
1. 3−1. 35
1. 4−1. 45
1. 5−1. 55
1. 6−1. 65
1. 7−1. 75
1. 8−1. 85
1. 9−1. 95
2−2. 05
2. 1−2. 15
2. 2−2. 25
2. 3−2. 35
2. 4−2. 45
2. 5−2. 55
2. 6−2. 65
2. 7−2. 75
2. 8−2. 85
2. 9−2. 95
>3
Ks value
Collinearity (*Ks* values) of all genes between *T. aestivum* and *B. distachyon*
Frequency of duplication events
8000
7000
6000
5000
4000
3000
2000
1000
0
0−0. 05
0. 1−0. 15
0. 2−0. 25
0. 3−0. 35
0. 4−0. 45
0. 5−0. 55
0. 6−0. 65
0. 7−0. 75
0. 8−0. 85
0. 9−0. 95
1−1. 05
1. 1−1. 15
1. 2−1. 25
1. 3−1. 35
1. 4−1. 45
1. 5−1. 55
1. 6−1. 65
1. 7−1. 75
1. 8−1. 85
1. 9−1. 95
2−2. 05
2. 1−2. 15
2. 2−2. 25
2. 3−2. 35
2. 4−2. 45
2. 5−2. 55
2. 6−2. 65
2. 7−2. 75
2. 8−2. 85
2. 9−2. 95
3−3. 05
Ks value

B

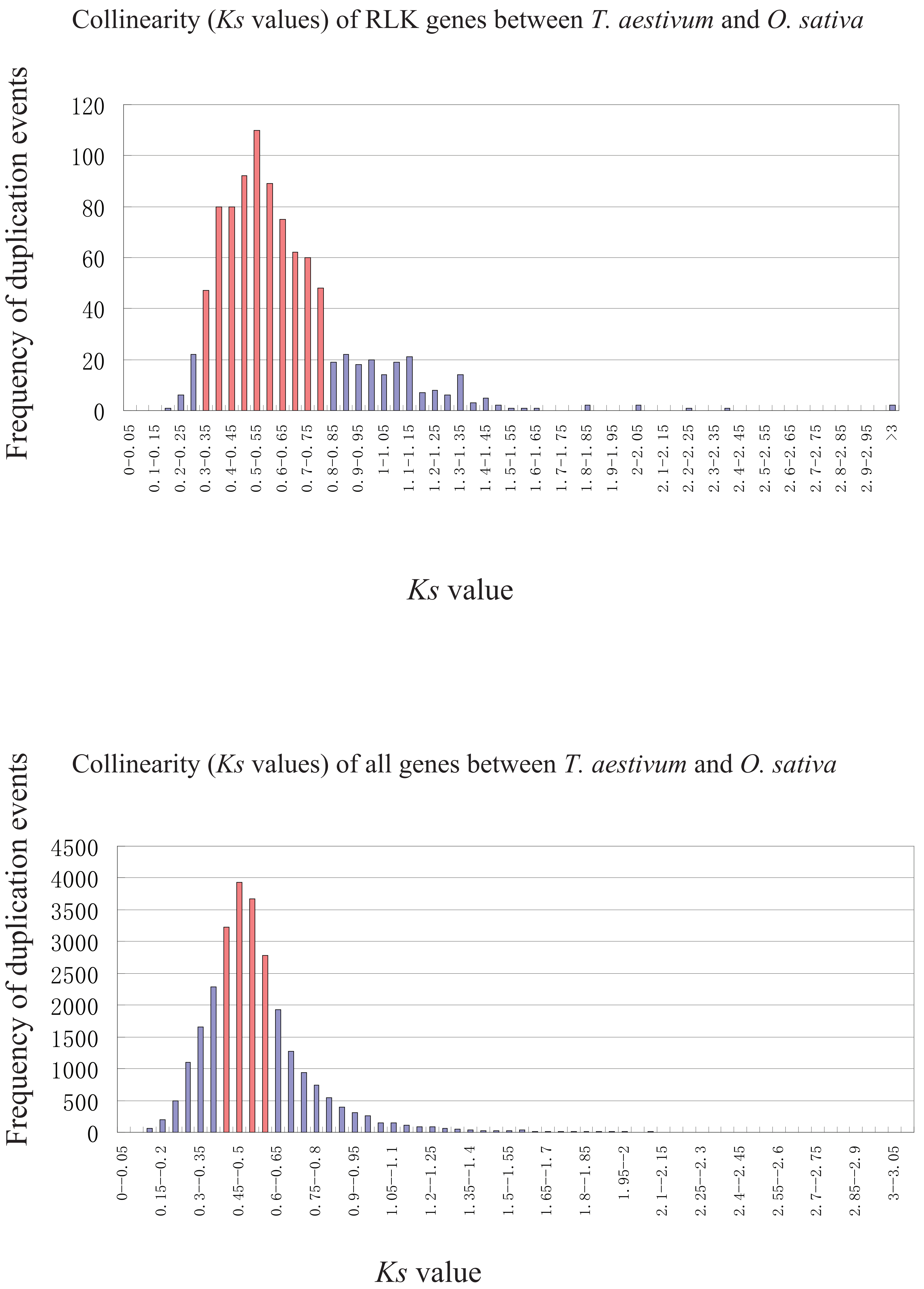
Collinearity (*Ks* values) of RLK genes between *T. aestivum* and *O. sativa*
Frequency of duplication events
0
20
40
60
80
100
120
Ks value
Collinearity (*Ks* values) of all genes between *T. aestivum* and *O. sativa*
Frequency of duplication events
0
500
1000
1500
2000
2500
3000
3500
4000
4500
Ks value
