# Supplementary material for: Phylogeny of the plant receptor-like kinase (RLK) gene family and expression analysis of wheat RLK genes in response to biotic and abiotic stresses
Source: BMC Genomics. 2023 May 1;24:224. doi: 10.1186/s12864-023-09303-7 (PMC10152718; doi:10.1186/s12864-023-09303-7)

TraesCS1B02G454000

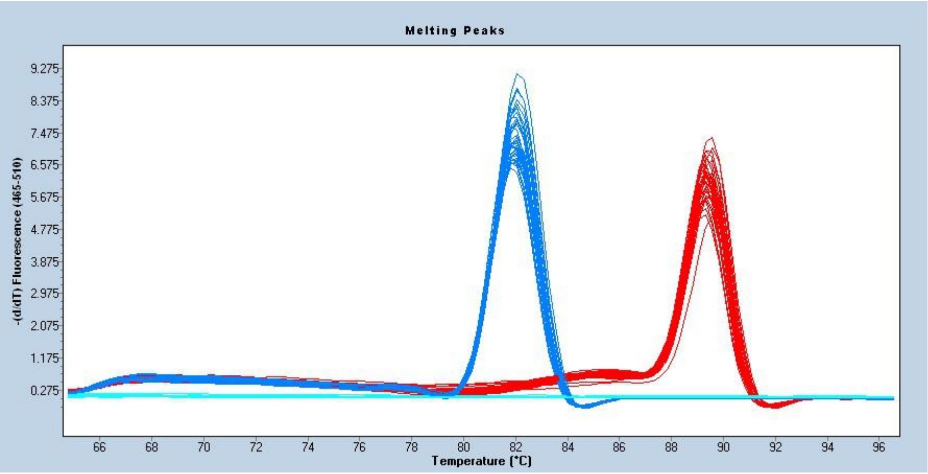

TraesCS2A02G079600

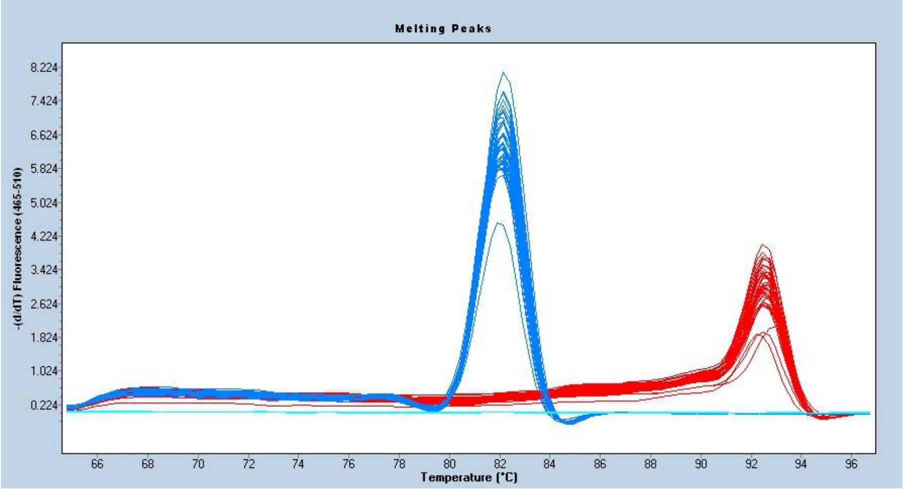

TraesCS2D02G419800

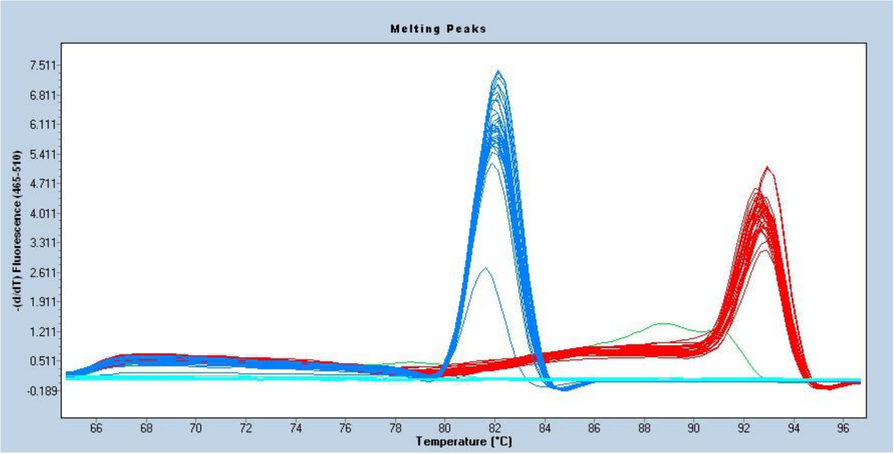

TraesCS3B02G049100

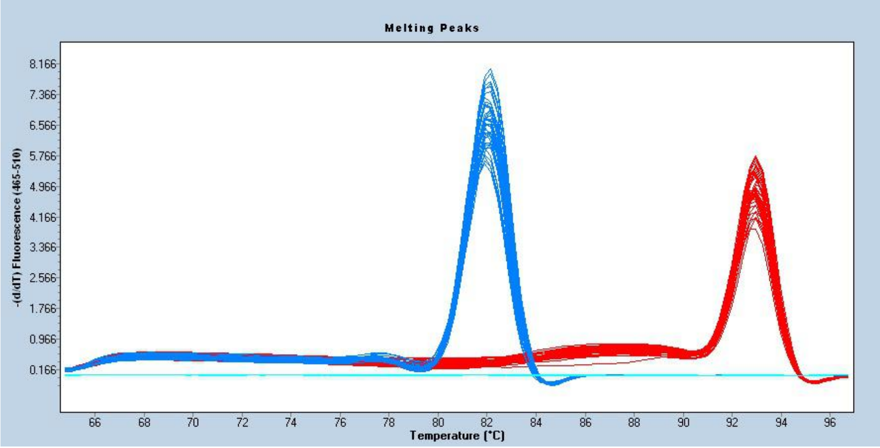

TraesCS3D02G097000

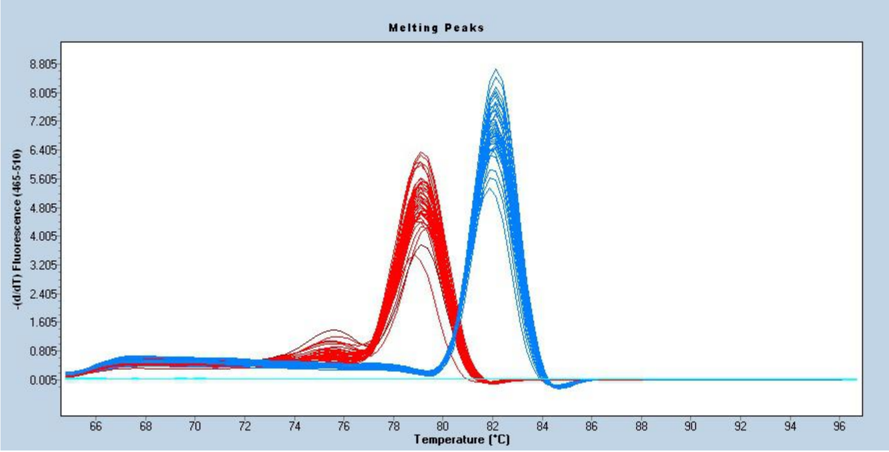

TraesCS5D02G247800

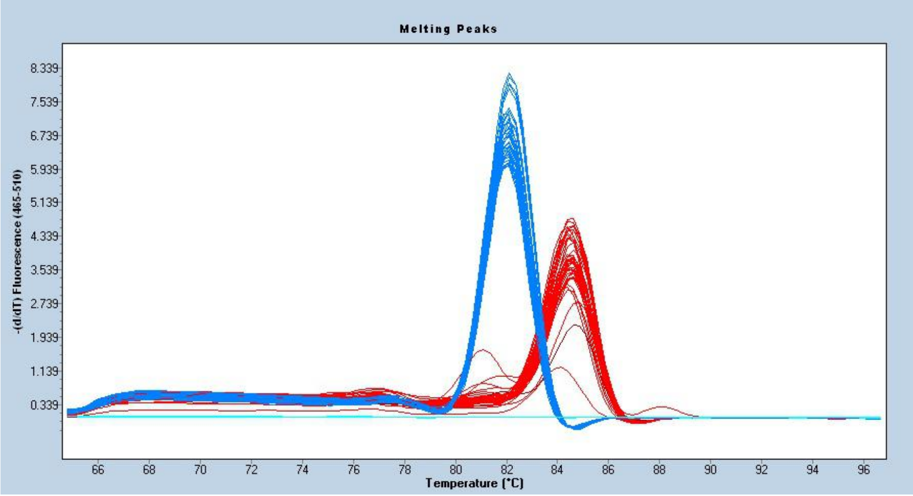

TraesCS5D02G437000

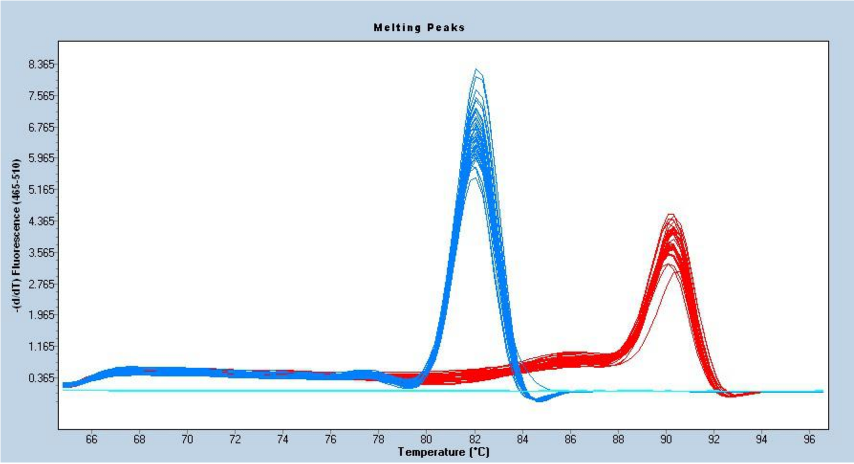

TraesCS6A02G390300

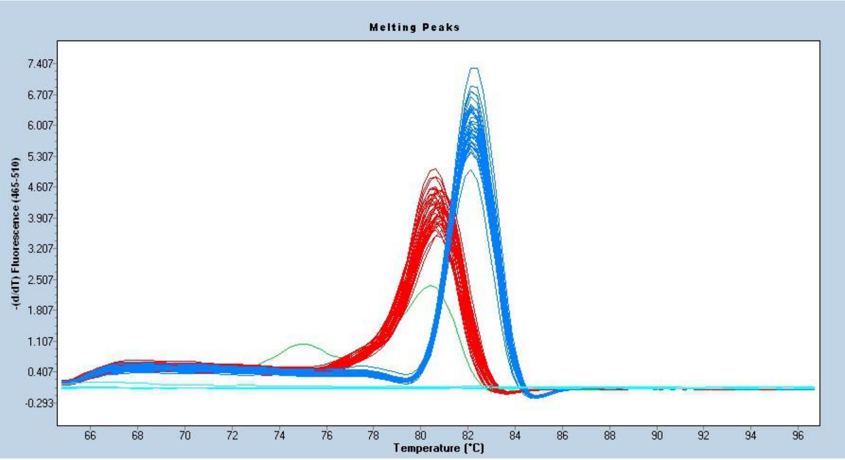

TraesCS7D02G355800

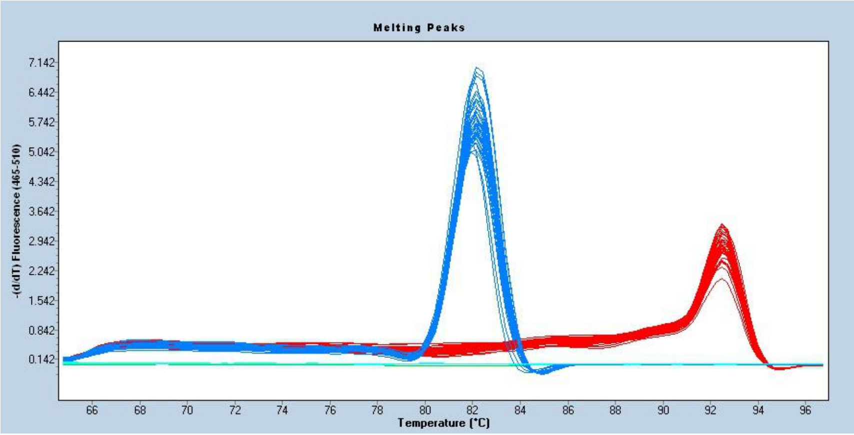

Supplement: Supplementary file 13 — Additional file 13: Figure S13. The melting curves of qRT‒PCR about 9 selected T. aestivum RLKs. [file 12864_2023_9303_MOESM13_ESM.pdf]
