# Supplementary material for: Phylogeny of the plant receptor-like kinase (RLK) gene family and expression analysis of wheat RLK genes in response to biotic and abiotic stresses
Source: BMC Genomics. 2023 May 1;24:224. doi: 10.1186/s12864-023-09303-7 (PMC10152718; doi:10.1186/s12864-023-09303-7)
Supplement: Supplementary file 2 — Additional file 2: Figure S2. Exon−intron and domain diagrams of RLKs in 15 plants. The descriptions of the domain and exon phases are the same as those in Fig. 2. The lengths of the boxes and lines are scaled based on the lengths of the genes. [file 12864_2023_9303_MOESM2_ESM.pdf]

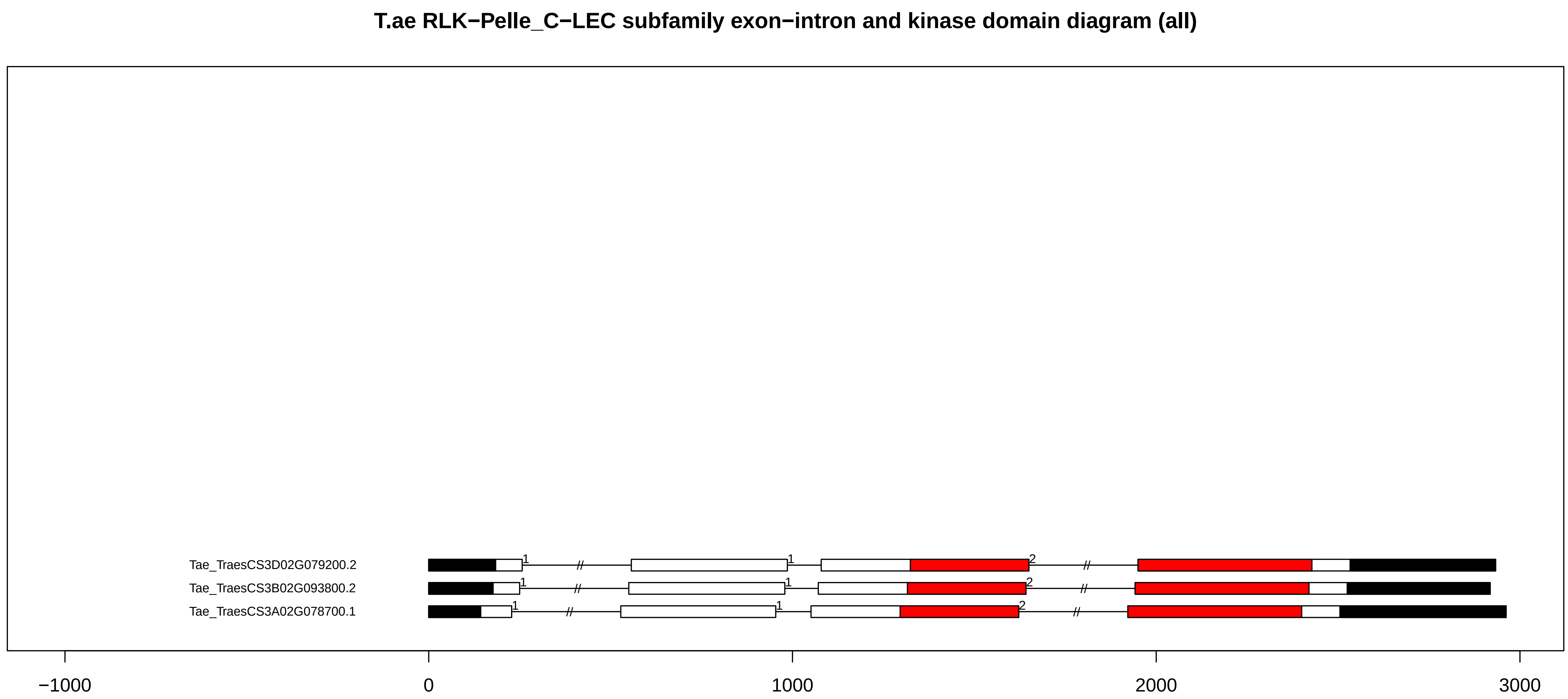
T.ae RLK−Pelle_C−LEC subfamily exon−intron and kinase domain diagram (all)
Tae_TraesCS3D02G079200.2
Tae_TraesCS3B02G093800.2
Tae_TraesCS3A02G078700.1
1
1
2
−1000
0
1000
2000
3000

T.ae RLK−Pelle_CR4L subfamily exon−intron and kinase domain diagram (all)

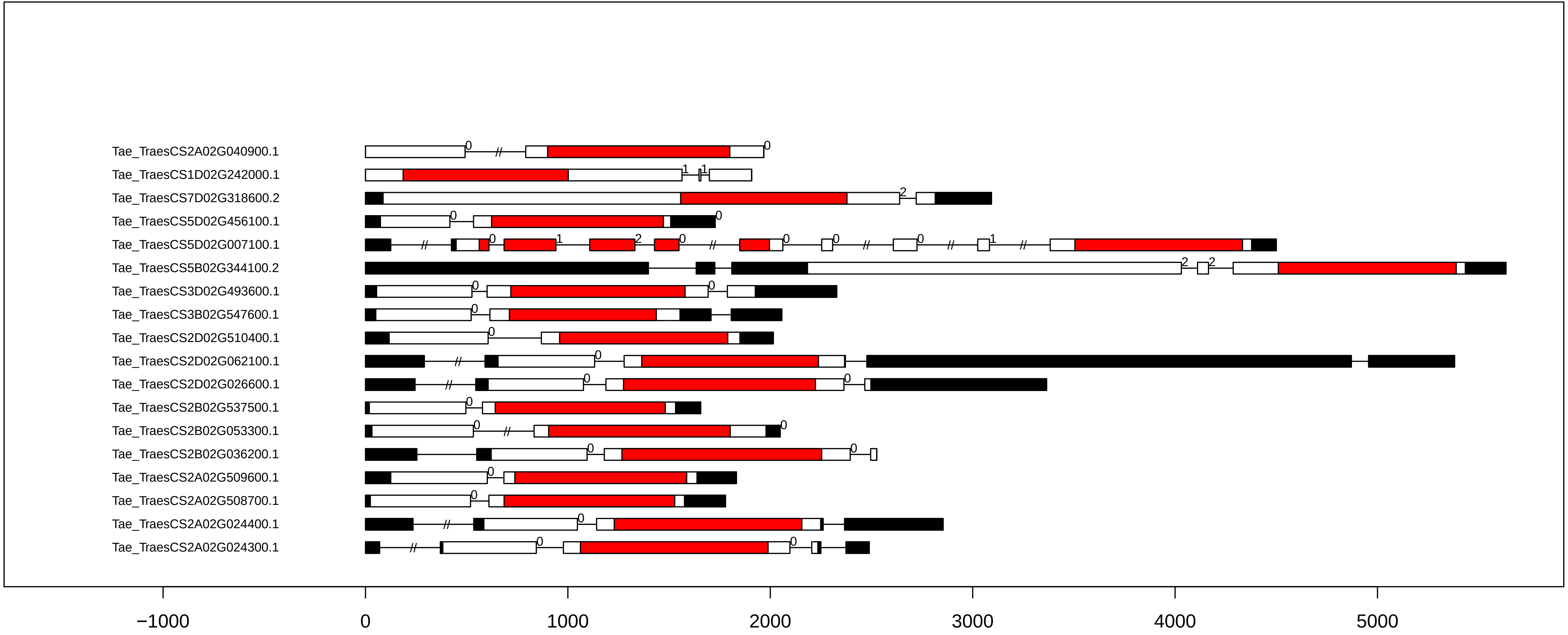

T.ae RLK−Pelle_CrRLK1L−1 subfamily exon−intron and kinase domain diagram (all)

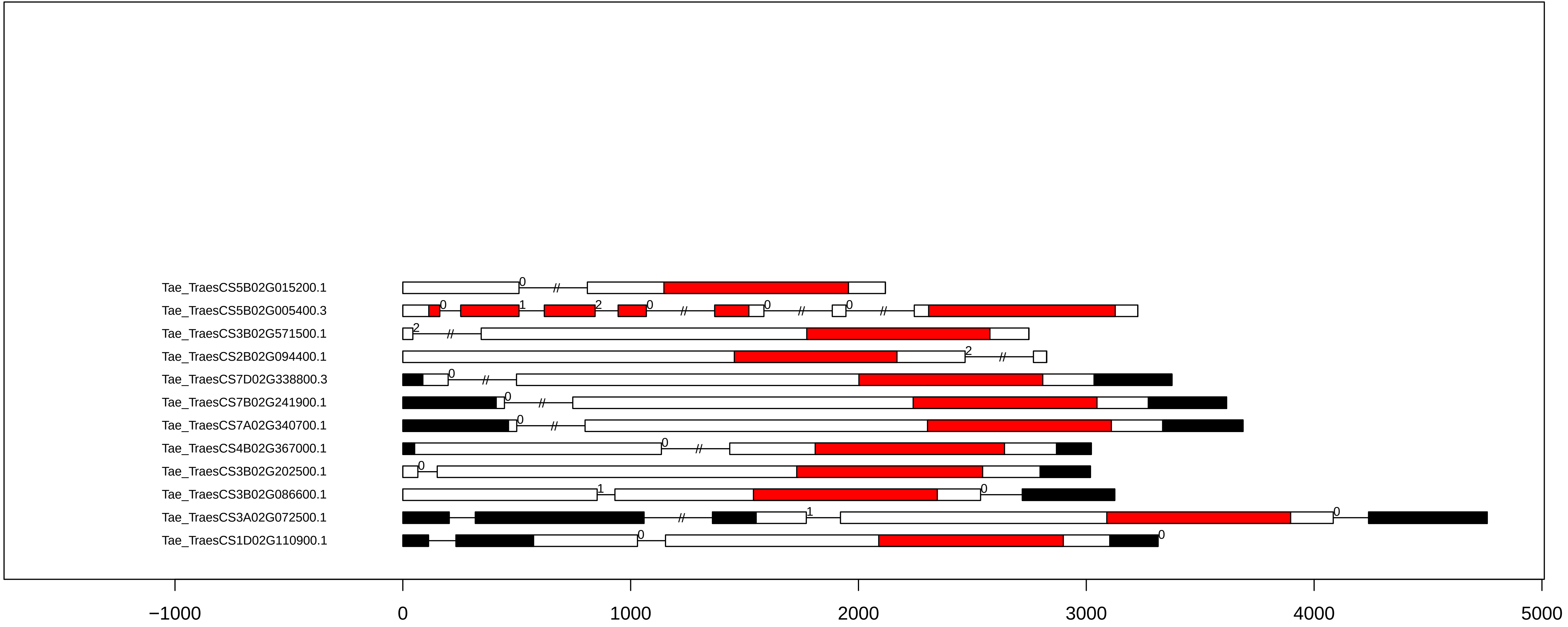

T.ae RLK−Pelle_DLSV subfamily exon−intron and kinase domain diagram (part 1)

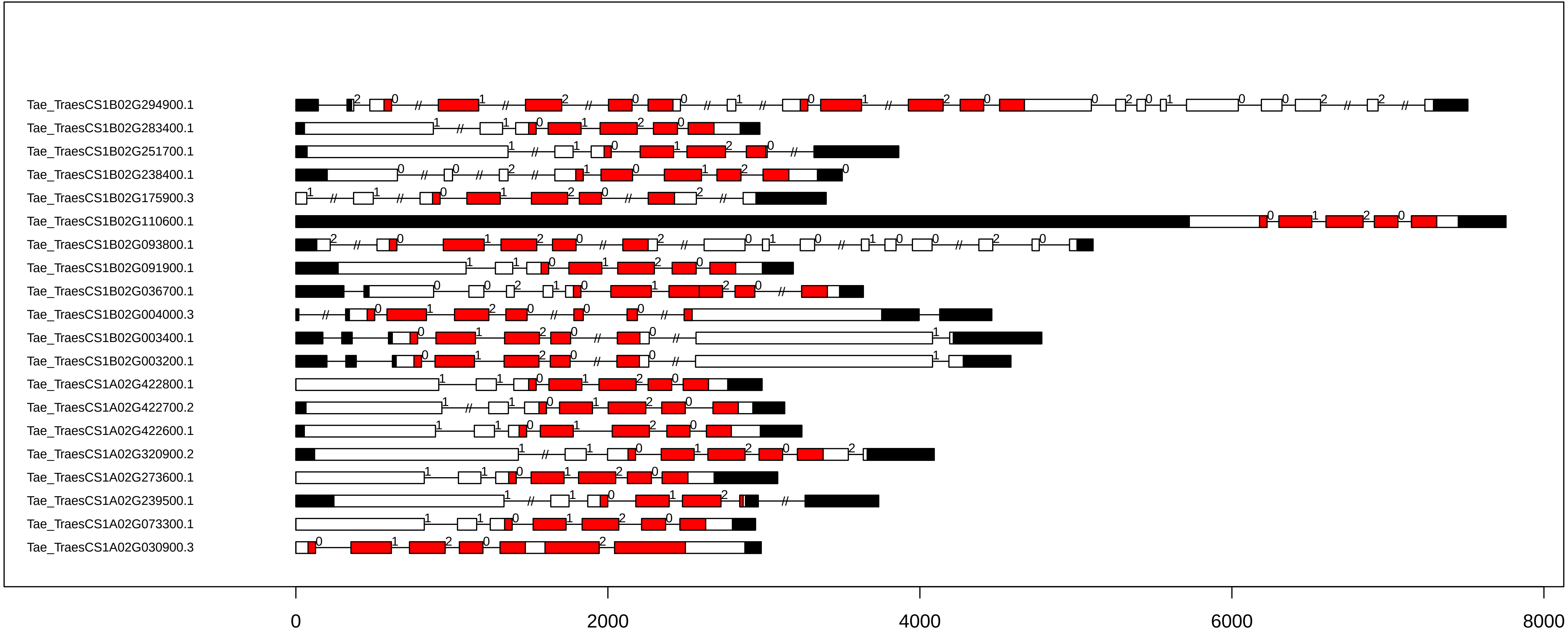

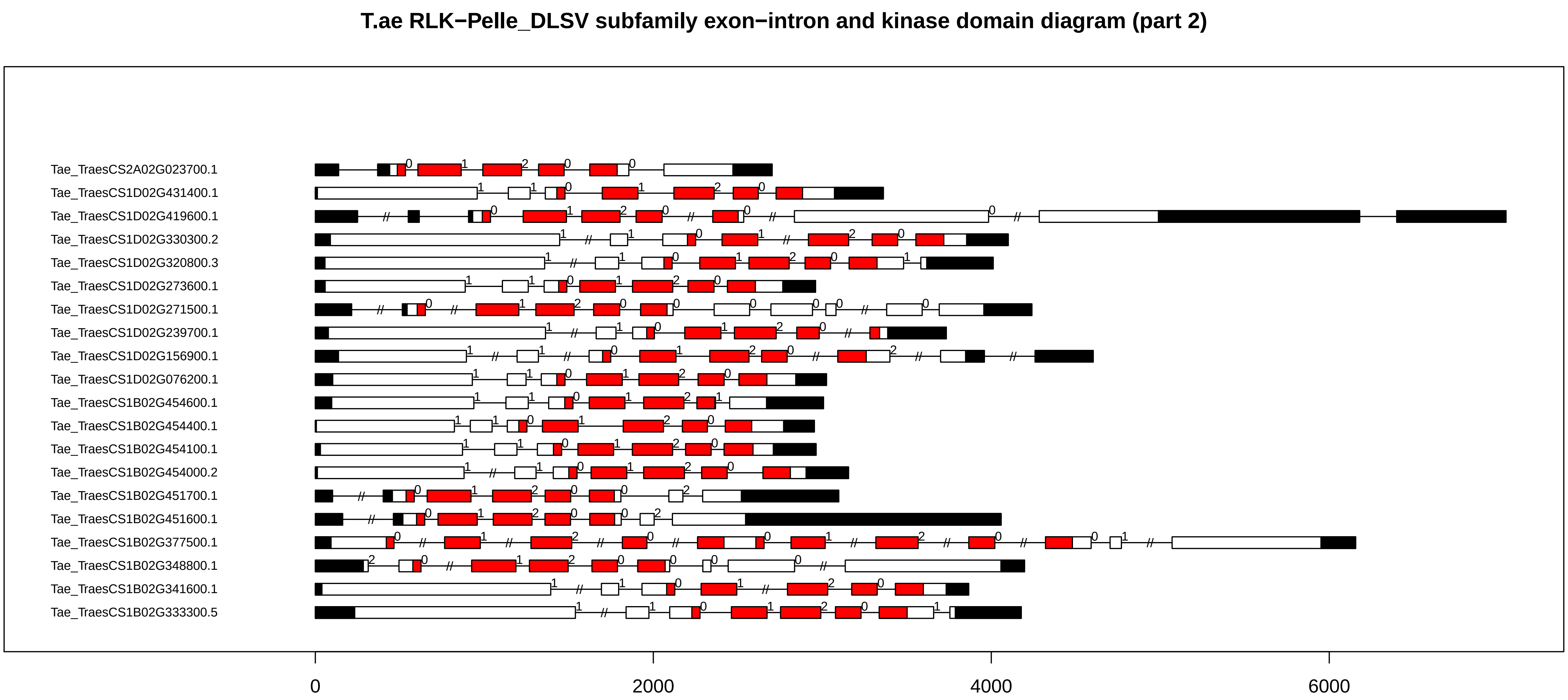
T.ae RLK−Pelle_DLSV subfamily exon−intron and kinase domain diagram (part 2)
Tae_TraesCS2A02G023700.1
Tae_TraesCS1D02G431400.1
Tae_TraesCS1D02G419600.1
Tae_TraesCS1D02G330300.2
Tae_TraesCS1D02G320800.3
Tae_TraesCS1D02G273600.1
Tae_TraesCS1D02G271500.1
Tae_TraesCS1D02G239700.1
Tae_TraesCS1D02G156900.1
Tae_TraesCS1D02G076200.1
Tae_TraesCS1B02G454600.1
Tae_TraesCS1B02G454400.1
Tae_TraesCS1B02G454100.1
Tae_TraesCS1B02G454000.2
Tae_TraesCS1B02G451700.1
Tae_TraesCS1B02G451600.1
Tae_TraesCS1B02G377500.1
Tae_TraesCS1B02G348800.1
Tae_TraesCS1B02G341600.1
Tae_TraesCS1B02G333300.5
0
2000
4000
6000

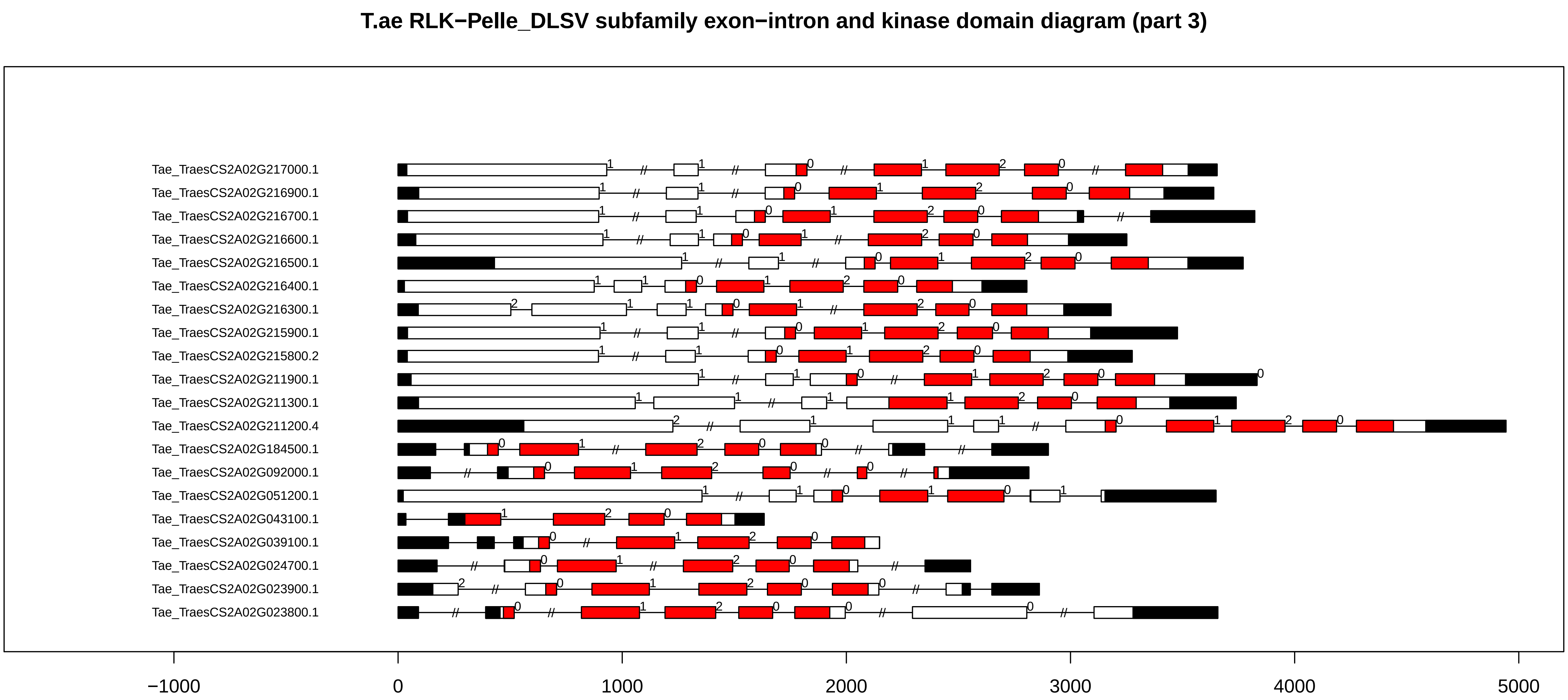
T.ae RLK−Pelle_DLSV subfamily exon−intron and kinase domain diagram (part 3)
Tae_TraesCS2A02G217000.1
Tae_TraesCS2A02G216900.1
Tae_TraesCS2A02G216700.1
Tae_TraesCS2A02G216600.1
Tae_TraesCS2A02G216500.1
Tae_TraesCS2A02G216400.1
Tae_TraesCS2A02G216300.1
Tae_TraesCS2A02G215900.1
Tae_TraesCS2A02G215800.2
Tae_TraesCS2A02G211900.1
Tae_TraesCS2A02G211300.1
Tae_TraesCS2A02G211200.4
Tae_TraesCS2A02G184500.1
Tae_TraesCS2A02G092000.1
Tae_TraesCS2A02G051200.1
Tae_TraesCS2A02G043100.1
Tae_TraesCS2A02G039100.1
Tae_TraesCS2A02G024700.1
Tae_TraesCS2A02G023900.1
Tae_TraesCS2A02G023800.1
−1000
0
1000
2000
3000
4000
5000

T.ae RLK−Pelle_DLSV subfamily exon−intron and kinase domain diagram (part 4)

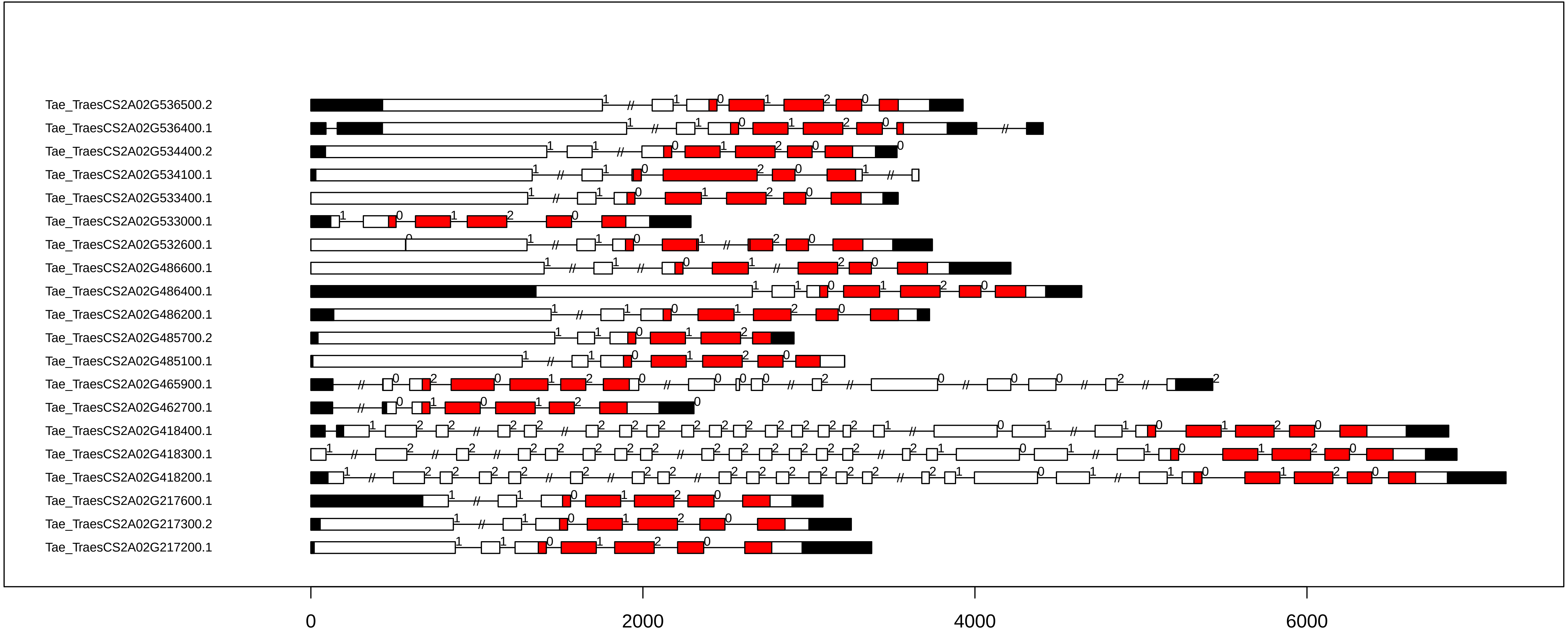

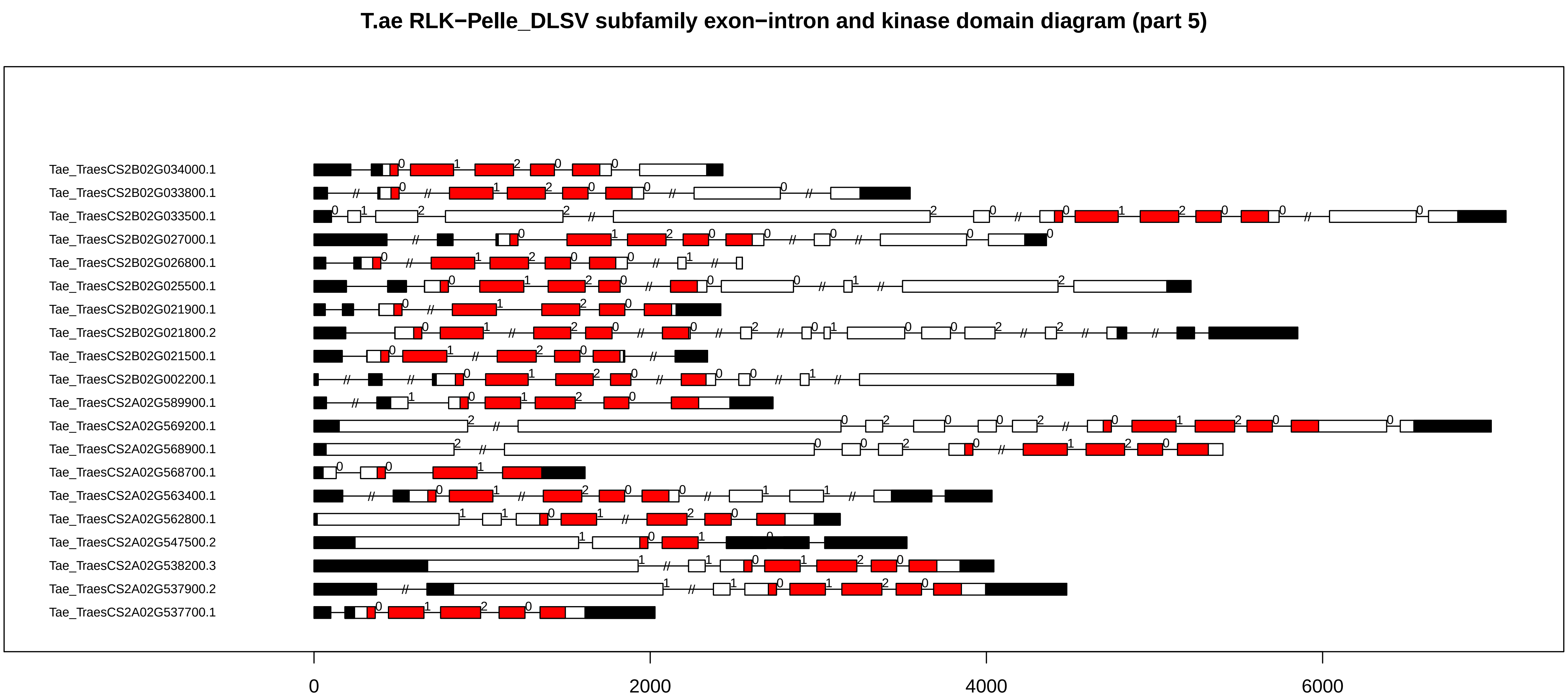
T.ae RLK−Pelle_DLSV subfamily exon−intron and kinase domain diagram (part 5)
Tae_TraesCS2B02G034000.1
Tae_TraesCS2B02G033800.1
Tae_TraesCS2B02G033500.1
Tae_TraesCS2B02G027000.1
Tae_TraesCS2B02G026800.1
Tae_TraesCS2B02G025500.1
Tae_TraesCS2B02G021900.1
Tae_TraesCS2B02G021800.2
Tae_TraesCS2B02G021500.1
Tae_TraesCS2B02G002200.1
Tae_TraesCS2A02G589900.1
Tae_TraesCS2A02G569200.1
Tae_TraesCS2A02G568900.1
Tae_TraesCS2A02G568700.1
Tae_TraesCS2A02G563400.1
Tae_TraesCS2A02G562800.1
Tae_TraesCS2A02G547500.2
Tae_TraesCS2A02G538200.3
Tae_TraesCS2A02G537900.2
Tae_TraesCS2A02G537700.1
0
2000
4000
6000

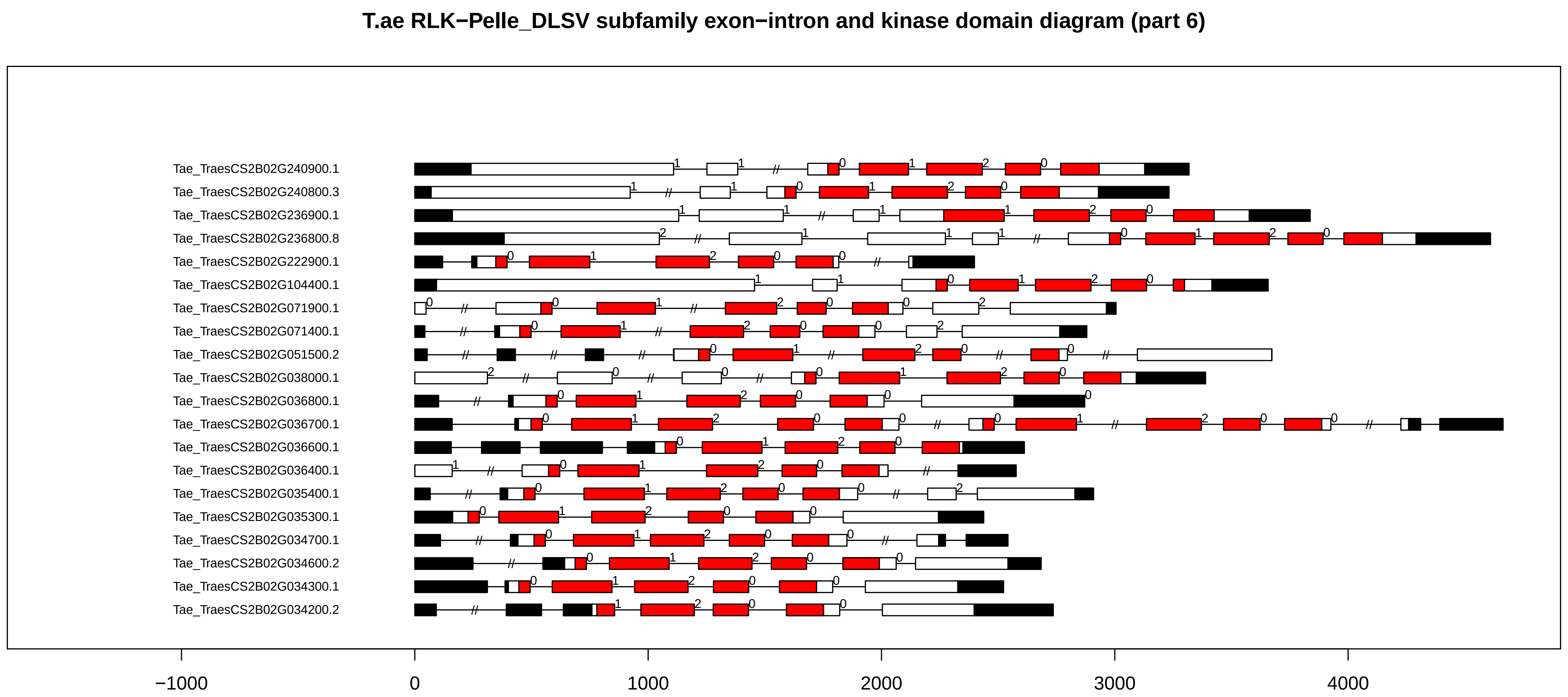
T.ae RLK−Pelle_DLSV subfamily exon−intron and kinase domain diagram (part 6)
Tae_TraesCS2B02G240900.1
Tae_TraesCS2B02G240800.3
Tae_TraesCS2B02G236900.1
Tae_TraesCS2B02G236800.8
Tae_TraesCS2B02G222900.1
Tae_TraesCS2B02G104400.1
Tae_TraesCS2B02G071900.1
Tae_TraesCS2B02G071400.1
Tae_TraesCS2B02G051500.2
Tae_TraesCS2B02G038000.1
Tae_TraesCS2B02G036800.1
Tae_TraesCS2B02G036700.1
Tae_TraesCS2B02G036600.1
Tae_TraesCS2B02G036400.1
Tae_TraesCS2B02G035400.1
Tae_TraesCS2B02G035300.1
Tae_TraesCS2B02G034700.1
Tae_TraesCS2B02G034600.2
Tae_TraesCS2B02G034300.1
Tae_TraesCS2B02G034200.2
−1000
0
1000
2000
3000
4000

T.ae RLK−Pelle_DLSV subfamily exon−intron and kinase domain diagram (part 7)

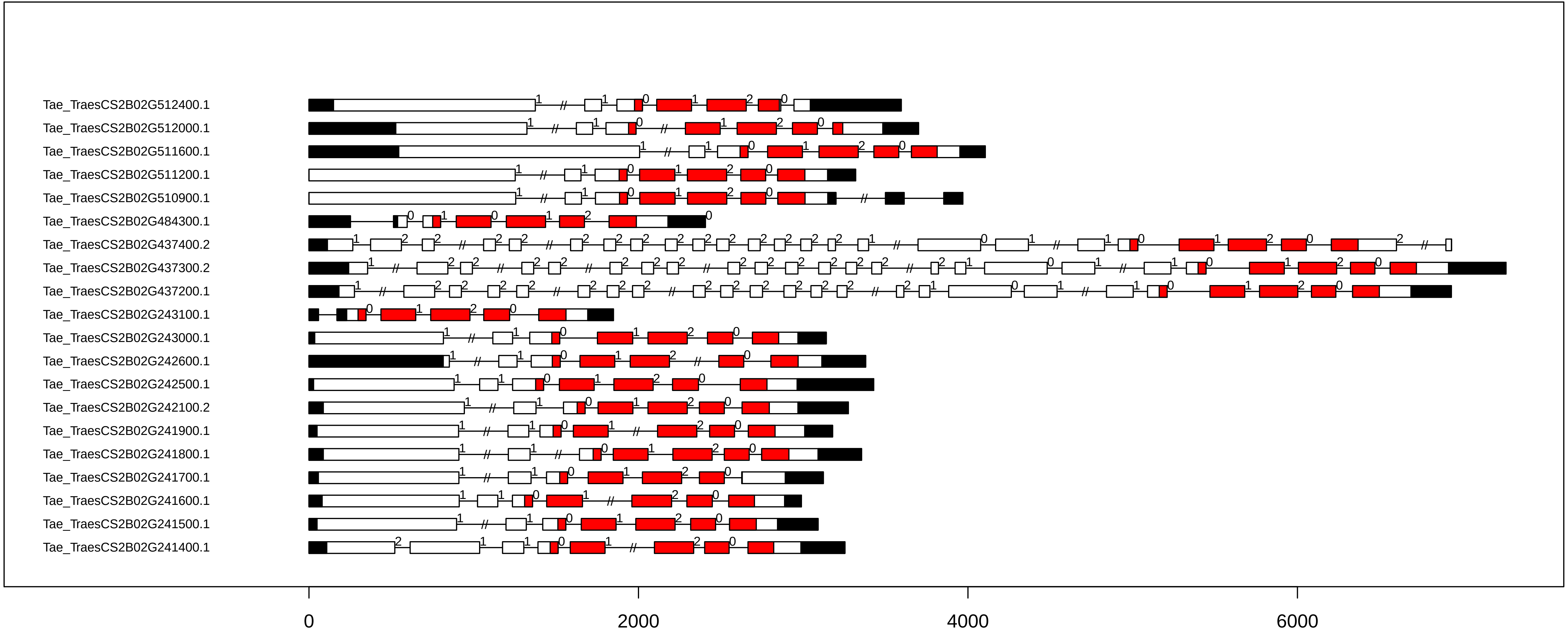

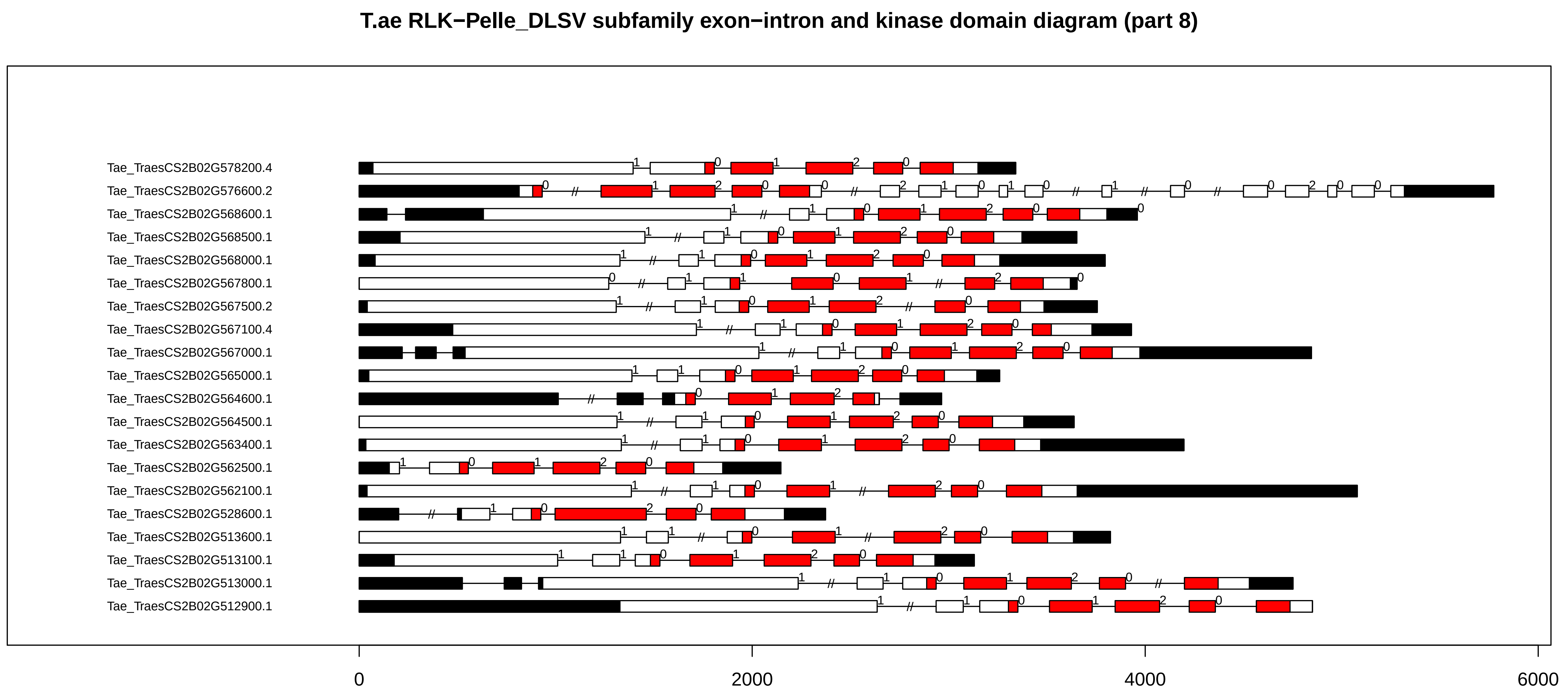
T.ae RLK−Pelle_DLSV subfamily exon−intron and kinase domain diagram (part 8)
Tae_TraesCS2B02G578200.4
Tae_TraesCS2B02G576600.2
Tae_TraesCS2B02G568600.1
Tae_TraesCS2B02G568500.1
Tae_TraesCS2B02G568000.1
Tae_TraesCS2B02G567800.1
Tae_TraesCS2B02G567500.2
Tae_TraesCS2B02G567100.4
Tae_TraesCS2B02G567000.1
Tae_TraesCS2B02G565000.1
Tae_TraesCS2B02G564600.1
Tae_TraesCS2B02G564500.1
Tae_TraesCS2B02G563400.1
Tae_TraesCS2B02G562500.1
Tae_TraesCS2B02G562100.1
Tae_TraesCS2B02G528600.1
Tae_TraesCS2B02G513600.1
Tae_TraesCS2B02G513100.1
Tae_TraesCS2B02G513000.1
Tae_TraesCS2B02G512900.1
0
2000
4000
6000

T.ae RLK−Pelle_DLSV subfamily exon−intron and kinase domain diagram (part 9)

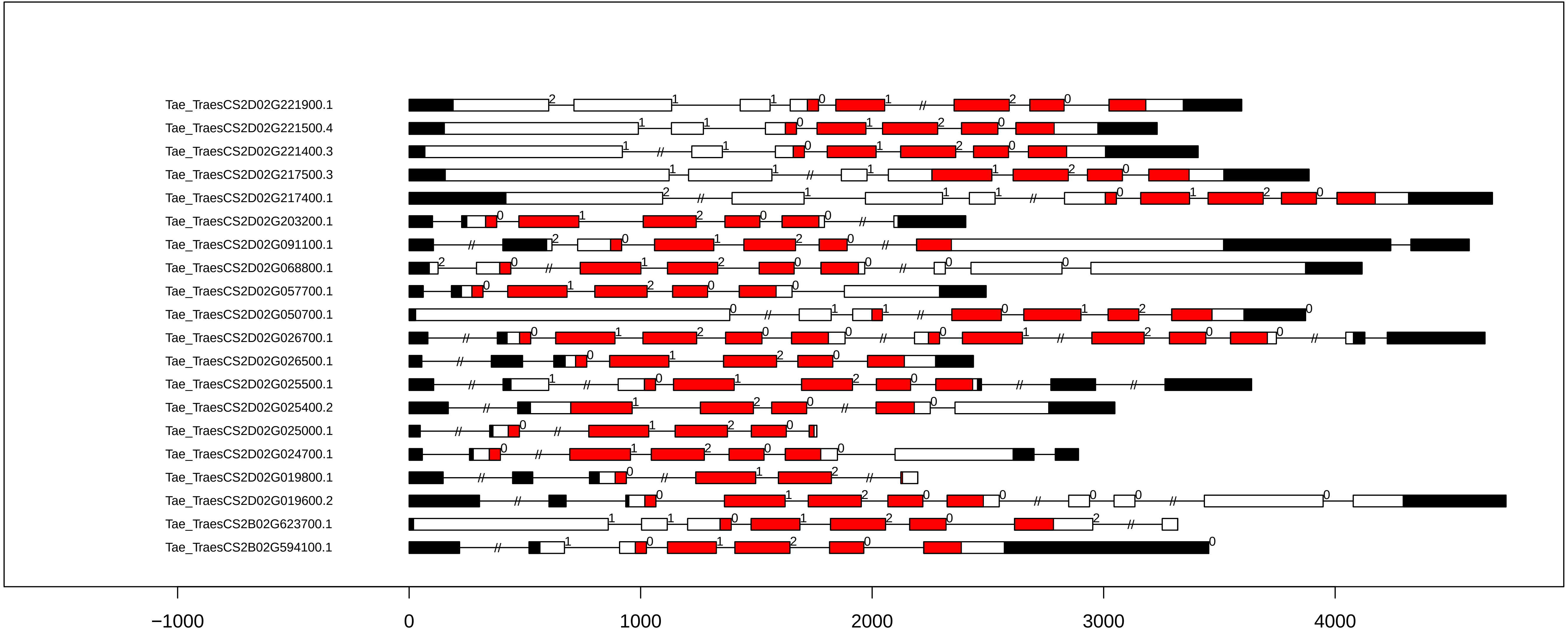

T.ae RLK−Pelle_DLSV subfamily exon−intron and kinase domain diagram (part 10)

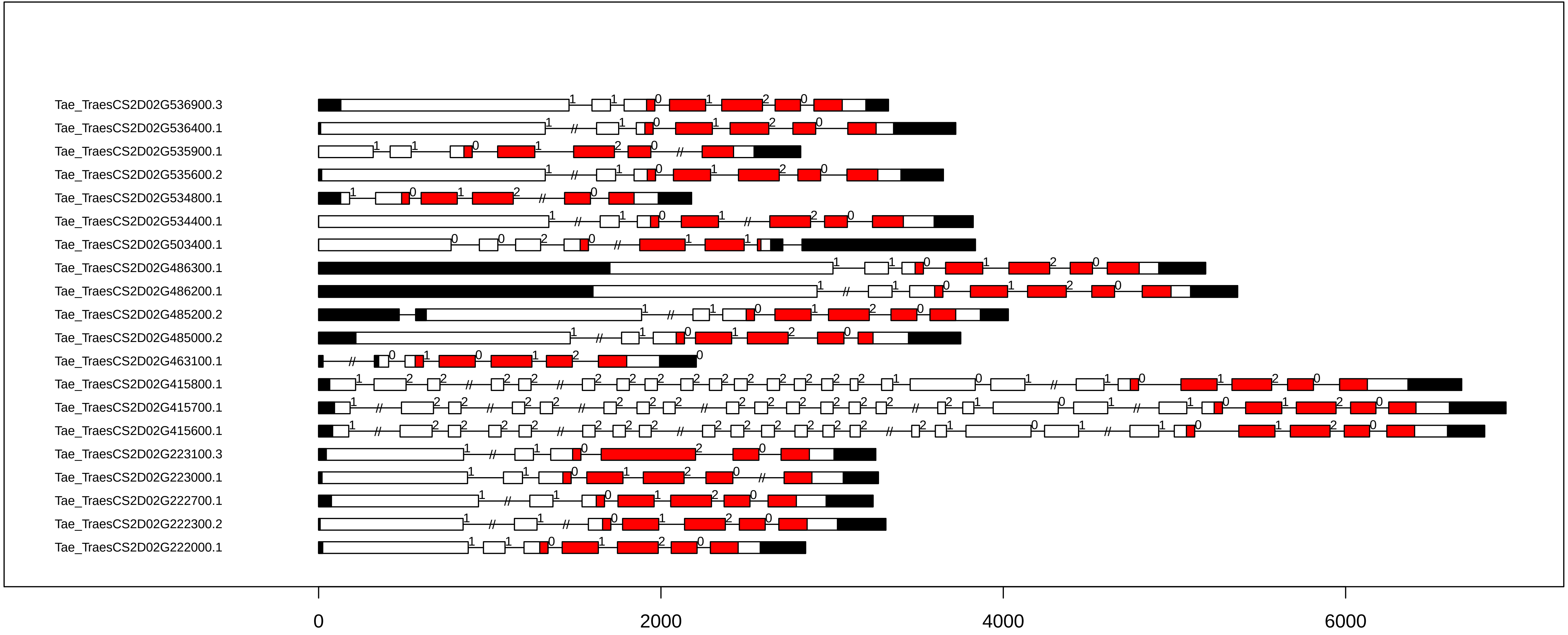

T.ae RLK−Pelle_DLSV subfamily exon−intron and kinase domain diagram (part 11)

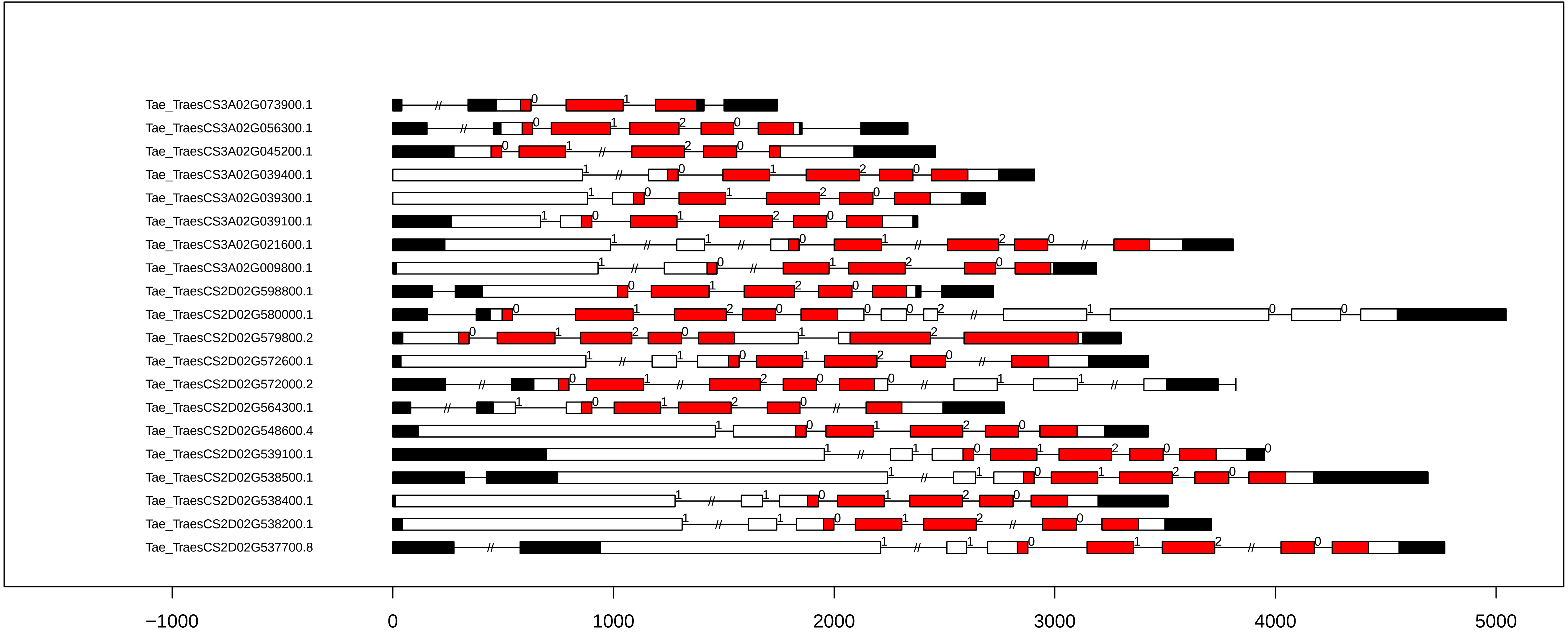

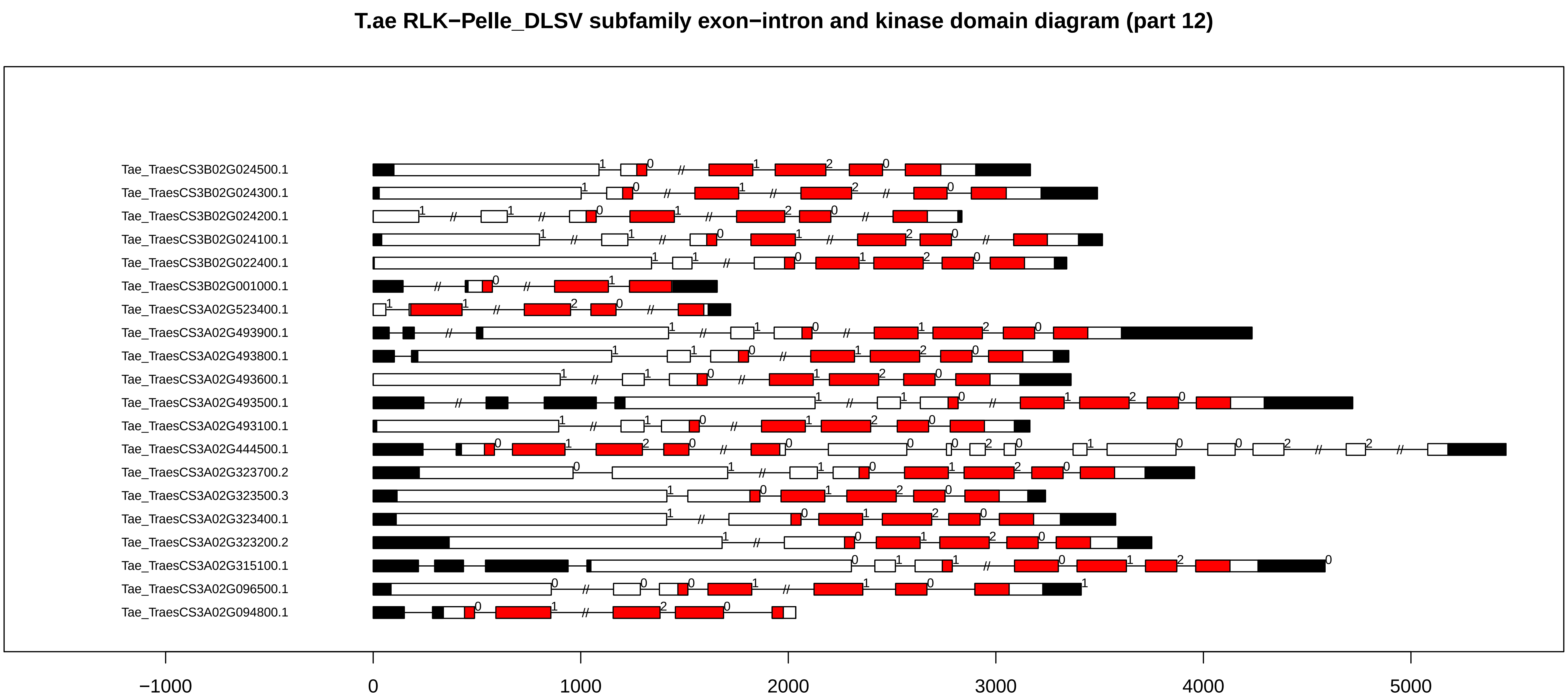
T.ae RLK−Pelle_DLSV subfamily exon−intron and kinase domain diagram (part 12)
Tae_TraesCS3B02G024500.1
Tae_TraesCS3B02G024300.1
Tae_TraesCS3B02G024200.1
Tae_TraesCS3B02G024100.1
Tae_TraesCS3B02G022400.1
Tae_TraesCS3B02G001000.1
Tae_TraesCS3A02G523400.1
Tae_TraesCS3A02G493900.1
Tae_TraesCS3A02G493800.1
Tae_TraesCS3A02G493600.1
Tae_TraesCS3A02G493500.1
Tae_TraesCS3A02G493100.1
Tae_TraesCS3A02G444500.1
Tae_TraesCS3A02G323700.2
Tae_TraesCS3A02G323500.3
Tae_TraesCS3A02G323400.1
Tae_TraesCS3A02G323200.2
Tae_TraesCS3A02G315100.1
Tae_TraesCS3A02G096500.1
Tae_TraesCS3A02G094800.1
−1000
0
1000
2000
3000
4000
5000

T.ae RLK−Pelle_DLSV subfamily exon−intron and kinase domain diagram (part 13)

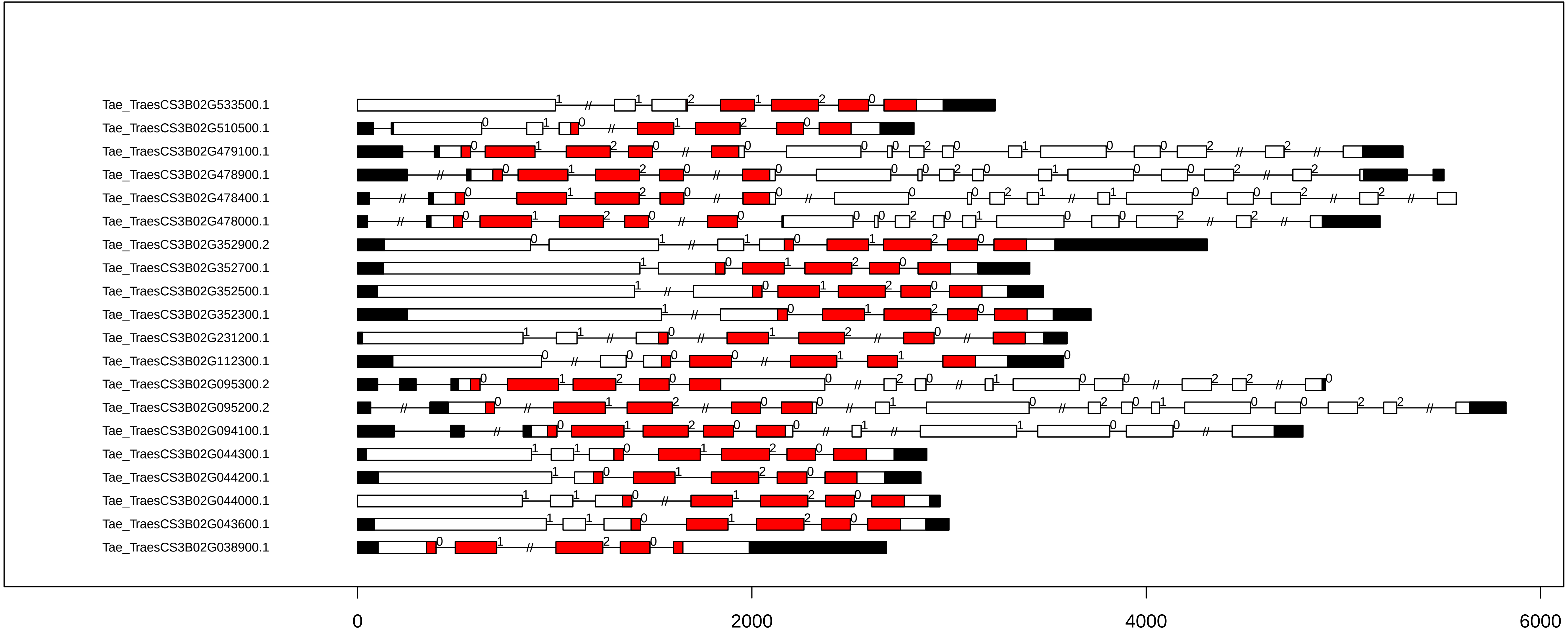

T.ae RLK−Pelle_DLSV subfamily exon−intron and kinase domain diagram (part 14)

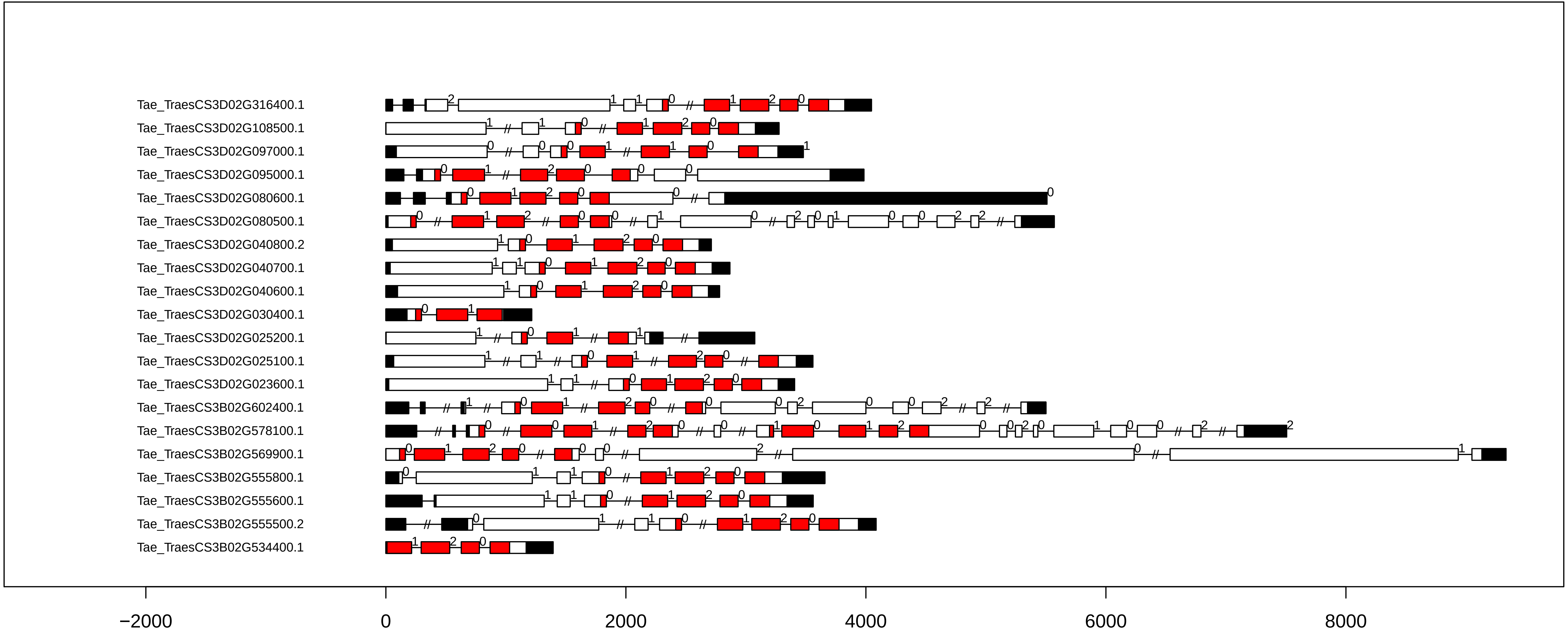

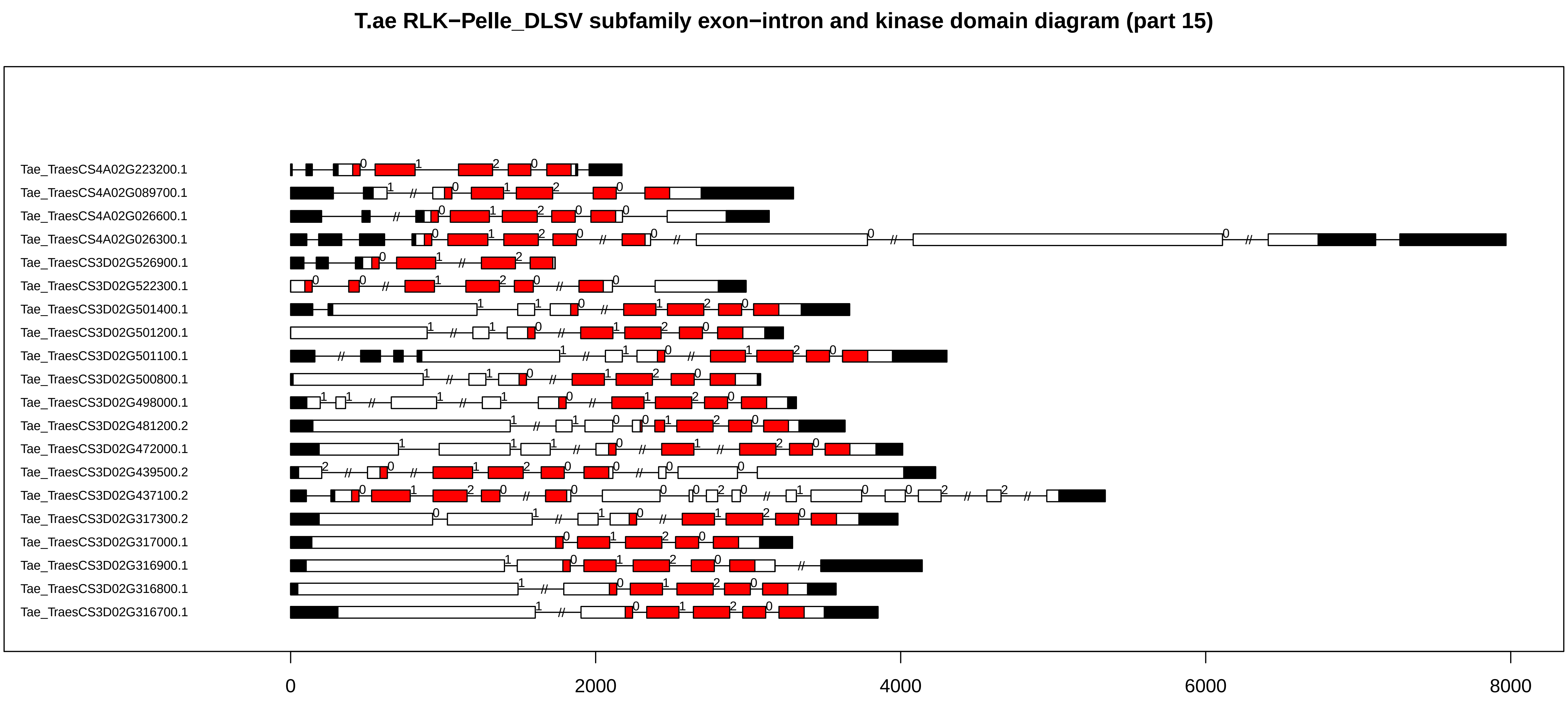
T.ae RLK−Pelle_DLSV subfamily exon−intron and kinase domain diagram (part 15)
Tae_TraesCS4A02G223200.1
Tae_TraesCS4A02G089700.1
Tae_TraesCS4A02G026600.1
Tae_TraesCS4A02G026300.1
Tae_TraesCS3D02G526900.1
Tae_TraesCS3D02G522300.1
Tae_TraesCS3D02G501400.1
Tae_TraesCS3D02G501200.1
Tae_TraesCS3D02G501100.1
Tae_TraesCS3D02G500800.1
Tae_TraesCS3D02G498000.1
Tae_TraesCS3D02G481200.2
Tae_TraesCS3D02G472000.1
Tae_TraesCS3D02G439500.2
Tae_TraesCS3D02G437100.2
Tae_TraesCS3D02G317300.2
Tae_TraesCS3D02G317000.1
Tae_TraesCS3D02G316900.1
Tae_TraesCS3D02G316800.1
Tae_TraesCS3D02G316700.1
0
2000
4000
6000
8000

T.ae RLK−Pelle_DLSV subfamily exon−intron and kinase domain diagram (part 16)

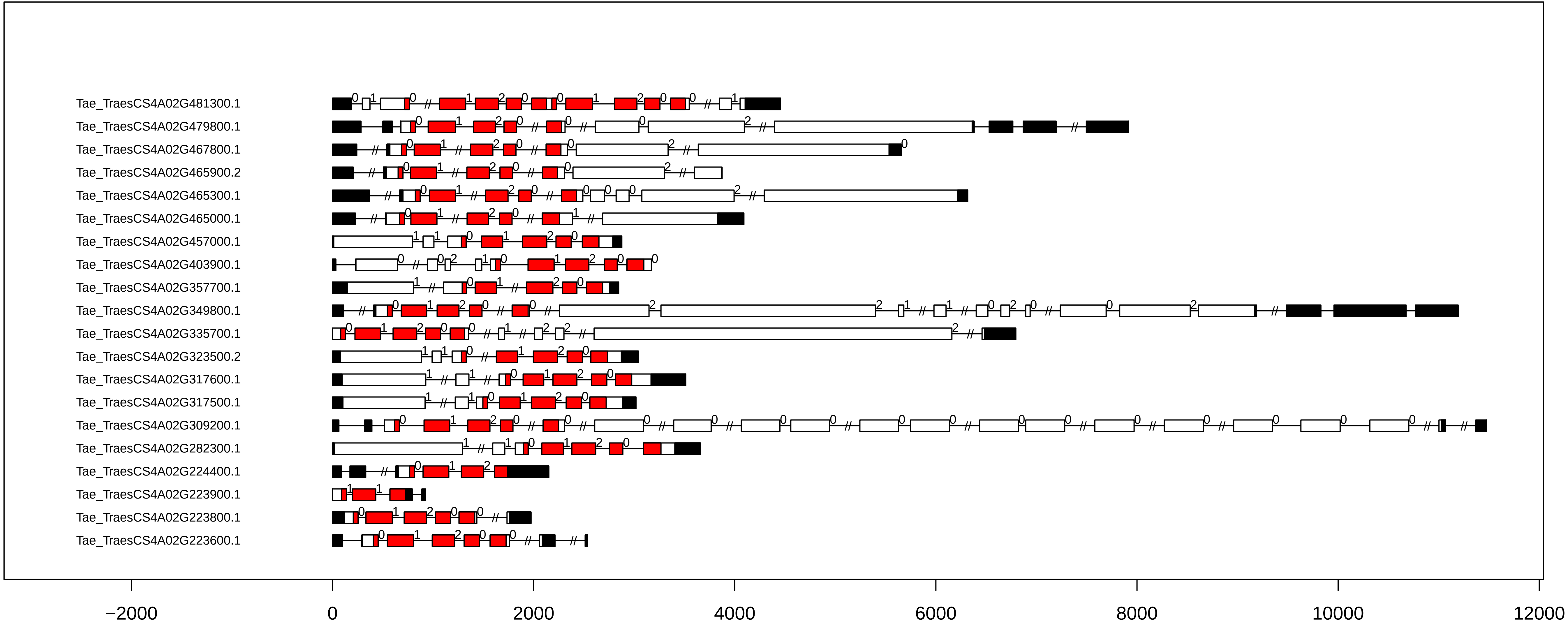

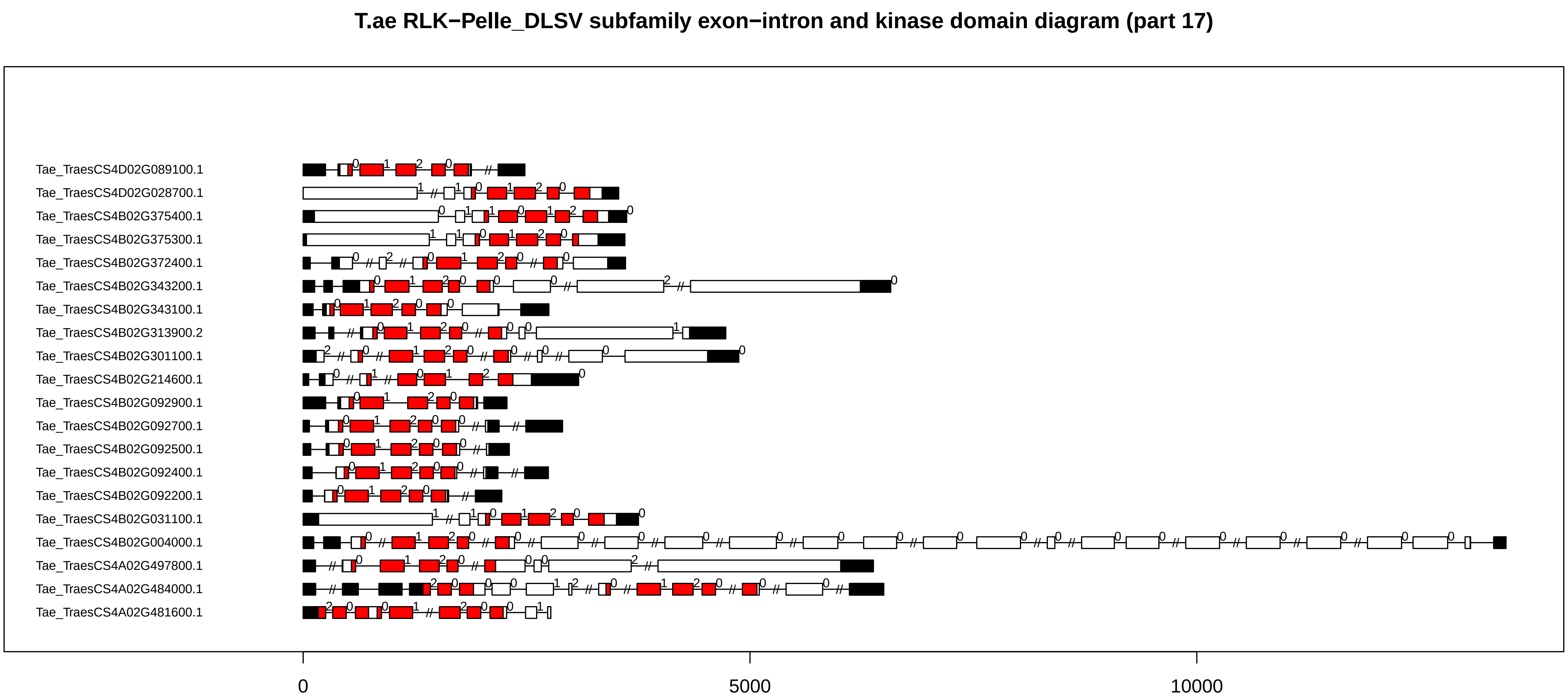
T.ae RLK−Pelle_DLSV subfamily exon−intron and kinase domain diagram (part 17)
Tae_TraesCS4D02G089100.1
Tae_TraesCS4D02G028700.1
Tae_TraesCS4B02G375400.1
Tae_TraesCS4B02G375300.1
Tae_TraesCS4B02G372400.1
Tae_TraesCS4B02G343200.1
Tae_TraesCS4B02G343100.1
Tae_TraesCS4B02G313900.2
Tae_TraesCS4B02G301100.1
Tae_TraesCS4B02G214600.1
Tae_TraesCS4B02G092900.1
Tae_TraesCS4B02G092700.1
Tae_TraesCS4B02G092500.1
Tae_TraesCS4B02G092400.1
Tae_TraesCS4B02G092200.1
Tae_TraesCS4B02G031100.1
Tae_TraesCS4B02G004000.1
Tae_TraesCS4A02G497800.1
Tae_TraesCS4A02G484000.1
Tae_TraesCS4A02G481600.1
0
5000
10000

T.ae RLK−Pelle_DLSV subfamily exon−intron and kinase domain diagram (part 18)

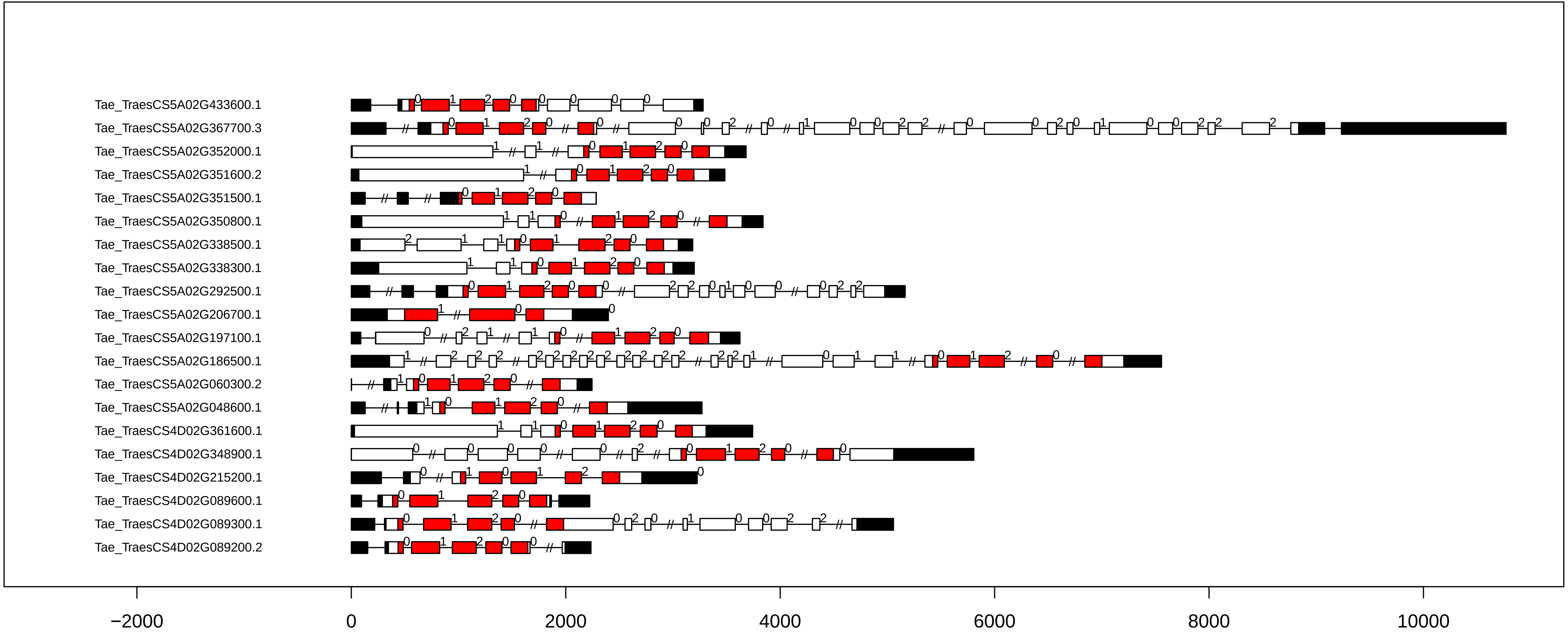

T.ae RLK−Pelle_DLSV subfamily exon−intron and kinase domain diagram (part 19)

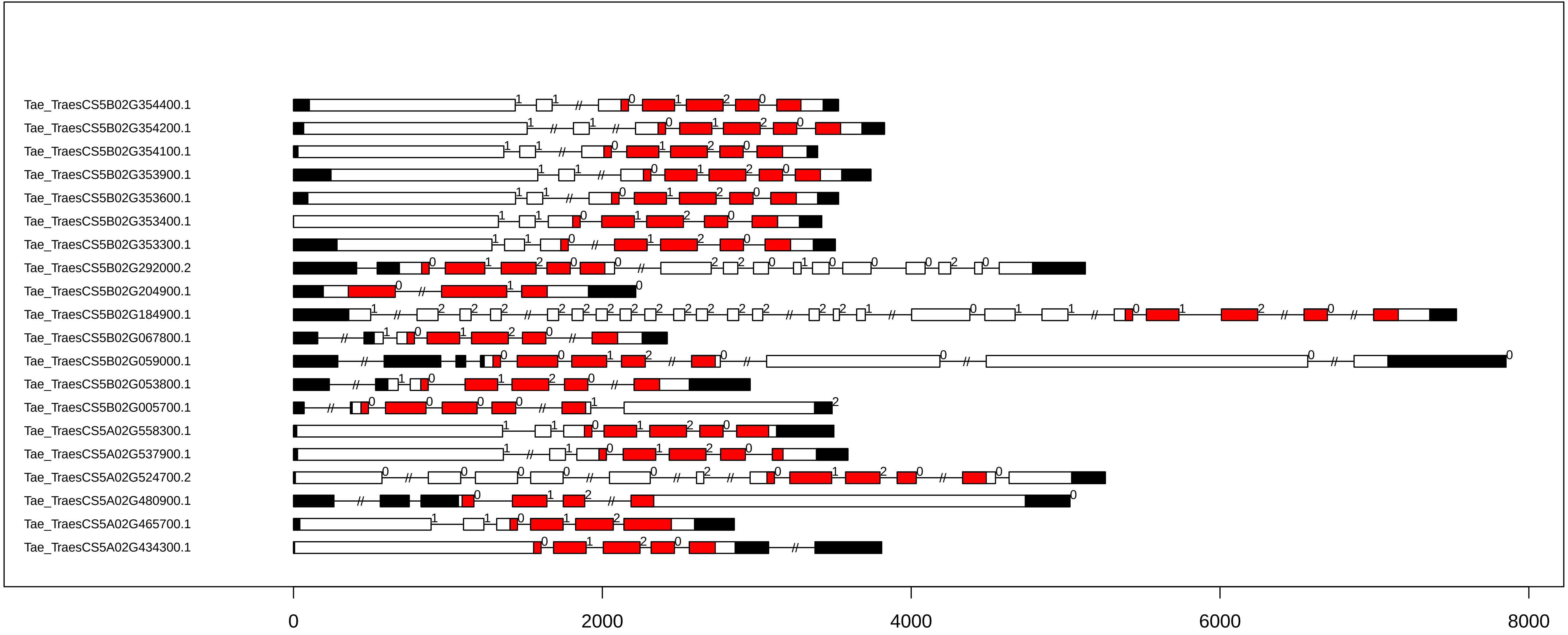

T.ae RLK−Pelle_DLSV subfamily exon−intron and kinase domain diagram (part 20)

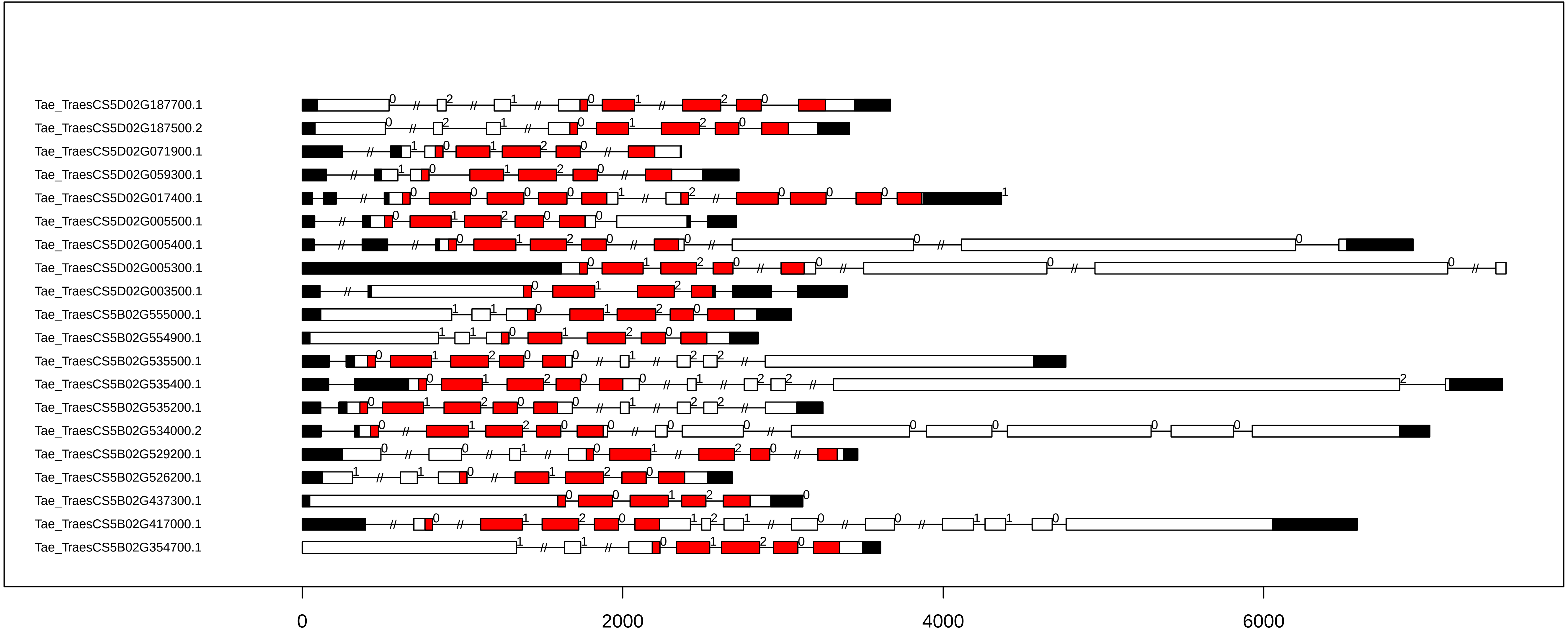

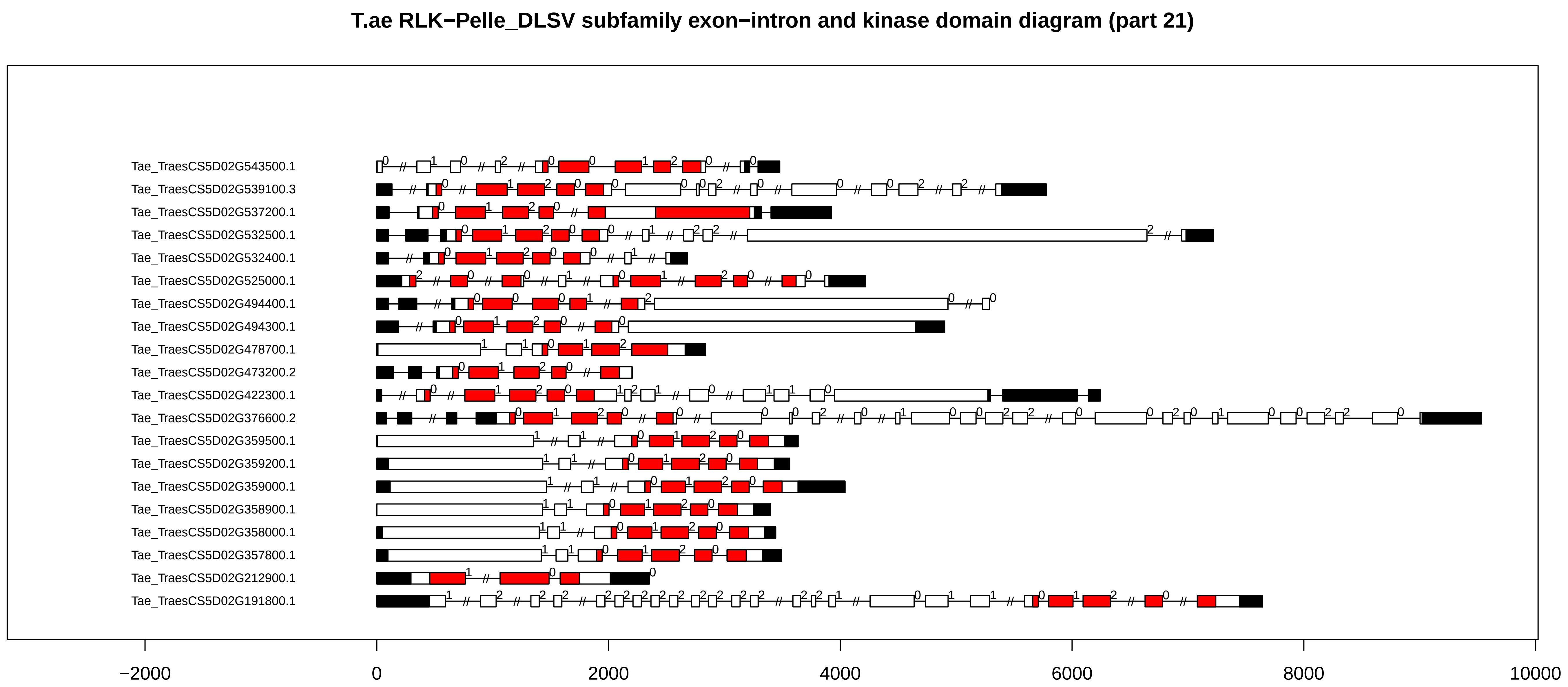
T.ae RLK−Pelle_DLSV subfamily exon−intron and kinase domain diagram (part 21)
Tae_TraesCS5D02G543500.1
Tae_TraesCS5D02G539100.3
Tae_TraesCS5D02G537200.1
Tae_TraesCS5D02G532500.1
Tae_TraesCS5D02G532400.1
Tae_TraesCS5D02G525000.1
Tae_TraesCS5D02G494400.1
Tae_TraesCS5D02G494300.1
Tae_TraesCS5D02G478700.1
Tae_TraesCS5D02G473200.2
Tae_TraesCS5D02G422300.1
Tae_TraesCS5D02G376600.2
Tae_TraesCS5D02G359500.1
Tae_TraesCS5D02G359200.1
Tae_TraesCS5D02G359000.1
Tae_TraesCS5D02G358900.1
Tae_TraesCS5D02G358000.1
Tae_TraesCS5D02G357800.1
Tae_TraesCS5D02G212900.1
Tae_TraesCS5D02G191800.1
−2000
0
2000
4000
6000
8000
10000

T.ae RLK−Pelle_DLSV subfamily exon−intron and kinase domain diagram (part 22)

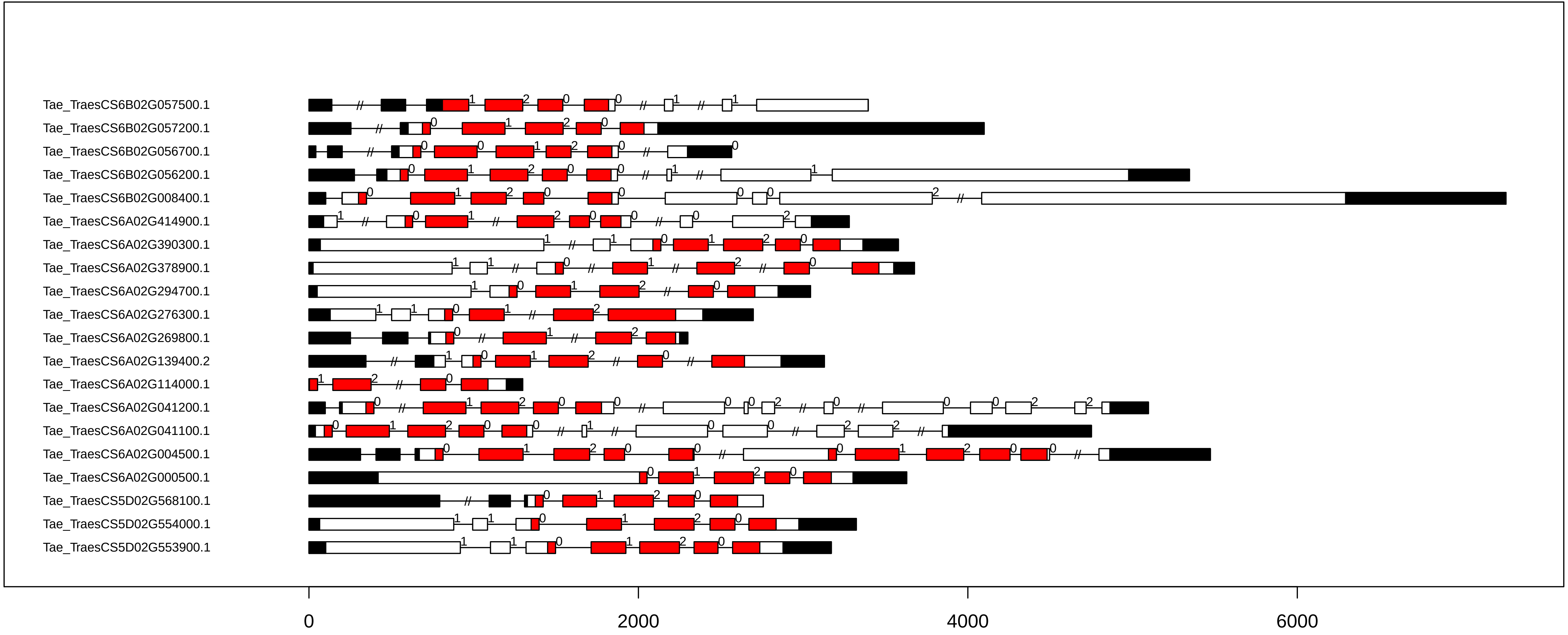

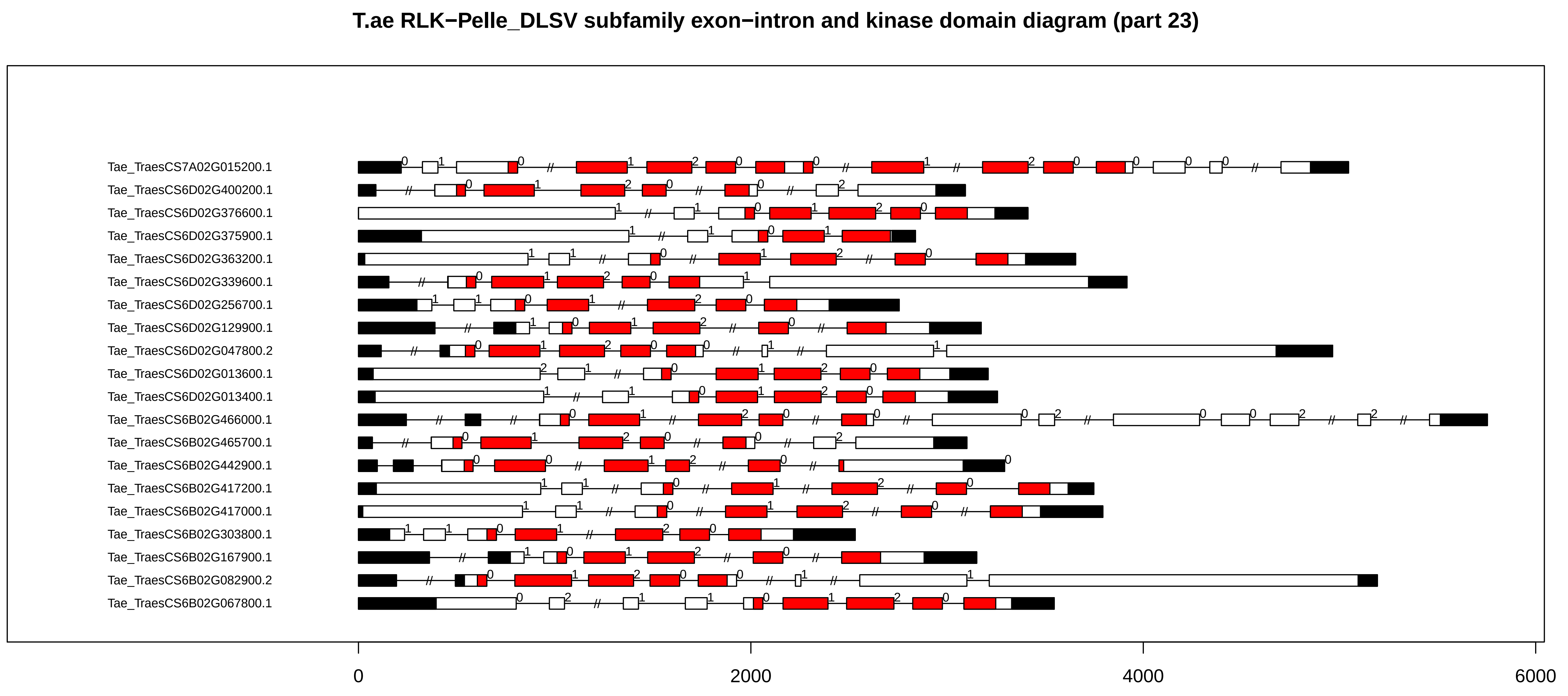
T.ae RLK−Pelle_DLSV subfamily exon−intron and kinase domain diagram (part 23)
Tae_TraesCS7A02G015200.1
Tae_TraesCS6D02G400200.1
Tae_TraesCS6D02G376600.1
Tae_TraesCS6D02G375900.1
Tae_TraesCS6D02G363200.1
Tae_TraesCS6D02G339600.1
Tae_TraesCS6D02G256700.1
Tae_TraesCS6D02G129900.1
Tae_TraesCS6D02G047800.2
Tae_TraesCS6D02G013600.1
Tae_TraesCS6D02G013400.1
Tae_TraesCS6B02G466000.1
Tae_TraesCS6B02G465700.1
Tae_TraesCS6B02G442900.1
Tae_TraesCS6B02G417200.1
Tae_TraesCS6B02G417000.1
Tae_TraesCS6B02G303800.1
Tae_TraesCS6B02G167900.1
Tae_TraesCS6B02G082900.2
Tae_TraesCS6B02G067800.1
0
2000
4000
6000

T.ae RLK−Pelle_DLSV subfamily exon−intron and kinase domain diagram (part 24)

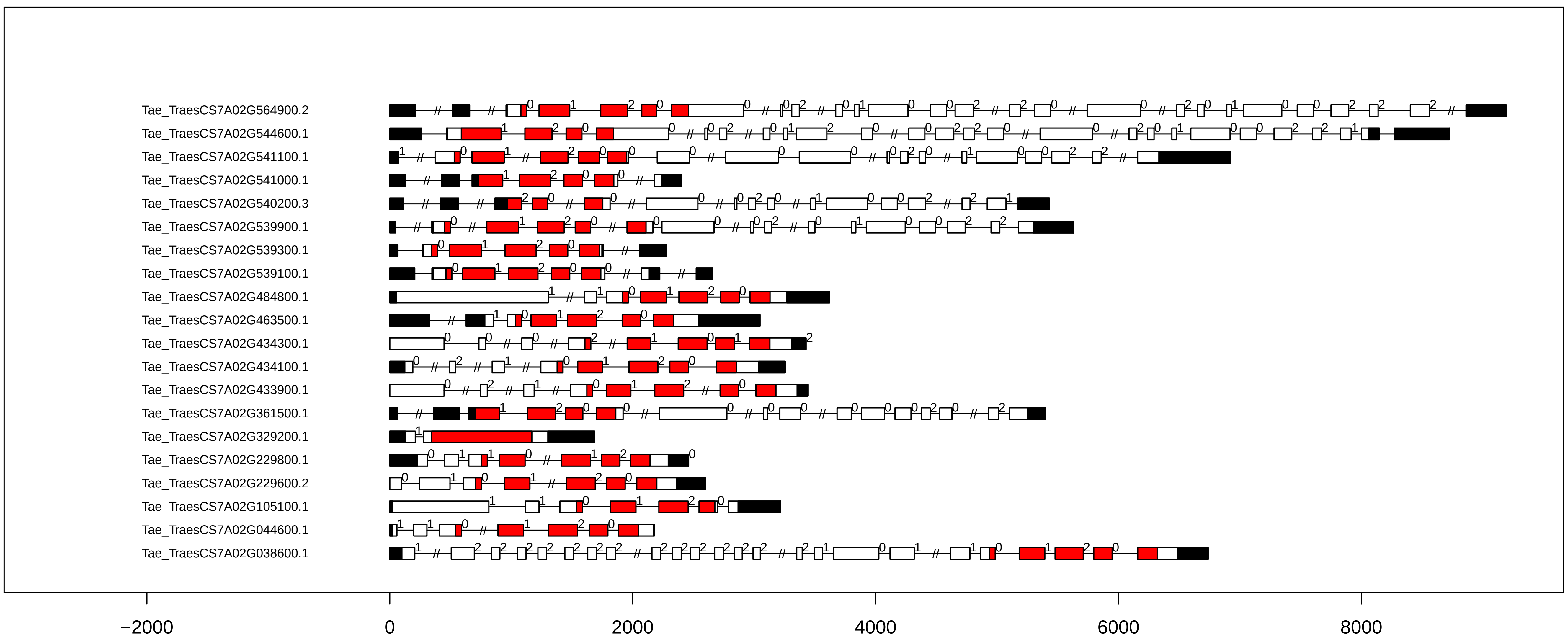

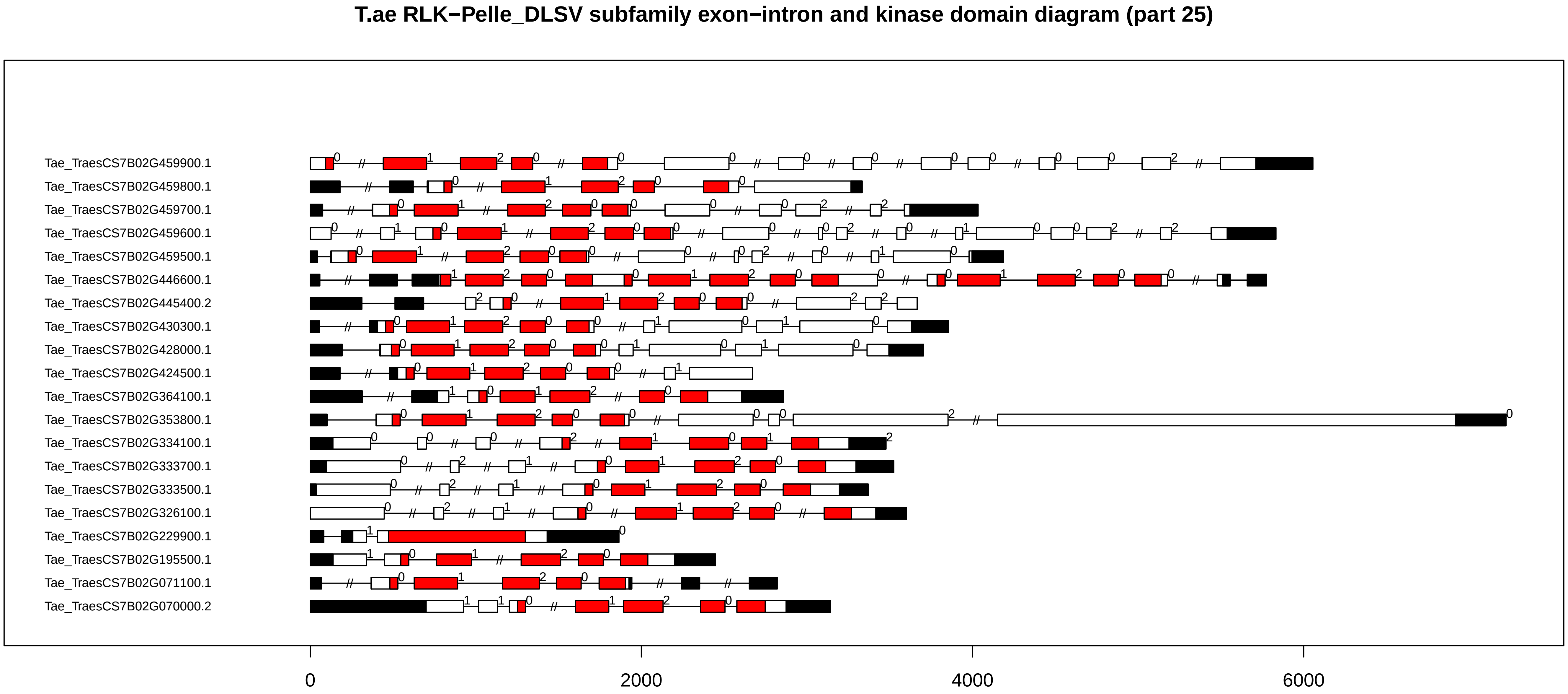

T.ae RLK−Pelle_DLSV subfamily exon−intron and kinase domain diagram (part 25)
Tae_TraesCS7B02G459900.1
Tae_TraesCS7B02G459800.1
Tae_TraesCS7B02G459700.1
Tae_TraesCS7B02G459600.1
Tae_TraesCS7B02G459500.1
Tae_TraesCS7B02G446600.1
Tae_TraesCS7B02G445400.2
Tae_TraesCS7B02G430300.1
Tae_TraesCS7B02G428000.1
Tae_TraesCS7B02G424500.1
Tae_TraesCS7B02G364100.1
Tae_TraesCS7B02G353800.1
Tae_TraesCS7B02G334100.1
Tae_TraesCS7B02G333700.1
Tae_TraesCS7B02G333500.1
Tae_TraesCS7B02G326100.1
Tae_TraesCS7B02G229900.1
Tae_TraesCS7B02G195500.1
Tae_TraesCS7B02G071100.1
Tae_TraesCS7B02G070000.2
0
2000
4000
6000

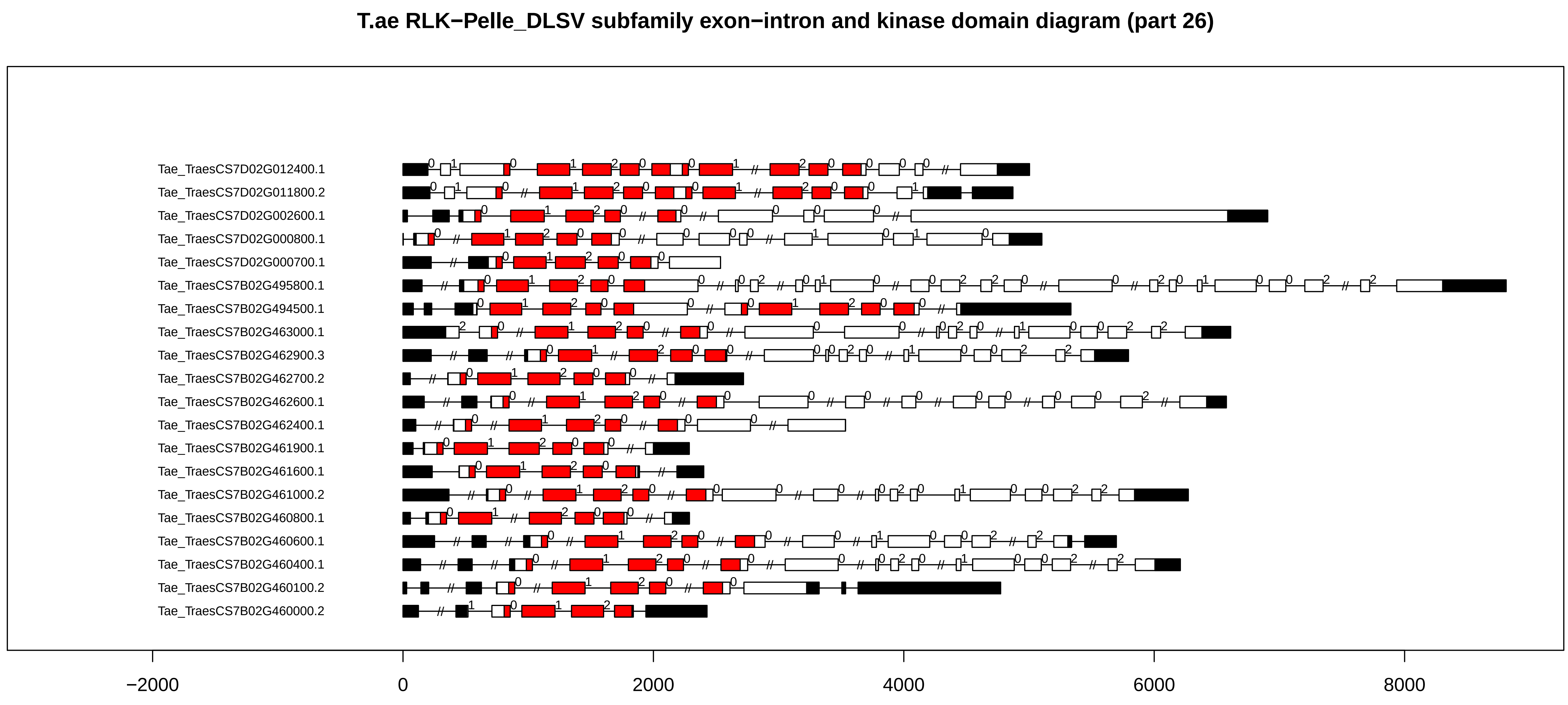
T.ae RLK−Pelle_DLSV subfamily exon−intron and kinase domain diagram (part 26)
Tae_TraesCS7D02G012400.1
Tae_TraesCS7D02G011800.2
Tae_TraesCS7D02G002600.1
Tae_TraesCS7D02G000800.1
Tae_TraesCS7D02G000700.1
Tae_TraesCS7B02G495800.1
Tae_TraesCS7B02G494500.1
Tae_TraesCS7B02G463000.1
Tae_TraesCS7B02G462900.3
Tae_TraesCS7B02G462700.2
Tae_TraesCS7B02G462600.1
Tae_TraesCS7B02G462400.1
Tae_TraesCS7B02G461900.1
Tae_TraesCS7B02G461600.1
Tae_TraesCS7B02G461000.2
Tae_TraesCS7B02G460800.1
Tae_TraesCS7B02G460600.1
Tae_TraesCS7B02G460400.1
Tae_TraesCS7B02G460100.2
Tae_TraesCS7B02G460000.2
−2000
0
2000
4000
6000
8000

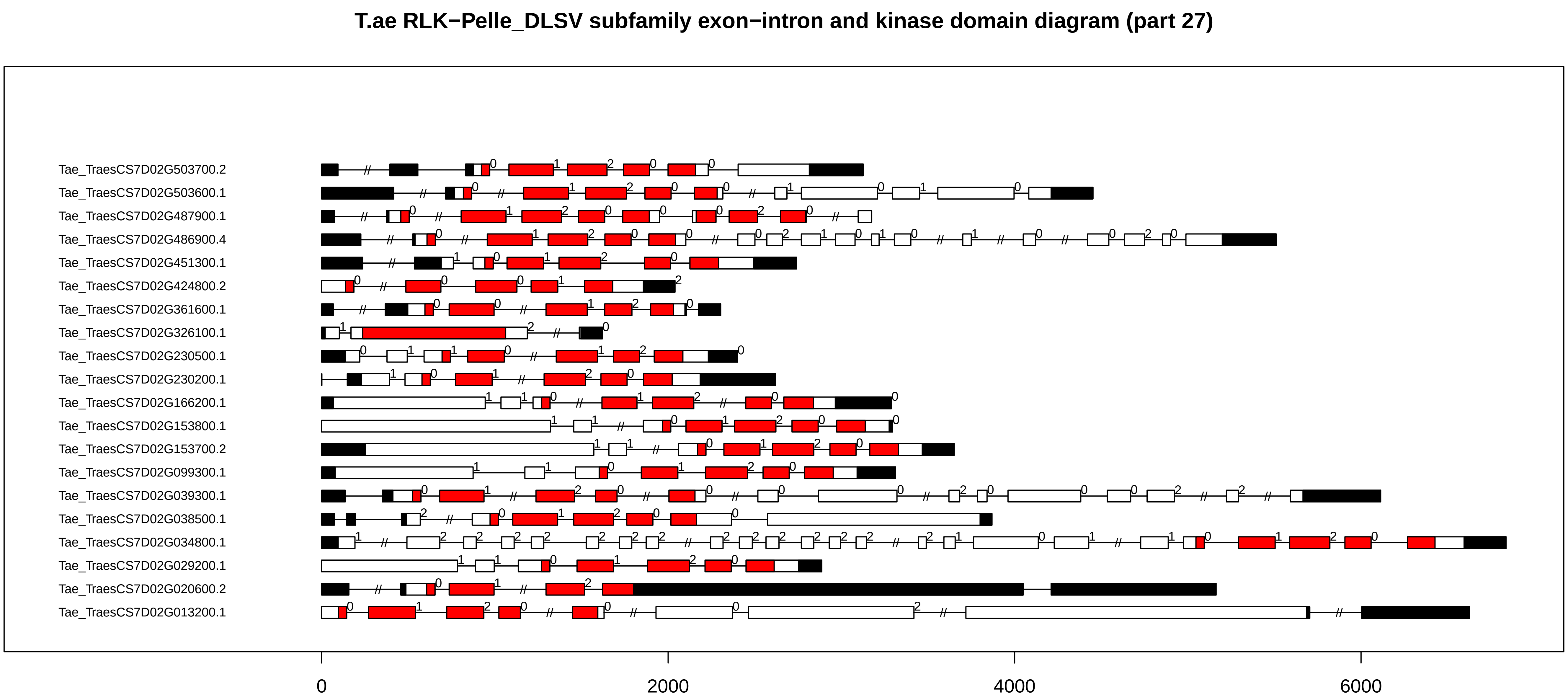
T.ae RLK−Pelle_DLSV subfamily exon−intron and kinase domain diagram (part 27)
Tae_TraesCS7D02G503700.2
Tae_TraesCS7D02G503600.1
Tae_TraesCS7D02G487900.1
Tae_TraesCS7D02G486900.4
Tae_TraesCS7D02G451300.1
Tae_TraesCS7D02G424800.2
Tae_TraesCS7D02G361600.1
Tae_TraesCS7D02G326100.1
Tae_TraesCS7D02G230500.1
Tae_TraesCS7D02G230200.1
Tae_TraesCS7D02G166200.1
Tae_TraesCS7D02G153800.1
Tae_TraesCS7D02G153700.2
Tae_TraesCS7D02G099300.1
Tae_TraesCS7D02G039300.1
Tae_TraesCS7D02G038500.1
Tae_TraesCS7D02G034800.1
Tae_TraesCS7D02G029200.1
Tae_TraesCS7D02G020600.2
Tae_TraesCS7D02G013200.1
0
2000
4000
6000

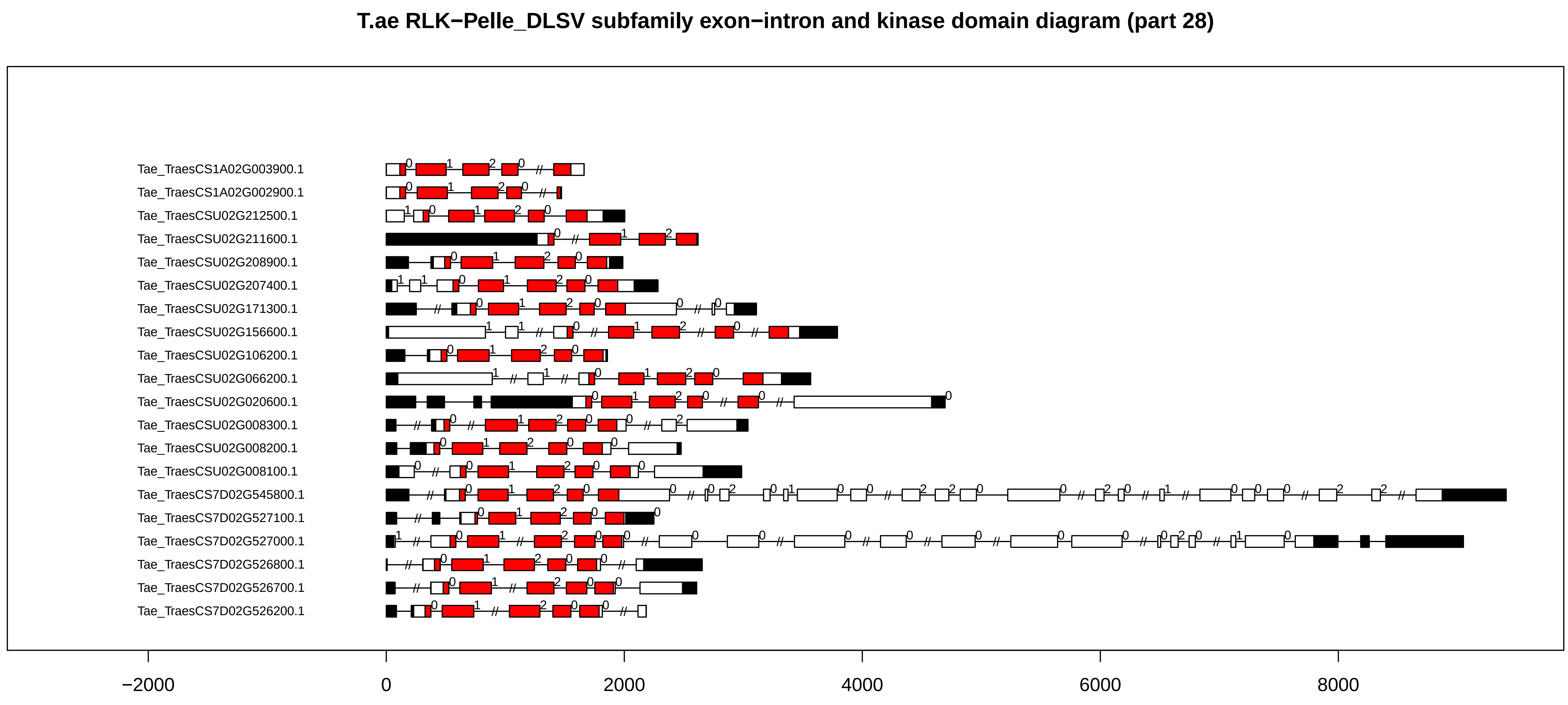
T.ae RLK−Pelle_DLSV subfamily exon−intron and kinase domain diagram (part 28)
Tae_TraesCS1A02G003900.1
Tae_TraesCS1A02G002900.1
Tae_TraesCSU02G212500.1
Tae_TraesCSU02G211600.1
Tae_TraesCSU02G208900.1
Tae_TraesCSU02G207400.1
Tae_TraesCSU02G171300.1
Tae_TraesCSU02G156600.1
Tae_TraesCSU02G106200.1
Tae_TraesCSU02G066200.1
Tae_TraesCSU02G020600.1
Tae_TraesCSU02G008300.1
Tae_TraesCSU02G008200.1
Tae_TraesCSU02G008100.1
Tae_TraesCS7D02G545800.1
Tae_TraesCS7D02G527100.1
Tae_TraesCS7D02G527000.1
Tae_TraesCS7D02G526800.1
Tae_TraesCS7D02G526700.1
Tae_TraesCS7D02G526200.1
−2000
0
2000
4000
6000
8000

T.ae RLK−Pelle_DLSV subfamily exon−intron and kinase domain diagram (part 29)

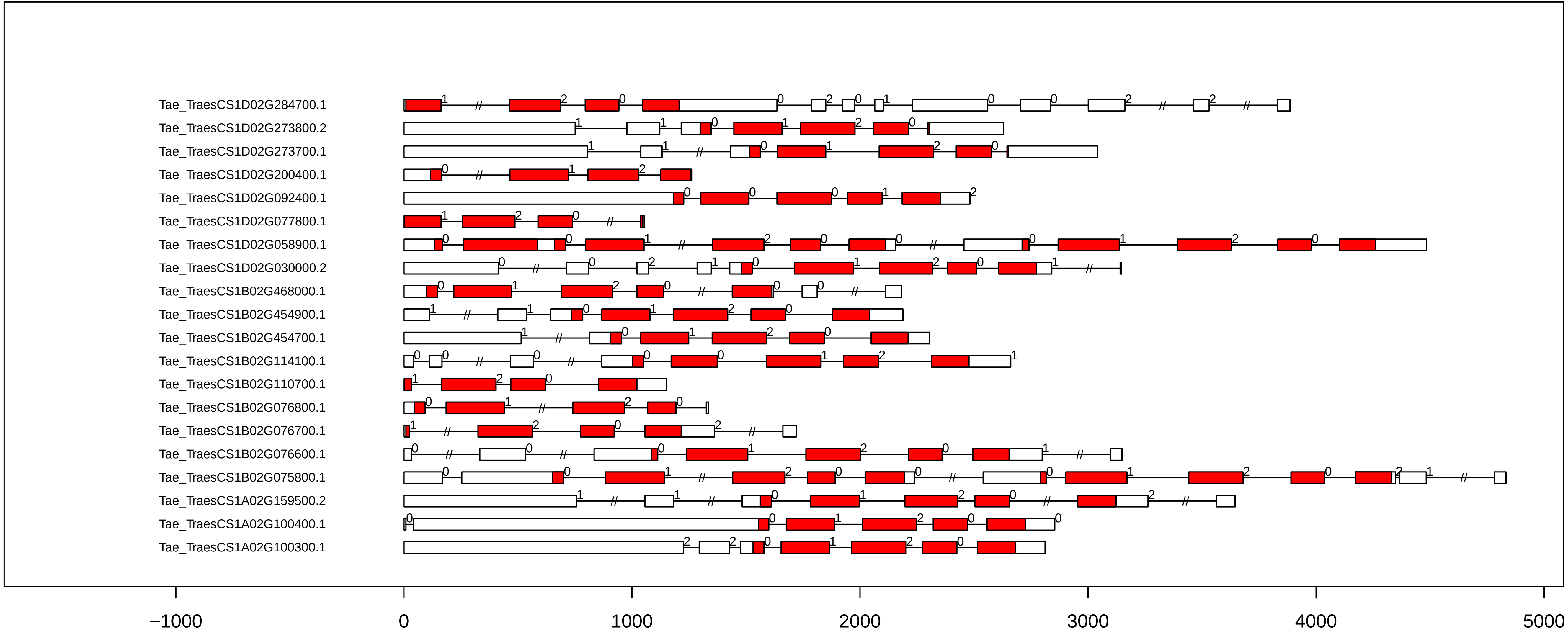

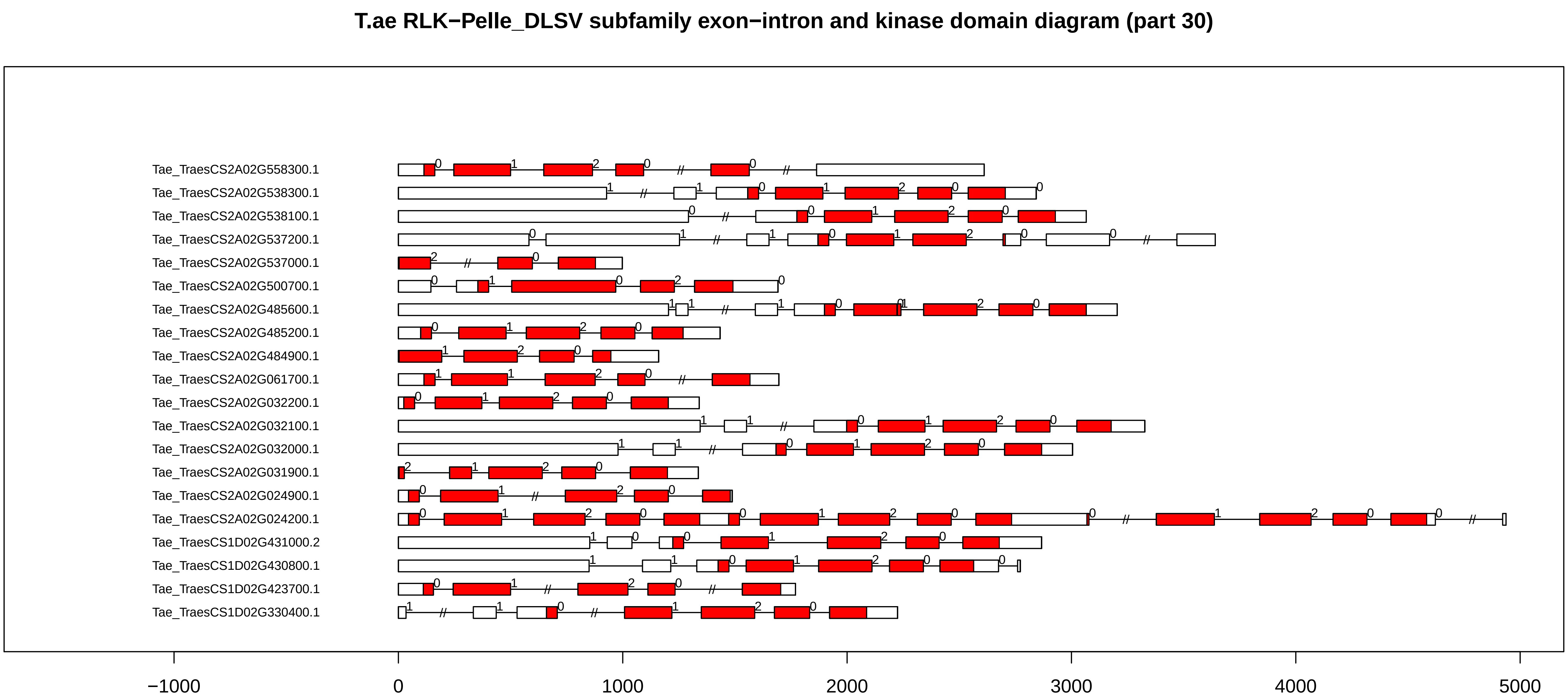

T.ae RLK−Pelle_DLSV subfamily exon−intron and kinase domain diagram (part 30)
Tae_TraesCS2A02G558300.1
Tae_TraesCS2A02G538300.1
Tae_TraesCS2A02G538100.1
Tae_TraesCS2A02G537200.1
Tae_TraesCS2A02G537000.1
Tae_TraesCS2A02G500700.1
Tae_TraesCS2A02G485600.1
Tae_TraesCS2A02G485200.1
Tae_TraesCS2A02G484900.1
Tae_TraesCS2A02G061700.1
Tae_TraesCS2A02G032200.1
Tae_TraesCS2A02G032100.1
Tae_TraesCS2A02G032000.1
Tae_TraesCS2A02G031900.1
Tae_TraesCS2A02G024900.1
Tae_TraesCS2A02G024200.1
Tae_TraesCS1D02G431000.2
Tae_TraesCS1D02G430800.1
Tae_TraesCS1D02G423700.1
Tae_TraesCS1D02G330400.1
−1000
0
1000
2000
3000
4000
5000

T.ae RLK−Pelle_DLSV subfamily exon−intron and kinase domain diagram (part 31)

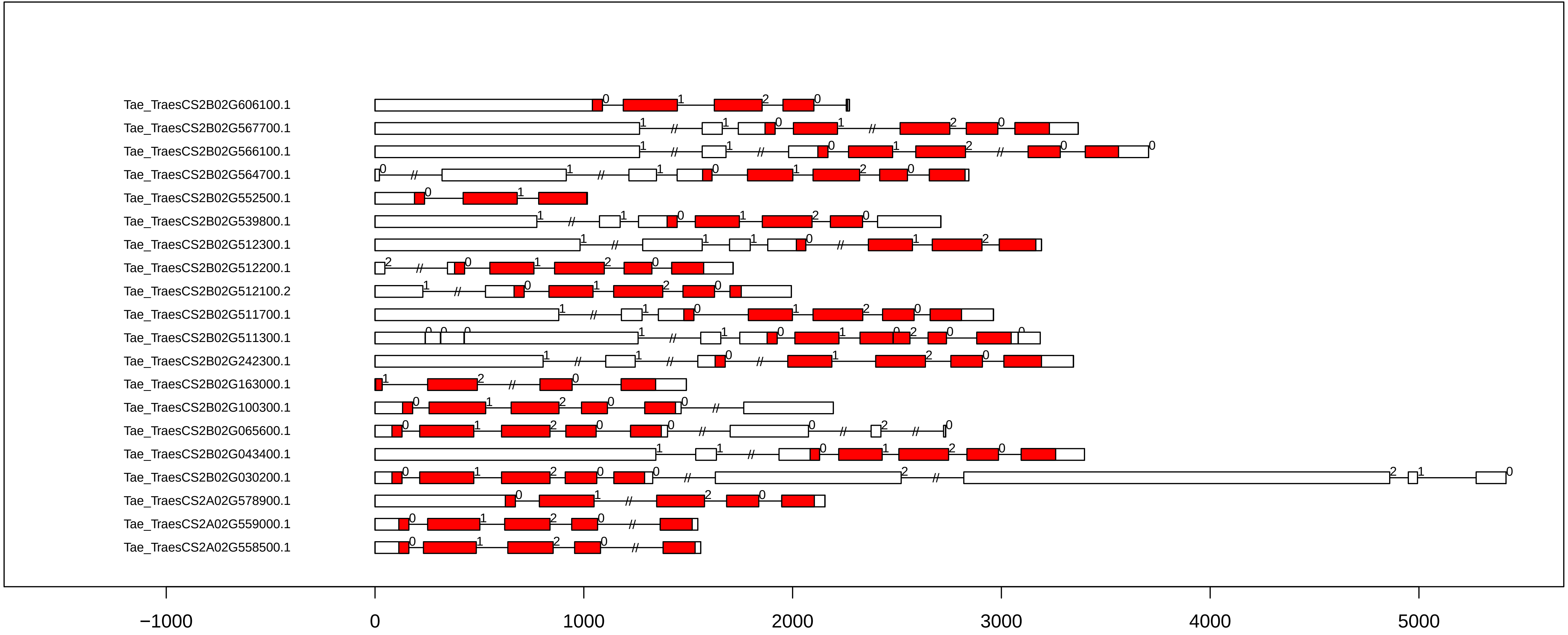

T.ae RLK−Pelle_DLSV subfamily exon−intron and kinase domain diagram (part 32)

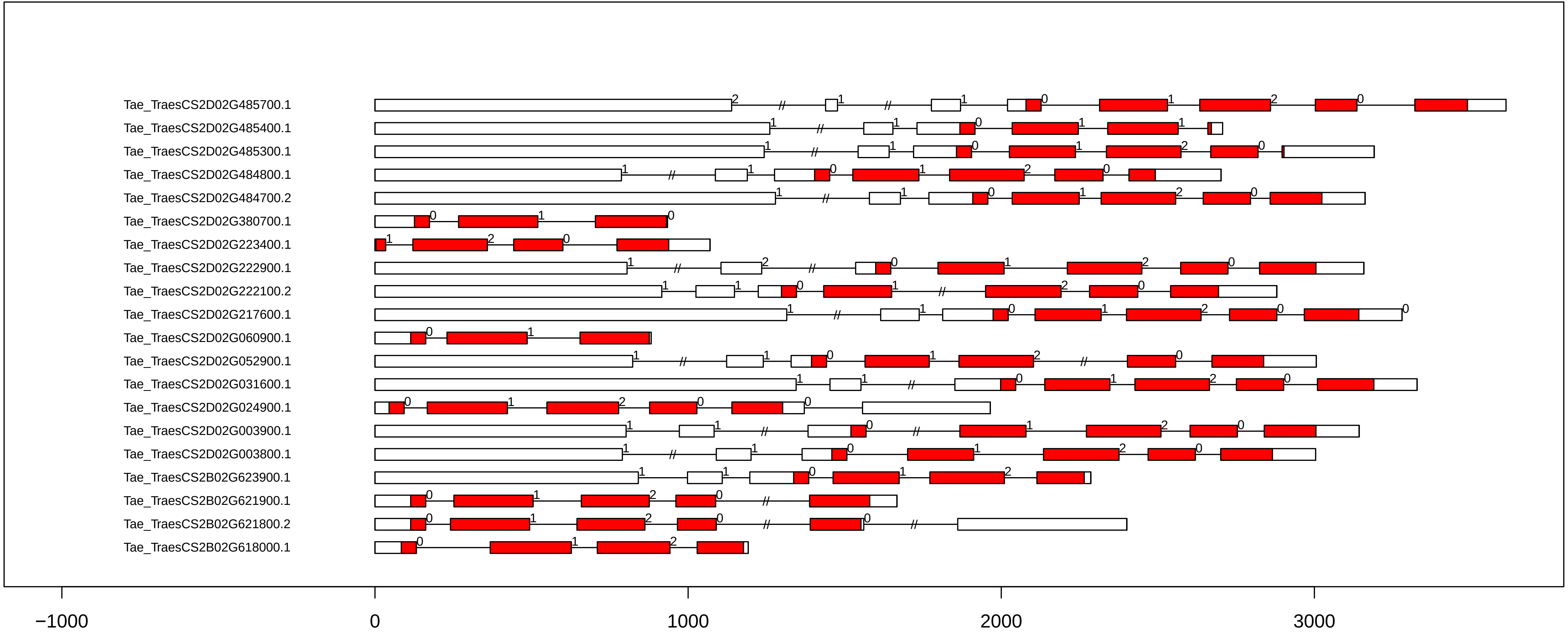

T.ae RLK−Pelle_DLSV subfamily exon−intron and kinase domain diagram (part 34)

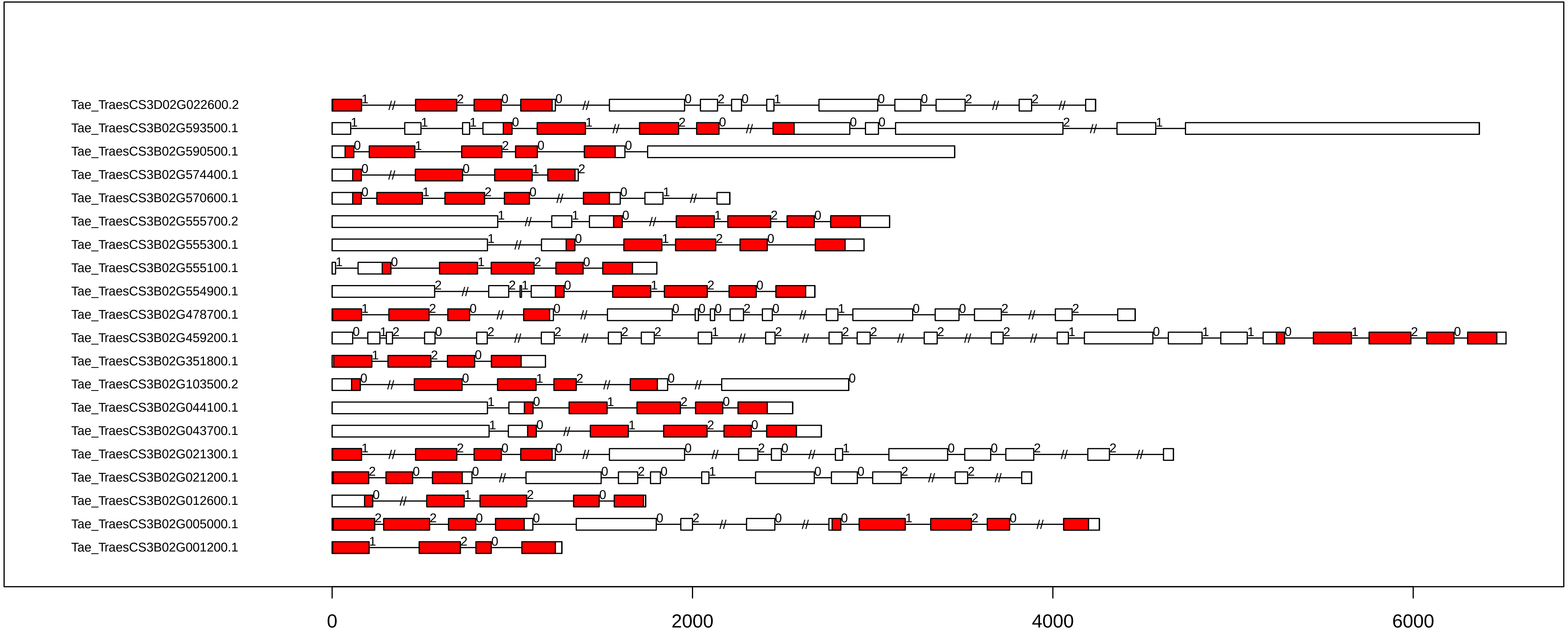

T.ae RLK−Pelle_DLSV subfamily exon−intron and kinase domain diagram (part 35)

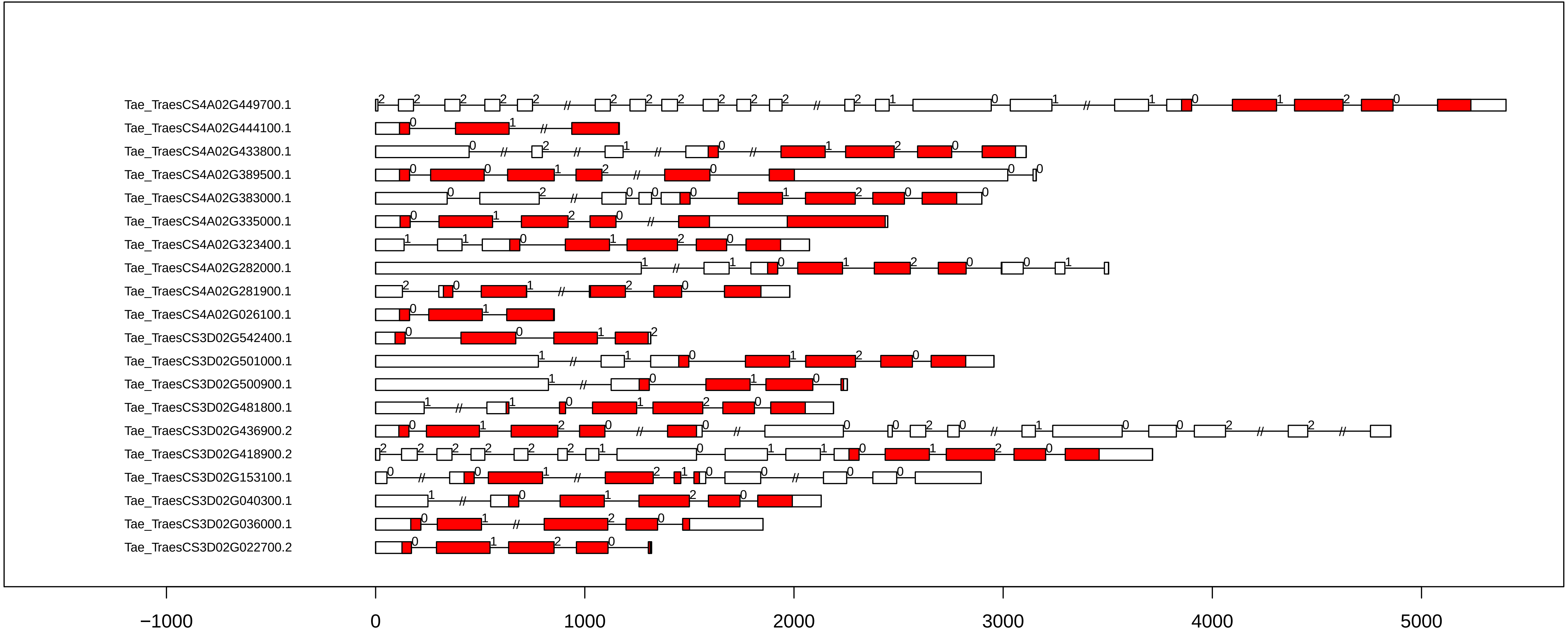

T.ae RLK−Pelle_DLSV subfamily exon−intron and kinase domain diagram (part 36)

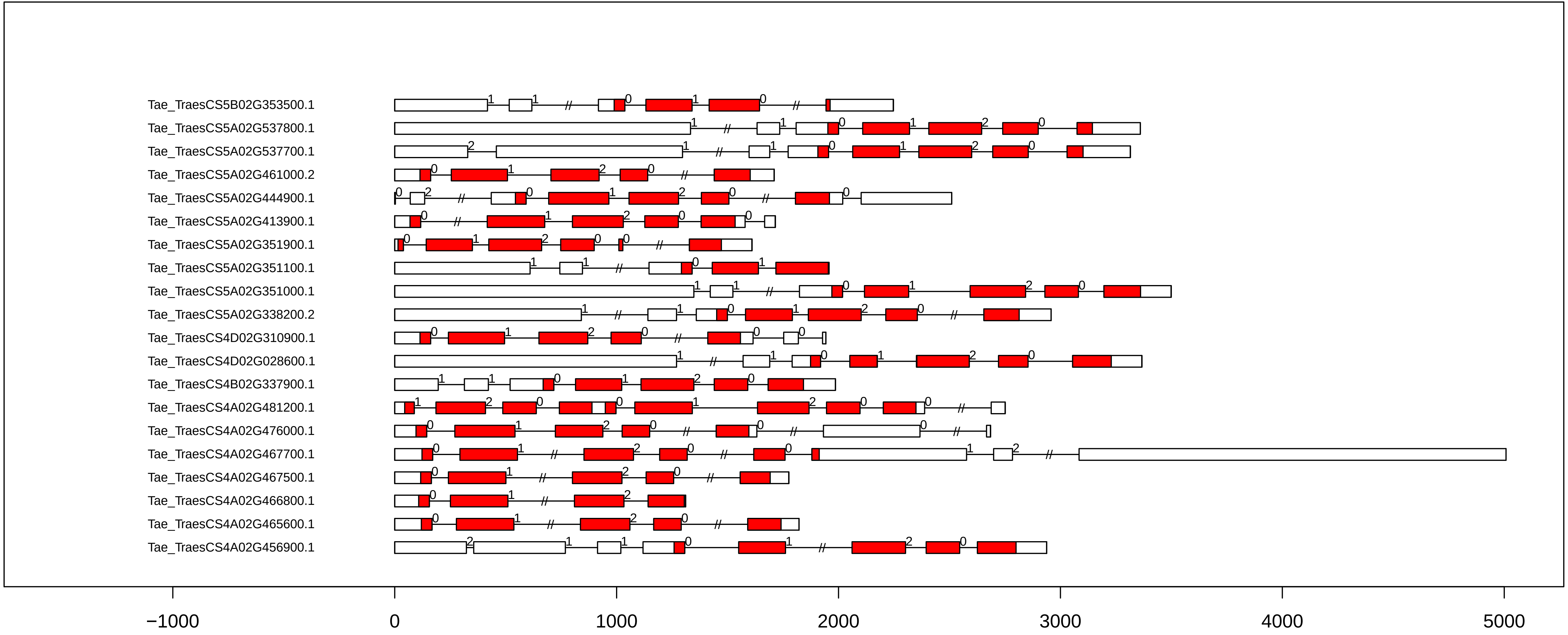

T.ae RLK−Pelle_DLSV subfamily exon−intron and kinase domain diagram (part 37)

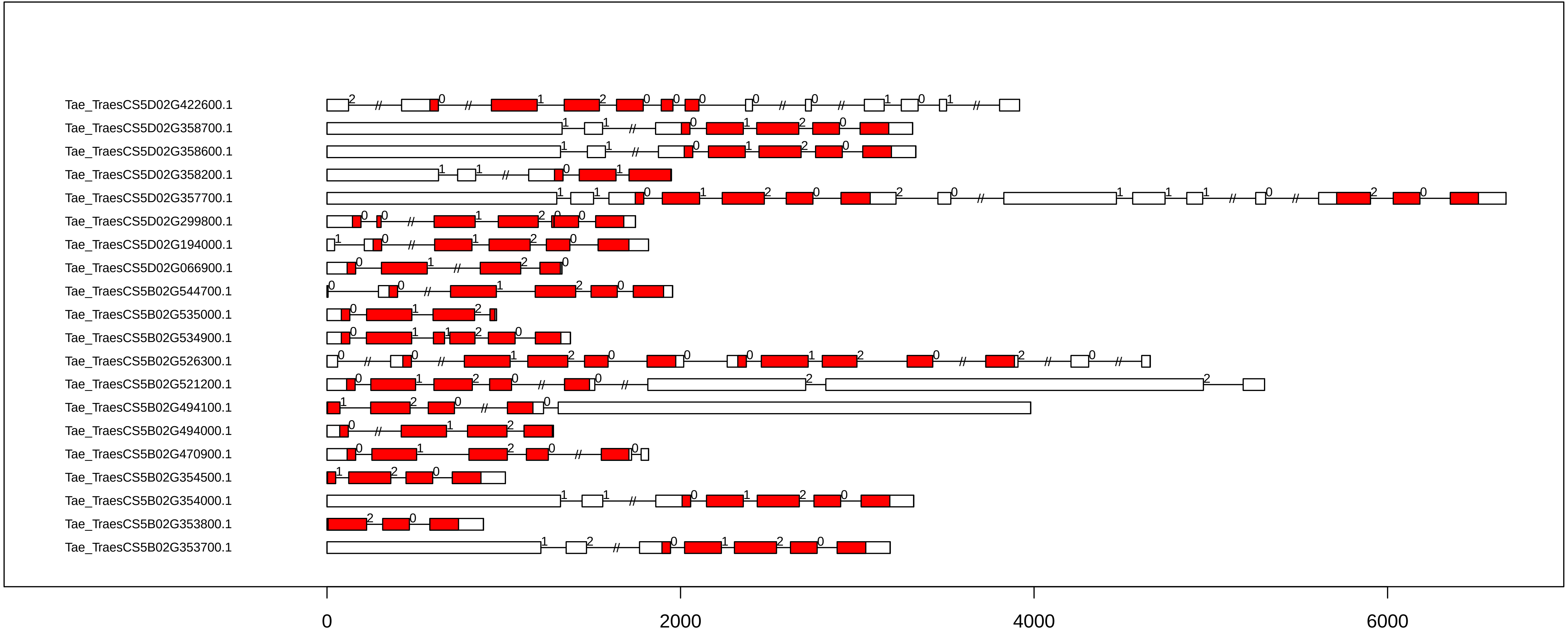

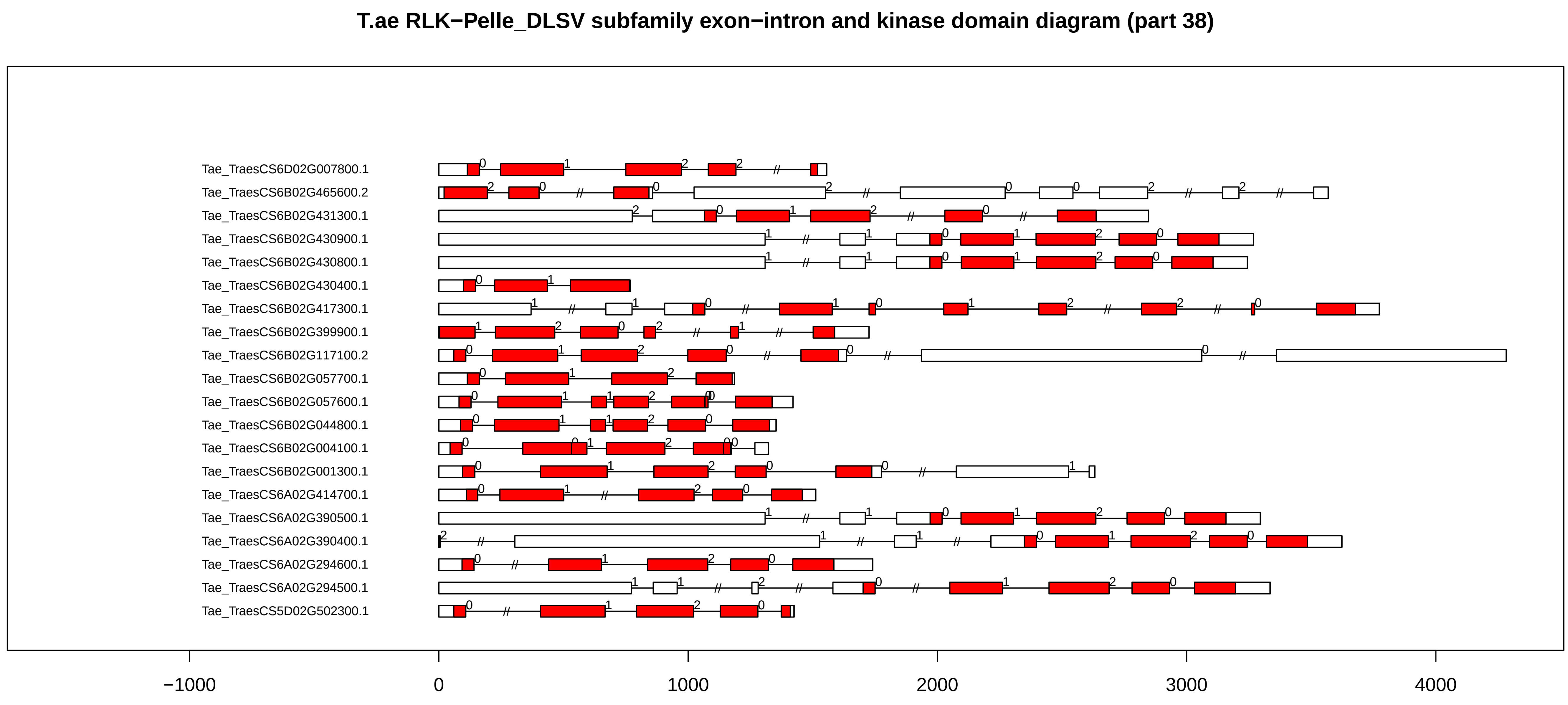

T.ae RLK−Pelle_DLSV subfamily exon−intron and kinase domain diagram (part 38)
Tae_TraesCS6D02G007800.1
Tae_TraesCS6B02G465600.2
Tae_TraesCS6B02G431300.1
Tae_TraesCS6B02G430900.1
Tae_TraesCS6B02G430800.1
Tae_TraesCS6B02G430400.1
Tae_TraesCS6B02G417300.1
Tae_TraesCS6B02G399900.1
Tae_TraesCS6B02G117100.2
Tae_TraesCS6B02G057700.1
Tae_TraesCS6B02G057600.1
Tae_TraesCS6B02G044800.1
Tae_TraesCS6B02G004100.1
Tae_TraesCS6B02G001300.1
Tae_TraesCS6A02G414700.1
Tae_TraesCS6A02G390500.1
Tae_TraesCS6A02G390400.1
Tae_TraesCS6A02G294600.1
Tae_TraesCS6A02G294500.1
Tae_TraesCS5D02G502300.1
−1000
0
1000
2000
3000
4000

T.ae RLK−Pelle_DLSV subfamily exon−intron and kinase domain diagram (part 39)

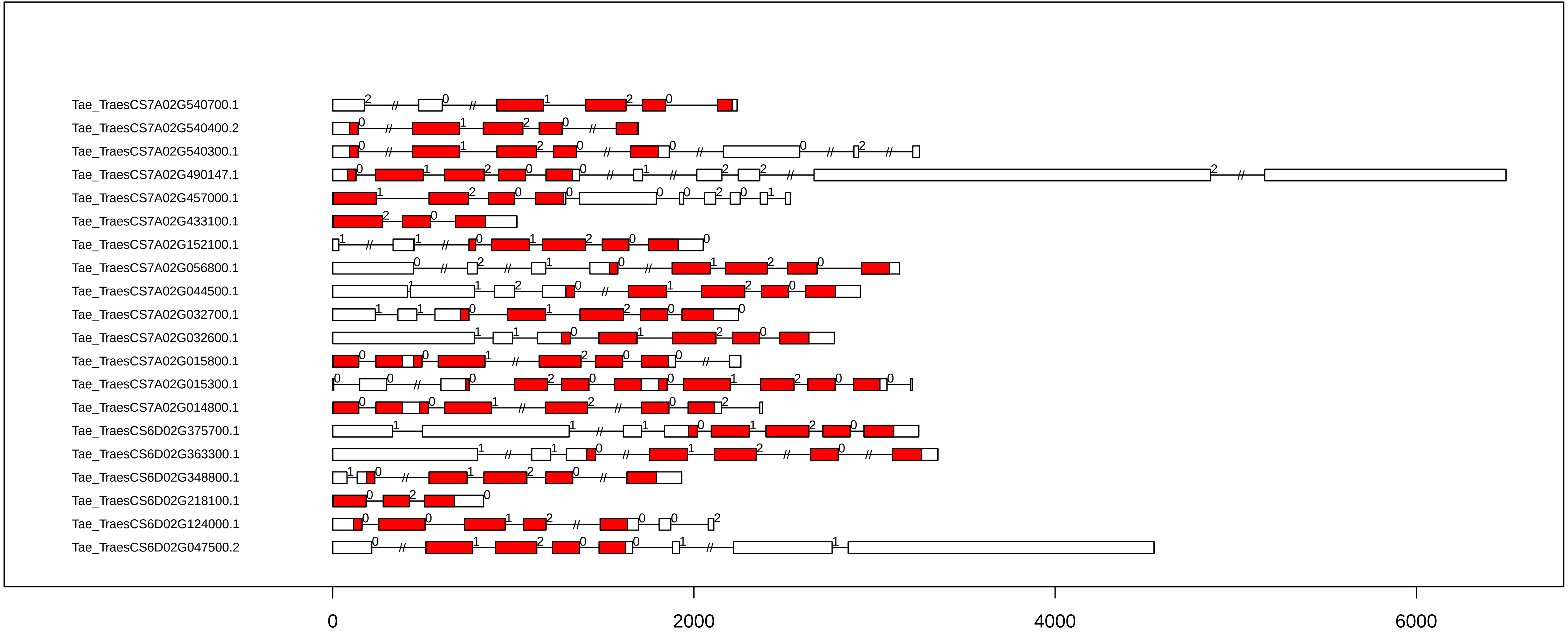

T.ae RLK−Pelle_DLSV subfamily exon−intron and kinase domain diagram (part 40)

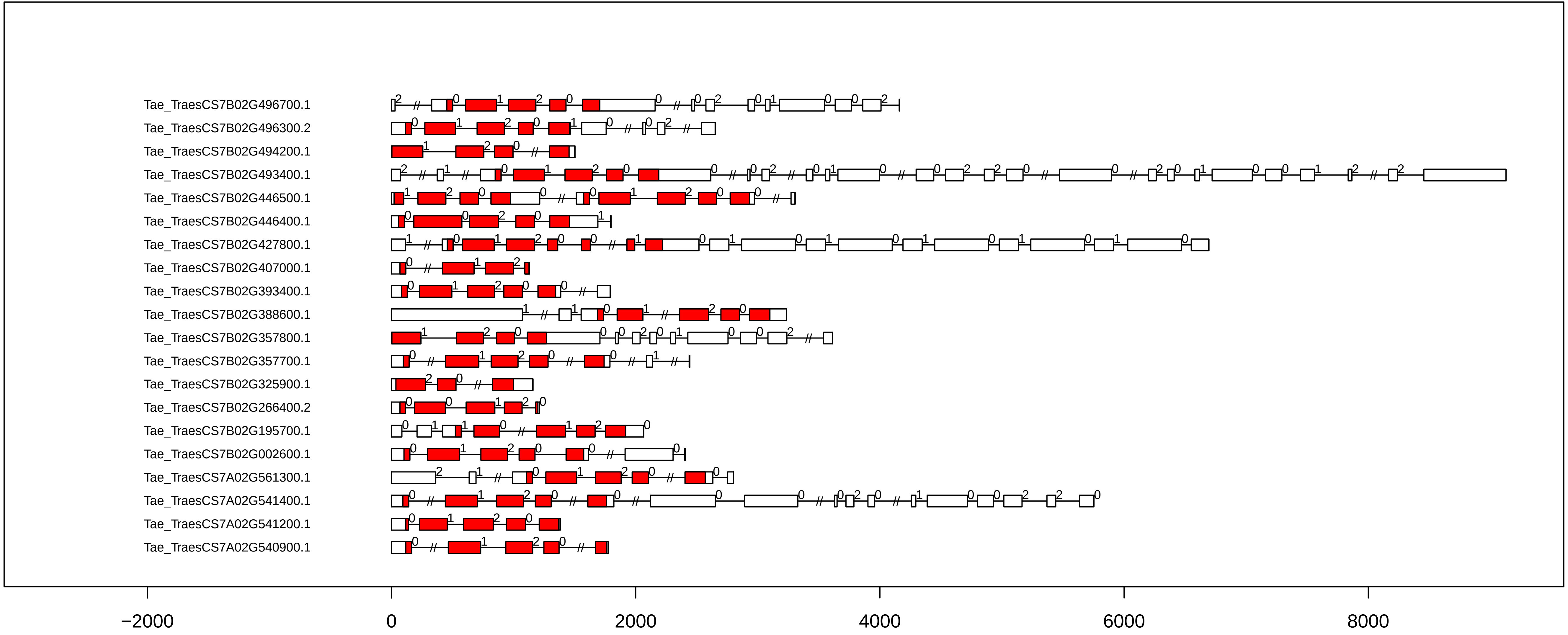

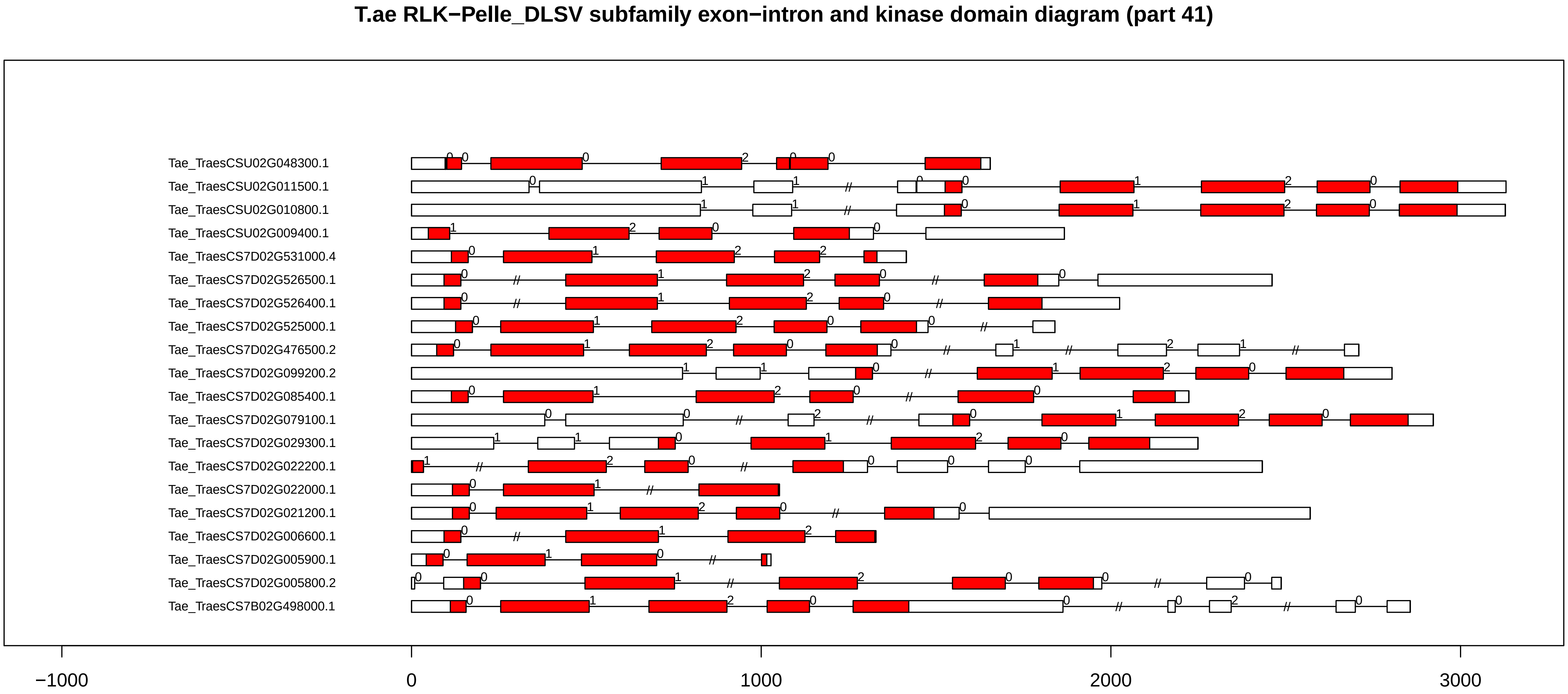
T.ae RLK−Pelle_DLSV subfamily exon−intron and kinase domain diagram (part 41)
Tae_TraesCSU02G048300.1
Tae_TraesCSU02G011500.1
Tae_TraesCSU02G010800.1
Tae_TraesCSU02G009400.1
Tae_TraesCS7D02G531000.4
Tae_TraesCS7D02G526500.1
Tae_TraesCS7D02G526400.1
Tae_TraesCS7D02G525000.1
Tae_TraesCS7D02G476500.2
Tae_TraesCS7D02G099200.2
Tae_TraesCS7D02G085400.1
Tae_TraesCS7D02G079100.1
Tae_TraesCS7D02G029300.1
Tae_TraesCS7D02G022200.1
Tae_TraesCS7D02G022000.1
Tae_TraesCS7D02G021200.1
Tae_TraesCS7D02G006600.1
Tae_TraesCS7D02G005900.1
Tae_TraesCS7D02G005800.2
Tae_TraesCS7B02G498000.1
−1000
0
1000
2000
3000

T.ae RLK−Pelle_DLSV subfamily exon−intron and kinase domain diagram (part 42)

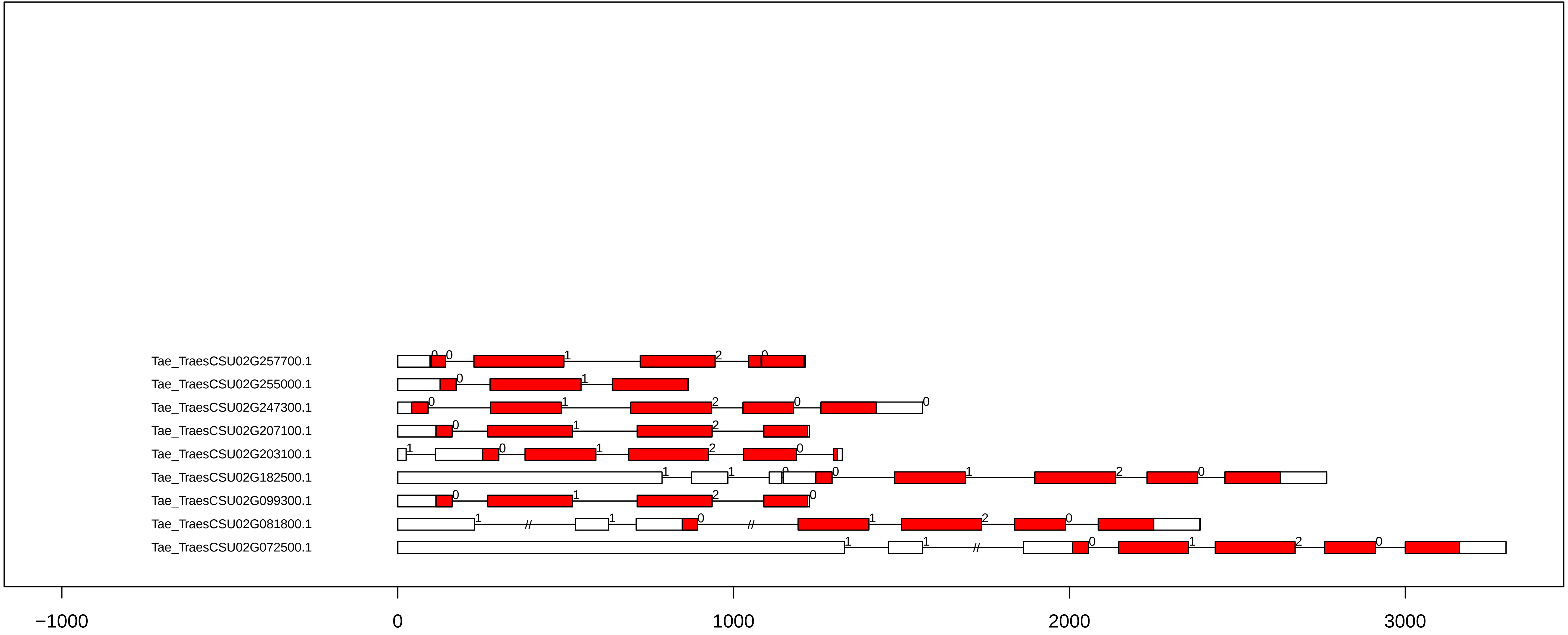

T.ae RLK−Pelle_Extensin subfamily exon−intron and kinase domain diagram (part 1)

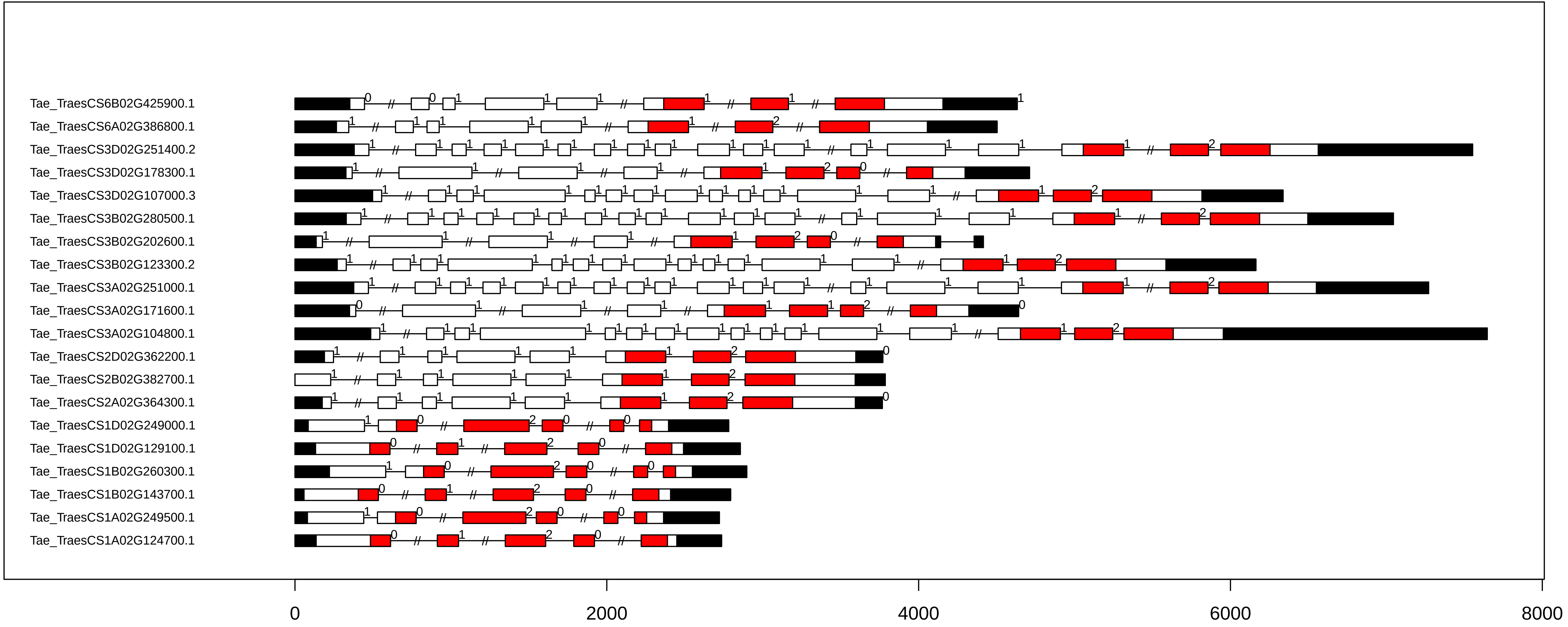

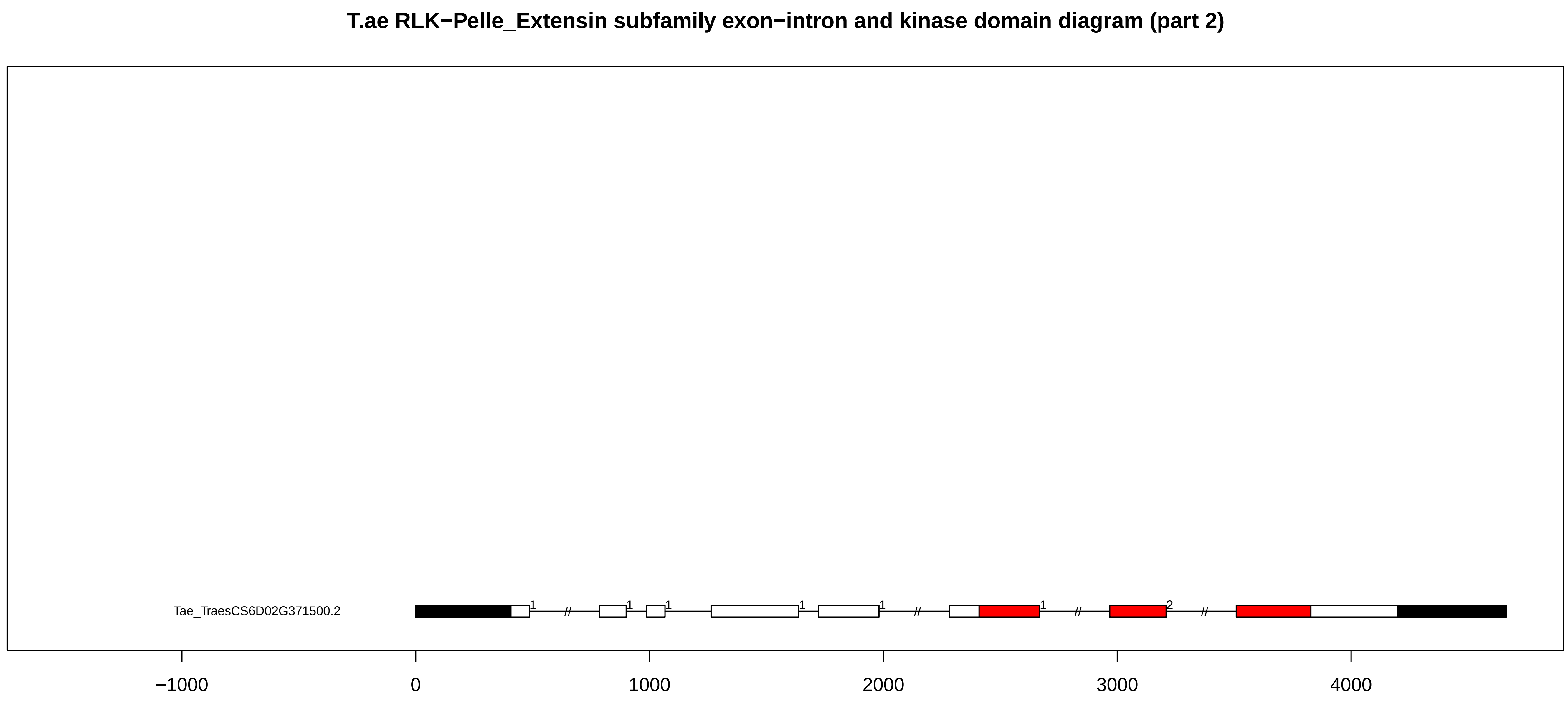

T.ae RLK−Pelle_Extensin subfamily exon−intron and kinase domain diagram (part 2)
Tae_TraesCS6D02G371500.2
1
1
1
1
1
1
2
−1000
0
1000
2000
3000
4000

T.ae RLK−Pelle_L−LEC subfamily exon−intron and kinase domain diagram (part 1)

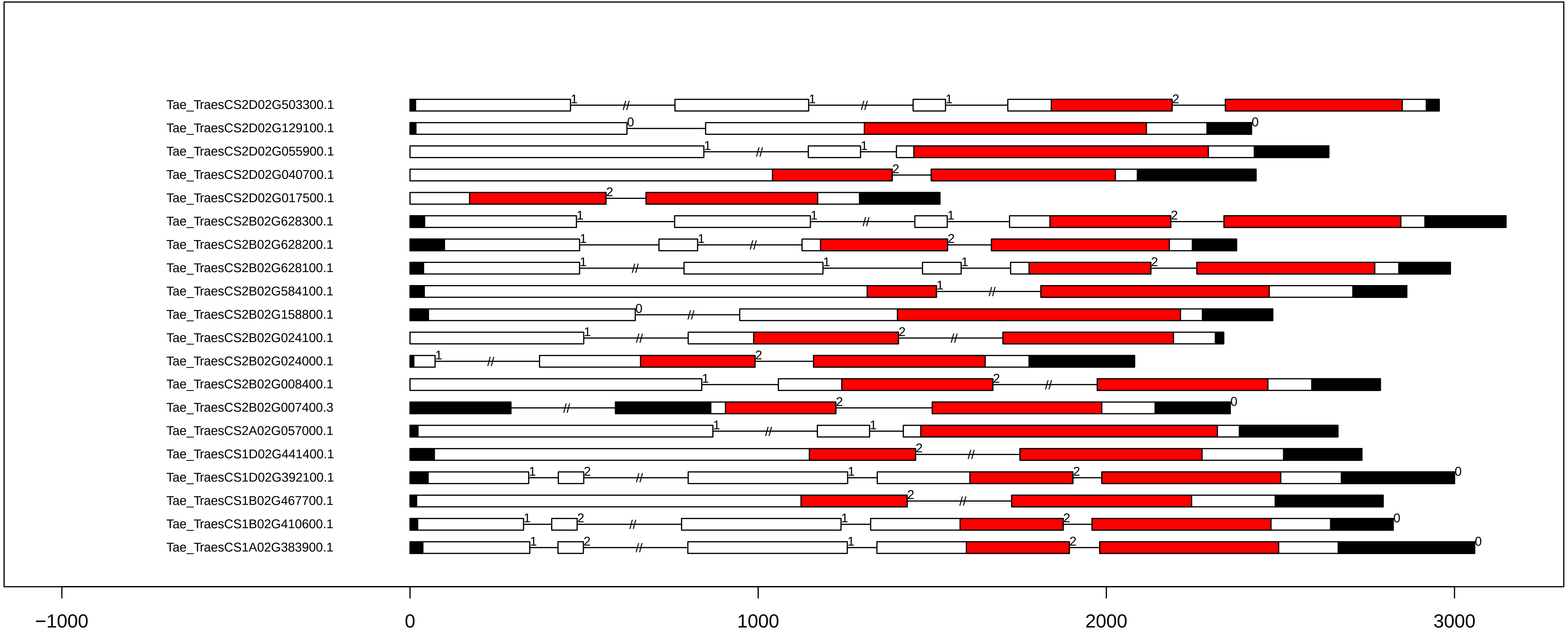

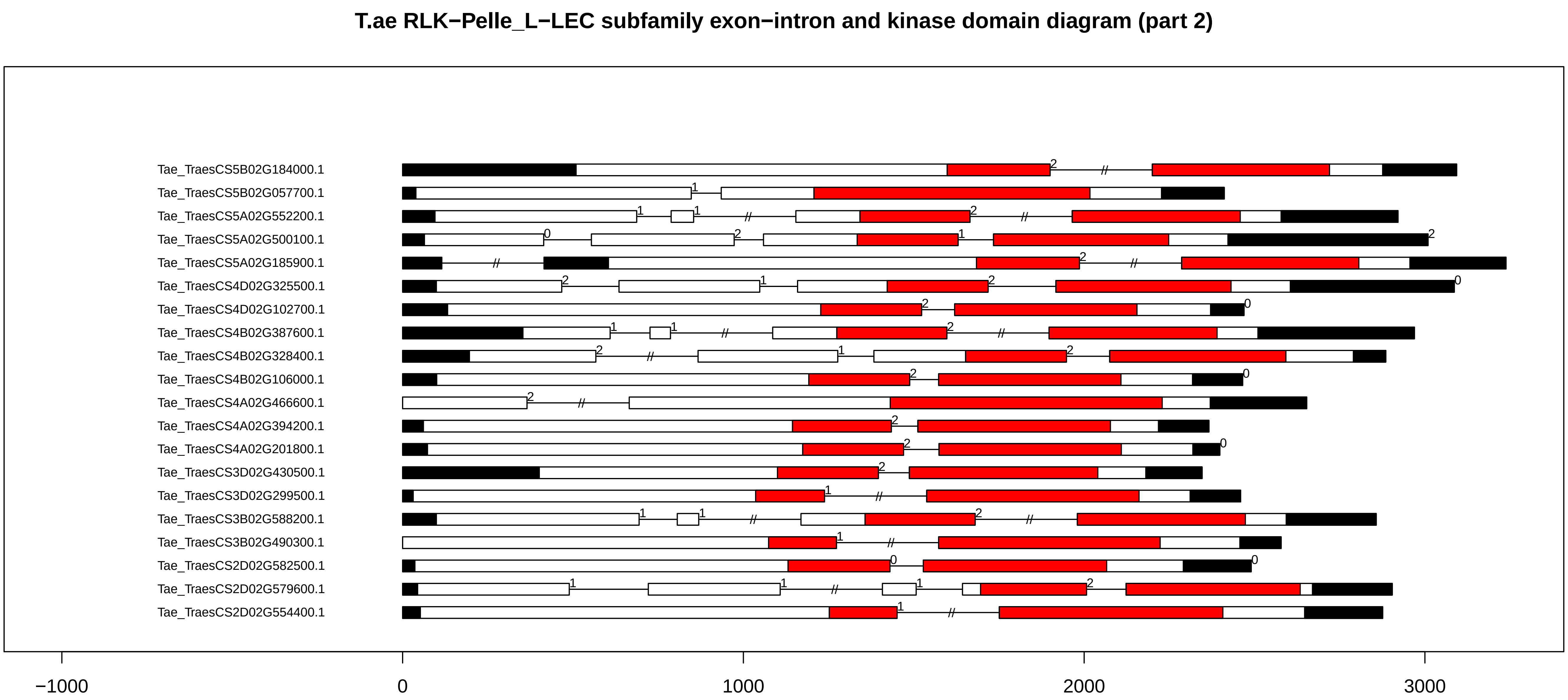
T.ae RLK−Pelle_L−LEC subfamily exon−intron and kinase domain diagram (part 2)
Tae_TraesCS5B02G184000.1
Tae_TraesCS5B02G057700.1
Tae_TraesCS5A02G552200.1
Tae_TraesCS5A02G500100.1
Tae_TraesCS5A02G185900.1
Tae_TraesCS4D02G325500.1
Tae_TraesCS4D02G102700.1
Tae_TraesCS4B02G387600.1
Tae_TraesCS4B02G328400.1
Tae_TraesCS4B02G106000.1
Tae_TraesCS4A02G466600.1
Tae_TraesCS4A02G394200.1
Tae_TraesCS4A02G201800.1
Tae_TraesCS3D02G430500.1
Tae_TraesCS3D02G299500.1
Tae_TraesCS3B02G588200.1
Tae_TraesCS3B02G490300.1
Tae_TraesCS2D02G582500.1
Tae_TraesCS2D02G579600.1
Tae_TraesCS2D02G554400.1
−1000
0
1000
2000
3000

T.ae RLK−Pelle_L−LEC subfamily exon−intron and kinase domain diagram (part 3)

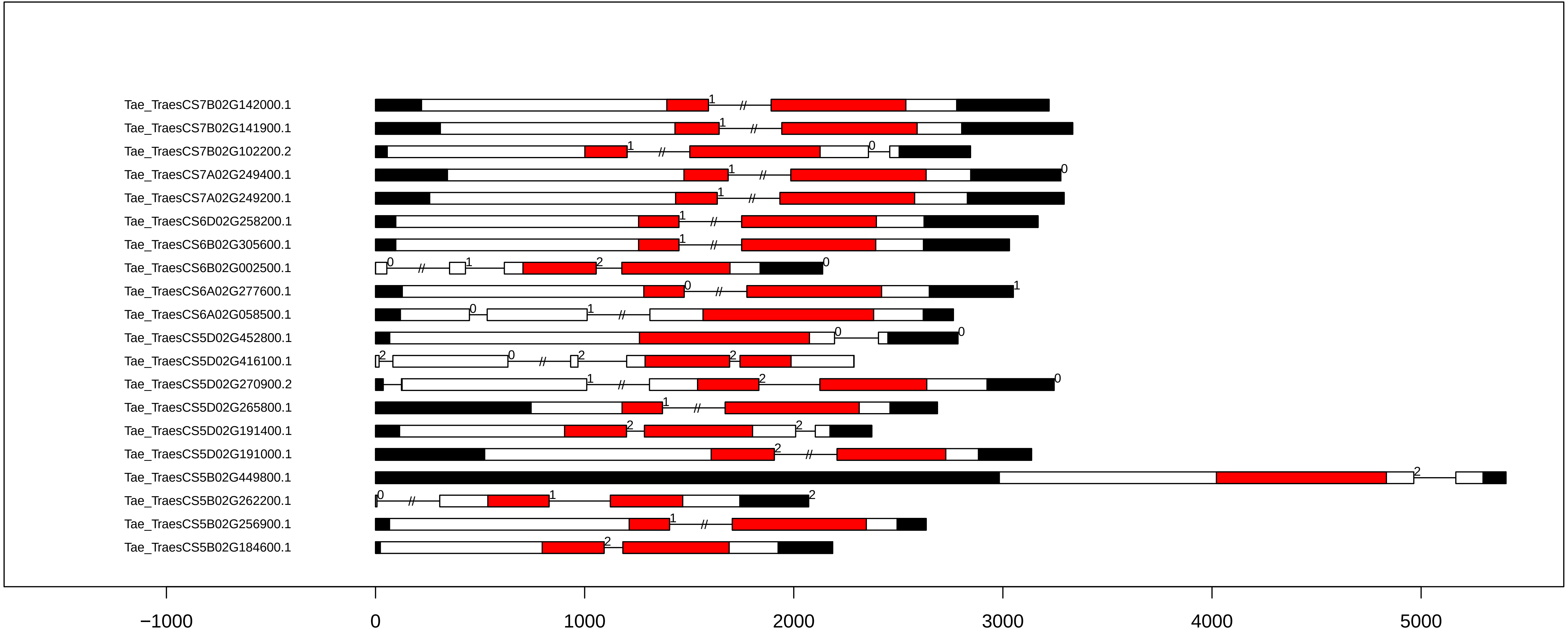

T.ae RLK−Pelle_L−LEC subfamily exon−intron and kinase domain diagram (part 4)

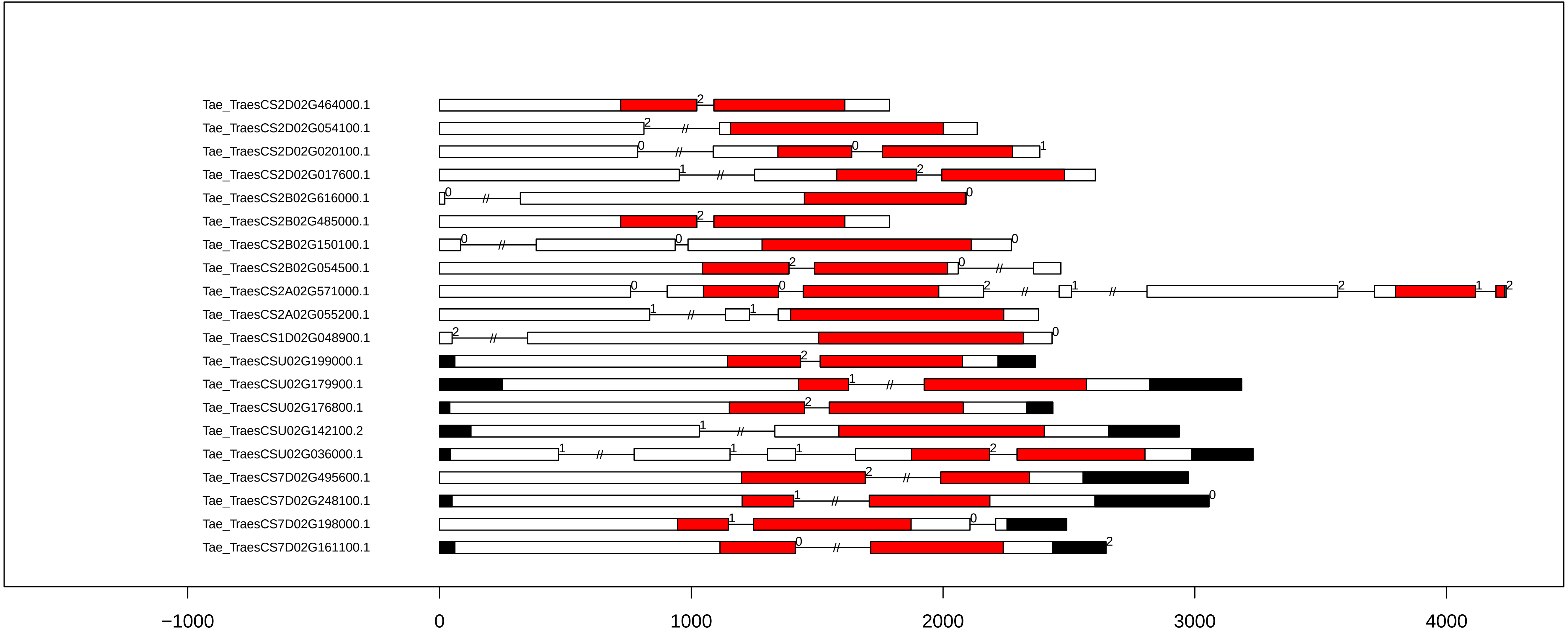

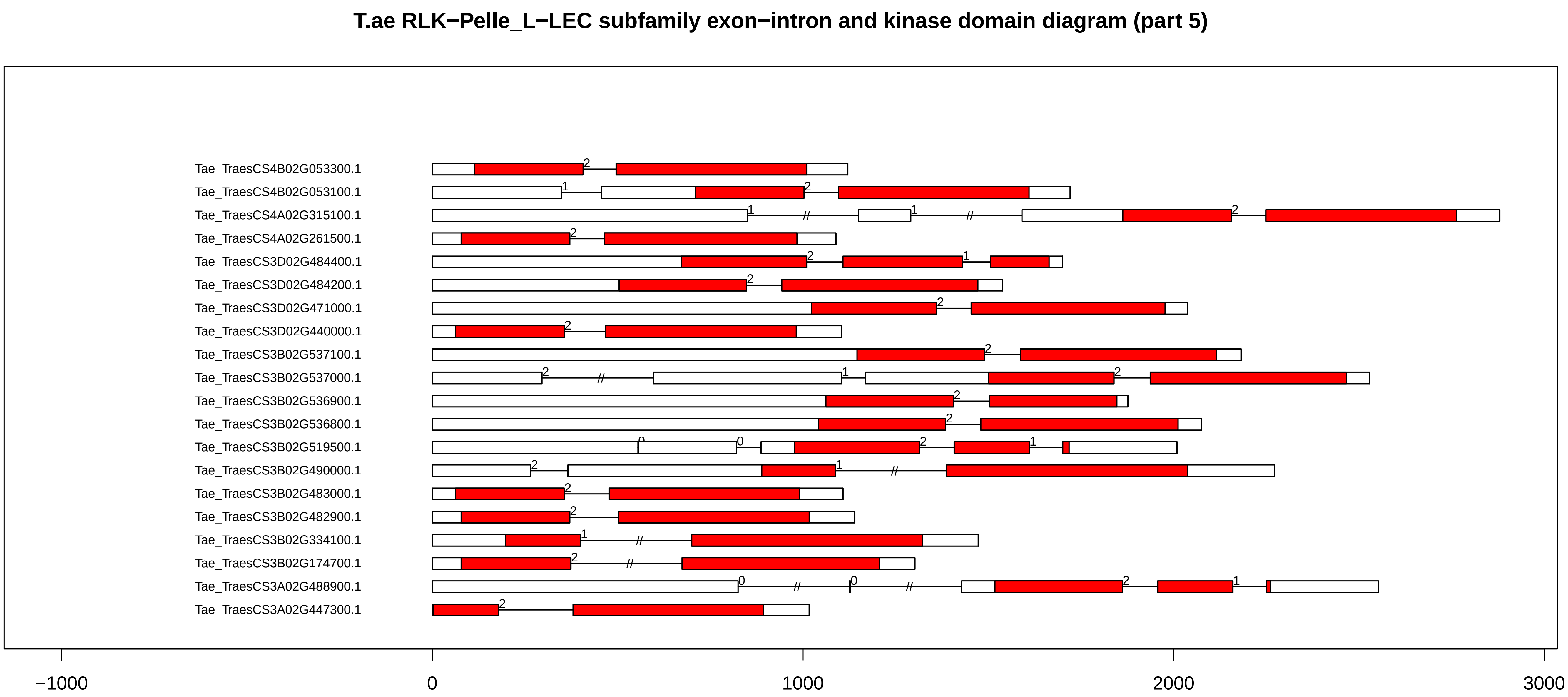
T.ae RLK−Pelle_L−LEC subfamily exon−intron and kinase domain diagram (part 5)
Tae_TraesCS4B02G053300.1
Tae_TraesCS4B02G053100.1
Tae_TraesCS4A02G315100.1
Tae_TraesCS4A02G261500.1
Tae_TraesCS3D02G484400.1
Tae_TraesCS3D02G484200.1
Tae_TraesCS3D02G471000.1
Tae_TraesCS3D02G440000.1
Tae_TraesCS3B02G537100.1
Tae_TraesCS3B02G537000.1
Tae_TraesCS3B02G536900.1
Tae_TraesCS3B02G536800.1
Tae_TraesCS3B02G519500.1
Tae_TraesCS3B02G490000.1
Tae_TraesCS3B02G483000.1
Tae_TraesCS3B02G482900.1
Tae_TraesCS3B02G334100.1
Tae_TraesCS3B02G174700.1
Tae_TraesCS3A02G488900.1
Tae_TraesCS3A02G447300.1
−1000
0
1000
2000
3000

T.ae RLK−Pelle_L−LEC subfamily exon−intron and kinase domain diagram (part 6)

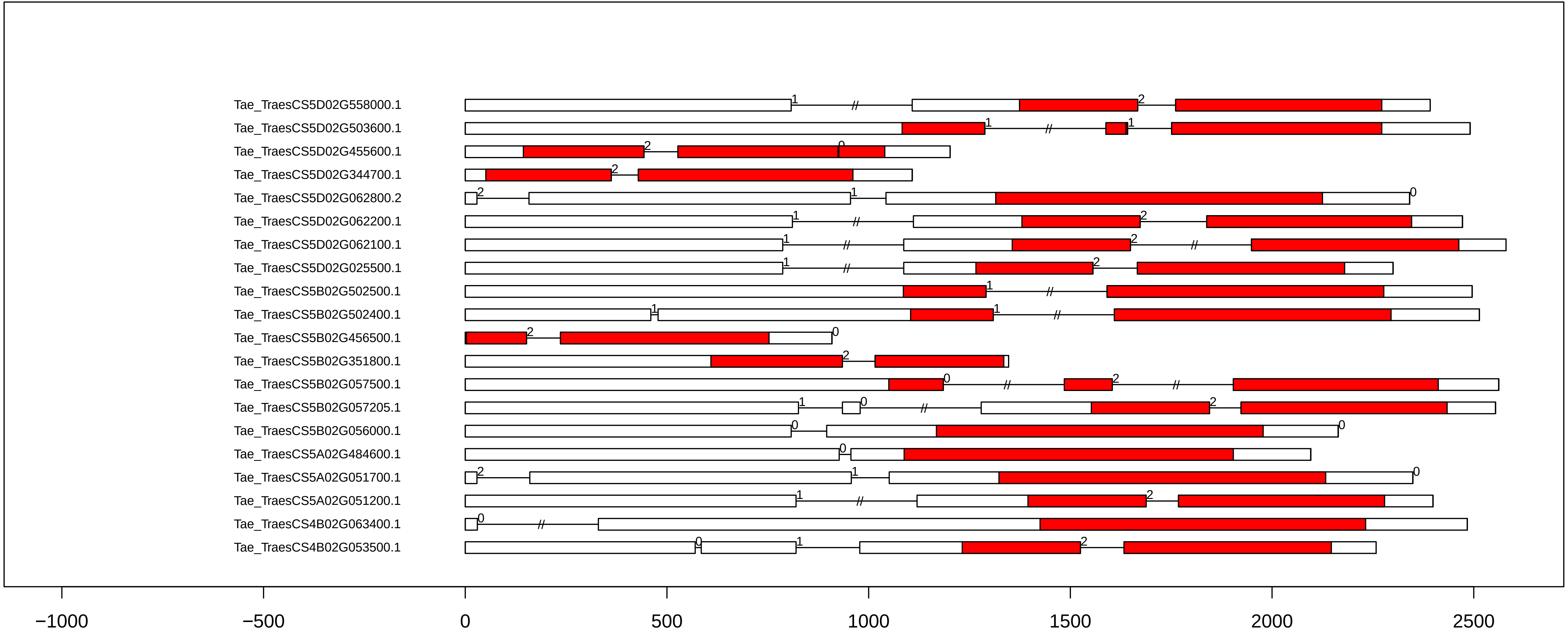

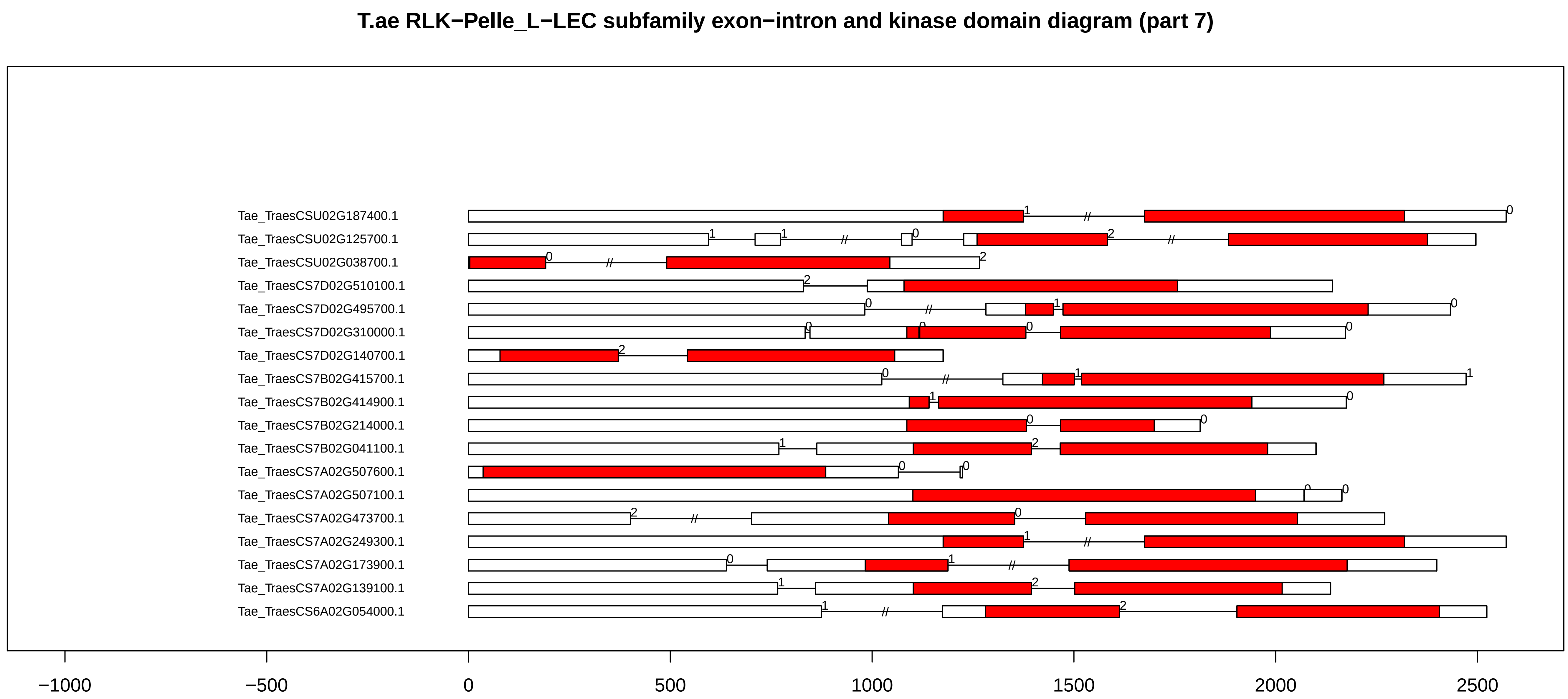
T.ae RLK−Pelle_L−LEC subfamily exon−intron and kinase domain diagram (part 7)
Tae_TraesCSU02G187400.1
Tae_TraesCSU02G125700.1
Tae_TraesCSU02G038700.1
Tae_TraesCS7D02G510100.1
Tae_TraesCS7D02G495700.1
Tae_TraesCS7D02G310000.1
Tae_TraesCS7D02G140700.1
Tae_TraesCS7B02G415700.1
Tae_TraesCS7B02G414900.1
Tae_TraesCS7B02G214000.1
Tae_TraesCS7B02G041100.1
Tae_TraesCS7A02G507600.1
Tae_TraesCS7A02G507100.1
Tae_TraesCS7A02G473700.1
Tae_TraesCS7A02G249300.1
Tae_TraesCS7A02G173900.1
Tae_TraesCS7A02G139100.1
Tae_TraesCS6A02G054000.1
−1000
−500
0
500
1000
1500
2000
2500

T.ae RLK−Pelle_LRK10L−2 subfamily exon−intron and kinase domain diagram (part 1)

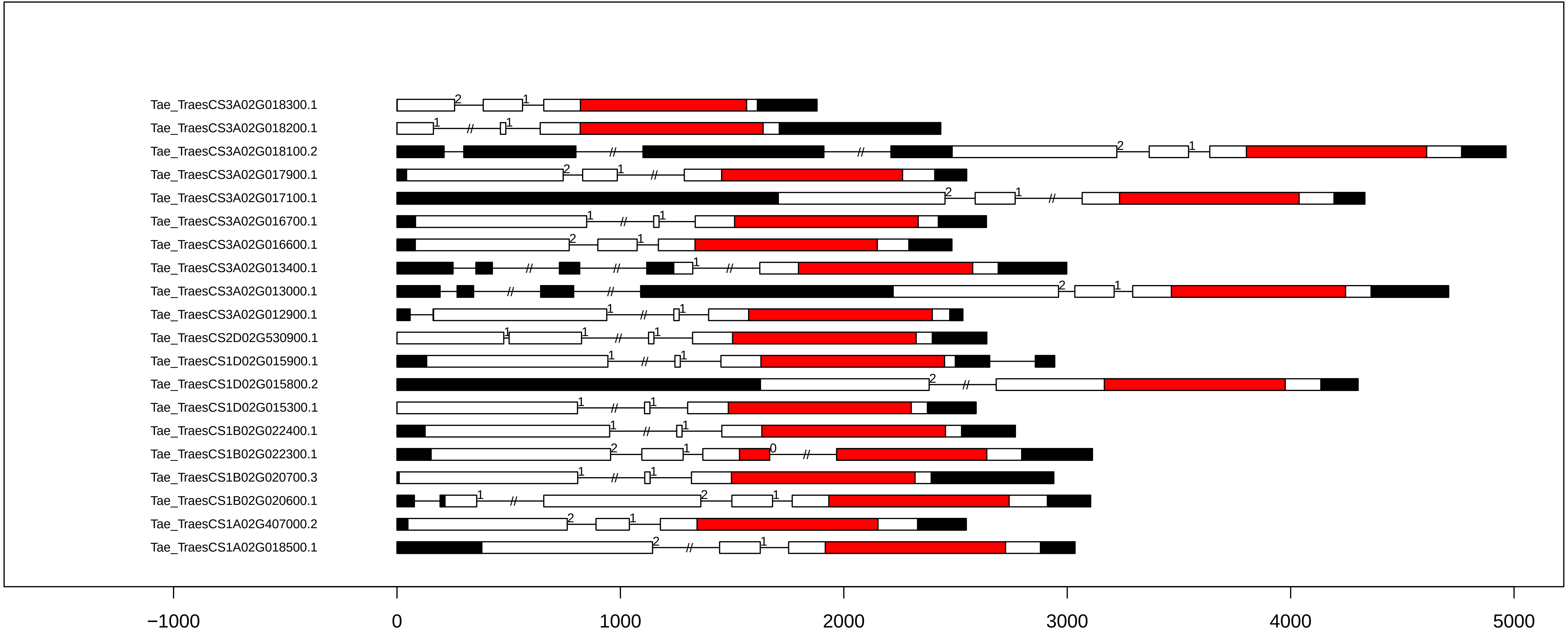

T.ae RLK−Pelle_LRK10L−2 subfamily exon−intron and kinase domain diagram (part 2)

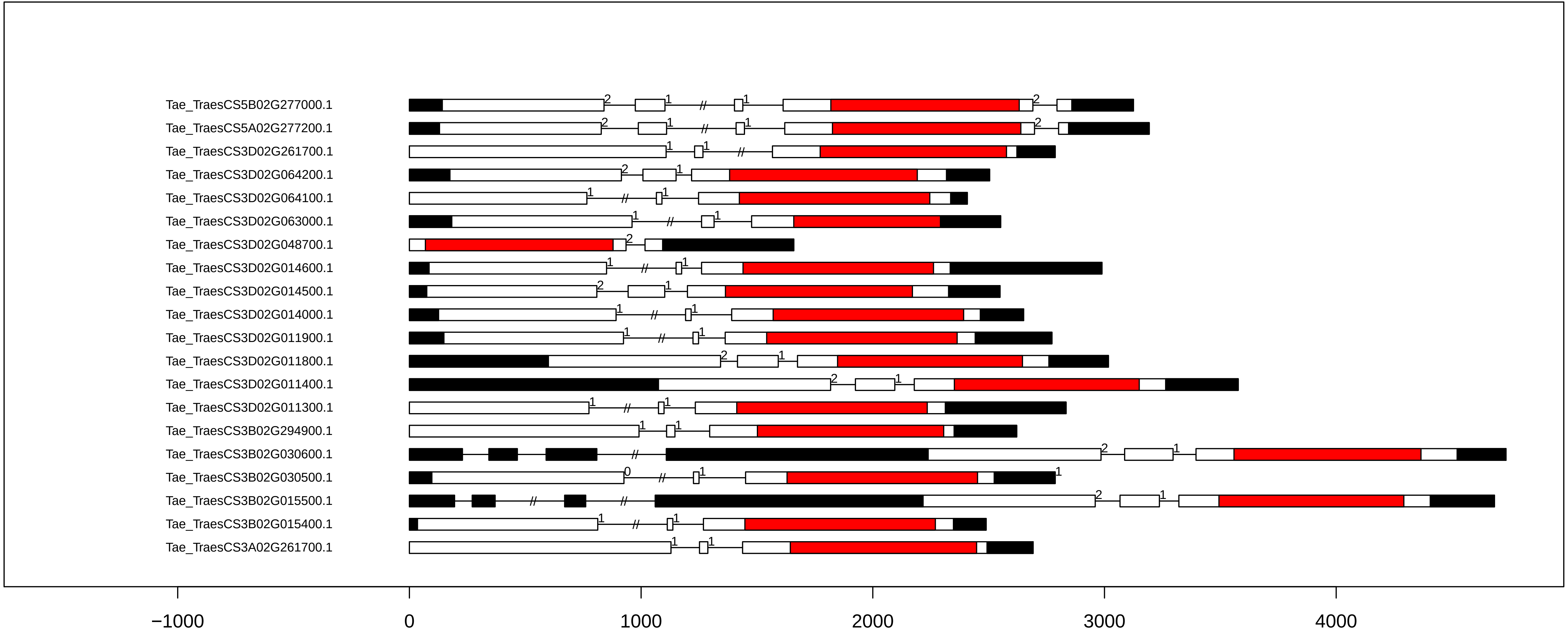

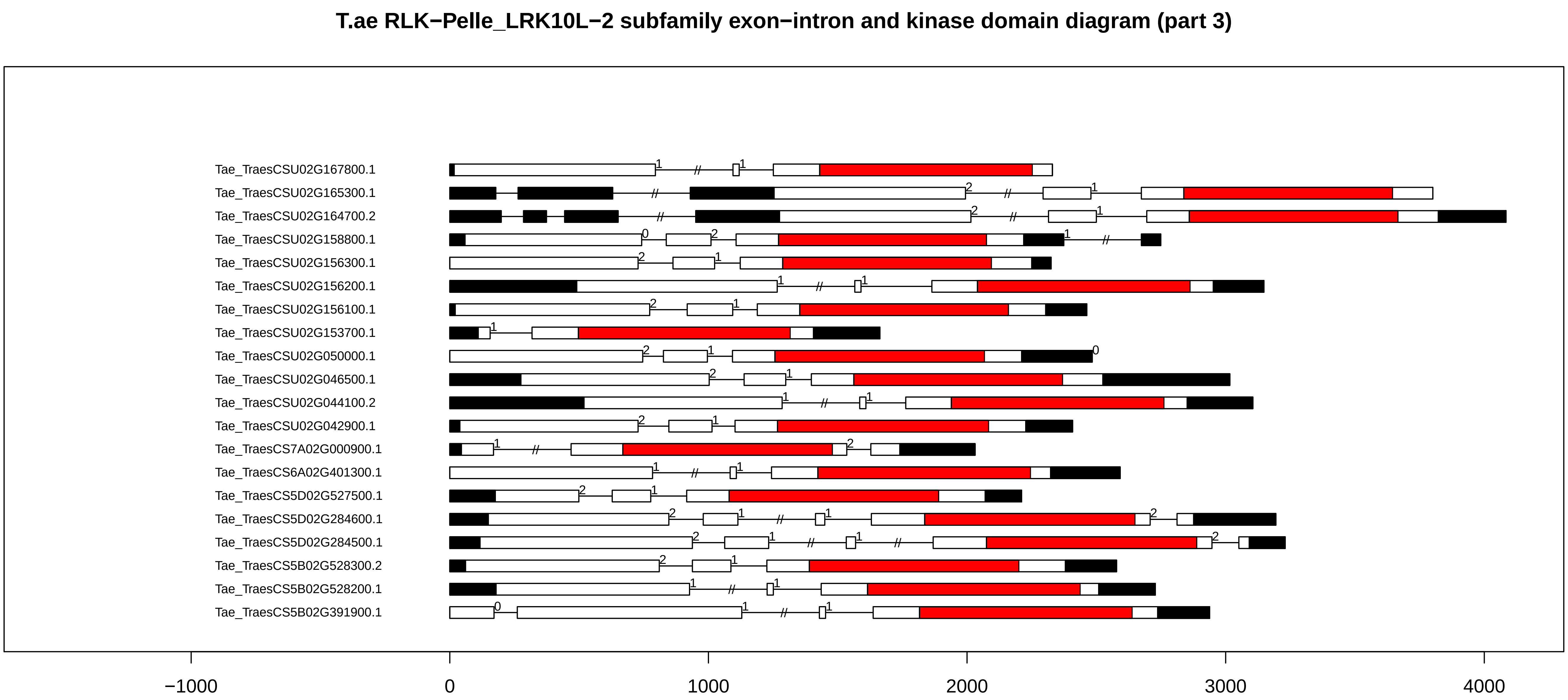

T.ae RLK−Pelle_LRK10L−2 subfamily exon−intron and kinase domain diagram (part 3)
Tae_TraesCSU02G167800.1
Tae_TraesCSU02G165300.1
Tae_TraesCSU02G164700.2
Tae_TraesCSU02G158800.1
Tae_TraesCSU02G156300.1
Tae_TraesCSU02G156200.1
Tae_TraesCSU02G156100.1
Tae_TraesCSU02G153700.1
Tae_TraesCSU02G050000.1
Tae_TraesCSU02G046500.1
Tae_TraesCSU02G044100.2
Tae_TraesCSU02G042900.1
Tae_TraesCS7A02G000900.1
Tae_TraesCS6A02G401300.1
Tae_TraesCS5D02G527500.1
Tae_TraesCS5D02G284600.1
Tae_TraesCS5D02G284500.1
Tae_TraesCS5B02G528300.2
Tae_TraesCS5B02G528200.1
Tae_TraesCS5B02G391900.1
−1000
0
1000
2000
3000
4000

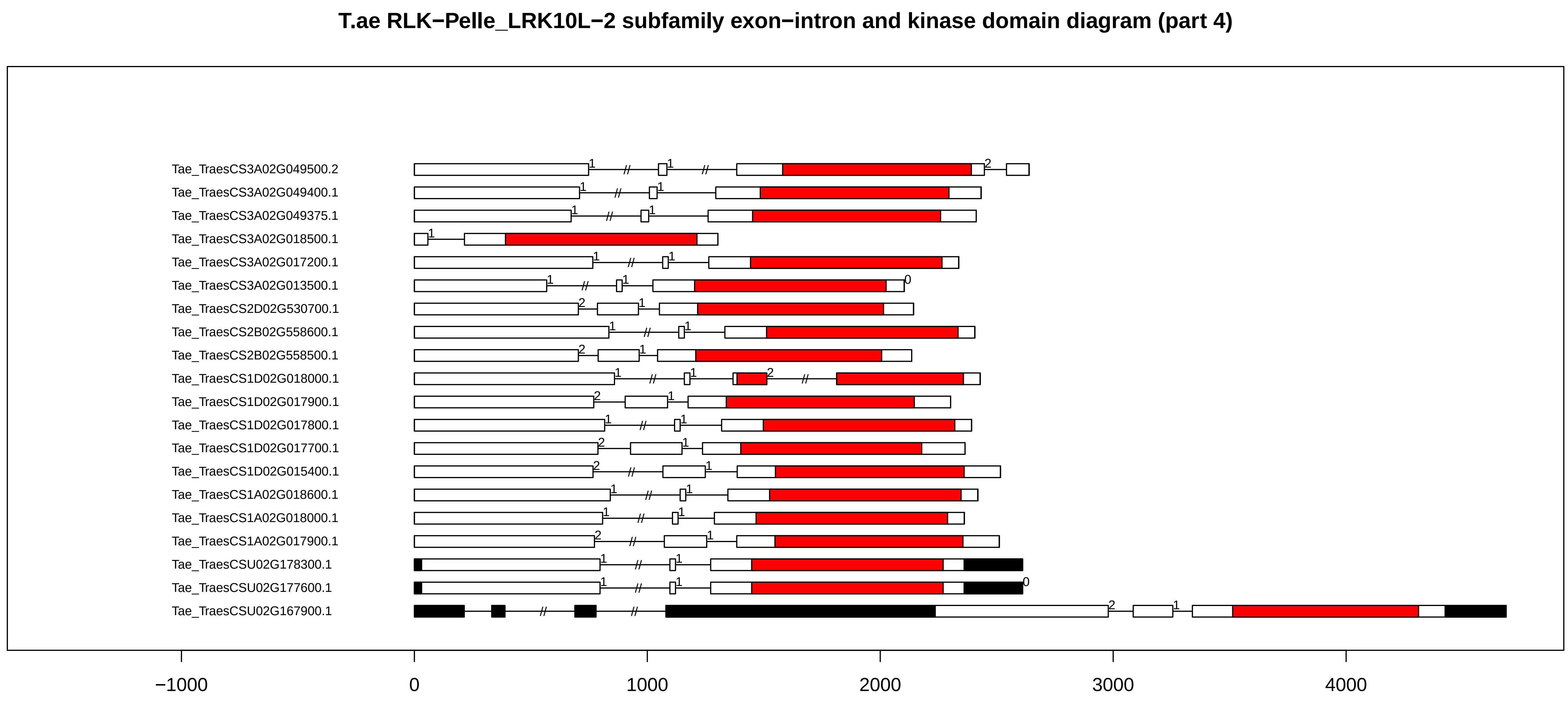

T.ae RLK−Pelle_LRK10L−2 subfamily exon−intron and kinase domain diagram (part 4)
Tae_TraesCS3A02G049500.2
Tae_TraesCS3A02G049400.1
Tae_TraesCS3A02G049375.1
Tae_TraesCS3A02G018500.1
Tae_TraesCS3A02G017200.1
Tae_TraesCS3A02G013500.1
Tae_TraesCS2D02G530700.1
Tae_TraesCS2B02G558600.1
Tae_TraesCS2B02G558500.1
Tae_TraesCS1D02G018000.1
Tae_TraesCS1D02G017900.1
Tae_TraesCS1D02G017800.1
Tae_TraesCS1D02G017700.1
Tae_TraesCS1D02G015400.1
Tae_TraesCS1A02G018600.1
Tae_TraesCS1A02G018000.1
Tae_TraesCS1A02G017900.1
Tae_TraesCSU02G178300.1
Tae_TraesCSU02G177600.1
Tae_TraesCSU02G167900.1
−1000
0
1000
2000
3000
4000

T.ae RLK−Pelle_LRK10L−2 subfamily exon−intron and kinase domain diagram (part 5)

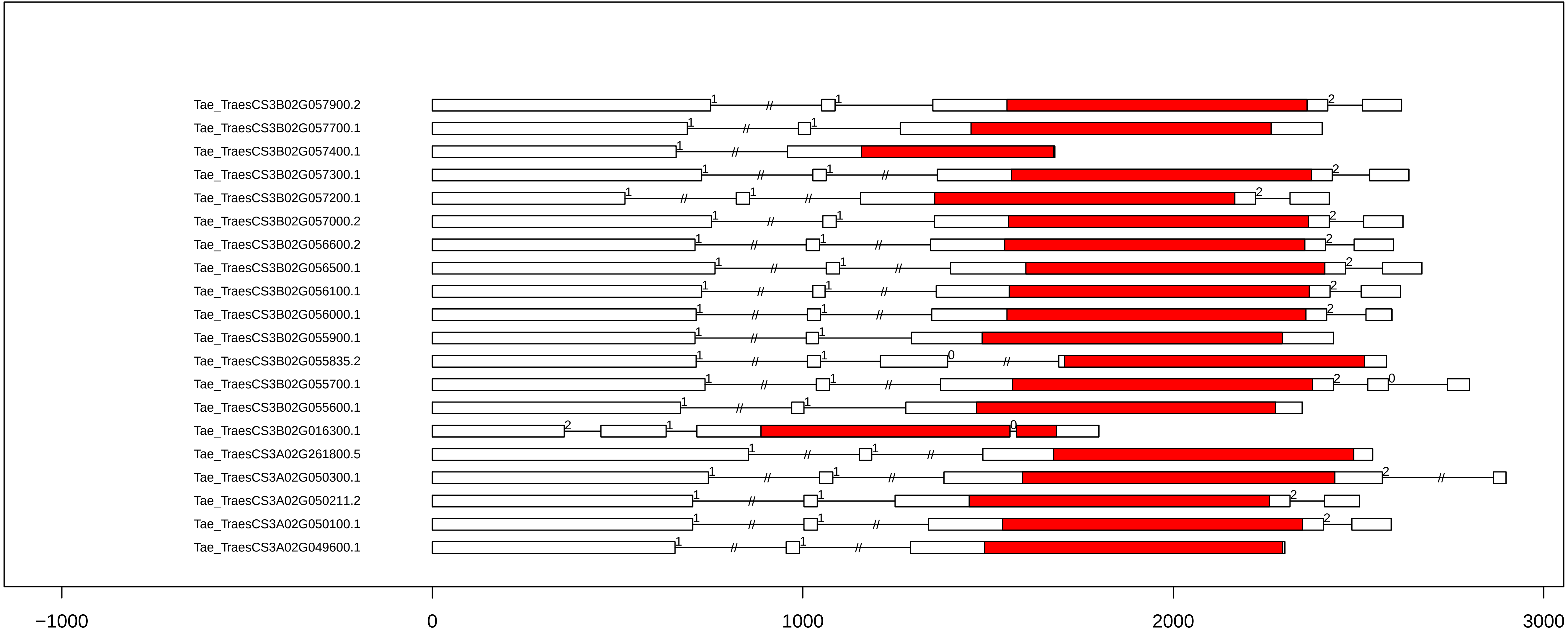

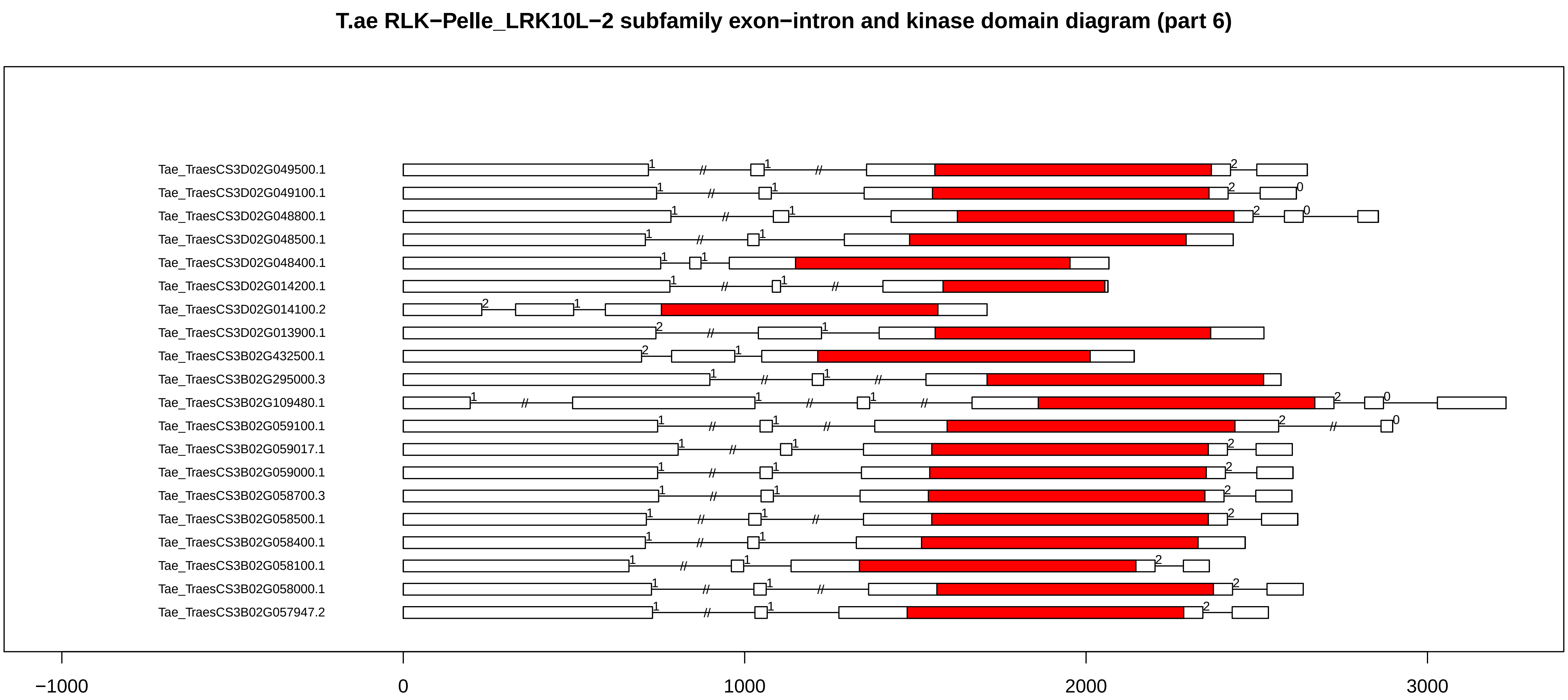
T.ae RLK−Pelle_LRK10L−2 subfamily exon−intron and kinase domain diagram (part 6)
Tae_TraesCS3D02G049500.1
Tae_TraesCS3D02G049100.1
Tae_TraesCS3D02G048800.1
Tae_TraesCS3D02G048500.1
Tae_TraesCS3D02G048400.1
Tae_TraesCS3D02G014200.1
Tae_TraesCS3D02G014100.2
Tae_TraesCS3D02G013900.1
Tae_TraesCS3B02G432500.1
Tae_TraesCS3B02G295000.3
Tae_TraesCS3B02G109480.1
Tae_TraesCS3B02G059100.1
Tae_TraesCS3B02G059017.1
Tae_TraesCS3B02G059000.1
Tae_TraesCS3B02G058700.3
Tae_TraesCS3B02G058500.1
Tae_TraesCS3B02G058400.1
Tae_TraesCS3B02G058100.1
Tae_TraesCS3B02G058000.1
Tae_TraesCS3B02G057947.2
−1000
0
1000
2000
3000

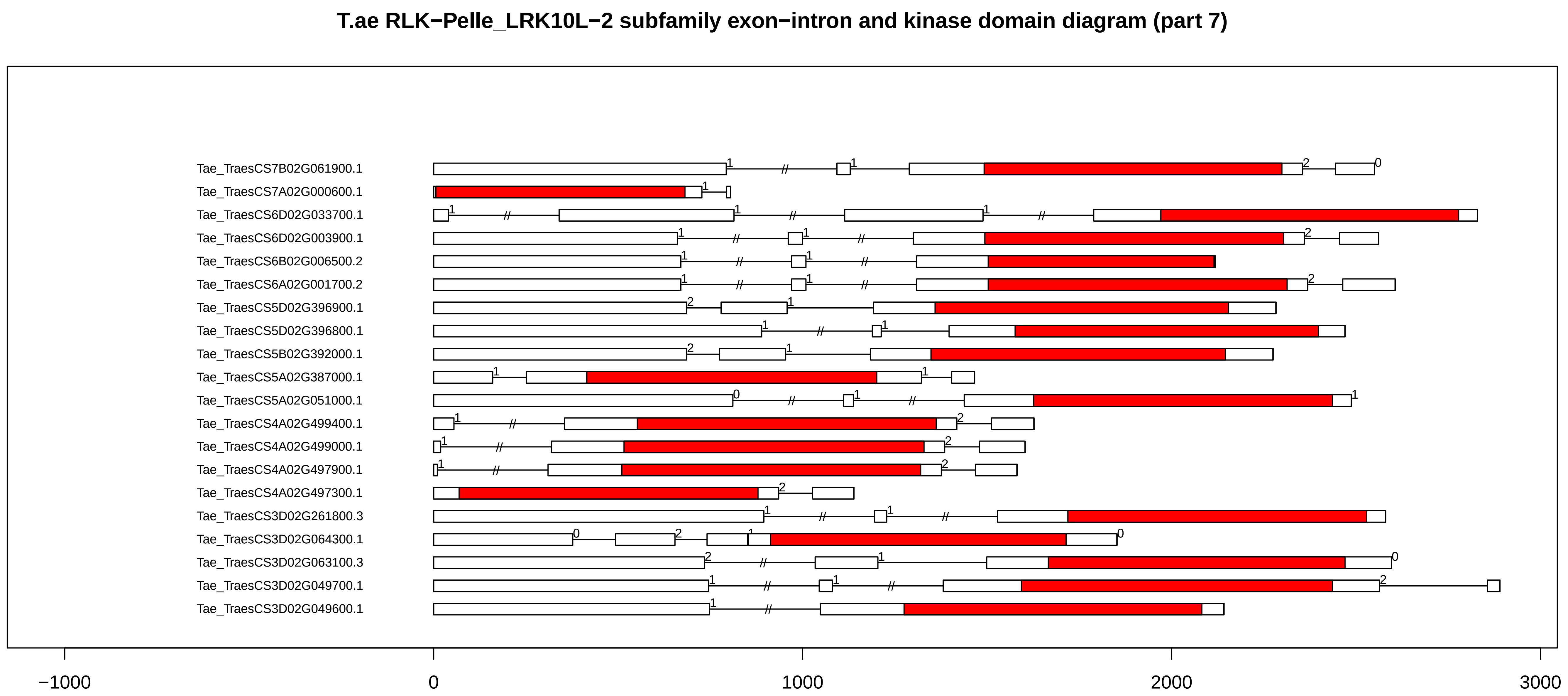
T.ae RLK−Pelle_LRK10L−2 subfamily exon−intron and kinase domain diagram (part 7)
Tae_TraesCS7B02G061900.1
Tae_TraesCS7A02G000600.1
Tae_TraesCS6D02G033700.1
Tae_TraesCS6D02G003900.1
Tae_TraesCS6B02G006500.2
Tae_TraesCS6A02G001700.2
Tae_TraesCS5D02G396900.1
Tae_TraesCS5D02G396800.1
Tae_TraesCS5B02G392000.1
Tae_TraesCS5A02G387000.1
Tae_TraesCS5A02G051000.1
Tae_TraesCS4A02G499400.1
Tae_TraesCS4A02G499000.1
Tae_TraesCS4A02G497900.1
Tae_TraesCS4A02G497300.1
Tae_TraesCS3D02G261800.3
Tae_TraesCS3D02G064300.1
Tae_TraesCS3D02G063100.3
Tae_TraesCS3D02G049700.1
Tae_TraesCS3D02G049600.1
−1000
0
1000
2000
3000

T.ae RLK−Pelle_LRK10L−2 subfamily exon−intron and kinase domain diagram (part 8)

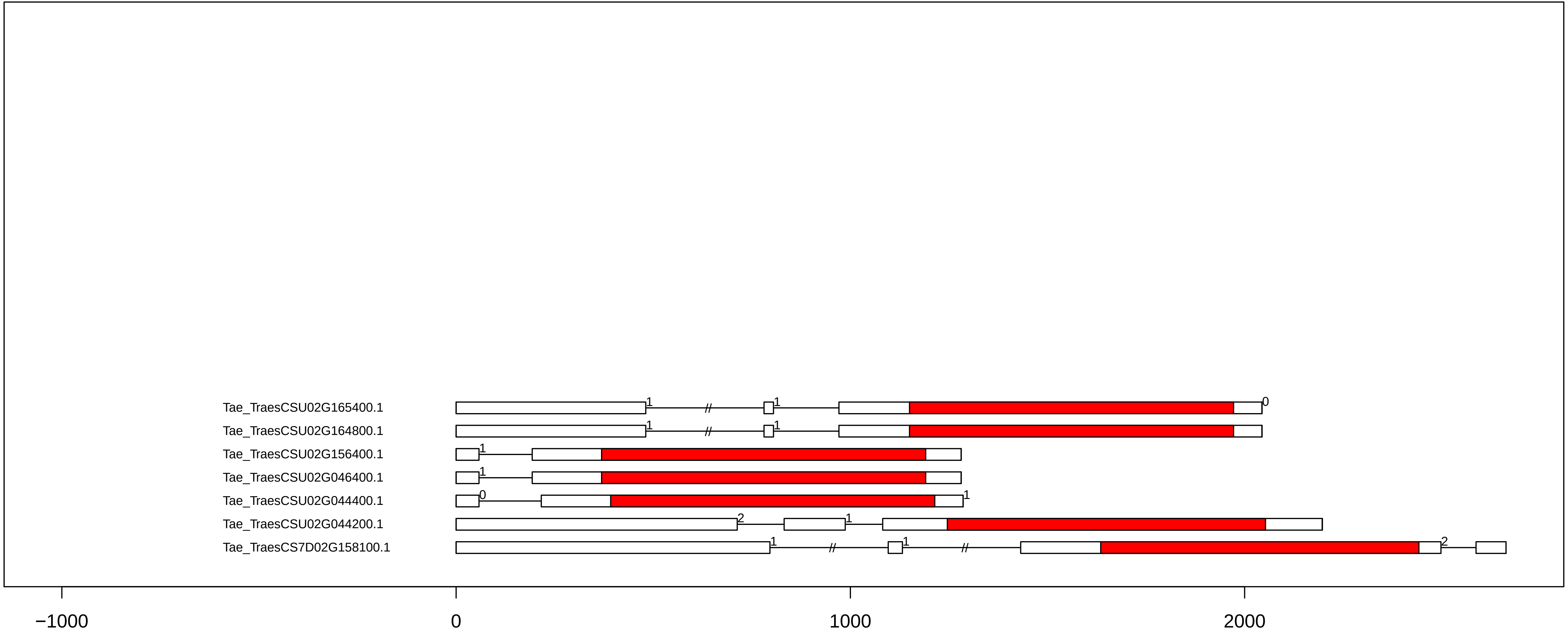

T.ae RLK−Pelle_LRR−I−1 subfamily exon−intron and kinase domain diagram (part 1)

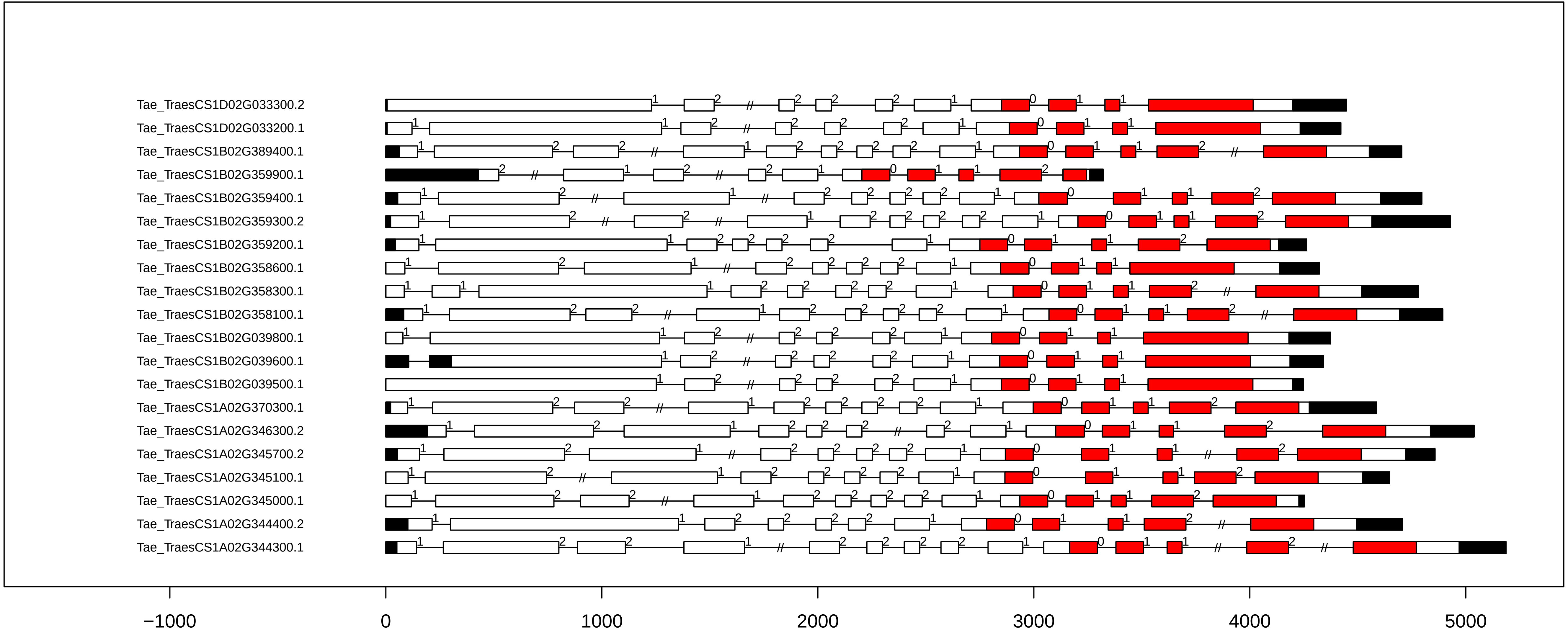

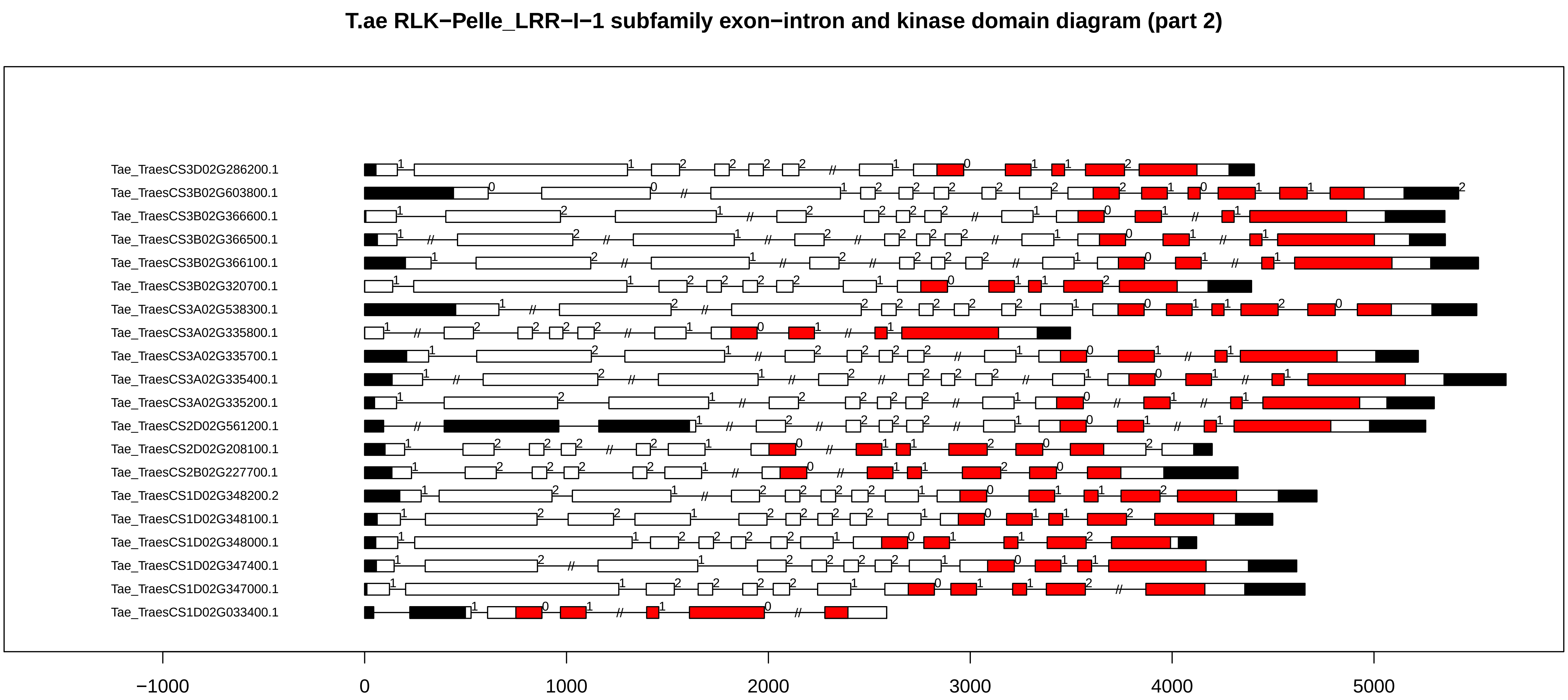

T.ae RLK−Pelle_LRR−I−1 subfamily exon−intron and kinase domain diagram (part 2)
Tae_TraesCS3D02G286200.1
Tae_TraesCS3B02G603800.1
Tae_TraesCS3B02G366600.1
Tae_TraesCS3B02G366500.1
Tae_TraesCS3B02G366100.1
Tae_TraesCS3B02G320700.1
Tae_TraesCS3A02G538300.1
Tae_TraesCS3A02G335800.1
Tae_TraesCS3A02G335700.1
Tae_TraesCS3A02G335400.1
Tae_TraesCS3A02G335200.1
Tae_TraesCS2D02G561200.1
Tae_TraesCS2D02G208100.1
Tae_TraesCS2B02G227700.1
Tae_TraesCS1D02G348200.2
Tae_TraesCS1D02G348100.1
Tae_TraesCS1D02G348000.1
Tae_TraesCS1D02G347400.1
Tae_TraesCS1D02G347000.1
Tae_TraesCS1D02G033400.1
−1000
0
1000
2000
3000
4000
5000

T.ae RLK−Pelle_LRR−I−1 subfamily exon−intron and kinase domain diagram (part 3)

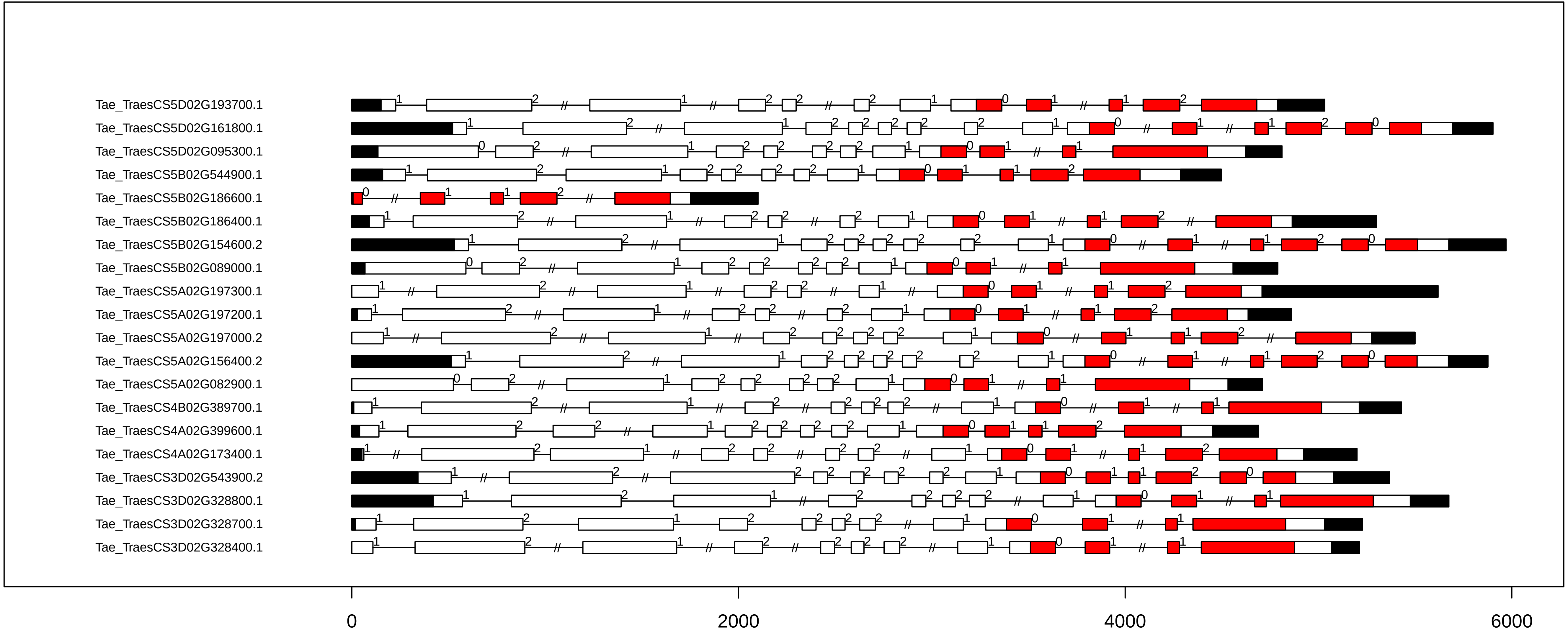

T.ae RLK−Pelle_LRR−I−1 subfamily exon−intron and kinase domain diagram (part 4)

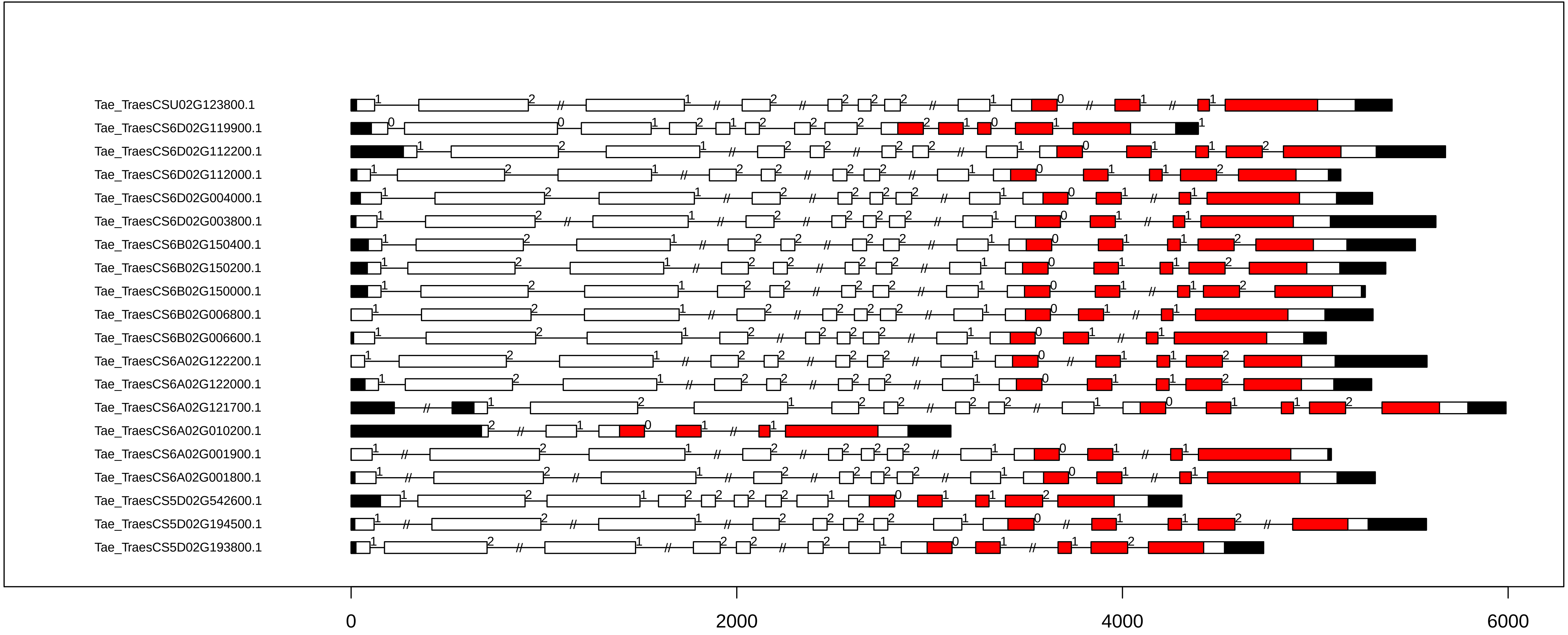

T.ae RLK−Pelle_LRR−I−1 subfamily exon−intron and kinase domain diagram (part 5)

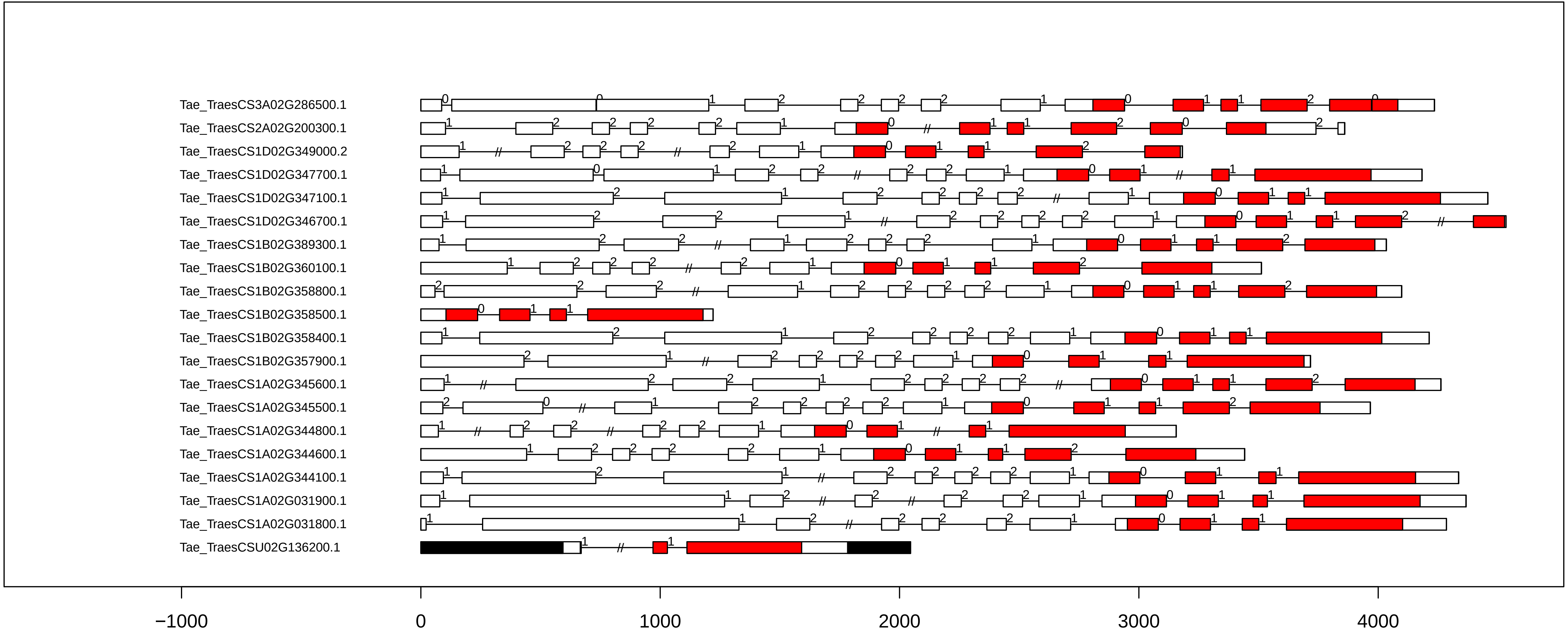

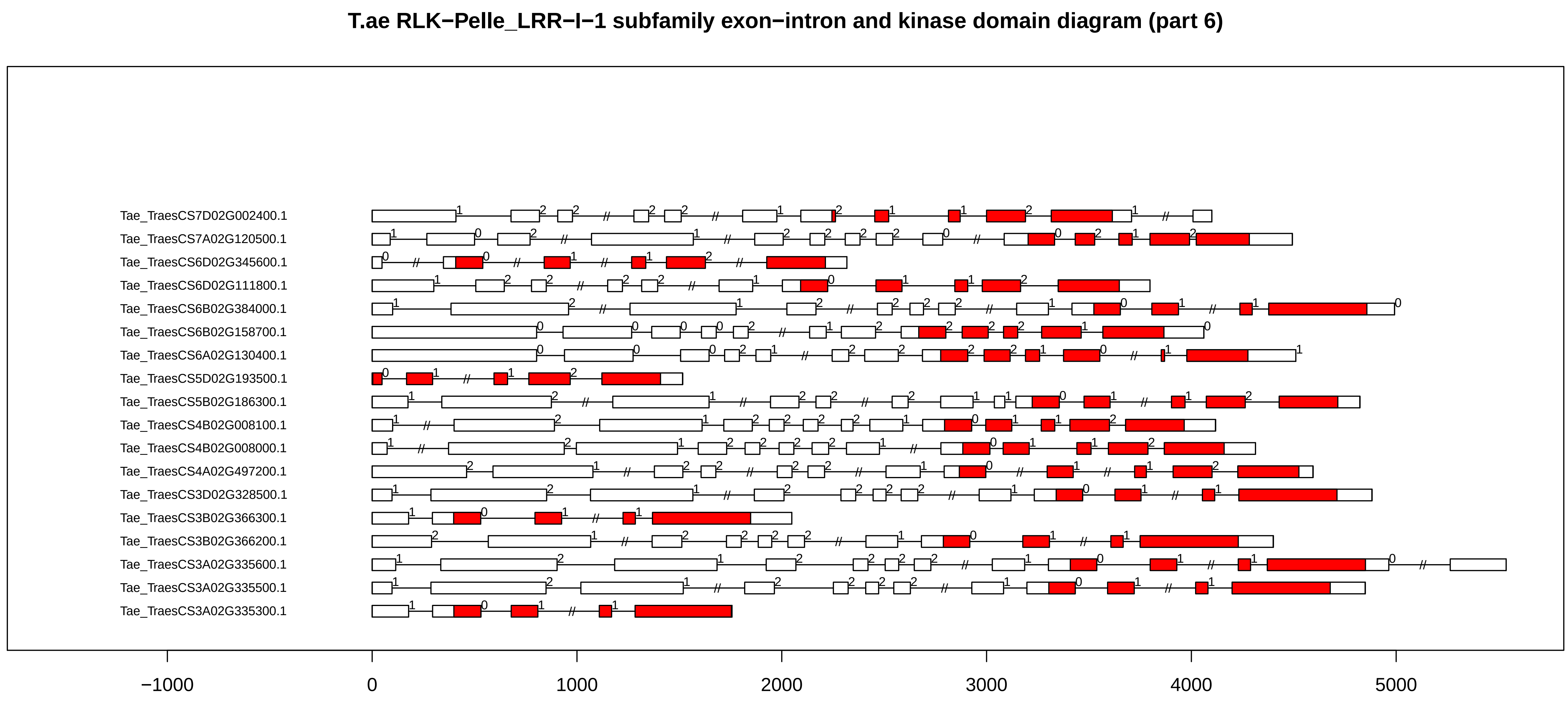
T.ae RLK−Pelle_LRR−I−1 subfamily exon−intron and kinase domain diagram (part 6)
Tae_TraesCS7D02G002400.1
Tae_TraesCS7A02G120500.1
Tae_TraesCS6D02G345600.1
Tae_TraesCS6D02G111800.1
Tae_TraesCS6B02G384000.1
Tae_TraesCS6B02G158700.1
Tae_TraesCS6A02G130400.1
Tae_TraesCS5D02G193500.1
Tae_TraesCS5B02G186300.1
Tae_TraesCS4B02G008100.1
Tae_TraesCS4B02G008000.1
Tae_TraesCS4A02G497200.1
Tae_TraesCS3D02G328500.1
Tae_TraesCS3B02G366300.1
Tae_TraesCS3B02G366200.1
Tae_TraesCS3A02G335600.1
Tae_TraesCS3A02G335500.1
Tae_TraesCS3A02G335300.1
−1000
0
1000
2000
3000
4000
5000

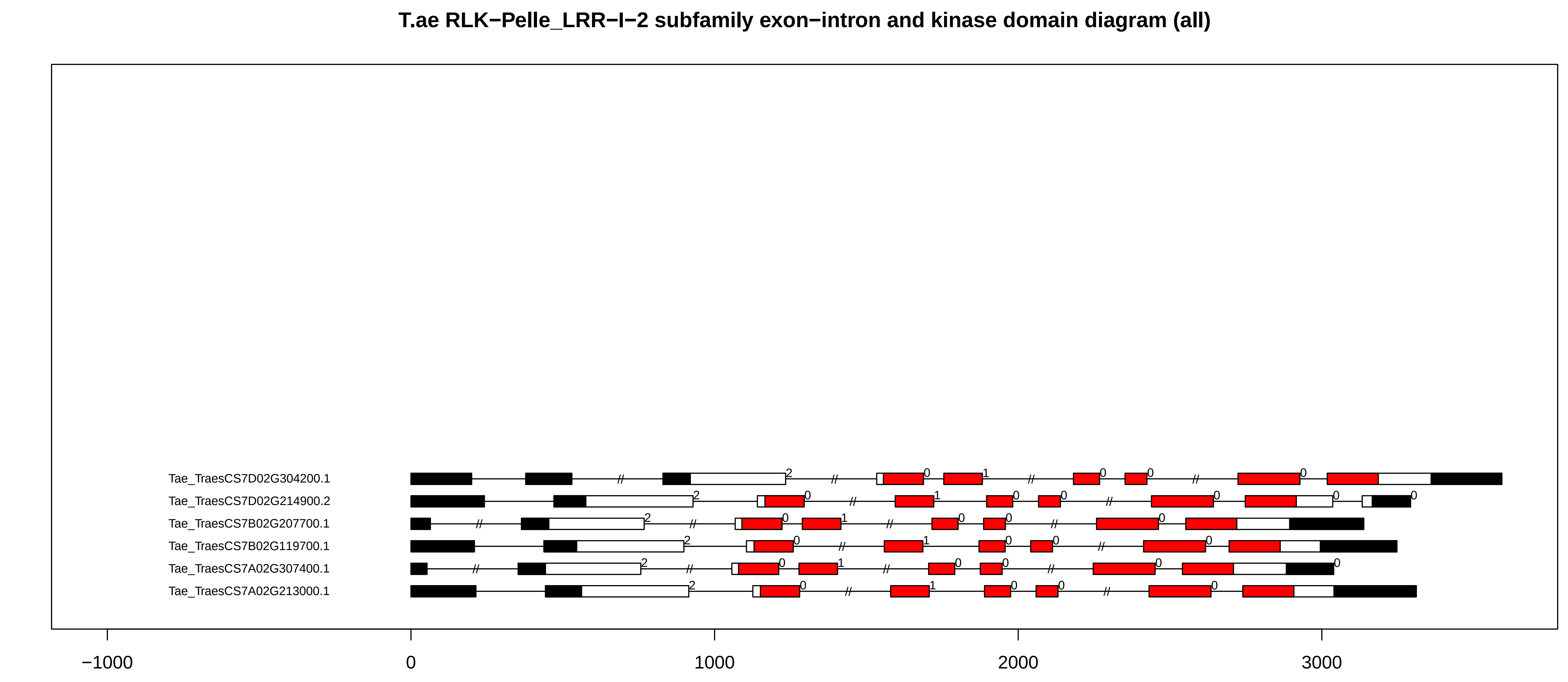

T.ae RLK−Pelle_LRR−I−2 subfamily exon−intron and kinase domain diagram (all)
Tae_TraesCS7D02G304200.1
Tae_TraesCS7D02G214900.2
Tae_TraesCS7B02G207700.1
Tae_TraesCS7B02G119700.1
Tae_TraesCS7A02G307400.1
Tae_TraesCS7A02G213000.1
−1000
0
1000
2000
3000

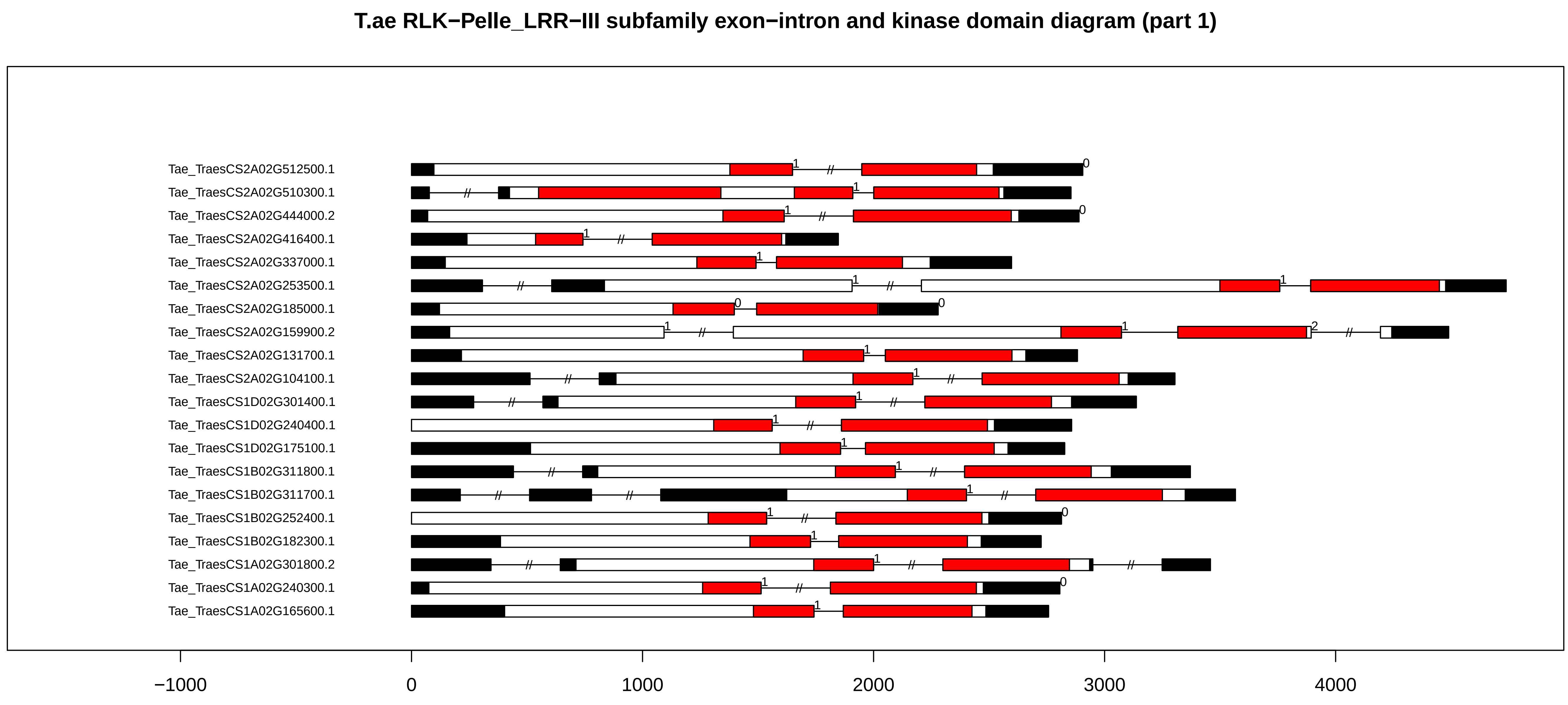
T.ae RLK−Pelle_LRR−III subfamily exon−intron and kinase domain diagram (part 1)
Tae_TraesCS2A02G512500.1
Tae_TraesCS2A02G510300.1
Tae_TraesCS2A02G444000.2
Tae_TraesCS2A02G416400.1
Tae_TraesCS2A02G337000.1
Tae_TraesCS2A02G253500.1
Tae_TraesCS2A02G185000.1
Tae_TraesCS2A02G159900.2
Tae_TraesCS2A02G131700.1
Tae_TraesCS2A02G104100.1
Tae_TraesCS1D02G301400.1
Tae_TraesCS1D02G240400.1
Tae_TraesCS1D02G175100.1
Tae_TraesCS1B02G311800.1
Tae_TraesCS1B02G311700.1
Tae_TraesCS1B02G252400.1
Tae_TraesCS1B02G182300.1
Tae_TraesCS1A02G301800.2
Tae_TraesCS1A02G240300.1
Tae_TraesCS1A02G165600.1
−1000
0
1000
2000
3000
4000

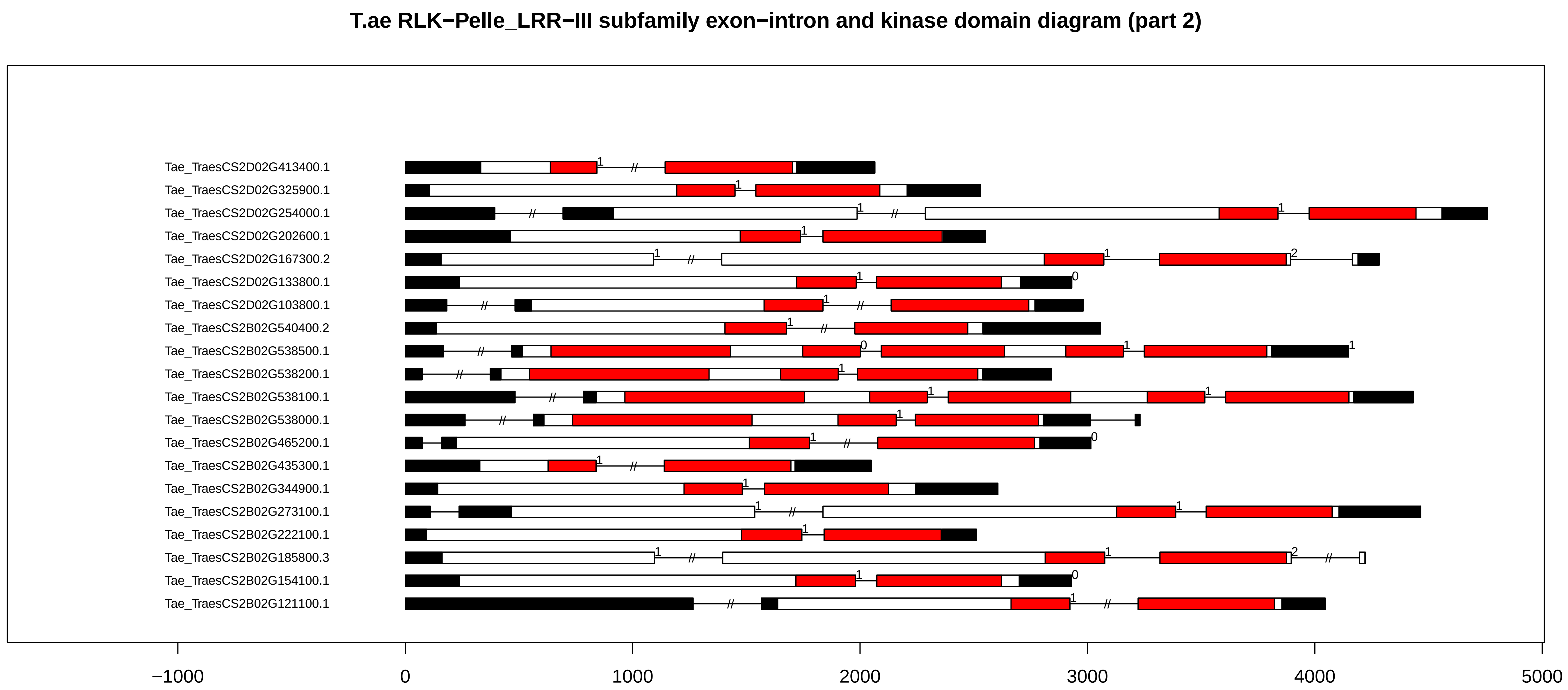
T.ae RLK−Pelle_LRR−III subfamily exon−intron and kinase domain diagram (part 2)
Tae_TraesCS2D02G413400.1
Tae_TraesCS2D02G325900.1
Tae_TraesCS2D02G254000.1
Tae_TraesCS2D02G202600.1
Tae_TraesCS2D02G167300.2
Tae_TraesCS2D02G133800.1
Tae_TraesCS2D02G103800.1
Tae_TraesCS2B02G540400.2
Tae_TraesCS2B02G538500.1
Tae_TraesCS2B02G538200.1
Tae_TraesCS2B02G538100.1
Tae_TraesCS2B02G538000.1
Tae_TraesCS2B02G465200.1
Tae_TraesCS2B02G435300.1
Tae_TraesCS2B02G344900.1
Tae_TraesCS2B02G273100.1
Tae_TraesCS2B02G222100.1
Tae_TraesCS2B02G185800.3
Tae_TraesCS2B02G154100.1
Tae_TraesCS2B02G121100.1
−1000
0
1000
2000
3000
4000
5000

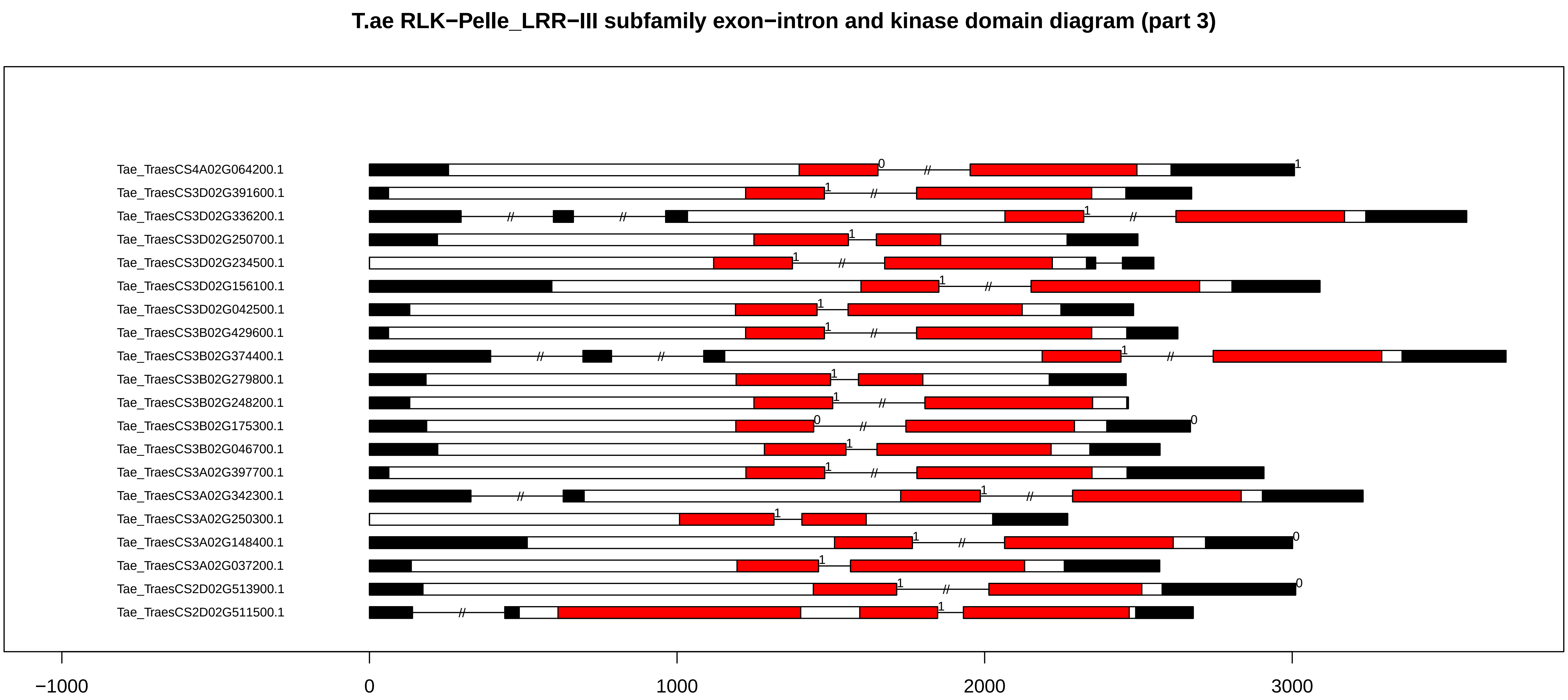
T.ae RLK−Pelle_LRR−III subfamily exon−intron and kinase domain diagram (part 3)
Tae_TraesCS4A02G064200.1
Tae_TraesCS3D02G391600.1
Tae_TraesCS3D02G336200.1
Tae_TraesCS3D02G250700.1
Tae_TraesCS3D02G234500.1
Tae_TraesCS3D02G156100.1
Tae_TraesCS3D02G042500.1
Tae_TraesCS3B02G429600.1
Tae_TraesCS3B02G374400.1
Tae_TraesCS3B02G279800.1
Tae_TraesCS3B02G248200.1
Tae_TraesCS3B02G175300.1
Tae_TraesCS3B02G046700.1
Tae_TraesCS3A02G397700.1
Tae_TraesCS3A02G342300.1
Tae_TraesCS3A02G250300.1
Tae_TraesCS3A02G148400.1
Tae_TraesCS3A02G037200.1
Tae_TraesCS2D02G513900.1
Tae_TraesCS2D02G511500.1
−1000
0
1000
2000
3000

T.ae RLK−Pelle_LRR−III subfamily exon−intron and kinase domain diagram (part 4)

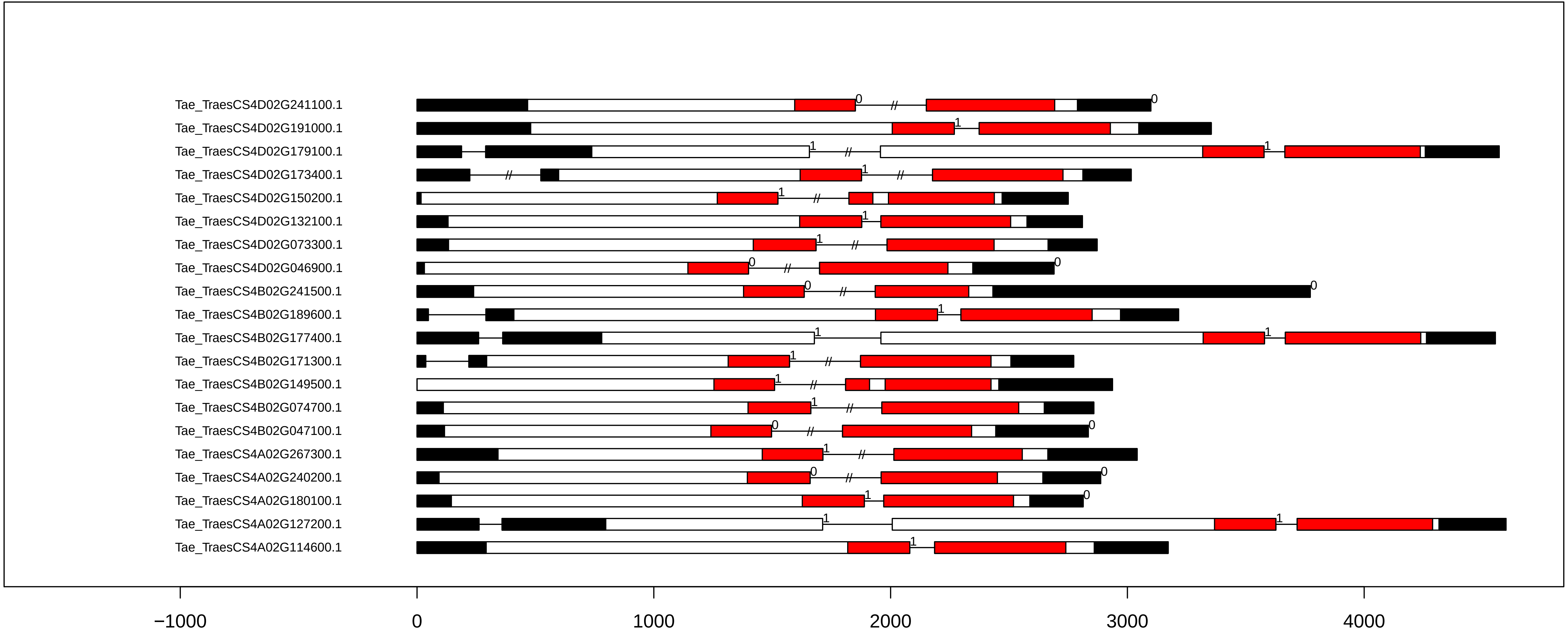

T.ae RLK−Pelle_LRR−III subfamily exon−intron and kinase domain diagram (part 5)

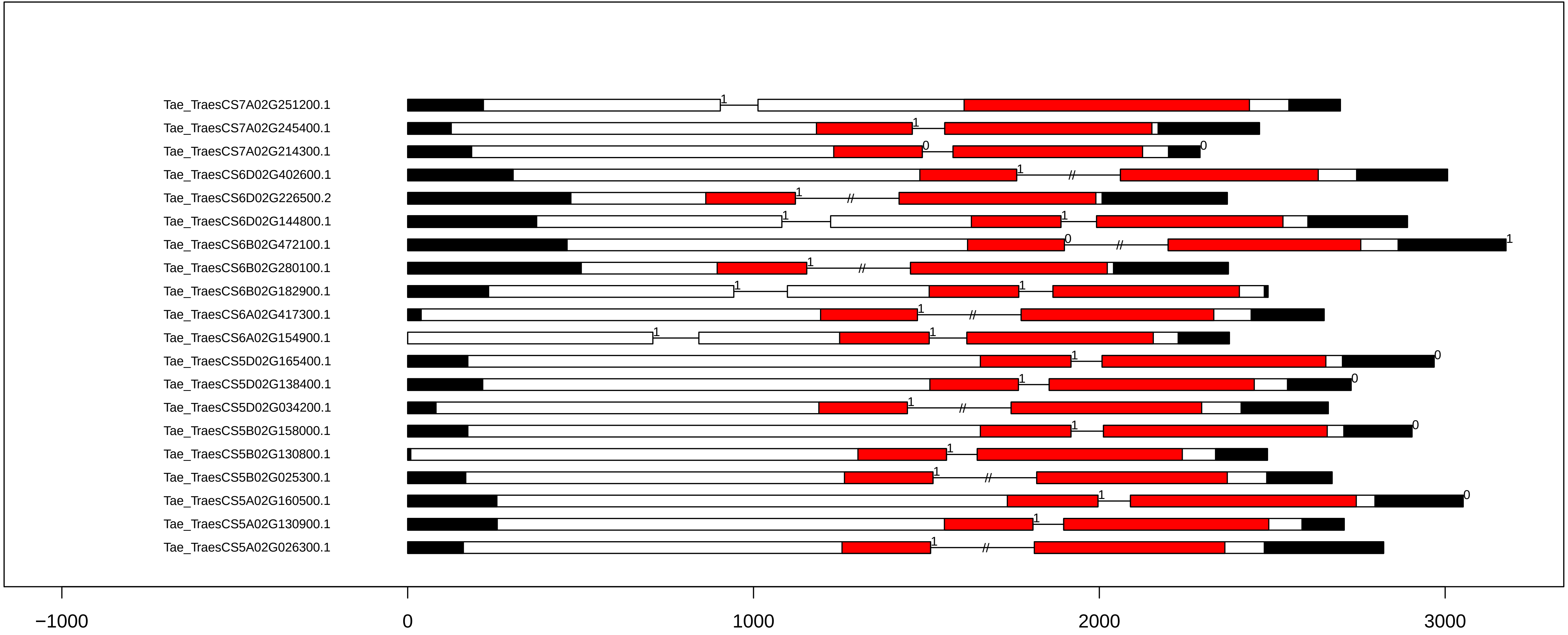

T.ae RLK−Pelle_LRR−III subfamily exon−intron and kinase domain diagram (part 6)

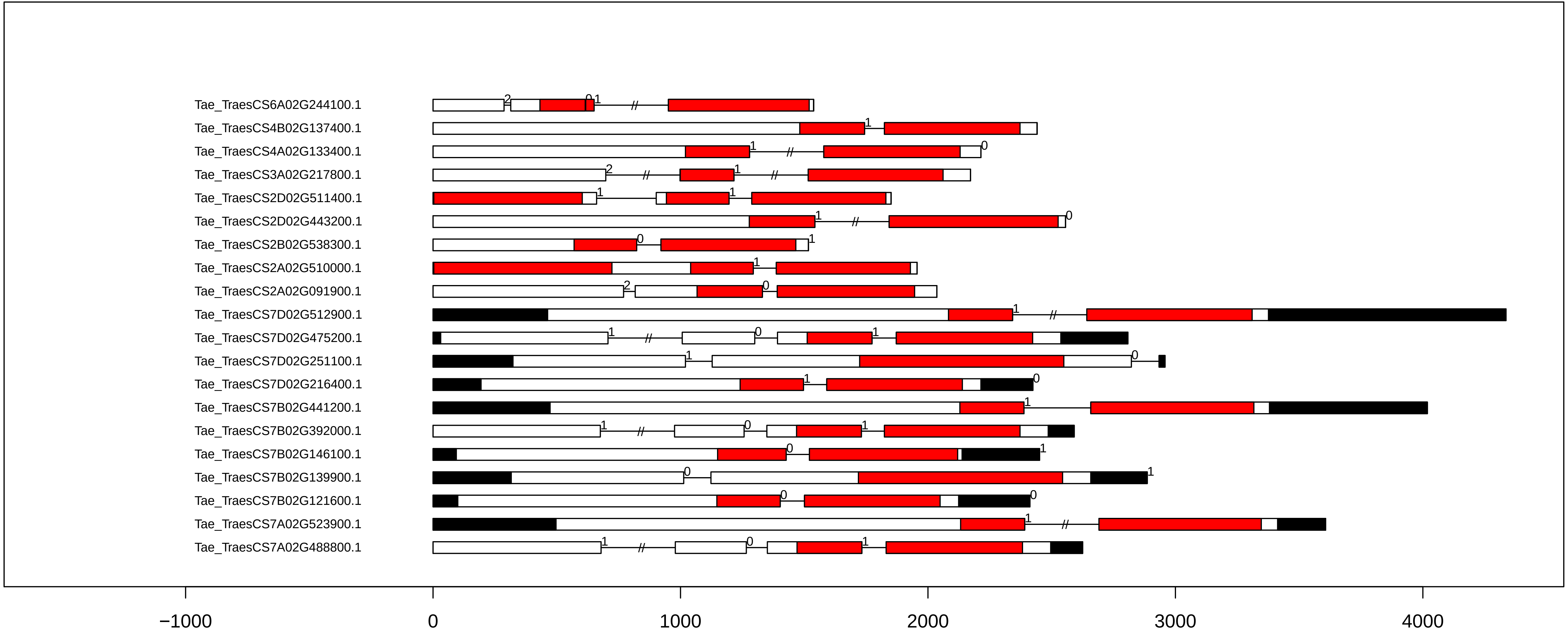

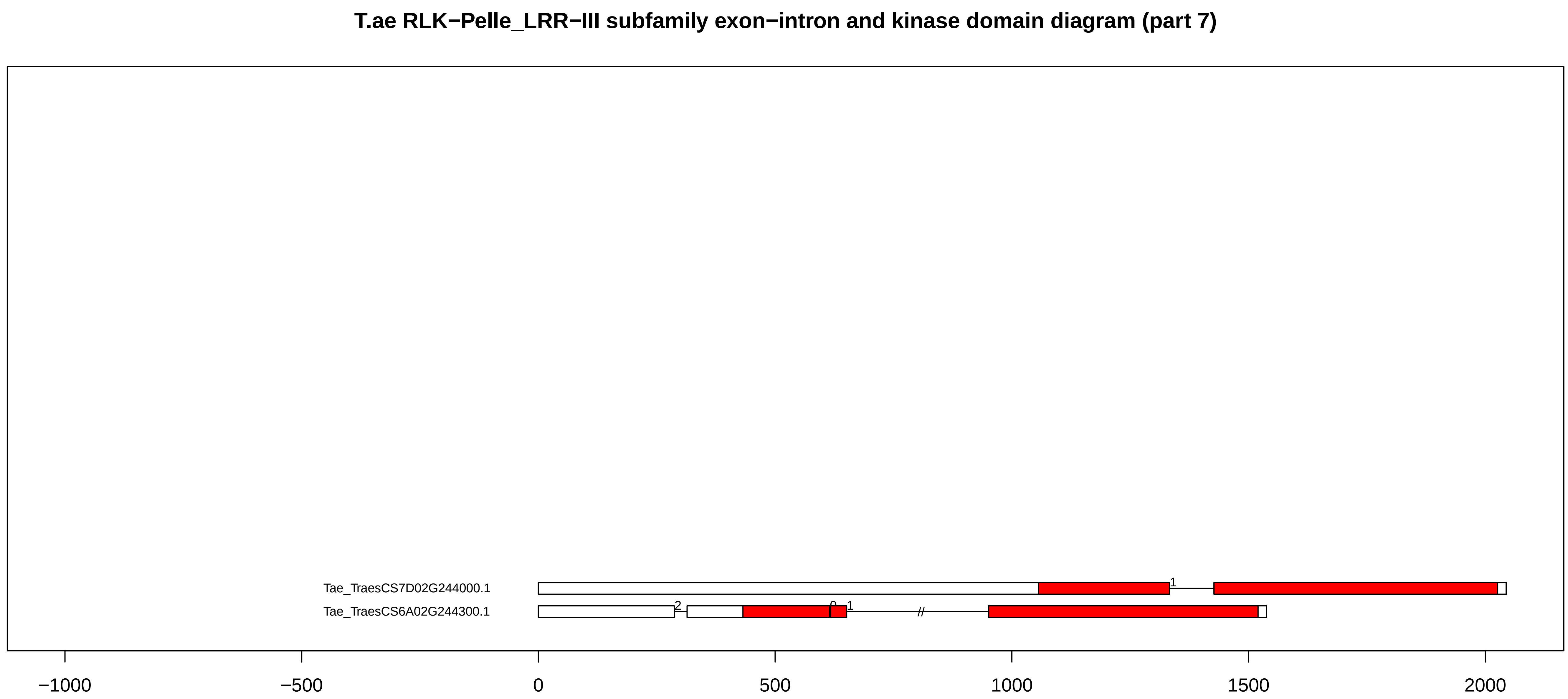
T.ae RLK−Pelle_LRR−III subfamily exon−intron and kinase domain diagram (part 7)
Tae_TraesCS7D02G244000.1
Tae_TraesCS6A02G244300.1
1
2
0
1
−1000
−500
0
500
1000
1500
2000

T.ae RLK−Pelle_LRR−II subfamily exon−intron and kinase domain diagram (part 1)

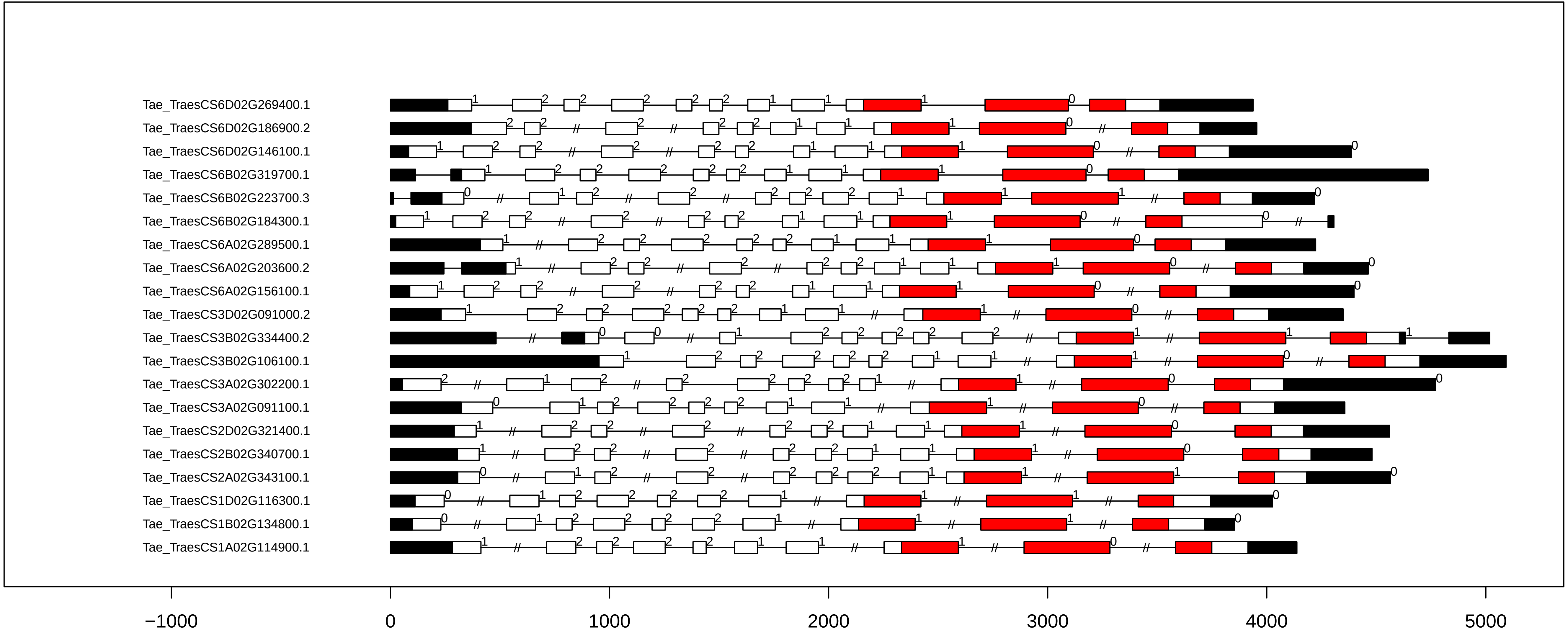

T.ae RLK−Pelle_LRR−II subfamily exon−intron and kinase domain diagram (part 2)

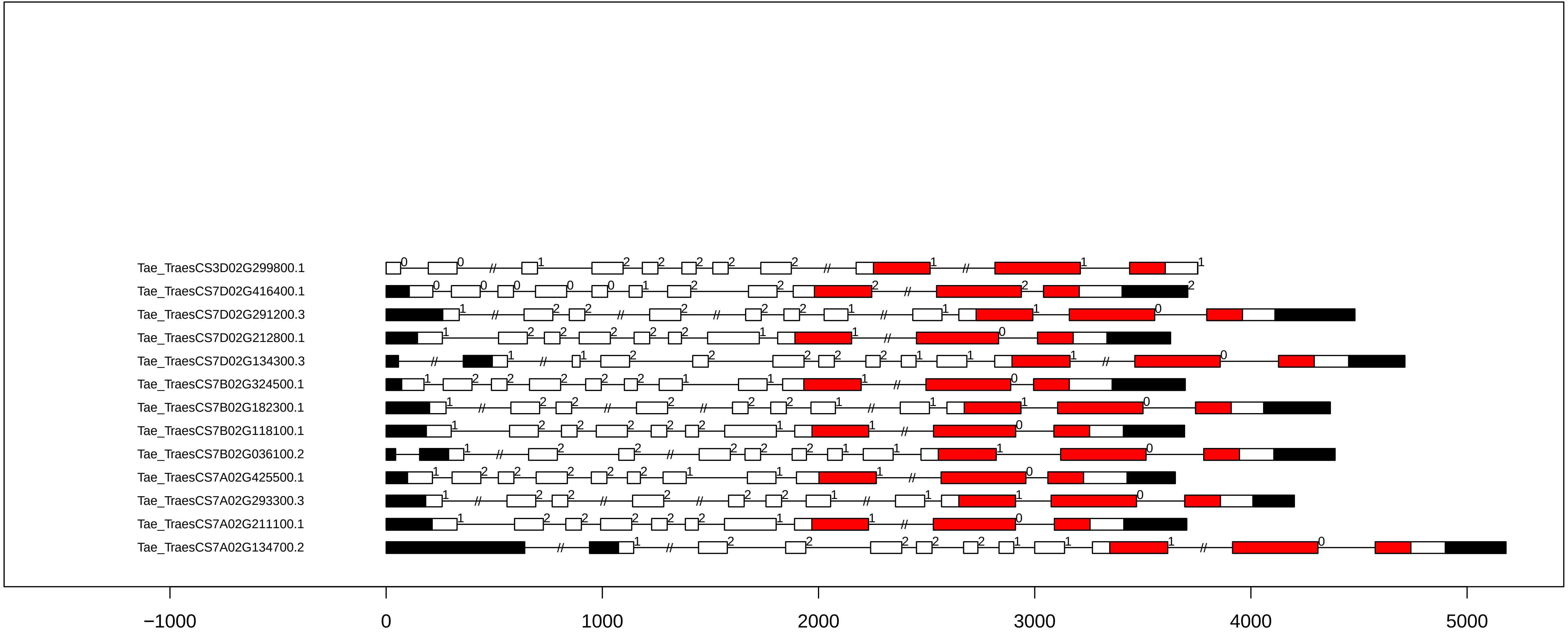

T.ae RLK−Pelle_LRR−IV subfamily exon−intron and kinase domain diagram (all)

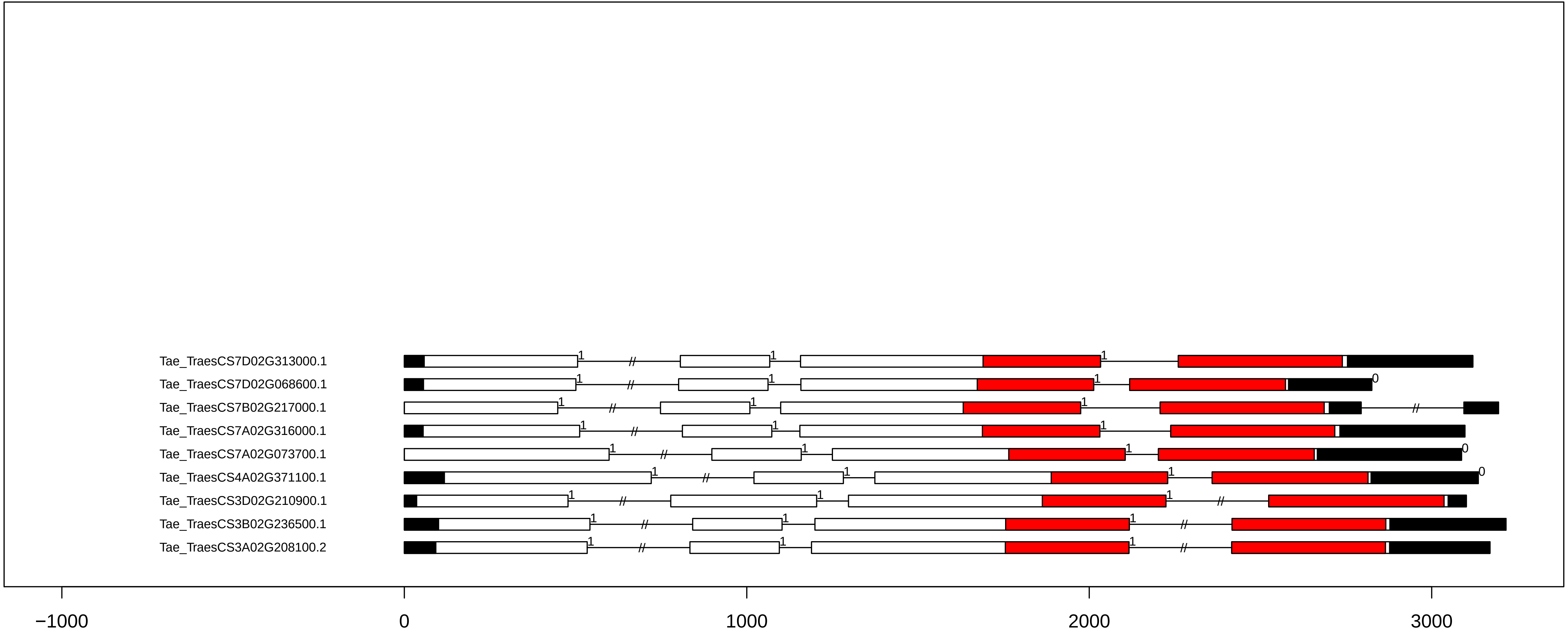

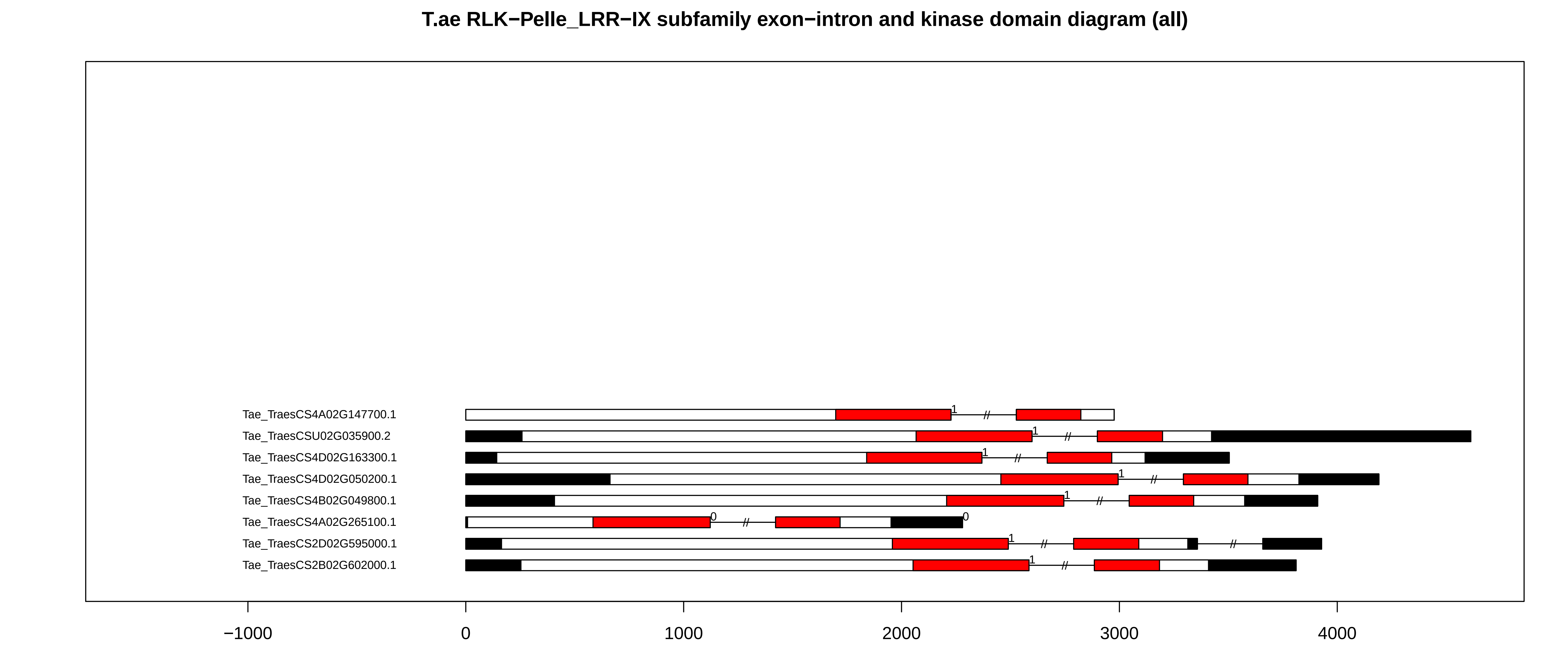

T.ae RLK−Pelle_LRR−IX subfamily exon−intron and kinase domain diagram (all)
Tae_TraesCS4A02G147700.1
Tae_TraesCSU02G035900.2
Tae_TraesCS4D02G163300.1
Tae_TraesCS4D02G050200.1
Tae_TraesCS4B02G049800.1
Tae_TraesCS4A02G265100.1
Tae_TraesCS2D02G595000.1
Tae_TraesCS2B02G602000.1
−1000
0
1000
2000
3000
4000

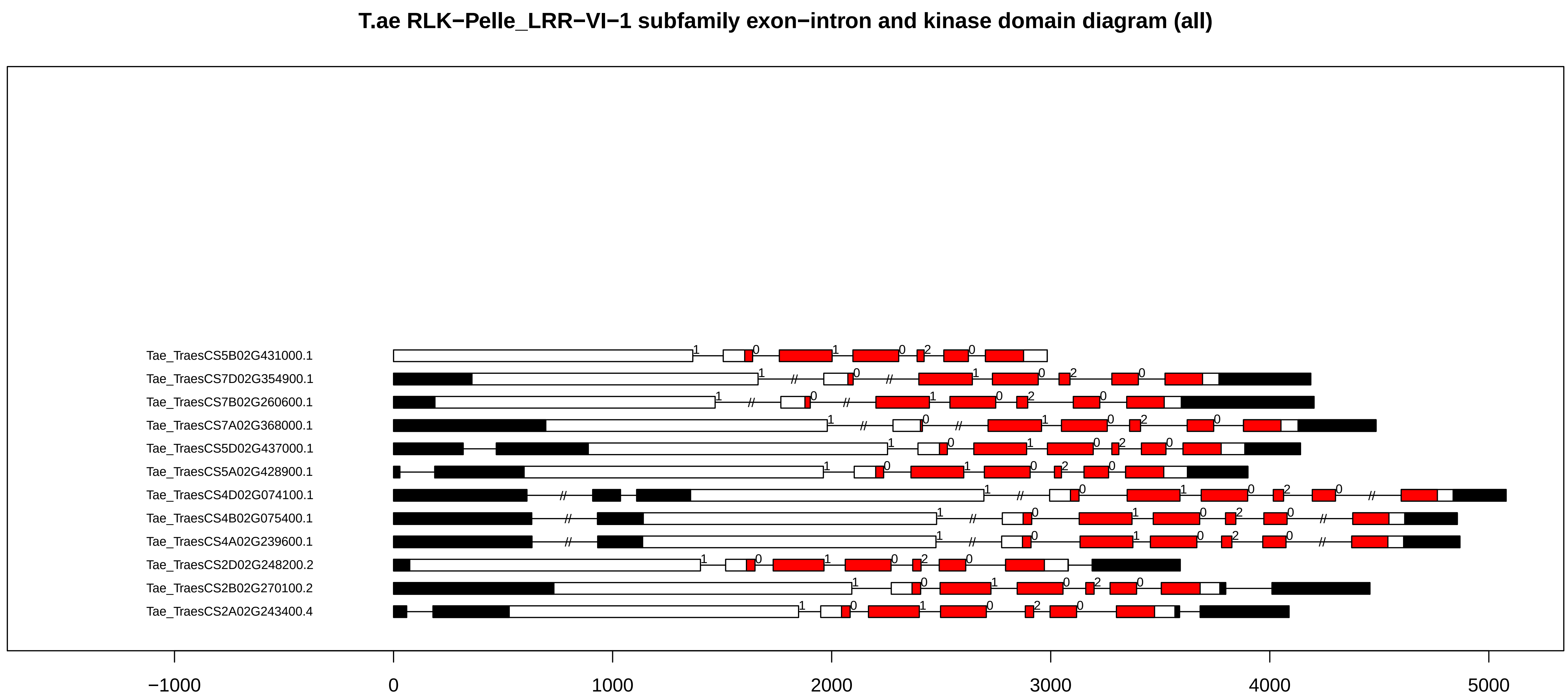
T.ae RLK−Pelle_LRR−VI−1 subfamily exon−intron and kinase domain diagram (all)
Tae_TraesCS5B02G431000.1
Tae_TraesCS7D02G354900.1
Tae_TraesCS7B02G260600.1
Tae_TraesCS7A02G368000.1
Tae_TraesCS5D02G437000.1
Tae_TraesCS5A02G428900.1
Tae_TraesCS4D02G074100.1
Tae_TraesCS4B02G075400.1
Tae_TraesCS4A02G239600.1
Tae_TraesCS2D02G248200.2
Tae_TraesCS2B02G270100.2
Tae_TraesCS2A02G243400.4
−1000
0
1000
2000
3000
4000
5000

T.ae RLK−Pelle_LRR−VI−2 subfamily exon−intron and kinase domain diagram (part 1)

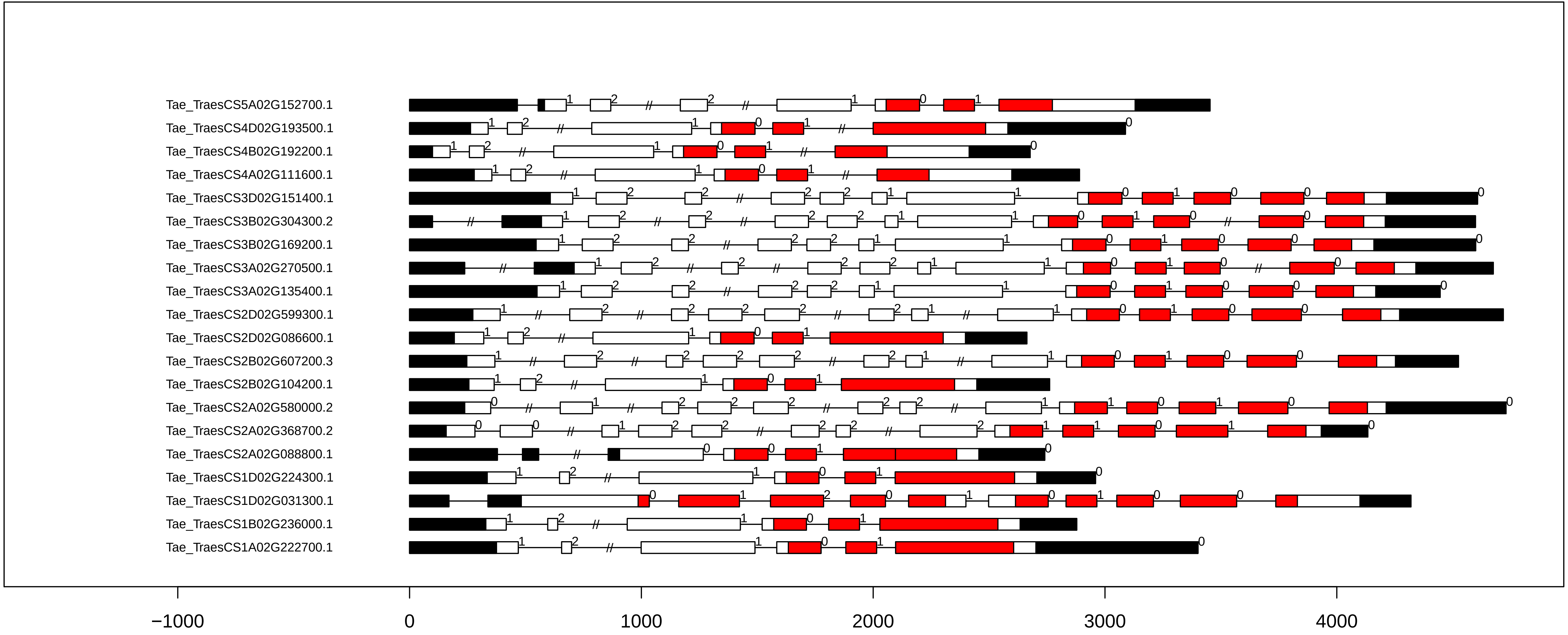

T.ae RLK−Pelle_LRR−VI−2 subfamily exon−intron and kinase domain diagram (part 2)

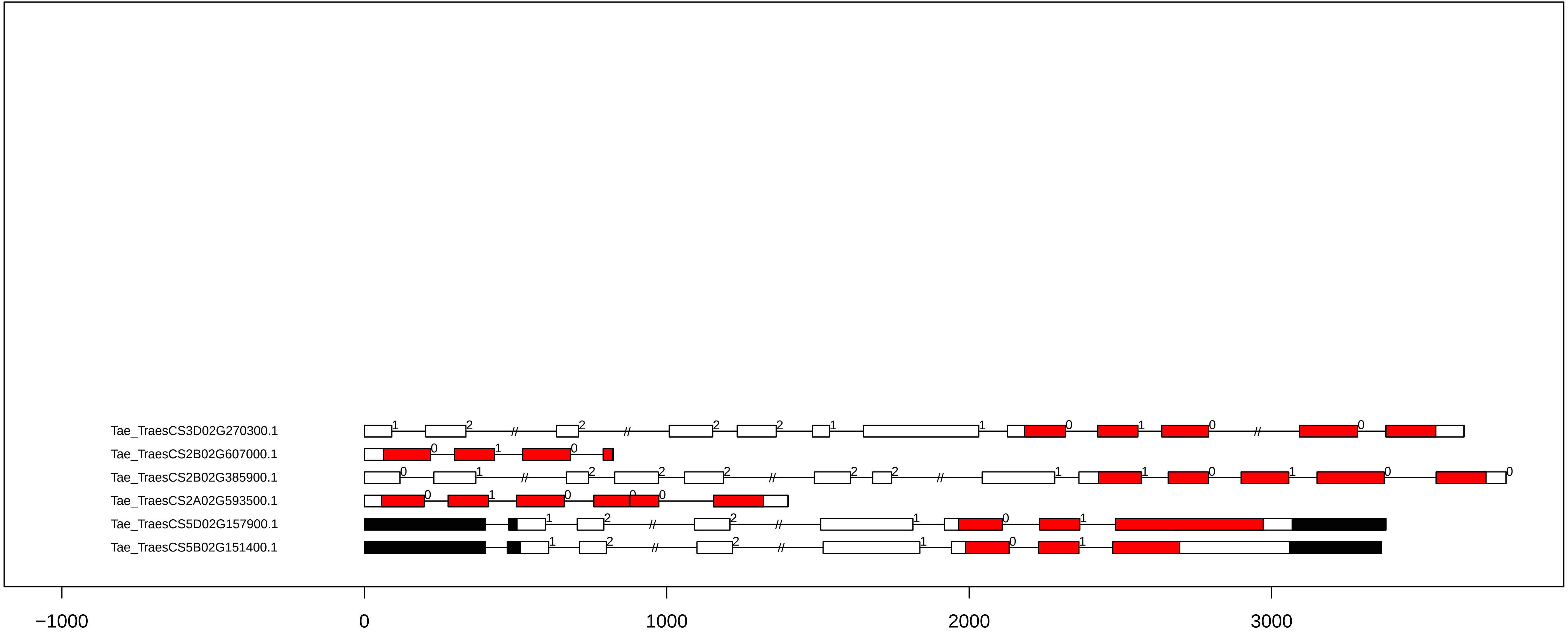

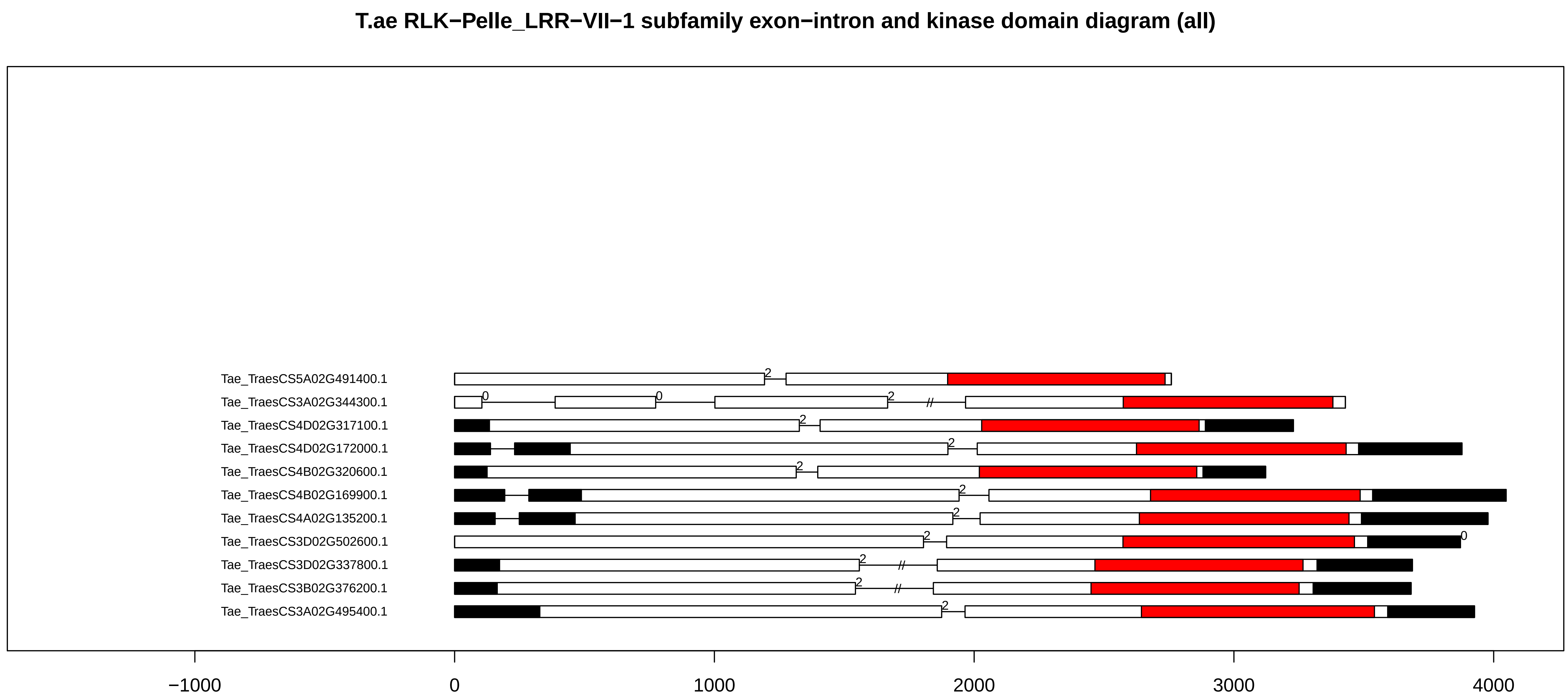
T.ae RLK−Pelle_LRR−VII−1 subfamily exon−intron and kinase domain diagram (all)
Tae_TraesCS5A02G491400.1
Tae_TraesCS3A02G344300.1
Tae_TraesCS4D02G317100.1
Tae_TraesCS4D02G172000.1
Tae_TraesCS4B02G320600.1
Tae_TraesCS4B02G169900.1
Tae_TraesCS4A02G135200.1
Tae_TraesCS3D02G502600.1
Tae_TraesCS3D02G337800.1
Tae_TraesCS3B02G376200.1
Tae_TraesCS3A02G495400.1
−1000
0
1000
2000
3000
4000

T.ae RLK−Pelle_LRR−VIII−1 subfamily exon−intron and kinase domain diagram (part 1)

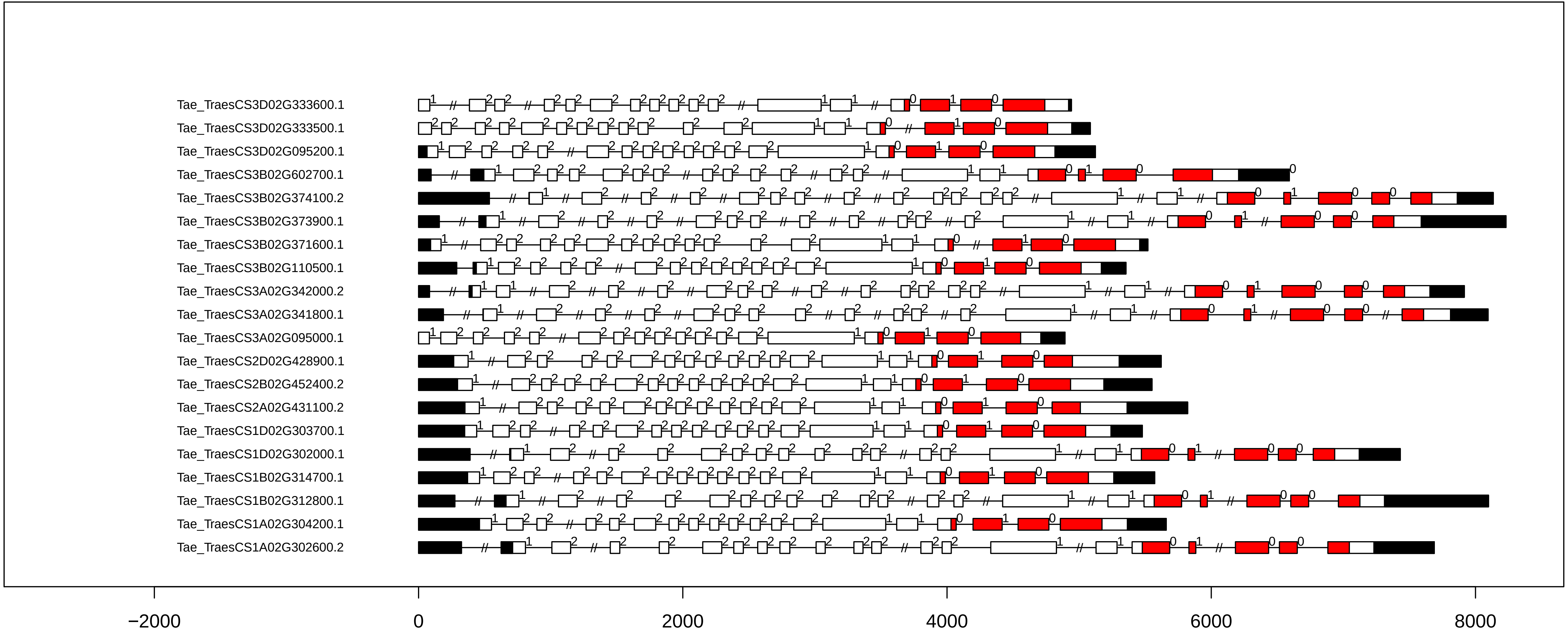

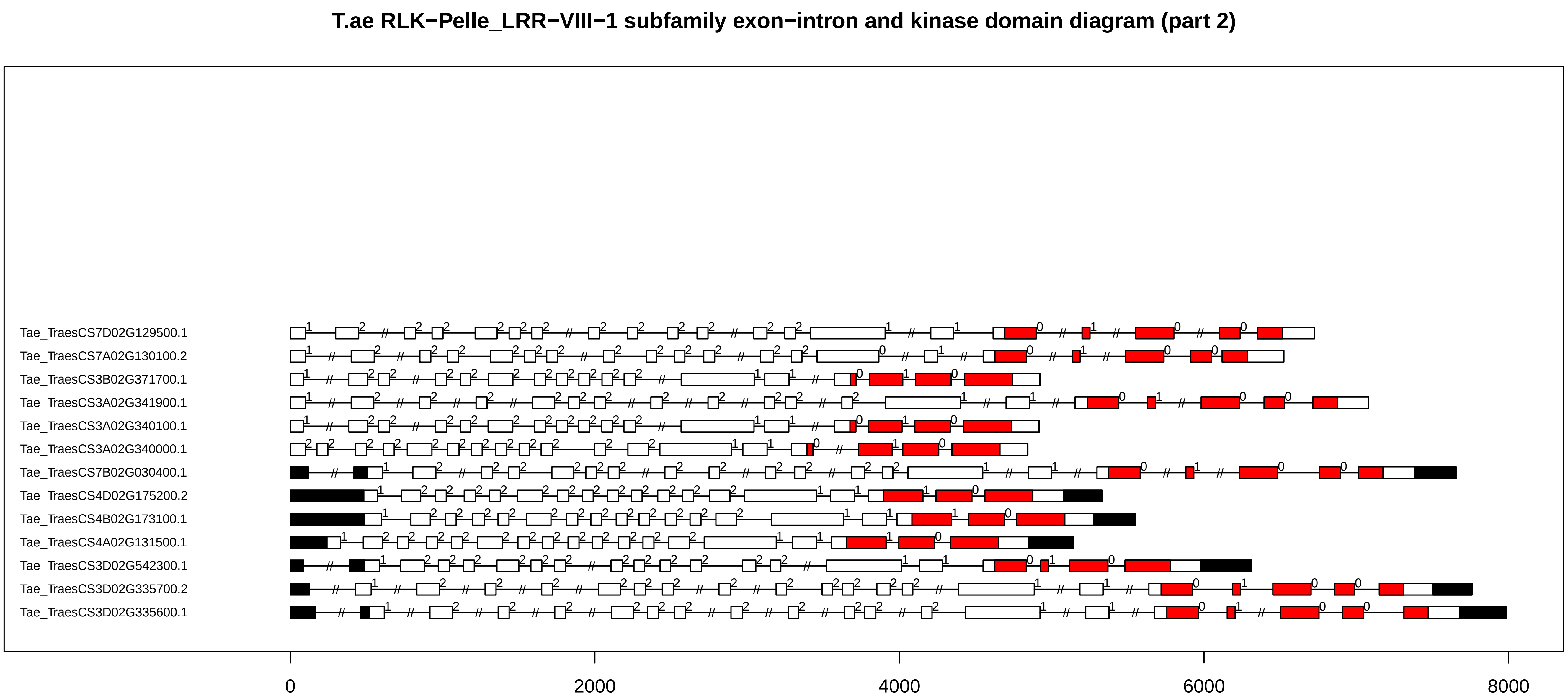

T.ae RLK−Pelle_LRR−VIII−1 subfamily exon−intron and kinase domain diagram (part 2)
Tae_TraesCS7D02G129500.1
Tae_TraesCS7A02G130100.2
Tae_TraesCS3B02G371700.1
Tae_TraesCS3A02G341900.1
Tae_TraesCS3A02G340100.1
Tae_TraesCS3A02G340000.1
Tae_TraesCS7B02G030400.1
Tae_TraesCS4D02G175200.2
Tae_TraesCS4B02G173100.1
Tae_TraesCS4A02G131500.1
Tae_TraesCS3D02G542300.1
Tae_TraesCS3D02G335700.2
Tae_TraesCS3D02G335600.1
0
2000
4000
6000
8000

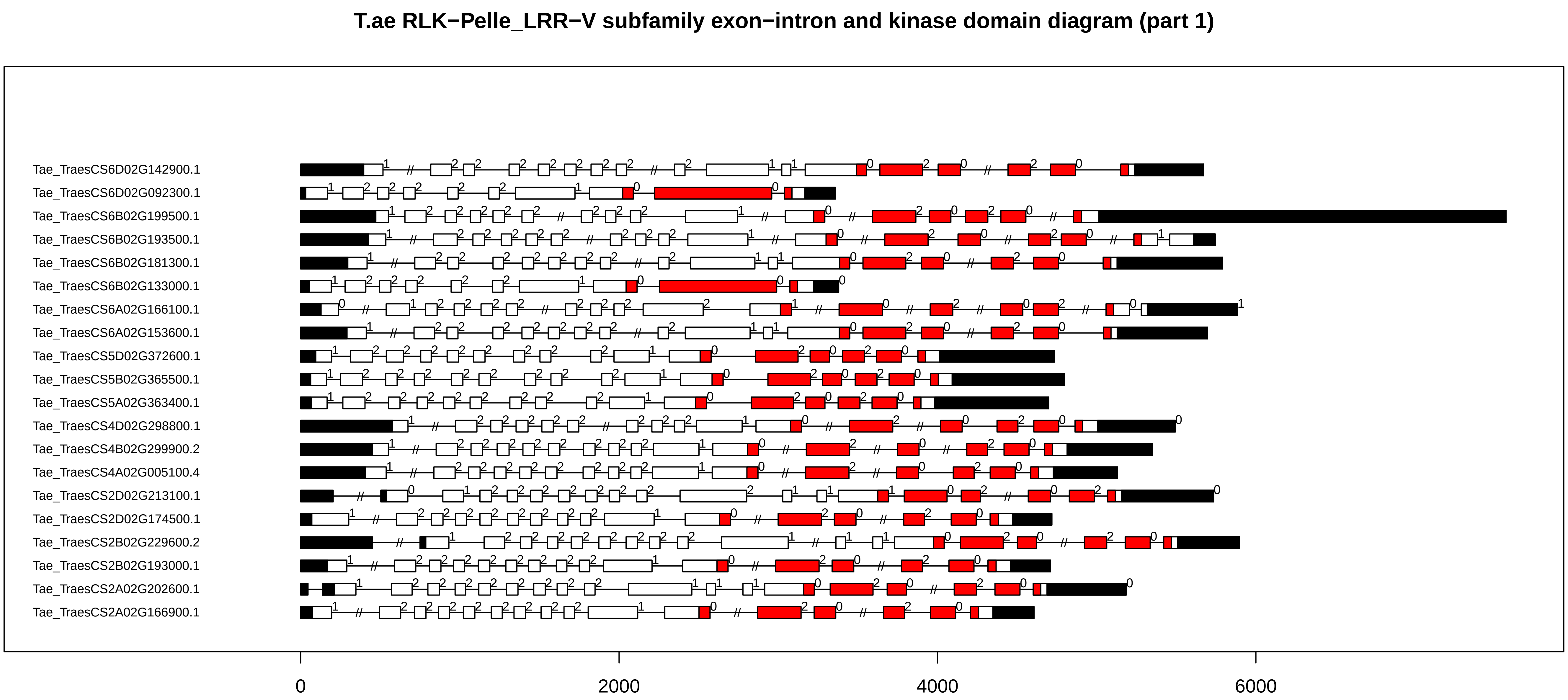
T.ae RLK−Pelle_LRR−V subfamily exon−intron and kinase domain diagram (part 1)
Tae_TraesCS6D02G142900.1
Tae_TraesCS6D02G092300.1
Tae_TraesCS6B02G199500.1
Tae_TraesCS6B02G193500.1
Tae_TraesCS6B02G181300.1
Tae_TraesCS6B02G133000.1
Tae_TraesCS6A02G166100.1
Tae_TraesCS6A02G153600.1
Tae_TraesCS5D02G372600.1
Tae_TraesCS5B02G365500.1
Tae_TraesCS5A02G363400.1
Tae_TraesCS4D02G298800.1
Tae_TraesCS4B02G299900.2
Tae_TraesCS4A02G005100.4
Tae_TraesCS2D02G213100.1
Tae_TraesCS2D02G174500.1
Tae_TraesCS2B02G229600.2
Tae_TraesCS2B02G193000.1
Tae_TraesCS2A02G202600.1
Tae_TraesCS2A02G166900.1
0
2000
4000
6000

T.ae RLK−Pelle_LRR−V subfamily exon−intron and kinase domain diagram (part 2)

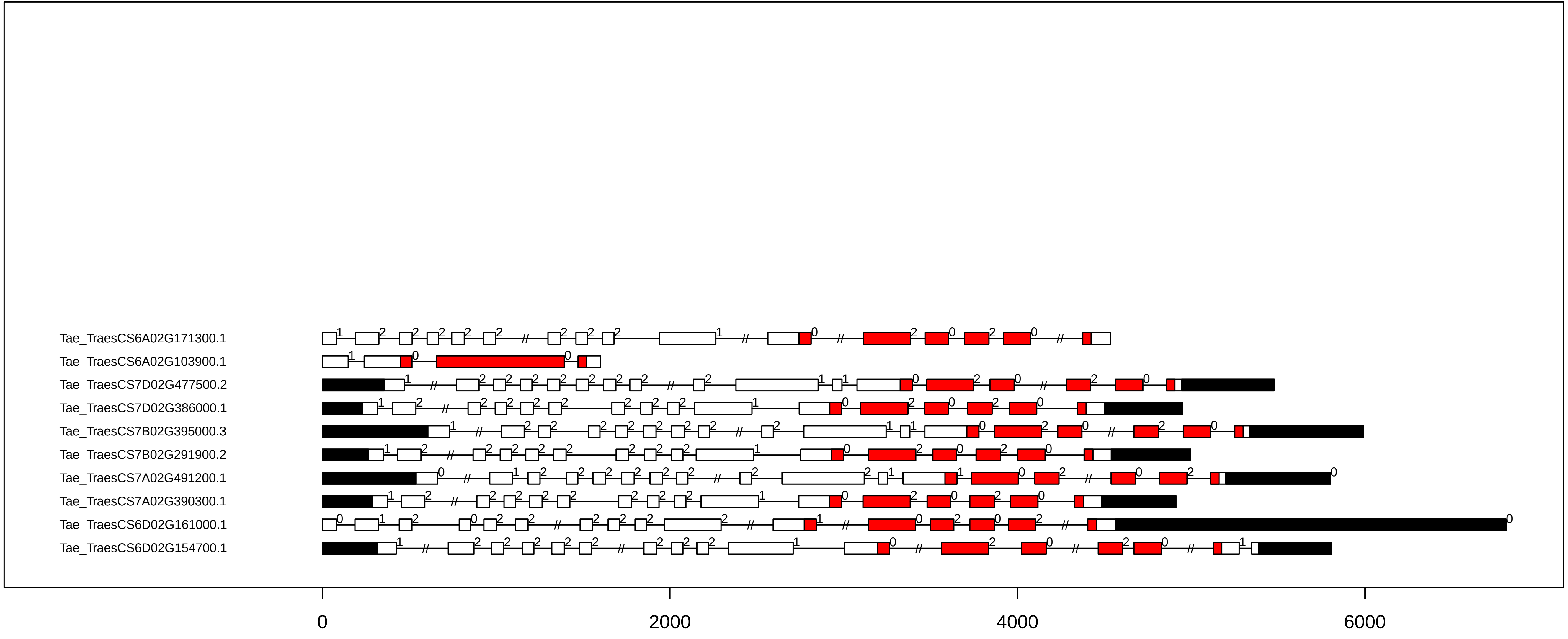

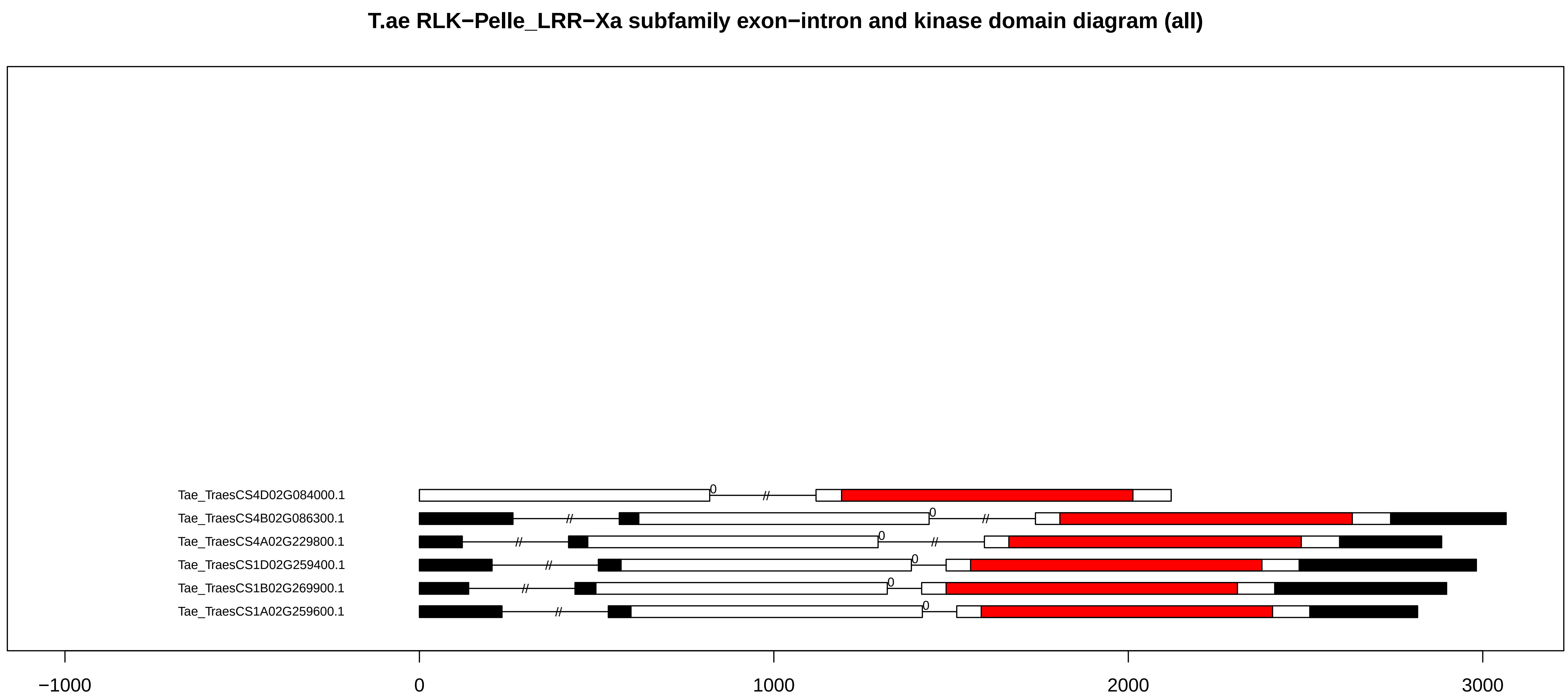
T.ae RLK−Pelle_LRR−Xa subfamily exon−intron and kinase domain diagram (all)
Tae_TraesCS4D02G084000.1
Tae_TraesCS4B02G086300.1
Tae_TraesCS4A02G229800.1
Tae_TraesCS1D02G259400.1
Tae_TraesCS1B02G269900.1
Tae_TraesCS1A02G259600.1
−1000
0
1000
2000
3000

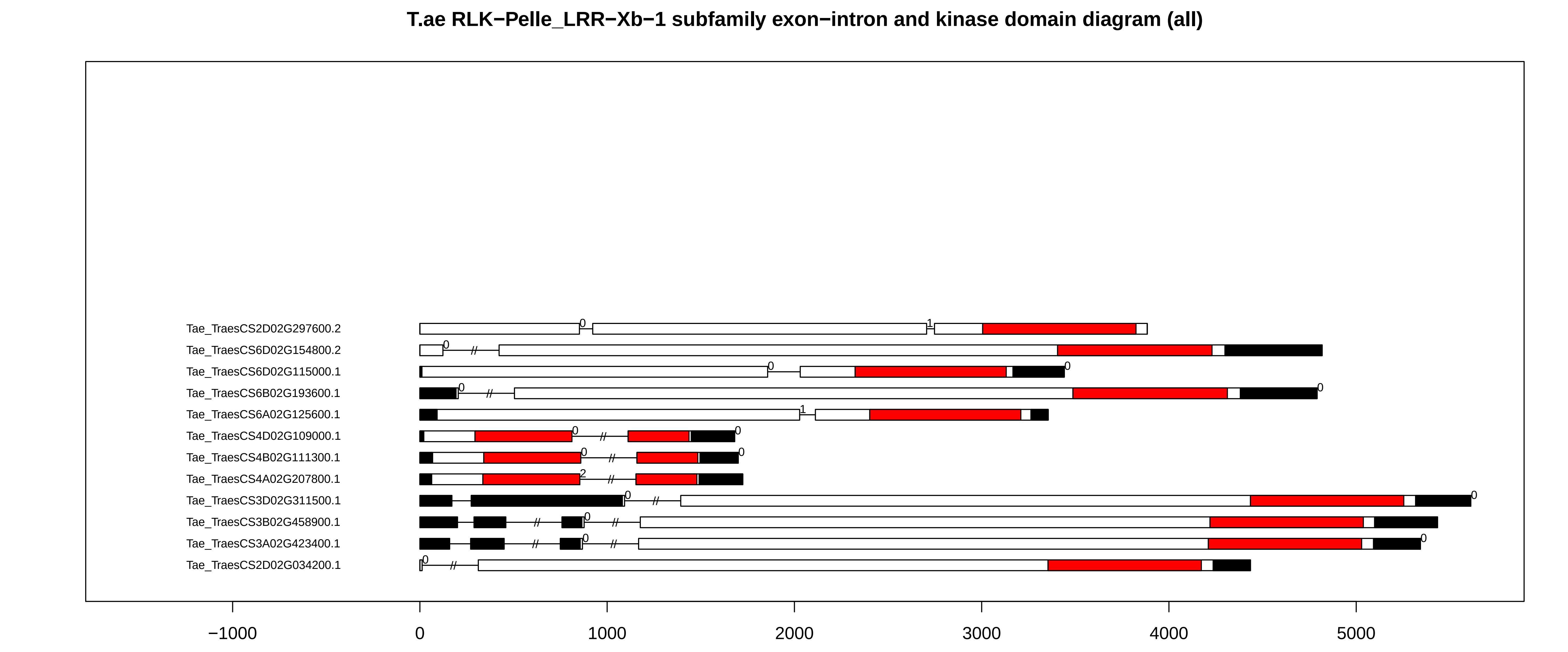
T.ae RLK−Pelle_LRR−Xb−1 subfamily exon−intron and kinase domain diagram (all)
Tae_TraesCS2D02G297600.2
Tae_TraesCS6D02G154800.2
Tae_TraesCS6D02G115000.1
Tae_TraesCS6B02G193600.1
Tae_TraesCS6A02G125600.1
Tae_TraesCS4D02G109000.1
Tae_TraesCS4B02G111300.1
Tae_TraesCS4A02G207800.1
Tae_TraesCS3D02G311500.1
Tae_TraesCS3B02G458900.1
Tae_TraesCS3A02G423400.1
Tae_TraesCS2D02G034200.1
−1000
0
1000
2000
3000
4000
5000

T.ae RLK−Pelle_LRR−Xb−2 subfamily exon−intron and kinase domain diagram (all)

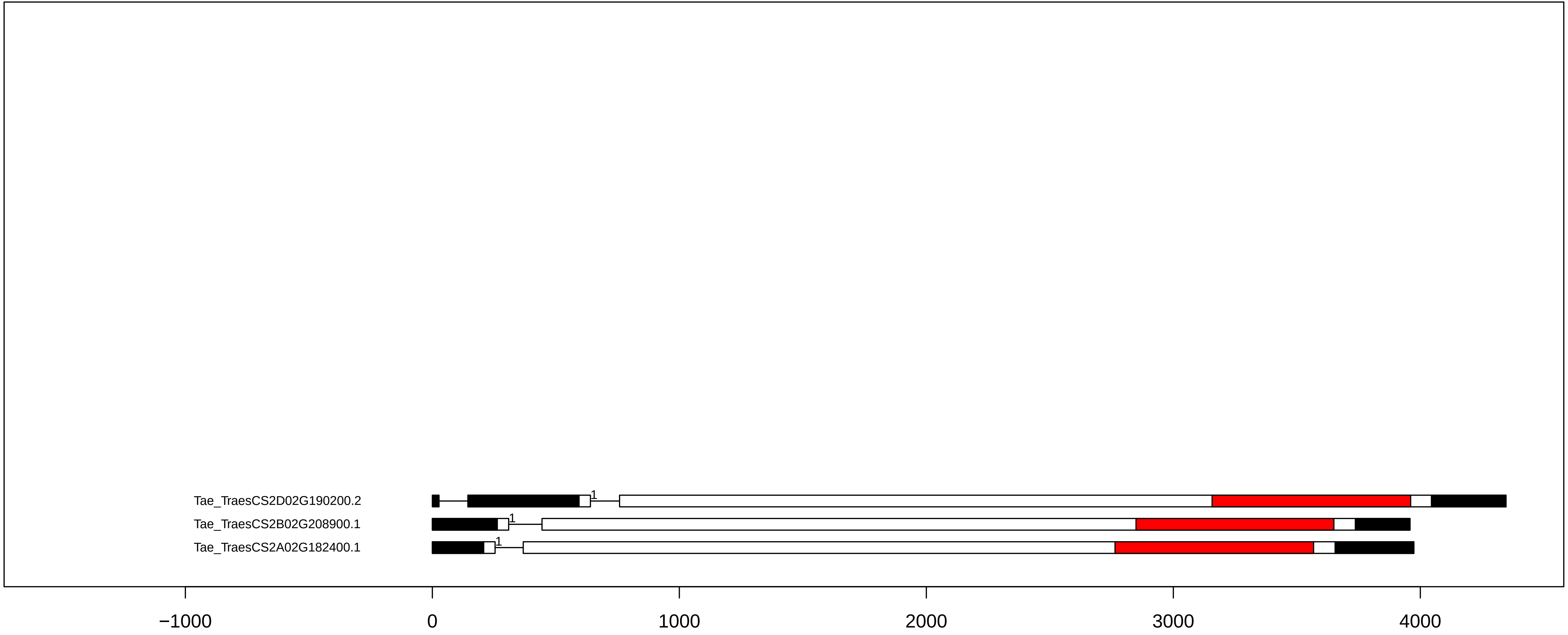

T.ae RLK−Pelle_LRR−XI−1 subfamily exon−intron and kinase domain diagram (part 1)

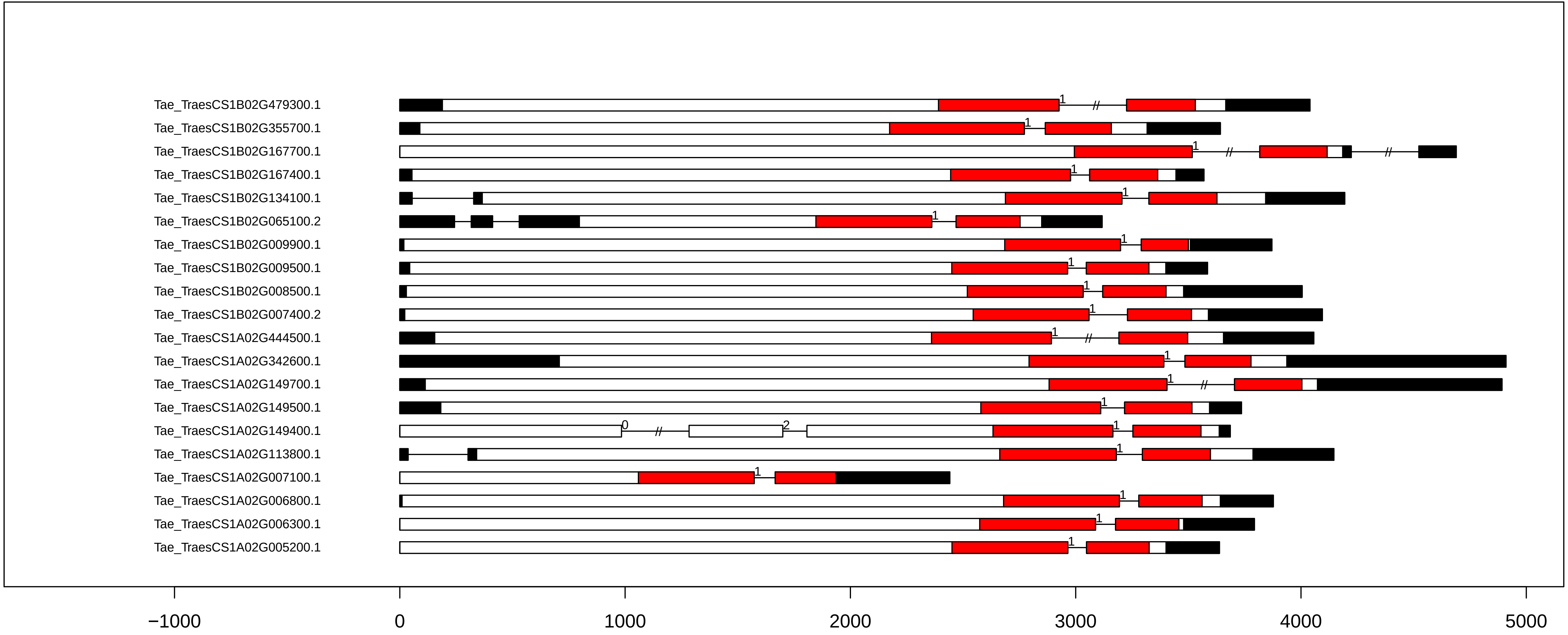

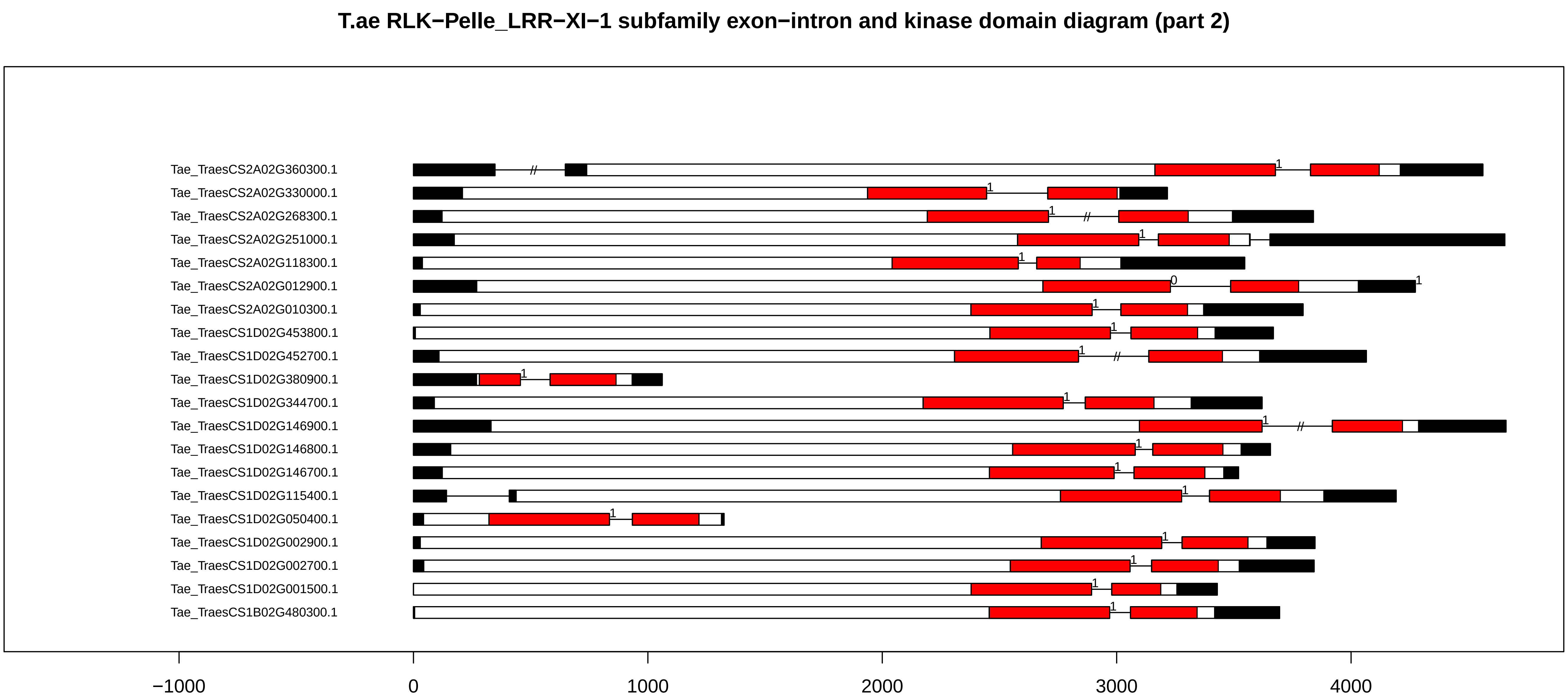
T.ae RLK−Pelle_LRR−XI−1 subfamily exon−intron and kinase domain diagram (part 2)
Tae_TraesCS2A02G360300.1
Tae_TraesCS2A02G330000.1
Tae_TraesCS2A02G268300.1
Tae_TraesCS2A02G251000.1
Tae_TraesCS2A02G118300.1
Tae_TraesCS2A02G012900.1
Tae_TraesCS2A02G010300.1
Tae_TraesCS1D02G453800.1
Tae_TraesCS1D02G452700.1
Tae_TraesCS1D02G380900.1
Tae_TraesCS1D02G344700.1
Tae_TraesCS1D02G146900.1
Tae_TraesCS1D02G146800.1
Tae_TraesCS1D02G146700.1
Tae_TraesCS1D02G115400.1
Tae_TraesCS1D02G050400.1
Tae_TraesCS1D02G002900.1
Tae_TraesCS1D02G002700.1
Tae_TraesCS1D02G001500.1
Tae_TraesCS1B02G480300.1
−1000
0
1000
2000
3000
4000

T.ae RLK−Pelle_LRR−XI−1 subfamily exon−intron and kinase domain diagram (part 3)

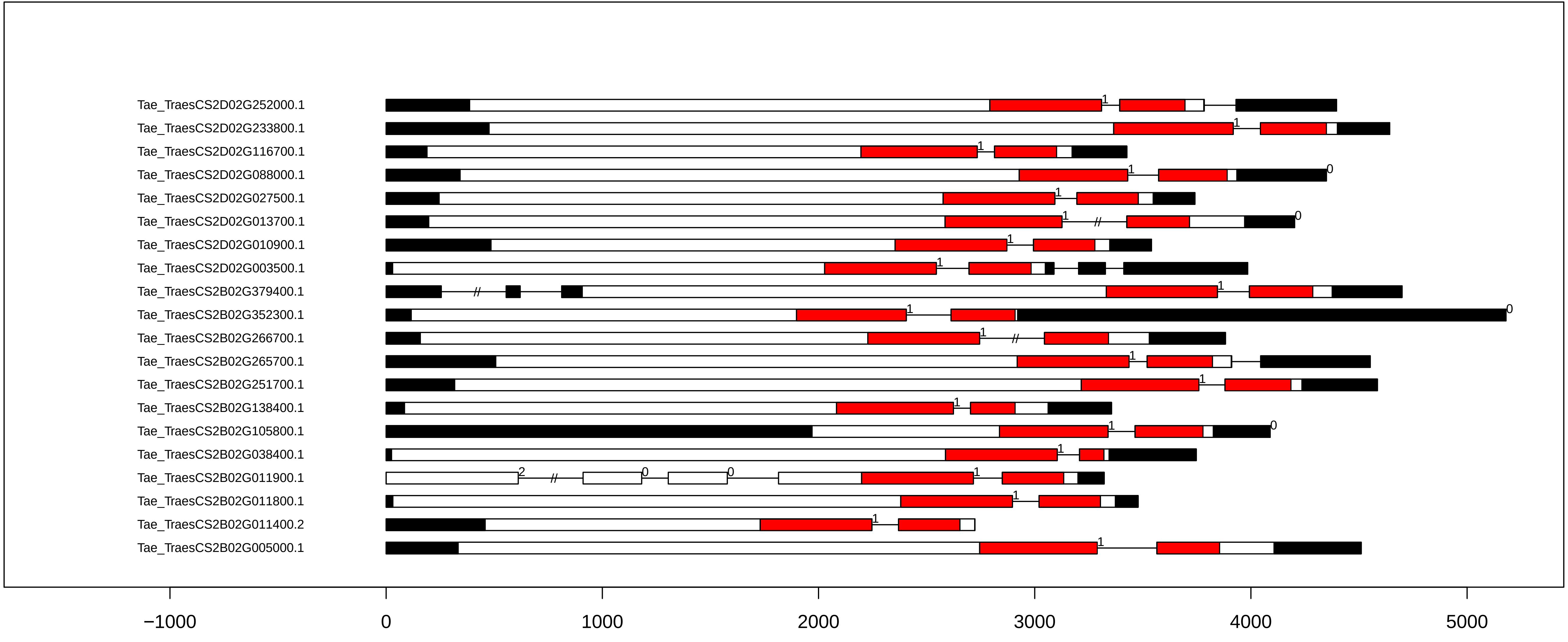

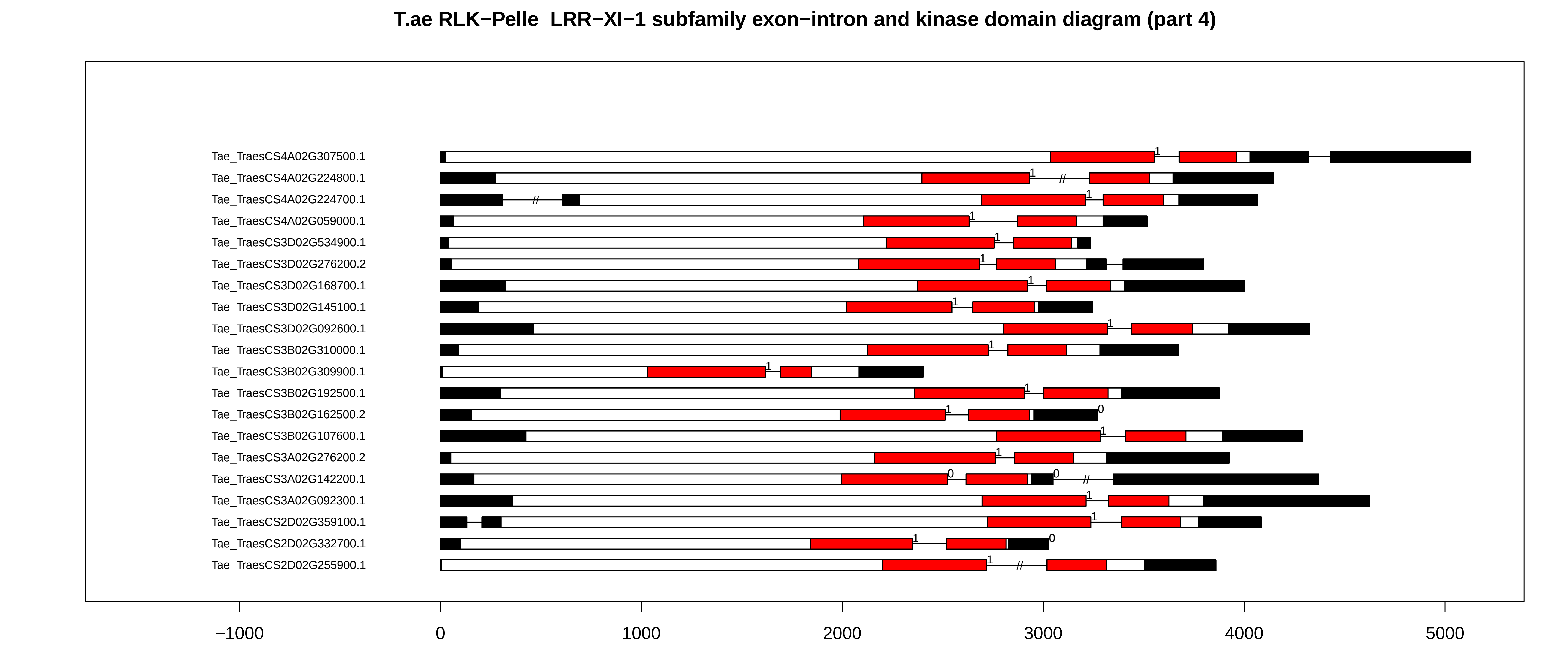

T.ae RLK−Pelle_LRR−XI−1 subfamily exon−intron and kinase domain diagram (part 4)
Tae_TraesCS4A02G307500.1
Tae_TraesCS4A02G224800.1
Tae_TraesCS4A02G224700.1
Tae_TraesCS4A02G059000.1
Tae_TraesCS3D02G534900.1
Tae_TraesCS3D02G276200.2
Tae_TraesCS3D02G168700.1
Tae_TraesCS3D02G145100.1
Tae_TraesCS3D02G092600.1
Tae_TraesCS3B02G310000.1
Tae_TraesCS3B02G309900.1
Tae_TraesCS3B02G192500.1
Tae_TraesCS3B02G162500.2
Tae_TraesCS3B02G107600.1
Tae_TraesCS3A02G276200.2
Tae_TraesCS3A02G142200.1
Tae_TraesCS3A02G092300.1
Tae_TraesCS2D02G359100.1
Tae_TraesCS2D02G332700.1
Tae_TraesCS2D02G255900.1
−1000
0
1000
2000
3000
4000
5000

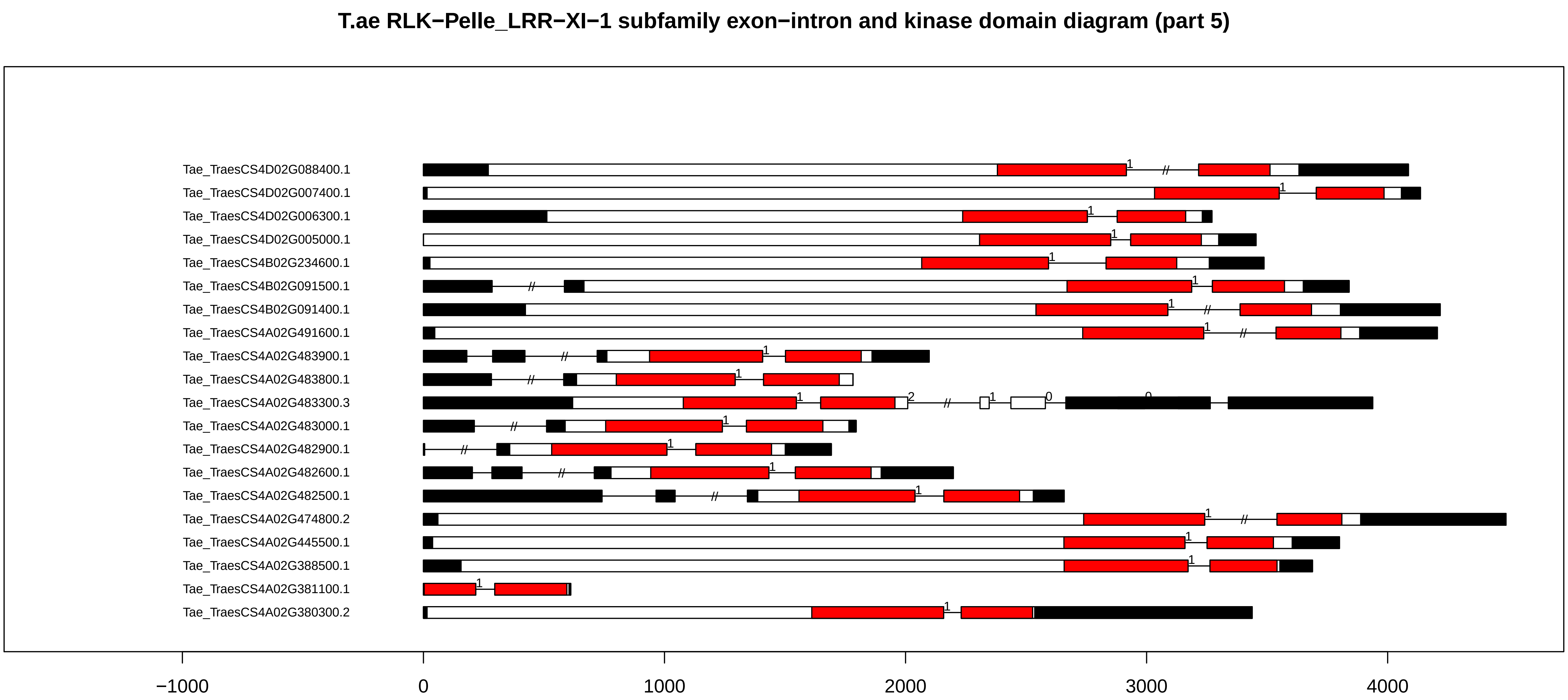

T.ae RLK−Pelle_LRR−XI−1 subfamily exon−intron and kinase domain diagram (part 5)
Tae_TraesCS4D02G088400.1
Tae_TraesCS4D02G007400.1
Tae_TraesCS4D02G006300.1
Tae_TraesCS4D02G005000.1
Tae_TraesCS4B02G234600.1
Tae_TraesCS4B02G091500.1
Tae_TraesCS4B02G091400.1
Tae_TraesCS4A02G491600.1
Tae_TraesCS4A02G483900.1
Tae_TraesCS4A02G483800.1
Tae_TraesCS4A02G483300.3
Tae_TraesCS4A02G483000.1
Tae_TraesCS4A02G482900.1
Tae_TraesCS4A02G482600.1
Tae_TraesCS4A02G482500.1
Tae_TraesCS4A02G474800.2
Tae_TraesCS4A02G445500.1
Tae_TraesCS4A02G388500.1
Tae_TraesCS4A02G381100.1
Tae_TraesCS4A02G380300.2
−1000
0
1000
2000
3000
4000

T.ae RLK−Pelle_LRR−XI−1 subfamily exon−intron and kinase domain diagram (part 6)

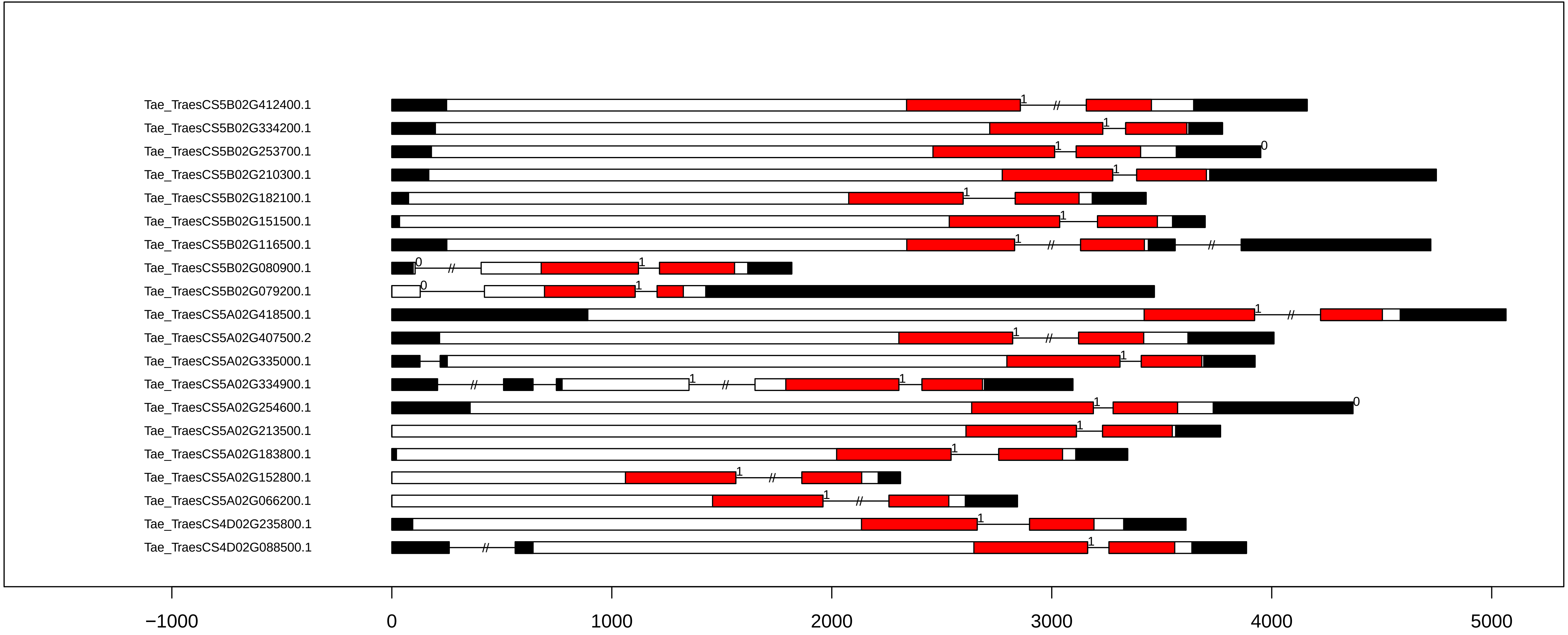

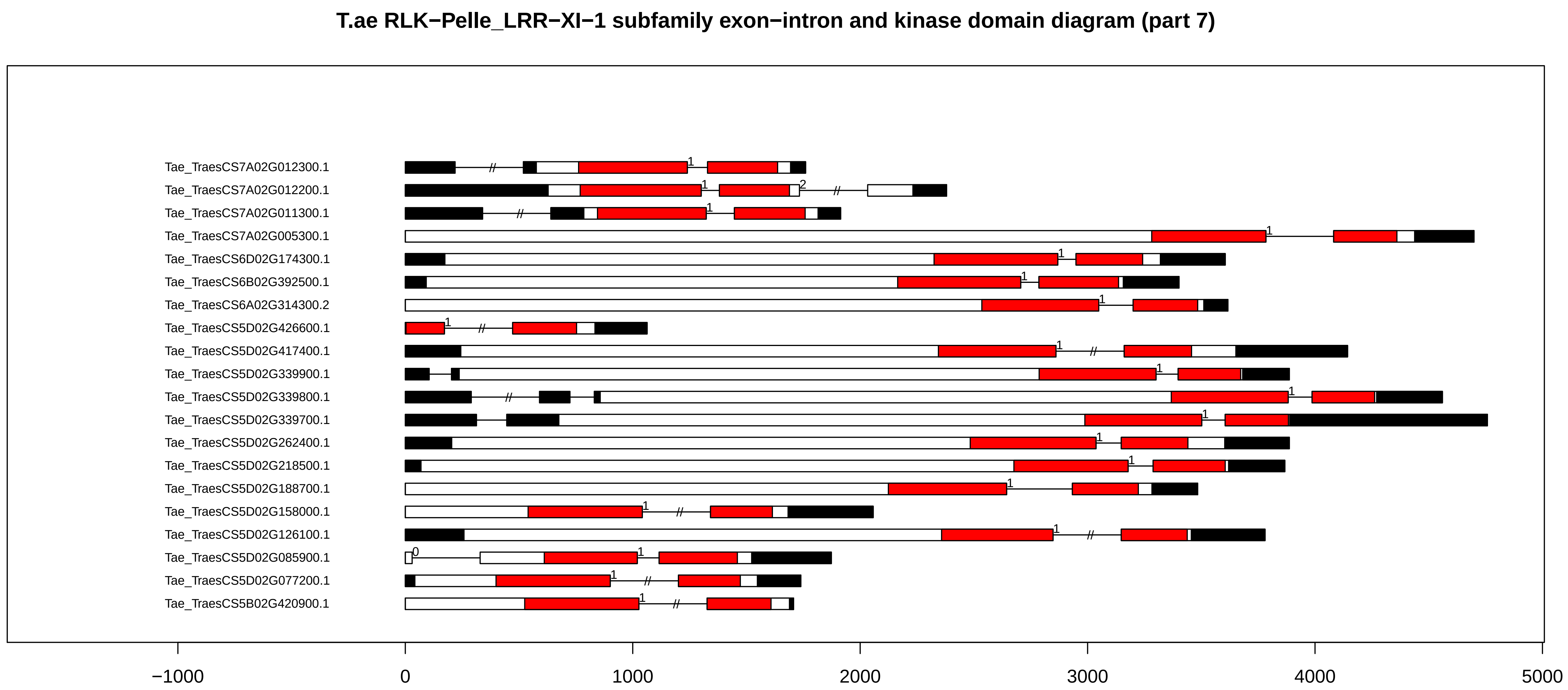

T.ae RLK−Pelle_LRR−XI−1 subfamily exon−intron and kinase domain diagram (part 7)
Tae_TraesCS7A02G012300.1
Tae_TraesCS7A02G012200.1
Tae_TraesCS7A02G011300.1
Tae_TraesCS7A02G005300.1
Tae_TraesCS6D02G174300.1
Tae_TraesCS6B02G392500.1
Tae_TraesCS6A02G314300.2
Tae_TraesCS5D02G426600.1
Tae_TraesCS5D02G417400.1
Tae_TraesCS5D02G339900.1
Tae_TraesCS5D02G339800.1
Tae_TraesCS5D02G339700.1
Tae_TraesCS5D02G262400.1
Tae_TraesCS5D02G218500.1
Tae_TraesCS5D02G188700.1
Tae_TraesCS5D02G158000.1
Tae_TraesCS5D02G126100.1
Tae_TraesCS5D02G085900.1
Tae_TraesCS5D02G077200.1
Tae_TraesCS5B02G420900.1
−1000
0
1000
2000
3000
4000
5000

T.ae RLK−Pelle_LRR−XI−1 subfamily exon−intron and kinase domain diagram (part 8)

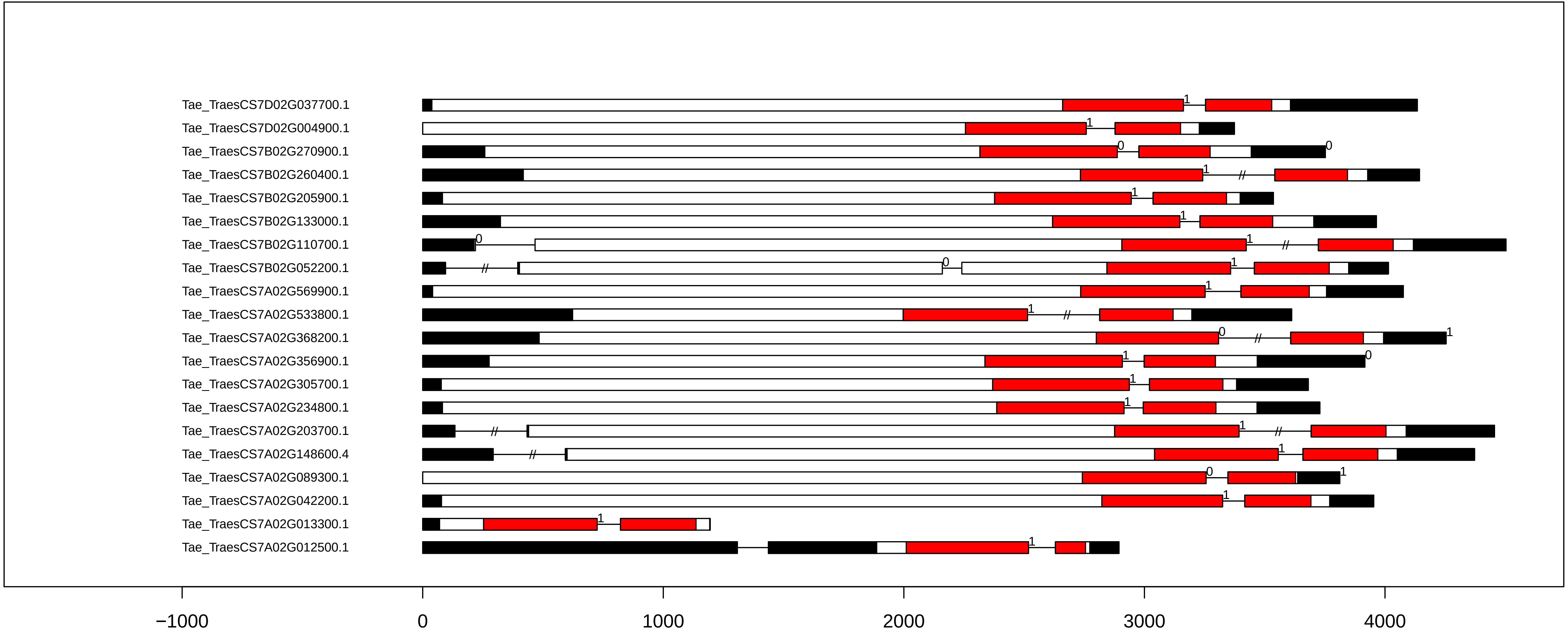

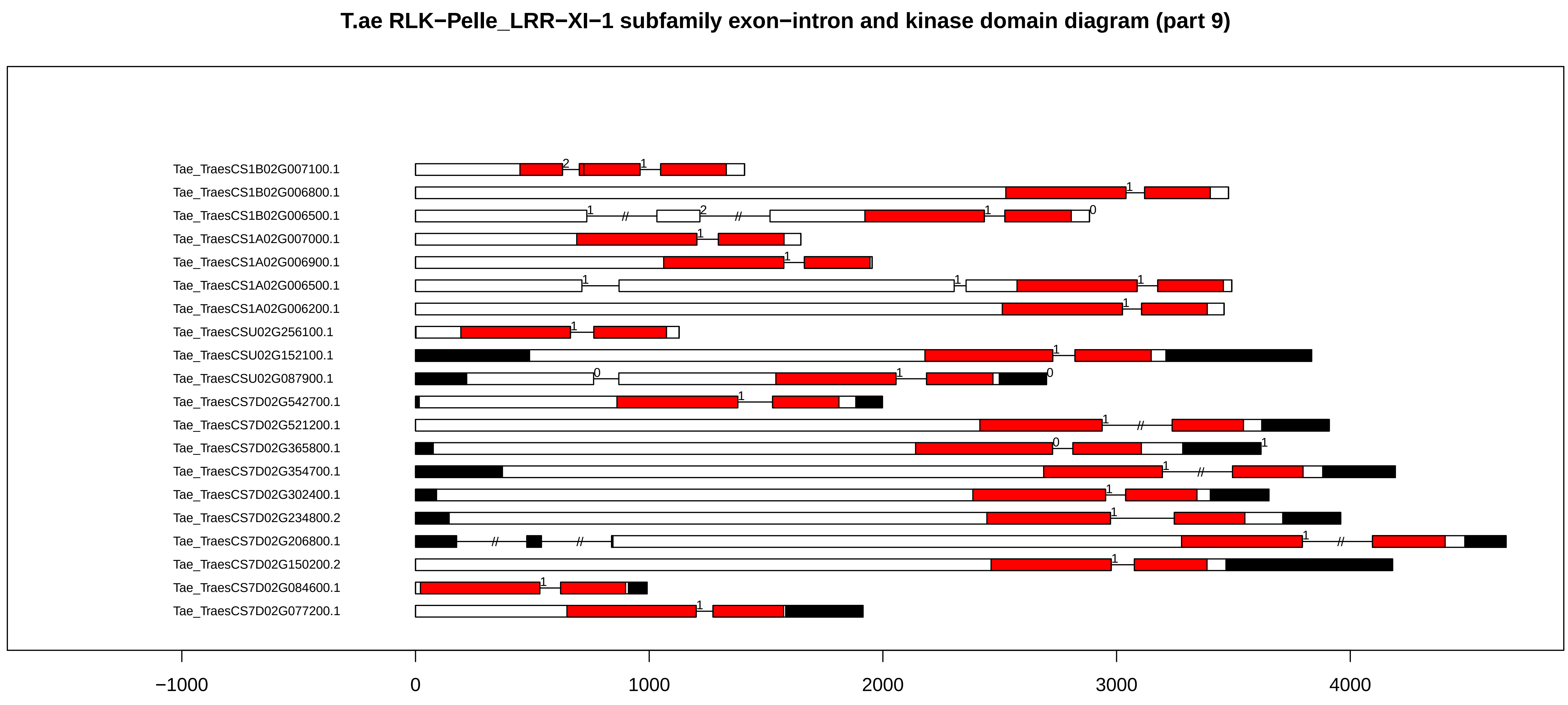

T.ae RLK−Pelle_LRR−XI−1 subfamily exon−intron and kinase domain diagram (part 9)
Tae_TraesCS1B02G007100.1
Tae_TraesCS1B02G006800.1
Tae_TraesCS1B02G006500.1
Tae_TraesCS1A02G007000.1
Tae_TraesCS1A02G006900.1
Tae_TraesCS1A02G006500.1
Tae_TraesCS1A02G006200.1
Tae_TraesCSU02G256100.1
Tae_TraesCSU02G152100.1
Tae_TraesCSU02G087900.1
Tae_TraesCS7D02G542700.1
Tae_TraesCS7D02G521200.1
Tae_TraesCS7D02G365800.1
Tae_TraesCS7D02G354700.1
Tae_TraesCS7D02G302400.1
Tae_TraesCS7D02G234800.2
Tae_TraesCS7D02G206800.1
Tae_TraesCS7D02G150200.2
Tae_TraesCS7D02G084600.1
Tae_TraesCS7D02G077200.1
−1000
0
1000
2000
3000
4000

T.ae RLK−Pelle_LRR−XI−1 subfamily exon−intron and kinase domain diagram (part 10)

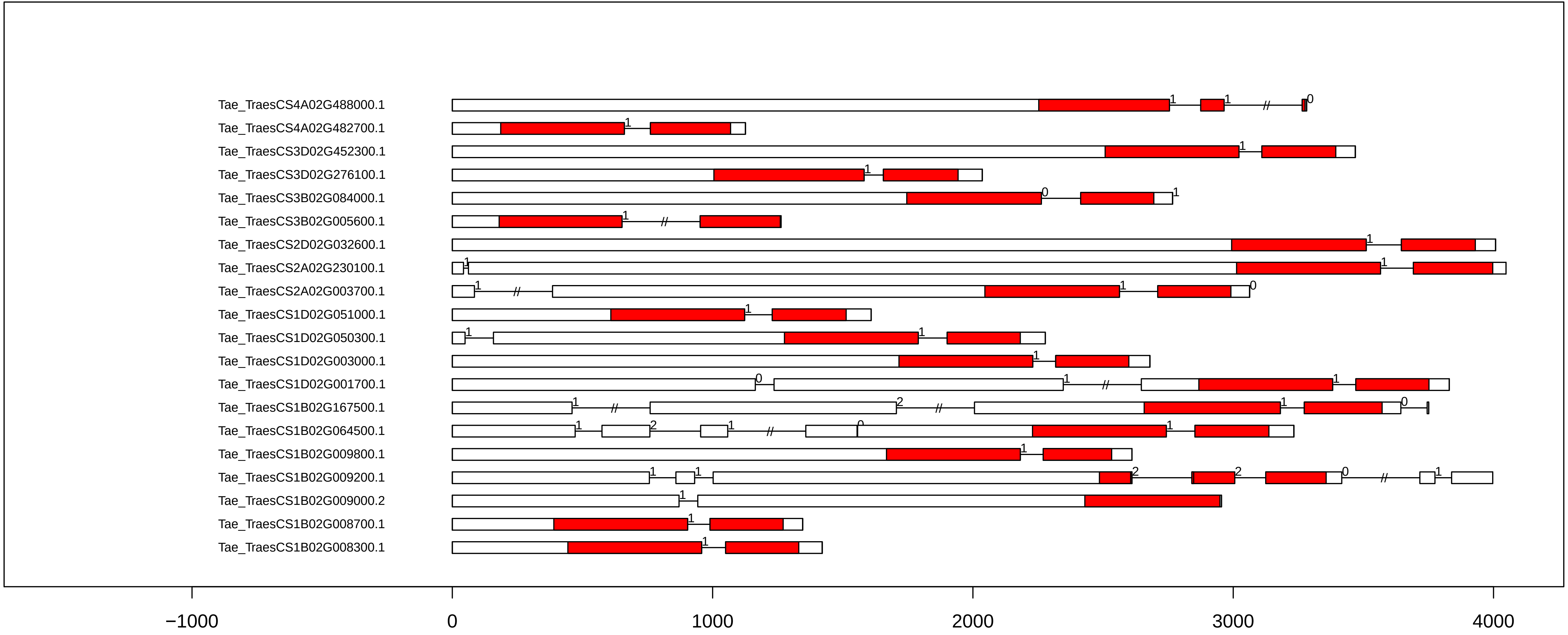

T.ae RLK−Pelle_LRR−XI−1 subfamily exon−intron and kinase domain diagram (part 11)

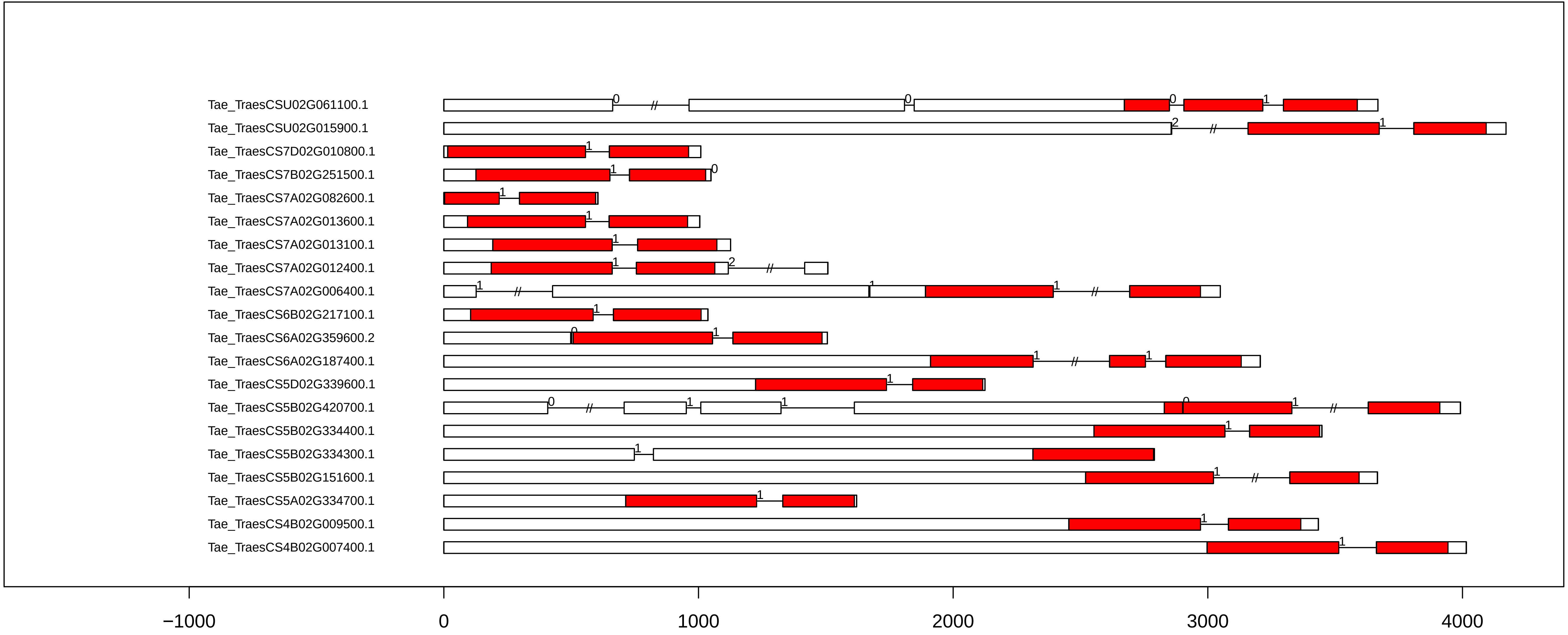

T.ae RLK−Pelle_LRR−XI−1 subfamily exon−intron and kinase domain diagram (part 12)

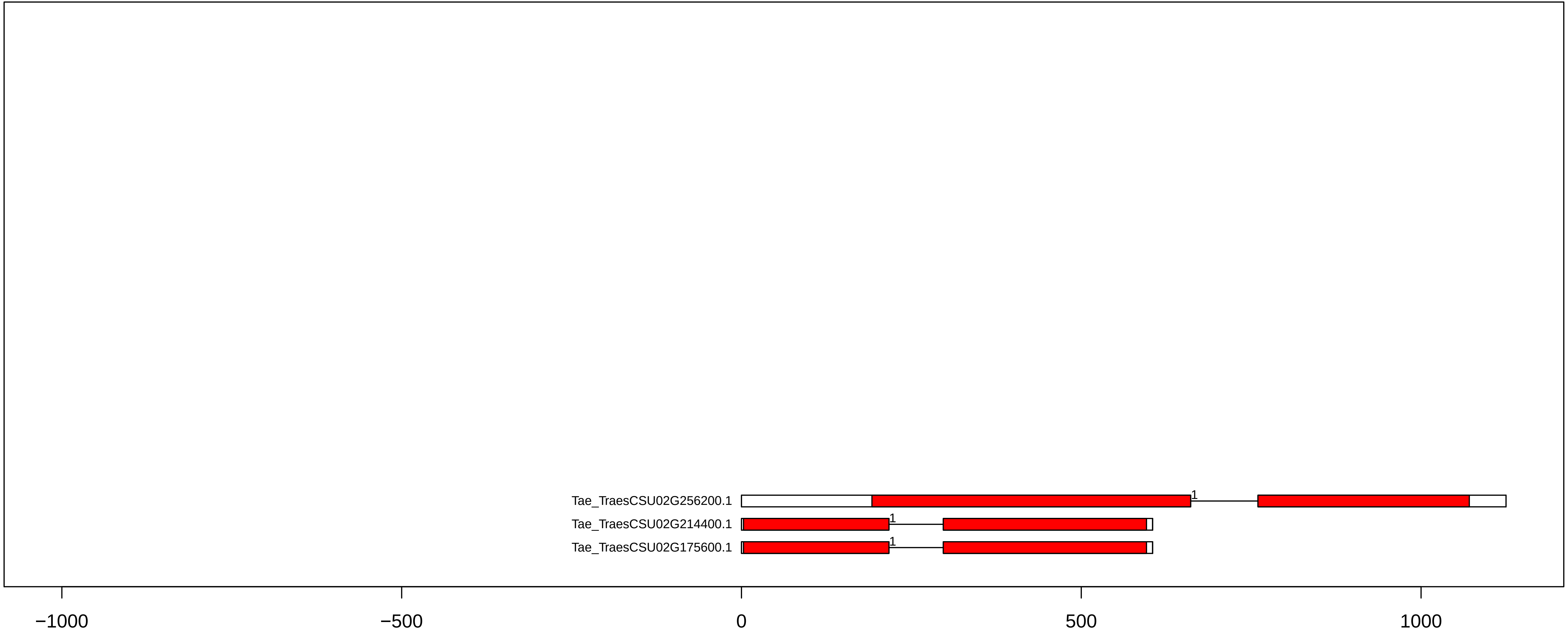

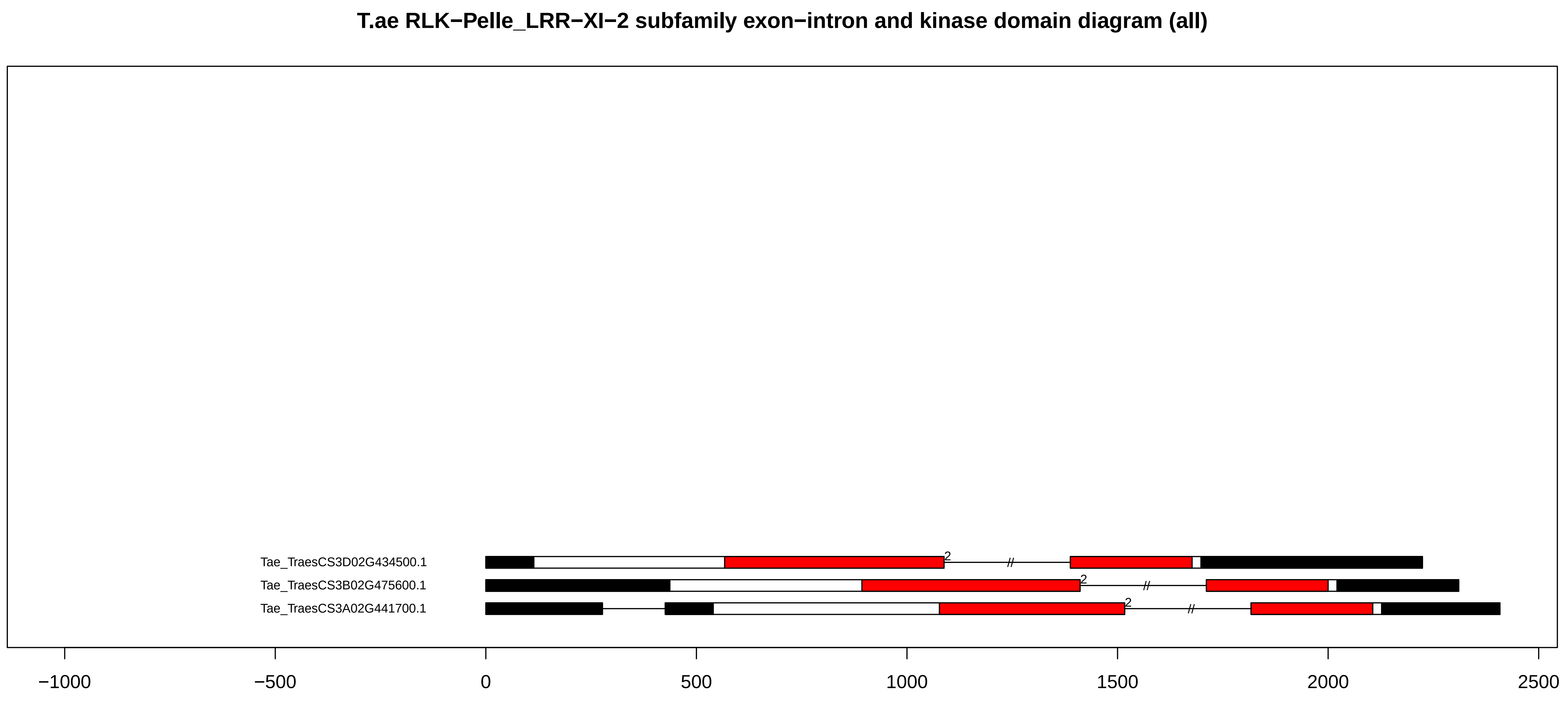

T.ae RLK−Pelle_LRR−XI−2 subfamily exon−intron and kinase domain diagram (all)
Tae_TraesCS3D02G434500.1
Tae_TraesCS3B02G475600.1
Tae_TraesCS3A02G441700.1
2
2
2
−1000
−500
0
500
1000
1500
2000
2500

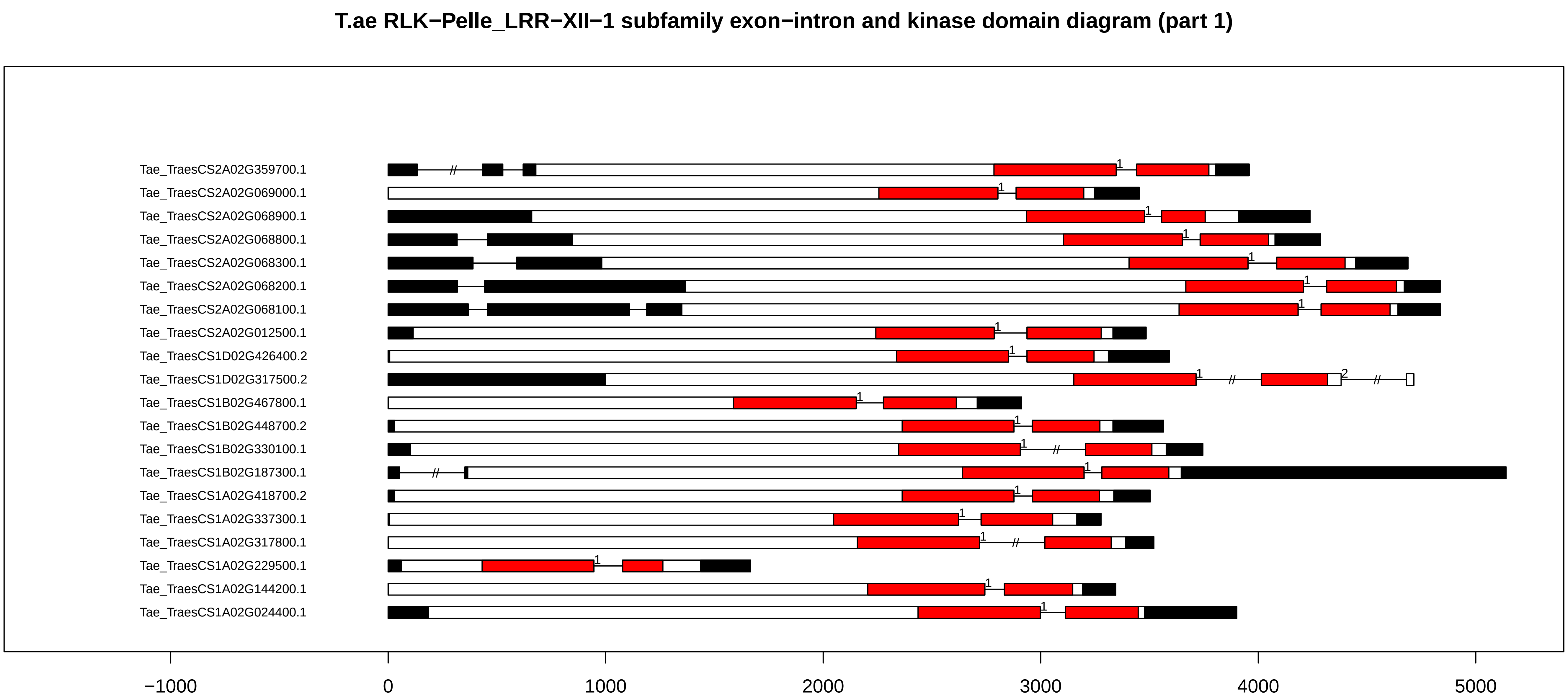
T.ae RLK−Pelle_LRR−XII−1 subfamily exon−intron and kinase domain diagram (part 1)
Tae_TraesCS2A02G359700.1
Tae_TraesCS2A02G069000.1
Tae_TraesCS2A02G068900.1
Tae_TraesCS2A02G068800.1
Tae_TraesCS2A02G068300.1
Tae_TraesCS2A02G068200.1
Tae_TraesCS2A02G068100.1
Tae_TraesCS2A02G012500.1
Tae_TraesCS1D02G426400.2
Tae_TraesCS1D02G317500.2
Tae_TraesCS1B02G467800.1
Tae_TraesCS1B02G448700.2
Tae_TraesCS1B02G330100.1
Tae_TraesCS1B02G187300.1
Tae_TraesCS1A02G418700.2
Tae_TraesCS1A02G337300.1
Tae_TraesCS1A02G317800.1
Tae_TraesCS1A02G229500.1
Tae_TraesCS1A02G144200.1
Tae_TraesCS1A02G024400.1
−1000
0
1000
2000
3000
4000
5000

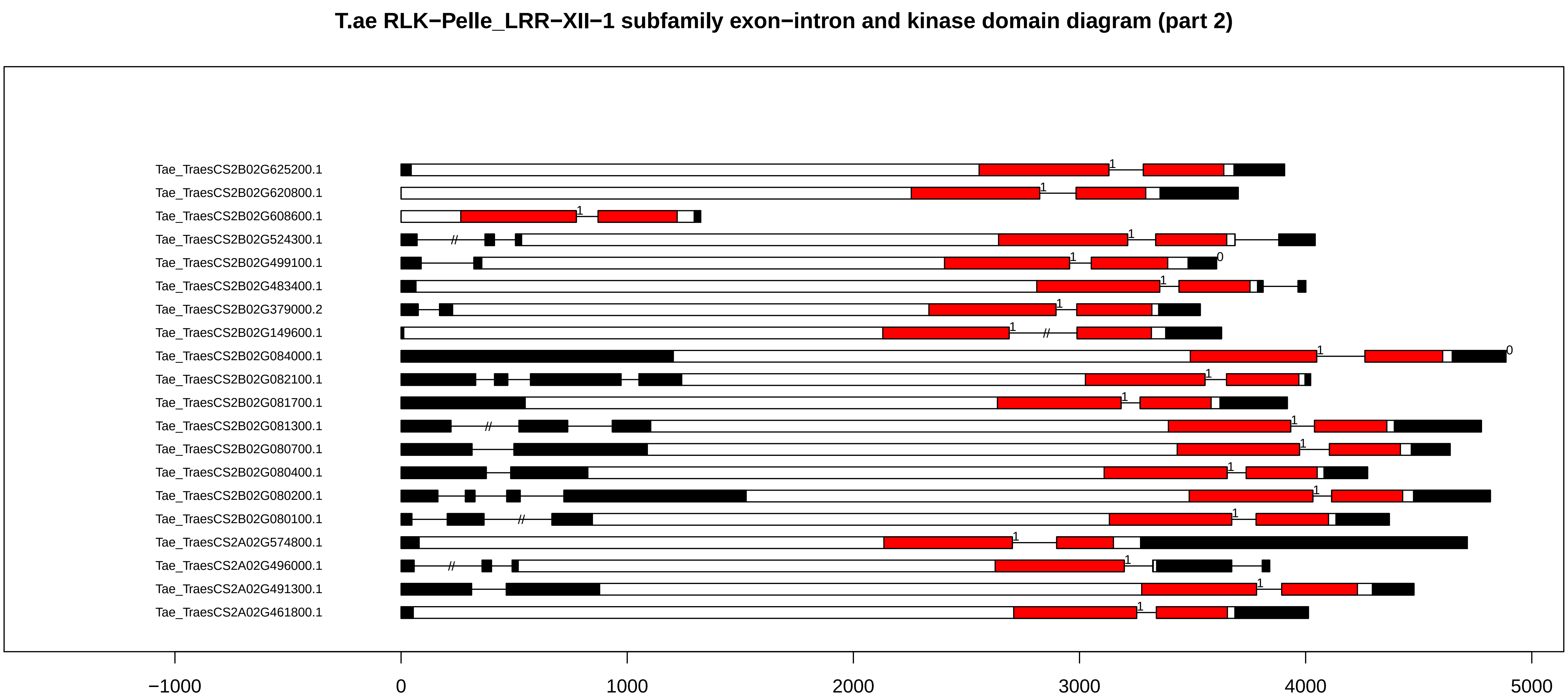

T.ae RLK−Pelle_LRR−XII−1 subfamily exon−intron and kinase domain diagram (part 2)
Tae_TraesCS2B02G625200.1
Tae_TraesCS2B02G620800.1
Tae_TraesCS2B02G608600.1
Tae_TraesCS2B02G524300.1
Tae_TraesCS2B02G499100.1
Tae_TraesCS2B02G483400.1
Tae_TraesCS2B02G379000.2
Tae_TraesCS2B02G149600.1
Tae_TraesCS2B02G084000.1
Tae_TraesCS2B02G082100.1
Tae_TraesCS2B02G081700.1
Tae_TraesCS2B02G081300.1
Tae_TraesCS2B02G080700.1
Tae_TraesCS2B02G080400.1
Tae_TraesCS2B02G080200.1
Tae_TraesCS2B02G080100.1
Tae_TraesCS2A02G574800.1
Tae_TraesCS2A02G496000.1
Tae_TraesCS2A02G491300.1
Tae_TraesCS2A02G461800.1
−1000
0
1000
2000
3000
4000
5000

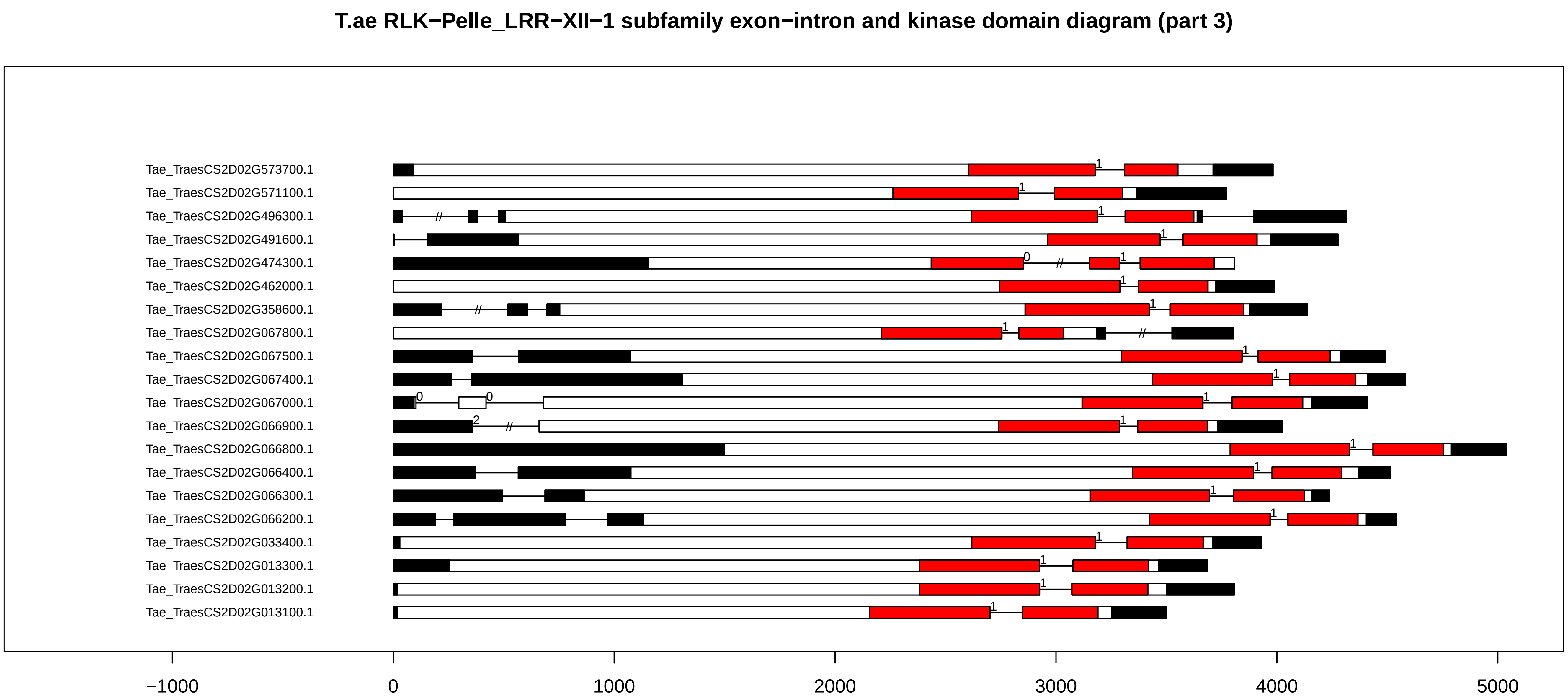

T.ae RLK−Pelle_LRR−XII−1 subfamily exon−intron and kinase domain diagram (part 3)
Tae_TraesCS2D02G573700.1
Tae_TraesCS2D02G571100.1
Tae_TraesCS2D02G496300.1
Tae_TraesCS2D02G491600.1
Tae_TraesCS2D02G474300.1
Tae_TraesCS2D02G462000.1
Tae_TraesCS2D02G358600.1
Tae_TraesCS2D02G067800.1
Tae_TraesCS2D02G067500.1
Tae_TraesCS2D02G067400.1
Tae_TraesCS2D02G067000.1
Tae_TraesCS2D02G066900.1
Tae_TraesCS2D02G066800.1
Tae_TraesCS2D02G066400.1
Tae_TraesCS2D02G066300.1
Tae_TraesCS2D02G066200.1
Tae_TraesCS2D02G033400.1
Tae_TraesCS2D02G013300.1
Tae_TraesCS2D02G013200.1
Tae_TraesCS2D02G013100.1
−1000
0
1000
2000
3000
4000
5000

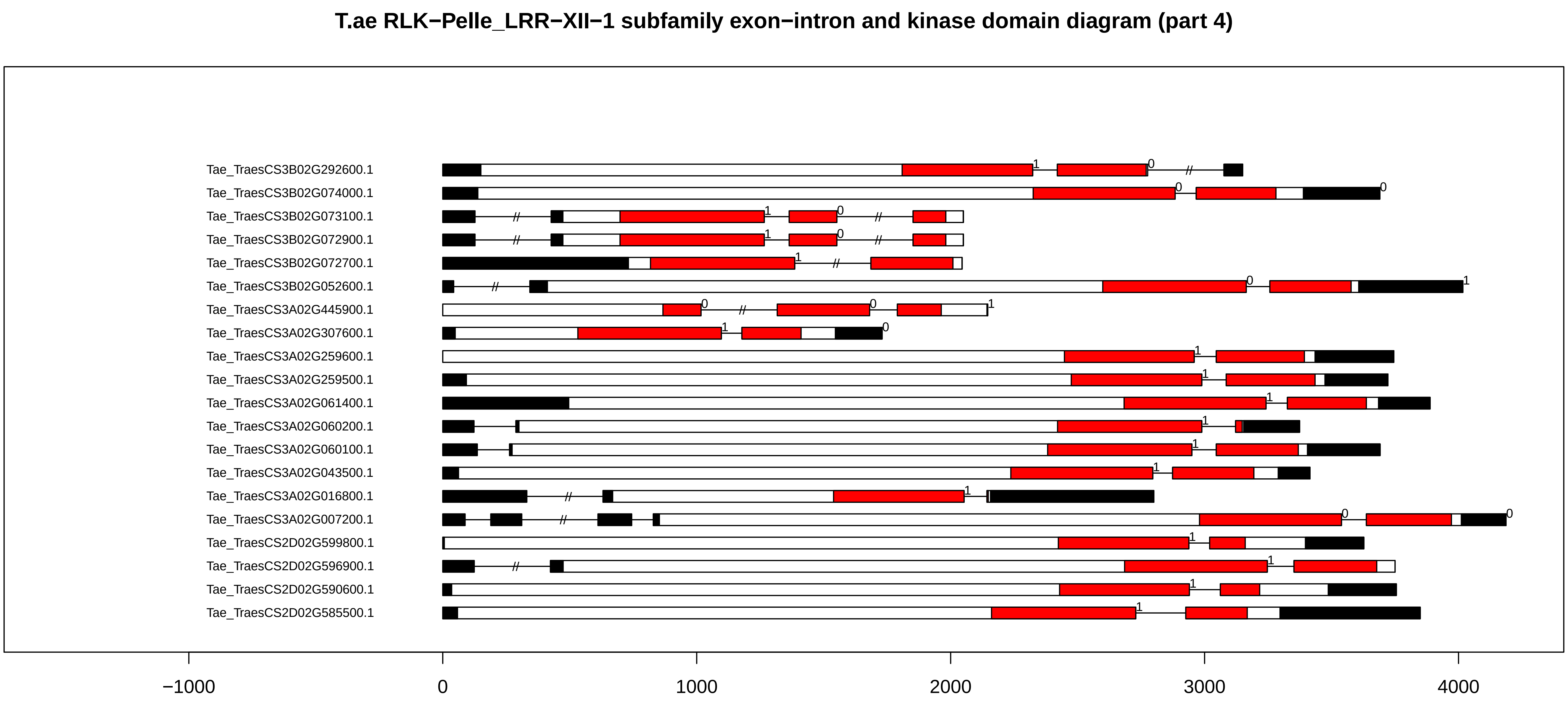

T.ae RLK−Pelle_LRR−XII−1 subfamily exon−intron and kinase domain diagram (part 4)
Tae_TraesCS3B02G292600.1
Tae_TraesCS3B02G074000.1
Tae_TraesCS3B02G073100.1
Tae_TraesCS3B02G072900.1
Tae_TraesCS3B02G072700.1
Tae_TraesCS3B02G052600.1
Tae_TraesCS3A02G445900.1
Tae_TraesCS3A02G307600.1
Tae_TraesCS3A02G259600.1
Tae_TraesCS3A02G259500.1
Tae_TraesCS3A02G061400.1
Tae_TraesCS3A02G060200.1
Tae_TraesCS3A02G060100.1
Tae_TraesCS3A02G043500.1
Tae_TraesCS3A02G016800.1
Tae_TraesCS3A02G007200.1
Tae_TraesCS2D02G599800.1
Tae_TraesCS2D02G596900.1
Tae_TraesCS2D02G590600.1
Tae_TraesCS2D02G585500.1
−1000
0
1000
2000
3000
4000

T.ae RLK−Pelle_LRR−XII−1 subfamily exon−intron and kinase domain diagram (part 5)

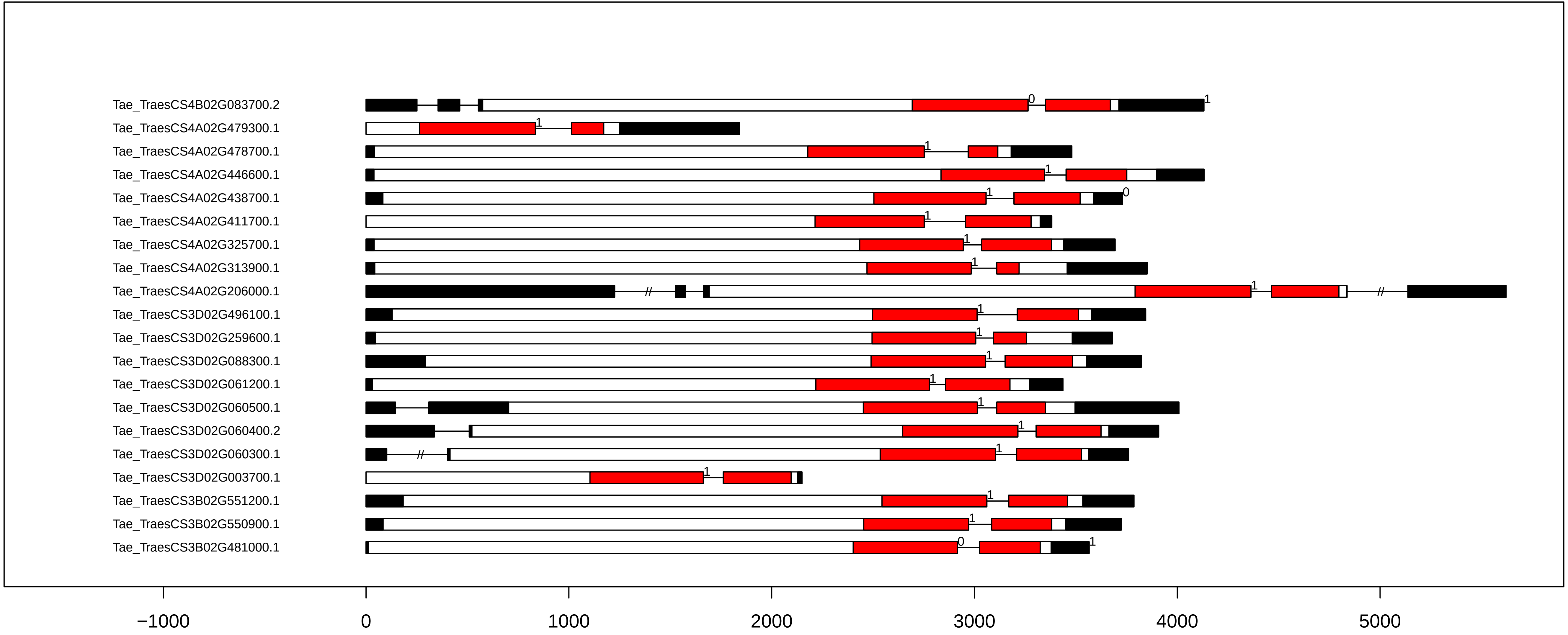

T.ae RLK−Pelle_LRR−XII−1 subfamily exon−intron and kinase domain diagram (part 6)

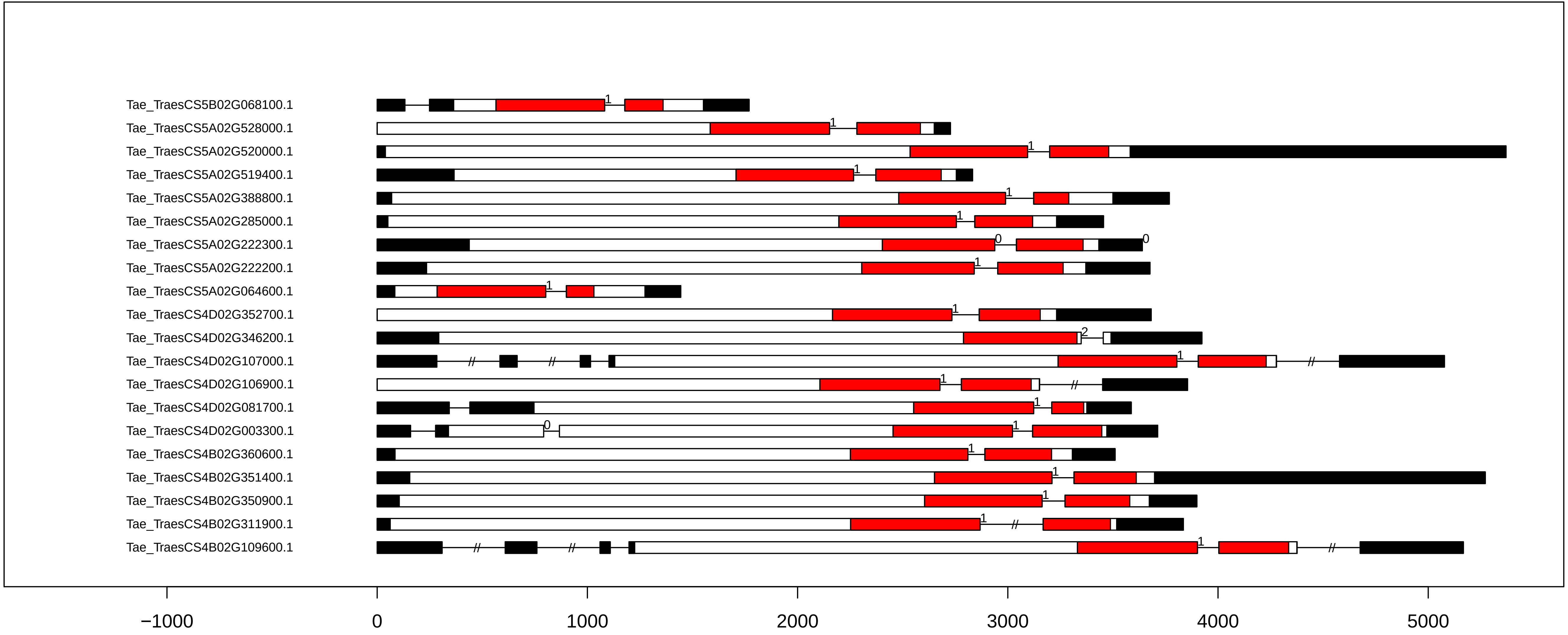

T.ae RLK−Pelle_LRR−XII−1 subfamily exon−intron and kinase domain diagram (part 7)

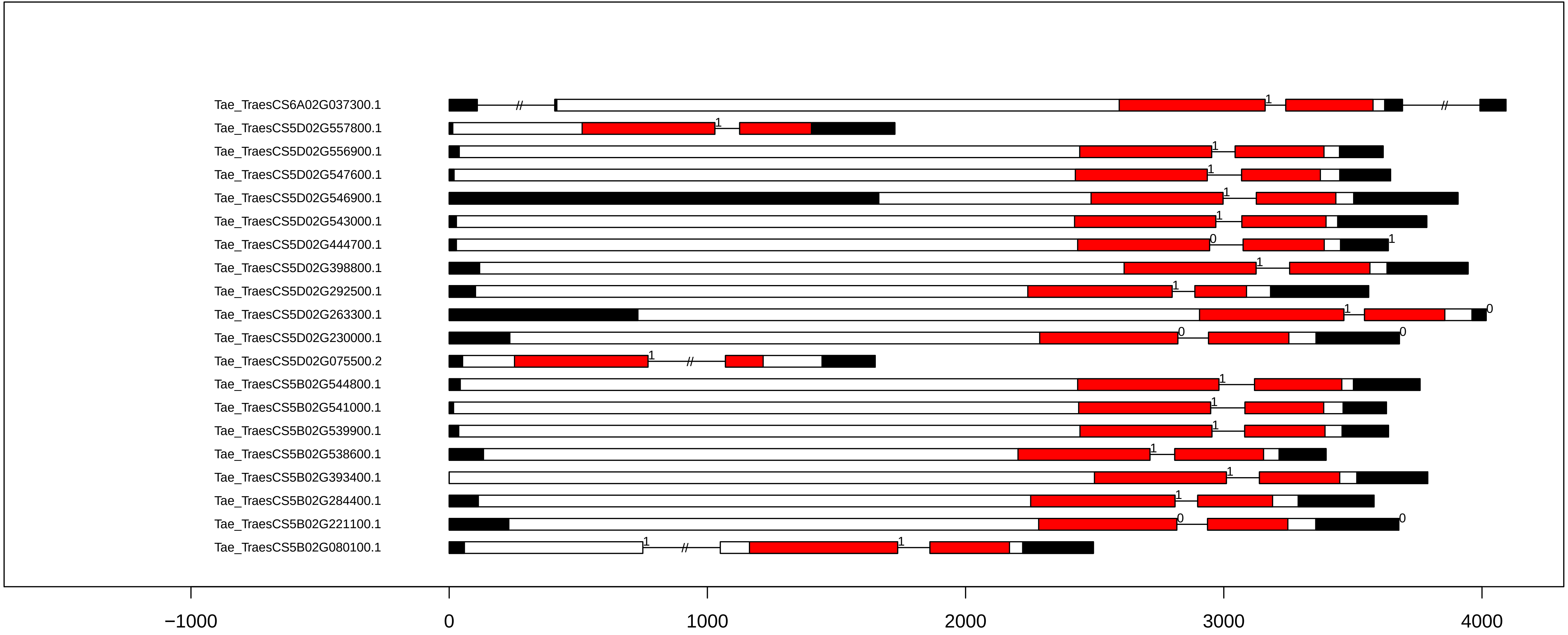

T.ae RLK−Pelle_LRR−XII−1 subfamily exon−intron and kinase domain diagram (part 8)

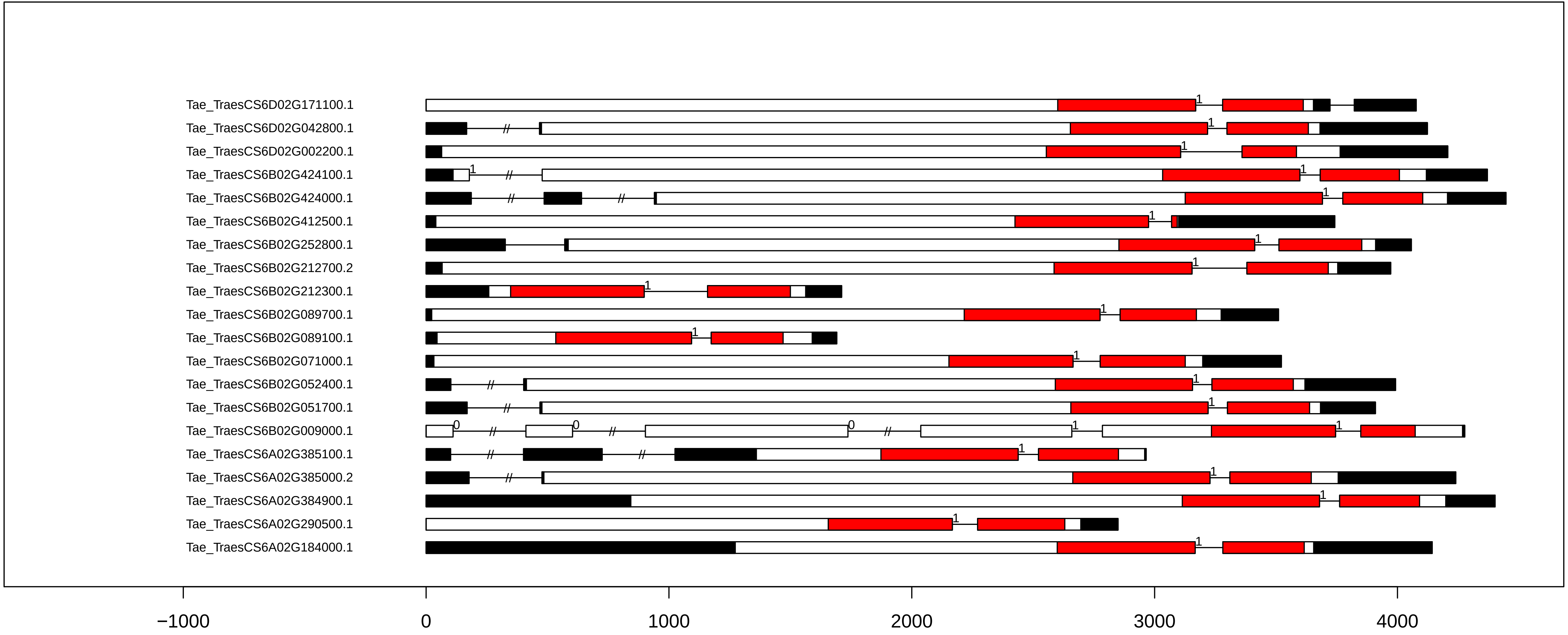

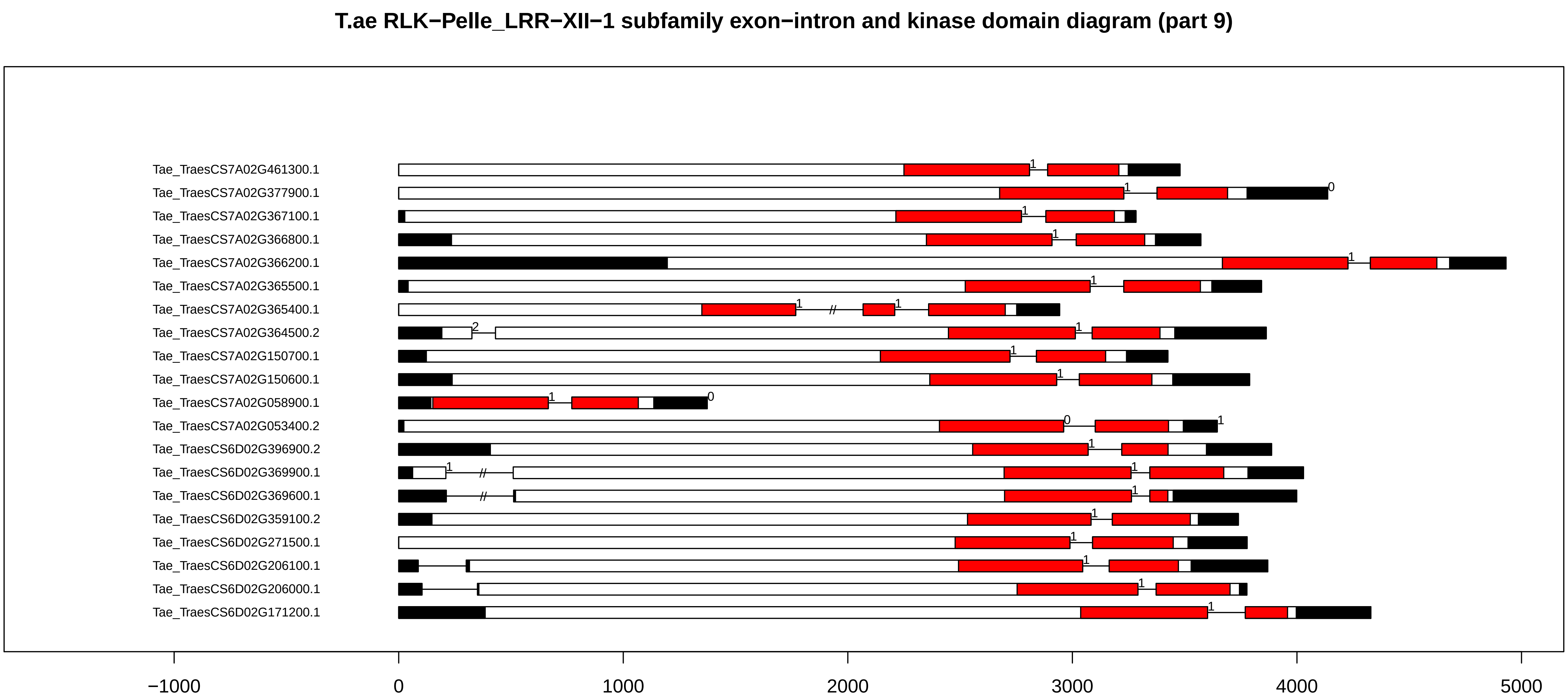
T.ae RLK−Pelle_LRR−XII−1 subfamily exon−intron and kinase domain diagram (part 9)
Tae_TraesCS7A02G461300.1
Tae_TraesCS7A02G377900.1
Tae_TraesCS7A02G367100.1
Tae_TraesCS7A02G366800.1
Tae_TraesCS7A02G366200.1
Tae_TraesCS7A02G365500.1
Tae_TraesCS7A02G365400.1
Tae_TraesCS7A02G364500.2
Tae_TraesCS7A02G150700.1
Tae_TraesCS7A02G150600.1
Tae_TraesCS7A02G058900.1
Tae_TraesCS7A02G053400.2
Tae_TraesCS6D02G396900.2
Tae_TraesCS6D02G369900.1
Tae_TraesCS6D02G369600.1
Tae_TraesCS6D02G359100.2
Tae_TraesCS6D02G271500.1
Tae_TraesCS6D02G206100.1
Tae_TraesCS6D02G206000.1
Tae_TraesCS6D02G171200.1
−1000
0
1000
2000
3000
4000
5000

T.ae RLK−Pelle_LRR−XII−1 subfamily exon−intron and kinase domain diagram (part 10)

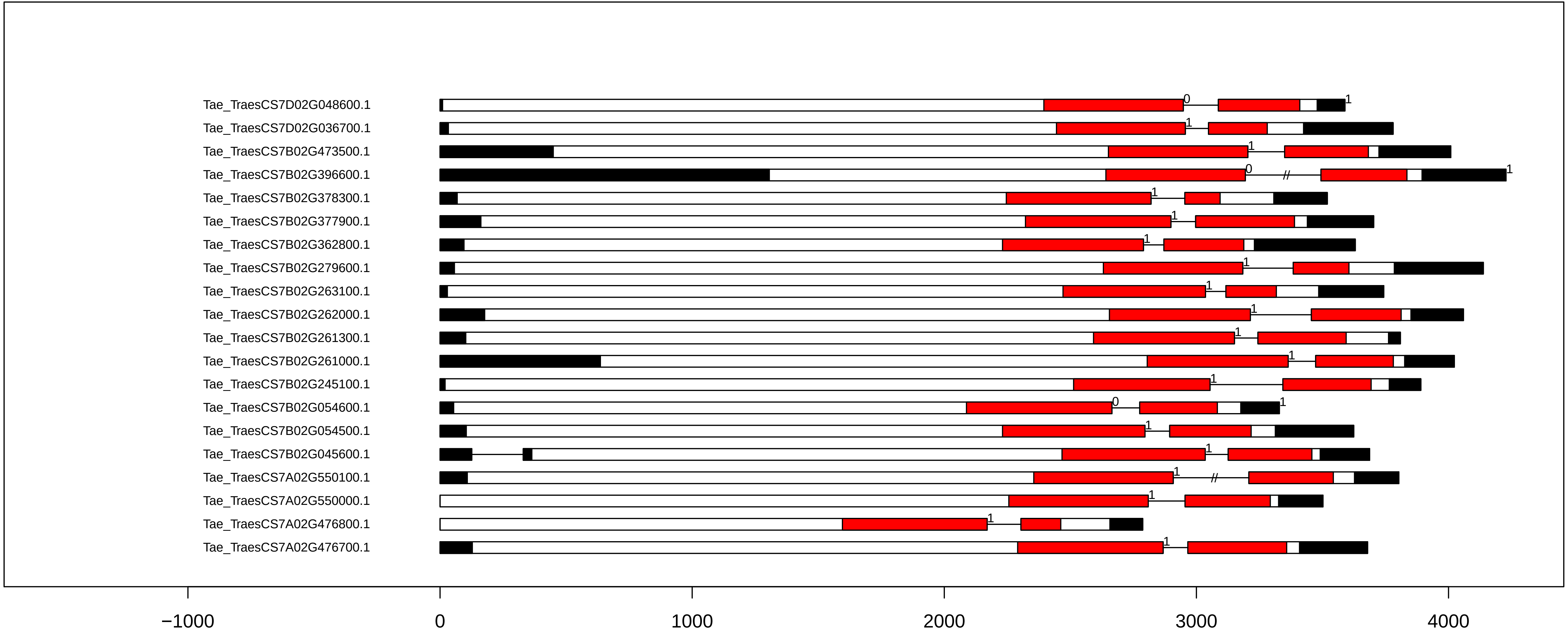

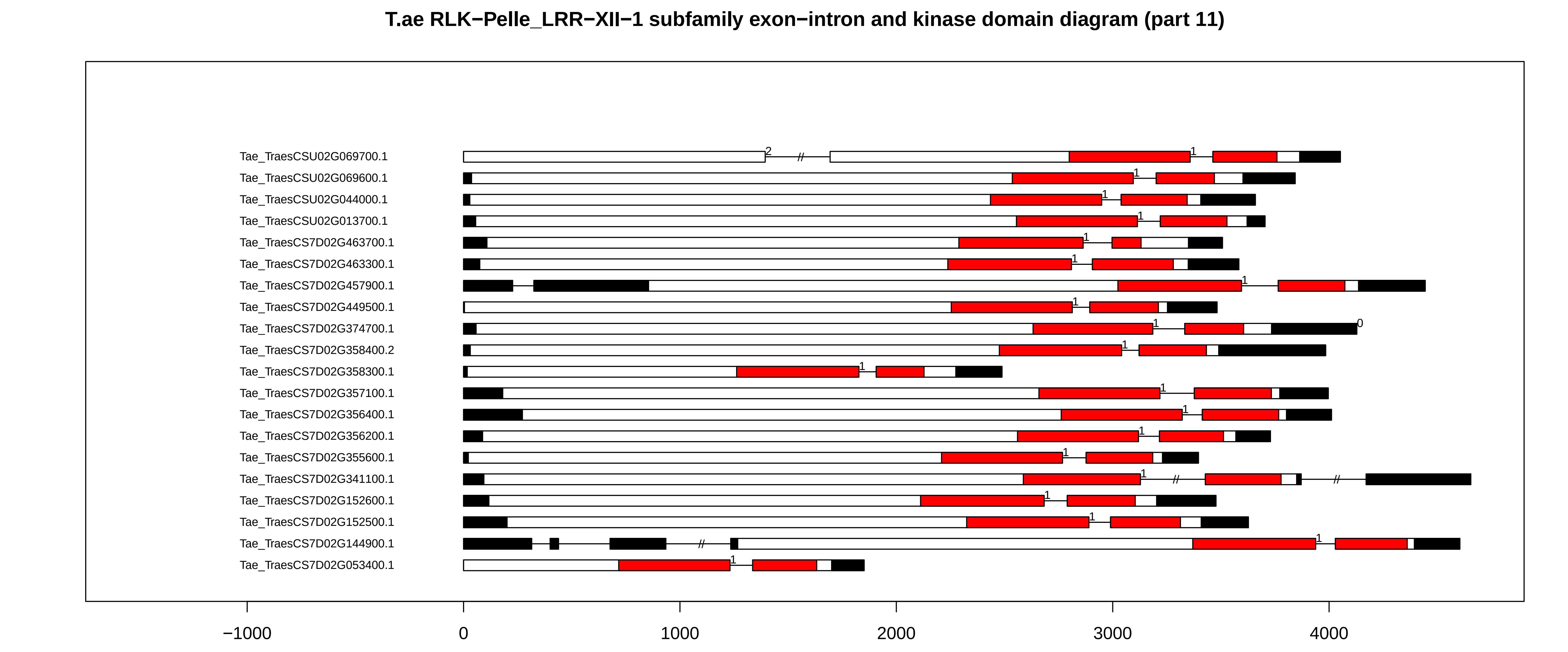

T.ae RLK−Pelle_LRR−XII−1 subfamily exon−intron and kinase domain diagram (part 11)
Tae_TraesCSU02G069700.1
Tae_TraesCSU02G069600.1
Tae_TraesCSU02G044000.1
Tae_TraesCSU02G013700.1
Tae_TraesCS7D02G463700.1
Tae_TraesCS7D02G463300.1
Tae_TraesCS7D02G457900.1
Tae_TraesCS7D02G449500.1
Tae_TraesCS7D02G374700.1
Tae_TraesCS7D02G358400.2
Tae_TraesCS7D02G358300.1
Tae_TraesCS7D02G357100.1
Tae_TraesCS7D02G356400.1
Tae_TraesCS7D02G356200.1
Tae_TraesCS7D02G355600.1
Tae_TraesCS7D02G341100.1
Tae_TraesCS7D02G152600.1
Tae_TraesCS7D02G152500.1
Tae_TraesCS7D02G144900.1
Tae_TraesCS7D02G053400.1
−1000
0
1000
2000
3000
4000

T.ae RLK−Pelle_LRR−XII−1 subfamily exon−intron and kinase domain diagram (part 12)

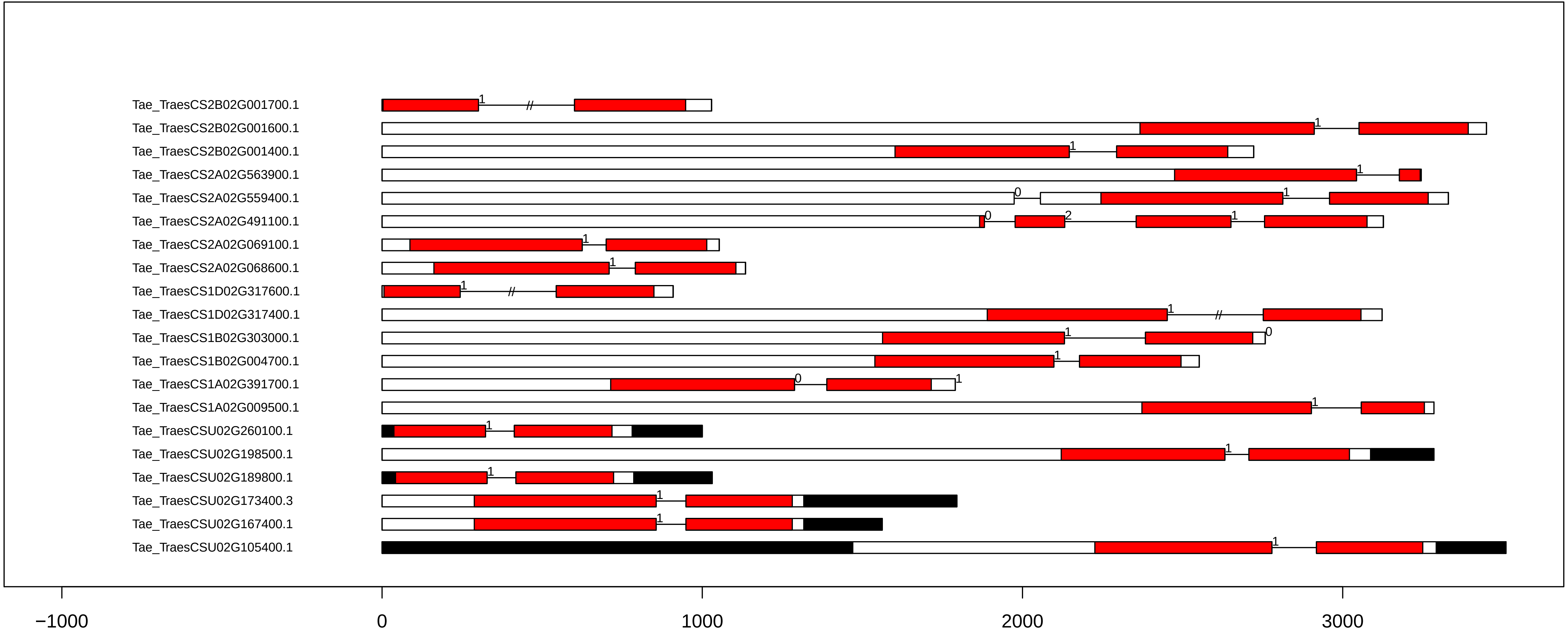

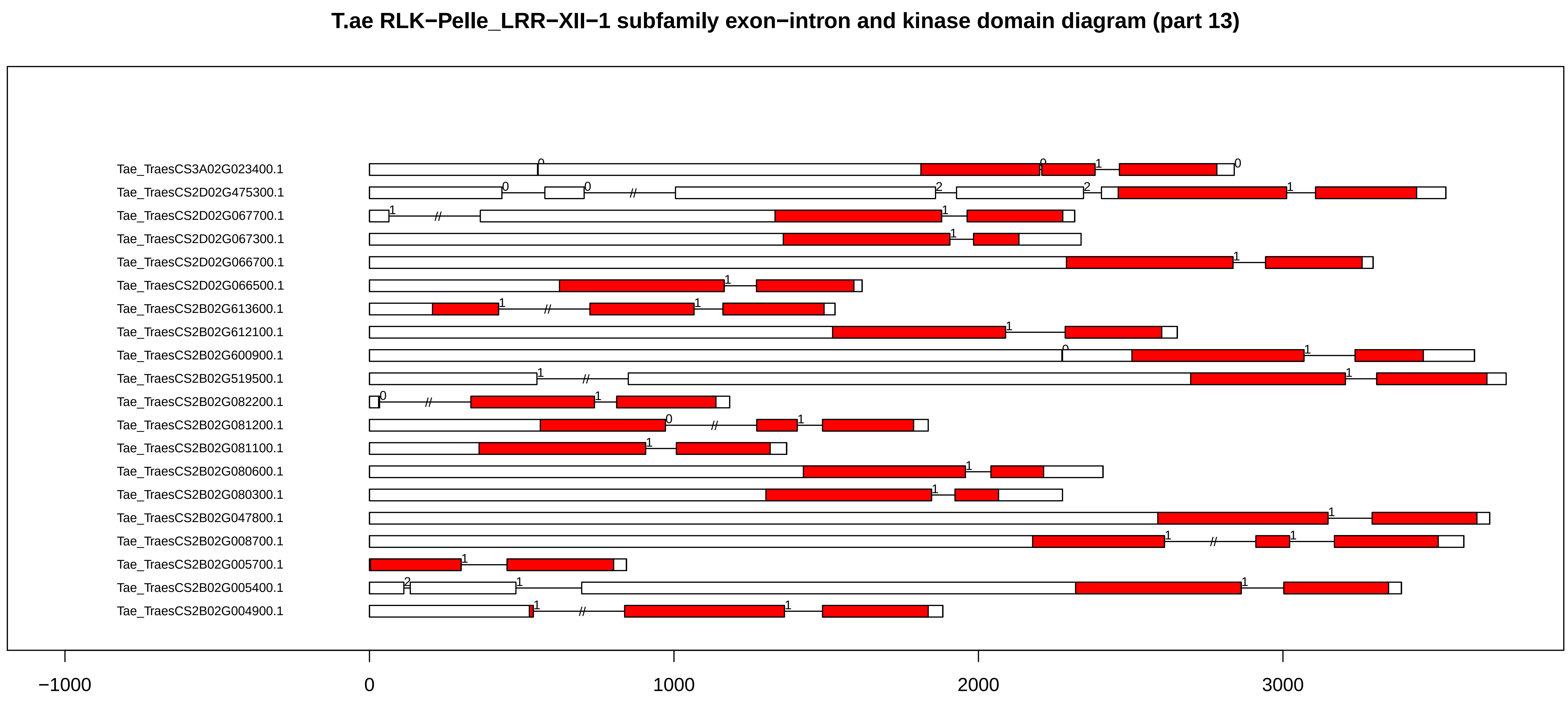
T.ae RLK−Pelle_LRR−XII−1 subfamily exon−intron and kinase domain diagram (part 13)
Tae_TraesCS3A02G023400.1
Tae_TraesCS2D02G475300.1
Tae_TraesCS2D02G067700.1
Tae_TraesCS2D02G067300.1
Tae_TraesCS2D02G066700.1
Tae_TraesCS2D02G066500.1
Tae_TraesCS2B02G613600.1
Tae_TraesCS2B02G612100.1
Tae_TraesCS2B02G600900.1
Tae_TraesCS2B02G519500.1
Tae_TraesCS2B02G082200.1
Tae_TraesCS2B02G081200.1
Tae_TraesCS2B02G081100.1
Tae_TraesCS2B02G080600.1
Tae_TraesCS2B02G080300.1
Tae_TraesCS2B02G047800.1
Tae_TraesCS2B02G008700.1
Tae_TraesCS2B02G005700.1
Tae_TraesCS2B02G005400.1
Tae_TraesCS2B02G004900.1
−1000
0
1000
2000
3000

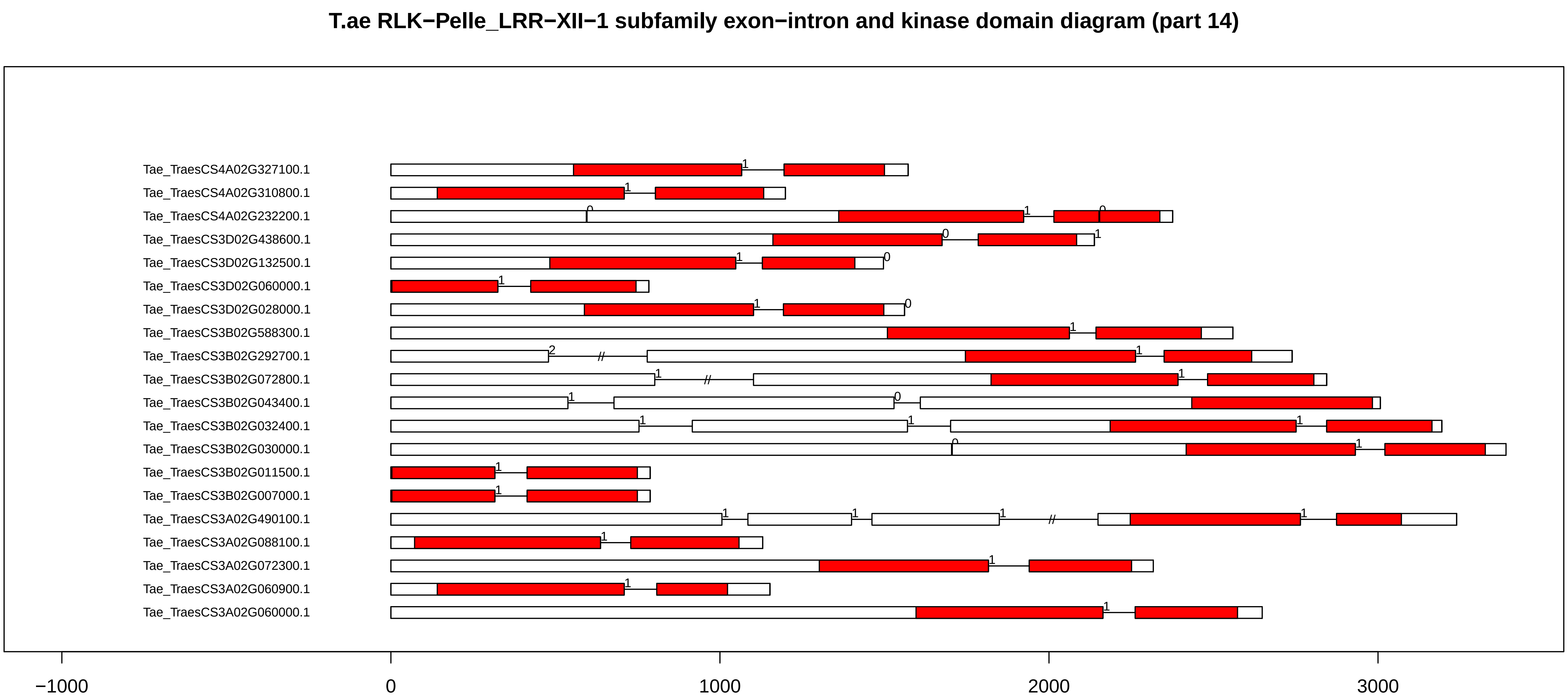
T.ae RLK−Pelle_LRR−XII−1 subfamily exon−intron and kinase domain diagram (part 14)
Tae_TraesCS4A02G327100.1
Tae_TraesCS4A02G310800.1
Tae_TraesCS4A02G232200.1
Tae_TraesCS3D02G438600.1
Tae_TraesCS3D02G132500.1
Tae_TraesCS3D02G060000.1
Tae_TraesCS3D02G028000.1
Tae_TraesCS3B02G588300.1
Tae_TraesCS3B02G292700.1
Tae_TraesCS3B02G072800.1
Tae_TraesCS3B02G043400.1
Tae_TraesCS3B02G032400.1
Tae_TraesCS3B02G030000.1
Tae_TraesCS3B02G011500.1
Tae_TraesCS3B02G007000.1
Tae_TraesCS3A02G490100.1
Tae_TraesCS3A02G088100.1
Tae_TraesCS3A02G072300.1
Tae_TraesCS3A02G060900.1
Tae_TraesCS3A02G060000.1
−1000
0
1000
2000
3000

T.ae RLK−Pelle_LRR−XII−1 subfamily exon−intron and kinase domain diagram (part 15)

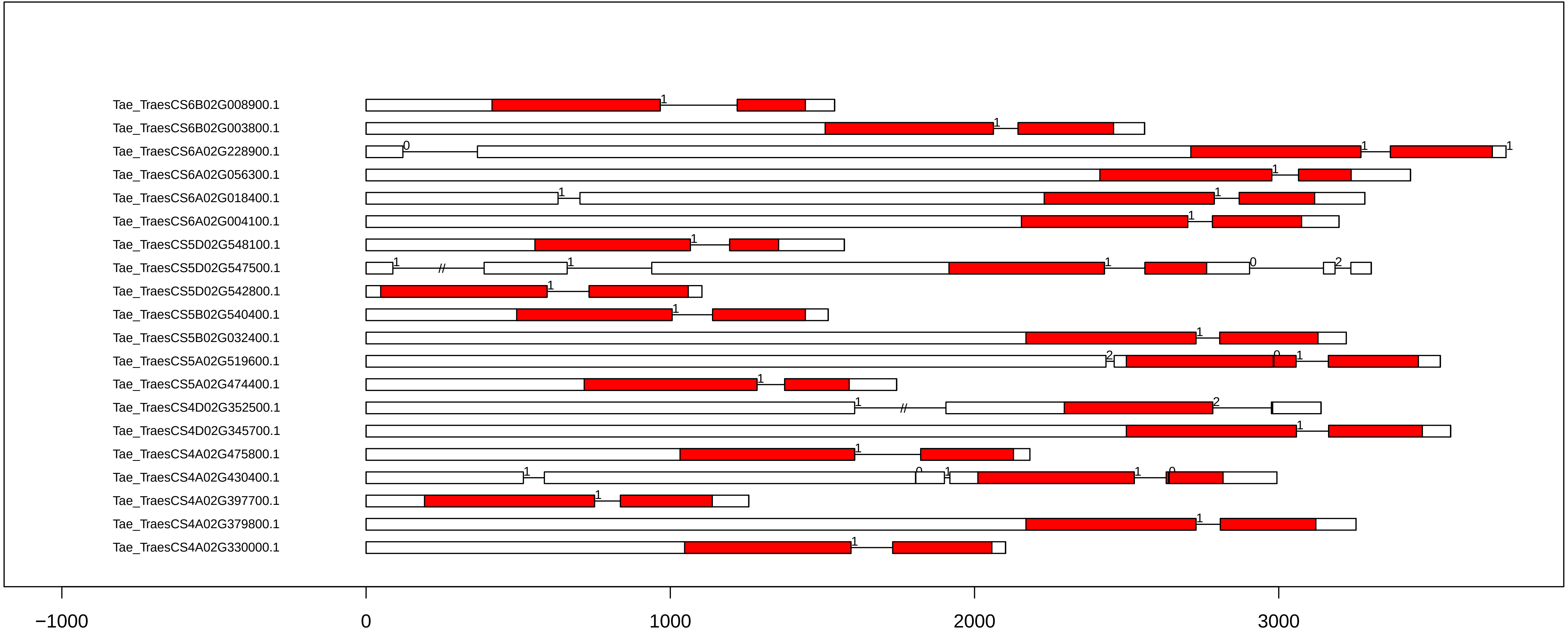

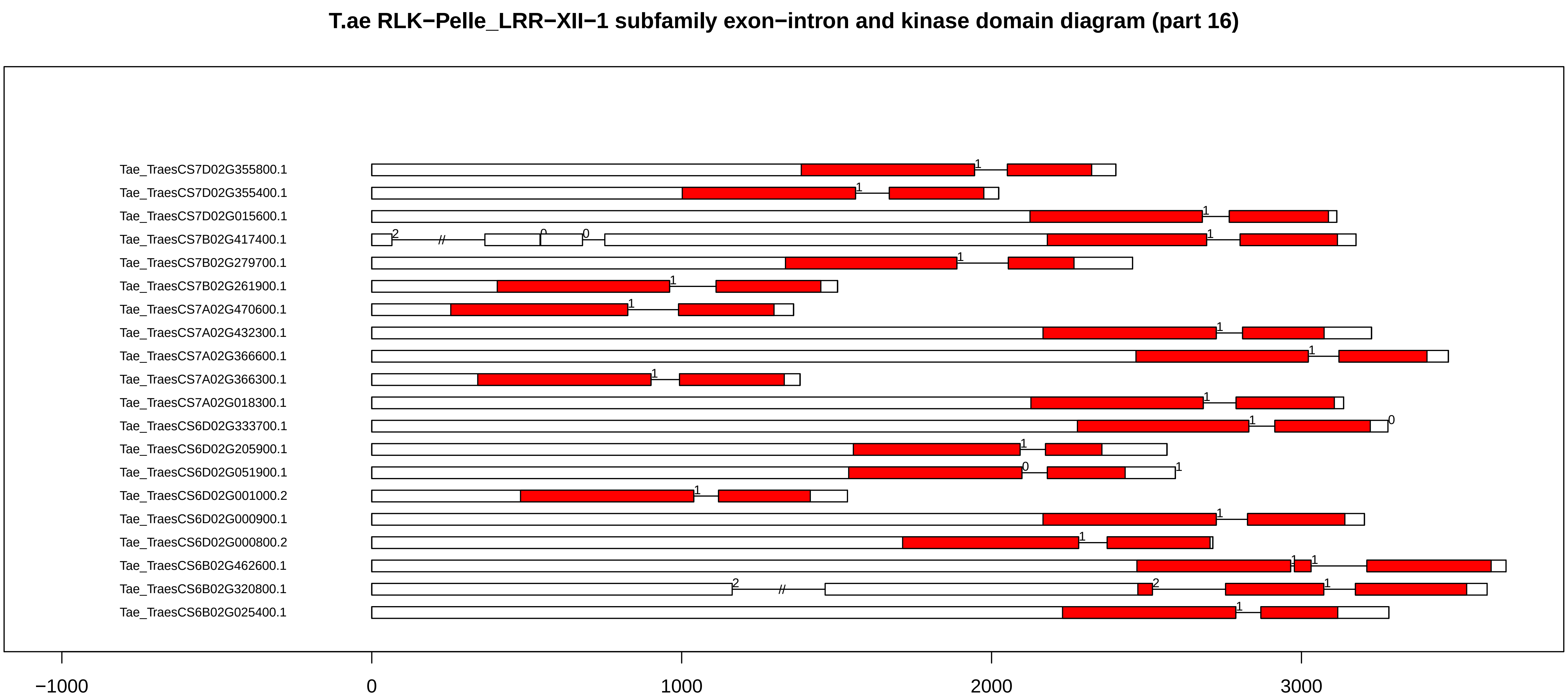
T.ae RLK−Pelle_LRR−XII−1 subfamily exon−intron and kinase domain diagram (part 16)
Tae_TraesCS7D02G355800.1
Tae_TraesCS7D02G355400.1
Tae_TraesCS7D02G015600.1
Tae_TraesCS7B02G417400.1
Tae_TraesCS7B02G279700.1
Tae_TraesCS7B02G261900.1
Tae_TraesCS7A02G470600.1
Tae_TraesCS7A02G432300.1
Tae_TraesCS7A02G366600.1
Tae_TraesCS7A02G366300.1
Tae_TraesCS7A02G018300.1
Tae_TraesCS6D02G333700.1
Tae_TraesCS6D02G205900.1
Tae_TraesCS6D02G051900.1
Tae_TraesCS6D02G001000.2
Tae_TraesCS6D02G000900.1
Tae_TraesCS6D02G000800.2
Tae_TraesCS6B02G462600.1
Tae_TraesCS6B02G320800.1
Tae_TraesCS6B02G025400.1
−1000
0
1000
2000
3000

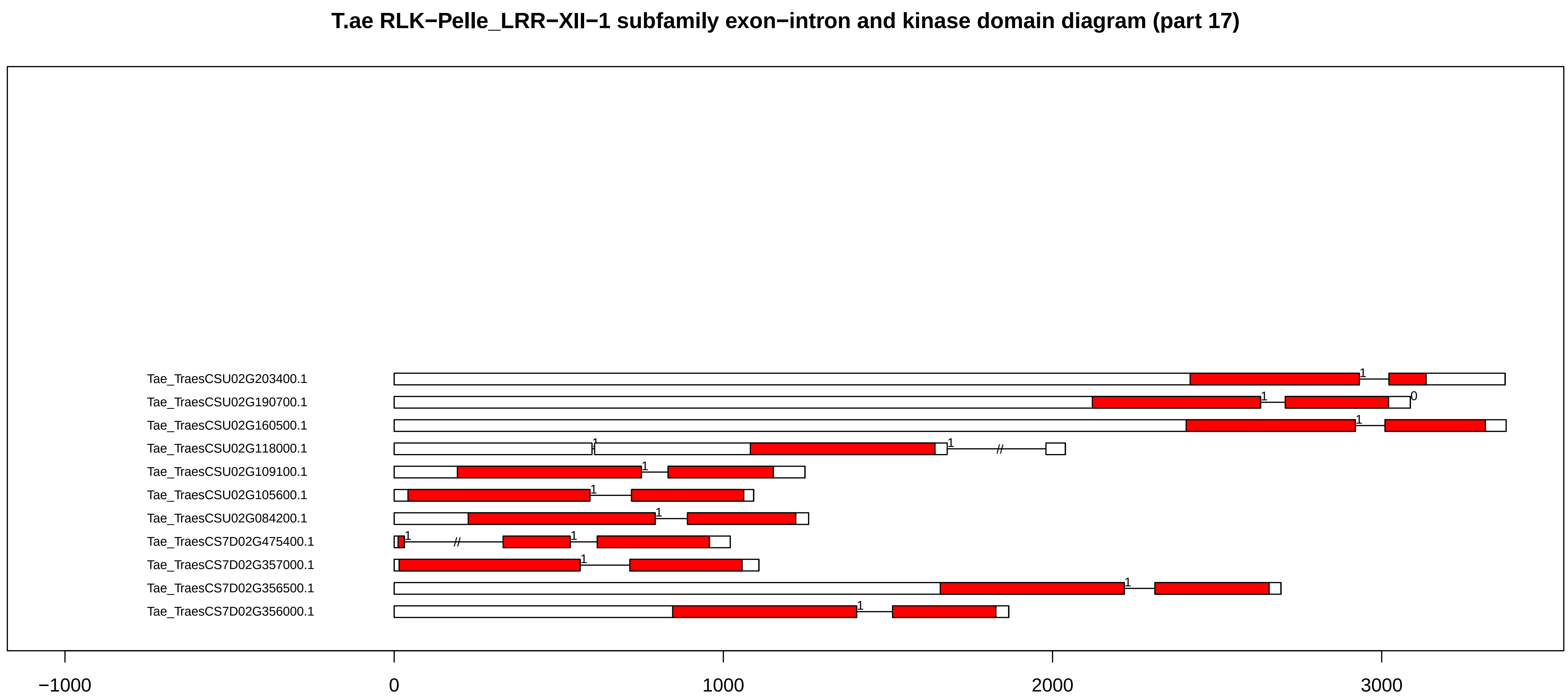

T.ae RLK−Pelle_LRR−XII−1 subfamily exon−intron and kinase domain diagram (part 17)
Tae_TraesCSU02G203400.1
Tae_TraesCSU02G190700.1
Tae_TraesCSU02G160500.1
Tae_TraesCSU02G118000.1
Tae_TraesCSU02G109100.1
Tae_TraesCSU02G105600.1
Tae_TraesCSU02G084200.1
Tae_TraesCS7D02G475400.1
Tae_TraesCS7D02G357000.1
Tae_TraesCS7D02G356500.1
Tae_TraesCS7D02G356000.1
−1000
0
1000
2000
3000

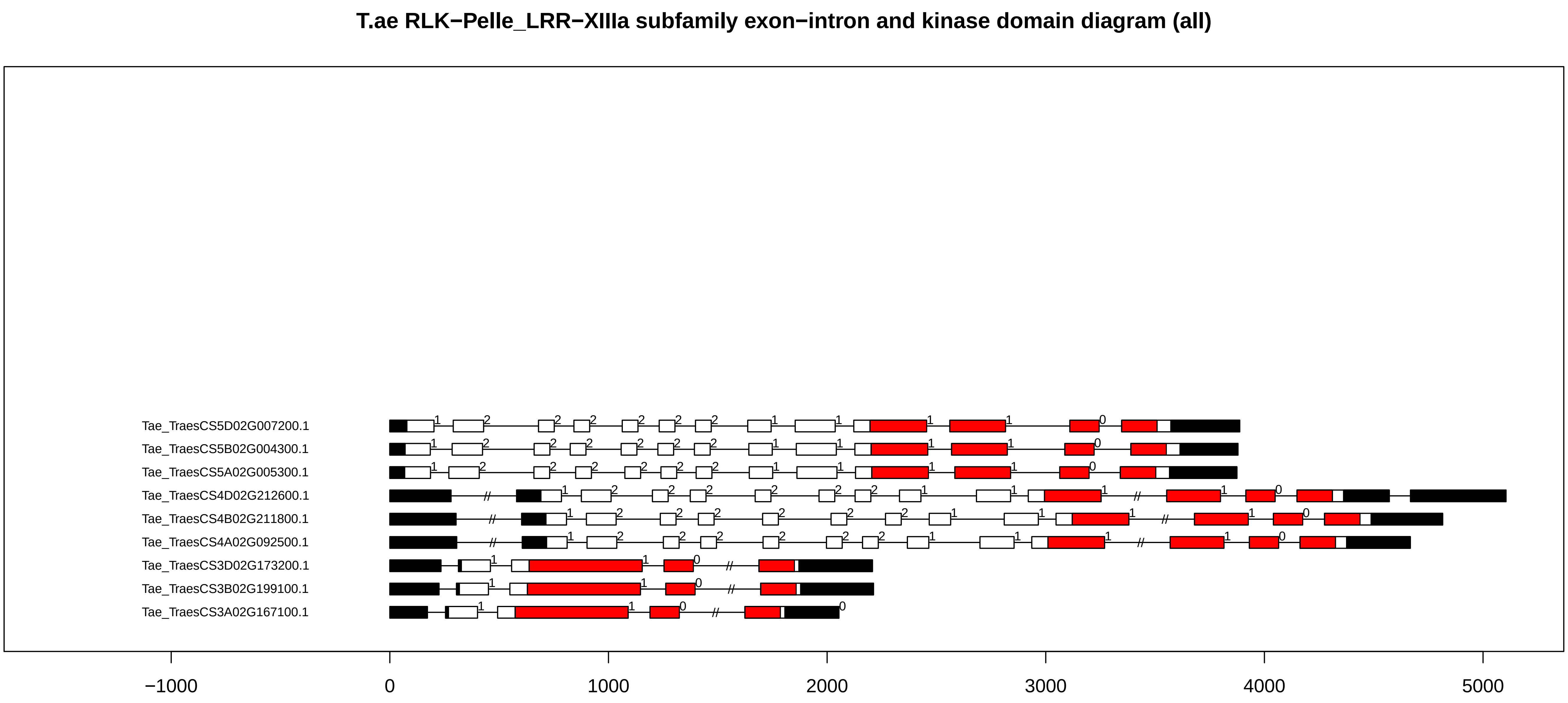
T.ae RLK−Pelle_LRR−XIIIa subfamily exon−intron and kinase domain diagram (all)
Tae_TraesCS5D02G007200.1
Tae_TraesCS5B02G004300.1
Tae_TraesCS5A02G005300.1
Tae_TraesCS4D02G212600.1
Tae_TraesCS4B02G211800.1
Tae_TraesCS4A02G092500.1
Tae_TraesCS3D02G173200.1
Tae_TraesCS3B02G199100.1
Tae_TraesCS3A02G167100.1
−1000
0
1000
2000
3000
4000
5000

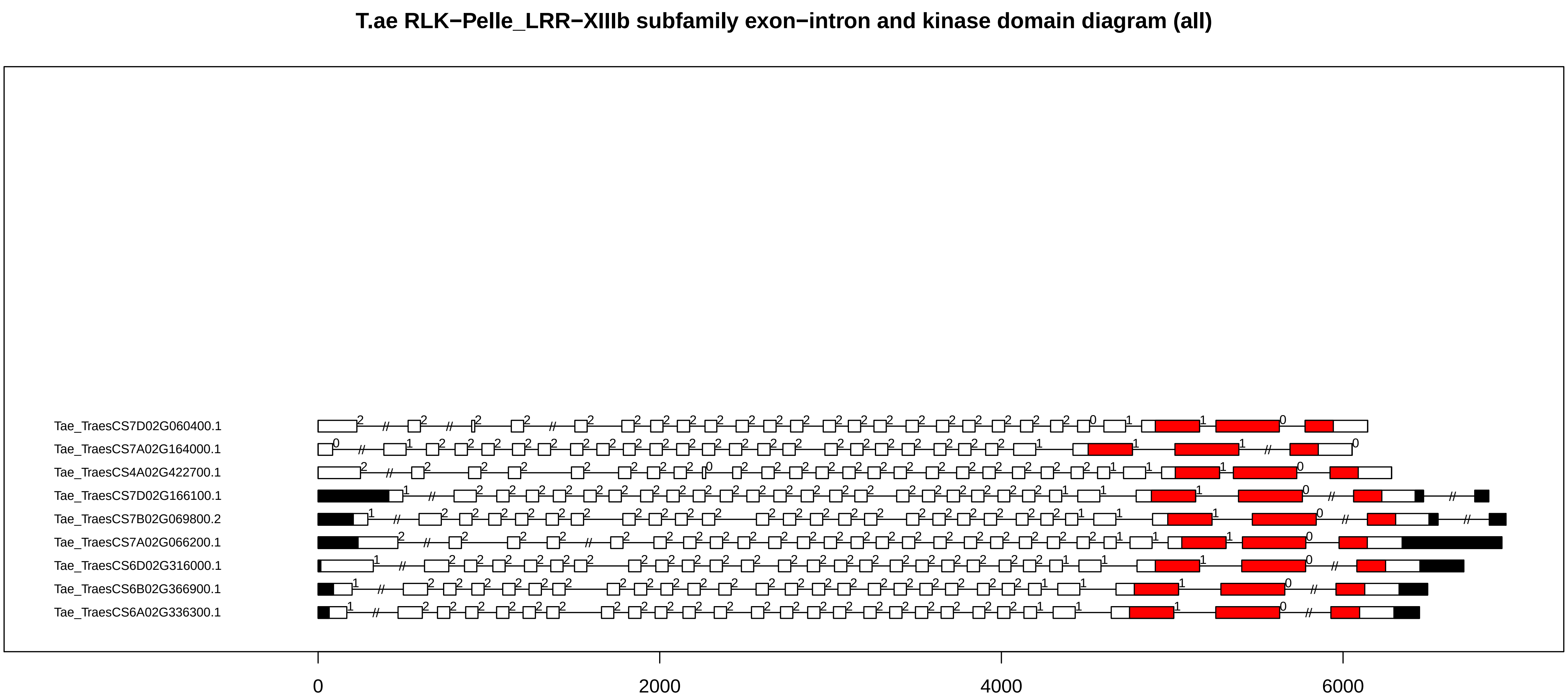

T.ae RLK−Pelle_LRR−XIIIb subfamily exon−intron and kinase domain diagram (all)
Tae_TraesCS7D02G060400.1
Tae_TraesCS7A02G164000.1
Tae_TraesCS4A02G422700.1
Tae_TraesCS7D02G166100.1
Tae_TraesCS7B02G069800.2
Tae_TraesCS7A02G066200.1
Tae_TraesCS6D02G316000.1
Tae_TraesCS6B02G366900.1
Tae_TraesCS6A02G336300.1
0
2000
4000
6000

T.ae RLK−Pelle_LRR−XIV subfamily exon−intron and kinase domain diagram (all)

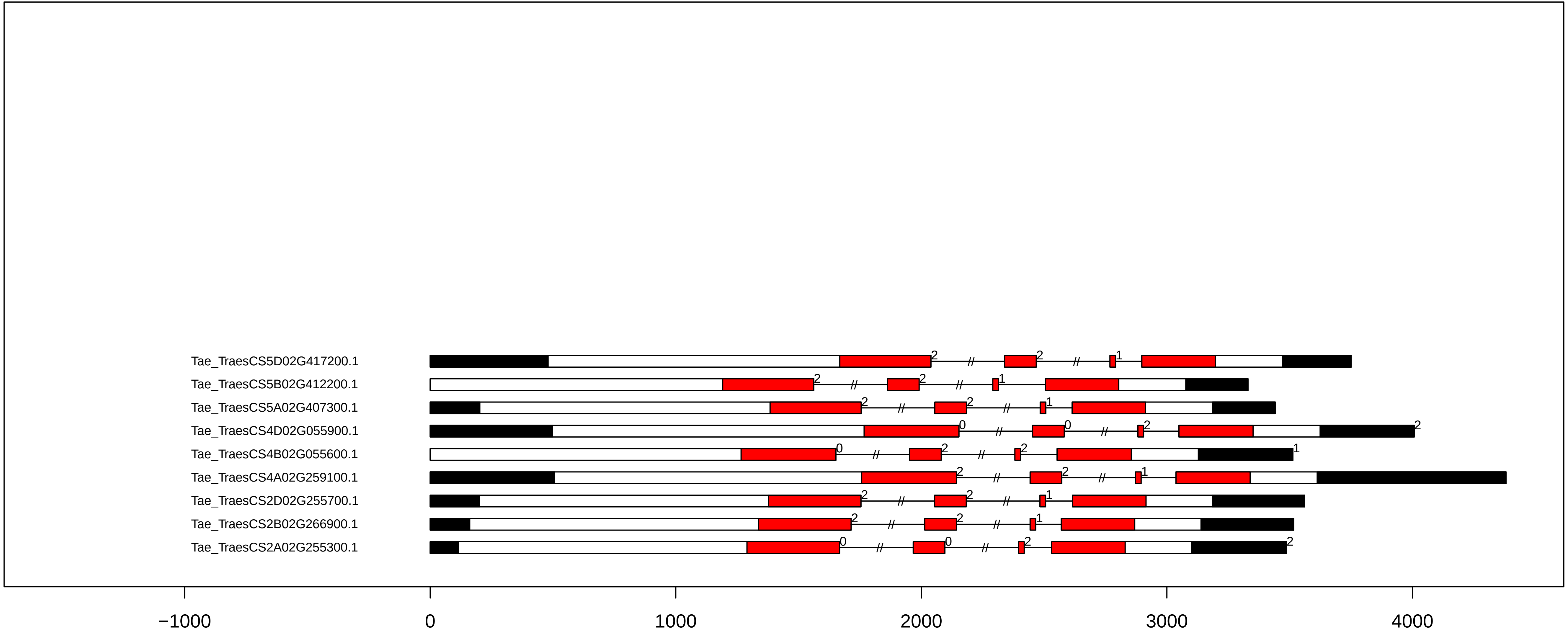

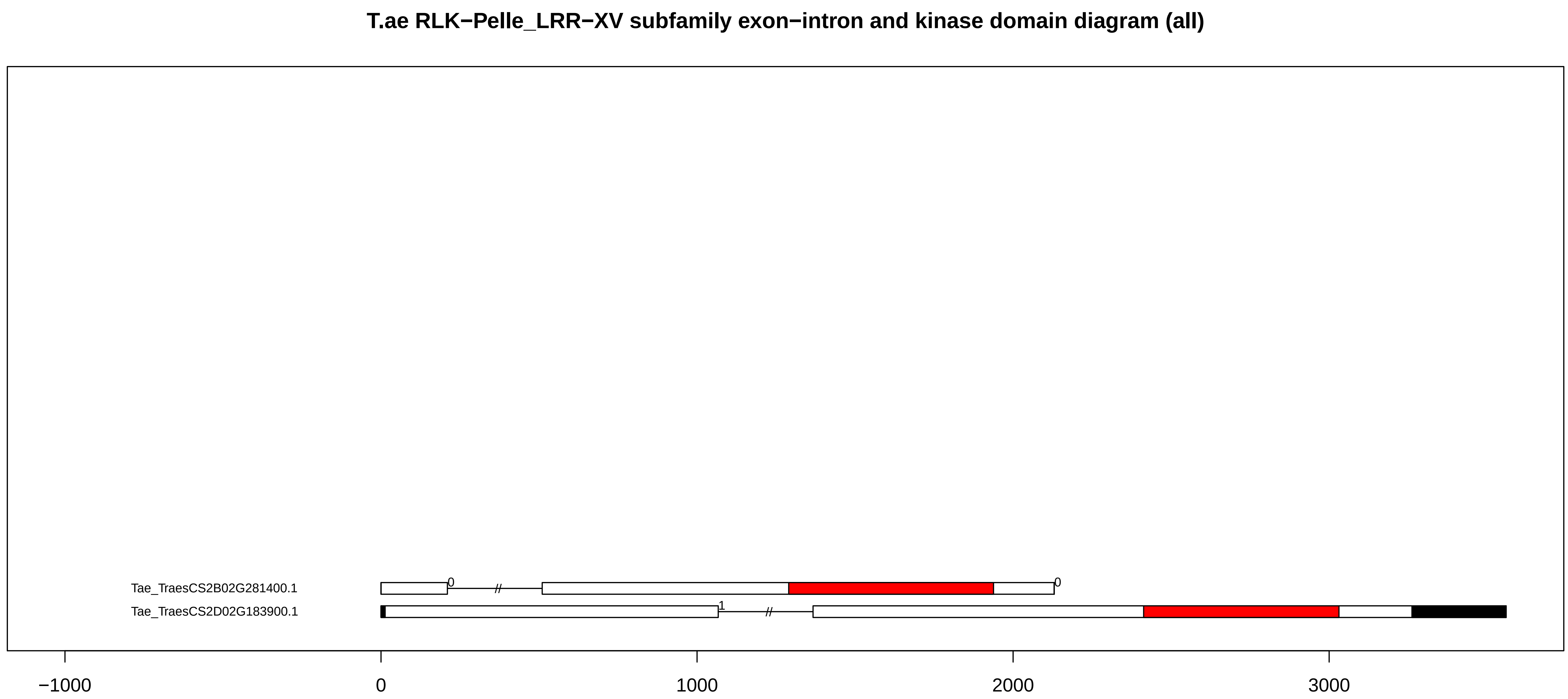
T.ae RLK−Pelle_LRR−XV subfamily exon−intron and kinase domain diagram (all)
Tae_TraesCS2B02G281400.1
Tae_TraesCS2D02G183900.1
0
0
1
−1000
0
1000
2000
3000

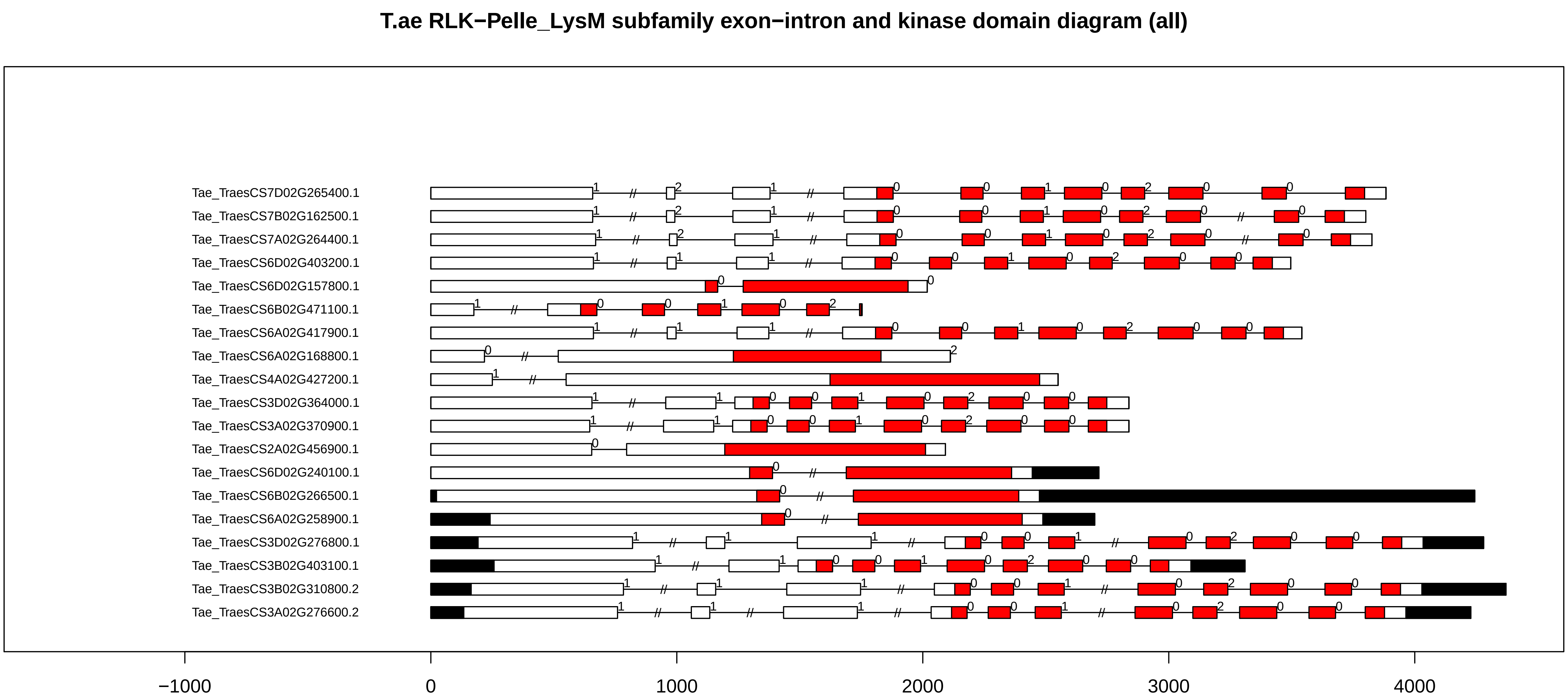
T.ae RLK−Pelle_LysM subfamily exon−intron and kinase domain diagram (all)
Tae_TraesCS7D02G265400.1
Tae_TraesCS7B02G162500.1
Tae_TraesCS7A02G264400.1
Tae_TraesCS6D02G403200.1
Tae_TraesCS6D02G157800.1
Tae_TraesCS6B02G471100.1
Tae_TraesCS6A02G417900.1
Tae_TraesCS6A02G168800.1
Tae_TraesCS4A02G427200.1
Tae_TraesCS3D02G364000.1
Tae_TraesCS3A02G370900.1
Tae_TraesCS2A02G456900.1
Tae_TraesCS6D02G240100.1
Tae_TraesCS6B02G266500.1
Tae_TraesCS6A02G258900.1
Tae_TraesCS3D02G276800.1
Tae_TraesCS3B02G403100.1
Tae_TraesCS3B02G310800.2
Tae_TraesCS3A02G276600.2
−1000
0
1000
2000
3000
4000

T.ae RLK−Pelle_PERK−1 subfamily exon−intron and kinase domain diagram (part 1)

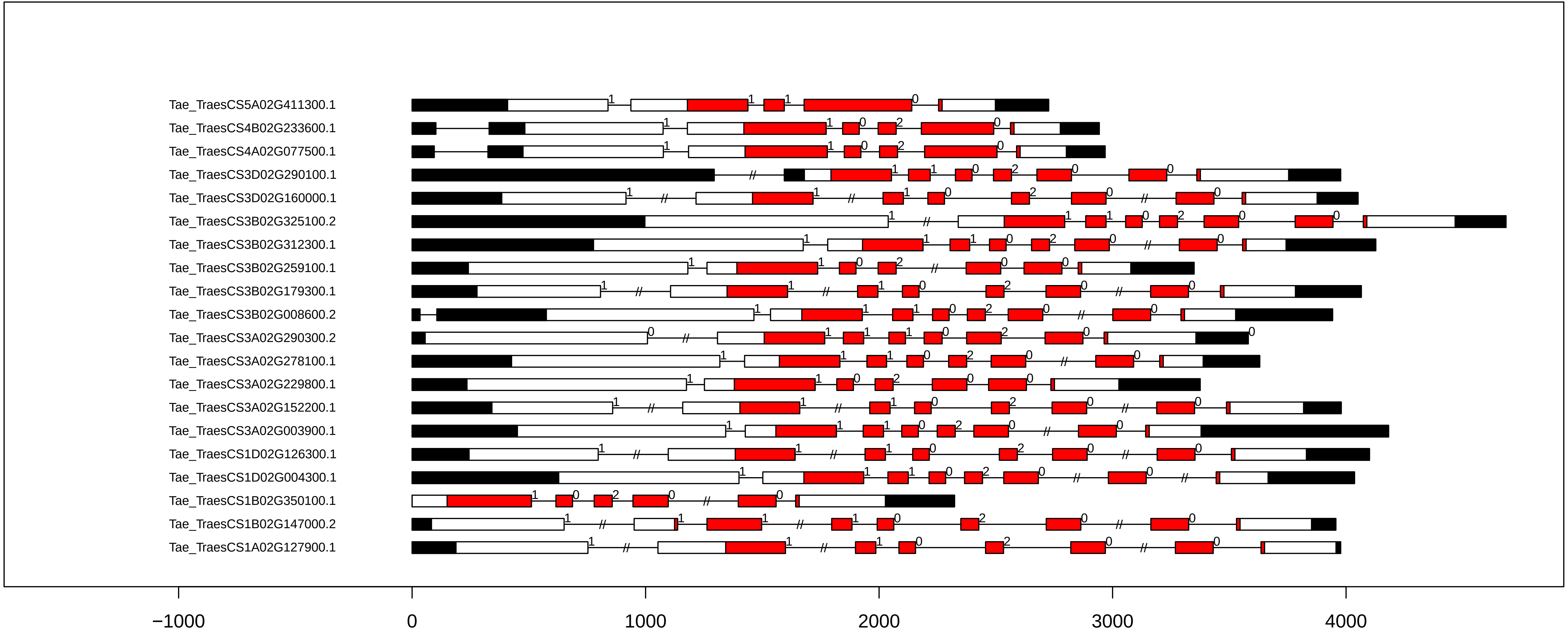

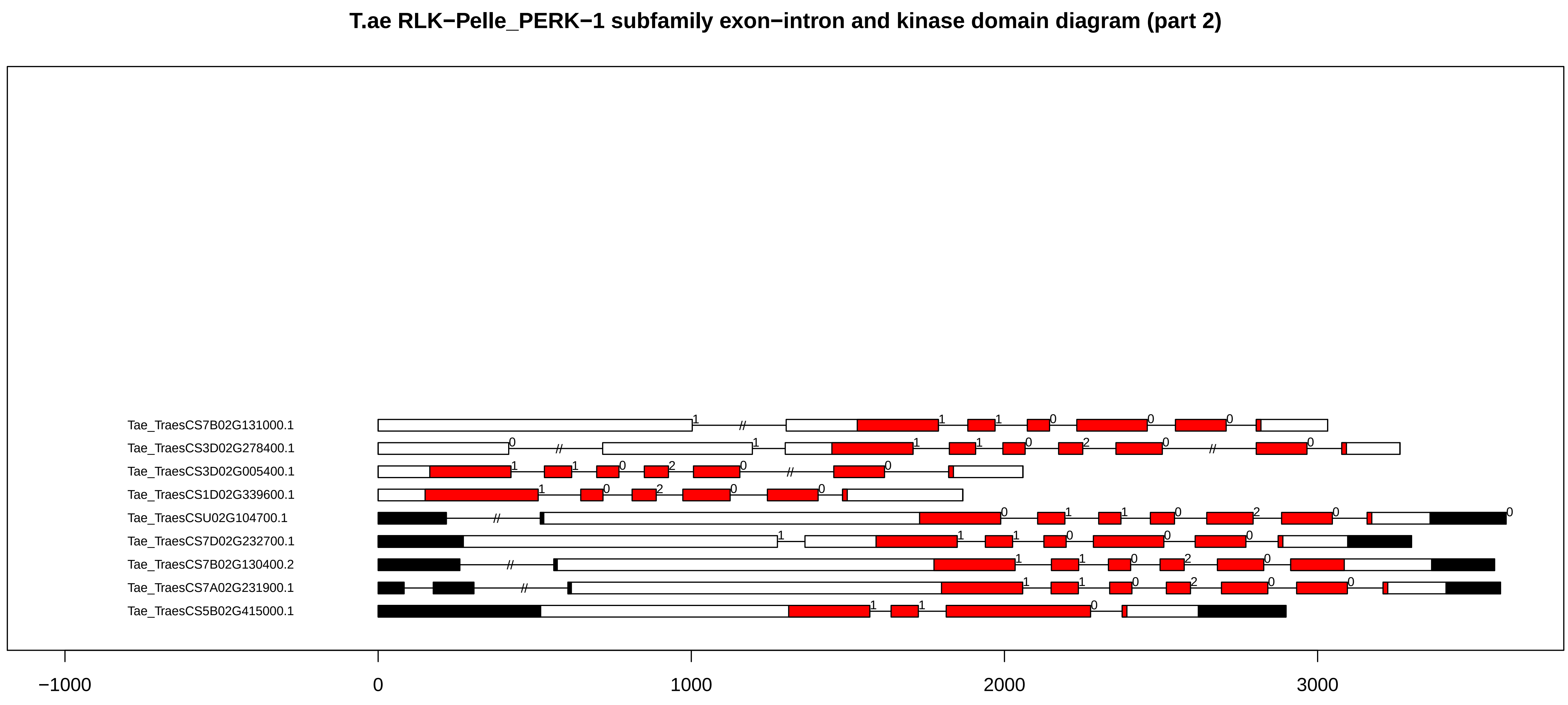
T.ae RLK−Pelle_PERK−1 subfamily exon−intron and kinase domain diagram (part 2)
Tae_TraesCS7B02G131000.1
Tae_TraesCS3D02G278400.1
Tae_TraesCS3D02G005400.1
Tae_TraesCS1D02G339600.1
Tae_TraesCSU02G104700.1
Tae_TraesCS7D02G232700.1
Tae_TraesCS7B02G130400.2
Tae_TraesCS7A02G231900.1
Tae_TraesCS5B02G415000.1
−1000
0
1000
2000
3000

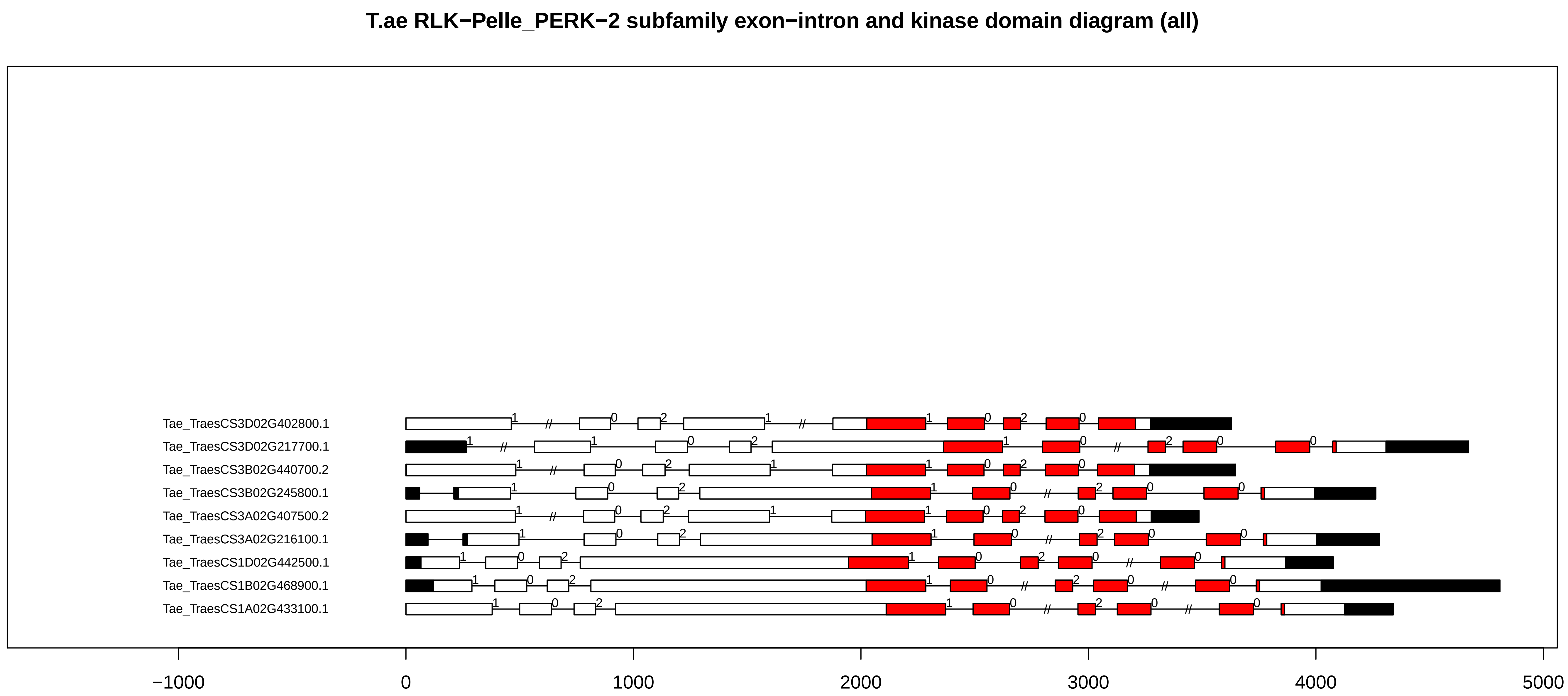
T.ae RLK−Pelle_PERK−2 subfamily exon−intron and kinase domain diagram (all)
Tae_TraesCS3D02G402800.1
Tae_TraesCS3D02G217700.1
Tae_TraesCS3B02G440700.2
Tae_TraesCS3B02G245800.1
Tae_TraesCS3A02G407500.2
Tae_TraesCS3A02G216100.1
Tae_TraesCS1D02G442500.1
Tae_TraesCS1B02G468900.1
Tae_TraesCS1A02G433100.1
−1000
0
1000
2000
3000
4000
5000

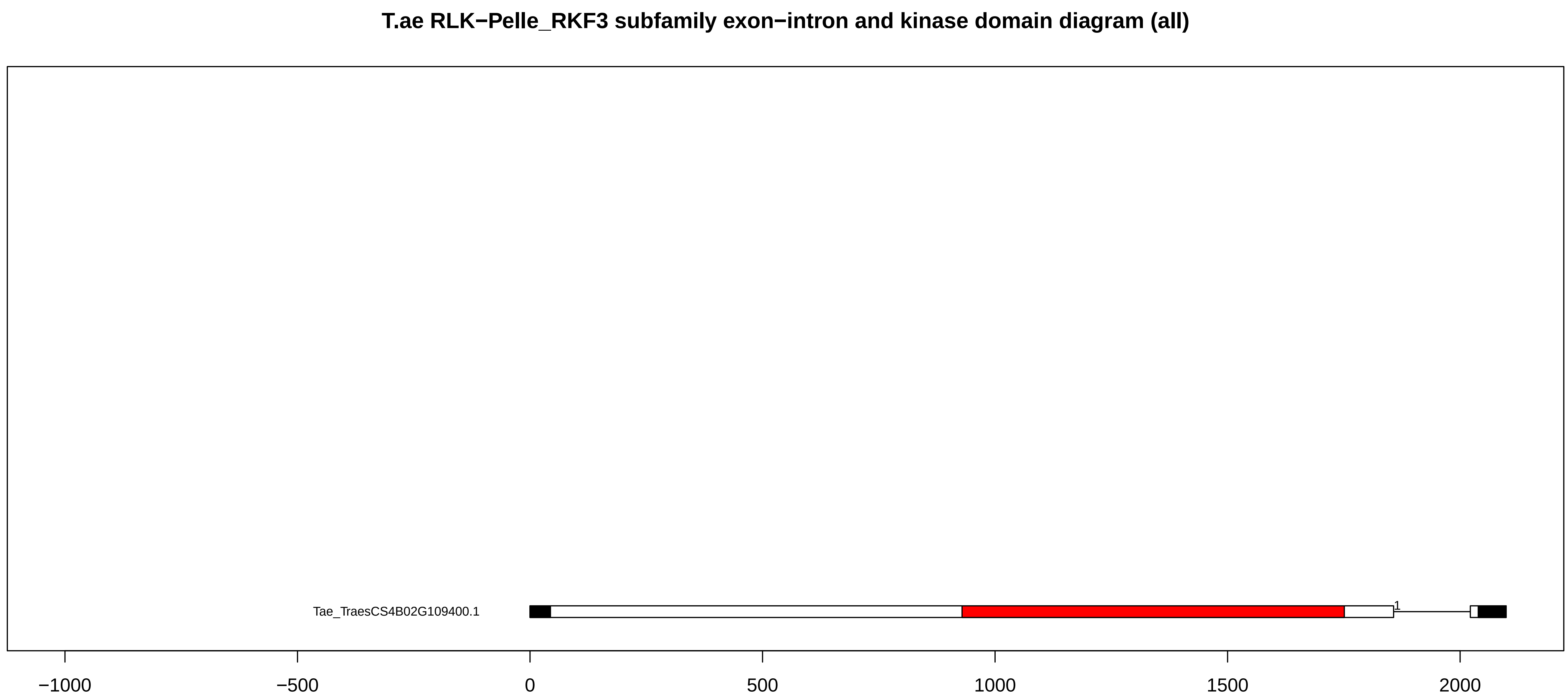
T.ae RLK−Pelle_RKF3 subfamily exon−intron and kinase domain diagram (all)
Tae_TraesCS4B02G109400.1
1
−1000
−500
0
500
1000
1500
2000

T.ae RLK−Pelle_RLCK−II subfamily exon−intron and kinase domain diagram (all)

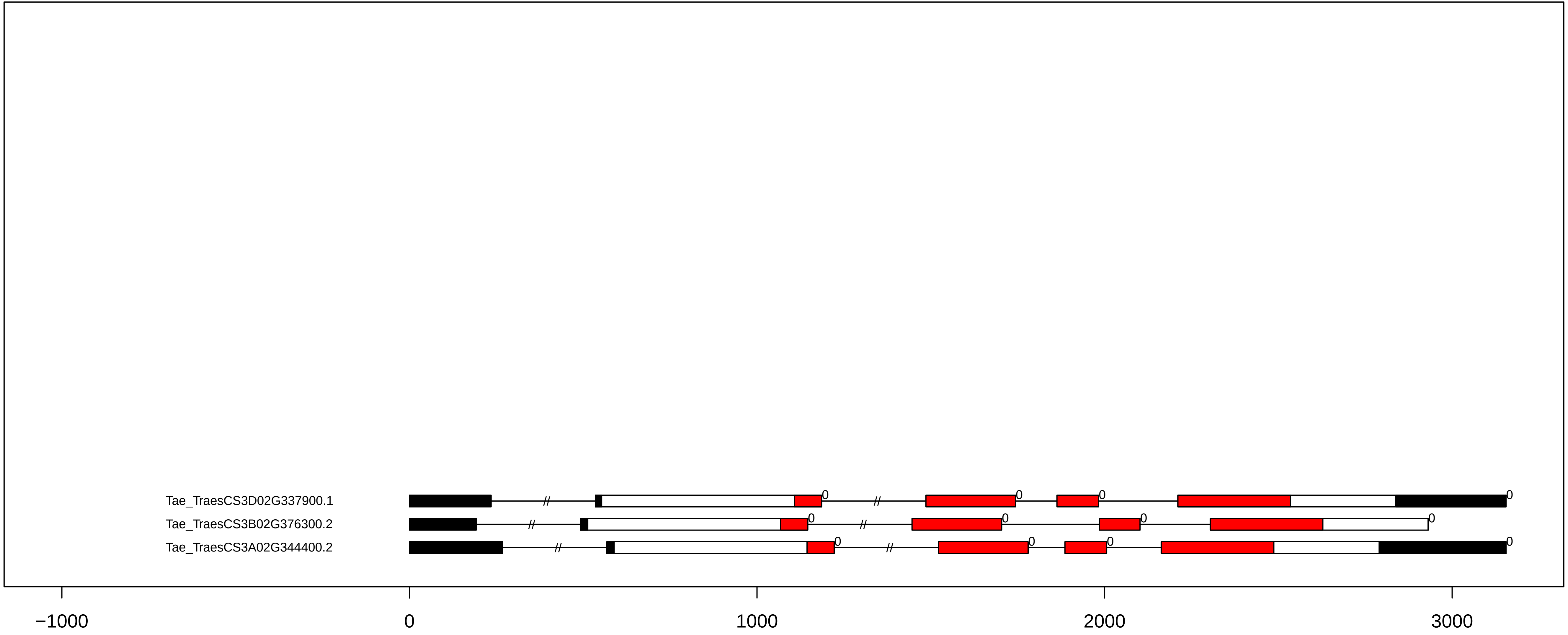

T.ae RLK−Pelle_RLCK−IV subfamily exon−intron and kinase domain diagram (all)

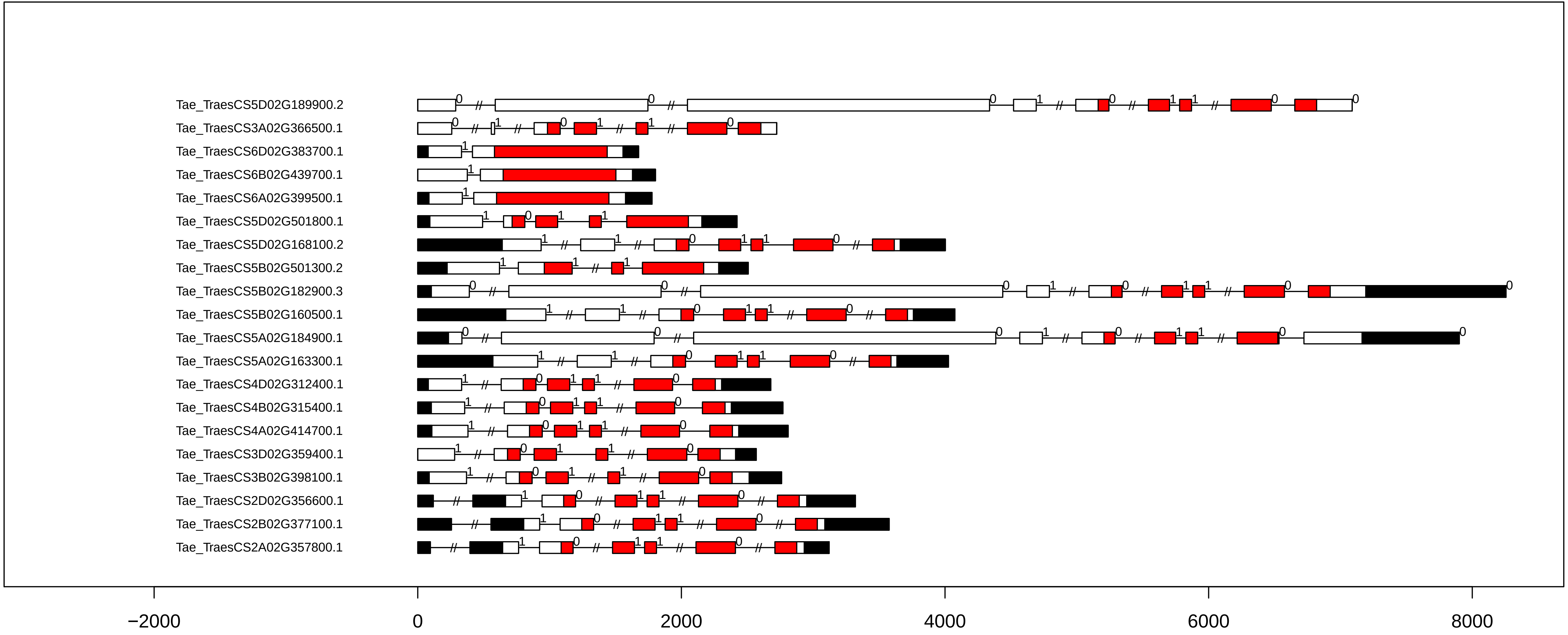

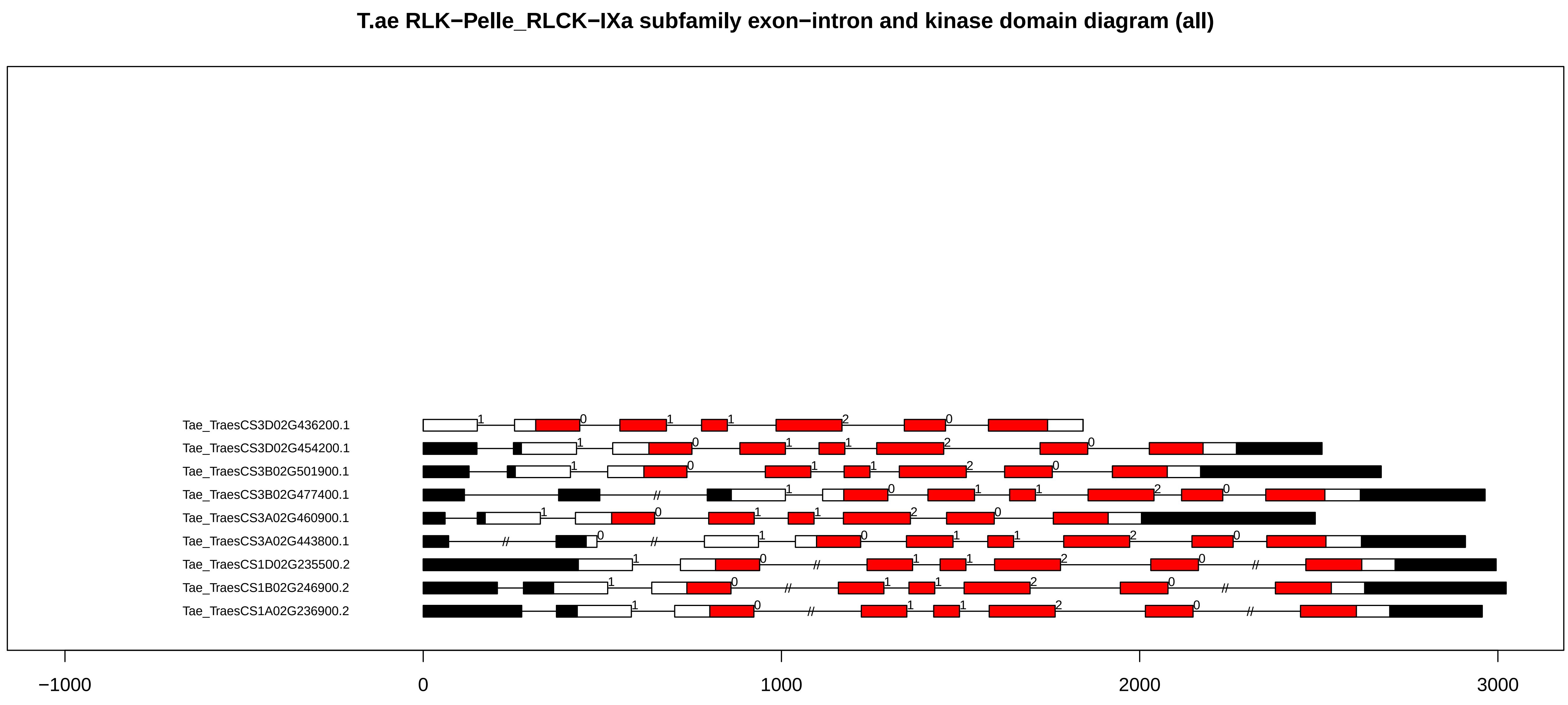

T.ae RLK−Pelle_RLCK−IXa subfamily exon−intron and kinase domain diagram (all)
Tae_TraesCS3D02G436200.1
Tae_TraesCS3D02G454200.1
Tae_TraesCS3B02G501900.1
Tae_TraesCS3B02G477400.1
Tae_TraesCS3A02G460900.1
Tae_TraesCS3A02G443800.1
Tae_TraesCS1D02G235500.2
Tae_TraesCS1B02G246900.2
Tae_TraesCS1A02G236900.2
−1000
0
1000
2000
3000

T.ae RLK−Pelle_RLCK−IXb subfamily exon−intron and kinase domain diagram (part 1)

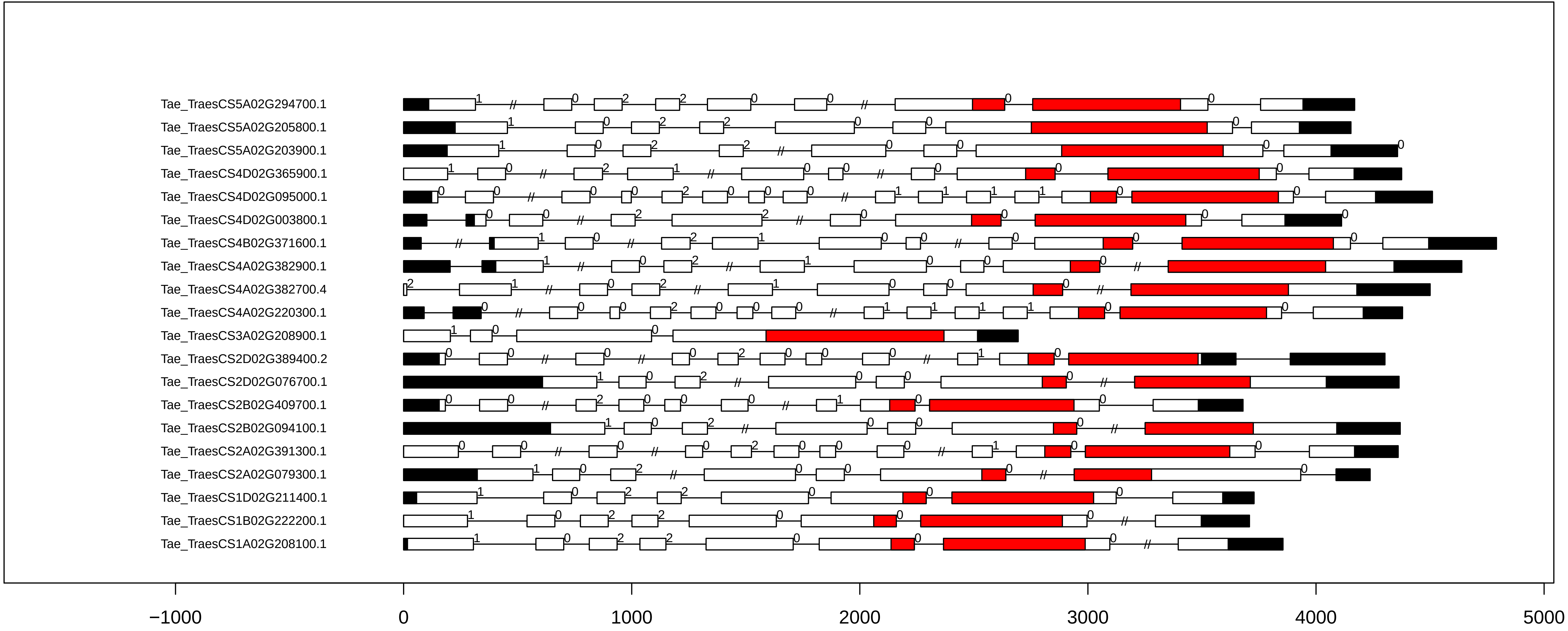

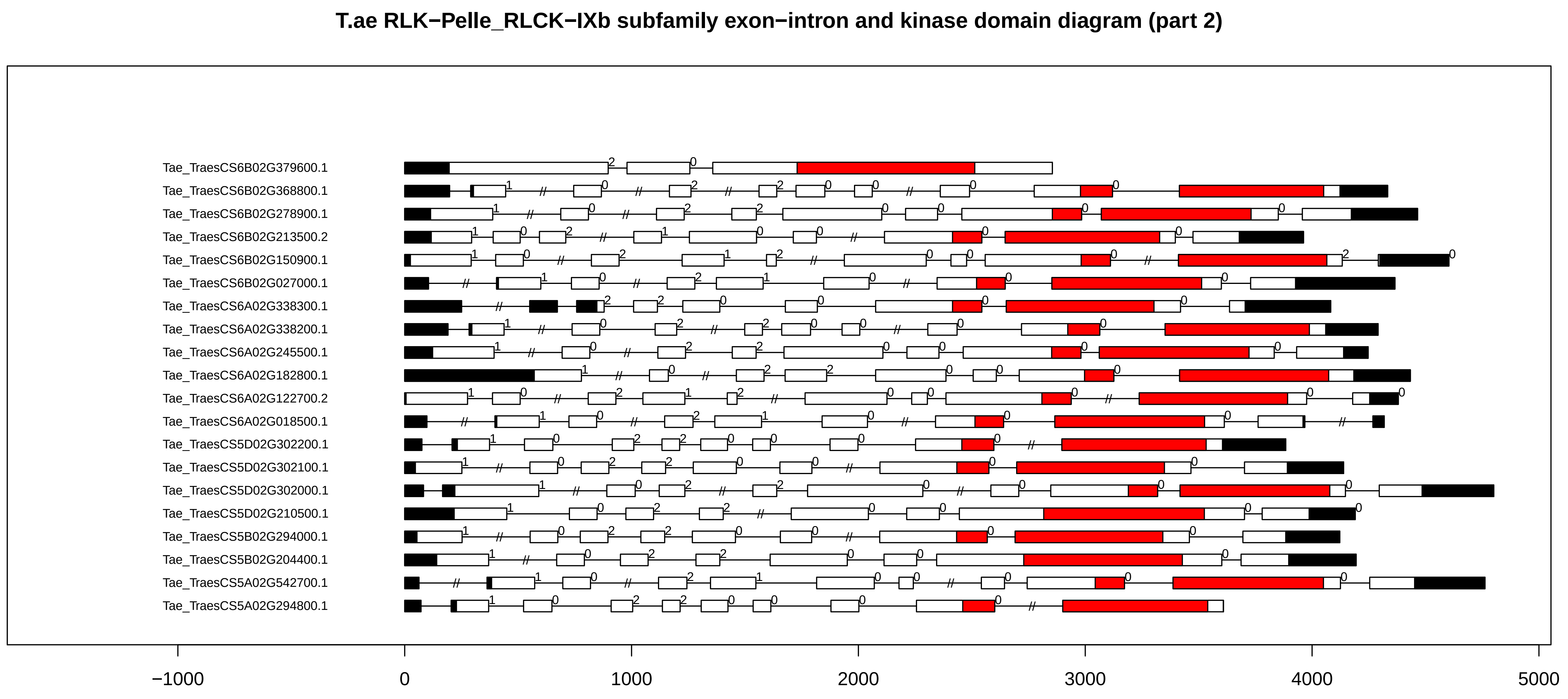

T.ae RLK−Pelle_RLCK−IXb subfamily exon−intron and kinase domain diagram (part 2)
Tae_TraesCS6B02G379600.1
Tae_TraesCS6B02G368800.1
Tae_TraesCS6B02G278900.1
Tae_TraesCS6B02G213500.2
Tae_TraesCS6B02G150900.1
Tae_TraesCS6B02G027000.1
Tae_TraesCS6A02G338300.1
Tae_TraesCS6A02G338200.1
Tae_TraesCS6A02G245500.1
Tae_TraesCS6A02G182800.1
Tae_TraesCS6A02G122700.2
Tae_TraesCS6A02G018500.1
Tae_TraesCS5D02G302200.1
Tae_TraesCS5D02G302100.1
Tae_TraesCS5D02G302000.1
Tae_TraesCS5D02G210500.1
Tae_TraesCS5B02G294000.1
Tae_TraesCS5B02G204400.1
Tae_TraesCS5A02G542700.1
Tae_TraesCS5A02G294800.1
−1000
0
1000
2000
3000
4000
5000

T.ae RLK−Pelle_RLCK−IXb subfamily exon−intron and kinase domain diagram (part 3)

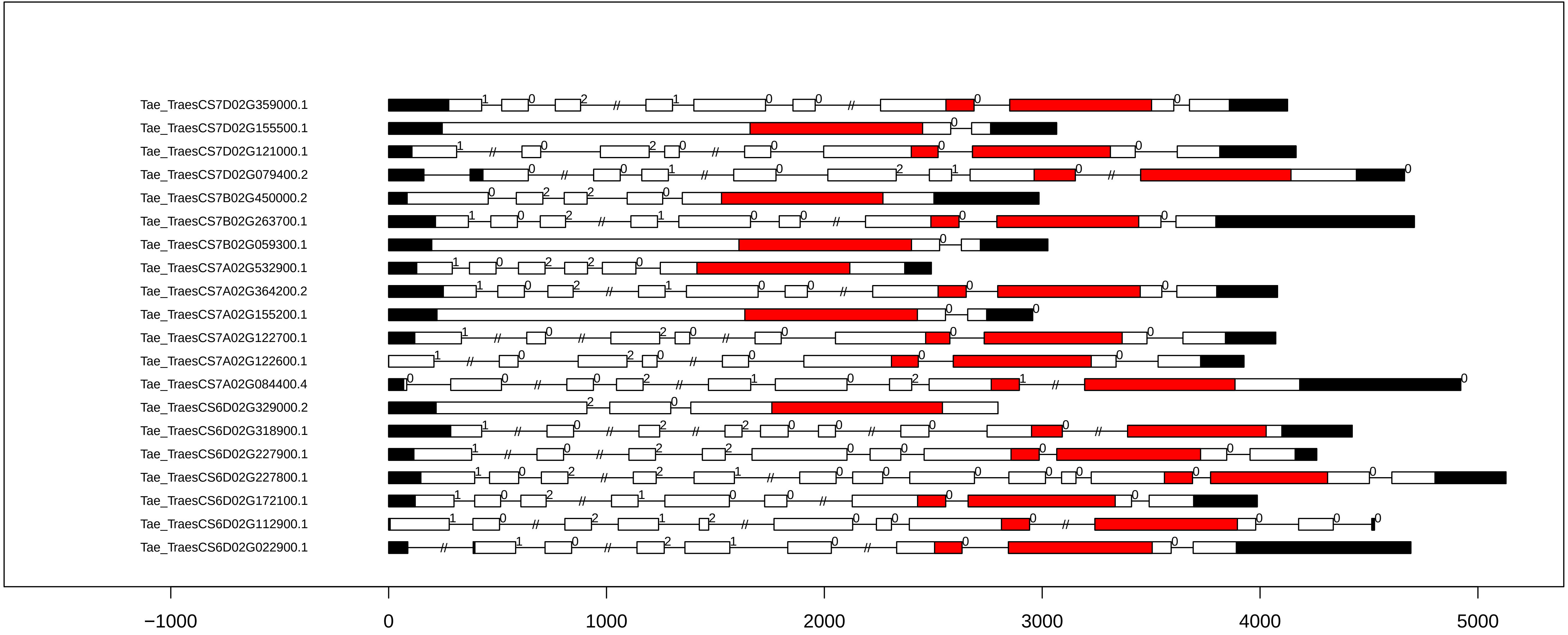

T.ae RLK−Pelle_RLCK−IXb subfamily exon−intron and kinase domain diagram (part 4)

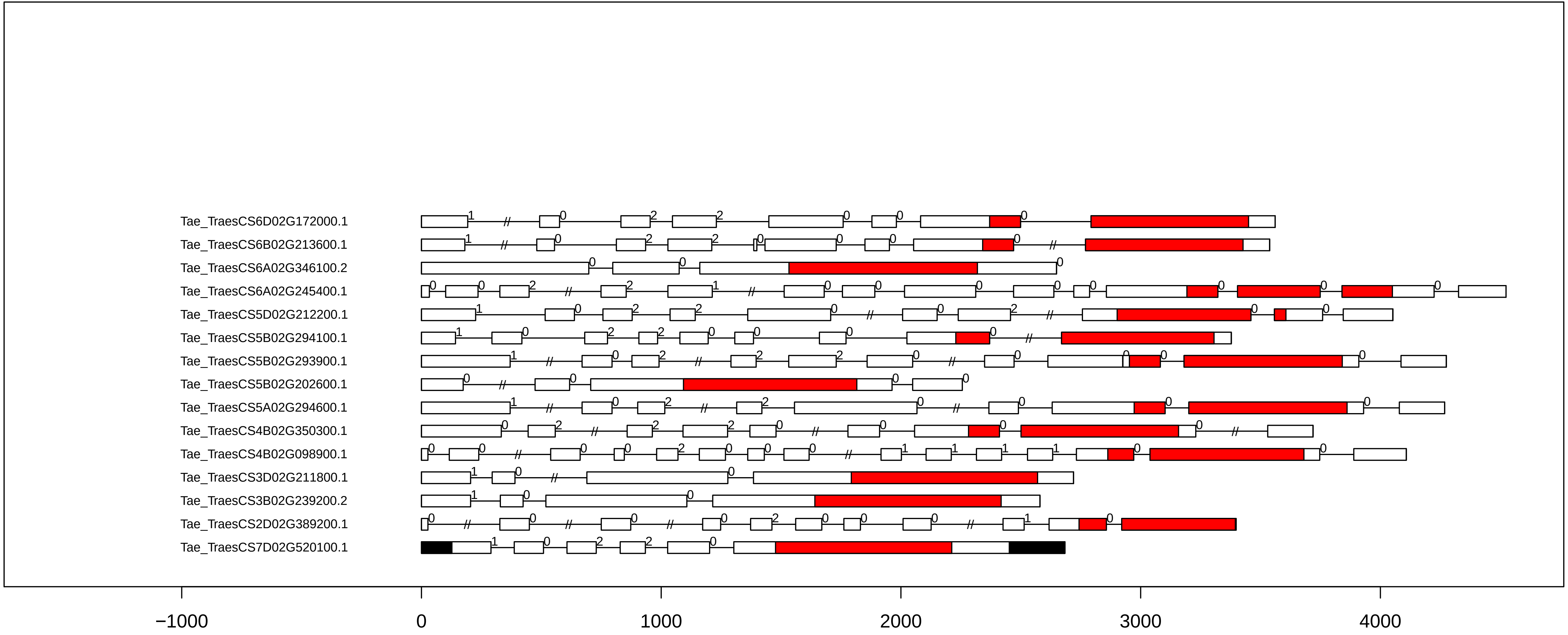

T.ae RLK−Pelle_RLCK−Os subfamily exon−intron and kinase domain diagram (all)

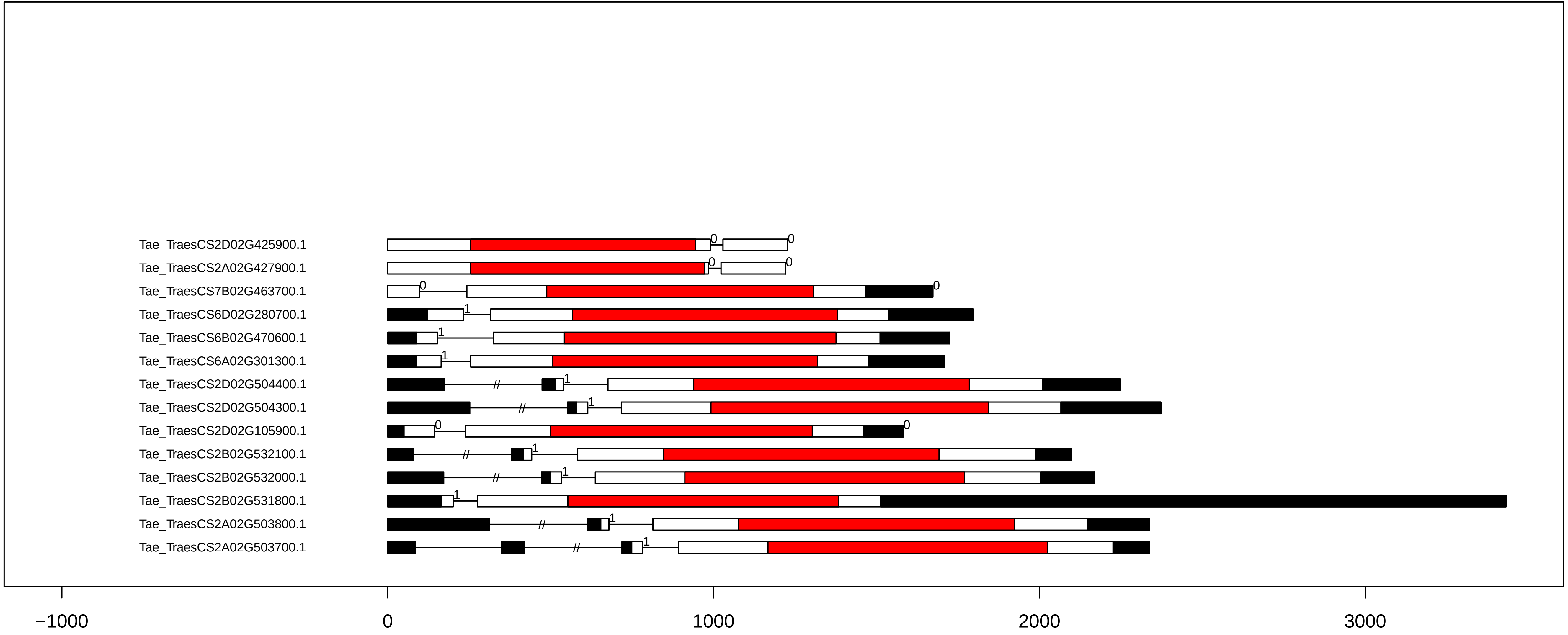

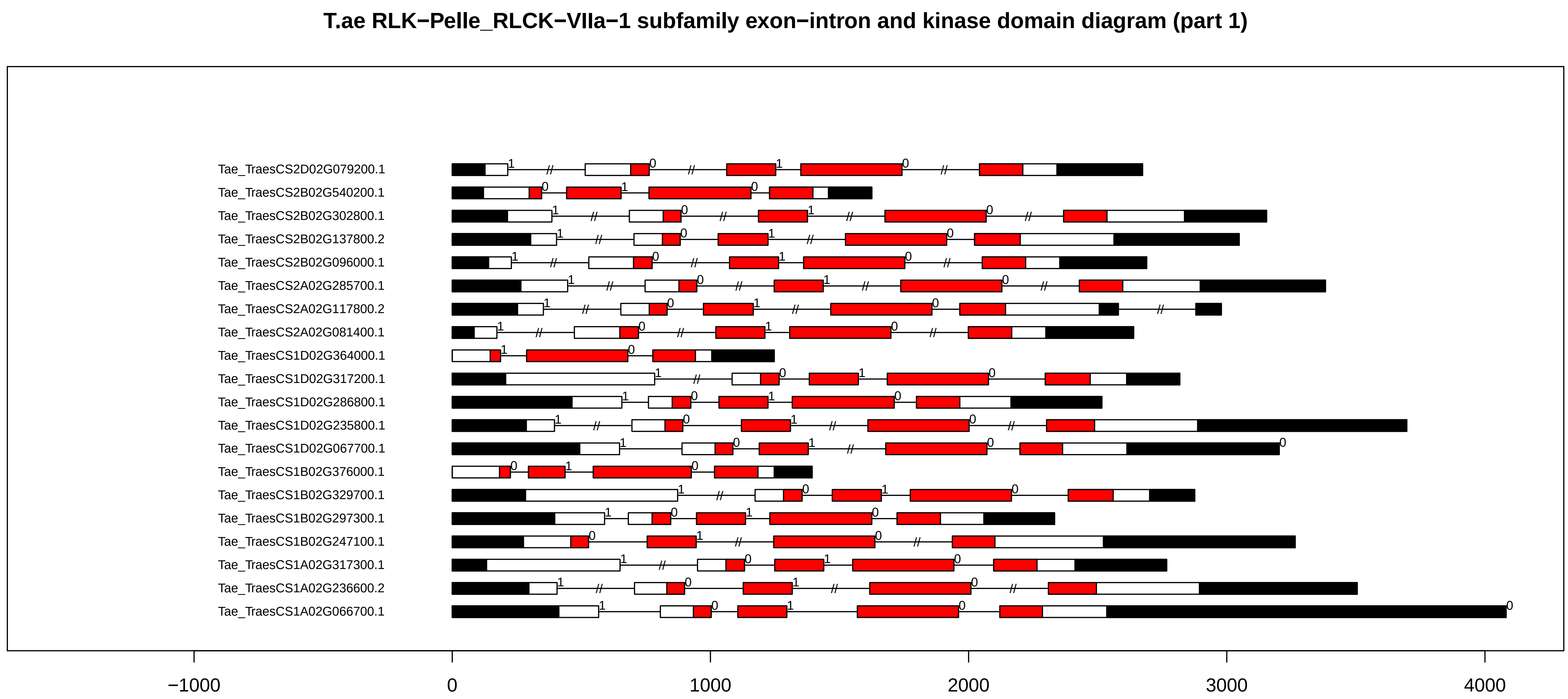

T.ae RLK−Pelle_RLCK−VIIa−1 subfamily exon−intron and kinase domain diagram (part 1)
Tae_TraesCS2D02G079200.1
Tae_TraesCS2B02G540200.1
Tae_TraesCS2B02G302800.1
Tae_TraesCS2B02G137800.2
Tae_TraesCS2B02G096000.1
Tae_TraesCS2A02G285700.1
Tae_TraesCS2A02G117800.2
Tae_TraesCS2A02G081400.1
Tae_TraesCS1D02G364000.1
Tae_TraesCS1D02G317200.1
Tae_TraesCS1D02G286800.1
Tae_TraesCS1D02G235800.1
Tae_TraesCS1D02G067700.1
Tae_TraesCS1B02G376000.1
Tae_TraesCS1B02G329700.1
Tae_TraesCS1B02G297300.1
Tae_TraesCS1B02G247100.1
Tae_TraesCS1A02G317300.1
Tae_TraesCS1A02G236600.2
Tae_TraesCS1A02G066700.1
−1000
0
1000
2000
3000
4000

T.ae RLK−Pelle_RLCK−VIIa−1 subfamily exon−intron and kinase domain diagram (part 2)

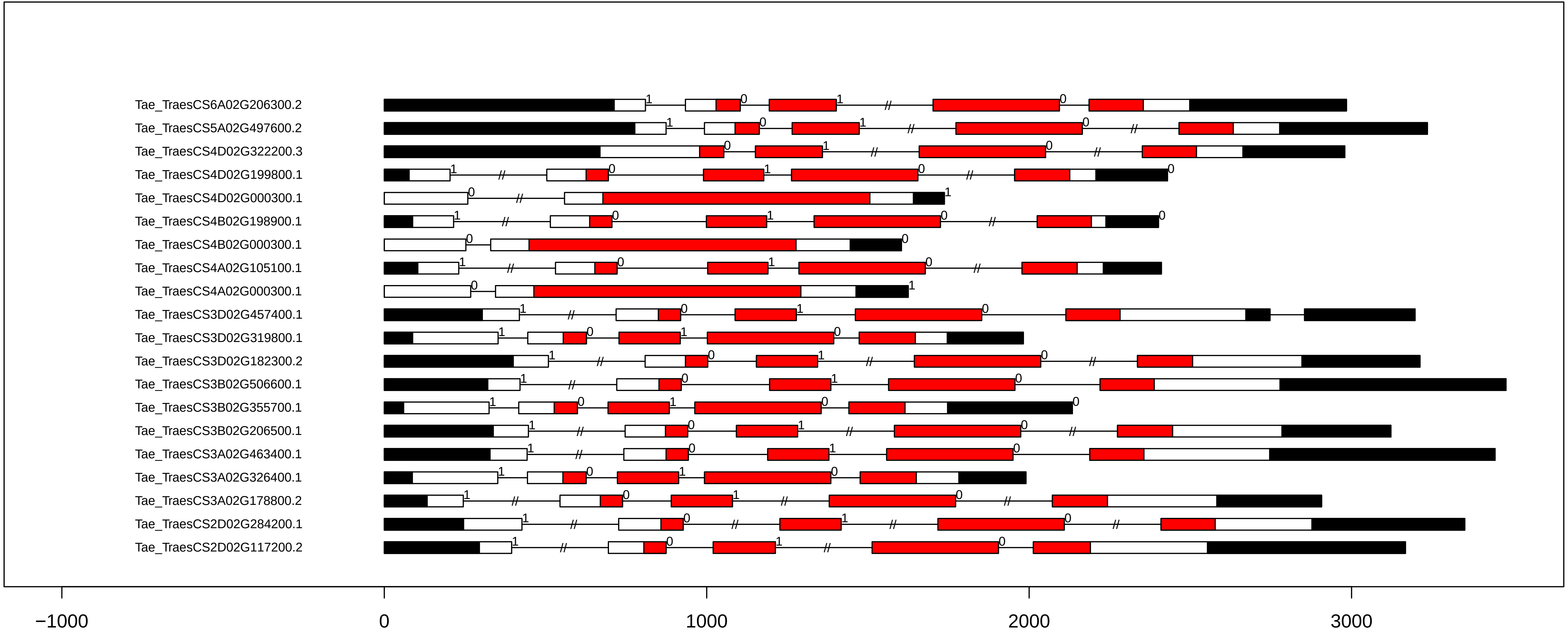

T.ae RLK−Pelle_RLCK−VIIa−1 subfamily exon−intron and kinase domain diagram (part 3)

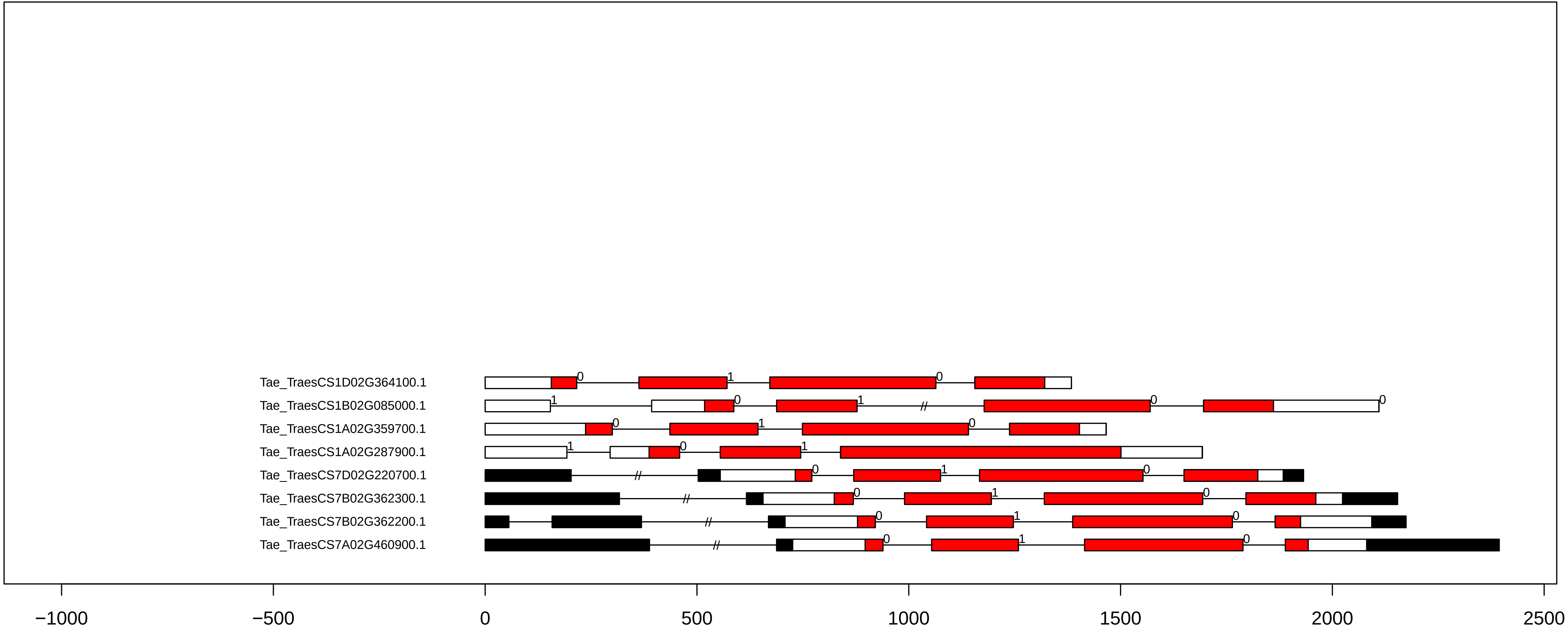

T.ae RLK−Pelle_RLCK−VIIa−2 subfamily exon−intron and kinase domain diagram (part 1)

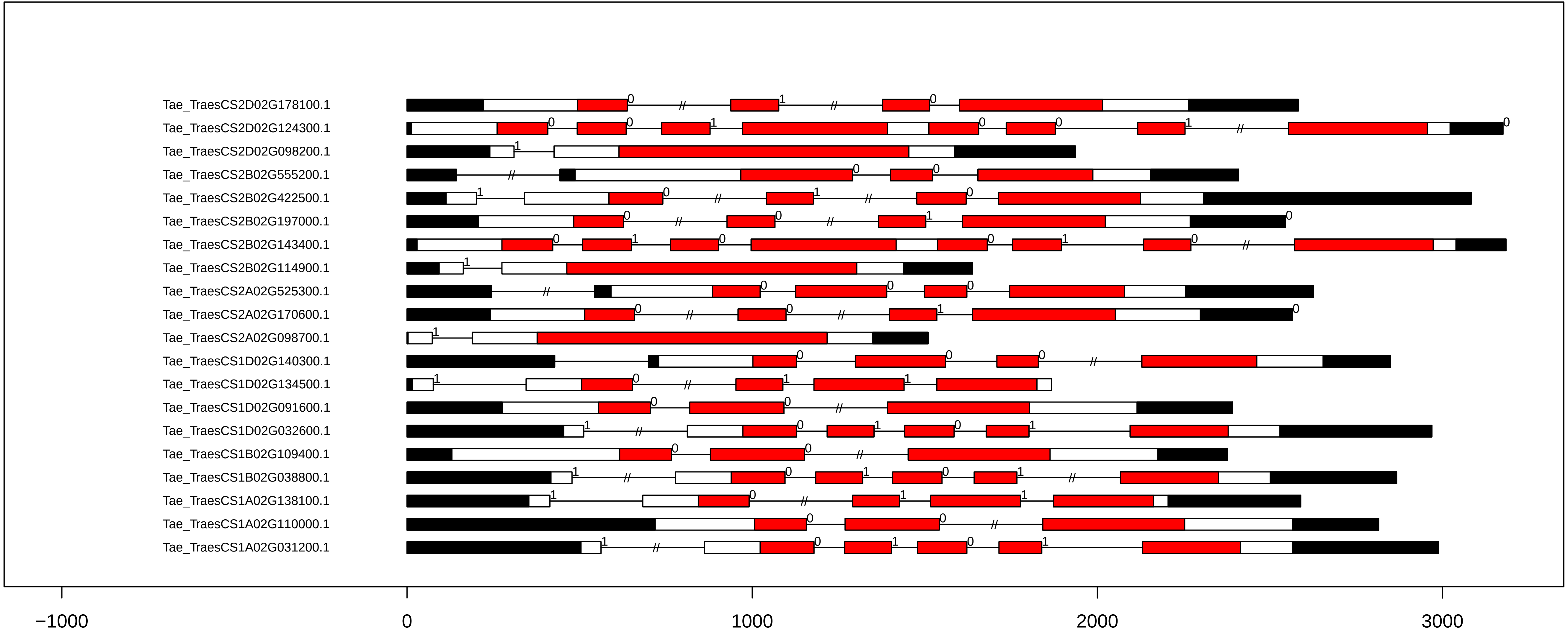

T.ae RLK−Pelle_RLCK−VIIa−2 subfamily exon−intron and kinase domain diagram (part 2)

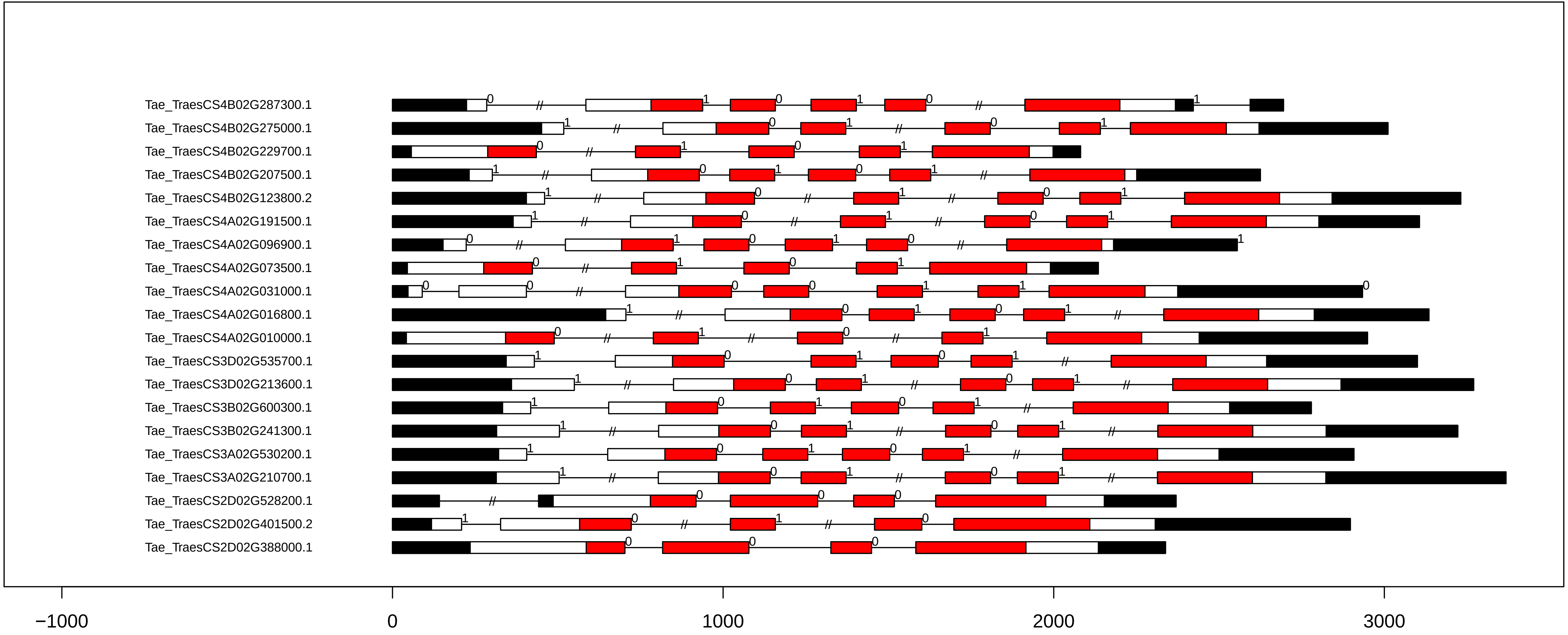

T.ae RLK−Pelle_RLCK−VIIa−2 subfamily exon−intron and kinase domain diagram (part 3)

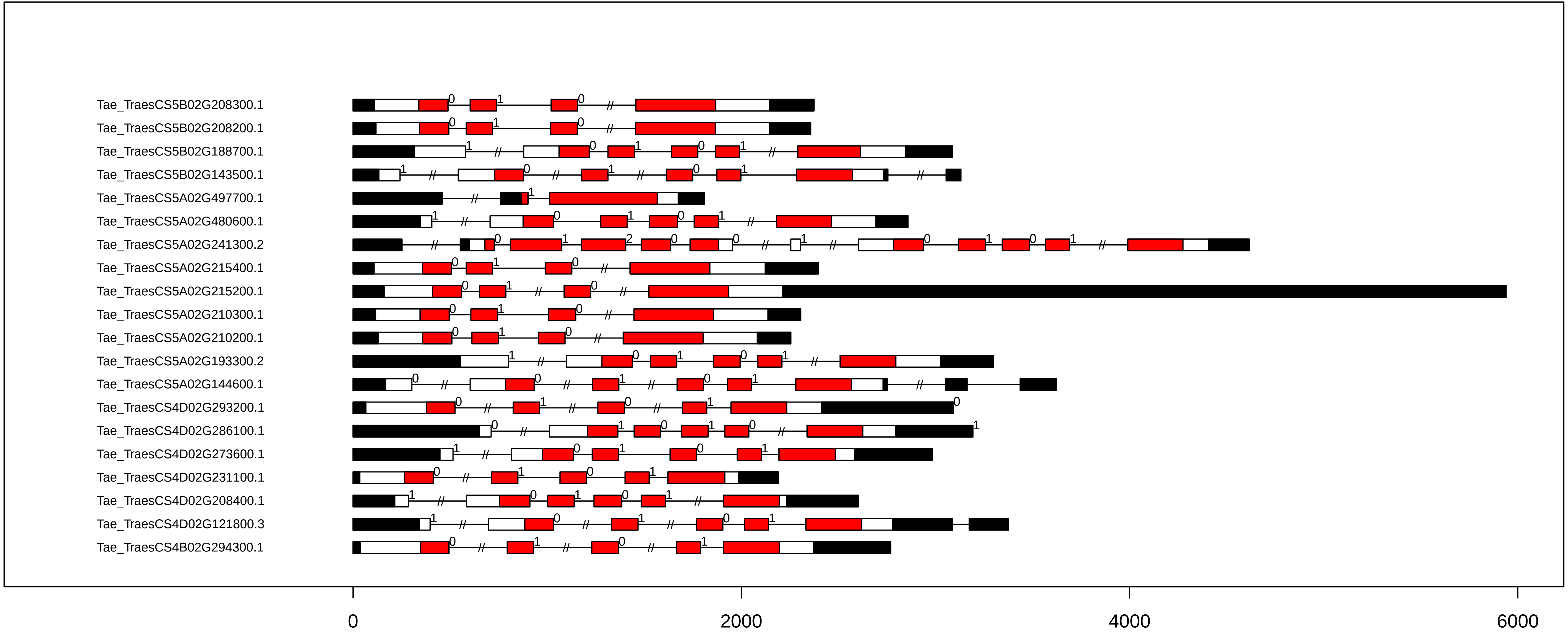

T.ae RLK−Pelle_RLCK−VIIa−2 subfamily exon−intron and kinase domain diagram (part 4)

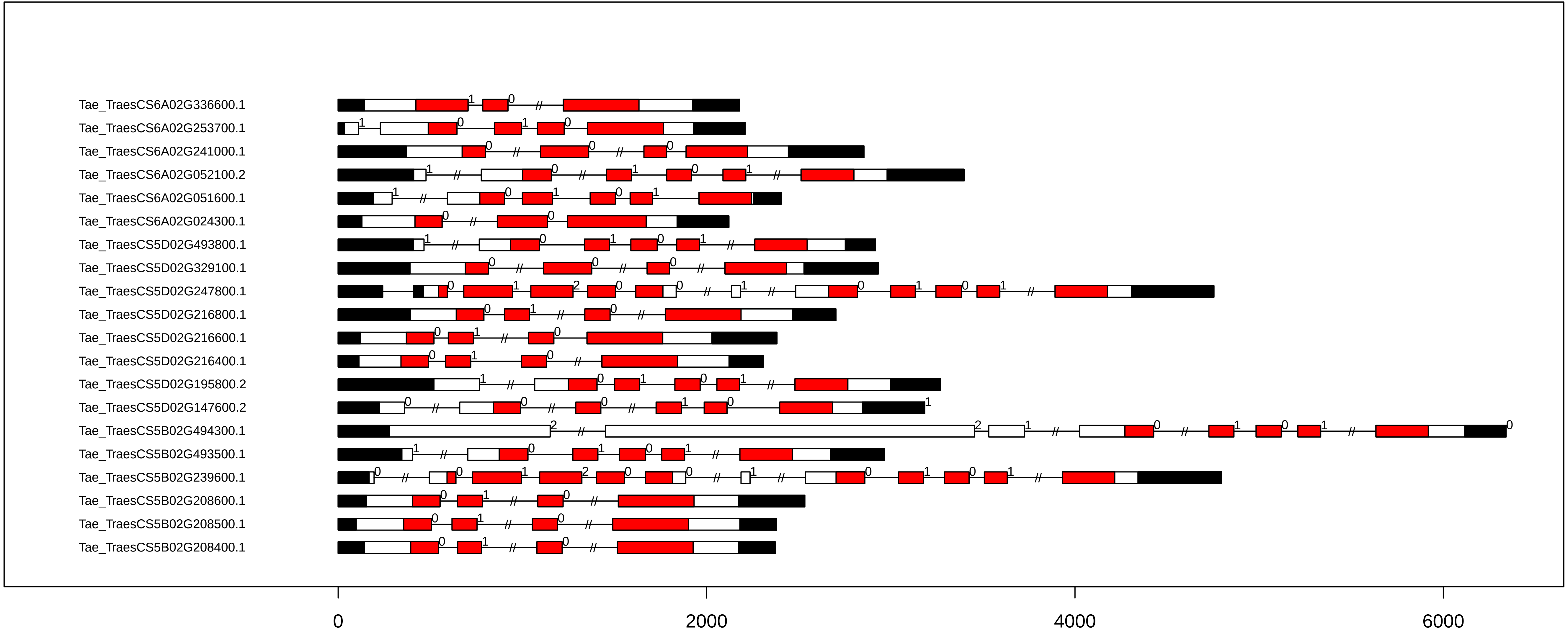

T.ae RLK−Pelle_RLCK−VIIa−2 subfamily exon−intron and kinase domain diagram (part 5)

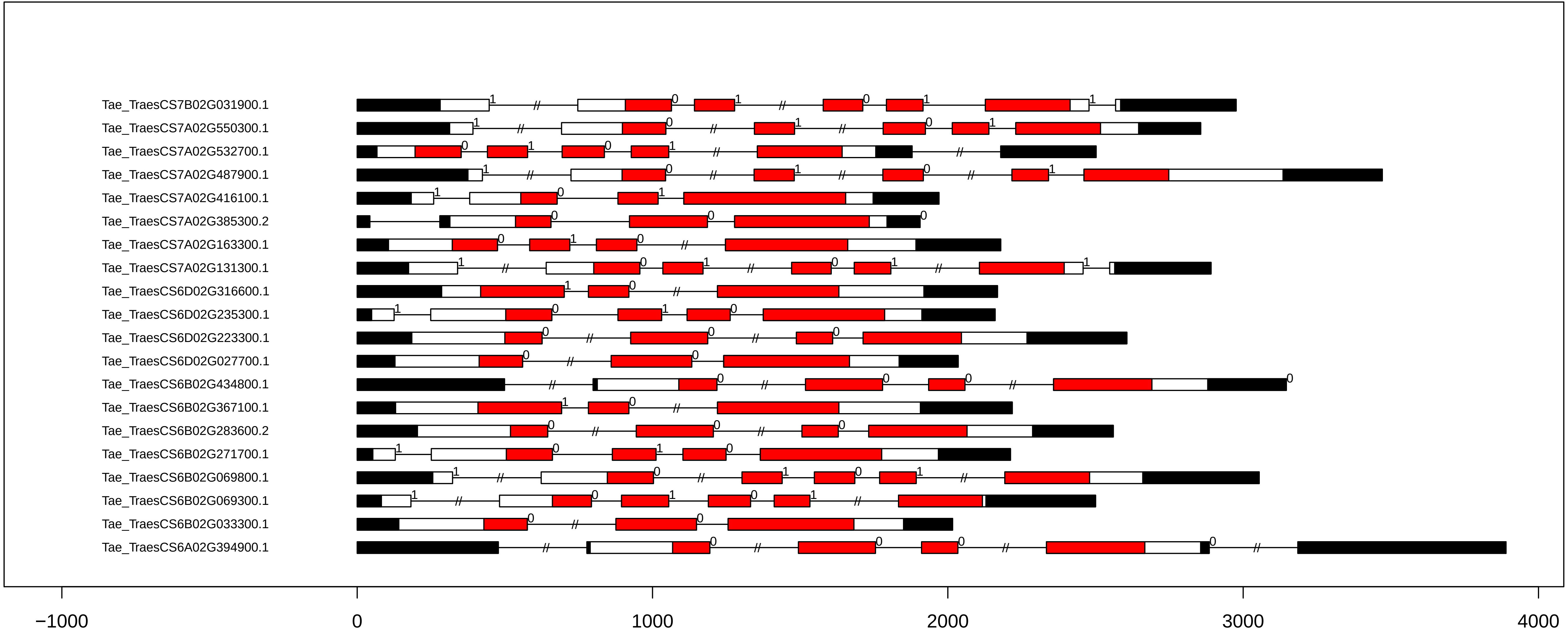

T.ae RLK−Pelle_RLCK−VIIa−2 subfamily exon−intron and kinase domain diagram (part 6)

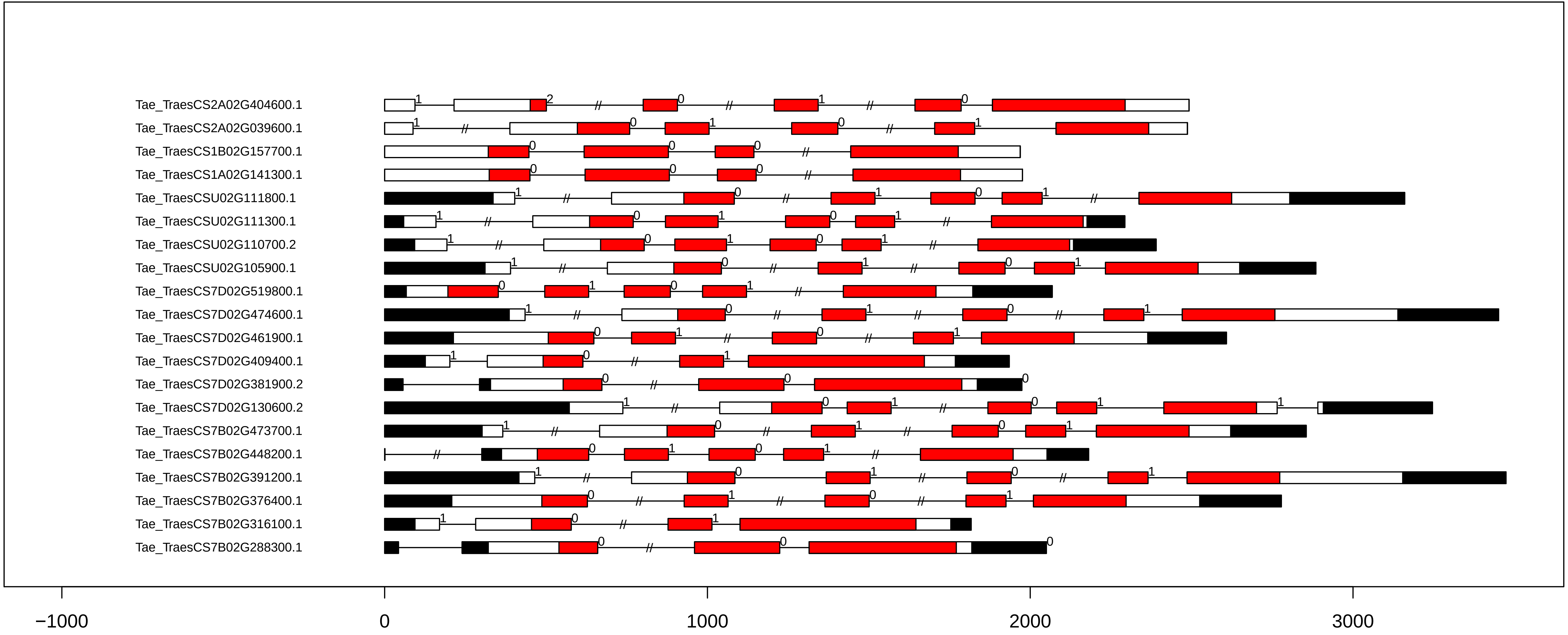

T.ae RLK−Pelle_RLCK−VIIa−2 subfamily exon−intron and kinase domain diagram (part 7)

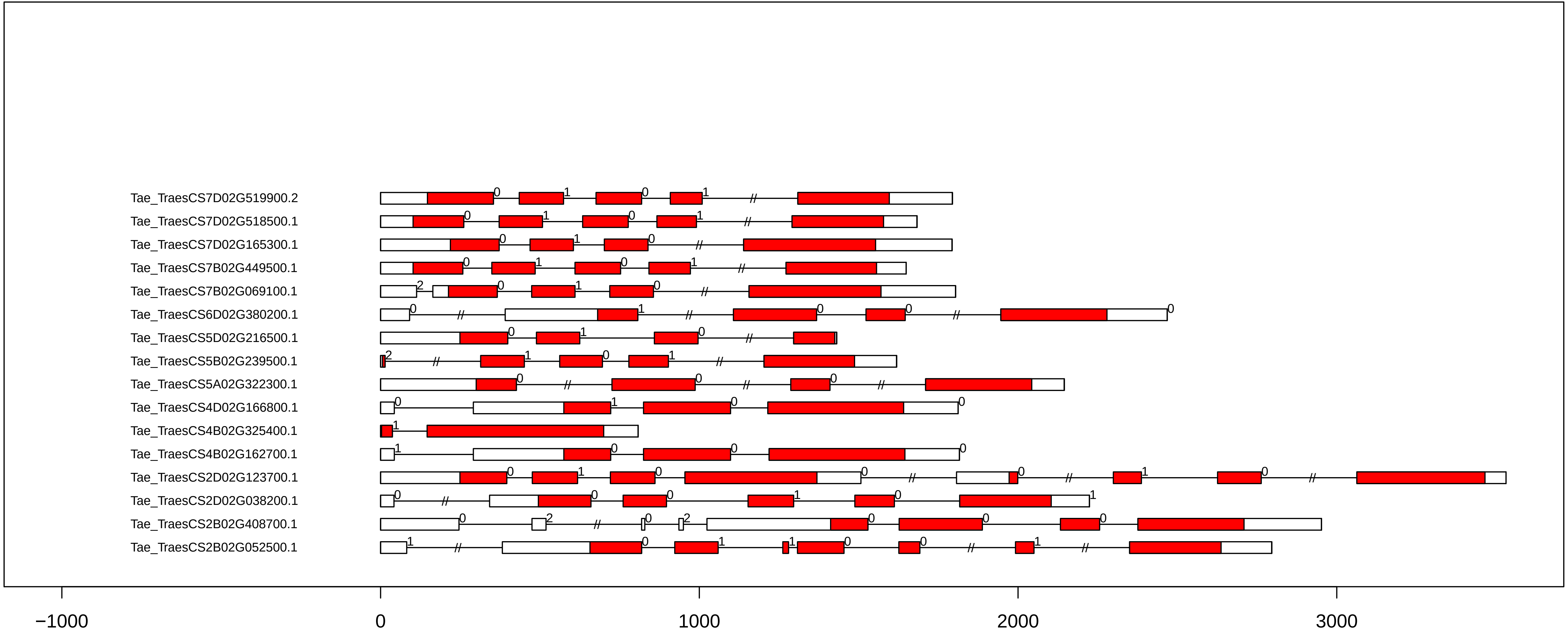

T.ae RLK−Pelle_RLCK−VIIb subfamily exon−intron and kinase domain diagram (all)

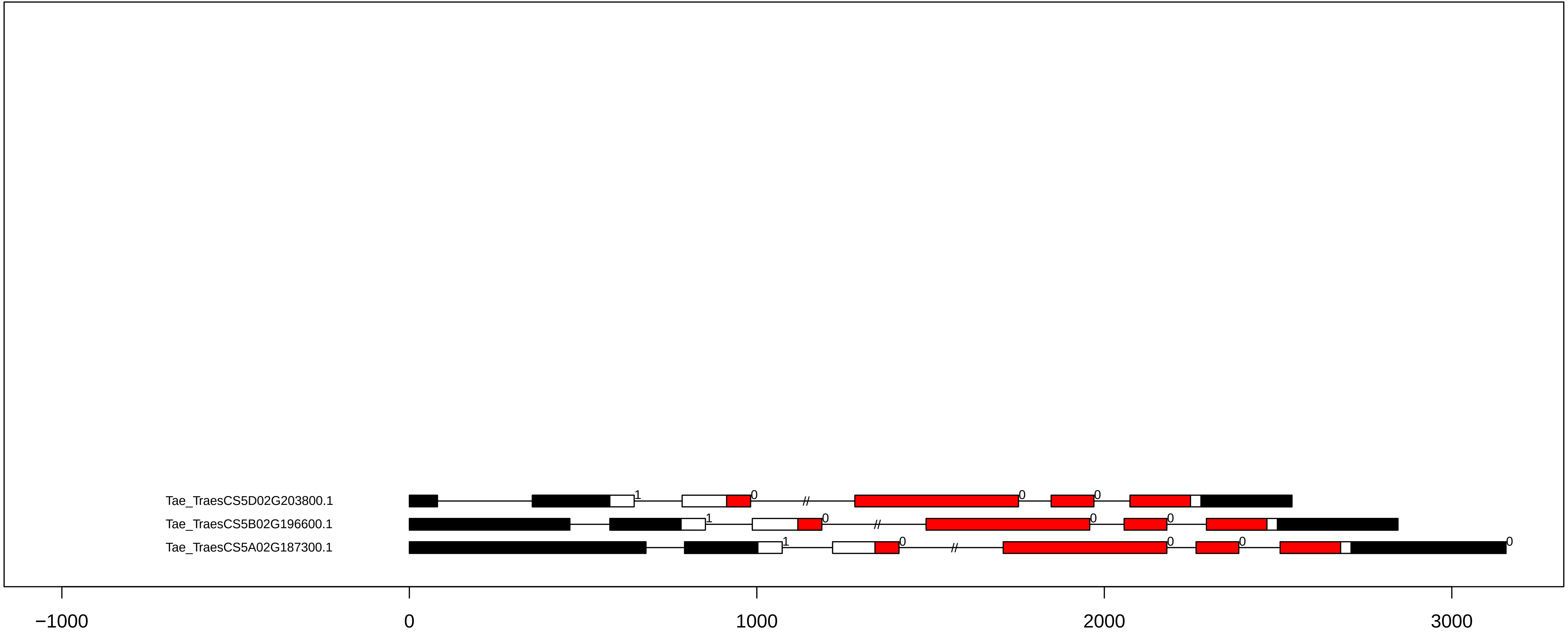

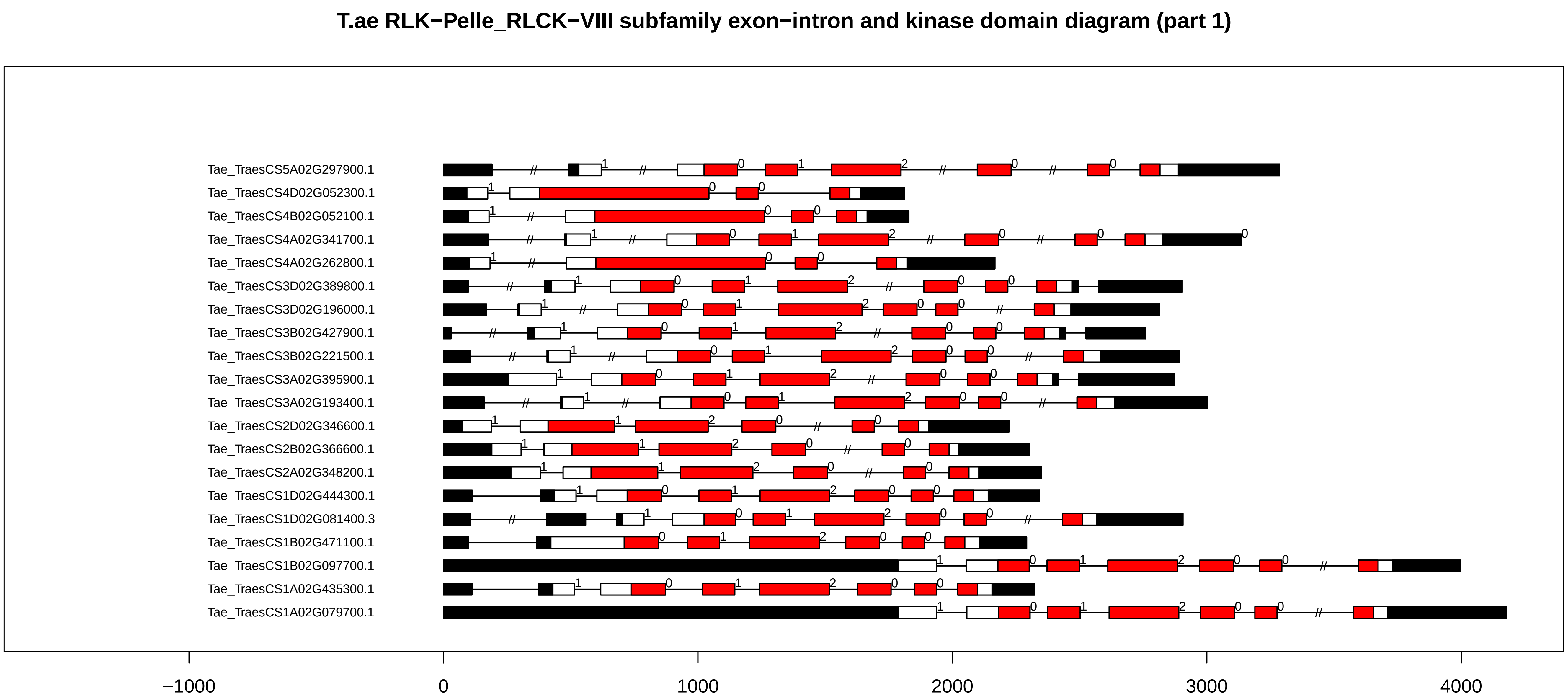
T.ae RLK−Pelle_RLCK−VIII subfamily exon−intron and kinase domain diagram (part 1)
Tae_TraesCS5A02G297900.1
Tae_TraesCS4D02G052300.1
Tae_TraesCS4B02G052100.1
Tae_TraesCS4A02G341700.1
Tae_TraesCS4A02G262800.1
Tae_TraesCS3D02G389800.1
Tae_TraesCS3D02G196000.1
Tae_TraesCS3B02G427900.1
Tae_TraesCS3B02G221500.1
Tae_TraesCS3A02G395900.1
Tae_TraesCS3A02G193400.1
Tae_TraesCS2D02G346600.1
Tae_TraesCS2B02G366600.1
Tae_TraesCS2A02G348200.1
Tae_TraesCS1D02G444300.1
Tae_TraesCS1D02G081400.3
Tae_TraesCS1B02G471100.1
Tae_TraesCS1B02G097700.1
Tae_TraesCS1A02G435300.1
Tae_TraesCS1A02G079700.1
−1000
0
1000
2000
3000
4000

T.ae RLK−Pelle_RLCK−VIII subfamily exon−intron and kinase domain diagram (part 2)

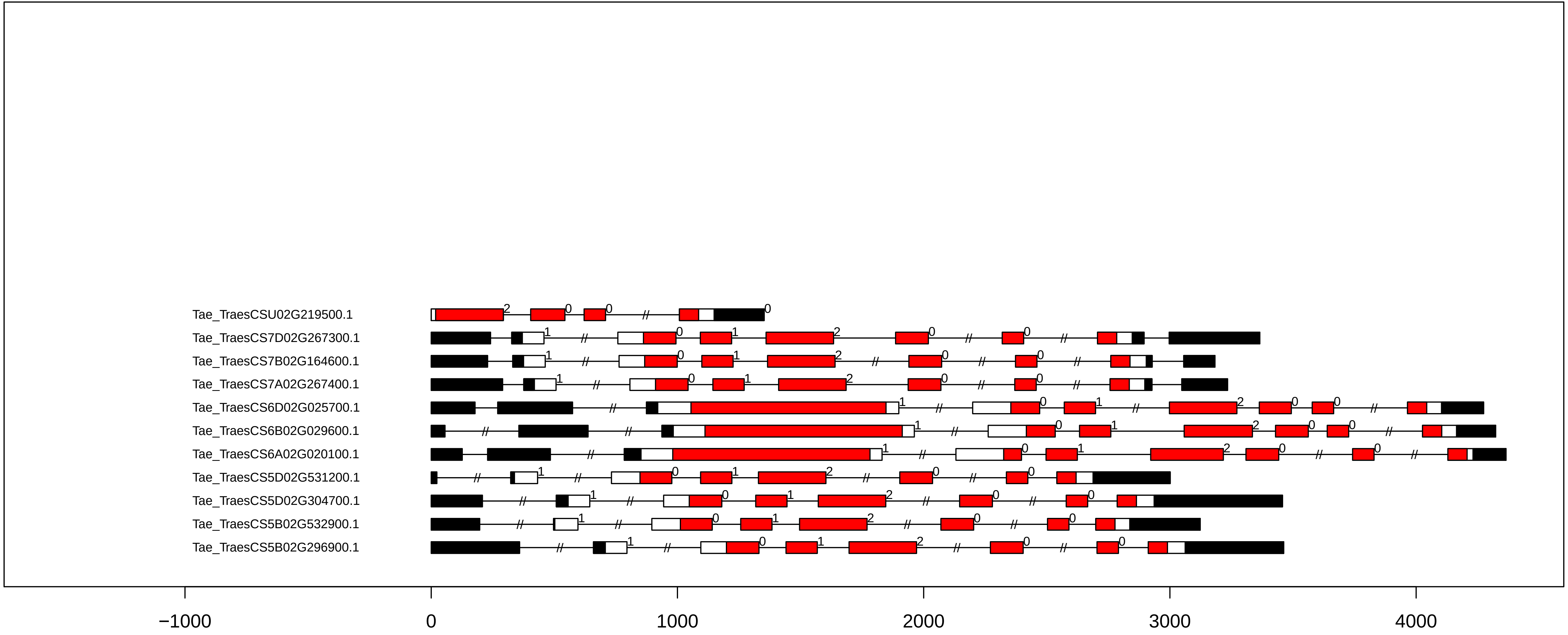

T.ae RLK−Pelle_RLCK−VI subfamily exon−intron and kinase domain diagram (part 1)

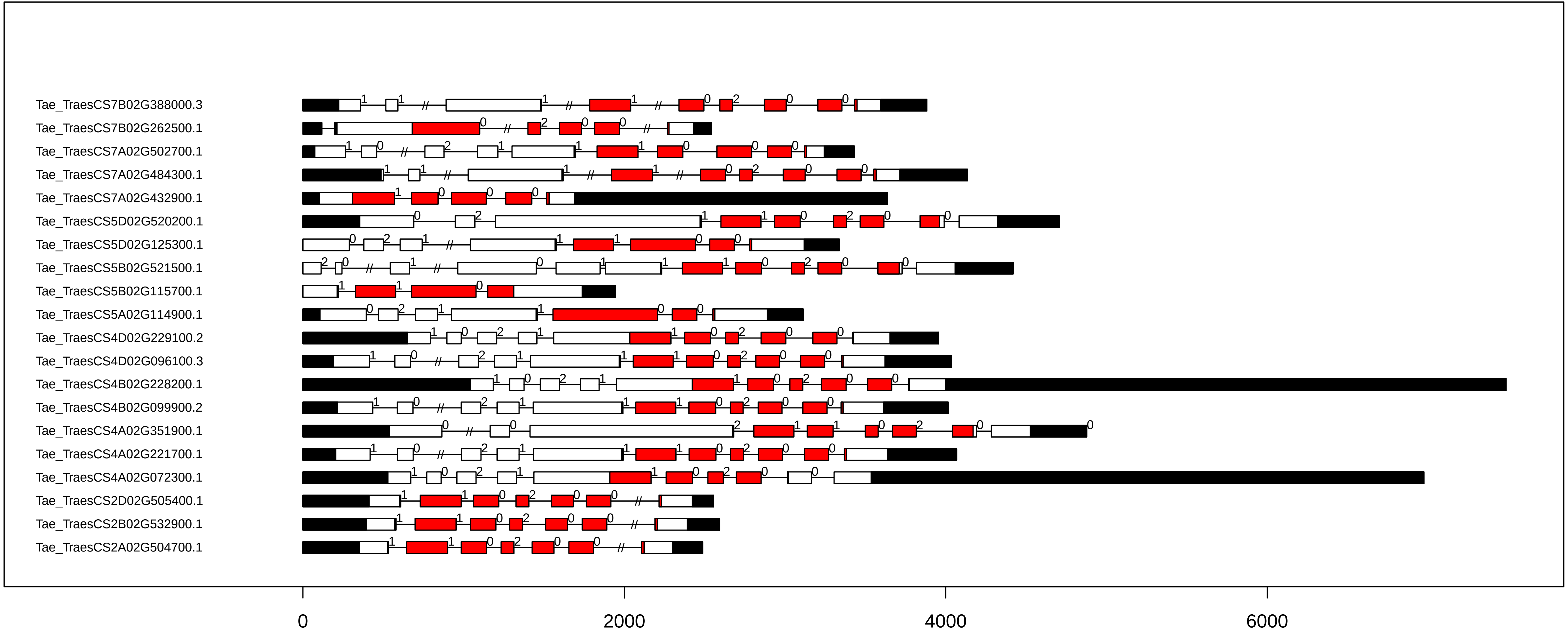

T.ae RLK−Pelle_RLCK−VI subfamily exon−intron and kinase domain diagram (part 2)

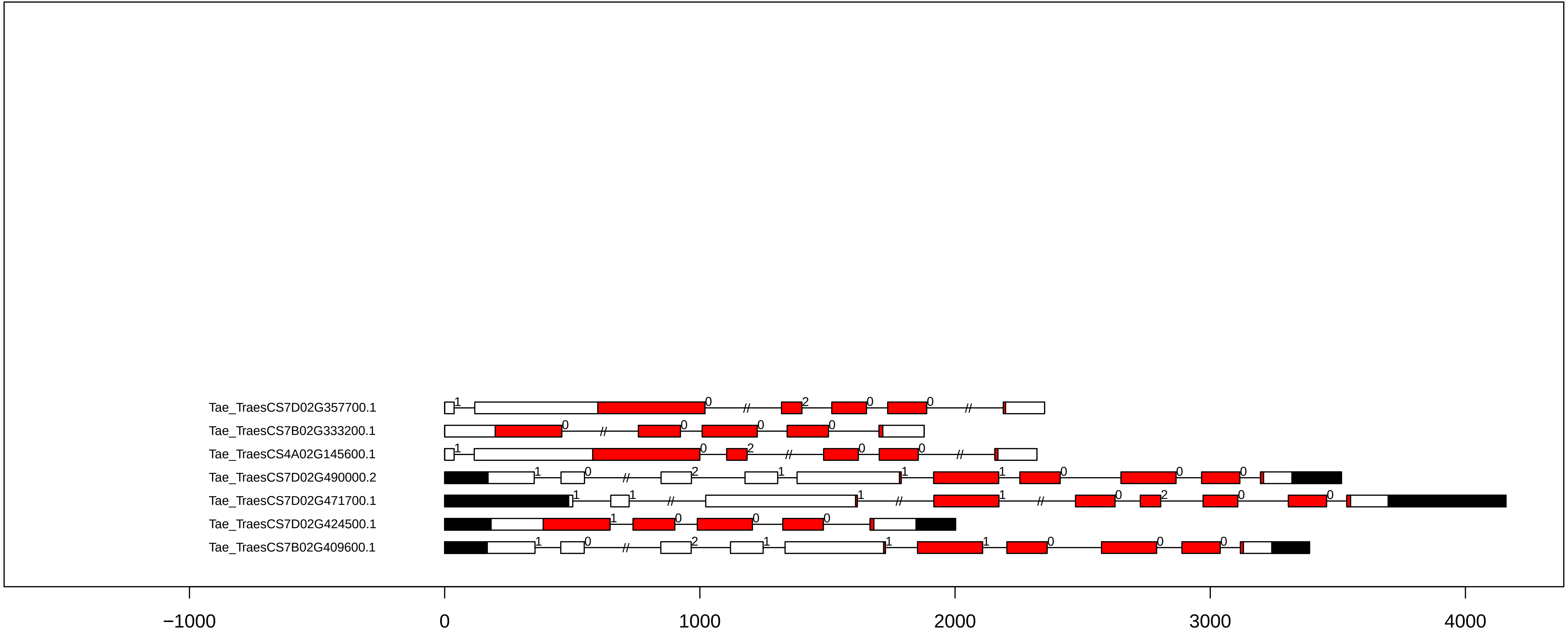

T.ae RLK−Pelle_RLCK−V subfamily exon−intron and kinase domain diagram (part 1)

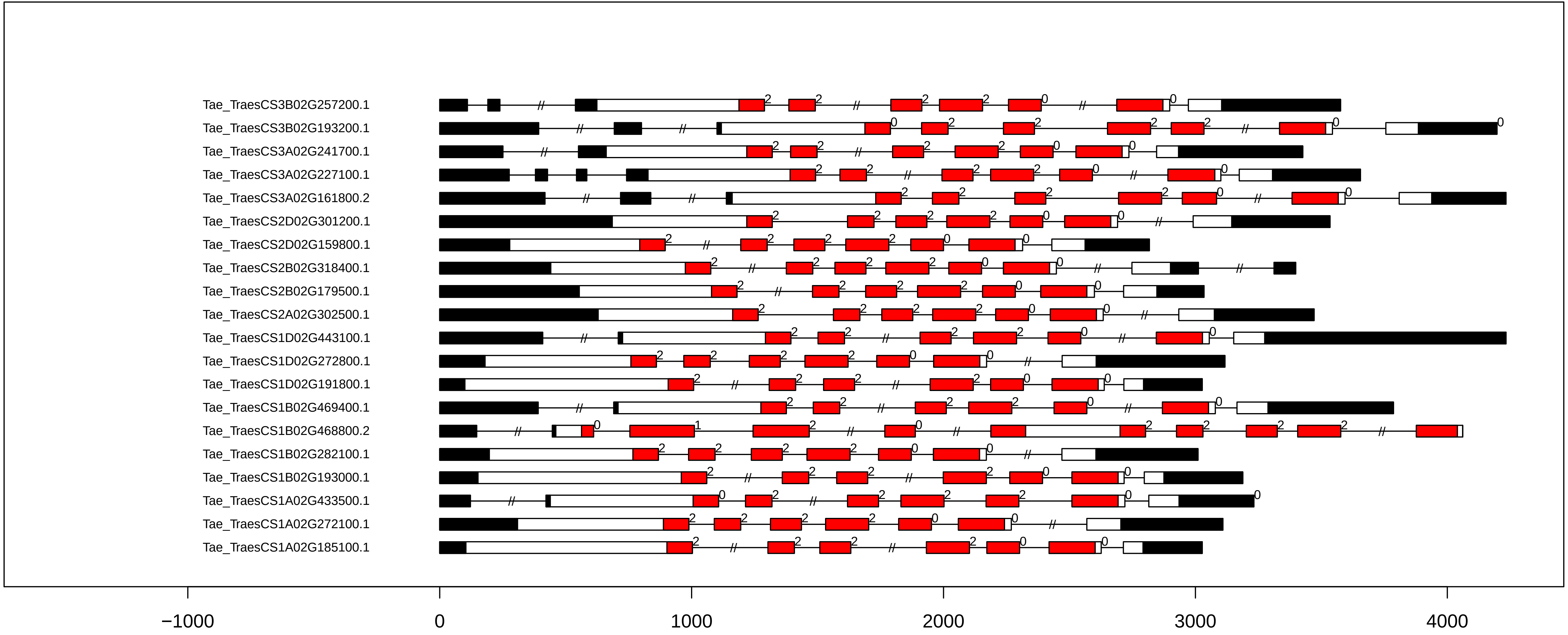

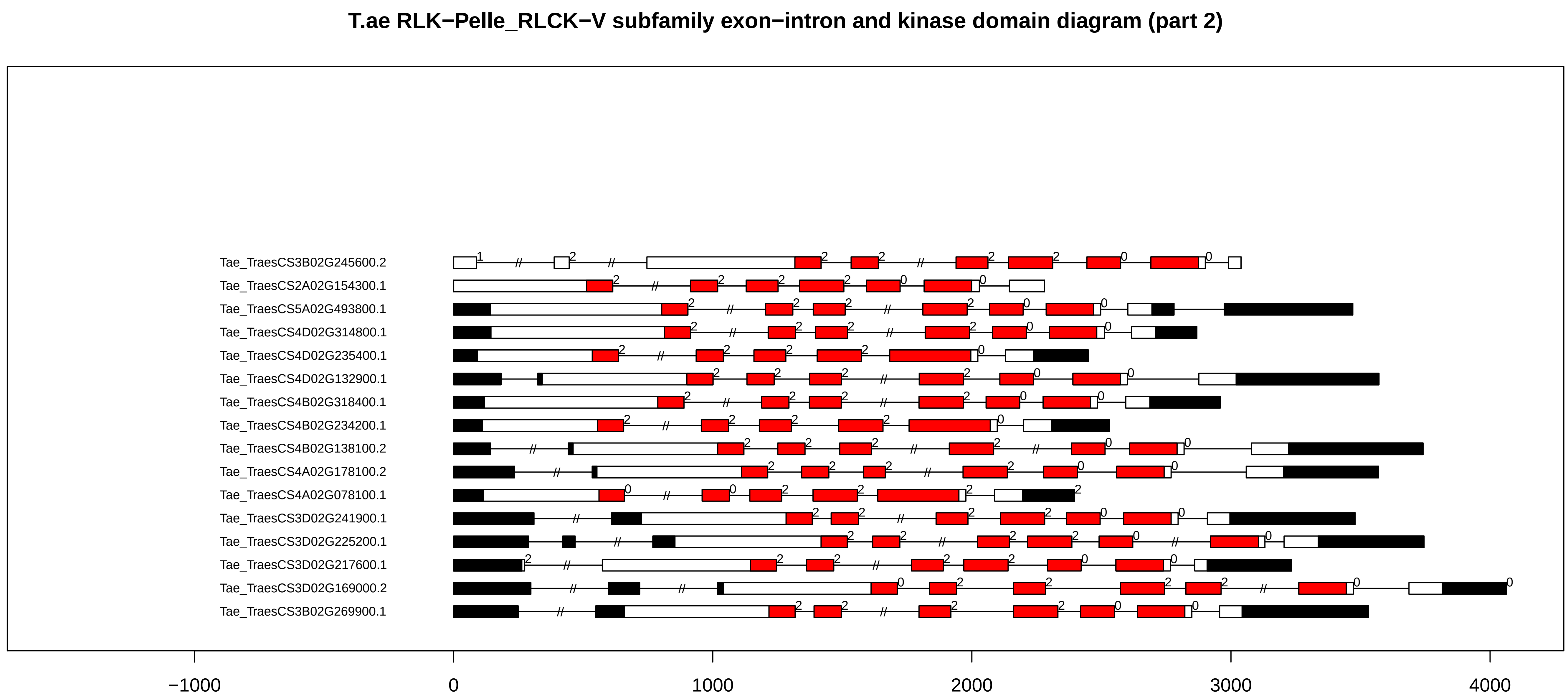

T.ae RLK−Pelle_RLCK−V subfamily exon−intron and kinase domain diagram (part 2)
Tae_TraesCS3B02G245600.2
Tae_TraesCS2A02G154300.1
Tae_TraesCS5A02G493800.1
Tae_TraesCS4D02G314800.1
Tae_TraesCS4D02G235400.1
Tae_TraesCS4D02G132900.1
Tae_TraesCS4B02G318400.1
Tae_TraesCS4B02G234200.1
Tae_TraesCS4B02G138100.2
Tae_TraesCS4A02G178100.2
Tae_TraesCS4A02G078100.1
Tae_TraesCS3D02G241900.1
Tae_TraesCS3D02G225200.1
Tae_TraesCS3D02G217600.1
Tae_TraesCS3D02G169000.2
Tae_TraesCS3B02G269900.1
−1000
0
1000
2000
3000
4000

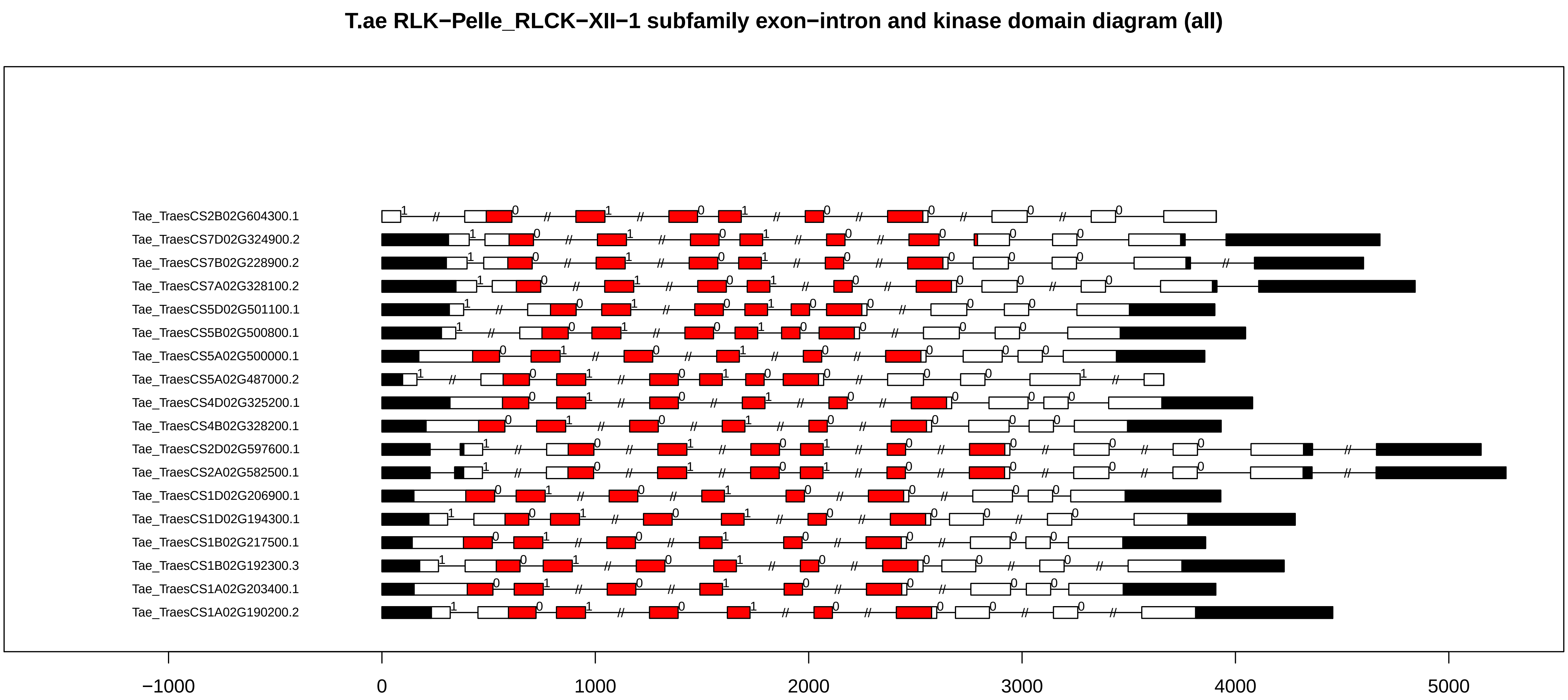
T.ae RLK−Pelle_RLCK−XII−1 subfamily exon−intron and kinase domain diagram (all)
Tae_TraesCS2B02G604300.1
Tae_TraesCS7D02G324900.2
Tae_TraesCS7B02G228900.2
Tae_TraesCS7A02G328100.2
Tae_TraesCS5D02G501100.1
Tae_TraesCS5B02G500800.1
Tae_TraesCS5A02G500000.1
Tae_TraesCS5A02G487000.2
Tae_TraesCS4D02G325200.1
Tae_TraesCS4B02G328200.1
Tae_TraesCS2D02G597600.1
Tae_TraesCS2A02G582500.1
Tae_TraesCS1D02G206900.1
Tae_TraesCS1D02G194300.1
Tae_TraesCS1B02G217500.1
Tae_TraesCS1B02G192300.3
Tae_TraesCS1A02G203400.1
Tae_TraesCS1A02G190200.2
−1000
0
1000
2000
3000
4000
5000

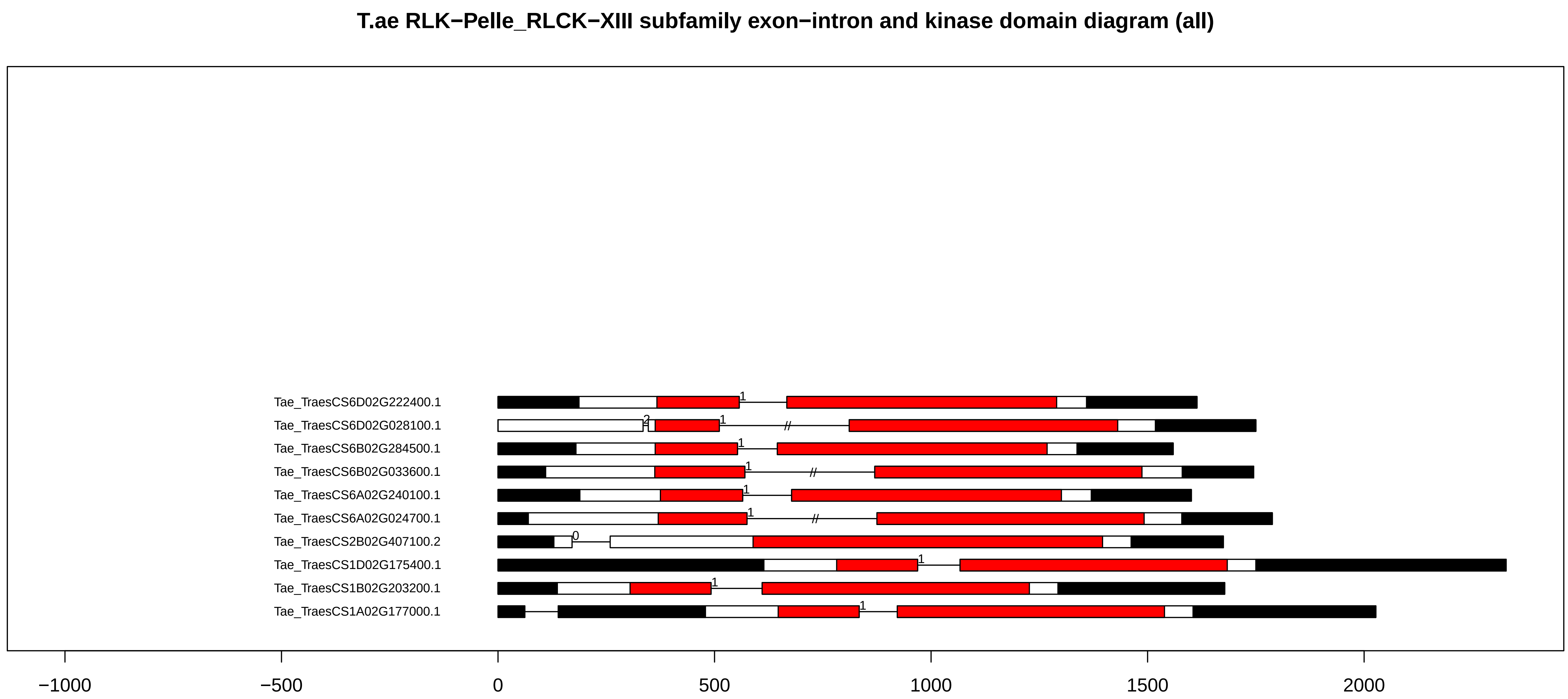

T.ae RLK−Pelle_RLCK−XIII subfamily exon−intron and kinase domain diagram (all)
Tae_TraesCS6D02G222400.1
Tae_TraesCS6D02G028100.1
Tae_TraesCS6B02G284500.1
Tae_TraesCS6B02G033600.1
Tae_TraesCS6A02G240100.1
Tae_TraesCS6A02G024700.1
Tae_TraesCS2B02G407100.2
Tae_TraesCS1D02G175400.1
Tae_TraesCS1B02G203200.1
Tae_TraesCS1A02G177000.1
−1000
−500
0
500
1000
1500
2000

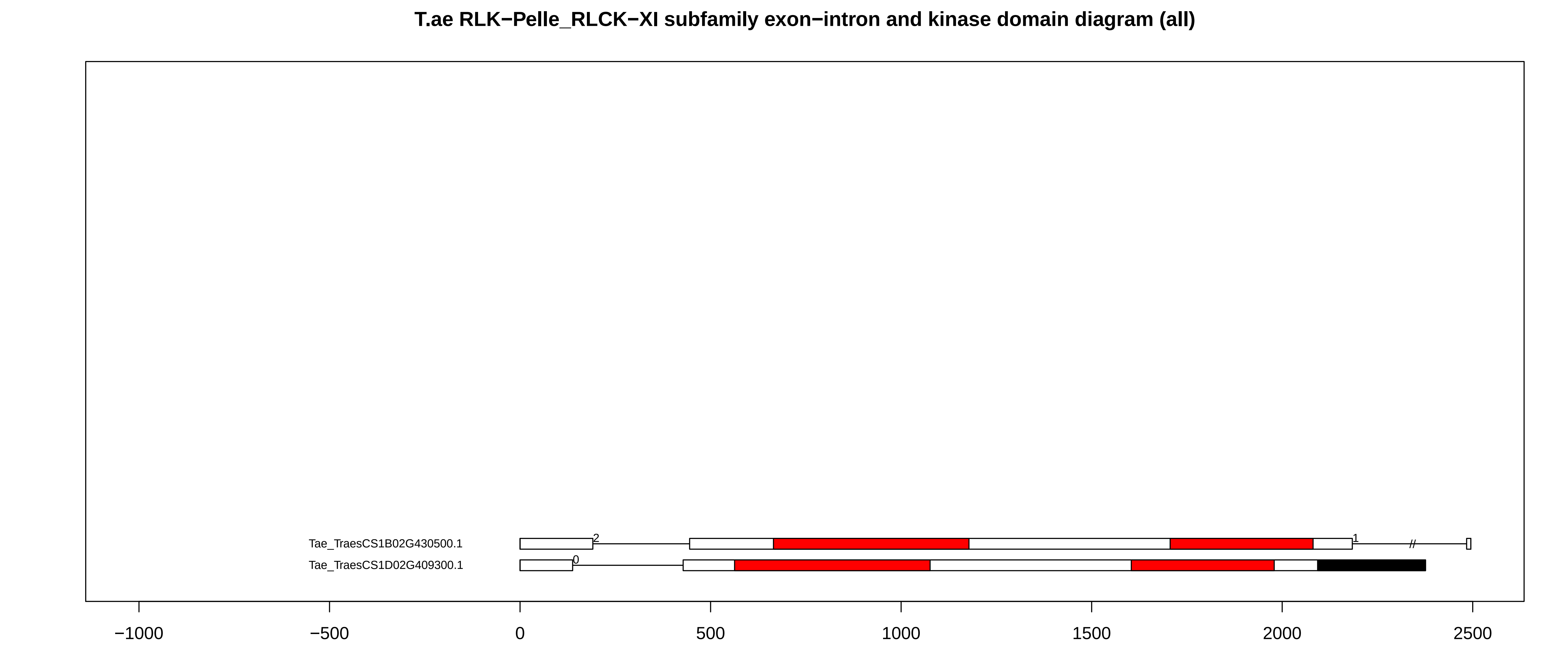
T.ae RLK−Pelle_RLCK−XI subfamily exon−intron and kinase domain diagram (all)
Tae_TraesCS1B02G430500.1
Tae_TraesCS1D02G409300.1
2
1
0
−1000
−500
0
500
1000
1500
2000
2500

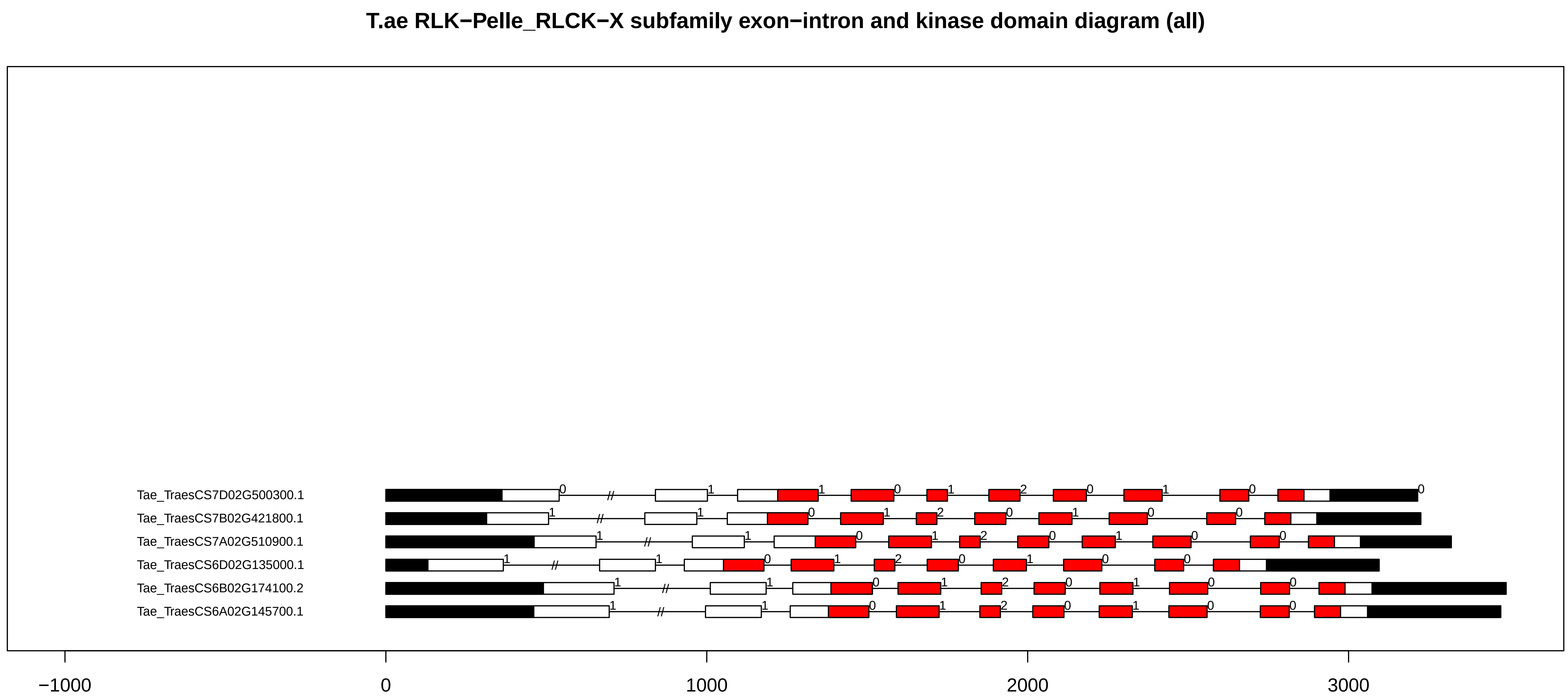

T.ae RLK−Pelle_RLCK−X subfamily exon−intron and kinase domain diagram (all)
Tae_TraesCS7D02G500300.1
Tae_TraesCS7B02G421800.1
Tae_TraesCS7A02G510900.1
Tae_TraesCS6D02G135000.1
Tae_TraesCS6B02G174100.2
Tae_TraesCS6A02G145700.1
−1000
0
1000
2000
3000

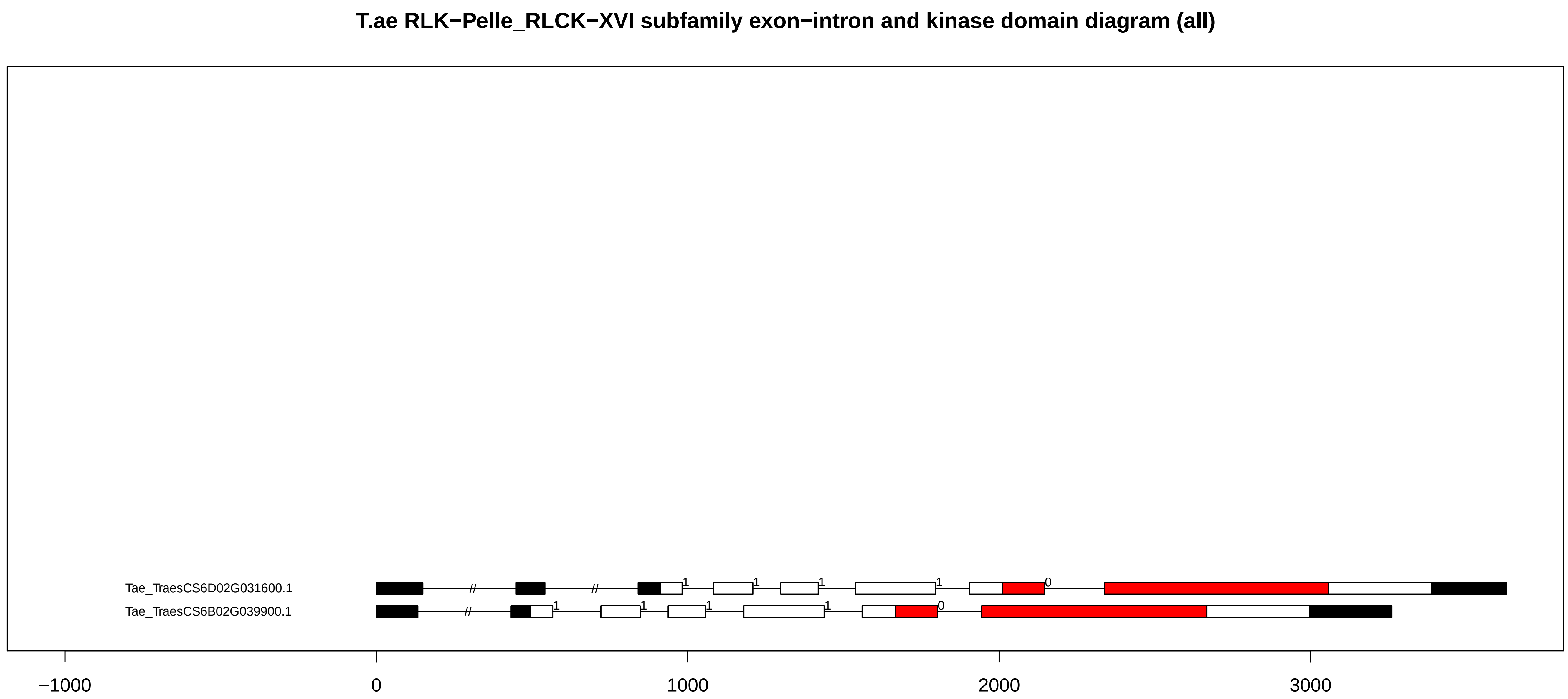

T.ae RLK−Pelle_RLCK−XVI subfamily exon−intron and kinase domain diagram (all)
Tae_TraesCS6D02G031600.1
Tae_TraesCS6B02G039900.1
1
1
1
1
0
1
1
1
1
0
−1000
0
1000
2000
3000

T.ae RLK−Pelle_RLCK−XV subfamily exon−intron and kinase domain diagram (all)

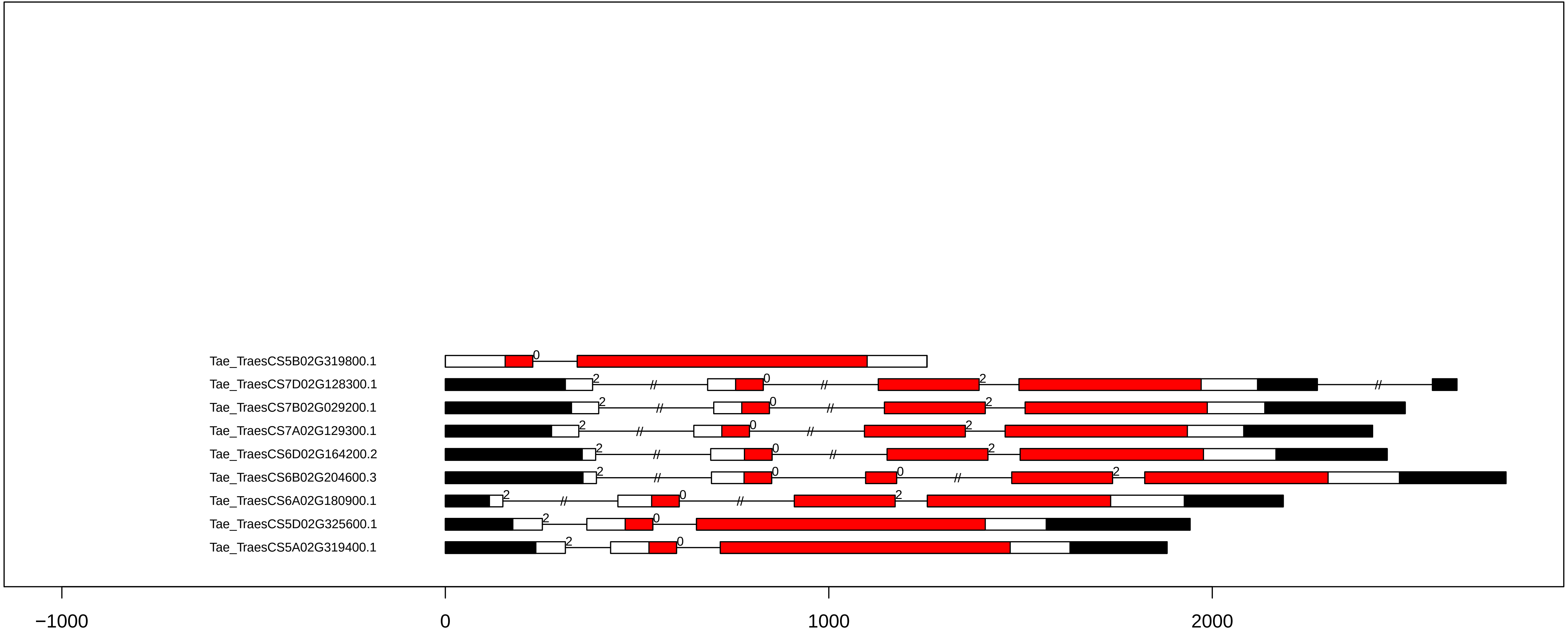

T.ae RLK−Pelle_SD−2b subfamily exon−intron and kinase domain diagram (part 1)

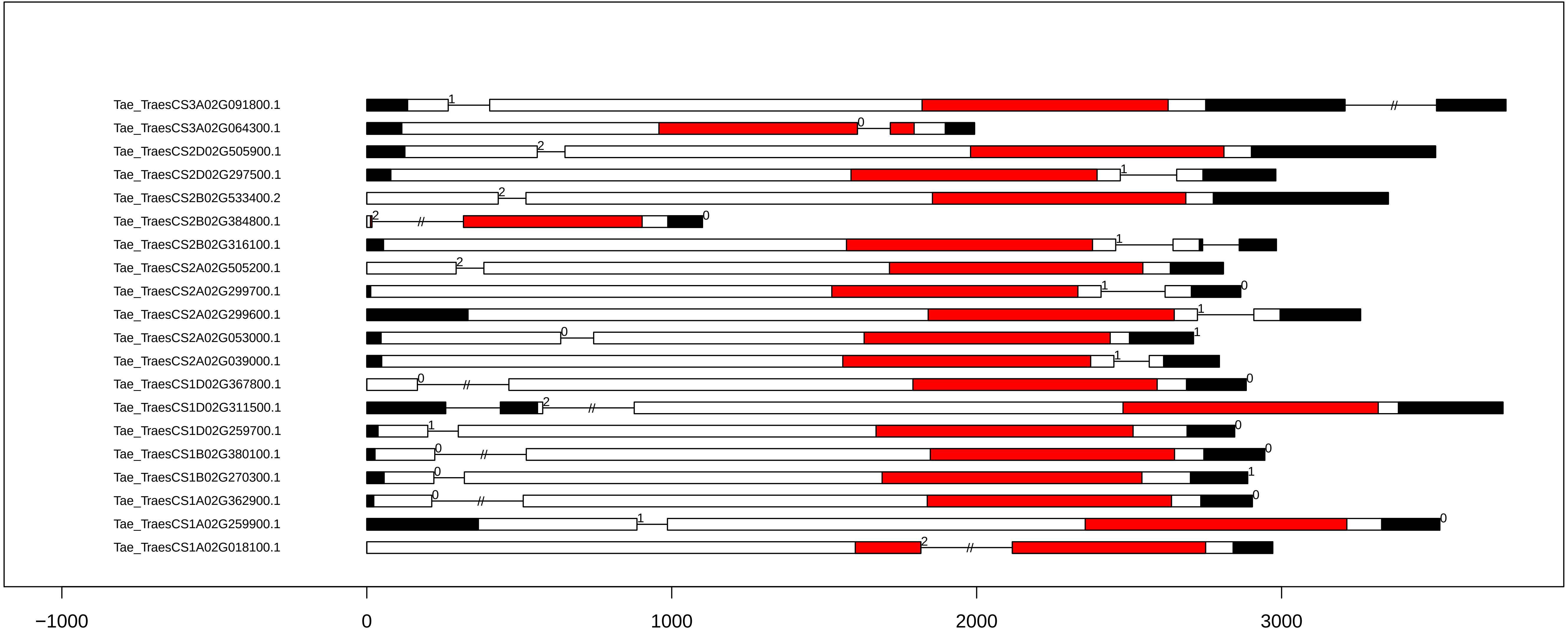

T.ae RLK−Pelle_SD−2b subfamily exon−intron and kinase domain diagram (part 2)

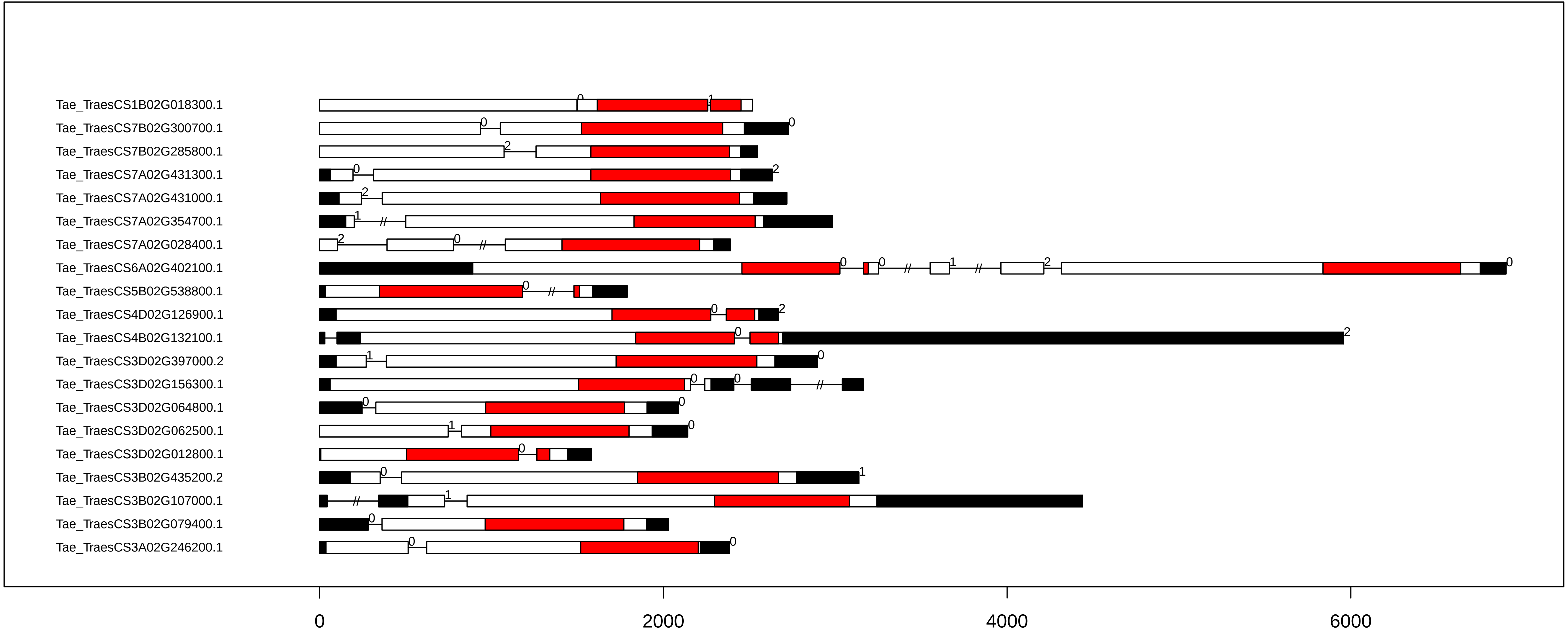

T.ae RLK−Pelle_SD−2b subfamily exon−intron and kinase domain diagram (part 3)

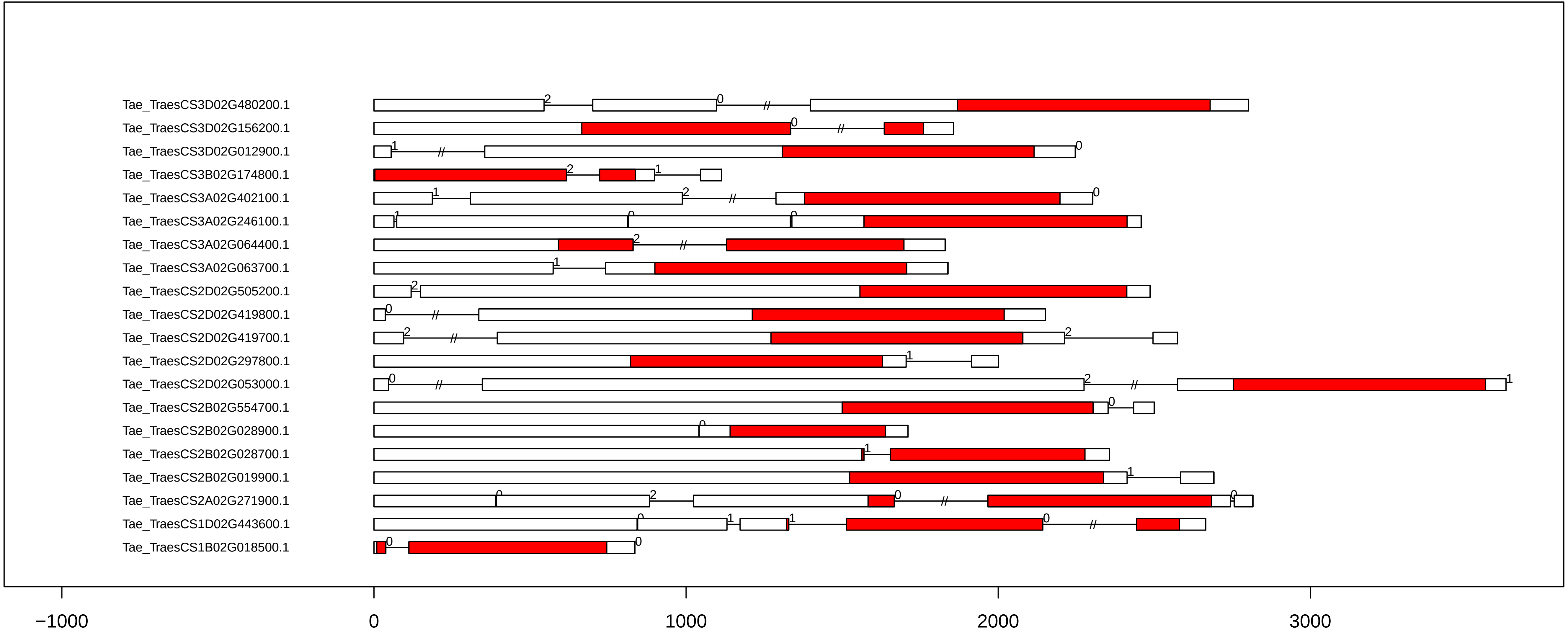

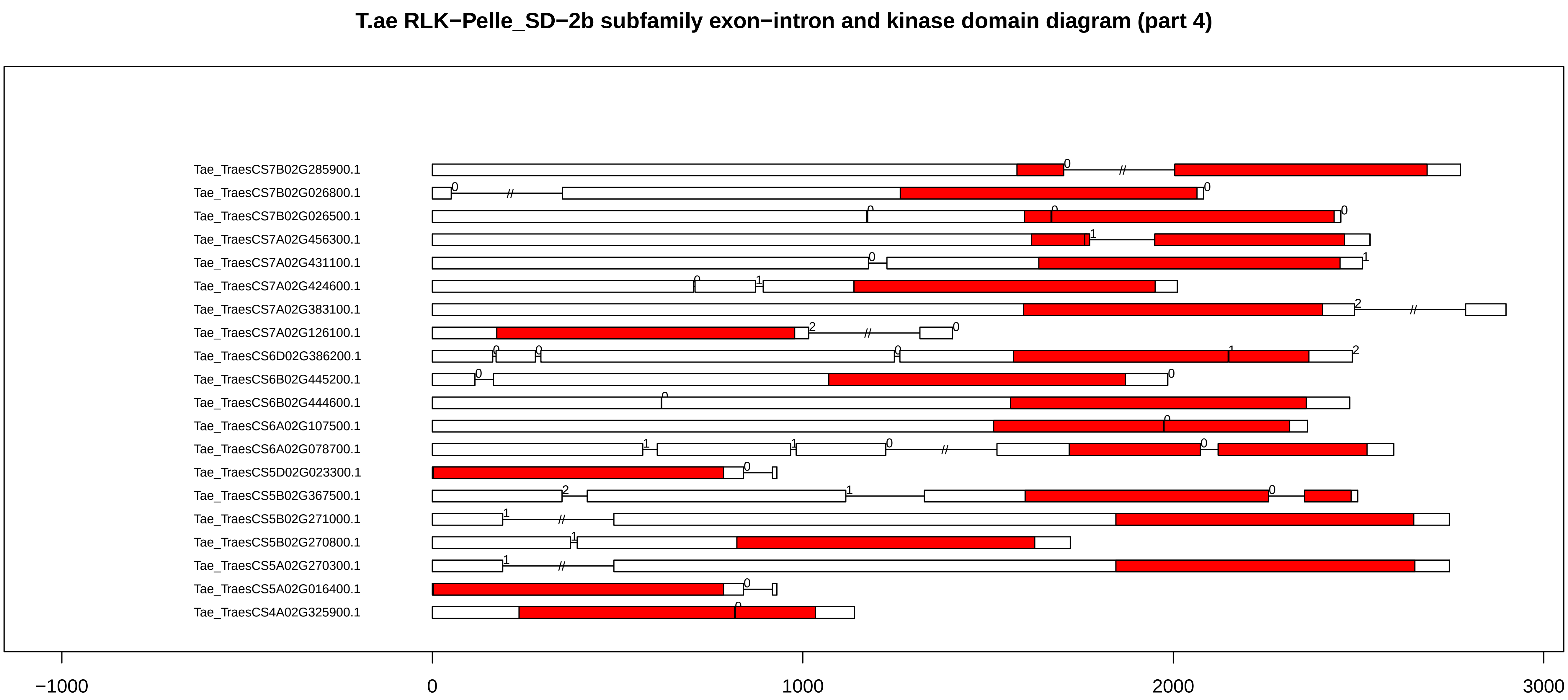
T.ae RLK−Pelle_SD−2b subfamily exon−intron and kinase domain diagram (part 4)
Tae_TraesCS7B02G285900.1
Tae_TraesCS7B02G026800.1
Tae_TraesCS7B02G026500.1
Tae_TraesCS7A02G456300.1
Tae_TraesCS7A02G431100.1
Tae_TraesCS7A02G424600.1
Tae_TraesCS7A02G383100.1
Tae_TraesCS7A02G126100.1
Tae_TraesCS6D02G386200.1
Tae_TraesCS6B02G445200.1
Tae_TraesCS6B02G444600.1
Tae_TraesCS6A02G107500.1
Tae_TraesCS6A02G078700.1
Tae_TraesCS5D02G023300.1
Tae_TraesCS5B02G367500.1
Tae_TraesCS5B02G271000.1
Tae_TraesCS5B02G270800.1
Tae_TraesCS5A02G270300.1
Tae_TraesCS5A02G016400.1
Tae_TraesCS4A02G325900.1
−1000
0
1000
2000
3000

T.ae RLK−Pelle_SD−2b subfamily exon−intron and kinase domain diagram (part 5)

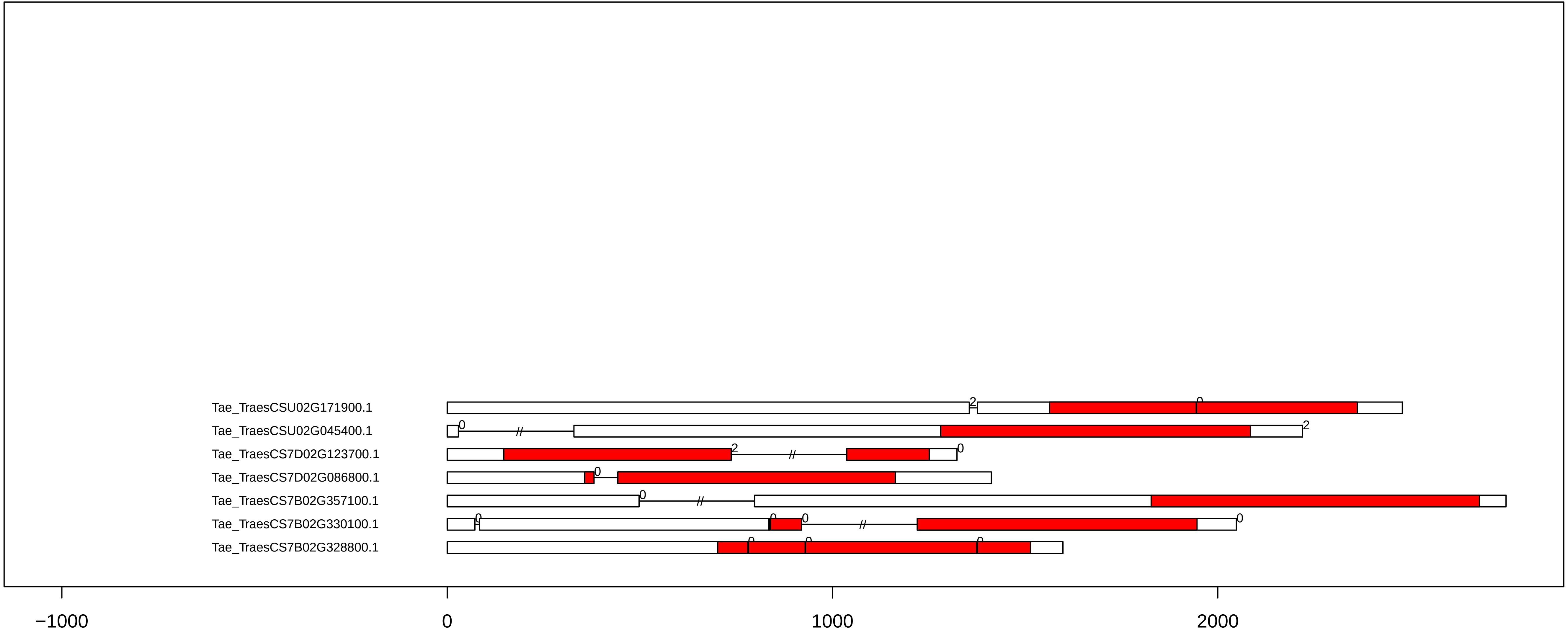

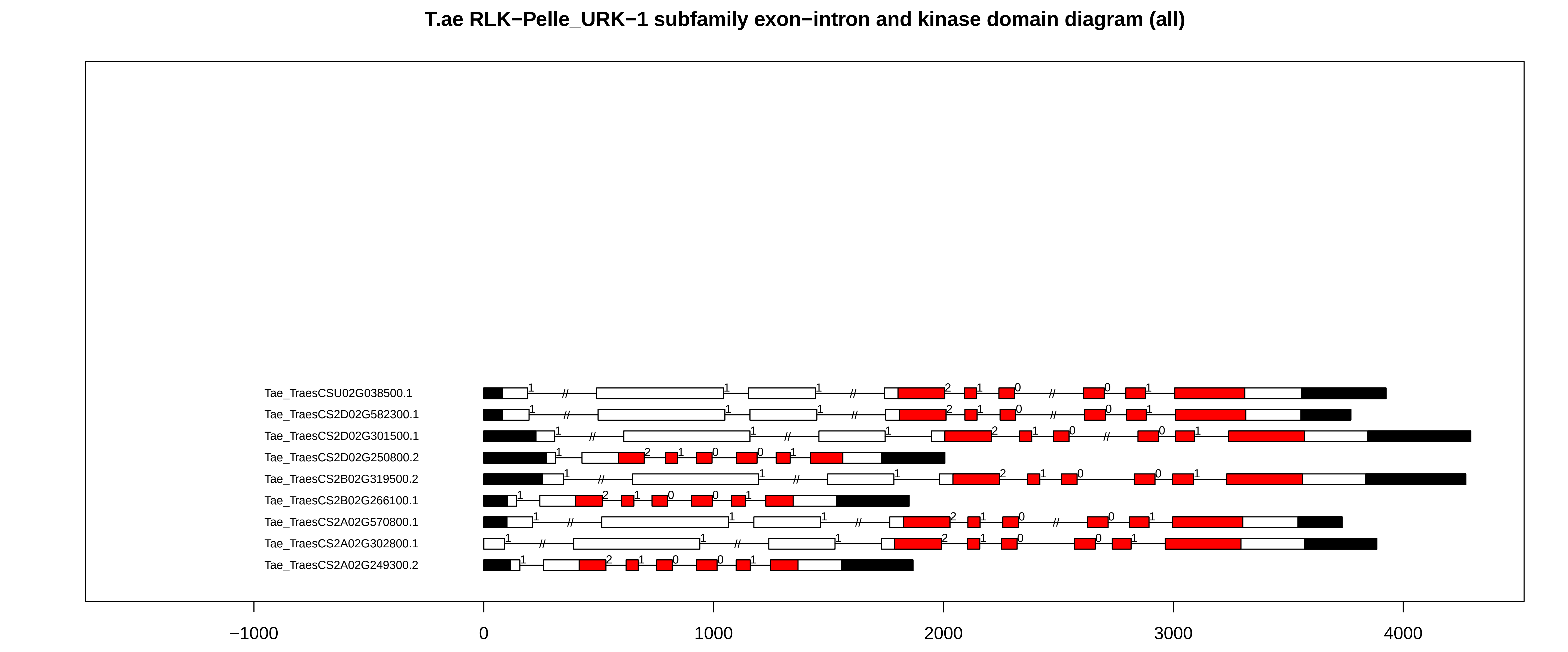
T.ae RLK−Pelle_URK−1 subfamily exon−intron and kinase domain diagram (all)
Tae_TraesCSU02G038500.1
Tae_TraesCS2D02G582300.1
Tae_TraesCS2D02G301500.1
Tae_TraesCS2D02G250800.2
Tae_TraesCS2B02G319500.2
Tae_TraesCS2B02G266100.1
Tae_TraesCS2A02G570800.1
Tae_TraesCS2A02G302800.1
Tae_TraesCS2A02G249300.2
−1000
0
1000
2000
3000
4000

T.ae RLK−Pelle_URK−2 subfamily exon−intron and kinase domain diagram (all)

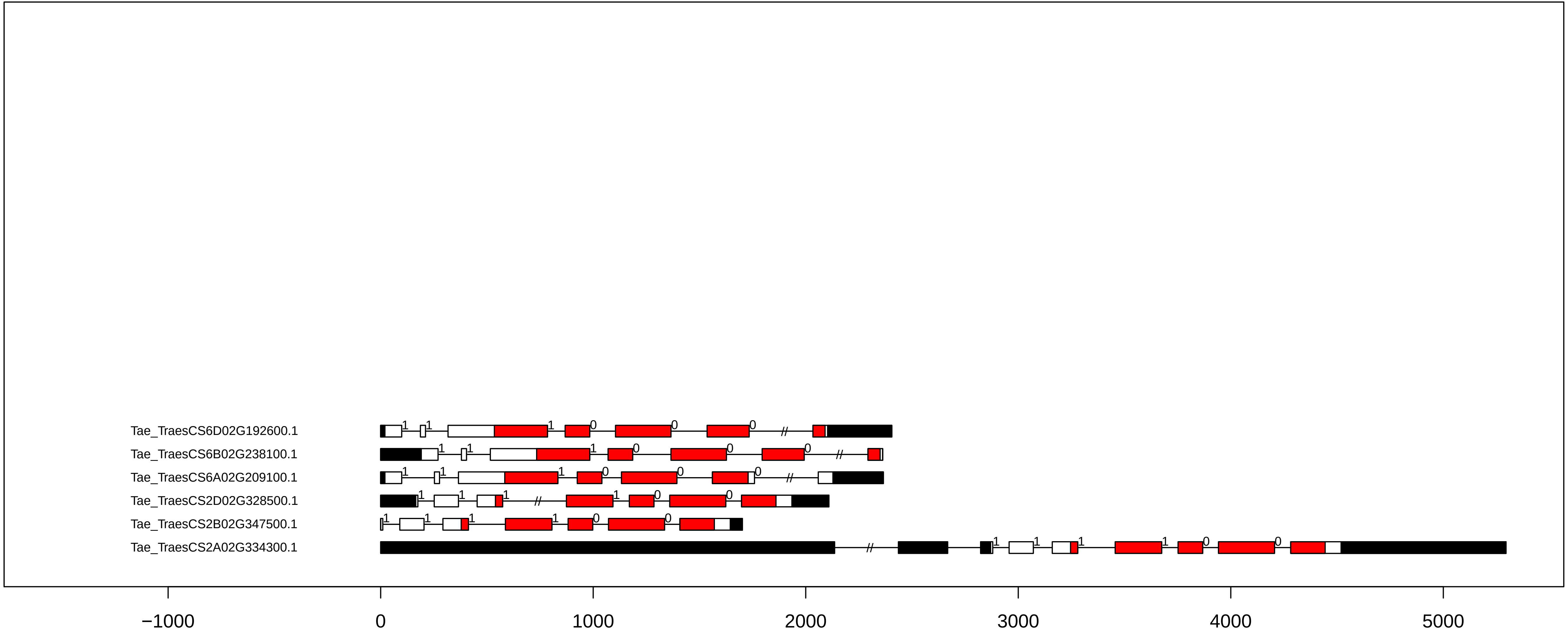

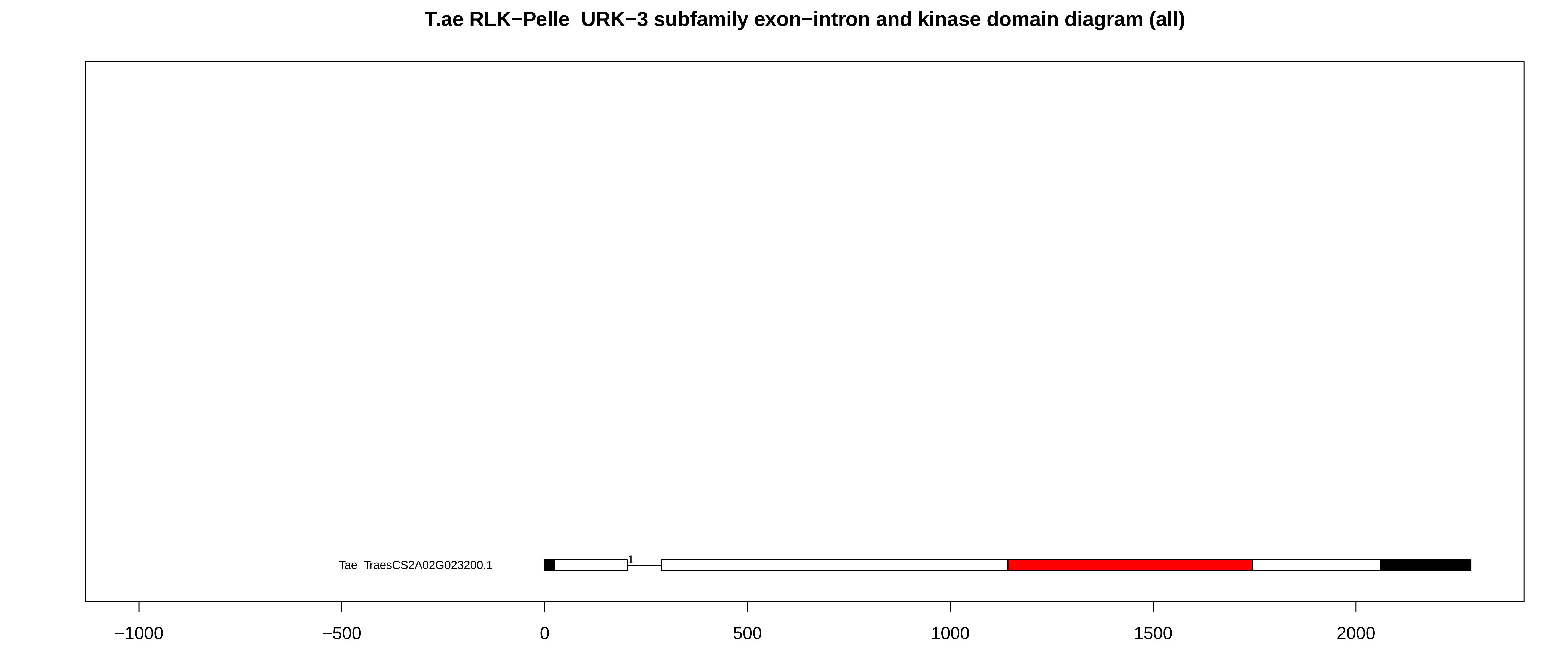
T.ae RLK−Pelle_URK−3 subfamily exon−intron and kinase domain diagram (all)
Tae_TraesCS2A02G023200.1
1
−1000
−500
0
500
1000
1500
2000

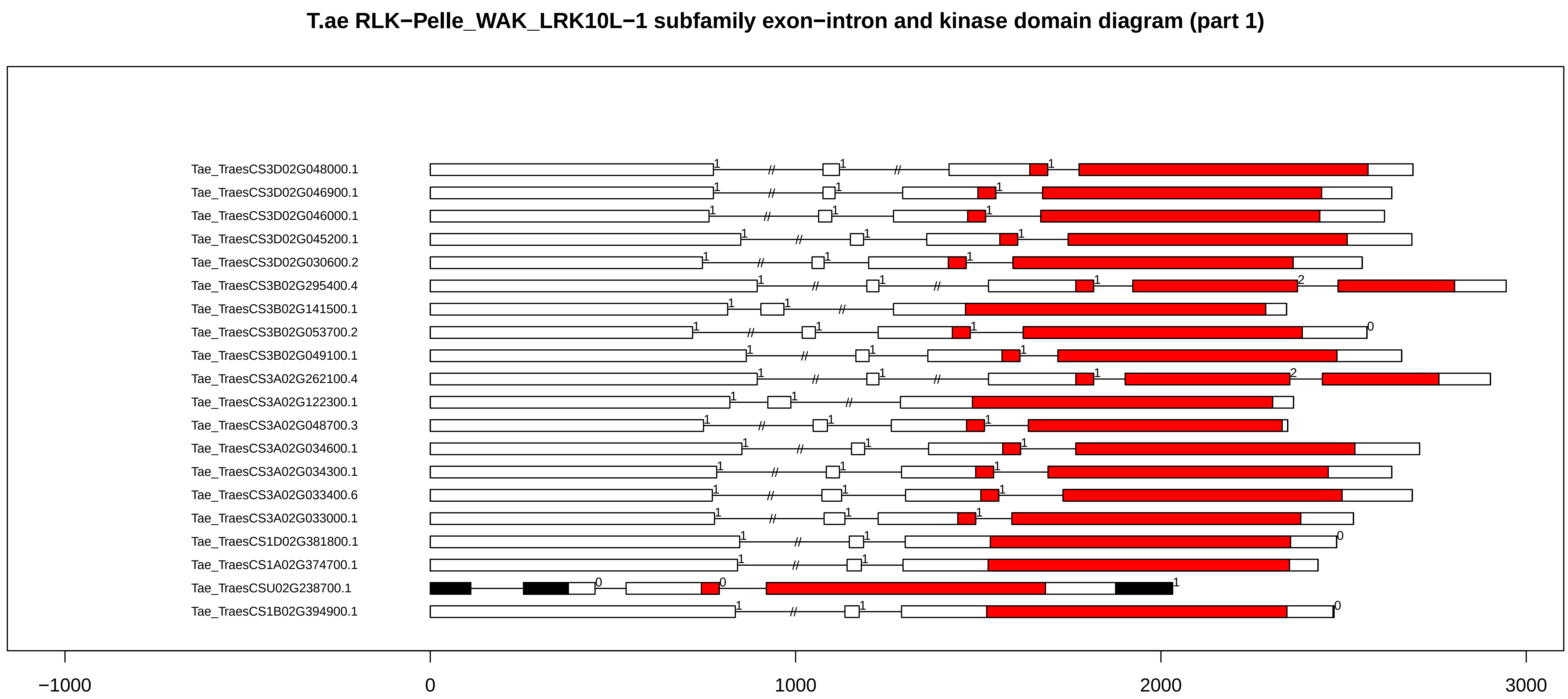
T.ae RLK−Pelle_WAK_LRK10L−1 subfamily exon−intron and kinase domain diagram (part 1)
Tae_TraesCS3D02G048000.1
Tae_TraesCS3D02G046900.1
Tae_TraesCS3D02G046000.1
Tae_TraesCS3D02G045200.1
Tae_TraesCS3D02G030600.2
Tae_TraesCS3B02G295400.4
Tae_TraesCS3B02G141500.1
Tae_TraesCS3B02G053700.2
Tae_TraesCS3B02G049100.1
Tae_TraesCS3A02G262100.4
Tae_TraesCS3A02G122300.1
Tae_TraesCS3A02G048700.3
Tae_TraesCS3A02G034600.1
Tae_TraesCS3A02G034300.1
Tae_TraesCS3A02G033400.6
Tae_TraesCS3A02G033000.1
Tae_TraesCS1D02G381800.1
Tae_TraesCS1A02G374700.1
Tae_TraesCSU02G238700.1
Tae_TraesCS1B02G394900.1
−1000
0
1000
2000
3000

T.ae RLK−Pelle_WAK_LRK10L−1 subfamily exon−intron and kinase domain diagram (part 2)

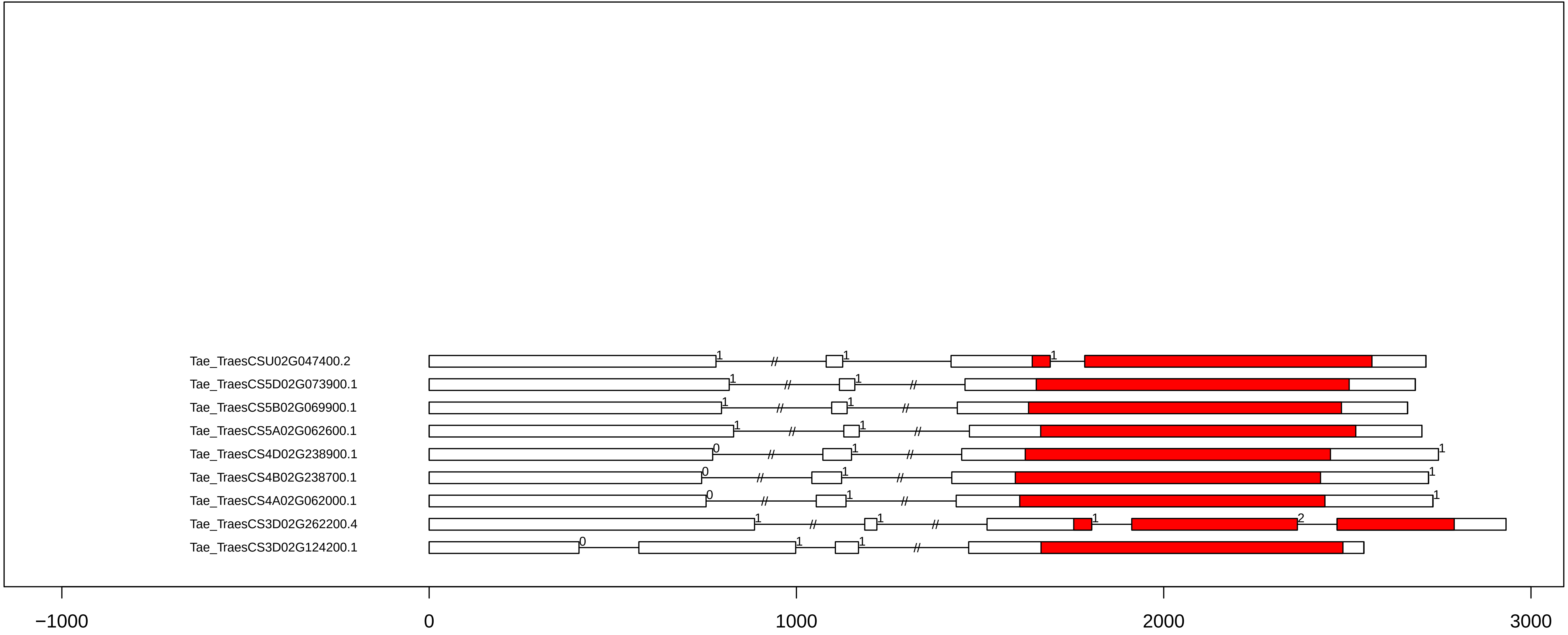

T.ae RLK−Pelle_WAK subfamily exon−intron and kinase domain diagram (part 1)

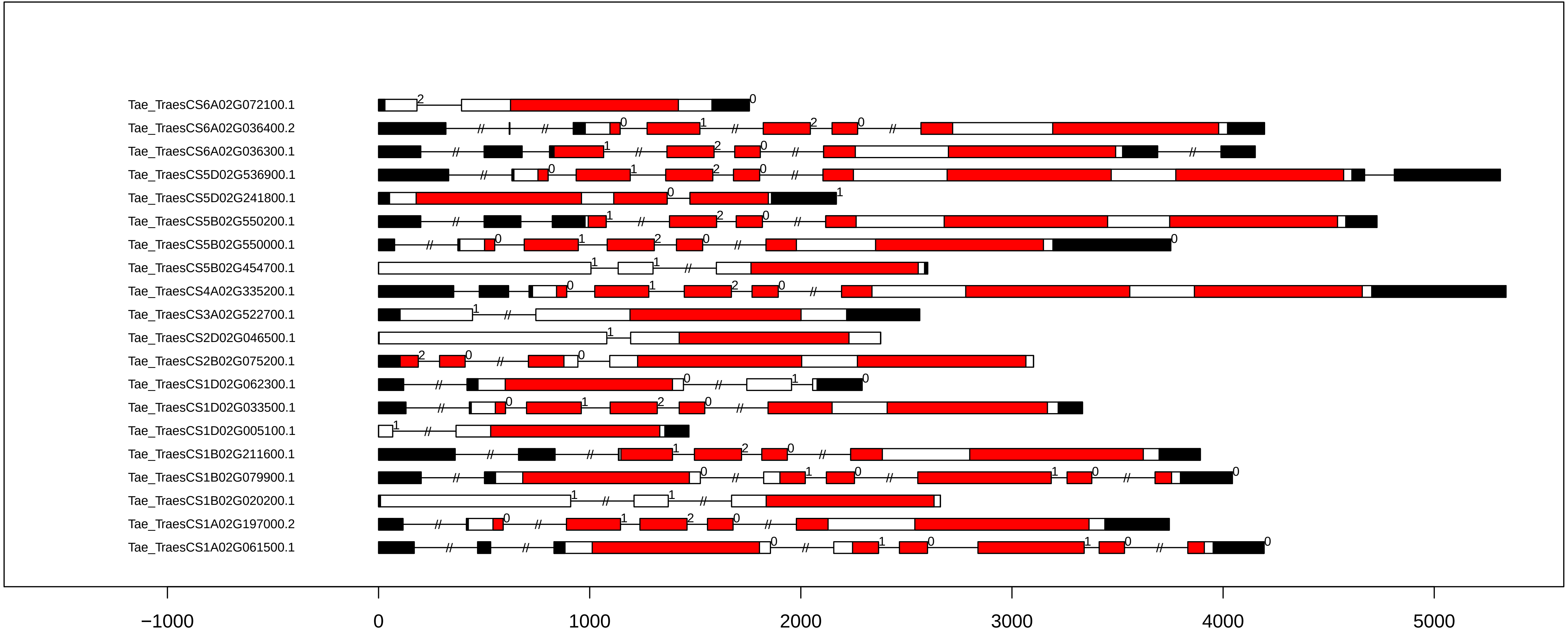

T.ae RLK−Pelle_WAK subfamily exon−intron and kinase domain diagram (part 2)

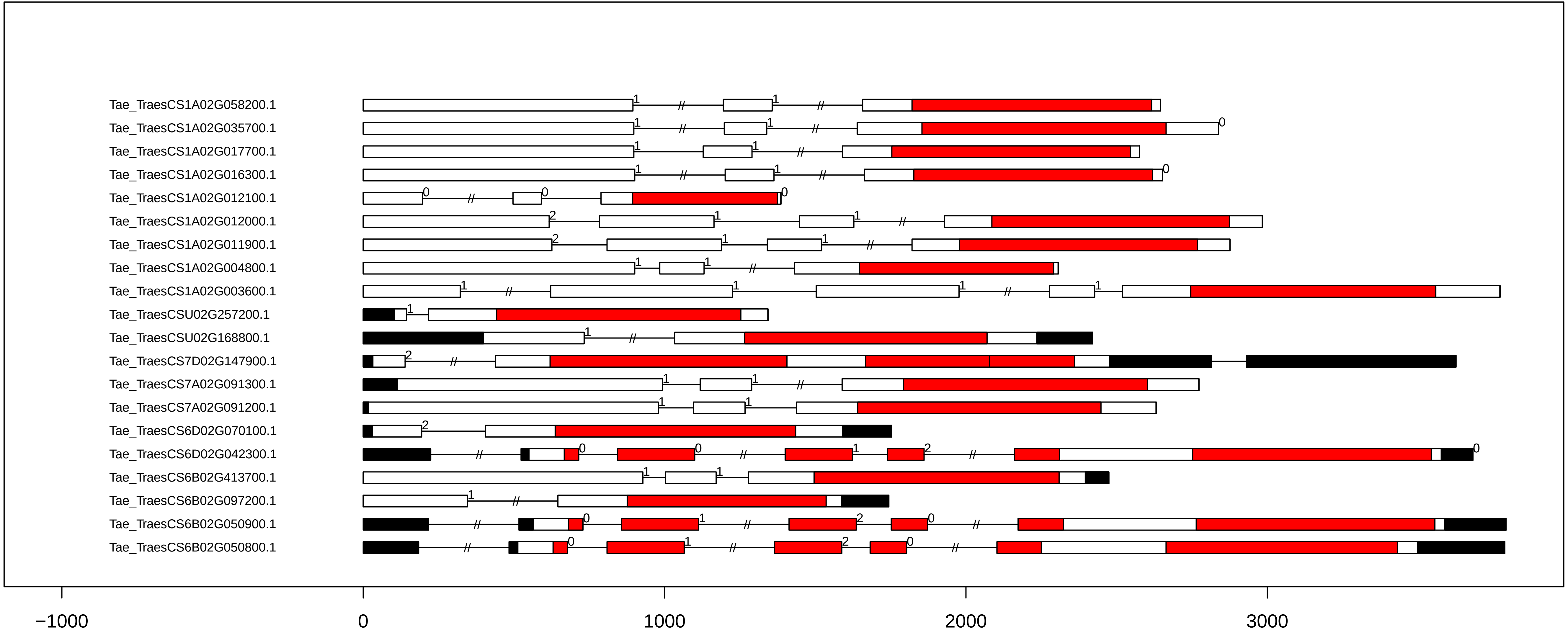

T.ae RLK−Pelle_WAK subfamily exon−intron and kinase domain diagram (part 3)

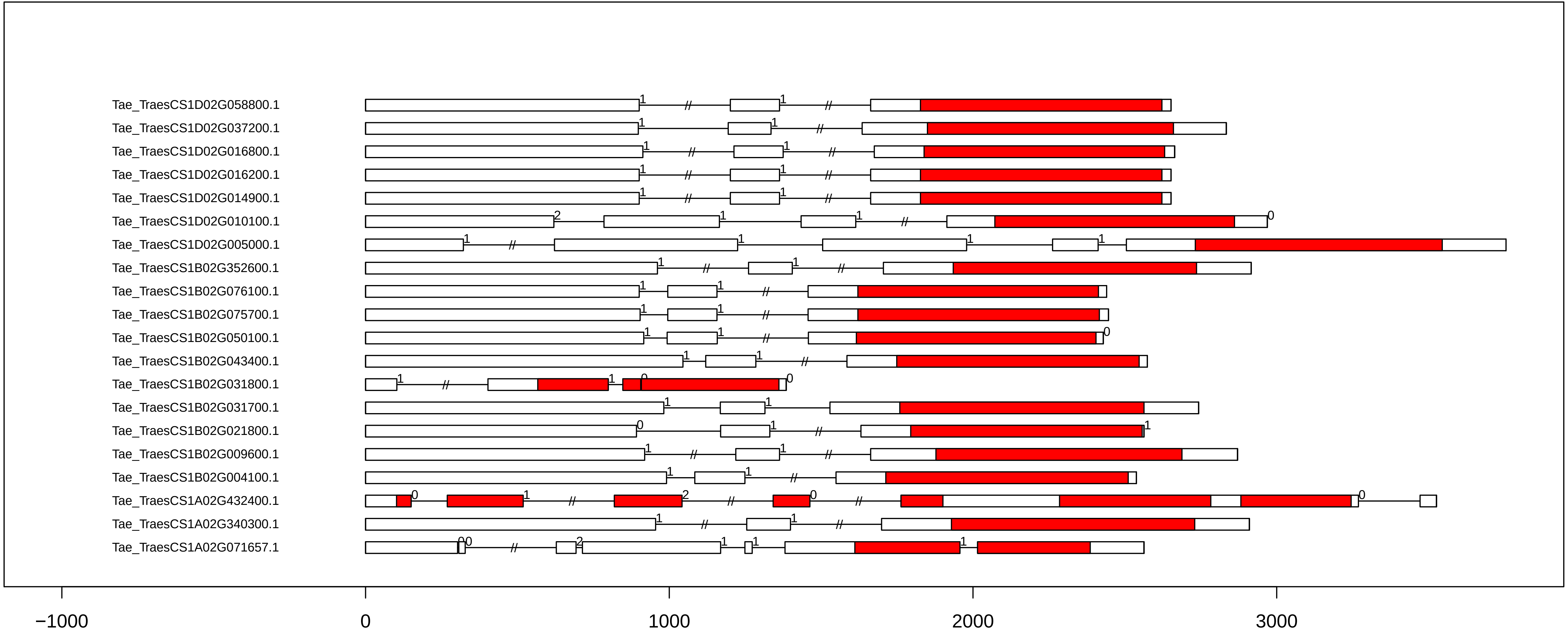

T.ae RLK−Pelle_WAK subfamily exon−intron and kinase domain diagram (part 4)

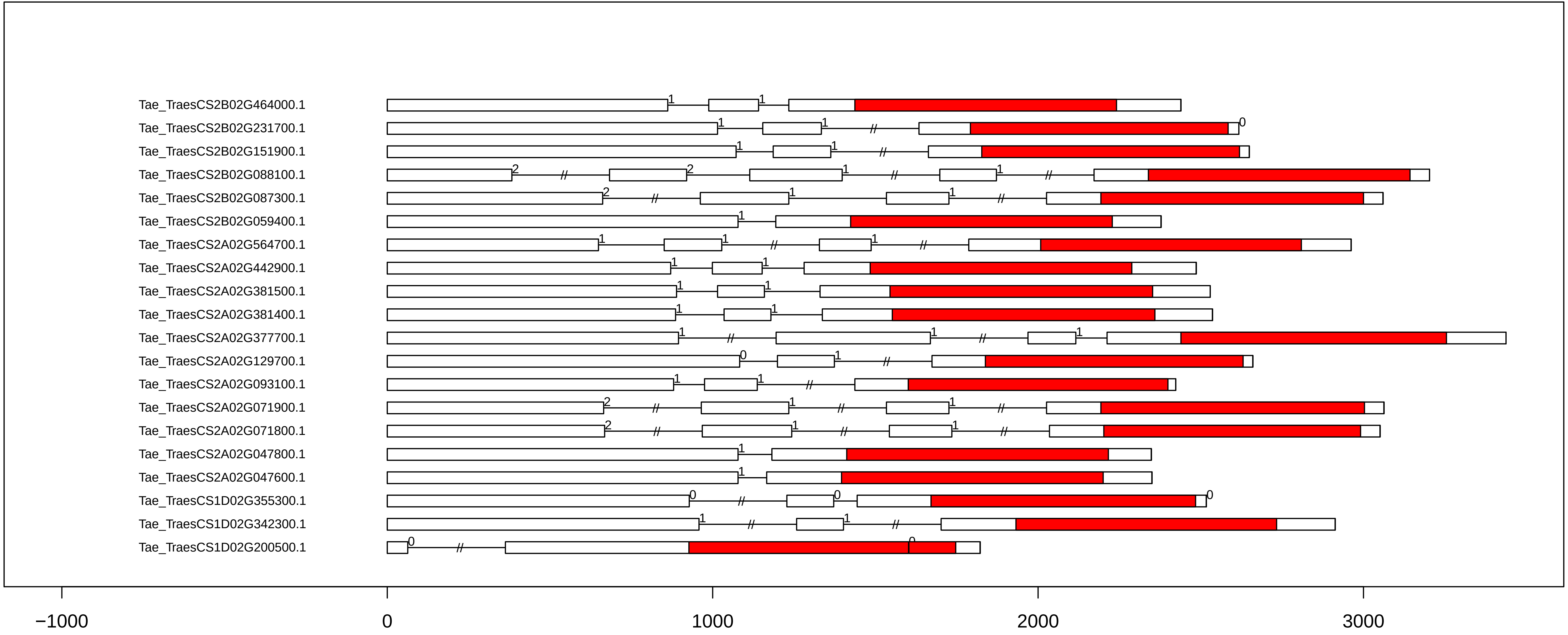

T.ae RLK−Pelle_WAK subfamily exon−intron and kinase domain diagram (part 5)

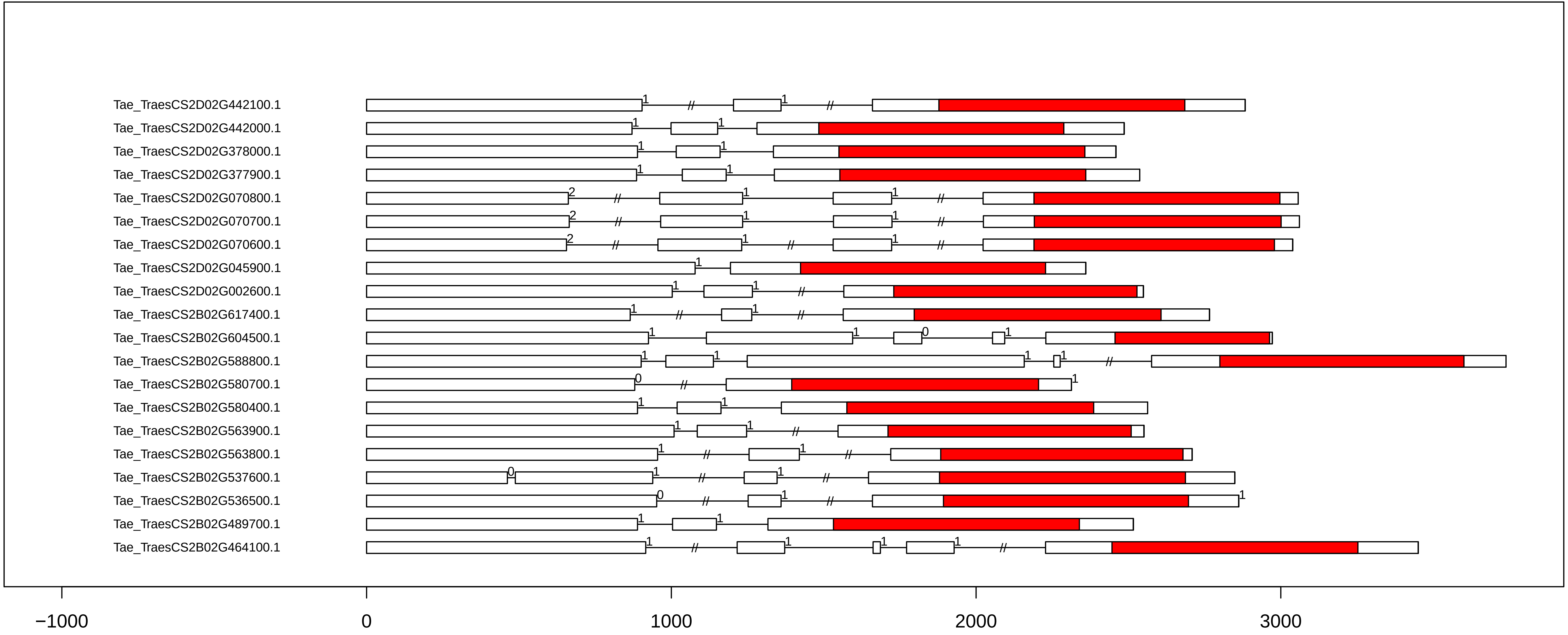

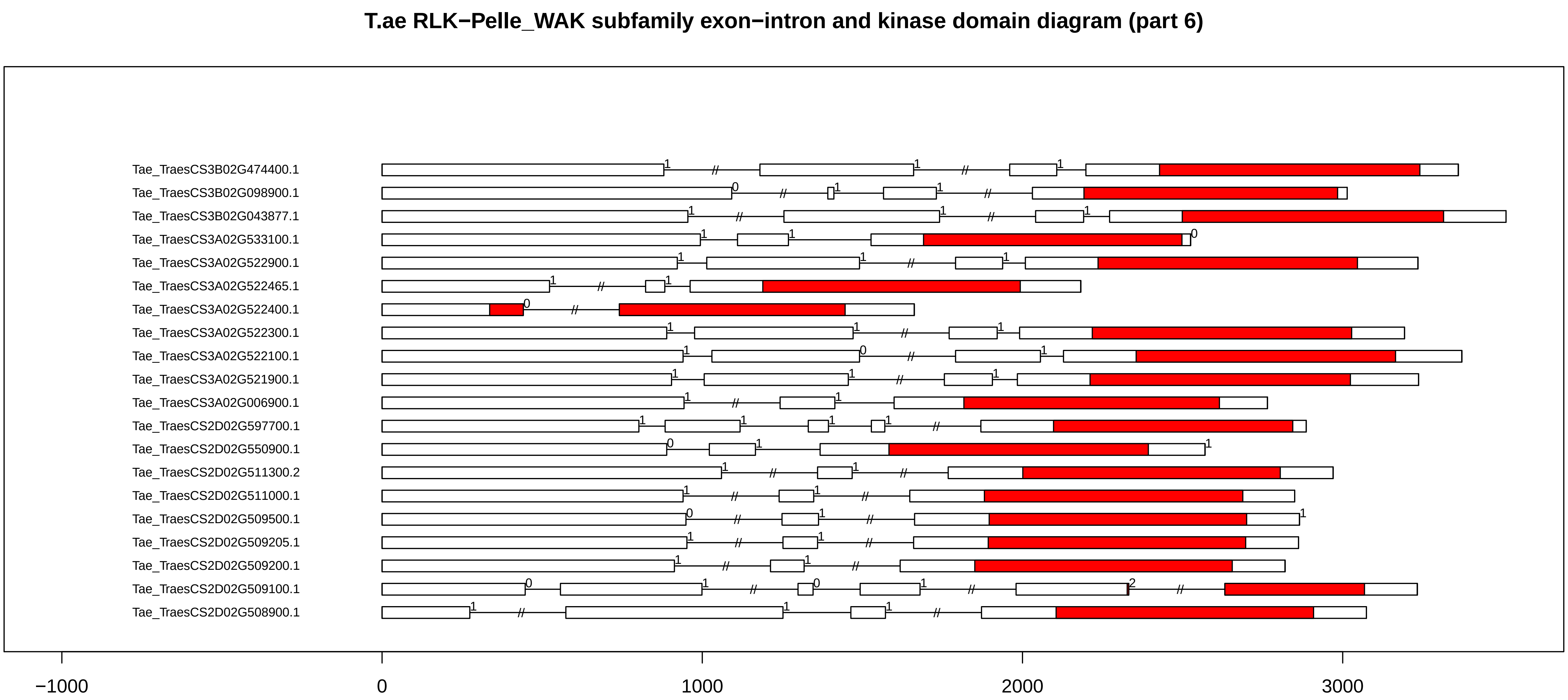
T.ae RLK−Pelle_WAK subfamily exon−intron and kinase domain diagram (part 6)
Tae_TraesCS3B02G474400.1
Tae_TraesCS3B02G098900.1
Tae_TraesCS3B02G043877.1
Tae_TraesCS3A02G533100.1
Tae_TraesCS3A02G522900.1
Tae_TraesCS3A02G522465.1
Tae_TraesCS3A02G522400.1
Tae_TraesCS3A02G522300.1
Tae_TraesCS3A02G522100.1
Tae_TraesCS3A02G521900.1
Tae_TraesCS3A02G006900.1
Tae_TraesCS2D02G597700.1
Tae_TraesCS2D02G550900.1
Tae_TraesCS2D02G511300.2
Tae_TraesCS2D02G511000.1
Tae_TraesCS2D02G509500.1
Tae_TraesCS2D02G509205.1
Tae_TraesCS2D02G509200.1
Tae_TraesCS2D02G509100.1
Tae_TraesCS2D02G508900.1
−1000
0
1000
2000
3000

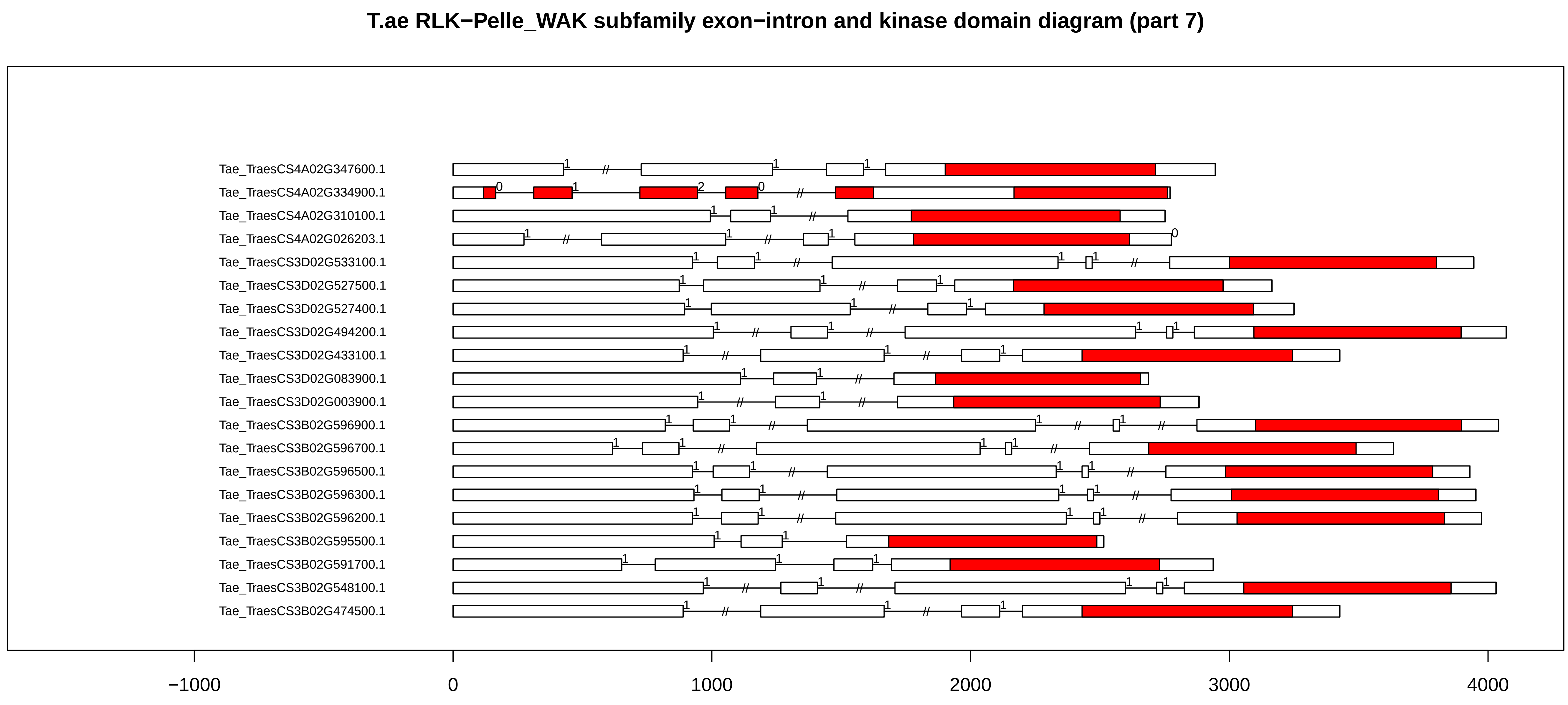
T.ae RLK−Pelle_WAK subfamily exon−intron and kinase domain diagram (part 7)
Tae_TraesCS4A02G347600.1
Tae_TraesCS4A02G334900.1
Tae_TraesCS4A02G310100.1
Tae_TraesCS4A02G026203.1
Tae_TraesCS3D02G533100.1
Tae_TraesCS3D02G527500.1
Tae_TraesCS3D02G527400.1
Tae_TraesCS3D02G494200.1
Tae_TraesCS3D02G433100.1
Tae_TraesCS3D02G083900.1
Tae_TraesCS3D02G003900.1
Tae_TraesCS3B02G596900.1
Tae_TraesCS3B02G596700.1
Tae_TraesCS3B02G596500.1
Tae_TraesCS3B02G596300.1
Tae_TraesCS3B02G596200.1
Tae_TraesCS3B02G595500.1
Tae_TraesCS3B02G591700.1
Tae_TraesCS3B02G548100.1
Tae_TraesCS3B02G474500.1
−1000
0
1000
2000
3000
4000

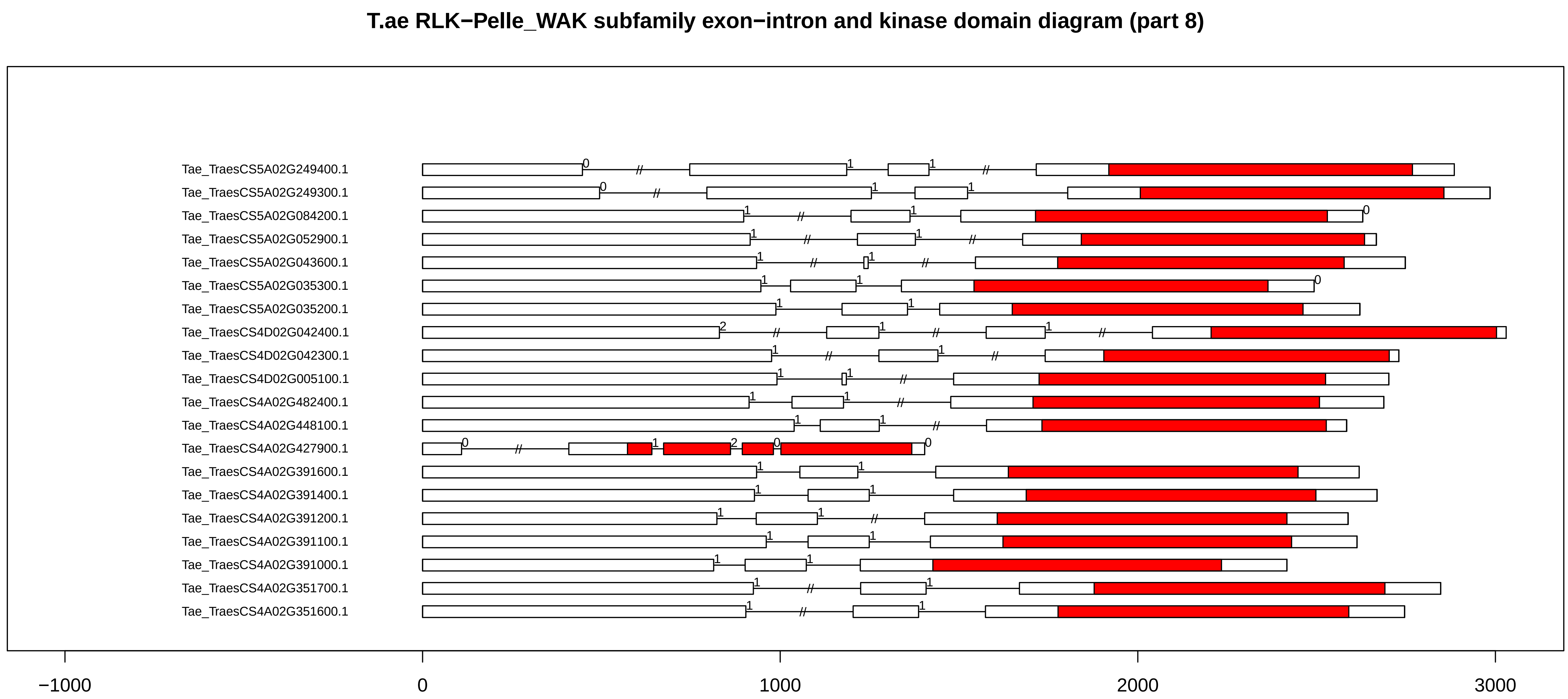
T.ae RLK−Pelle_WAK subfamily exon−intron and kinase domain diagram (part 8)
Tae_TraesCS5A02G249400.1
Tae_TraesCS5A02G249300.1
Tae_TraesCS5A02G084200.1
Tae_TraesCS5A02G052900.1
Tae_TraesCS5A02G043600.1
Tae_TraesCS5A02G035300.1
Tae_TraesCS5A02G035200.1
Tae_TraesCS4D02G042400.1
Tae_TraesCS4D02G042300.1
Tae_TraesCS4D02G005100.1
Tae_TraesCS4A02G482400.1
Tae_TraesCS4A02G448100.1
Tae_TraesCS4A02G427900.1
Tae_TraesCS4A02G391600.1
Tae_TraesCS4A02G391400.1
Tae_TraesCS4A02G391200.1
Tae_TraesCS4A02G391100.1
Tae_TraesCS4A02G391000.1
Tae_TraesCS4A02G351700.1
Tae_TraesCS4A02G351600.1
−1000
0
1000
2000
3000

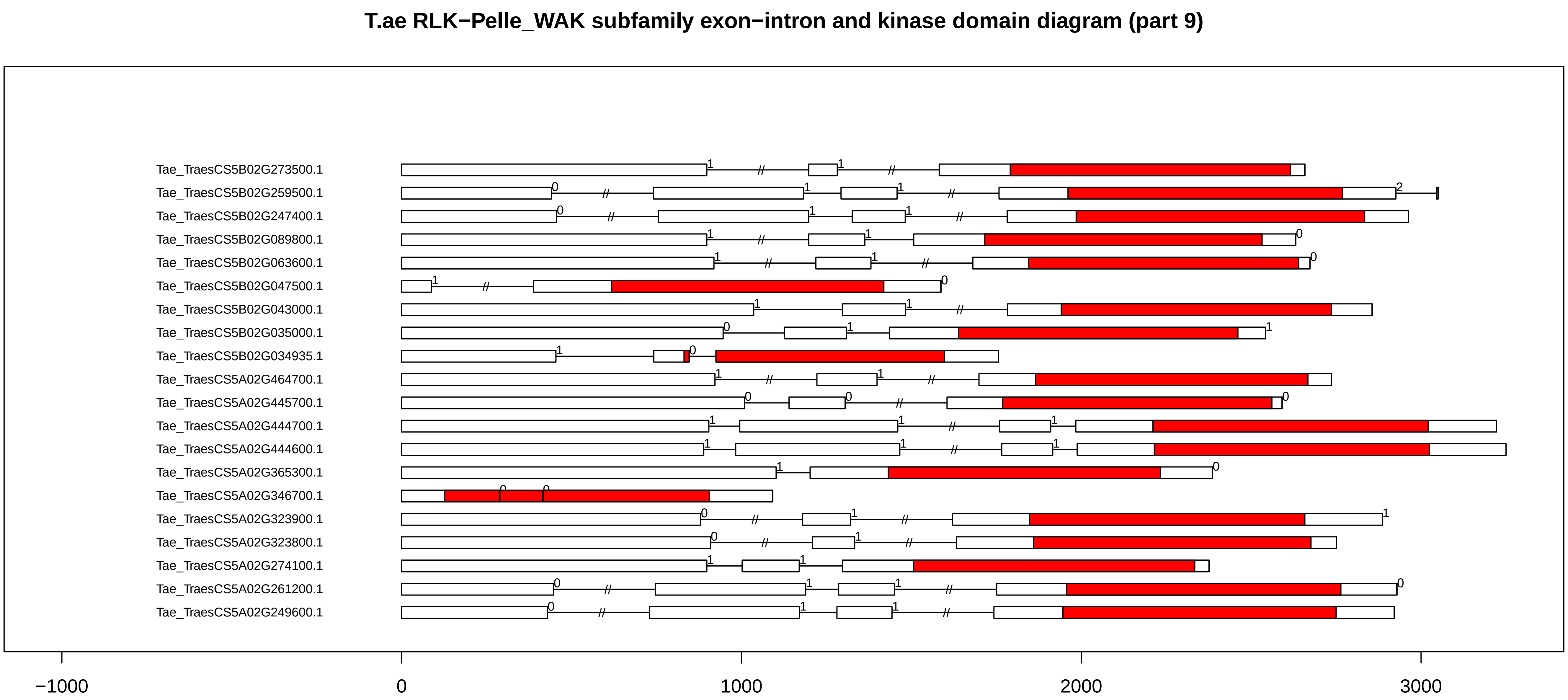
T.ae RLK−Pelle_WAK subfamily exon−intron and kinase domain diagram (part 9)
Tae_TraesCS5B02G273500.1
Tae_TraesCS5B02G259500.1
Tae_TraesCS5B02G247400.1
Tae_TraesCS5B02G089800.1
Tae_TraesCS5B02G063600.1
Tae_TraesCS5B02G047500.1
Tae_TraesCS5B02G043000.1
Tae_TraesCS5B02G035000.1
Tae_TraesCS5B02G034935.1
Tae_TraesCS5A02G464700.1
Tae_TraesCS5A02G445700.1
Tae_TraesCS5A02G444700.1
Tae_TraesCS5A02G444600.1
Tae_TraesCS5A02G365300.1
Tae_TraesCS5A02G346700.1
Tae_TraesCS5A02G323900.1
Tae_TraesCS5A02G323800.1
Tae_TraesCS5A02G274100.1
Tae_TraesCS5A02G261200.1
Tae_TraesCS5A02G249600.1
−1000
0
1000
2000
3000

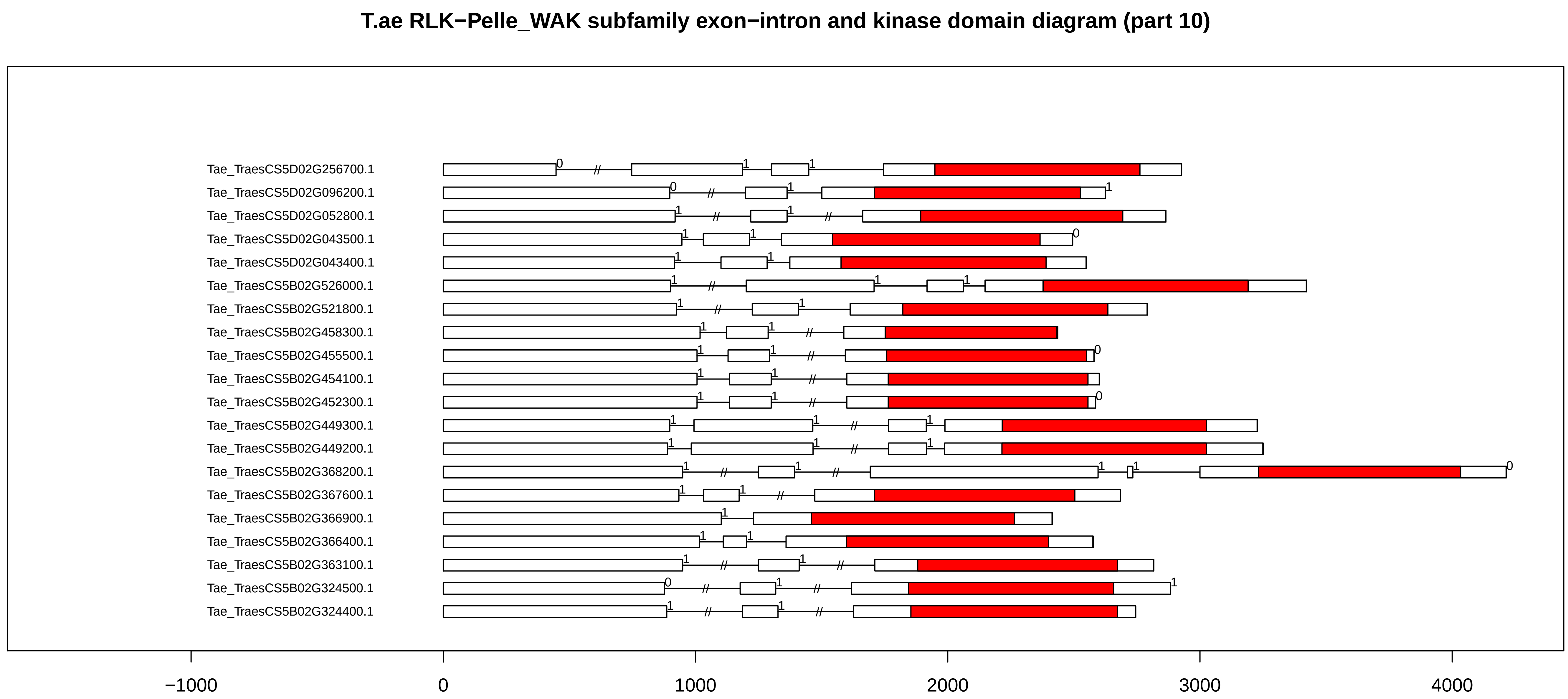
T.ae RLK−Pelle_WAK subfamily exon−intron and kinase domain diagram (part 10)
Tae_TraesCS5D02G256700.1
Tae_TraesCS5D02G096200.1
Tae_TraesCS5D02G052800.1
Tae_TraesCS5D02G043500.1
Tae_TraesCS5D02G043400.1
Tae_TraesCS5B02G526000.1
Tae_TraesCS5B02G521800.1
Tae_TraesCS5B02G458300.1
Tae_TraesCS5B02G455500.1
Tae_TraesCS5B02G454100.1
Tae_TraesCS5B02G452300.1
Tae_TraesCS5B02G449300.1
Tae_TraesCS5B02G449200.1
Tae_TraesCS5B02G368200.1
Tae_TraesCS5B02G367600.1
Tae_TraesCS5B02G366900.1
Tae_TraesCS5B02G366400.1
Tae_TraesCS5B02G363100.1
Tae_TraesCS5B02G324500.1
Tae_TraesCS5B02G324400.1
−1000
0
1000
2000
3000
4000

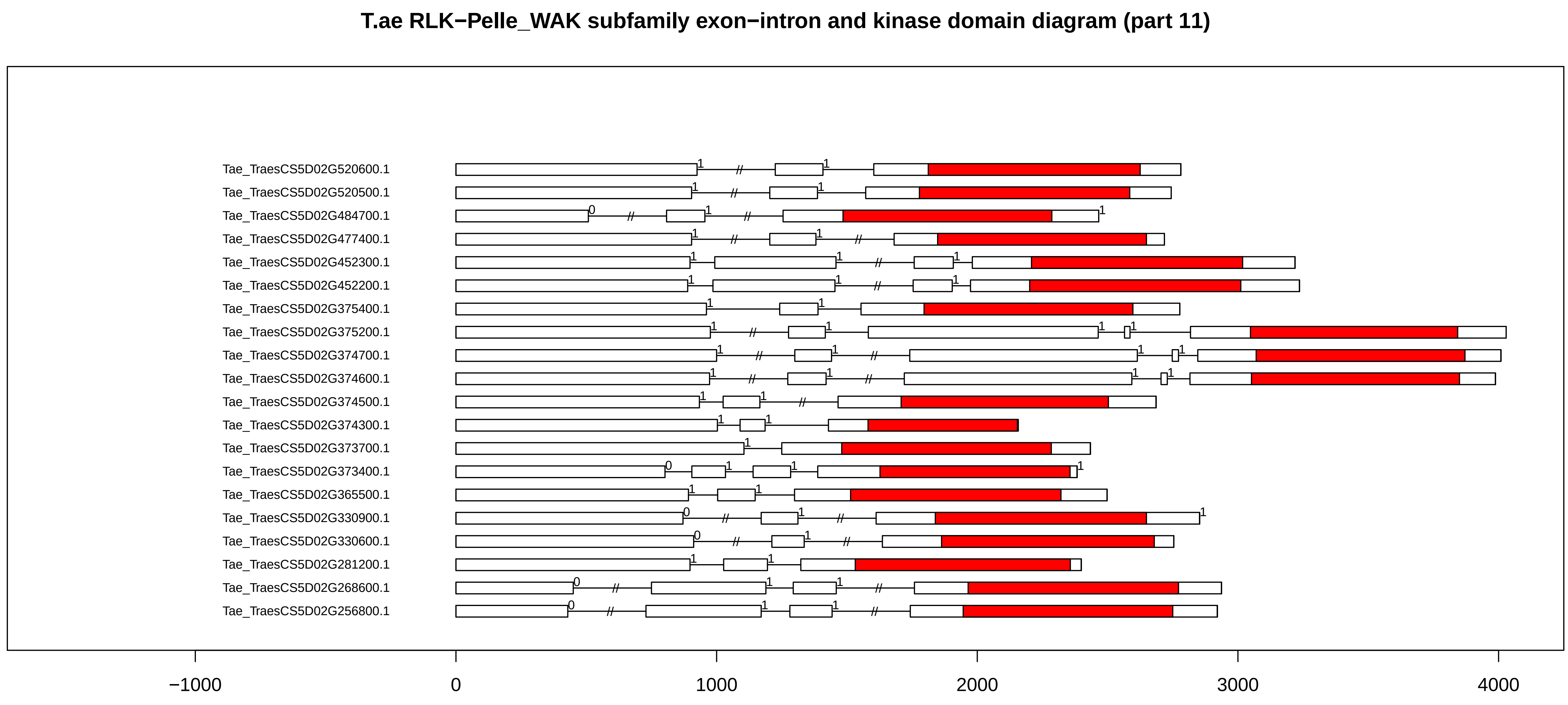
T.ae RLK−Pelle_WAK subfamily exon−intron and kinase domain diagram (part 11)
Tae_TraesCS5D02G520600.1
Tae_TraesCS5D02G520500.1
Tae_TraesCS5D02G484700.1
Tae_TraesCS5D02G477400.1
Tae_TraesCS5D02G452300.1
Tae_TraesCS5D02G452200.1
Tae_TraesCS5D02G375400.1
Tae_TraesCS5D02G375200.1
Tae_TraesCS5D02G374700.1
Tae_TraesCS5D02G374600.1
Tae_TraesCS5D02G374500.1
Tae_TraesCS5D02G374300.1
Tae_TraesCS5D02G373700.1
Tae_TraesCS5D02G373400.1
Tae_TraesCS5D02G365500.1
Tae_TraesCS5D02G330900.1
Tae_TraesCS5D02G330600.1
Tae_TraesCS5D02G281200.1
Tae_TraesCS5D02G268600.1
Tae_TraesCS5D02G256800.1
−1000
0
1000
2000
3000
4000

T.ae RLK−Pelle_WAK subfamily exon−intron and kinase domain diagram (part 12)

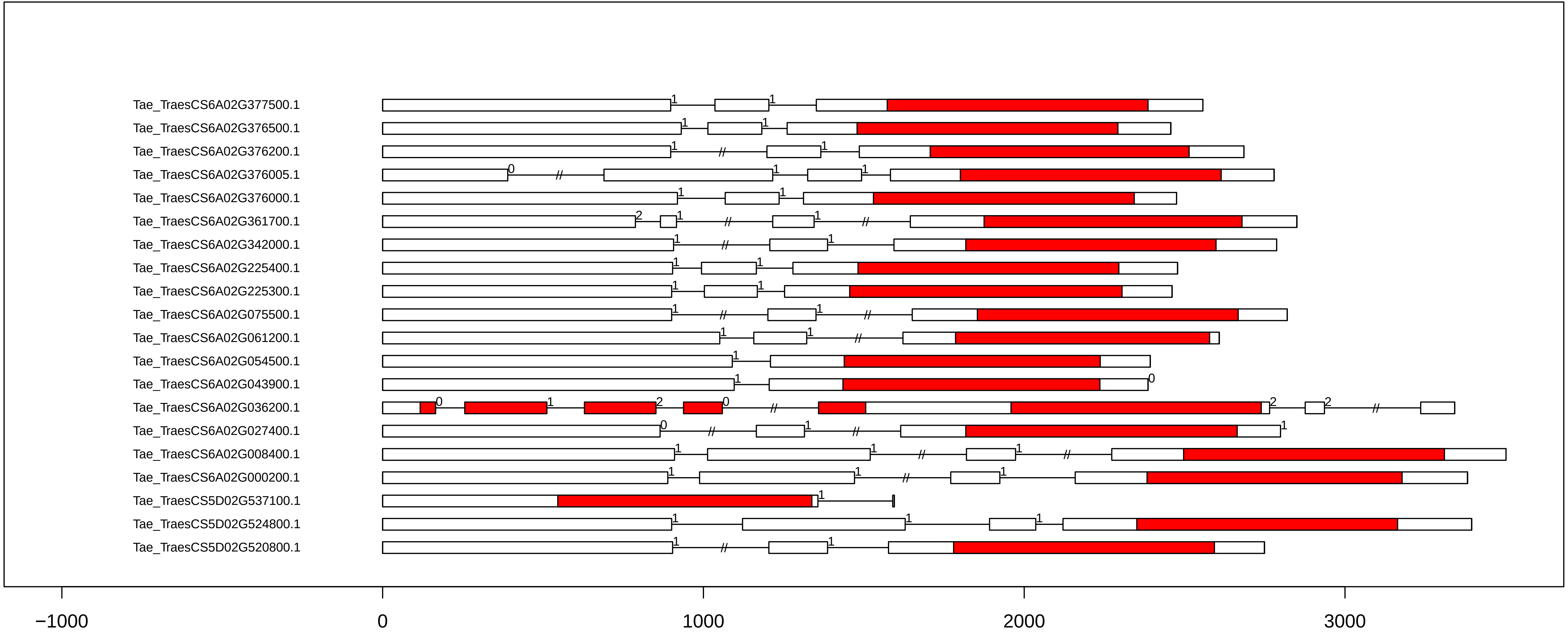

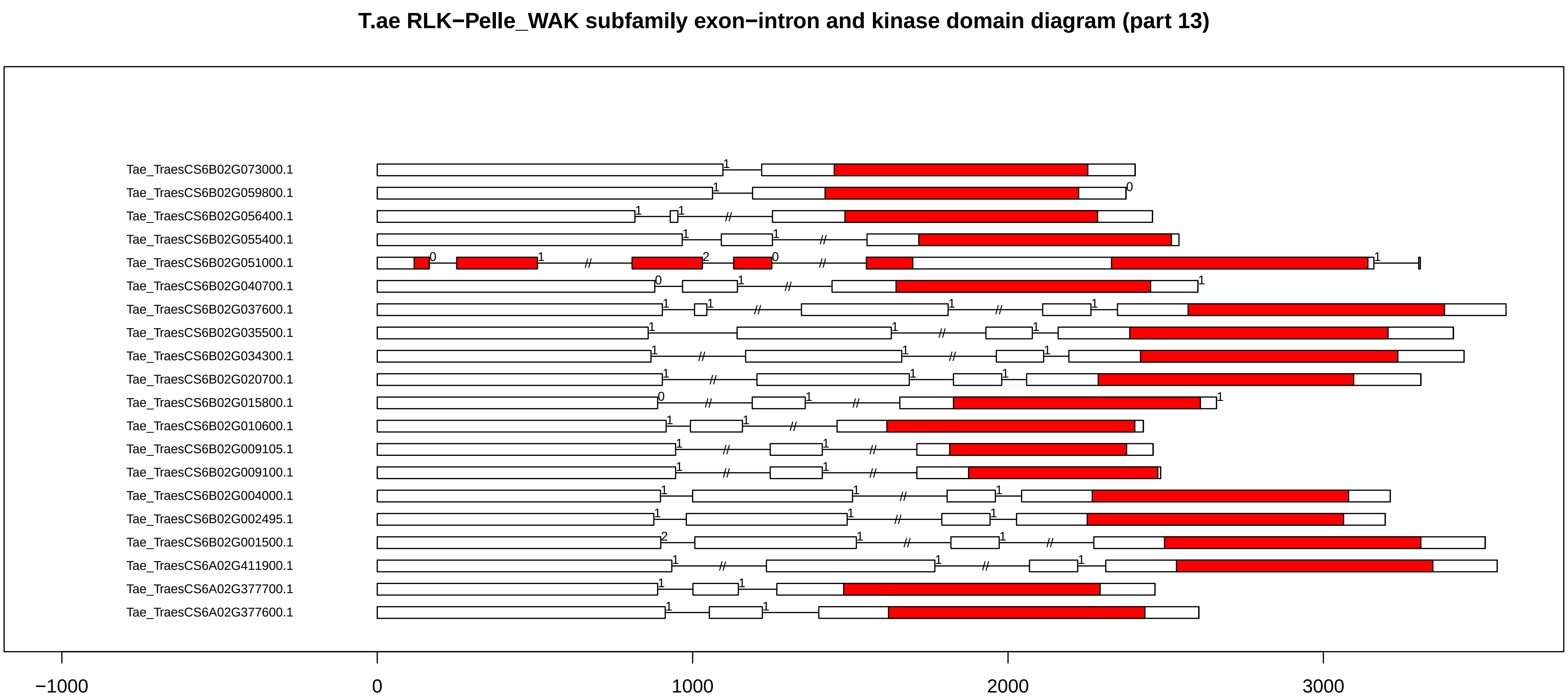

T.ae RLK−Pelle_WAK subfamily exon−intron and kinase domain diagram (part 13)
Tae_TraesCS6B02G073000.1
Tae_TraesCS6B02G059800.1
Tae_TraesCS6B02G056400.1
Tae_TraesCS6B02G055400.1
Tae_TraesCS6B02G051000.1
Tae_TraesCS6B02G040700.1
Tae_TraesCS6B02G037600.1
Tae_TraesCS6B02G035500.1
Tae_TraesCS6B02G034300.1
Tae_TraesCS6B02G020700.1
Tae_TraesCS6B02G015800.1
Tae_TraesCS6B02G010600.1
Tae_TraesCS6B02G009105.1
Tae_TraesCS6B02G009100.1
Tae_TraesCS6B02G004000.1
Tae_TraesCS6B02G002495.1
Tae_TraesCS6B02G001500.1
Tae_TraesCS6A02G411900.1
Tae_TraesCS6A02G377700.1
Tae_TraesCS6A02G377600.1
−1000
0
1000
2000
3000

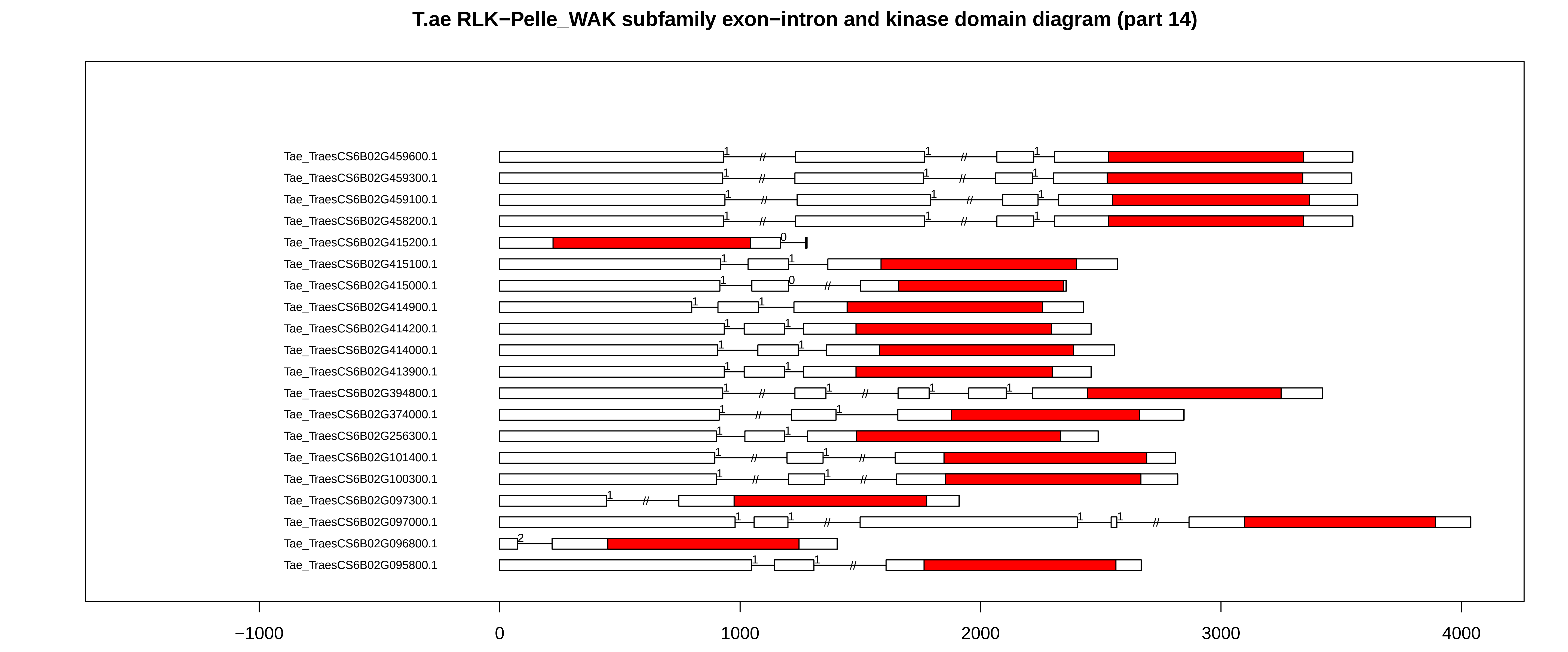
T.ae RLK−Pelle_WAK subfamily exon−intron and kinase domain diagram (part 14)
Tae_TraesCS6B02G459600.1
Tae_TraesCS6B02G459300.1
Tae_TraesCS6B02G459100.1
Tae_TraesCS6B02G458200.1
Tae_TraesCS6B02G415200.1
Tae_TraesCS6B02G415100.1
Tae_TraesCS6B02G415000.1
Tae_TraesCS6B02G414900.1
Tae_TraesCS6B02G414200.1
Tae_TraesCS6B02G414000.1
Tae_TraesCS6B02G413900.1
Tae_TraesCS6B02G394800.1
Tae_TraesCS6B02G374000.1
Tae_TraesCS6B02G256300.1
Tae_TraesCS6B02G101400.1
Tae_TraesCS6B02G100300.1
Tae_TraesCS6B02G097300.1
Tae_TraesCS6B02G097000.1
Tae_TraesCS6B02G096800.1
Tae_TraesCS6B02G095800.1
−1000
0
1000
2000
3000
4000

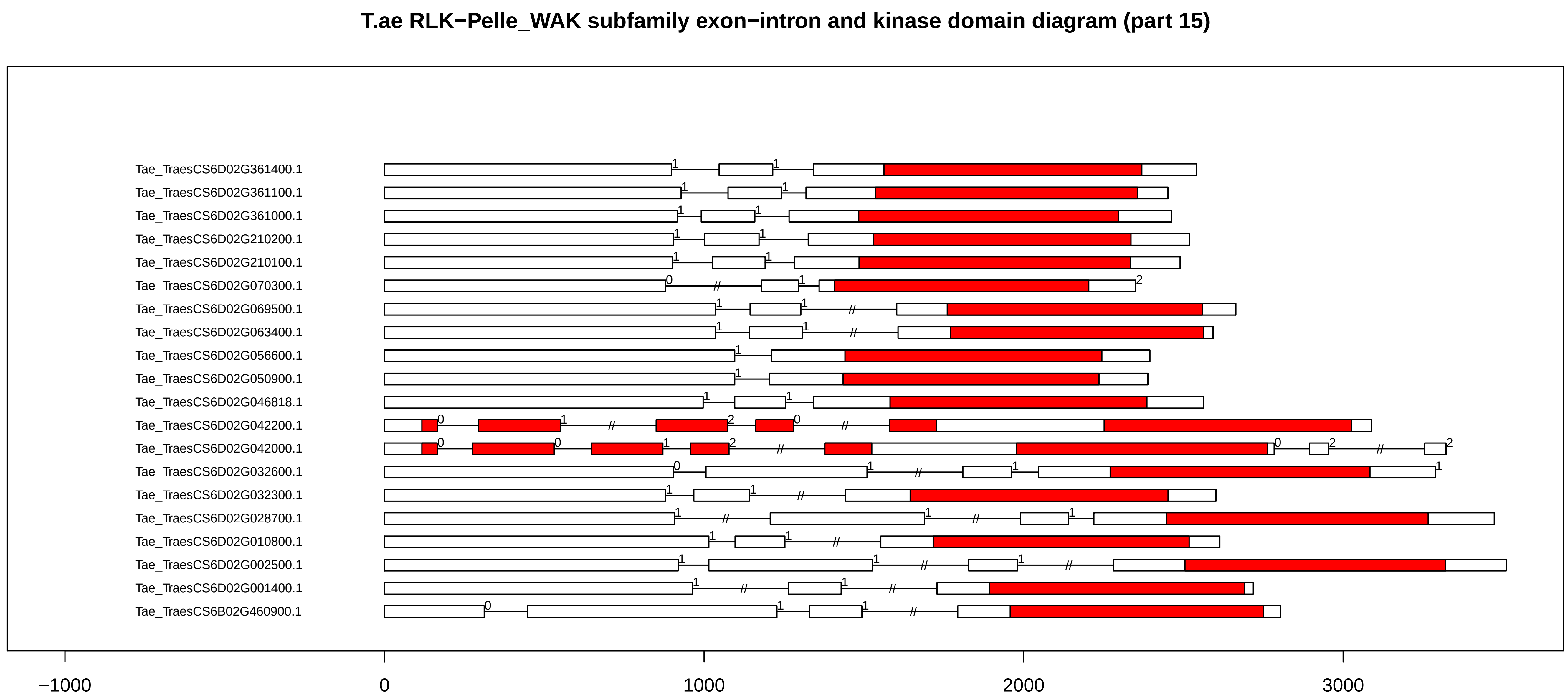
T.ae RLK−Pelle_WAK subfamily exon−intron and kinase domain diagram (part 15)
Tae_TraesCS6D02G361400.1
Tae_TraesCS6D02G361100.1
Tae_TraesCS6D02G361000.1
Tae_TraesCS6D02G210200.1
Tae_TraesCS6D02G210100.1
Tae_TraesCS6D02G070300.1
Tae_TraesCS6D02G069500.1
Tae_TraesCS6D02G063400.1
Tae_TraesCS6D02G056600.1
Tae_TraesCS6D02G050900.1
Tae_TraesCS6D02G046818.1
Tae_TraesCS6D02G042200.1
Tae_TraesCS6D02G042000.1
Tae_TraesCS6D02G032600.1
Tae_TraesCS6D02G032300.1
Tae_TraesCS6D02G028700.1
Tae_TraesCS6D02G010800.1
Tae_TraesCS6D02G002500.1
Tae_TraesCS6D02G001400.1
Tae_TraesCS6B02G460900.1
−1000
0
1000
2000
3000

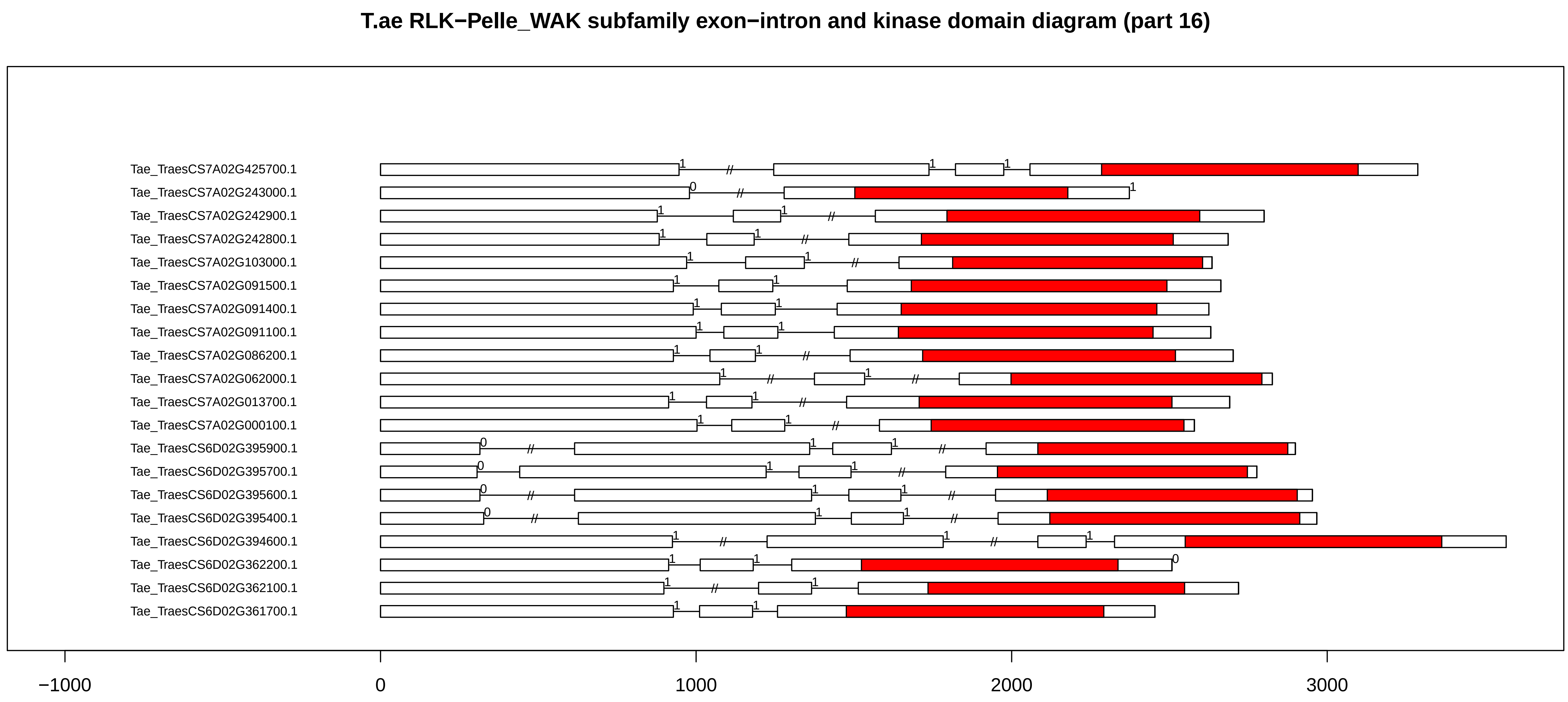
T.ae RLK−Pelle_WAK subfamily exon−intron and kinase domain diagram (part 16)
Tae_TraesCS7A02G425700.1
Tae_TraesCS7A02G243000.1
Tae_TraesCS7A02G242900.1
Tae_TraesCS7A02G242800.1
Tae_TraesCS7A02G103000.1
Tae_TraesCS7A02G091500.1
Tae_TraesCS7A02G091400.1
Tae_TraesCS7A02G091100.1
Tae_TraesCS7A02G086200.1
Tae_TraesCS7A02G062000.1
Tae_TraesCS7A02G013700.1
Tae_TraesCS7A02G000100.1
Tae_TraesCS6D02G395900.1
Tae_TraesCS6D02G395700.1
Tae_TraesCS6D02G395600.1
Tae_TraesCS6D02G395400.1
Tae_TraesCS6D02G394600.1
Tae_TraesCS6D02G362200.1
Tae_TraesCS6D02G362100.1
Tae_TraesCS6D02G361700.1
−1000
0
1000
2000
3000

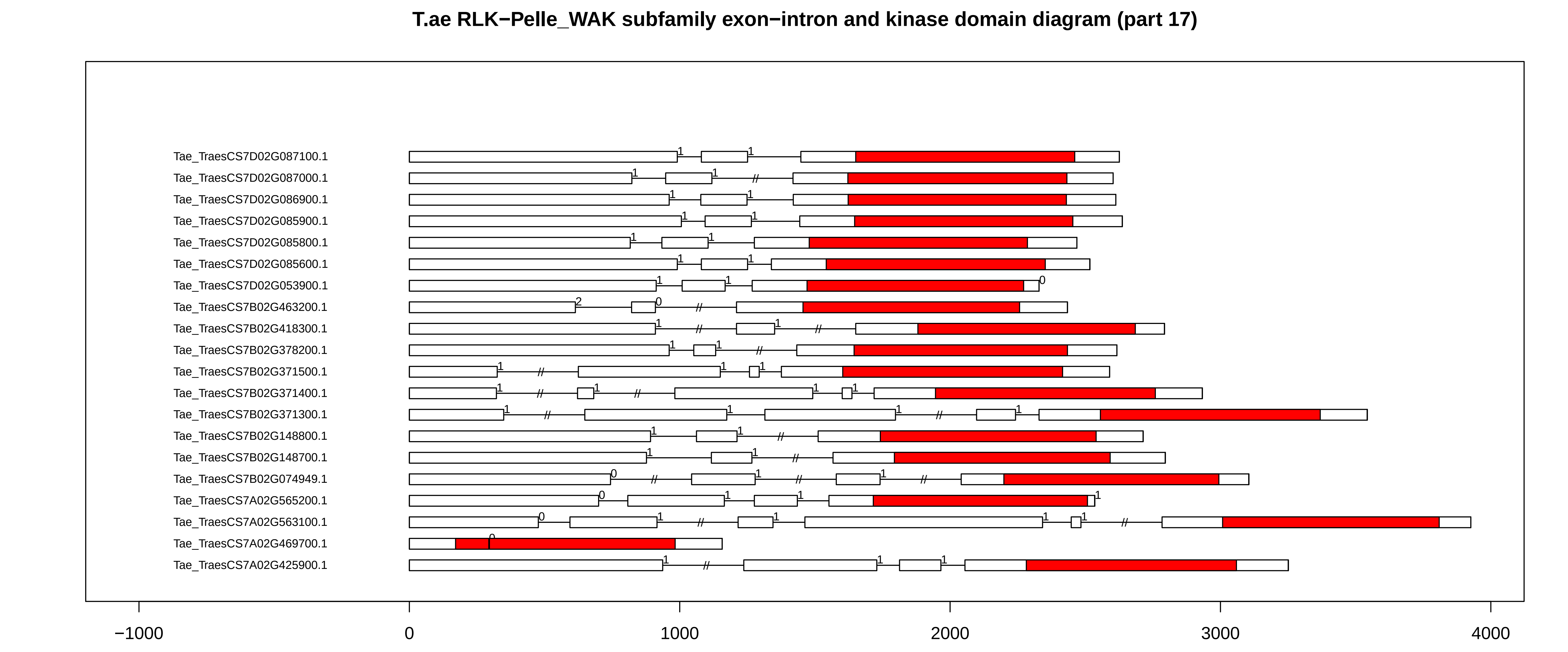
T.ae RLK−Pelle_WAK subfamily exon−intron and kinase domain diagram (part 17)
Tae_TraesCS7D02G087100.1
Tae_TraesCS7D02G087000.1
Tae_TraesCS7D02G086900.1
Tae_TraesCS7D02G085900.1
Tae_TraesCS7D02G085800.1
Tae_TraesCS7D02G085600.1
Tae_TraesCS7D02G053900.1
Tae_TraesCS7B02G463200.1
Tae_TraesCS7B02G418300.1
Tae_TraesCS7B02G378200.1
Tae_TraesCS7B02G371500.1
Tae_TraesCS7B02G371400.1
Tae_TraesCS7B02G371300.1
Tae_TraesCS7B02G148800.1
Tae_TraesCS7B02G148700.1
Tae_TraesCS7B02G074949.1
Tae_TraesCS7A02G565200.1
Tae_TraesCS7A02G563100.1
Tae_TraesCS7A02G469700.1
Tae_TraesCS7A02G425900.1
−1000
0
1000
2000
3000
4000

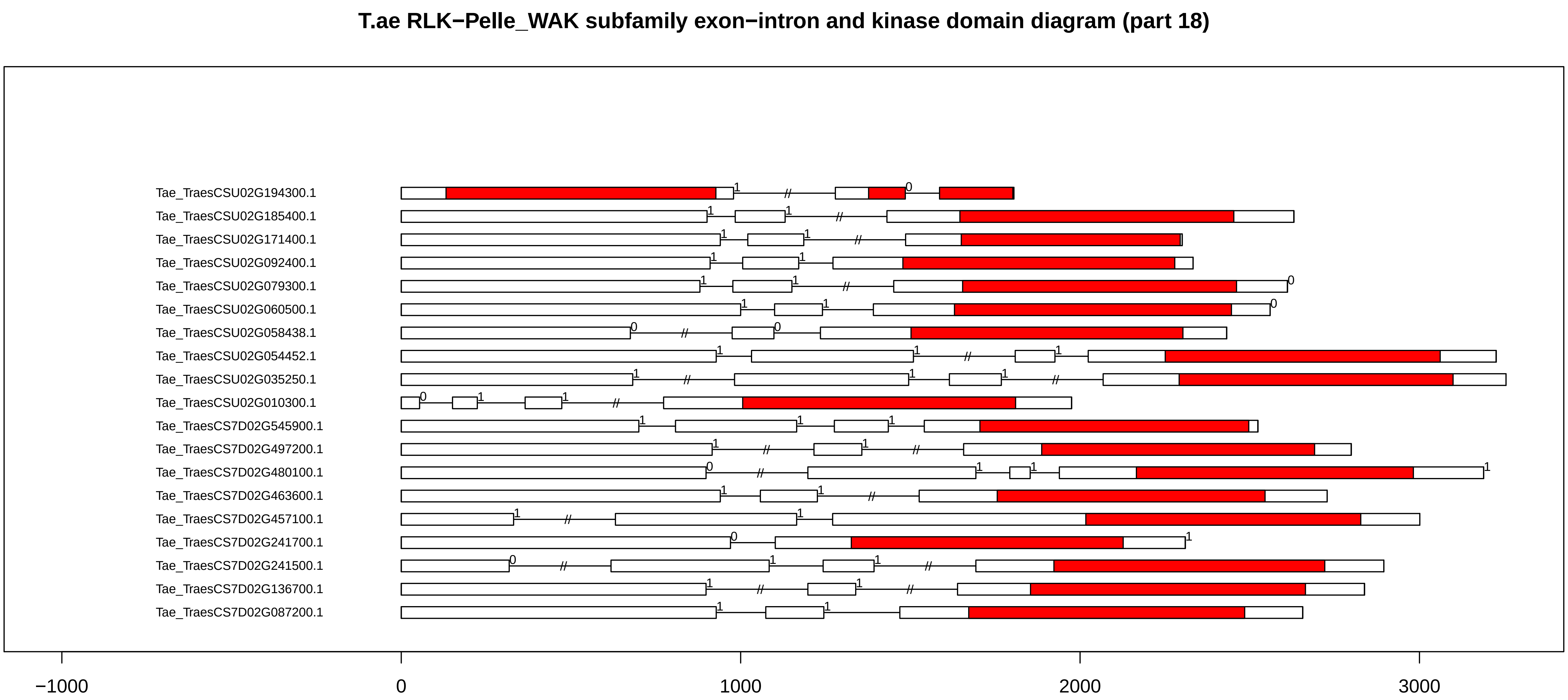
T.ae RLK−Pelle_WAK subfamily exon−intron and kinase domain diagram (part 18)
Tae_TraesCSU02G194300.1
Tae_TraesCSU02G185400.1
Tae_TraesCSU02G171400.1
Tae_TraesCSU02G092400.1
Tae_TraesCSU02G079300.1
Tae_TraesCSU02G060500.1
Tae_TraesCSU02G058438.1
Tae_TraesCSU02G054452.1
Tae_TraesCSU02G035250.1
Tae_TraesCSU02G010300.1
Tae_TraesCS7D02G545900.1
Tae_TraesCS7D02G497200.1
Tae_TraesCS7D02G480100.1
Tae_TraesCS7D02G463600.1
Tae_TraesCS7D02G457100.1
Tae_TraesCS7D02G241700.1
Tae_TraesCS7D02G241500.1
Tae_TraesCS7D02G136700.1
Tae_TraesCS7D02G087200.1
−1000
0
1000
2000
3000

T.sp RLK−Pelle_C−LEC subfamily exon−intron and kinase domain diagram (all)

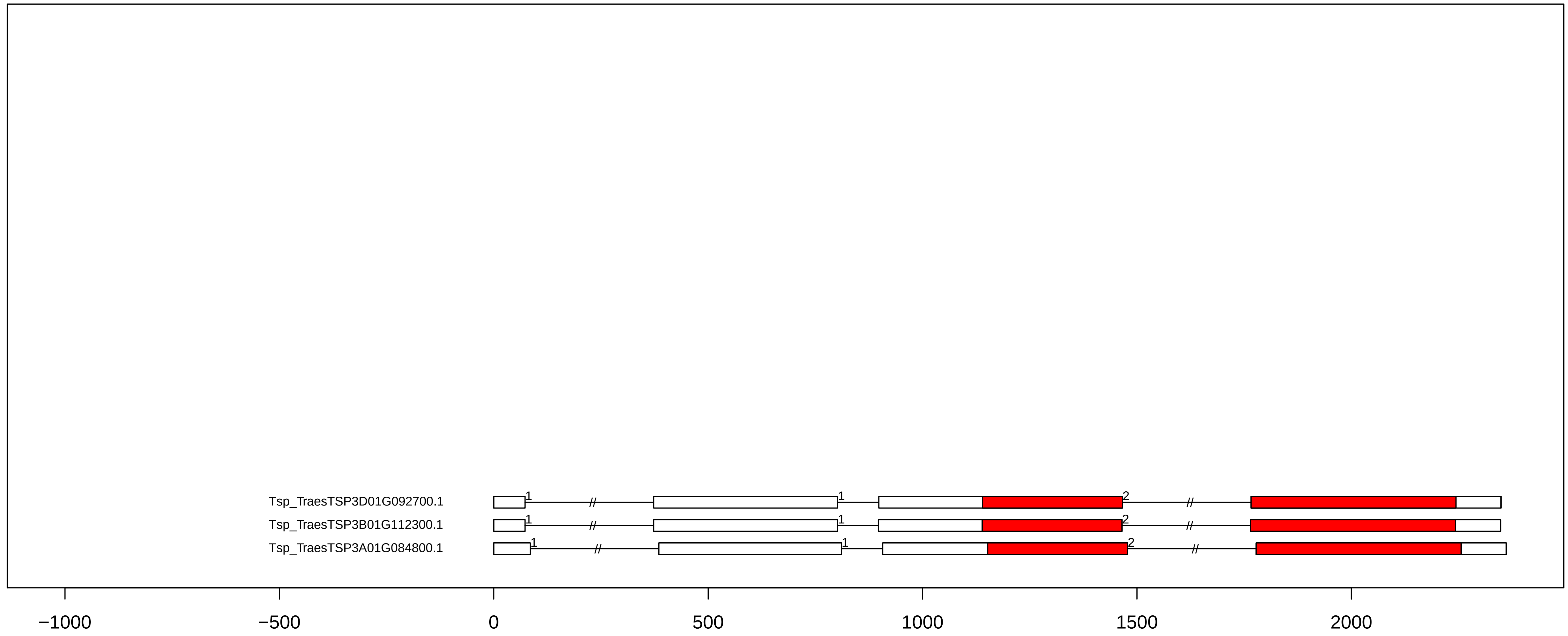

T.sp RLK−Pelle_CR4L subfamily exon−intron and kinase domain diagram (part 1)

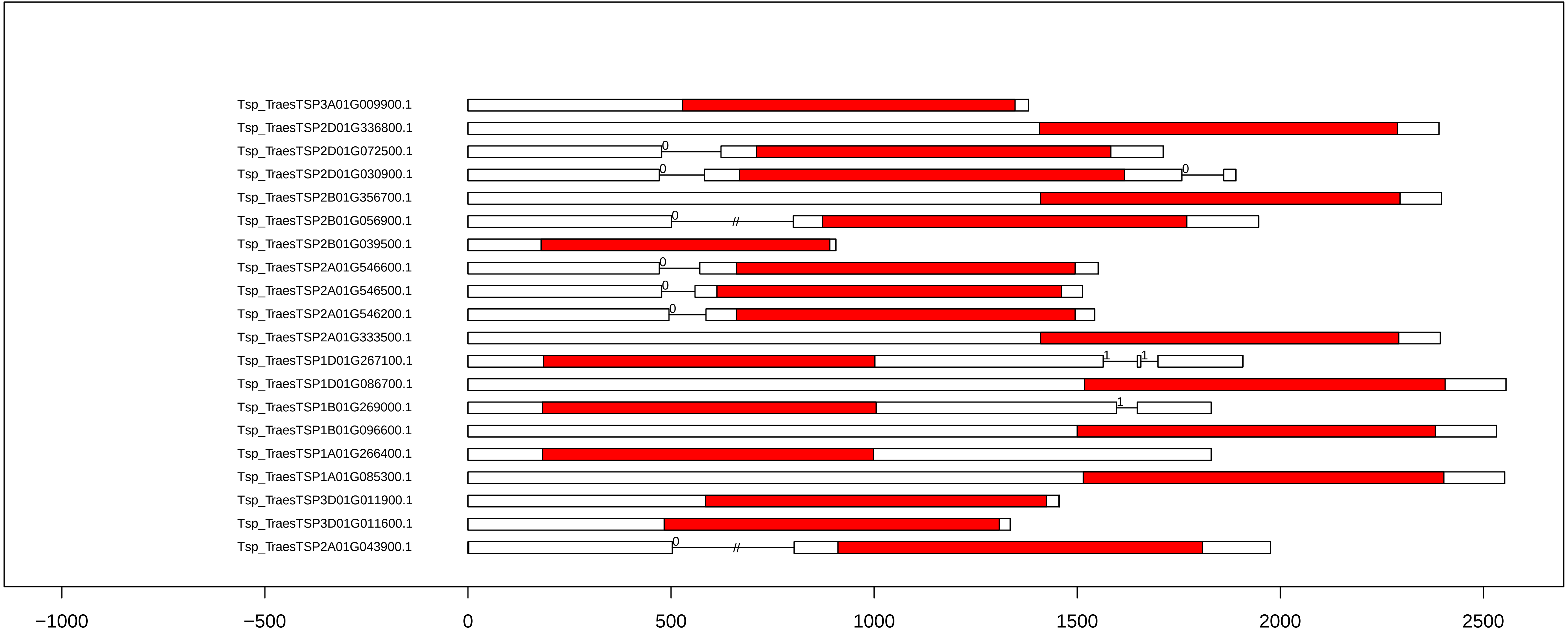

T.sp RLK−Pelle_CR4L subfamily exon−intron and kinase domain diagram (part 2)

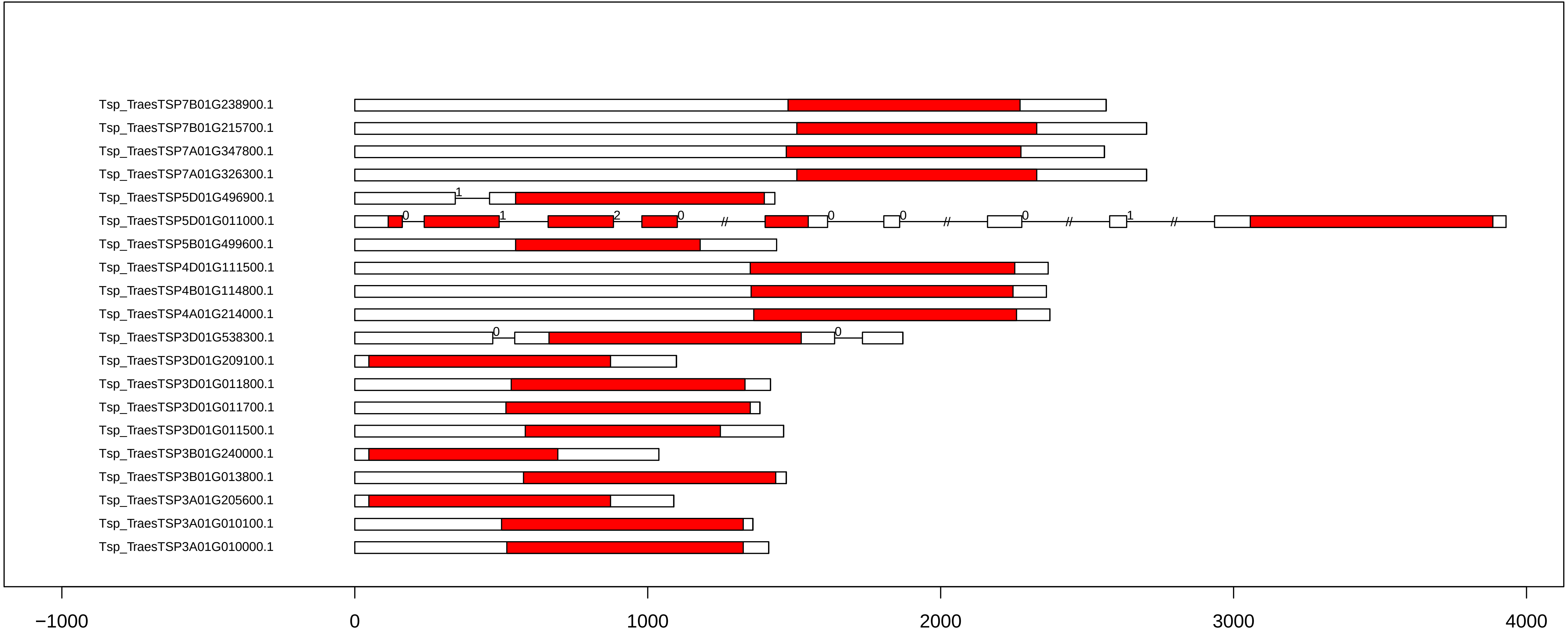

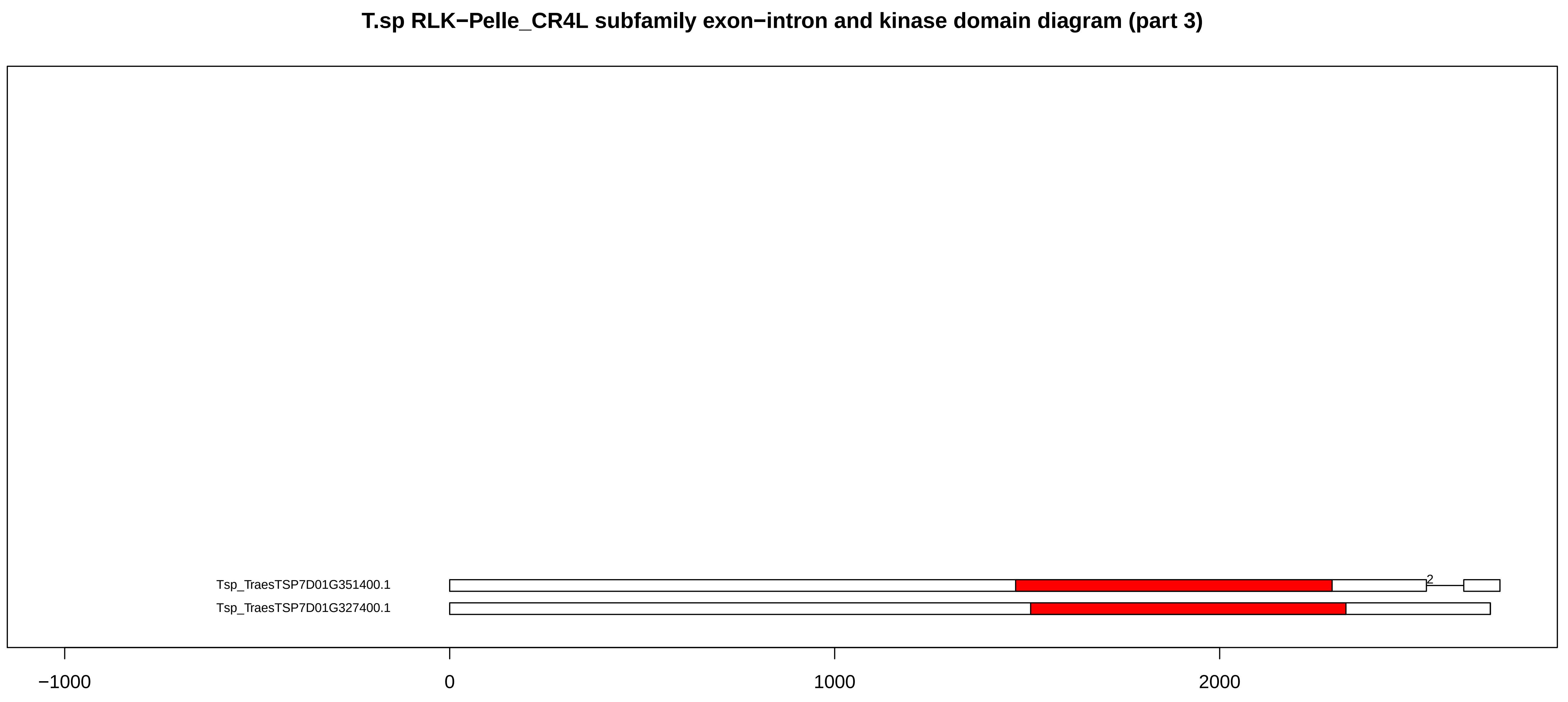

T.sp RLK−Pelle_CR4L subfamily exon−intron and kinase domain diagram (part 3)
Tsp_TraesTSP7D01G351400.1
Tsp_TraesTSP7D01G327400.1
2
−1000
0
1000
2000

T.sp RLK−Pelle_CrRLK1L−1 subfamily exon−intron and kinase domain diagram (part 1)

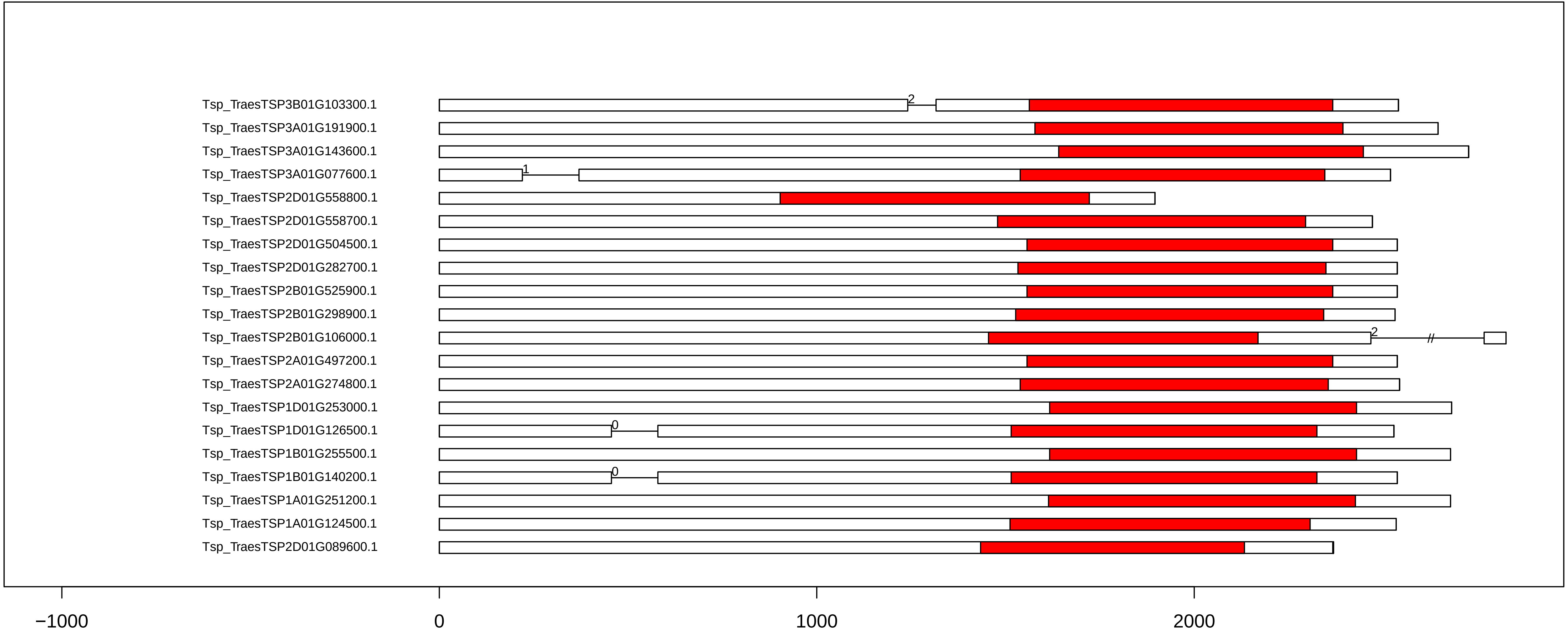

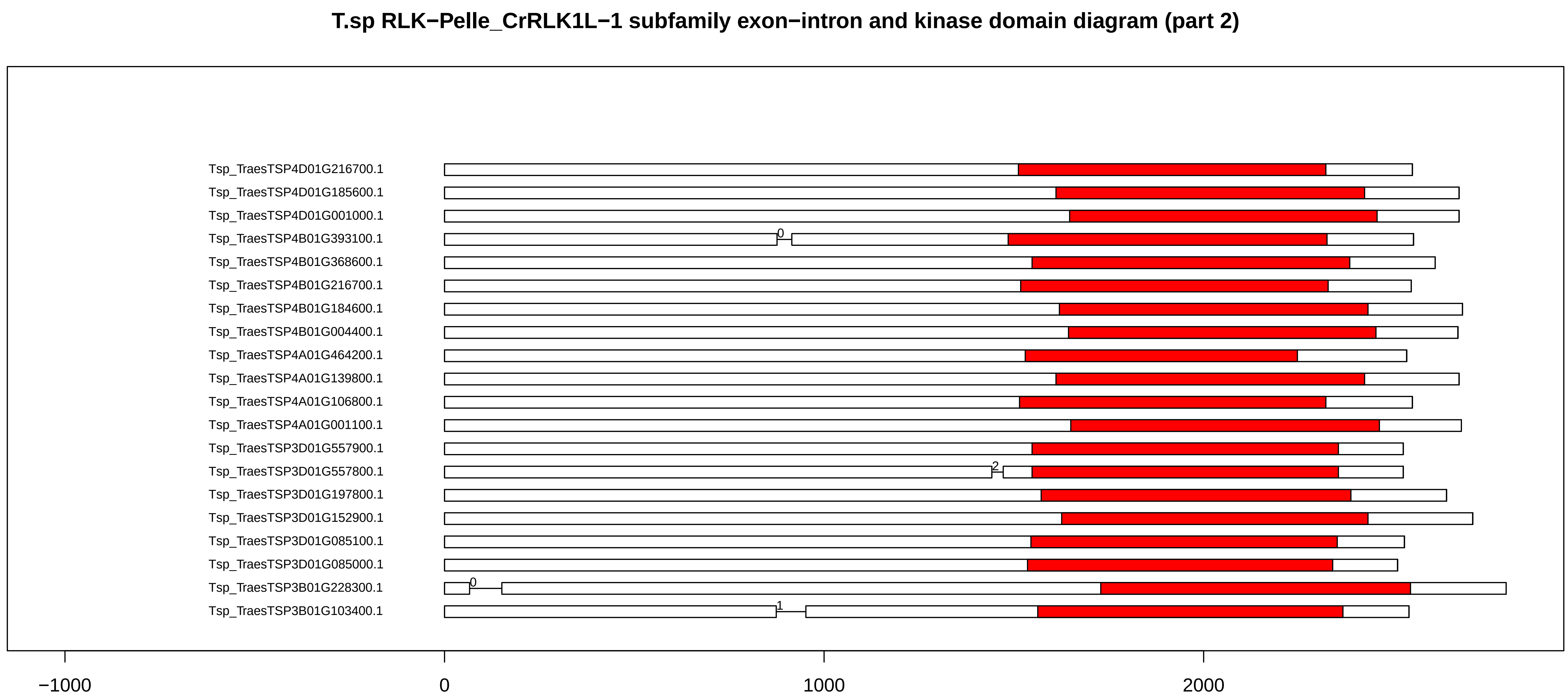

T.sp RLK−Pelle_CrRLK1L−1 subfamily exon−intron and kinase domain diagram (part 2)
Tsp_TraesTSP4D01G216700.1
Tsp_TraesTSP4D01G185600.1
Tsp_TraesTSP4D01G001000.1
Tsp_TraesTSP4B01G393100.1
Tsp_TraesTSP4B01G368600.1
Tsp_TraesTSP4B01G216700.1
Tsp_TraesTSP4B01G184600.1
Tsp_TraesTSP4B01G004400.1
Tsp_TraesTSP4A01G464200.1
Tsp_TraesTSP4A01G139800.1
Tsp_TraesTSP4A01G106800.1
Tsp_TraesTSP4A01G001100.1
Tsp_TraesTSP3D01G557900.1
Tsp_TraesTSP3D01G557800.1
Tsp_TraesTSP3D01G197800.1
Tsp_TraesTSP3D01G152900.1
Tsp_TraesTSP3D01G085100.1
Tsp_TraesTSP3D01G085000.1
Tsp_TraesTSP3B01G228300.1
Tsp_TraesTSP3B01G103400.1
0
2
0
1
−1000
0
1000
2000

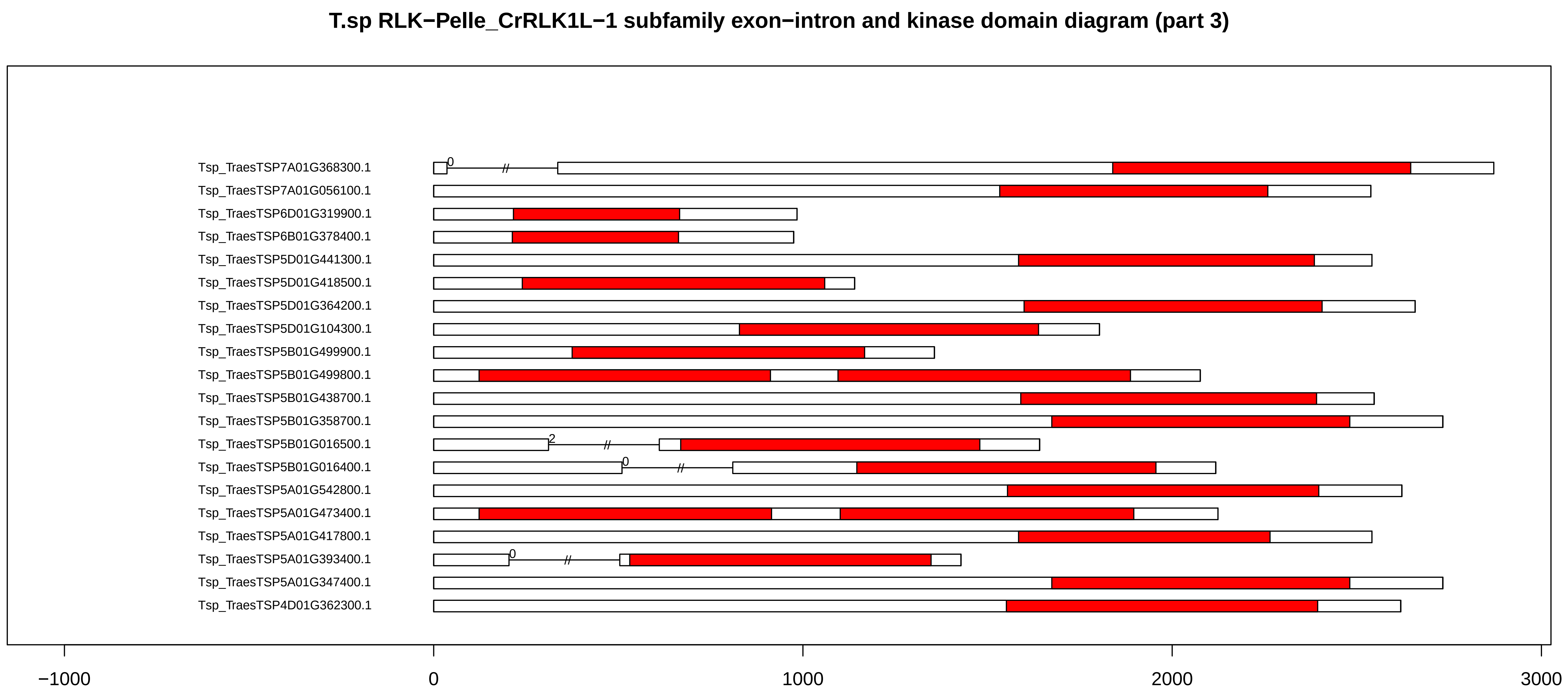
T.sp RLK−Pelle_CrRLK1L−1 subfamily exon−intron and kinase domain diagram (part 3)
Tsp_TraesTSP7A01G368300.1
Tsp_TraesTSP7A01G056100.1
Tsp_TraesTSP6D01G319900.1
Tsp_TraesTSP6B01G378400.1
Tsp_TraesTSP5D01G441300.1
Tsp_TraesTSP5D01G418500.1
Tsp_TraesTSP5D01G364200.1
Tsp_TraesTSP5D01G104300.1
Tsp_TraesTSP5B01G499900.1
Tsp_TraesTSP5B01G499800.1
Tsp_TraesTSP5B01G438700.1
Tsp_TraesTSP5B01G358700.1
Tsp_TraesTSP5B01G016500.1
Tsp_TraesTSP5B01G016400.1
Tsp_TraesTSP5A01G542800.1
Tsp_TraesTSP5A01G473400.1
Tsp_TraesTSP5A01G417800.1
Tsp_TraesTSP5A01G393400.1
Tsp_TraesTSP5A01G347400.1
Tsp_TraesTSP4D01G362300.1
0
2
0
0
−1000
0
1000
2000
3000

T.sp RLK−Pelle_CrRLK1L−1 subfamily exon−intron and kinase domain diagram (part 4)

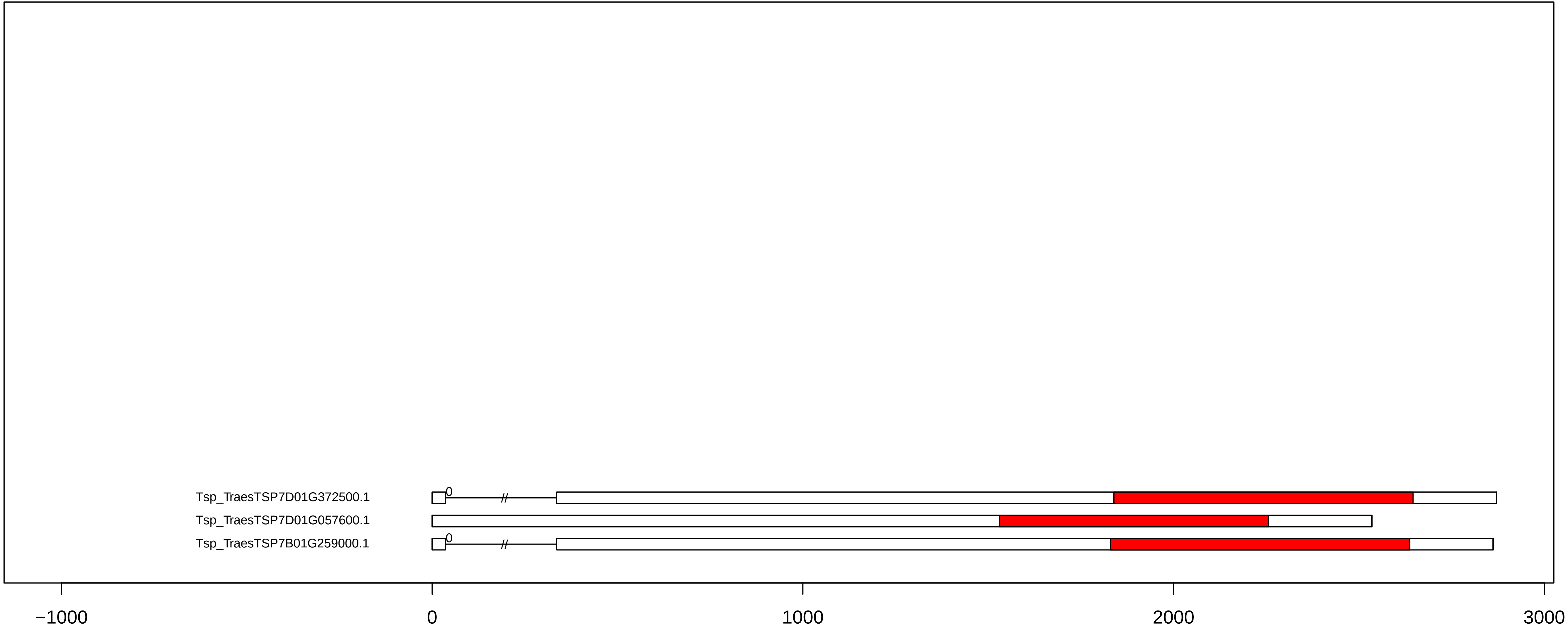

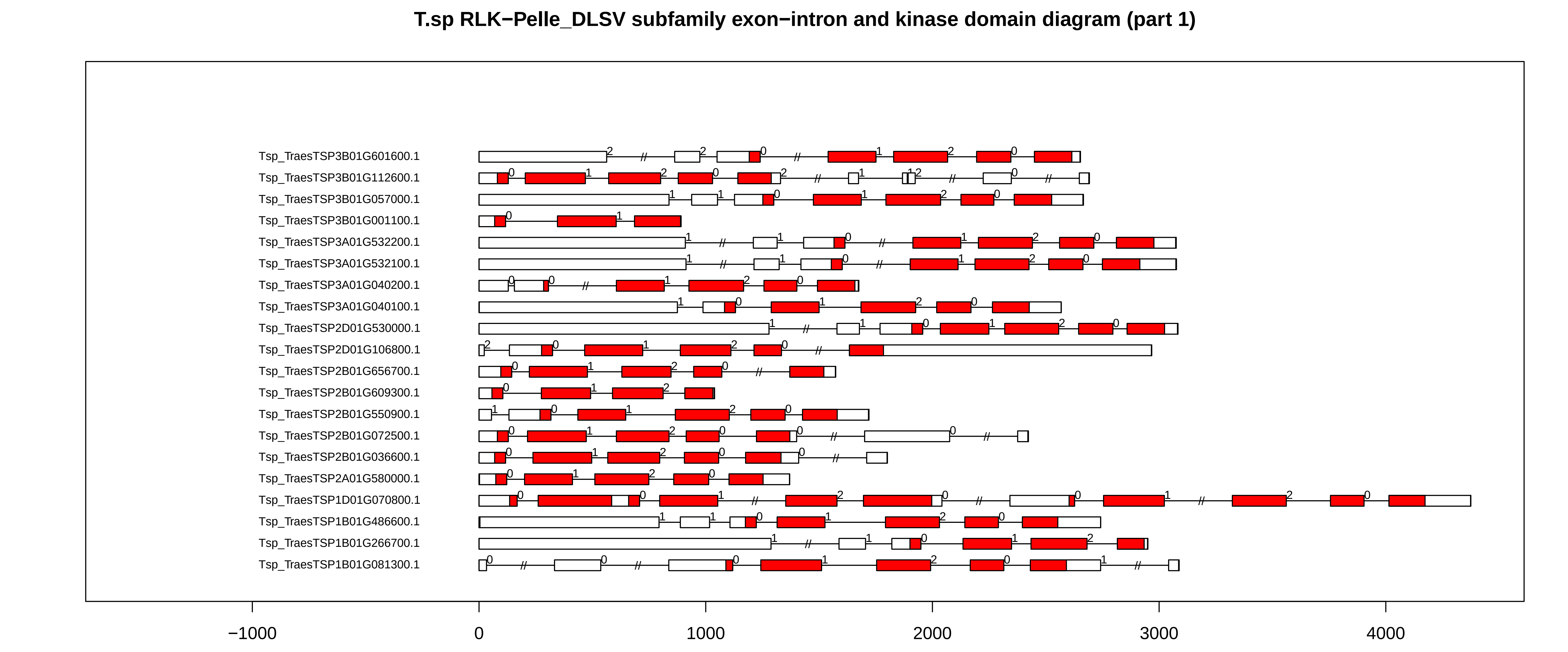
T.sp RLK−Pelle_DLSV subfamily exon−intron and kinase domain diagram (part 1)
Tsp_TraesTSP3B01G601600.1
Tsp_TraesTSP3B01G112600.1
Tsp_TraesTSP3B01G057000.1
Tsp_TraesTSP3B01G001100.1
Tsp_TraesTSP3A01G532200.1
Tsp_TraesTSP3A01G532100.1
Tsp_TraesTSP3A01G040200.1
Tsp_TraesTSP3A01G040100.1
Tsp_TraesTSP2D01G530000.1
Tsp_TraesTSP2D01G106800.1
Tsp_TraesTSP2B01G656700.1
Tsp_TraesTSP2B01G609300.1
Tsp_TraesTSP2B01G550900.1
Tsp_TraesTSP2B01G072500.1
Tsp_TraesTSP2B01G036600.1
Tsp_TraesTSP2A01G580000.1
Tsp_TraesTSP1D01G070800.1
Tsp_TraesTSP1B01G486600.1
Tsp_TraesTSP1B01G266700.1
Tsp_TraesTSP1B01G081300.1
−1000
0
1000
2000
3000
4000

T.sp RLK−Pelle_DLSV subfamily exon−intron and kinase domain diagram (part 2)

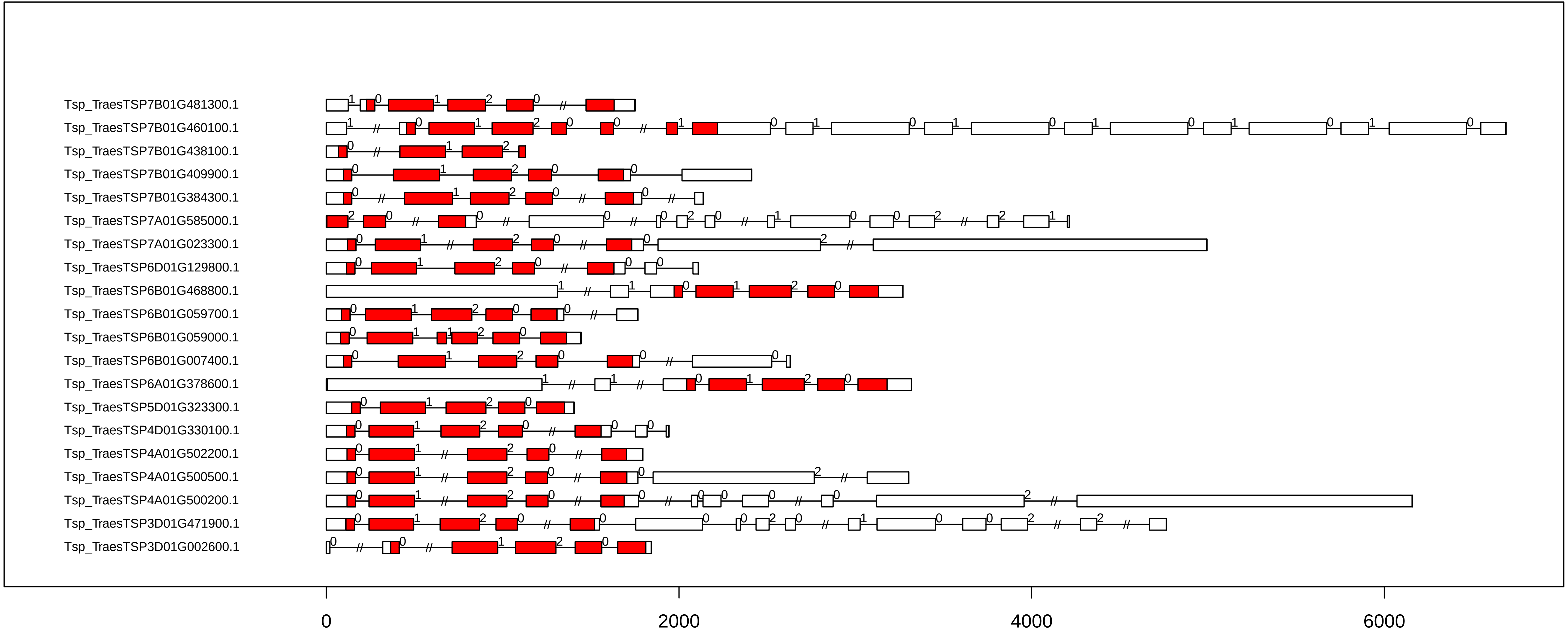

T.sp RLK−Pelle_DLSV subfamily exon−intron and kinase domain diagram (part 3)

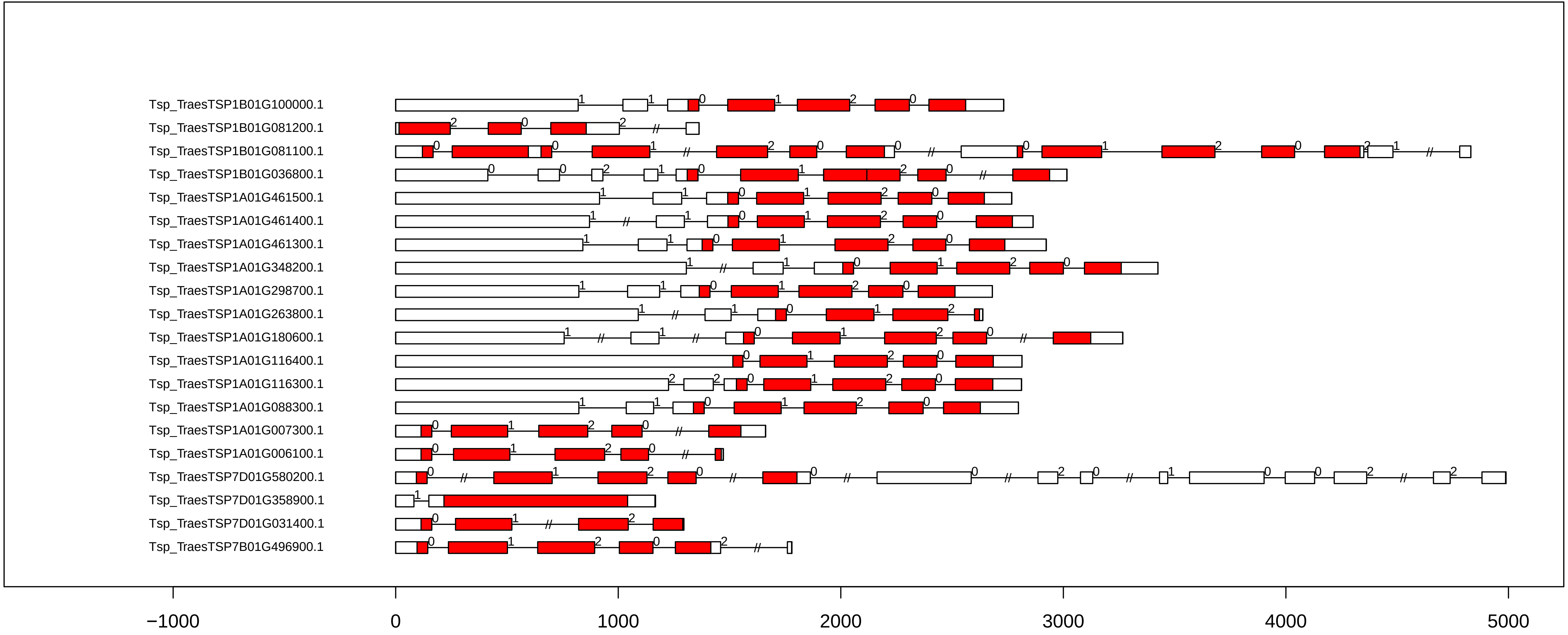

T.sp RLK−Pelle_DLSV subfamily exon−intron and kinase domain diagram (part 4)

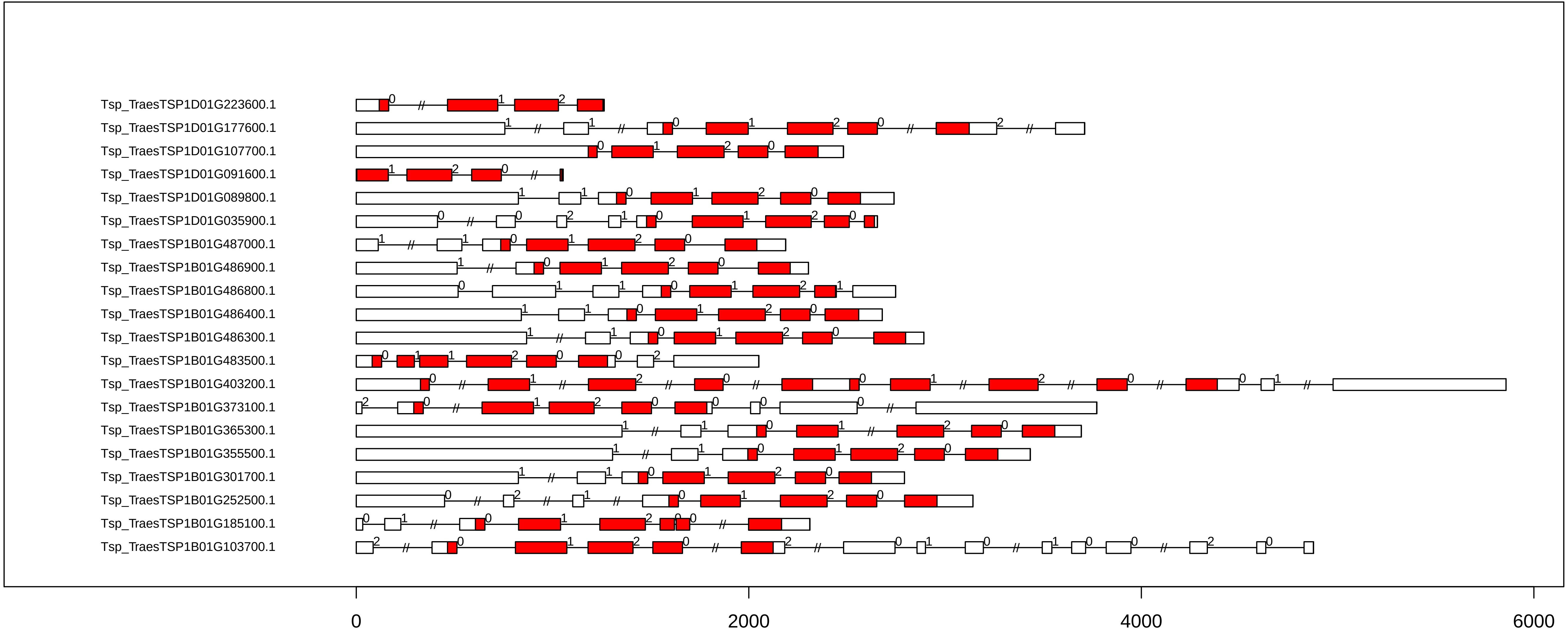

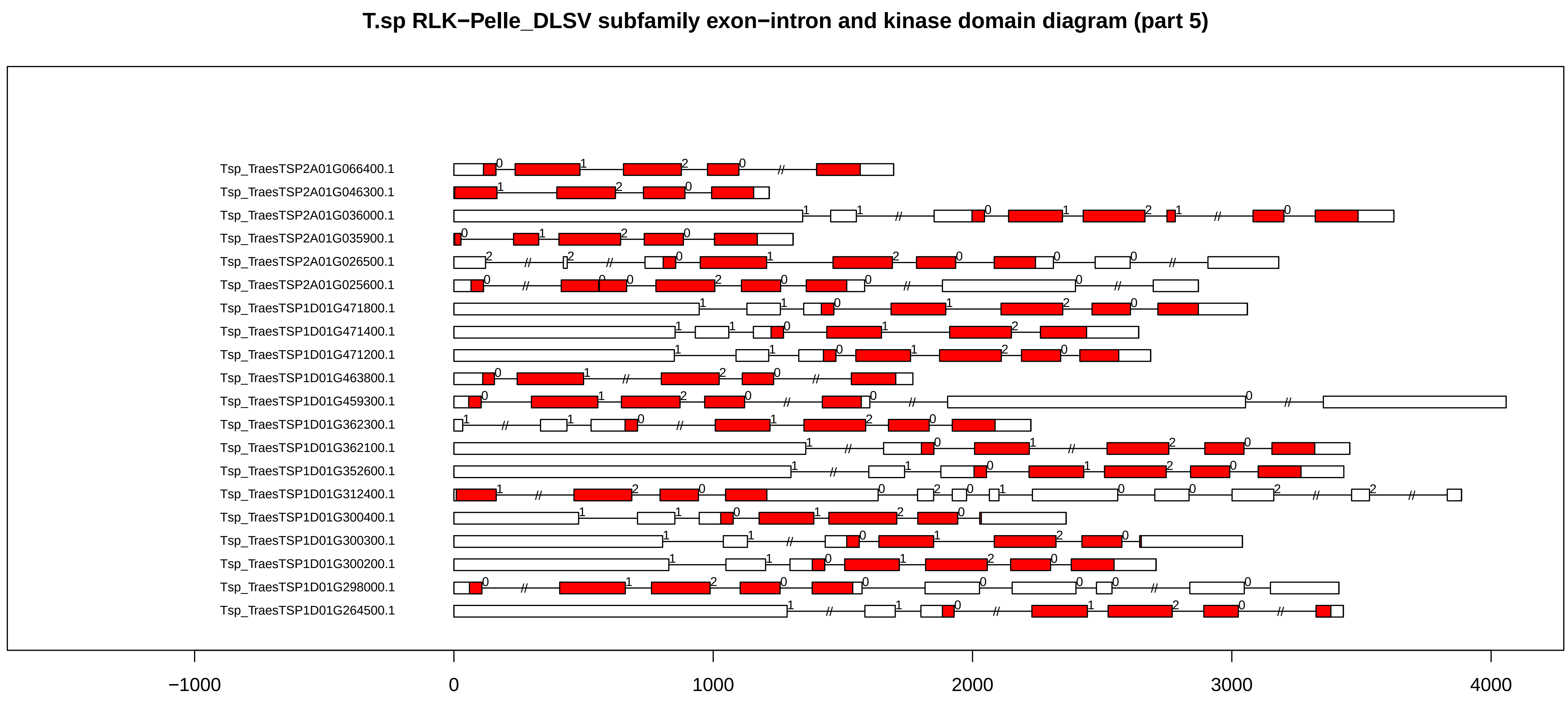
T.sp RLK−Pelle_DLSV subfamily exon−intron and kinase domain diagram (part 5)
Tsp_TraesTSP2A01G066400.1
Tsp_TraesTSP2A01G046300.1
Tsp_TraesTSP2A01G036000.1
Tsp_TraesTSP2A01G035900.1
Tsp_TraesTSP2A01G026500.1
Tsp_TraesTSP2A01G025600.1
Tsp_TraesTSP1D01G471800.1
Tsp_TraesTSP1D01G471400.1
Tsp_TraesTSP1D01G471200.1
Tsp_TraesTSP1D01G463800.1
Tsp_TraesTSP1D01G459300.1
Tsp_TraesTSP1D01G362300.1
Tsp_TraesTSP1D01G362100.1
Tsp_TraesTSP1D01G352600.1
Tsp_TraesTSP1D01G312400.1
Tsp_TraesTSP1D01G300400.1
Tsp_TraesTSP1D01G300300.1
Tsp_TraesTSP1D01G300200.1
Tsp_TraesTSP1D01G298000.1
Tsp_TraesTSP1D01G264500.1
−1000
0
1000
2000
3000
4000

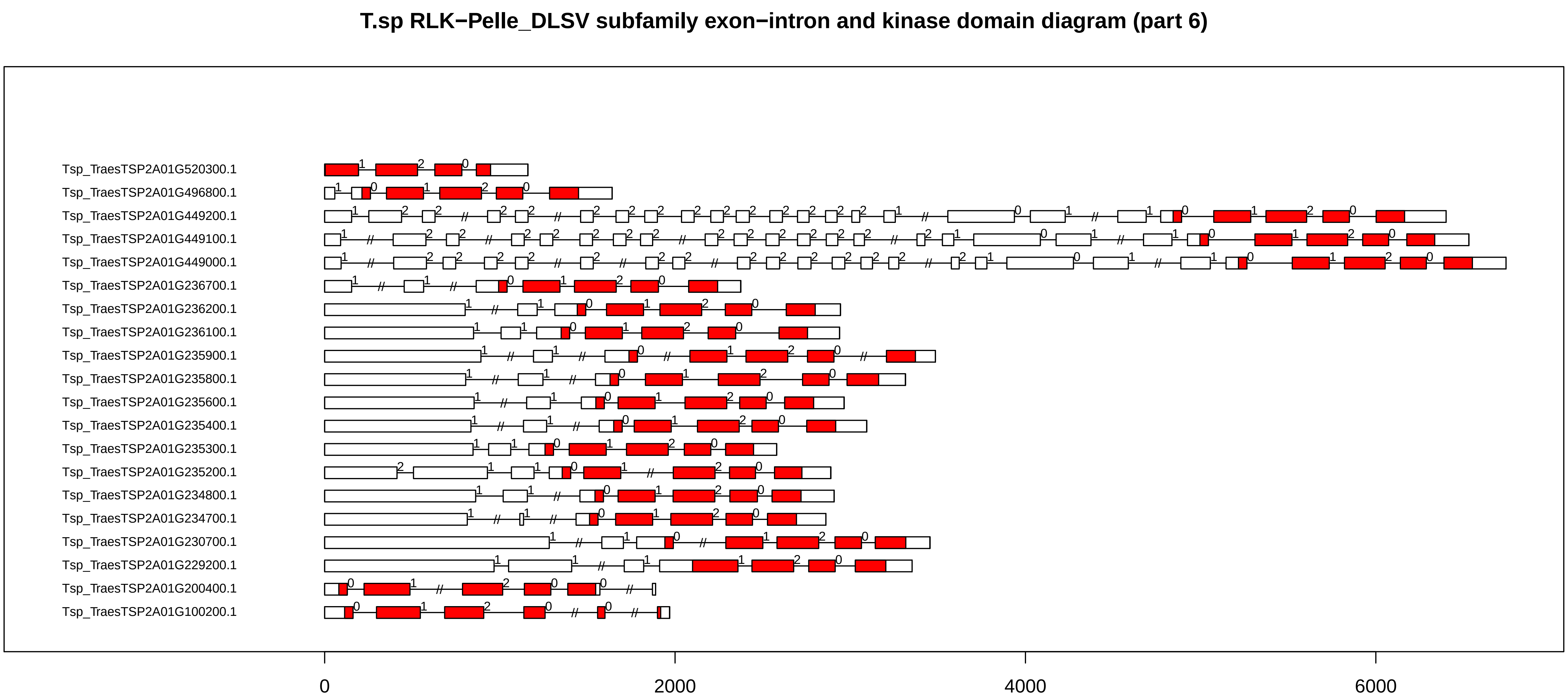

T.sp RLK−Pelle_DLSV subfamily exon−intron and kinase domain diagram (part 6)
Tsp_TraesTSP2A01G520300.1
Tsp_TraesTSP2A01G496800.1
Tsp_TraesTSP2A01G449200.1
Tsp_TraesTSP2A01G449100.1
Tsp_TraesTSP2A01G449000.1
Tsp_TraesTSP2A01G236700.1
Tsp_TraesTSP2A01G236200.1
Tsp_TraesTSP2A01G236100.1
Tsp_TraesTSP2A01G235900.1
Tsp_TraesTSP2A01G235800.1
Tsp_TraesTSP2A01G235600.1
Tsp_TraesTSP2A01G235400.1
Tsp_TraesTSP2A01G235300.1
Tsp_TraesTSP2A01G235200.1
Tsp_TraesTSP2A01G234800.1
Tsp_TraesTSP2A01G234700.1
Tsp_TraesTSP2A01G230700.1
Tsp_TraesTSP2A01G229200.1
Tsp_TraesTSP2A01G200400.1
Tsp_TraesTSP2A01G100200.1
0
2000
4000
6000

T.sp RLK−Pelle_DLSV subfamily exon−intron and kinase domain diagram (part 7)

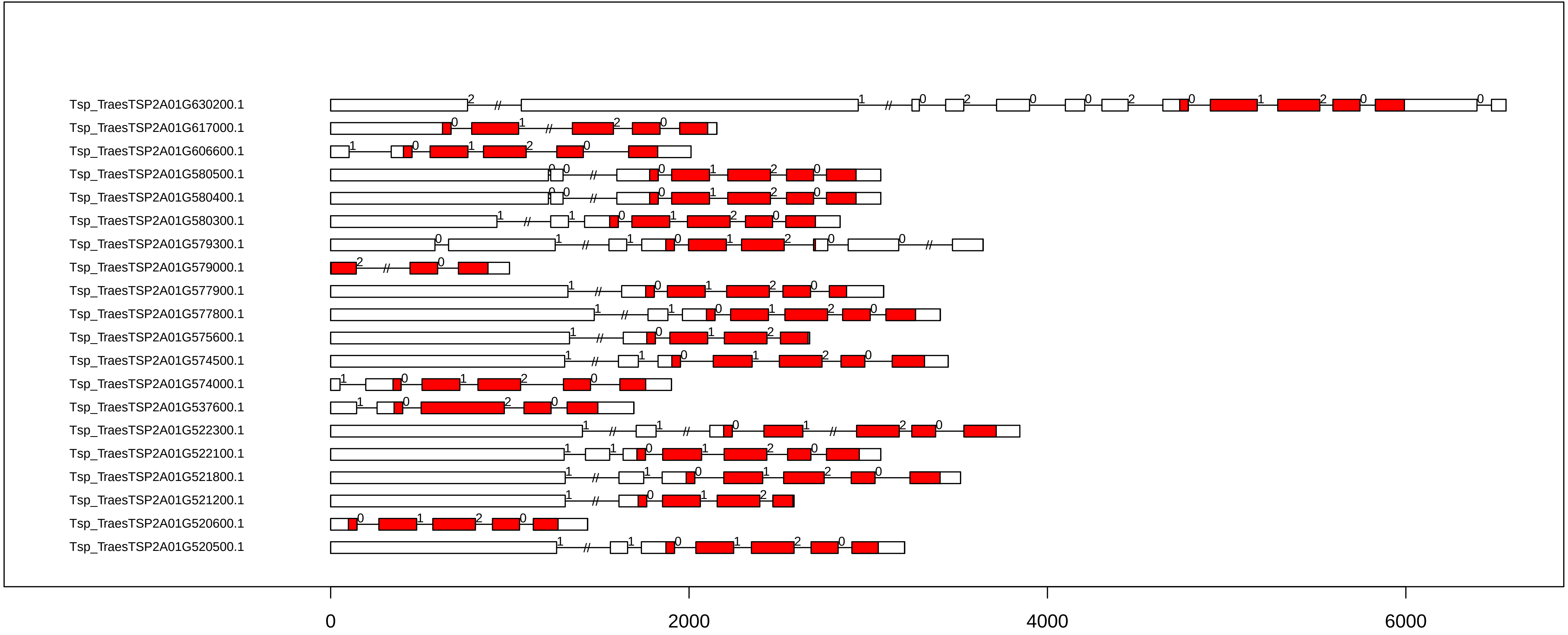

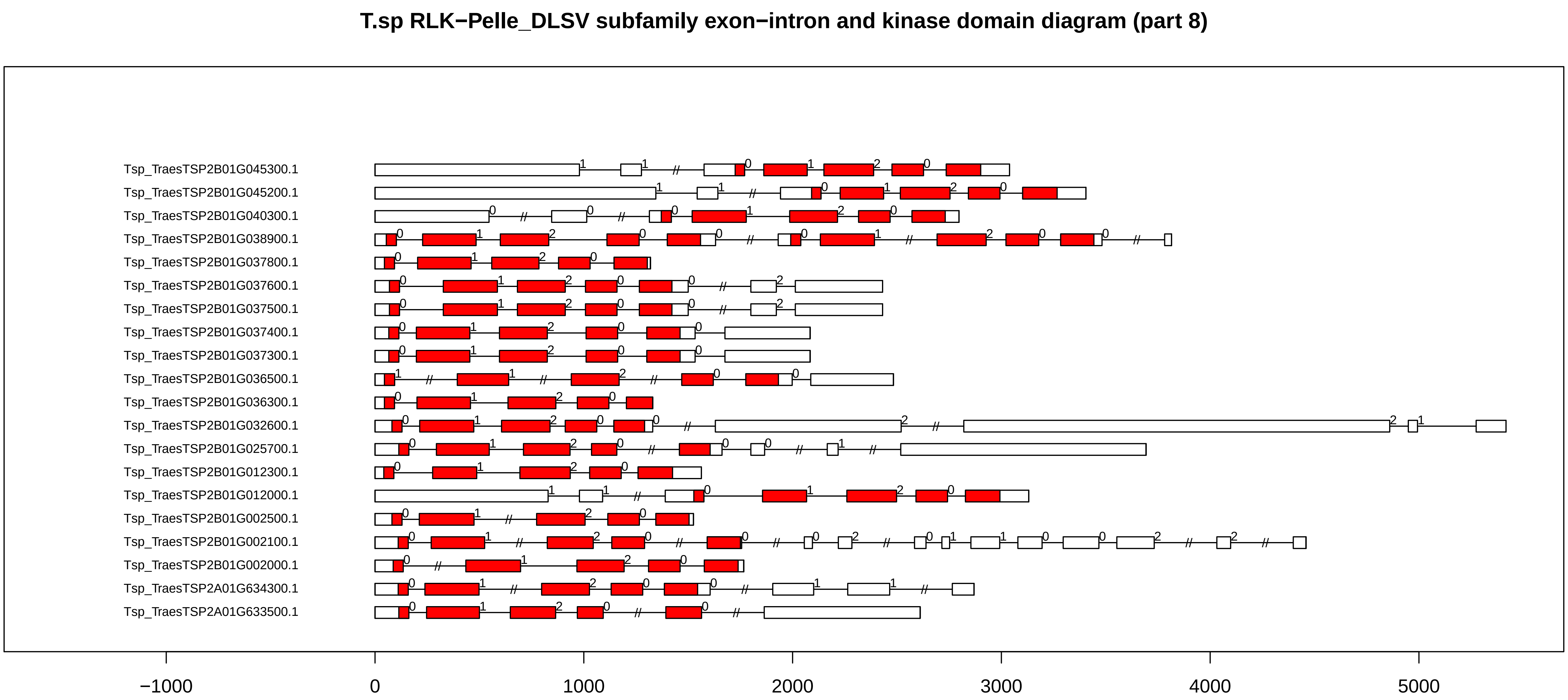

T.sp RLK−Pelle_DLSV subfamily exon−intron and kinase domain diagram (part 8)
Tsp_TraesTSP2B01G045300.1
Tsp_TraesTSP2B01G045200.1
Tsp_TraesTSP2B01G040300.1
Tsp_TraesTSP2B01G038900.1
Tsp_TraesTSP2B01G037800.1
Tsp_TraesTSP2B01G037600.1
Tsp_TraesTSP2B01G037500.1
Tsp_TraesTSP2B01G037400.1
Tsp_TraesTSP2B01G037300.1
Tsp_TraesTSP2B01G036500.1
Tsp_TraesTSP2B01G036300.1
Tsp_TraesTSP2B01G032600.1
Tsp_TraesTSP2B01G025700.1
Tsp_TraesTSP2B01G012300.1
Tsp_TraesTSP2B01G012000.1
Tsp_TraesTSP2B01G002500.1
Tsp_TraesTSP2B01G002100.1
Tsp_TraesTSP2B01G002000.1
Tsp_TraesTSP2A01G634300.1
Tsp_TraesTSP2A01G633500.1
−1000
0
1000
2000
3000
4000
5000

T.sp RLK−Pelle_DLSV subfamily exon−intron and kinase domain diagram (part 9)

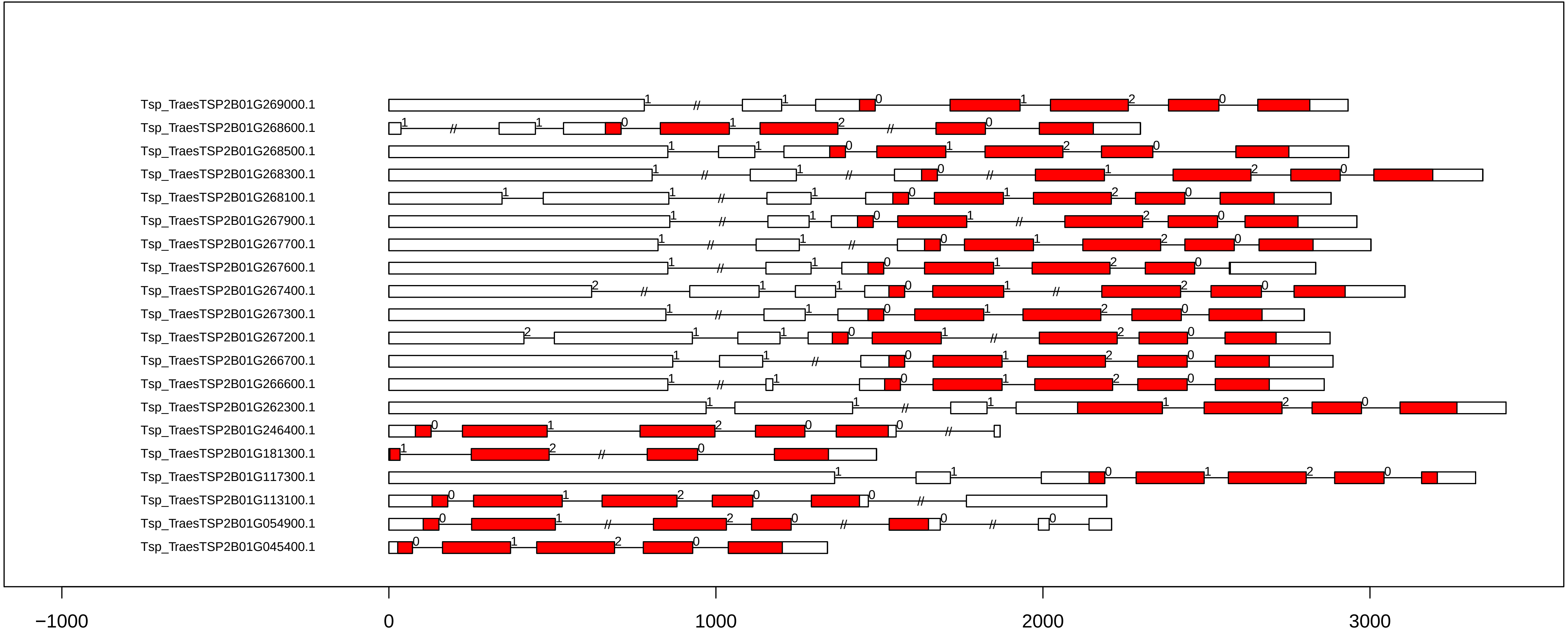

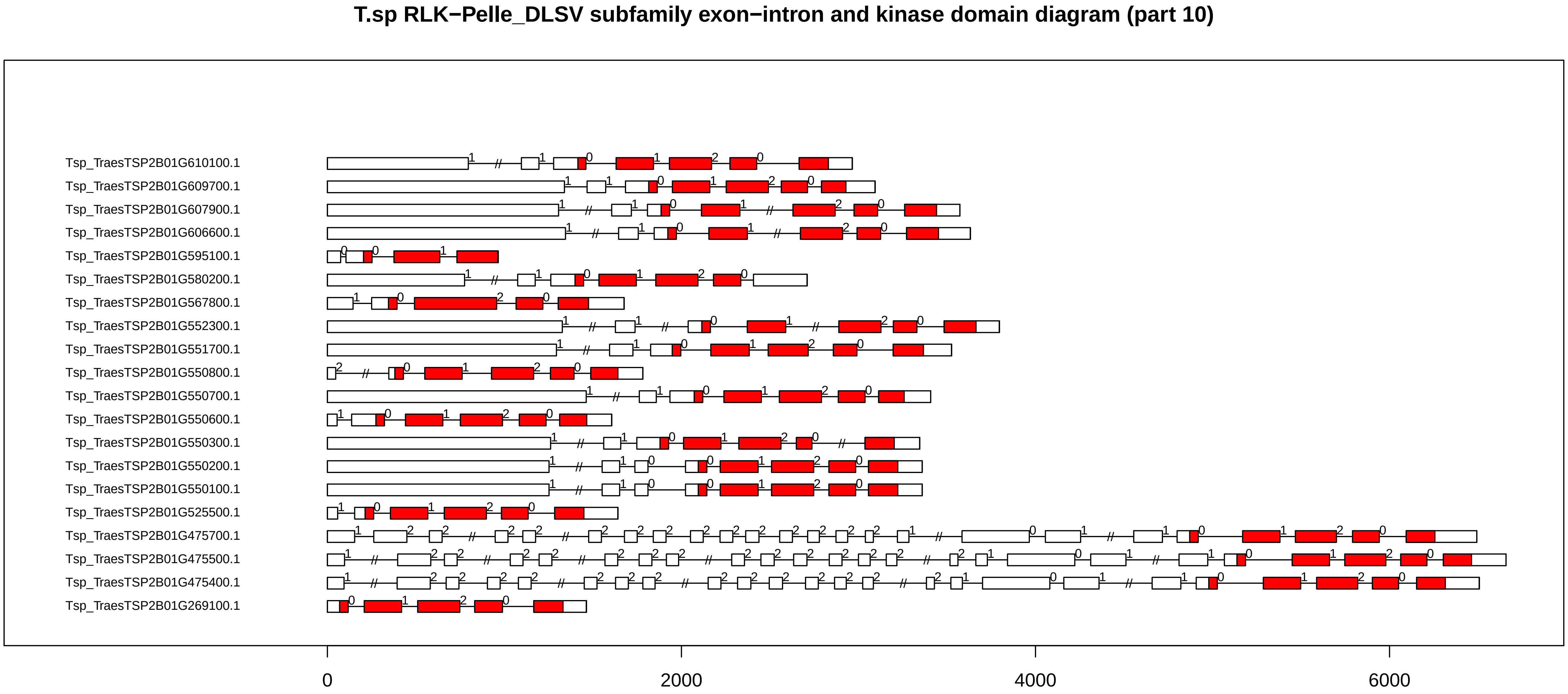

T.sp RLK−Pelle_DLSV subfamily exon−intron and kinase domain diagram (part 10)
Tsp_TraesTSP2B01G610100.1
Tsp_TraesTSP2B01G609700.1
Tsp_TraesTSP2B01G607900.1
Tsp_TraesTSP2B01G606600.1
Tsp_TraesTSP2B01G595100.1
Tsp_TraesTSP2B01G580200.1
Tsp_TraesTSP2B01G567800.1
Tsp_TraesTSP2B01G552300.1
Tsp_TraesTSP2B01G551700.1
Tsp_TraesTSP2B01G550800.1
Tsp_TraesTSP2B01G550700.1
Tsp_TraesTSP2B01G550600.1
Tsp_TraesTSP2B01G550300.1
Tsp_TraesTSP2B01G550200.1
Tsp_TraesTSP2B01G550100.1
Tsp_TraesTSP2B01G525500.1
Tsp_TraesTSP2B01G475700.1
Tsp_TraesTSP2B01G475500.1
Tsp_TraesTSP2B01G475400.1
Tsp_TraesTSP2B01G269100.1
0
2000
4000
6000

T.sp RLK−Pelle_DLSV subfamily exon−intron and kinase domain diagram (part 11)

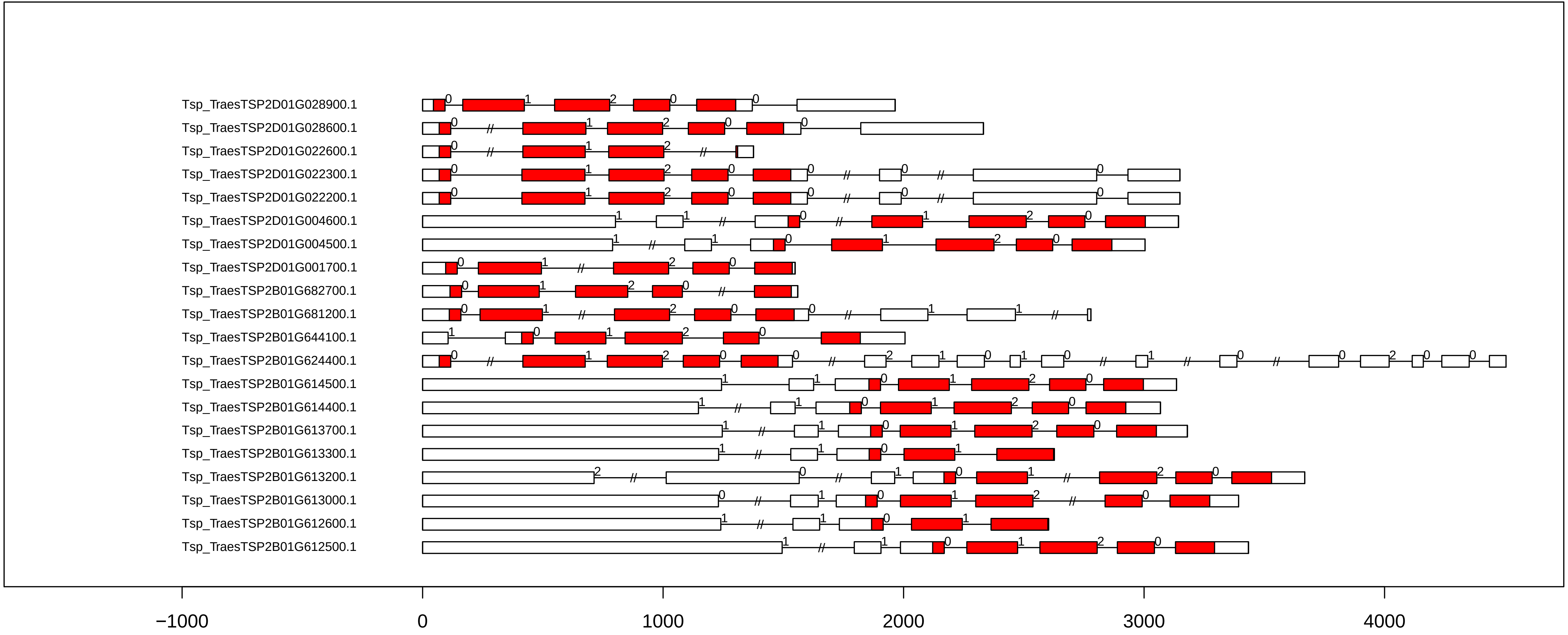

T.sp RLK−Pelle_DLSV subfamily exon−intron and kinase domain diagram (part 12)

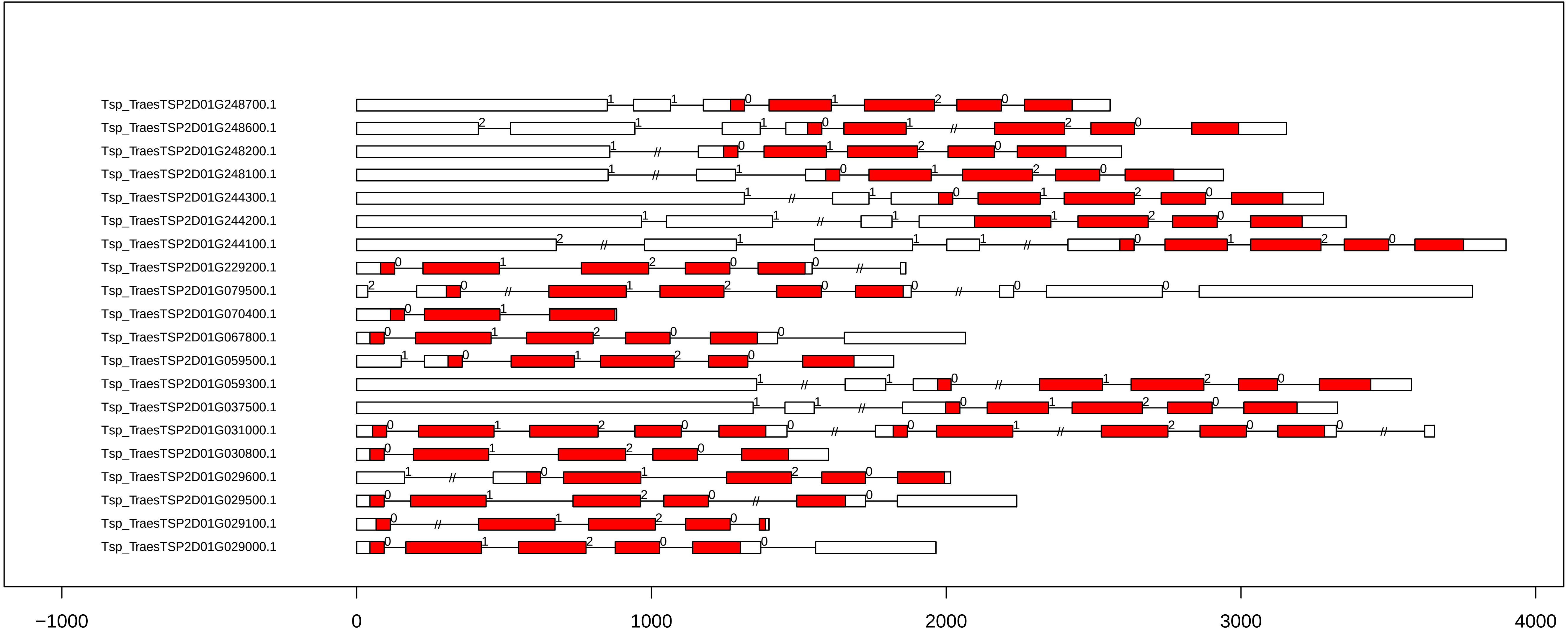

T.sp RLK−Pelle_DLSV subfamily exon−intron and kinase domain diagram (part 13)

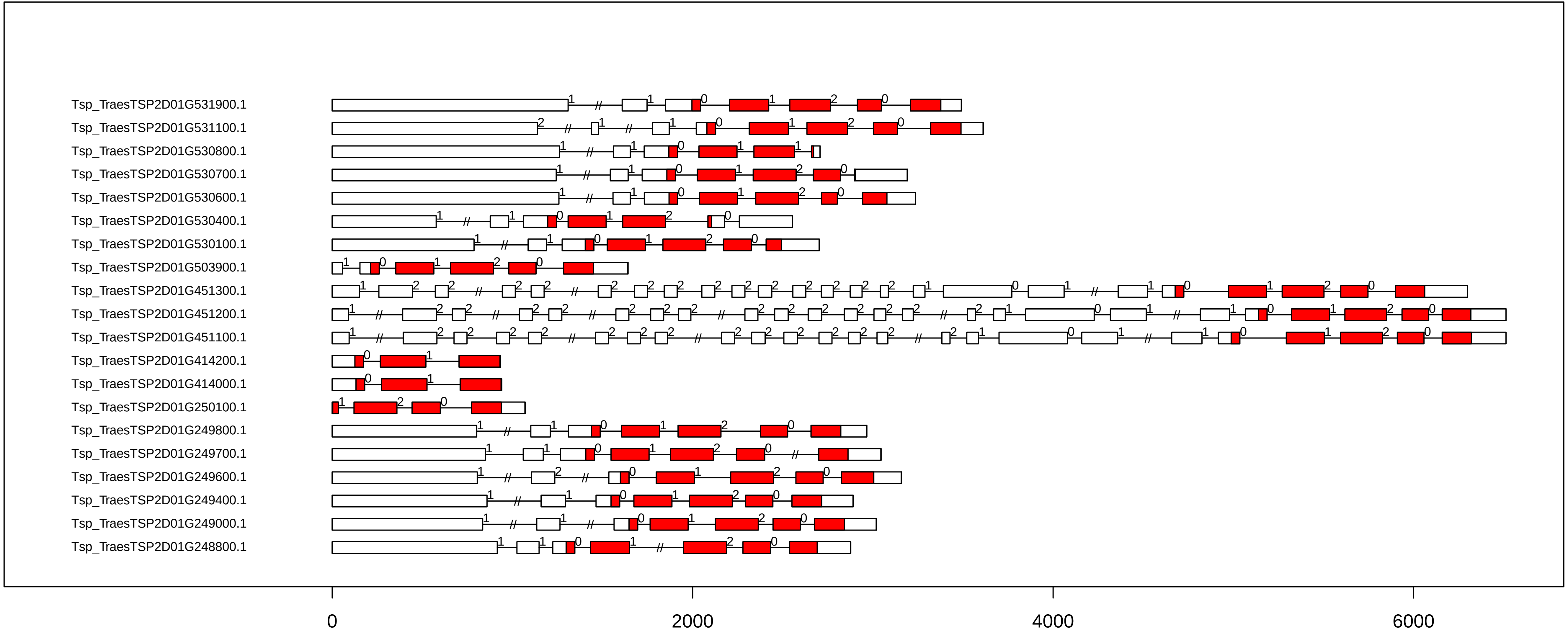

T.sp RLK−Pelle_DLSV subfamily exon−intron and kinase domain diagram (part 14)

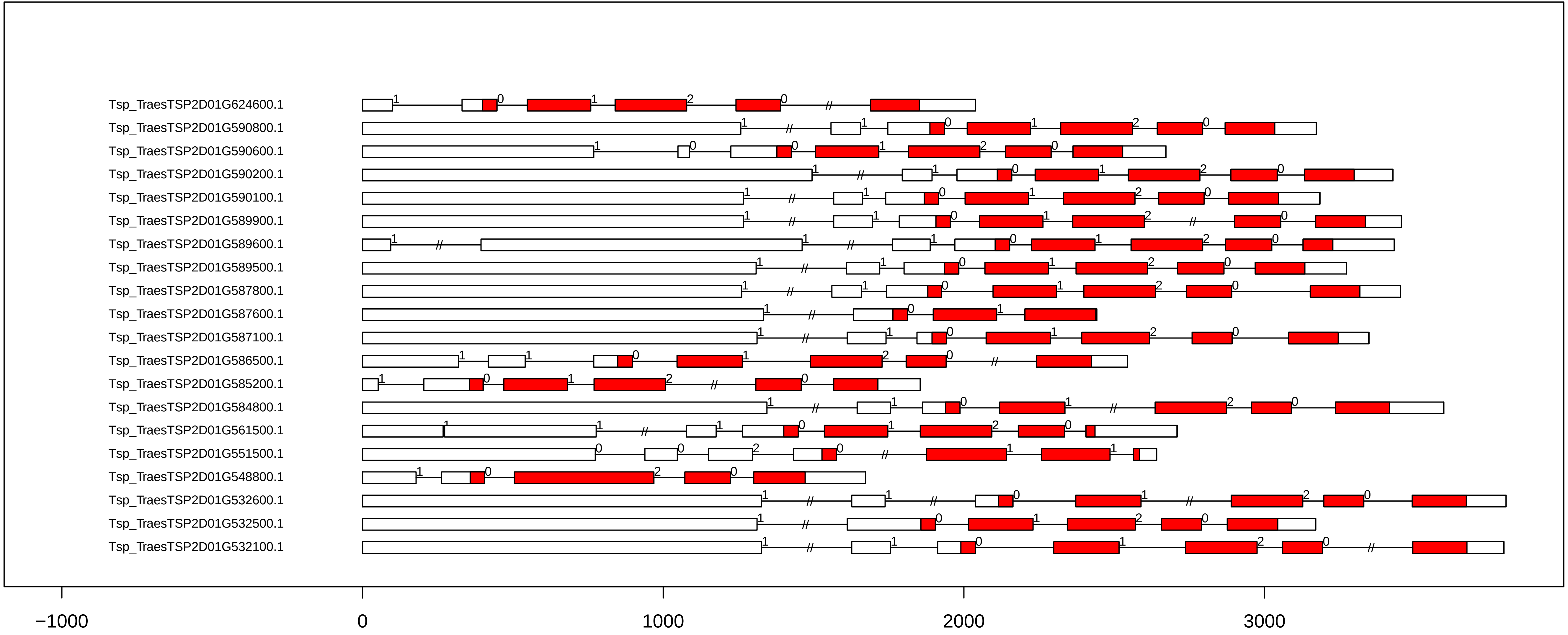

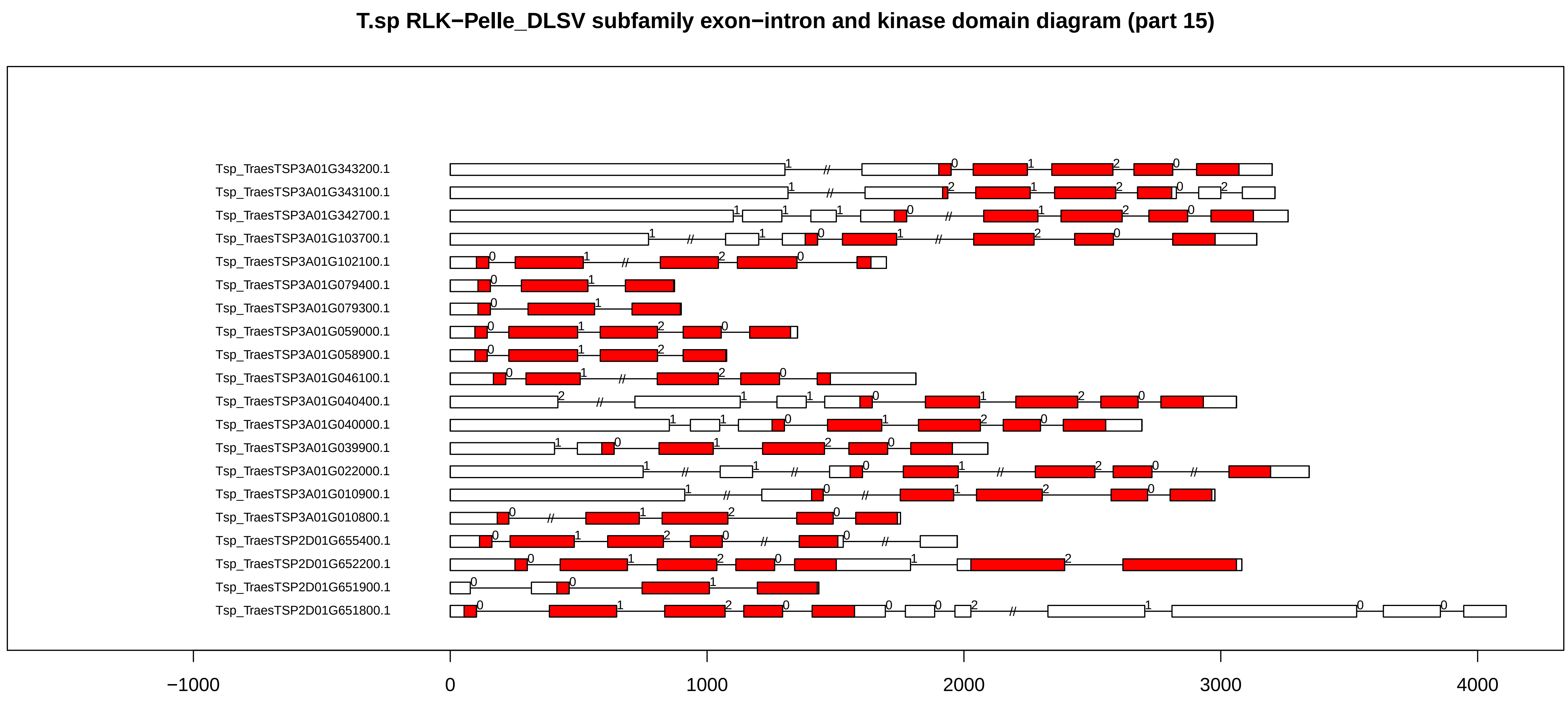

T.sp RLK−Pelle_DLSV subfamily exon−intron and kinase domain diagram (part 15)
Tsp_TraesTSP3A01G343200.1
Tsp_TraesTSP3A01G343100.1
Tsp_TraesTSP3A01G342700.1
Tsp_TraesTSP3A01G103700.1
Tsp_TraesTSP3A01G102100.1
Tsp_TraesTSP3A01G079400.1
Tsp_TraesTSP3A01G079300.1
Tsp_TraesTSP3A01G059000.1
Tsp_TraesTSP3A01G058900.1
Tsp_TraesTSP3A01G046100.1
Tsp_TraesTSP3A01G040400.1
Tsp_TraesTSP3A01G040000.1
Tsp_TraesTSP3A01G039900.1
Tsp_TraesTSP3A01G022000.1
Tsp_TraesTSP3A01G010900.1
Tsp_TraesTSP3A01G010800.1
Tsp_TraesTSP2D01G655400.1
Tsp_TraesTSP2D01G652200.1
Tsp_TraesTSP2D01G651900.1
Tsp_TraesTSP2D01G651800.1
−1000
0
1000
2000
3000
4000

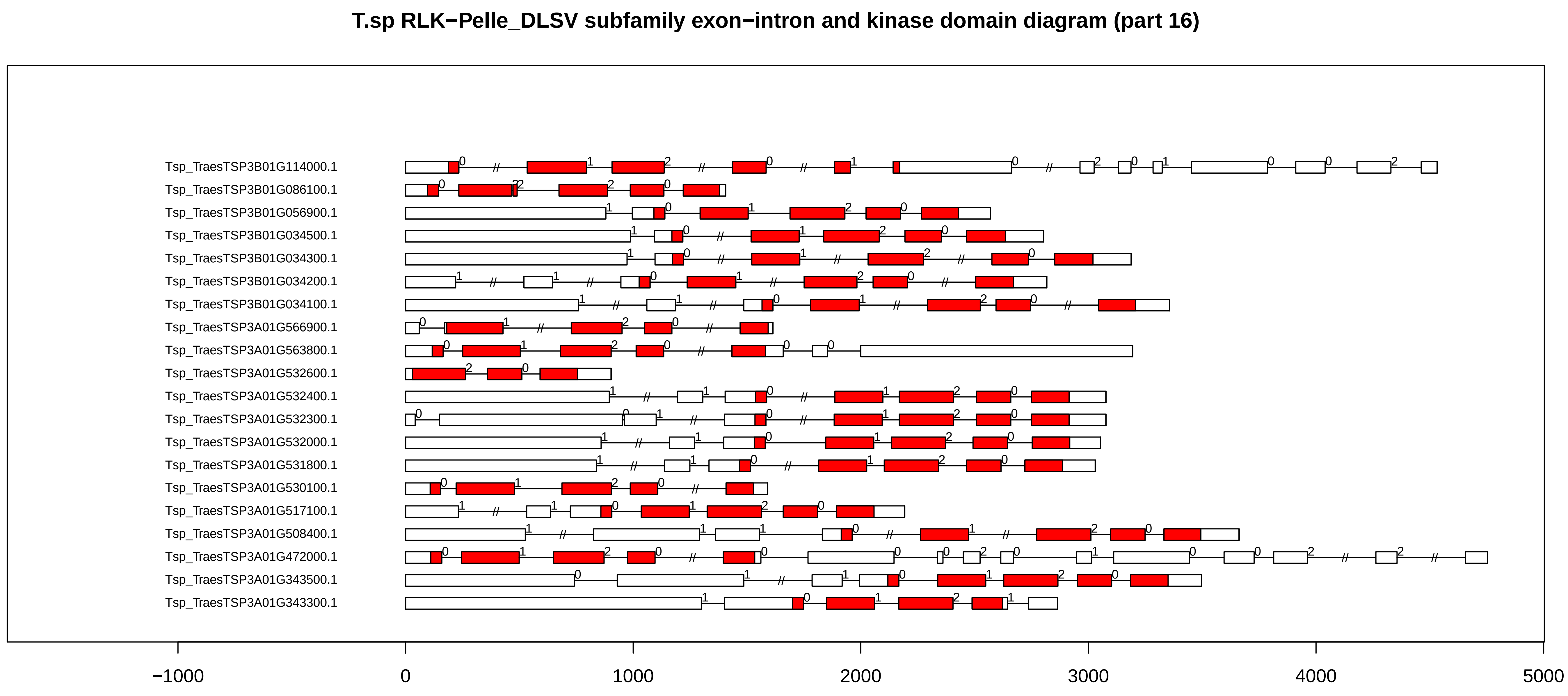
T.sp RLK−Pelle_DLSV subfamily exon−intron and kinase domain diagram (part 16)
Tsp_TraesTSP3B01G114000.1
Tsp_TraesTSP3B01G086100.1
Tsp_TraesTSP3B01G056900.1
Tsp_TraesTSP3B01G034500.1
Tsp_TraesTSP3B01G034300.1
Tsp_TraesTSP3B01G034200.1
Tsp_TraesTSP3B01G034100.1
Tsp_TraesTSP3A01G566900.1
Tsp_TraesTSP3A01G563800.1
Tsp_TraesTSP3A01G532600.1
Tsp_TraesTSP3A01G532400.1
Tsp_TraesTSP3A01G532300.1
Tsp_TraesTSP3A01G532000.1
Tsp_TraesTSP3A01G531800.1
Tsp_TraesTSP3A01G530100.1
Tsp_TraesTSP3A01G517100.1
Tsp_TraesTSP3A01G508400.1
Tsp_TraesTSP3A01G472000.1
Tsp_TraesTSP3A01G343500.1
Tsp_TraesTSP3A01G343300.1
−1000
0
1000
2000
3000
4000
5000

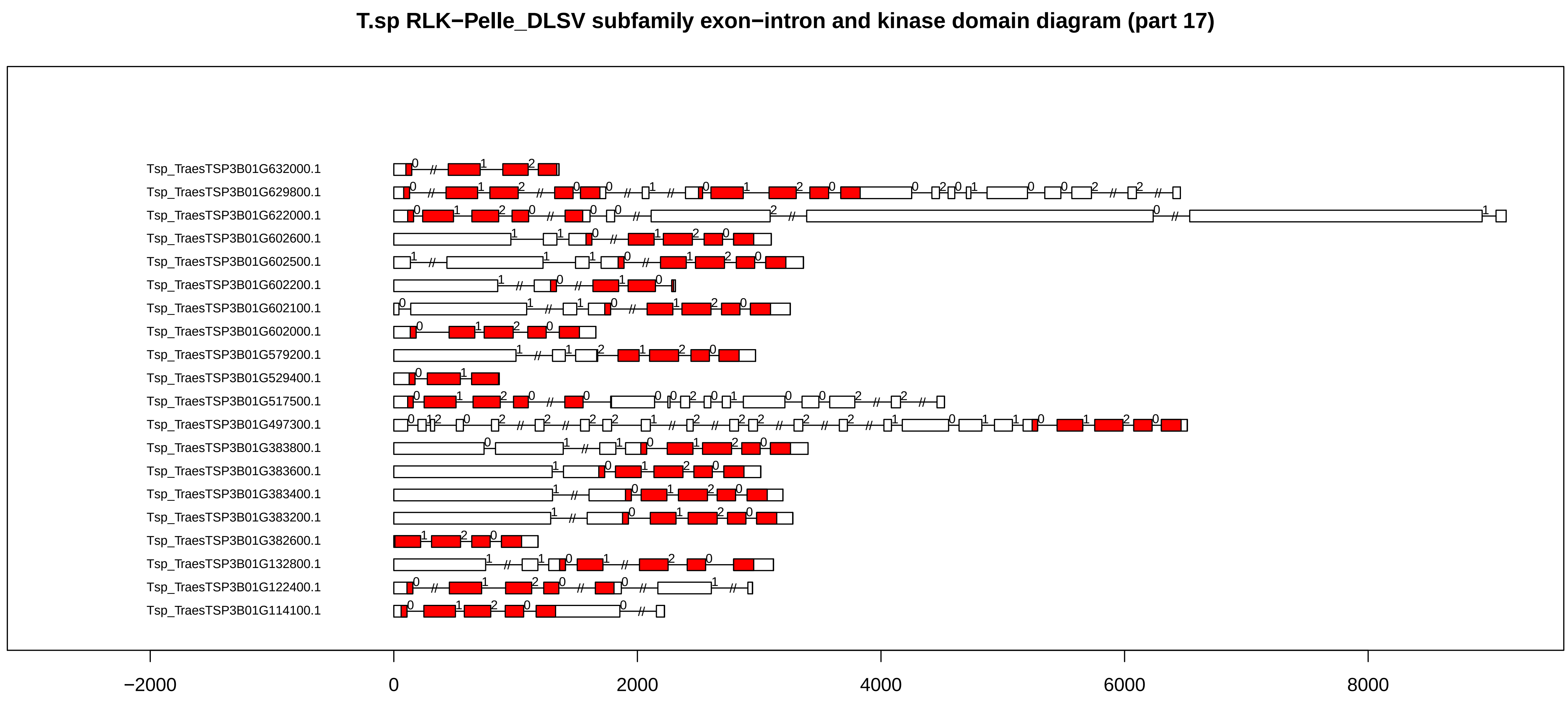

T.sp RLK−Pelle_DLSV subfamily exon−intron and kinase domain diagram (part 17)
Tsp_TraesTSP3B01G632000.1
Tsp_TraesTSP3B01G629800.1
Tsp_TraesTSP3B01G622000.1
Tsp_TraesTSP3B01G602600.1
Tsp_TraesTSP3B01G602500.1
Tsp_TraesTSP3B01G602200.1
Tsp_TraesTSP3B01G602100.1
Tsp_TraesTSP3B01G602000.1
Tsp_TraesTSP3B01G579200.1
Tsp_TraesTSP3B01G529400.1
Tsp_TraesTSP3B01G517500.1
Tsp_TraesTSP3B01G497300.1
Tsp_TraesTSP3B01G383800.1
Tsp_TraesTSP3B01G383600.1
Tsp_TraesTSP3B01G383400.1
Tsp_TraesTSP3B01G383200.1
Tsp_TraesTSP3B01G382600.1
Tsp_TraesTSP3B01G132800.1
Tsp_TraesTSP3B01G122400.1
Tsp_TraesTSP3B01G114100.1
−2000
0
2000
4000
6000
8000

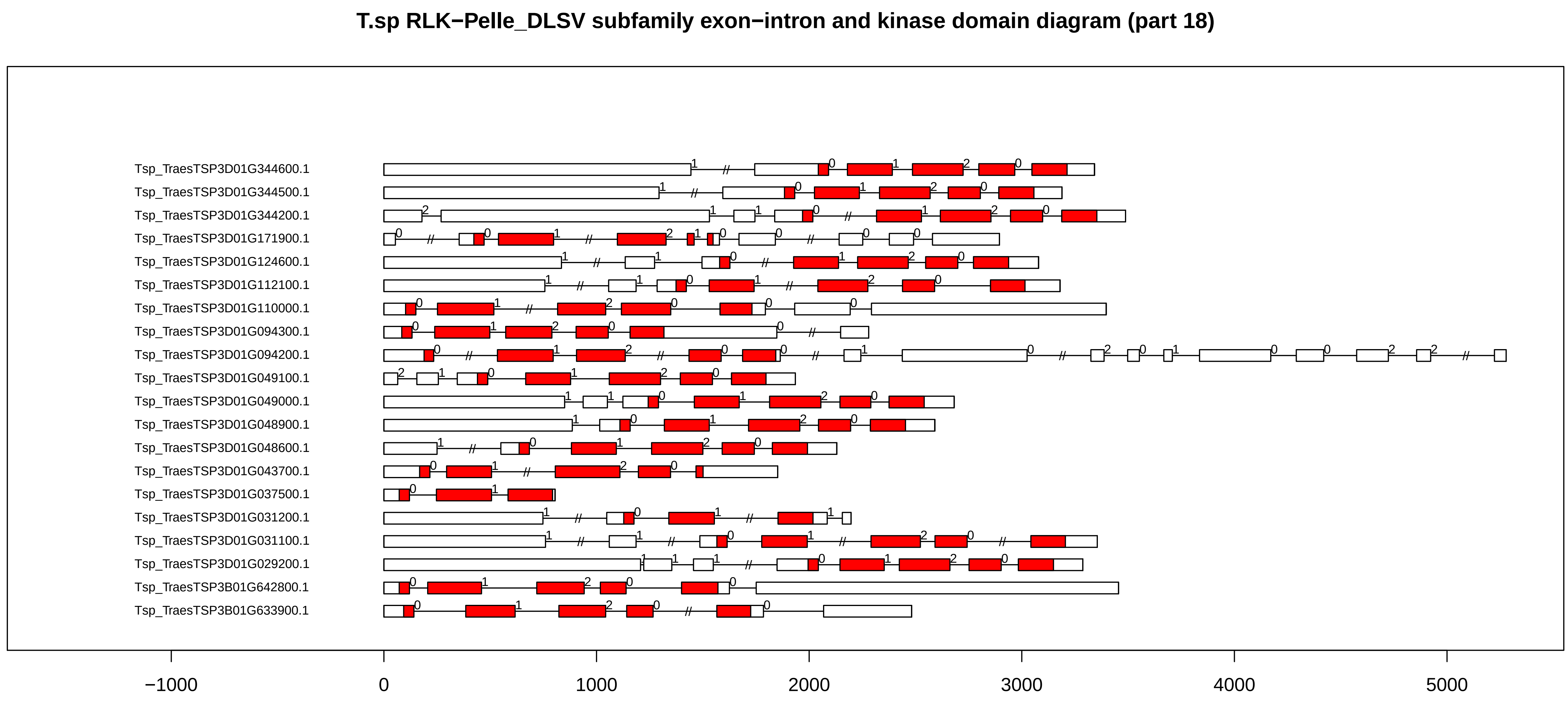
T.sp RLK−Pelle_DLSV subfamily exon−intron and kinase domain diagram (part 18)
Tsp_TraesTSP3D01G344600.1
Tsp_TraesTSP3D01G344500.1
Tsp_TraesTSP3D01G344200.1
Tsp_TraesTSP3D01G171900.1
Tsp_TraesTSP3D01G124600.1
Tsp_TraesTSP3D01G112100.1
Tsp_TraesTSP3D01G110000.1
Tsp_TraesTSP3D01G094300.1
Tsp_TraesTSP3D01G094200.1
Tsp_TraesTSP3D01G049100.1
Tsp_TraesTSP3D01G049000.1
Tsp_TraesTSP3D01G048900.1
Tsp_TraesTSP3D01G048600.1
Tsp_TraesTSP3D01G043700.1
Tsp_TraesTSP3D01G037500.1
Tsp_TraesTSP3D01G031200.1
Tsp_TraesTSP3D01G031100.1
Tsp_TraesTSP3D01G029200.1
Tsp_TraesTSP3B01G642800.1
Tsp_TraesTSP3B01G633900.1
−1000
0
1000
2000
3000
4000
5000

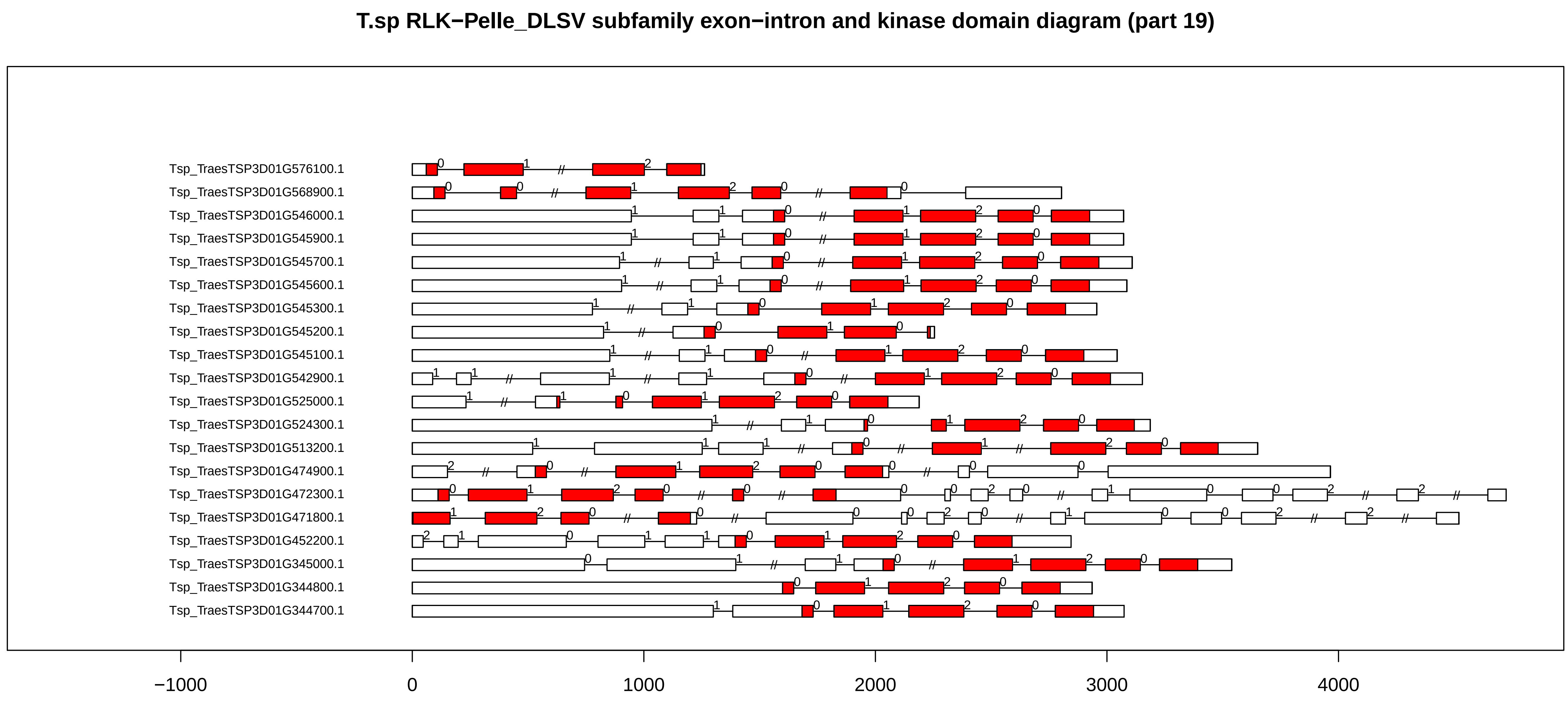
T.sp RLK−Pelle_DLSV subfamily exon−intron and kinase domain diagram (part 19)
Tsp_TraesTSP3D01G576100.1
Tsp_TraesTSP3D01G568900.1
Tsp_TraesTSP3D01G546000.1
Tsp_TraesTSP3D01G545900.1
Tsp_TraesTSP3D01G545700.1
Tsp_TraesTSP3D01G545600.1
Tsp_TraesTSP3D01G545300.1
Tsp_TraesTSP3D01G545200.1
Tsp_TraesTSP3D01G545100.1
Tsp_TraesTSP3D01G542900.1
Tsp_TraesTSP3D01G525000.1
Tsp_TraesTSP3D01G524300.1
Tsp_TraesTSP3D01G513200.1
Tsp_TraesTSP3D01G474900.1
Tsp_TraesTSP3D01G472300.1
Tsp_TraesTSP3D01G471800.1
Tsp_TraesTSP3D01G452200.1
Tsp_TraesTSP3D01G345000.1
Tsp_TraesTSP3D01G344800.1
Tsp_TraesTSP3D01G344700.1
−1000
0
1000
2000
3000
4000

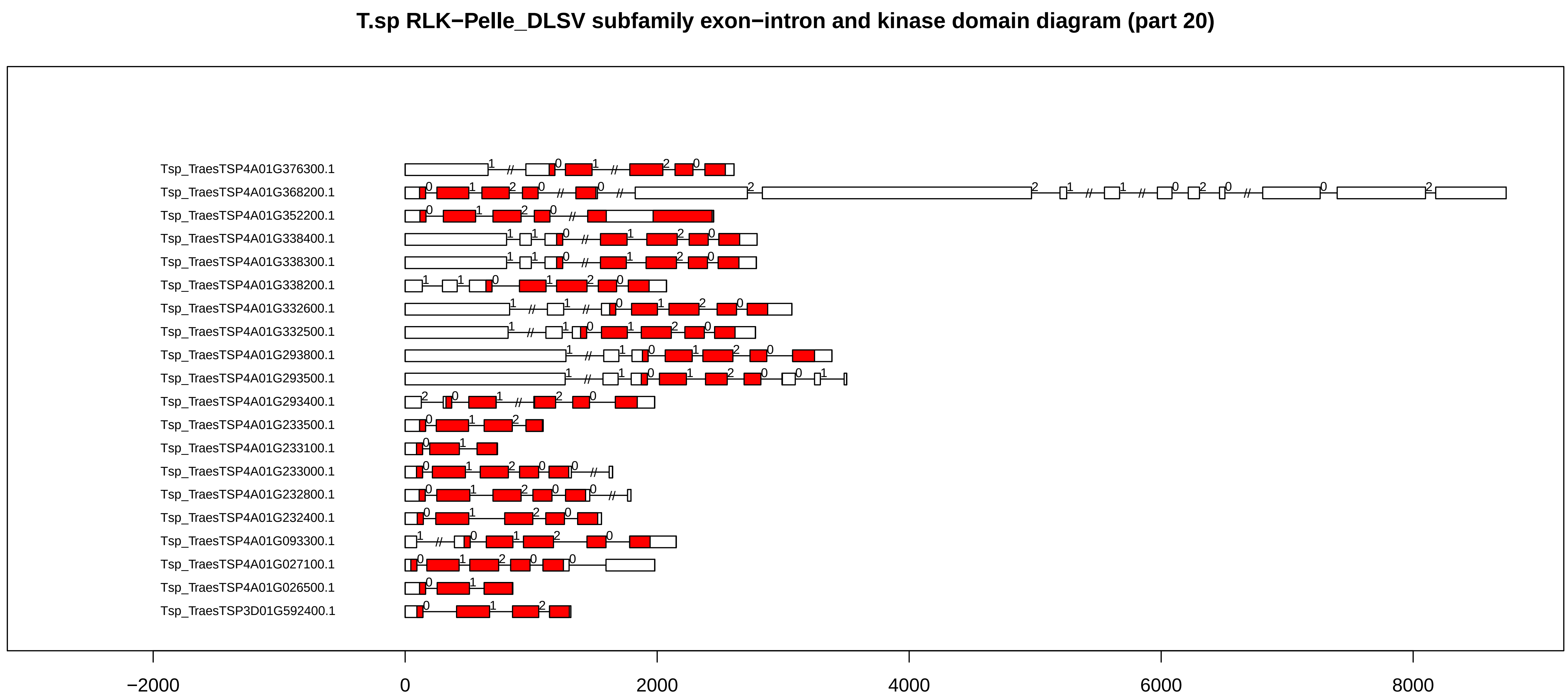
T.sp RLK−Pelle_DLSV subfamily exon−intron and kinase domain diagram (part 20)
Tsp_TraesTSP4A01G376300.1
Tsp_TraesTSP4A01G368200.1
Tsp_TraesTSP4A01G352200.1
Tsp_TraesTSP4A01G338400.1
Tsp_TraesTSP4A01G338300.1
Tsp_TraesTSP4A01G338200.1
Tsp_TraesTSP4A01G332600.1
Tsp_TraesTSP4A01G332500.1
Tsp_TraesTSP4A01G293800.1
Tsp_TraesTSP4A01G293500.1
Tsp_TraesTSP4A01G293400.1
Tsp_TraesTSP4A01G233500.1
Tsp_TraesTSP4A01G233100.1
Tsp_TraesTSP4A01G233000.1
Tsp_TraesTSP4A01G232800.1
Tsp_TraesTSP4A01G232400.1
Tsp_TraesTSP4A01G093300.1
Tsp_TraesTSP4A01G027100.1
Tsp_TraesTSP4A01G026500.1
Tsp_TraesTSP3D01G592400.1
−2000
0
2000
4000
6000
8000

T.sp RLK−Pelle_DLSV subfamily exon−intron and kinase domain diagram (part 21)

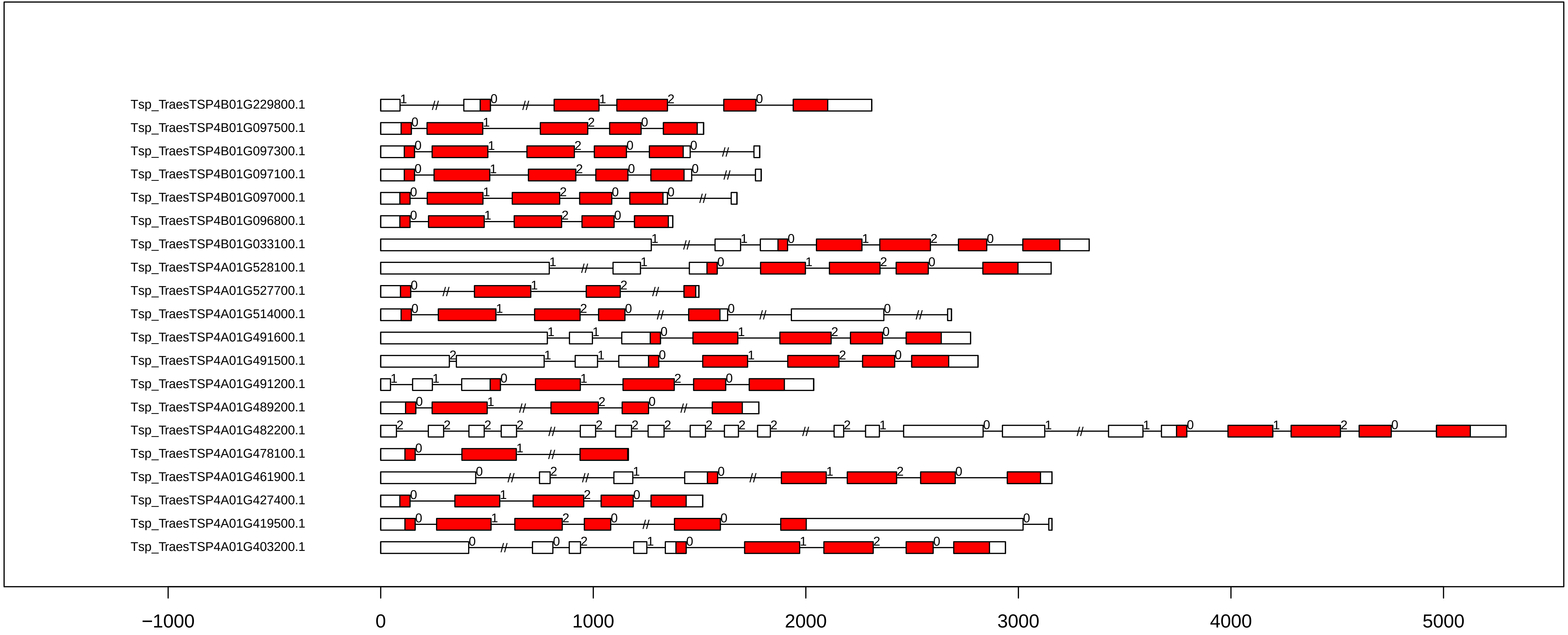

T.sp RLK−Pelle_DLSV subfamily exon−intron and kinase domain diagram (part 22)

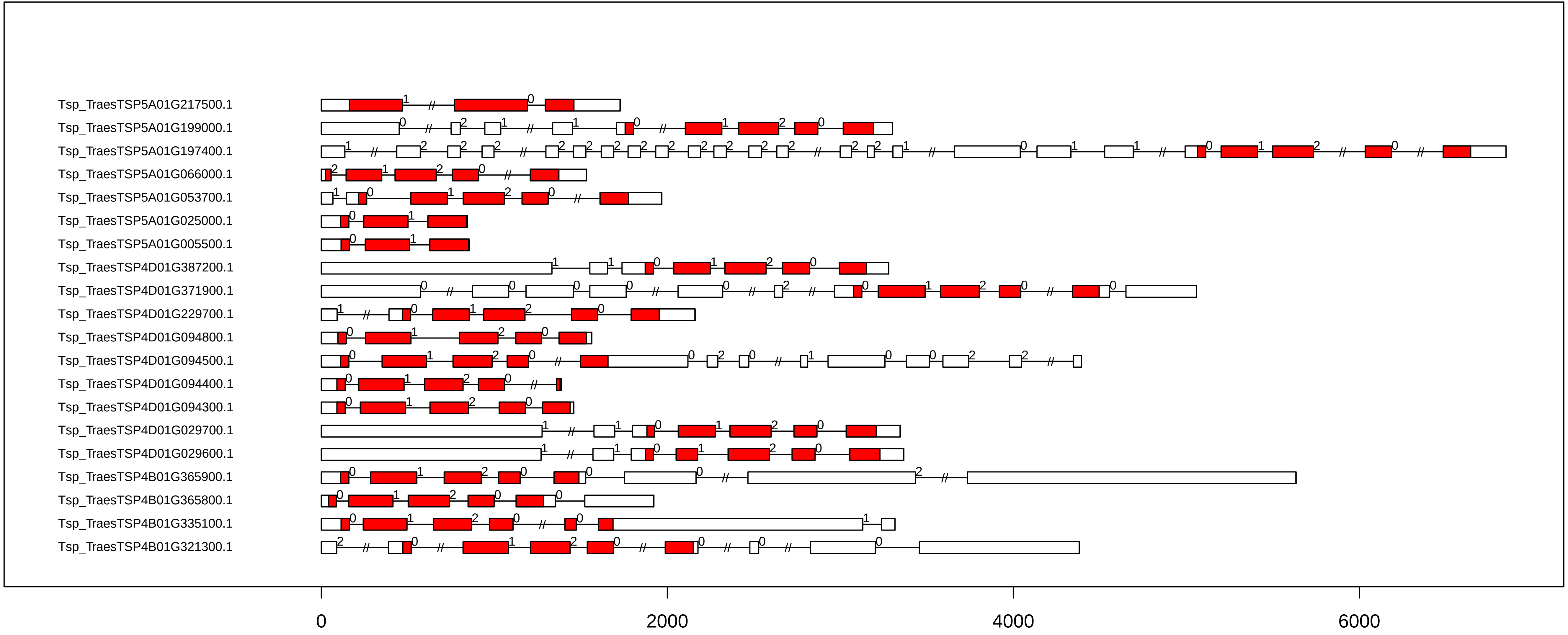

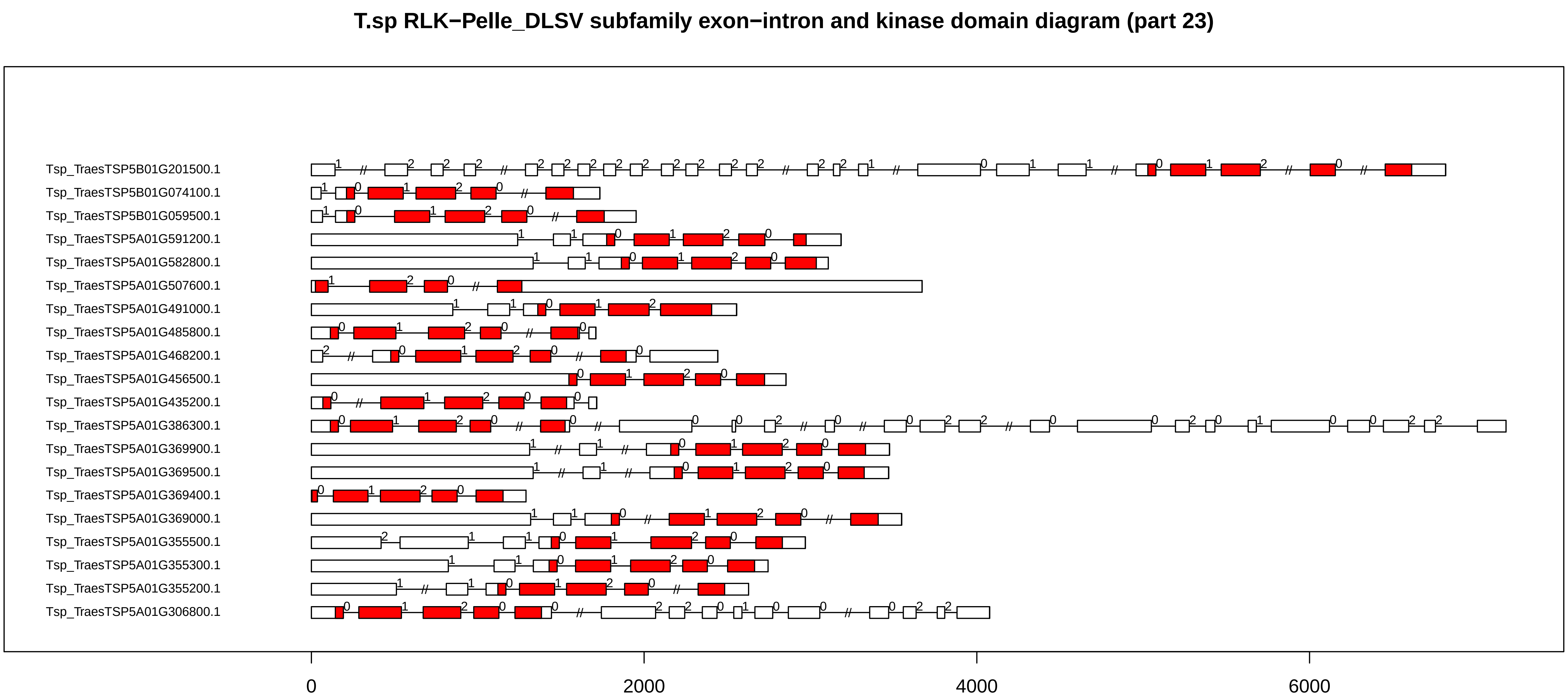
T.sp RLK−Pelle_DLSV subfamily exon−intron and kinase domain diagram (part 23)
Tsp_TraesTSP5B01G201500.1
Tsp_TraesTSP5B01G074100.1
Tsp_TraesTSP5B01G059500.1
Tsp_TraesTSP5A01G591200.1
Tsp_TraesTSP5A01G582800.1
Tsp_TraesTSP5A01G507600.1
Tsp_TraesTSP5A01G491000.1
Tsp_TraesTSP5A01G485800.1
Tsp_TraesTSP5A01G468200.1
Tsp_TraesTSP5A01G456500.1
Tsp_TraesTSP5A01G435200.1
Tsp_TraesTSP5A01G386300.1
Tsp_TraesTSP5A01G369900.1
Tsp_TraesTSP5A01G369500.1
Tsp_TraesTSP5A01G369400.1
Tsp_TraesTSP5A01G369000.1
Tsp_TraesTSP5A01G355500.1
Tsp_TraesTSP5A01G355300.1
Tsp_TraesTSP5A01G355200.1
Tsp_TraesTSP5A01G306800.1
0
2000
4000
6000

T.sp RLK−Pelle_DLSV subfamily exon−intron and kinase domain diagram (part 24)

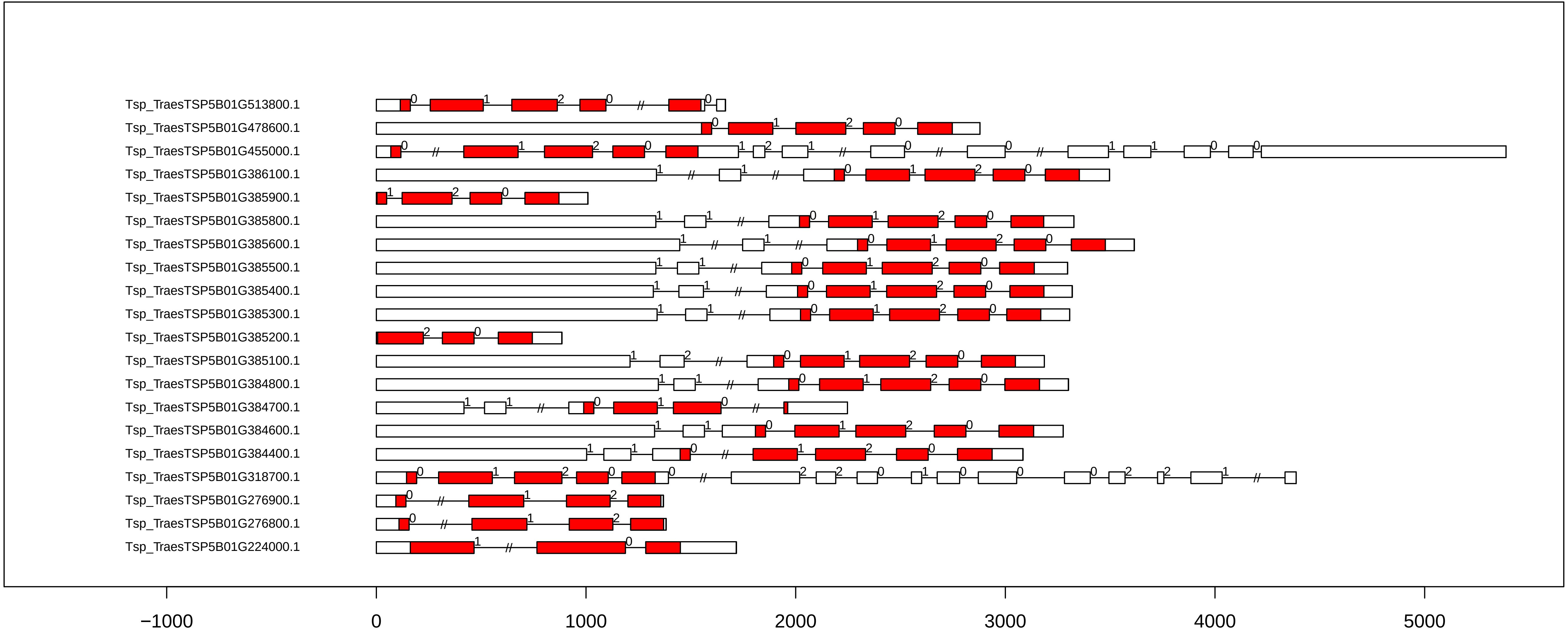

T.sp RLK−Pelle_DLSV subfamily exon−intron and kinase domain diagram (part 25)

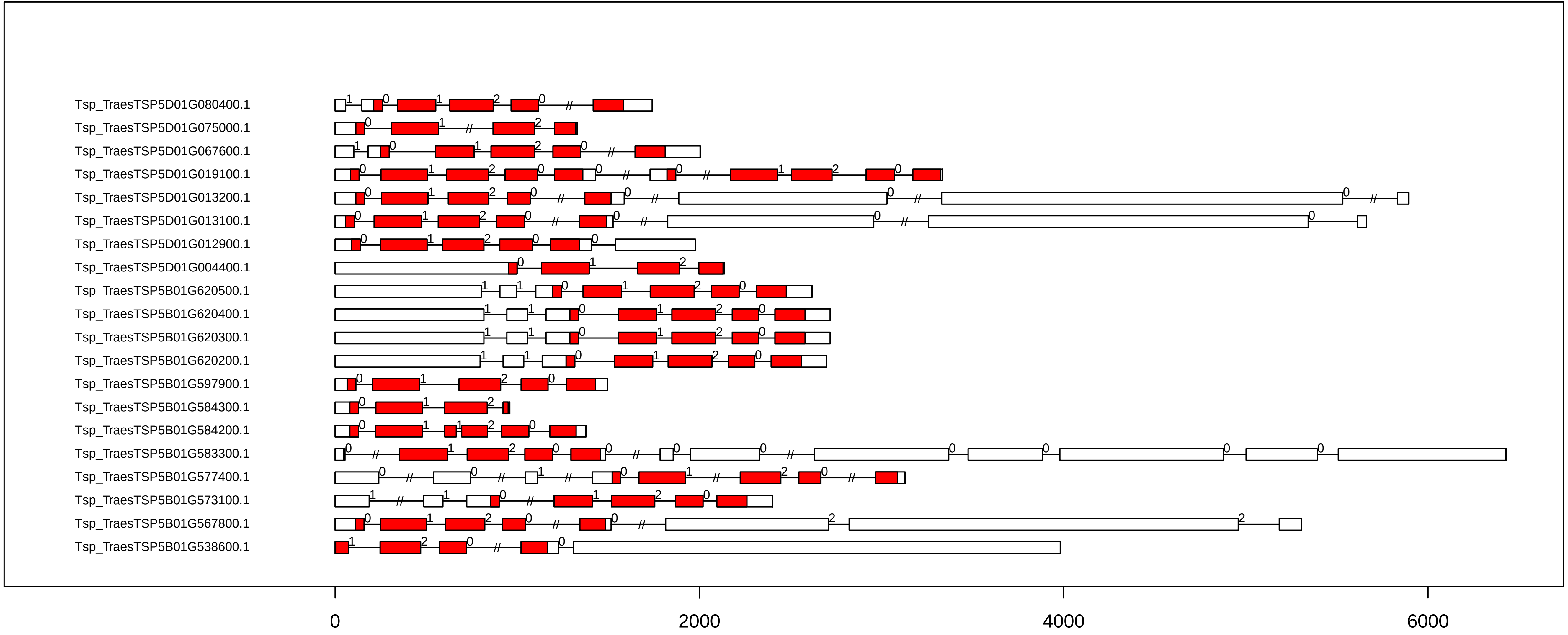

T.sp RLK−Pelle_DLSV subfamily exon−intron and kinase domain diagram (part 26)

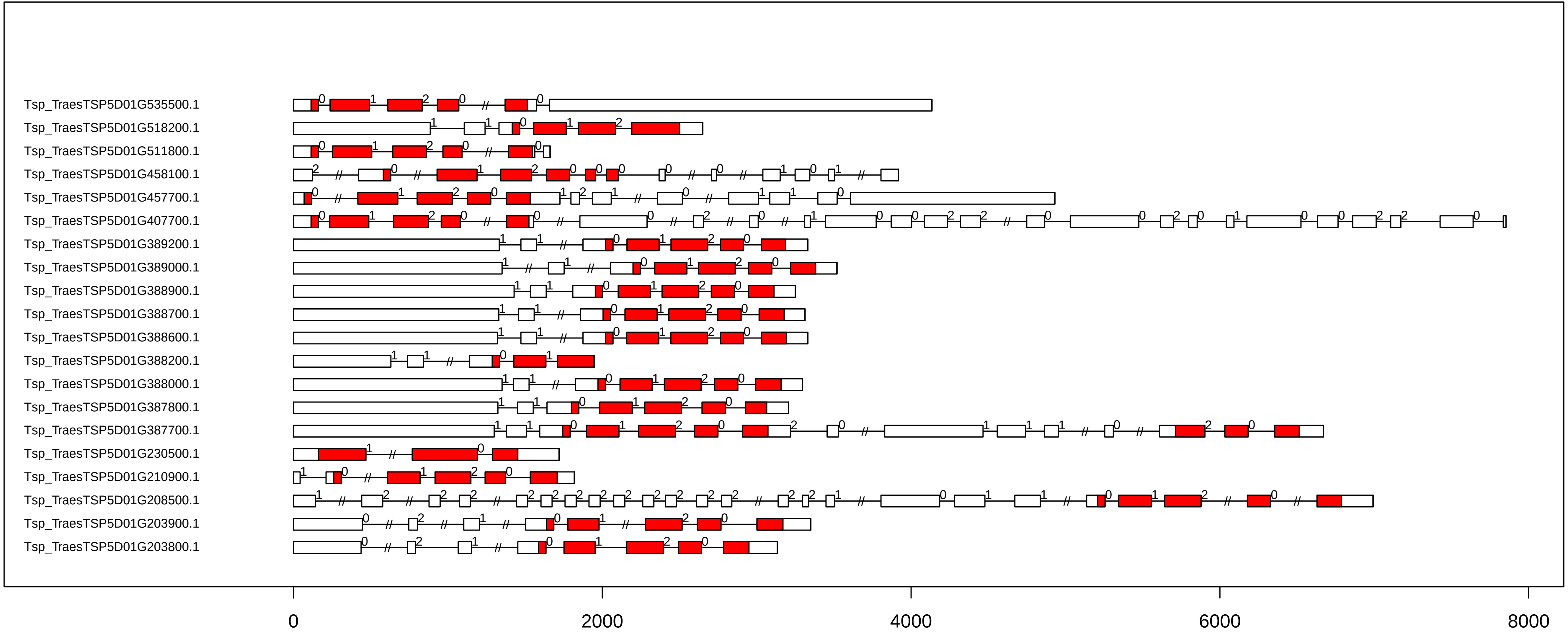

T.sp RLK−Pelle_DLSV subfamily exon−intron and kinase domain diagram (part 27)

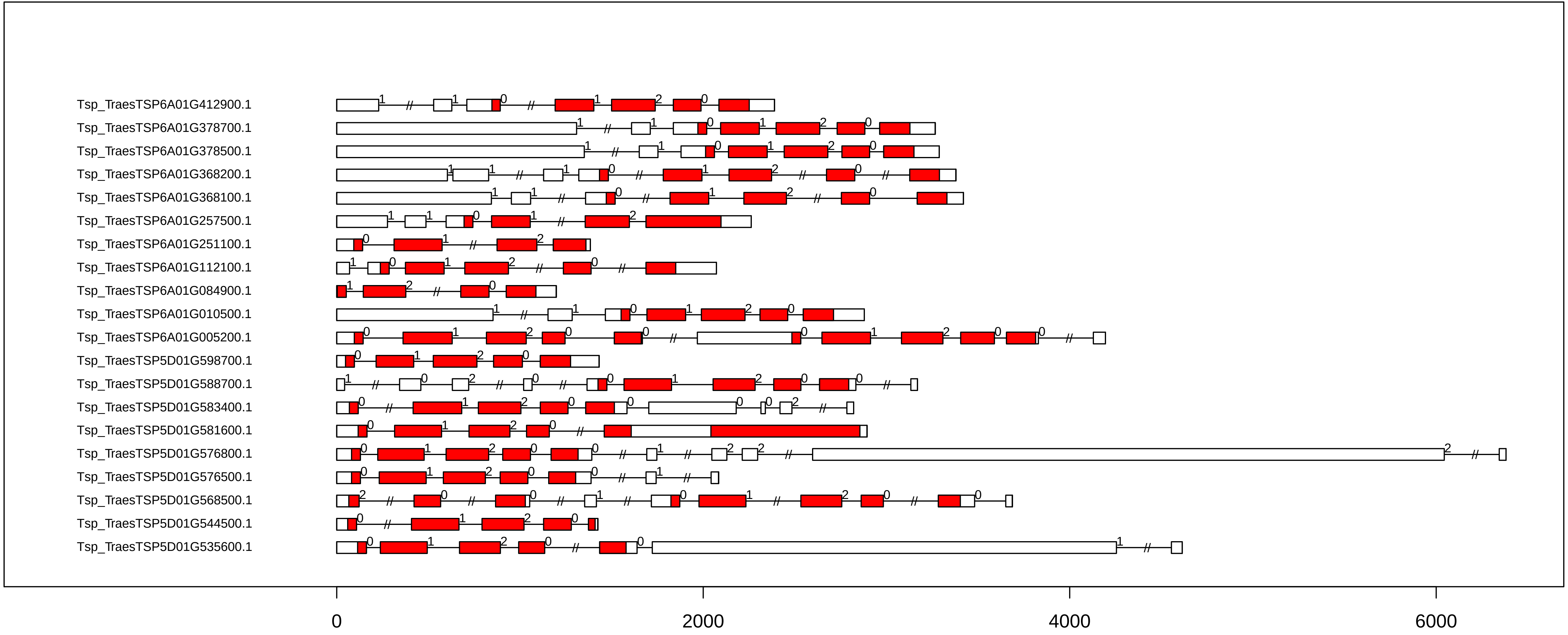

T.sp RLK−Pelle_DLSV subfamily exon−intron and kinase domain diagram (part 28)

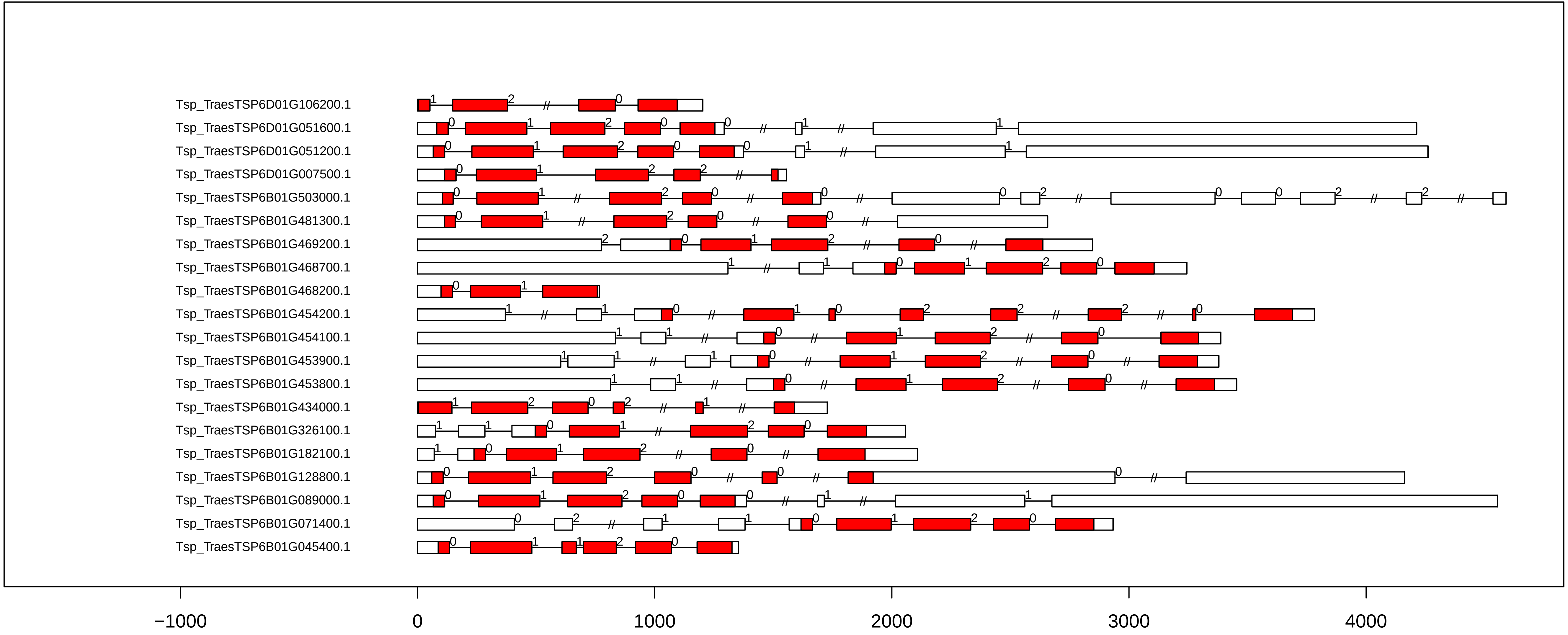

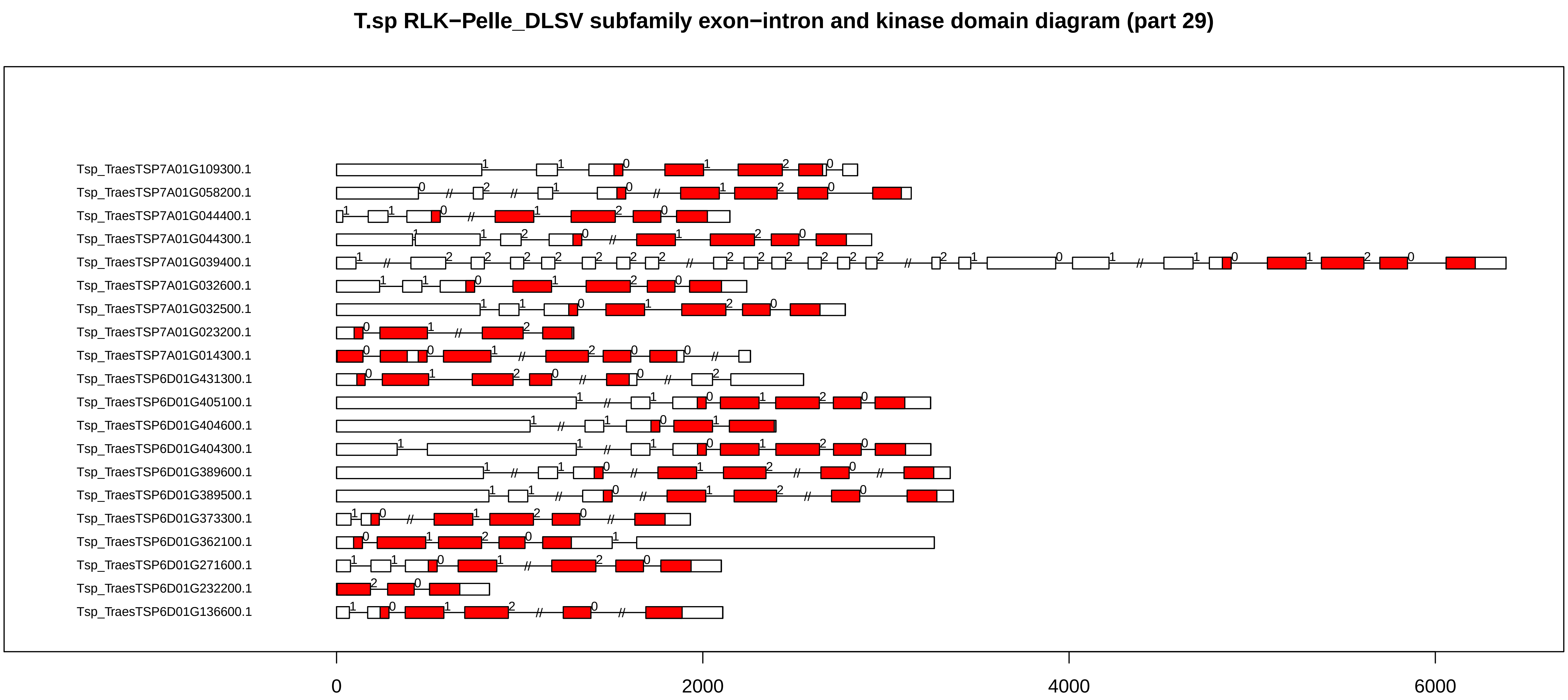
T.sp RLK−Pelle_DLSV subfamily exon−intron and kinase domain diagram (part 29)
Tsp_TraesTSP7A01G109300.1
Tsp_TraesTSP7A01G058200.1
Tsp_TraesTSP7A01G044400.1
Tsp_TraesTSP7A01G044300.1
Tsp_TraesTSP7A01G039400.1
Tsp_TraesTSP7A01G032600.1
Tsp_TraesTSP7A01G032500.1
Tsp_TraesTSP7A01G023200.1
Tsp_TraesTSP7A01G014300.1
Tsp_TraesTSP6D01G431300.1
Tsp_TraesTSP6D01G405100.1
Tsp_TraesTSP6D01G404600.1
Tsp_TraesTSP6D01G404300.1
Tsp_TraesTSP6D01G389600.1
Tsp_TraesTSP6D01G389500.1
Tsp_TraesTSP6D01G373300.1
Tsp_TraesTSP6D01G362100.1
Tsp_TraesTSP6D01G271600.1
Tsp_TraesTSP6D01G232200.1
Tsp_TraesTSP6D01G136600.1
0
2000
4000
6000

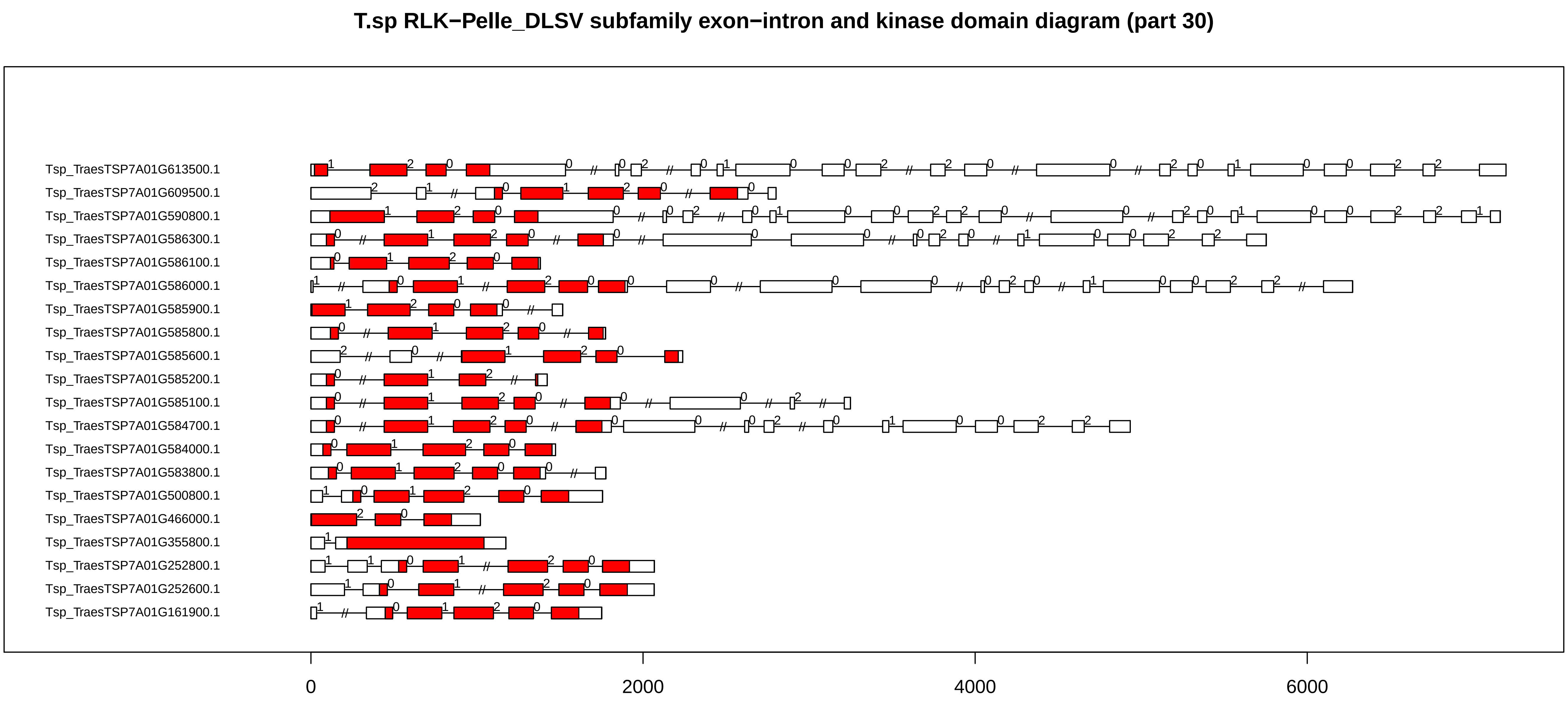
T.sp RLK−Pelle_DLSV subfamily exon−intron and kinase domain diagram (part 30)
Tsp_TraesTSP7A01G613500.1
Tsp_TraesTSP7A01G609500.1
Tsp_TraesTSP7A01G590800.1
Tsp_TraesTSP7A01G586300.1
Tsp_TraesTSP7A01G586100.1
Tsp_TraesTSP7A01G586000.1
Tsp_TraesTSP7A01G585900.1
Tsp_TraesTSP7A01G585800.1
Tsp_TraesTSP7A01G585600.1
Tsp_TraesTSP7A01G585200.1
Tsp_TraesTSP7A01G585100.1
Tsp_TraesTSP7A01G584700.1
Tsp_TraesTSP7A01G584000.1
Tsp_TraesTSP7A01G583800.1
Tsp_TraesTSP7A01G500800.1
Tsp_TraesTSP7A01G466000.1
Tsp_TraesTSP7A01G355800.1
Tsp_TraesTSP7A01G252800.1
Tsp_TraesTSP7A01G252600.1
Tsp_TraesTSP7A01G161900.1
0
2000
4000
6000

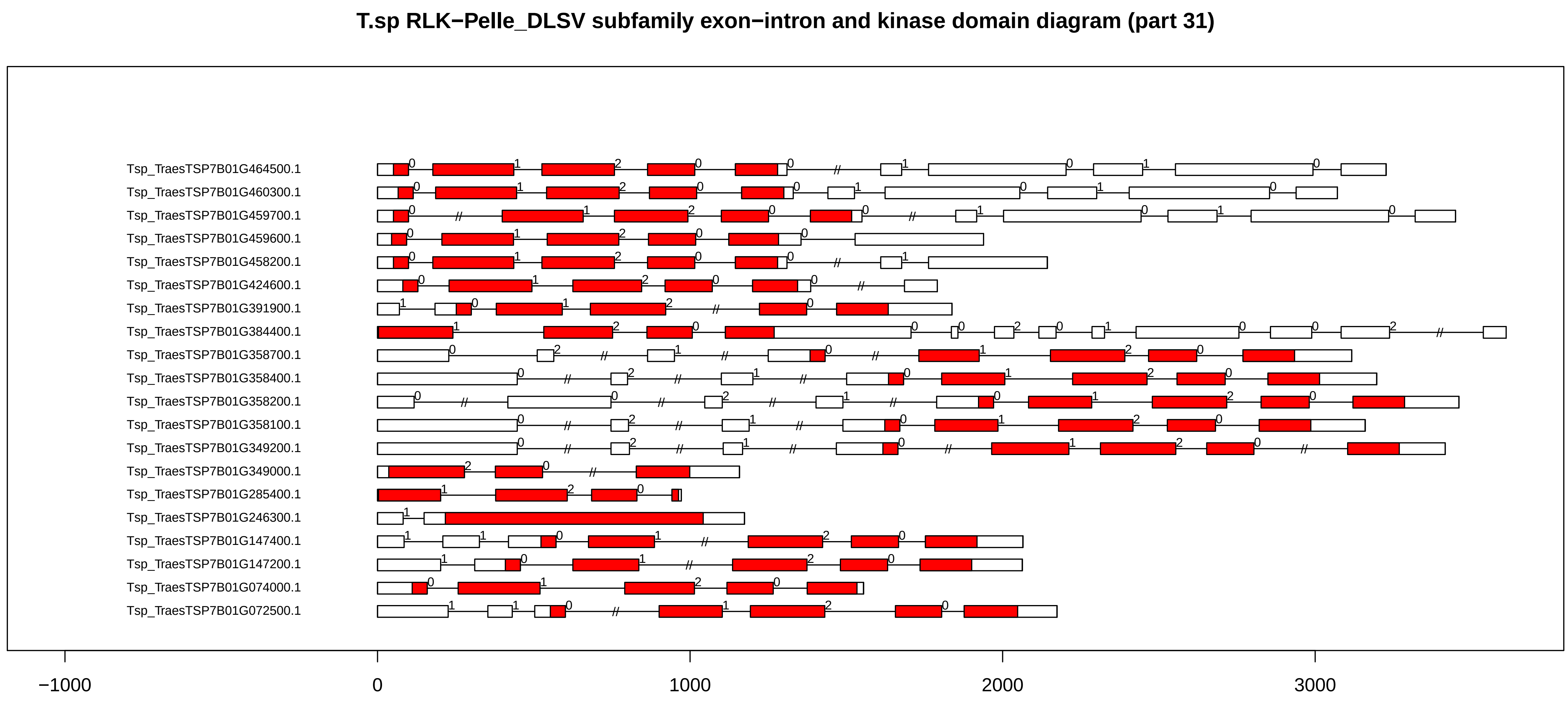
T.sp RLK−Pelle_DLSV subfamily exon−intron and kinase domain diagram (part 31)
Tsp_TraesTSP7B01G464500.1
Tsp_TraesTSP7B01G460300.1
Tsp_TraesTSP7B01G459700.1
Tsp_TraesTSP7B01G459600.1
Tsp_TraesTSP7B01G458200.1
Tsp_TraesTSP7B01G424600.1
Tsp_TraesTSP7B01G391900.1
Tsp_TraesTSP7B01G384400.1
Tsp_TraesTSP7B01G358700.1
Tsp_TraesTSP7B01G358400.1
Tsp_TraesTSP7B01G358200.1
Tsp_TraesTSP7B01G358100.1
Tsp_TraesTSP7B01G349200.1
Tsp_TraesTSP7B01G349000.1
Tsp_TraesTSP7B01G285400.1
Tsp_TraesTSP7B01G246300.1
Tsp_TraesTSP7B01G147400.1
Tsp_TraesTSP7B01G147200.1
Tsp_TraesTSP7B01G074000.1
Tsp_TraesTSP7B01G072500.1
−1000
0
1000
2000
3000

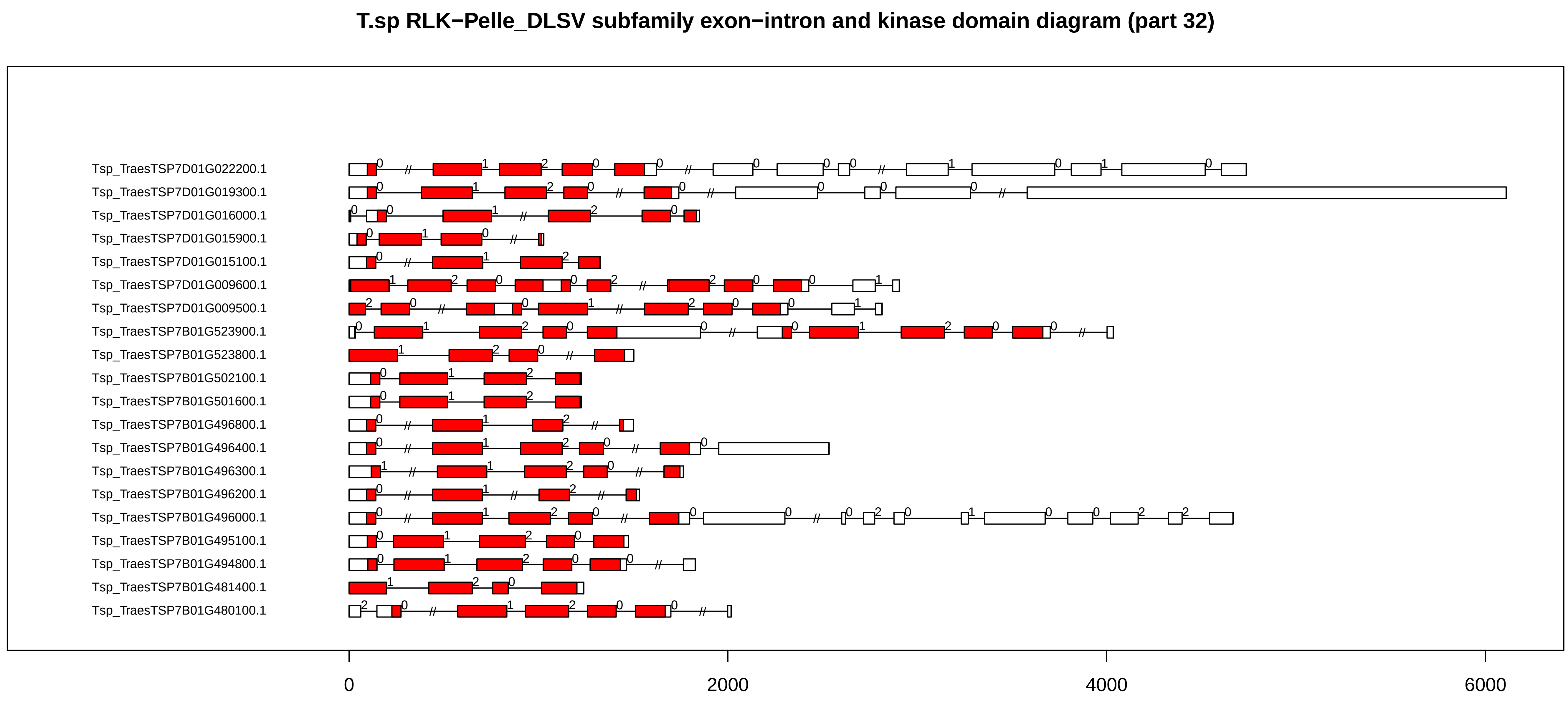
T.sp RLK−Pelle_DLSV subfamily exon−intron and kinase domain diagram (part 32)
Tsp_TraesTSP7D01G022200.1
Tsp_TraesTSP7D01G019300.1
Tsp_TraesTSP7D01G016000.1
Tsp_TraesTSP7D01G015900.1
Tsp_TraesTSP7D01G015100.1
Tsp_TraesTSP7D01G009600.1
Tsp_TraesTSP7D01G009500.1
Tsp_TraesTSP7B01G523900.1
Tsp_TraesTSP7B01G523800.1
Tsp_TraesTSP7B01G502100.1
Tsp_TraesTSP7B01G501600.1
Tsp_TraesTSP7B01G496800.1
Tsp_TraesTSP7B01G496400.1
Tsp_TraesTSP7B01G496300.1
Tsp_TraesTSP7B01G496200.1
Tsp_TraesTSP7B01G496000.1
Tsp_TraesTSP7B01G495100.1
Tsp_TraesTSP7B01G494800.1
Tsp_TraesTSP7B01G481400.1
Tsp_TraesTSP7B01G480100.1
0
2000
4000
6000

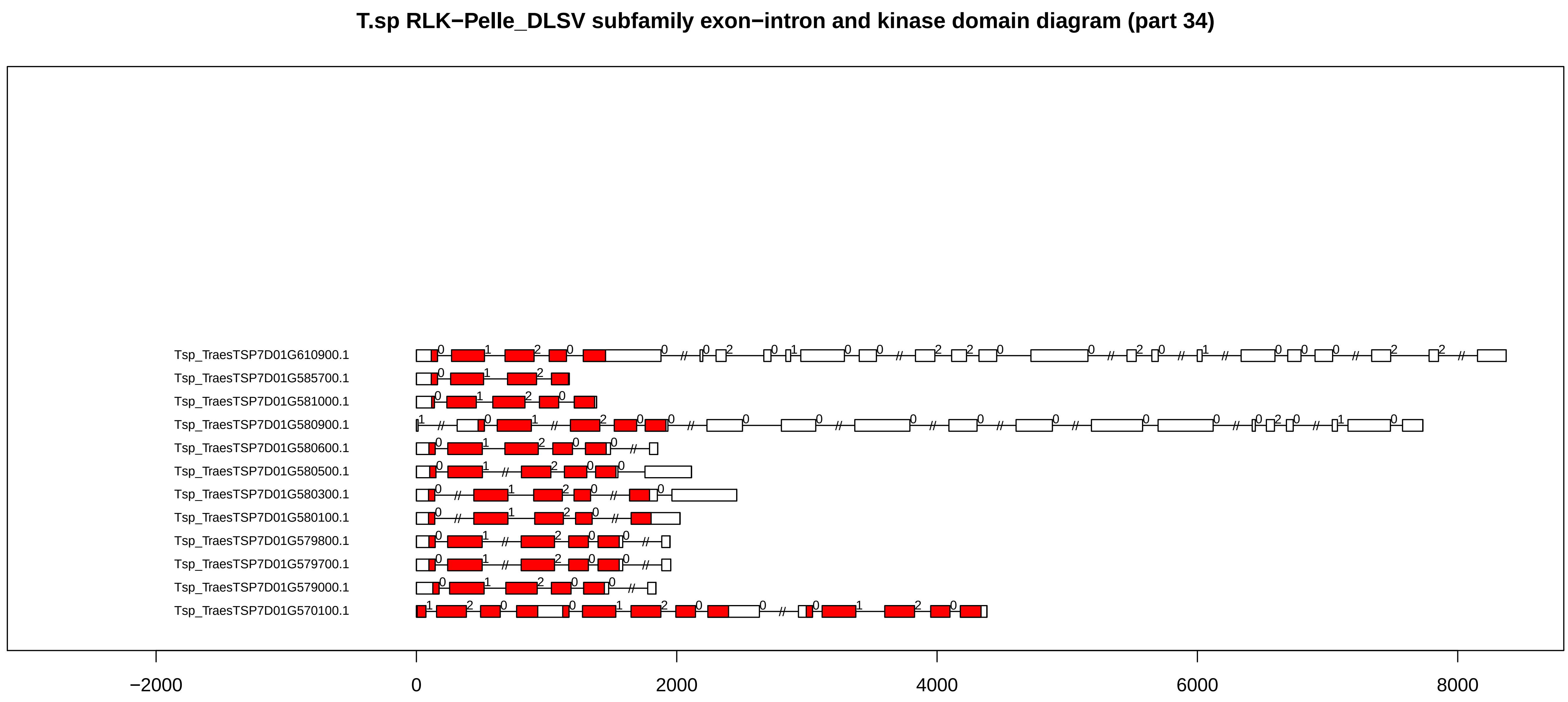
T.sp RLK−Pelle_DLSV subfamily exon−intron and kinase domain diagram (part 34)
Tsp_TraesTSP7D01G610900.1
Tsp_TraesTSP7D01G585700.1
Tsp_TraesTSP7D01G581000.1
Tsp_TraesTSP7D01G580900.1
Tsp_TraesTSP7D01G580600.1
Tsp_TraesTSP7D01G580500.1
Tsp_TraesTSP7D01G580300.1
Tsp_TraesTSP7D01G580100.1
Tsp_TraesTSP7D01G579800.1
Tsp_TraesTSP7D01G579700.1
Tsp_TraesTSP7D01G579000.1
Tsp_TraesTSP7D01G570100.1
−2000
0
2000
4000
6000
8000

T.sp RLK−Pelle_Extensin subfamily exon−intron and kinase domain diagram (all)

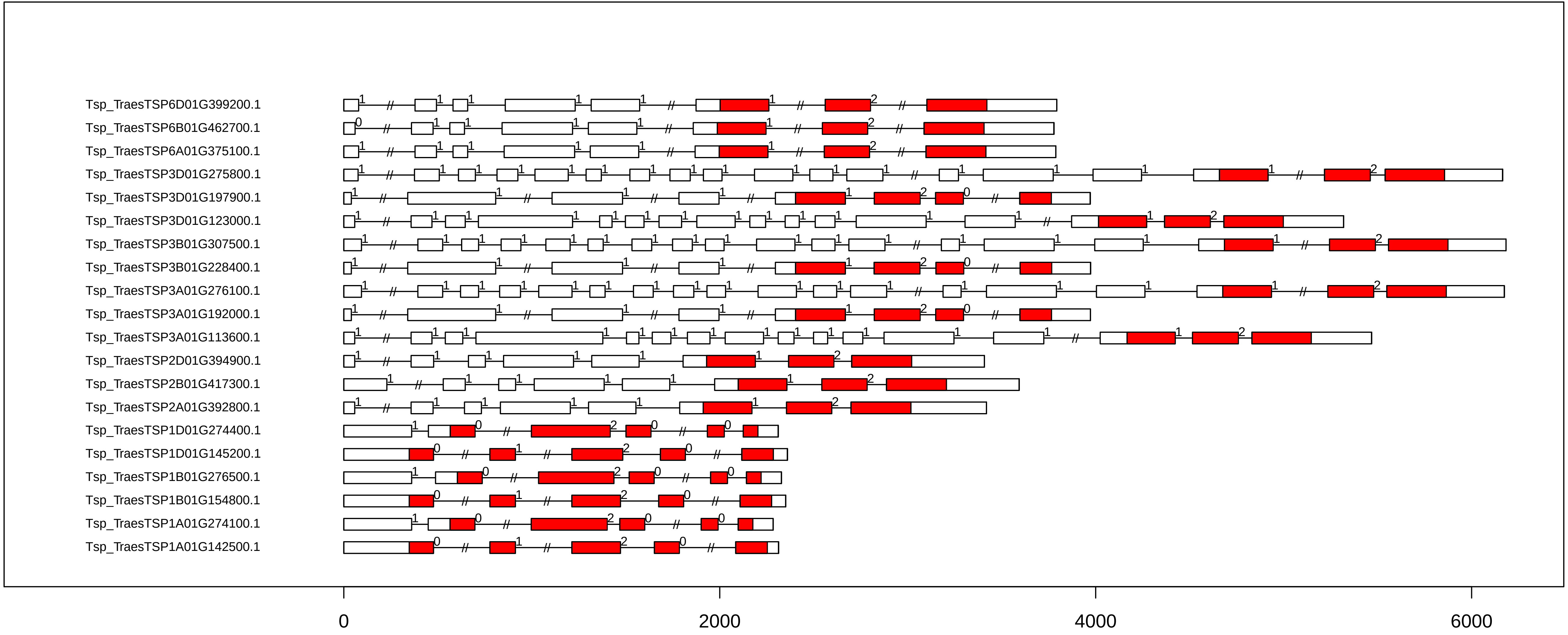

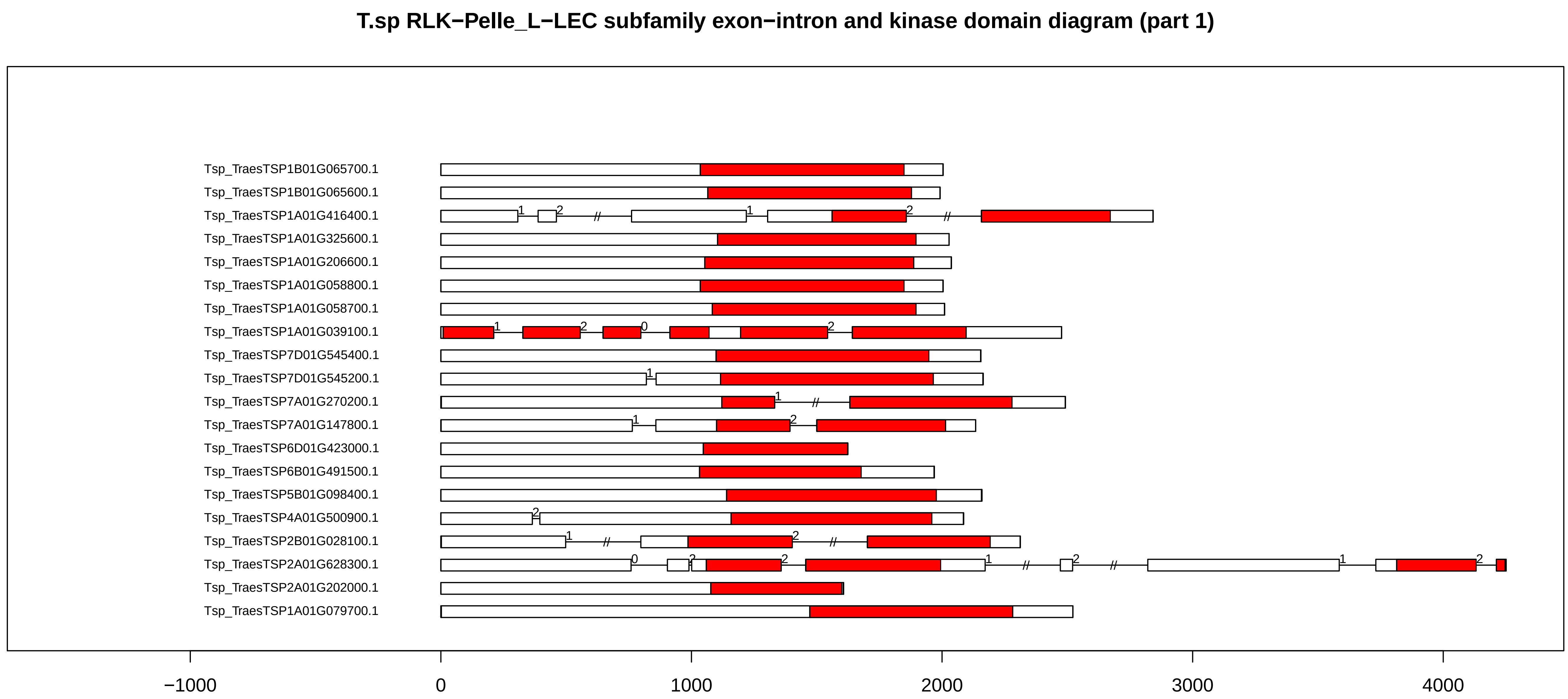

T.sp RLK−Pelle_L−LEC subfamily exon−intron and kinase domain diagram (part 1)
Tsp_TraesTSP1B01G065700.1
Tsp_TraesTSP1B01G065600.1
Tsp_TraesTSP1A01G416400.1
Tsp_TraesTSP1A01G325600.1
Tsp_TraesTSP1A01G206600.1
Tsp_TraesTSP1A01G058800.1
Tsp_TraesTSP1A01G058700.1
Tsp_TraesTSP1A01G039100.1
Tsp_TraesTSP7D01G545400.1
Tsp_TraesTSP7D01G545200.1
Tsp_TraesTSP7A01G270200.1
Tsp_TraesTSP7A01G147800.1
Tsp_TraesTSP6D01G423000.1
Tsp_TraesTSP6B01G491500.1
Tsp_TraesTSP5B01G098400.1
Tsp_TraesTSP4A01G500900.1
Tsp_TraesTSP2B01G028100.1
Tsp_TraesTSP2A01G628300.1
Tsp_TraesTSP2A01G202000.1
Tsp_TraesTSP1A01G079700.1
−1000
0
1000
2000
3000
4000

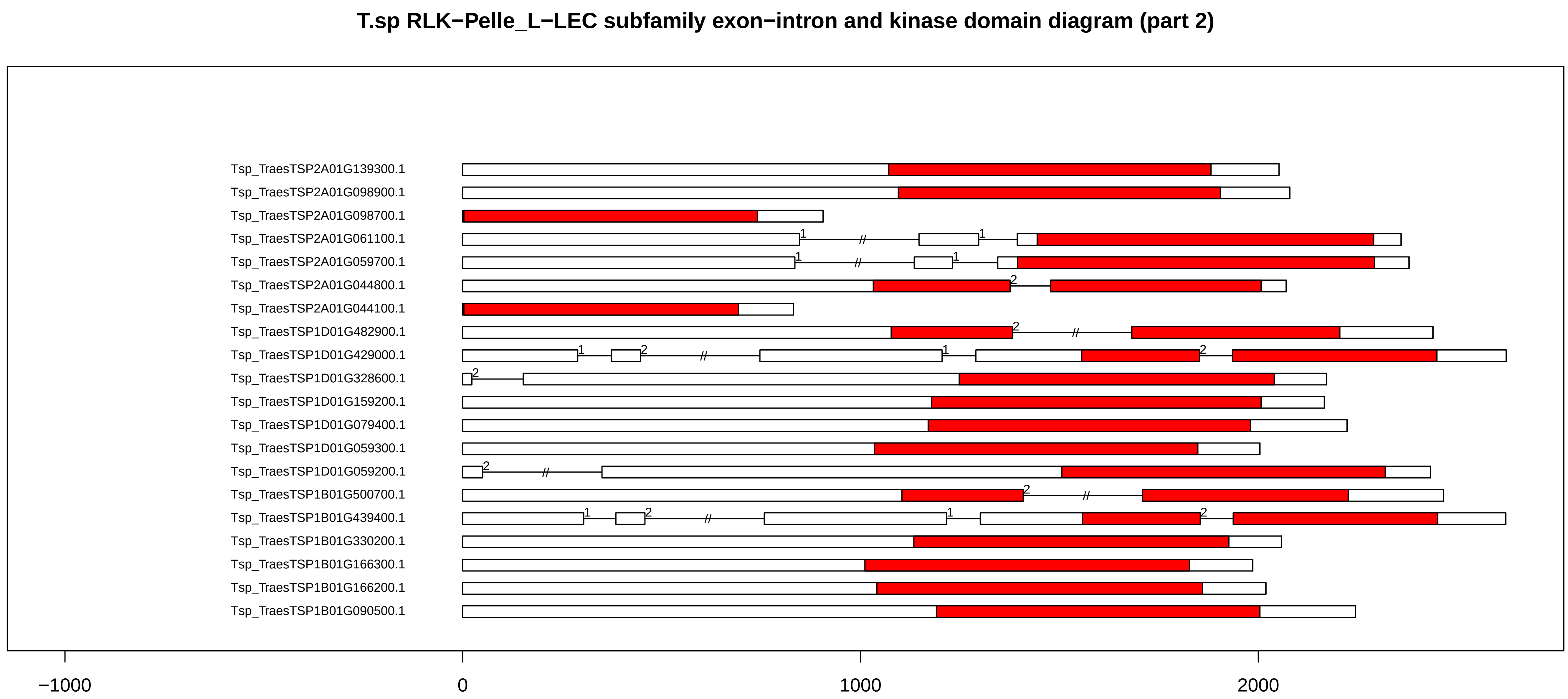
T.sp RLK−Pelle_L−LEC subfamily exon−intron and kinase domain diagram (part 2)
Tsp_TraesTSP2A01G139300.1
Tsp_TraesTSP2A01G098900.1
Tsp_TraesTSP2A01G098700.1
Tsp_TraesTSP2A01G061100.1
Tsp_TraesTSP2A01G059700.1
Tsp_TraesTSP2A01G044800.1
Tsp_TraesTSP2A01G044100.1
Tsp_TraesTSP1D01G482900.1
Tsp_TraesTSP1D01G429000.1
Tsp_TraesTSP1D01G328600.1
Tsp_TraesTSP1D01G159200.1
Tsp_TraesTSP1D01G079400.1
Tsp_TraesTSP1D01G059300.1
Tsp_TraesTSP1D01G059200.1
Tsp_TraesTSP1B01G500700.1
Tsp_TraesTSP1B01G439400.1
Tsp_TraesTSP1B01G330200.1
Tsp_TraesTSP1B01G166300.1
Tsp_TraesTSP1B01G166200.1
Tsp_TraesTSP1B01G090500.1
−1000
0
1000
2000

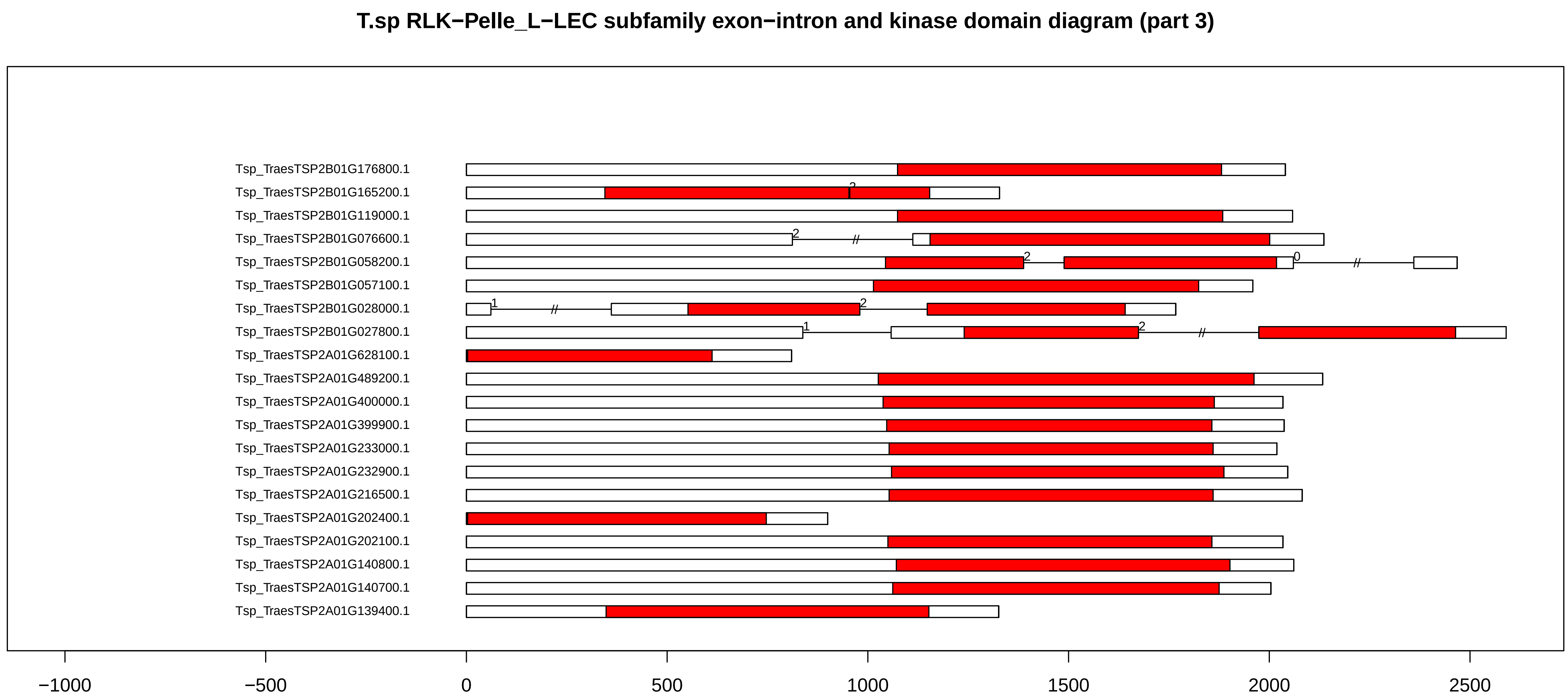
T.sp RLK−Pelle_L−LEC subfamily exon−intron and kinase domain diagram (part 3)
Tsp_TraesTSP2B01G176800.1
Tsp_TraesTSP2B01G165200.1
Tsp_TraesTSP2B01G119000.1
Tsp_TraesTSP2B01G076600.1
Tsp_TraesTSP2B01G058200.1
Tsp_TraesTSP2B01G057100.1
Tsp_TraesTSP2B01G028000.1
Tsp_TraesTSP2B01G027800.1
Tsp_TraesTSP2A01G628100.1
Tsp_TraesTSP2A01G489200.1
Tsp_TraesTSP2A01G400000.1
Tsp_TraesTSP2A01G399900.1
Tsp_TraesTSP2A01G233000.1
Tsp_TraesTSP2A01G232900.1
Tsp_TraesTSP2A01G216500.1
Tsp_TraesTSP2A01G202400.1
Tsp_TraesTSP2A01G202100.1
Tsp_TraesTSP2A01G140800.1
Tsp_TraesTSP2A01G140700.1
Tsp_TraesTSP2A01G139400.1
−1000
−500
0
500
1000
1500
2000
2500

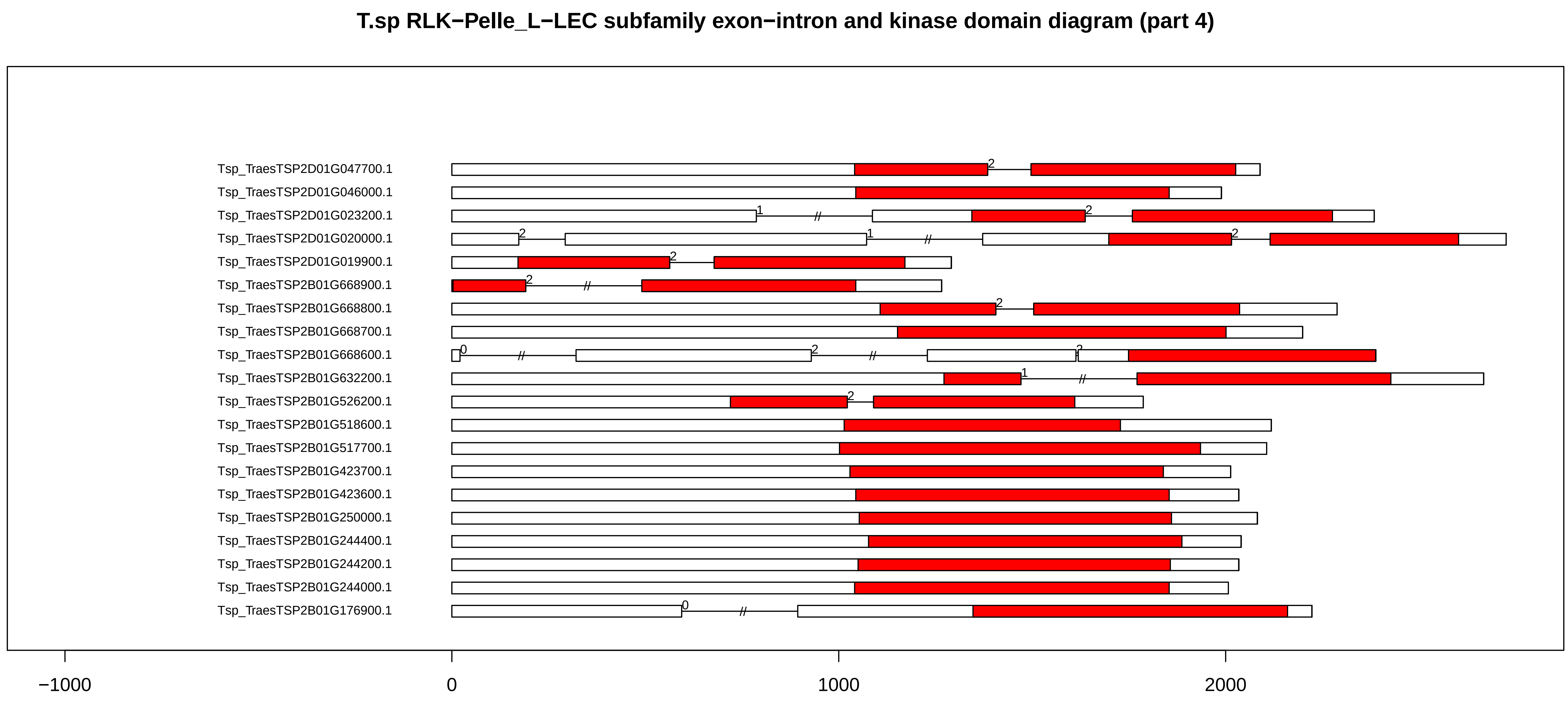
T.sp RLK−Pelle_L−LEC subfamily exon−intron and kinase domain diagram (part 4)
Tsp_TraesTSP2D01G047700.1
Tsp_TraesTSP2D01G046000.1
Tsp_TraesTSP2D01G023200.1
Tsp_TraesTSP2D01G020000.1
Tsp_TraesTSP2D01G019900.1
Tsp_TraesTSP2B01G668900.1
Tsp_TraesTSP2B01G668800.1
Tsp_TraesTSP2B01G668700.1
Tsp_TraesTSP2B01G668600.1
Tsp_TraesTSP2B01G632200.1
Tsp_TraesTSP2B01G526200.1
Tsp_TraesTSP2B01G518600.1
Tsp_TraesTSP2B01G517700.1
Tsp_TraesTSP2B01G423700.1
Tsp_TraesTSP2B01G423600.1
Tsp_TraesTSP2B01G250000.1
Tsp_TraesTSP2B01G244400.1
Tsp_TraesTSP2B01G244200.1
Tsp_TraesTSP2B01G244000.1
Tsp_TraesTSP2B01G176900.1
−1000
0
1000
2000

T.sp RLK−Pelle_L−LEC subfamily exon−intron and kinase domain diagram (part 5)

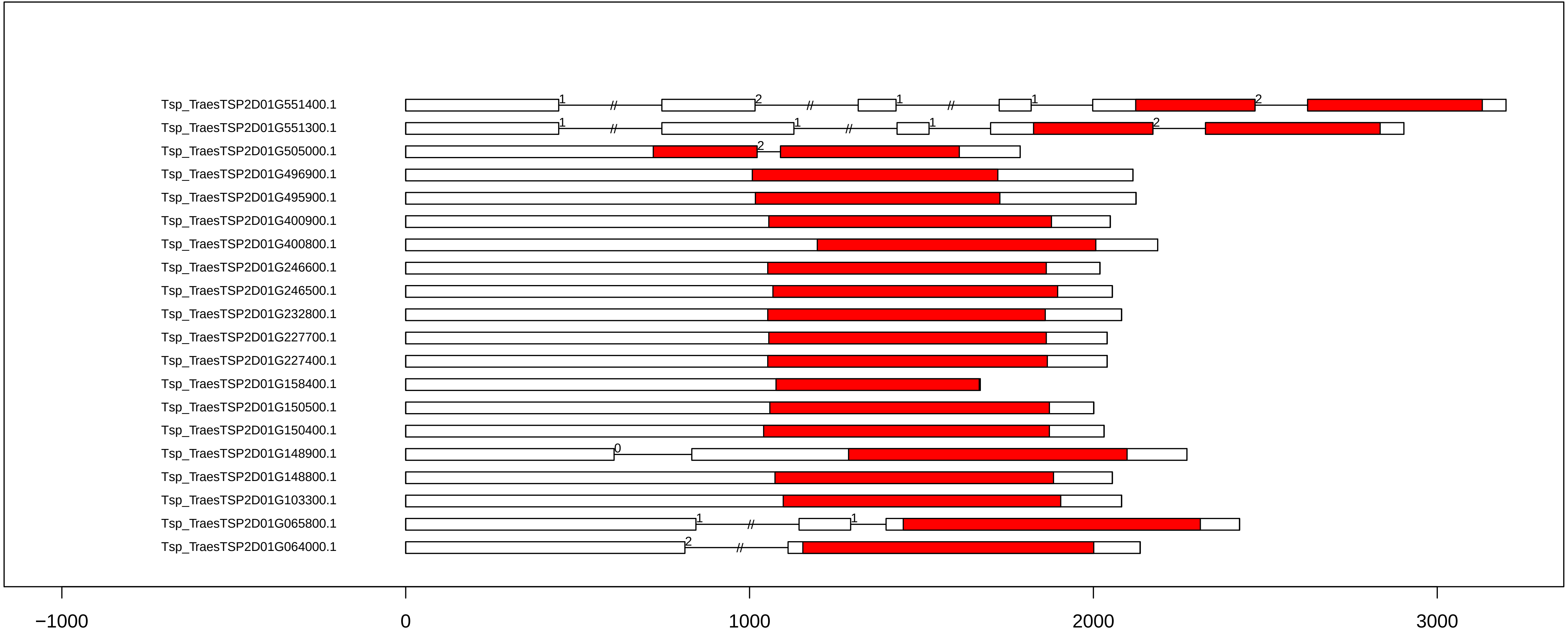

T.sp RLK−Pelle_L−LEC subfamily exon−intron and kinase domain diagram (part 6)

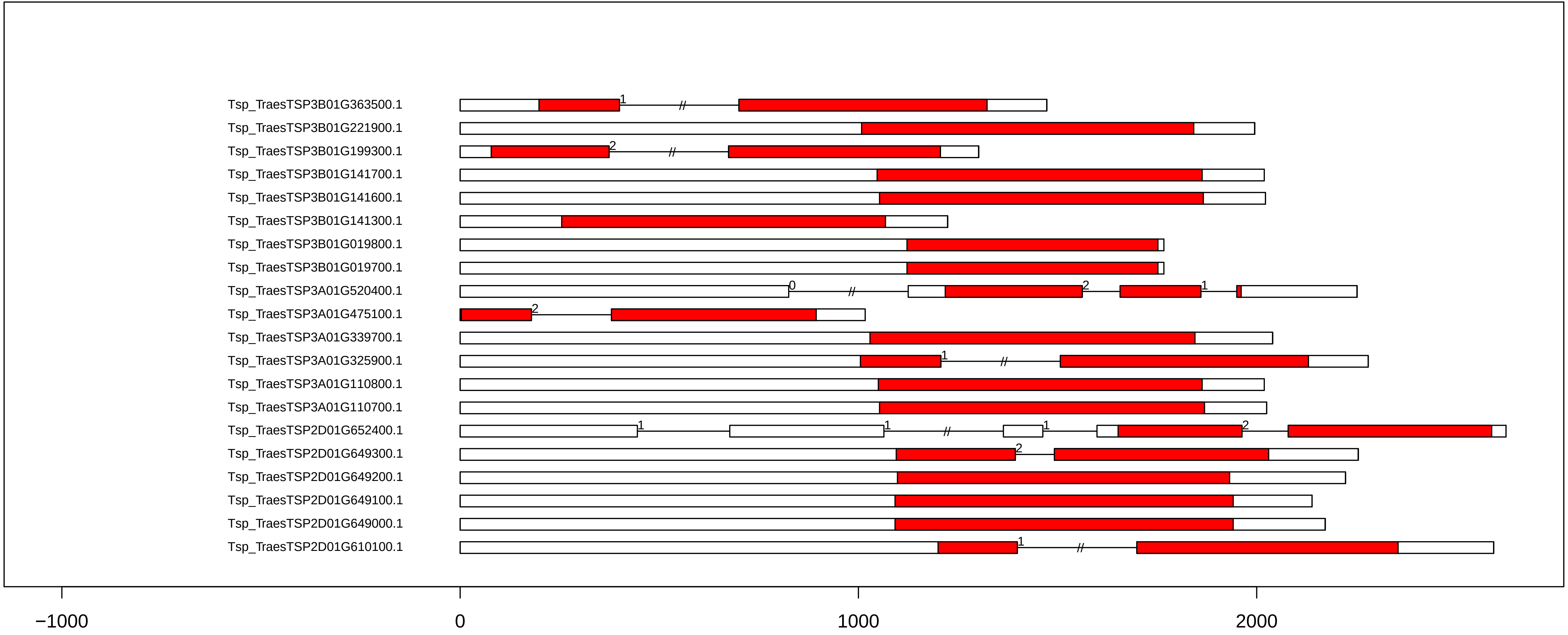

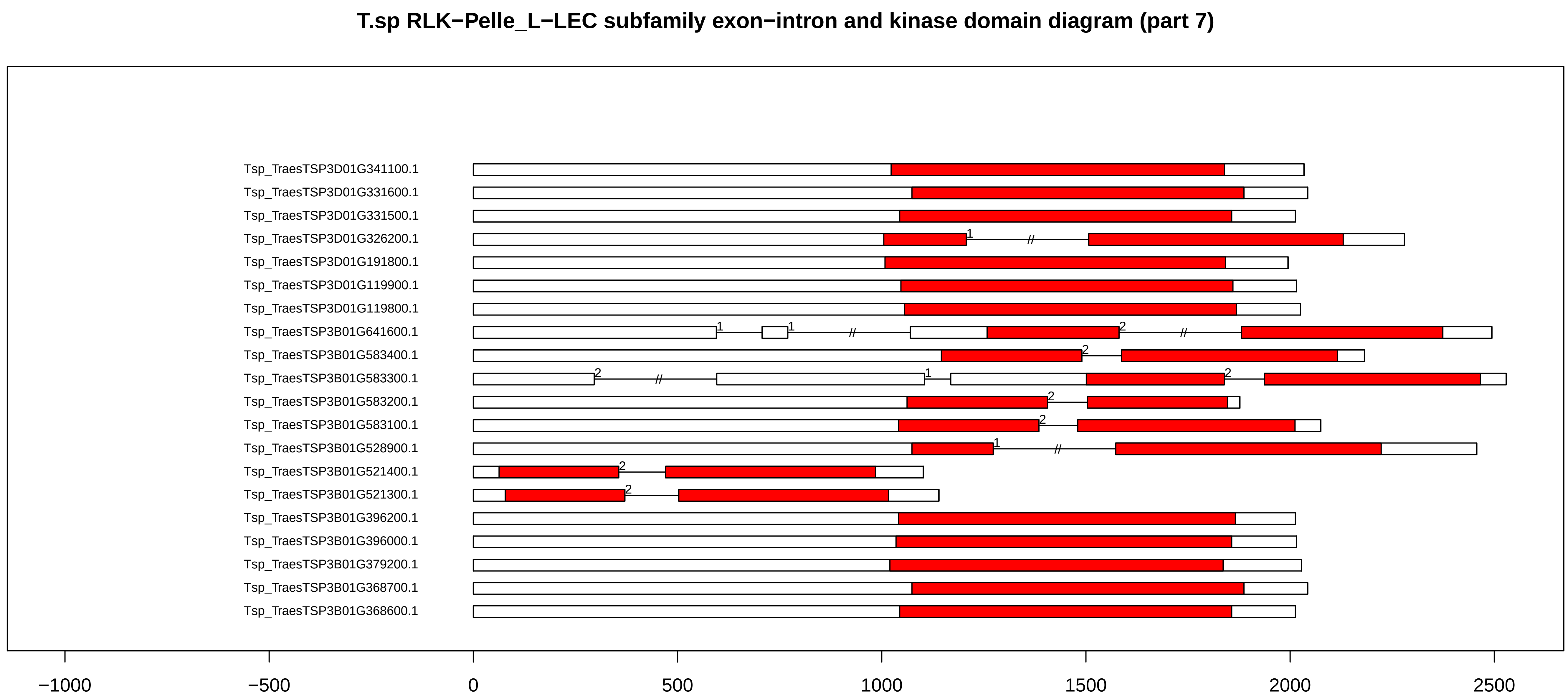

T.sp RLK−Pelle_L−LEC subfamily exon−intron and kinase domain diagram (part 7)
Tsp_TraesTSP3D01G341100.1
Tsp_TraesTSP3D01G331600.1
Tsp_TraesTSP3D01G331500.1
Tsp_TraesTSP3D01G326200.1
Tsp_TraesTSP3D01G191800.1
Tsp_TraesTSP3D01G119900.1
Tsp_TraesTSP3D01G119800.1
Tsp_TraesTSP3B01G641600.1
Tsp_TraesTSP3B01G583400.1
Tsp_TraesTSP3B01G583300.1
Tsp_TraesTSP3B01G583200.1
Tsp_TraesTSP3B01G583100.1
Tsp_TraesTSP3B01G528900.1
Tsp_TraesTSP3B01G521400.1
Tsp_TraesTSP3B01G521300.1
Tsp_TraesTSP3B01G396200.1
Tsp_TraesTSP3B01G396000.1
Tsp_TraesTSP3B01G379200.1
Tsp_TraesTSP3B01G368700.1
Tsp_TraesTSP3B01G368600.1
−1000
−500
0
500
1000
1500
2000
2500

T.sp RLK−Pelle_L−LEC subfamily exon−intron and kinase domain diagram (part 8)

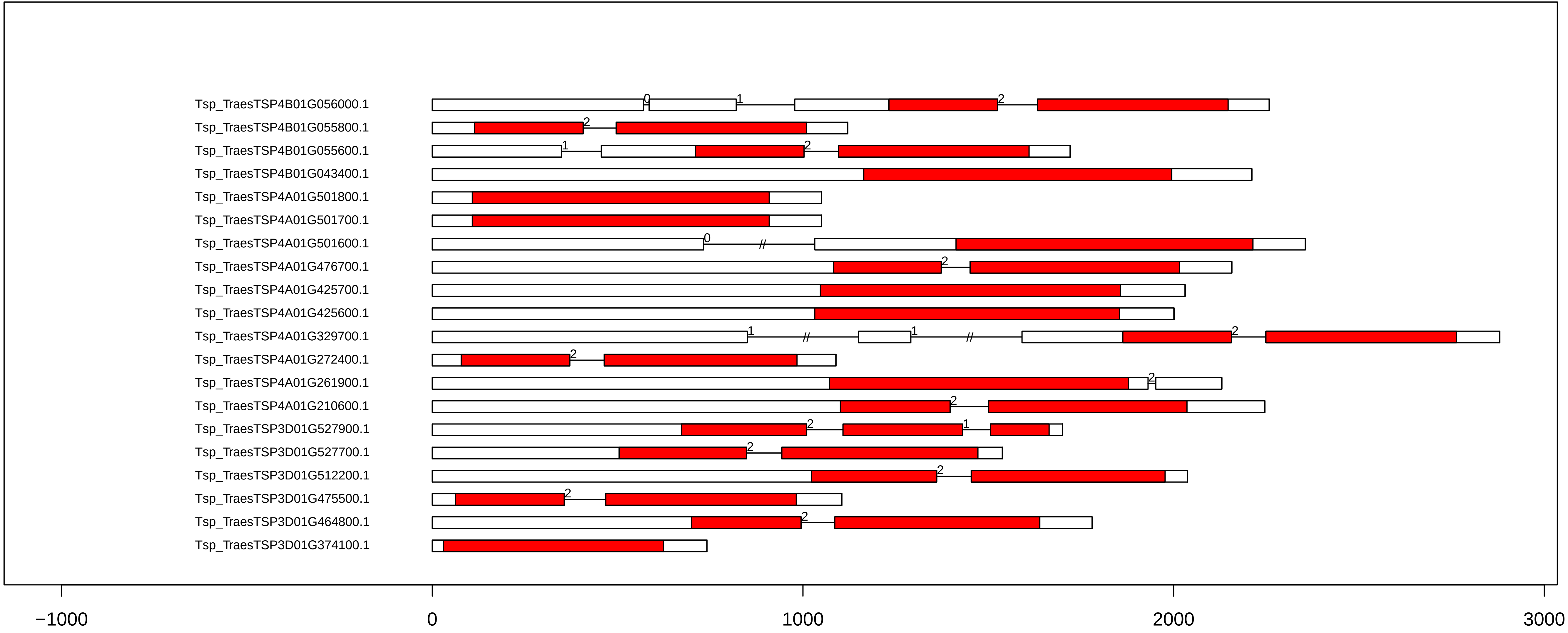

T.sp RLK−Pelle_L−LEC subfamily exon−intron and kinase domain diagram (part 9)

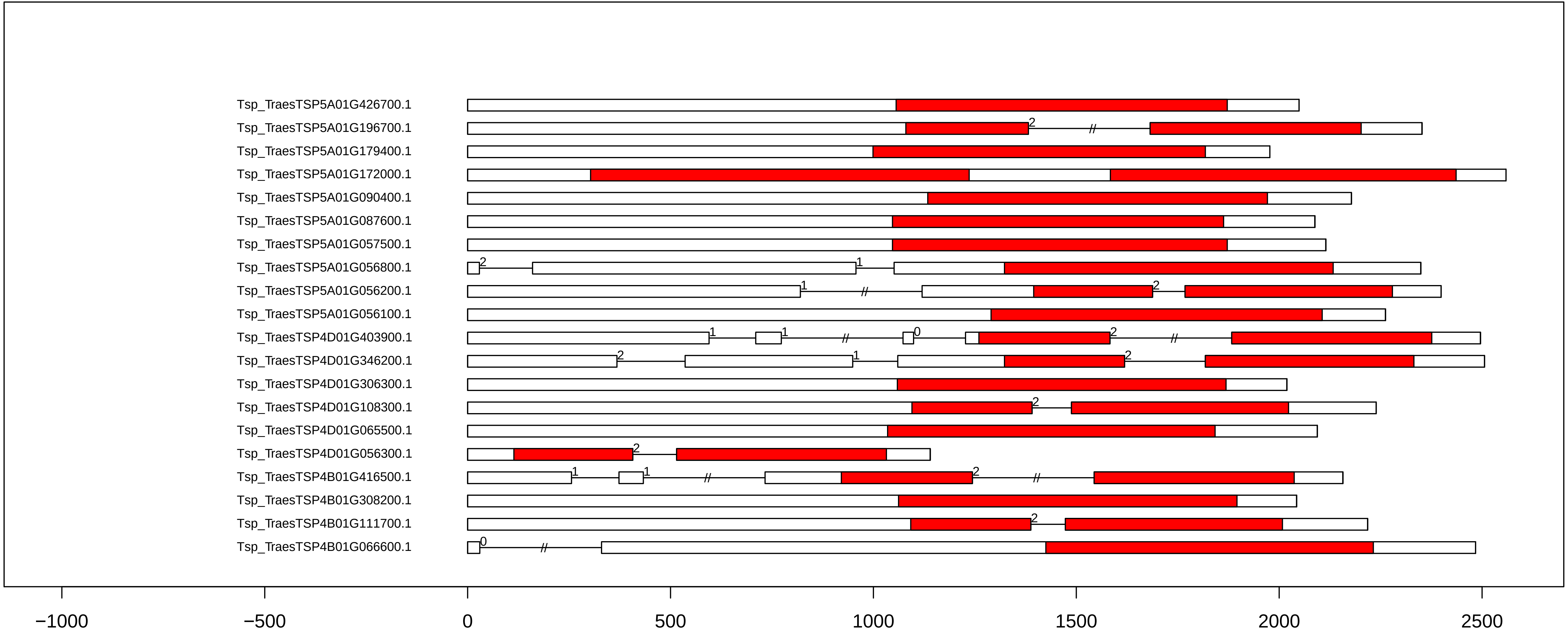

T.sp RLK−Pelle_L−LEC subfamily exon−intron and kinase domain diagram (part 10)

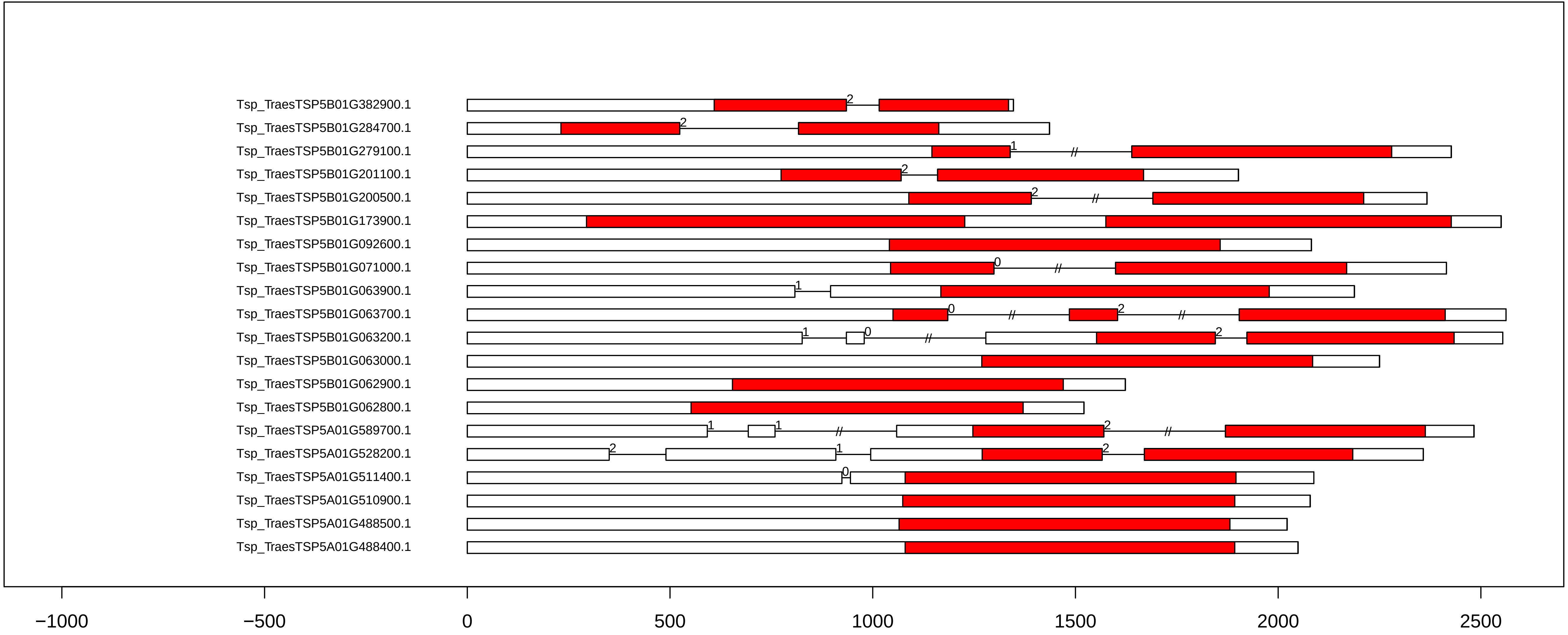

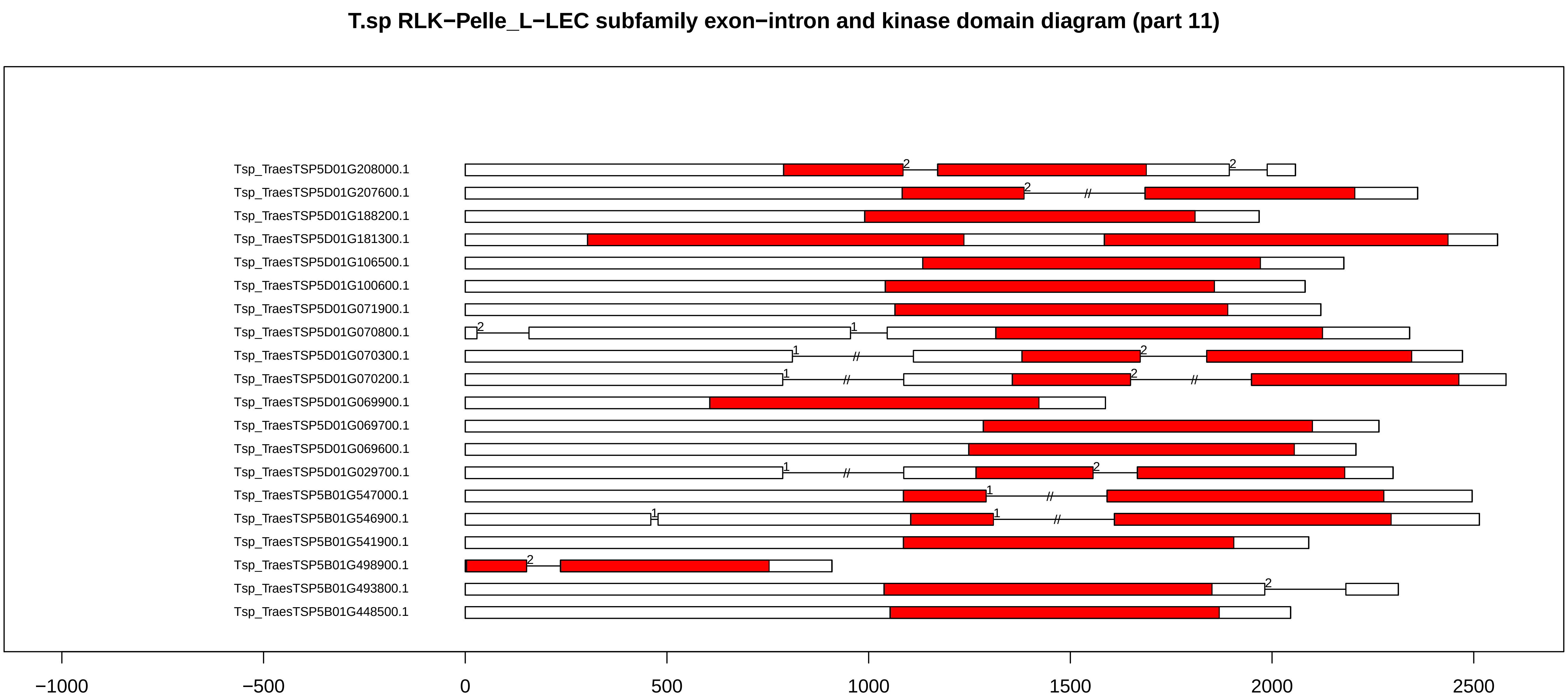
T.sp RLK−Pelle_L−LEC subfamily exon−intron and kinase domain diagram (part 11)
Tsp_TraesTSP5D01G208000.1
Tsp_TraesTSP5D01G207600.1
Tsp_TraesTSP5D01G188200.1
Tsp_TraesTSP5D01G181300.1
Tsp_TraesTSP5D01G106500.1
Tsp_TraesTSP5D01G100600.1
Tsp_TraesTSP5D01G071900.1
Tsp_TraesTSP5D01G070800.1
Tsp_TraesTSP5D01G070300.1
Tsp_TraesTSP5D01G070200.1
Tsp_TraesTSP5D01G069900.1
Tsp_TraesTSP5D01G069700.1
Tsp_TraesTSP5D01G069600.1
Tsp_TraesTSP5D01G029700.1
Tsp_TraesTSP5B01G547000.1
Tsp_TraesTSP5B01G546900.1
Tsp_TraesTSP5B01G541900.1
Tsp_TraesTSP5B01G498900.1
Tsp_TraesTSP5B01G493800.1
Tsp_TraesTSP5B01G448500.1
−1000
−500
0
500
1000
1500
2000
2500

T.sp RLK−Pelle_L−LEC subfamily exon−intron and kinase domain diagram (part 12)

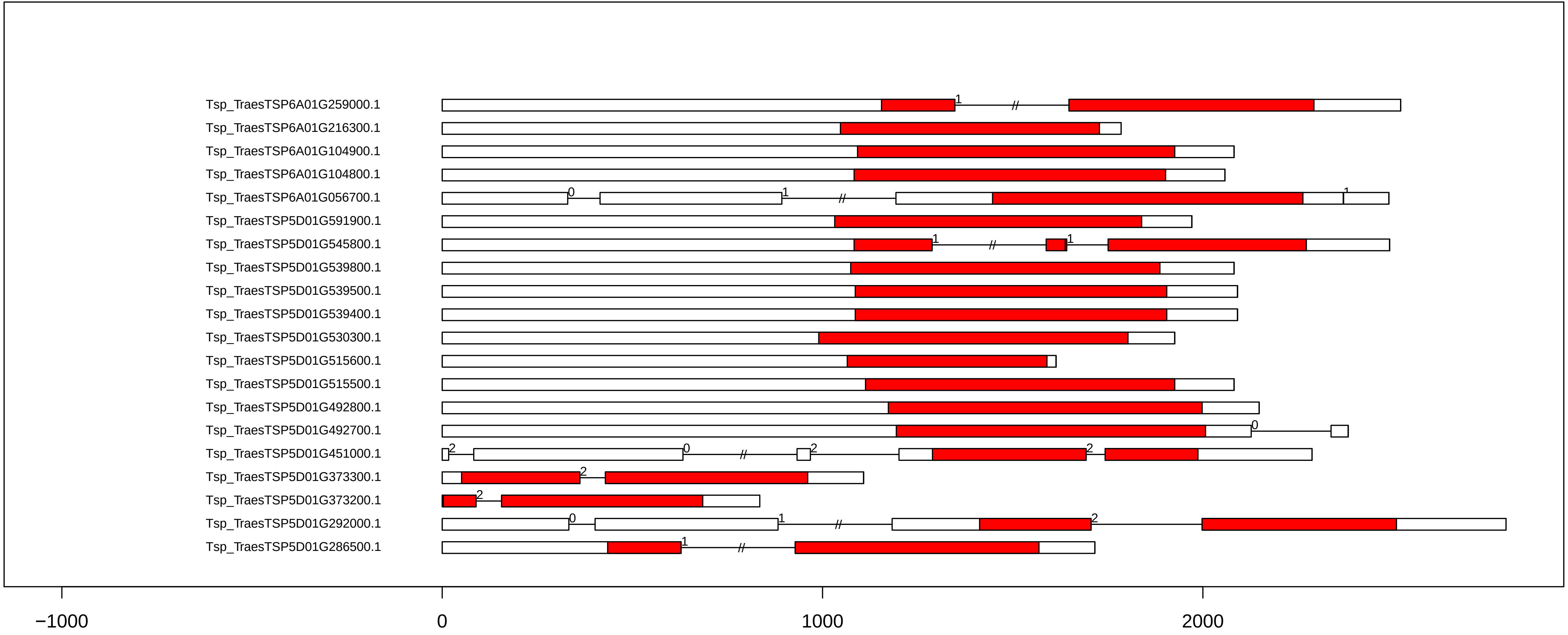

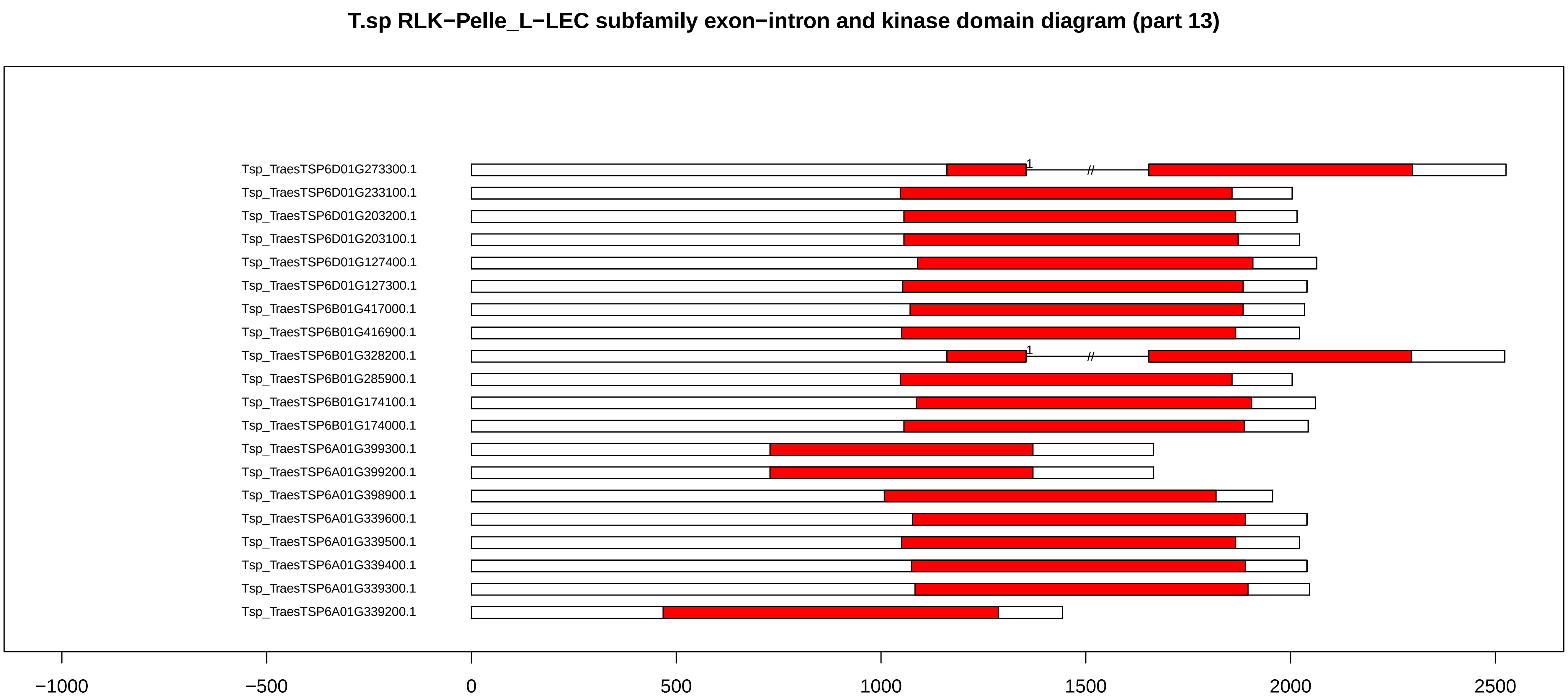
T.sp RLK−Pelle_L−LEC subfamily exon−intron and kinase domain diagram (part 13)
Tsp_TraesTSP6D01G273300.1
Tsp_TraesTSP6D01G233100.1
Tsp_TraesTSP6D01G203200.1
Tsp_TraesTSP6D01G203100.1
Tsp_TraesTSP6D01G127400.1
Tsp_TraesTSP6D01G127300.1
Tsp_TraesTSP6B01G417000.1
Tsp_TraesTSP6B01G416900.1
Tsp_TraesTSP6B01G328200.1
Tsp_TraesTSP6B01G285900.1
Tsp_TraesTSP6B01G174100.1
Tsp_TraesTSP6B01G174000.1
Tsp_TraesTSP6A01G399300.1
Tsp_TraesTSP6A01G399200.1
Tsp_TraesTSP6A01G398900.1
Tsp_TraesTSP6A01G339600.1
Tsp_TraesTSP6A01G339500.1
Tsp_TraesTSP6A01G339400.1
Tsp_TraesTSP6A01G339300.1
Tsp_TraesTSP6A01G339200.1
1
1
−1000
−500
0
500
1000
1500
2000
2500

T.sp RLK−Pelle_L−LEC subfamily exon−intron and kinase domain diagram (part 14)

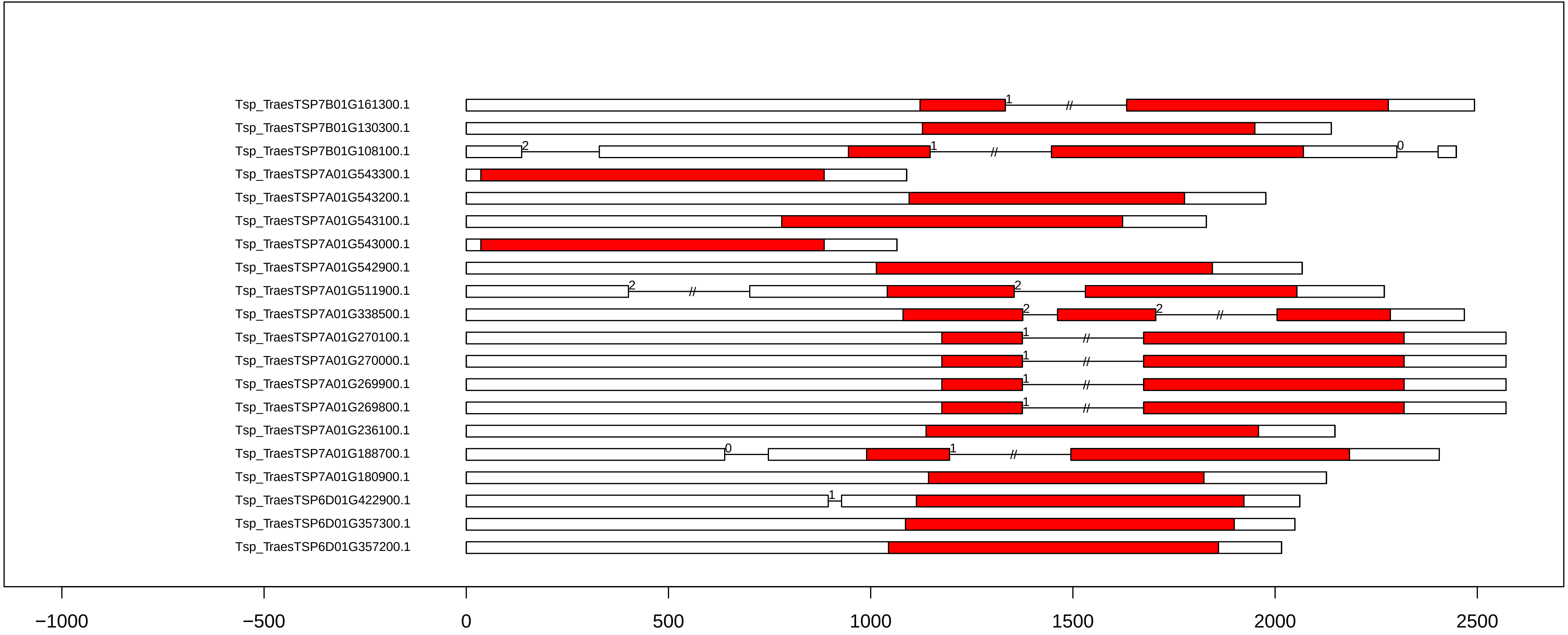

T.sp RLK−Pelle_L−LEC subfamily exon−intron and kinase domain diagram (part 15)

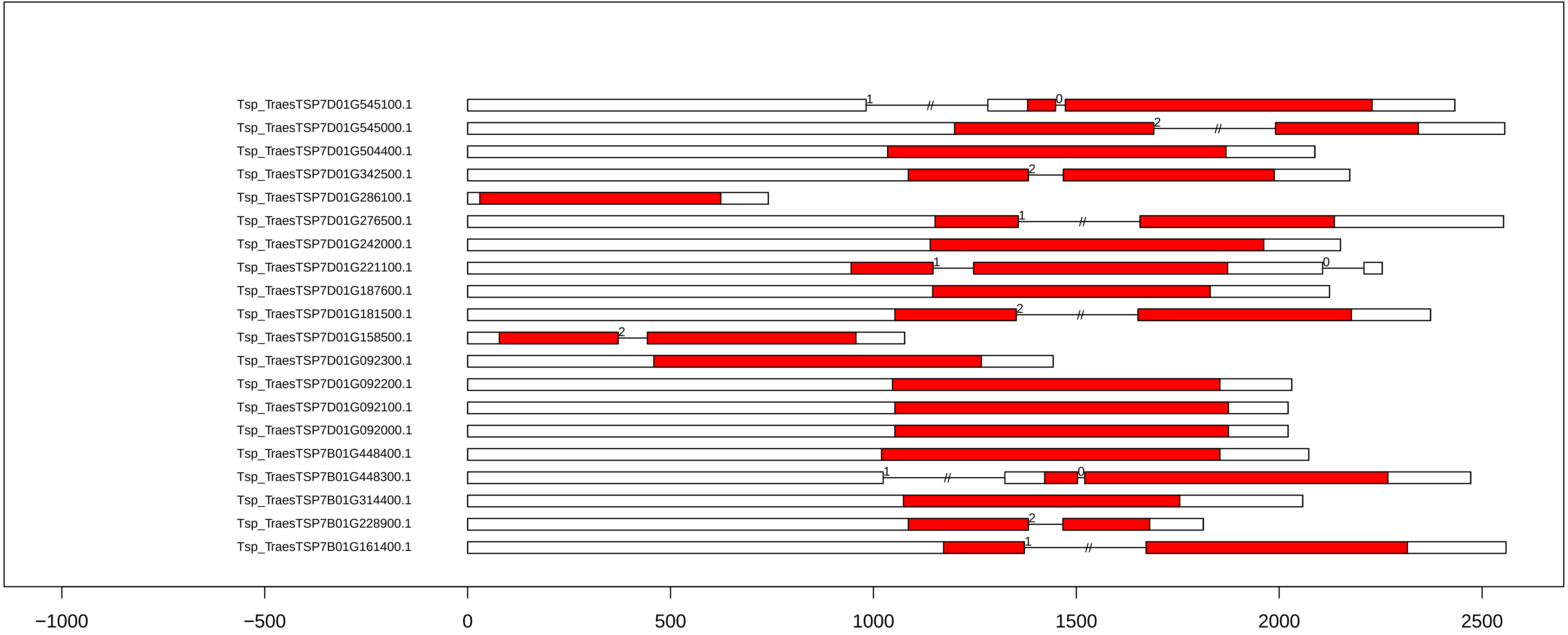

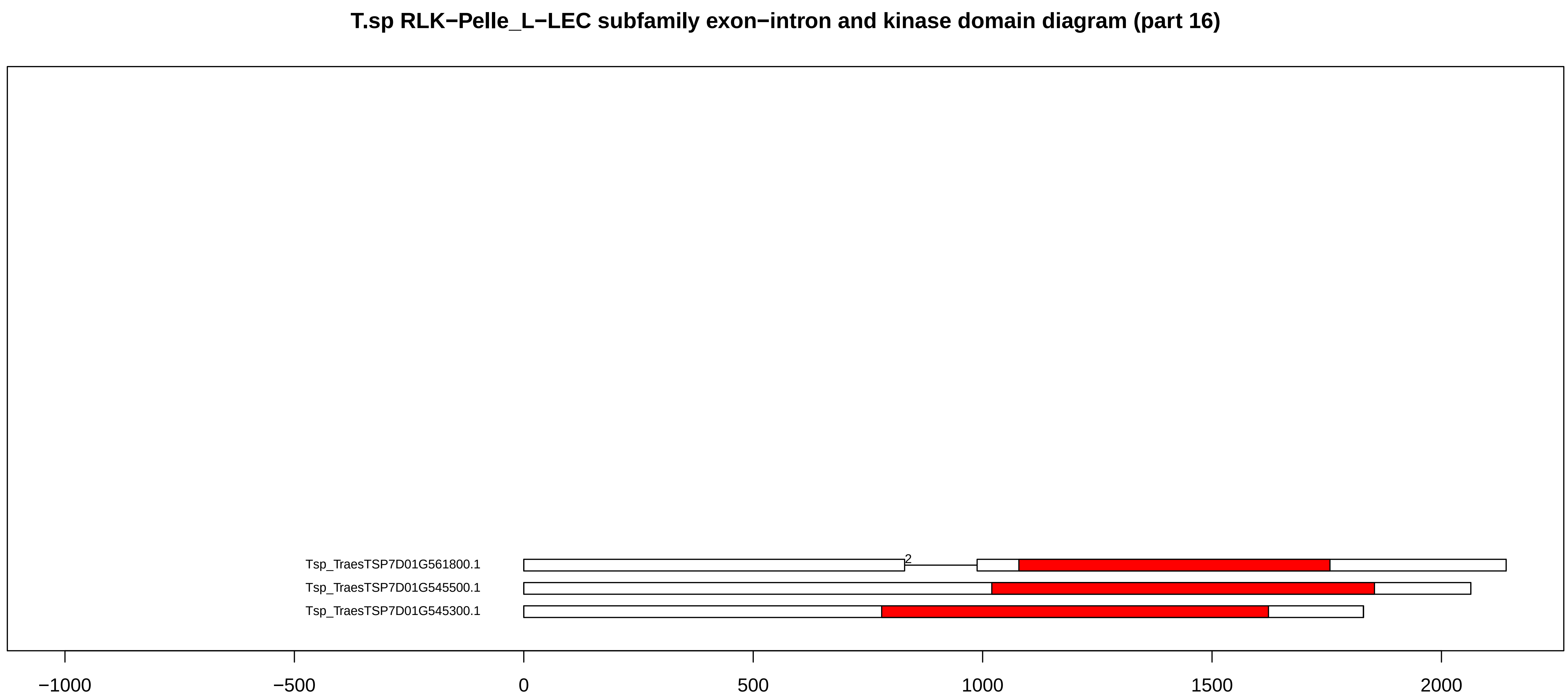
T.sp RLK−Pelle_L−LEC subfamily exon−intron and kinase domain diagram (part 16)
Tsp_TraesTSP7D01G561800.1
2
Tsp_TraesTSP7D01G545500.1
Tsp_TraesTSP7D01G545300.1
−1000
−500
0
500
1000
1500
2000

T.sp RLK−Pelle_LRK10L−2 subfamily exon−intron and kinase domain diagram (part 1)

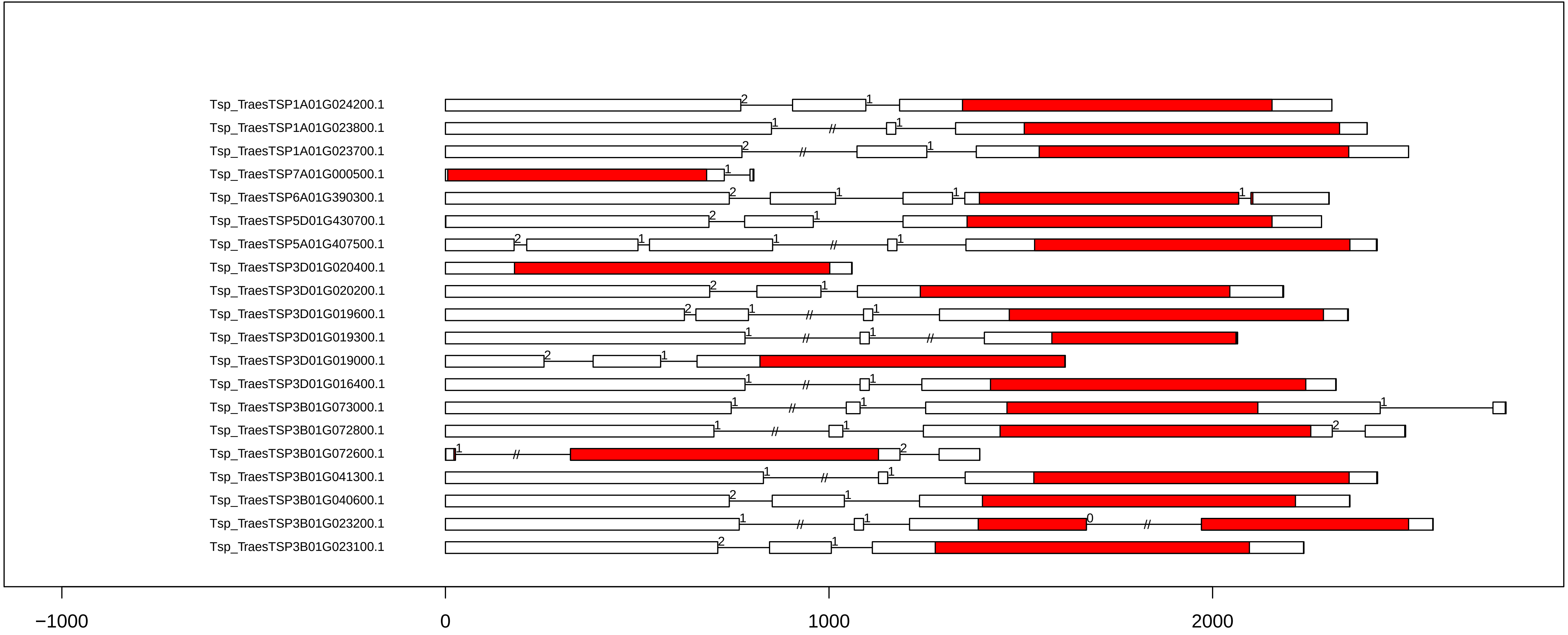

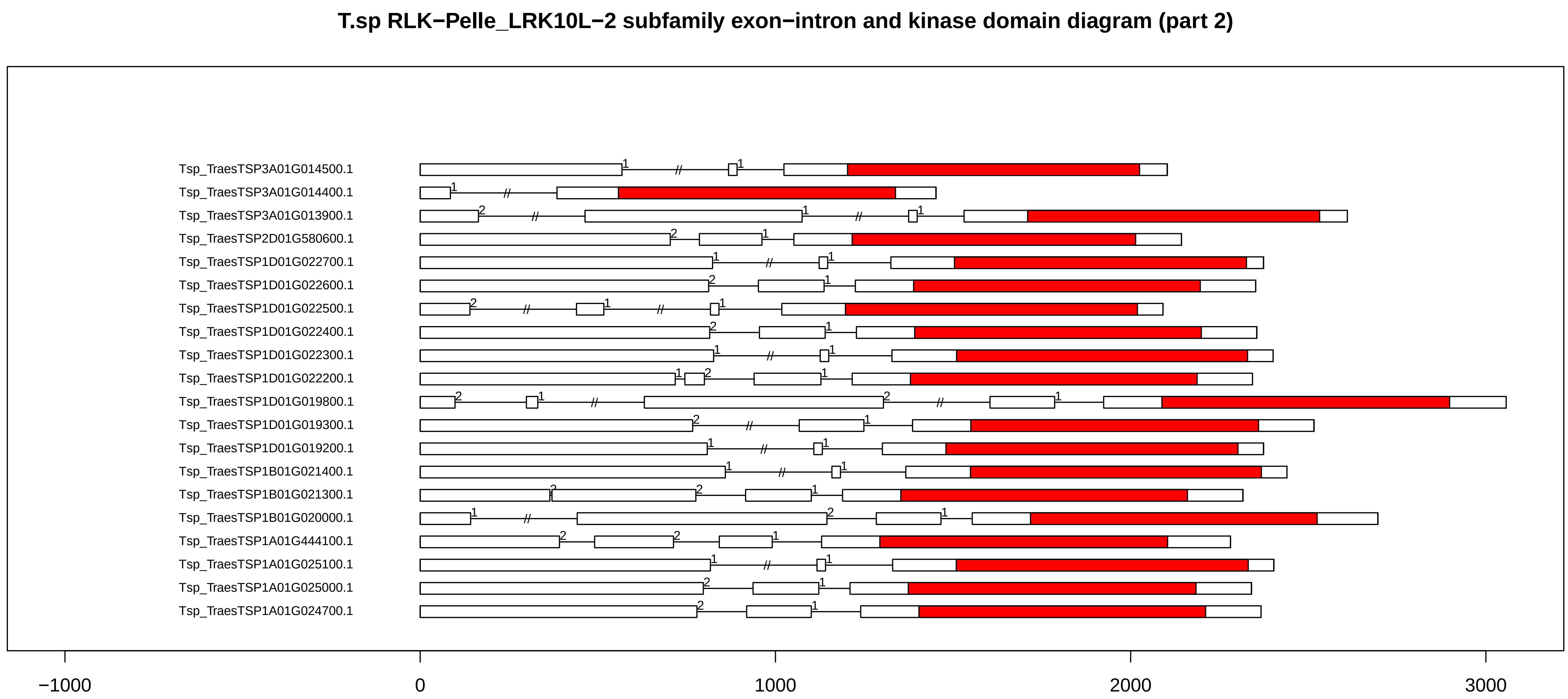
T.sp RLK−Pelle_LRK10L−2 subfamily exon−intron and kinase domain diagram (part 2)
Tsp_TraesTSP3A01G014500.1
Tsp_TraesTSP3A01G014400.1
Tsp_TraesTSP3A01G013900.1
Tsp_TraesTSP2D01G580600.1
Tsp_TraesTSP1D01G022700.1
Tsp_TraesTSP1D01G022600.1
Tsp_TraesTSP1D01G022500.1
Tsp_TraesTSP1D01G022400.1
Tsp_TraesTSP1D01G022300.1
Tsp_TraesTSP1D01G022200.1
Tsp_TraesTSP1D01G019800.1
Tsp_TraesTSP1D01G019300.1
Tsp_TraesTSP1D01G019200.1
Tsp_TraesTSP1B01G021400.1
Tsp_TraesTSP1B01G021300.1
Tsp_TraesTSP1B01G020000.1
Tsp_TraesTSP1A01G444100.1
Tsp_TraesTSP1A01G025100.1
Tsp_TraesTSP1A01G025000.1
Tsp_TraesTSP1A01G024700.1
−1000
0
1000
2000
3000

T.sp RLK−Pelle_LRK10L−2 subfamily exon−intron and kinase domain diagram (part 3)

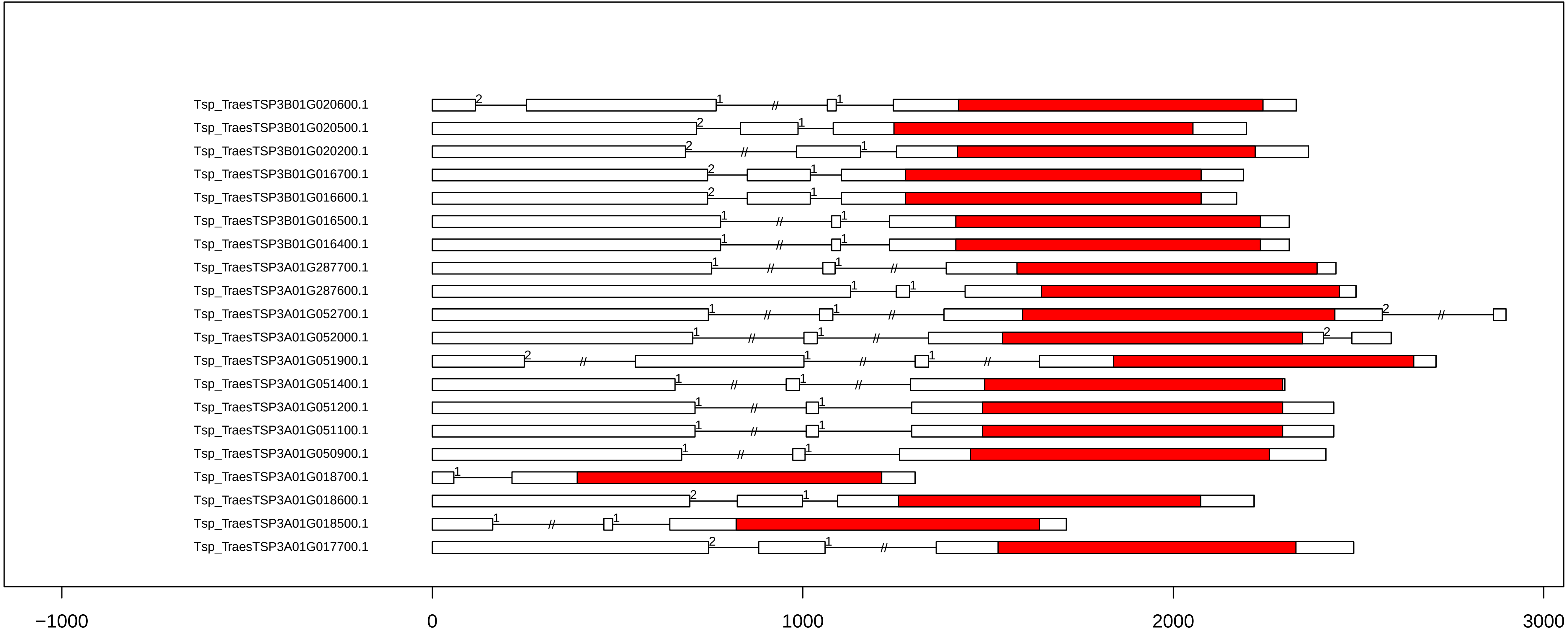

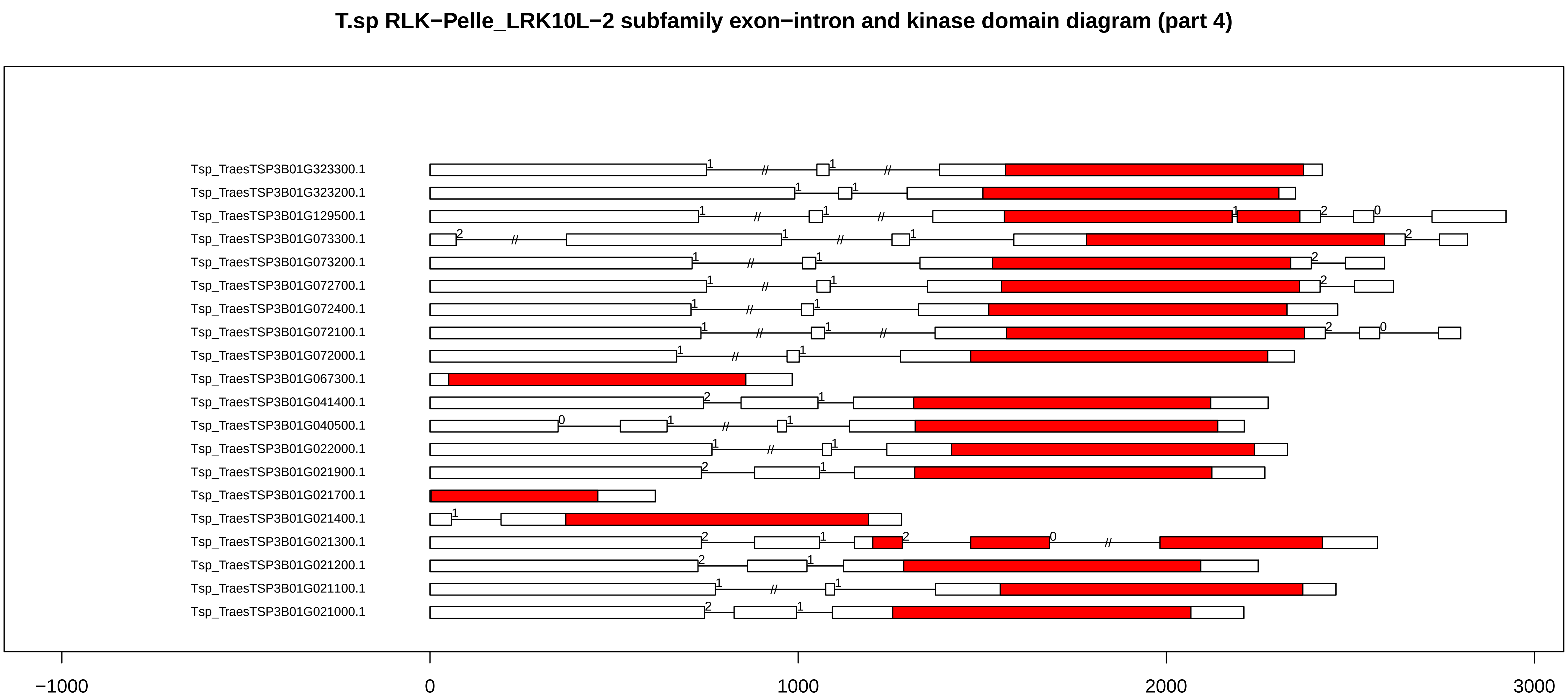
T.sp RLK−Pelle_LRK10L−2 subfamily exon−intron and kinase domain diagram (part 4)
Tsp_TraesTSP3B01G323300.1
Tsp_TraesTSP3B01G323200.1
Tsp_TraesTSP3B01G129500.1
Tsp_TraesTSP3B01G073300.1
Tsp_TraesTSP3B01G073200.1
Tsp_TraesTSP3B01G072700.1
Tsp_TraesTSP3B01G072400.1
Tsp_TraesTSP3B01G072100.1
Tsp_TraesTSP3B01G072000.1
Tsp_TraesTSP3B01G067300.1
Tsp_TraesTSP3B01G041400.1
Tsp_TraesTSP3B01G040500.1
Tsp_TraesTSP3B01G022000.1
Tsp_TraesTSP3B01G021900.1
Tsp_TraesTSP3B01G021700.1
Tsp_TraesTSP3B01G021400.1
Tsp_TraesTSP3B01G021300.1
Tsp_TraesTSP3B01G021200.1
Tsp_TraesTSP3B01G021100.1
Tsp_TraesTSP3B01G021000.1
−1000
0
1000
2000
3000

T.sp RLK−Pelle_LRK10L−2 subfamily exon−intron and kinase domain diagram (part 5)

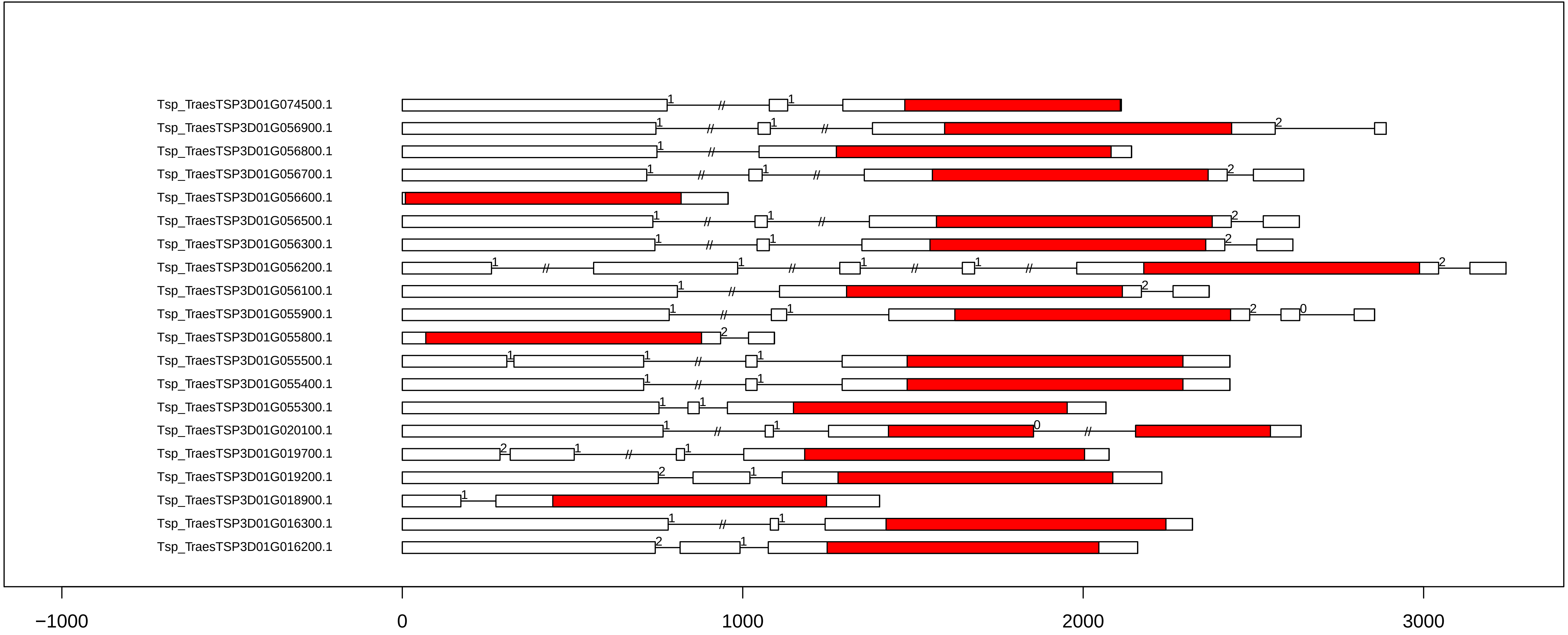

T.sp RLK−Pelle_LRK10L−2 subfamily exon−intron and kinase domain diagram (part 6)

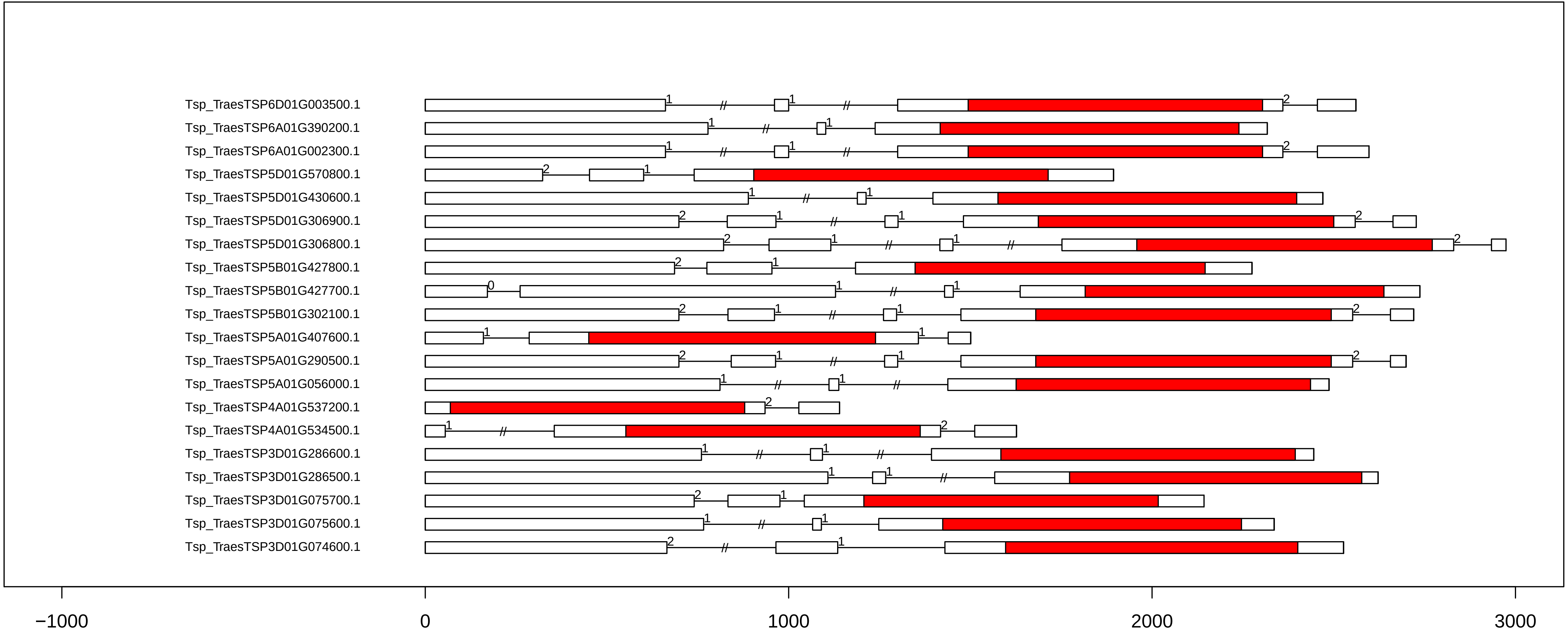

T.sp RLK−Pelle_LRK10L−2 subfamily exon−intron and kinase domain diagram (part 7)

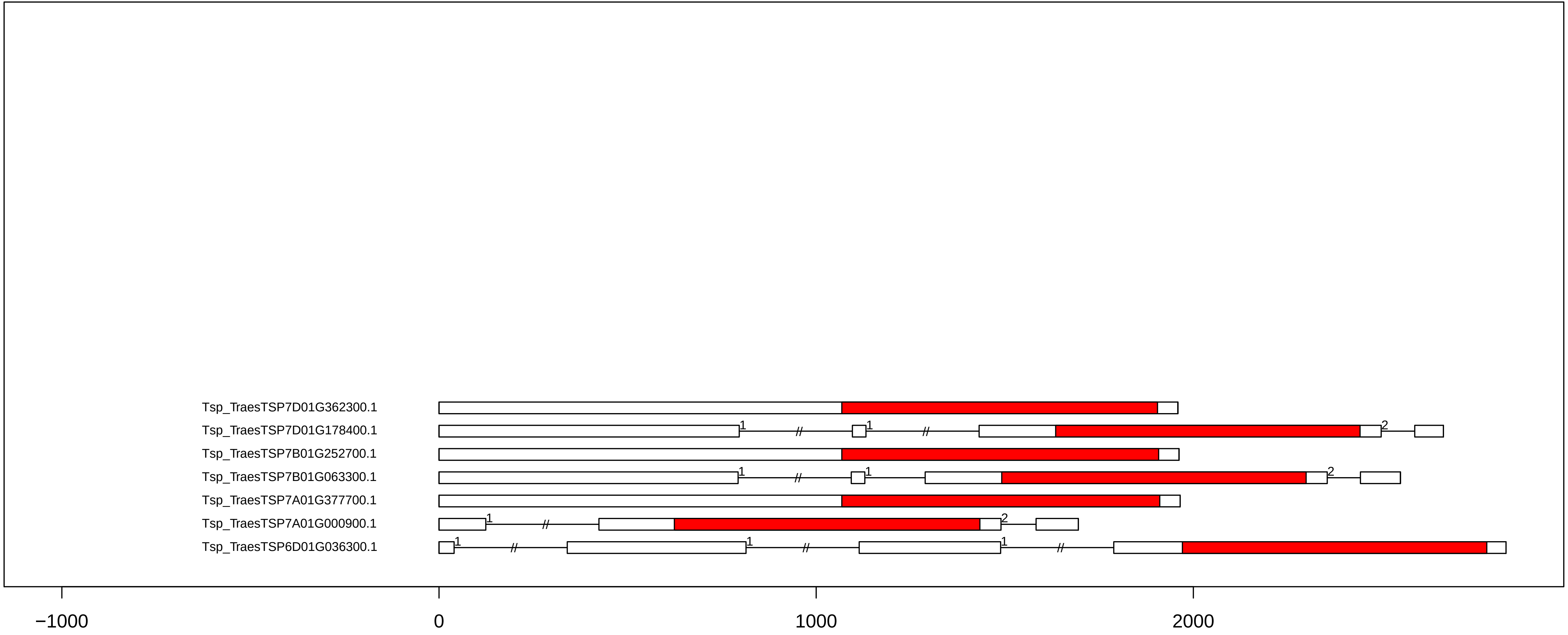

T.sp RLK−Pelle_LRR−I−1 subfamily exon−intron and kinase domain diagram (part 1)

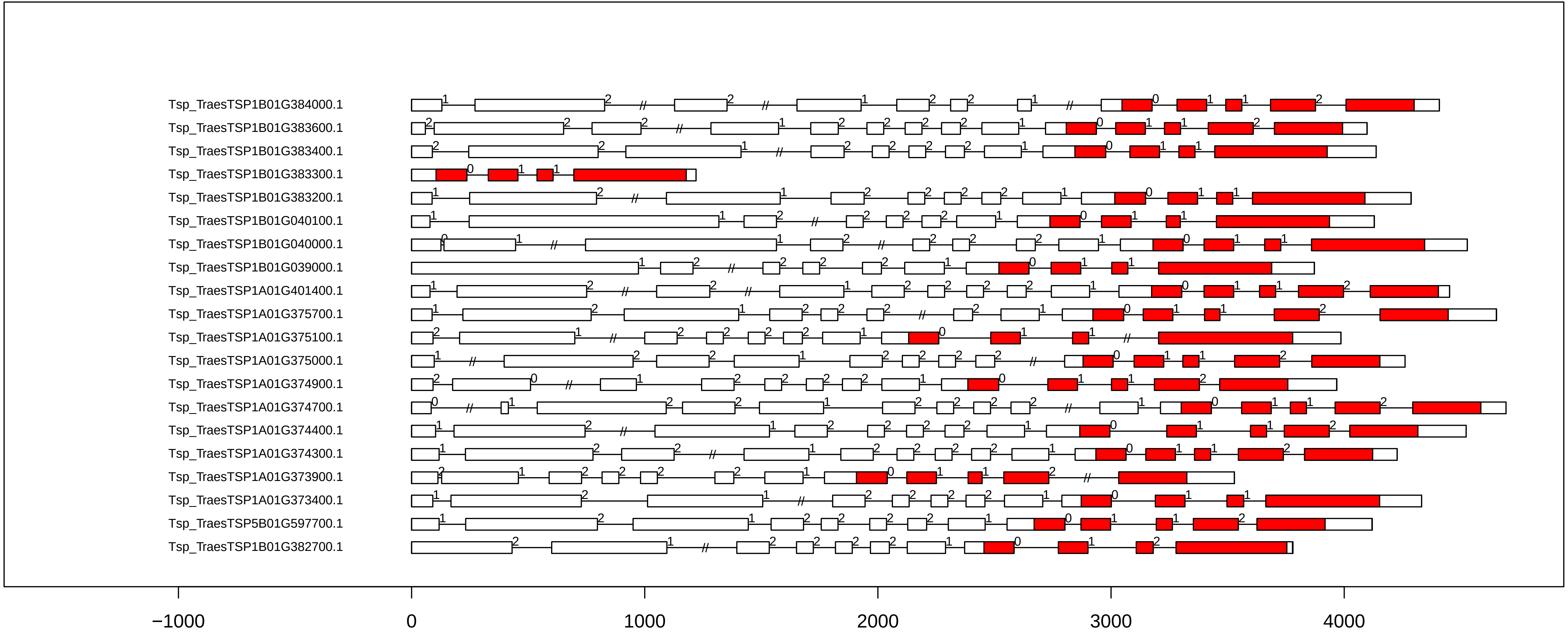

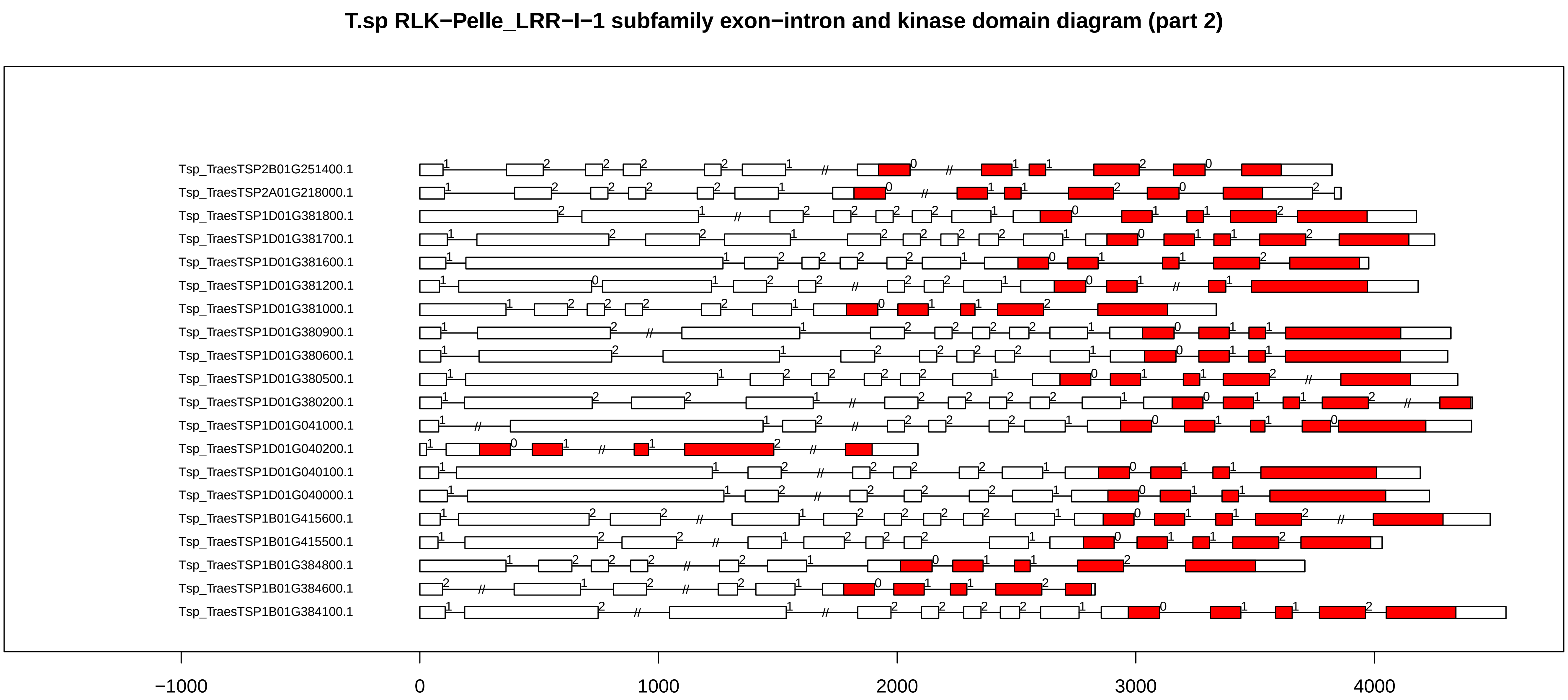
T.sp RLK−Pelle_LRR−I−1 subfamily exon−intron and kinase domain diagram (part 2)
Tsp_TraesTSP2B01G251400.1
Tsp_TraesTSP2A01G218000.1
Tsp_TraesTSP1D01G381800.1
Tsp_TraesTSP1D01G381700.1
Tsp_TraesTSP1D01G381600.1
Tsp_TraesTSP1D01G381200.1
Tsp_TraesTSP1D01G381000.1
Tsp_TraesTSP1D01G380900.1
Tsp_TraesTSP1D01G380600.1
Tsp_TraesTSP1D01G380500.1
Tsp_TraesTSP1D01G380200.1
Tsp_TraesTSP1D01G041000.1
Tsp_TraesTSP1D01G040200.1
Tsp_TraesTSP1D01G040100.1
Tsp_TraesTSP1D01G040000.1
Tsp_TraesTSP1B01G415600.1
Tsp_TraesTSP1B01G415500.1
Tsp_TraesTSP1B01G384800.1
Tsp_TraesTSP1B01G384600.1
Tsp_TraesTSP1B01G384100.1
−1000
0
1000
2000
3000
4000

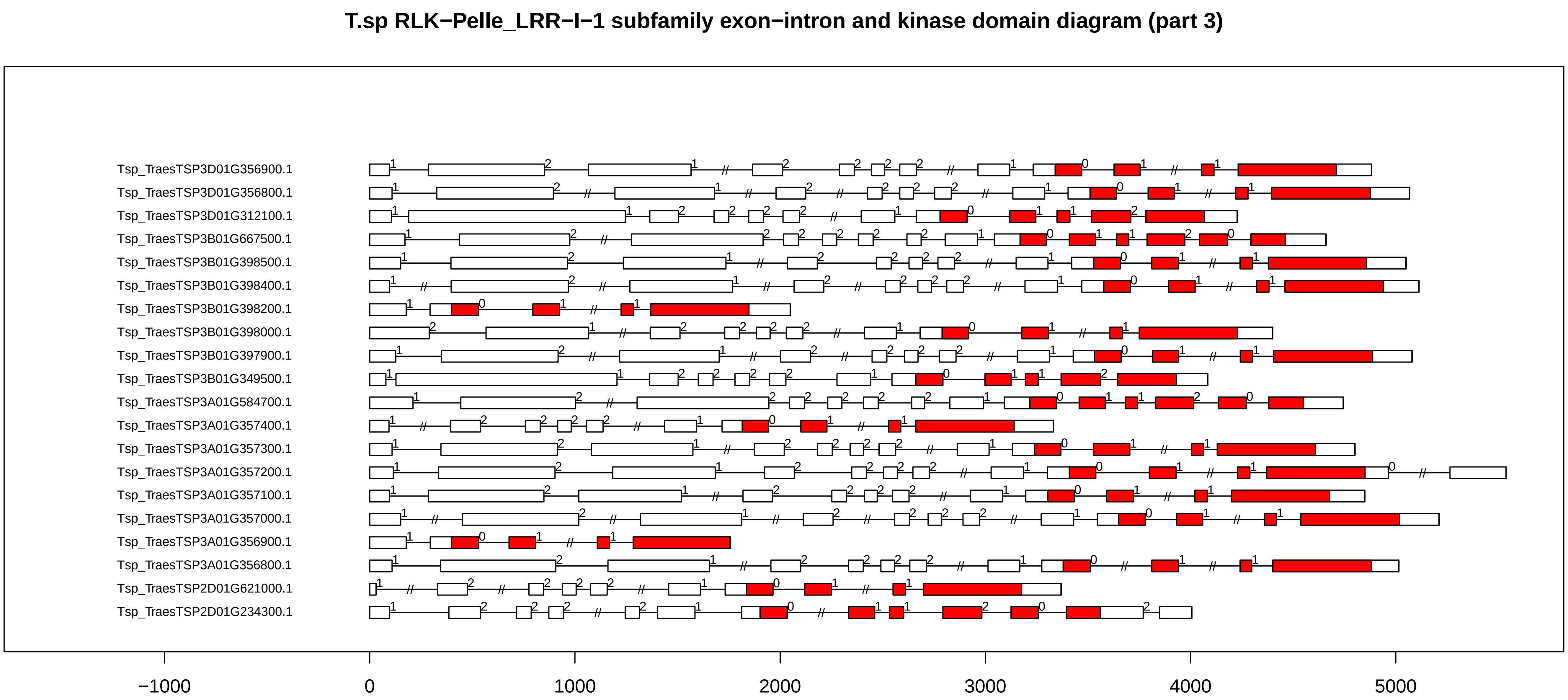

T.sp RLK−Pelle_LRR−I−1 subfamily exon−intron and kinase domain diagram (part 3)
Tsp_TraesTSP3D01G356900.1
Tsp_TraesTSP3D01G356800.1
Tsp_TraesTSP3D01G312100.1
Tsp_TraesTSP3B01G667500.1
Tsp_TraesTSP3B01G398500.1
Tsp_TraesTSP3B01G398400.1
Tsp_TraesTSP3B01G398200.1
Tsp_TraesTSP3B01G398000.1
Tsp_TraesTSP3B01G397900.1
Tsp_TraesTSP3B01G349500.1
Tsp_TraesTSP3A01G584700.1
Tsp_TraesTSP3A01G357400.1
Tsp_TraesTSP3A01G357300.1
Tsp_TraesTSP3A01G357200.1
Tsp_TraesTSP3A01G357100.1
Tsp_TraesTSP3A01G357000.1
Tsp_TraesTSP3A01G356900.1
Tsp_TraesTSP3A01G356800.1
Tsp_TraesTSP2D01G621000.1
Tsp_TraesTSP2D01G234300.1
−1000
0
1000
2000
3000
4000
5000

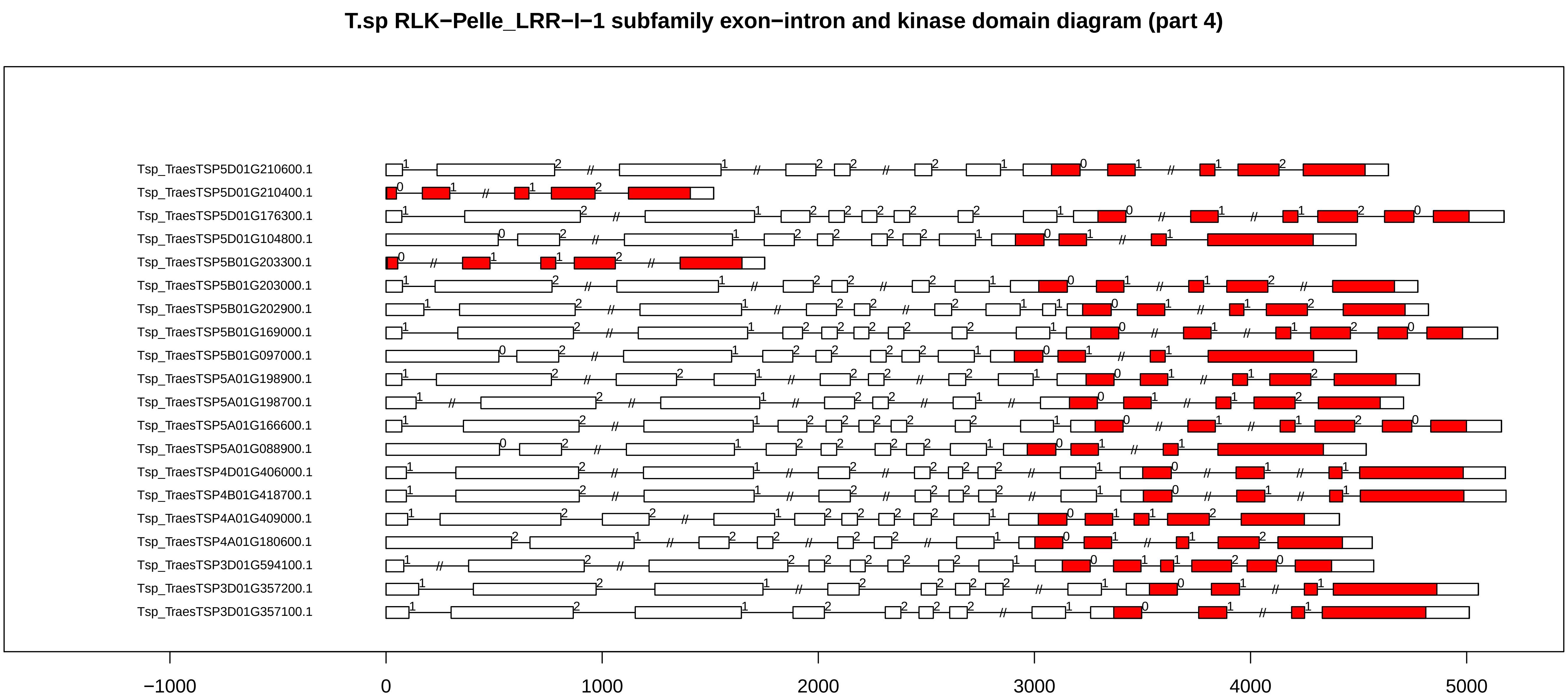
T.sp RLK−Pelle_LRR−I−1 subfamily exon−intron and kinase domain diagram (part 4)
Tsp_TraesTSP5D01G210600.1
Tsp_TraesTSP5D01G210400.1
Tsp_TraesTSP5D01G176300.1
Tsp_TraesTSP5D01G104800.1
Tsp_TraesTSP5B01G203300.1
Tsp_TraesTSP5B01G203000.1
Tsp_TraesTSP5B01G202900.1
Tsp_TraesTSP5B01G169000.1
Tsp_TraesTSP5B01G097000.1
Tsp_TraesTSP5A01G198900.1
Tsp_TraesTSP5A01G198700.1
Tsp_TraesTSP5A01G166600.1
Tsp_TraesTSP5A01G088900.1
Tsp_TraesTSP4D01G406000.1
Tsp_TraesTSP4B01G418700.1
Tsp_TraesTSP4A01G409000.1
Tsp_TraesTSP4A01G180600.1
Tsp_TraesTSP3D01G594100.1
Tsp_TraesTSP3D01G357200.1
Tsp_TraesTSP3D01G357100.1
−1000
0
1000
2000
3000
4000
5000

T.sp RLK−Pelle_LRR−I−1 subfamily exon−intron and kinase domain diagram (part 5)

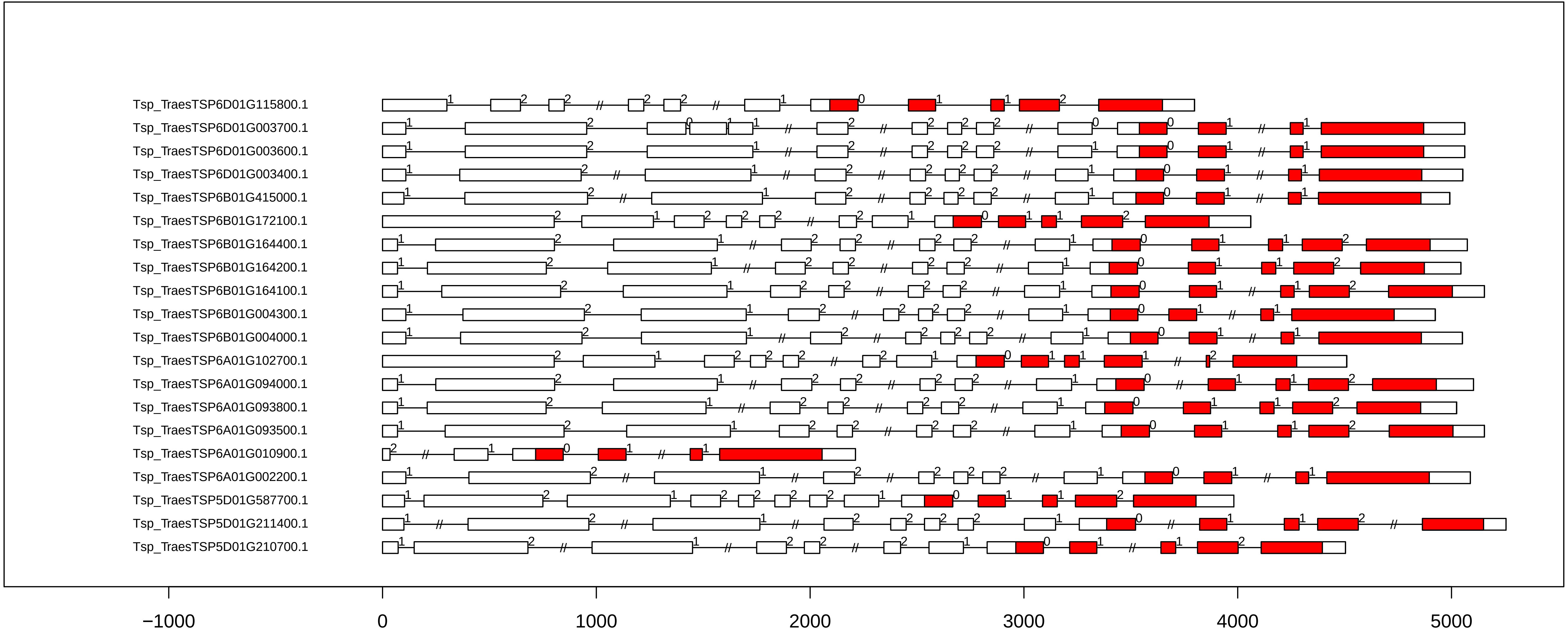

T.sp RLK−Pelle_LRR−I−1 subfamily exon−intron and kinase domain diagram (part 6)

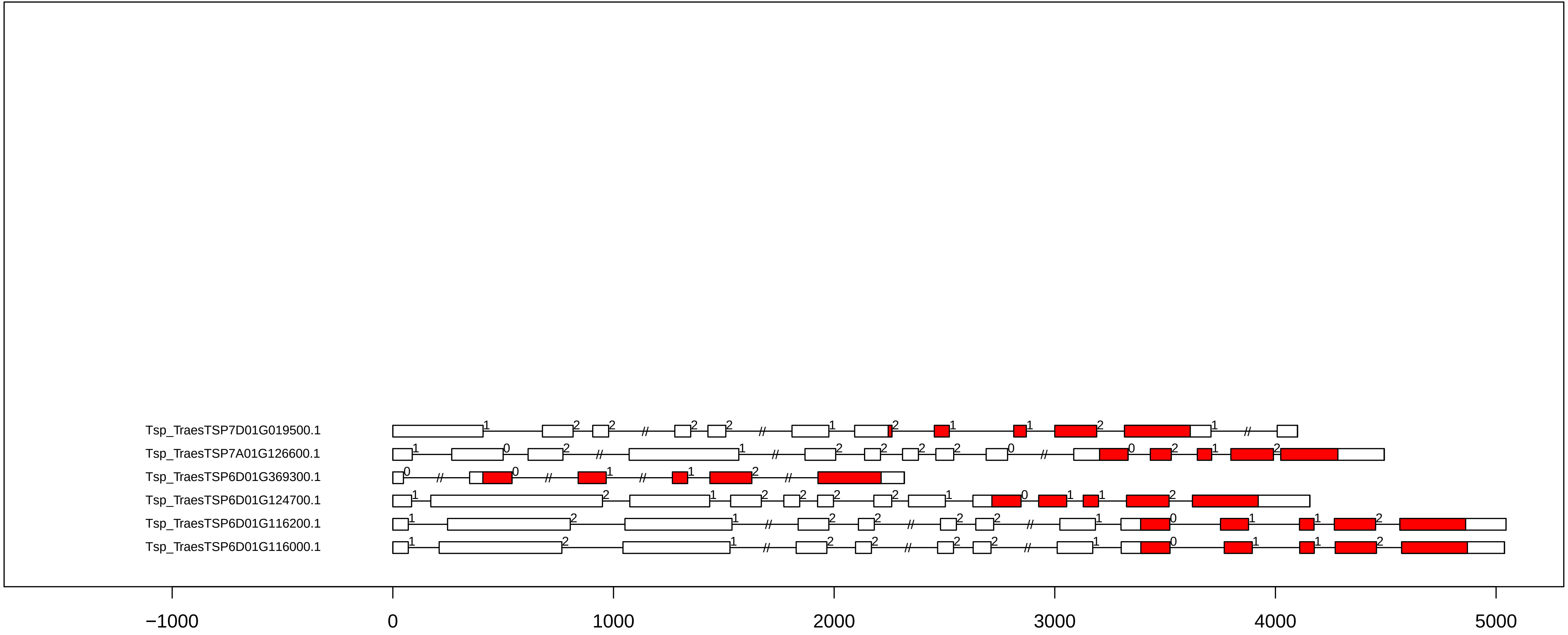

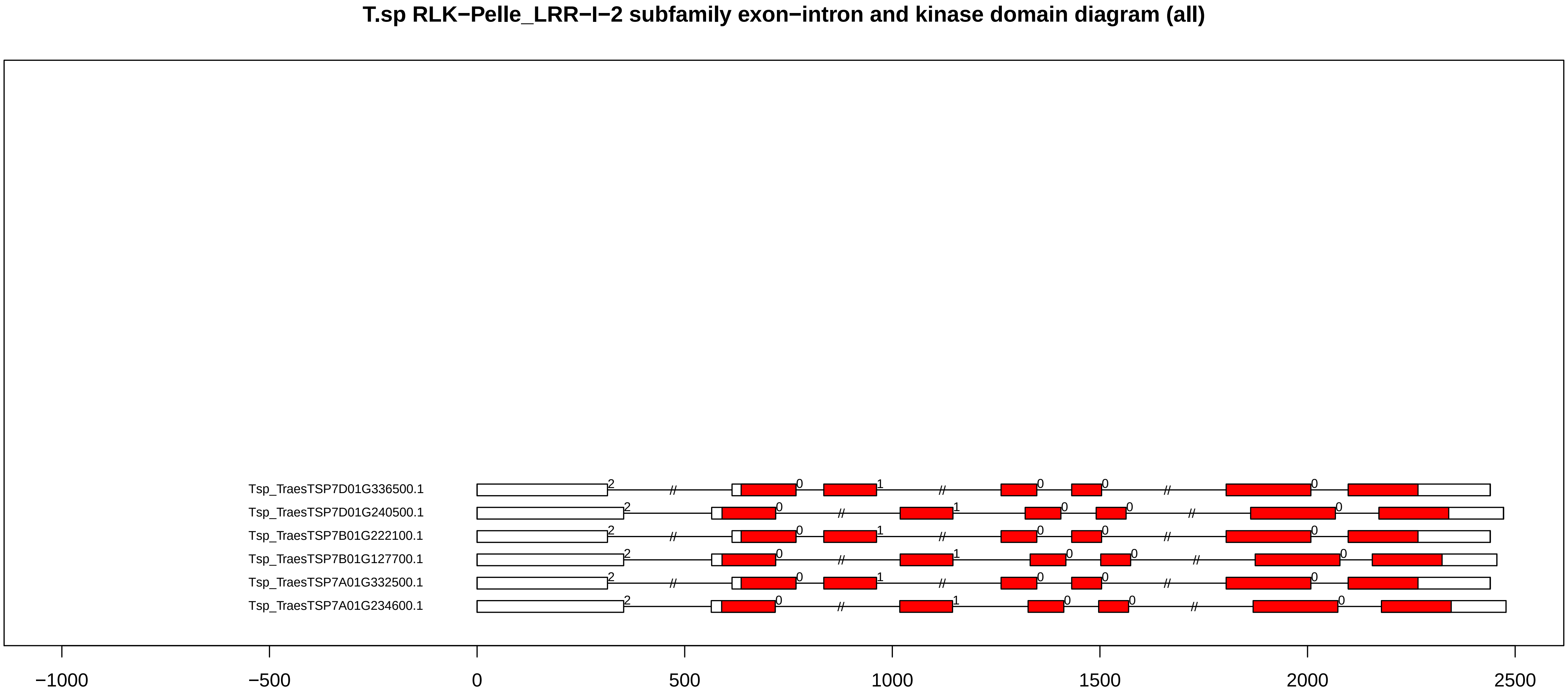

T.sp RLK−Pelle_LRR−I−2 subfamily exon−intron and kinase domain diagram (all)
Tsp_TraesTSP7D01G336500.1
Tsp_TraesTSP7D01G240500.1
Tsp_TraesTSP7B01G222100.1
Tsp_TraesTSP7B01G127700.1
Tsp_TraesTSP7A01G332500.1
Tsp_TraesTSP7A01G234600.1
−1000
−500
0
500
1000
1500
2000
2500

T.sp RLK−Pelle_LRR−III subfamily exon−intron and kinase domain diagram (part 1)

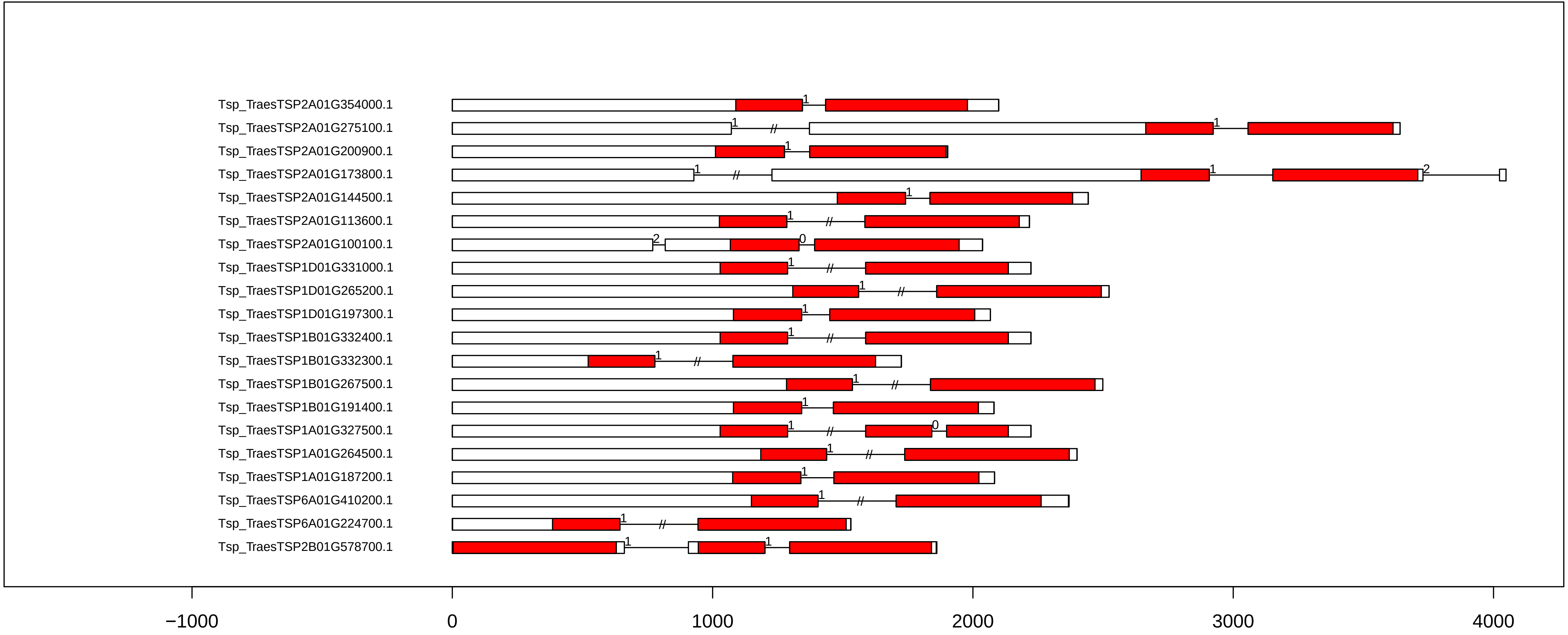

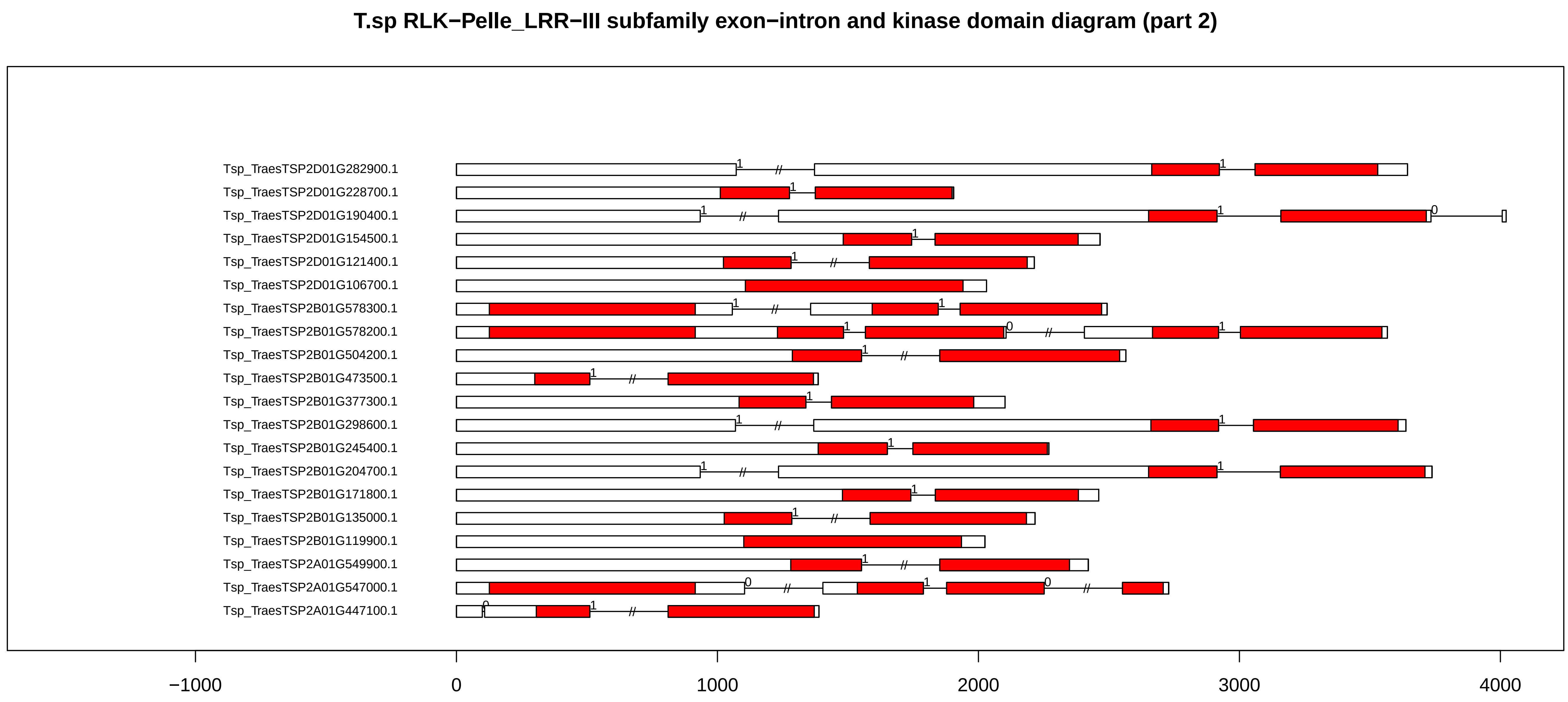
T.sp RLK−Pelle_LRR−III subfamily exon−intron and kinase domain diagram (part 2)
Tsp_TraesTSP2D01G282900.1
Tsp_TraesTSP2D01G228700.1
Tsp_TraesTSP2D01G190400.1
Tsp_TraesTSP2D01G154500.1
Tsp_TraesTSP2D01G121400.1
Tsp_TraesTSP2D01G106700.1
Tsp_TraesTSP2B01G578300.1
Tsp_TraesTSP2B01G578200.1
Tsp_TraesTSP2B01G504200.1
Tsp_TraesTSP2B01G473500.1
Tsp_TraesTSP2B01G377300.1
Tsp_TraesTSP2B01G298600.1
Tsp_TraesTSP2B01G245400.1
Tsp_TraesTSP2B01G204700.1
Tsp_TraesTSP2B01G171800.1
Tsp_TraesTSP2B01G135000.1
Tsp_TraesTSP2B01G119900.1
Tsp_TraesTSP2A01G549900.1
Tsp_TraesTSP2A01G547000.1
Tsp_TraesTSP2A01G447100.1
−1000
0
1000
2000
3000
4000

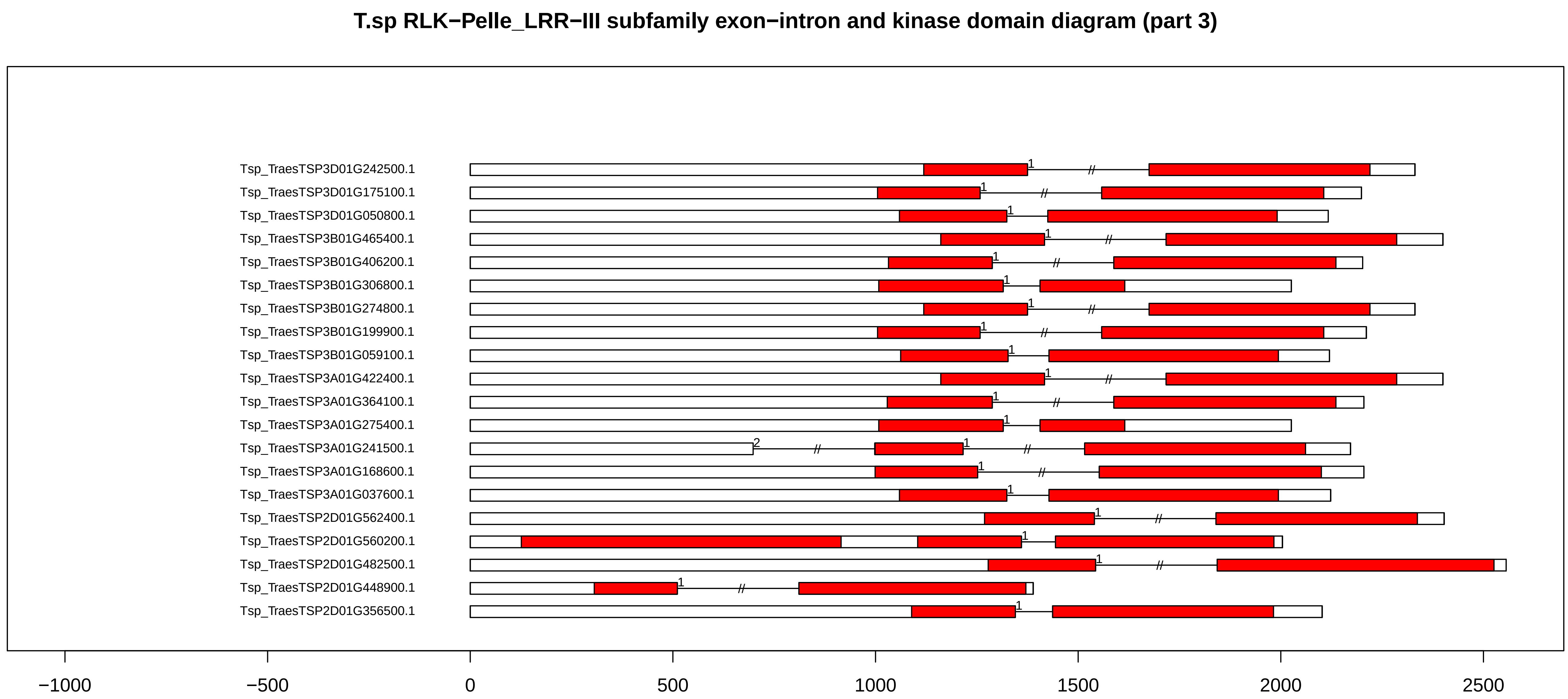

T.sp RLK−Pelle_LRR−III subfamily exon−intron and kinase domain diagram (part 3)
Tsp_TraesTSP3D01G242500.1
Tsp_TraesTSP3D01G175100.1
Tsp_TraesTSP3D01G050800.1
Tsp_TraesTSP3B01G465400.1
Tsp_TraesTSP3B01G406200.1
Tsp_TraesTSP3B01G306800.1
Tsp_TraesTSP3B01G274800.1
Tsp_TraesTSP3B01G199900.1
Tsp_TraesTSP3B01G059100.1
Tsp_TraesTSP3A01G422400.1
Tsp_TraesTSP3A01G364100.1
Tsp_TraesTSP3A01G275400.1
Tsp_TraesTSP3A01G241500.1
Tsp_TraesTSP3A01G168600.1
Tsp_TraesTSP3A01G037600.1
Tsp_TraesTSP2D01G562400.1
Tsp_TraesTSP2D01G560200.1
Tsp_TraesTSP2D01G482500.1
Tsp_TraesTSP2D01G448900.1
Tsp_TraesTSP2D01G356500.1
−1000
−500
0
500
1000
1500
2000
2500

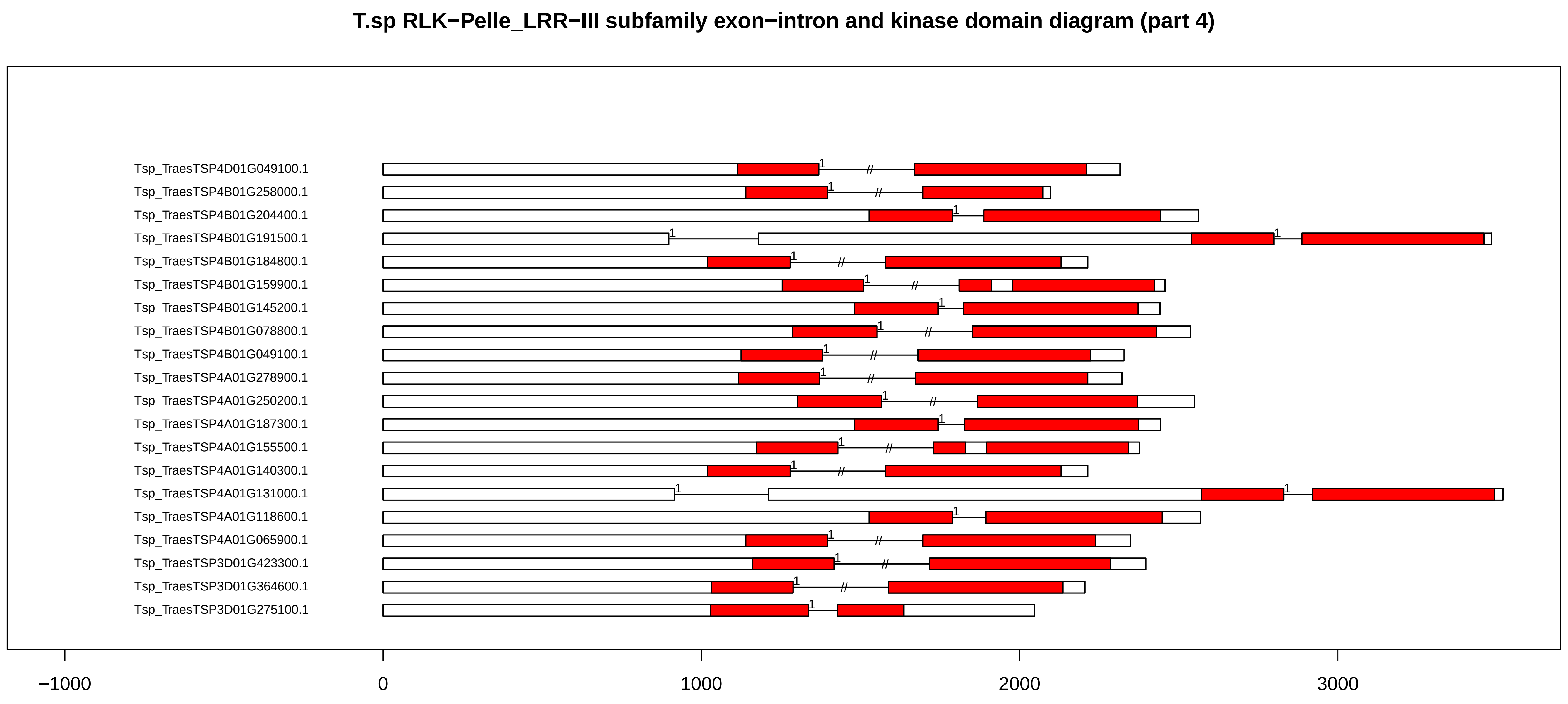

T.sp RLK−Pelle_LRR−III subfamily exon−intron and kinase domain diagram (part 4)
Tsp_TraesTSP4D01G049100.1
Tsp_TraesTSP4B01G258000.1
Tsp_TraesTSP4B01G204400.1
Tsp_TraesTSP4B01G191500.1
Tsp_TraesTSP4B01G184800.1
Tsp_TraesTSP4B01G159900.1
Tsp_TraesTSP4B01G145200.1
Tsp_TraesTSP4B01G078800.1
Tsp_TraesTSP4B01G049100.1
Tsp_TraesTSP4A01G278900.1
Tsp_TraesTSP4A01G250200.1
Tsp_TraesTSP4A01G187300.1
Tsp_TraesTSP4A01G155500.1
Tsp_TraesTSP4A01G140300.1
Tsp_TraesTSP4A01G131000.1
Tsp_TraesTSP4A01G118600.1
Tsp_TraesTSP4A01G065900.1
Tsp_TraesTSP3D01G423300.1
Tsp_TraesTSP3D01G364600.1
Tsp_TraesTSP3D01G275100.1
−1000
0
1000
2000
3000

T.sp RLK−Pelle_LRR−III subfamily exon−intron and kinase domain diagram (part 5)

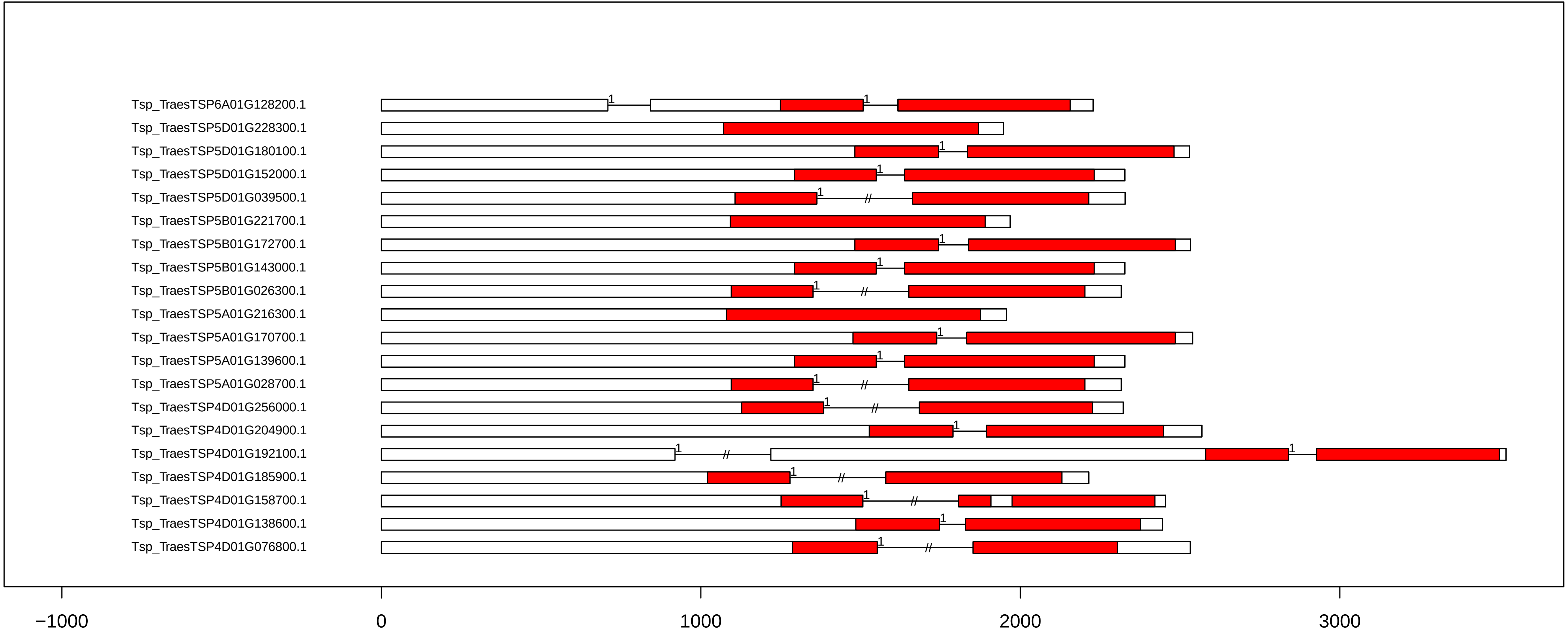

T.sp RLK−Pelle_LRR−III subfamily exon−intron and kinase domain diagram (part 6)

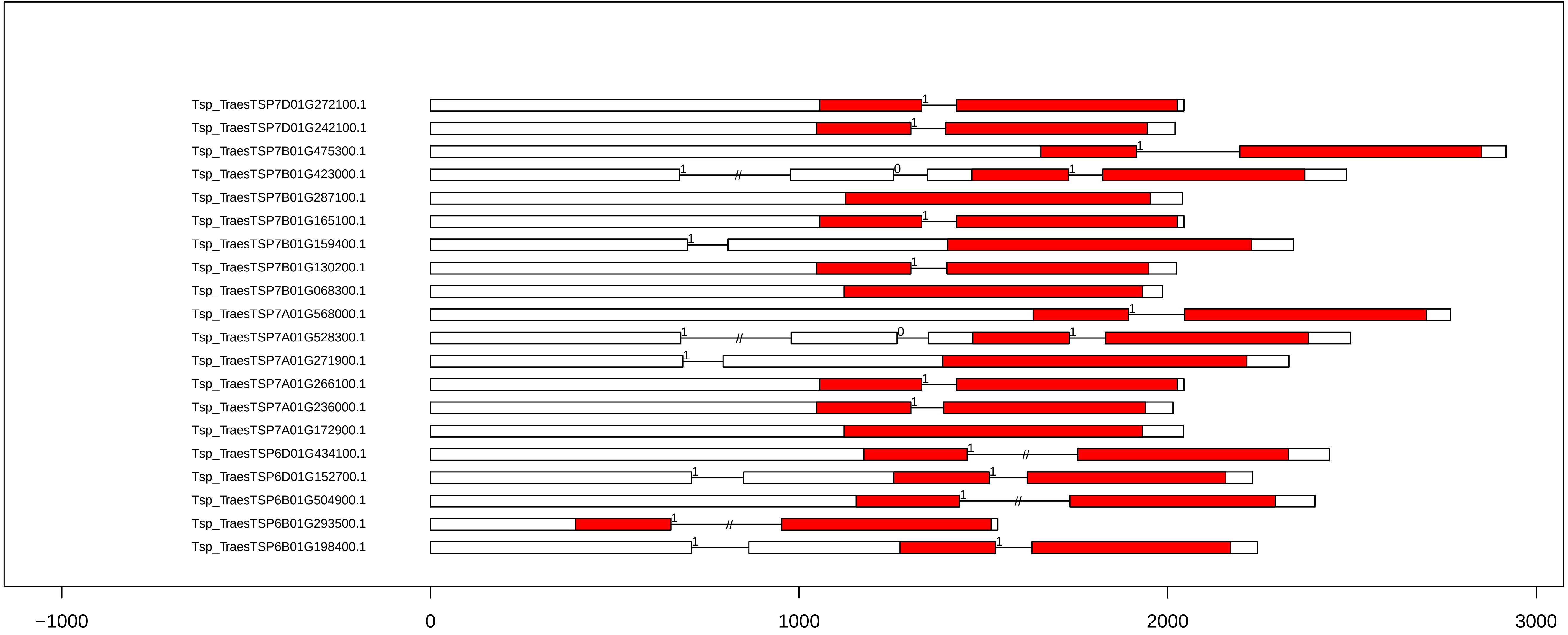

T.sp RLK−Pelle_LRR−III subfamily exon−intron and kinase domain diagram (part 7)

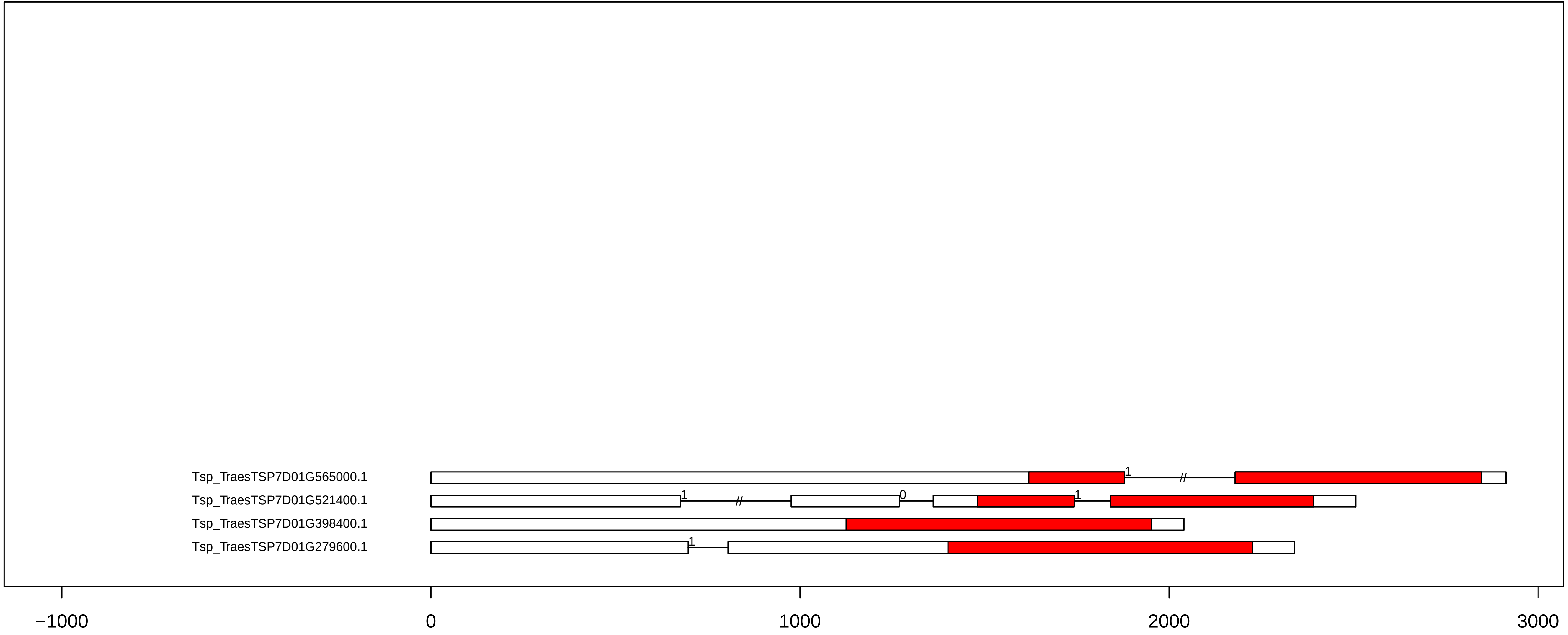

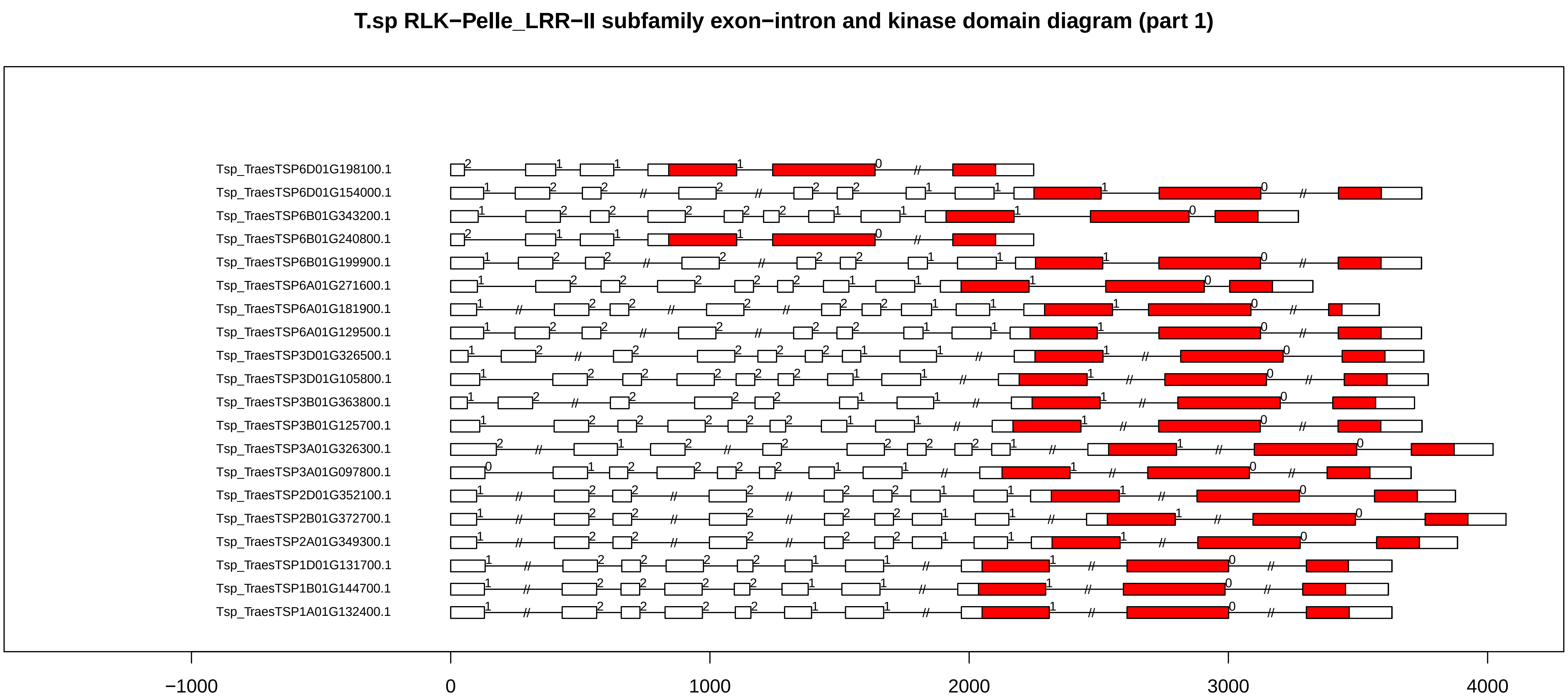
T.sp RLK−Pelle_LRR−II subfamily exon−intron and kinase domain diagram (part 1)
Tsp_TraesTSP6D01G198100.1
Tsp_TraesTSP6D01G154000.1
Tsp_TraesTSP6B01G343200.1
Tsp_TraesTSP6B01G240800.1
Tsp_TraesTSP6B01G199900.1
Tsp_TraesTSP6A01G271600.1
Tsp_TraesTSP6A01G181900.1
Tsp_TraesTSP6A01G129500.1
Tsp_TraesTSP3D01G326500.1
Tsp_TraesTSP3D01G105800.1
Tsp_TraesTSP3B01G363800.1
Tsp_TraesTSP3B01G125700.1
Tsp_TraesTSP3A01G326300.1
Tsp_TraesTSP3A01G097800.1
Tsp_TraesTSP2D01G352100.1
Tsp_TraesTSP2B01G372700.1
Tsp_TraesTSP2A01G349300.1
Tsp_TraesTSP1D01G131700.1
Tsp_TraesTSP1B01G144700.1
Tsp_TraesTSP1A01G132400.1
−1000
0
1000
2000
3000
4000

T.sp RLK−Pelle_LRR−II subfamily exon−intron and kinase domain diagram (part 2)

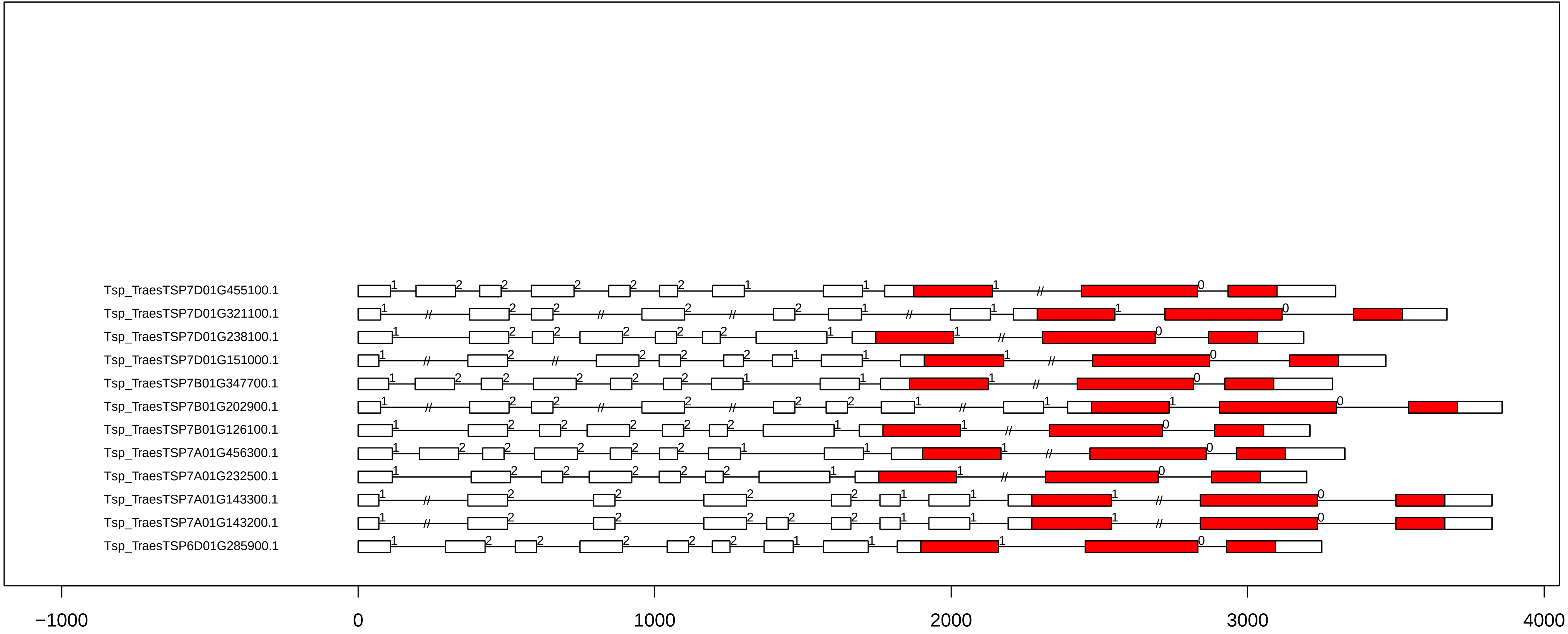

T.sp RLK−Pelle_LRR−IV subfamily exon−intron and kinase domain diagram (all)

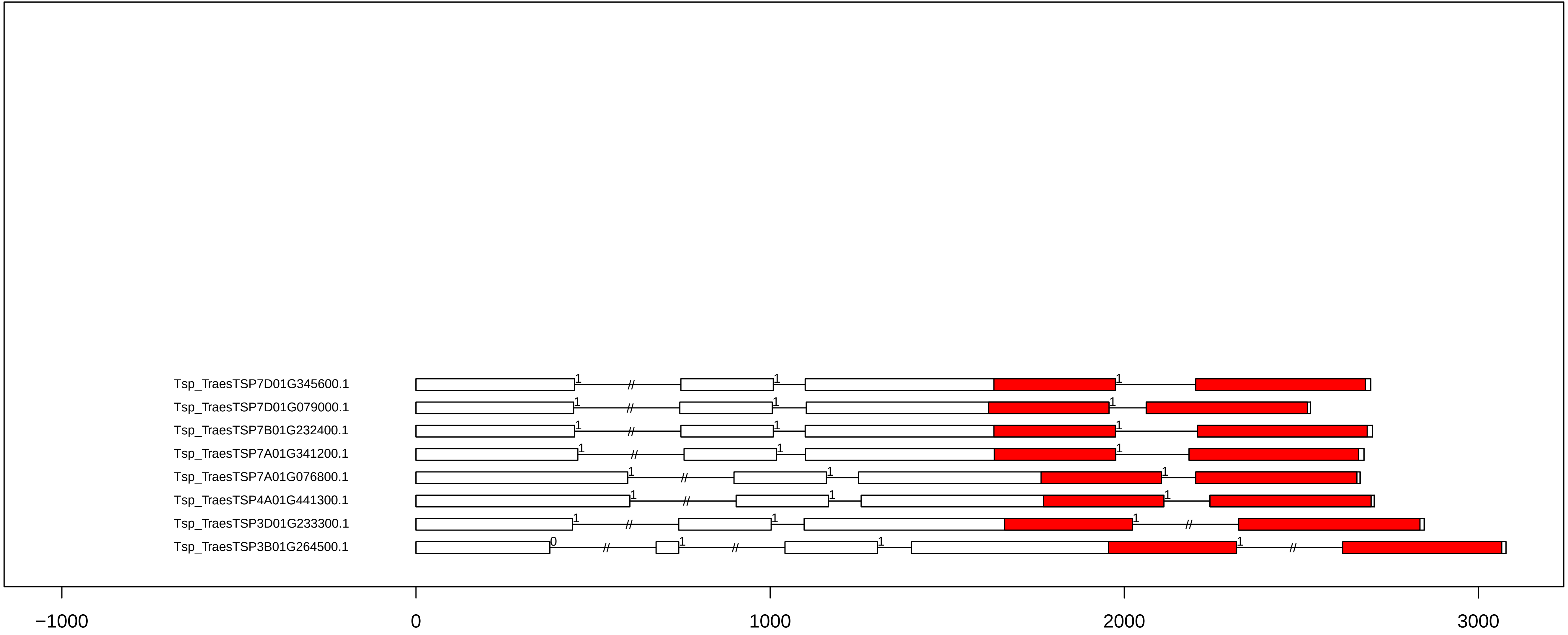

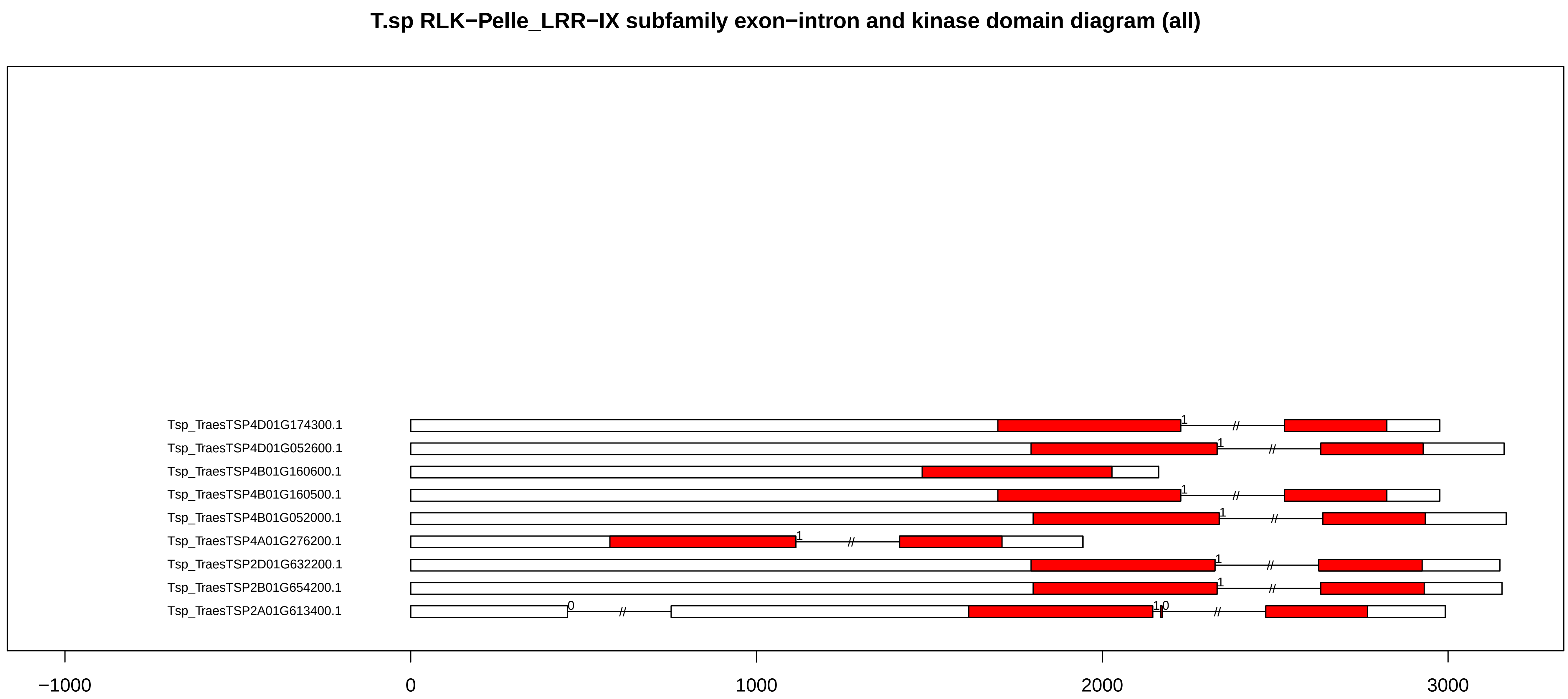

T.sp RLK−Pelle_LRR−IX subfamily exon−intron and kinase domain diagram (all)
Tsp_TraesTSP4D01G174300.1
Tsp_TraesTSP4D01G052600.1
Tsp_TraesTSP4B01G160600.1
Tsp_TraesTSP4B01G160500.1
Tsp_TraesTSP4B01G052000.1
Tsp_TraesTSP4A01G276200.1
Tsp_TraesTSP2D01G632200.1
Tsp_TraesTSP2B01G654200.1
Tsp_TraesTSP2A01G613400.1
−1000
0
1000
2000
3000

T.sp RLK−Pelle_LRR−VI−1 subfamily exon−intron and kinase domain diagram (all)

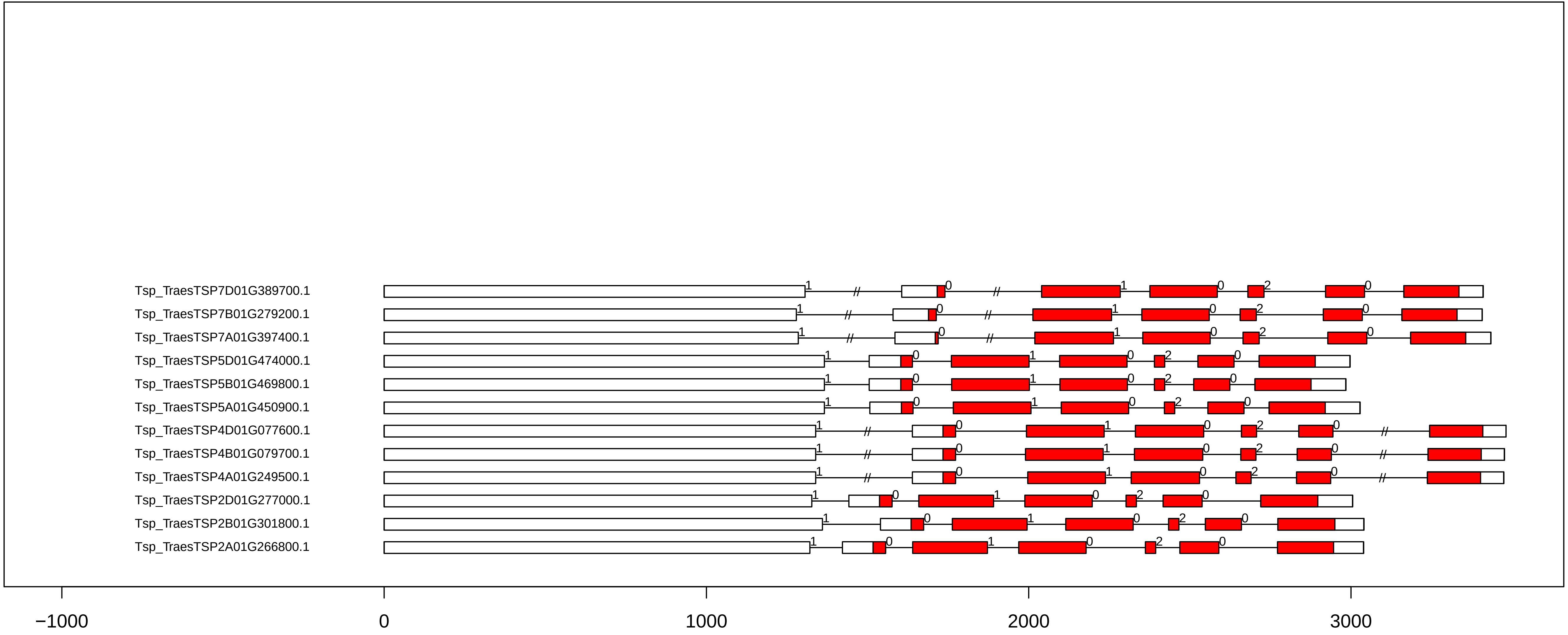

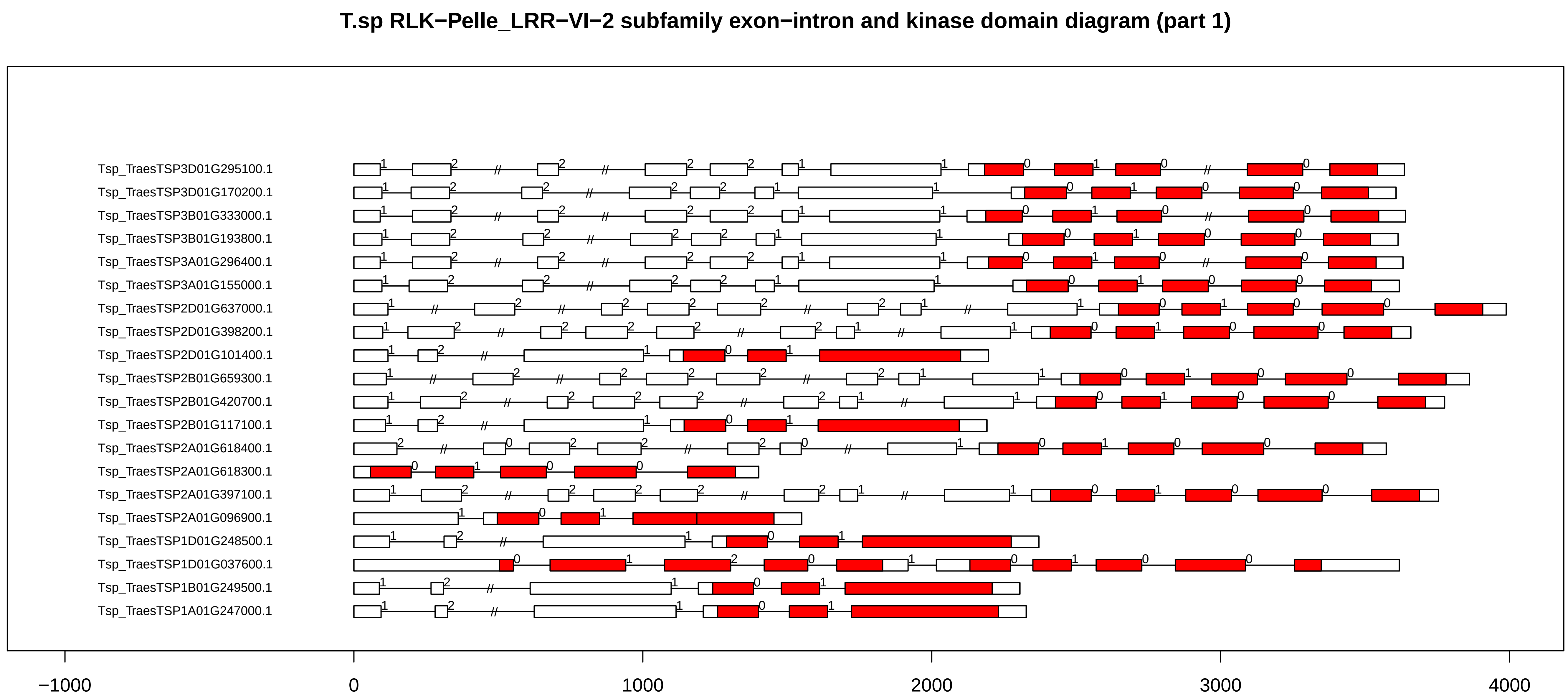

T.sp RLK−Pelle_LRR−VI−2 subfamily exon−intron and kinase domain diagram (part 1)
Tsp_TraesTSP3D01G295100.1
Tsp_TraesTSP3D01G170200.1
Tsp_TraesTSP3B01G333000.1
Tsp_TraesTSP3B01G193800.1
Tsp_TraesTSP3A01G296400.1
Tsp_TraesTSP3A01G155000.1
Tsp_TraesTSP2D01G637000.1
Tsp_TraesTSP2D01G398200.1
Tsp_TraesTSP2D01G101400.1
Tsp_TraesTSP2B01G659300.1
Tsp_TraesTSP2B01G420700.1
Tsp_TraesTSP2B01G117100.1
Tsp_TraesTSP2A01G618400.1
Tsp_TraesTSP2A01G618300.1
Tsp_TraesTSP2A01G397100.1
Tsp_TraesTSP2A01G096900.1
Tsp_TraesTSP1D01G248500.1
Tsp_TraesTSP1D01G037600.1
Tsp_TraesTSP1B01G249500.1
Tsp_TraesTSP1A01G247000.1
−1000
0
1000
2000
3000
4000

T.sp RLK−Pelle_LRR−VI−2 subfamily exon−intron and kinase domain diagram (part 2)

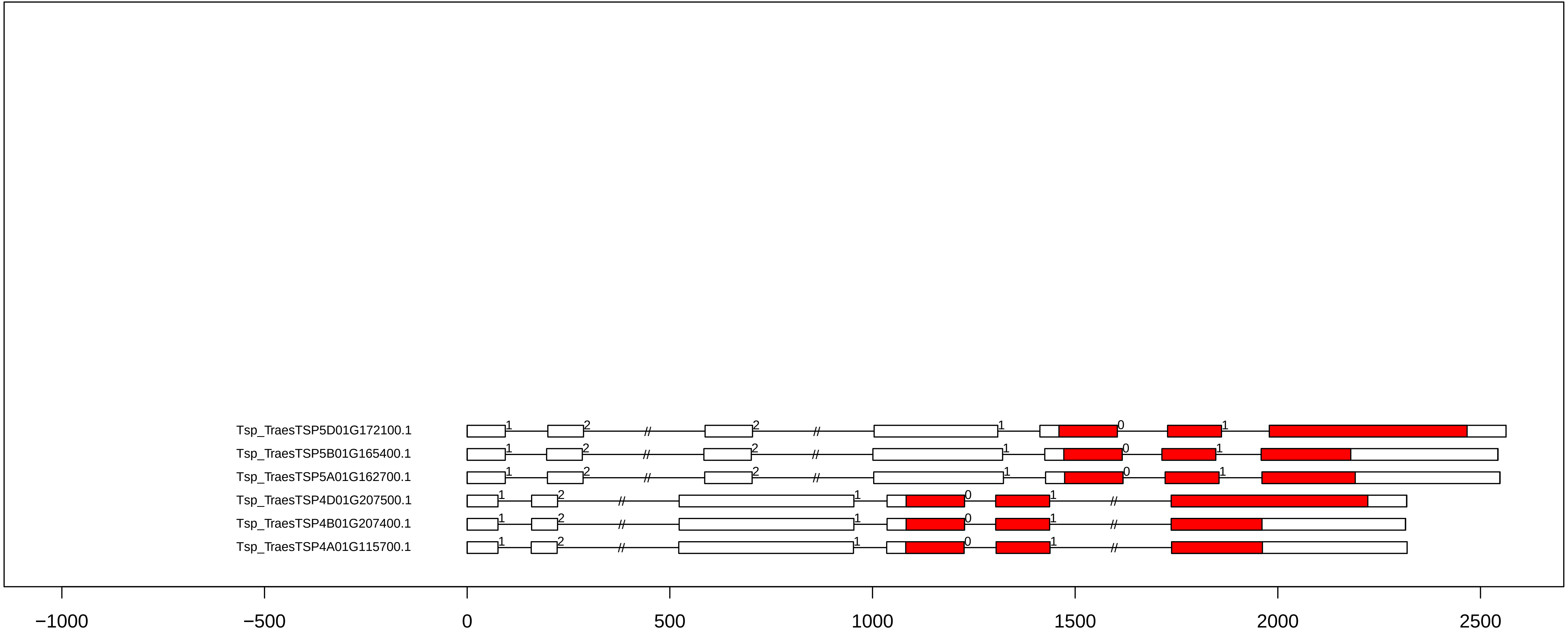

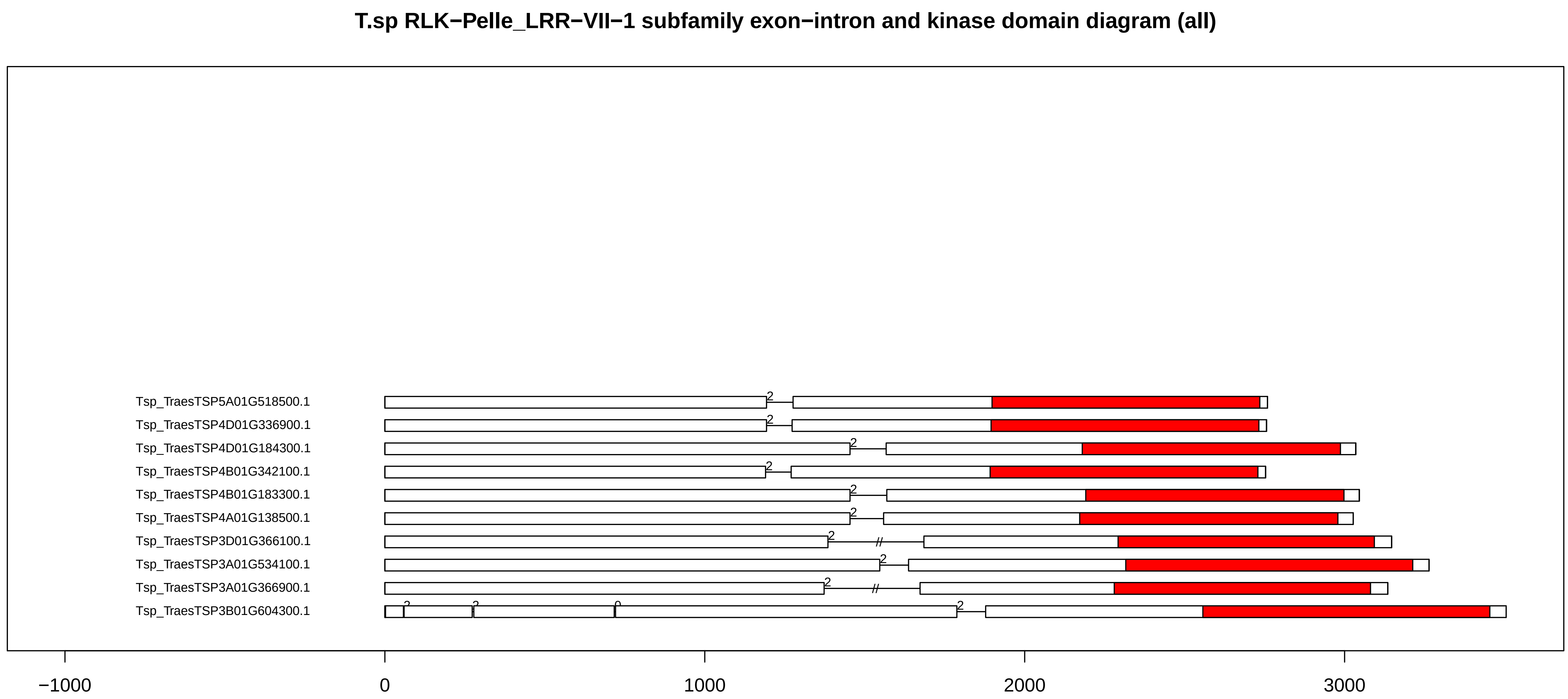
T.sp RLK−Pelle_LRR−VII−1 subfamily exon−intron and kinase domain diagram (all)
Tsp_TraesTSP5A01G518500.1
Tsp_TraesTSP4D01G336900.1
Tsp_TraesTSP4D01G184300.1
Tsp_TraesTSP4B01G342100.1
Tsp_TraesTSP4B01G183300.1
Tsp_TraesTSP4A01G138500.1
Tsp_TraesTSP3D01G366100.1
Tsp_TraesTSP3A01G534100.1
Tsp_TraesTSP3A01G366900.1
Tsp_TraesTSP3B01G604300.1
−1000
0
1000
2000
3000

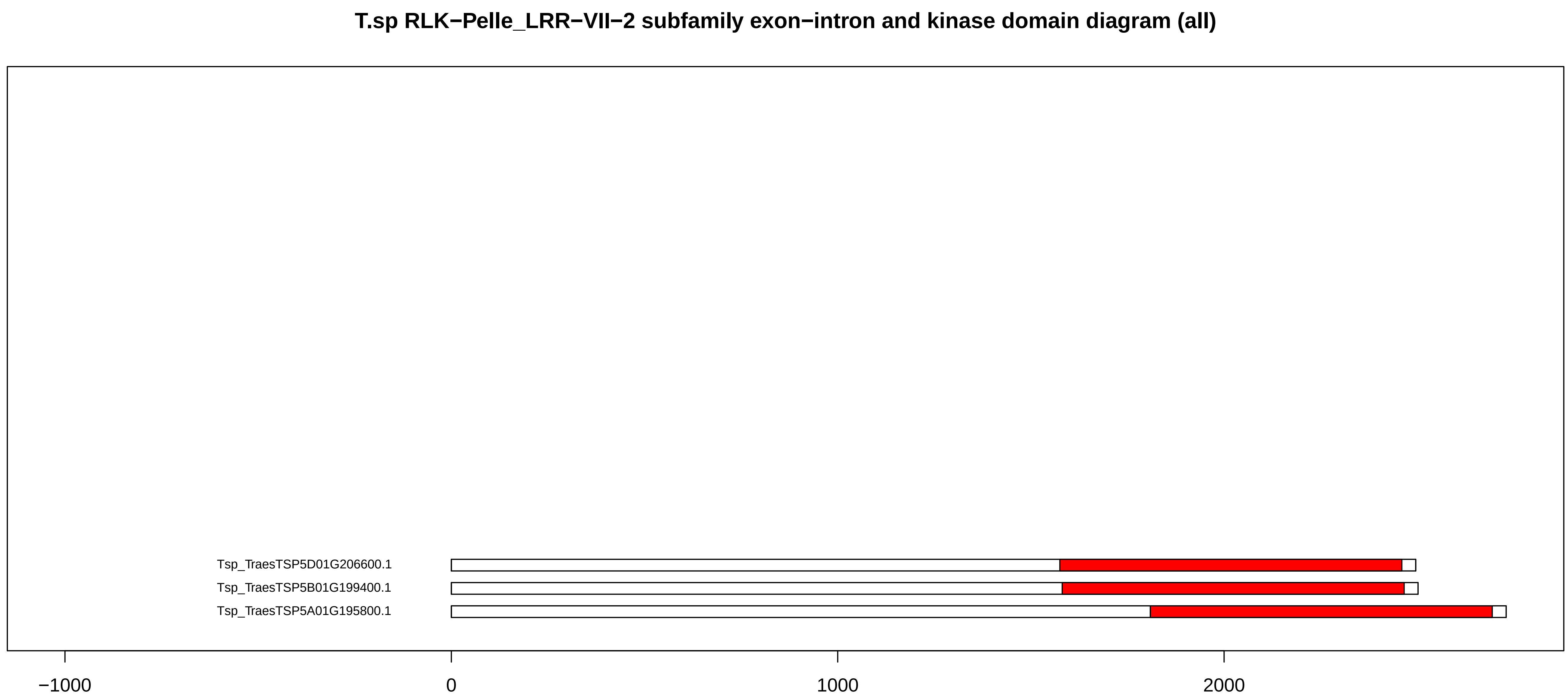
T.sp RLK−Pelle_LRR−VII−2 subfamily exon−intron and kinase domain diagram (all)
Tsp_TraesTSP5D01G206600.1
Tsp_TraesTSP5B01G199400.1
Tsp_TraesTSP5A01G195800.1
−1000
0
1000
2000

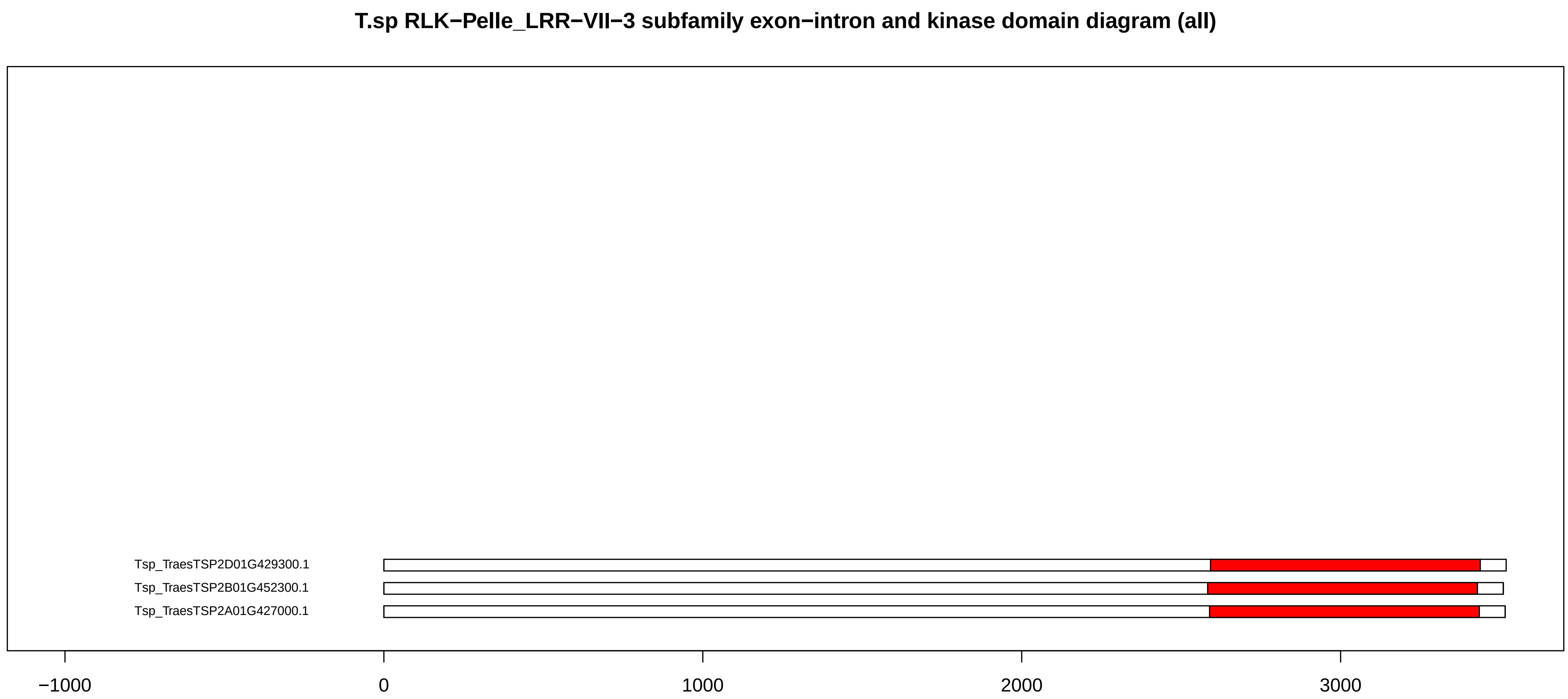
T.sp RLK−Pelle_LRR−VII−3 subfamily exon−intron and kinase domain diagram (all)
Tsp_TraesTSP2D01G429300.1
Tsp_TraesTSP2B01G452300.1
Tsp_TraesTSP2A01G427000.1
−1000
0
1000
2000
3000

T.sp RLK−Pelle_LRR−VIII−1 subfamily exon−intron and kinase domain diagram (part 1)

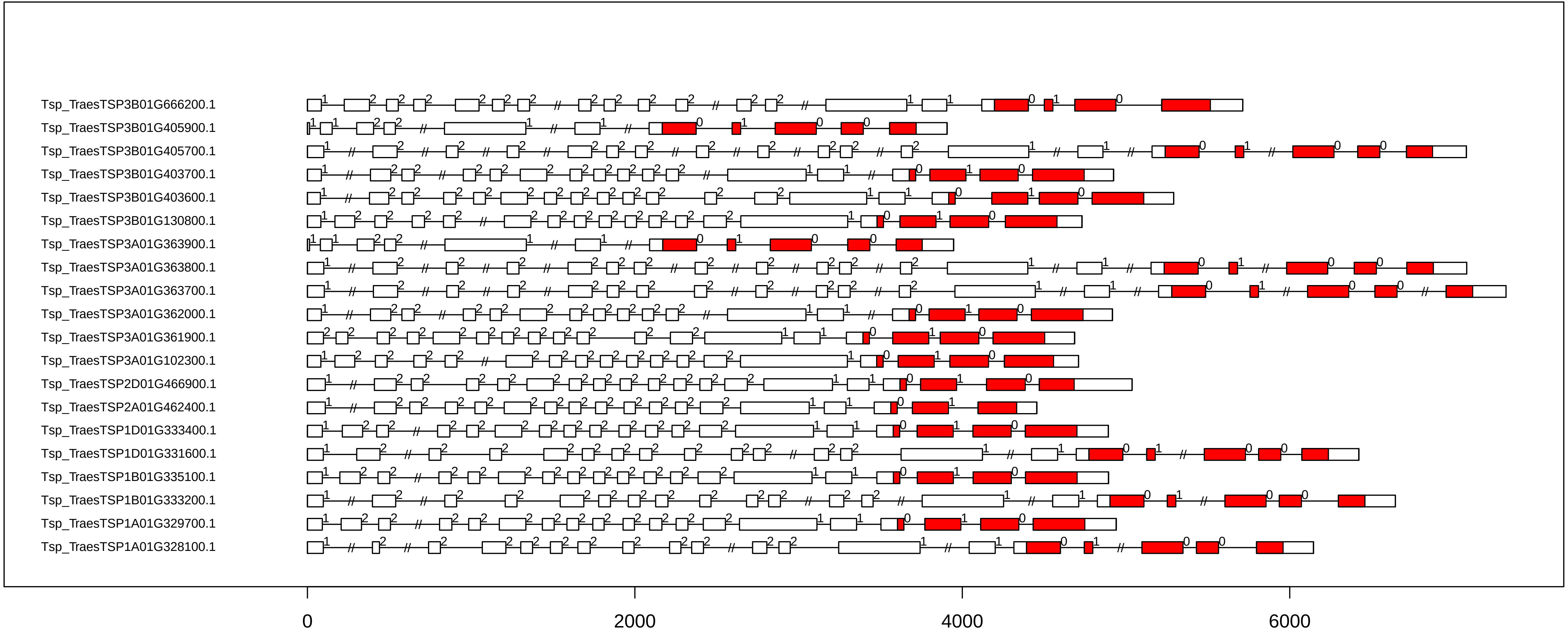

T.sp RLK−Pelle_LRR−VIII−1 subfamily exon−intron and kinase domain diagram (part 2)

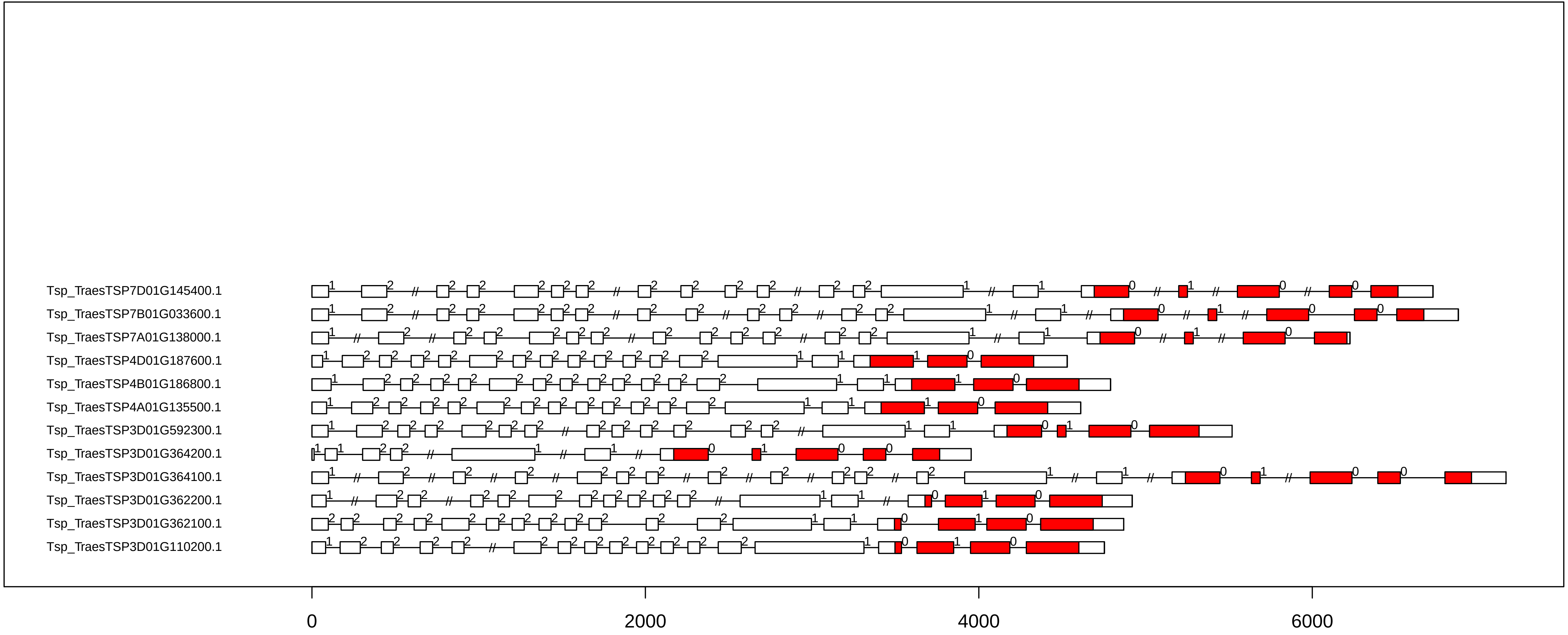

T.sp RLK−Pelle_LRR−V subfamily exon−intron and kinase domain diagram (part 1)

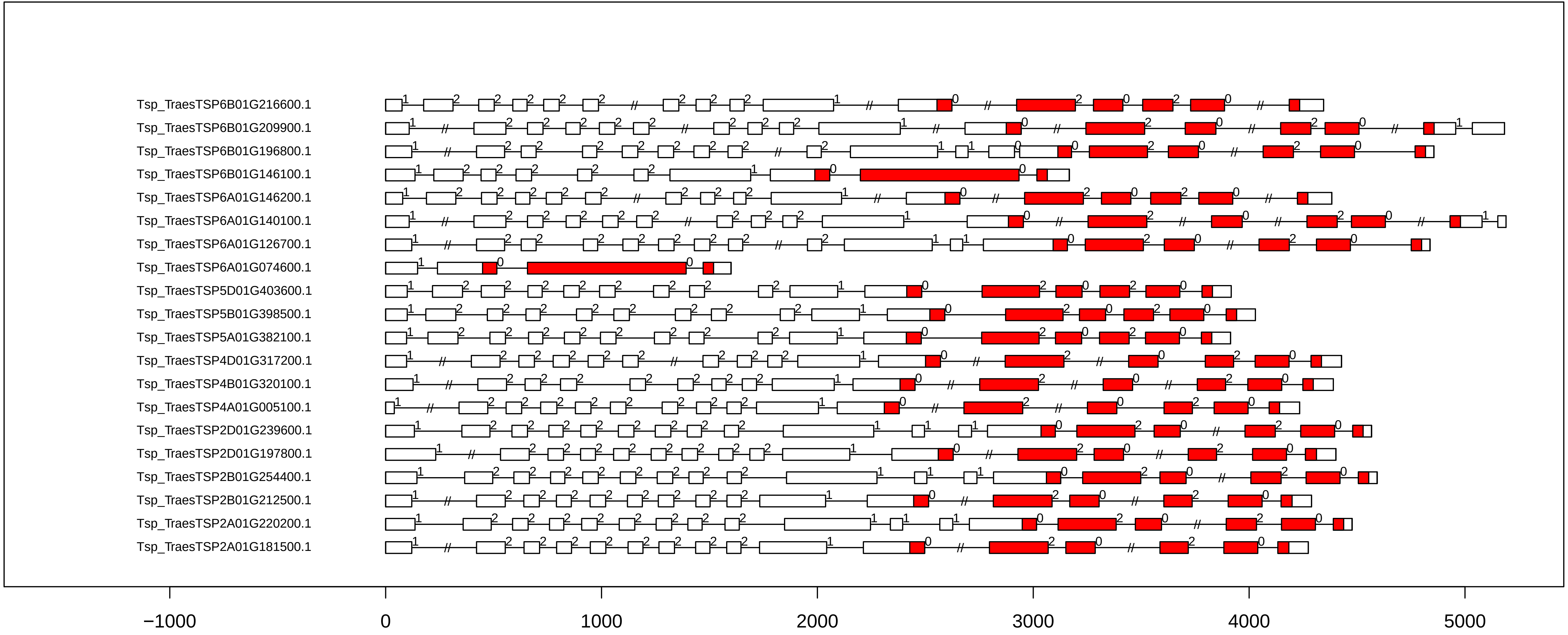

T.sp RLK−Pelle_LRR−V subfamily exon−intron and kinase domain diagram (part 2)

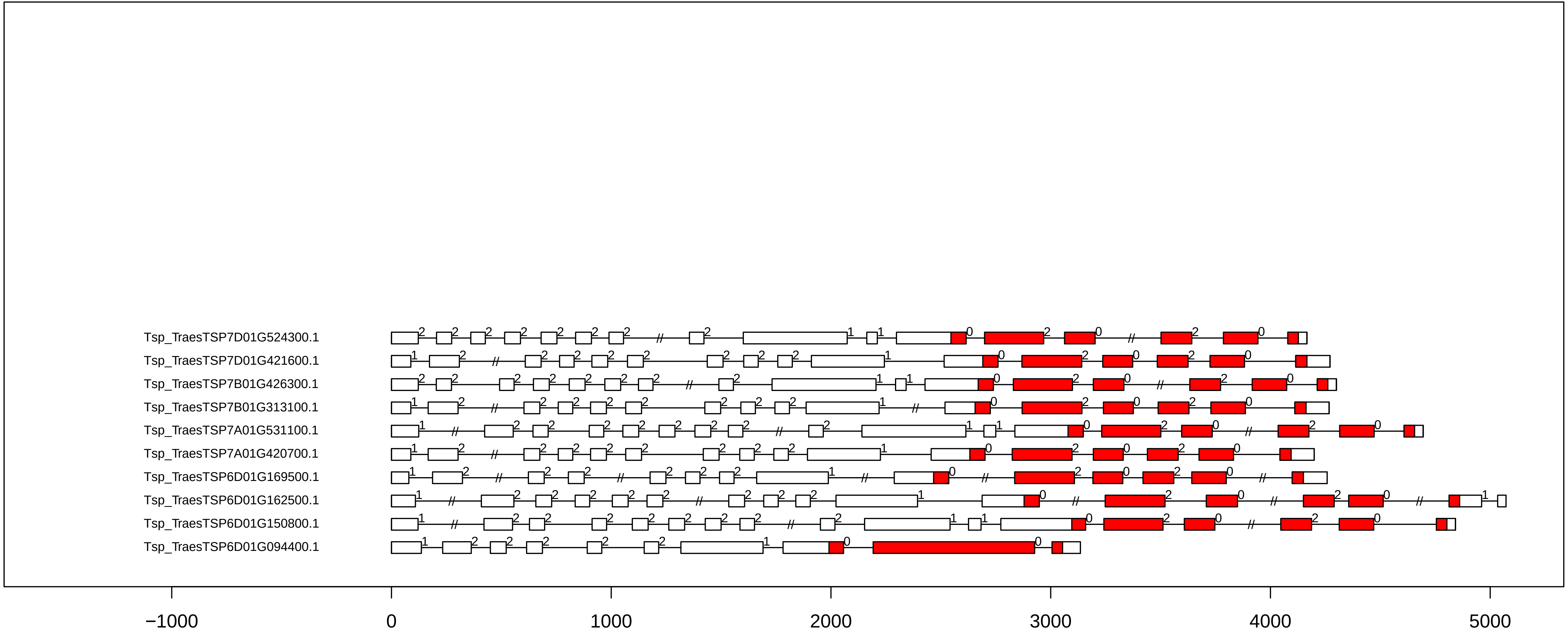

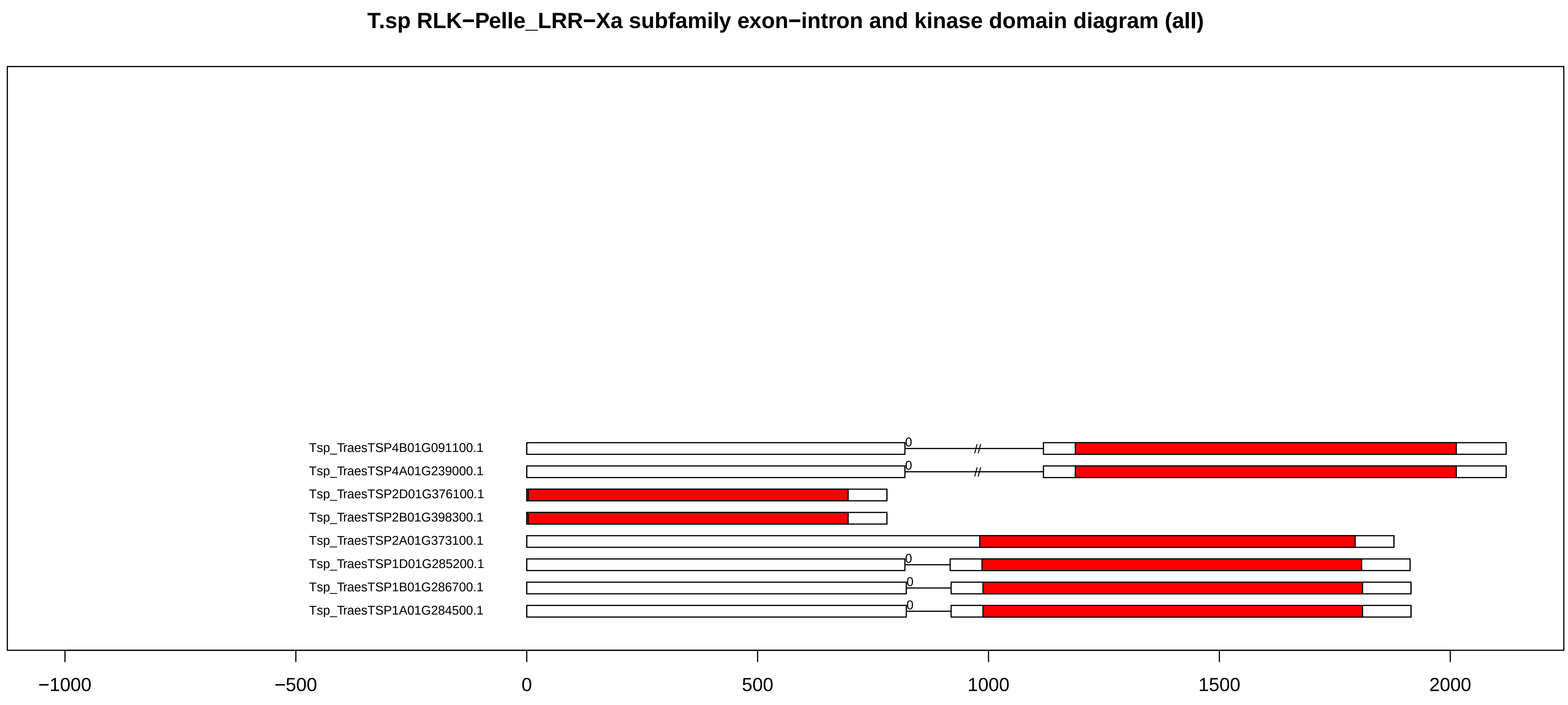

T.sp RLK−Pelle_LRR−Xa subfamily exon−intron and kinase domain diagram (all)
Tsp_TraesTSP4B01G091100.1
Tsp_TraesTSP4A01G239000.1
Tsp_TraesTSP2D01G376100.1
Tsp_TraesTSP2B01G398300.1
Tsp_TraesTSP2A01G373100.1
Tsp_TraesTSP1D01G285200.1
Tsp_TraesTSP1B01G286700.1
Tsp_TraesTSP1A01G284500.1
0
0
0
0
0
−1000
−500
0
500
1000
1500
2000

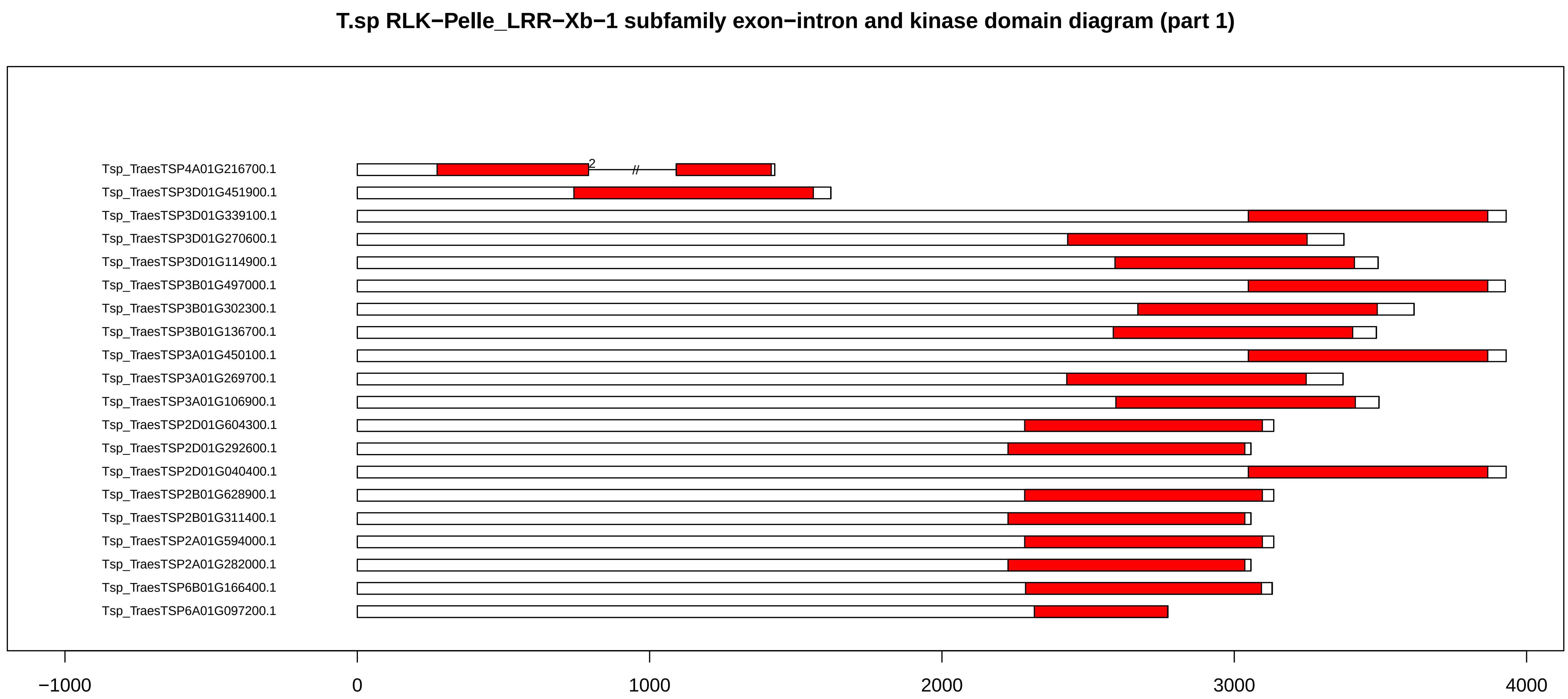

T.sp RLK−Pelle_LRR−Xb−1 subfamily exon−intron and kinase domain diagram (part 1)
Tsp_TraesTSP4A01G216700.1
2
//
Tsp_TraesTSP3D01G451900.1
Tsp_TraesTSP3D01G339100.1
Tsp_TraesTSP3D01G270600.1
Tsp_TraesTSP3D01G114900.1
Tsp_TraesTSP3B01G497000.1
Tsp_TraesTSP3B01G302300.1
Tsp_TraesTSP3B01G136700.1
Tsp_TraesTSP3A01G450100.1
Tsp_TraesTSP3A01G269700.1
Tsp_TraesTSP3A01G106900.1
Tsp_TraesTSP2D01G604300.1
Tsp_TraesTSP2D01G292600.1
Tsp_TraesTSP2D01G040400.1
Tsp_TraesTSP2B01G628900.1
Tsp_TraesTSP2B01G311400.1
Tsp_TraesTSP2A01G594000.1
Tsp_TraesTSP2A01G282000.1
Tsp_TraesTSP6B01G166400.1
Tsp_TraesTSP6A01G097200.1
−1000
0
1000
2000
3000
4000

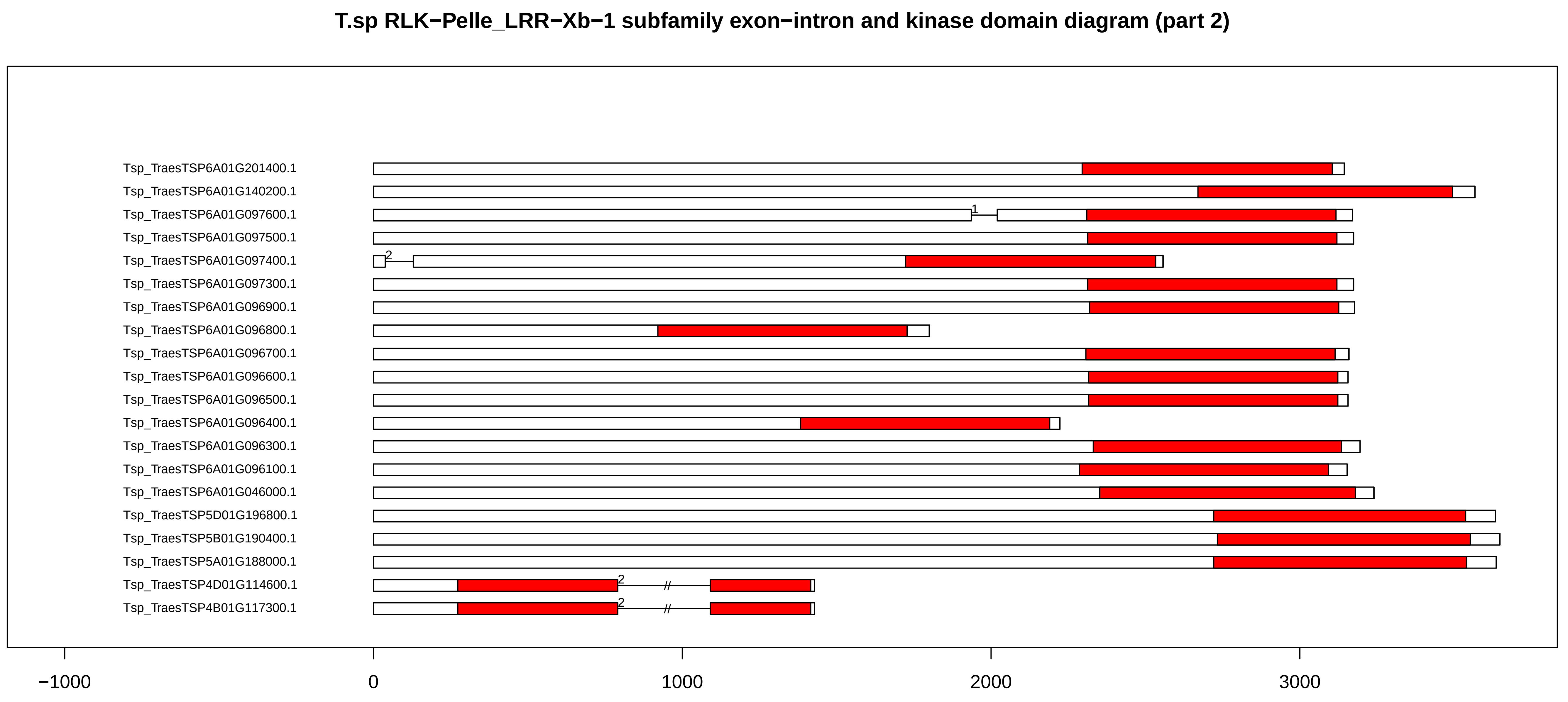
T.sp RLK−Pelle_LRR−Xb−1 subfamily exon−intron and kinase domain diagram (part 2)
Tsp_TraesTSP6A01G201400.1
Tsp_TraesTSP6A01G140200.1
Tsp_TraesTSP6A01G097600.1
Tsp_TraesTSP6A01G097500.1
Tsp_TraesTSP6A01G097400.1
Tsp_TraesTSP6A01G097300.1
Tsp_TraesTSP6A01G096900.1
Tsp_TraesTSP6A01G096800.1
Tsp_TraesTSP6A01G096700.1
Tsp_TraesTSP6A01G096600.1
Tsp_TraesTSP6A01G096500.1
Tsp_TraesTSP6A01G096400.1
Tsp_TraesTSP6A01G096300.1
Tsp_TraesTSP6A01G096100.1
Tsp_TraesTSP6A01G046000.1
Tsp_TraesTSP5D01G196800.1
Tsp_TraesTSP5B01G190400.1
Tsp_TraesTSP5A01G188000.1
Tsp_TraesTSP4D01G114600.1
Tsp_TraesTSP4B01G117300.1
1
2
2
2
−1000
0
1000
2000
3000

T.sp RLK−Pelle_LRR−Xb−1 subfamily exon−intron and kinase domain diagram (part 3)

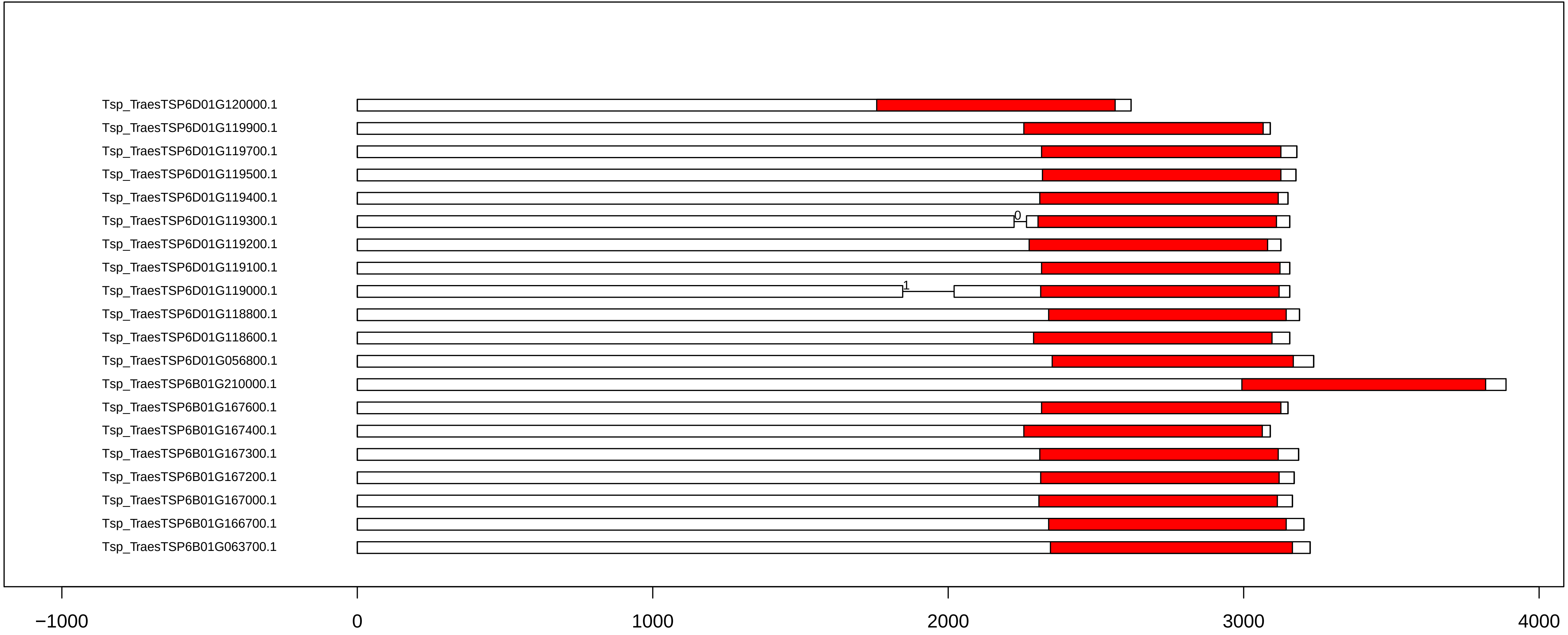

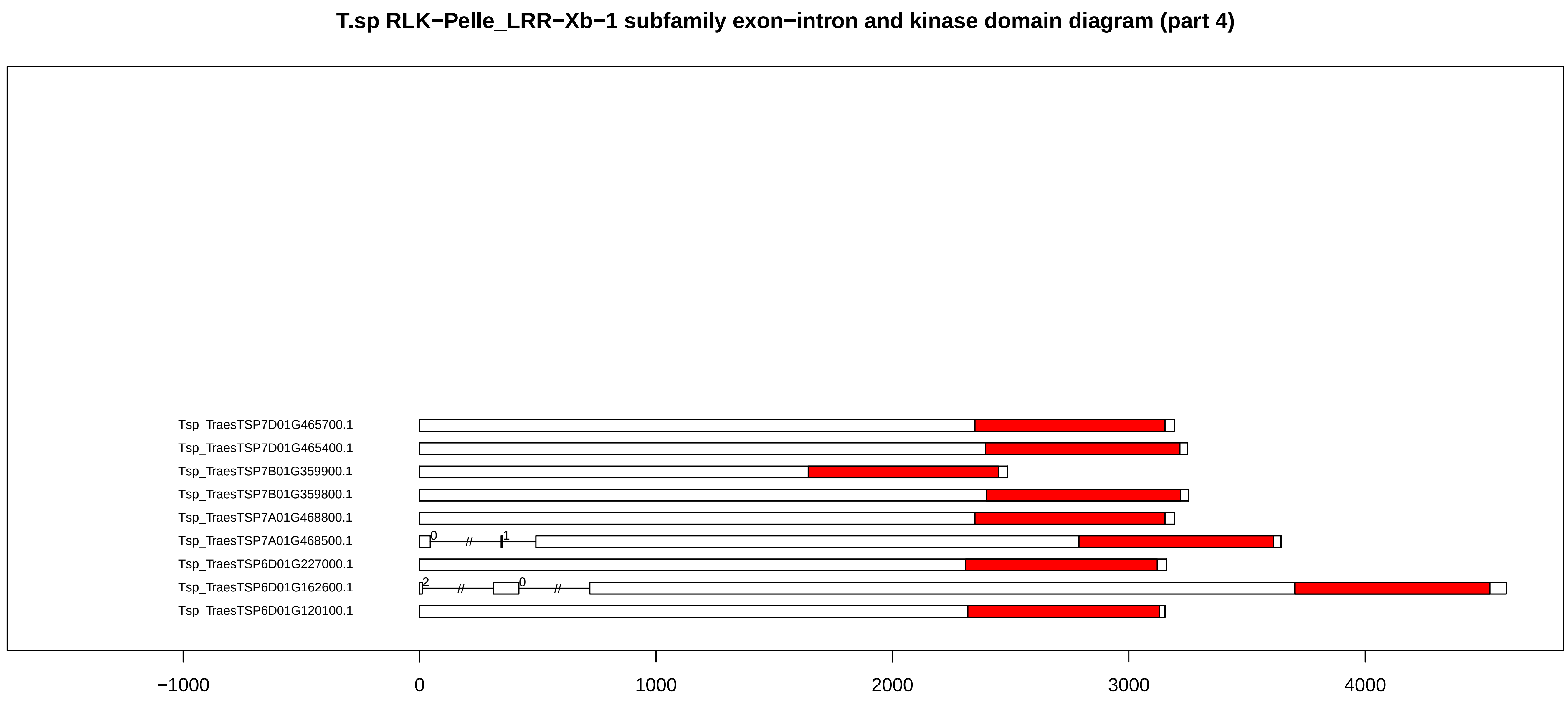
T.sp RLK−Pelle_LRR−Xb−1 subfamily exon−intron and kinase domain diagram (part 4)
Tsp_TraesTSP7D01G465700.1
Tsp_TraesTSP7D01G465400.1
Tsp_TraesTSP7B01G359900.1
Tsp_TraesTSP7B01G359800.1
Tsp_TraesTSP7A01G468800.1
Tsp_TraesTSP7A01G468500.1
Tsp_TraesTSP6D01G227000.1
Tsp_TraesTSP6D01G162600.1
Tsp_TraesTSP6D01G120100.1
0
1
2
0
−1000
0
1000
2000
3000
4000

T.sp RLK−Pelle_LRR−Xb−2 subfamily exon−intron and kinase domain diagram (all)

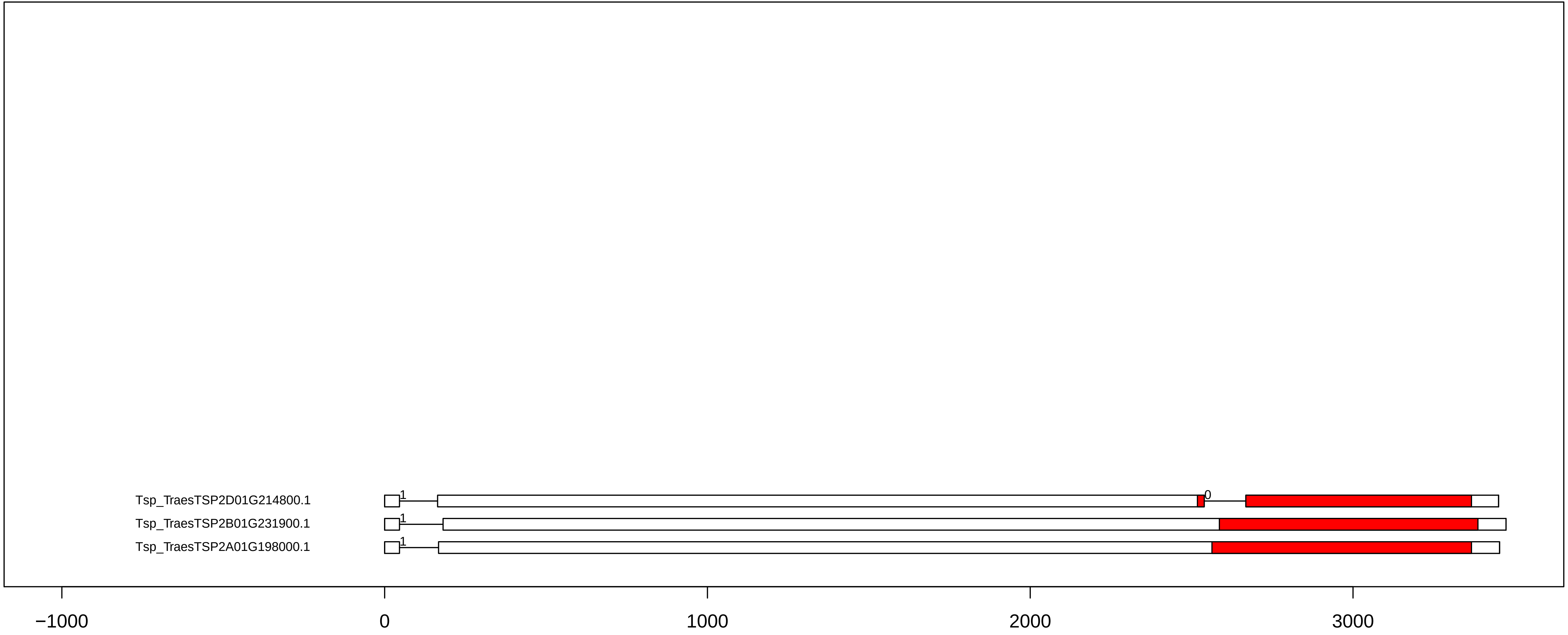

T.sp RLK−Pelle_LRR−XI−1 subfamily exon−intron and kinase domain diagram (part 1)

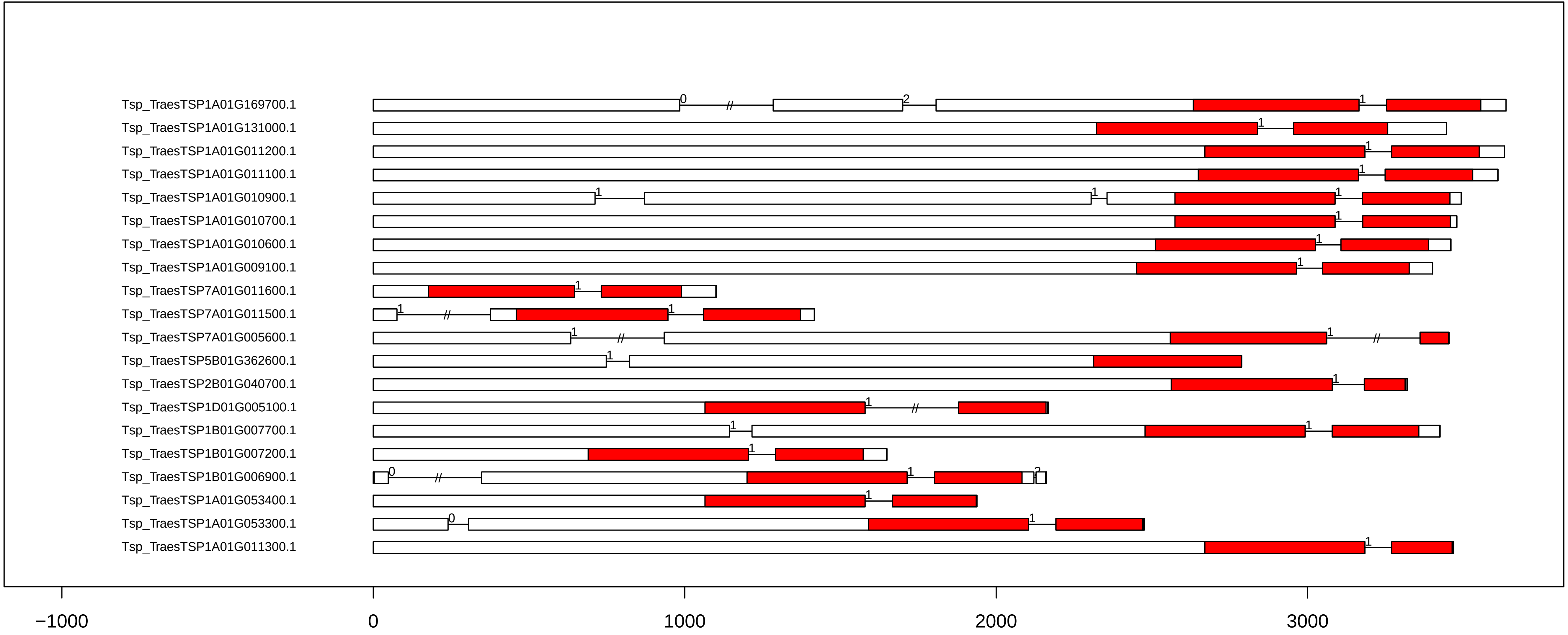

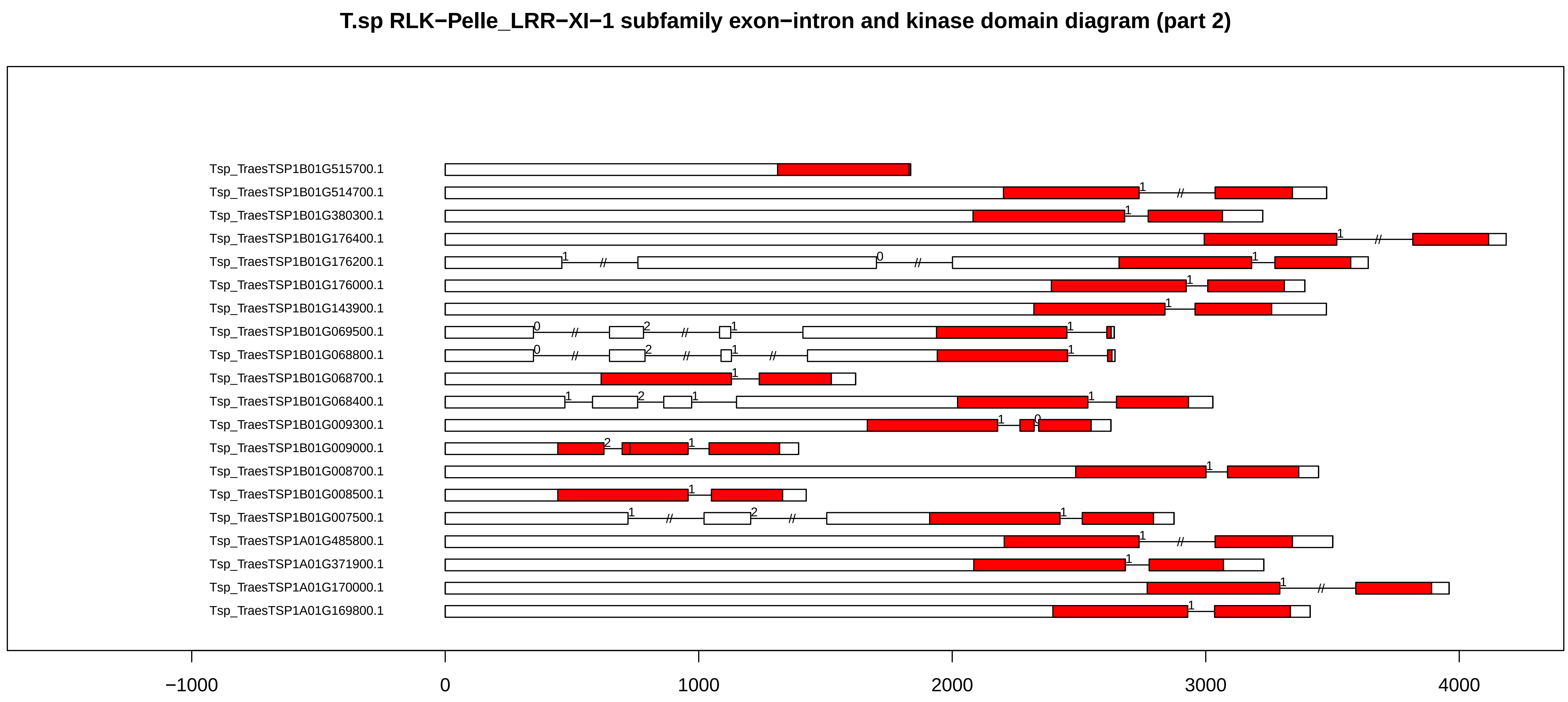

T.sp RLK−Pelle_LRR−XI−1 subfamily exon−intron and kinase domain diagram (part 2)
Tsp_TraesTSP1B01G515700.1
Tsp_TraesTSP1B01G514700.1
Tsp_TraesTSP1B01G380300.1
Tsp_TraesTSP1B01G176400.1
Tsp_TraesTSP1B01G176200.1
Tsp_TraesTSP1B01G176000.1
Tsp_TraesTSP1B01G143900.1
Tsp_TraesTSP1B01G069500.1
Tsp_TraesTSP1B01G068800.1
Tsp_TraesTSP1B01G068700.1
Tsp_TraesTSP1B01G068400.1
Tsp_TraesTSP1B01G009300.1
Tsp_TraesTSP1B01G009000.1
Tsp_TraesTSP1B01G008700.1
Tsp_TraesTSP1B01G008500.1
Tsp_TraesTSP1B01G007500.1
Tsp_TraesTSP1A01G485800.1
Tsp_TraesTSP1A01G371900.1
Tsp_TraesTSP1A01G170000.1
Tsp_TraesTSP1A01G169800.1
−1000
0
1000
2000
3000
4000

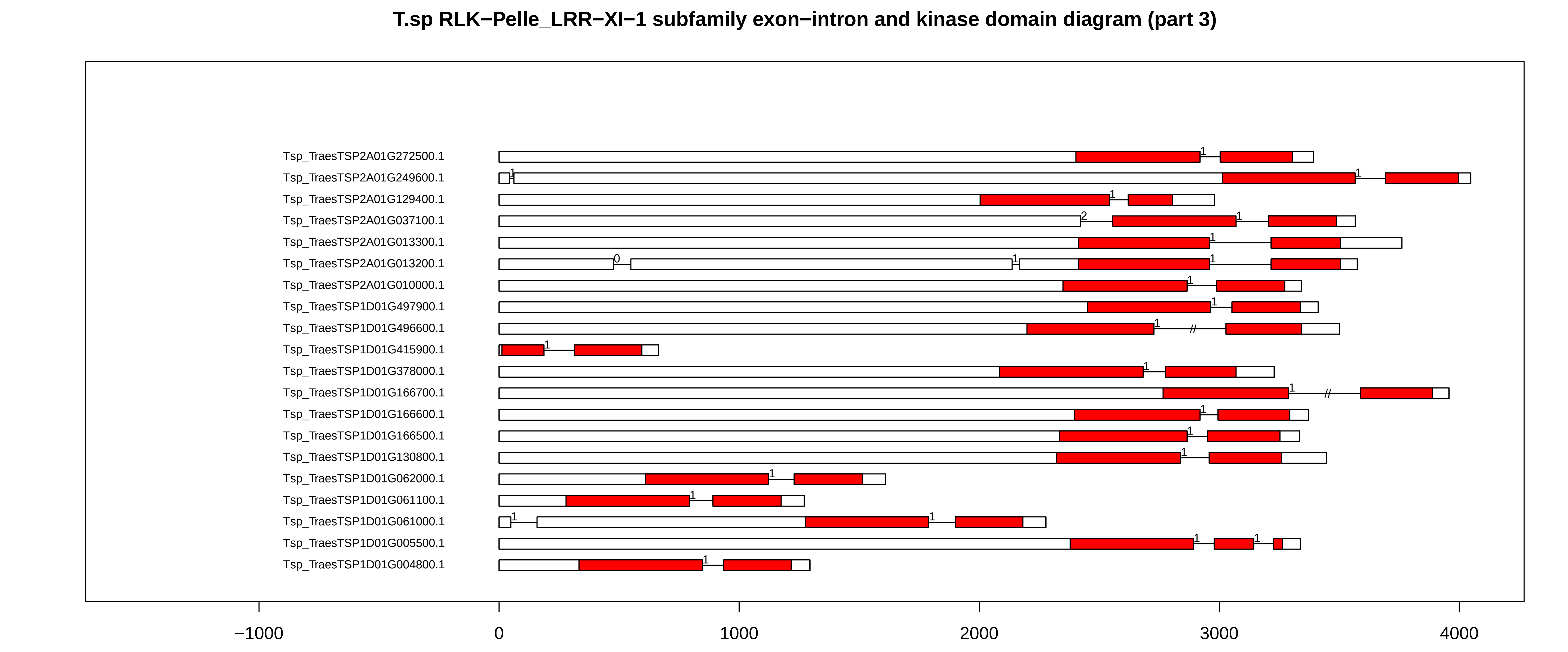

T.sp RLK−Pelle_LRR−XI−1 subfamily exon−intron and kinase domain diagram (part 3)
Tsp_TraesTSP2A01G272500.1
Tsp_TraesTSP2A01G249600.1
Tsp_TraesTSP2A01G129400.1
Tsp_TraesTSP2A01G037100.1
Tsp_TraesTSP2A01G013300.1
Tsp_TraesTSP2A01G013200.1
Tsp_TraesTSP2A01G010000.1
Tsp_TraesTSP1D01G497900.1
Tsp_TraesTSP1D01G496600.1
Tsp_TraesTSP1D01G415900.1
Tsp_TraesTSP1D01G378000.1
Tsp_TraesTSP1D01G166700.1
Tsp_TraesTSP1D01G166600.1
Tsp_TraesTSP1D01G166500.1
Tsp_TraesTSP1D01G130800.1
Tsp_TraesTSP1D01G062000.1
Tsp_TraesTSP1D01G061100.1
Tsp_TraesTSP1D01G061000.1
Tsp_TraesTSP1D01G005500.1
Tsp_TraesTSP1D01G004800.1
−1000
0
1000
2000
3000
4000

T.sp RLK−Pelle_LRR−XI−1 subfamily exon−intron and kinase domain diagram (part 4)

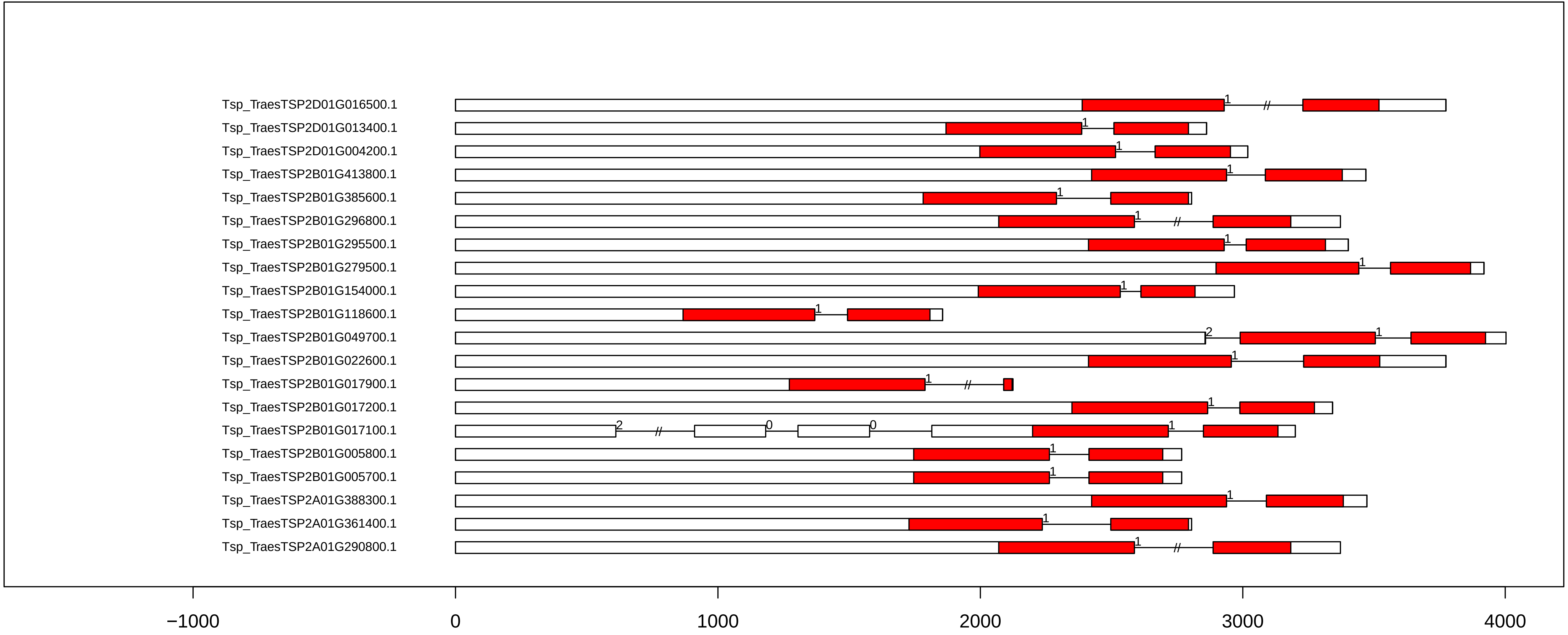

T.sp RLK−Pelle_LRR−XI−1 subfamily exon−intron and kinase domain diagram (part 5)

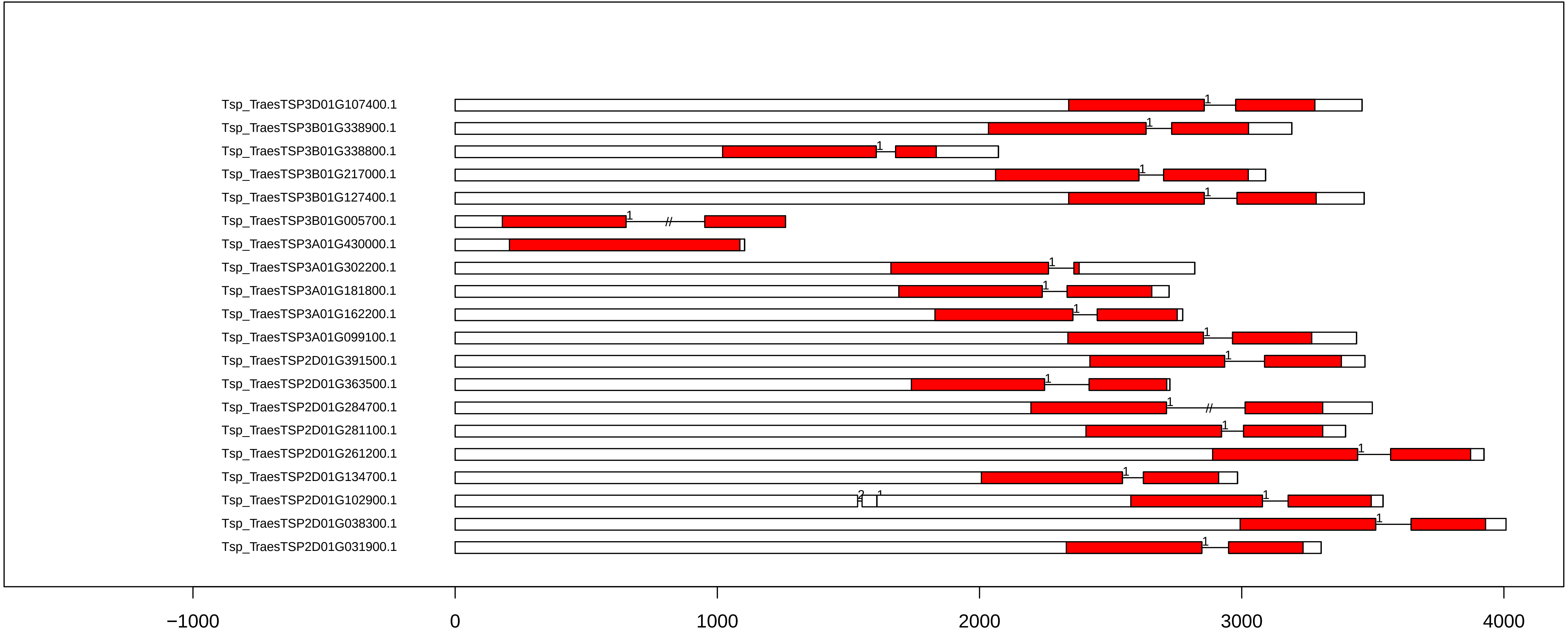

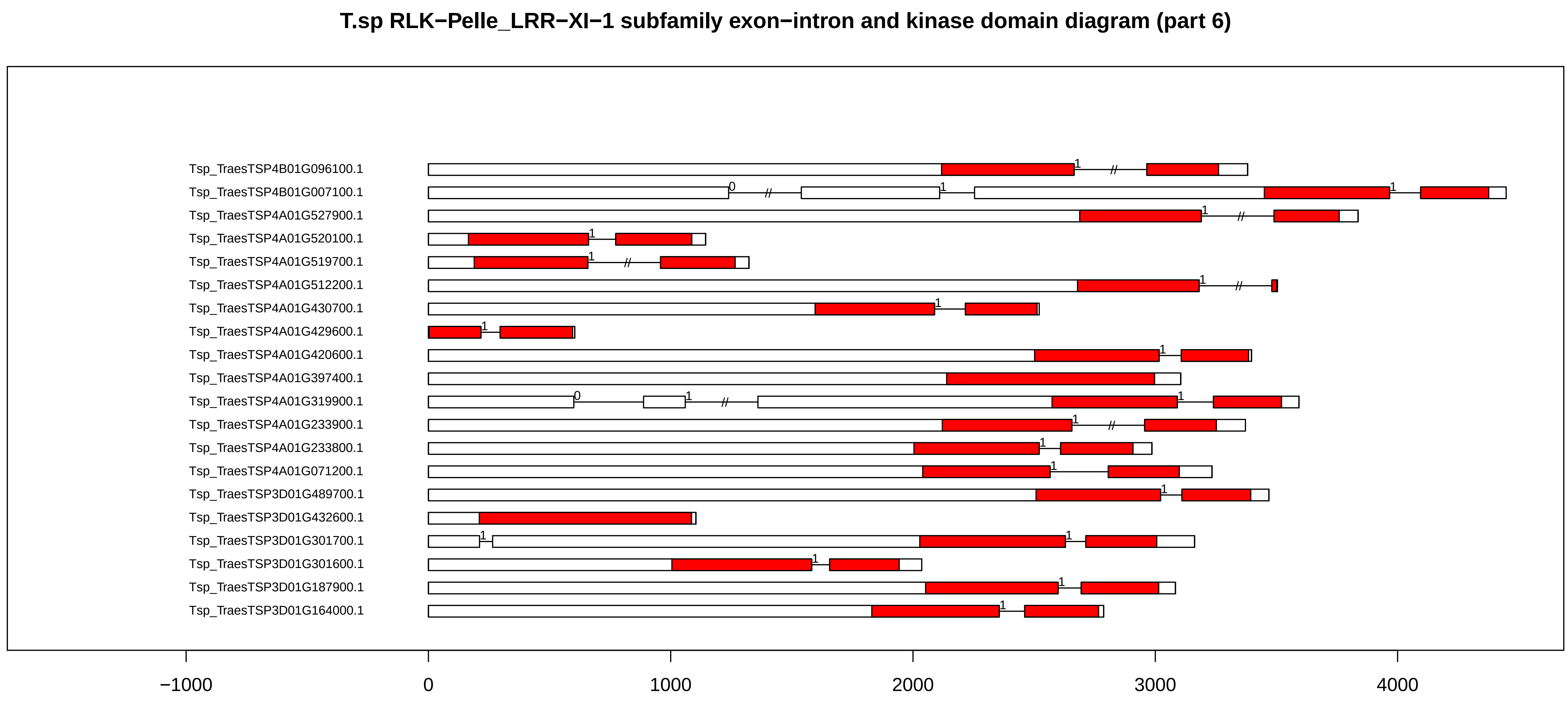
T.sp RLK−Pelle_LRR−XI−1 subfamily exon−intron and kinase domain diagram (part 6)
Tsp_TraesTSP4B01G096100.1
Tsp_TraesTSP4B01G007100.1
Tsp_TraesTSP4A01G527900.1
Tsp_TraesTSP4A01G520100.1
Tsp_TraesTSP4A01G519700.1
Tsp_TraesTSP4A01G512200.1
Tsp_TraesTSP4A01G430700.1
Tsp_TraesTSP4A01G429600.1
Tsp_TraesTSP4A01G420600.1
Tsp_TraesTSP4A01G397400.1
Tsp_TraesTSP4A01G319900.1
Tsp_TraesTSP4A01G233900.1
Tsp_TraesTSP4A01G233800.1
Tsp_TraesTSP4A01G071200.1
Tsp_TraesTSP3D01G489700.1
Tsp_TraesTSP3D01G432600.1
Tsp_TraesTSP3D01G301700.1
Tsp_TraesTSP3D01G301600.1
Tsp_TraesTSP3D01G187900.1
Tsp_TraesTSP3D01G164000.1
−1000
0
1000
2000
3000
4000

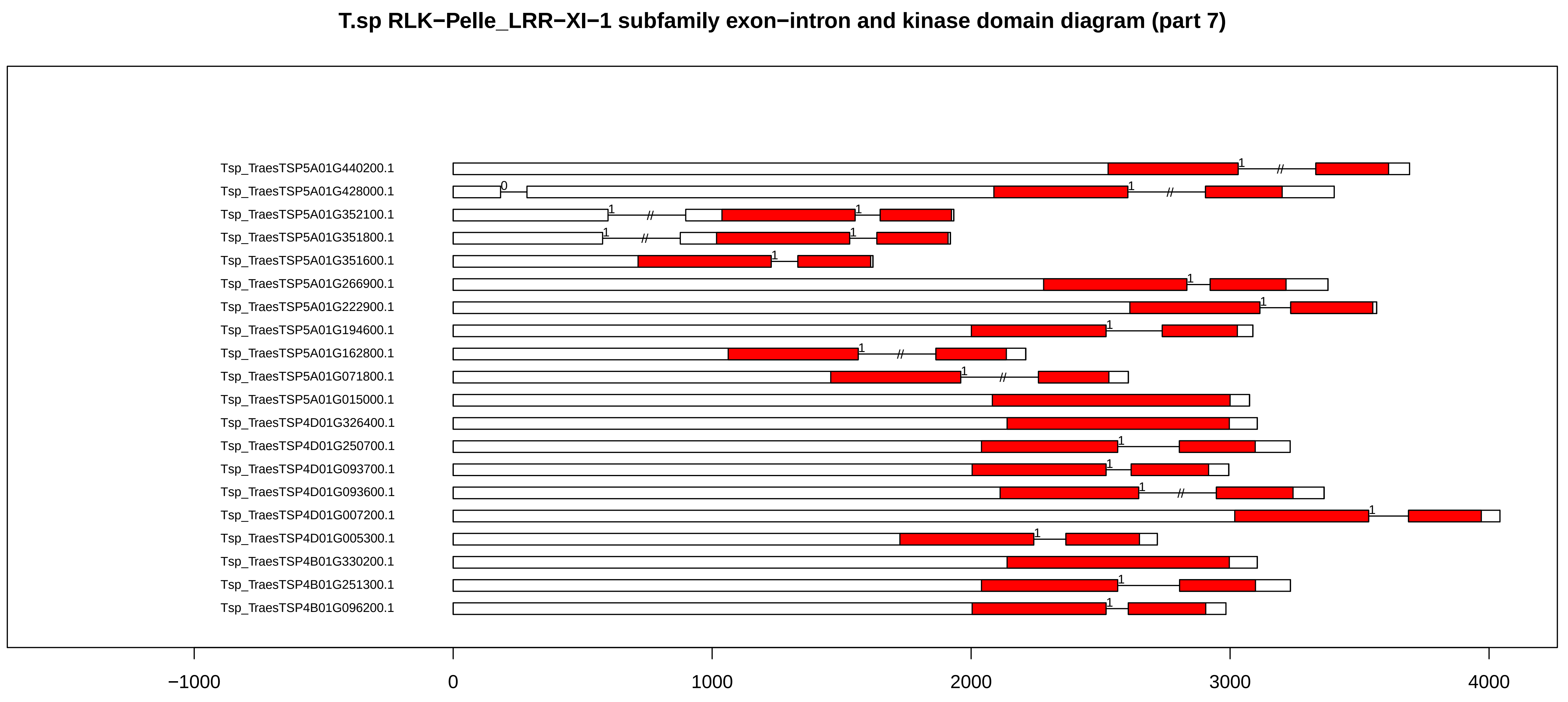

T.sp RLK−Pelle_LRR−XI−1 subfamily exon−intron and kinase domain diagram (part 7)
Tsp_TraesTSP5A01G440200.1
Tsp_TraesTSP5A01G428000.1
Tsp_TraesTSP5A01G352100.1
Tsp_TraesTSP5A01G351800.1
Tsp_TraesTSP5A01G351600.1
Tsp_TraesTSP5A01G266900.1
Tsp_TraesTSP5A01G222900.1
Tsp_TraesTSP5A01G194600.1
Tsp_TraesTSP5A01G162800.1
Tsp_TraesTSP5A01G071800.1
Tsp_TraesTSP5A01G015000.1
Tsp_TraesTSP4D01G326400.1
Tsp_TraesTSP4D01G250700.1
Tsp_TraesTSP4D01G093700.1
Tsp_TraesTSP4D01G093600.1
Tsp_TraesTSP4D01G007200.1
Tsp_TraesTSP4D01G005300.1
Tsp_TraesTSP4B01G330200.1
Tsp_TraesTSP4B01G251300.1
Tsp_TraesTSP4B01G096200.1
−1000
0
1000
2000
3000
4000

T.sp RLK−Pelle_LRR−XI−1 subfamily exon−intron and kinase domain diagram (part 8)

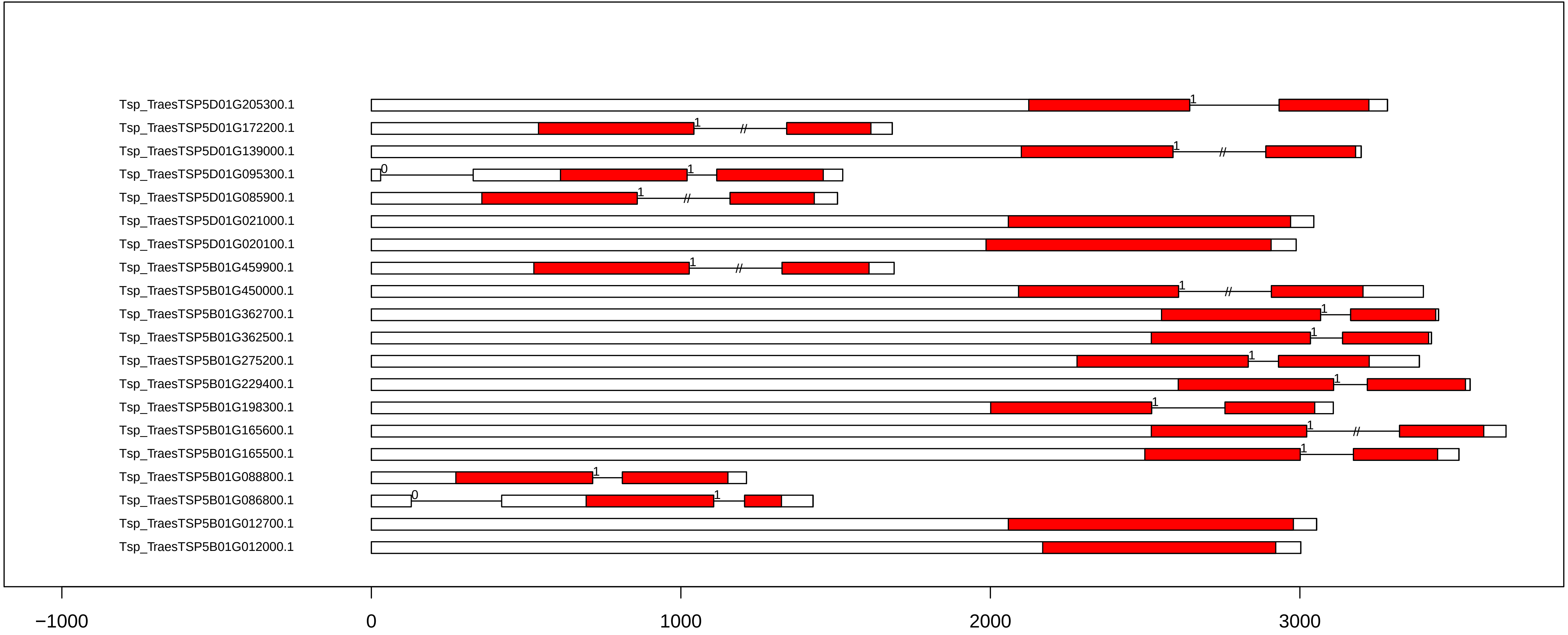

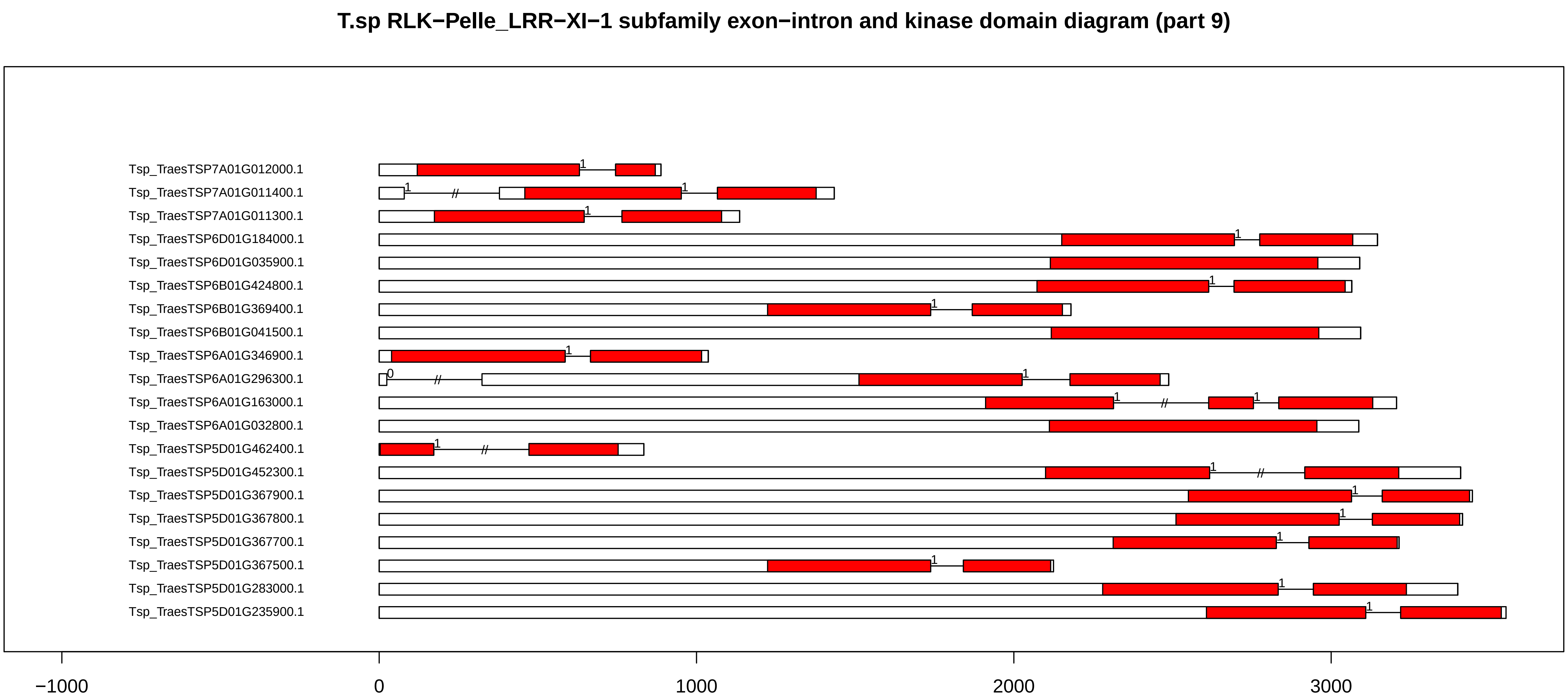

T.sp RLK−Pelle_LRR−XI−1 subfamily exon−intron and kinase domain diagram (part 9)
Tsp_TraesTSP7A01G012000.1
Tsp_TraesTSP7A01G011400.1
Tsp_TraesTSP7A01G011300.1
Tsp_TraesTSP6D01G184000.1
Tsp_TraesTSP6D01G035900.1
Tsp_TraesTSP6B01G424800.1
Tsp_TraesTSP6B01G369400.1
Tsp_TraesTSP6B01G041500.1
Tsp_TraesTSP6A01G346900.1
Tsp_TraesTSP6A01G296300.1
Tsp_TraesTSP6A01G163000.1
Tsp_TraesTSP6A01G032800.1
Tsp_TraesTSP5D01G462400.1
Tsp_TraesTSP5D01G452300.1
Tsp_TraesTSP5D01G367900.1
Tsp_TraesTSP5D01G367800.1
Tsp_TraesTSP5D01G367700.1
Tsp_TraesTSP5D01G367500.1
Tsp_TraesTSP5D01G283000.1
Tsp_TraesTSP5D01G235900.1
−1000
0
1000
2000
3000

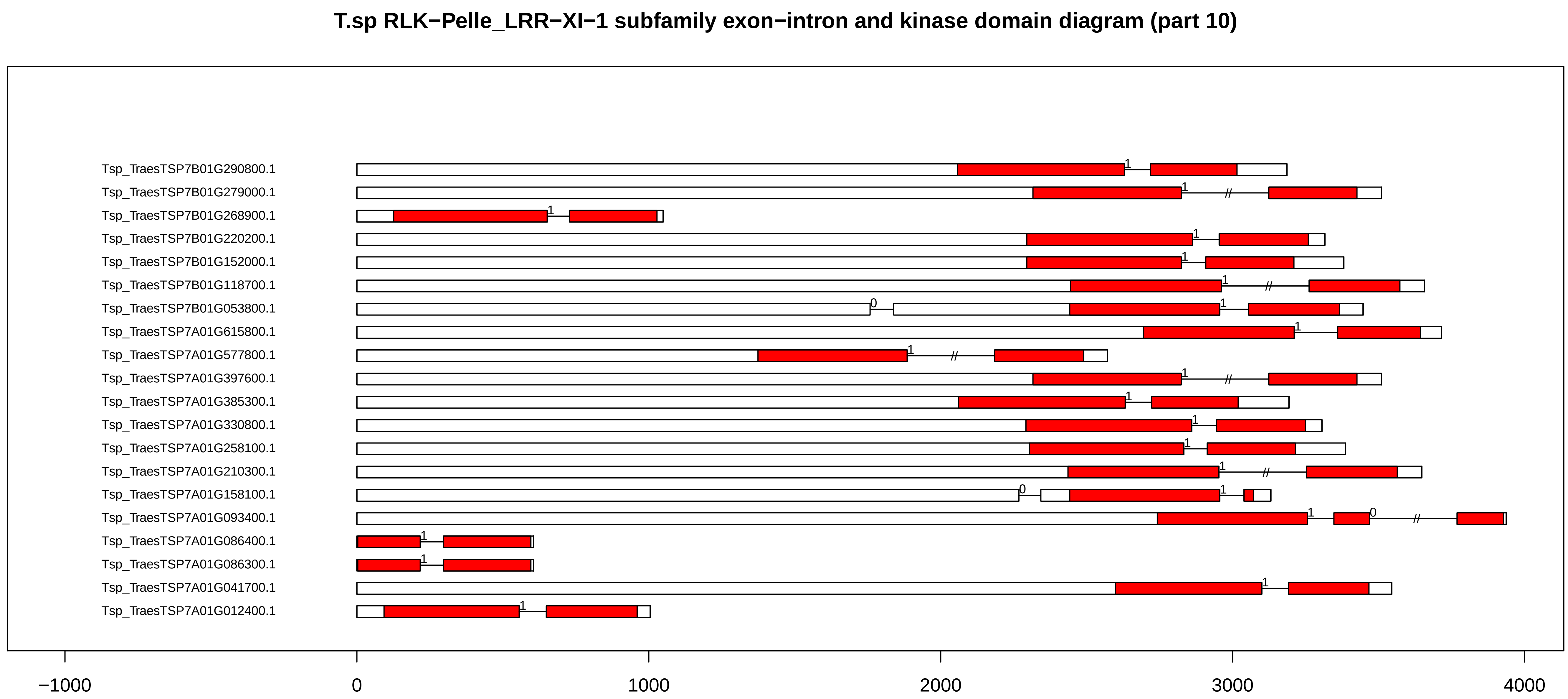
T.sp RLK−Pelle_LRR−XI−1 subfamily exon−intron and kinase domain diagram (part 10)
Tsp_TraesTSP7B01G290800.1
Tsp_TraesTSP7B01G279000.1
Tsp_TraesTSP7B01G268900.1
Tsp_TraesTSP7B01G220200.1
Tsp_TraesTSP7B01G152000.1
Tsp_TraesTSP7B01G118700.1
Tsp_TraesTSP7B01G053800.1
Tsp_TraesTSP7A01G615800.1
Tsp_TraesTSP7A01G577800.1
Tsp_TraesTSP7A01G397600.1
Tsp_TraesTSP7A01G385300.1
Tsp_TraesTSP7A01G330800.1
Tsp_TraesTSP7A01G258100.1
Tsp_TraesTSP7A01G210300.1
Tsp_TraesTSP7A01G158100.1
Tsp_TraesTSP7A01G093400.1
Tsp_TraesTSP7A01G086400.1
Tsp_TraesTSP7A01G086300.1
Tsp_TraesTSP7A01G041700.1
Tsp_TraesTSP7A01G012400.1
−1000
0
1000
2000
3000
4000

T.sp RLK−Pelle_LRR−XI−1 subfamily exon−intron and kinase domain diagram (part 11)

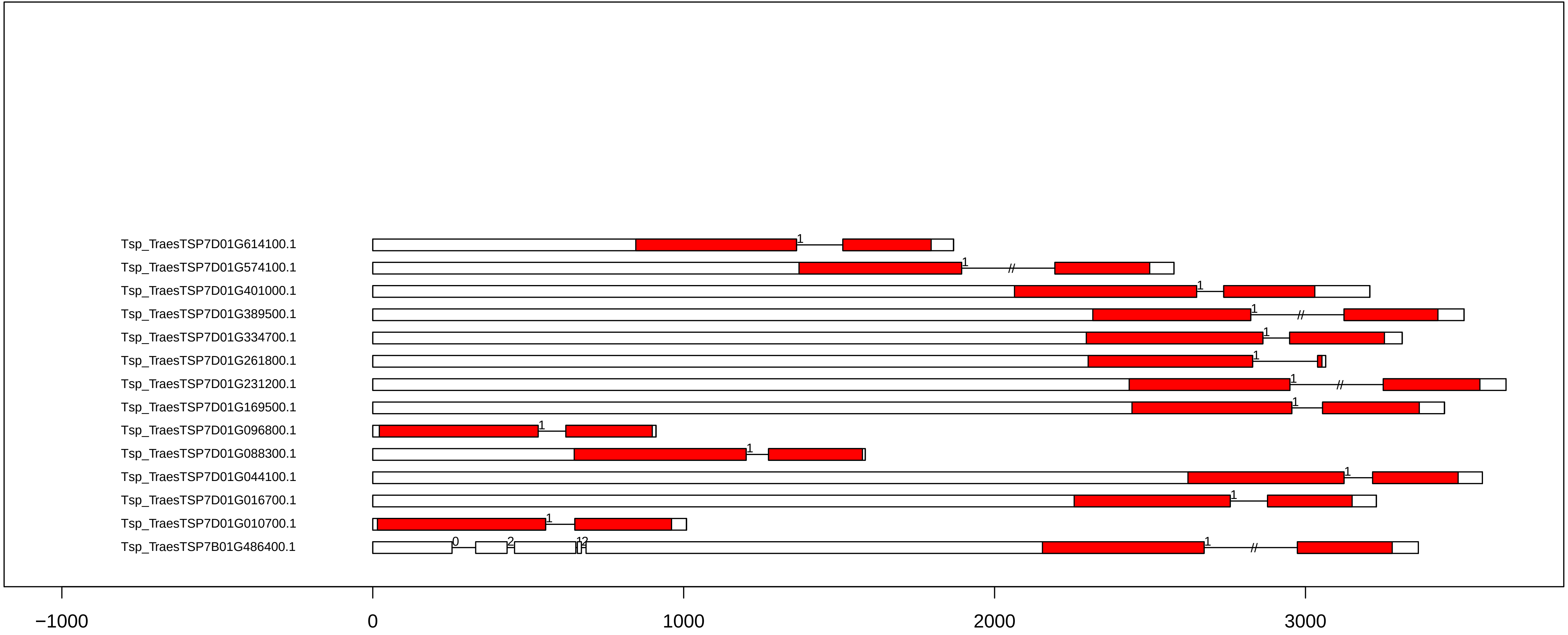

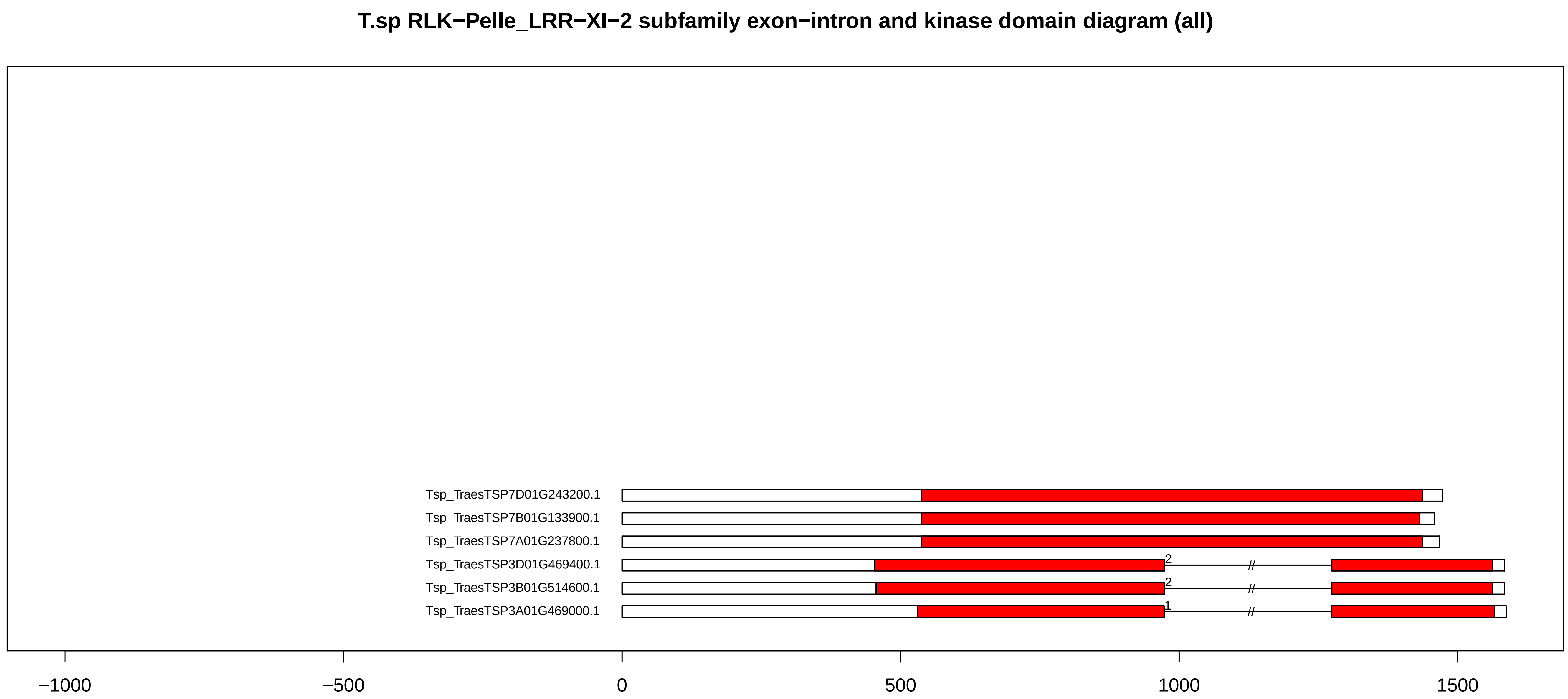
T.sp RLK−Pelle_LRR−XI−2 subfamily exon−intron and kinase domain diagram (all)
Tsp_TraesTSP7D01G243200.1
Tsp_TraesTSP7B01G133900.1
Tsp_TraesTSP7A01G237800.1
Tsp_TraesTSP3D01G469400.1
Tsp_TraesTSP3B01G514600.1
Tsp_TraesTSP3A01G469000.1
2
2
1
−1000
−500
0
500
1000
1500

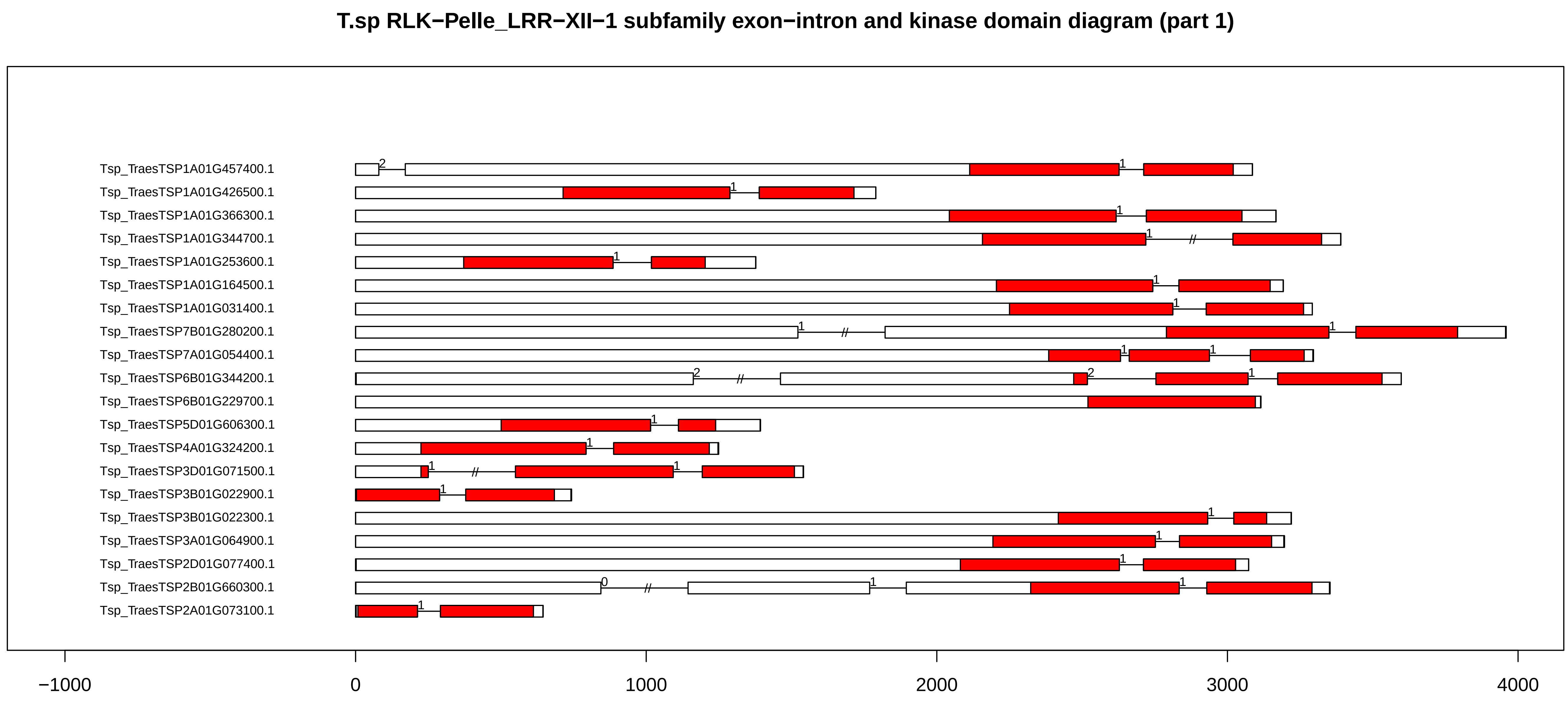

T.sp RLK−Pelle_LRR−XII−1 subfamily exon−intron and kinase domain diagram (part 1)
Tsp_TraesTSP1A01G457400.1
Tsp_TraesTSP1A01G426500.1
Tsp_TraesTSP1A01G366300.1
Tsp_TraesTSP1A01G344700.1
Tsp_TraesTSP1A01G253600.1
Tsp_TraesTSP1A01G164500.1
Tsp_TraesTSP1A01G031400.1
Tsp_TraesTSP7B01G280200.1
Tsp_TraesTSP7A01G054400.1
Tsp_TraesTSP6B01G344200.1
Tsp_TraesTSP6B01G229700.1
Tsp_TraesTSP5D01G606300.1
Tsp_TraesTSP4A01G324200.1
Tsp_TraesTSP3D01G071500.1
Tsp_TraesTSP3B01G022900.1
Tsp_TraesTSP3B01G022300.1
Tsp_TraesTSP3A01G064900.1
Tsp_TraesTSP2D01G077400.1
Tsp_TraesTSP2B01G660300.1
Tsp_TraesTSP2A01G073100.1
−1000
0
1000
2000
3000
4000

T.sp RLK−Pelle_LRR−XII−1 subfamily exon−intron and kinase domain diagram (part 2)

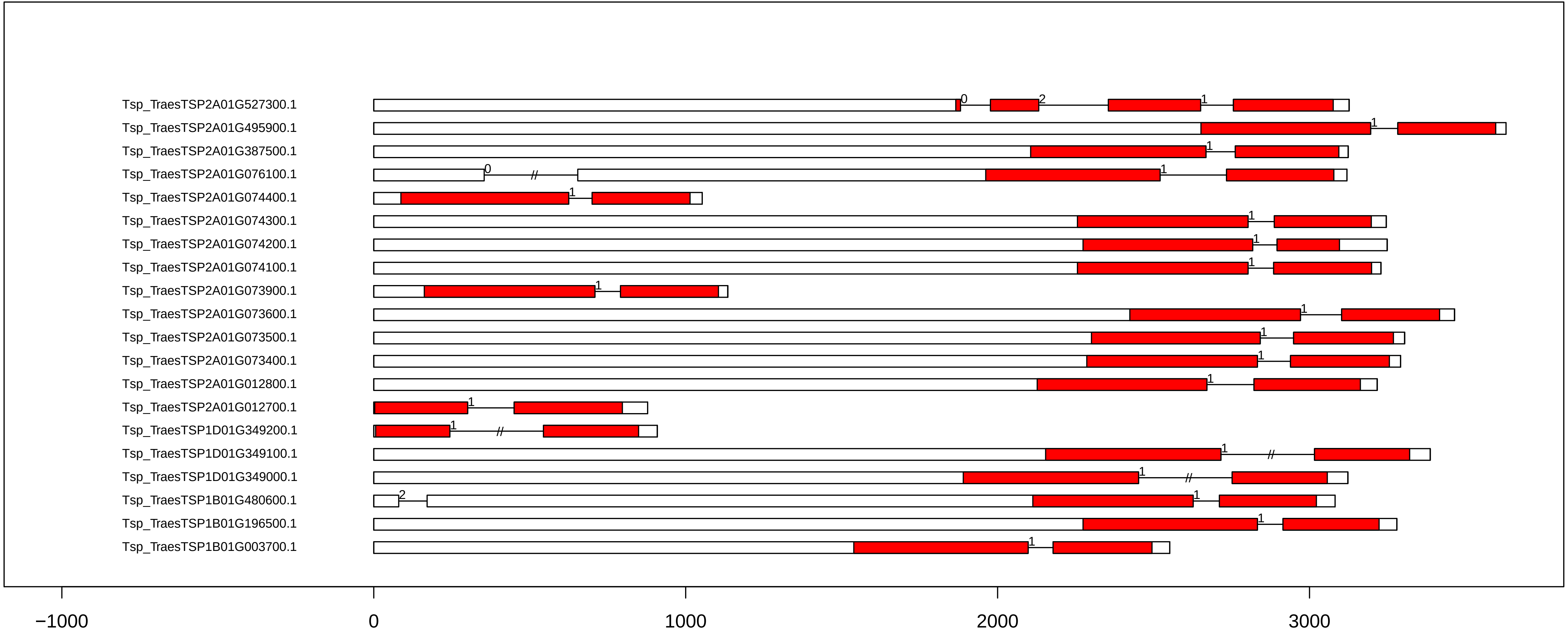

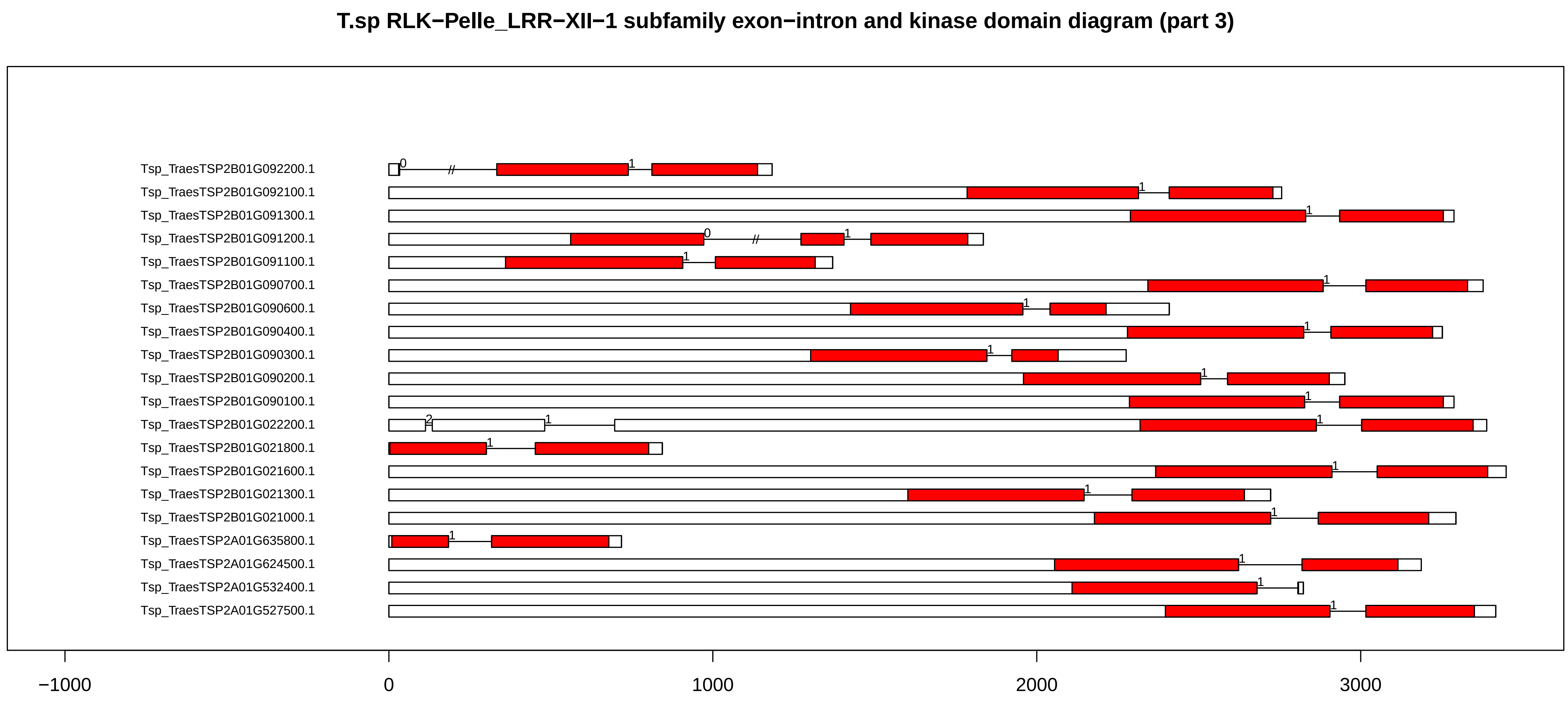
T.sp RLK−Pelle_LRR−XII−1 subfamily exon−intron and kinase domain diagram (part 3)
Tsp_TraesTSP2B01G092200.1
Tsp_TraesTSP2B01G092100.1
Tsp_TraesTSP2B01G091300.1
Tsp_TraesTSP2B01G091200.1
Tsp_TraesTSP2B01G091100.1
Tsp_TraesTSP2B01G090700.1
Tsp_TraesTSP2B01G090600.1
Tsp_TraesTSP2B01G090400.1
Tsp_TraesTSP2B01G090300.1
Tsp_TraesTSP2B01G090200.1
Tsp_TraesTSP2B01G090100.1
Tsp_TraesTSP2B01G022200.1
Tsp_TraesTSP2B01G021800.1
Tsp_TraesTSP2B01G021600.1
Tsp_TraesTSP2B01G021300.1
Tsp_TraesTSP2B01G021000.1
Tsp_TraesTSP2A01G635800.1
Tsp_TraesTSP2A01G624500.1
Tsp_TraesTSP2A01G532400.1
Tsp_TraesTSP2A01G527500.1
−1000
0
1000
2000
3000

T.sp RLK−Pelle_LRR−XII−1 subfamily exon−intron and kinase domain diagram (part 4)

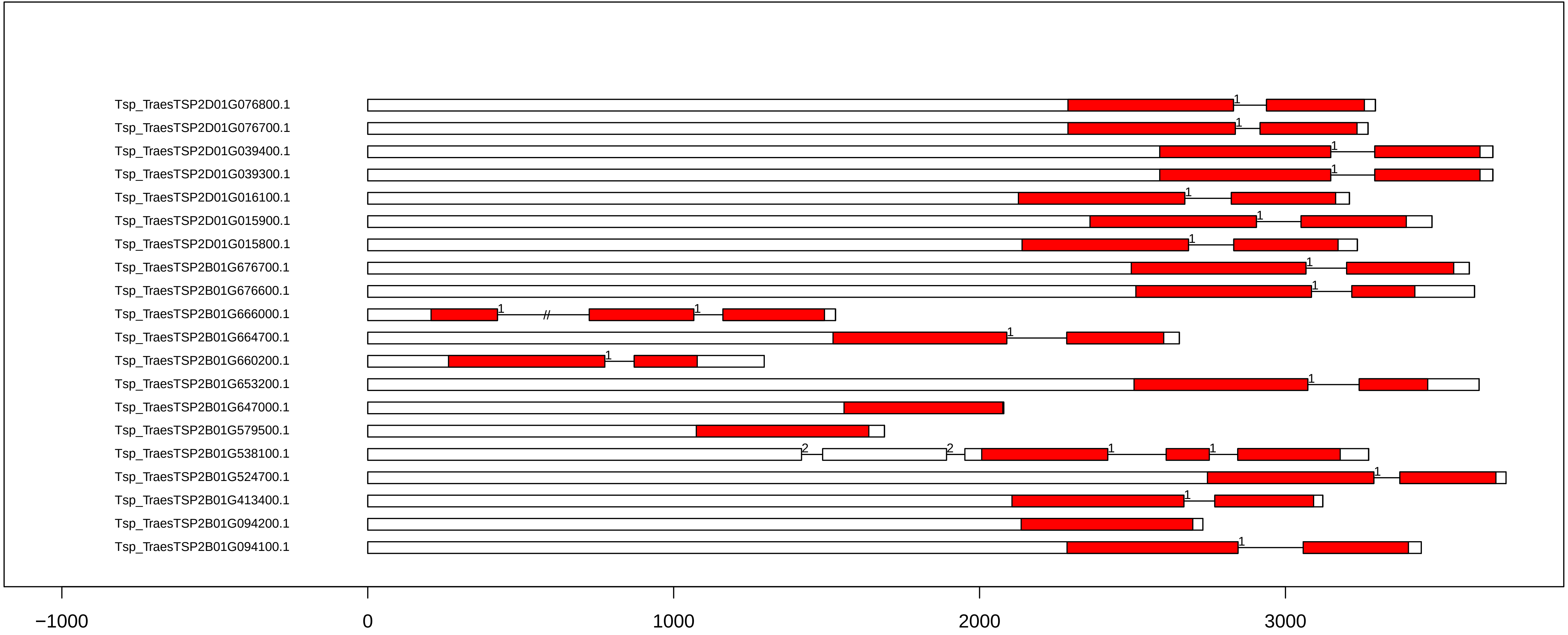

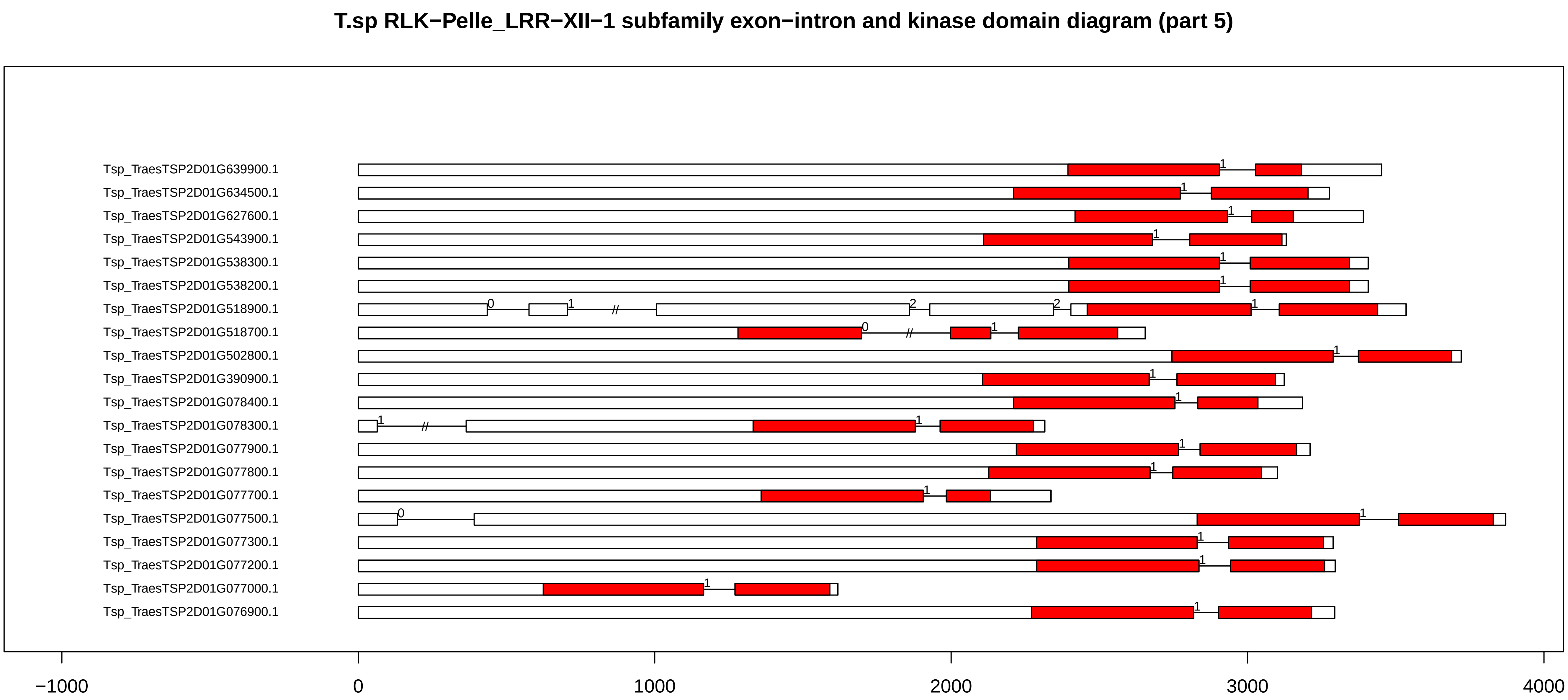

T.sp RLK−Pelle_LRR−XII−1 subfamily exon−intron and kinase domain diagram (part 5)
Tsp_TraesTSP2D01G639900.1
Tsp_TraesTSP2D01G634500.1
Tsp_TraesTSP2D01G627600.1
Tsp_TraesTSP2D01G543900.1
Tsp_TraesTSP2D01G538300.1
Tsp_TraesTSP2D01G538200.1
Tsp_TraesTSP2D01G518900.1
Tsp_TraesTSP2D01G518700.1
Tsp_TraesTSP2D01G502800.1
Tsp_TraesTSP2D01G390900.1
Tsp_TraesTSP2D01G078400.1
Tsp_TraesTSP2D01G078300.1
Tsp_TraesTSP2D01G077900.1
Tsp_TraesTSP2D01G077800.1
Tsp_TraesTSP2D01G077700.1
Tsp_TraesTSP2D01G077500.1
Tsp_TraesTSP2D01G077300.1
Tsp_TraesTSP2D01G077200.1
Tsp_TraesTSP2D01G077000.1
Tsp_TraesTSP2D01G076900.1
−1000
0
1000
2000
3000
4000

T.sp RLK−Pelle_LRR−XII−1 subfamily exon−intron and kinase domain diagram (part 6)

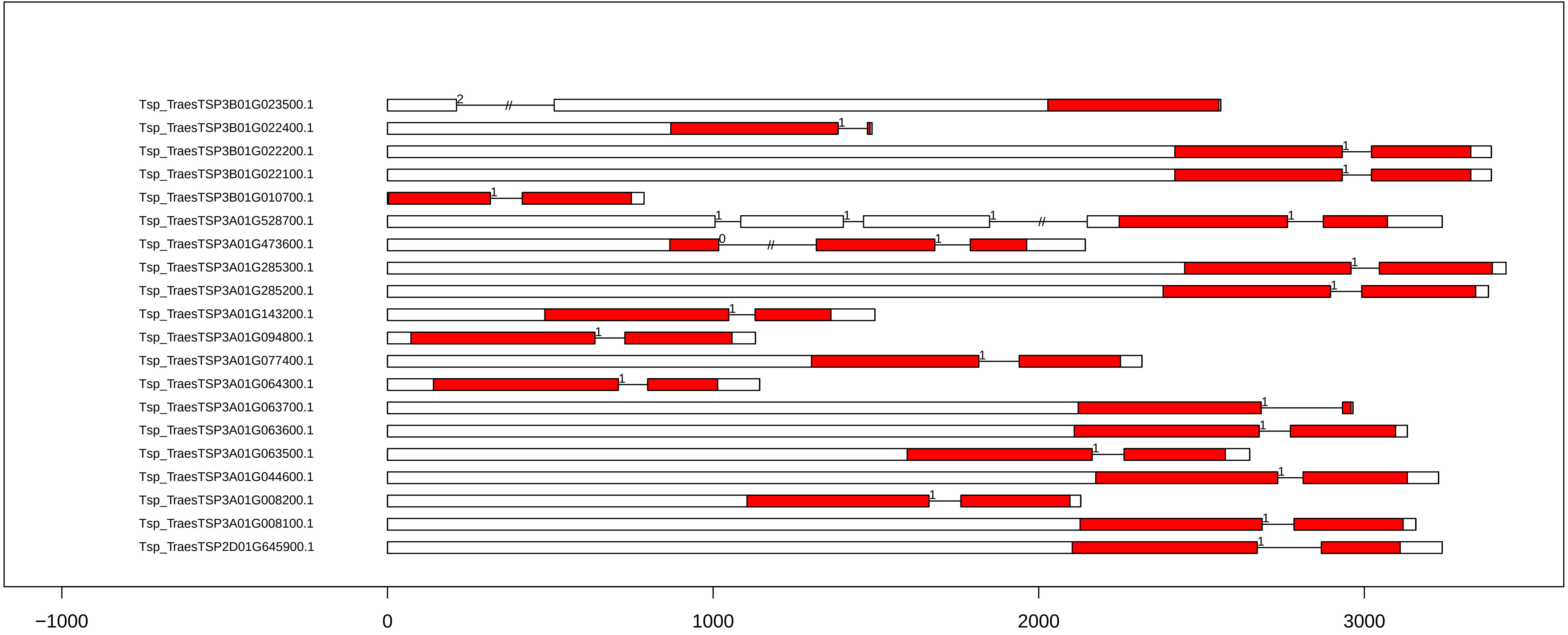

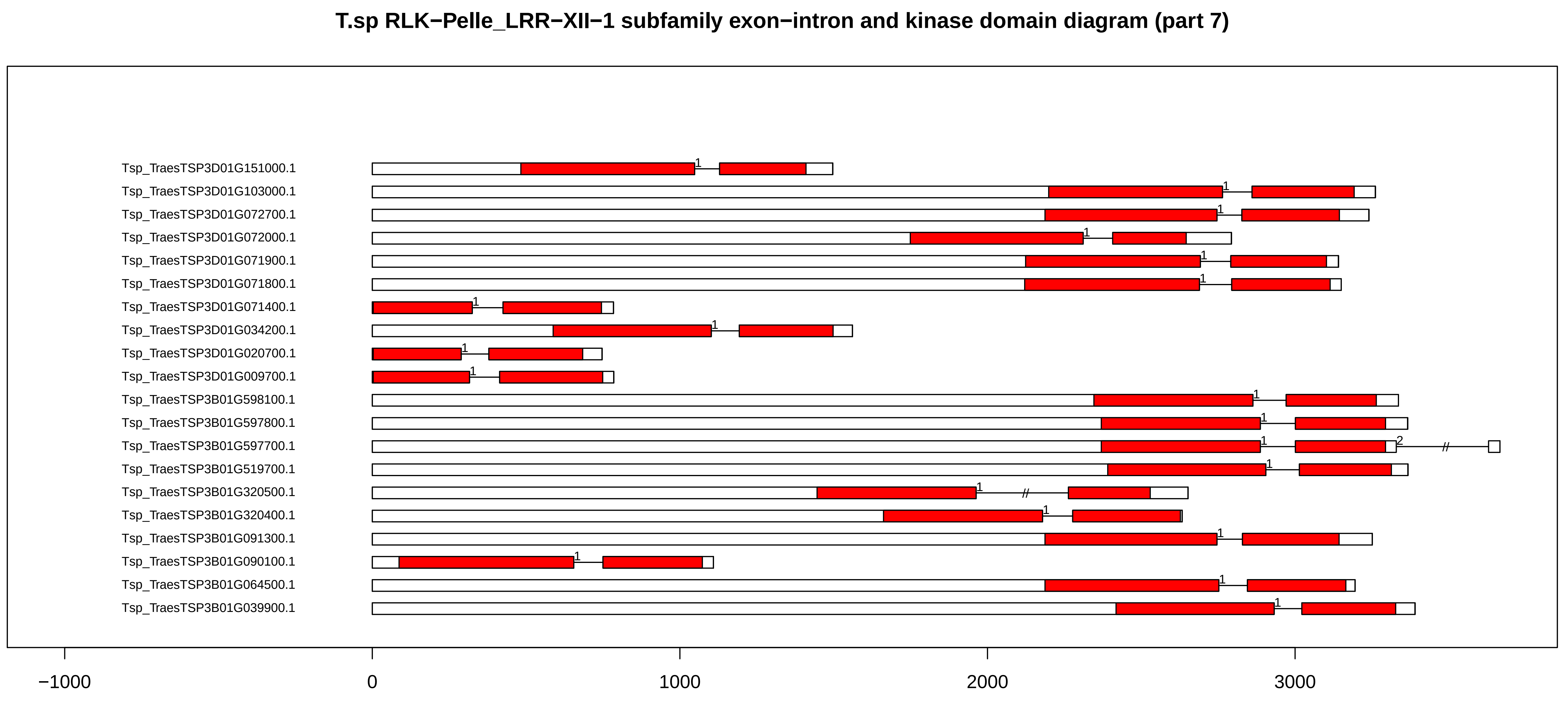

T.sp RLK−Pelle_LRR−XII−1 subfamily exon−intron and kinase domain diagram (part 7)
Tsp_TraesTSP3D01G151000.1
Tsp_TraesTSP3D01G103000.1
Tsp_TraesTSP3D01G072700.1
Tsp_TraesTSP3D01G072000.1
Tsp_TraesTSP3D01G071900.1
Tsp_TraesTSP3D01G071800.1
Tsp_TraesTSP3D01G071400.1
Tsp_TraesTSP3D01G034200.1
Tsp_TraesTSP3D01G020700.1
Tsp_TraesTSP3D01G009700.1
Tsp_TraesTSP3B01G598100.1
Tsp_TraesTSP3B01G597800.1
Tsp_TraesTSP3B01G597700.1
Tsp_TraesTSP3B01G519700.1
Tsp_TraesTSP3B01G320500.1
Tsp_TraesTSP3B01G320400.1
Tsp_TraesTSP3B01G091300.1
Tsp_TraesTSP3B01G090100.1
Tsp_TraesTSP3B01G064500.1
Tsp_TraesTSP3B01G039900.1
−1000
0
1000
2000
3000

T.sp RLK−Pelle_LRR−XII−1 subfamily exon−intron and kinase domain diagram (part 8)

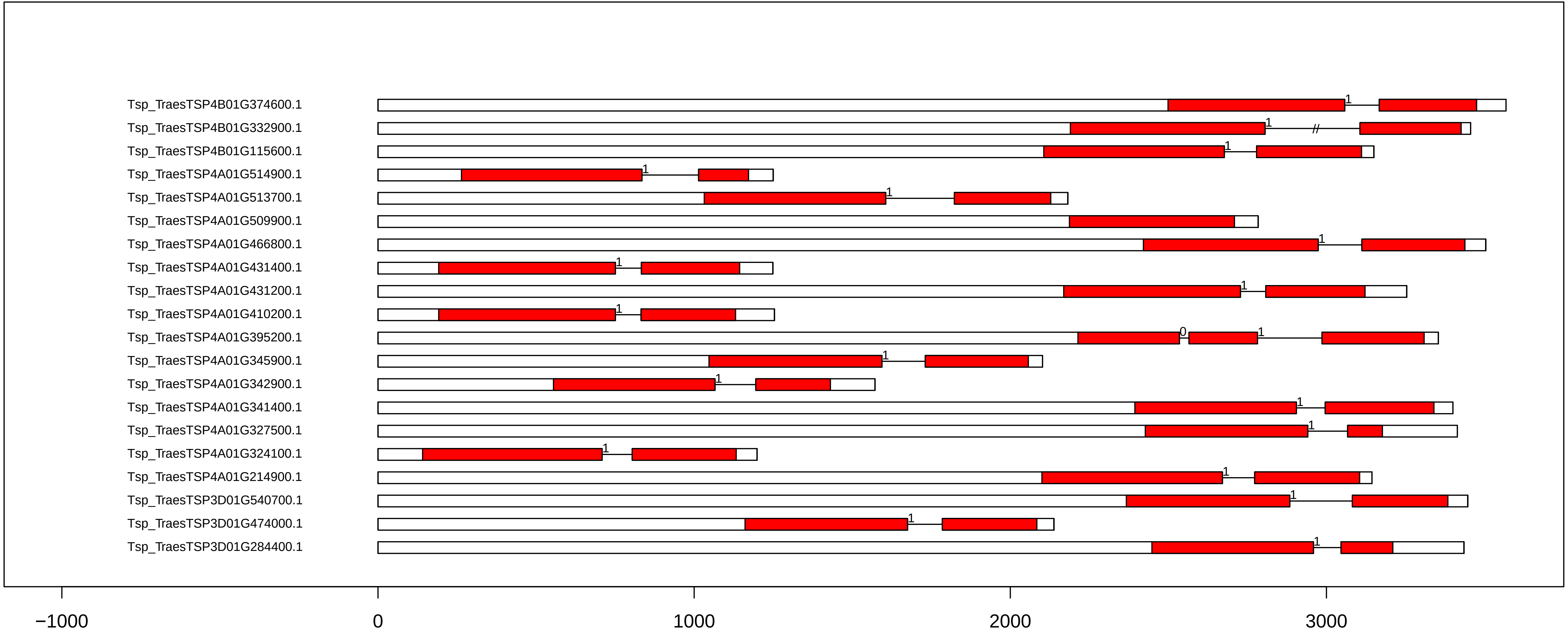

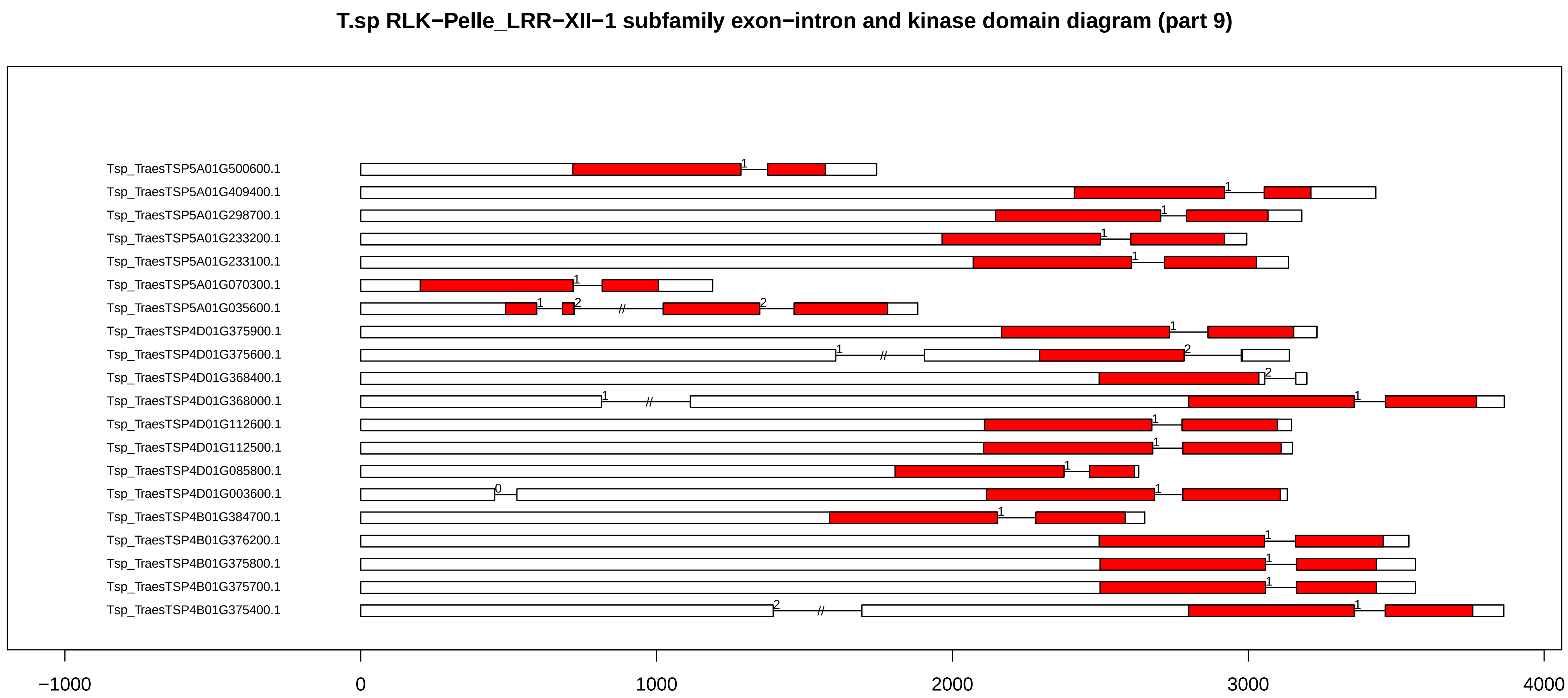
T.sp RLK−Pelle_LRR−XII−1 subfamily exon−intron and kinase domain diagram (part 9)
Tsp_TraesTSP5A01G500600.1
Tsp_TraesTSP5A01G409400.1
Tsp_TraesTSP5A01G298700.1
Tsp_TraesTSP5A01G233200.1
Tsp_TraesTSP5A01G233100.1
Tsp_TraesTSP5A01G070300.1
Tsp_TraesTSP5A01G035600.1
Tsp_TraesTSP4D01G375900.1
Tsp_TraesTSP4D01G375600.1
Tsp_TraesTSP4D01G368400.1
Tsp_TraesTSP4D01G368000.1
Tsp_TraesTSP4D01G112600.1
Tsp_TraesTSP4D01G112500.1
Tsp_TraesTSP4D01G085800.1
Tsp_TraesTSP4D01G003600.1
Tsp_TraesTSP4B01G384700.1
Tsp_TraesTSP4B01G376200.1
Tsp_TraesTSP4B01G375800.1
Tsp_TraesTSP4B01G375700.1
Tsp_TraesTSP4B01G375400.1
−1000
0
1000
2000
3000
4000

T.sp RLK−Pelle_LRR−XII−1 subfamily exon−intron and kinase domain diagram (part 10)

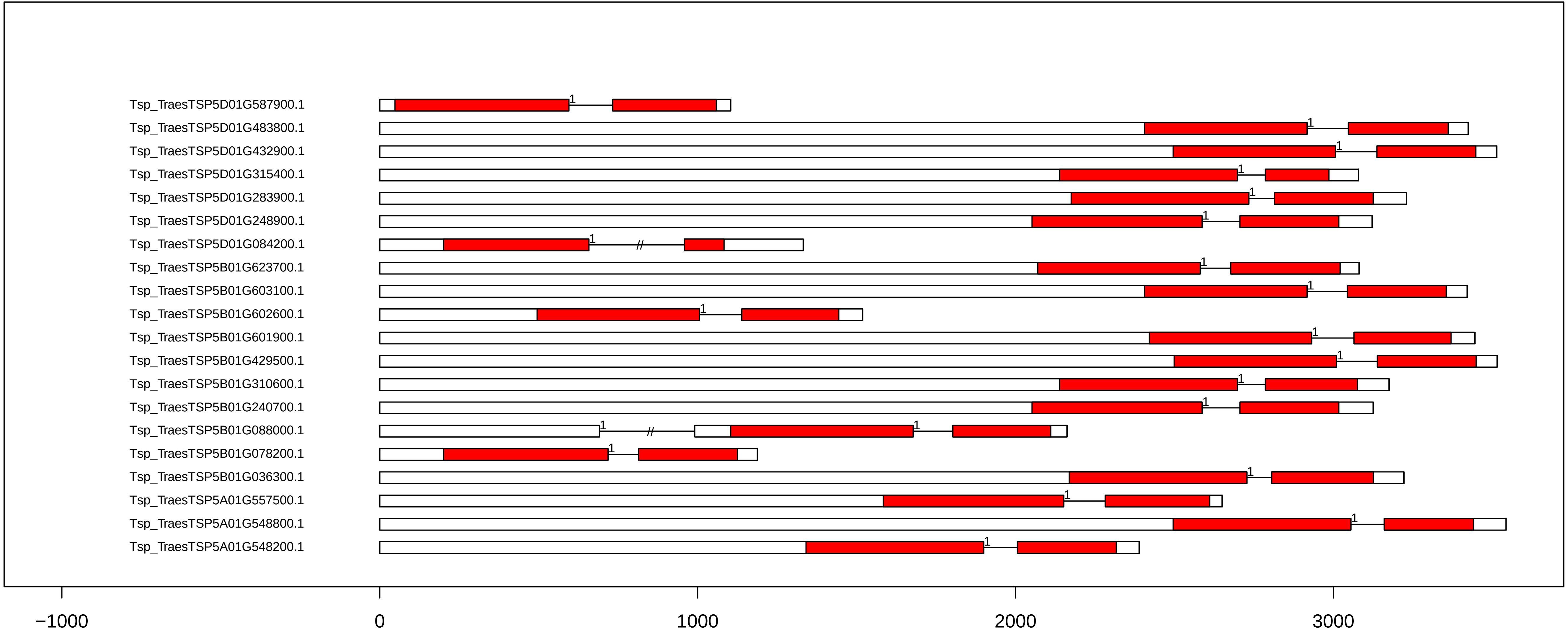

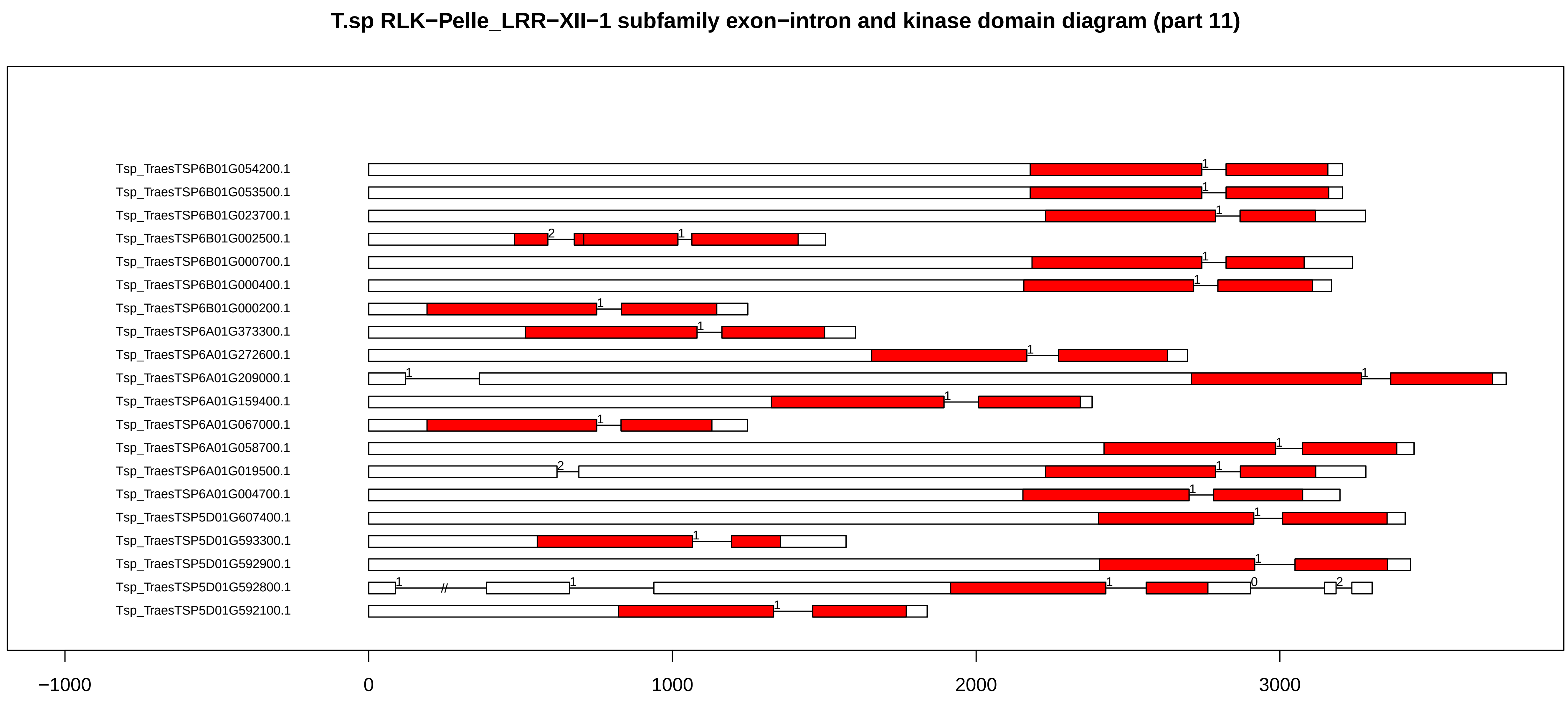

T.sp RLK−Pelle_LRR−XII−1 subfamily exon−intron and kinase domain diagram (part 11)
Tsp_TraesTSP6B01G054200.1
Tsp_TraesTSP6B01G053500.1
Tsp_TraesTSP6B01G023700.1
Tsp_TraesTSP6B01G002500.1
Tsp_TraesTSP6B01G000700.1
Tsp_TraesTSP6B01G000400.1
Tsp_TraesTSP6B01G000200.1
Tsp_TraesTSP6A01G373300.1
Tsp_TraesTSP6A01G272600.1
Tsp_TraesTSP6A01G209000.1
Tsp_TraesTSP6A01G159400.1
Tsp_TraesTSP6A01G067000.1
Tsp_TraesTSP6A01G058700.1
Tsp_TraesTSP6A01G019500.1
Tsp_TraesTSP6A01G004700.1
Tsp_TraesTSP5D01G607400.1
Tsp_TraesTSP5D01G593300.1
Tsp_TraesTSP5D01G592900.1
Tsp_TraesTSP5D01G592800.1
Tsp_TraesTSP5D01G592100.1
−1000
0
1000
2000
3000

T.sp RLK−Pelle_LRR−XII−1 subfamily exon−intron and kinase domain diagram (part 12)

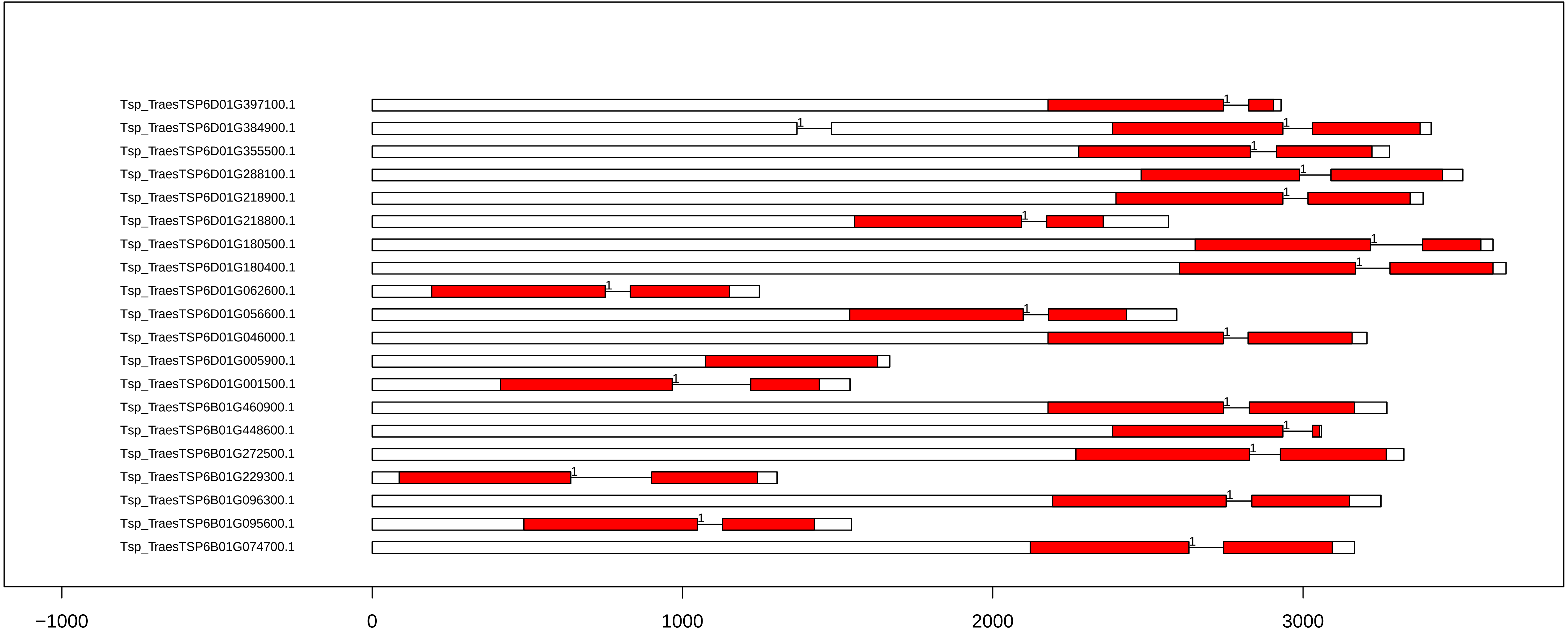

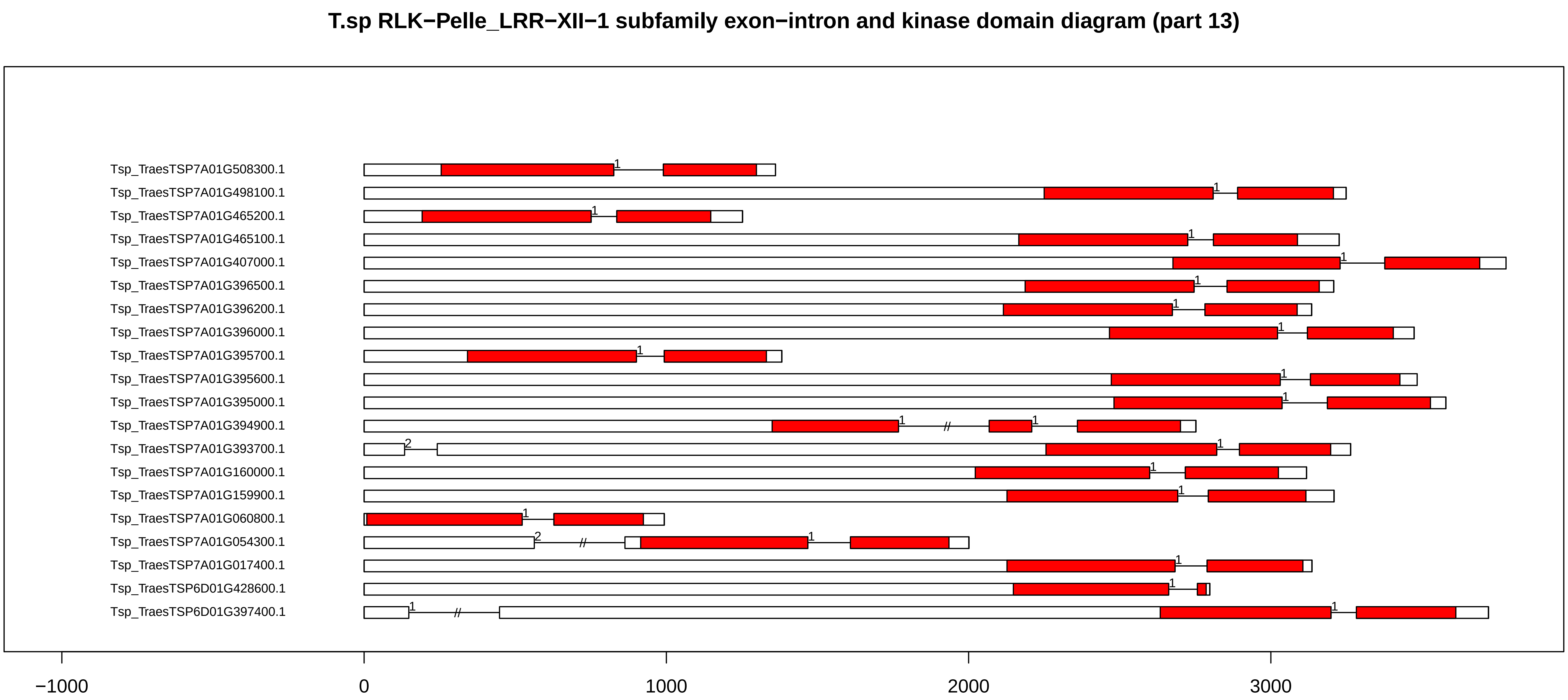

T.sp RLK−Pelle_LRR−XII−1 subfamily exon−intron and kinase domain diagram (part 13)
Tsp_TraesTSP7A01G508300.1
Tsp_TraesTSP7A01G498100.1
Tsp_TraesTSP7A01G465200.1
Tsp_TraesTSP7A01G465100.1
Tsp_TraesTSP7A01G407000.1
Tsp_TraesTSP7A01G396500.1
Tsp_TraesTSP7A01G396200.1
Tsp_TraesTSP7A01G396000.1
Tsp_TraesTSP7A01G395700.1
Tsp_TraesTSP7A01G395600.1
Tsp_TraesTSP7A01G395000.1
Tsp_TraesTSP7A01G394900.1
Tsp_TraesTSP7A01G393700.1
Tsp_TraesTSP7A01G160000.1
Tsp_TraesTSP7A01G159900.1
Tsp_TraesTSP7A01G060800.1
Tsp_TraesTSP7A01G054300.1
Tsp_TraesTSP7A01G017400.1
Tsp_TraesTSP6D01G428600.1
Tsp_TraesTSP6D01G397400.1
1
2
−1000
0
1000
2000
3000

T.sp RLK−Pelle_LRR−XII−1 subfamily exon−intron and kinase domain diagram (part 14)

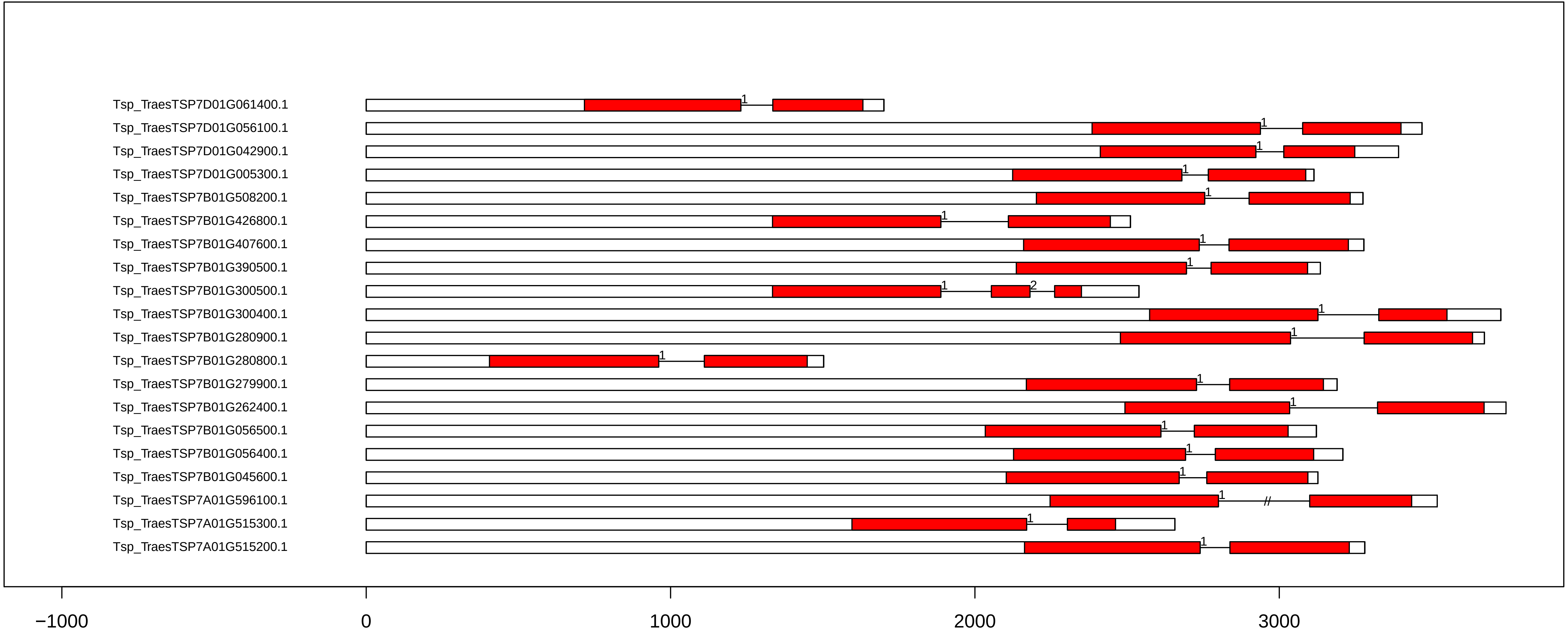

T.sp RLK−Pelle_LRR−XII−1 subfamily exon−intron and kinase domain diagram (part 15)

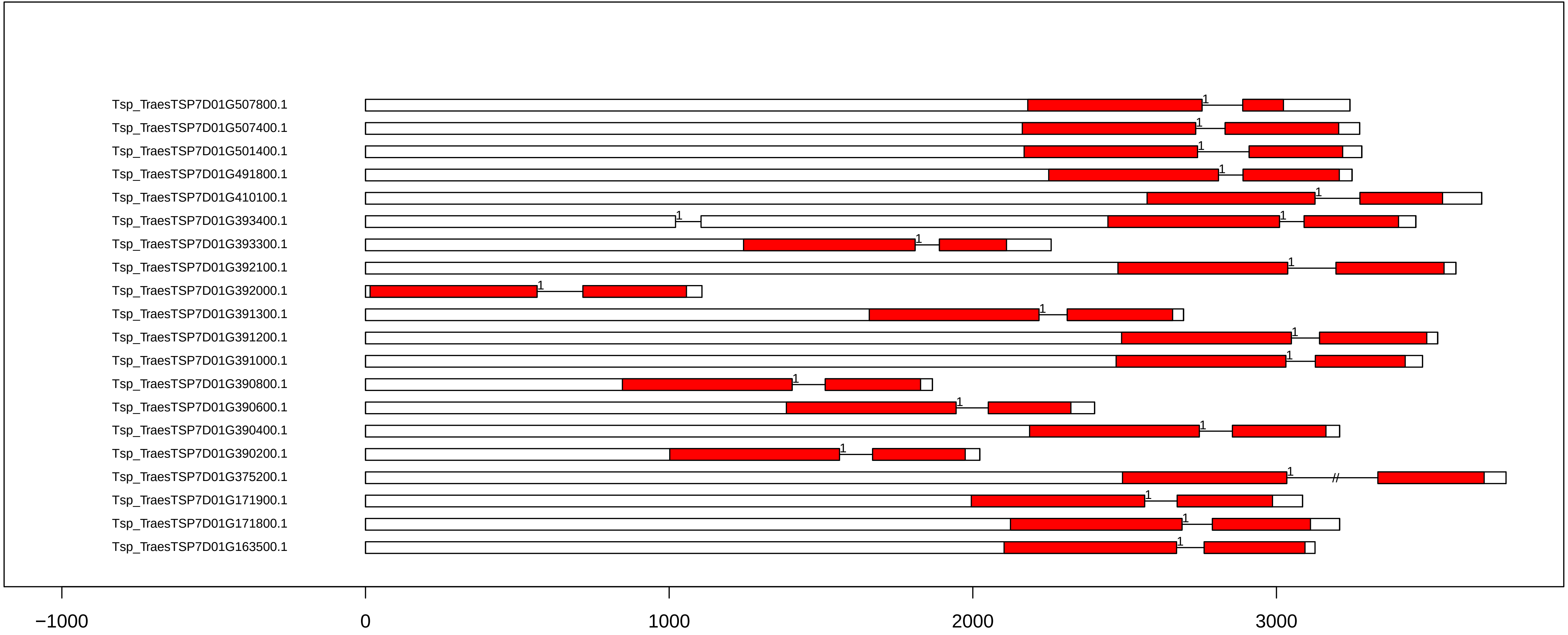

T.sp RLK−Pelle_LRR−XII−1 subfamily exon−intron and kinase domain diagram (part 16)

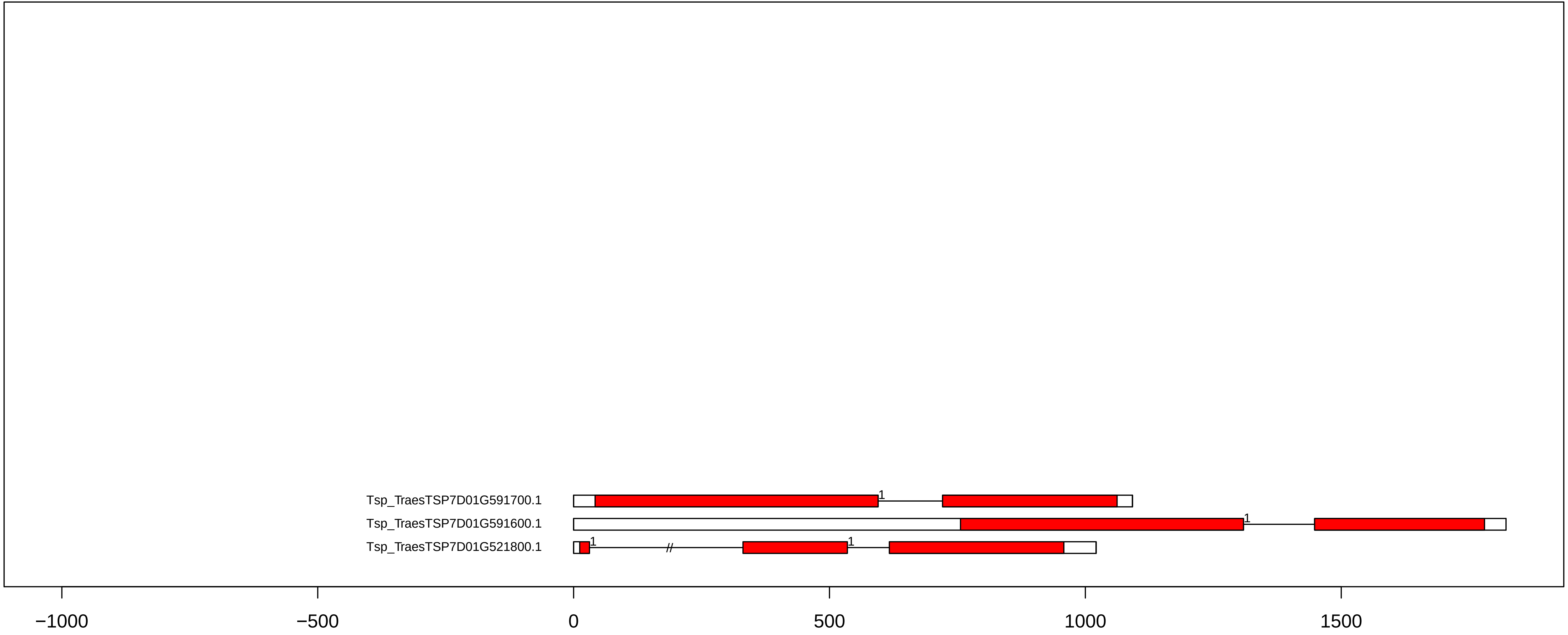

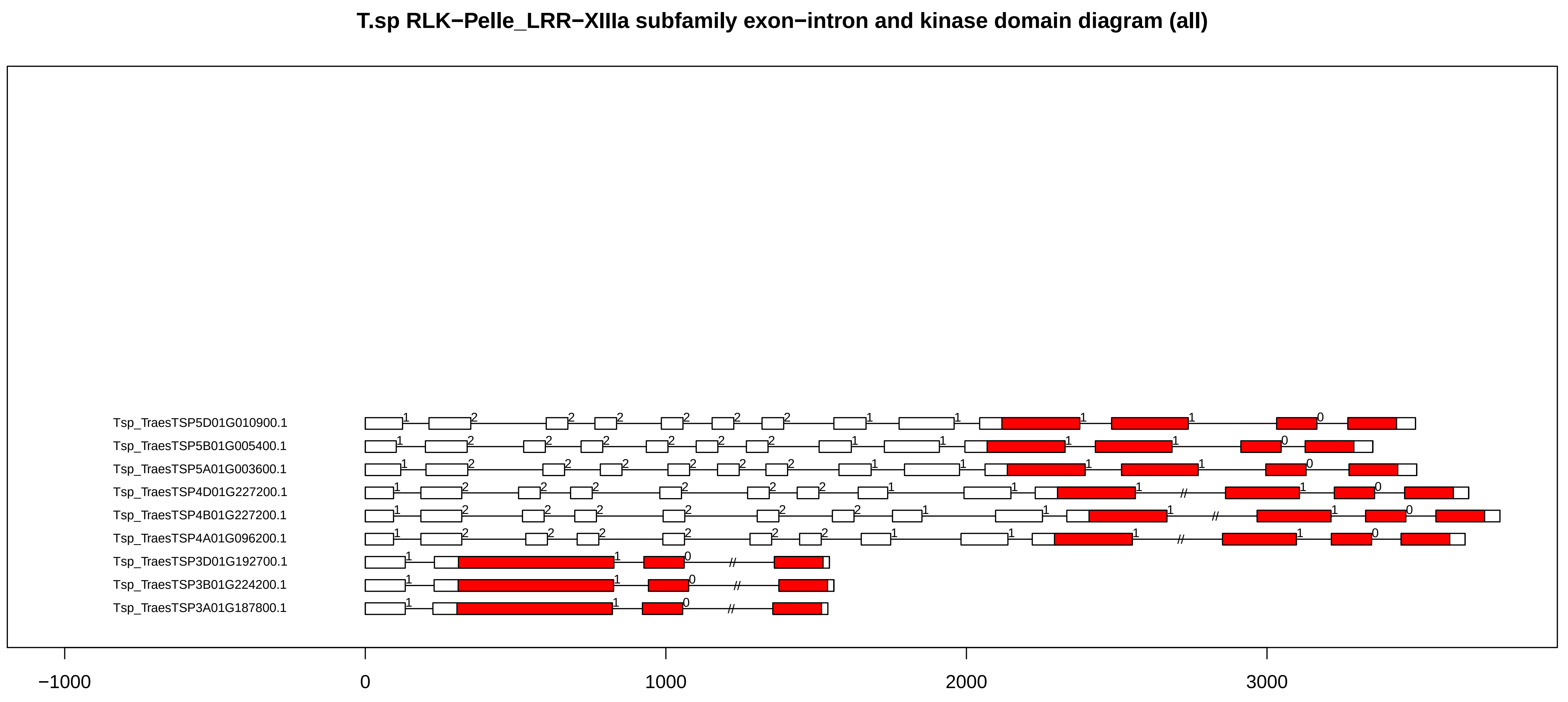
T.sp RLK−Pelle_LRR−XIIIa subfamily exon−intron and kinase domain diagram (all)
Tsp_TraesTSP5D01G010900.1
Tsp_TraesTSP5B01G005400.1
Tsp_TraesTSP5A01G003600.1
Tsp_TraesTSP4D01G227200.1
Tsp_TraesTSP4B01G227200.1
Tsp_TraesTSP4A01G096200.1
Tsp_TraesTSP3D01G192700.1
Tsp_TraesTSP3B01G224200.1
Tsp_TraesTSP3A01G187800.1
−1000
0
1000
2000
3000

T.sp RLK−Pelle_LRR−XIIIb subfamily exon−intron and kinase domain diagram (all)

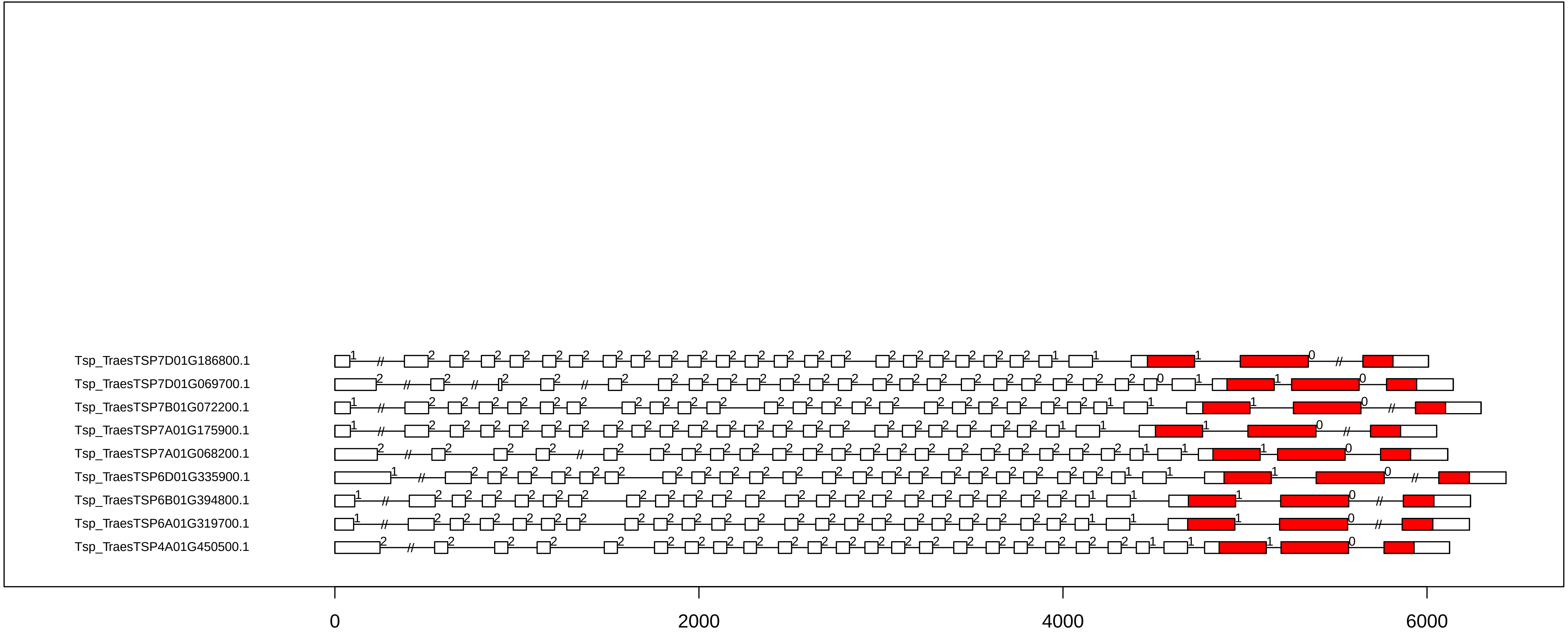

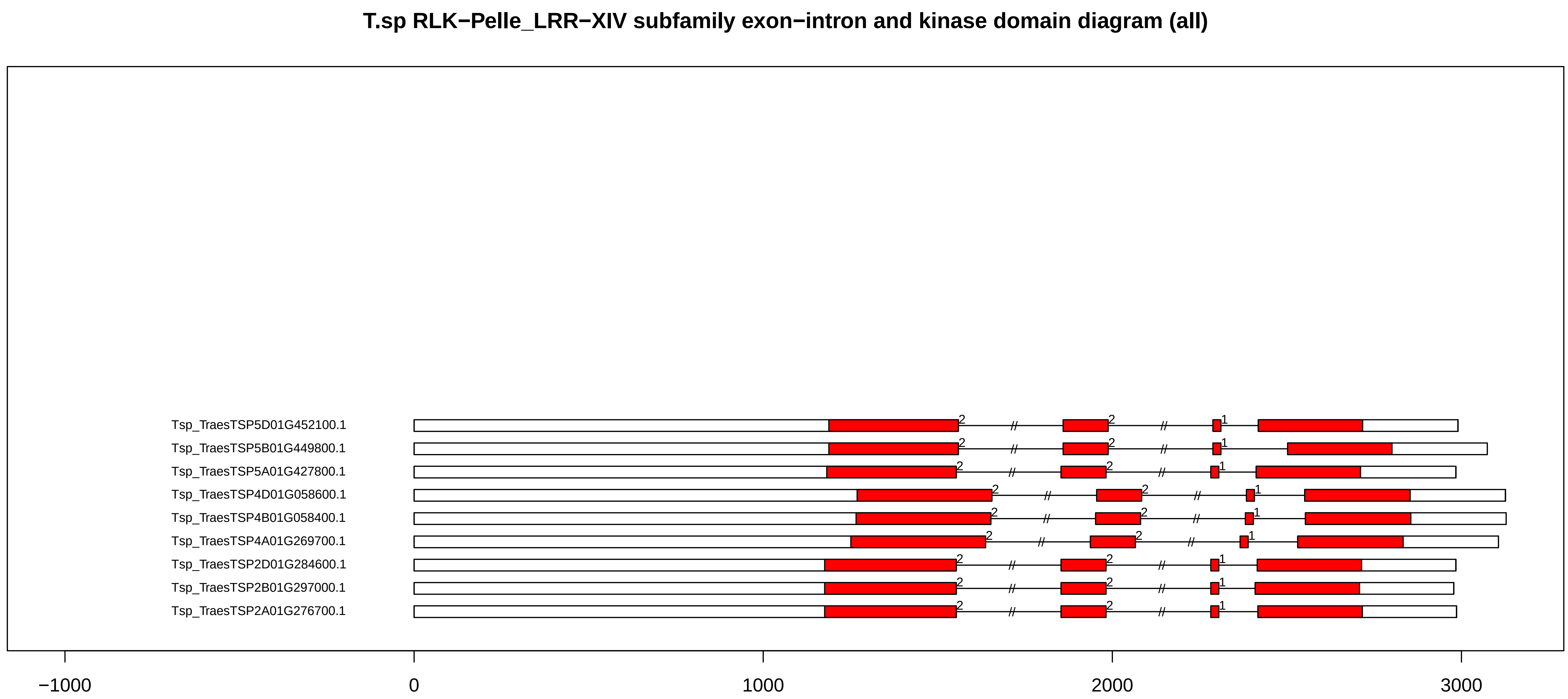

T.sp RLK−Pelle_LRR−XIV subfamily exon−intron and kinase domain diagram (all)
Tsp_TraesTSP5D01G452100.1
Tsp_TraesTSP5B01G449800.1
Tsp_TraesTSP5A01G427800.1
Tsp_TraesTSP4D01G058600.1
Tsp_TraesTSP4B01G058400.1
Tsp_TraesTSP4A01G269700.1
Tsp_TraesTSP2D01G284600.1
Tsp_TraesTSP2B01G297000.1
Tsp_TraesTSP2A01G276700.1
−1000
0
1000
2000
3000

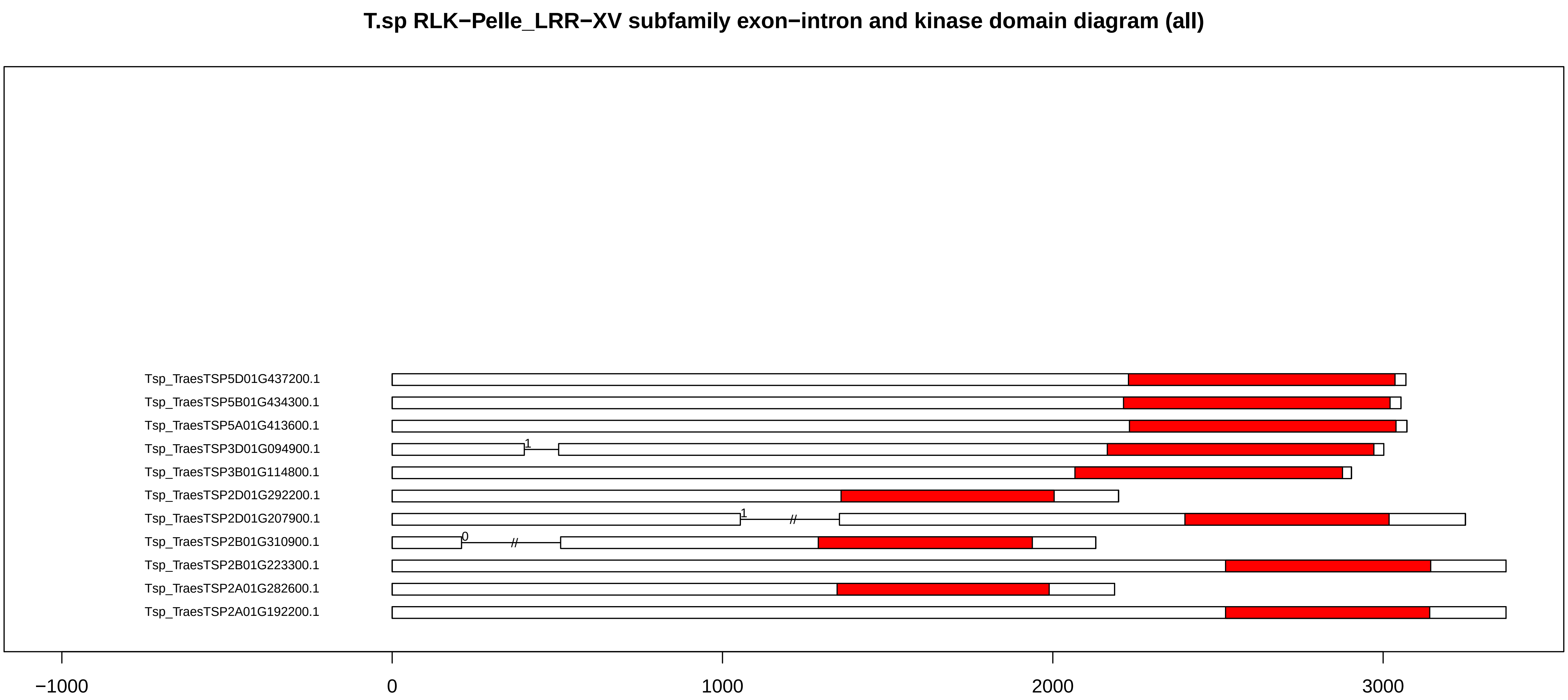
T.sp RLK−Pelle_LRR−XV subfamily exon−intron and kinase domain diagram (all)
Tsp_TraesTSP5D01G437200.1
Tsp_TraesTSP5B01G434300.1
Tsp_TraesTSP5A01G413600.1
Tsp_TraesTSP3D01G094900.1
Tsp_TraesTSP3B01G114800.1
Tsp_TraesTSP2D01G292200.1
Tsp_TraesTSP2D01G207900.1
Tsp_TraesTSP2B01G310900.1
Tsp_TraesTSP2B01G223300.1
Tsp_TraesTSP2A01G282600.1
Tsp_TraesTSP2A01G192200.1
1
1
0
−1000
0
1000
2000
3000

T.sp RLK−Pelle_LysM subfamily exon−intron and kinase domain diagram (part 1)

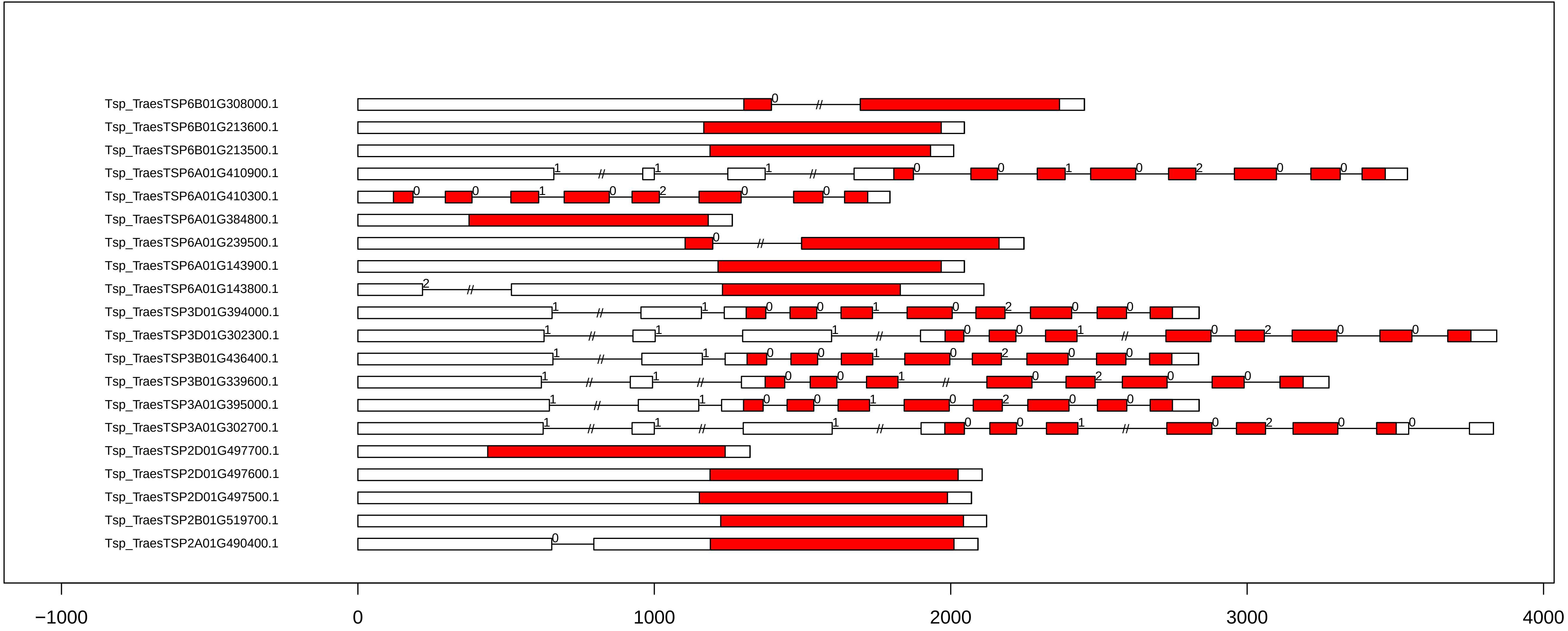

T.sp RLK−Pelle_LysM subfamily exon−intron and kinase domain diagram (part 2)

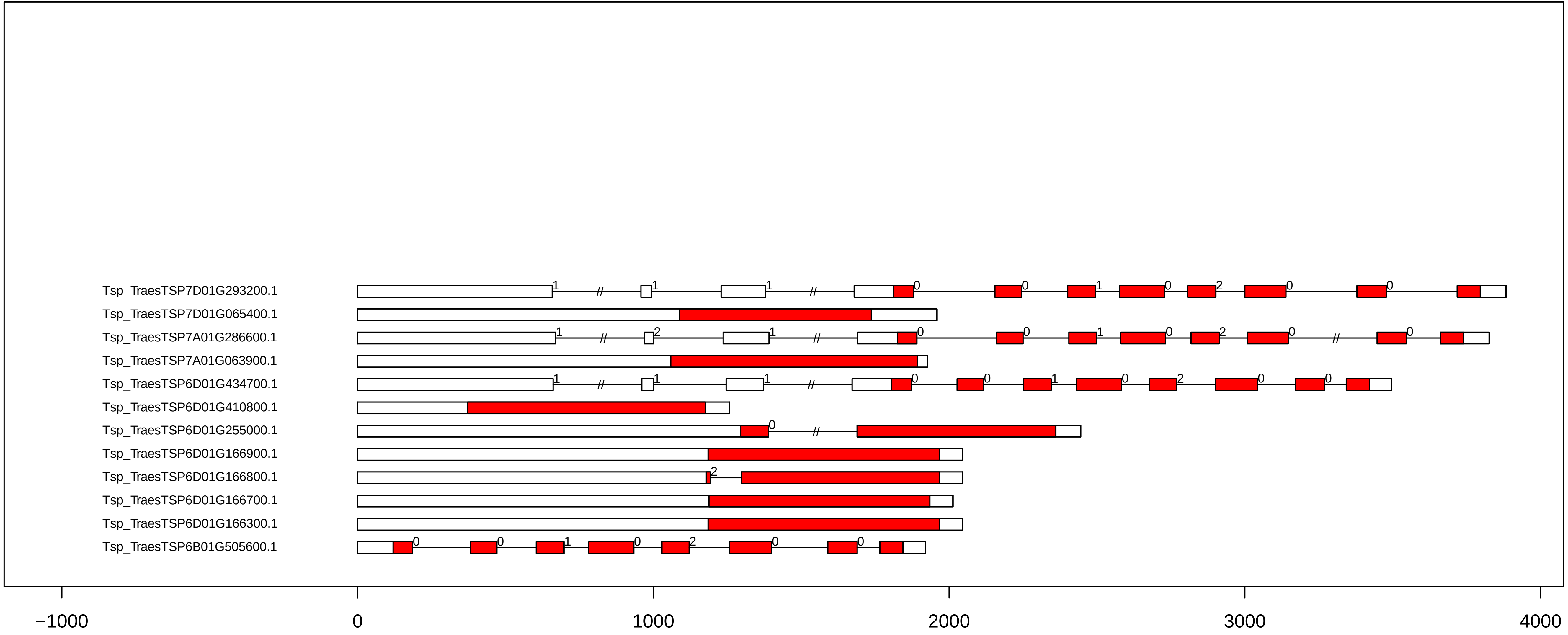

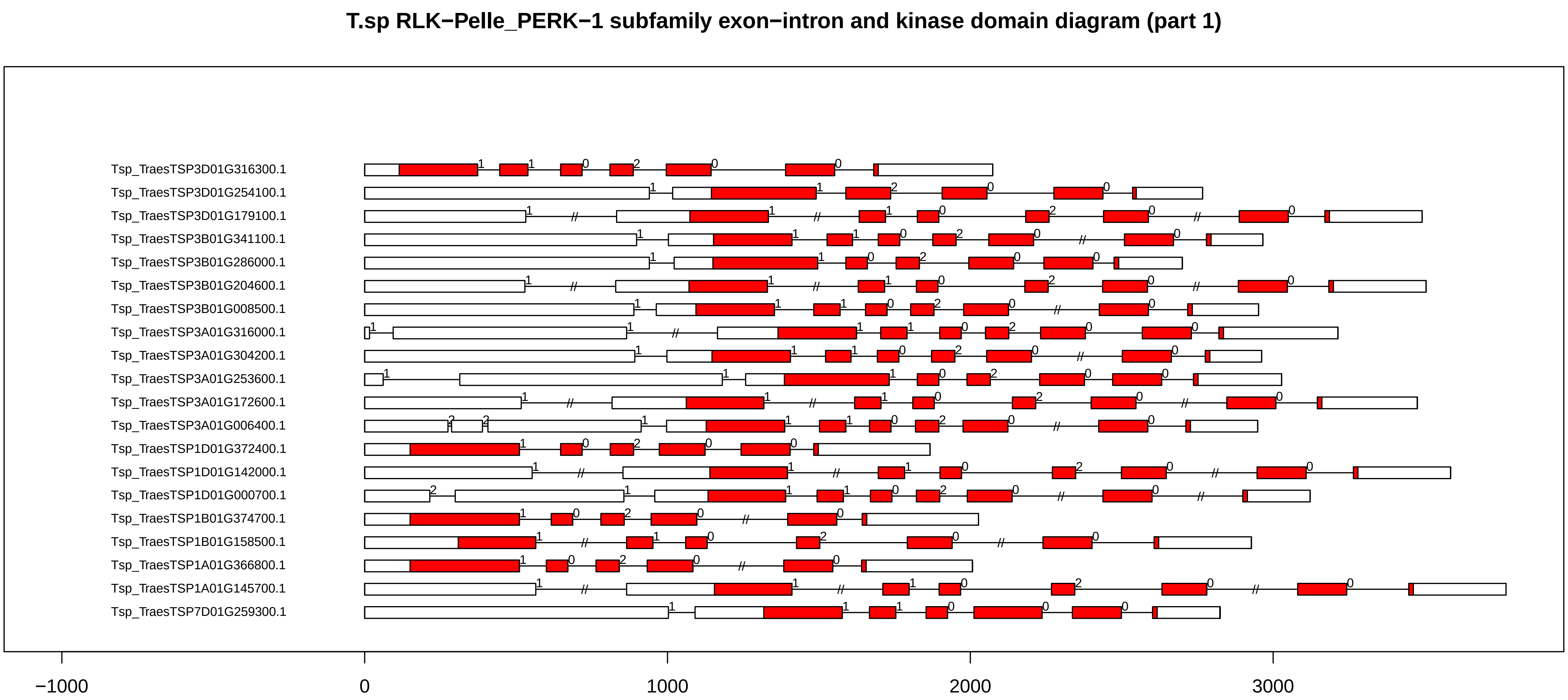

T.sp RLK−Pelle_PERK−1 subfamily exon−intron and kinase domain diagram (part 1)
Tsp_TraesTSP3D01G316300.1
Tsp_TraesTSP3D01G254100.1
Tsp_TraesTSP3D01G179100.1
Tsp_TraesTSP3B01G341100.1
Tsp_TraesTSP3B01G286000.1
Tsp_TraesTSP3B01G204600.1
Tsp_TraesTSP3B01G008500.1
Tsp_TraesTSP3A01G316000.1
Tsp_TraesTSP3A01G304200.1
Tsp_TraesTSP3A01G253600.1
Tsp_TraesTSP3A01G172600.1
Tsp_TraesTSP3A01G006400.1
Tsp_TraesTSP1D01G372400.1
Tsp_TraesTSP1D01G142000.1
Tsp_TraesTSP1D01G000700.1
Tsp_TraesTSP1B01G374700.1
Tsp_TraesTSP1B01G158500.1
Tsp_TraesTSP1A01G366800.1
Tsp_TraesTSP1A01G145700.1
Tsp_TraesTSP7D01G259300.1
−1000
0
1000
2000
3000

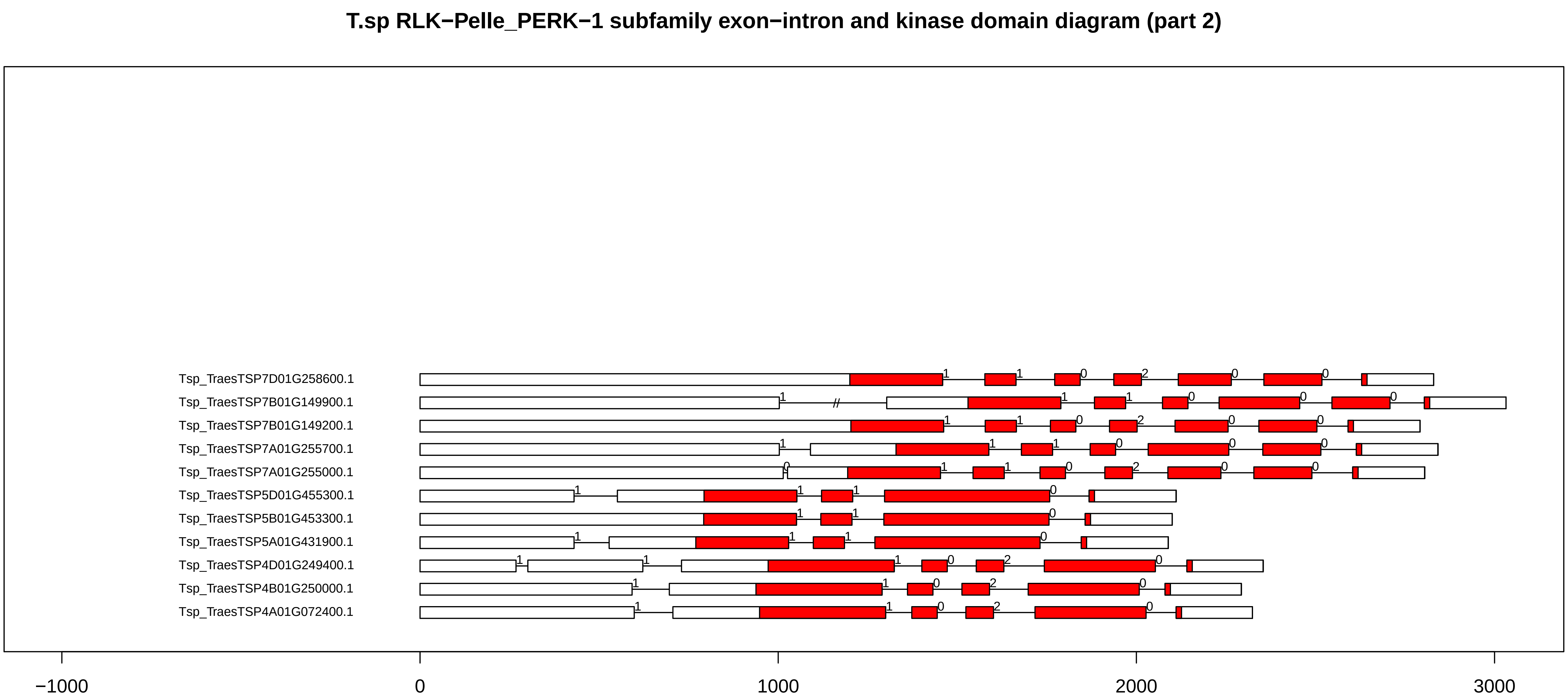

T.sp RLK−Pelle_PERK−1 subfamily exon−intron and kinase domain diagram (part 2)
Tsp_TraesTSP7D01G258600.1
Tsp_TraesTSP7B01G149900.1
Tsp_TraesTSP7B01G149200.1
Tsp_TraesTSP7A01G255700.1
Tsp_TraesTSP7A01G255000.1
Tsp_TraesTSP5D01G455300.1
Tsp_TraesTSP5B01G453300.1
Tsp_TraesTSP5A01G431900.1
Tsp_TraesTSP4D01G249400.1
Tsp_TraesTSP4B01G250000.1
Tsp_TraesTSP4A01G072400.1
−1000
0
1000
2000
3000

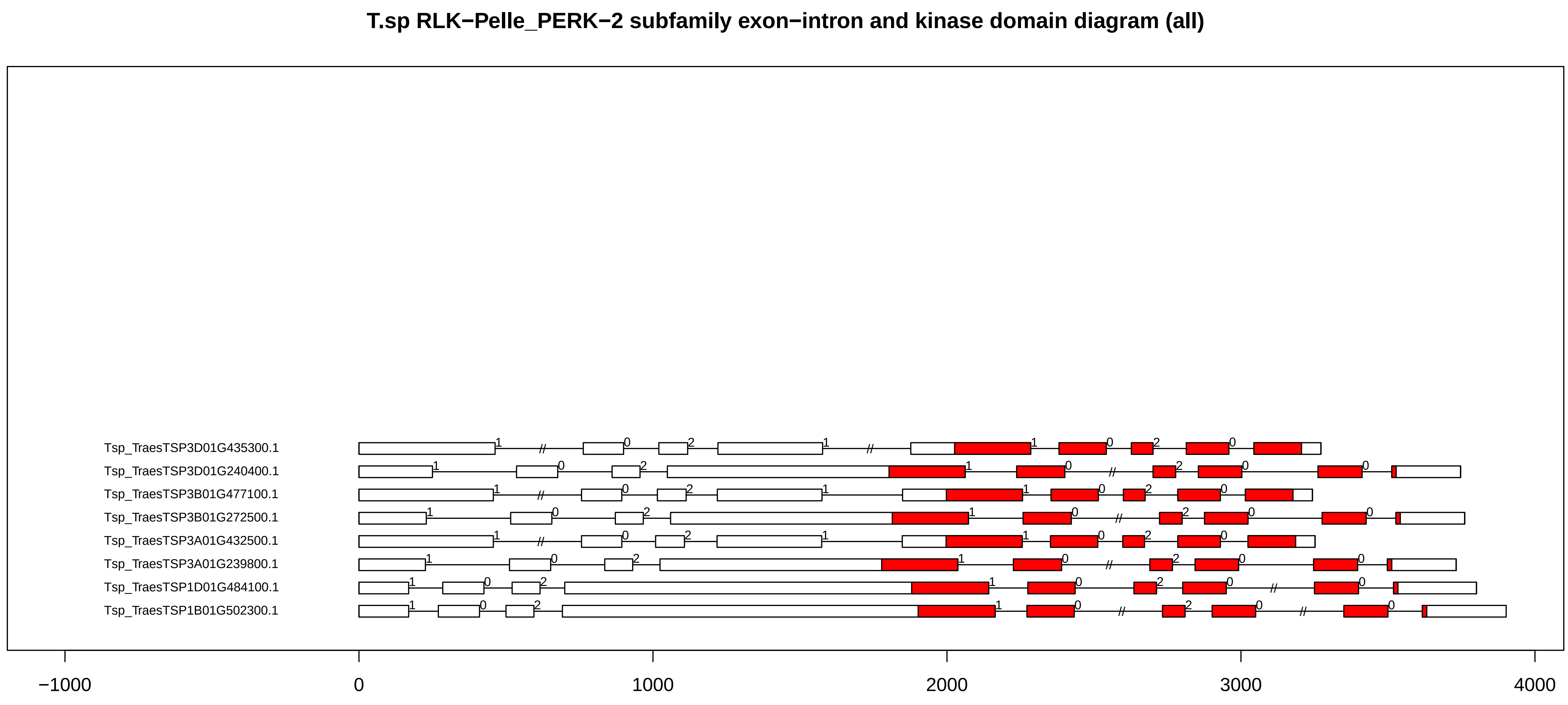

T.sp RLK−Pelle_PERK−2 subfamily exon−intron and kinase domain diagram (all)
Tsp_TraesTSP3D01G435300.1
Tsp_TraesTSP3D01G240400.1
Tsp_TraesTSP3B01G477100.1
Tsp_TraesTSP3B01G272500.1
Tsp_TraesTSP3A01G432500.1
Tsp_TraesTSP3A01G239800.1
Tsp_TraesTSP1D01G484100.1
Tsp_TraesTSP1B01G502300.1
−1000
0
1000
2000
3000
4000

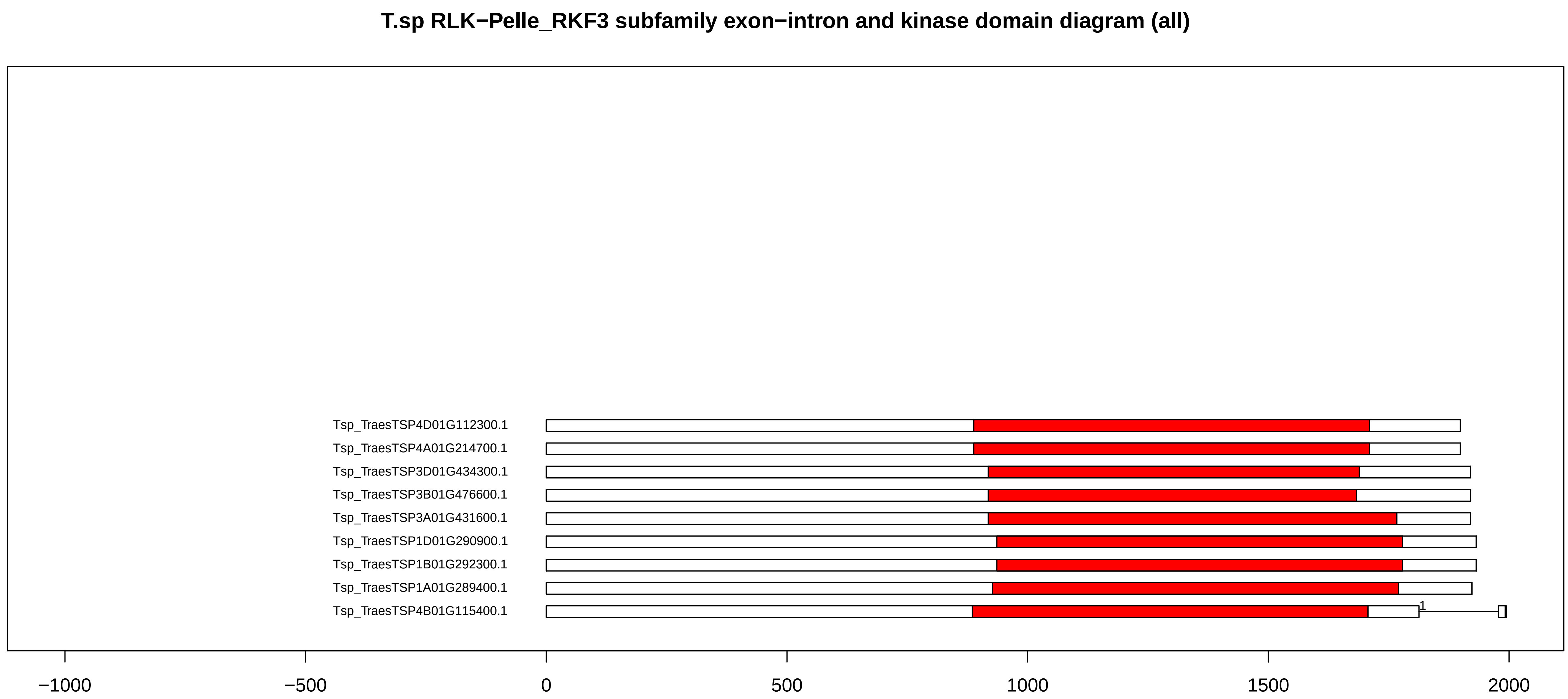

T.sp RLK−Pelle_RKF3 subfamily exon−intron and kinase domain diagram (all)
Tsp_TraesTSP4D01G112300.1
Tsp_TraesTSP4A01G214700.1
Tsp_TraesTSP3D01G434300.1
Tsp_TraesTSP3B01G476600.1
Tsp_TraesTSP3A01G431600.1
Tsp_TraesTSP1D01G290900.1
Tsp_TraesTSP1B01G292300.1
Tsp_TraesTSP1A01G289400.1
Tsp_TraesTSP4B01G115400.1
1
−1000
−500
0
500
1000
1500
2000

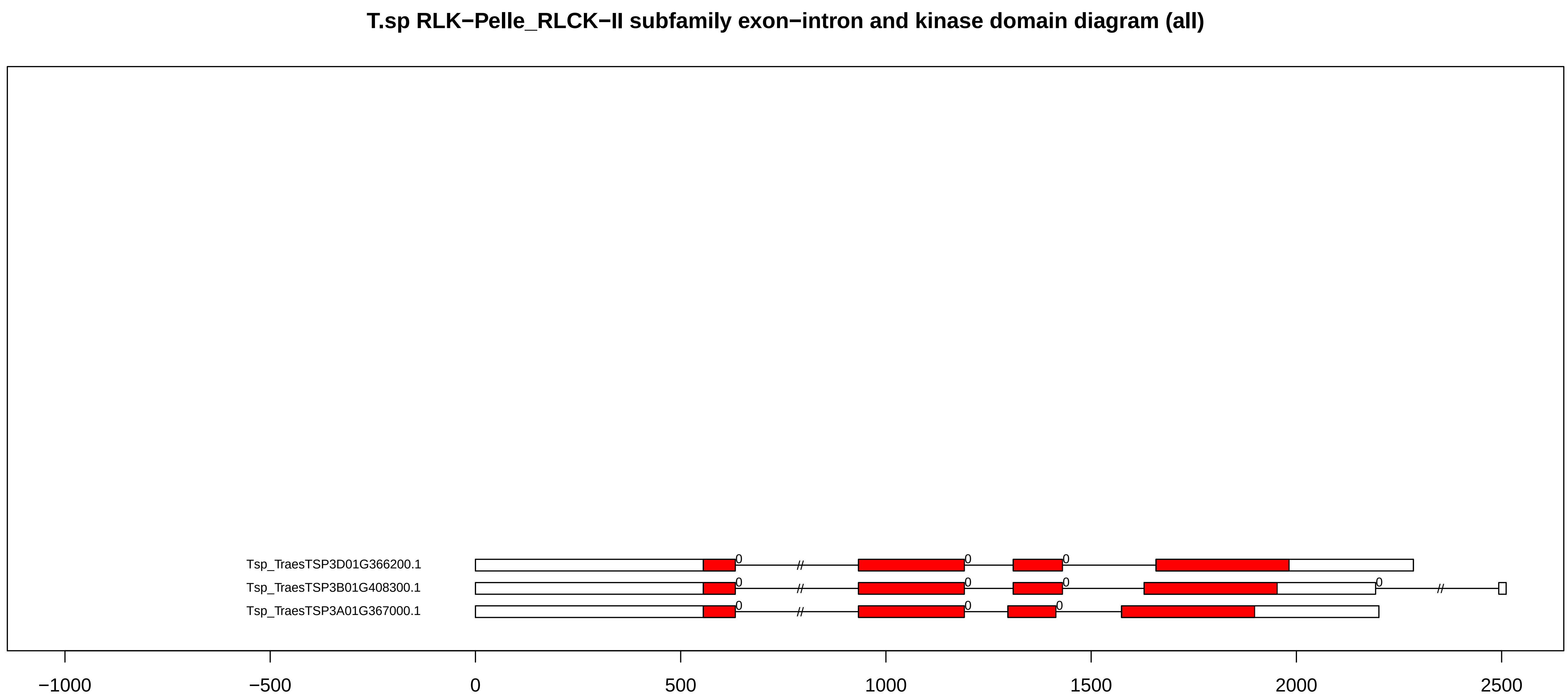
T.sp RLK−Pelle_RLCK−II subfamily exon−intron and kinase domain diagram (all)
Tsp_TraesTSP3D01G366200.1
Tsp_TraesTSP3B01G408300.1
Tsp_TraesTSP3A01G367000.1
−1000
−500
0
500
1000
1500
2000
2500

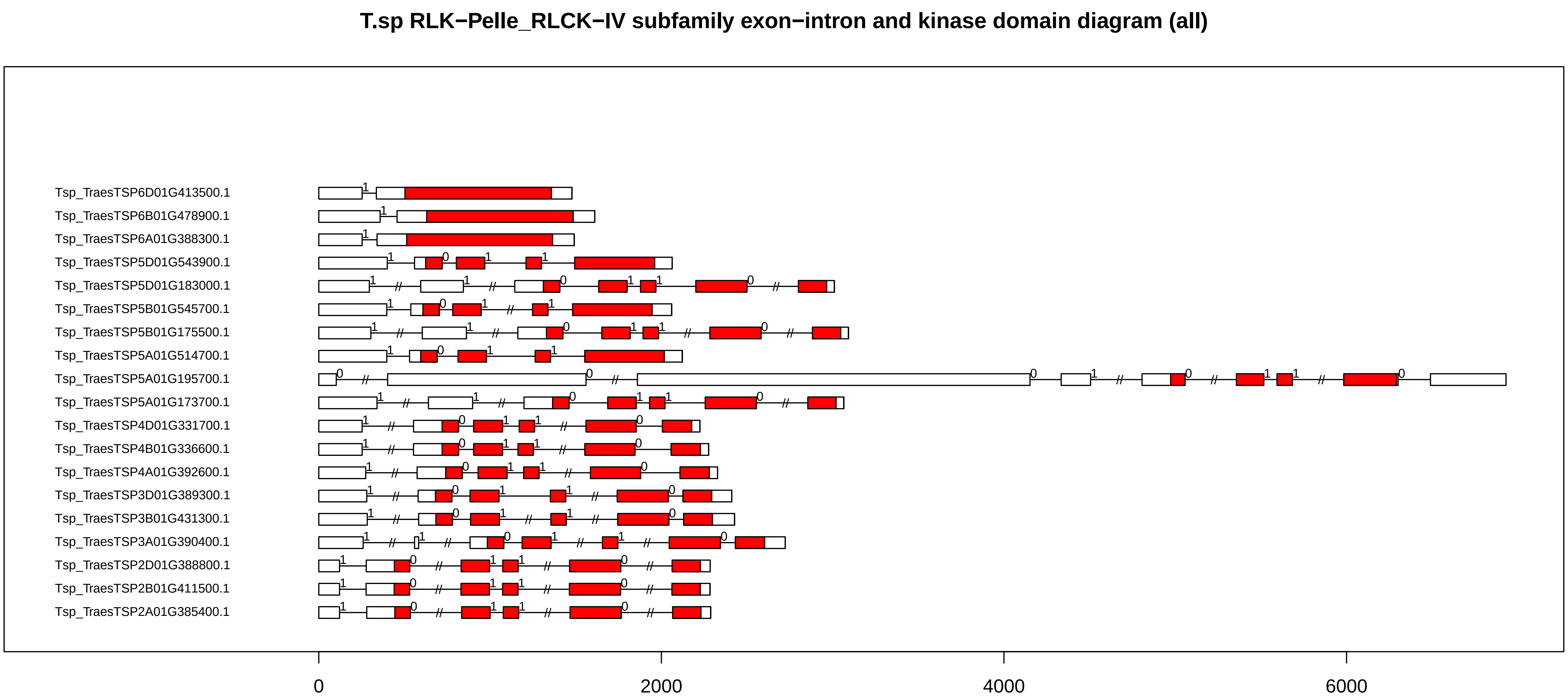
T.sp RLK−Pelle_RLCK−IV subfamily exon−intron and kinase domain diagram (all)
Tsp_TraesTSP6D01G413500.1
Tsp_TraesTSP6B01G478900.1
Tsp_TraesTSP6A01G388300.1
Tsp_TraesTSP5D01G543900.1
Tsp_TraesTSP5D01G183000.1
Tsp_TraesTSP5B01G545700.1
Tsp_TraesTSP5B01G175500.1
Tsp_TraesTSP5A01G514700.1
Tsp_TraesTSP5A01G195700.1
Tsp_TraesTSP5A01G173700.1
Tsp_TraesTSP4D01G331700.1
Tsp_TraesTSP4B01G336600.1
Tsp_TraesTSP4A01G392600.1
Tsp_TraesTSP3D01G389300.1
Tsp_TraesTSP3B01G431300.1
Tsp_TraesTSP3A01G390400.1
Tsp_TraesTSP2D01G388800.1
Tsp_TraesTSP2B01G411500.1
Tsp_TraesTSP2A01G385400.1
0
2000
4000
6000

T.sp RLK−Pelle_RLCK−IXa subfamily exon−intron and kinase domain diagram (all)

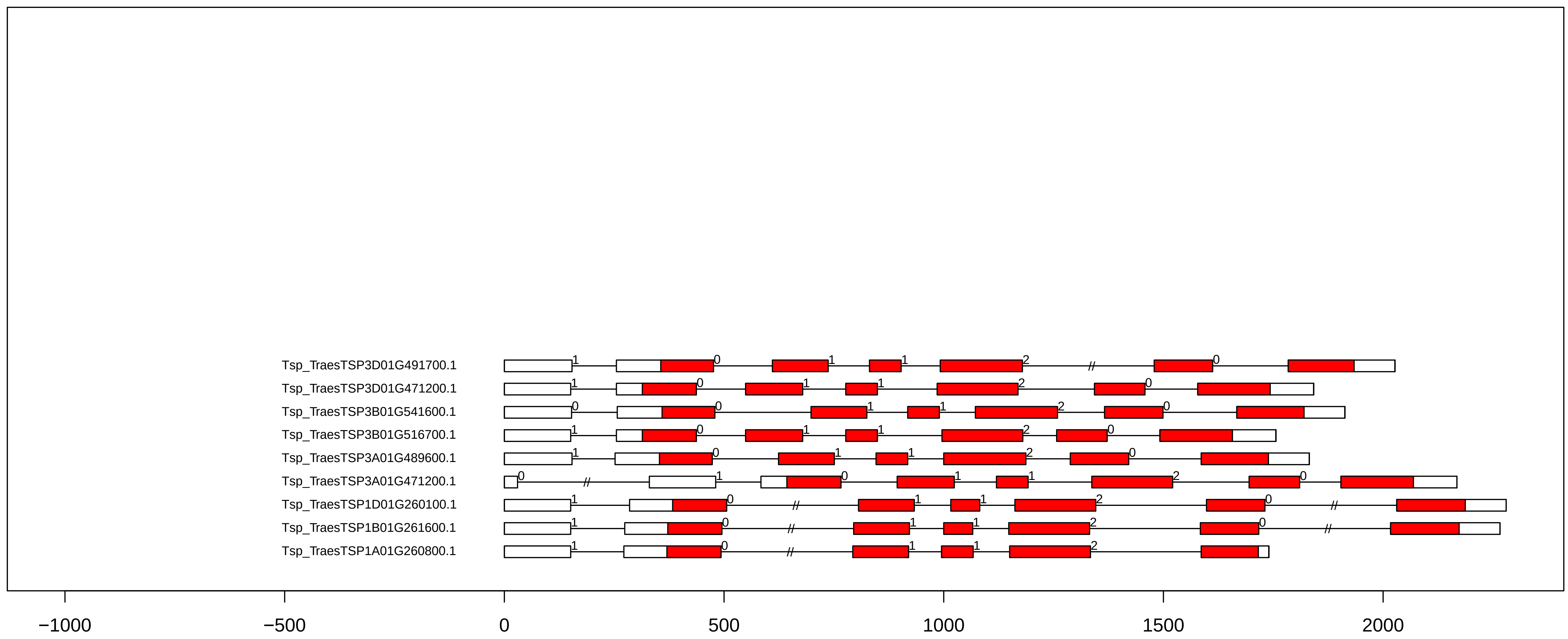

T.sp RLK−Pelle_RLCK−IXb subfamily exon−intron and kinase domain diagram (part 1)

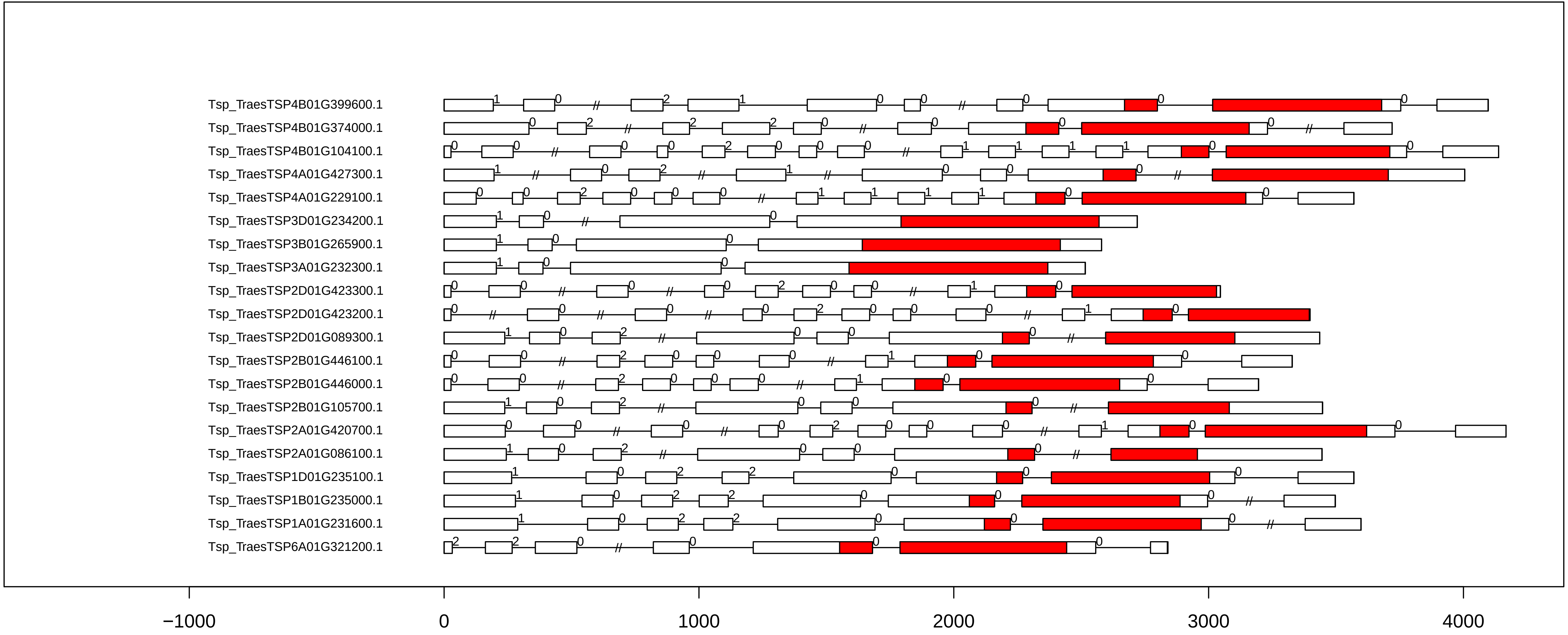

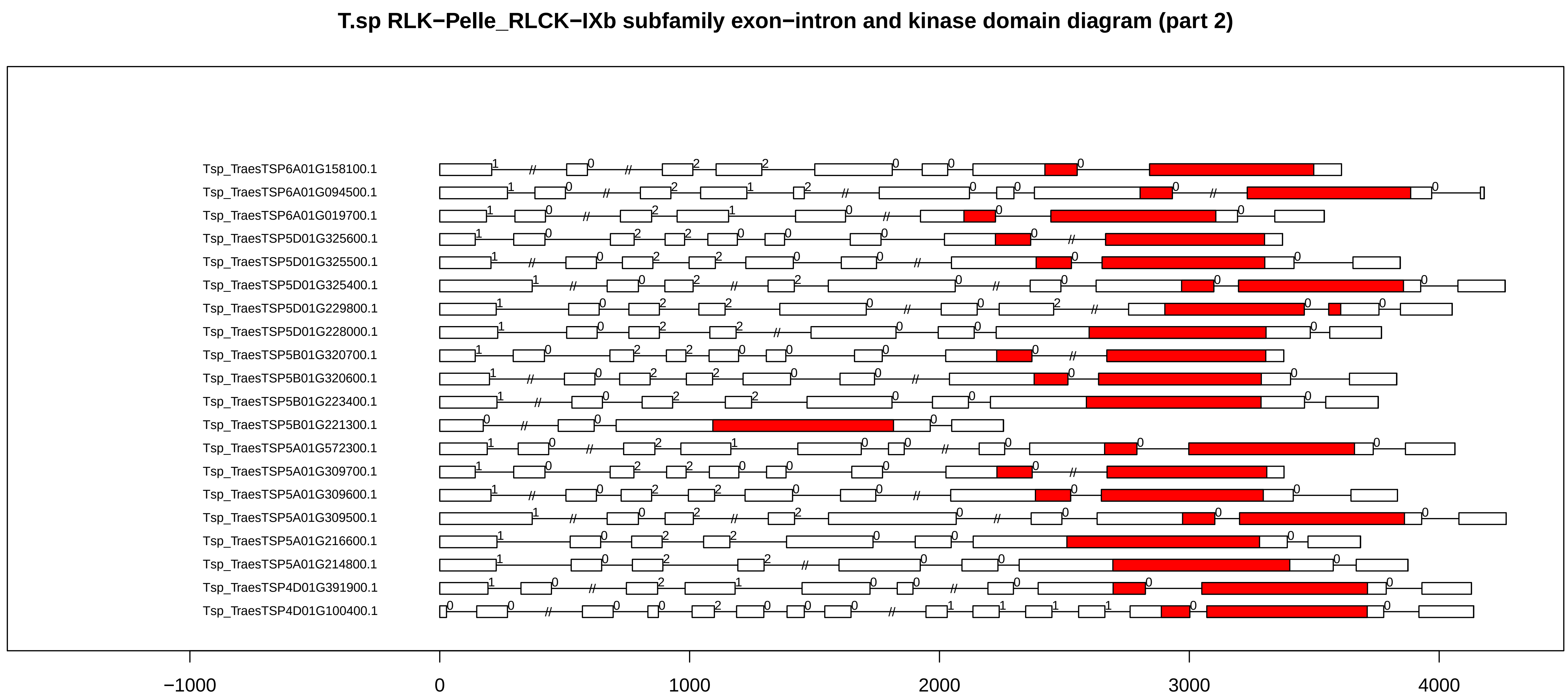

T.sp RLK−Pelle_RLCK−IXb subfamily exon−intron and kinase domain diagram (part 2)
Tsp_TraesTSP6A01G158100.1
Tsp_TraesTSP6A01G094500.1
Tsp_TraesTSP6A01G019700.1
Tsp_TraesTSP5D01G325600.1
Tsp_TraesTSP5D01G325500.1
Tsp_TraesTSP5D01G325400.1
Tsp_TraesTSP5D01G229800.1
Tsp_TraesTSP5D01G228000.1
Tsp_TraesTSP5B01G320700.1
Tsp_TraesTSP5B01G320600.1
Tsp_TraesTSP5B01G223400.1
Tsp_TraesTSP5B01G221300.1
Tsp_TraesTSP5A01G572300.1
Tsp_TraesTSP5A01G309700.1
Tsp_TraesTSP5A01G309600.1
Tsp_TraesTSP5A01G309500.1
Tsp_TraesTSP5A01G216600.1
Tsp_TraesTSP5A01G214800.1
Tsp_TraesTSP4D01G391900.1
Tsp_TraesTSP4D01G100400.1
−1000
0
1000
2000
3000
4000

T.sp RLK−Pelle_RLCK−IXb subfamily exon−intron and kinase domain diagram (part 3)

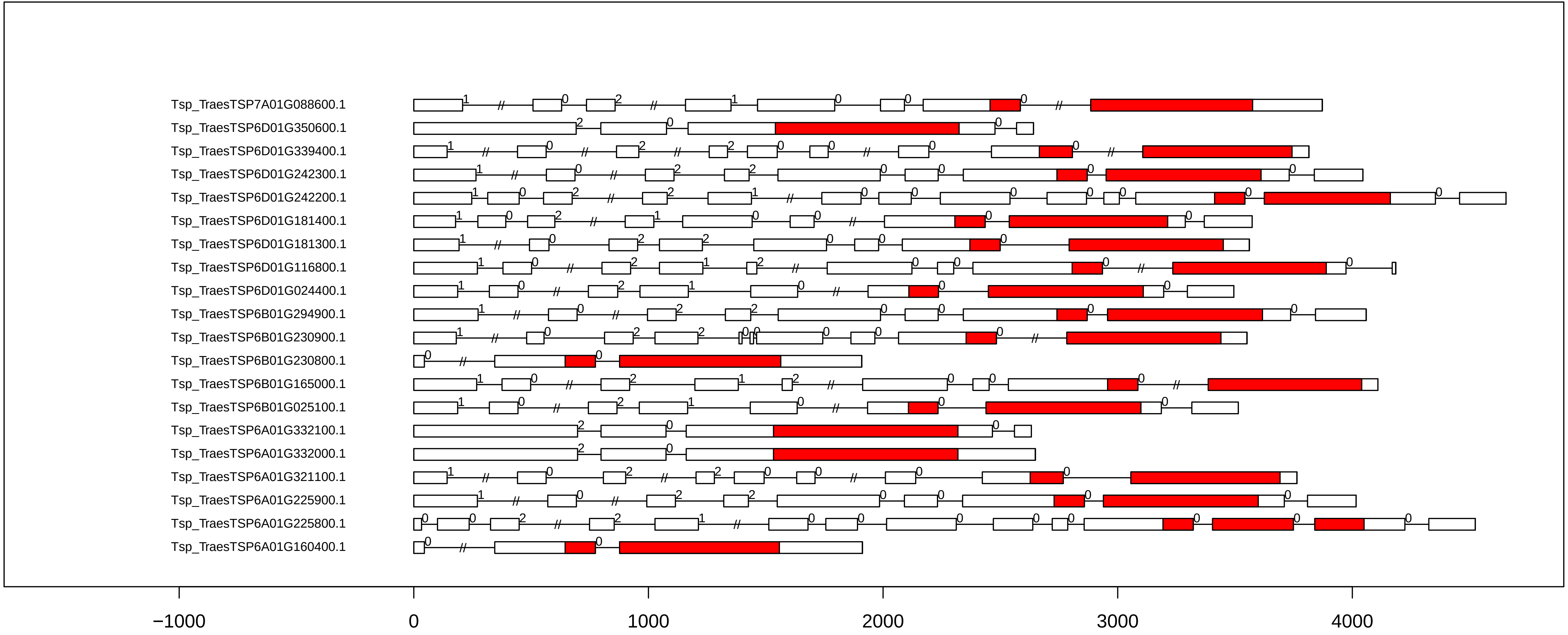

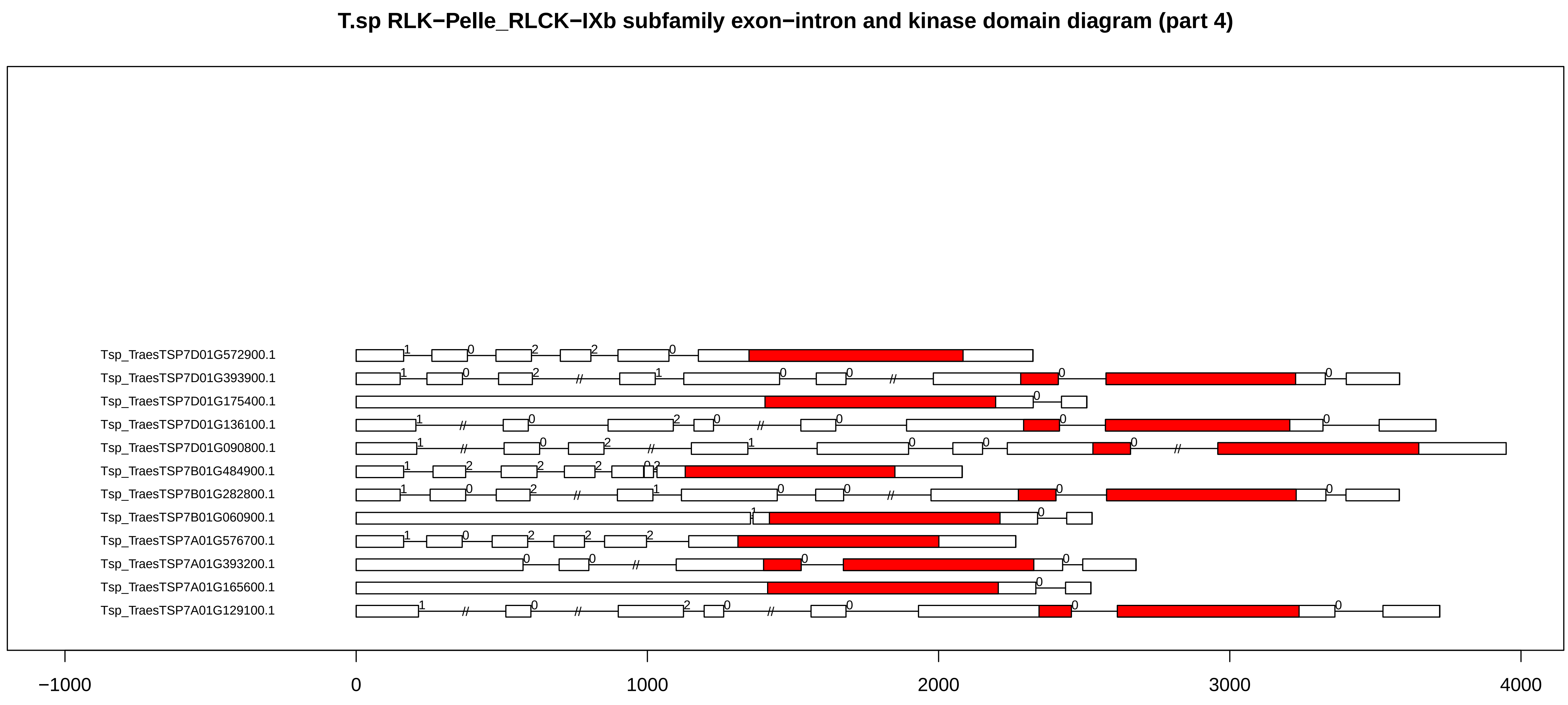

T.sp RLK−Pelle_RLCK−IXb subfamily exon−intron and kinase domain diagram (part 4)
Tsp_TraesTSP7D01G572900.1
Tsp_TraesTSP7D01G393900.1
Tsp_TraesTSP7D01G175400.1
Tsp_TraesTSP7D01G136100.1
Tsp_TraesTSP7D01G090800.1
Tsp_TraesTSP7B01G484900.1
Tsp_TraesTSP7B01G282800.1
Tsp_TraesTSP7B01G060900.1
Tsp_TraesTSP7A01G576700.1
Tsp_TraesTSP7A01G393200.1
Tsp_TraesTSP7A01G165600.1
Tsp_TraesTSP7A01G129100.1
−1000
0
1000
2000
3000
4000

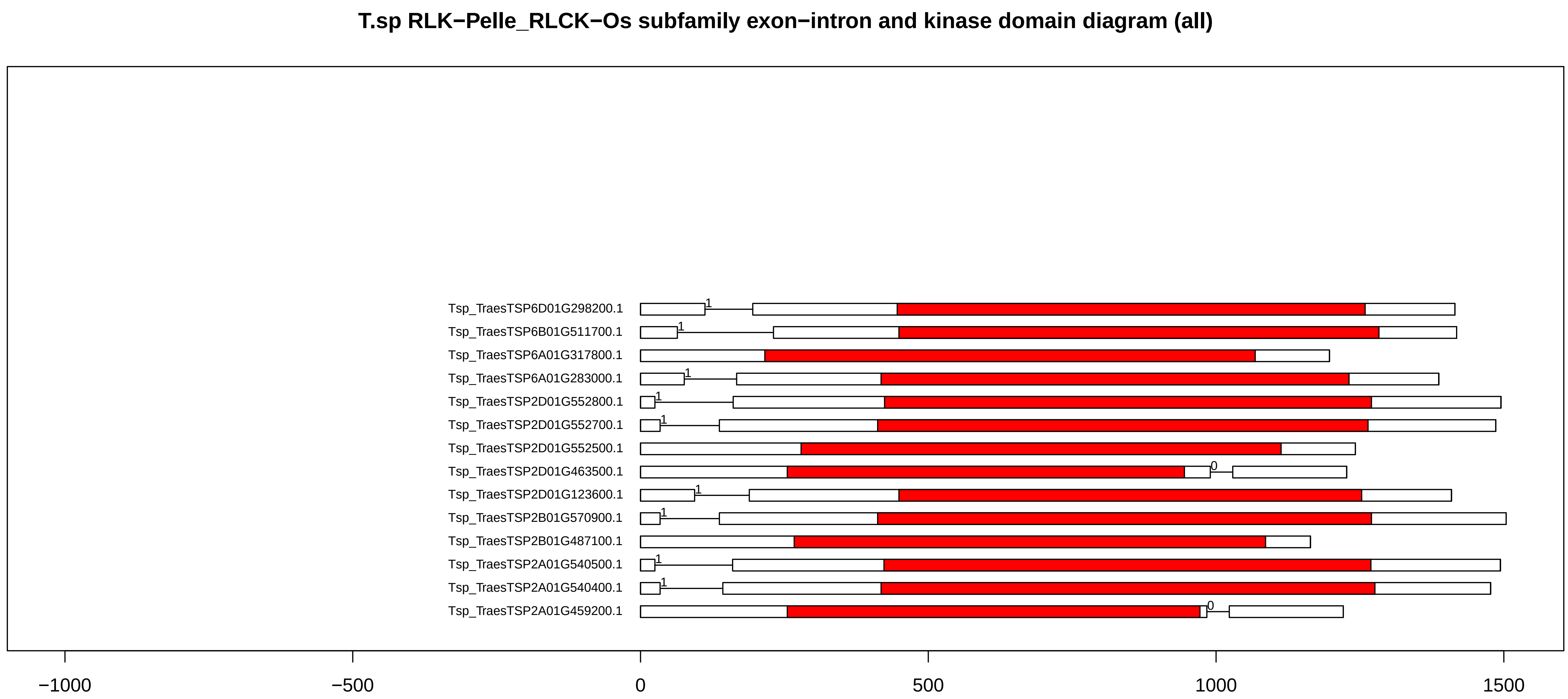

T.sp RLK−Pelle_RLCK−Os subfamily exon−intron and kinase domain diagram (all)
Tsp_TraesTSP6D01G298200.1
Tsp_TraesTSP6B01G511700.1
Tsp_TraesTSP6A01G317800.1
Tsp_TraesTSP6A01G283000.1
Tsp_TraesTSP2D01G552800.1
Tsp_TraesTSP2D01G552700.1
Tsp_TraesTSP2D01G552500.1
Tsp_TraesTSP2D01G463500.1
Tsp_TraesTSP2D01G123600.1
Tsp_TraesTSP2B01G570900.1
Tsp_TraesTSP2B01G487100.1
Tsp_TraesTSP2A01G540500.1
Tsp_TraesTSP2A01G540400.1
Tsp_TraesTSP2A01G459200.1
1
0
−1000
−500
0
500
1000
1500

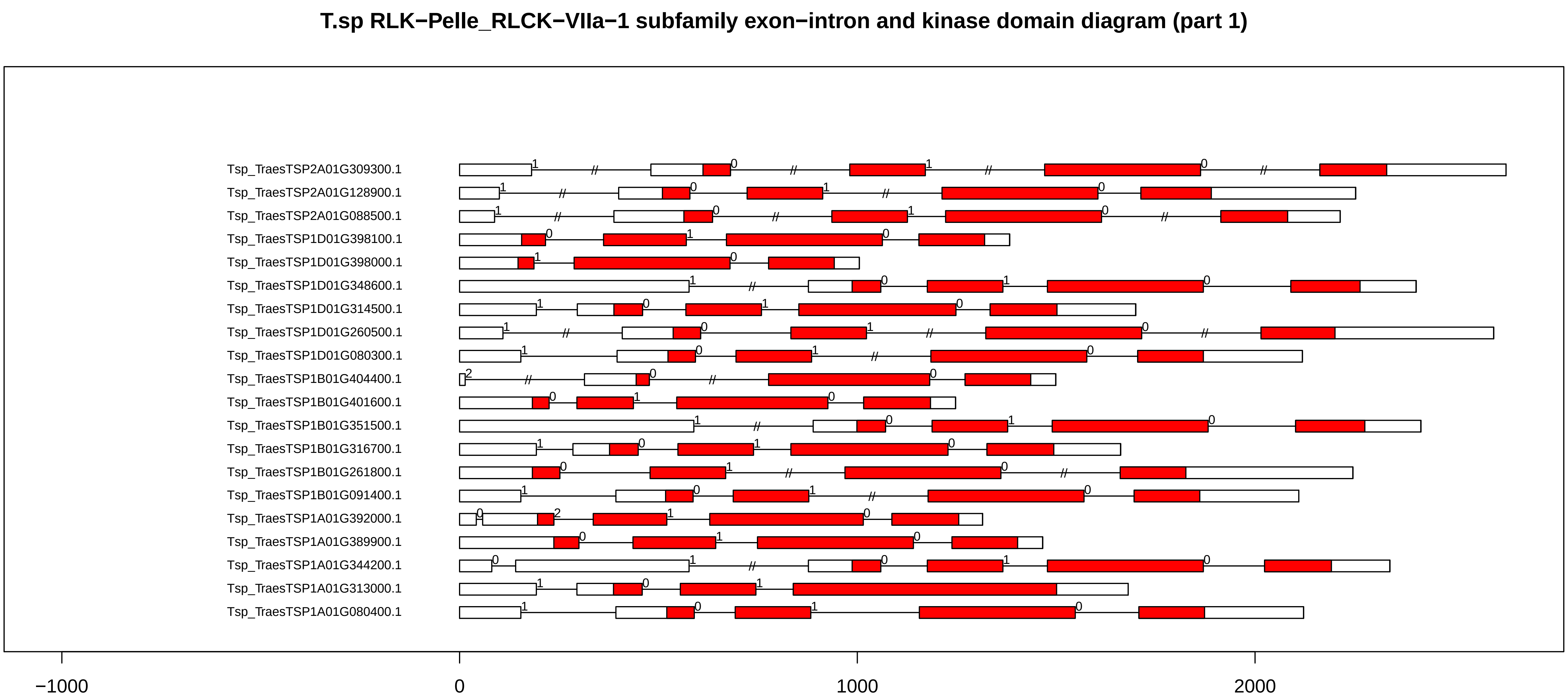
T.sp RLK−Pelle_RLCK−VIIa−1 subfamily exon−intron and kinase domain diagram (part 1)
Tsp_TraesTSP2A01G309300.1
Tsp_TraesTSP2A01G128900.1
Tsp_TraesTSP2A01G088500.1
Tsp_TraesTSP1D01G398100.1
Tsp_TraesTSP1D01G398000.1
Tsp_TraesTSP1D01G348600.1
Tsp_TraesTSP1D01G314500.1
Tsp_TraesTSP1D01G260500.1
Tsp_TraesTSP1D01G080300.1
Tsp_TraesTSP1B01G404400.1
Tsp_TraesTSP1B01G401600.1
Tsp_TraesTSP1B01G351500.1
Tsp_TraesTSP1B01G316700.1
Tsp_TraesTSP1B01G261800.1
Tsp_TraesTSP1B01G091400.1
Tsp_TraesTSP1A01G392000.1
Tsp_TraesTSP1A01G389900.1
Tsp_TraesTSP1A01G344200.1
Tsp_TraesTSP1A01G313000.1
Tsp_TraesTSP1A01G080400.1
−1000
0
1000
2000

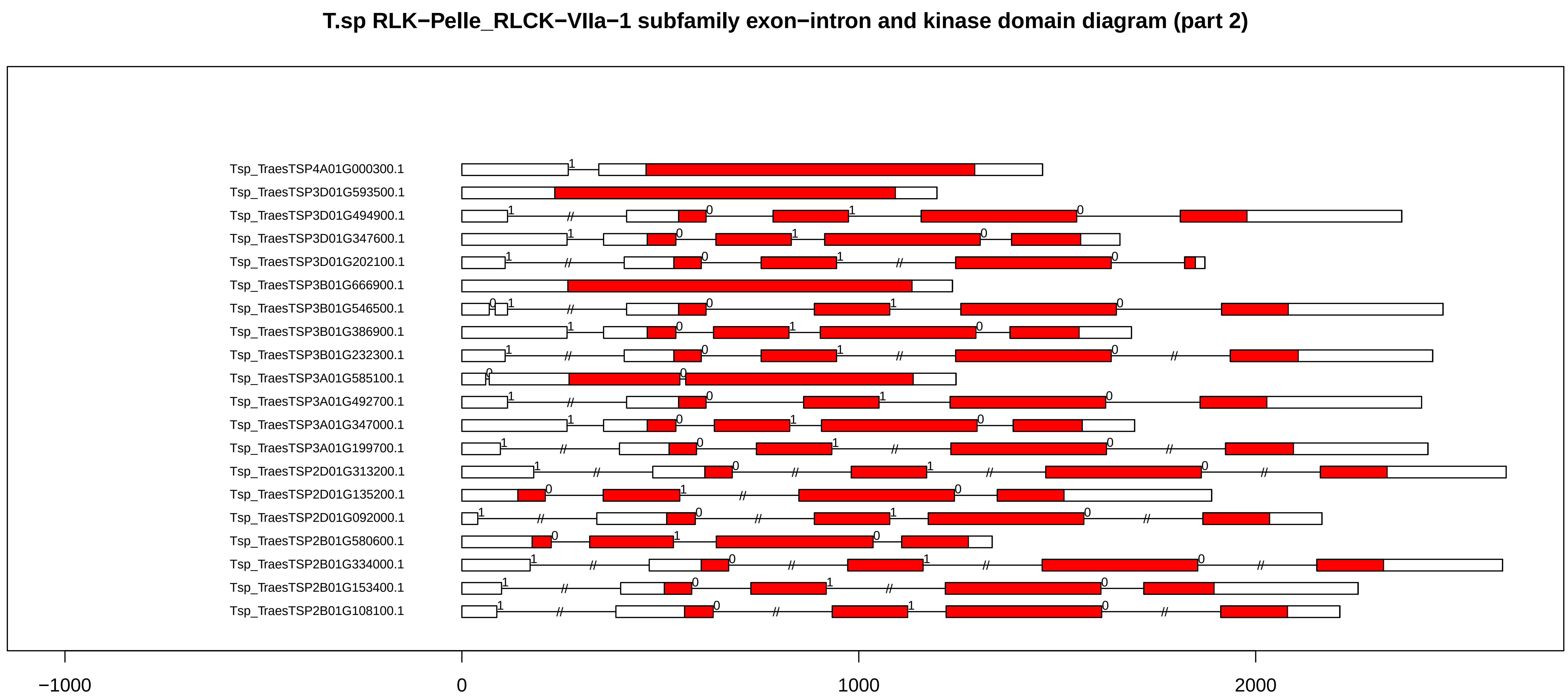

T.sp RLK−Pelle_RLCK−VIIa−1 subfamily exon−intron and kinase domain diagram (part 2)
Tsp_TraesTSP4A01G000300.1
Tsp_TraesTSP3D01G593500.1
Tsp_TraesTSP3D01G494900.1
Tsp_TraesTSP3D01G347600.1
Tsp_TraesTSP3D01G202100.1
Tsp_TraesTSP3B01G666900.1
Tsp_TraesTSP3B01G546500.1
Tsp_TraesTSP3B01G386900.1
Tsp_TraesTSP3B01G232300.1
Tsp_TraesTSP3A01G585100.1
Tsp_TraesTSP3A01G492700.1
Tsp_TraesTSP3A01G347000.1
Tsp_TraesTSP3A01G199700.1
Tsp_TraesTSP2D01G313200.1
Tsp_TraesTSP2D01G135200.1
Tsp_TraesTSP2D01G092000.1
Tsp_TraesTSP2B01G580600.1
Tsp_TraesTSP2B01G334000.1
Tsp_TraesTSP2B01G153400.1
Tsp_TraesTSP2B01G108100.1
−1000
0
1000
2000

T.sp RLK−Pelle_RLCK−VIIa−1 subfamily exon−intron and kinase domain diagram (part 3)

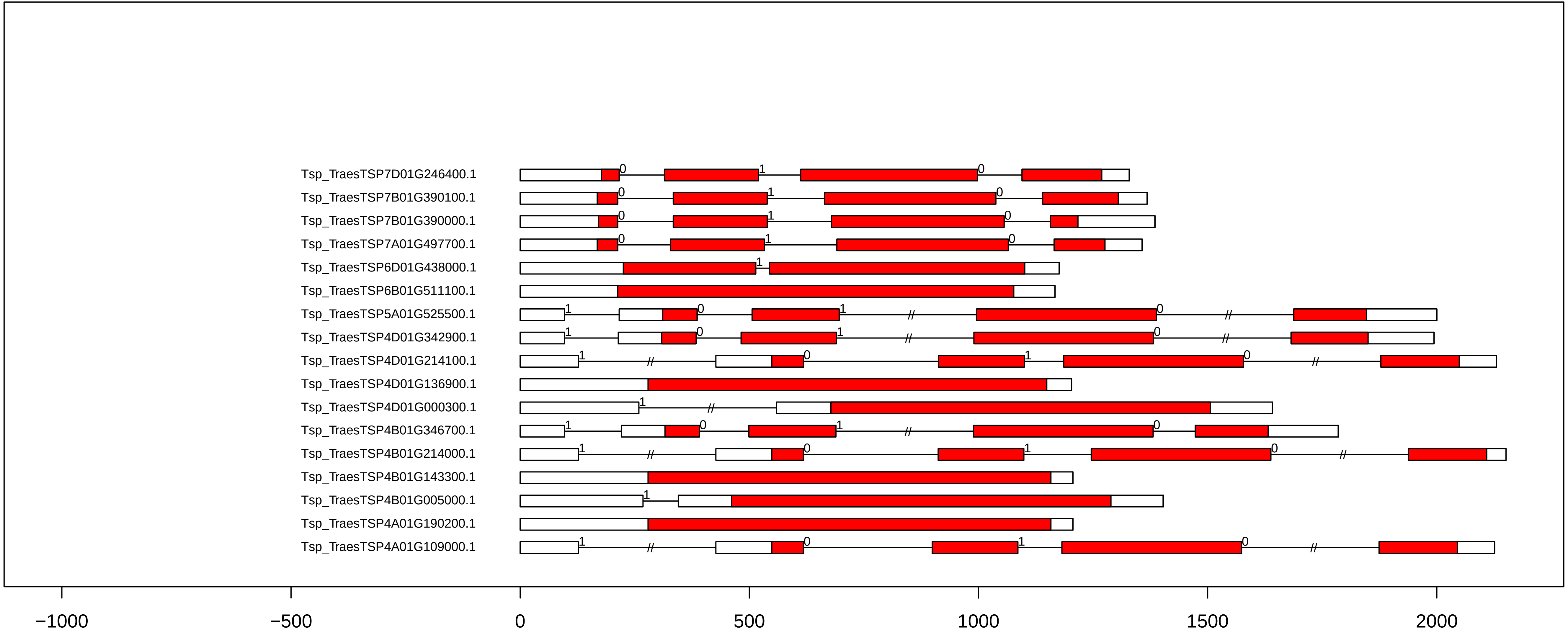

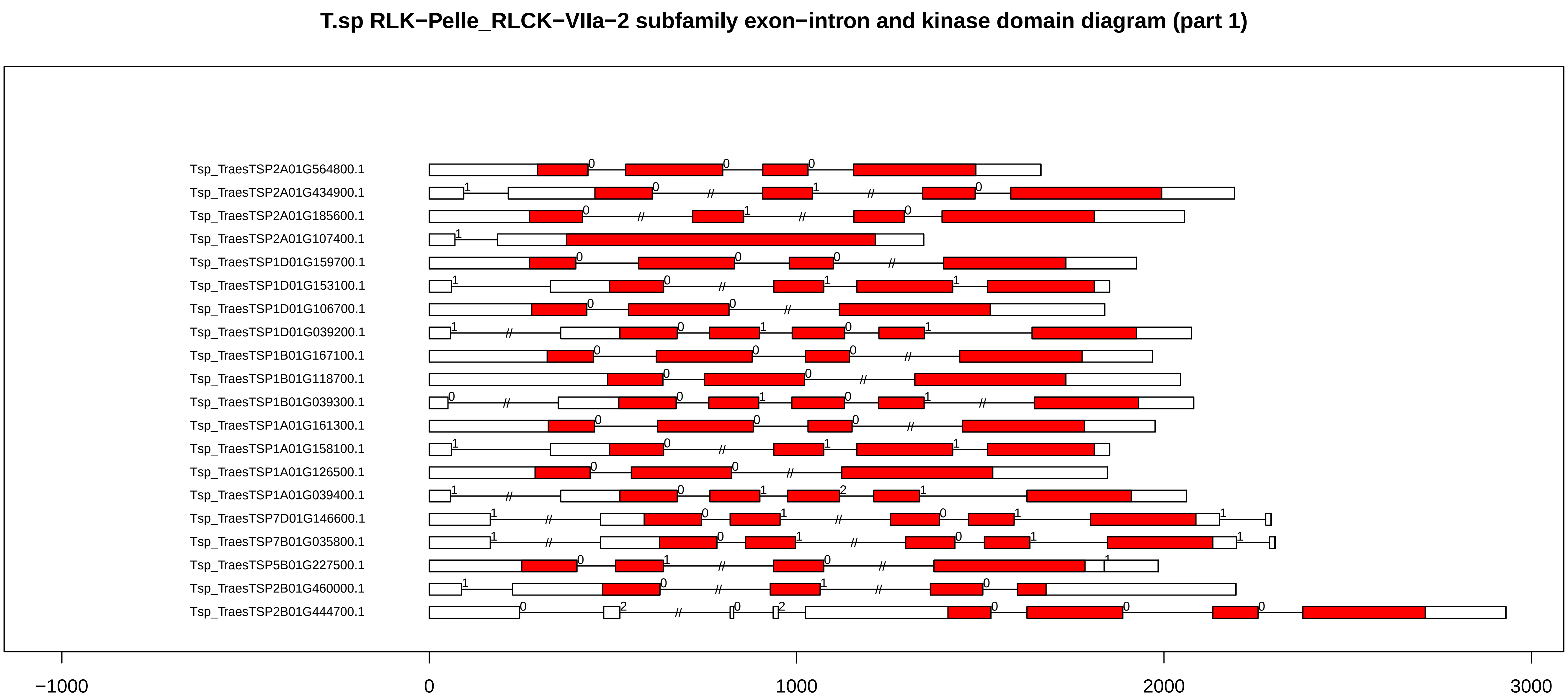
T.sp RLK−Pelle_RLCK−VIIa−2 subfamily exon−intron and kinase domain diagram (part 1)
Tsp_TraesTSP2A01G564800.1
Tsp_TraesTSP2A01G434900.1
Tsp_TraesTSP2A01G185600.1
Tsp_TraesTSP2A01G107400.1
Tsp_TraesTSP1D01G159700.1
Tsp_TraesTSP1D01G153100.1
Tsp_TraesTSP1D01G106700.1
Tsp_TraesTSP1D01G039200.1
Tsp_TraesTSP1B01G167100.1
Tsp_TraesTSP1B01G118700.1
Tsp_TraesTSP1B01G039300.1
Tsp_TraesTSP1A01G161300.1
Tsp_TraesTSP1A01G158100.1
Tsp_TraesTSP1A01G126500.1
Tsp_TraesTSP1A01G039400.1
Tsp_TraesTSP7D01G146600.1
Tsp_TraesTSP7B01G035800.1
Tsp_TraesTSP5B01G227500.1
Tsp_TraesTSP2B01G460000.1
Tsp_TraesTSP2B01G444700.1
−1000
0
1000
2000
3000

T.sp RLK−Pelle_RLCK−VIIa−2 subfamily exon−intron and kinase domain diagram (part 2)

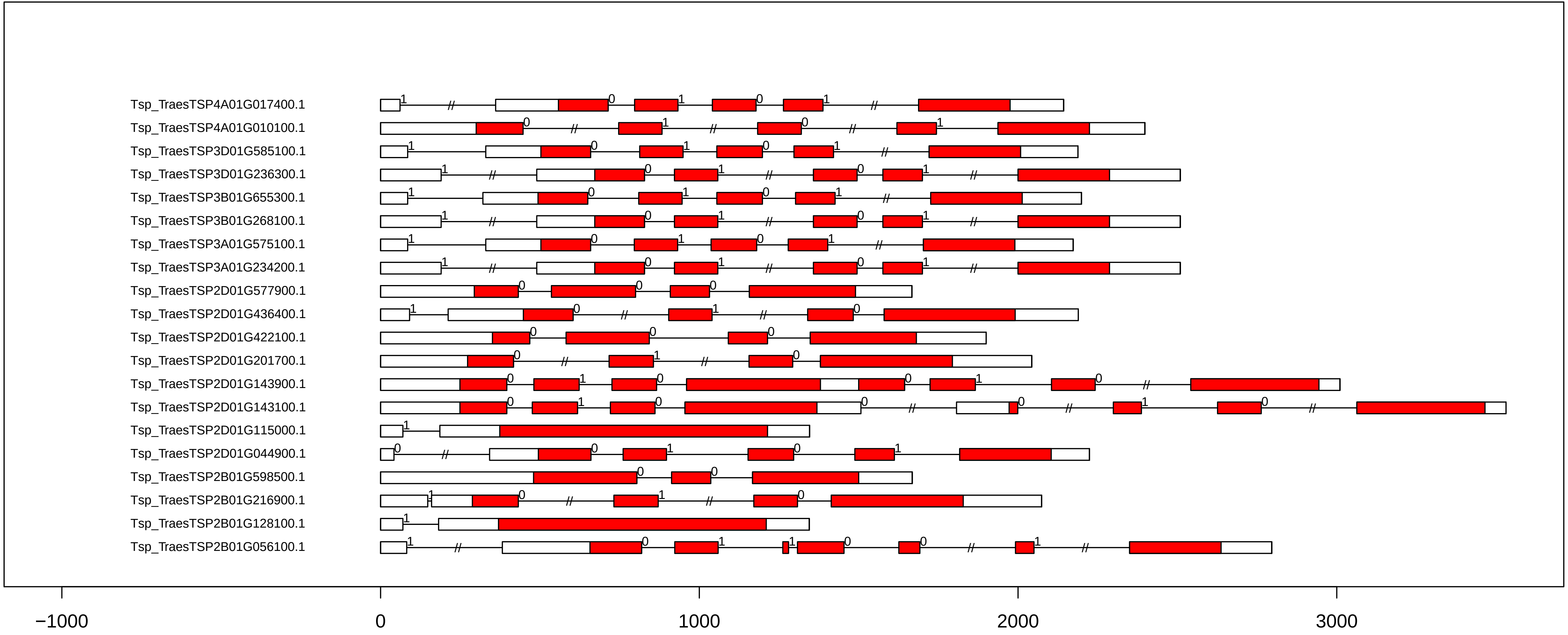

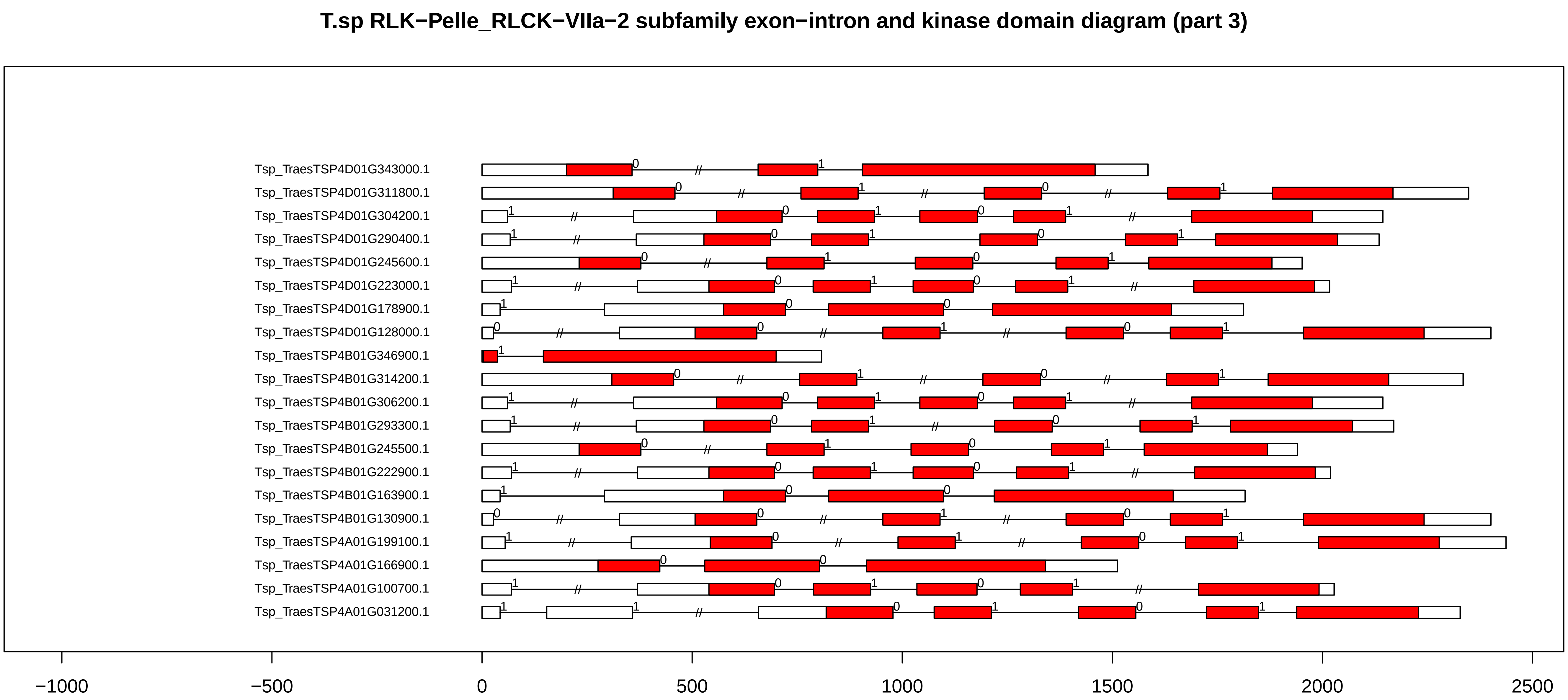
T.sp RLK−Pelle_RLCK−VIIa−2 subfamily exon−intron and kinase domain diagram (part 3)
Tsp_TraesTSP4D01G343000.1
Tsp_TraesTSP4D01G311800.1
Tsp_TraesTSP4D01G304200.1
Tsp_TraesTSP4D01G290400.1
Tsp_TraesTSP4D01G245600.1
Tsp_TraesTSP4D01G223000.1
Tsp_TraesTSP4D01G178900.1
Tsp_TraesTSP4D01G128000.1
Tsp_TraesTSP4B01G346900.1
Tsp_TraesTSP4B01G314200.1
Tsp_TraesTSP4B01G306200.1
Tsp_TraesTSP4B01G293300.1
Tsp_TraesTSP4B01G245500.1
Tsp_TraesTSP4B01G222900.1
Tsp_TraesTSP4B01G163900.1
Tsp_TraesTSP4B01G130900.1
Tsp_TraesTSP4A01G199100.1
Tsp_TraesTSP4A01G166900.1
Tsp_TraesTSP4A01G100700.1
Tsp_TraesTSP4A01G031200.1
−1000
−500
0
500
1000
1500
2000
2500

T.sp RLK−Pelle_RLCK−VIIa−2 subfamily exon−intron and kinase domain diagram (part 4)

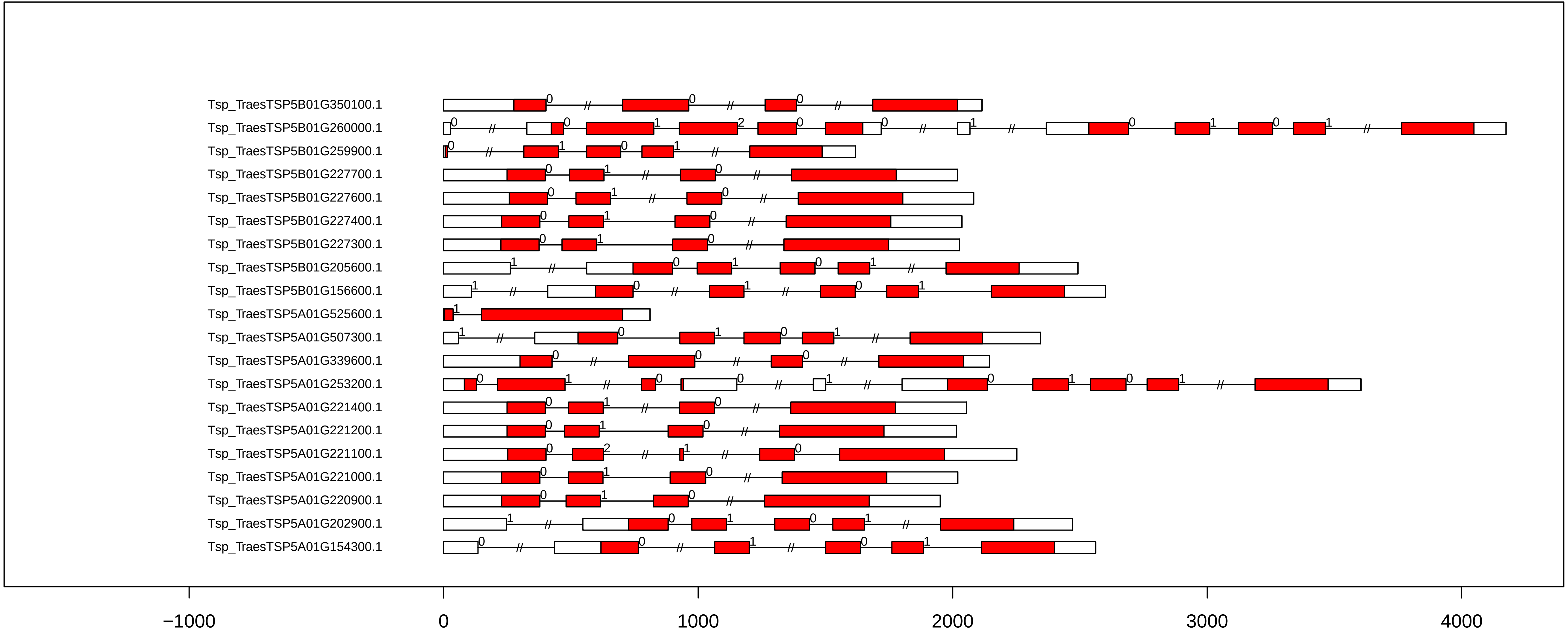

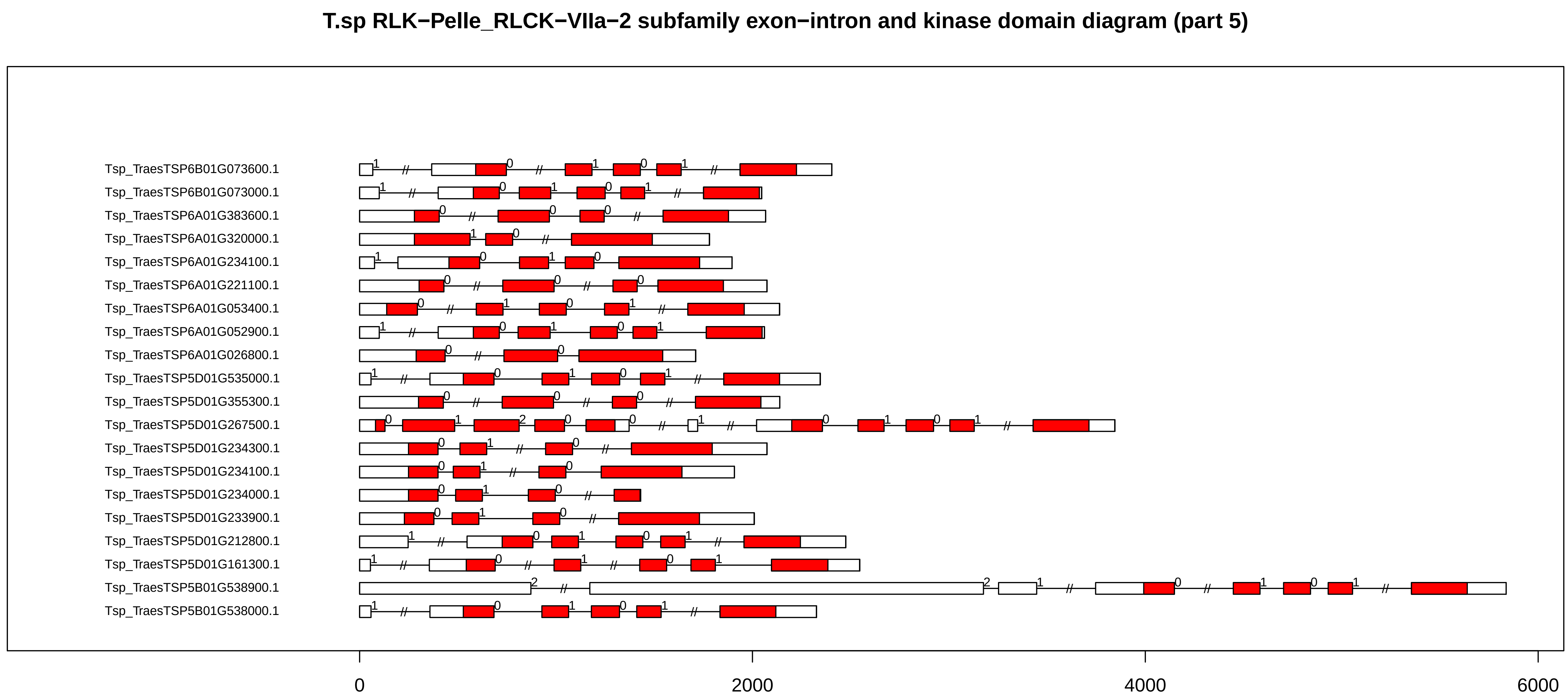
T.sp RLK−Pelle_RLCK−VIIa−2 subfamily exon−intron and kinase domain diagram (part 5)
Tsp_TraesTSP6B01G073600.1
Tsp_TraesTSP6B01G073000.1
Tsp_TraesTSP6A01G383600.1
Tsp_TraesTSP6A01G320000.1
Tsp_TraesTSP6A01G234100.1
Tsp_TraesTSP6A01G221100.1
Tsp_TraesTSP6A01G053400.1
Tsp_TraesTSP6A01G052900.1
Tsp_TraesTSP6A01G026800.1
Tsp_TraesTSP5D01G535000.1
Tsp_TraesTSP5D01G355300.1
Tsp_TraesTSP5D01G267500.1
Tsp_TraesTSP5D01G234300.1
Tsp_TraesTSP5D01G234100.1
Tsp_TraesTSP5D01G234000.1
Tsp_TraesTSP5D01G233900.1
Tsp_TraesTSP5D01G212800.1
Tsp_TraesTSP5D01G161300.1
Tsp_TraesTSP5B01G538900.1
Tsp_TraesTSP5B01G538000.1
0
2000
4000
6000

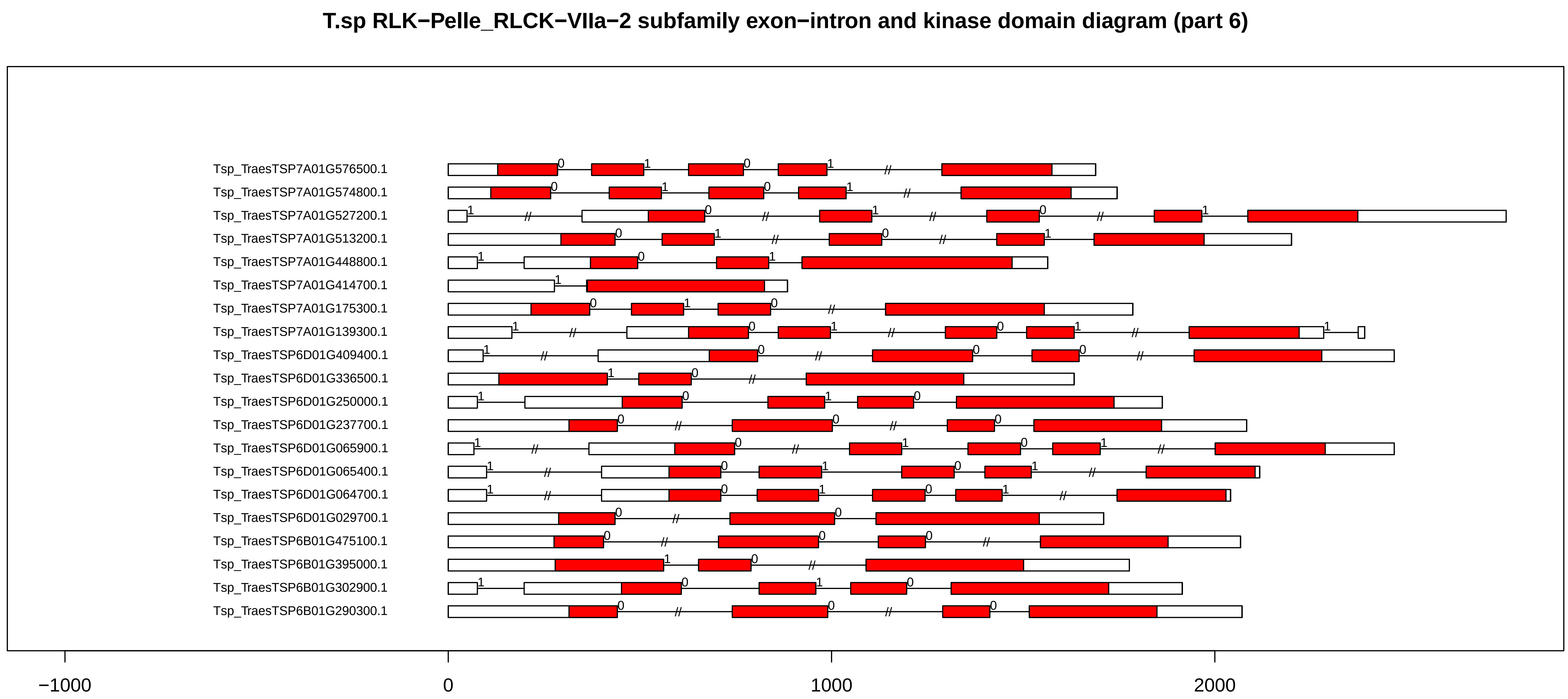
T.sp RLK−Pelle_RLCK−VIIa−2 subfamily exon−intron and kinase domain diagram (part 6)
Tsp_TraesTSP7A01G576500.1
Tsp_TraesTSP7A01G574800.1
Tsp_TraesTSP7A01G527200.1
Tsp_TraesTSP7A01G513200.1
Tsp_TraesTSP7A01G448800.1
Tsp_TraesTSP7A01G414700.1
Tsp_TraesTSP7A01G175300.1
Tsp_TraesTSP7A01G139300.1
Tsp_TraesTSP6D01G409400.1
Tsp_TraesTSP6D01G336500.1
Tsp_TraesTSP6D01G250000.1
Tsp_TraesTSP6D01G237700.1
Tsp_TraesTSP6D01G065900.1
Tsp_TraesTSP6D01G065400.1
Tsp_TraesTSP6D01G064700.1
Tsp_TraesTSP6D01G029700.1
Tsp_TraesTSP6B01G475100.1
Tsp_TraesTSP6B01G395000.1
Tsp_TraesTSP6B01G302900.1
Tsp_TraesTSP6B01G290300.1
−1000
0
1000
2000

T.sp RLK−Pelle_RLCK−VIIa−2 subfamily exon−intron and kinase domain diagram (part 7)

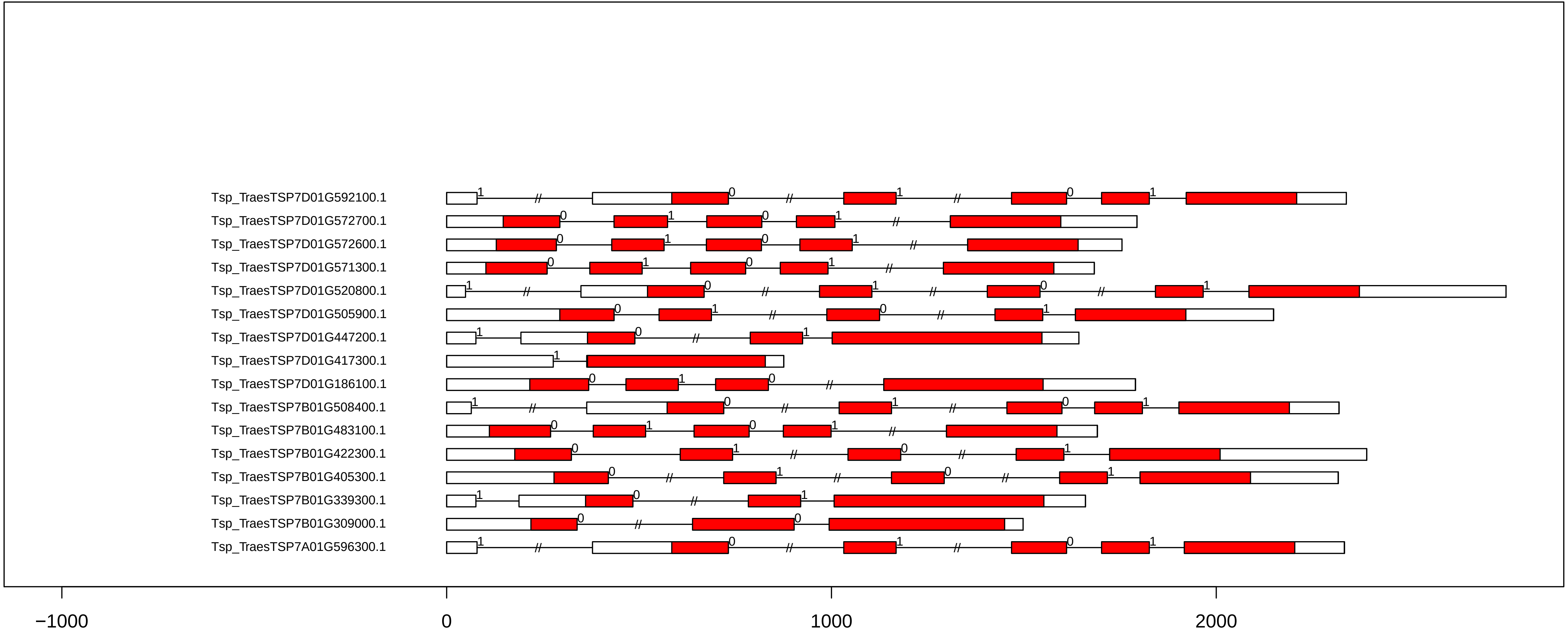

T.sp RLK−Pelle_RLCK−VIIb subfamily exon−intron and kinase domain diagram (all)

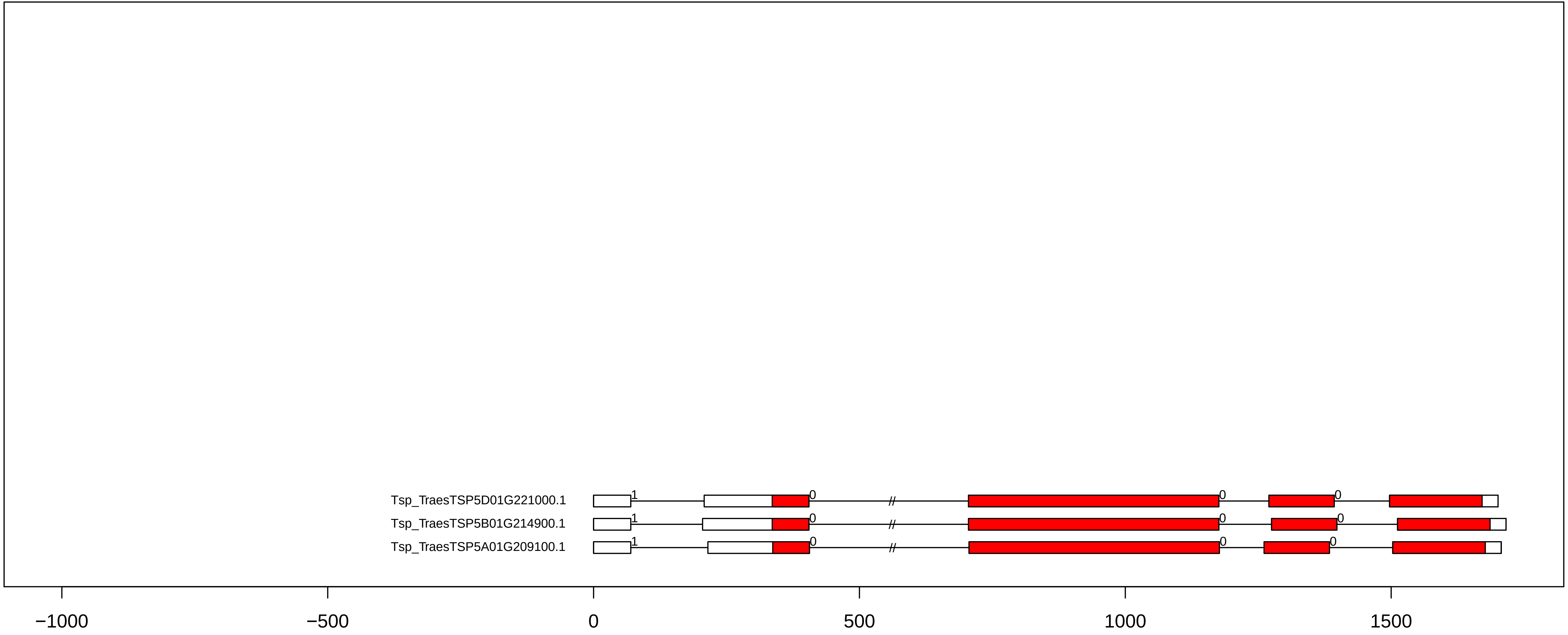

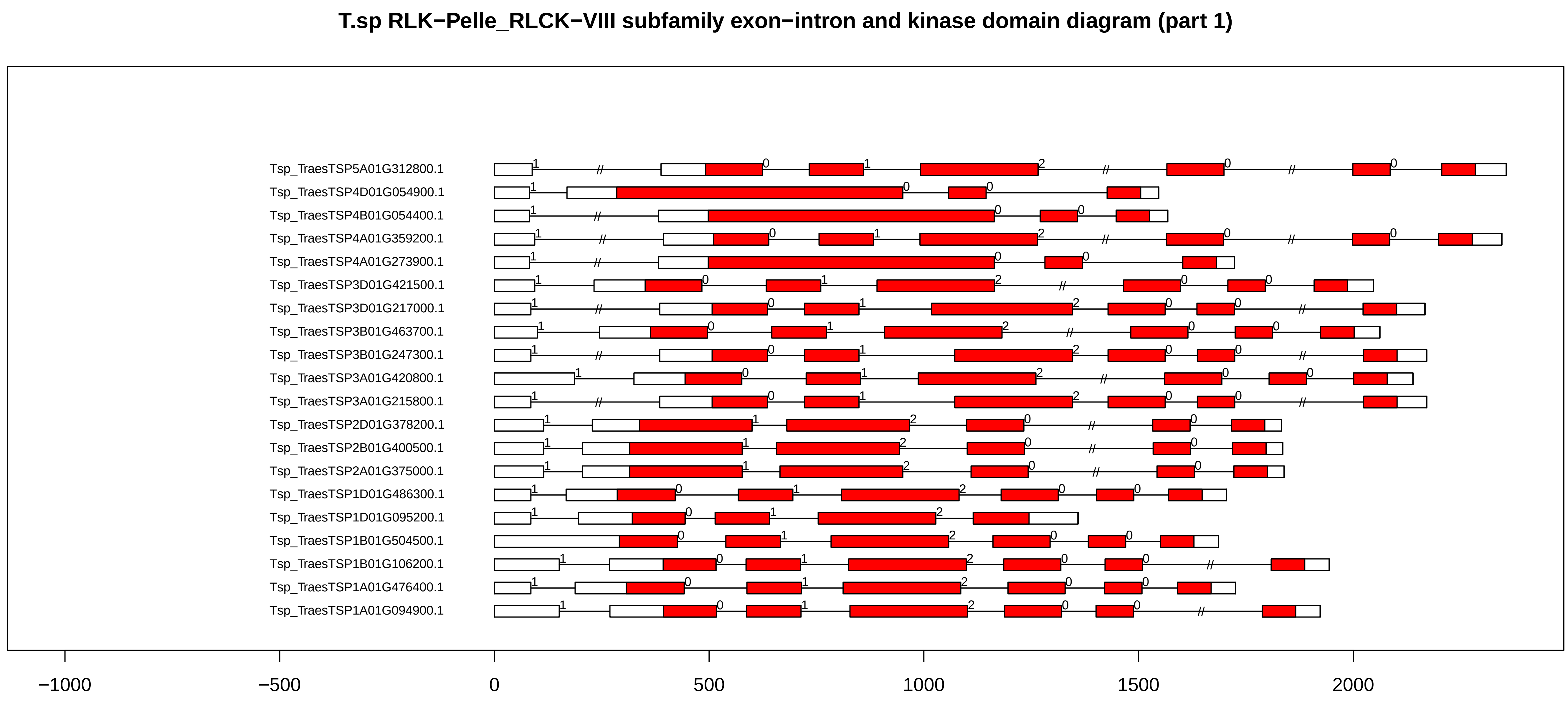
T.sp RLK−Pelle_RLCK−VIII subfamily exon−intron and kinase domain diagram (part 1)
Tsp_TraesTSP5A01G312800.1
Tsp_TraesTSP4D01G054900.1
Tsp_TraesTSP4B01G054400.1
Tsp_TraesTSP4A01G359200.1
Tsp_TraesTSP4A01G273900.1
Tsp_TraesTSP3D01G421500.1
Tsp_TraesTSP3D01G217000.1
Tsp_TraesTSP3B01G463700.1
Tsp_TraesTSP3B01G247300.1
Tsp_TraesTSP3A01G420800.1
Tsp_TraesTSP3A01G215800.1
Tsp_TraesTSP2D01G378200.1
Tsp_TraesTSP2B01G400500.1
Tsp_TraesTSP2A01G375000.1
Tsp_TraesTSP1D01G486300.1
Tsp_TraesTSP1D01G095200.1
Tsp_TraesTSP1B01G504500.1
Tsp_TraesTSP1B01G106200.1
Tsp_TraesTSP1A01G476400.1
Tsp_TraesTSP1A01G094900.1
−1000
−500
0
500
1000
1500
2000

T.sp RLK−Pelle_RLCK−VIII subfamily exon−intron and kinase domain diagram (part 2)

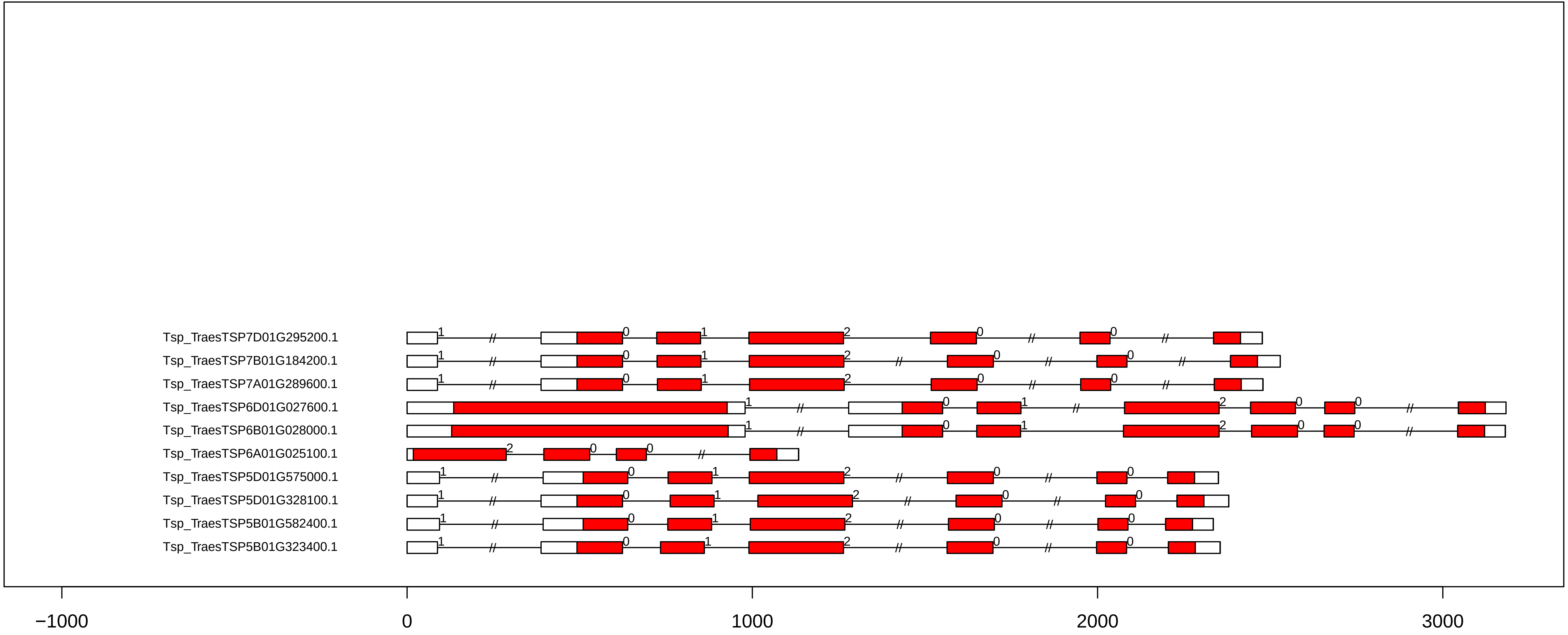

T.sp RLK−Pelle_RLCK−VI subfamily exon−intron and kinase domain diagram (part 1)

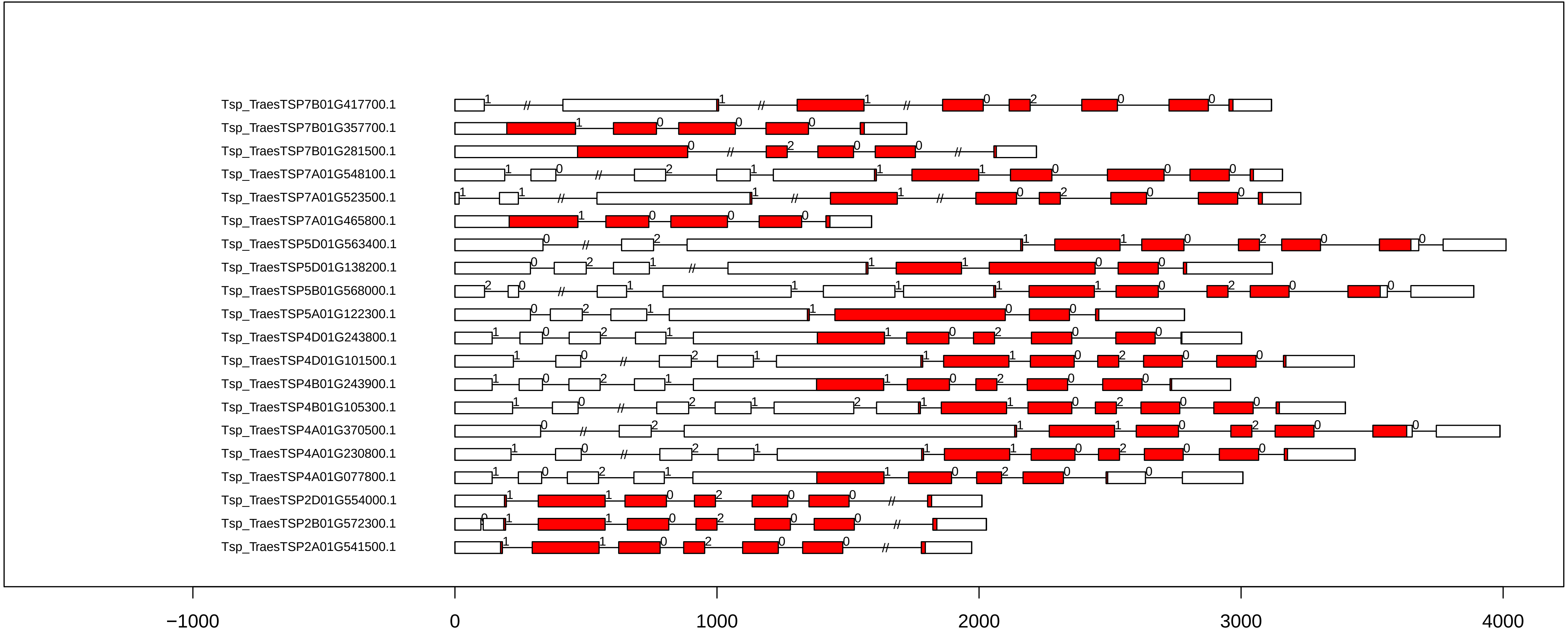

T.sp RLK−Pelle_RLCK−VI subfamily exon−intron and kinase domain diagram (part 2)

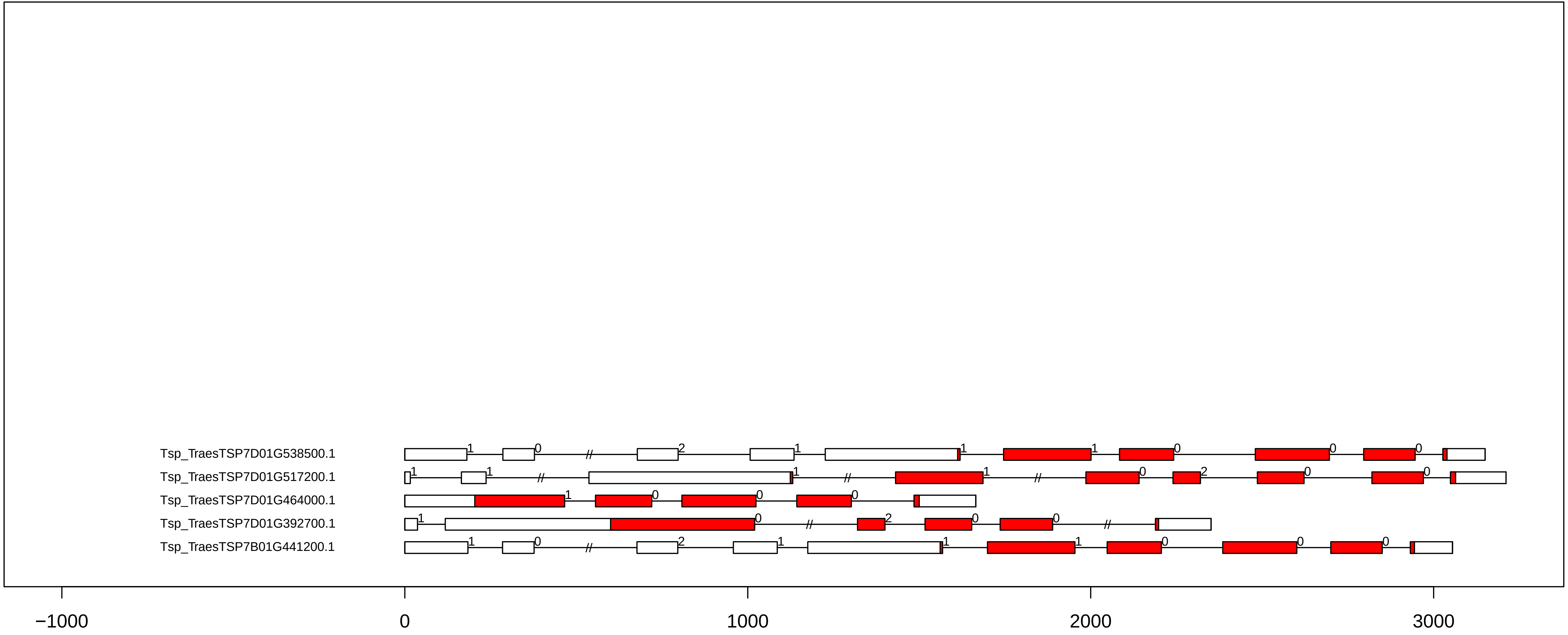

T.sp RLK−Pelle_RLCK−V subfamily exon−intron and kinase domain diagram (part 1)

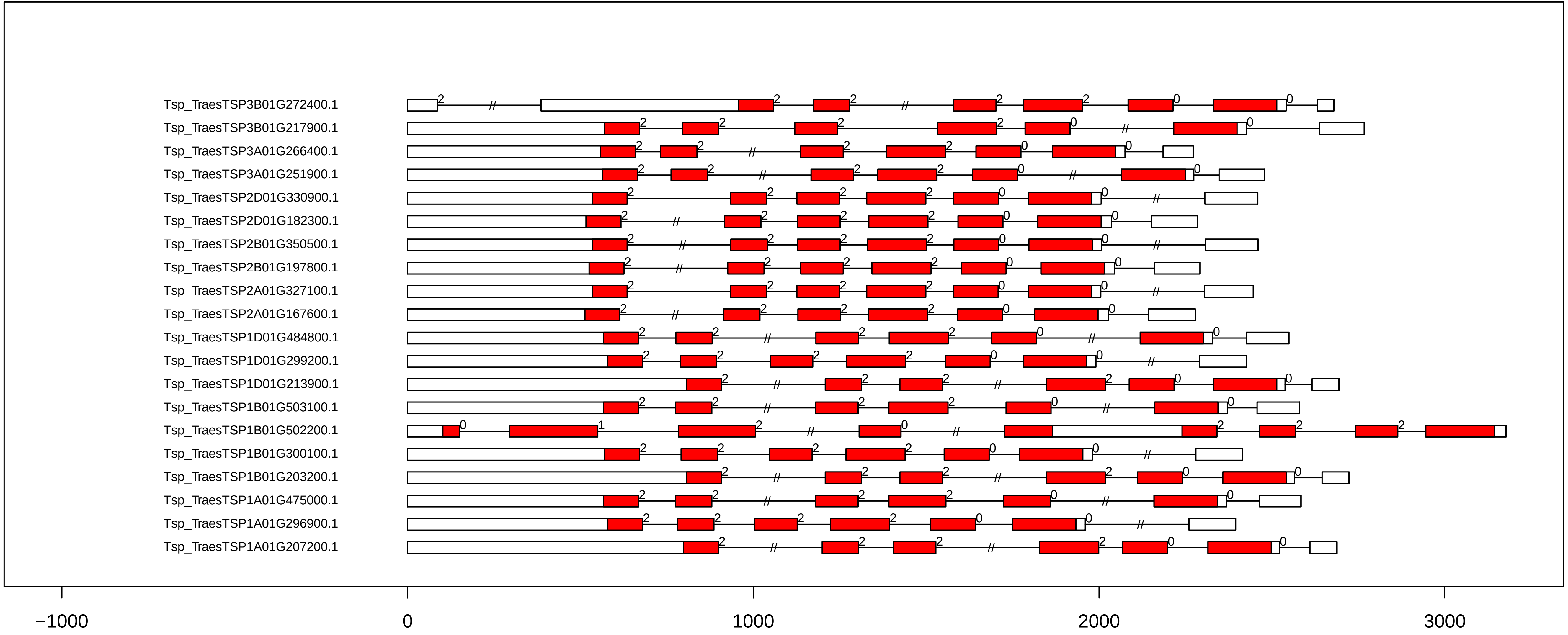

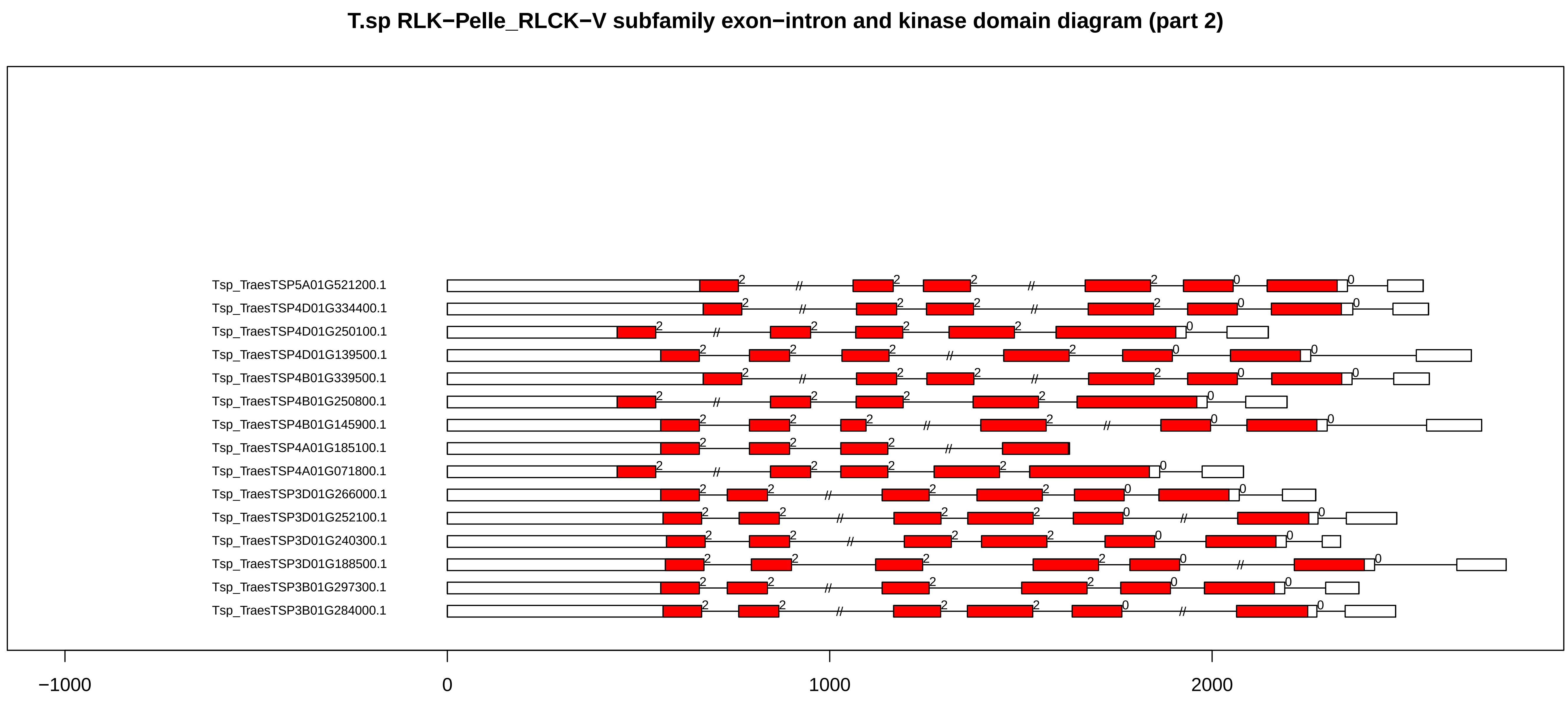
T.sp RLK−Pelle_RLCK−V subfamily exon−intron and kinase domain diagram (part 2)
Tsp_TraesTSP5A01G521200.1
Tsp_TraesTSP4D01G334400.1
Tsp_TraesTSP4D01G250100.1
Tsp_TraesTSP4D01G139500.1
Tsp_TraesTSP4B01G339500.1
Tsp_TraesTSP4B01G250800.1
Tsp_TraesTSP4B01G145900.1
Tsp_TraesTSP4A01G185100.1
Tsp_TraesTSP4A01G071800.1
Tsp_TraesTSP3D01G266000.1
Tsp_TraesTSP3D01G252100.1
Tsp_TraesTSP3D01G240300.1
Tsp_TraesTSP3D01G188500.1
Tsp_TraesTSP3B01G297300.1
Tsp_TraesTSP3B01G284000.1
−1000
0
1000
2000

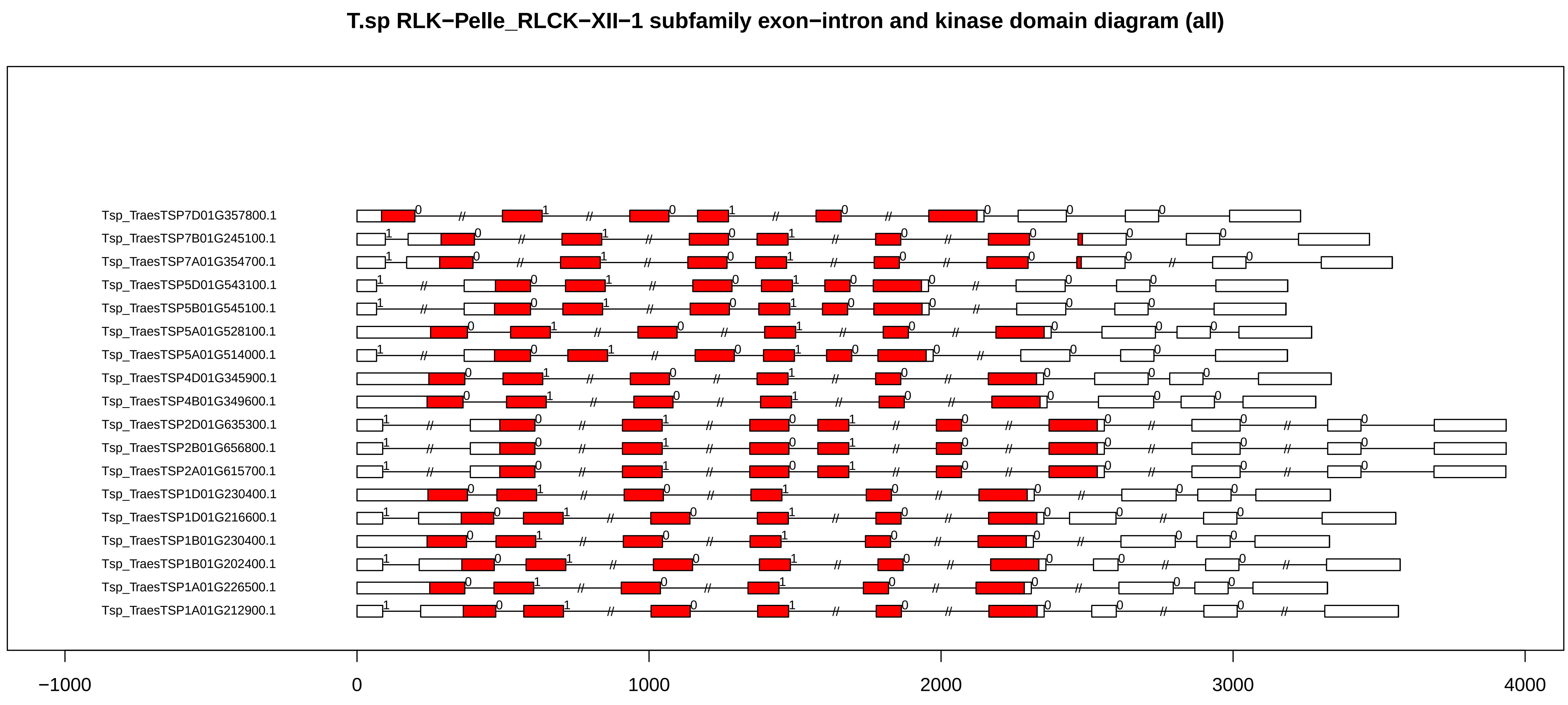
T.sp RLK−Pelle_RLCK−XII−1 subfamily exon−intron and kinase domain diagram (all)
Tsp_TraesTSP7D01G357800.1
Tsp_TraesTSP7B01G245100.1
Tsp_TraesTSP7A01G354700.1
Tsp_TraesTSP5D01G543100.1
Tsp_TraesTSP5B01G545100.1
Tsp_TraesTSP5A01G528100.1
Tsp_TraesTSP5A01G514000.1
Tsp_TraesTSP4D01G345900.1
Tsp_TraesTSP4B01G349600.1
Tsp_TraesTSP2D01G635300.1
Tsp_TraesTSP2B01G656800.1
Tsp_TraesTSP2A01G615700.1
Tsp_TraesTSP1D01G230400.1
Tsp_TraesTSP1D01G216600.1
Tsp_TraesTSP1B01G230400.1
Tsp_TraesTSP1B01G202400.1
Tsp_TraesTSP1A01G226500.1
Tsp_TraesTSP1A01G212900.1
−1000
0
1000
2000
3000
4000

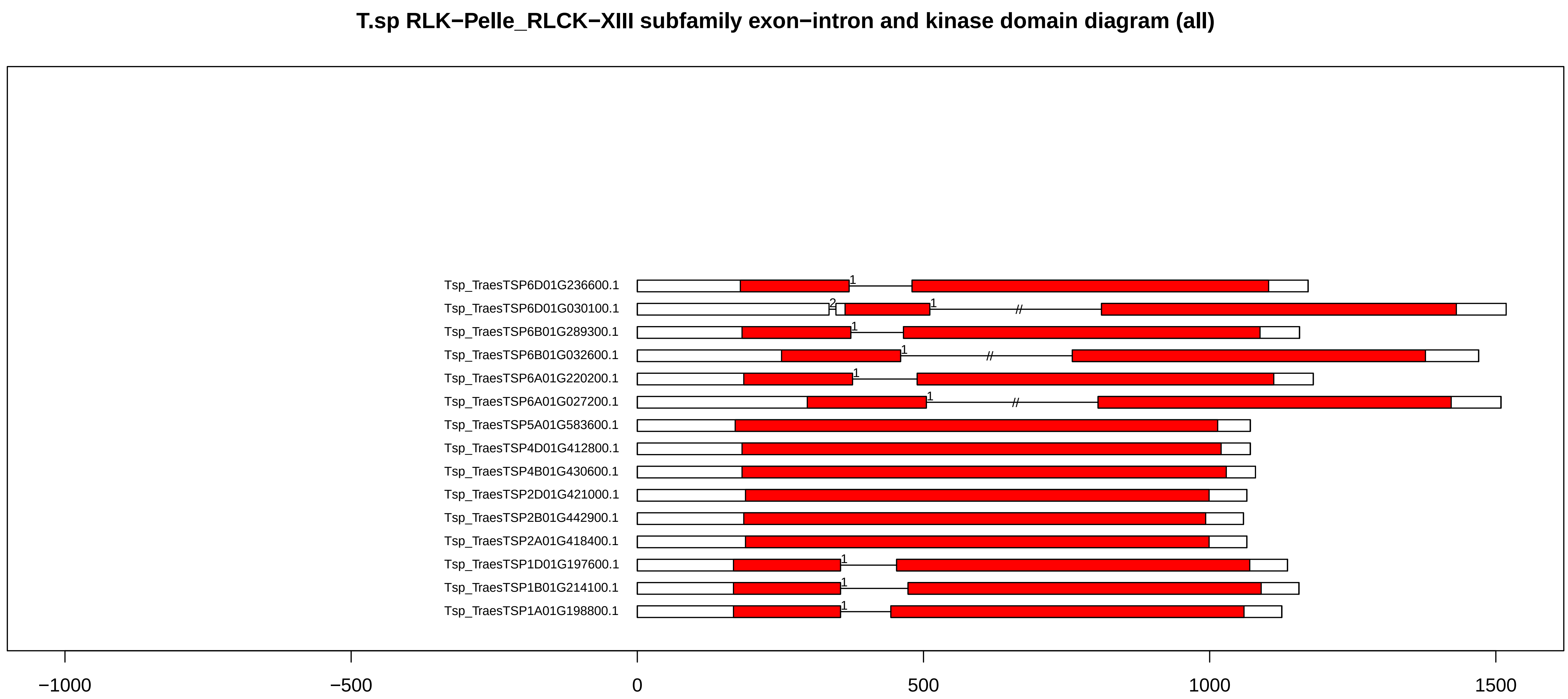

T.sp RLK−Pelle_RLCK−XIII subfamily exon−intron and kinase domain diagram (all)
Tsp_TraesTSP6D01G236600.1
Tsp_TraesTSP6D01G030100.1
Tsp_TraesTSP6B01G289300.1
Tsp_TraesTSP6B01G032600.1
Tsp_TraesTSP6A01G220200.1
Tsp_TraesTSP6A01G027200.1
Tsp_TraesTSP5A01G583600.1
Tsp_TraesTSP4D01G412800.1
Tsp_TraesTSP4B01G430600.1
Tsp_TraesTSP2D01G421000.1
Tsp_TraesTSP2B01G442900.1
Tsp_TraesTSP2A01G418400.1
Tsp_TraesTSP1D01G197600.1
Tsp_TraesTSP1B01G214100.1
Tsp_TraesTSP1A01G198800.1
−1000
−500
0
500
1000
1500

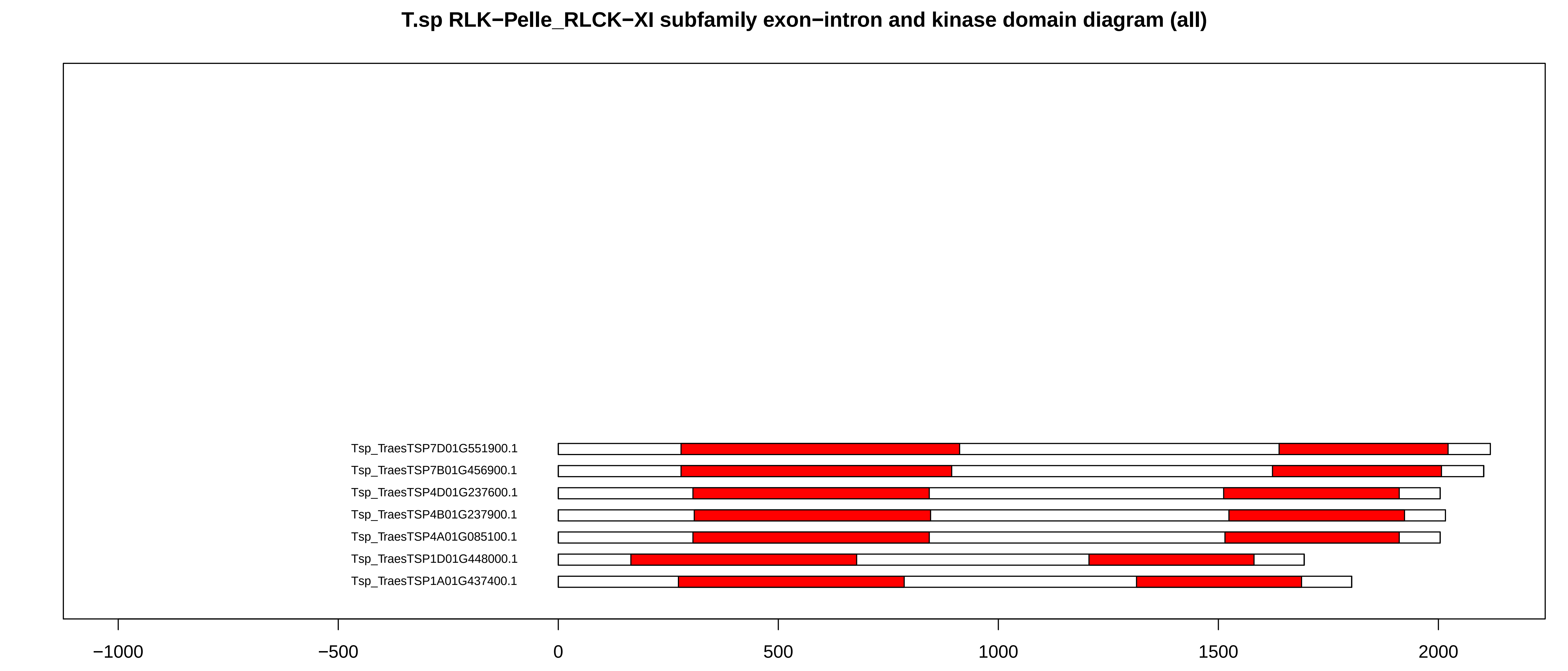

T.sp RLK−Pelle_RLCK−XI subfamily exon−intron and kinase domain diagram (all)
Tsp_TraesTSP7D01G551900.1
Tsp_TraesTSP7B01G456900.1
Tsp_TraesTSP4D01G237600.1
Tsp_TraesTSP4B01G237900.1
Tsp_TraesTSP4A01G085100.1
Tsp_TraesTSP1D01G448000.1
Tsp_TraesTSP1A01G437400.1
−1000
−500
0
500
1000
1500
2000

T.sp RLK−Pelle_RLCK−X subfamily exon−intron and kinase domain diagram (all)

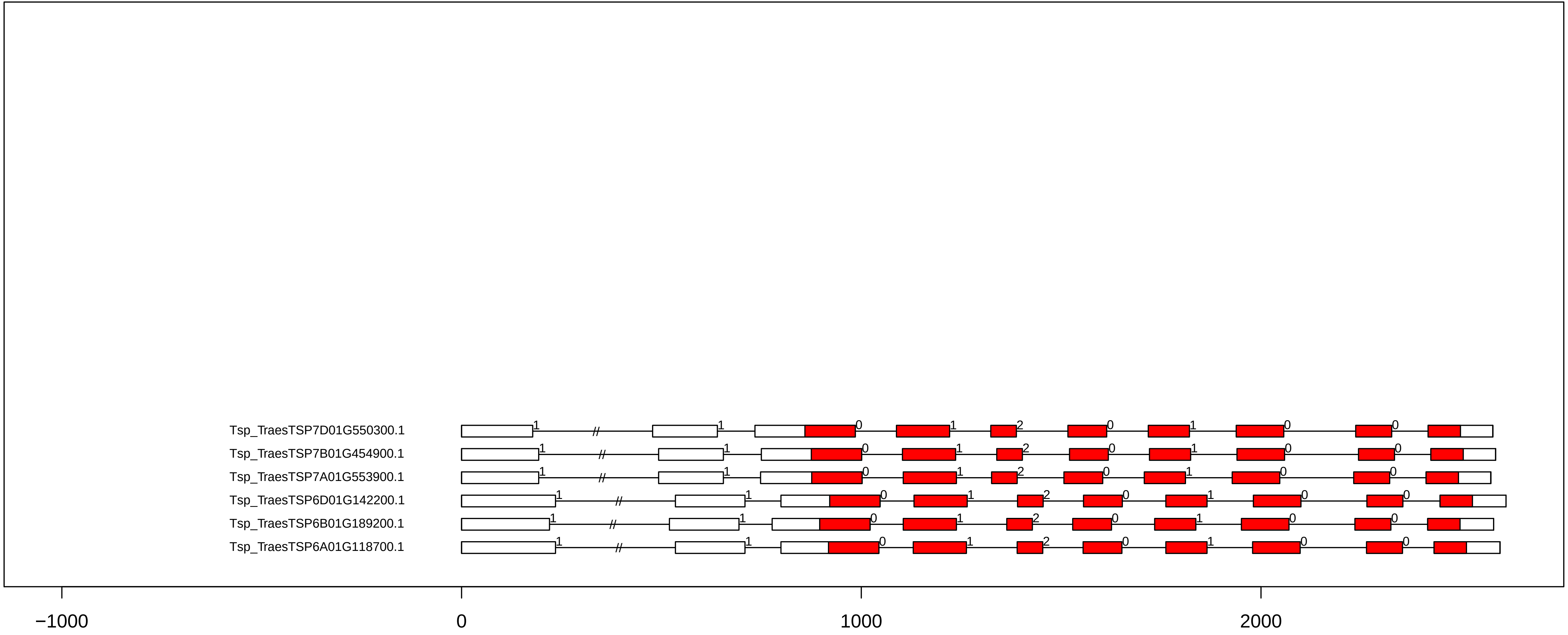

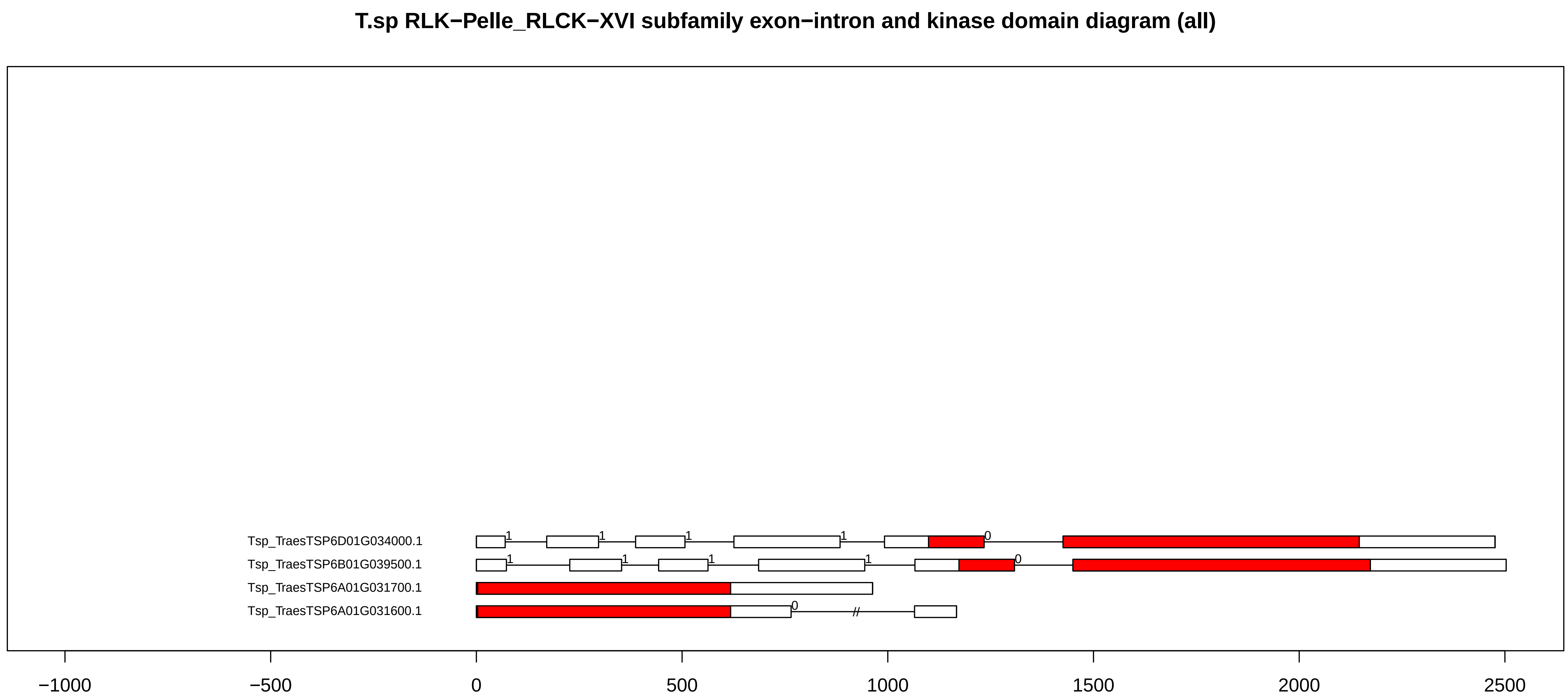
T.sp RLK−Pelle_RLCK−XVI subfamily exon−intron and kinase domain diagram (all)
Tsp_TraesTSP6D01G034000.1
Tsp_TraesTSP6B01G039500.1
Tsp_TraesTSP6A01G031700.1
Tsp_TraesTSP6A01G031600.1
1
1
1
1
0
1
1
1
1
0
0
−1000
−500
0
500
1000
1500
2000
2500

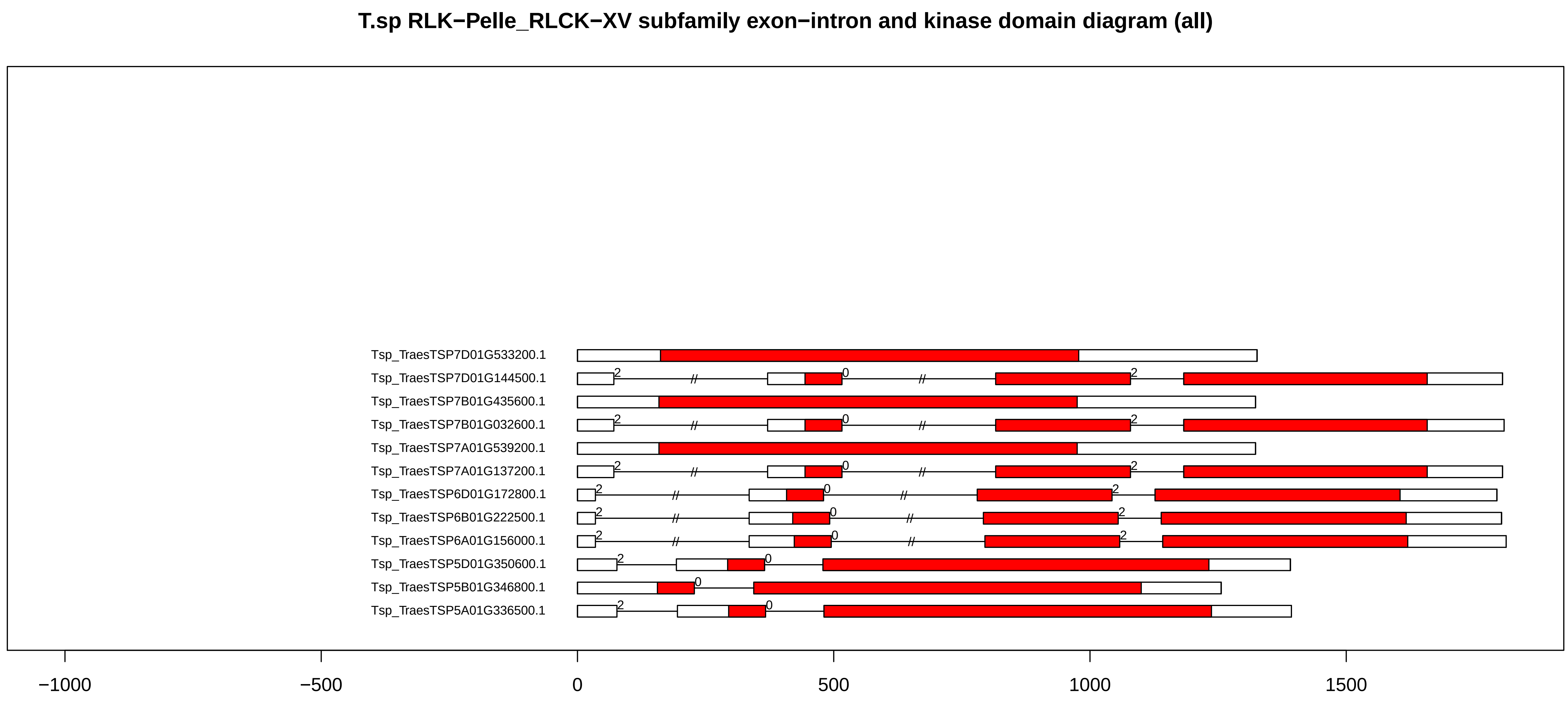
T.sp RLK−Pelle_RLCK−XV subfamily exon−intron and kinase domain diagram (all)
Tsp_TraesTSP7D01G533200.1
Tsp_TraesTSP7D01G144500.1
Tsp_TraesTSP7B01G435600.1
Tsp_TraesTSP7B01G032600.1
Tsp_TraesTSP7A01G539200.1
Tsp_TraesTSP7A01G137200.1
Tsp_TraesTSP6D01G172800.1
Tsp_TraesTSP6B01G222500.1
Tsp_TraesTSP6A01G156000.1
Tsp_TraesTSP5D01G350600.1
Tsp_TraesTSP5B01G346800.1
Tsp_TraesTSP5A01G336500.1
−1000
−500
0
500
1000
1500

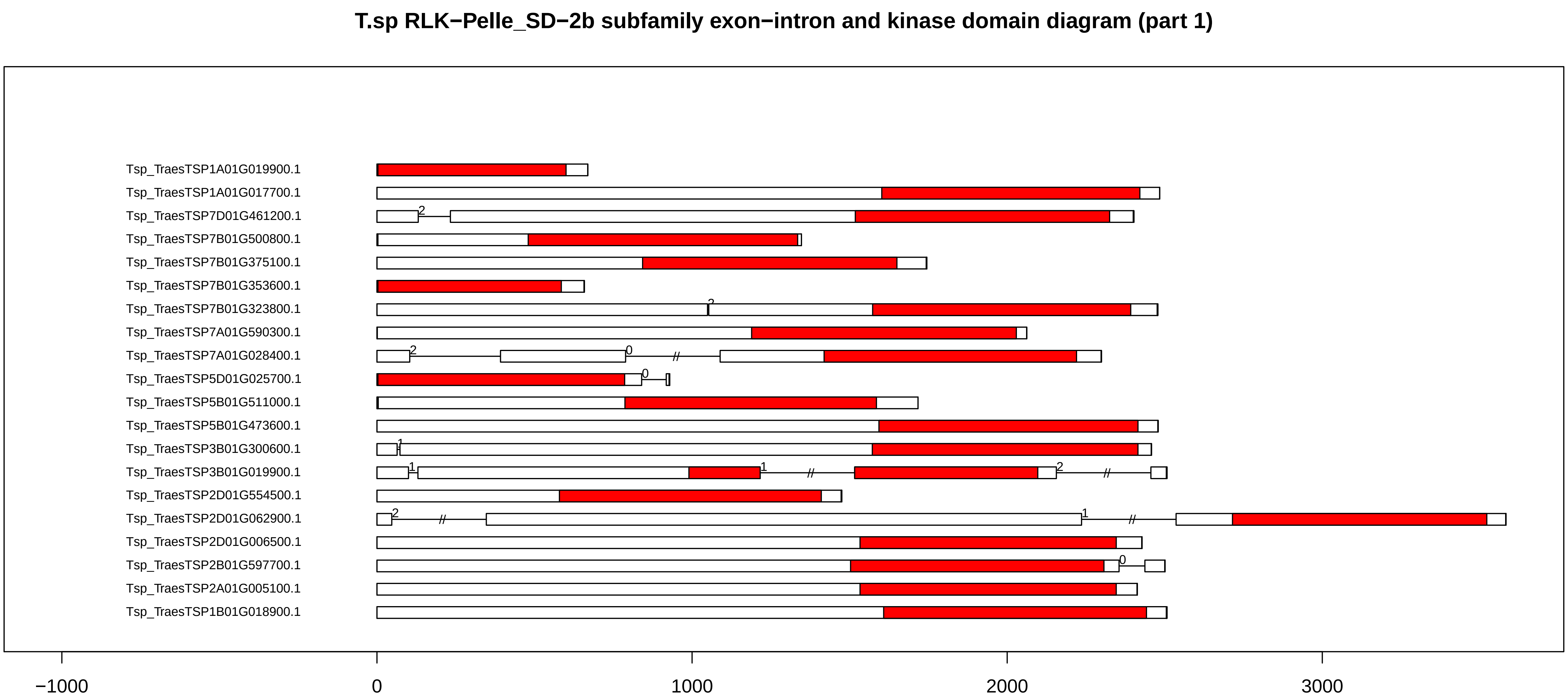
T.sp RLK−Pelle_SD−2b subfamily exon−intron and kinase domain diagram (part 1)
Tsp_TraesTSP1A01G019900.1
Tsp_TraesTSP1A01G017700.1
Tsp_TraesTSP7D01G461200.1
Tsp_TraesTSP7B01G500800.1
Tsp_TraesTSP7B01G375100.1
Tsp_TraesTSP7B01G353600.1
Tsp_TraesTSP7B01G323800.1
Tsp_TraesTSP7A01G590300.1
Tsp_TraesTSP7A01G028400.1
Tsp_TraesTSP5D01G025700.1
Tsp_TraesTSP5B01G511000.1
Tsp_TraesTSP5B01G473600.1
Tsp_TraesTSP3B01G300600.1
Tsp_TraesTSP3B01G019900.1
Tsp_TraesTSP2D01G554500.1
Tsp_TraesTSP2D01G062900.1
Tsp_TraesTSP2D01G006500.1
Tsp_TraesTSP2B01G597700.1
Tsp_TraesTSP2A01G005100.1
Tsp_TraesTSP1B01G018900.1
−1000
0
1000
2000
3000

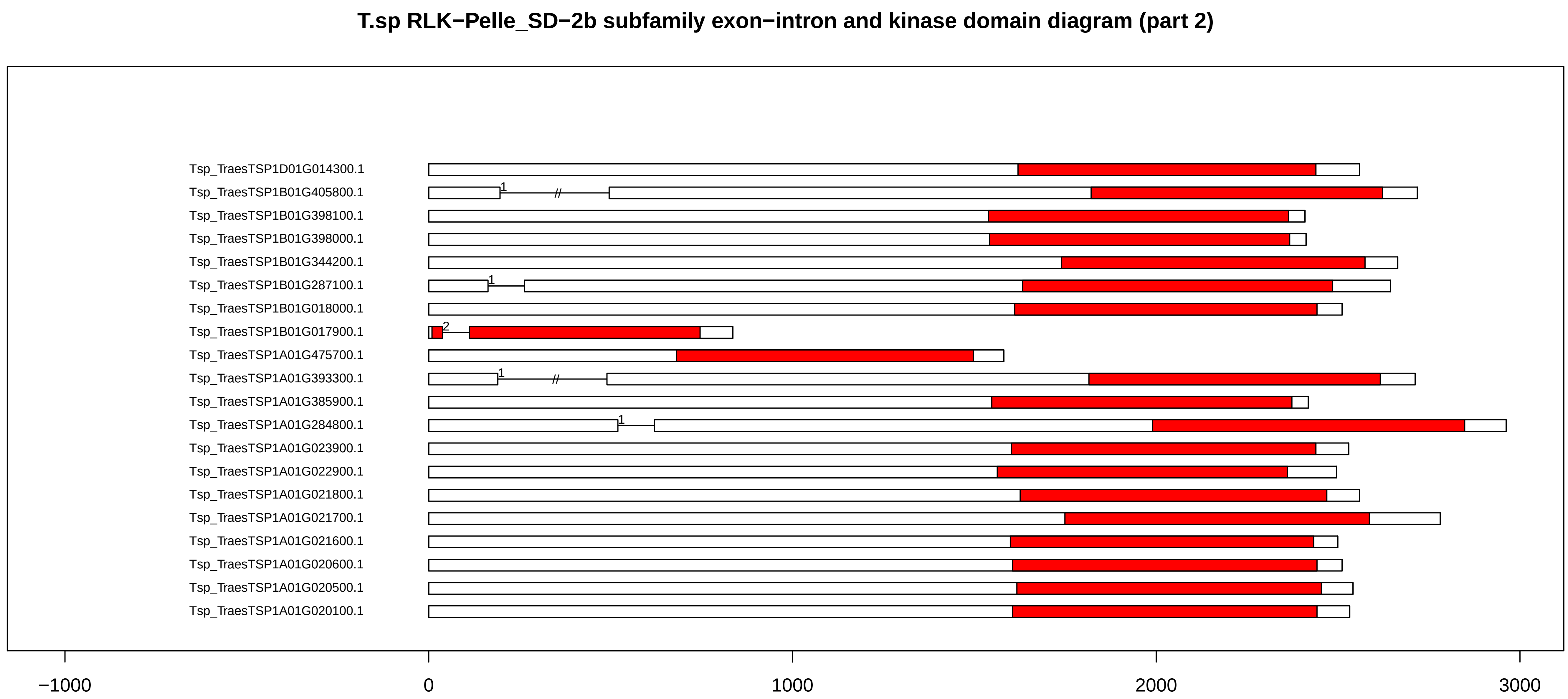

T.sp RLK−Pelle_SD−2b subfamily exon−intron and kinase domain diagram (part 2)
Tsp_TraesTSP1D01G014300.1
Tsp_TraesTSP1B01G405800.1
Tsp_TraesTSP1B01G398100.1
Tsp_TraesTSP1B01G398000.1
Tsp_TraesTSP1B01G344200.1
Tsp_TraesTSP1B01G287100.1
Tsp_TraesTSP1B01G018000.1
Tsp_TraesTSP1B01G017900.1
Tsp_TraesTSP1A01G475700.1
Tsp_TraesTSP1A01G393300.1
Tsp_TraesTSP1A01G385900.1
Tsp_TraesTSP1A01G284800.1
Tsp_TraesTSP1A01G023900.1
Tsp_TraesTSP1A01G022900.1
Tsp_TraesTSP1A01G021800.1
Tsp_TraesTSP1A01G021700.1
Tsp_TraesTSP1A01G021600.1
Tsp_TraesTSP1A01G020600.1
Tsp_TraesTSP1A01G020500.1
Tsp_TraesTSP1A01G020100.1
1
1
2
1
1
−1000
0
1000
2000
3000

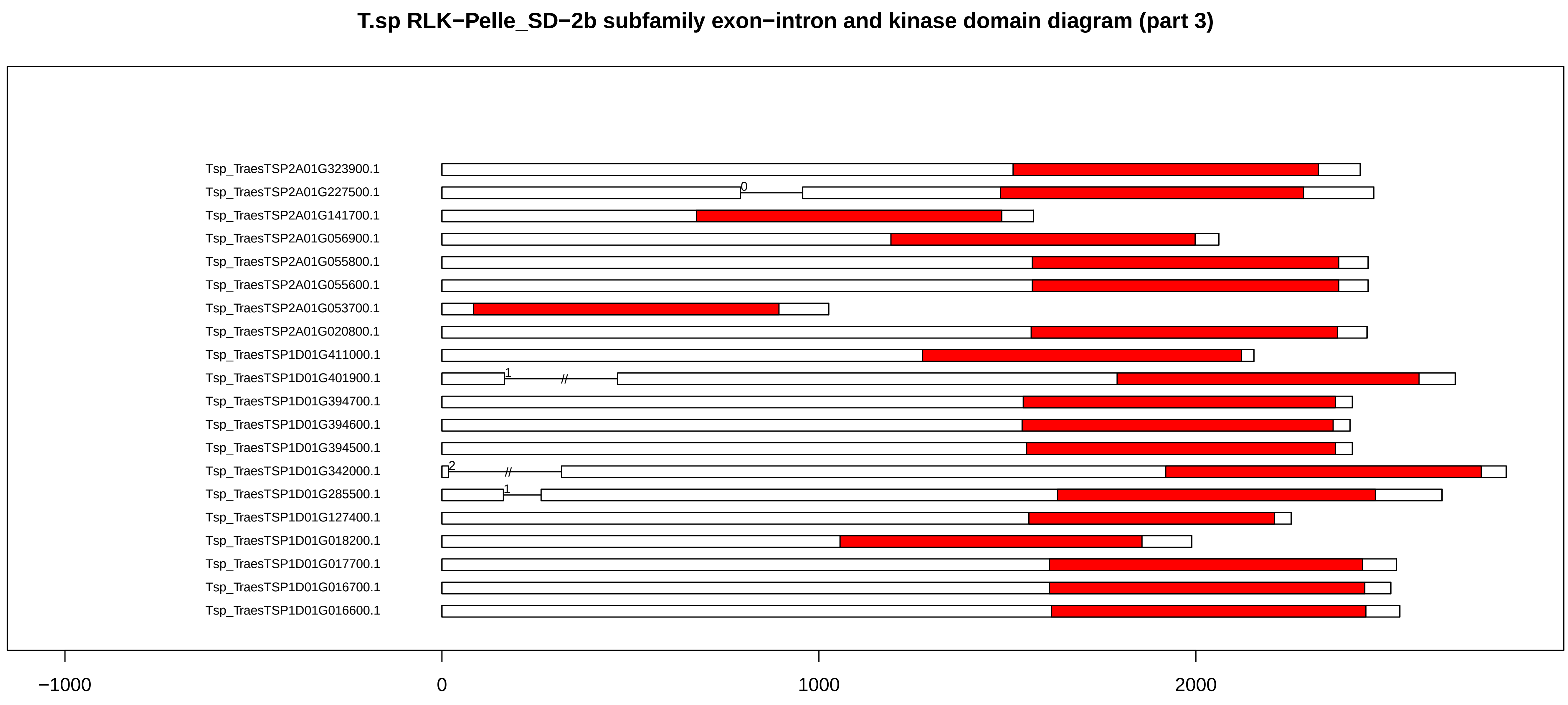

T.sp RLK−Pelle_SD−2b subfamily exon−intron and kinase domain diagram (part 3)
Tsp_TraesTSP2A01G323900.1
Tsp_TraesTSP2A01G227500.1
Tsp_TraesTSP2A01G141700.1
Tsp_TraesTSP2A01G056900.1
Tsp_TraesTSP2A01G055800.1
Tsp_TraesTSP2A01G055600.1
Tsp_TraesTSP2A01G053700.1
Tsp_TraesTSP2A01G020800.1
Tsp_TraesTSP1D01G411000.1
Tsp_TraesTSP1D01G401900.1
Tsp_TraesTSP1D01G394700.1
Tsp_TraesTSP1D01G394600.1
Tsp_TraesTSP1D01G394500.1
Tsp_TraesTSP1D01G342000.1
Tsp_TraesTSP1D01G285500.1
Tsp_TraesTSP1D01G127400.1
Tsp_TraesTSP1D01G018200.1
Tsp_TraesTSP1D01G017700.1
Tsp_TraesTSP1D01G016700.1
Tsp_TraesTSP1D01G016600.1
0
1
2
1
−1000
0
1000
2000

T.sp RLK−Pelle_SD−2b subfamily exon−intron and kinase domain diagram (part 4)

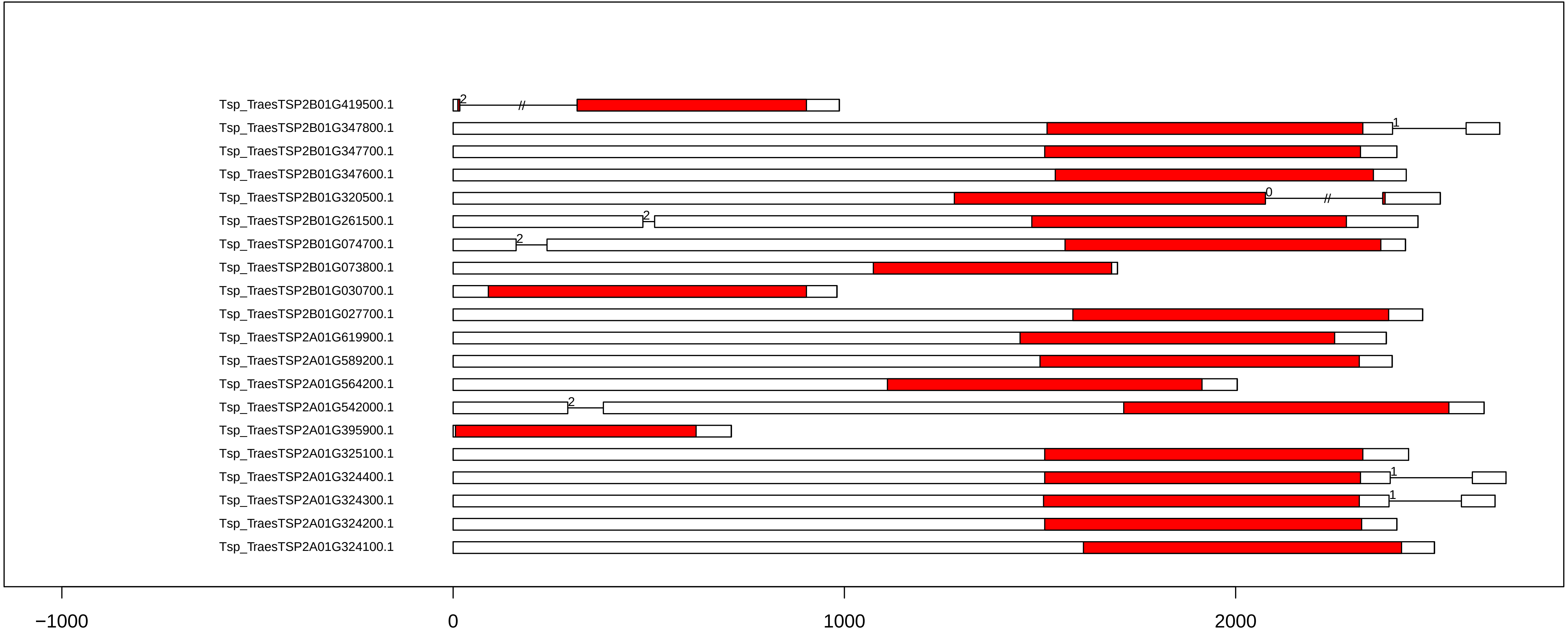

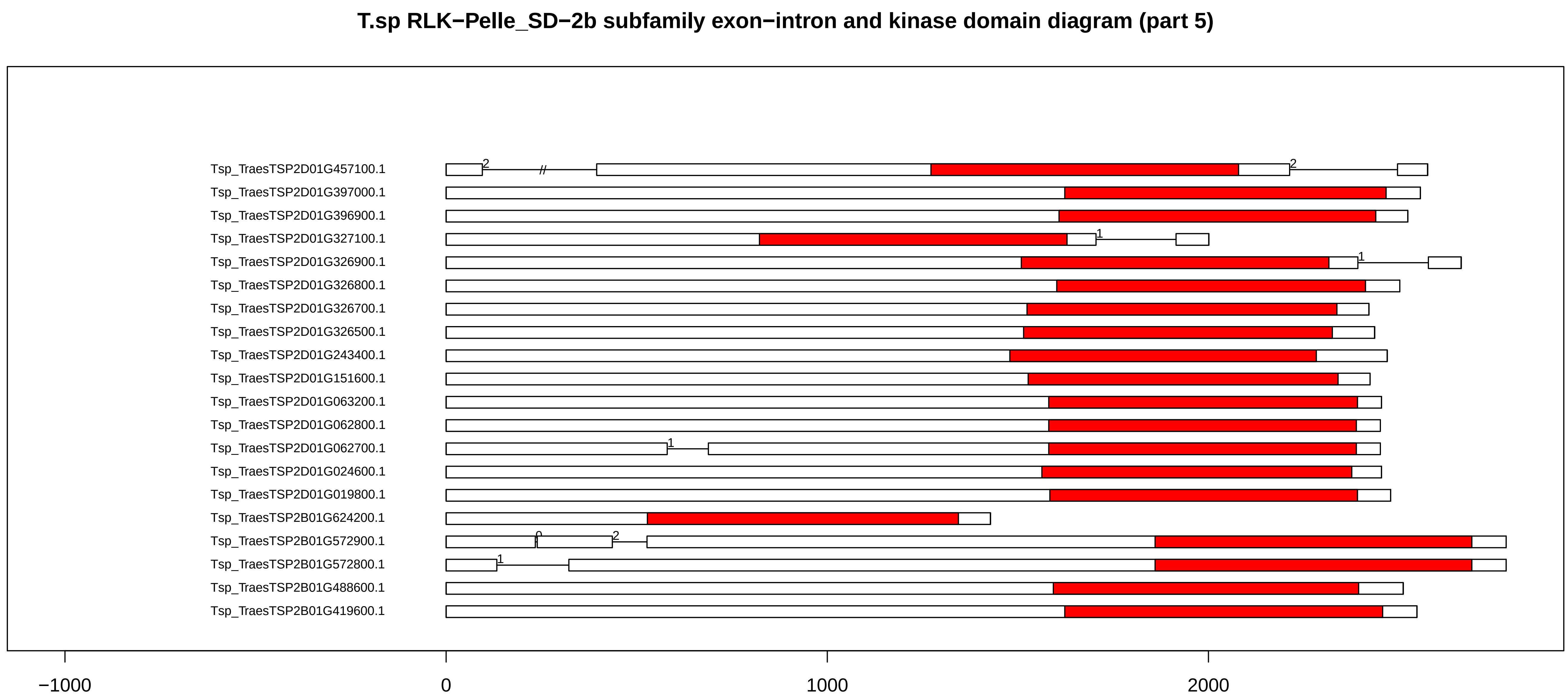
T.sp RLK−Pelle_SD−2b subfamily exon−intron and kinase domain diagram (part 5)
Tsp_TraesTSP2D01G457100.1
Tsp_TraesTSP2D01G397000.1
Tsp_TraesTSP2D01G396900.1
Tsp_TraesTSP2D01G327100.1
Tsp_TraesTSP2D01G326900.1
Tsp_TraesTSP2D01G326800.1
Tsp_TraesTSP2D01G326700.1
Tsp_TraesTSP2D01G326500.1
Tsp_TraesTSP2D01G243400.1
Tsp_TraesTSP2D01G151600.1
Tsp_TraesTSP2D01G063200.1
Tsp_TraesTSP2D01G062800.1
Tsp_TraesTSP2D01G062700.1
Tsp_TraesTSP2D01G024600.1
Tsp_TraesTSP2D01G019800.1
Tsp_TraesTSP2B01G624200.1
Tsp_TraesTSP2B01G572900.1
Tsp_TraesTSP2B01G572800.1
Tsp_TraesTSP2B01G488600.1
Tsp_TraesTSP2B01G419600.1
−1000
0
1000
2000

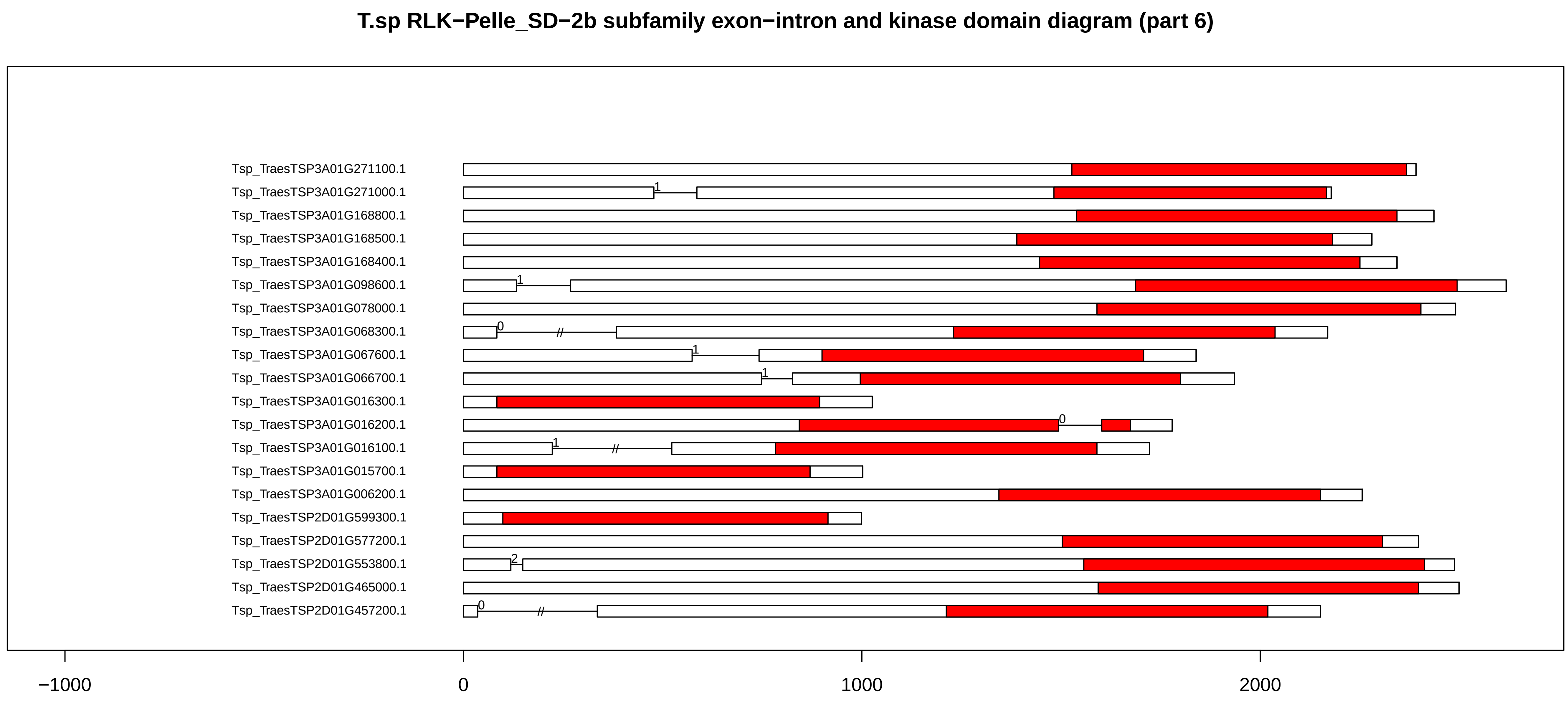

T.sp RLK−Pelle_SD−2b subfamily exon−intron and kinase domain diagram (part 6)
Tsp_TraesTSP3A01G271100.1
Tsp_TraesTSP3A01G271000.1
Tsp_TraesTSP3A01G168800.1
Tsp_TraesTSP3A01G168500.1
Tsp_TraesTSP3A01G168400.1
Tsp_TraesTSP3A01G098600.1
Tsp_TraesTSP3A01G078000.1
Tsp_TraesTSP3A01G068300.1
Tsp_TraesTSP3A01G067600.1
Tsp_TraesTSP3A01G066700.1
Tsp_TraesTSP3A01G016300.1
Tsp_TraesTSP3A01G016200.1
Tsp_TraesTSP3A01G016100.1
Tsp_TraesTSP3A01G015700.1
Tsp_TraesTSP3A01G006200.1
Tsp_TraesTSP2D01G599300.1
Tsp_TraesTSP2D01G577200.1
Tsp_TraesTSP2D01G553800.1
Tsp_TraesTSP2D01G465000.1
Tsp_TraesTSP2D01G457200.1
−1000
0
1000
2000

T.sp RLK−Pelle_SD−2b subfamily exon−intron and kinase domain diagram (part 7)

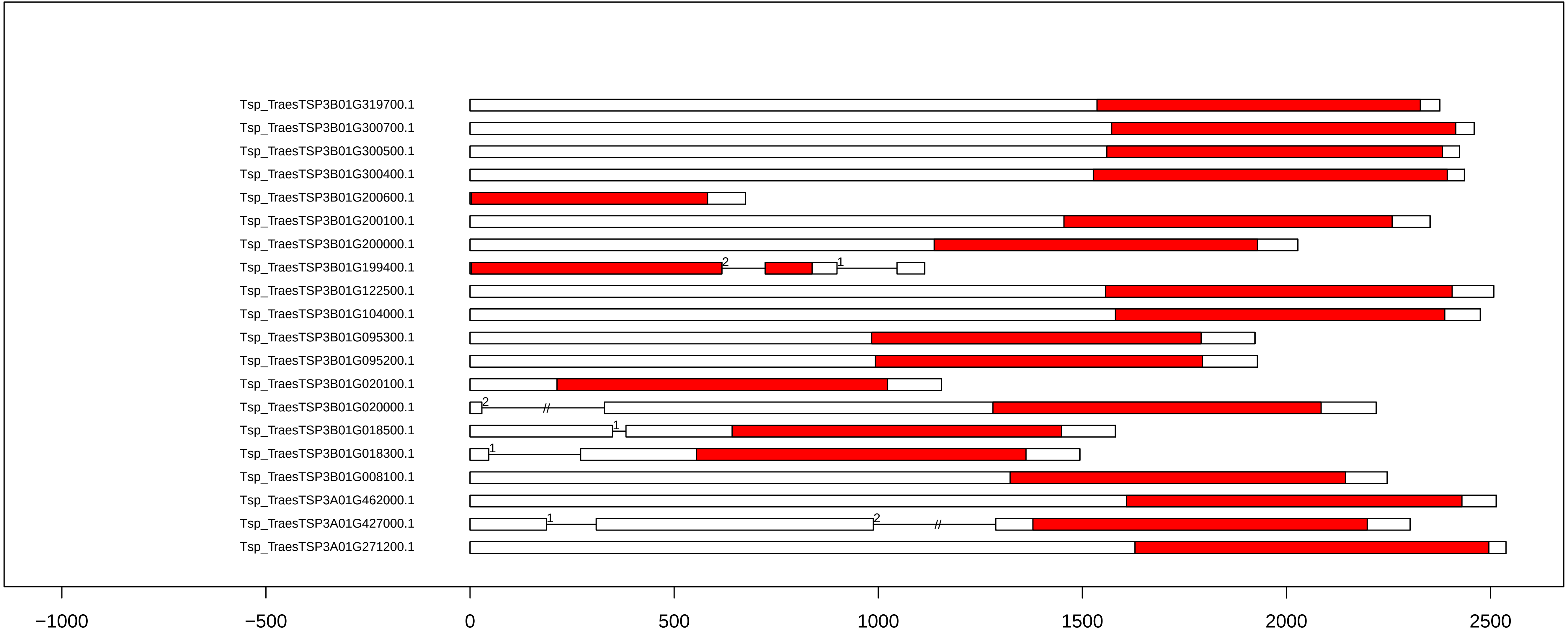

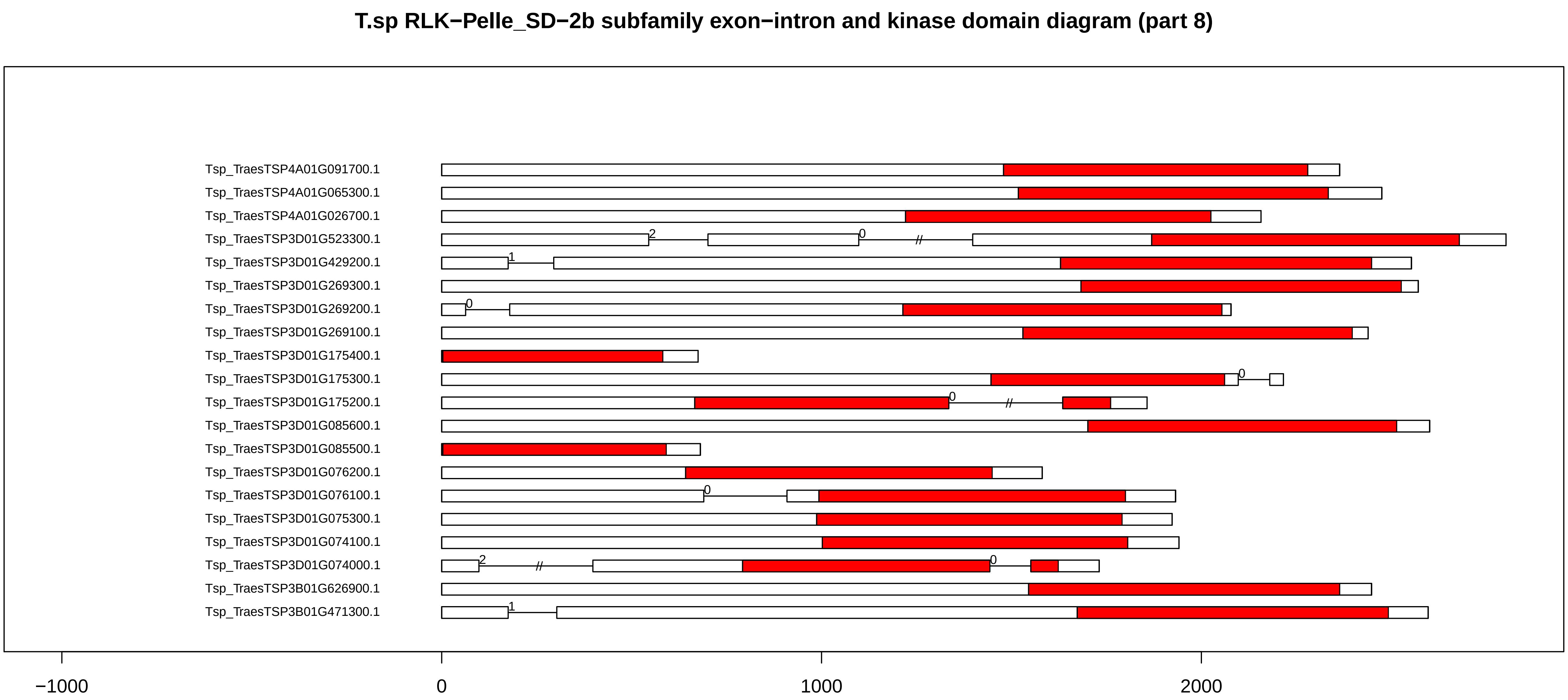

T.sp RLK−Pelle_SD−2b subfamily exon−intron and kinase domain diagram (part 8)
Tsp_TraesTSP4A01G091700.1
Tsp_TraesTSP4A01G065300.1
Tsp_TraesTSP4A01G026700.1
Tsp_TraesTSP3D01G523300.1
Tsp_TraesTSP3D01G429200.1
Tsp_TraesTSP3D01G269300.1
Tsp_TraesTSP3D01G269200.1
Tsp_TraesTSP3D01G269100.1
Tsp_TraesTSP3D01G175400.1
Tsp_TraesTSP3D01G175300.1
Tsp_TraesTSP3D01G175200.1
Tsp_TraesTSP3D01G085600.1
Tsp_TraesTSP3D01G085500.1
Tsp_TraesTSP3D01G076200.1
Tsp_TraesTSP3D01G076100.1
Tsp_TraesTSP3D01G075300.1
Tsp_TraesTSP3D01G074100.1
Tsp_TraesTSP3D01G074000.1
Tsp_TraesTSP3B01G626900.1
Tsp_TraesTSP3B01G471300.1
−1000
0
1000
2000

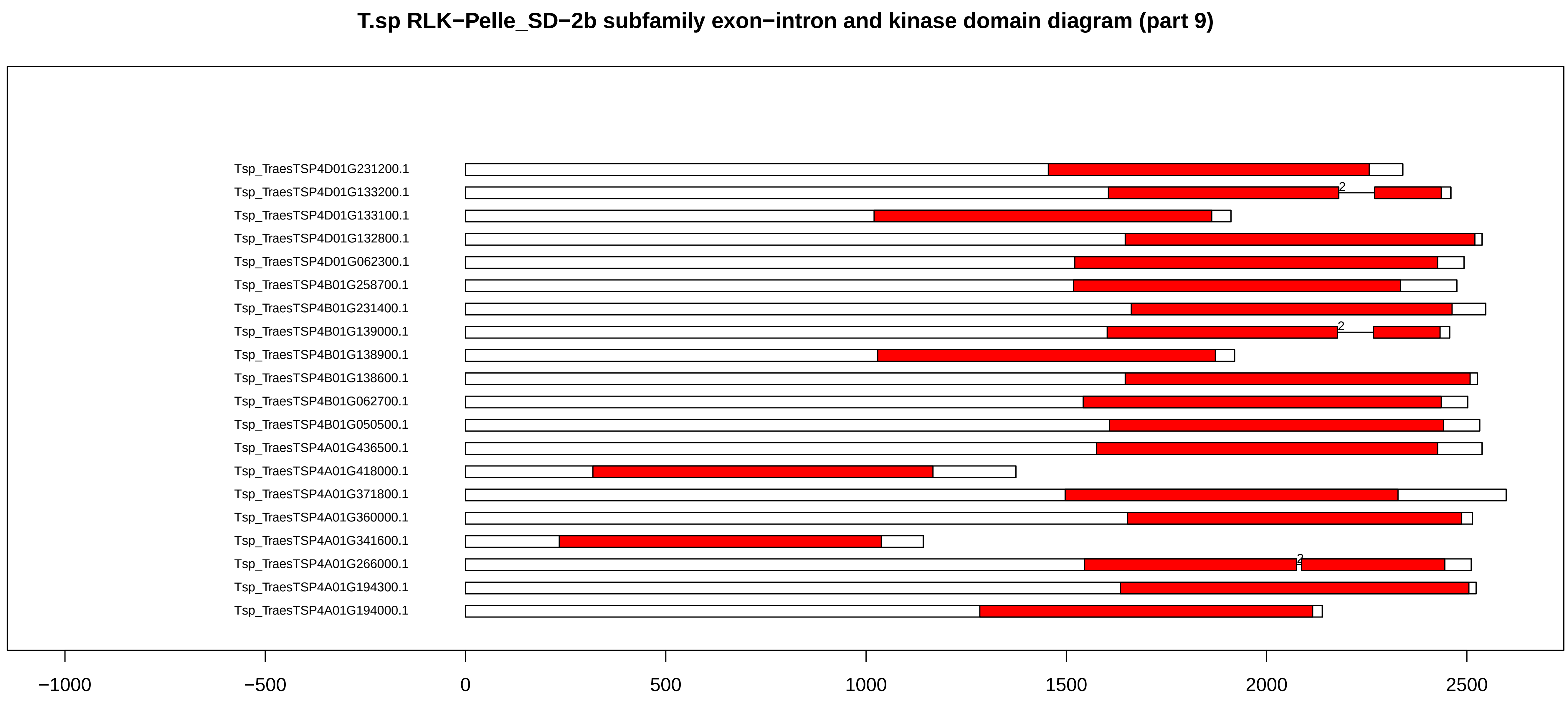

T.sp RLK−Pelle_SD−2b subfamily exon−intron and kinase domain diagram (part 9)
Tsp_TraesTSP4D01G231200.1
Tsp_TraesTSP4D01G133200.1
Tsp_TraesTSP4D01G133100.1
Tsp_TraesTSP4D01G132800.1
Tsp_TraesTSP4D01G062300.1
Tsp_TraesTSP4B01G258700.1
Tsp_TraesTSP4B01G231400.1
Tsp_TraesTSP4B01G139000.1
Tsp_TraesTSP4B01G138900.1
Tsp_TraesTSP4B01G138600.1
Tsp_TraesTSP4B01G062700.1
Tsp_TraesTSP4B01G050500.1
Tsp_TraesTSP4A01G436500.1
Tsp_TraesTSP4A01G418000.1
Tsp_TraesTSP4A01G371800.1
Tsp_TraesTSP4A01G360000.1
Tsp_TraesTSP4A01G341600.1
Tsp_TraesTSP4A01G266000.1
Tsp_TraesTSP4A01G194300.1
Tsp_TraesTSP4A01G194000.1
2
2
2
−1000
−500
0
500
1000
1500
2000
2500

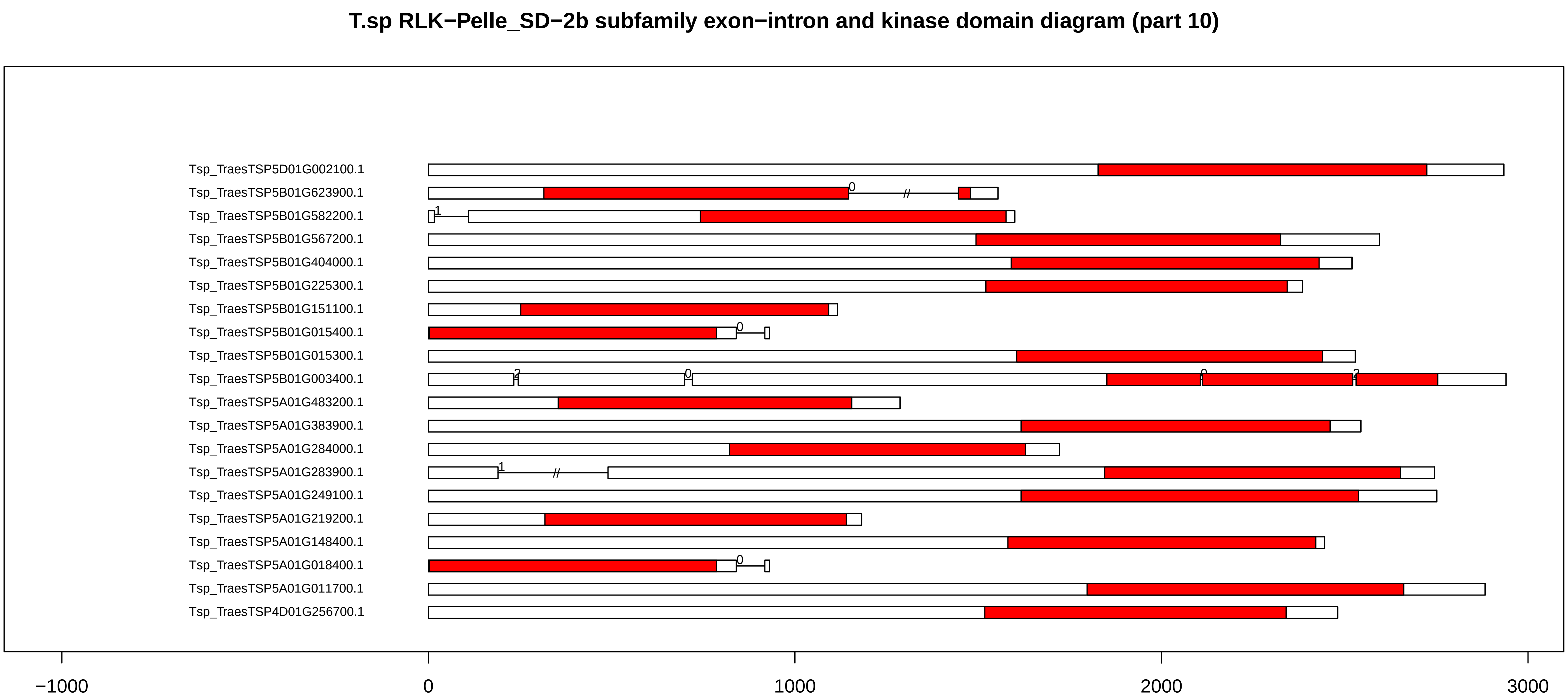

T.sp RLK−Pelle_SD−2b subfamily exon−intron and kinase domain diagram (part 10)
Tsp_TraesTSP5D01G002100.1
Tsp_TraesTSP5B01G623900.1
Tsp_TraesTSP5B01G582200.1
Tsp_TraesTSP5B01G567200.1
Tsp_TraesTSP5B01G404000.1
Tsp_TraesTSP5B01G225300.1
Tsp_TraesTSP5B01G151100.1
Tsp_TraesTSP5B01G015400.1
Tsp_TraesTSP5B01G015300.1
Tsp_TraesTSP5B01G003400.1
Tsp_TraesTSP5A01G483200.1
Tsp_TraesTSP5A01G383900.1
Tsp_TraesTSP5A01G284000.1
Tsp_TraesTSP5A01G283900.1
Tsp_TraesTSP5A01G249100.1
Tsp_TraesTSP5A01G219200.1
Tsp_TraesTSP5A01G148400.1
Tsp_TraesTSP5A01G018400.1
Tsp_TraesTSP5A01G011700.1
Tsp_TraesTSP4D01G256700.1
−1000
0
1000
2000
3000

T.sp RLK−Pelle_SD−2b subfamily exon−intron and kinase domain diagram (part 11)

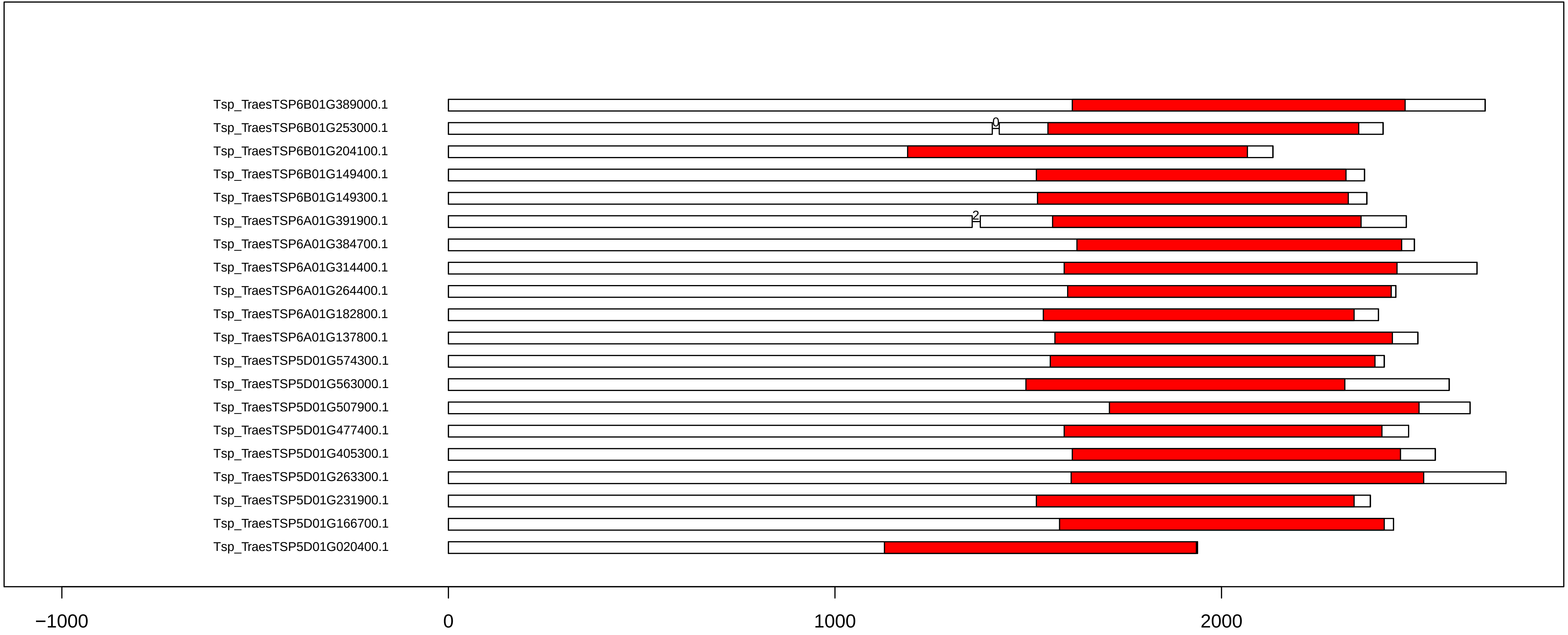

T.sp RLK−Pelle_SD−2b subfamily exon−intron and kinase domain diagram (part 12)

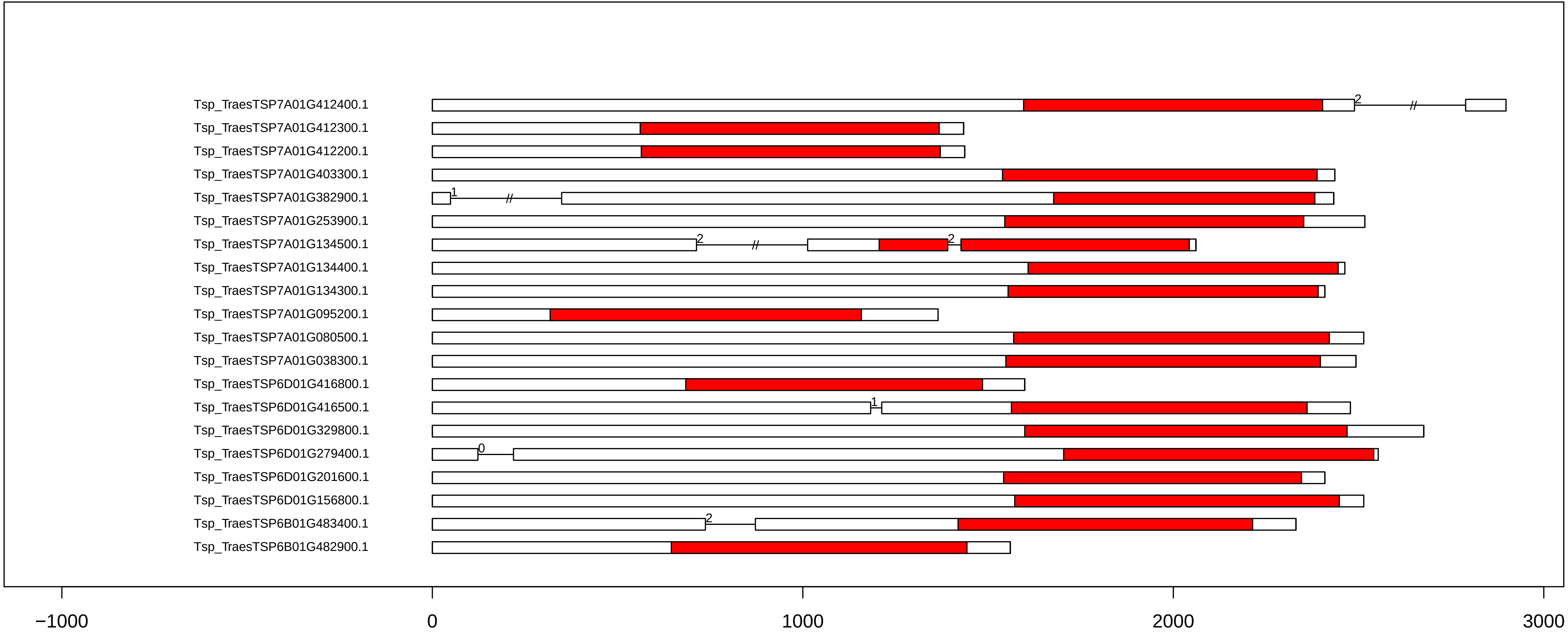

T.sp RLK−Pelle_SD−2b subfamily exon−intron and kinase domain diagram (part 13)

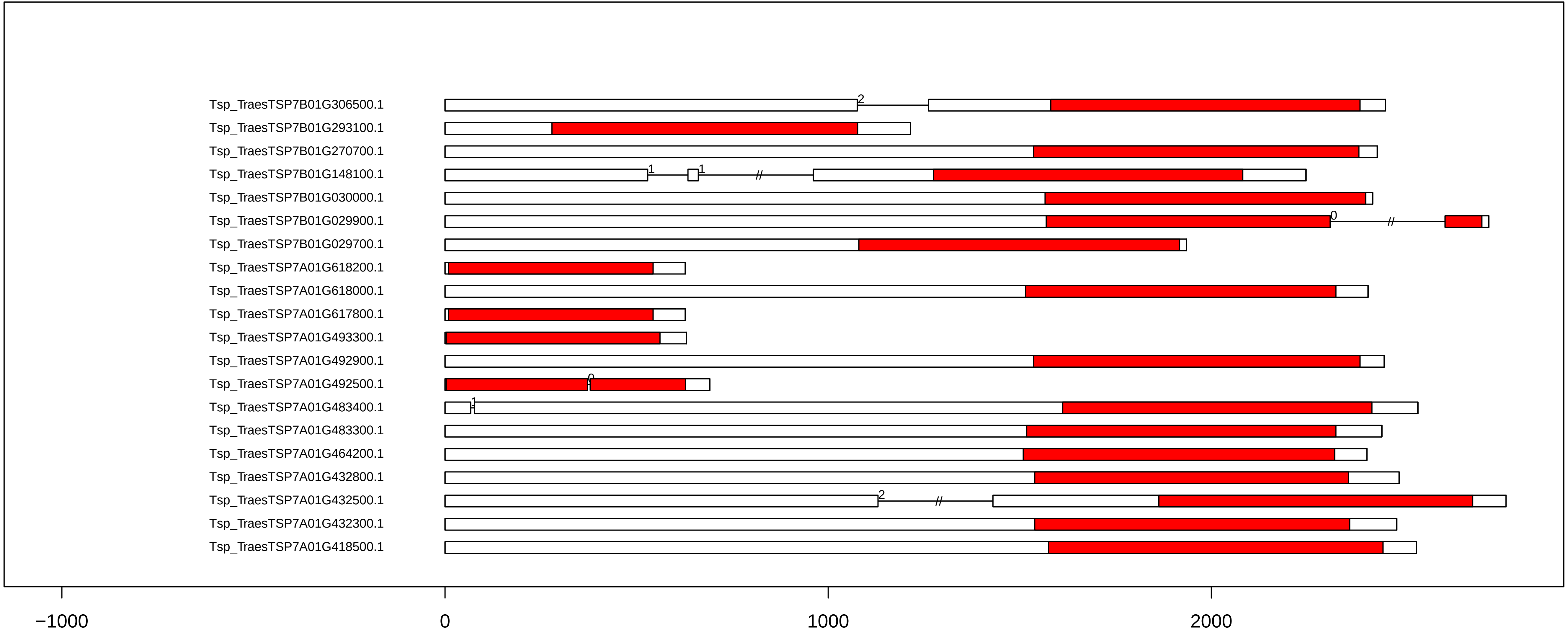

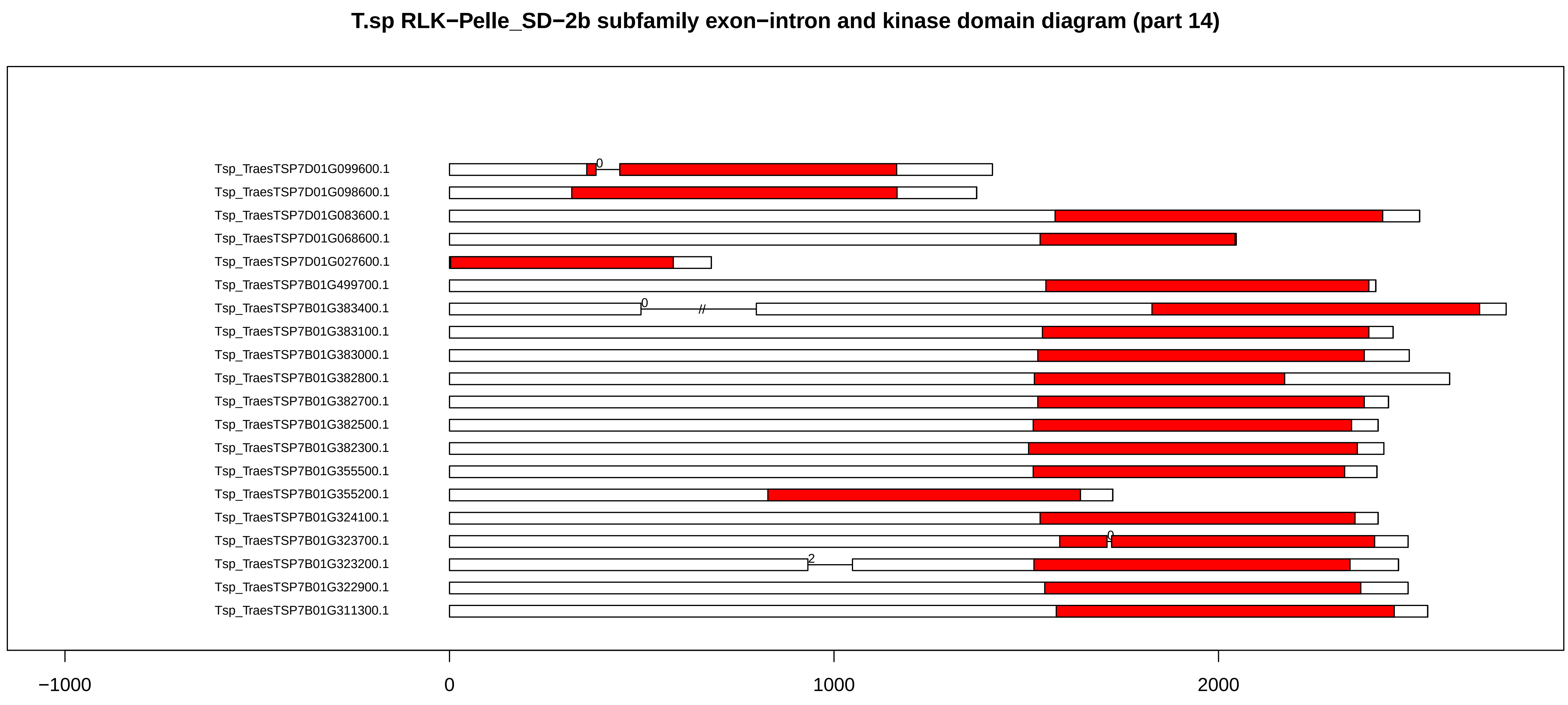
T.sp RLK−Pelle_SD−2b subfamily exon−intron and kinase domain diagram (part 14)
Tsp_TraesTSP7D01G099600.1
Tsp_TraesTSP7D01G098600.1
Tsp_TraesTSP7D01G083600.1
Tsp_TraesTSP7D01G068600.1
Tsp_TraesTSP7D01G027600.1
Tsp_TraesTSP7B01G499700.1
Tsp_TraesTSP7B01G383400.1
Tsp_TraesTSP7B01G383100.1
Tsp_TraesTSP7B01G383000.1
Tsp_TraesTSP7B01G382800.1
Tsp_TraesTSP7B01G382700.1
Tsp_TraesTSP7B01G382500.1
Tsp_TraesTSP7B01G382300.1
Tsp_TraesTSP7B01G355500.1
Tsp_TraesTSP7B01G355200.1
Tsp_TraesTSP7B01G324100.1
Tsp_TraesTSP7B01G323700.1
Tsp_TraesTSP7B01G323200.1
Tsp_TraesTSP7B01G322900.1
Tsp_TraesTSP7B01G311300.1
0
0
0
2
−1000
0
1000
2000

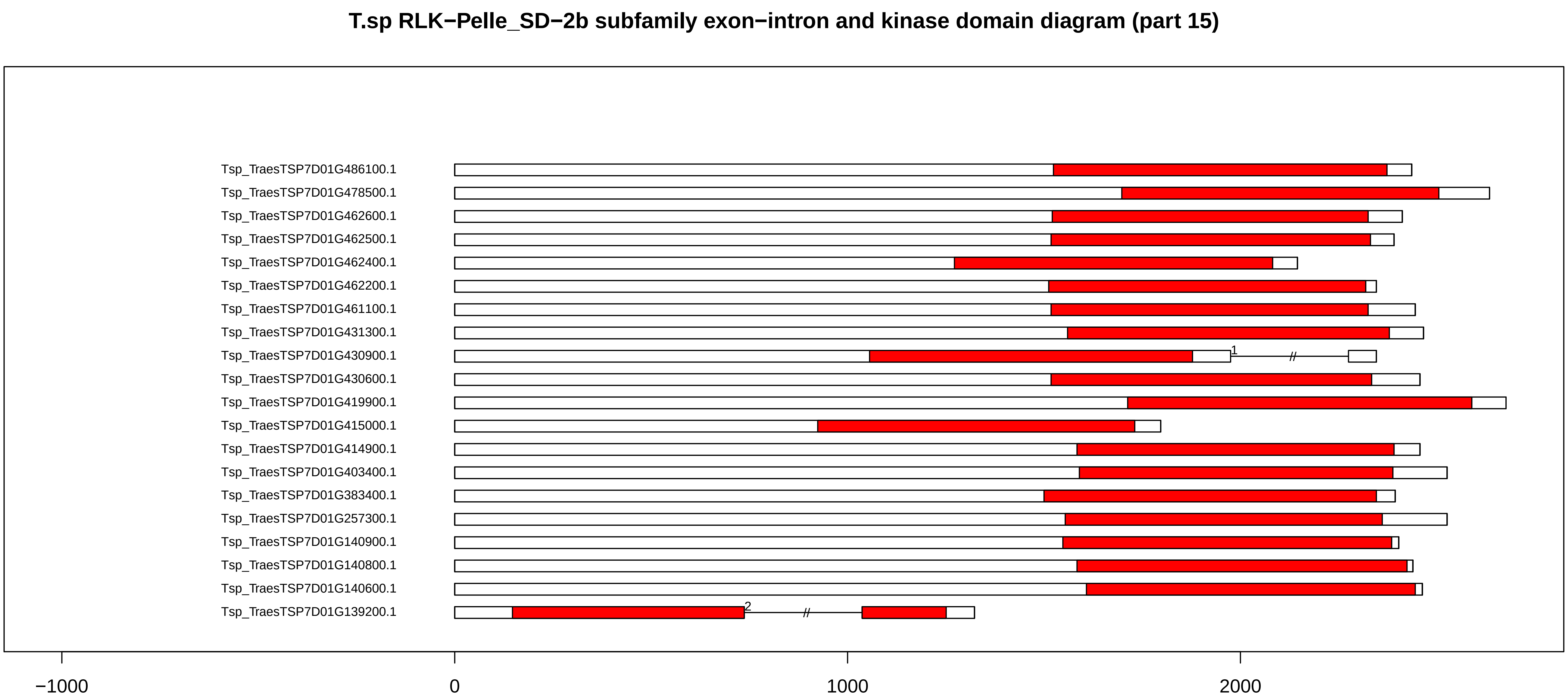
T.sp RLK−Pelle_SD−2b subfamily exon−intron and kinase domain diagram (part 15)
Tsp_TraesTSP7D01G486100.1
Tsp_TraesTSP7D01G478500.1
Tsp_TraesTSP7D01G462600.1
Tsp_TraesTSP7D01G462500.1
Tsp_TraesTSP7D01G462400.1
Tsp_TraesTSP7D01G462200.1
Tsp_TraesTSP7D01G461100.1
Tsp_TraesTSP7D01G431300.1
Tsp_TraesTSP7D01G430900.1
1
Tsp_TraesTSP7D01G430600.1
Tsp_TraesTSP7D01G419900.1
Tsp_TraesTSP7D01G415000.1
Tsp_TraesTSP7D01G414900.1
Tsp_TraesTSP7D01G403400.1
Tsp_TraesTSP7D01G383400.1
Tsp_TraesTSP7D01G257300.1
Tsp_TraesTSP7D01G140900.1
Tsp_TraesTSP7D01G140800.1
Tsp_TraesTSP7D01G140600.1
Tsp_TraesTSP7D01G139200.1
2
−1000
0
1000
2000

T.sp RLK−Pelle_SD−2b subfamily exon−intron and kinase domain diagram (part 16)

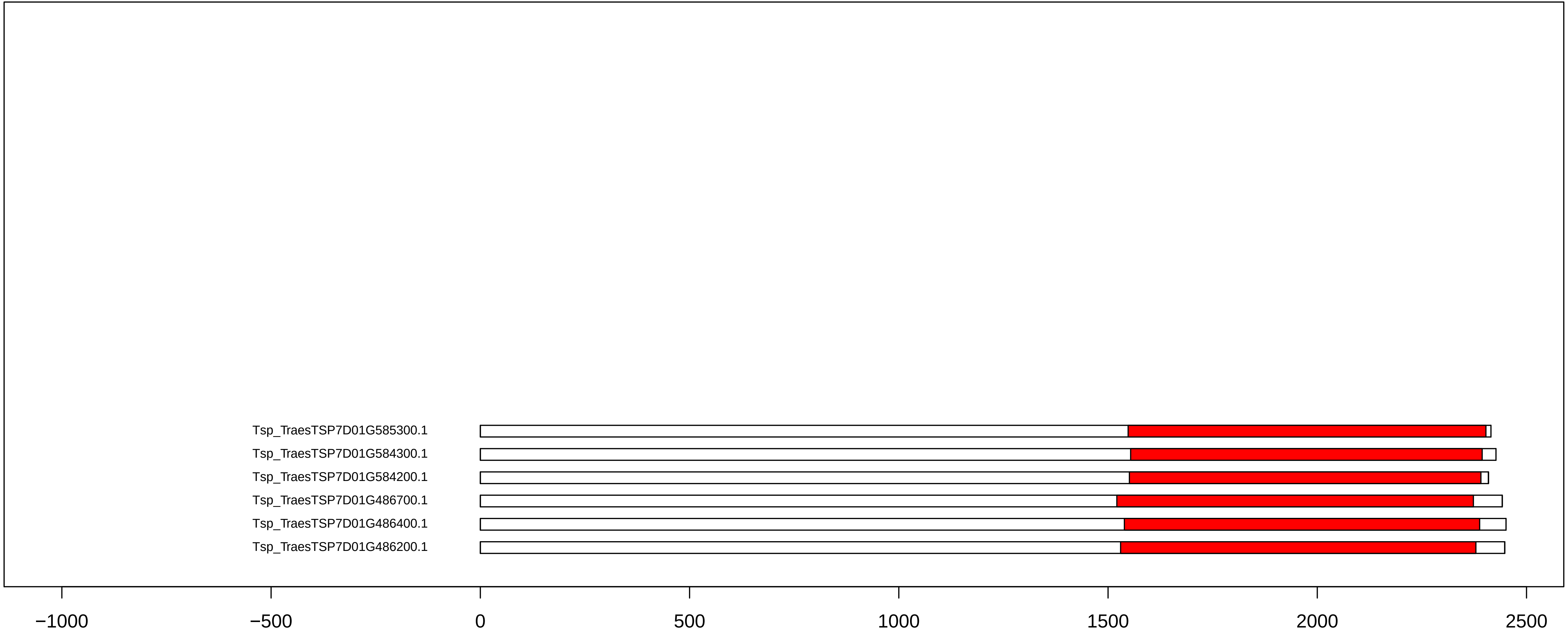

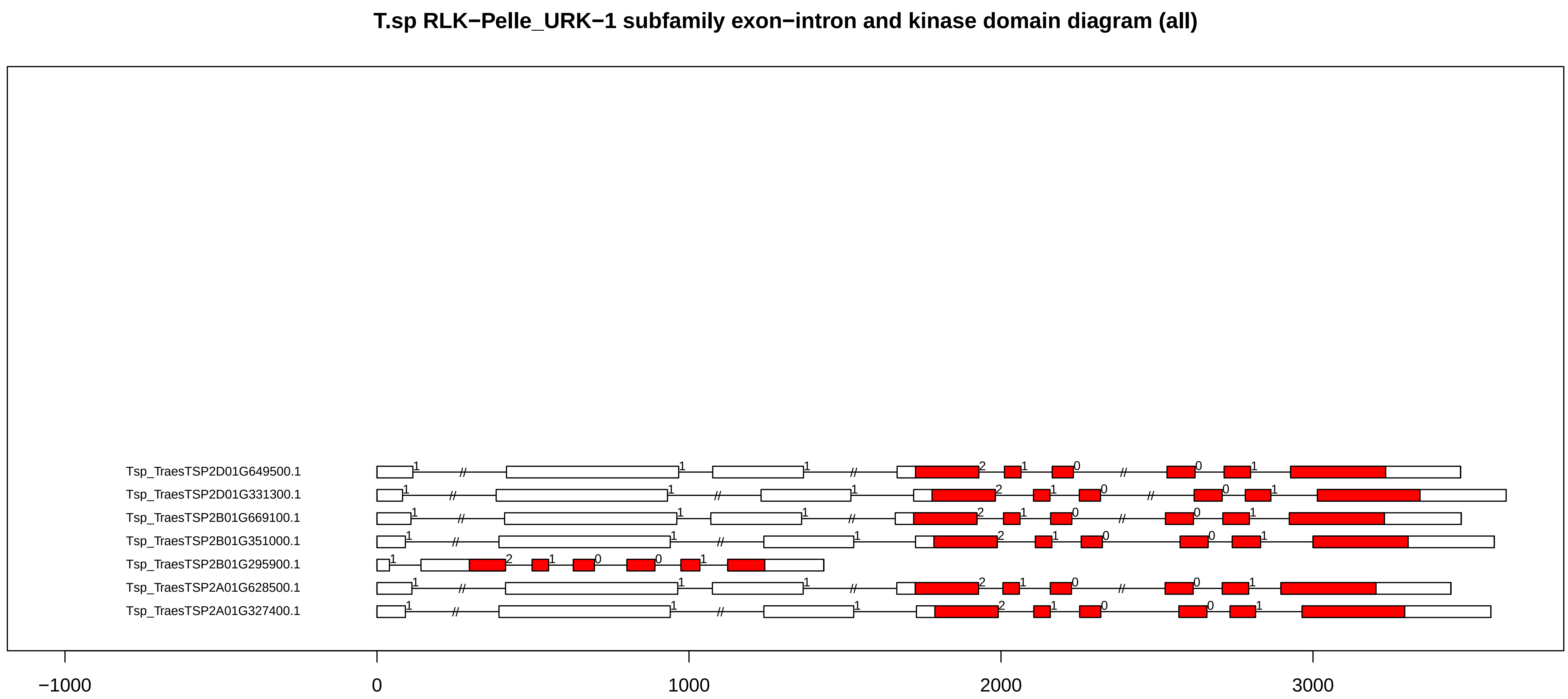
T.sp RLK−Pelle_URK−1 subfamily exon−intron and kinase domain diagram (all)
Tsp_TraesTSP2D01G649500.1
Tsp_TraesTSP2D01G331300.1
Tsp_TraesTSP2B01G669100.1
Tsp_TraesTSP2B01G351000.1
Tsp_TraesTSP2B01G295900.1
Tsp_TraesTSP2A01G628500.1
Tsp_TraesTSP2A01G327400.1
−1000
0
1000
2000
3000

T.sp RLK−Pelle_URK−2 subfamily exon−intron and kinase domain diagram (all)

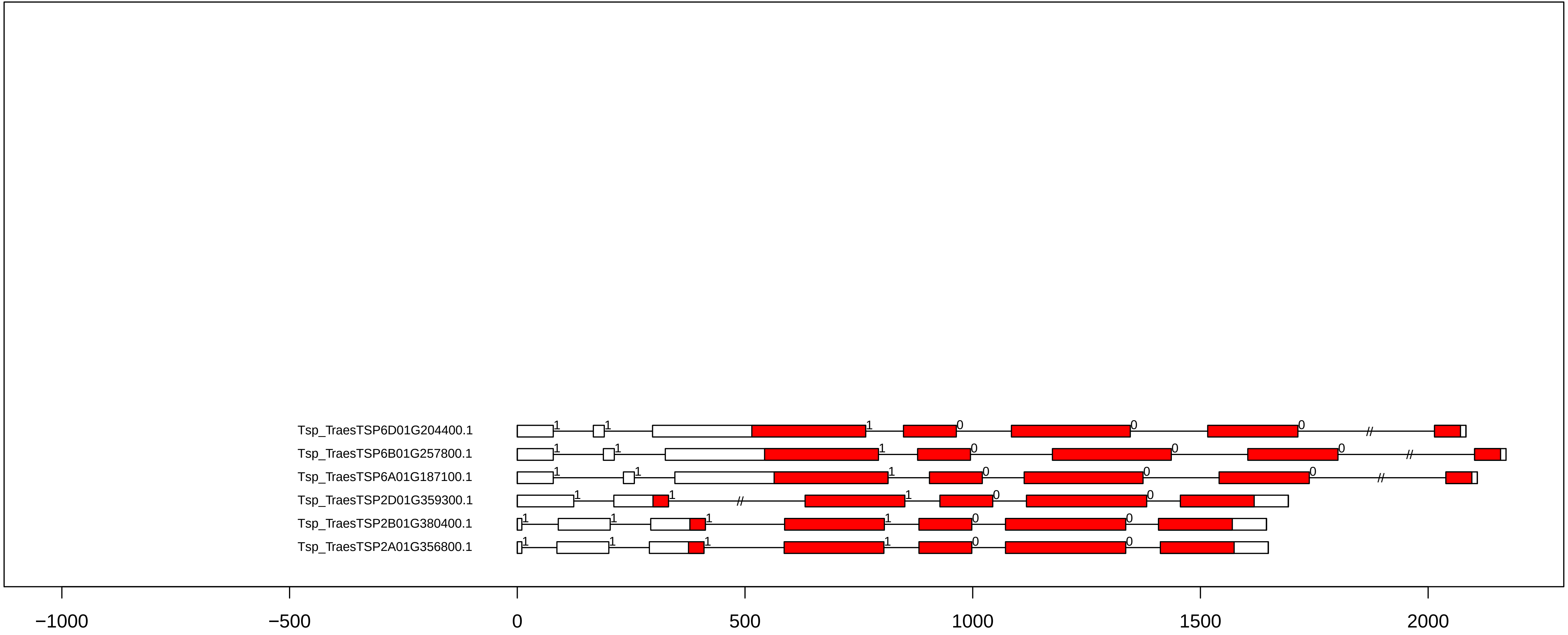

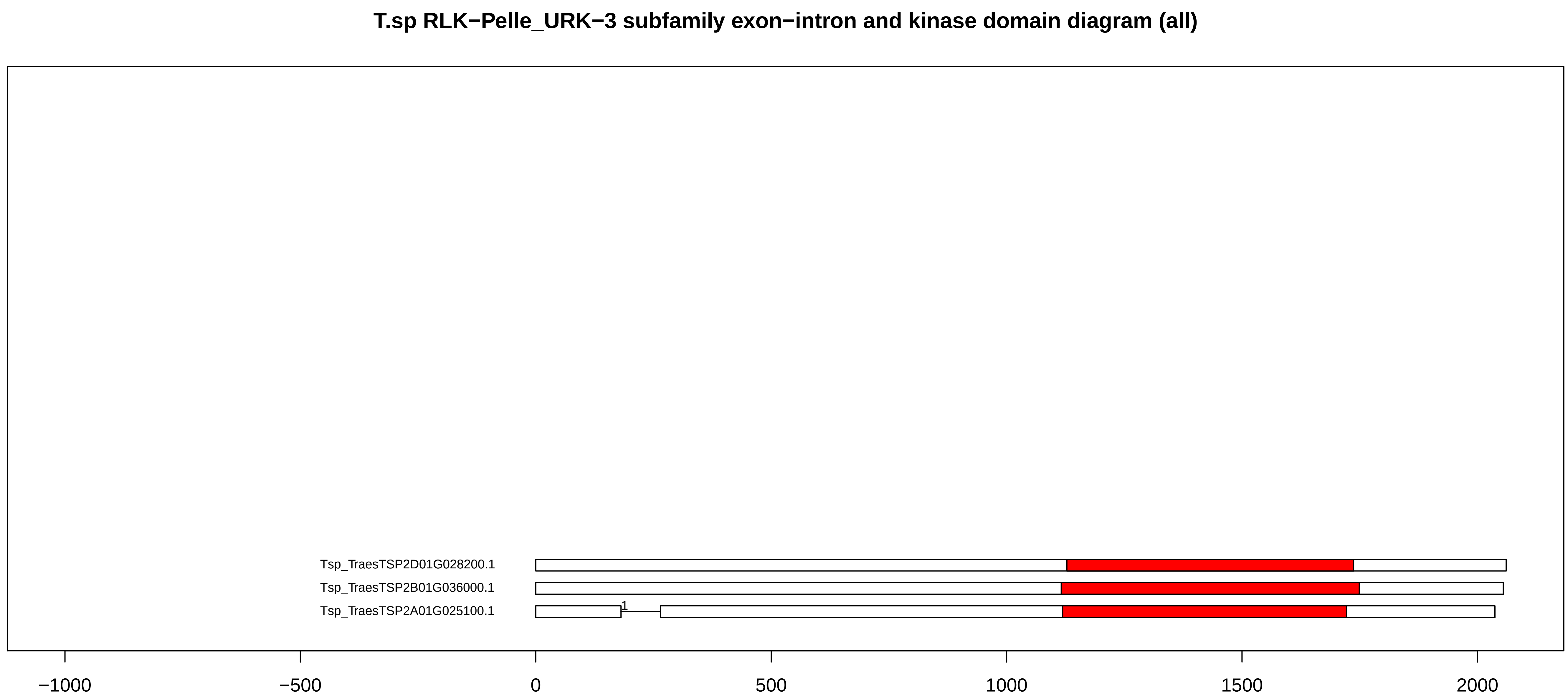
T.sp RLK−Pelle_URK−3 subfamily exon−intron and kinase domain diagram (all)
Tsp_TraesTSP2D01G028200.1
Tsp_TraesTSP2B01G036000.1
Tsp_TraesTSP2A01G025100.1
1
−1000
−500
0
500
1000
1500
2000

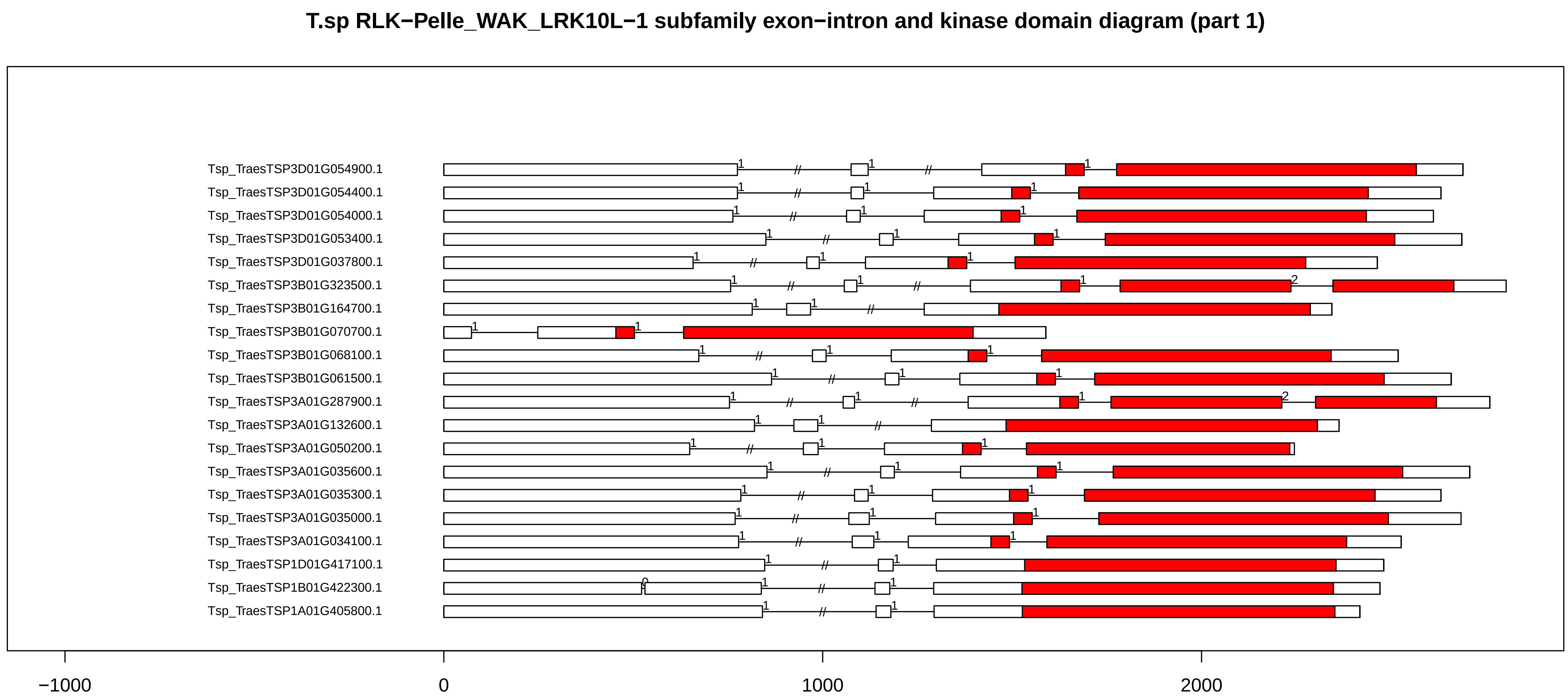
T.sp RLK−Pelle_WAK_LRK10L−1 subfamily exon−intron and kinase domain diagram (part 1)
Tsp_TraesTSP3D01G054900.1
Tsp_TraesTSP3D01G054400.1
Tsp_TraesTSP3D01G054000.1
Tsp_TraesTSP3D01G053400.1
Tsp_TraesTSP3D01G037800.1
Tsp_TraesTSP3B01G323500.1
Tsp_TraesTSP3B01G164700.1
Tsp_TraesTSP3B01G070700.1
Tsp_TraesTSP3B01G068100.1
Tsp_TraesTSP3B01G061500.1
Tsp_TraesTSP3A01G287900.1
Tsp_TraesTSP3A01G132600.1
Tsp_TraesTSP3A01G050200.1
Tsp_TraesTSP3A01G035600.1
Tsp_TraesTSP3A01G035300.1
Tsp_TraesTSP3A01G035000.1
Tsp_TraesTSP3A01G034100.1
Tsp_TraesTSP1D01G417100.1
Tsp_TraesTSP1B01G422300.1
Tsp_TraesTSP1A01G405800.1
−1000
0
1000
2000

T.sp RLK−Pelle_WAK_LRK10L−1 subfamily exon−intron and kinase domain diagram (part 2)

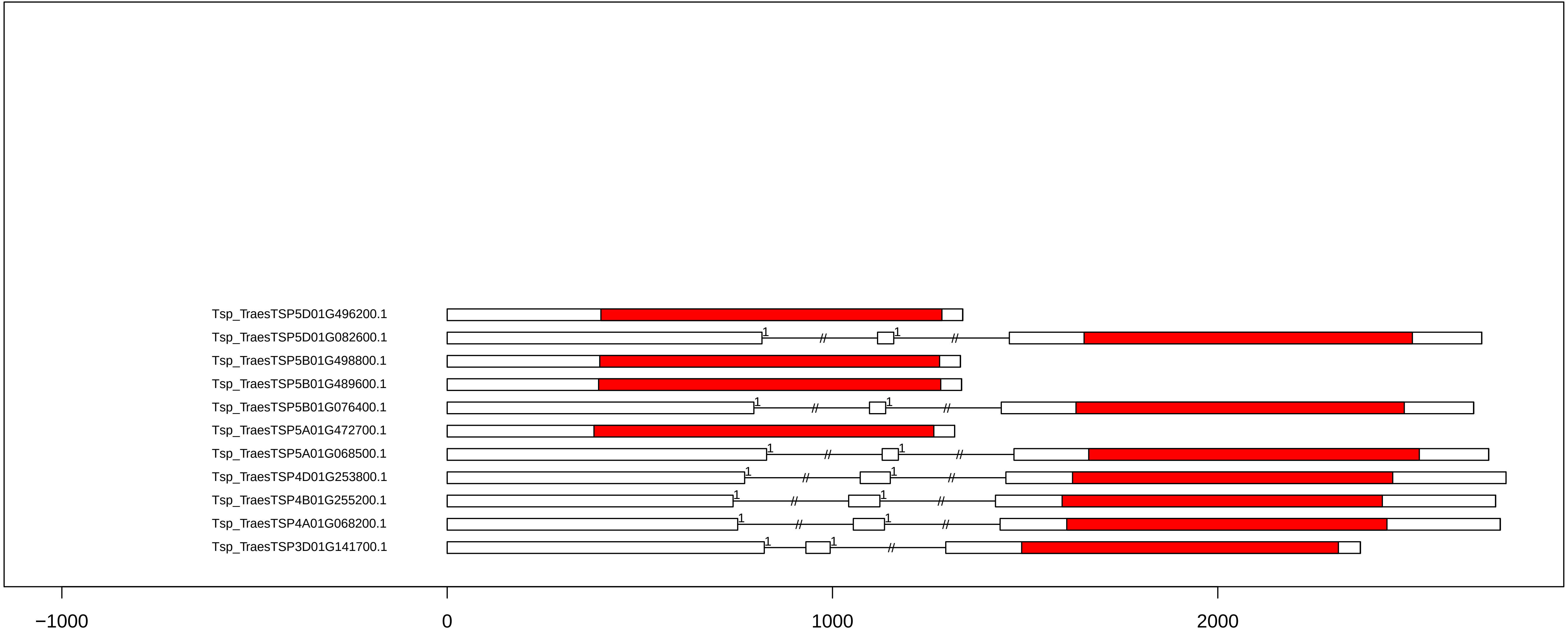

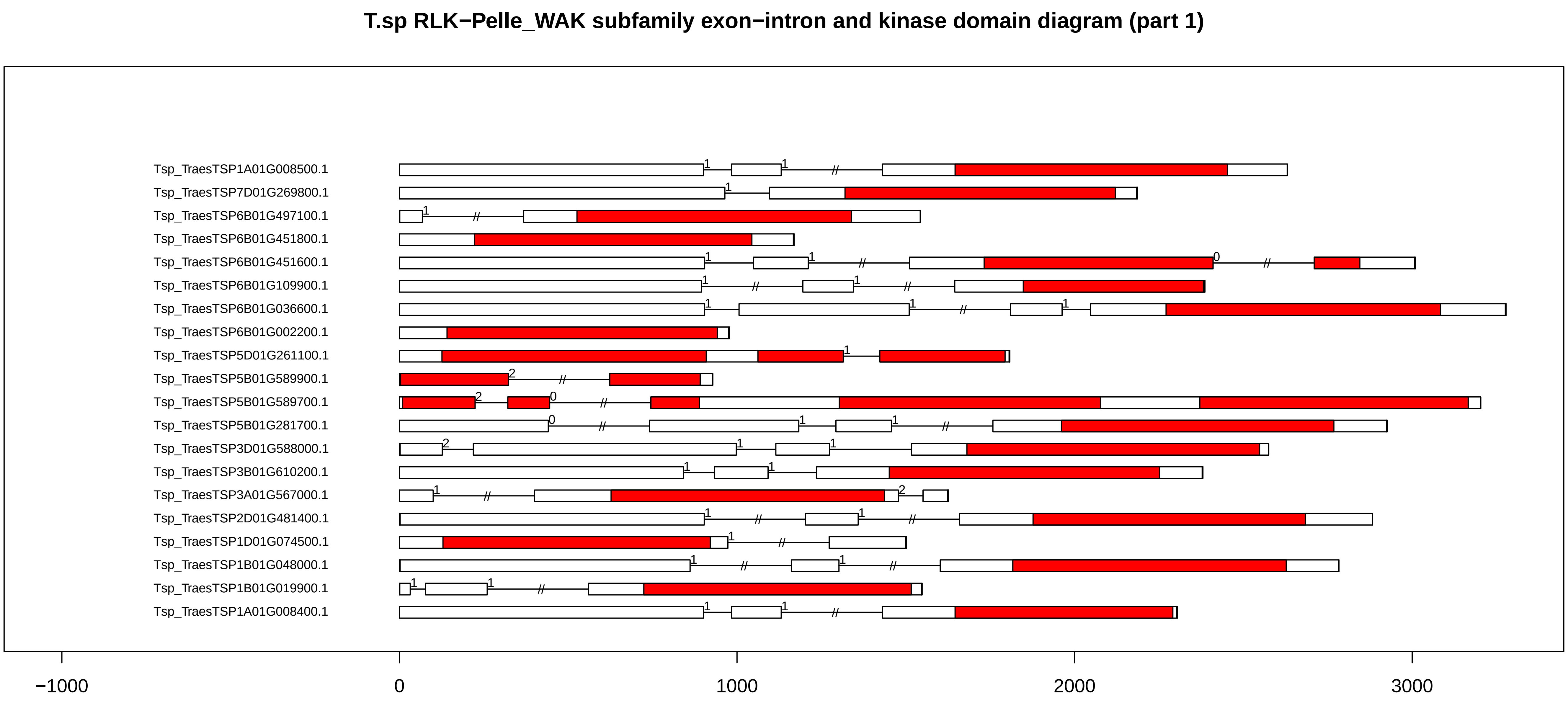

T.sp RLK−Pelle_WAK subfamily exon−intron and kinase domain diagram (part 1)
Tsp_TraesTSP1A01G008500.1
Tsp_TraesTSP7D01G269800.1
Tsp_TraesTSP6B01G497100.1
Tsp_TraesTSP6B01G451800.1
Tsp_TraesTSP6B01G451600.1
Tsp_TraesTSP6B01G109900.1
Tsp_TraesTSP6B01G036600.1
Tsp_TraesTSP6B01G002200.1
Tsp_TraesTSP5D01G261100.1
Tsp_TraesTSP5B01G589900.1
Tsp_TraesTSP5B01G589700.1
Tsp_TraesTSP5B01G281700.1
Tsp_TraesTSP3D01G588000.1
Tsp_TraesTSP3B01G610200.1
Tsp_TraesTSP3A01G567000.1
Tsp_TraesTSP2D01G481400.1
Tsp_TraesTSP1D01G074500.1
Tsp_TraesTSP1B01G048000.1
Tsp_TraesTSP1B01G019900.1
Tsp_TraesTSP1A01G008400.1
−1000
0
1000
2000
3000

T.sp RLK−Pelle_WAK subfamily exon−intron and kinase domain diagram (part 2)

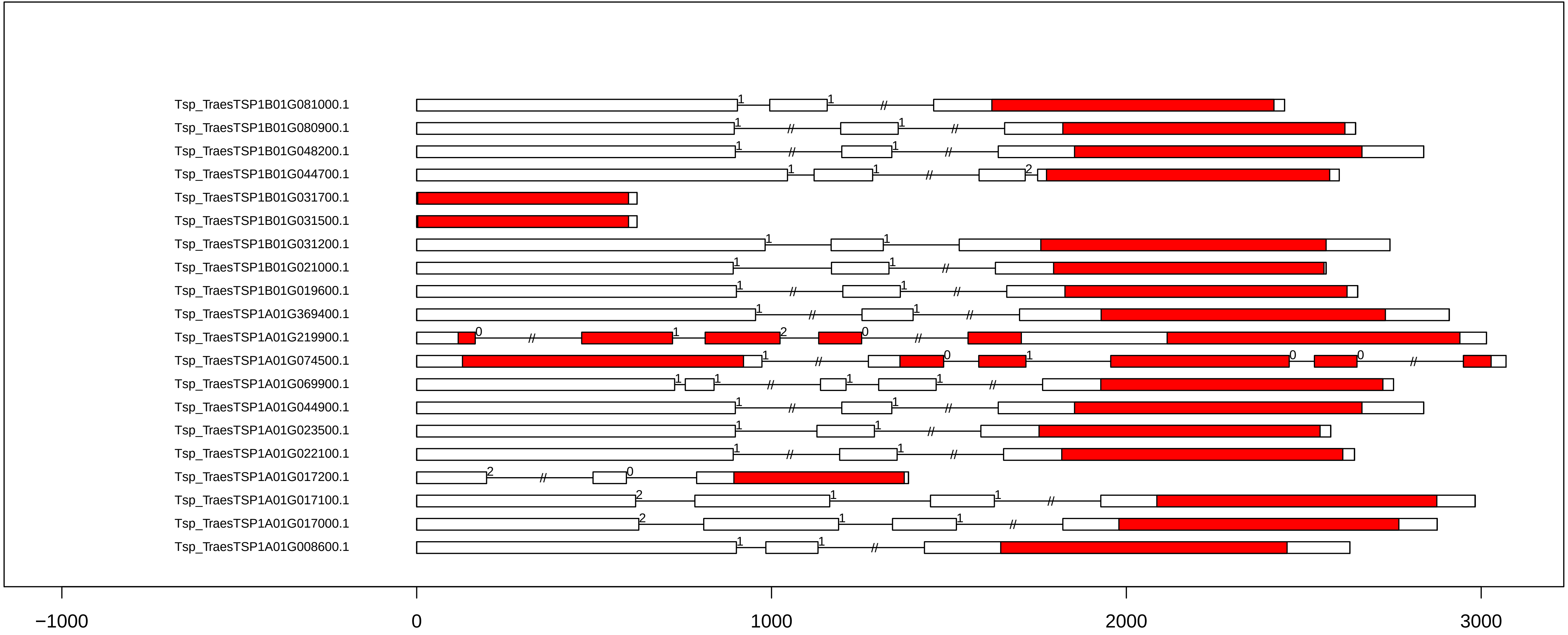

T.sp RLK−Pelle_WAK subfamily exon−intron and kinase domain diagram (part 3)

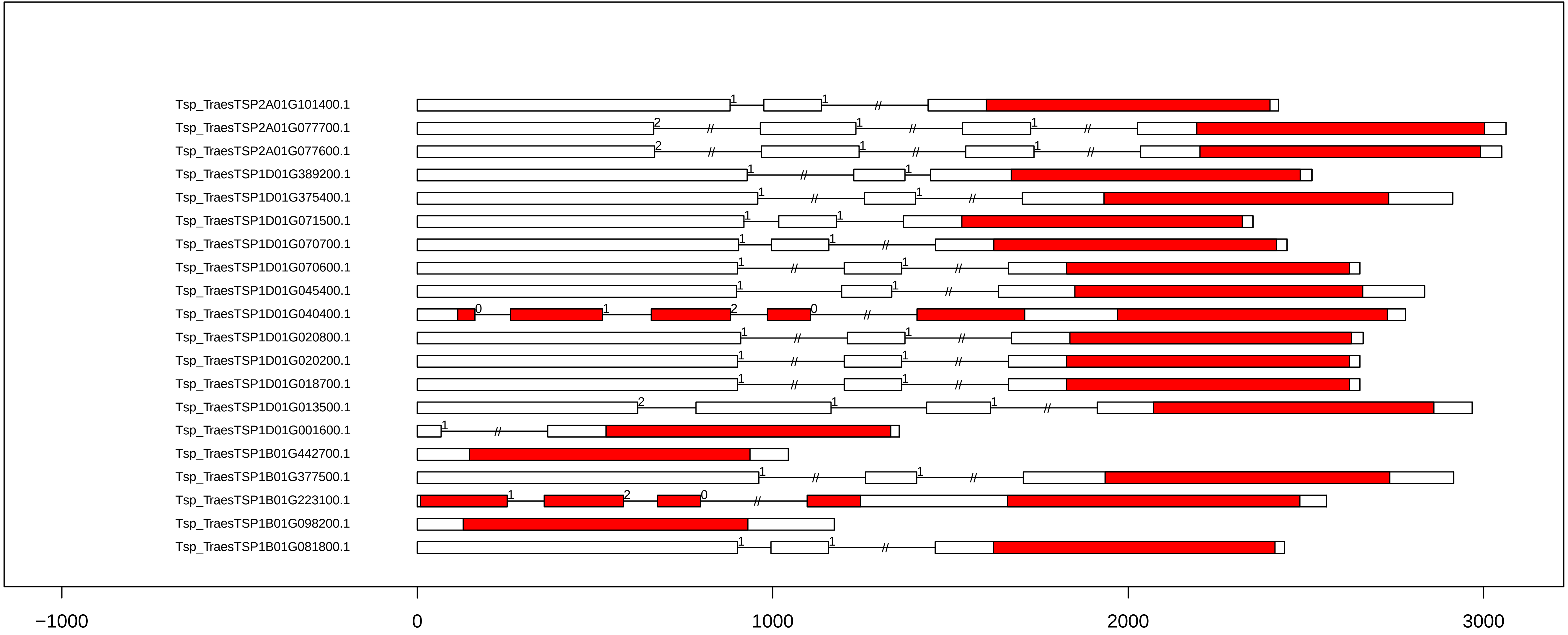

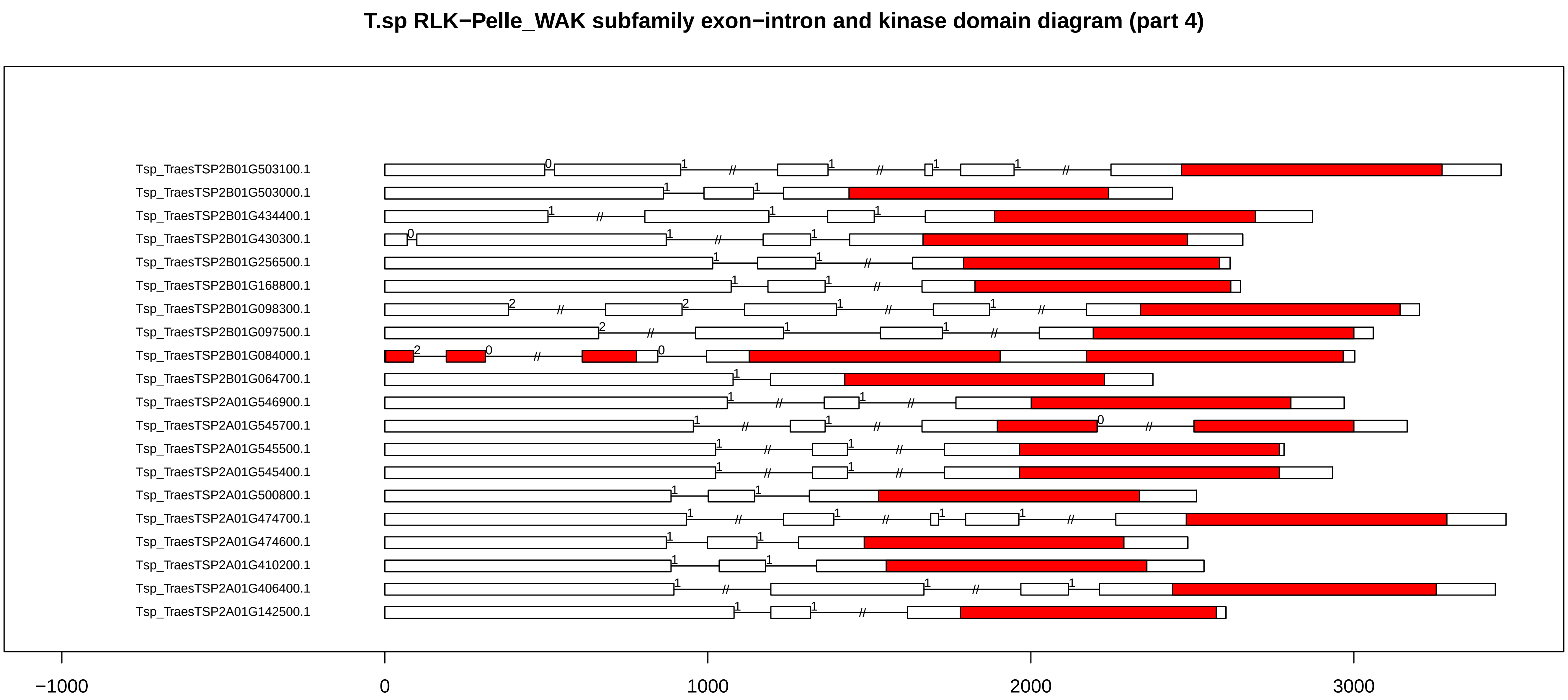

T.sp RLK−Pelle_WAK subfamily exon−intron and kinase domain diagram (part 4)
Tsp_TraesTSP2B01G503100.1
Tsp_TraesTSP2B01G503000.1
Tsp_TraesTSP2B01G434400.1
Tsp_TraesTSP2B01G430300.1
Tsp_TraesTSP2B01G256500.1
Tsp_TraesTSP2B01G168800.1
Tsp_TraesTSP2B01G098300.1
Tsp_TraesTSP2B01G097500.1
Tsp_TraesTSP2B01G084000.1
Tsp_TraesTSP2B01G064700.1
Tsp_TraesTSP2A01G546900.1
Tsp_TraesTSP2A01G545700.1
Tsp_TraesTSP2A01G545500.1
Tsp_TraesTSP2A01G545400.1
Tsp_TraesTSP2A01G500800.1
Tsp_TraesTSP2A01G474700.1
Tsp_TraesTSP2A01G474600.1
Tsp_TraesTSP2A01G410200.1
Tsp_TraesTSP2A01G406400.1
Tsp_TraesTSP2A01G142500.1
−1000
0
1000
2000
3000

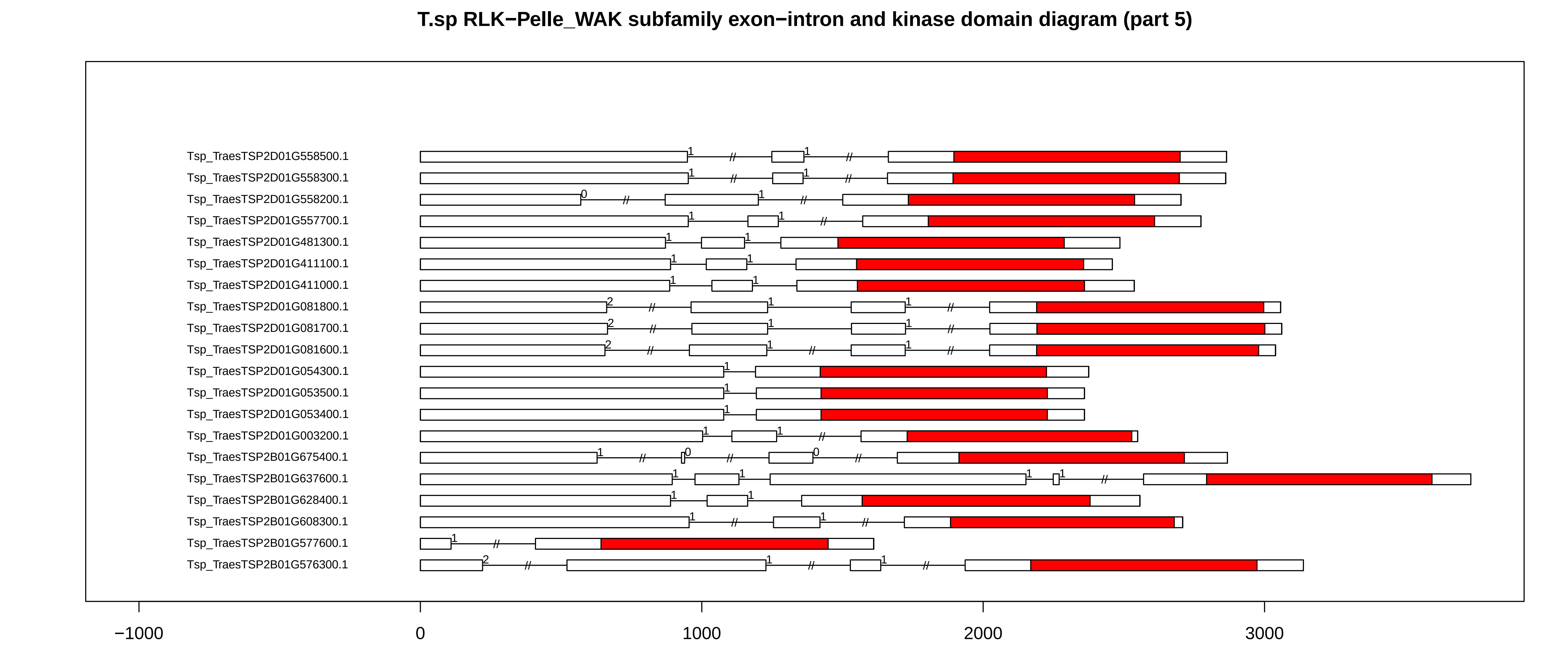
T.sp RLK−Pelle_WAK subfamily exon−intron and kinase domain diagram (part 5)
Tsp_TraesTSP2D01G558500.1
Tsp_TraesTSP2D01G558300.1
Tsp_TraesTSP2D01G558200.1
Tsp_TraesTSP2D01G557700.1
Tsp_TraesTSP2D01G481300.1
Tsp_TraesTSP2D01G411100.1
Tsp_TraesTSP2D01G411000.1
Tsp_TraesTSP2D01G081800.1
Tsp_TraesTSP2D01G081700.1
Tsp_TraesTSP2D01G081600.1
Tsp_TraesTSP2D01G054300.1
Tsp_TraesTSP2D01G053500.1
Tsp_TraesTSP2D01G053400.1
Tsp_TraesTSP2D01G003200.1
Tsp_TraesTSP2B01G675400.1
Tsp_TraesTSP2B01G637600.1
Tsp_TraesTSP2B01G628400.1
Tsp_TraesTSP2B01G608300.1
Tsp_TraesTSP2B01G577600.1
Tsp_TraesTSP2B01G576300.1
−1000
0
1000
2000
3000

T.sp RLK−Pelle_WAK subfamily exon−intron and kinase domain diagram (part 6)

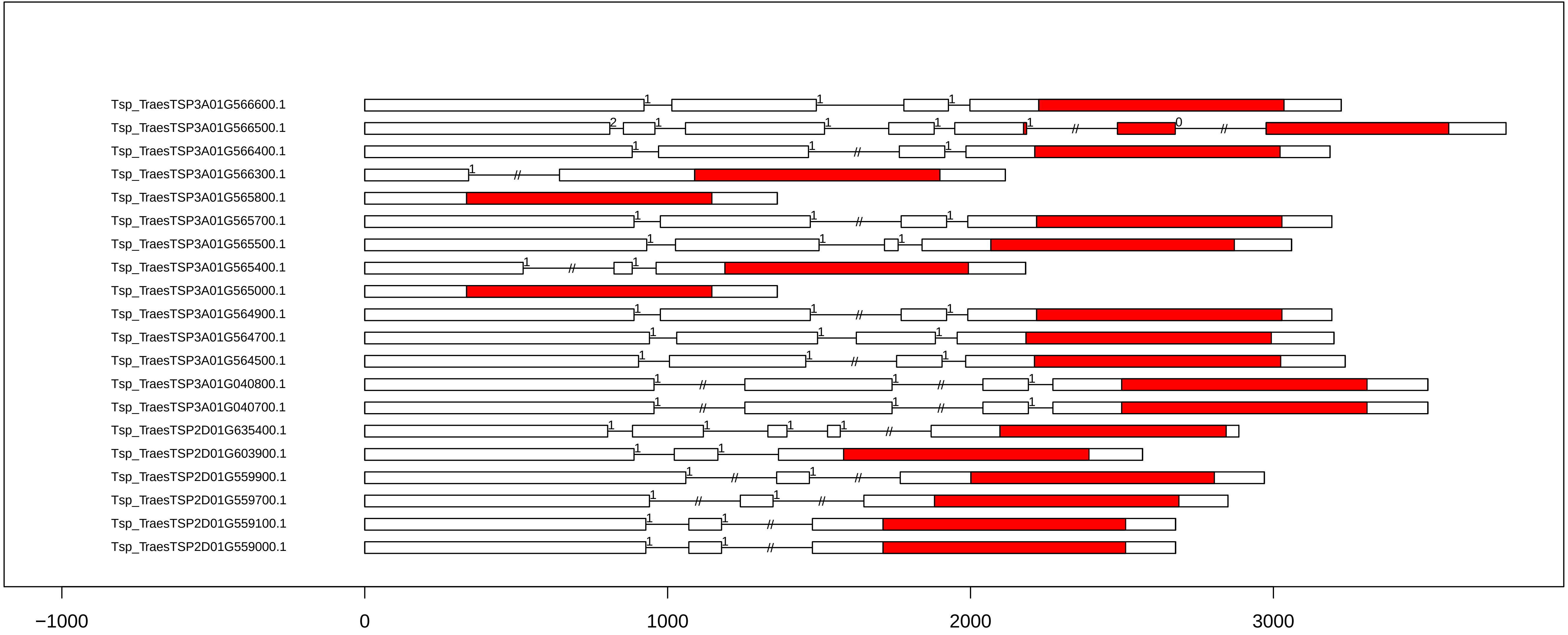

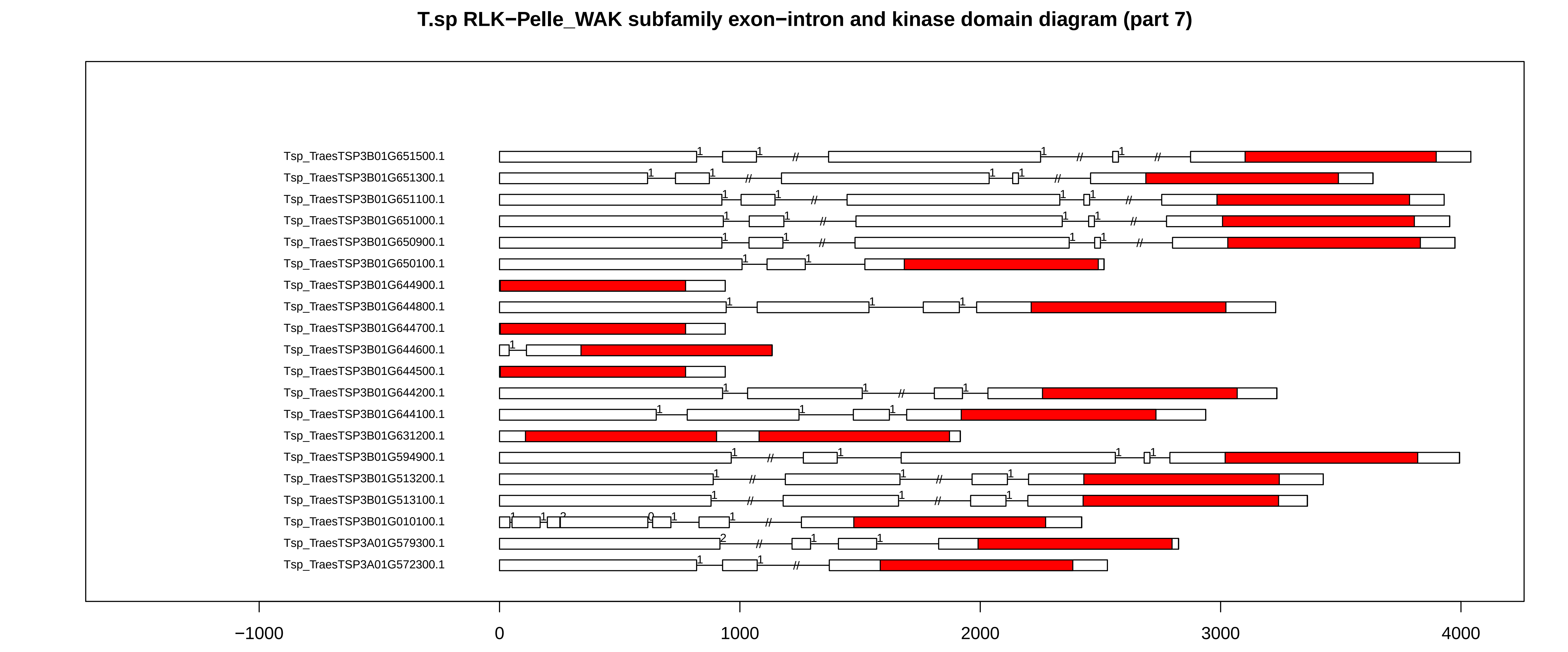

T.sp RLK−Pelle_WAK subfamily exon−intron and kinase domain diagram (part 7)
Tsp_TraesTSP3B01G651500.1
Tsp_TraesTSP3B01G651300.1
Tsp_TraesTSP3B01G651100.1
Tsp_TraesTSP3B01G651000.1
Tsp_TraesTSP3B01G650900.1
Tsp_TraesTSP3B01G650100.1
Tsp_TraesTSP3B01G644900.1
Tsp_TraesTSP3B01G644800.1
Tsp_TraesTSP3B01G644700.1
Tsp_TraesTSP3B01G644600.1
Tsp_TraesTSP3B01G644500.1
Tsp_TraesTSP3B01G644200.1
Tsp_TraesTSP3B01G644100.1
Tsp_TraesTSP3B01G631200.1
Tsp_TraesTSP3B01G594900.1
Tsp_TraesTSP3B01G513200.1
Tsp_TraesTSP3B01G513100.1
Tsp_TraesTSP3B01G010100.1
Tsp_TraesTSP3A01G579300.1
Tsp_TraesTSP3A01G572300.1
−1000
0
1000
2000
3000
4000

T.sp RLK−Pelle_WAK subfamily exon−intron and kinase domain diagram (part 8)

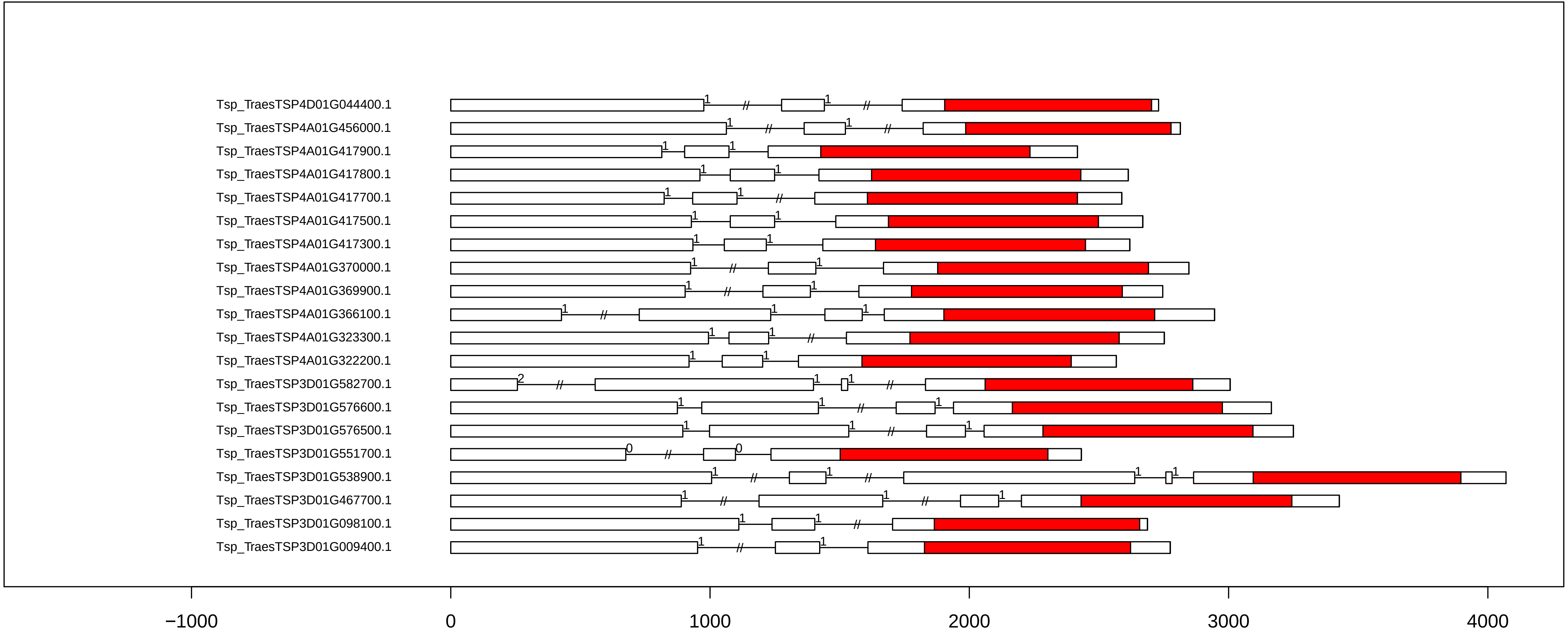

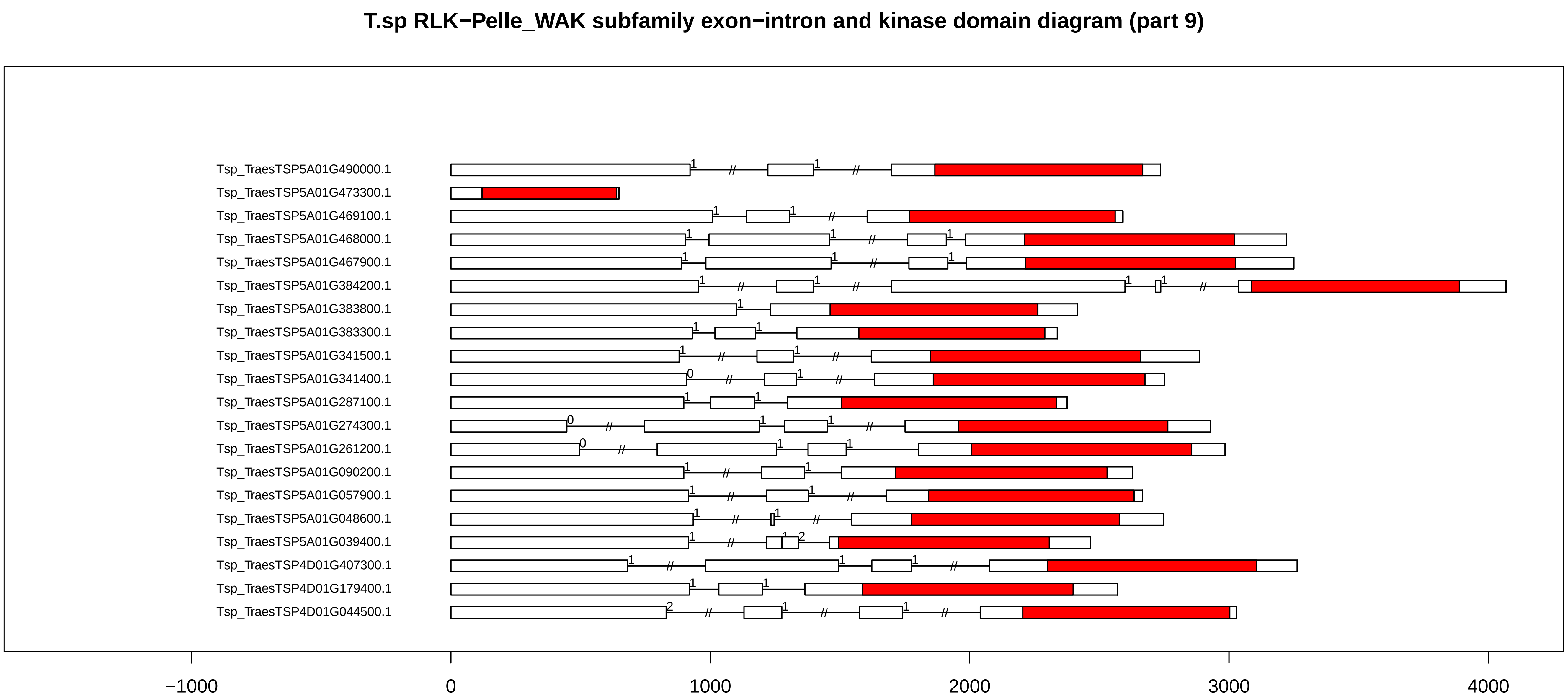

T.sp RLK−Pelle_WAK subfamily exon−intron and kinase domain diagram (part 9)
Tsp_TraesTSP5A01G490000.1
Tsp_TraesTSP5A01G473300.1
Tsp_TraesTSP5A01G469100.1
Tsp_TraesTSP5A01G468000.1
Tsp_TraesTSP5A01G467900.1
Tsp_TraesTSP5A01G384200.1
Tsp_TraesTSP5A01G383800.1
Tsp_TraesTSP5A01G383300.1
Tsp_TraesTSP5A01G341500.1
Tsp_TraesTSP5A01G341400.1
Tsp_TraesTSP5A01G287100.1
Tsp_TraesTSP5A01G274300.1
Tsp_TraesTSP5A01G261200.1
Tsp_TraesTSP5A01G090200.1
Tsp_TraesTSP5A01G057900.1
Tsp_TraesTSP5A01G048600.1
Tsp_TraesTSP5A01G039400.1
Tsp_TraesTSP4D01G407300.1
Tsp_TraesTSP4D01G179400.1
Tsp_TraesTSP4D01G044500.1
−1000
0
1000
2000
3000
4000

T.sp RLK−Pelle_WAK subfamily exon−intron and kinase domain diagram (part 10)

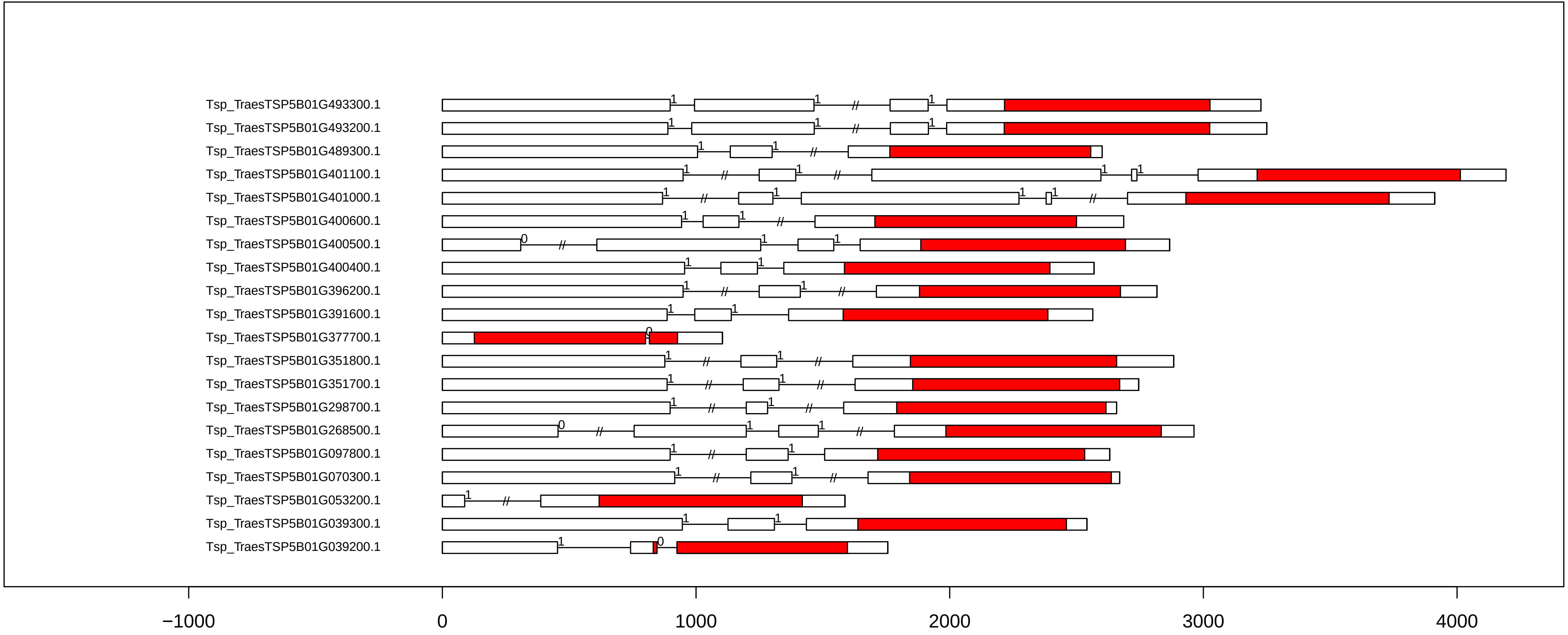

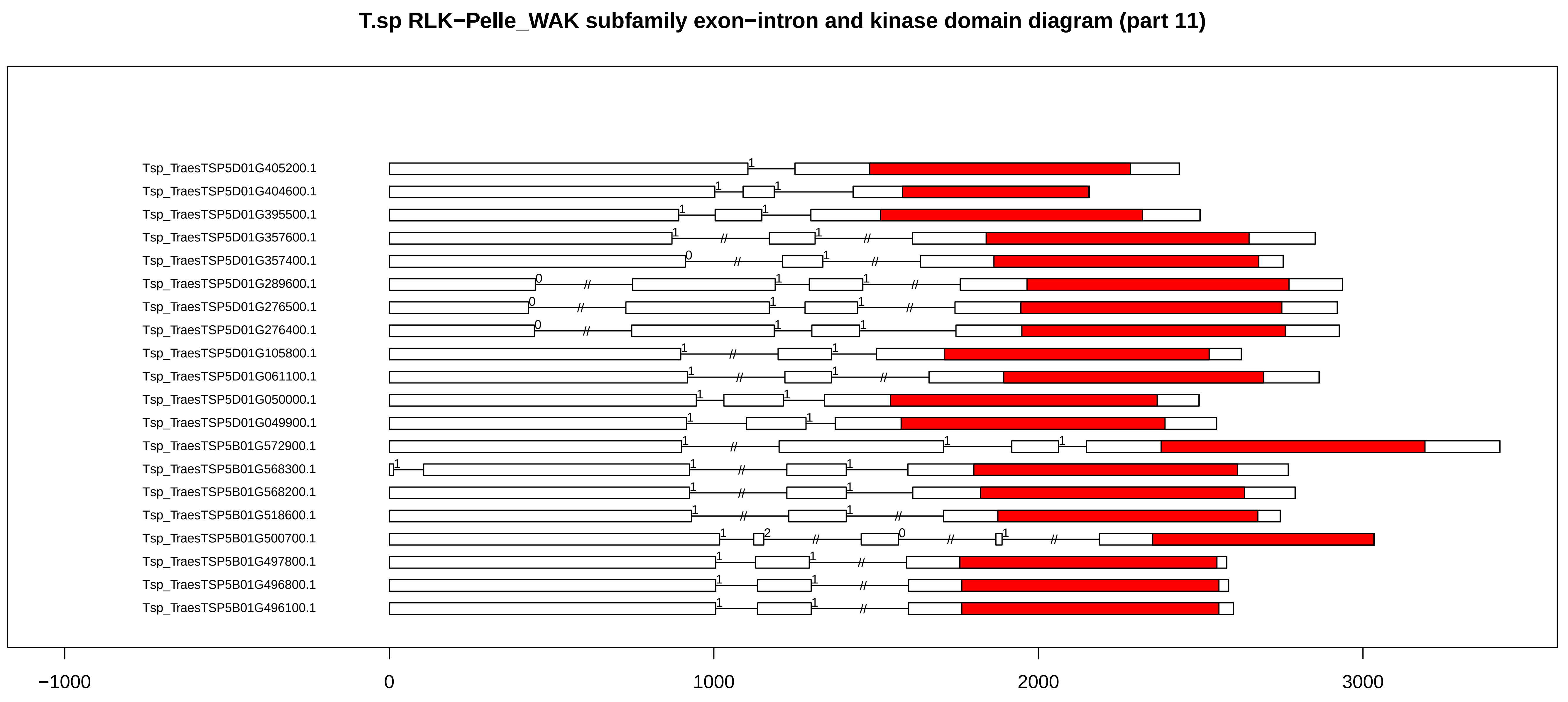
T.sp RLK−Pelle_WAK subfamily exon−intron and kinase domain diagram (part 11)
Tsp_TraesTSP5D01G405200.1
Tsp_TraesTSP5D01G404600.1
Tsp_TraesTSP5D01G395500.1
Tsp_TraesTSP5D01G357600.1
Tsp_TraesTSP5D01G357400.1
Tsp_TraesTSP5D01G289600.1
Tsp_TraesTSP5D01G276500.1
Tsp_TraesTSP5D01G276400.1
Tsp_TraesTSP5D01G105800.1
Tsp_TraesTSP5D01G061100.1
Tsp_TraesTSP5D01G050000.1
Tsp_TraesTSP5D01G049900.1
Tsp_TraesTSP5B01G572900.1
Tsp_TraesTSP5B01G568300.1
Tsp_TraesTSP5B01G568200.1
Tsp_TraesTSP5B01G518600.1
Tsp_TraesTSP5B01G500700.1
Tsp_TraesTSP5B01G497800.1
Tsp_TraesTSP5B01G496800.1
Tsp_TraesTSP5B01G496100.1
−1000
0
1000
2000
3000

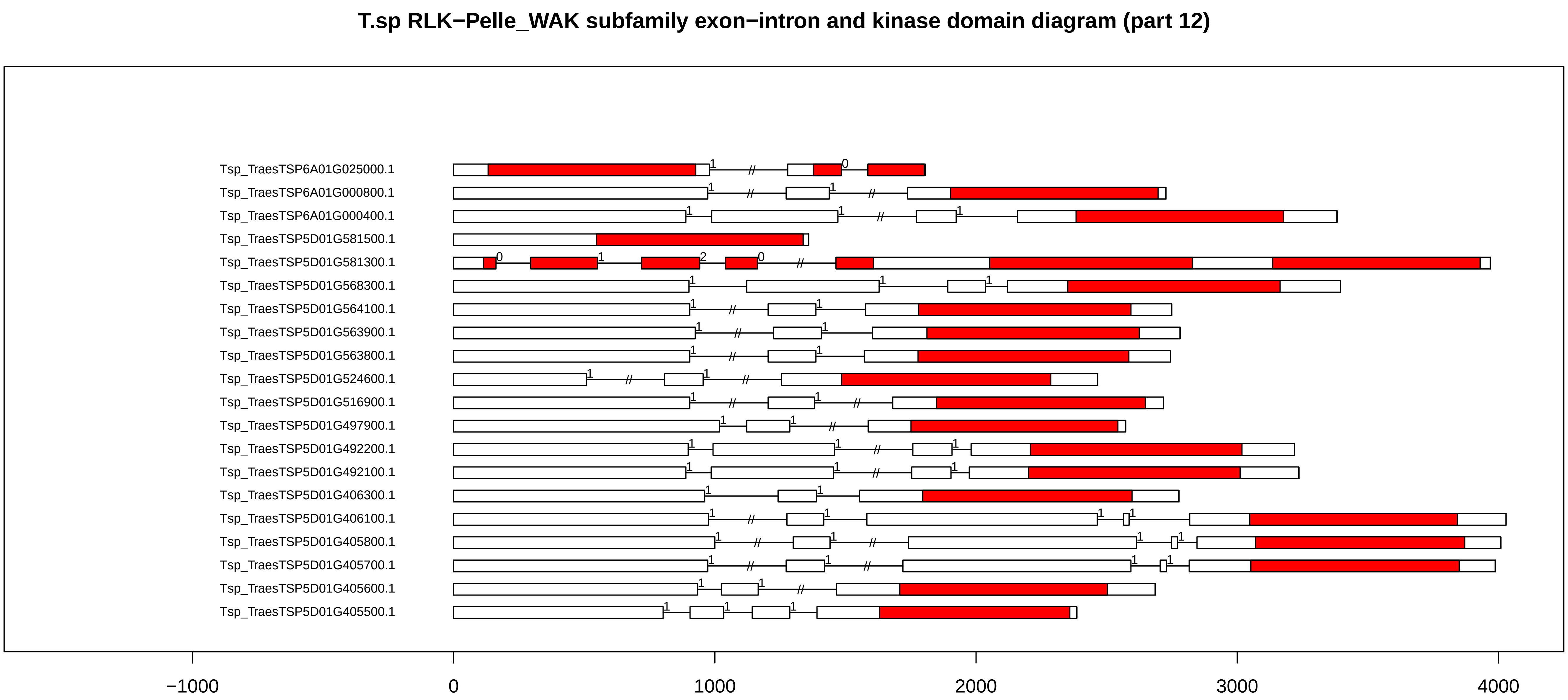
T.sp RLK−Pelle_WAK subfamily exon−intron and kinase domain diagram (part 12)
Tsp_TraesTSP6A01G025000.1
Tsp_TraesTSP6A01G000800.1
Tsp_TraesTSP6A01G000400.1
Tsp_TraesTSP5D01G581500.1
Tsp_TraesTSP5D01G581300.1
Tsp_TraesTSP5D01G568300.1
Tsp_TraesTSP5D01G564100.1
Tsp_TraesTSP5D01G563900.1
Tsp_TraesTSP5D01G563800.1
Tsp_TraesTSP5D01G524600.1
Tsp_TraesTSP5D01G516900.1
Tsp_TraesTSP5D01G497900.1
Tsp_TraesTSP5D01G492200.1
Tsp_TraesTSP5D01G492100.1
Tsp_TraesTSP5D01G406300.1
Tsp_TraesTSP5D01G406100.1
Tsp_TraesTSP5D01G405800.1
Tsp_TraesTSP5D01G405700.1
Tsp_TraesTSP5D01G405600.1
Tsp_TraesTSP5D01G405500.1
−1000
0
1000
2000
3000
4000

T.sp RLK−Pelle_WAK subfamily exon−intron and kinase domain diagram (part 13)

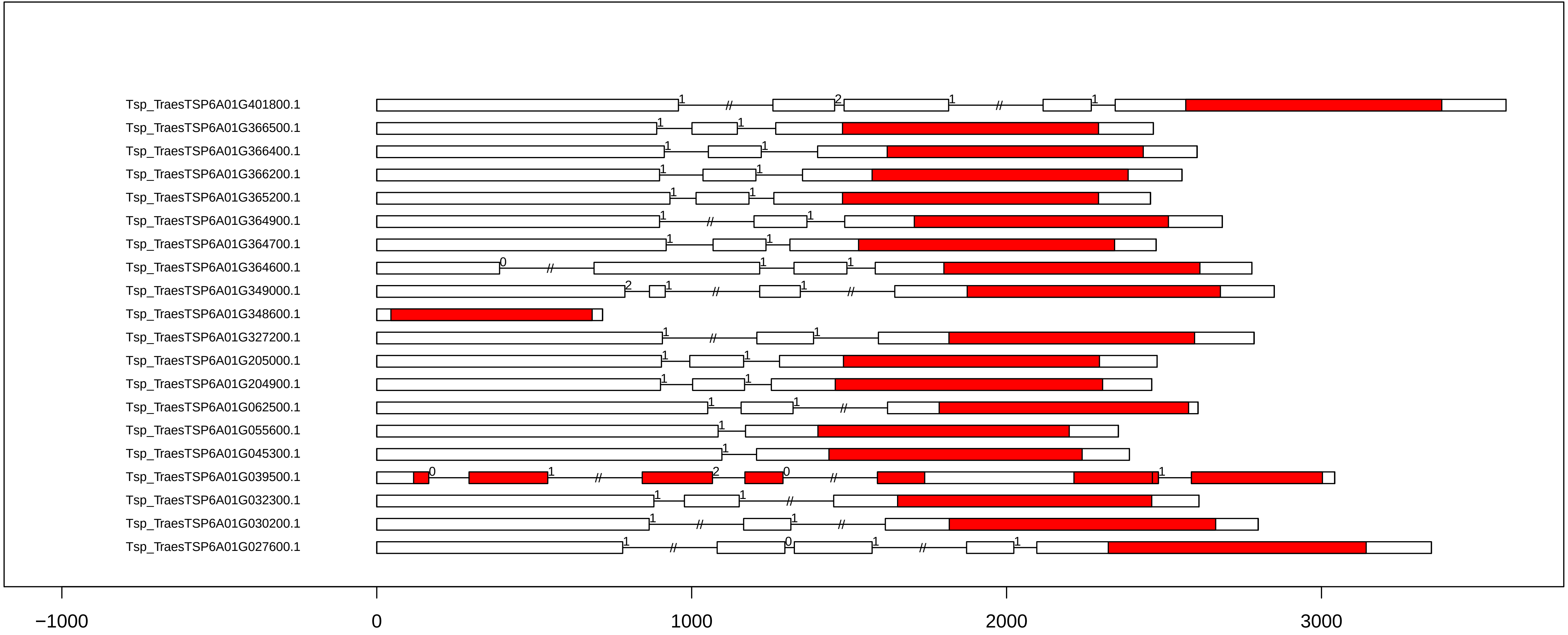

T.sp RLK−Pelle_WAK subfamily exon−intron and kinase domain diagram (part 14)

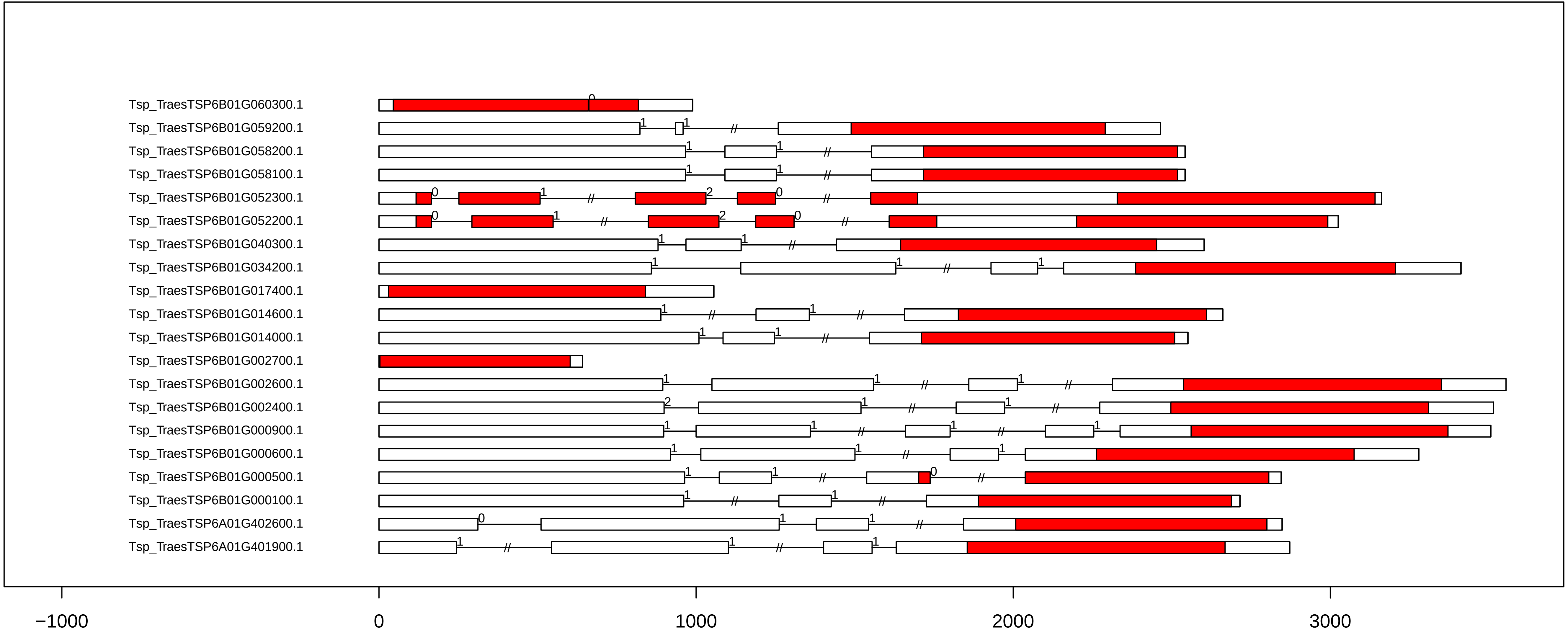

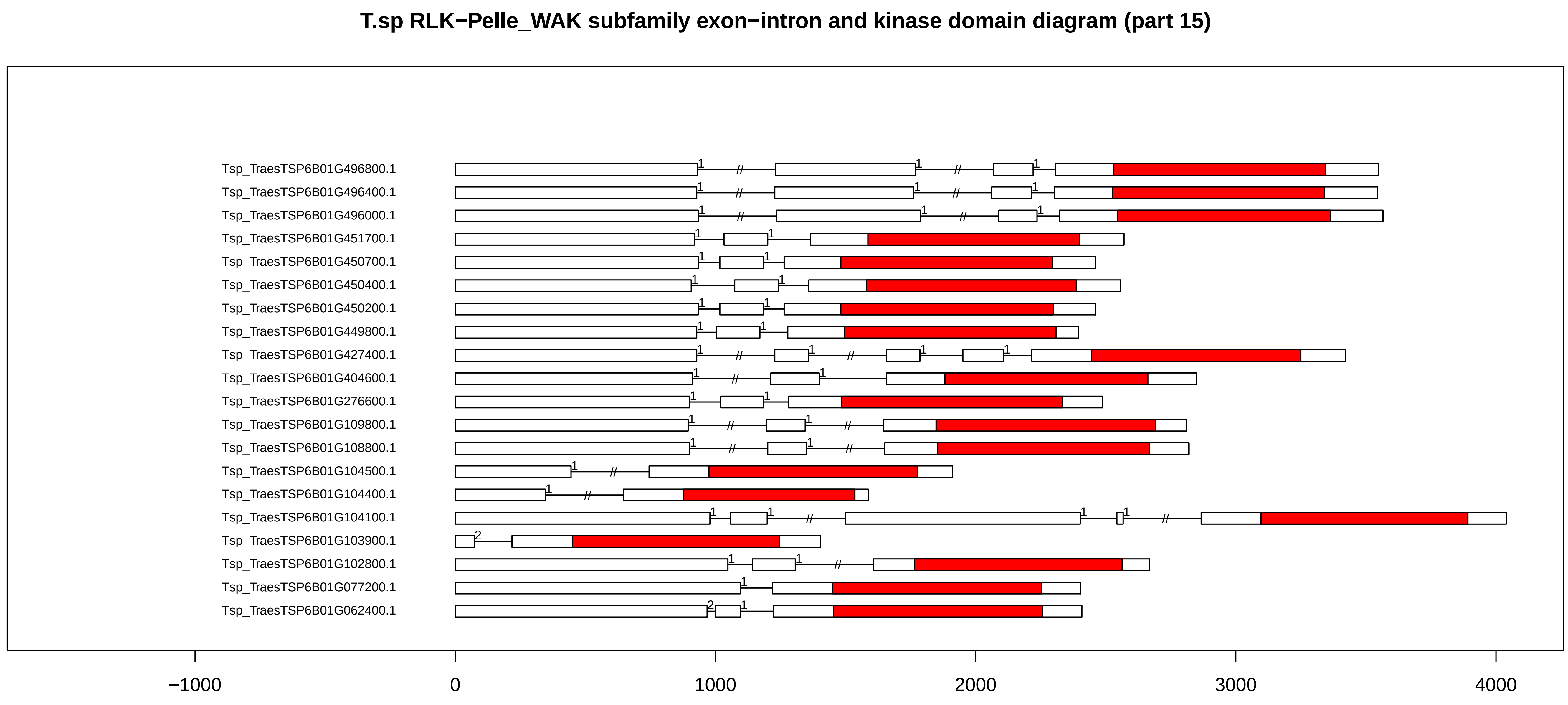
T.sp RLK−Pelle_WAK subfamily exon−intron and kinase domain diagram (part 15)
Tsp_TraesTSP6B01G496800.1
Tsp_TraesTSP6B01G496400.1
Tsp_TraesTSP6B01G496000.1
Tsp_TraesTSP6B01G451700.1
Tsp_TraesTSP6B01G450700.1
Tsp_TraesTSP6B01G450400.1
Tsp_TraesTSP6B01G450200.1
Tsp_TraesTSP6B01G449800.1
Tsp_TraesTSP6B01G427400.1
Tsp_TraesTSP6B01G404600.1
Tsp_TraesTSP6B01G276600.1
Tsp_TraesTSP6B01G109800.1
Tsp_TraesTSP6B01G108800.1
Tsp_TraesTSP6B01G104500.1
Tsp_TraesTSP6B01G104400.1
Tsp_TraesTSP6B01G104100.1
Tsp_TraesTSP6B01G103900.1
Tsp_TraesTSP6B01G102800.1
Tsp_TraesTSP6B01G077200.1
Tsp_TraesTSP6B01G062400.1
−1000
0
1000
2000
3000
4000

T.sp RLK−Pelle_WAK subfamily exon−intron and kinase domain diagram (part 16)

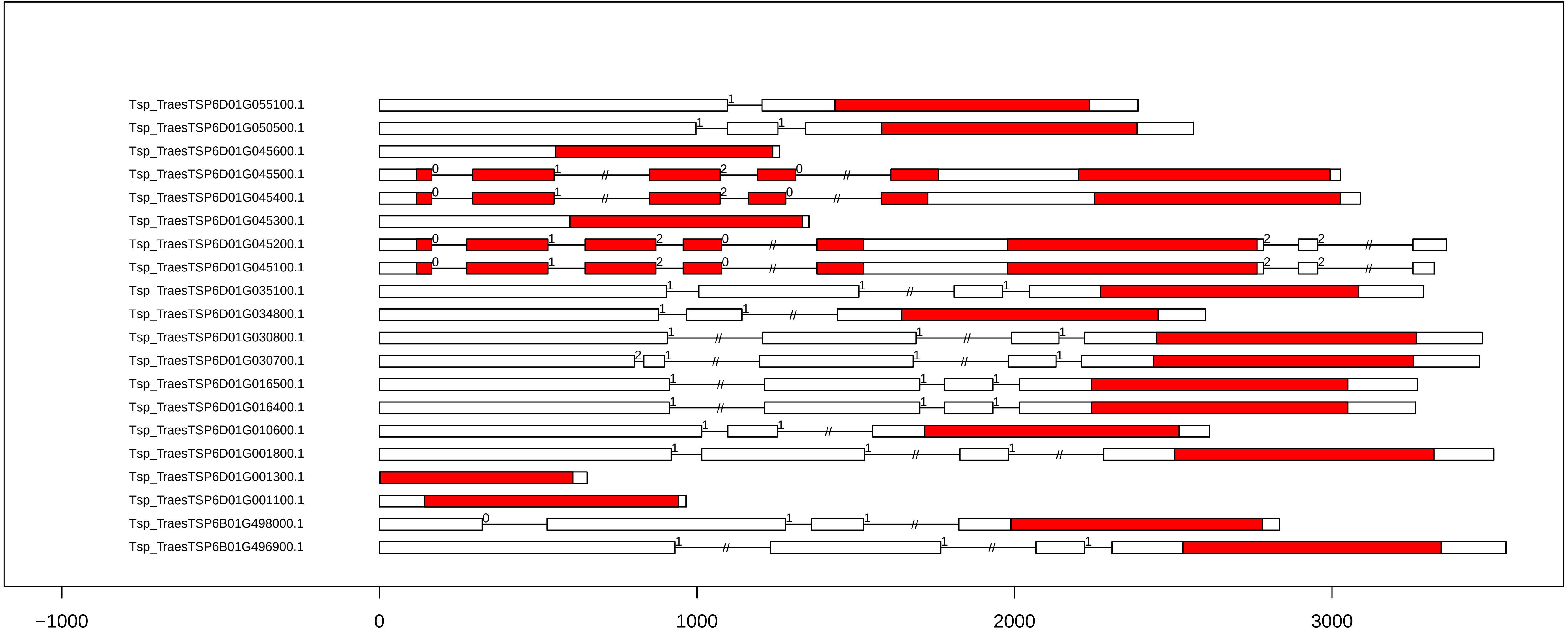

T.sp RLK−Pelle_WAK subfamily exon−intron and kinase domain diagram (part 17)

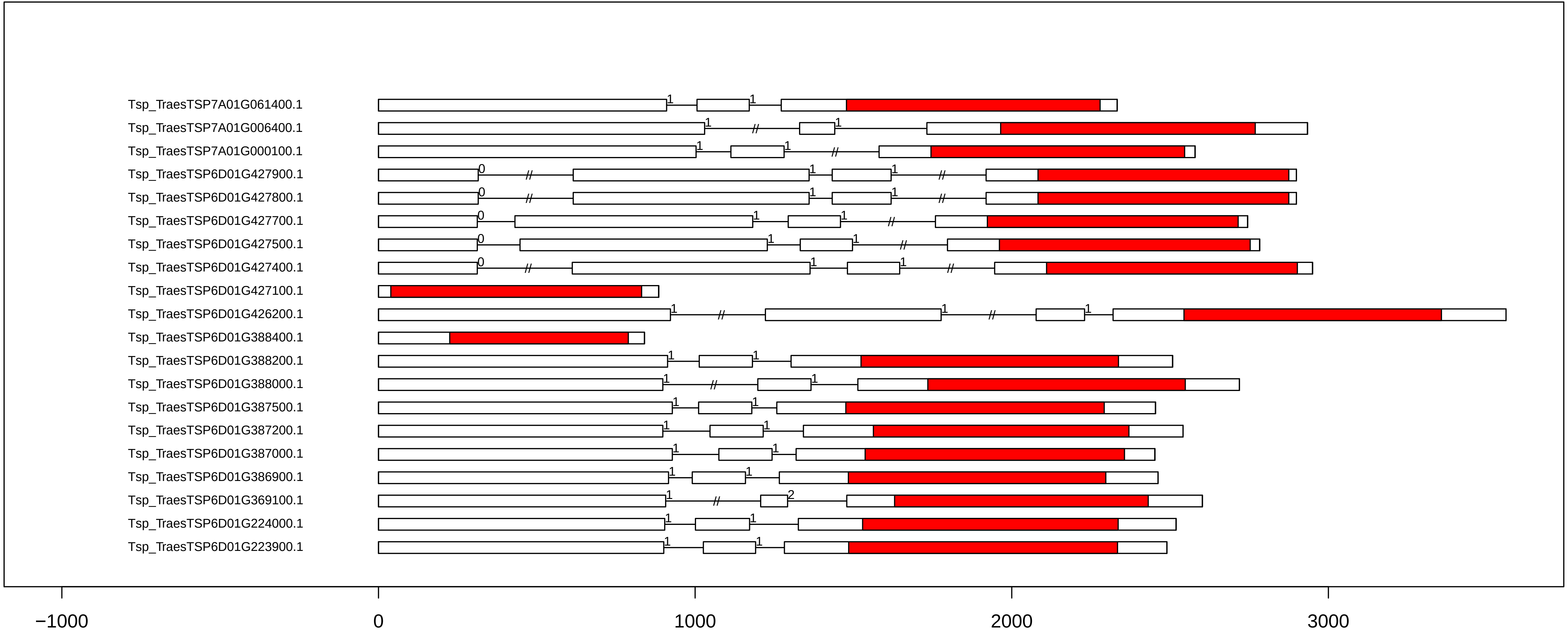

T.sp RLK−Pelle_WAK subfamily exon−intron and kinase domain diagram (part 18)

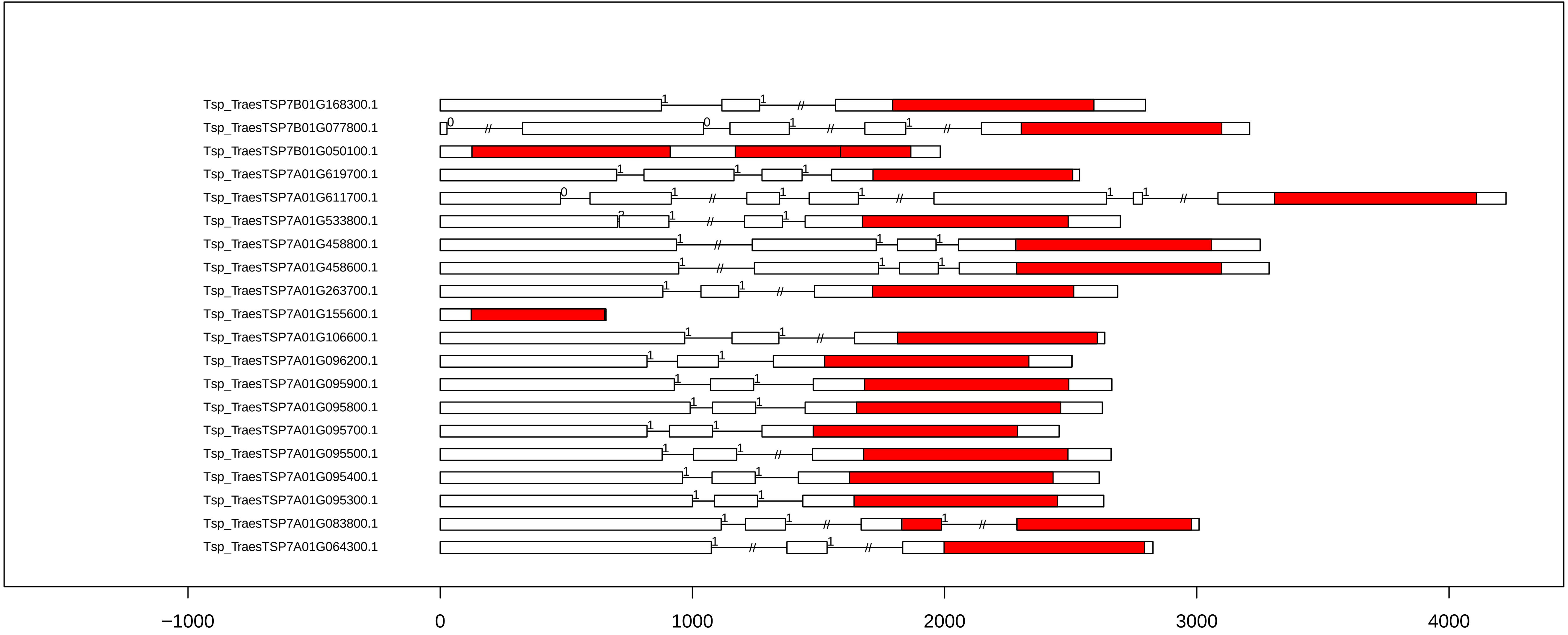

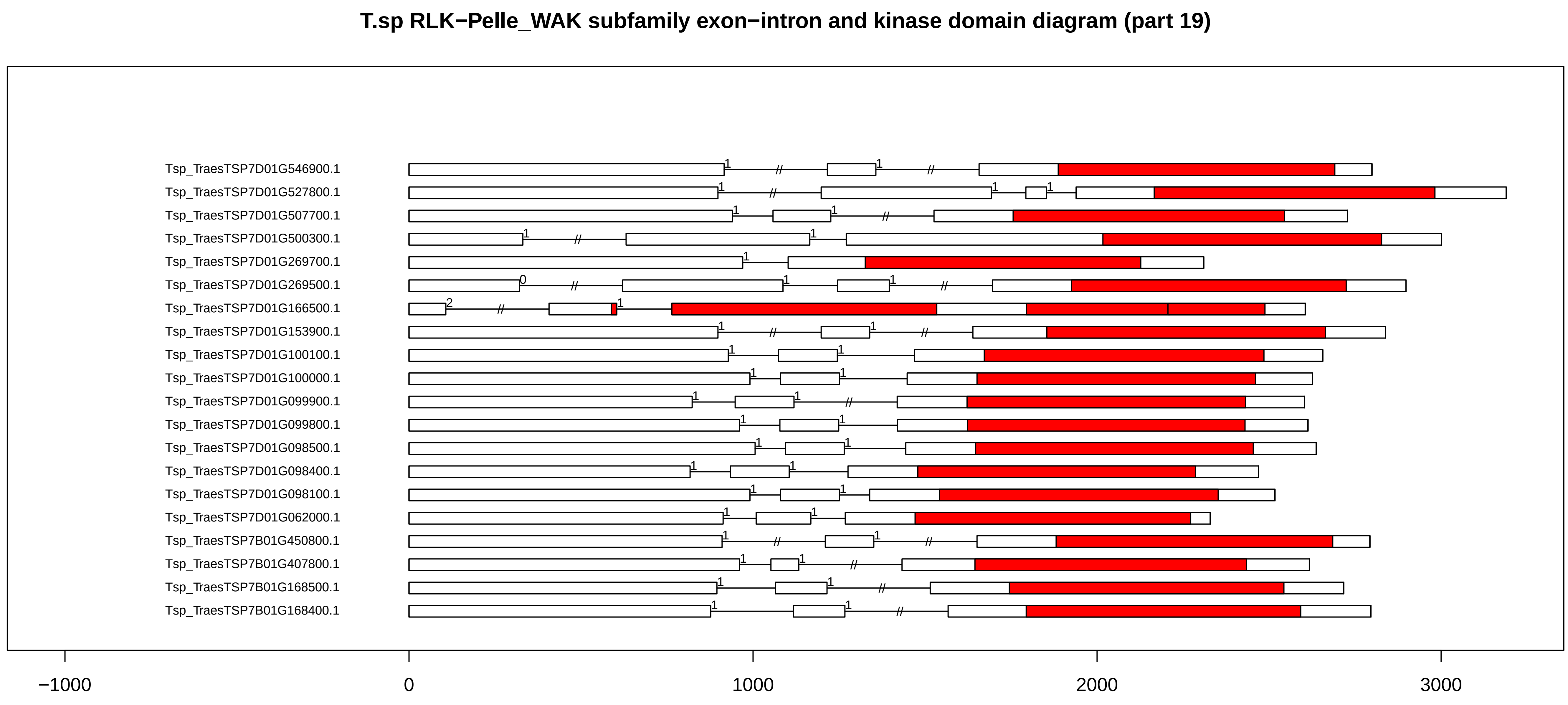

T.sp RLK−Pelle_WAK subfamily exon−intron and kinase domain diagram (part 19)
Tsp_TraesTSP7D01G546900.1
Tsp_TraesTSP7D01G527800.1
Tsp_TraesTSP7D01G507700.1
Tsp_TraesTSP7D01G500300.1
Tsp_TraesTSP7D01G269700.1
Tsp_TraesTSP7D01G269500.1
Tsp_TraesTSP7D01G166500.1
Tsp_TraesTSP7D01G153900.1
Tsp_TraesTSP7D01G100100.1
Tsp_TraesTSP7D01G100000.1
Tsp_TraesTSP7D01G099900.1
Tsp_TraesTSP7D01G099800.1
Tsp_TraesTSP7D01G098500.1
Tsp_TraesTSP7D01G098400.1
Tsp_TraesTSP7D01G098100.1
Tsp_TraesTSP7D01G062000.1
Tsp_TraesTSP7B01G450800.1
Tsp_TraesTSP7B01G407800.1
Tsp_TraesTSP7B01G168500.1
Tsp_TraesTSP7B01G168400.1
−1000
0
1000
2000
3000

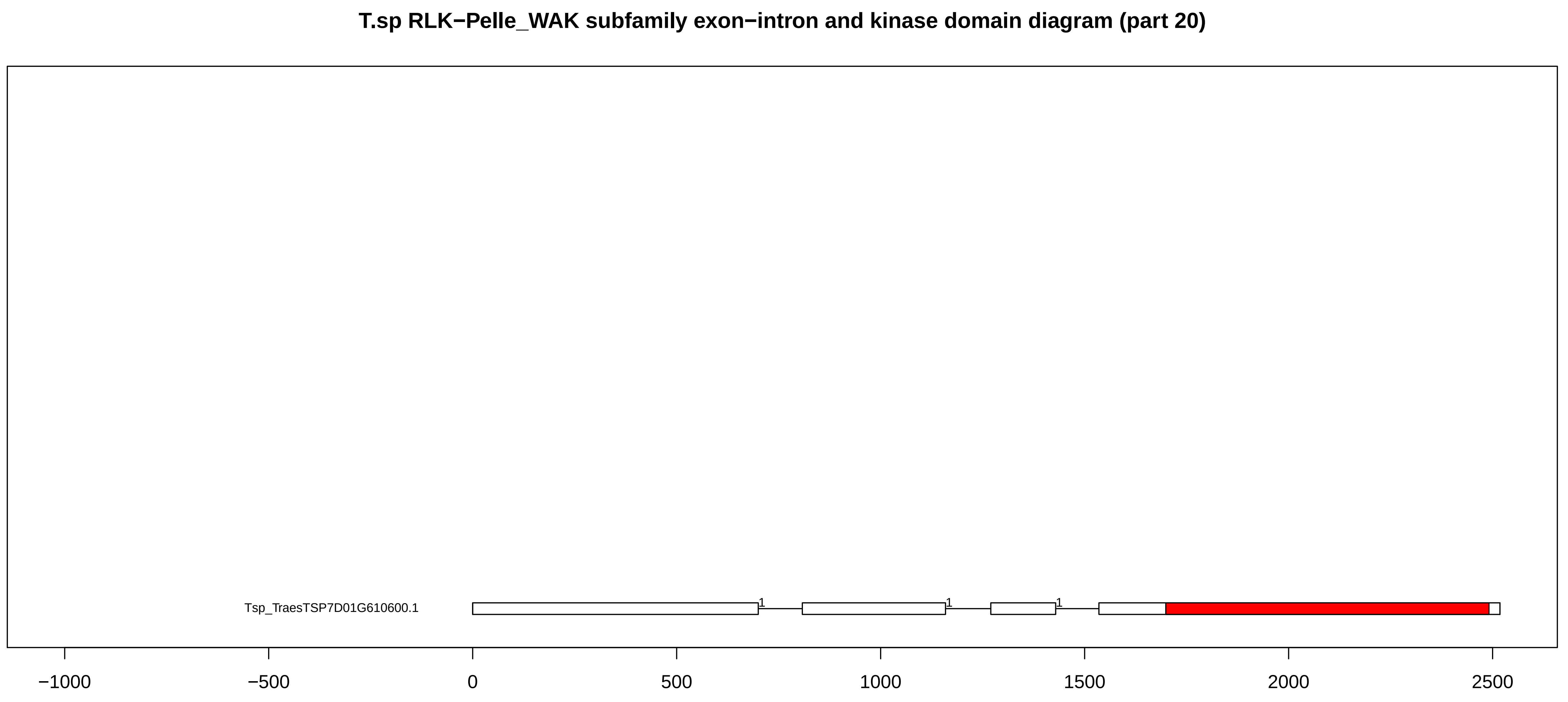
T.sp RLK−Pelle_WAK subfamily exon−intron and kinase domain diagram (part 20)
Tsp_TraesTSP7D01G610600.1
1
1
1
−1000
−500
0
500
1000
1500
2000
2500

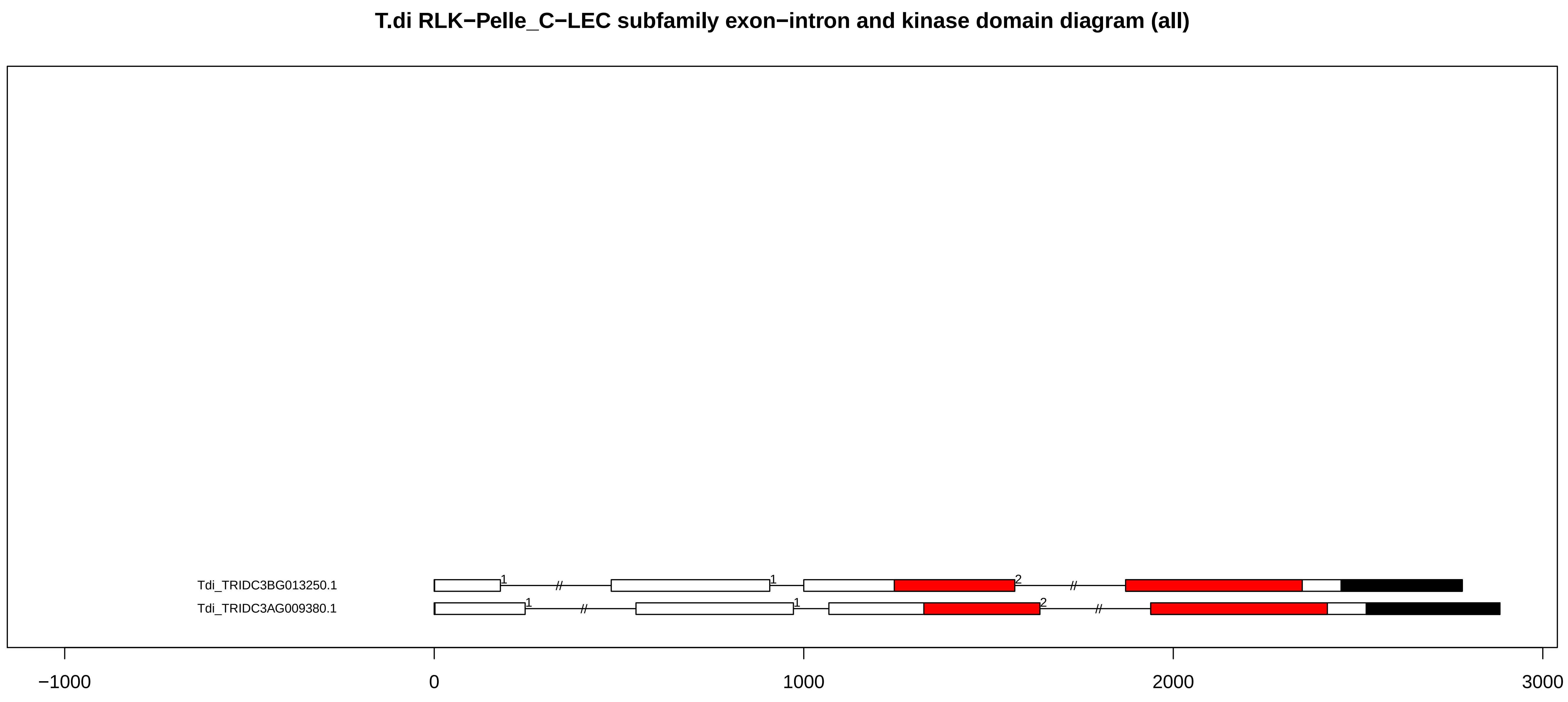

T.di RLK−Pelle_C−LEC subfamily exon−intron and kinase domain diagram (all)
Tdi_TRIDC3BG013250.1
Tdi_TRIDC3AG009380.1
1
1
2
1
1
2
−1000
0
1000
2000
3000

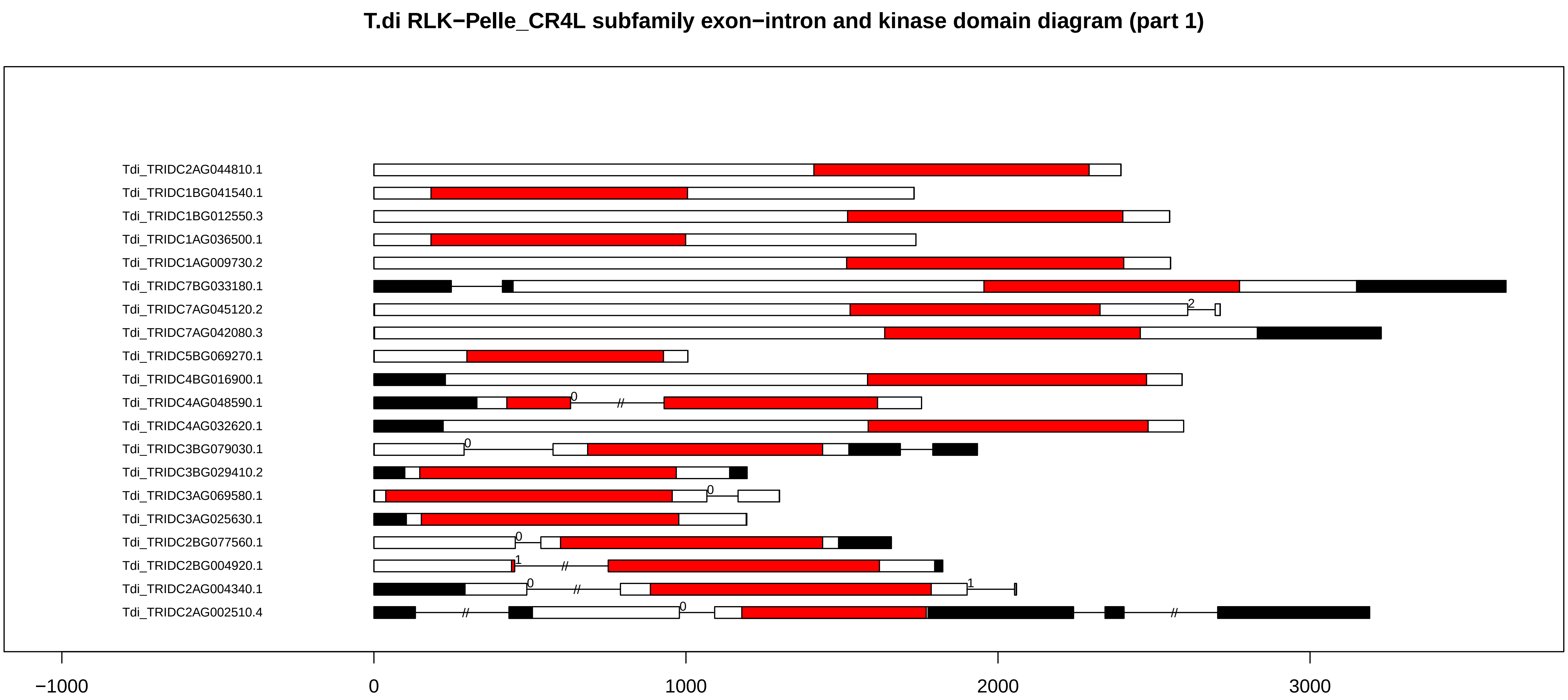

T.di RLK−Pelle_CR4L subfamily exon−intron and kinase domain diagram (part 1)
Tdi_TRIDC2AG044810.1
Tdi_TRIDC1BG041540.1
Tdi_TRIDC1BG012550.3
Tdi_TRIDC1AG036500.1
Tdi_TRIDC1AG009730.2
Tdi_TRIDC7BG033180.1
Tdi_TRIDC7AG045120.2
Tdi_TRIDC7AG042080.3
Tdi_TRIDC5BG069270.1
Tdi_TRIDC4BG016900.1
Tdi_TRIDC4AG048590.1
Tdi_TRIDC4AG032620.1
Tdi_TRIDC3BG079030.1
Tdi_TRIDC3BG029410.2
Tdi_TRIDC3AG069580.1
Tdi_TRIDC3AG025630.1
Tdi_TRIDC2BG077560.1
Tdi_TRIDC2BG004920.1
Tdi_TRIDC2AG004340.1
Tdi_TRIDC2AG002510.4
−1000
0
1000
2000
3000

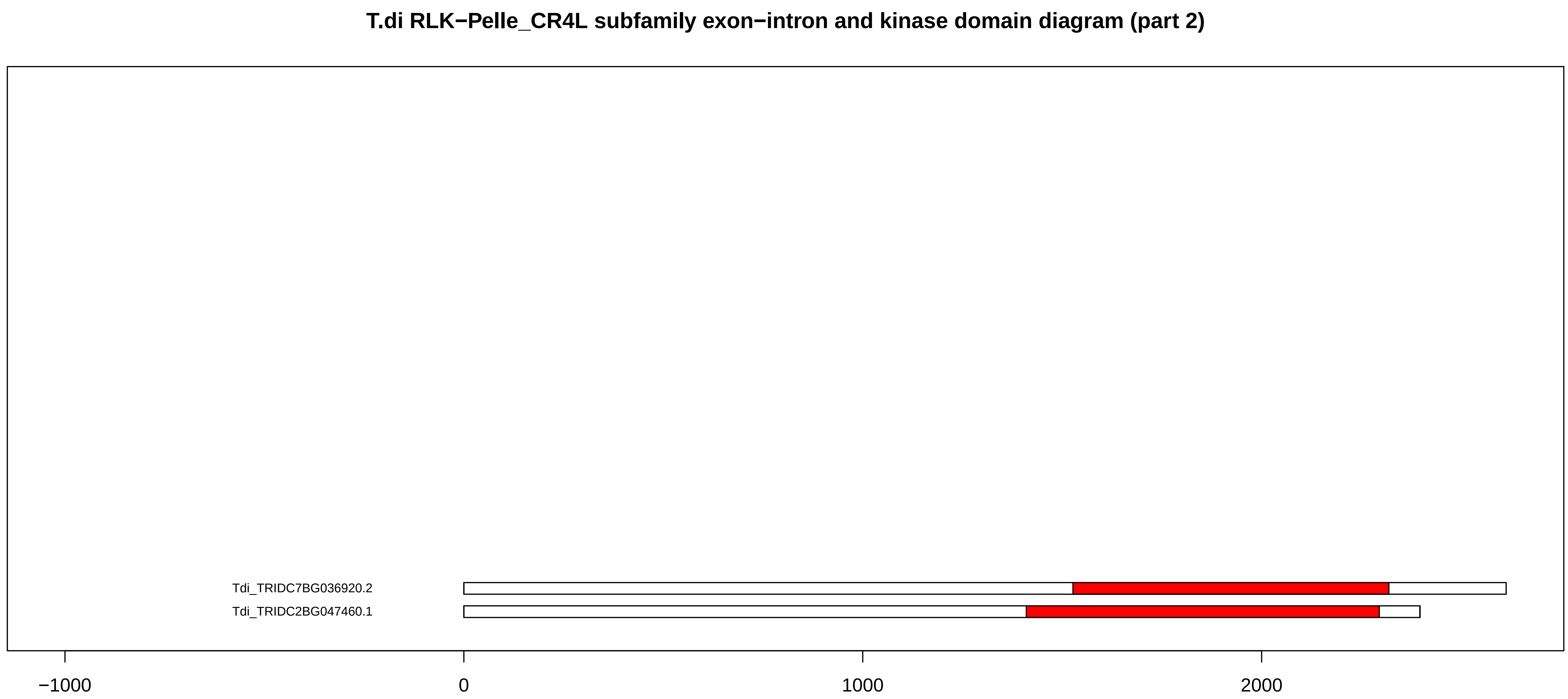
T.di RLK−Pelle_CR4L subfamily exon−intron and kinase domain diagram (part 2)
Tdi_TRIDC7BG036920.2
Tdi_TRIDC2BG047460.1
−1000
0
1000
2000

T.di RLK−Pelle_CrRLK1L−1 subfamily exon−intron and kinase domain diagram (part 1)

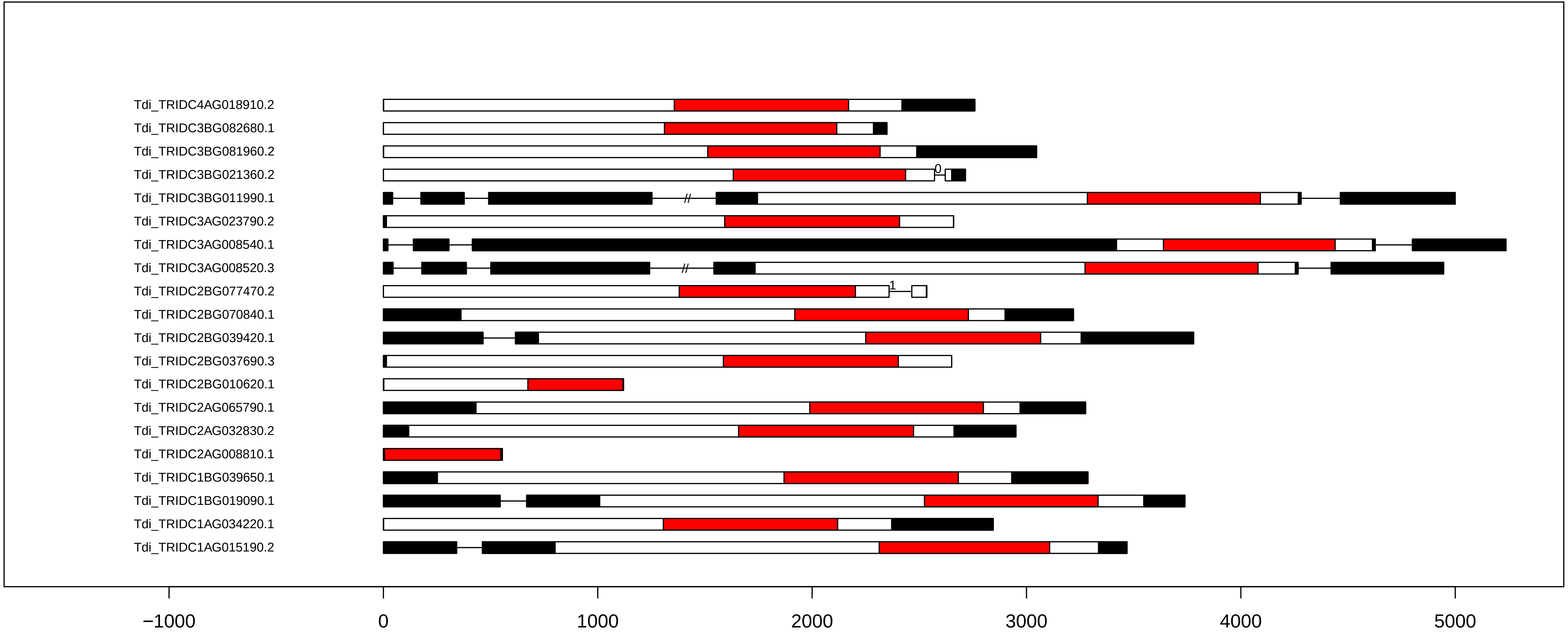

T.di RLK−Pelle_CrRLK1L−1 subfamily exon−intron and kinase domain diagram (part 2)

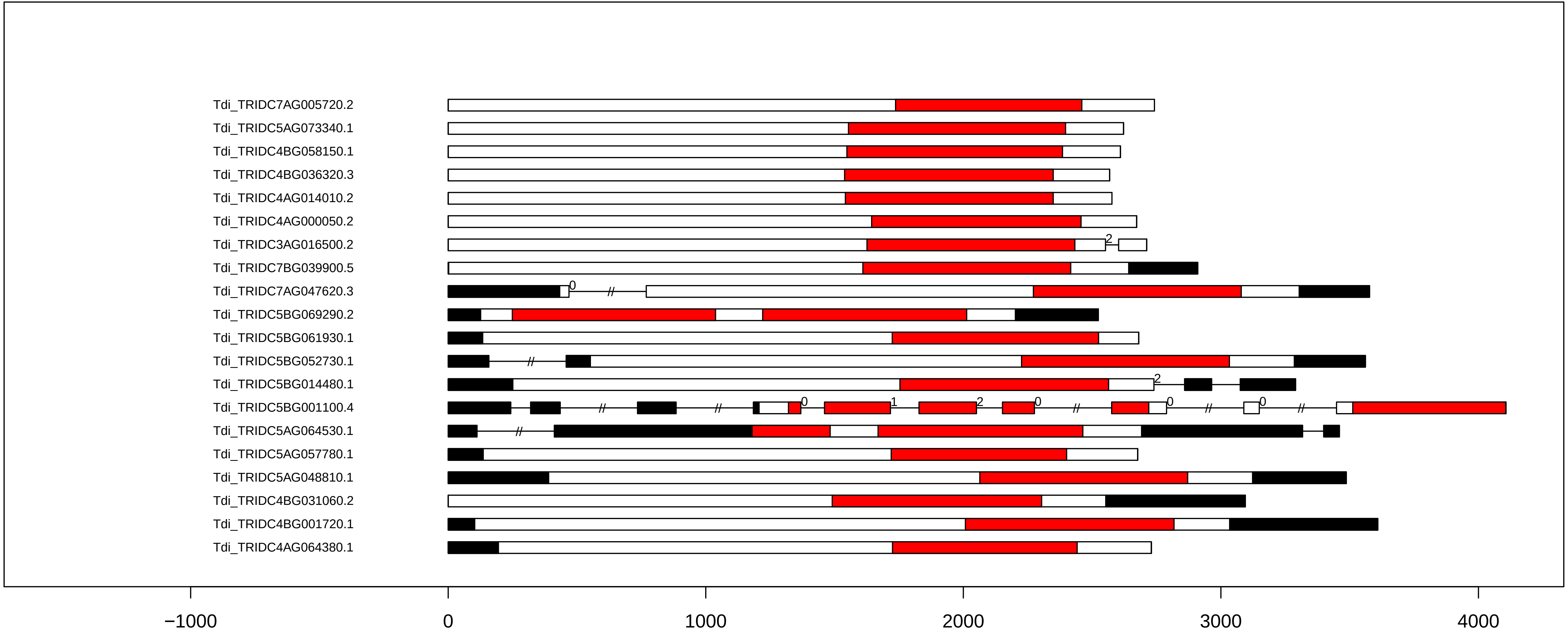

T.di RLK−Pelle_DLSV subfamily exon−intron and kinase domain diagram (part 1)

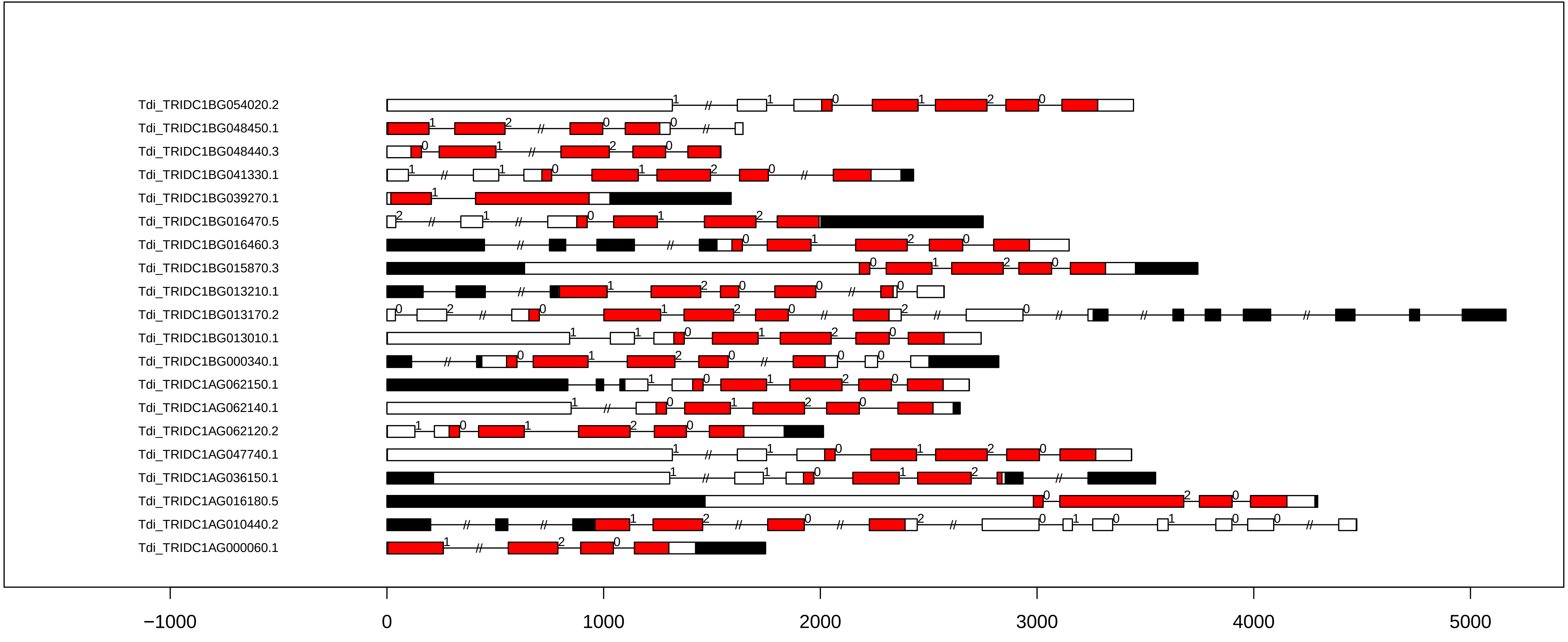

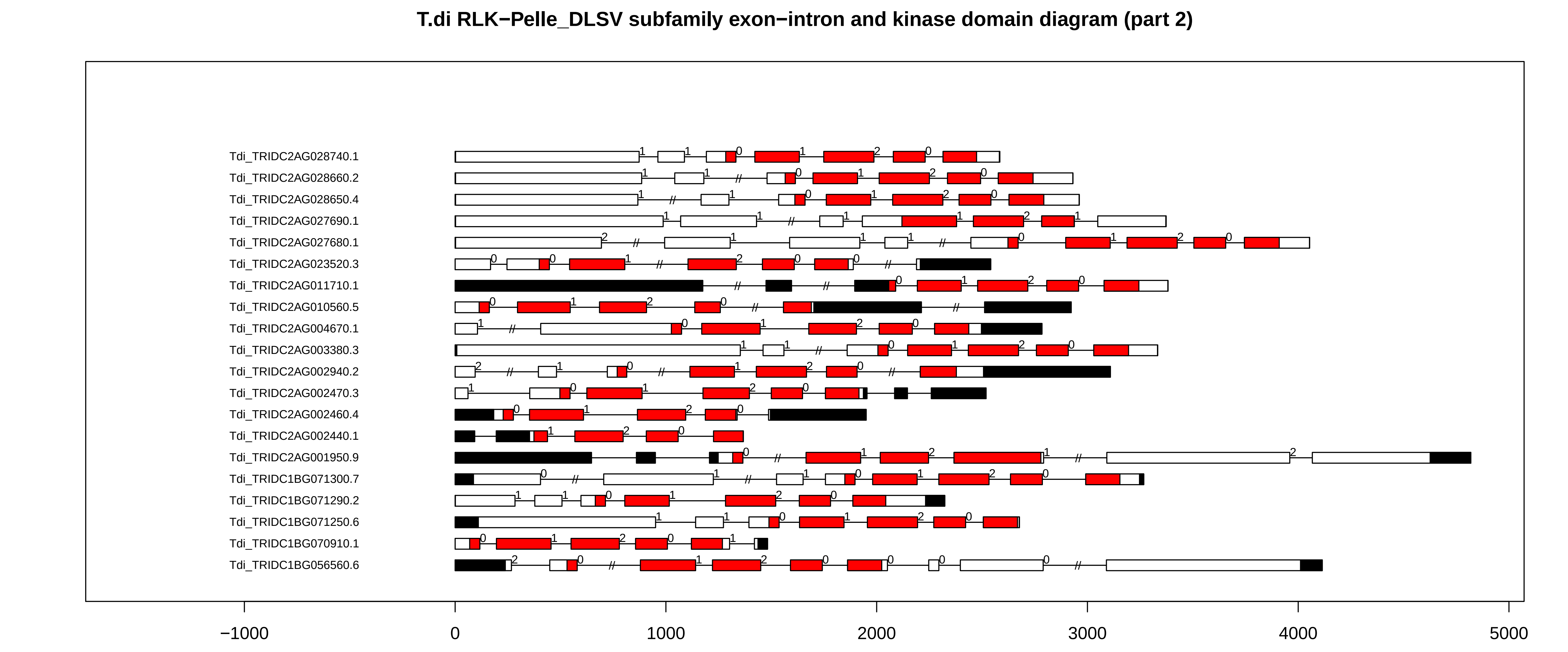
T.di RLK−Pelle_DLSV subfamily exon−intron and kinase domain diagram (part 2)
Tdi_TRIDC2AG028740.1
Tdi_TRIDC2AG028660.2
Tdi_TRIDC2AG028650.4
Tdi_TRIDC2AG027690.1
Tdi_TRIDC2AG027680.1
Tdi_TRIDC2AG023520.3
Tdi_TRIDC2AG011710.1
Tdi_TRIDC2AG010560.5
Tdi_TRIDC2AG004670.1
Tdi_TRIDC2AG003380.3
Tdi_TRIDC2AG002940.2
Tdi_TRIDC2AG002470.3
Tdi_TRIDC2AG002460.4
Tdi_TRIDC2AG002440.1
Tdi_TRIDC2AG001950.9
Tdi_TRIDC1BG071300.7
Tdi_TRIDC1BG071290.2
Tdi_TRIDC1BG071250.6
Tdi_TRIDC1BG070910.1
Tdi_TRIDC1BG056560.6
−1000
0
1000
2000
3000
4000
5000

T.di RLK−Pelle_DLSV subfamily exon−intron and kinase domain diagram (part 3)

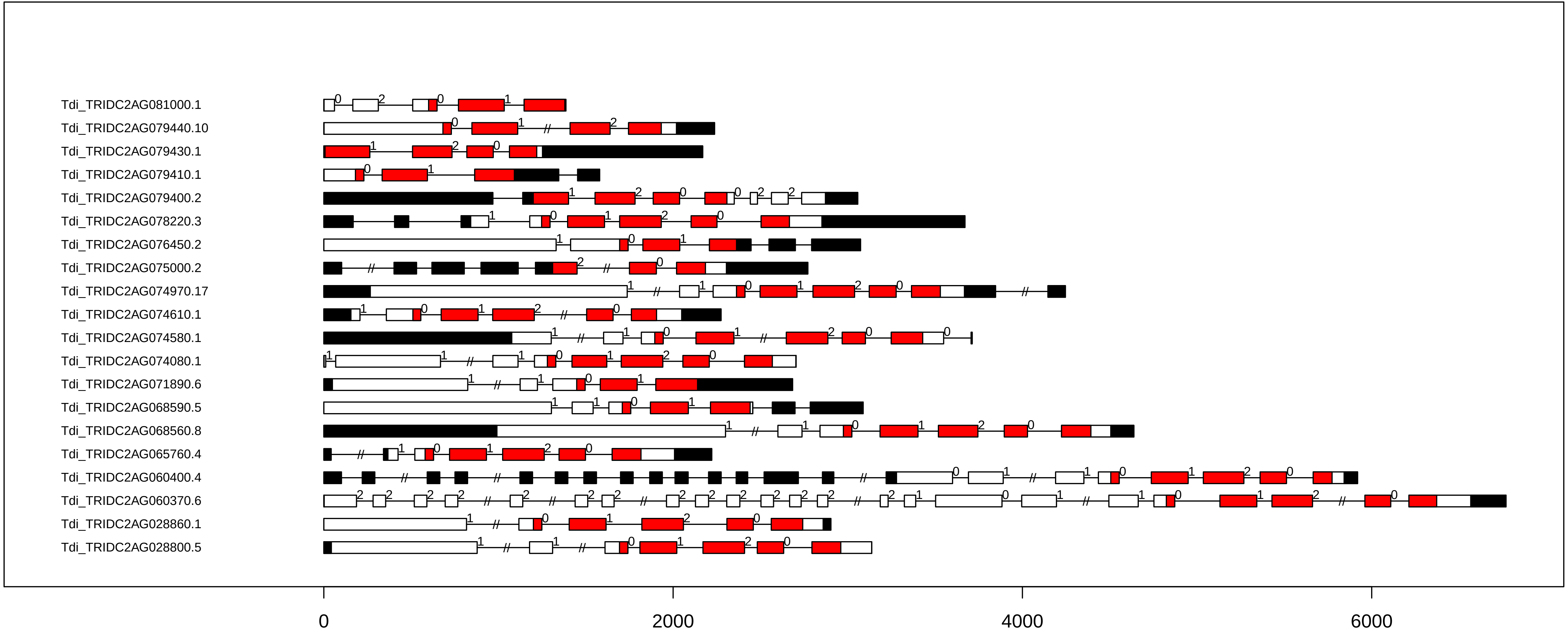

T.di RLK−Pelle_DLSV subfamily exon−intron and kinase domain diagram (part 4)

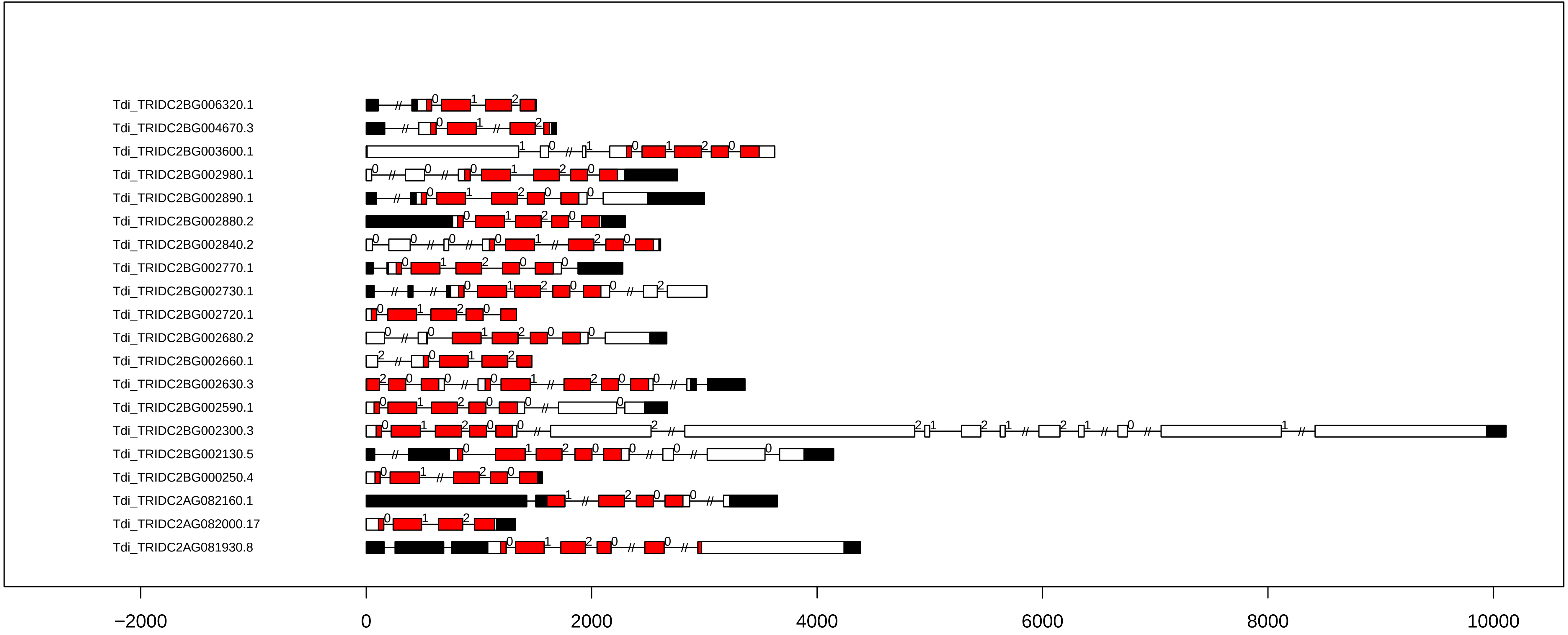

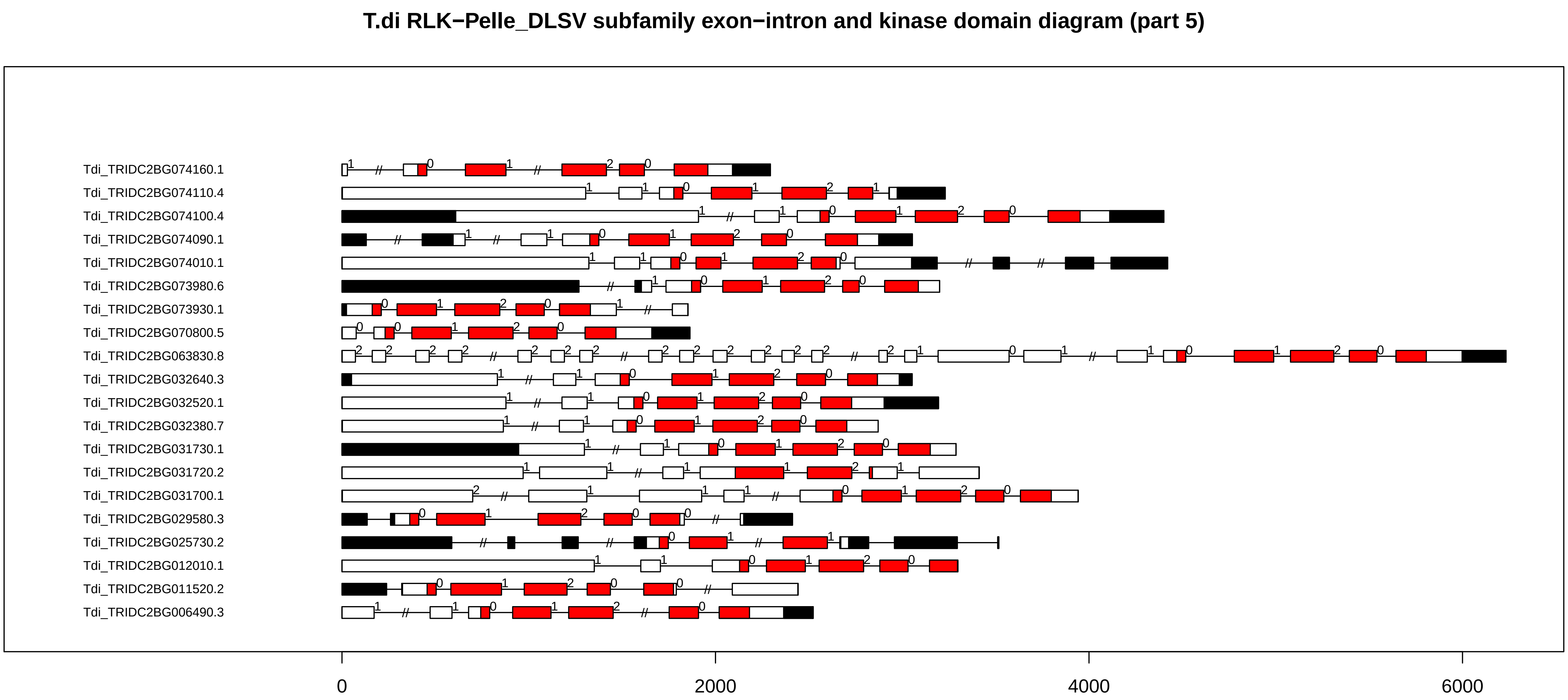
T.di RLK−Pelle_DLSV subfamily exon−intron and kinase domain diagram (part 5)
Tdi_TRIDC2BG074160.1
Tdi_TRIDC2BG074110.4
Tdi_TRIDC2BG074100.4
Tdi_TRIDC2BG074090.1
Tdi_TRIDC2BG074010.1
Tdi_TRIDC2BG073980.6
Tdi_TRIDC2BG073930.1
Tdi_TRIDC2BG070800.5
Tdi_TRIDC2BG063830.8
Tdi_TRIDC2BG032640.3
Tdi_TRIDC2BG032520.1
Tdi_TRIDC2BG032380.7
Tdi_TRIDC2BG031730.1
Tdi_TRIDC2BG031720.2
Tdi_TRIDC2BG031700.1
Tdi_TRIDC2BG029580.3
Tdi_TRIDC2BG025730.2
Tdi_TRIDC2BG012010.1
Tdi_TRIDC2BG011520.2
Tdi_TRIDC2BG006490.3
0
2000
4000
6000

T.di RLK−Pelle_DLSV subfamily exon−intron and kinase domain diagram (part 6)

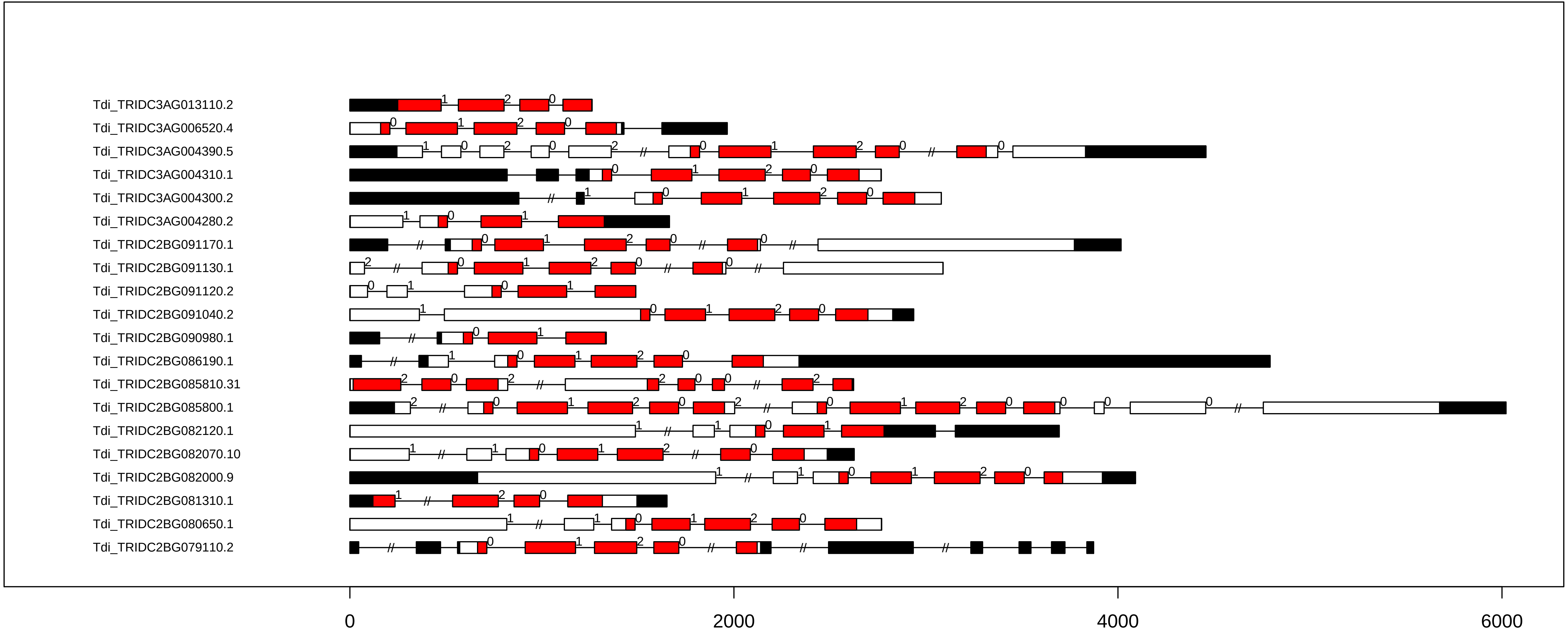

T.di RLK−Pelle_DLSV subfamily exon−intron and kinase domain diagram (part 7)

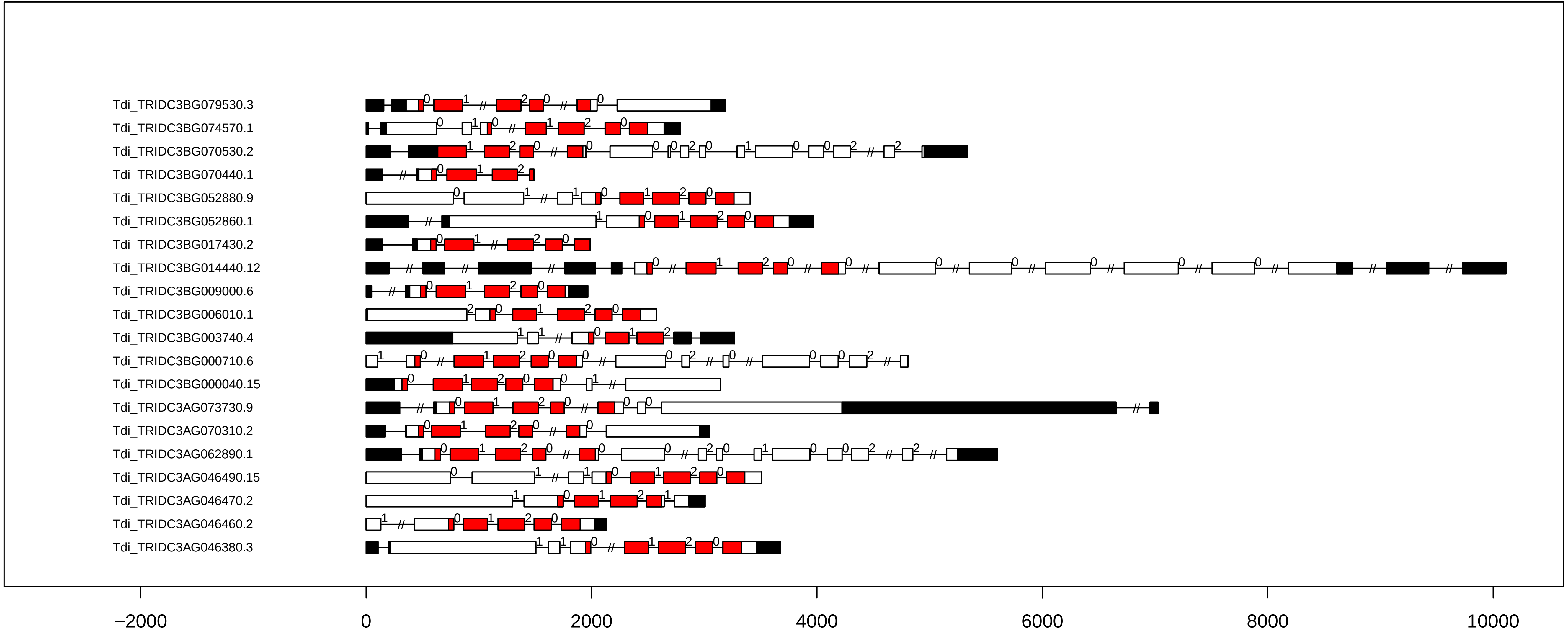

T.di RLK−Pelle_DLSV subfamily exon−intron and kinase domain diagram (part 8)

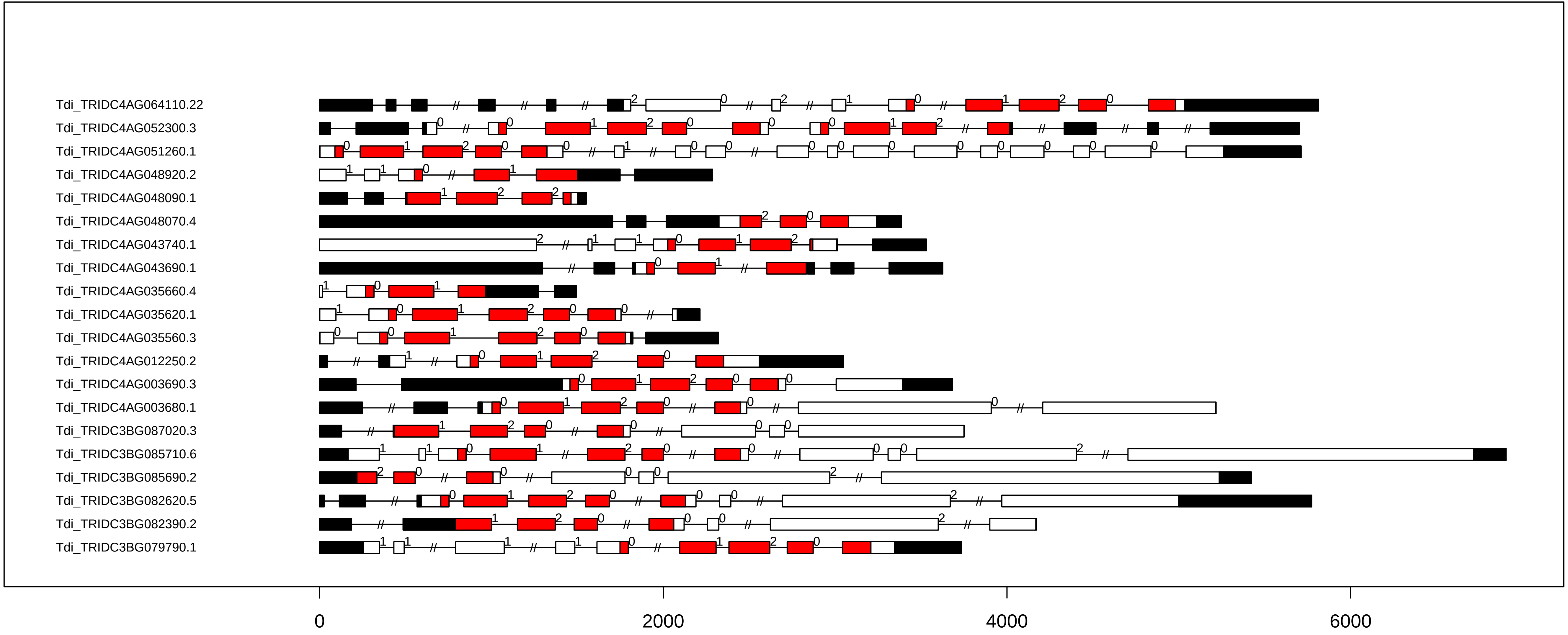

T.di RLK−Pelle_DLSV subfamily exon−intron and kinase domain diagram (part 9)

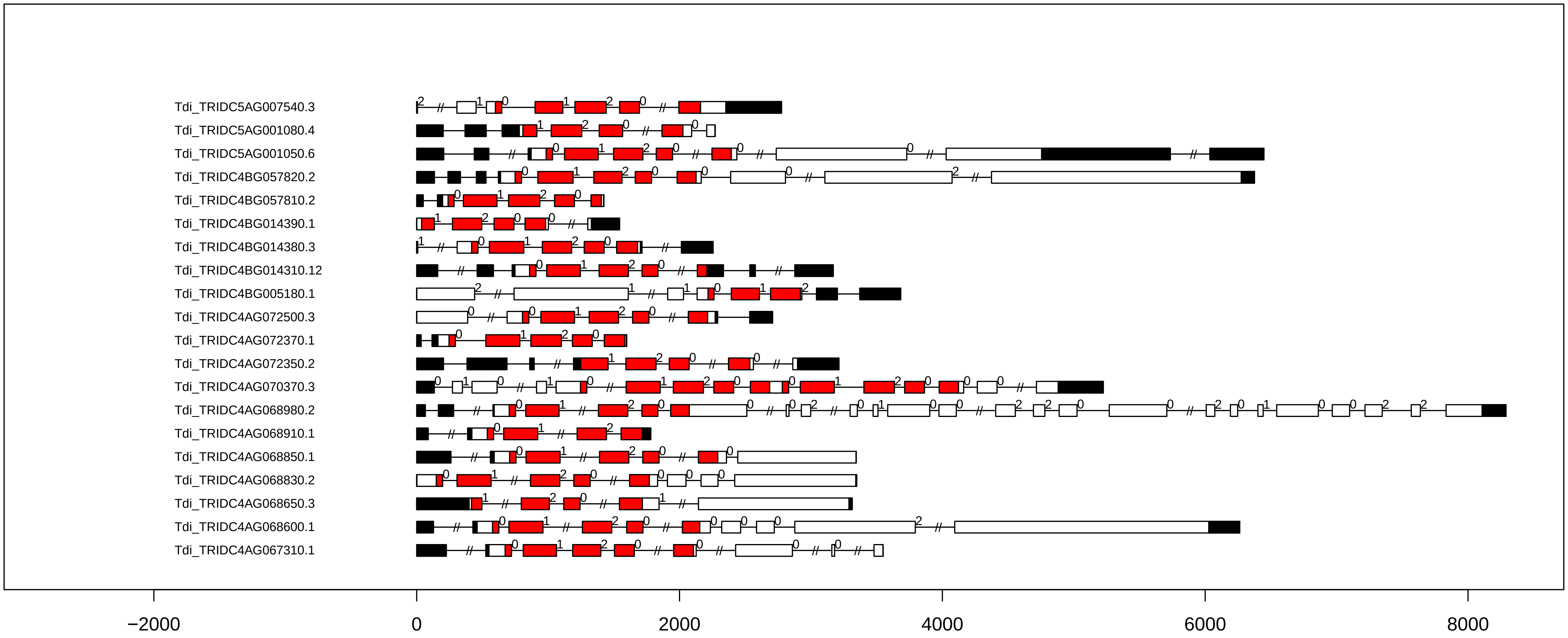

T.di RLK−Pelle_DLSV subfamily exon−intron and kinase domain diagram (part 10)

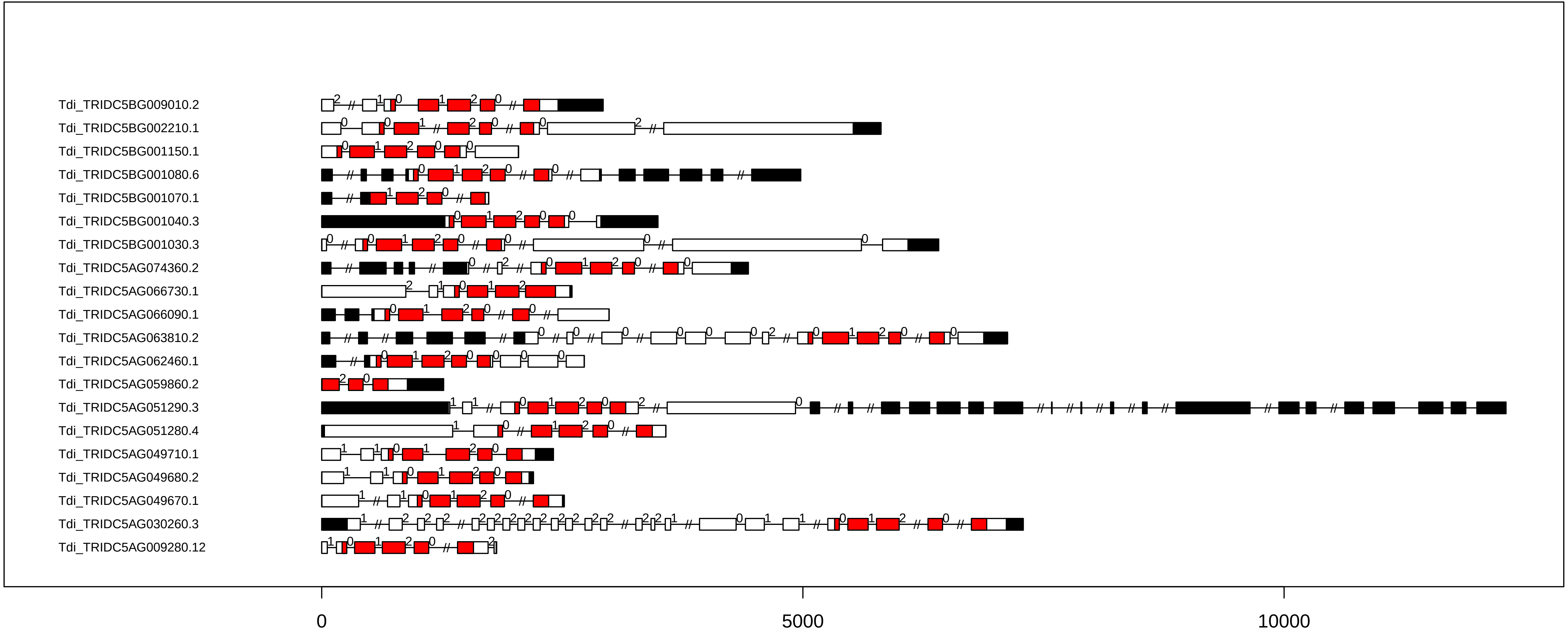

T.di RLK−Pelle_DLSV subfamily exon−intron and kinase domain diagram (part 11)

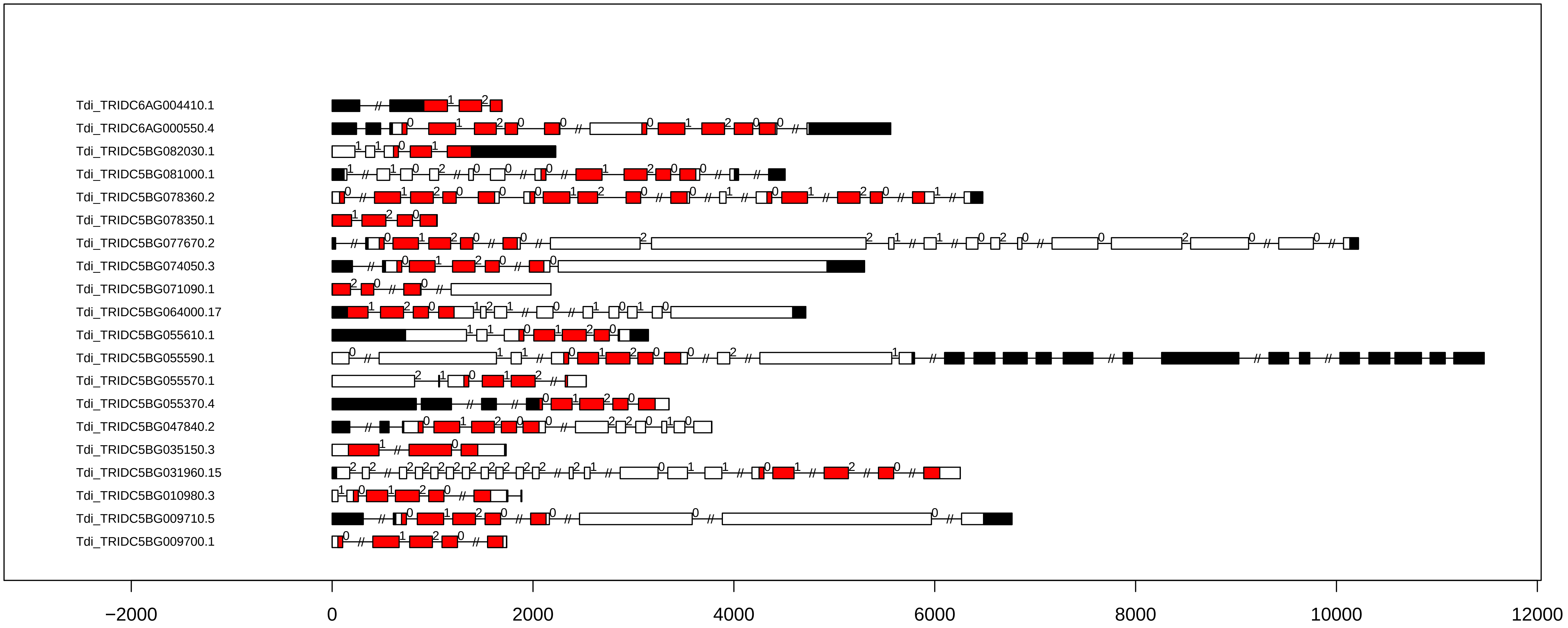

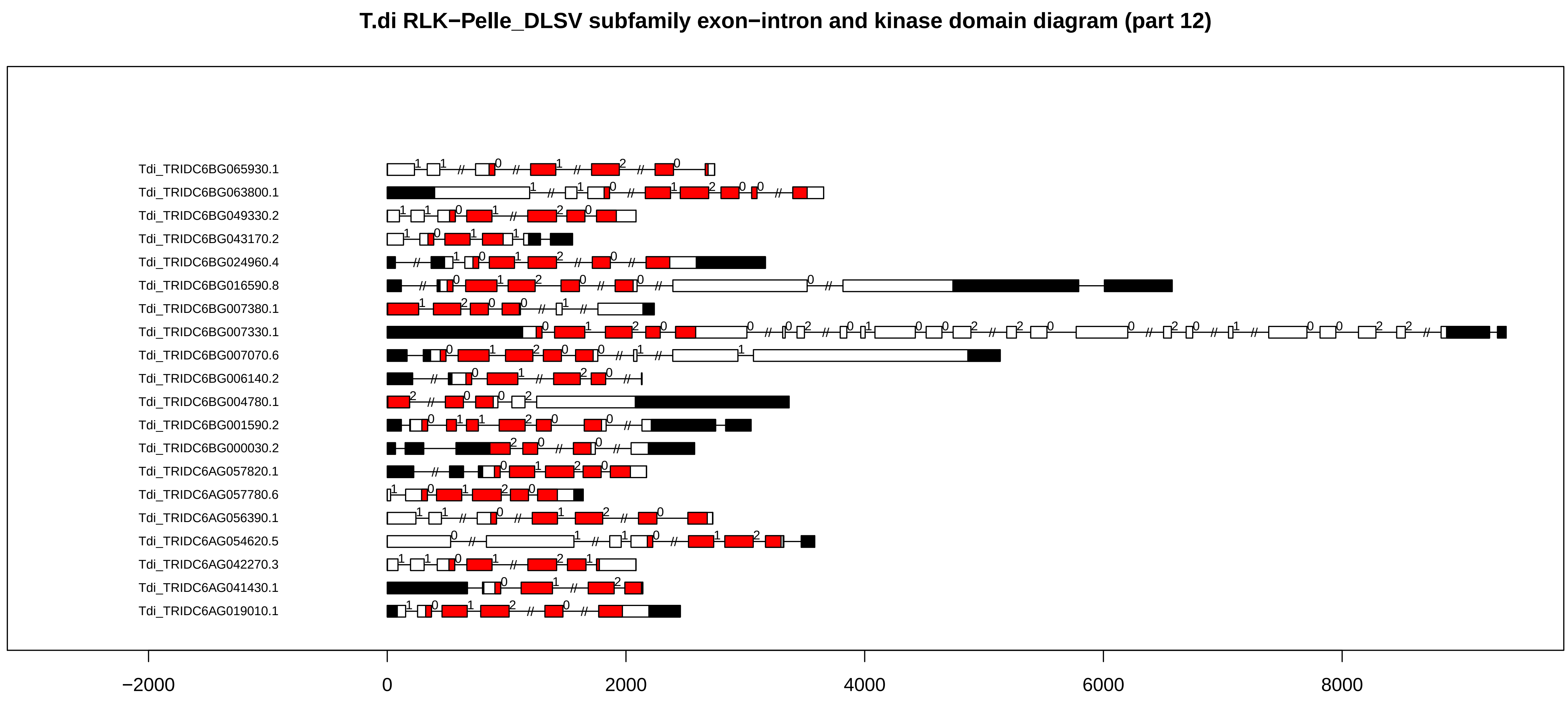
T.di RLK−Pelle_DLSV subfamily exon−intron and kinase domain diagram (part 12)
Tdi_TRIDC6BG065930.1
Tdi_TRIDC6BG063800.1
Tdi_TRIDC6BG049330.2
Tdi_TRIDC6BG043170.2
Tdi_TRIDC6BG024960.4
Tdi_TRIDC6BG016590.8
Tdi_TRIDC6BG007380.1
Tdi_TRIDC6BG007330.1
Tdi_TRIDC6BG007070.6
Tdi_TRIDC6BG006140.2
Tdi_TRIDC6BG004780.1
Tdi_TRIDC6BG001590.2
Tdi_TRIDC6BG000030.2
Tdi_TRIDC6AG057820.1
Tdi_TRIDC6AG057780.6
Tdi_TRIDC6AG056390.1
Tdi_TRIDC6AG054620.5
Tdi_TRIDC6AG042270.3
Tdi_TRIDC6AG041430.1
Tdi_TRIDC6AG019010.1
−2000
0
2000
4000
6000
8000

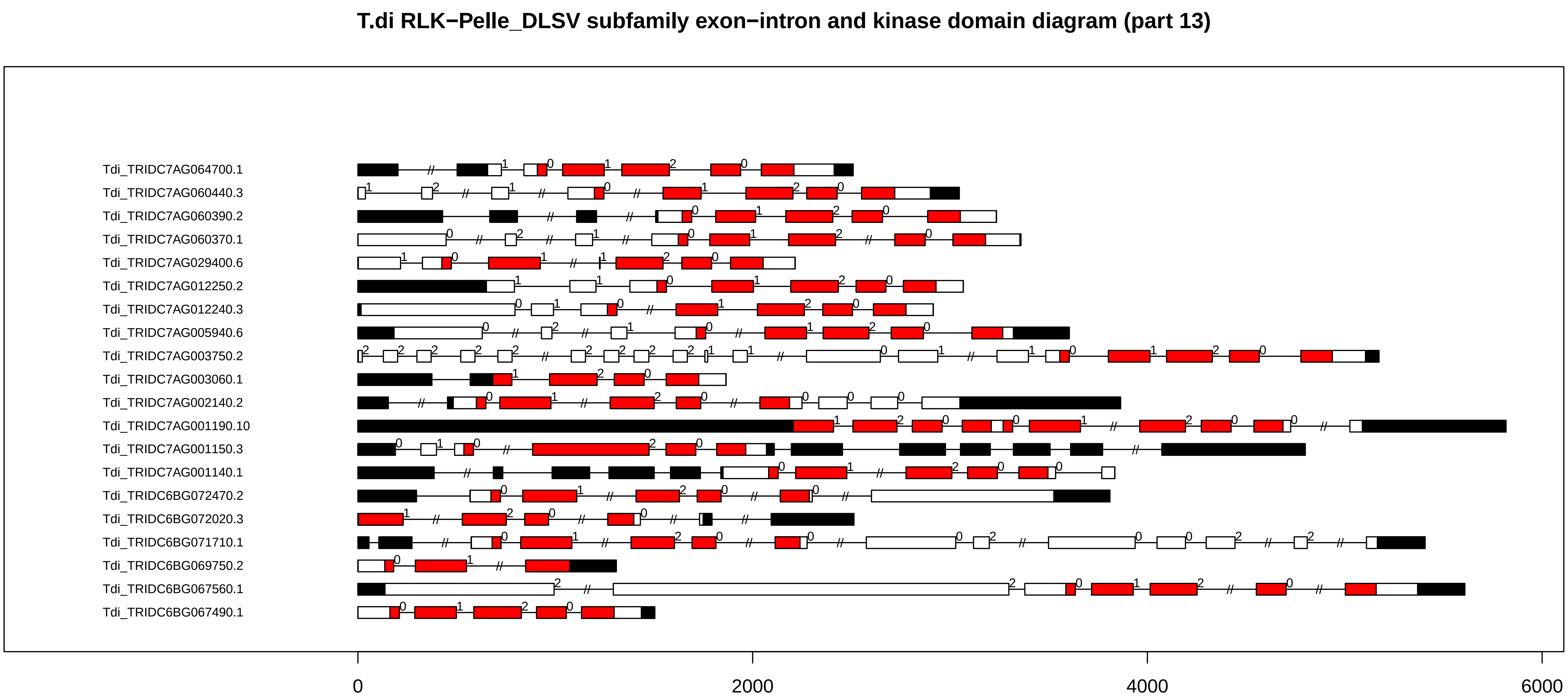

T.di RLK−Pelle_DLSV subfamily exon−intron and kinase domain diagram (part 13)
Tdi_TRIDC7AG064700.1
Tdi_TRIDC7AG060440.3
Tdi_TRIDC7AG060390.2
Tdi_TRIDC7AG060370.1
Tdi_TRIDC7AG029400.6
Tdi_TRIDC7AG012250.2
Tdi_TRIDC7AG012240.3
Tdi_TRIDC7AG005940.6
Tdi_TRIDC7AG003750.2
Tdi_TRIDC7AG003060.1
Tdi_TRIDC7AG002140.2
Tdi_TRIDC7AG001190.10
Tdi_TRIDC7AG001150.3
Tdi_TRIDC7AG001140.1
Tdi_TRIDC6BG072470.2
Tdi_TRIDC6BG072020.3
Tdi_TRIDC6BG071710.1
Tdi_TRIDC6BG069750.2
Tdi_TRIDC6BG067560.1
Tdi_TRIDC6BG067490.1
0
2000
4000
6000

T.di RLK−Pelle_DLSV subfamily exon−intron and kinase domain diagram (part 14)

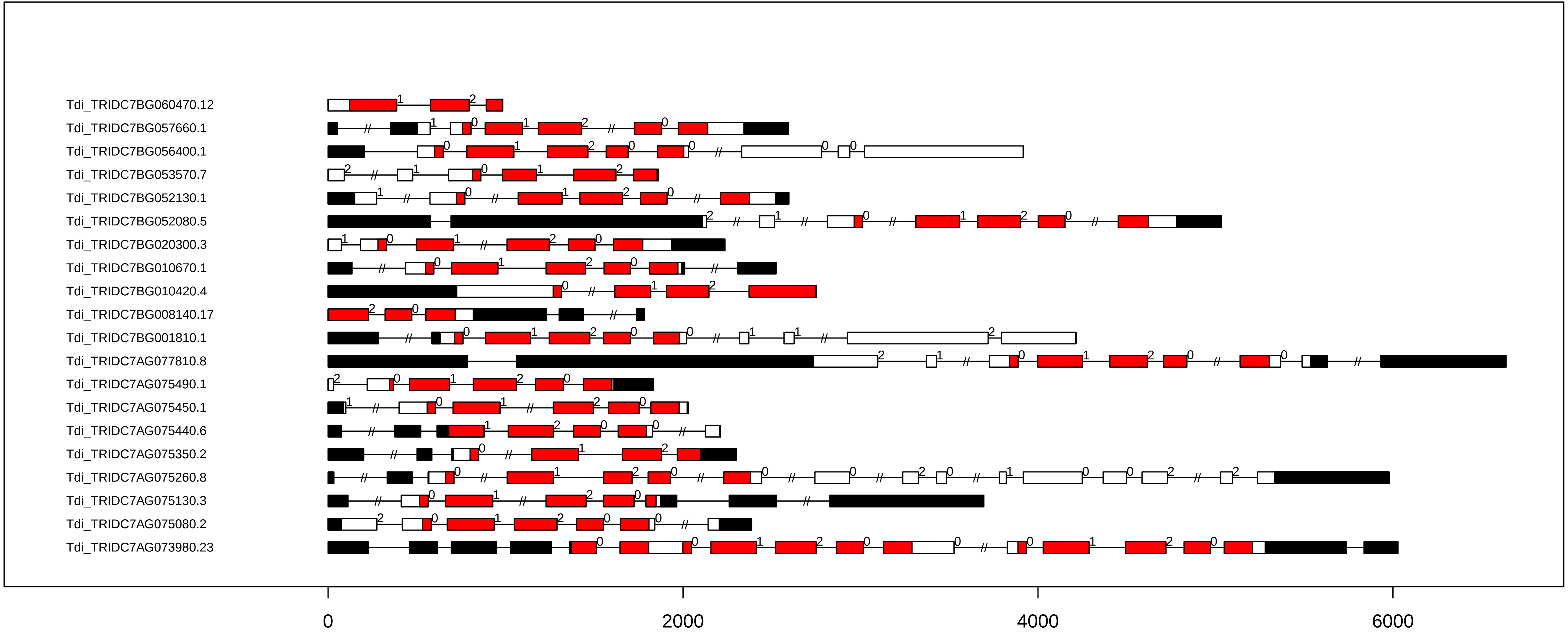

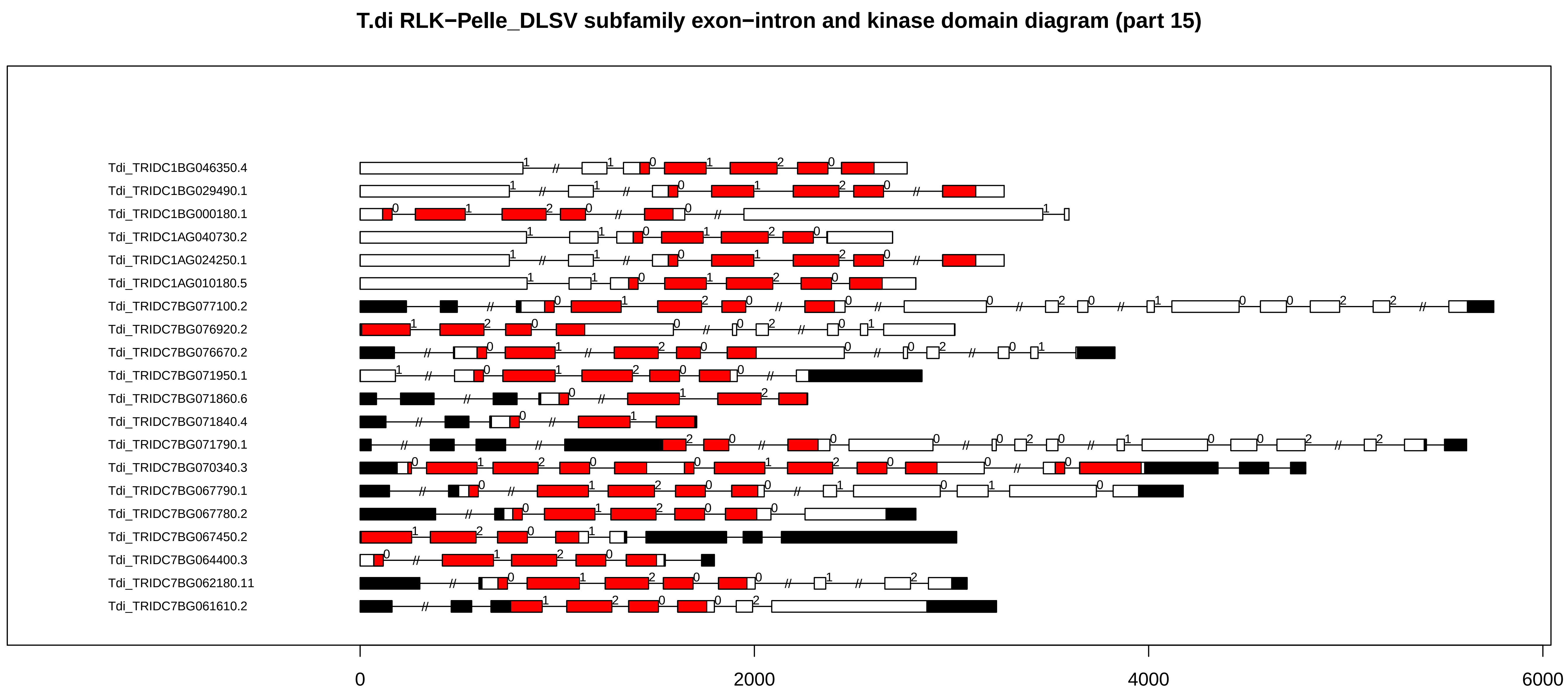
T.di RLK−Pelle_DLSV subfamily exon−intron and kinase domain diagram (part 15)
Tdi_TRIDC1BG046350.4
Tdi_TRIDC1BG029490.1
Tdi_TRIDC1BG000180.1
Tdi_TRIDC1AG040730.2
Tdi_TRIDC1AG024250.1
Tdi_TRIDC1AG010180.5
Tdi_TRIDC7BG077100.2
Tdi_TRIDC7BG076920.2
Tdi_TRIDC7BG076670.2
Tdi_TRIDC7BG071950.1
Tdi_TRIDC7BG071860.6
Tdi_TRIDC7BG071840.4
Tdi_TRIDC7BG071790.1
Tdi_TRIDC7BG070340.3
Tdi_TRIDC7BG067790.1
Tdi_TRIDC7BG067780.2
Tdi_TRIDC7BG067450.2
Tdi_TRIDC7BG064400.3
Tdi_TRIDC7BG062180.11
Tdi_TRIDC7BG061610.2
0
2000
4000
6000

T.di RLK−Pelle_DLSV subfamily exon−intron and kinase domain diagram (part 16)

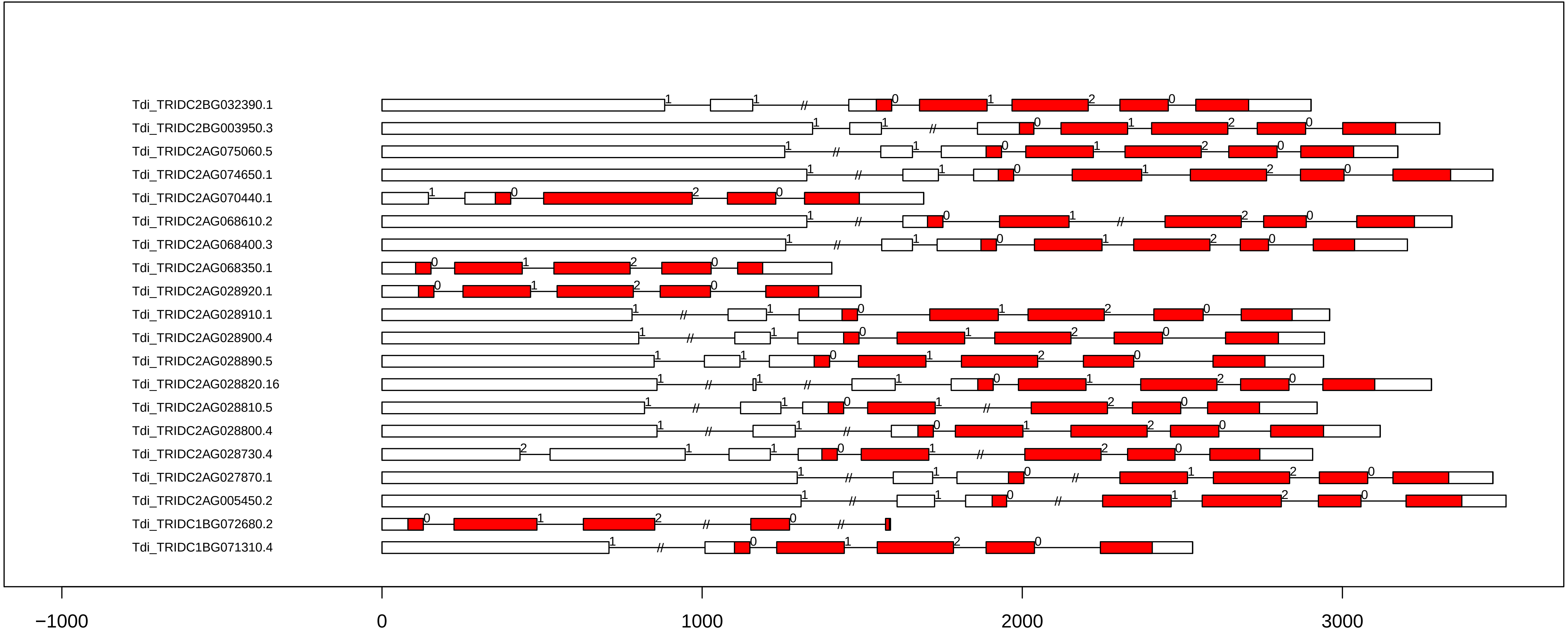

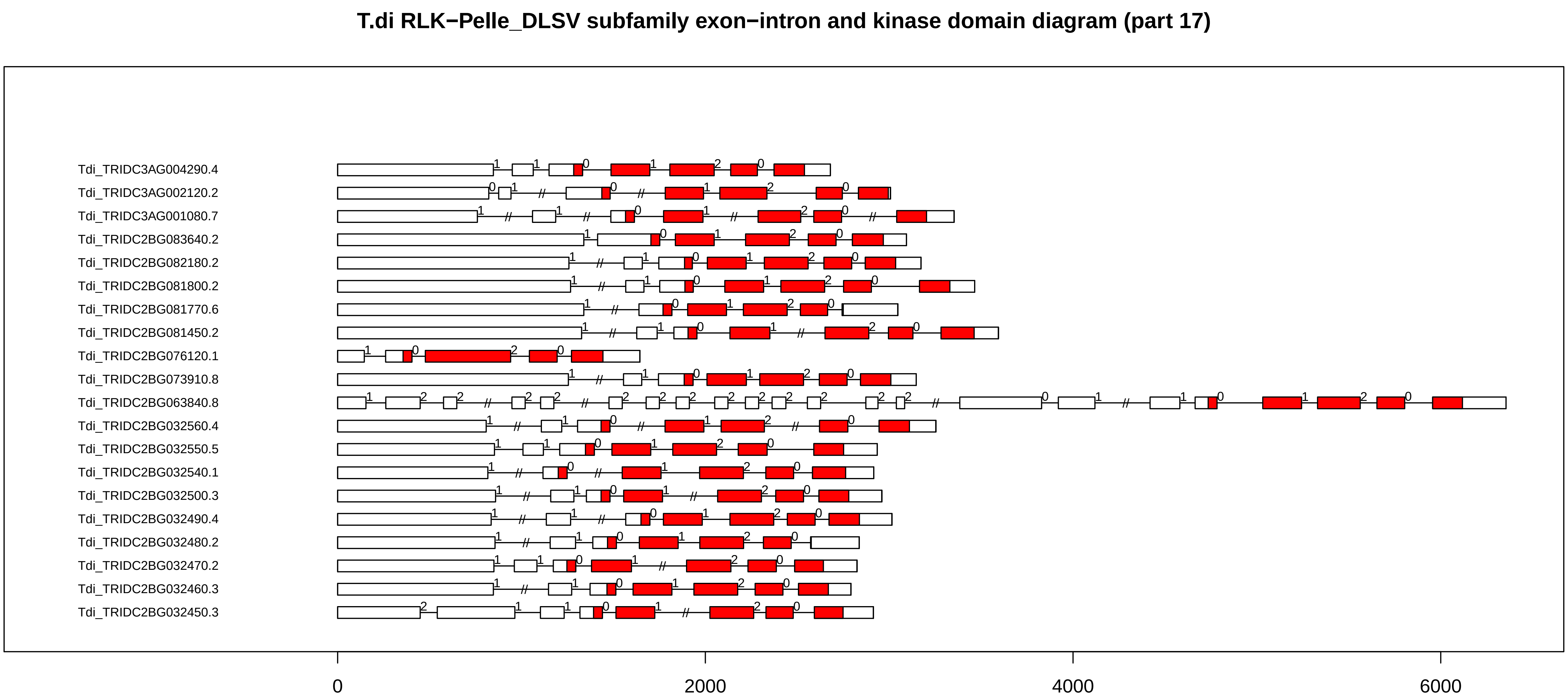

T.di RLK−Pelle_DLSV subfamily exon−intron and kinase domain diagram (part 17)
Tdi_TRIDC3AG004290.4
Tdi_TRIDC3AG002120.2
Tdi_TRIDC3AG001080.7
Tdi_TRIDC2BG083640.2
Tdi_TRIDC2BG082180.2
Tdi_TRIDC2BG081800.2
Tdi_TRIDC2BG081770.6
Tdi_TRIDC2BG081450.2
Tdi_TRIDC2BG076120.1
Tdi_TRIDC2BG073910.8
Tdi_TRIDC2BG063840.8
Tdi_TRIDC2BG032560.4
Tdi_TRIDC2BG032550.5
Tdi_TRIDC2BG032540.1
Tdi_TRIDC2BG032500.3
Tdi_TRIDC2BG032490.4
Tdi_TRIDC2BG032480.2
Tdi_TRIDC2BG032470.2
Tdi_TRIDC2BG032460.3
Tdi_TRIDC2BG032450.3
0
2000
4000
6000

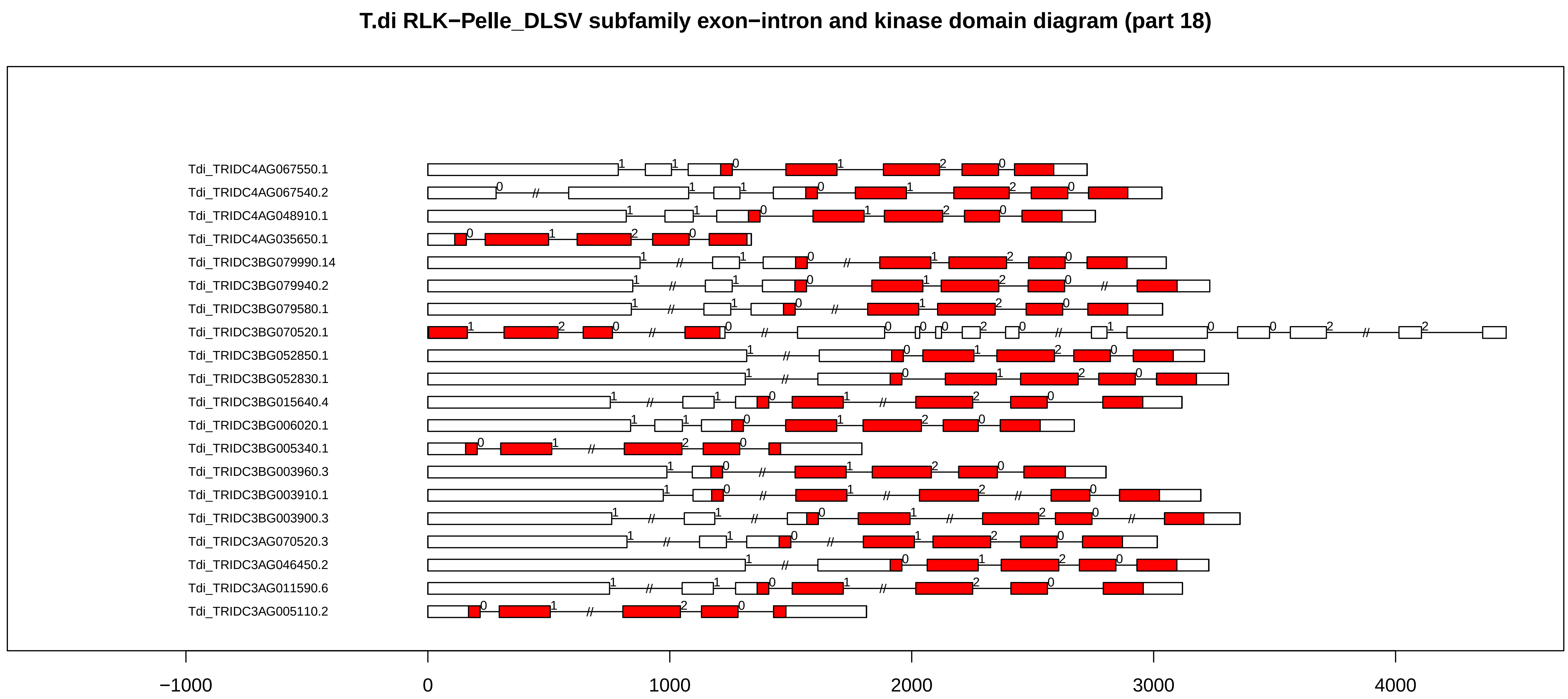
T.di RLK−Pelle_DLSV subfamily exon−intron and kinase domain diagram (part 18)
Tdi_TRIDC4AG067550.1
Tdi_TRIDC4AG067540.2
Tdi_TRIDC4AG048910.1
Tdi_TRIDC4AG035650.1
Tdi_TRIDC3BG079990.14
Tdi_TRIDC3BG079940.2
Tdi_TRIDC3BG079580.1
Tdi_TRIDC3BG070520.1
Tdi_TRIDC3BG052850.1
Tdi_TRIDC3BG052830.1
Tdi_TRIDC3BG015640.4
Tdi_TRIDC3BG006020.1
Tdi_TRIDC3BG005340.1
Tdi_TRIDC3BG003960.3
Tdi_TRIDC3BG003910.1
Tdi_TRIDC3BG003900.3
Tdi_TRIDC3AG070520.3
Tdi_TRIDC3AG046450.2
Tdi_TRIDC3AG011590.6
Tdi_TRIDC3AG005110.2
−1000
0
1000
2000
3000
4000

T.di RLK−Pelle_DLSV subfamily exon−intron and kinase domain diagram (part 19)

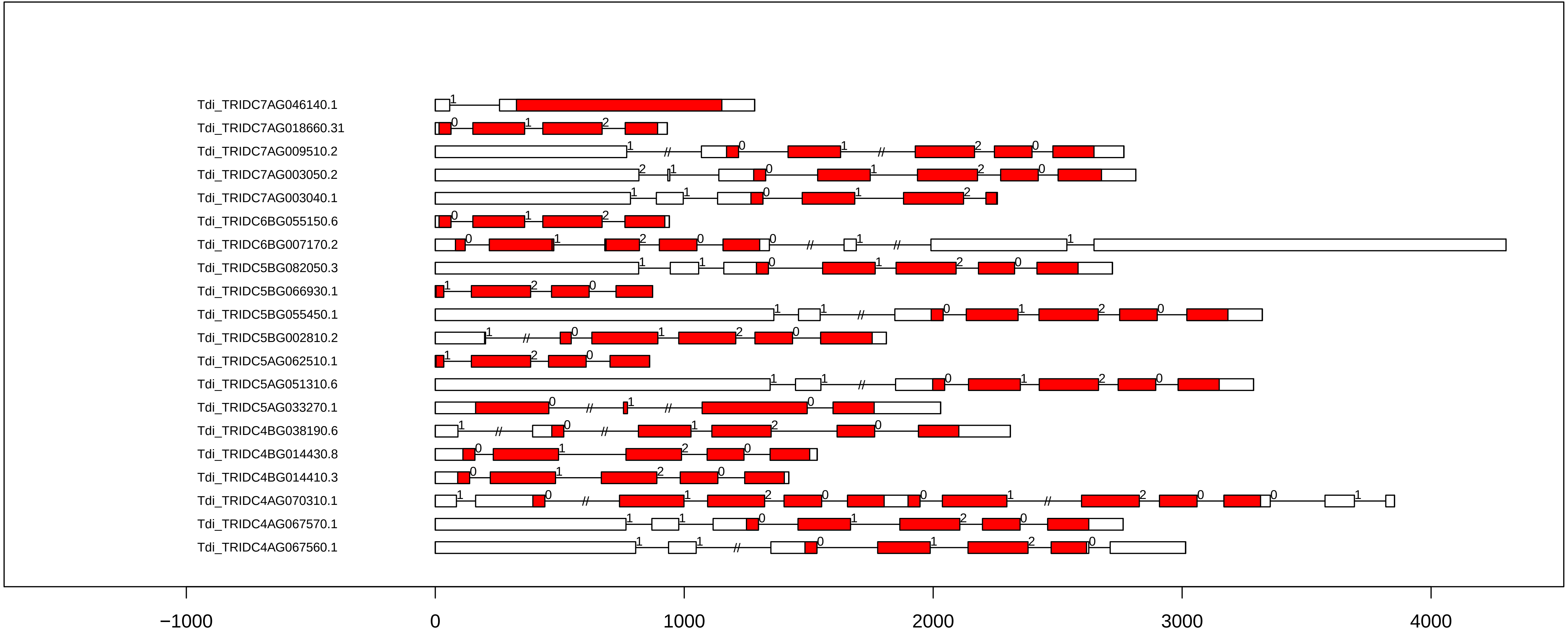

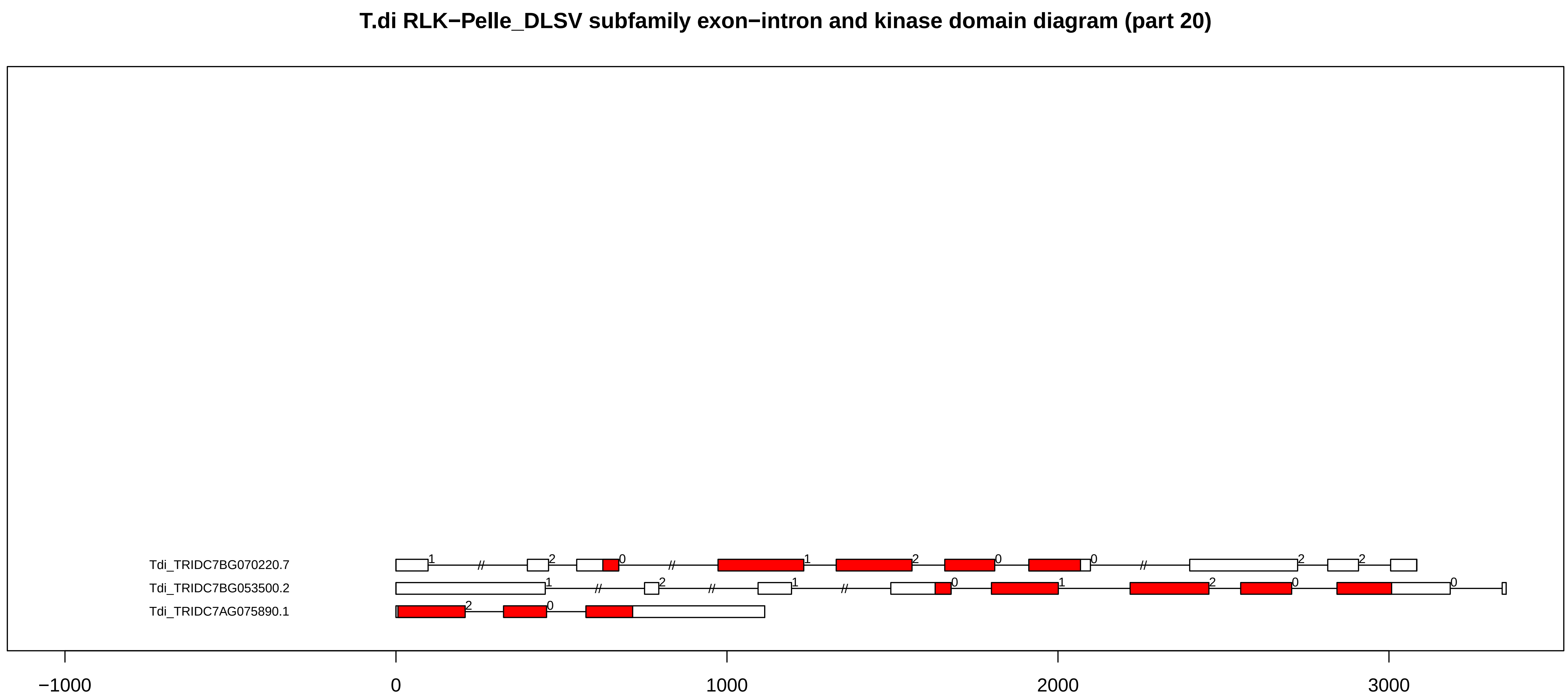
T.di RLK−Pelle_DLSV subfamily exon−intron and kinase domain diagram (part 20)
Tdi_TRIDC7BG070220.7
Tdi_TRIDC7BG053500.2
Tdi_TRIDC7AG075890.1
1
2
0
1
2
0
0
2
2
1
2
1
0
1
2
0
0
2
0
−1000
0
1000
2000
3000

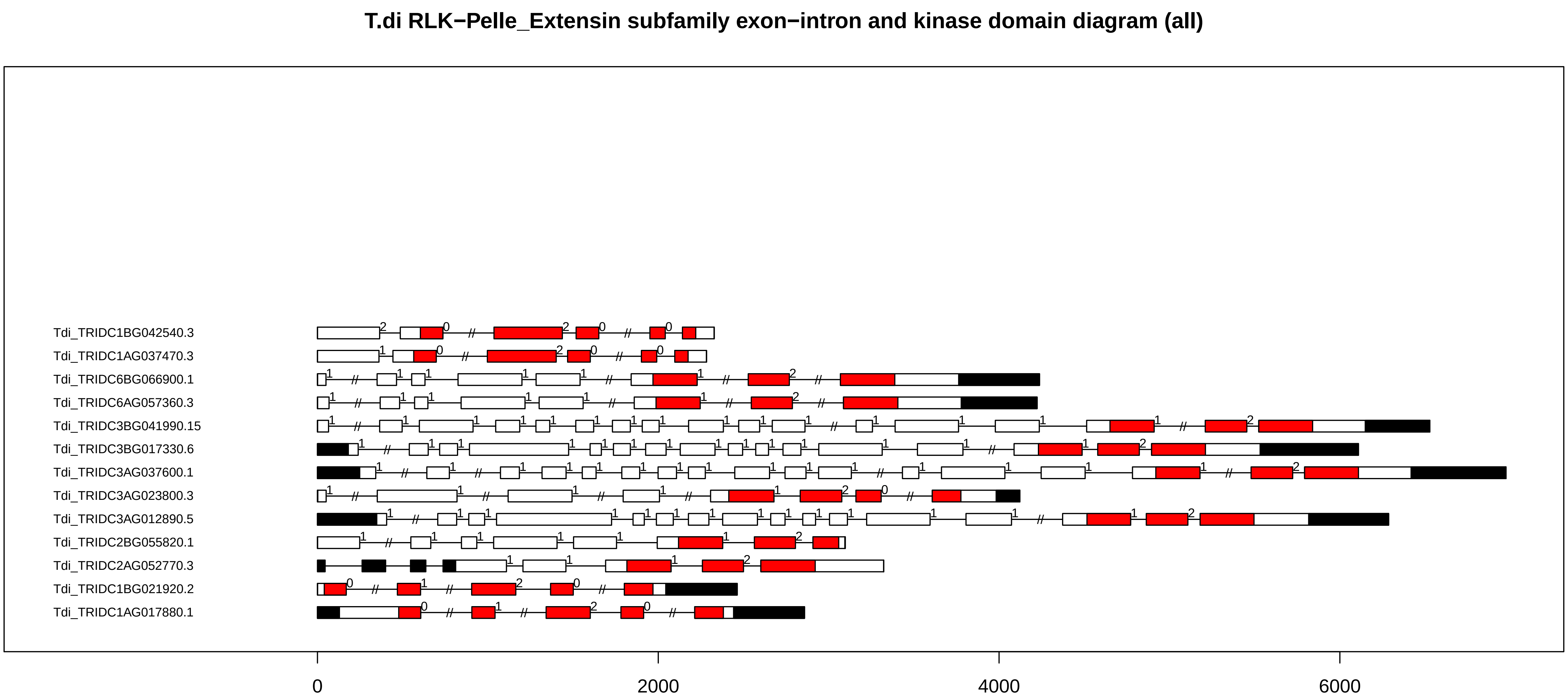
T.di RLK−Pelle_Extensin subfamily exon−intron and kinase domain diagram (all)
Tdi_TRIDC1BG042540.3
Tdi_TRIDC1AG037470.3
Tdi_TRIDC6BG066900.1
Tdi_TRIDC6AG057360.3
Tdi_TRIDC3BG041990.15
Tdi_TRIDC3BG017330.6
Tdi_TRIDC3AG037600.1
Tdi_TRIDC3AG023800.3
Tdi_TRIDC3AG012890.5
Tdi_TRIDC2BG055820.1
Tdi_TRIDC2AG052770.3
Tdi_TRIDC1BG021920.2
Tdi_TRIDC1AG017880.1
0
2000
4000
6000

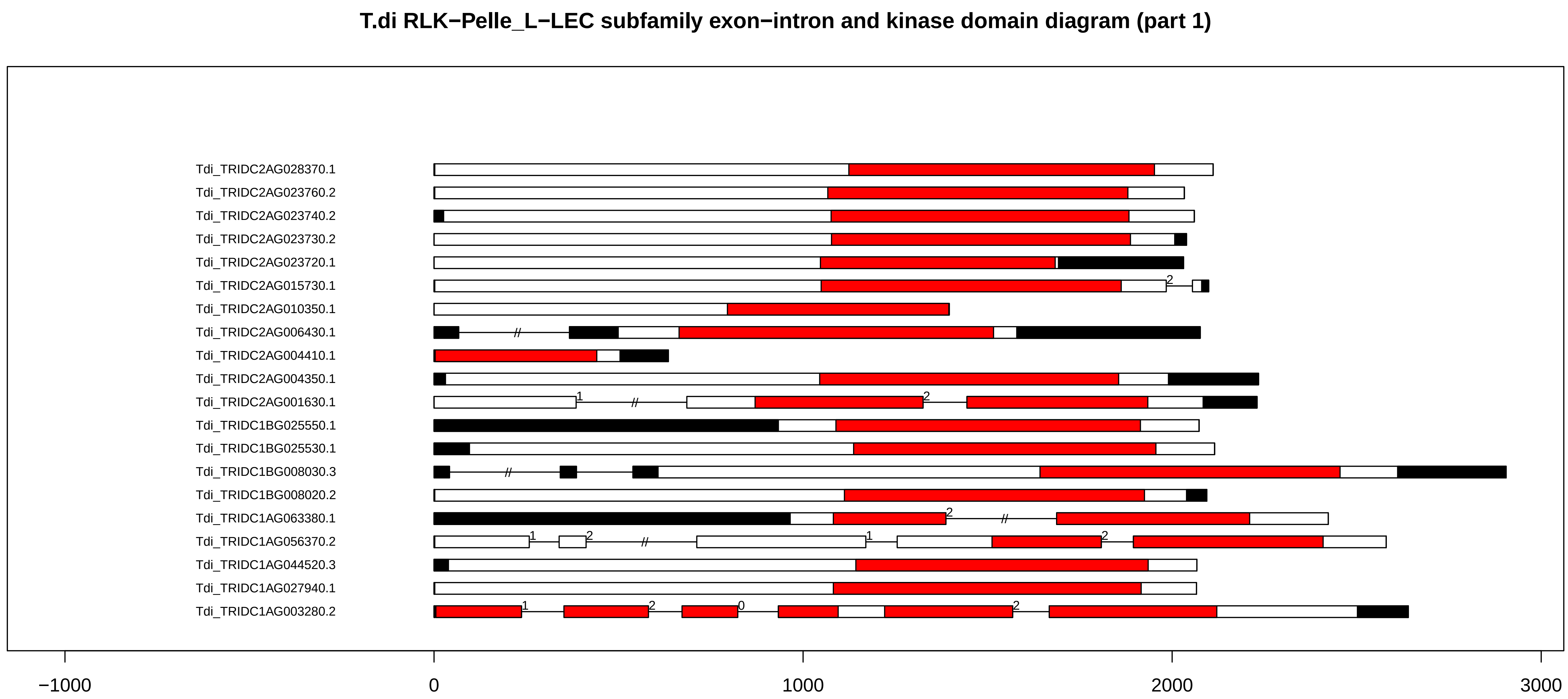
T.di RLK−Pelle_L−LEC subfamily exon−intron and kinase domain diagram (part 1)
Tdi_TRIDC2AG028370.1
Tdi_TRIDC2AG023760.2
Tdi_TRIDC2AG023740.2
Tdi_TRIDC2AG023730.2
Tdi_TRIDC2AG023720.1
Tdi_TRIDC2AG015730.1
Tdi_TRIDC2AG010350.1
Tdi_TRIDC2AG006430.1
Tdi_TRIDC2AG004410.1
Tdi_TRIDC2AG004350.1
Tdi_TRIDC2AG001630.1
Tdi_TRIDC1BG025550.1
Tdi_TRIDC1BG025530.1
Tdi_TRIDC1BG008030.3
Tdi_TRIDC1BG008020.2
Tdi_TRIDC1AG063380.1
Tdi_TRIDC1AG056370.2
Tdi_TRIDC1AG044520.3
Tdi_TRIDC1AG027940.1
Tdi_TRIDC1AG003280.2
−1000
0
1000
2000
3000

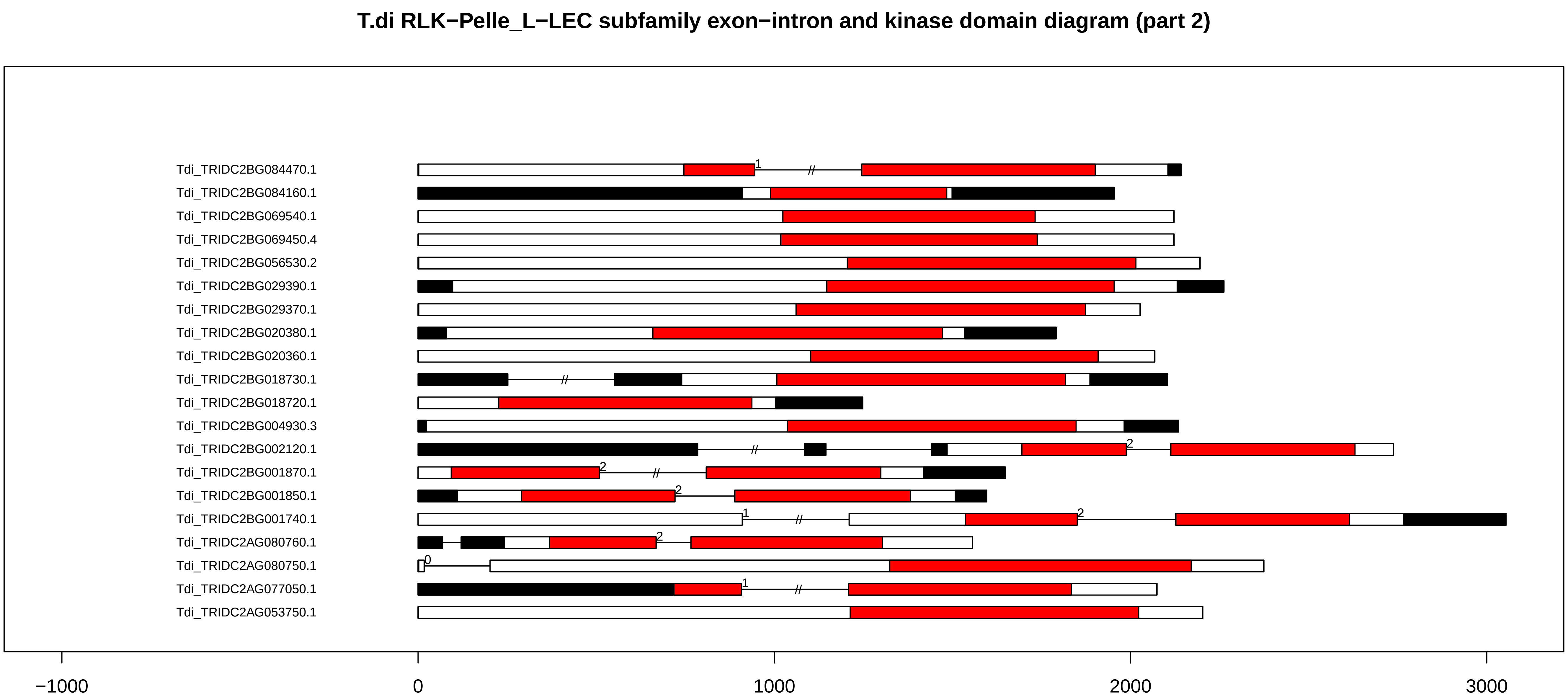

T.di RLK−Pelle_L−LEC subfamily exon−intron and kinase domain diagram (part 2)
Tdi_TRIDC2BG084470.1
Tdi_TRIDC2BG084160.1
Tdi_TRIDC2BG069540.1
Tdi_TRIDC2BG069450.4
Tdi_TRIDC2BG056530.2
Tdi_TRIDC2BG029390.1
Tdi_TRIDC2BG029370.1
Tdi_TRIDC2BG020380.1
Tdi_TRIDC2BG020360.1
Tdi_TRIDC2BG018730.1
Tdi_TRIDC2BG018720.1
Tdi_TRIDC2BG004930.3
Tdi_TRIDC2BG002120.1
Tdi_TRIDC2BG001870.1
Tdi_TRIDC2BG001850.1
Tdi_TRIDC2BG001740.1
Tdi_TRIDC2AG080760.1
Tdi_TRIDC2AG080750.1
Tdi_TRIDC2AG077050.1
Tdi_TRIDC2AG053750.1
−1000
0
1000
2000
3000

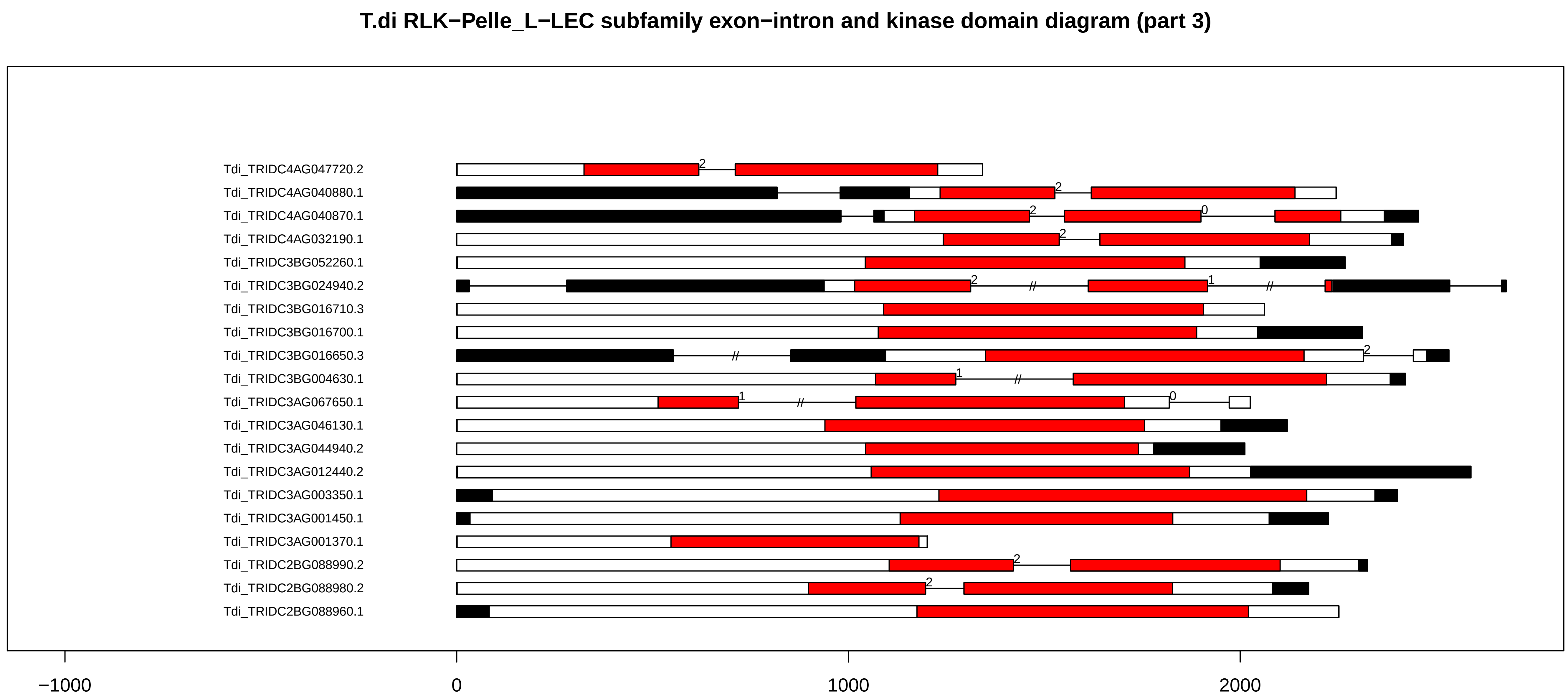
T.di RLK−Pelle_L−LEC subfamily exon−intron and kinase domain diagram (part 3)
Tdi_TRIDC4AG047720.2
Tdi_TRIDC4AG040880.1
Tdi_TRIDC4AG040870.1
Tdi_TRIDC4AG032190.1
Tdi_TRIDC3BG052260.1
Tdi_TRIDC3BG024940.2
Tdi_TRIDC3BG016710.3
Tdi_TRIDC3BG016700.1
Tdi_TRIDC3BG016650.3
Tdi_TRIDC3BG004630.1
Tdi_TRIDC3AG067650.1
Tdi_TRIDC3AG046130.1
Tdi_TRIDC3AG044940.2
Tdi_TRIDC3AG012440.2
Tdi_TRIDC3AG003350.1
Tdi_TRIDC3AG001450.1
Tdi_TRIDC3AG001370.1
Tdi_TRIDC2BG088990.2
Tdi_TRIDC2BG088980.2
Tdi_TRIDC2BG088960.1
−1000
0
1000
2000

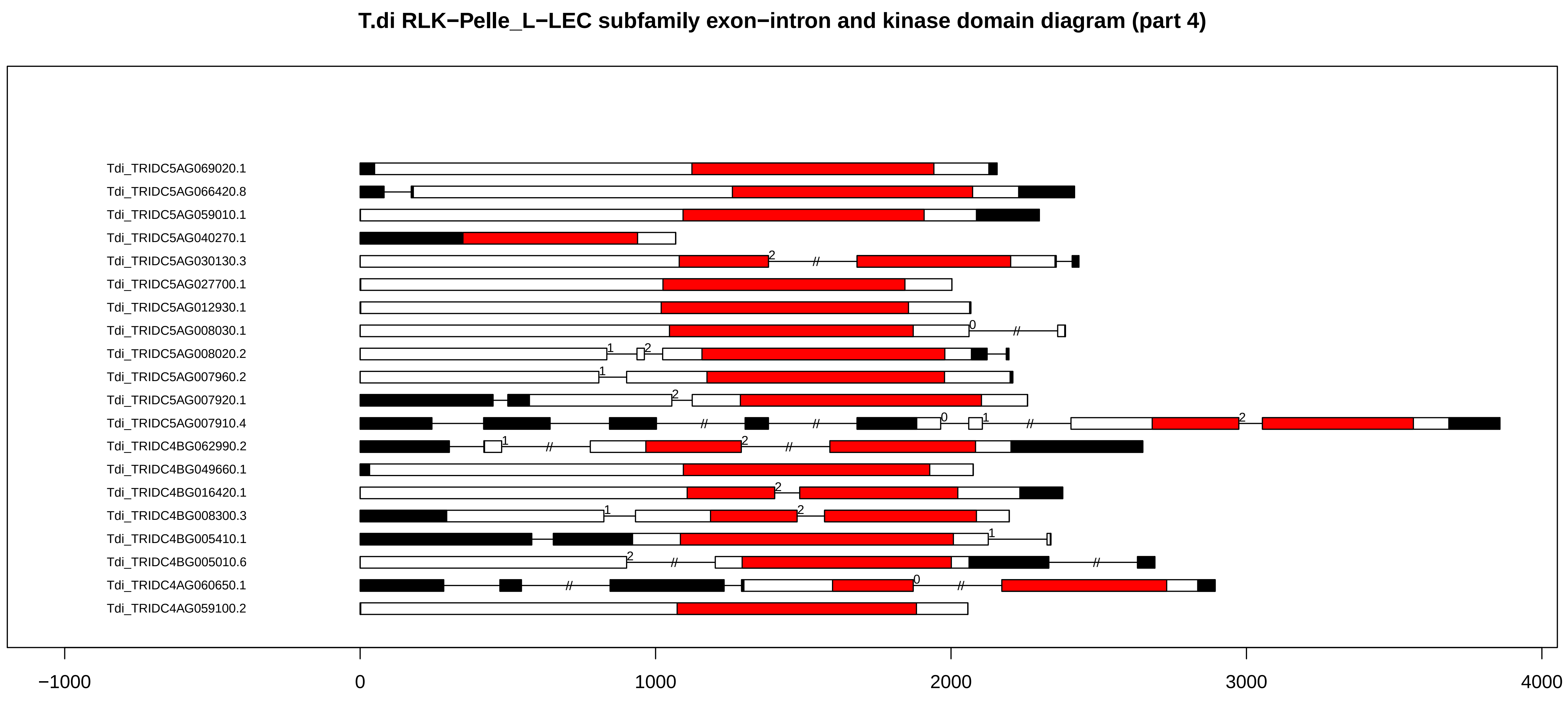
T.di RLK−Pelle_L−LEC subfamily exon−intron and kinase domain diagram (part 4)
Tdi_TRIDC5AG069020.1
Tdi_TRIDC5AG066420.8
Tdi_TRIDC5AG059010.1
Tdi_TRIDC5AG040270.1
Tdi_TRIDC5AG030130.3
Tdi_TRIDC5AG027700.1
Tdi_TRIDC5AG012930.1
Tdi_TRIDC5AG008030.1
Tdi_TRIDC5AG008020.2
Tdi_TRIDC5AG007960.2
Tdi_TRIDC5AG007920.1
Tdi_TRIDC5AG007910.4
Tdi_TRIDC4BG062990.2
Tdi_TRIDC4BG049660.1
Tdi_TRIDC4BG016420.1
Tdi_TRIDC4BG008300.3
Tdi_TRIDC4BG005410.1
Tdi_TRIDC4BG005010.6
Tdi_TRIDC4AG060650.1
Tdi_TRIDC4AG059100.2
−1000
0
1000
2000
3000
4000

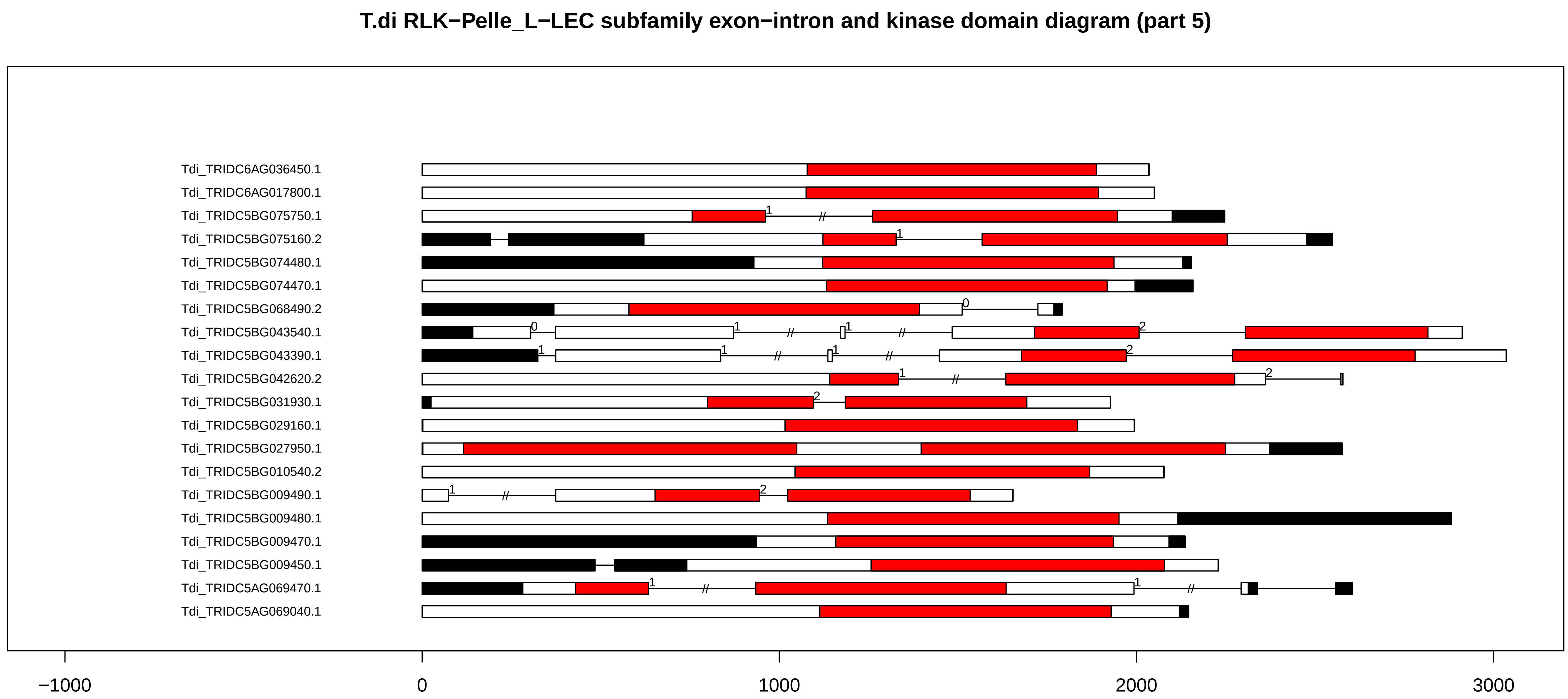
T.di RLK−Pelle_L−LEC subfamily exon−intron and kinase domain diagram (part 5)
Tdi_TRIDC6AG036450.1
Tdi_TRIDC6AG017800.1
Tdi_TRIDC5BG075750.1
Tdi_TRIDC5BG075160.2
Tdi_TRIDC5BG074480.1
Tdi_TRIDC5BG074470.1
Tdi_TRIDC5BG068490.2
Tdi_TRIDC5BG043540.1
Tdi_TRIDC5BG043390.1
Tdi_TRIDC5BG042620.2
Tdi_TRIDC5BG031930.1
Tdi_TRIDC5BG029160.1
Tdi_TRIDC5BG027950.1
Tdi_TRIDC5BG010540.2
Tdi_TRIDC5BG009490.1
Tdi_TRIDC5BG009480.1
Tdi_TRIDC5BG009470.1
Tdi_TRIDC5BG009450.1
Tdi_TRIDC5AG069470.1
Tdi_TRIDC5AG069040.1
−1000
0
1000
2000
3000

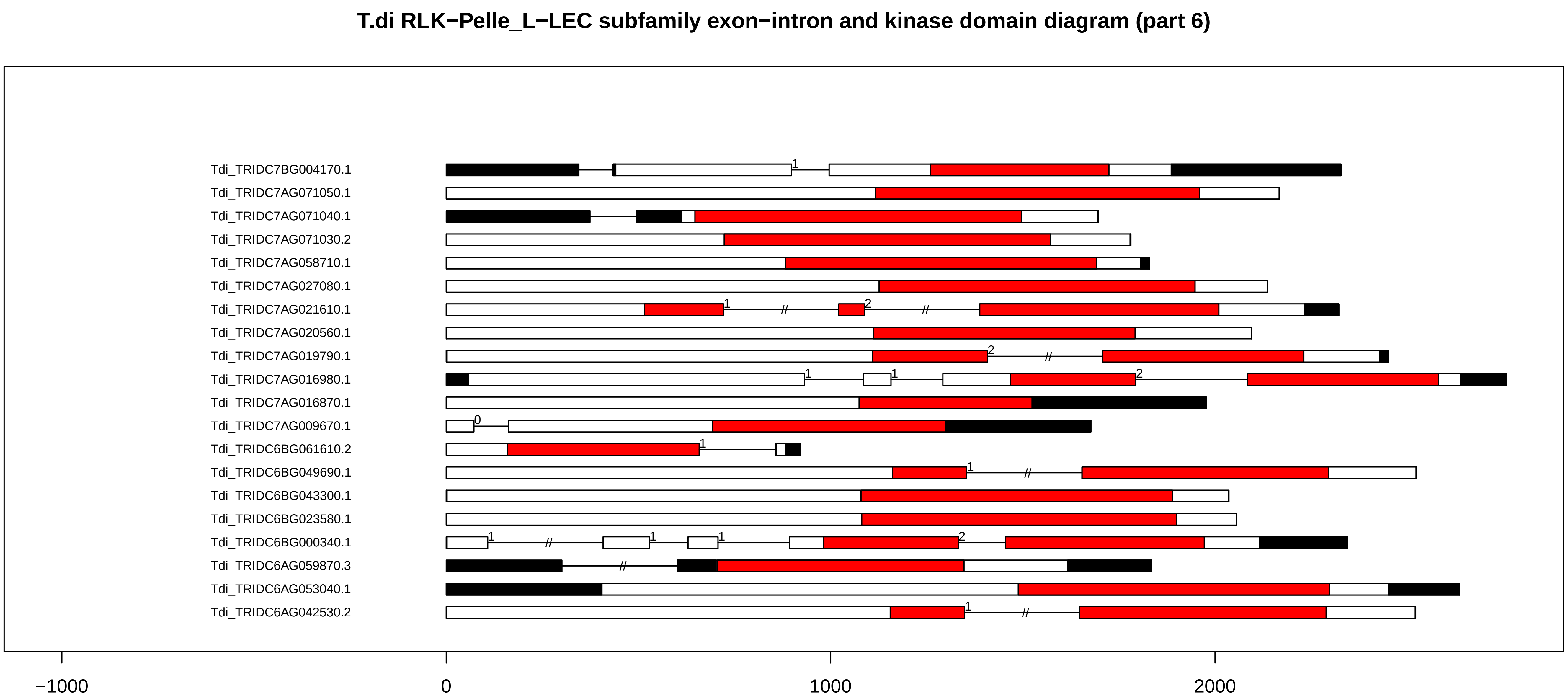

T.di RLK−Pelle_L−LEC subfamily exon−intron and kinase domain diagram (part 6)
Tdi_TRIDC7BG004170.1
Tdi_TRIDC7AG071050.1
Tdi_TRIDC7AG071040.1
Tdi_TRIDC7AG071030.2
Tdi_TRIDC7AG058710.1
Tdi_TRIDC7AG027080.1
Tdi_TRIDC7AG021610.1
Tdi_TRIDC7AG020560.1
Tdi_TRIDC7AG019790.1
Tdi_TRIDC7AG016980.1
Tdi_TRIDC7AG016870.1
Tdi_TRIDC7AG009670.1
Tdi_TRIDC6BG061610.2
Tdi_TRIDC6BG049690.1
Tdi_TRIDC6BG043300.1
Tdi_TRIDC6BG023580.1
Tdi_TRIDC6BG000340.1
Tdi_TRIDC6AG059870.3
Tdi_TRIDC6AG053040.1
Tdi_TRIDC6AG042530.2
−1000
0
1000
2000

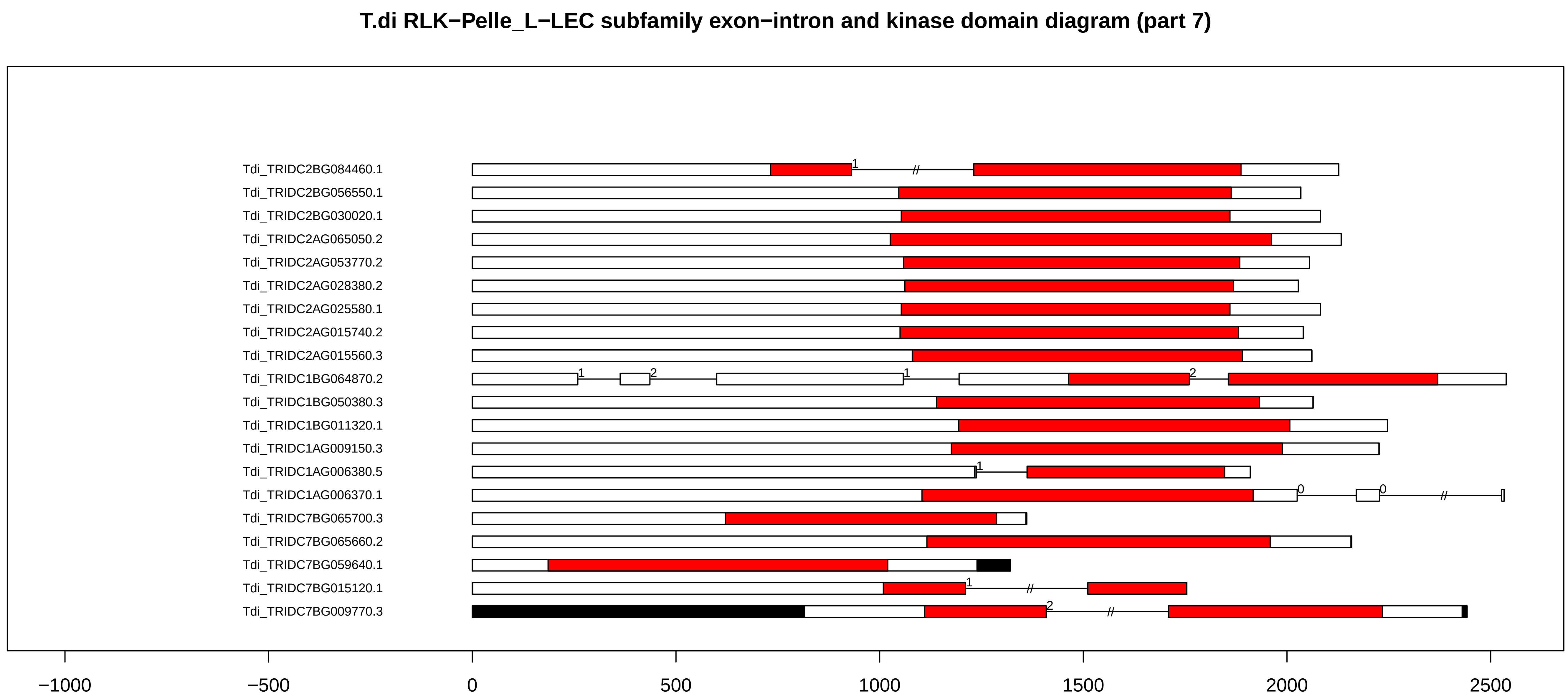

T.di RLK−Pelle_L−LEC subfamily exon−intron and kinase domain diagram (part 7)
Tdi_TRIDC2BG084460.1
Tdi_TRIDC2BG056550.1
Tdi_TRIDC2BG030020.1
Tdi_TRIDC2AG065050.2
Tdi_TRIDC2AG053770.2
Tdi_TRIDC2AG028380.2
Tdi_TRIDC2AG025580.1
Tdi_TRIDC2AG015740.2
Tdi_TRIDC2AG015560.3
Tdi_TRIDC1BG064870.2
Tdi_TRIDC1BG050380.3
Tdi_TRIDC1BG011320.1
Tdi_TRIDC1AG009150.3
Tdi_TRIDC1AG006380.5
Tdi_TRIDC1AG006370.1
Tdi_TRIDC7BG065700.3
Tdi_TRIDC7BG065660.2
Tdi_TRIDC7BG059640.1
Tdi_TRIDC7BG015120.1
Tdi_TRIDC7BG009770.3
−1000
−500
0
500
1000
1500
2000
2500

T.di RLK−Pelle_L−LEC subfamily exon−intron and kinase domain diagram (part 8)

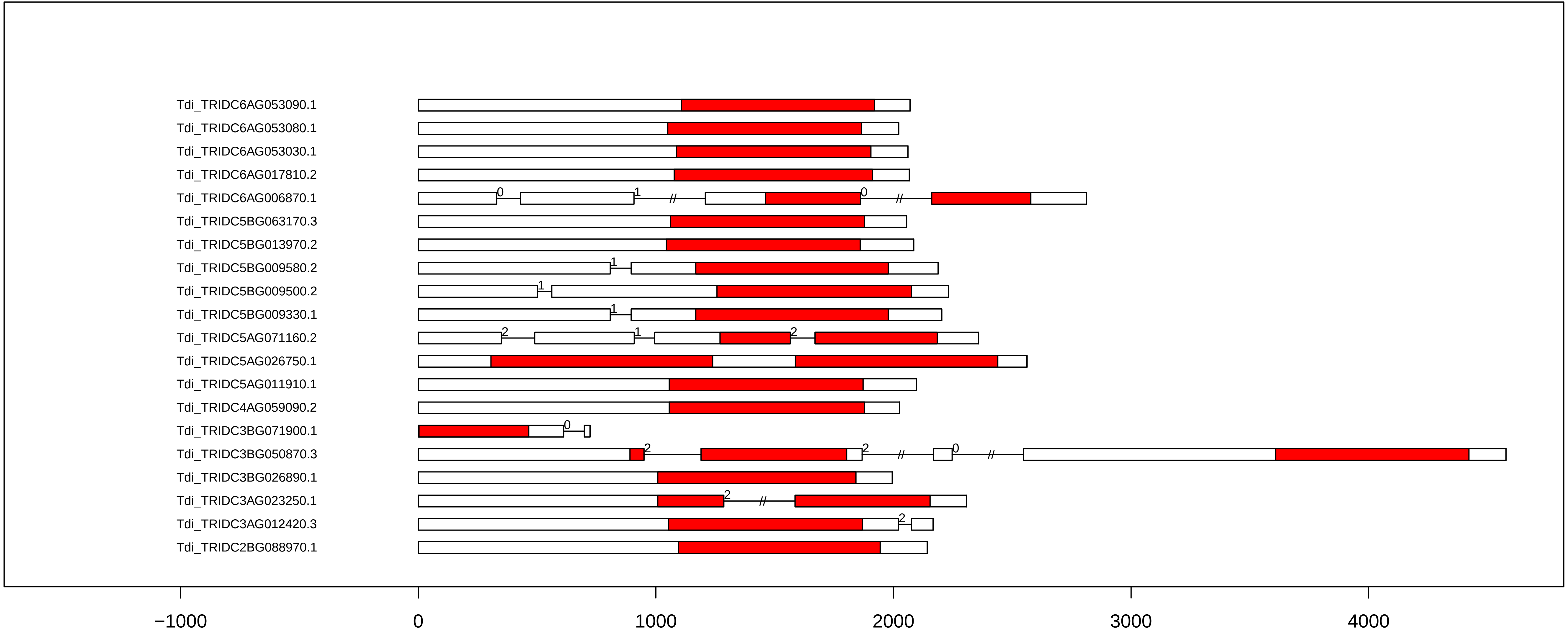

T.di RLK−Pelle_L−LEC subfamily exon−intron and kinase domain diagram (part 9)

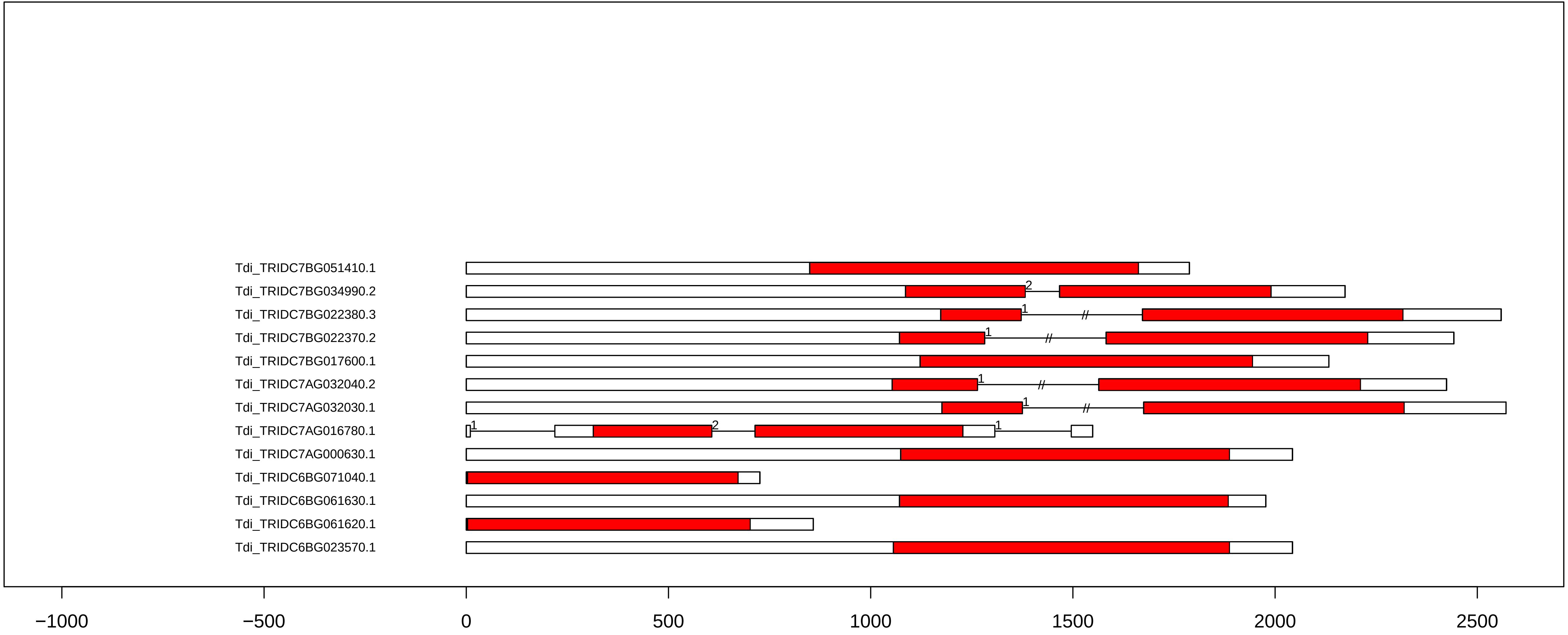

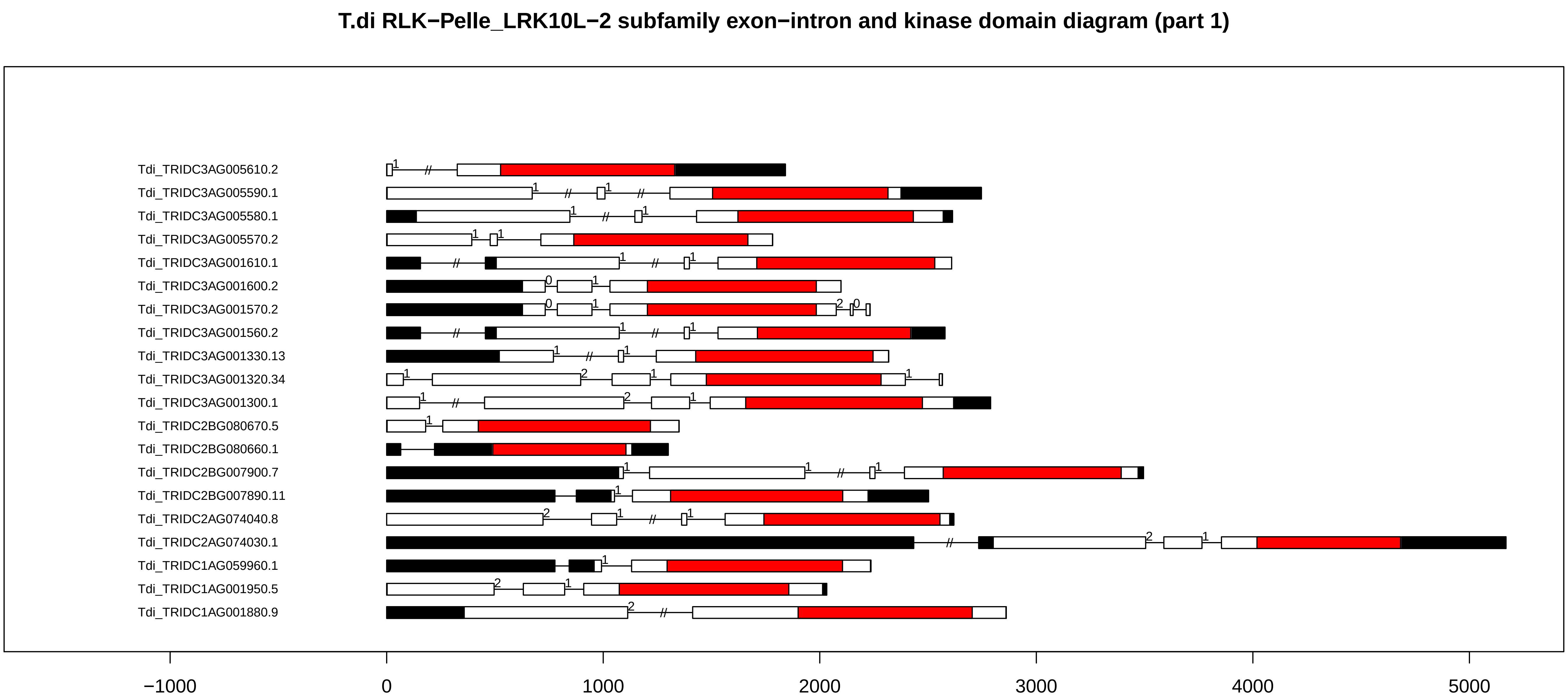
T.di RLK−Pelle_LRK10L−2 subfamily exon−intron and kinase domain diagram (part 1)
Tdi_TRIDC3AG005610.2
Tdi_TRIDC3AG005590.1
Tdi_TRIDC3AG005580.1
Tdi_TRIDC3AG005570.2
Tdi_TRIDC3AG001610.1
Tdi_TRIDC3AG001600.2
Tdi_TRIDC3AG001570.2
Tdi_TRIDC3AG001560.2
Tdi_TRIDC3AG001330.13
Tdi_TRIDC3AG001320.34
Tdi_TRIDC3AG001300.1
Tdi_TRIDC2BG080670.5
Tdi_TRIDC2BG080660.1
Tdi_TRIDC2BG007900.7
Tdi_TRIDC2BG007890.11
Tdi_TRIDC2AG074040.8
Tdi_TRIDC2AG074030.1
Tdi_TRIDC1AG059960.1
Tdi_TRIDC1AG001950.5
Tdi_TRIDC1AG001880.9
−1000
0
1000
2000
3000
4000
5000

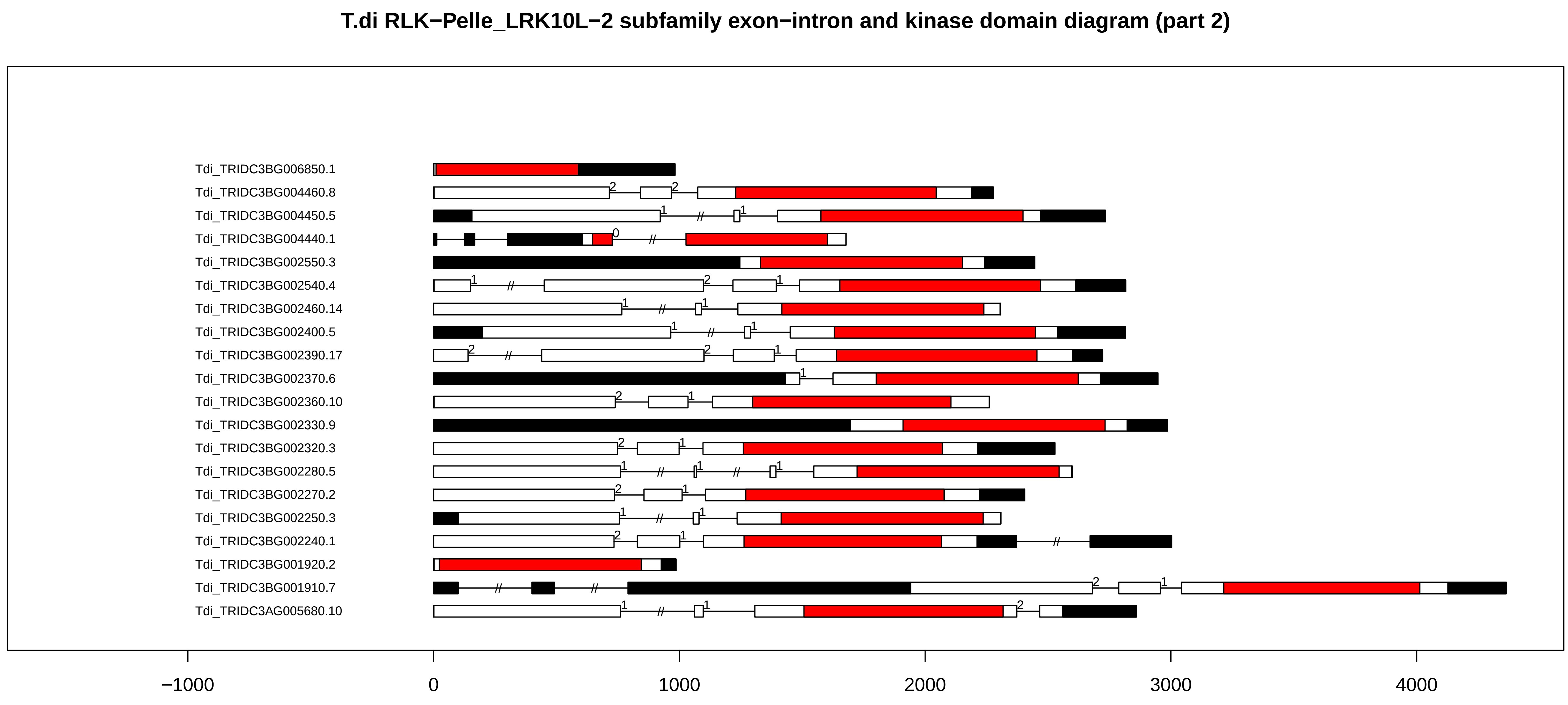
T.di RLK−Pelle_LRK10L−2 subfamily exon−intron and kinase domain diagram (part 2)
Tdi_TRIDC3BG006850.1
Tdi_TRIDC3BG004460.8
Tdi_TRIDC3BG004450.5
Tdi_TRIDC3BG004440.1
Tdi_TRIDC3BG002550.3
Tdi_TRIDC3BG002540.4
Tdi_TRIDC3BG002460.14
Tdi_TRIDC3BG002400.5
Tdi_TRIDC3BG002390.17
Tdi_TRIDC3BG002370.6
Tdi_TRIDC3BG002360.10
Tdi_TRIDC3BG002330.9
Tdi_TRIDC3BG002320.3
Tdi_TRIDC3BG002280.5
Tdi_TRIDC3BG002270.2
Tdi_TRIDC3BG002250.3
Tdi_TRIDC3BG002240.1
Tdi_TRIDC3BG001920.2
Tdi_TRIDC3BG001910.7
Tdi_TRIDC3AG005680.10
−1000
0
1000
2000
3000
4000

T.di RLK−Pelle_LRK10L−2 subfamily exon−intron and kinase domain diagram (part 3)

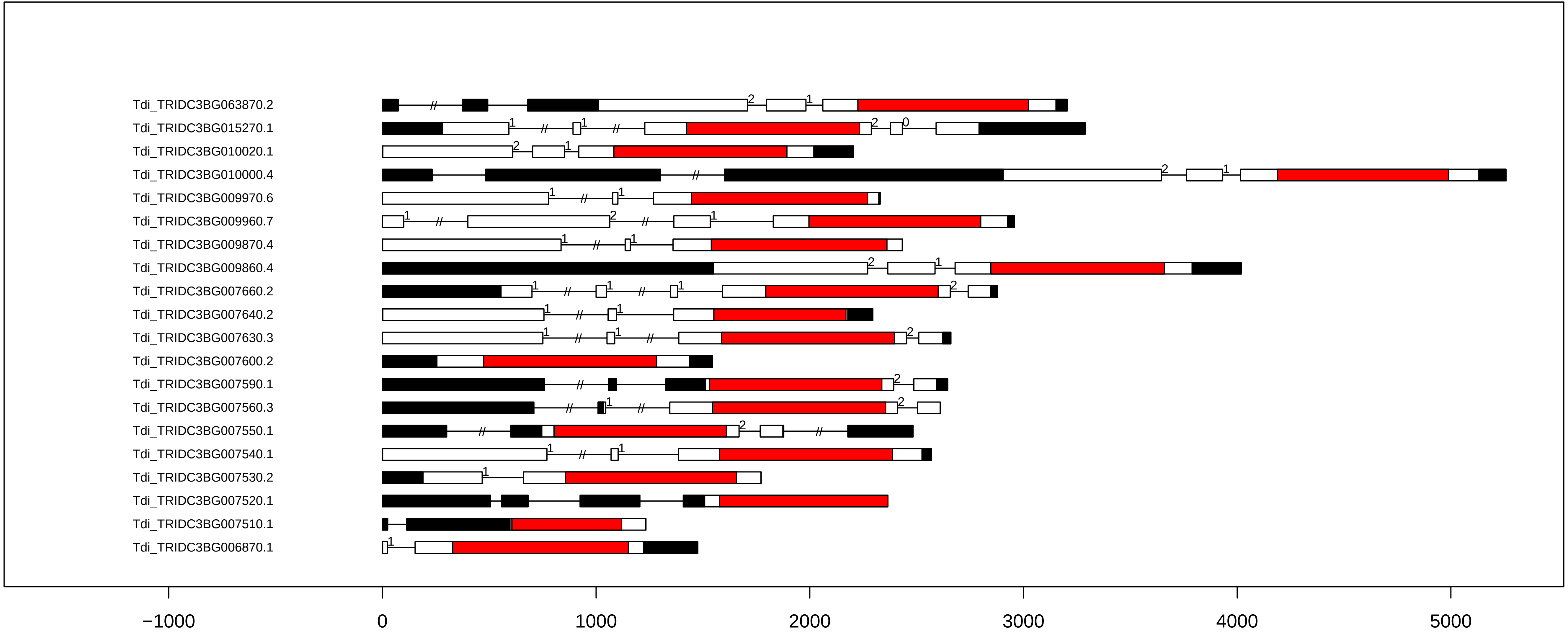

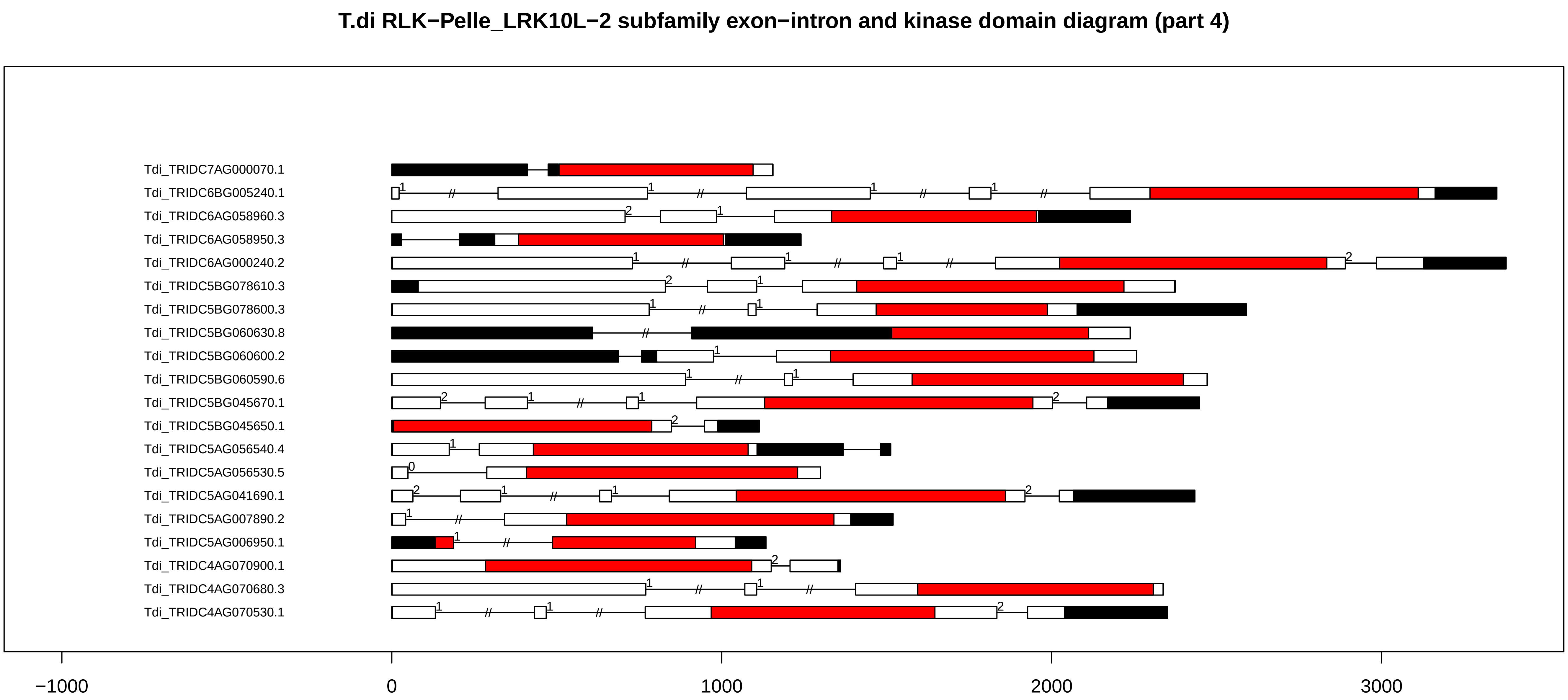
T.di RLK−Pelle_LRK10L−2 subfamily exon−intron and kinase domain diagram (part 4)
Tdi_TRIDC7AG000070.1
Tdi_TRIDC6BG005240.1
Tdi_TRIDC6AG058960.3
Tdi_TRIDC6AG058950.3
Tdi_TRIDC6AG000240.2
Tdi_TRIDC5BG078610.3
Tdi_TRIDC5BG078600.3
Tdi_TRIDC5BG060630.8
Tdi_TRIDC5BG060600.2
Tdi_TRIDC5BG060590.6
Tdi_TRIDC5BG045670.1
Tdi_TRIDC5BG045650.1
Tdi_TRIDC5AG056540.4
Tdi_TRIDC5AG056530.5
Tdi_TRIDC5AG041690.1
Tdi_TRIDC5AG007890.2
Tdi_TRIDC5AG006950.1
Tdi_TRIDC4AG070900.1
Tdi_TRIDC4AG070680.3
Tdi_TRIDC4AG070530.1
−1000
0
1000
2000
3000

T.di RLK−Pelle_LRK10L−2 subfamily exon−intron and kinase domain diagram (part 5)

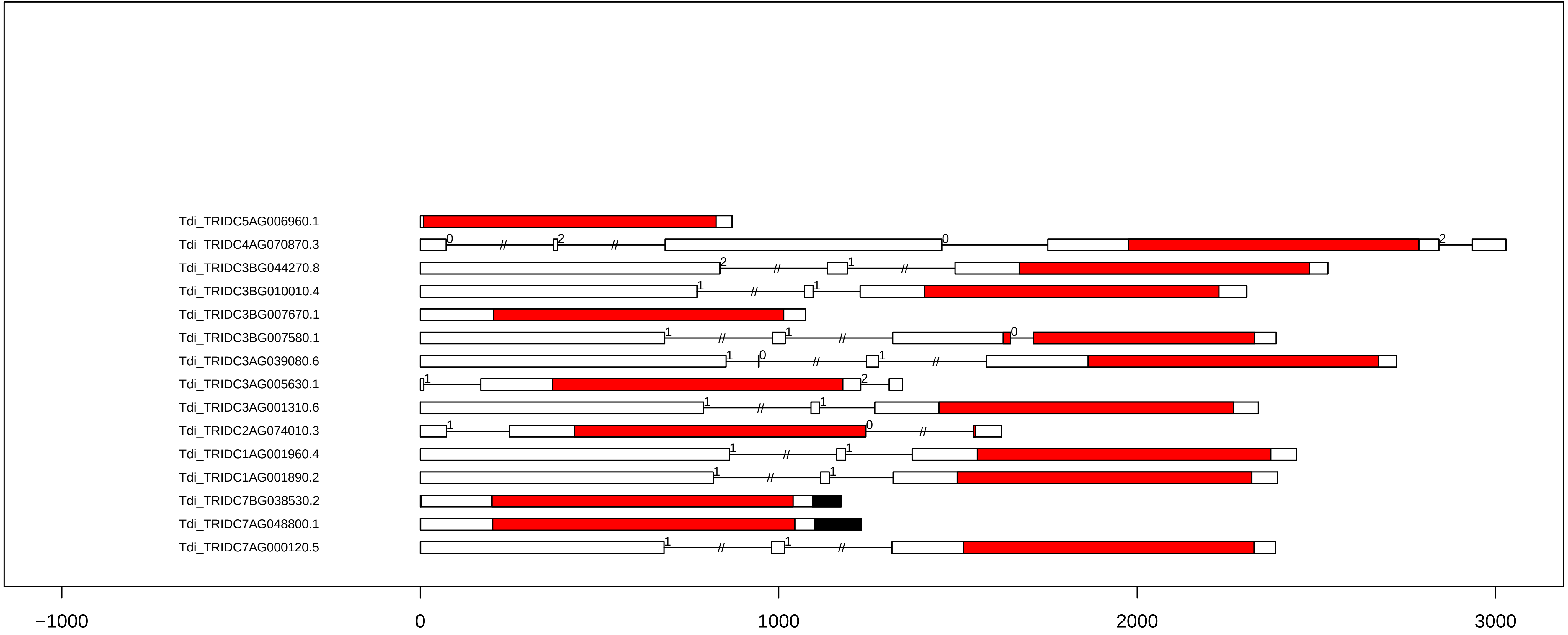

T.di RLK−Pelle_LRR−I−1 subfamily exon−intron and kinase domain diagram (part 1)

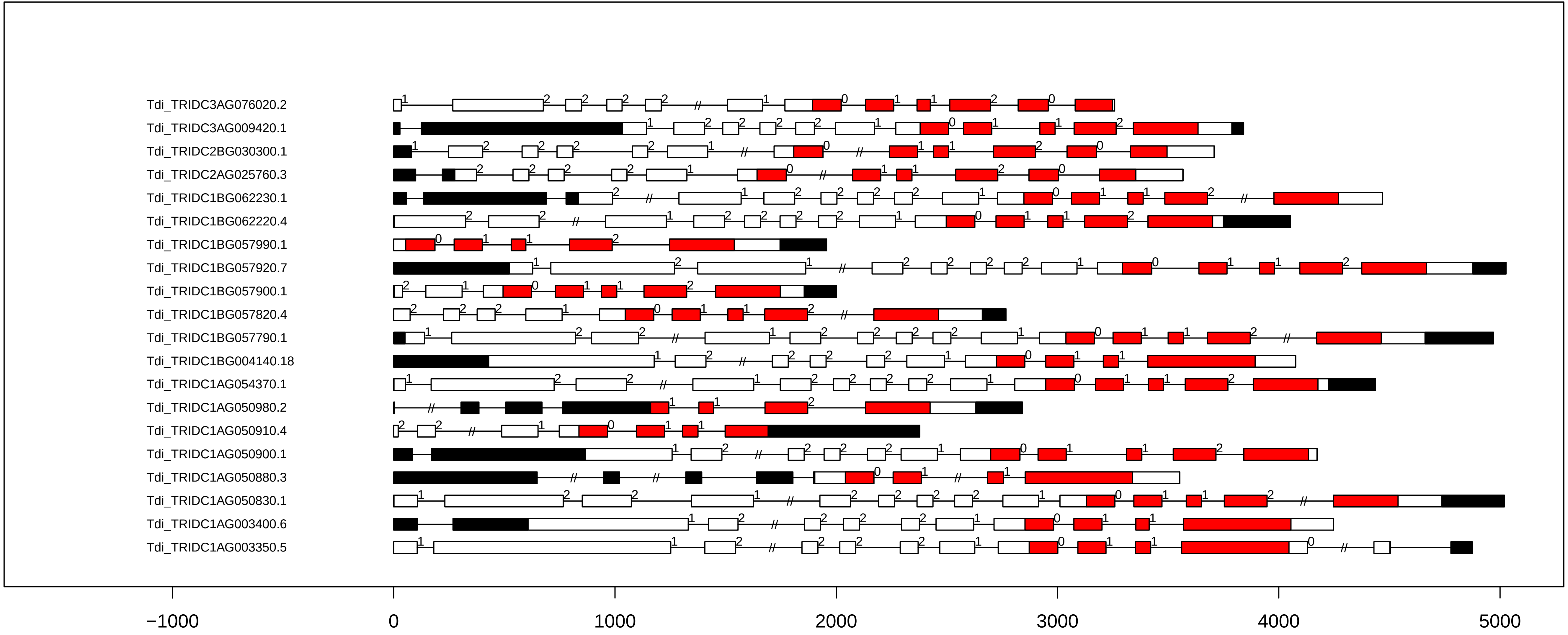

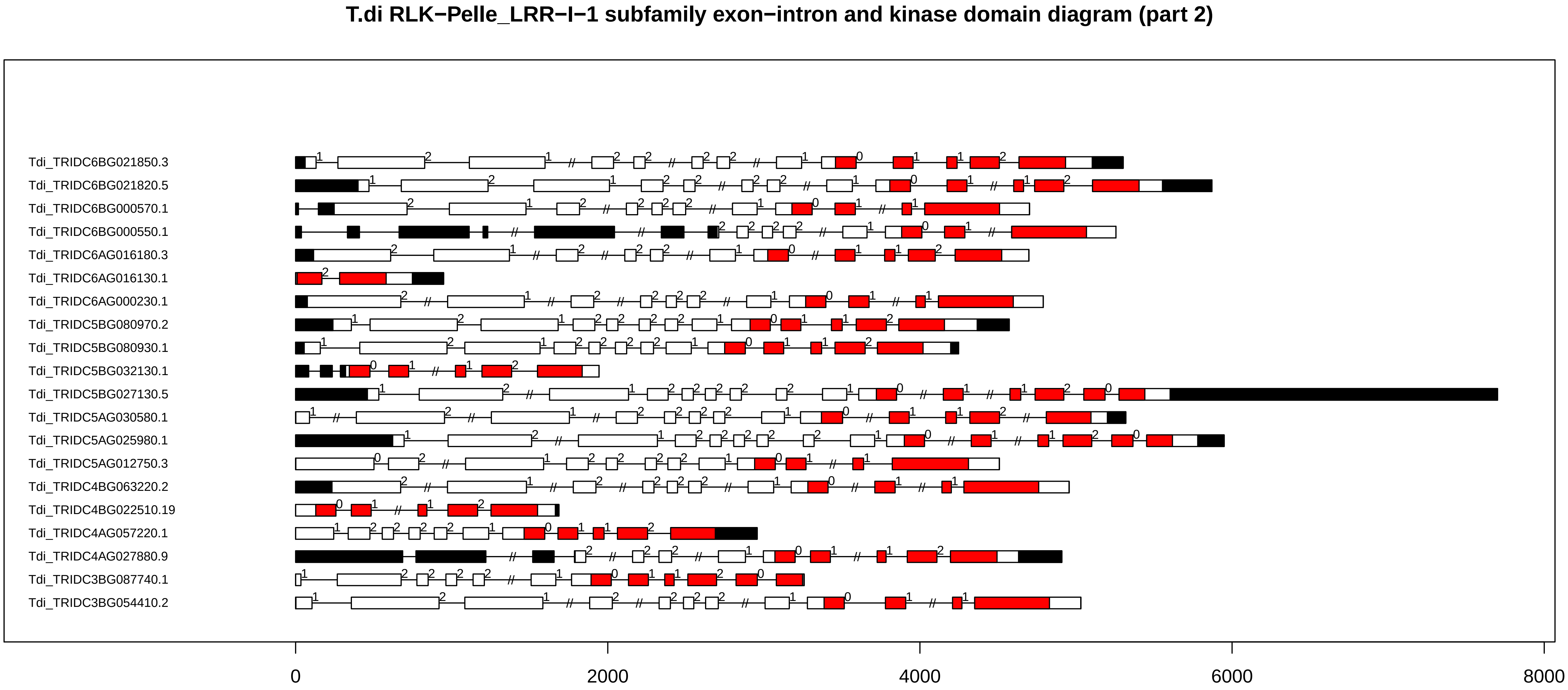
T.di RLK−Pelle_LRR−I−1 subfamily exon−intron and kinase domain diagram (part 2)
Tdi_TRIDC6BG021850.3
Tdi_TRIDC6BG021820.5
Tdi_TRIDC6BG000570.1
Tdi_TRIDC6BG000550.1
Tdi_TRIDC6AG016180.3
Tdi_TRIDC6AG016130.1
Tdi_TRIDC6AG000230.1
Tdi_TRIDC5BG080970.2
Tdi_TRIDC5BG080930.1
Tdi_TRIDC5BG032130.1
Tdi_TRIDC5BG027130.5
Tdi_TRIDC5AG030580.1
Tdi_TRIDC5AG025980.1
Tdi_TRIDC5AG012750.3
Tdi_TRIDC4BG063220.2
Tdi_TRIDC4BG022510.19
Tdi_TRIDC4AG057220.1
Tdi_TRIDC4AG027880.9
Tdi_TRIDC3BG087740.1
Tdi_TRIDC3BG054410.2
0
2000
4000
6000
8000

T.di RLK−Pelle_LRR−I−1 subfamily exon−intron and kinase domain diagram (part 3)

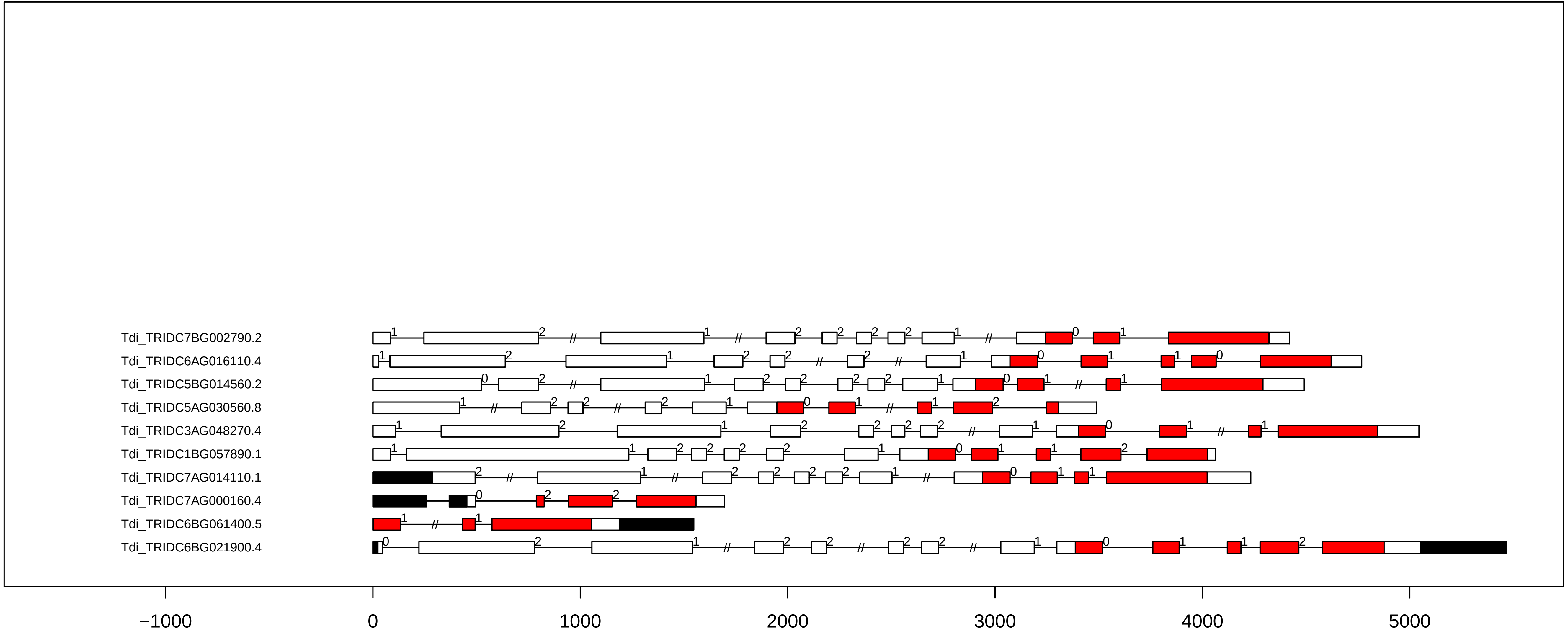

T.di RLK−Pelle_LRR−I−2 subfamily exon−intron and kinase domain diagram (all)

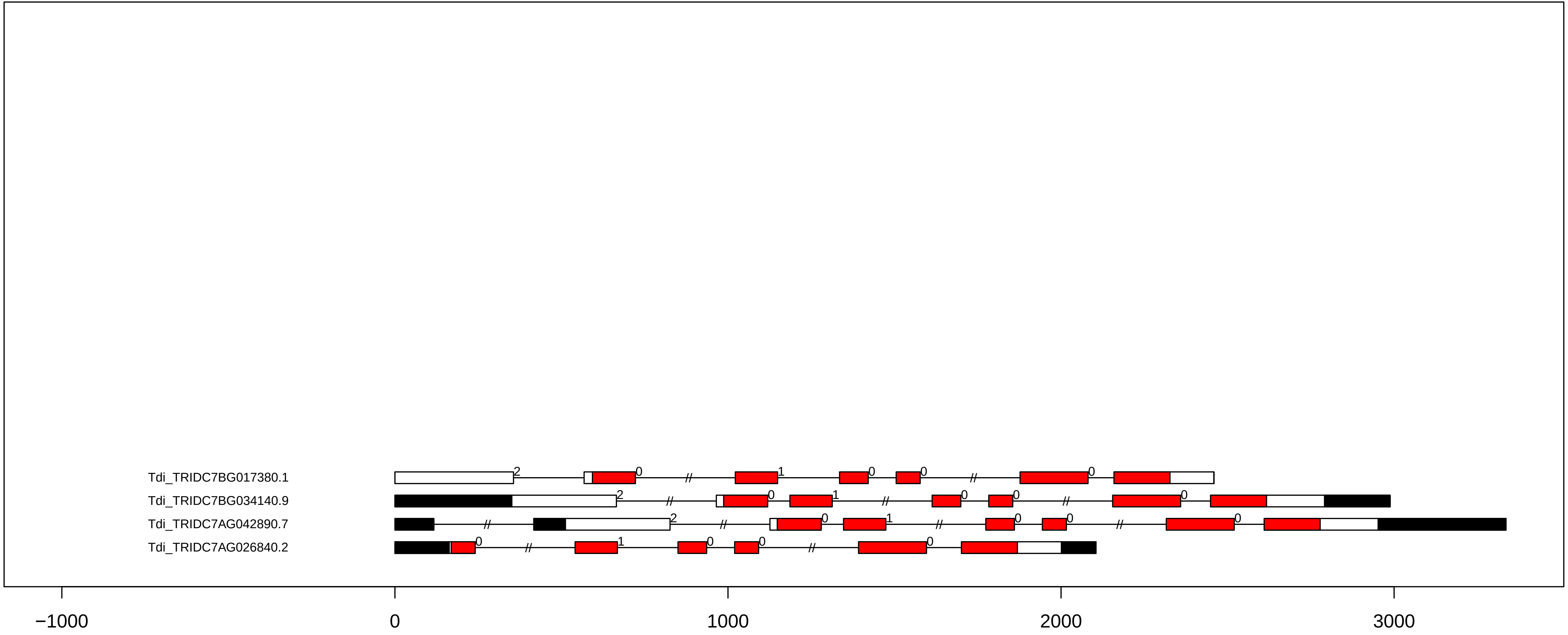

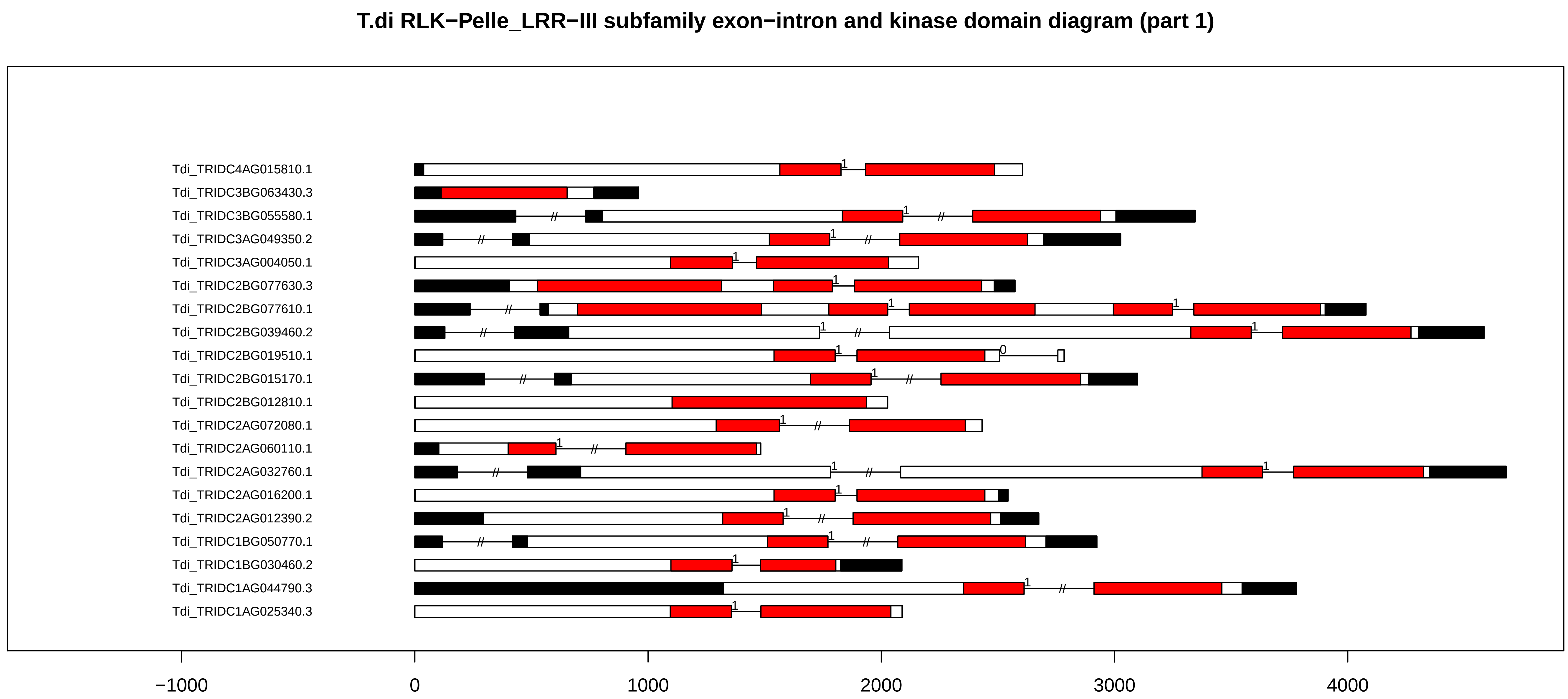

T.di RLK−Pelle_LRR−III subfamily exon−intron and kinase domain diagram (part 1)
Tdi_TRIDC4AG015810.1
Tdi_TRIDC3BG063430.3
Tdi_TRIDC3BG055580.1
Tdi_TRIDC3AG049350.2
Tdi_TRIDC3AG004050.1
Tdi_TRIDC2BG077630.3
Tdi_TRIDC2BG077610.1
Tdi_TRIDC2BG039460.2
Tdi_TRIDC2BG019510.1
Tdi_TRIDC2BG015170.1
Tdi_TRIDC2BG012810.1
Tdi_TRIDC2AG072080.1
Tdi_TRIDC2AG060110.1
Tdi_TRIDC2AG032760.1
Tdi_TRIDC2AG016200.1
Tdi_TRIDC2AG012390.2
Tdi_TRIDC1BG050770.1
Tdi_TRIDC1BG030460.2
Tdi_TRIDC1AG044790.3
Tdi_TRIDC1AG025340.3
−1000
0
1000
2000
3000
4000

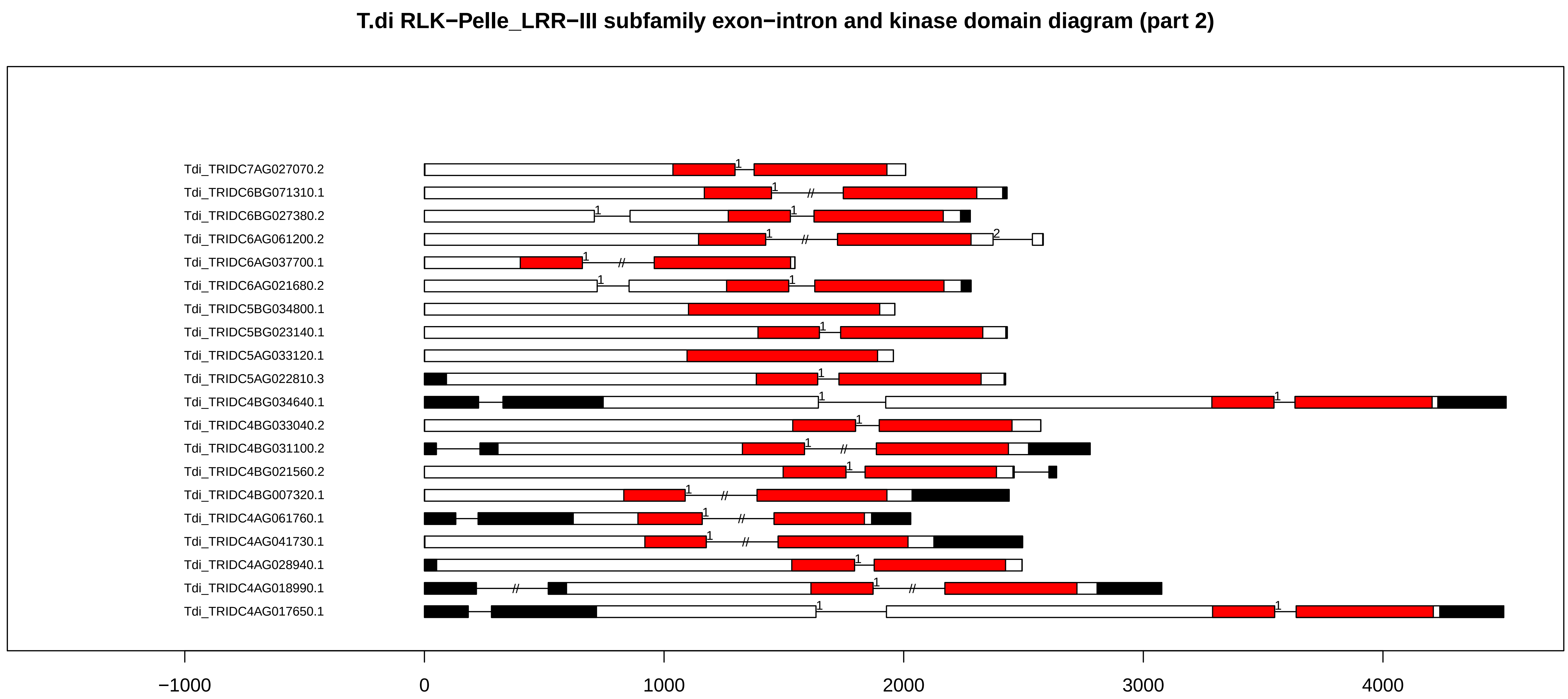
T.di RLK−Pelle_LRR−III subfamily exon−intron and kinase domain diagram (part 2)
Tdi_TRIDC7AG027070.2
Tdi_TRIDC6BG071310.1
Tdi_TRIDC6BG027380.2
Tdi_TRIDC6AG061200.2
Tdi_TRIDC6AG037700.1
Tdi_TRIDC6AG021680.2
Tdi_TRIDC5BG034800.1
Tdi_TRIDC5BG023140.1
Tdi_TRIDC5AG033120.1
Tdi_TRIDC5AG022810.3
Tdi_TRIDC4BG034640.1
Tdi_TRIDC4BG033040.2
Tdi_TRIDC4BG031100.2
Tdi_TRIDC4BG021560.2
Tdi_TRIDC4BG007320.1
Tdi_TRIDC4AG061760.1
Tdi_TRIDC4AG041730.1
Tdi_TRIDC4AG028940.1
Tdi_TRIDC4AG018990.1
Tdi_TRIDC4AG017650.1
−1000
0
1000
2000
3000
4000

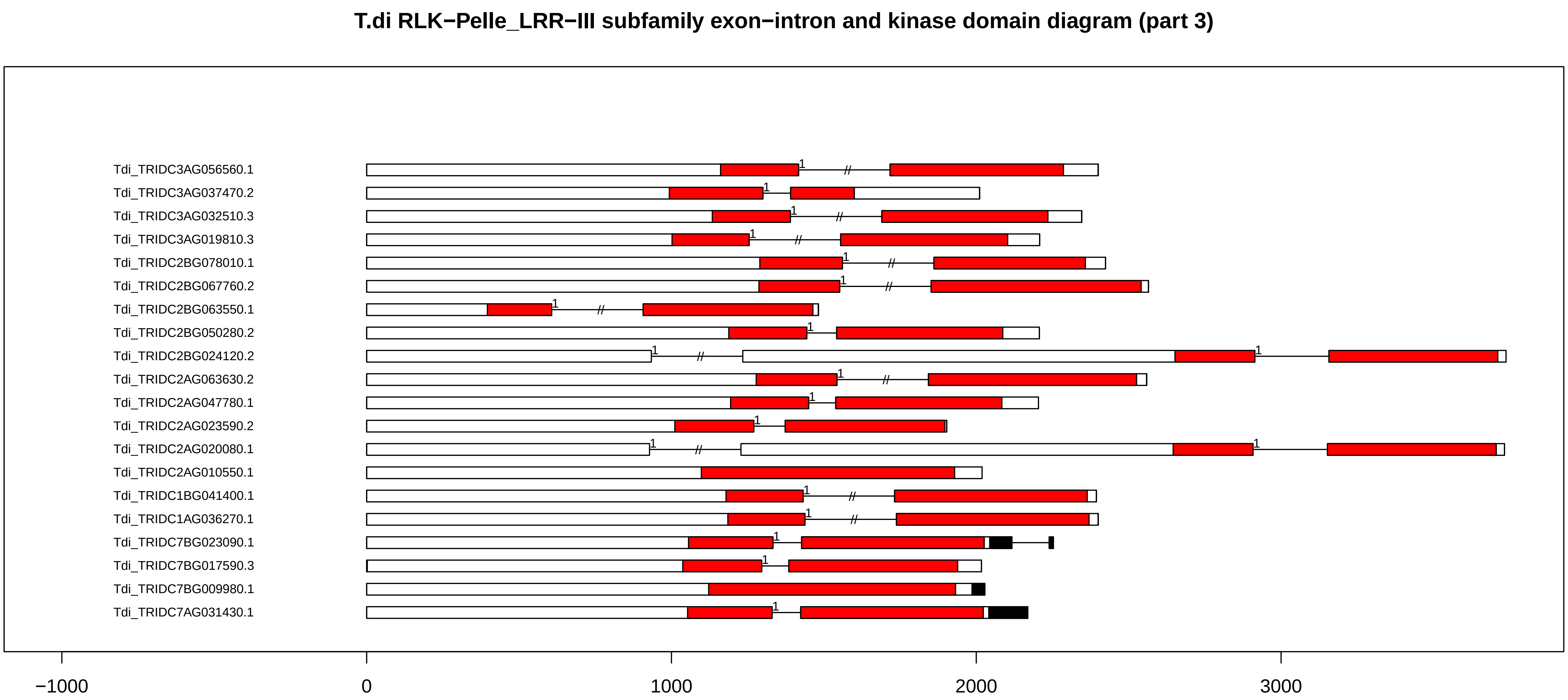
T.di RLK−Pelle_LRR−III subfamily exon−intron and kinase domain diagram (part 3)
Tdi_TRIDC3AG056560.1
Tdi_TRIDC3AG037470.2
Tdi_TRIDC3AG032510.3
Tdi_TRIDC3AG019810.3
Tdi_TRIDC2BG078010.1
Tdi_TRIDC2BG067760.2
Tdi_TRIDC2BG063550.1
Tdi_TRIDC2BG050280.2
Tdi_TRIDC2BG024120.2
Tdi_TRIDC2AG063630.2
Tdi_TRIDC2AG047780.1
Tdi_TRIDC2AG023590.2
Tdi_TRIDC2AG020080.1
Tdi_TRIDC2AG010550.1
Tdi_TRIDC1BG041400.1
Tdi_TRIDC1AG036270.1
Tdi_TRIDC7BG023090.1
Tdi_TRIDC7BG017590.3
Tdi_TRIDC7BG009980.1
Tdi_TRIDC7AG031430.1
−1000
0
1000
2000
3000

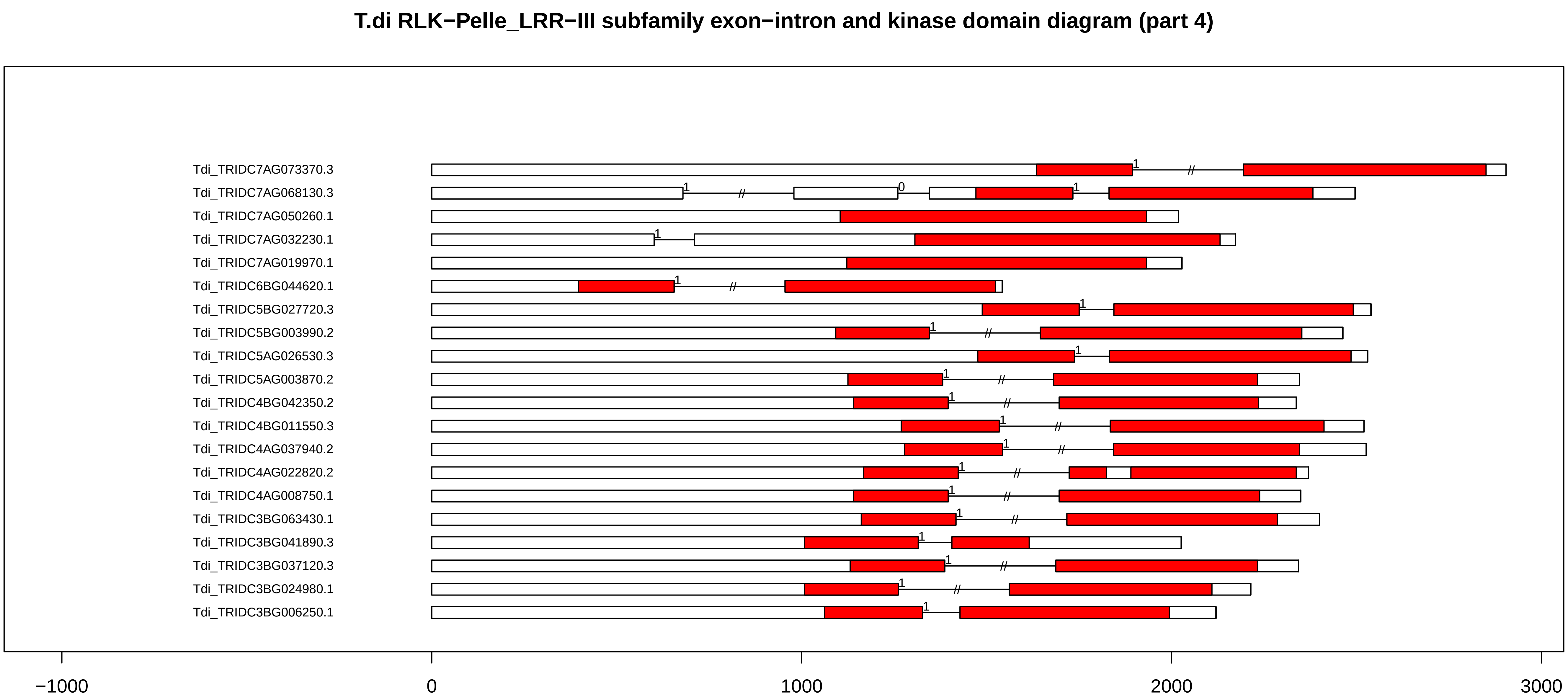
T.di RLK−Pelle_LRR−III subfamily exon−intron and kinase domain diagram (part 4)
Tdi_TRIDC7AG073370.3
Tdi_TRIDC7AG068130.3
Tdi_TRIDC7AG050260.1
Tdi_TRIDC7AG032230.1
Tdi_TRIDC7AG019970.1
Tdi_TRIDC6BG044620.1
Tdi_TRIDC5BG027720.3
Tdi_TRIDC5BG003990.2
Tdi_TRIDC5AG026530.3
Tdi_TRIDC5AG003870.2
Tdi_TRIDC4BG042350.2
Tdi_TRIDC4BG011550.3
Tdi_TRIDC4AG037940.2
Tdi_TRIDC4AG022820.2
Tdi_TRIDC4AG008750.1
Tdi_TRIDC3BG063430.1
Tdi_TRIDC3BG041890.3
Tdi_TRIDC3BG037120.3
Tdi_TRIDC3BG024980.1
Tdi_TRIDC3BG006250.1
−1000
0
1000
2000
3000

T.di RLK−Pelle_LRR−III subfamily exon−intron and kinase domain diagram (part 5)

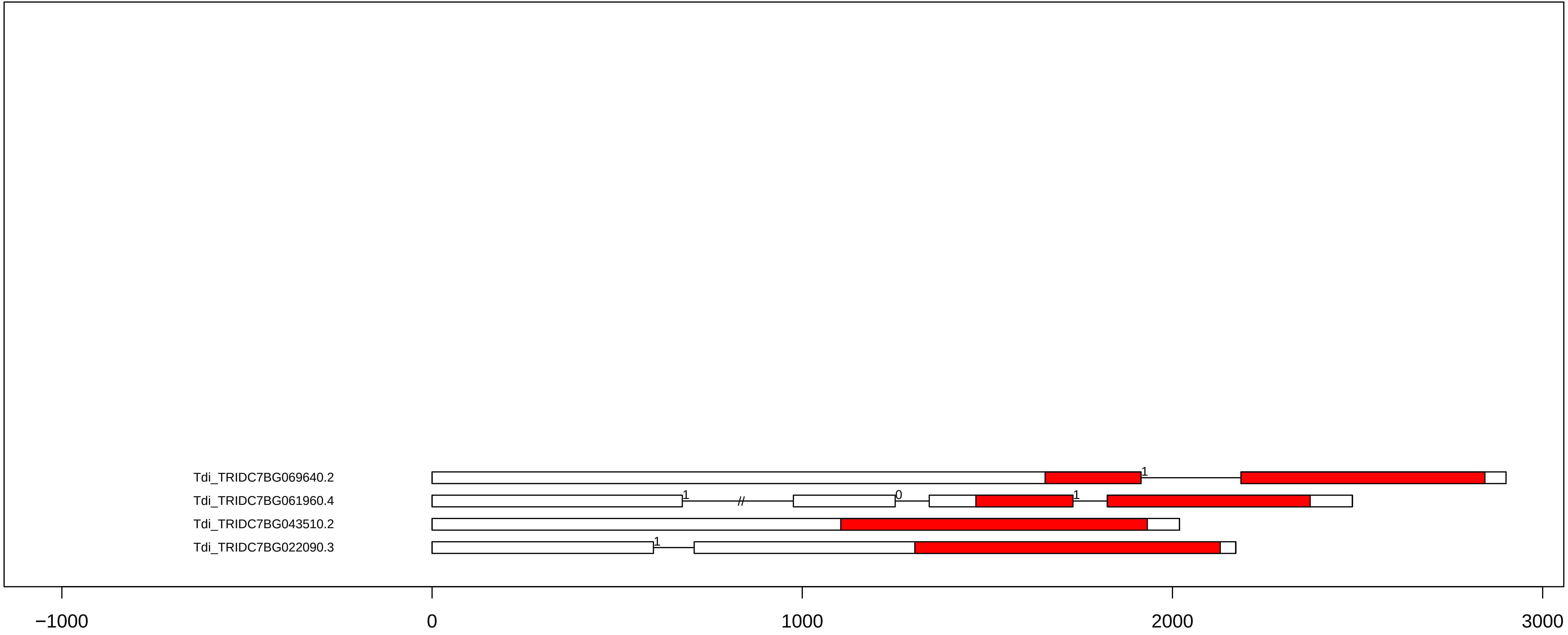

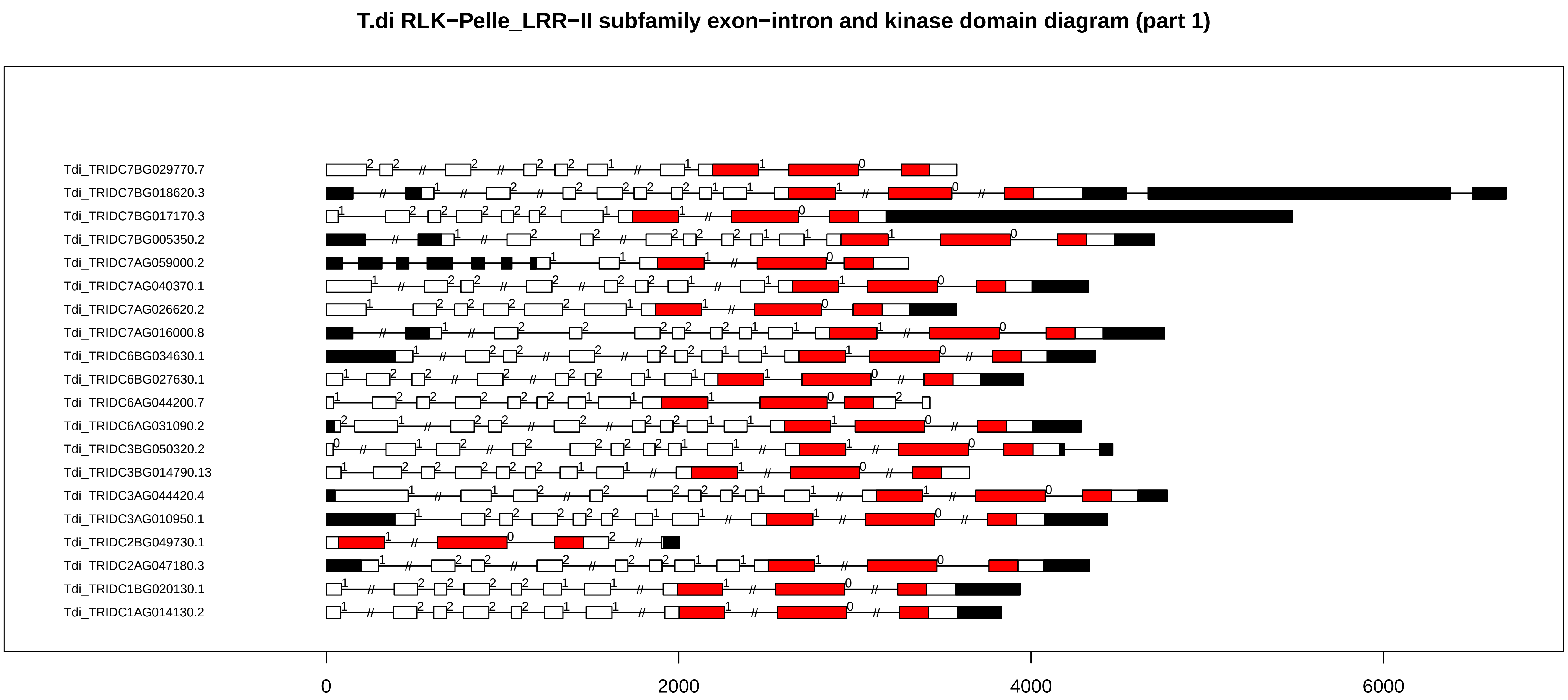
T.di RLK−Pelle_LRR−II subfamily exon−intron and kinase domain diagram (part 1)
Tdi_TRIDC7BG029770.7
Tdi_TRIDC7BG018620.3
Tdi_TRIDC7BG017170.3
Tdi_TRIDC7BG005350.2
Tdi_TRIDC7AG059000.2
Tdi_TRIDC7AG040370.1
Tdi_TRIDC7AG026620.2
Tdi_TRIDC7AG016000.8
Tdi_TRIDC6BG034630.1
Tdi_TRIDC6BG027630.1
Tdi_TRIDC6AG044200.7
Tdi_TRIDC6AG031090.2
Tdi_TRIDC3BG050320.2
Tdi_TRIDC3BG014790.13
Tdi_TRIDC3AG044420.4
Tdi_TRIDC3AG010950.1
Tdi_TRIDC2BG049730.1
Tdi_TRIDC2AG047180.3
Tdi_TRIDC1BG020130.1
Tdi_TRIDC1AG014130.2
0
2000
4000
6000

T.di RLK−Pelle_LRR−II subfamily exon−intron and kinase domain diagram (part 2)

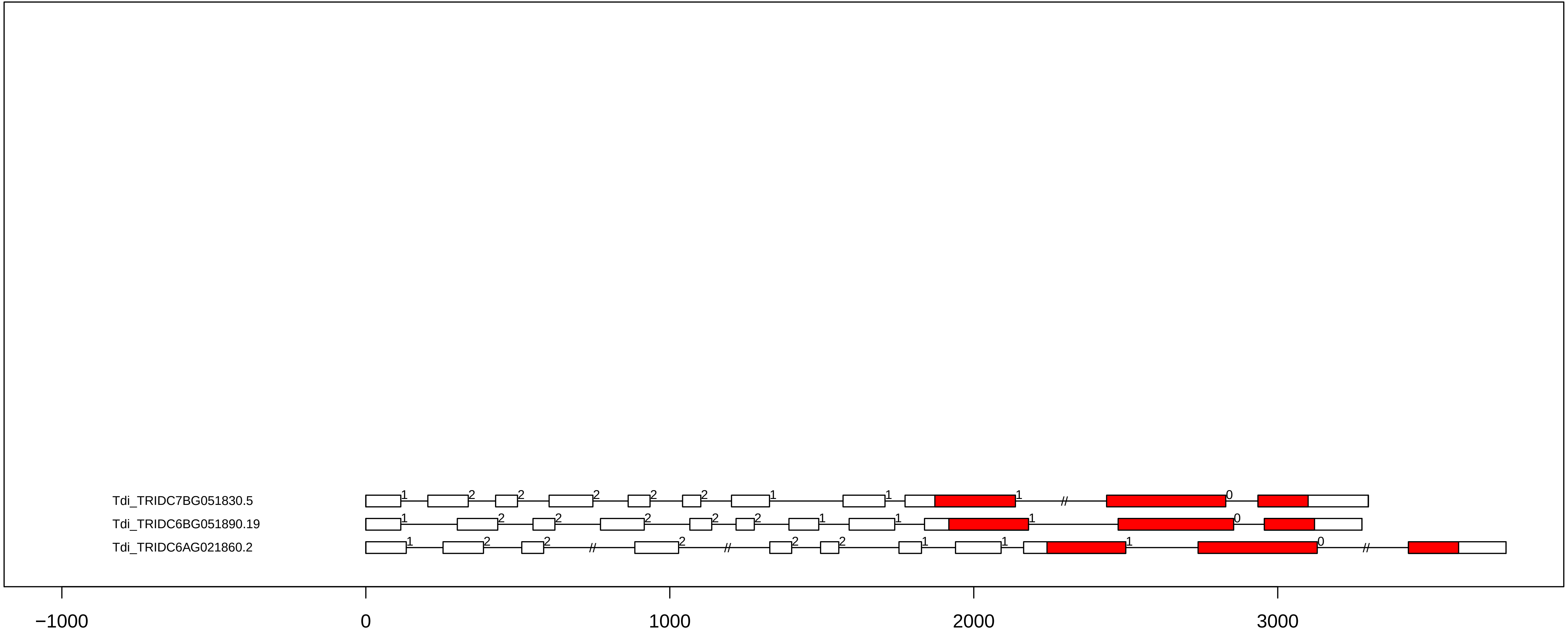

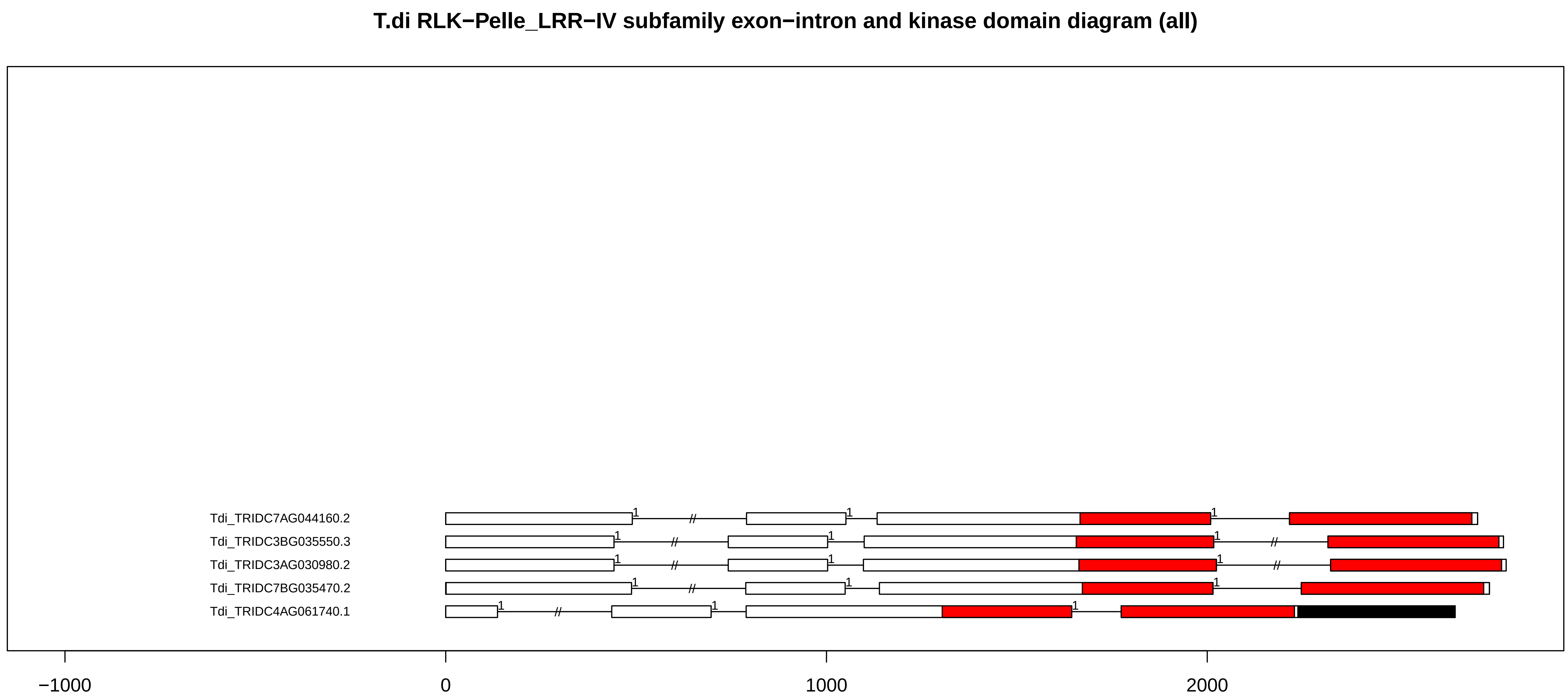

T.di RLK−Pelle_LRR−IV subfamily exon−intron and kinase domain diagram (all)
Tdi_TRIDC7AG044160.2
Tdi_TRIDC3BG035550.3
Tdi_TRIDC3AG030980.2
Tdi_TRIDC7BG035470.2
Tdi_TRIDC4AG061740.1
−1000
0
1000
2000

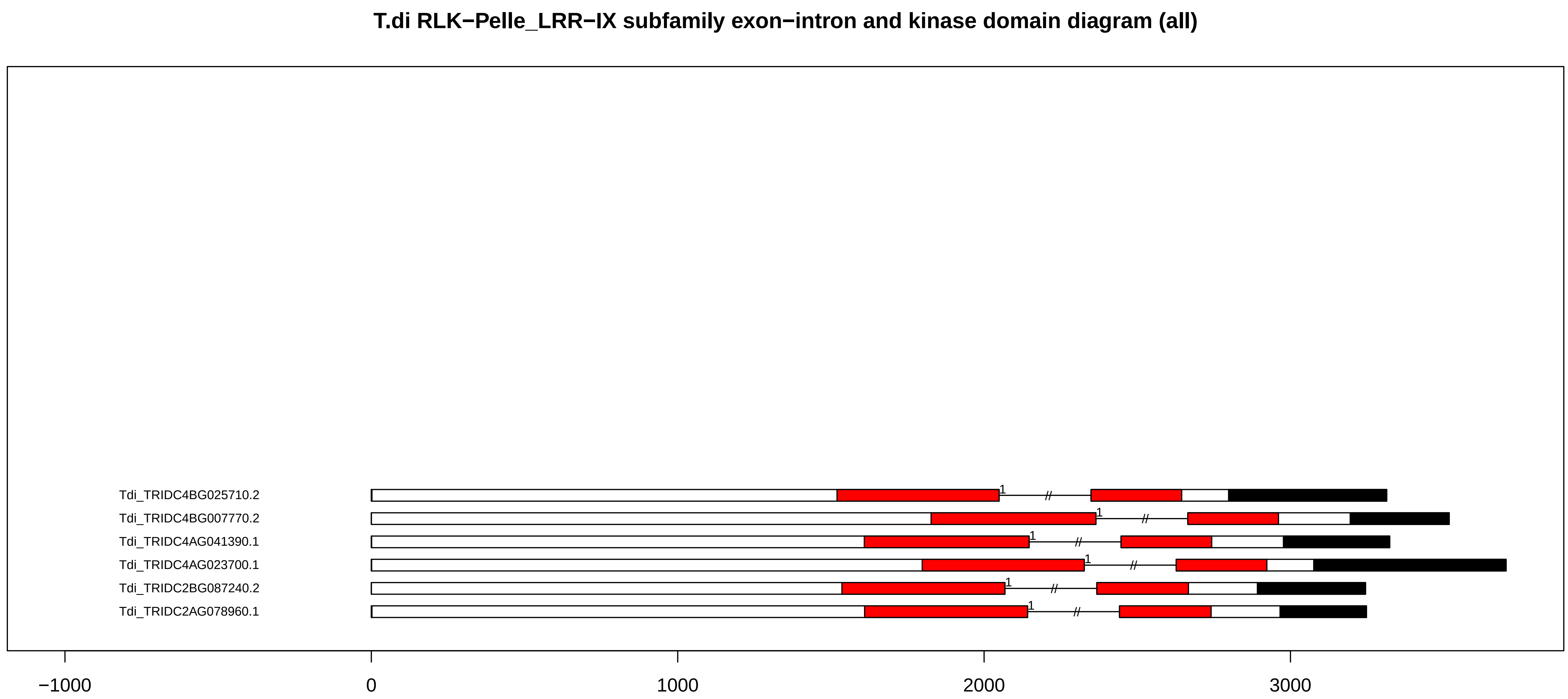
T.di RLK−Pelle_LRR−IX subfamily exon−intron and kinase domain diagram (all)
Tdi_TRIDC4BG025710.2
Tdi_TRIDC4BG007770.2
Tdi_TRIDC4AG041390.1
Tdi_TRIDC4AG023700.1
Tdi_TRIDC2BG087240.2
Tdi_TRIDC2AG078960.1
1
−1000
0
1000
2000
3000

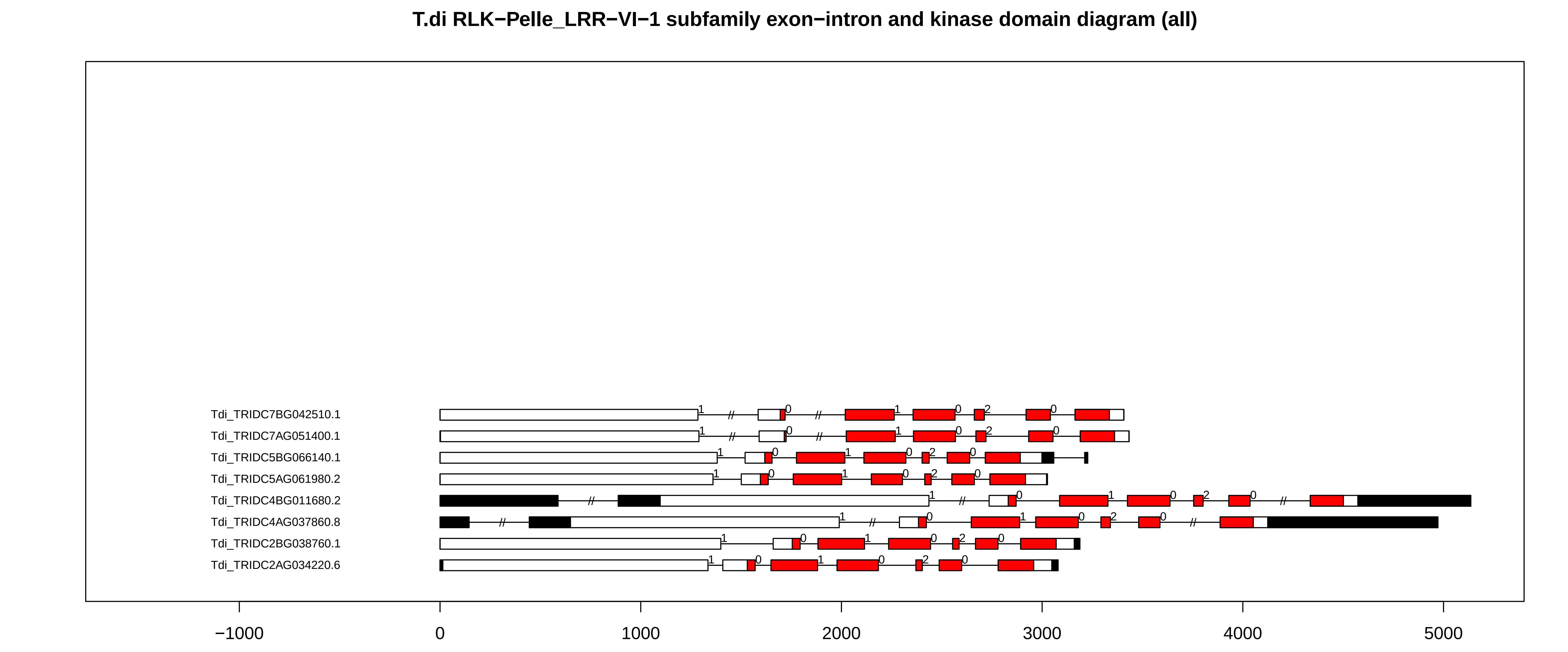
T.di RLK−Pelle_LRR−VI−1 subfamily exon−intron and kinase domain diagram (all)
Tdi_TRIDC7BG042510.1
Tdi_TRIDC7AG051400.1
Tdi_TRIDC5BG066140.1
Tdi_TRIDC5AG061980.2
Tdi_TRIDC4BG011680.2
Tdi_TRIDC4AG037860.8
Tdi_TRIDC2BG038760.1
Tdi_TRIDC2AG034220.6
−1000
0
1000
2000
3000
4000
5000

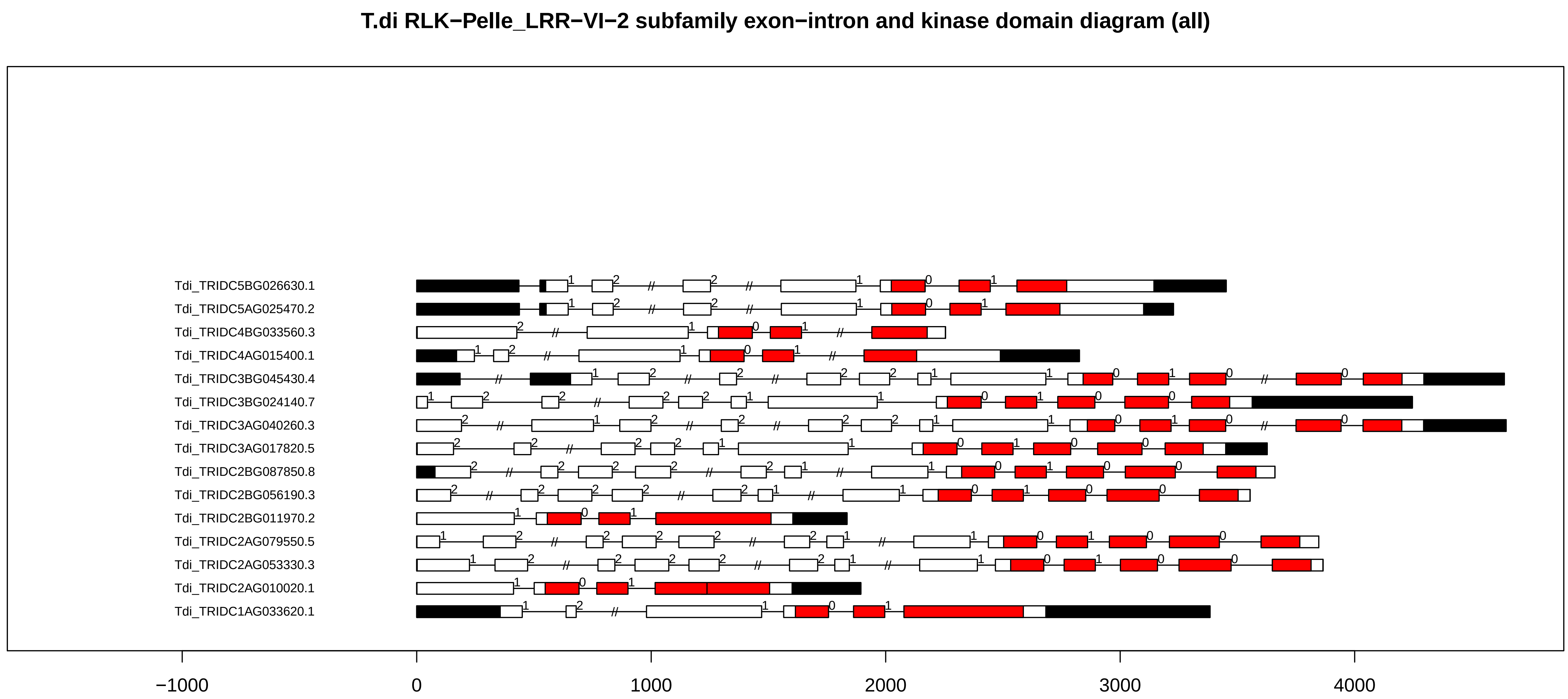

T.di RLK−Pelle_LRR−VI−2 subfamily exon−intron and kinase domain diagram (all)
Tdi_TRIDC5BG026630.1
Tdi_TRIDC5AG025470.2
Tdi_TRIDC4BG033560.3
Tdi_TRIDC4AG015400.1
Tdi_TRIDC3BG045430.4
Tdi_TRIDC3BG024140.7
Tdi_TRIDC3AG040260.3
Tdi_TRIDC3AG017820.5
Tdi_TRIDC2BG087850.8
Tdi_TRIDC2BG056190.3
Tdi_TRIDC2BG011970.2
Tdi_TRIDC2AG079550.5
Tdi_TRIDC2AG053330.3
Tdi_TRIDC2AG010020.1
Tdi_TRIDC1AG033620.1
−1000
0
1000
2000
3000
4000

T.di RLK−Pelle_LRR−VII−1 subfamily exon−intron and kinase domain diagram (all)

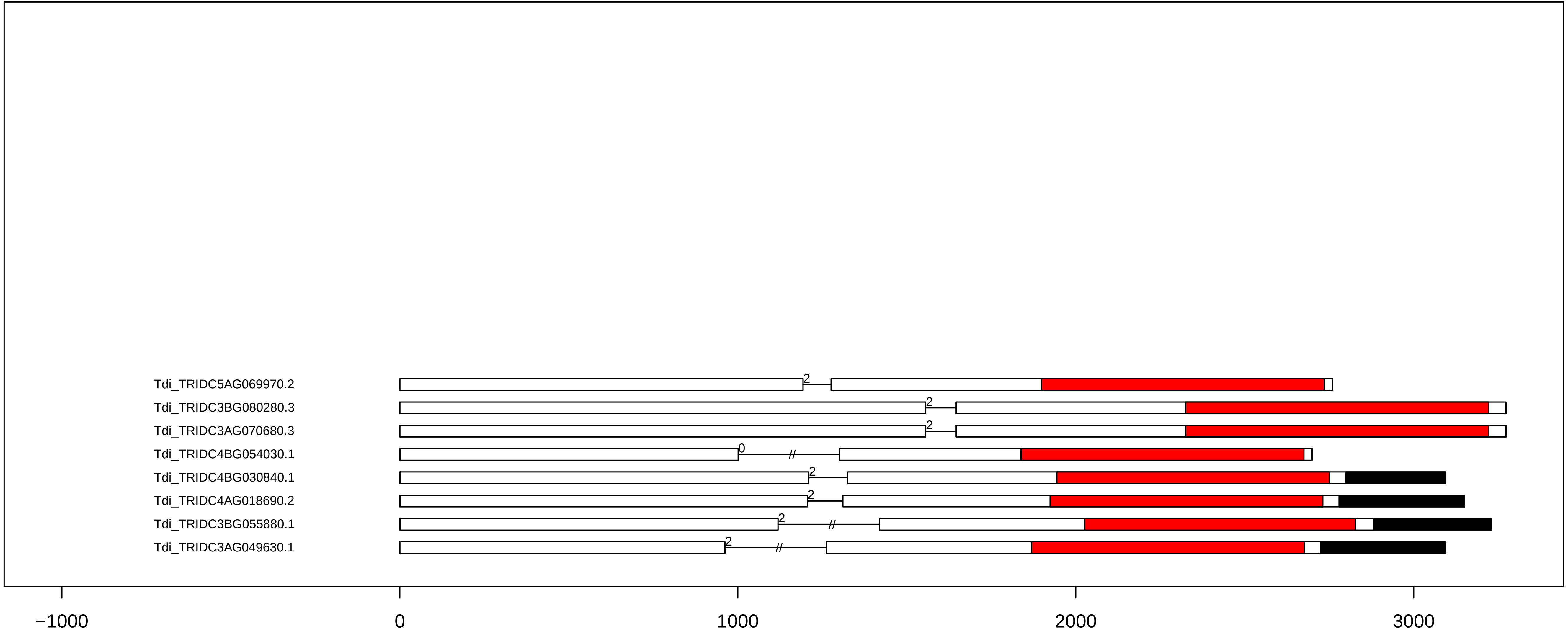

T.di RLK−Pelle_LRR−VII−2 subfamily exon−intron and kinase domain diagram (all)

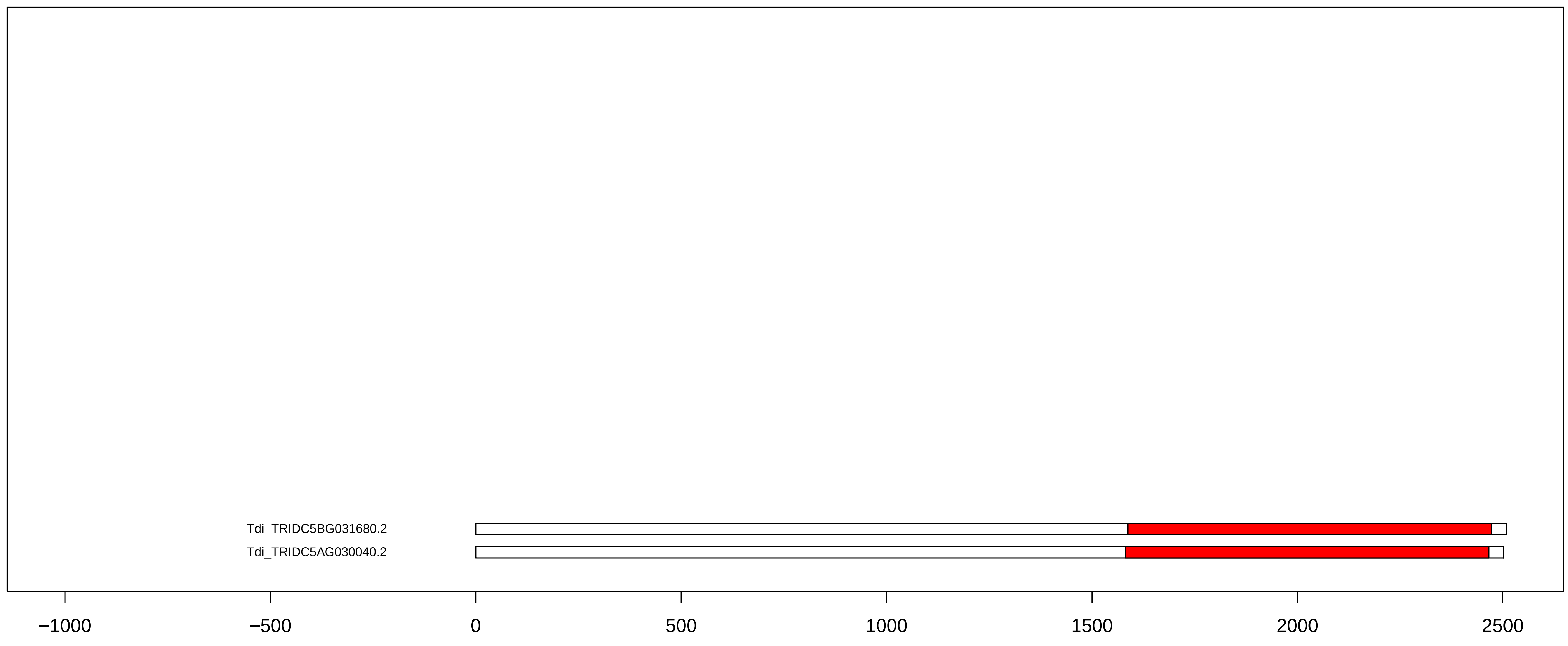

T.di RLK−Pelle_LRR−VII−3 subfamily exon−intron and kinase domain diagram (all)

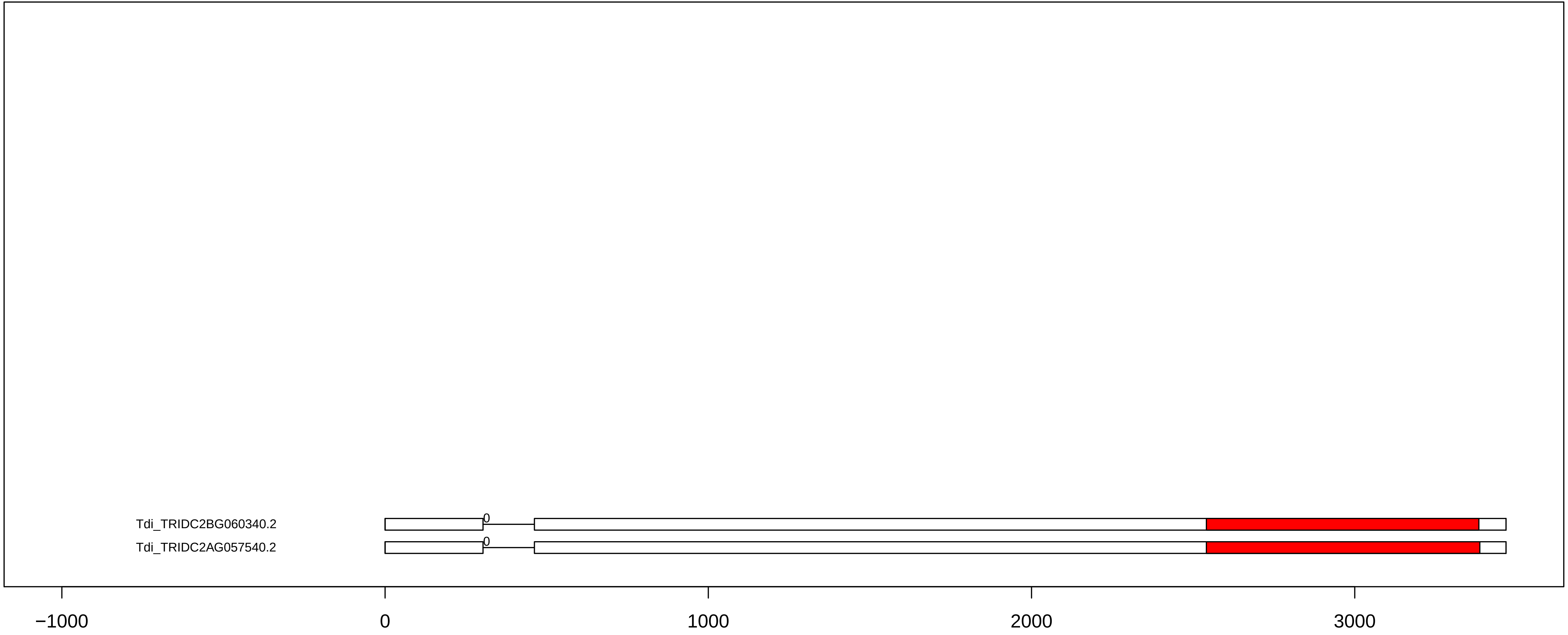

T.di RLK−Pelle_LRR−VIII−1 subfamily exon−intron and kinase domain diagram (all)

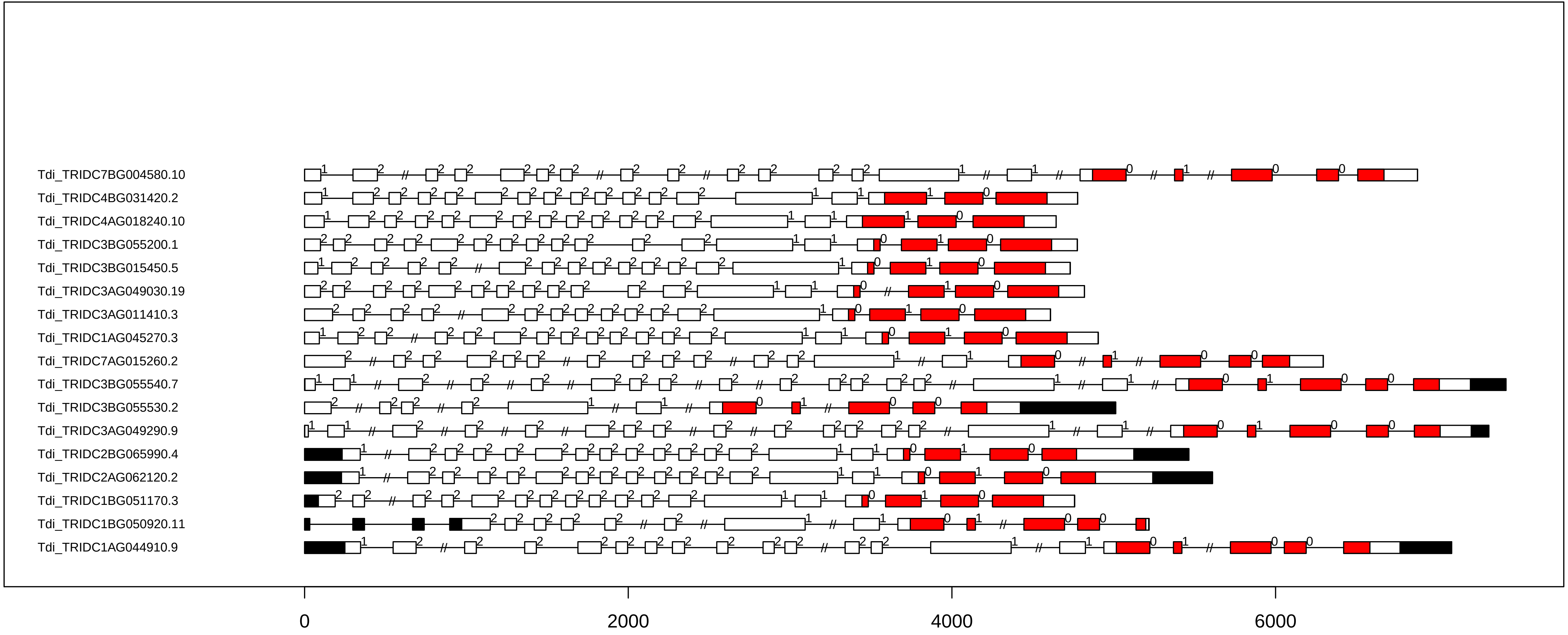

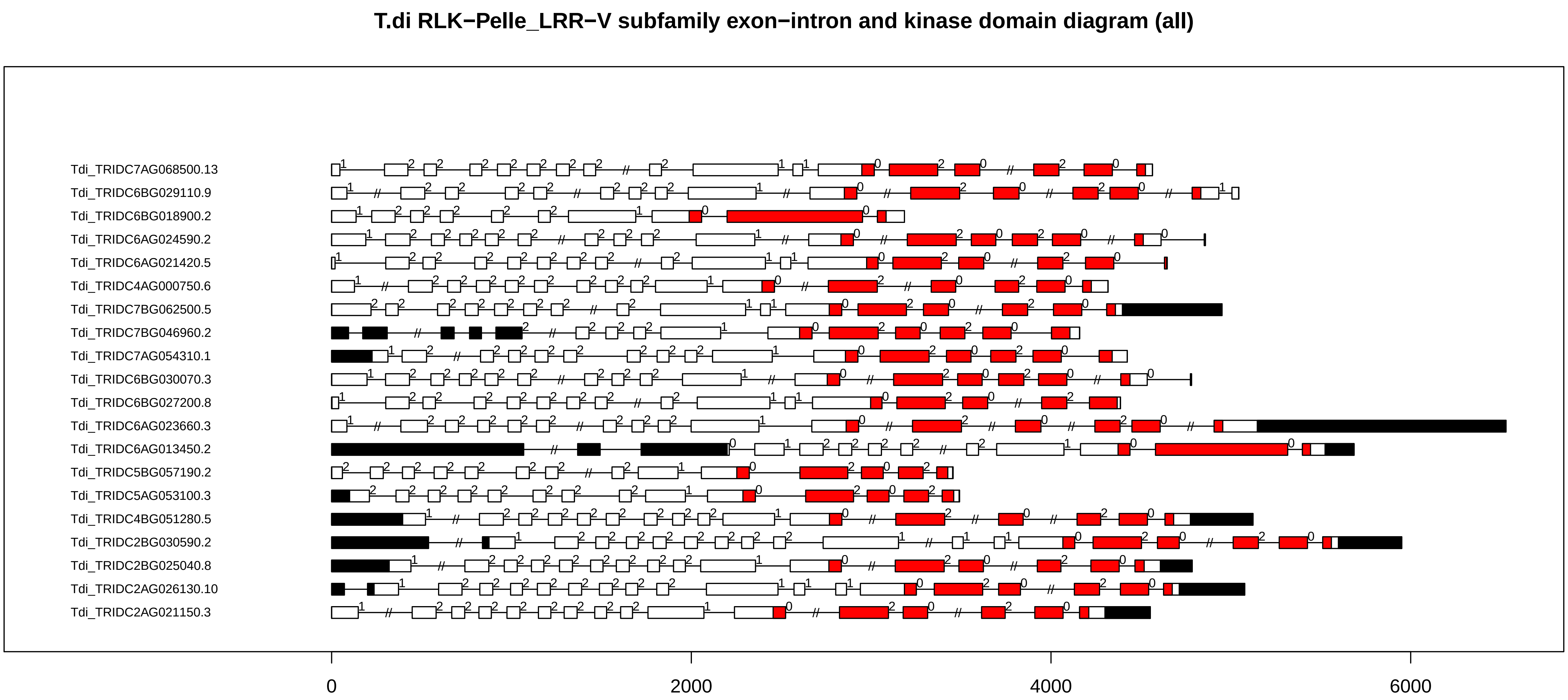
T.di RLK−Pelle_LRR−V subfamily exon−intron and kinase domain diagram (all)
Tdi_TRIDC7AG068500.13
Tdi_TRIDC6BG029110.9
Tdi_TRIDC6BG018900.2
Tdi_TRIDC6AG024590.2
Tdi_TRIDC6AG021420.5
Tdi_TRIDC4AG000750.6
Tdi_TRIDC7BG062500.5
Tdi_TRIDC7BG046960.2
Tdi_TRIDC7AG054310.1
Tdi_TRIDC6BG030070.3
Tdi_TRIDC6BG027200.8
Tdi_TRIDC6AG023660.3
Tdi_TRIDC6AG013450.2
Tdi_TRIDC5BG057190.2
Tdi_TRIDC5AG053100.3
Tdi_TRIDC4BG051280.5
Tdi_TRIDC2BG030590.2
Tdi_TRIDC2BG025040.8
Tdi_TRIDC2AG026130.10
Tdi_TRIDC2AG021150.3
0
2000
4000
6000

T.di RLK−Pelle_LRR−Xa subfamily exon−intron and kinase domain diagram (all)

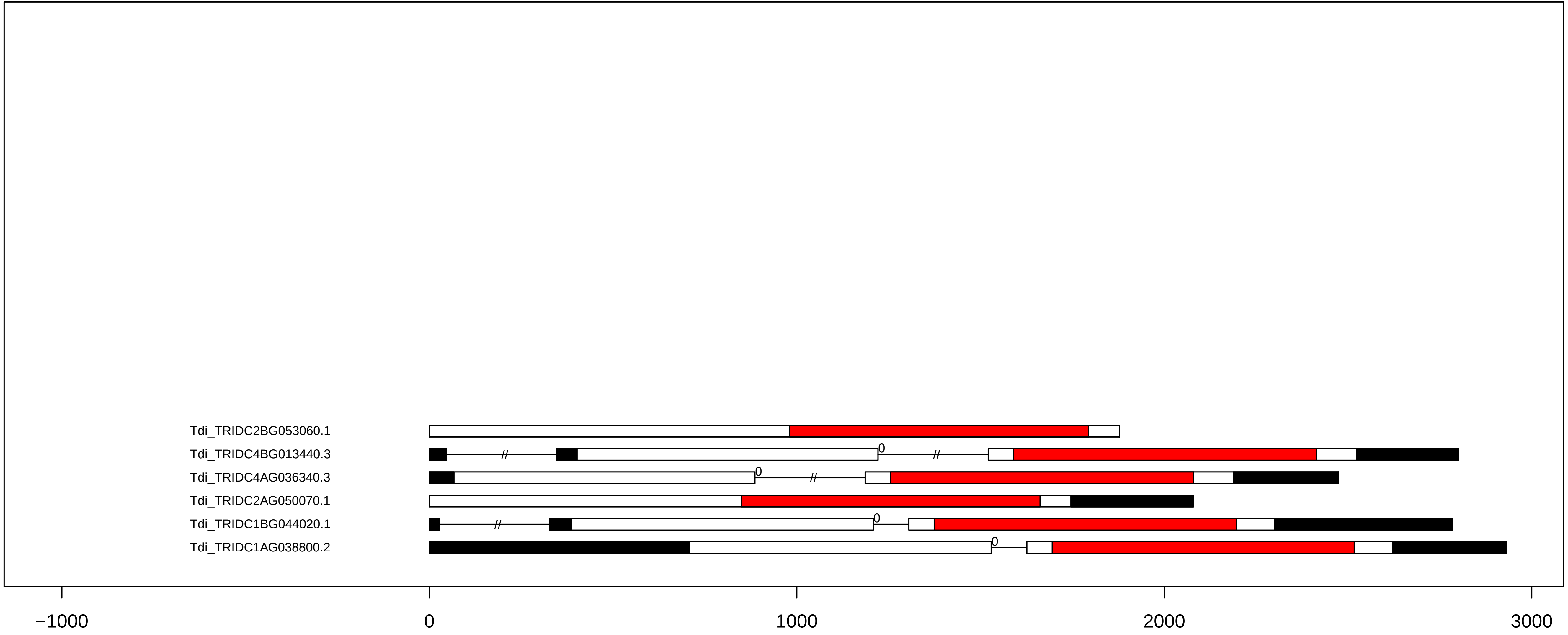

T.di RLK−Pelle_LRR−Xb−1 subfamily exon−intron and kinase domain diagram (part 1)

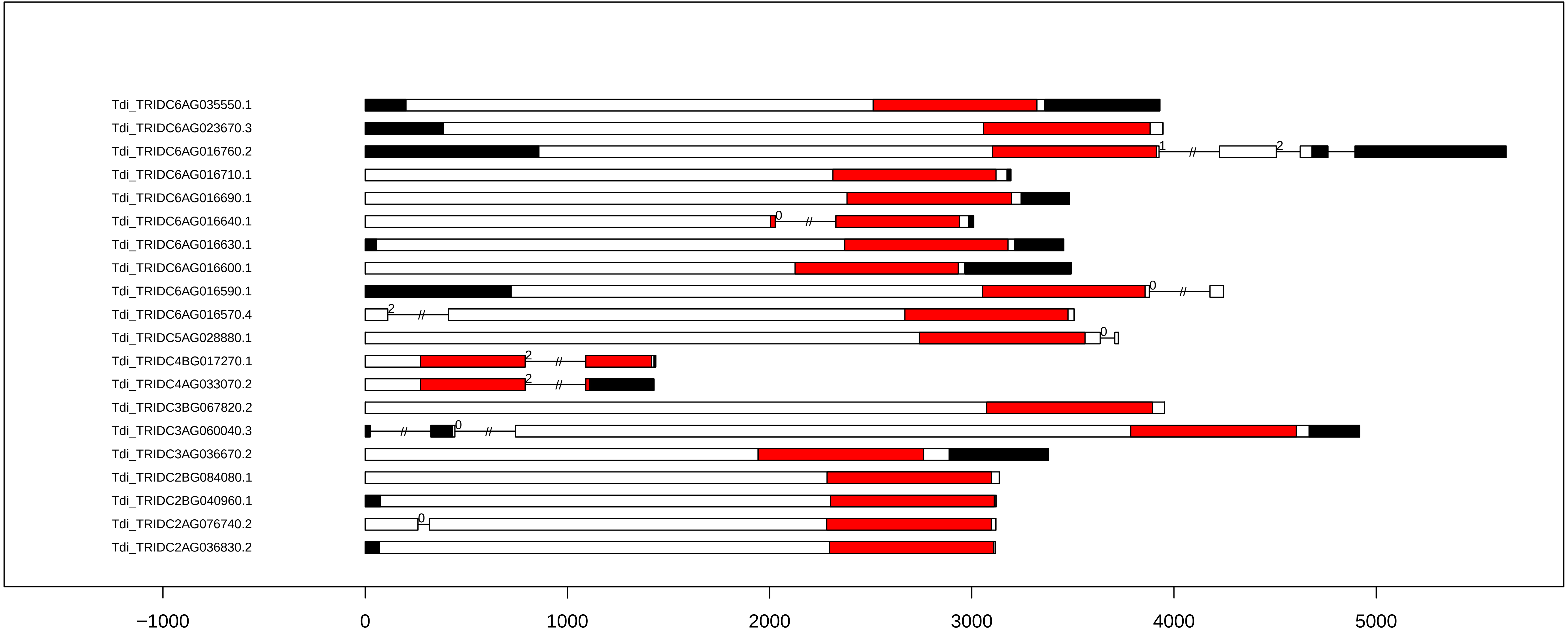

T.di RLK−Pelle_LRR−Xb−1 subfamily exon−intron and kinase domain diagram (part 2)

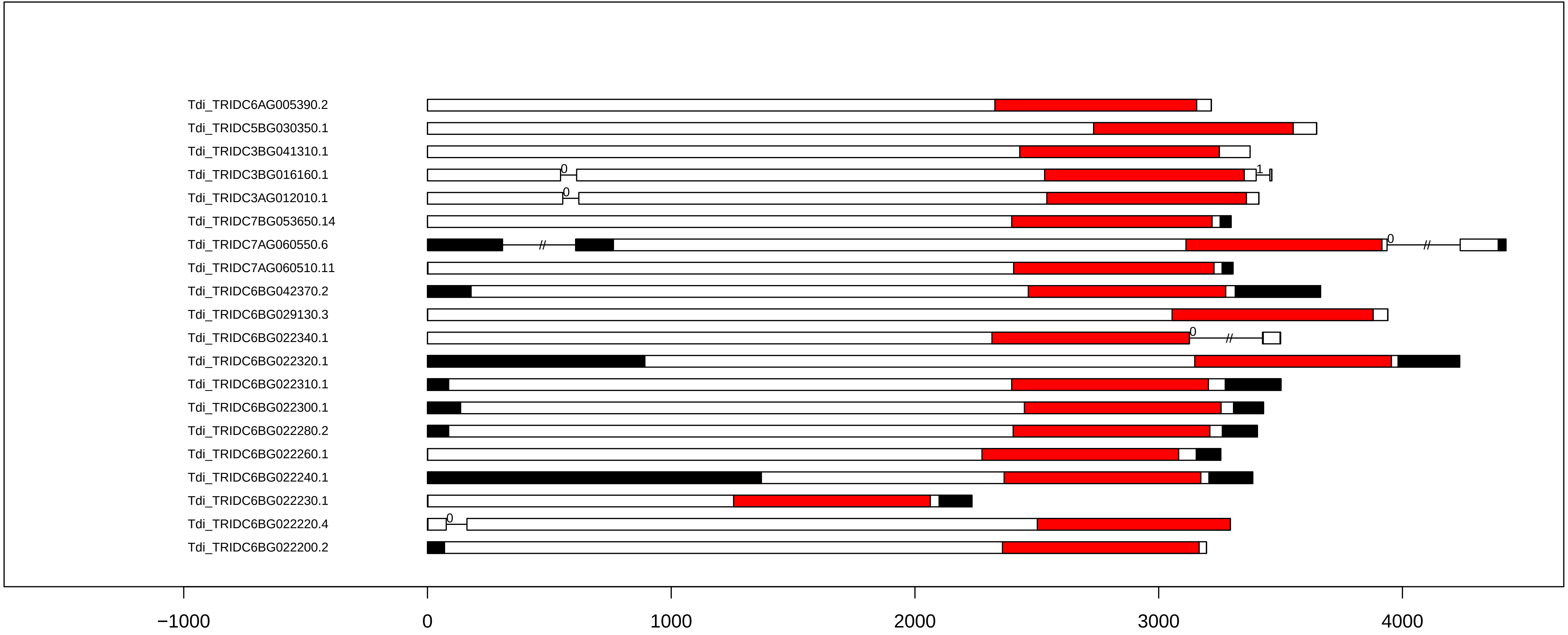

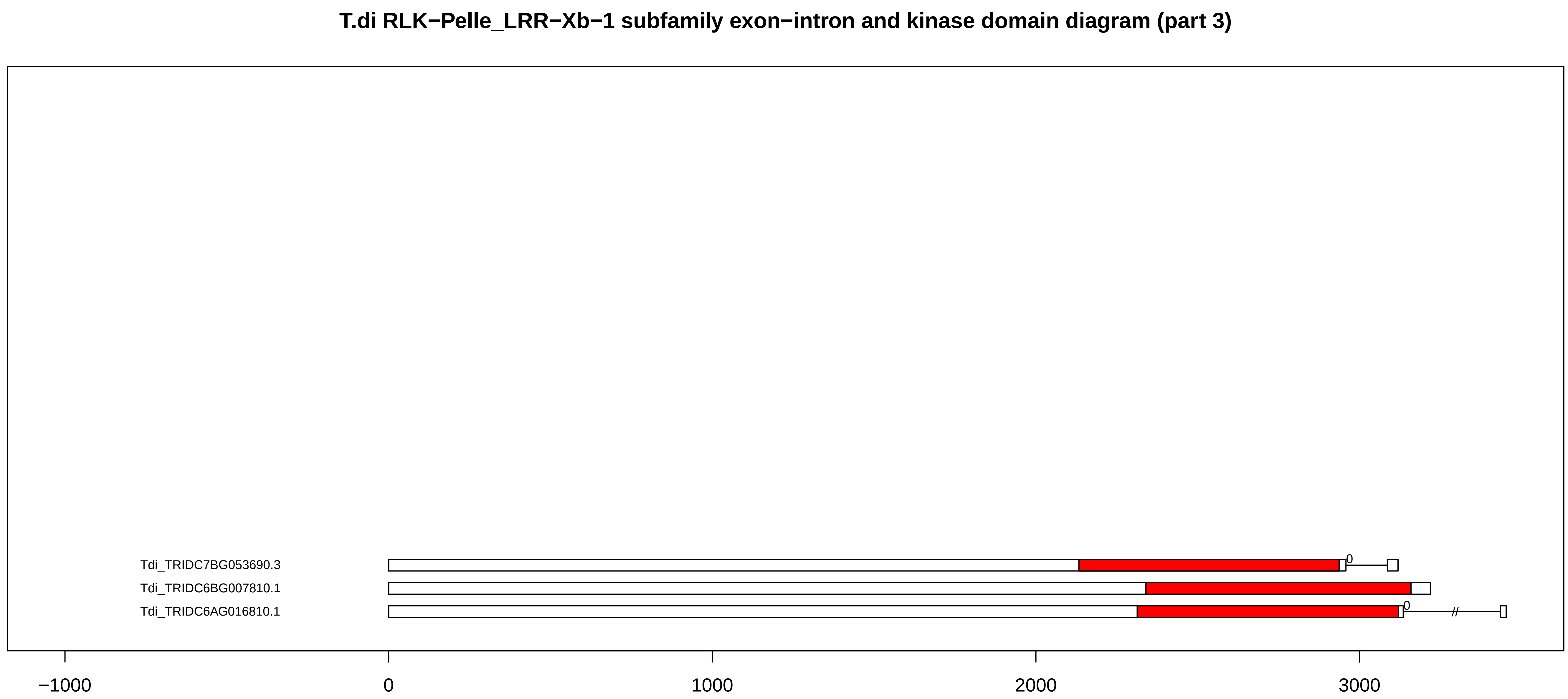
T.di RLK−Pelle_LRR−Xb−1 subfamily exon−intron and kinase domain diagram (part 3)
Tdi_TRIDC7BG053690.3
Tdi_TRIDC6BG007810.1
Tdi_TRIDC6AG016810.1
0
0
−1000
0
1000
2000
3000

T.di RLK−Pelle_LRR−Xb−2 subfamily exon−intron and kinase domain diagram (all)

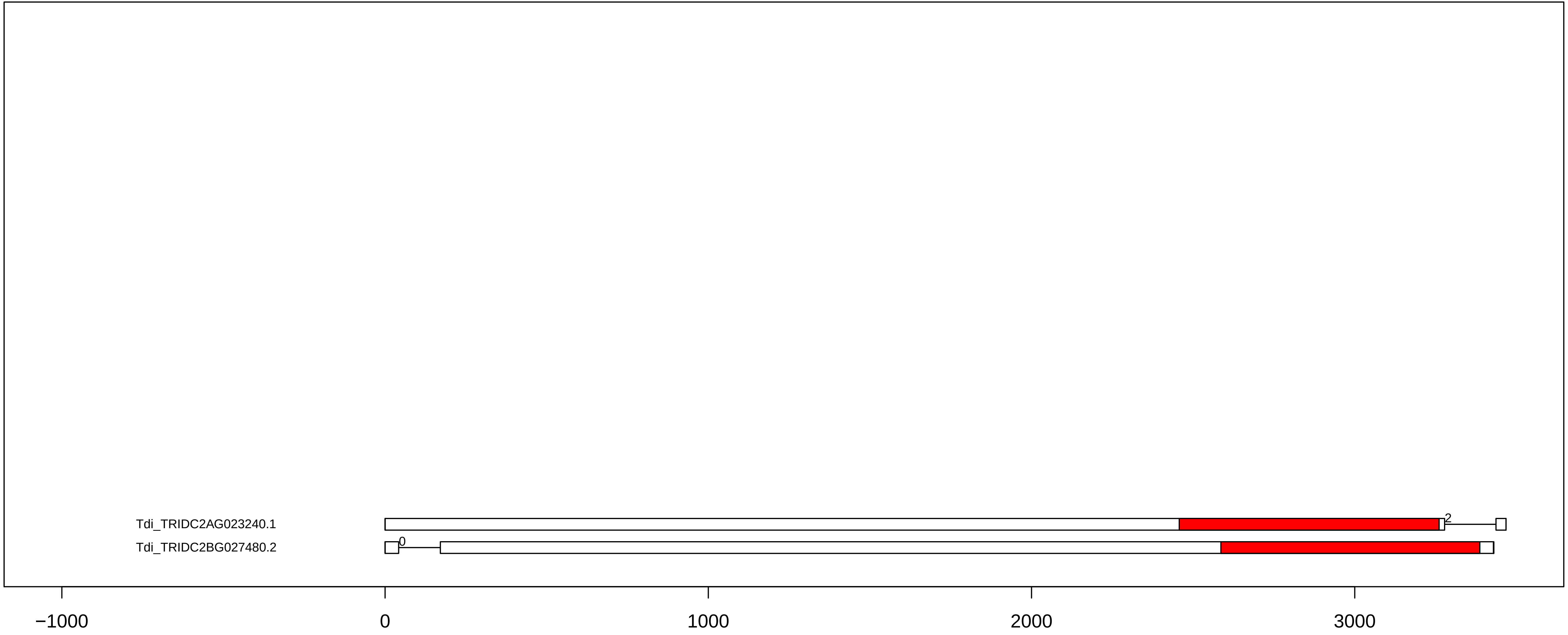

T.di RLK−Pelle_LRR−XI−1 subfamily exon−intron and kinase domain diagram (part 1)

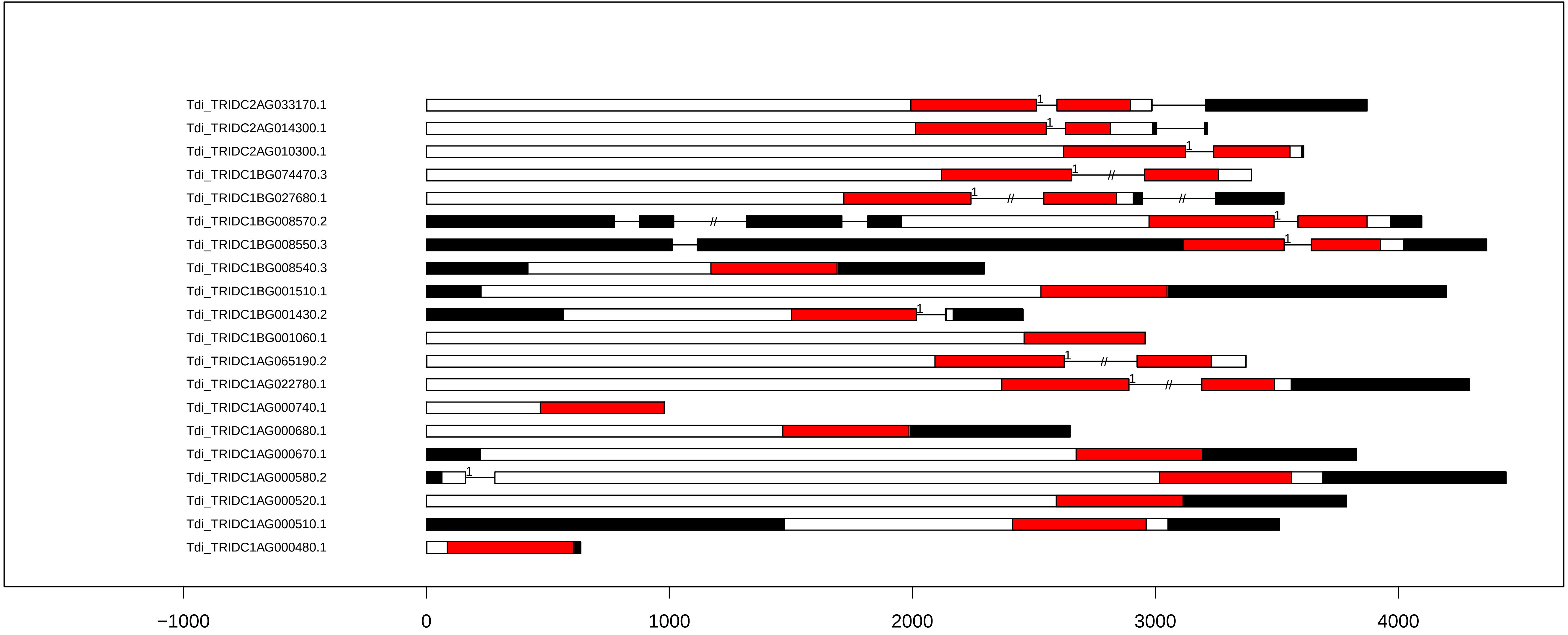

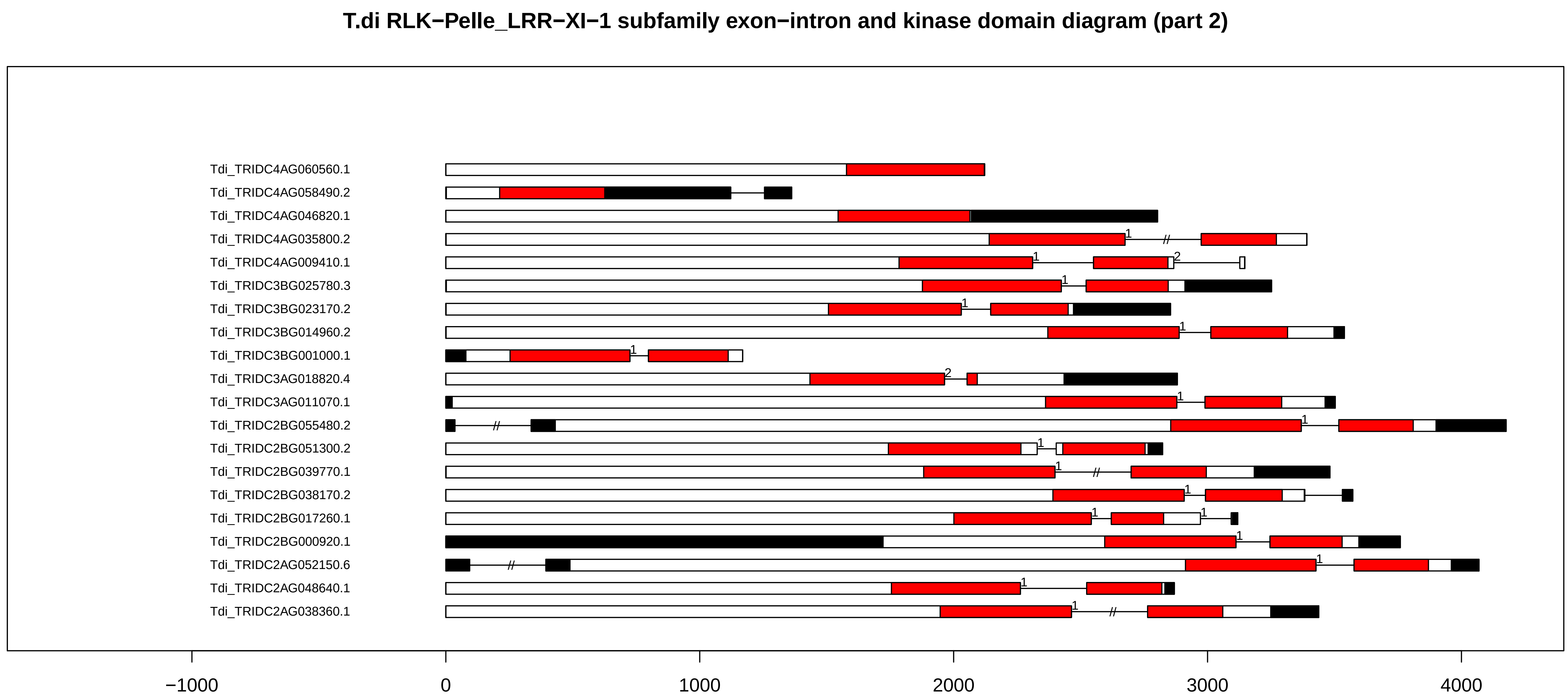
T.di RLK−Pelle_LRR−XI−1 subfamily exon−intron and kinase domain diagram (part 2)
Tdi_TRIDC4AG060560.1
Tdi_TRIDC4AG058490.2
Tdi_TRIDC4AG046820.1
Tdi_TRIDC4AG035800.2
Tdi_TRIDC4AG009410.1
Tdi_TRIDC3BG025780.3
Tdi_TRIDC3BG023170.2
Tdi_TRIDC3BG014960.2
Tdi_TRIDC3BG001000.1
Tdi_TRIDC3AG018820.4
Tdi_TRIDC3AG011070.1
Tdi_TRIDC2BG055480.2
Tdi_TRIDC2BG051300.2
Tdi_TRIDC2BG039770.1
Tdi_TRIDC2BG038170.2
Tdi_TRIDC2BG017260.1
Tdi_TRIDC2BG000920.1
Tdi_TRIDC2AG052150.6
Tdi_TRIDC2AG048640.1
Tdi_TRIDC2AG038360.1
−1000
0
1000
2000
3000
4000

T.di RLK−Pelle_LRR−XI−1 subfamily exon−intron and kinase domain diagram (part 3)

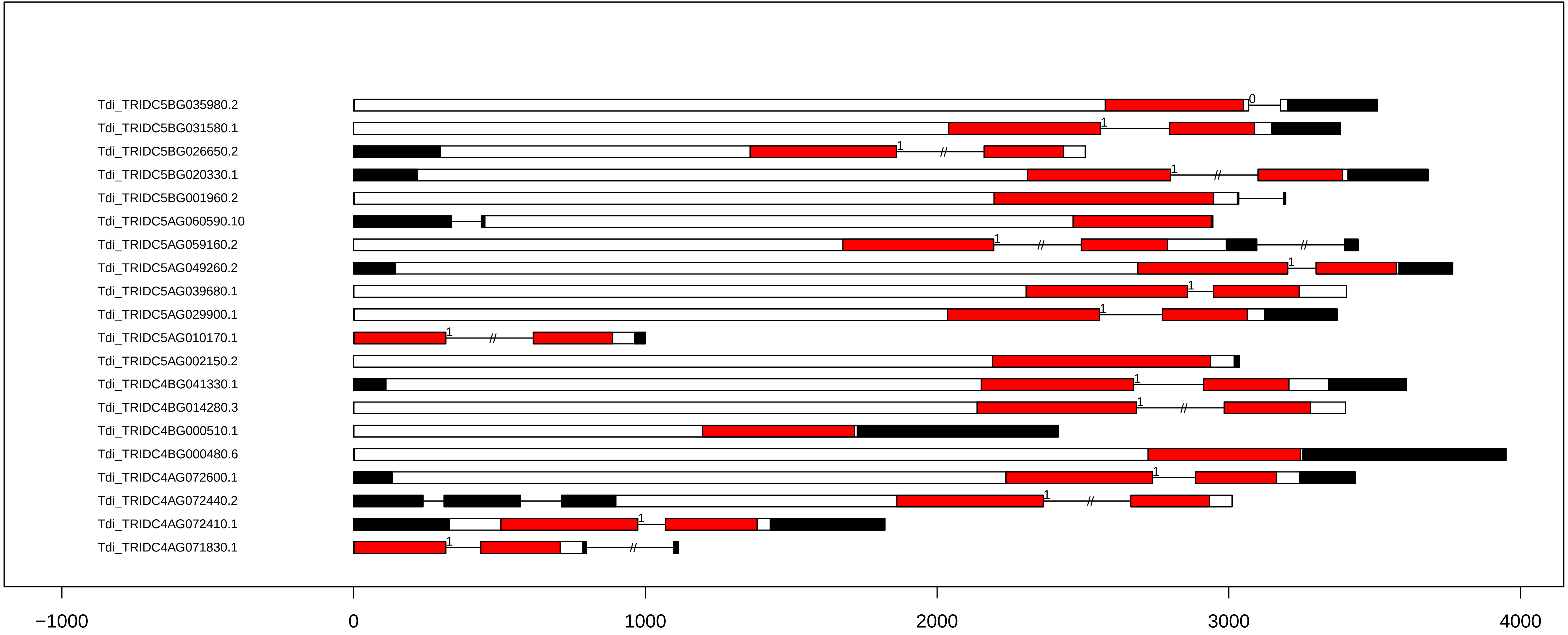

T.di RLK−Pelle_LRR−XI−1 subfamily exon−intron and kinase domain diagram (part 4)

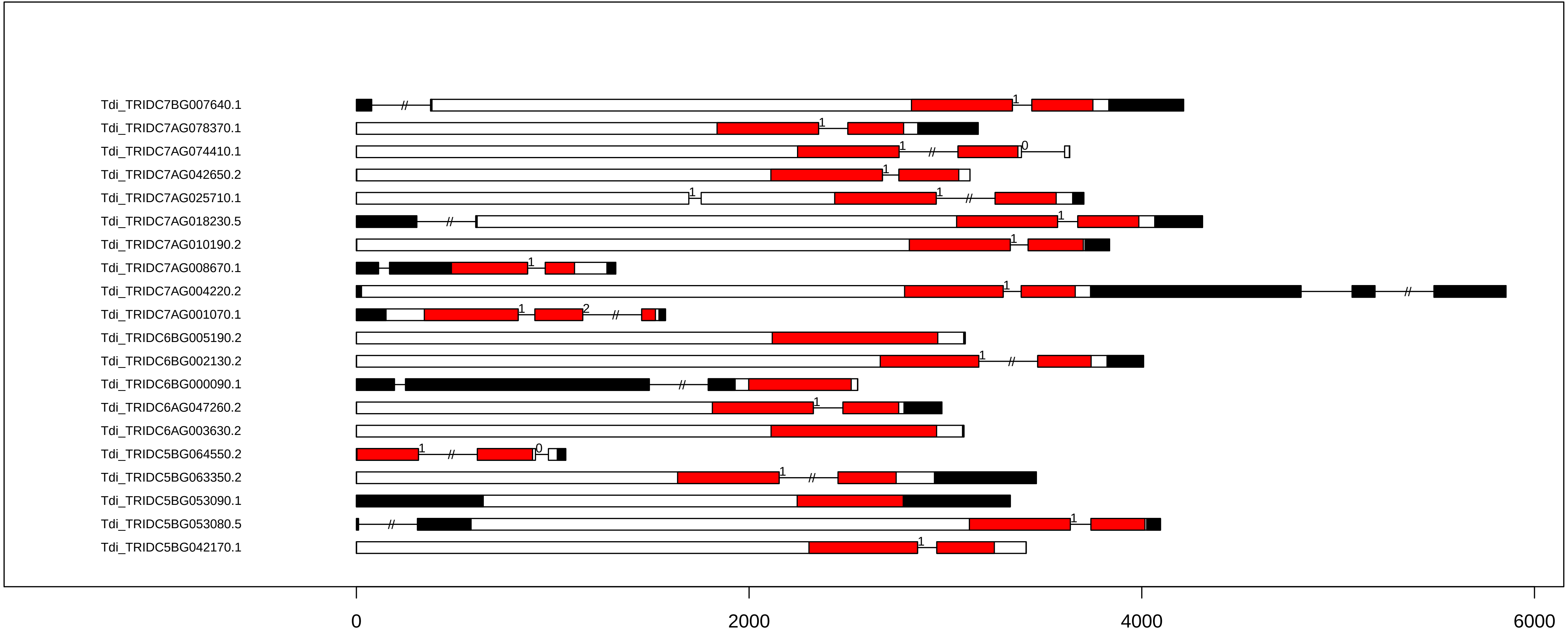

T.di RLK−Pelle_LRR−XI−1 subfamily exon−intron and kinase domain diagram (part 5)

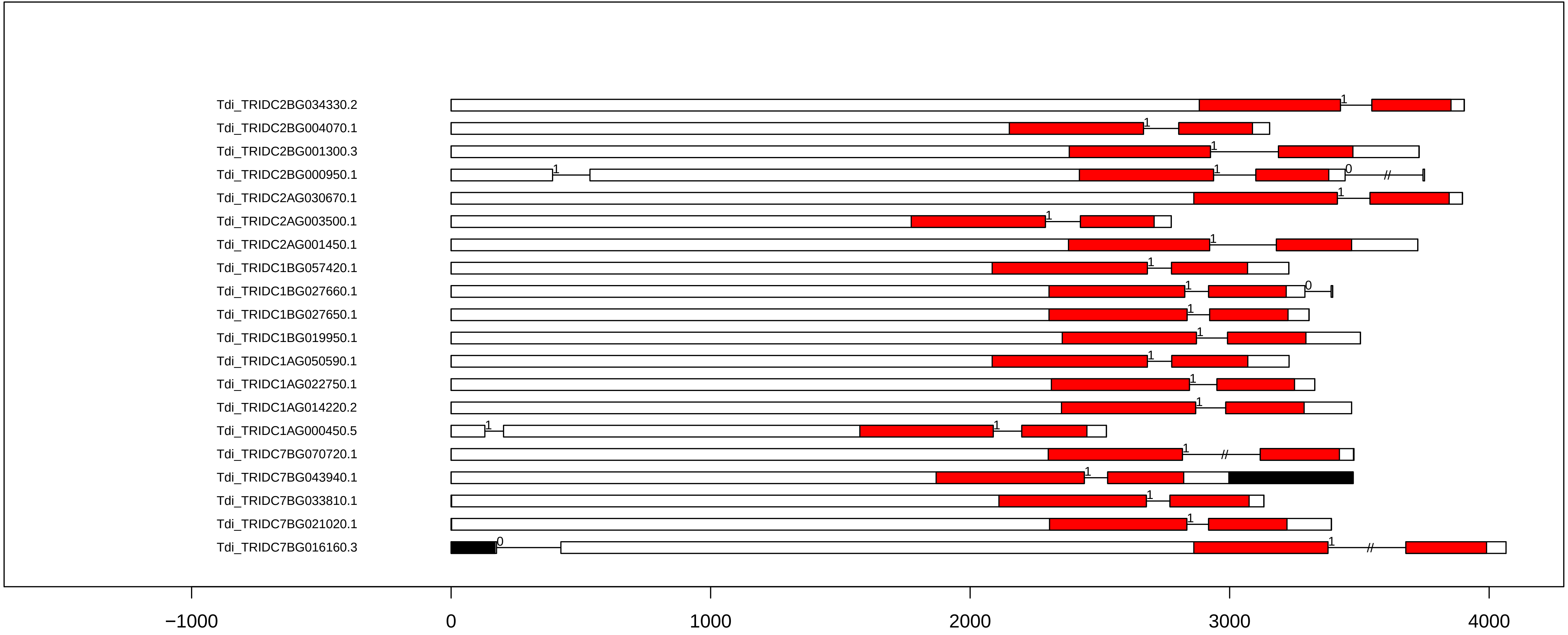

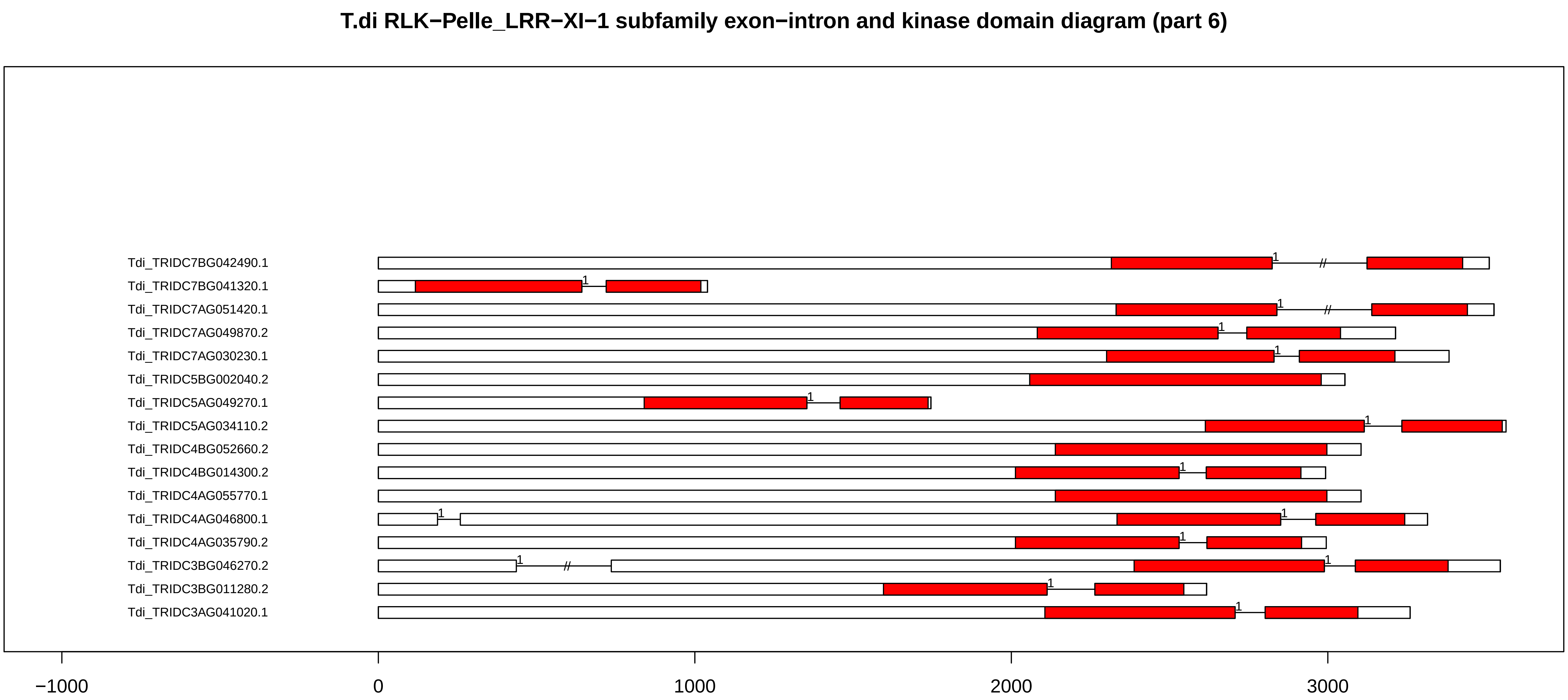
T.di RLK−Pelle_LRR−XI−1 subfamily exon−intron and kinase domain diagram (part 6)
Tdi_TRIDC7BG042490.1
Tdi_TRIDC7BG041320.1
Tdi_TRIDC7AG051420.1
Tdi_TRIDC7AG049870.2
Tdi_TRIDC7AG030230.1
Tdi_TRIDC5BG002040.2
Tdi_TRIDC5AG049270.1
Tdi_TRIDC5AG034110.2
Tdi_TRIDC4BG052660.2
Tdi_TRIDC4BG014300.2
Tdi_TRIDC4AG055770.1
Tdi_TRIDC4AG046800.1
Tdi_TRIDC4AG035790.2
Tdi_TRIDC3BG046270.2
Tdi_TRIDC3BG011280.2
Tdi_TRIDC3AG041020.1
−1000
0
1000
2000
3000

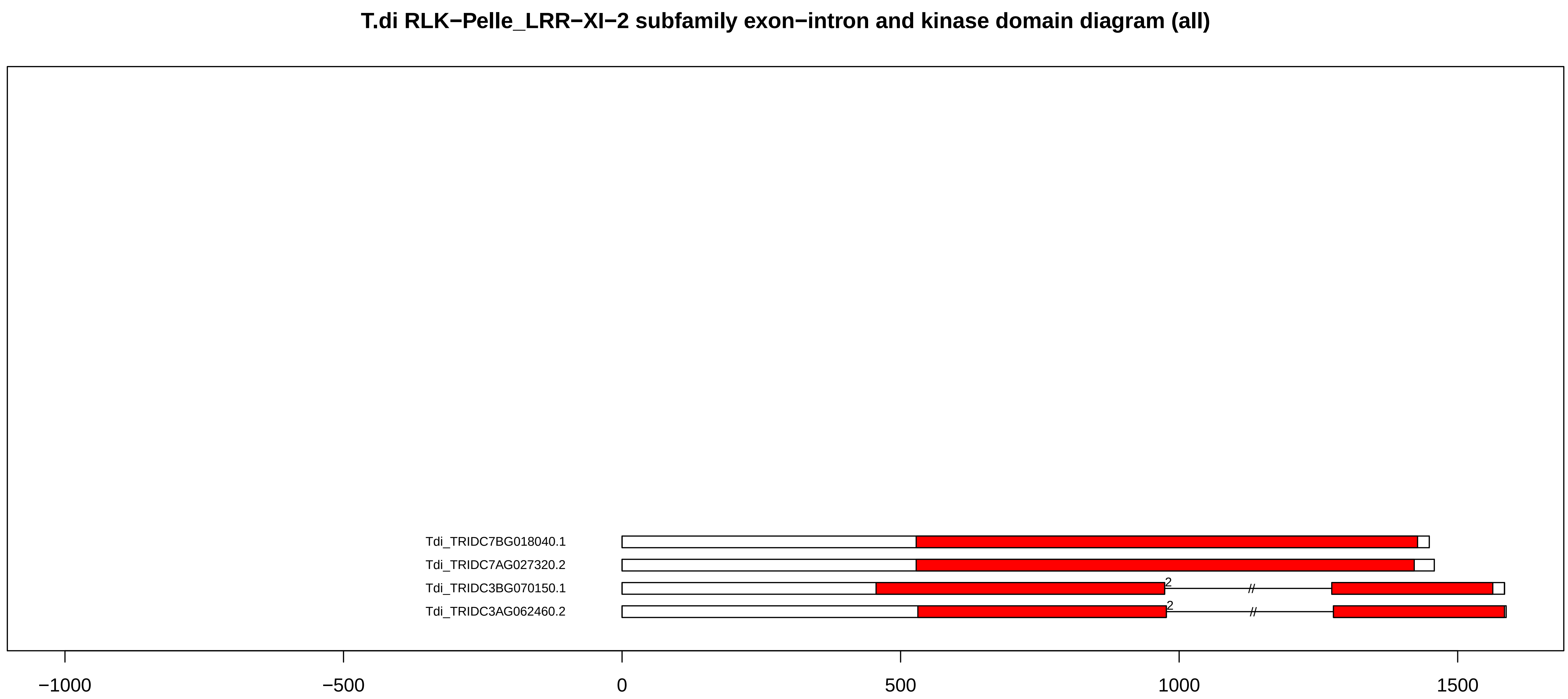
T.di RLK−Pelle_LRR−XI−2 subfamily exon−intron and kinase domain diagram (all)
Tdi_TRIDC7BG018040.1
Tdi_TRIDC7AG027320.2
Tdi_TRIDC3BG070150.1
Tdi_TRIDC3AG062460.2
2
2
−1000
−500
0
500
1000
1500

T.di RLK−Pelle_LRR−XII−1 subfamily exon−intron and kinase domain diagram (part 1)

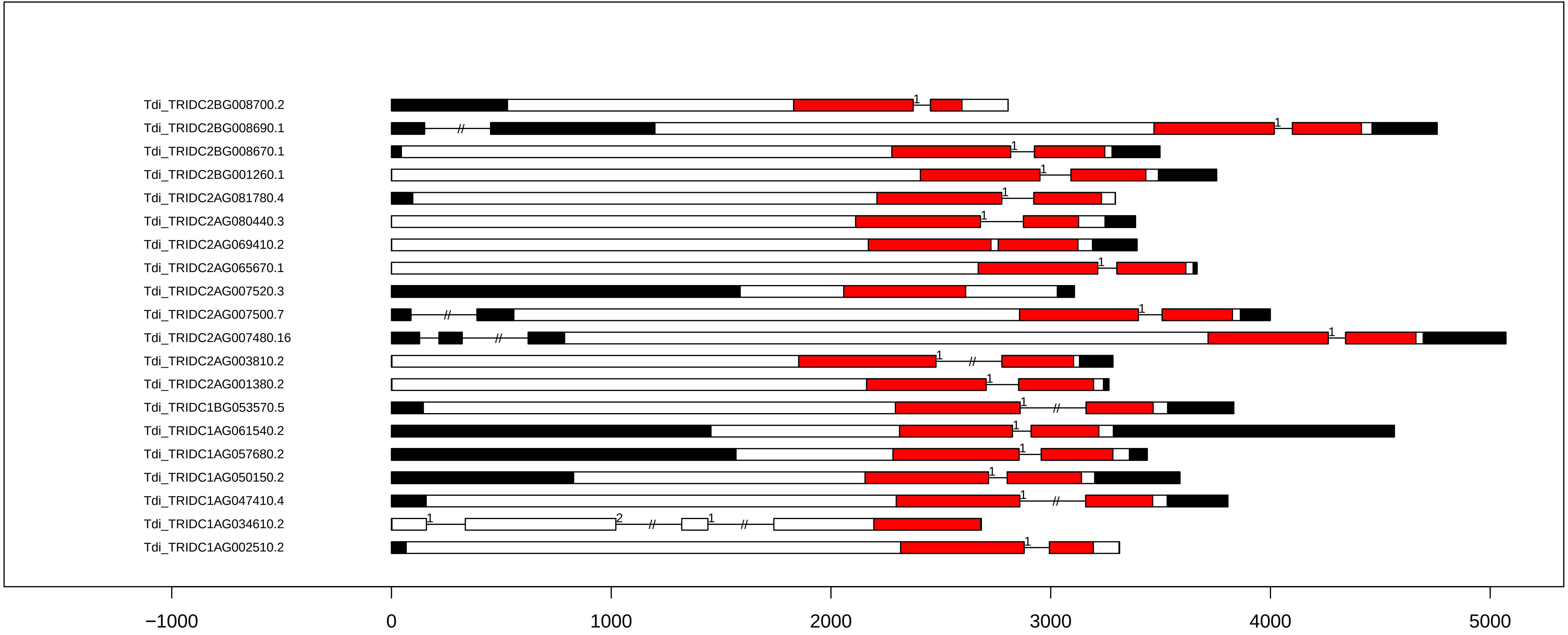

T.di RLK−Pelle_LRR−XII−1 subfamily exon−intron and kinase domain diagram (part 2)

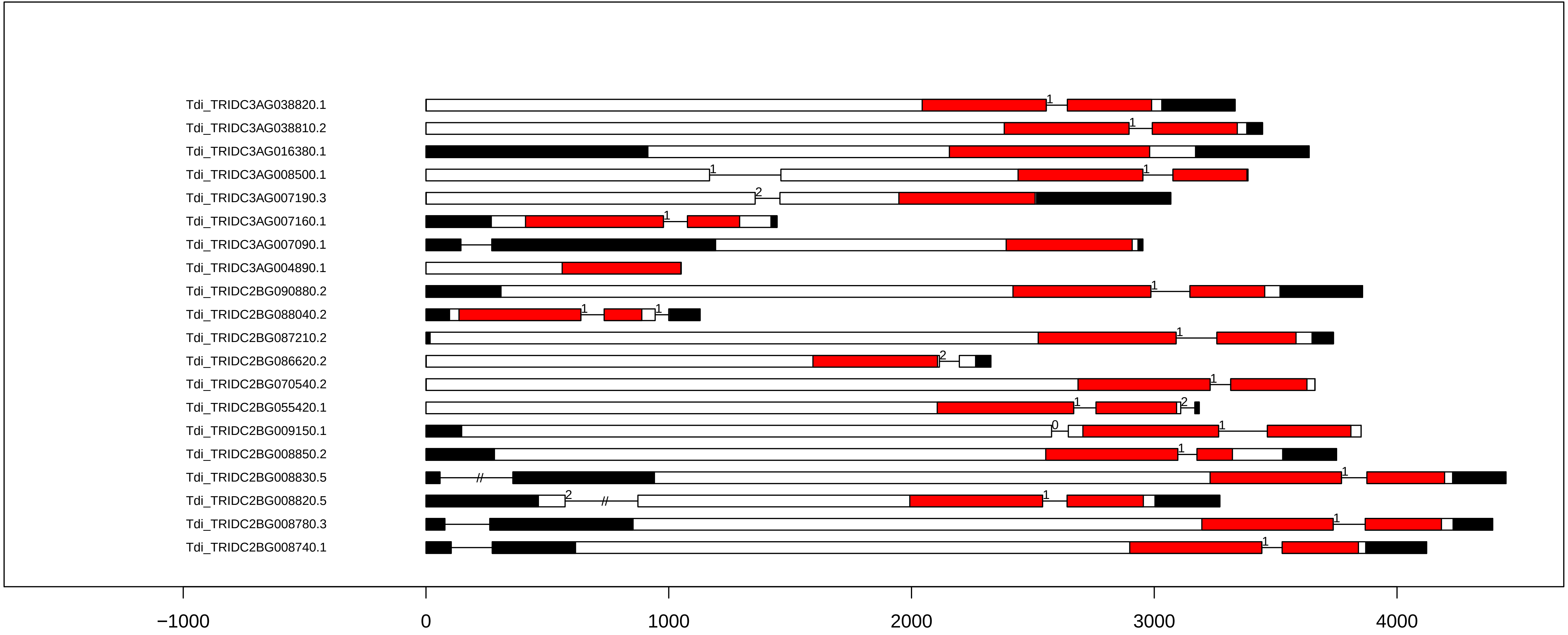

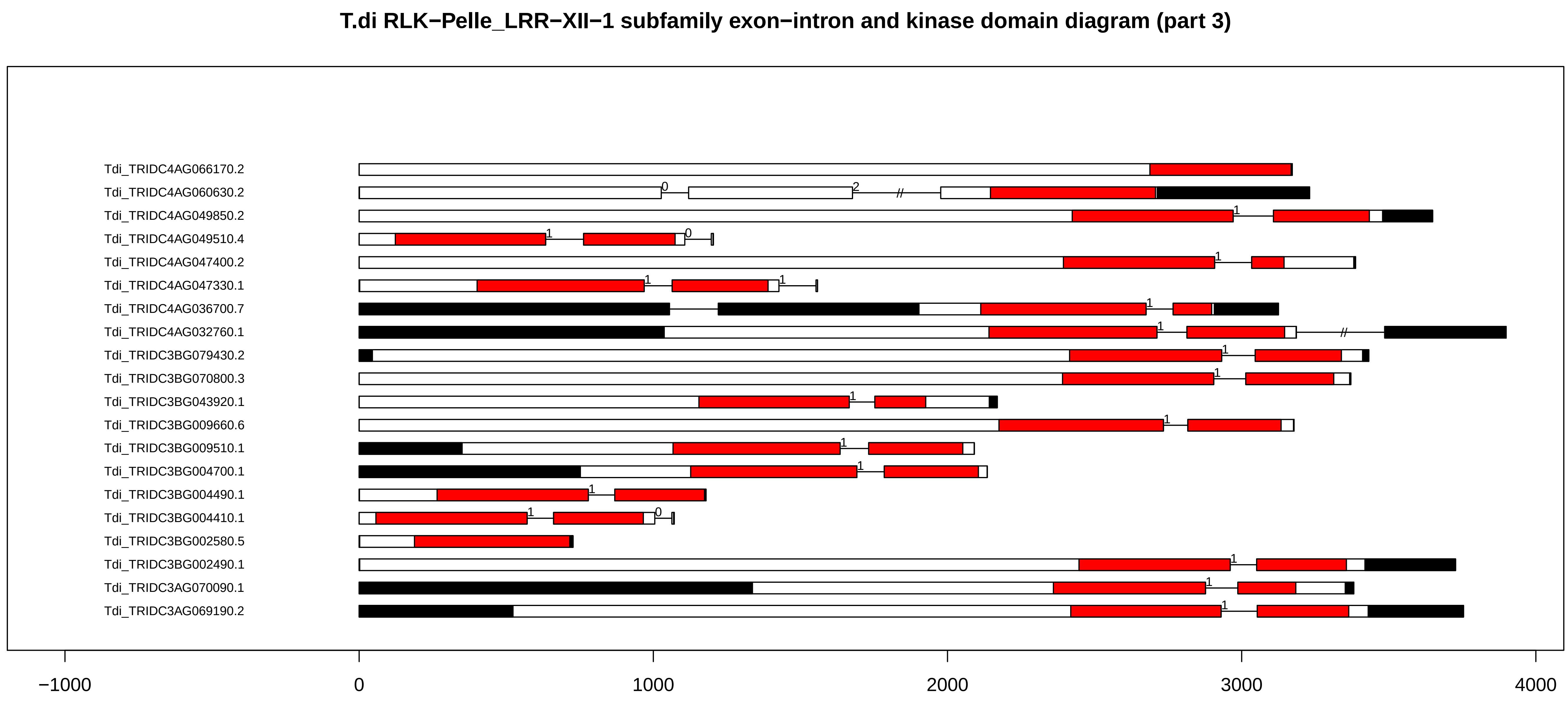
T.di RLK−Pelle_LRR−XII−1 subfamily exon−intron and kinase domain diagram (part 3)
Tdi_TRIDC4AG066170.2
Tdi_TRIDC4AG060630.2
Tdi_TRIDC4AG049850.2
Tdi_TRIDC4AG049510.4
Tdi_TRIDC4AG047400.2
Tdi_TRIDC4AG047330.1
Tdi_TRIDC4AG036700.7
Tdi_TRIDC4AG032760.1
Tdi_TRIDC3BG079430.2
Tdi_TRIDC3BG070800.3
Tdi_TRIDC3BG043920.1
Tdi_TRIDC3BG009660.6
Tdi_TRIDC3BG009510.1
Tdi_TRIDC3BG004700.1
Tdi_TRIDC3BG004490.1
Tdi_TRIDC3BG004410.1
Tdi_TRIDC3BG002580.5
Tdi_TRIDC3BG002490.1
Tdi_TRIDC3AG070090.1
Tdi_TRIDC3AG069190.2
−1000
0
1000
2000
3000
4000

T.di RLK−Pelle_LRR−XII−1 subfamily exon−intron and kinase domain diagram (part 4)

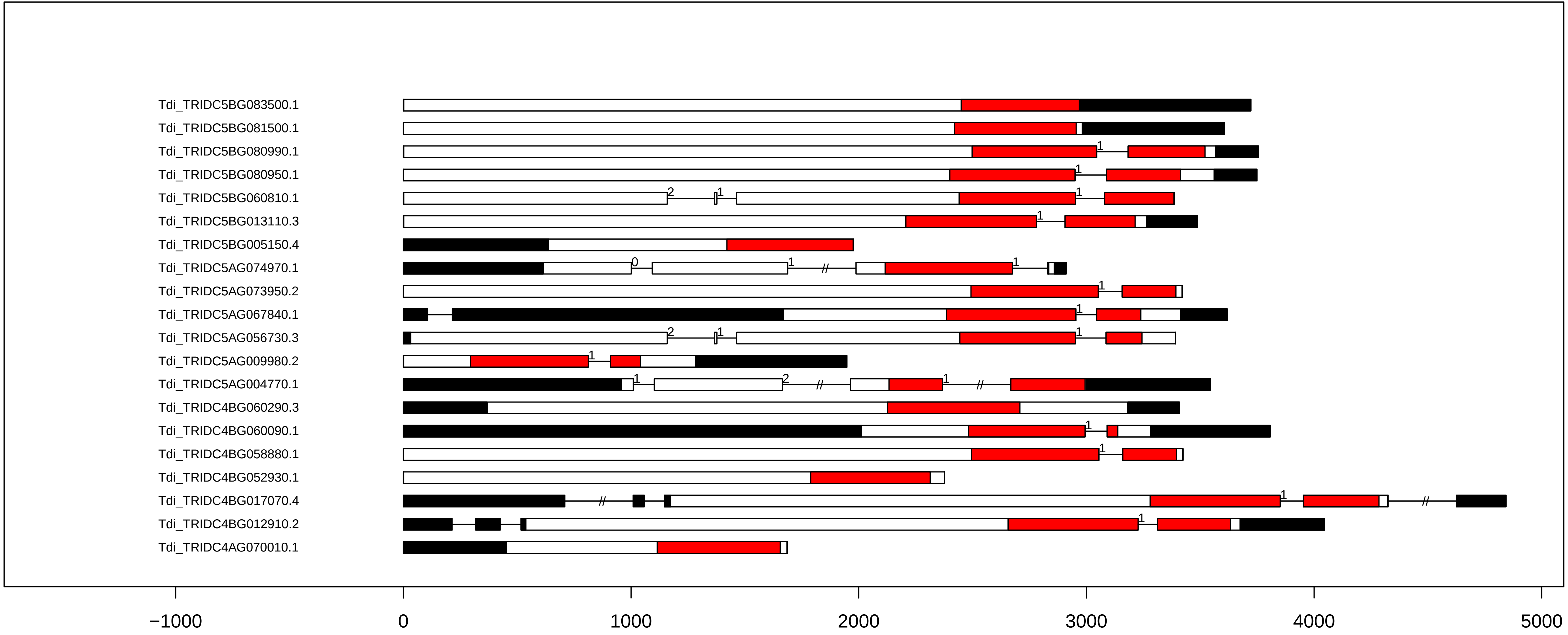

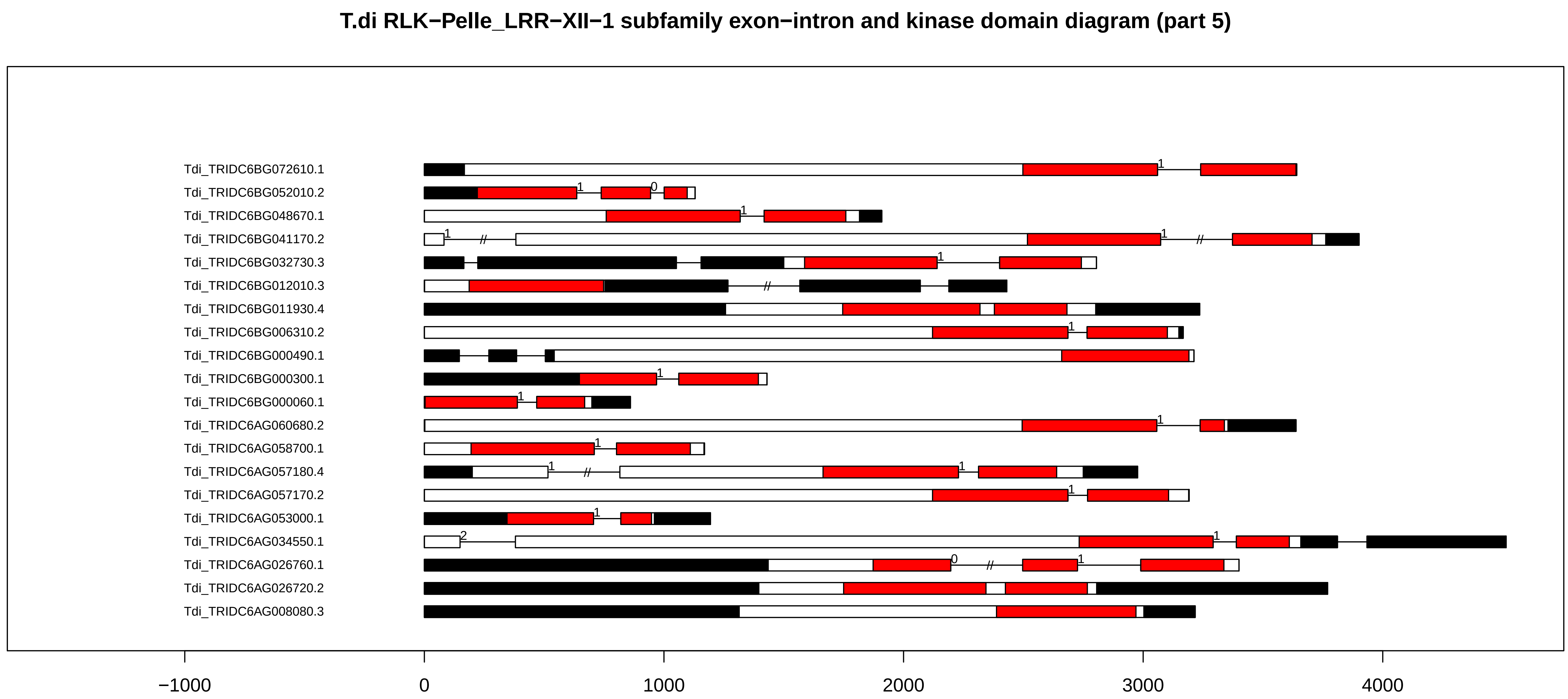
T.di RLK−Pelle_LRR−XII−1 subfamily exon−intron and kinase domain diagram (part 5)
Tdi_TRIDC6BG072610.1
Tdi_TRIDC6BG052010.2
Tdi_TRIDC6BG048670.1
Tdi_TRIDC6BG041170.2
Tdi_TRIDC6BG032730.3
Tdi_TRIDC6BG012010.3
Tdi_TRIDC6BG011930.4
Tdi_TRIDC6BG006310.2
Tdi_TRIDC6BG000490.1
Tdi_TRIDC6BG000300.1
Tdi_TRIDC6BG000060.1
Tdi_TRIDC6AG060680.2
Tdi_TRIDC6AG058700.1
Tdi_TRIDC6AG057180.4
Tdi_TRIDC6AG057170.2
Tdi_TRIDC6AG053000.1
Tdi_TRIDC6AG034550.1
Tdi_TRIDC6AG026760.1
Tdi_TRIDC6AG026720.2
Tdi_TRIDC6AG008080.3
−1000
0
1000
2000
3000
4000

T.di RLK−Pelle_LRR−XII−1 subfamily exon−intron and kinase domain diagram (part 6)

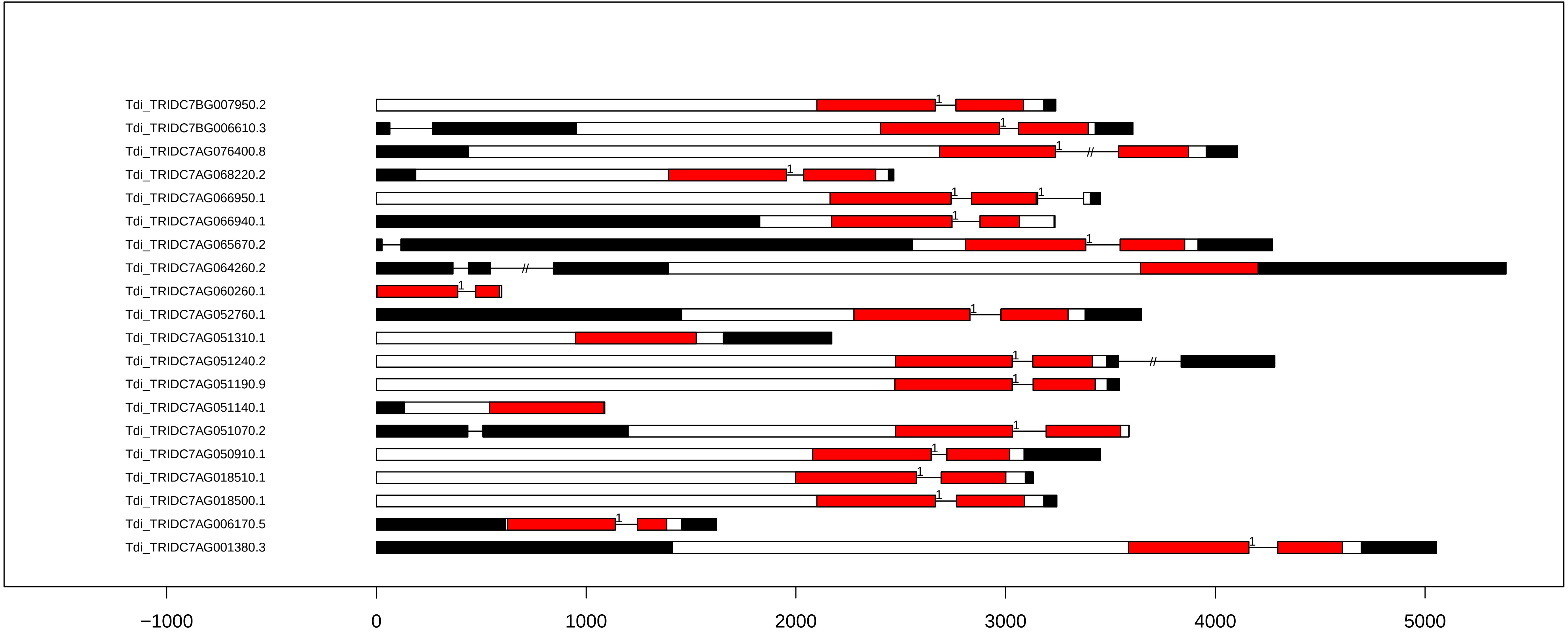

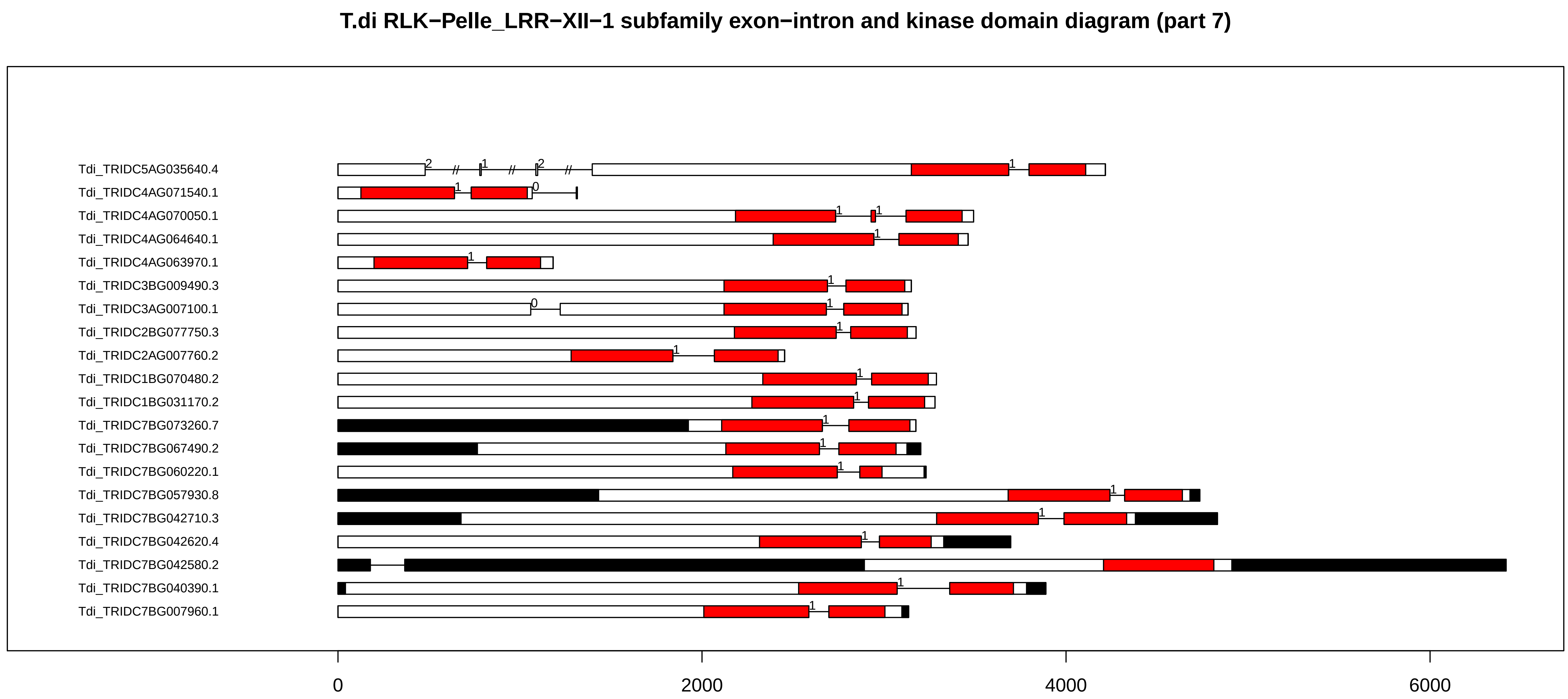
T.di RLK−Pelle_LRR−XII−1 subfamily exon−intron and kinase domain diagram (part 7)
Tdi_TRIDC5AG035640.4
Tdi_TRIDC4AG071540.1
Tdi_TRIDC4AG070050.1
Tdi_TRIDC4AG064640.1
Tdi_TRIDC4AG063970.1
Tdi_TRIDC3BG009490.3
Tdi_TRIDC3AG007100.1
Tdi_TRIDC2BG077750.3
Tdi_TRIDC2AG007760.2
Tdi_TRIDC1BG070480.2
Tdi_TRIDC1BG031170.2
Tdi_TRIDC7BG073260.7
Tdi_TRIDC7BG067490.2
Tdi_TRIDC7BG060220.1
Tdi_TRIDC7BG057930.8
Tdi_TRIDC7BG042710.3
Tdi_TRIDC7BG042620.4
Tdi_TRIDC7BG042580.2
Tdi_TRIDC7BG040390.1
Tdi_TRIDC7BG007960.1
0
2000
4000
6000

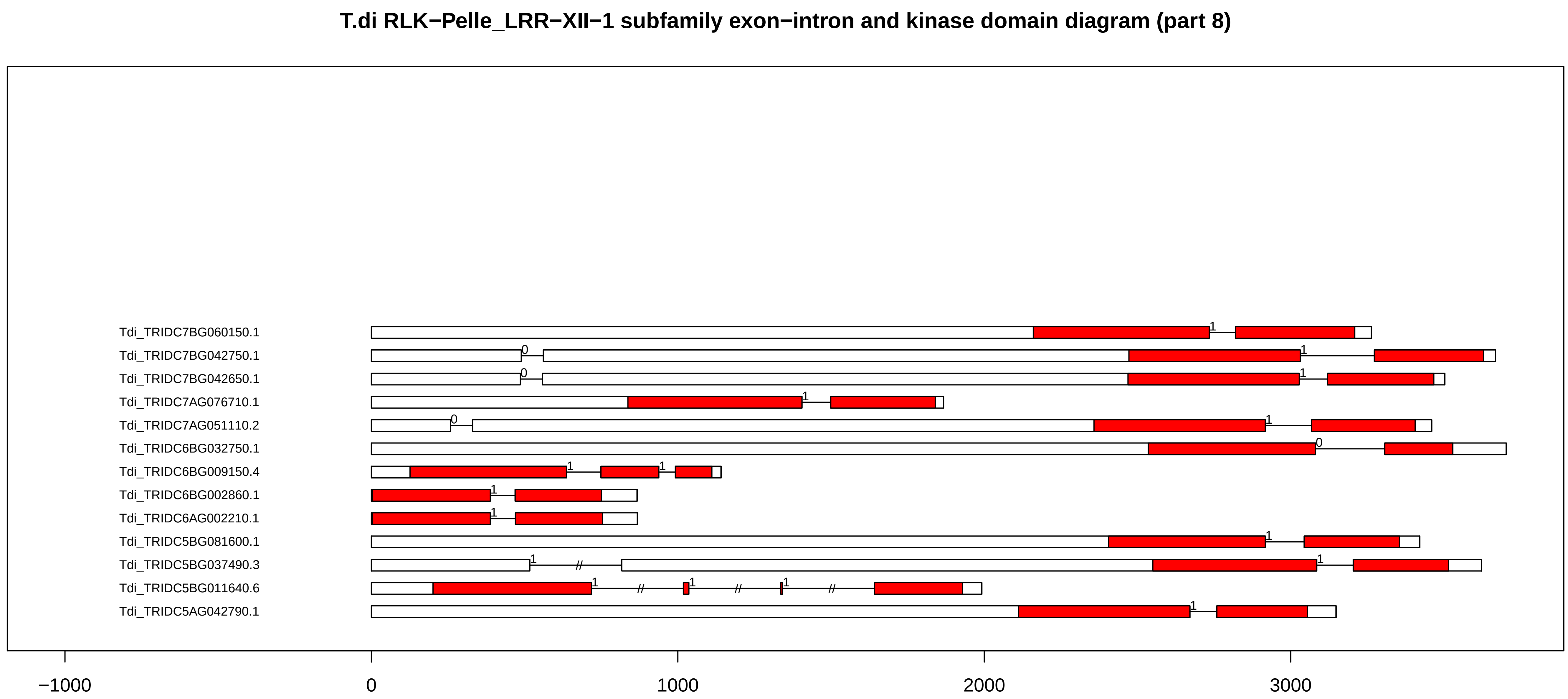
T.di RLK−Pelle_LRR−XII−1 subfamily exon−intron and kinase domain diagram (part 8)
Tdi_TRIDC7BG060150.1
Tdi_TRIDC7BG042750.1
Tdi_TRIDC7BG042650.1
Tdi_TRIDC7AG076710.1
Tdi_TRIDC7AG051110.2
Tdi_TRIDC6BG032750.1
Tdi_TRIDC6BG009150.4
Tdi_TRIDC6BG002860.1
Tdi_TRIDC6AG002210.1
Tdi_TRIDC5BG081600.1
Tdi_TRIDC5BG037490.3
Tdi_TRIDC5BG011640.6
Tdi_TRIDC5AG042790.1
−1000
0
1000
2000
3000

T.di RLK−Pelle_LRR−XIIIa subfamily exon−intron and kinase domain diagram (all)

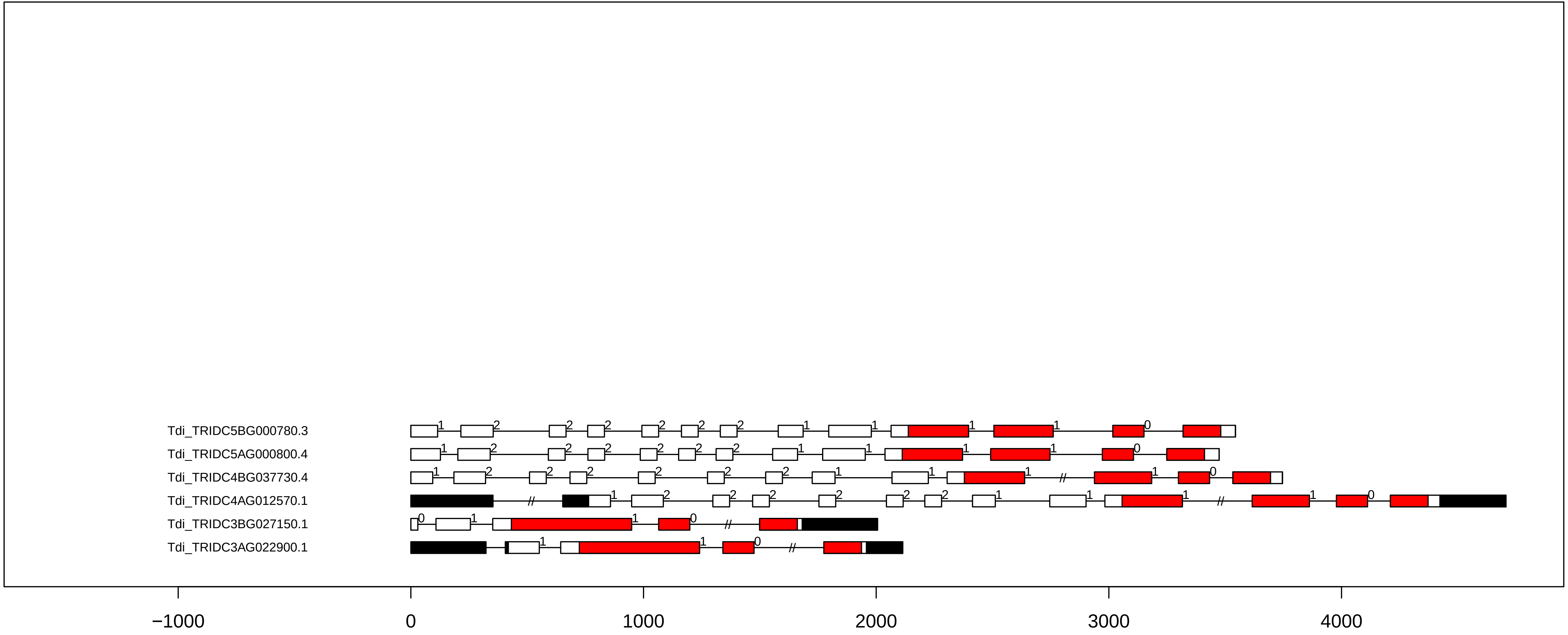

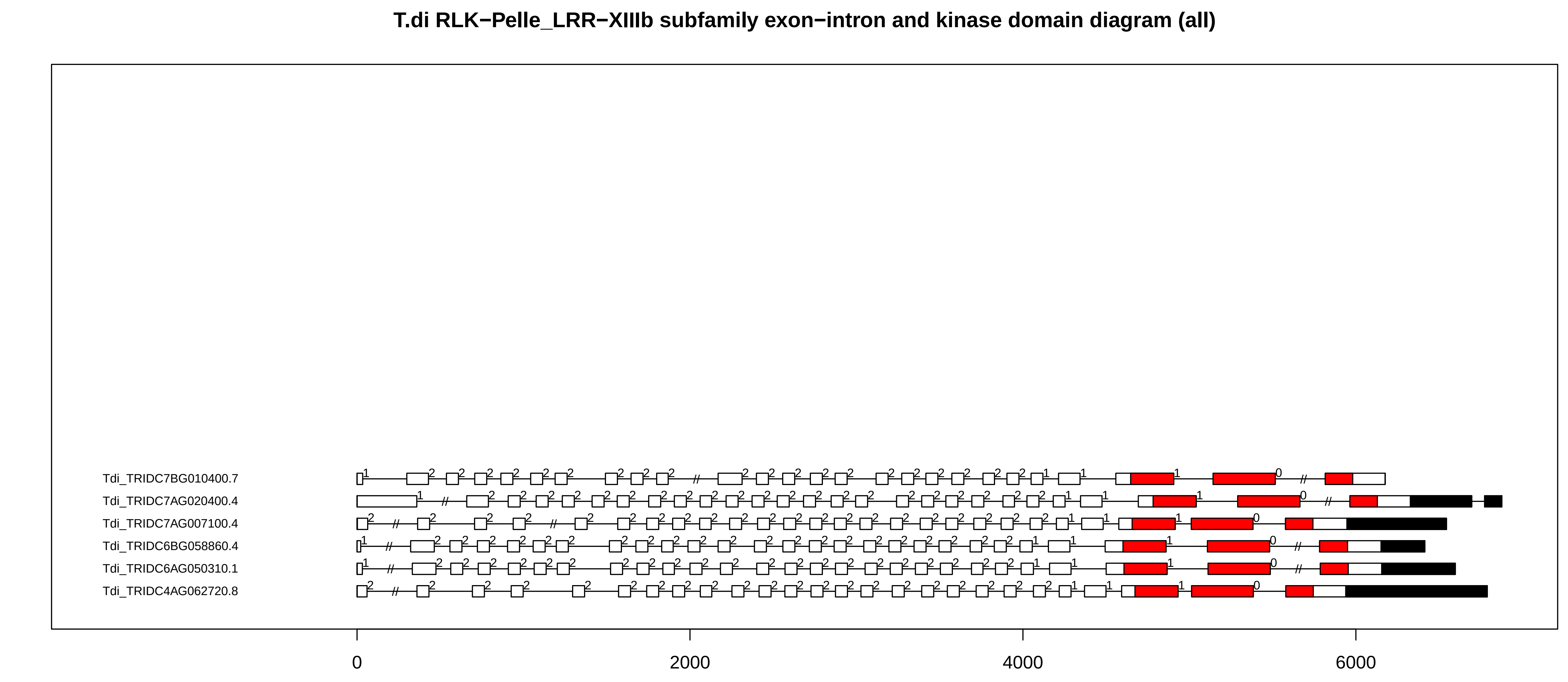
T.di RLK−Pelle_LRR−XIIIb subfamily exon−intron and kinase domain diagram (all)
Tdi_TRIDC7BG010400.7
Tdi_TRIDC7AG020400.4
Tdi_TRIDC7AG007100.4
Tdi_TRIDC6BG058860.4
Tdi_TRIDC6AG050310.1
Tdi_TRIDC4AG062720.8
0
2000
4000
6000

T.di RLK−Pelle_LRR−XIV subfamily exon−intron and kinase domain diagram (all)

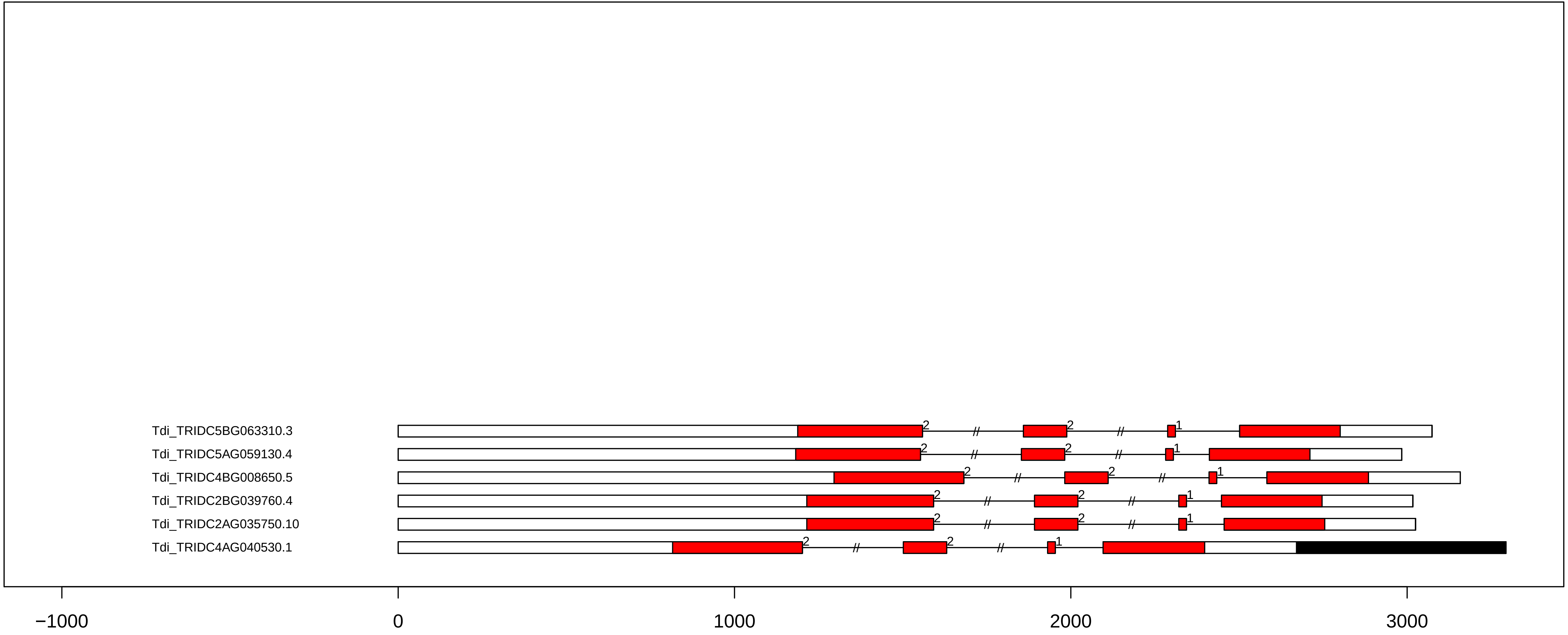

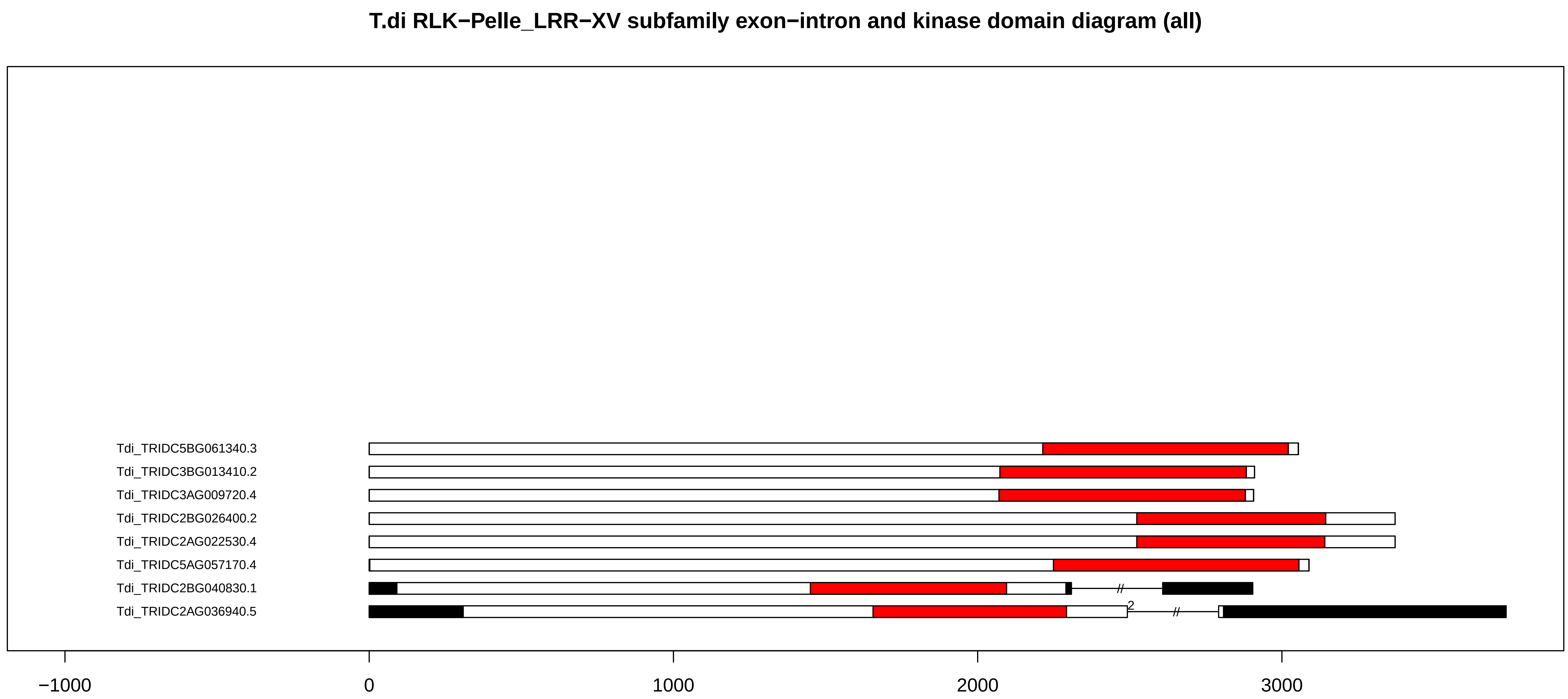
T.di RLK−Pelle_LRR−XV subfamily exon−intron and kinase domain diagram (all)
Tdi_TRIDC5BG061340.3
Tdi_TRIDC3BG013410.2
Tdi_TRIDC3AG009720.4
Tdi_TRIDC2BG026400.2
Tdi_TRIDC2AG022530.4
Tdi_TRIDC5AG057170.4
Tdi_TRIDC2BG040830.1
Tdi_TRIDC2AG036940.5
2
−1000
0
1000
2000
3000

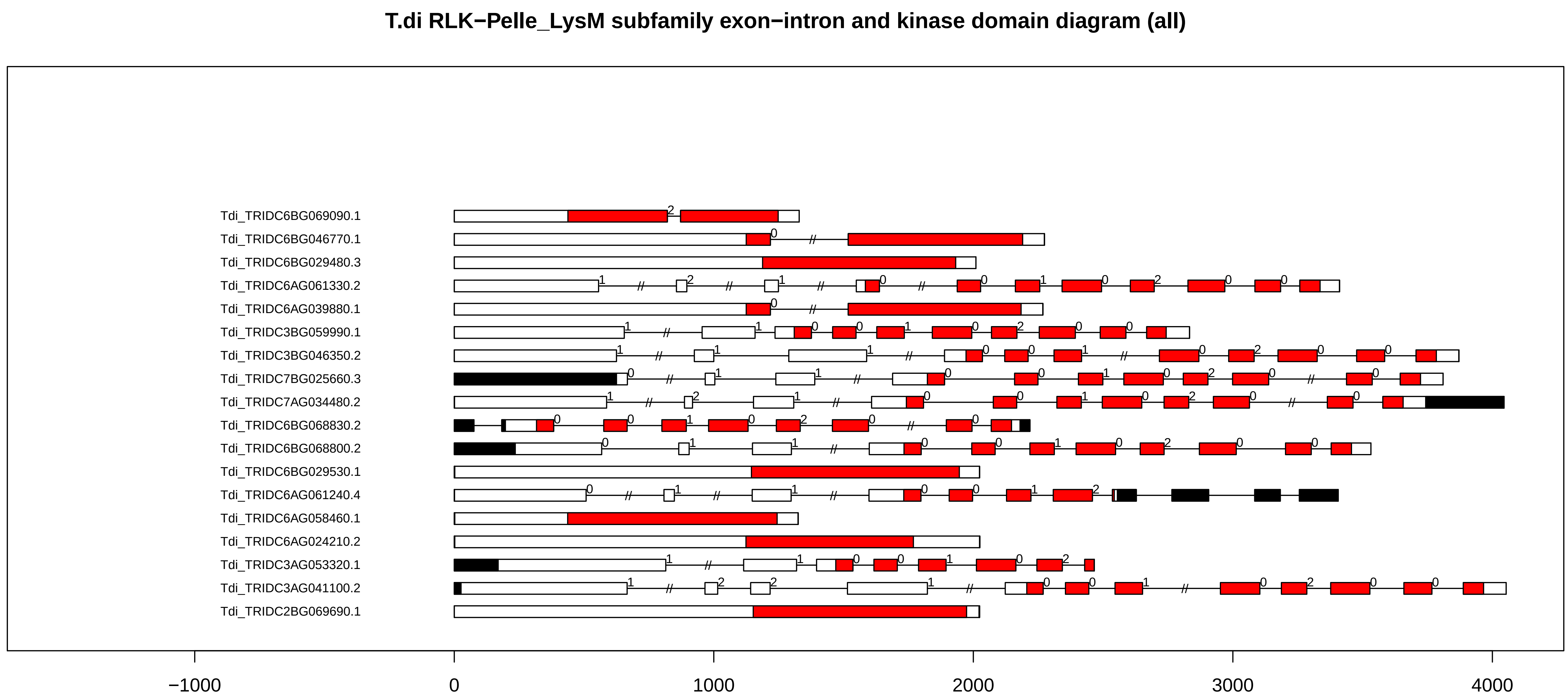
T.di RLK−Pelle_LysM subfamily exon−intron and kinase domain diagram (all)
Tdi_TRIDC6BG069090.1
Tdi_TRIDC6BG046770.1
Tdi_TRIDC6BG029480.3
Tdi_TRIDC6AG061330.2
Tdi_TRIDC6AG039880.1
Tdi_TRIDC3BG059990.1
Tdi_TRIDC3BG046350.2
Tdi_TRIDC7BG025660.3
Tdi_TRIDC7AG034480.2
Tdi_TRIDC6BG068830.2
Tdi_TRIDC6BG068800.2
Tdi_TRIDC6BG029530.1
Tdi_TRIDC6AG061240.4
Tdi_TRIDC6AG058460.1
Tdi_TRIDC6AG024210.2
Tdi_TRIDC3AG053320.1
Tdi_TRIDC3AG041100.2
Tdi_TRIDC2BG069690.1
−1000
0
1000
2000
3000
4000

T.di RLK−Pelle_PERK−1 subfamily exon−intron and kinase domain diagram (part 1)

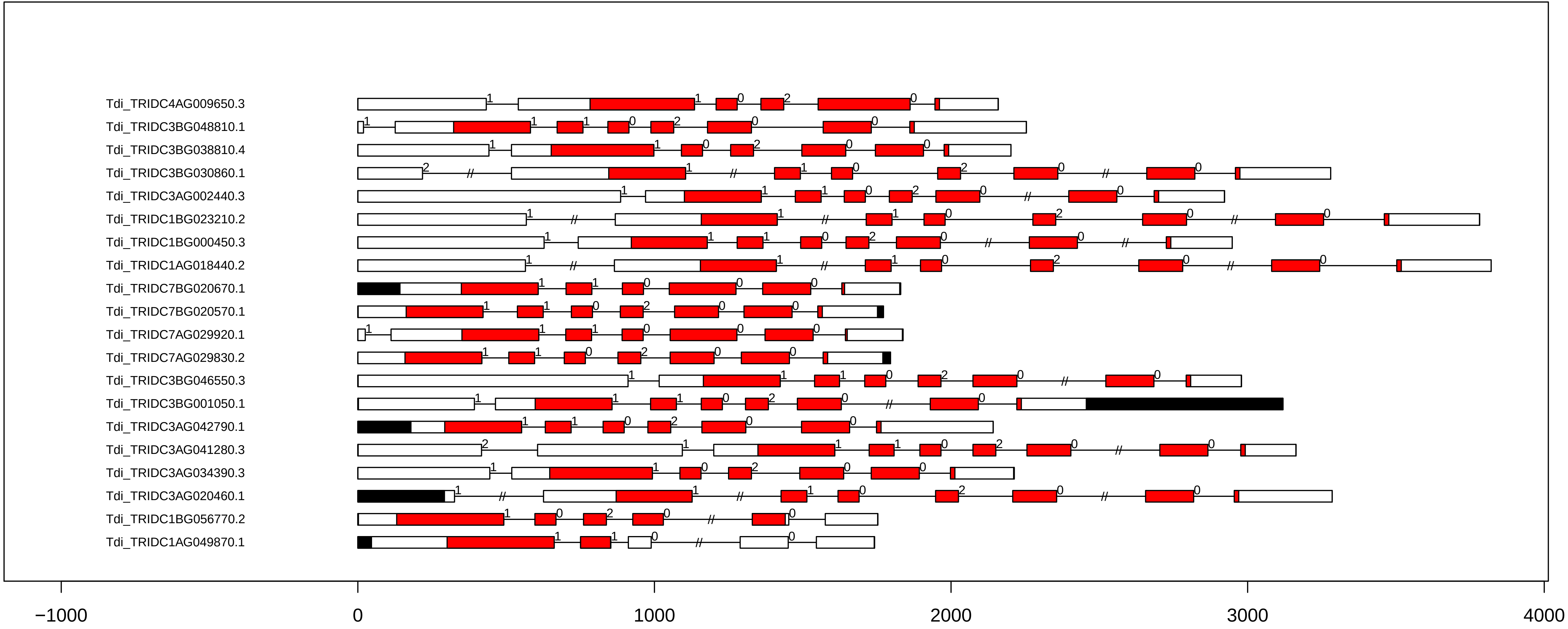

T.di RLK−Pelle_PERK−1 subfamily exon−intron and kinase domain diagram (part 2)

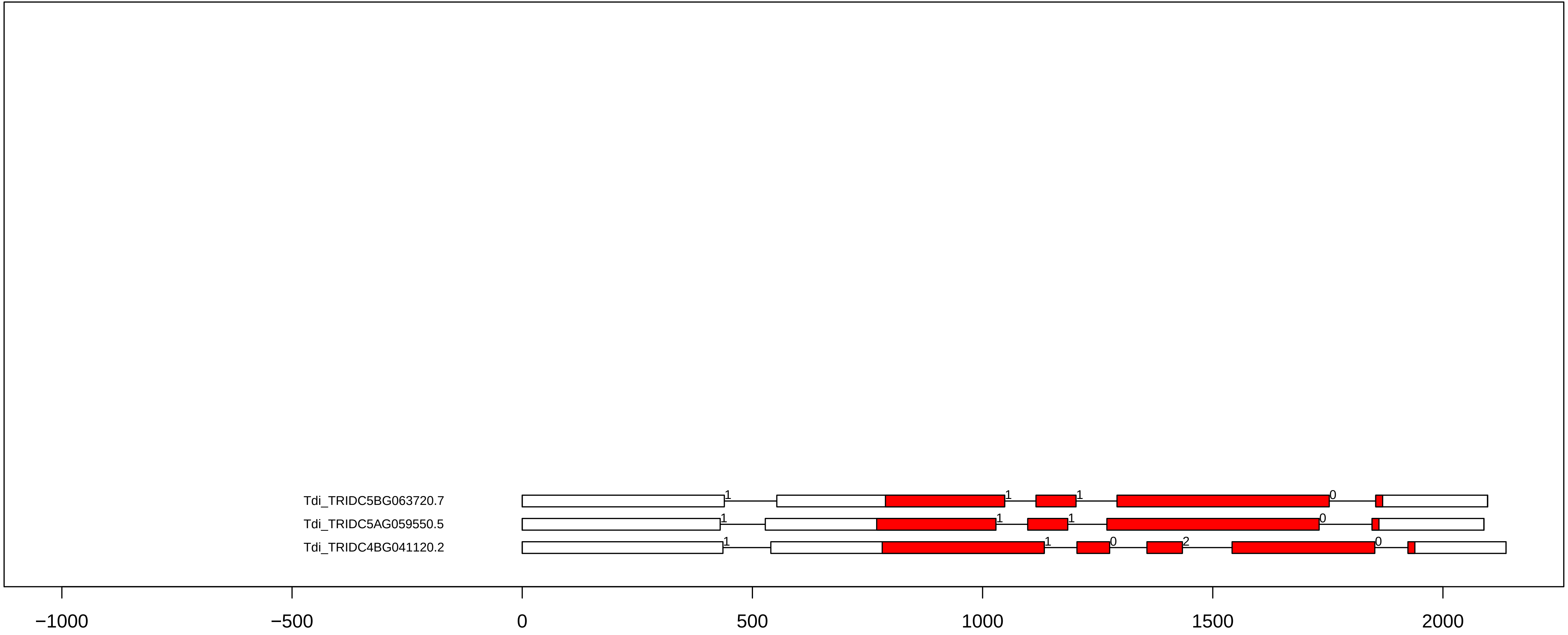

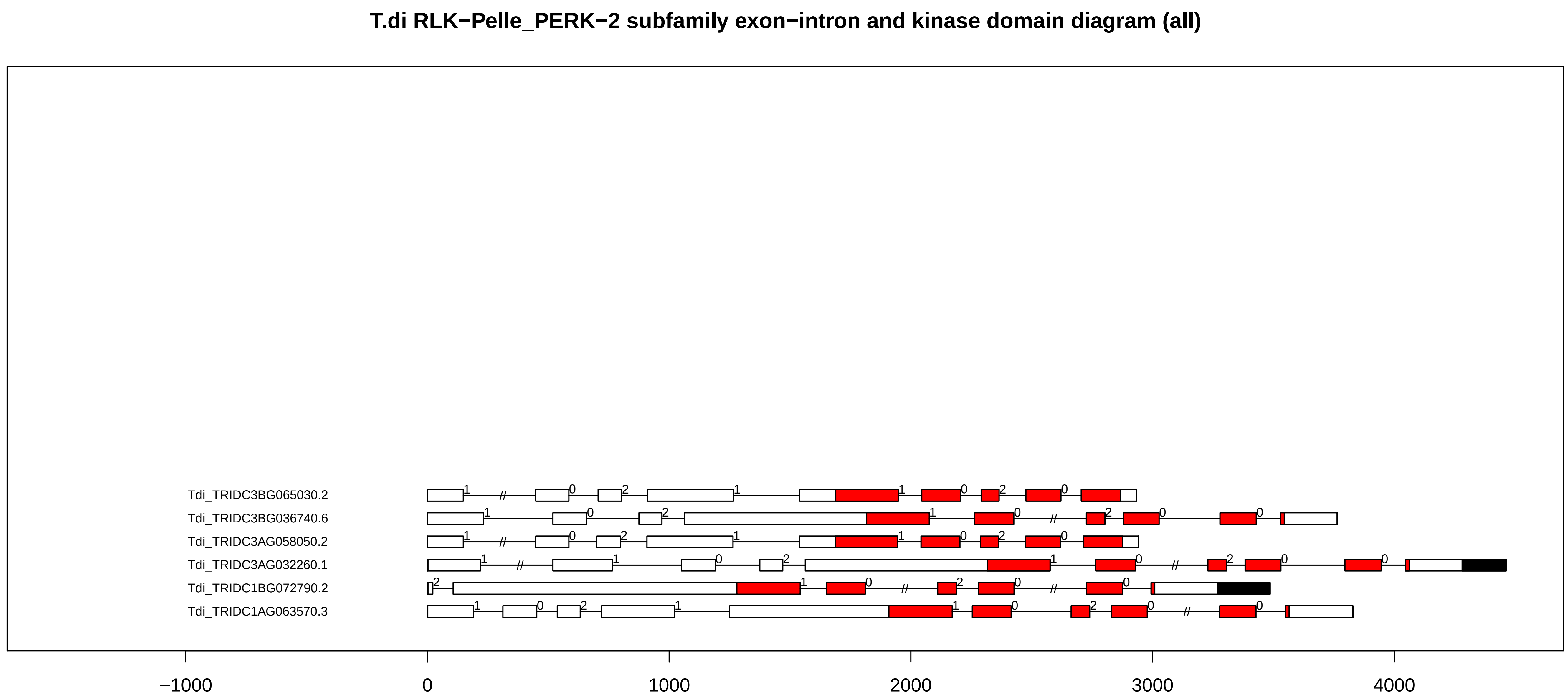
T.di RLK−Pelle_PERK−2 subfamily exon−intron and kinase domain diagram (all)
Tdi_TRIDC3BG065030.2
Tdi_TRIDC3BG036740.6
Tdi_TRIDC3AG058050.2
Tdi_TRIDC3AG032260.1
Tdi_TRIDC1BG072790.2
Tdi_TRIDC1AG063570.3
−1000
0
1000
2000
3000
4000

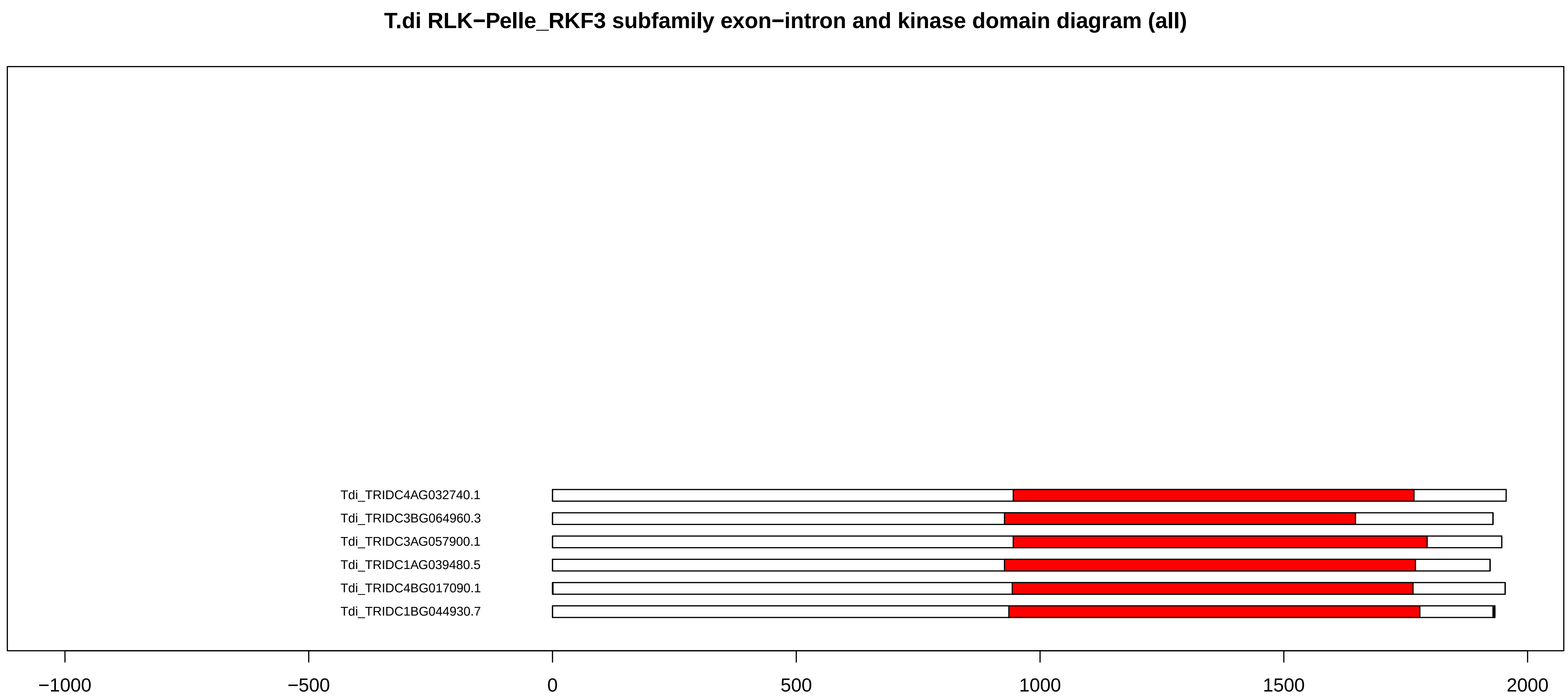
T.di RLK−Pelle_RKF3 subfamily exon−intron and kinase domain diagram (all)
Tdi_TRIDC4AG032740.1
Tdi_TRIDC3BG064960.3
Tdi_TRIDC3AG057900.1
Tdi_TRIDC1AG039480.5
Tdi_TRIDC4BG017090.1
Tdi_TRIDC1BG044930.7
−1000
−500
0
500
1000
1500
2000

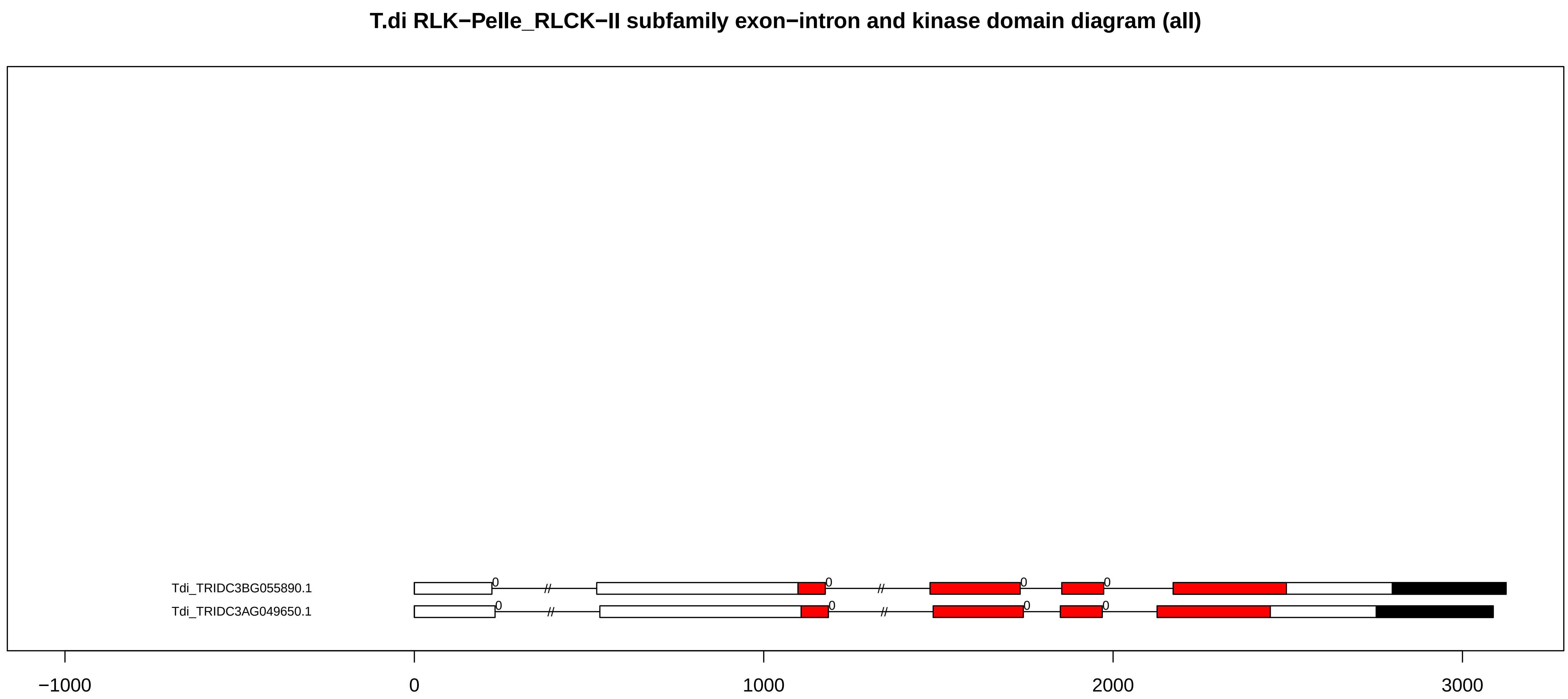

T.di RLK−Pelle_RLCK−II subfamily exon−intron and kinase domain diagram (all)
Tdi_TRIDC3BG055890.1
Tdi_TRIDC3AG049650.1
−1000
0
1000
2000
3000

T.di RLK−Pelle_RLCK−IV subfamily exon−intron and kinase domain diagram (all)

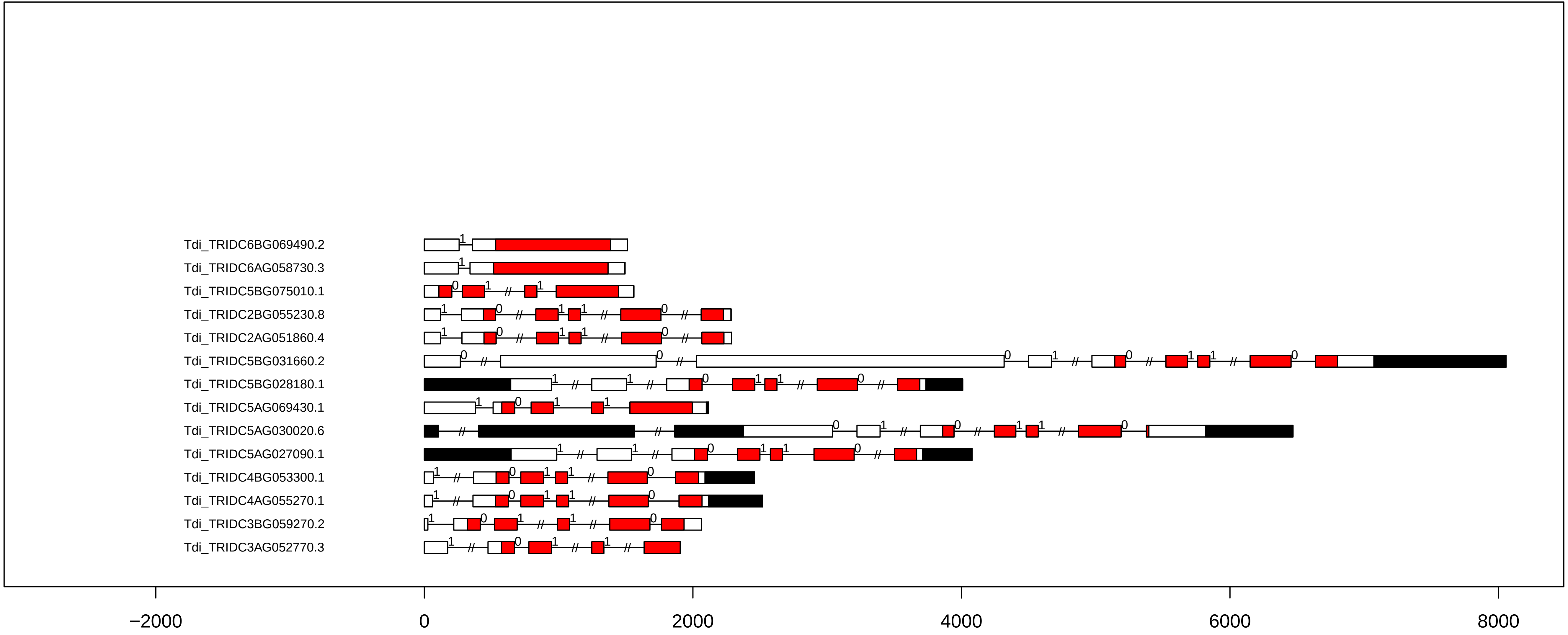

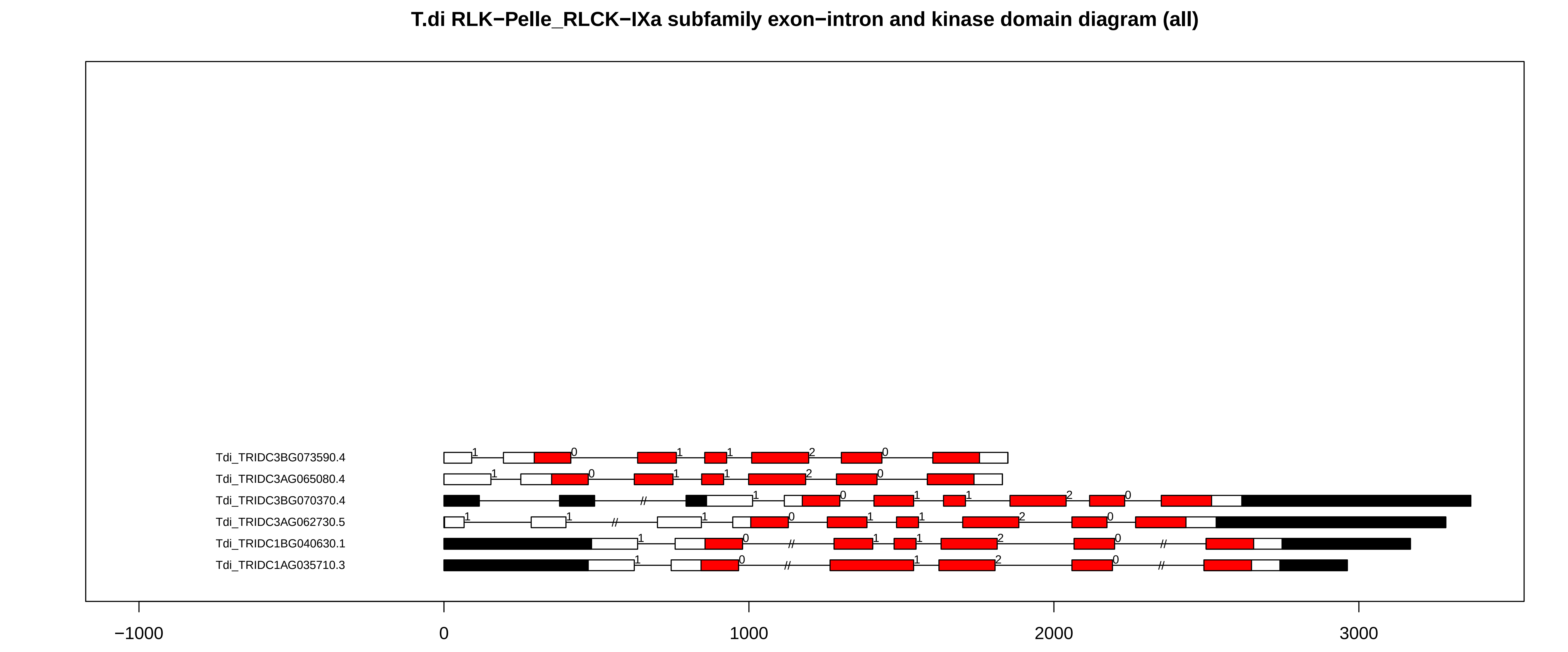
T.di RLK−Pelle_RLCK−IXa subfamily exon−intron and kinase domain diagram (all)
Tdi_TRIDC3BG073590.4
Tdi_TRIDC3AG065080.4
Tdi_TRIDC3BG070370.4
Tdi_TRIDC3AG062730.5
Tdi_TRIDC1BG040630.1
Tdi_TRIDC1AG035710.3
−1000
0
1000
2000
3000

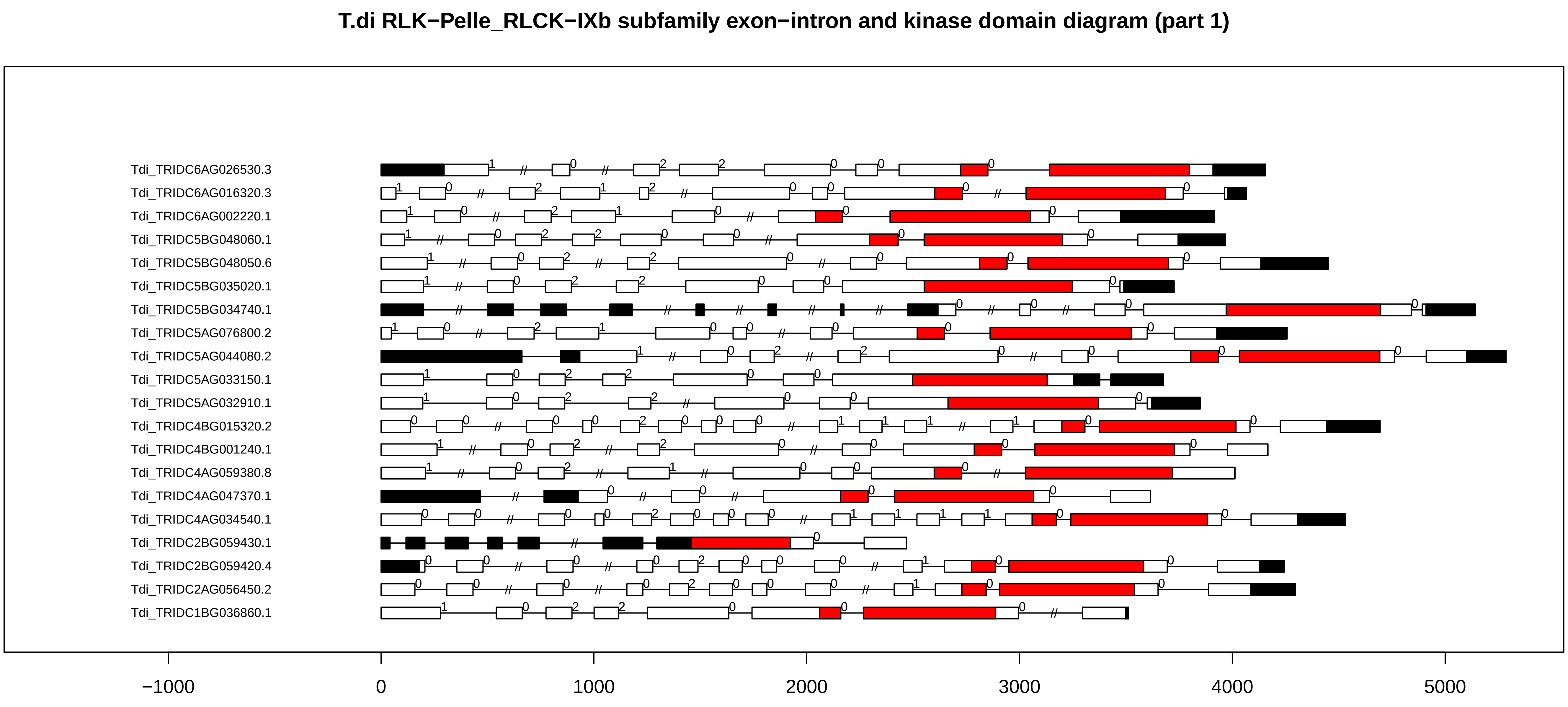

T.di RLK−Pelle_RLCK−IXb subfamily exon−intron and kinase domain diagram (part 1)
Tdi_TRIDC6AG026530.3
Tdi_TRIDC6AG016320.3
Tdi_TRIDC6AG002220.1
Tdi_TRIDC5BG048060.1
Tdi_TRIDC5BG048050.6
Tdi_TRIDC5BG035020.1
Tdi_TRIDC5BG034740.1
Tdi_TRIDC5AG076800.2
Tdi_TRIDC5AG044080.2
Tdi_TRIDC5AG033150.1
Tdi_TRIDC5AG032910.1
Tdi_TRIDC4BG015320.2
Tdi_TRIDC4BG001240.1
Tdi_TRIDC4AG059380.8
Tdi_TRIDC4AG047370.1
Tdi_TRIDC4AG034540.1
Tdi_TRIDC2BG059430.1
Tdi_TRIDC2BG059420.4
Tdi_TRIDC2AG056450.2
Tdi_TRIDC1BG036860.1
−1000
0
1000
2000
3000
4000
5000

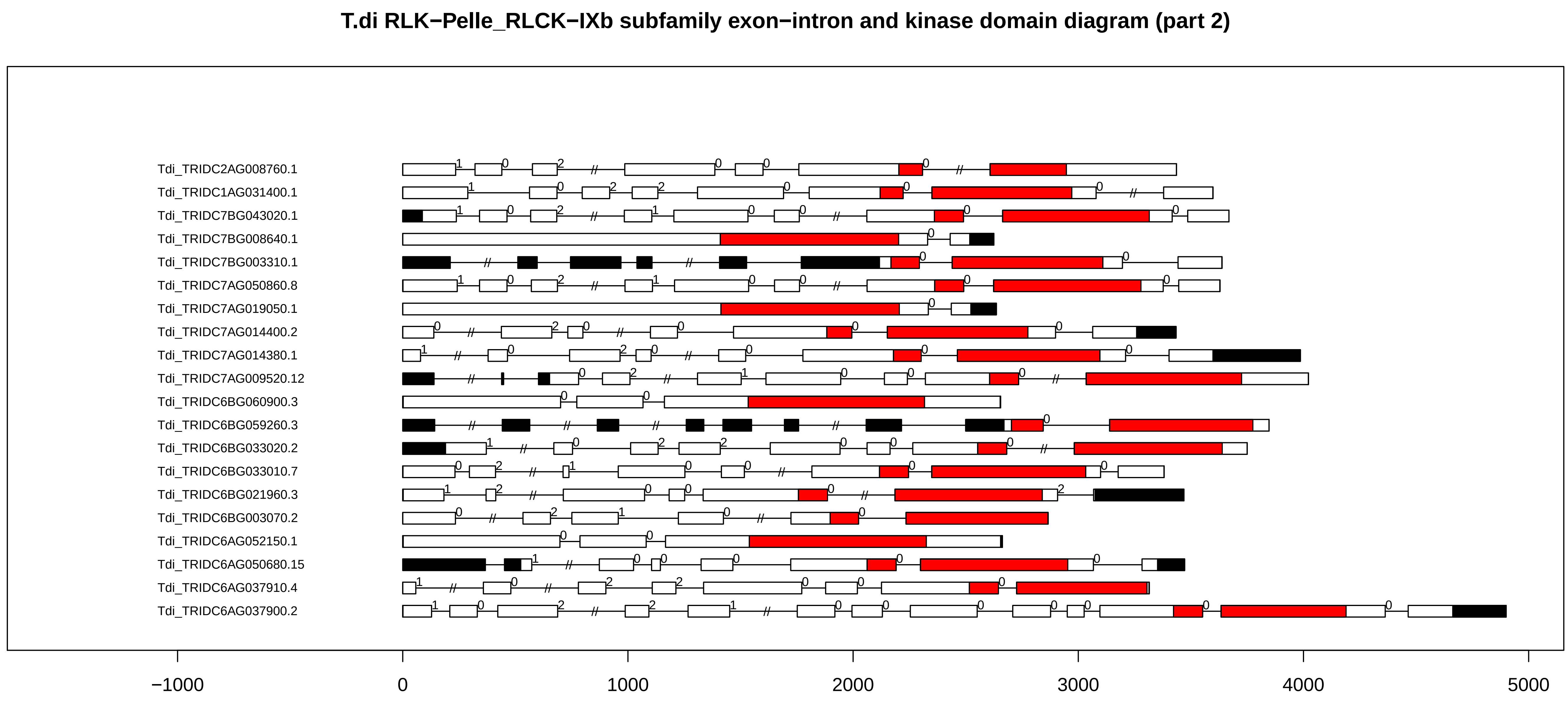
T.di RLK−Pelle_RLCK−IXb subfamily exon−intron and kinase domain diagram (part 2)
Tdi_TRIDC2AG008760.1
Tdi_TRIDC1AG031400.1
Tdi_TRIDC7BG043020.1
Tdi_TRIDC7BG008640.1
Tdi_TRIDC7BG003310.1
Tdi_TRIDC7AG050860.8
Tdi_TRIDC7AG019050.1
Tdi_TRIDC7AG014400.2
Tdi_TRIDC7AG014380.1
Tdi_TRIDC7AG009520.12
Tdi_TRIDC6BG060900.3
Tdi_TRIDC6BG059260.3
Tdi_TRIDC6BG033020.2
Tdi_TRIDC6BG033010.7
Tdi_TRIDC6BG021960.3
Tdi_TRIDC6BG003070.2
Tdi_TRIDC6AG052150.1
Tdi_TRIDC6AG050680.15
Tdi_TRIDC6AG037910.4
Tdi_TRIDC6AG037900.2
−1000
0
1000
2000
3000
4000
5000

T.di RLK−Pelle_RLCK−IXb subfamily exon−intron and kinase domain diagram (part 3)

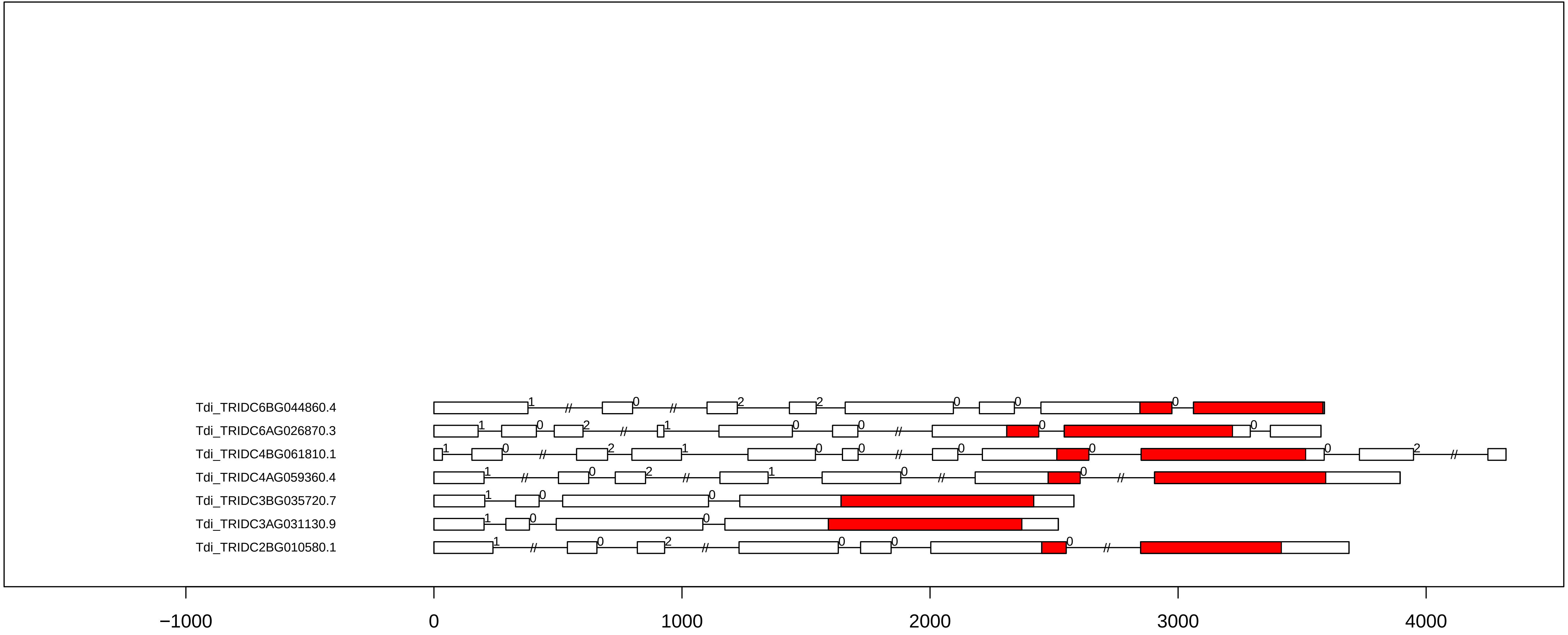

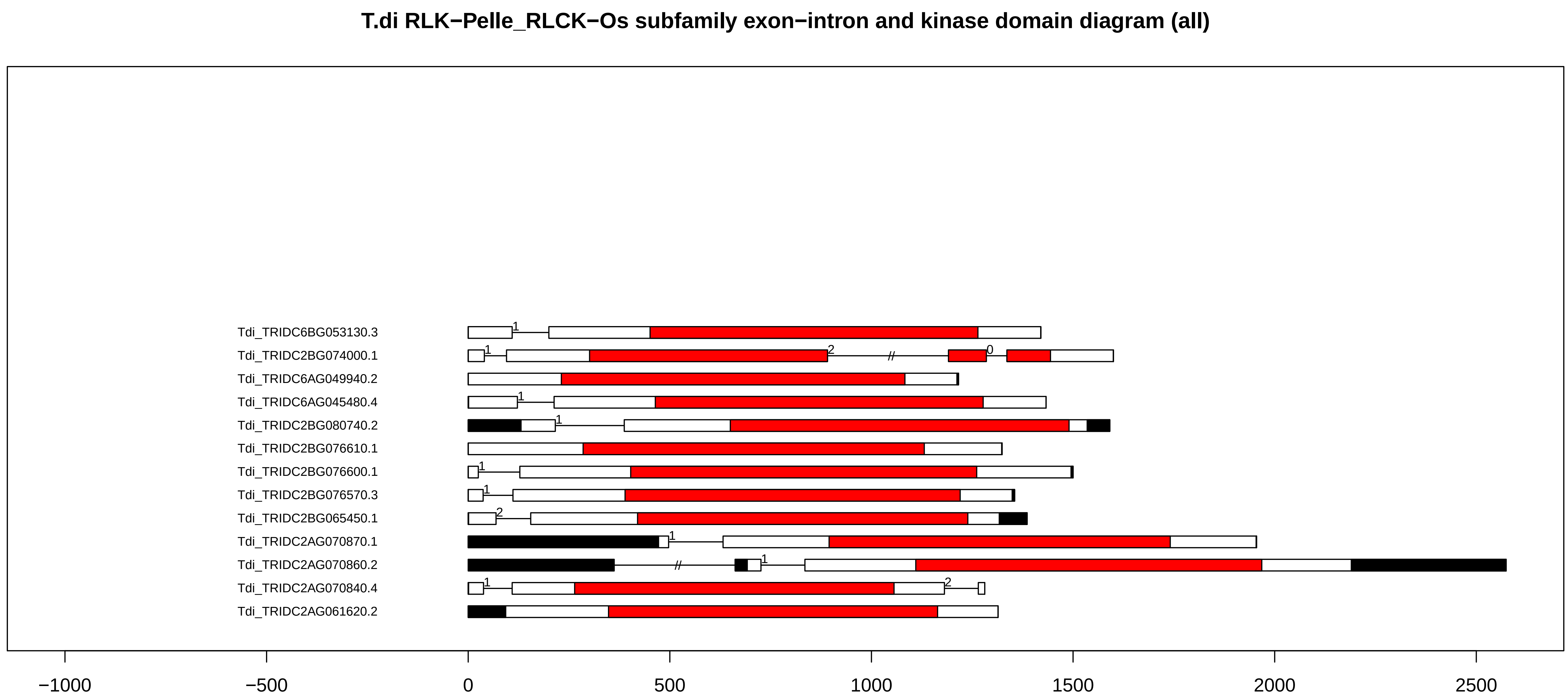
T.di RLK−Pelle_RLCK−Os subfamily exon−intron and kinase domain diagram (all)
Tdi_TRIDC6BG053130.3
Tdi_TRIDC2BG074000.1
Tdi_TRIDC6AG049940.2
Tdi_TRIDC6AG045480.4
Tdi_TRIDC2BG080740.2
Tdi_TRIDC2BG076610.1
Tdi_TRIDC2BG076600.1
Tdi_TRIDC2BG076570.3
Tdi_TRIDC2BG065450.1
Tdi_TRIDC2AG070870.1
Tdi_TRIDC2AG070860.2
Tdi_TRIDC2AG070840.4
Tdi_TRIDC2AG061620.2
−1000
−500
0
500
1000
1500
2000
2500

T.di RLK−Pelle_RLCK−VIIa−1 subfamily exon−intron and kinase domain diagram (part 1)

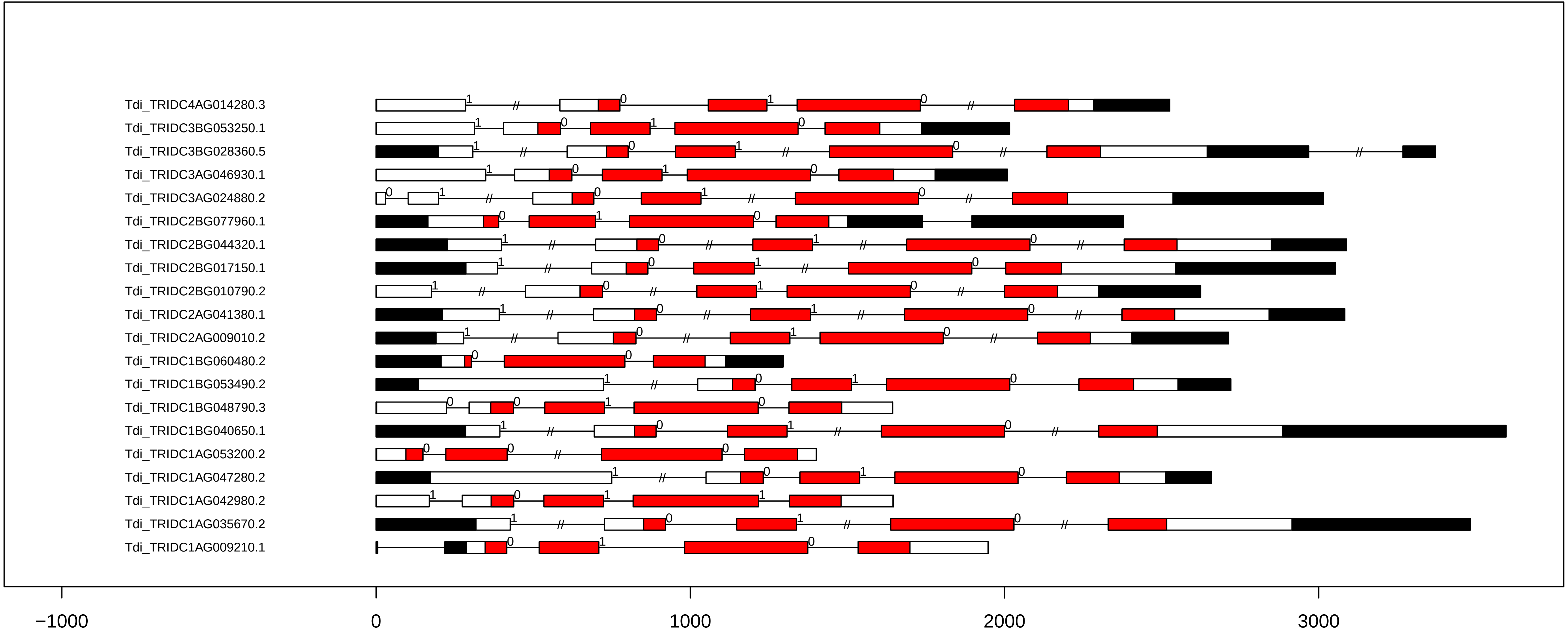

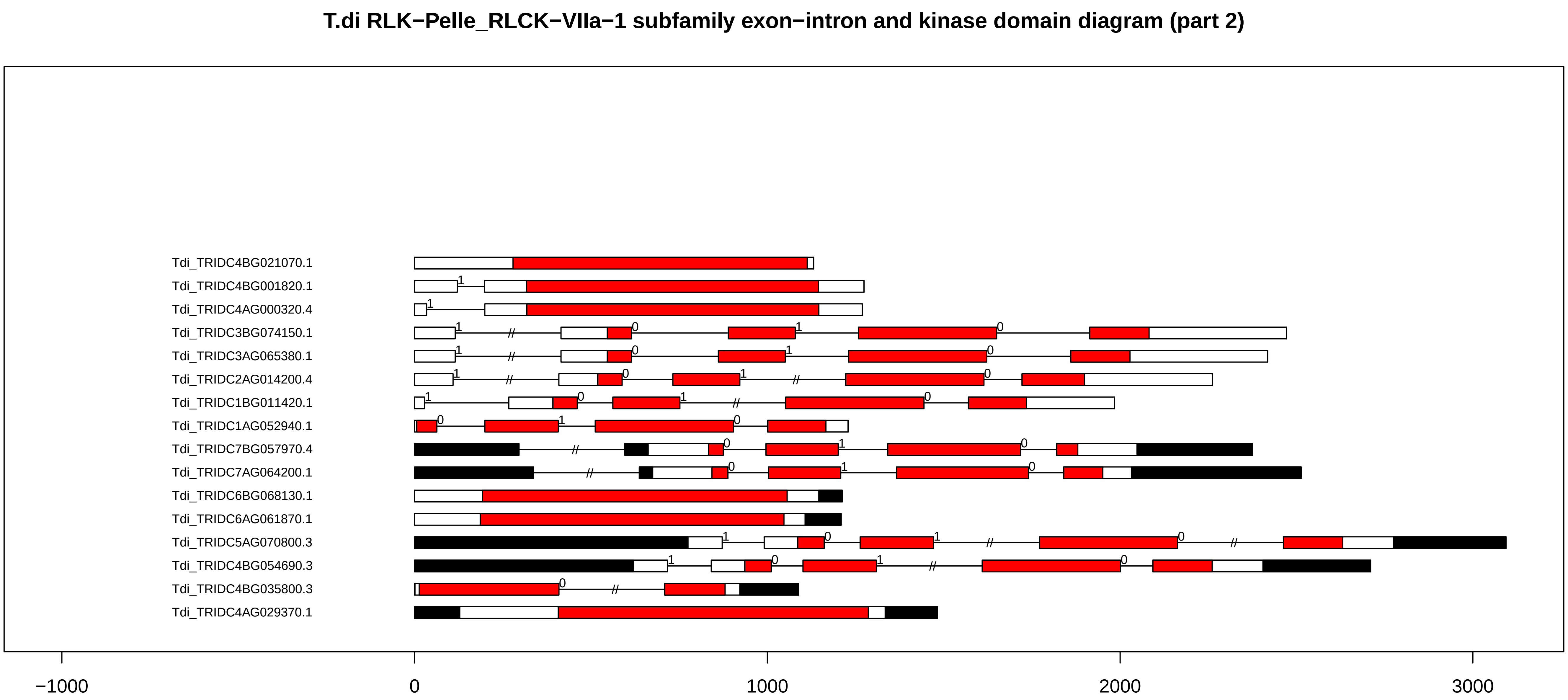
T.di RLK−Pelle_RLCK−VIIa−1 subfamily exon−intron and kinase domain diagram (part 2)
Tdi_TRIDC4BG021070.1
Tdi_TRIDC4BG001820.1
Tdi_TRIDC4AG000320.4
Tdi_TRIDC3BG074150.1
Tdi_TRIDC3AG065380.1
Tdi_TRIDC2AG014200.4
Tdi_TRIDC1BG011420.1
Tdi_TRIDC1AG052940.1
Tdi_TRIDC7BG057970.4
Tdi_TRIDC7AG064200.1
Tdi_TRIDC6BG068130.1
Tdi_TRIDC6AG061870.1
Tdi_TRIDC5AG070800.3
Tdi_TRIDC4BG054690.3
Tdi_TRIDC4BG035800.3
Tdi_TRIDC4AG029370.1
−1000
0
1000
2000
3000

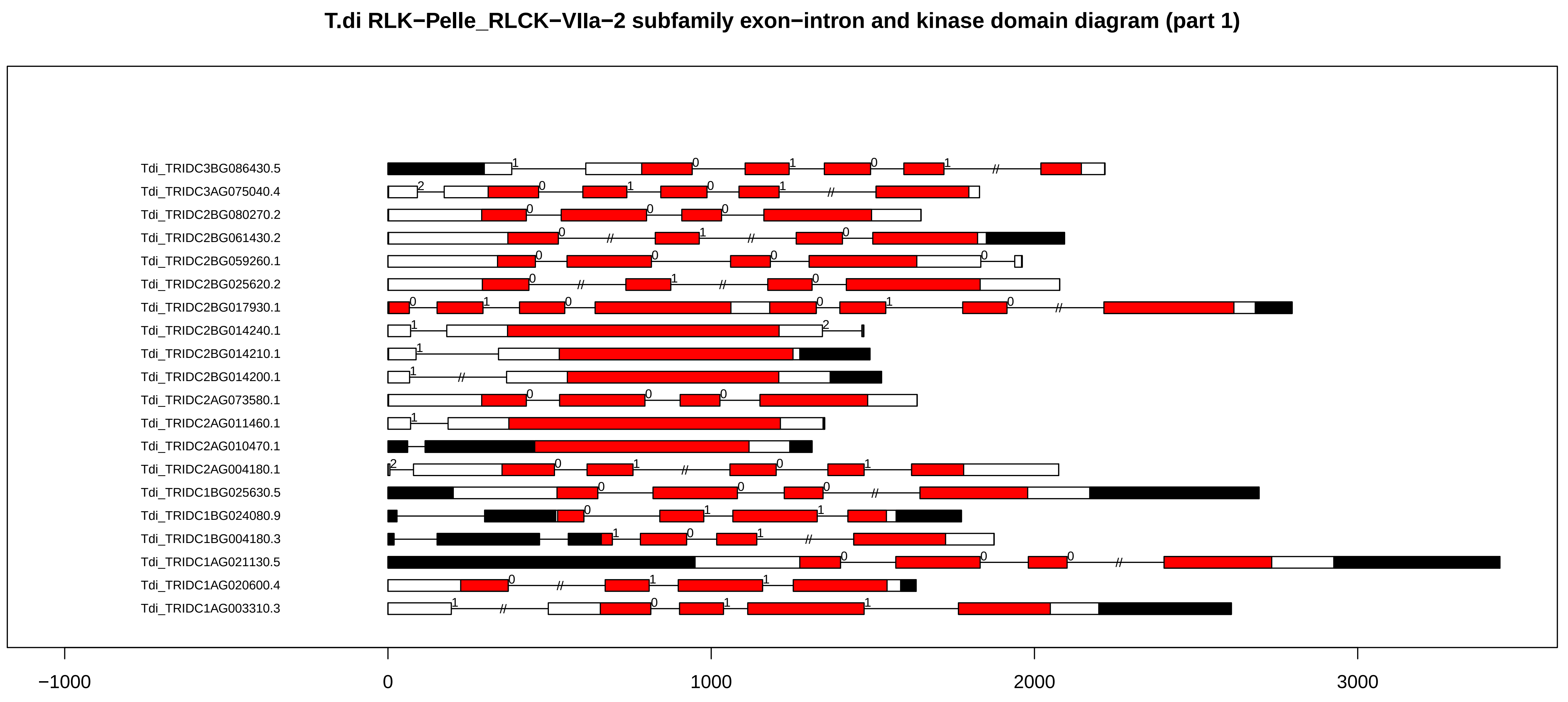

T.di RLK−Pelle_RLCK−VIIa−2 subfamily exon−intron and kinase domain diagram (part 1)
Tdi_TRIDC3BG086430.5
Tdi_TRIDC3AG075040.4
Tdi_TRIDC2BG080270.2
Tdi_TRIDC2BG061430.2
Tdi_TRIDC2BG059260.1
Tdi_TRIDC2BG025620.2
Tdi_TRIDC2BG017930.1
Tdi_TRIDC2BG014240.1
Tdi_TRIDC2BG014210.1
Tdi_TRIDC2BG014200.1
Tdi_TRIDC2AG073580.1
Tdi_TRIDC2AG011460.1
Tdi_TRIDC2AG010470.1
Tdi_TRIDC2AG004180.1
Tdi_TRIDC1BG025630.5
Tdi_TRIDC1BG024080.9
Tdi_TRIDC1BG004180.3
Tdi_TRIDC1AG021130.5
Tdi_TRIDC1AG020600.4
Tdi_TRIDC1AG003310.3
−1000
0
1000
2000
3000

T.di RLK−Pelle_RLCK−VIIa−2 subfamily exon−intron and kinase domain diagram (part 2)

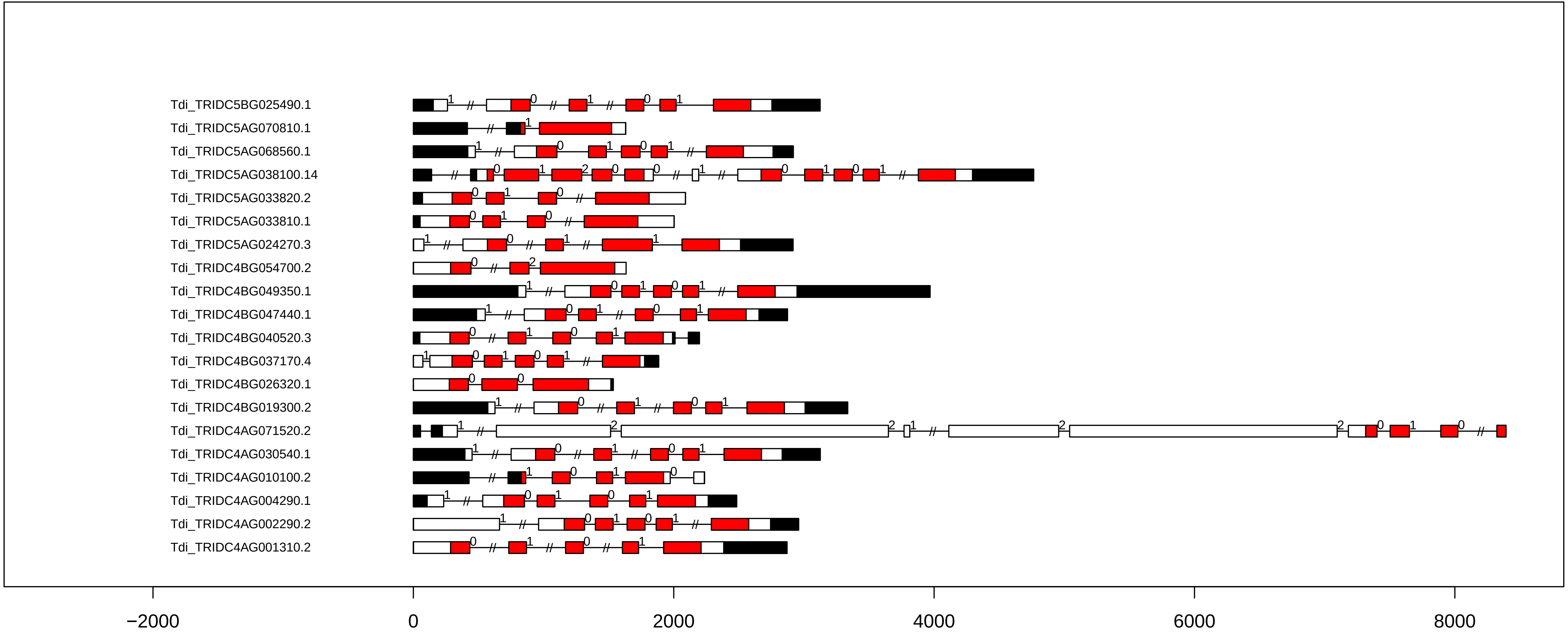

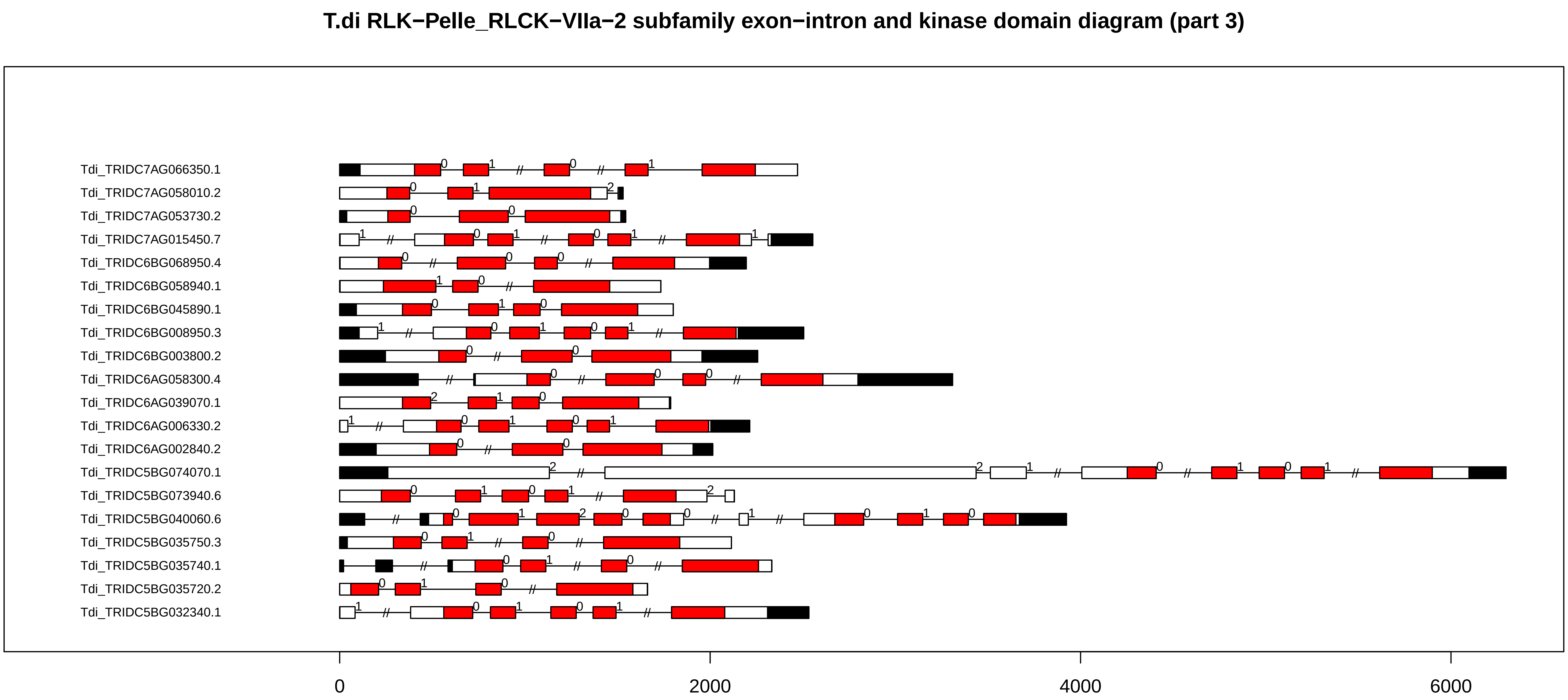

T.di RLK−Pelle_RLCK−VIIa−2 subfamily exon−intron and kinase domain diagram (part 3)
Tdi_TRIDC7AG066350.1
Tdi_TRIDC7AG058010.2
Tdi_TRIDC7AG053730.2
Tdi_TRIDC7AG015450.7
Tdi_TRIDC6BG068950.4
Tdi_TRIDC6BG058940.1
Tdi_TRIDC6BG045890.1
Tdi_TRIDC6BG008950.3
Tdi_TRIDC6BG003800.2
Tdi_TRIDC6AG058300.4
Tdi_TRIDC6AG039070.1
Tdi_TRIDC6AG006330.2
Tdi_TRIDC6AG002840.2
Tdi_TRIDC5BG074070.1
Tdi_TRIDC5BG073940.6
Tdi_TRIDC5BG040060.6
Tdi_TRIDC5BG035750.3
Tdi_TRIDC5BG035740.1
Tdi_TRIDC5BG035720.2
Tdi_TRIDC5BG032340.1
0
2000
4000
6000

T.di RLK−Pelle_RLCK−VIIa−2 subfamily exon−intron and kinase domain diagram (part 4)

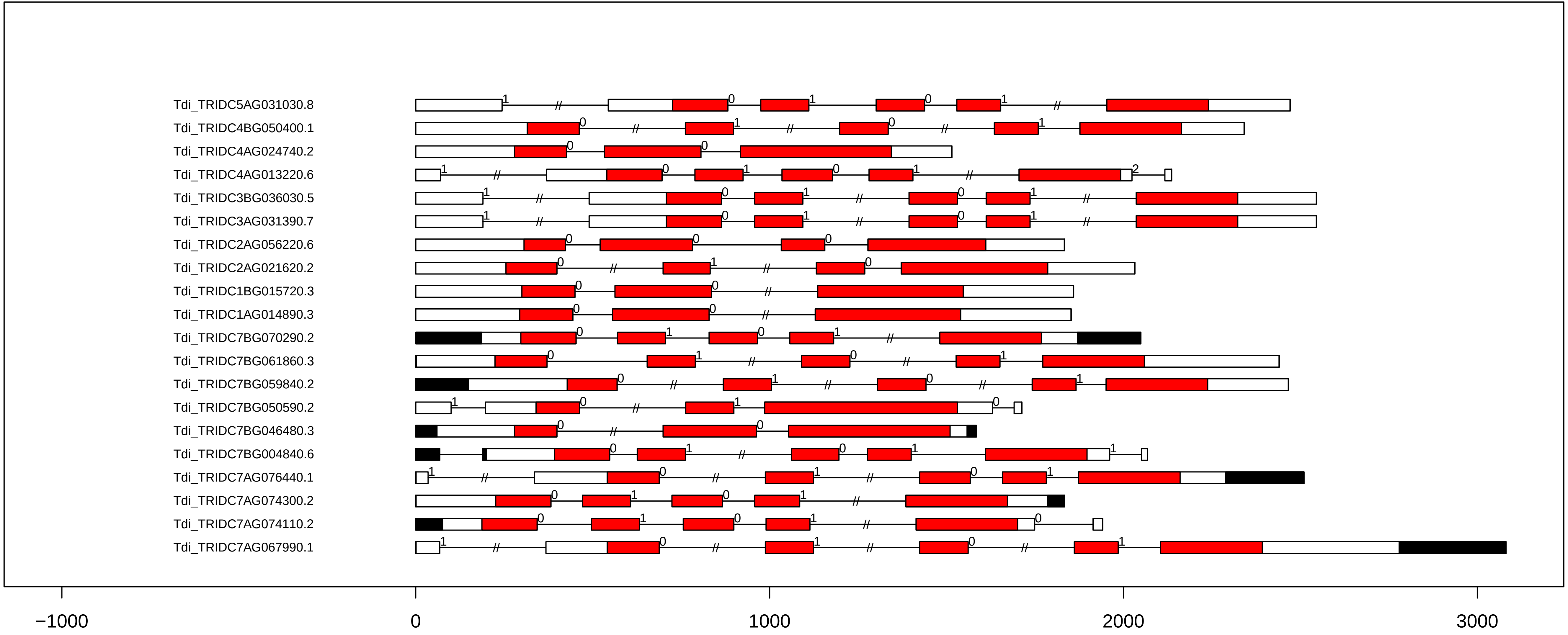

T.di RLK−Pelle_RLCK−VIIa−2 subfamily exon−intron and kinase domain diagram (part 5)

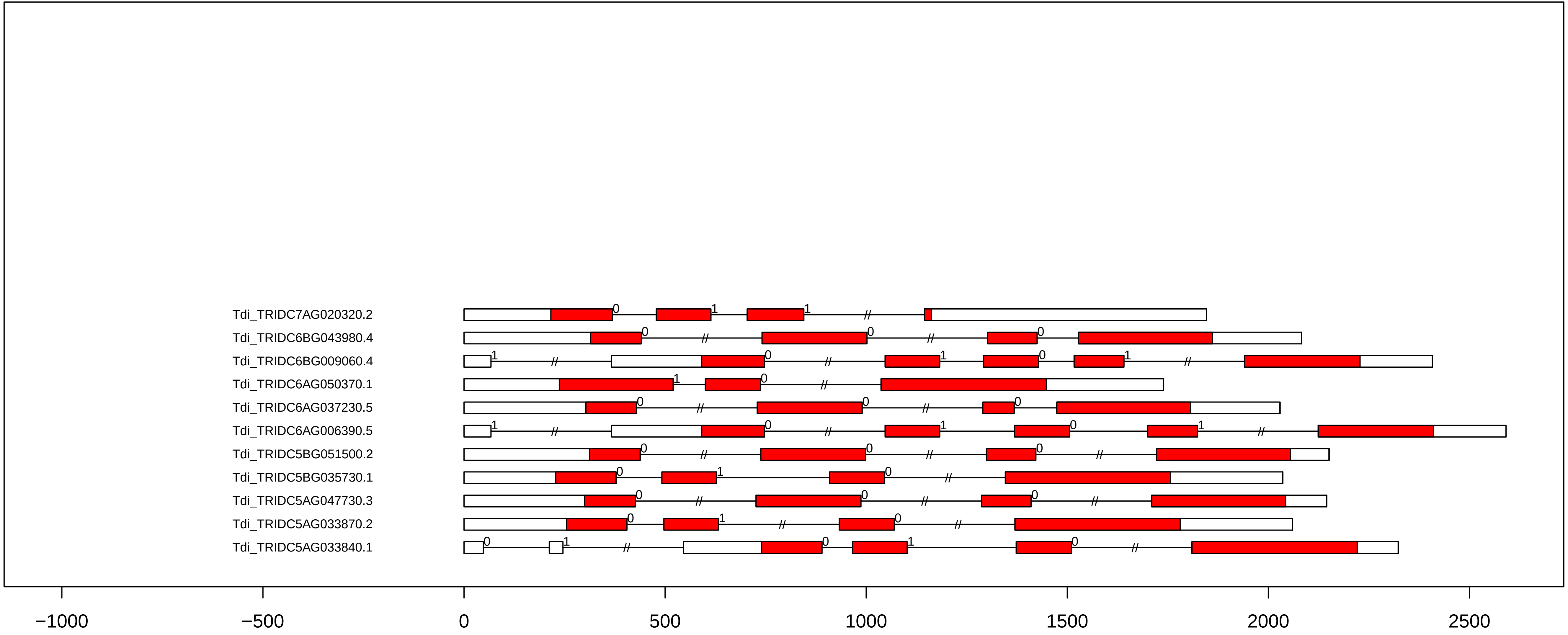

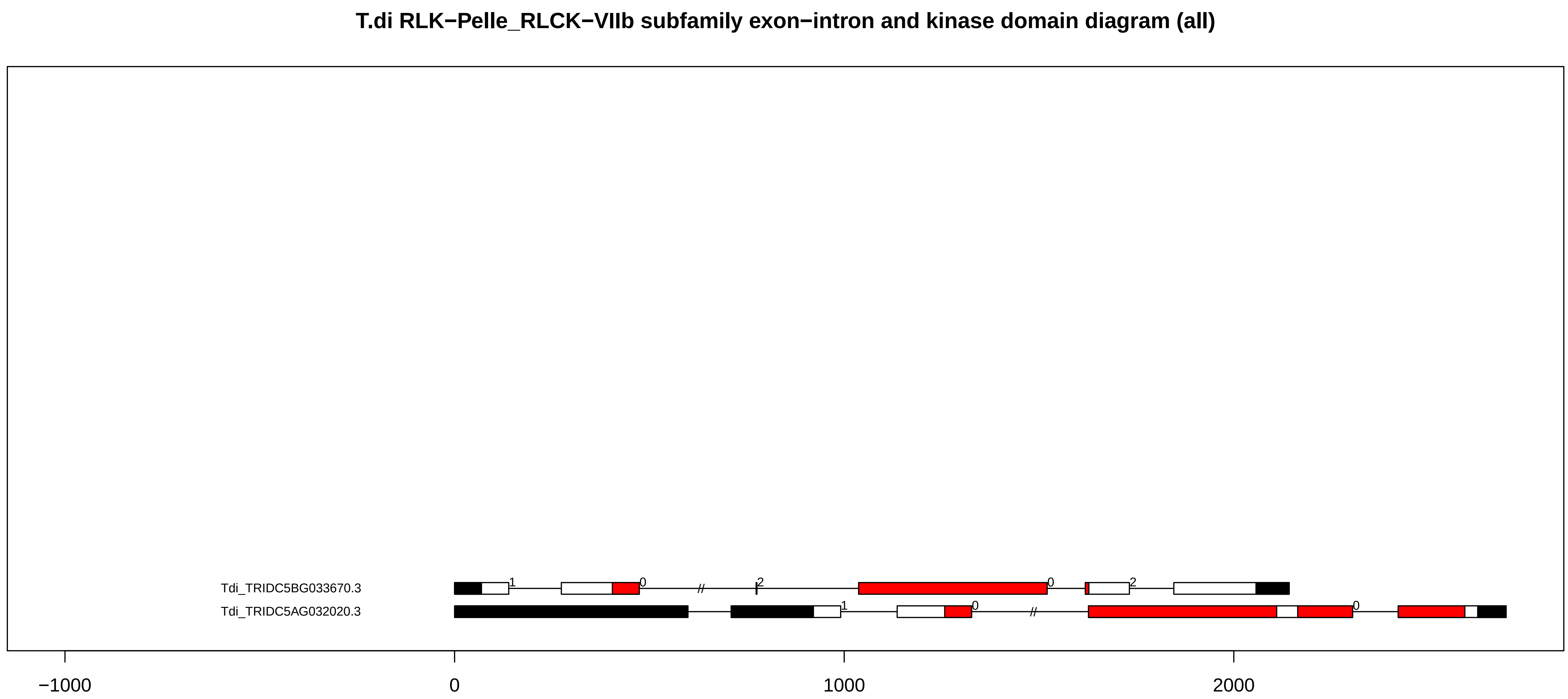

T.di RLK−Pelle_RLCK−VIIb subfamily exon−intron and kinase domain diagram (all)
Tdi_TRIDC5BG033670.3
Tdi_TRIDC5AG032020.3
1
0
2
0
2
1
0
0
−1000
0
1000
2000

T.di RLK−Pelle_RLCK−VIII subfamily exon−intron and kinase domain diagram (all)

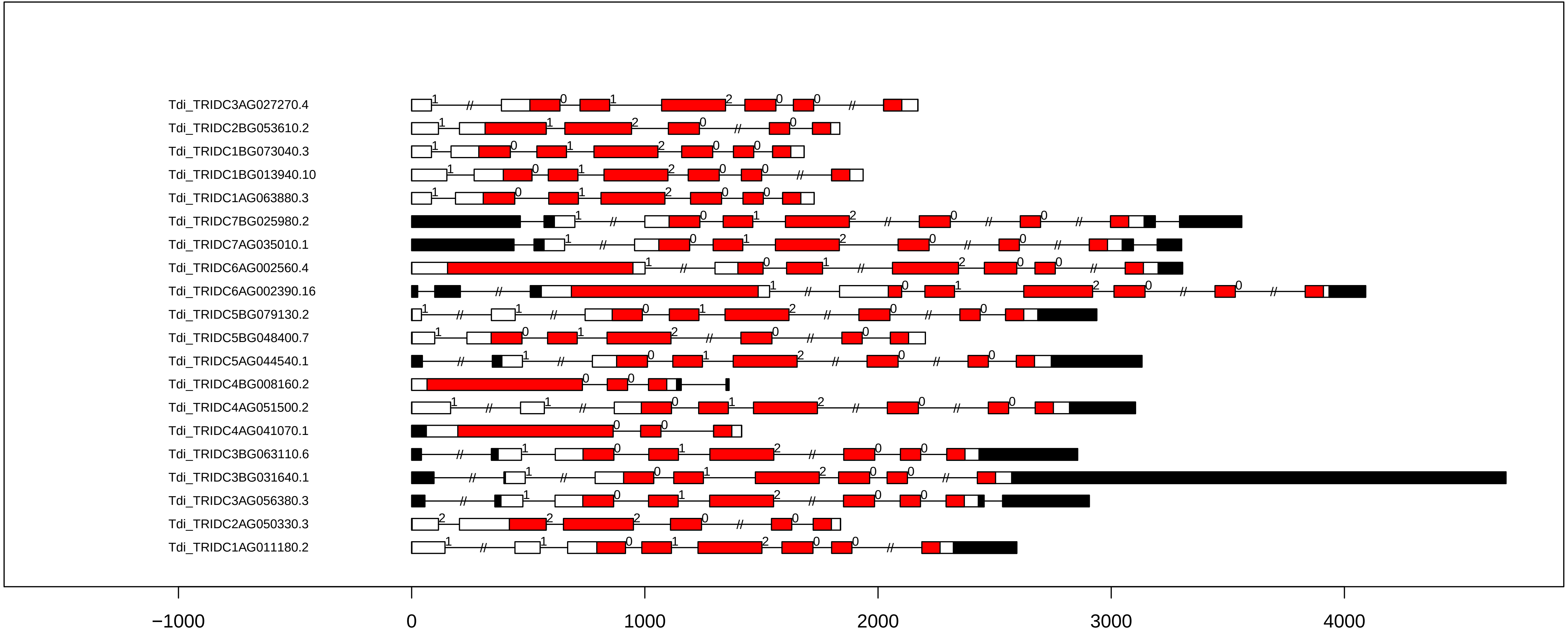

T.di RLK−Pelle_RLCK−VI subfamily exon−intron and kinase domain diagram (all)

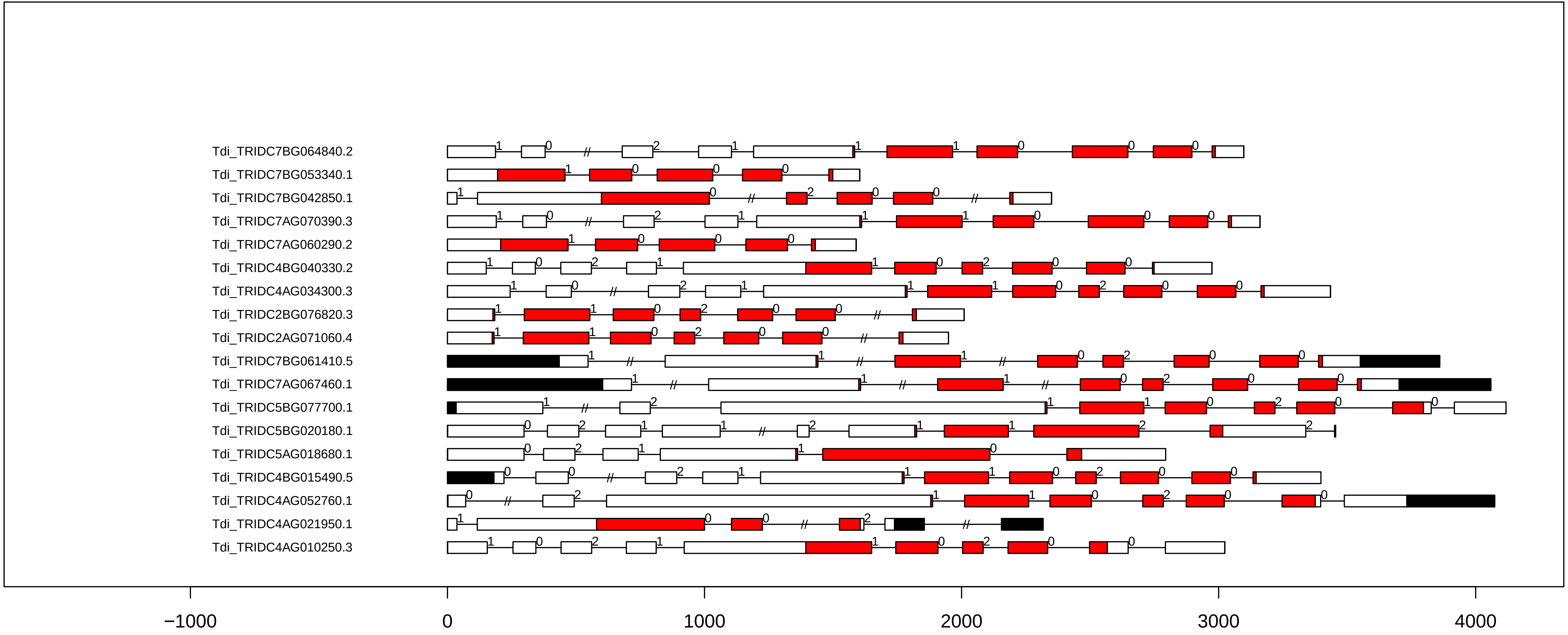

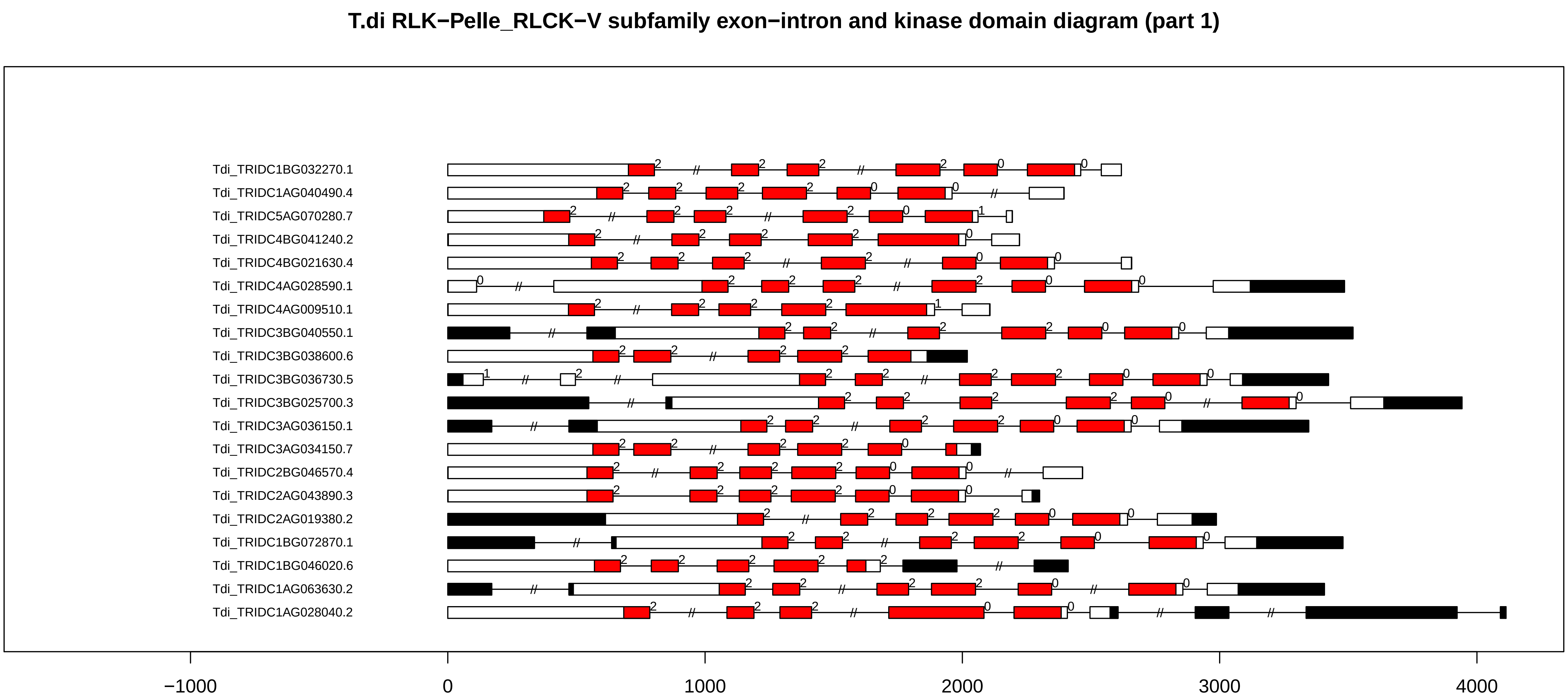
T.di RLK−Pelle_RLCK−V subfamily exon−intron and kinase domain diagram (part 1)
Tdi_TRIDC1BG032270.1
Tdi_TRIDC1AG040490.4
Tdi_TRIDC5AG070280.7
Tdi_TRIDC4BG041240.2
Tdi_TRIDC4BG021630.4
Tdi_TRIDC4AG028590.1
Tdi_TRIDC4AG009510.1
Tdi_TRIDC3BG040550.1
Tdi_TRIDC3BG038600.6
Tdi_TRIDC3BG036730.5
Tdi_TRIDC3BG025700.3
Tdi_TRIDC3AG036150.1
Tdi_TRIDC3AG034150.7
Tdi_TRIDC2BG046570.4
Tdi_TRIDC2AG043890.3
Tdi_TRIDC2AG019380.2
Tdi_TRIDC1BG072870.1
Tdi_TRIDC1BG046020.6
Tdi_TRIDC1AG063630.2
Tdi_TRIDC1AG028040.2
−1000
0
1000
2000
3000
4000

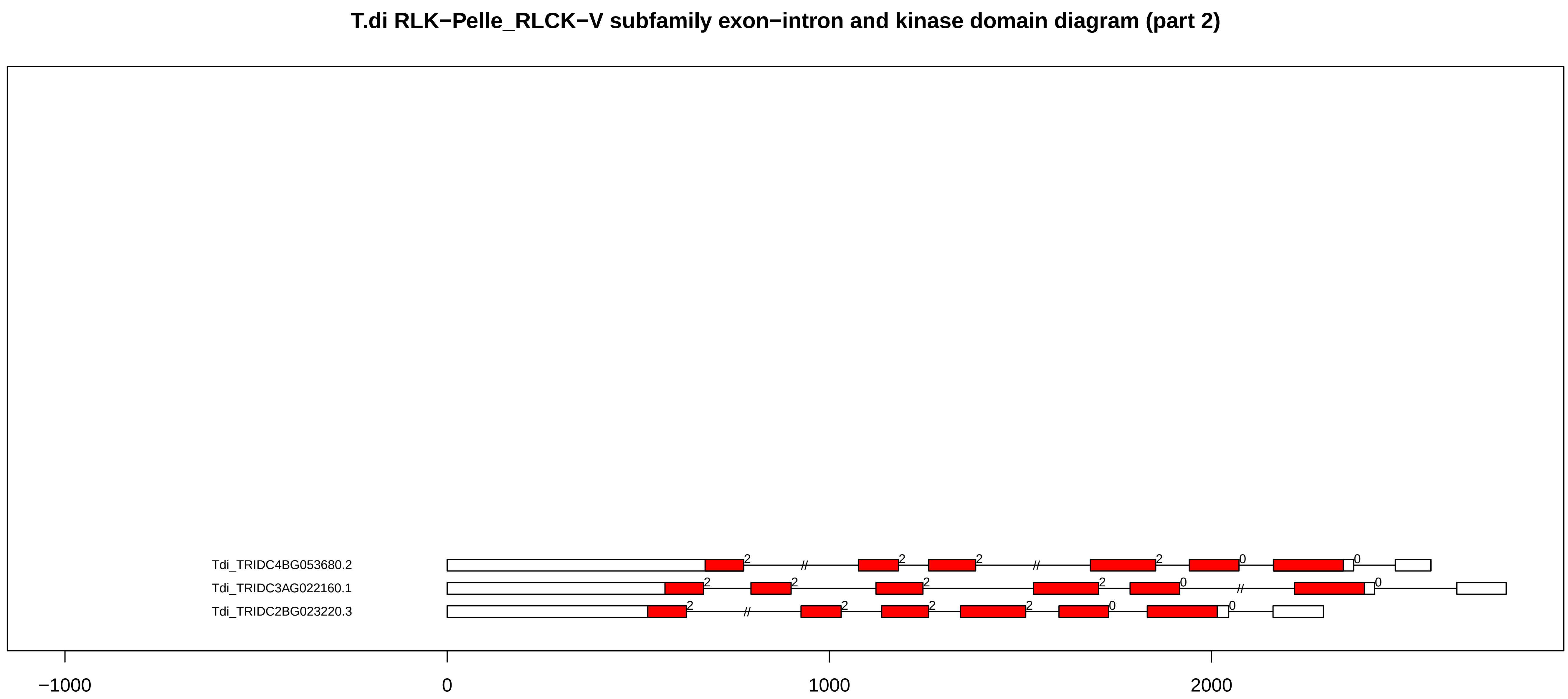
T.di RLK−Pelle_RLCK−V subfamily exon−intron and kinase domain diagram (part 2)
Tdi_TRIDC4BG053680.2
Tdi_TRIDC3AG022160.1
Tdi_TRIDC2BG023220.3
−1000
0
1000
2000

T.di RLK−Pelle_RLCK−XII−1 subfamily exon−intron and kinase domain diagram (all)

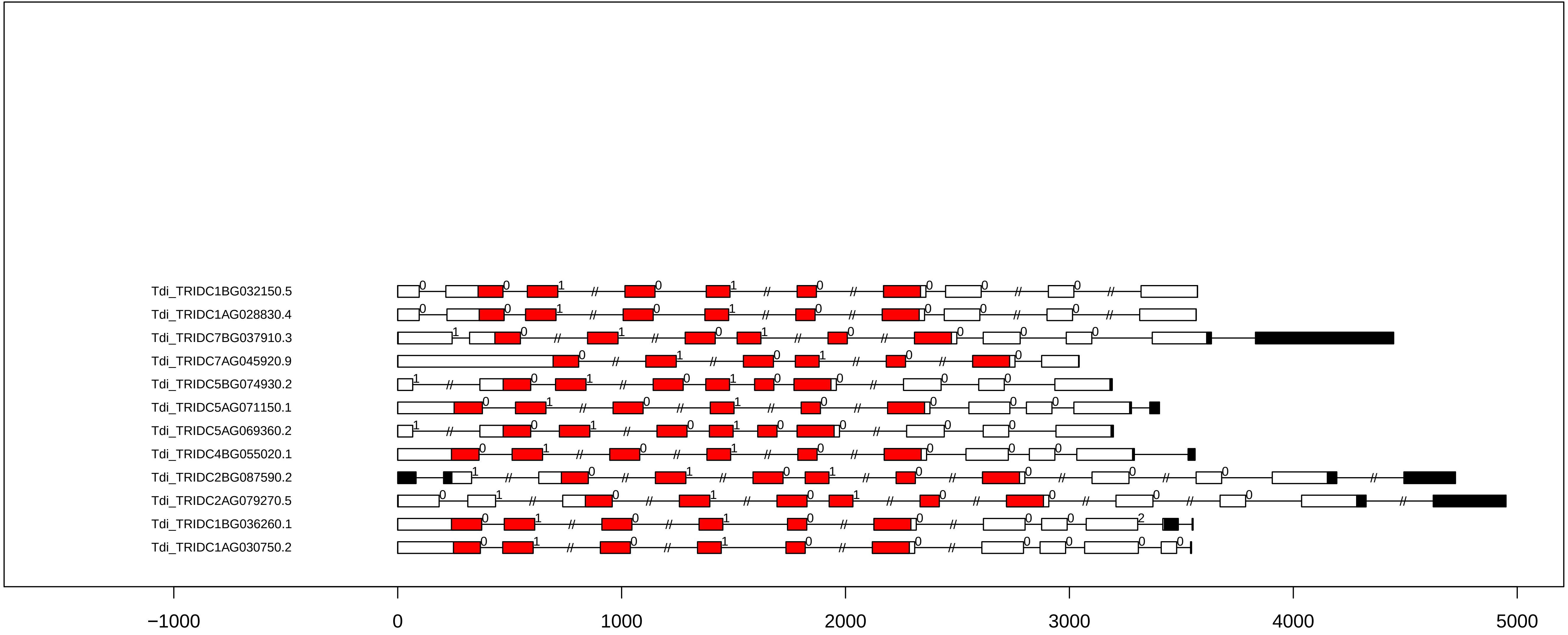

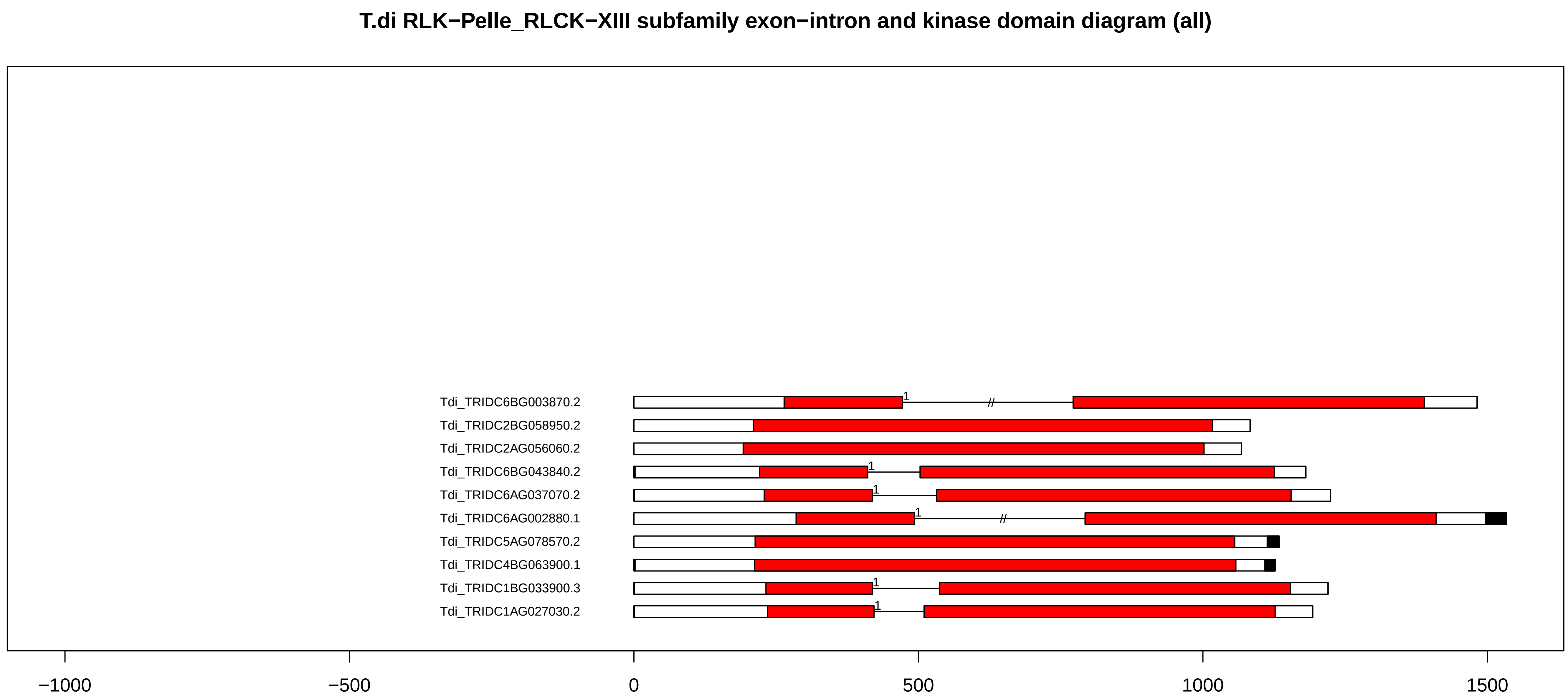
T.di RLK−Pelle_RLCK−XIII subfamily exon−intron and kinase domain diagram (all)
Tdi_TRIDC6BG003870.2
Tdi_TRIDC2BG058950.2
Tdi_TRIDC2AG056060.2
Tdi_TRIDC6BG043840.2
Tdi_TRIDC6AG037070.2
Tdi_TRIDC6AG002880.1
Tdi_TRIDC5AG078570.2
Tdi_TRIDC4BG063900.1
Tdi_TRIDC1BG033900.3
Tdi_TRIDC1AG027030.2
1
1
1
1
1
1
−1000
−500
0
500
1000
1500

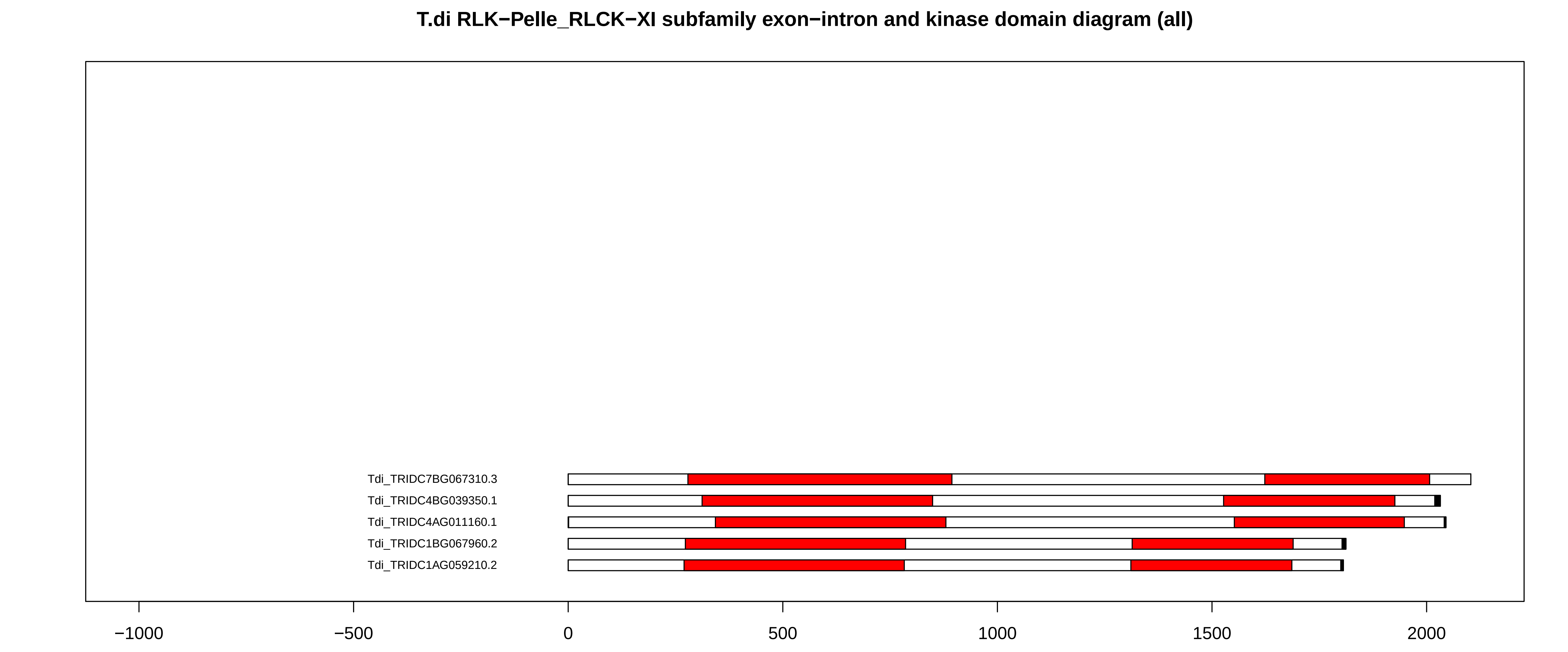
T.di RLK−Pelle_RLCK−XI subfamily exon−intron and kinase domain diagram (all)
Tdi_TRIDC7BG067310.3
Tdi_TRIDC4BG039350.1
Tdi_TRIDC4AG011160.1
Tdi_TRIDC1BG067960.2
Tdi_TRIDC1AG059210.2
−1000
−500
0
500
1000
1500
2000

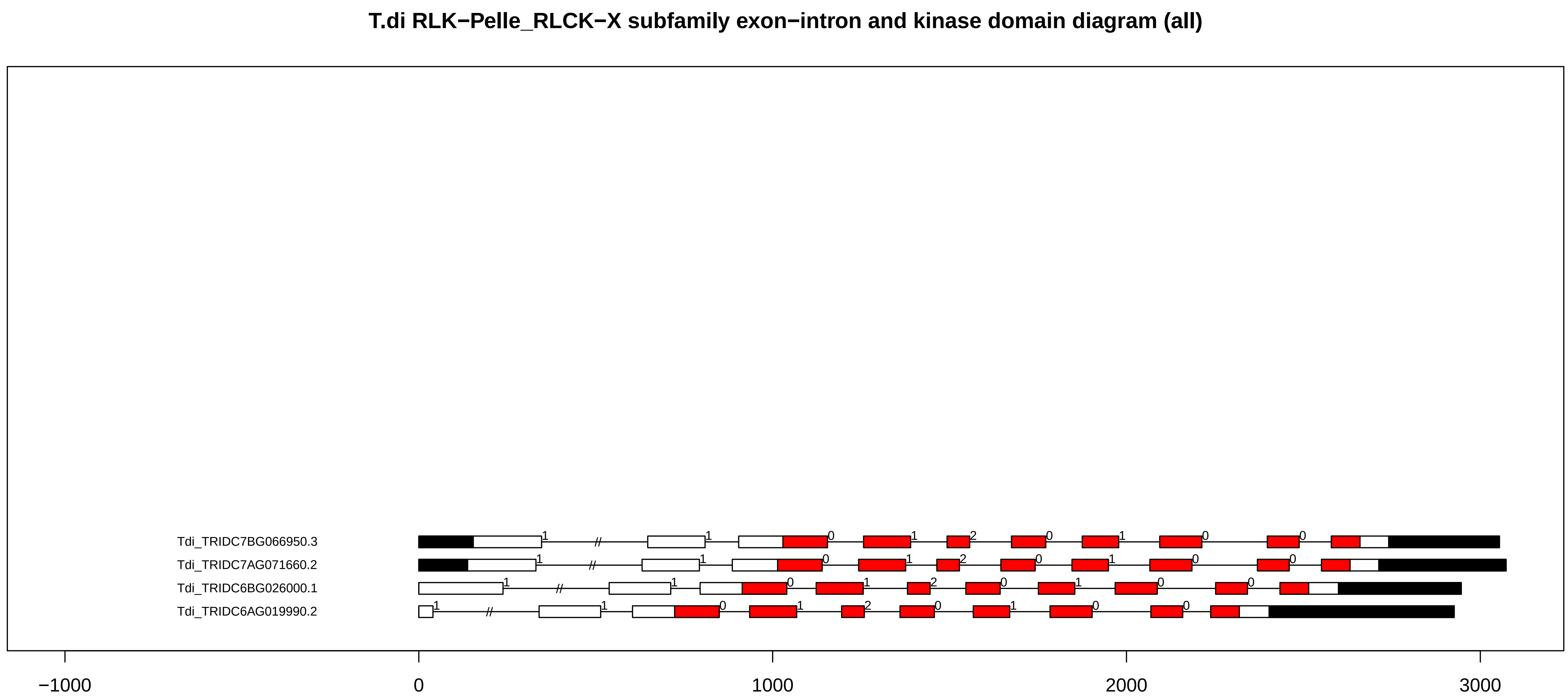
T.di RLK−Pelle_RLCK−X subfamily exon−intron and kinase domain diagram (all)
Tdi_TRIDC7BG066950.3
Tdi_TRIDC7AG071660.2
Tdi_TRIDC6BG026000.1
Tdi_TRIDC6AG019990.2
−1000
0
1000
2000
3000

T.di RLK−Pelle_RLCK−XVI subfamily exon−intron and kinase domain diagram (all)

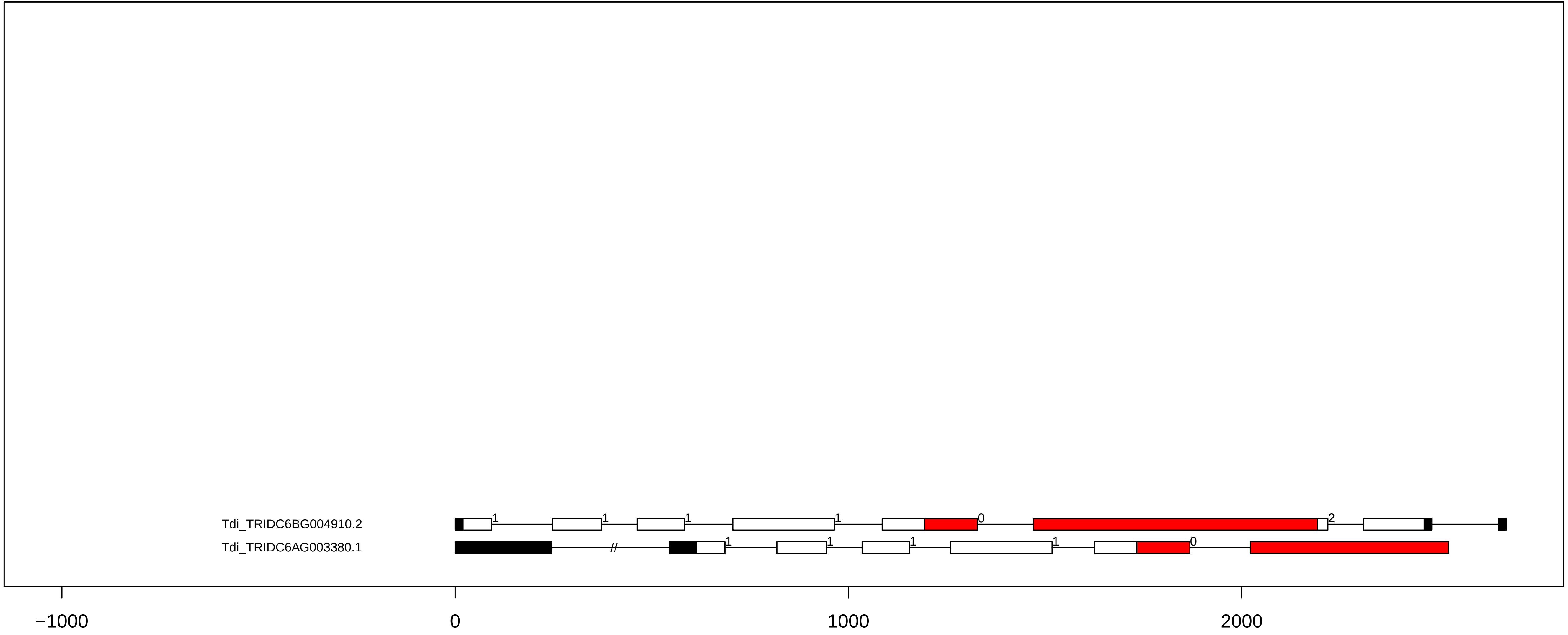

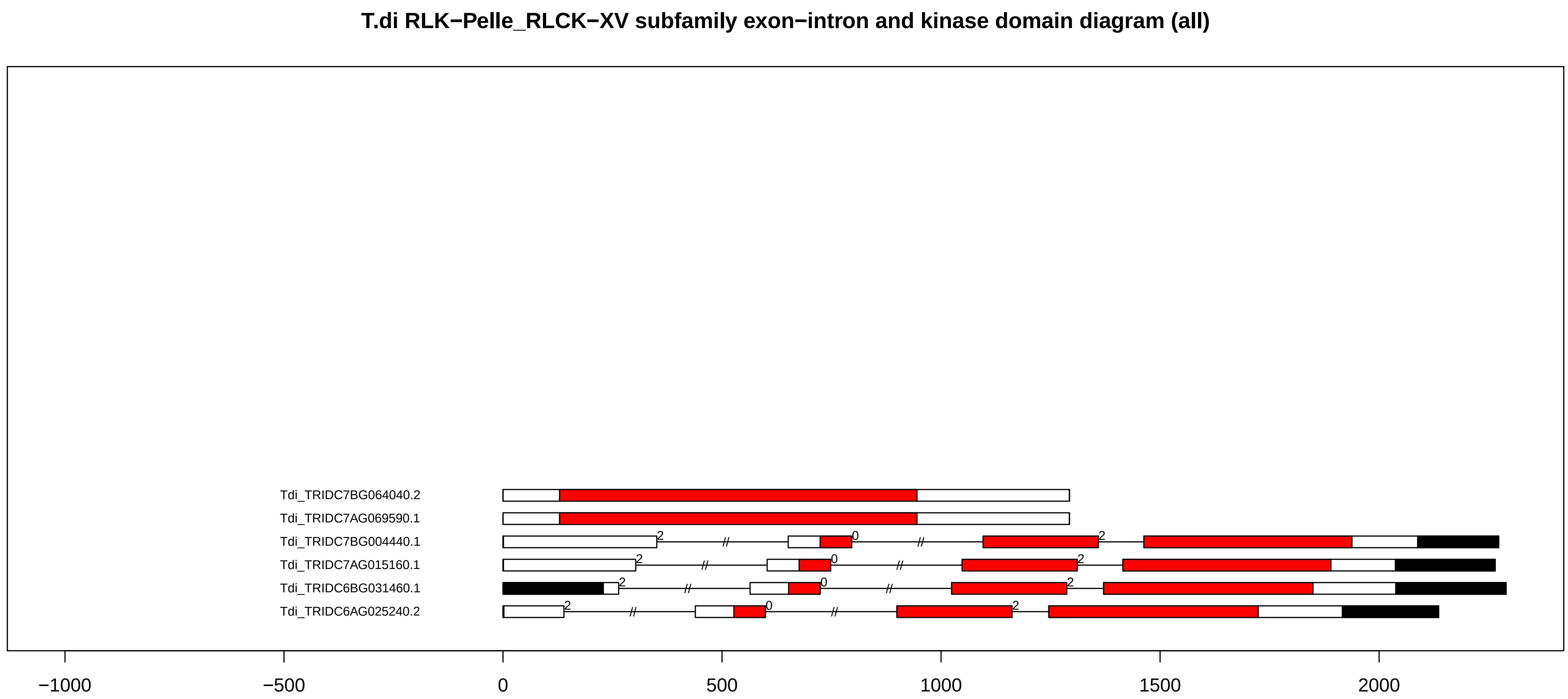

T.di RLK−Pelle_RLCK−XV subfamily exon−intron and kinase domain diagram (all)
Tdi_TRIDC7BG064040.2
Tdi_TRIDC7AG069590.1
Tdi_TRIDC7BG004440.1
Tdi_TRIDC7AG015160.1
Tdi_TRIDC6BG031460.1
Tdi_TRIDC6AG025240.2
−1000
−500
0
500
1000
1500
2000

T.di RLK−Pelle_SD−2b subfamily exon−intron and kinase domain diagram (part 1)

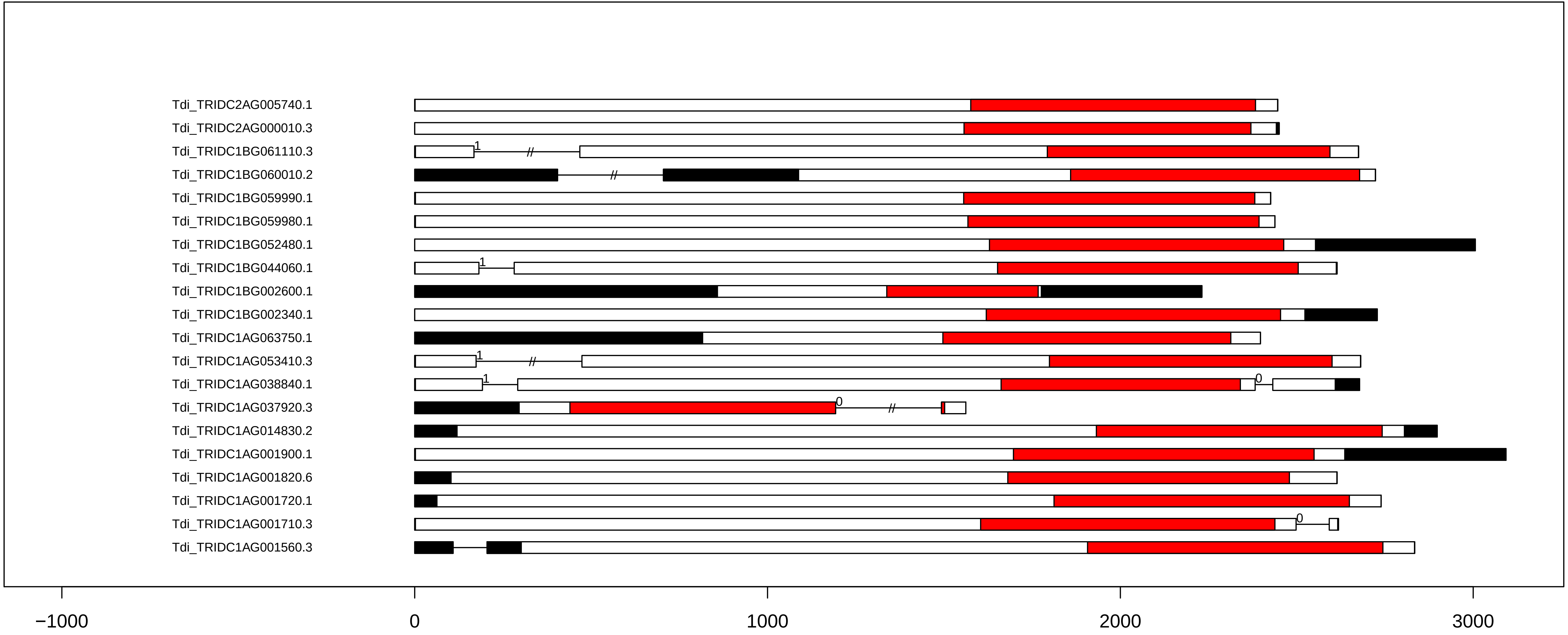

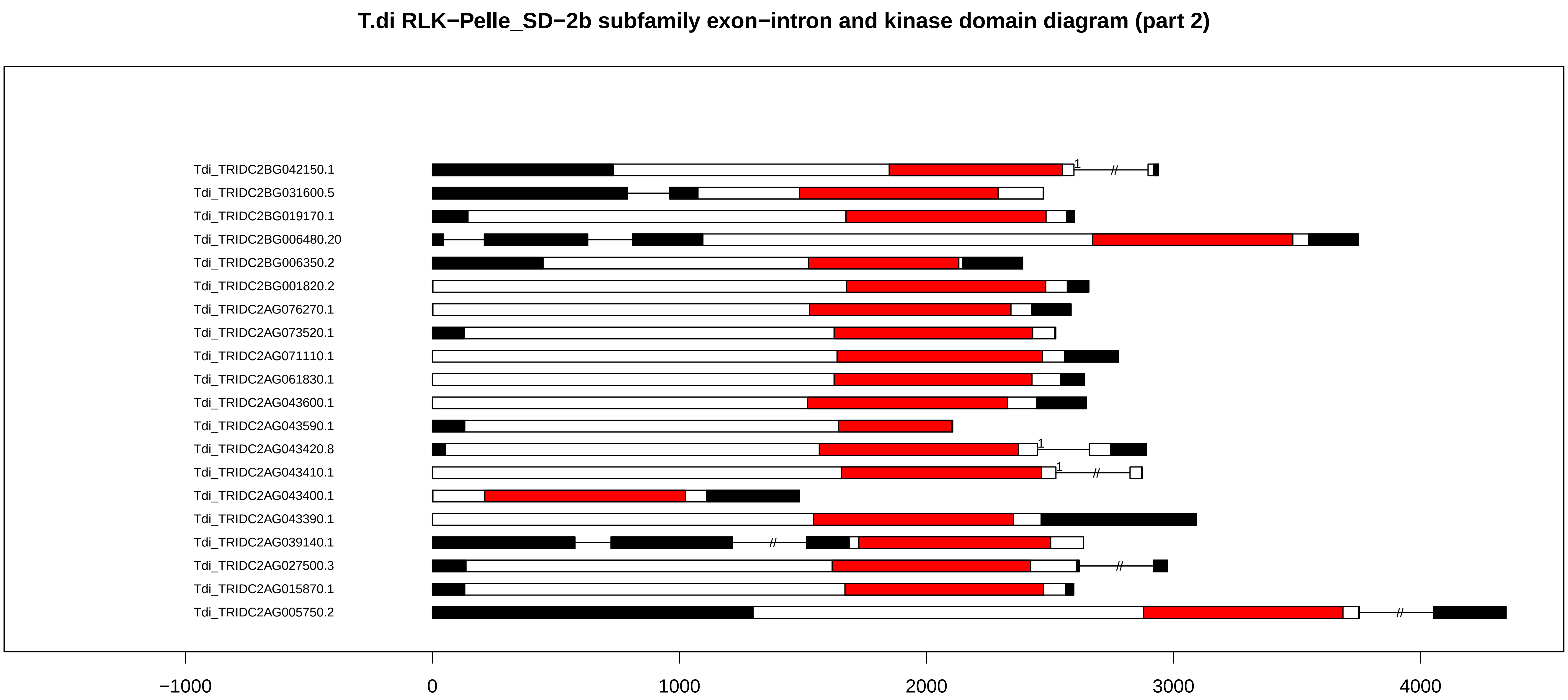
T.di RLK−Pelle_SD−2b subfamily exon−intron and kinase domain diagram (part 2)
Tdi_TRIDC2BG042150.1
Tdi_TRIDC2BG031600.5
Tdi_TRIDC2BG019170.1
Tdi_TRIDC2BG006480.20
Tdi_TRIDC2BG006350.2
Tdi_TRIDC2BG001820.2
Tdi_TRIDC2AG076270.1
Tdi_TRIDC2AG073520.1
Tdi_TRIDC2AG071110.1
Tdi_TRIDC2AG061830.1
Tdi_TRIDC2AG043600.1
Tdi_TRIDC2AG043590.1
Tdi_TRIDC2AG043420.8
Tdi_TRIDC2AG043410.1
Tdi_TRIDC2AG043400.1
Tdi_TRIDC2AG043390.1
Tdi_TRIDC2AG039140.1
Tdi_TRIDC2AG027500.3
Tdi_TRIDC2AG015870.1
Tdi_TRIDC2AG005750.2
−1000
0
1000
2000
3000
4000

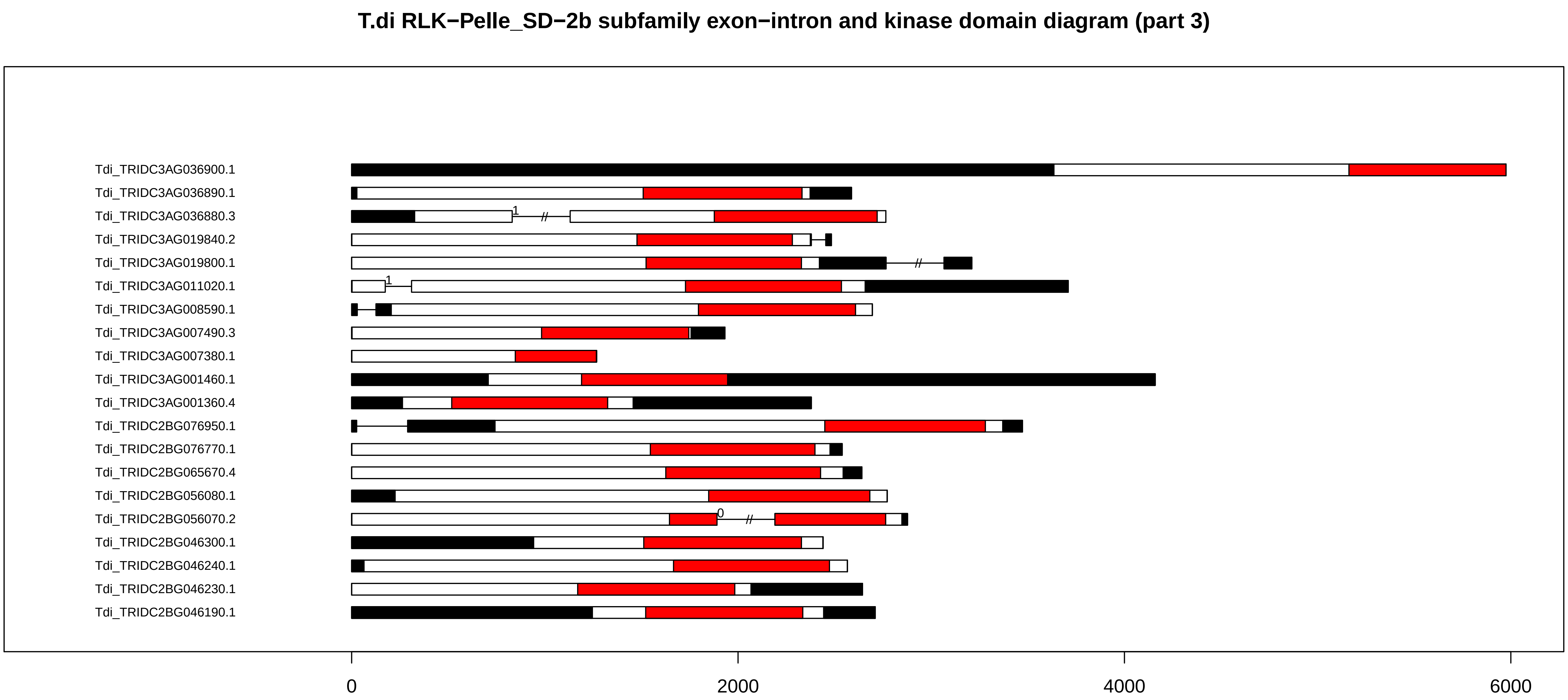

T.di RLK−Pelle_SD−2b subfamily exon−intron and kinase domain diagram (part 3)
Tdi_TRIDC3AG036900.1
Tdi_TRIDC3AG036890.1
Tdi_TRIDC3AG036880.3
Tdi_TRIDC3AG019840.2
Tdi_TRIDC3AG019800.1
Tdi_TRIDC3AG011020.1
Tdi_TRIDC3AG008590.1
Tdi_TRIDC3AG007490.3
Tdi_TRIDC3AG007380.1
Tdi_TRIDC3AG001460.1
Tdi_TRIDC3AG001360.4
Tdi_TRIDC2BG076950.1
Tdi_TRIDC2BG076770.1
Tdi_TRIDC2BG065670.4
Tdi_TRIDC2BG056080.1
Tdi_TRIDC2BG056070.2
Tdi_TRIDC2BG046300.1
Tdi_TRIDC2BG046240.1
Tdi_TRIDC2BG046230.1
Tdi_TRIDC2BG046190.1
0
2000
4000
6000

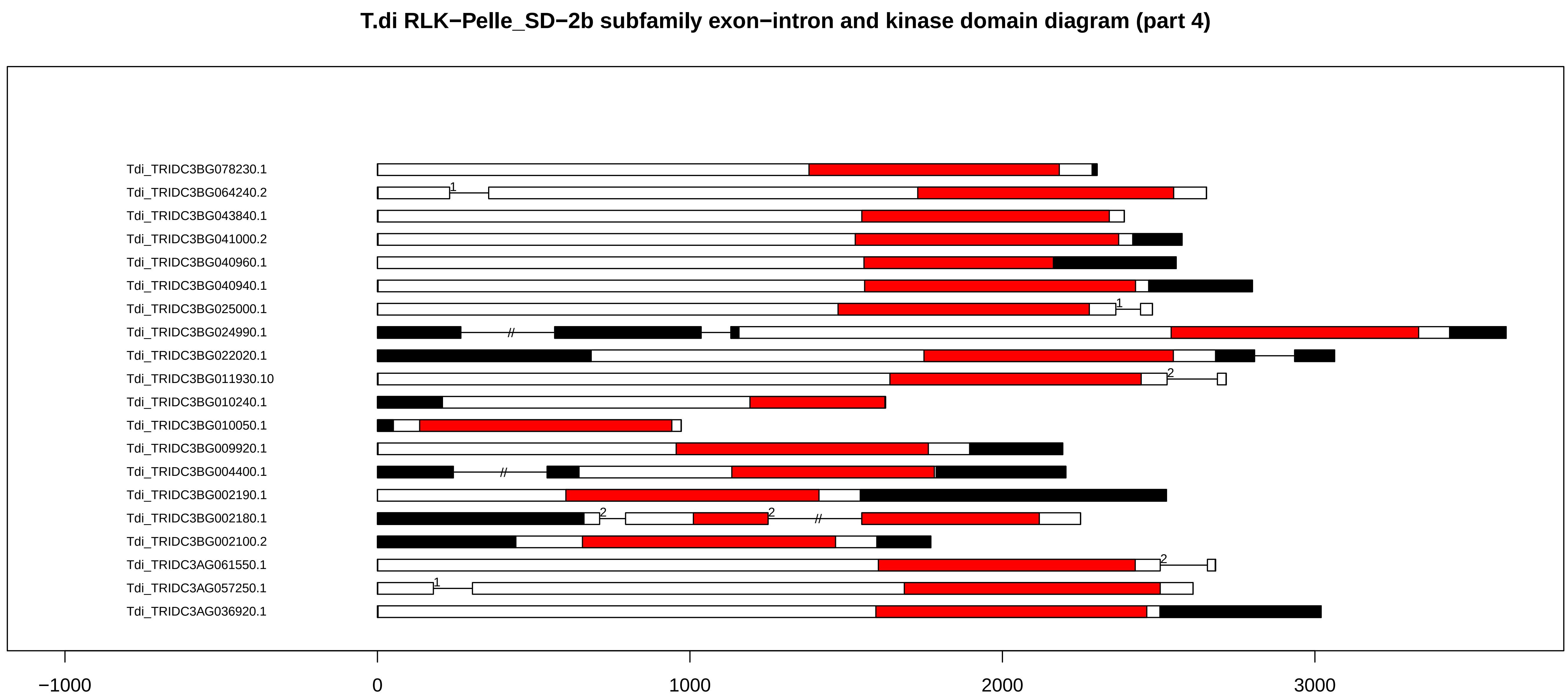
T.di RLK−Pelle_SD−2b subfamily exon−intron and kinase domain diagram (part 4)
Tdi_TRIDC3BG078230.1
Tdi_TRIDC3BG064240.2
Tdi_TRIDC3BG043840.1
Tdi_TRIDC3BG041000.2
Tdi_TRIDC3BG040960.1
Tdi_TRIDC3BG040940.1
Tdi_TRIDC3BG025000.1
Tdi_TRIDC3BG024990.1
Tdi_TRIDC3BG022020.1
Tdi_TRIDC3BG011930.10
Tdi_TRIDC3BG010240.1
Tdi_TRIDC3BG010050.1
Tdi_TRIDC3BG009920.1
Tdi_TRIDC3BG004400.1
Tdi_TRIDC3BG002190.1
Tdi_TRIDC3BG002180.1
Tdi_TRIDC3BG002100.2
Tdi_TRIDC3AG061550.1
Tdi_TRIDC3AG057250.1
Tdi_TRIDC3AG036920.1
−1000
0
1000
2000
3000

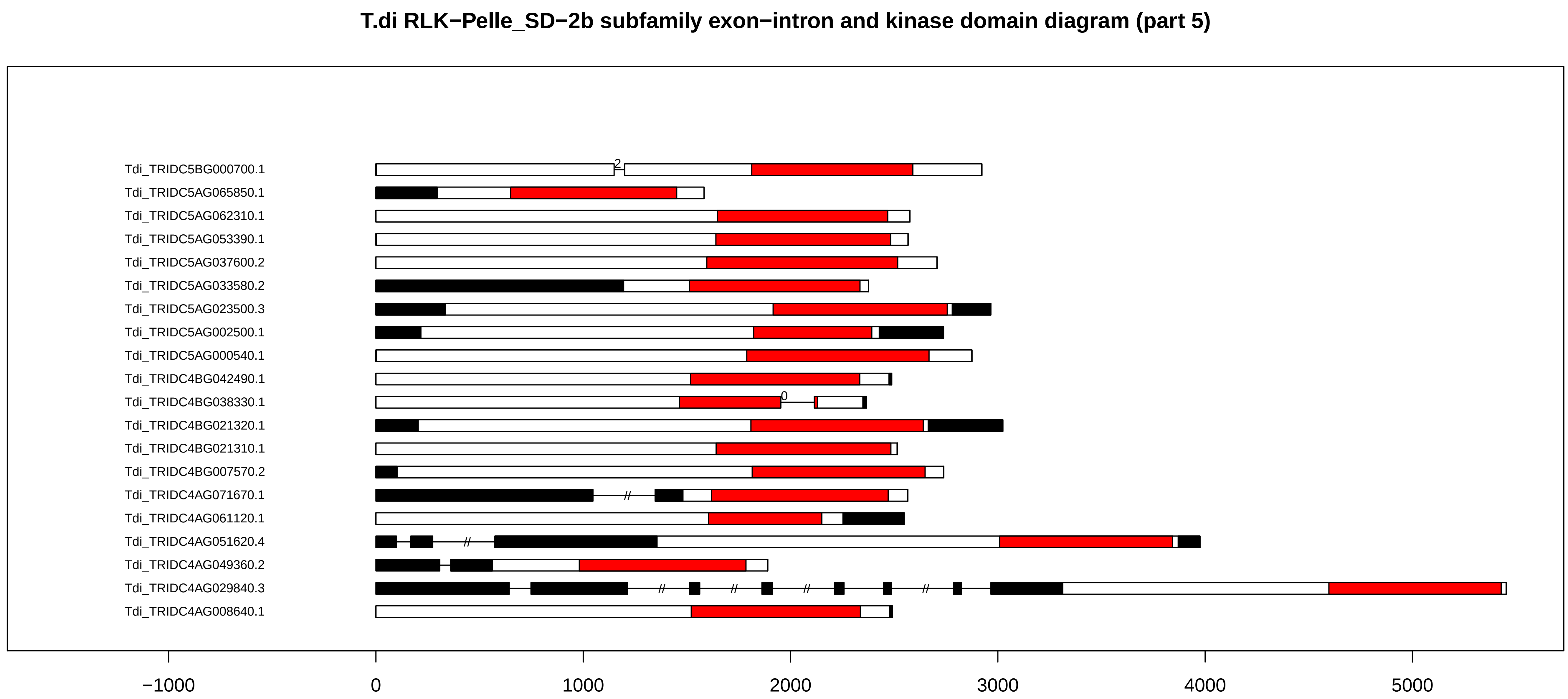

T.di RLK−Pelle_SD−2b subfamily exon−intron and kinase domain diagram (part 5)
Tdi_TRIDC5BG000700.1
Tdi_TRIDC5AG065850.1
Tdi_TRIDC5AG062310.1
Tdi_TRIDC5AG053390.1
Tdi_TRIDC5AG037600.2
Tdi_TRIDC5AG033580.2
Tdi_TRIDC5AG023500.3
Tdi_TRIDC5AG002500.1
Tdi_TRIDC5AG000540.1
Tdi_TRIDC4BG042490.1
Tdi_TRIDC4BG038330.1
Tdi_TRIDC4BG021320.1
Tdi_TRIDC4BG021310.1
Tdi_TRIDC4BG007570.2
Tdi_TRIDC4AG071670.1
Tdi_TRIDC4AG061120.1
Tdi_TRIDC4AG051620.4
Tdi_TRIDC4AG049360.2
Tdi_TRIDC4AG029840.3
Tdi_TRIDC4AG008640.1
2
0
−1000
0
1000
2000
3000
4000
5000

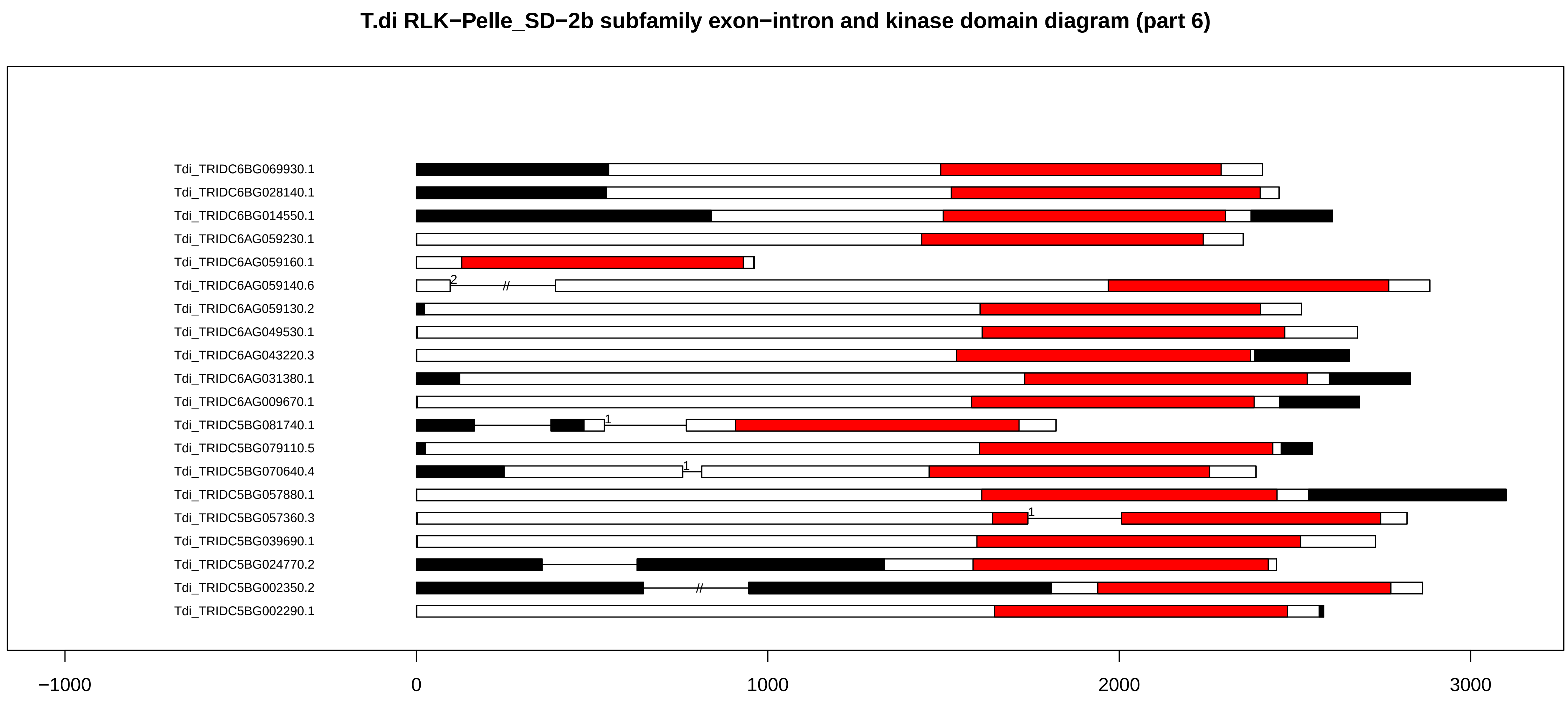

T.di RLK−Pelle_SD−2b subfamily exon−intron and kinase domain diagram (part 6)
Tdi_TRIDC6BG069930.1
Tdi_TRIDC6BG028140.1
Tdi_TRIDC6BG014550.1
Tdi_TRIDC6AG059230.1
Tdi_TRIDC6AG059160.1
Tdi_TRIDC6AG059140.6
Tdi_TRIDC6AG059130.2
Tdi_TRIDC6AG049530.1
Tdi_TRIDC6AG043220.3
Tdi_TRIDC6AG031380.1
Tdi_TRIDC6AG009670.1
Tdi_TRIDC5BG081740.1
Tdi_TRIDC5BG079110.5
Tdi_TRIDC5BG070640.4
Tdi_TRIDC5BG057880.1
Tdi_TRIDC5BG057360.3
Tdi_TRIDC5BG039690.1
Tdi_TRIDC5BG024770.2
Tdi_TRIDC5BG002350.2
Tdi_TRIDC5BG002290.1
2
1
1
1
−1000
0
1000
2000
3000

T.di RLK−Pelle_SD−2b subfamily exon−intron and kinase domain diagram (part 7)

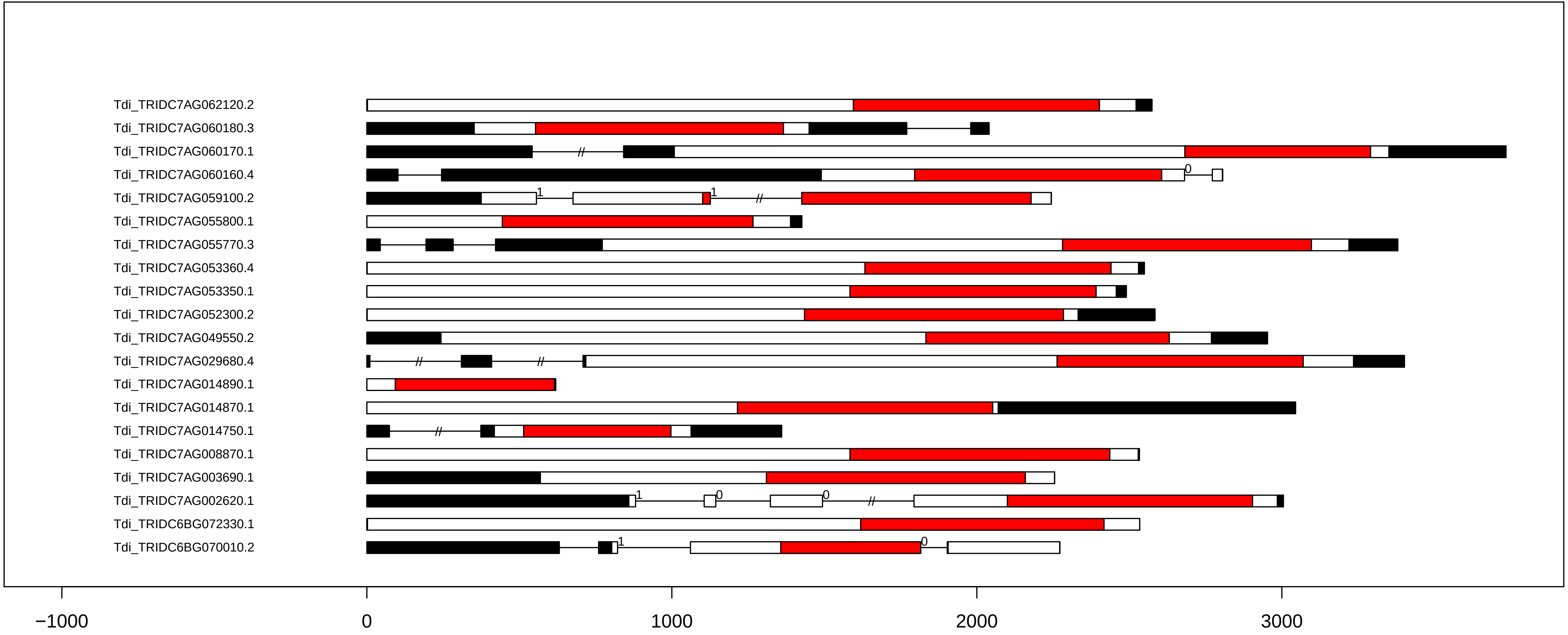

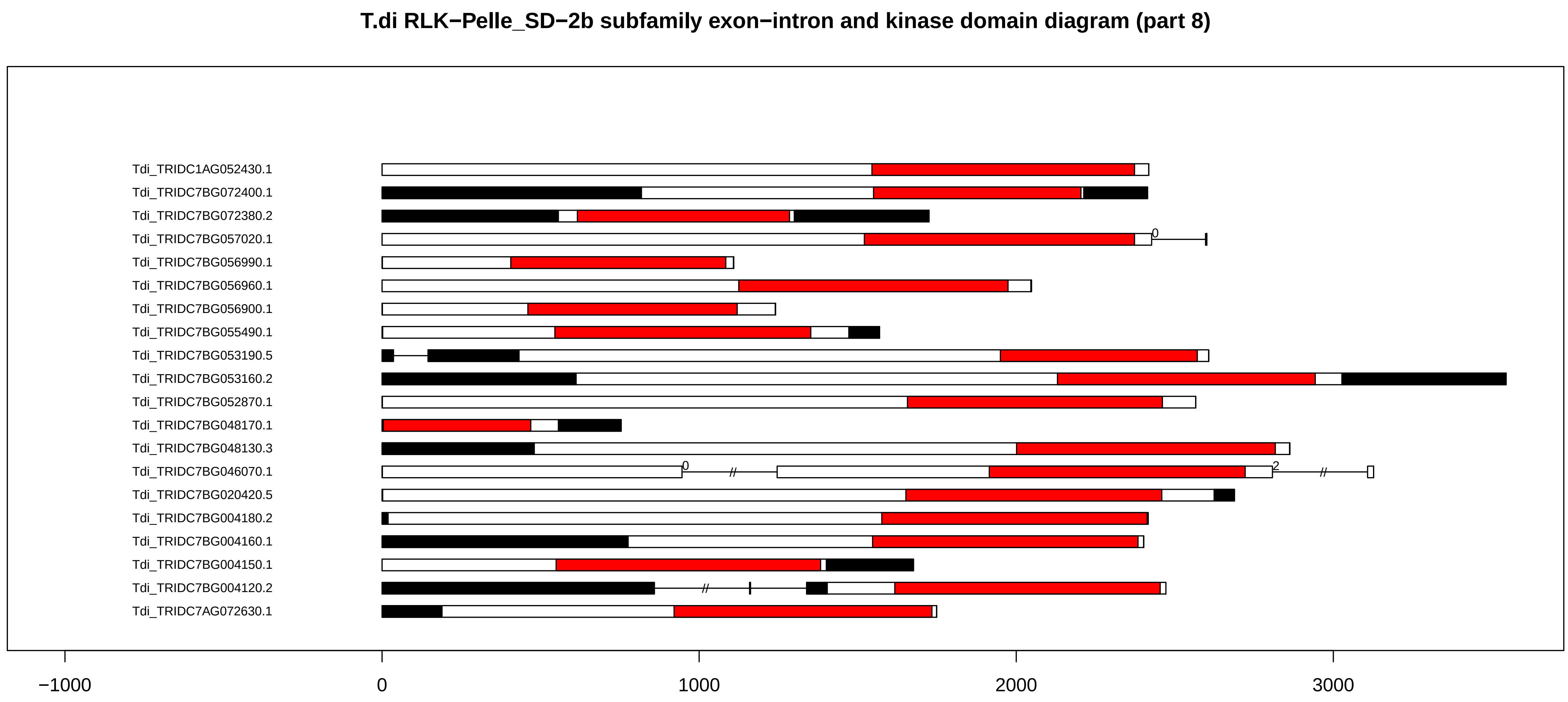
T.di RLK−Pelle_SD−2b subfamily exon−intron and kinase domain diagram (part 8)
Tdi_TRIDC1AG052430.1
Tdi_TRIDC7BG072400.1
Tdi_TRIDC7BG072380.2
Tdi_TRIDC7BG057020.1
Tdi_TRIDC7BG056990.1
Tdi_TRIDC7BG056960.1
Tdi_TRIDC7BG056900.1
Tdi_TRIDC7BG055490.1
Tdi_TRIDC7BG053190.5
Tdi_TRIDC7BG053160.2
Tdi_TRIDC7BG052870.1
Tdi_TRIDC7BG048170.1
Tdi_TRIDC7BG048130.3
Tdi_TRIDC7BG046070.1
Tdi_TRIDC7BG020420.5
Tdi_TRIDC7BG004180.2
Tdi_TRIDC7BG004160.1
Tdi_TRIDC7BG004150.1
Tdi_TRIDC7BG004120.2
Tdi_TRIDC7AG072630.1
0
0
2
−1000
0
1000
2000
3000

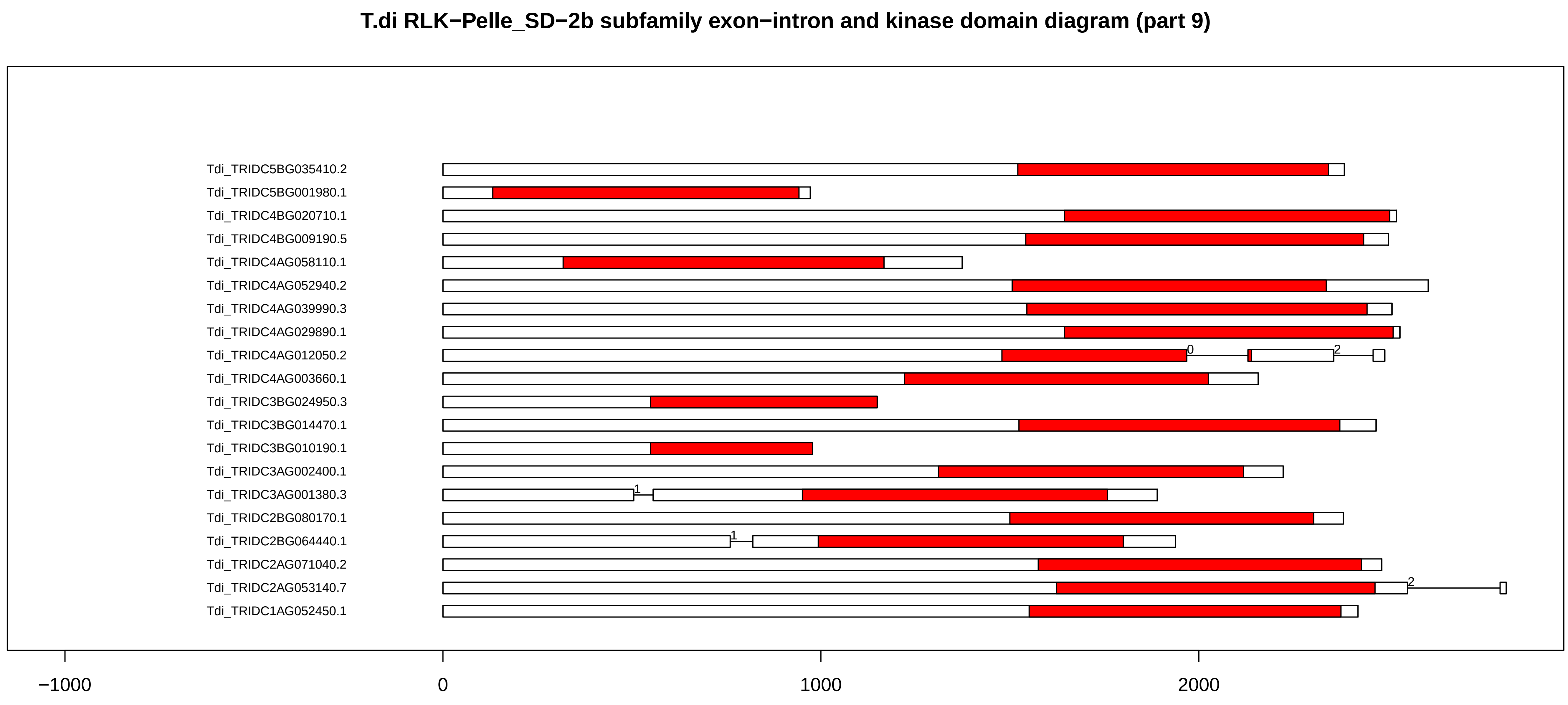
T.di RLK−Pelle_SD−2b subfamily exon−intron and kinase domain diagram (part 9)
Tdi_TRIDC5BG035410.2
Tdi_TRIDC5BG001980.1
Tdi_TRIDC4BG020710.1
Tdi_TRIDC4BG009190.5
Tdi_TRIDC4AG058110.1
Tdi_TRIDC4AG052940.2
Tdi_TRIDC4AG039990.3
Tdi_TRIDC4AG029890.1
Tdi_TRIDC4AG012050.2
Tdi_TRIDC4AG003660.1
Tdi_TRIDC3BG024950.3
Tdi_TRIDC3BG014470.1
Tdi_TRIDC3BG010190.1
Tdi_TRIDC3AG002400.1
Tdi_TRIDC3AG001380.3
Tdi_TRIDC2BG080170.1
Tdi_TRIDC2BG064440.1
Tdi_TRIDC2AG071040.2
Tdi_TRIDC2AG053140.7
Tdi_TRIDC1AG052450.1
0
2
1
1
2
−1000
0
1000
2000

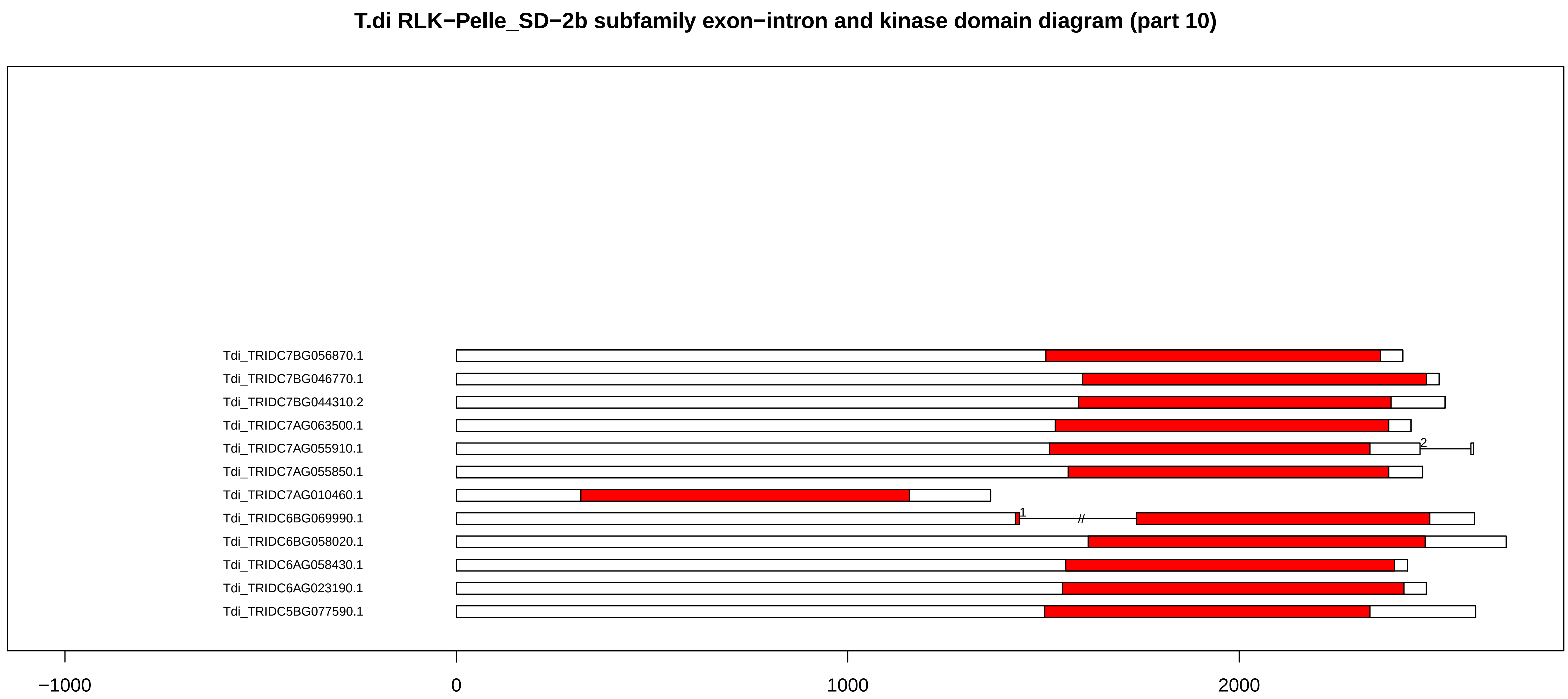
T.di RLK−Pelle_SD−2b subfamily exon−intron and kinase domain diagram (part 10)
Tdi_TRIDC7BG056870.1
Tdi_TRIDC7BG046770.1
Tdi_TRIDC7BG044310.2
Tdi_TRIDC7AG063500.1
Tdi_TRIDC7AG055910.1
2
Tdi_TRIDC7AG055850.1
Tdi_TRIDC7AG010460.1
Tdi_TRIDC6BG069990.1
1
//
Tdi_TRIDC6BG058020.1
Tdi_TRIDC6AG058430.1
Tdi_TRIDC6AG023190.1
Tdi_TRIDC5BG077590.1
−1000
0
1000
2000

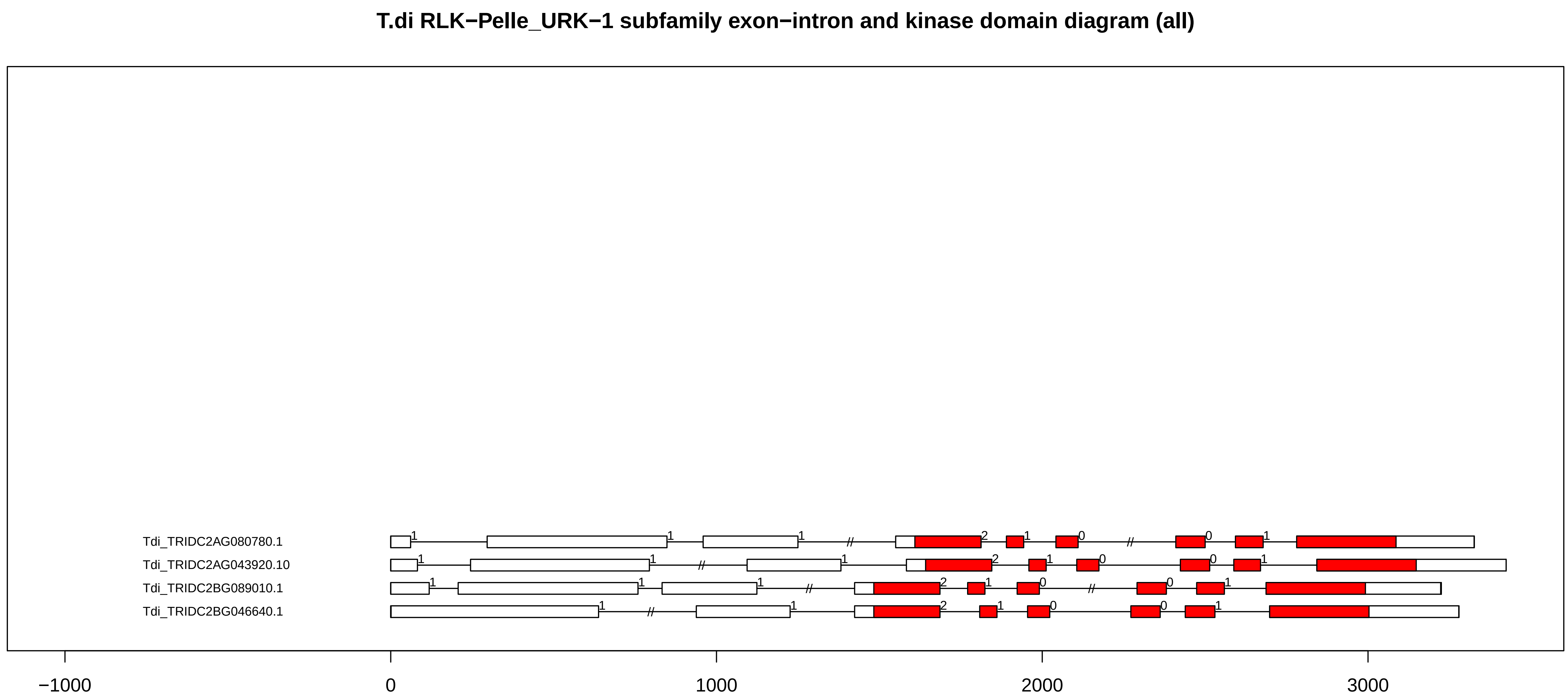
T.di RLK−Pelle_URK−1 subfamily exon−intron and kinase domain diagram (all)
Tdi_TRIDC2AG080780.1
Tdi_TRIDC2AG043920.10
Tdi_TRIDC2BG089010.1
Tdi_TRIDC2BG046640.1
−1000
0
1000
2000
3000

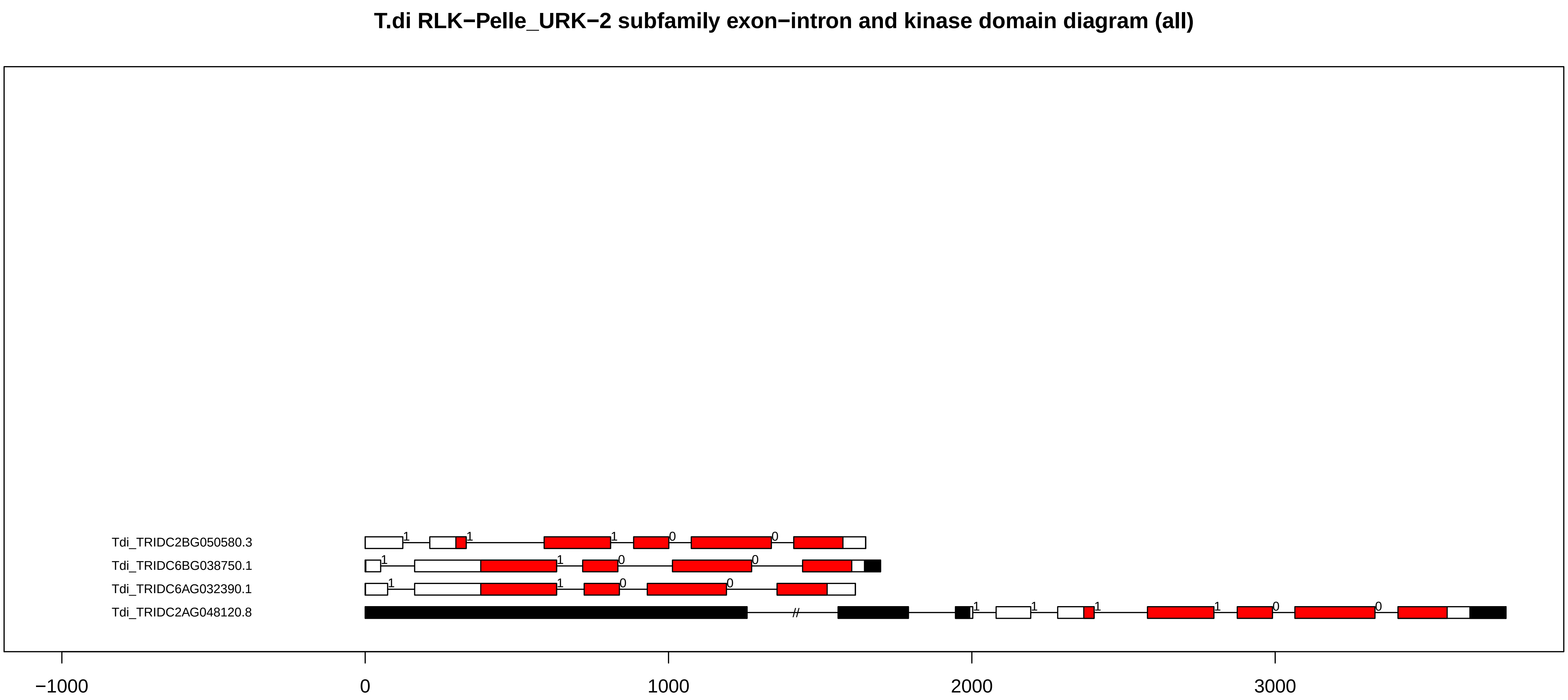
T.di RLK−Pelle_URK−2 subfamily exon−intron and kinase domain diagram (all)
Tdi_TRIDC2BG050580.3
Tdi_TRIDC6BG038750.1
Tdi_TRIDC6AG032390.1
Tdi_TRIDC2AG048120.8
−1000
0
1000
2000
3000

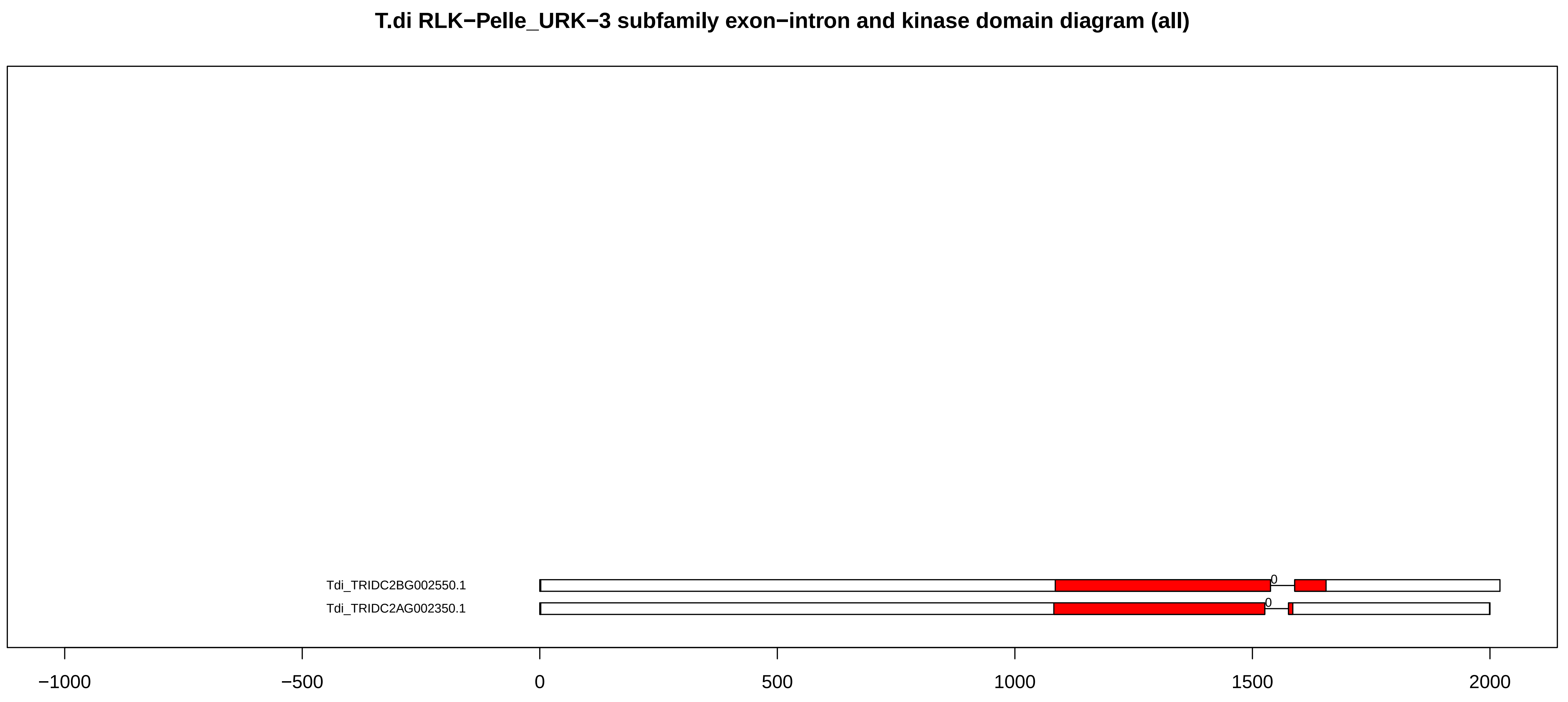
T.di RLK−Pelle_URK−3 subfamily exon−intron and kinase domain diagram (all)
Tdi_TRIDC2BG002550.1
Tdi_TRIDC2AG002350.1
0
0
−1000
−500
0
500
1000
1500
2000

T.di RLK−Pelle_WAK_LRK10L−1 subfamily exon−intron and kinase domain diagram (all)

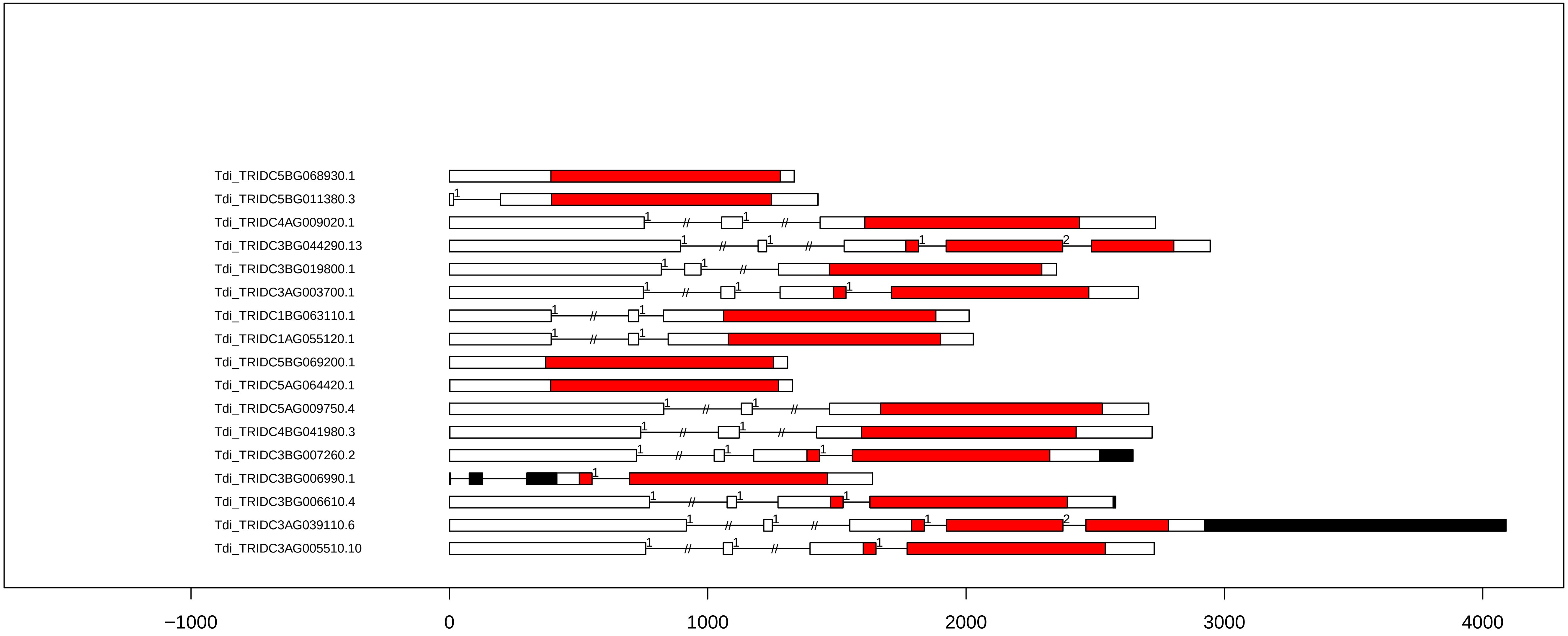

T.di RLK−Pelle_WAK subfamily exon−intron and kinase domain diagram (part 1)

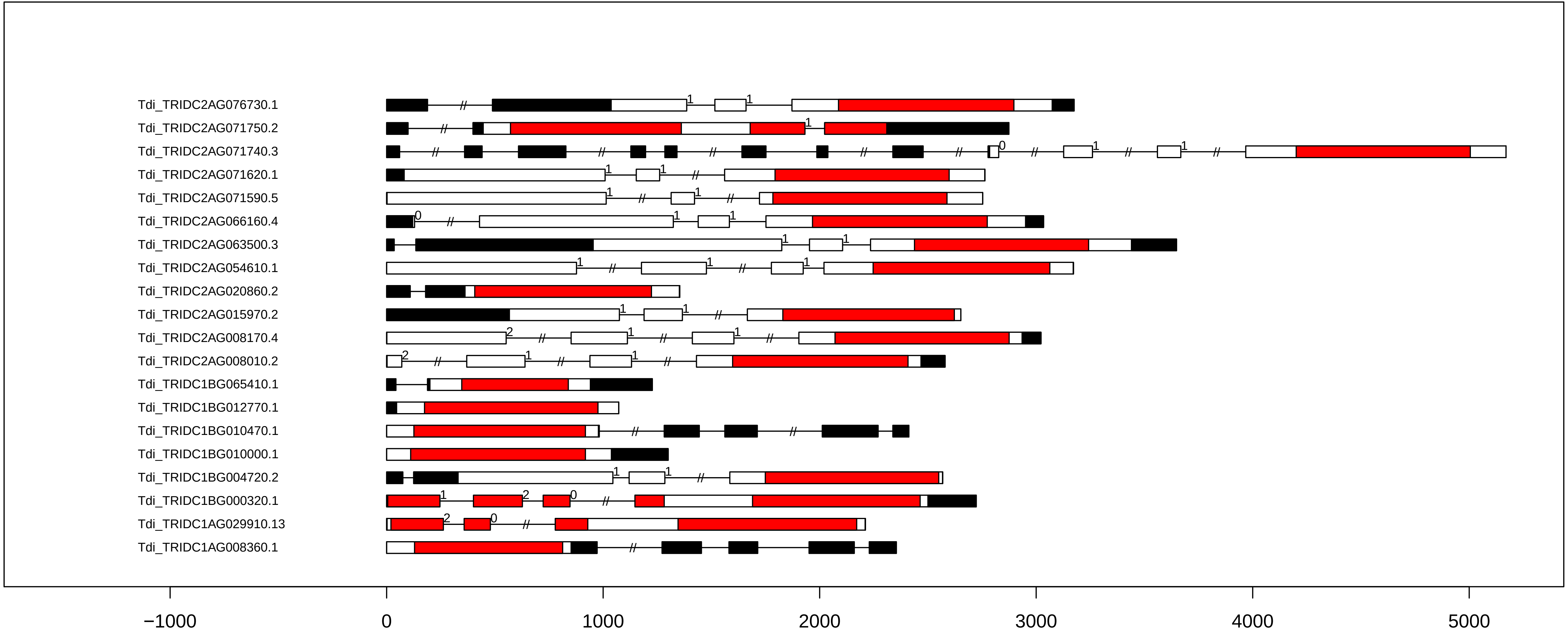

T.di RLK−Pelle_WAK subfamily exon−intron and kinase domain diagram (part 2)

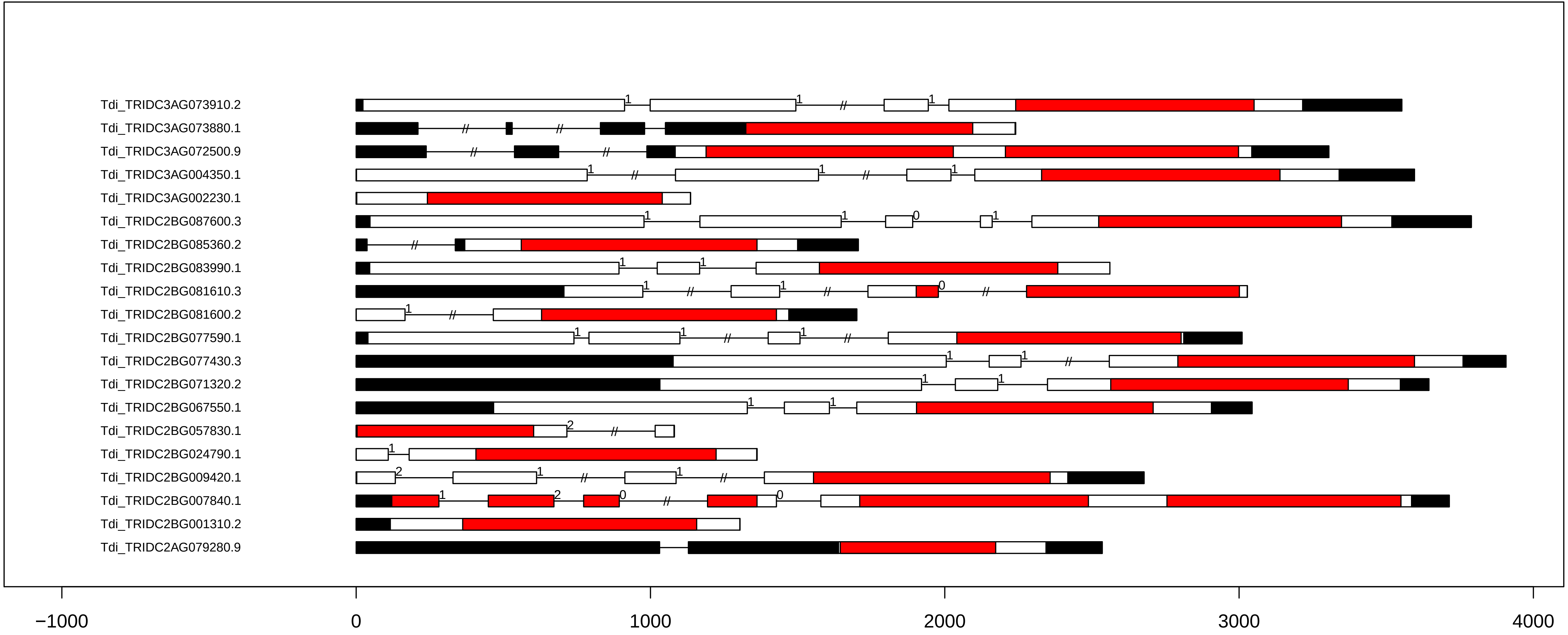

T.di RLK−Pelle_WAK subfamily exon−intron and kinase domain diagram (part 3)

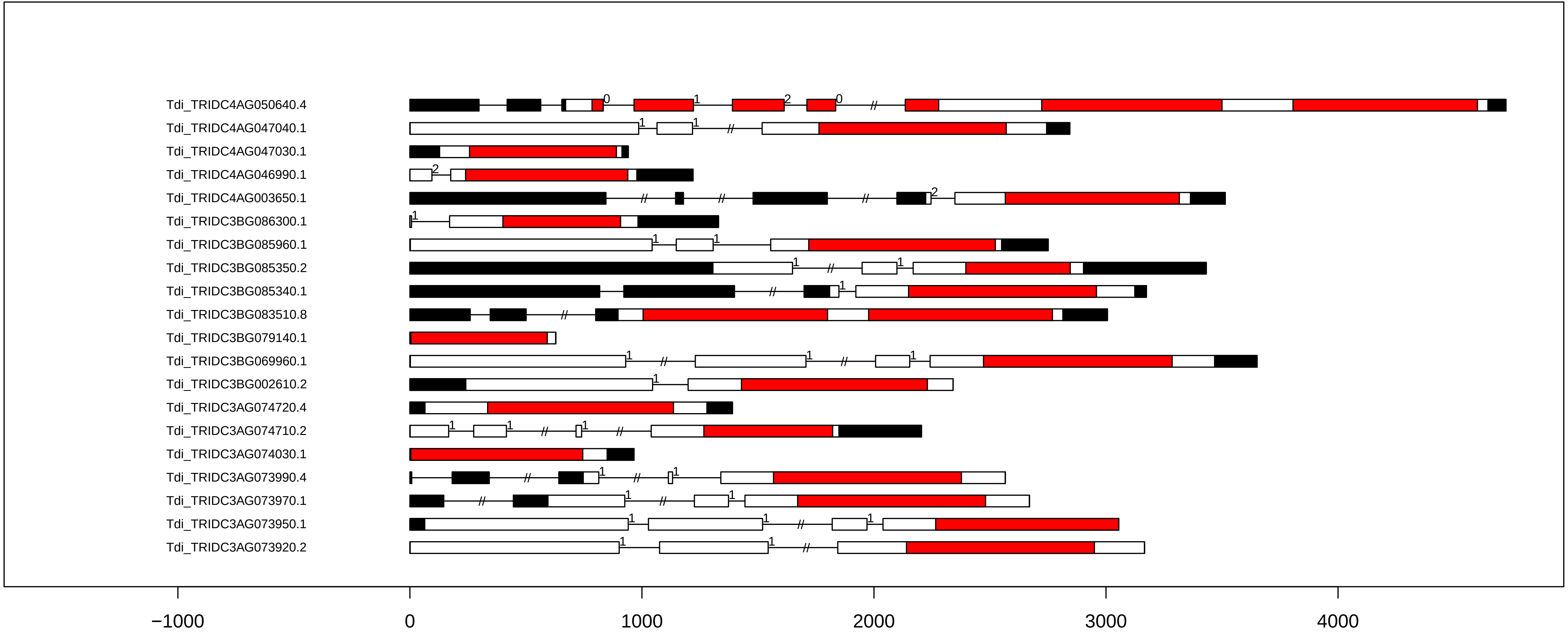

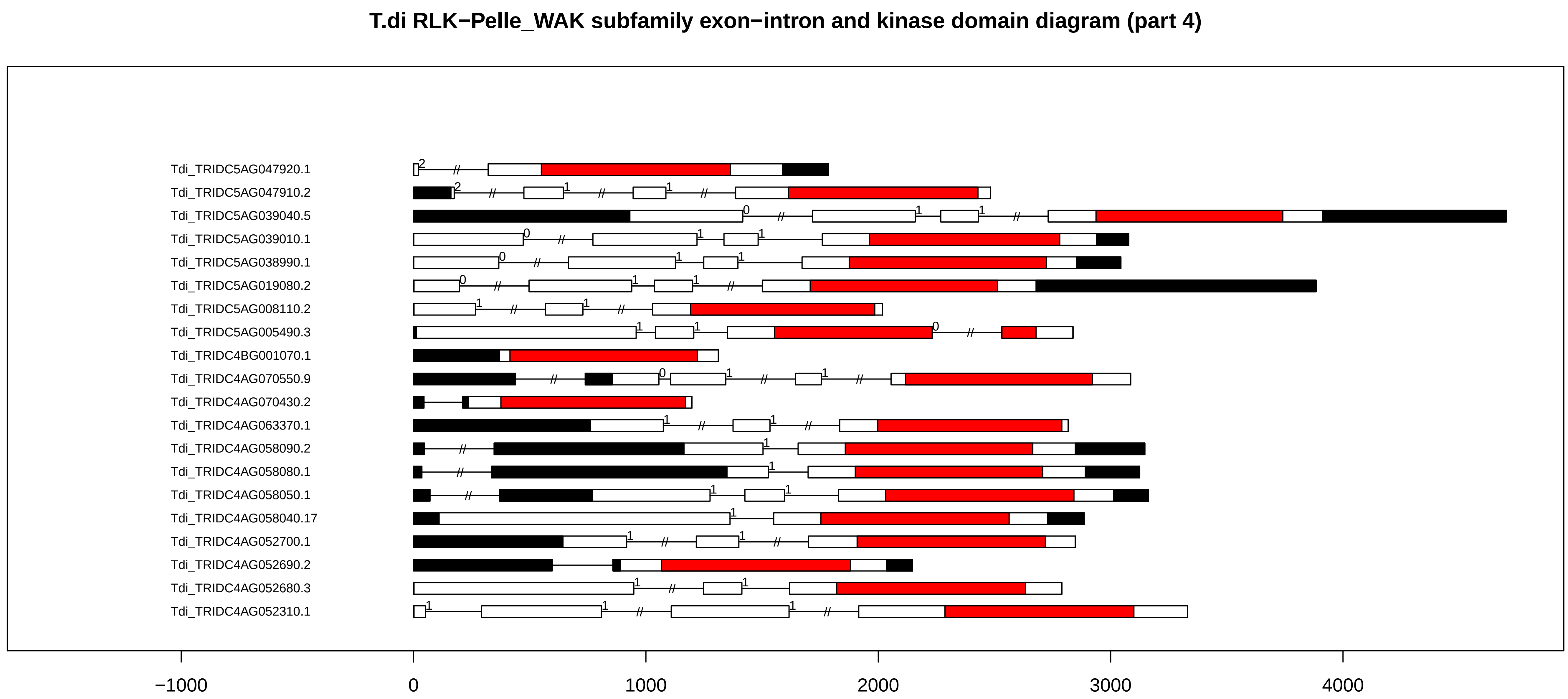
T.di RLK−Pelle_WAK subfamily exon−intron and kinase domain diagram (part 4)
Tdi_TRIDC5AG047920.1
Tdi_TRIDC5AG047910.2
Tdi_TRIDC5AG039040.5
Tdi_TRIDC5AG039010.1
Tdi_TRIDC5AG038990.1
Tdi_TRIDC5AG019080.2
Tdi_TRIDC5AG008110.2
Tdi_TRIDC5AG005490.3
Tdi_TRIDC4BG001070.1
Tdi_TRIDC4AG070550.9
Tdi_TRIDC4AG070430.2
Tdi_TRIDC4AG063370.1
Tdi_TRIDC4AG058090.2
Tdi_TRIDC4AG058080.1
Tdi_TRIDC4AG058050.1
Tdi_TRIDC4AG058040.17
Tdi_TRIDC4AG052700.1
Tdi_TRIDC4AG052690.2
Tdi_TRIDC4AG052680.3
Tdi_TRIDC4AG052310.1
−1000
0
1000
2000
3000
4000

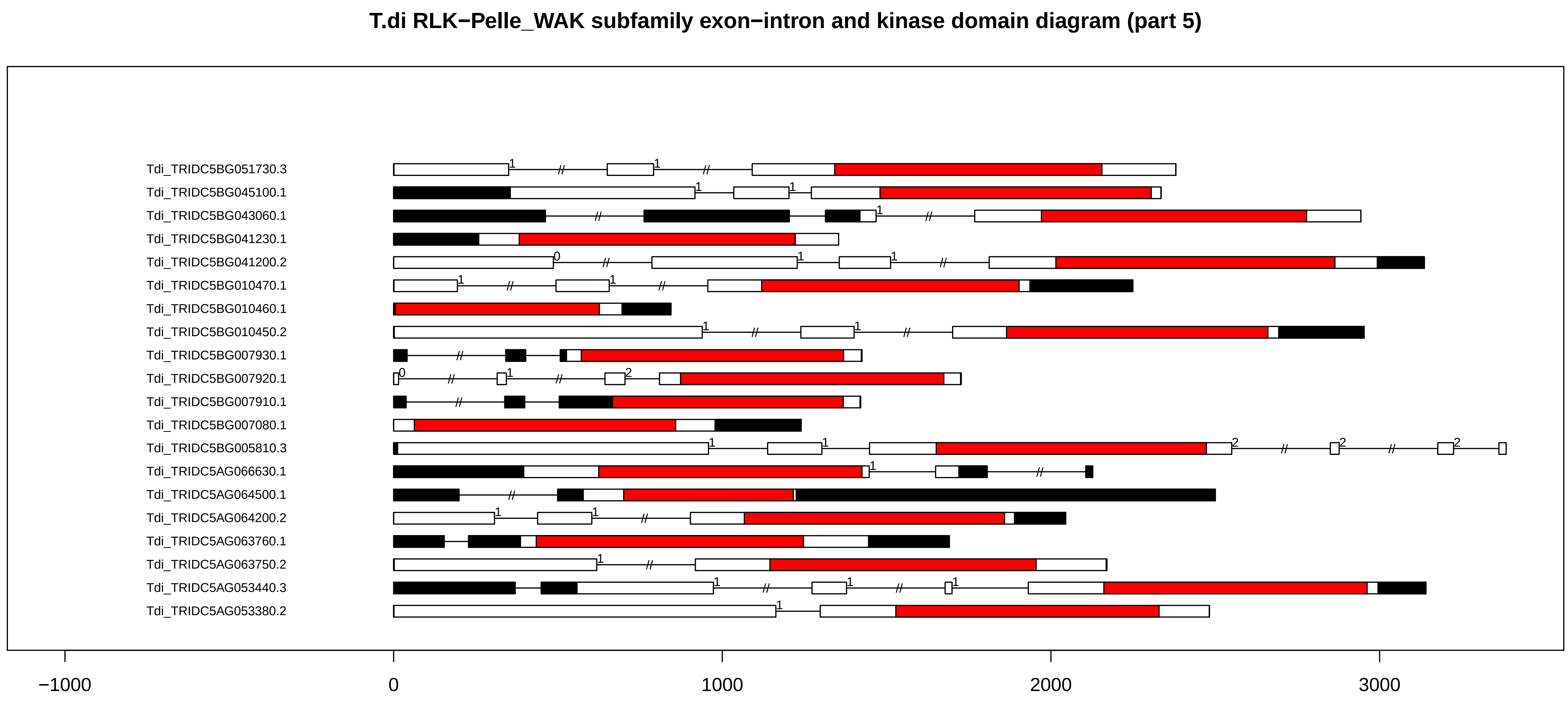
T.di RLK−Pelle_WAK subfamily exon−intron and kinase domain diagram (part 5)
Tdi_TRIDC5BG051730.3
Tdi_TRIDC5BG045100.1
Tdi_TRIDC5BG043060.1
Tdi_TRIDC5BG041230.1
Tdi_TRIDC5BG041200.2
Tdi_TRIDC5BG010470.1
Tdi_TRIDC5BG010460.1
Tdi_TRIDC5BG010450.2
Tdi_TRIDC5BG007930.1
Tdi_TRIDC5BG007920.1
Tdi_TRIDC5BG007910.1
Tdi_TRIDC5BG007080.1
Tdi_TRIDC5BG005810.3
Tdi_TRIDC5AG066630.1
Tdi_TRIDC5AG064500.1
Tdi_TRIDC5AG064200.2
Tdi_TRIDC5AG063760.1
Tdi_TRIDC5AG063750.2
Tdi_TRIDC5AG053440.3
Tdi_TRIDC5AG053380.2
−1000
0
1000
2000
3000

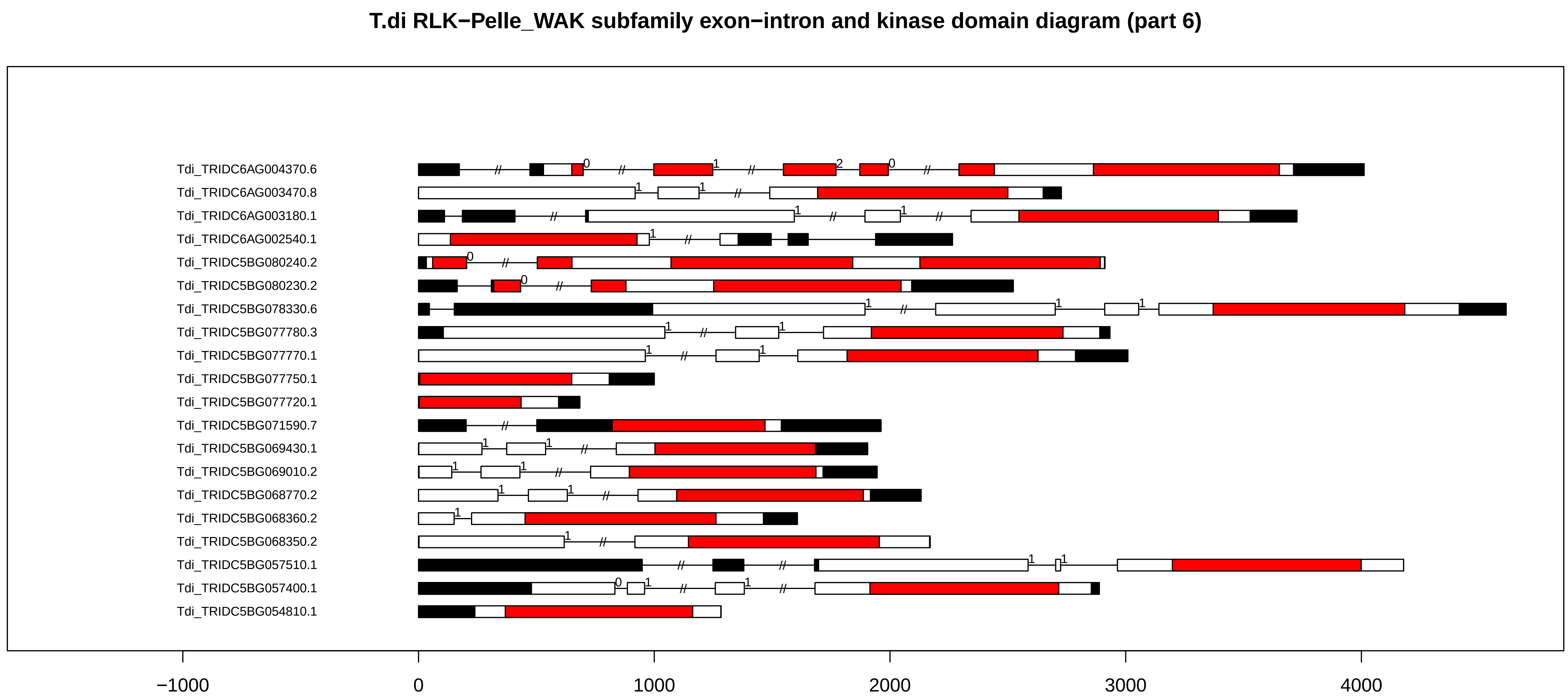
T.di RLK−Pelle_WAK subfamily exon−intron and kinase domain diagram (part 6)
Tdi_TRIDC6AG004370.6
Tdi_TRIDC6AG003470.8
Tdi_TRIDC6AG003180.1
Tdi_TRIDC6AG002540.1
Tdi_TRIDC5BG080240.2
Tdi_TRIDC5BG080230.2
Tdi_TRIDC5BG078330.6
Tdi_TRIDC5BG077780.3
Tdi_TRIDC5BG077770.1
Tdi_TRIDC5BG077750.1
Tdi_TRIDC5BG077720.1
Tdi_TRIDC5BG071590.7
Tdi_TRIDC5BG069430.1
Tdi_TRIDC5BG069010.2
Tdi_TRIDC5BG068770.2
Tdi_TRIDC5BG068360.2
Tdi_TRIDC5BG068350.2
Tdi_TRIDC5BG057510.1
Tdi_TRIDC5BG057400.1
Tdi_TRIDC5BG054810.1
−1000
0
1000
2000
3000
4000

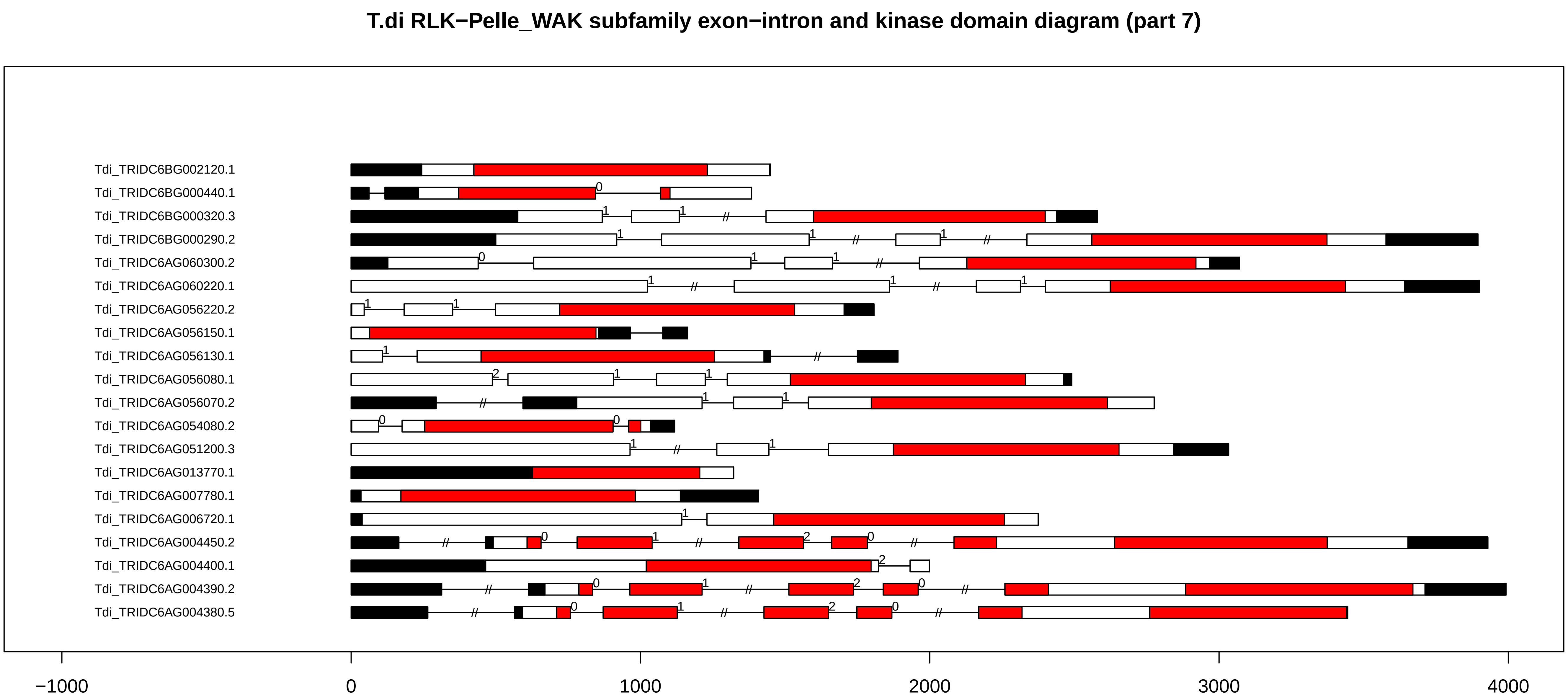
T.di RLK−Pelle_WAK subfamily exon−intron and kinase domain diagram (part 7)
Tdi_TRIDC6BG002120.1
Tdi_TRIDC6BG000440.1
Tdi_TRIDC6BG000320.3
Tdi_TRIDC6BG000290.2
Tdi_TRIDC6AG060300.2
Tdi_TRIDC6AG060220.1
Tdi_TRIDC6AG056220.2
Tdi_TRIDC6AG056150.1
Tdi_TRIDC6AG056130.1
Tdi_TRIDC6AG056080.1
Tdi_TRIDC6AG056070.2
Tdi_TRIDC6AG054080.2
Tdi_TRIDC6AG051200.3
Tdi_TRIDC6AG013770.1
Tdi_TRIDC6AG007780.1
Tdi_TRIDC6AG006720.1
Tdi_TRIDC6AG004450.2
Tdi_TRIDC6AG004400.1
Tdi_TRIDC6AG004390.2
Tdi_TRIDC6AG004380.5
−1000
0
1000
2000
3000
4000

T.di RLK−Pelle_WAK subfamily exon−intron and kinase domain diagram (part 8)

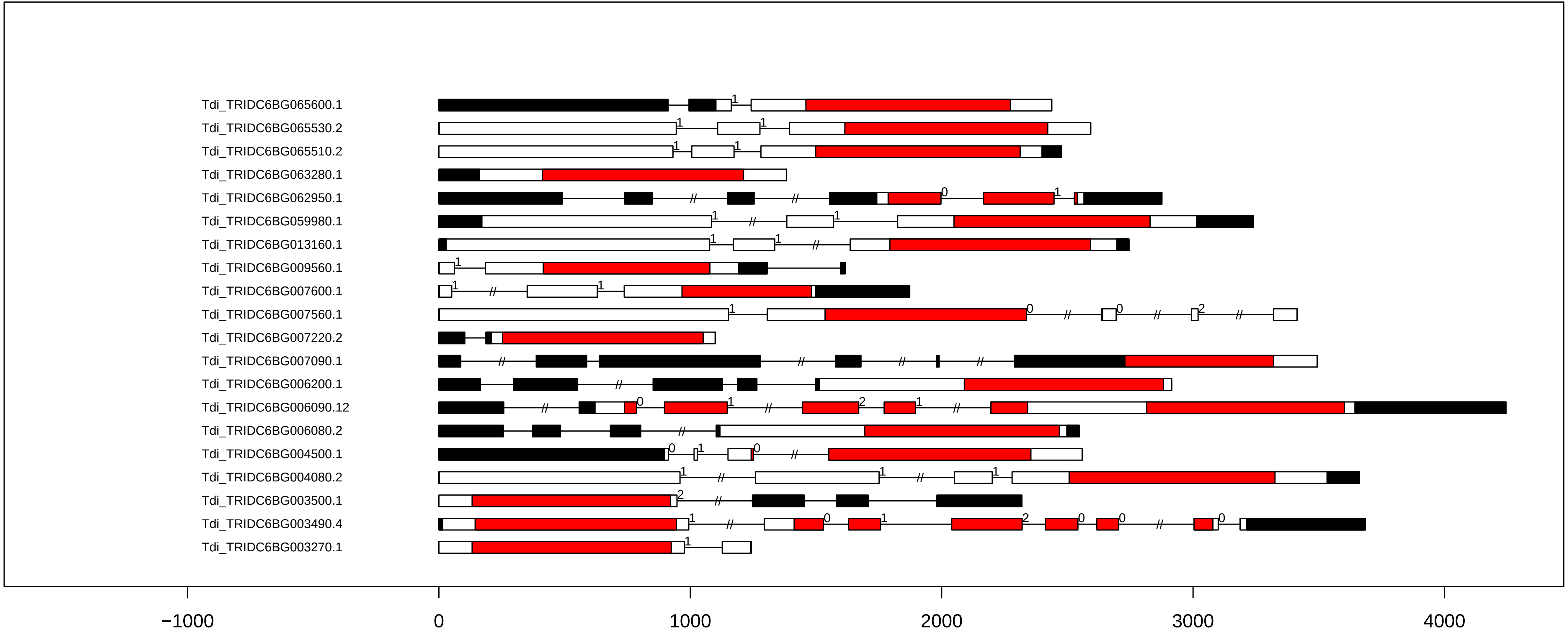

T.di RLK−Pelle_WAK subfamily exon−intron and kinase domain diagram (part 9)

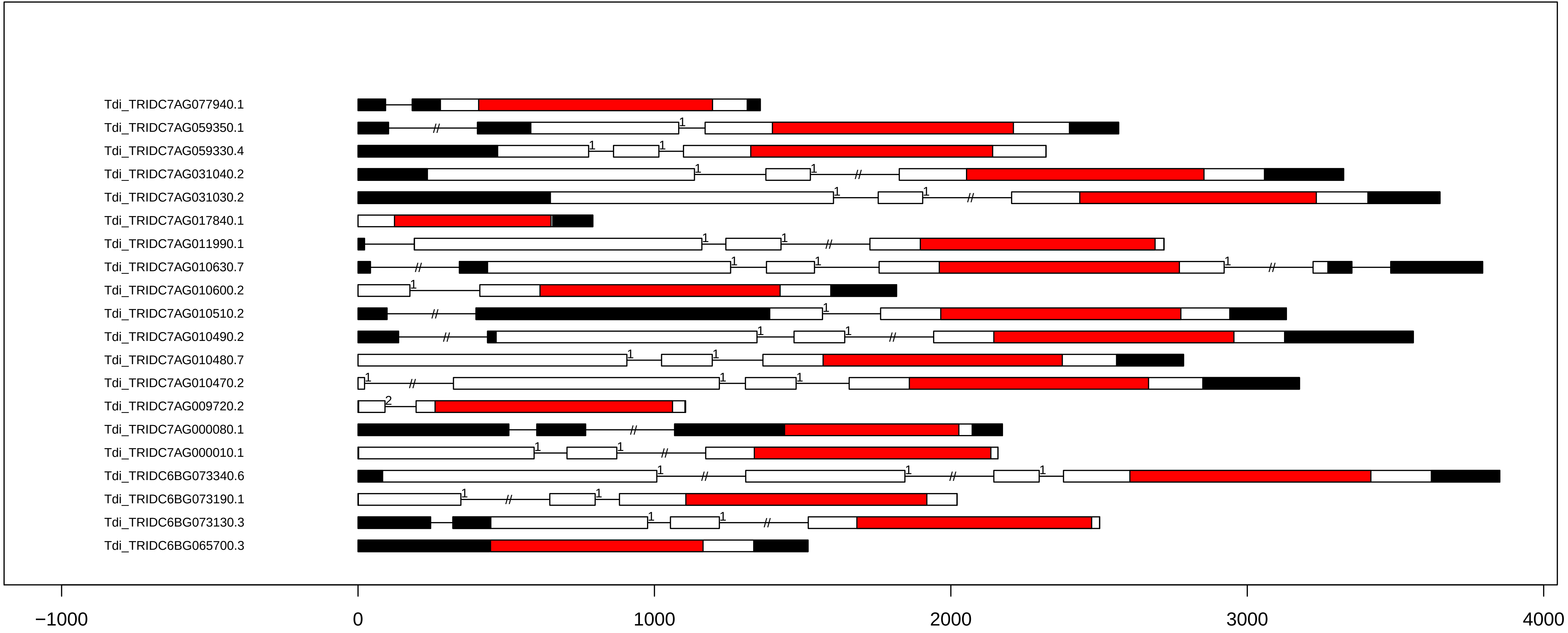

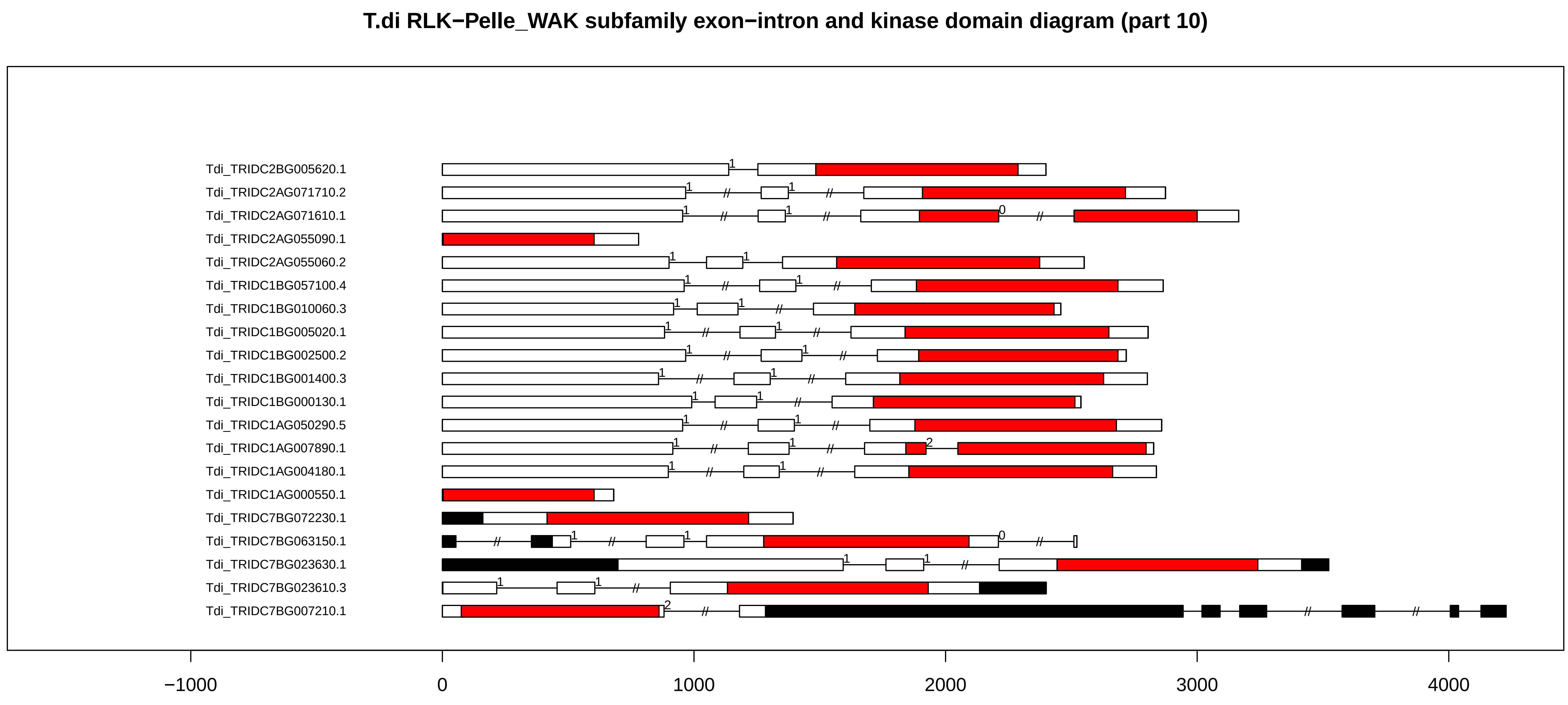

T.di RLK−Pelle_WAK subfamily exon−intron and kinase domain diagram (part 10)
Tdi_TRIDC2BG005620.1
Tdi_TRIDC2AG071710.2
Tdi_TRIDC2AG071610.1
Tdi_TRIDC2AG055090.1
Tdi_TRIDC2AG055060.2
Tdi_TRIDC1BG057100.4
Tdi_TRIDC1BG010060.3
Tdi_TRIDC1BG005020.1
Tdi_TRIDC1BG002500.2
Tdi_TRIDC1BG001400.3
Tdi_TRIDC1BG000130.1
Tdi_TRIDC1AG050290.5
Tdi_TRIDC1AG007890.1
Tdi_TRIDC1AG004180.1
Tdi_TRIDC1AG000550.1
Tdi_TRIDC7BG072230.1
Tdi_TRIDC7BG063150.1
Tdi_TRIDC7BG023630.1
Tdi_TRIDC7BG023610.3
Tdi_TRIDC7BG007210.1
−1000
0
1000
2000
3000
4000

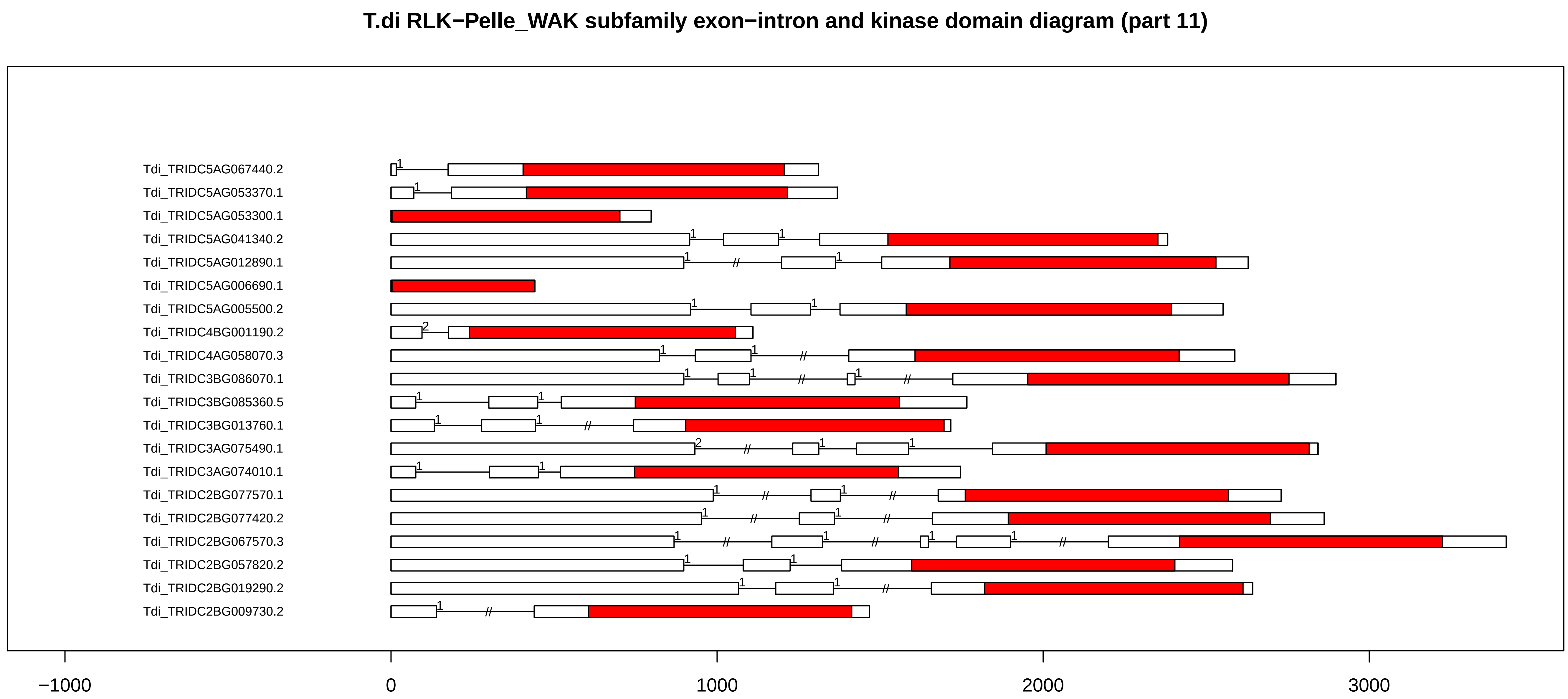

T.di RLK−Pelle_WAK subfamily exon−intron and kinase domain diagram (part 11)
Tdi_TRIDC5AG067440.2
Tdi_TRIDC5AG053370.1
Tdi_TRIDC5AG053300.1
Tdi_TRIDC5AG041340.2
Tdi_TRIDC5AG012890.1
Tdi_TRIDC5AG006690.1
Tdi_TRIDC5AG005500.2
Tdi_TRIDC4BG001190.2
Tdi_TRIDC4AG058070.3
Tdi_TRIDC3BG086070.1
Tdi_TRIDC3BG085360.5
Tdi_TRIDC3BG013760.1
Tdi_TRIDC3AG075490.1
Tdi_TRIDC3AG074010.1
Tdi_TRIDC2BG077570.1
Tdi_TRIDC2BG077420.2
Tdi_TRIDC2BG067570.3
Tdi_TRIDC2BG057820.2
Tdi_TRIDC2BG019290.2
Tdi_TRIDC2BG009730.2
−1000
0
1000
2000
3000

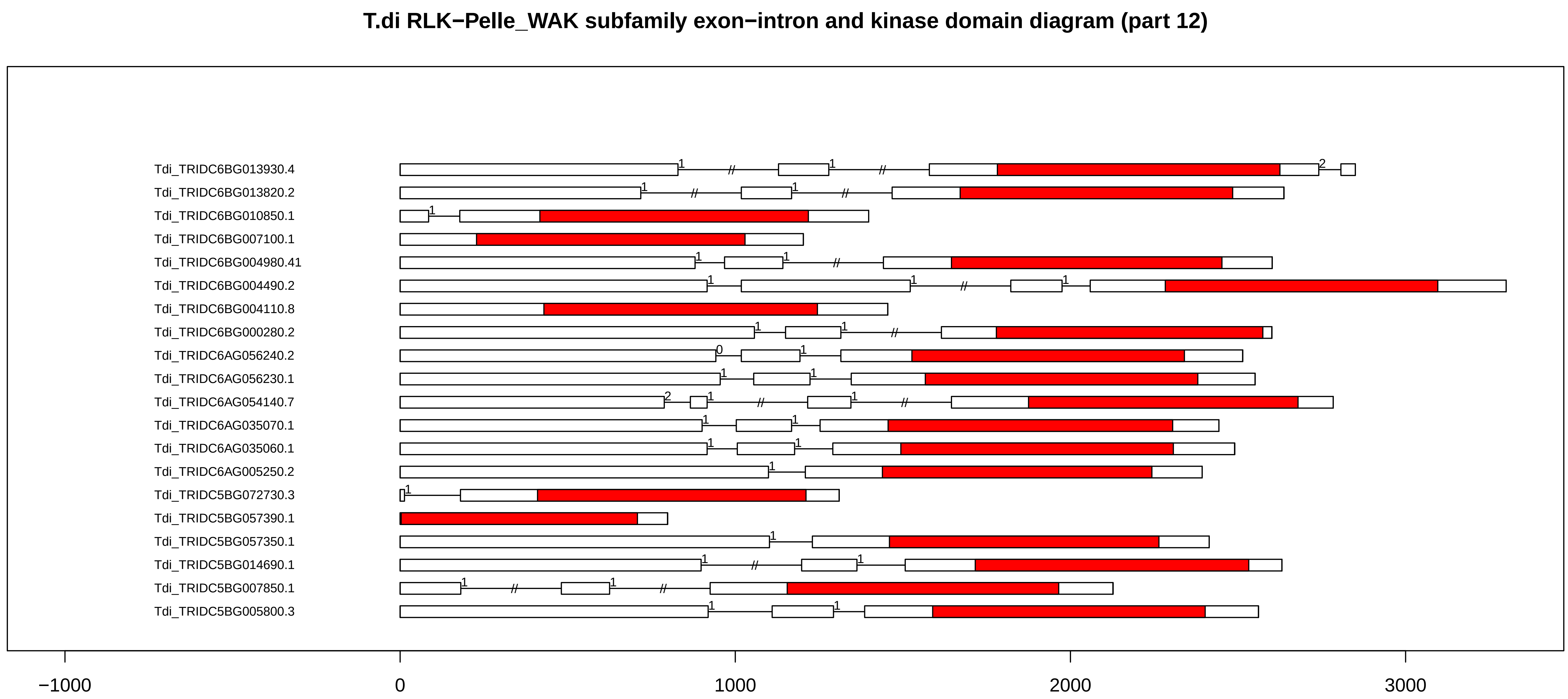
T.di RLK−Pelle_WAK subfamily exon−intron and kinase domain diagram (part 12)
Tdi_TRIDC6BG013930.4
Tdi_TRIDC6BG013820.2
Tdi_TRIDC6BG010850.1
Tdi_TRIDC6BG007100.1
Tdi_TRIDC6BG004980.41
Tdi_TRIDC6BG004490.2
Tdi_TRIDC6BG004110.8
Tdi_TRIDC6BG000280.2
Tdi_TRIDC6AG056240.2
Tdi_TRIDC6AG056230.1
Tdi_TRIDC6AG054140.7
Tdi_TRIDC6AG035070.1
Tdi_TRIDC6AG035060.1
Tdi_TRIDC6AG005250.2
Tdi_TRIDC5BG072730.3
Tdi_TRIDC5BG057390.1
Tdi_TRIDC5BG057350.1
Tdi_TRIDC5BG014690.1
Tdi_TRIDC5BG007850.1
Tdi_TRIDC5BG005800.3
−1000
0
1000
2000
3000

T.di RLK−Pelle_WAK subfamily exon−intron and kinase domain diagram (part 13)

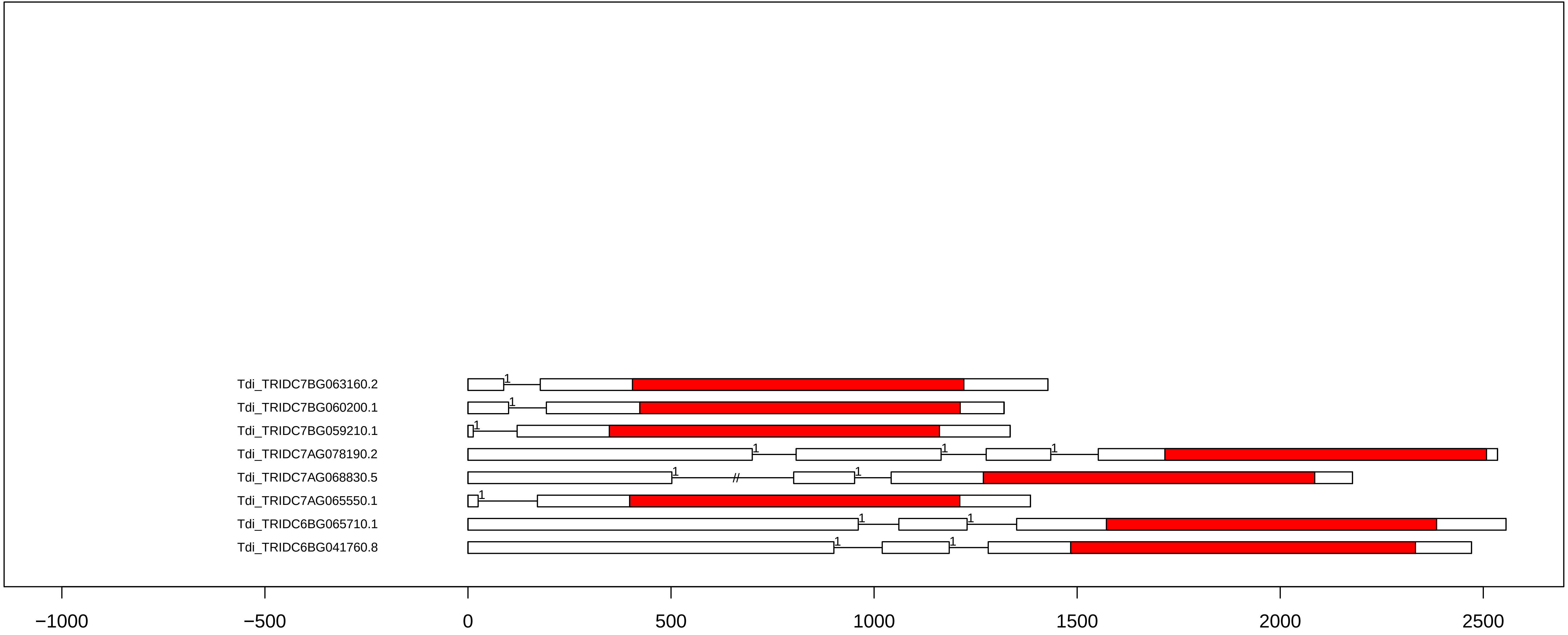

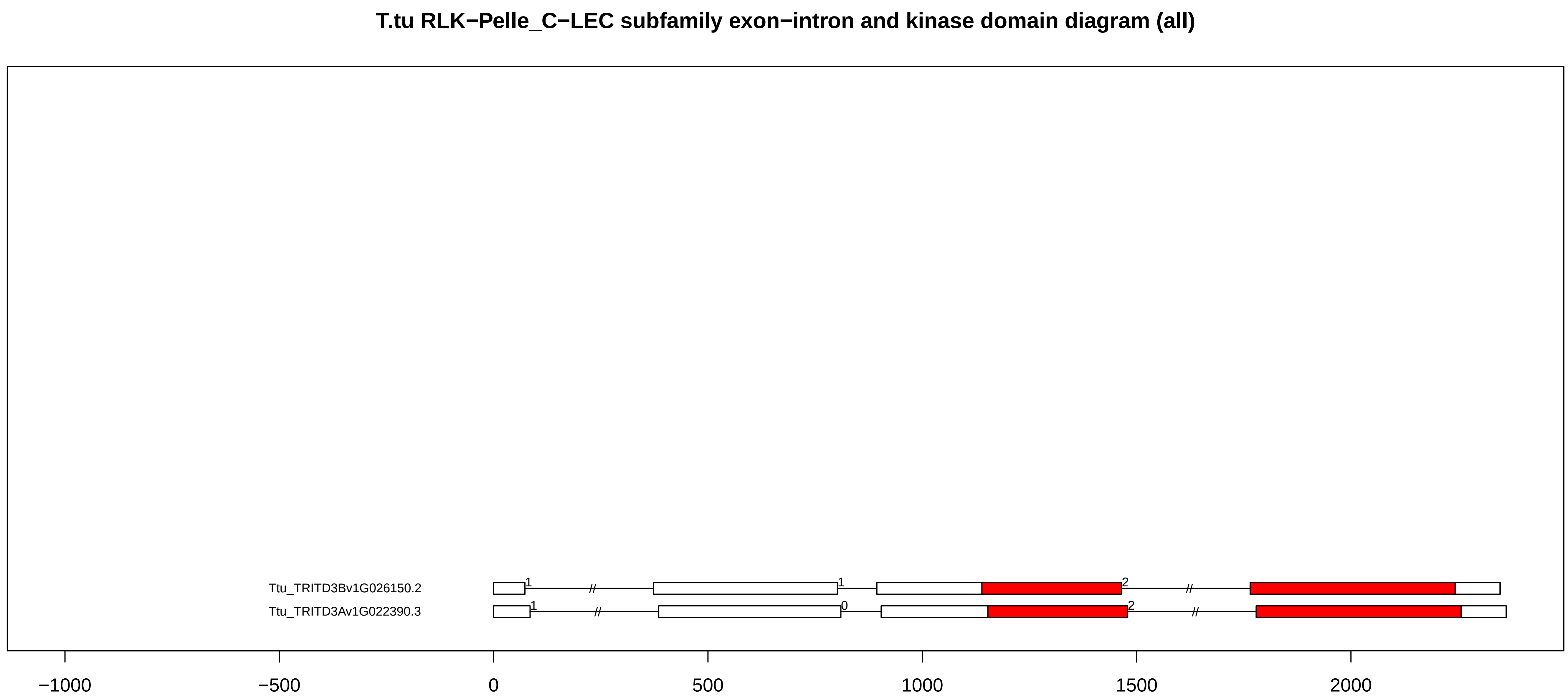

T.tu RLK−Pelle_C−LEC subfamily exon−intron and kinase domain diagram (all)
Ttu_TRITD3Bv1G026150.2
Ttu_TRITD3Av1G022390.3
1
1
2
1
0
2
−1000
−500
0
500
1000
1500
2000

T.tu RLK−Pelle_CR4L subfamily exon−intron and kinase domain diagram (part 1)

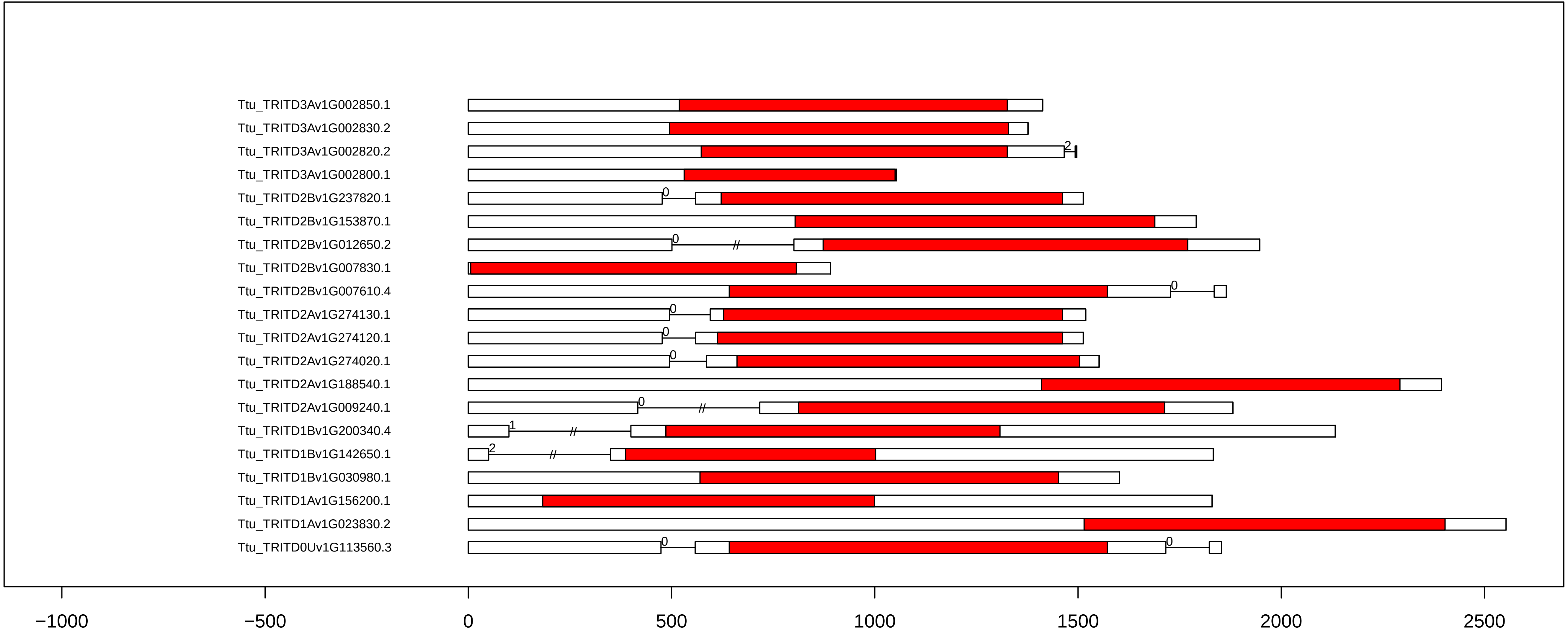

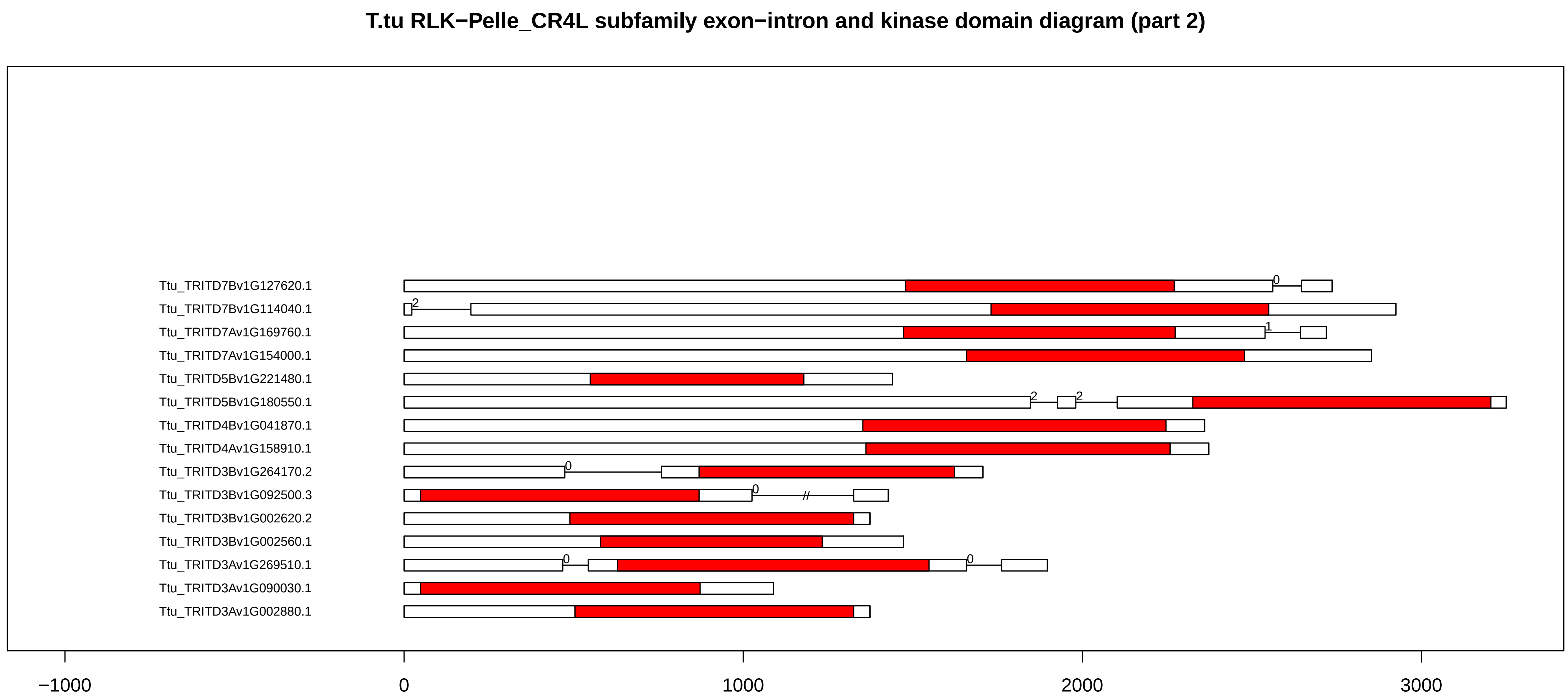

T.tu RLK−Pelle_CR4L subfamily exon−intron and kinase domain diagram (part 2)
Ttu_TRITD7Bv1G127620.1
Ttu_TRITD7Bv1G114040.1
Ttu_TRITD7Av1G169760.1
Ttu_TRITD7Av1G154000.1
Ttu_TRITD5Bv1G221480.1
Ttu_TRITD5Bv1G180550.1
Ttu_TRITD4Bv1G041870.1
Ttu_TRITD4Av1G158910.1
Ttu_TRITD3Bv1G264170.2
Ttu_TRITD3Bv1G092500.3
Ttu_TRITD3Bv1G002620.2
Ttu_TRITD3Bv1G002560.1
Ttu_TRITD3Av1G269510.1
Ttu_TRITD3Av1G090030.1
Ttu_TRITD3Av1G002880.1
−1000
0
1000
2000
3000

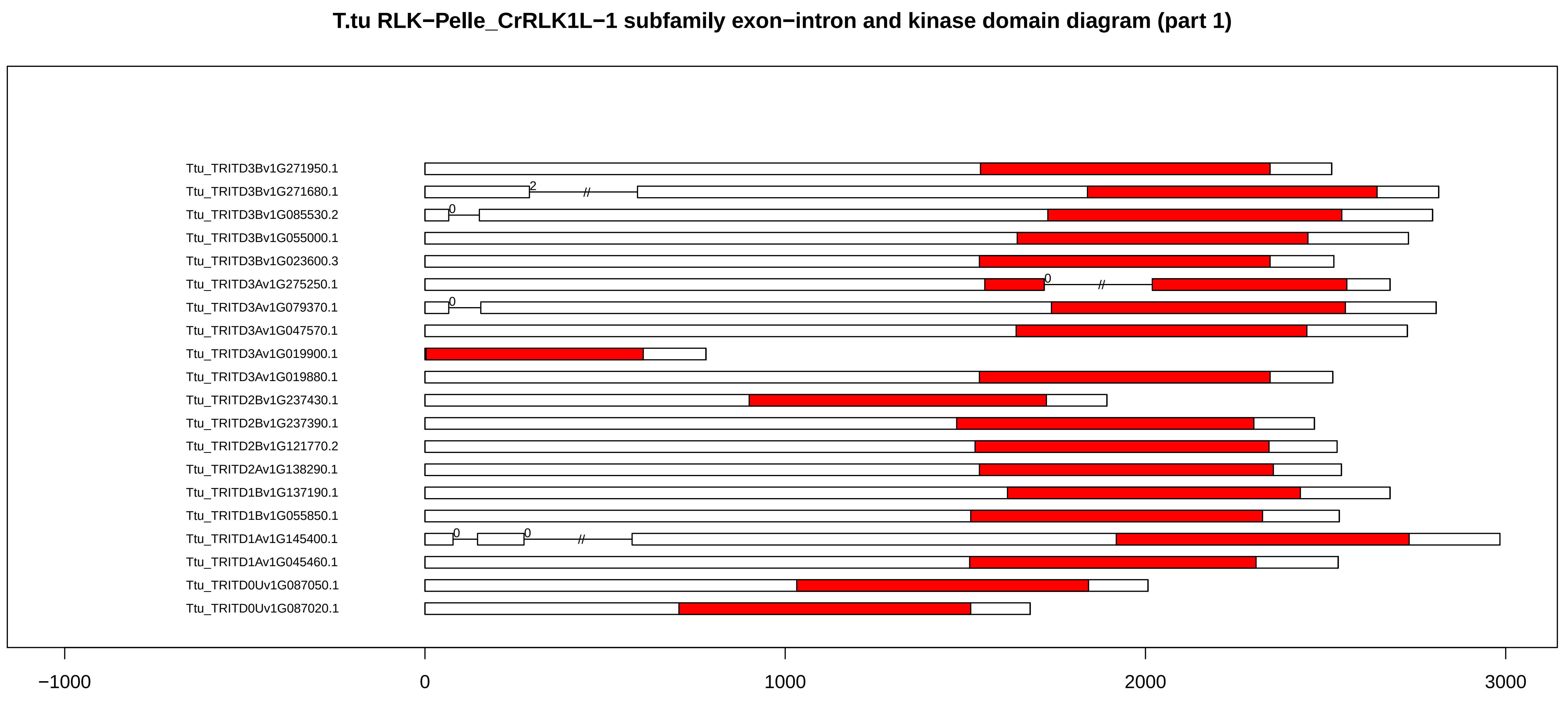
T.tu RLK−Pelle_CrRLK1L−1 subfamily exon−intron and kinase domain diagram (part 1)
Ttu_TRITD3Bv1G271950.1
Ttu_TRITD3Bv1G271680.1
Ttu_TRITD3Bv1G085530.2
Ttu_TRITD3Bv1G055000.1
Ttu_TRITD3Bv1G023600.3
Ttu_TRITD3Av1G275250.1
Ttu_TRITD3Av1G079370.1
Ttu_TRITD3Av1G047570.1
Ttu_TRITD3Av1G019900.1
Ttu_TRITD3Av1G019880.1
Ttu_TRITD2Bv1G237430.1
Ttu_TRITD2Bv1G237390.1
Ttu_TRITD2Bv1G121770.2
Ttu_TRITD2Av1G138290.1
Ttu_TRITD1Bv1G137190.1
Ttu_TRITD1Bv1G055850.1
Ttu_TRITD1Av1G145400.1
Ttu_TRITD1Av1G045460.1
Ttu_TRITD0Uv1G087050.1
Ttu_TRITD0Uv1G087020.1
−1000
0
1000
2000
3000

T.tu RLK−Pelle_CrRLK1L−1 subfamily exon−intron and kinase domain diagram (part 2)

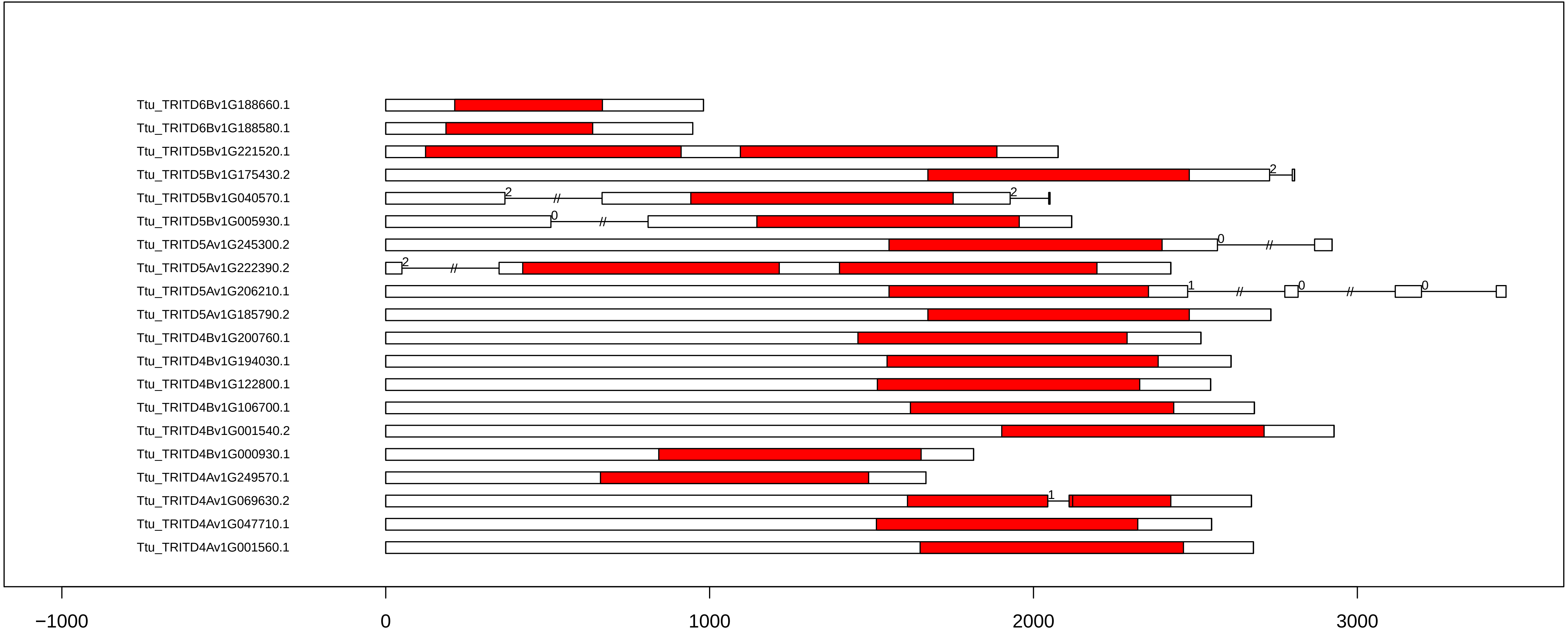

T.tu RLK−Pelle_CrRLK1L−1 subfamily exon−intron and kinase domain diagram (part 3)

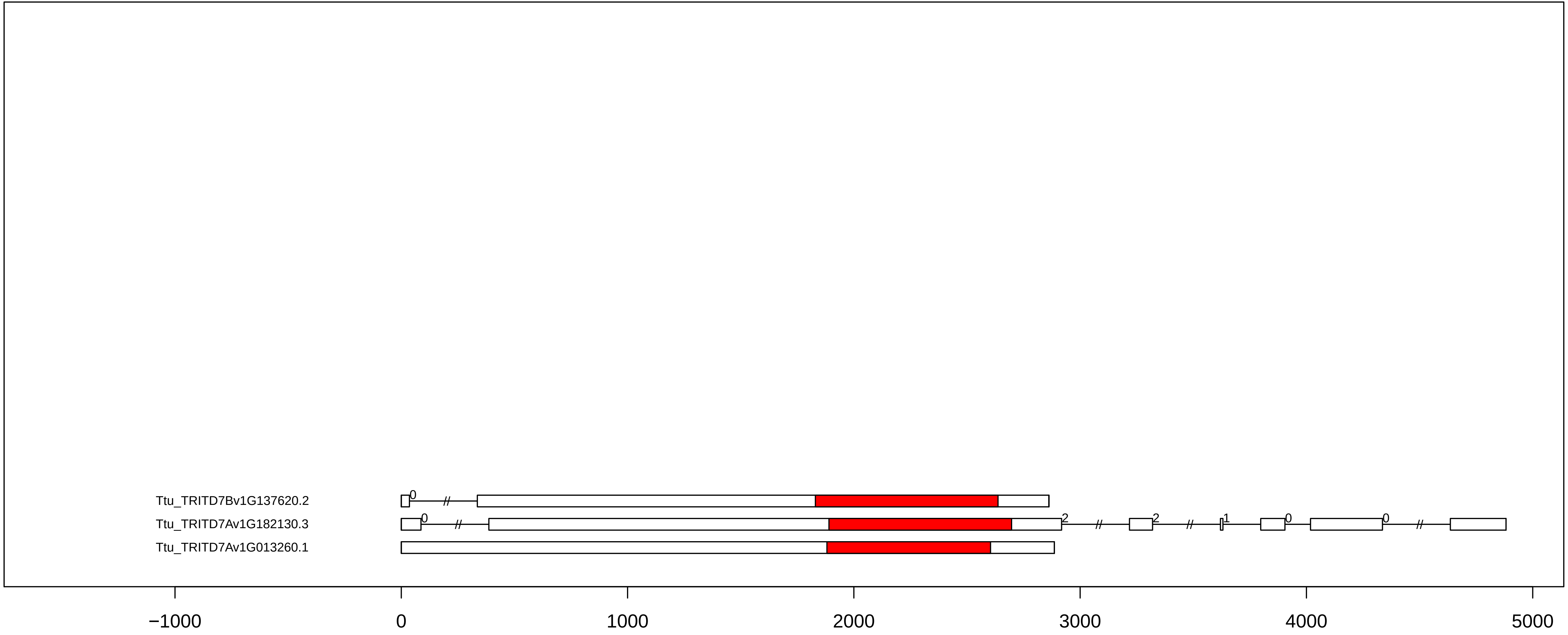

T.tu RLK−Pelle_DLSV subfamily exon−intron and kinase domain diagram (part 1)

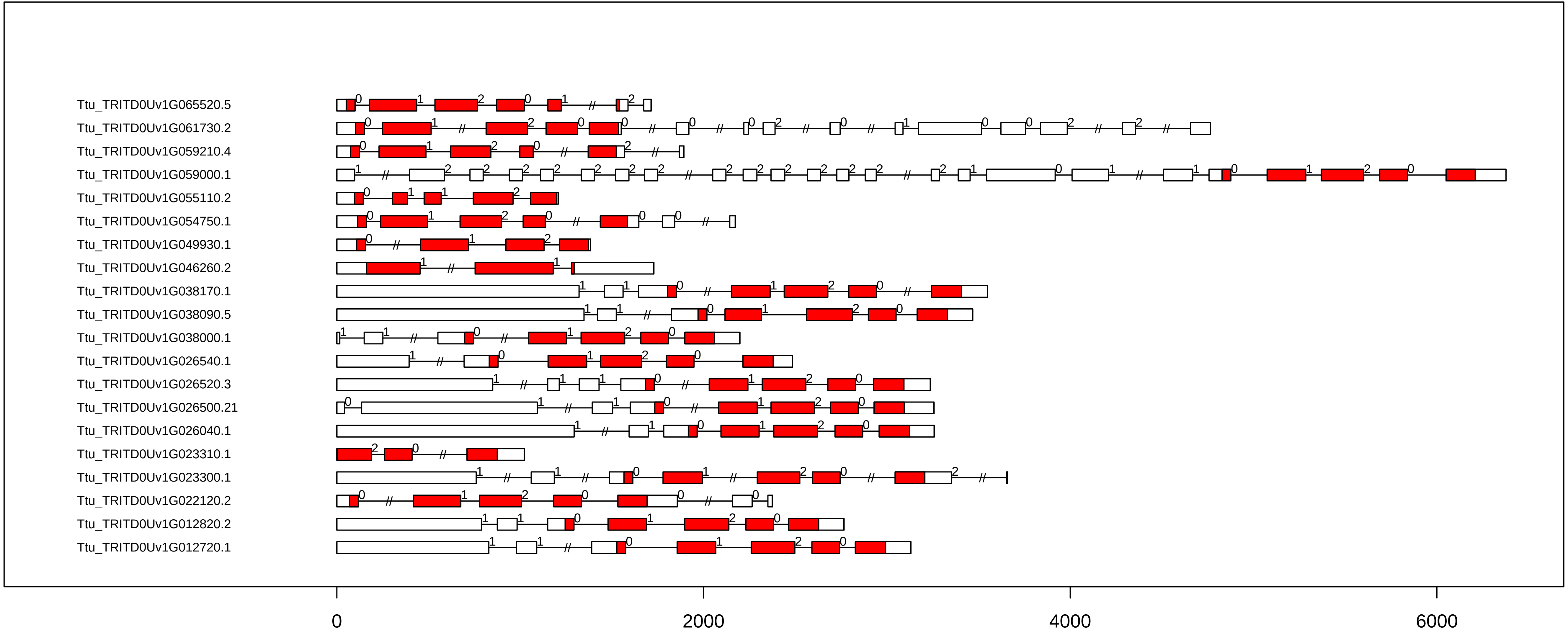

T.tu RLK−Pelle_DLSV subfamily exon−intron and kinase domain diagram (part 2)

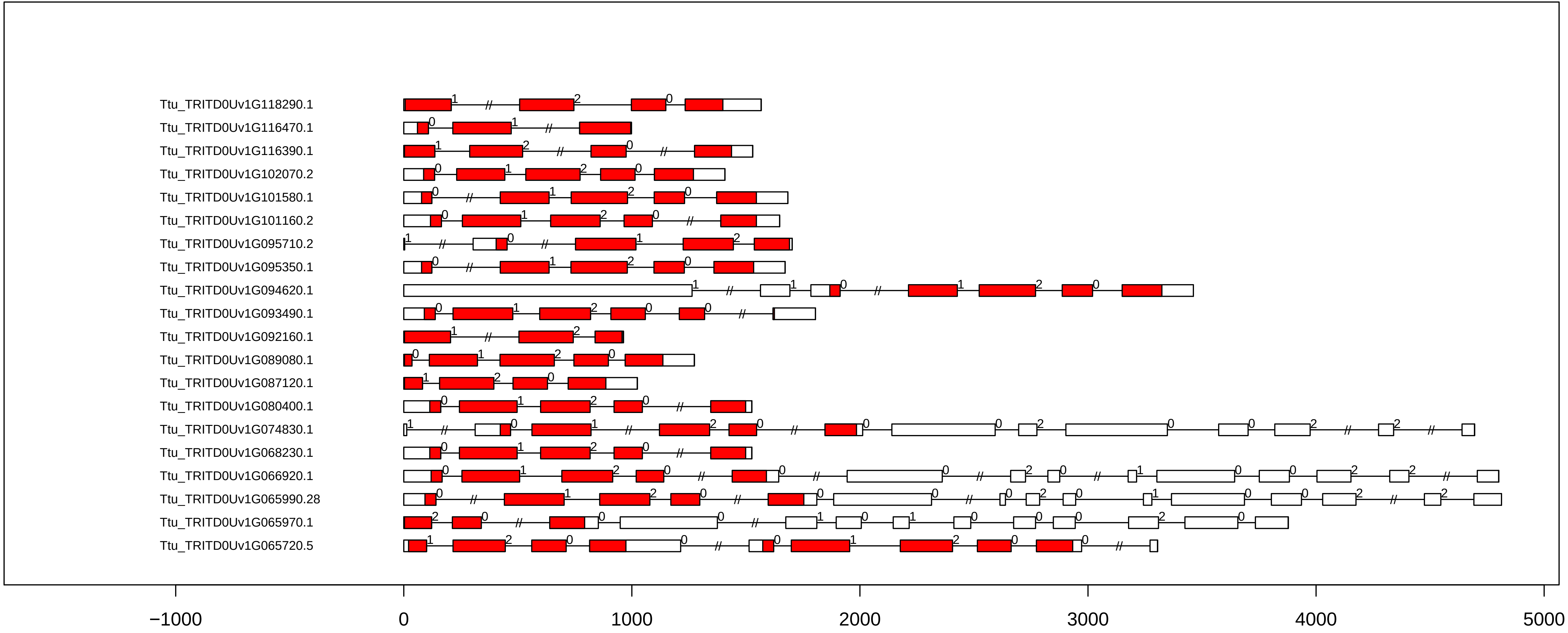

T.tu RLK−Pelle_DLSV subfamily exon−intron and kinase domain diagram (part 3)

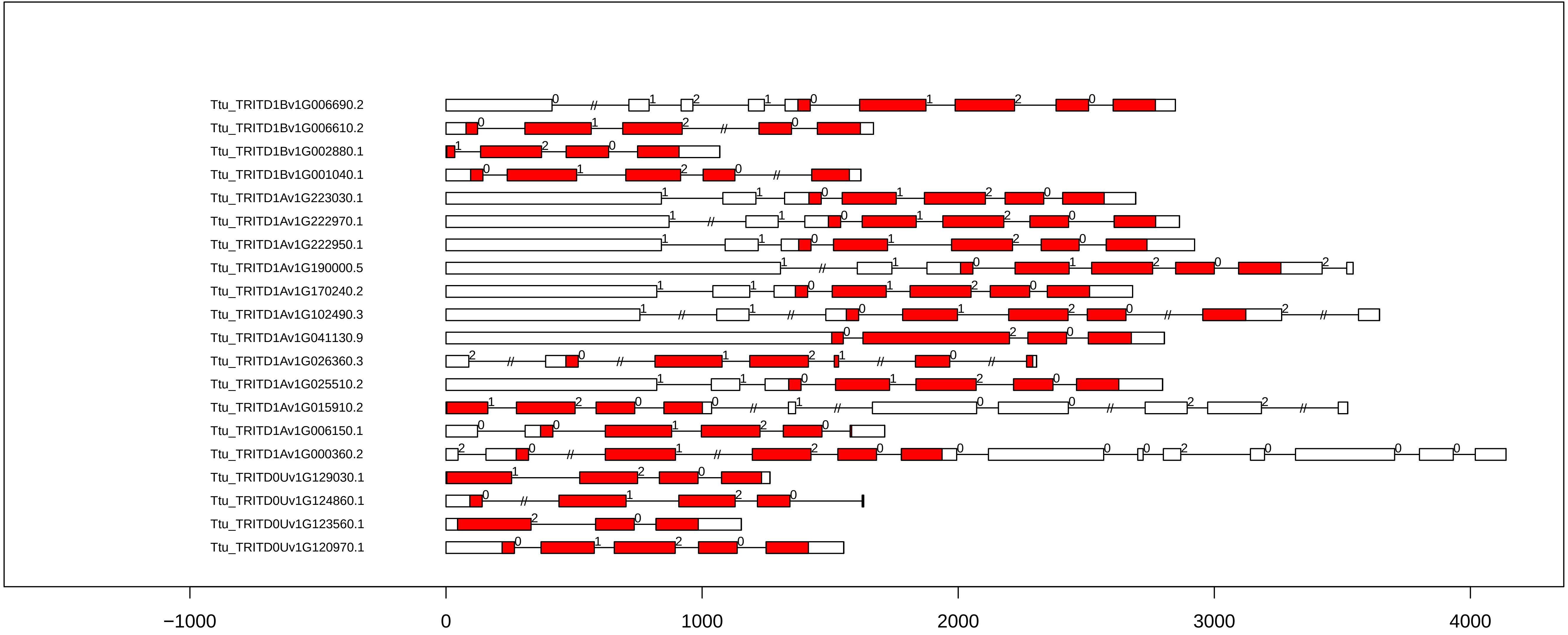

T.tu RLK−Pelle_DLSV subfamily exon−intron and kinase domain diagram (part 4)

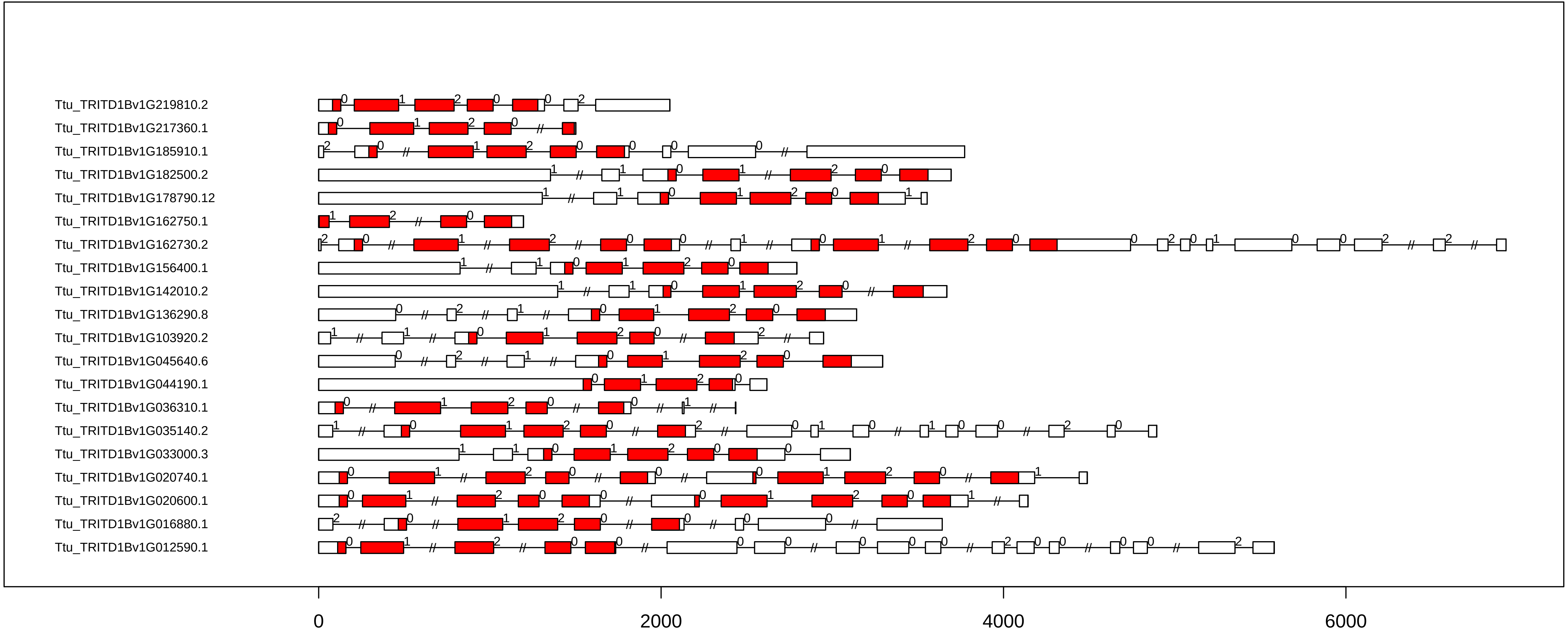

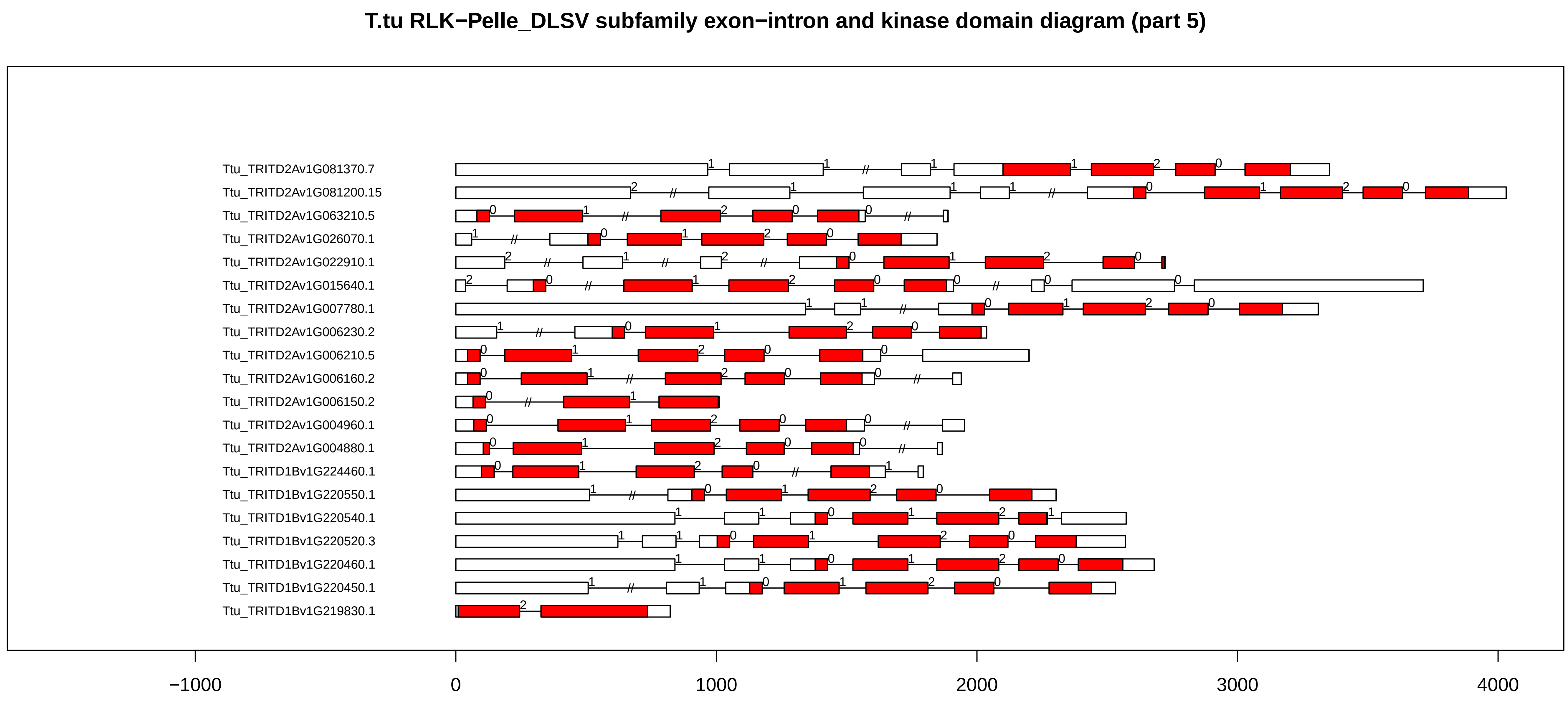
T.tu RLK−Pelle_DLSV subfamily exon−intron and kinase domain diagram (part 5)
Ttu_TRITD2Av1G081370.7
Ttu_TRITD2Av1G081200.15
Ttu_TRITD2Av1G063210.5
Ttu_TRITD2Av1G026070.1
Ttu_TRITD2Av1G022910.1
Ttu_TRITD2Av1G015640.1
Ttu_TRITD2Av1G007780.1
Ttu_TRITD2Av1G006230.2
Ttu_TRITD2Av1G006210.5
Ttu_TRITD2Av1G006160.2
Ttu_TRITD2Av1G006150.2
Ttu_TRITD2Av1G004960.1
Ttu_TRITD2Av1G004880.1
Ttu_TRITD1Bv1G224460.1
Ttu_TRITD1Bv1G220550.1
Ttu_TRITD1Bv1G220540.1
Ttu_TRITD1Bv1G220520.3
Ttu_TRITD1Bv1G220460.1
Ttu_TRITD1Bv1G220450.1
Ttu_TRITD1Bv1G219830.1
−1000
0
1000
2000
3000
4000

T.tu RLK−Pelle_DLSV subfamily exon−intron and kinase domain diagram (part 6)

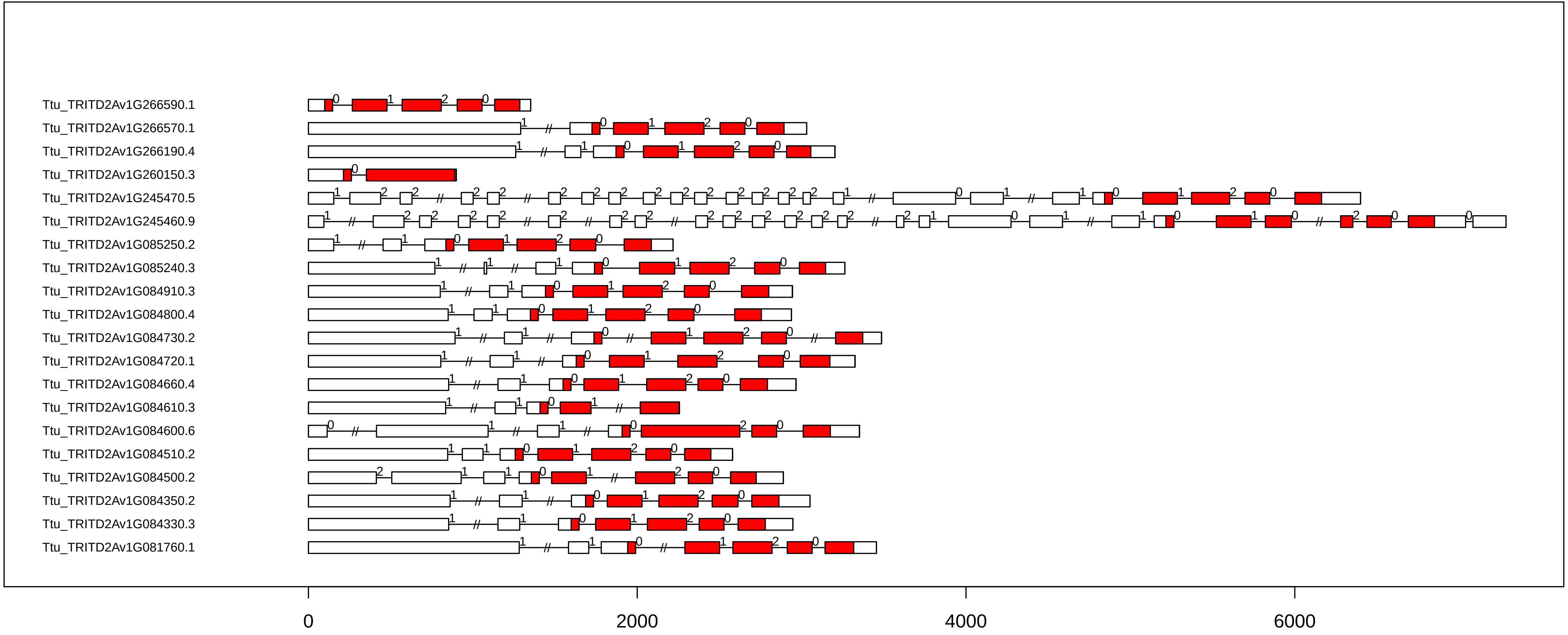

T.tu RLK−Pelle_DLSV subfamily exon−intron and kinase domain diagram (part 7)

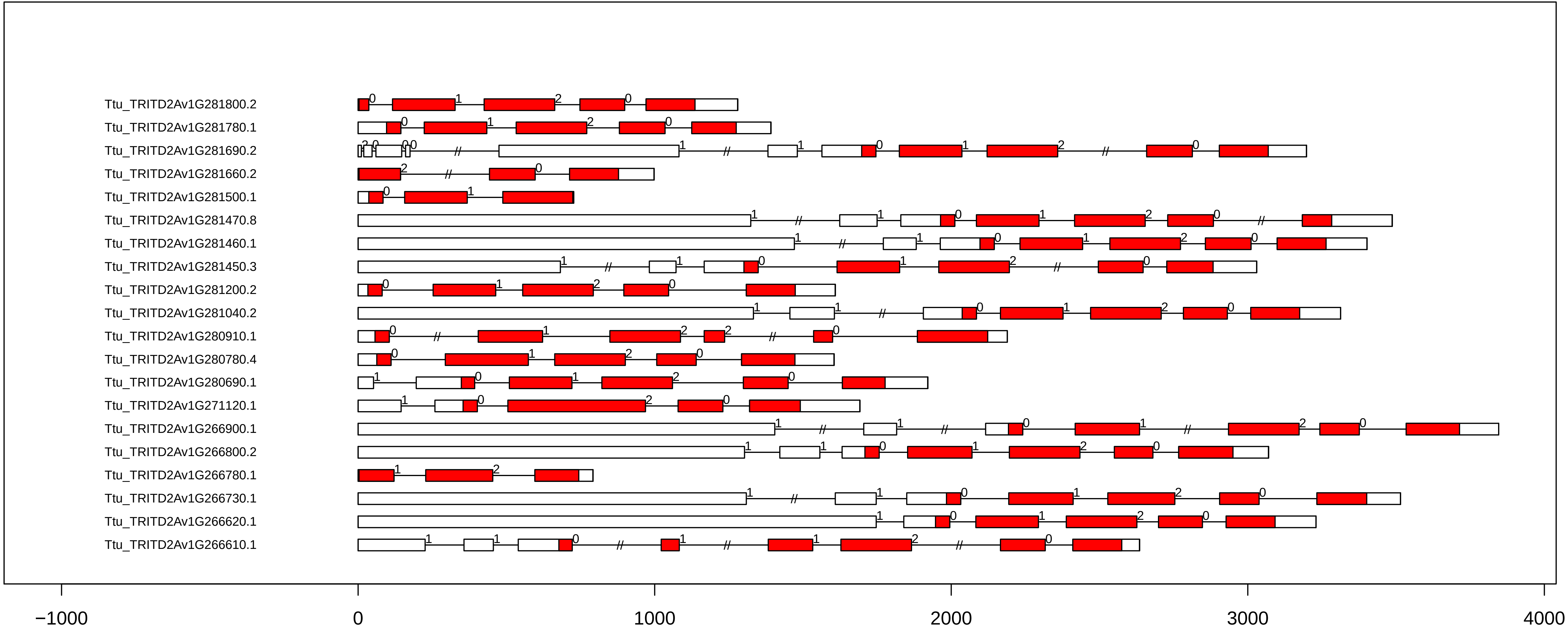

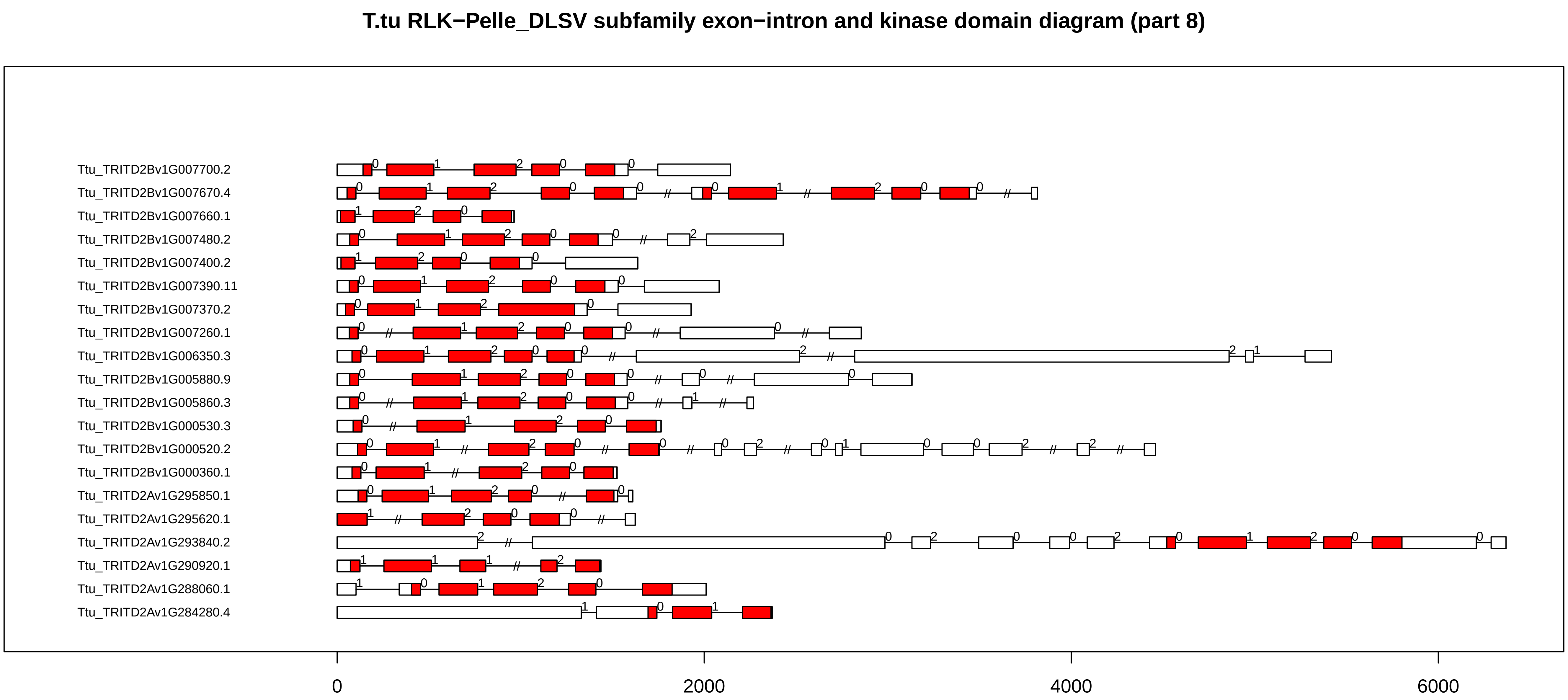
T.tu RLK−Pelle_DLSV subfamily exon−intron and kinase domain diagram (part 8)
Ttu_TRITD2Bv1G007700.2
Ttu_TRITD2Bv1G007670.4
Ttu_TRITD2Bv1G007660.1
Ttu_TRITD2Bv1G007480.2
Ttu_TRITD2Bv1G007400.2
Ttu_TRITD2Bv1G007390.11
Ttu_TRITD2Bv1G007370.2
Ttu_TRITD2Bv1G007260.1
Ttu_TRITD2Bv1G006350.3
Ttu_TRITD2Bv1G005880.9
Ttu_TRITD2Bv1G005860.3
Ttu_TRITD2Bv1G000530.3
Ttu_TRITD2Bv1G000520.2
Ttu_TRITD2Bv1G000360.1
Ttu_TRITD2Av1G295850.1
Ttu_TRITD2Av1G295620.1
Ttu_TRITD2Av1G293840.2
Ttu_TRITD2Av1G290920.1
Ttu_TRITD2Av1G288060.1
Ttu_TRITD2Av1G284280.4
0
2000
4000
6000

T.tu RLK−Pelle_DLSV subfamily exon−intron and kinase domain diagram (part 9)

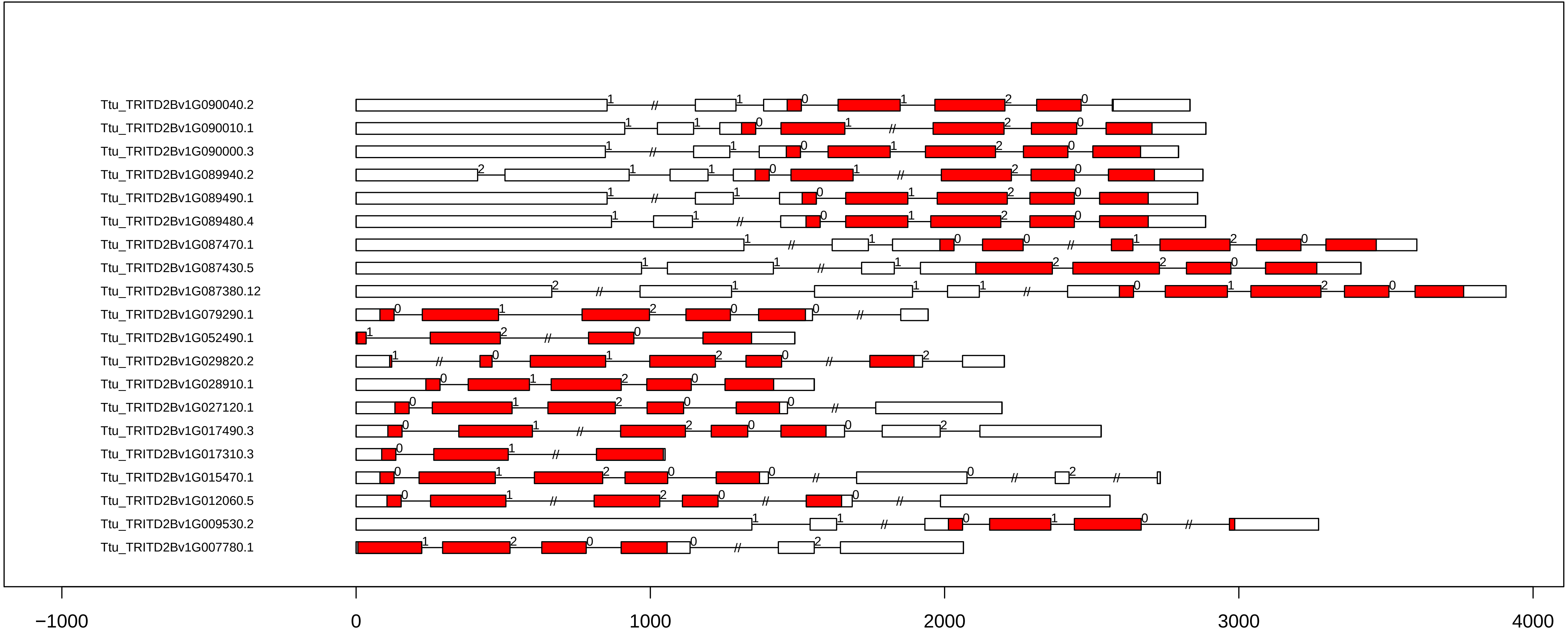

T.tu RLK−Pelle_DLSV subfamily exon−intron and kinase domain diagram (part 10)

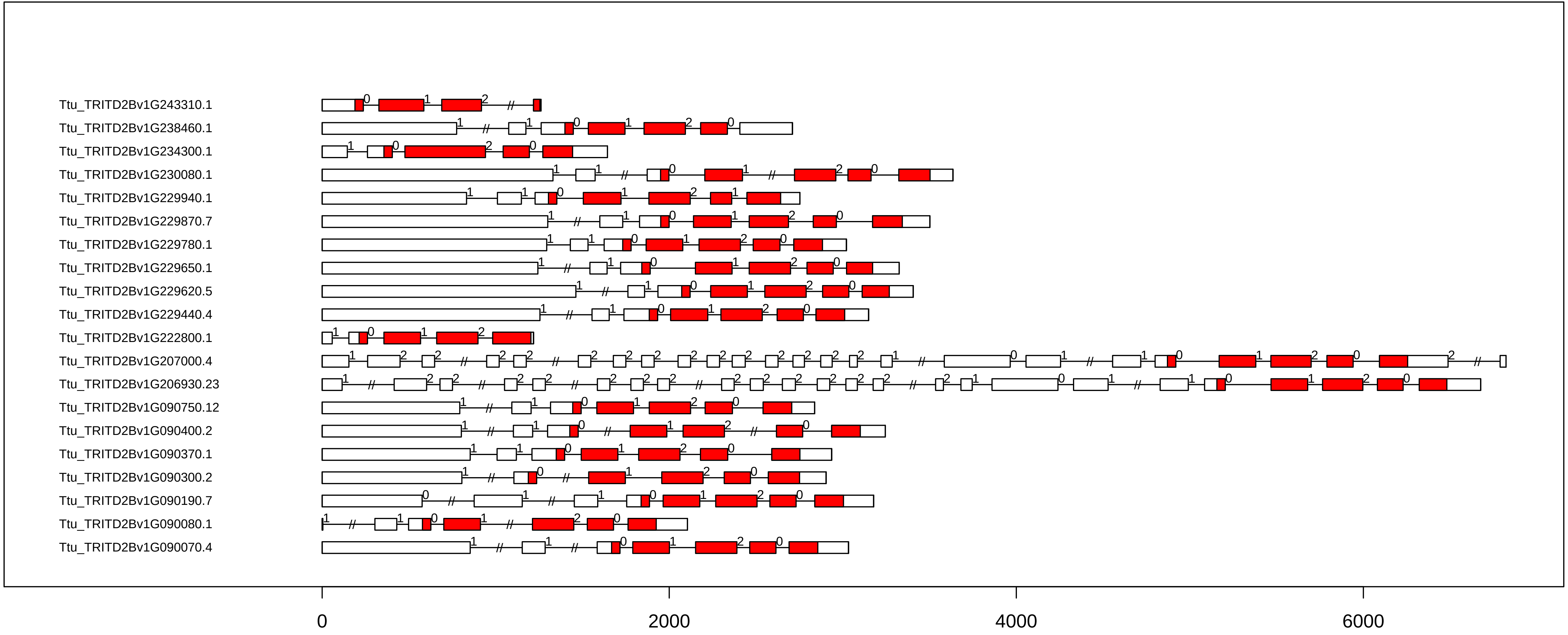

T.tu RLK−Pelle_DLSV subfamily exon−intron and kinase domain diagram (part 11)

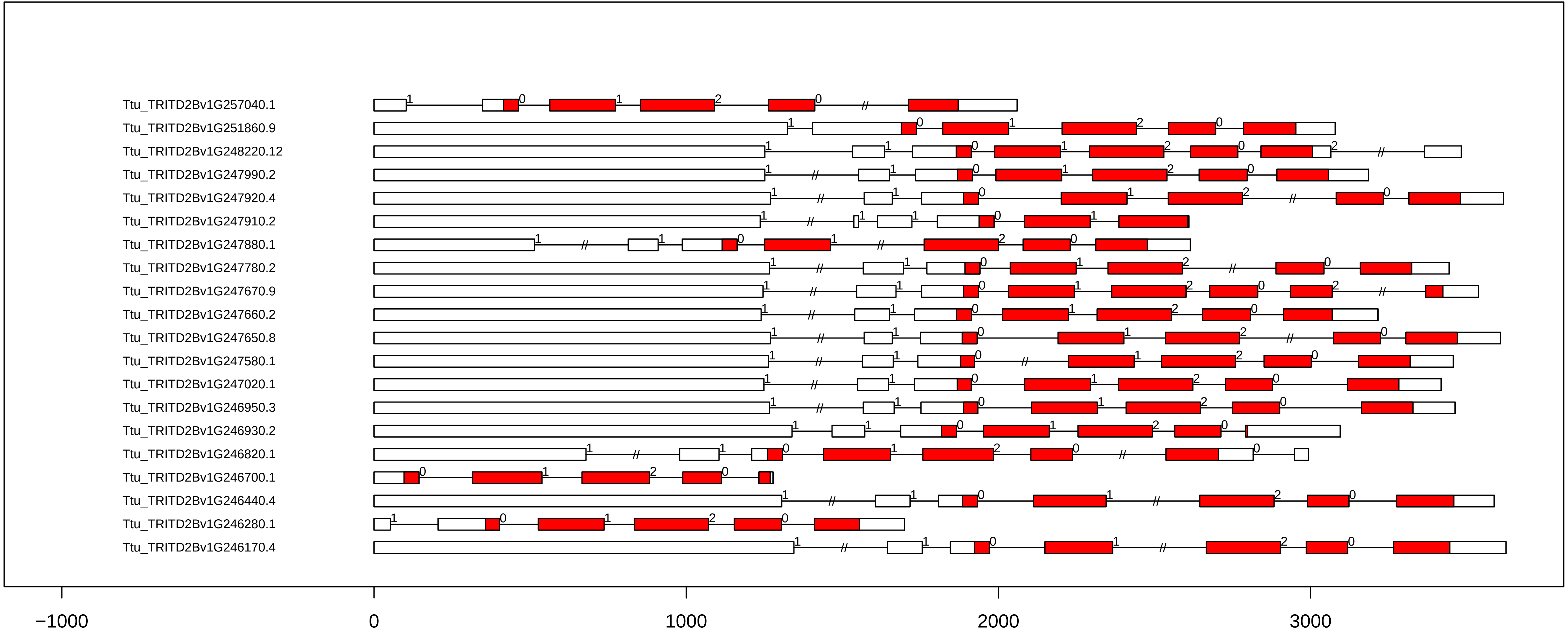

T.tu RLK−Pelle_DLSV subfamily exon−intron and kinase domain diagram (part 12)

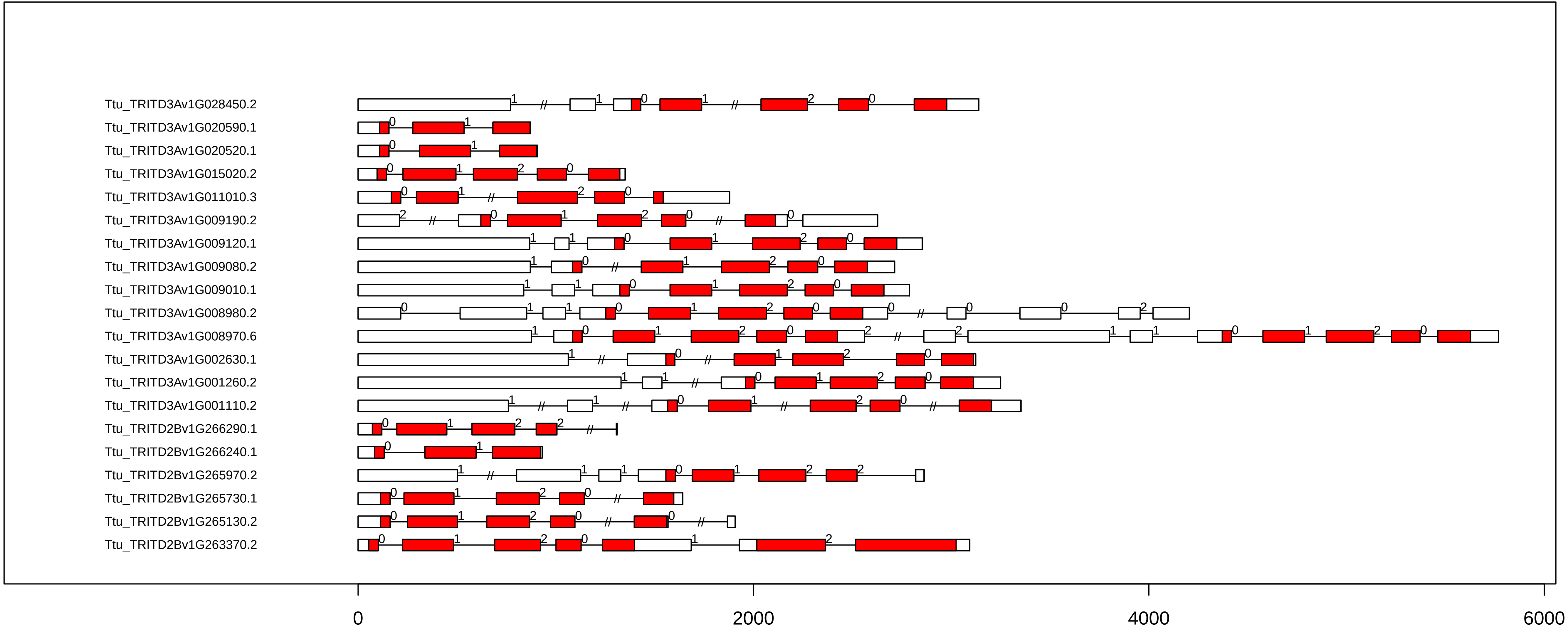

T.tu RLK−Pelle_DLSV subfamily exon−intron and kinase domain diagram (part 13)

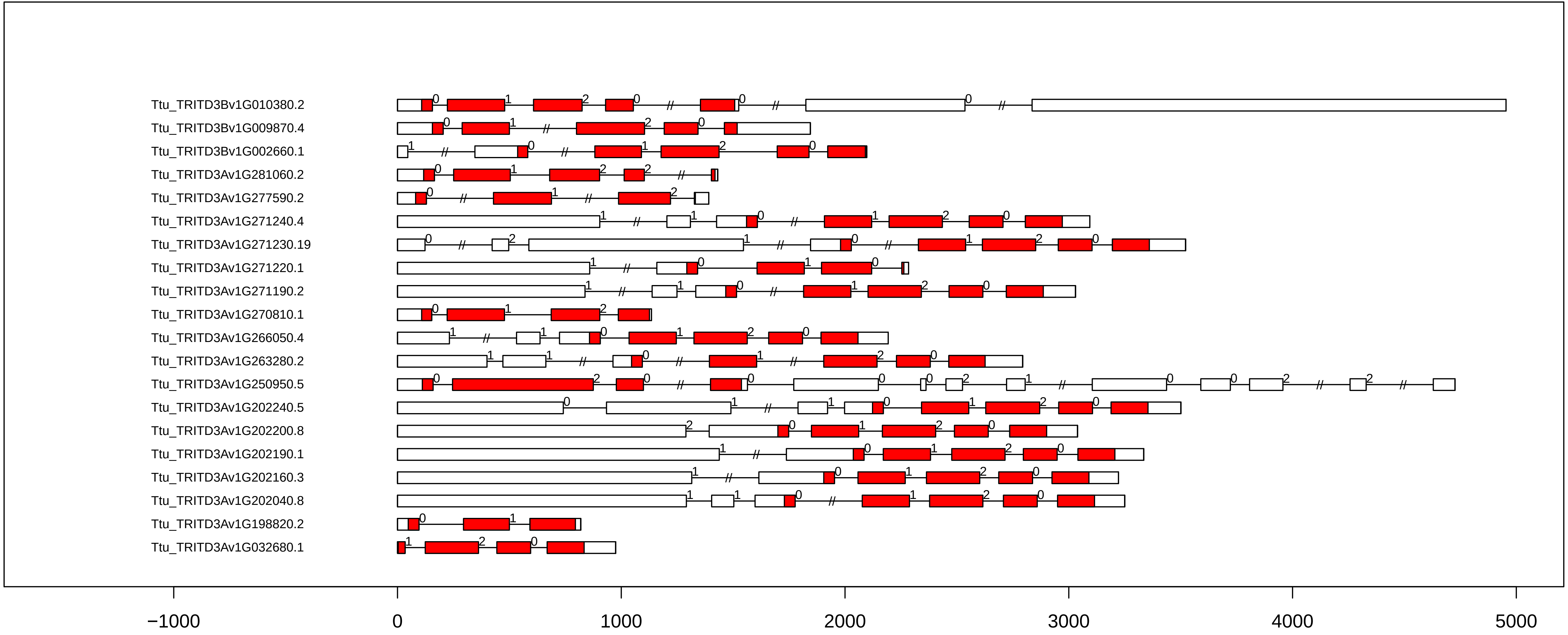

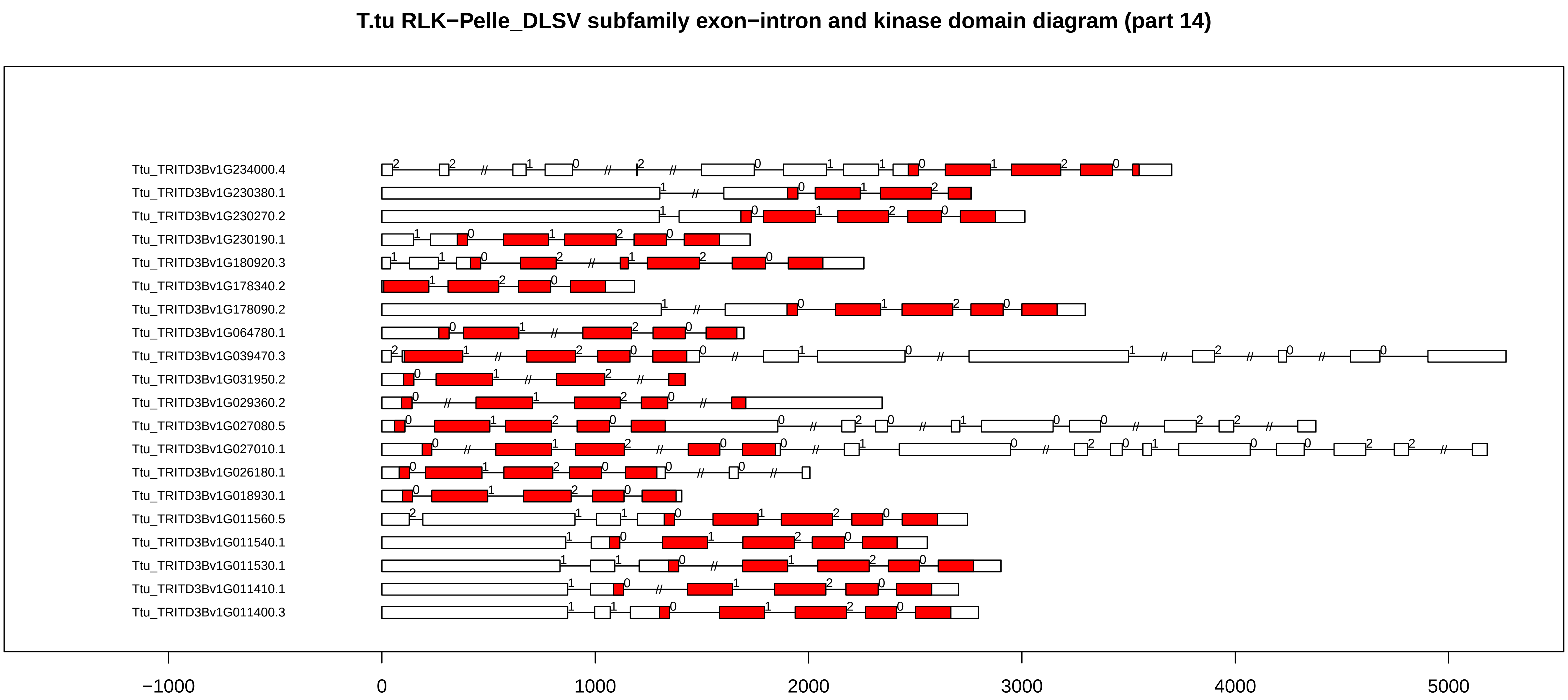
T.tu RLK−Pelle_DLSV subfamily exon−intron and kinase domain diagram (part 14)
Ttu_TRITD3Bv1G234000.4
Ttu_TRITD3Bv1G230380.1
Ttu_TRITD3Bv1G230270.2
Ttu_TRITD3Bv1G230190.1
Ttu_TRITD3Bv1G180920.3
Ttu_TRITD3Bv1G178340.2
Ttu_TRITD3Bv1G178090.2
Ttu_TRITD3Bv1G064780.1
Ttu_TRITD3Bv1G039470.3
Ttu_TRITD3Bv1G031950.2
Ttu_TRITD3Bv1G029360.2
Ttu_TRITD3Bv1G027080.5
Ttu_TRITD3Bv1G027010.1
Ttu_TRITD3Bv1G026180.1
Ttu_TRITD3Bv1G018930.1
Ttu_TRITD3Bv1G011560.5
Ttu_TRITD3Bv1G011540.1
Ttu_TRITD3Bv1G011530.1
Ttu_TRITD3Bv1G011410.1
Ttu_TRITD3Bv1G011400.3
−1000
0
1000
2000
3000
4000
5000

T.tu RLK−Pelle_DLSV subfamily exon−intron and kinase domain diagram (part 15)

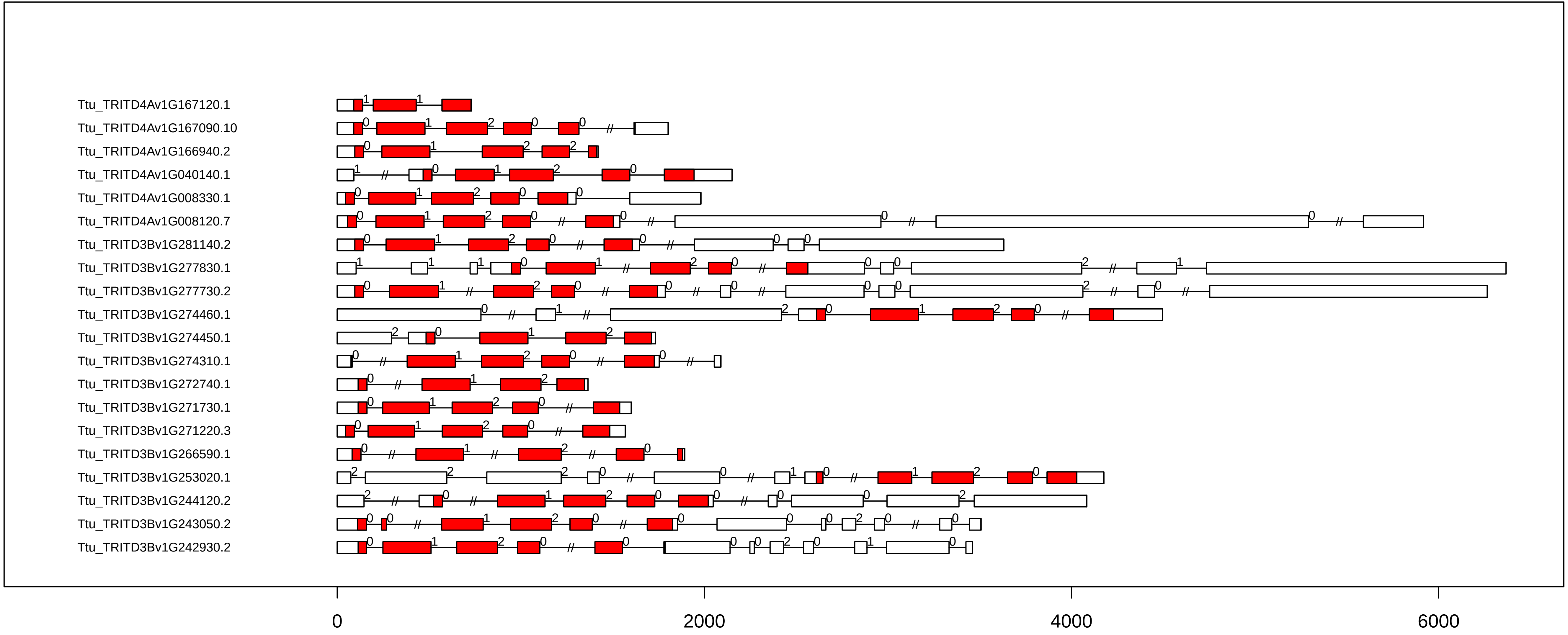

T.tu RLK−Pelle_DLSV subfamily exon−intron and kinase domain diagram (part 16)

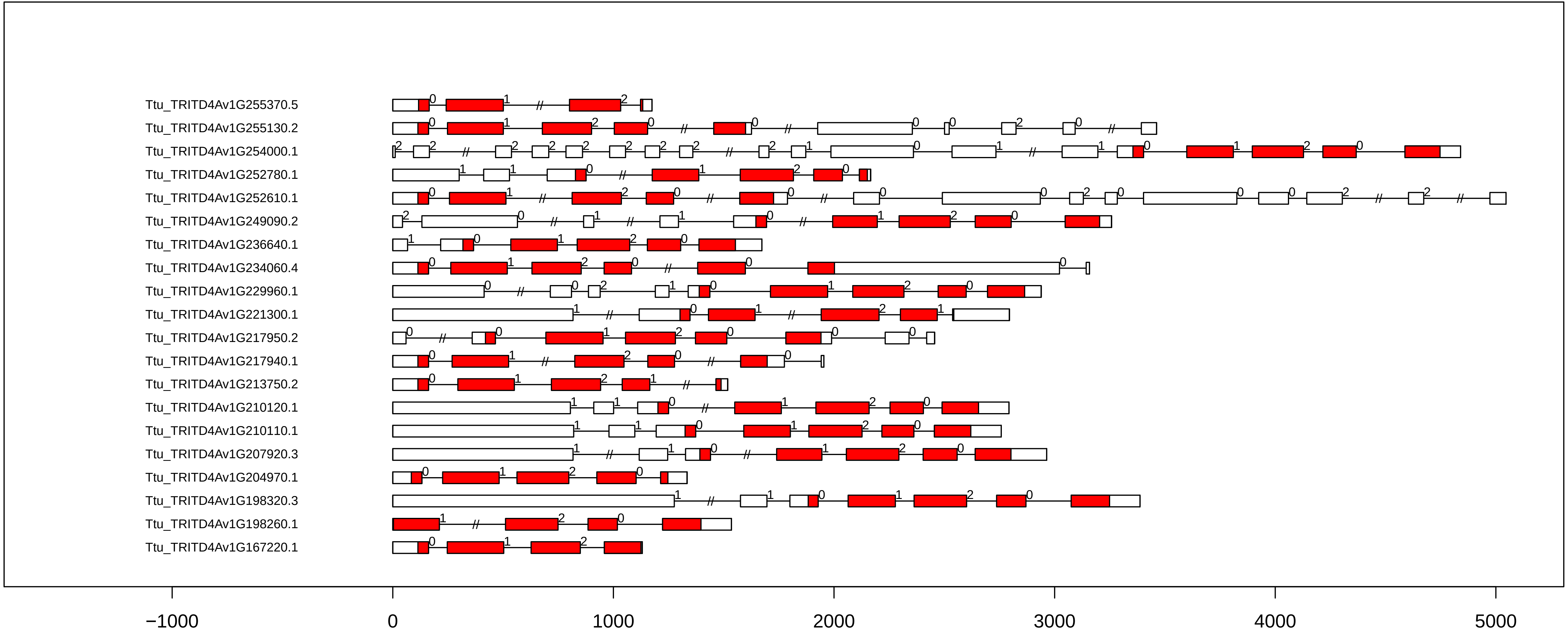

T.tu RLK−Pelle_DLSV subfamily exon−intron and kinase domain diagram (part 17)

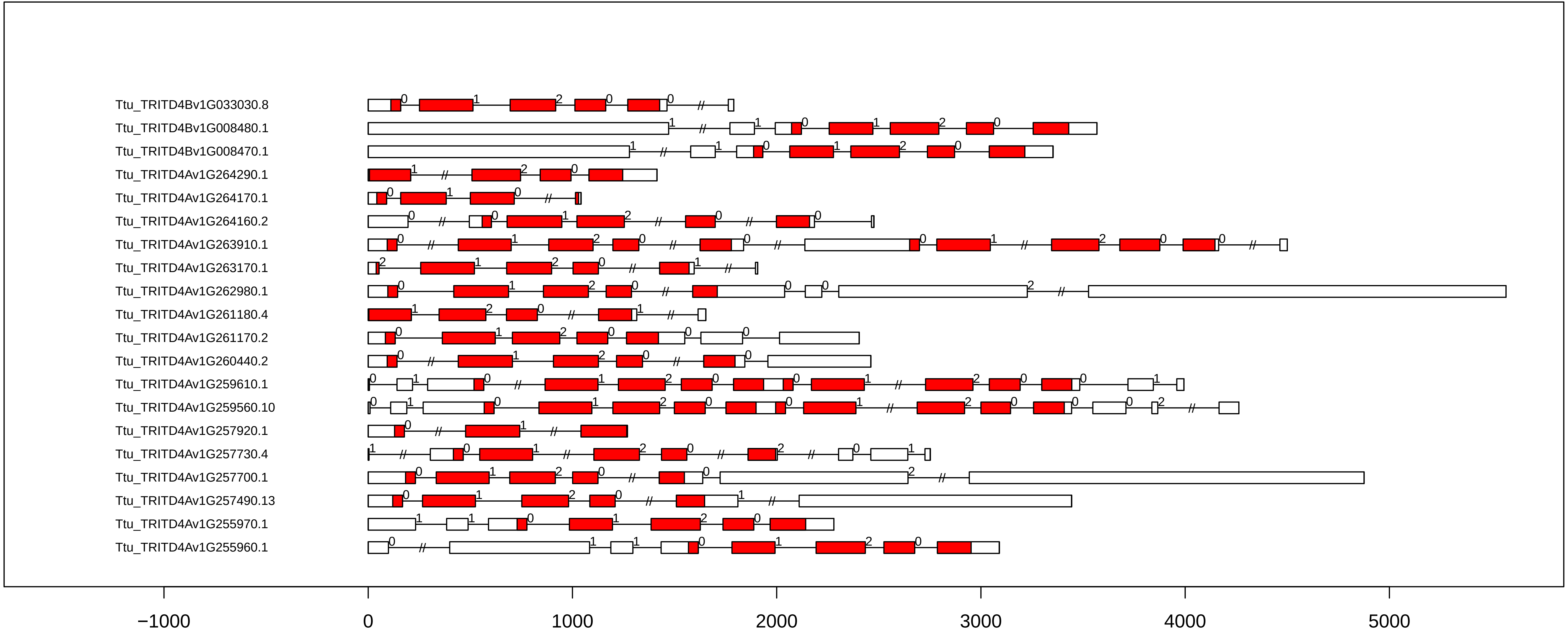

T.tu RLK−Pelle_DLSV subfamily exon−intron and kinase domain diagram (part 18)

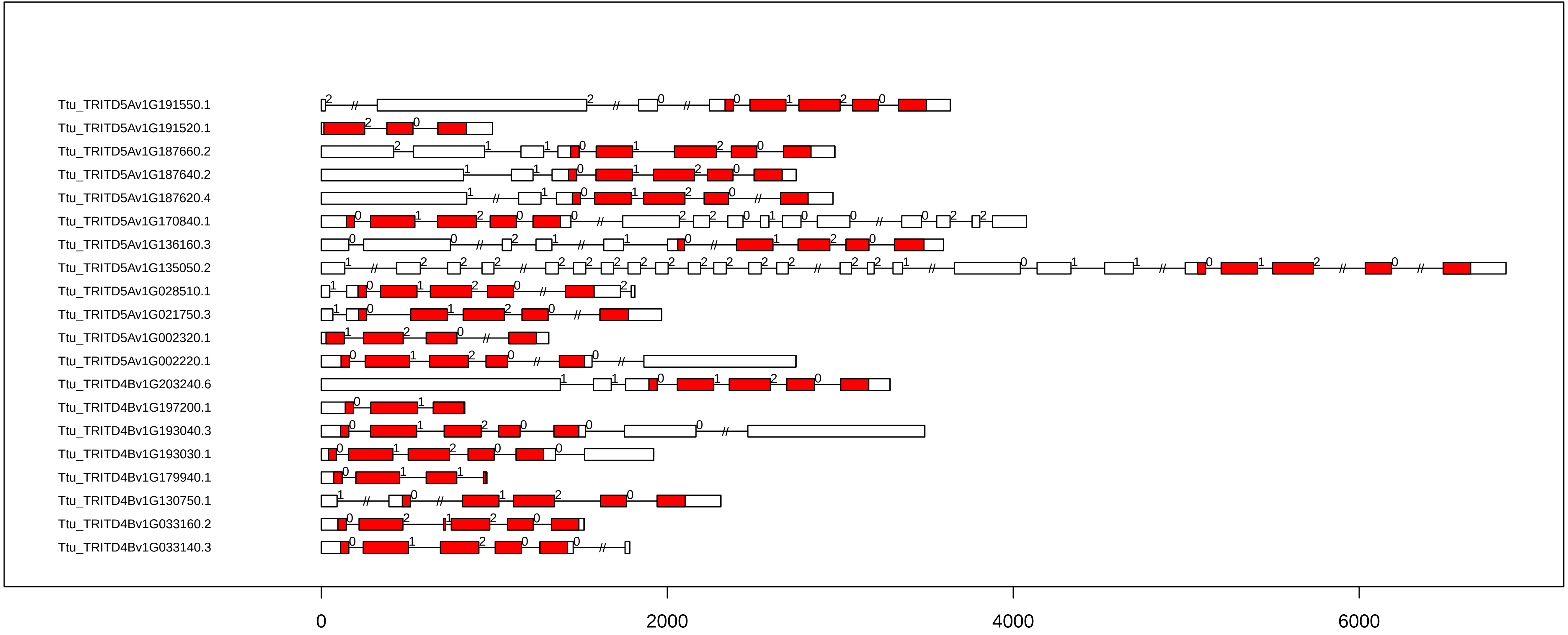

T.tu RLK−Pelle_DLSV subfamily exon−intron and kinase domain diagram (part 19)

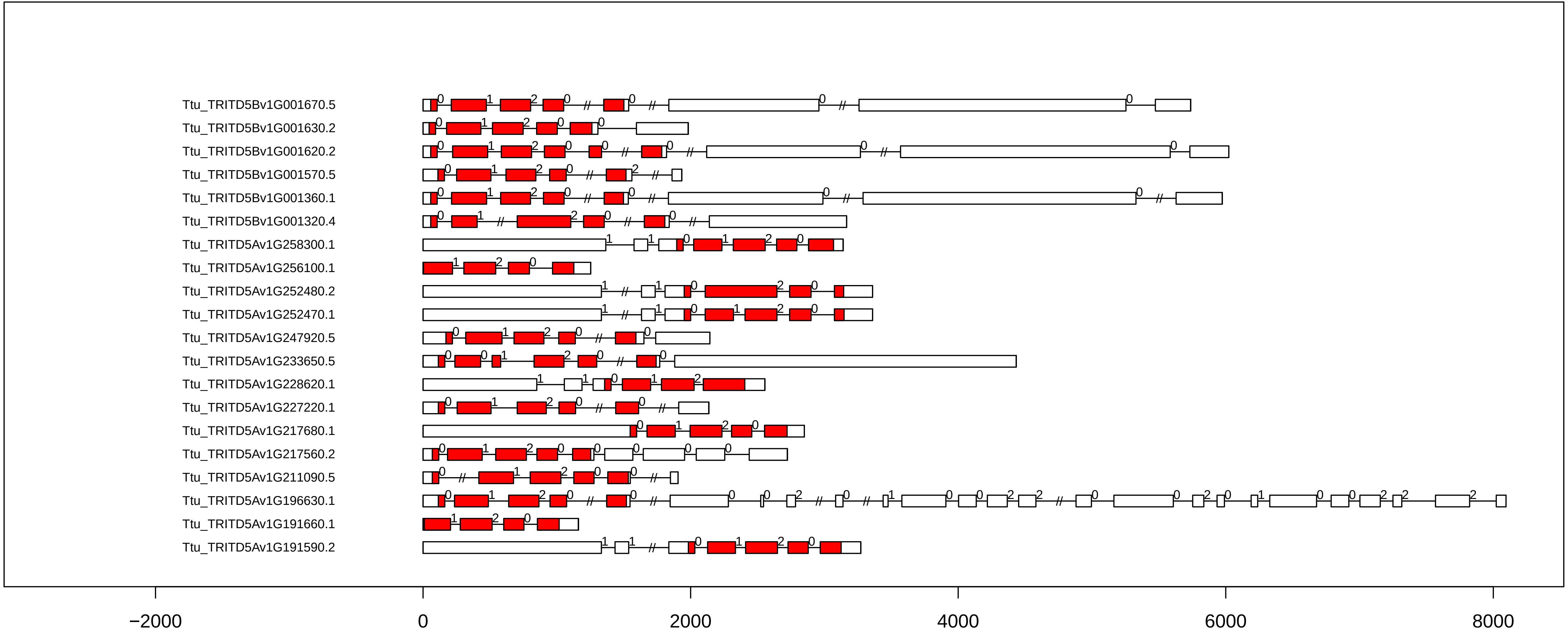

T.tu RLK−Pelle_DLSV subfamily exon−intron and kinase domain diagram (part 20)

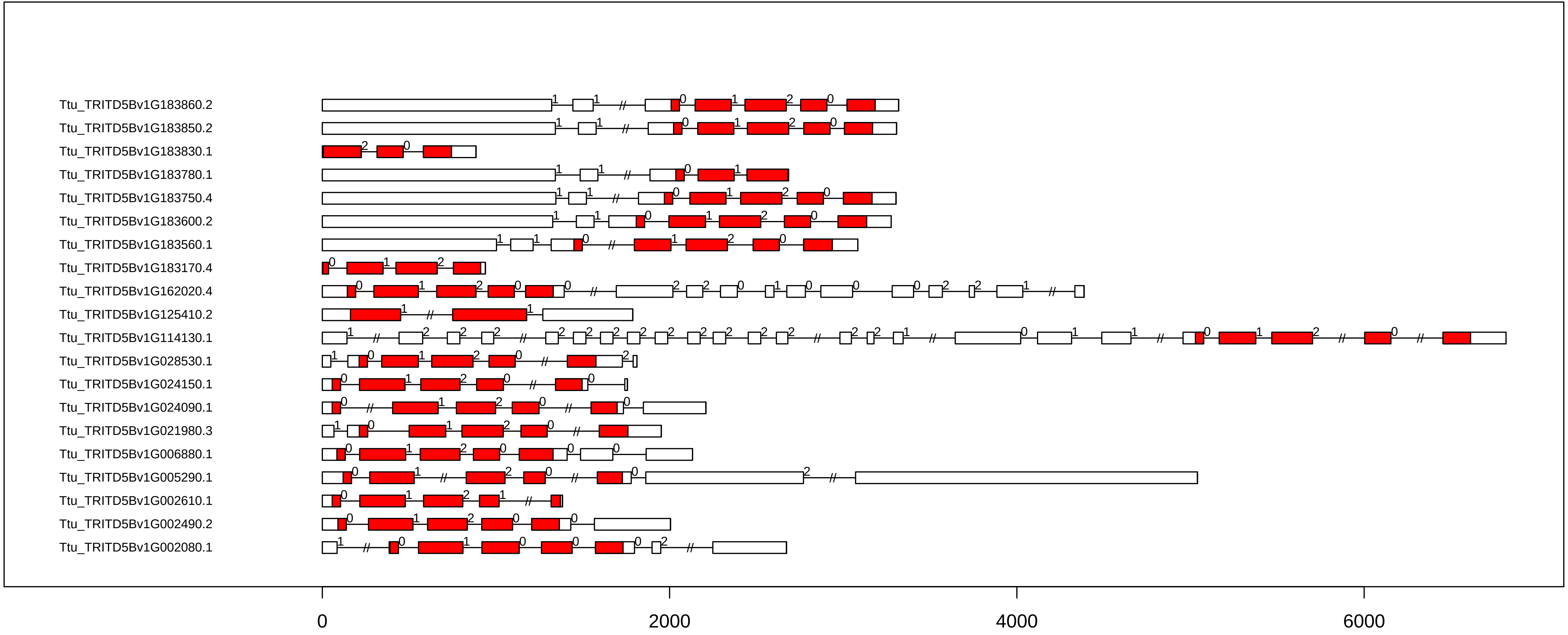

T.tu RLK−Pelle_DLSV subfamily exon−intron and kinase domain diagram (part 21)

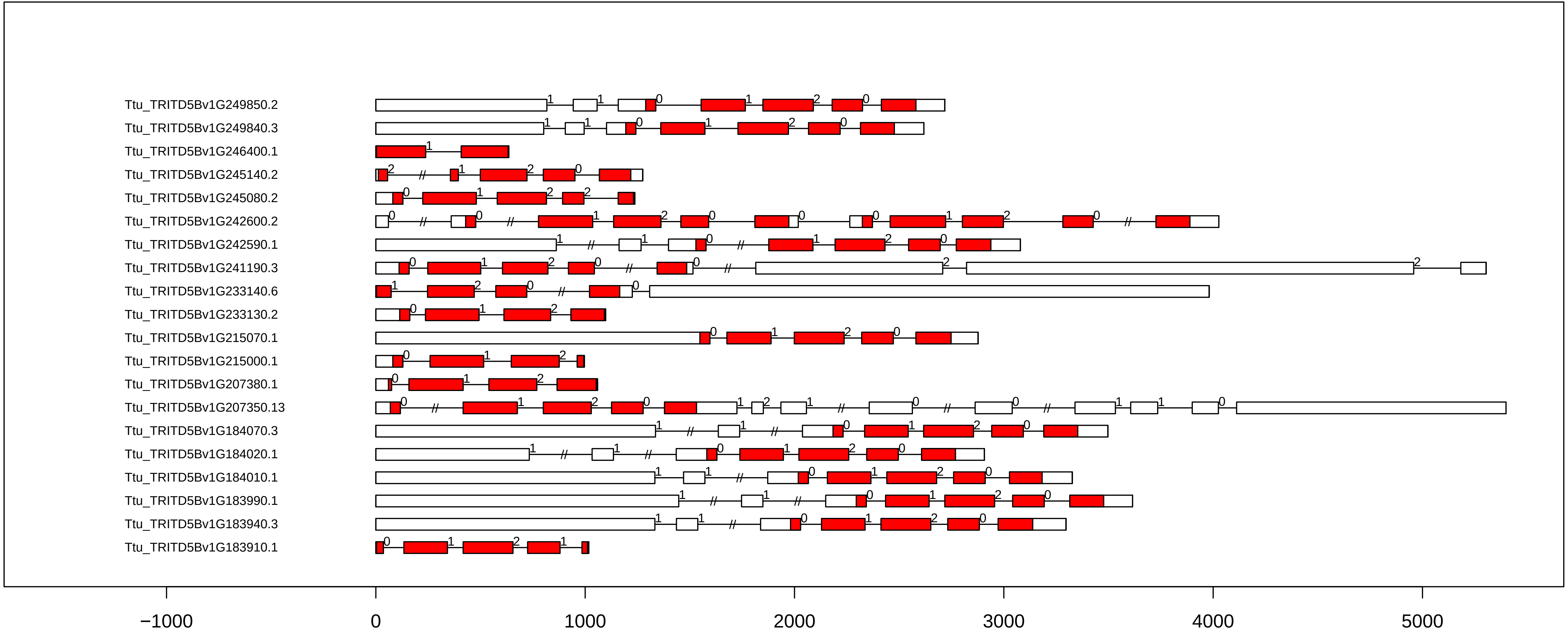

T.tu RLK−Pelle_DLSV subfamily exon−intron and kinase domain diagram (part 22)

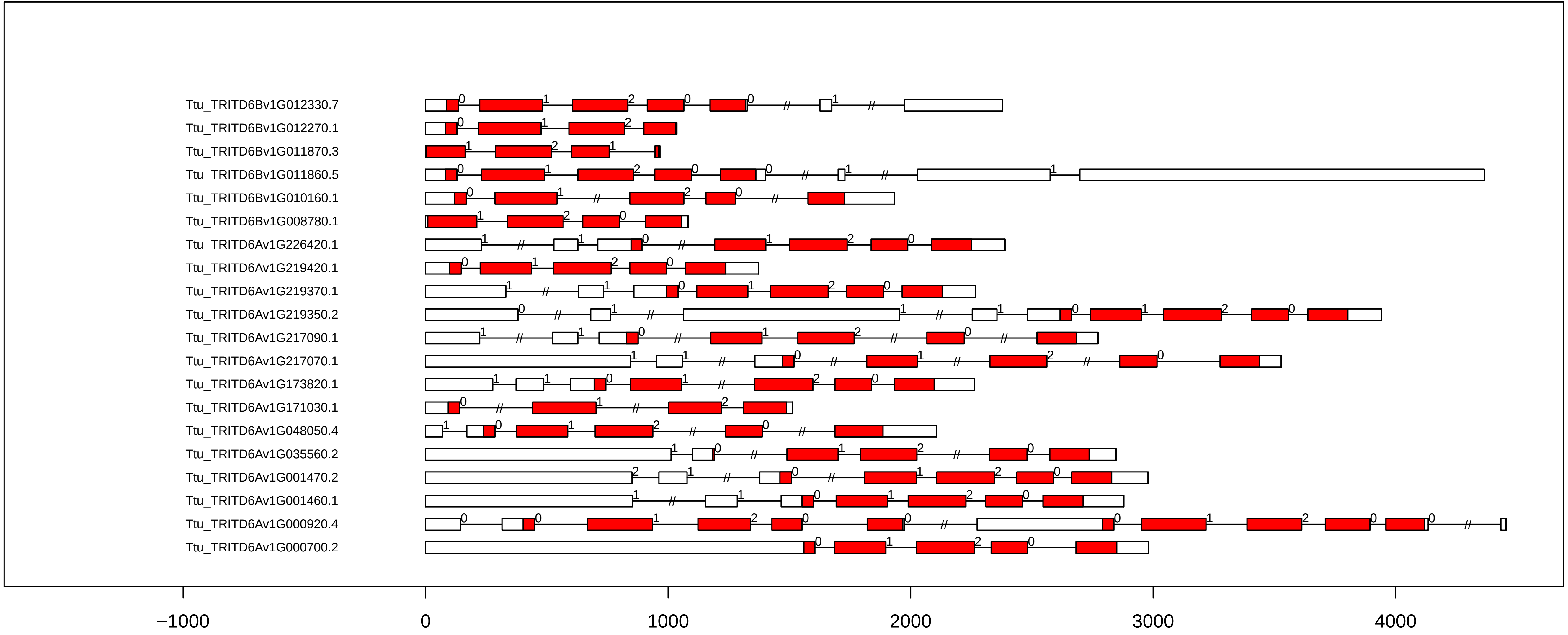

T.tu RLK−Pelle_DLSV subfamily exon−intron and kinase domain diagram (part 23)

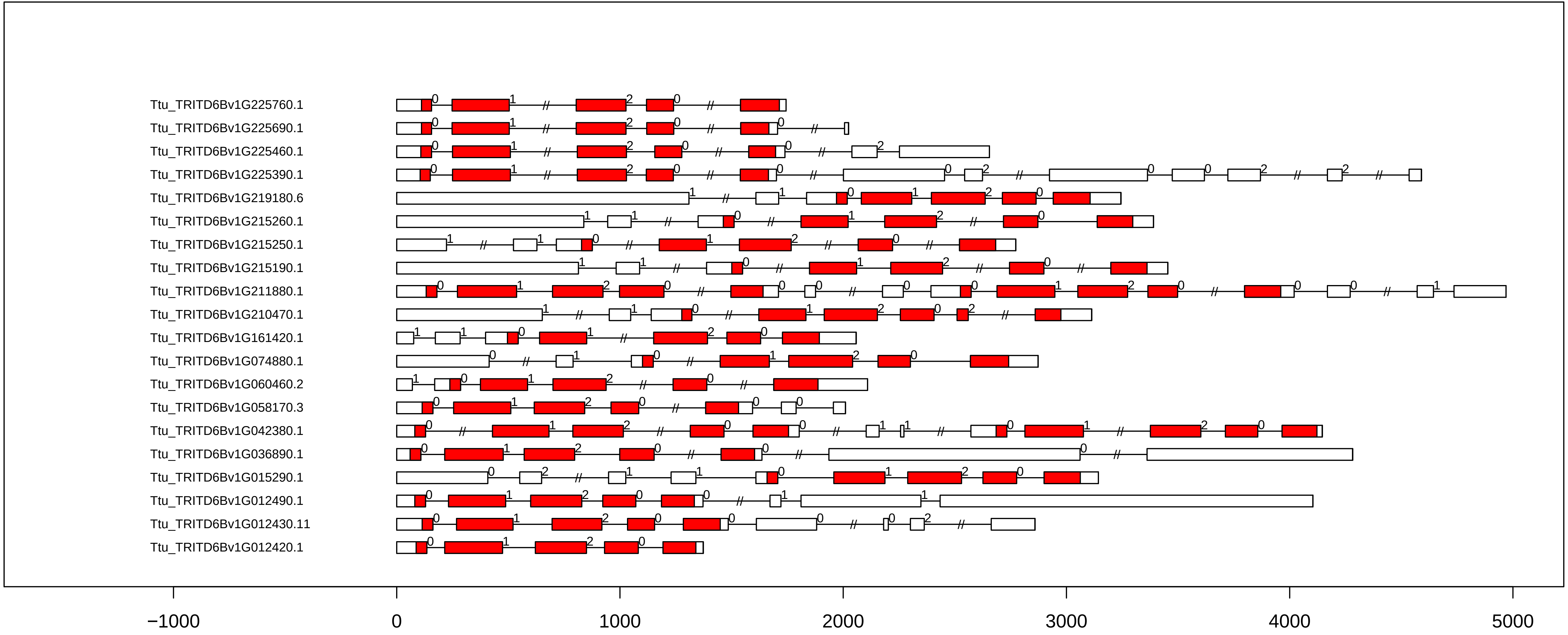

T.tu RLK−Pelle_DLSV subfamily exon−intron and kinase domain diagram (part 24)

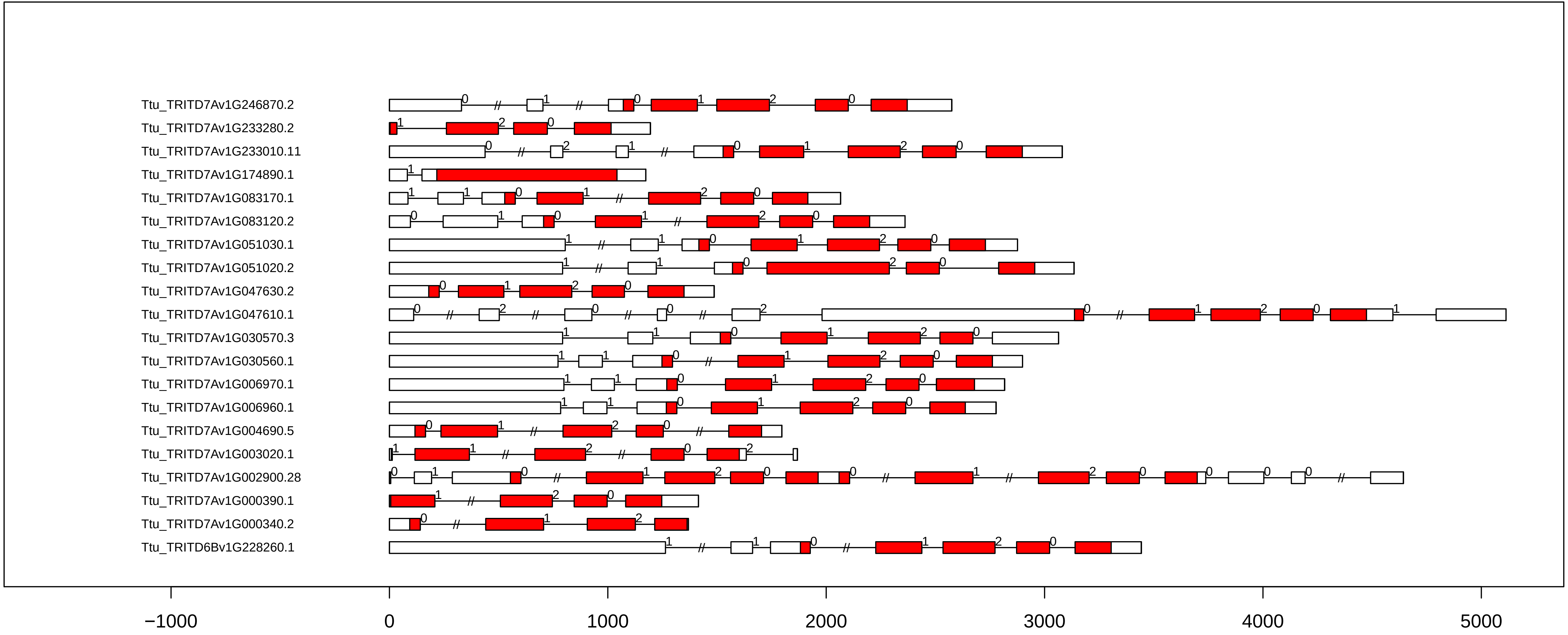

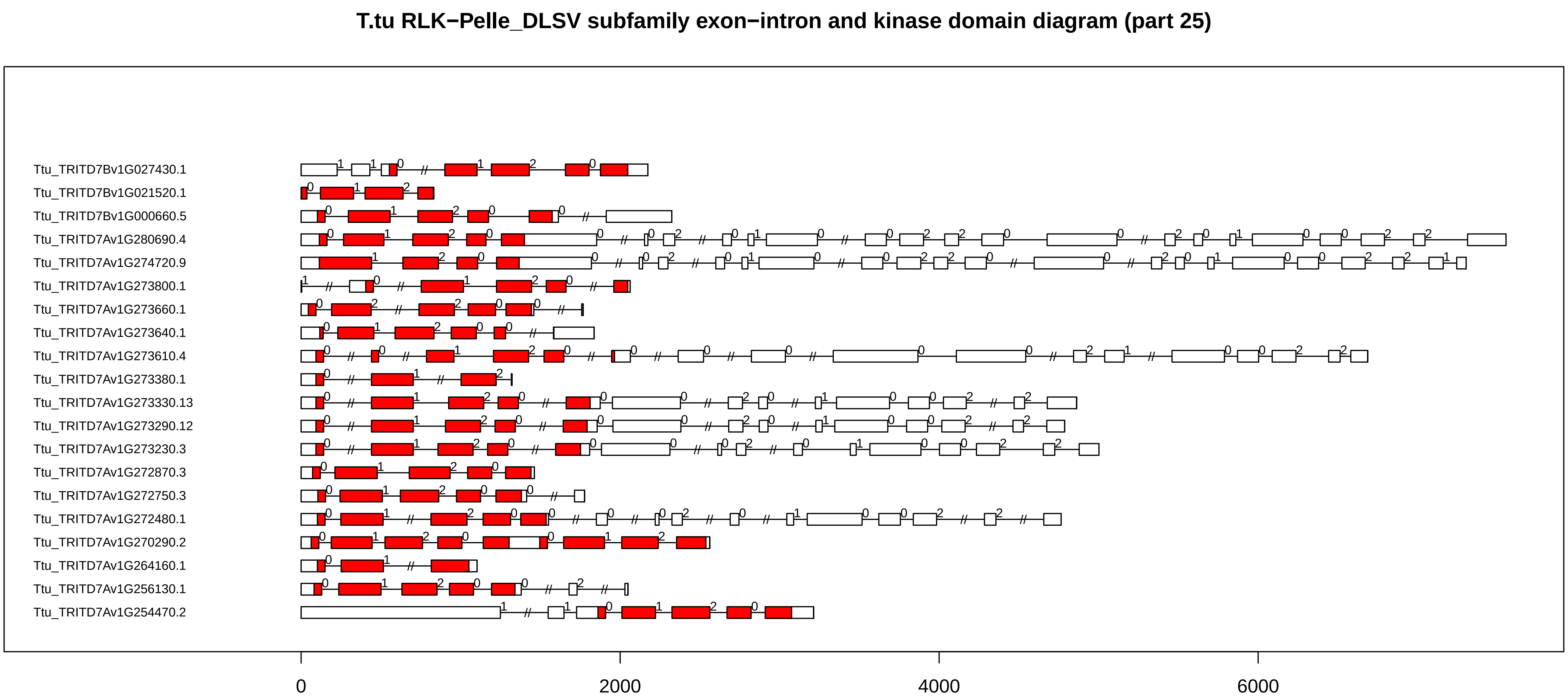
T.tu RLK−Pelle_DLSV subfamily exon−intron and kinase domain diagram (part 25)
Ttu_TRITD7Bv1G027430.1
Ttu_TRITD7Bv1G021520.1
Ttu_TRITD7Bv1G000660.5
Ttu_TRITD7Av1G280690.4
Ttu_TRITD7Av1G274720.9
Ttu_TRITD7Av1G273800.1
Ttu_TRITD7Av1G273660.1
Ttu_TRITD7Av1G273640.1
Ttu_TRITD7Av1G273610.4
Ttu_TRITD7Av1G273380.1
Ttu_TRITD7Av1G273330.13
Ttu_TRITD7Av1G273290.12
Ttu_TRITD7Av1G273230.3
Ttu_TRITD7Av1G272870.3
Ttu_TRITD7Av1G272750.3
Ttu_TRITD7Av1G272480.1
Ttu_TRITD7Av1G270290.2
Ttu_TRITD7Av1G264160.1
Ttu_TRITD7Av1G256130.1
Ttu_TRITD7Av1G254470.2
0
2000
4000
6000

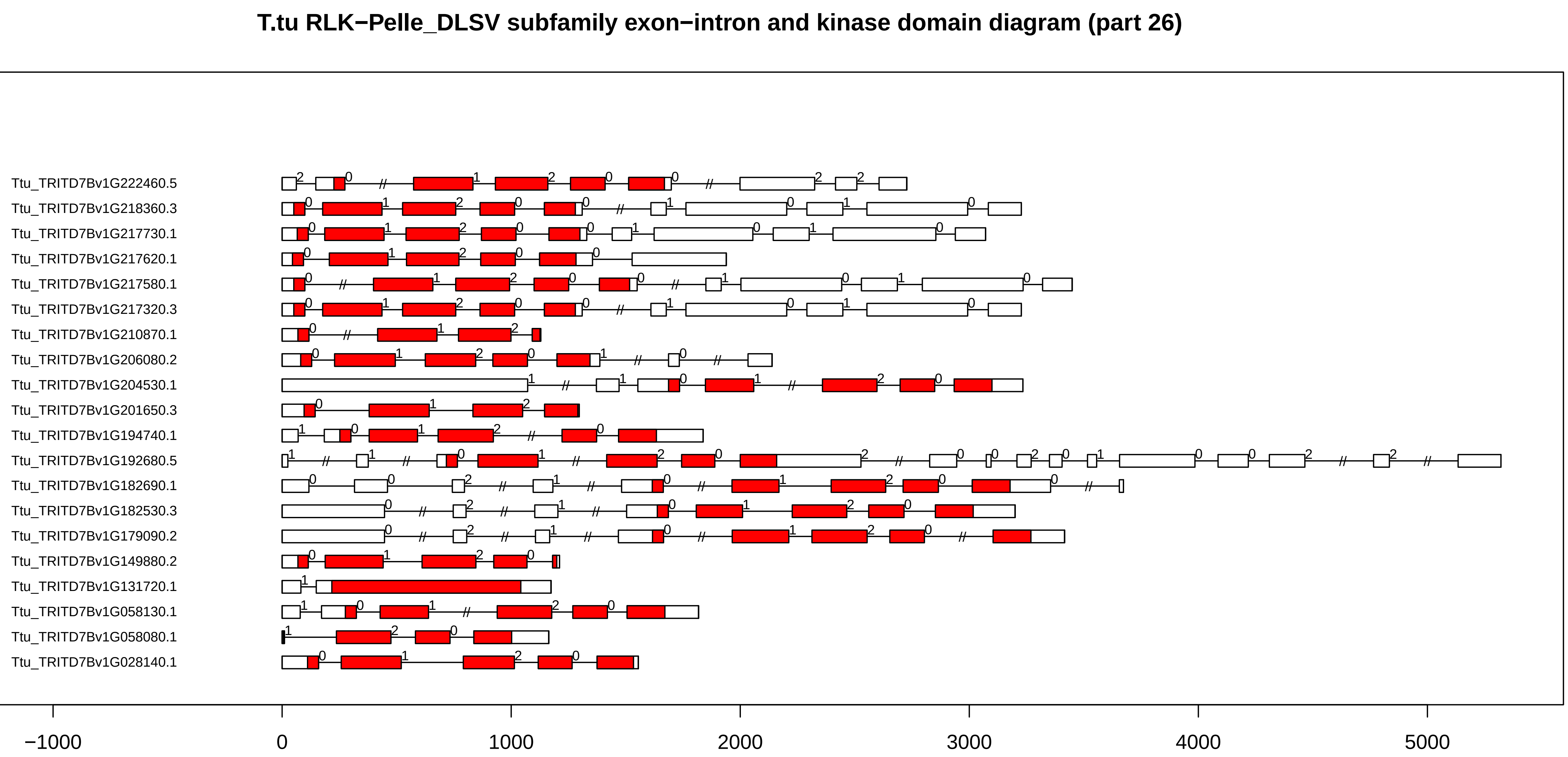
T.tu RLK−Pelle_DLSV subfamily exon−intron and kinase domain diagram (part 26)
Ttu_TRITD7Bv1G222460.5
Ttu_TRITD7Bv1G218360.3
Ttu_TRITD7Bv1G217730.1
Ttu_TRITD7Bv1G217620.1
Ttu_TRITD7Bv1G217580.1
Ttu_TRITD7Bv1G217320.3
Ttu_TRITD7Bv1G210870.1
Ttu_TRITD7Bv1G206080.2
Ttu_TRITD7Bv1G204530.1
Ttu_TRITD7Bv1G201650.3
Ttu_TRITD7Bv1G194740.1
Ttu_TRITD7Bv1G192680.5
Ttu_TRITD7Bv1G182690.1
Ttu_TRITD7Bv1G182530.3
Ttu_TRITD7Bv1G179090.2
Ttu_TRITD7Bv1G149880.2
Ttu_TRITD7Bv1G131720.1
Ttu_TRITD7Bv1G058130.1
Ttu_TRITD7Bv1G058080.1
Ttu_TRITD7Bv1G028140.1
−1000
0
1000
2000
3000
4000
5000

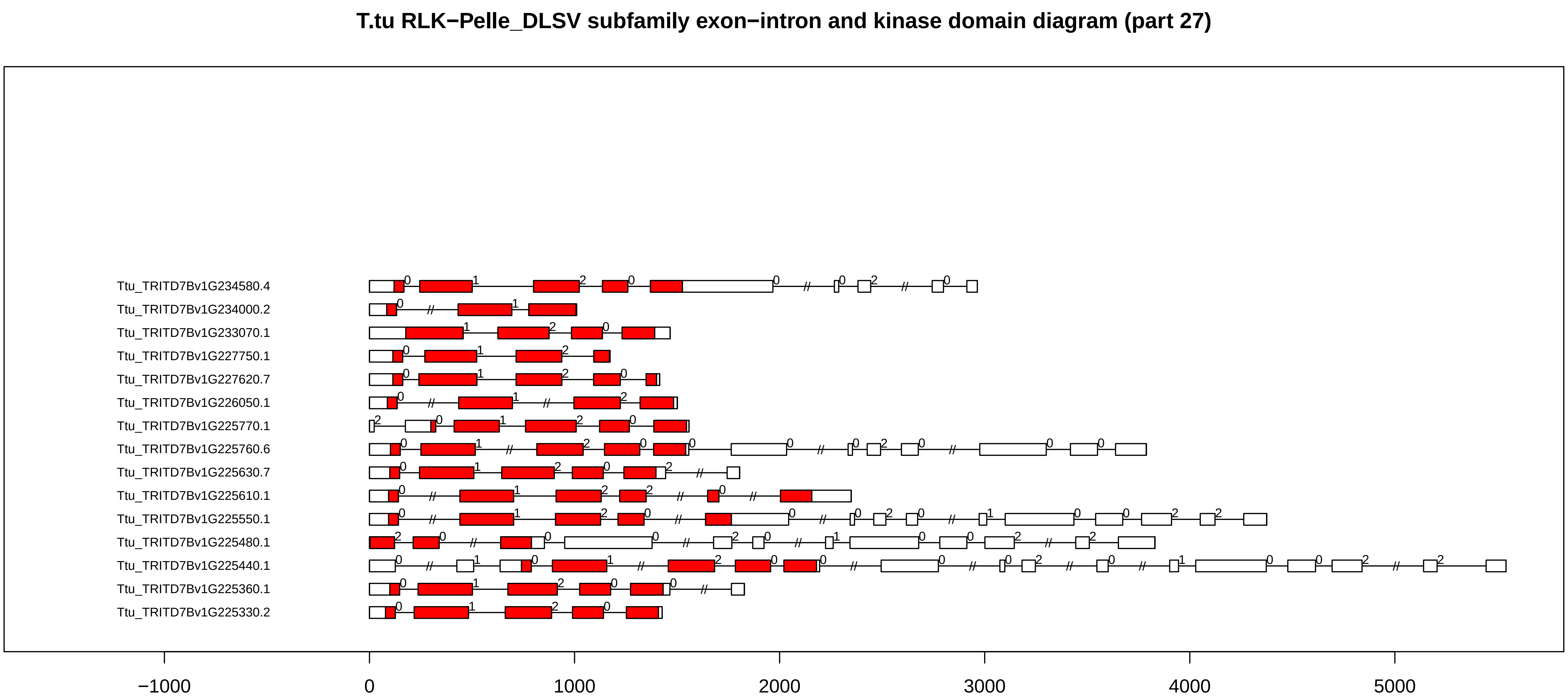
T.tu RLK−Pelle_DLSV subfamily exon−intron and kinase domain diagram (part 27)
Ttu_TRITD7Bv1G234580.4
Ttu_TRITD7Bv1G234000.2
Ttu_TRITD7Bv1G233070.1
Ttu_TRITD7Bv1G227750.1
Ttu_TRITD7Bv1G227620.7
Ttu_TRITD7Bv1G226050.1
Ttu_TRITD7Bv1G225770.1
Ttu_TRITD7Bv1G225760.6
Ttu_TRITD7Bv1G225630.7
Ttu_TRITD7Bv1G225610.1
Ttu_TRITD7Bv1G225550.1
Ttu_TRITD7Bv1G225480.1
Ttu_TRITD7Bv1G225440.1
Ttu_TRITD7Bv1G225360.1
Ttu_TRITD7Bv1G225330.2
−1000
0
1000
2000
3000
4000
5000

T.tu RLK−Pelle_Extensin subfamily exon−intron and kinase domain diagram (all)

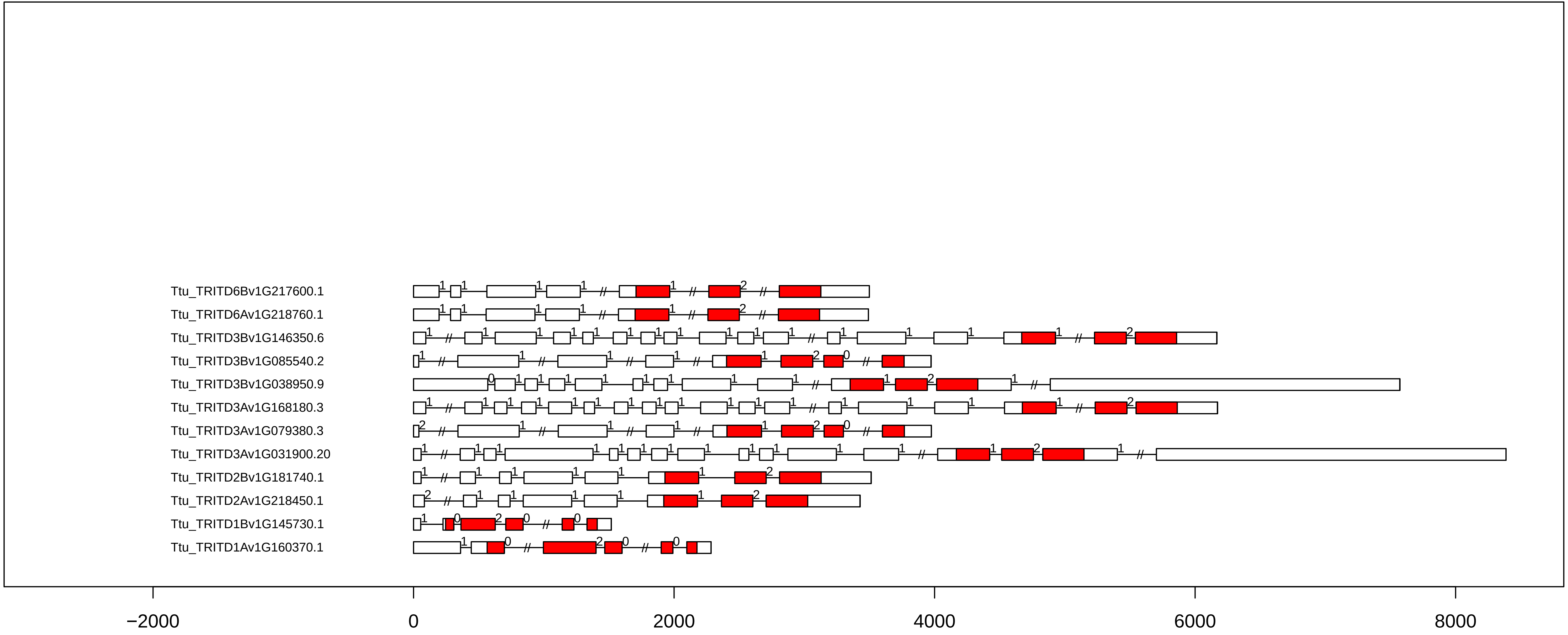

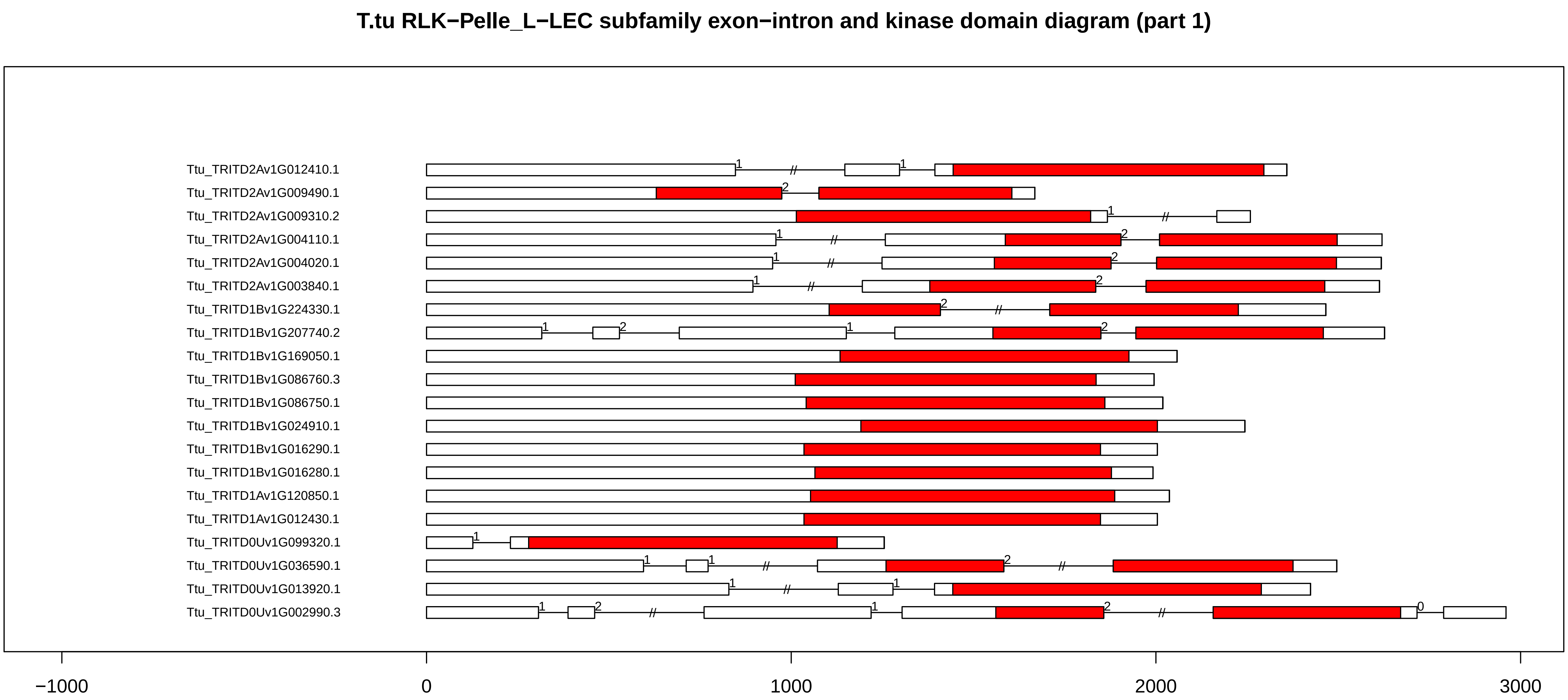
T.tu RLK−Pelle_L−LEC subfamily exon−intron and kinase domain diagram (part 1)
Ttu_TRITD2Av1G012410.1
Ttu_TRITD2Av1G009490.1
Ttu_TRITD2Av1G009310.2
Ttu_TRITD2Av1G004110.1
Ttu_TRITD2Av1G004020.1
Ttu_TRITD2Av1G003840.1
Ttu_TRITD1Bv1G224330.1
Ttu_TRITD1Bv1G207740.2
Ttu_TRITD1Bv1G169050.1
Ttu_TRITD1Bv1G086760.3
Ttu_TRITD1Bv1G086750.1
Ttu_TRITD1Bv1G024910.1
Ttu_TRITD1Bv1G016290.1
Ttu_TRITD1Bv1G016280.1
Ttu_TRITD1Av1G120850.1
Ttu_TRITD1Av1G012430.1
Ttu_TRITD0Uv1G099320.1
Ttu_TRITD0Uv1G036590.1
Ttu_TRITD0Uv1G013920.1
Ttu_TRITD0Uv1G002990.3
−1000
0
1000
2000
3000

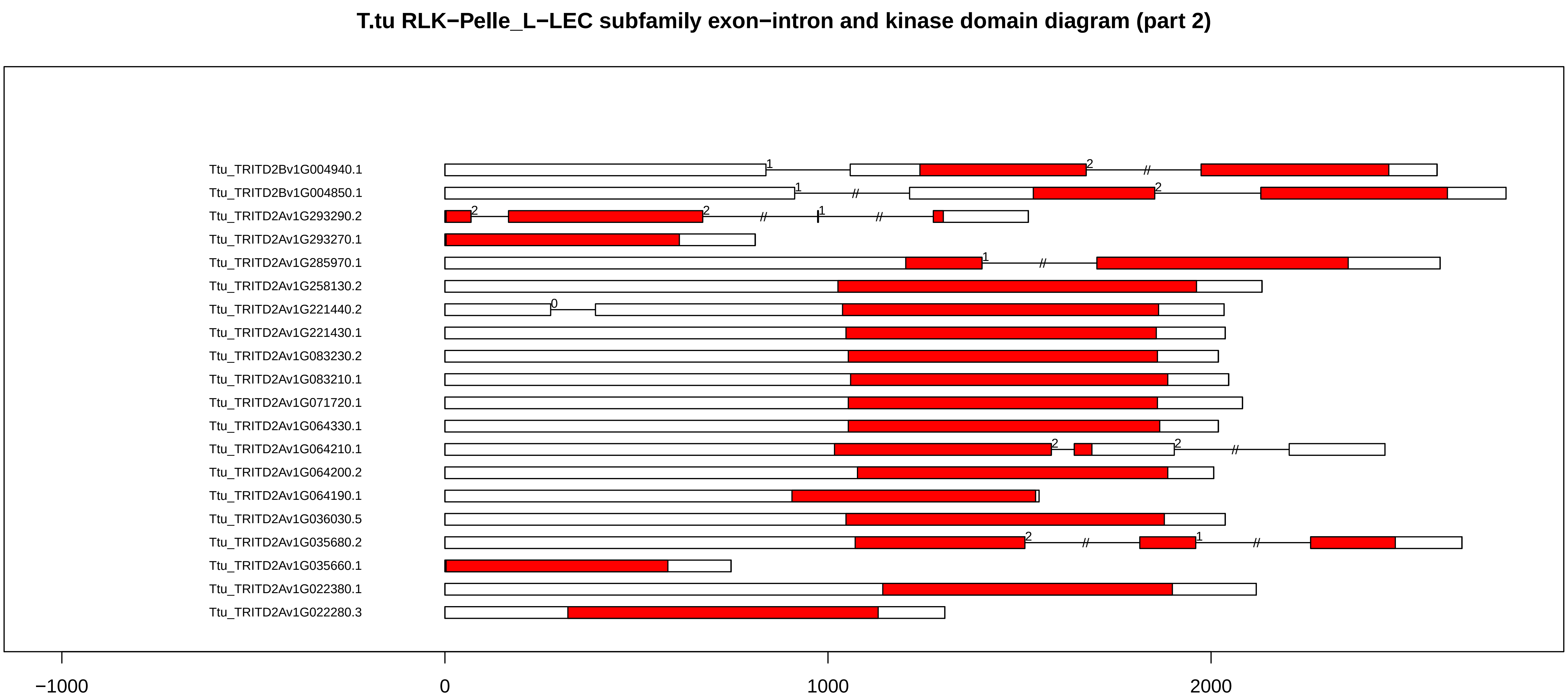
T.tu RLK−Pelle_L−LEC subfamily exon−intron and kinase domain diagram (part 2)
Ttu_TRITD2Bv1G004940.1
Ttu_TRITD2Bv1G004850.1
Ttu_TRITD2Av1G293290.2
Ttu_TRITD2Av1G293270.1
Ttu_TRITD2Av1G285970.1
Ttu_TRITD2Av1G258130.2
Ttu_TRITD2Av1G221440.2
Ttu_TRITD2Av1G221430.1
Ttu_TRITD2Av1G083230.2
Ttu_TRITD2Av1G083210.1
Ttu_TRITD2Av1G071720.1
Ttu_TRITD2Av1G064330.1
Ttu_TRITD2Av1G064210.1
Ttu_TRITD2Av1G064200.2
Ttu_TRITD2Av1G064190.1
Ttu_TRITD2Av1G036030.5
Ttu_TRITD2Av1G035680.2
Ttu_TRITD2Av1G035660.1
Ttu_TRITD2Av1G022380.1
Ttu_TRITD2Av1G022280.3
−1000
0
1000
2000

T.tu RLK−Pelle_L−LEC subfamily exon−intron and kinase domain diagram (part 3)

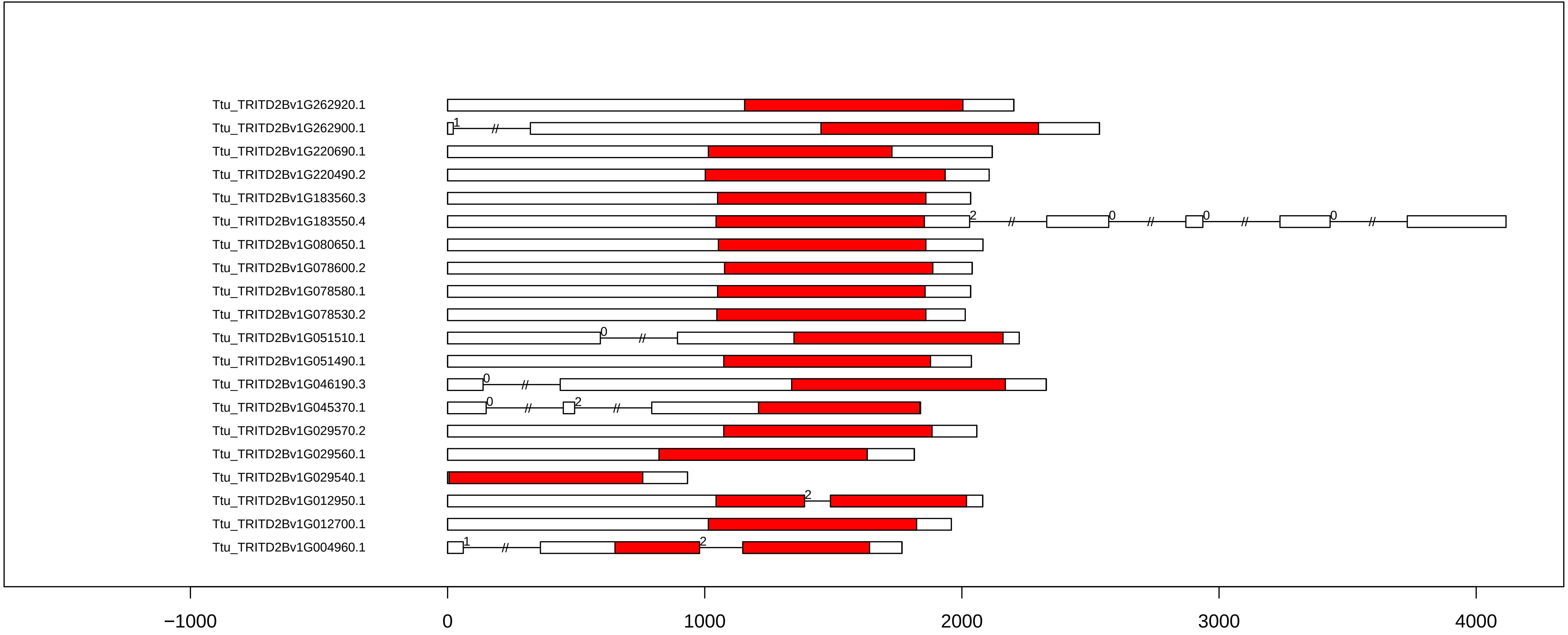

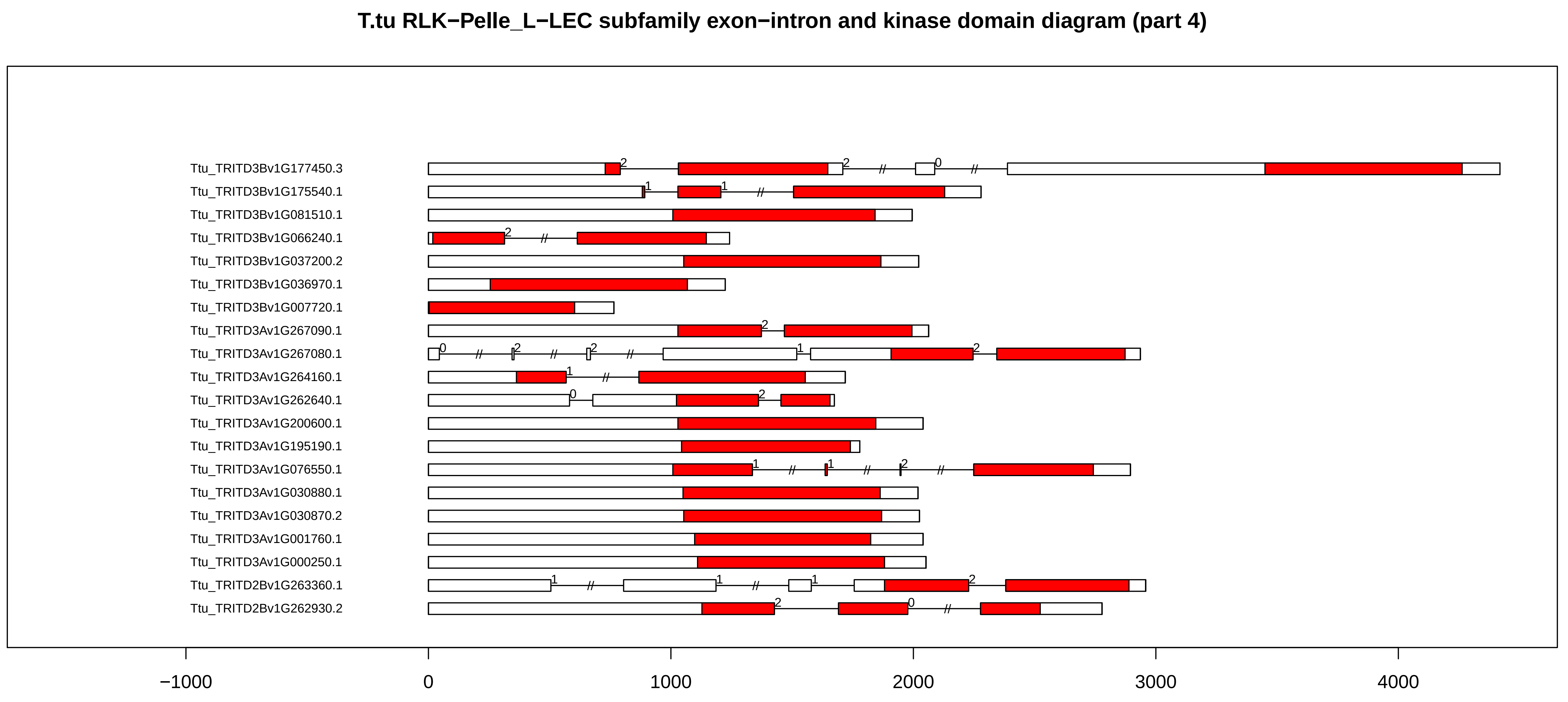
T.tu RLK−Pelle_L−LEC subfamily exon−intron and kinase domain diagram (part 4)
Ttu_TRITD3Bv1G177450.3
Ttu_TRITD3Bv1G175540.1
Ttu_TRITD3Bv1G081510.1
Ttu_TRITD3Bv1G066240.1
Ttu_TRITD3Bv1G037200.2
Ttu_TRITD3Bv1G036970.1
Ttu_TRITD3Bv1G007720.1
Ttu_TRITD3Av1G267090.1
Ttu_TRITD3Av1G267080.1
Ttu_TRITD3Av1G264160.1
Ttu_TRITD3Av1G262640.1
Ttu_TRITD3Av1G200600.1
Ttu_TRITD3Av1G195190.1
Ttu_TRITD3Av1G076550.1
Ttu_TRITD3Av1G030880.1
Ttu_TRITD3Av1G030870.2
Ttu_TRITD3Av1G001760.1
Ttu_TRITD3Av1G000250.1
Ttu_TRITD2Bv1G263360.1
Ttu_TRITD2Bv1G262930.2
−1000
0
1000
2000
3000
4000

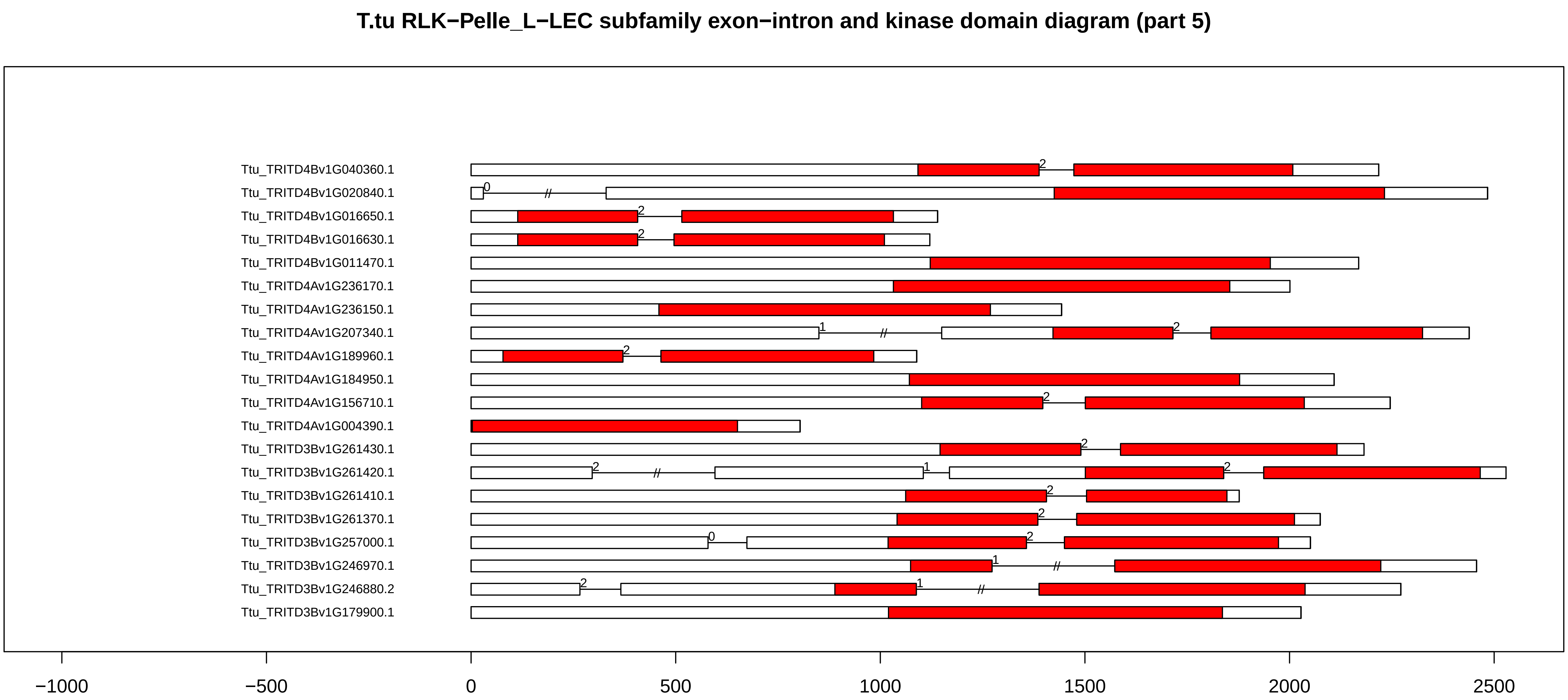
T.tu RLK−Pelle_L−LEC subfamily exon−intron and kinase domain diagram (part 5)
Ttu_TRITD4Bv1G040360.1
Ttu_TRITD4Bv1G020840.1
Ttu_TRITD4Bv1G016650.1
Ttu_TRITD4Bv1G016630.1
Ttu_TRITD4Bv1G011470.1
Ttu_TRITD4Av1G236170.1
Ttu_TRITD4Av1G236150.1
Ttu_TRITD4Av1G207340.1
Ttu_TRITD4Av1G189960.1
Ttu_TRITD4Av1G184950.1
Ttu_TRITD4Av1G156710.1
Ttu_TRITD4Av1G004390.1
Ttu_TRITD3Bv1G261430.1
Ttu_TRITD3Bv1G261420.1
Ttu_TRITD3Bv1G261410.1
Ttu_TRITD3Bv1G261370.1
Ttu_TRITD3Bv1G257000.1
Ttu_TRITD3Bv1G246970.1
Ttu_TRITD3Bv1G246880.2
Ttu_TRITD3Bv1G179900.1
−1000
−500
0
500
1000
1500
2000
2500

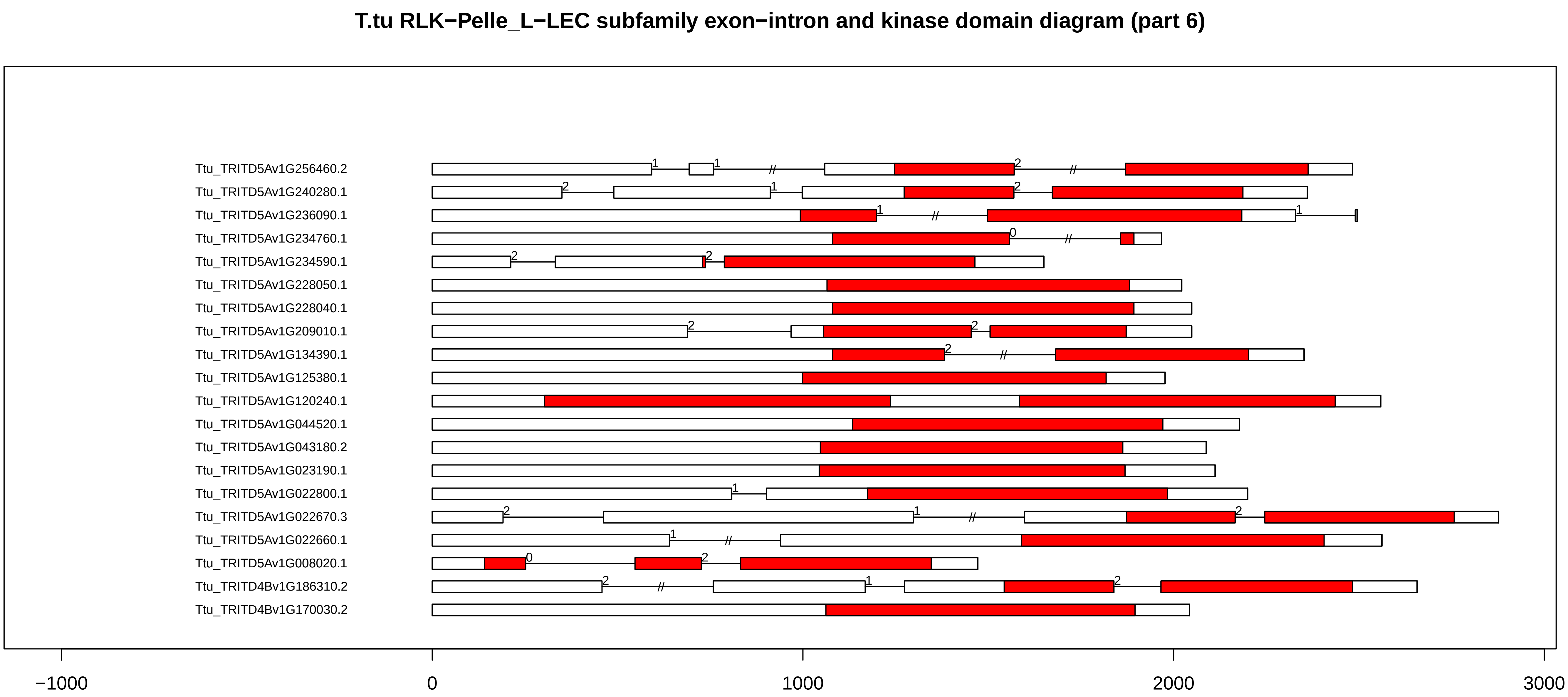
T.tu RLK−Pelle_L−LEC subfamily exon−intron and kinase domain diagram (part 6)
Ttu_TRITD5Av1G256460.2
Ttu_TRITD5Av1G240280.1
Ttu_TRITD5Av1G236090.1
Ttu_TRITD5Av1G234760.1
Ttu_TRITD5Av1G234590.1
Ttu_TRITD5Av1G228050.1
Ttu_TRITD5Av1G228040.1
Ttu_TRITD5Av1G209010.1
Ttu_TRITD5Av1G134390.1
Ttu_TRITD5Av1G125380.1
Ttu_TRITD5Av1G120240.1
Ttu_TRITD5Av1G044520.1
Ttu_TRITD5Av1G043180.2
Ttu_TRITD5Av1G023190.1
Ttu_TRITD5Av1G022800.1
Ttu_TRITD5Av1G022670.3
Ttu_TRITD5Av1G022660.1
Ttu_TRITD5Av1G008020.1
Ttu_TRITD4Bv1G186310.2
Ttu_TRITD4Bv1G170030.2
−1000
0
1000
2000
3000

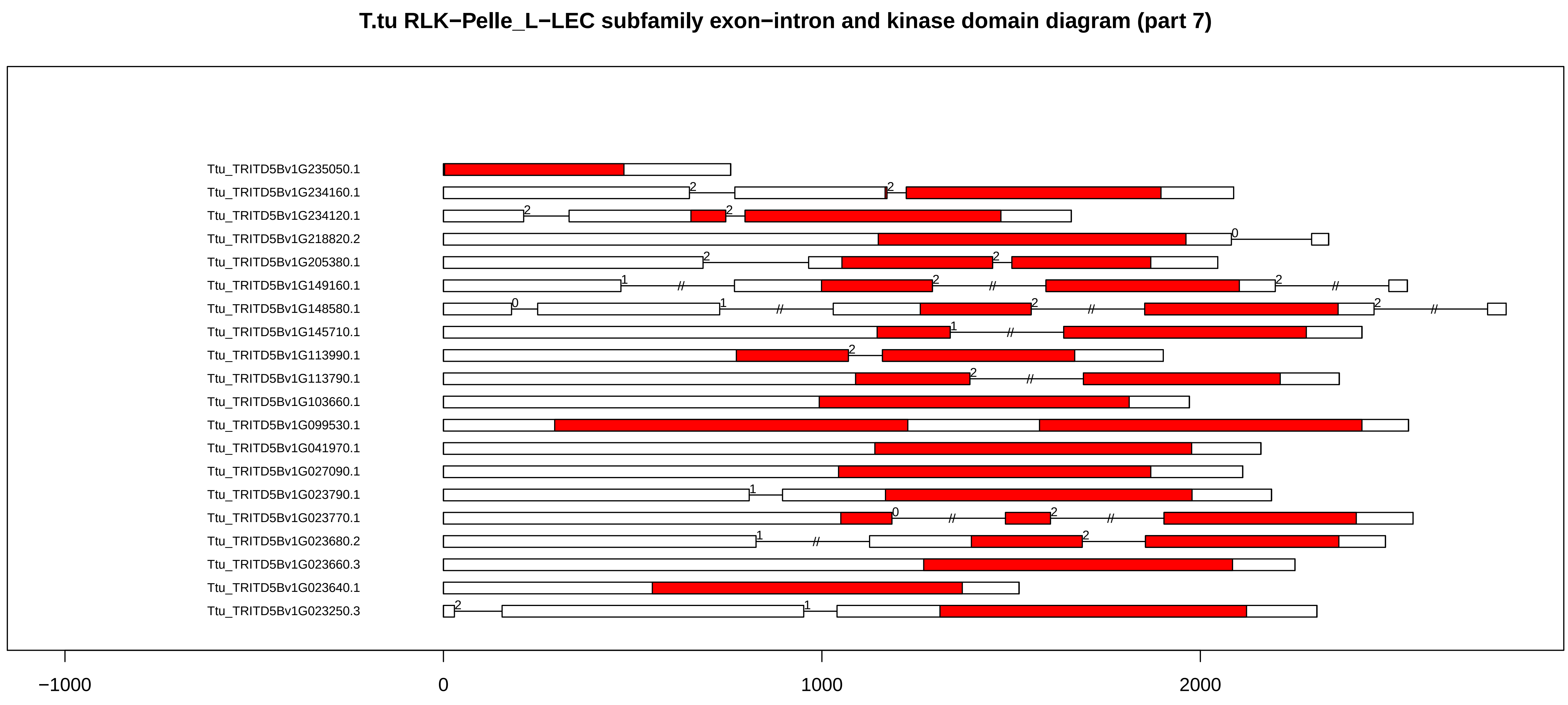

T.tu RLK−Pelle_L−LEC subfamily exon−intron and kinase domain diagram (part 7)
Ttu_TRITD5Bv1G235050.1
Ttu_TRITD5Bv1G234160.1
Ttu_TRITD5Bv1G234120.1
Ttu_TRITD5Bv1G218820.2
Ttu_TRITD5Bv1G205380.1
Ttu_TRITD5Bv1G149160.1
Ttu_TRITD5Bv1G148580.1
Ttu_TRITD5Bv1G145710.1
Ttu_TRITD5Bv1G113990.1
Ttu_TRITD5Bv1G113790.1
Ttu_TRITD5Bv1G103660.1
Ttu_TRITD5Bv1G099530.1
Ttu_TRITD5Bv1G041970.1
Ttu_TRITD5Bv1G027090.1
Ttu_TRITD5Bv1G023790.1
Ttu_TRITD5Bv1G023770.1
Ttu_TRITD5Bv1G023680.2
Ttu_TRITD5Bv1G023660.3
Ttu_TRITD5Bv1G023640.1
Ttu_TRITD5Bv1G023250.3
−1000
0
1000
2000

T.tu RLK−Pelle_L−LEC subfamily exon−intron and kinase domain diagram (part 8)

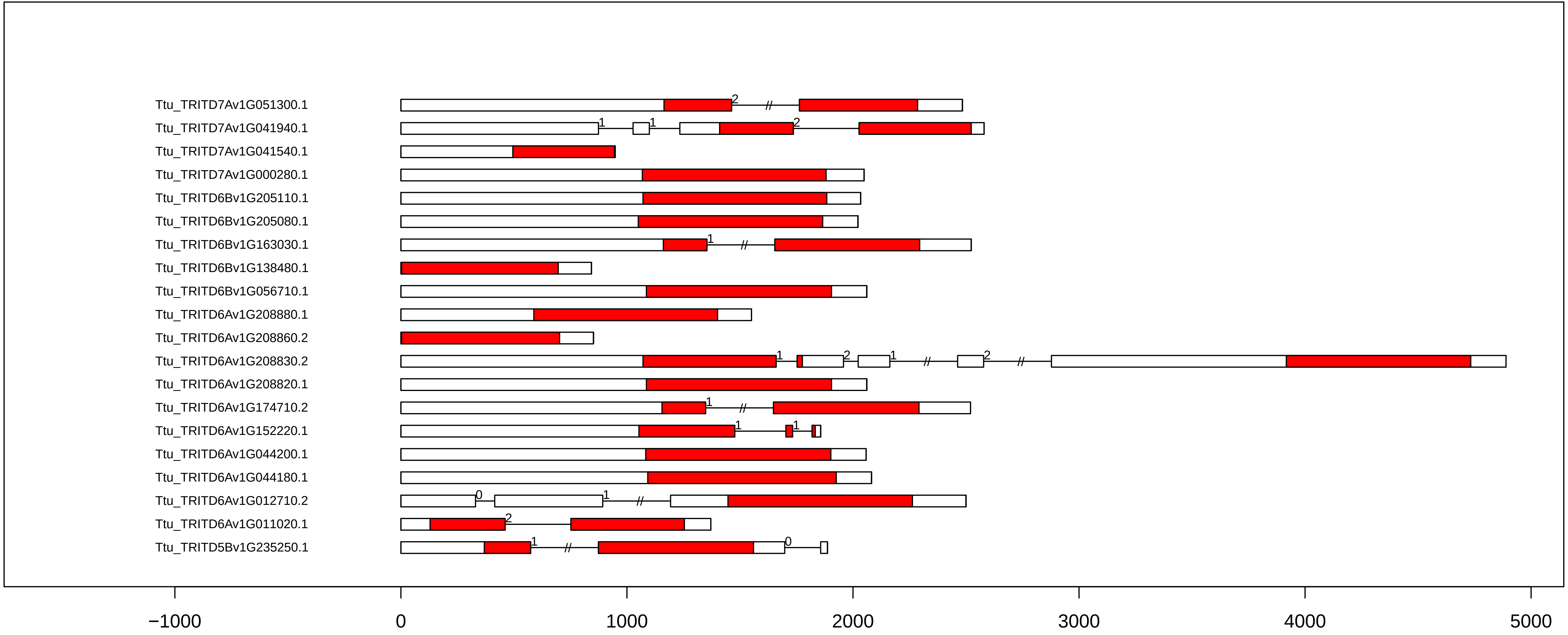

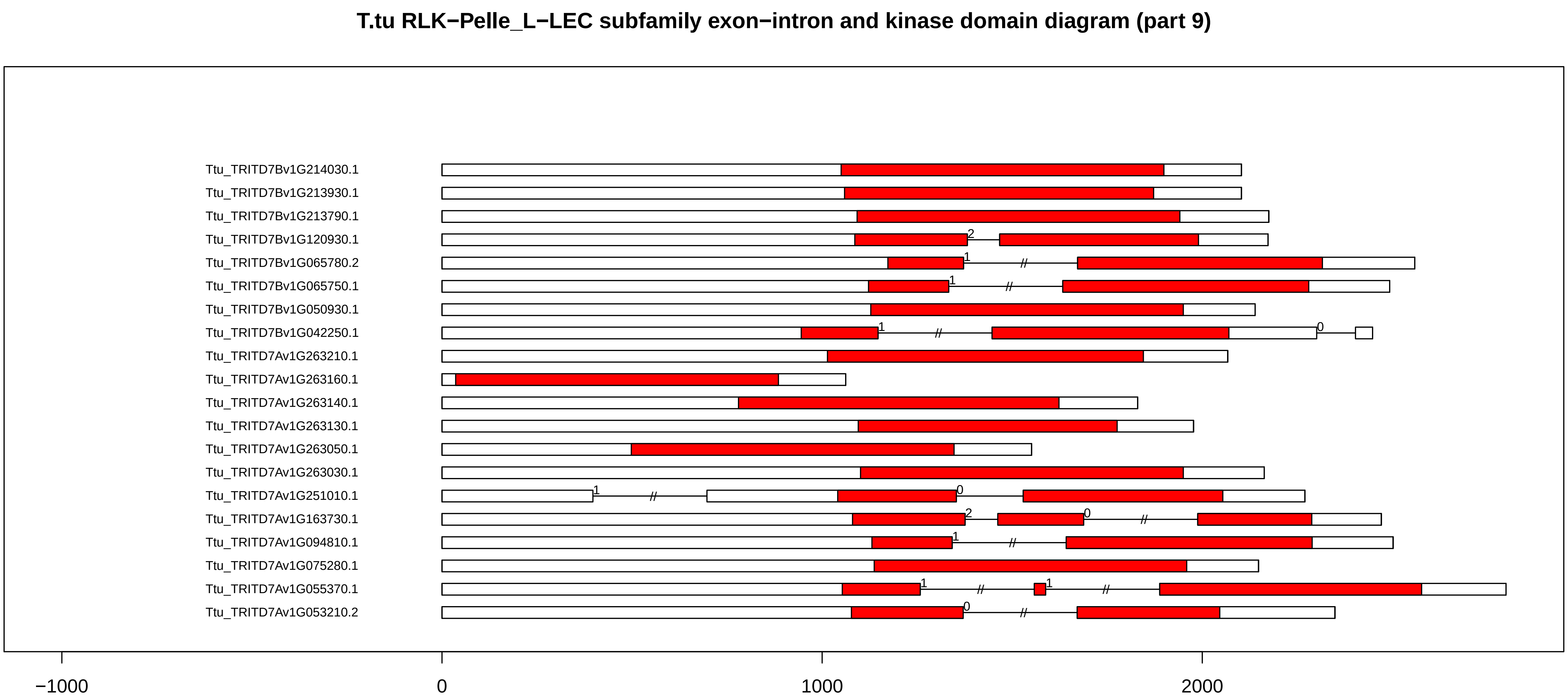
T.tu RLK−Pelle_L−LEC subfamily exon−intron and kinase domain diagram (part 9)
Ttu_TRITD7Bv1G214030.1
Ttu_TRITD7Bv1G213930.1
Ttu_TRITD7Bv1G213790.1
Ttu_TRITD7Bv1G120930.1
Ttu_TRITD7Bv1G065780.2
Ttu_TRITD7Bv1G065750.1
Ttu_TRITD7Bv1G050930.1
Ttu_TRITD7Bv1G042250.1
Ttu_TRITD7Av1G263210.1
Ttu_TRITD7Av1G263160.1
Ttu_TRITD7Av1G263140.1
Ttu_TRITD7Av1G263130.1
Ttu_TRITD7Av1G263050.1
Ttu_TRITD7Av1G263030.1
Ttu_TRITD7Av1G251010.1
Ttu_TRITD7Av1G163730.1
Ttu_TRITD7Av1G094810.1
Ttu_TRITD7Av1G075280.1
Ttu_TRITD7Av1G055370.1
Ttu_TRITD7Av1G053210.2
−1000
0
1000
2000

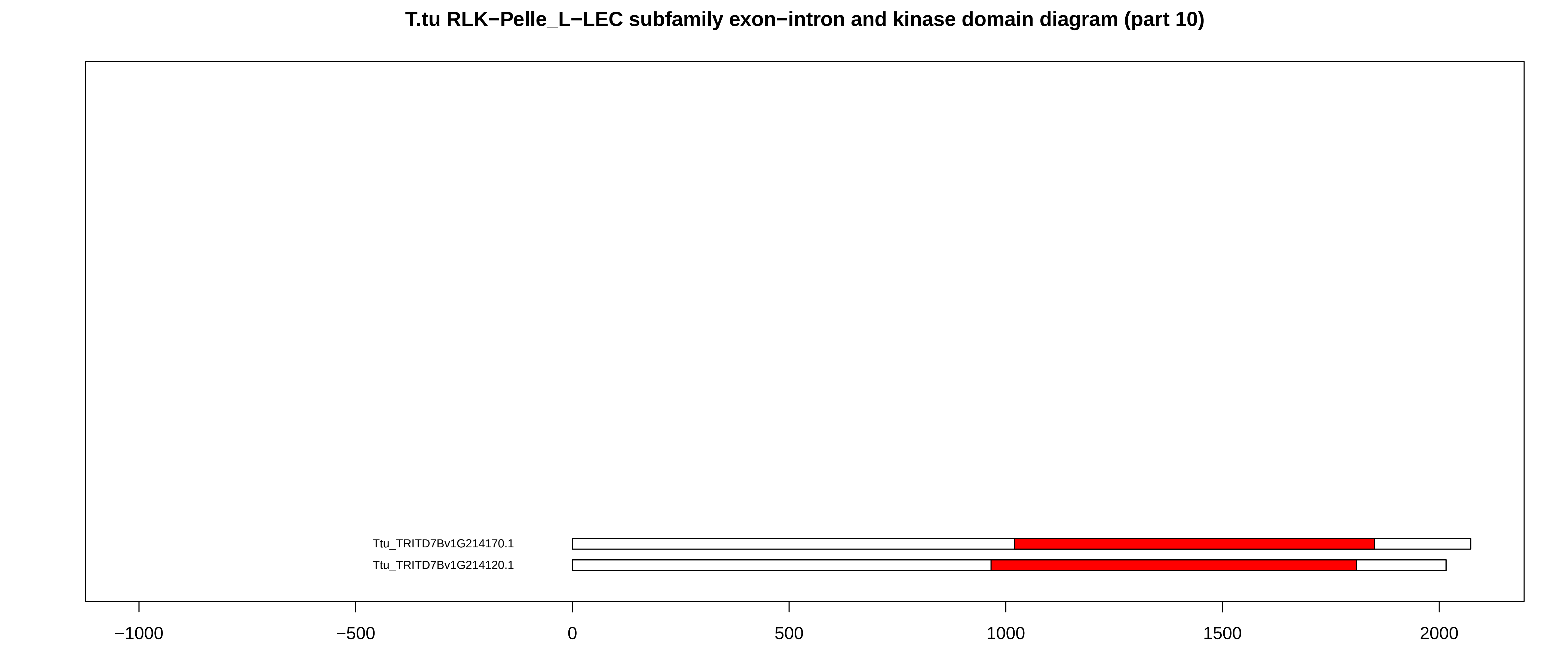
T.tu RLK−Pelle_L−LEC subfamily exon−intron and kinase domain diagram (part 10)
Ttu_TRITD7Bv1G214170.1
Ttu_TRITD7Bv1G214120.1
−1000
−500
0
500
1000
1500
2000

T.tu RLK−Pelle_LRK10L−2 subfamily exon−intron and kinase domain diagram (part 1)

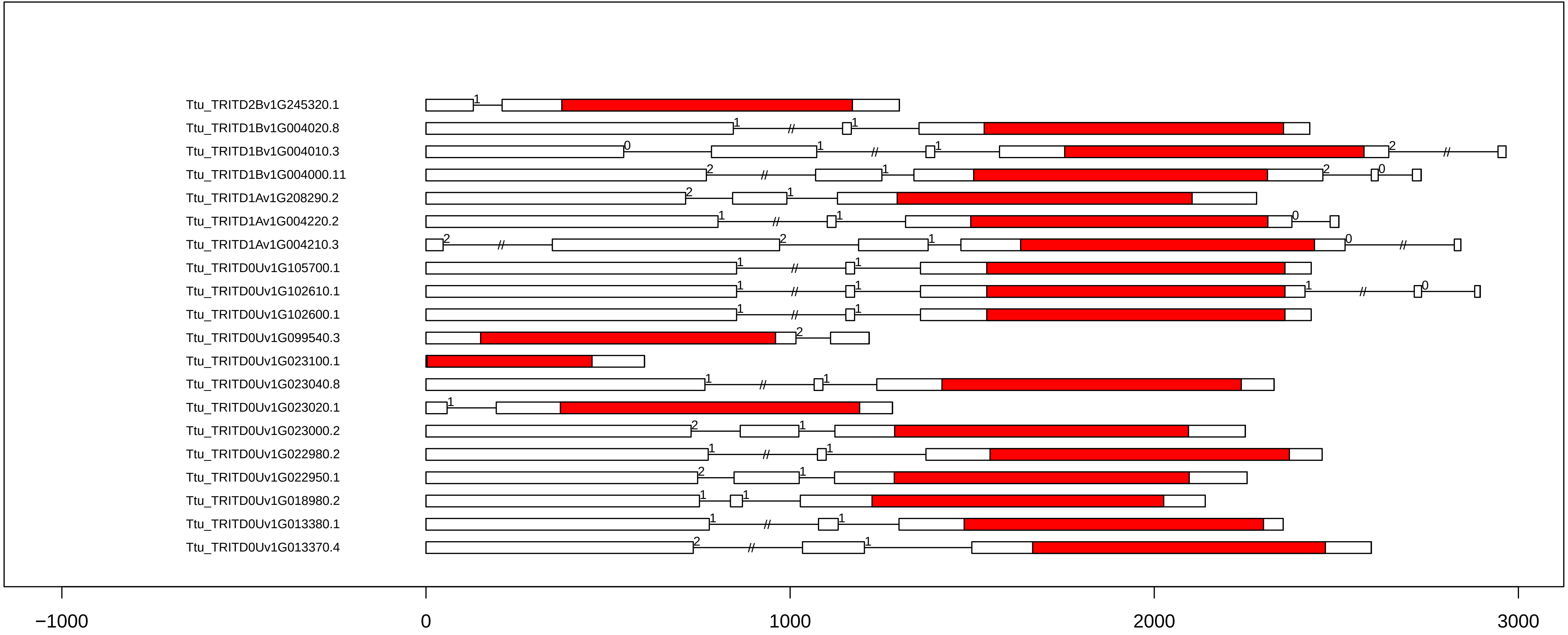

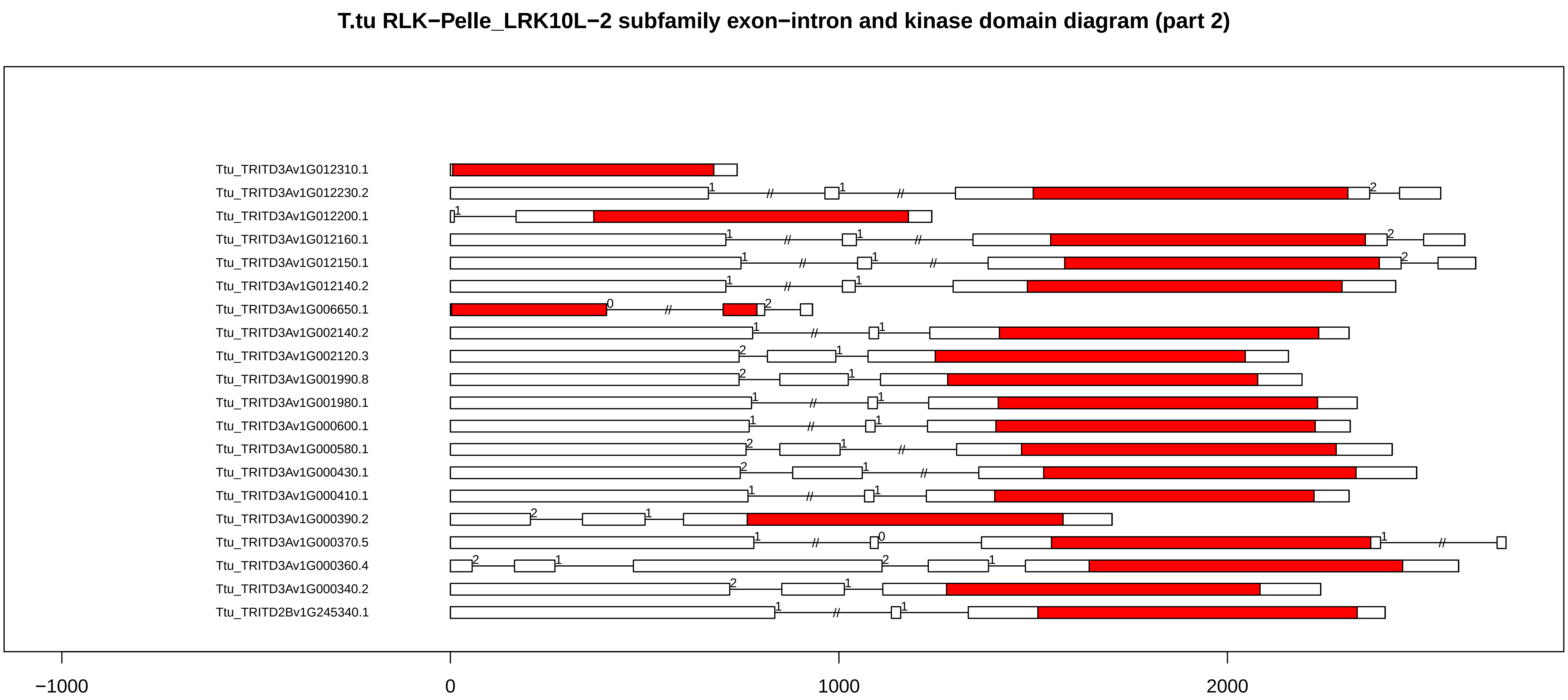
T.tu RLK−Pelle_LRK10L−2 subfamily exon−intron and kinase domain diagram (part 2)
Ttu_TRITD3Av1G012310.1
Ttu_TRITD3Av1G012230.2
Ttu_TRITD3Av1G012200.1
Ttu_TRITD3Av1G012160.1
Ttu_TRITD3Av1G012150.1
Ttu_TRITD3Av1G012140.2
Ttu_TRITD3Av1G006650.1
Ttu_TRITD3Av1G002140.2
Ttu_TRITD3Av1G002120.3
Ttu_TRITD3Av1G001990.8
Ttu_TRITD3Av1G001980.1
Ttu_TRITD3Av1G000600.1
Ttu_TRITD3Av1G000580.1
Ttu_TRITD3Av1G000430.1
Ttu_TRITD3Av1G000410.1
Ttu_TRITD3Av1G000390.2
Ttu_TRITD3Av1G000370.5
Ttu_TRITD3Av1G000360.4
Ttu_TRITD3Av1G000340.2
Ttu_TRITD2Bv1G245340.1
−1000
0
1000
2000

T.tu RLK−Pelle_LRK10L−2 subfamily exon−intron and kinase domain diagram (part 3)

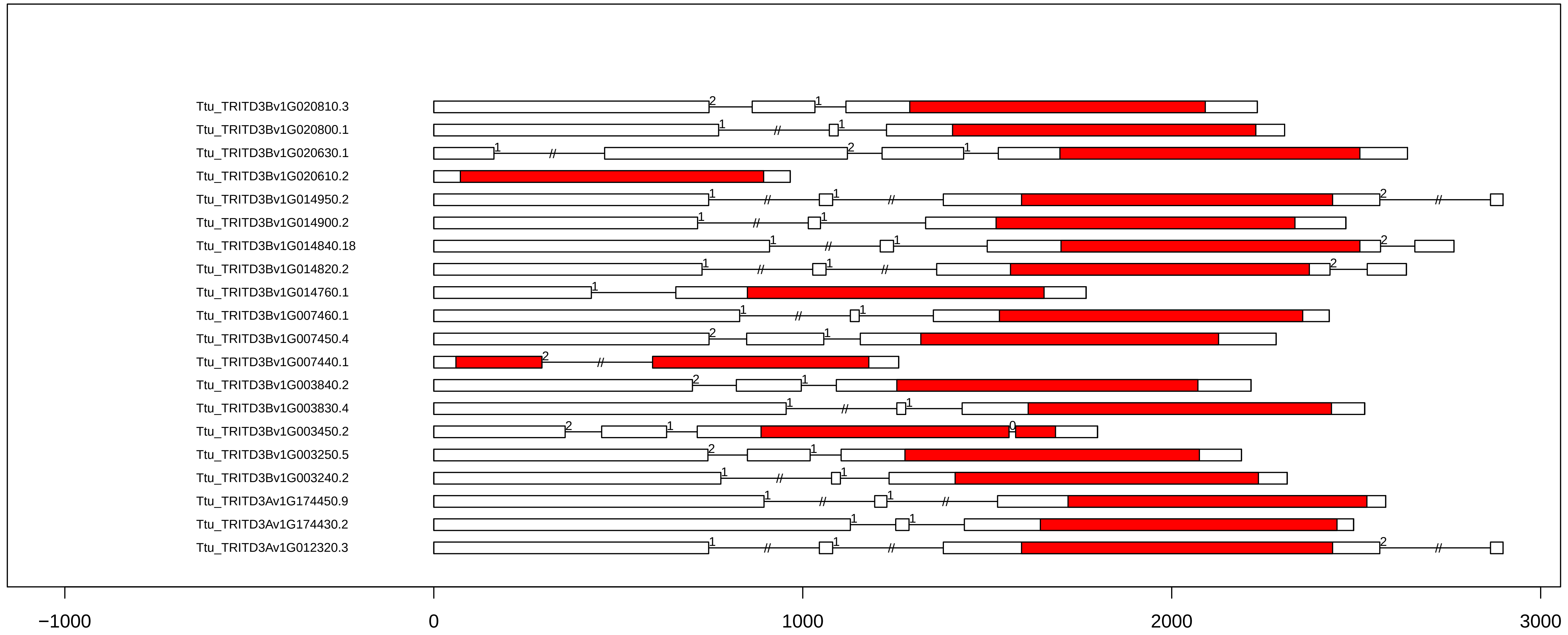

T.tu RLK−Pelle_LRK10L−2 subfamily exon−intron and kinase domain diagram (part 4)

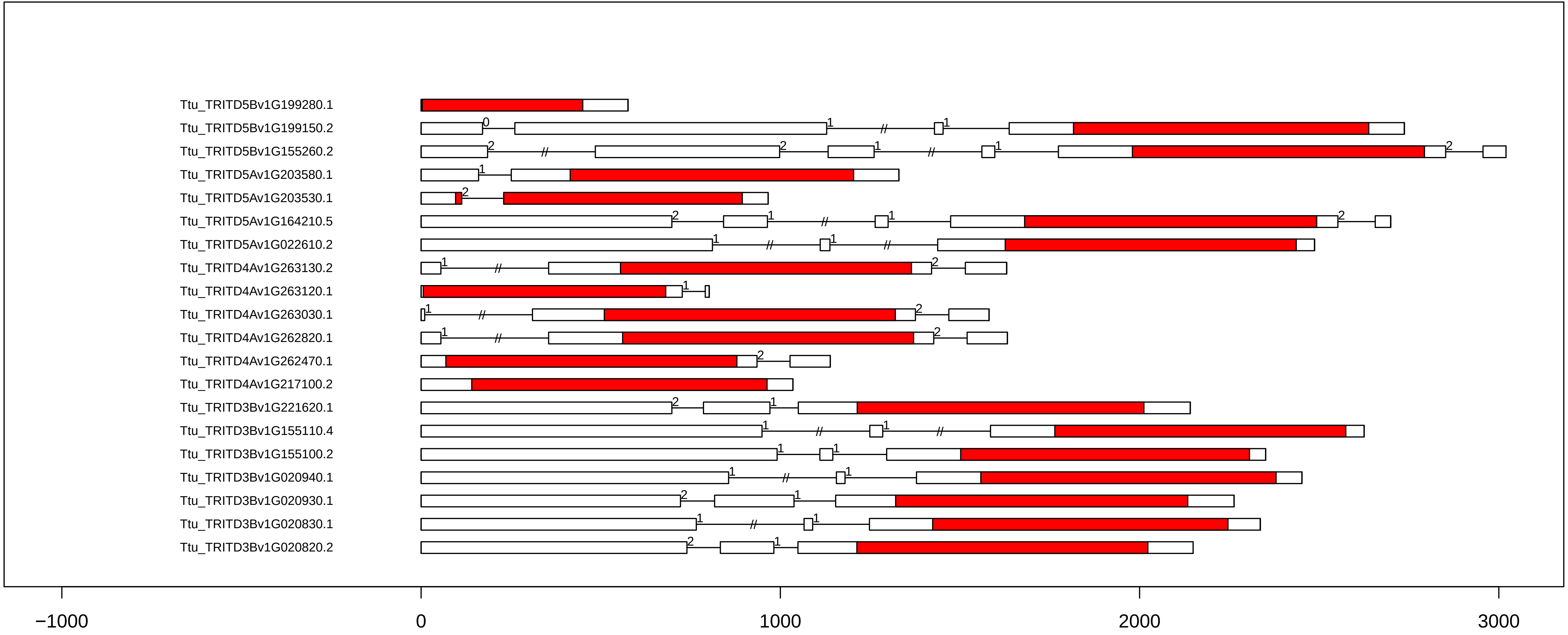

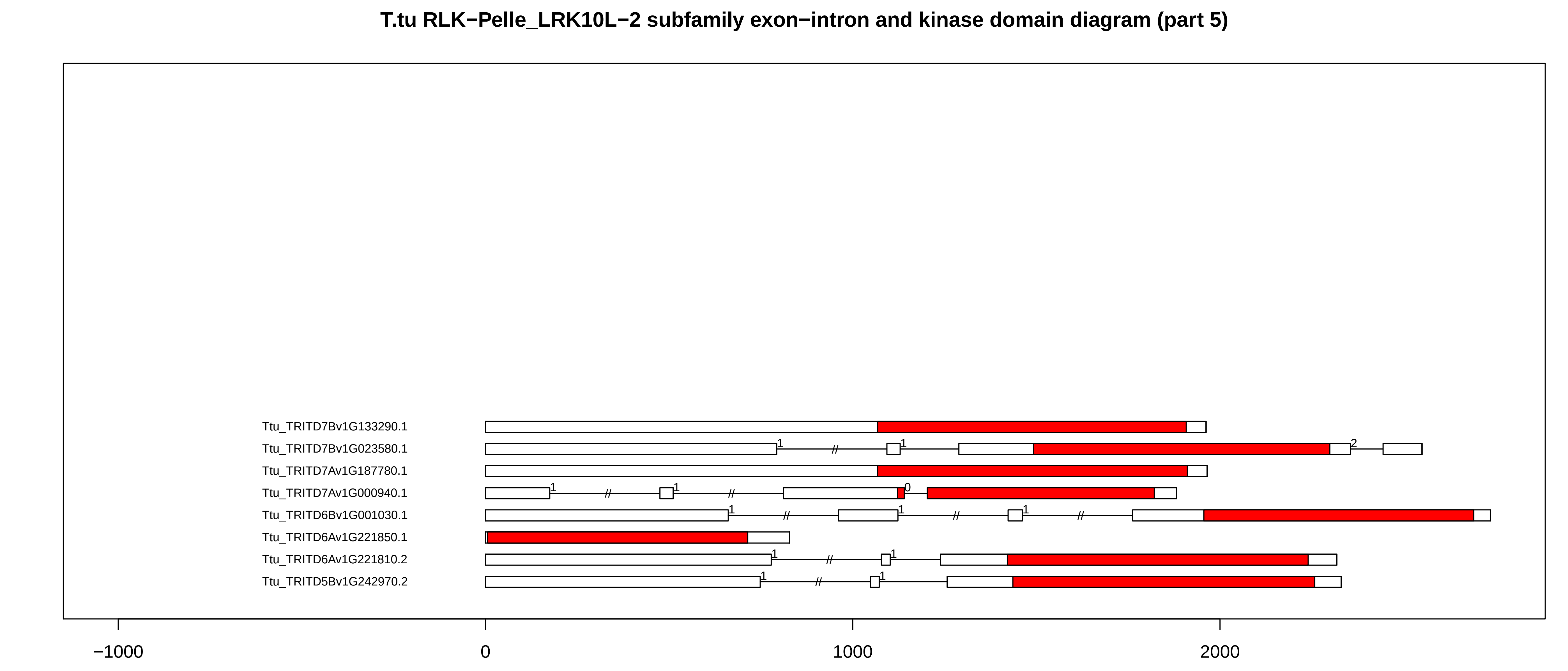
T.tu RLK−Pelle_LRK10L−2 subfamily exon−intron and kinase domain diagram (part 5)
Ttu_TRITD7Bv1G133290.1
Ttu_TRITD7Bv1G023580.1
Ttu_TRITD7Av1G187780.1
Ttu_TRITD7Av1G000940.1
Ttu_TRITD6Bv1G001030.1
Ttu_TRITD6Av1G221850.1
Ttu_TRITD6Av1G221810.2
Ttu_TRITD5Bv1G242970.2
−1000
0
1000
2000

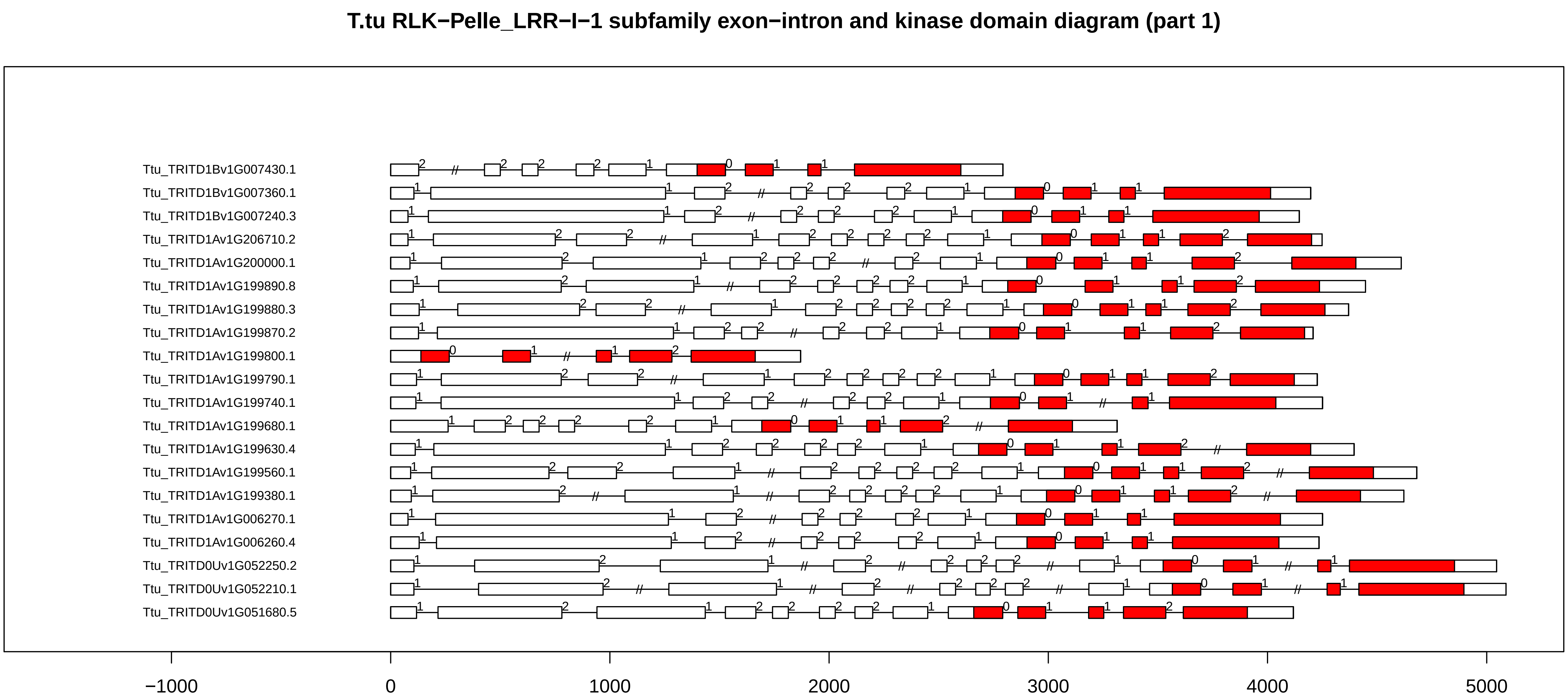

T.tu RLK−Pelle_LRR−I−1 subfamily exon−intron and kinase domain diagram (part 1)
Ttu_TRITD1Bv1G007430.1
Ttu_TRITD1Bv1G007360.1
Ttu_TRITD1Bv1G007240.3
Ttu_TRITD1Av1G206710.2
Ttu_TRITD1Av1G200000.1
Ttu_TRITD1Av1G199890.8
Ttu_TRITD1Av1G199880.3
Ttu_TRITD1Av1G199870.2
Ttu_TRITD1Av1G199800.1
Ttu_TRITD1Av1G199790.1
Ttu_TRITD1Av1G199740.1
Ttu_TRITD1Av1G199680.1
Ttu_TRITD1Av1G199630.4
Ttu_TRITD1Av1G199560.1
Ttu_TRITD1Av1G199380.1
Ttu_TRITD1Av1G006270.1
Ttu_TRITD1Av1G006260.4
Ttu_TRITD0Uv1G052250.2
Ttu_TRITD0Uv1G052210.1
Ttu_TRITD0Uv1G051680.5
−1000
0
1000
2000
3000
4000
5000

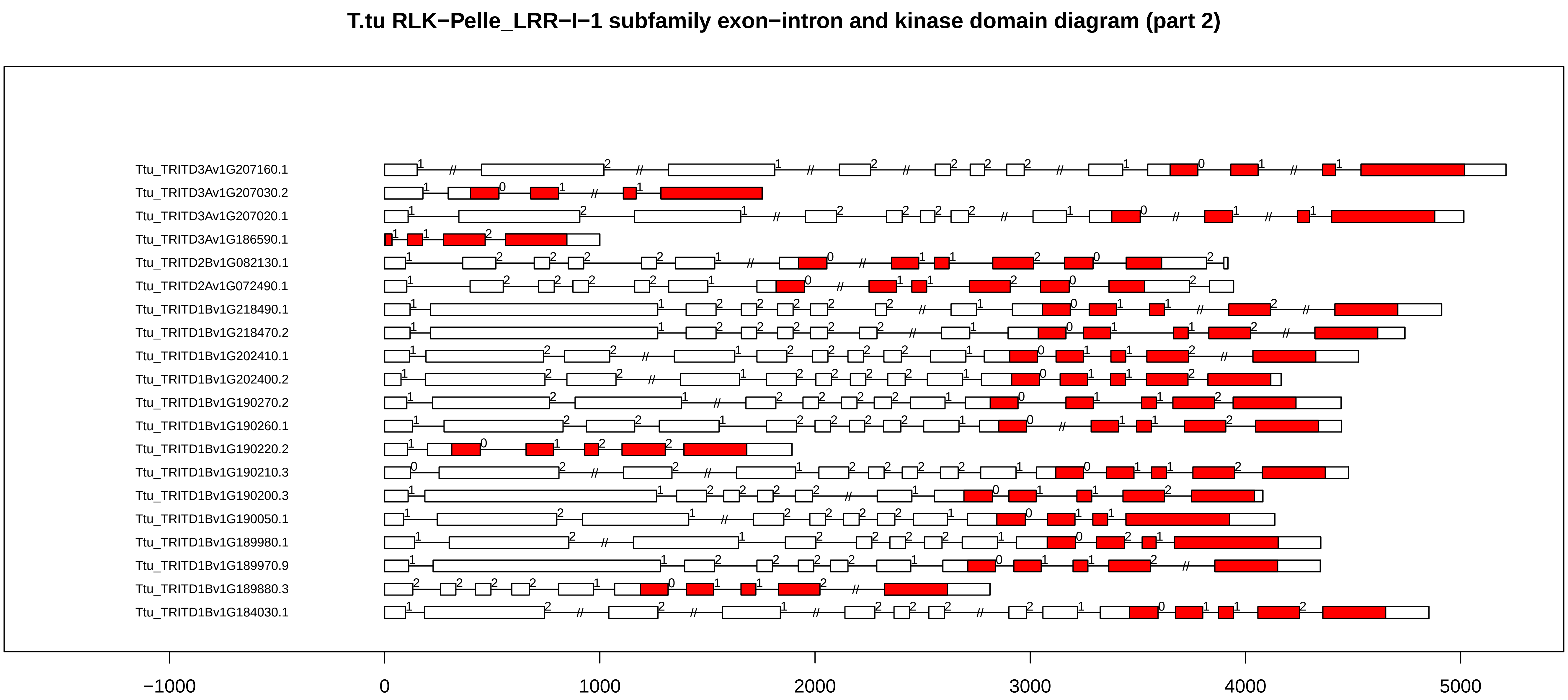
T.tu RLK−Pelle_LRR−I−1 subfamily exon−intron and kinase domain diagram (part 2)
Ttu_TRITD3Av1G207160.1
Ttu_TRITD3Av1G207030.2
Ttu_TRITD3Av1G207020.1
Ttu_TRITD3Av1G186590.1
Ttu_TRITD2Bv1G082130.1
Ttu_TRITD2Av1G072490.1
Ttu_TRITD1Bv1G218490.1
Ttu_TRITD1Bv1G218470.2
Ttu_TRITD1Bv1G202410.1
Ttu_TRITD1Bv1G202400.2
Ttu_TRITD1Bv1G190270.2
Ttu_TRITD1Bv1G190260.1
Ttu_TRITD1Bv1G190220.2
Ttu_TRITD1Bv1G190210.3
Ttu_TRITD1Bv1G190200.3
Ttu_TRITD1Bv1G190050.1
Ttu_TRITD1Bv1G189980.1
Ttu_TRITD1Bv1G189970.9
Ttu_TRITD1Bv1G189880.3
Ttu_TRITD1Bv1G184030.1
−1000
0
1000
2000
3000
4000
5000

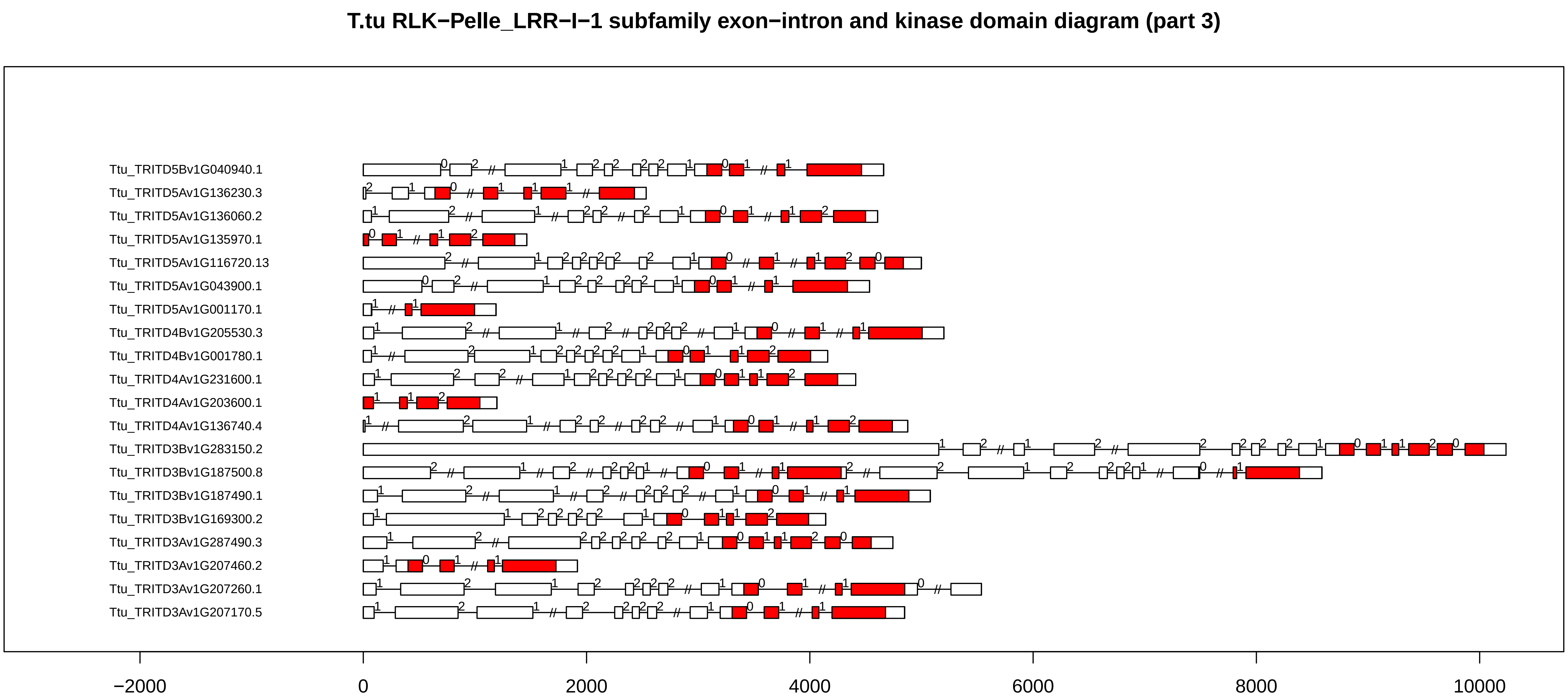
T.tu RLK−Pelle_LRR−I−1 subfamily exon−intron and kinase domain diagram (part 3)
Ttu_TRITD5Bv1G040940.1
Ttu_TRITD5Av1G136230.3
Ttu_TRITD5Av1G136060.2
Ttu_TRITD5Av1G135970.1
Ttu_TRITD5Av1G116720.13
Ttu_TRITD5Av1G043900.1
Ttu_TRITD5Av1G001170.1
Ttu_TRITD4Bv1G205530.3
Ttu_TRITD4Bv1G001780.1
Ttu_TRITD4Av1G231600.1
Ttu_TRITD4Av1G203600.1
Ttu_TRITD4Av1G136740.4
Ttu_TRITD3Bv1G283150.2
Ttu_TRITD3Bv1G187500.8
Ttu_TRITD3Bv1G187490.1
Ttu_TRITD3Bv1G169300.2
Ttu_TRITD3Av1G287490.3
Ttu_TRITD3Av1G207460.2
Ttu_TRITD3Av1G207260.1
Ttu_TRITD3Av1G207170.5
−2000
0
2000
4000
6000
8000
10000

T.tu RLK−Pelle_LRR−I−1 subfamily exon−intron and kinase domain diagram (part 4)

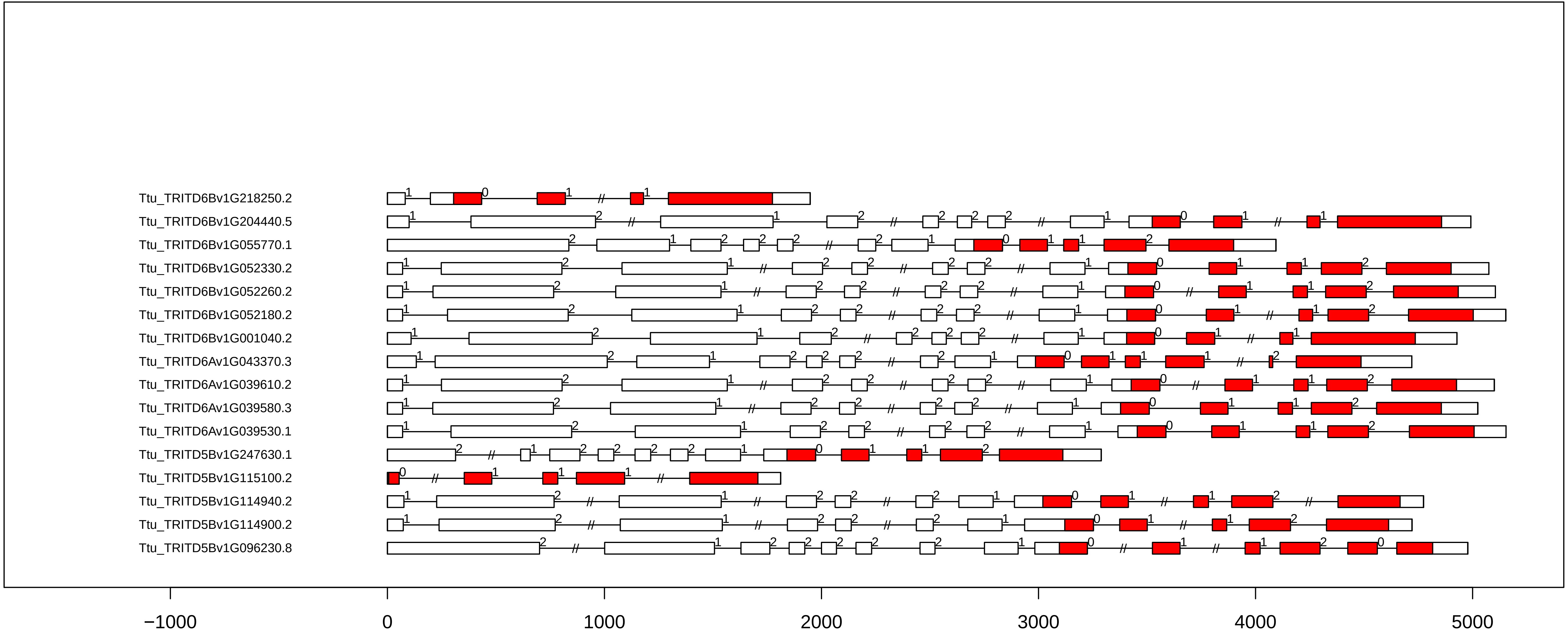

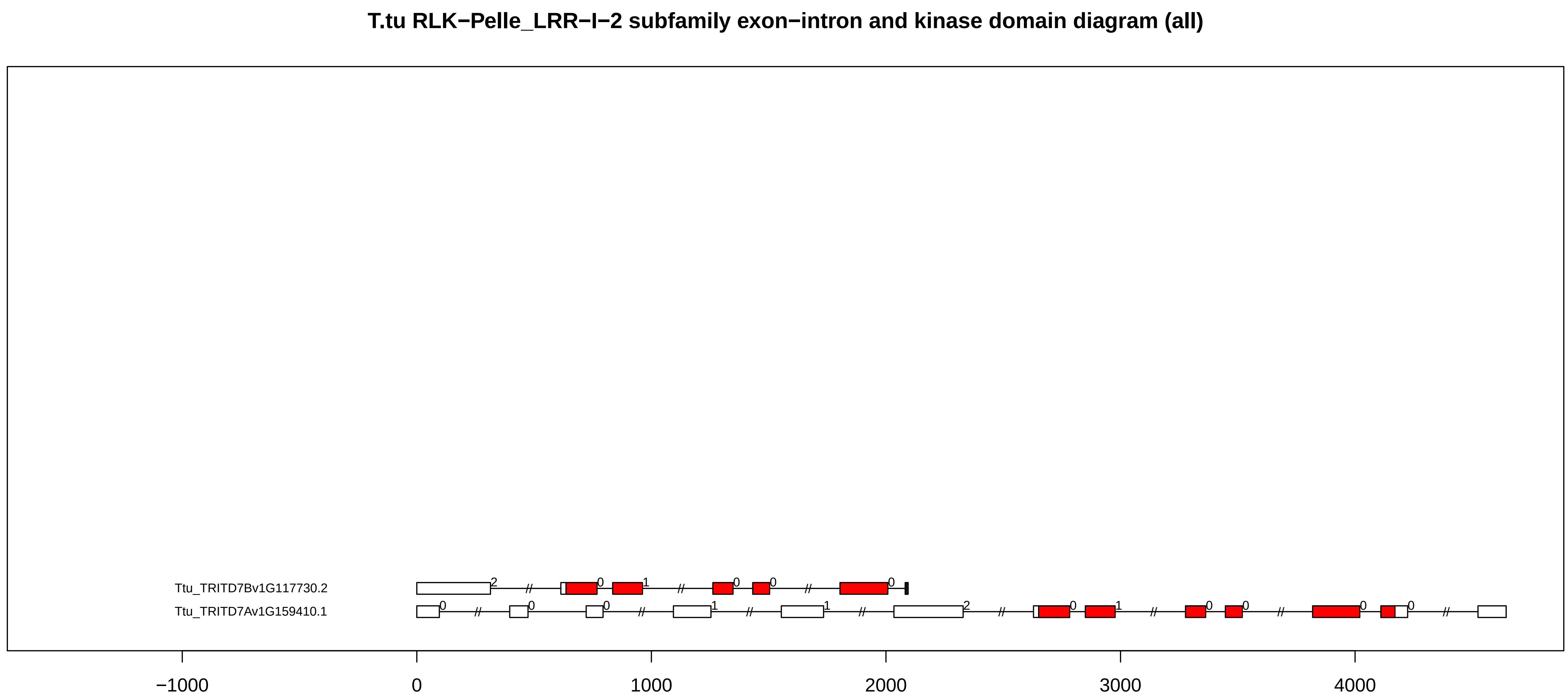

T.tu RLK−Pelle_LRR−I−2 subfamily exon−intron and kinase domain diagram (all)
Ttu_TRITD7Bv1G117730.2
Ttu_TRITD7Av1G159410.1
2
0
1
0
0
0
0
0
0
0
1
1
2
0
1
0
0
0
0
−1000
0
1000
2000
3000
4000

T.tu RLK−Pelle_LRR−III subfamily exon−intron and kinase domain diagram (part 1)

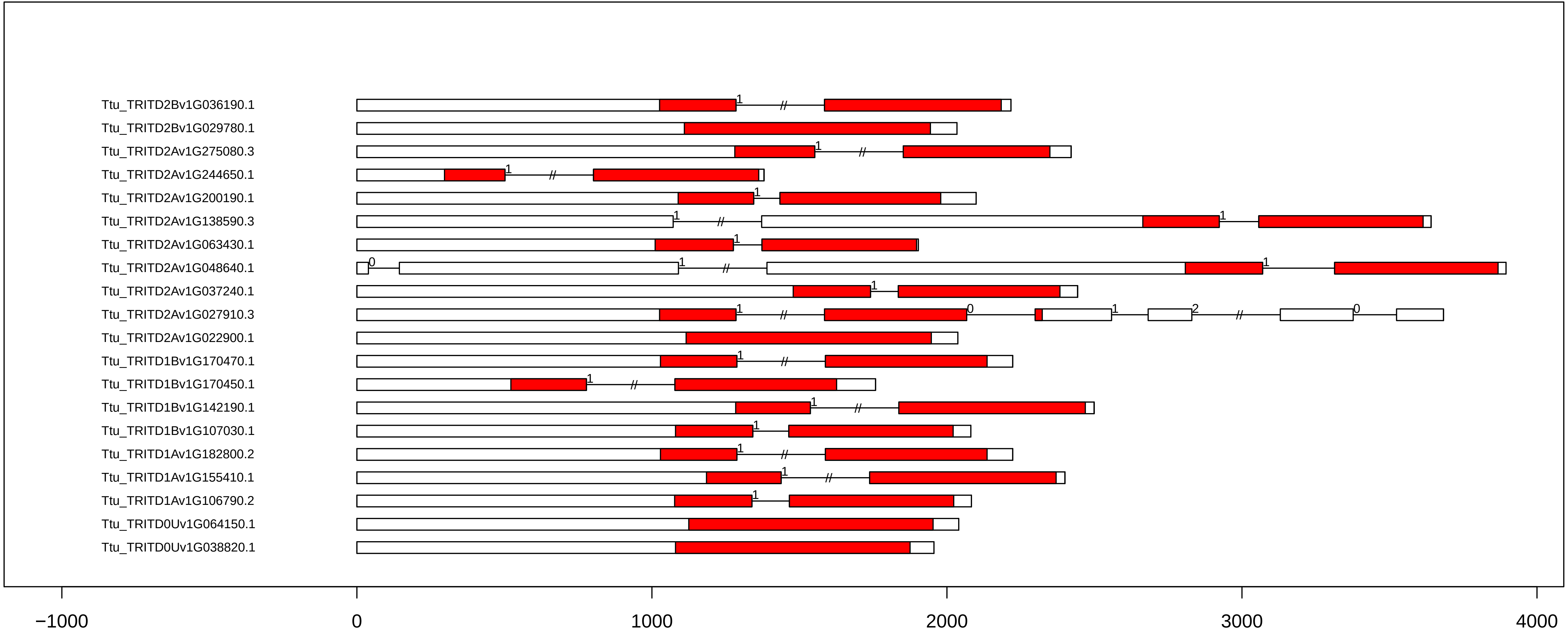

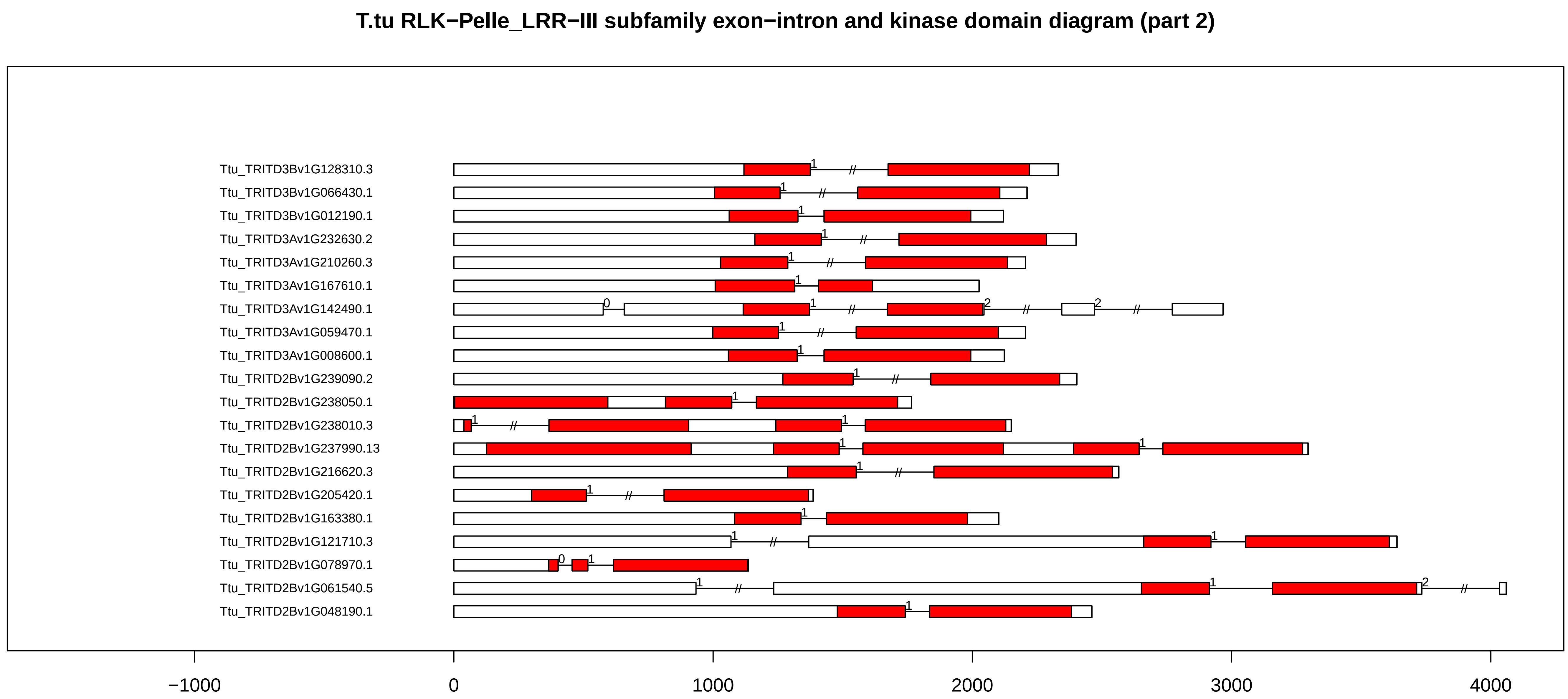

T.tu RLK−Pelle_LRR−III subfamily exon−intron and kinase domain diagram (part 2)
Ttu_TRITD3Bv1G128310.3
Ttu_TRITD3Bv1G066430.1
Ttu_TRITD3Bv1G012190.1
Ttu_TRITD3Av1G232630.2
Ttu_TRITD3Av1G210260.3
Ttu_TRITD3Av1G167610.1
Ttu_TRITD3Av1G142490.1
Ttu_TRITD3Av1G059470.1
Ttu_TRITD3Av1G008600.1
Ttu_TRITD2Bv1G239090.2
Ttu_TRITD2Bv1G238050.1
Ttu_TRITD2Bv1G238010.3
Ttu_TRITD2Bv1G237990.13
Ttu_TRITD2Bv1G216620.3
Ttu_TRITD2Bv1G205420.1
Ttu_TRITD2Bv1G163380.1
Ttu_TRITD2Bv1G121710.3
Ttu_TRITD2Bv1G078970.1
Ttu_TRITD2Bv1G061540.5
Ttu_TRITD2Bv1G048190.1
−1000
0
1000
2000
3000
4000

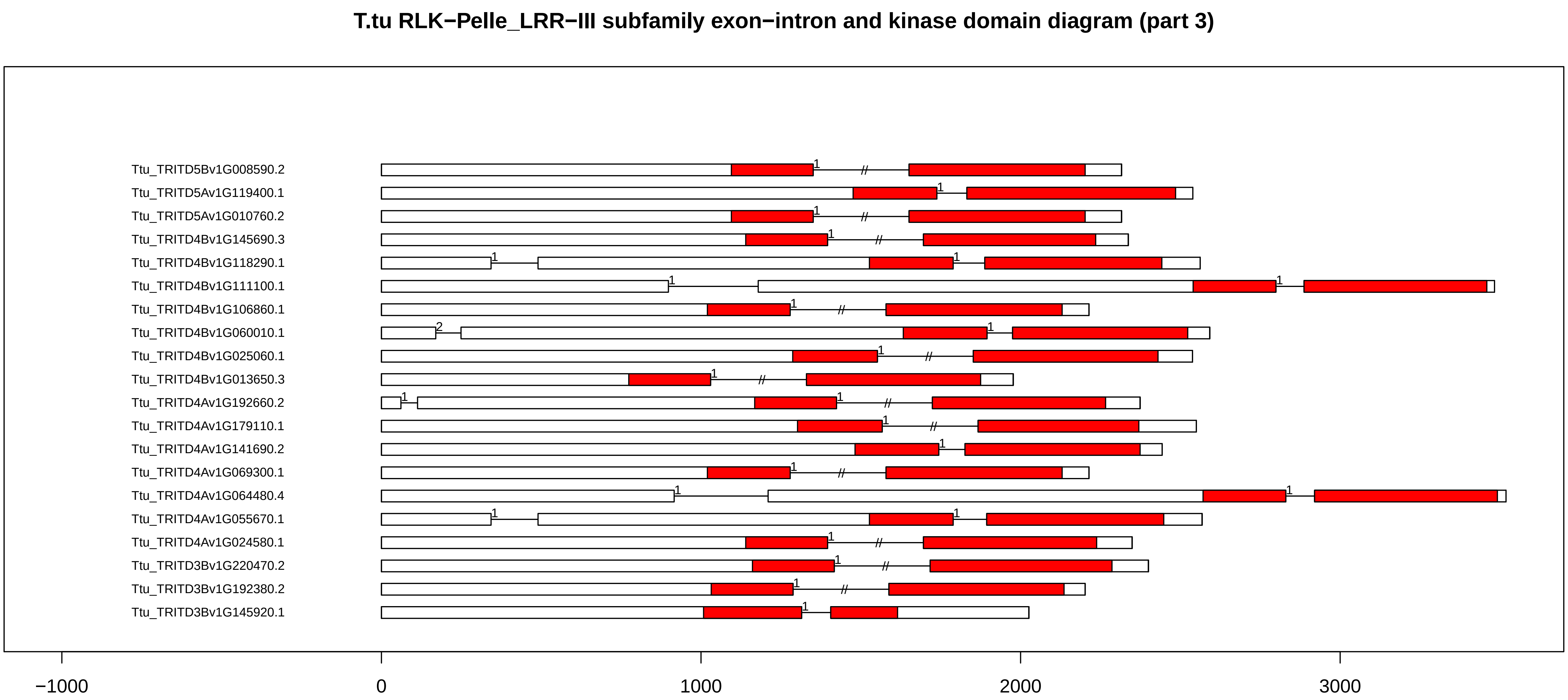
T.tu RLK−Pelle_LRR−III subfamily exon−intron and kinase domain diagram (part 3)
Ttu_TRITD5Bv1G008590.2
Ttu_TRITD5Av1G119400.1
Ttu_TRITD5Av1G010760.2
Ttu_TRITD4Bv1G145690.3
Ttu_TRITD4Bv1G118290.1
Ttu_TRITD4Bv1G111100.1
Ttu_TRITD4Bv1G106860.1
Ttu_TRITD4Bv1G060010.1
Ttu_TRITD4Bv1G025060.1
Ttu_TRITD4Bv1G013650.3
Ttu_TRITD4Av1G192660.2
Ttu_TRITD4Av1G179110.1
Ttu_TRITD4Av1G141690.2
Ttu_TRITD4Av1G069300.1
Ttu_TRITD4Av1G064480.4
Ttu_TRITD4Av1G055670.1
Ttu_TRITD4Av1G024580.1
Ttu_TRITD3Bv1G220470.2
Ttu_TRITD3Bv1G192380.2
Ttu_TRITD3Bv1G145920.1
−1000
0
1000
2000
3000

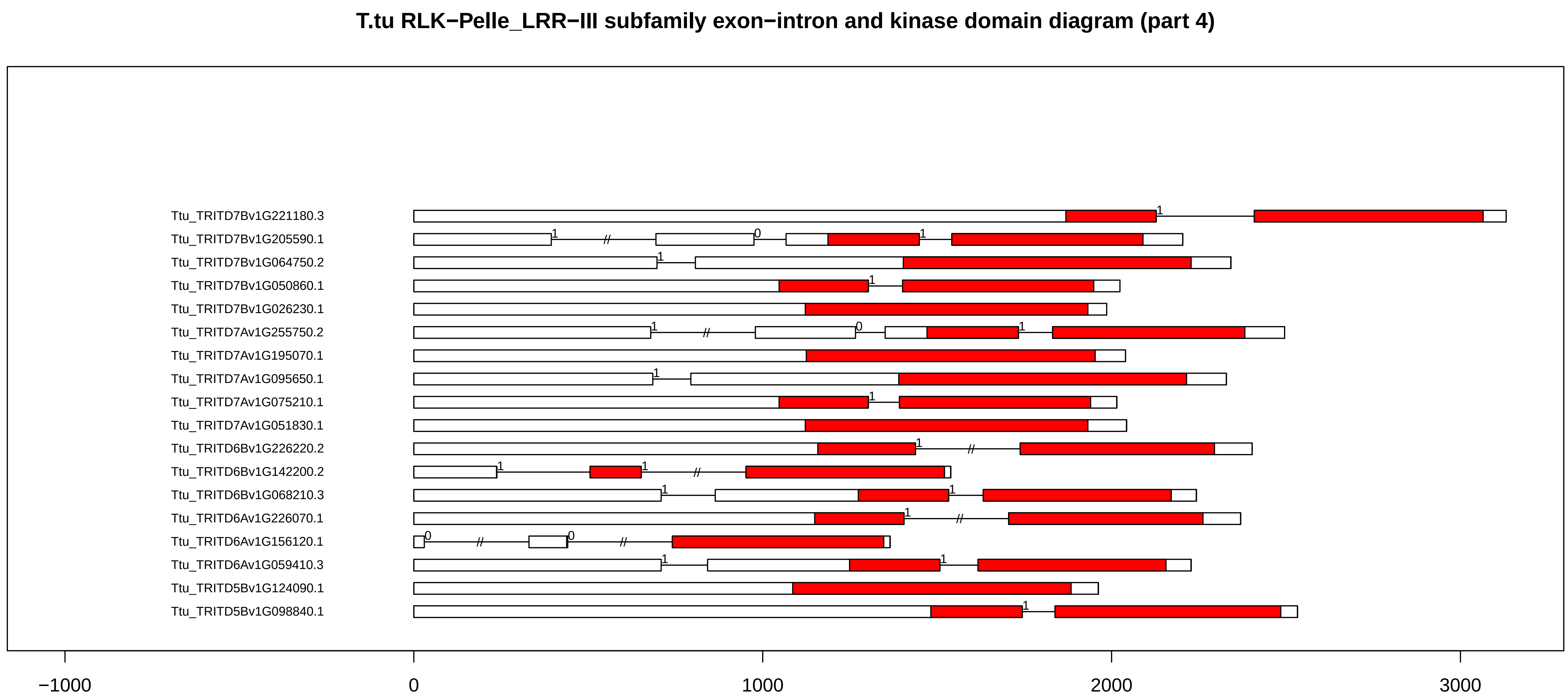
T.tu RLK−Pelle_LRR−III subfamily exon−intron and kinase domain diagram (part 4)
Ttu_TRITD7Bv1G221180.3
Ttu_TRITD7Bv1G205590.1
Ttu_TRITD7Bv1G064750.2
Ttu_TRITD7Bv1G050860.1
Ttu_TRITD7Bv1G026230.1
Ttu_TRITD7Av1G255750.2
Ttu_TRITD7Av1G195070.1
Ttu_TRITD7Av1G095650.1
Ttu_TRITD7Av1G075210.1
Ttu_TRITD7Av1G051830.1
Ttu_TRITD6Bv1G226220.2
Ttu_TRITD6Bv1G142200.2
Ttu_TRITD6Bv1G068210.3
Ttu_TRITD6Av1G226070.1
Ttu_TRITD6Av1G156120.1
Ttu_TRITD6Av1G059410.3
Ttu_TRITD5Bv1G124090.1
Ttu_TRITD5Bv1G098840.1
−1000
0
1000
2000
3000

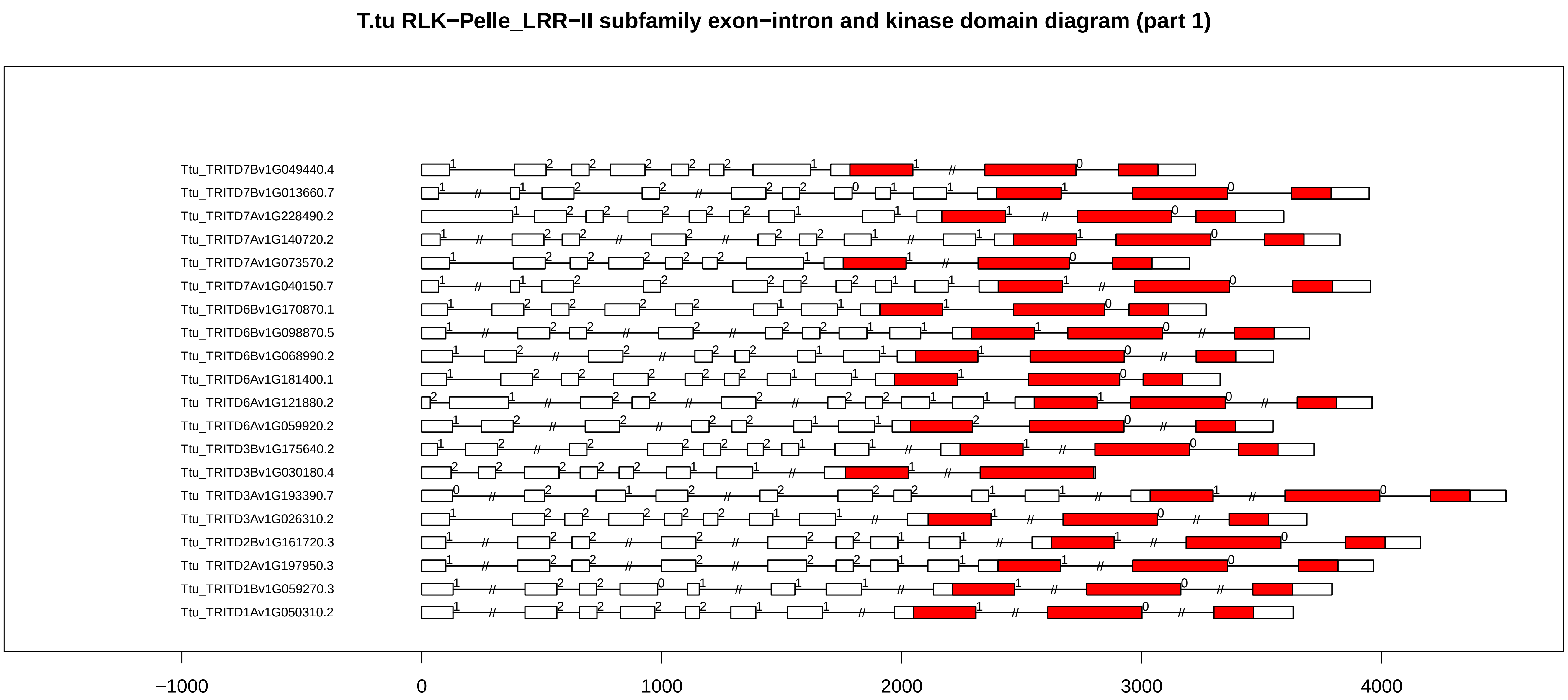
T.tu RLK−Pelle_LRR−II subfamily exon−intron and kinase domain diagram (part 1)
Ttu_TRITD7Bv1G049440.4
Ttu_TRITD7Bv1G013660.7
Ttu_TRITD7Av1G228490.2
Ttu_TRITD7Av1G140720.2
Ttu_TRITD7Av1G073570.2
Ttu_TRITD7Av1G040150.7
Ttu_TRITD6Bv1G170870.1
Ttu_TRITD6Bv1G098870.5
Ttu_TRITD6Bv1G068990.2
Ttu_TRITD6Av1G181400.1
Ttu_TRITD6Av1G121880.2
Ttu_TRITD6Av1G059920.2
Ttu_TRITD3Bv1G175640.2
Ttu_TRITD3Bv1G030180.4
Ttu_TRITD3Av1G193390.7
Ttu_TRITD3Av1G026310.2
Ttu_TRITD2Bv1G161720.3
Ttu_TRITD2Av1G197950.3
Ttu_TRITD1Bv1G059270.3
Ttu_TRITD1Av1G050310.2
−1000
0
1000
2000
3000
4000

T.tu RLK−Pelle_LRR−II subfamily exon−intron and kinase domain diagram (part 2)

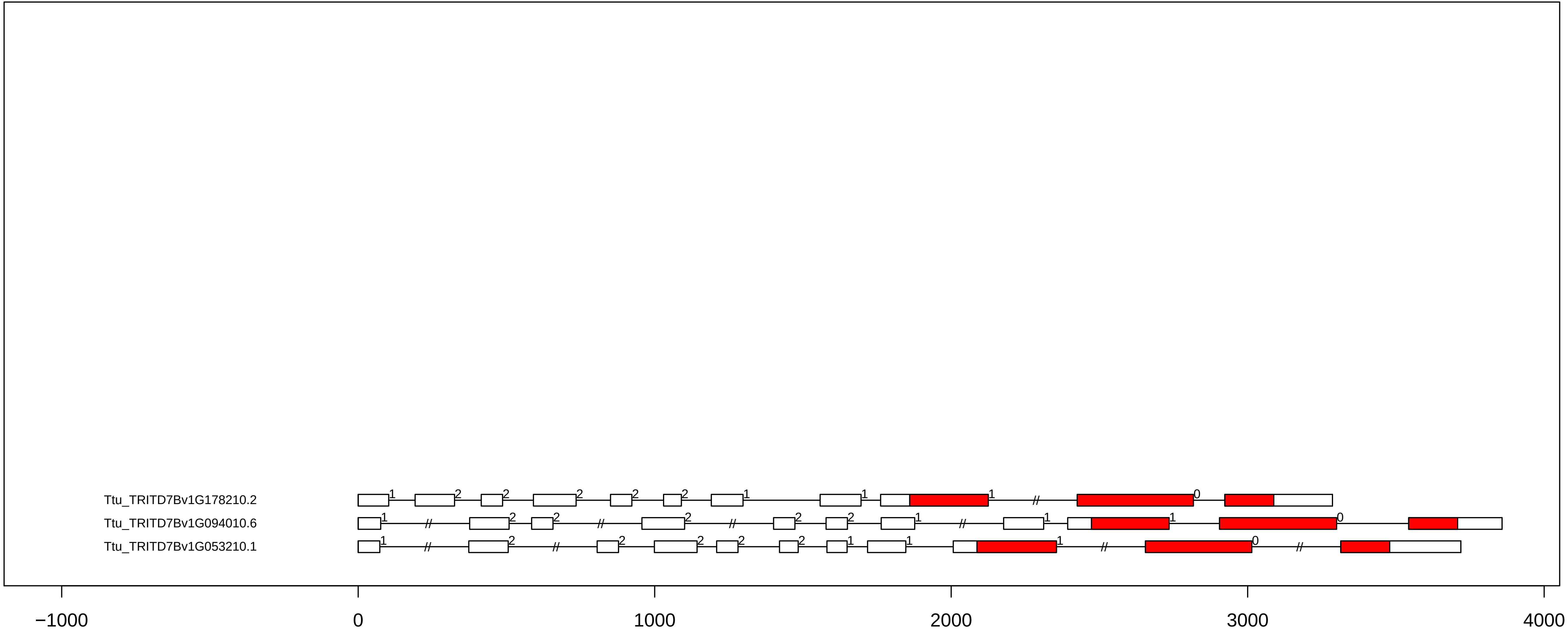

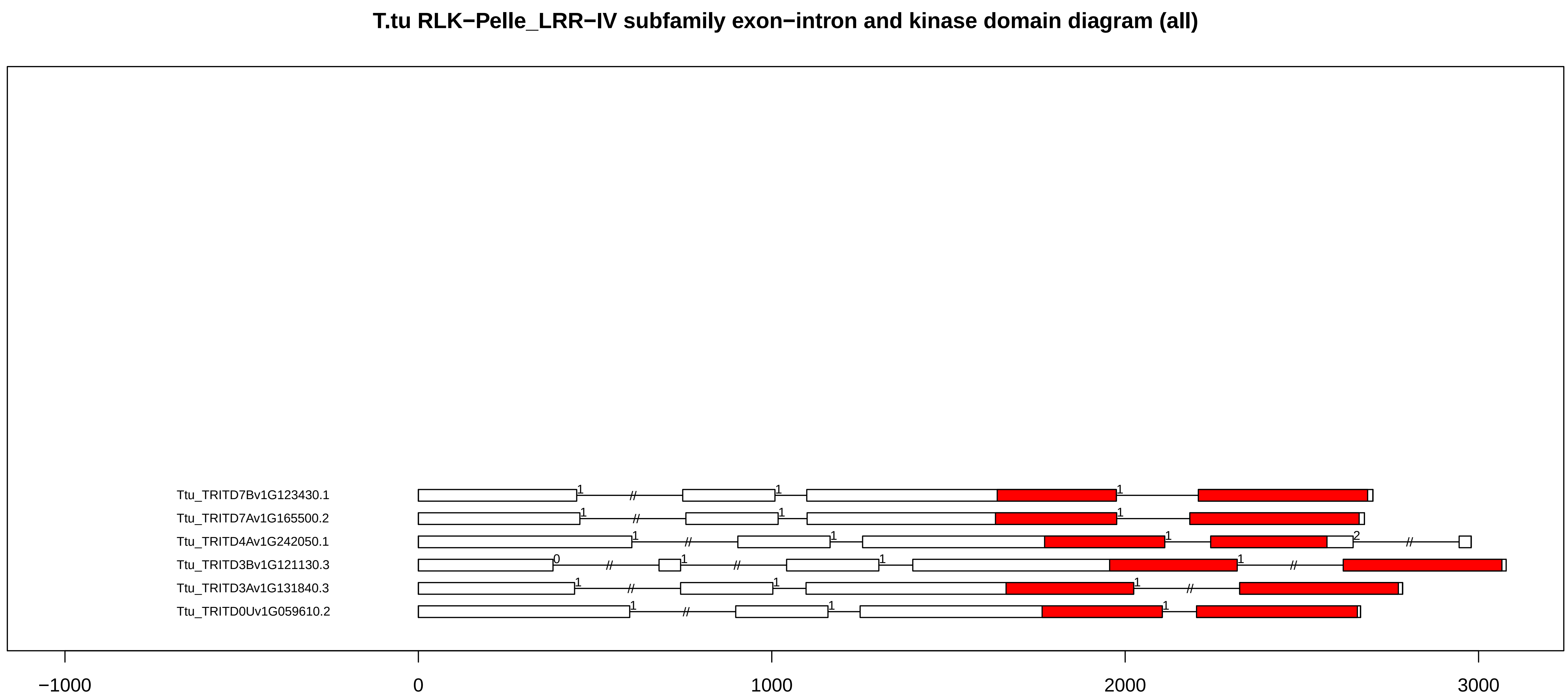

T.tu RLK−Pelle_LRR−IV subfamily exon−intron and kinase domain diagram (all)
Ttu_TRITD7Bv1G123430.1
Ttu_TRITD7Av1G165500.2
Ttu_TRITD4Av1G242050.1
Ttu_TRITD3Bv1G121130.3
Ttu_TRITD3Av1G131840.3
Ttu_TRITD0Uv1G059610.2
−1000
0
1000
2000
3000

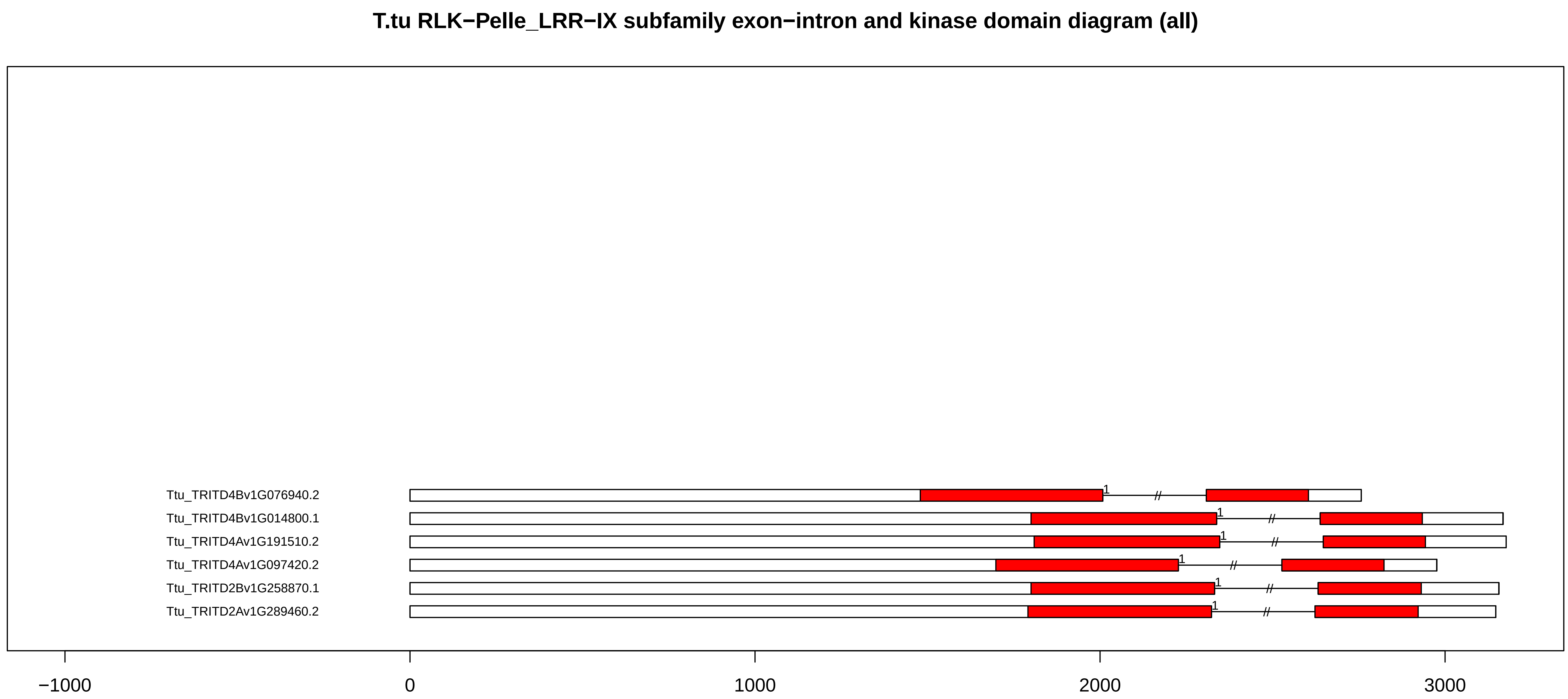
T.tu RLK−Pelle_LRR−IX subfamily exon−intron and kinase domain diagram (all)
Ttu_TRITD4Bv1G076940.2
Ttu_TRITD4Bv1G014800.1
Ttu_TRITD4Av1G191510.2
Ttu_TRITD4Av1G097420.2
Ttu_TRITD2Bv1G258870.1
Ttu_TRITD2Av1G289460.2
−1000
0
1000
2000
3000

T.tu RLK−Pelle_LRR−VI−1 subfamily exon−intron and kinase domain diagram (all)

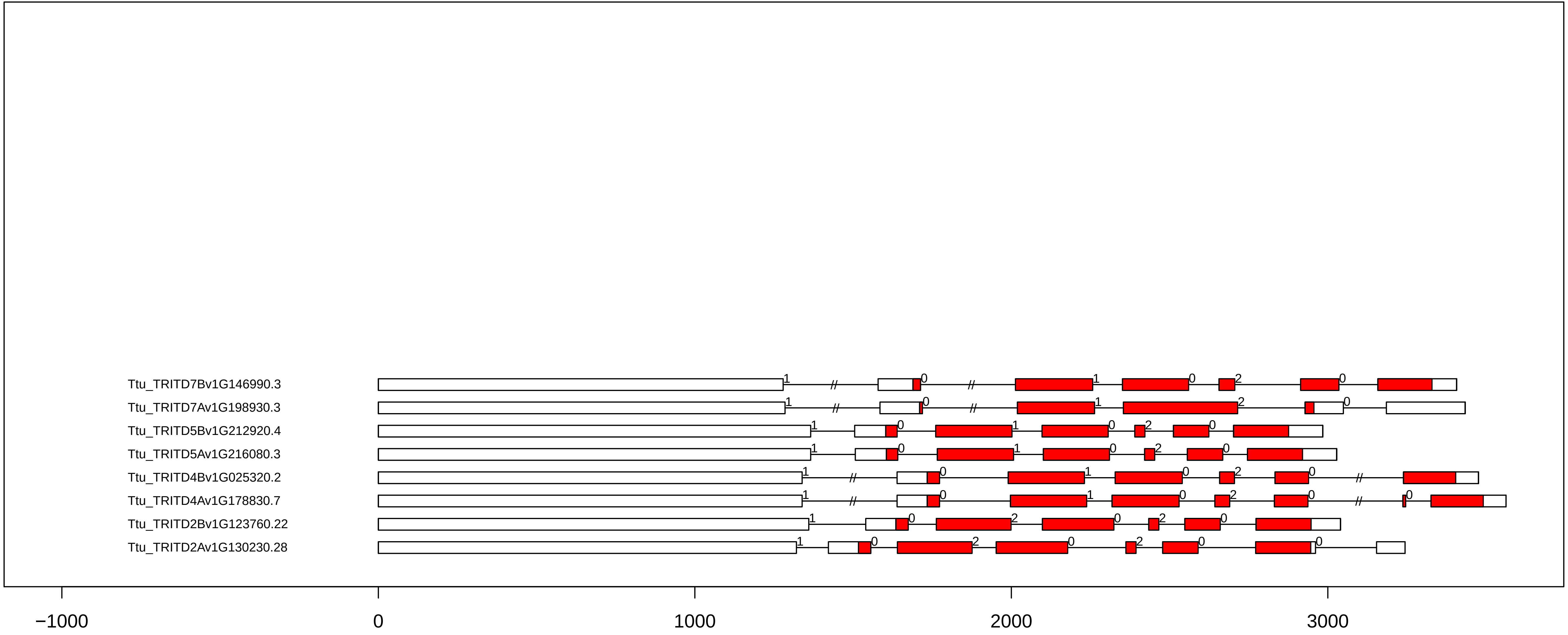

T.tu RLK−Pelle_LRR−VI−2 subfamily exon−intron and kinase domain diagram (all)

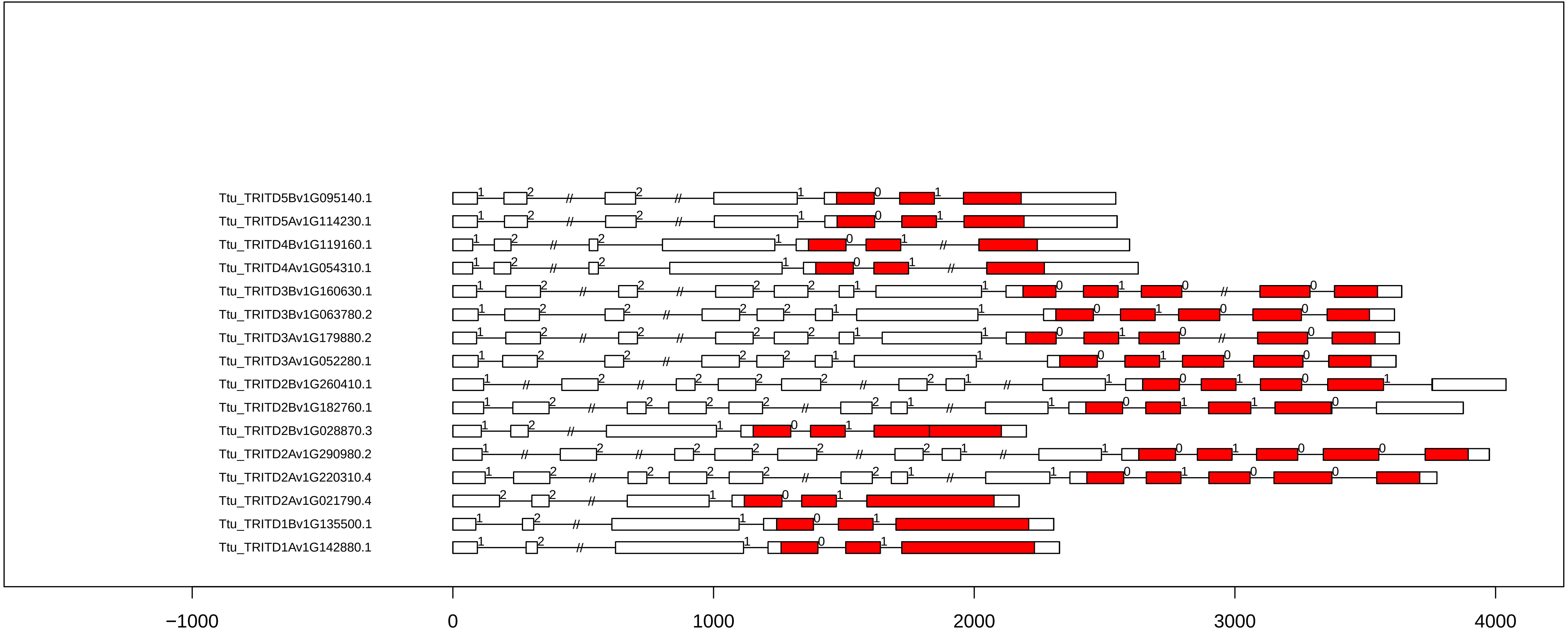

T.tu RLK−Pelle_LRR−VII−1 subfamily exon−intron and kinase domain diagram (all)

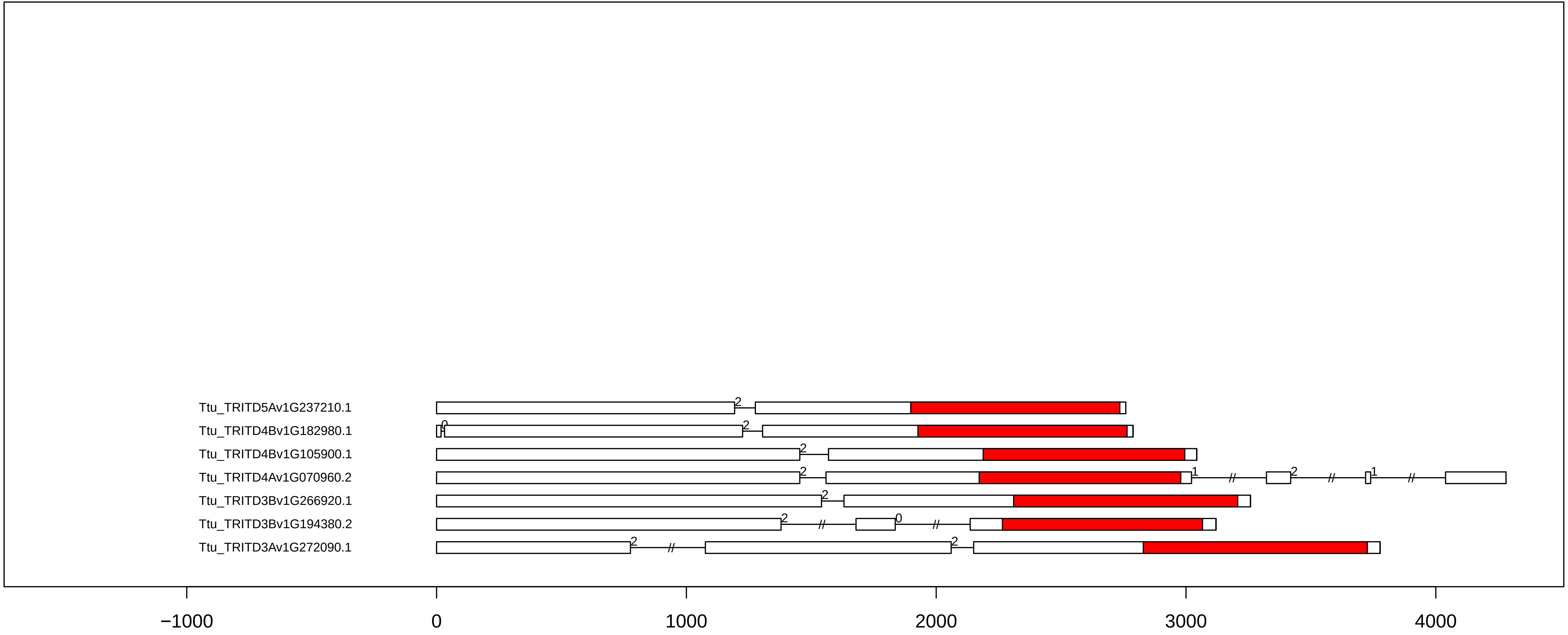

T.tu RLK−Pelle_LRR−VII−2 subfamily exon−intron and kinase domain diagram (all)

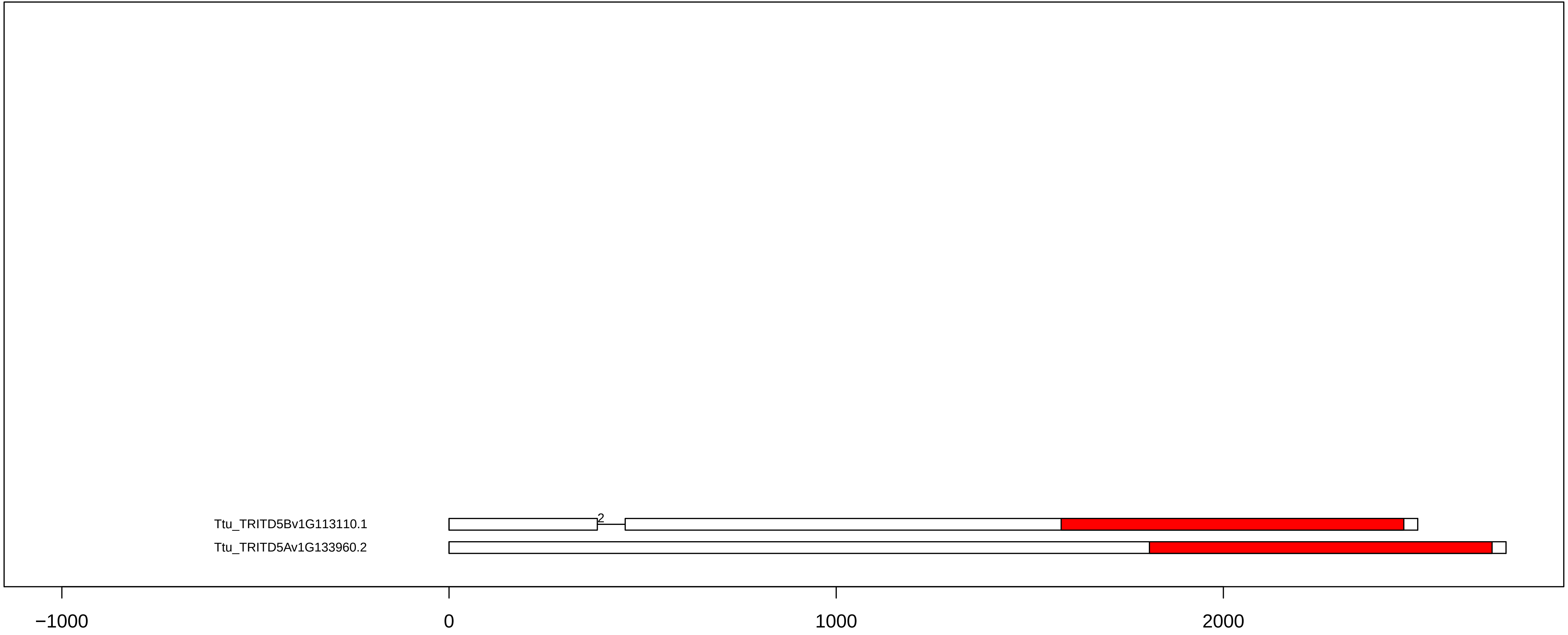

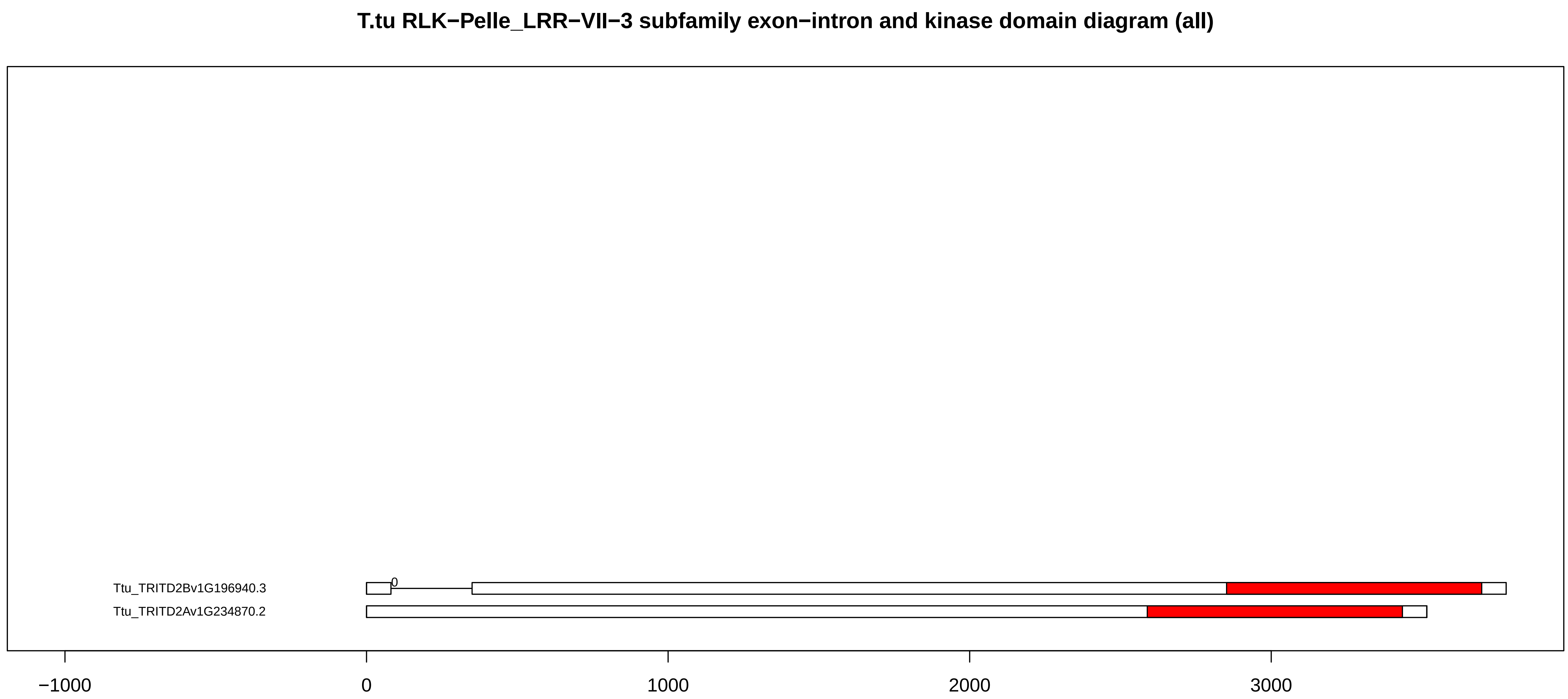
T.tu RLK−Pelle_LRR−VII−3 subfamily exon−intron and kinase domain diagram (all)
Ttu_TRITD2Bv1G196940.3
0
Ttu_TRITD2Av1G234870.2
−1000
0
1000
2000
3000

T.tu RLK−Pelle_LRR−VIII−1 subfamily exon−intron and kinase domain diagram (part 1)

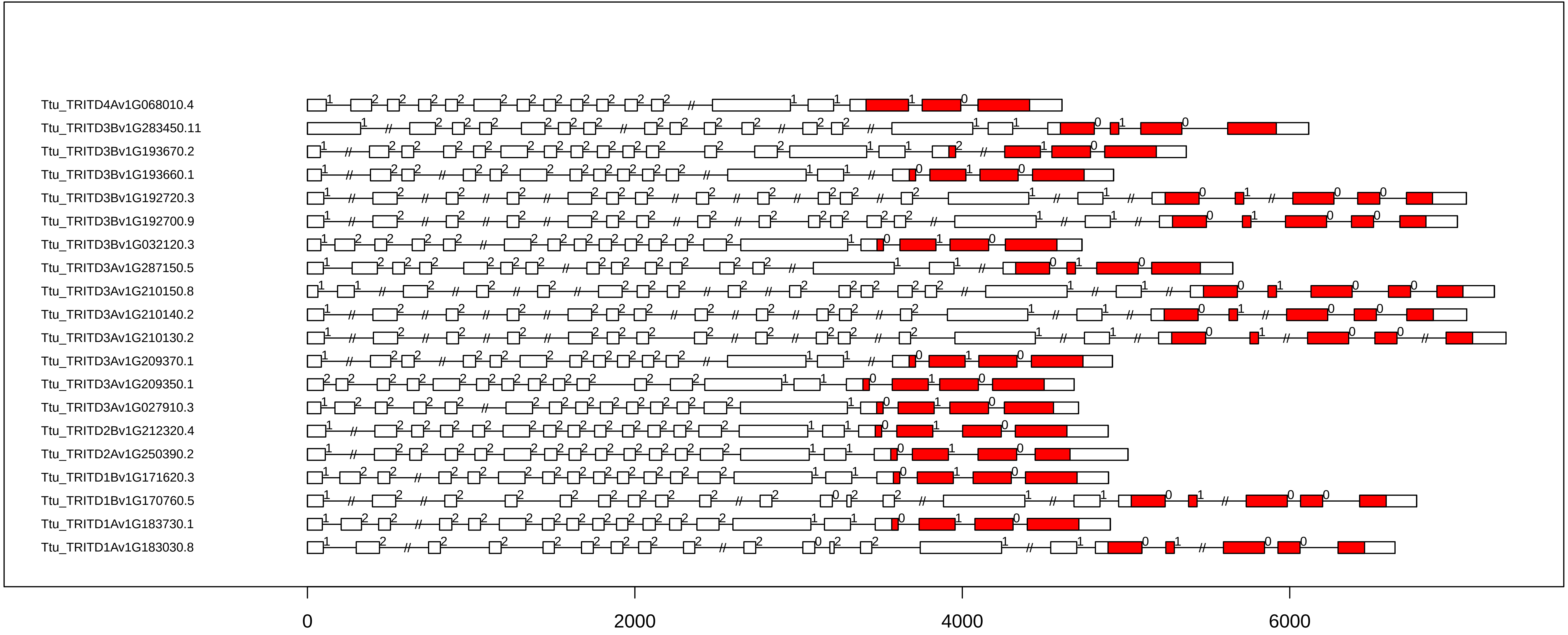

T.tu RLK−Pelle_LRR−VIII−1 subfamily exon−intron and kinase domain diagram (part 2)

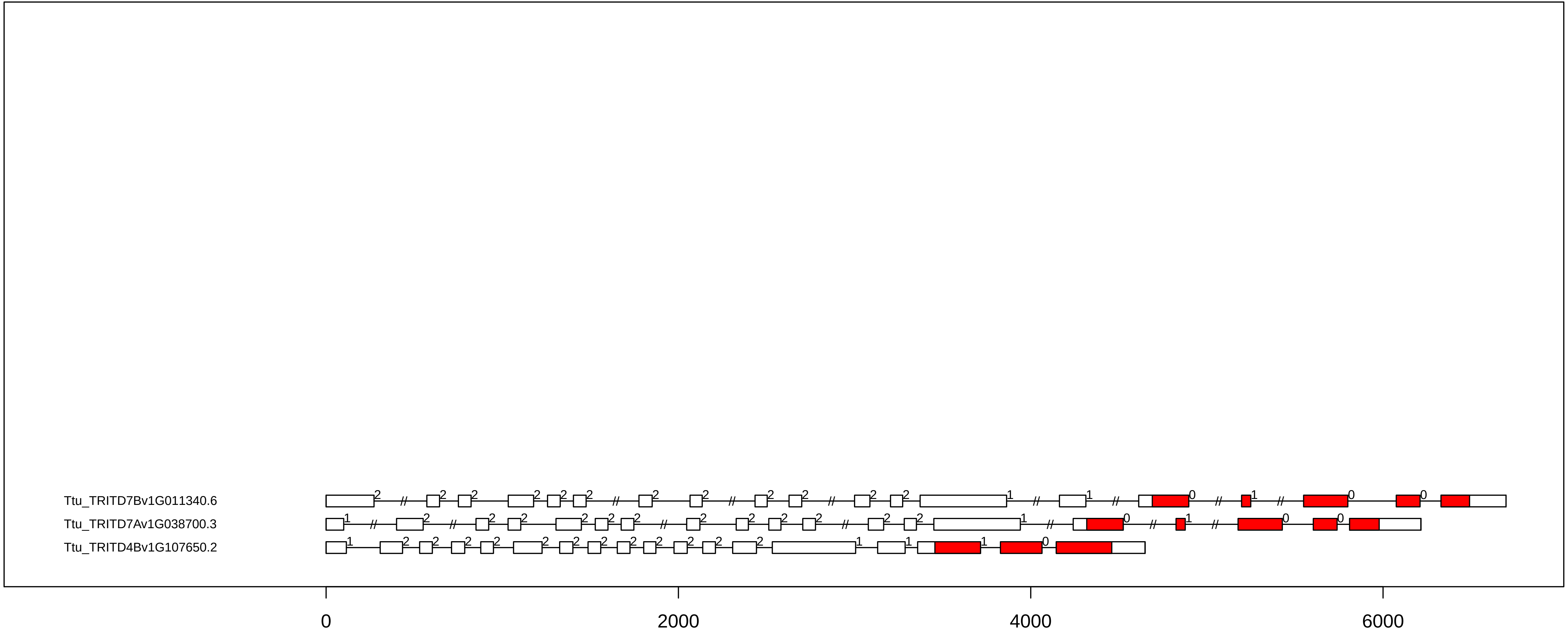

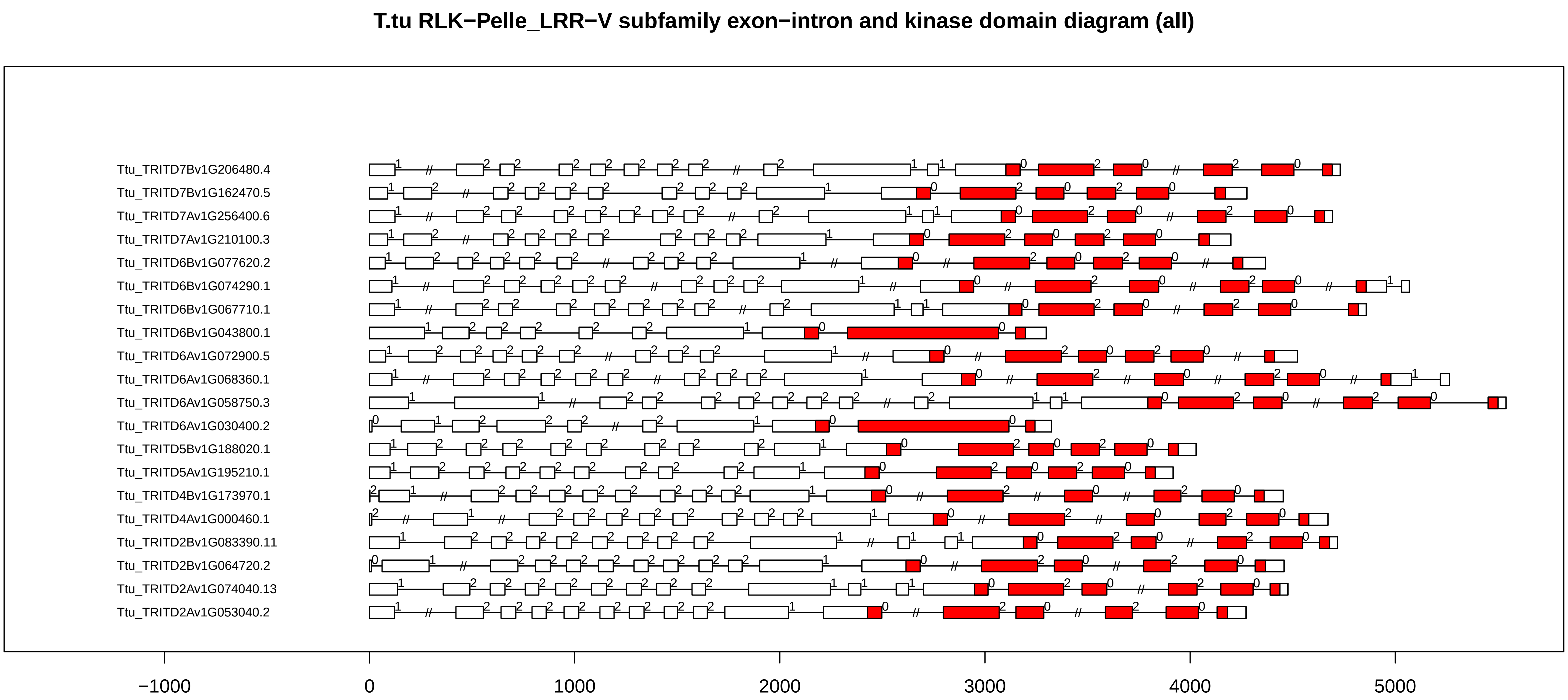
T.tu RLK−Pelle_LRR−V subfamily exon−intron and kinase domain diagram (all)
Ttu_TRITD7Bv1G206480.4
Ttu_TRITD7Bv1G162470.5
Ttu_TRITD7Av1G256400.6
Ttu_TRITD7Av1G210100.3
Ttu_TRITD6Bv1G077620.2
Ttu_TRITD6Bv1G074290.1
Ttu_TRITD6Bv1G067710.1
Ttu_TRITD6Bv1G043800.1
Ttu_TRITD6Av1G072900.5
Ttu_TRITD6Av1G068360.1
Ttu_TRITD6Av1G058750.3
Ttu_TRITD6Av1G030400.2
Ttu_TRITD5Bv1G188020.1
Ttu_TRITD5Av1G195210.1
Ttu_TRITD4Bv1G173970.1
Ttu_TRITD4Av1G000460.1
Ttu_TRITD2Bv1G083390.11
Ttu_TRITD2Bv1G064720.2
Ttu_TRITD2Av1G074040.13
Ttu_TRITD2Av1G053040.2
−1000
0
1000
2000
3000
4000
5000

T.tu RLK−Pelle_LRR−Xa subfamily exon−intron and kinase domain diagram (all)

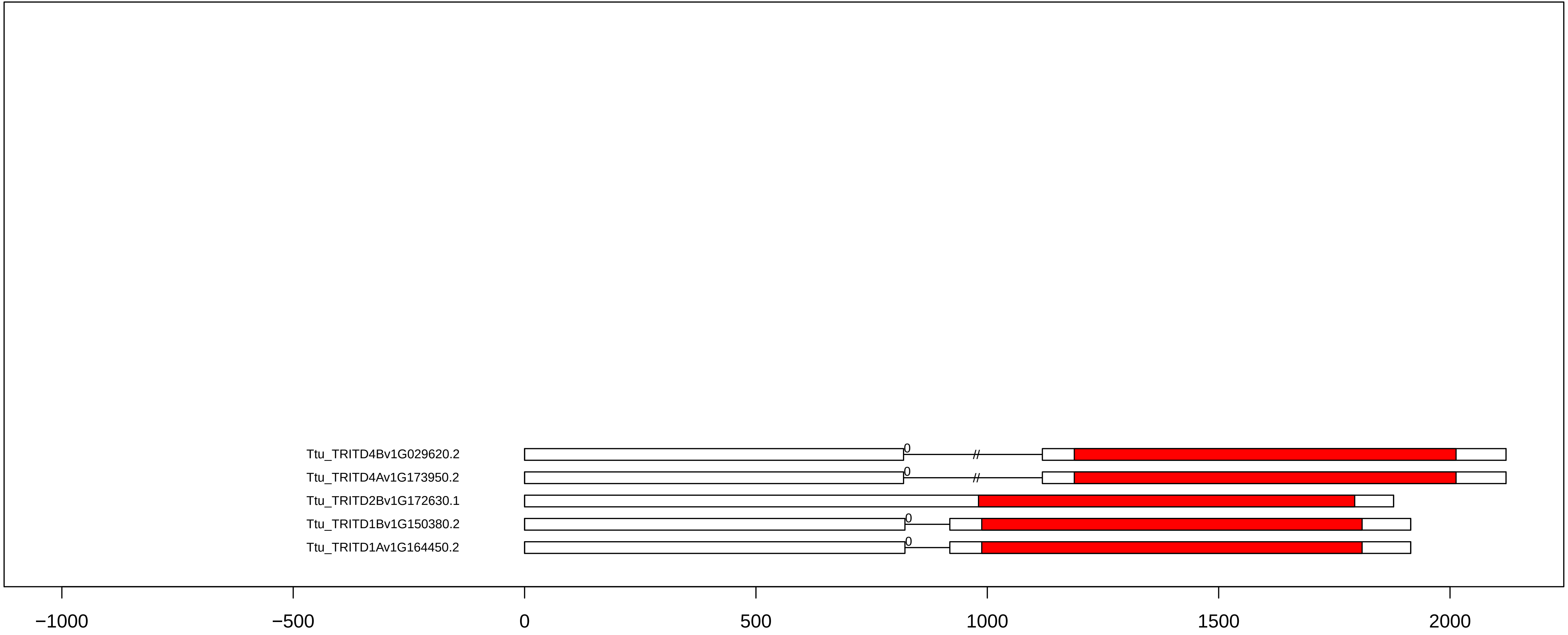

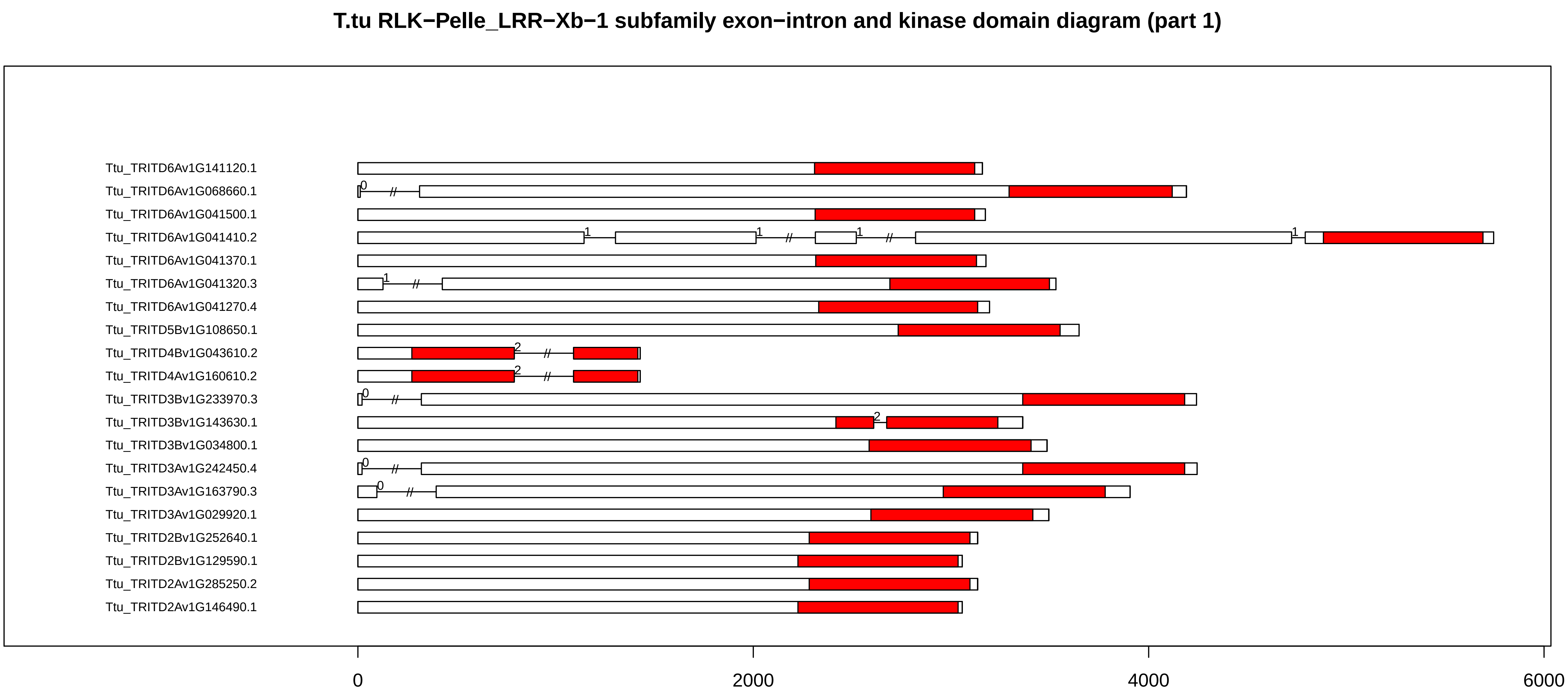

T.tu RLK−Pelle_LRR−Xb−1 subfamily exon−intron and kinase domain diagram (part 1)
Ttu_TRITD6Av1G141120.1
Ttu_TRITD6Av1G068660.1
Ttu_TRITD6Av1G041500.1
Ttu_TRITD6Av1G041410.2
Ttu_TRITD6Av1G041370.1
Ttu_TRITD6Av1G041320.3
Ttu_TRITD6Av1G041270.4
Ttu_TRITD5Bv1G108650.1
Ttu_TRITD4Bv1G043610.2
Ttu_TRITD4Av1G160610.2
Ttu_TRITD3Bv1G233970.3
Ttu_TRITD3Bv1G143630.1
Ttu_TRITD3Bv1G034800.1
Ttu_TRITD3Av1G242450.4
Ttu_TRITD3Av1G163790.3
Ttu_TRITD3Av1G029920.1
Ttu_TRITD2Bv1G252640.1
Ttu_TRITD2Bv1G129590.1
Ttu_TRITD2Av1G285250.2
Ttu_TRITD2Av1G146490.1
0
2000
4000
6000

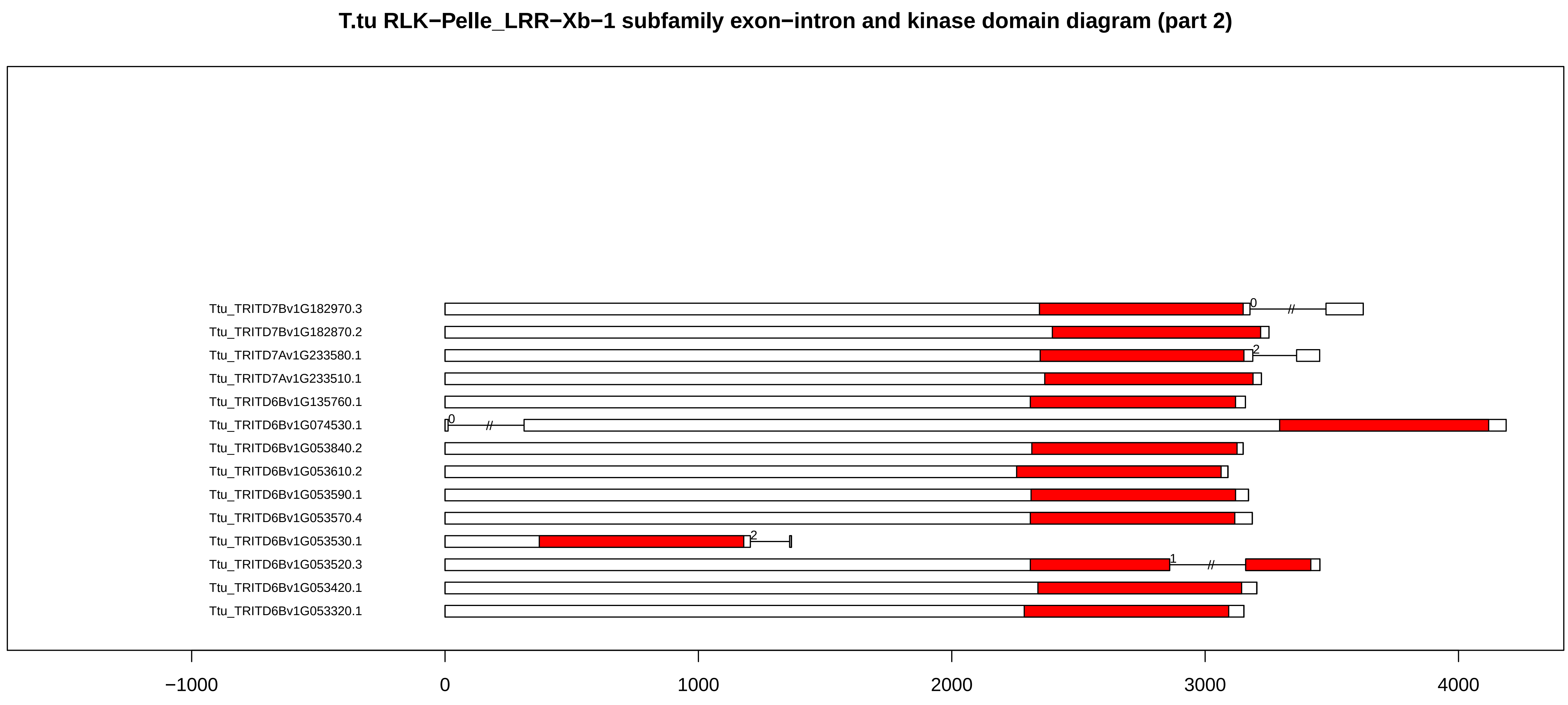
T.tu RLK−Pelle_LRR−Xb−1 subfamily exon−intron and kinase domain diagram (part 2)
Ttu_TRITD7Bv1G182970.3
Ttu_TRITD7Bv1G182870.2
Ttu_TRITD7Av1G233580.1
Ttu_TRITD7Av1G233510.1
Ttu_TRITD6Bv1G135760.1
Ttu_TRITD6Bv1G074530.1
Ttu_TRITD6Bv1G053840.2
Ttu_TRITD6Bv1G053610.2
Ttu_TRITD6Bv1G053590.1
Ttu_TRITD6Bv1G053570.4
Ttu_TRITD6Bv1G053530.1
Ttu_TRITD6Bv1G053520.3
Ttu_TRITD6Bv1G053420.1
Ttu_TRITD6Bv1G053320.1
0
2
0
2
1
−1000
0
1000
2000
3000
4000

T.tu RLK−Pelle_LRR−Xb−2 subfamily exon−intron and kinase domain diagram (all)

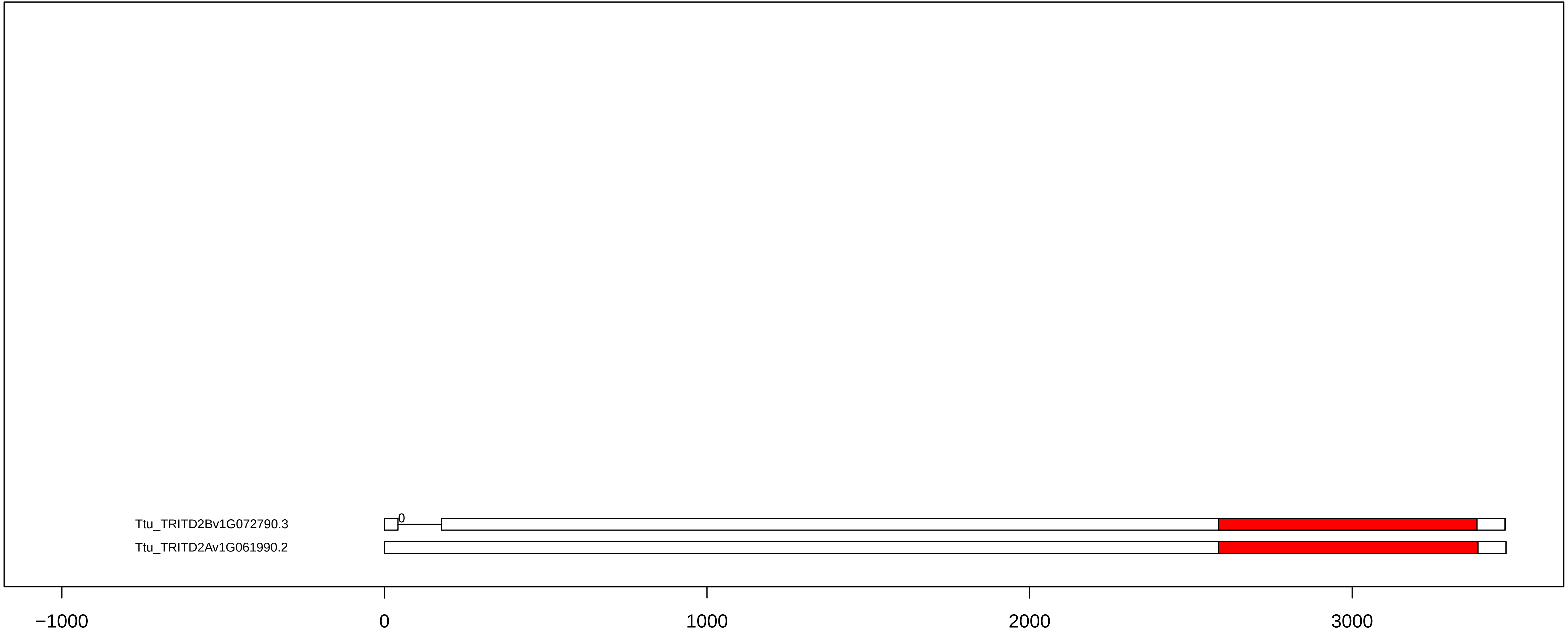

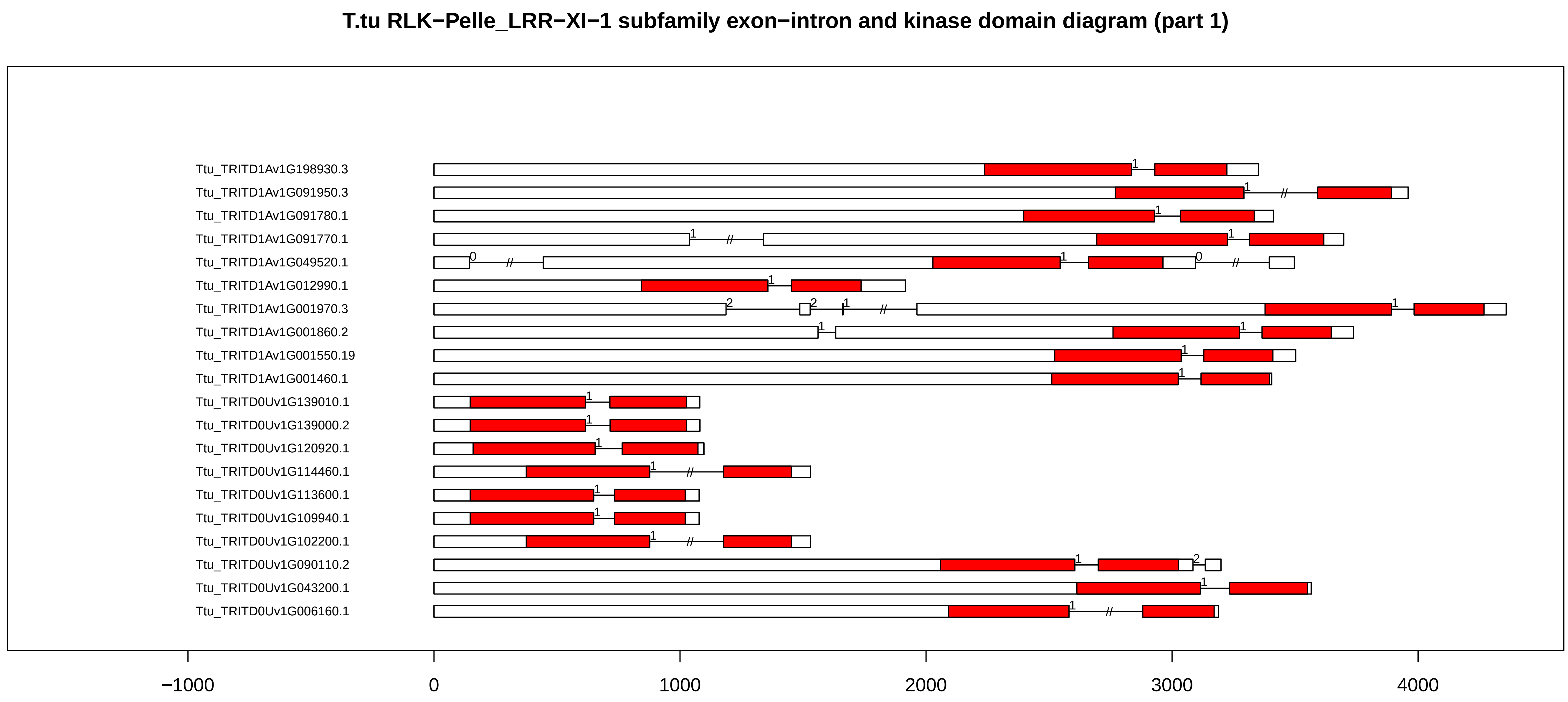
T.tu RLK−Pelle_LRR−XI−1 subfamily exon−intron and kinase domain diagram (part 1)
Ttu_TRITD1Av1G198930.3
Ttu_TRITD1Av1G091950.3
Ttu_TRITD1Av1G091780.1
Ttu_TRITD1Av1G091770.1
Ttu_TRITD1Av1G049520.1
Ttu_TRITD1Av1G012990.1
Ttu_TRITD1Av1G001970.3
Ttu_TRITD1Av1G001860.2
Ttu_TRITD1Av1G001550.19
Ttu_TRITD1Av1G001460.1
Ttu_TRITD0Uv1G139010.1
Ttu_TRITD0Uv1G139000.2
Ttu_TRITD0Uv1G120920.1
Ttu_TRITD0Uv1G114460.1
Ttu_TRITD0Uv1G113600.1
Ttu_TRITD0Uv1G109940.1
Ttu_TRITD0Uv1G102200.1
Ttu_TRITD0Uv1G090110.2
Ttu_TRITD0Uv1G043200.1
Ttu_TRITD0Uv1G006160.1
−1000
0
1000
2000
3000
4000

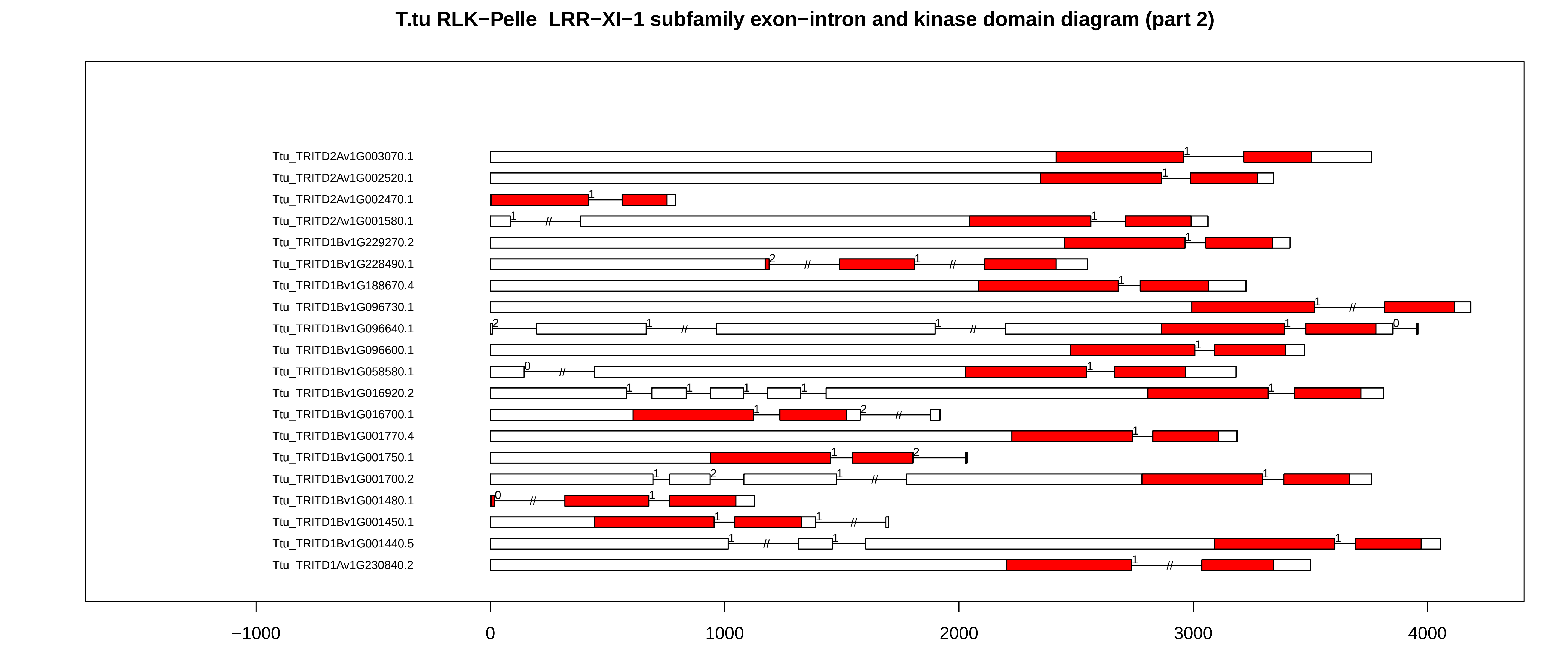

T.tu RLK−Pelle_LRR−XI−1 subfamily exon−intron and kinase domain diagram (part 2)
Ttu_TRITD2Av1G003070.1
Ttu_TRITD2Av1G002520.1
Ttu_TRITD2Av1G002470.1
Ttu_TRITD2Av1G001580.1
Ttu_TRITD1Bv1G229270.2
Ttu_TRITD1Bv1G228490.1
Ttu_TRITD1Bv1G188670.4
Ttu_TRITD1Bv1G096730.1
Ttu_TRITD1Bv1G096640.1
Ttu_TRITD1Bv1G096600.1
Ttu_TRITD1Bv1G058580.1
Ttu_TRITD1Bv1G016920.2
Ttu_TRITD1Bv1G016700.1
Ttu_TRITD1Bv1G001770.4
Ttu_TRITD1Bv1G001750.1
Ttu_TRITD1Bv1G001700.2
Ttu_TRITD1Bv1G001480.1
Ttu_TRITD1Bv1G001450.1
Ttu_TRITD1Bv1G001440.5
Ttu_TRITD1Av1G230840.2
−1000
0
1000
2000
3000
4000

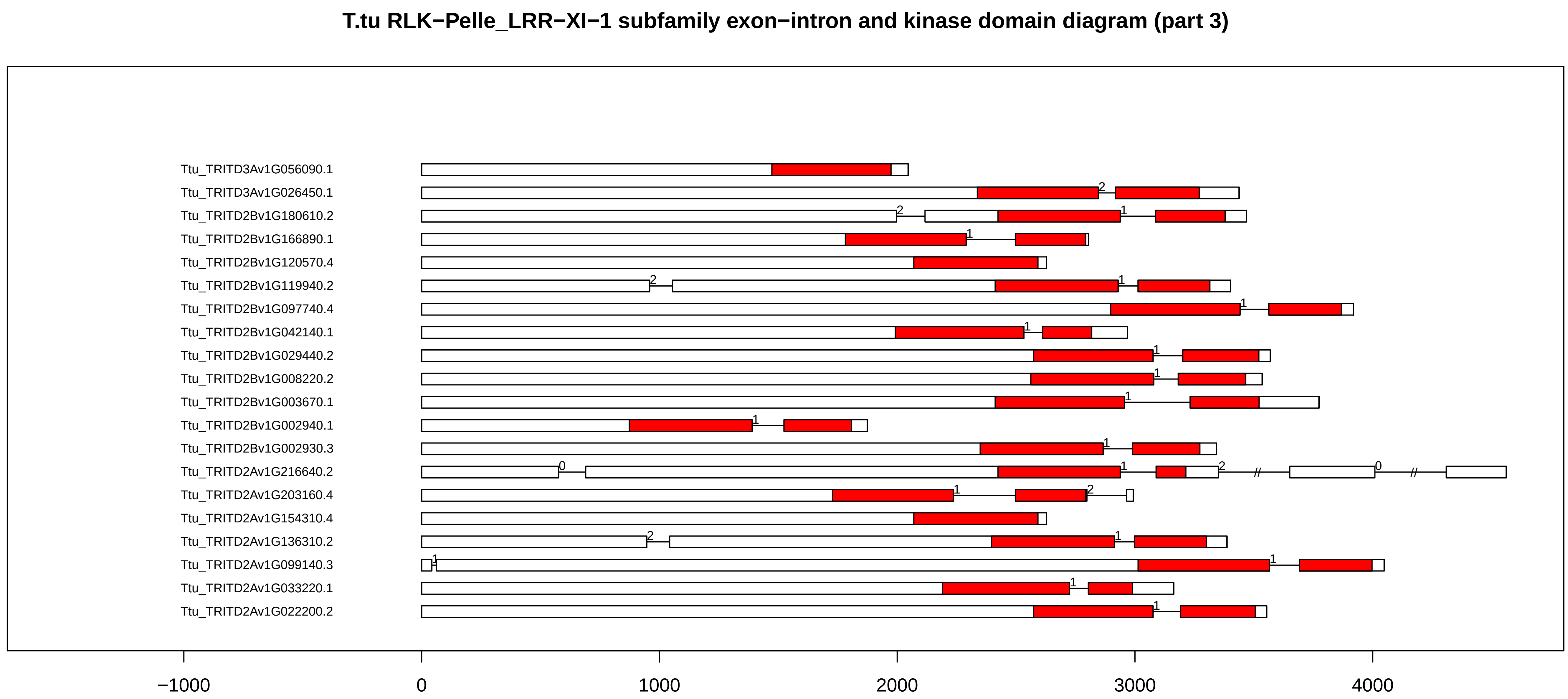
T.tu RLK−Pelle_LRR−XI−1 subfamily exon−intron and kinase domain diagram (part 3)
Ttu_TRITD3Av1G056090.1
Ttu_TRITD3Av1G026450.1
Ttu_TRITD2Bv1G180610.2
Ttu_TRITD2Bv1G166890.1
Ttu_TRITD2Bv1G120570.4
Ttu_TRITD2Bv1G119940.2
Ttu_TRITD2Bv1G097740.4
Ttu_TRITD2Bv1G042140.1
Ttu_TRITD2Bv1G029440.2
Ttu_TRITD2Bv1G008220.2
Ttu_TRITD2Bv1G003670.1
Ttu_TRITD2Bv1G002940.1
Ttu_TRITD2Bv1G002930.3
Ttu_TRITD2Av1G216640.2
Ttu_TRITD2Av1G203160.4
Ttu_TRITD2Av1G154310.4
Ttu_TRITD2Av1G136310.2
Ttu_TRITD2Av1G099140.3
Ttu_TRITD2Av1G033220.1
Ttu_TRITD2Av1G022200.2
−1000
0
1000
2000
3000
4000

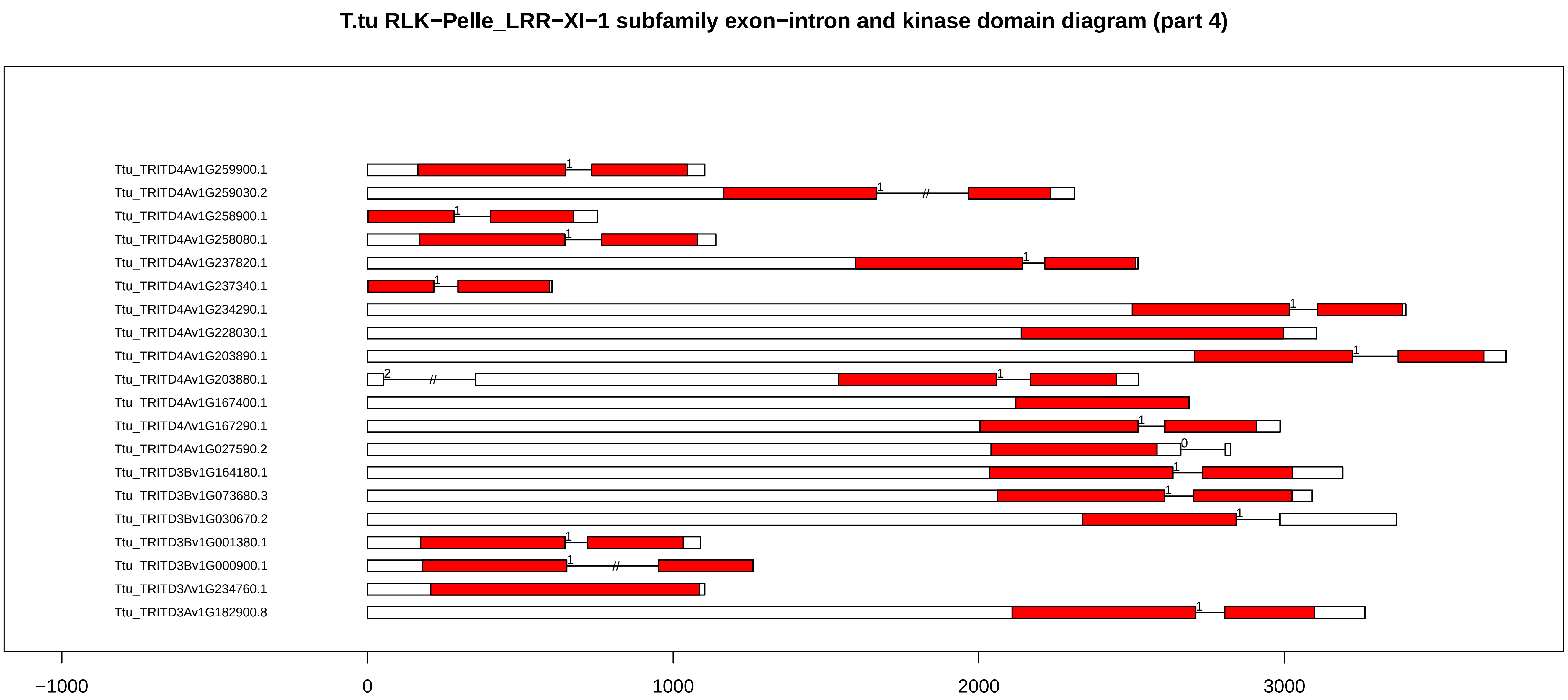
T.tu RLK−Pelle_LRR−XI−1 subfamily exon−intron and kinase domain diagram (part 4)
Ttu_TRITD4Av1G259900.1
Ttu_TRITD4Av1G259030.2
Ttu_TRITD4Av1G258900.1
Ttu_TRITD4Av1G258080.1
Ttu_TRITD4Av1G237820.1
Ttu_TRITD4Av1G237340.1
Ttu_TRITD4Av1G234290.1
Ttu_TRITD4Av1G228030.1
Ttu_TRITD4Av1G203890.1
Ttu_TRITD4Av1G203880.1
Ttu_TRITD4Av1G167400.1
Ttu_TRITD4Av1G167290.1
Ttu_TRITD4Av1G027590.2
Ttu_TRITD3Bv1G164180.1
Ttu_TRITD3Bv1G073680.3
Ttu_TRITD3Bv1G030670.2
Ttu_TRITD3Bv1G001380.1
Ttu_TRITD3Bv1G000900.1
Ttu_TRITD3Av1G234760.1
Ttu_TRITD3Av1G182900.8
−1000
0
1000
2000
3000

T.tu RLK−Pelle_LRR−XI−1 subfamily exon−intron and kinase domain diagram (part 5)

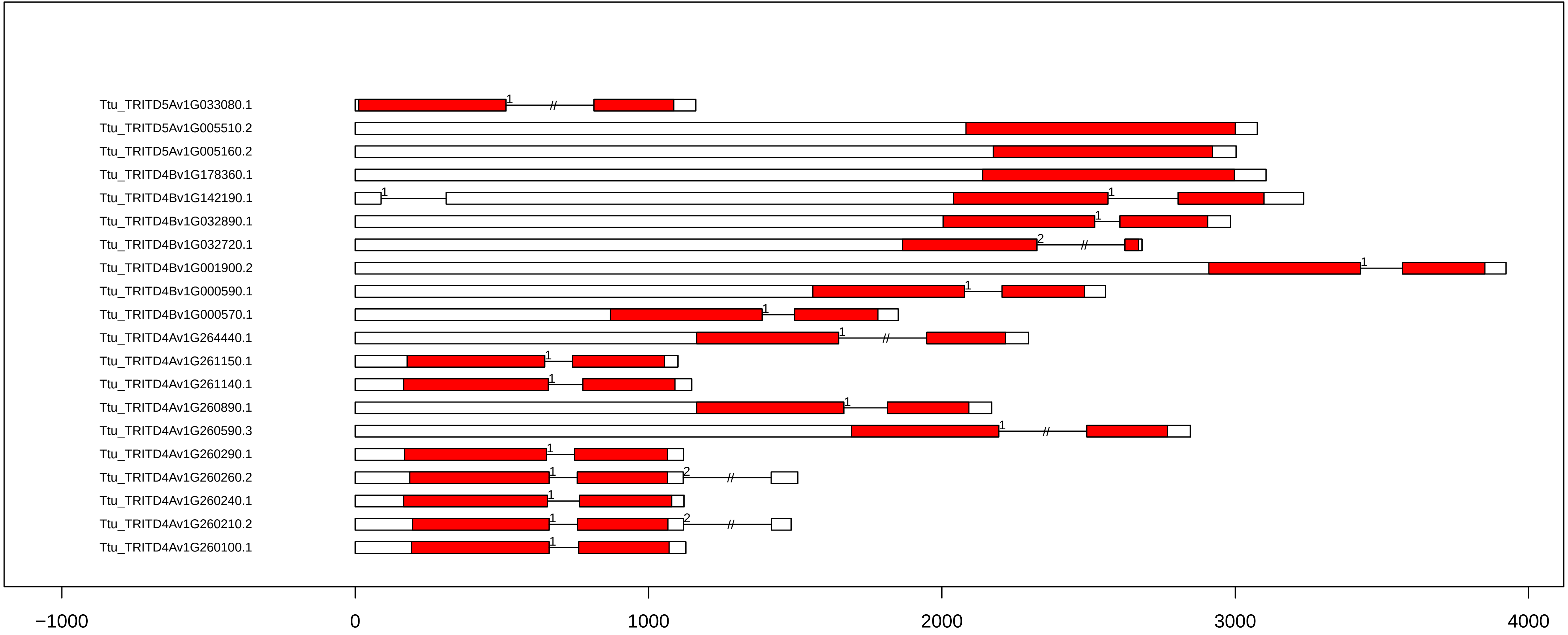

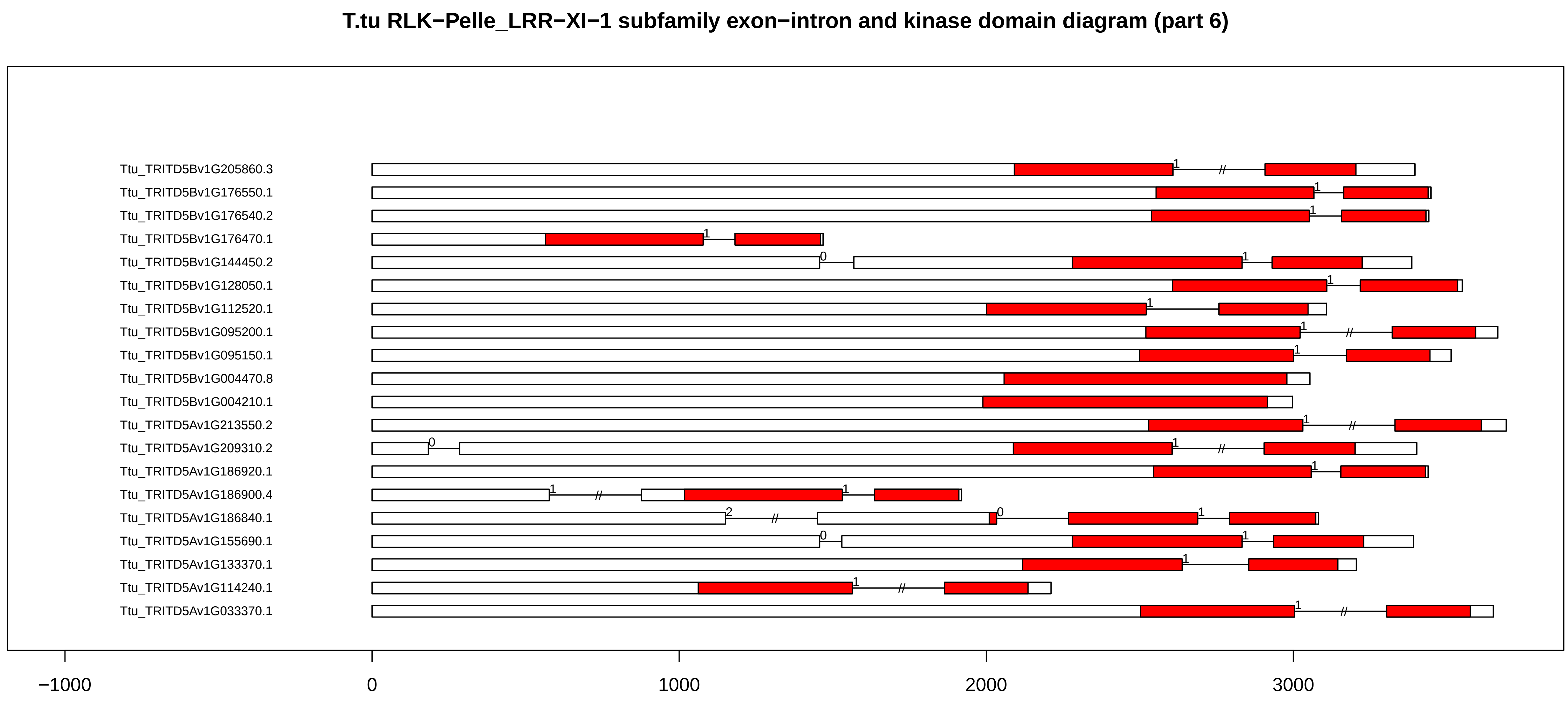
T.tu RLK−Pelle_LRR−XI−1 subfamily exon−intron and kinase domain diagram (part 6)
Ttu_TRITD5Bv1G205860.3
Ttu_TRITD5Bv1G176550.1
Ttu_TRITD5Bv1G176540.2
Ttu_TRITD5Bv1G176470.1
Ttu_TRITD5Bv1G144450.2
Ttu_TRITD5Bv1G128050.1
Ttu_TRITD5Bv1G112520.1
Ttu_TRITD5Bv1G095200.1
Ttu_TRITD5Bv1G095150.1
Ttu_TRITD5Bv1G004470.8
Ttu_TRITD5Bv1G004210.1
Ttu_TRITD5Av1G213550.2
Ttu_TRITD5Av1G209310.2
Ttu_TRITD5Av1G186920.1
Ttu_TRITD5Av1G186900.4
Ttu_TRITD5Av1G186840.1
Ttu_TRITD5Av1G155690.1
Ttu_TRITD5Av1G133370.1
Ttu_TRITD5Av1G114240.1
Ttu_TRITD5Av1G033370.1
−1000
0
1000
2000
3000

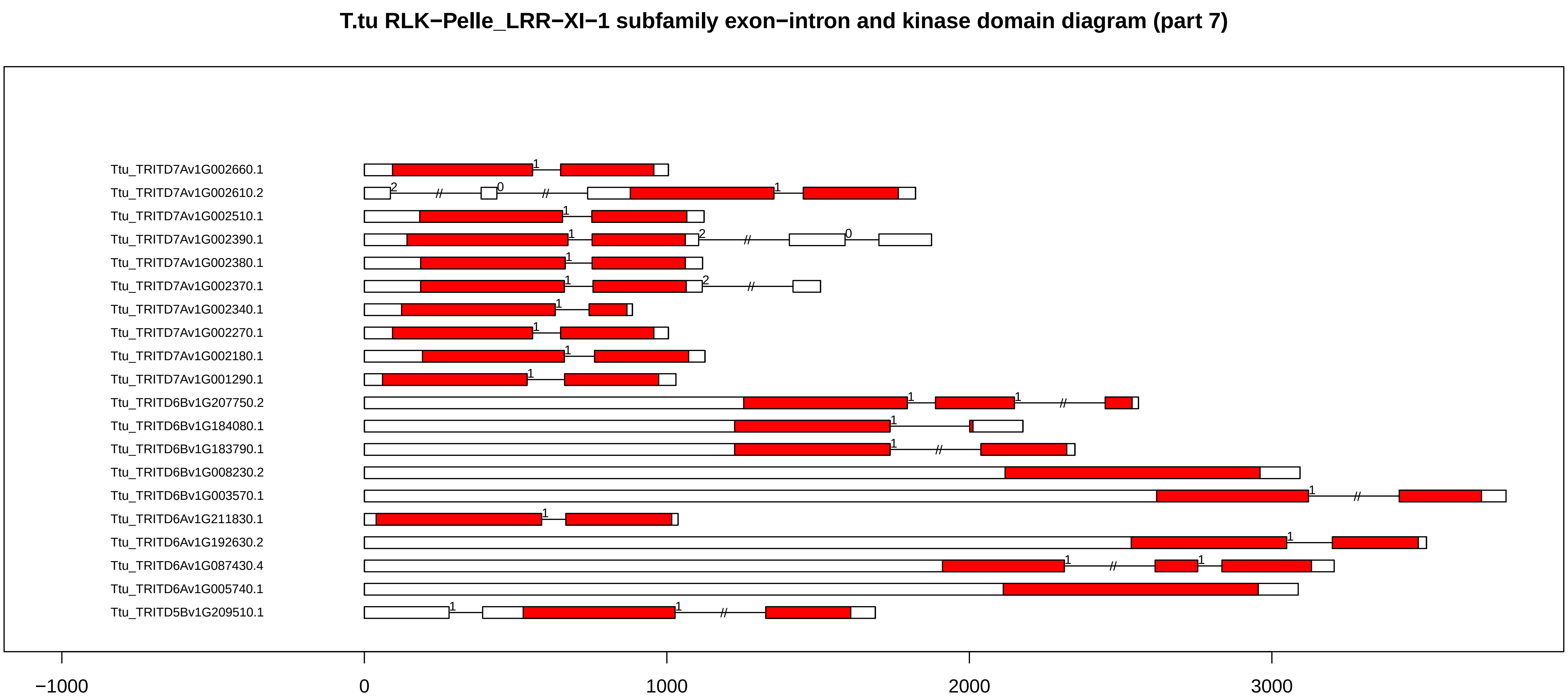

T.tu RLK−Pelle_LRR−XI−1 subfamily exon−intron and kinase domain diagram (part 7)
Ttu_TRITD7Av1G002660.1
Ttu_TRITD7Av1G002610.2
Ttu_TRITD7Av1G002510.1
Ttu_TRITD7Av1G002390.1
Ttu_TRITD7Av1G002380.1
Ttu_TRITD7Av1G002370.1
Ttu_TRITD7Av1G002340.1
Ttu_TRITD7Av1G002270.1
Ttu_TRITD7Av1G002180.1
Ttu_TRITD7Av1G001290.1
Ttu_TRITD6Bv1G207750.2
Ttu_TRITD6Bv1G184080.1
Ttu_TRITD6Bv1G183790.1
Ttu_TRITD6Bv1G008230.2
Ttu_TRITD6Bv1G003570.1
Ttu_TRITD6Av1G211830.1
Ttu_TRITD6Av1G192630.2
Ttu_TRITD6Av1G087430.4
Ttu_TRITD6Av1G005740.1
Ttu_TRITD5Bv1G209510.1
−1000
0
1000
2000
3000

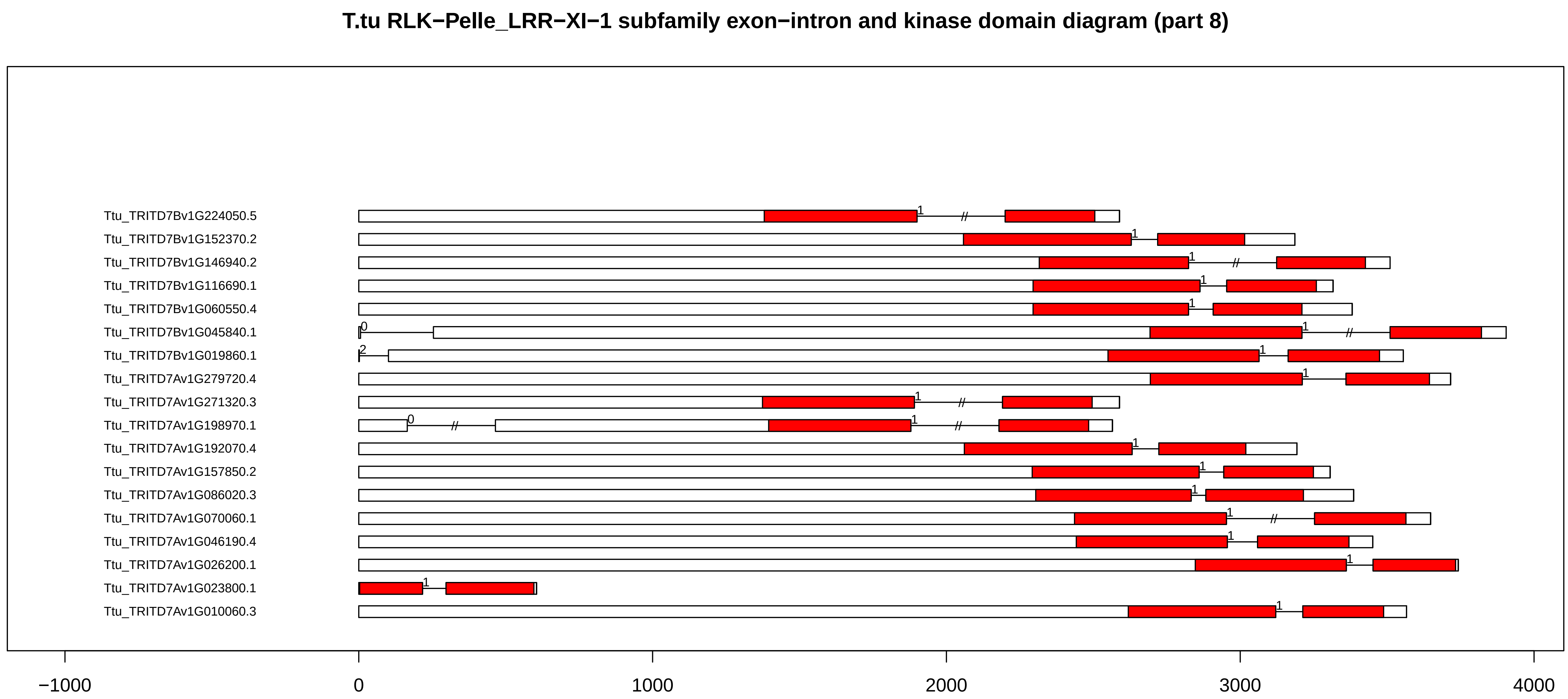
T.tu RLK−Pelle_LRR−XI−1 subfamily exon−intron and kinase domain diagram (part 8)
Ttu_TRITD7Bv1G224050.5
Ttu_TRITD7Bv1G152370.2
Ttu_TRITD7Bv1G146940.2
Ttu_TRITD7Bv1G116690.1
Ttu_TRITD7Bv1G060550.4
Ttu_TRITD7Bv1G045840.1
Ttu_TRITD7Bv1G019860.1
Ttu_TRITD7Av1G279720.4
Ttu_TRITD7Av1G271320.3
Ttu_TRITD7Av1G198970.1
Ttu_TRITD7Av1G192070.4
Ttu_TRITD7Av1G157850.2
Ttu_TRITD7Av1G086020.3
Ttu_TRITD7Av1G070060.1
Ttu_TRITD7Av1G046190.4
Ttu_TRITD7Av1G026200.1
Ttu_TRITD7Av1G023800.1
Ttu_TRITD7Av1G010060.3
−1000
0
1000
2000
3000
4000

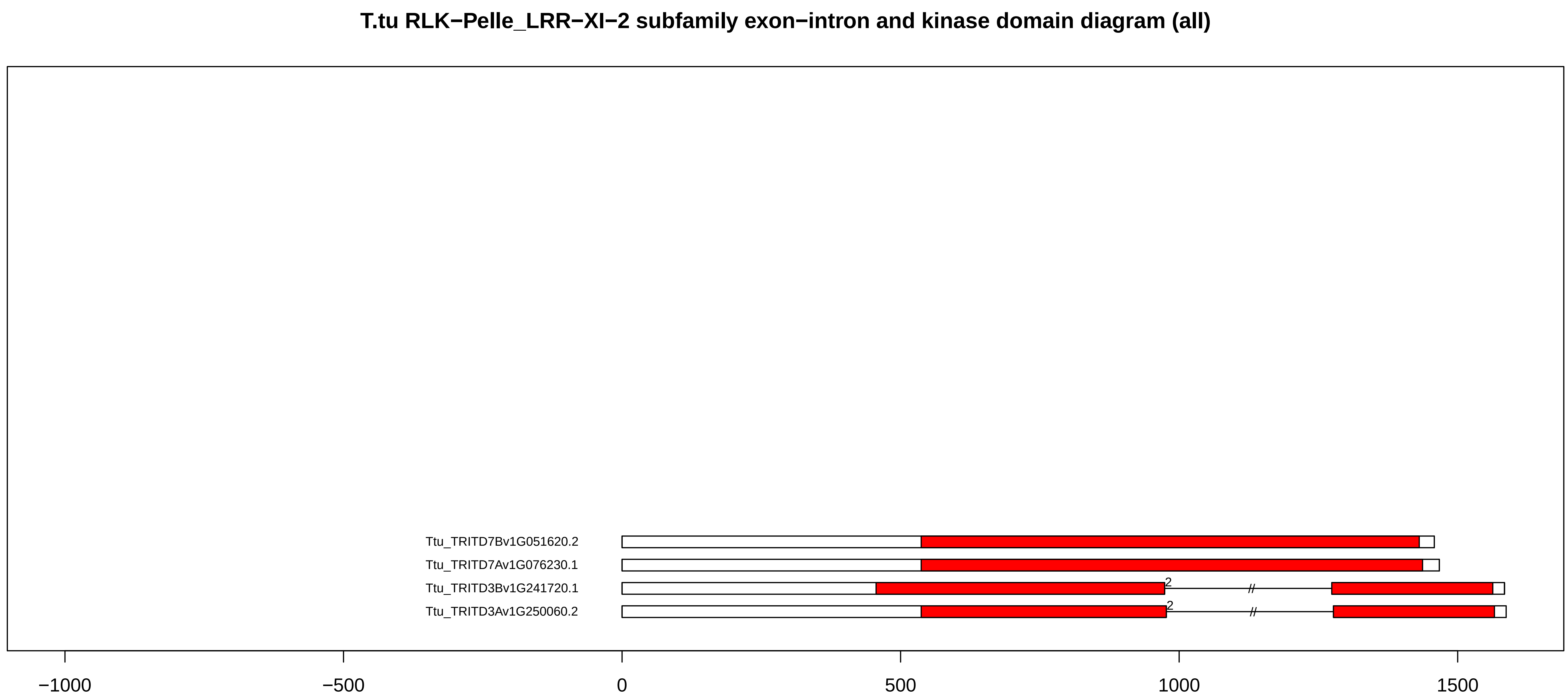
T.tu RLK−Pelle_LRR−XI−2 subfamily exon−intron and kinase domain diagram (all)
Ttu_TRITD7Bv1G051620.2
Ttu_TRITD7Av1G076230.1
Ttu_TRITD3Bv1G241720.1
Ttu_TRITD3Av1G250060.2
2
2
−1000
−500
0
500
1000
1500

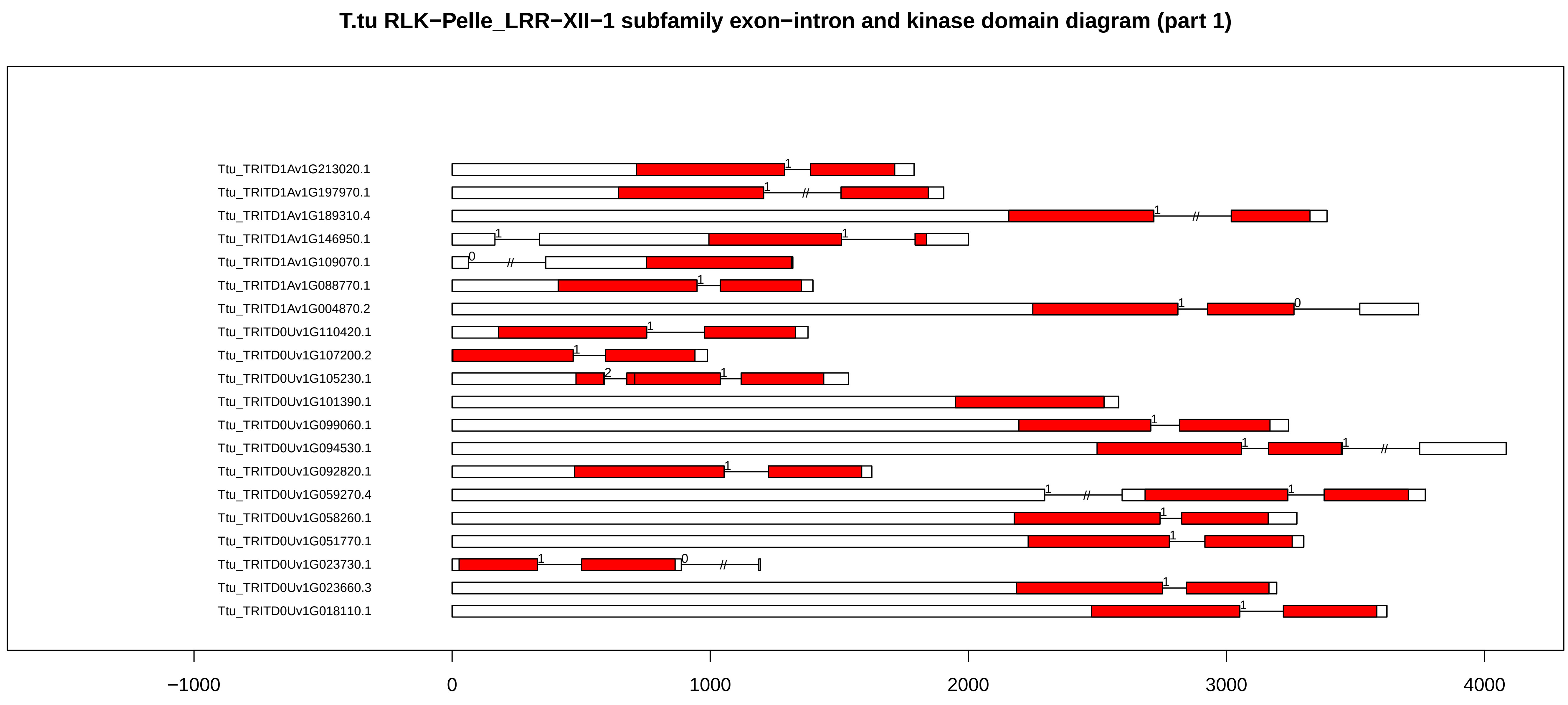
T.tu RLK−Pelle_LRR−XII−1 subfamily exon−intron and kinase domain diagram (part 1)
Ttu_TRITD1Av1G213020.1
Ttu_TRITD1Av1G197970.1
Ttu_TRITD1Av1G189310.4
Ttu_TRITD1Av1G146950.1
Ttu_TRITD1Av1G109070.1
Ttu_TRITD1Av1G088770.1
Ttu_TRITD1Av1G004870.2
Ttu_TRITD0Uv1G110420.1
Ttu_TRITD0Uv1G107200.2
Ttu_TRITD0Uv1G105230.1
Ttu_TRITD0Uv1G101390.1
Ttu_TRITD0Uv1G099060.1
Ttu_TRITD0Uv1G094530.1
Ttu_TRITD0Uv1G092820.1
Ttu_TRITD0Uv1G059270.4
Ttu_TRITD0Uv1G058260.1
Ttu_TRITD0Uv1G051770.1
Ttu_TRITD0Uv1G023730.1
Ttu_TRITD0Uv1G023660.3
Ttu_TRITD0Uv1G018110.1
−1000
0
1000
2000
3000
4000

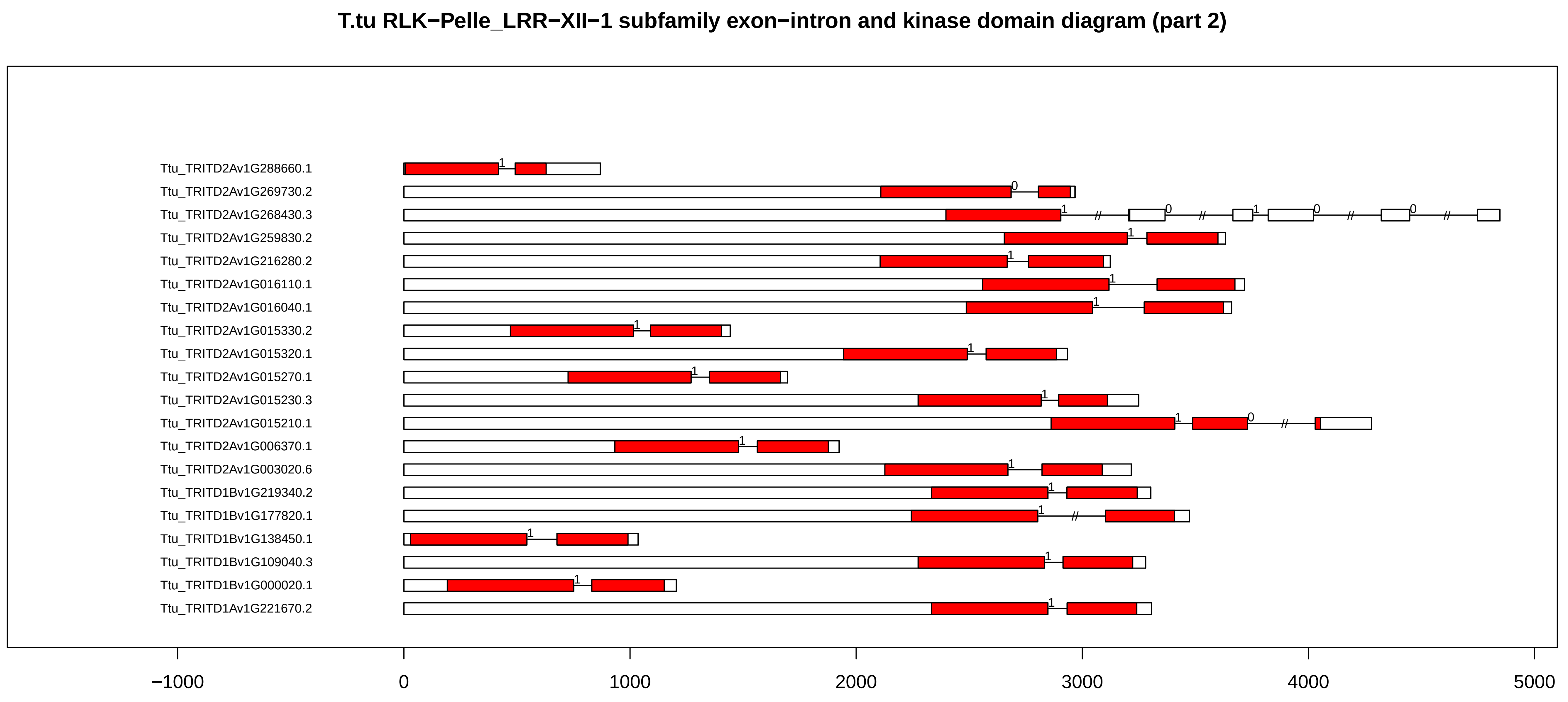
T.tu RLK−Pelle_LRR−XII−1 subfamily exon−intron and kinase domain diagram (part 2)
Ttu_TRITD2Av1G288660.1
Ttu_TRITD2Av1G269730.2
Ttu_TRITD2Av1G268430.3
Ttu_TRITD2Av1G259830.2
Ttu_TRITD2Av1G216280.2
Ttu_TRITD2Av1G016110.1
Ttu_TRITD2Av1G016040.1
Ttu_TRITD2Av1G015330.2
Ttu_TRITD2Av1G015320.1
Ttu_TRITD2Av1G015270.1
Ttu_TRITD2Av1G015230.3
Ttu_TRITD2Av1G015210.1
Ttu_TRITD2Av1G006370.1
Ttu_TRITD2Av1G003020.6
Ttu_TRITD1Bv1G219340.2
Ttu_TRITD1Bv1G177820.1
Ttu_TRITD1Bv1G138450.1
Ttu_TRITD1Bv1G109040.3
Ttu_TRITD1Bv1G000020.1
Ttu_TRITD1Av1G221670.2
−1000
0
1000
2000
3000
4000
5000

T.tu RLK−Pelle_LRR−XII−1 subfamily exon−intron and kinase domain diagram (part 3)

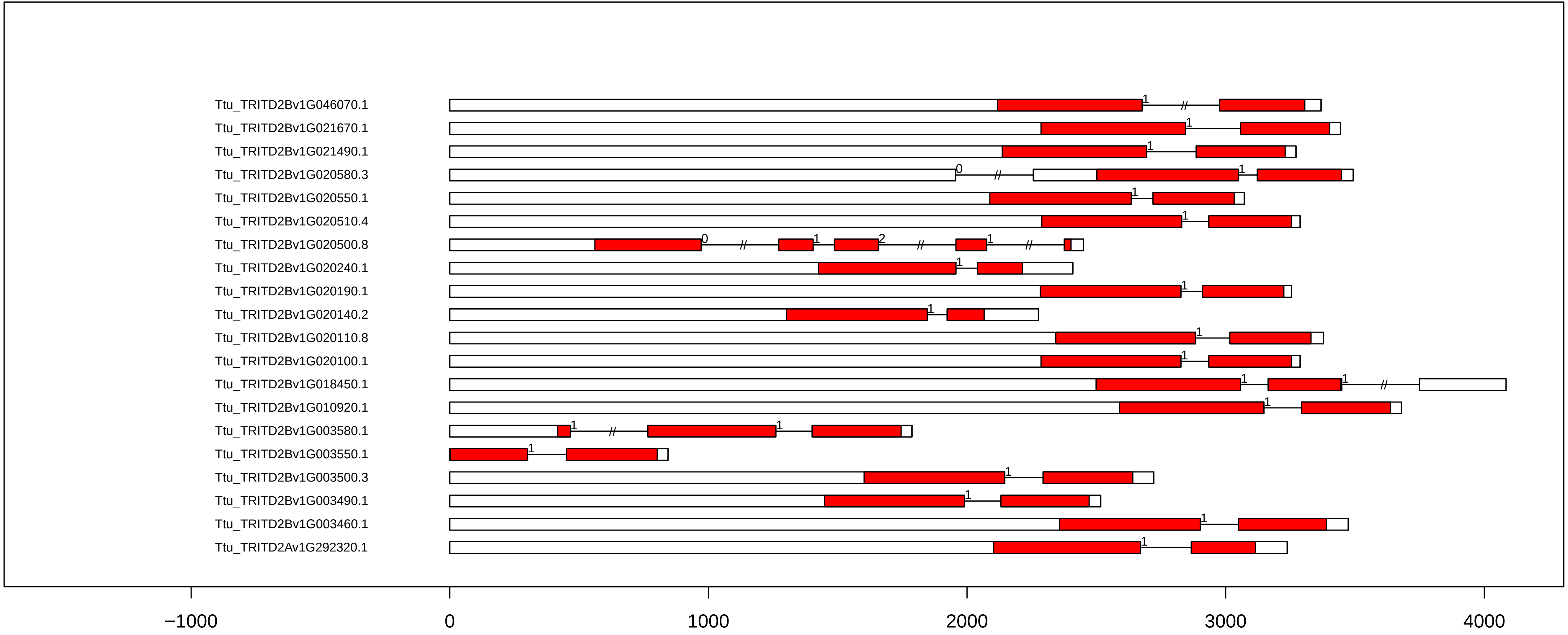

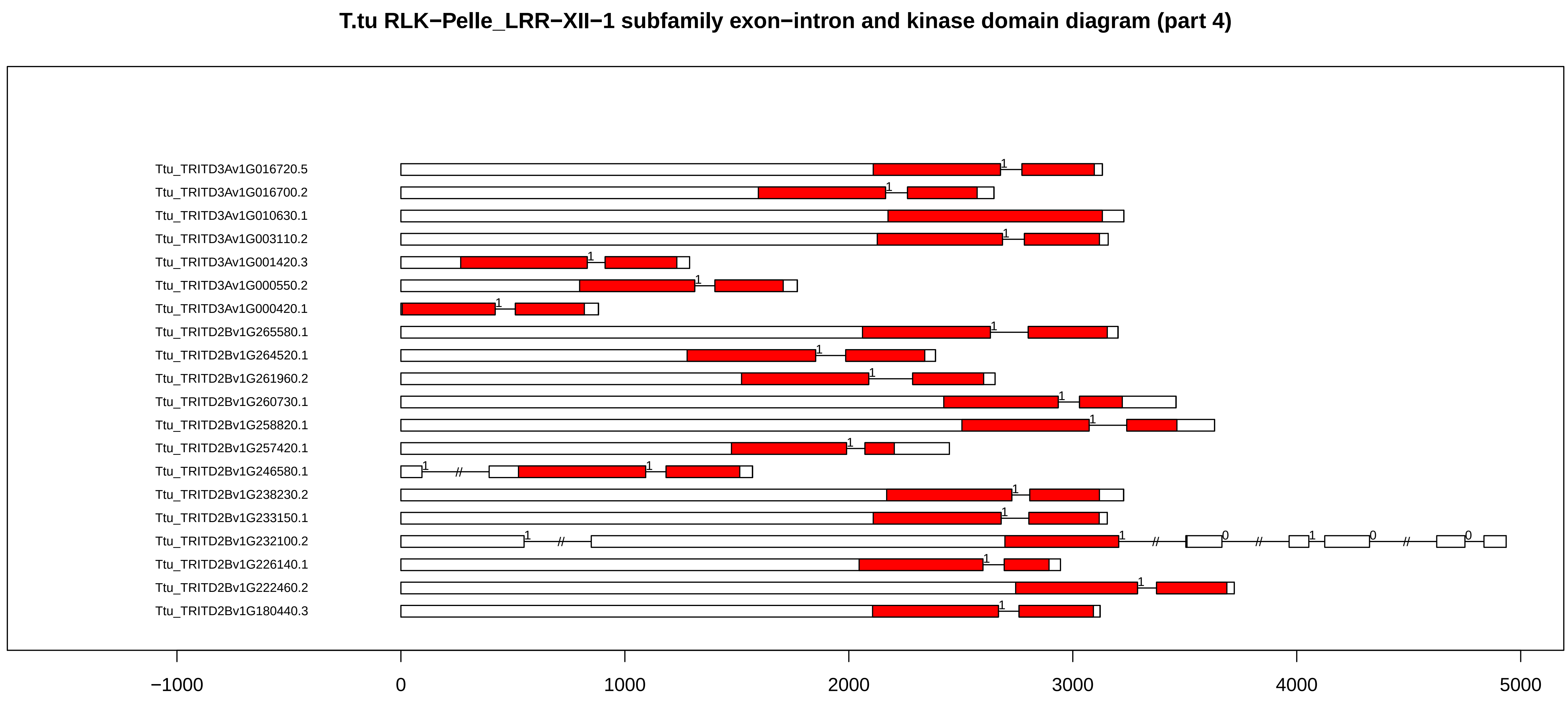
T.tu RLK−Pelle_LRR−XII−1 subfamily exon−intron and kinase domain diagram (part 4)
Ttu_TRITD3Av1G016720.5
Ttu_TRITD3Av1G016700.2
Ttu_TRITD3Av1G010630.1
Ttu_TRITD3Av1G003110.2
Ttu_TRITD3Av1G001420.3
Ttu_TRITD3Av1G000550.2
Ttu_TRITD3Av1G000420.1
Ttu_TRITD2Bv1G265580.1
Ttu_TRITD2Bv1G264520.1
Ttu_TRITD2Bv1G261960.2
Ttu_TRITD2Bv1G260730.1
Ttu_TRITD2Bv1G258820.1
Ttu_TRITD2Bv1G257420.1
Ttu_TRITD2Bv1G246580.1
Ttu_TRITD2Bv1G238230.2
Ttu_TRITD2Bv1G233150.1
Ttu_TRITD2Bv1G232100.2
Ttu_TRITD2Bv1G226140.1
Ttu_TRITD2Bv1G222460.2
Ttu_TRITD2Bv1G180440.3
−1000
0
1000
2000
3000
4000
5000

T.tu RLK−Pelle_LRR−XII−1 subfamily exon−intron and kinase domain diagram (part 5)

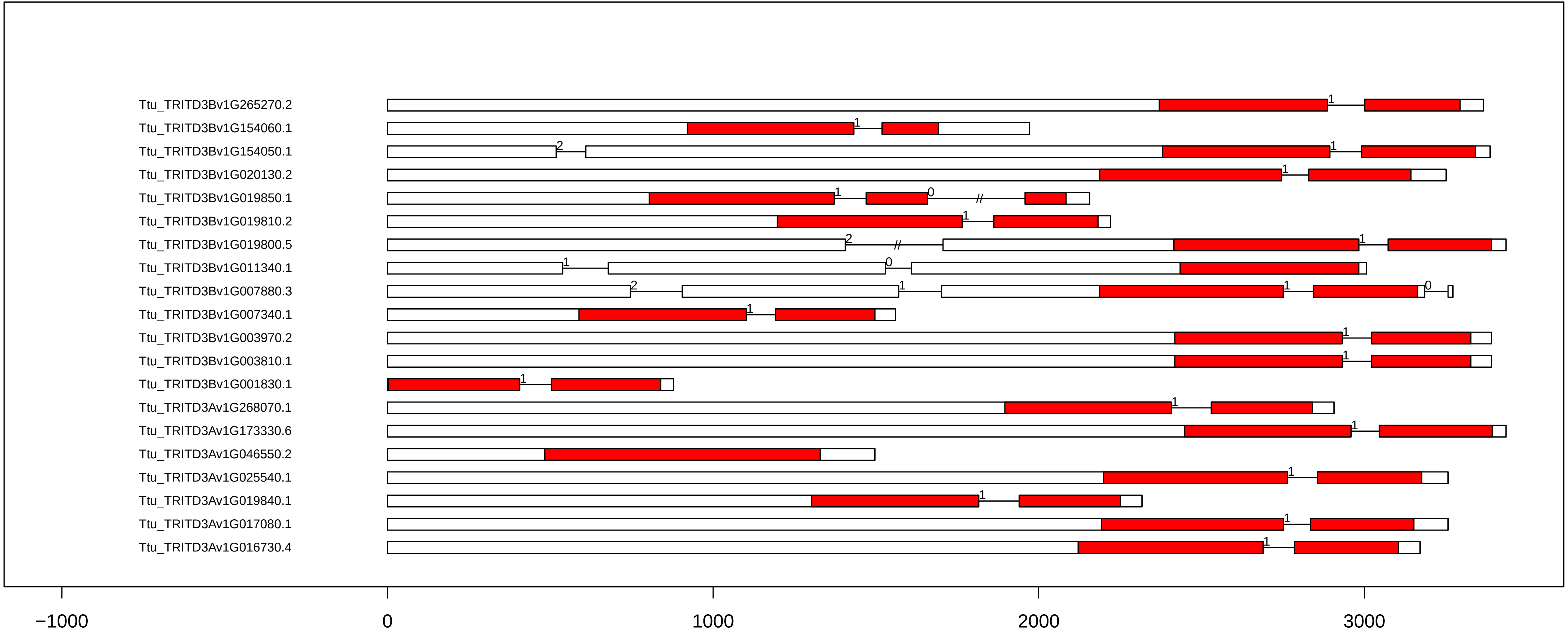

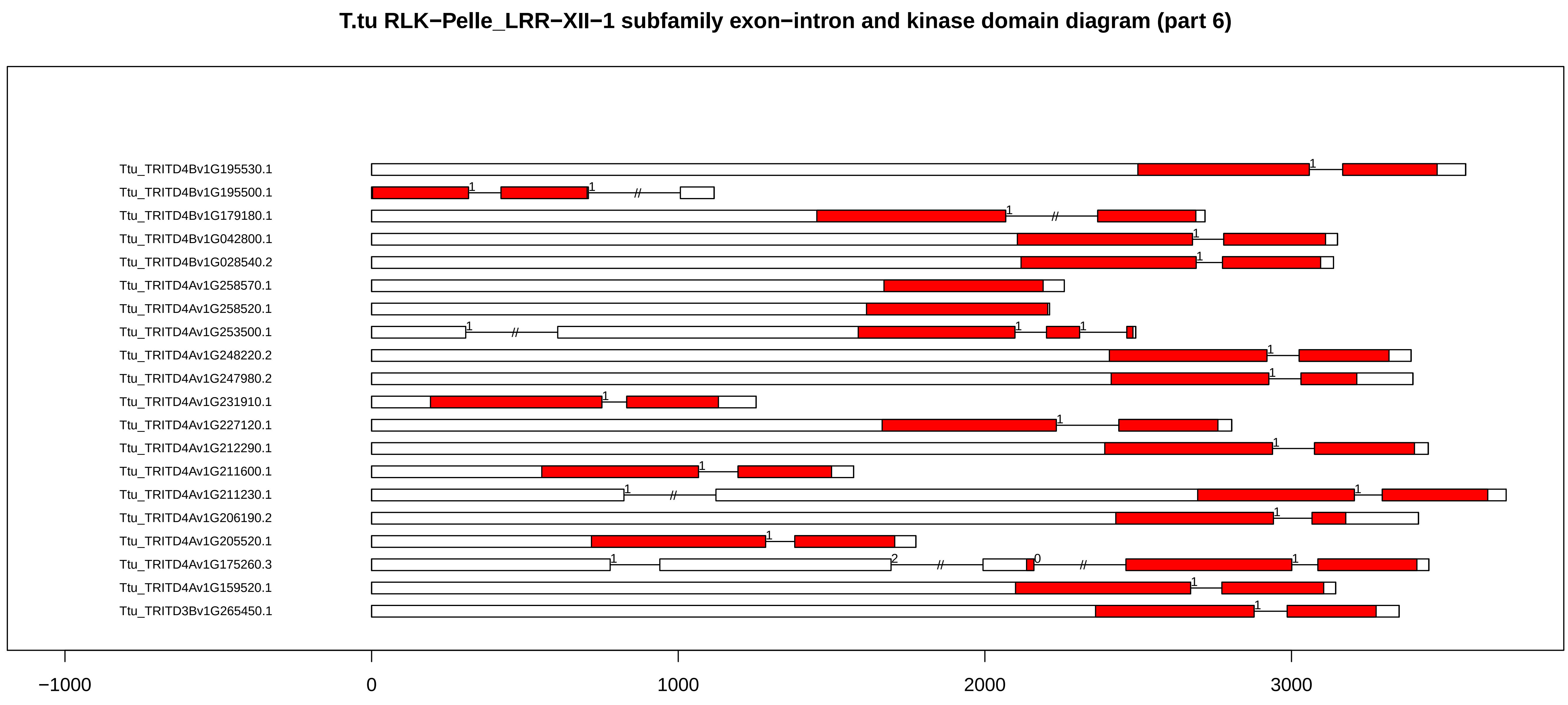

T.tu RLK−Pelle_LRR−XII−1 subfamily exon−intron and kinase domain diagram (part 6)
Ttu_TRITD4Bv1G195530.1
Ttu_TRITD4Bv1G195500.1
Ttu_TRITD4Bv1G179180.1
Ttu_TRITD4Bv1G042800.1
Ttu_TRITD4Bv1G028540.2
Ttu_TRITD4Av1G258570.1
Ttu_TRITD4Av1G258520.1
Ttu_TRITD4Av1G253500.1
Ttu_TRITD4Av1G248220.2
Ttu_TRITD4Av1G247980.2
Ttu_TRITD4Av1G231910.1
Ttu_TRITD4Av1G227120.1
Ttu_TRITD4Av1G212290.1
Ttu_TRITD4Av1G211600.1
Ttu_TRITD4Av1G211230.1
Ttu_TRITD4Av1G206190.2
Ttu_TRITD4Av1G205520.1
Ttu_TRITD4Av1G175260.3
Ttu_TRITD4Av1G159520.1
Ttu_TRITD3Bv1G265450.1
−1000
0
1000
2000
3000

T.tu RLK−Pelle_LRR−XII−1 subfamily exon−intron and kinase domain diagram (part 7)

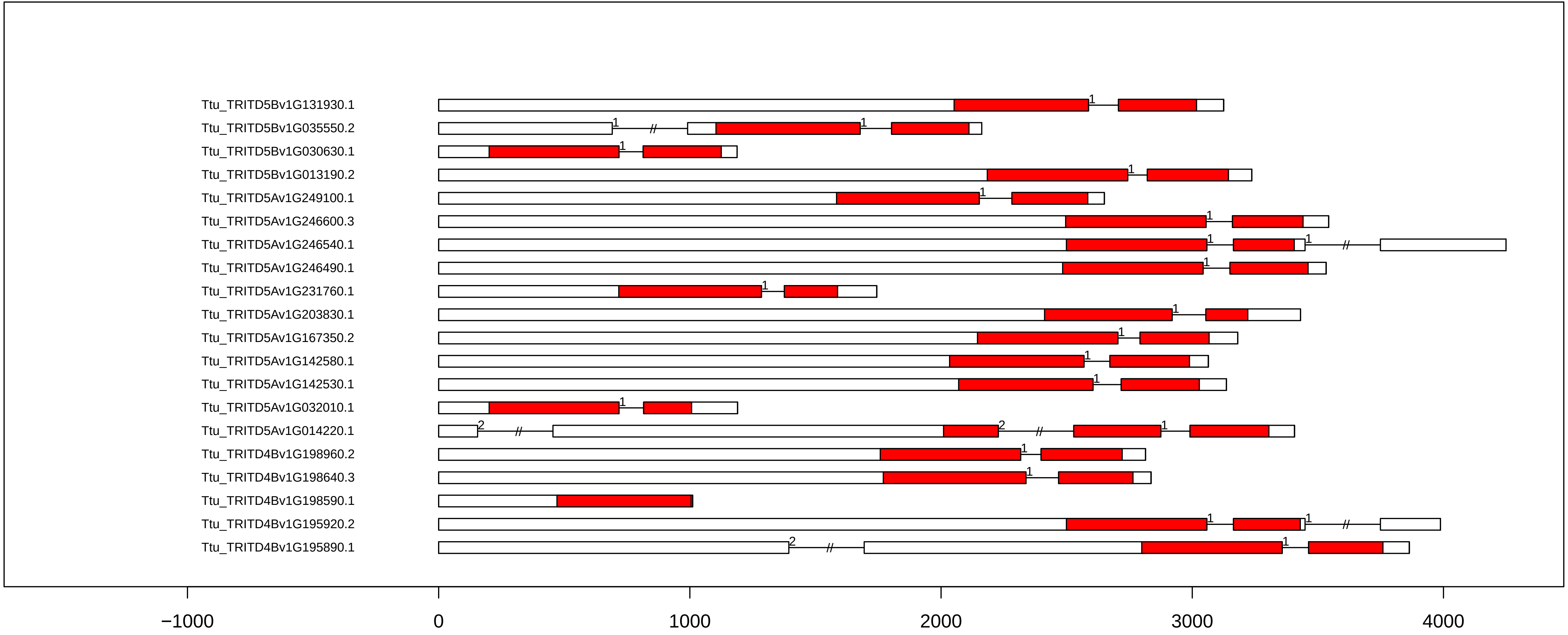

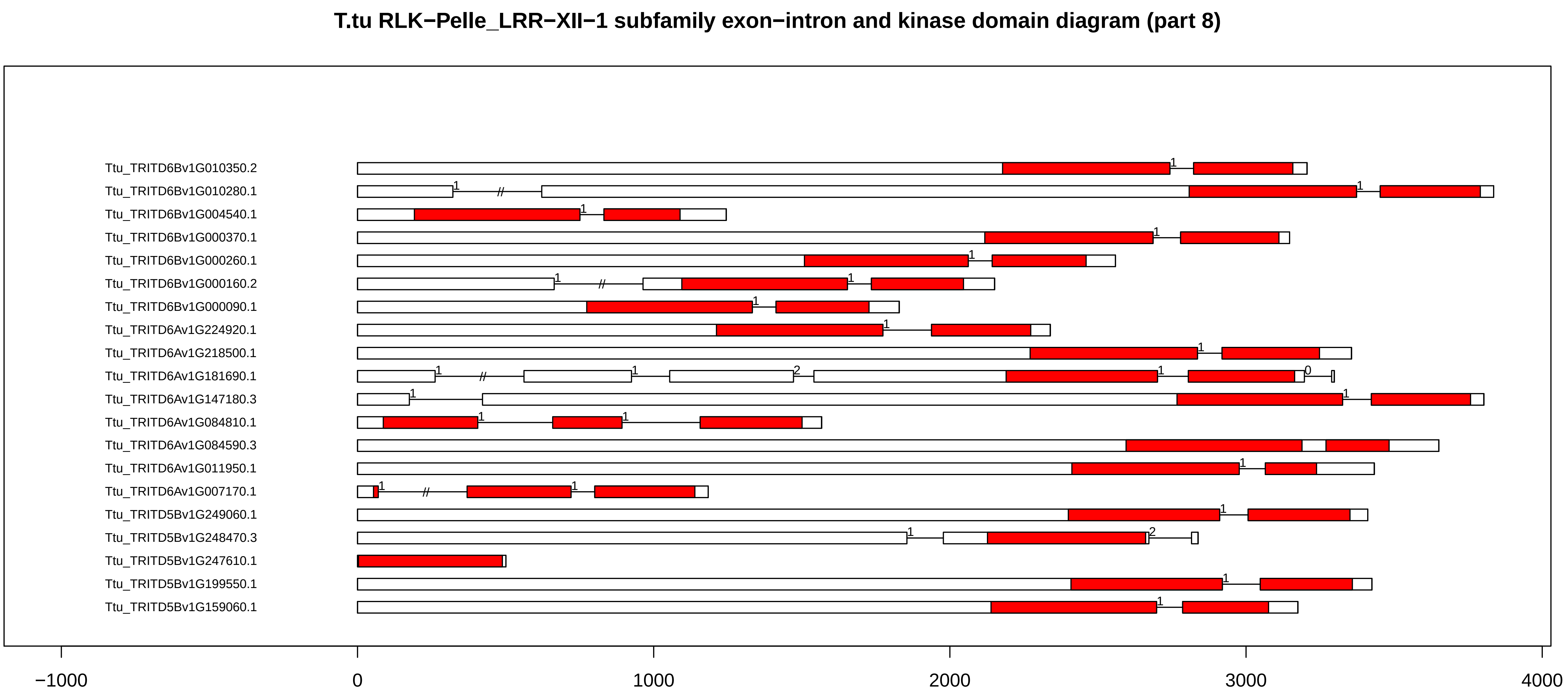

T.tu RLK−Pelle_LRR−XII−1 subfamily exon−intron and kinase domain diagram (part 8)
Ttu_TRITD6Bv1G010350.2
Ttu_TRITD6Bv1G010280.1
Ttu_TRITD6Bv1G004540.1
Ttu_TRITD6Bv1G000370.1
Ttu_TRITD6Bv1G000260.1
Ttu_TRITD6Bv1G000160.2
Ttu_TRITD6Bv1G000090.1
Ttu_TRITD6Av1G224920.1
Ttu_TRITD6Av1G218500.1
Ttu_TRITD6Av1G181690.1
Ttu_TRITD6Av1G147180.3
Ttu_TRITD6Av1G084810.1
Ttu_TRITD6Av1G084590.3
Ttu_TRITD6Av1G011950.1
Ttu_TRITD6Av1G007170.1
Ttu_TRITD5Bv1G249060.1
Ttu_TRITD5Bv1G248470.3
Ttu_TRITD5Bv1G247610.1
Ttu_TRITD5Bv1G199550.1
Ttu_TRITD5Bv1G159060.1
−1000
0
1000
2000
3000
4000

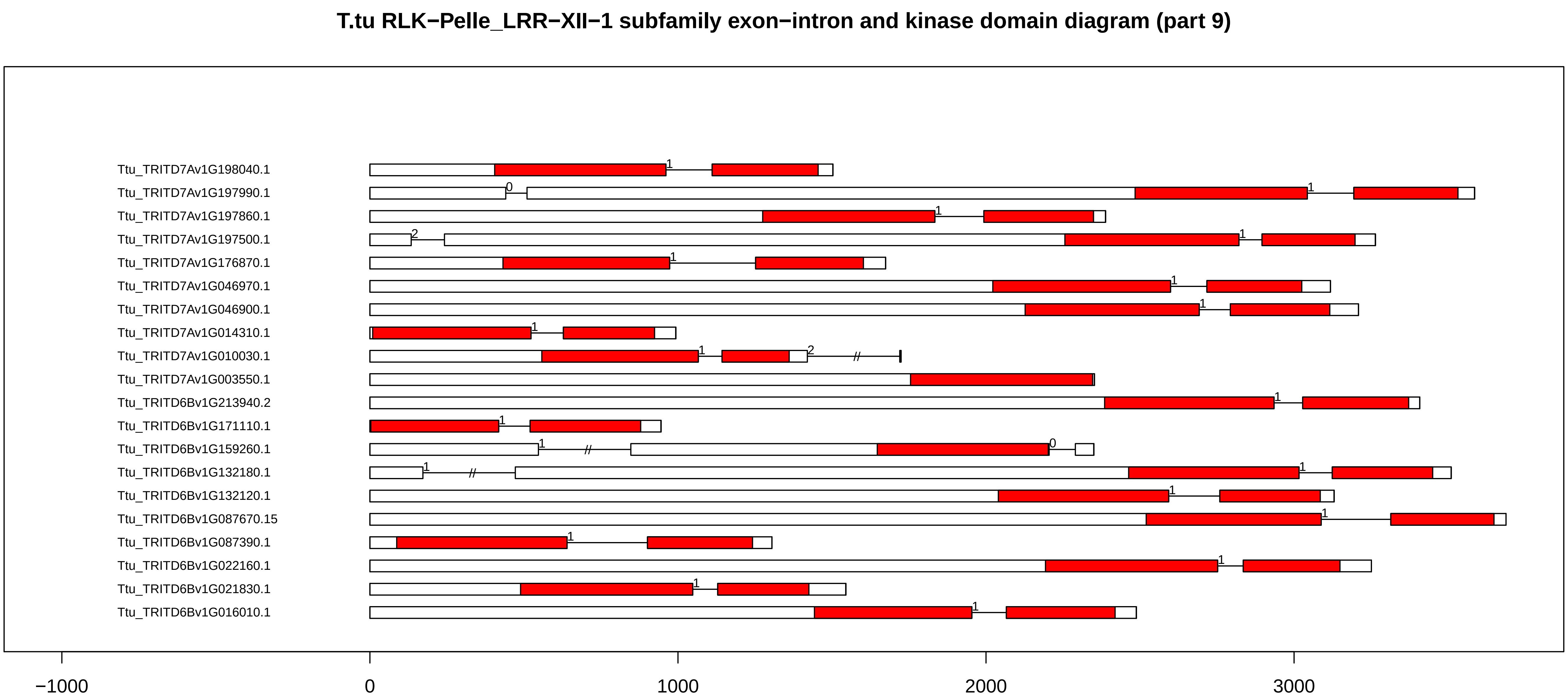

T.tu RLK−Pelle_LRR−XII−1 subfamily exon−intron and kinase domain diagram (part 9)
Ttu_TRITD7Av1G198040.1
Ttu_TRITD7Av1G197990.1
Ttu_TRITD7Av1G197860.1
Ttu_TRITD7Av1G197500.1
Ttu_TRITD7Av1G176870.1
Ttu_TRITD7Av1G046970.1
Ttu_TRITD7Av1G046900.1
Ttu_TRITD7Av1G014310.1
Ttu_TRITD7Av1G010030.1
Ttu_TRITD7Av1G003550.1
Ttu_TRITD6Bv1G213940.2
Ttu_TRITD6Bv1G171110.1
Ttu_TRITD6Bv1G159260.1
Ttu_TRITD6Bv1G132180.1
Ttu_TRITD6Bv1G132120.1
Ttu_TRITD6Bv1G087670.15
Ttu_TRITD6Bv1G087390.1
Ttu_TRITD6Bv1G022160.1
Ttu_TRITD6Bv1G021830.1
Ttu_TRITD6Bv1G016010.1
−1000
0
1000
2000
3000

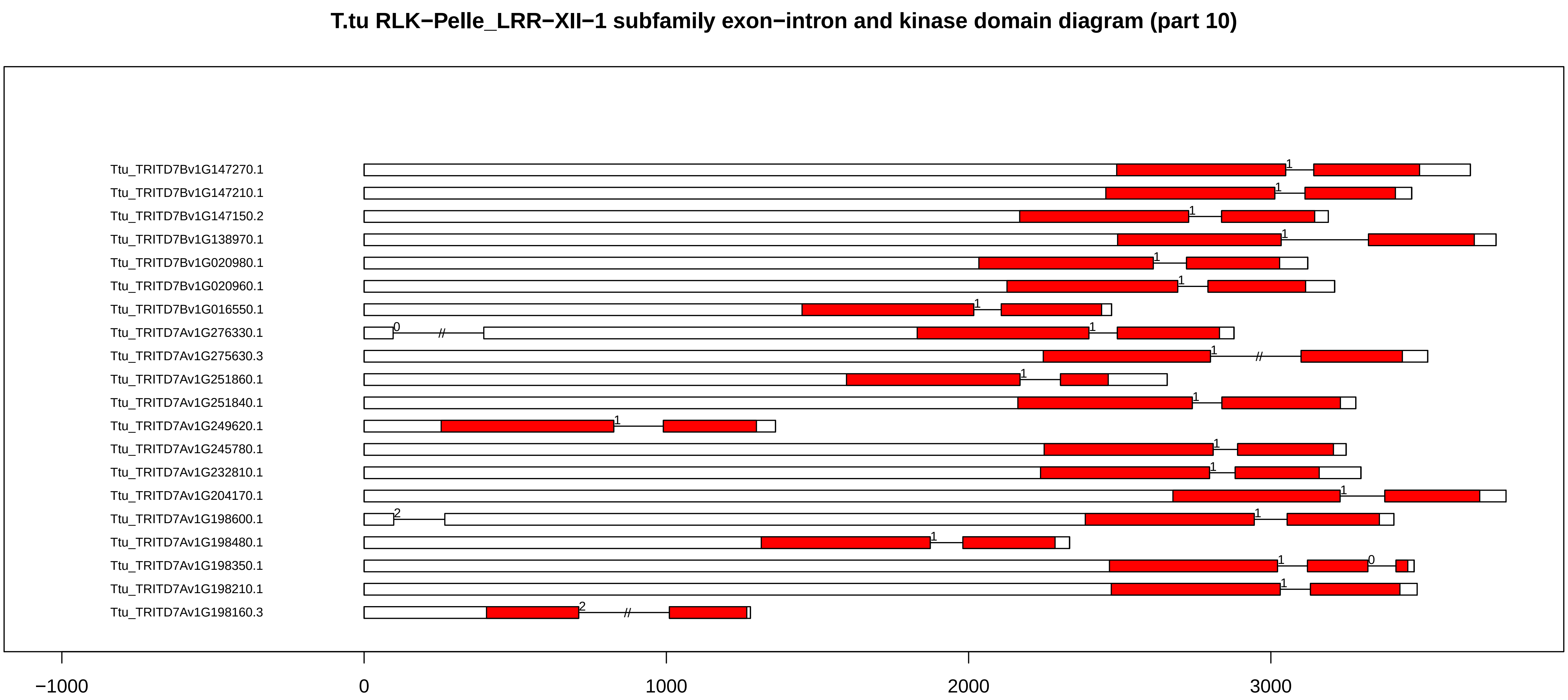

T.tu RLK−Pelle_LRR−XII−1 subfamily exon−intron and kinase domain diagram (part 10)
Ttu_TRITD7Bv1G147270.1
Ttu_TRITD7Bv1G147210.1
Ttu_TRITD7Bv1G147150.2
Ttu_TRITD7Bv1G138970.1
Ttu_TRITD7Bv1G020980.1
Ttu_TRITD7Bv1G020960.1
Ttu_TRITD7Bv1G016550.1
Ttu_TRITD7Av1G276330.1
Ttu_TRITD7Av1G275630.3
Ttu_TRITD7Av1G251860.1
Ttu_TRITD7Av1G251840.1
Ttu_TRITD7Av1G249620.1
Ttu_TRITD7Av1G245780.1
Ttu_TRITD7Av1G232810.1
Ttu_TRITD7Av1G204170.1
Ttu_TRITD7Av1G198600.1
Ttu_TRITD7Av1G198480.1
Ttu_TRITD7Av1G198350.1
Ttu_TRITD7Av1G198210.1
Ttu_TRITD7Av1G198160.3
−1000
0
1000
2000
3000

T.tu RLK−Pelle_LRR−XII−1 subfamily exon−intron and kinase domain diagram (part 11)

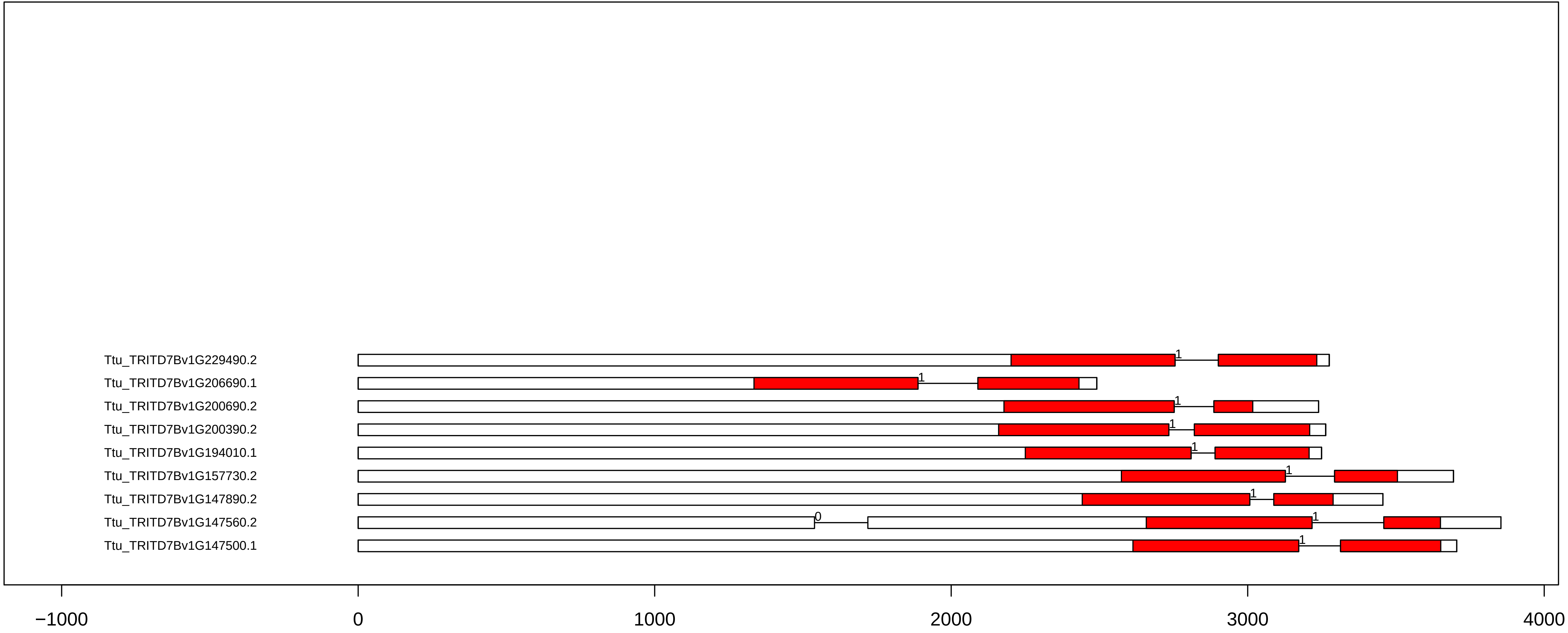

T.tu RLK−Pelle_LRR−XIIIa subfamily exon−intron and kinase domain diagram (all)

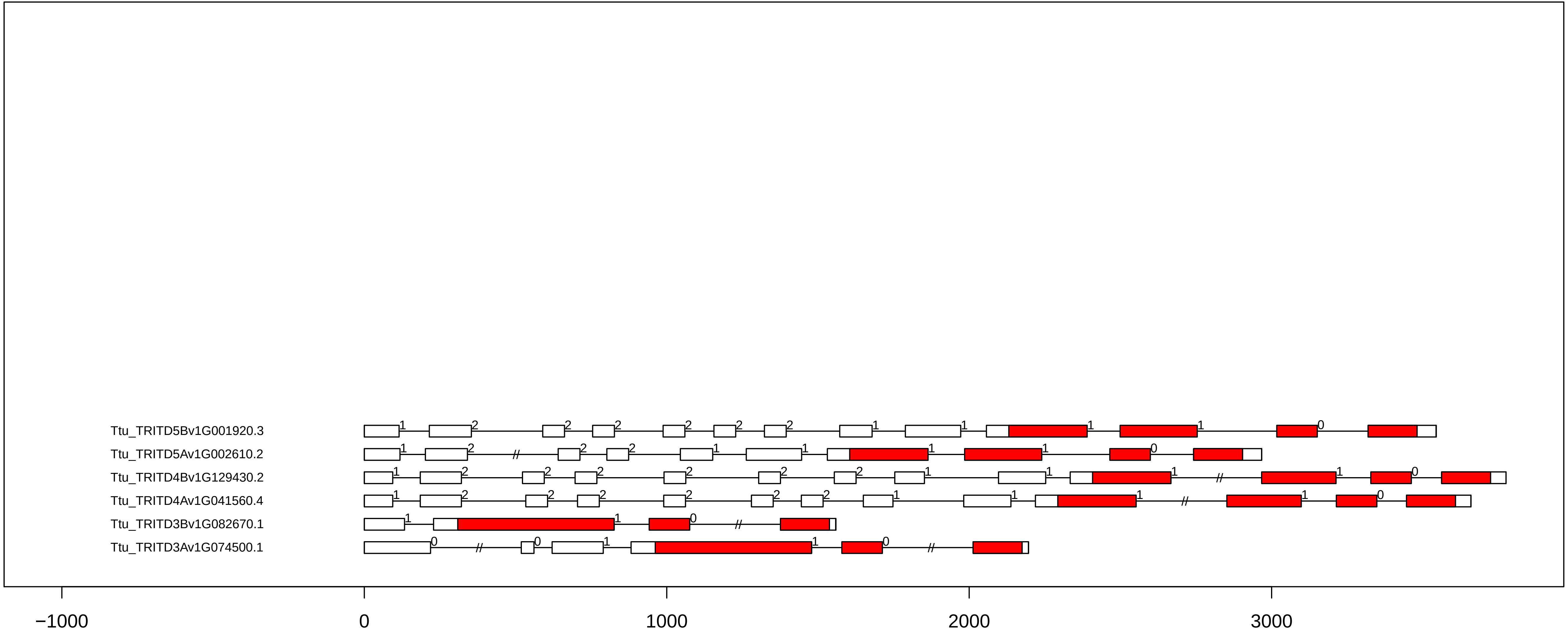

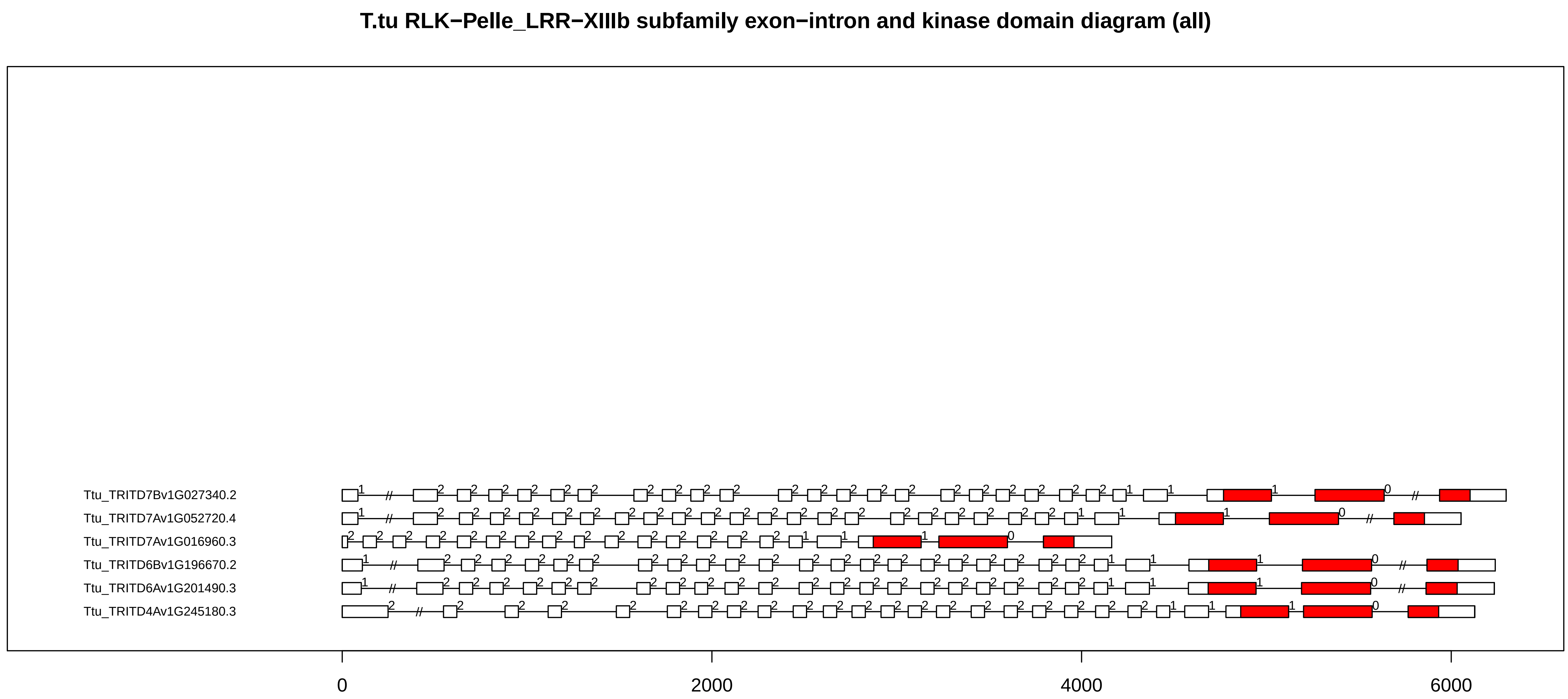
T.tu RLK−Pelle_LRR−XIIIb subfamily exon−intron and kinase domain diagram (all)
Ttu_TRITD7Bv1G027340.2
Ttu_TRITD7Av1G052720.4
Ttu_TRITD7Av1G016960.3
Ttu_TRITD6Bv1G196670.2
Ttu_TRITD6Av1G201490.3
Ttu_TRITD4Av1G245180.3
0
2000
4000
6000

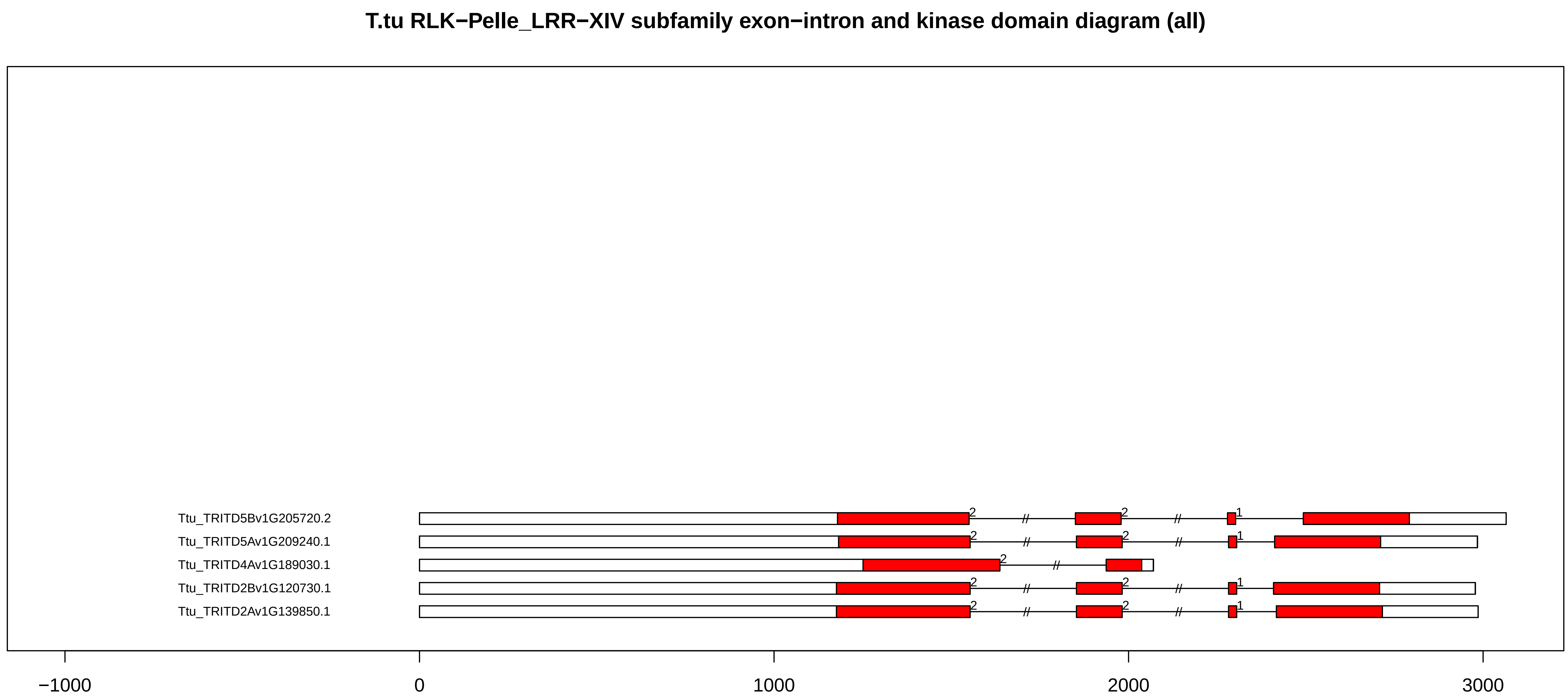
T.tu RLK−Pelle_LRR−XIV subfamily exon−intron and kinase domain diagram (all)
Ttu_TRITD5Bv1G205720.2
Ttu_TRITD5Av1G209240.1
Ttu_TRITD4Av1G189030.1
Ttu_TRITD2Bv1G120730.1
Ttu_TRITD2Av1G139850.1
−1000
0
1000
2000
3000

T.tu RLK−Pelle_LRR−XV subfamily exon−intron and kinase domain diagram (all)

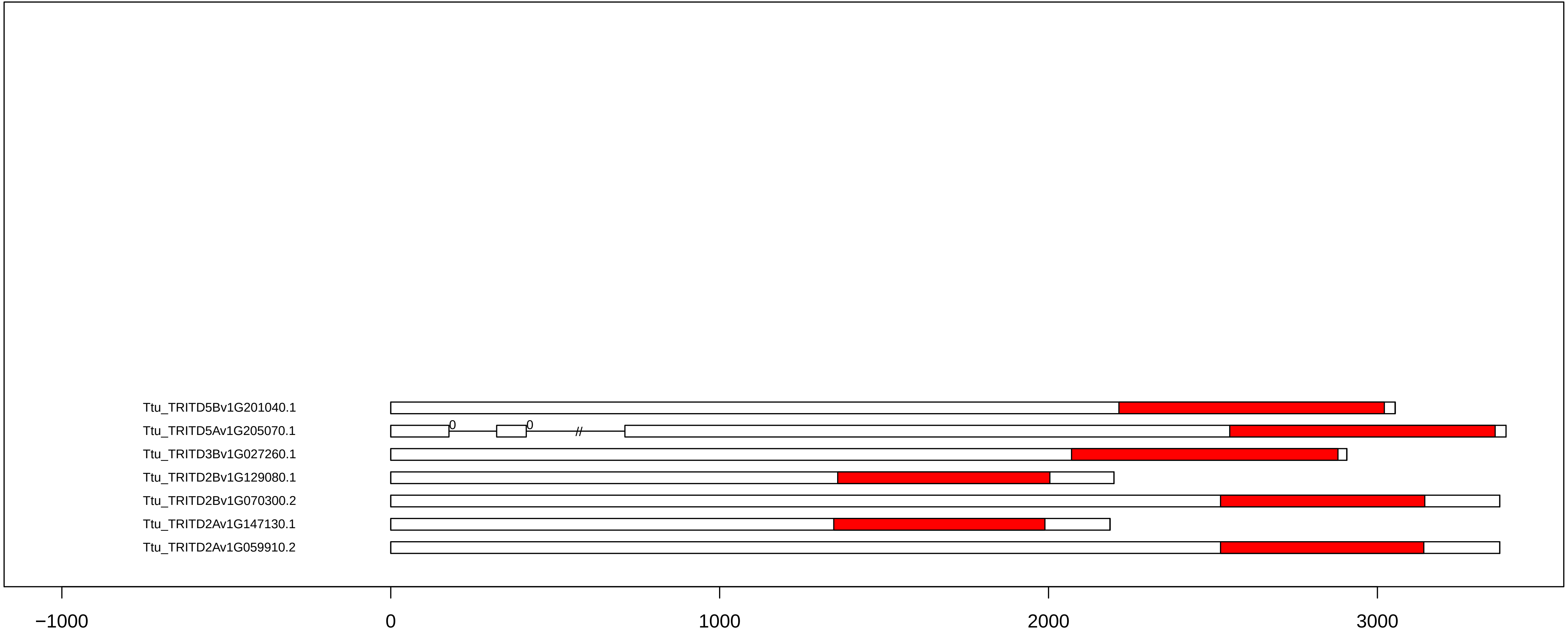

T.tu RLK−Pelle_LysM subfamily exon−intron and kinase domain diagram (all)

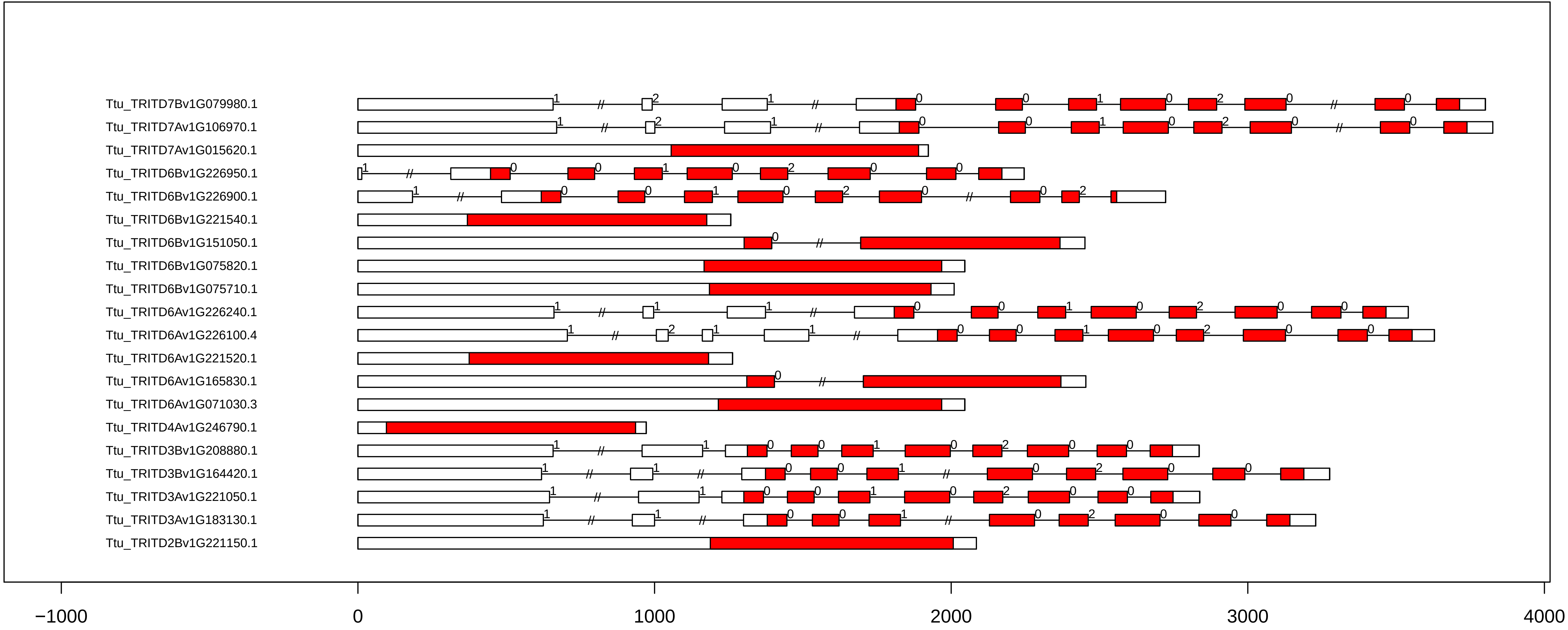

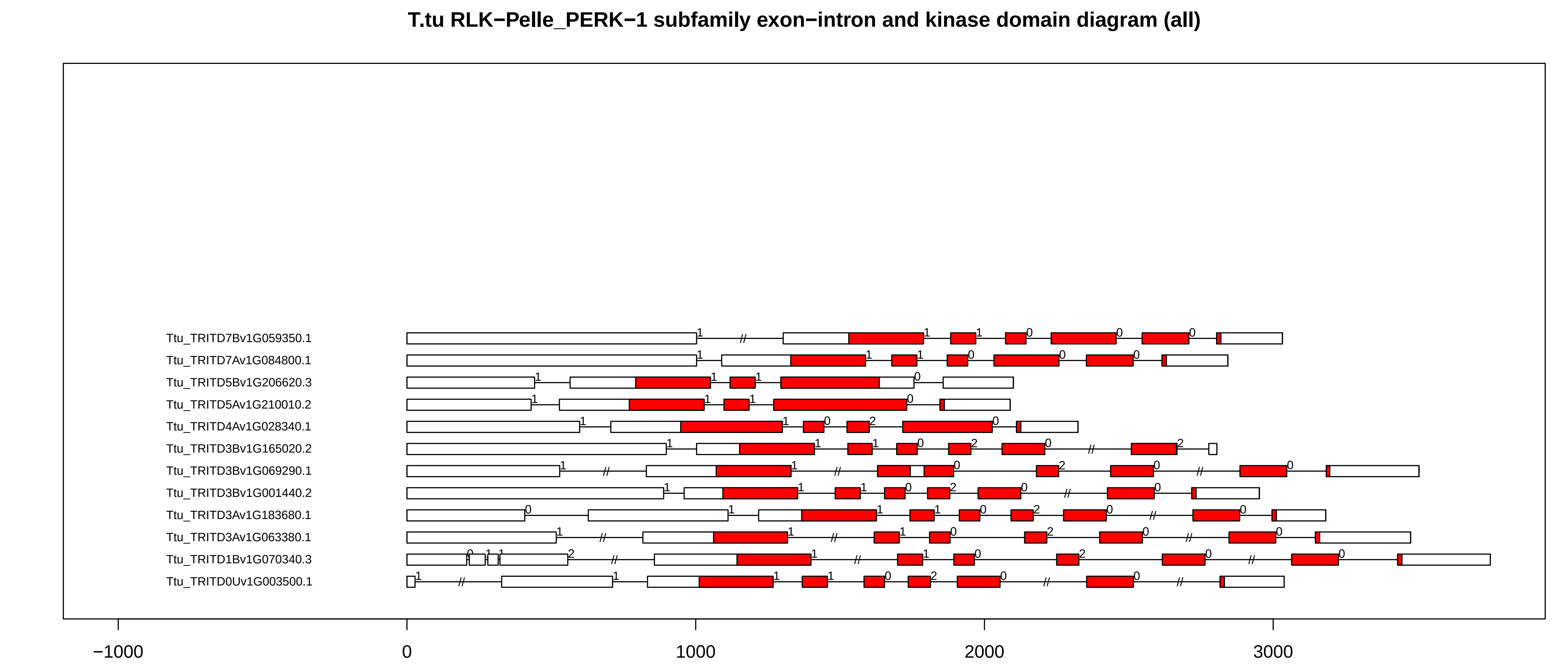

T.tu RLK−Pelle_PERK−1 subfamily exon−intron and kinase domain diagram (all)
Ttu_TRITD7Bv1G059350.1
Ttu_TRITD7Av1G084800.1
Ttu_TRITD5Bv1G206620.3
Ttu_TRITD5Av1G210010.2
Ttu_TRITD4Av1G028340.1
Ttu_TRITD3Bv1G165020.2
Ttu_TRITD3Bv1G069290.1
Ttu_TRITD3Bv1G001440.2
Ttu_TRITD3Av1G183680.1
Ttu_TRITD3Av1G063380.1
Ttu_TRITD1Bv1G070340.3
Ttu_TRITD0Uv1G003500.1
−1000
0
1000
2000
3000

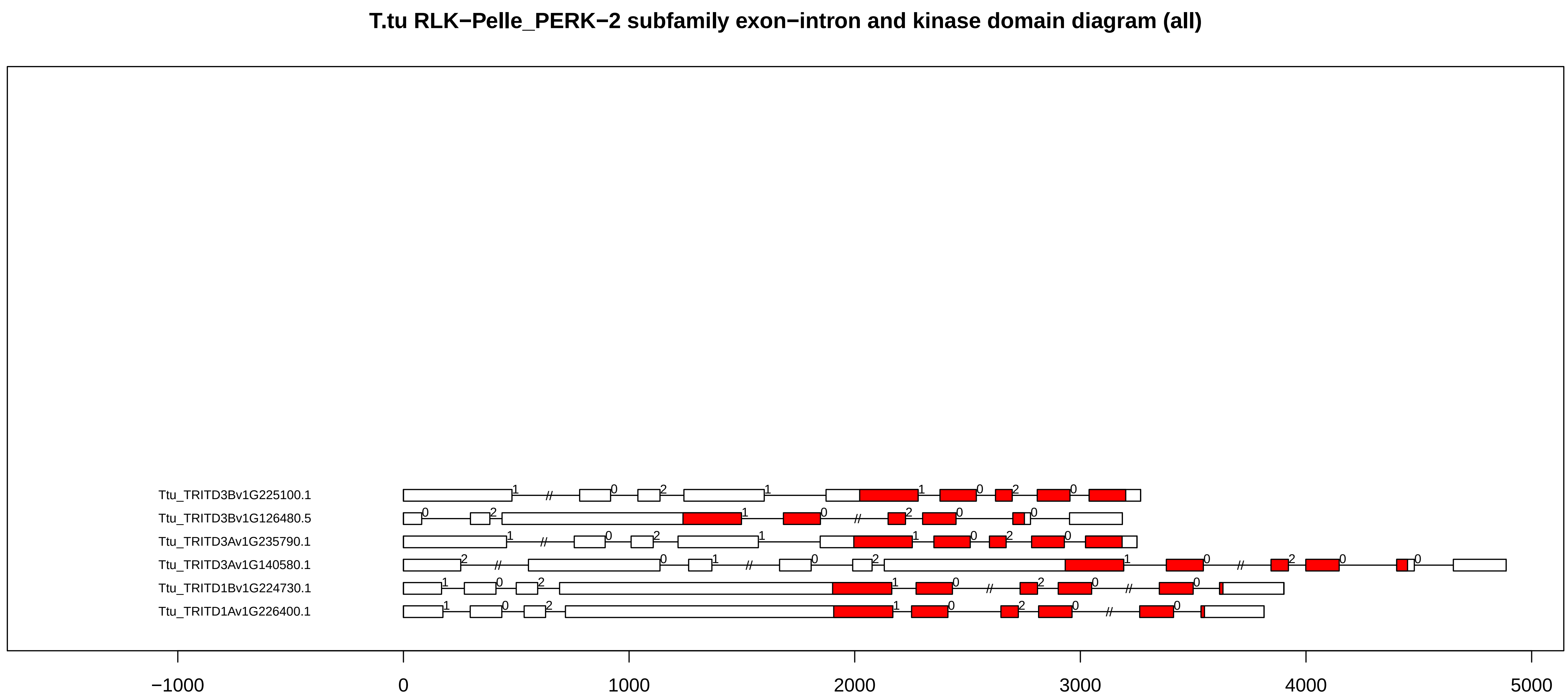
T.tu RLK−Pelle_PERK−2 subfamily exon−intron and kinase domain diagram (all)
Ttu_TRITD3Bv1G225100.1
Ttu_TRITD3Bv1G126480.5
Ttu_TRITD3Av1G235790.1
Ttu_TRITD3Av1G140580.1
Ttu_TRITD1Bv1G224730.1
Ttu_TRITD1Av1G226400.1
−1000
0
1000
2000
3000
4000
5000

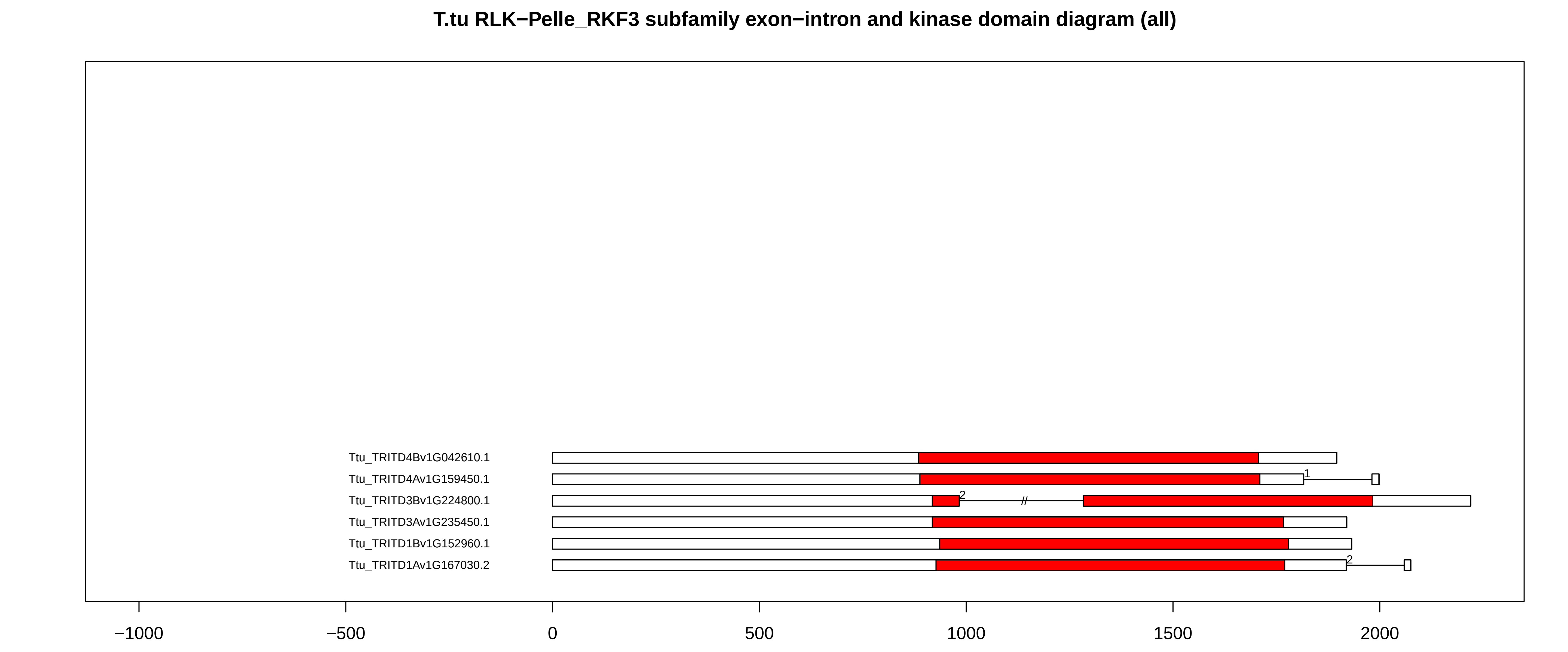
T.tu RLK−Pelle_RKF3 subfamily exon−intron and kinase domain diagram (all)
Ttu_TRITD4Bv1G042610.1
Ttu_TRITD4Av1G159450.1
Ttu_TRITD3Bv1G224800.1
Ttu_TRITD3Av1G235450.1
Ttu_TRITD1Bv1G152960.1
Ttu_TRITD1Av1G167030.2
1
2
2
−1000
−500
0
500
1000
1500
2000

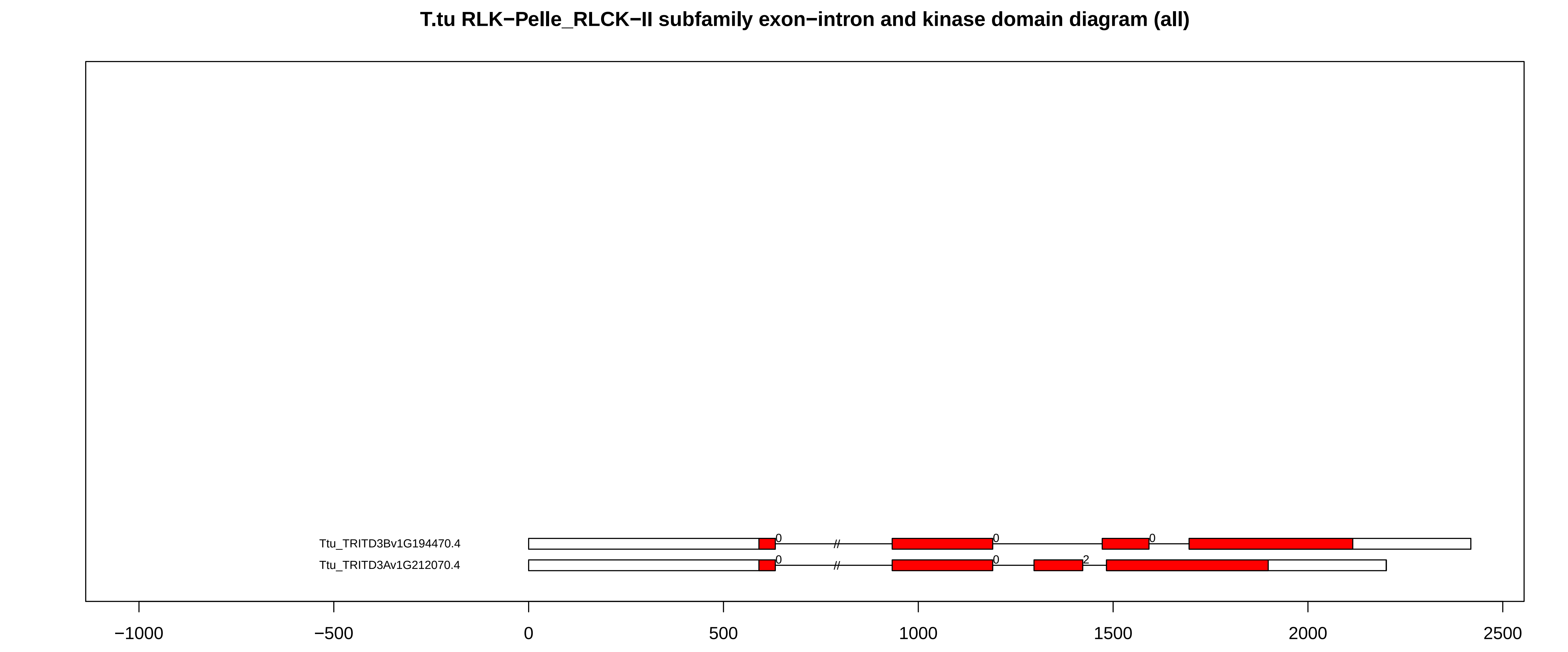
T.tu RLK−Pelle_RLCK−II subfamily exon−intron and kinase domain diagram (all)
Ttu_TRITD3Bv1G194470.4
Ttu_TRITD3Av1G212070.4
0
0
0
0
0
2
−1000
−500
0
500
1000
1500
2000
2500

T.tu RLK−Pelle_RLCK−IV subfamily exon−intron and kinase domain diagram (all)

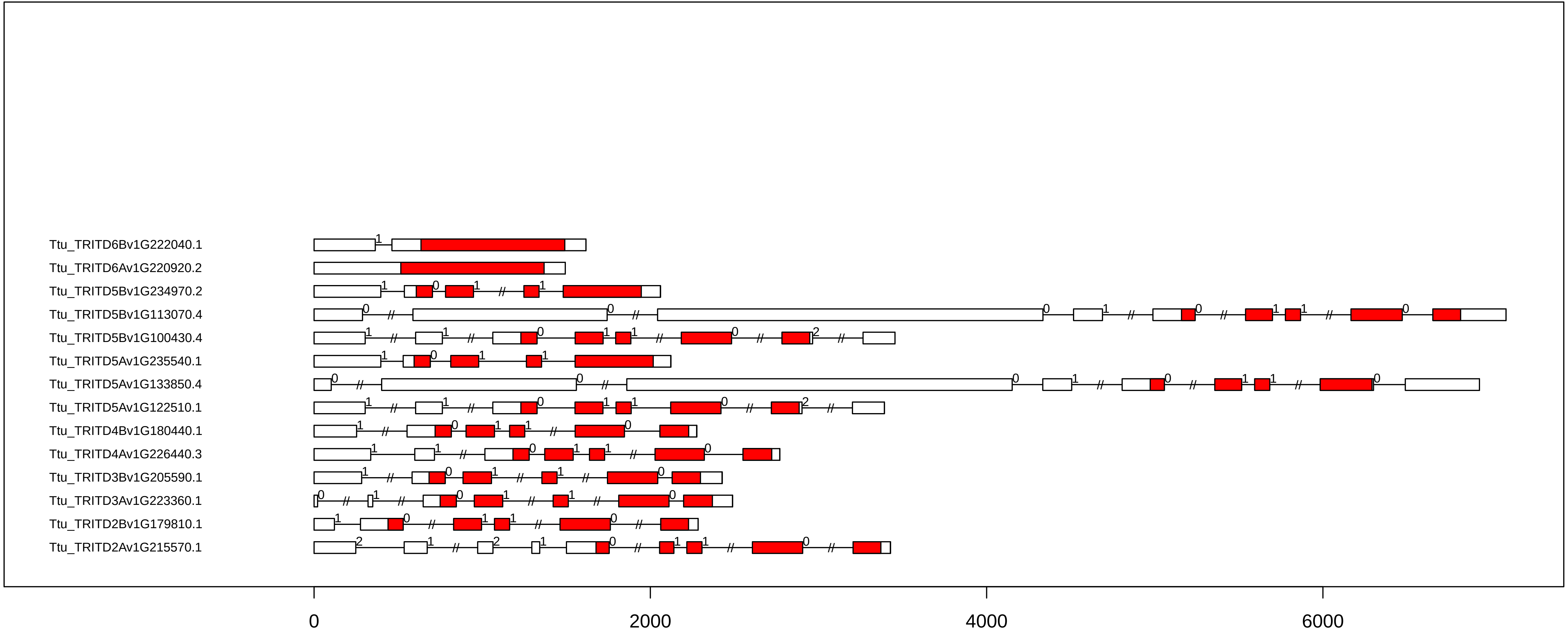

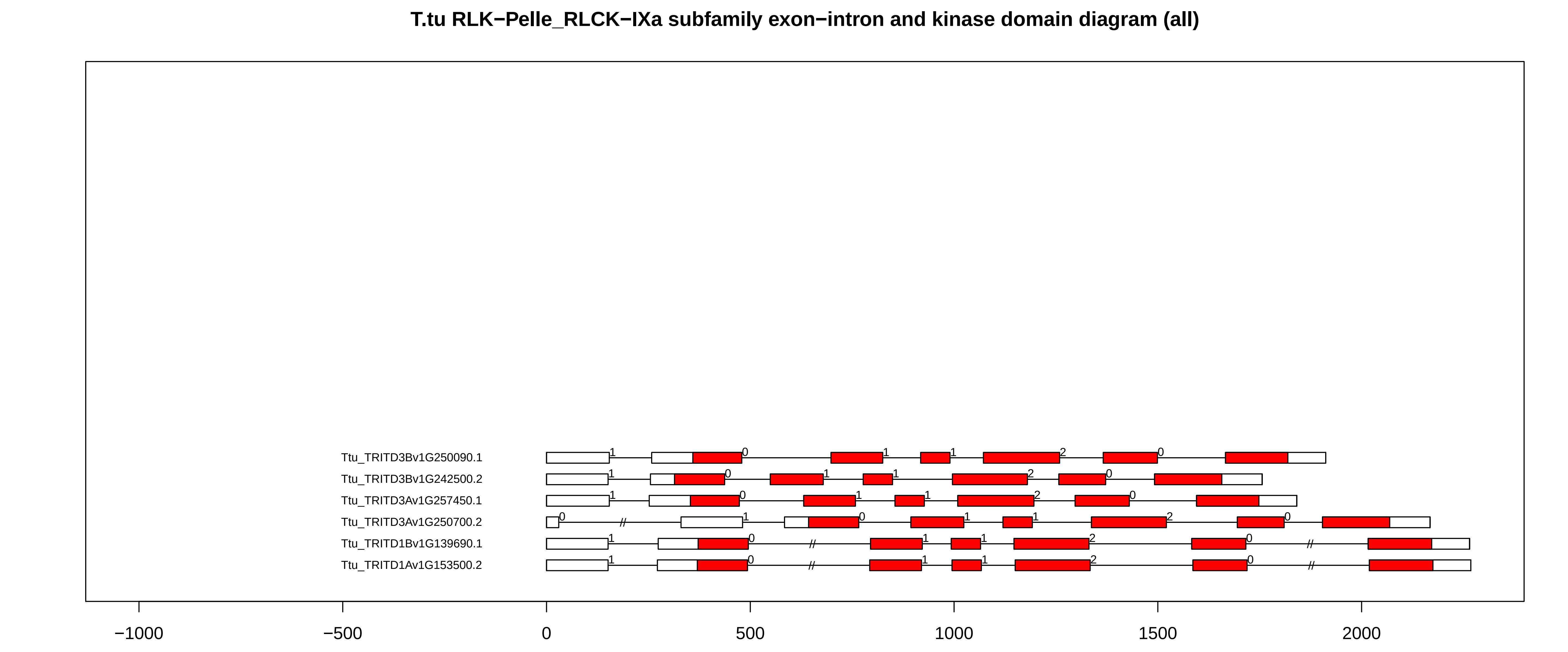
T.tu RLK−Pelle_RLCK−IXa subfamily exon−intron and kinase domain diagram (all)
Ttu_TRITD3Bv1G250090.1
Ttu_TRITD3Bv1G242500.2
Ttu_TRITD3Av1G257450.1
Ttu_TRITD3Av1G250700.2
Ttu_TRITD1Bv1G139690.1
Ttu_TRITD1Av1G153500.2
−1000
−500
0
500
1000
1500
2000

T.tu RLK−Pelle_RLCK−IXb subfamily exon−intron and kinase domain diagram (part 1)

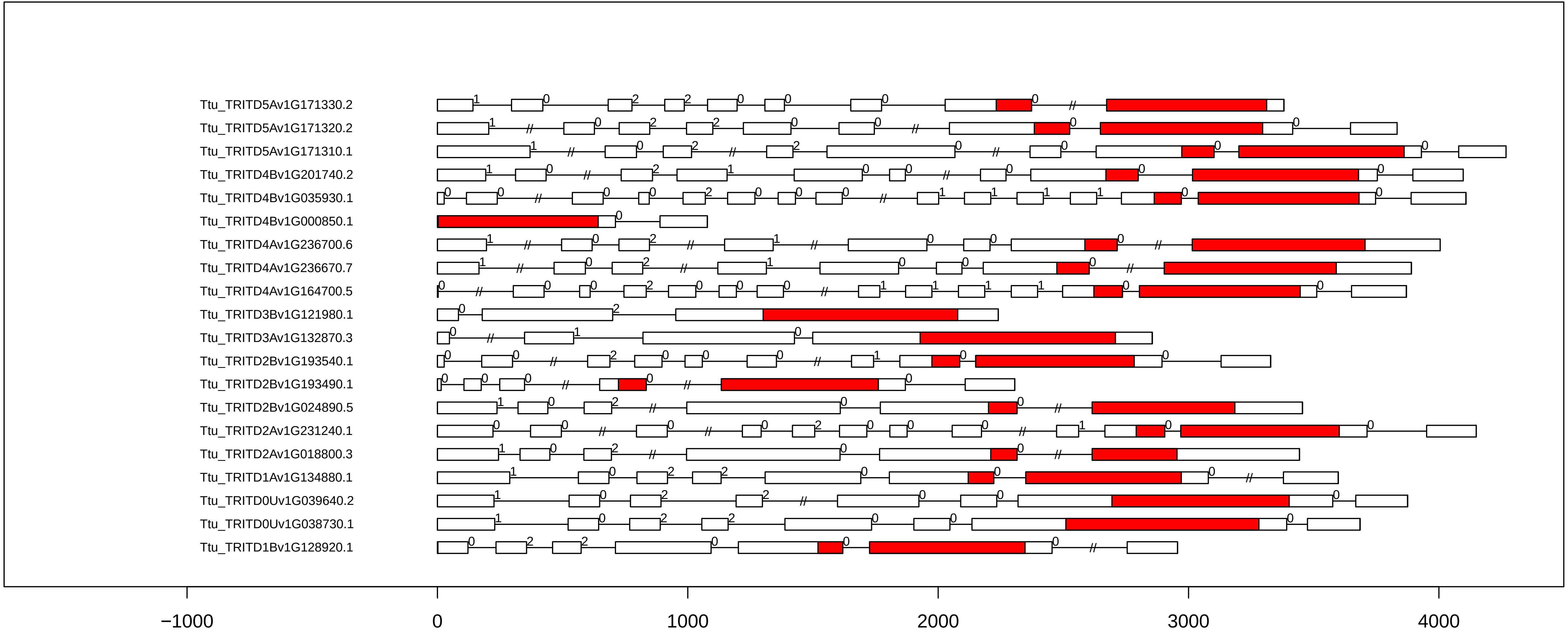

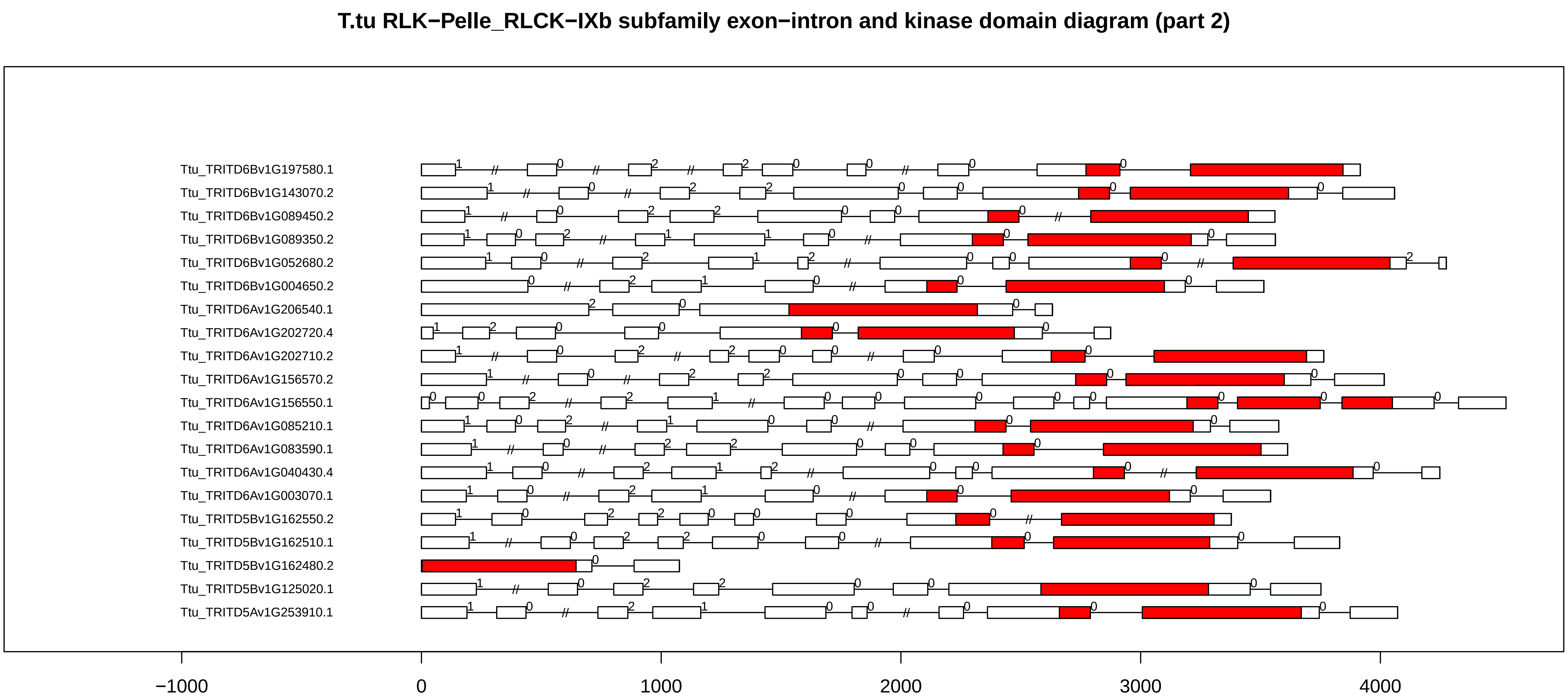
T.tu RLK−Pelle_RLCK−IXb subfamily exon−intron and kinase domain diagram (part 2)
Ttu_TRITD6Bv1G197580.1
Ttu_TRITD6Bv1G143070.2
Ttu_TRITD6Bv1G089450.2
Ttu_TRITD6Bv1G089350.2
Ttu_TRITD6Bv1G052680.2
Ttu_TRITD6Bv1G004650.2
Ttu_TRITD6Av1G206540.1
Ttu_TRITD6Av1G202720.4
Ttu_TRITD6Av1G202710.2
Ttu_TRITD6Av1G156570.2
Ttu_TRITD6Av1G156550.1
Ttu_TRITD6Av1G085210.1
Ttu_TRITD6Av1G083590.1
Ttu_TRITD6Av1G040430.4
Ttu_TRITD6Av1G003070.1
Ttu_TRITD5Bv1G162550.2
Ttu_TRITD5Bv1G162510.1
Ttu_TRITD5Bv1G162480.2
Ttu_TRITD5Bv1G125020.1
Ttu_TRITD5Av1G253910.1
−1000
0
1000
2000
3000
4000

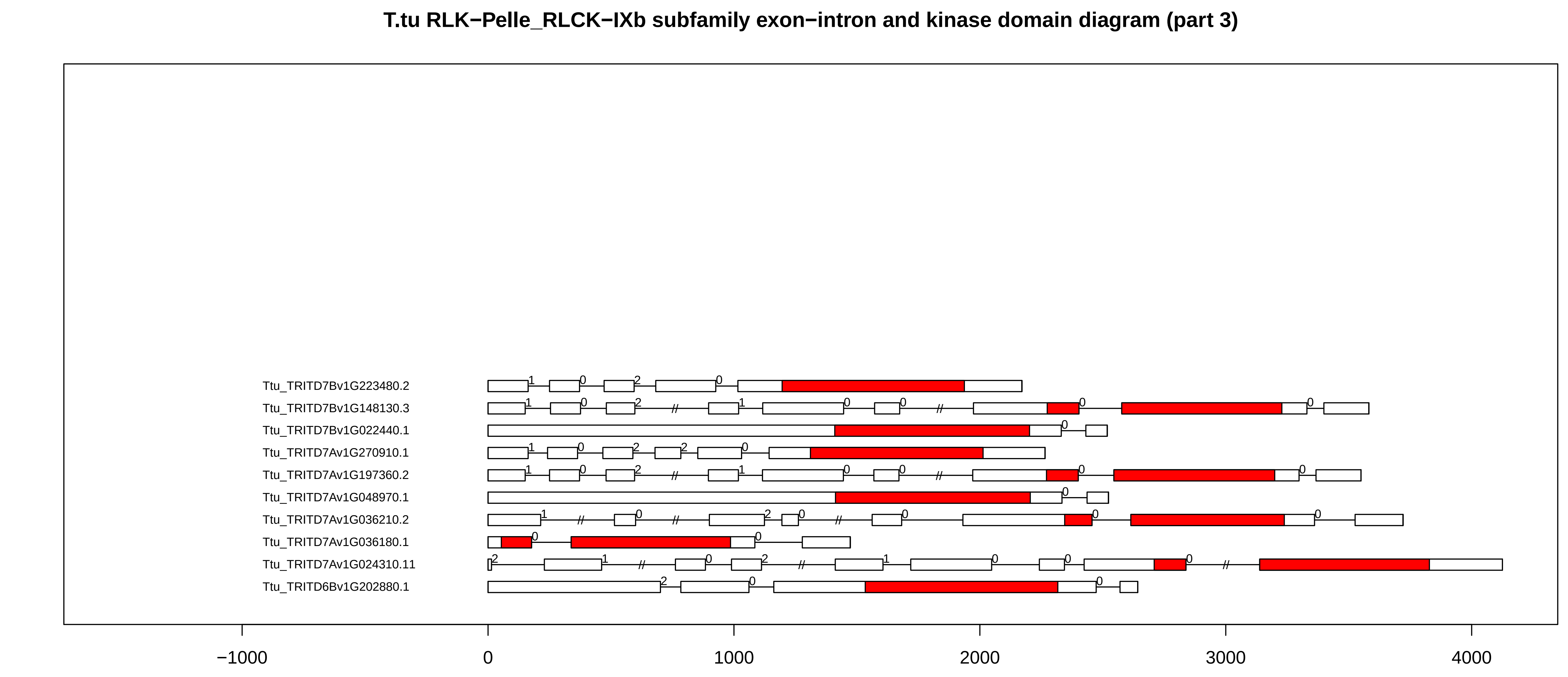

T.tu RLK−Pelle_RLCK−IXb subfamily exon−intron and kinase domain diagram (part 3)
Ttu_TRITD7Bv1G223480.2
Ttu_TRITD7Bv1G148130.3
Ttu_TRITD7Bv1G022440.1
Ttu_TRITD7Av1G270910.1
Ttu_TRITD7Av1G197360.2
Ttu_TRITD7Av1G048970.1
Ttu_TRITD7Av1G036210.2
Ttu_TRITD7Av1G036180.1
Ttu_TRITD7Av1G024310.11
Ttu_TRITD6Bv1G202880.1
−1000
0
1000
2000
3000
4000

T.tu RLK−Pelle_RLCK−Os subfamily exon−intron and kinase domain diagram (all)

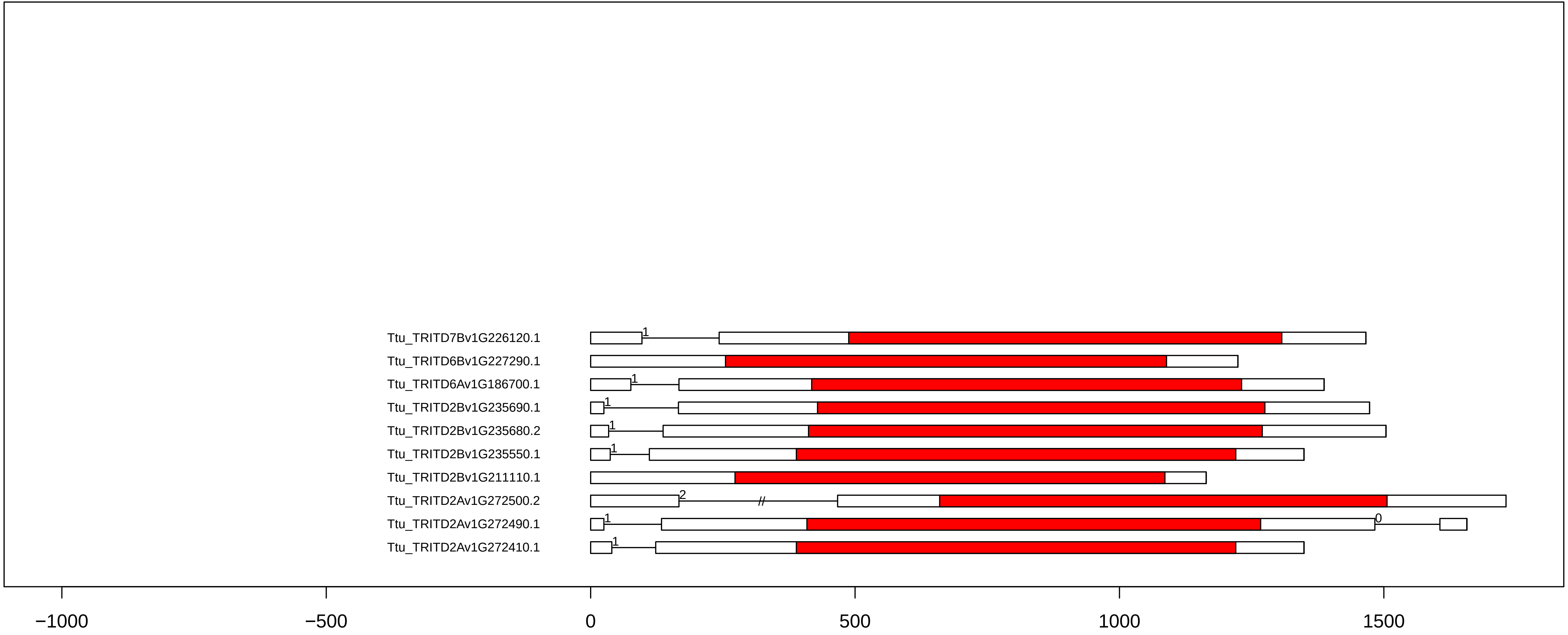

T.tu RLK−Pelle_RLCK−VIIa−1 subfamily exon−intron and kinase domain diagram (part 1)

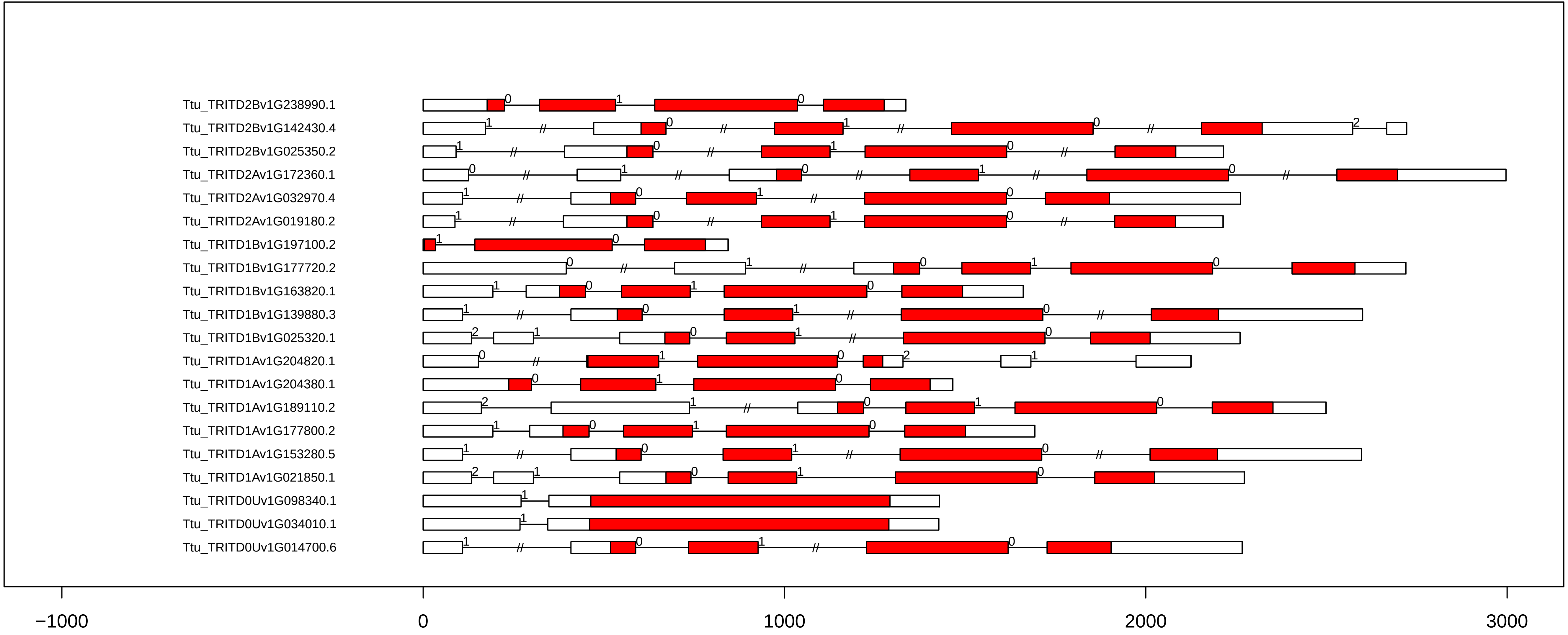

T.tu RLK−Pelle_RLCK−VIIa−1 subfamily exon−intron and kinase domain diagram (part 2)

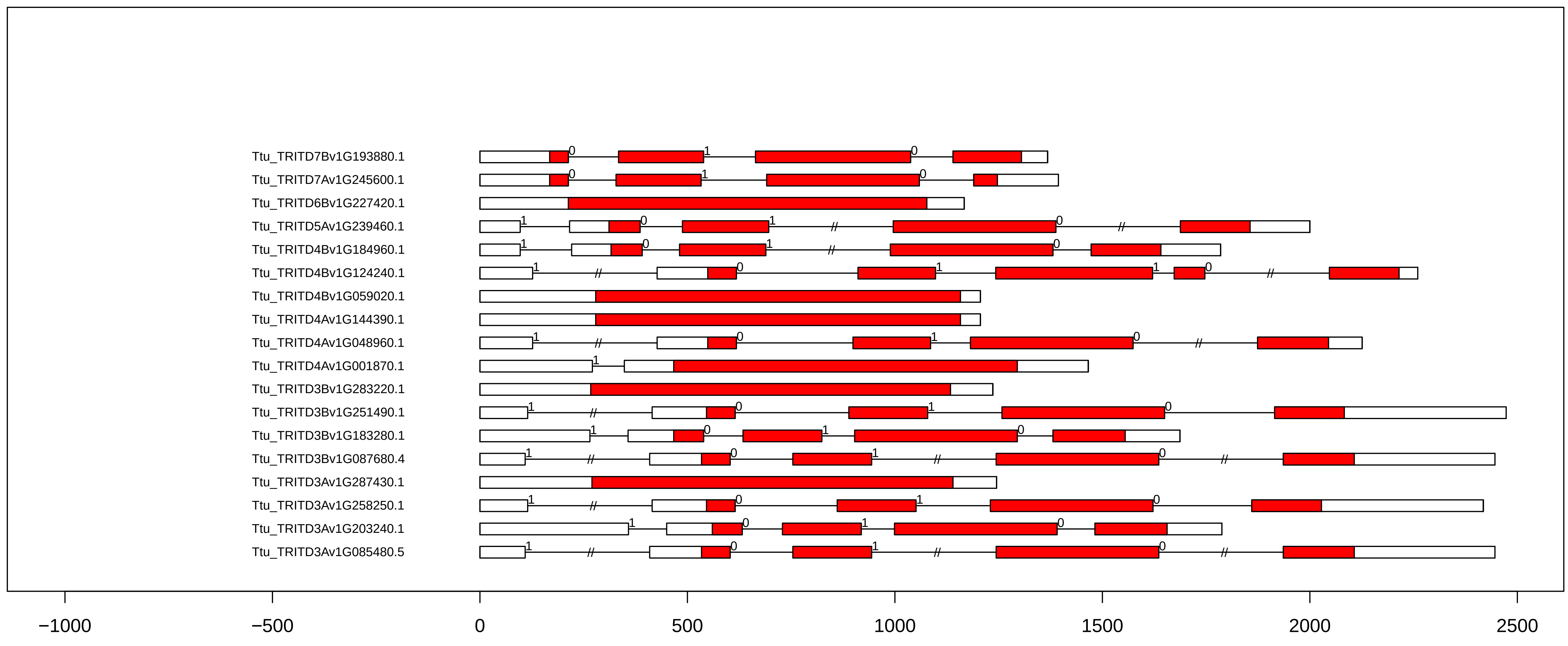

T.tu RLK−Pelle_RLCK−VIIa−2 subfamily exon−intron and kinase domain diagram (part 1)

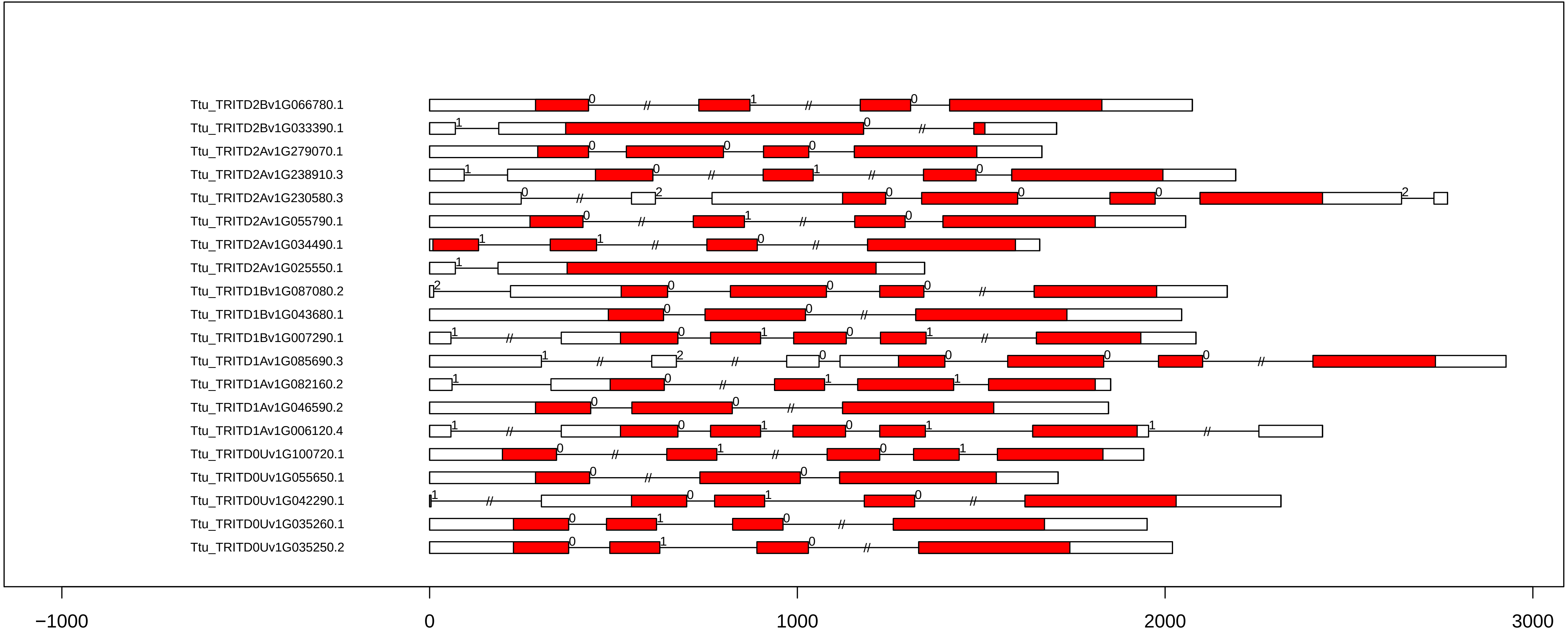

T.tu RLK−Pelle_RLCK−VIIa−2 subfamily exon−intron and kinase domain diagram (part 2)

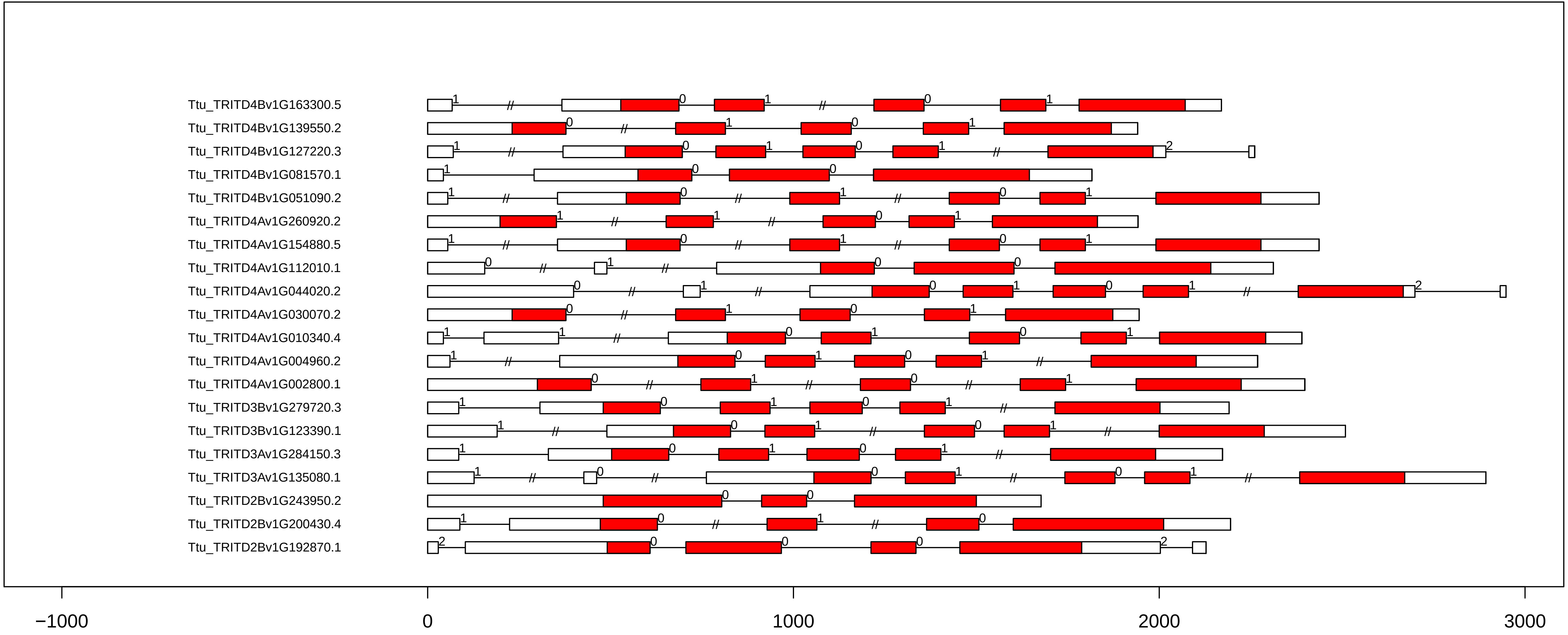

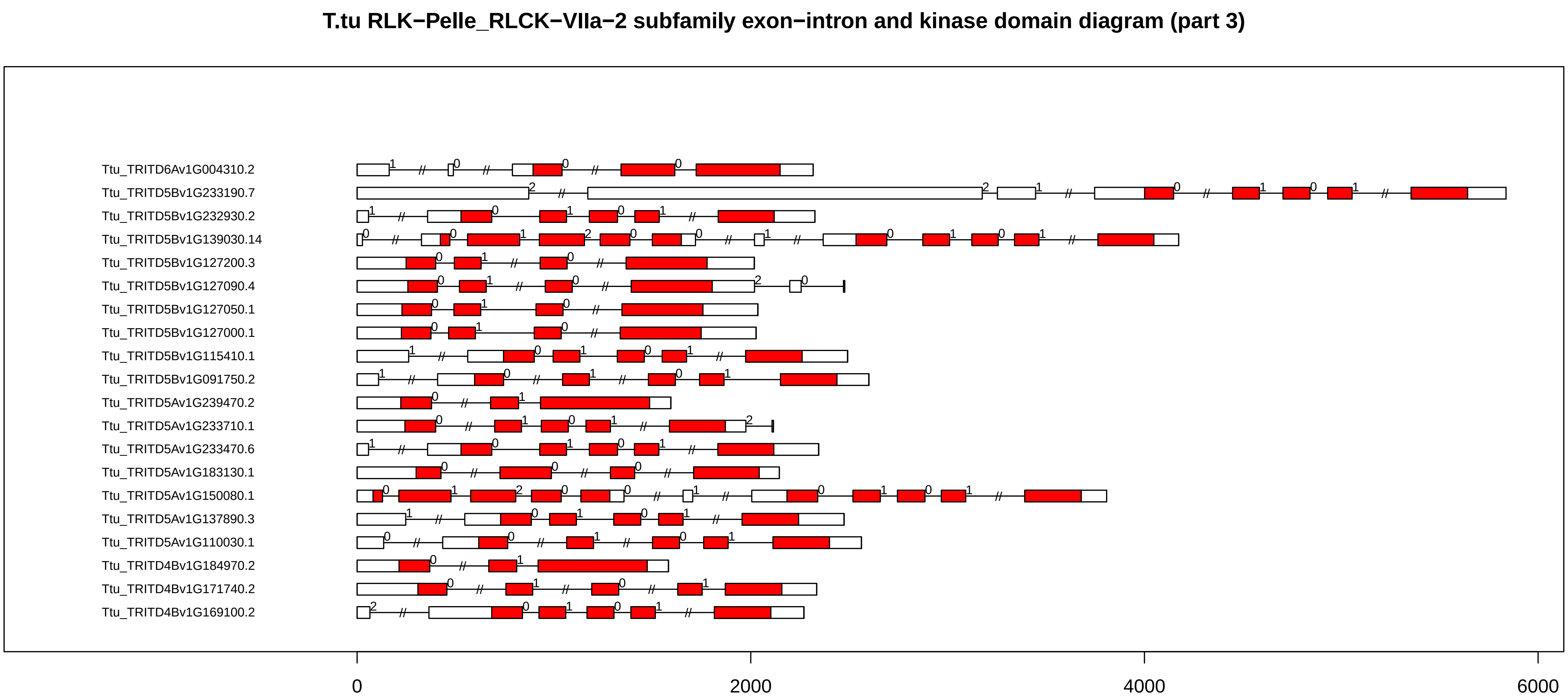
T.tu RLK−Pelle_RLCK−VIIa−2 subfamily exon−intron and kinase domain diagram (part 3)
Ttu_TRITD6Av1G004310.2
Ttu_TRITD5Bv1G233190.7
Ttu_TRITD5Bv1G232930.2
Ttu_TRITD5Bv1G139030.14
Ttu_TRITD5Bv1G127200.3
Ttu_TRITD5Bv1G127090.4
Ttu_TRITD5Bv1G127050.1
Ttu_TRITD5Bv1G127000.1
Ttu_TRITD5Bv1G115410.1
Ttu_TRITD5Bv1G091750.2
Ttu_TRITD5Av1G239470.2
Ttu_TRITD5Av1G233710.1
Ttu_TRITD5Av1G233470.6
Ttu_TRITD5Av1G183130.1
Ttu_TRITD5Av1G150080.1
Ttu_TRITD5Av1G137890.3
Ttu_TRITD5Av1G110030.1
Ttu_TRITD4Bv1G184970.2
Ttu_TRITD4Bv1G171740.2
Ttu_TRITD4Bv1G169100.2
0
2000
4000
6000

T.tu RLK−Pelle_RLCK−VIIa−2 subfamily exon−intron and kinase domain diagram (part 4)

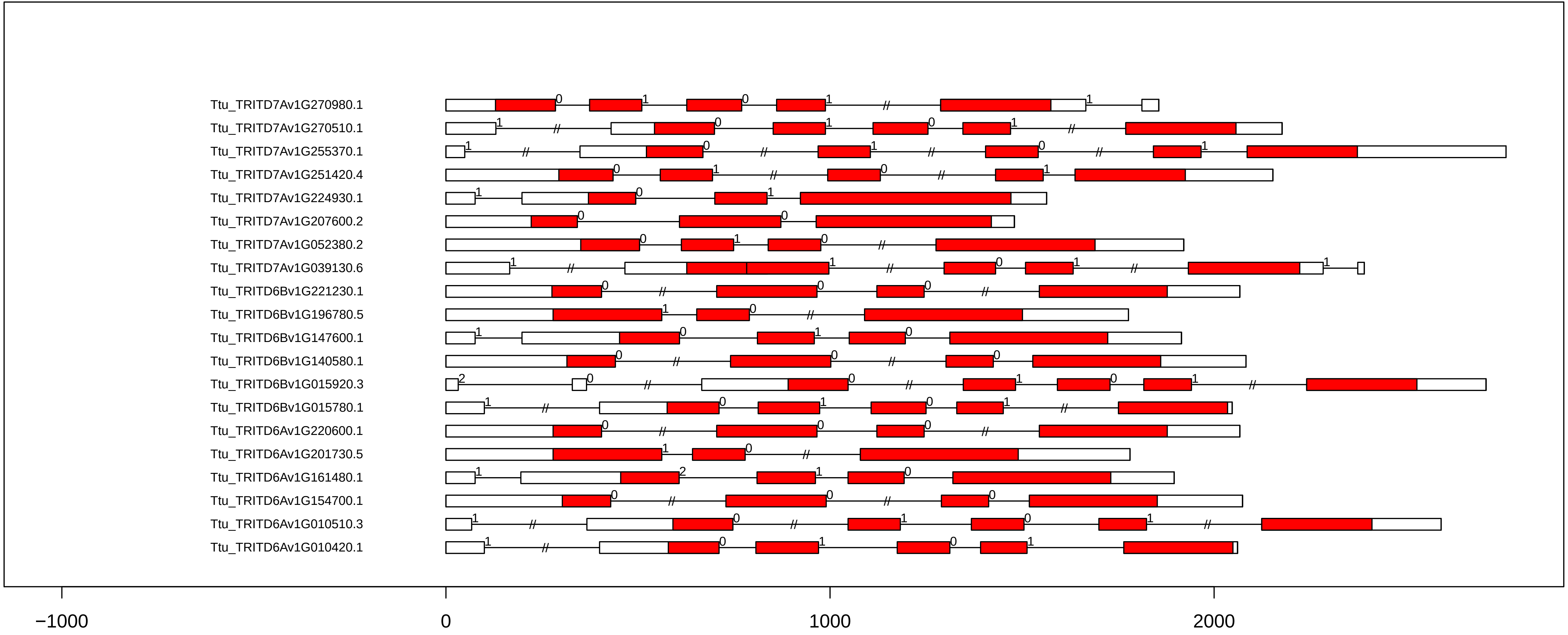

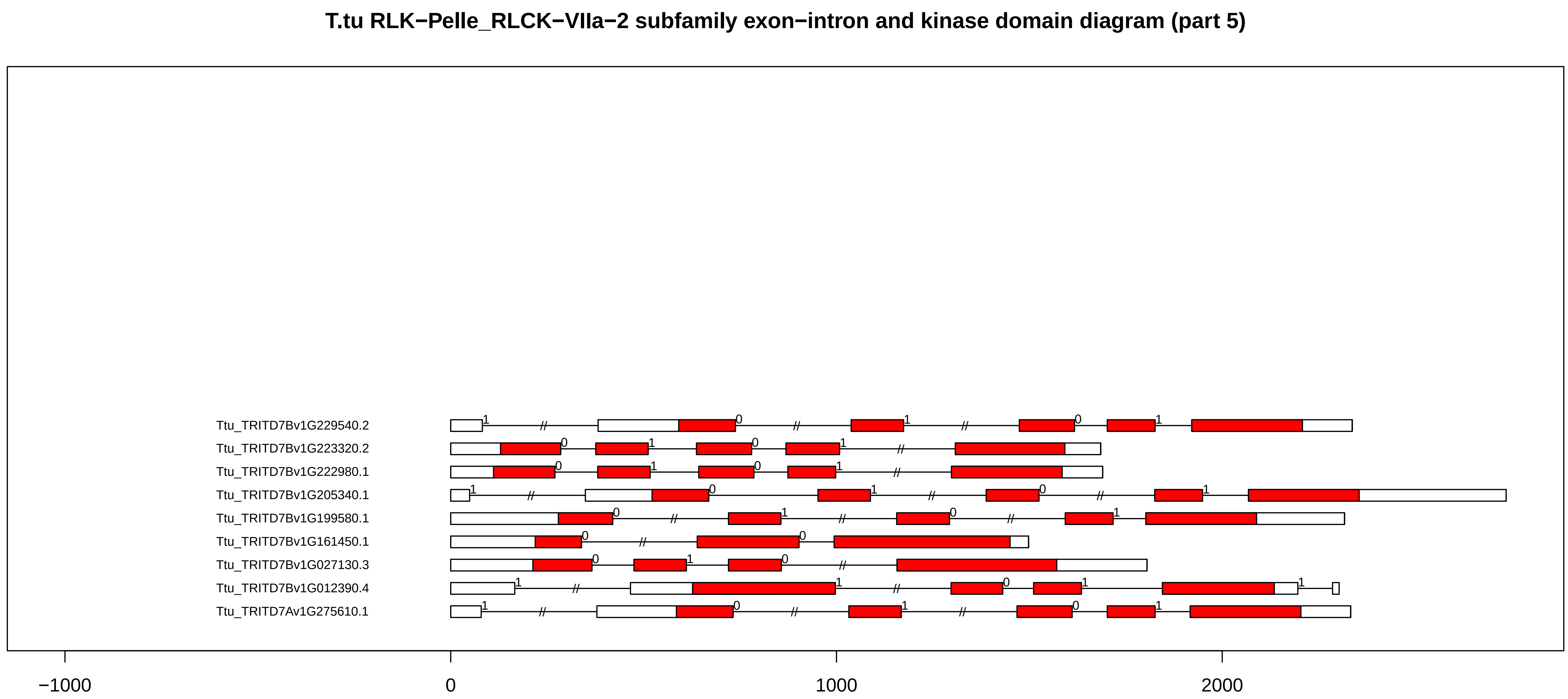
T.tu RLK−Pelle_RLCK−VIIa−2 subfamily exon−intron and kinase domain diagram (part 5)
Ttu_TRITD7Bv1G229540.2
Ttu_TRITD7Bv1G223320.2
Ttu_TRITD7Bv1G222980.1
Ttu_TRITD7Bv1G205340.1
Ttu_TRITD7Bv1G199580.1
Ttu_TRITD7Bv1G161450.1
Ttu_TRITD7Bv1G027130.3
Ttu_TRITD7Bv1G012390.4
Ttu_TRITD7Av1G275610.1
−1000
0
1000
2000

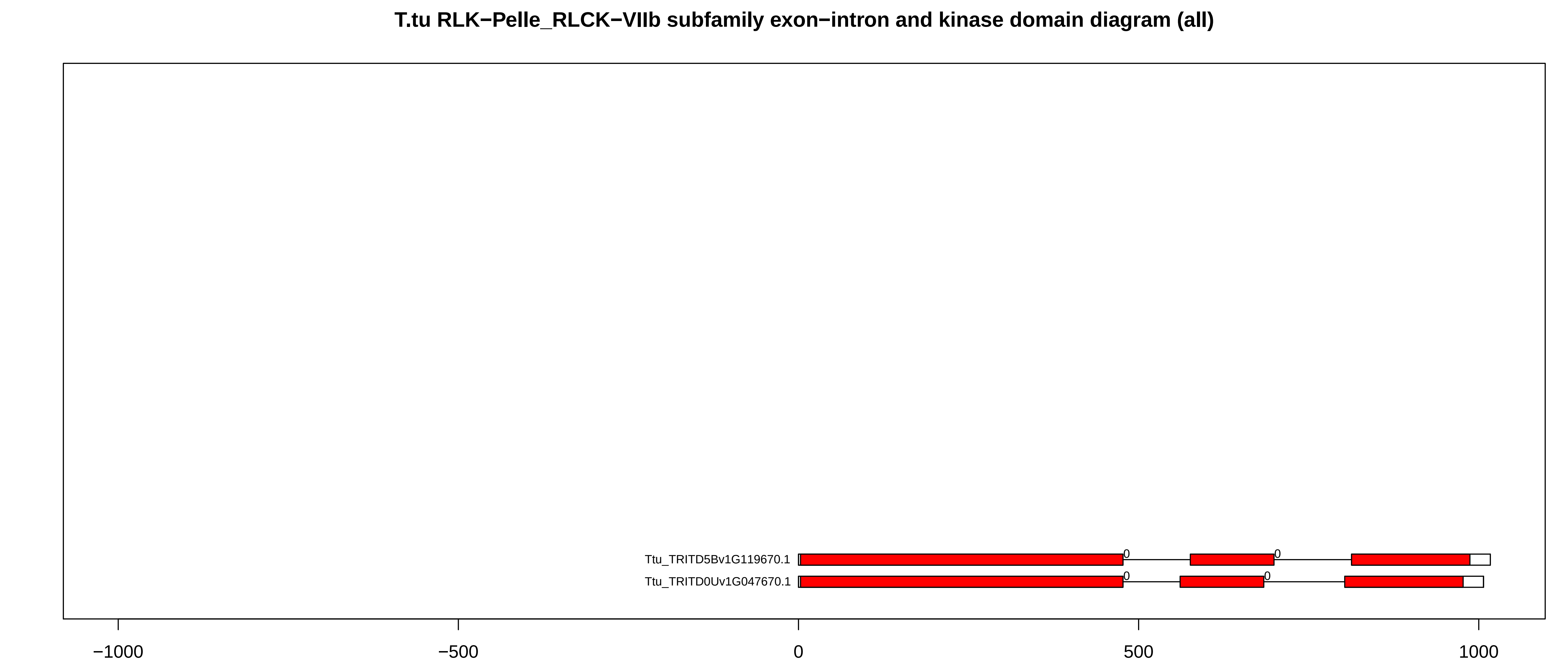
T.tu RLK−Pelle_RLCK−VIIb subfamily exon−intron and kinase domain diagram (all)
Ttu_TRITD5Bv1G119670.1
Ttu_TRITD0Uv1G047670.1
0
0
0
0
−1000
−500
0
500
1000

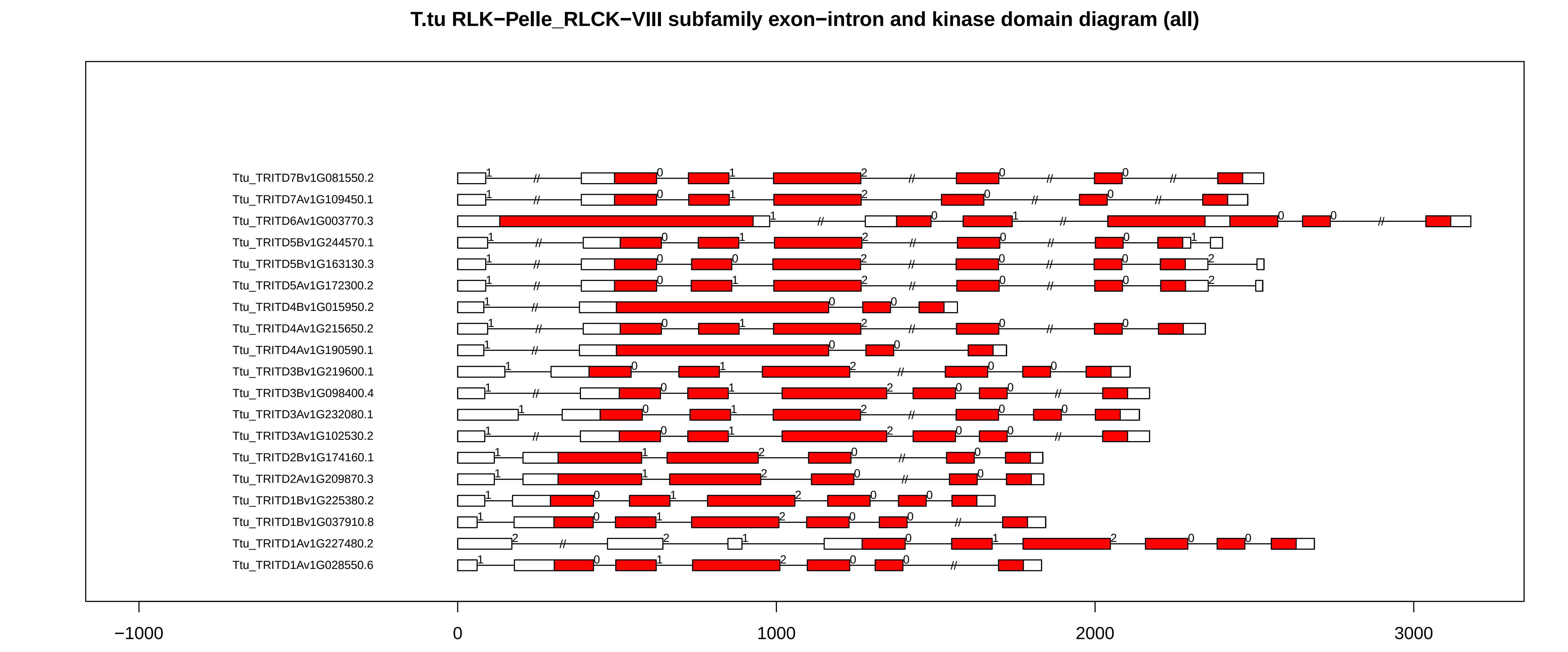
T.tu RLK−Pelle_RLCK−VIII subfamily exon−intron and kinase domain diagram (all)
Ttu_TRITD7Bv1G081550.2
Ttu_TRITD7Av1G109450.1
Ttu_TRITD6Av1G003770.3
Ttu_TRITD5Bv1G244570.1
Ttu_TRITD5Bv1G163130.3
Ttu_TRITD5Av1G172300.2
Ttu_TRITD4Bv1G015950.2
Ttu_TRITD4Av1G215650.2
Ttu_TRITD4Av1G190590.1
Ttu_TRITD3Bv1G219600.1
Ttu_TRITD3Bv1G098400.4
Ttu_TRITD3Av1G232080.1
Ttu_TRITD3Av1G102530.2
Ttu_TRITD2Bv1G174160.1
Ttu_TRITD2Av1G209870.3
Ttu_TRITD1Bv1G225380.2
Ttu_TRITD1Bv1G037910.8
Ttu_TRITD1Av1G227480.2
Ttu_TRITD1Av1G028550.6
−1000
0
1000
2000
3000

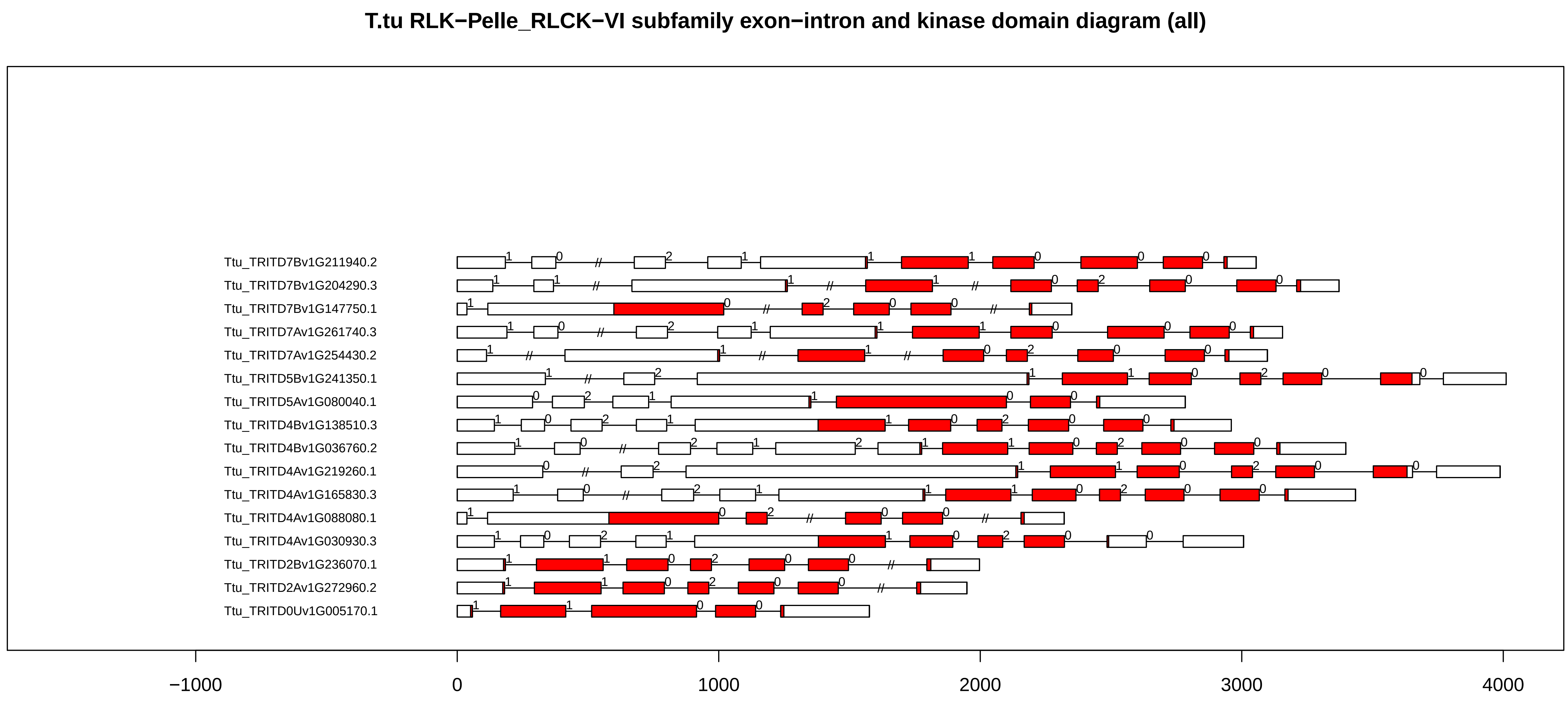
T.tu RLK−Pelle_RLCK−VI subfamily exon−intron and kinase domain diagram (all)
Ttu_TRITD7Bv1G211940.2
Ttu_TRITD7Bv1G204290.3
Ttu_TRITD7Bv1G147750.1
Ttu_TRITD7Av1G261740.3
Ttu_TRITD7Av1G254430.2
Ttu_TRITD5Bv1G241350.1
Ttu_TRITD5Av1G080040.1
Ttu_TRITD4Bv1G138510.3
Ttu_TRITD4Bv1G036760.2
Ttu_TRITD4Av1G219260.1
Ttu_TRITD4Av1G165830.3
Ttu_TRITD4Av1G088080.1
Ttu_TRITD4Av1G030930.3
Ttu_TRITD2Bv1G236070.1
Ttu_TRITD2Av1G272960.2
Ttu_TRITD0Uv1G005170.1
−1000
0
1000
2000
3000
4000

T.tu RLK−Pelle_RLCK−V subfamily exon−intron and kinase domain diagram (part 1)

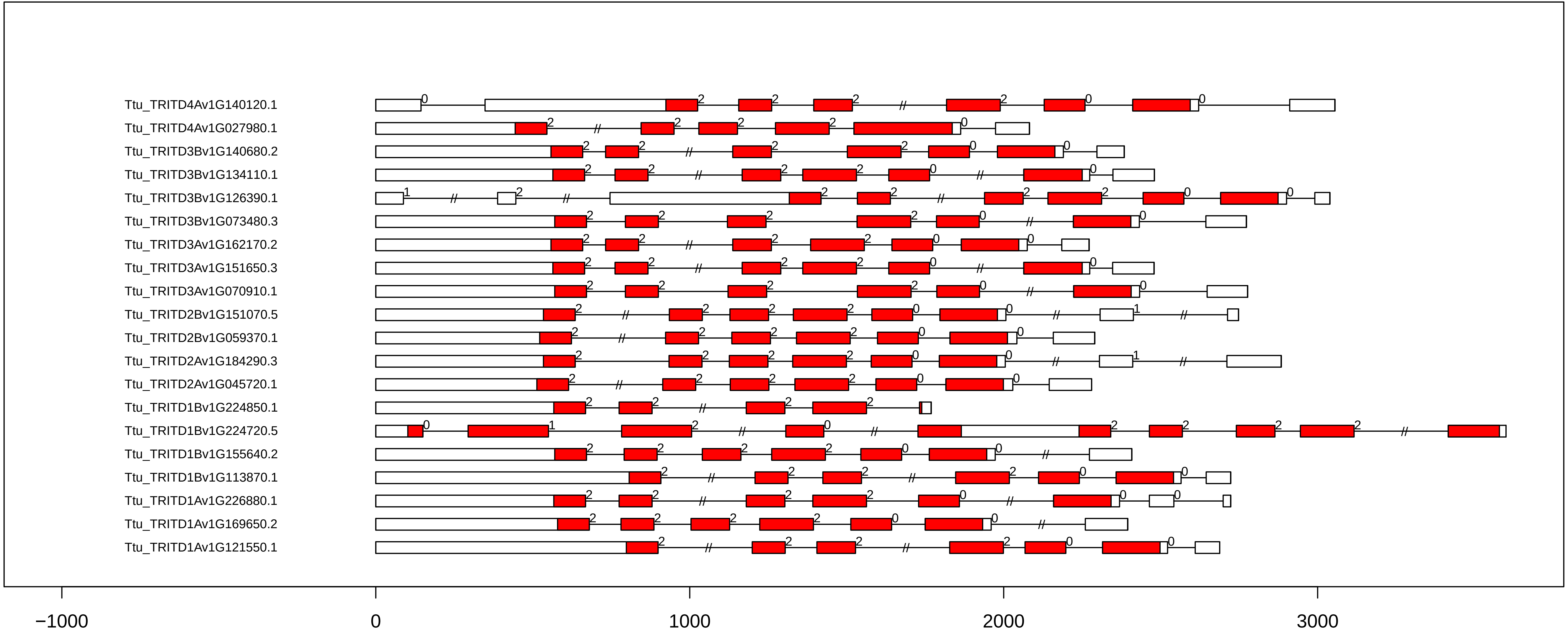

T.tu RLK−Pelle_RLCK−V subfamily exon−intron and kinase domain diagram (part 2)

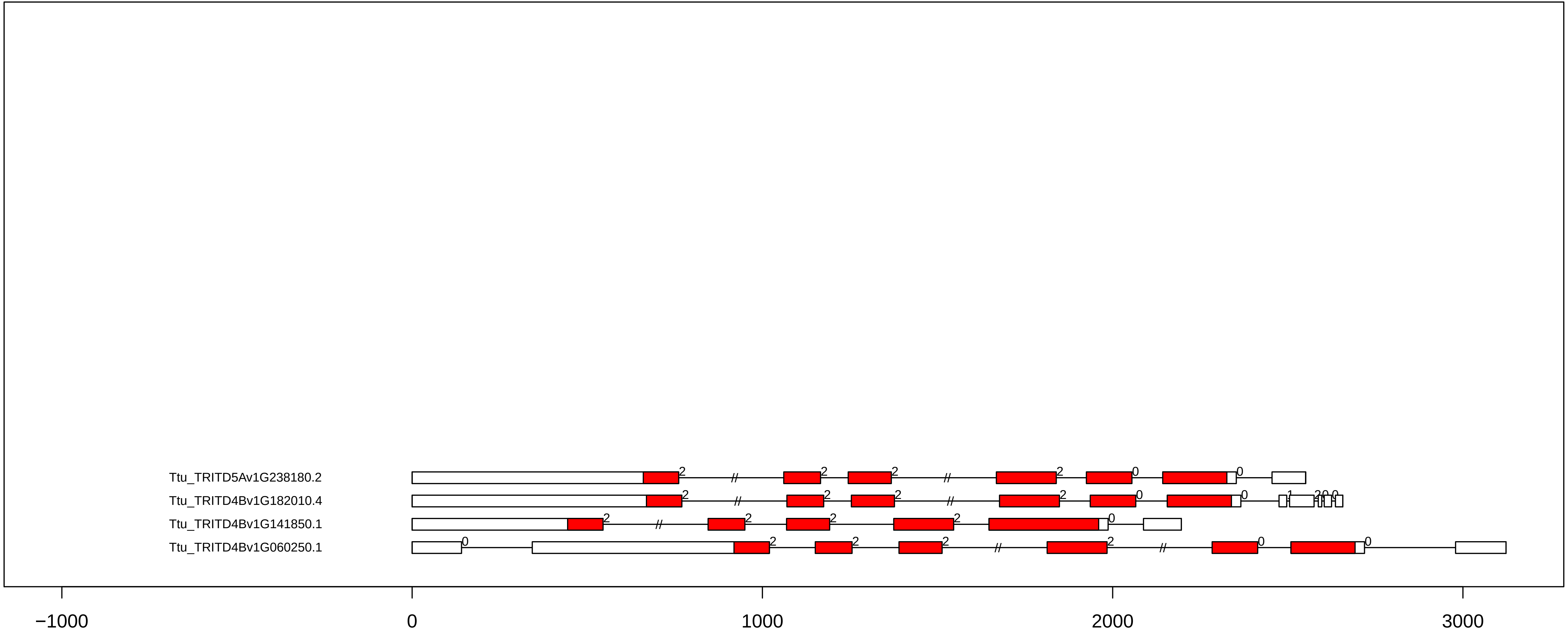

T.tu RLK−Pelle_RLCK−XII−1 subfamily exon−intron and kinase domain diagram (all)

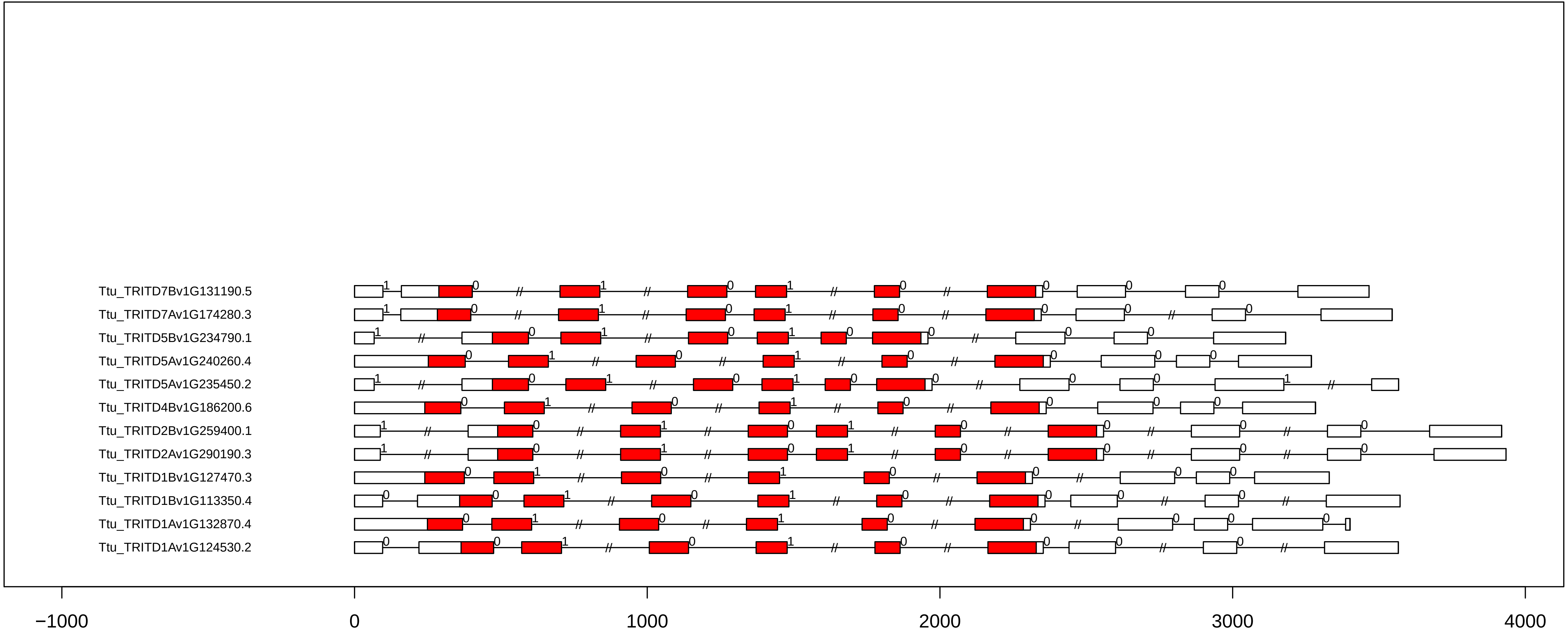

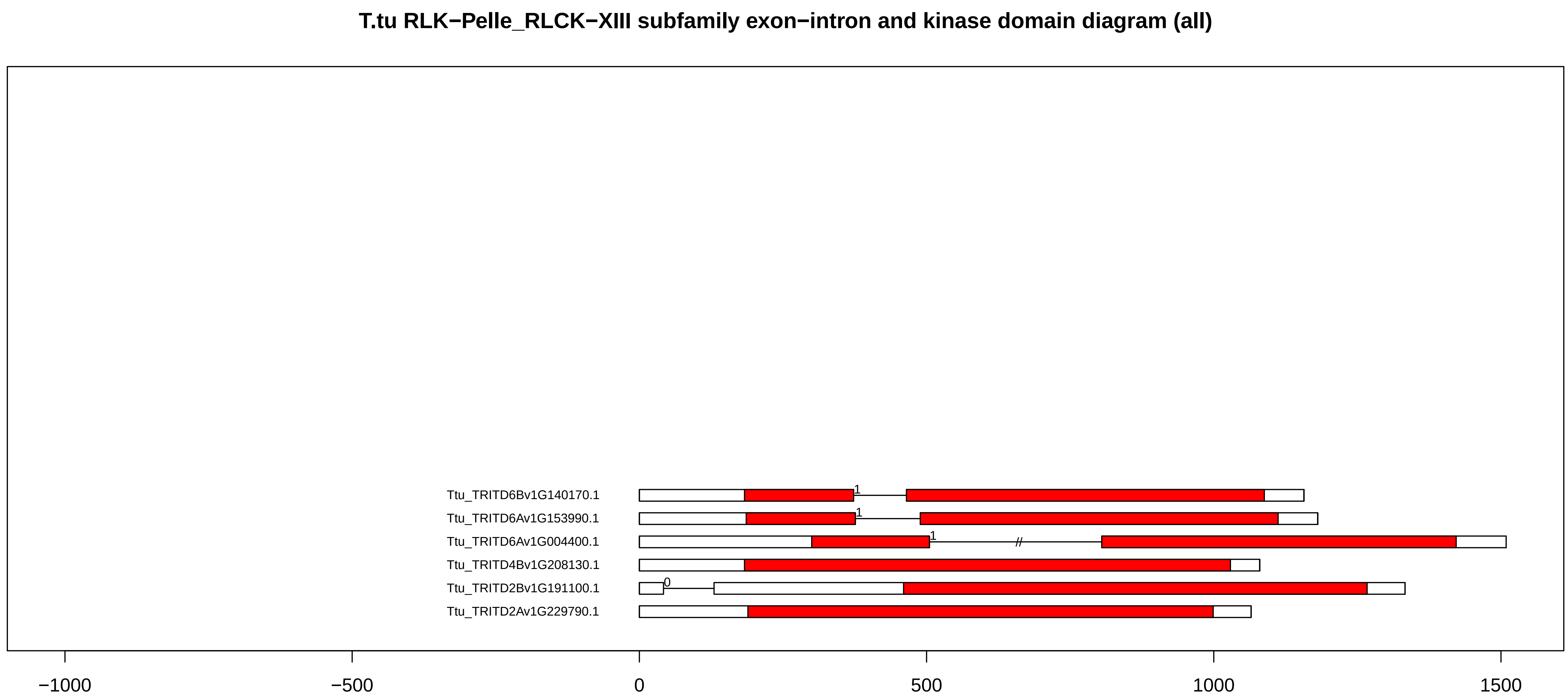

T.tu RLK−Pelle_RLCK−XIII subfamily exon−intron and kinase domain diagram (all)
Ttu_TRITD6Bv1G140170.1
Ttu_TRITD6Av1G153990.1
Ttu_TRITD6Av1G004400.1
Ttu_TRITD4Bv1G208130.1
Ttu_TRITD2Bv1G191100.1
Ttu_TRITD2Av1G229790.1
1
1
1
0
−1000
−500
0
500
1000
1500

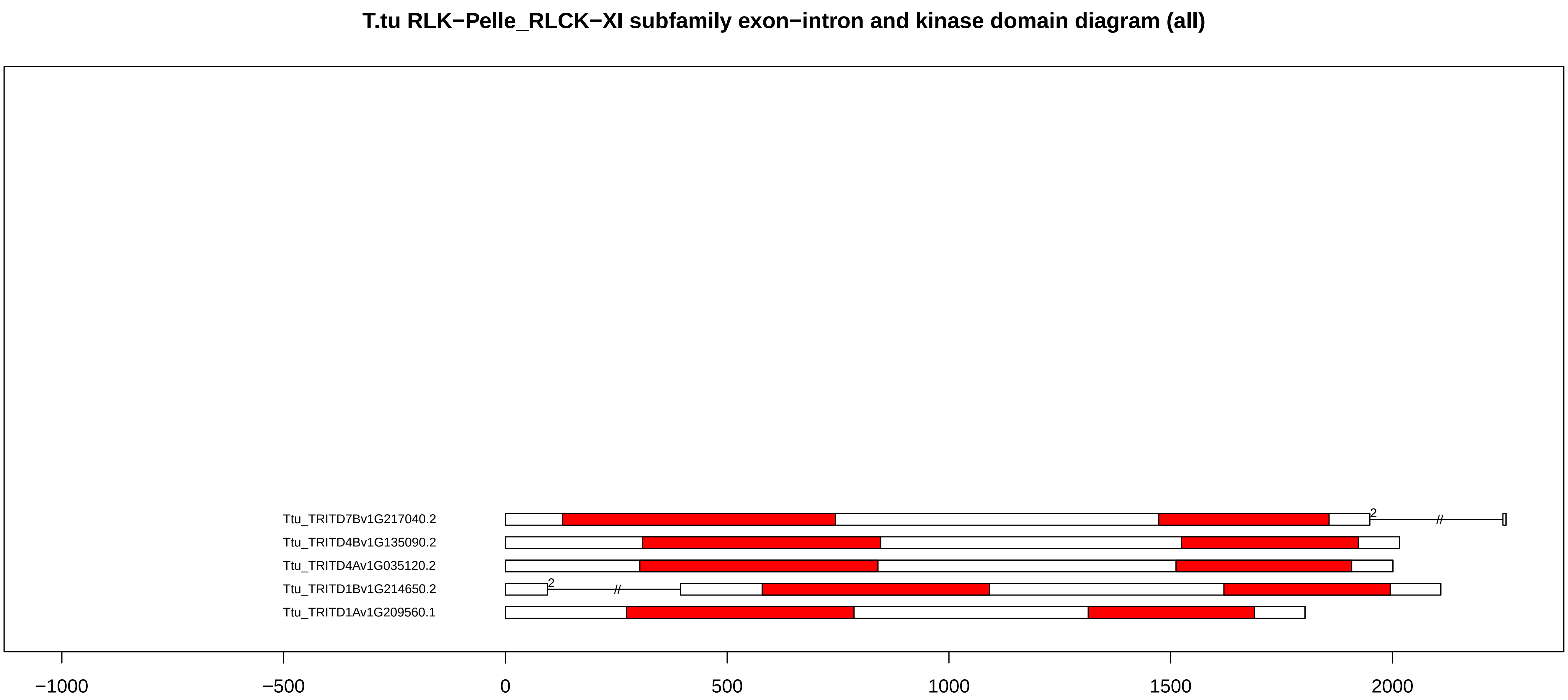

T.tu RLK−Pelle_RLCK−XI subfamily exon−intron and kinase domain diagram (all)
Ttu_TRITD7Bv1G217040.2
Ttu_TRITD4Bv1G135090.2
Ttu_TRITD4Av1G035120.2
Ttu_TRITD1Bv1G214650.2
Ttu_TRITD1Av1G209560.1
2
2
−1000
−500
0
500
1000
1500
2000

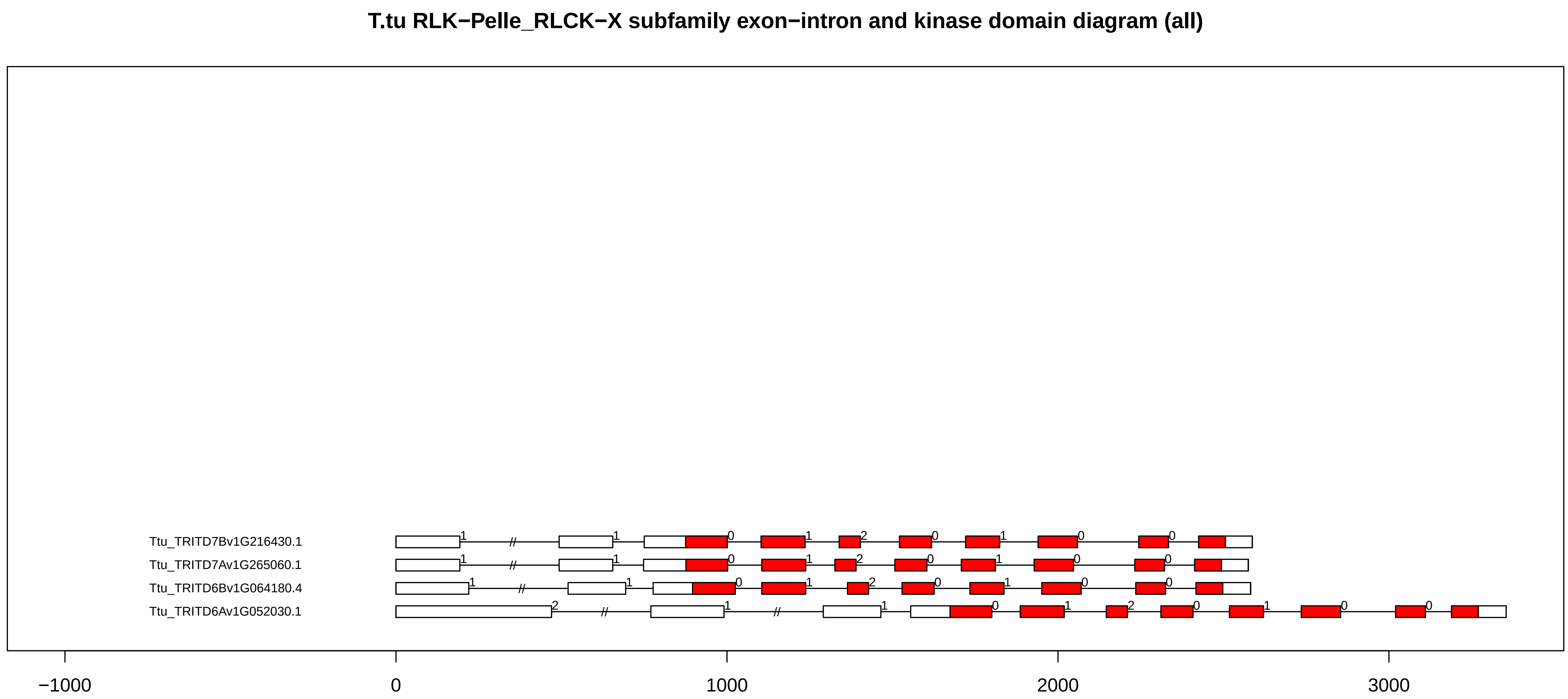
T.tu RLK−Pelle_RLCK−X subfamily exon−intron and kinase domain diagram (all)
Ttu_TRITD7Bv1G216430.1
Ttu_TRITD7Av1G265060.1
Ttu_TRITD6Bv1G064180.4
Ttu_TRITD6Av1G052030.1
−1000
0
1000
2000
3000

T.tu RLK−Pelle_RLCK−XVI subfamily exon−intron and kinase domain diagram (all)

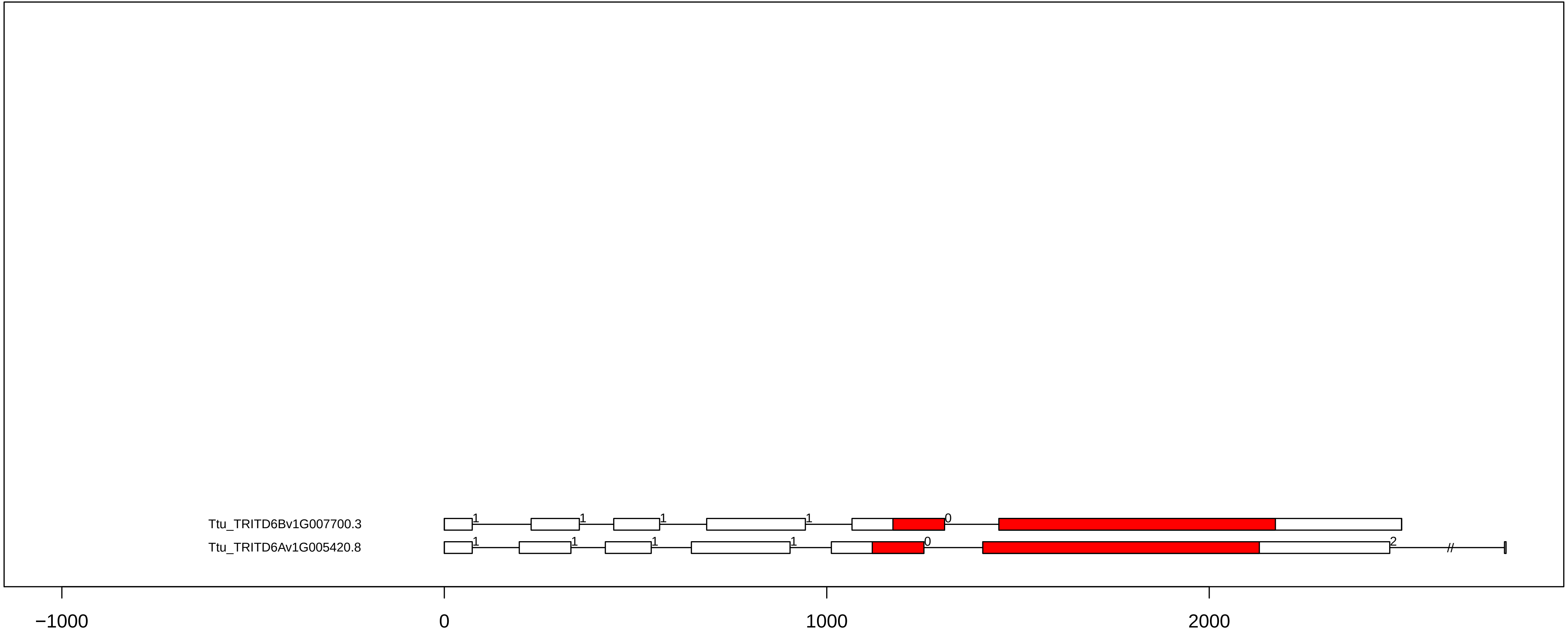

T.tu RLK−Pelle_RLCK−XV subfamily exon−intron and kinase domain diagram (all)

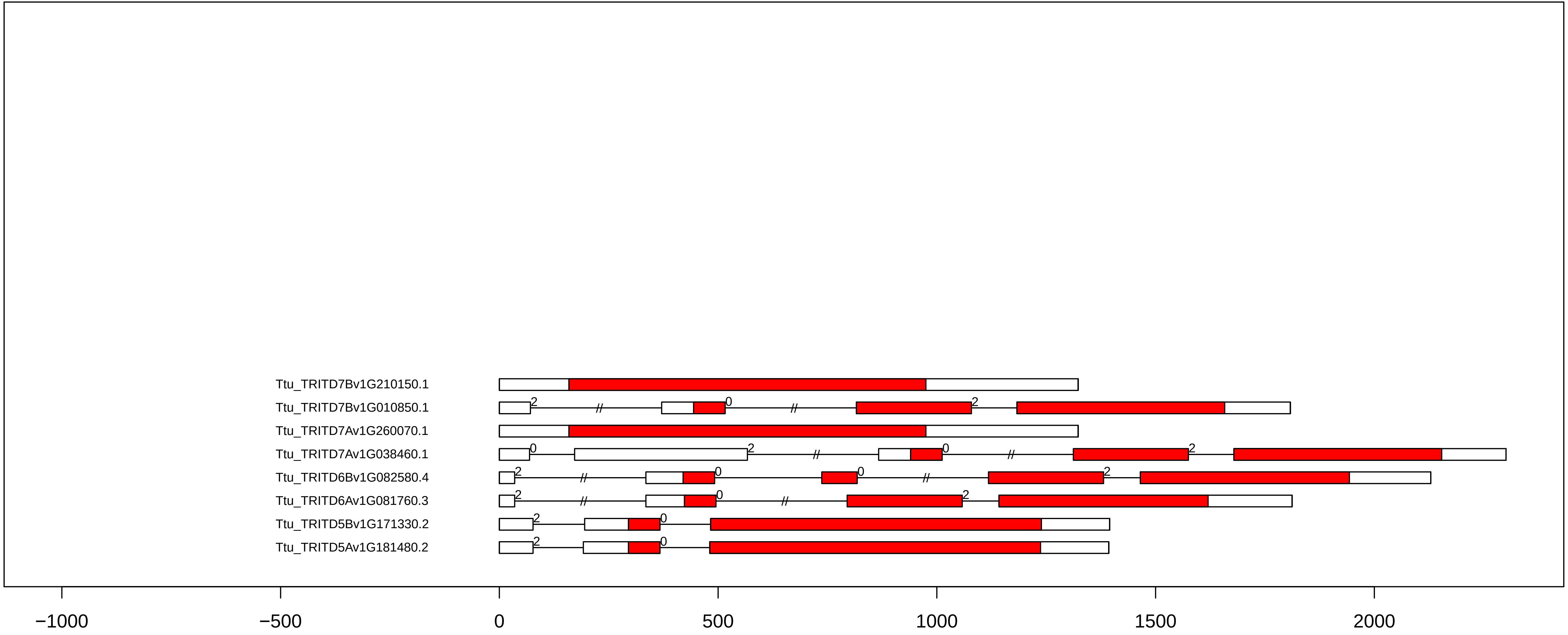

T.tu RLK−Pelle_SD−2b subfamily exon−intron and kinase domain diagram (part 1)

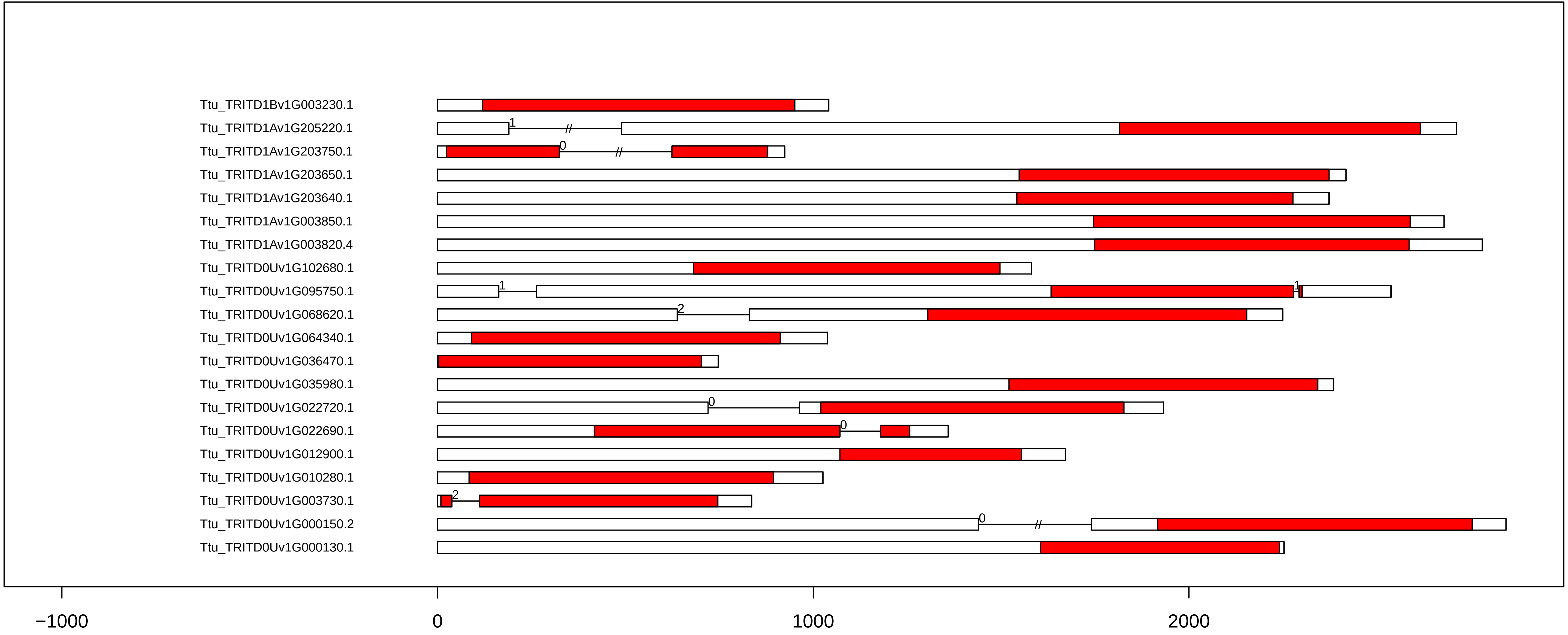

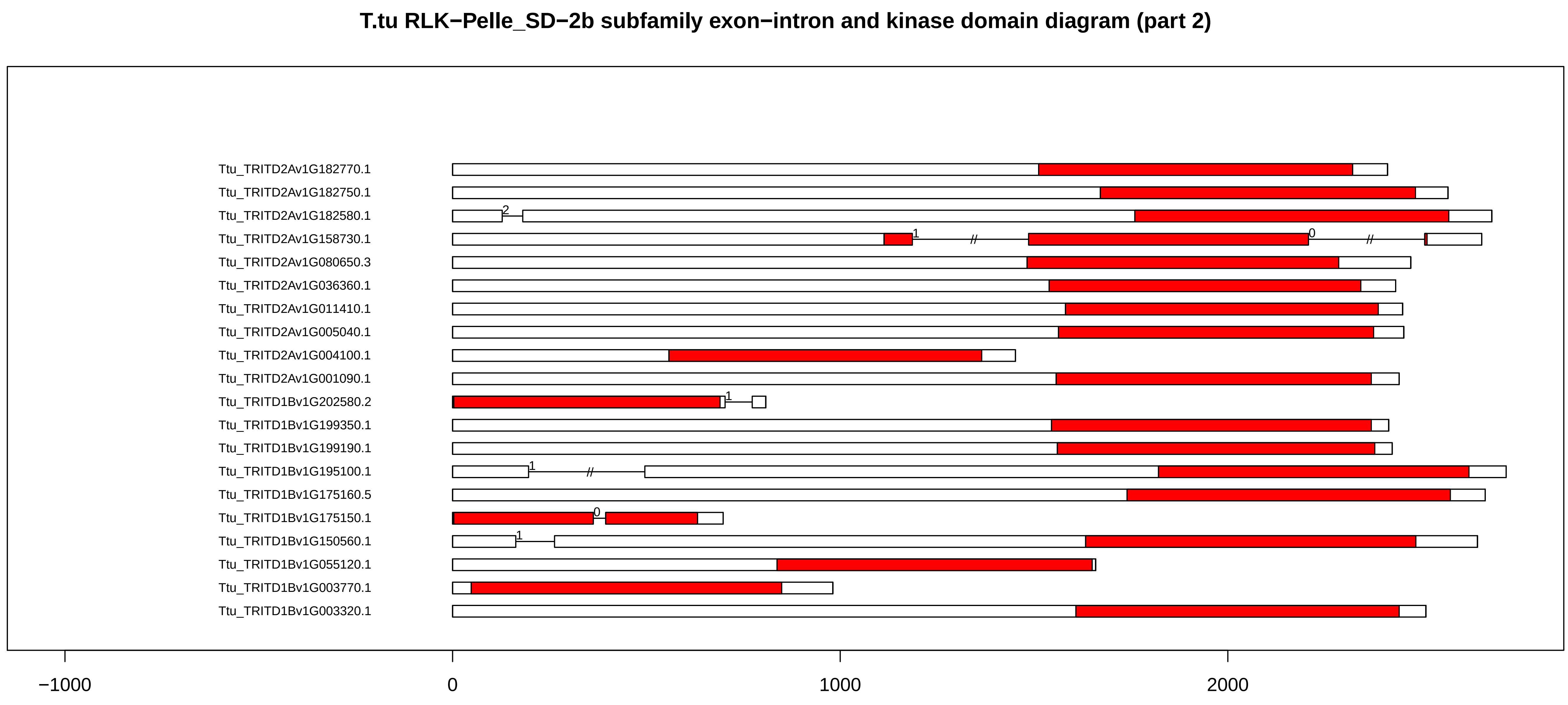

T.tu RLK−Pelle_SD−2b subfamily exon−intron and kinase domain diagram (part 2)
Ttu_TRITD2Av1G182770.1
Ttu_TRITD2Av1G182750.1
Ttu_TRITD2Av1G182580.1
Ttu_TRITD2Av1G158730.1
Ttu_TRITD2Av1G080650.3
Ttu_TRITD2Av1G036360.1
Ttu_TRITD2Av1G011410.1
Ttu_TRITD2Av1G005040.1
Ttu_TRITD2Av1G004100.1
Ttu_TRITD2Av1G001090.1
Ttu_TRITD1Bv1G202580.2
Ttu_TRITD1Bv1G199350.1
Ttu_TRITD1Bv1G199190.1
Ttu_TRITD1Bv1G195100.1
Ttu_TRITD1Bv1G175160.5
Ttu_TRITD1Bv1G175150.1
Ttu_TRITD1Bv1G150560.1
Ttu_TRITD1Bv1G055120.1
Ttu_TRITD1Bv1G003770.1
Ttu_TRITD1Bv1G003320.1
2
1
0
1
1
0
1
−1000
0
1000
2000

T.tu RLK−Pelle_SD−2b subfamily exon−intron and kinase domain diagram (part 3)

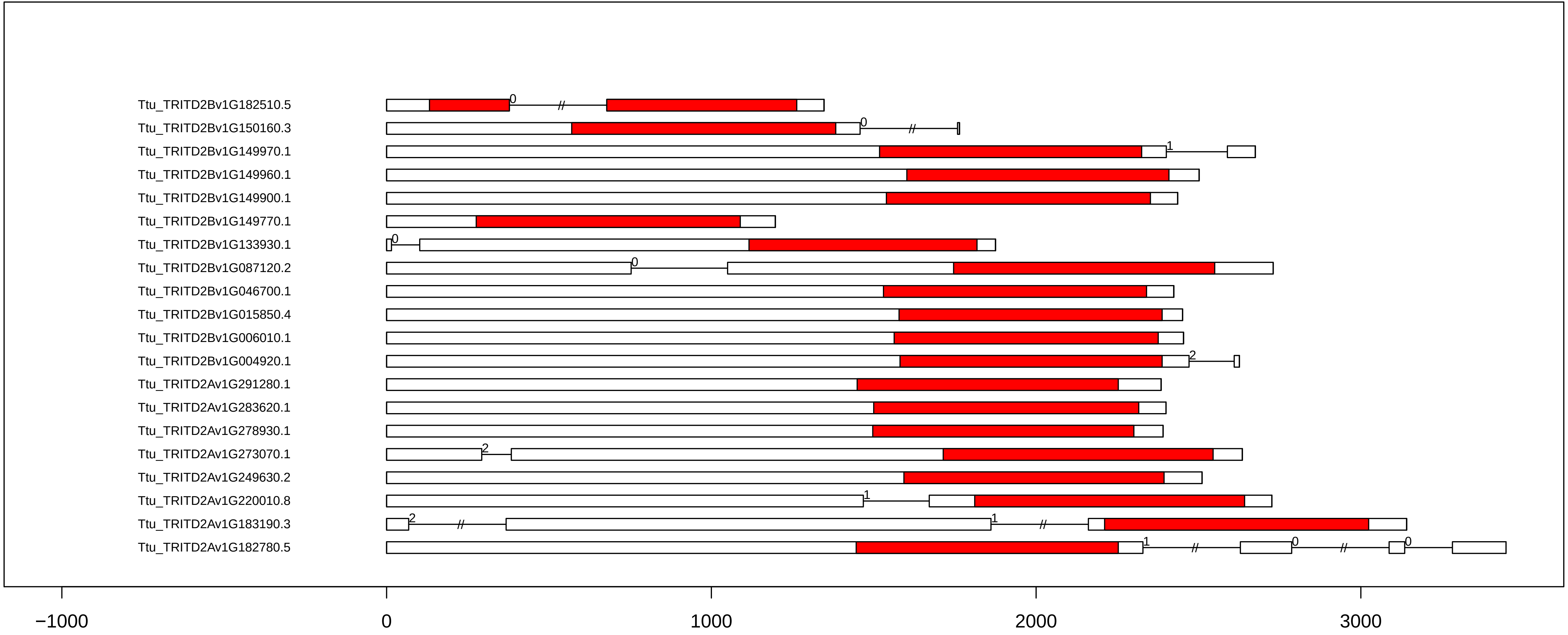

T.tu RLK−Pelle_SD−2b subfamily exon−intron and kinase domain diagram (part 4)

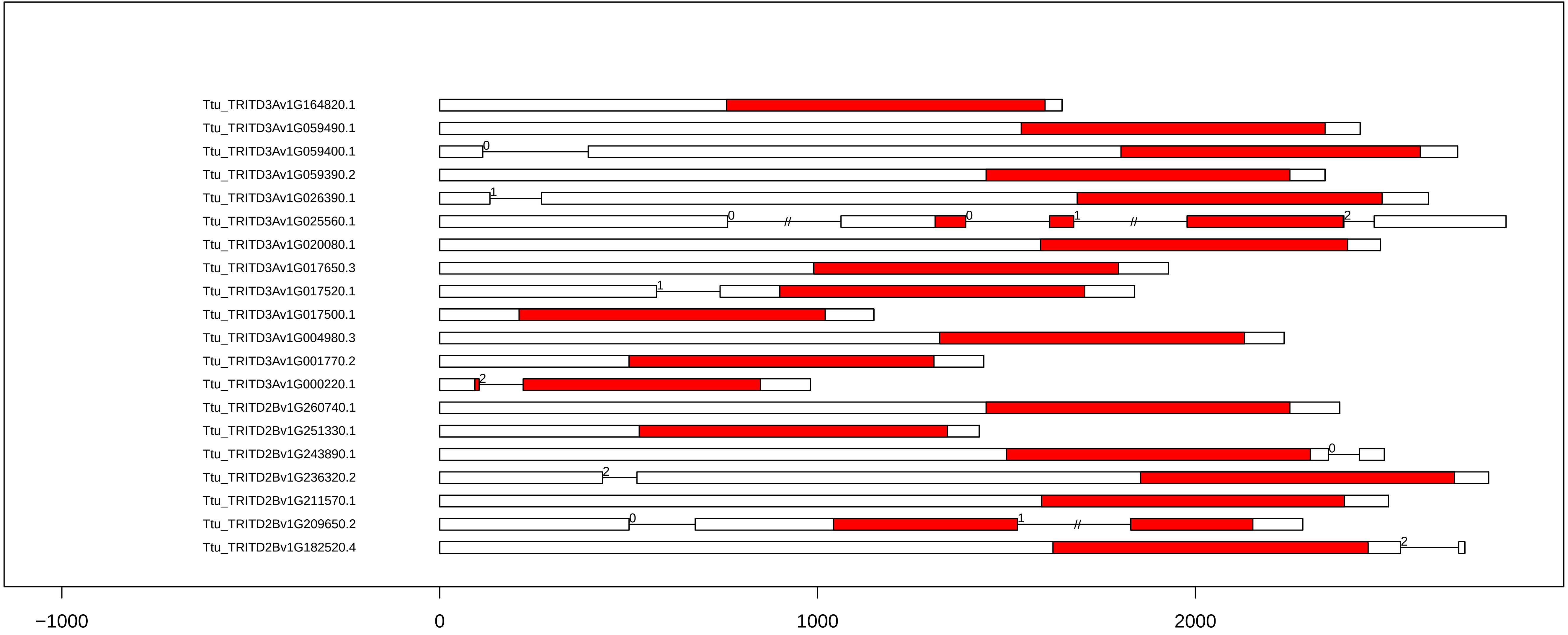

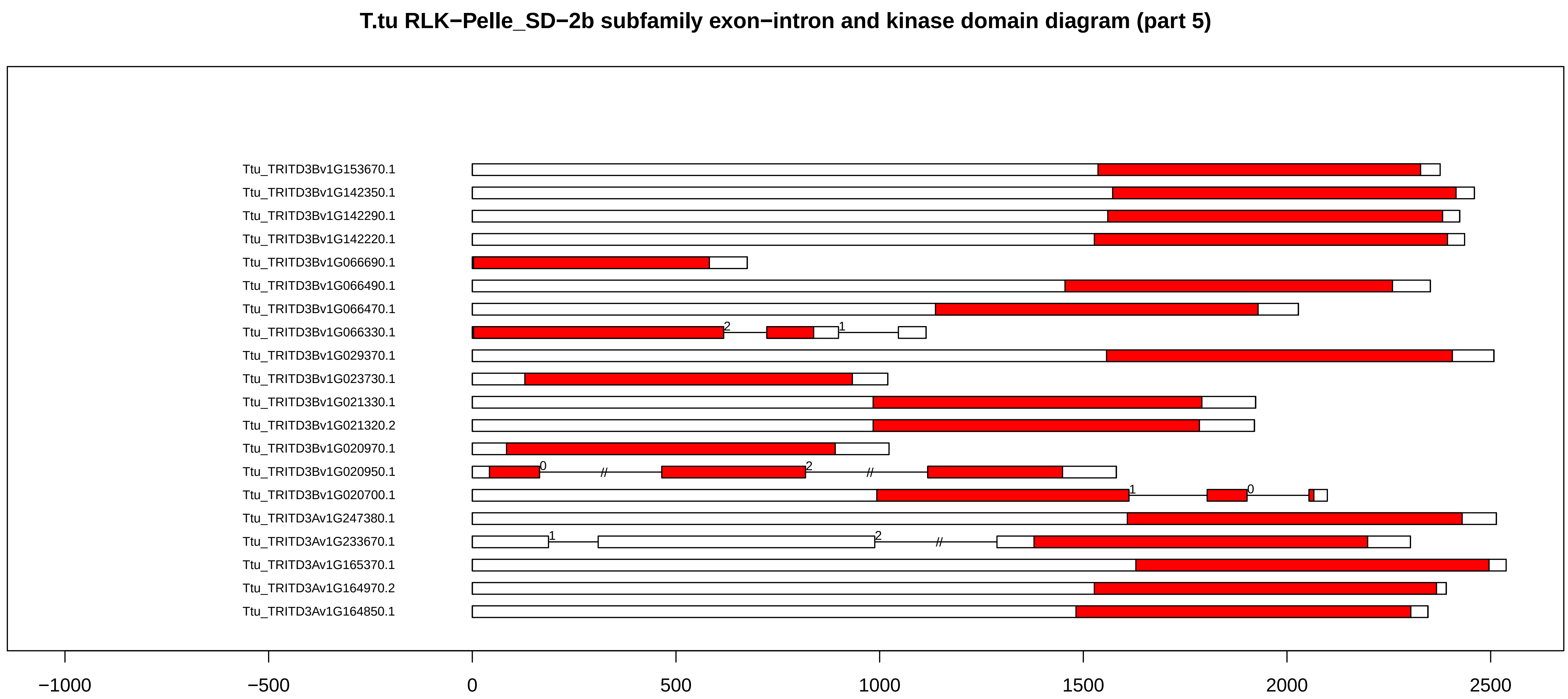
T.tu RLK−Pelle_SD−2b subfamily exon−intron and kinase domain diagram (part 5)
Ttu_TRITD3Bv1G153670.1
Ttu_TRITD3Bv1G142350.1
Ttu_TRITD3Bv1G142290.1
Ttu_TRITD3Bv1G142220.1
Ttu_TRITD3Bv1G066690.1
Ttu_TRITD3Bv1G066490.1
Ttu_TRITD3Bv1G066470.1
Ttu_TRITD3Bv1G066330.1
Ttu_TRITD3Bv1G029370.1
Ttu_TRITD3Bv1G023730.1
Ttu_TRITD3Bv1G021330.1
Ttu_TRITD3Bv1G021320.2
Ttu_TRITD3Bv1G020970.1
Ttu_TRITD3Bv1G020950.1
Ttu_TRITD3Bv1G020700.1
Ttu_TRITD3Av1G247380.1
Ttu_TRITD3Av1G233670.1
Ttu_TRITD3Av1G165370.1
Ttu_TRITD3Av1G164970.2
Ttu_TRITD3Av1G164850.1
−1000
−500
0
500
1000
1500
2000
2500

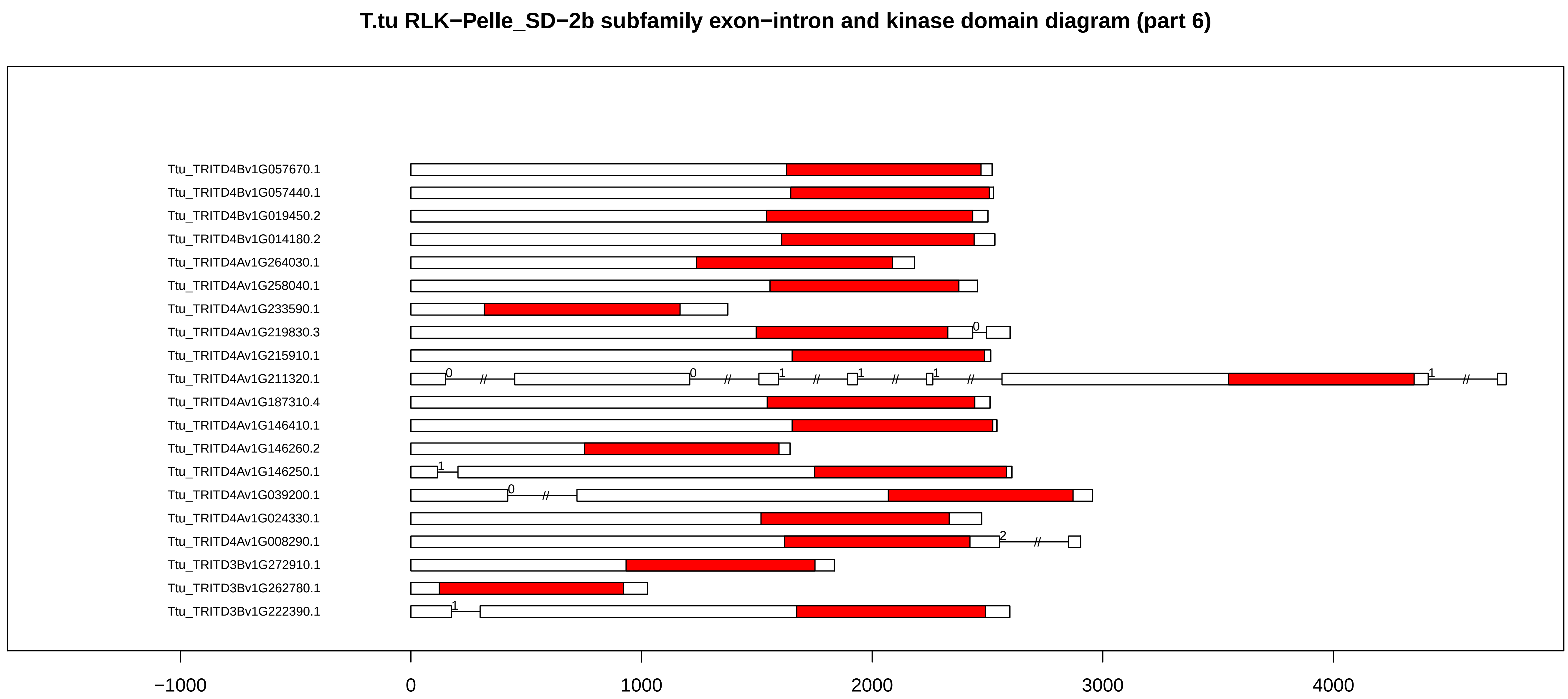

T.tu RLK−Pelle_SD−2b subfamily exon−intron and kinase domain diagram (part 6)
Ttu_TRITD4Bv1G057670.1
Ttu_TRITD4Bv1G057440.1
Ttu_TRITD4Bv1G019450.2
Ttu_TRITD4Bv1G014180.2
Ttu_TRITD4Av1G264030.1
Ttu_TRITD4Av1G258040.1
Ttu_TRITD4Av1G233590.1
Ttu_TRITD4Av1G219830.3
Ttu_TRITD4Av1G215910.1
Ttu_TRITD4Av1G211320.1
Ttu_TRITD4Av1G187310.4
Ttu_TRITD4Av1G146410.1
Ttu_TRITD4Av1G146260.2
Ttu_TRITD4Av1G146250.1
Ttu_TRITD4Av1G039200.1
Ttu_TRITD4Av1G024330.1
Ttu_TRITD4Av1G008290.1
Ttu_TRITD3Bv1G272910.1
Ttu_TRITD3Bv1G262780.1
Ttu_TRITD3Bv1G222390.1
−1000
0
1000
2000
3000
4000

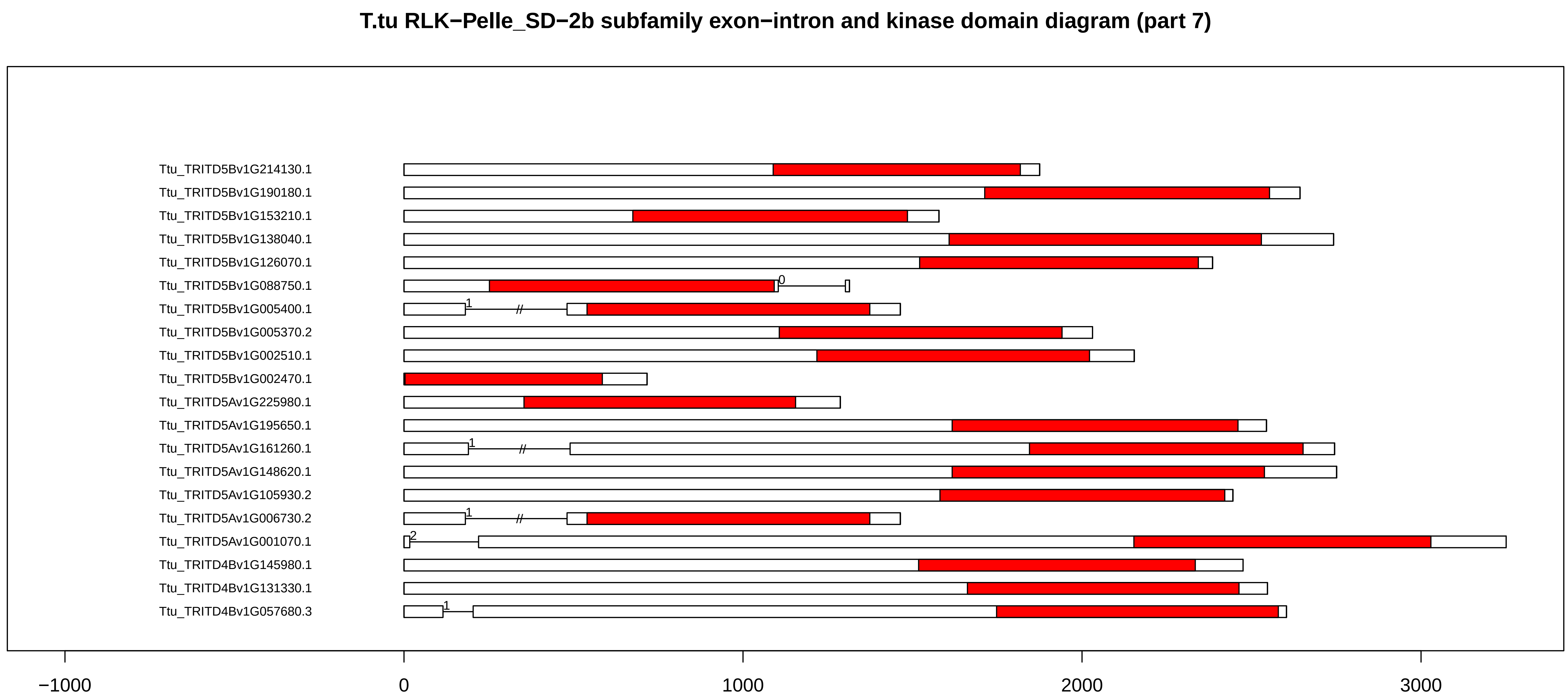
T.tu RLK−Pelle_SD−2b subfamily exon−intron and kinase domain diagram (part 7)
Ttu_TRITD5Bv1G214130.1
Ttu_TRITD5Bv1G190180.1
Ttu_TRITD5Bv1G153210.1
Ttu_TRITD5Bv1G138040.1
Ttu_TRITD5Bv1G126070.1
Ttu_TRITD5Bv1G088750.1
Ttu_TRITD5Bv1G005400.1
Ttu_TRITD5Bv1G005370.2
Ttu_TRITD5Bv1G002510.1
Ttu_TRITD5Bv1G002470.1
Ttu_TRITD5Av1G225980.1
Ttu_TRITD5Av1G195650.1
Ttu_TRITD5Av1G161260.1
Ttu_TRITD5Av1G148620.1
Ttu_TRITD5Av1G105930.2
Ttu_TRITD5Av1G006730.2
Ttu_TRITD5Av1G001070.1
Ttu_TRITD4Bv1G145980.1
Ttu_TRITD4Bv1G131330.1
Ttu_TRITD4Bv1G057680.3
0
1
1
1
2
1
−1000
0
1000
2000
3000

T.tu RLK−Pelle_SD−2b subfamily exon−intron and kinase domain diagram (part 8)

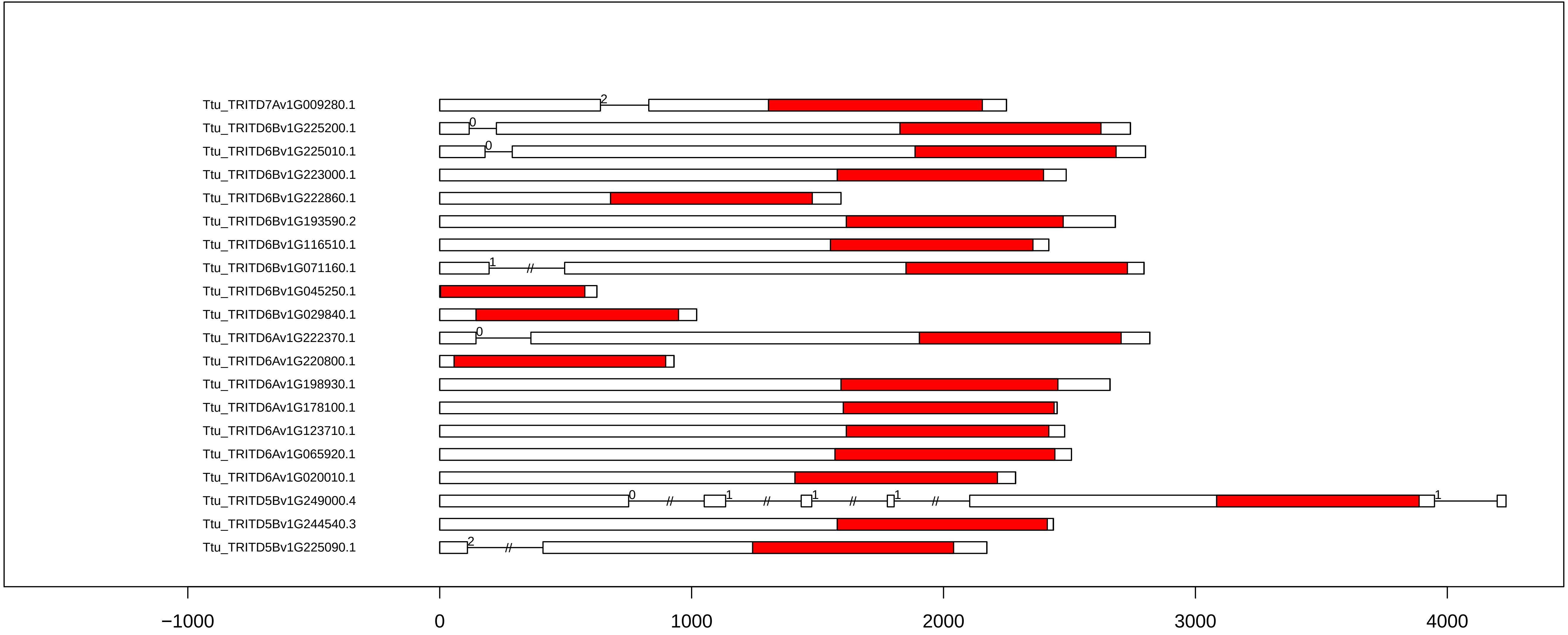

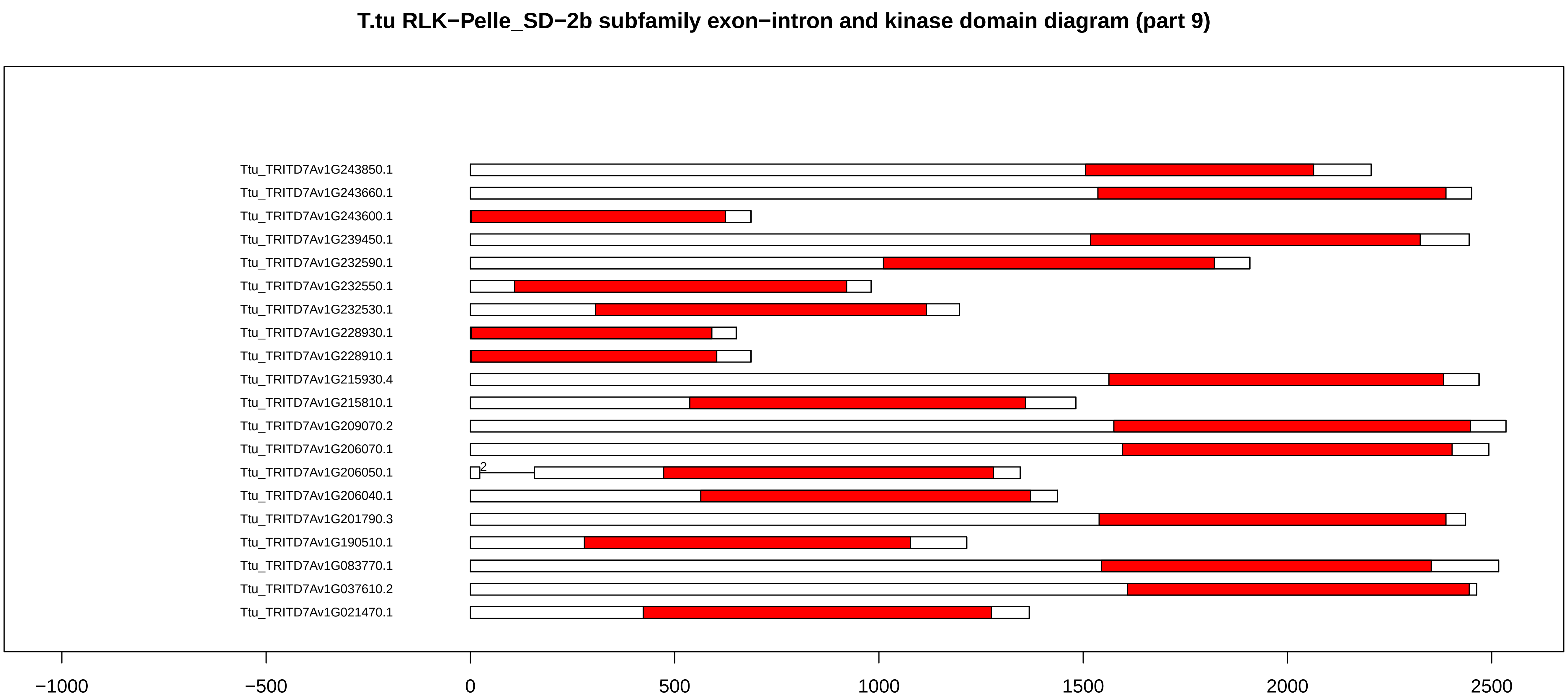
T.tu RLK−Pelle_SD−2b subfamily exon−intron and kinase domain diagram (part 9)
Ttu_TRITD7Av1G243850.1
Ttu_TRITD7Av1G243660.1
Ttu_TRITD7Av1G243600.1
Ttu_TRITD7Av1G239450.1
Ttu_TRITD7Av1G232590.1
Ttu_TRITD7Av1G232550.1
Ttu_TRITD7Av1G232530.1
Ttu_TRITD7Av1G228930.1
Ttu_TRITD7Av1G228910.1
Ttu_TRITD7Av1G215930.4
Ttu_TRITD7Av1G215810.1
Ttu_TRITD7Av1G209070.2
Ttu_TRITD7Av1G206070.1
Ttu_TRITD7Av1G206050.1
2
Ttu_TRITD7Av1G206040.1
Ttu_TRITD7Av1G201790.3
Ttu_TRITD7Av1G190510.1
Ttu_TRITD7Av1G083770.1
Ttu_TRITD7Av1G037610.2
Ttu_TRITD7Av1G021470.1
−1000
−500
0
500
1000
1500
2000
2500

T.tu RLK−Pelle_SD−2b subfamily exon−intron and kinase domain diagram (part 10)

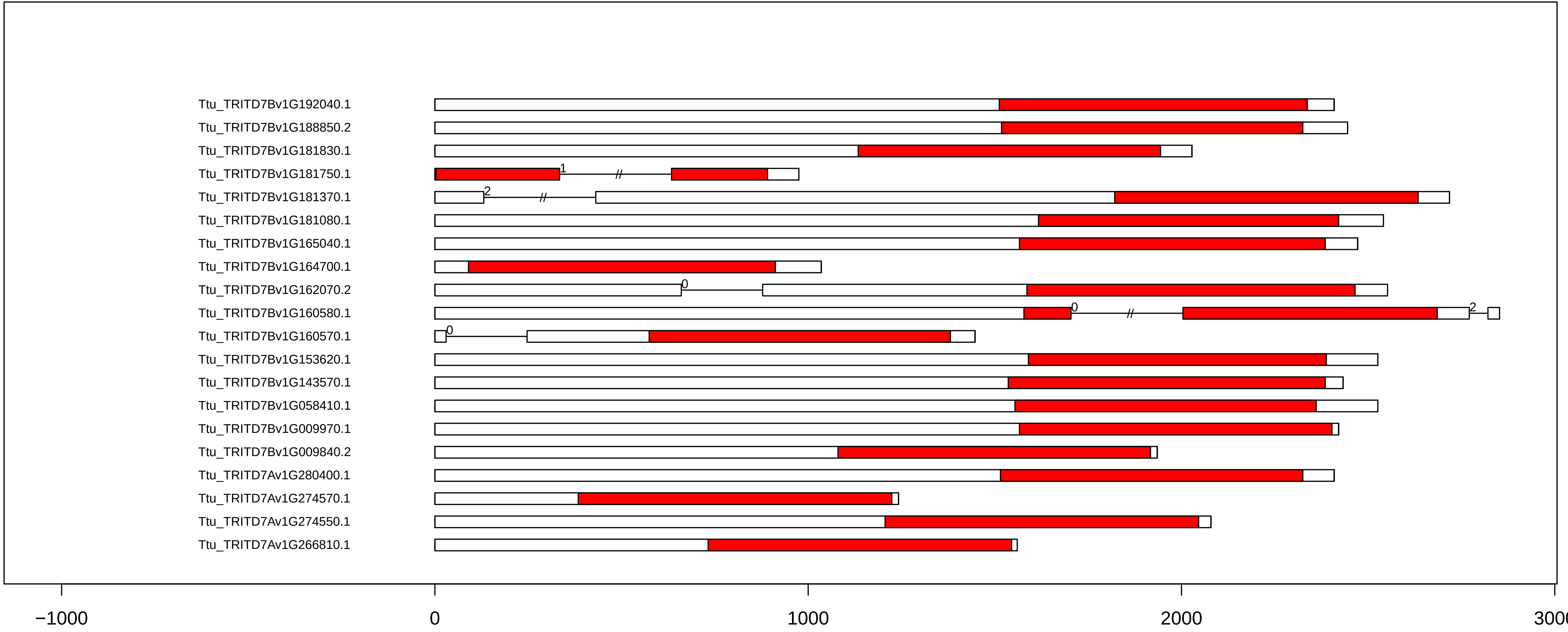

T.tu RLK−Pelle_SD−2b subfamily exon−intron and kinase domain diagram (part 11)

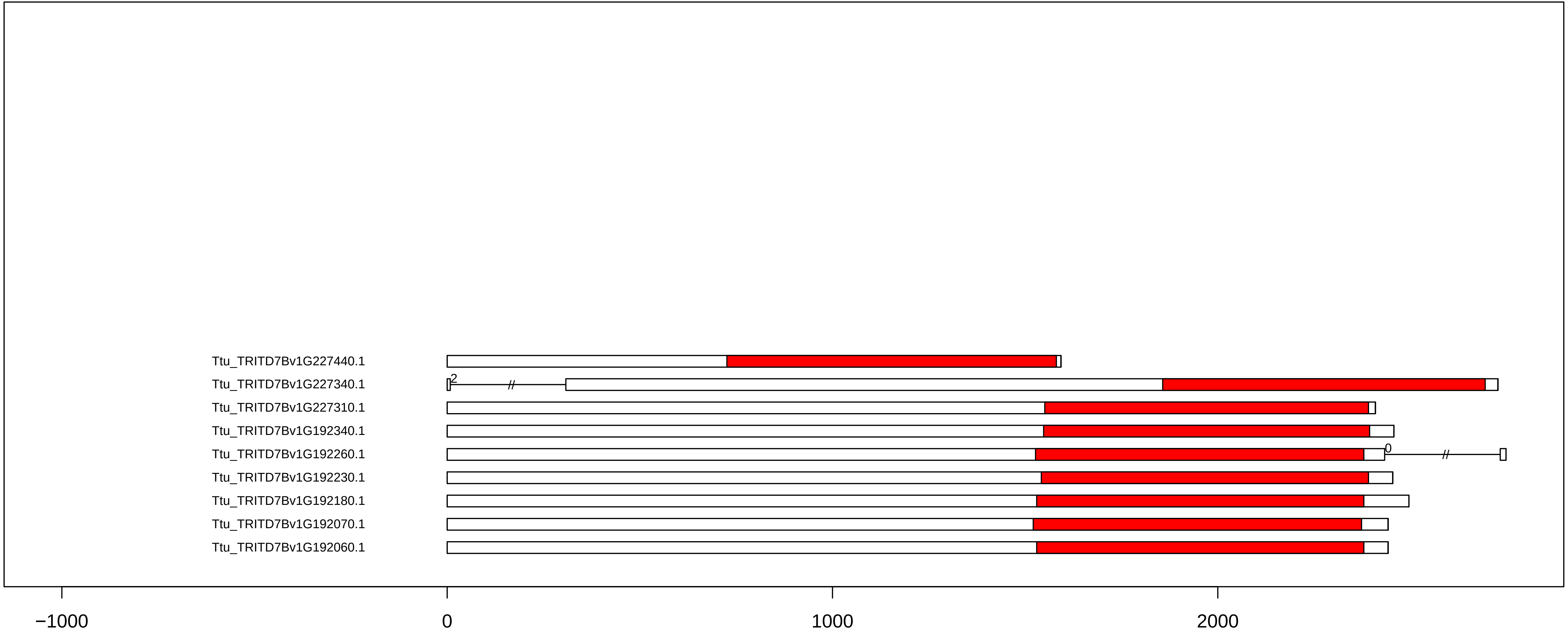

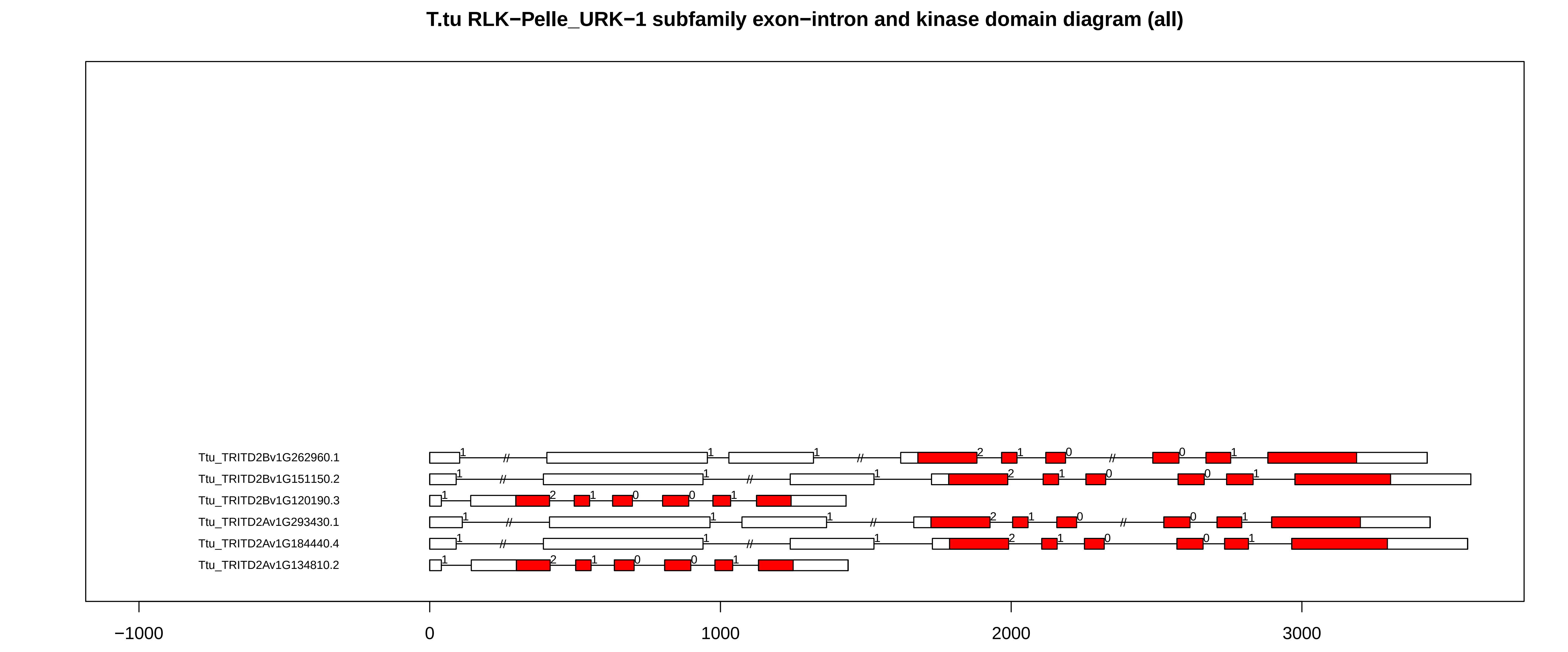
T.tu RLK−Pelle_URK−1 subfamily exon−intron and kinase domain diagram (all)
Ttu_TRITD2Bv1G262960.1
Ttu_TRITD2Bv1G151150.2
Ttu_TRITD2Bv1G120190.3
Ttu_TRITD2Av1G293430.1
Ttu_TRITD2Av1G184440.4
Ttu_TRITD2Av1G134810.2
−1000
0
1000
2000
3000

T.tu RLK−Pelle_URK−2 subfamily exon−intron and kinase domain diagram (all)

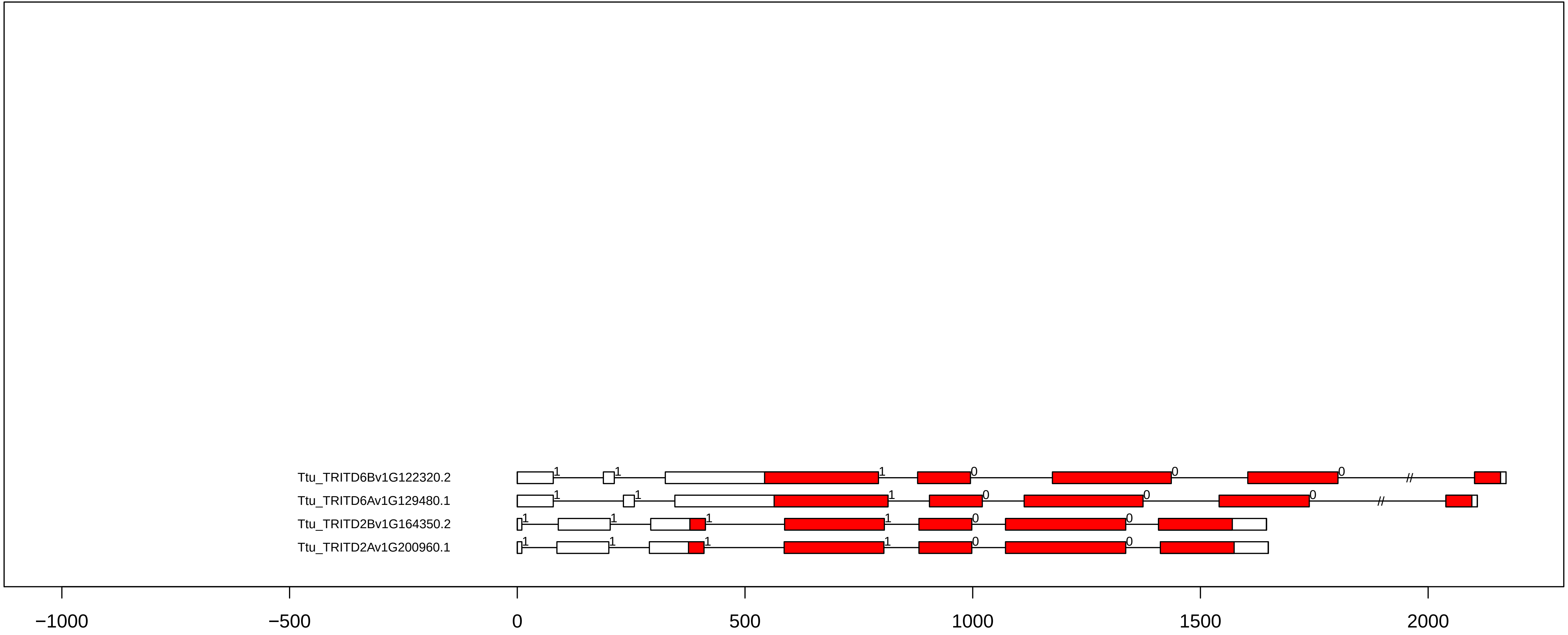

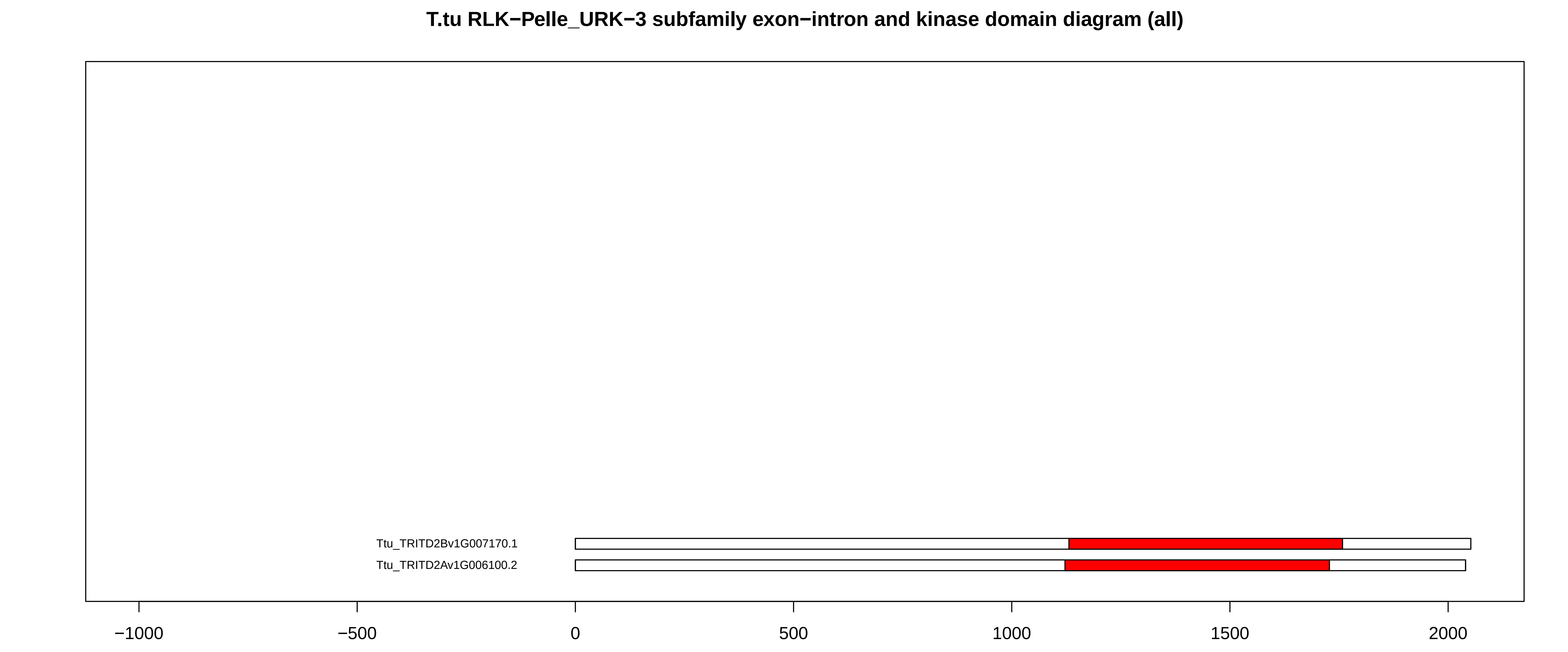
T.tu RLK−Pelle_URK−3 subfamily exon−intron and kinase domain diagram (all)
Ttu_TRITD2Bv1G007170.1
Ttu_TRITD2Av1G006100.2
−1000
−500
0
500
1000
1500
2000

T.tu RLK−Pelle_WAK_LRK10L−1 subfamily exon−intron and kinase domain diagram (all)

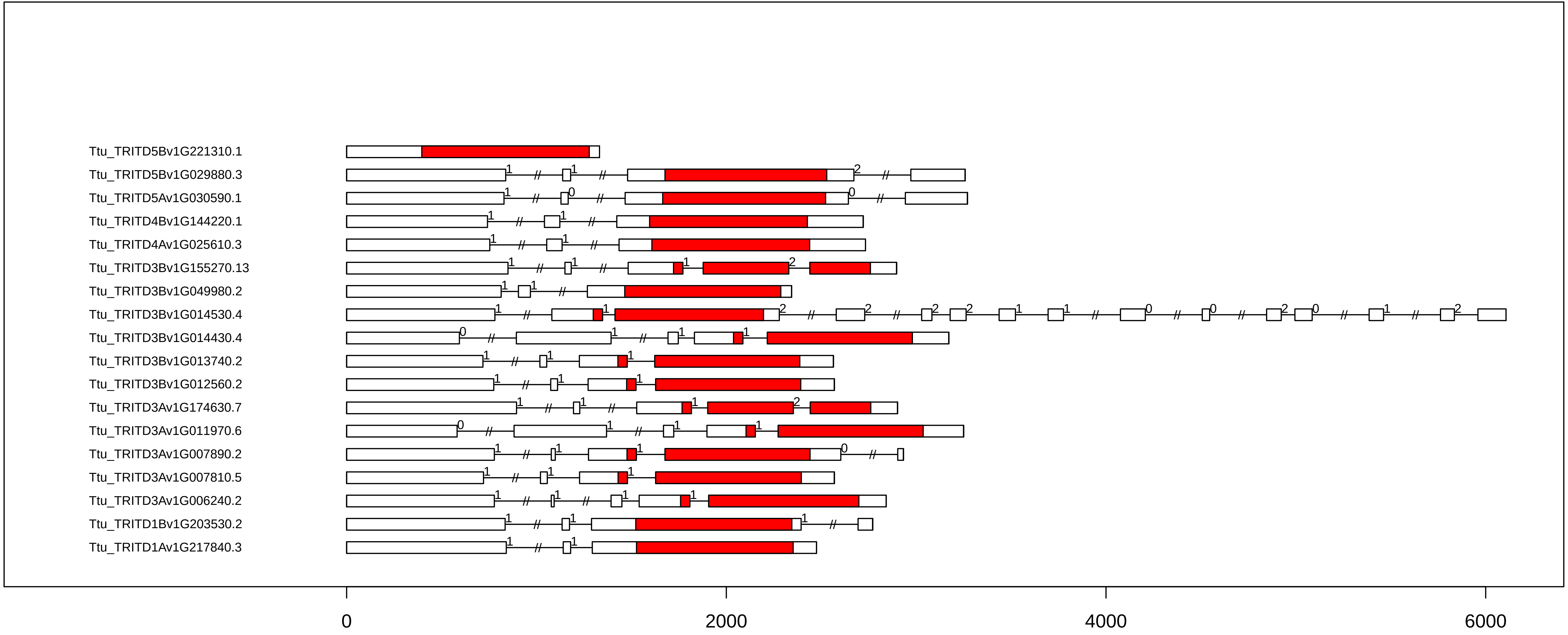

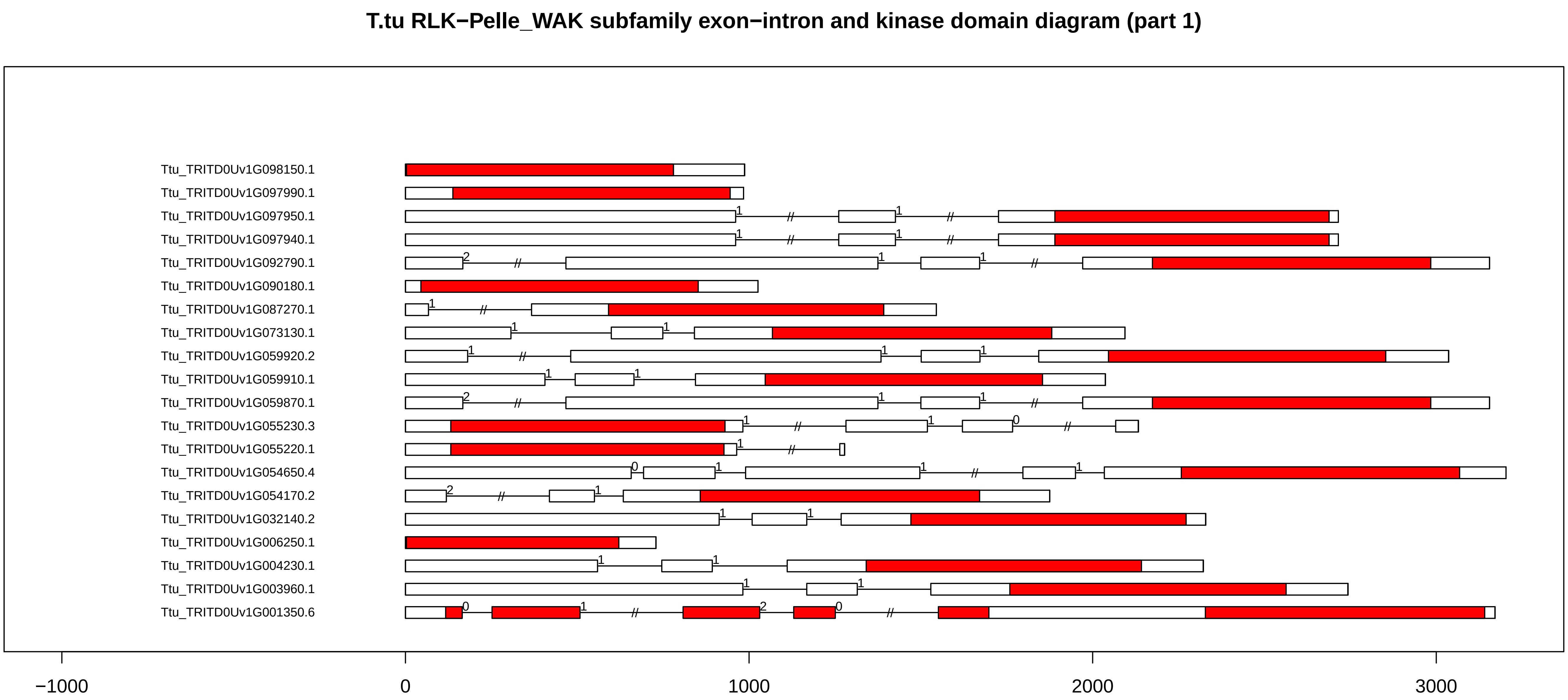

T.tu RLK−Pelle_WAK subfamily exon−intron and kinase domain diagram (part 1)
Ttu_TRITD0Uv1G098150.1
Ttu_TRITD0Uv1G097990.1
Ttu_TRITD0Uv1G097950.1
Ttu_TRITD0Uv1G097940.1
Ttu_TRITD0Uv1G092790.1
Ttu_TRITD0Uv1G090180.1
Ttu_TRITD0Uv1G087270.1
Ttu_TRITD0Uv1G073130.1
Ttu_TRITD0Uv1G059920.2
Ttu_TRITD0Uv1G059910.1
Ttu_TRITD0Uv1G059870.1
Ttu_TRITD0Uv1G055230.3
Ttu_TRITD0Uv1G055220.1
Ttu_TRITD0Uv1G054650.4
Ttu_TRITD0Uv1G054170.2
Ttu_TRITD0Uv1G032140.2
Ttu_TRITD0Uv1G006250.1
Ttu_TRITD0Uv1G004230.1
Ttu_TRITD0Uv1G003960.1
Ttu_TRITD0Uv1G001350.6
−1000
0
1000
2000
3000

T.tu RLK−Pelle_WAK subfamily exon−intron and kinase domain diagram (part 2)

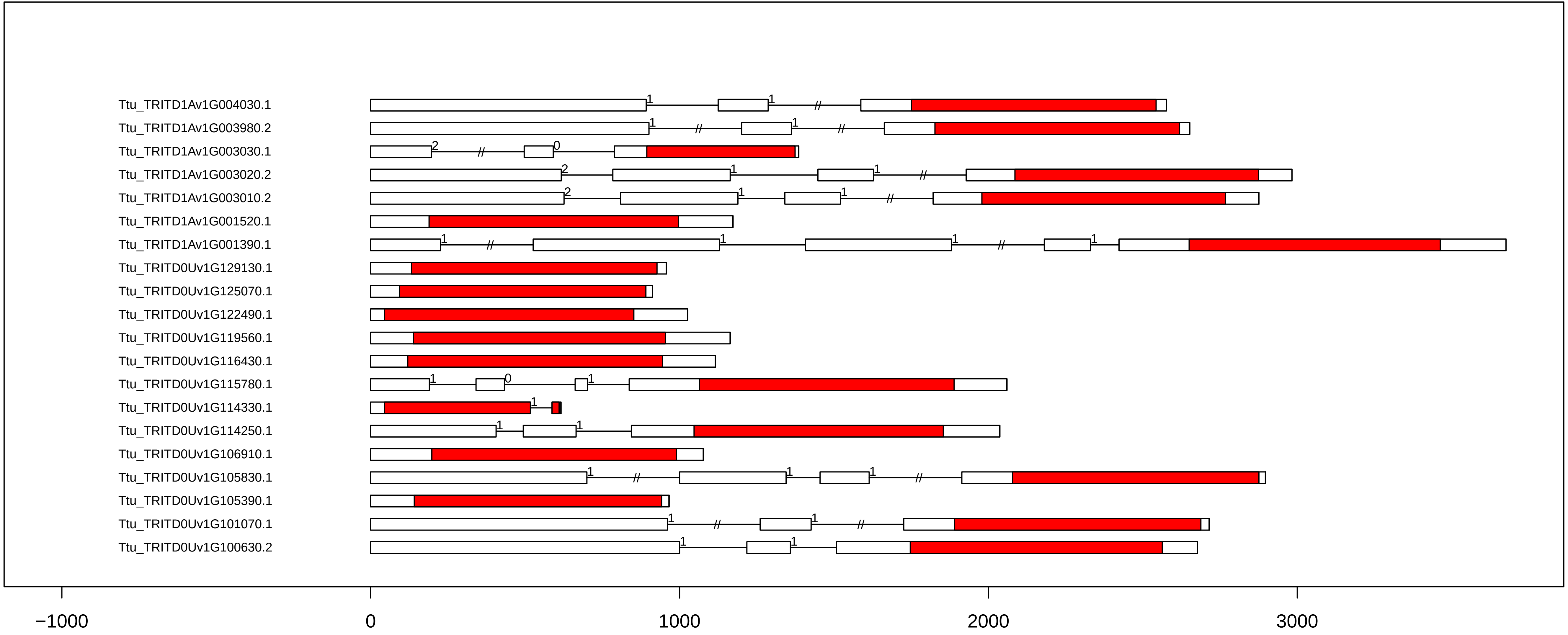

T.tu RLK−Pelle_WAK subfamily exon−intron and kinase domain diagram (part 3)

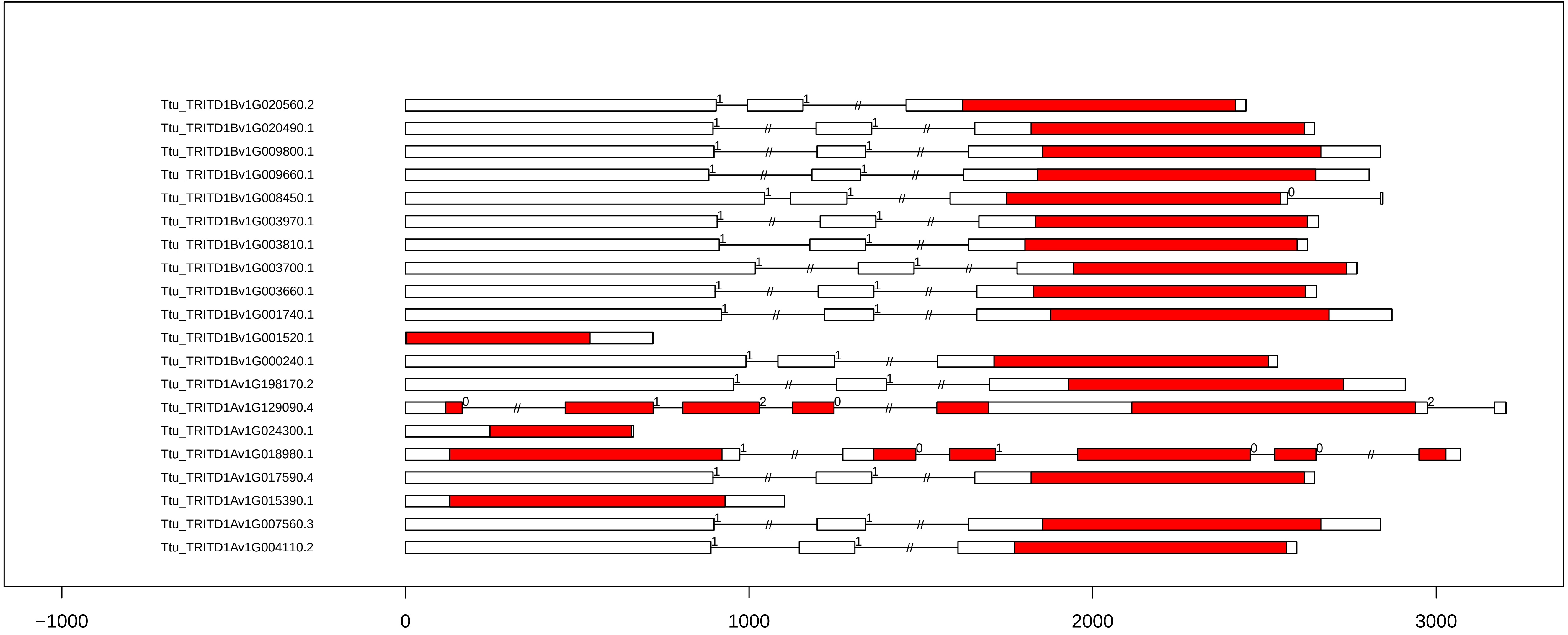

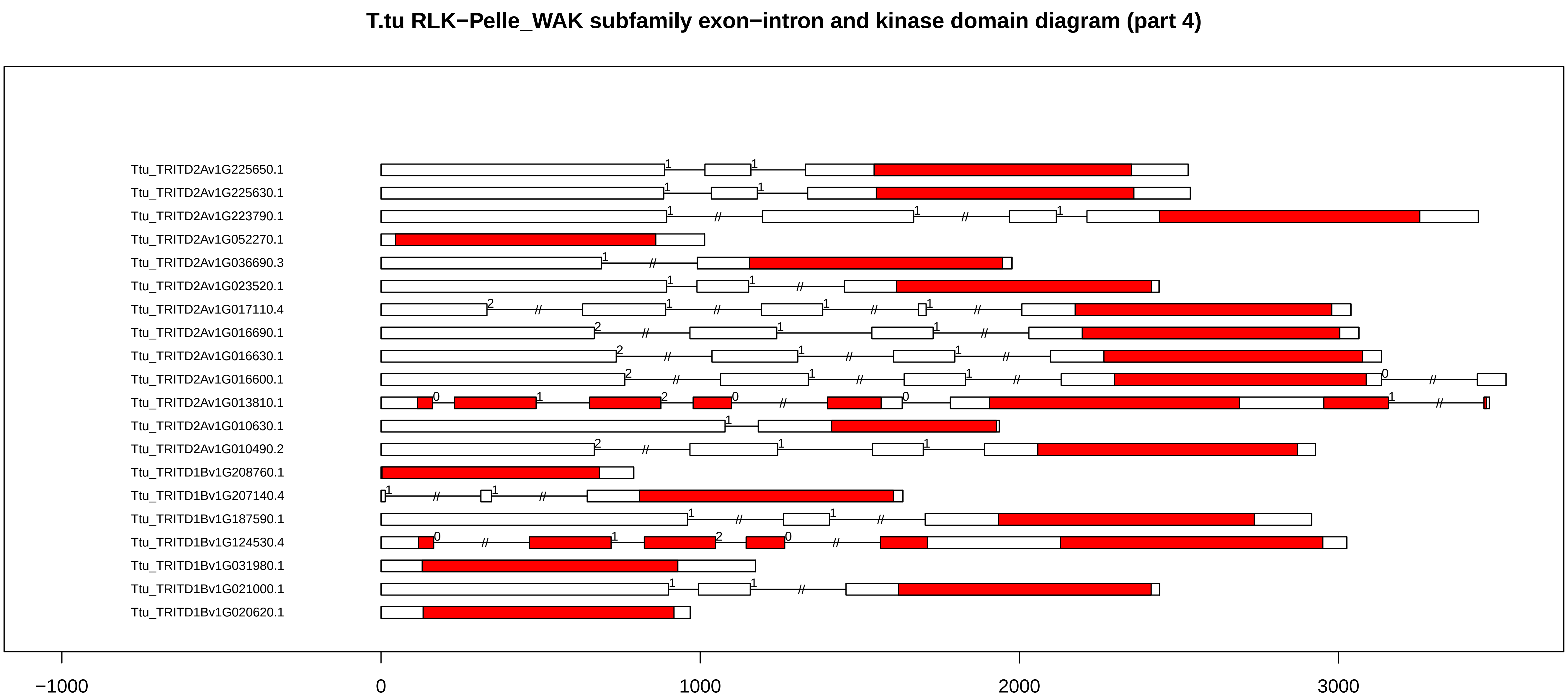
T.tu RLK−Pelle_WAK subfamily exon−intron and kinase domain diagram (part 4)
Ttu_TRITD2Av1G225650.1
Ttu_TRITD2Av1G225630.1
Ttu_TRITD2Av1G223790.1
Ttu_TRITD2Av1G052270.1
Ttu_TRITD2Av1G036690.3
Ttu_TRITD2Av1G023520.1
Ttu_TRITD2Av1G017110.4
Ttu_TRITD2Av1G016690.1
Ttu_TRITD2Av1G016630.1
Ttu_TRITD2Av1G016600.1
Ttu_TRITD2Av1G013810.1
Ttu_TRITD2Av1G010630.1
Ttu_TRITD2Av1G010490.2
Ttu_TRITD1Bv1G208760.1
Ttu_TRITD1Bv1G207140.4
Ttu_TRITD1Bv1G187590.1
Ttu_TRITD1Bv1G124530.4
Ttu_TRITD1Bv1G031980.1
Ttu_TRITD1Bv1G021000.1
Ttu_TRITD1Bv1G020620.1
−1000
0
1000
2000
3000

T.tu RLK−Pelle_WAK subfamily exon−intron and kinase domain diagram (part 5)

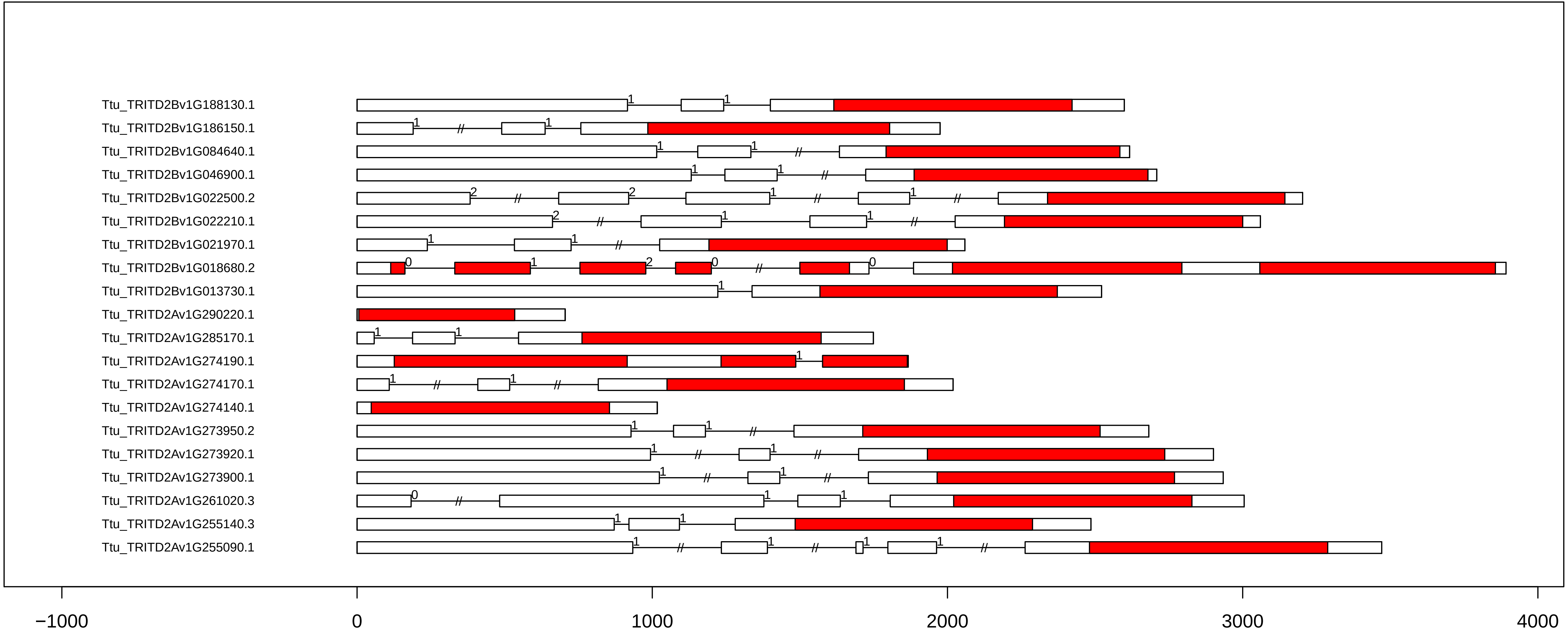

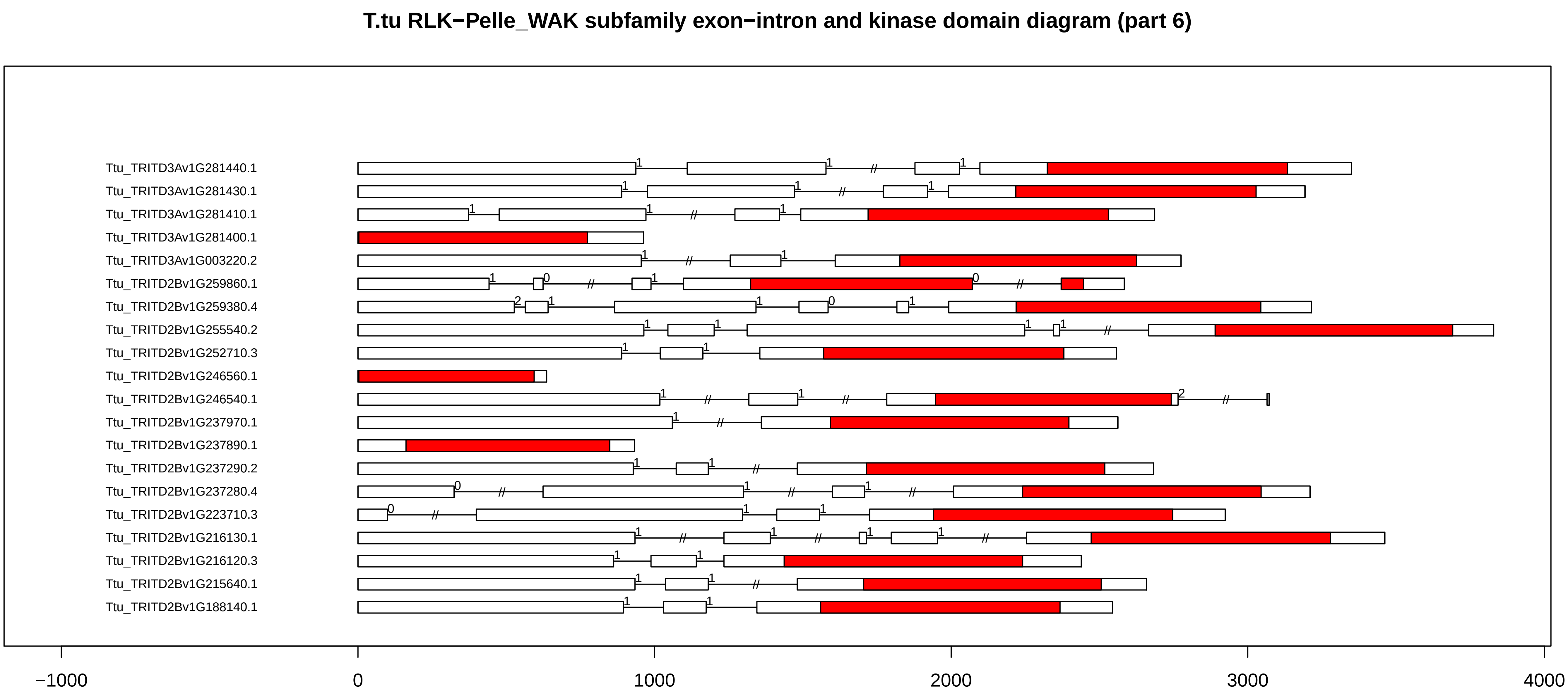
T.tu RLK−Pelle_WAK subfamily exon−intron and kinase domain diagram (part 6)
Ttu_TRITD3Av1G281440.1
Ttu_TRITD3Av1G281430.1
Ttu_TRITD3Av1G281410.1
Ttu_TRITD3Av1G281400.1
Ttu_TRITD3Av1G003220.2
Ttu_TRITD2Bv1G259860.1
Ttu_TRITD2Bv1G259380.4
Ttu_TRITD2Bv1G255540.2
Ttu_TRITD2Bv1G252710.3
Ttu_TRITD2Bv1G246560.1
Ttu_TRITD2Bv1G246540.1
Ttu_TRITD2Bv1G237970.1
Ttu_TRITD2Bv1G237890.1
Ttu_TRITD2Bv1G237290.2
Ttu_TRITD2Bv1G237280.4
Ttu_TRITD2Bv1G223710.3
Ttu_TRITD2Bv1G216130.1
Ttu_TRITD2Bv1G216120.3
Ttu_TRITD2Bv1G215640.1
Ttu_TRITD2Bv1G188140.1
−1000
0
1000
2000
3000
4000

T.tu RLK−Pelle_WAK subfamily exon−intron and kinase domain diagram (part 7)

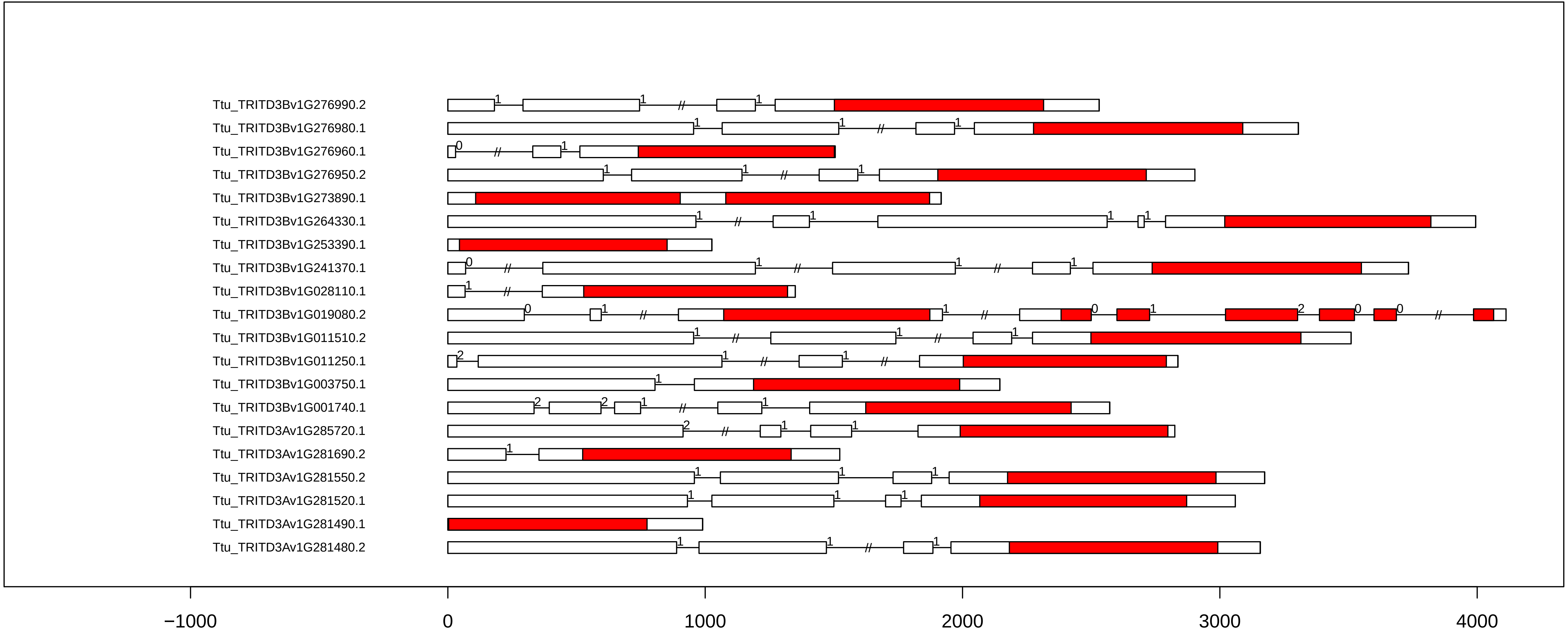

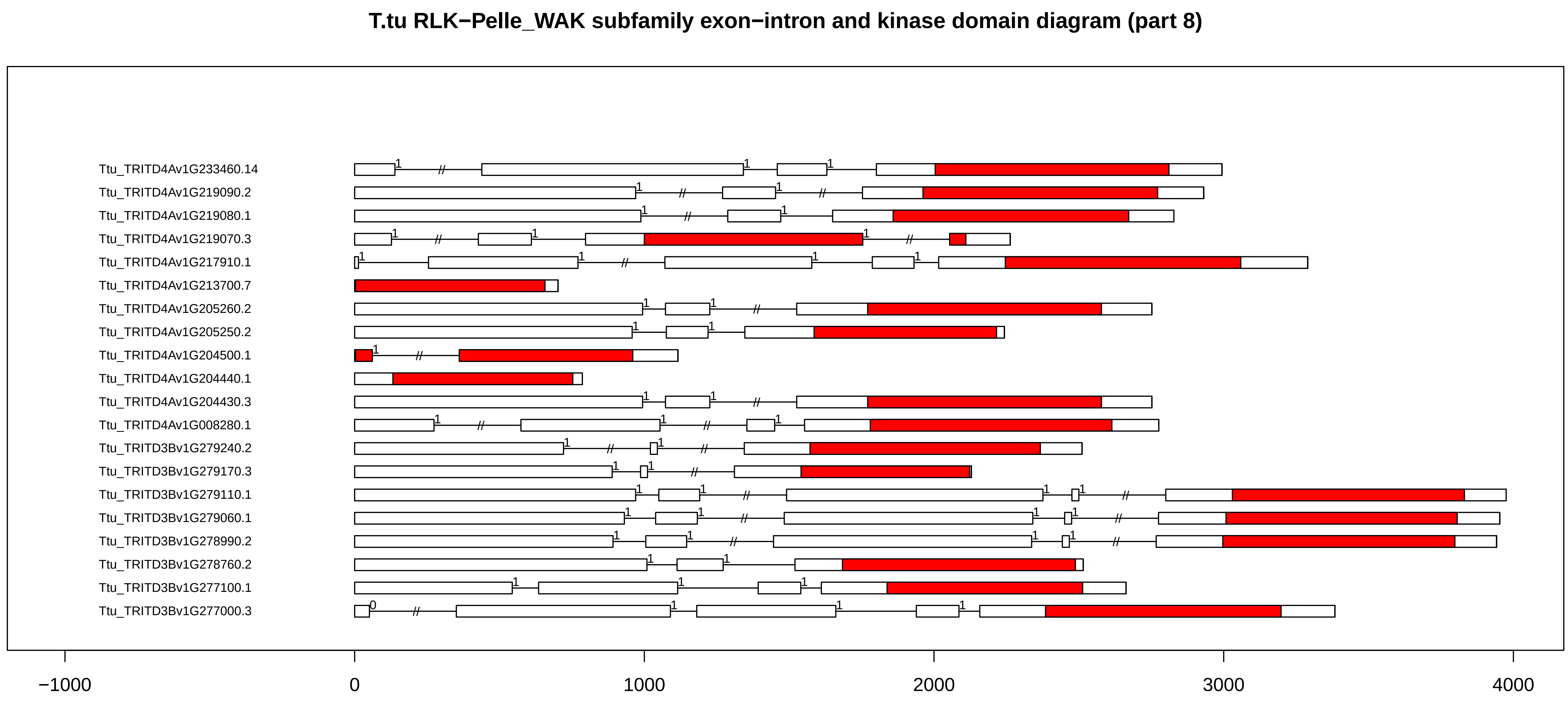
T.tu RLK−Pelle_WAK subfamily exon−intron and kinase domain diagram (part 8)
Ttu_TRITD4Av1G233460.14
Ttu_TRITD4Av1G219090.2
Ttu_TRITD4Av1G219080.1
Ttu_TRITD4Av1G219070.3
Ttu_TRITD4Av1G217910.1
Ttu_TRITD4Av1G213700.7
Ttu_TRITD4Av1G205260.2
Ttu_TRITD4Av1G205250.2
Ttu_TRITD4Av1G204500.1
Ttu_TRITD4Av1G204440.1
Ttu_TRITD4Av1G204430.3
Ttu_TRITD4Av1G008280.1
Ttu_TRITD3Bv1G279240.2
Ttu_TRITD3Bv1G279170.3
Ttu_TRITD3Bv1G279110.1
Ttu_TRITD3Bv1G279060.1
Ttu_TRITD3Bv1G278990.2
Ttu_TRITD3Bv1G278760.2
Ttu_TRITD3Bv1G277100.1
Ttu_TRITD3Bv1G277000.3
−1000
0
1000
2000
3000
4000

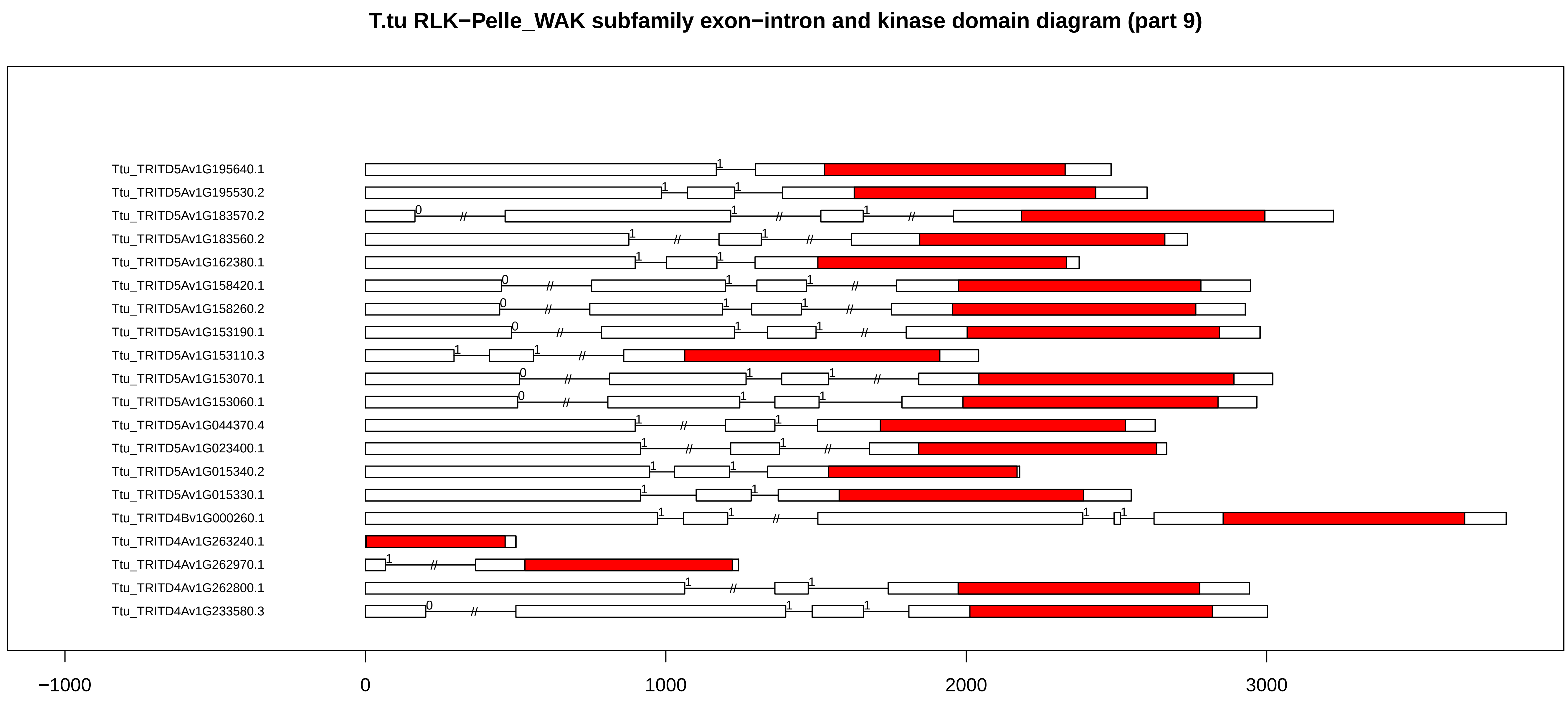

T.tu RLK−Pelle_WAK subfamily exon−intron and kinase domain diagram (part 9)
Ttu_TRITD5Av1G195640.1
Ttu_TRITD5Av1G195530.2
Ttu_TRITD5Av1G183570.2
Ttu_TRITD5Av1G183560.2
Ttu_TRITD5Av1G162380.1
Ttu_TRITD5Av1G158420.1
Ttu_TRITD5Av1G158260.2
Ttu_TRITD5Av1G153190.1
Ttu_TRITD5Av1G153110.3
Ttu_TRITD5Av1G153070.1
Ttu_TRITD5Av1G153060.1
Ttu_TRITD5Av1G044370.4
Ttu_TRITD5Av1G023400.1
Ttu_TRITD5Av1G015340.2
Ttu_TRITD5Av1G015330.1
Ttu_TRITD4Bv1G000260.1
Ttu_TRITD4Av1G263240.1
Ttu_TRITD4Av1G262970.1
Ttu_TRITD4Av1G262800.1
Ttu_TRITD4Av1G233580.3
−1000
0
1000
2000
3000

T.tu RLK−Pelle_WAK subfamily exon−intron and kinase domain diagram (part 10)

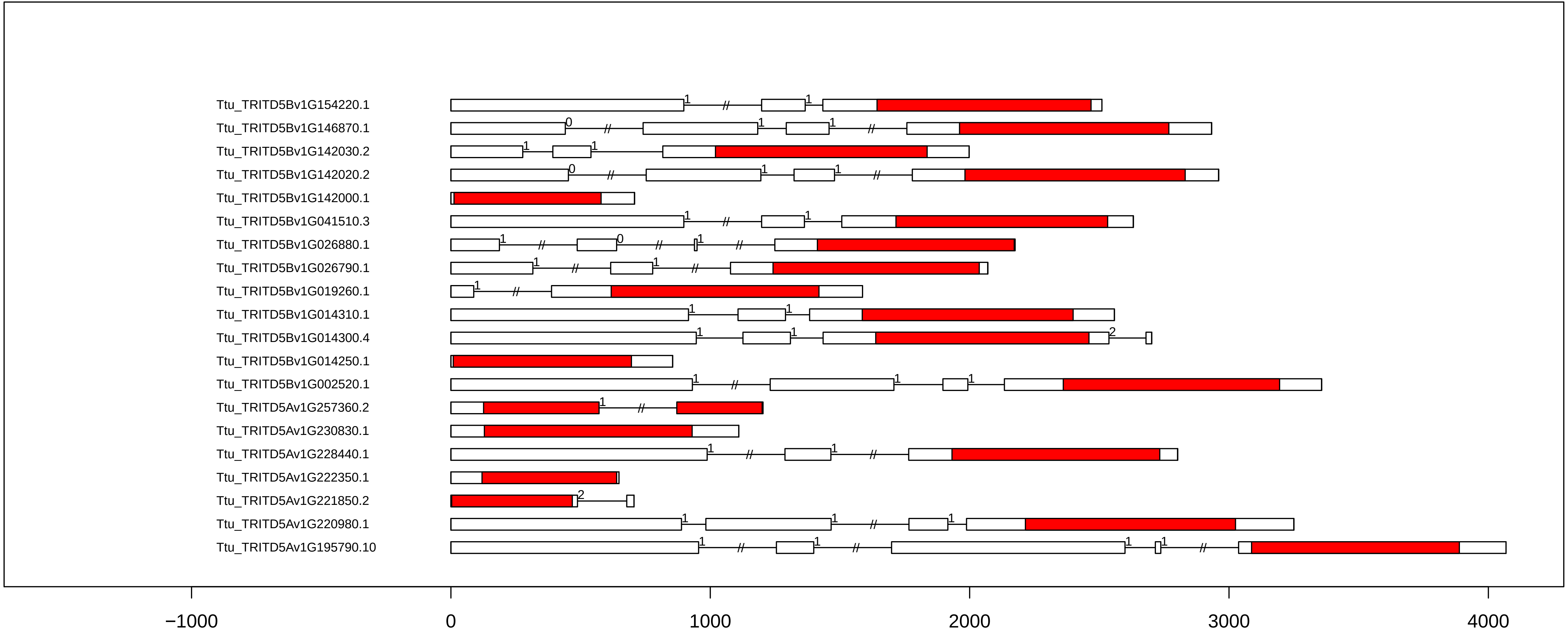

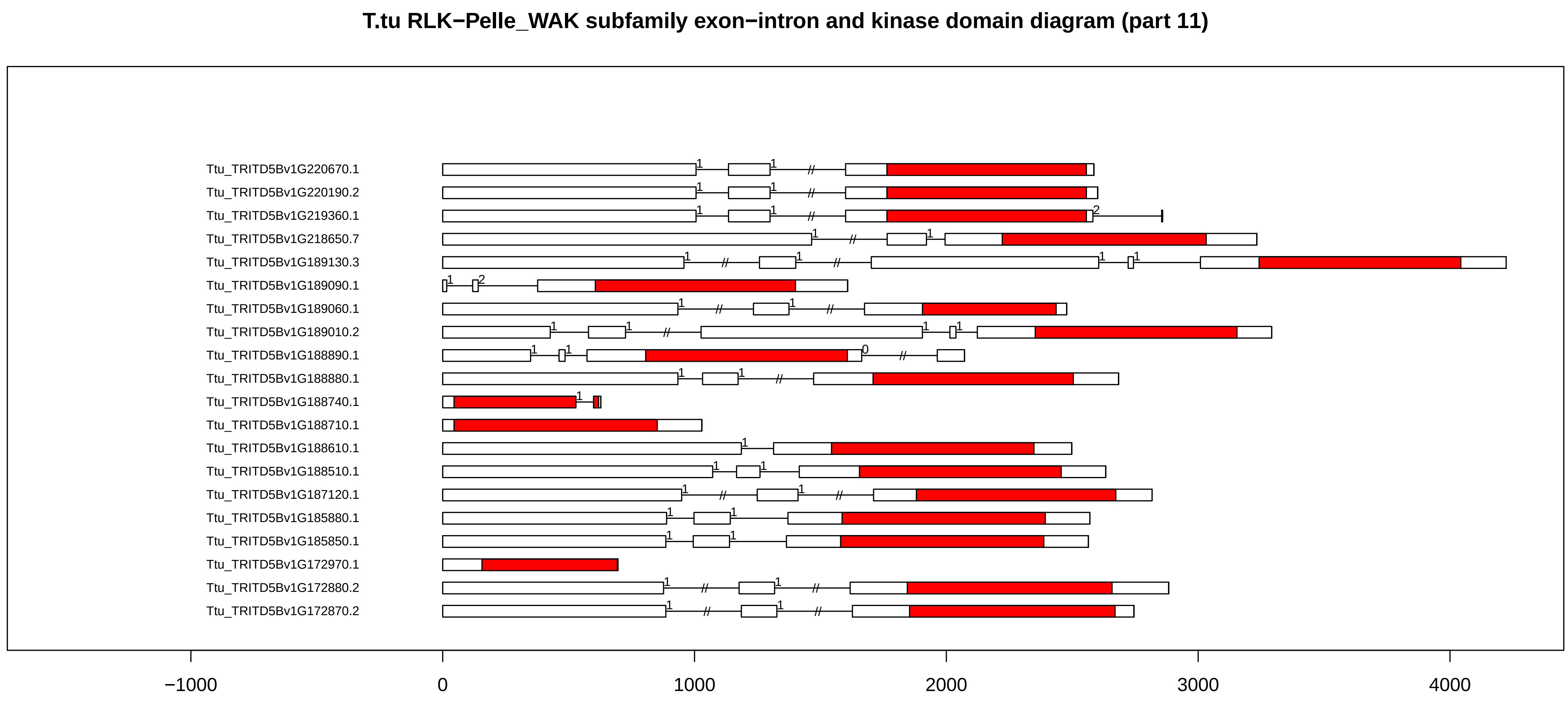
T.tu RLK−Pelle_WAK subfamily exon−intron and kinase domain diagram (part 11)
Ttu_TRITD5Bv1G220670.1
Ttu_TRITD5Bv1G220190.2
Ttu_TRITD5Bv1G219360.1
Ttu_TRITD5Bv1G218650.7
Ttu_TRITD5Bv1G189130.3
Ttu_TRITD5Bv1G189090.1
Ttu_TRITD5Bv1G189060.1
Ttu_TRITD5Bv1G189010.2
Ttu_TRITD5Bv1G188890.1
Ttu_TRITD5Bv1G188880.1
Ttu_TRITD5Bv1G188740.1
Ttu_TRITD5Bv1G188710.1
Ttu_TRITD5Bv1G188610.1
Ttu_TRITD5Bv1G188510.1
Ttu_TRITD5Bv1G187120.1
Ttu_TRITD5Bv1G185880.1
Ttu_TRITD5Bv1G185850.1
Ttu_TRITD5Bv1G172970.1
Ttu_TRITD5Bv1G172880.2
Ttu_TRITD5Bv1G172870.2
−1000
0
1000
2000
3000
4000

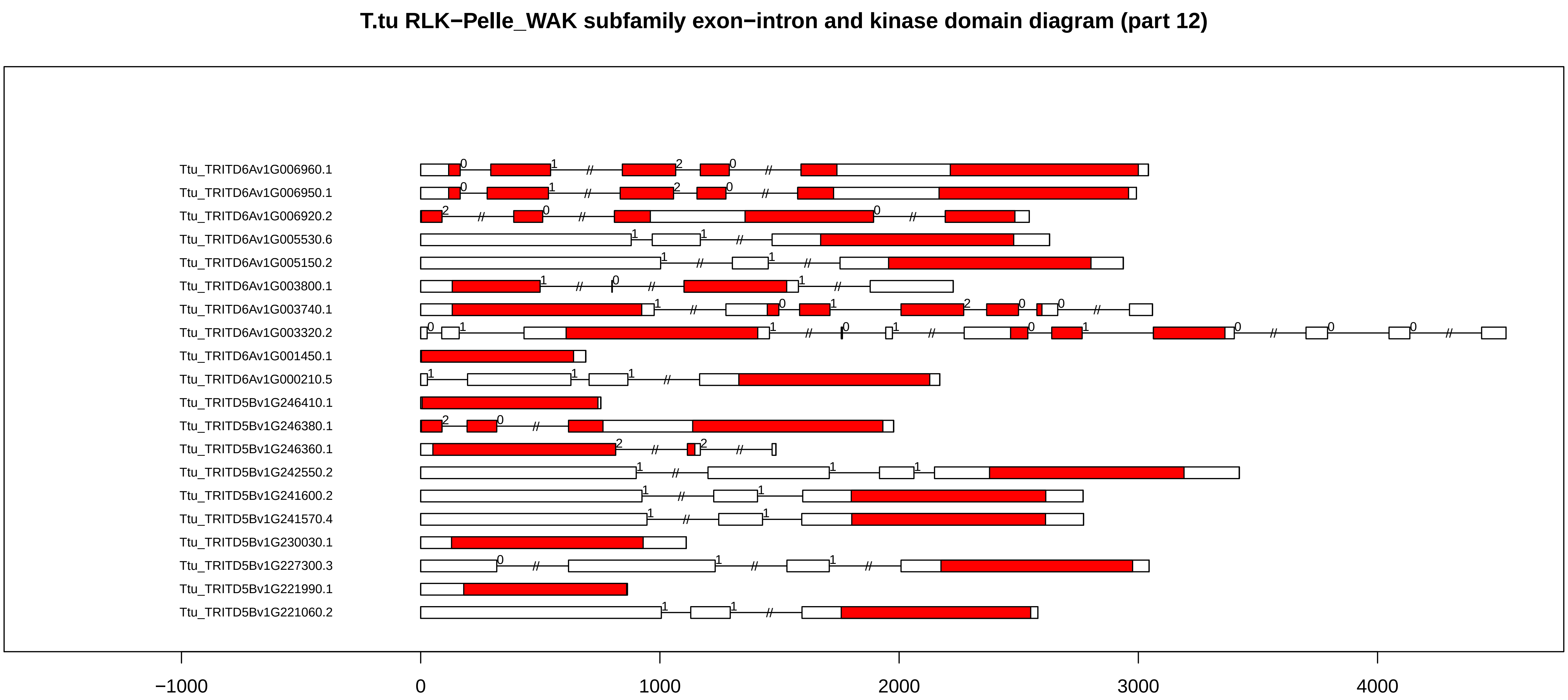

T.tu RLK−Pelle_WAK subfamily exon−intron and kinase domain diagram (part 12)
Ttu_TRITD6Av1G006960.1
Ttu_TRITD6Av1G006950.1
Ttu_TRITD6Av1G006920.2
Ttu_TRITD6Av1G005530.6
Ttu_TRITD6Av1G005150.2
Ttu_TRITD6Av1G003800.1
Ttu_TRITD6Av1G003740.1
Ttu_TRITD6Av1G003320.2
Ttu_TRITD6Av1G001450.1
Ttu_TRITD6Av1G000210.5
Ttu_TRITD5Bv1G246410.1
Ttu_TRITD5Bv1G246380.1
Ttu_TRITD5Bv1G246360.1
Ttu_TRITD5Bv1G242550.2
Ttu_TRITD5Bv1G241600.2
Ttu_TRITD5Bv1G241570.4
Ttu_TRITD5Bv1G230030.1
Ttu_TRITD5Bv1G227300.3
Ttu_TRITD5Bv1G221990.1
Ttu_TRITD5Bv1G221060.2
−1000
0
1000
2000
3000
4000

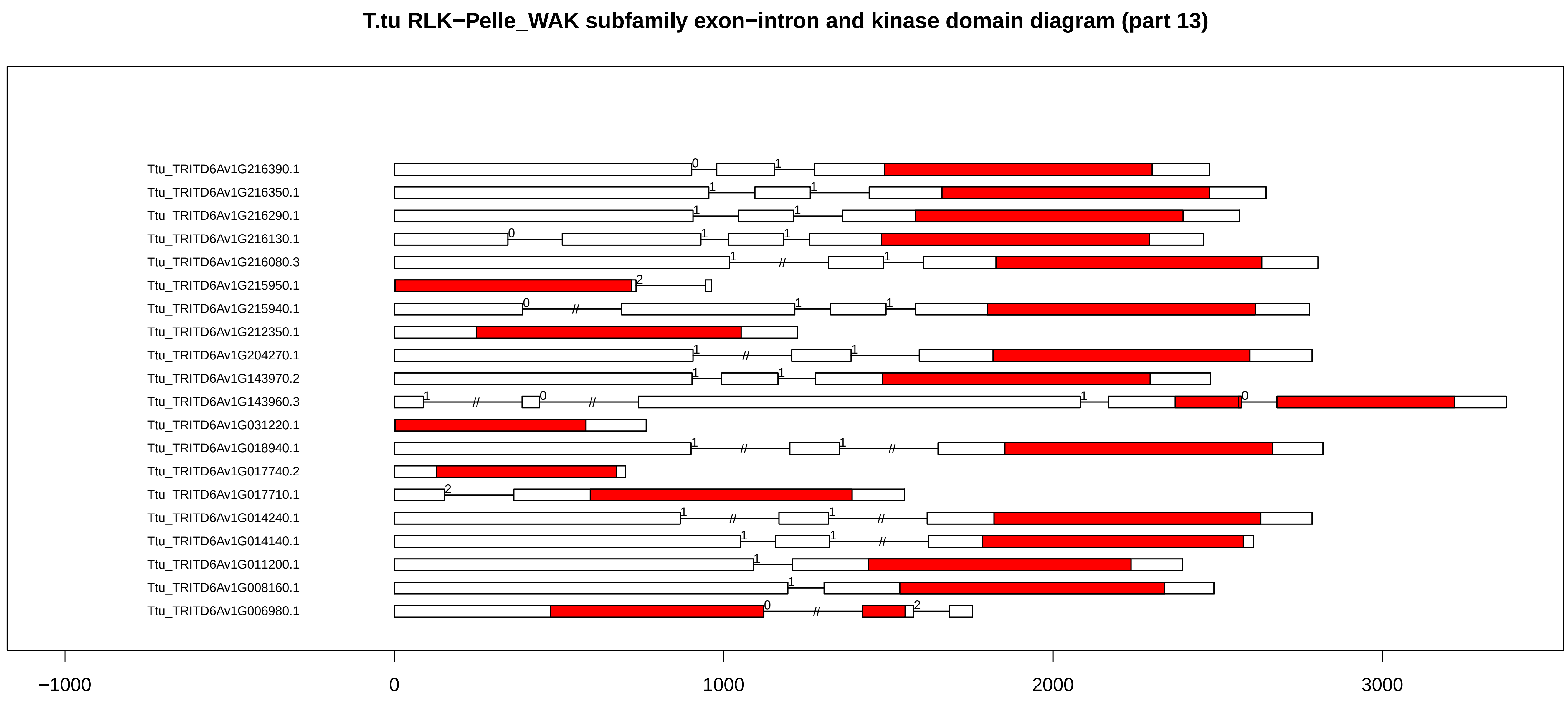

T.tu RLK−Pelle_WAK subfamily exon−intron and kinase domain diagram (part 13)
Ttu_TRITD6Av1G216390.1
Ttu_TRITD6Av1G216350.1
Ttu_TRITD6Av1G216290.1
Ttu_TRITD6Av1G216130.1
Ttu_TRITD6Av1G216080.3
Ttu_TRITD6Av1G215950.1
Ttu_TRITD6Av1G215940.1
Ttu_TRITD6Av1G212350.1
Ttu_TRITD6Av1G204270.1
Ttu_TRITD6Av1G143970.2
Ttu_TRITD6Av1G143960.3
Ttu_TRITD6Av1G031220.1
Ttu_TRITD6Av1G018940.1
Ttu_TRITD6Av1G017740.2
Ttu_TRITD6Av1G017710.1
Ttu_TRITD6Av1G014240.1
Ttu_TRITD6Av1G014140.1
Ttu_TRITD6Av1G011200.1
Ttu_TRITD6Av1G008160.1
Ttu_TRITD6Av1G006980.1
−1000
0
1000
2000
3000

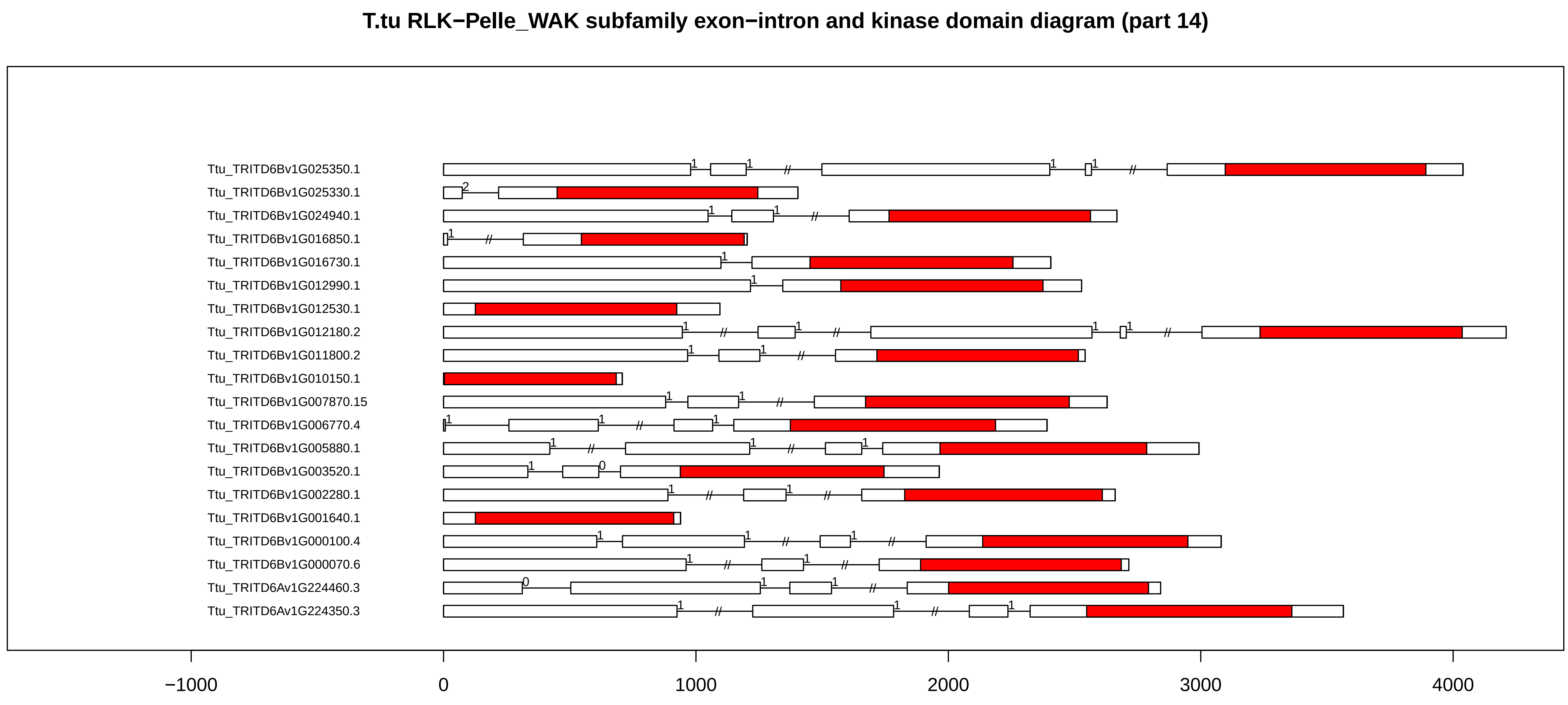

T.tu RLK−Pelle_WAK subfamily exon−intron and kinase domain diagram (part 14)
Ttu_TRITD6Bv1G025350.1
Ttu_TRITD6Bv1G025330.1
Ttu_TRITD6Bv1G024940.1
Ttu_TRITD6Bv1G016850.1
Ttu_TRITD6Bv1G016730.1
Ttu_TRITD6Bv1G012990.1
Ttu_TRITD6Bv1G012530.1
Ttu_TRITD6Bv1G012180.2
Ttu_TRITD6Bv1G011800.2
Ttu_TRITD6Bv1G010150.1
Ttu_TRITD6Bv1G007870.15
Ttu_TRITD6Bv1G006770.4
Ttu_TRITD6Bv1G005880.1
Ttu_TRITD6Bv1G003520.1
Ttu_TRITD6Bv1G002280.1
Ttu_TRITD6Bv1G001640.1
Ttu_TRITD6Bv1G000100.4
Ttu_TRITD6Bv1G000070.6
Ttu_TRITD6Av1G224460.3
Ttu_TRITD6Av1G224350.3
−1000
0
1000
2000
3000
4000

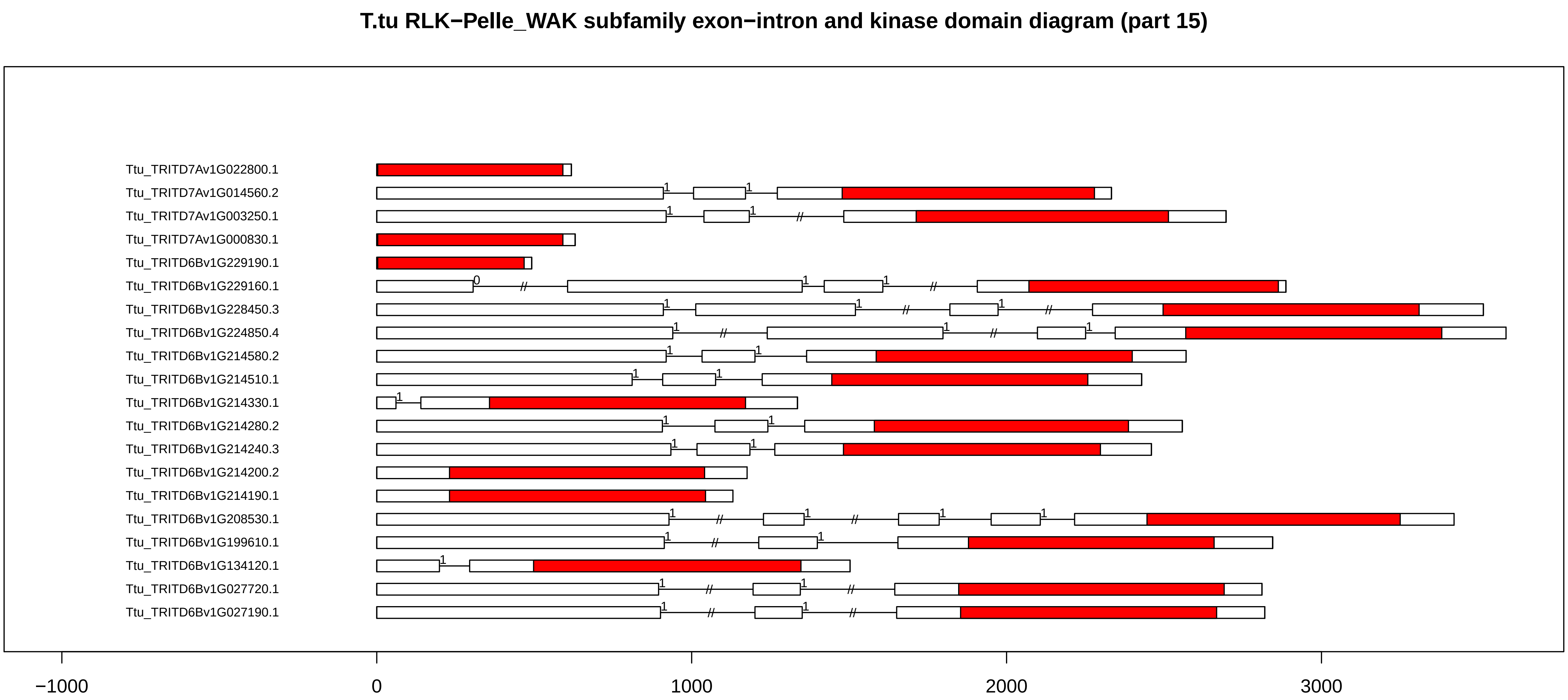

T.tu RLK−Pelle_WAK subfamily exon−intron and kinase domain diagram (part 15)
Ttu_TRITD7Av1G022800.1
Ttu_TRITD7Av1G014560.2
Ttu_TRITD7Av1G003250.1
Ttu_TRITD7Av1G000830.1
Ttu_TRITD6Bv1G229190.1
Ttu_TRITD6Bv1G229160.1
Ttu_TRITD6Bv1G228450.3
Ttu_TRITD6Bv1G224850.4
Ttu_TRITD6Bv1G214580.2
Ttu_TRITD6Bv1G214510.1
Ttu_TRITD6Bv1G214330.1
Ttu_TRITD6Bv1G214280.2
Ttu_TRITD6Bv1G214240.3
Ttu_TRITD6Bv1G214200.2
Ttu_TRITD6Bv1G214190.1
Ttu_TRITD6Bv1G208530.1
Ttu_TRITD6Bv1G199610.1
Ttu_TRITD6Bv1G134120.1
Ttu_TRITD6Bv1G027720.1
Ttu_TRITD6Bv1G027190.1
−1000
0
1000
2000
3000

T.tu RLK−Pelle_WAK subfamily exon−intron and kinase domain diagram (part 16)

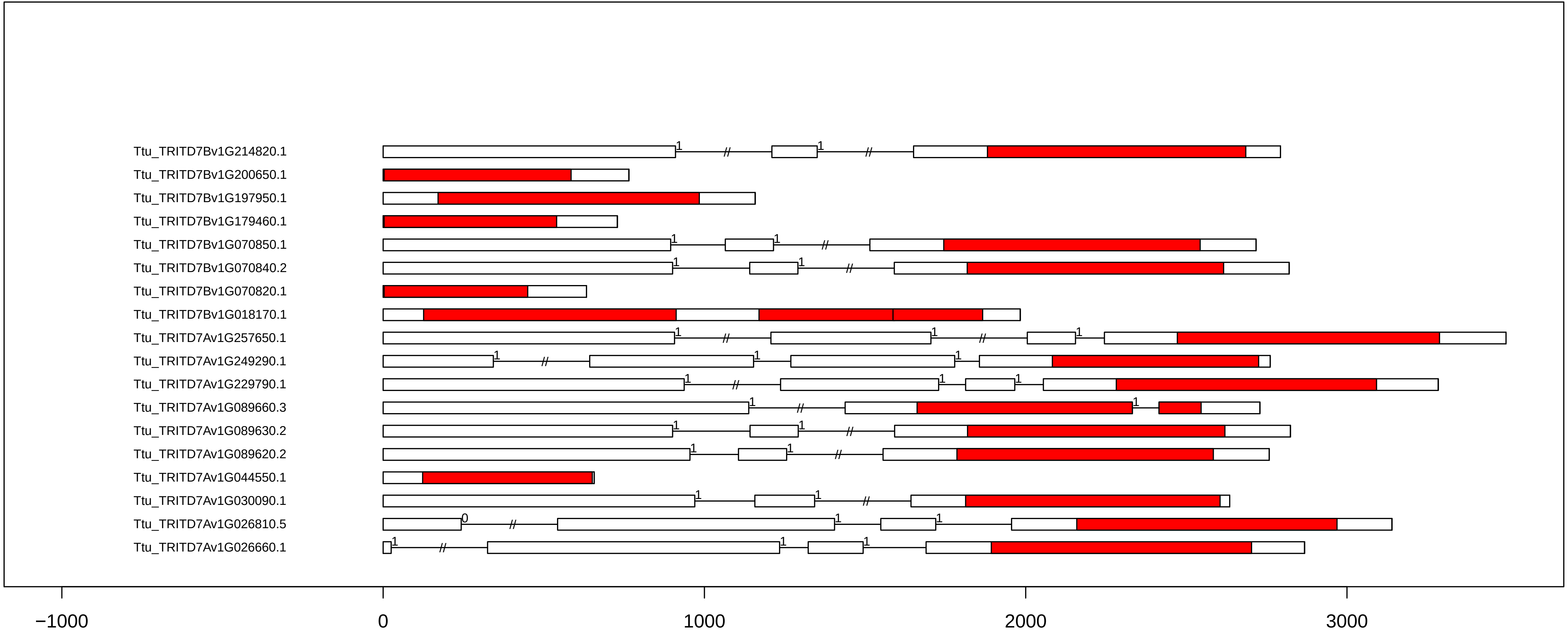

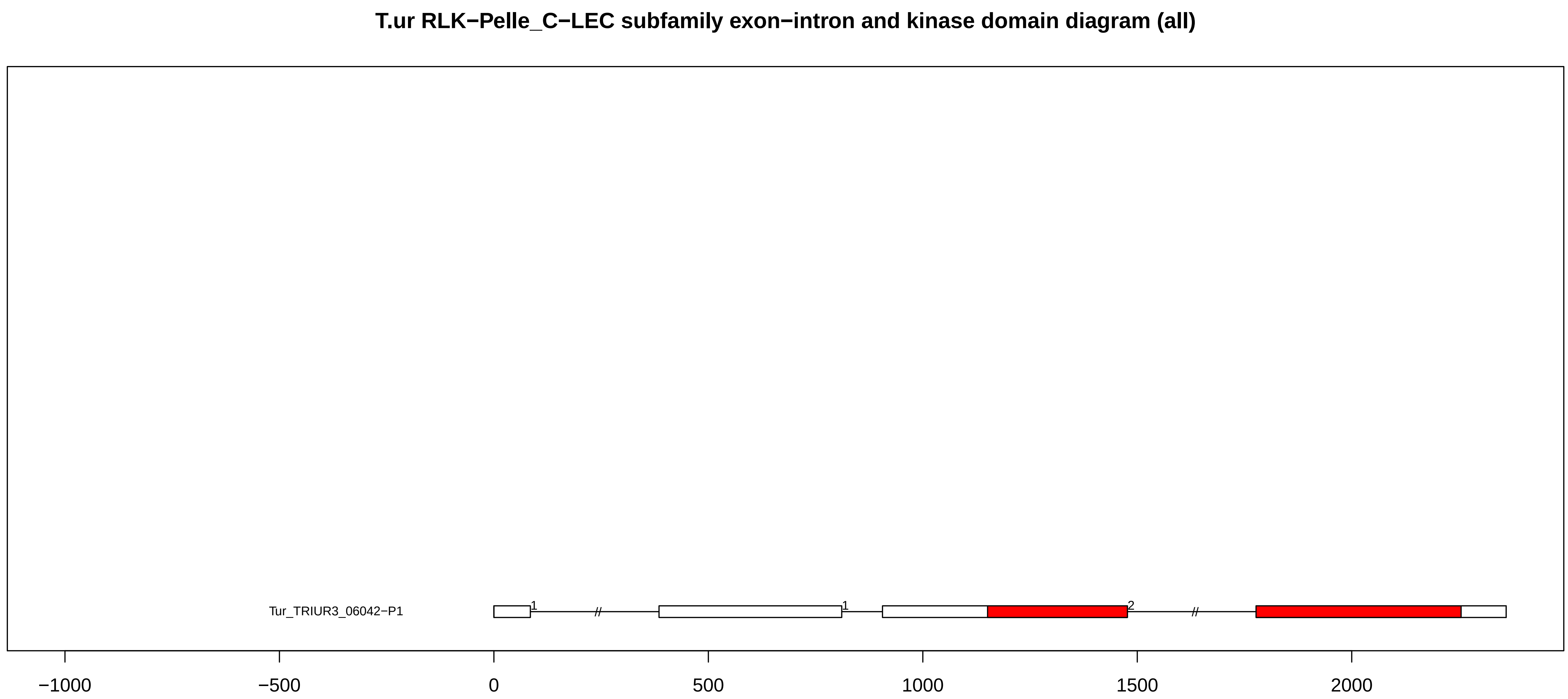
T.ur RLK−Pelle_C−LEC subfamily exon−intron and kinase domain diagram (all)
Tur_TRIUR3_06042−P1
1
1
2
−1000
−500
0
500
1000
1500
2000

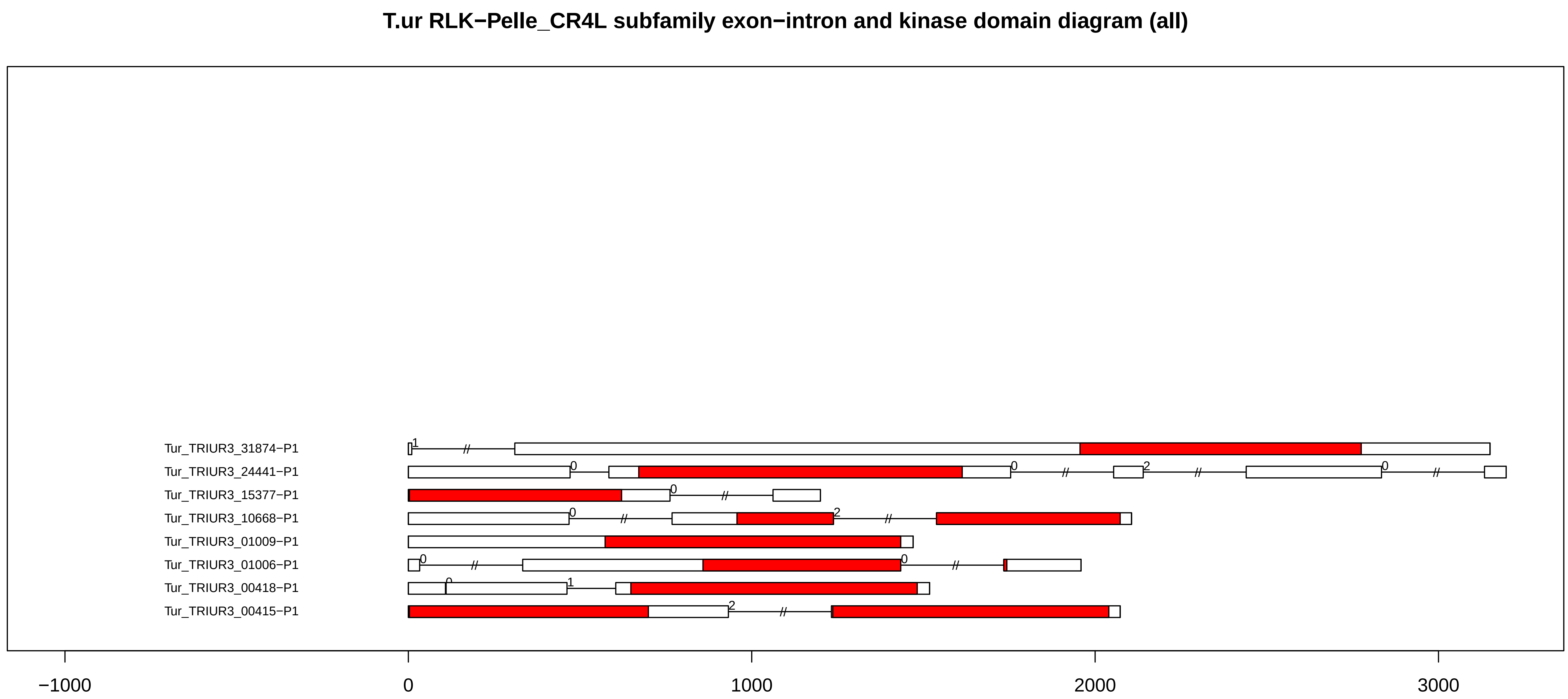
T.ur RLK−Pelle_CR4L subfamily exon−intron and kinase domain diagram (all)
Tur_TRIUR3_31874−P1
Tur_TRIUR3_24441−P1
Tur_TRIUR3_15377−P1
Tur_TRIUR3_10668−P1
Tur_TRIUR3_01009−P1
Tur_TRIUR3_01006−P1
Tur_TRIUR3_00418−P1
Tur_TRIUR3_00415−P1
−1000
0
1000
2000
3000

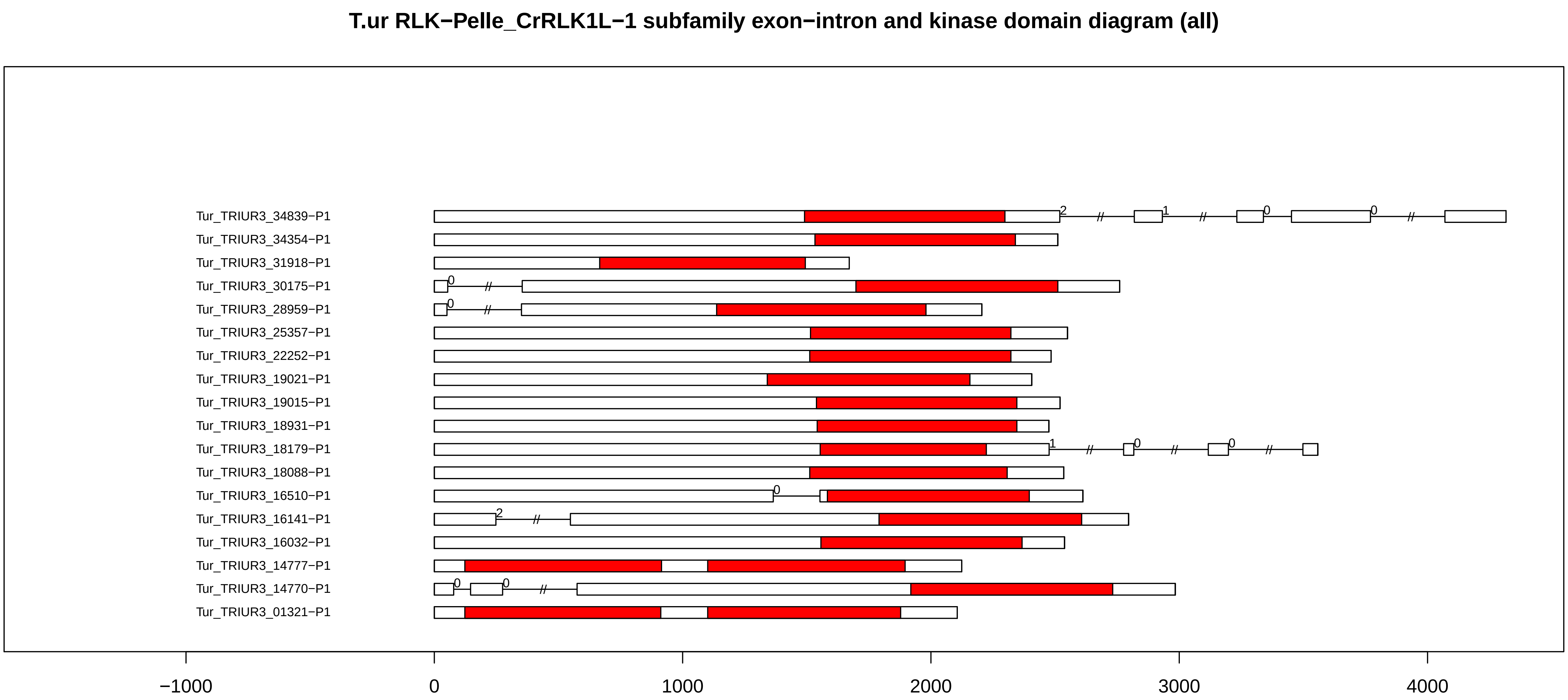

T.ur RLK−Pelle_CrRLK1L−1 subfamily exon−intron and kinase domain diagram (all)
Tur_TRIUR3_34839−P1
Tur_TRIUR3_34354−P1
Tur_TRIUR3_31918−P1
Tur_TRIUR3_30175−P1
Tur_TRIUR3_28959−P1
Tur_TRIUR3_25357−P1
Tur_TRIUR3_22252−P1
Tur_TRIUR3_19021−P1
Tur_TRIUR3_19015−P1
Tur_TRIUR3_18931−P1
Tur_TRIUR3_18179−P1
Tur_TRIUR3_18088−P1
Tur_TRIUR3_16510−P1
Tur_TRIUR3_16141−P1
Tur_TRIUR3_16032−P1
Tur_TRIUR3_14777−P1
Tur_TRIUR3_14770−P1
Tur_TRIUR3_01321−P1
−1000
0
1000
2000
3000
4000

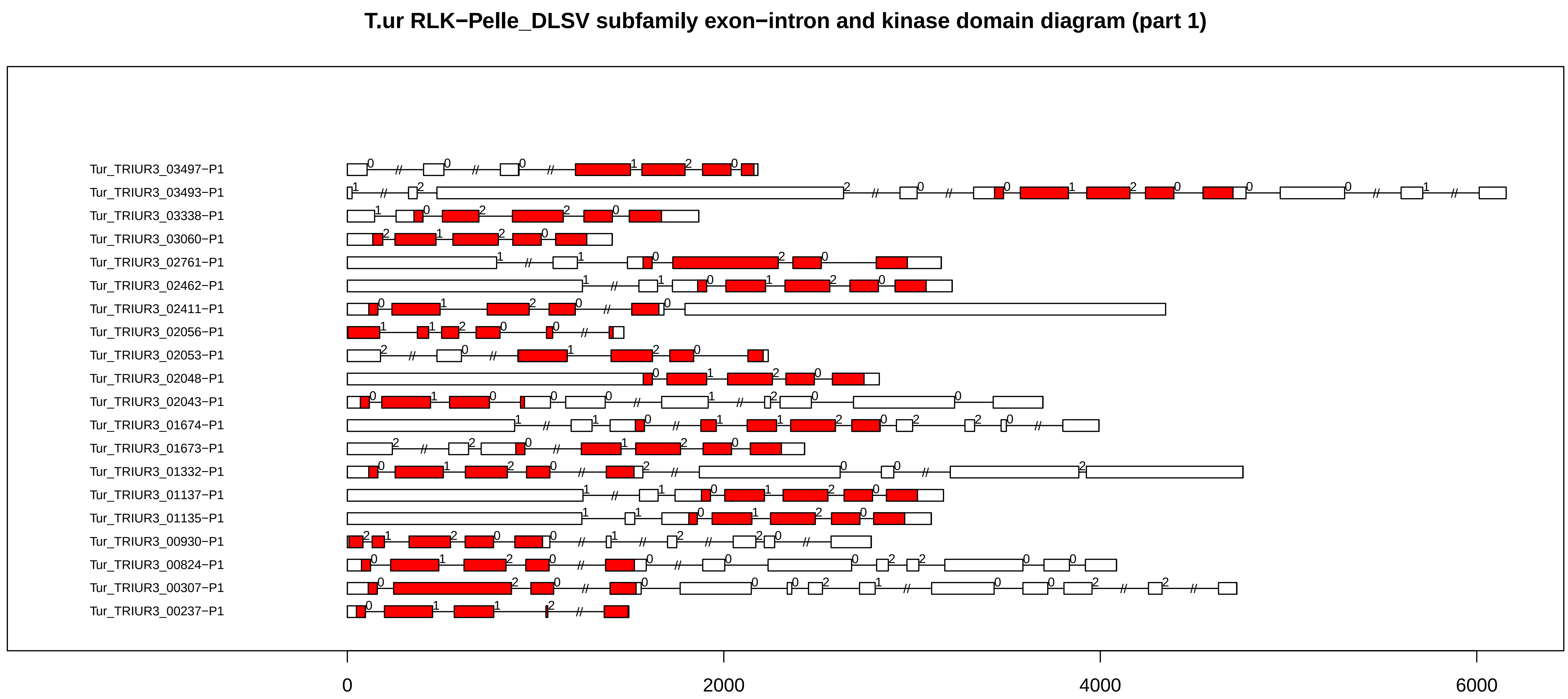
T.ur RLK−Pelle_DLSV subfamily exon−intron and kinase domain diagram (part 1)
Tur_TRIUR3_03497−P1
Tur_TRIUR3_03493−P1
Tur_TRIUR3_03338−P1
Tur_TRIUR3_03060−P1
Tur_TRIUR3_02761−P1
Tur_TRIUR3_02462−P1
Tur_TRIUR3_02411−P1
Tur_TRIUR3_02056−P1
Tur_TRIUR3_02053−P1
Tur_TRIUR3_02048−P1
Tur_TRIUR3_02043−P1
Tur_TRIUR3_01674−P1
Tur_TRIUR3_01673−P1
Tur_TRIUR3_01332−P1
Tur_TRIUR3_01137−P1
Tur_TRIUR3_01135−P1
Tur_TRIUR3_00930−P1
Tur_TRIUR3_00824−P1
Tur_TRIUR3_00307−P1
Tur_TRIUR3_00237−P1
0
2000
4000
6000

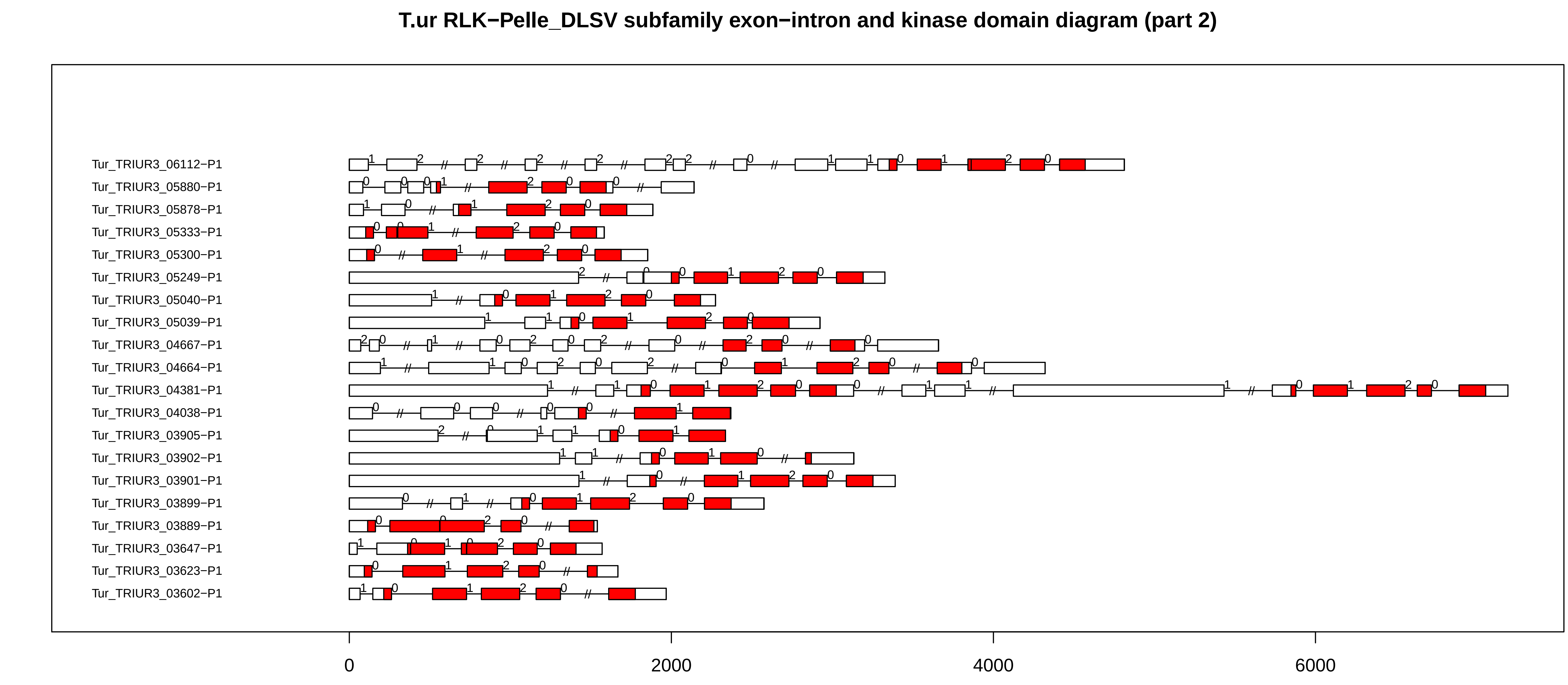
T.ur RLK−Pelle_DLSV subfamily exon−intron and kinase domain diagram (part 2)
Tur_TRIUR3_06112−P1
Tur_TRIUR3_05880−P1
Tur_TRIUR3_05878−P1
Tur_TRIUR3_05333−P1
Tur_TRIUR3_05300−P1
Tur_TRIUR3_05249−P1
Tur_TRIUR3_05040−P1
Tur_TRIUR3_05039−P1
Tur_TRIUR3_04667−P1
Tur_TRIUR3_04664−P1
Tur_TRIUR3_04381−P1
Tur_TRIUR3_04038−P1
Tur_TRIUR3_03905−P1
Tur_TRIUR3_03902−P1
Tur_TRIUR3_03901−P1
Tur_TRIUR3_03899−P1
Tur_TRIUR3_03889−P1
Tur_TRIUR3_03647−P1
Tur_TRIUR3_03623−P1
Tur_TRIUR3_03602−P1
0
2000
4000
6000

T.ur RLK−Pelle_DLSV subfamily exon−intron and kinase domain diagram (part 3)

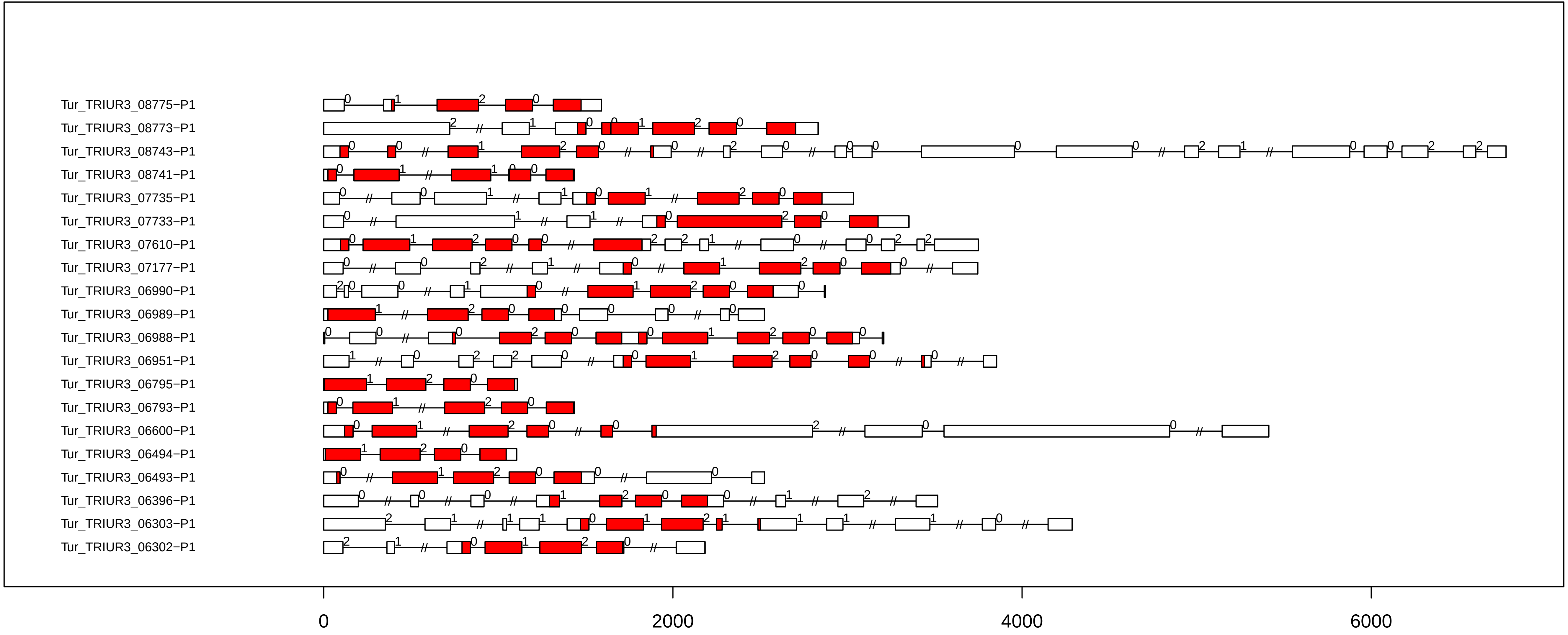

T.ur RLK−Pelle_DLSV subfamily exon−intron and kinase domain diagram (part 4)

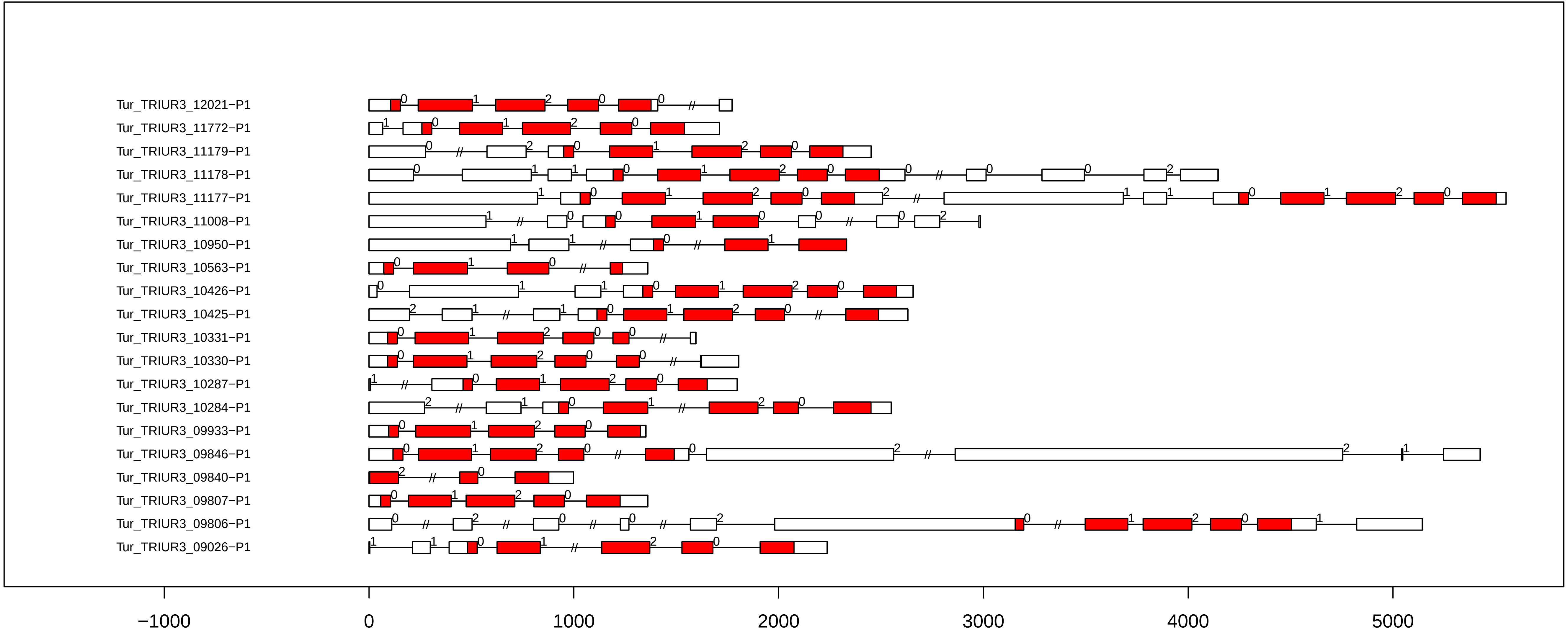

T.ur RLK−Pelle_DLSV subfamily exon−intron and kinase domain diagram (part 5)

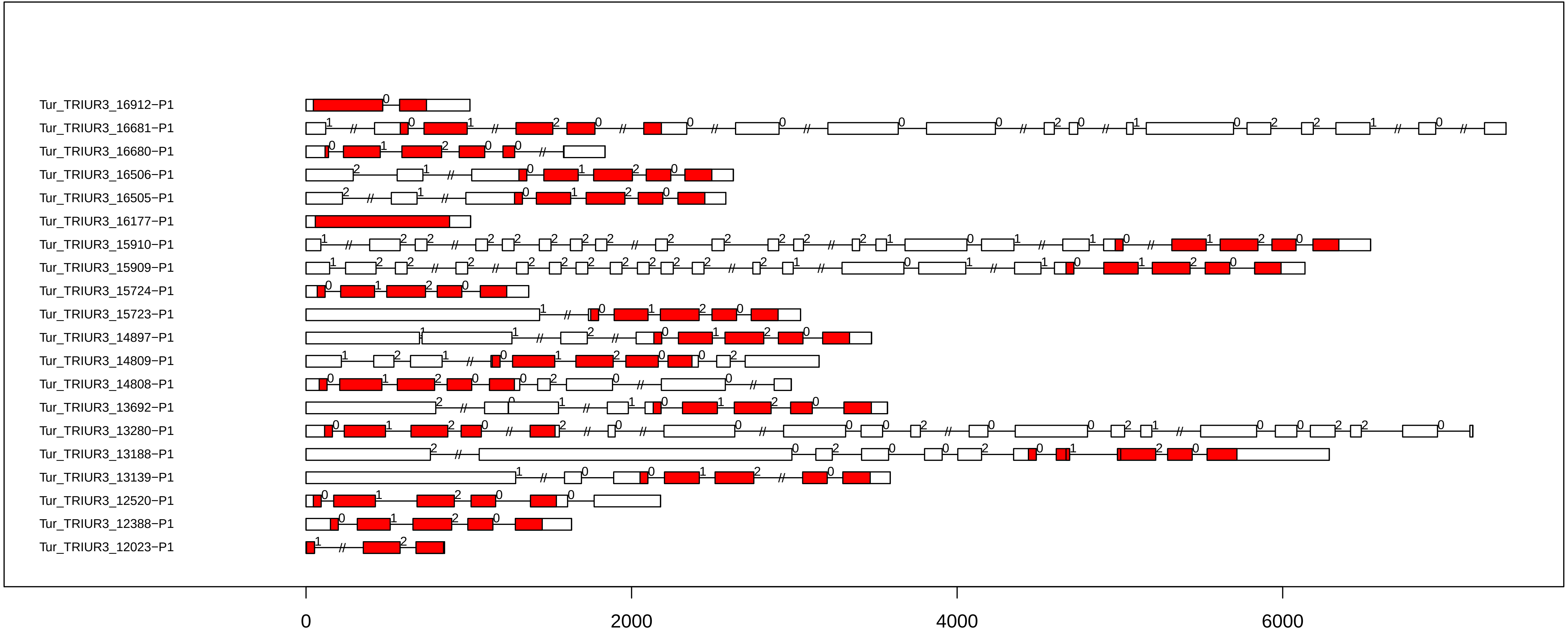

T.ur RLK−Pelle_DLSV subfamily exon−intron and kinase domain diagram (part 6)

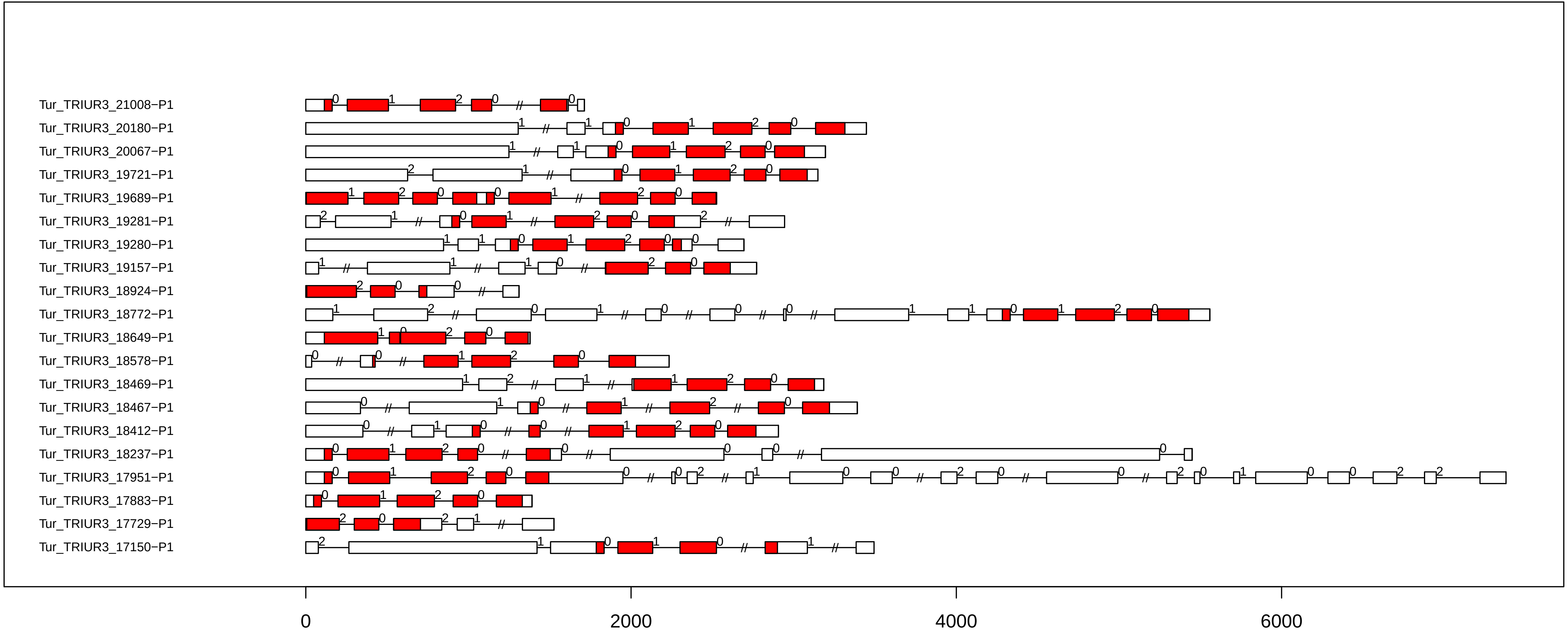

T.ur RLK−Pelle_DLSV subfamily exon−intron and kinase domain diagram (part 7)

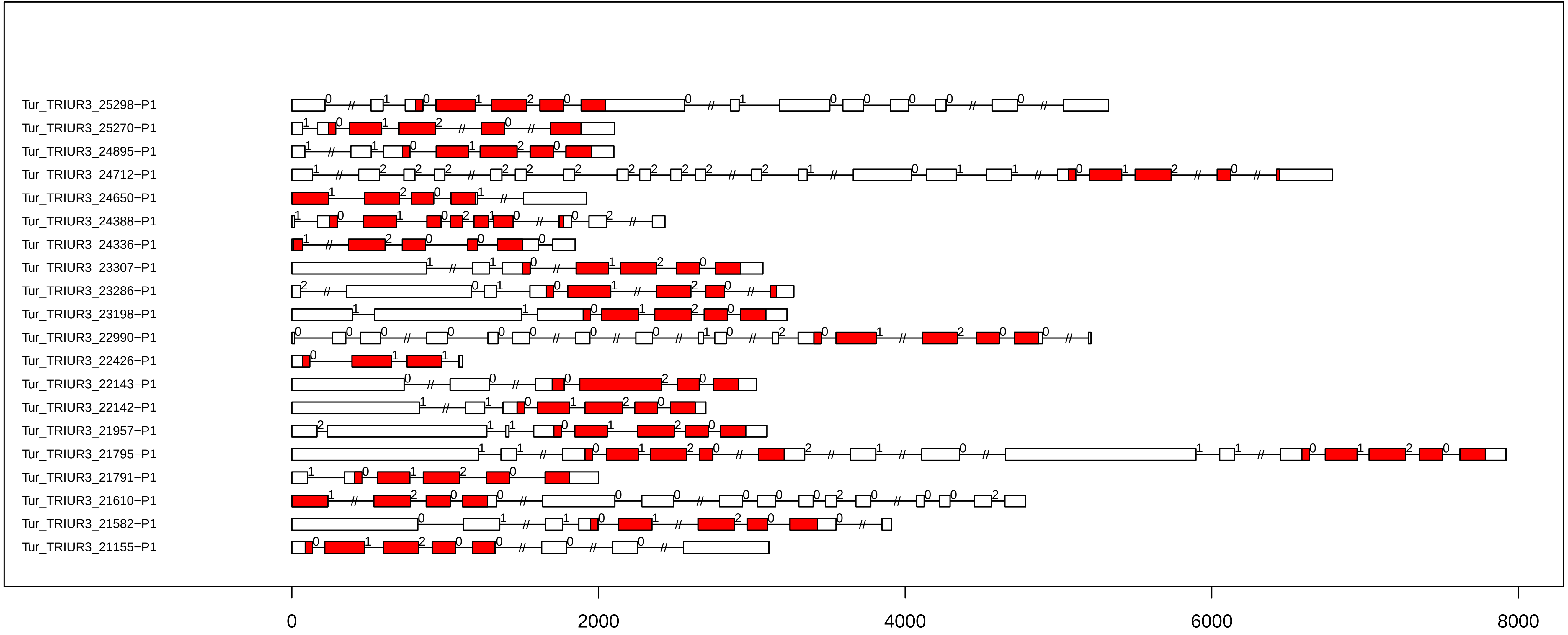

T.ur RLK−Pelle_DLSV subfamily exon−intron and kinase domain diagram (part 8)

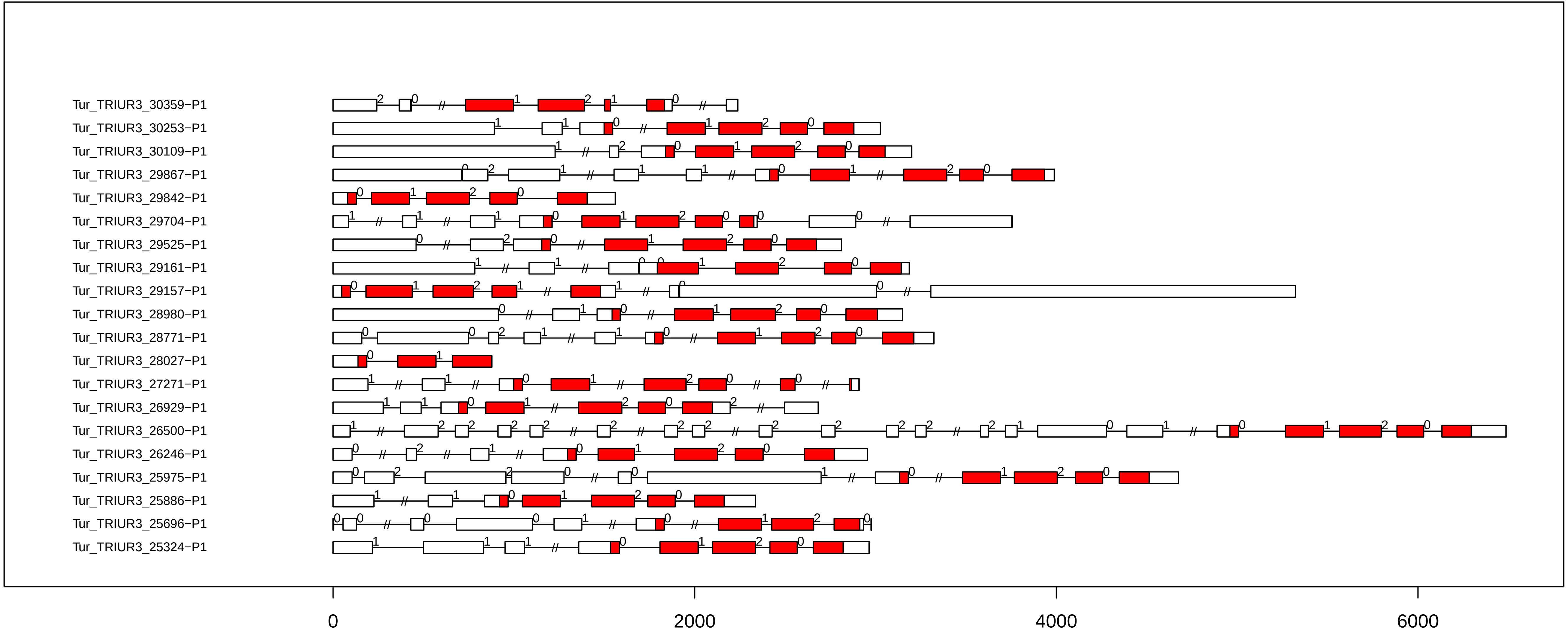

T.ur RLK−Pelle_DLSV subfamily exon−intron and kinase domain diagram (part 9)

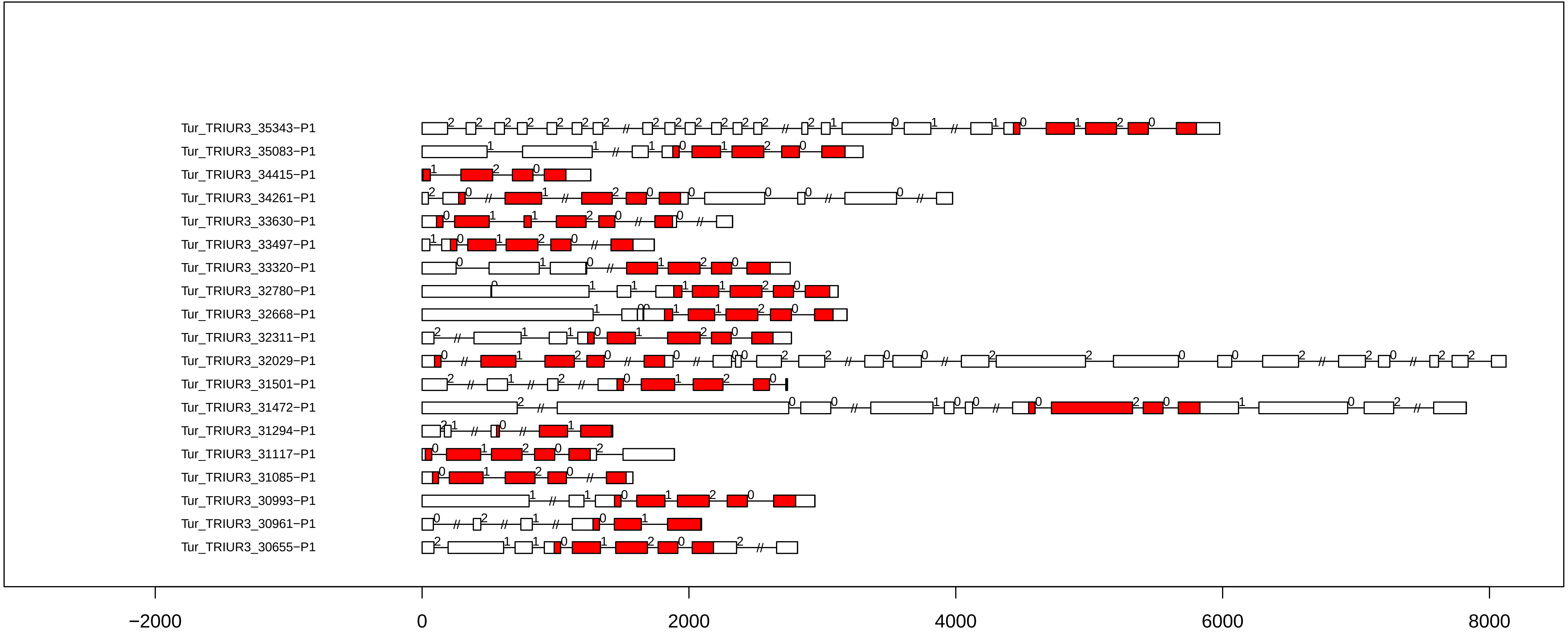

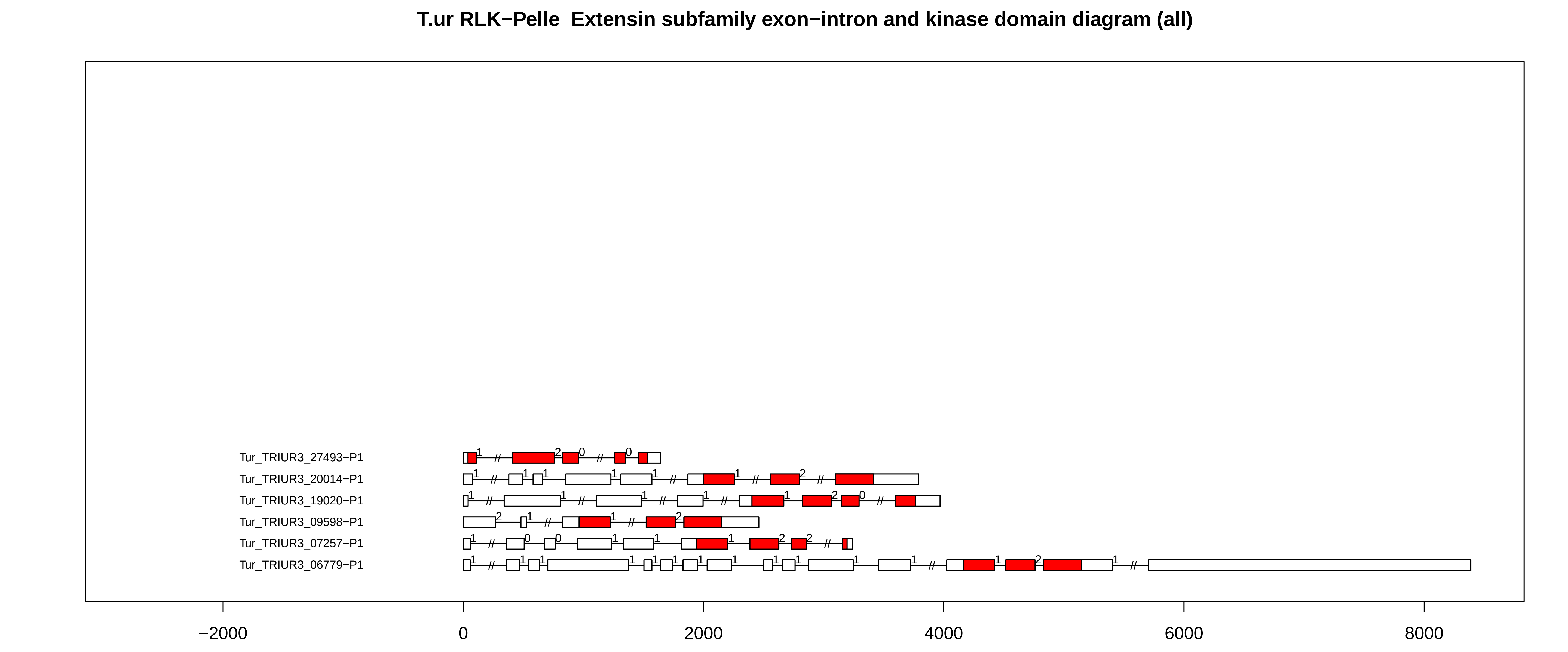
T.ur RLK−Pelle_Extensin subfamily exon−intron and kinase domain diagram (all)
Tur_TRIUR3_27493−P1
Tur_TRIUR3_20014−P1
Tur_TRIUR3_19020−P1
Tur_TRIUR3_09598−P1
Tur_TRIUR3_07257−P1
Tur_TRIUR3_06779−P1
−2000
0
2000
4000
6000
8000

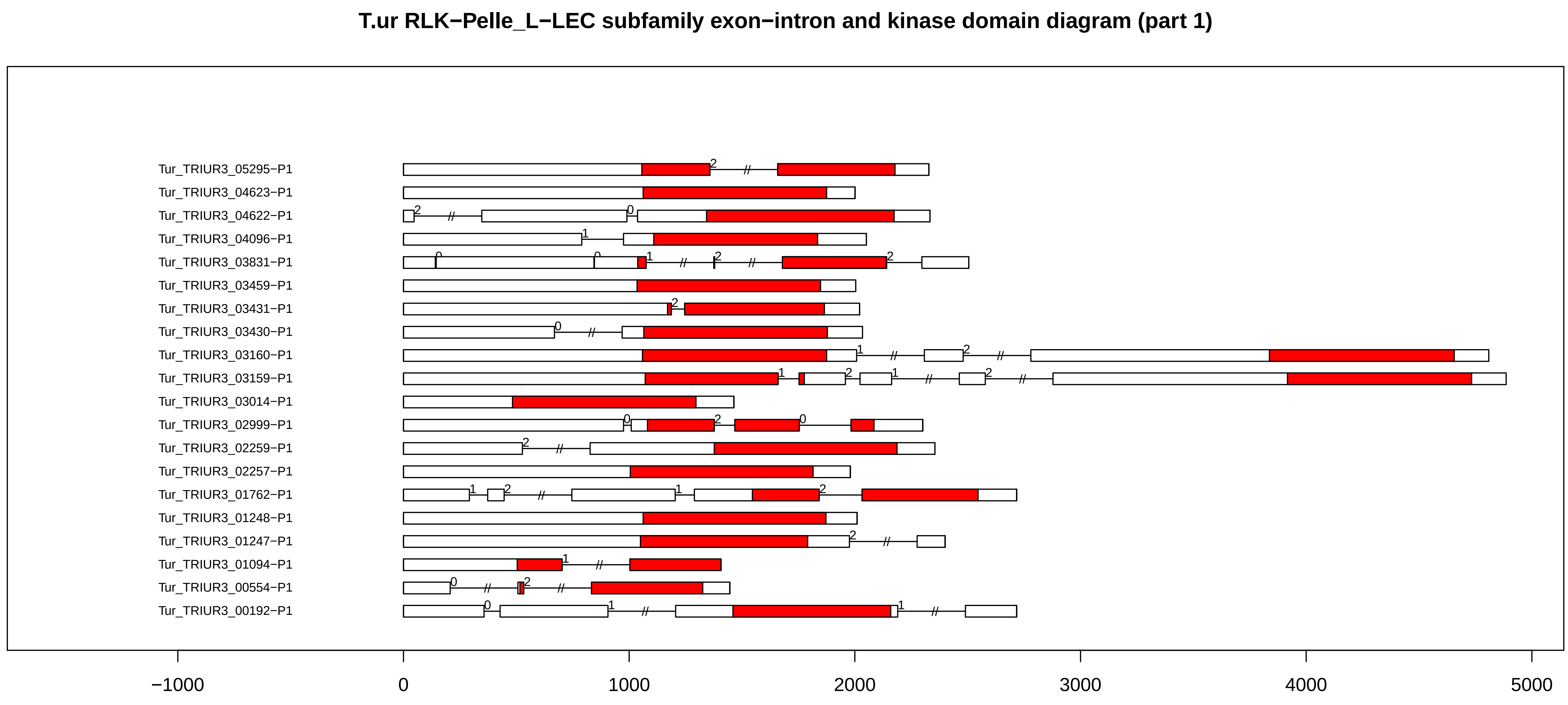
T.ur RLK−Pelle_L−LEC subfamily exon−intron and kinase domain diagram (part 1)
Tur_TRIUR3_05295−P1
Tur_TRIUR3_04623−P1
Tur_TRIUR3_04622−P1
Tur_TRIUR3_04096−P1
Tur_TRIUR3_03831−P1
Tur_TRIUR3_03459−P1
Tur_TRIUR3_03431−P1
Tur_TRIUR3_03430−P1
Tur_TRIUR3_03160−P1
Tur_TRIUR3_03159−P1
Tur_TRIUR3_03014−P1
Tur_TRIUR3_02999−P1
Tur_TRIUR3_02259−P1
Tur_TRIUR3_02257−P1
Tur_TRIUR3_01762−P1
Tur_TRIUR3_01248−P1
Tur_TRIUR3_01247−P1
Tur_TRIUR3_01094−P1
Tur_TRIUR3_00554−P1
Tur_TRIUR3_00192−P1
−1000
0
1000
2000
3000
4000
5000

T.ur RLK−Pelle_L−LEC subfamily exon−intron and kinase domain diagram (part 2)

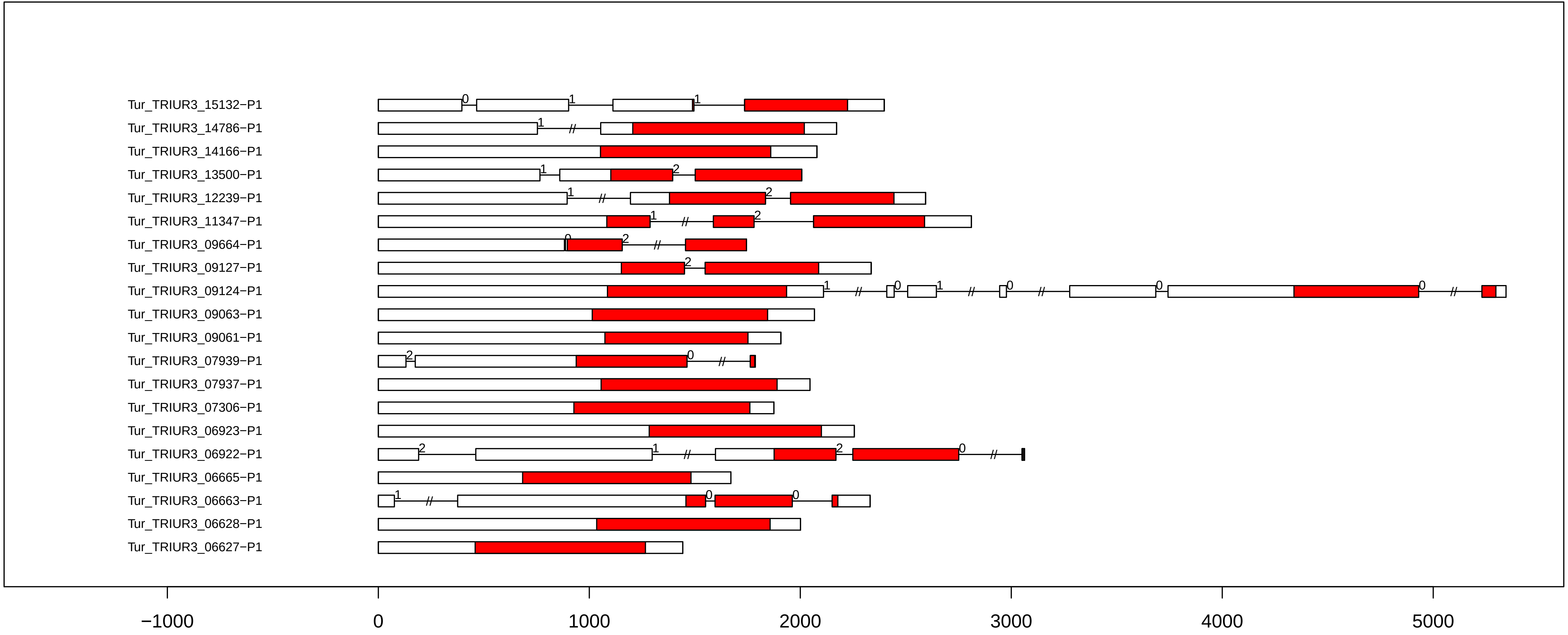

T.ur RLK−Pelle_L−LEC subfamily exon−intron and kinase domain diagram (part 3)

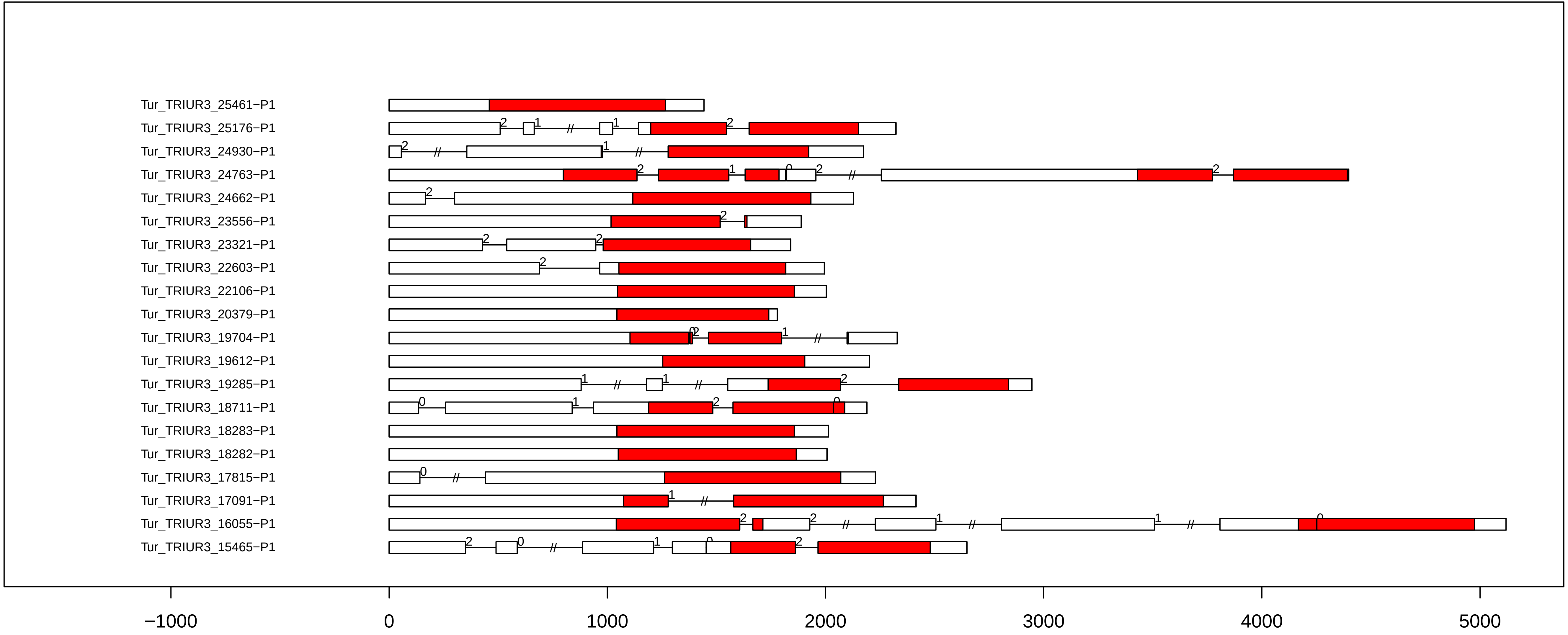

T.ur RLK−Pelle_L−LEC subfamily exon−intron and kinase domain diagram (part 4)

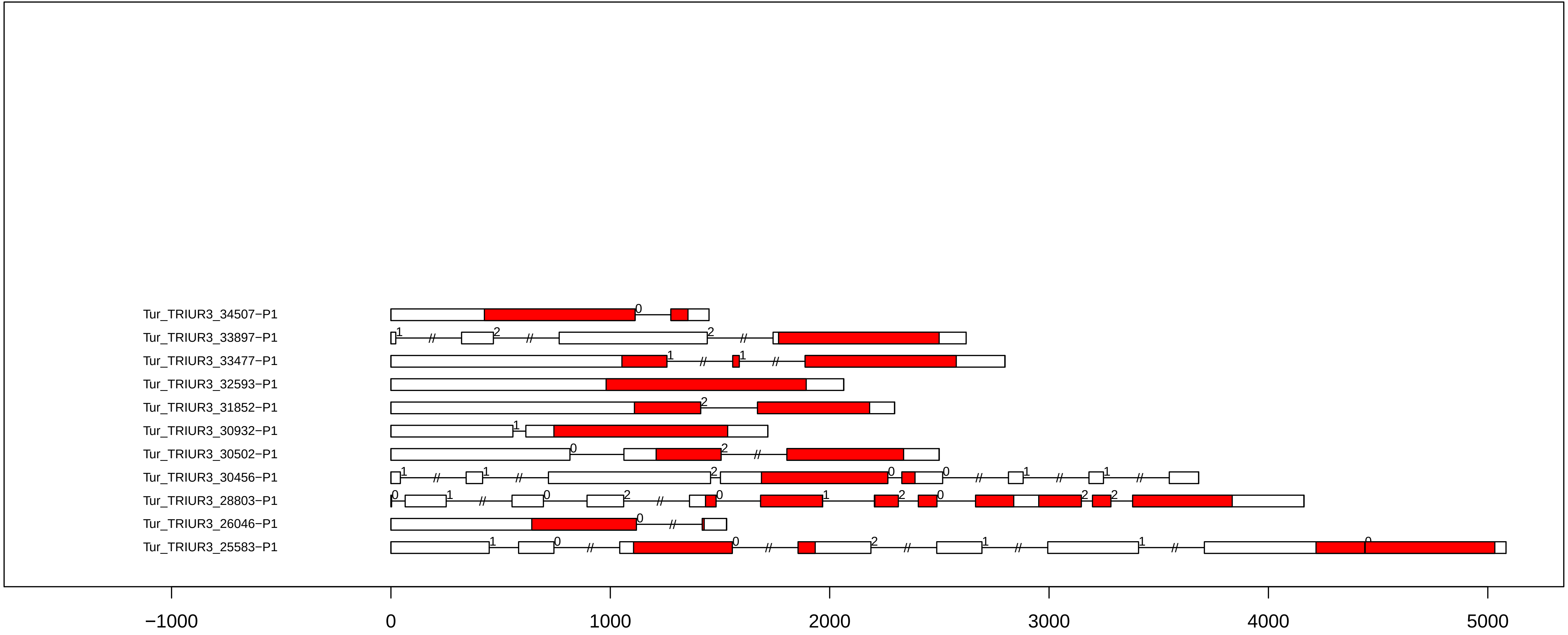

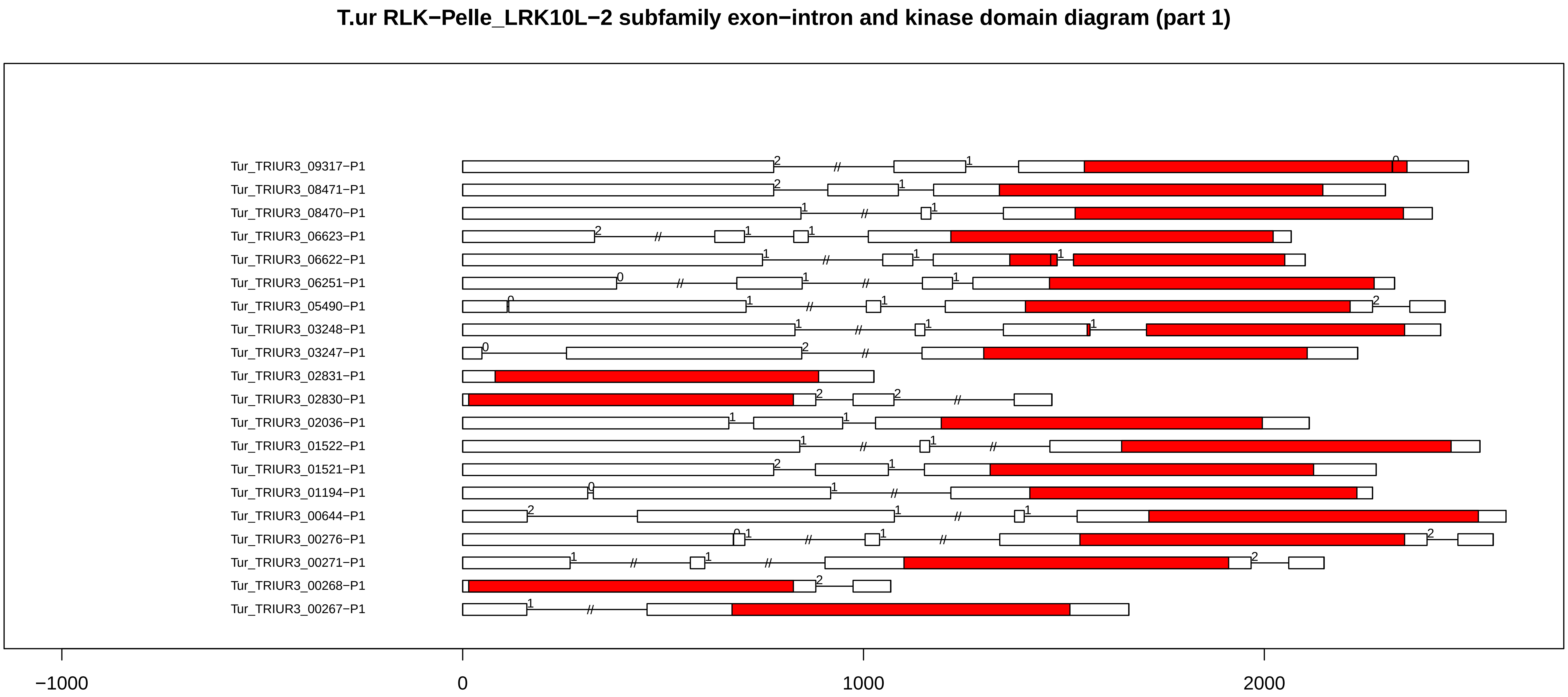

T.ur RLK−Pelle_LRK10L−2 subfamily exon−intron and kinase domain diagram (part 1)
Tur_TRIUR3_09317−P1
Tur_TRIUR3_08471−P1
Tur_TRIUR3_08470−P1
Tur_TRIUR3_06623−P1
Tur_TRIUR3_06622−P1
Tur_TRIUR3_06251−P1
Tur_TRIUR3_05490−P1
Tur_TRIUR3_03248−P1
Tur_TRIUR3_03247−P1
Tur_TRIUR3_02831−P1
Tur_TRIUR3_02830−P1
Tur_TRIUR3_02036−P1
Tur_TRIUR3_01522−P1
Tur_TRIUR3_01521−P1
Tur_TRIUR3_01194−P1
Tur_TRIUR3_00644−P1
Tur_TRIUR3_00276−P1
Tur_TRIUR3_00271−P1
Tur_TRIUR3_00268−P1
Tur_TRIUR3_00267−P1
−1000
0
1000
2000

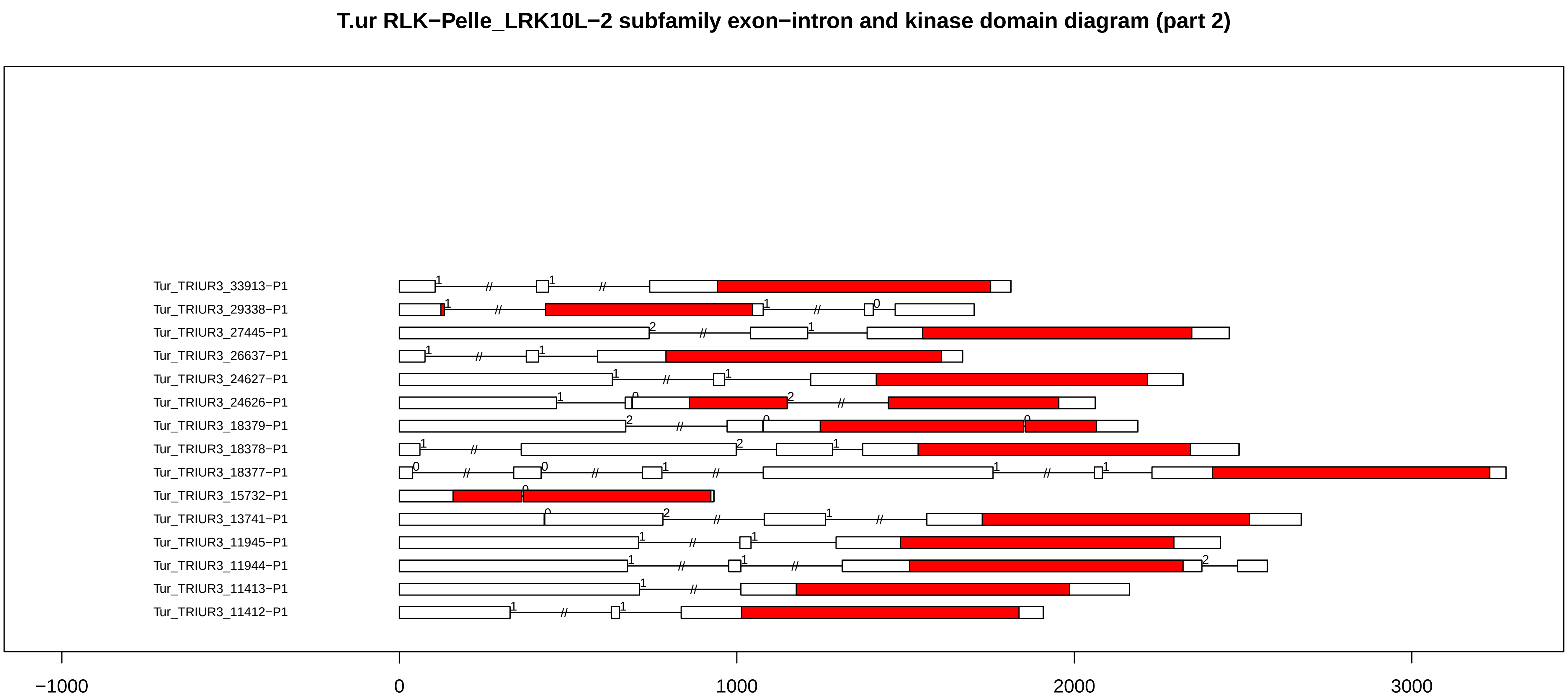

T.ur RLK−Pelle_LRK10L−2 subfamily exon−intron and kinase domain diagram (part 2)
Tur_TRIUR3_33913−P1
Tur_TRIUR3_29338−P1
Tur_TRIUR3_27445−P1
Tur_TRIUR3_26637−P1
Tur_TRIUR3_24627−P1
Tur_TRIUR3_24626−P1
Tur_TRIUR3_18379−P1
Tur_TRIUR3_18378−P1
Tur_TRIUR3_18377−P1
Tur_TRIUR3_15732−P1
Tur_TRIUR3_13741−P1
Tur_TRIUR3_11945−P1
Tur_TRIUR3_11944−P1
Tur_TRIUR3_11413−P1
Tur_TRIUR3_11412−P1
−1000
0
1000
2000
3000

T.ur RLK−Pelle_LRR−I−1 subfamily exon−intron and kinase domain diagram (part 1)

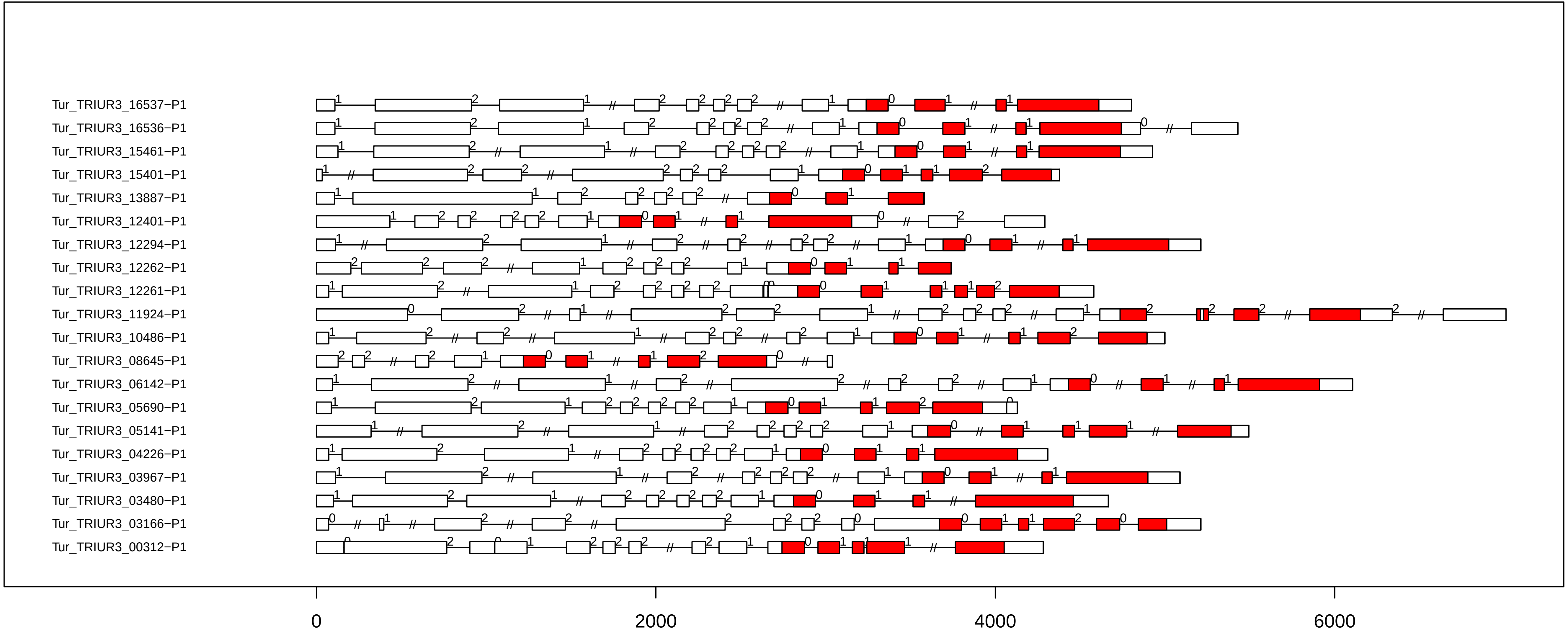

T.ur RLK−Pelle_LRR−I−1 subfamily exon−intron and kinase domain diagram (part 2)

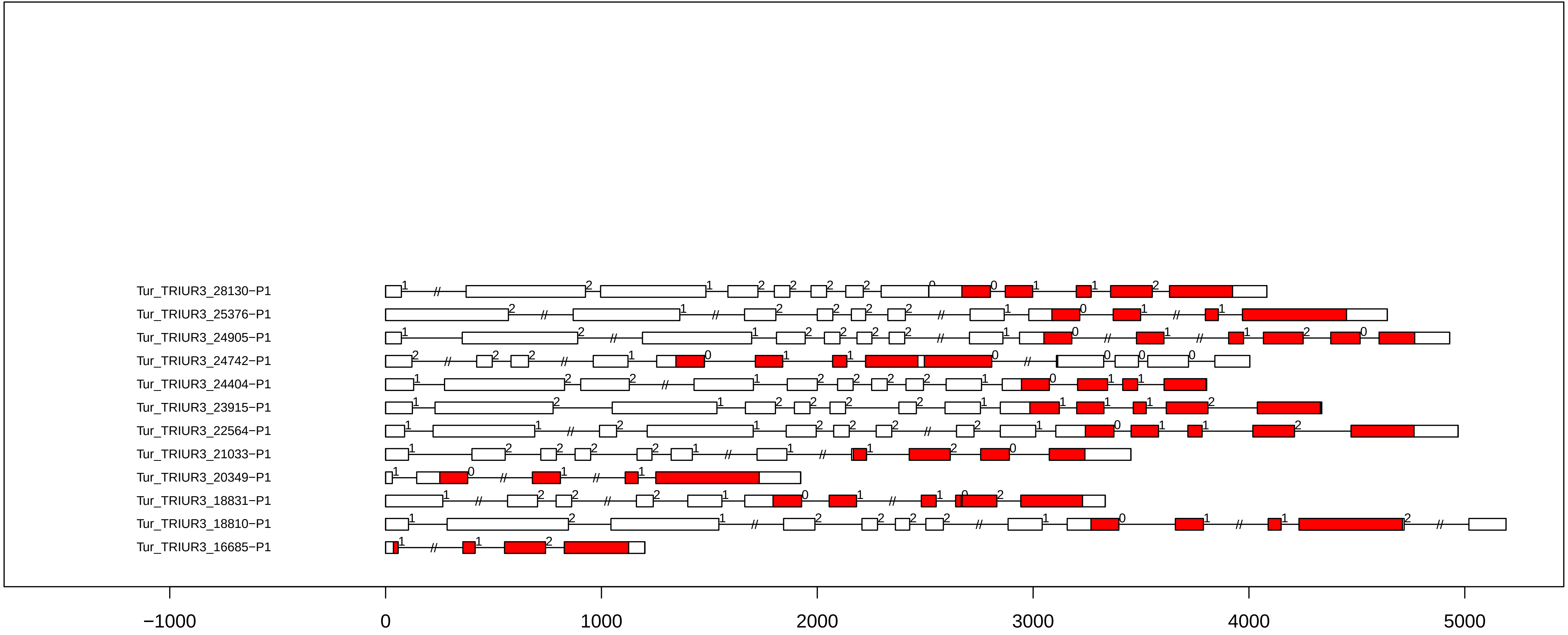

T.ur RLK−Pelle_LRR−I−2 subfamily exon−intron and kinase domain diagram (all)

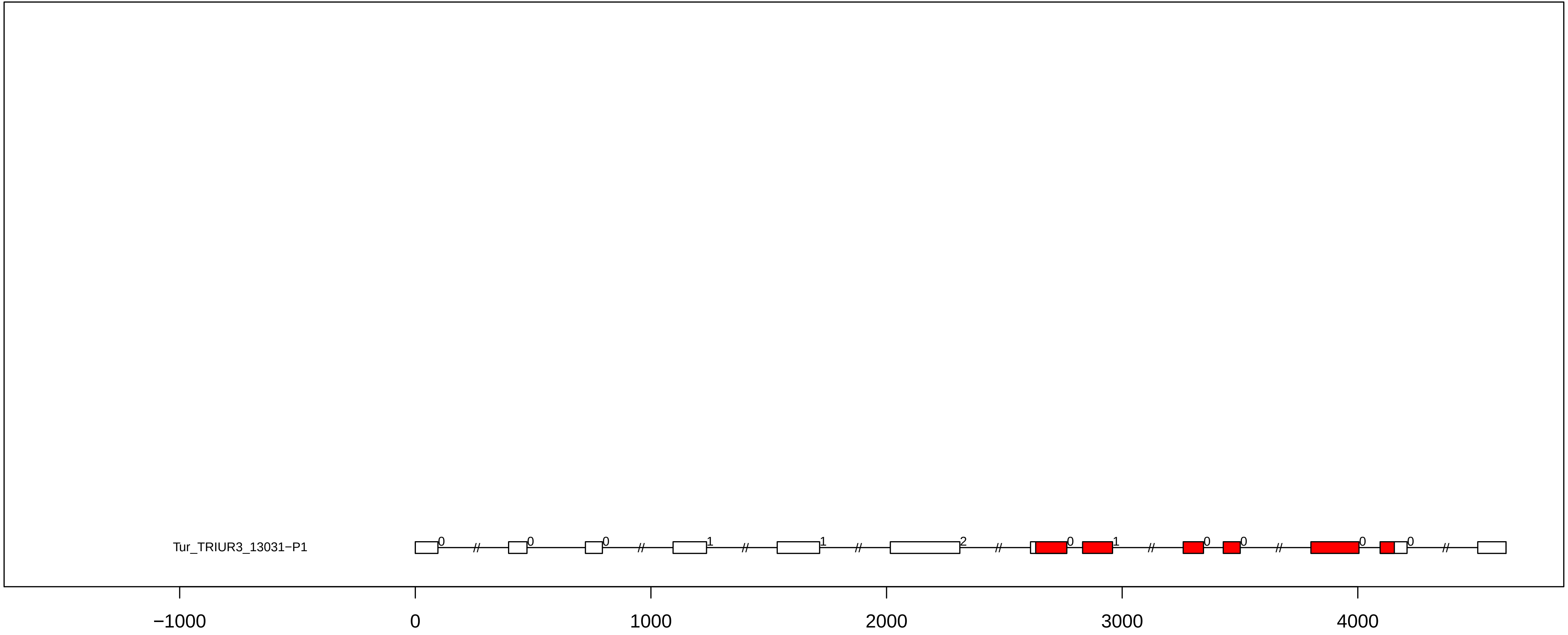

T.ur RLK−Pelle_LRR−III subfamily exon−intron and kinase domain diagram (part 1)

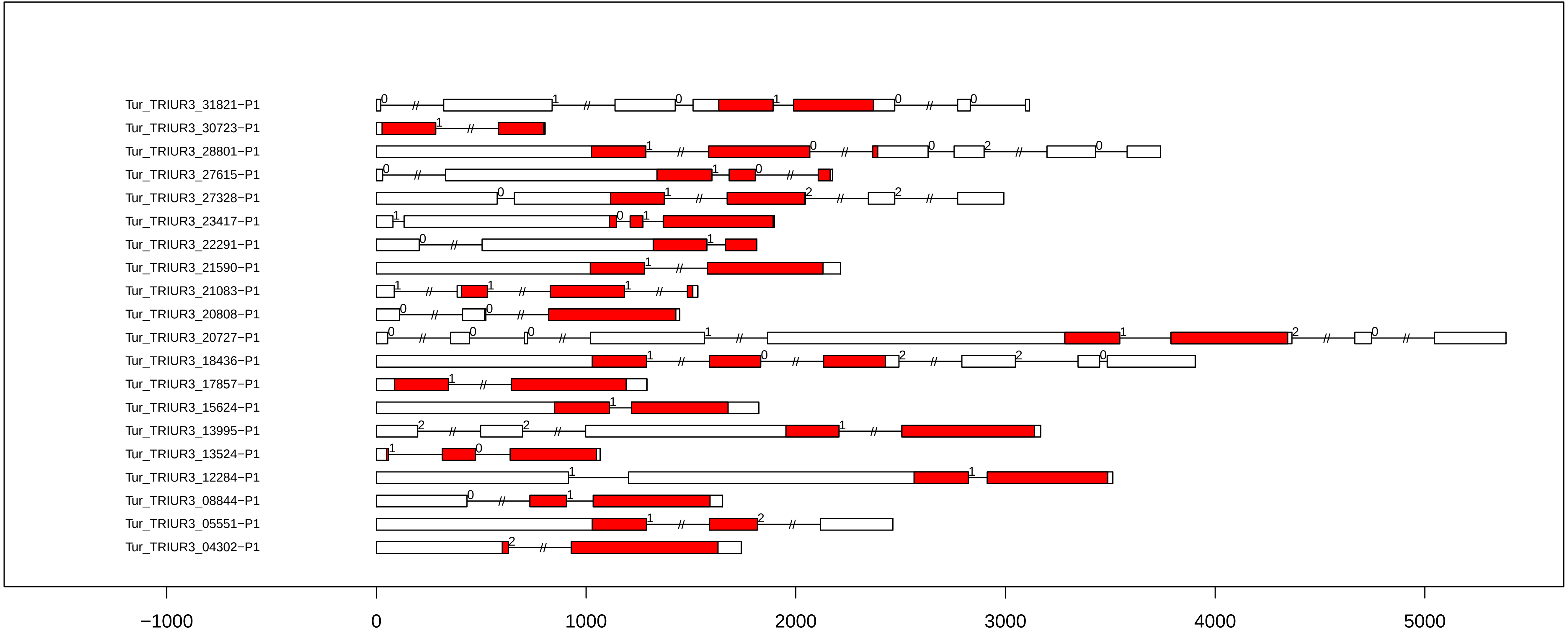

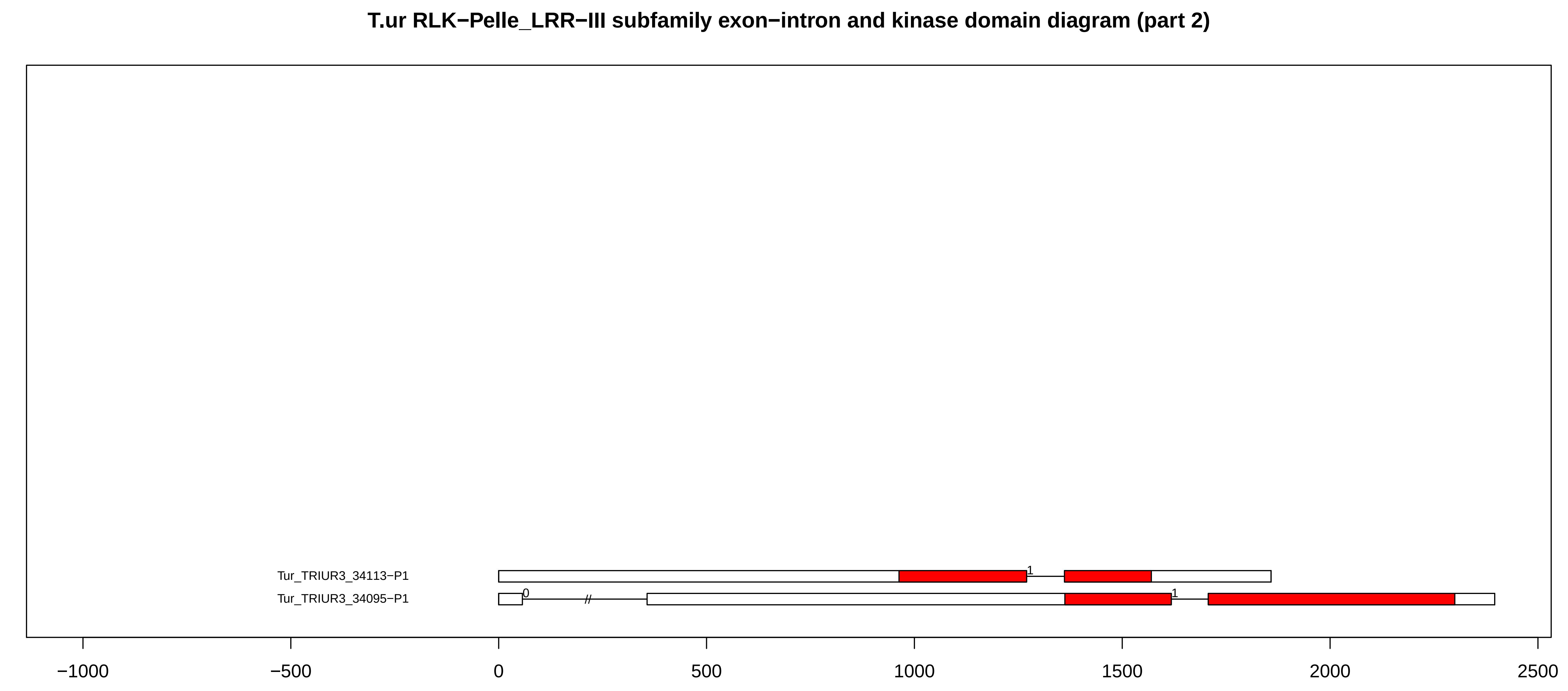
T.ur RLK−Pelle_LRR−III subfamily exon−intron and kinase domain diagram (part 2)
Tur_TRIUR3_34113−P1
Tur_TRIUR3_34095−P1
1
0
1
−1000
−500
0
500
1000
1500
2000
2500

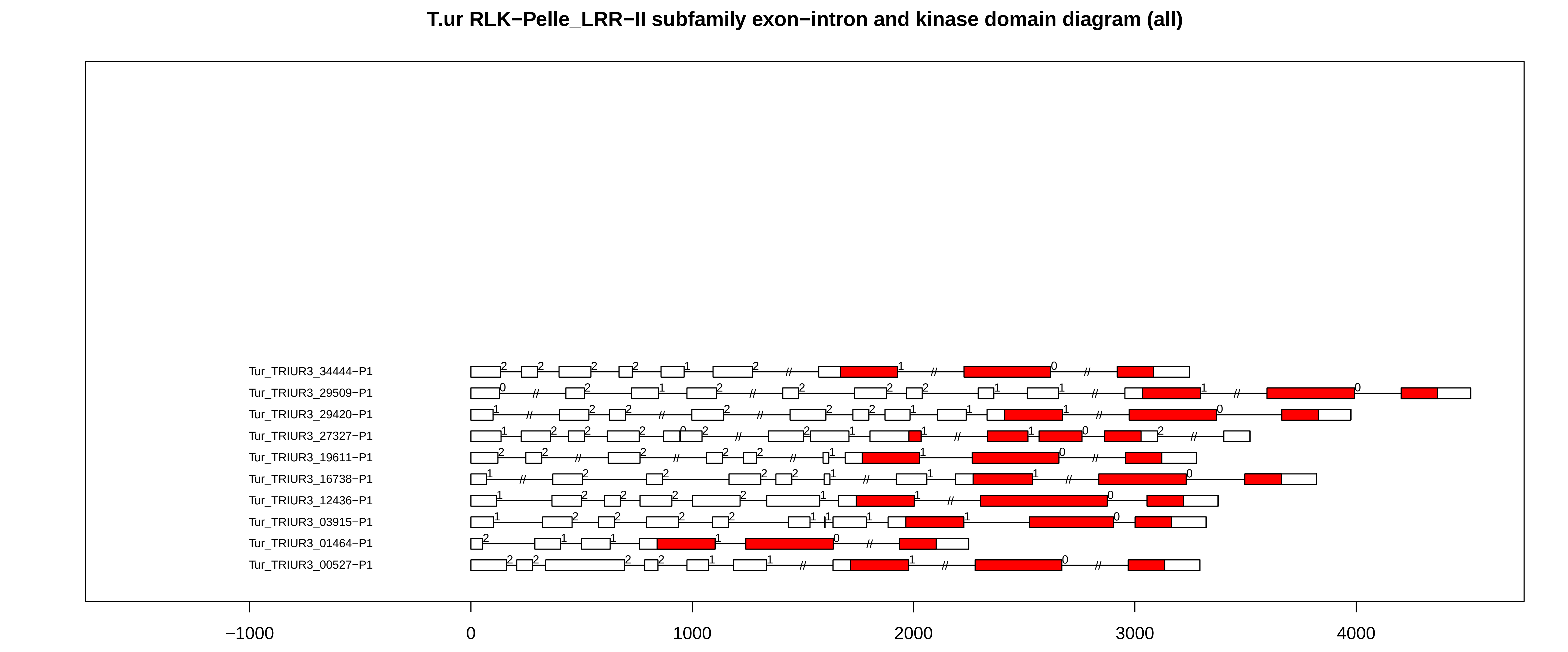
T.ur RLK−Pelle_LRR−II subfamily exon−intron and kinase domain diagram (all)
Tur_TRIUR3_34444−P1
Tur_TRIUR3_29509−P1
Tur_TRIUR3_29420−P1
Tur_TRIUR3_27327−P1
Tur_TRIUR3_19611−P1
Tur_TRIUR3_16738−P1
Tur_TRIUR3_12436−P1
Tur_TRIUR3_03915−P1
Tur_TRIUR3_01464−P1
Tur_TRIUR3_00527−P1
−1000
0
1000
2000
3000
4000

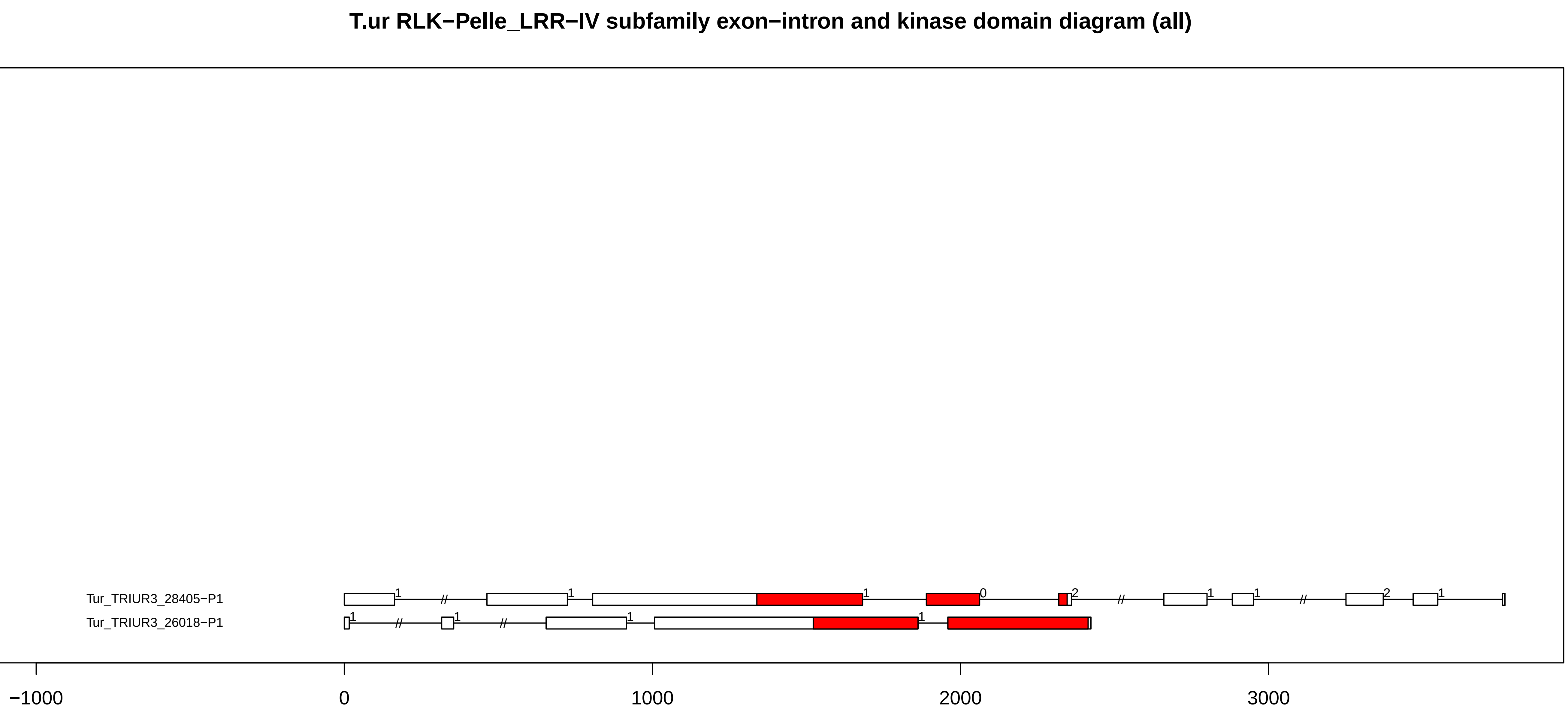
T.ur RLK−Pelle_LRR−IV subfamily exon−intron and kinase domain diagram (all)
Tur_TRIUR3_28405−P1
Tur_TRIUR3_26018−P1
−1000
0
1000
2000
3000

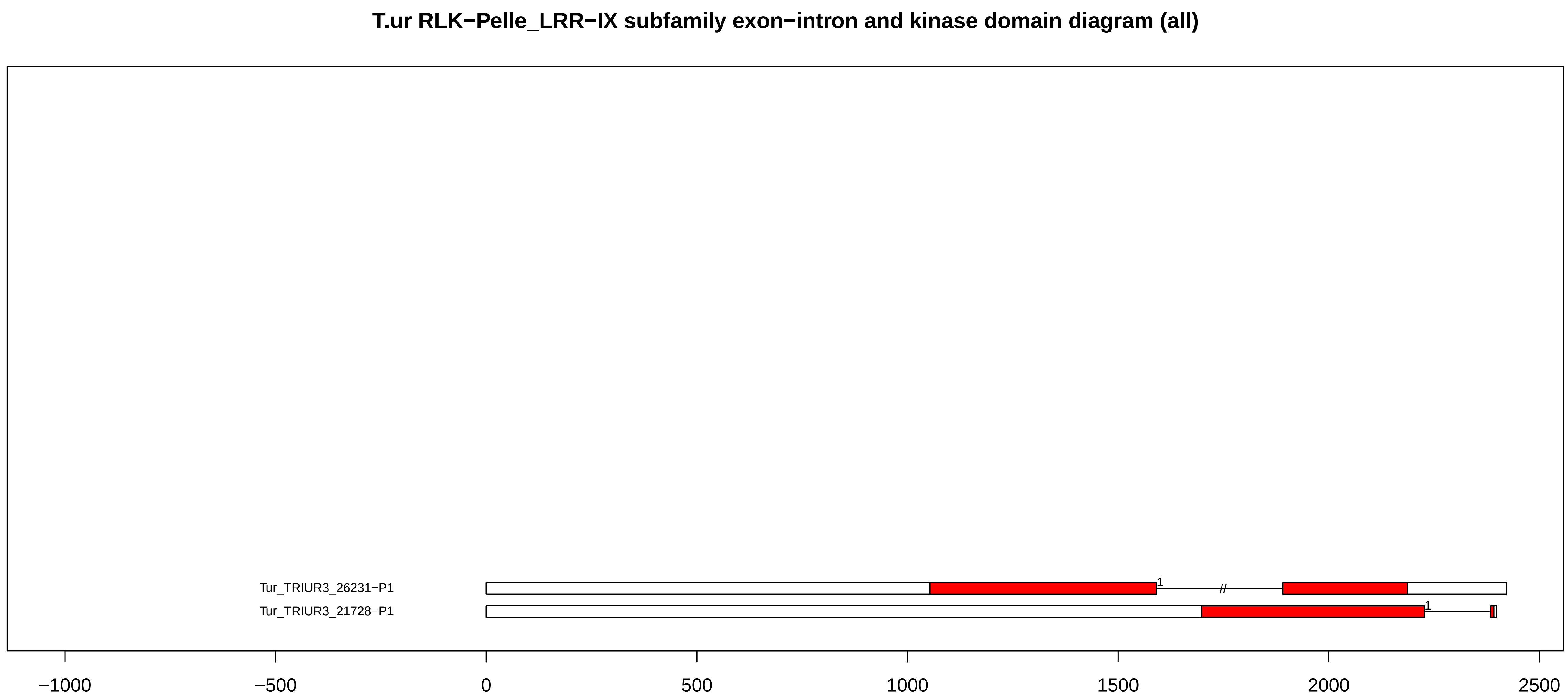
T.ur RLK−Pelle_LRR−IX subfamily exon−intron and kinase domain diagram (all)
Tur_TRIUR3_26231−P1
Tur_TRIUR3_21728−P1
1
1
−1000
−500
0
500
1000
1500
2000
2500

T.ur RLK−Pelle_LRR−VI−1 subfamily exon−intron and kinase domain diagram (all)

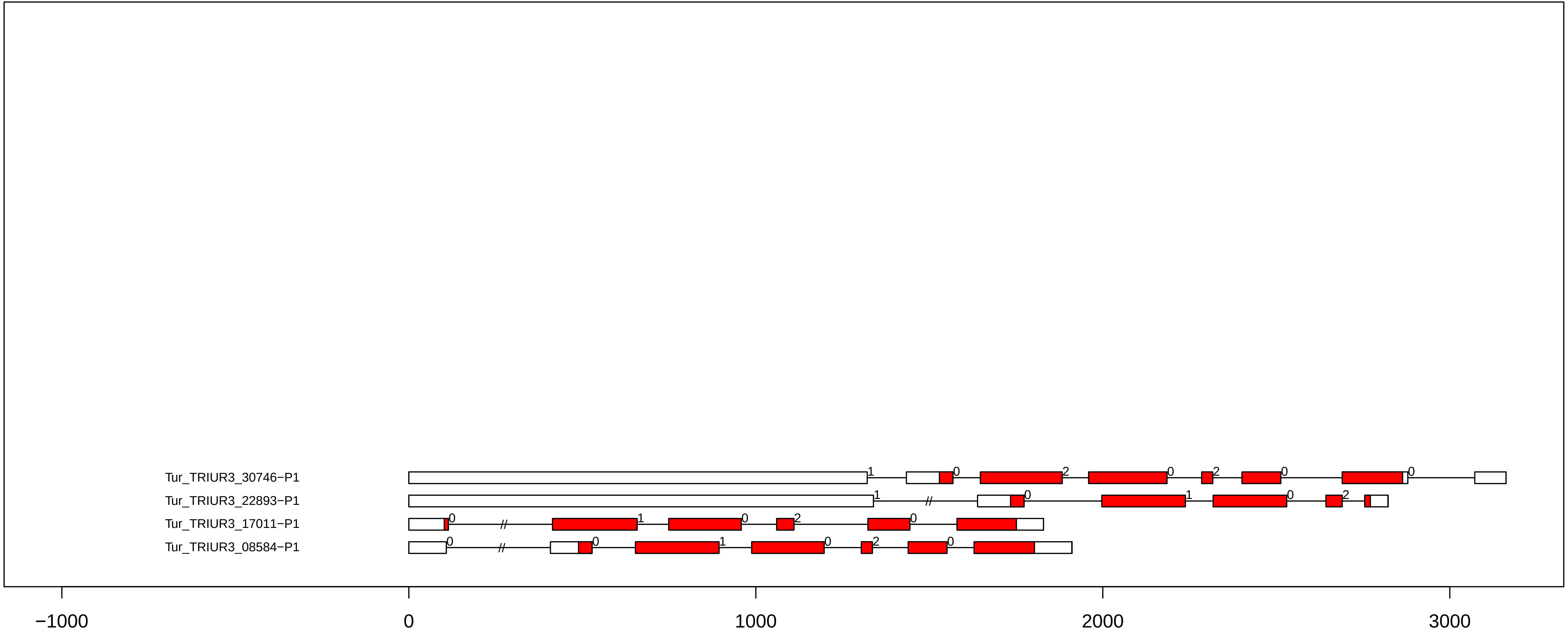

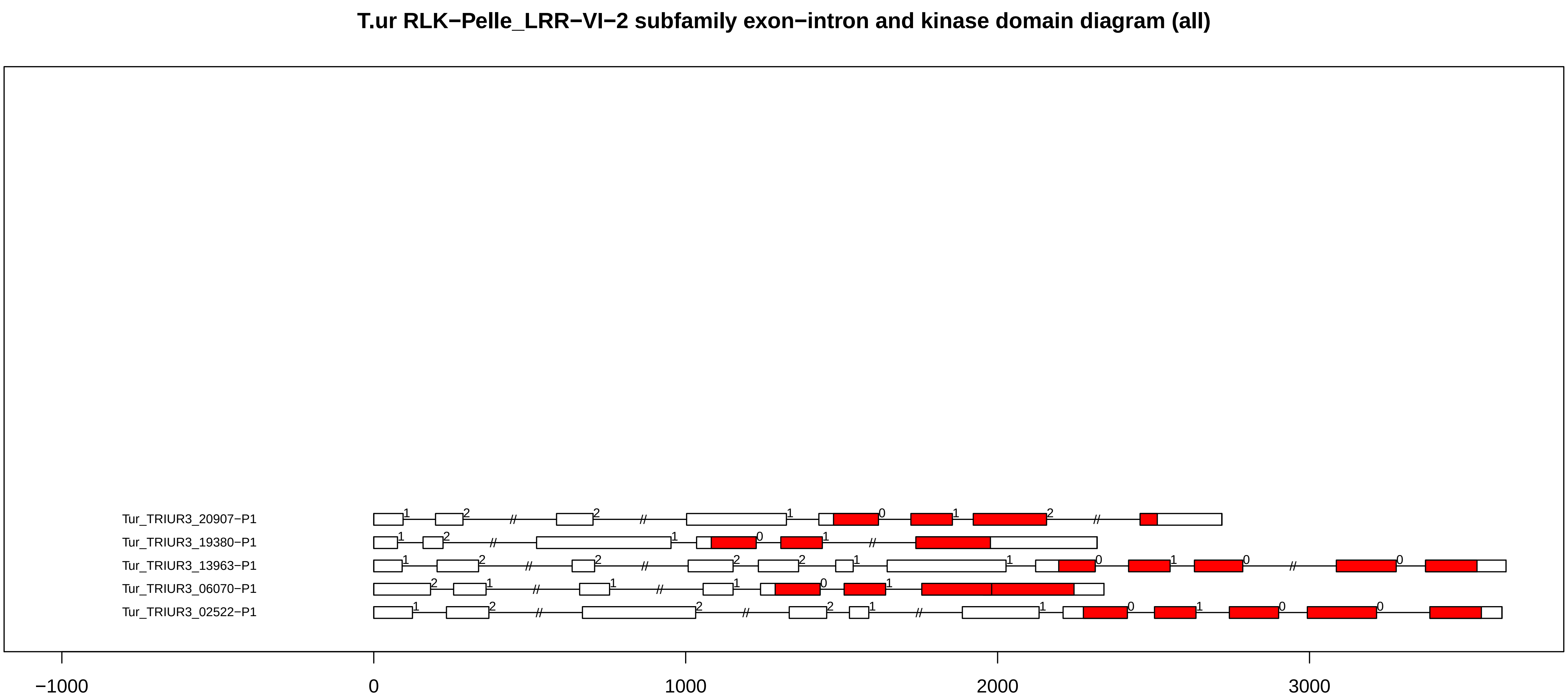

T.ur RLK−Pelle_LRR−VI−2 subfamily exon−intron and kinase domain diagram (all)
Tur_TRIUR3_20907−P1
Tur_TRIUR3_19380−P1
Tur_TRIUR3_13963−P1
Tur_TRIUR3_06070−P1
Tur_TRIUR3_02522−P1
−1000
0
1000
2000
3000

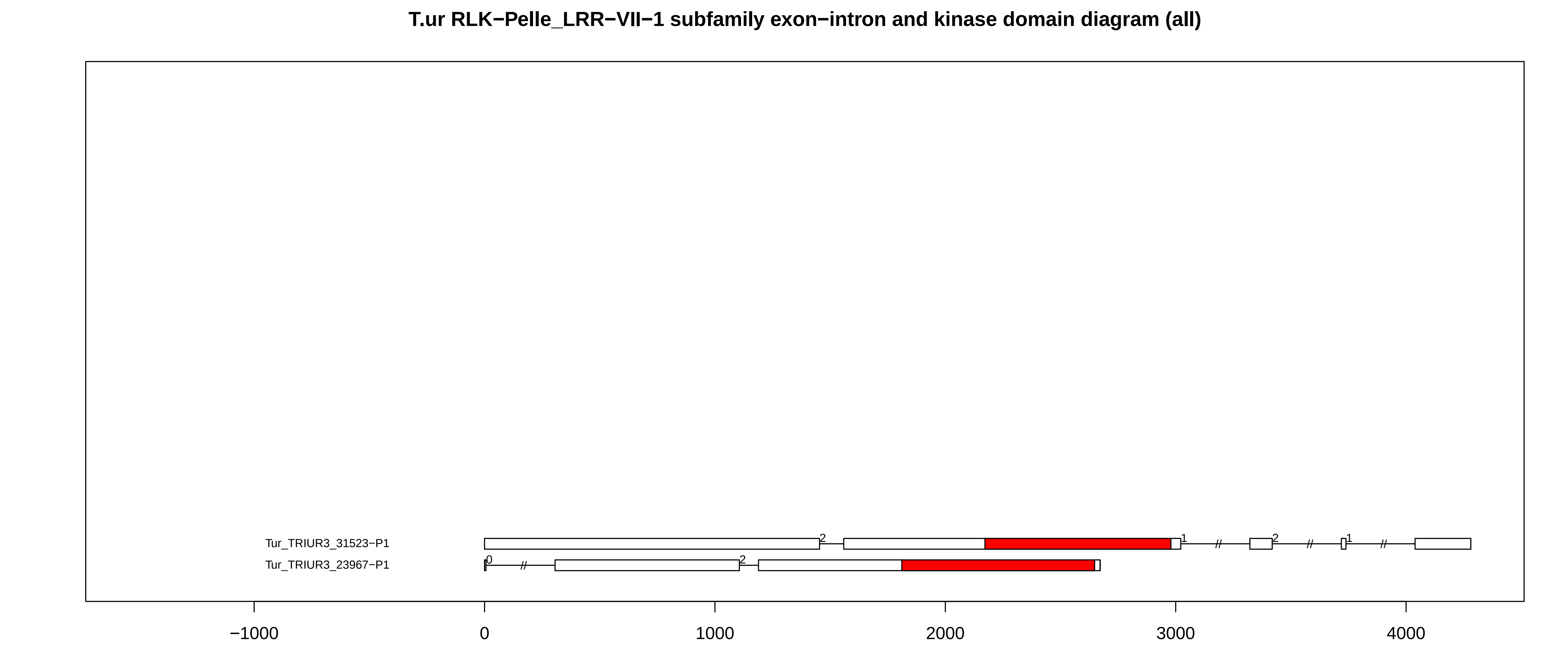

T.ur RLK−Pelle_LRR−VII−1 subfamily exon−intron and kinase domain diagram (all)
Tur_TRIUR3_31523−P1
Tur_TRIUR3_23967−P1
2
1
2
1
0
2
−1000
0
1000
2000
3000
4000

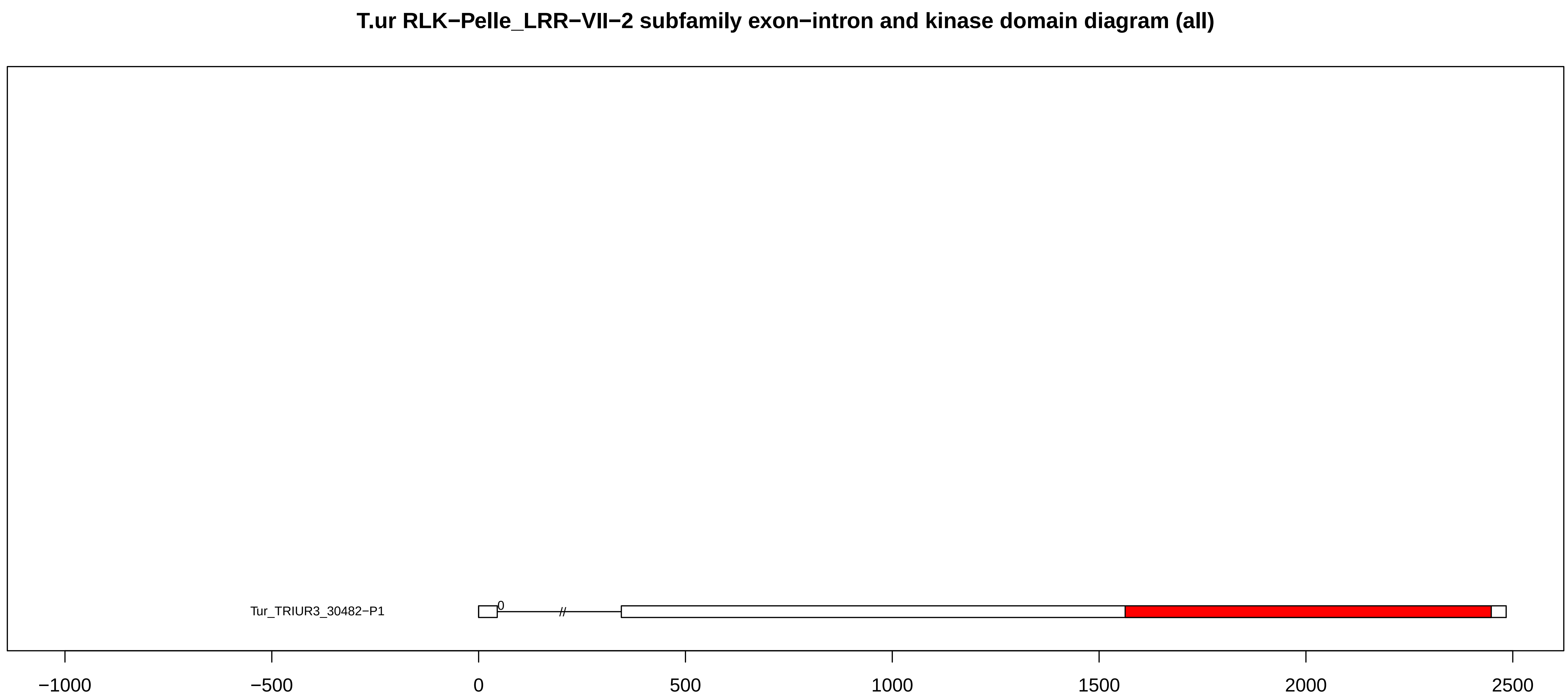
T.ur RLK−Pelle_LRR−VII−2 subfamily exon−intron and kinase domain diagram (all)
Tur_TRIUR3_30482−P1
0
−1000
−500
0
500
1000
1500
2000
2500

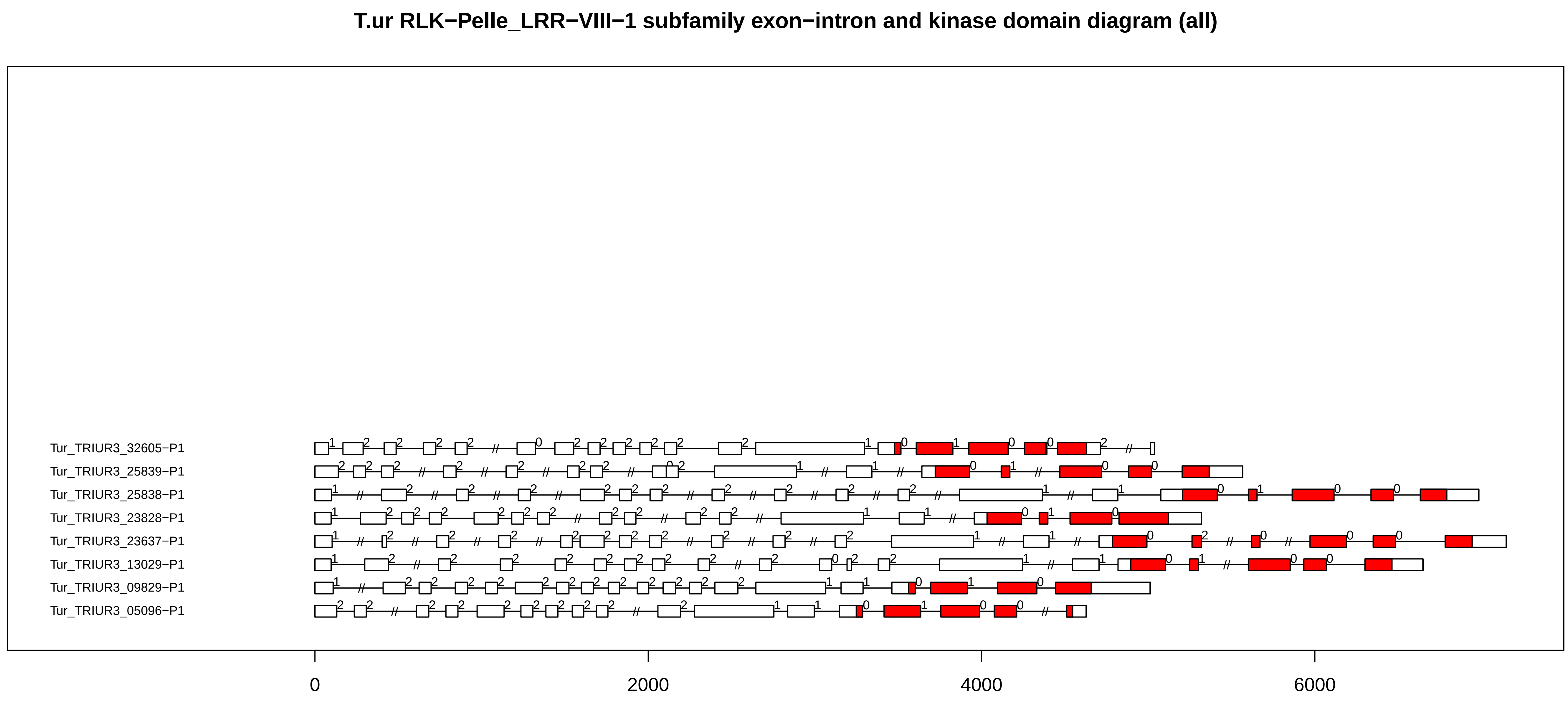
T.ur RLK−Pelle_LRR−VIII−1 subfamily exon−intron and kinase domain diagram (all)
Tur_TRIUR3_32605−P1
Tur_TRIUR3_25839−P1
Tur_TRIUR3_25838−P1
Tur_TRIUR3_23828−P1
Tur_TRIUR3_23637−P1
Tur_TRIUR3_13029−P1
Tur_TRIUR3_09829−P1
Tur_TRIUR3_05096−P1
0
2000
4000
6000

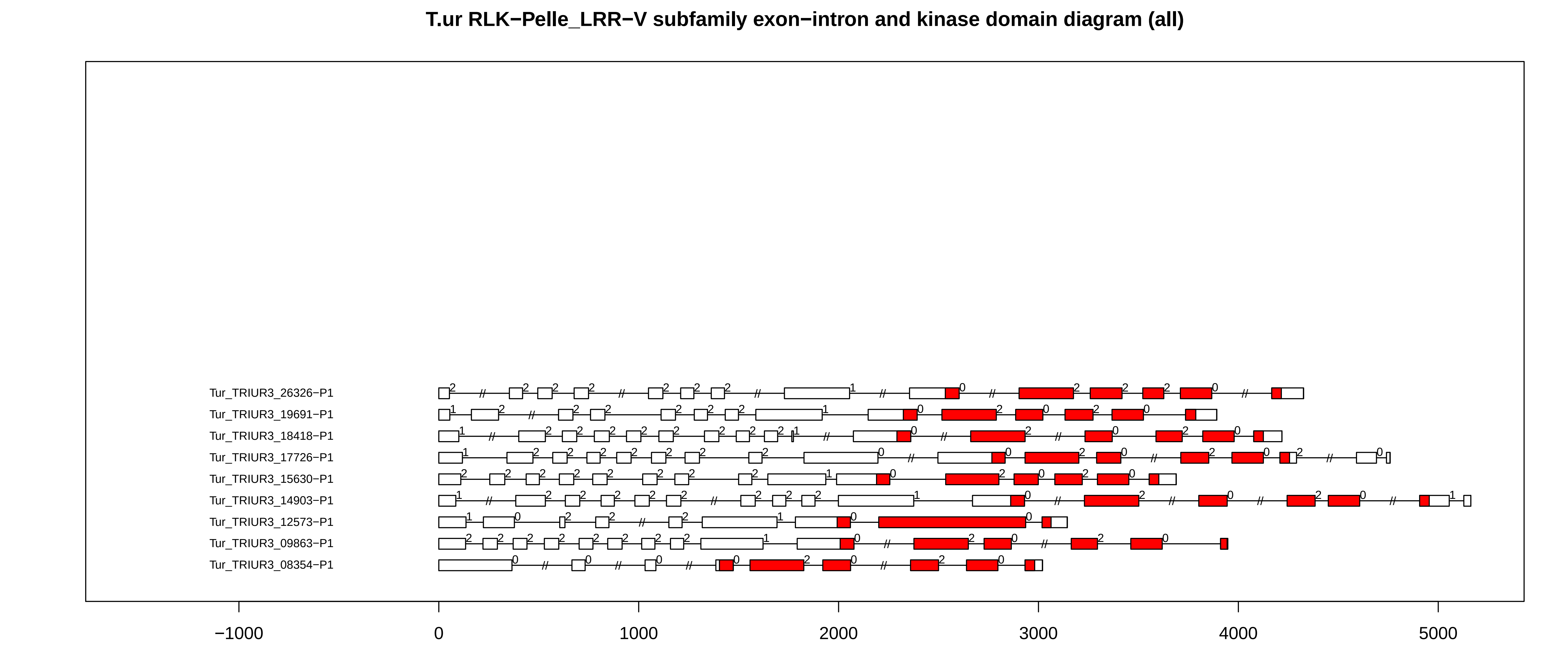

T.ur RLK−Pelle_LRR−V subfamily exon−intron and kinase domain diagram (all)
Tur_TRIUR3_26326−P1
Tur_TRIUR3_19691−P1
Tur_TRIUR3_18418−P1
Tur_TRIUR3_17726−P1
Tur_TRIUR3_15630−P1
Tur_TRIUR3_14903−P1
Tur_TRIUR3_12573−P1
Tur_TRIUR3_09863−P1
Tur_TRIUR3_08354−P1
−1000
0
1000
2000
3000
4000
5000

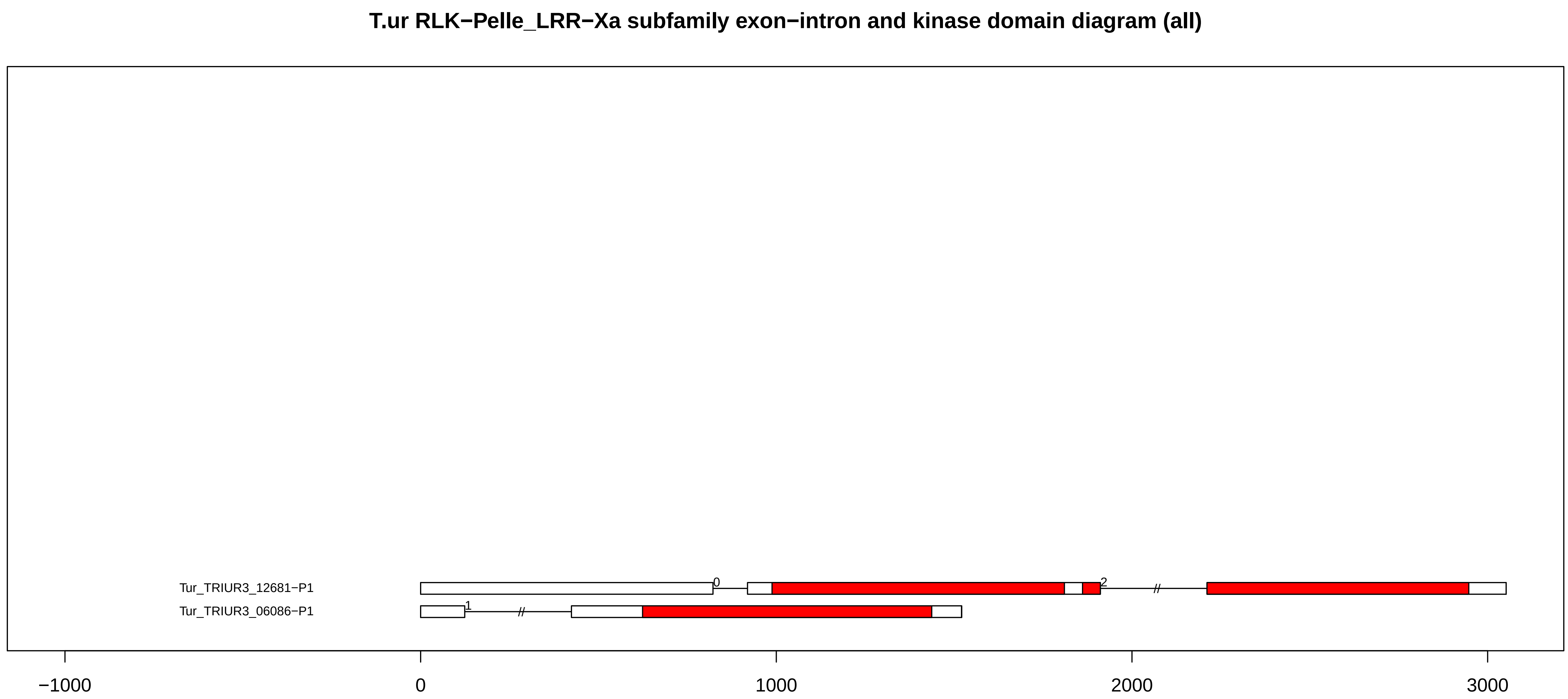
T.ur RLK−Pelle_LRR−Xa subfamily exon−intron and kinase domain diagram (all)
Tur_TRIUR3_12681−P1
Tur_TRIUR3_06086−P1
0
2
1
−1000
0
1000
2000
3000

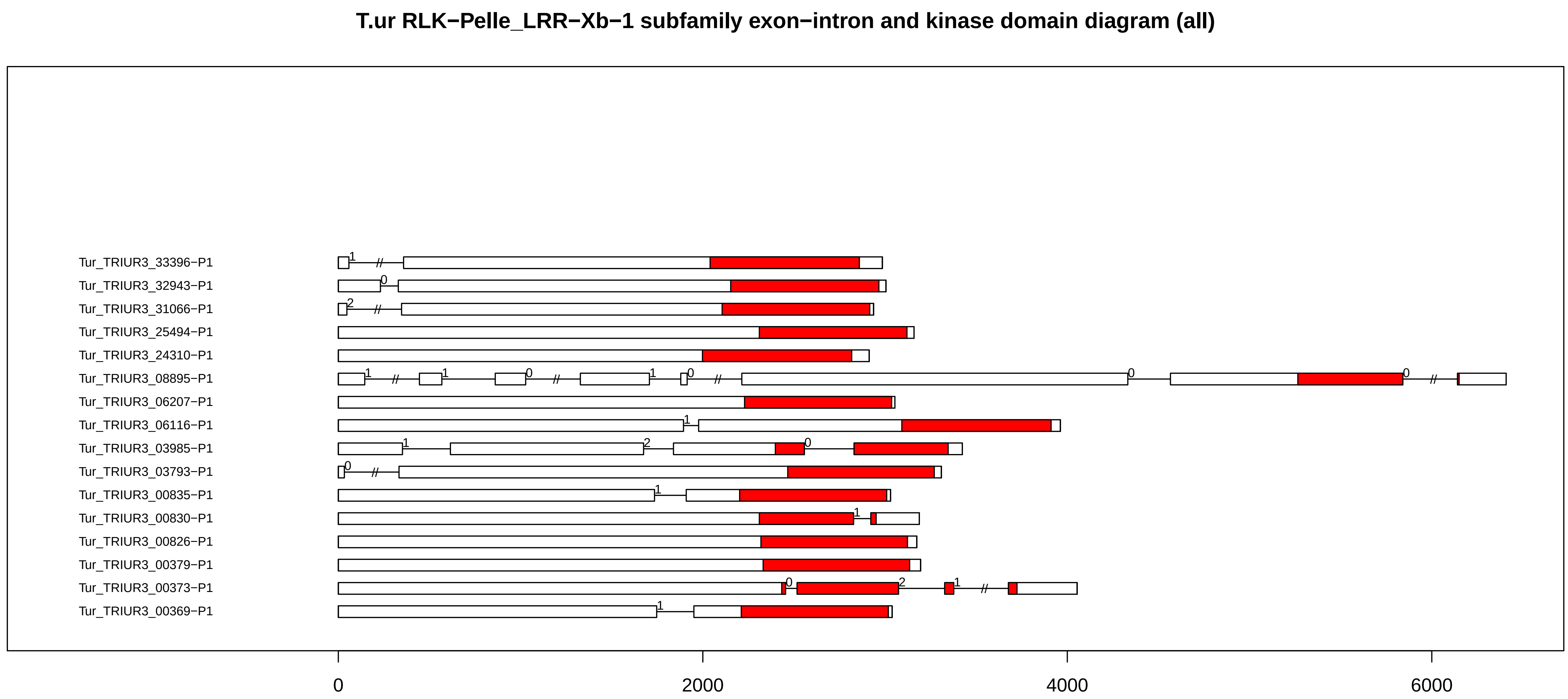

T.ur RLK−Pelle_LRR−Xb−1 subfamily exon−intron and kinase domain diagram (all)
Tur_TRIUR3_33396−P1
Tur_TRIUR3_32943−P1
Tur_TRIUR3_31066−P1
Tur_TRIUR3_25494−P1
Tur_TRIUR3_24310−P1
Tur_TRIUR3_08895−P1
Tur_TRIUR3_06207−P1
Tur_TRIUR3_06116−P1
Tur_TRIUR3_03985−P1
Tur_TRIUR3_03793−P1
Tur_TRIUR3_00835−P1
Tur_TRIUR3_00830−P1
Tur_TRIUR3_00826−P1
Tur_TRIUR3_00379−P1
Tur_TRIUR3_00373−P1
Tur_TRIUR3_00369−P1
0
2000
4000
6000

T.ur RLK−Pelle_LRR−XI−1 subfamily exon−intron and kinase domain diagram (part 1)

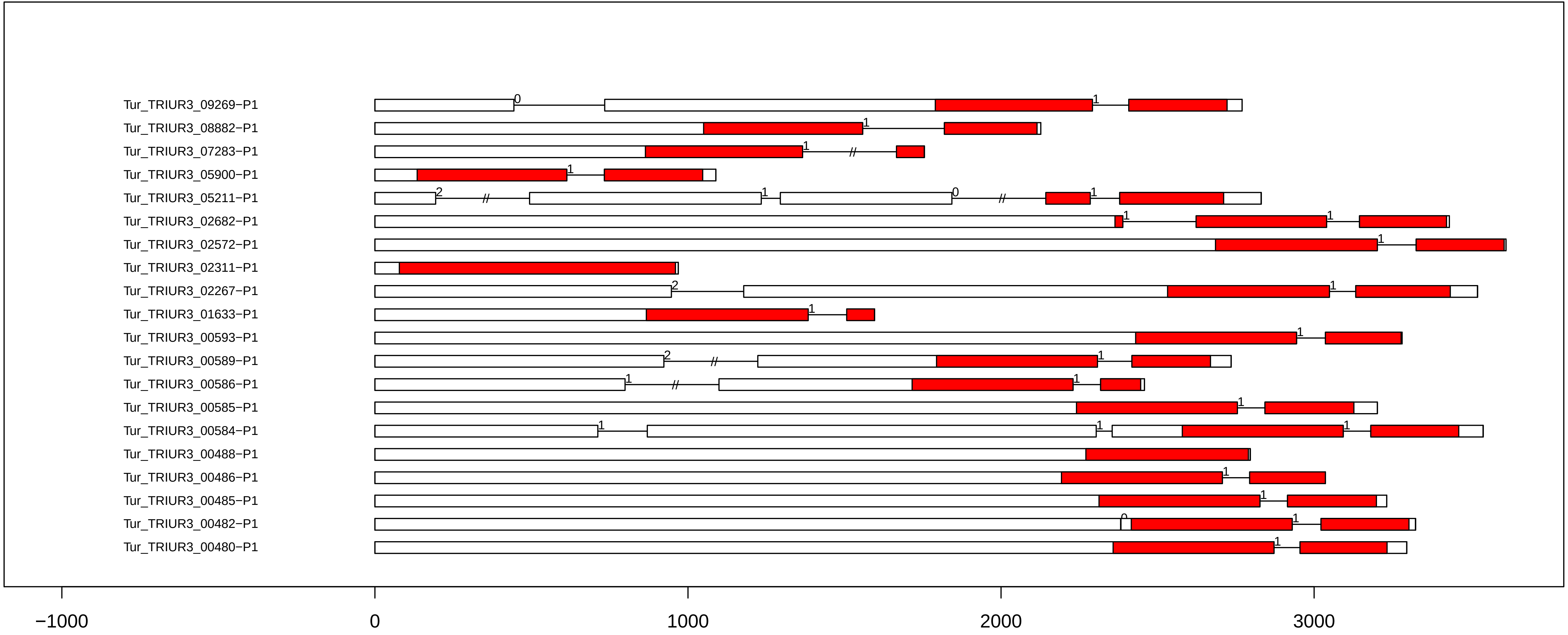

T.ur RLK−Pelle_LRR−XI−1 subfamily exon−intron and kinase domain diagram (part 2)

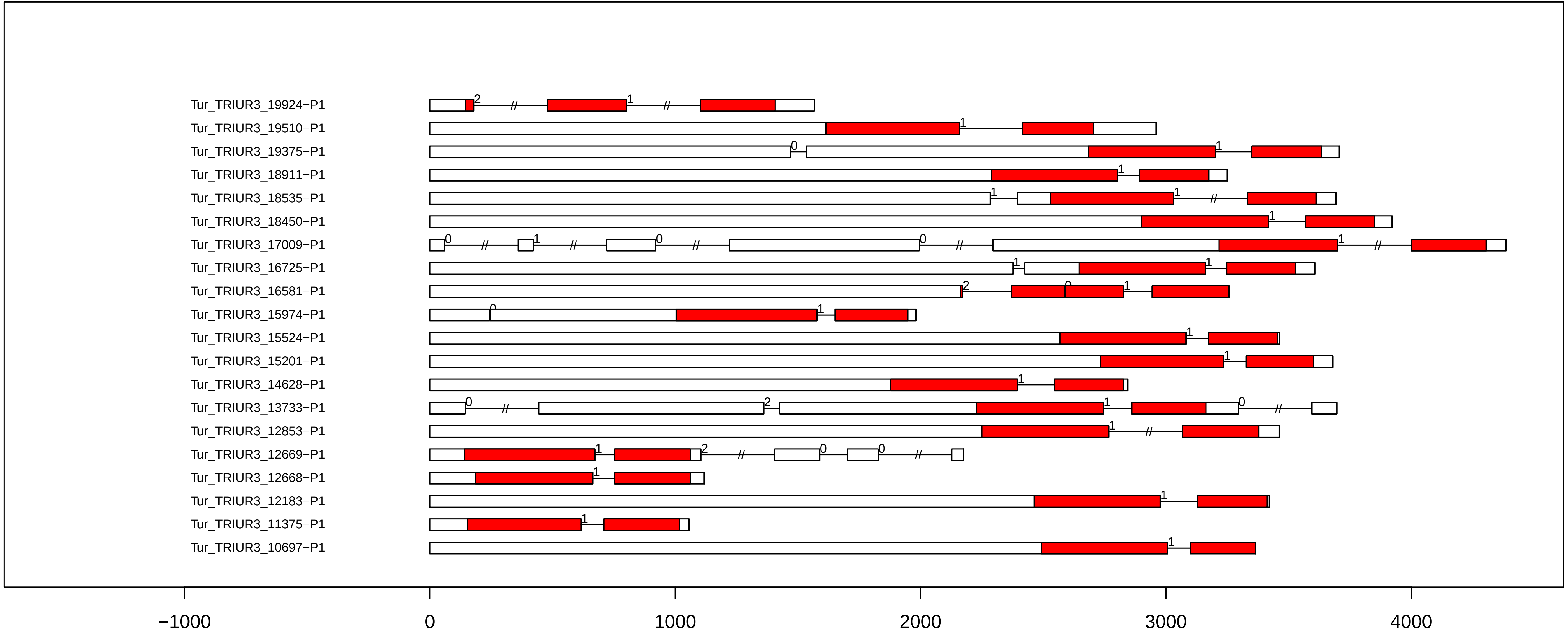

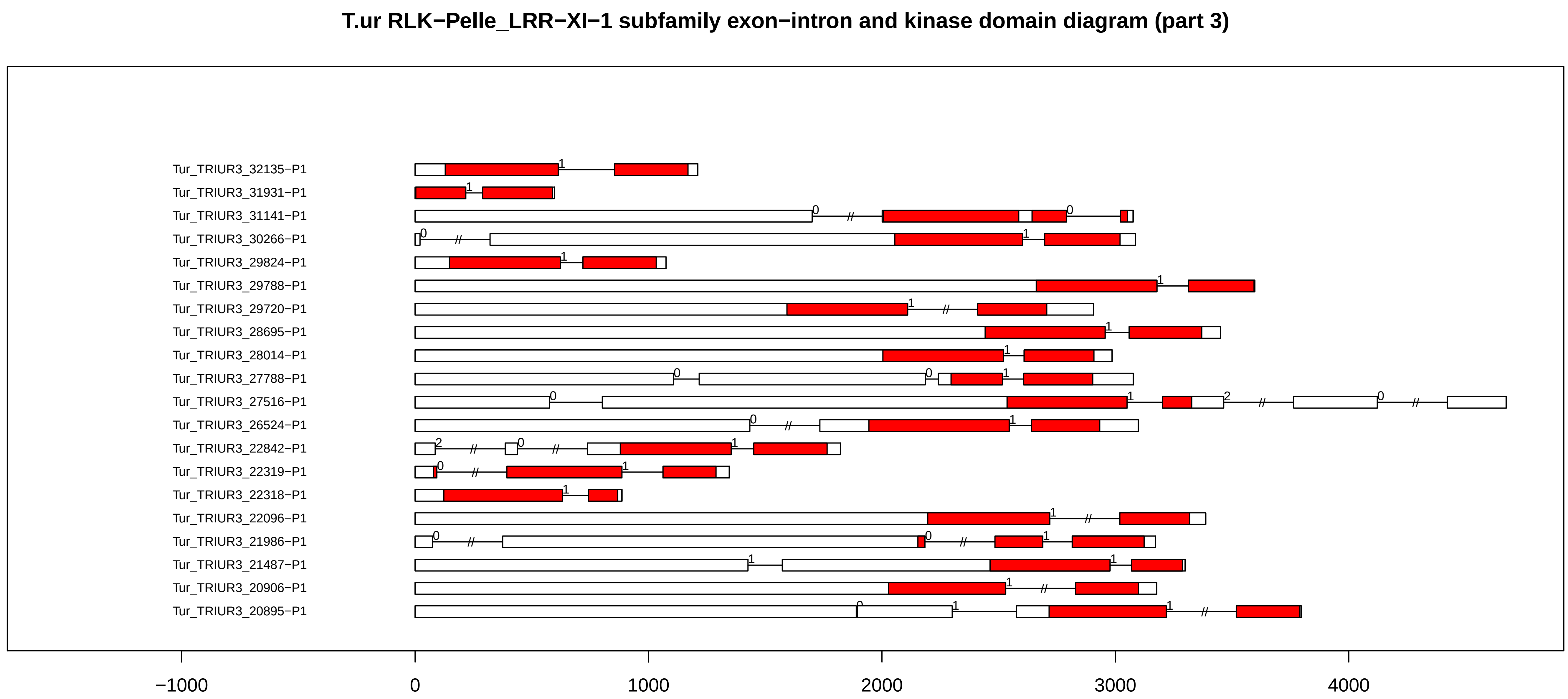
T.ur RLK−Pelle_LRR−XI−1 subfamily exon−intron and kinase domain diagram (part 3)
Tur_TRIUR3_32135−P1
Tur_TRIUR3_31931−P1
Tur_TRIUR3_31141−P1
Tur_TRIUR3_30266−P1
Tur_TRIUR3_29824−P1
Tur_TRIUR3_29788−P1
Tur_TRIUR3_29720−P1
Tur_TRIUR3_28695−P1
Tur_TRIUR3_28014−P1
Tur_TRIUR3_27788−P1
Tur_TRIUR3_27516−P1
Tur_TRIUR3_26524−P1
Tur_TRIUR3_22842−P1
Tur_TRIUR3_22319−P1
Tur_TRIUR3_22318−P1
Tur_TRIUR3_22096−P1
Tur_TRIUR3_21986−P1
Tur_TRIUR3_21487−P1
Tur_TRIUR3_20906−P1
Tur_TRIUR3_20895−P1
−1000
0
1000
2000
3000
4000

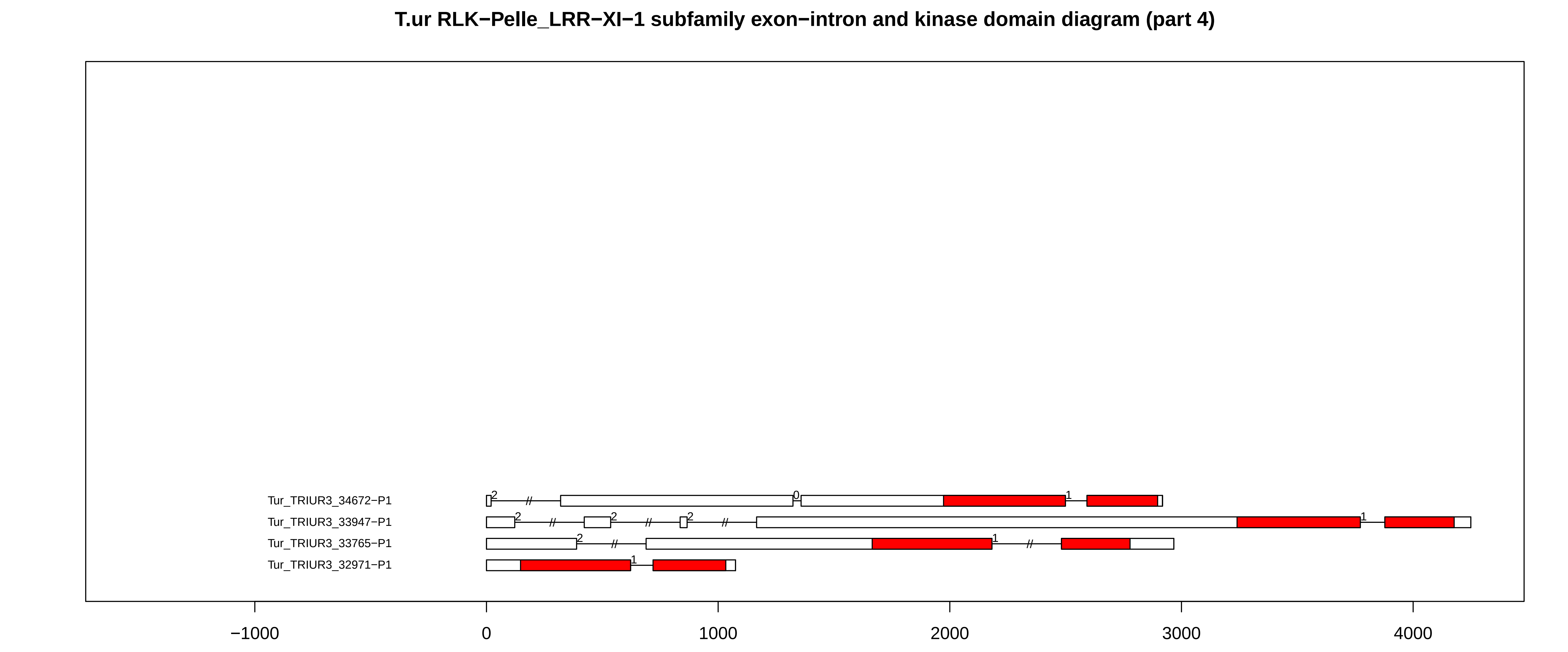
T.ur RLK−Pelle_LRR−XI−1 subfamily exon−intron and kinase domain diagram (part 4)
Tur_TRIUR3_34672−P1
Tur_TRIUR3_33947−P1
Tur_TRIUR3_33765−P1
Tur_TRIUR3_32971−P1
−1000
0
1000
2000
3000
4000

T.ur RLK−Pelle_LRR−XI−2 subfamily exon−intron and kinase domain diagram (all)

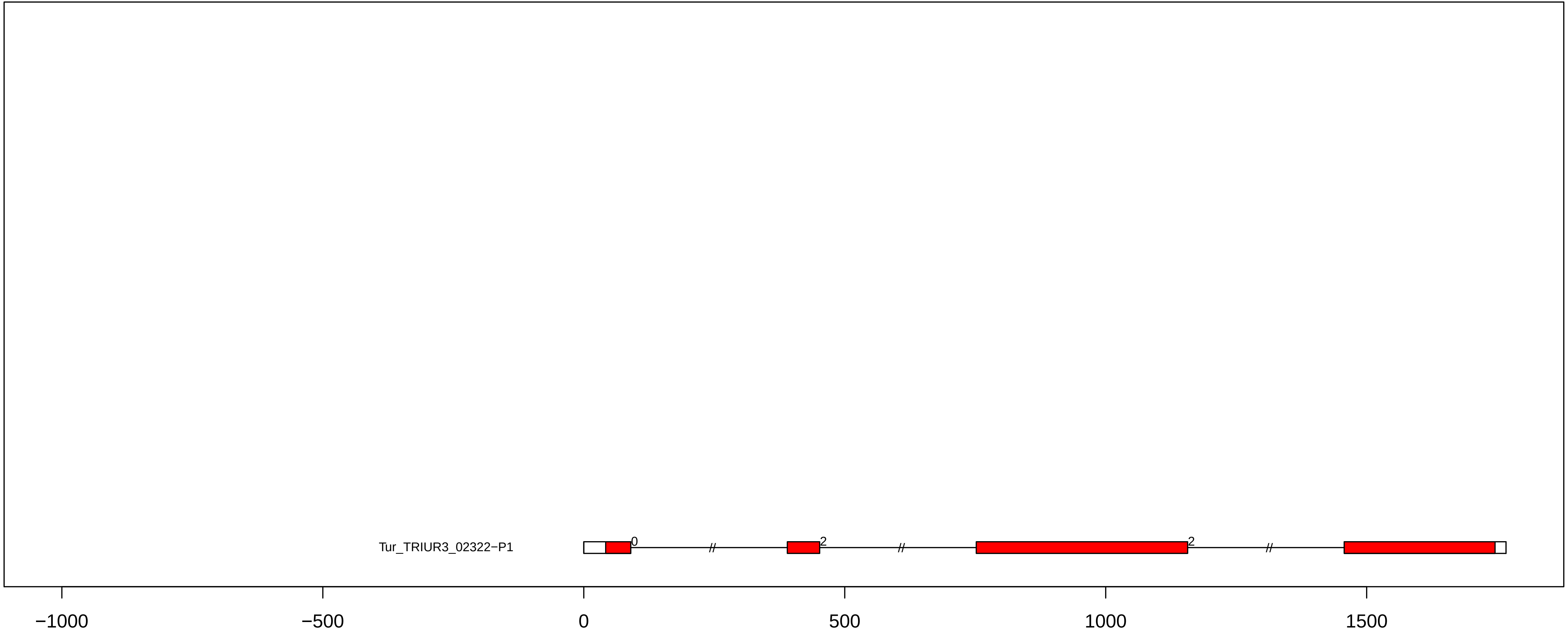

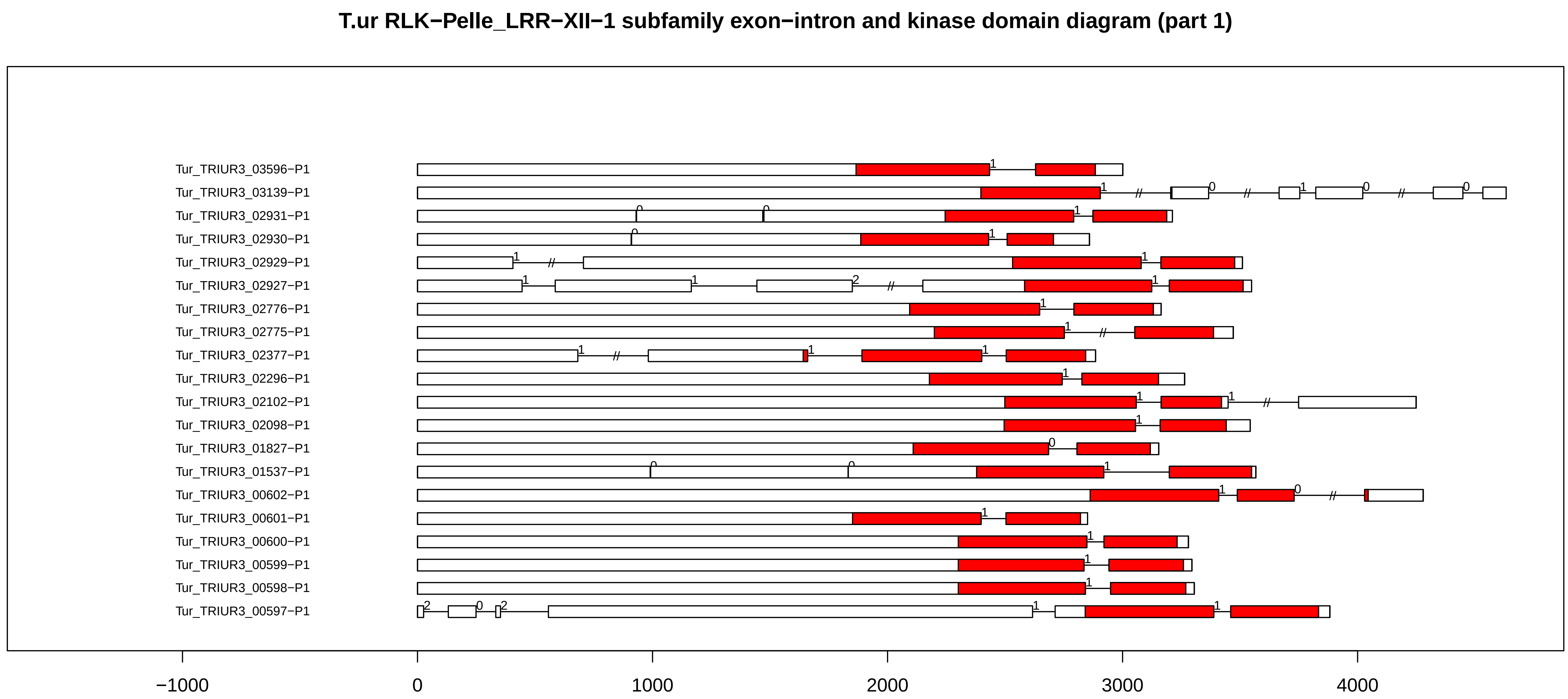
T.ur RLK−Pelle_LRR−XII−1 subfamily exon−intron and kinase domain diagram (part 1)
Tur_TRIUR3_03596−P1
Tur_TRIUR3_03139−P1
Tur_TRIUR3_02931−P1
Tur_TRIUR3_02930−P1
Tur_TRIUR3_02929−P1
Tur_TRIUR3_02927−P1
Tur_TRIUR3_02776−P1
Tur_TRIUR3_02775−P1
Tur_TRIUR3_02377−P1
Tur_TRIUR3_02296−P1
Tur_TRIUR3_02102−P1
Tur_TRIUR3_02098−P1
Tur_TRIUR3_01827−P1
Tur_TRIUR3_01537−P1
Tur_TRIUR3_00602−P1
Tur_TRIUR3_00601−P1
Tur_TRIUR3_00600−P1
Tur_TRIUR3_00599−P1
Tur_TRIUR3_00598−P1
Tur_TRIUR3_00597−P1
−1000
0
1000
2000
3000
4000

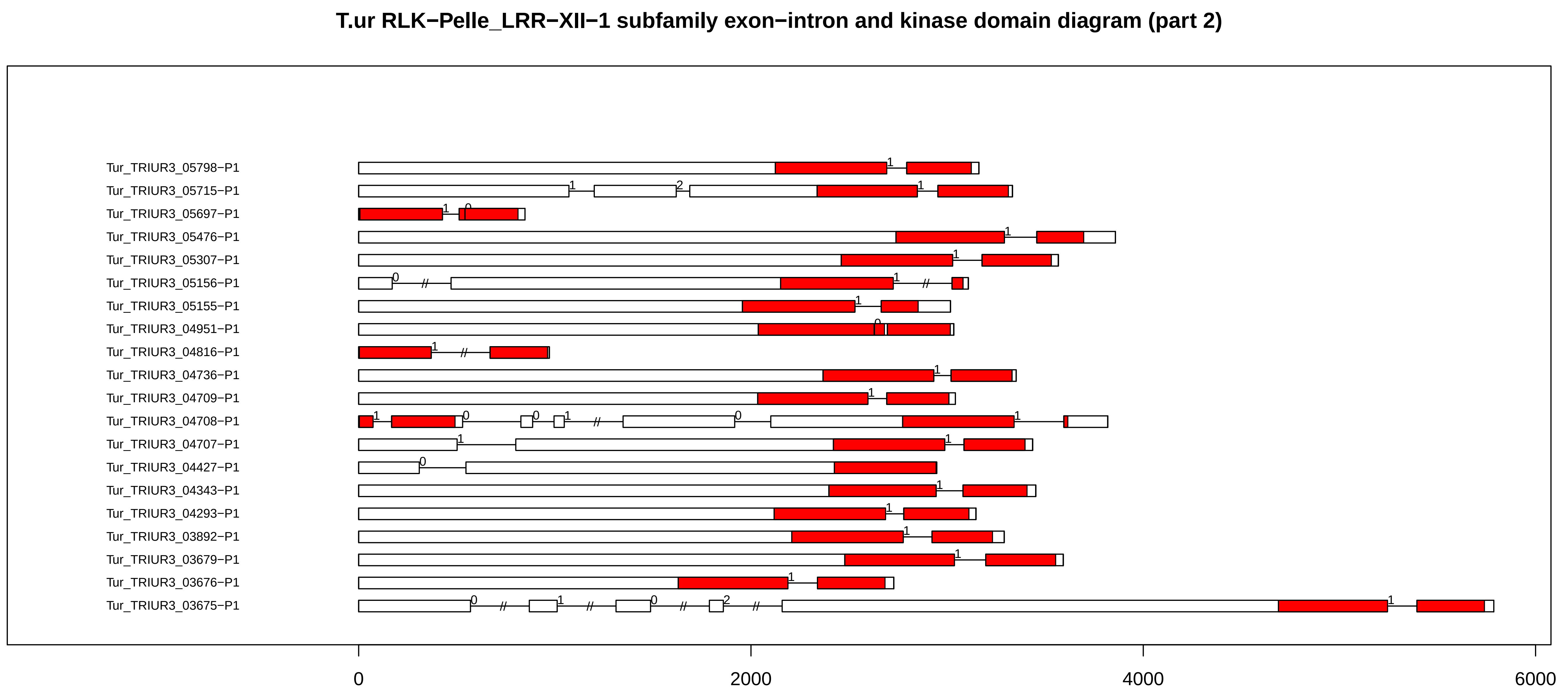

T.ur RLK−Pelle_LRR−XII−1 subfamily exon−intron and kinase domain diagram (part 2)
Tur_TRIUR3_05798−P1
Tur_TRIUR3_05715−P1
Tur_TRIUR3_05697−P1
Tur_TRIUR3_05476−P1
Tur_TRIUR3_05307−P1
Tur_TRIUR3_05156−P1
Tur_TRIUR3_05155−P1
Tur_TRIUR3_04951−P1
Tur_TRIUR3_04816−P1
Tur_TRIUR3_04736−P1
Tur_TRIUR3_04709−P1
Tur_TRIUR3_04708−P1
Tur_TRIUR3_04707−P1
Tur_TRIUR3_04427−P1
Tur_TRIUR3_04343−P1
Tur_TRIUR3_04293−P1
Tur_TRIUR3_03892−P1
Tur_TRIUR3_03679−P1
Tur_TRIUR3_03676−P1
Tur_TRIUR3_03675−P1
0
2000
4000
6000

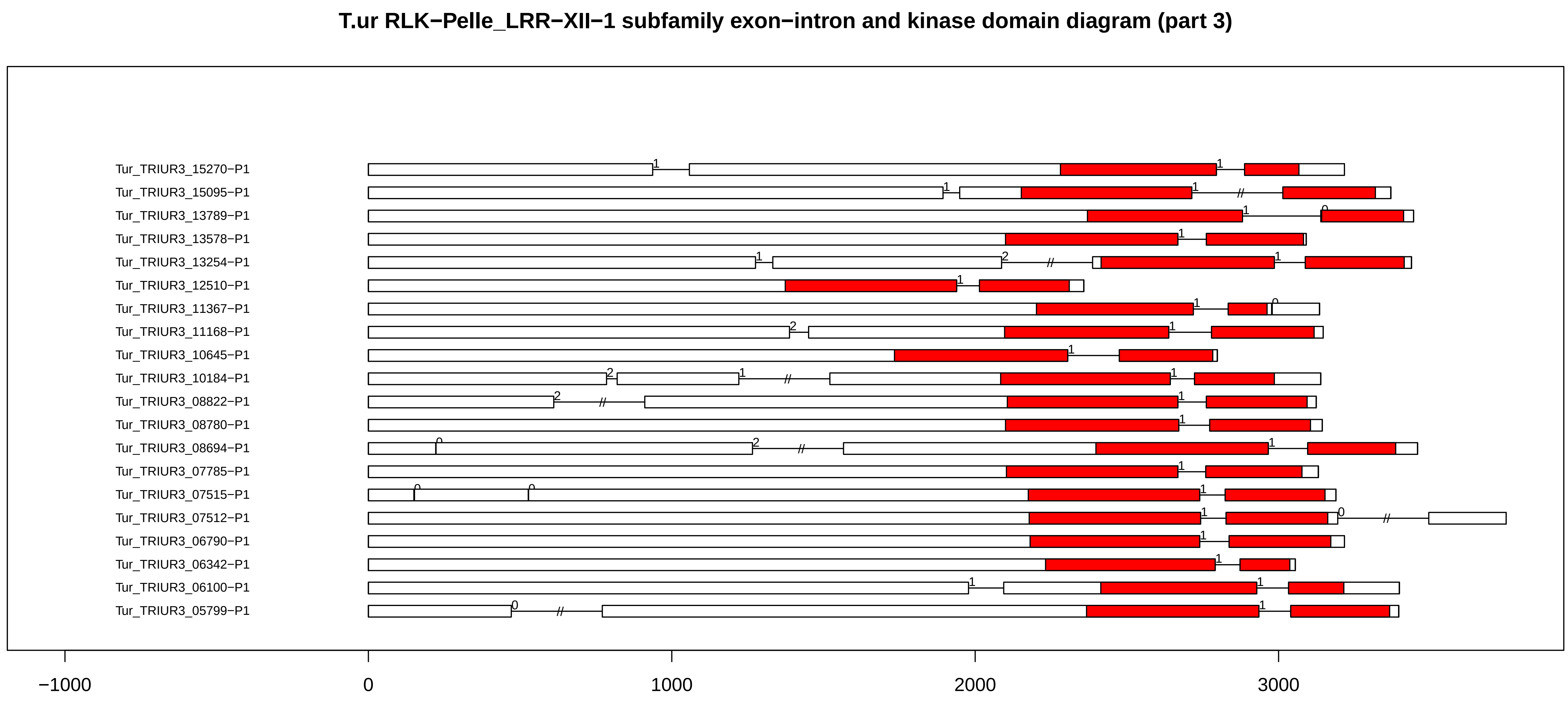
T.ur RLK−Pelle_LRR−XII−1 subfamily exon−intron and kinase domain diagram (part 3)
Tur_TRIUR3_15270−P1
Tur_TRIUR3_15095−P1
Tur_TRIUR3_13789−P1
Tur_TRIUR3_13578−P1
Tur_TRIUR3_13254−P1
Tur_TRIUR3_12510−P1
Tur_TRIUR3_11367−P1
Tur_TRIUR3_11168−P1
Tur_TRIUR3_10645−P1
Tur_TRIUR3_10184−P1
Tur_TRIUR3_08822−P1
Tur_TRIUR3_08780−P1
Tur_TRIUR3_08694−P1
Tur_TRIUR3_07785−P1
Tur_TRIUR3_07515−P1
Tur_TRIUR3_07512−P1
Tur_TRIUR3_06790−P1
Tur_TRIUR3_06342−P1
Tur_TRIUR3_06100−P1
Tur_TRIUR3_05799−P1
−1000
0
1000
2000
3000

T.ur RLK−Pelle_LRR−XII−1 subfamily exon−intron and kinase domain diagram (part 4)

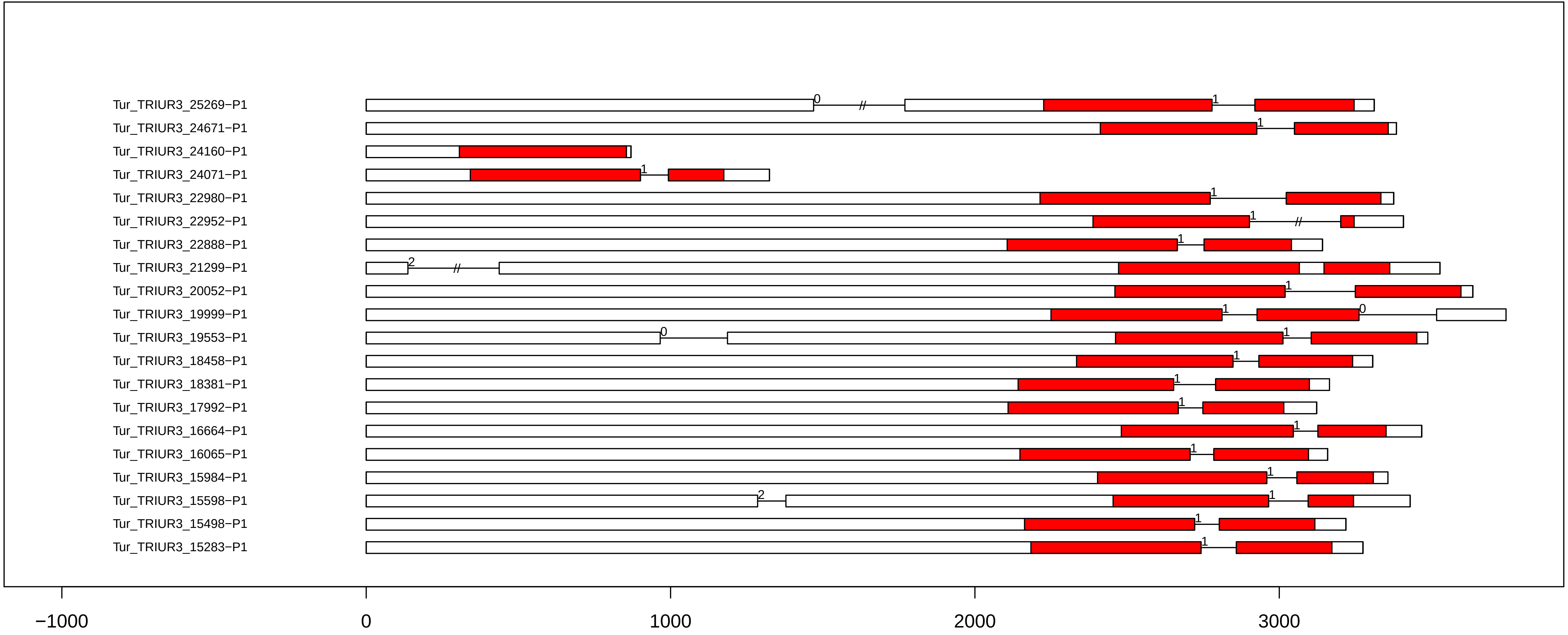

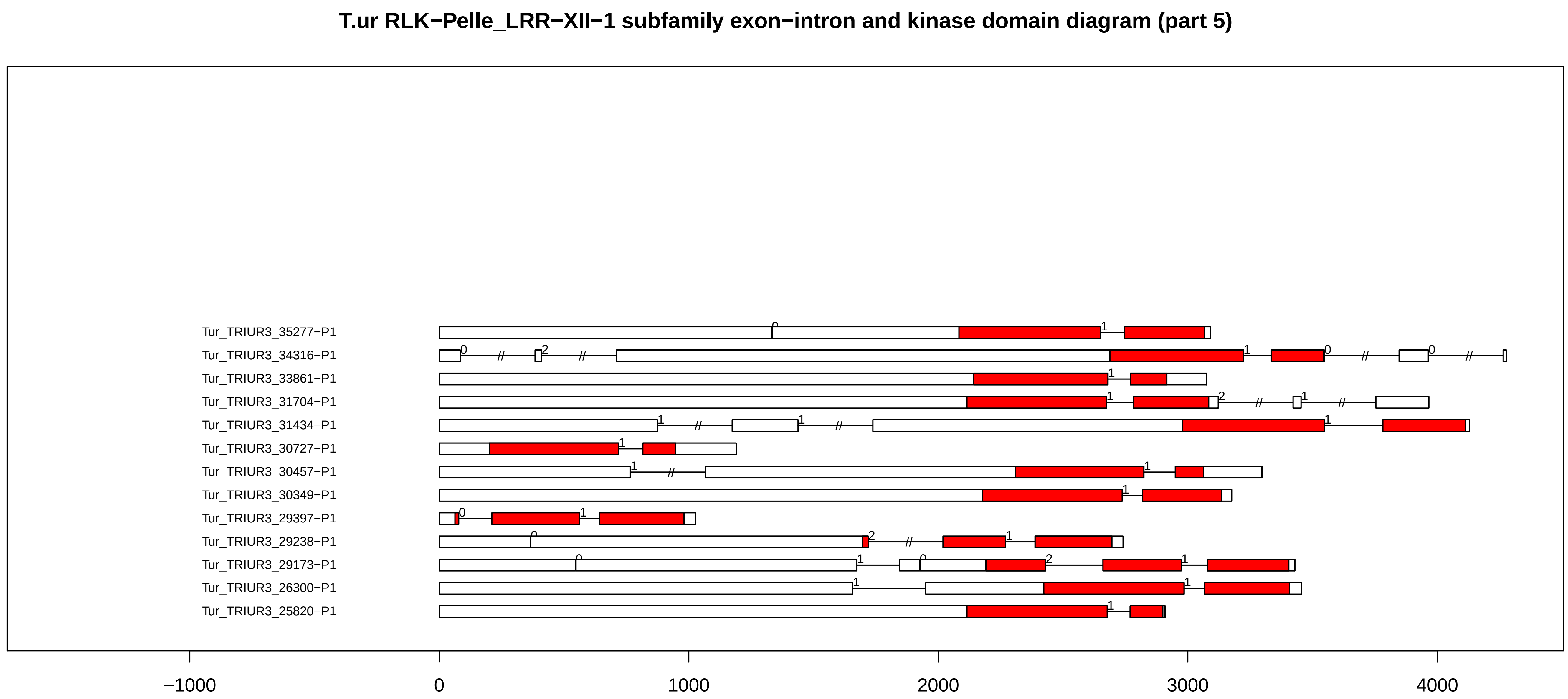
T.ur RLK−Pelle_LRR−XII−1 subfamily exon−intron and kinase domain diagram (part 5)
Tur_TRIUR3_35277−P1
Tur_TRIUR3_34316−P1
Tur_TRIUR3_33861−P1
Tur_TRIUR3_31704−P1
Tur_TRIUR3_31434−P1
Tur_TRIUR3_30727−P1
Tur_TRIUR3_30457−P1
Tur_TRIUR3_30349−P1
Tur_TRIUR3_29397−P1
Tur_TRIUR3_29238−P1
Tur_TRIUR3_29173−P1
Tur_TRIUR3_26300−P1
Tur_TRIUR3_25820−P1
−1000
0
1000
2000
3000
4000

T.ur RLK−Pelle_LRR−XIIIa subfamily exon−intron and kinase domain diagram (all)

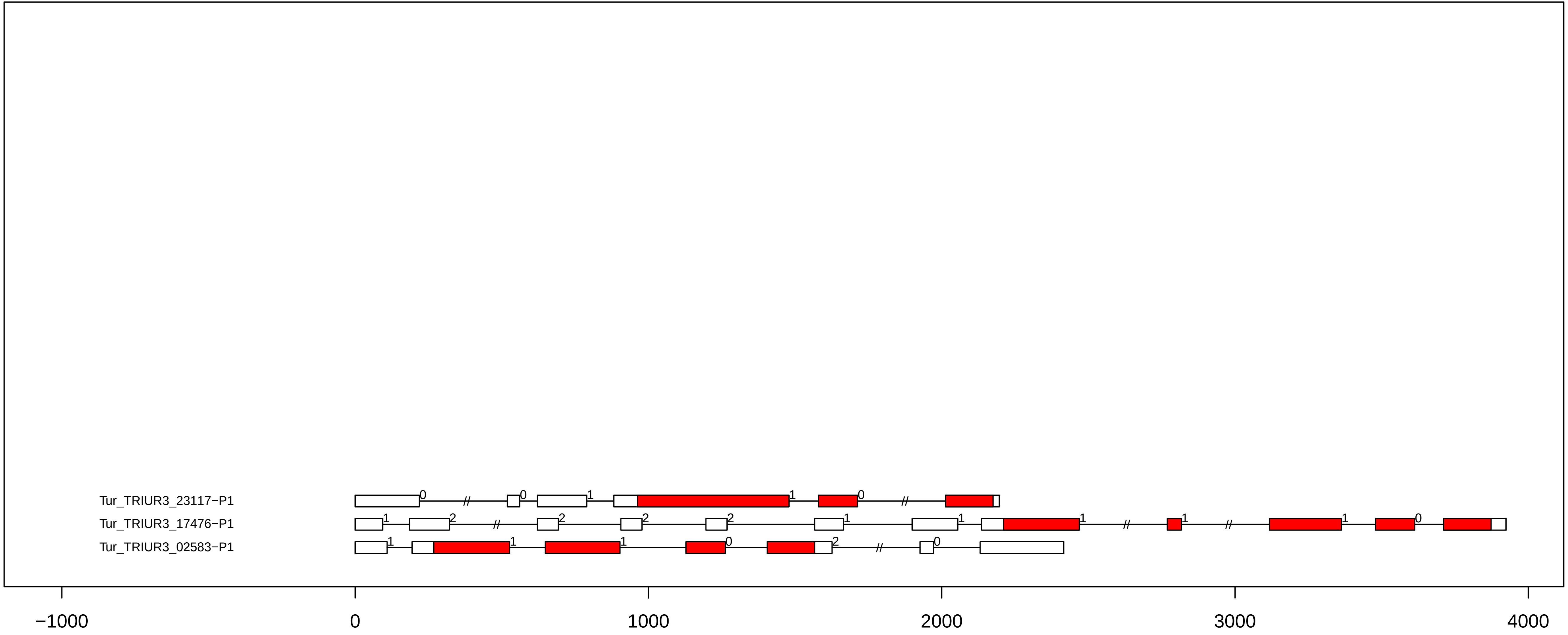

T.ur RLK−Pelle_LRR−XIIIb subfamily exon−intron and kinase domain diagram (all)

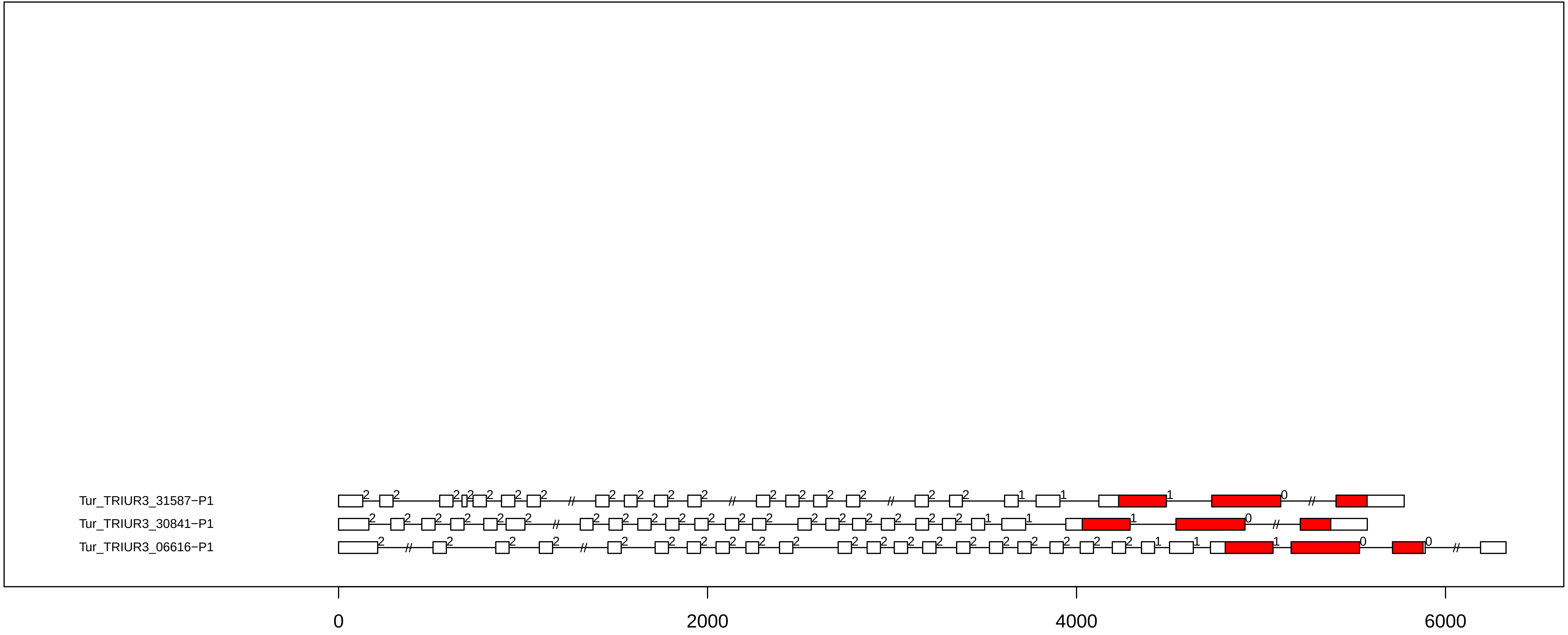

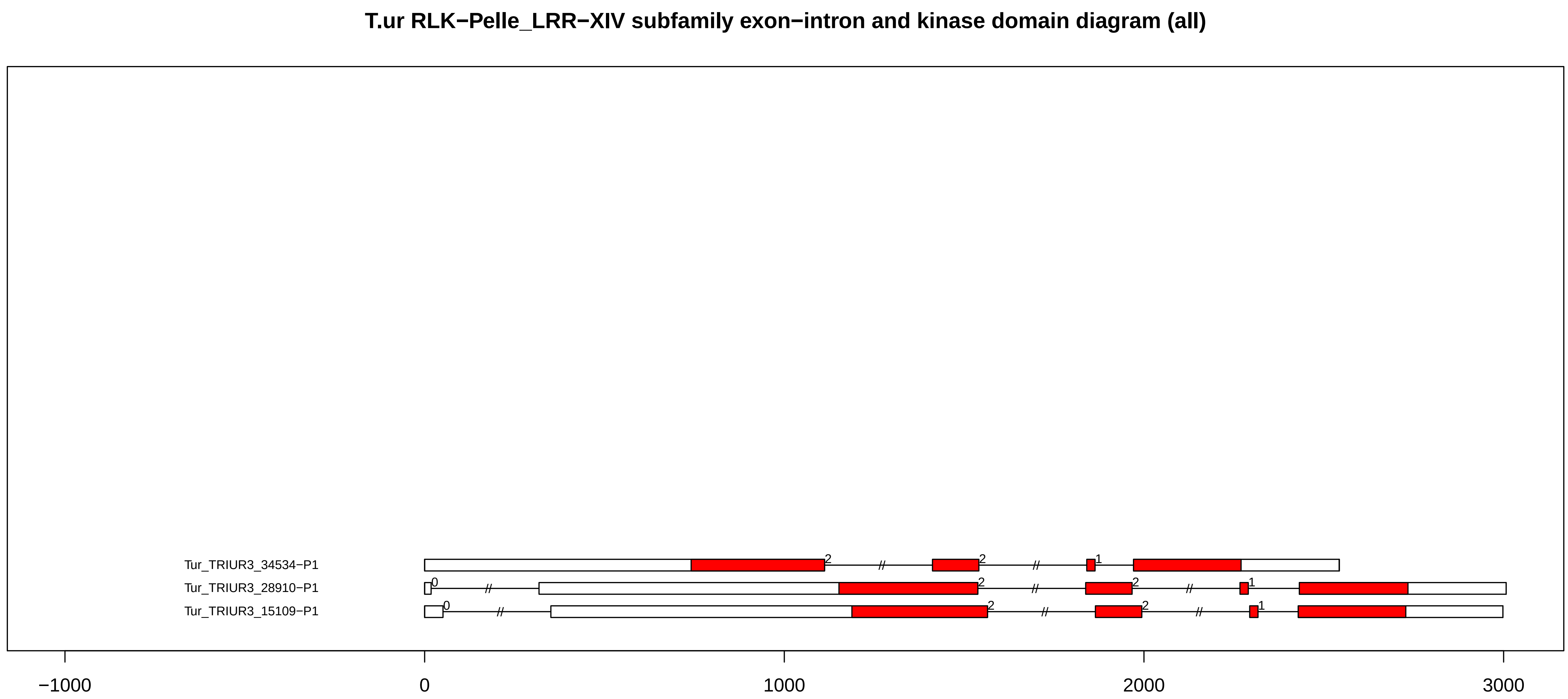

T.ur RLK−Pelle_LRR−XIV subfamily exon−intron and kinase domain diagram (all)
Tur_TRIUR3_34534−P1
Tur_TRIUR3_28910−P1
Tur_TRIUR3_15109−P1
2
2
1
0
2
2
1
0
2
2
1
−1000
0
1000
2000
3000

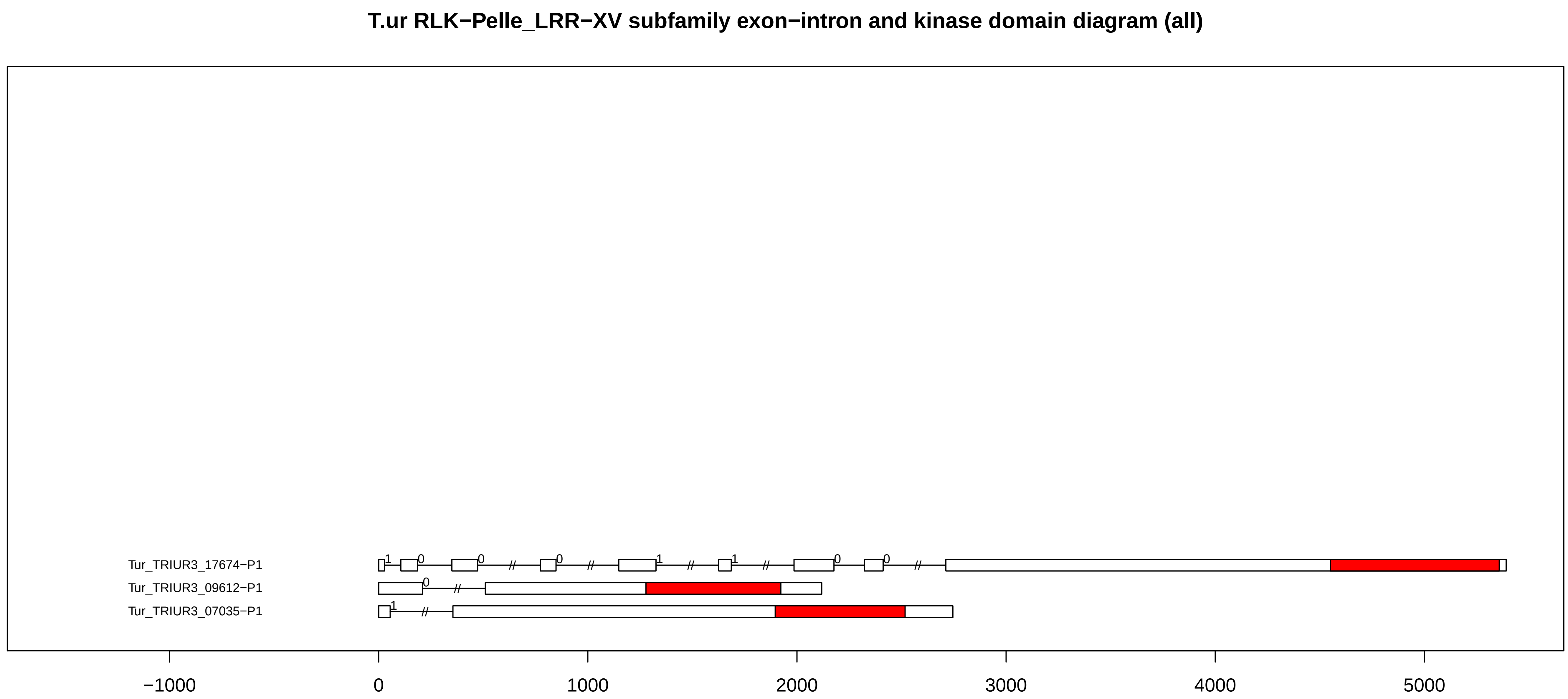

T.ur RLK−Pelle_LRR−XV subfamily exon−intron and kinase domain diagram (all)
Tur_TRIUR3_17674−P1
Tur_TRIUR3_09612−P1
Tur_TRIUR3_07035−P1
1
0
0
0
1
1
0
0
0
1
−1000
0
1000
2000
3000
4000
5000

T.ur RLK−Pelle_LysM subfamily exon−intron and kinase domain diagram (all)

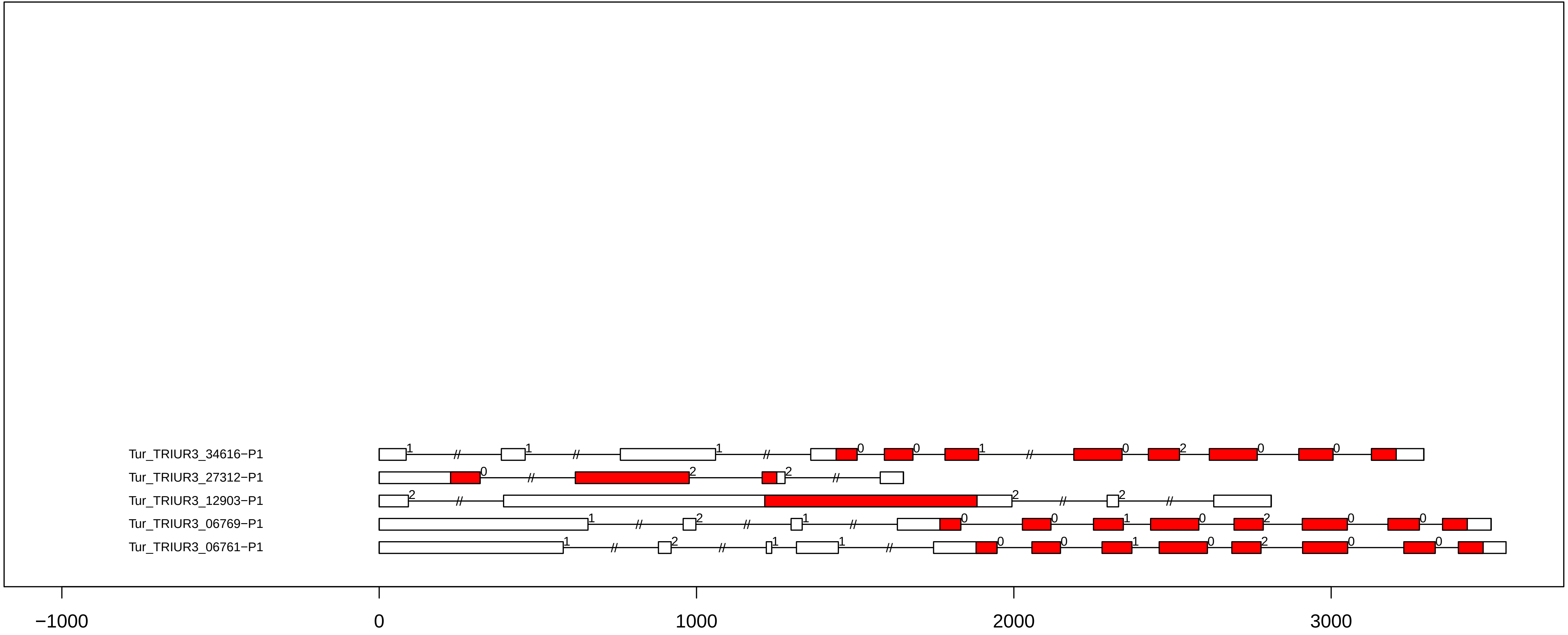

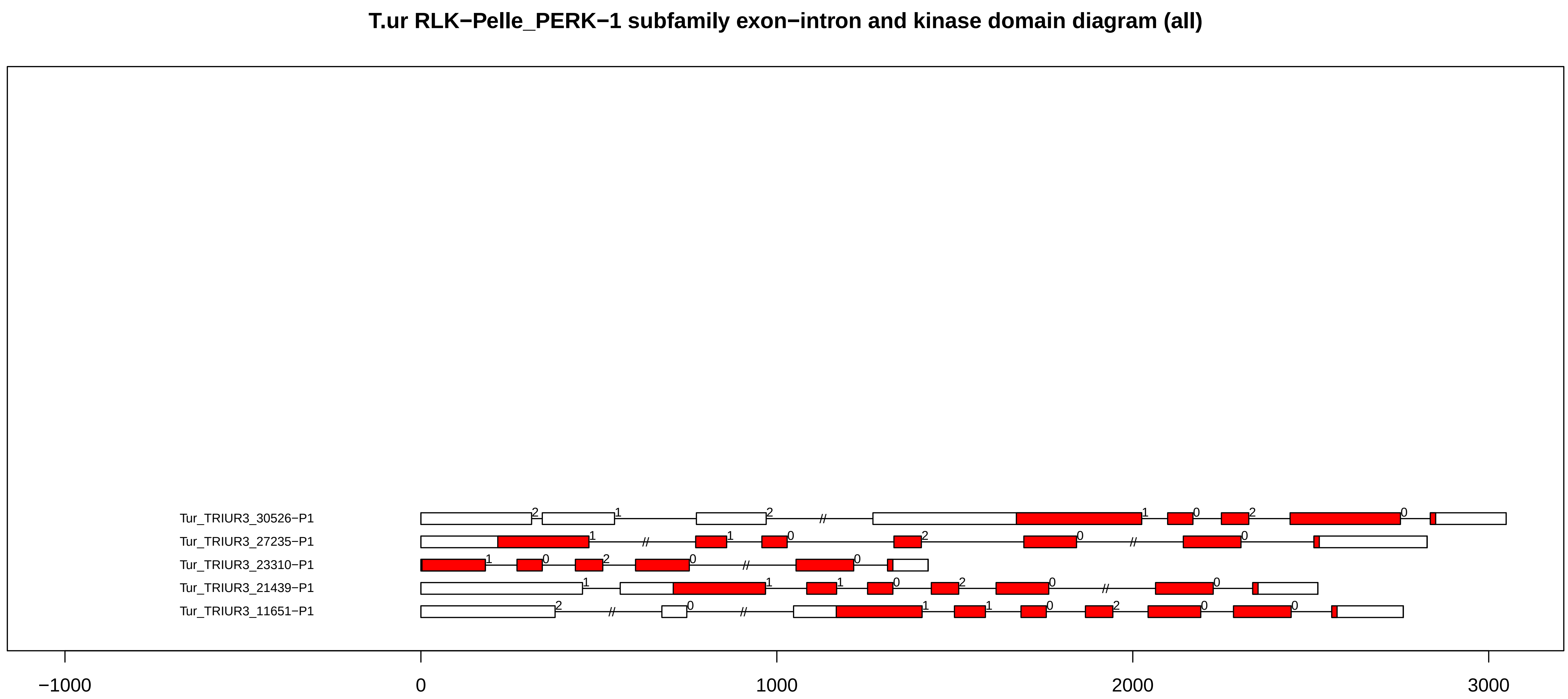
T.ur RLK−Pelle_PERK−1 subfamily exon−intron and kinase domain diagram (all)
Tur_TRIUR3_30526−P1
Tur_TRIUR3_27235−P1
Tur_TRIUR3_23310−P1
Tur_TRIUR3_21439−P1
Tur_TRIUR3_11651−P1
−1000
0
1000
2000
3000

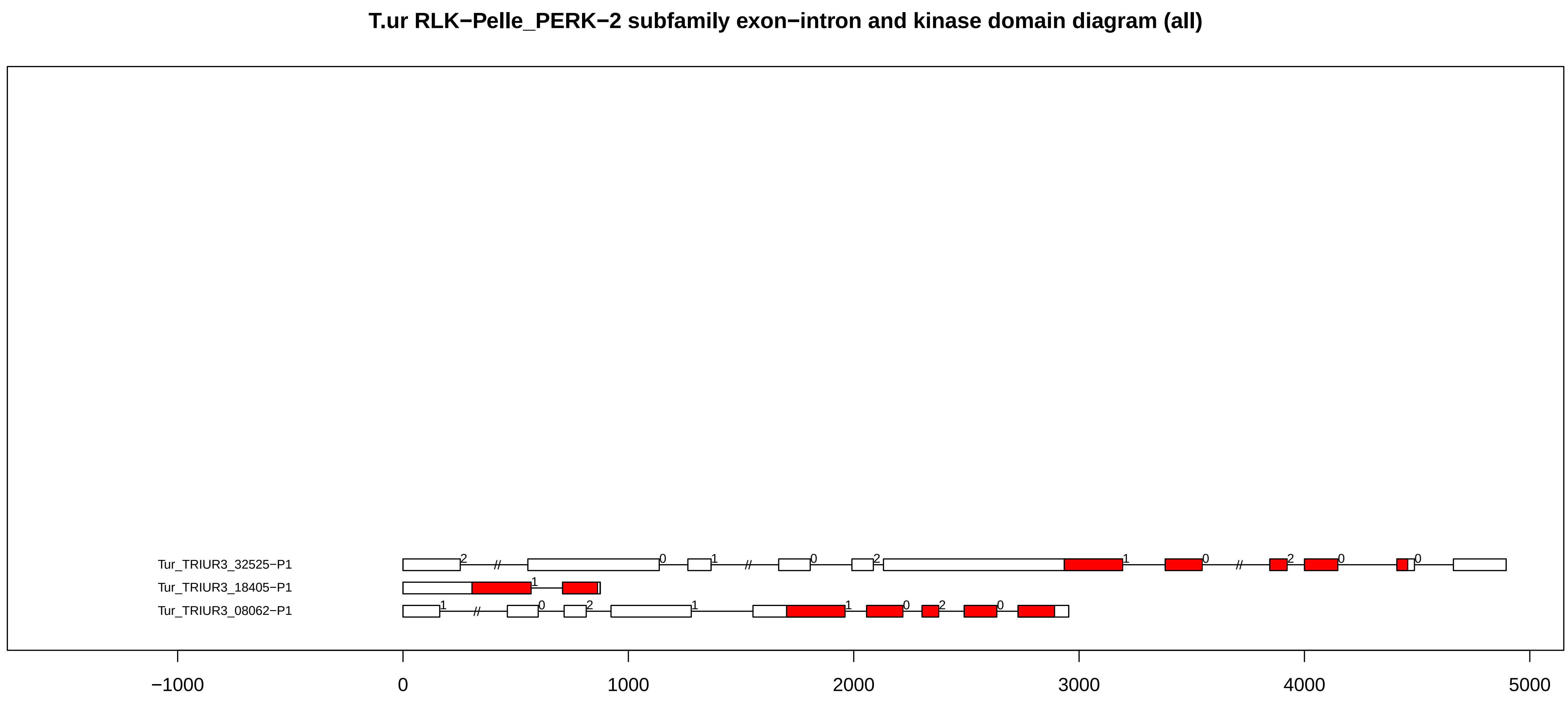
T.ur RLK−Pelle_PERK−2 subfamily exon−intron and kinase domain diagram (all)
Tur_TRIUR3_32525−P1
Tur_TRIUR3_18405−P1
Tur_TRIUR3_08062−P1
−1000
0
1000
2000
3000
4000
5000

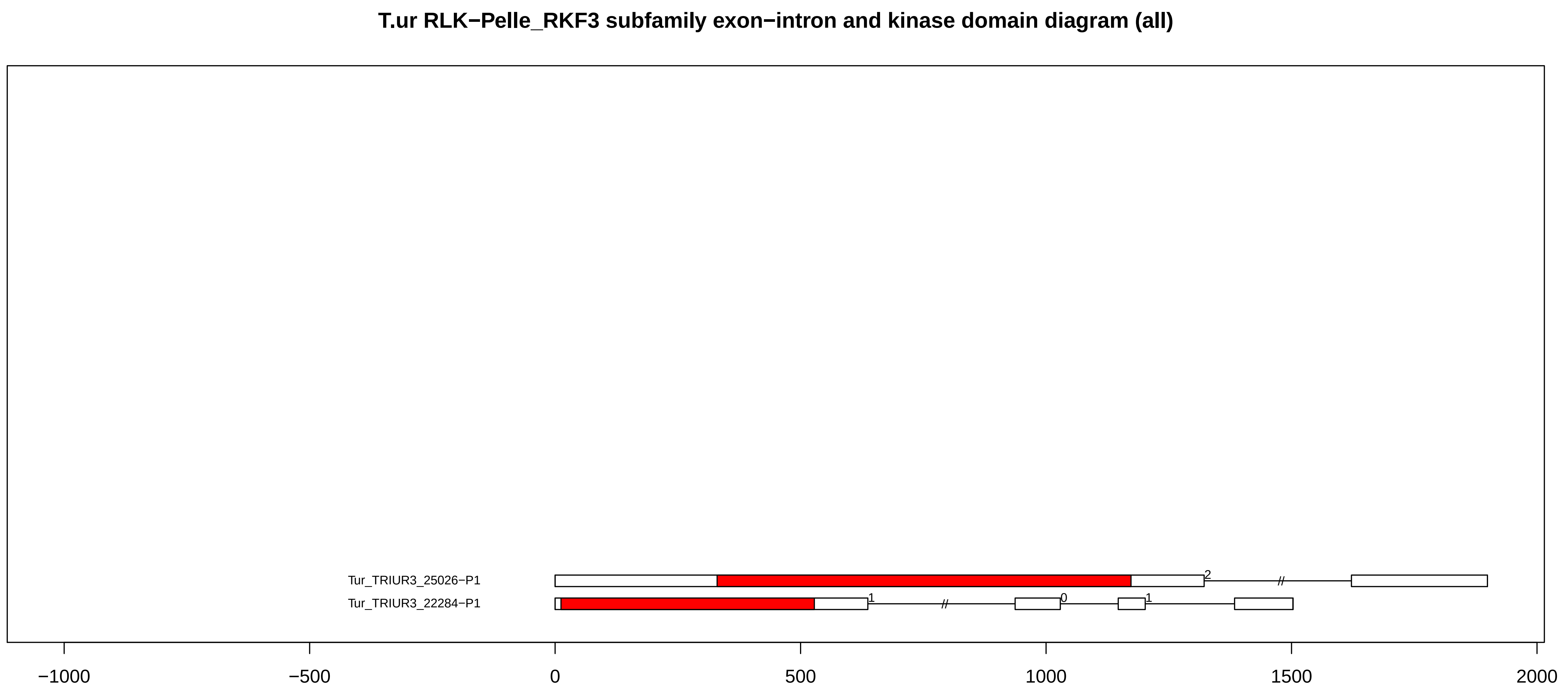

T.ur RLK−Pelle_RKF3 subfamily exon−intron and kinase domain diagram (all)
Tur_TRIUR3_25026−P1
Tur_TRIUR3_22284−P1
2
1
0
1
−1000
−500
0
500
1000
1500
2000

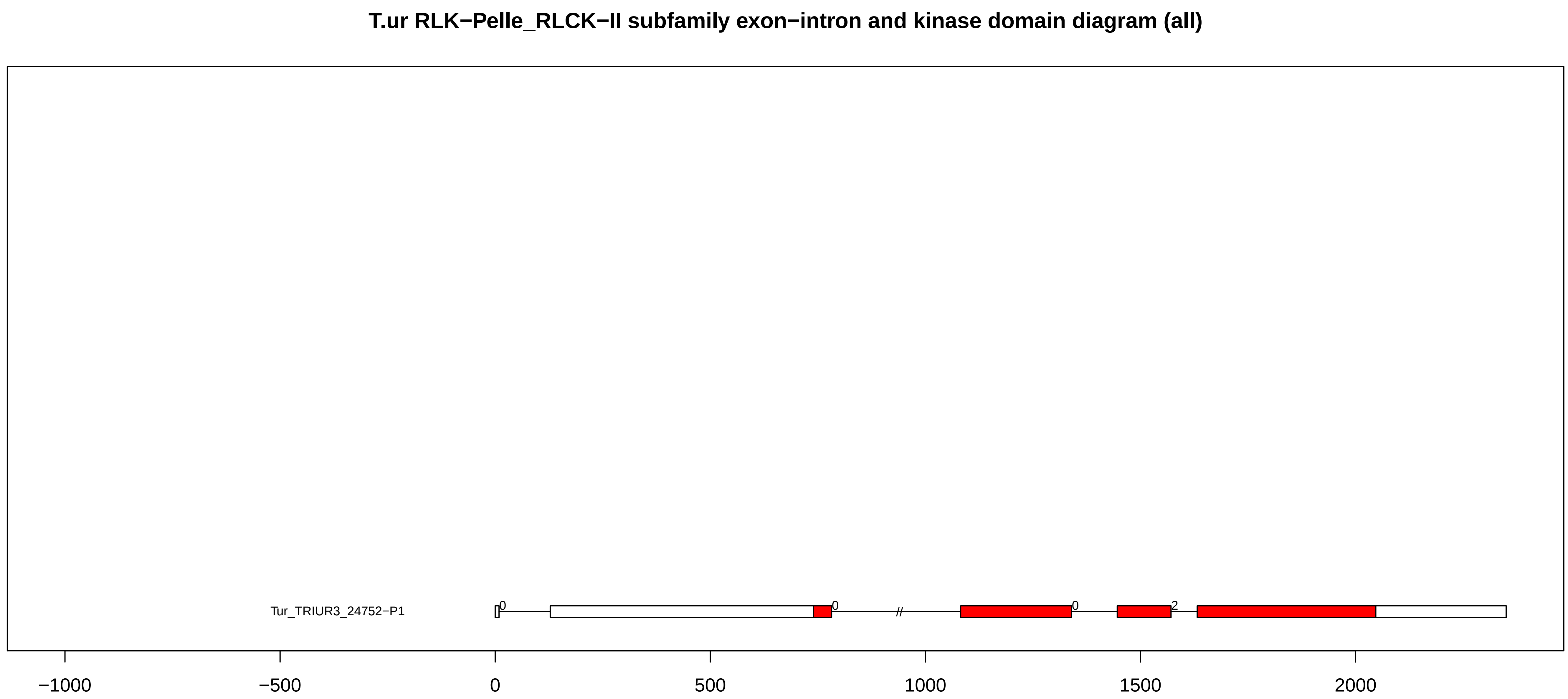
T.ur RLK−Pelle_RLCK−II subfamily exon−intron and kinase domain diagram (all)
Tur_TRIUR3_24752−P1
0
0
0
2
−1000
−500
0
500
1000
1500
2000

T.ur RLK−Pelle_RLCK−IV subfamily exon−intron and kinase domain diagram (all)

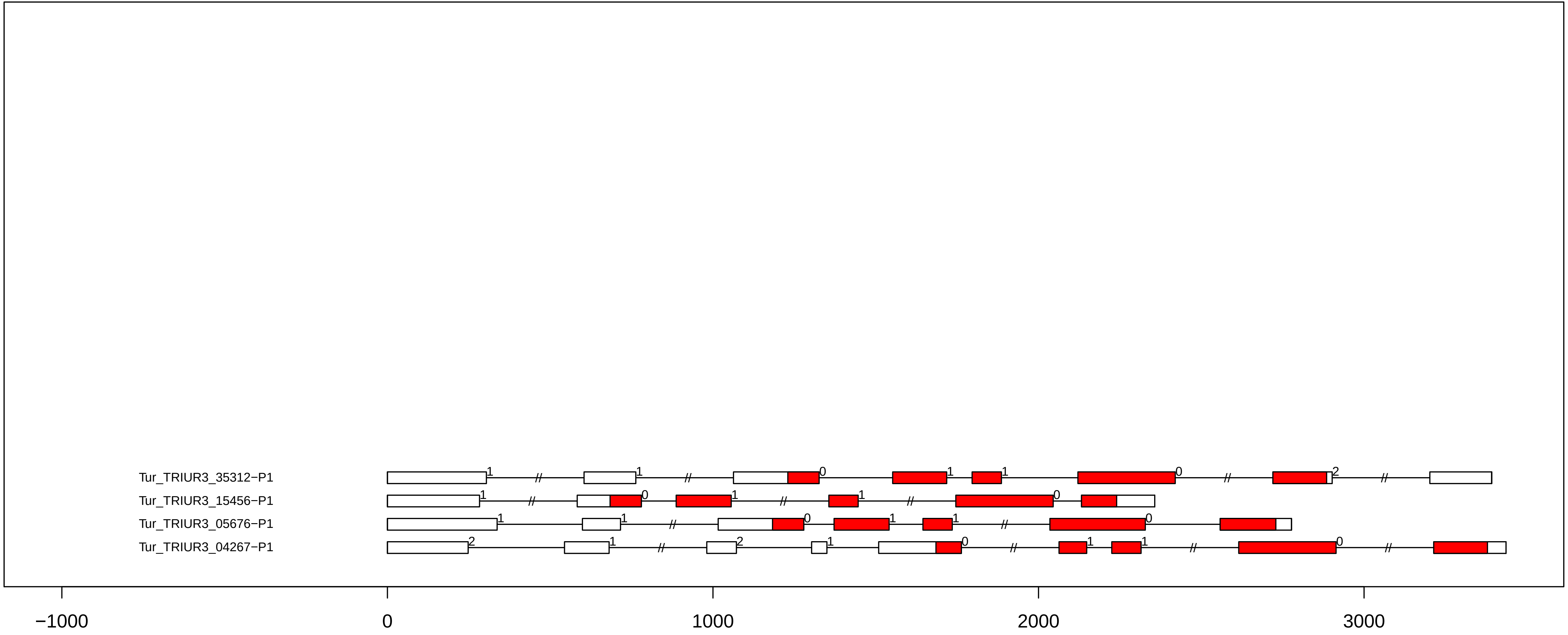

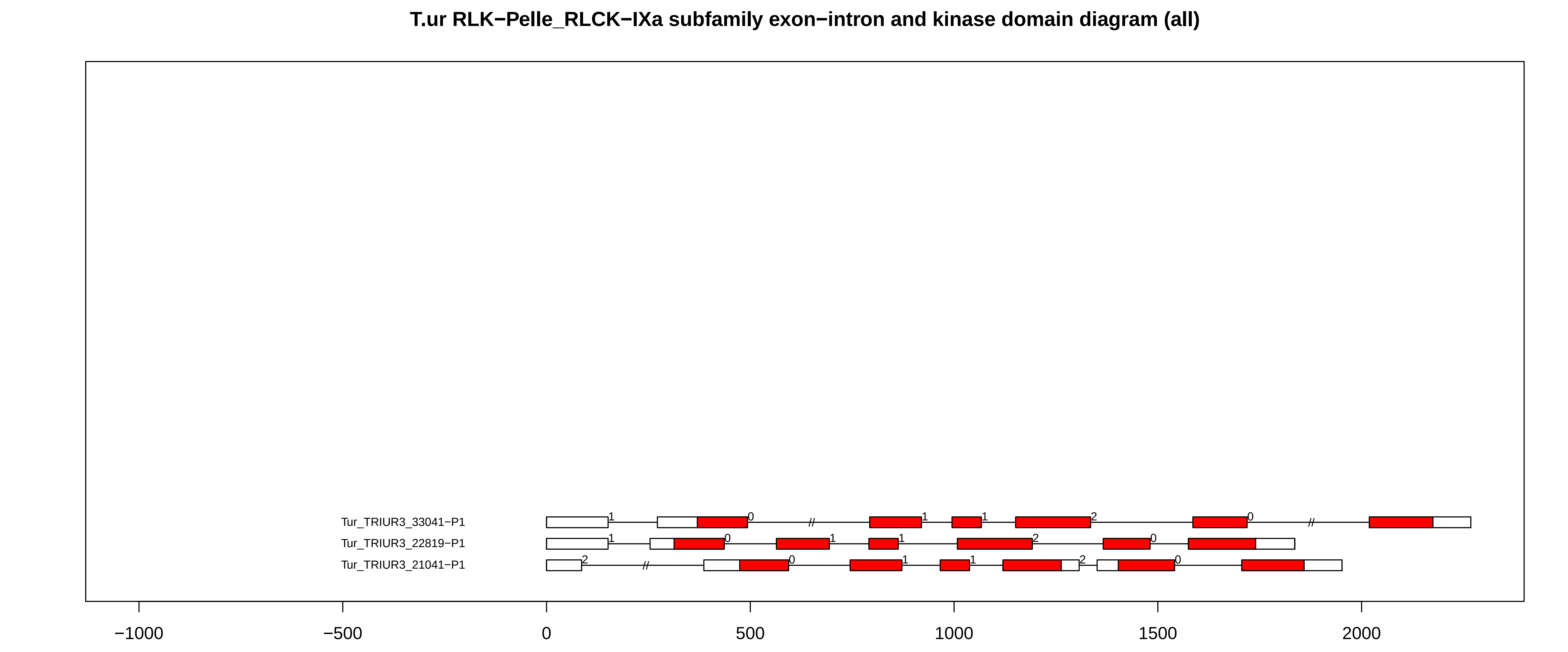
T.ur RLK−Pelle_RLCK−IXa subfamily exon−intron and kinase domain diagram (all)
Tur_TRIUR3_33041−P1
Tur_TRIUR3_22819−P1
Tur_TRIUR3_21041−P1
−1000
−500
0
500
1000
1500
2000

T.ur RLK−Pelle_RLCK−IXb subfamily exon−intron and kinase domain diagram (part 1)

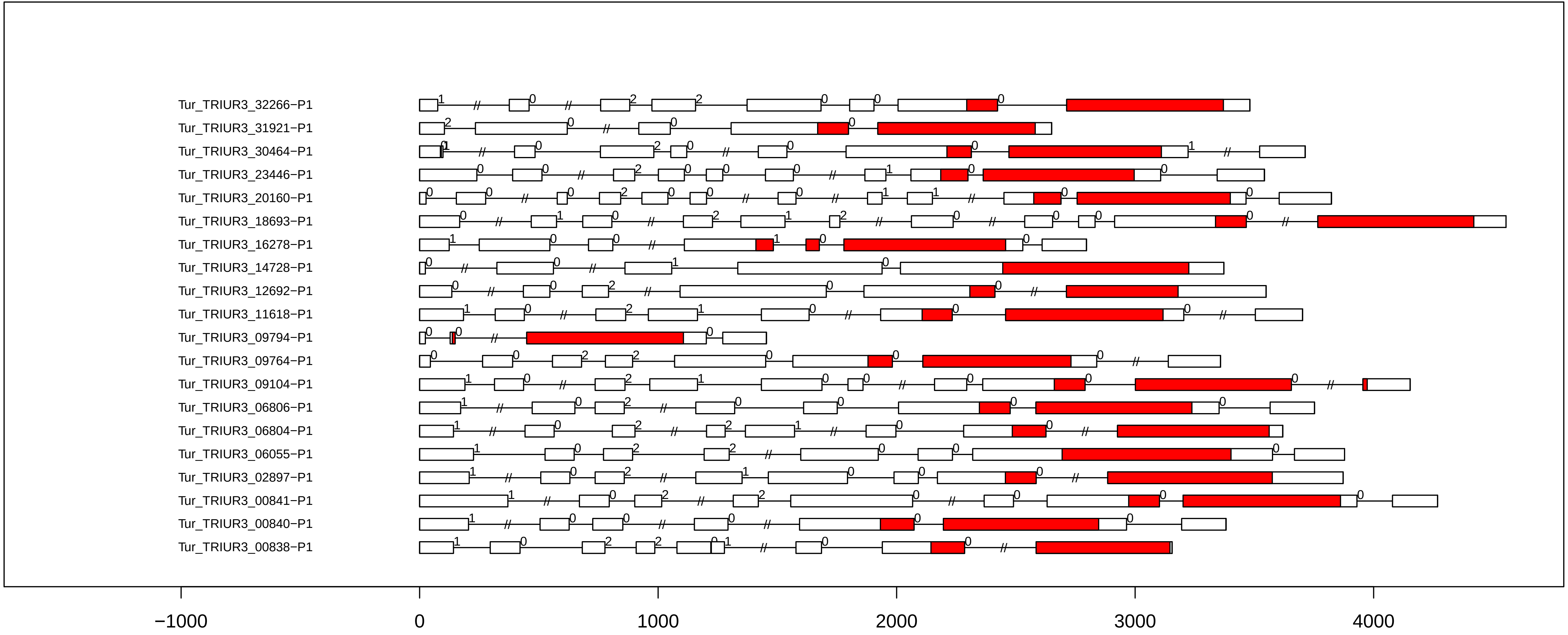

T.ur RLK−Pelle_RLCK−IXb subfamily exon−intron and kinase domain diagram (part 2)

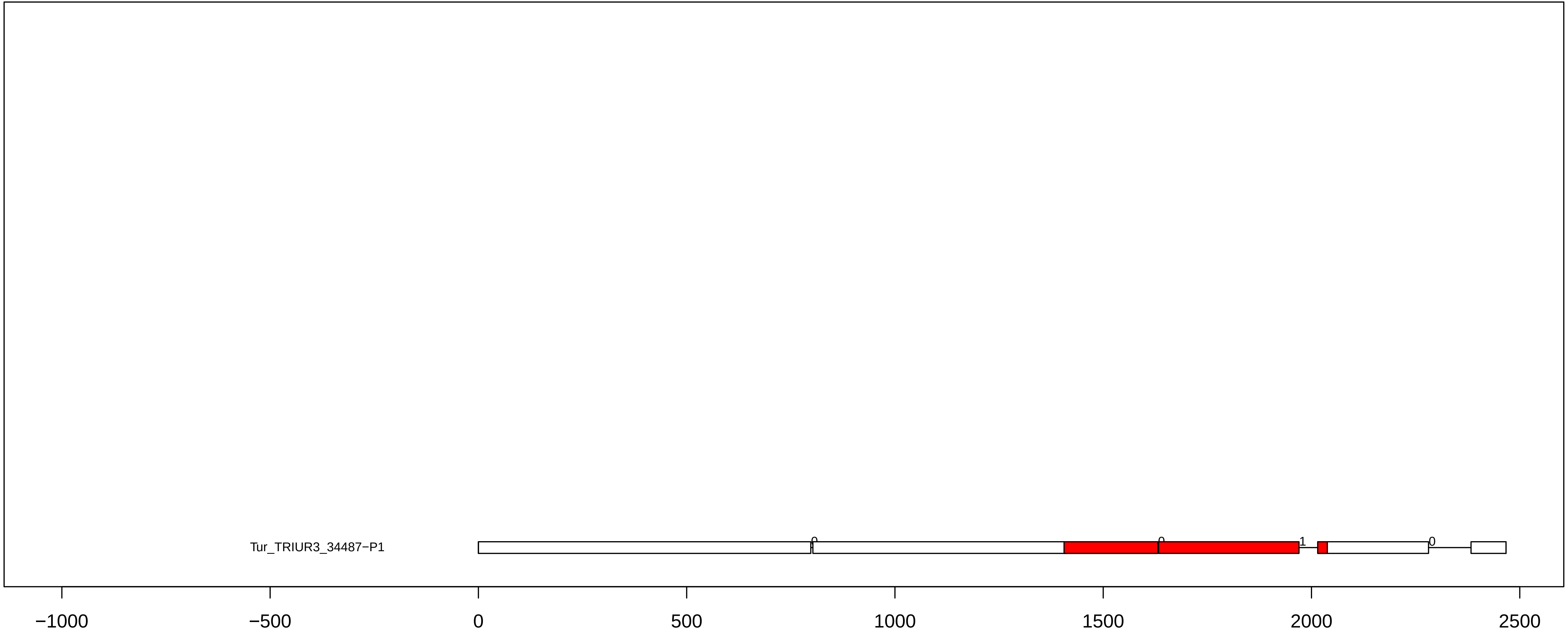

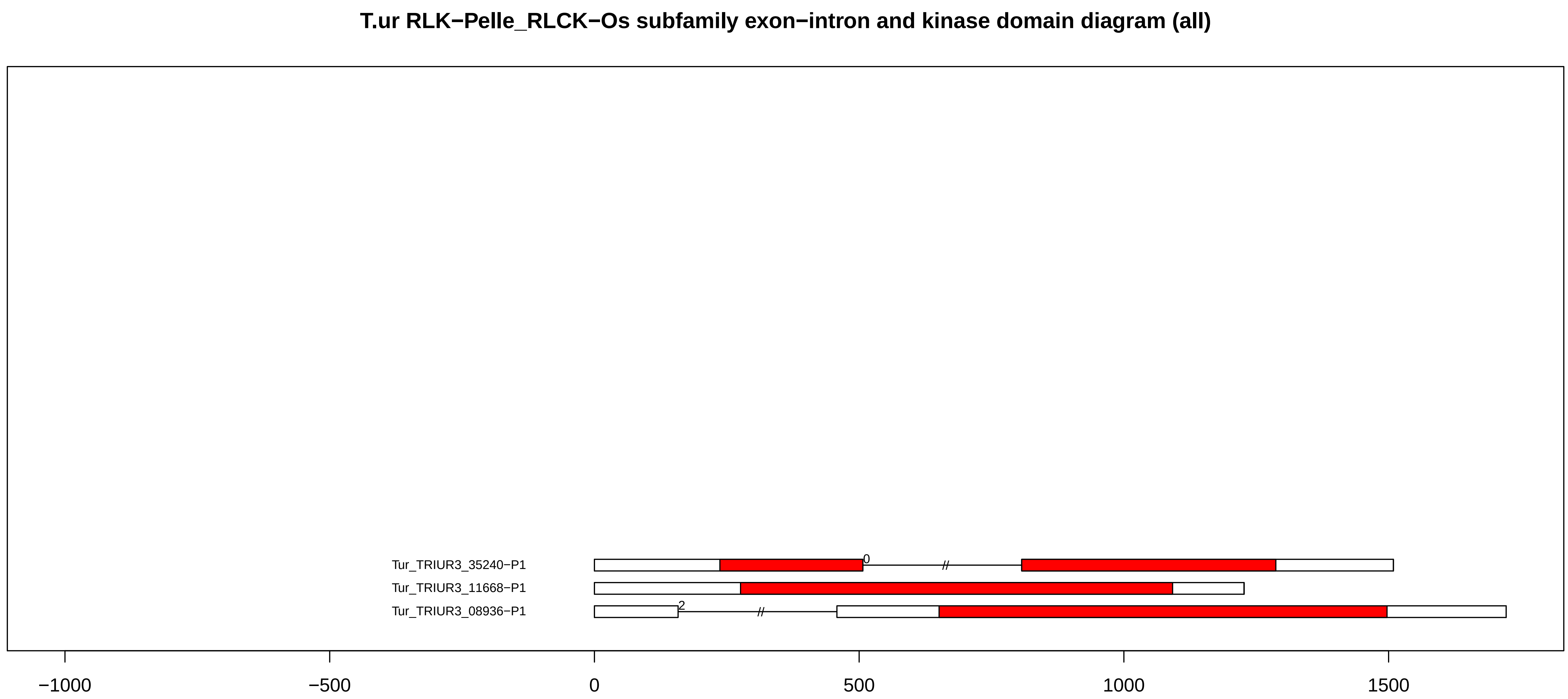

T.ur RLK−Pelle_RLCK−Os subfamily exon−intron and kinase domain diagram (all)
Tur_TRIUR3_35240−P1
Tur_TRIUR3_11668−P1
Tur_TRIUR3_08936−P1
0
2
−1000
−500
0
500
1000
1500

T.ur RLK−Pelle_RLCK−VIIa−1 subfamily exon−intron and kinase domain diagram (all)

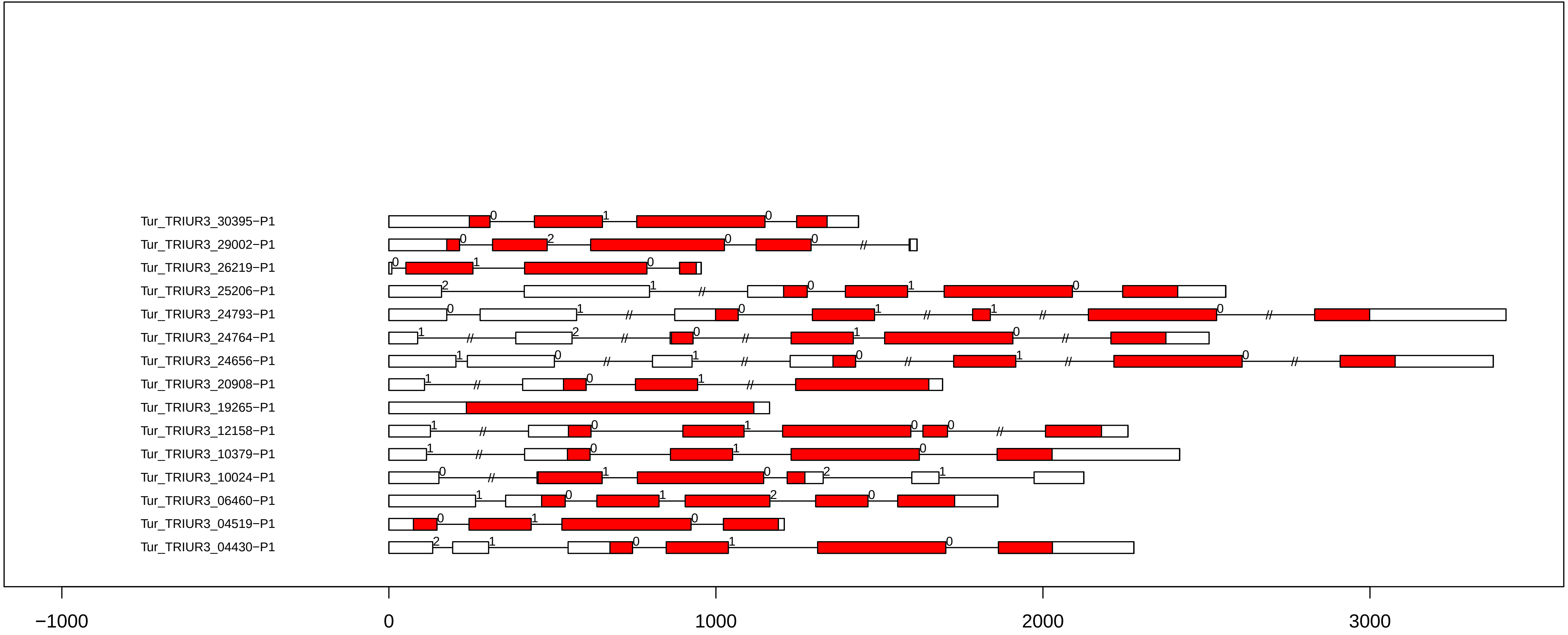

T.ur RLK−Pelle_RLCK−VIIa−2 subfamily exon−intron and kinase domain diagram (part 1)

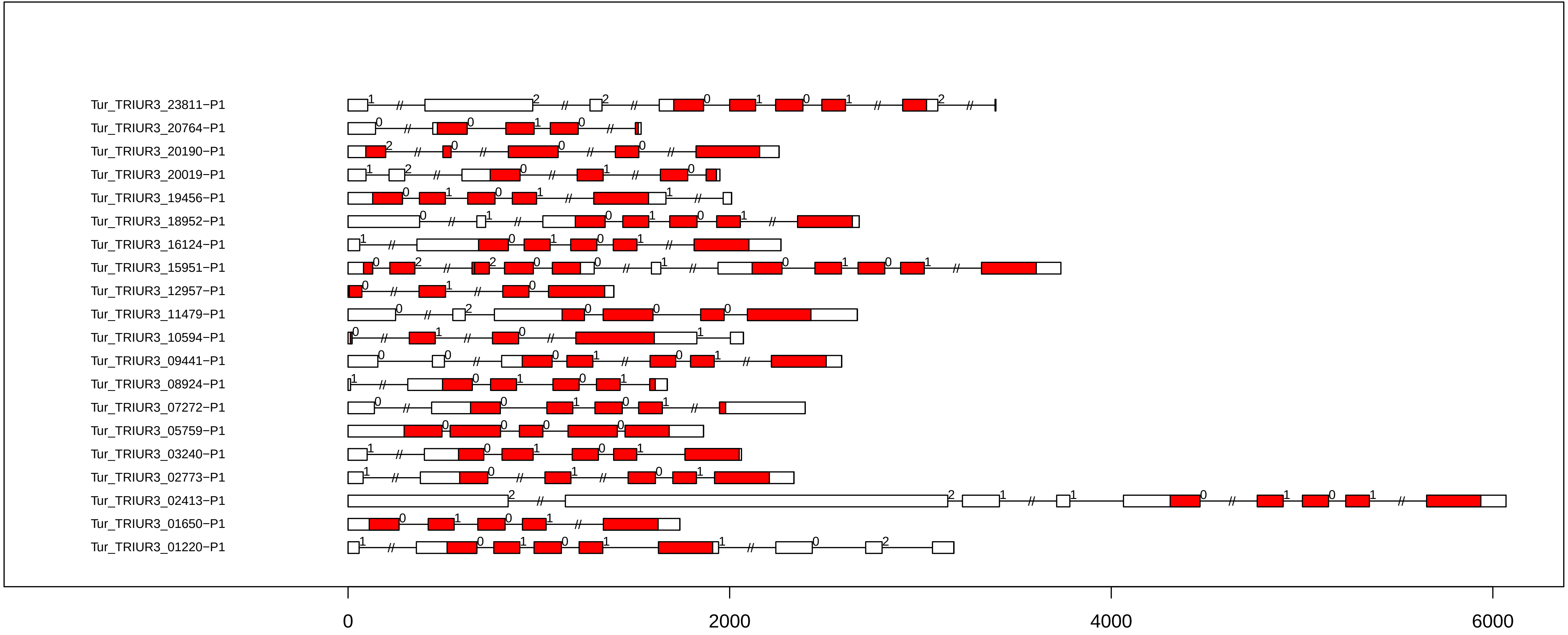

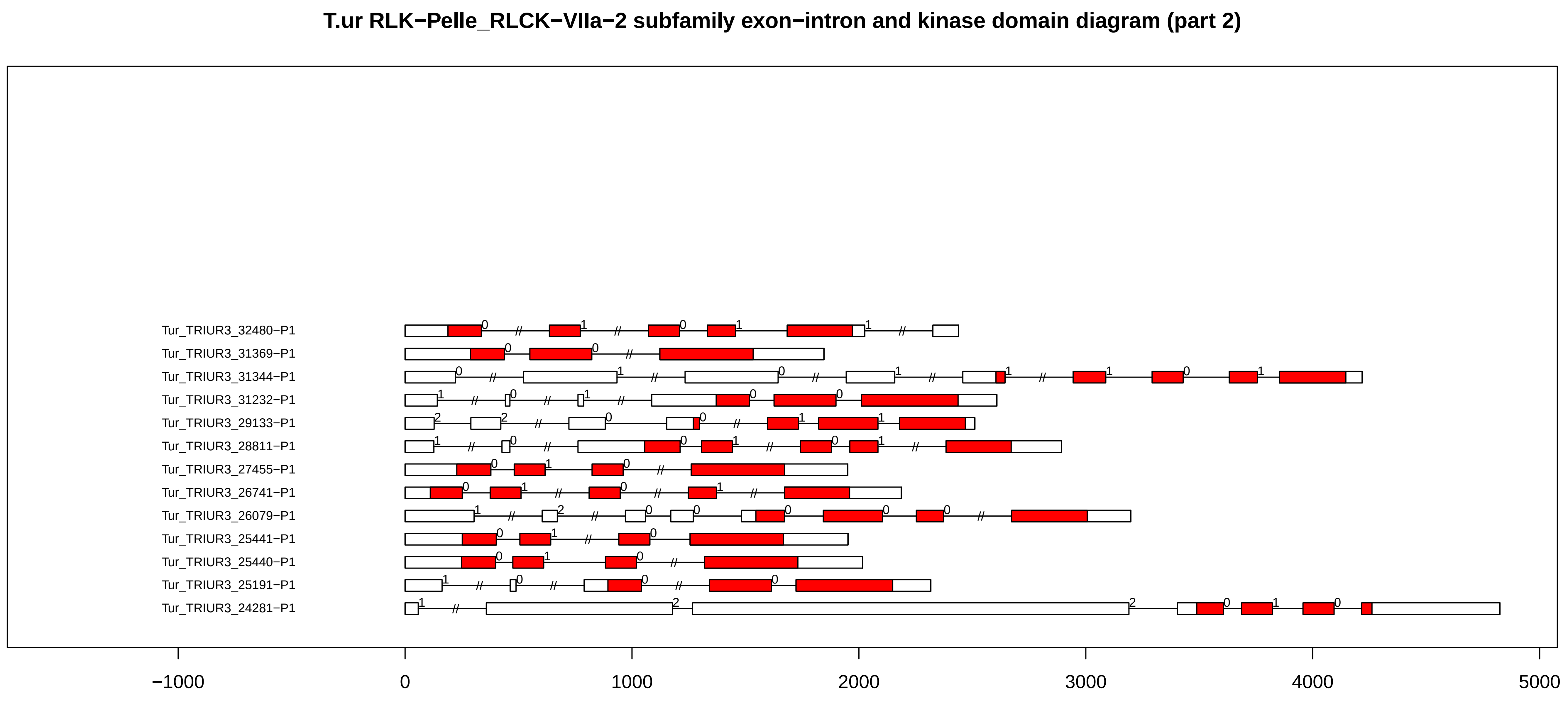
T.ur RLK−Pelle_RLCK−VIIa−2 subfamily exon−intron and kinase domain diagram (part 2)
Tur_TRIUR3_32480−P1
Tur_TRIUR3_31369−P1
Tur_TRIUR3_31344−P1
Tur_TRIUR3_31232−P1
Tur_TRIUR3_29133−P1
Tur_TRIUR3_28811−P1
Tur_TRIUR3_27455−P1
Tur_TRIUR3_26741−P1
Tur_TRIUR3_26079−P1
Tur_TRIUR3_25441−P1
Tur_TRIUR3_25440−P1
Tur_TRIUR3_25191−P1
Tur_TRIUR3_24281−P1
−1000
0
1000
2000
3000
4000
5000

T.ur RLK−Pelle_RLCK−VIIb subfamily exon−intron and kinase domain diagram (all)

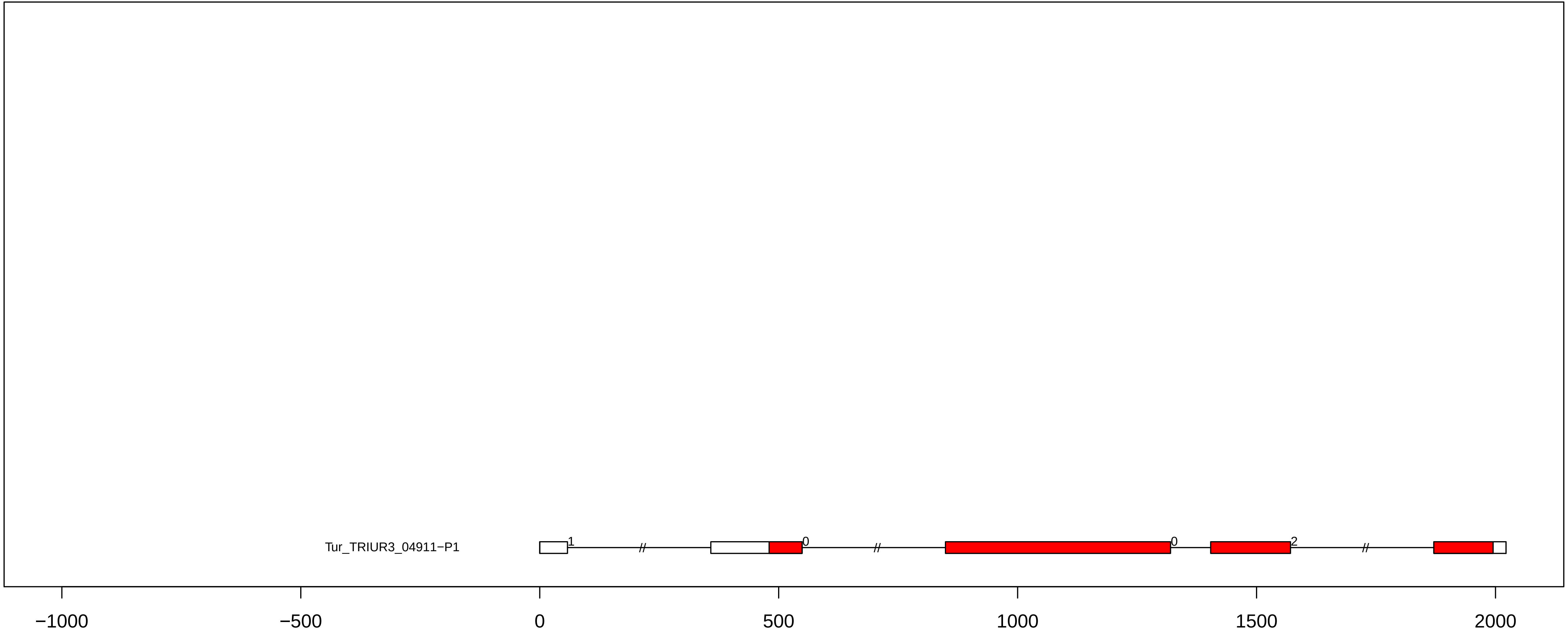

T.ur RLK−Pelle_RLCK−VIII subfamily exon−intron and kinase domain diagram (all)

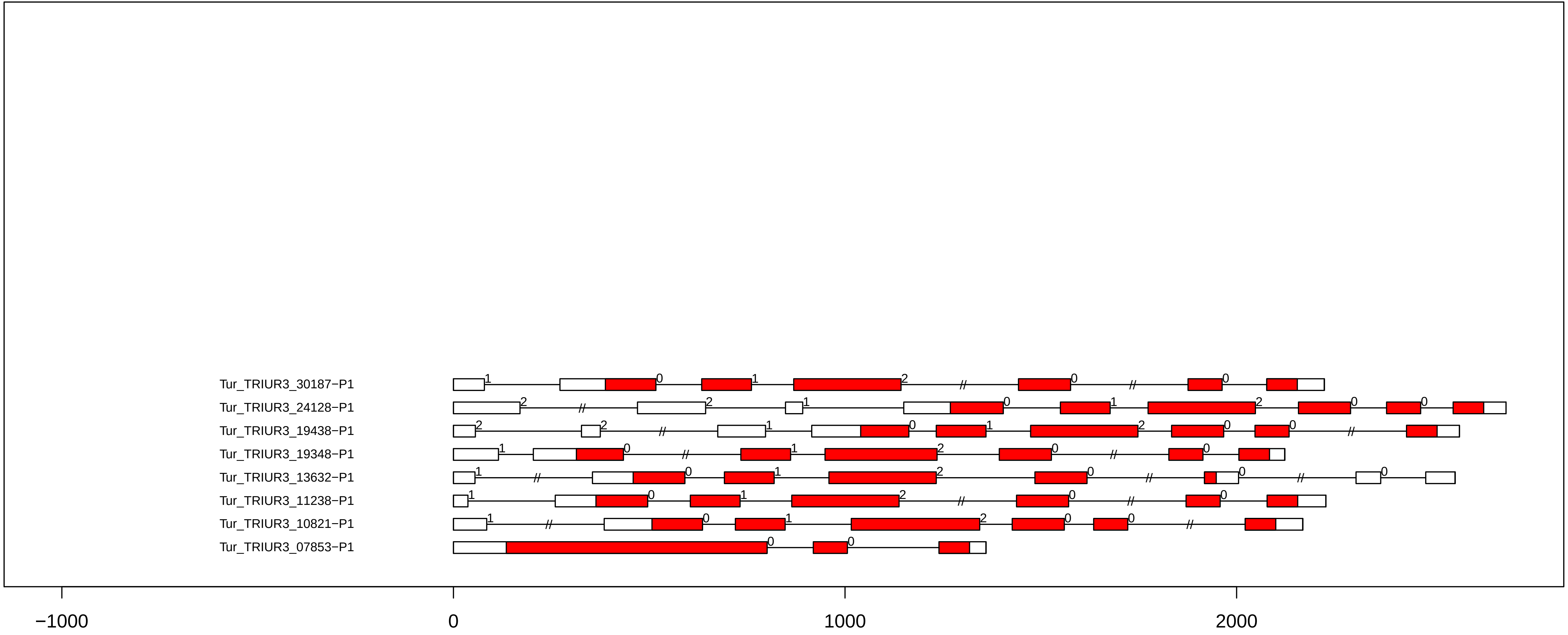

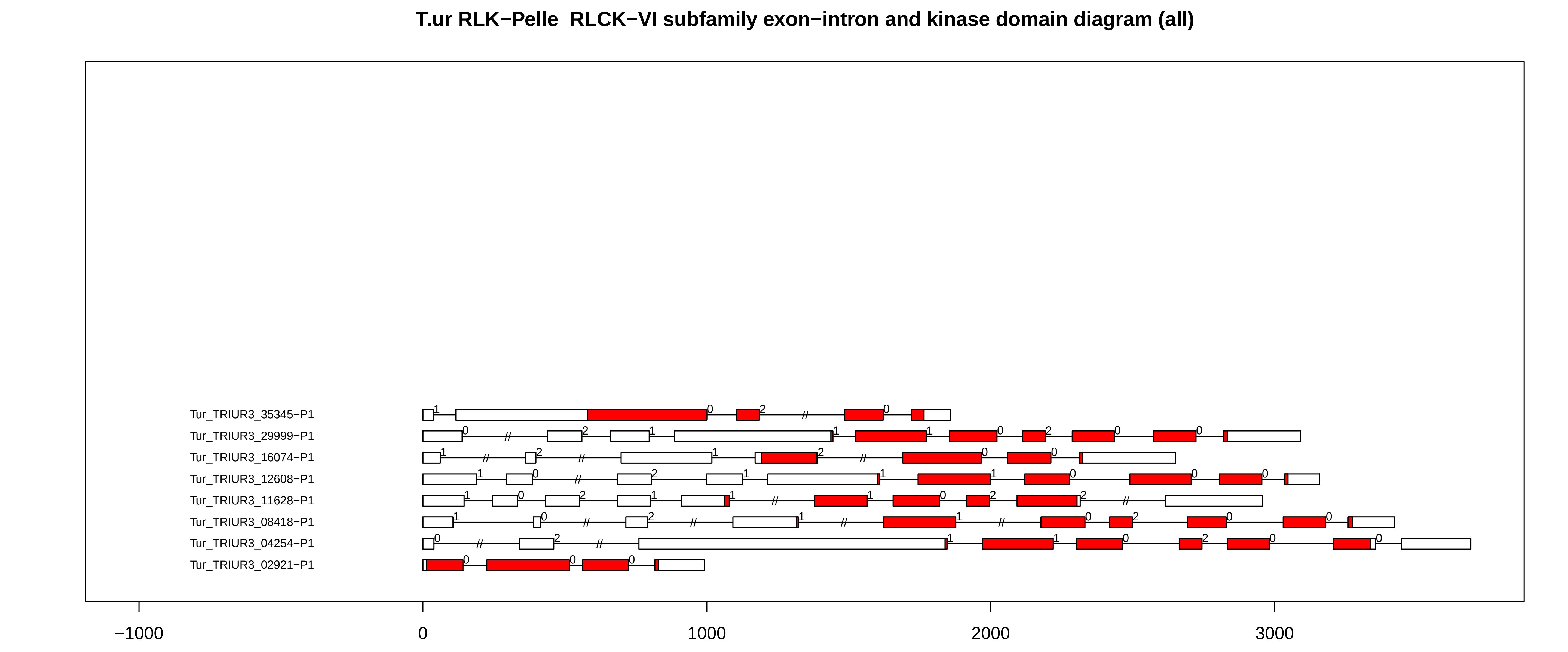
T.ur RLK−Pelle_RLCK−VI subfamily exon−intron and kinase domain diagram (all)
Tur_TRIUR3_35345−P1
Tur_TRIUR3_29999−P1
Tur_TRIUR3_16074−P1
Tur_TRIUR3_12608−P1
Tur_TRIUR3_11628−P1
Tur_TRIUR3_08418−P1
Tur_TRIUR3_04254−P1
Tur_TRIUR3_02921−P1
−1000
0
1000
2000
3000

T.ur RLK−Pelle_RLCK−V subfamily exon−intron and kinase domain diagram (all)

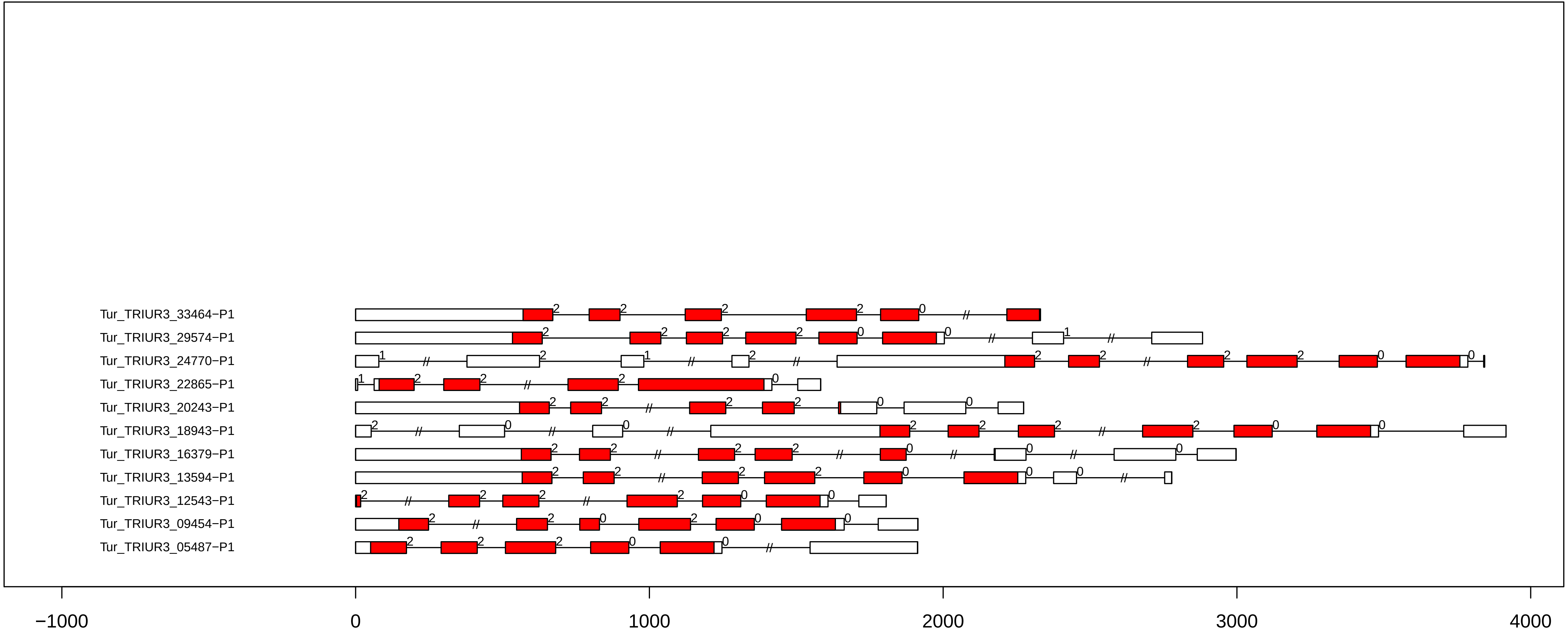

T.ur RLK−Pelle_RLCK−XII−1 subfamily exon−intron and kinase domain diagram (all)

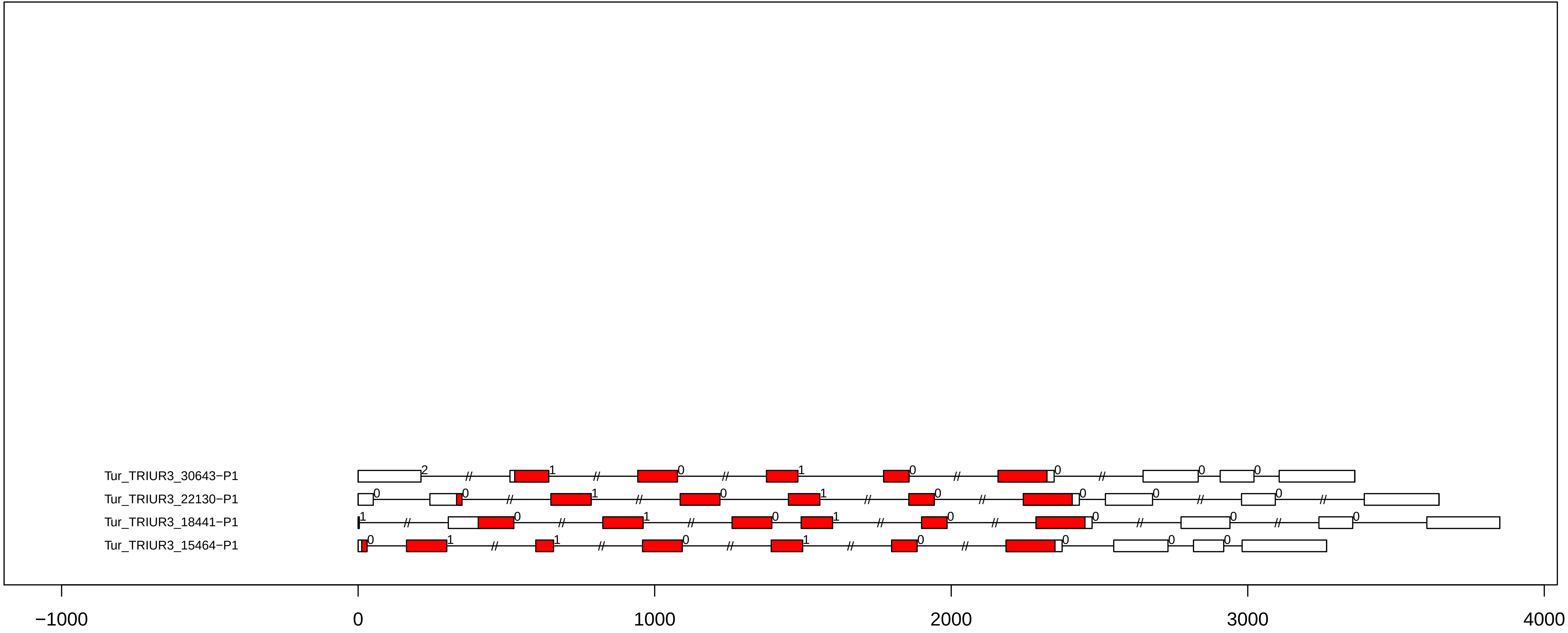

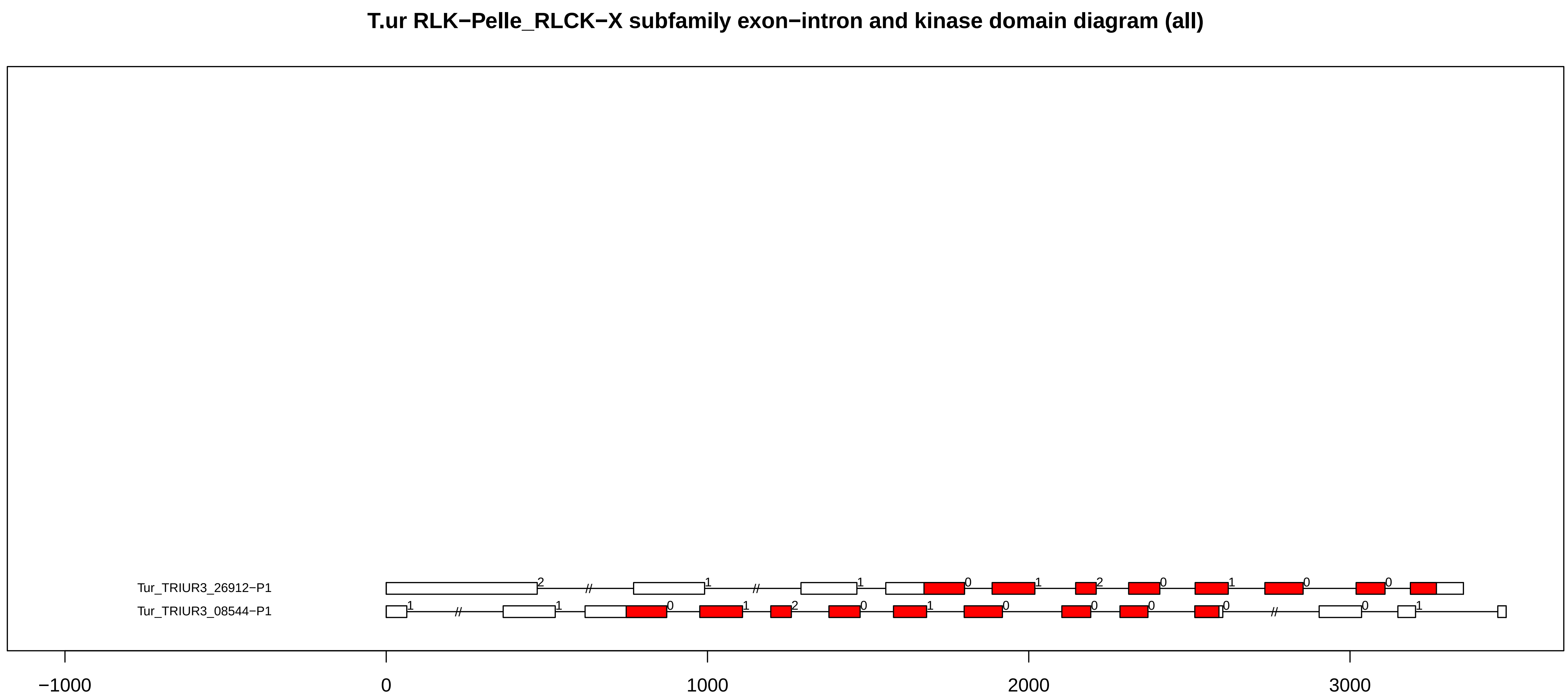
T.ur RLK−Pelle_RLCK−X subfamily exon−intron and kinase domain diagram (all)
Tur_TRIUR3_26912−P1
Tur_TRIUR3_08544−P1
2
1
1
0
1
2
0
1
0
0
1
1
0
1
2
0
1
0
0
0
0
0
1
−1000
0
1000
2000
3000

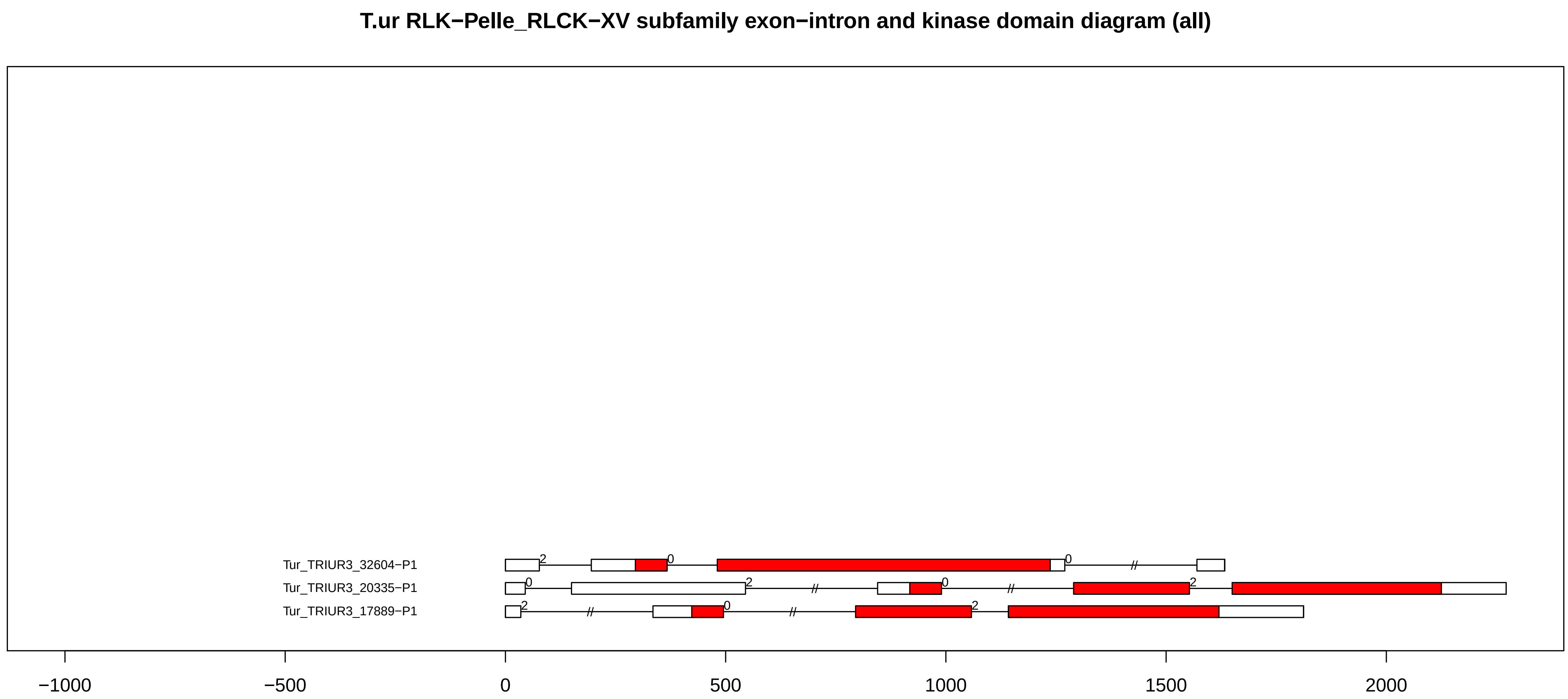

T.ur RLK−Pelle_RLCK−XV subfamily exon−intron and kinase domain diagram (all)
Tur_TRIUR3_32604−P1
Tur_TRIUR3_20335−P1
Tur_TRIUR3_17889−P1
−1000
−500
0
500
1000
1500
2000

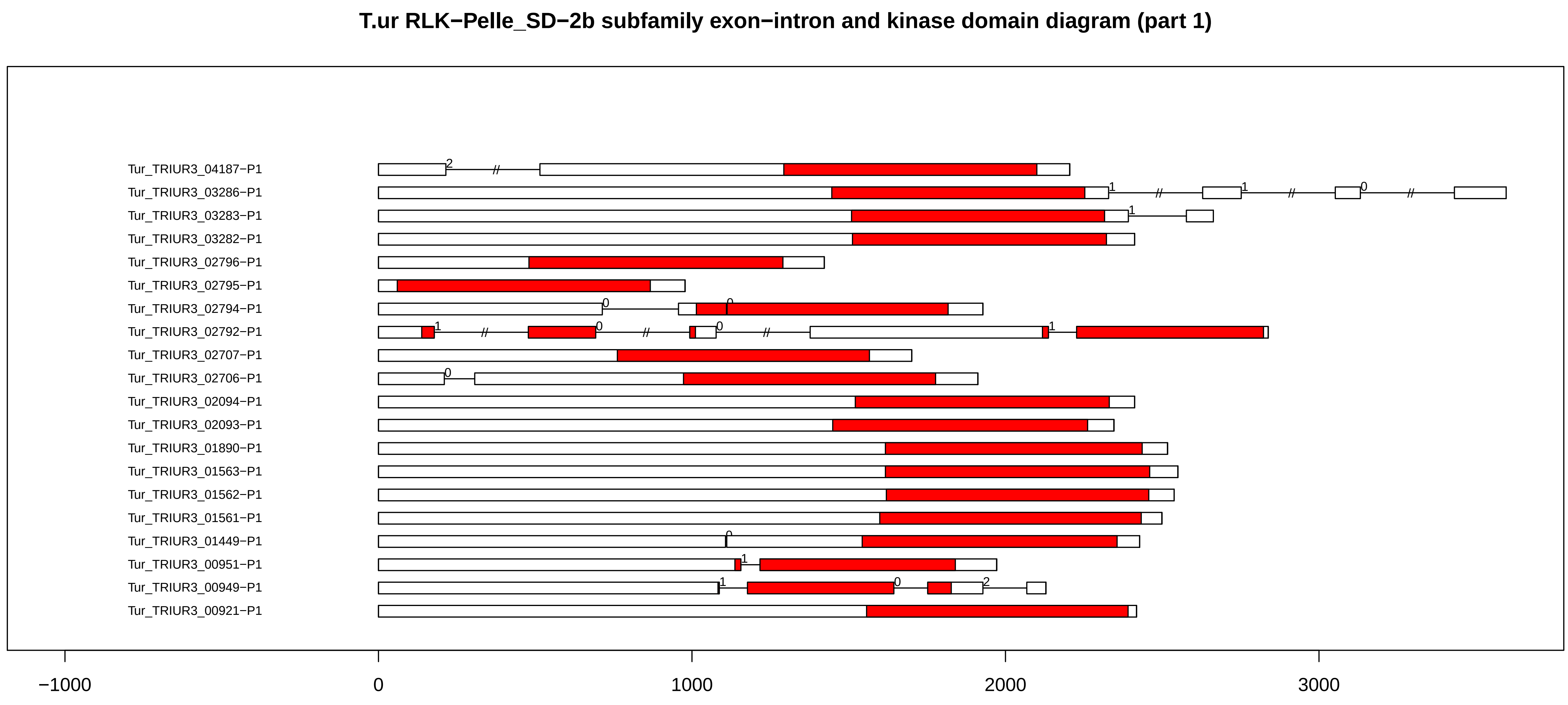
T.ur RLK−Pelle_SD−2b subfamily exon−intron and kinase domain diagram (part 1)
Tur_TRIUR3_04187−P1
Tur_TRIUR3_03286−P1
Tur_TRIUR3_03283−P1
Tur_TRIUR3_03282−P1
Tur_TRIUR3_02796−P1
Tur_TRIUR3_02795−P1
Tur_TRIUR3_02794−P1
Tur_TRIUR3_02792−P1
Tur_TRIUR3_02707−P1
Tur_TRIUR3_02706−P1
Tur_TRIUR3_02094−P1
Tur_TRIUR3_02093−P1
Tur_TRIUR3_01890−P1
Tur_TRIUR3_01563−P1
Tur_TRIUR3_01562−P1
Tur_TRIUR3_01561−P1
Tur_TRIUR3_01449−P1
Tur_TRIUR3_00951−P1
Tur_TRIUR3_00949−P1
Tur_TRIUR3_00921−P1
−1000
0
1000
2000
3000

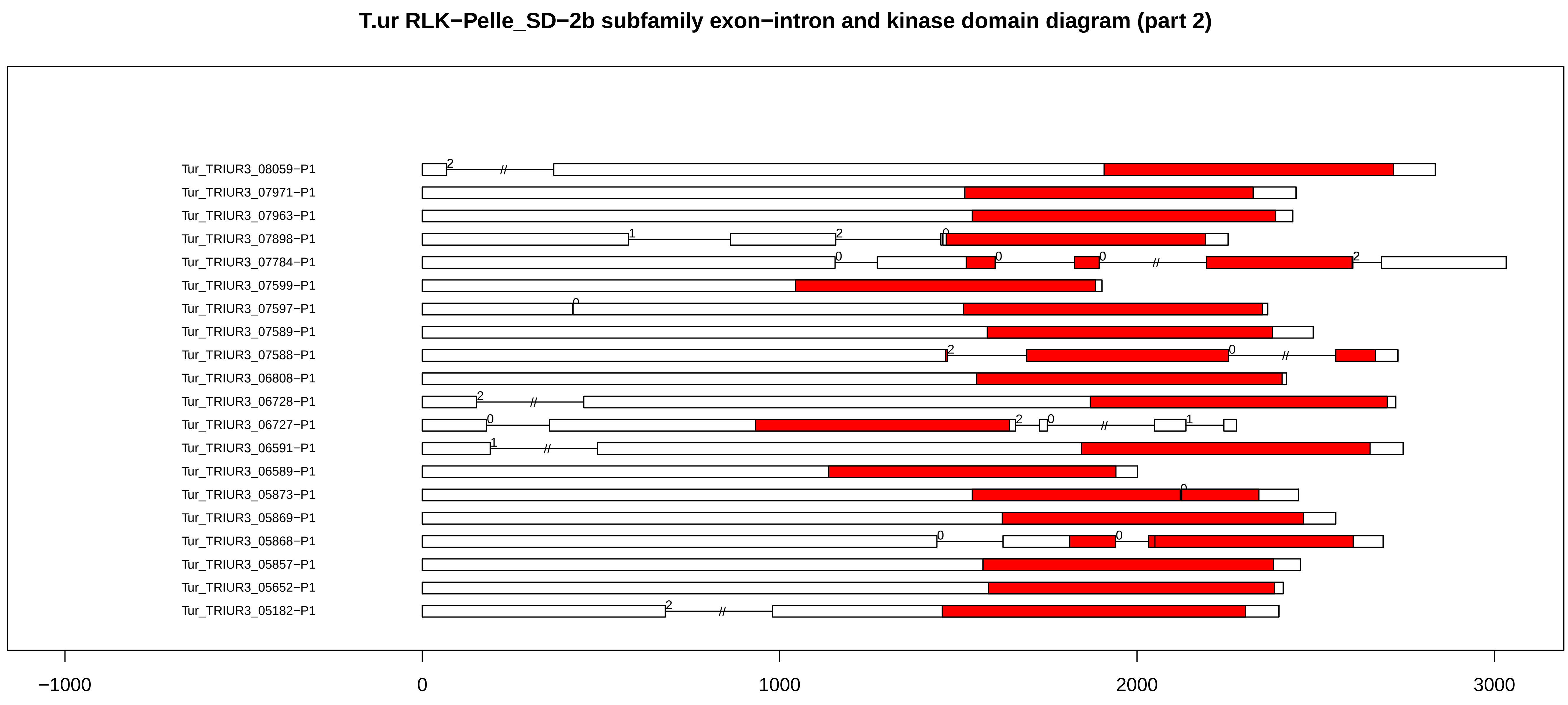
T.ur RLK−Pelle_SD−2b subfamily exon−intron and kinase domain diagram (part 2)
Tur_TRIUR3_08059−P1
Tur_TRIUR3_07971−P1
Tur_TRIUR3_07963−P1
Tur_TRIUR3_07898−P1
Tur_TRIUR3_07784−P1
Tur_TRIUR3_07599−P1
Tur_TRIUR3_07597−P1
Tur_TRIUR3_07589−P1
Tur_TRIUR3_07588−P1
Tur_TRIUR3_06808−P1
Tur_TRIUR3_06728−P1
Tur_TRIUR3_06727−P1
Tur_TRIUR3_06591−P1
Tur_TRIUR3_06589−P1
Tur_TRIUR3_05873−P1
Tur_TRIUR3_05869−P1
Tur_TRIUR3_05868−P1
Tur_TRIUR3_05857−P1
Tur_TRIUR3_05652−P1
Tur_TRIUR3_05182−P1
−1000
0
1000
2000
3000

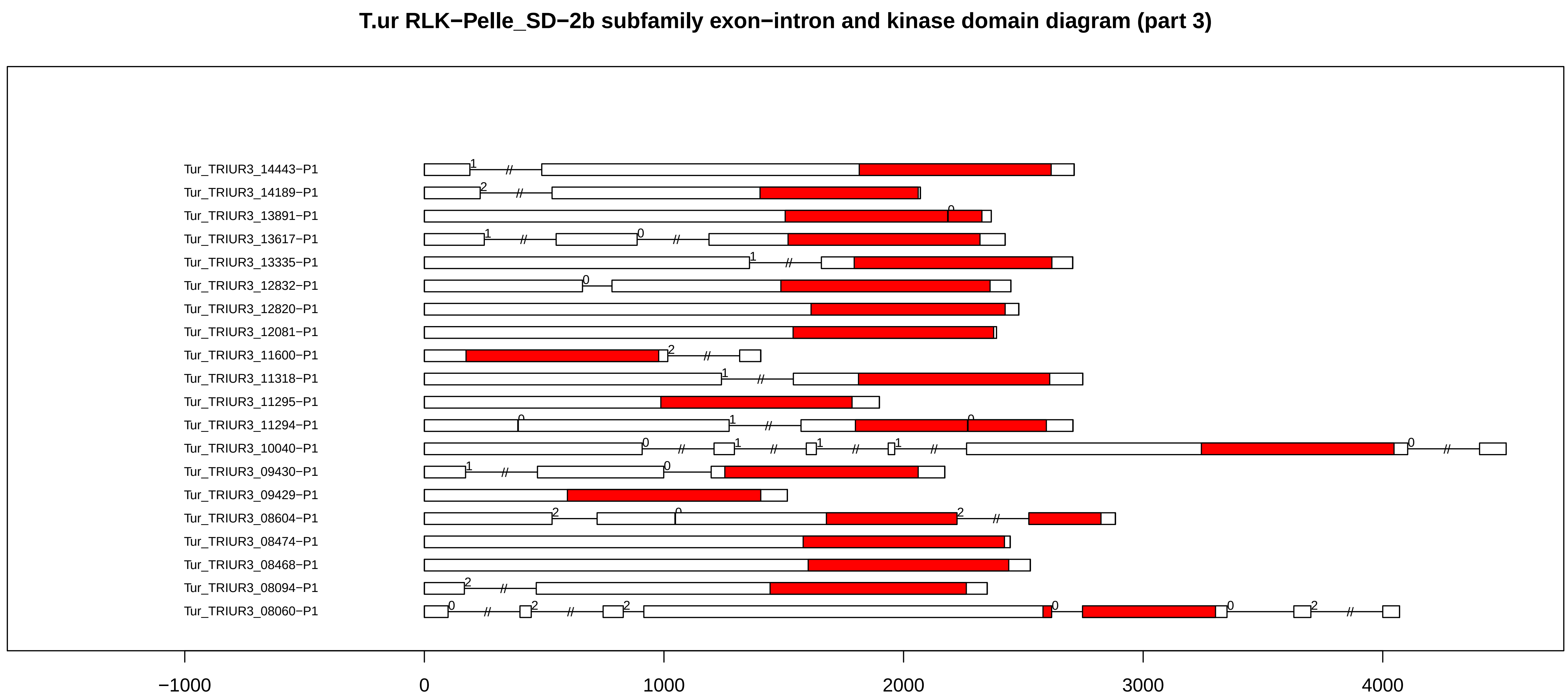
T.ur RLK−Pelle_SD−2b subfamily exon−intron and kinase domain diagram (part 3)
Tur_TRIUR3_14443−P1
Tur_TRIUR3_14189−P1
Tur_TRIUR3_13891−P1
Tur_TRIUR3_13617−P1
Tur_TRIUR3_13335−P1
Tur_TRIUR3_12832−P1
Tur_TRIUR3_12820−P1
Tur_TRIUR3_12081−P1
Tur_TRIUR3_11600−P1
Tur_TRIUR3_11318−P1
Tur_TRIUR3_11295−P1
Tur_TRIUR3_11294−P1
Tur_TRIUR3_10040−P1
Tur_TRIUR3_09430−P1
Tur_TRIUR3_09429−P1
Tur_TRIUR3_08604−P1
Tur_TRIUR3_08474−P1
Tur_TRIUR3_08468−P1
Tur_TRIUR3_08094−P1
Tur_TRIUR3_08060−P1
−1000
0
1000
2000
3000
4000

T.ur RLK−Pelle_SD−2b subfamily exon−intron and kinase domain diagram (part 4)

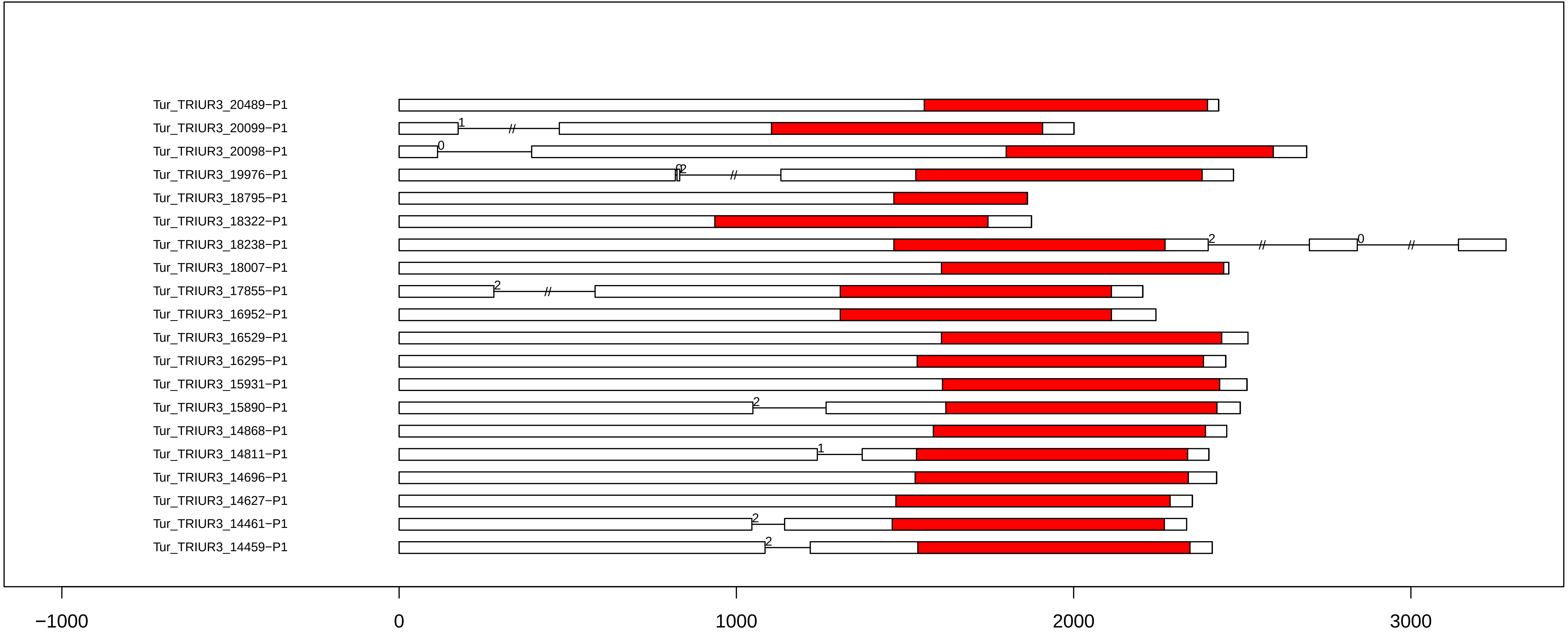

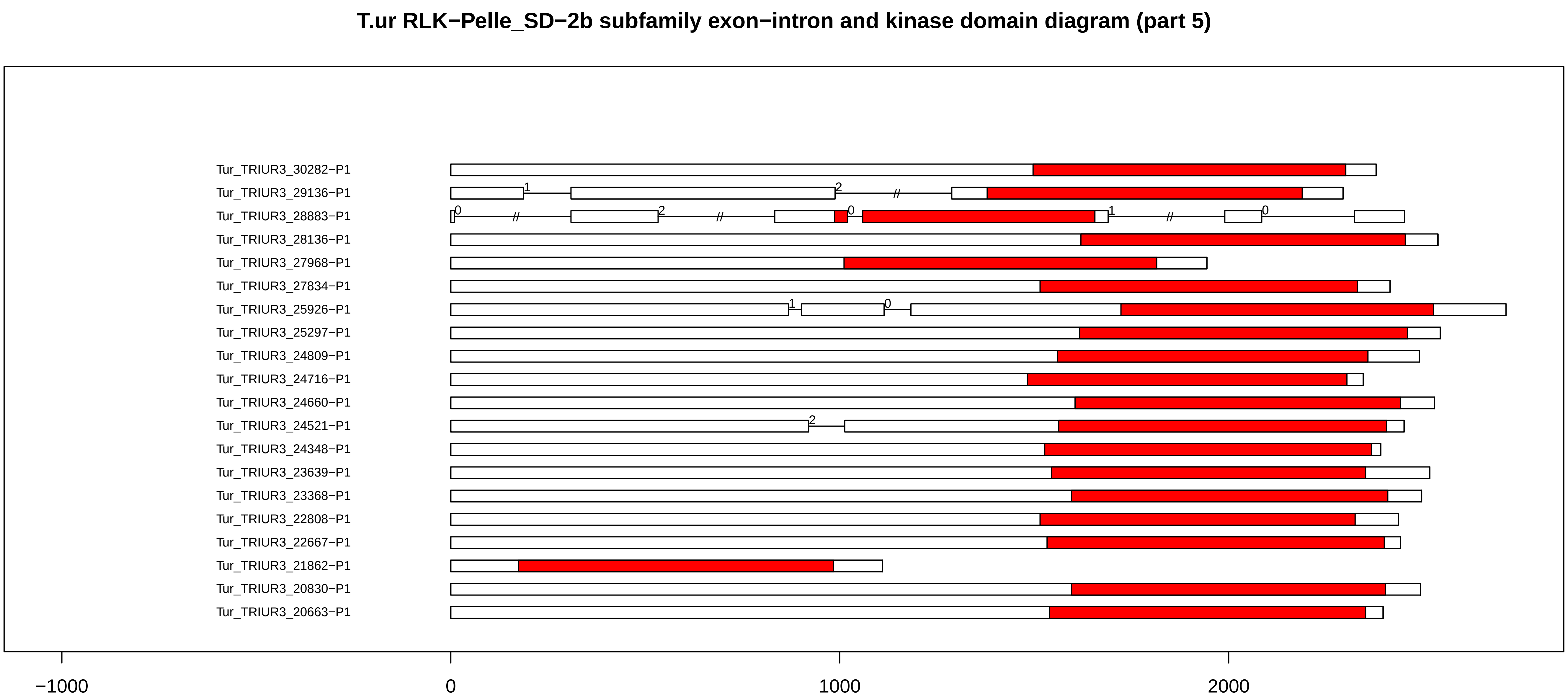

T.ur RLK−Pelle_SD−2b subfamily exon−intron and kinase domain diagram (part 5)
Tur_TRIUR3_30282−P1
Tur_TRIUR3_29136−P1
Tur_TRIUR3_28883−P1
Tur_TRIUR3_28136−P1
Tur_TRIUR3_27968−P1
Tur_TRIUR3_27834−P1
Tur_TRIUR3_25926−P1
Tur_TRIUR3_25297−P1
Tur_TRIUR3_24809−P1
Tur_TRIUR3_24716−P1
Tur_TRIUR3_24660−P1
Tur_TRIUR3_24521−P1
Tur_TRIUR3_24348−P1
Tur_TRIUR3_23639−P1
Tur_TRIUR3_23368−P1
Tur_TRIUR3_22808−P1
Tur_TRIUR3_22667−P1
Tur_TRIUR3_21862−P1
Tur_TRIUR3_20830−P1
Tur_TRIUR3_20663−P1
−1000
0
1000
2000

T.ur RLK−Pelle_SD−2b subfamily exon−intron and kinase domain diagram (part 6)

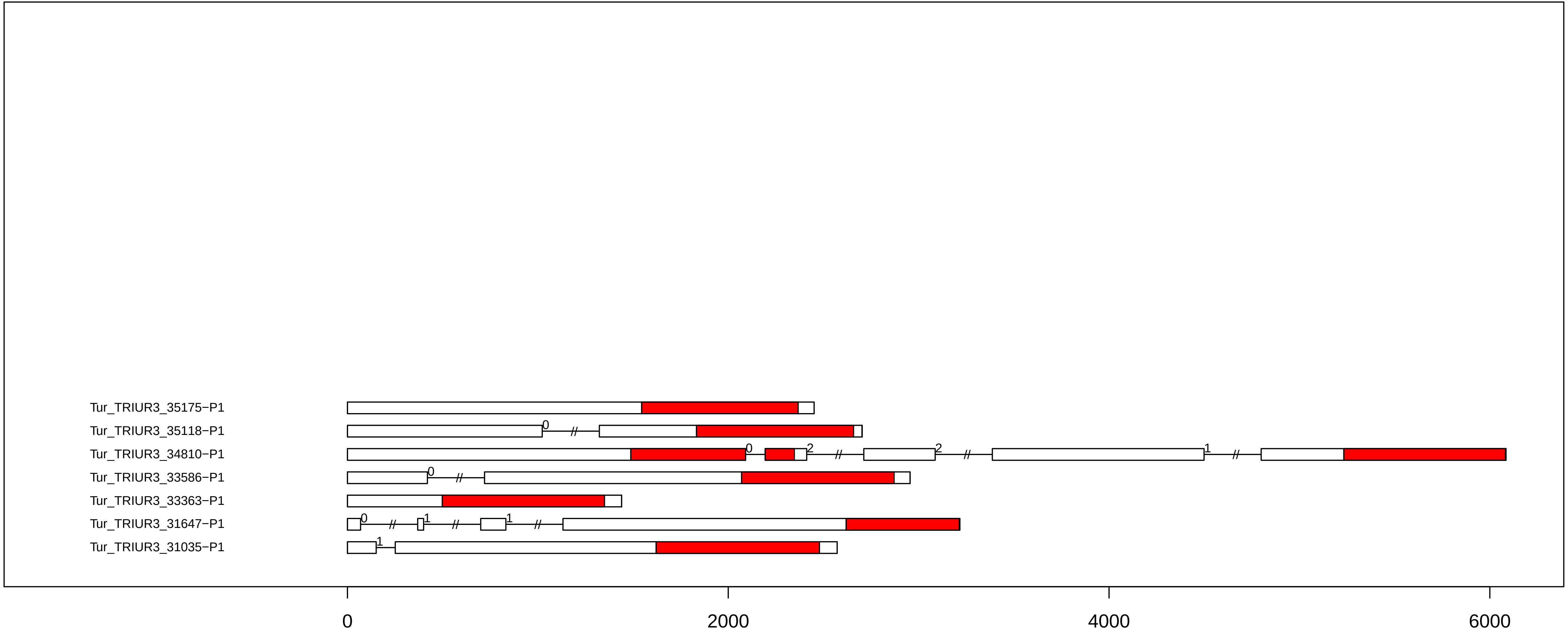

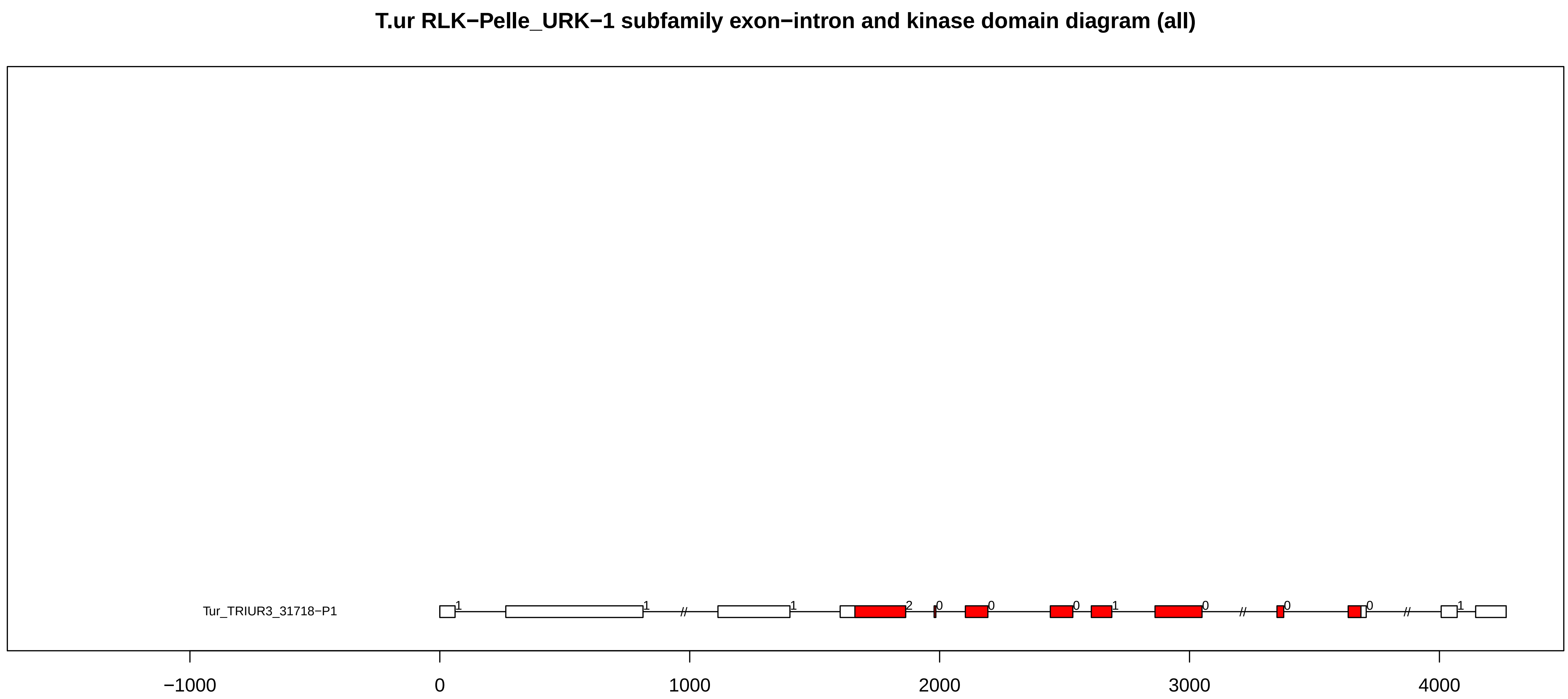

T.ur RLK−Pelle_URK−1 subfamily exon−intron and kinase domain diagram (all)
Tur_TRIUR3_31718−P1
1
1
1
2
0
0
0
1
0
0
0
1
−1000
0
1000
2000
3000
4000

T.ur RLK−Pelle_URK−2 subfamily exon−intron and kinase domain diagram (all)

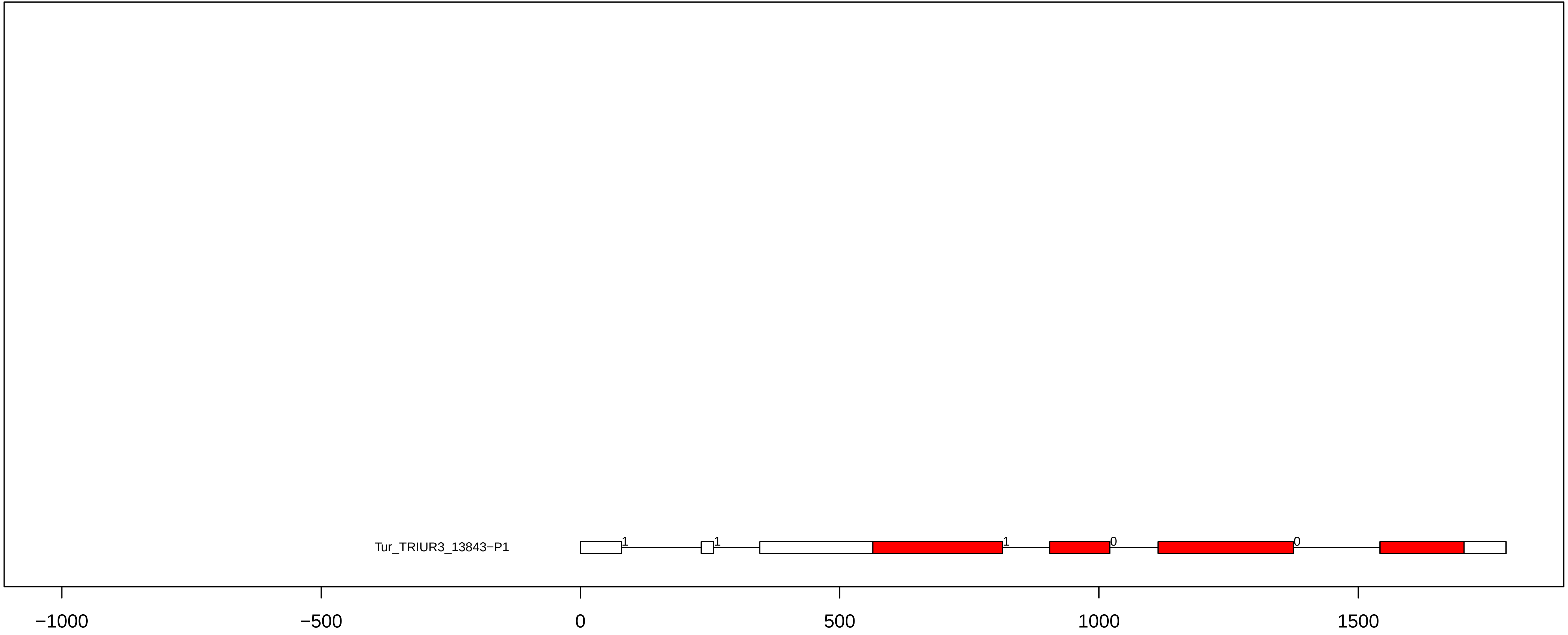

T.ur RLK−Pelle_WAK_LRK10L−1 subfamily exon−intron and kinase domain diagram (all)

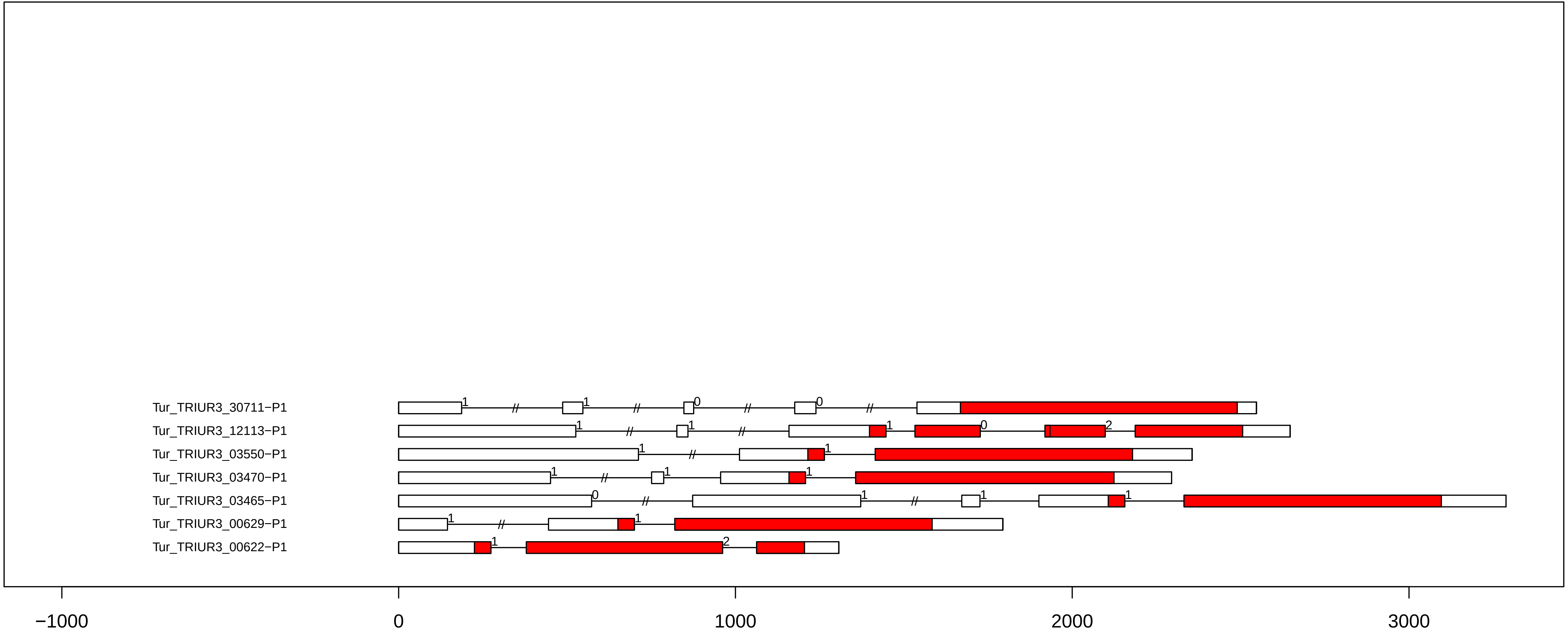

T.ur RLK−Pelle_WAK subfamily exon−intron and kinase domain diagram (part 1)

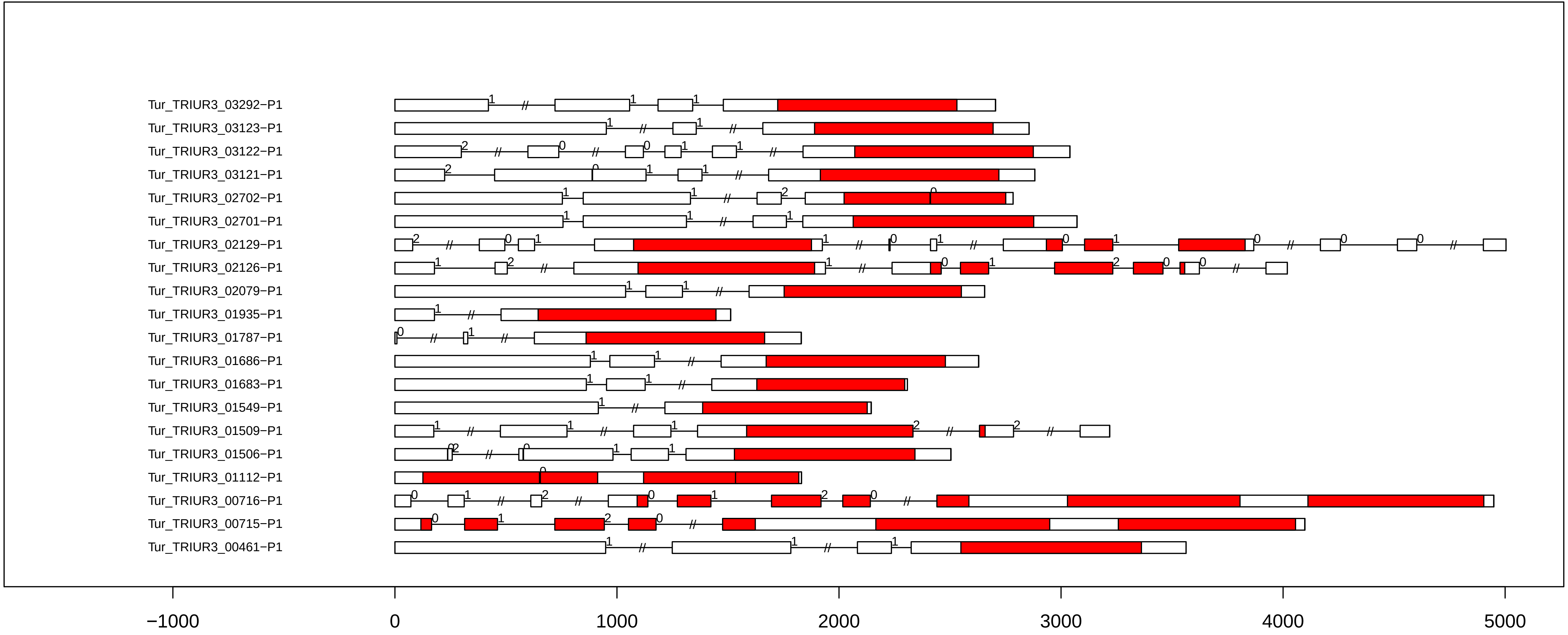

T.ur RLK−Pelle_WAK subfamily exon−intron and kinase domain diagram (part 2)

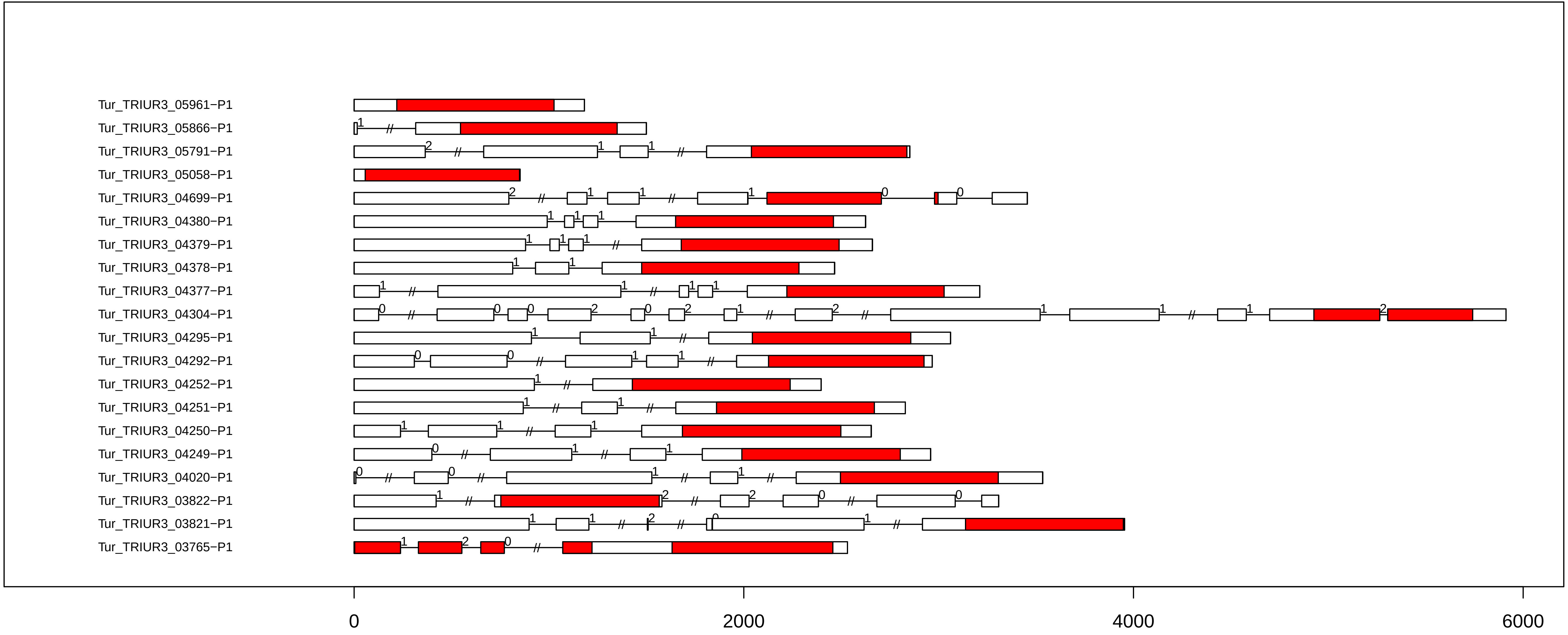

T.ur RLK−Pelle_WAK subfamily exon−intron and kinase domain diagram (part 3)

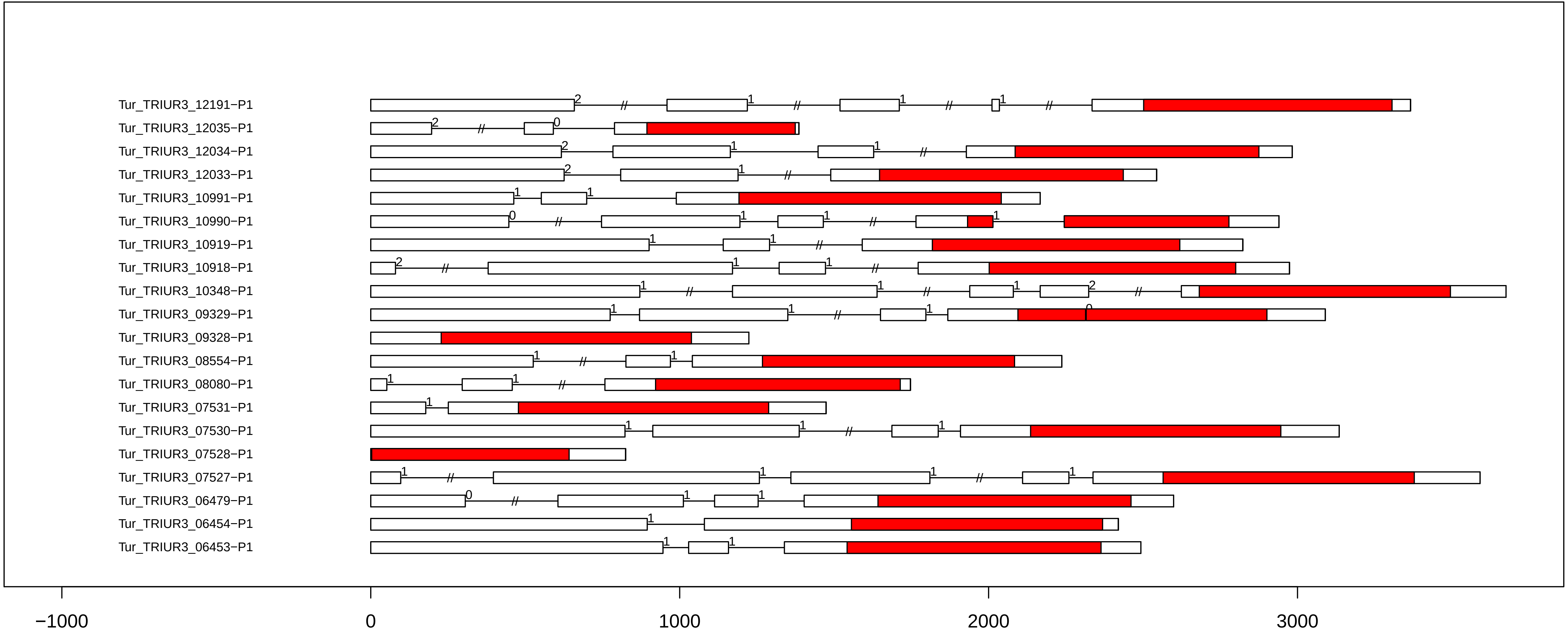

T.ur RLK−Pelle_WAK subfamily exon−intron and kinase domain diagram (part 4)

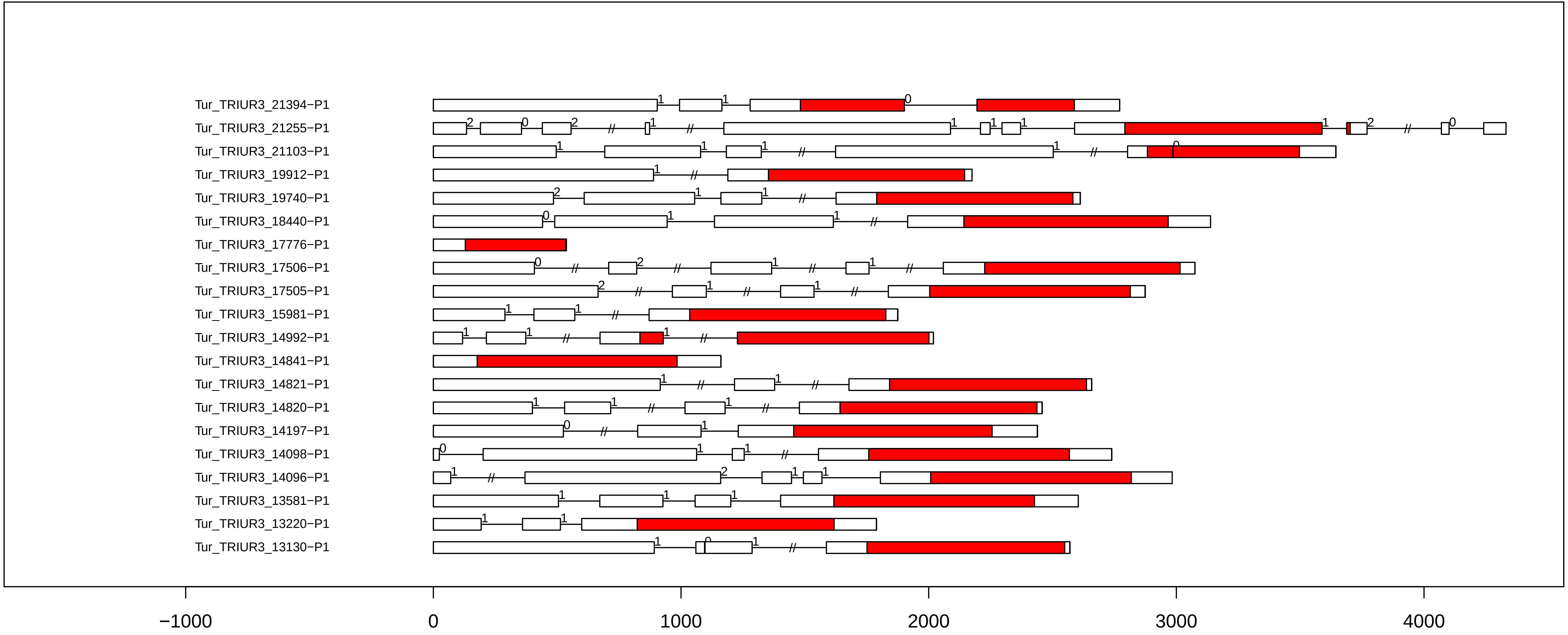

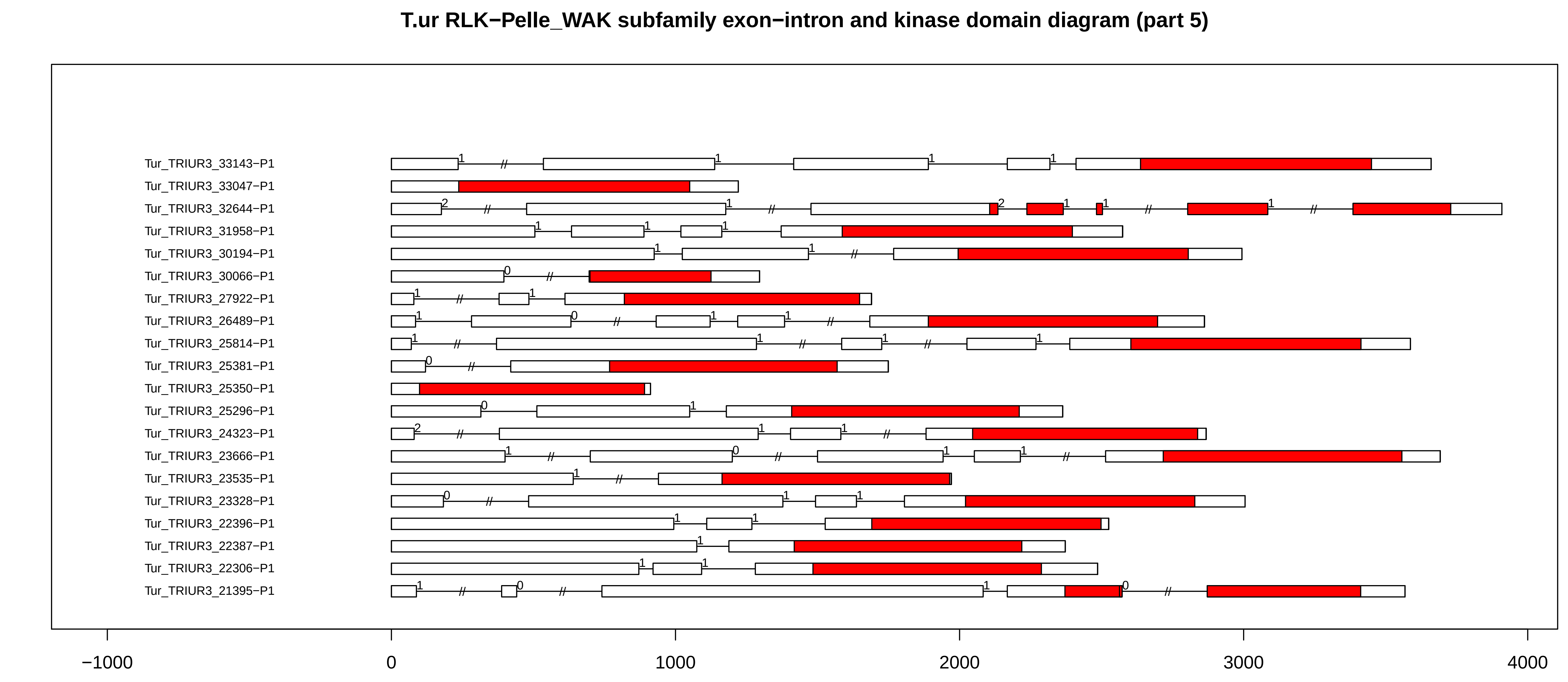
T.ur RLK−Pelle_WAK subfamily exon−intron and kinase domain diagram (part 5)
Tur_TRIUR3_33143−P1
Tur_TRIUR3_33047−P1
Tur_TRIUR3_32644−P1
Tur_TRIUR3_31958−P1
Tur_TRIUR3_30194−P1
Tur_TRIUR3_30066−P1
Tur_TRIUR3_27922−P1
Tur_TRIUR3_26489−P1
Tur_TRIUR3_25814−P1
Tur_TRIUR3_25381−P1
Tur_TRIUR3_25350−P1
Tur_TRIUR3_25296−P1
Tur_TRIUR3_24323−P1
Tur_TRIUR3_23666−P1
Tur_TRIUR3_23535−P1
Tur_TRIUR3_23328−P1
Tur_TRIUR3_22396−P1
Tur_TRIUR3_22387−P1
Tur_TRIUR3_22306−P1
Tur_TRIUR3_21395−P1
−1000
0
1000
2000
3000
4000

T.ur RLK−Pelle_WAK subfamily exon−intron and kinase domain diagram (part 6)

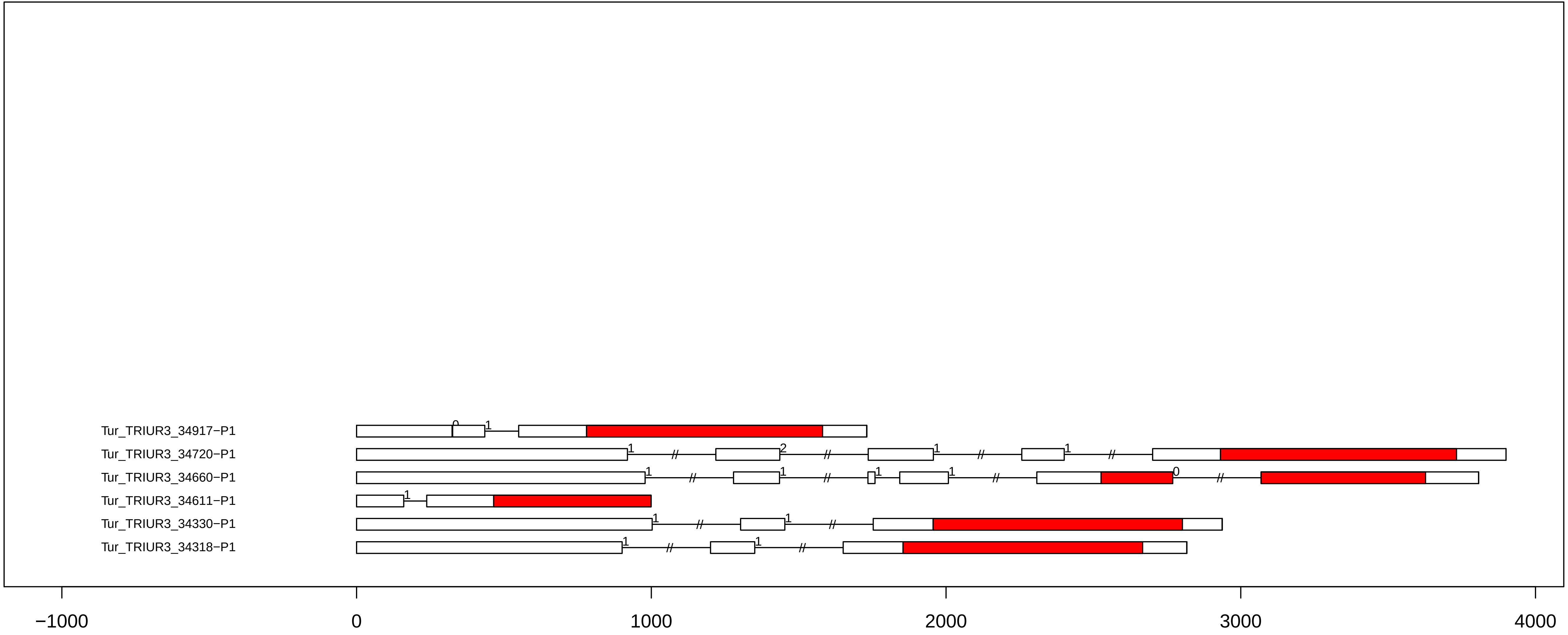

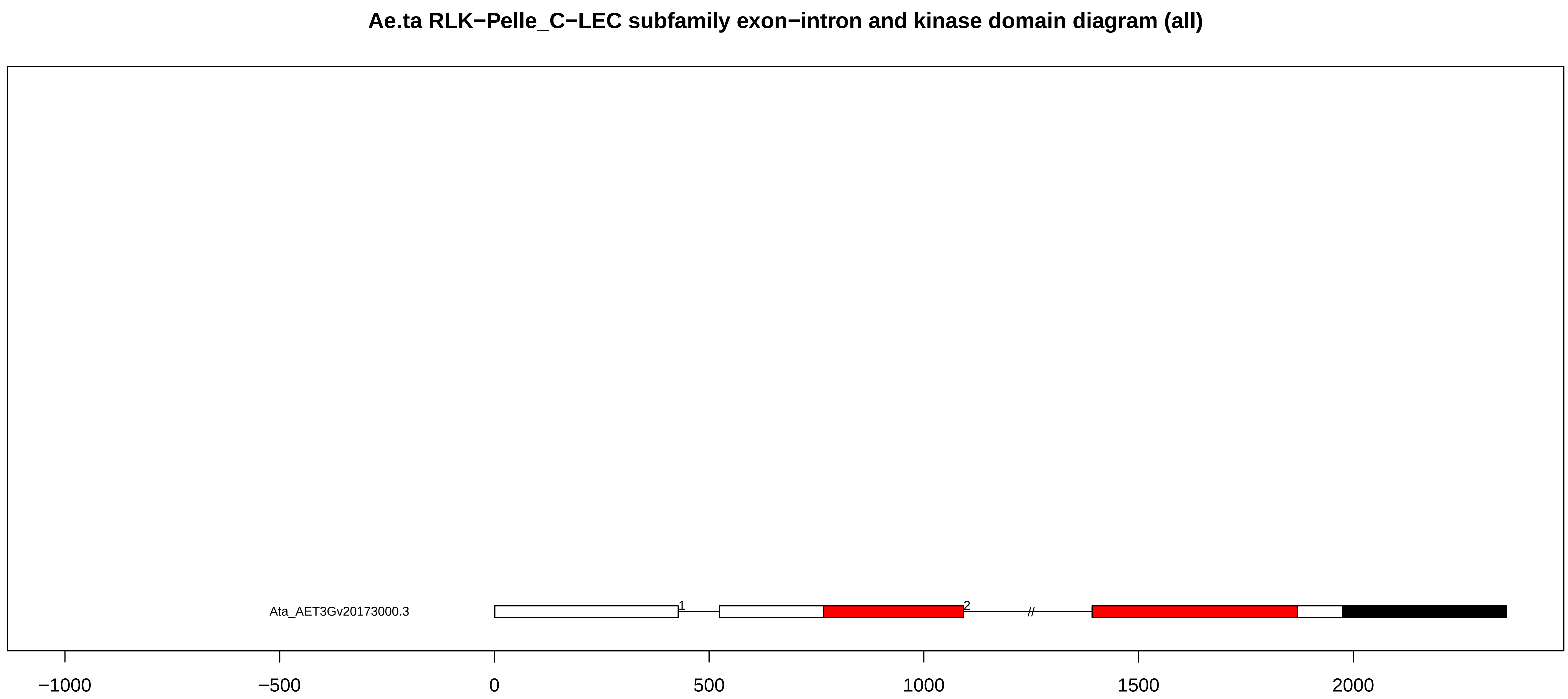
Ae.ta RLK−Pelle_C−LEC subfamily exon−intron and kinase domain diagram (all)
Ata_AET3Gv20173000.3
1
2
−1000
−500
0
500
1000
1500
2000

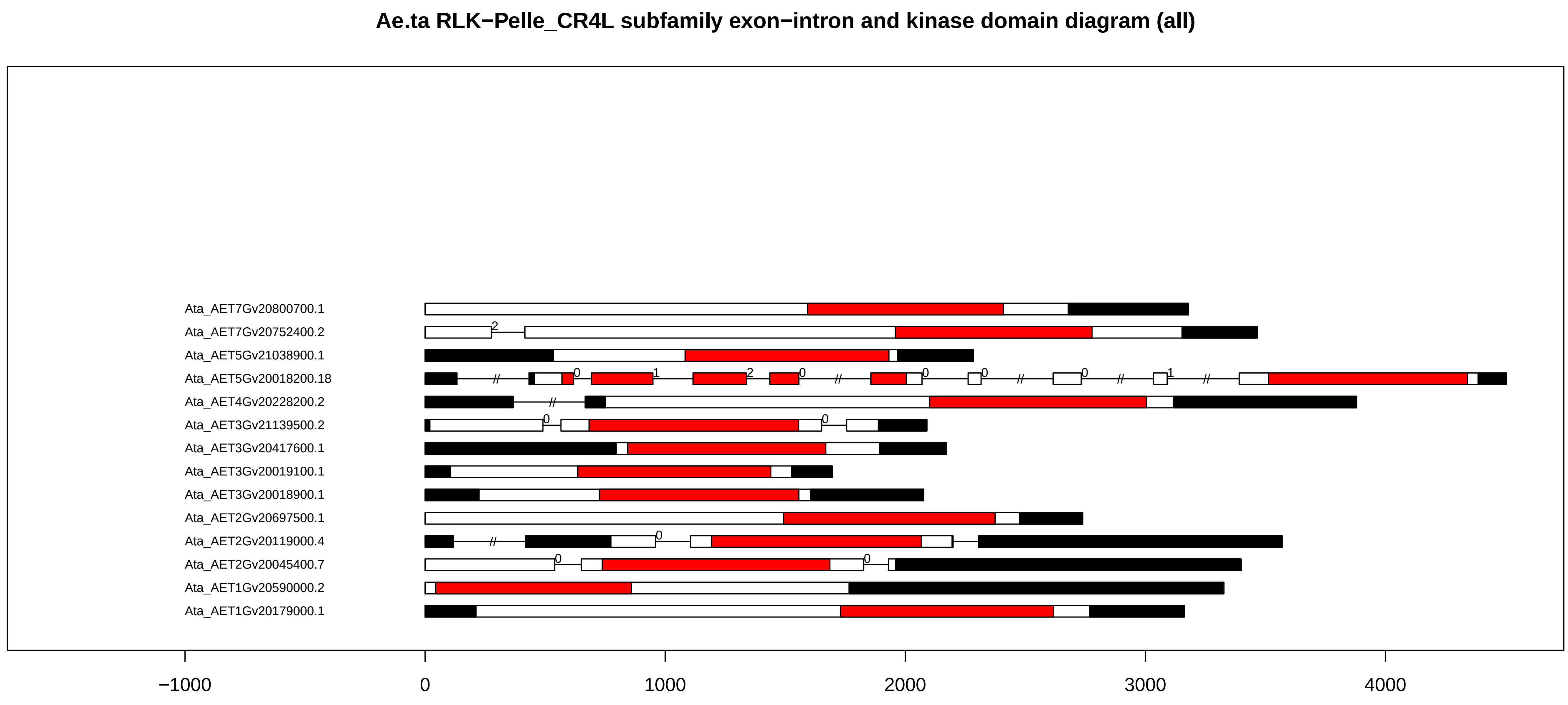

Ae.ta RLK−Pelle_CR4L subfamily exon−intron and kinase domain diagram (all)
Ata_AET7Gv20800700.1
Ata_AET7Gv20752400.2
Ata_AET5Gv21038900.1
Ata_AET5Gv20018200.18
Ata_AET4Gv20228200.2
Ata_AET3Gv21139500.2
Ata_AET3Gv20417600.1
Ata_AET3Gv20019100.1
Ata_AET3Gv20018900.1
Ata_AET2Gv20697500.1
Ata_AET2Gv20119000.4
Ata_AET2Gv20045400.7
Ata_AET1Gv20590000.2
Ata_AET1Gv20179000.1
−1000
0
1000
2000
3000
4000

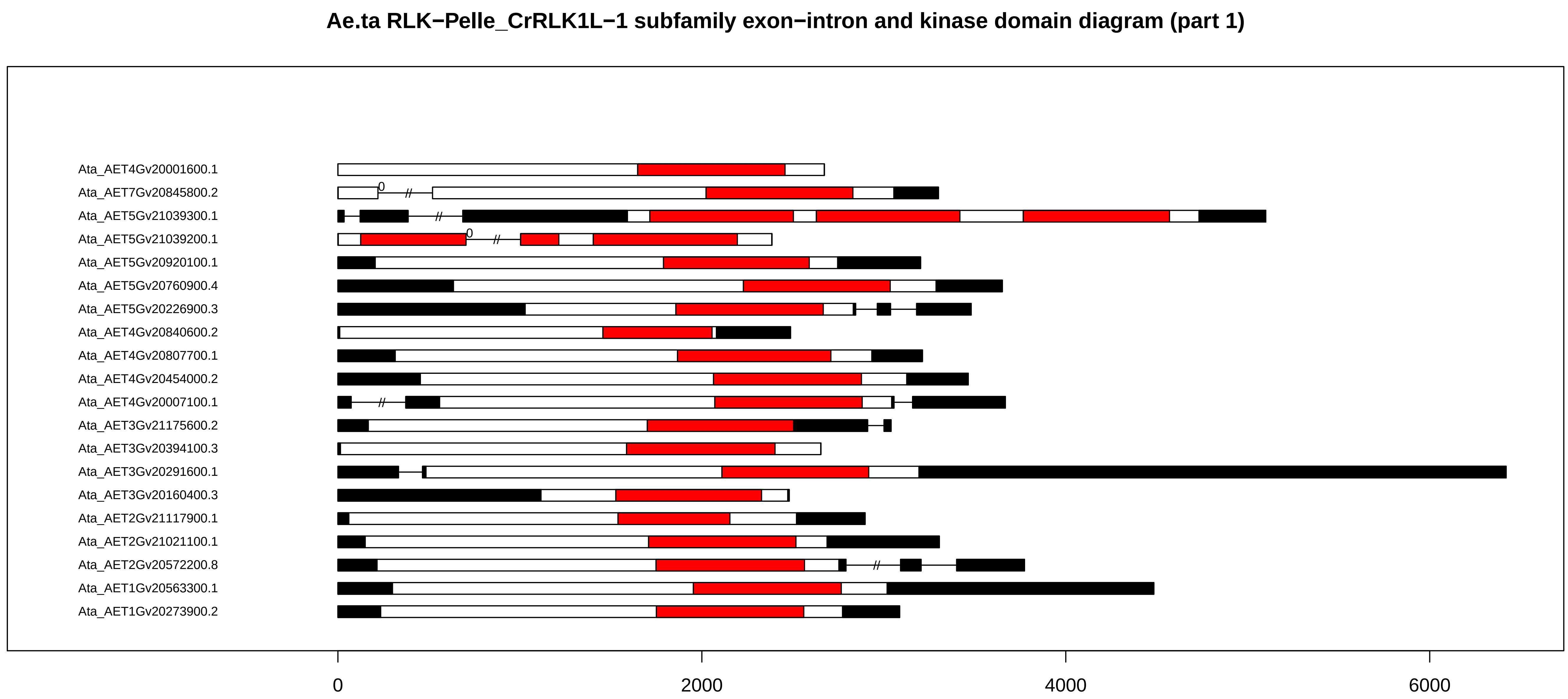

Ae.ta RLK−Pelle_CrRLK1L−1 subfamily exon−intron and kinase domain diagram (part 1)
Ata_AET4Gv20001600.1
Ata_AET7Gv20845800.2
Ata_AET5Gv21039300.1
Ata_AET5Gv21039200.1
Ata_AET5Gv20920100.1
Ata_AET5Gv20760900.4
Ata_AET5Gv20226900.3
Ata_AET4Gv20840600.2
Ata_AET4Gv20807700.1
Ata_AET4Gv20454000.2
Ata_AET4Gv20007100.1
Ata_AET3Gv21175600.2
Ata_AET3Gv20394100.3
Ata_AET3Gv20291600.1
Ata_AET3Gv20160400.3
Ata_AET2Gv21117900.1
Ata_AET2Gv21021100.1
Ata_AET2Gv20572200.8
Ata_AET1Gv20563300.1
Ata_AET1Gv20273900.2
0
0
0
2000
4000
6000

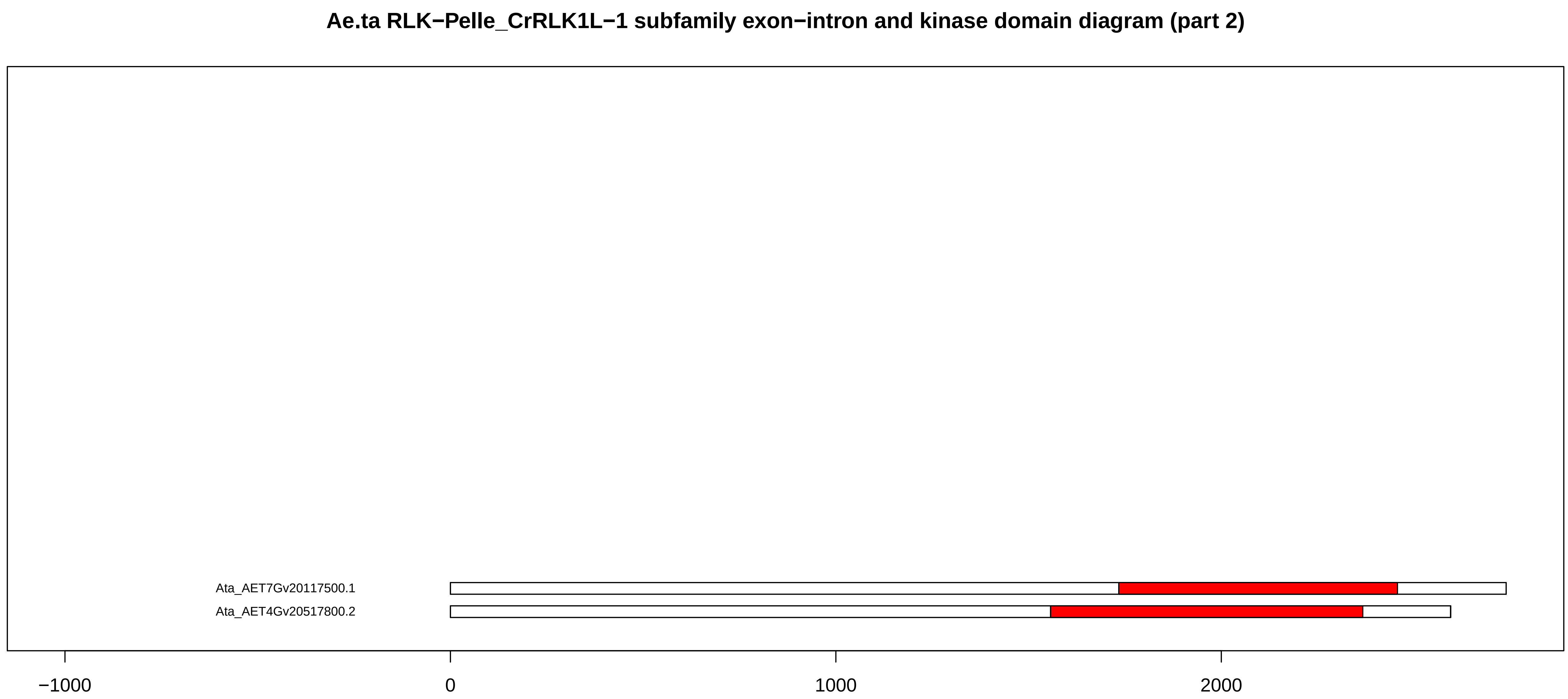
Ae.ta RLK−Pelle_CrRLK1L−1 subfamily exon−intron and kinase domain diagram (part 2)
Ata_AET7Gv20117500.1
Ata_AET4Gv20517800.2
−1000
0
1000
2000

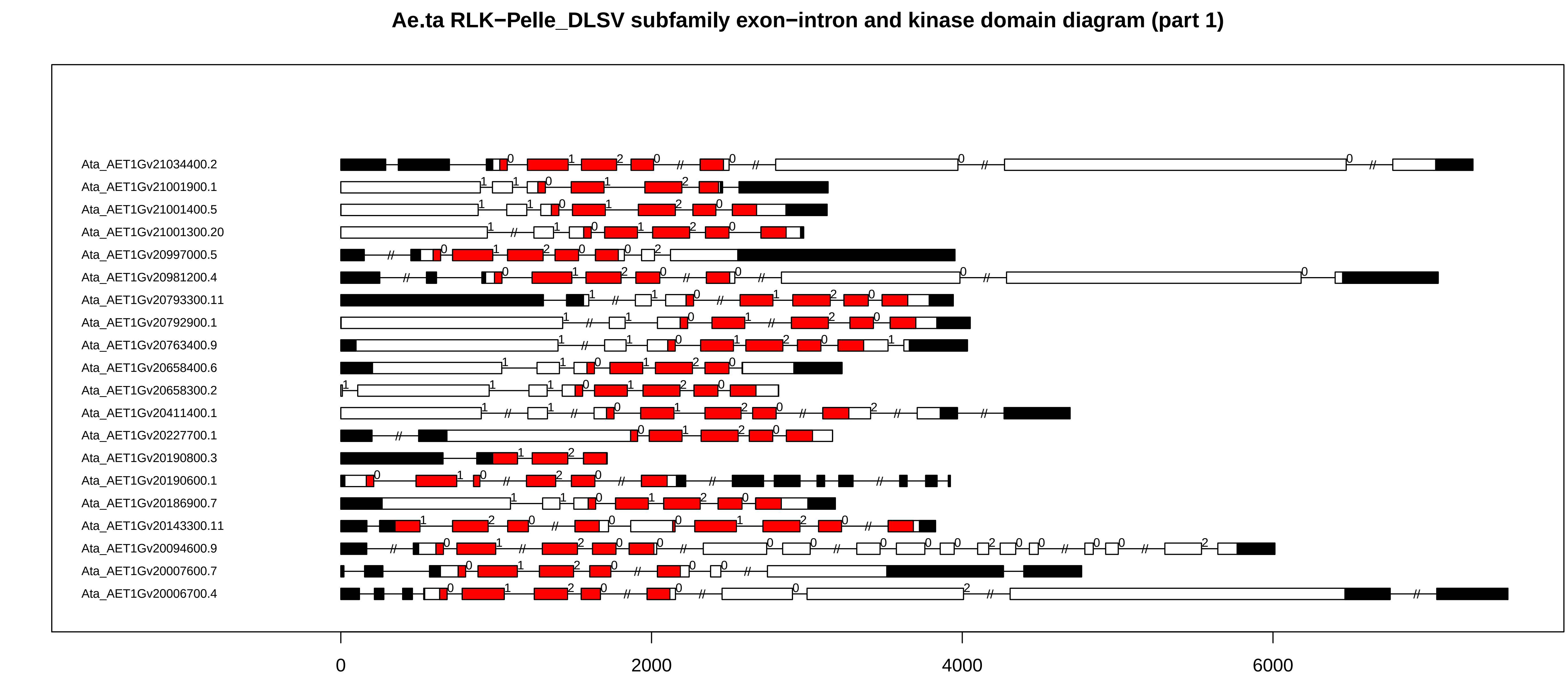

Ae.ta RLK−Pelle_DLSV subfamily exon−intron and kinase domain diagram (part 1)
Ata_AET1Gv21034400.2
Ata_AET1Gv21001900.1
Ata_AET1Gv21001400.5
Ata_AET1Gv21001300.20
Ata_AET1Gv20997000.5
Ata_AET1Gv20981200.4
Ata_AET1Gv20793300.11
Ata_AET1Gv20792900.1
Ata_AET1Gv20763400.9
Ata_AET1Gv20658400.6
Ata_AET1Gv20658300.2
Ata_AET1Gv20411400.1
Ata_AET1Gv20227700.1
Ata_AET1Gv20190800.3
Ata_AET1Gv20190600.1
Ata_AET1Gv20186900.7
Ata_AET1Gv20143300.11
Ata_AET1Gv20094600.9
Ata_AET1Gv20007600.7
Ata_AET1Gv20006700.4
0
2000
4000
6000

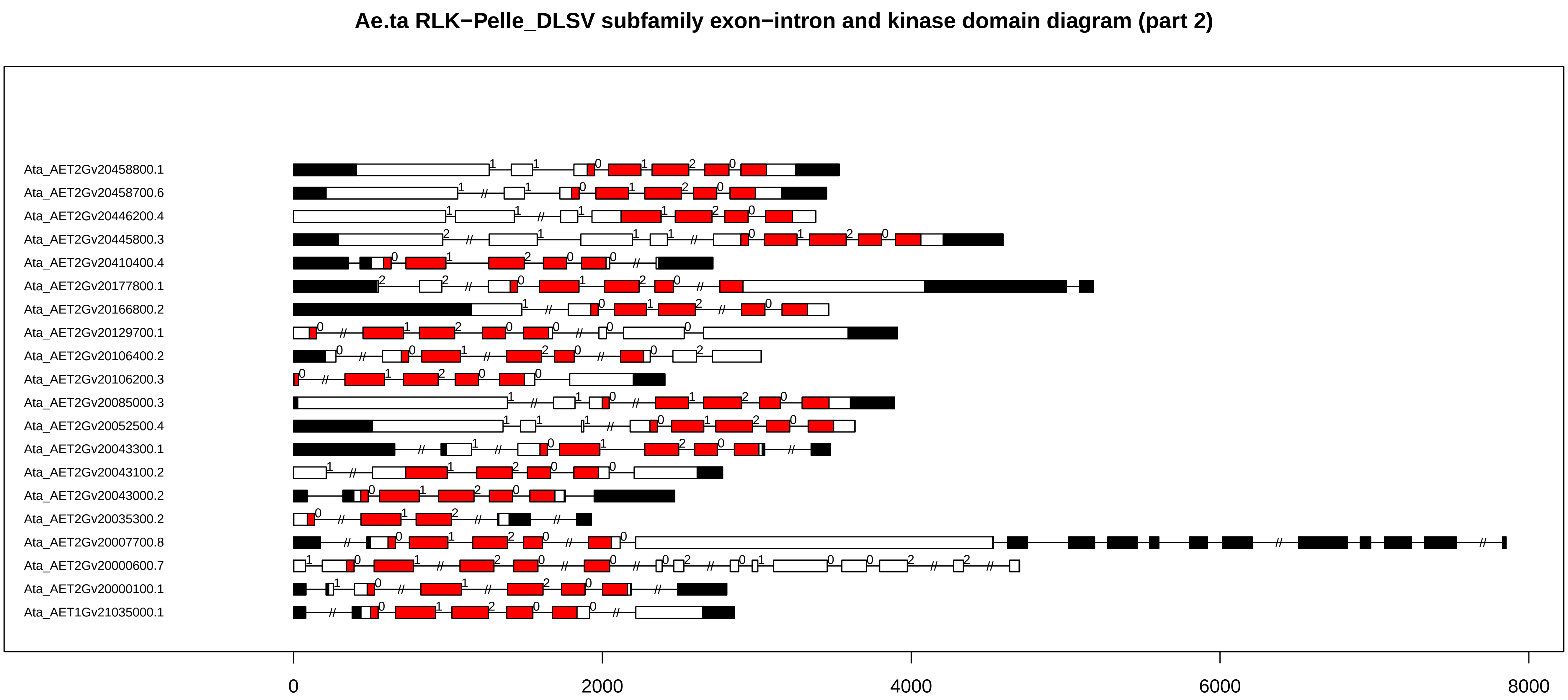

Ae.ta RLK−Pelle_DLSV subfamily exon−intron and kinase domain diagram (part 2)
Ata_AET2Gv20458800.1
Ata_AET2Gv20458700.6
Ata_AET2Gv20446200.4
Ata_AET2Gv20445800.3
Ata_AET2Gv20410400.4
Ata_AET2Gv20177800.1
Ata_AET2Gv20166800.2
Ata_AET2Gv20129700.1
Ata_AET2Gv20106400.2
Ata_AET2Gv20106200.3
Ata_AET2Gv20085000.3
Ata_AET2Gv20052500.4
Ata_AET2Gv20043300.1
Ata_AET2Gv20043100.2
Ata_AET2Gv20043000.2
Ata_AET2Gv20035300.2
Ata_AET2Gv20007700.8
Ata_AET2Gv20000600.7
Ata_AET2Gv20000100.1
Ata_AET1Gv21035000.1
0
2000
4000
6000
8000

Ae.ta RLK−Pelle_DLSV subfamily exon−intron and kinase domain diagram (part 3)

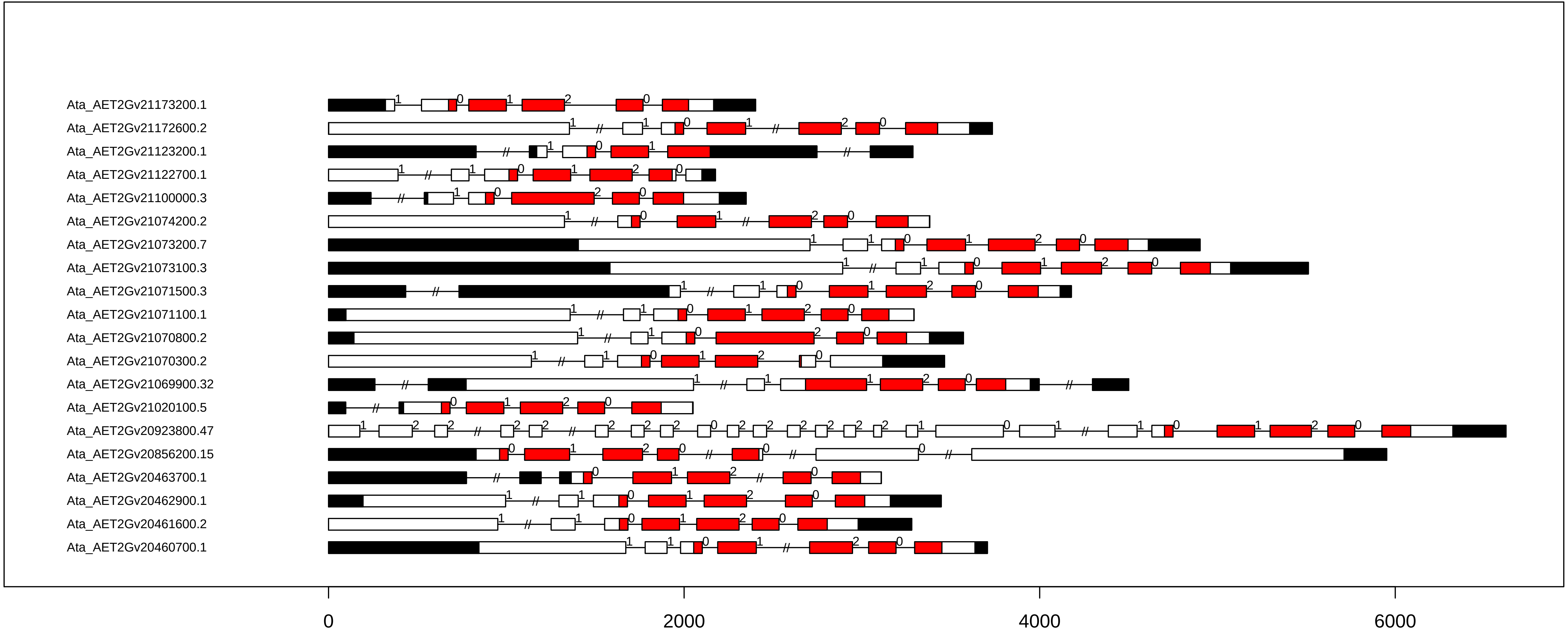

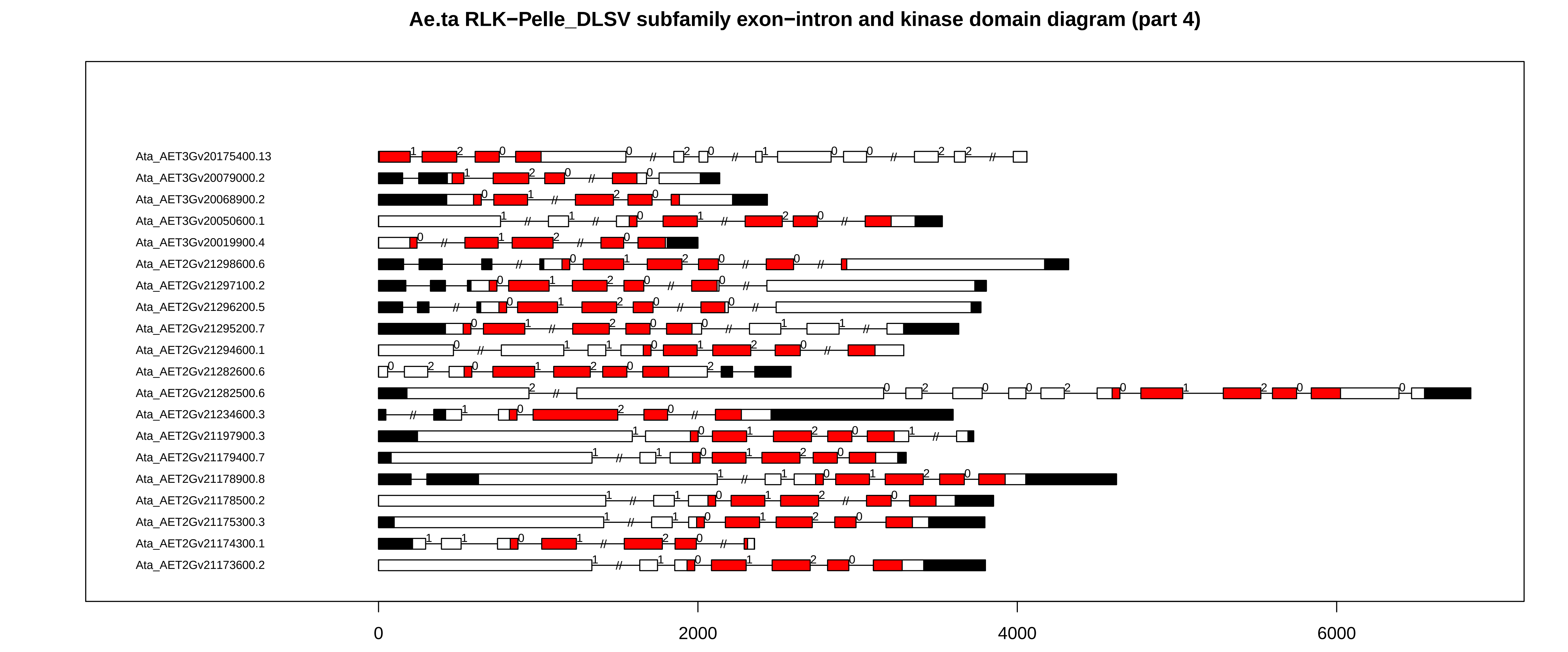

Ae.ta RLK−Pelle_DLSV subfamily exon−intron and kinase domain diagram (part 4)
Ata_AET3Gv20175400.13
Ata_AET3Gv20079000.2
Ata_AET3Gv20068900.2
Ata_AET3Gv20050600.1
Ata_AET3Gv20019900.4
Ata_AET2Gv21298600.6
Ata_AET2Gv21297100.2
Ata_AET2Gv21296200.5
Ata_AET2Gv21295200.7
Ata_AET2Gv21294600.1
Ata_AET2Gv21282600.6
Ata_AET2Gv21282500.6
Ata_AET2Gv21234600.3
Ata_AET2Gv21197900.3
Ata_AET2Gv21179400.7
Ata_AET2Gv21178900.8
Ata_AET2Gv21178500.2
Ata_AET2Gv21175300.3
Ata_AET2Gv21174300.1
Ata_AET2Gv21173600.2
0
2000
4000
6000

Ae.ta RLK−Pelle_DLSV subfamily exon−intron and kinase domain diagram (part 5)

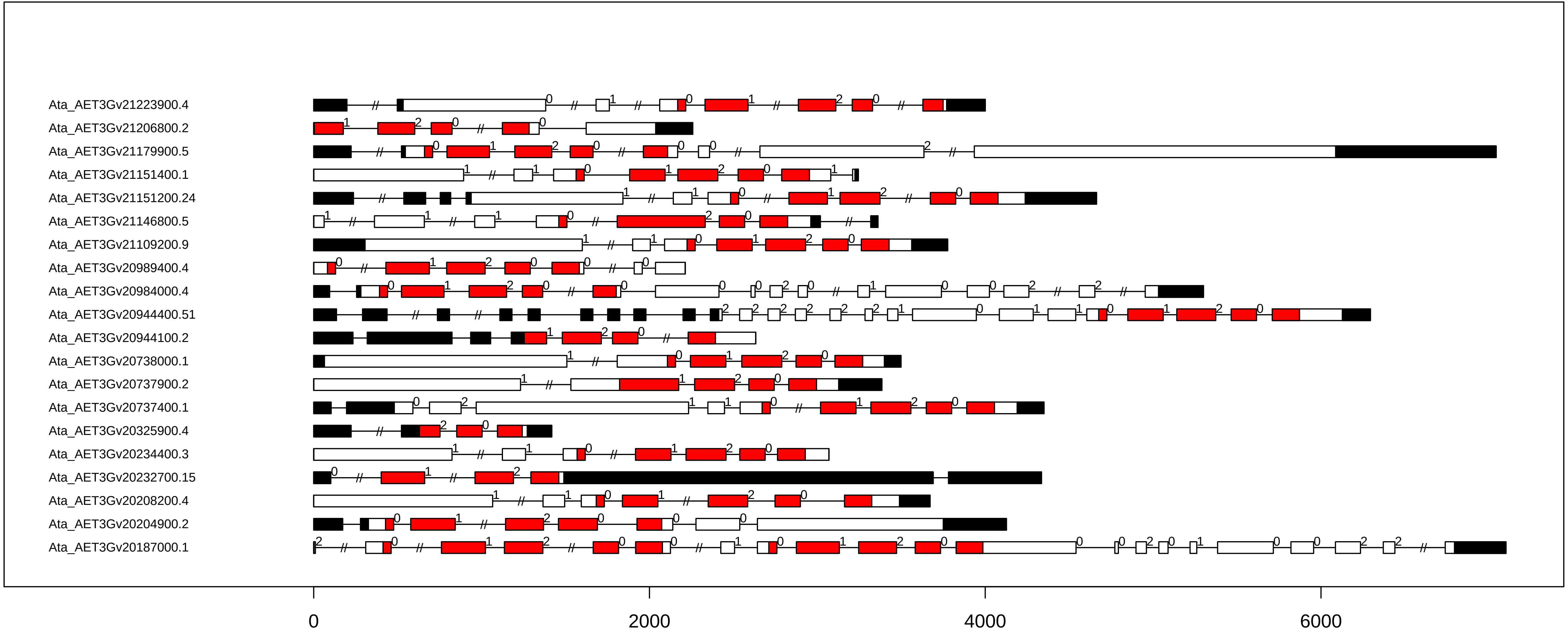

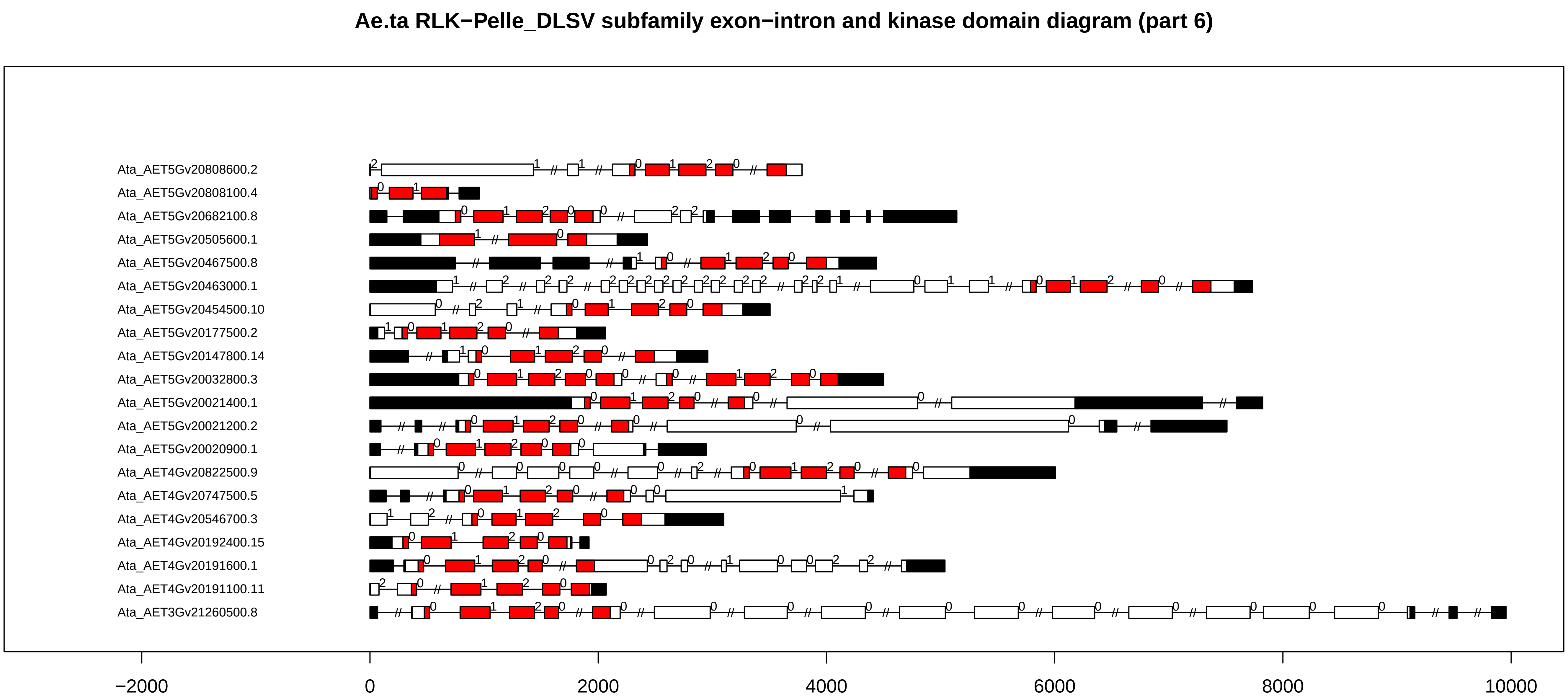
Ae.ta RLK−Pelle_DLSV subfamily exon−intron and kinase domain diagram (part 6)
Ata_AET5Gv20808600.2
Ata_AET5Gv20808100.4
Ata_AET5Gv20682100.8
Ata_AET5Gv20505600.1
Ata_AET5Gv20467500.8
Ata_AET5Gv20463000.1
Ata_AET5Gv20454500.10
Ata_AET5Gv20177500.2
Ata_AET5Gv20147800.14
Ata_AET5Gv20032800.3
Ata_AET5Gv20021400.1
Ata_AET5Gv20021200.2
Ata_AET5Gv20020900.1
Ata_AET4Gv20822500.9
Ata_AET4Gv20747500.5
Ata_AET4Gv20546700.3
Ata_AET4Gv20192400.15
Ata_AET4Gv20191600.1
Ata_AET4Gv20191100.11
Ata_AET3Gv21260500.8
−2000
0
2000
4000
6000
8000
10000

Ae.ta RLK−Pelle_DLSV subfamily exon−intron and kinase domain diagram (part 7)

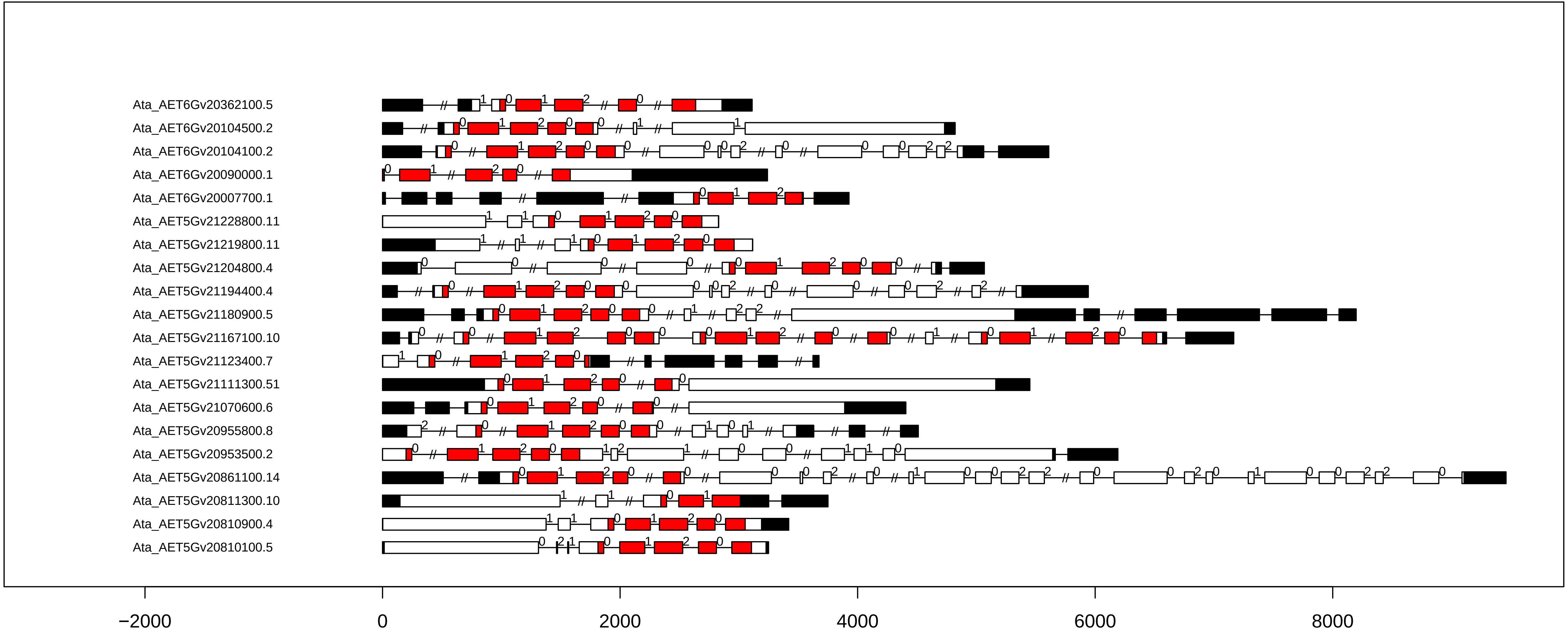

Ae.ta RLK−Pelle_DLSV subfamily exon−intron and kinase domain diagram (part 8)

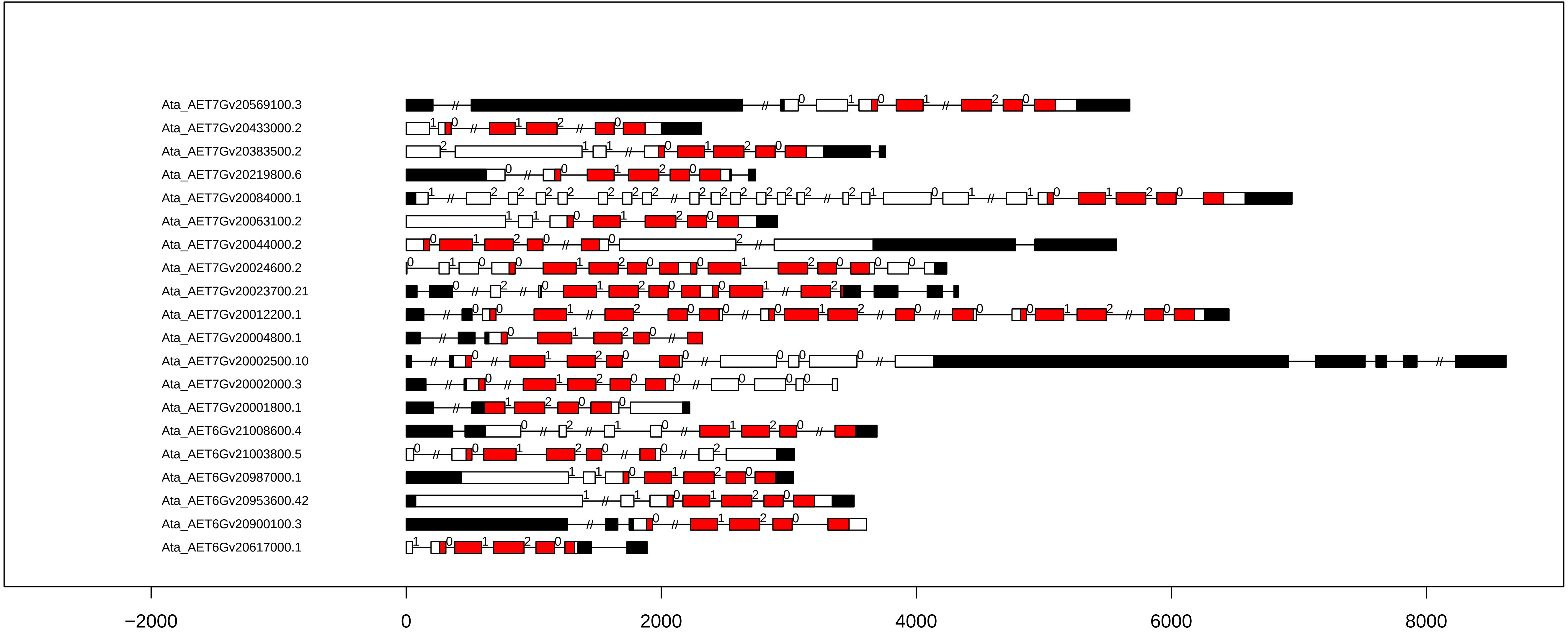

Ae.ta RLK−Pelle_DLSV subfamily exon−intron and kinase domain diagram (part 9)

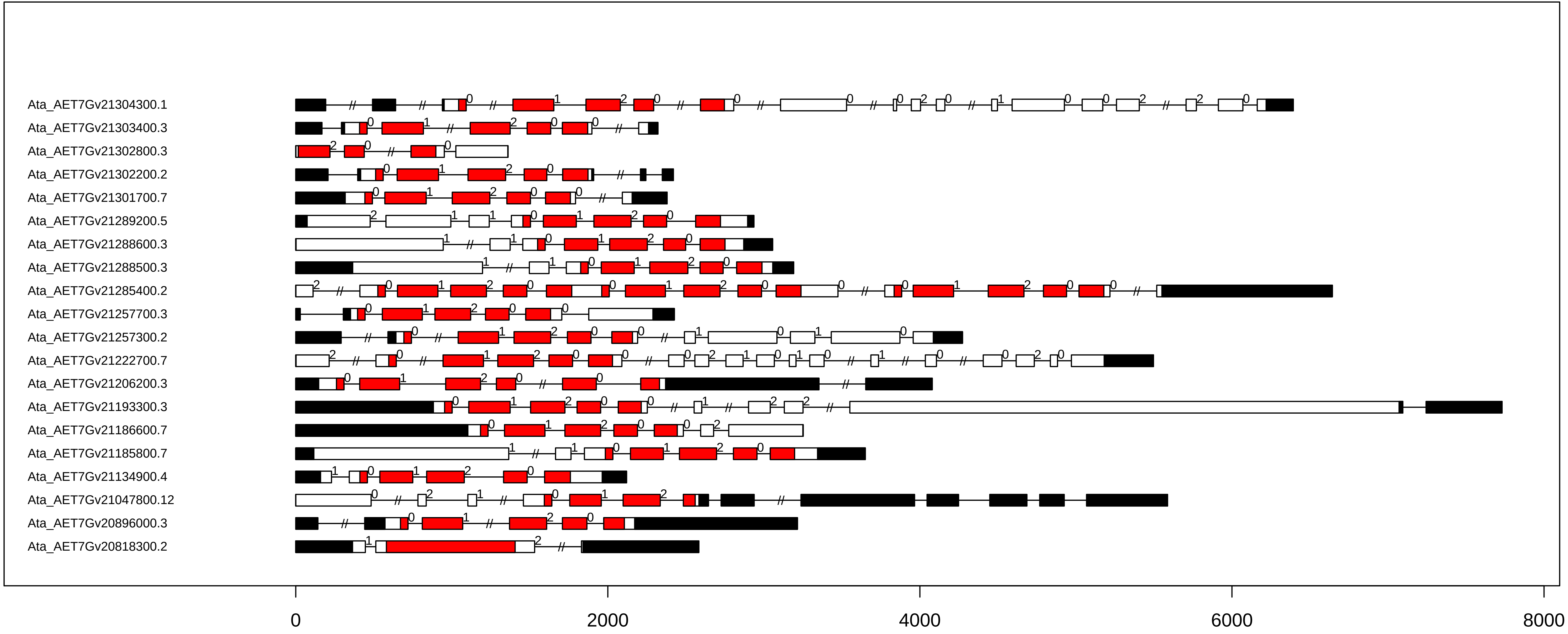

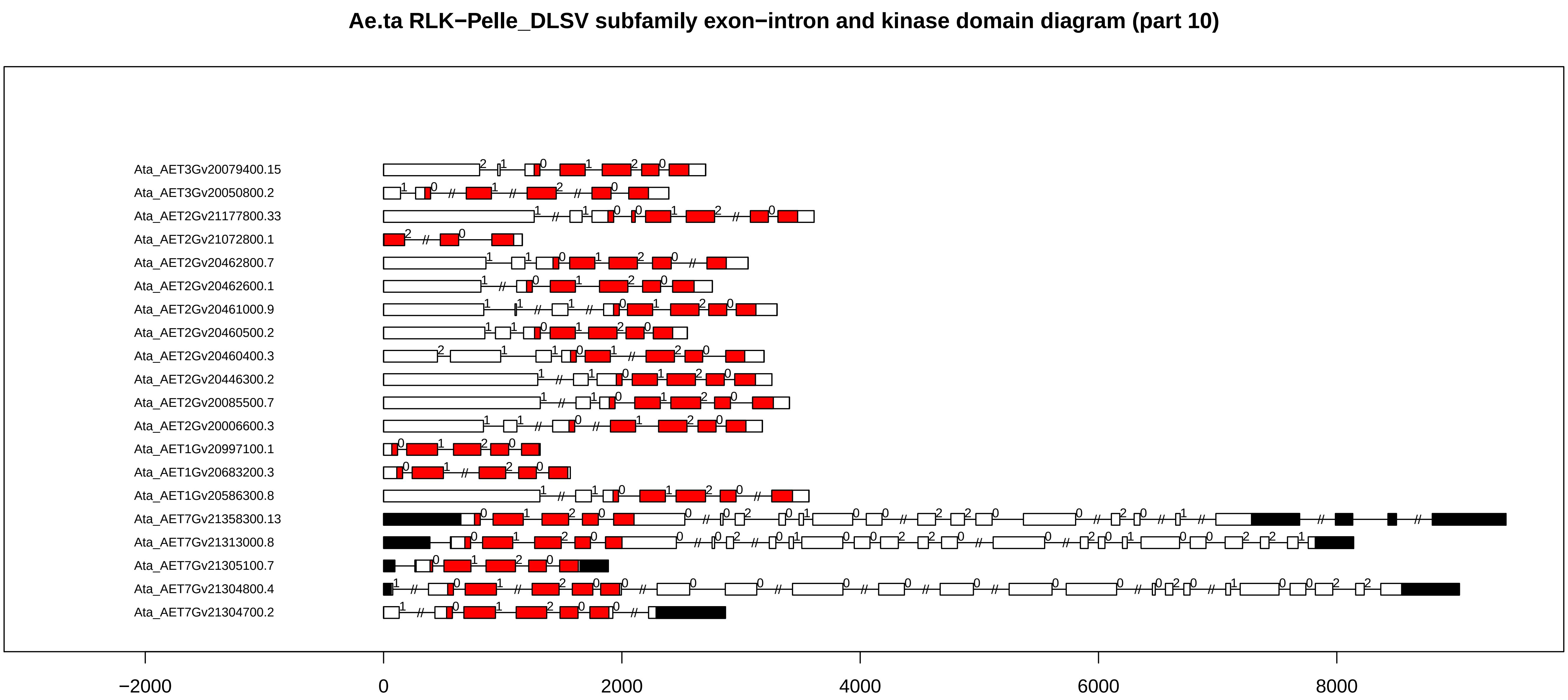
Ae.ta RLK−Pelle_DLSV subfamily exon−intron and kinase domain diagram (part 10)
Ata_AET3Gv20079400.15
Ata_AET3Gv20050800.2
Ata_AET2Gv21177800.33
Ata_AET2Gv21072800.1
Ata_AET2Gv20462800.7
Ata_AET2Gv20462600.1
Ata_AET2Gv20461000.9
Ata_AET2Gv20460500.2
Ata_AET2Gv20460400.3
Ata_AET2Gv20446300.2
Ata_AET2Gv20085500.7
Ata_AET2Gv20006600.3
Ata_AET1Gv20997100.1
Ata_AET1Gv20683200.3
Ata_AET1Gv20586300.8
Ata_AET7Gv21358300.13
Ata_AET7Gv21313000.8
Ata_AET7Gv21305100.7
Ata_AET7Gv21304800.4
Ata_AET7Gv21304700.2
−2000
0
2000
4000
6000
8000

Ae.ta RLK−Pelle_DLSV subfamily exon−intron and kinase domain diagram (part 11)

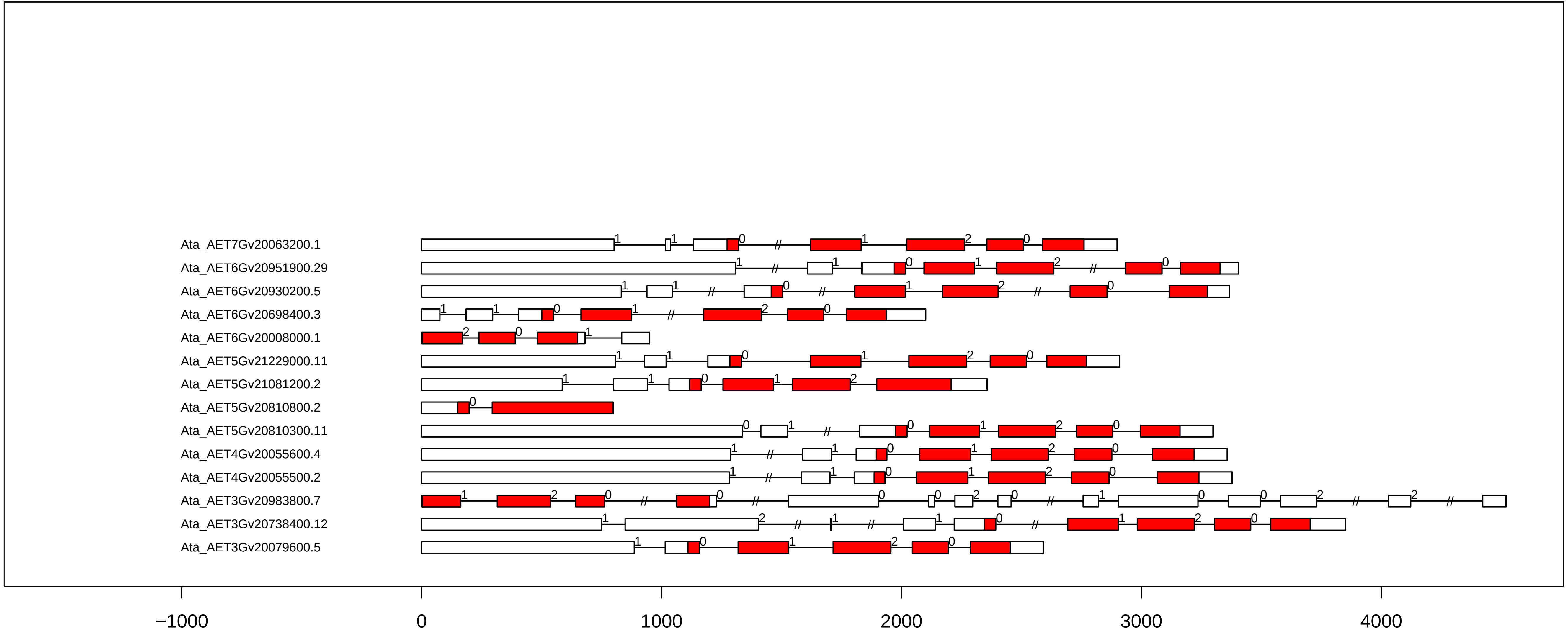

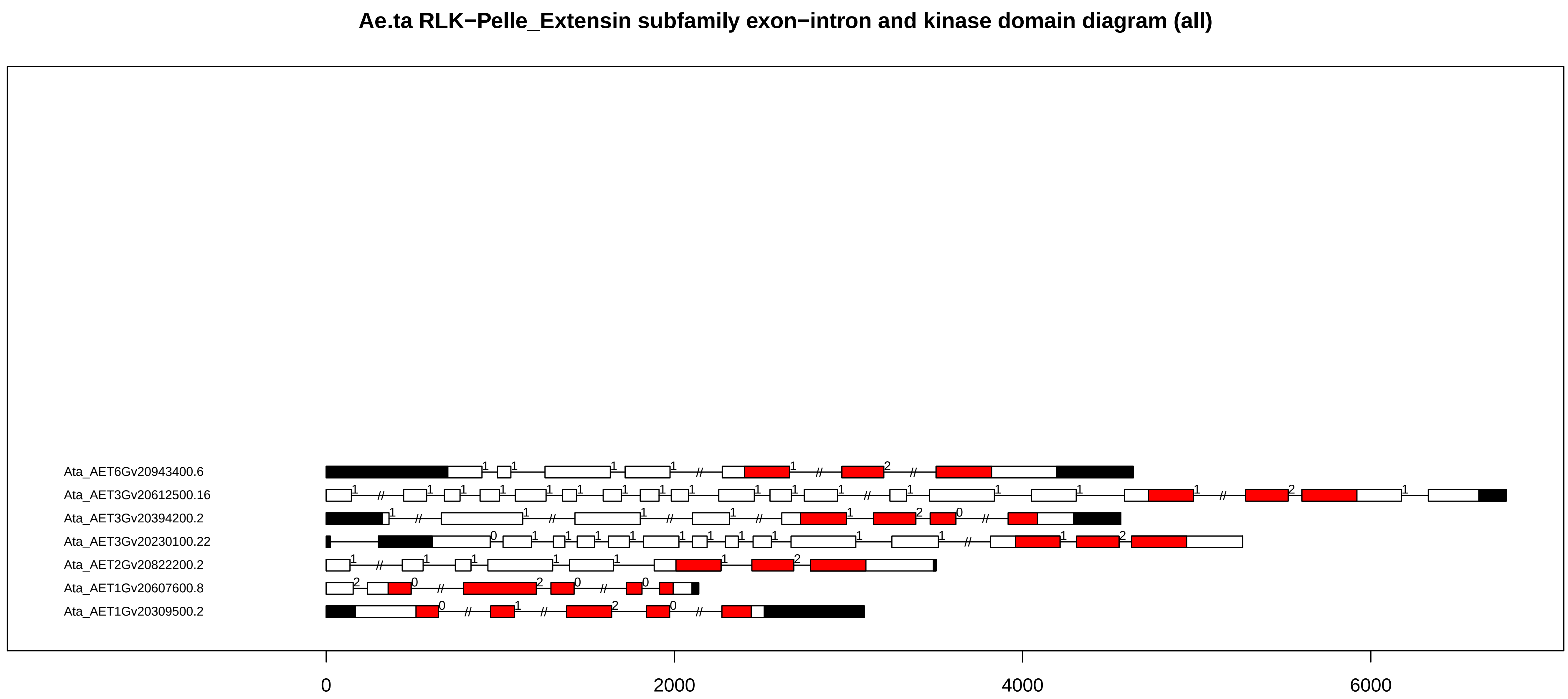

Ae.ta RLK−Pelle_Extensin subfamily exon−intron and kinase domain diagram (all)
Ata_AET6Gv20943400.6
Ata_AET3Gv20612500.16
Ata_AET3Gv20394200.2
Ata_AET3Gv20230100.22
Ata_AET2Gv20822200.2
Ata_AET1Gv20607600.8
Ata_AET1Gv20309500.2
0
2000
4000
6000

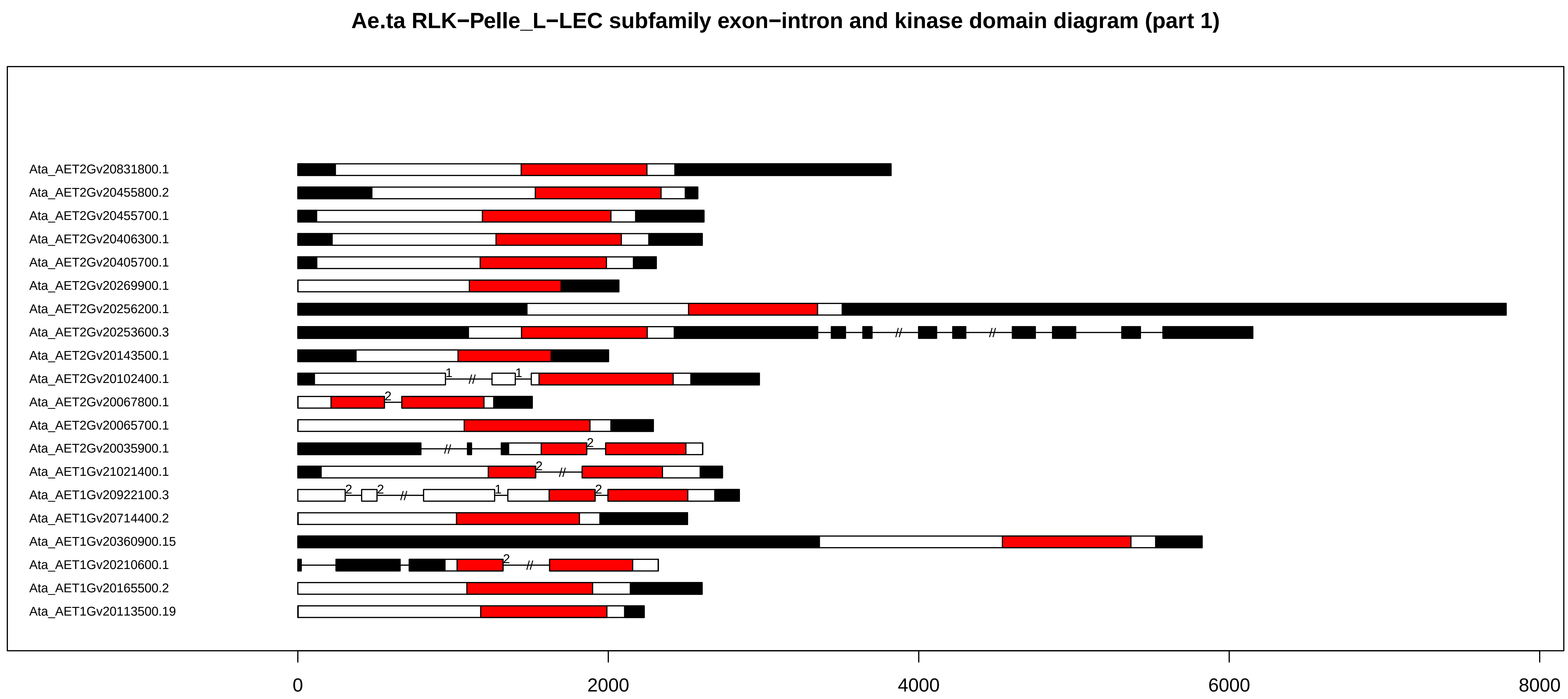

Ae.ta RLK−Pelle_L−LEC subfamily exon−intron and kinase domain diagram (part 1)
Ata_AET2Gv20831800.1
Ata_AET2Gv20455800.2
Ata_AET2Gv20455700.1
Ata_AET2Gv20406300.1
Ata_AET2Gv20405700.1
Ata_AET2Gv20269900.1
Ata_AET2Gv20256200.1
Ata_AET2Gv20253600.3
Ata_AET2Gv20143500.1
Ata_AET2Gv20102400.1
Ata_AET2Gv20067800.1
Ata_AET2Gv20065700.1
Ata_AET2Gv20035900.1
Ata_AET1Gv21021400.1
Ata_AET1Gv20922100.3
Ata_AET1Gv20714400.2
Ata_AET1Gv20360900.15
Ata_AET1Gv20210600.1
Ata_AET1Gv20165500.2
Ata_AET1Gv20113500.19
0
2000
4000
6000
8000

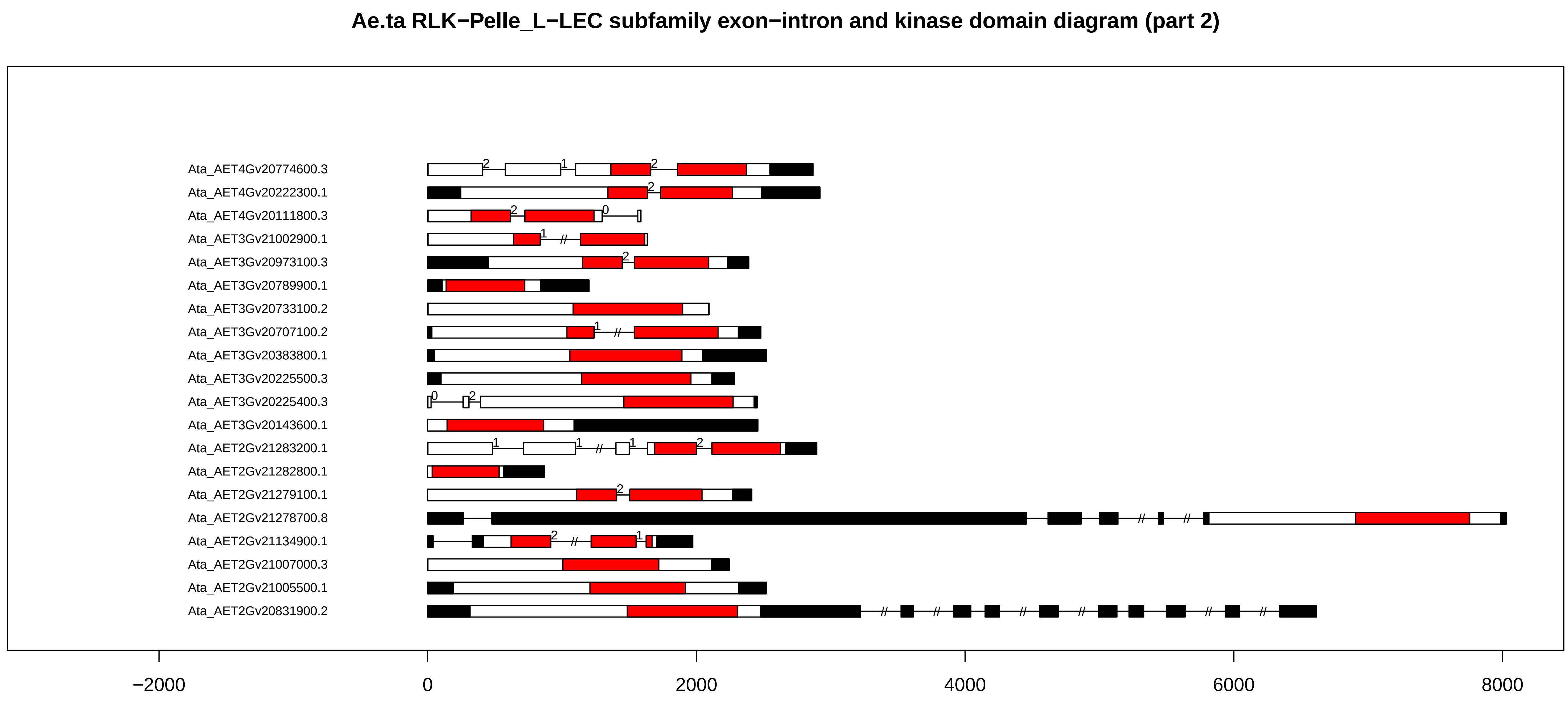

Ae.ta RLK−Pelle_L−LEC subfamily exon−intron and kinase domain diagram (part 2)
Ata_AET4Gv20774600.3
Ata_AET4Gv20222300.1
Ata_AET4Gv20111800.3
Ata_AET3Gv21002900.1
Ata_AET3Gv20973100.3
Ata_AET3Gv20789900.1
Ata_AET3Gv20733100.2
Ata_AET3Gv20707100.2
Ata_AET3Gv20383800.1
Ata_AET3Gv20225500.3
Ata_AET3Gv20225400.3
Ata_AET3Gv20143600.1
Ata_AET2Gv21283200.1
Ata_AET2Gv21282800.1
Ata_AET2Gv21279100.1
Ata_AET2Gv21278700.8
Ata_AET2Gv21134900.1
Ata_AET2Gv21007000.3
Ata_AET2Gv21005500.1
Ata_AET2Gv20831900.2
−2000
0
2000
4000
6000
8000

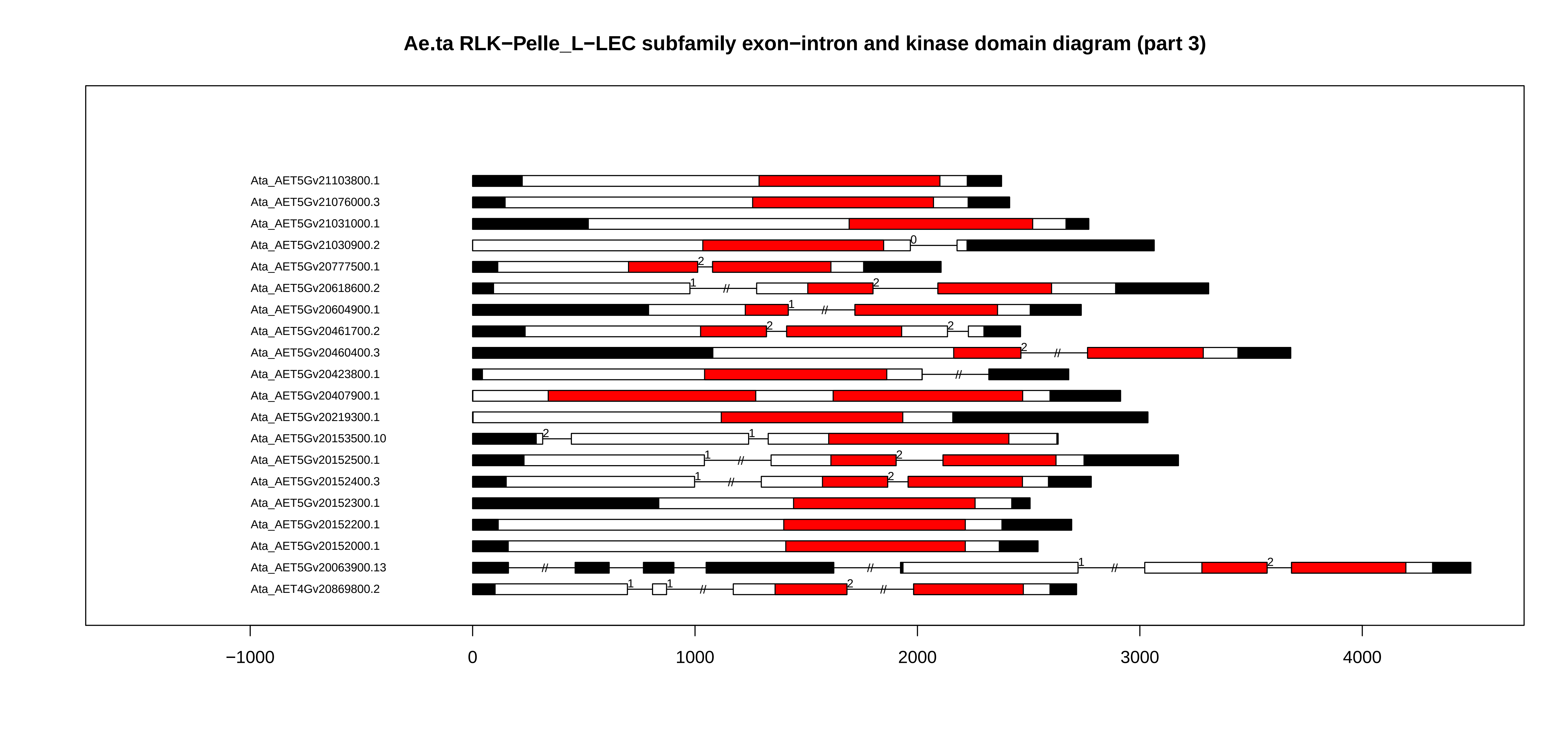

Ae.ta RLK−Pelle_L−LEC subfamily exon−intron and kinase domain diagram (part 3)
Ata_AET5Gv21103800.1
Ata_AET5Gv21076000.3
Ata_AET5Gv21031000.1
Ata_AET5Gv21030900.2
Ata_AET5Gv20777500.1
Ata_AET5Gv20618600.2
Ata_AET5Gv20604900.1
Ata_AET5Gv20461700.2
Ata_AET5Gv20460400.3
Ata_AET5Gv20423800.1
Ata_AET5Gv20407900.1
Ata_AET5Gv20219300.1
Ata_AET5Gv20153500.10
Ata_AET5Gv20152500.1
Ata_AET5Gv20152400.3
Ata_AET5Gv20152300.1
Ata_AET5Gv20152200.1
Ata_AET5Gv20152000.1
Ata_AET5Gv20063900.13
Ata_AET4Gv20869800.2
−1000
0
1000
2000
3000
4000

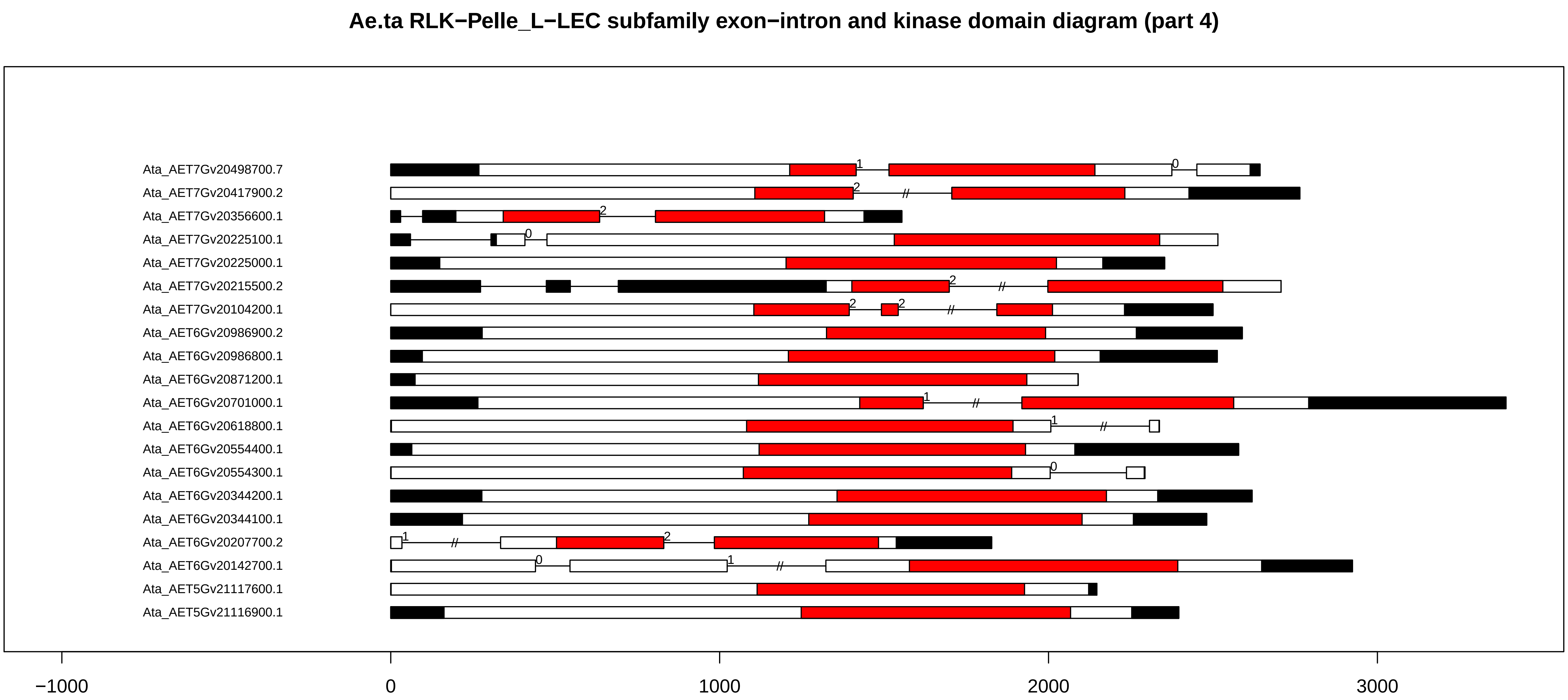
Ae.ta RLK−Pelle_L−LEC subfamily exon−intron and kinase domain diagram (part 4)
Ata_AET7Gv20498700.7
Ata_AET7Gv20417900.2
Ata_AET7Gv20356600.1
Ata_AET7Gv20225100.1
Ata_AET7Gv20225000.1
Ata_AET7Gv20215500.2
Ata_AET7Gv20104200.1
Ata_AET6Gv20986900.2
Ata_AET6Gv20986800.1
Ata_AET6Gv20871200.1
Ata_AET6Gv20701000.1
Ata_AET6Gv20618800.1
Ata_AET6Gv20554400.1
Ata_AET6Gv20554300.1
Ata_AET6Gv20344200.1
Ata_AET6Gv20344100.1
Ata_AET6Gv20207700.2
Ata_AET6Gv20142700.1
Ata_AET5Gv21117600.1
Ata_AET5Gv21116900.1
−1000
0
1000
2000
3000

Ae.ta RLK−Pelle_L−LEC subfamily exon−intron and kinase domain diagram (part 5)

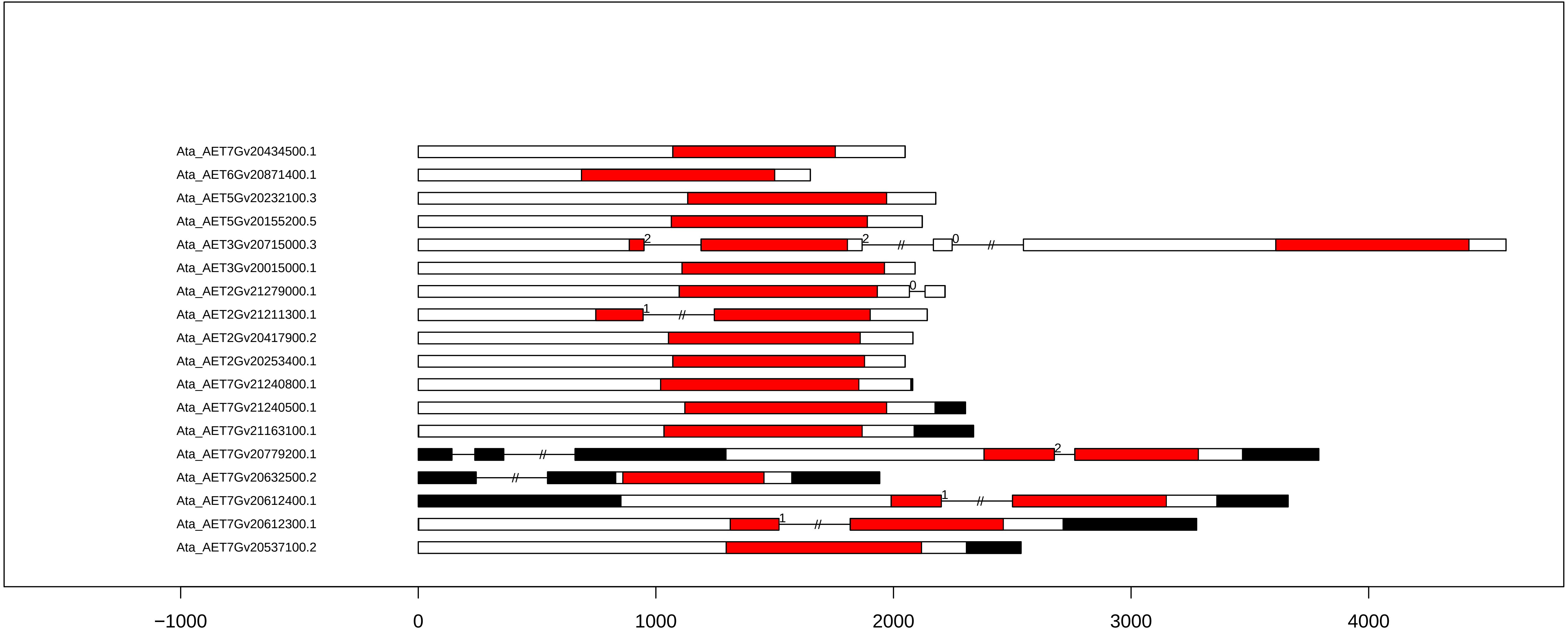

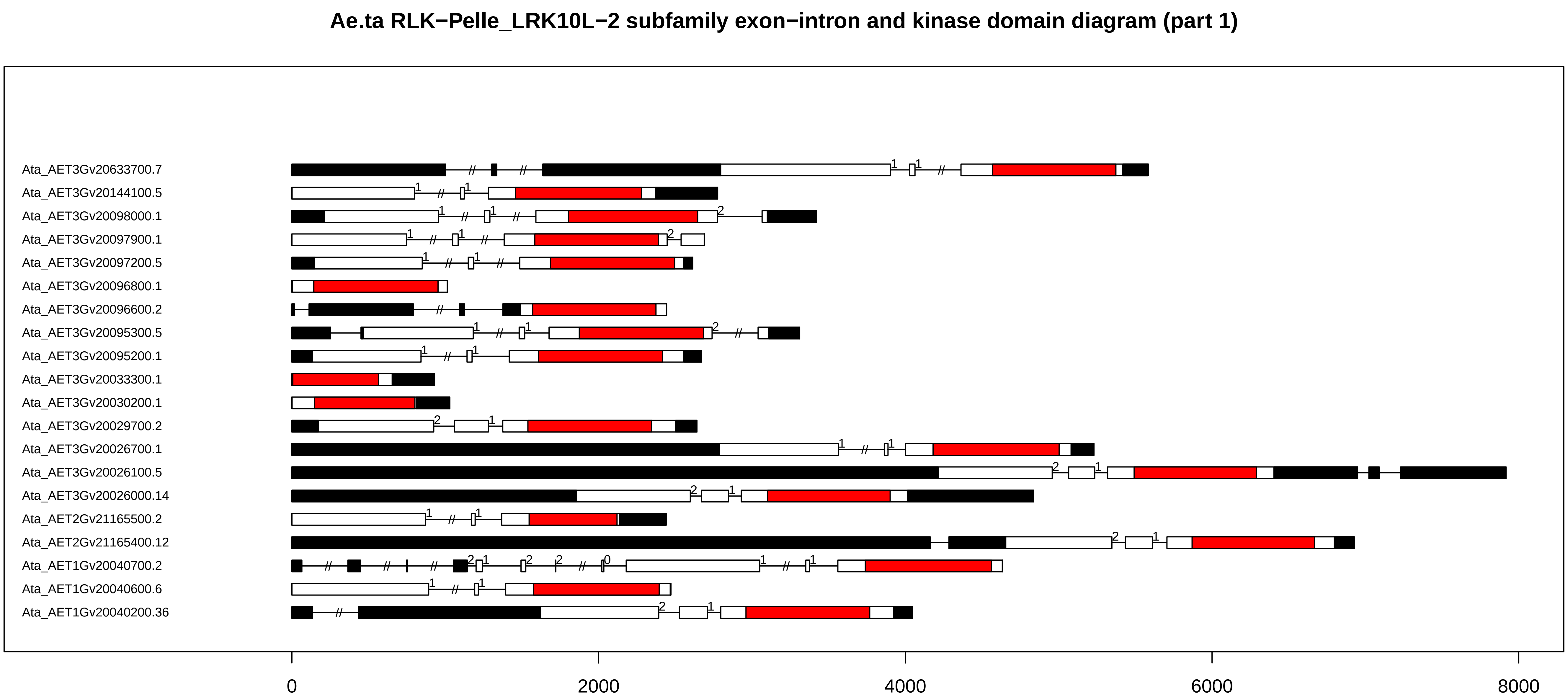
Ae.ta RLK−Pelle_LRK10L−2 subfamily exon−intron and kinase domain diagram (part 1)
Ata_AET3Gv20633700.7
Ata_AET3Gv20144100.5
Ata_AET3Gv20098000.1
Ata_AET3Gv20097900.1
Ata_AET3Gv20097200.5
Ata_AET3Gv20096800.1
Ata_AET3Gv20096600.2
Ata_AET3Gv20095300.5
Ata_AET3Gv20095200.1
Ata_AET3Gv20033300.1
Ata_AET3Gv20030200.1
Ata_AET3Gv20029700.2
Ata_AET3Gv20026700.1
Ata_AET3Gv20026100.5
Ata_AET3Gv20026000.14
Ata_AET2Gv21165500.2
Ata_AET2Gv21165400.12
Ata_AET1Gv20040700.2
Ata_AET1Gv20040600.6
Ata_AET1Gv20040200.36
0
2000
4000
6000
8000

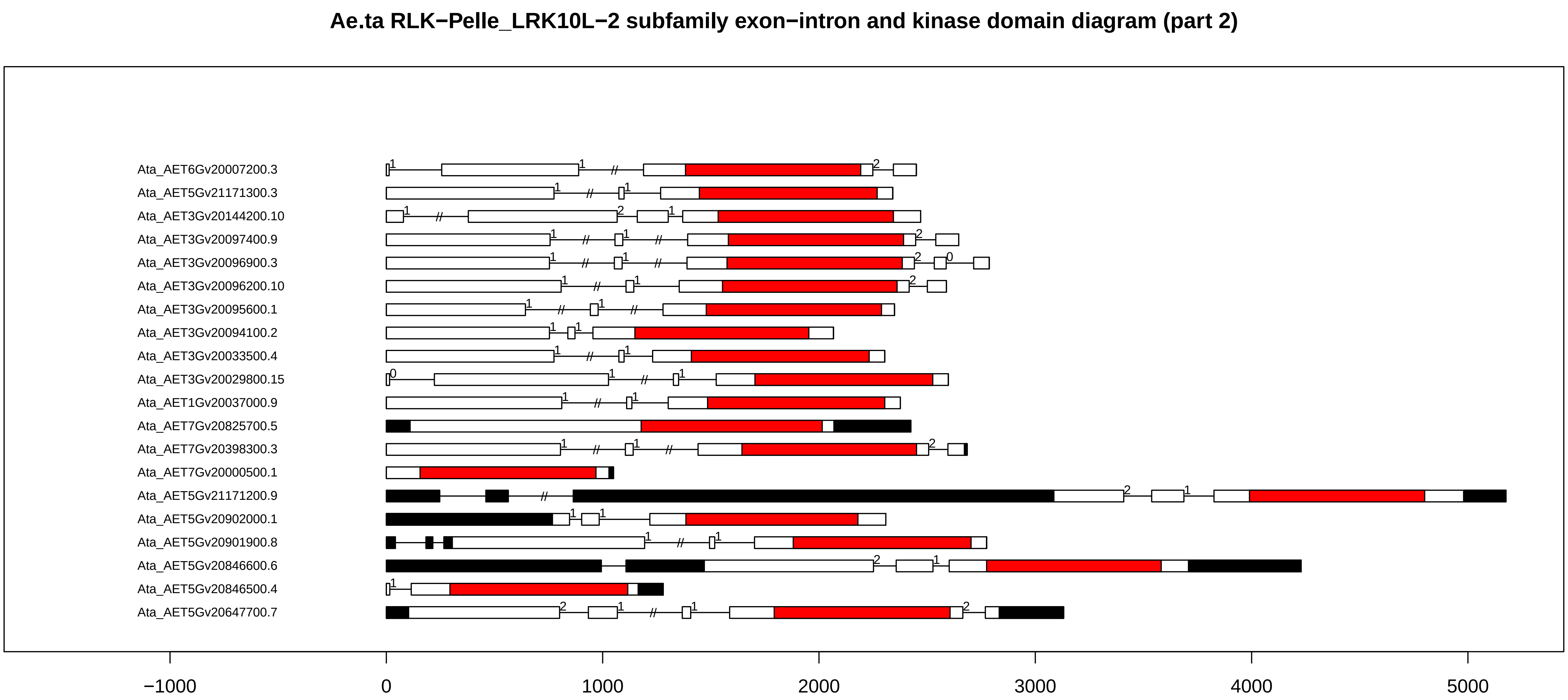
Ae.ta RLK−Pelle_LRK10L−2 subfamily exon−intron and kinase domain diagram (part 2)
Ata_AET6Gv20007200.3
Ata_AET5Gv21171300.3
Ata_AET3Gv20144200.10
Ata_AET3Gv20097400.9
Ata_AET3Gv20096900.3
Ata_AET3Gv20096200.10
Ata_AET3Gv20095600.1
Ata_AET3Gv20094100.2
Ata_AET3Gv20033500.4
Ata_AET3Gv20029800.15
Ata_AET1Gv20037000.9
Ata_AET7Gv20825700.5
Ata_AET7Gv20398300.3
Ata_AET7Gv20000500.1
Ata_AET5Gv21171200.9
Ata_AET5Gv20902000.1
Ata_AET5Gv20901900.8
Ata_AET5Gv20846600.6
Ata_AET5Gv20846500.4
Ata_AET5Gv20647700.7
−1000
0
1000
2000
3000
4000
5000

Ae.ta RLK−Pelle_LRR−I−1 subfamily exon−intron and kinase domain diagram (part 1)

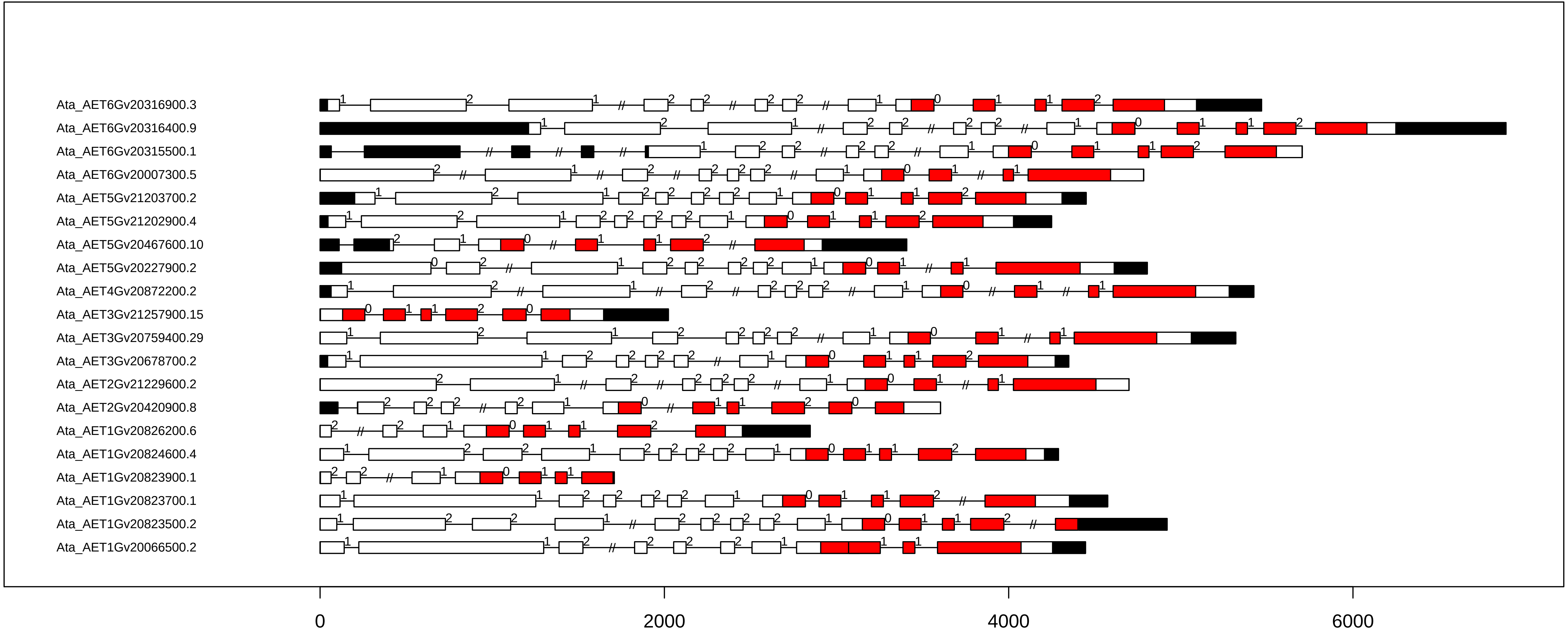

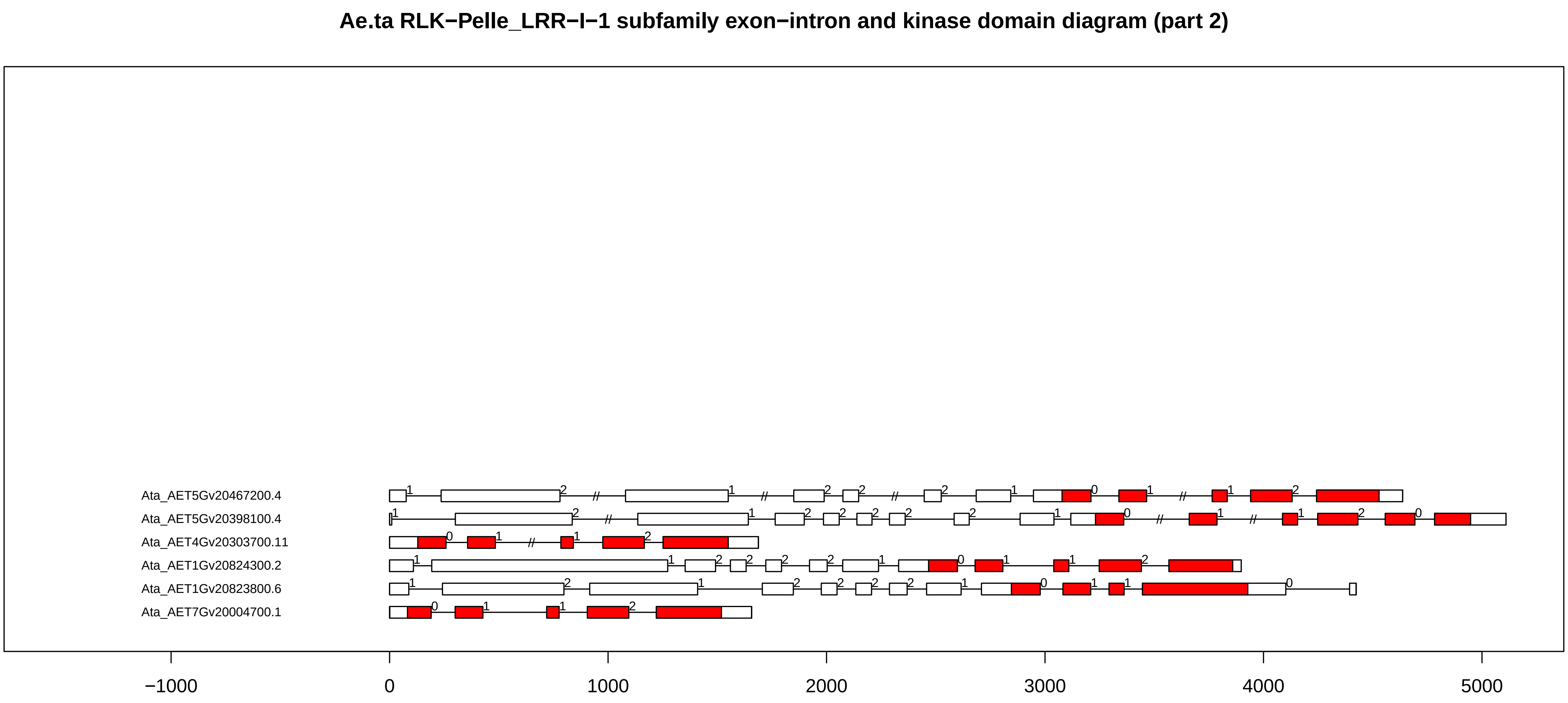
Ae.ta RLK−Pelle_LRR−I−1 subfamily exon−intron and kinase domain diagram (part 2)
Ata_AET5Gv20467200.4
Ata_AET5Gv20398100.4
Ata_AET4Gv20303700.11
Ata_AET1Gv20824300.2
Ata_AET1Gv20823800.6
Ata_AET7Gv20004700.1
−1000
0
1000
2000
3000
4000
5000

Ae.ta RLK−Pelle_LRR−I−2 subfamily exon−intron and kinase domain diagram (all)

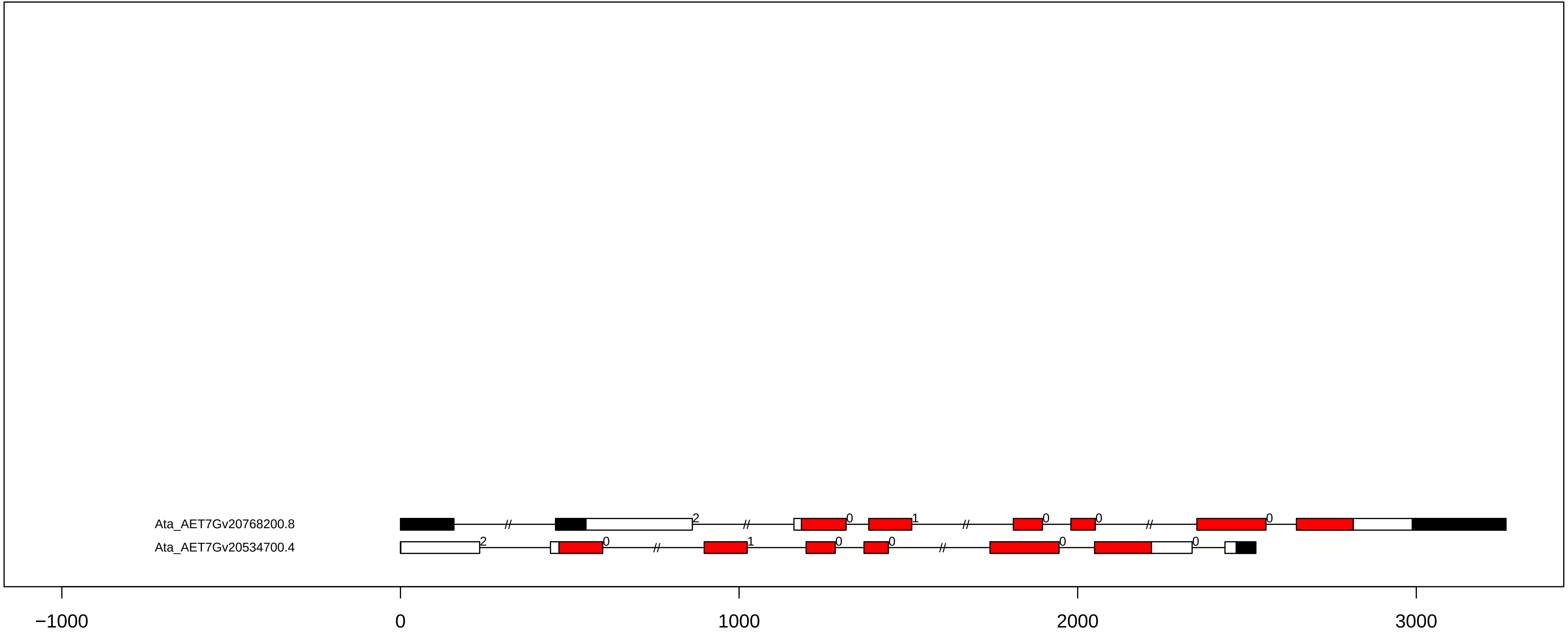

Ae.ta RLK−Pelle_LRR−III subfamily exon−intron and kinase domain diagram (part 1)

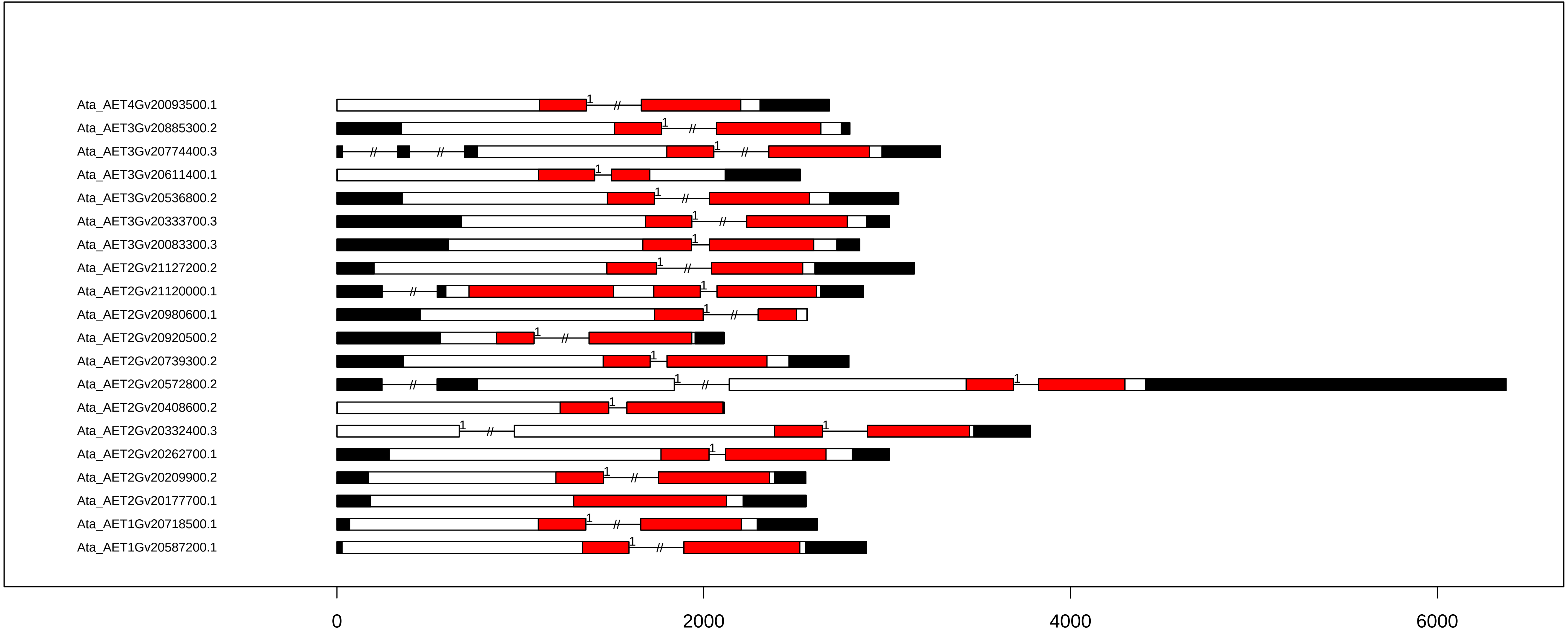

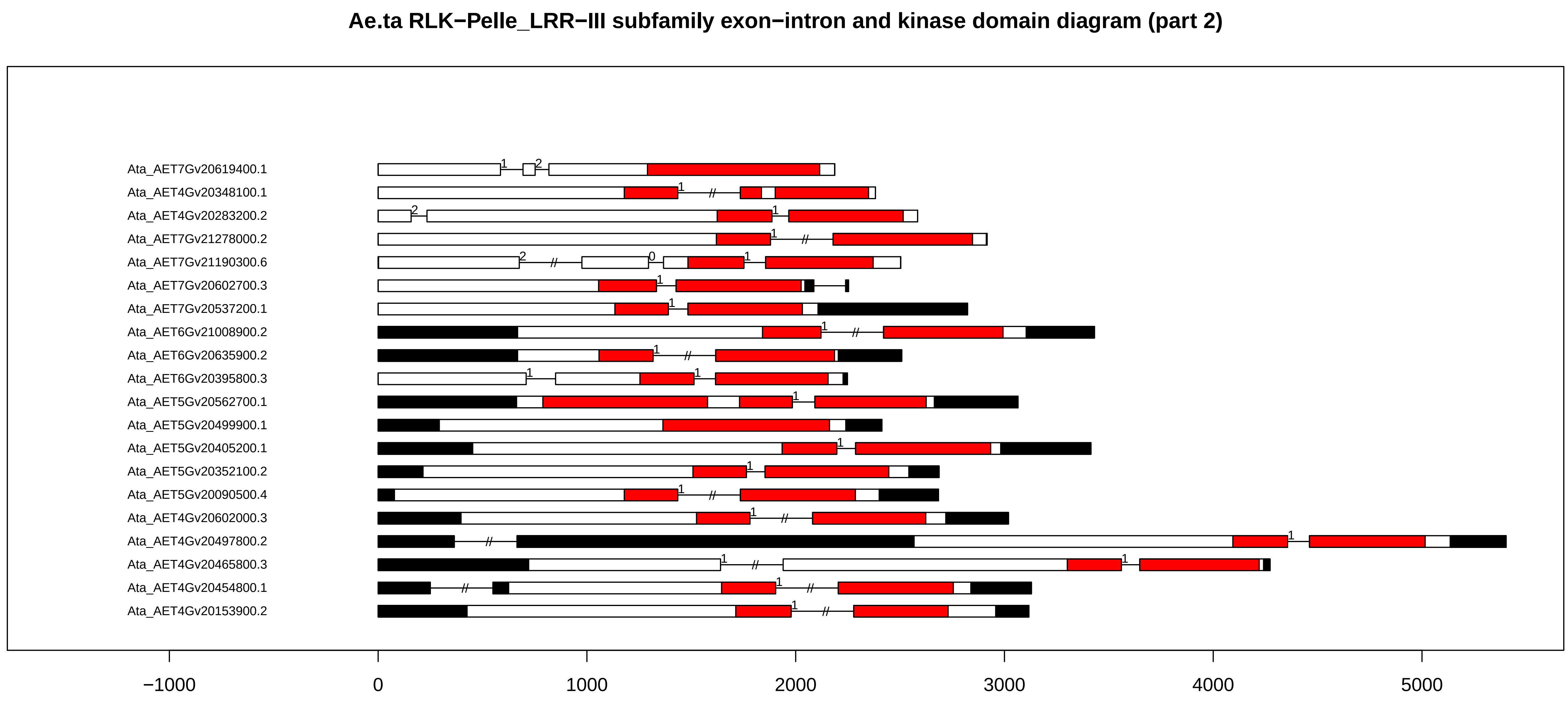

Ae.ta RLK−Pelle_LRR−III subfamily exon−intron and kinase domain diagram (part 2)
Ata_AET7Gv20619400.1
Ata_AET4Gv20348100.1
Ata_AET4Gv20283200.2
Ata_AET7Gv21278000.2
Ata_AET7Gv21190300.6
Ata_AET7Gv20602700.3
Ata_AET7Gv20537200.1
Ata_AET6Gv21008900.2
Ata_AET6Gv20635900.2
Ata_AET6Gv20395800.3
Ata_AET5Gv20562700.1
Ata_AET5Gv20499900.1
Ata_AET5Gv20405200.1
Ata_AET5Gv20352100.2
Ata_AET5Gv20090500.4
Ata_AET4Gv20602000.3
Ata_AET4Gv20497800.2
Ata_AET4Gv20465800.3
Ata_AET4Gv20454800.1
Ata_AET4Gv20153900.2
−1000
0
1000
2000
3000
4000
5000

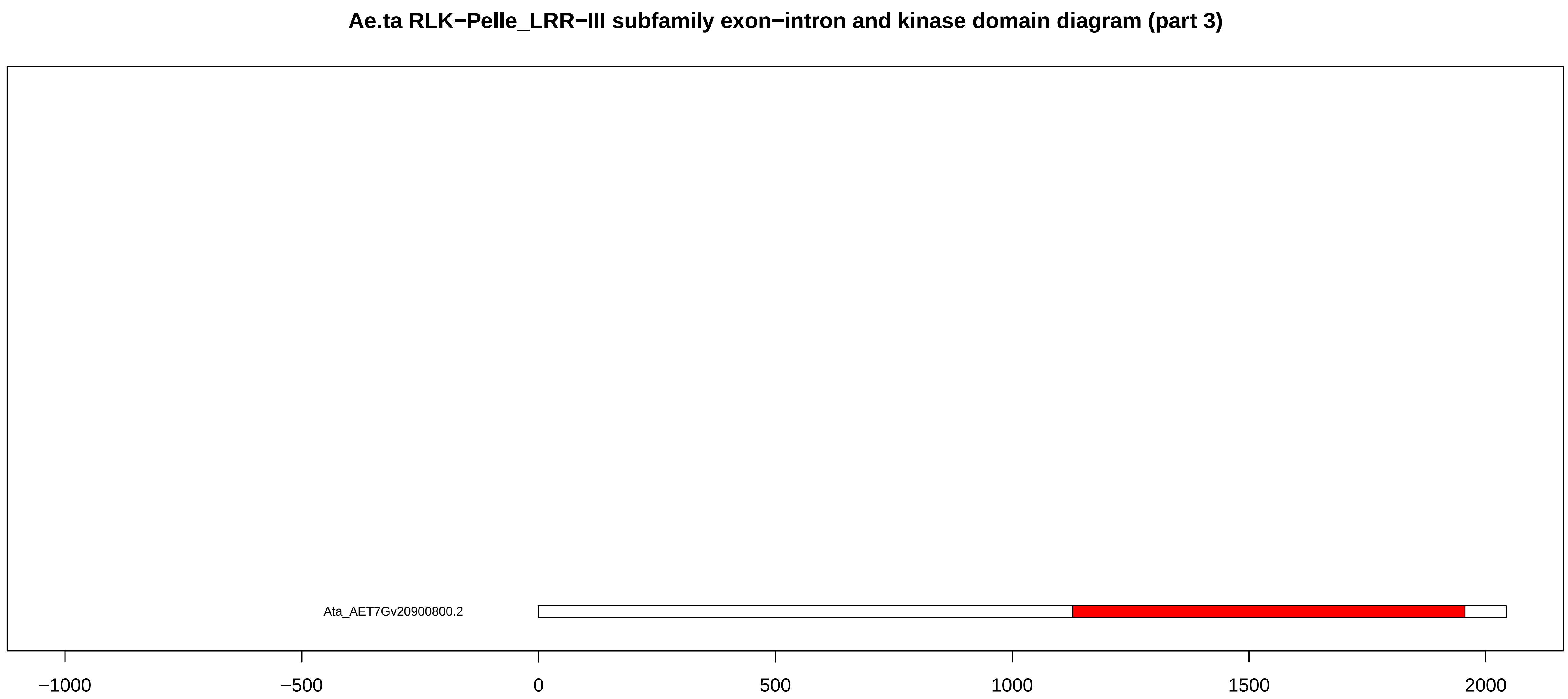
Ae.ta RLK−Pelle_LRR−III subfamily exon−intron and kinase domain diagram (part 3)
Ata_AET7Gv20900800.2
−1000
−500
0
500
1000
1500
2000

Ae.ta RLK−Pelle_LRR−II subfamily exon−intron and kinase domain diagram (all)

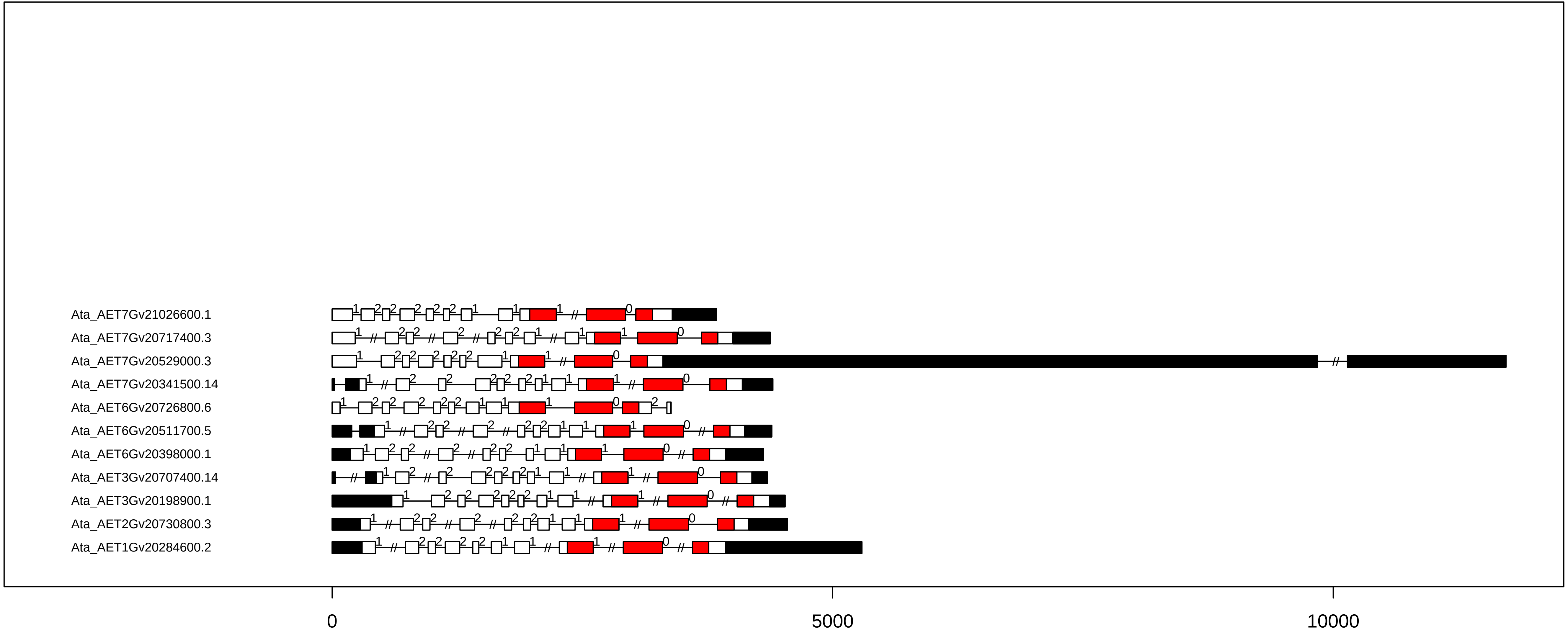

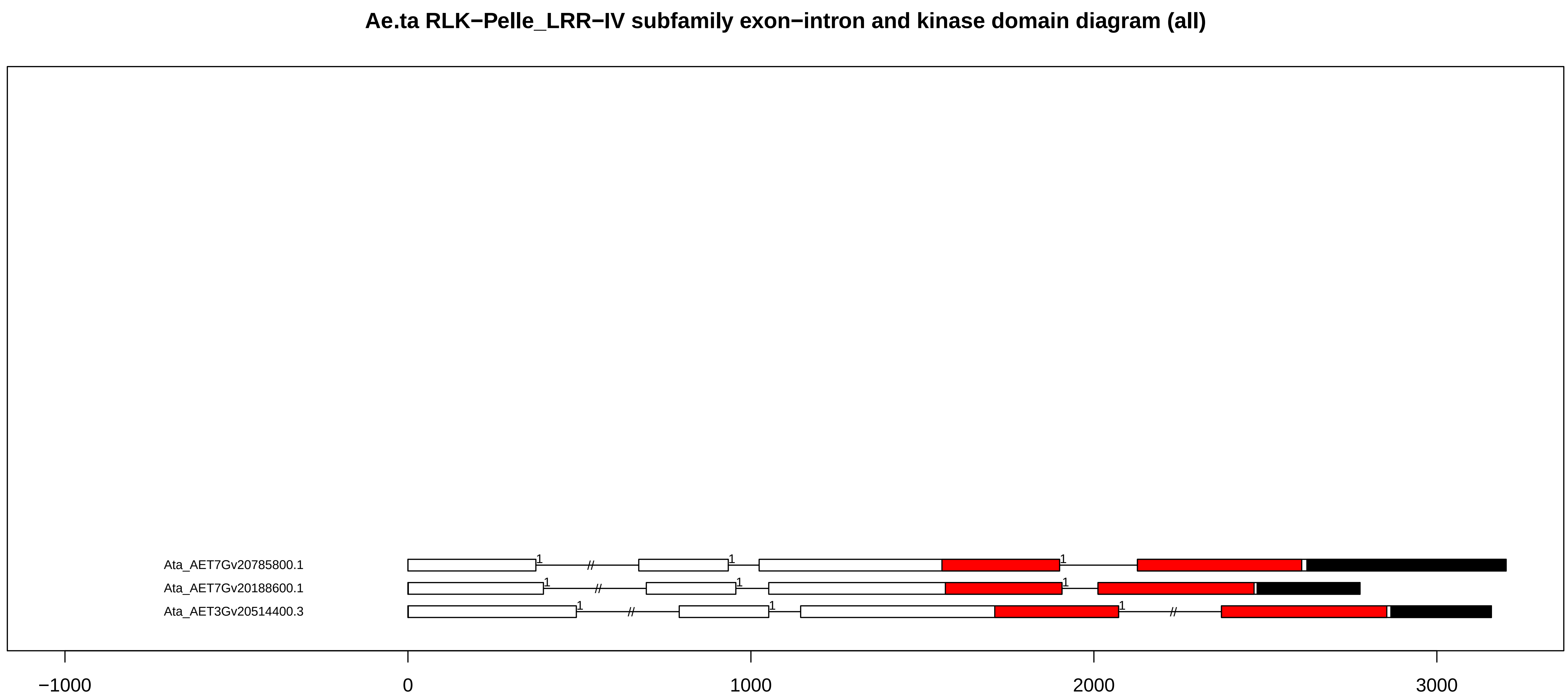

Ae.ta RLK−Pelle_LRR−IV subfamily exon−intron and kinase domain diagram (all)
Ata_AET7Gv20785800.1
Ata_AET7Gv20188600.1
Ata_AET3Gv20514400.3
−1000
0
1000
2000
3000

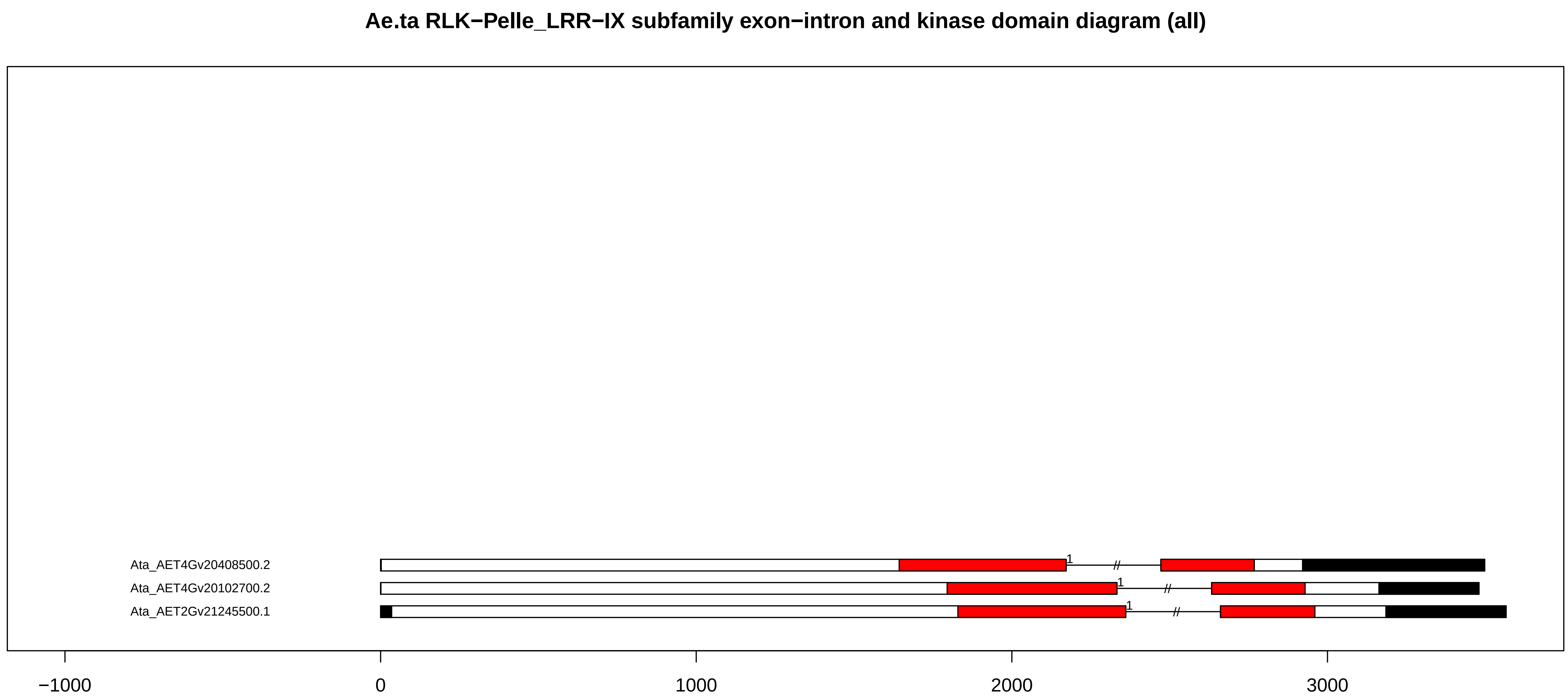

Ae.ta RLK−Pelle_LRR−IX subfamily exon−intron and kinase domain diagram (all)
Ata_AET4Gv20408500.2
Ata_AET4Gv20102700.2
Ata_AET2Gv21245500.1
1
1
1
−1000
0
1000
2000
3000

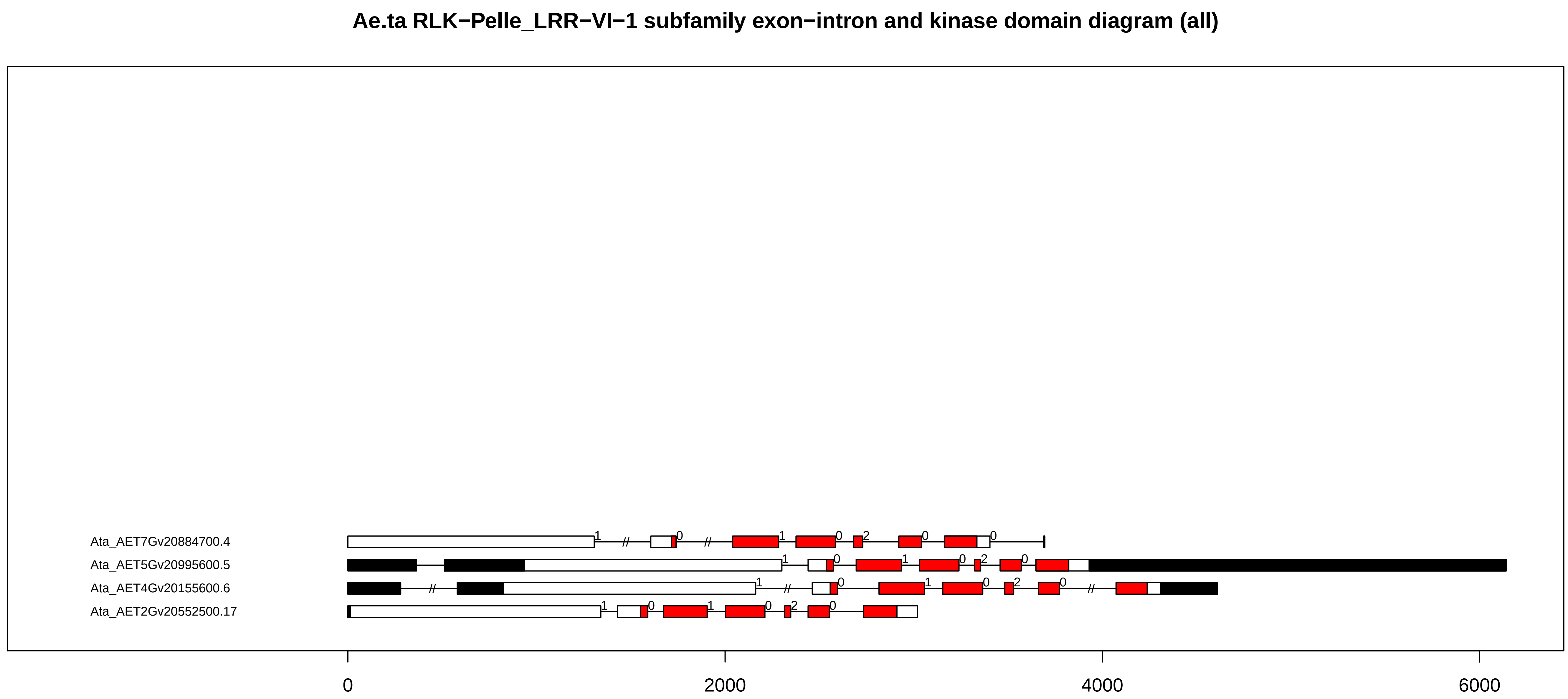

Ae.ta RLK−Pelle_LRR−VI−1 subfamily exon−intron and kinase domain diagram (all)
Ata_AET7Gv20884700.4
Ata_AET5Gv20995600.5
Ata_AET4Gv20155600.6
Ata_AET2Gv20552500.17
0
2000
4000
6000

Ae.ta RLK−Pelle_LRR−VI−2 subfamily exon−intron and kinase domain diagram (all)

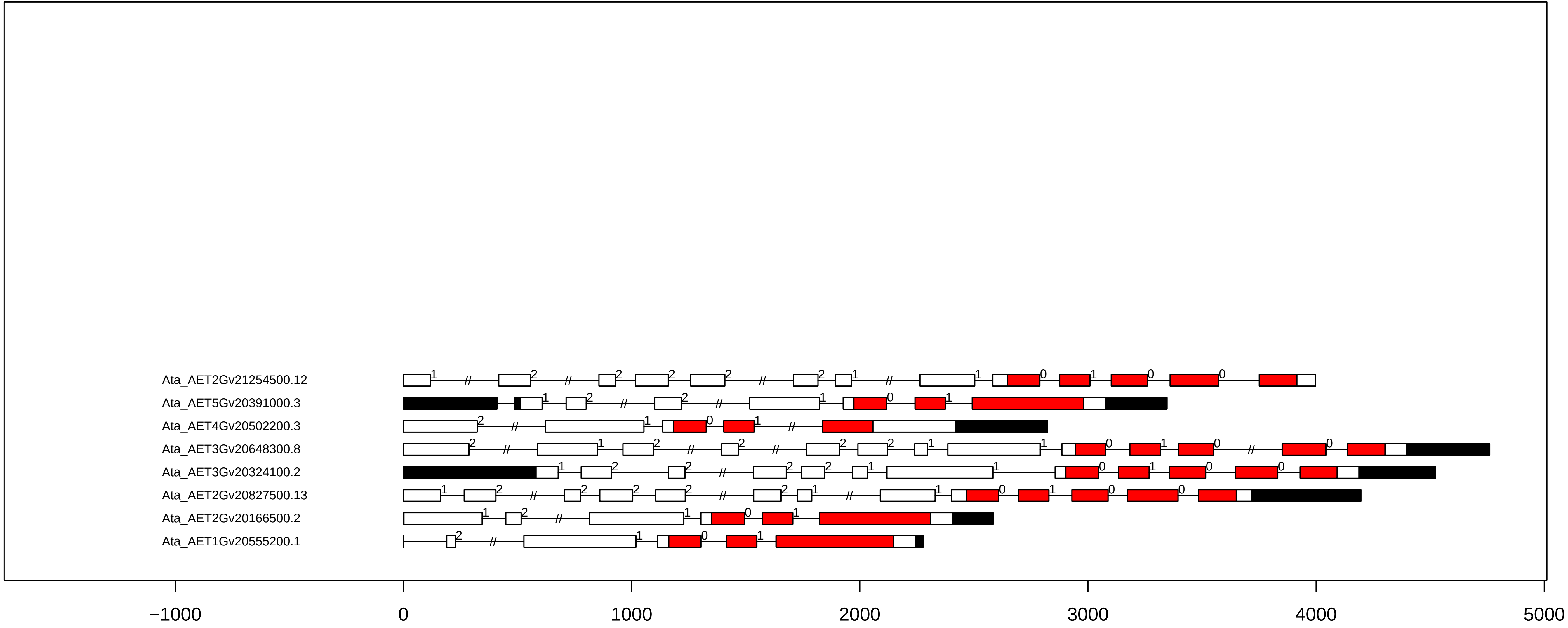

Ae.ta RLK−Pelle_LRR−VII−1 subfamily exon−intron and kinase domain diagram (all)

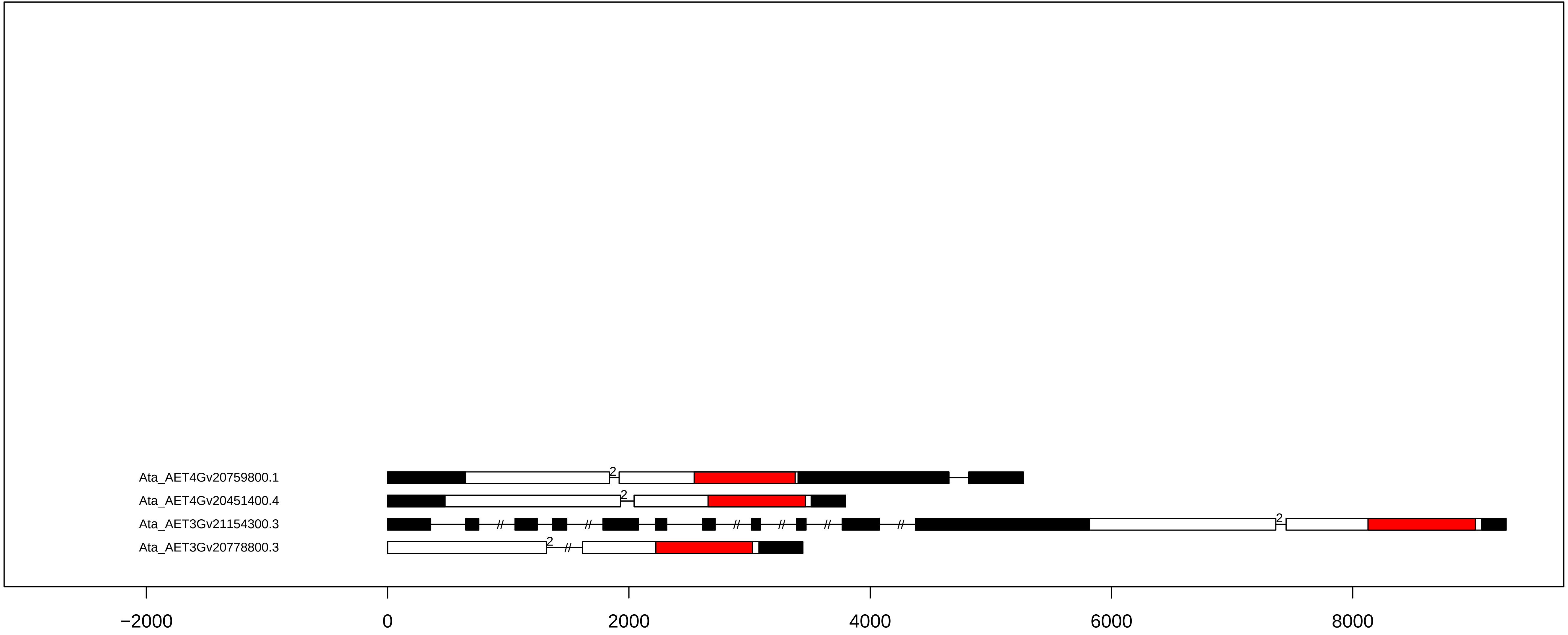

Ae.ta RLK−Pelle_LRR−VII−2 subfamily exon−intron and kinase domain diagram (all)

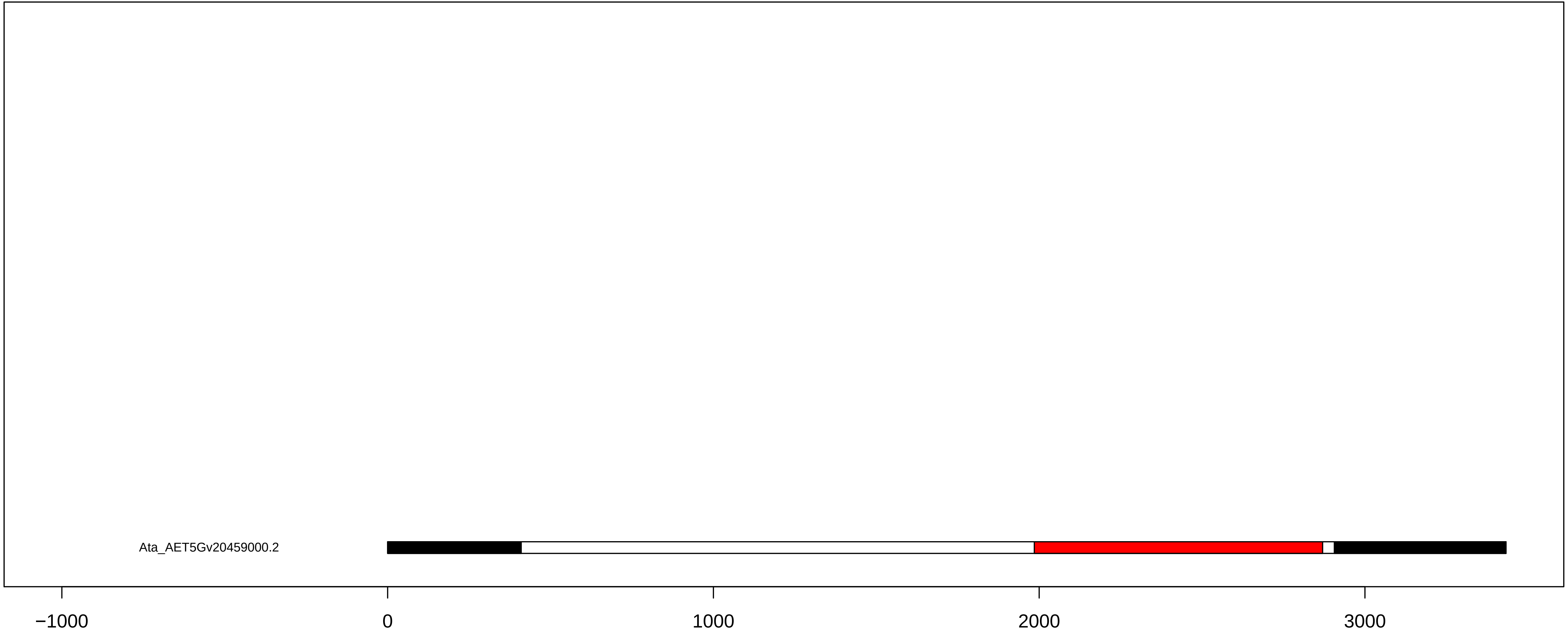

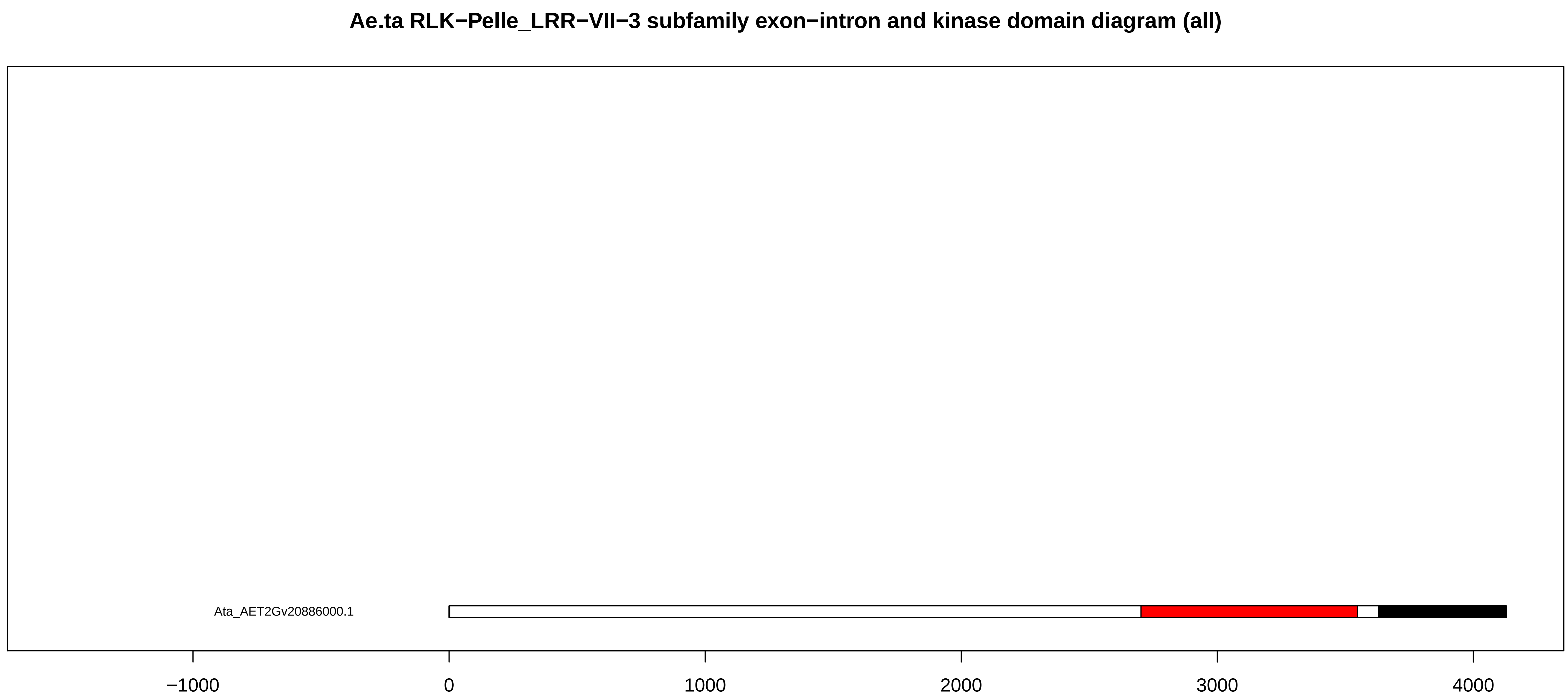
Ae.ta RLK−Pelle_LRR−VII−3 subfamily exon−intron and kinase domain diagram (all)
Ata_AET2Gv20886000.1
−1000
0
1000
2000
3000
4000

Ae.ta RLK−Pelle_LRR−VIII−1 subfamily exon−intron and kinase domain diagram (all)

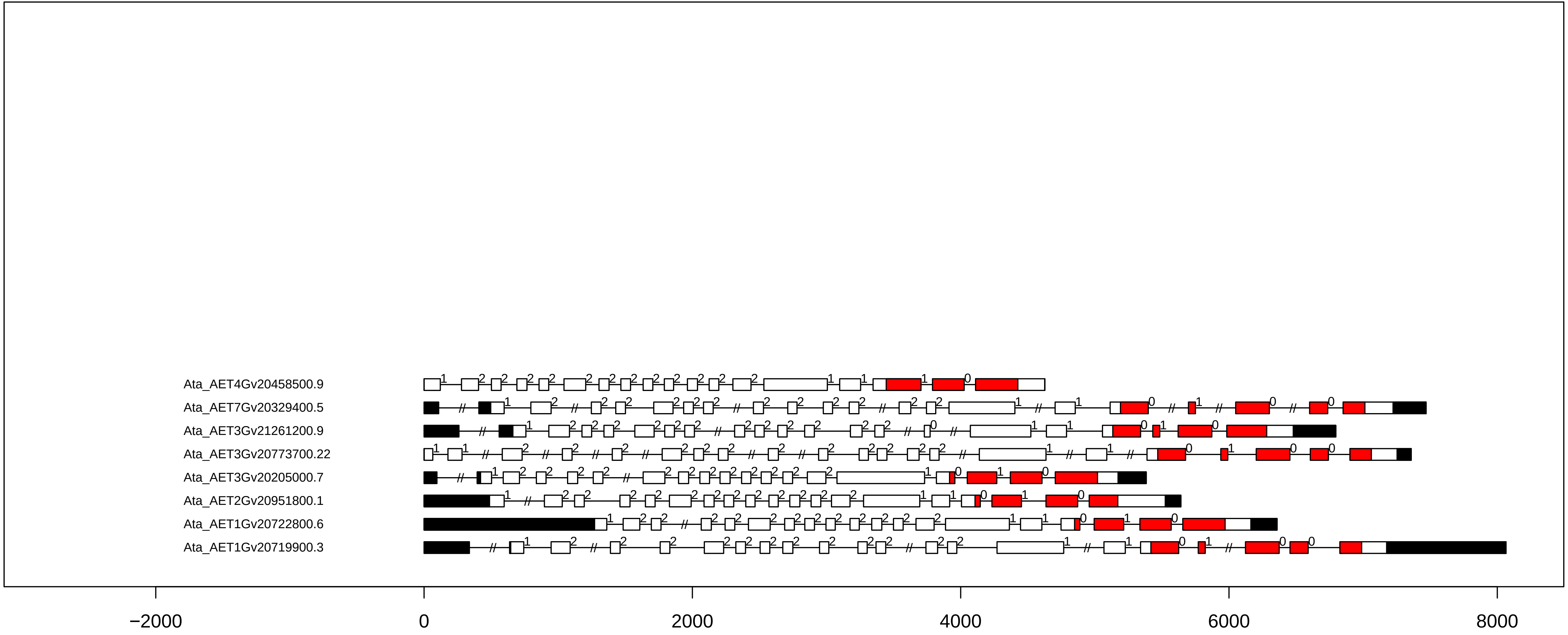

Ae.ta RLK−Pelle_LRR−V subfamily exon−intron and kinase domain diagram (all)

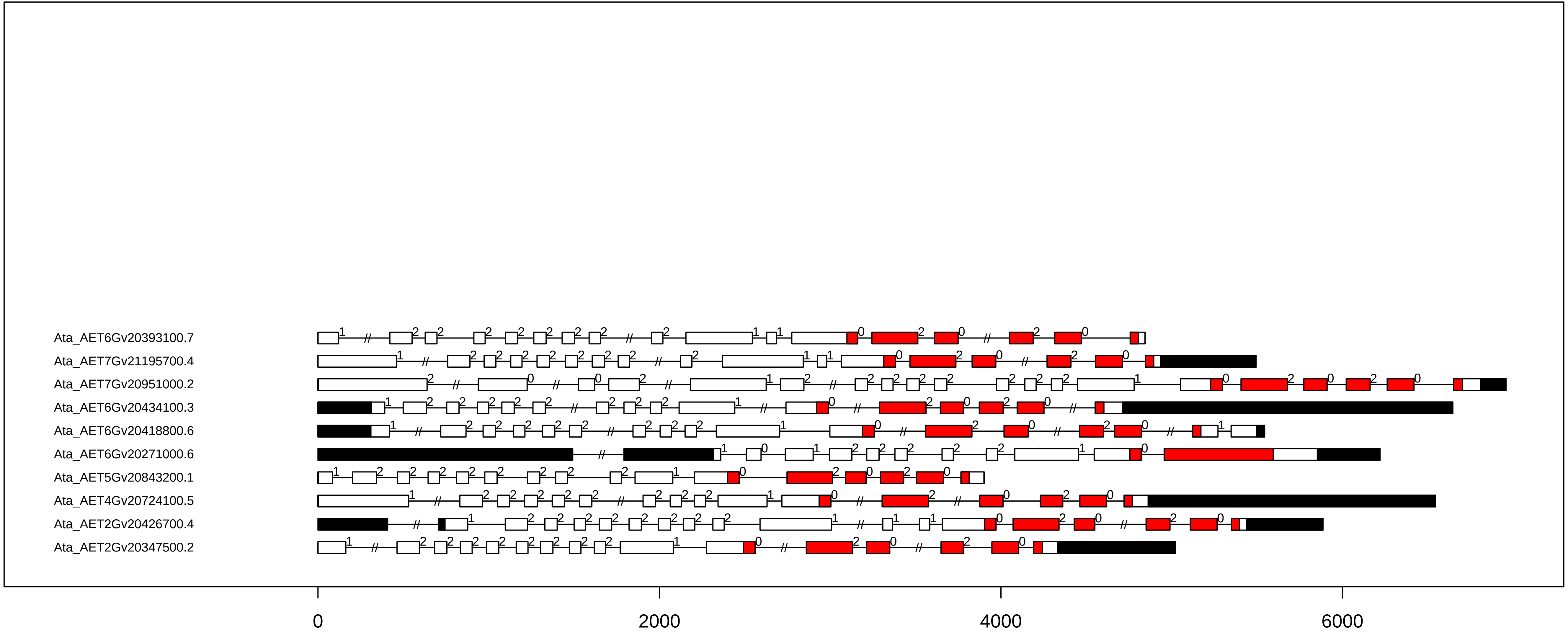

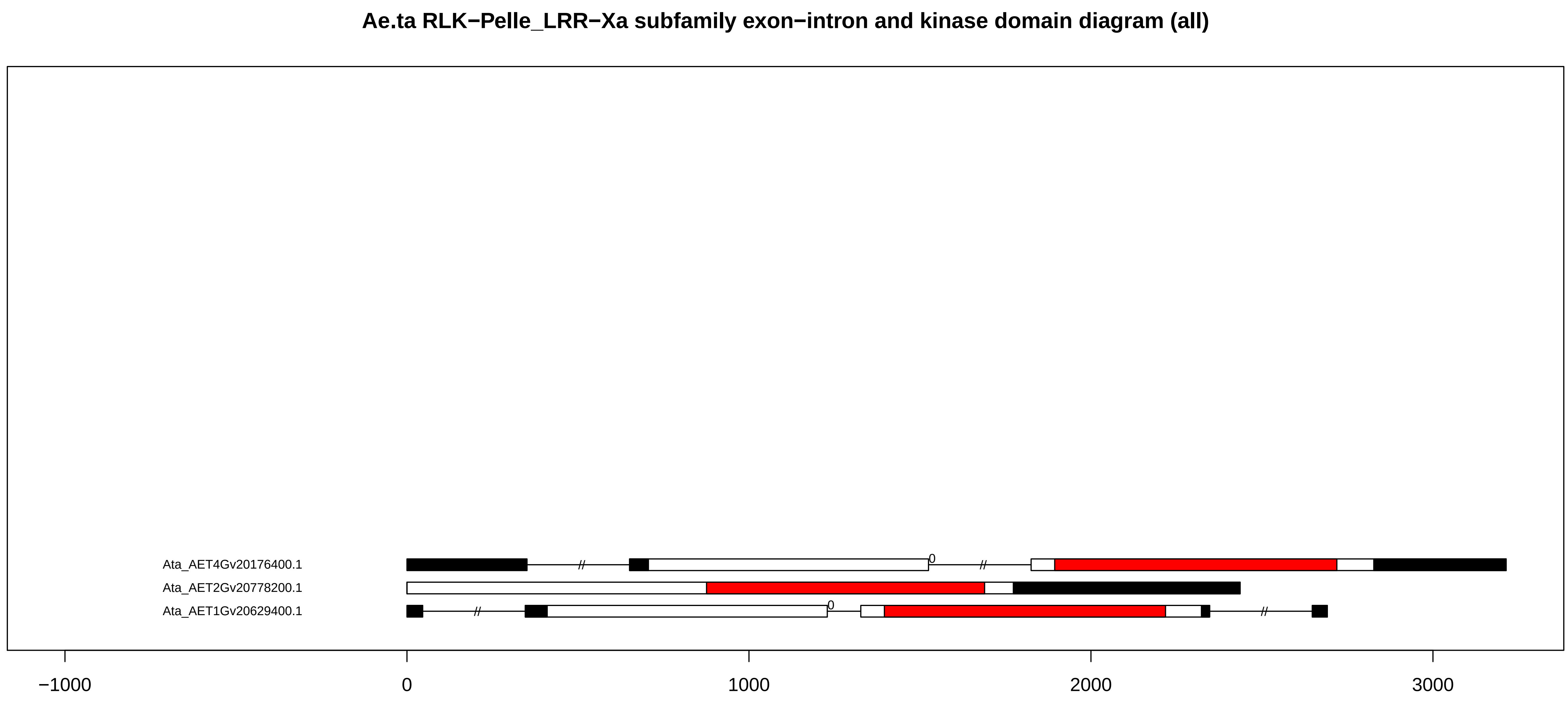
Ae.ta RLK−Pelle_LRR−Xa subfamily exon−intron and kinase domain diagram (all)
Ata_AET4Gv20176400.1
Ata_AET2Gv20778200.1
Ata_AET1Gv20629400.1
0
0
−1000
0
1000
2000
3000

Ae.ta RLK−Pelle_LRR−Xb−1 subfamily exon−intron and kinase domain diagram (part 1)

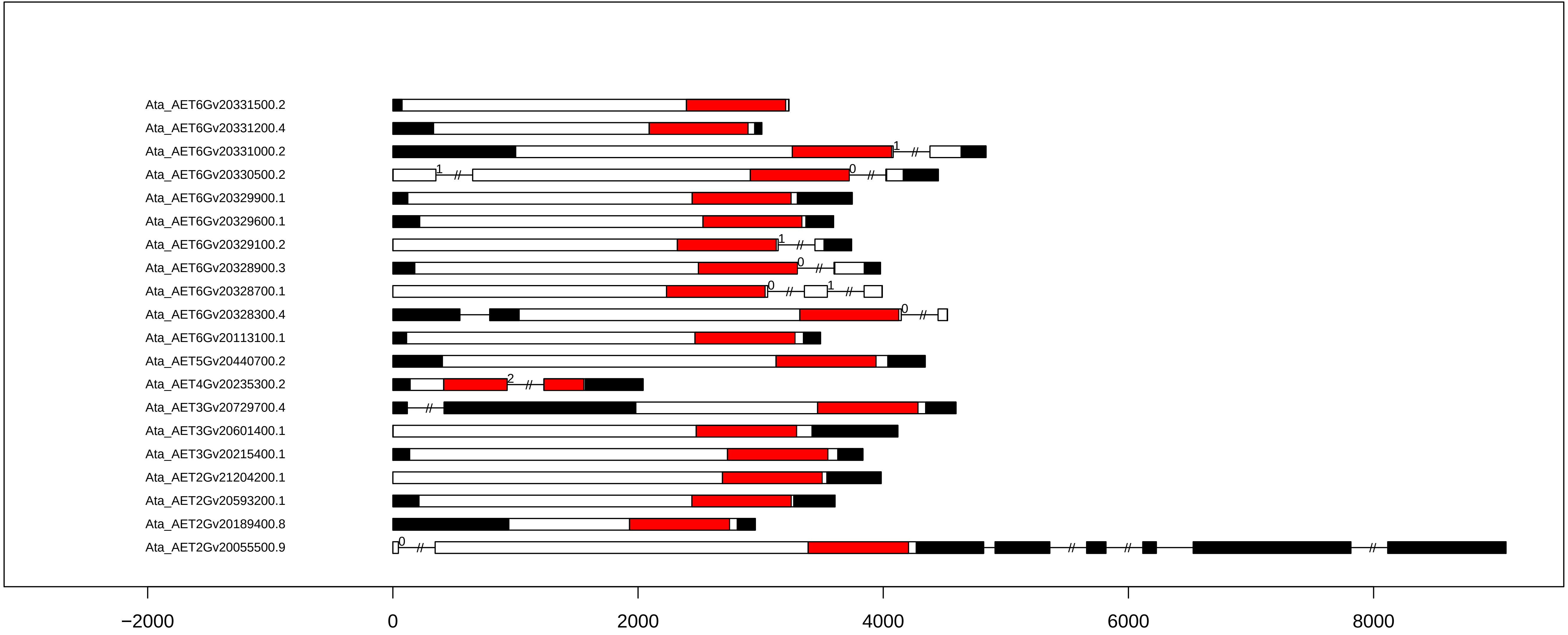

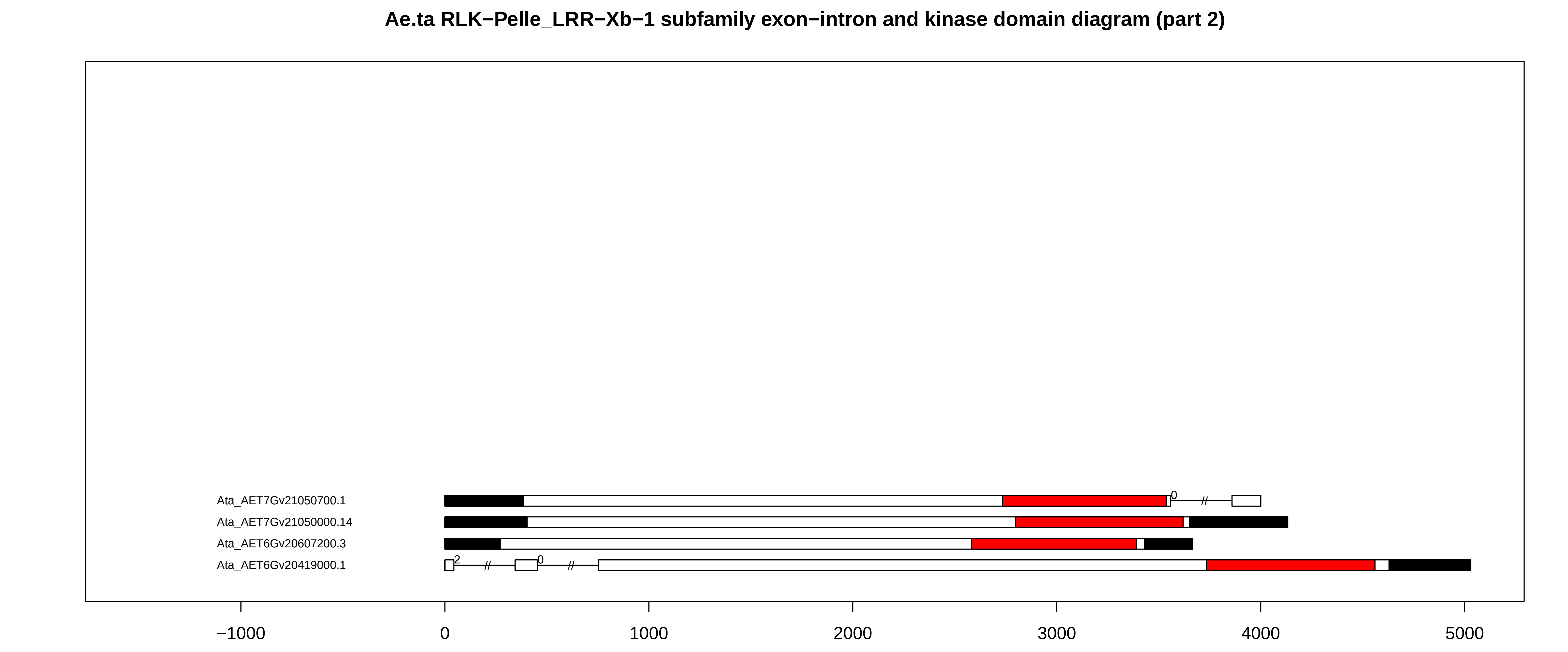
Ae.ta RLK−Pelle_LRR−Xb−1 subfamily exon−intron and kinase domain diagram (part 2)
Ata_AET7Gv21050700.1
Ata_AET7Gv21050000.14
Ata_AET6Gv20607200.3
Ata_AET6Gv20419000.1
0
2
0
−1000
0
1000
2000
3000
4000
5000

Ae.ta RLK−Pelle_LRR−Xb−2 subfamily exon−intron and kinase domain diagram (all)

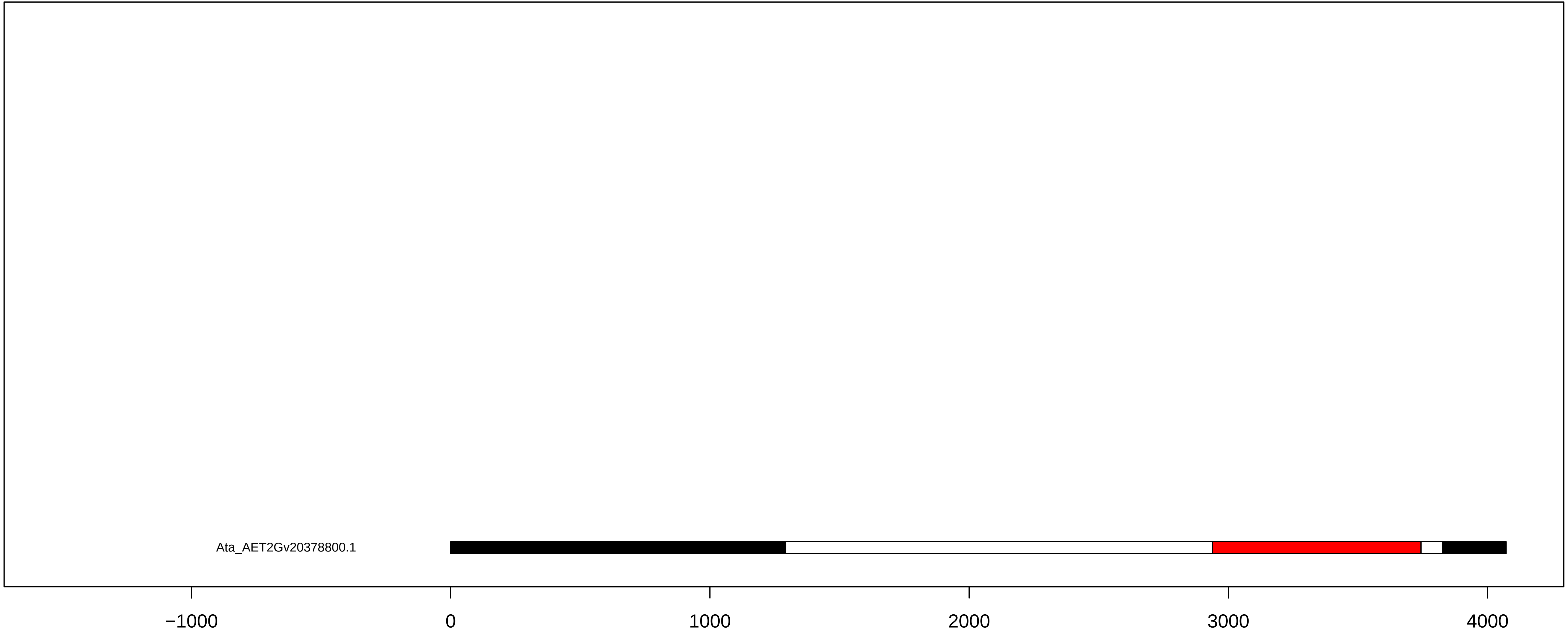

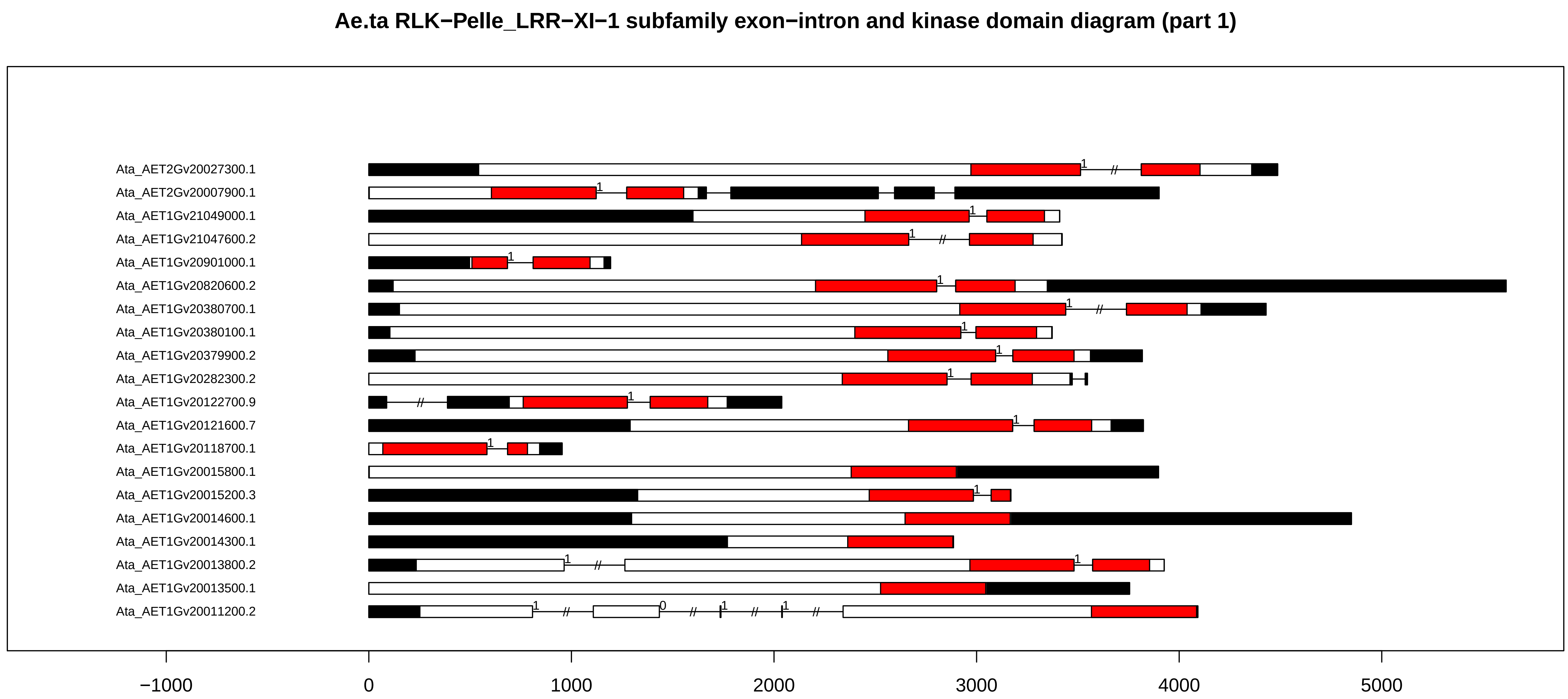

Ae.ta RLK−Pelle_LRR−XI−1 subfamily exon−intron and kinase domain diagram (part 1)
Ata_AET2Gv20027300.1
Ata_AET2Gv20007900.1
Ata_AET1Gv21049000.1
Ata_AET1Gv21047600.2
Ata_AET1Gv20901000.1
Ata_AET1Gv20820600.2
Ata_AET1Gv20380700.1
Ata_AET1Gv20380100.1
Ata_AET1Gv20379900.2
Ata_AET1Gv20282300.2
Ata_AET1Gv20122700.9
Ata_AET1Gv20121600.7
Ata_AET1Gv20118700.1
Ata_AET1Gv20015800.1
Ata_AET1Gv20015200.3
Ata_AET1Gv20014600.1
Ata_AET1Gv20014300.1
Ata_AET1Gv20013800.2
Ata_AET1Gv20013500.1
Ata_AET1Gv20011200.2
−1000
0
1000
2000
3000
4000
5000

Ae.ta RLK−Pelle_LRR−XI−1 subfamily exon−intron and kinase domain diagram (part 2)

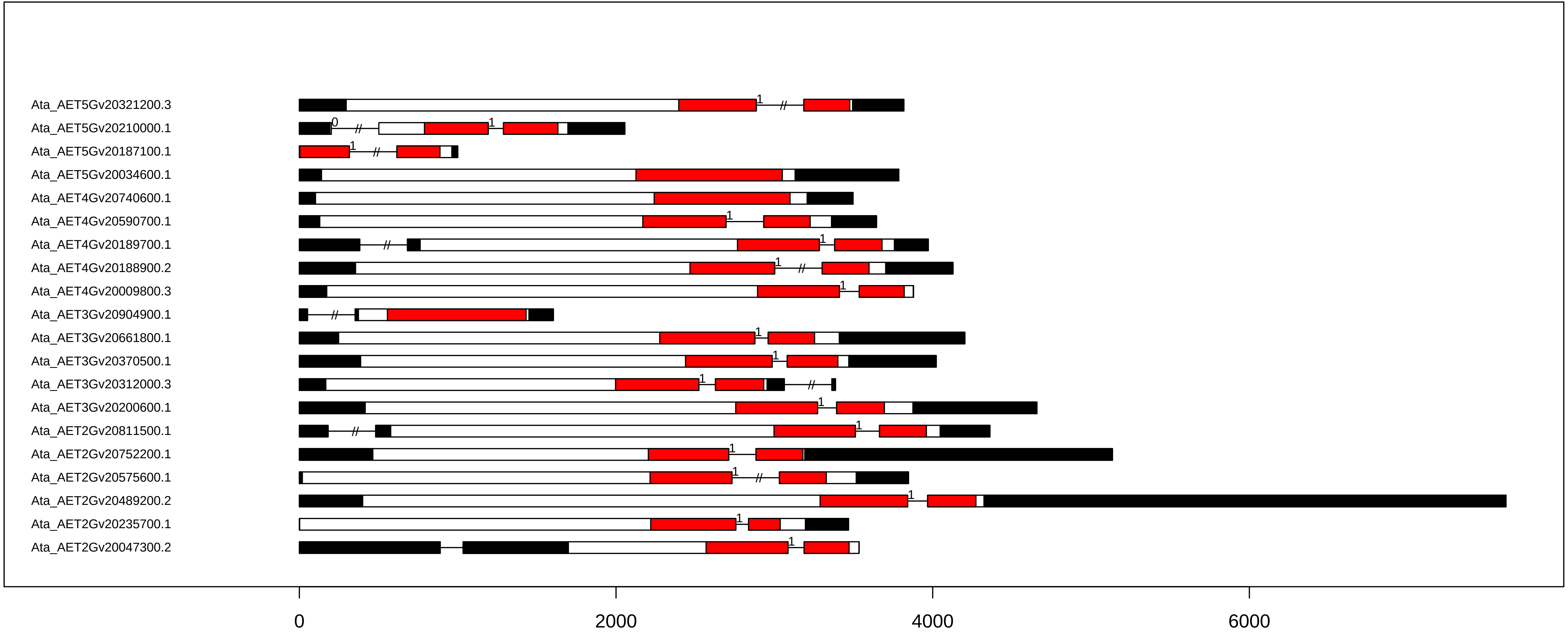

Ae.ta RLK−Pelle_LRR−XI−1 subfamily exon−intron and kinase domain diagram (part 3)

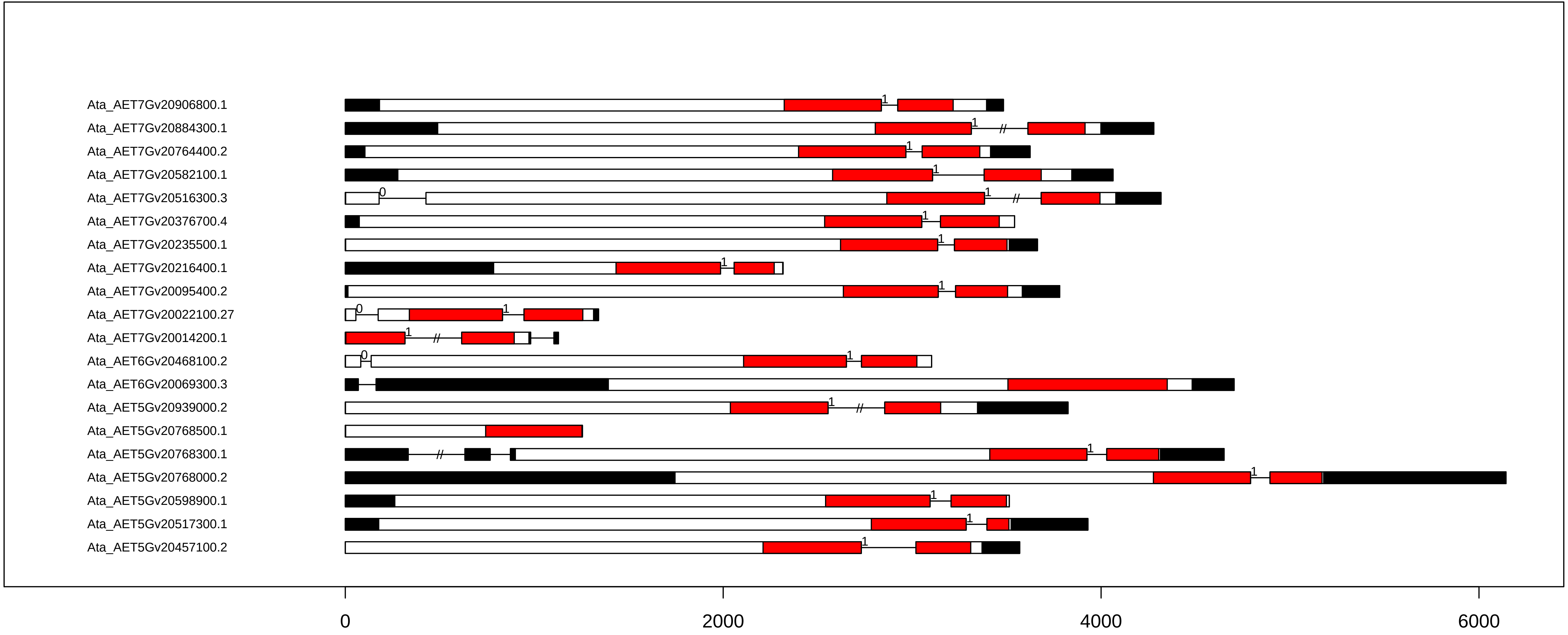

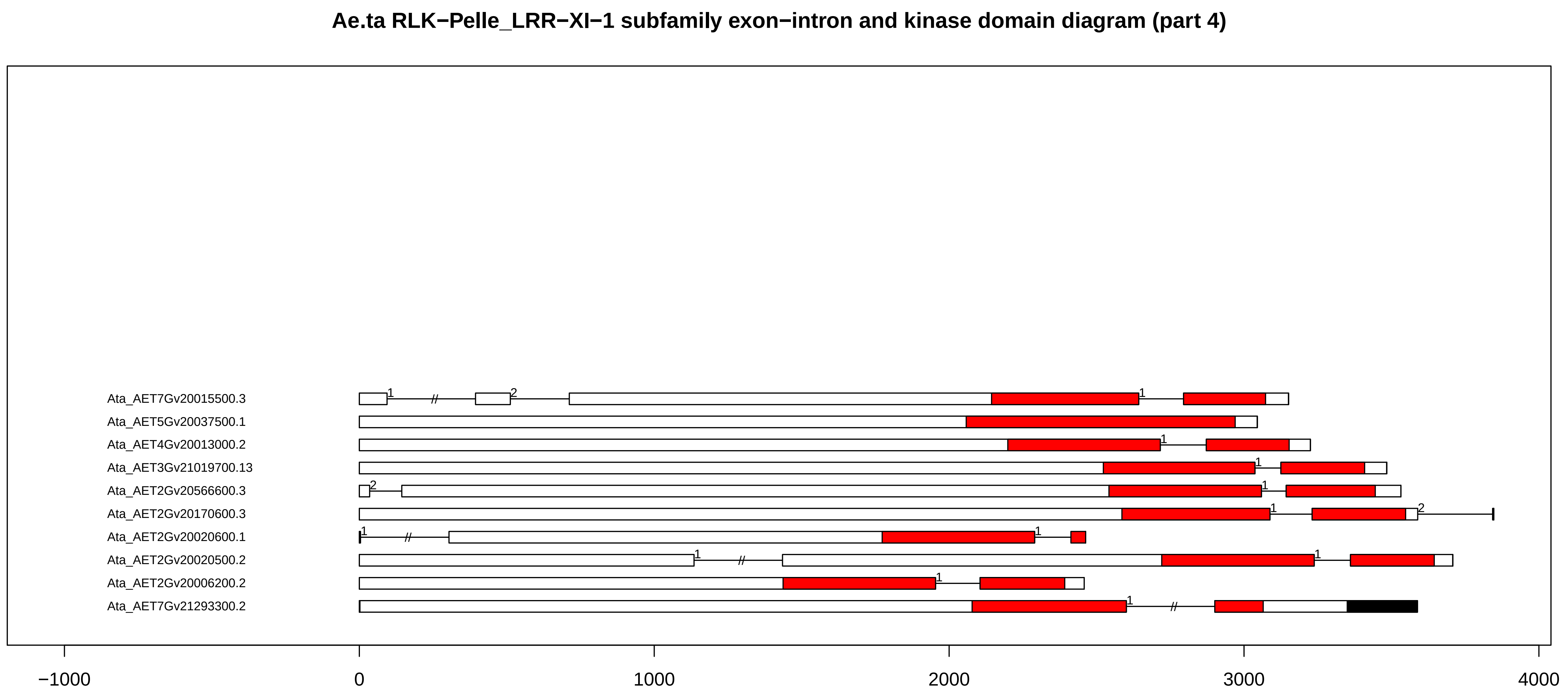

Ae.ta RLK−Pelle_LRR−XI−1 subfamily exon−intron and kinase domain diagram (part 4)
Ata_AET7Gv20015500.3
Ata_AET5Gv20037500.1
Ata_AET4Gv20013000.2
Ata_AET3Gv21019700.13
Ata_AET2Gv20566600.3
Ata_AET2Gv20170600.3
Ata_AET2Gv20020600.1
Ata_AET2Gv20020500.2
Ata_AET2Gv20006200.2
Ata_AET7Gv21293300.2
−1000
0
1000
2000
3000
4000

Ae.ta RLK−Pelle_LRR−XI−2 subfamily exon−intron and kinase domain diagram (all)

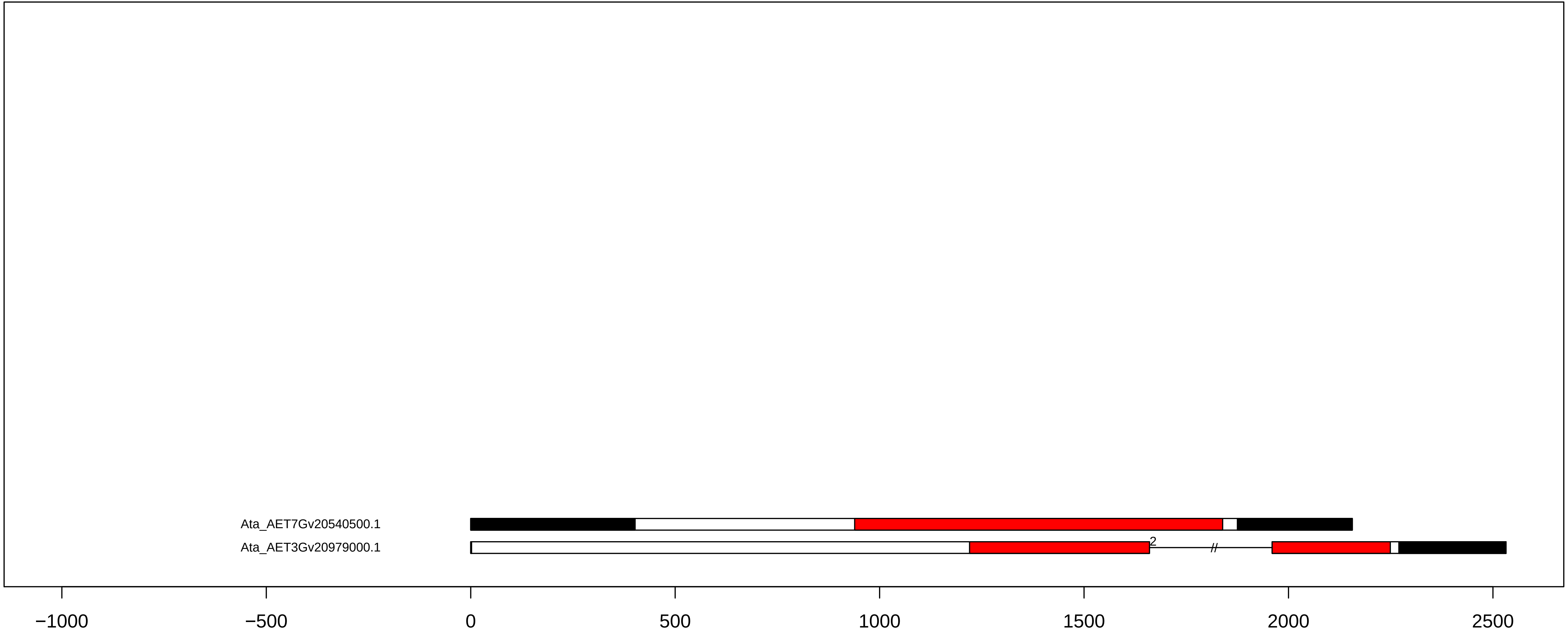

Ae.ta RLK−Pelle_LRR−XII−1 subfamily exon−intron and kinase domain diagram (part 1)

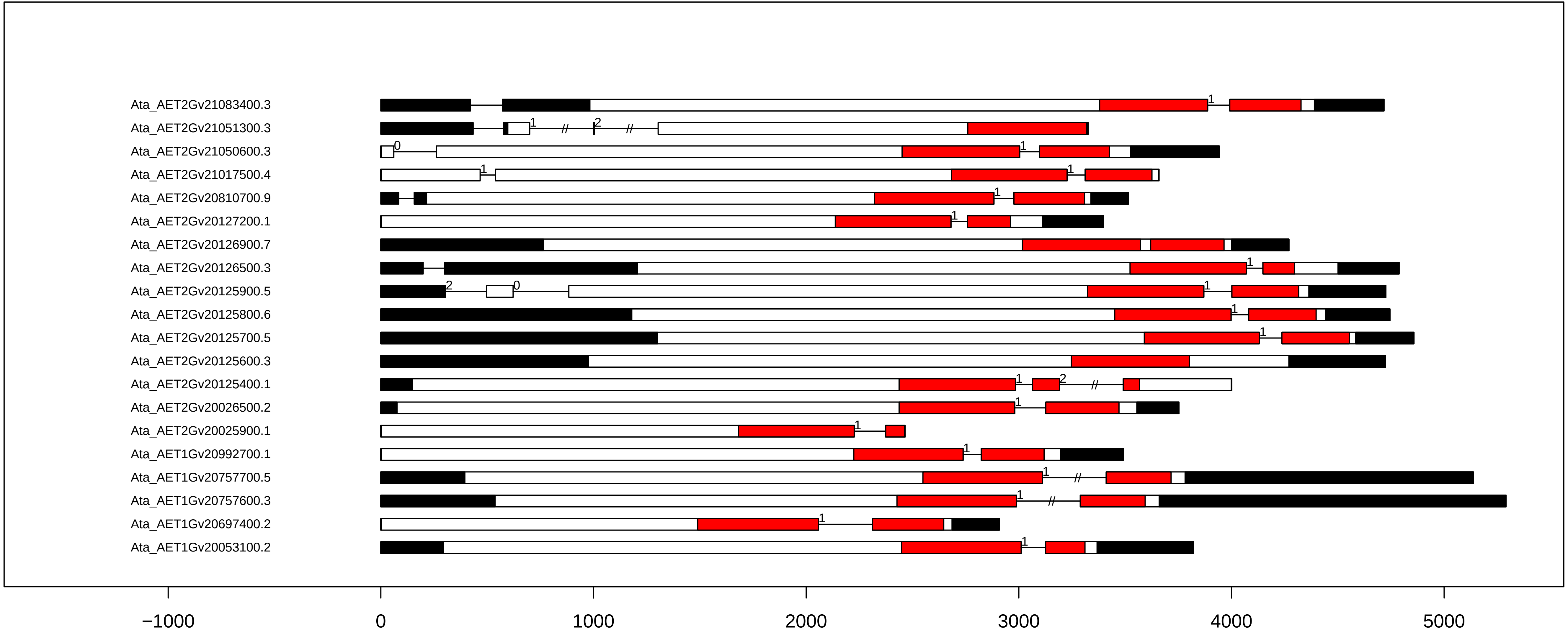

Ae.ta RLK−Pelle_LRR−XII−1 subfamily exon−intron and kinase domain diagram (part 2)

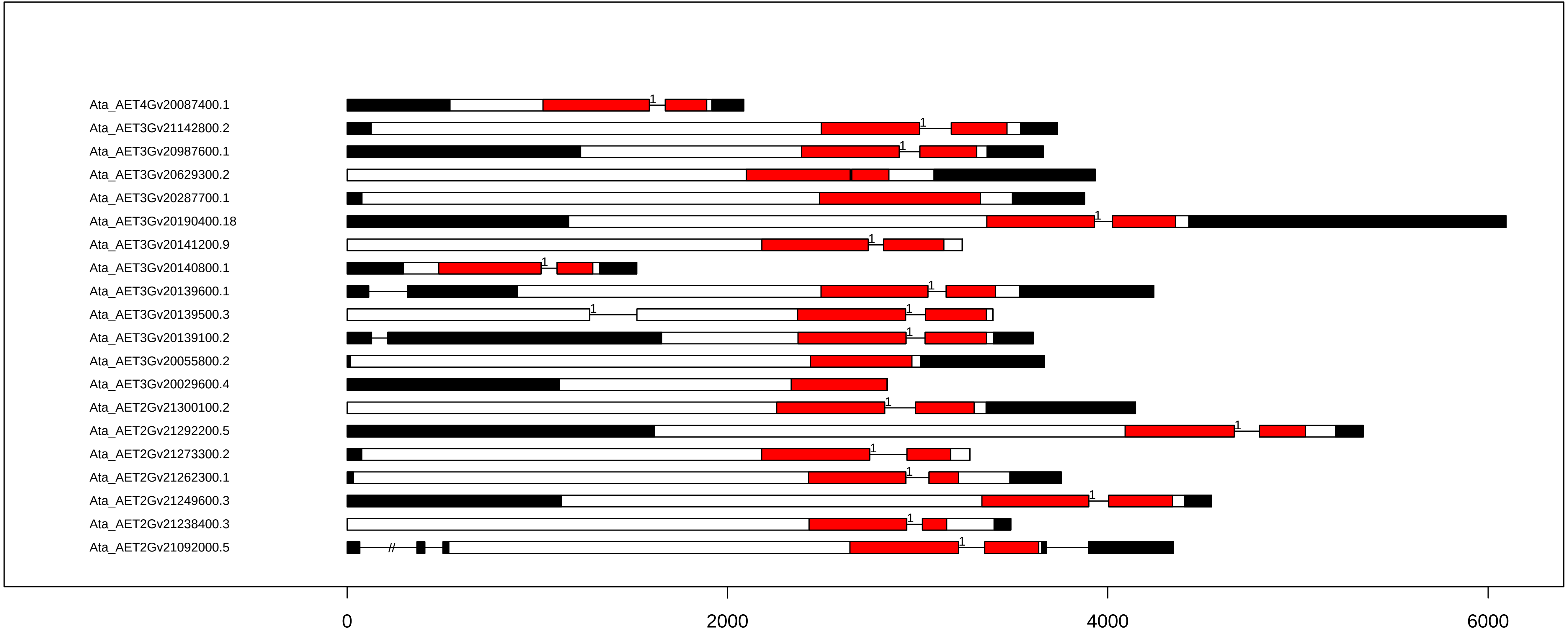

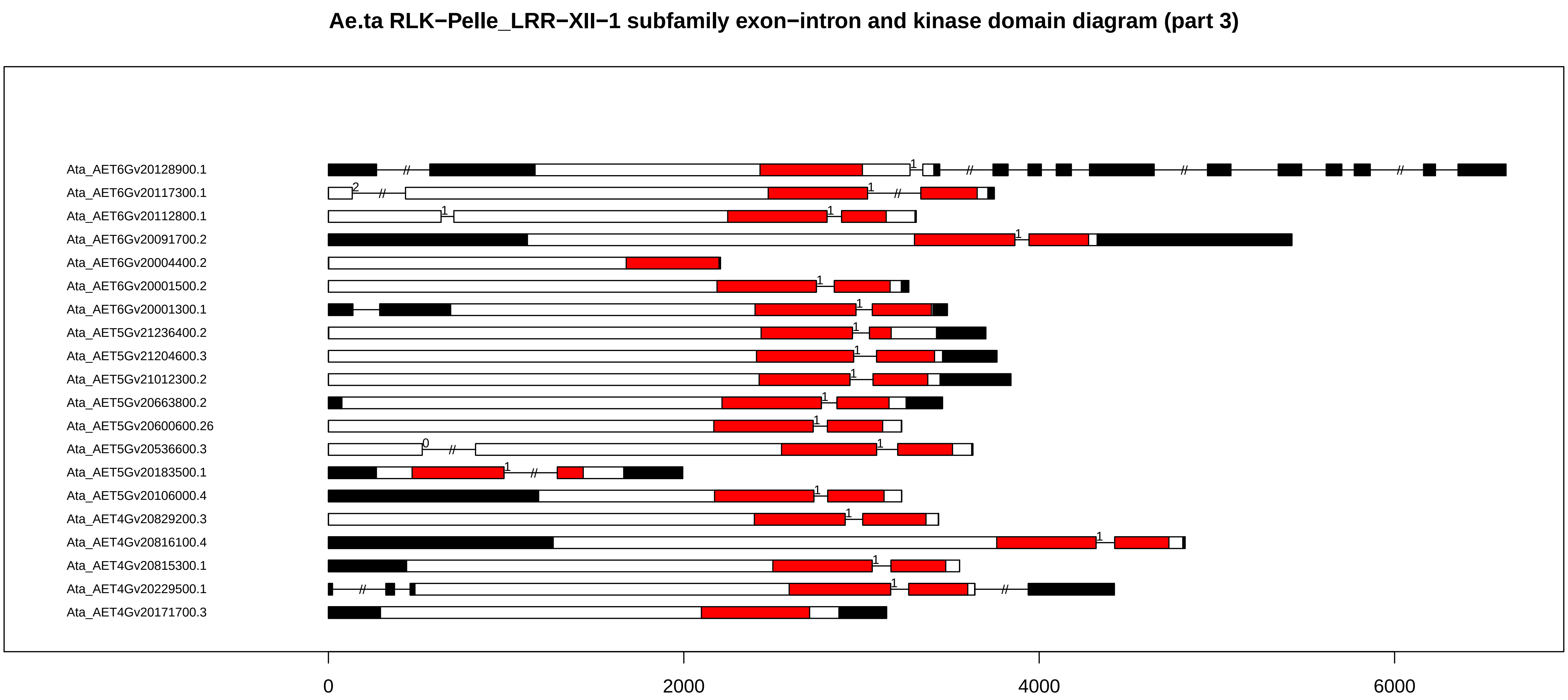

Ae.ta RLK−Pelle_LRR−XII−1 subfamily exon−intron and kinase domain diagram (part 3)
Ata_AET6Gv20128900.1
Ata_AET6Gv20117300.1
Ata_AET6Gv20112800.1
Ata_AET6Gv20091700.2
Ata_AET6Gv20004400.2
Ata_AET6Gv20001500.2
Ata_AET6Gv20001300.1
Ata_AET5Gv21236400.2
Ata_AET5Gv21204600.3
Ata_AET5Gv21012300.2
Ata_AET5Gv20663800.2
Ata_AET5Gv20600600.26
Ata_AET5Gv20536600.3
Ata_AET5Gv20183500.1
Ata_AET5Gv20106000.4
Ata_AET4Gv20829200.3
Ata_AET4Gv20816100.4
Ata_AET4Gv20815300.1
Ata_AET4Gv20229500.1
Ata_AET4Gv20171700.3
0
2000
4000
6000

Ae.ta RLK−Pelle_LRR−XII−1 subfamily exon−intron and kinase domain diagram (part 4)

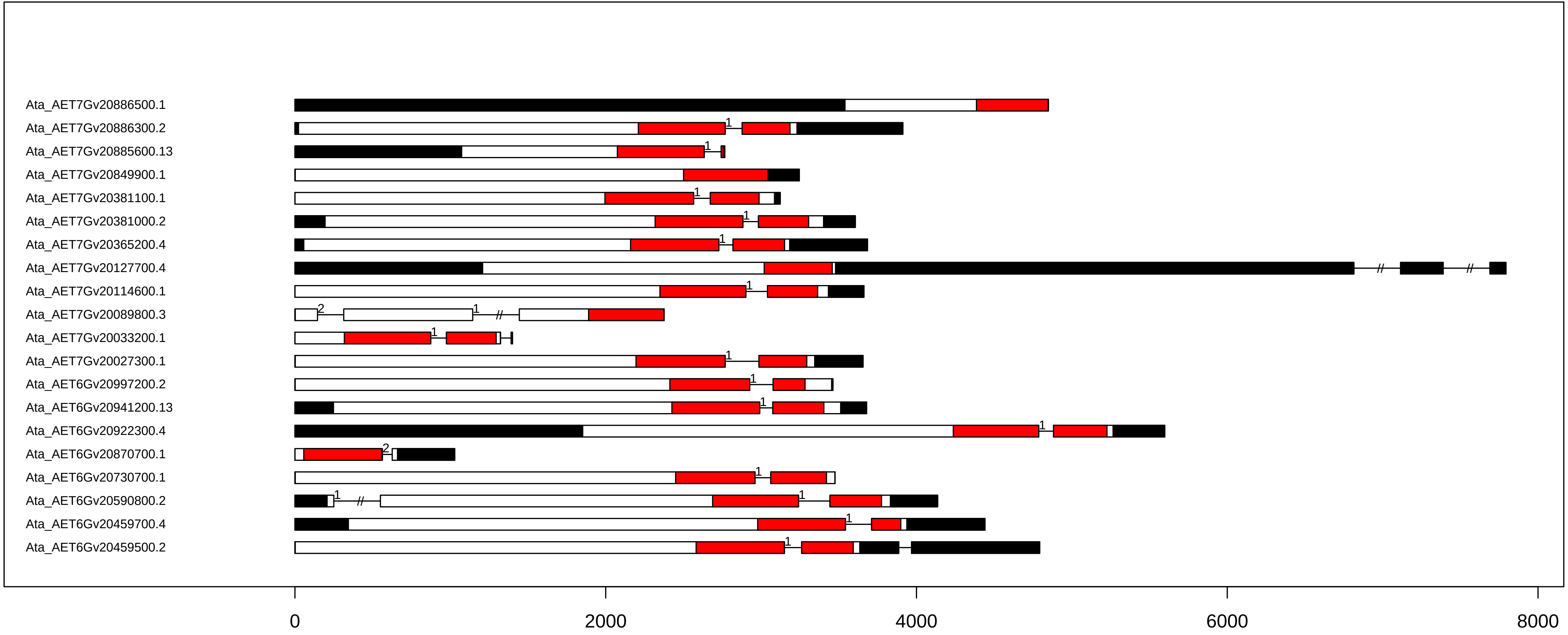

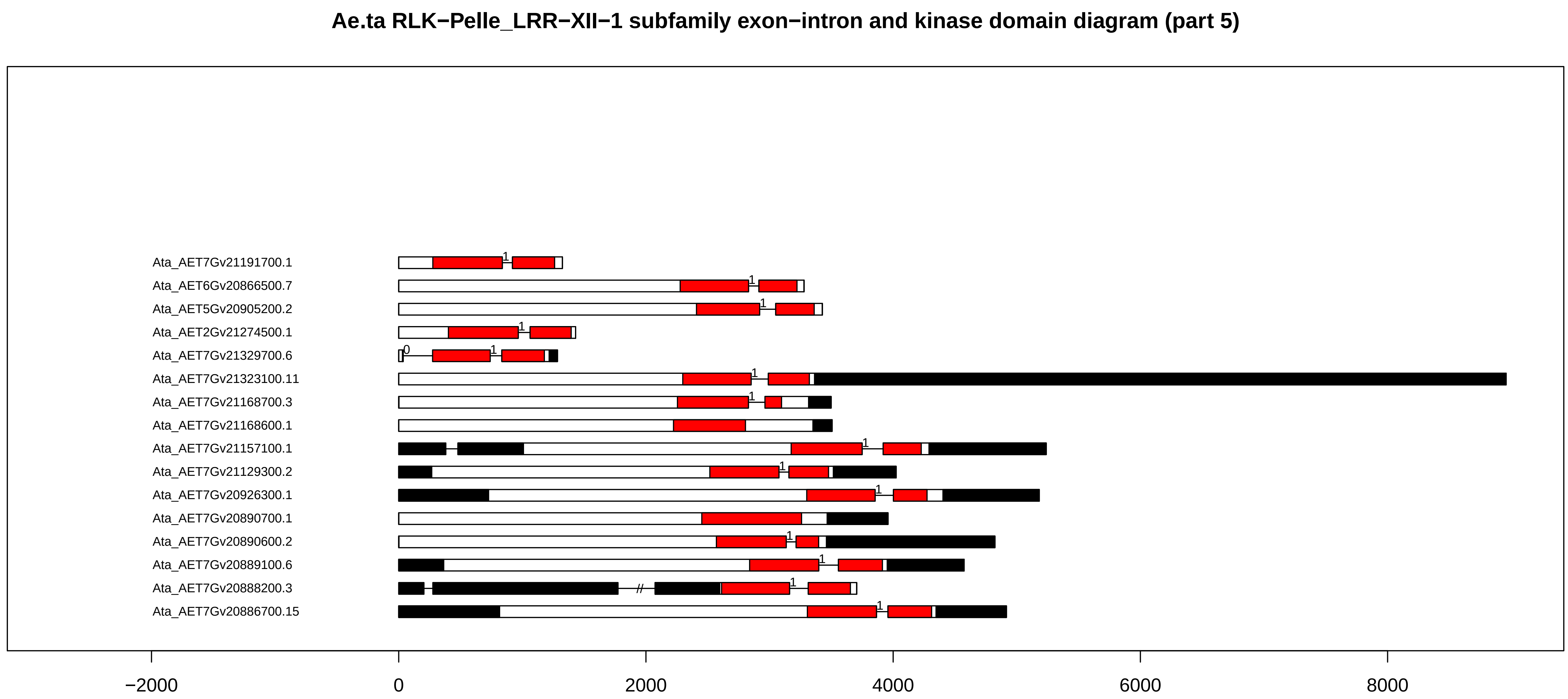

Ae.ta RLK−Pelle_LRR−XII−1 subfamily exon−intron and kinase domain diagram (part 5)
Ata_AET7Gv21191700.1
Ata_AET6Gv20866500.7
Ata_AET5Gv20905200.2
Ata_AET2Gv21274500.1
Ata_AET7Gv21329700.6
Ata_AET7Gv21323100.11
Ata_AET7Gv21168700.3
Ata_AET7Gv21168600.1
Ata_AET7Gv21157100.1
Ata_AET7Gv21129300.2
Ata_AET7Gv20926300.1
Ata_AET7Gv20890700.1
Ata_AET7Gv20890600.2
Ata_AET7Gv20889100.6
Ata_AET7Gv20888200.3
Ata_AET7Gv20886700.15
−2000
0
2000
4000
6000
8000

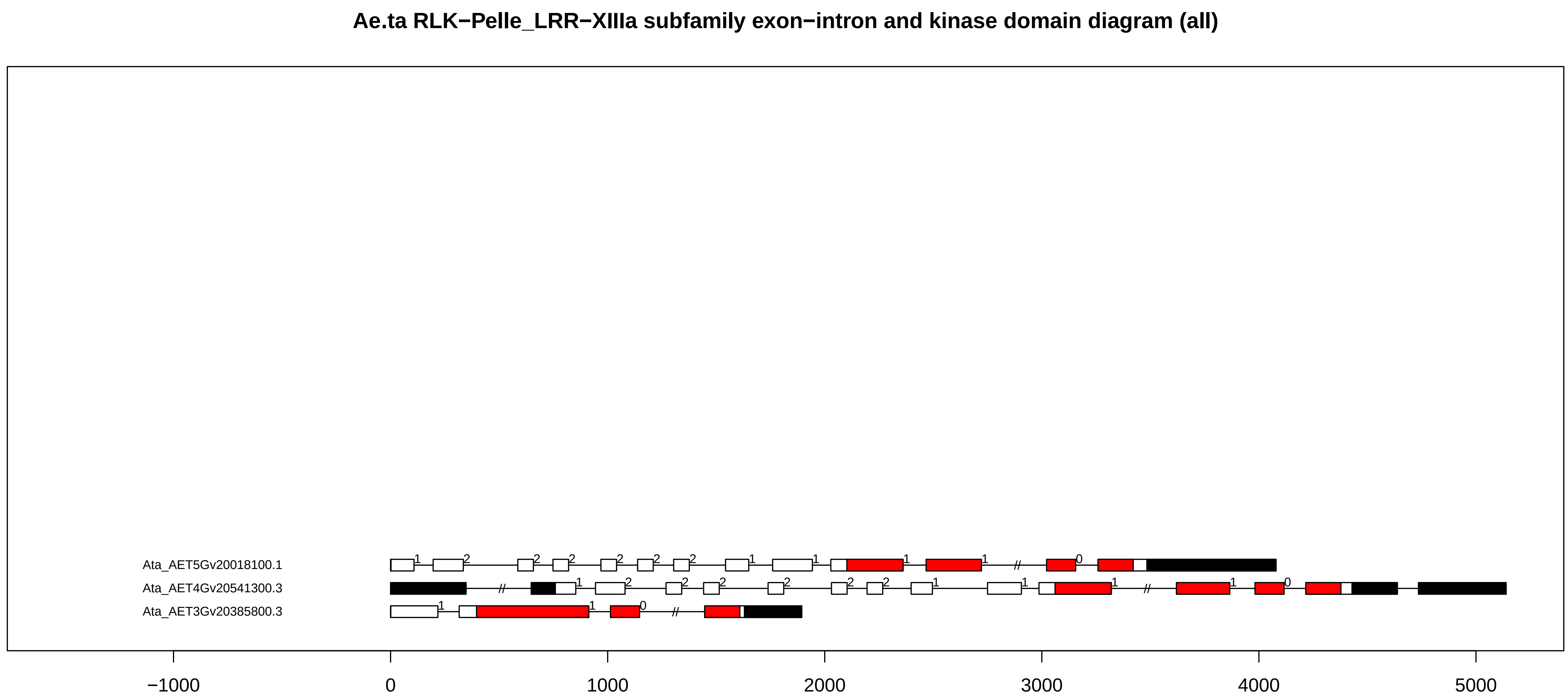

Ae.ta RLK−Pelle_LRR−XIIIa subfamily exon−intron and kinase domain diagram (all)
Ata_AET5Gv20018100.1
Ata_AET4Gv20541300.3
Ata_AET3Gv20385800.3
−1000
0
1000
2000
3000
4000
5000

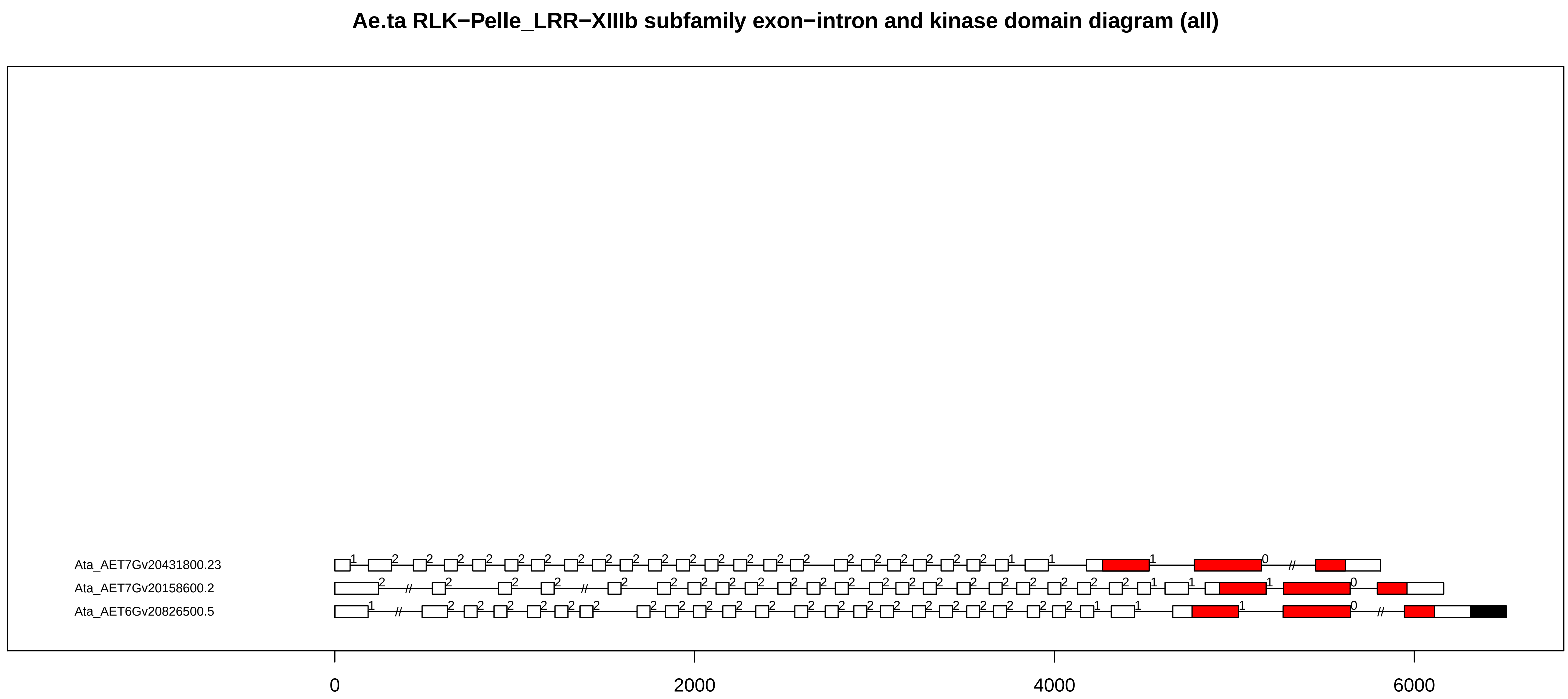

Ae.ta RLK−Pelle_LRR−XIIIb subfamily exon−intron and kinase domain diagram (all)
Ata_AET7Gv20431800.23
Ata_AET7Gv20158600.2
Ata_AET6Gv20826500.5
0
2000
4000
6000

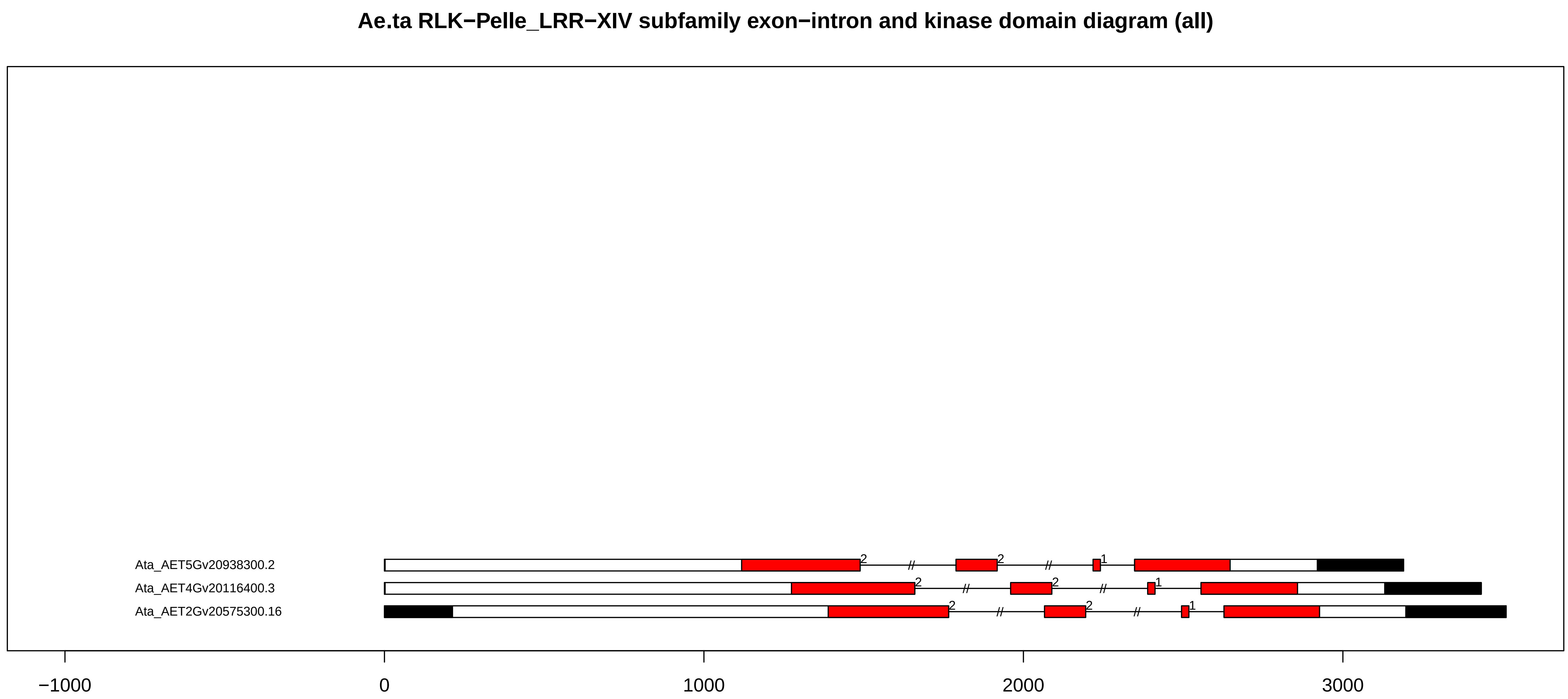
Ae.ta RLK−Pelle_LRR−XIV subfamily exon−intron and kinase domain diagram (all)
Ata_AET5Gv20938300.2
Ata_AET4Gv20116400.3
Ata_AET2Gv20575300.16
2
2
1
−1000
0
1000
2000
3000

Ae.ta RLK−Pelle_LRR−XV subfamily exon−intron and kinase domain diagram (all)

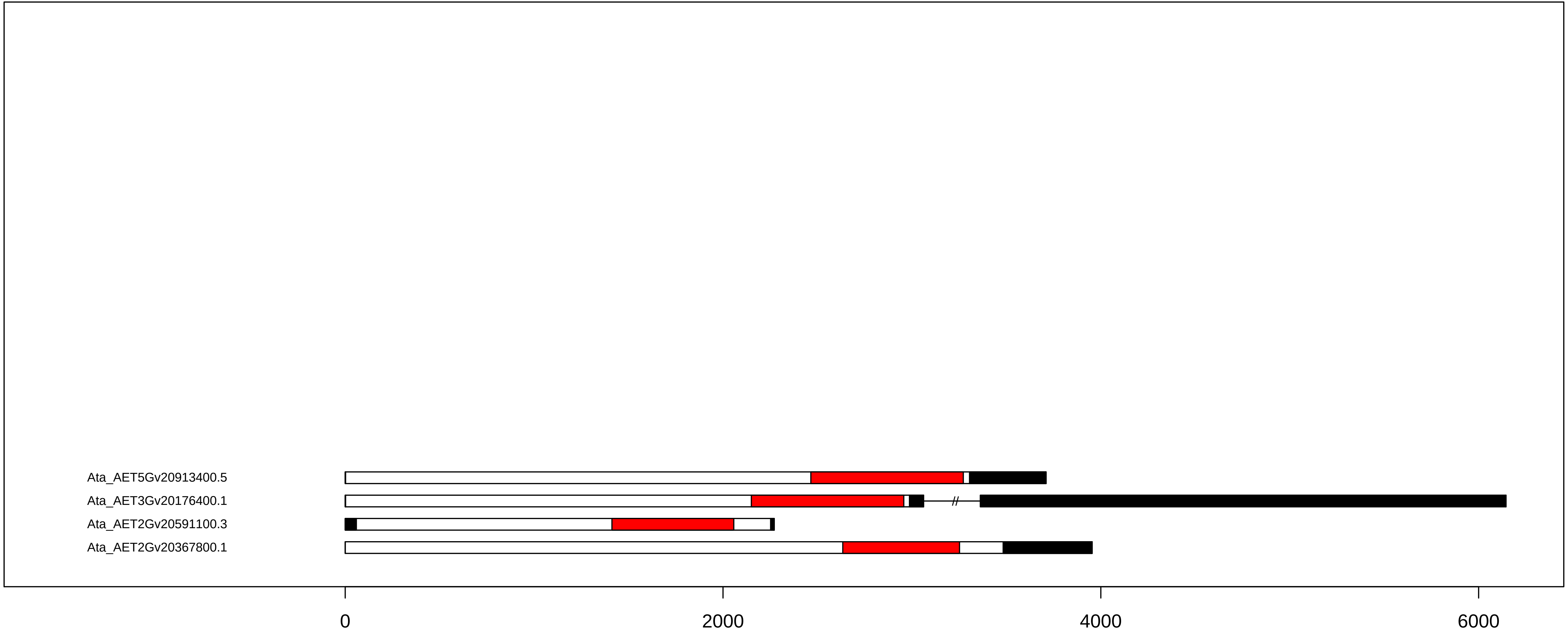

Ae.ta RLK−Pelle_LysM subfamily exon−intron and kinase domain diagram (all)

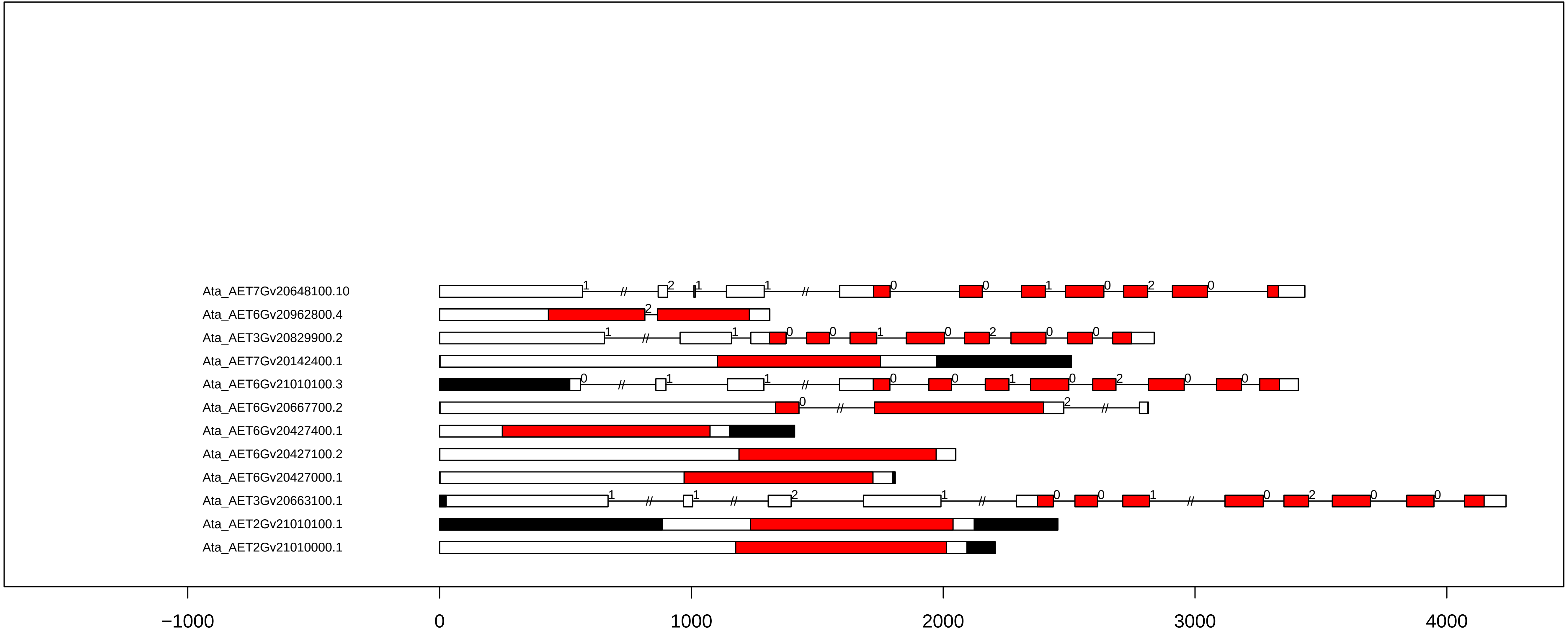

Ae.ta RLK−Pelle_PERK−1 subfamily exon−intron and kinase domain diagram (all)

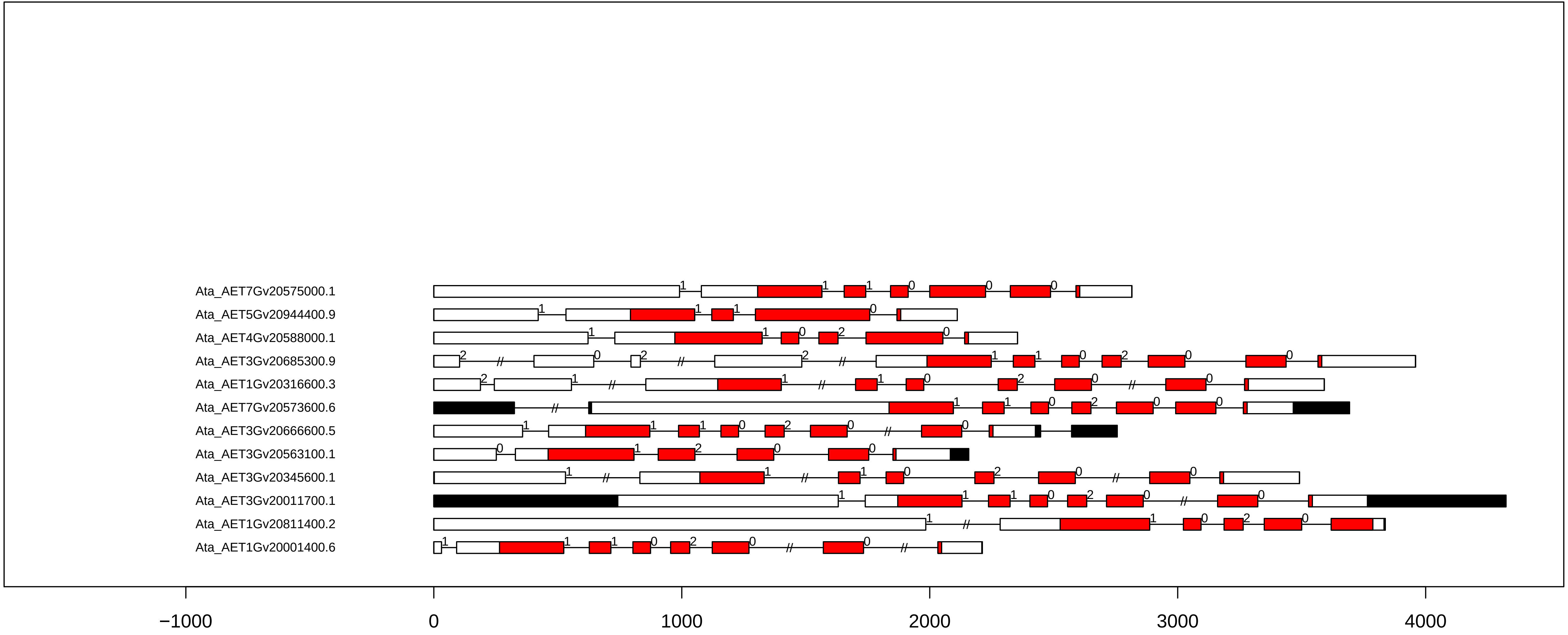

Ae.ta RLK−Pelle_PERK−2 subfamily exon−intron and kinase domain diagram (all)

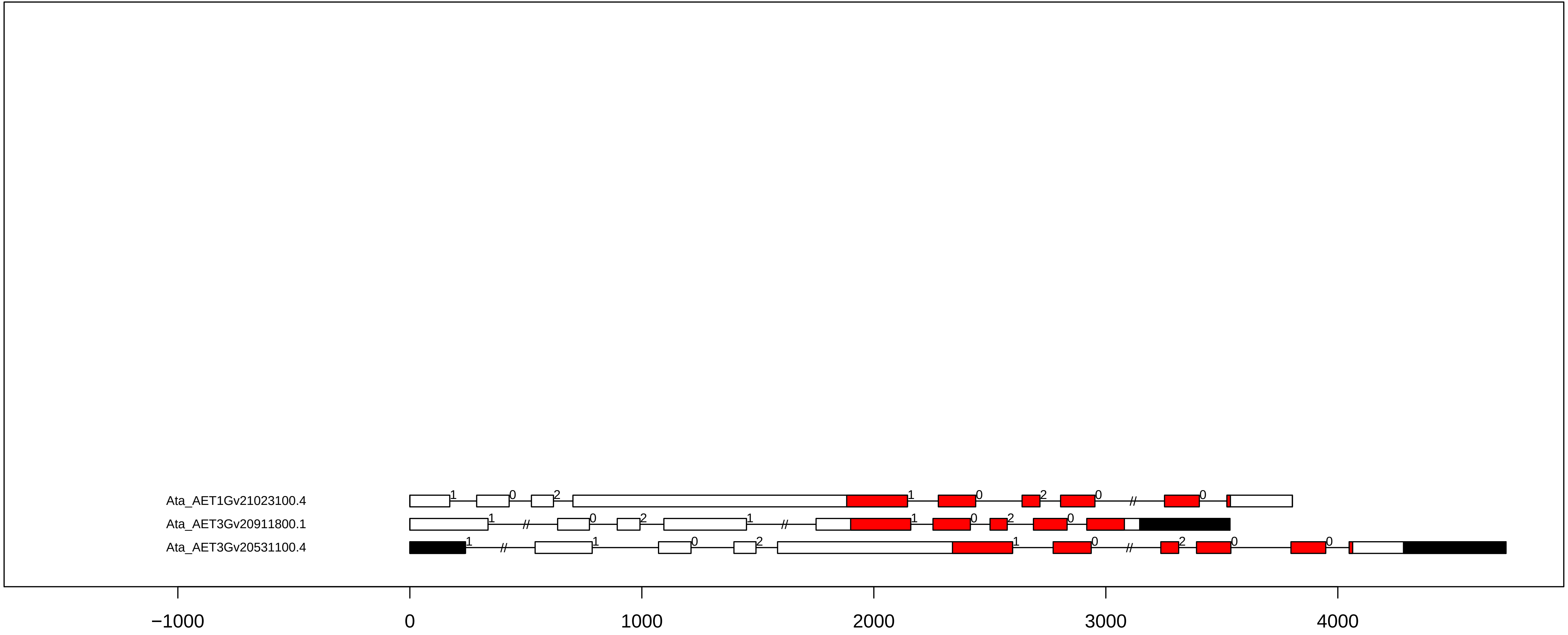

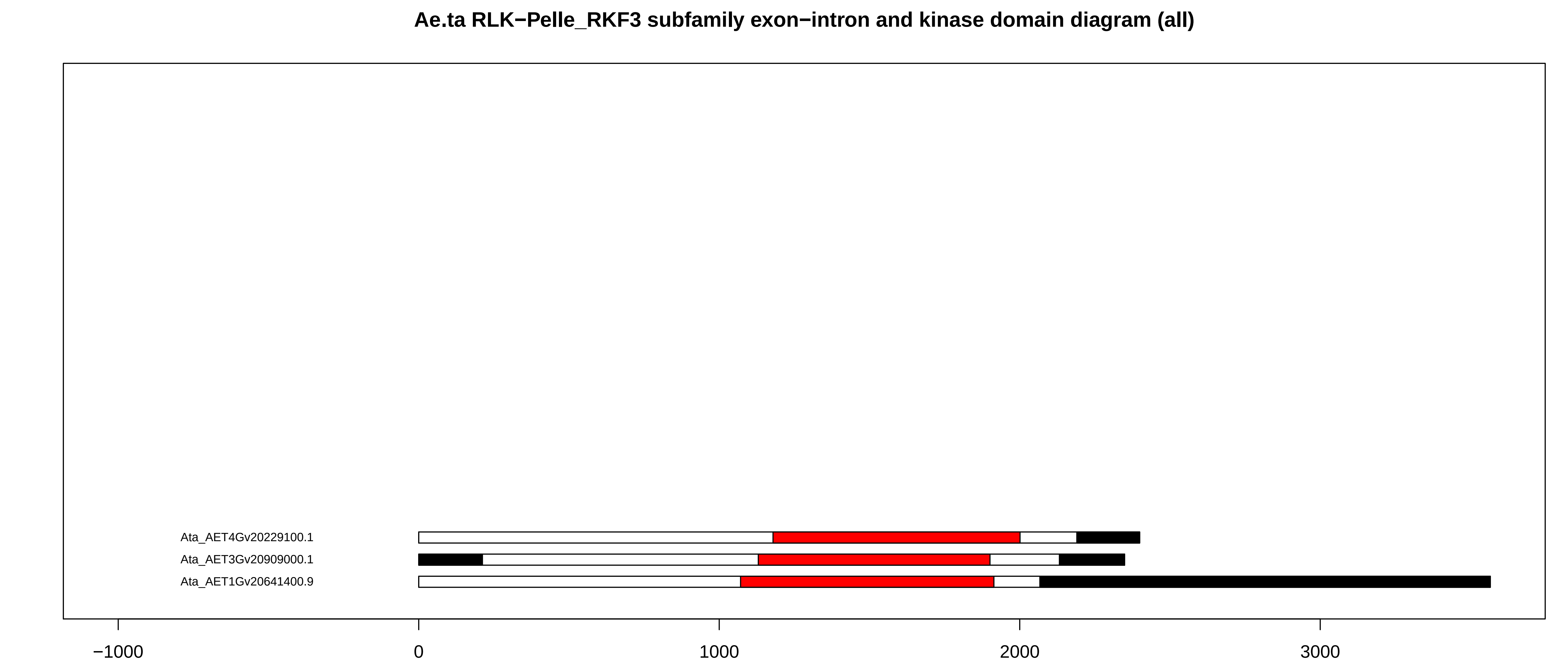

Ae.ta RLK−Pelle_RKF3 subfamily exon−intron and kinase domain diagram (all)
Ata_AET4Gv20229100.1
Ata_AET3Gv20909000.1
Ata_AET1Gv20641400.9
−1000
0
1000
2000
3000

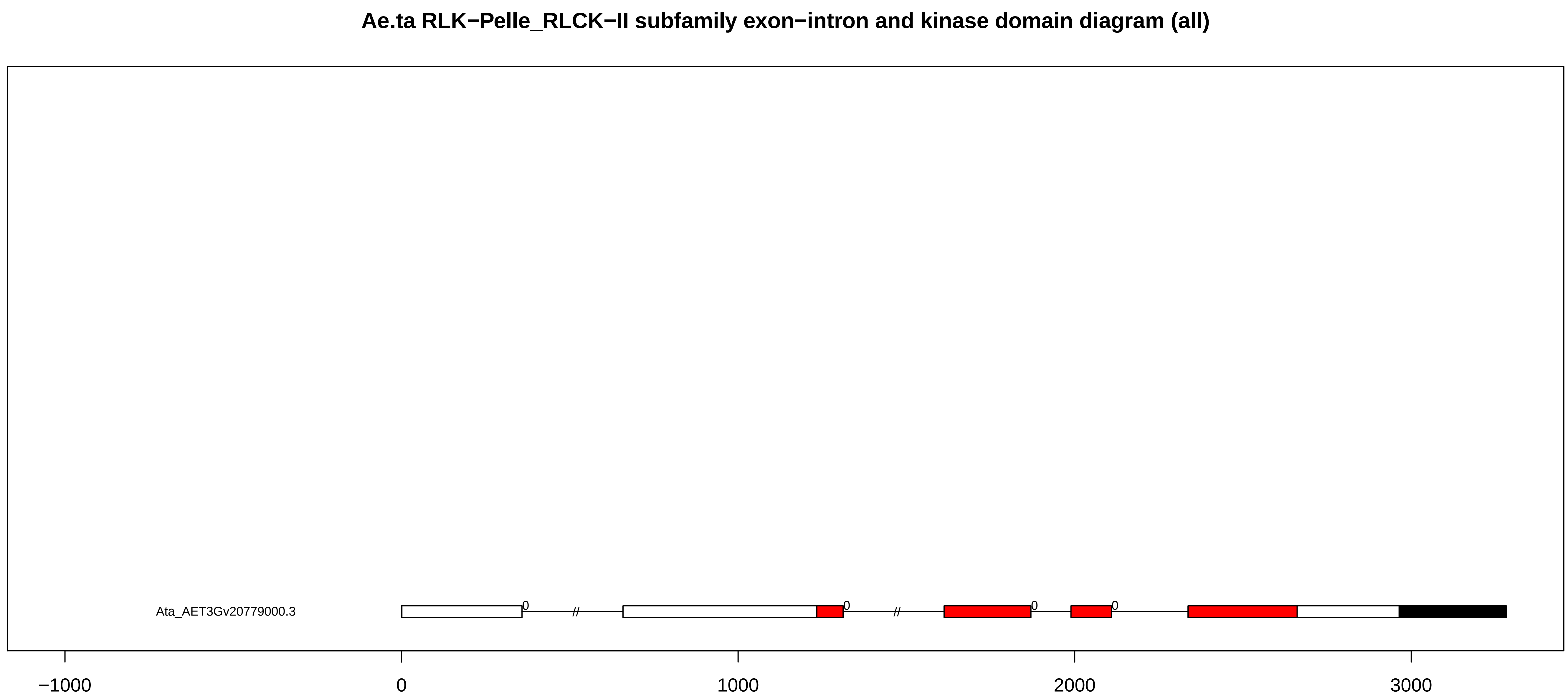

Ae.ta RLK−Pelle_RLCK−II subfamily exon−intron and kinase domain diagram (all)
Ata_AET3Gv20779000.3
0
0
0
0
−1000
0
1000
2000
3000

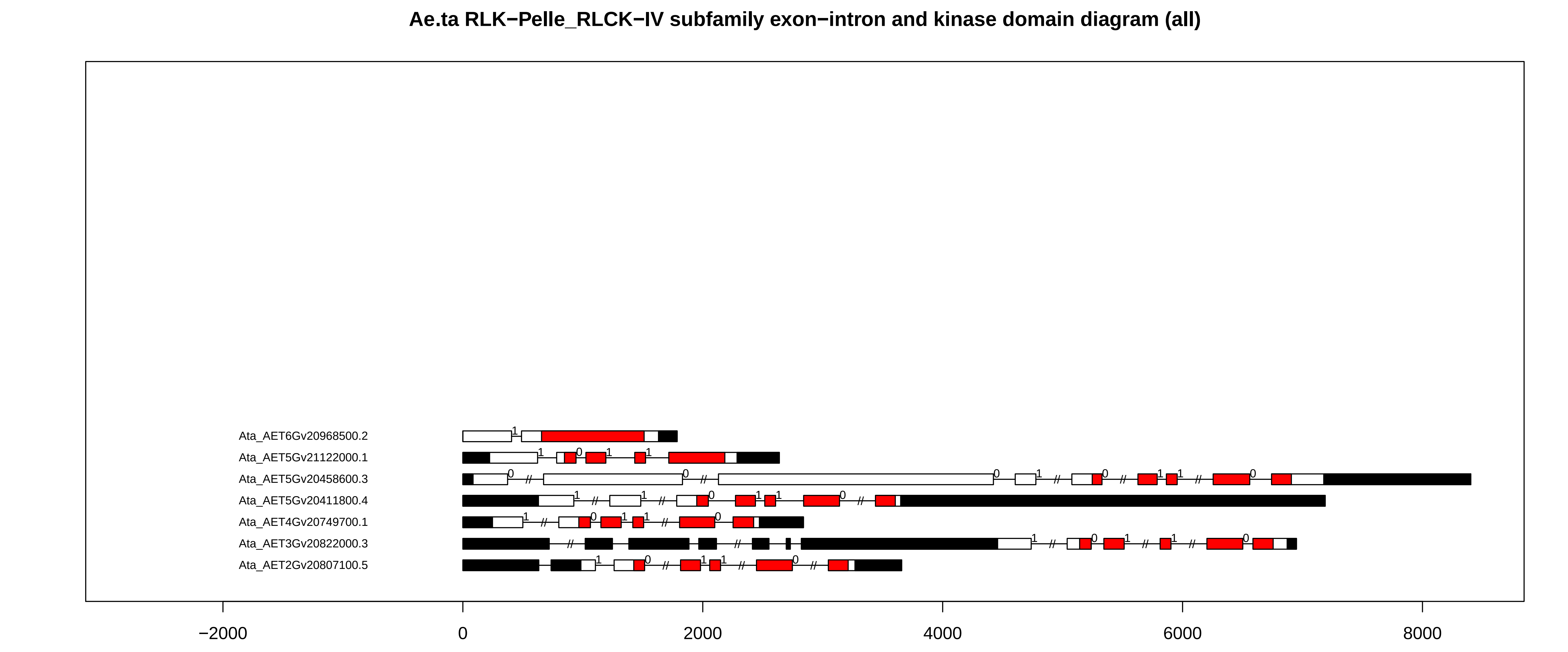

Ae.ta RLK−Pelle_RLCK−IV subfamily exon−intron and kinase domain diagram (all)
Ata_AET6Gv20968500.2
Ata_AET5Gv21122000.1
Ata_AET5Gv20458600.3
Ata_AET5Gv20411800.4
Ata_AET4Gv20749700.1
Ata_AET3Gv20822000.3
Ata_AET2Gv20807100.5
−2000
0
2000
4000
6000
8000

Ae.ta RLK−Pelle_RLCK−IXa subfamily exon−intron and kinase domain diagram (all)

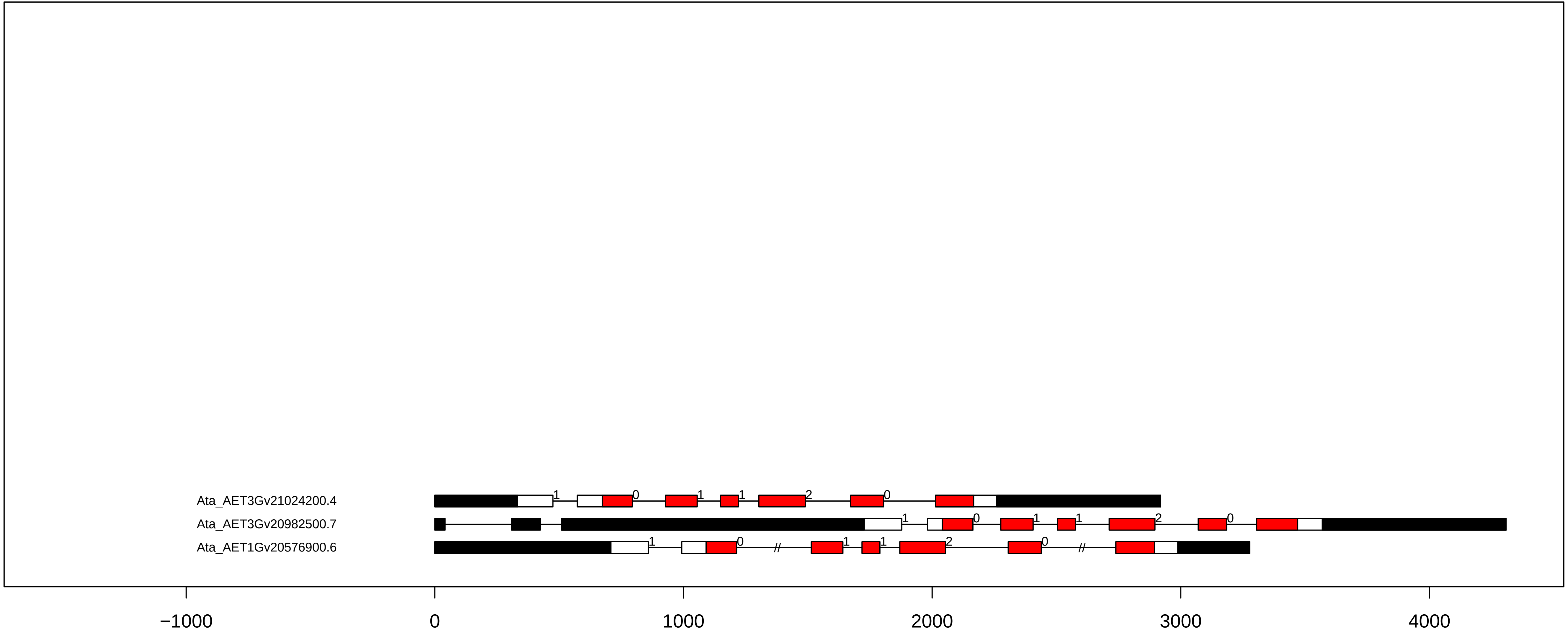

Ae.ta RLK−Pelle_RLCK−IXb subfamily exon−intron and kinase domain diagram (part 1)

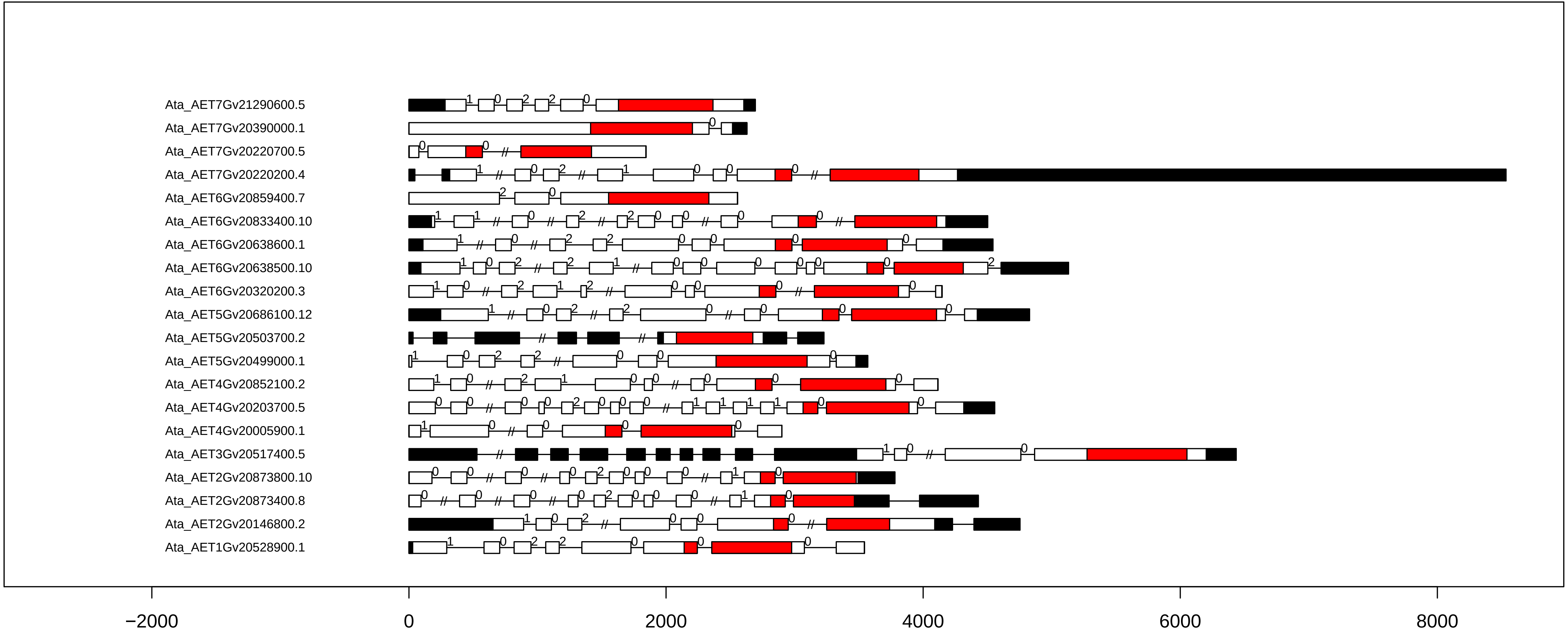

Ae.ta RLK−Pelle_RLCK−IXb subfamily exon−intron and kinase domain diagram (part 2)

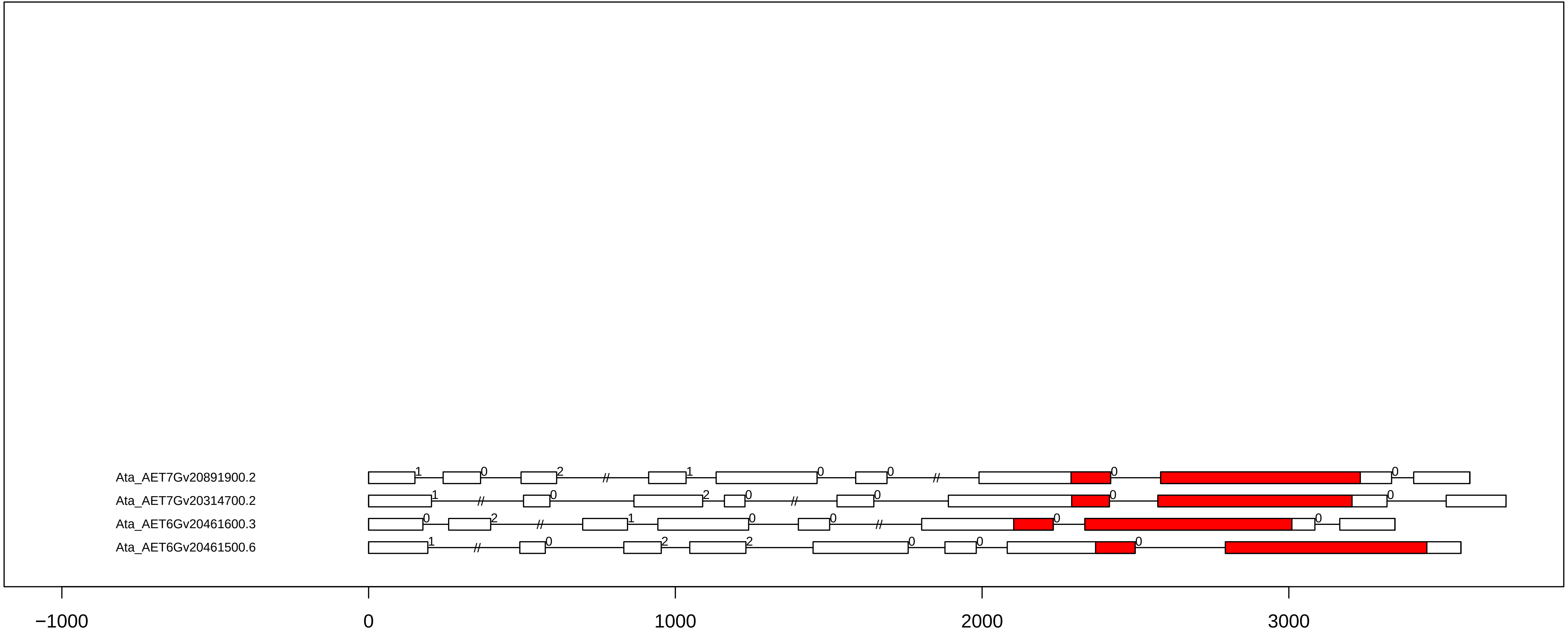

Ae.ta RLK−Pelle_RLCK−Os subfamily exon−intron and kinase domain diagram (all)

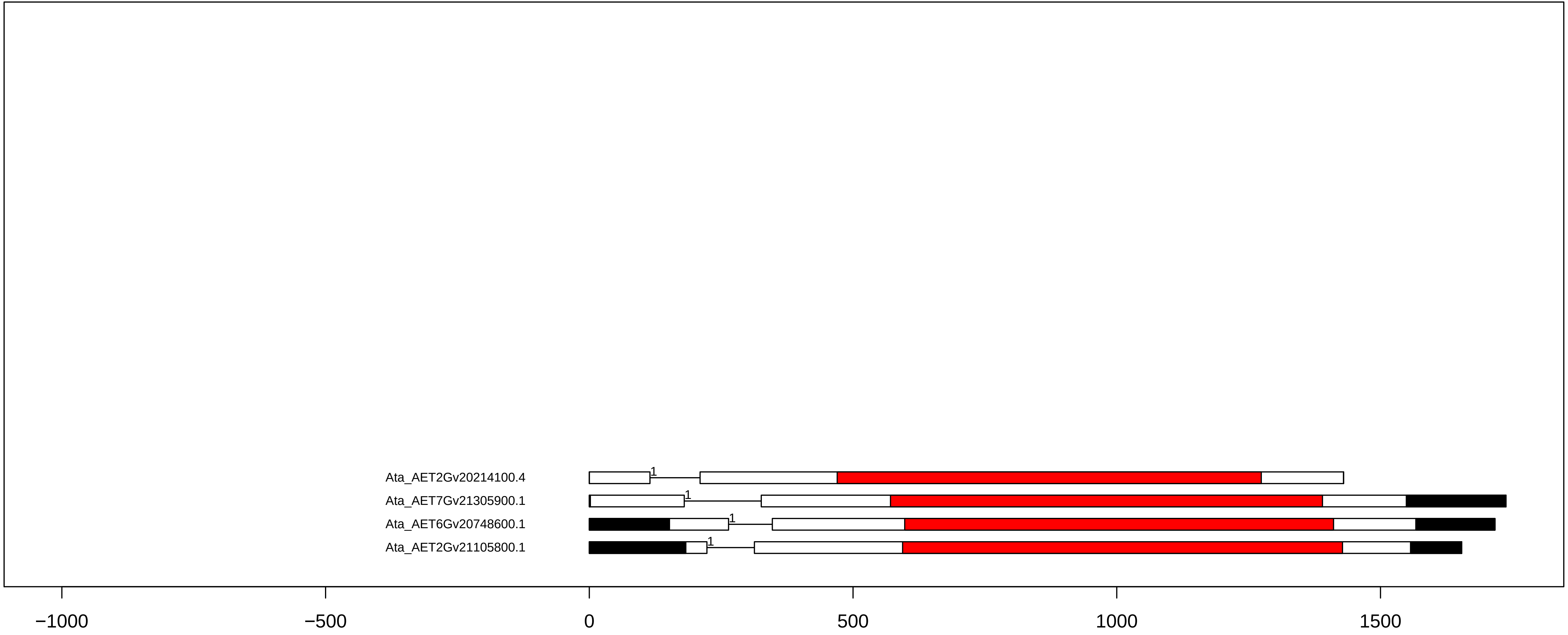

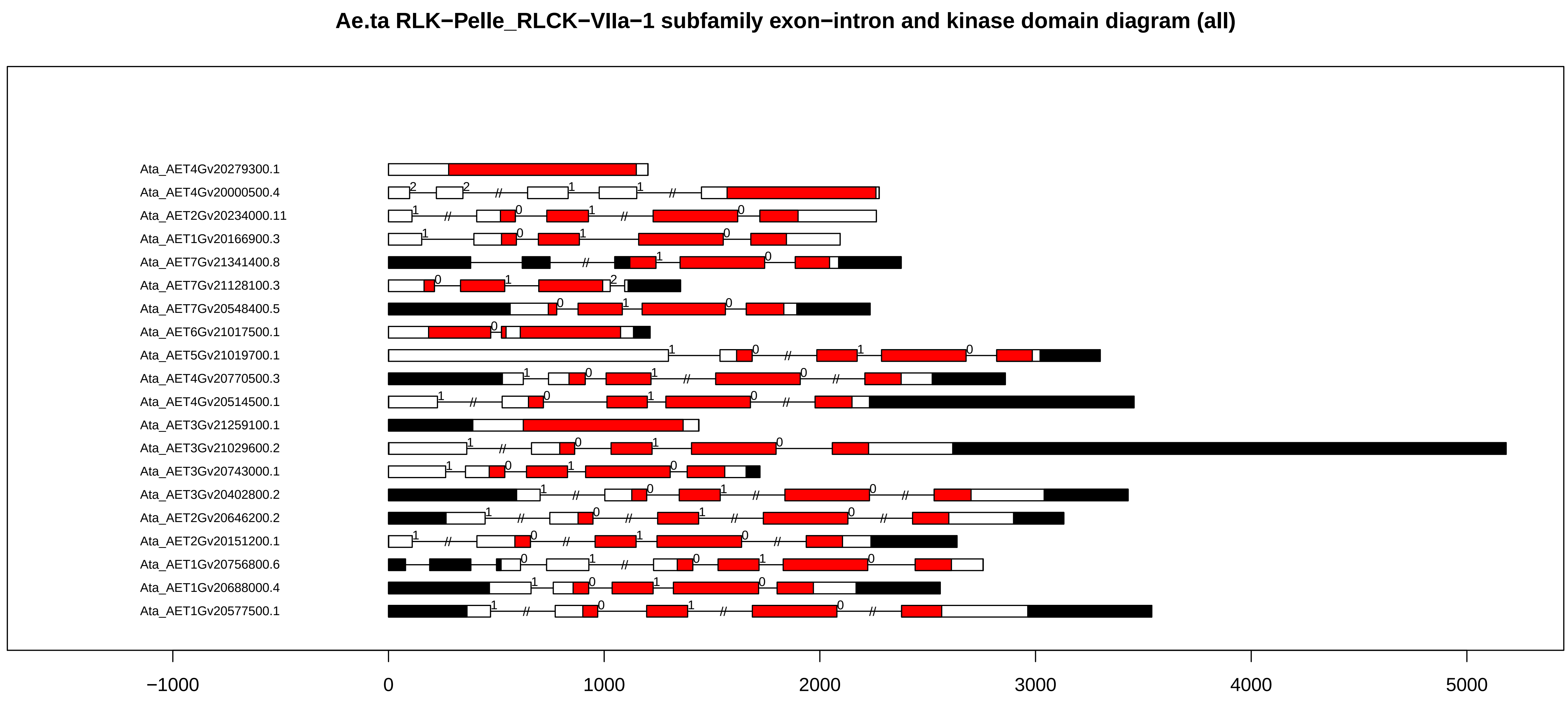
Ae.ta RLK−Pelle_RLCK−VIIa−1 subfamily exon−intron and kinase domain diagram (all)
Ata_AET4Gv20279300.1
Ata_AET4Gv20000500.4
Ata_AET2Gv20234000.11
Ata_AET1Gv20166900.3
Ata_AET7Gv21341400.8
Ata_AET7Gv21128100.3
Ata_AET7Gv20548400.5
Ata_AET6Gv21017500.1
Ata_AET5Gv21019700.1
Ata_AET4Gv20770500.3
Ata_AET4Gv20514500.1
Ata_AET3Gv21259100.1
Ata_AET3Gv21029600.2
Ata_AET3Gv20743000.1
Ata_AET3Gv20402800.2
Ata_AET2Gv20646200.2
Ata_AET2Gv20151200.1
Ata_AET1Gv20756800.6
Ata_AET1Gv20688000.4
Ata_AET1Gv20577500.1
−1000
0
1000
2000
3000
4000
5000

Ae.ta RLK−Pelle_RLCK−VIIa−2 subfamily exon−intron and kinase domain diagram (part 1)

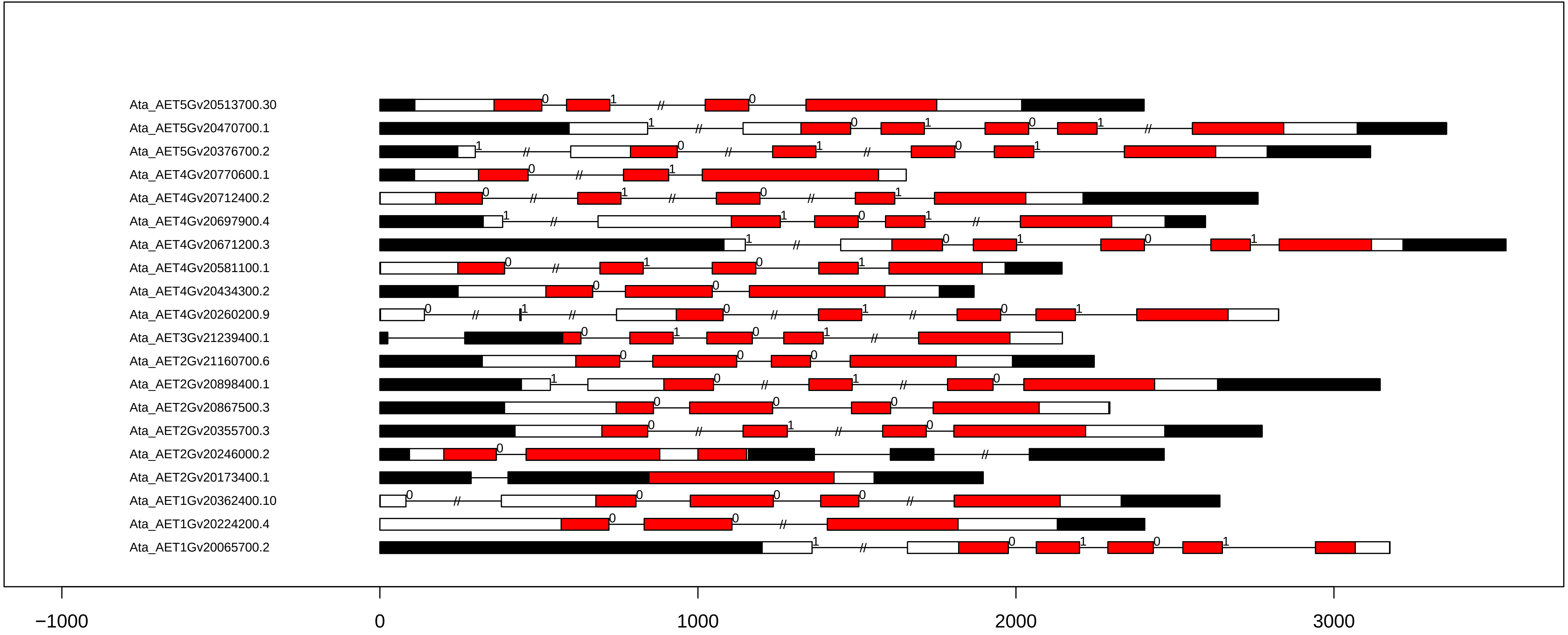

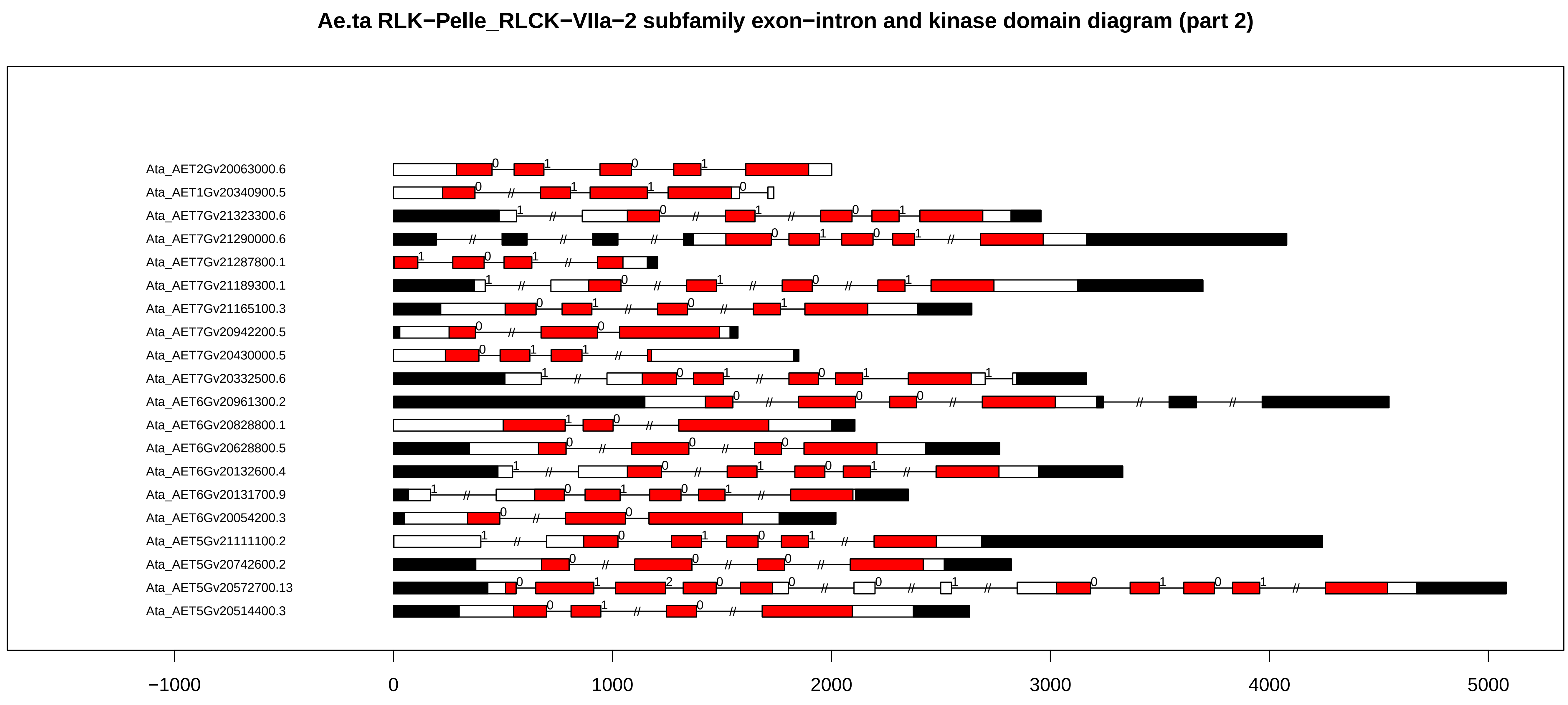

Ae.ta RLK−Pelle_RLCK−VIIa−2 subfamily exon−intron and kinase domain diagram (part 2)
Ata_AET2Gv20063000.6
Ata_AET1Gv20340900.5
Ata_AET7Gv21323300.6
Ata_AET7Gv21290000.6
Ata_AET7Gv21287800.1
Ata_AET7Gv21189300.1
Ata_AET7Gv21165100.3
Ata_AET7Gv20942200.5
Ata_AET7Gv20430000.5
Ata_AET7Gv20332500.6
Ata_AET6Gv20961300.2
Ata_AET6Gv20828800.1
Ata_AET6Gv20628800.5
Ata_AET6Gv20132600.4
Ata_AET6Gv20131700.9
Ata_AET6Gv20054200.3
Ata_AET5Gv21111100.2
Ata_AET5Gv20742600.2
Ata_AET5Gv20572700.13
Ata_AET5Gv20514400.3
−1000
0
1000
2000
3000
4000
5000

Ae.ta RLK−Pelle_RLCK−VIIa−2 subfamily exon−intron and kinase domain diagram (part 3)

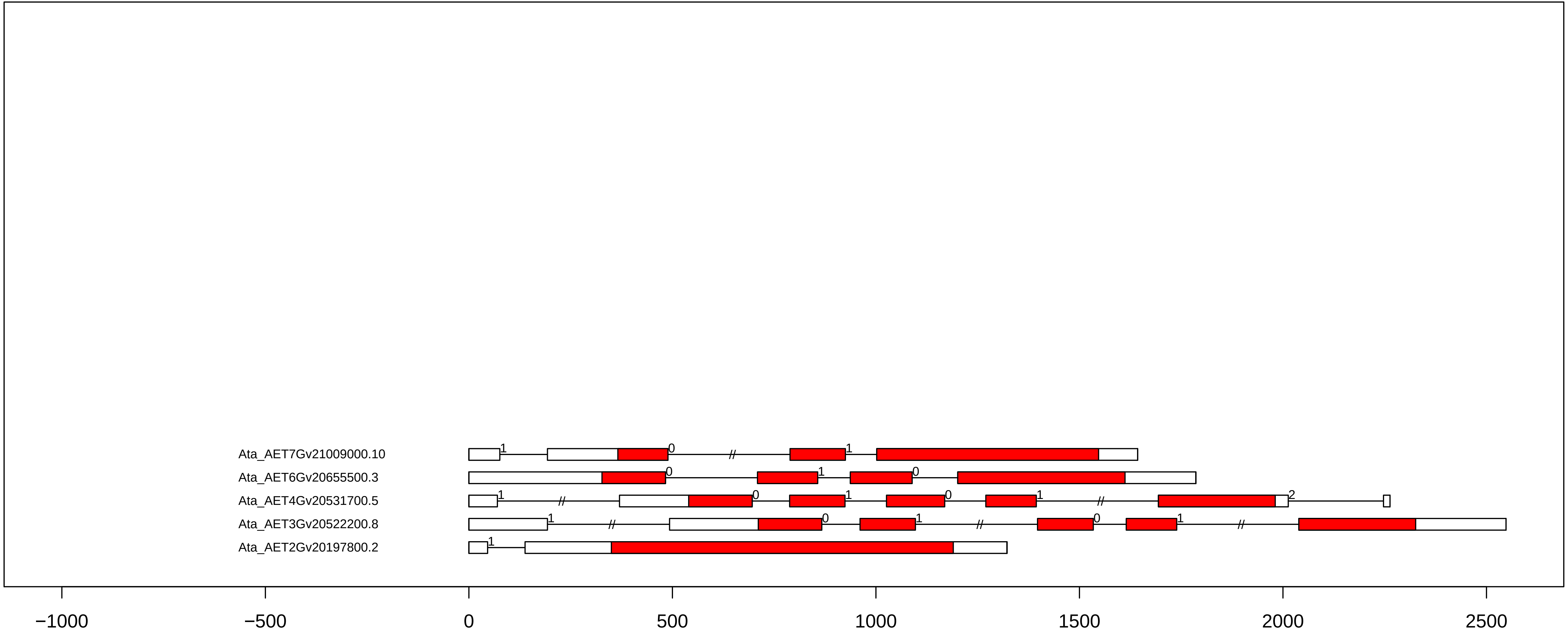

Ae.ta RLK−Pelle_RLCK−VIIb subfamily exon−intron and kinase domain diagram (all)

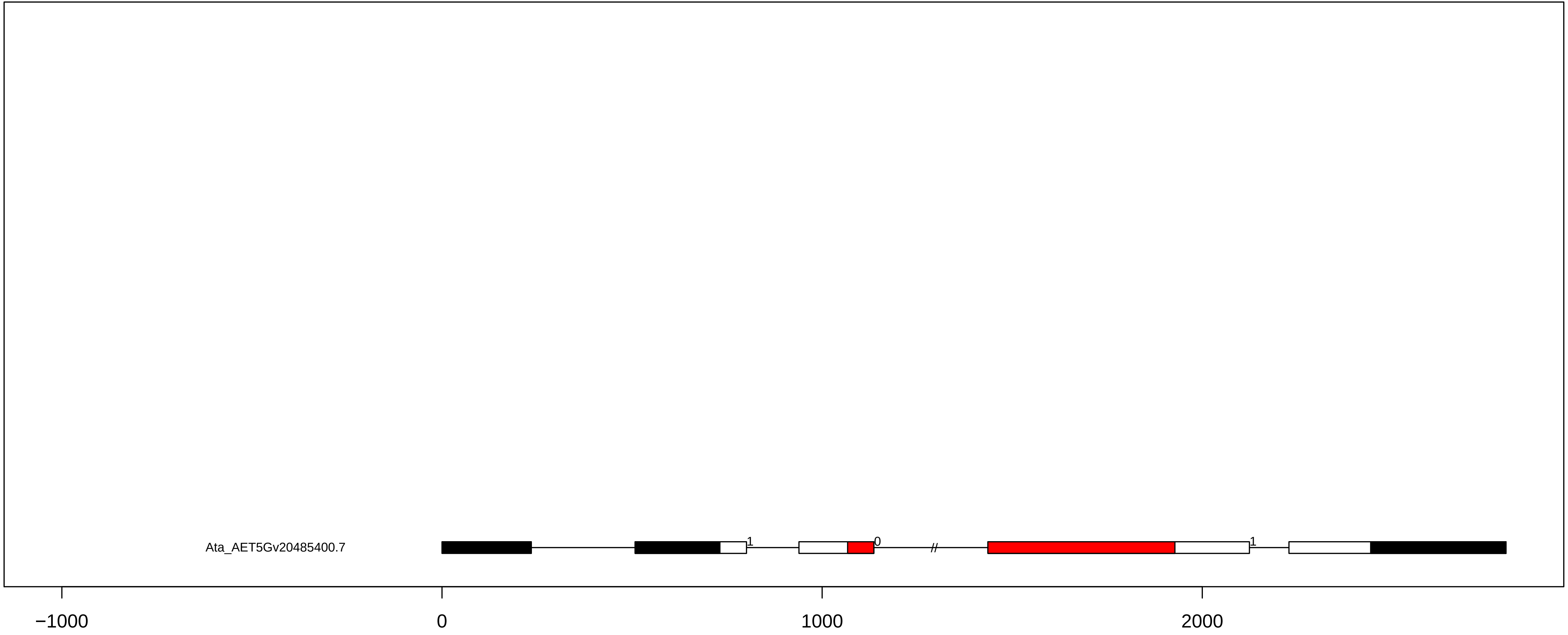

Ae.ta RLK−Pelle_RLCK−VIII subfamily exon−intron and kinase domain diagram (all)

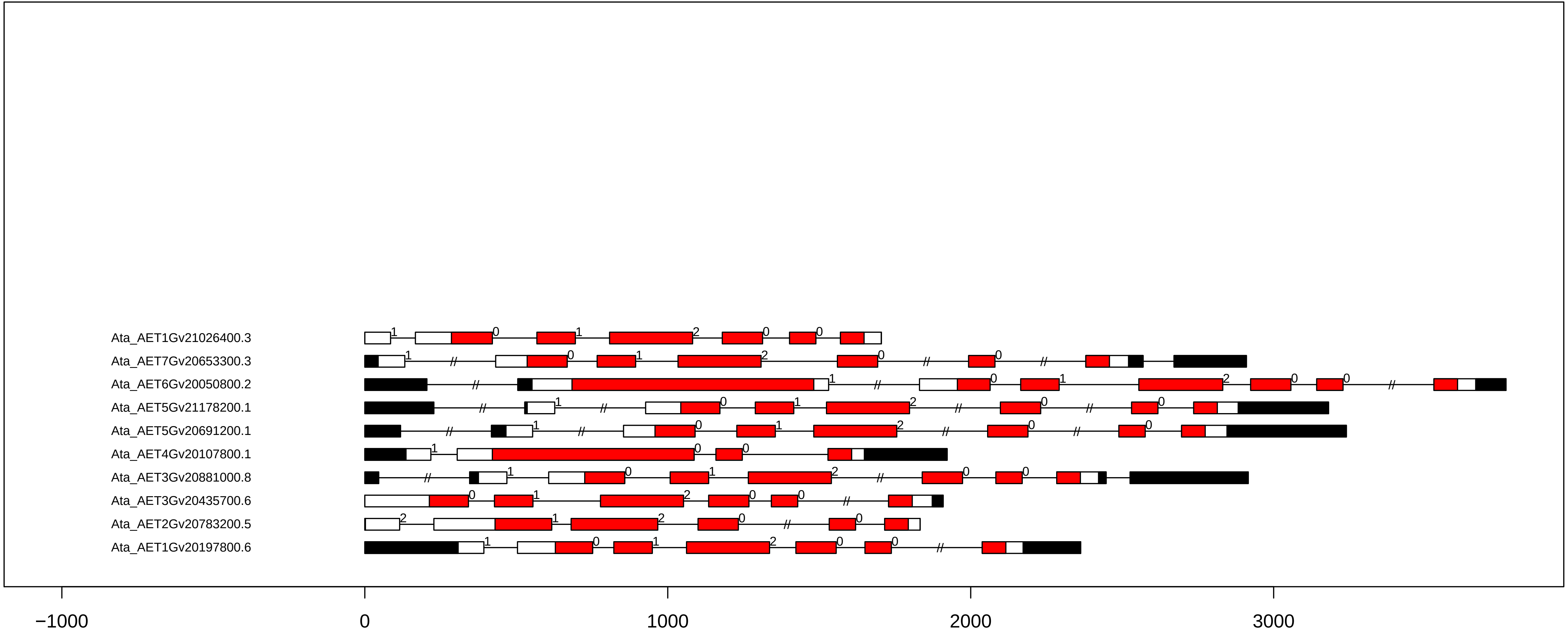

Ae.ta RLK−Pelle_RLCK−VI subfamily exon−intron and kinase domain diagram (all)

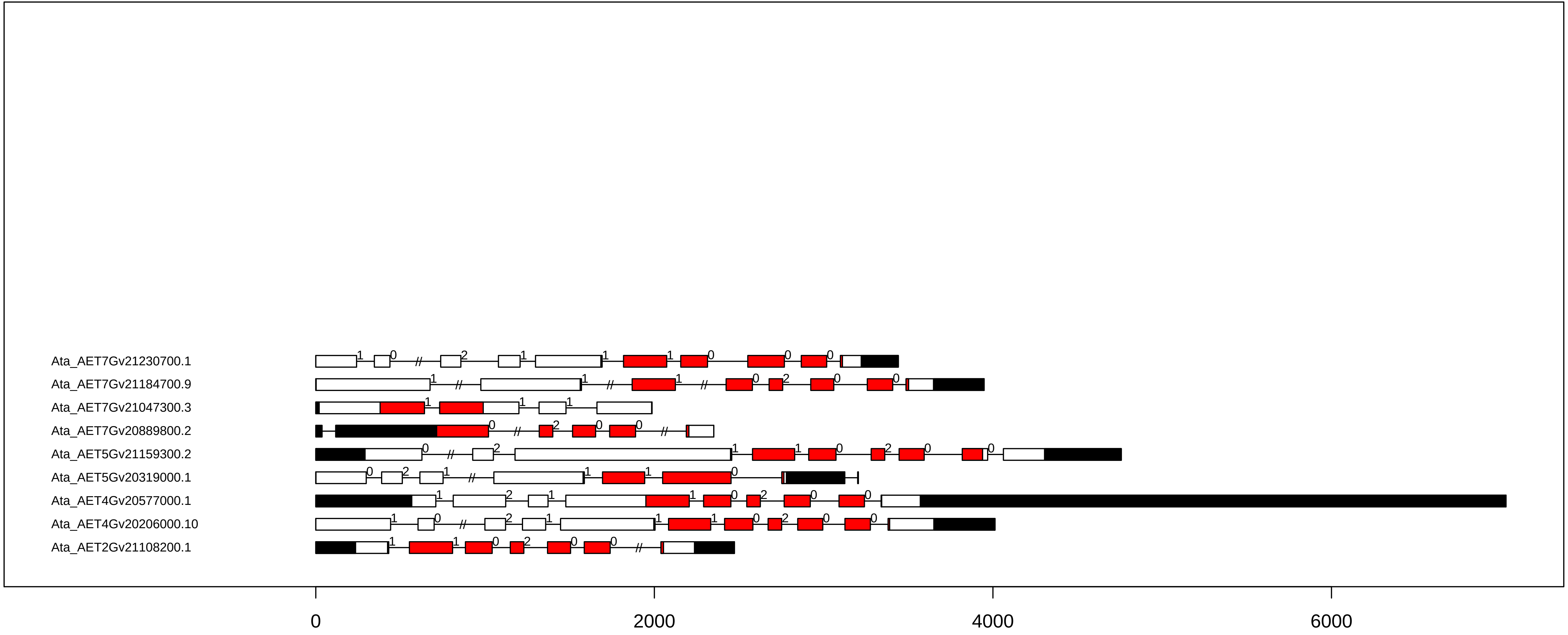

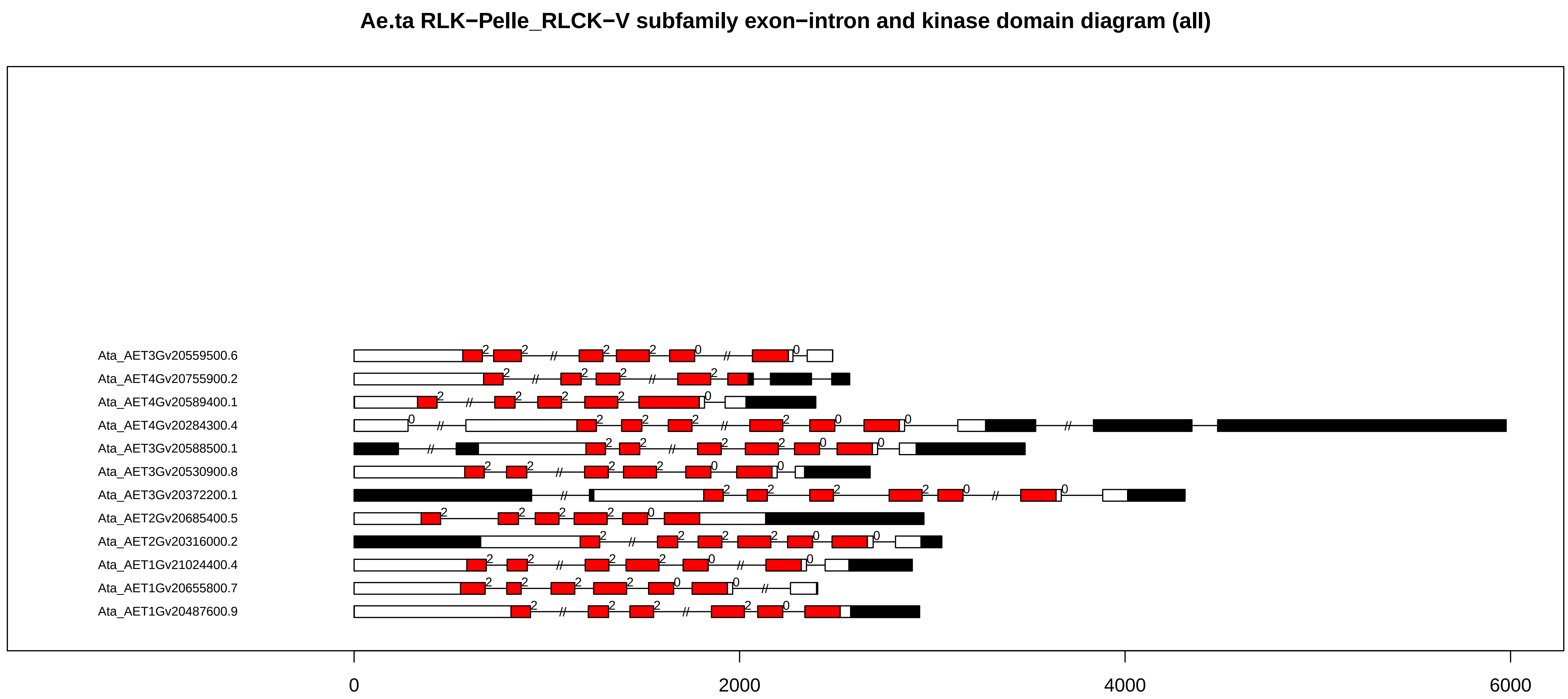

Ae.ta RLK−Pelle_RLCK−V subfamily exon−intron and kinase domain diagram (all)
Ata_AET3Gv20559500.6
Ata_AET4Gv20755900.2
Ata_AET4Gv20589400.1
Ata_AET4Gv20284300.4
Ata_AET3Gv20588500.1
Ata_AET3Gv20530900.8
Ata_AET3Gv20372200.1
Ata_AET2Gv20685400.5
Ata_AET2Gv20316000.2
Ata_AET1Gv21024400.4
Ata_AET1Gv20655800.7
Ata_AET1Gv20487600.9
0
2000
4000
6000

Ae.ta RLK−Pelle_RLCK−XII−1 subfamily exon−intron and kinase domain diagram (all)

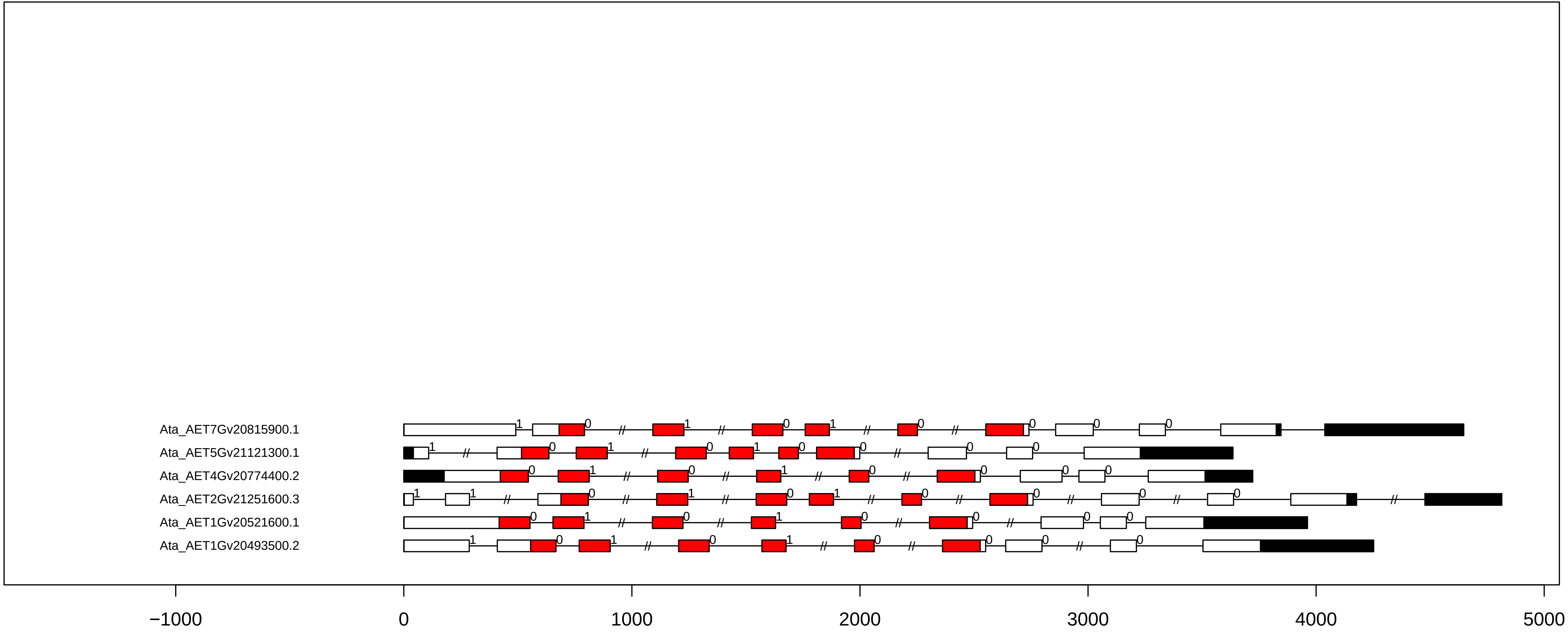

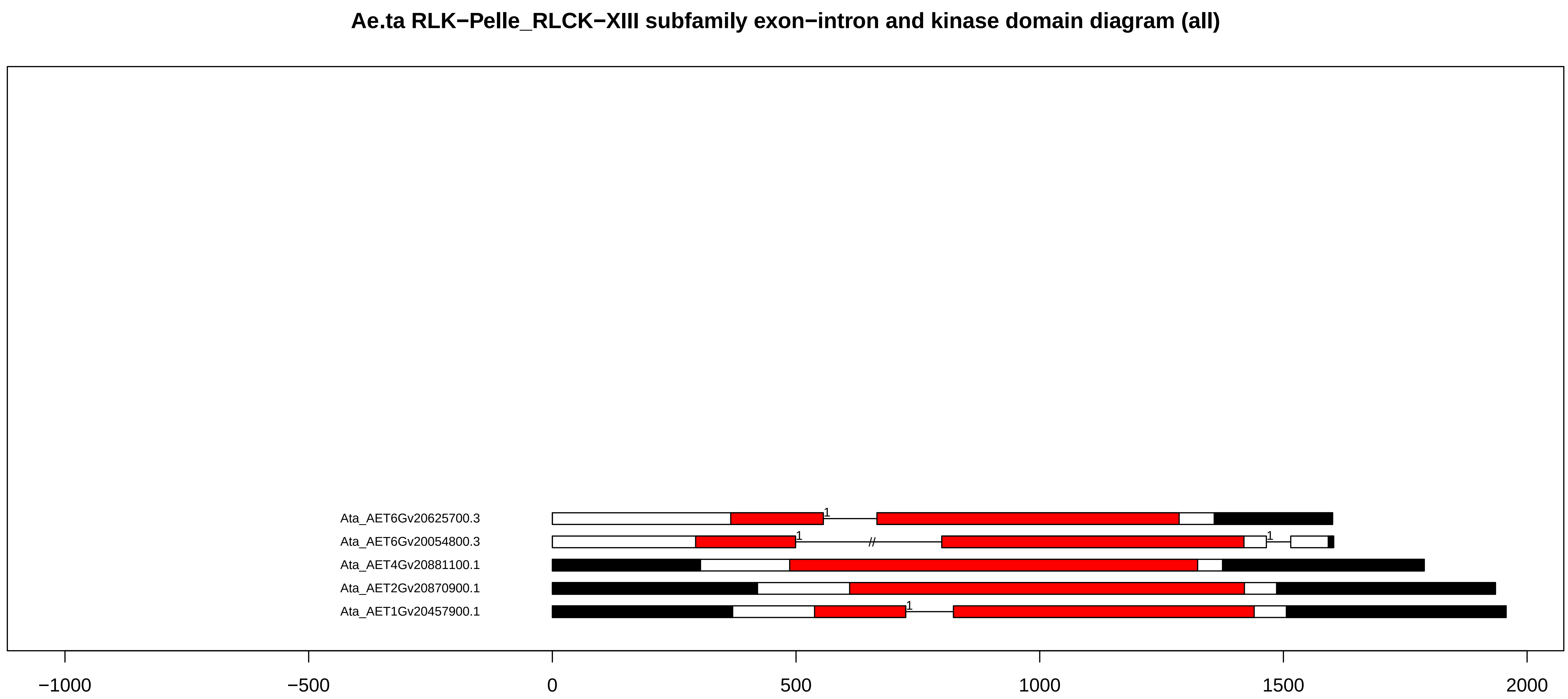

Ae.ta RLK−Pelle_RLCK−XIII subfamily exon−intron and kinase domain diagram (all)
Ata_AET6Gv20625700.3
Ata_AET6Gv20054800.3
Ata_AET4Gv20881100.1
Ata_AET2Gv20870900.1
Ata_AET1Gv20457900.1
1
1
//
1
1
−1000
−500
0
500
1000
1500
2000

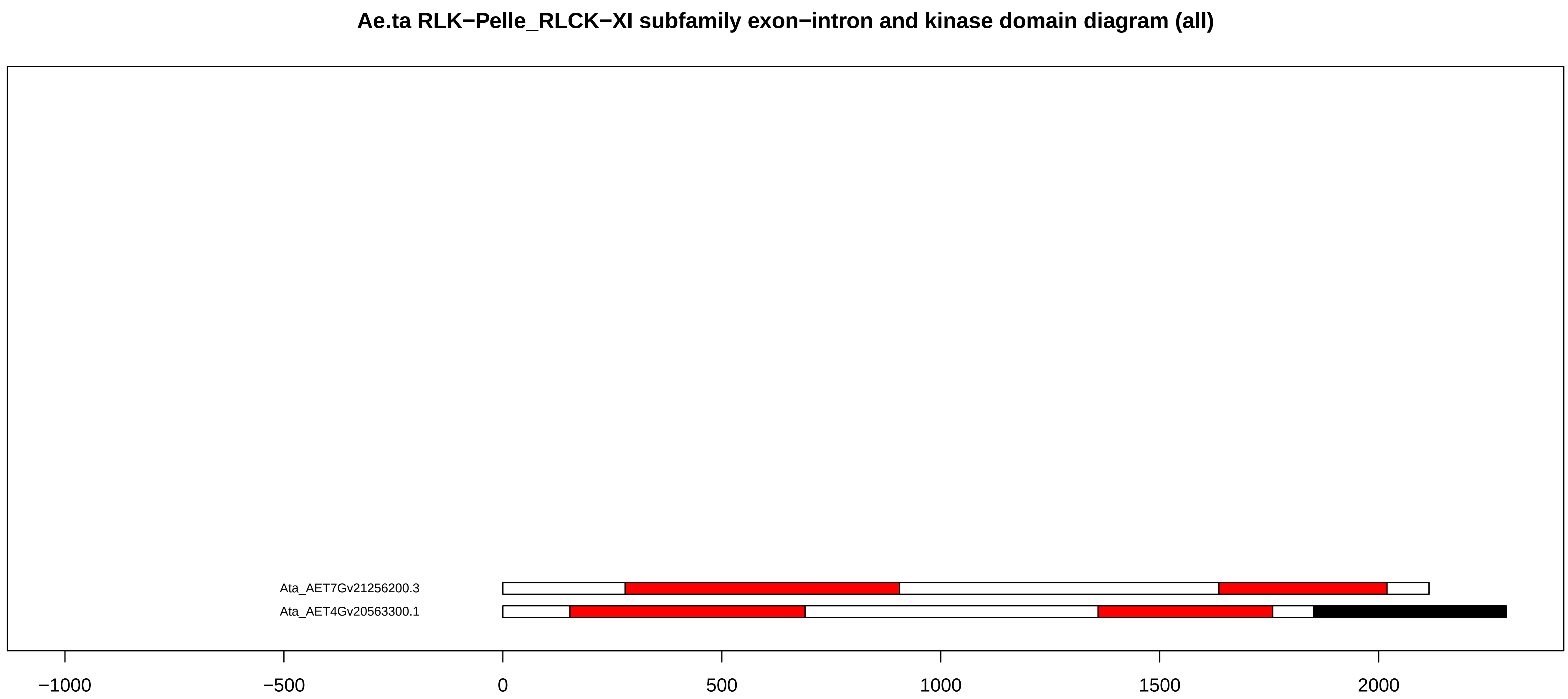
Ae.ta RLK−Pelle_RLCK−XI subfamily exon−intron and kinase domain diagram (all)
Ata_AET7Gv21256200.3
Ata_AET4Gv20563300.1
−1000
−500
0
500
1000
1500
2000

Ae.ta RLK−Pelle_RLCK−X subfamily exon−intron and kinase domain diagram (all)

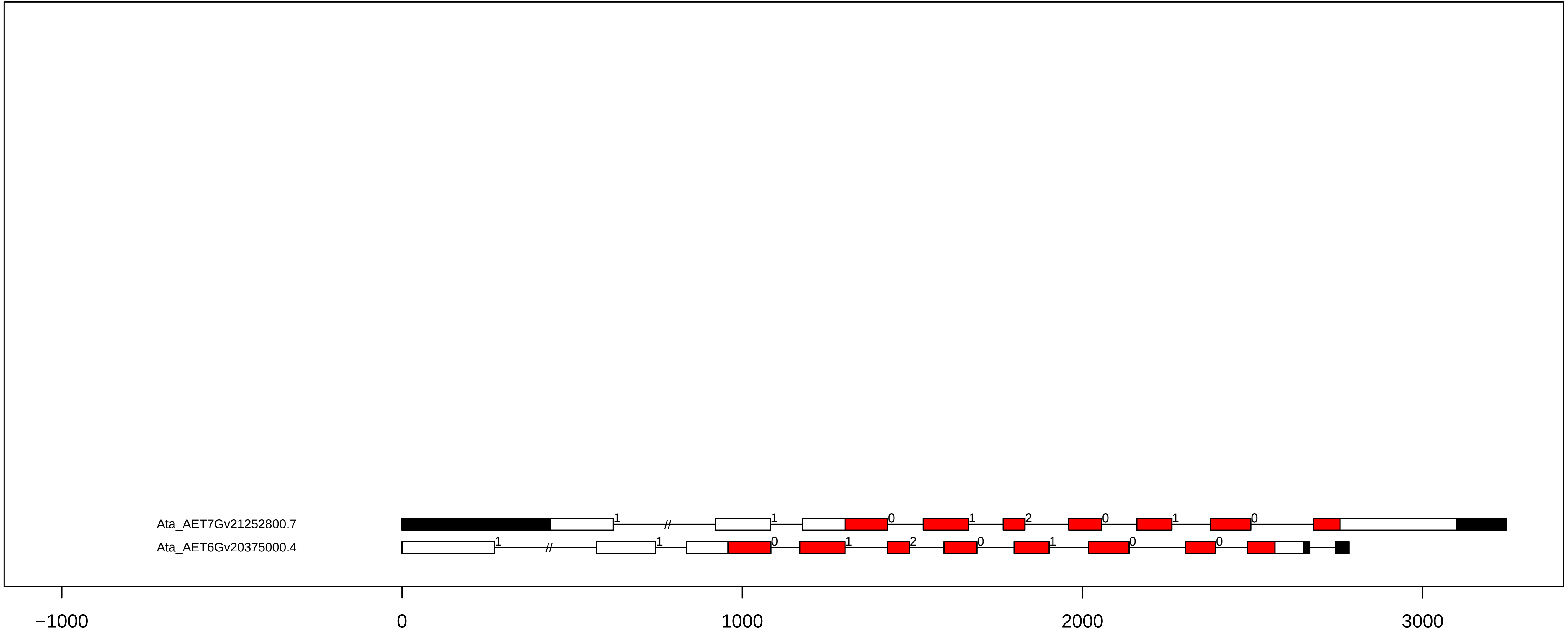

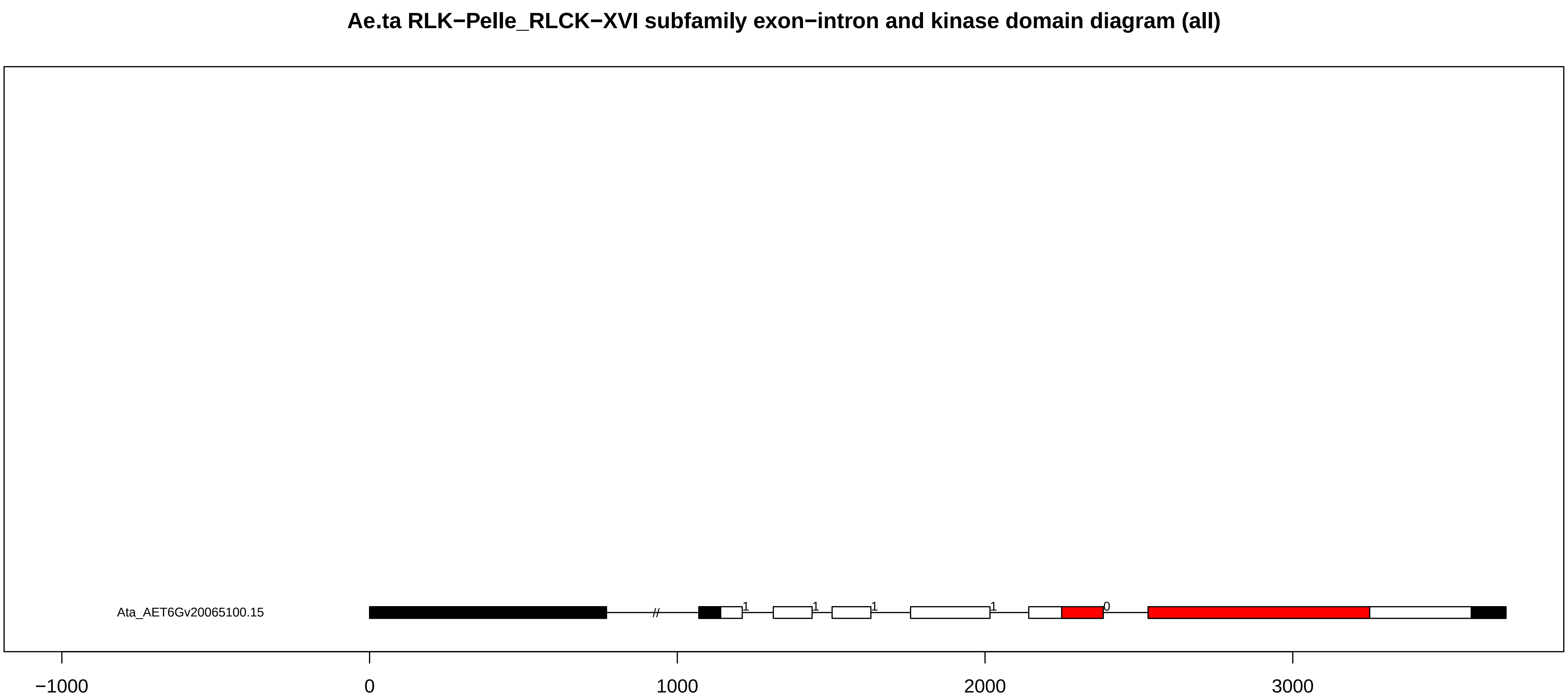
Ae.ta RLK−Pelle_RLCK−XVI subfamily exon−intron and kinase domain diagram (all)
Ata_AET6Gv20065100.15
//
1
1
1
1
0
−1000
0
1000
2000
3000

Ae.ta RLK−Pelle_RLCK−XV subfamily exon−intron and kinase domain diagram (all)

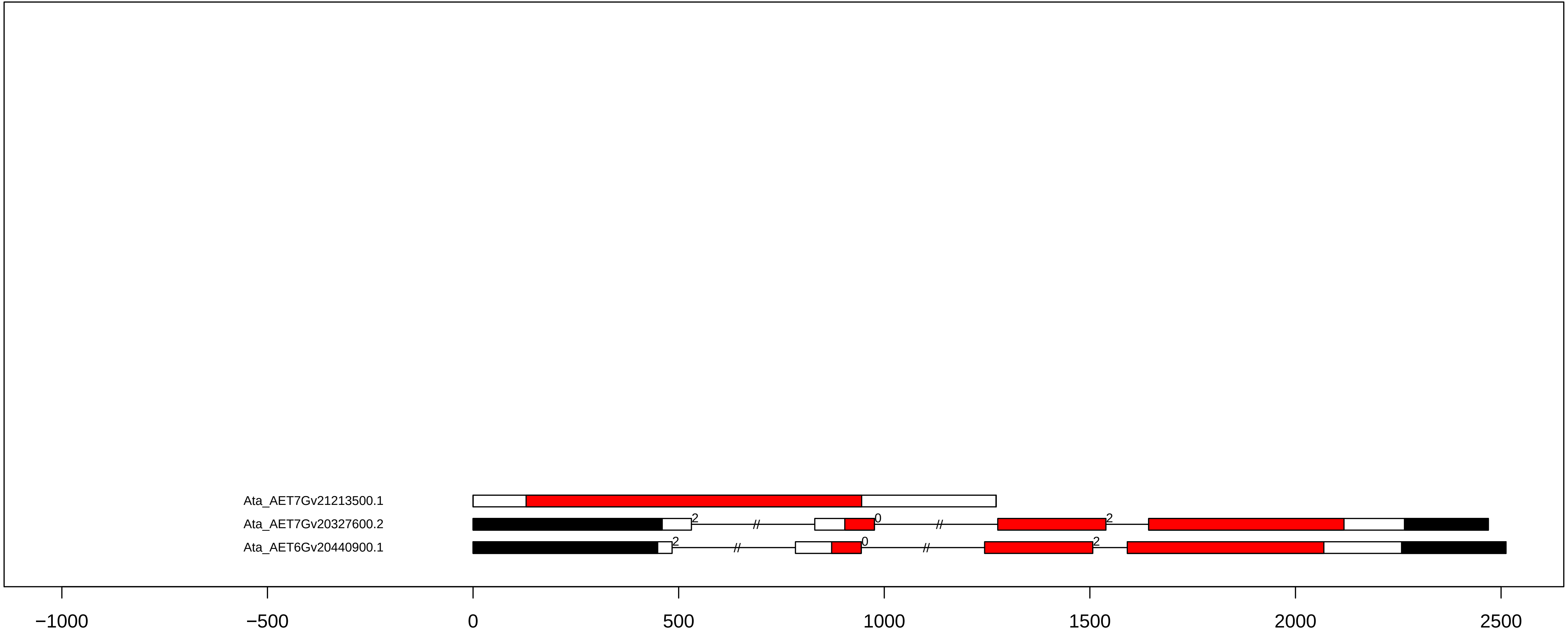

Ae.ta RLK−Pelle_SD−2b subfamily exon−intron and kinase domain diagram (part 1)

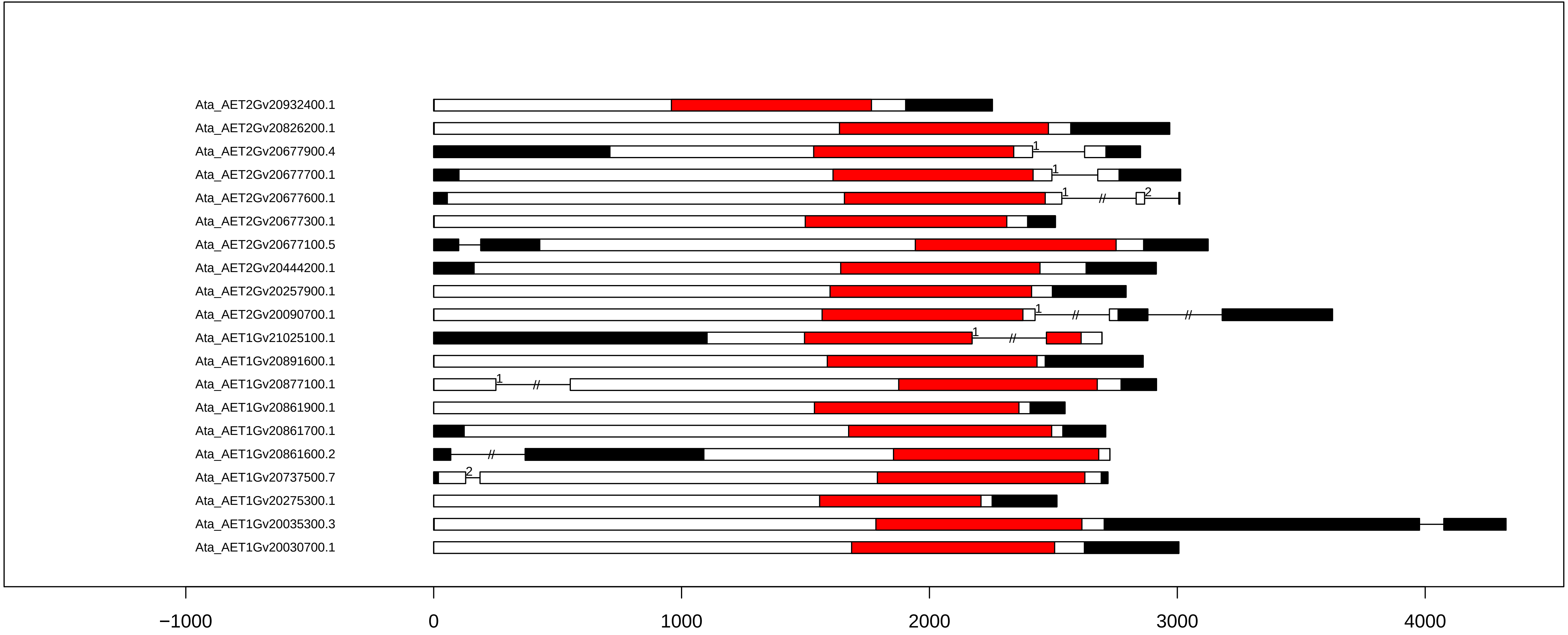

Ae.ta RLK−Pelle_SD−2b subfamily exon−intron and kinase domain diagram (part 2)

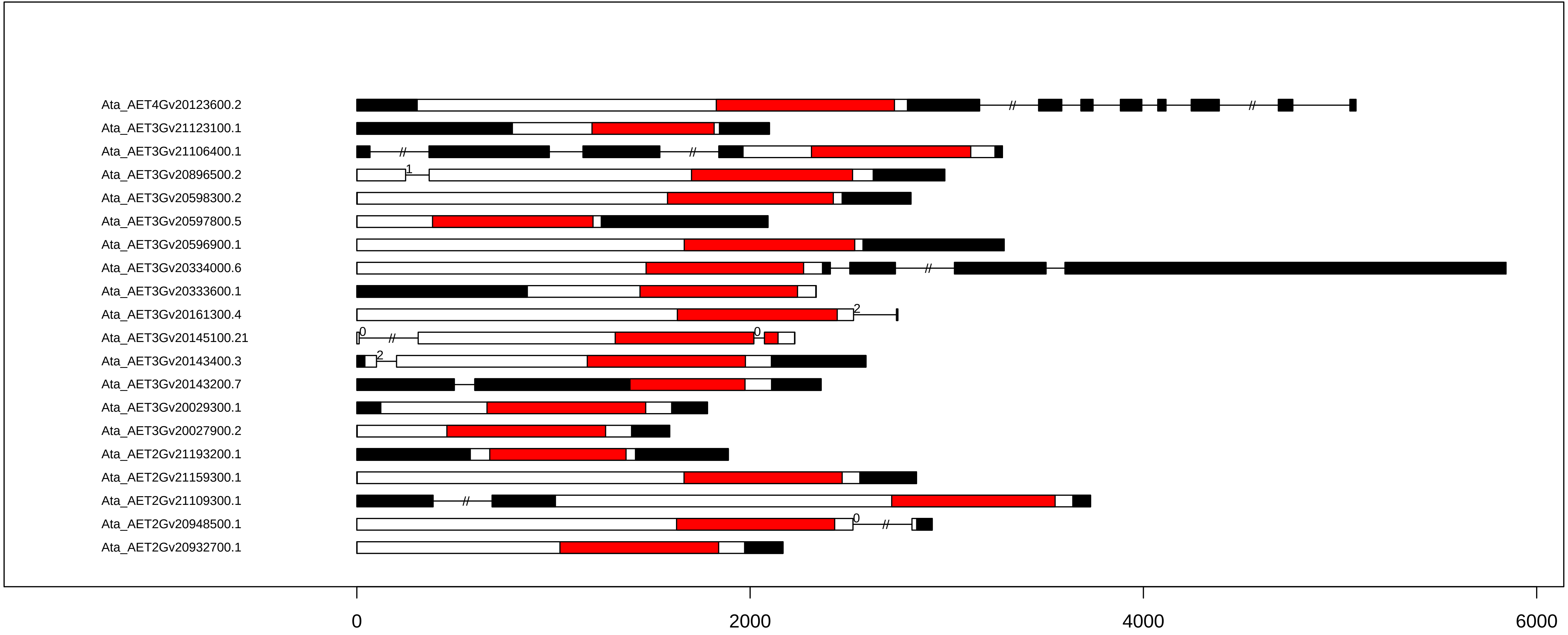

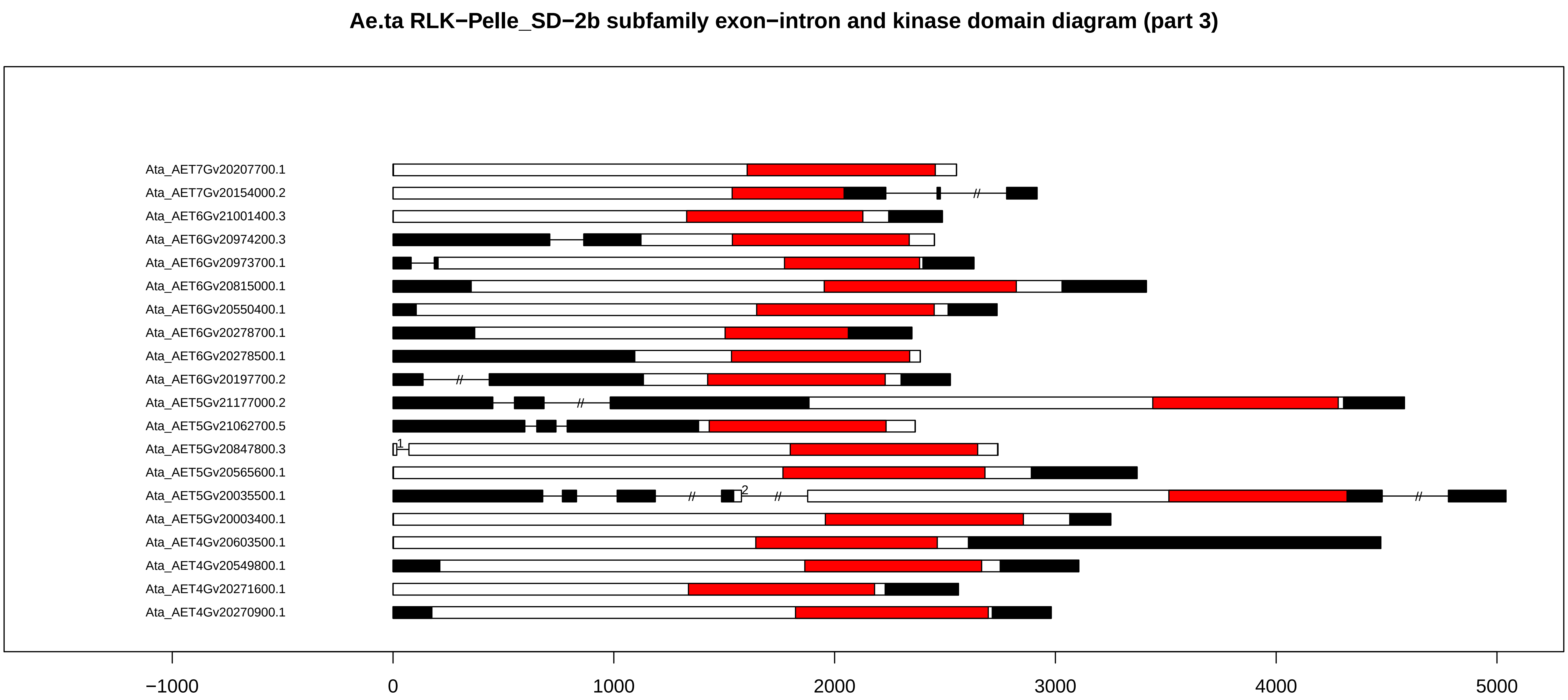

Ae.ta RLK−Pelle_SD−2b subfamily exon−intron and kinase domain diagram (part 3)
Ata_AET7Gv20207700.1
Ata_AET7Gv20154000.2
Ata_AET6Gv21001400.3
Ata_AET6Gv20974200.3
Ata_AET6Gv20973700.1
Ata_AET6Gv20815000.1
Ata_AET6Gv20550400.1
Ata_AET6Gv20278700.1
Ata_AET6Gv20278500.1
Ata_AET6Gv20197700.2
Ata_AET5Gv21177000.2
Ata_AET5Gv21062700.5
Ata_AET5Gv20847800.3
Ata_AET5Gv20565600.1
Ata_AET5Gv20035500.1
Ata_AET5Gv20003400.1
Ata_AET4Gv20603500.1
Ata_AET4Gv20549800.1
Ata_AET4Gv20271600.1
Ata_AET4Gv20270900.1
1
2
−1000
0
1000
2000
3000
4000
5000

Ae.ta RLK−Pelle_SD−2b subfamily exon−intron and kinase domain diagram (part 4)

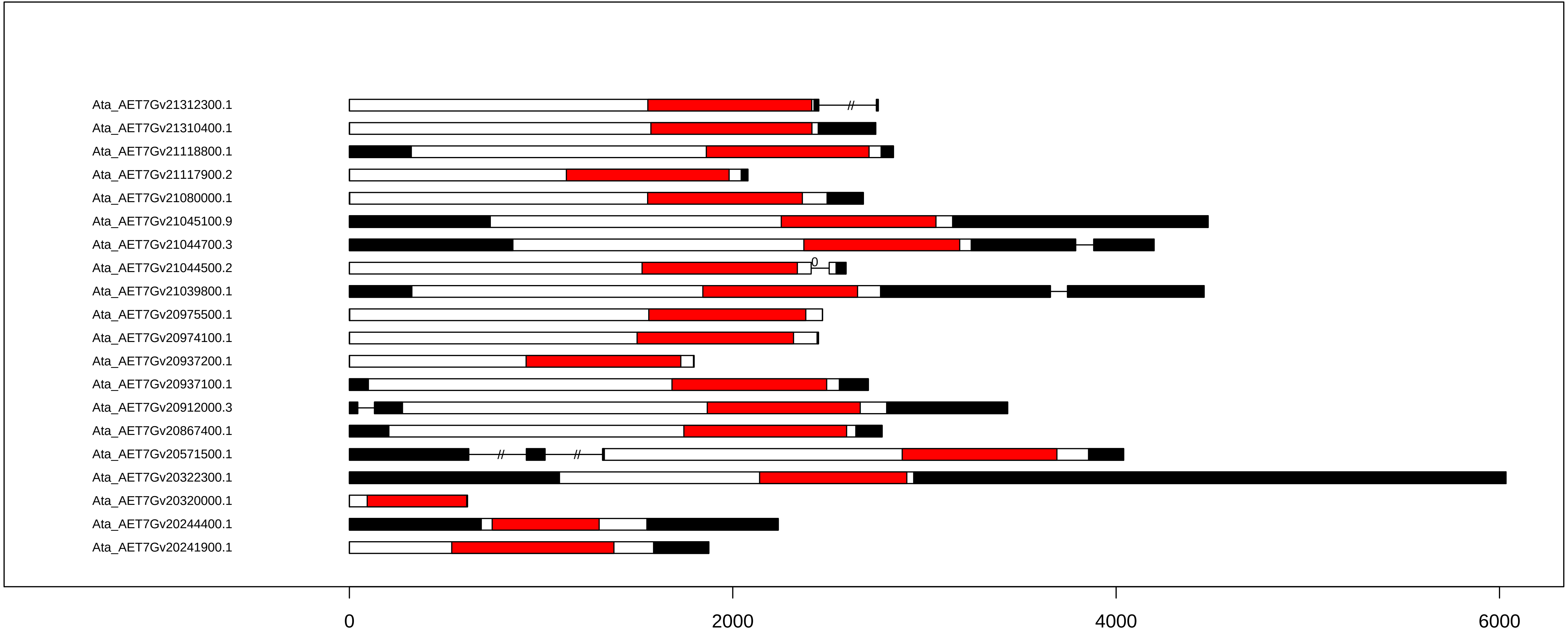

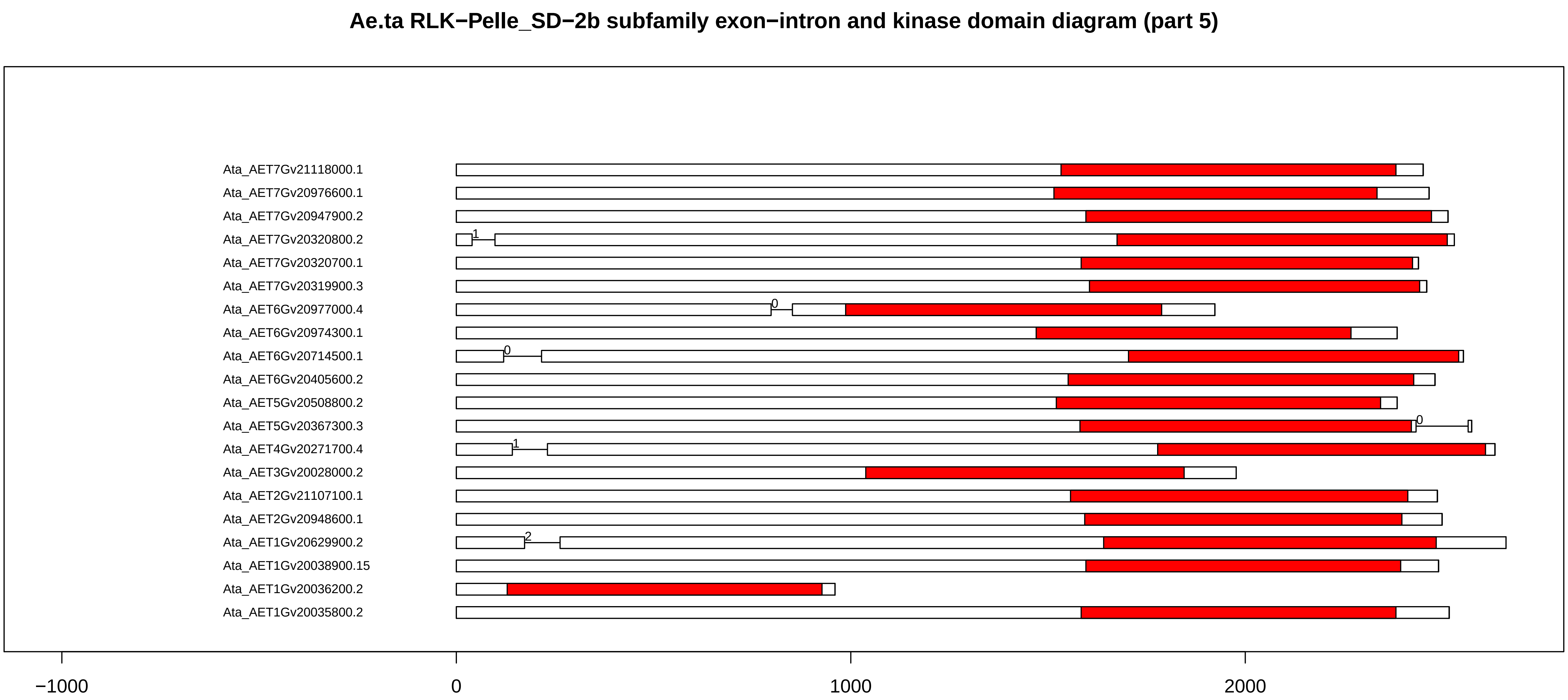
Ae.ta RLK−Pelle_SD−2b subfamily exon−intron and kinase domain diagram (part 5)
Ata_AET7Gv21118000.1
Ata_AET7Gv20976600.1
Ata_AET7Gv20947900.2
Ata_AET7Gv20320800.2
Ata_AET7Gv20320700.1
Ata_AET7Gv20319900.3
Ata_AET6Gv20977000.4
Ata_AET6Gv20974300.1
Ata_AET6Gv20714500.1
Ata_AET6Gv20405600.2
Ata_AET5Gv20508800.2
Ata_AET5Gv20367300.3
Ata_AET4Gv20271700.4
Ata_AET3Gv20028000.2
Ata_AET2Gv21107100.1
Ata_AET2Gv20948600.1
Ata_AET1Gv20629900.2
Ata_AET1Gv20038900.15
Ata_AET1Gv20036200.2
Ata_AET1Gv20035800.2
1
0
0
0
1
2
−1000
0
1000
2000

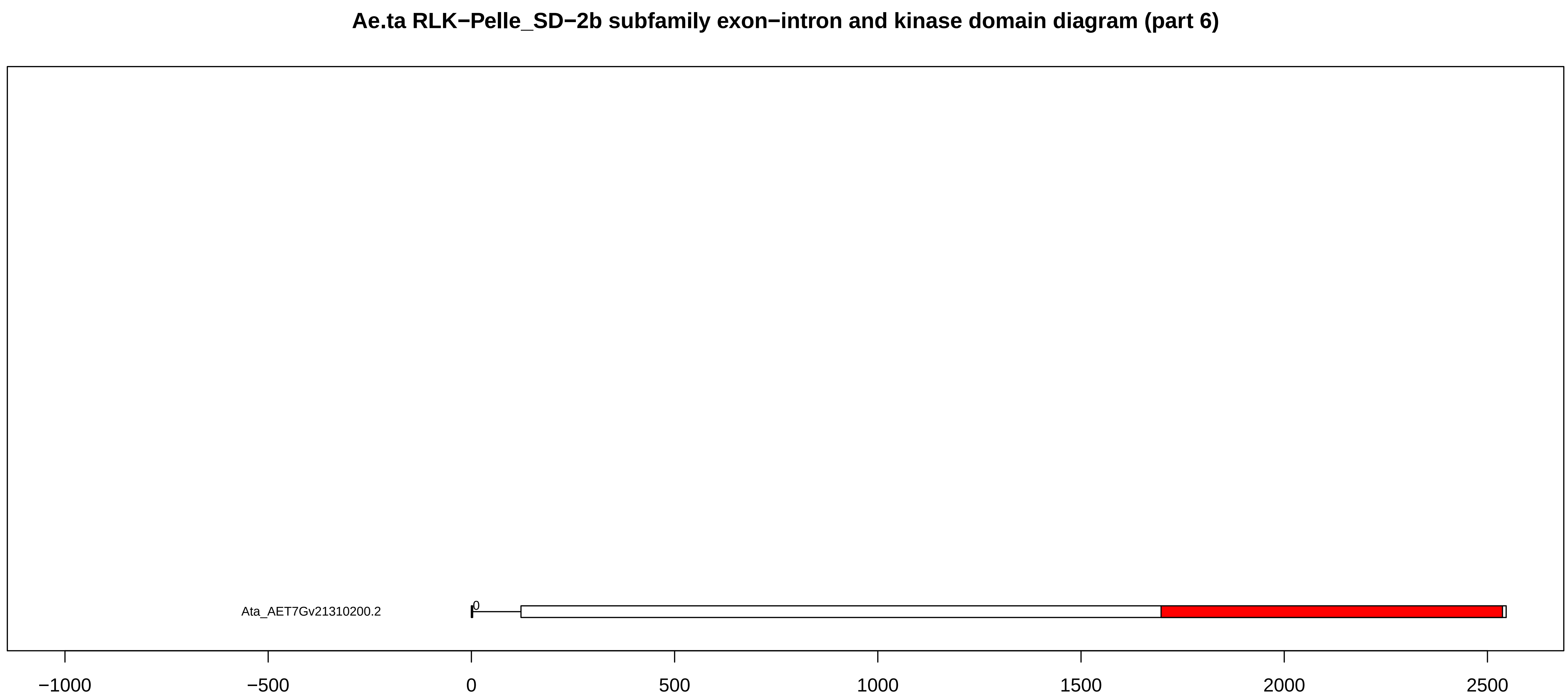

Ae.ta RLK−Pelle_SD−2b subfamily exon−intron and kinase domain diagram (part 6)
Ata_AET7Gv21310200.2
0
−1000
−500
0
500
1000
1500
2000
2500

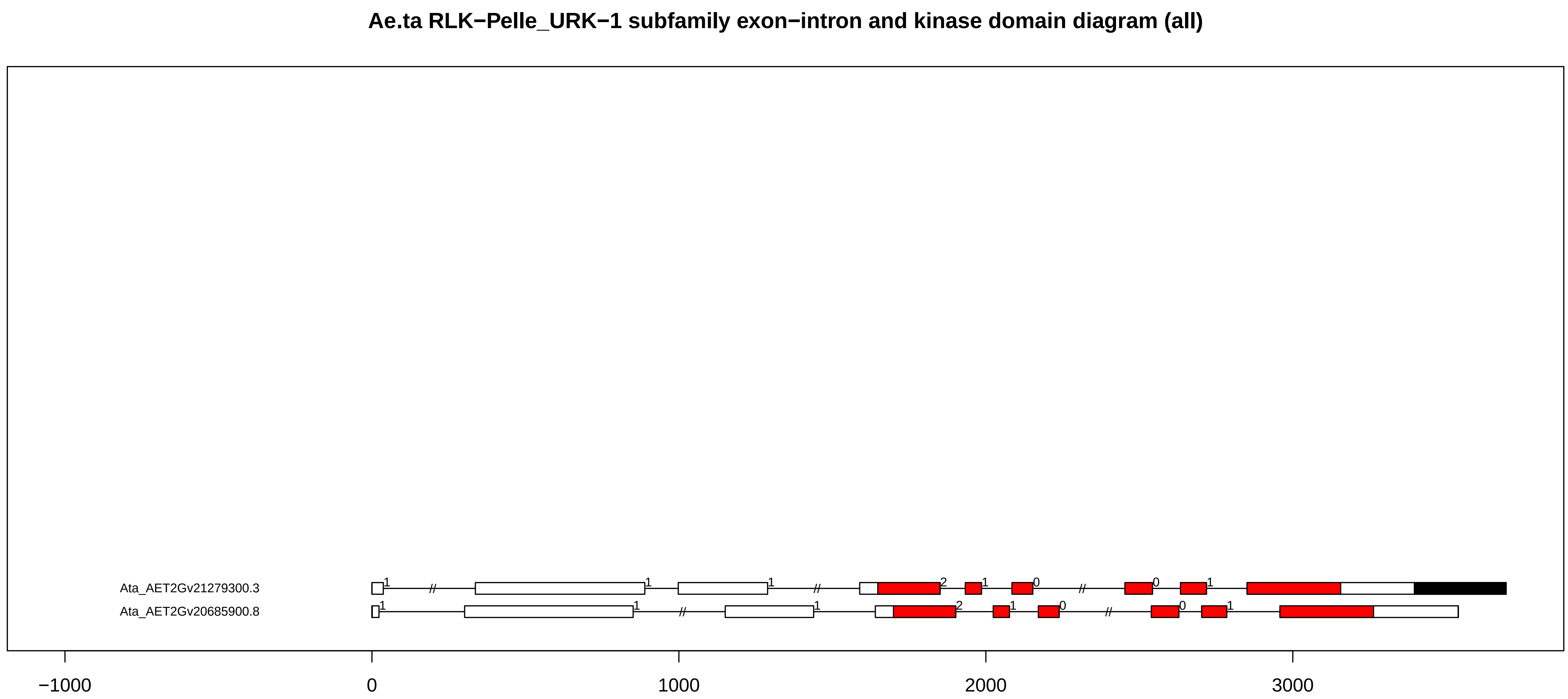

Ae.ta RLK−Pelle_URK−1 subfamily exon−intron and kinase domain diagram (all)
Ata_AET2Gv21279300.3
Ata_AET2Gv20685900.8
1
1
1
2
1
0
0
1
1
1
1
2
1
0
0
1
−1000
0
1000
2000
3000

Ae.ta RLK−Pelle_URK−2 subfamily exon−intron and kinase domain diagram (all)

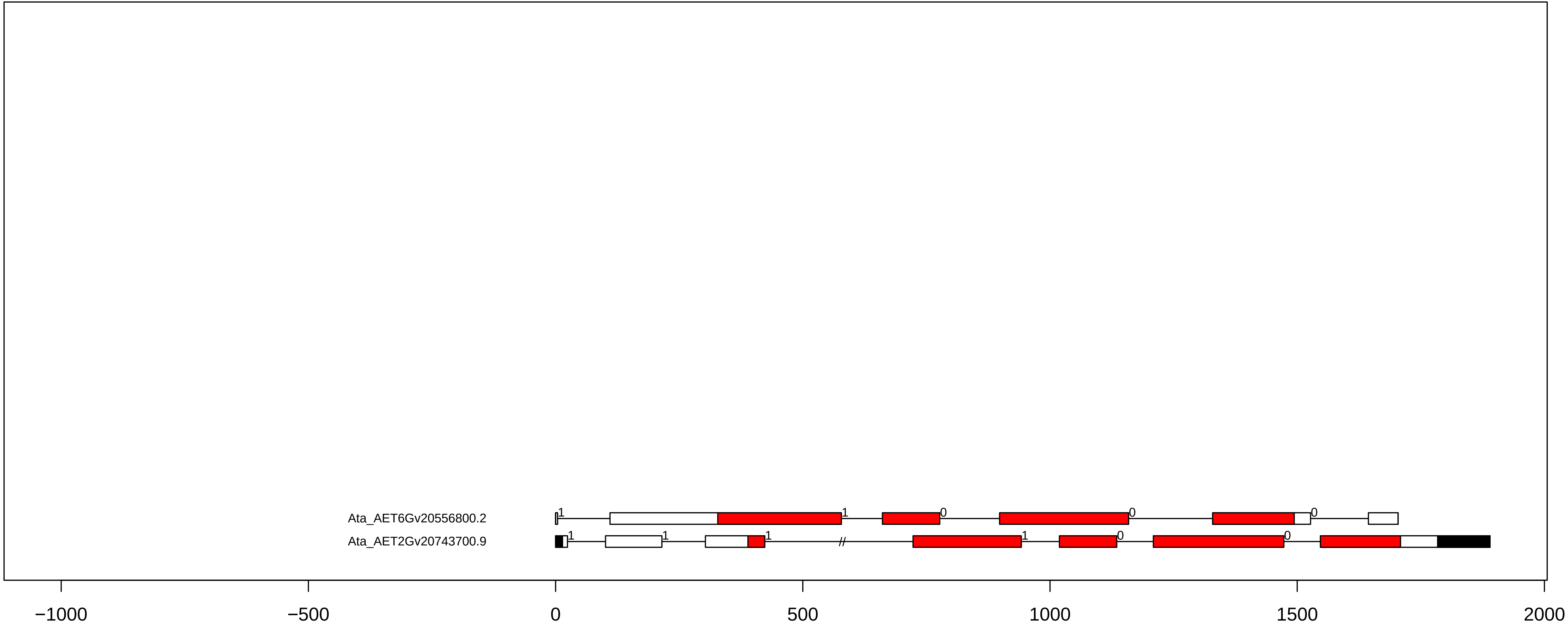

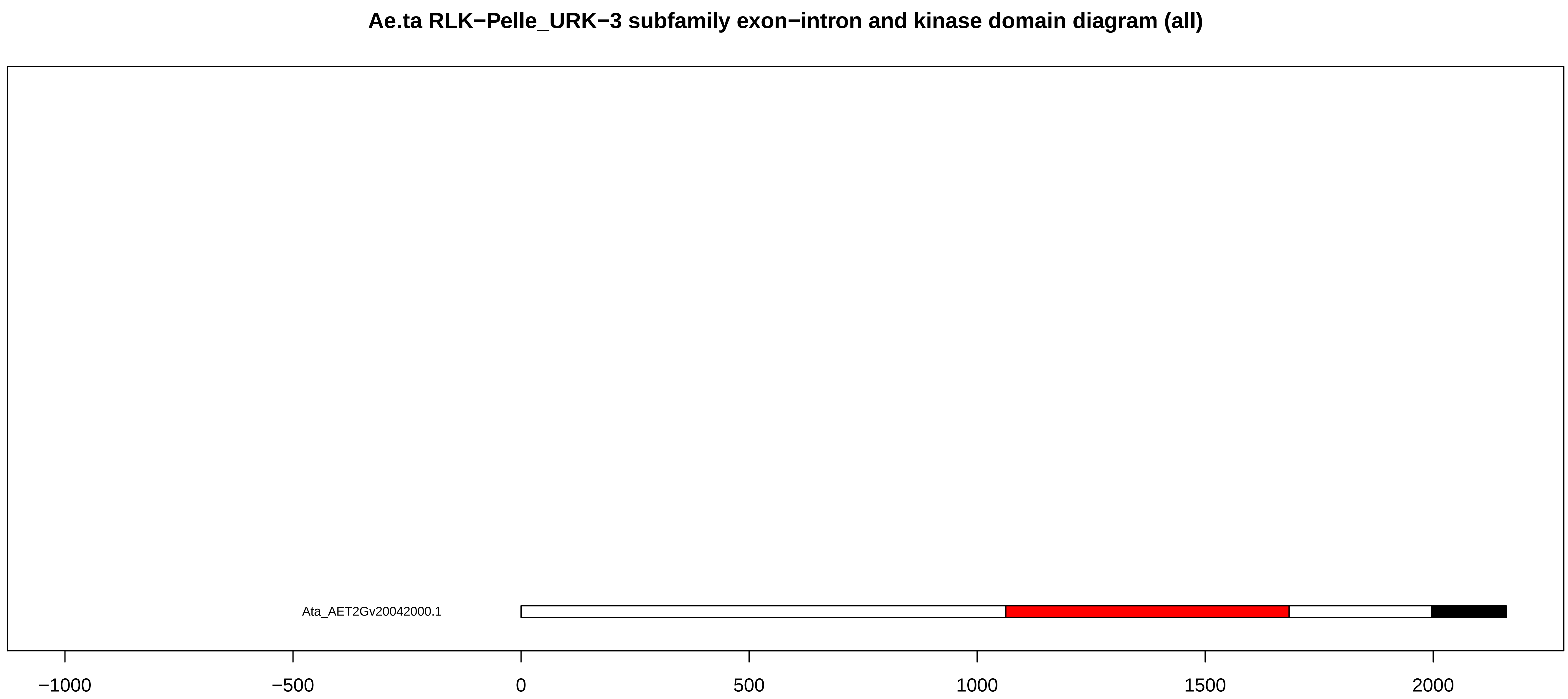

Ae.ta RLK−Pelle_URK−3 subfamily exon−intron and kinase domain diagram (all)
Ata_AET2Gv20042000.1
−1000
−500
0
500
1000
1500
2000

Ae.ta RLK−Pelle_WAK_LRK10L−1 subfamily exon−intron and kinase domain diagram (all)

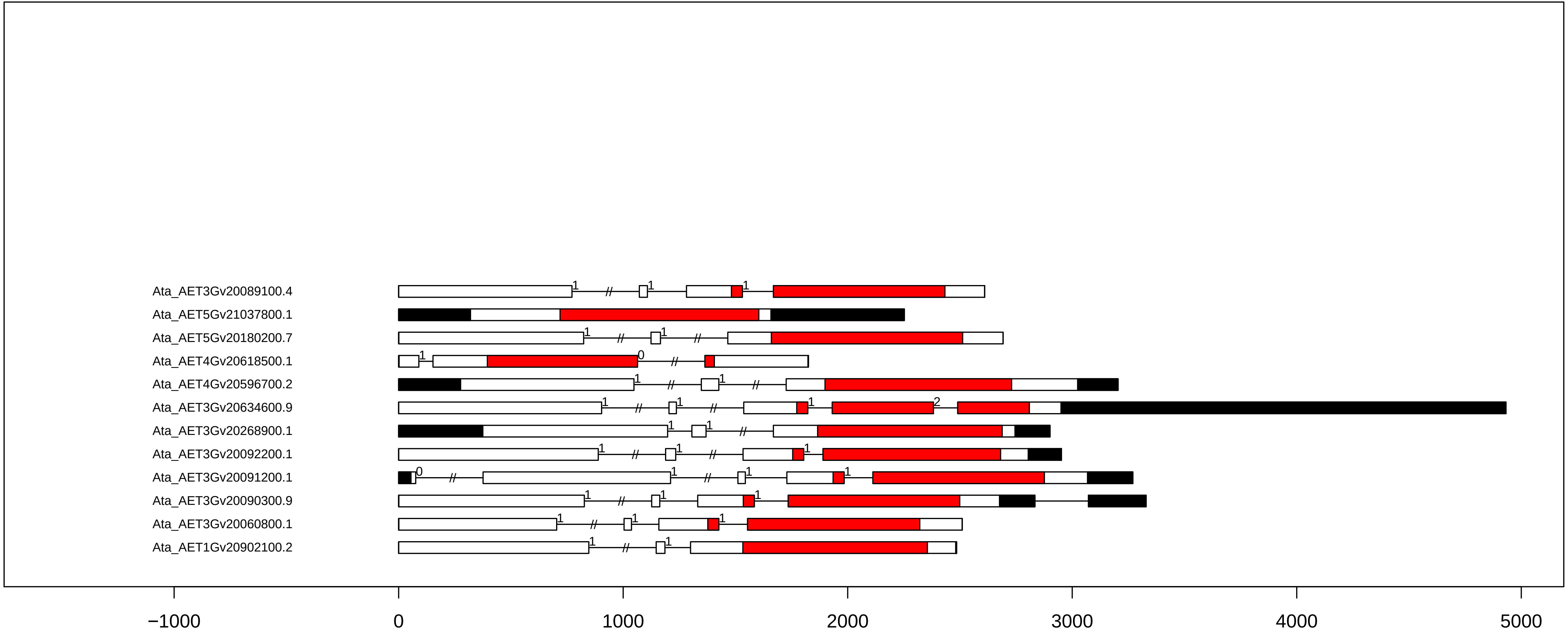

Ae.ta RLK−Pelle_WAK subfamily exon−intron and kinase domain diagram (part 1)

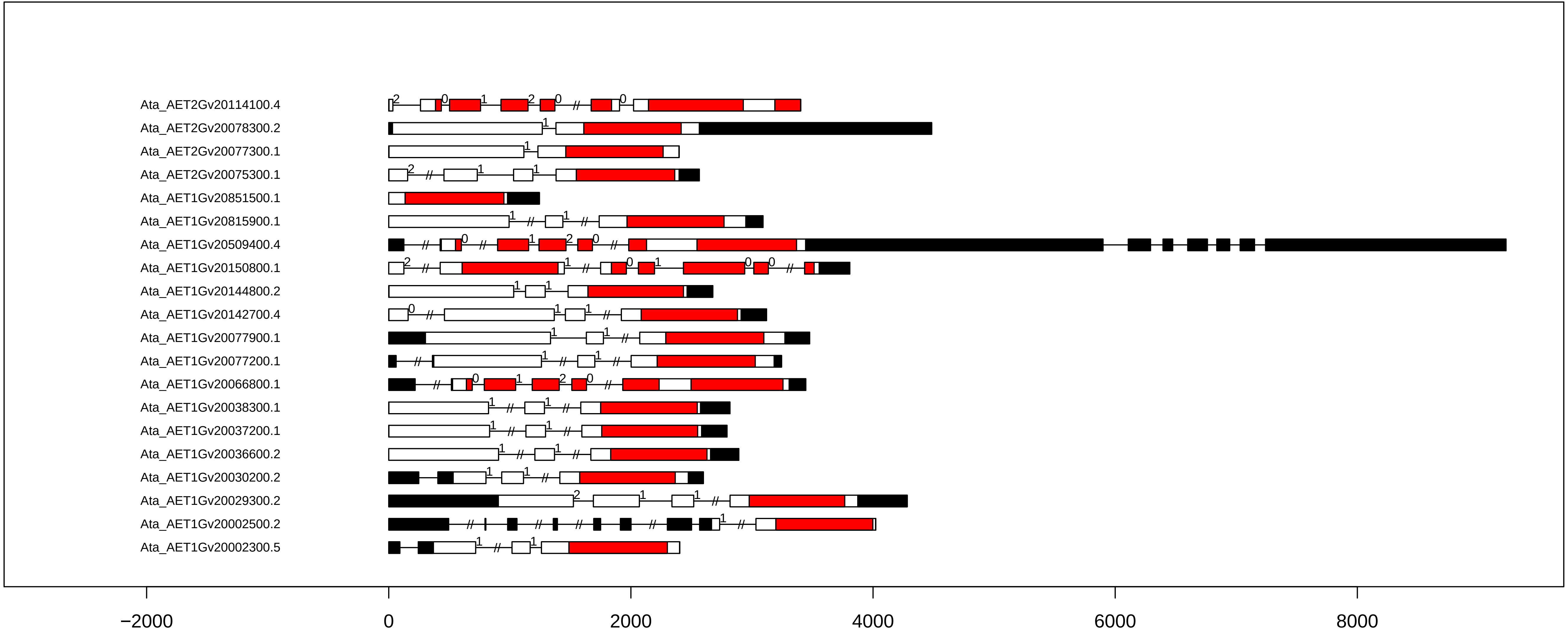

Ae.ta RLK−Pelle_WAK subfamily exon−intron and kinase domain diagram (part 2)

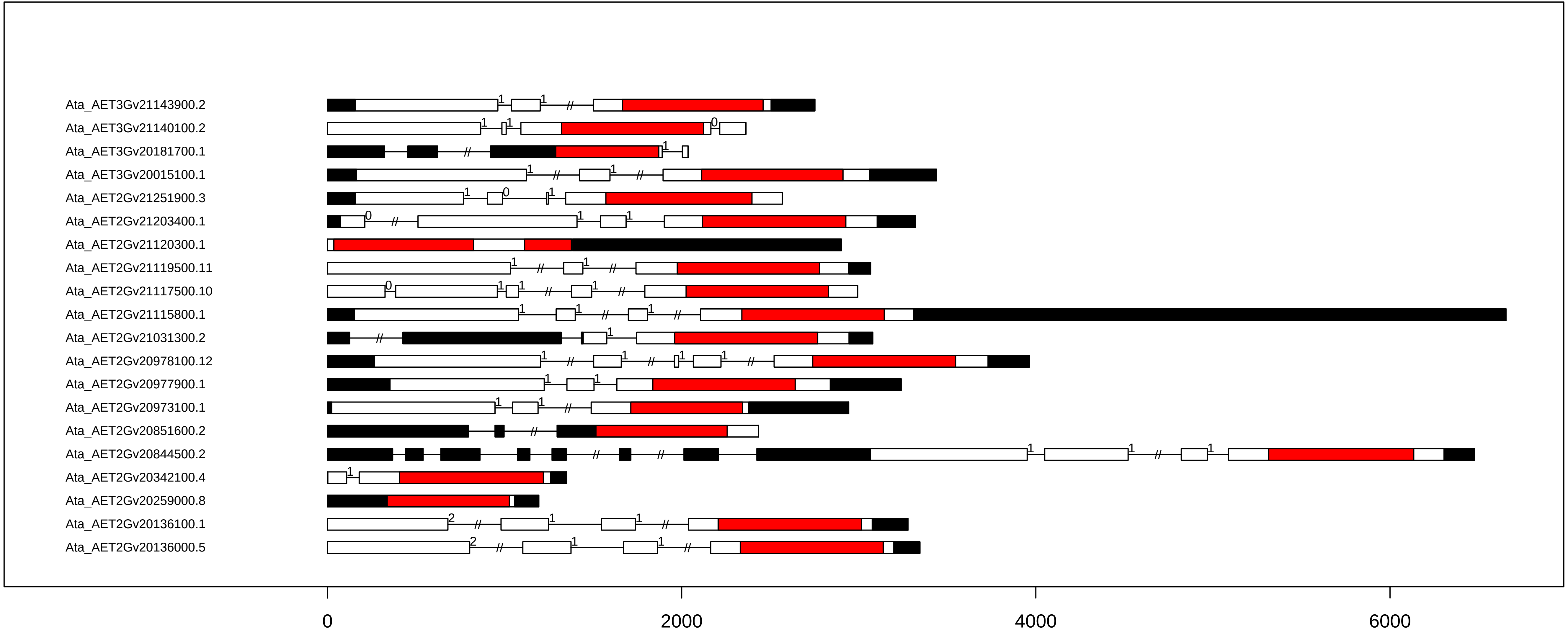

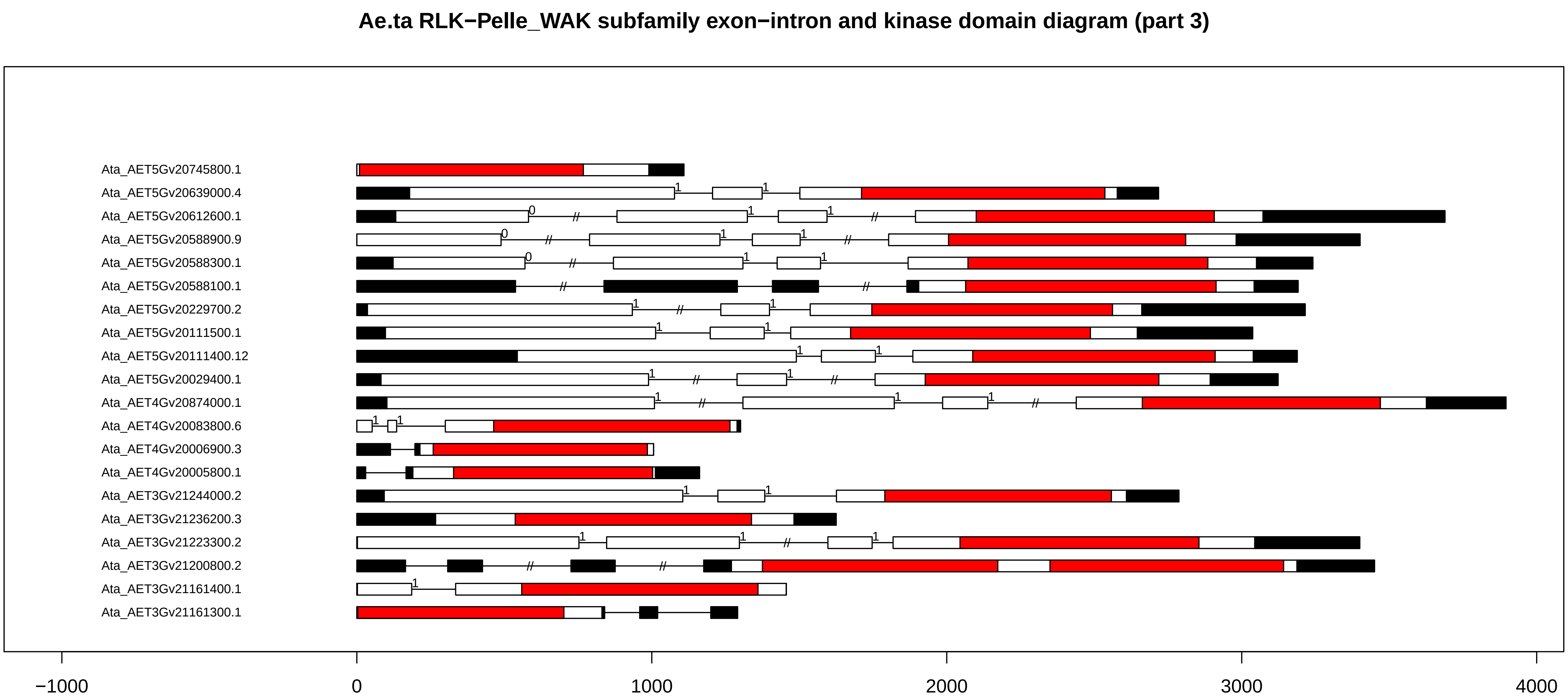
Ae.ta RLK−Pelle_WAK subfamily exon−intron and kinase domain diagram (part 3)
Ata_AET5Gv20745800.1
Ata_AET5Gv20639000.4
Ata_AET5Gv20612600.1
Ata_AET5Gv20588900.9
Ata_AET5Gv20588300.1
Ata_AET5Gv20588100.1
Ata_AET5Gv20229700.2
Ata_AET5Gv20111500.1
Ata_AET5Gv20111400.12
Ata_AET5Gv20029400.1
Ata_AET4Gv20874000.1
Ata_AET4Gv20083800.6
Ata_AET4Gv20006900.3
Ata_AET4Gv20005800.1
Ata_AET3Gv21244000.2
Ata_AET3Gv21236200.3
Ata_AET3Gv21223300.2
Ata_AET3Gv21200800.2
Ata_AET3Gv21161400.1
Ata_AET3Gv21161300.1
−1000
0
1000
2000
3000
4000

Ae.ta RLK−Pelle_WAK subfamily exon−intron and kinase domain diagram (part 4)

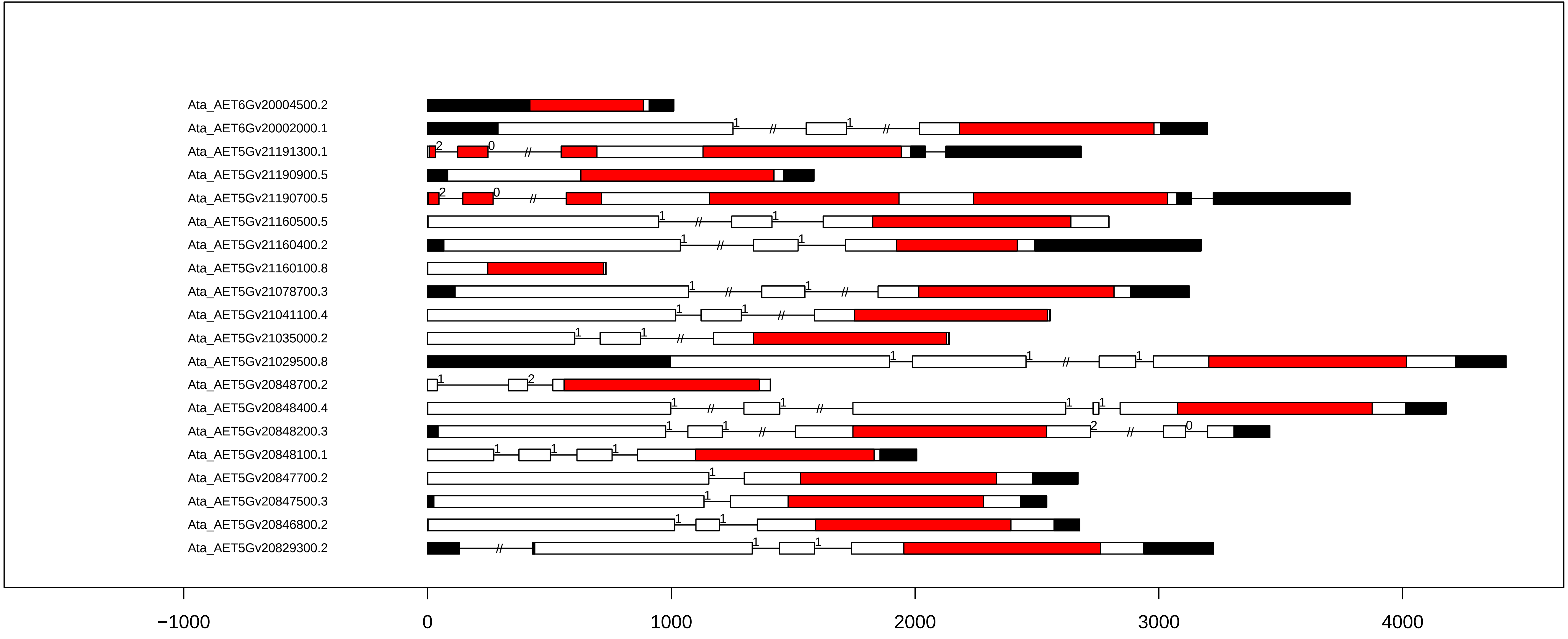

Ae.ta RLK−Pelle_WAK subfamily exon−intron and kinase domain diagram (part 5)

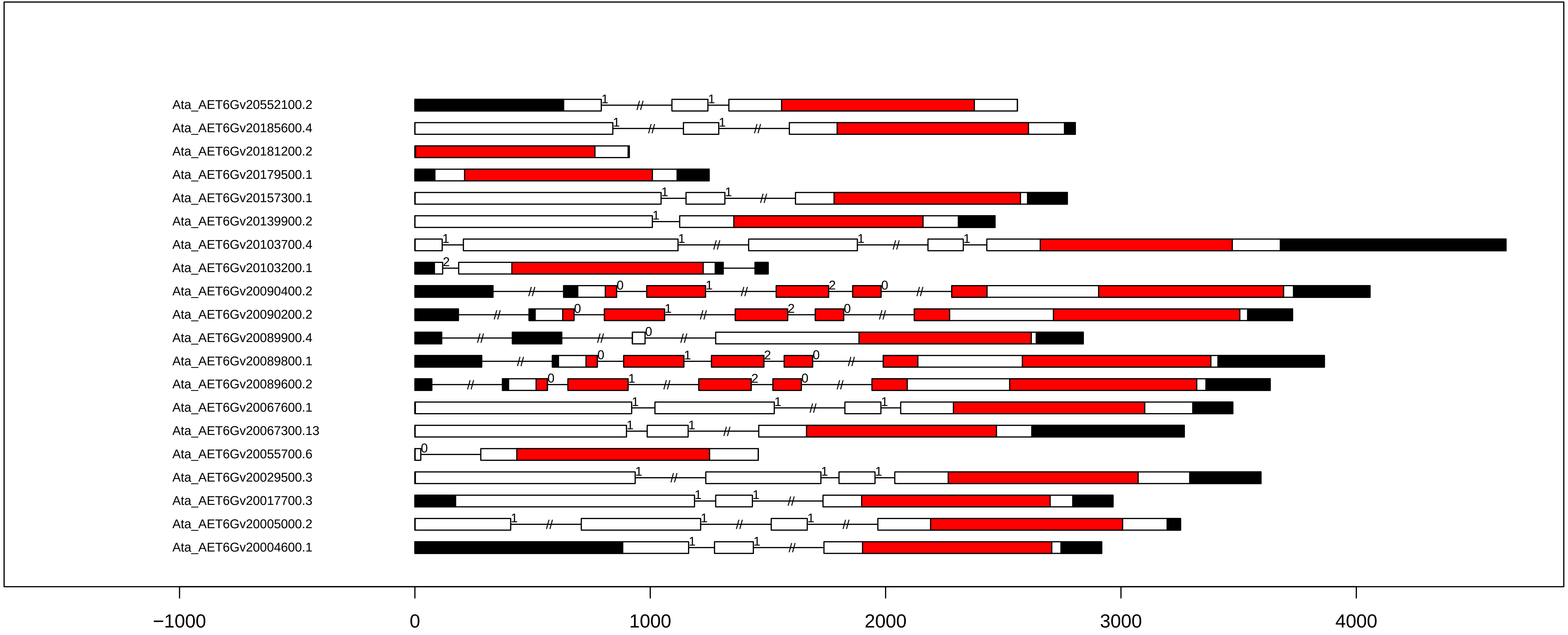

Ae.ta RLK−Pelle_WAK subfamily exon−intron and kinase domain diagram (part 6)

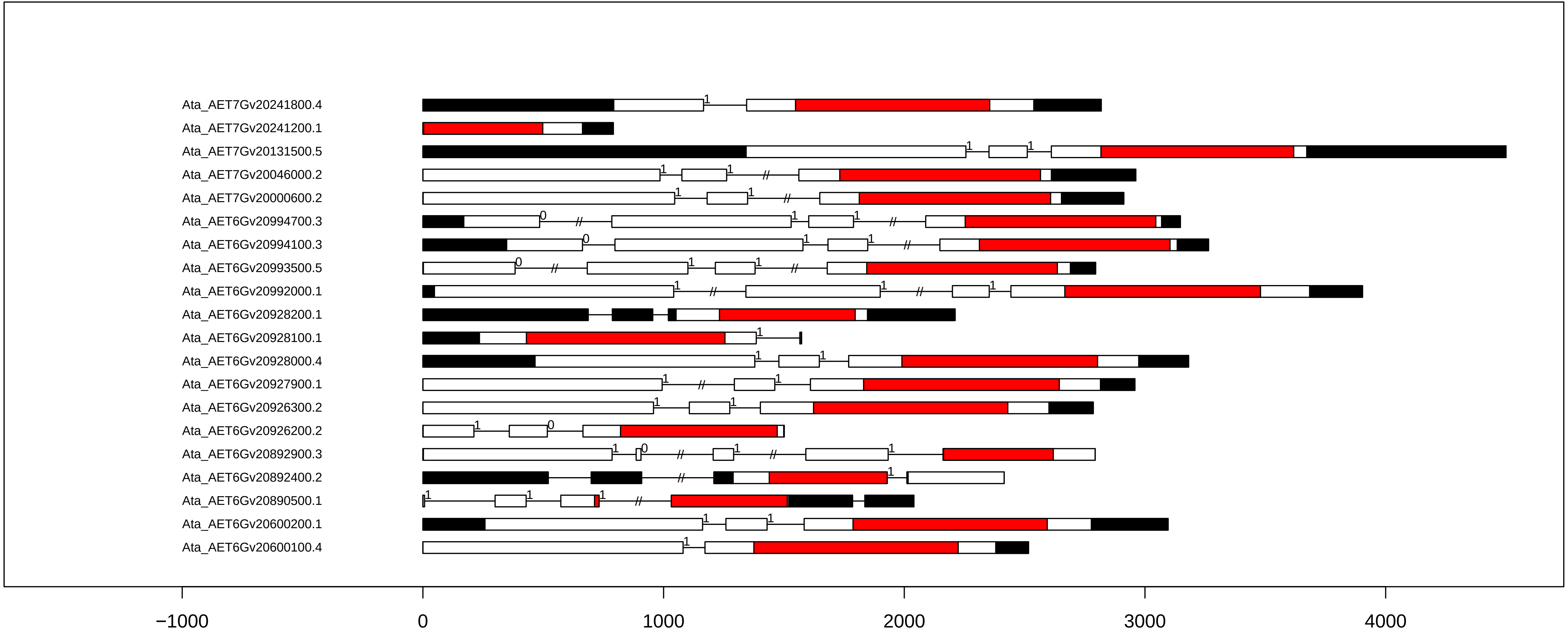

Ae.ta RLK−Pelle_WAK subfamily exon−intron and kinase domain diagram (part 7)

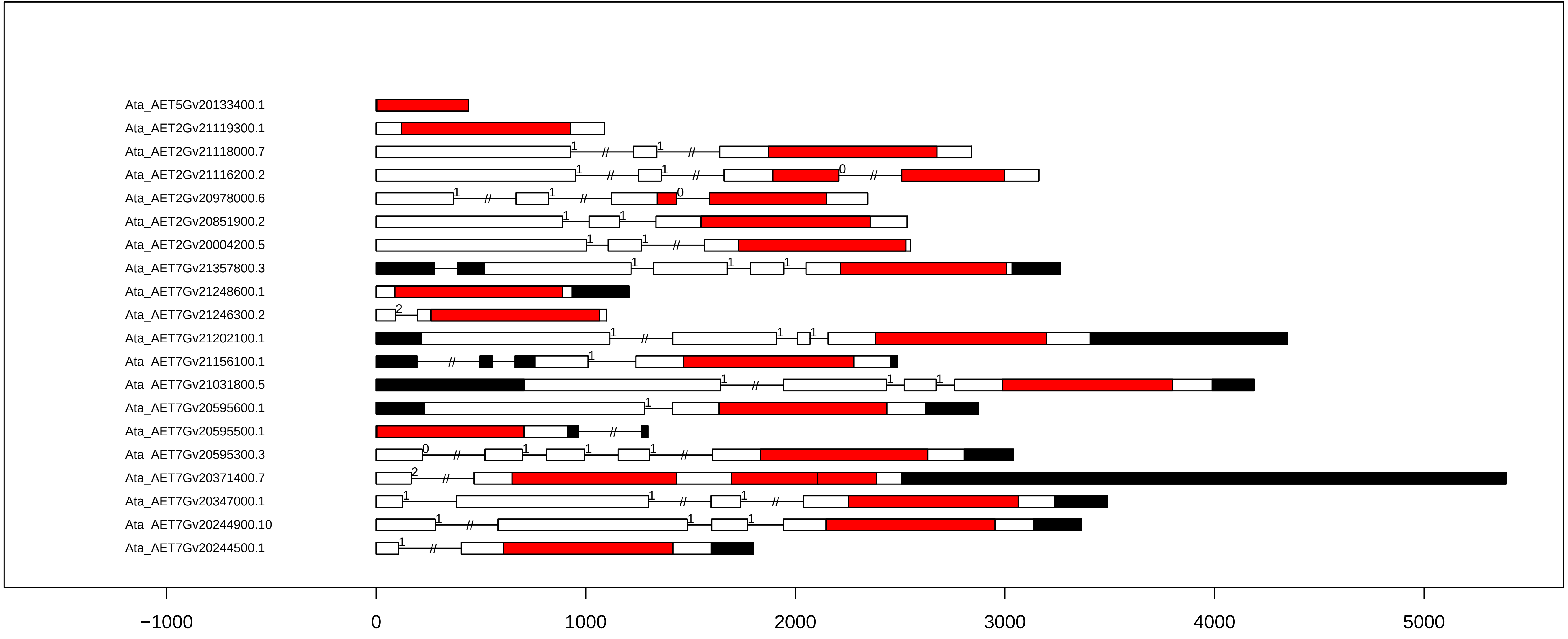

Ae.ta RLK−Pelle_WAK subfamily exon−intron and kinase domain diagram (part 8)

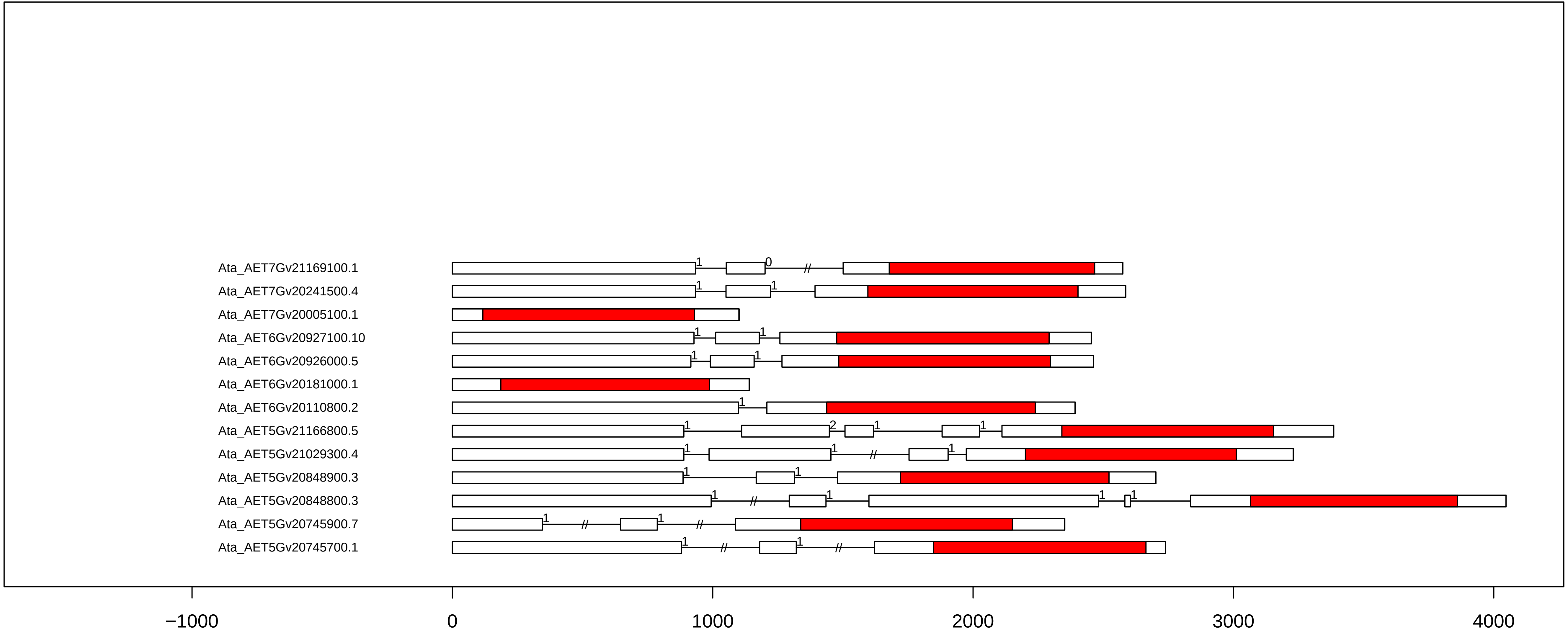

B.di RLK−Pelle_C−LEC subfamily exon−intron and kinase domain diagram (all)

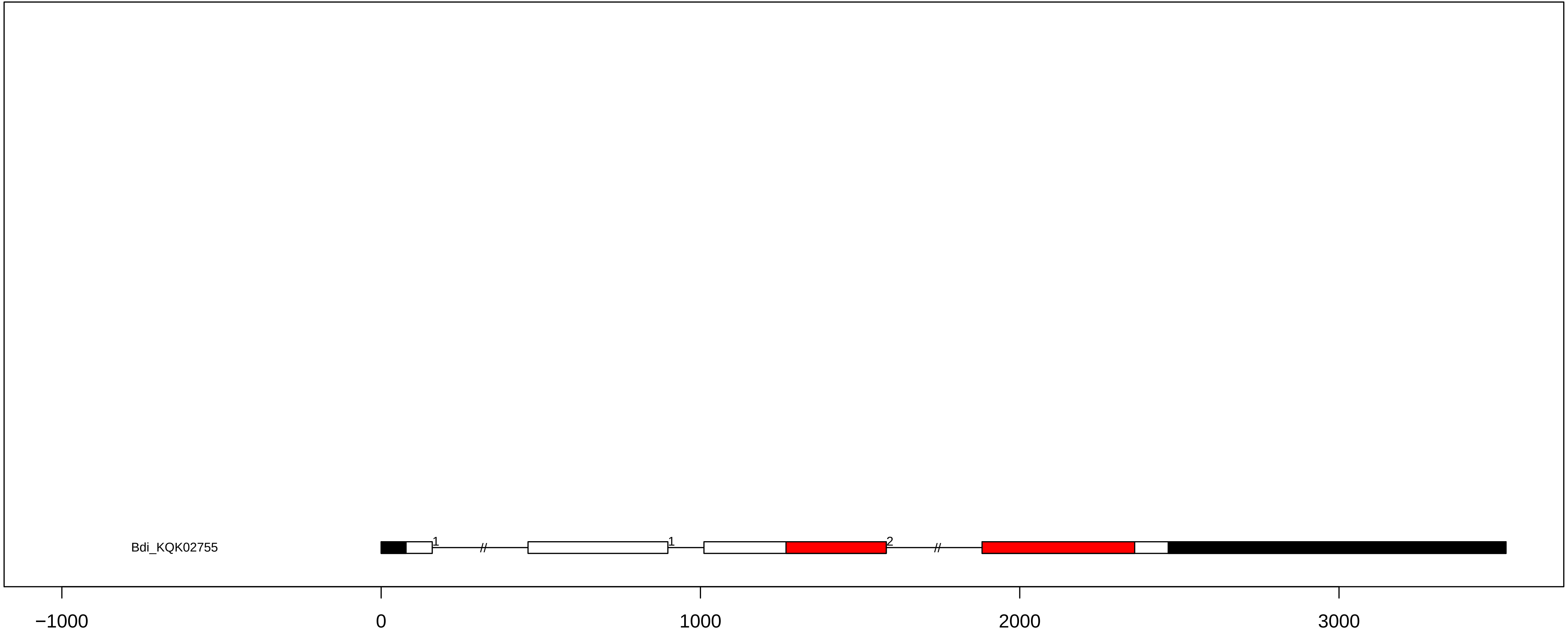

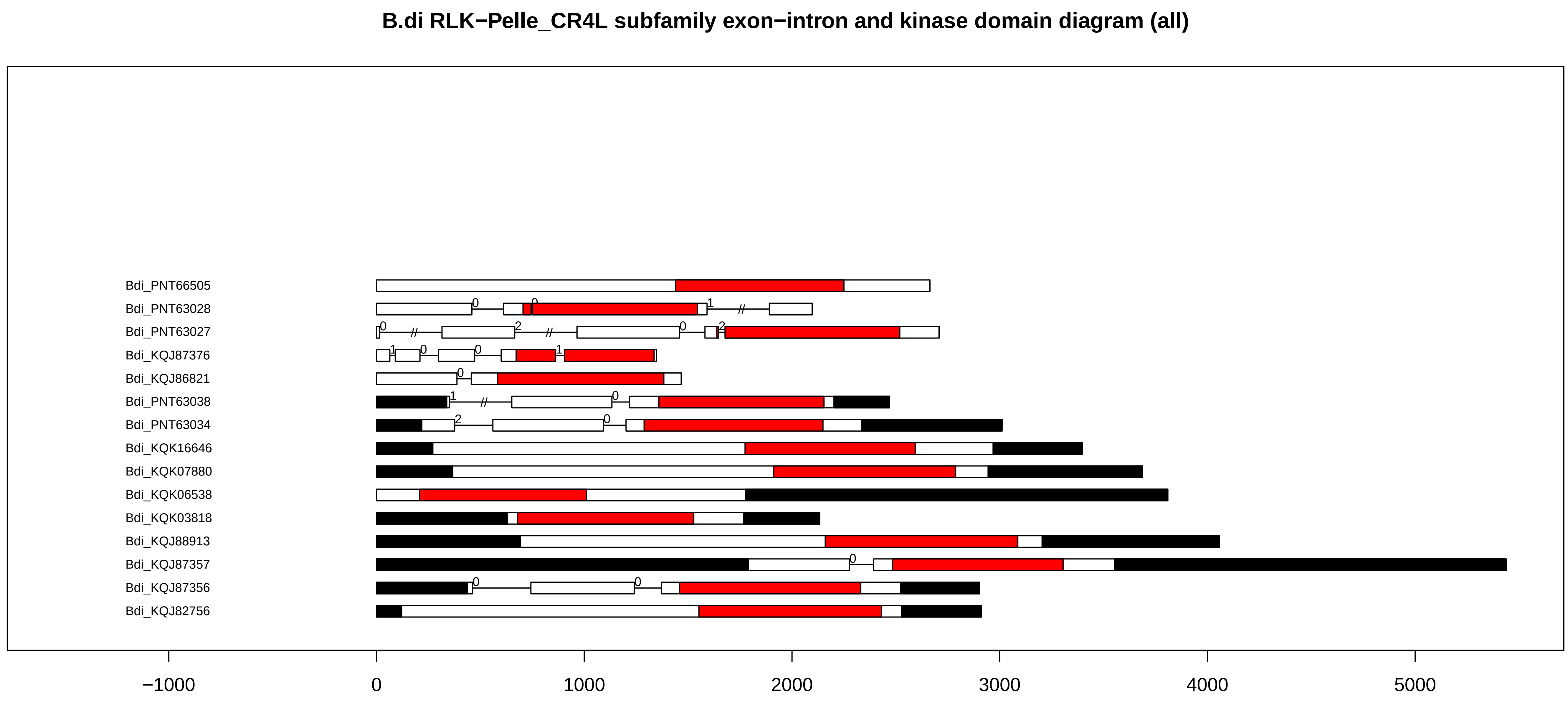

B.di RLK−Pelle_CR4L subfamily exon−intron and kinase domain diagram (all)
Bdi_PNT66505
Bdi_PNT63028
Bdi_PNT63027
Bdi_KQJ87376
Bdi_KQJ86821
Bdi_PNT63038
Bdi_PNT63034
Bdi_KQK16646
Bdi_KQK07880
Bdi_KQK06538
Bdi_KQK03818
Bdi_KQJ88913
Bdi_KQJ87357
Bdi_KQJ87356
Bdi_KQJ82756
−1000
0
1000
2000
3000
4000
5000

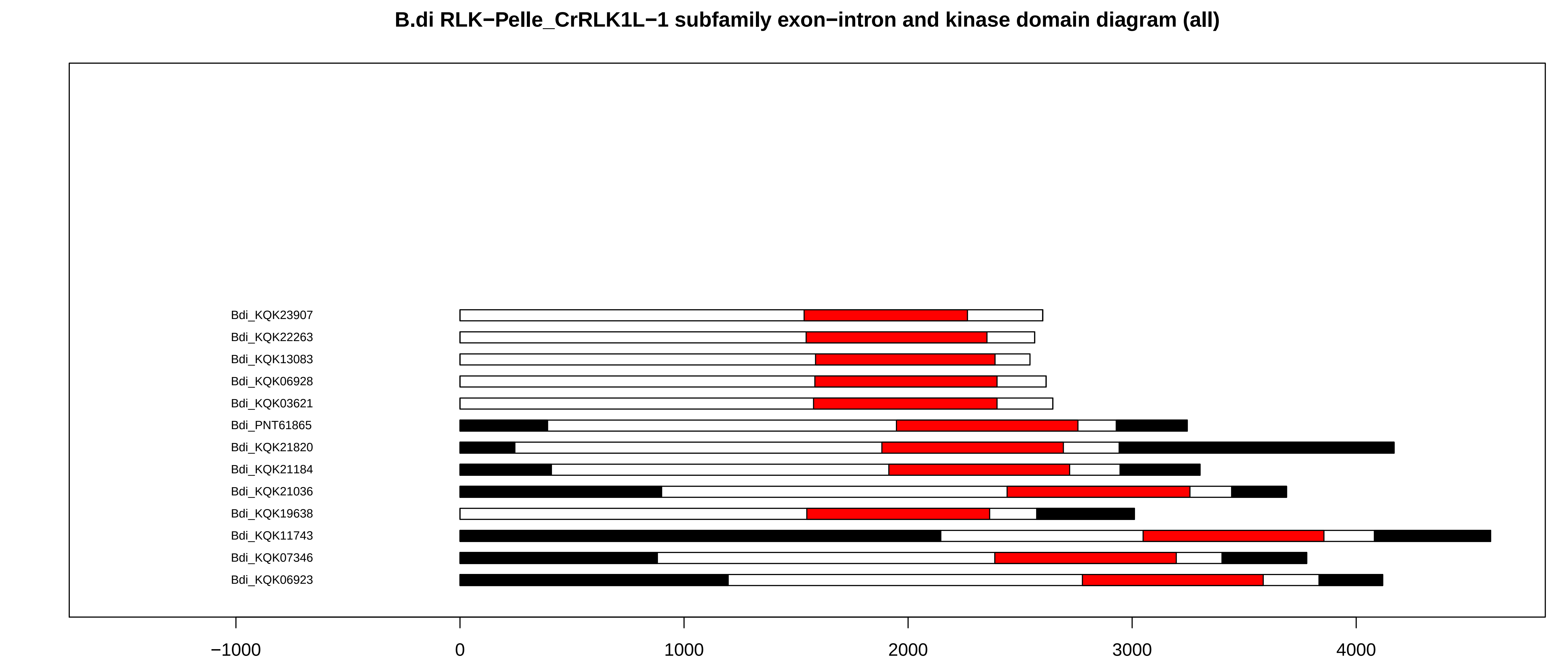
B.di RLK−Pelle_CrRLK1L−1 subfamily exon−intron and kinase domain diagram (all)
Bdi_KQK23907
Bdi_KQK22263
Bdi_KQK13083
Bdi_KQK06928
Bdi_KQK03621
Bdi_PNT61865
Bdi_KQK21820
Bdi_KQK21184
Bdi_KQK21036
Bdi_KQK19638
Bdi_KQK11743
Bdi_KQK07346
Bdi_KQK06923
−1000
0
1000
2000
3000
4000

B.di RLK−Pelle_DLSV subfamily exon−intron and kinase domain diagram (part 1)

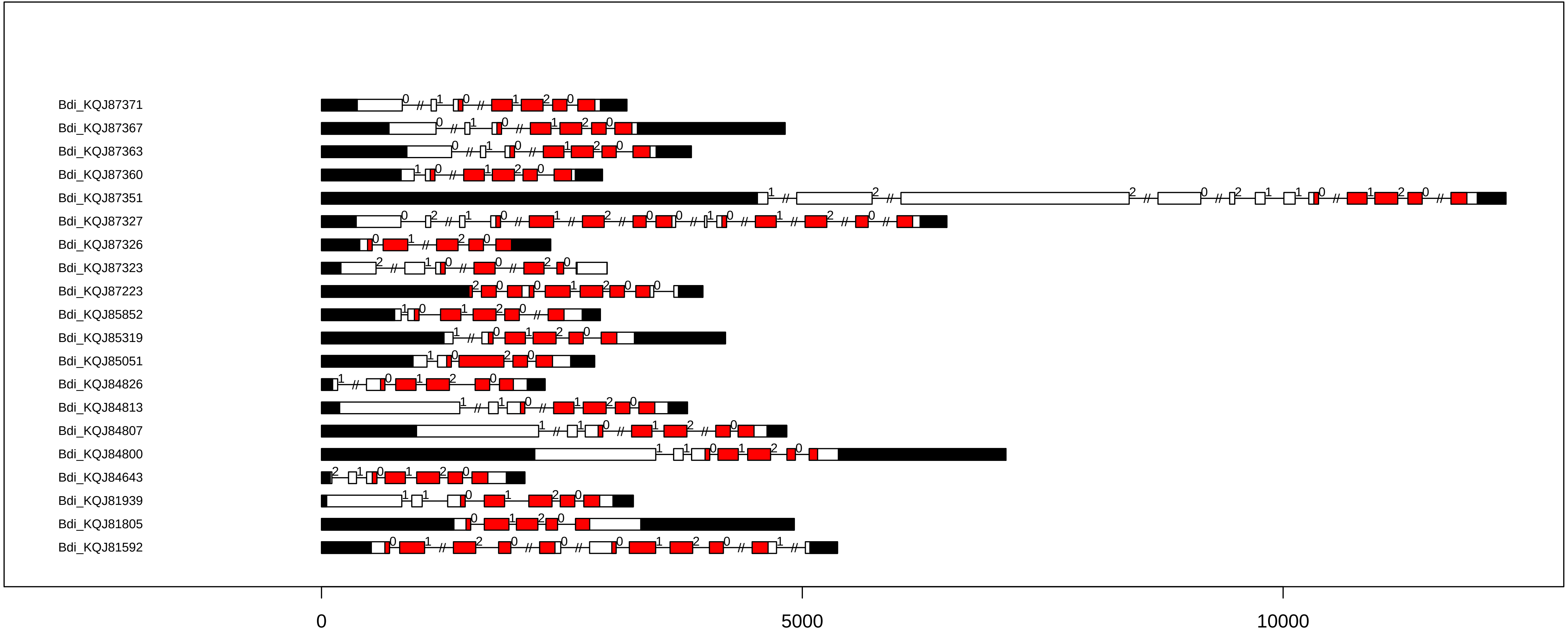

B.di RLK−Pelle_DLSV subfamily exon−intron and kinase domain diagram (part 2)

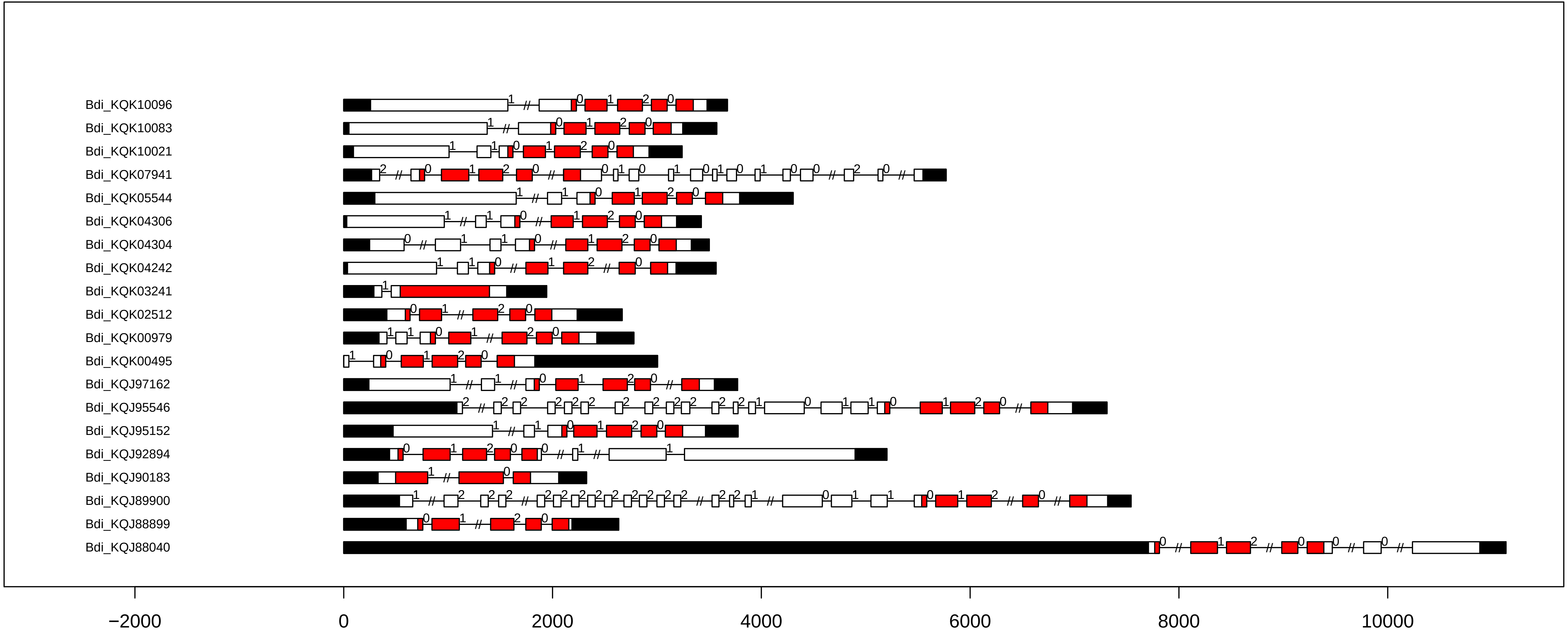

B.di RLK−Pelle_DLSV subfamily exon−intron and kinase domain diagram (part 3)

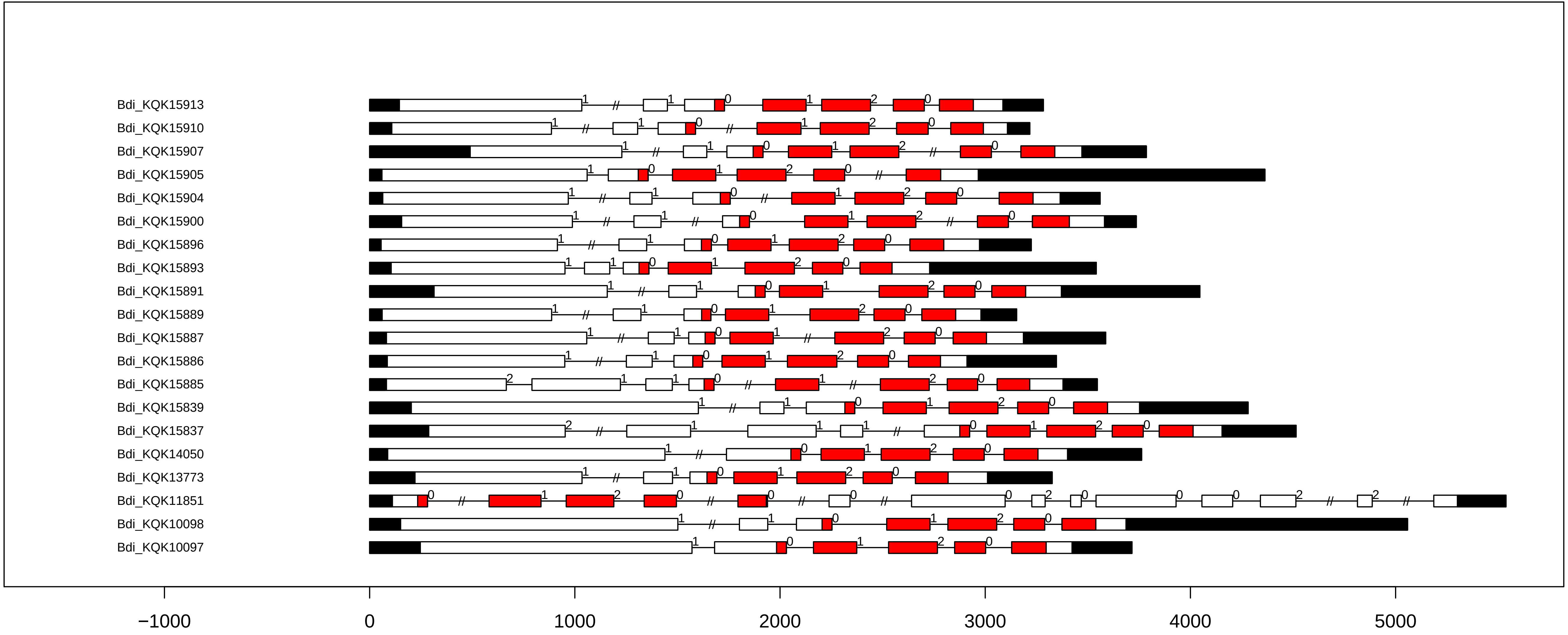

B.di RLK−Pelle_DLSV subfamily exon−intron and kinase domain diagram (part 4)

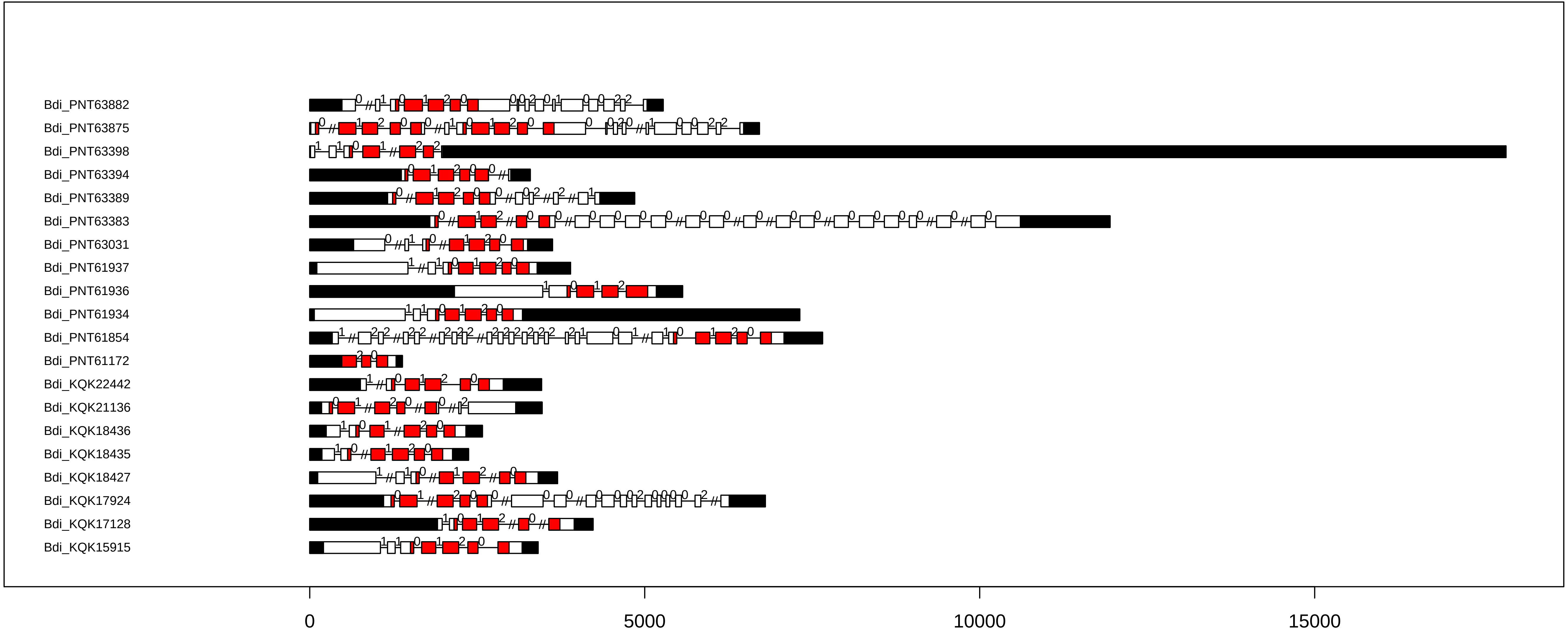

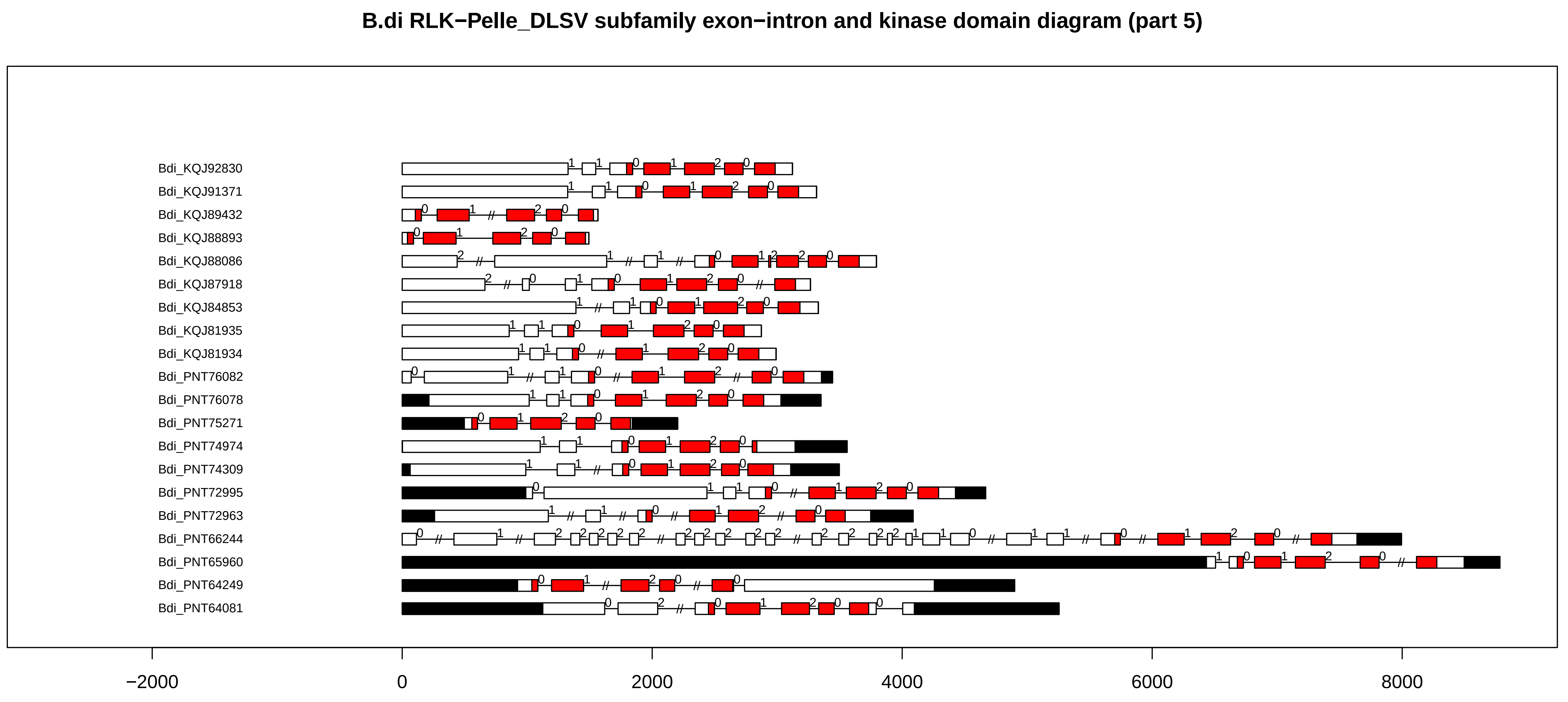
B.di RLK−Pelle_DLSV subfamily exon−intron and kinase domain diagram (part 5)
Bdi_KQJ92830
Bdi_KQJ91371
Bdi_KQJ89432
Bdi_KQJ88893
Bdi_KQJ88086
Bdi_KQJ87918
Bdi_KQJ84853
Bdi_KQJ81935
Bdi_KQJ81934
Bdi_PNT76082
Bdi_PNT76078
Bdi_PNT75271
Bdi_PNT74974
Bdi_PNT74309
Bdi_PNT72995
Bdi_PNT72963
Bdi_PNT66244
Bdi_PNT65960
Bdi_PNT64249
Bdi_PNT64081
−2000
0
2000
4000
6000
8000

B.di RLK−Pelle_DLSV subfamily exon−intron and kinase domain diagram (part 6)

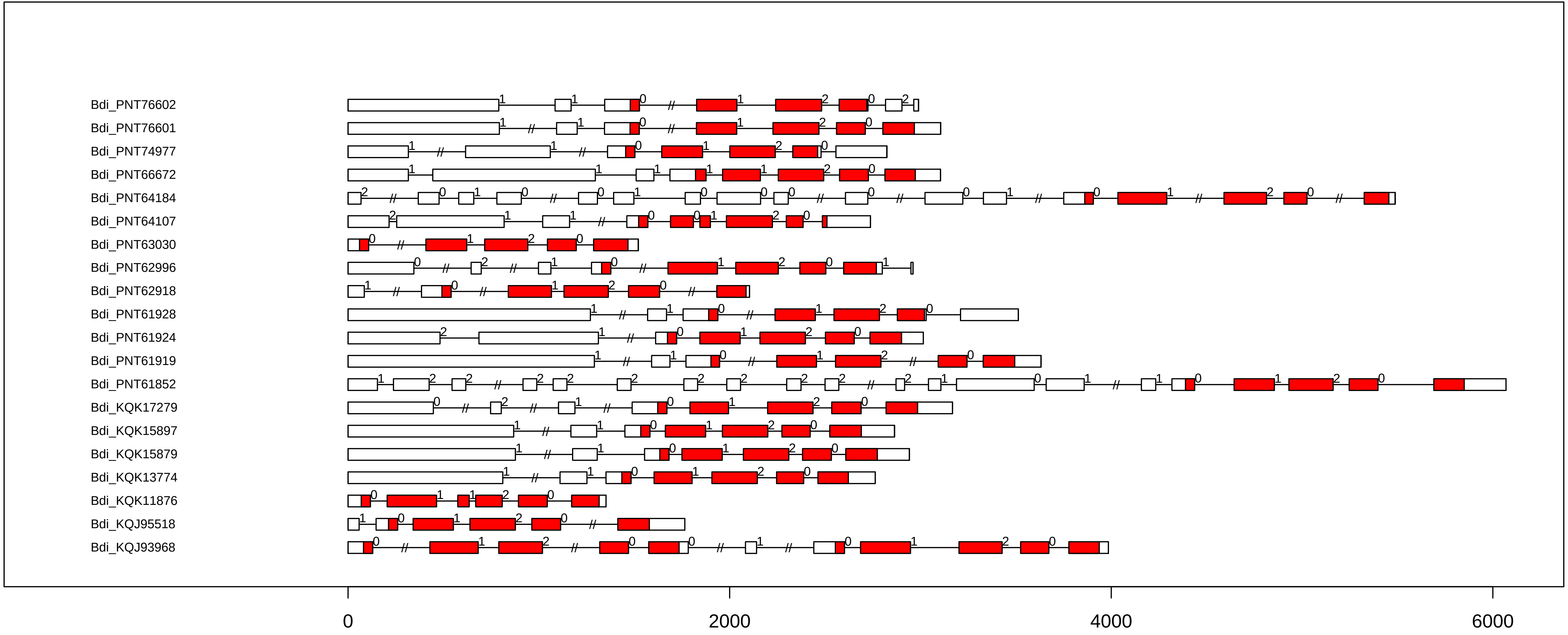

B.di RLK−Pelle_Extensin subfamily exon−intron and kinase domain diagram (all)

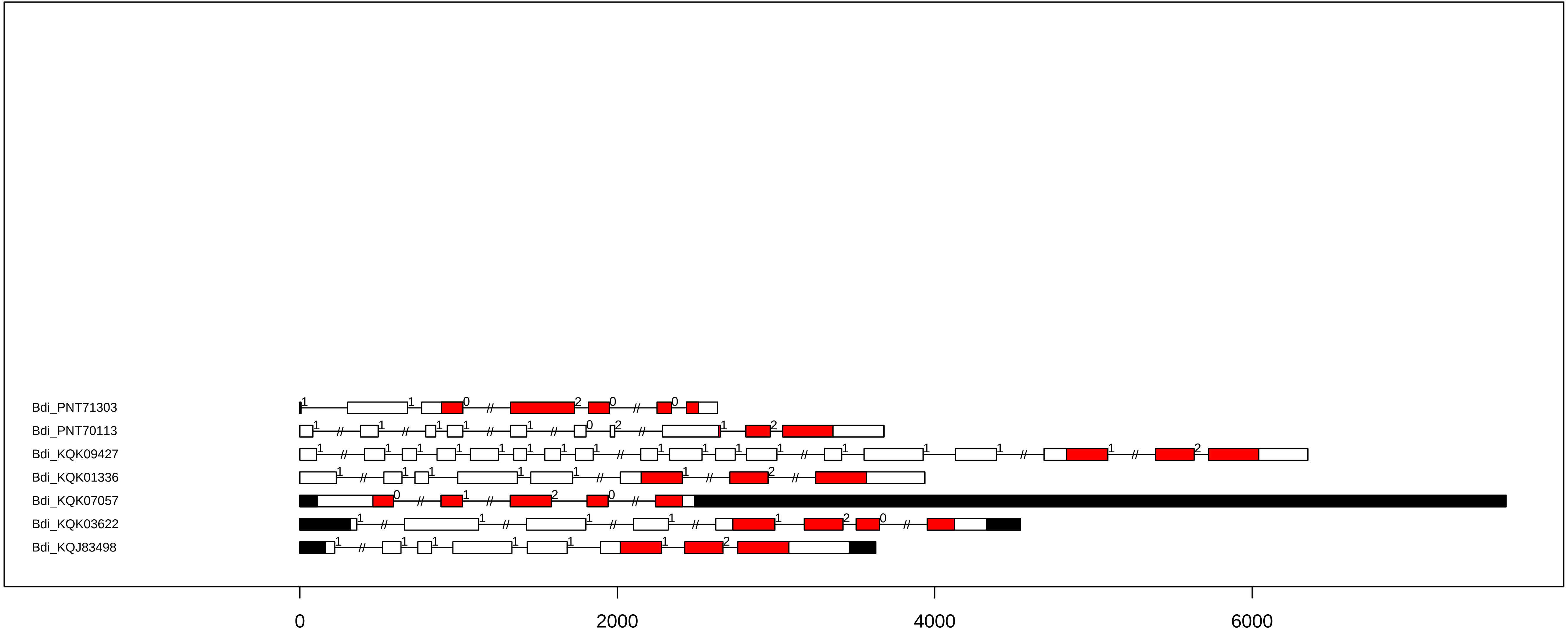

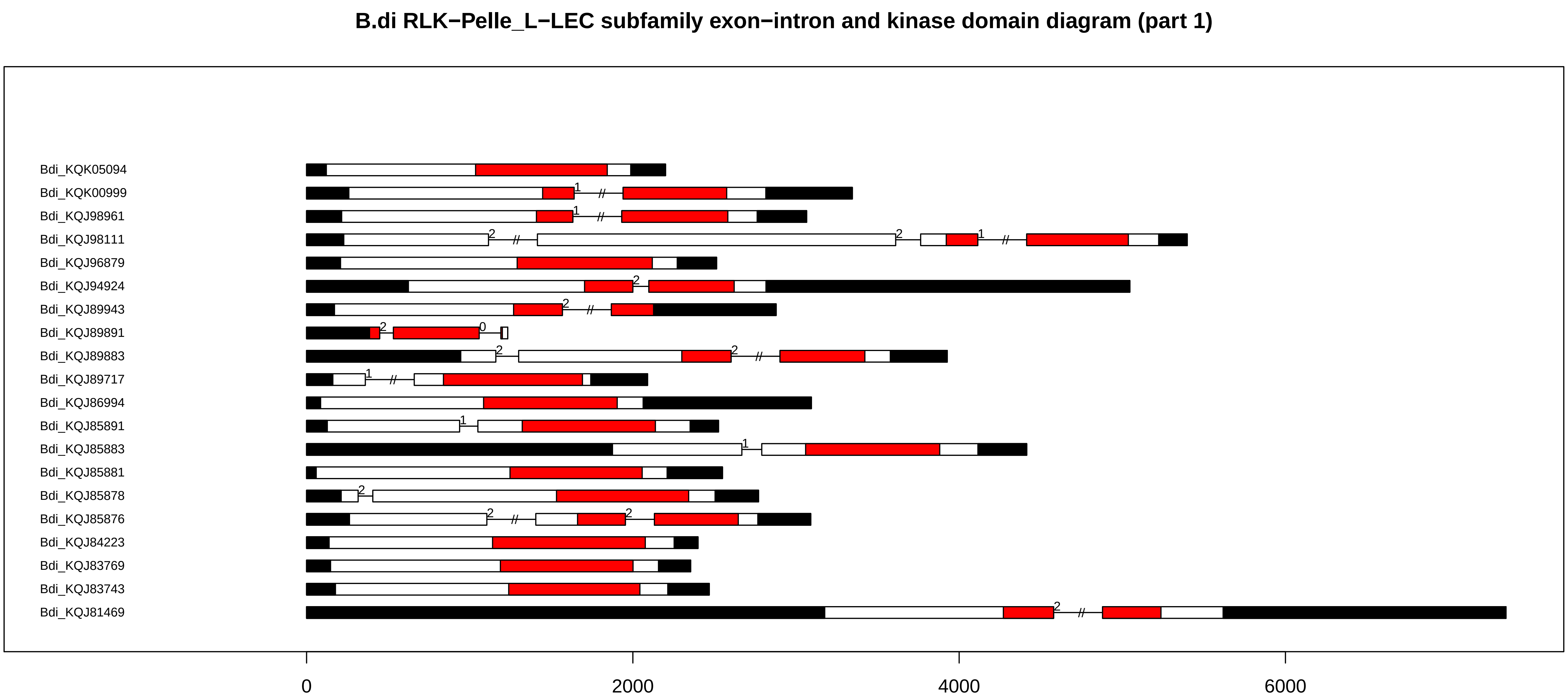

B.di RLK−Pelle_L−LEC subfamily exon−intron and kinase domain diagram (part 1)
Bdi_KQK05094
Bdi_KQK00999
Bdi_KQJ98961
Bdi_KQJ98111
Bdi_KQJ96879
Bdi_KQJ94924
Bdi_KQJ89943
Bdi_KQJ89891
Bdi_KQJ89883
Bdi_KQJ89717
Bdi_KQJ86994
Bdi_KQJ85891
Bdi_KQJ85883
Bdi_KQJ85881
Bdi_KQJ85878
Bdi_KQJ85876
Bdi_KQJ84223
Bdi_KQJ83769
Bdi_KQJ83743
Bdi_KQJ81469
0
2000
4000
6000

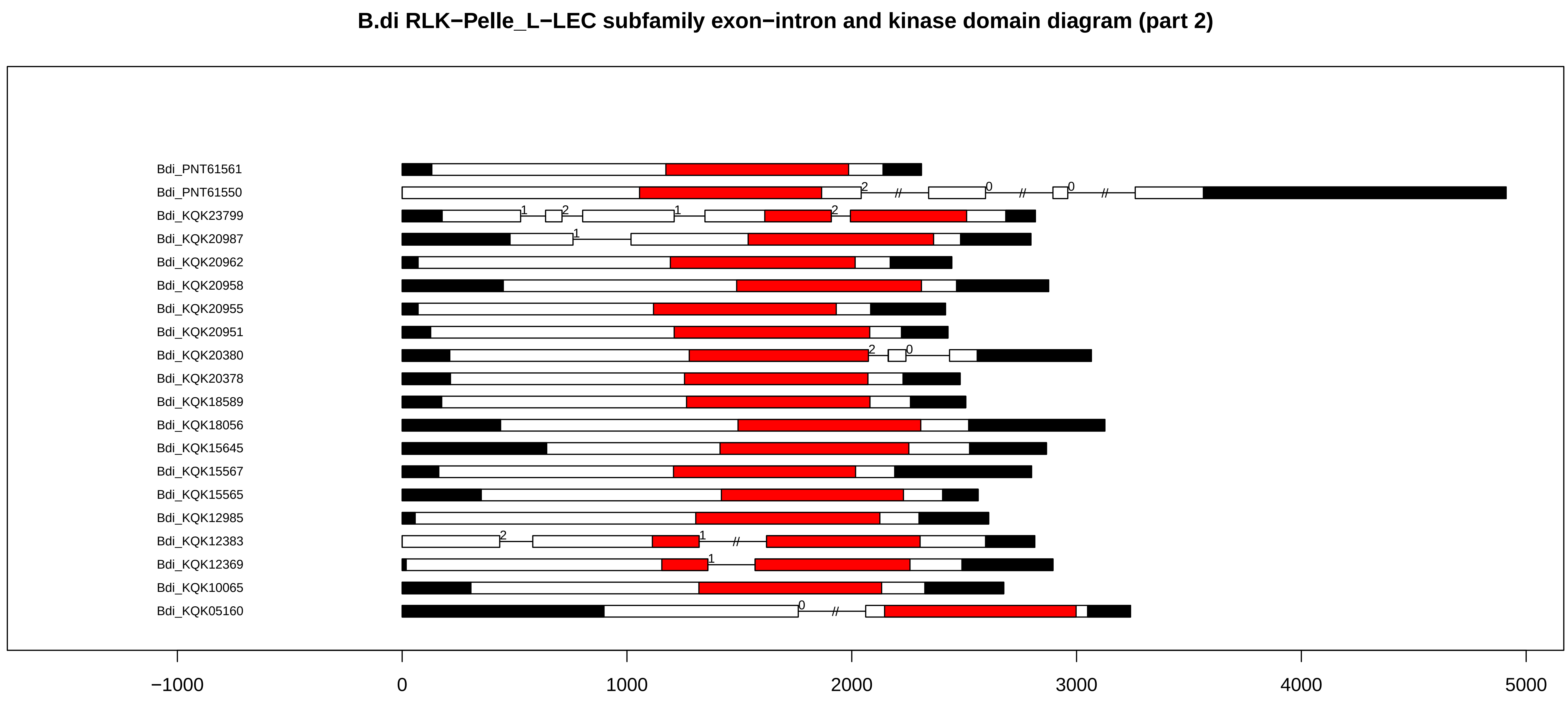
B.di RLK−Pelle_L−LEC subfamily exon−intron and kinase domain diagram (part 2)
Bdi_PNT61561
Bdi_PNT61550
Bdi_KQK23799
Bdi_KQK20987
Bdi_KQK20962
Bdi_KQK20958
Bdi_KQK20955
Bdi_KQK20951
Bdi_KQK20380
Bdi_KQK20378
Bdi_KQK18589
Bdi_KQK18056
Bdi_KQK15645
Bdi_KQK15567
Bdi_KQK15565
Bdi_KQK12985
Bdi_KQK12383
Bdi_KQK12369
Bdi_KQK10065
Bdi_KQK05160
−1000
0
1000
2000
3000
4000
5000

B.di RLK−Pelle_L−LEC subfamily exon−intron and kinase domain diagram (part 3)

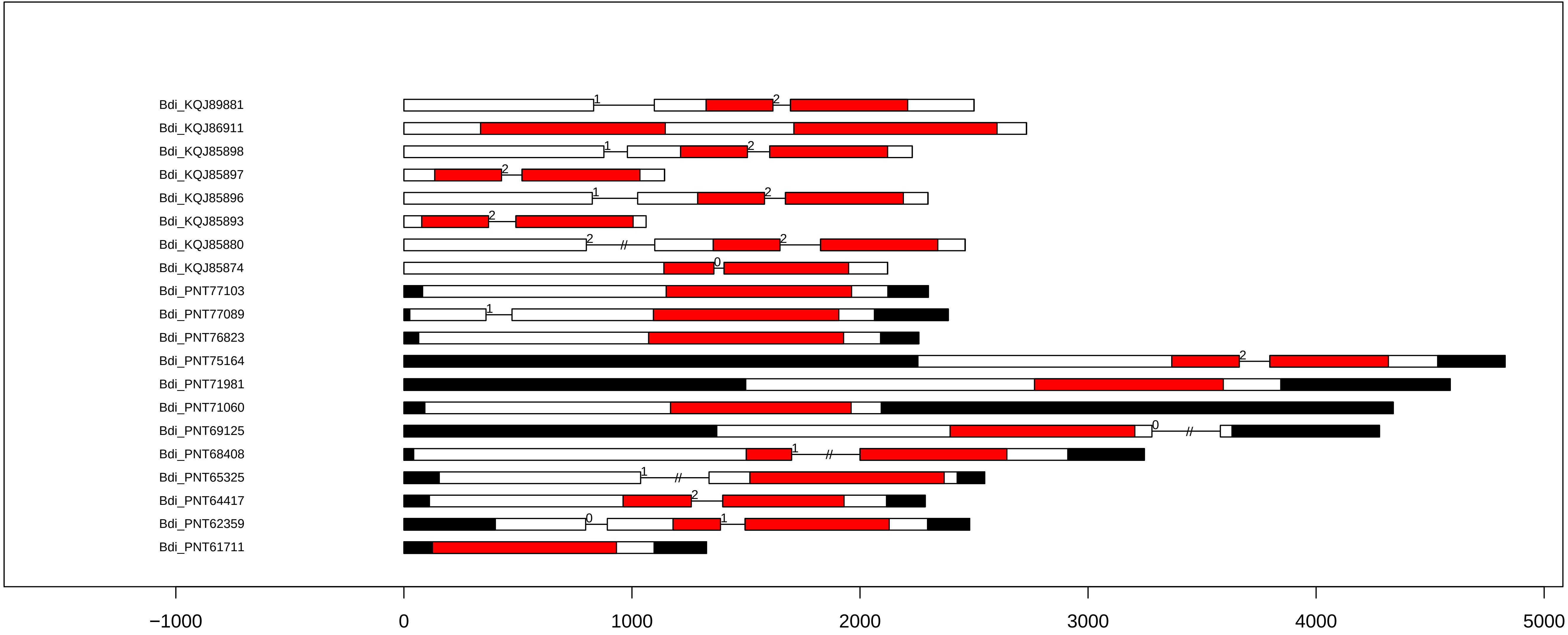

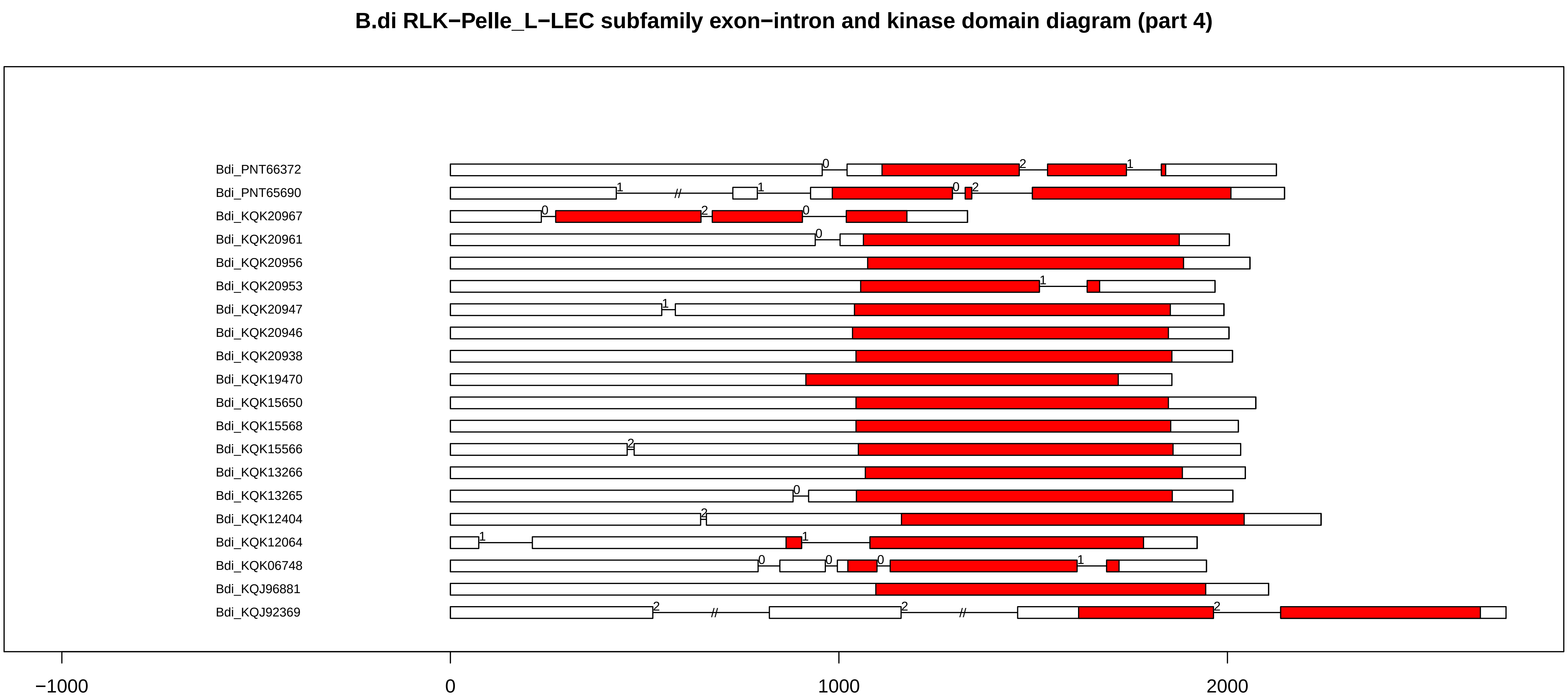
B.di RLK−Pelle_L−LEC subfamily exon−intron and kinase domain diagram (part 4)
Bdi_PNT66372
Bdi_PNT65690
Bdi_KQK20967
Bdi_KQK20961
Bdi_KQK20956
Bdi_KQK20953
Bdi_KQK20947
Bdi_KQK20946
Bdi_KQK20938
Bdi_KQK19470
Bdi_KQK15650
Bdi_KQK15568
Bdi_KQK15566
Bdi_KQK13266
Bdi_KQK13265
Bdi_KQK12404
Bdi_KQK12064
Bdi_KQK06748
Bdi_KQJ96881
Bdi_KQJ92369
−1000
0
1000
2000

B.di RLK−Pelle_L−LEC subfamily exon−intron and kinase domain diagram (part 5)

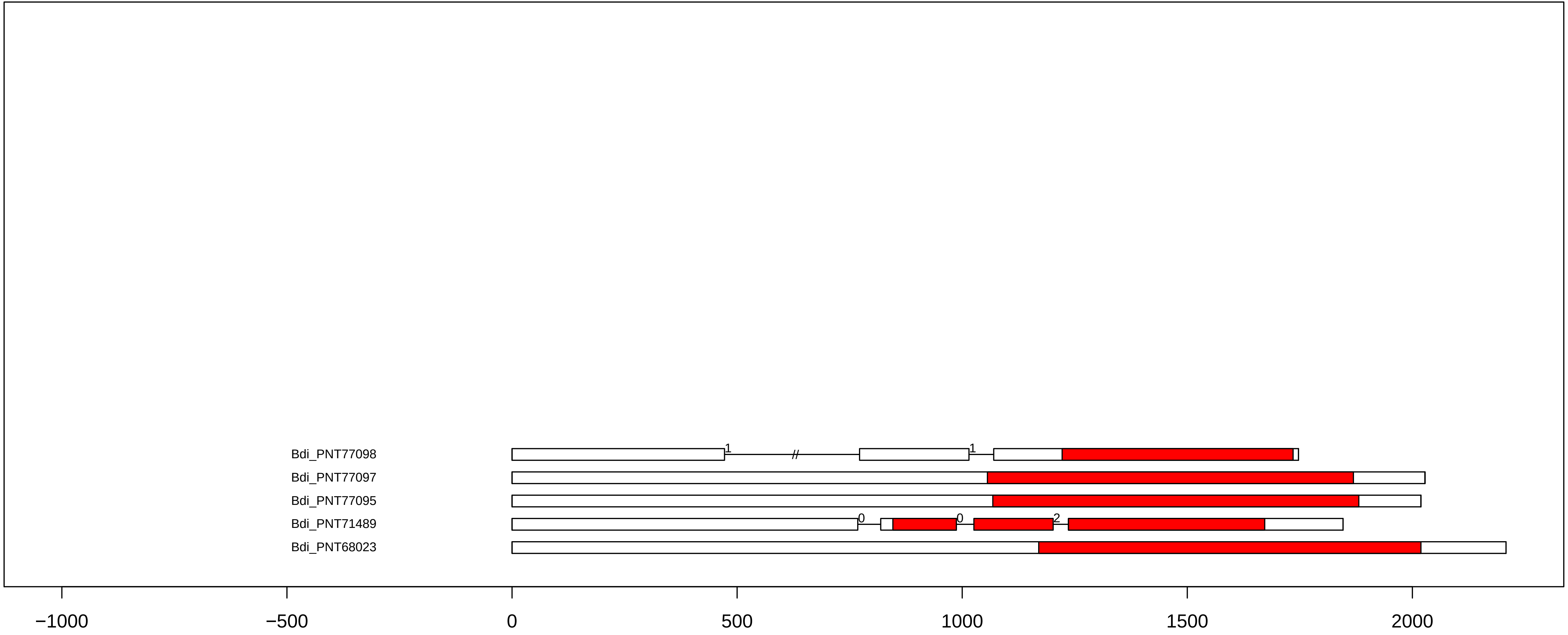

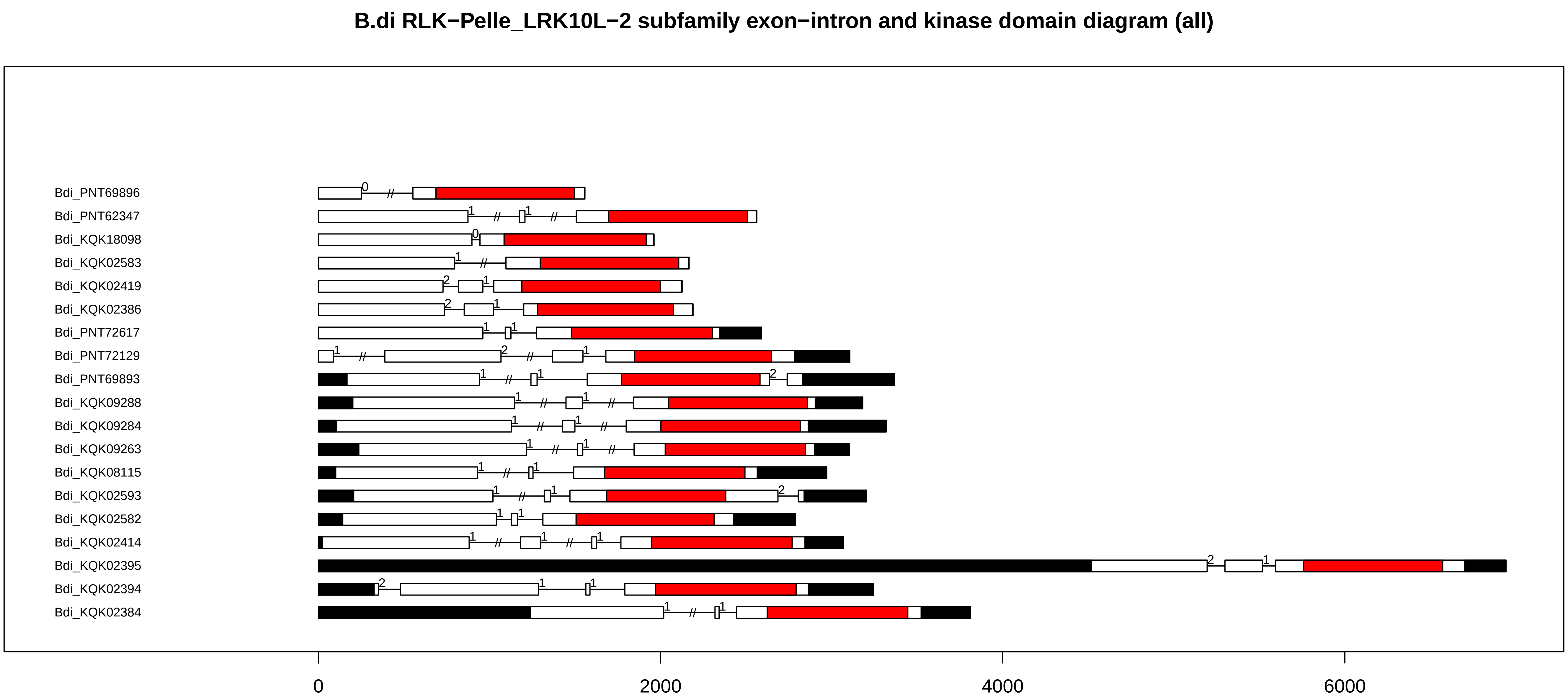
B.di RLK−Pelle_LRK10L−2 subfamily exon−intron and kinase domain diagram (all)
Bdi_PNT69896
Bdi_PNT62347
Bdi_KQK18098
Bdi_KQK02583
Bdi_KQK02419
Bdi_KQK02386
Bdi_PNT72617
Bdi_PNT72129
Bdi_PNT69893
Bdi_KQK09288
Bdi_KQK09284
Bdi_KQK09263
Bdi_KQK08115
Bdi_KQK02593
Bdi_KQK02582
Bdi_KQK02414
Bdi_KQK02395
Bdi_KQK02394
Bdi_KQK02384
0
2000
4000
6000

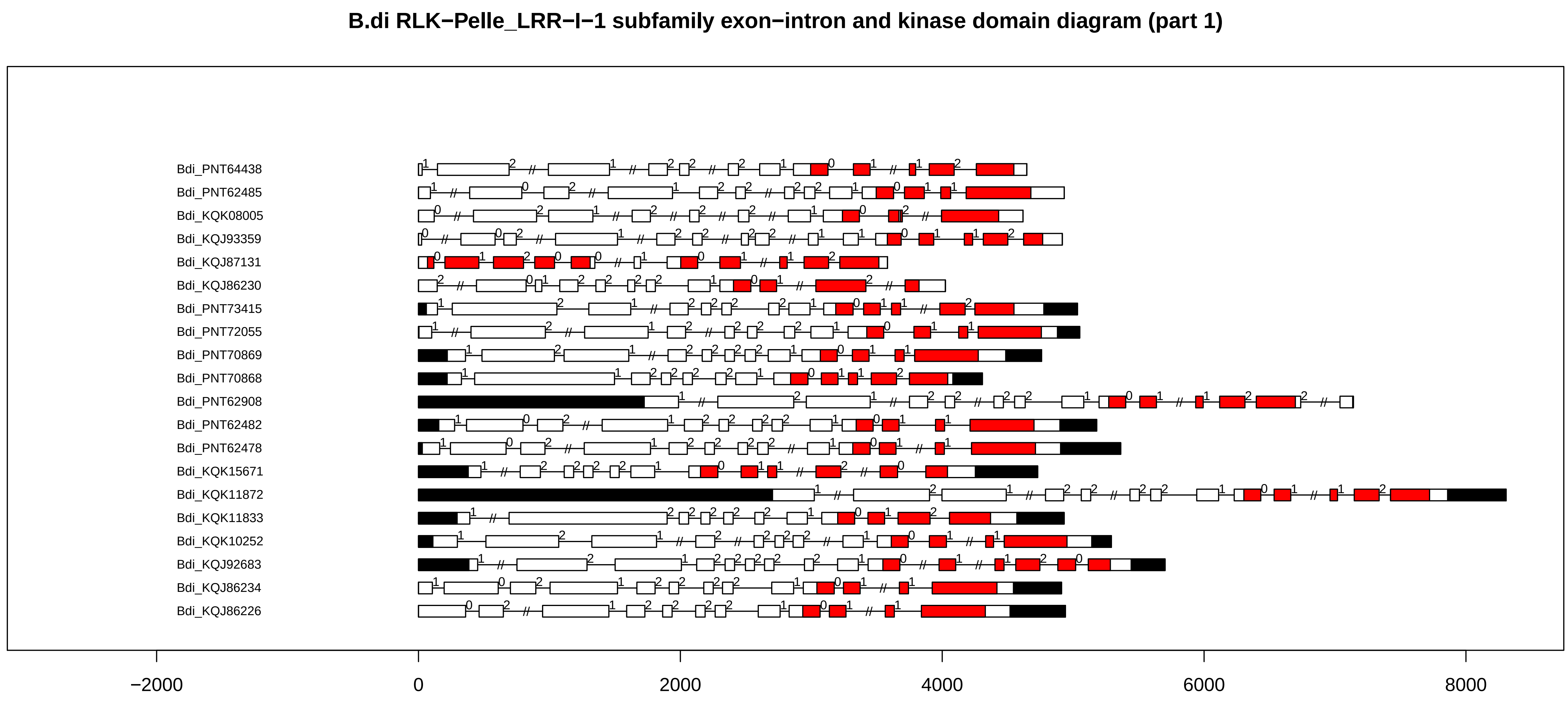

B.di RLK−Pelle_LRR−I−1 subfamily exon−intron and kinase domain diagram (part 1)
Bdi_PNT64438
Bdi_PNT62485
Bdi_KQK08005
Bdi_KQJ93359
Bdi_KQJ87131
Bdi_KQJ86230
Bdi_PNT73415
Bdi_PNT72055
Bdi_PNT70869
Bdi_PNT70868
Bdi_PNT62908
Bdi_PNT62482
Bdi_PNT62478
Bdi_KQK15671
Bdi_KQK11872
Bdi_KQK11833
Bdi_KQK10252
Bdi_KQJ92683
Bdi_KQJ86234
Bdi_KQJ86226
−2000
0
2000
4000
6000
8000

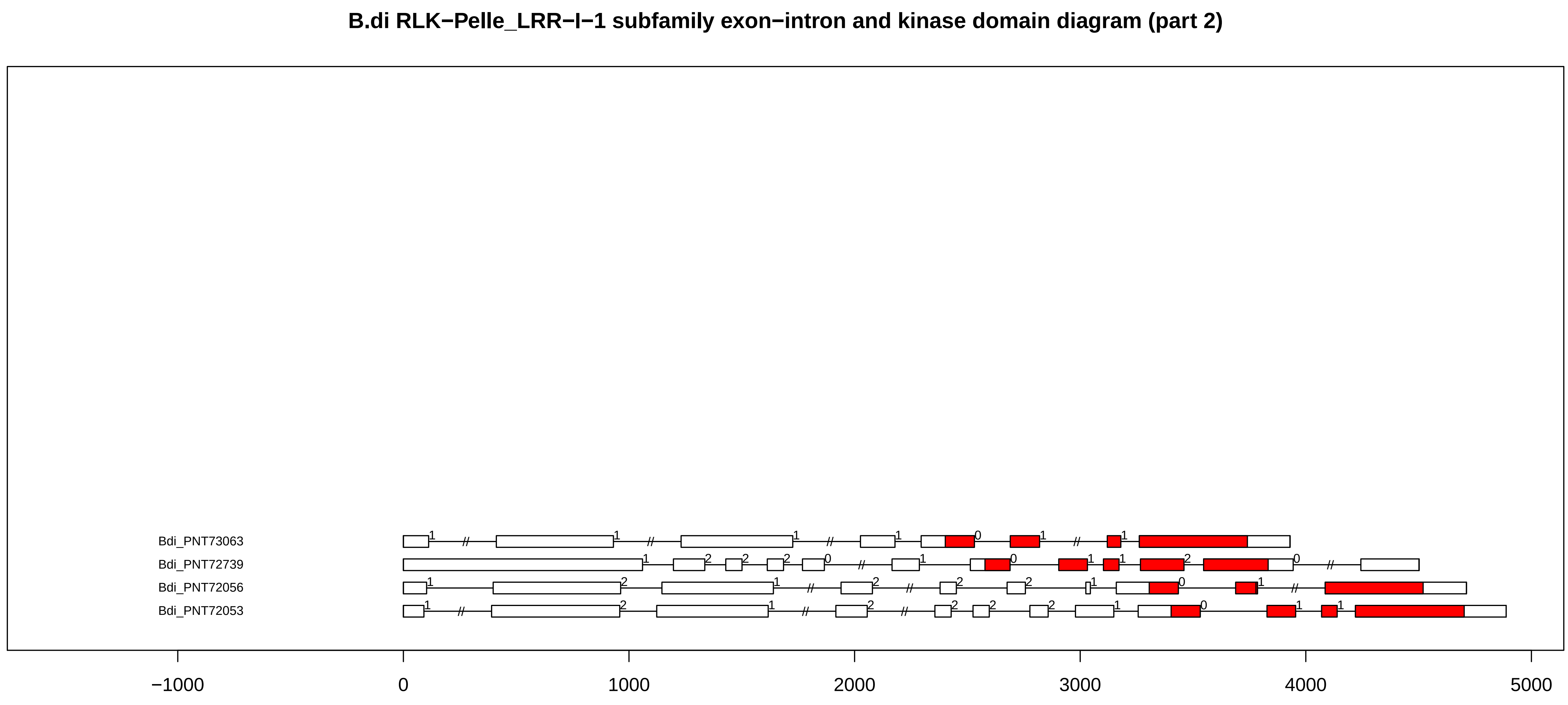

B.di RLK−Pelle_LRR−I−1 subfamily exon−intron and kinase domain diagram (part 2)
Bdi_PNT73063
Bdi_PNT72739
Bdi_PNT72056
Bdi_PNT72053
−1000
0
1000
2000
3000
4000
5000

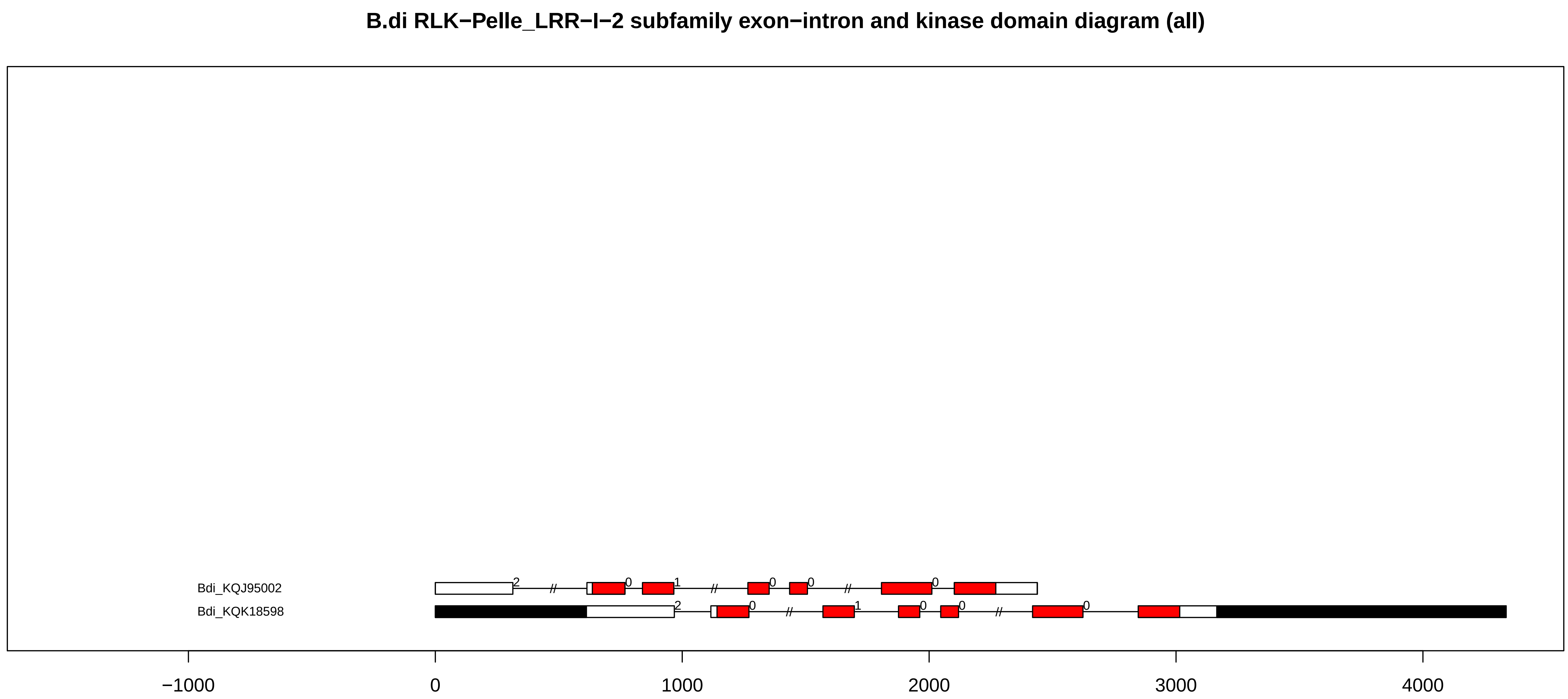
B.di RLK−Pelle_LRR−I−2 subfamily exon−intron and kinase domain diagram (all)
Bdi_KQJ95002
Bdi_KQK18598
2
0
1
0
0
0
2
0
1
0
0
0
−1000
0
1000
2000
3000
4000

B.di RLK−Pelle_LRR−III subfamily exon−intron and kinase domain diagram (part 1)

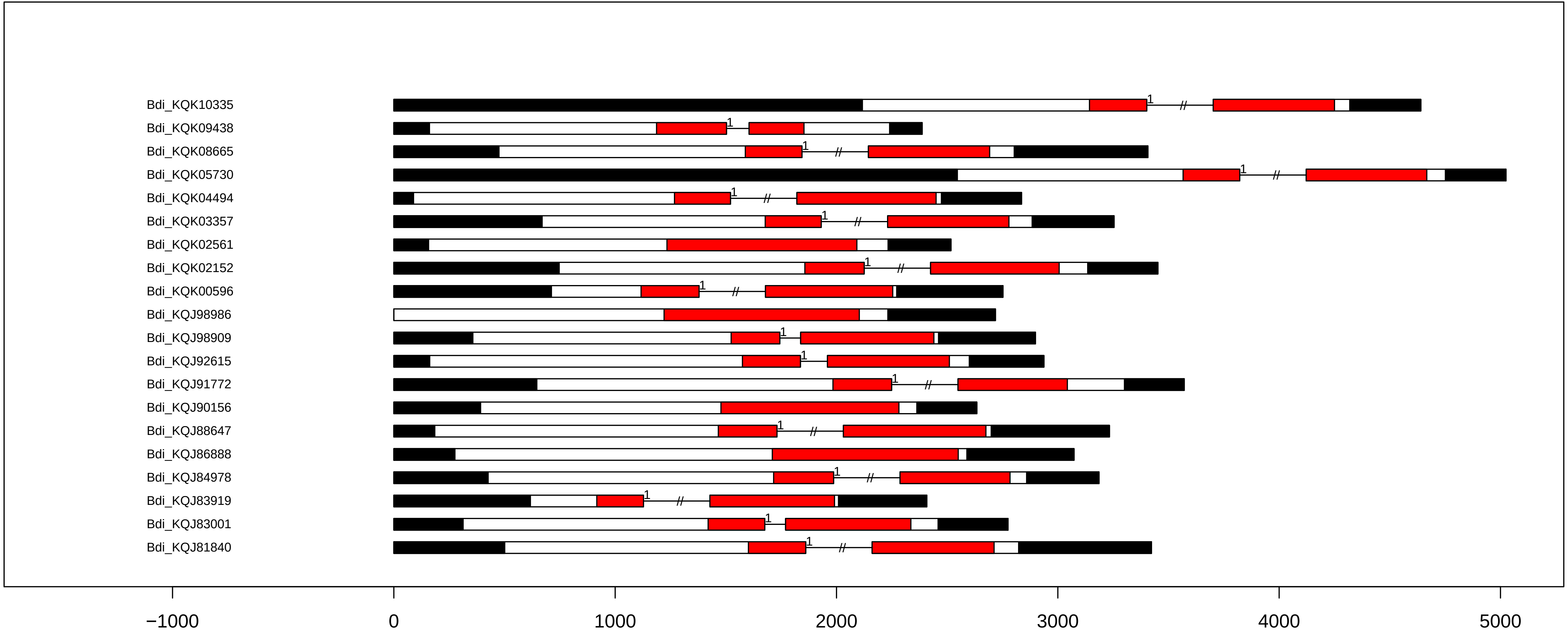

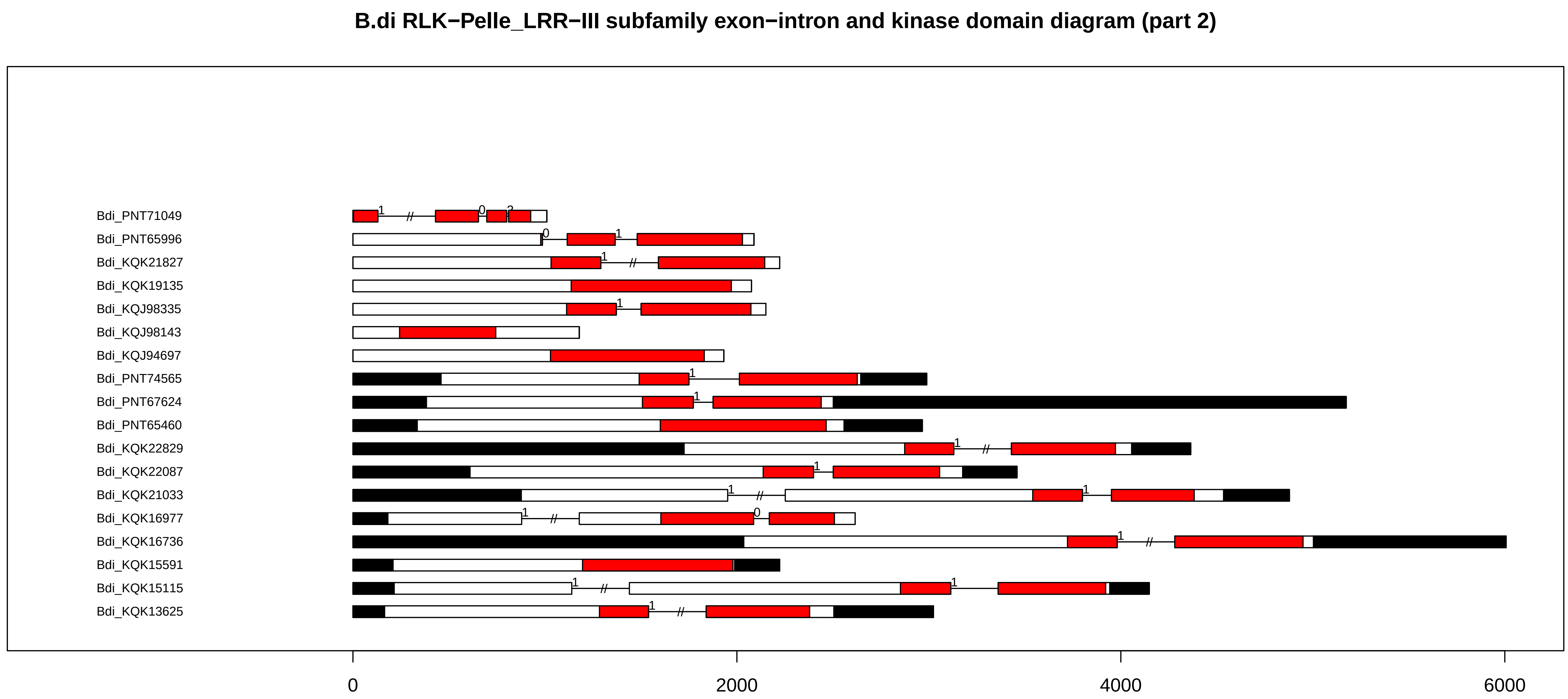
B.di RLK−Pelle_LRR−III subfamily exon−intron and kinase domain diagram (part 2)
Bdi_PNT71049
Bdi_PNT65996
Bdi_KQK21827
Bdi_KQK19135
Bdi_KQJ98335
Bdi_KQJ98143
Bdi_KQJ94697
Bdi_PNT74565
Bdi_PNT67624
Bdi_PNT65460
Bdi_KQK22829
Bdi_KQK22087
Bdi_KQK21033
Bdi_KQK16977
Bdi_KQK16736
Bdi_KQK15591
Bdi_KQK15115
Bdi_KQK13625
0
2000
4000
6000

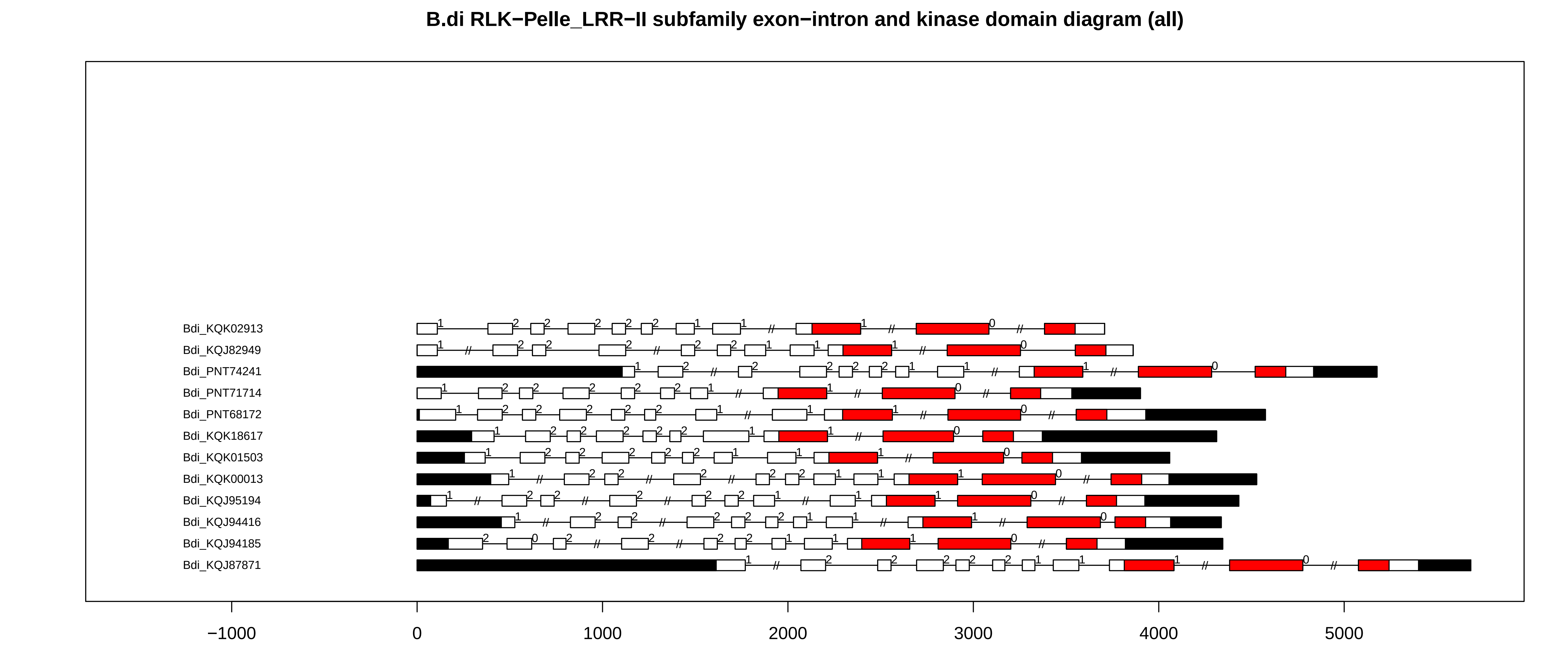

B.di RLK−Pelle_LRR−II subfamily exon−intron and kinase domain diagram (all)
Bdi_KQK02913
Bdi_KQJ82949
Bdi_PNT74241
Bdi_PNT71714
Bdi_PNT68172
Bdi_KQK18617
Bdi_KQK01503
Bdi_KQK00013
Bdi_KQJ95194
Bdi_KQJ94416
Bdi_KQJ94185
Bdi_KQJ87871
−1000
0
1000
2000
3000
4000
5000

B.di RLK−Pelle_LRR−IV subfamily exon−intron and kinase domain diagram (all)

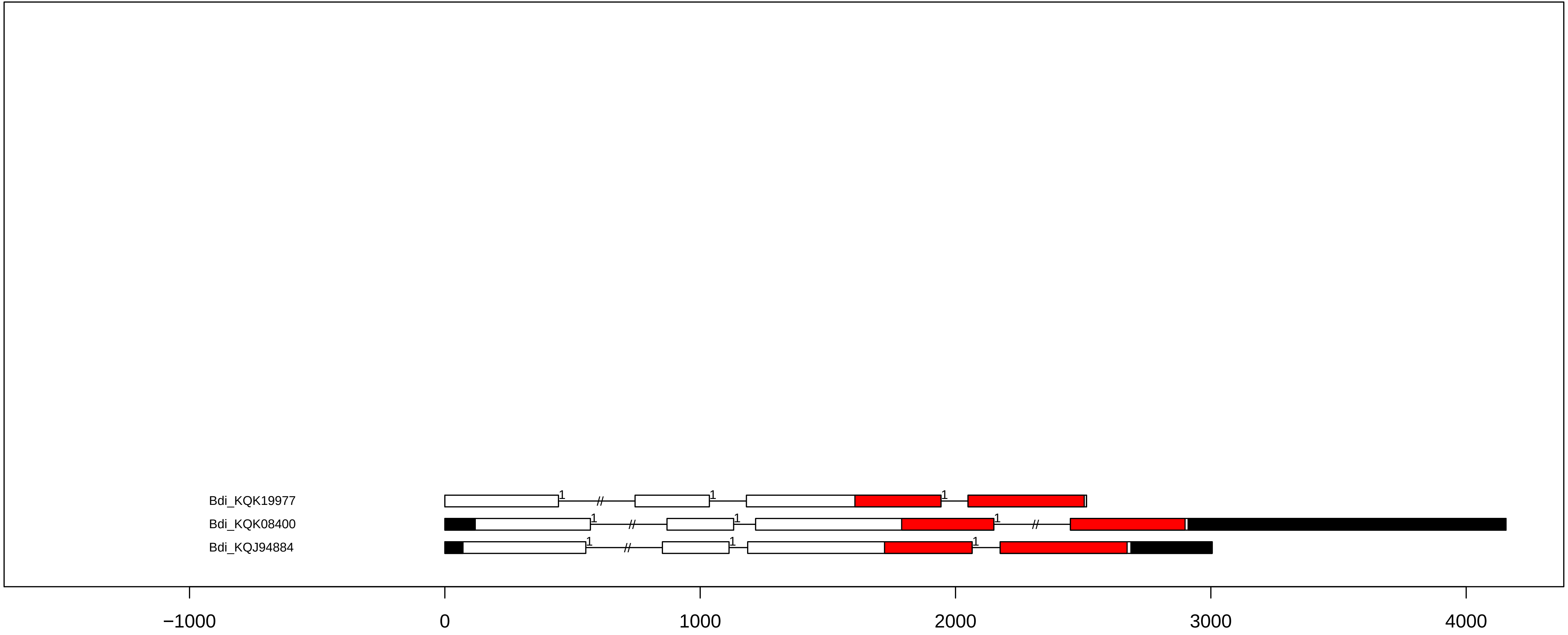

B.di RLK−Pelle_LRR−IX subfamily exon−intron and kinase domain diagram (all)

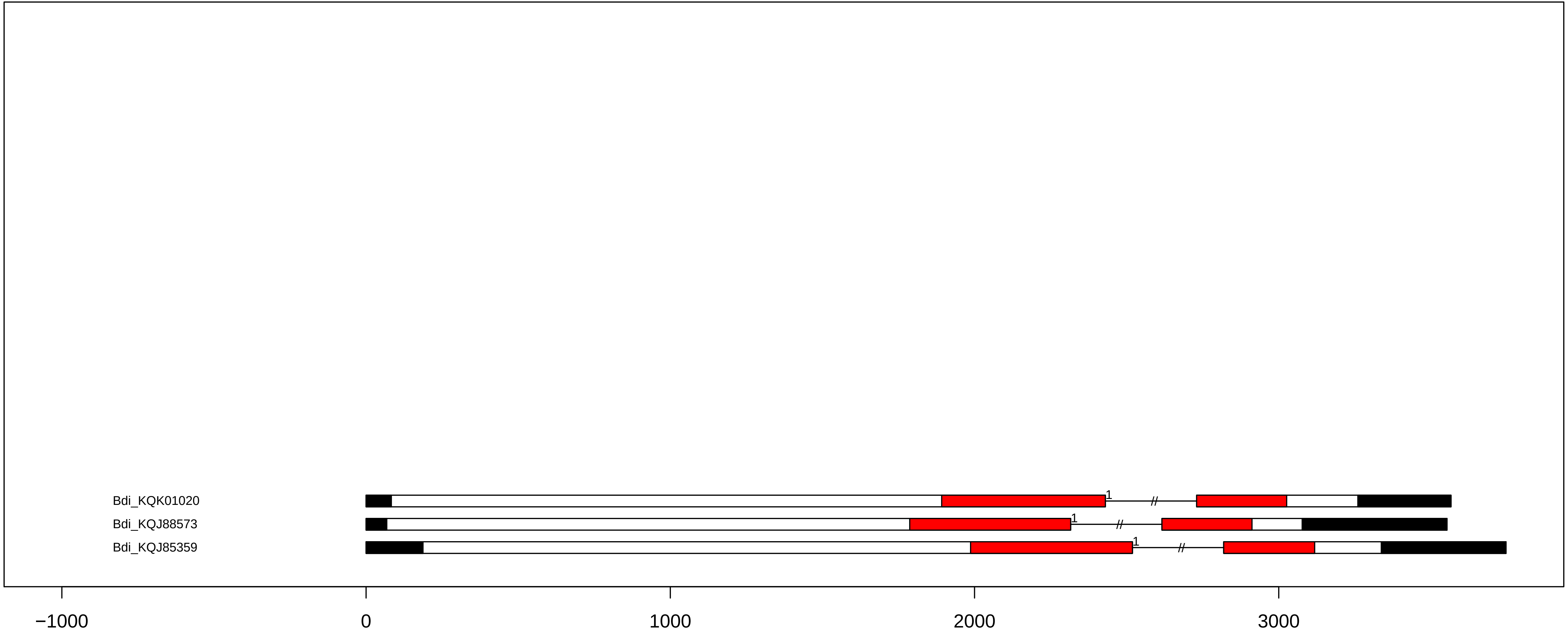

B.di RLK−Pelle_LRR−VI−1 subfamily exon−intron and kinase domain diagram (all)

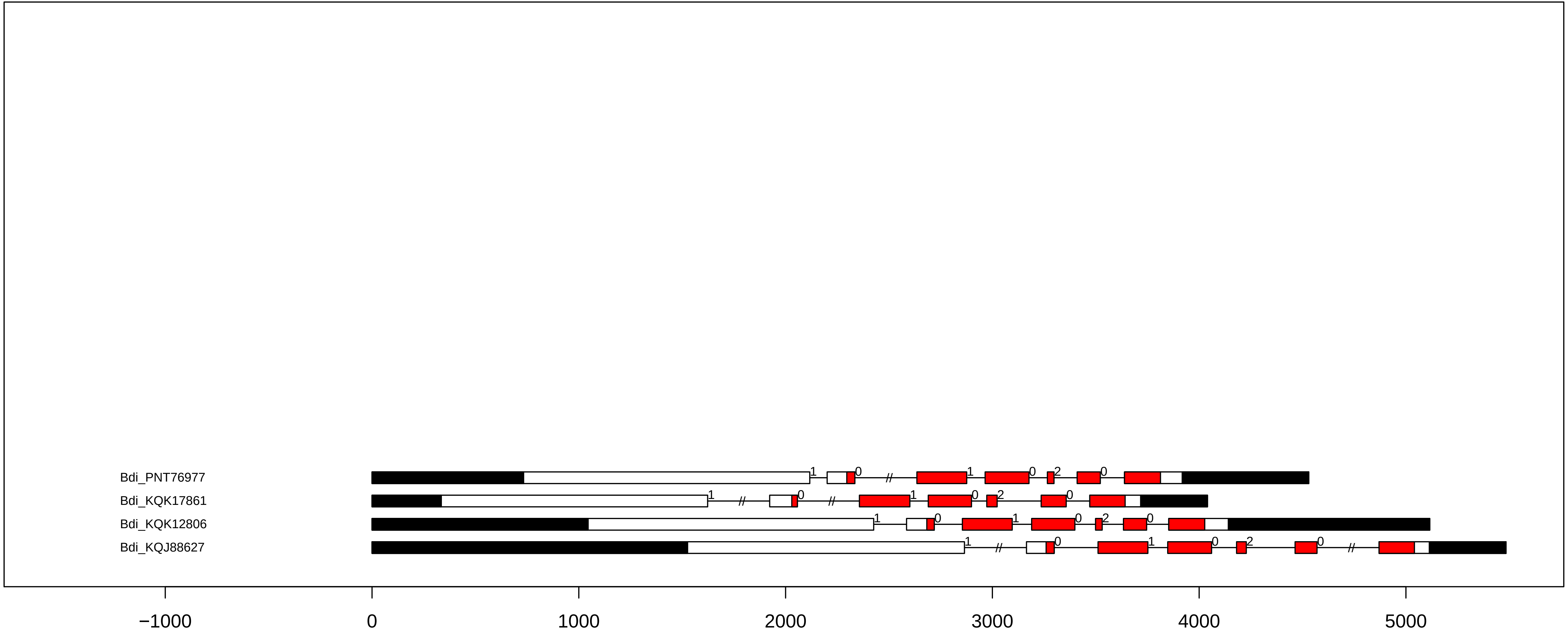

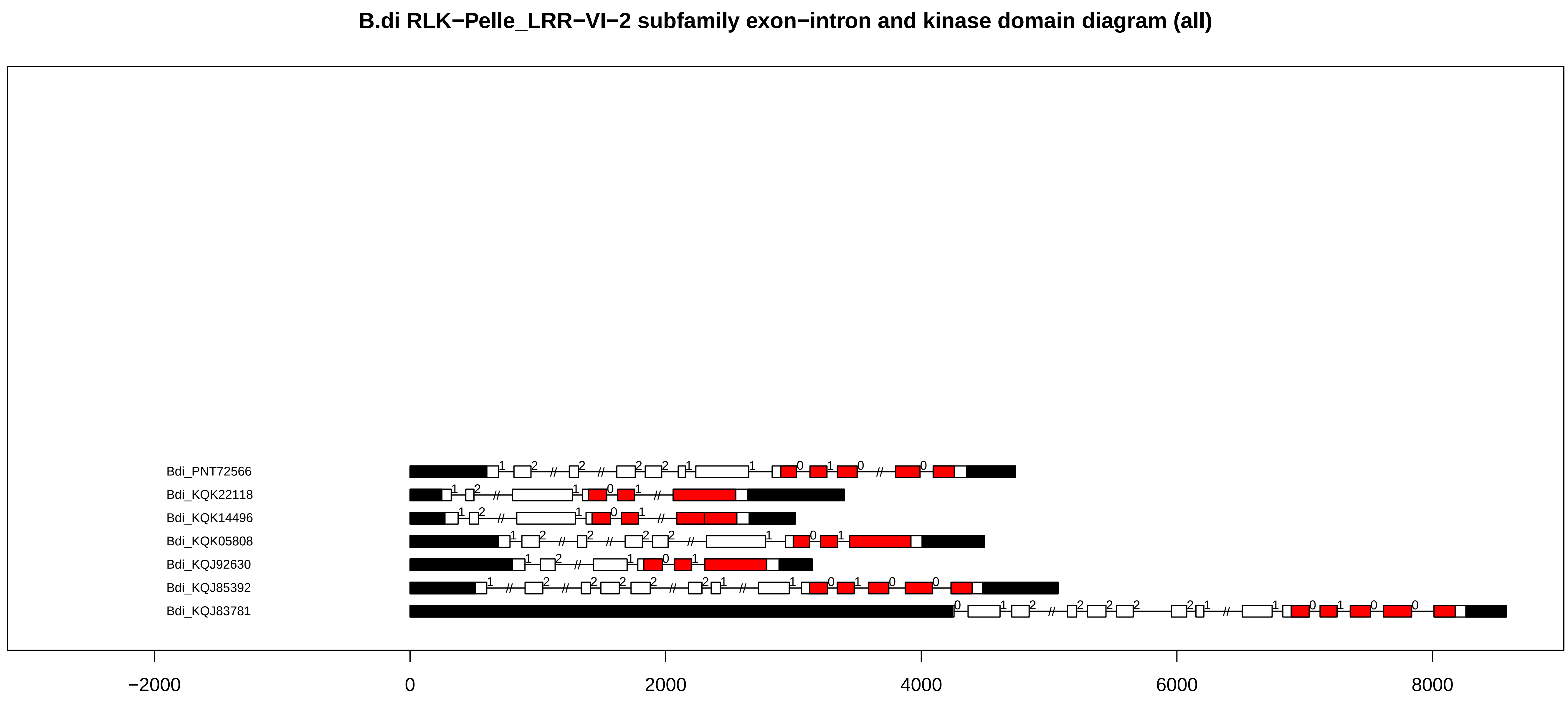

B.di RLK−Pelle_LRR−VI−2 subfamily exon−intron and kinase domain diagram (all)
Bdi_PNT72566
Bdi_KQK22118
Bdi_KQK14496
Bdi_KQK05808
Bdi_KQJ92630
Bdi_KQJ85392
Bdi_KQJ83781
−2000
0
2000
4000
6000
8000

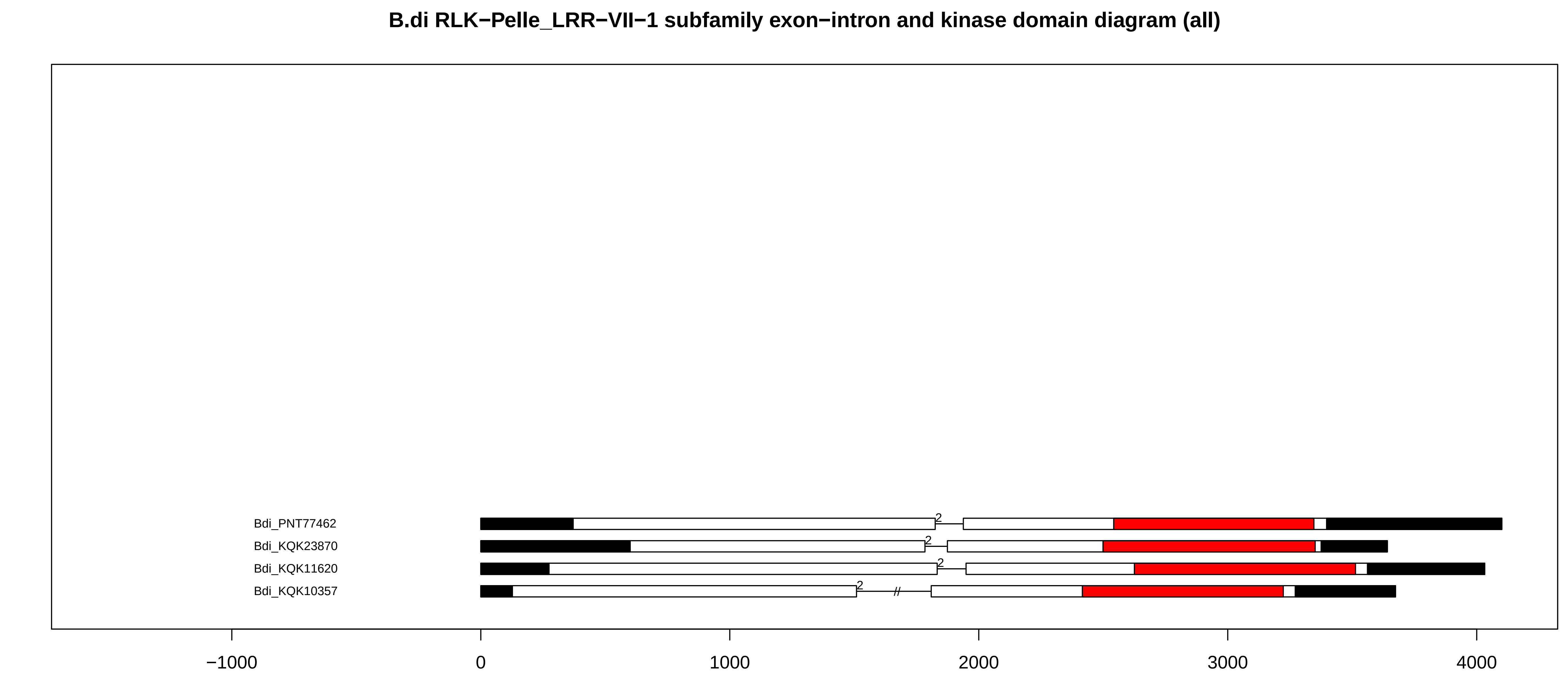

B.di RLK−Pelle_LRR−VII−1 subfamily exon−intron and kinase domain diagram (all)
Bdi_PNT77462
Bdi_KQK23870
Bdi_KQK11620
Bdi_KQK10357
2
2
2
2
−1000
0
1000
2000
3000
4000

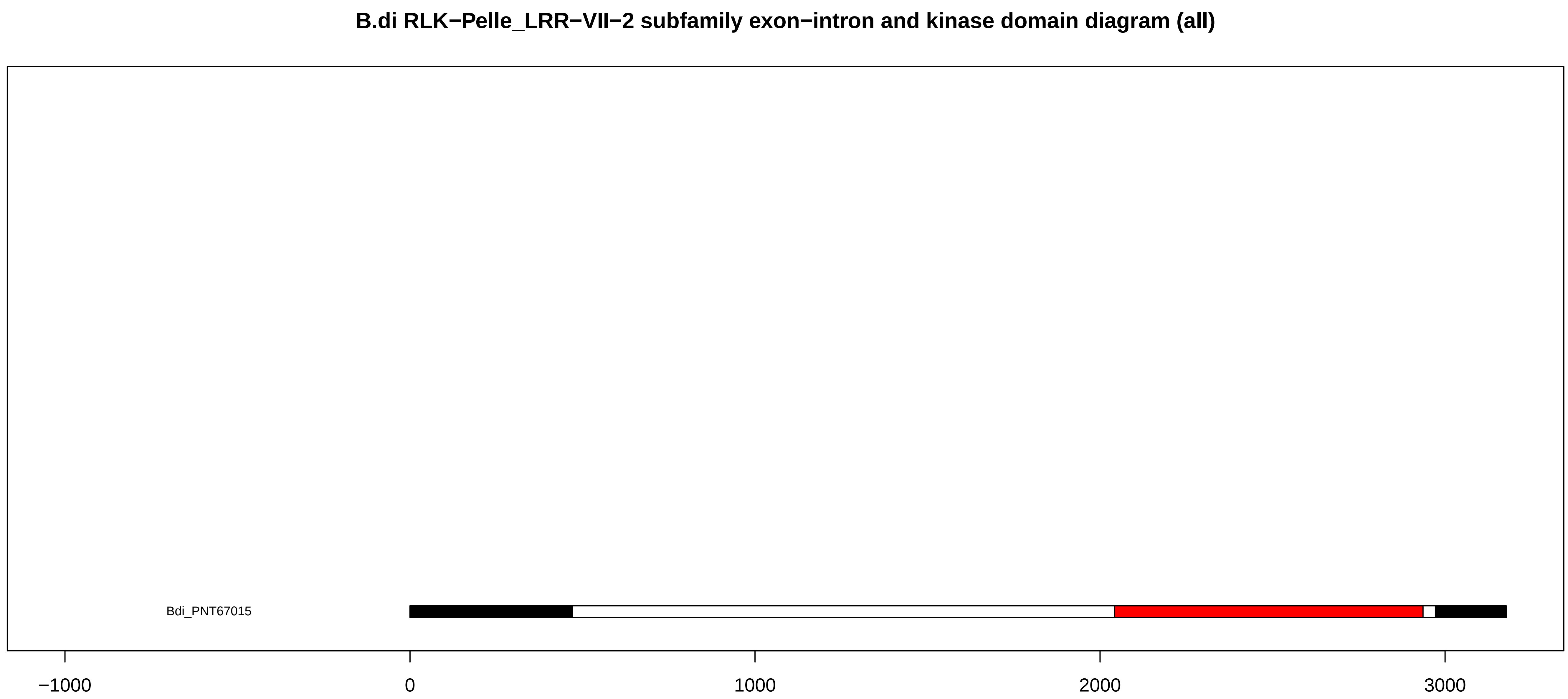
B.di RLK−Pelle_LRR−VII−2 subfamily exon−intron and kinase domain diagram (all)
Bdi_PNT67015
−1000
0
1000
2000
3000

B.di RLK−Pelle_LRR−VII−3 subfamily exon−intron and kinase domain diagram (all)

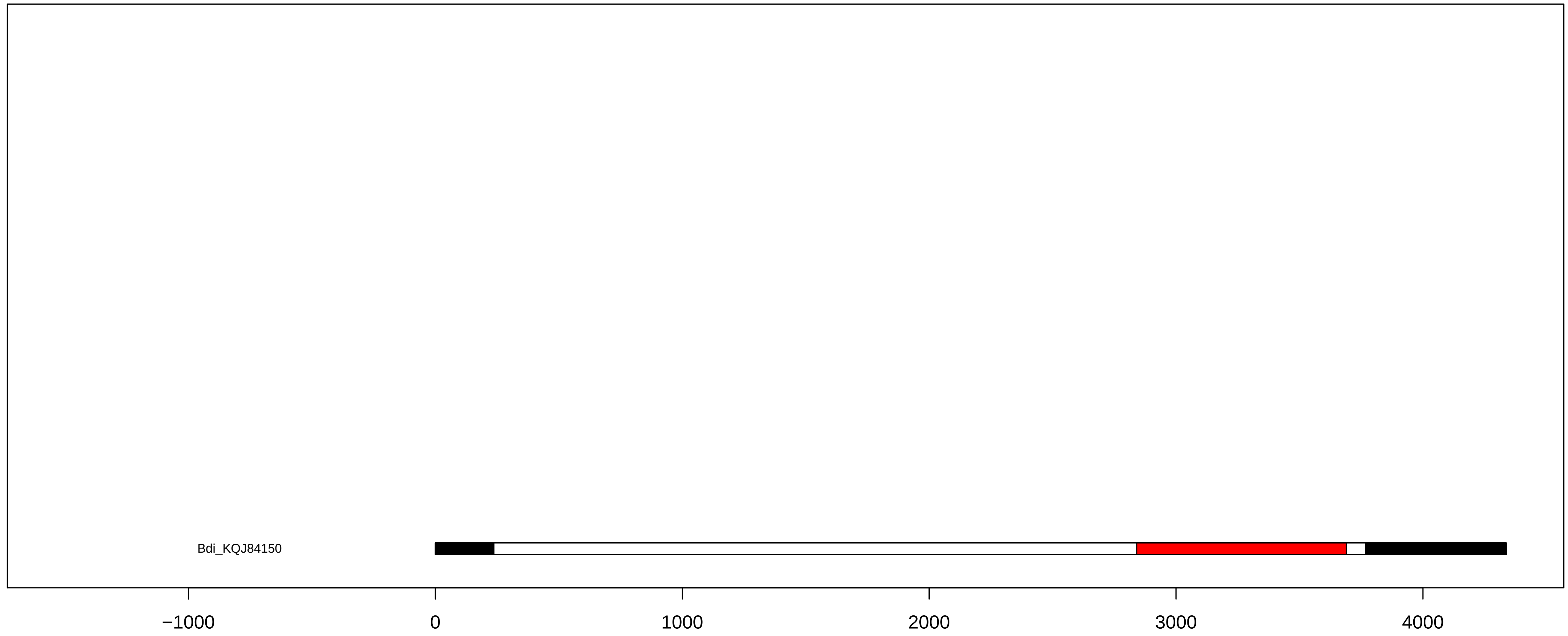

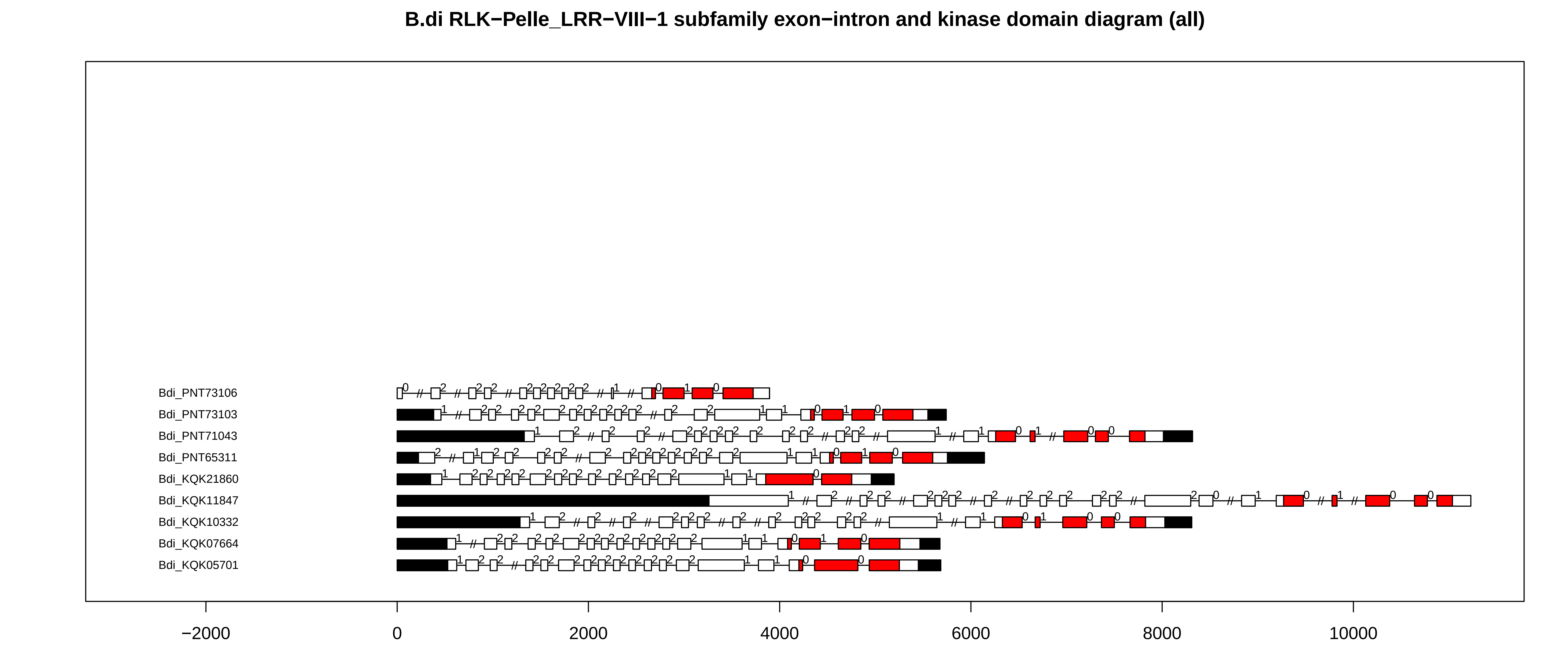

B.di RLK−Pelle_LRR−VIII−1 subfamily exon−intron and kinase domain diagram (all)
Bdi_PNT73106
Bdi_PNT73103
Bdi_PNT71043
Bdi_PNT65311
Bdi_KQK21860
Bdi_KQK11847
Bdi_KQK10332
Bdi_KQK07664
Bdi_KQK05701
−2000
0
2000
4000
6000
8000
10000

B.di RLK−Pelle_LRR−V subfamily exon−intron and kinase domain diagram (all)

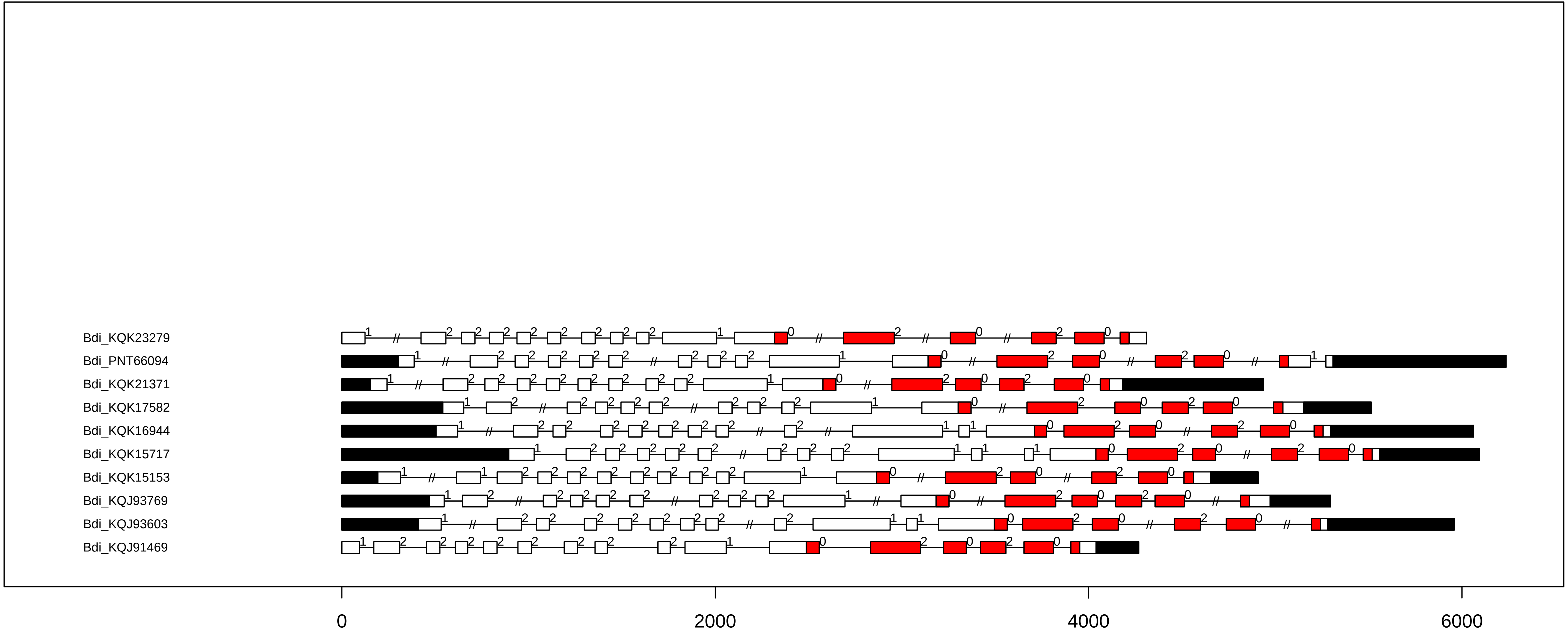

B.di RLK−Pelle_LRR−Xa subfamily exon−intron and kinase domain diagram (all)

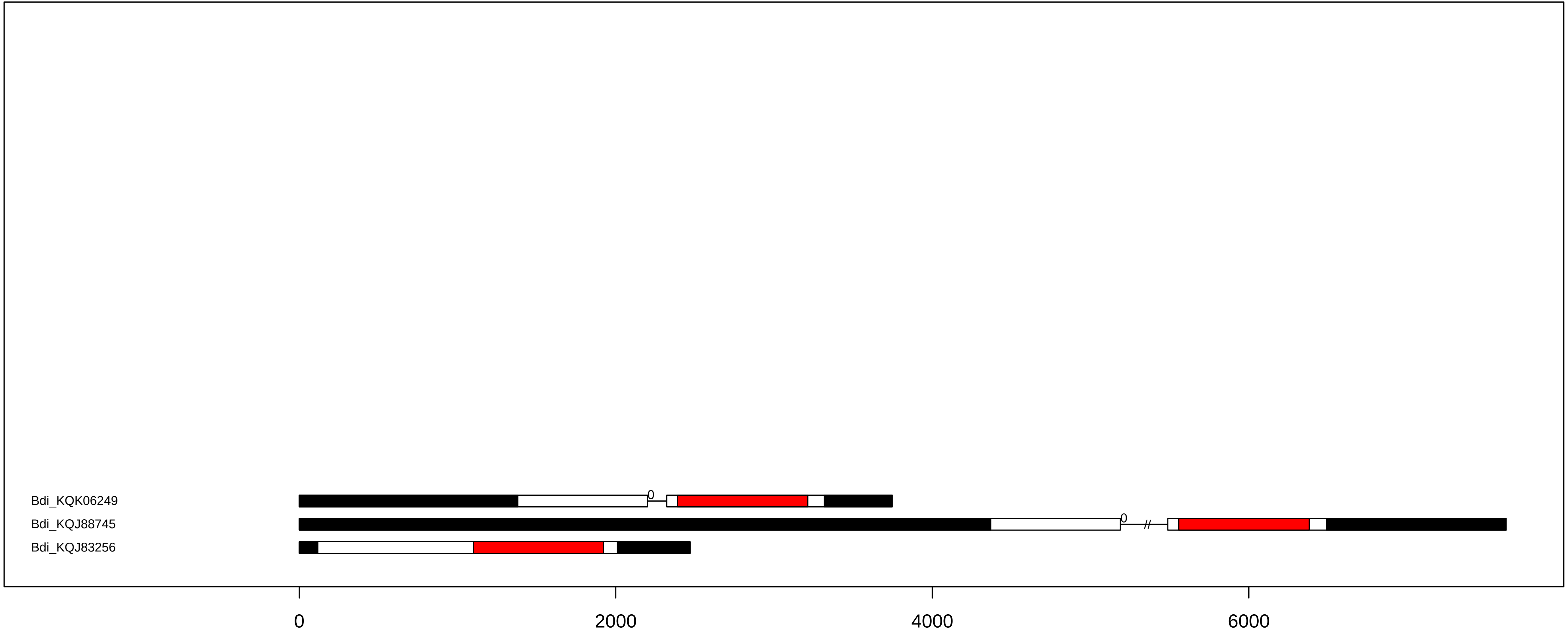

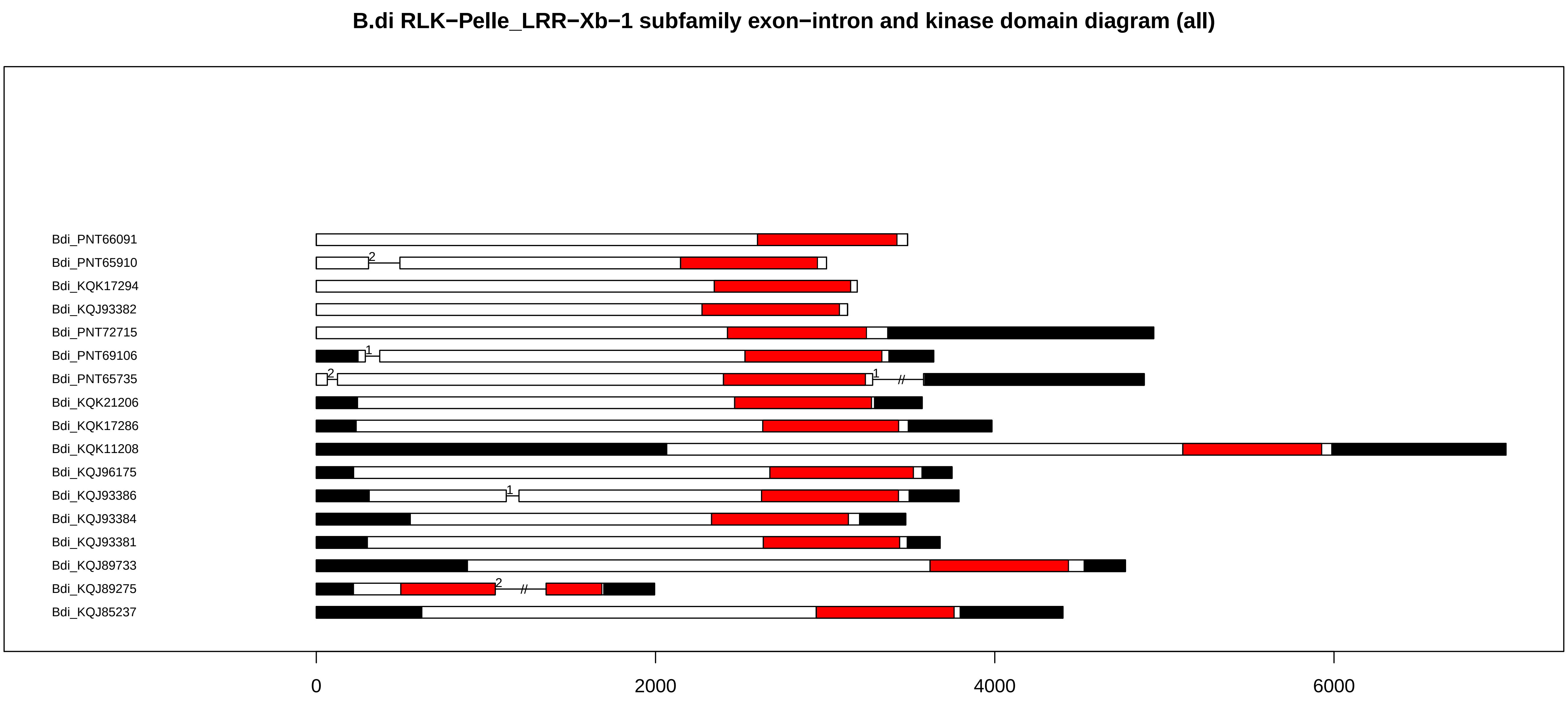
B.di RLK−Pelle_LRR−Xb−1 subfamily exon−intron and kinase domain diagram (all)
Bdi_PNT66091
Bdi_PNT65910
Bdi_KQK17294
Bdi_KQJ93382
Bdi_PNT72715
Bdi_PNT69106
Bdi_PNT65735
Bdi_KQK21206
Bdi_KQK17286
Bdi_KQK11208
Bdi_KQJ96175
Bdi_KQJ93386
Bdi_KQJ93384
Bdi_KQJ93381
Bdi_KQJ89733
Bdi_KQJ89275
Bdi_KQJ85237
0
2000
4000
6000

B.di RLK−Pelle_LRR−Xb−2 subfamily exon−intron and kinase domain diagram (all)

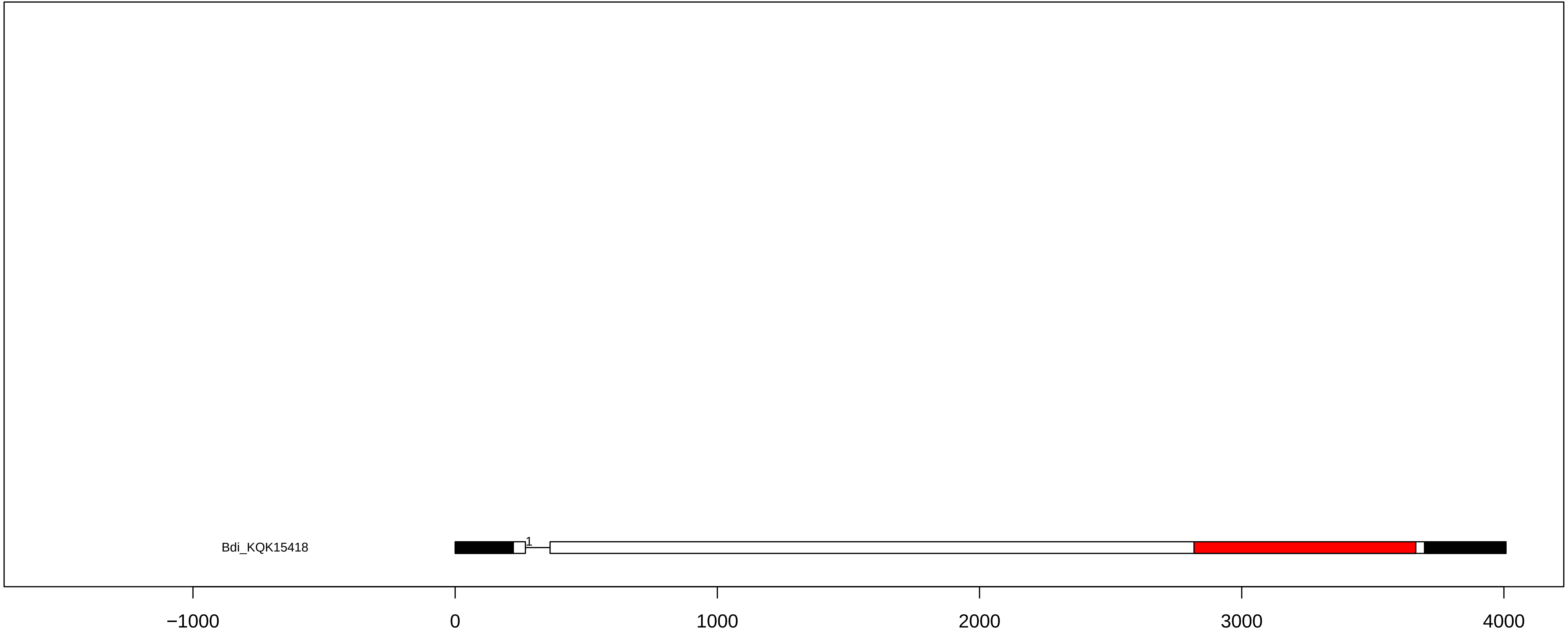

B.di RLK−Pelle_LRR−XI−1 subfamily exon−intron and kinase domain diagram (part 1)

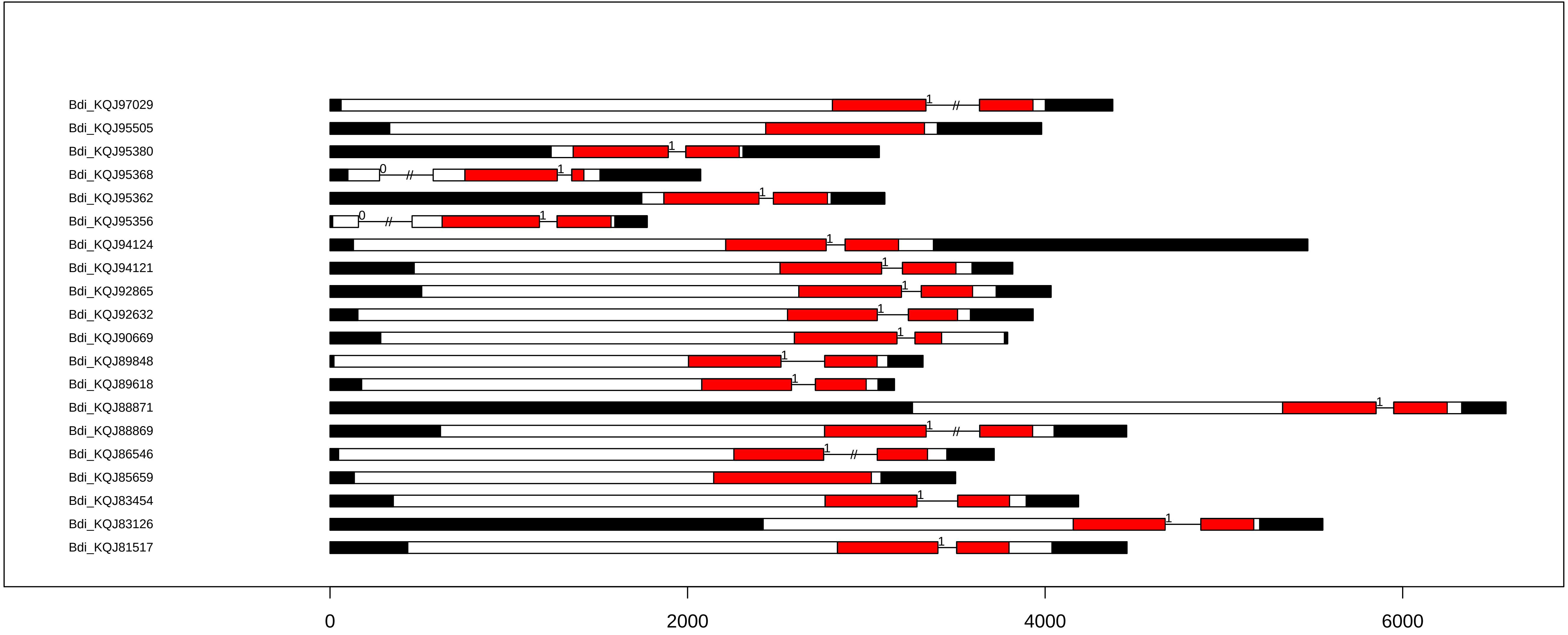

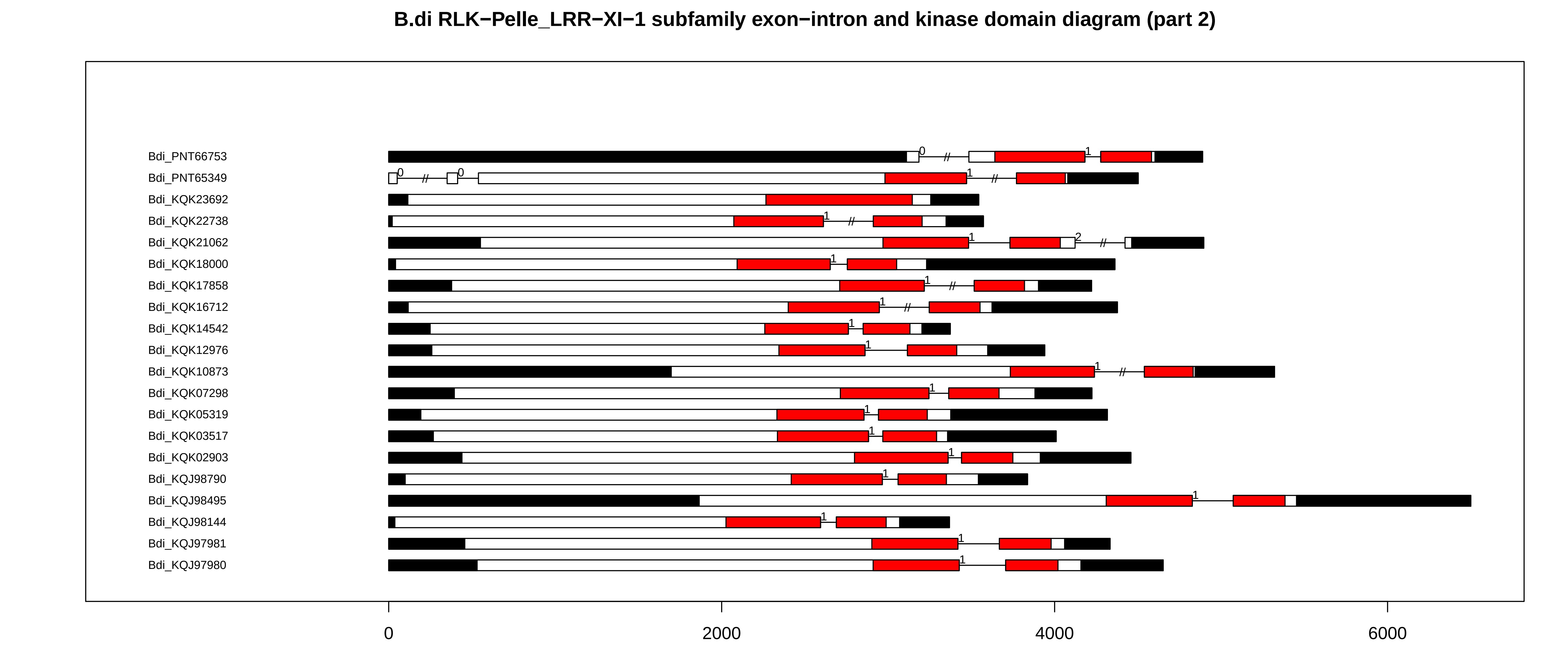
B.di RLK−Pelle_LRR−XI−1 subfamily exon−intron and kinase domain diagram (part 2)
Bdi_PNT66753
Bdi_PNT65349
Bdi_KQK23692
Bdi_KQK22738
Bdi_KQK21062
Bdi_KQK18000
Bdi_KQK17858
Bdi_KQK16712
Bdi_KQK14542
Bdi_KQK12976
Bdi_KQK10873
Bdi_KQK07298
Bdi_KQK05319
Bdi_KQK03517
Bdi_KQK02903
Bdi_KQJ98790
Bdi_KQJ98495
Bdi_KQJ98144
Bdi_KQJ97981
Bdi_KQJ97980
0
2000
4000
6000

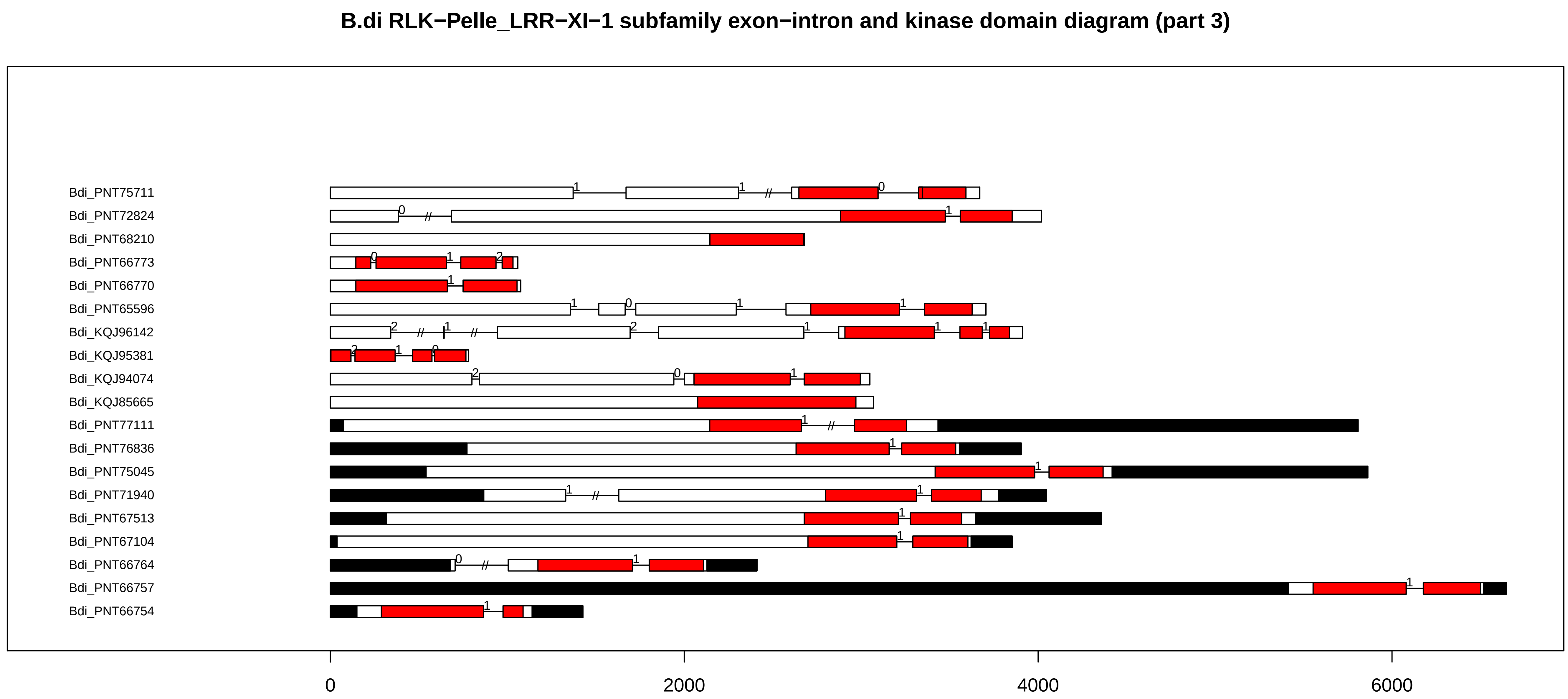

B.di RLK−Pelle_LRR−XI−1 subfamily exon−intron and kinase domain diagram (part 3)
Bdi_PNT75711
Bdi_PNT72824
Bdi_PNT68210
Bdi_PNT66773
Bdi_PNT66770
Bdi_PNT65596
Bdi_KQJ96142
Bdi_KQJ95381
Bdi_KQJ94074
Bdi_KQJ85665
Bdi_PNT77111
Bdi_PNT76836
Bdi_PNT75045
Bdi_PNT71940
Bdi_PNT67513
Bdi_PNT67104
Bdi_PNT66764
Bdi_PNT66757
Bdi_PNT66754
0
2000
4000
6000

B.di RLK−Pelle_LRR−XI−2 subfamily exon−intron and kinase domain diagram (all)

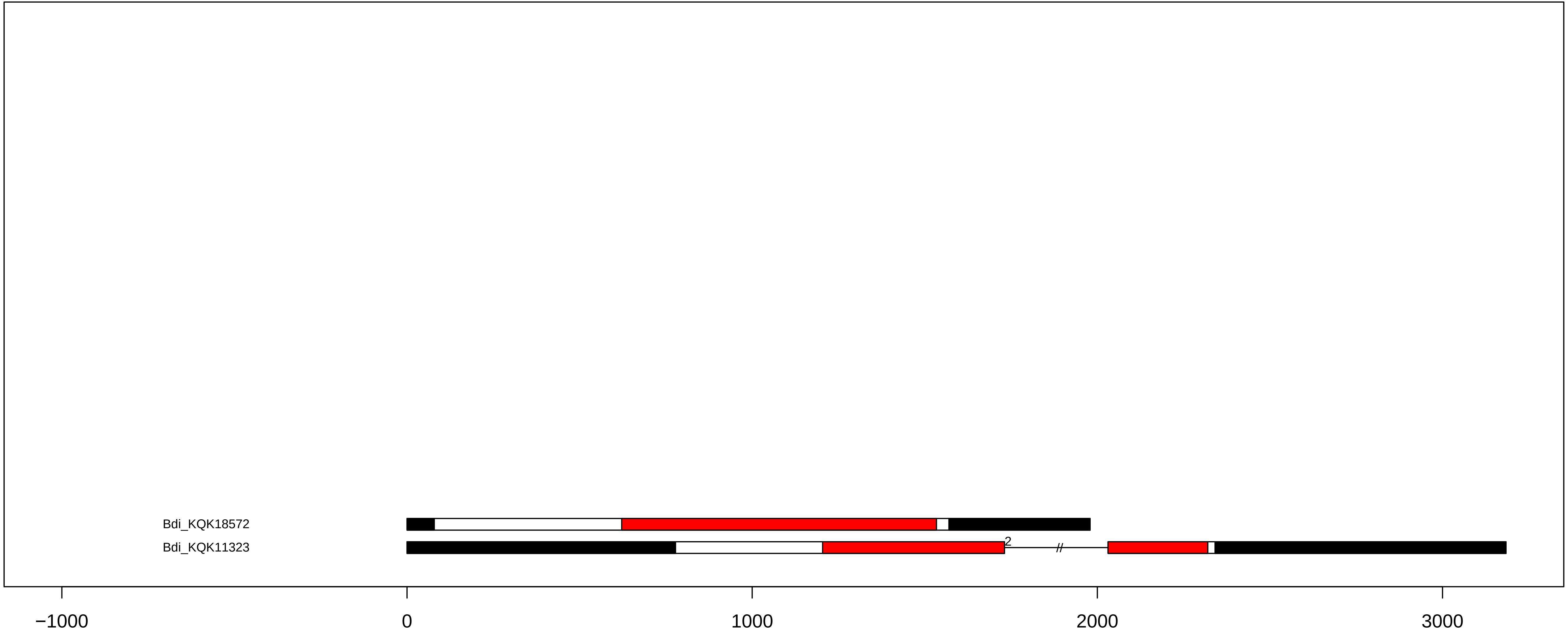

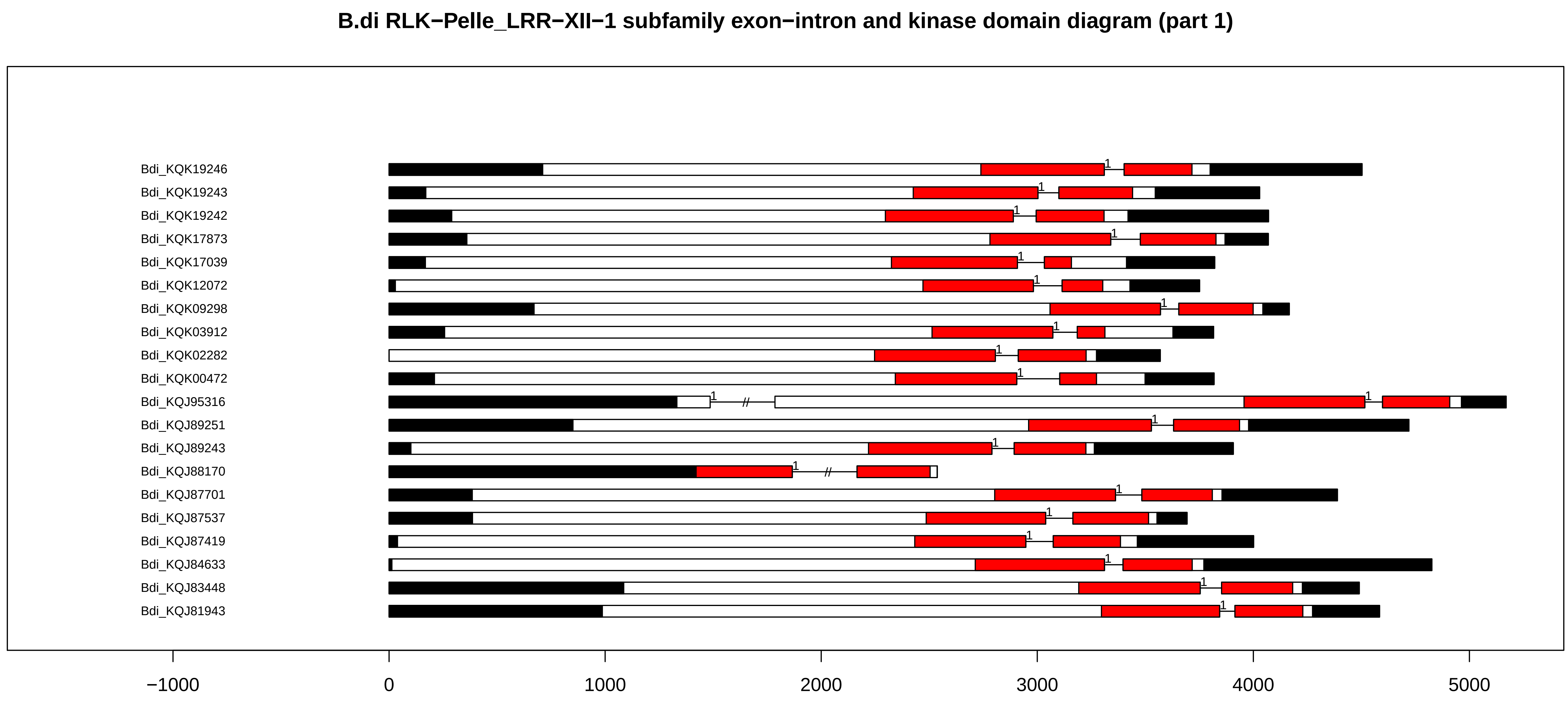
B.di RLK−Pelle_LRR−XII−1 subfamily exon−intron and kinase domain diagram (part 1)
Bdi_KQK19246
Bdi_KQK19243
Bdi_KQK19242
Bdi_KQK17873
Bdi_KQK17039
Bdi_KQK12072
Bdi_KQK09298
Bdi_KQK03912
Bdi_KQK02282
Bdi_KQK00472
Bdi_KQJ95316
Bdi_KQJ89251
Bdi_KQJ89243
Bdi_KQJ88170
Bdi_KQJ87701
Bdi_KQJ87537
Bdi_KQJ87419
Bdi_KQJ84633
Bdi_KQJ83448
Bdi_KQJ81943
−1000
0
1000
2000
3000
4000
5000

B.di RLK−Pelle_LRR−XII−1 subfamily exon−intron and kinase domain diagram (part 2)

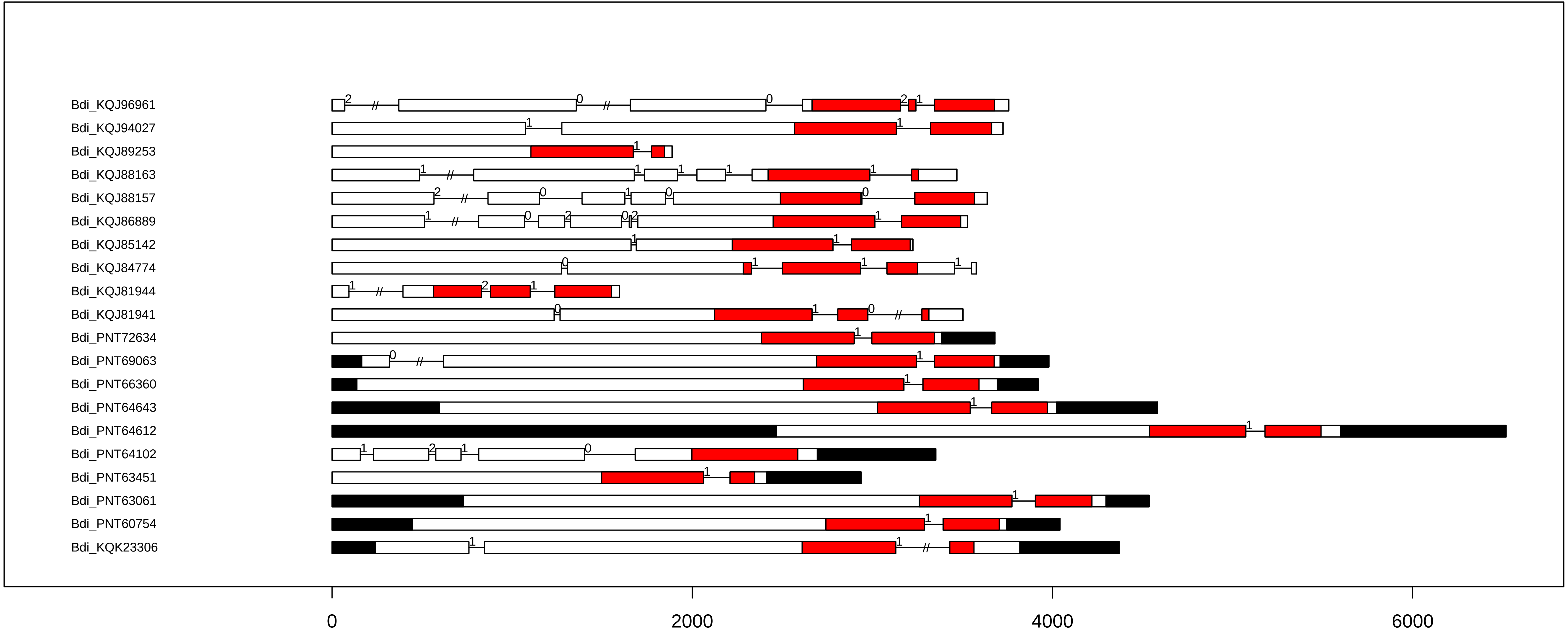

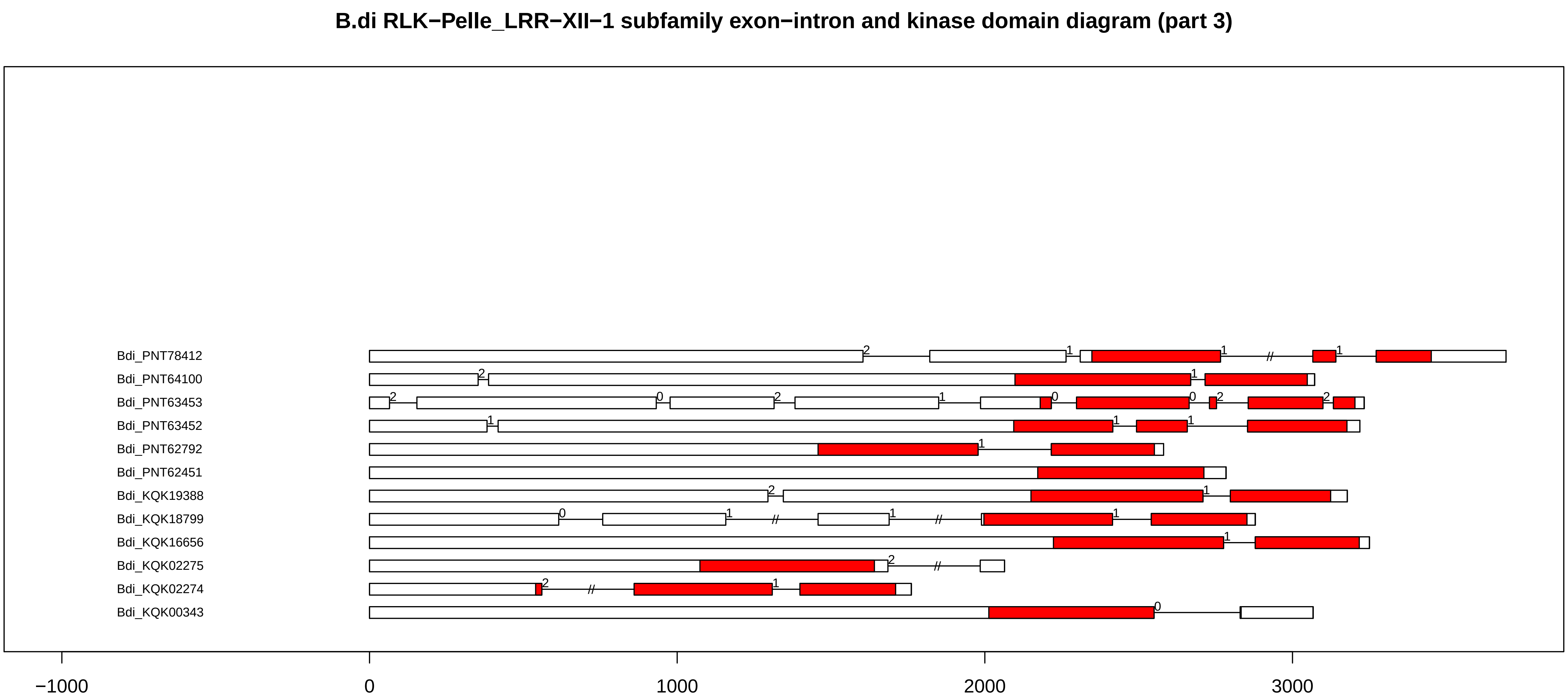
B.di RLK−Pelle_LRR−XII−1 subfamily exon−intron and kinase domain diagram (part 3)
Bdi_PNT78412
Bdi_PNT64100
Bdi_PNT63453
Bdi_PNT63452
Bdi_PNT62792
Bdi_PNT62451
Bdi_KQK19388
Bdi_KQK18799
Bdi_KQK16656
Bdi_KQK02275
Bdi_KQK02274
Bdi_KQK00343
−1000
0
1000
2000
3000

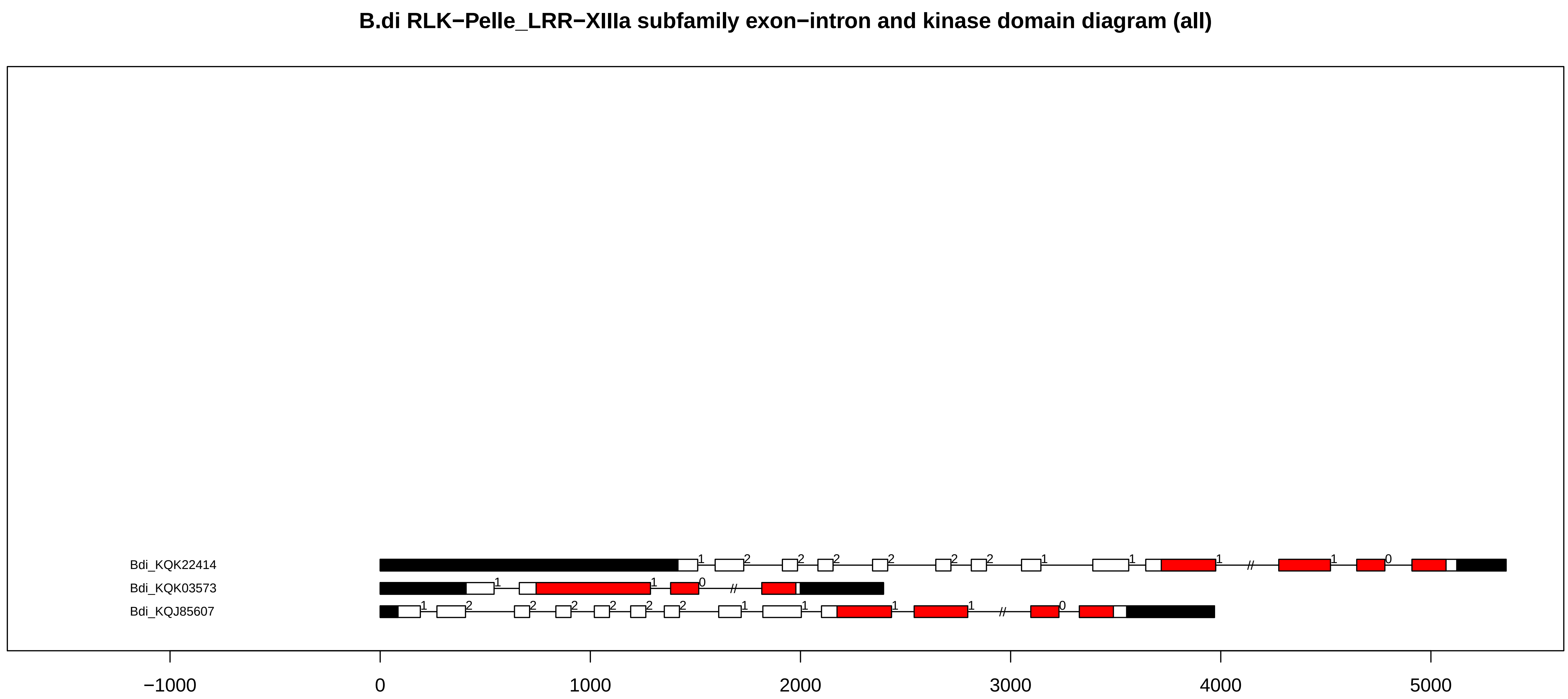
B.di RLK−Pelle_LRR−XIIIa subfamily exon−intron and kinase domain diagram (all)
Bdi_KQK22414
Bdi_KQK03573
Bdi_KQJ85607
−1000
0
1000
2000
3000
4000
5000

B.di RLK−Pelle_LRR−XIIIb subfamily exon−intron and kinase domain diagram (all)

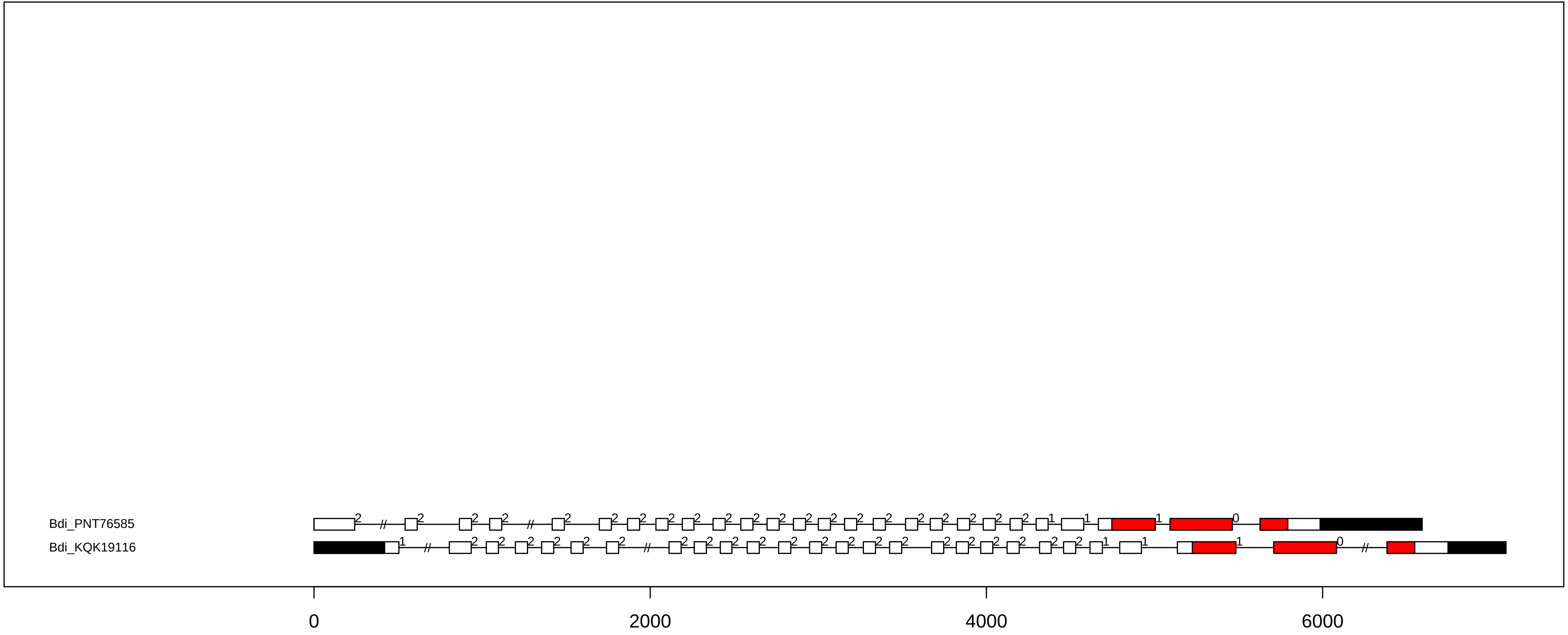

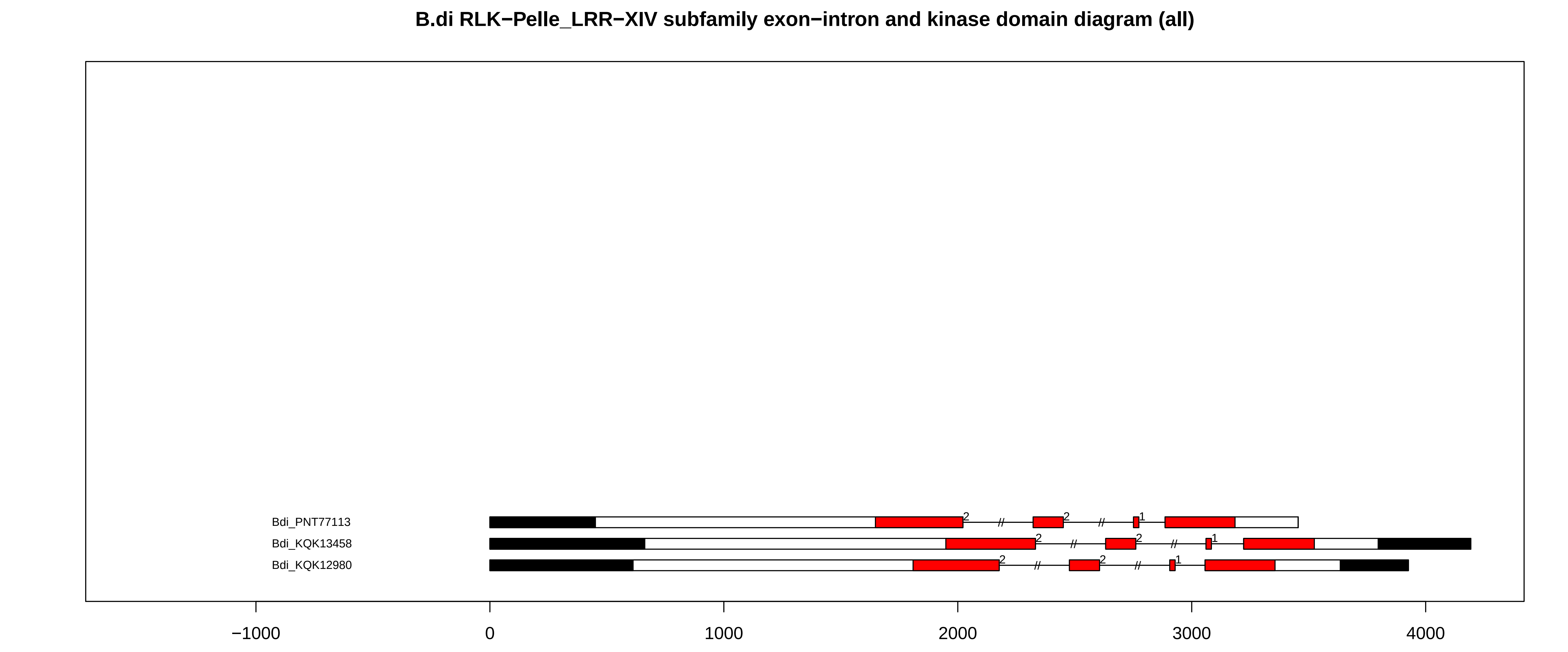
B.di RLK−Pelle_LRR−XIV subfamily exon−intron and kinase domain diagram (all)
Bdi_PNT77113
Bdi_KQK13458
Bdi_KQK12980
2
2
1
−1000
0
1000
2000
3000
4000

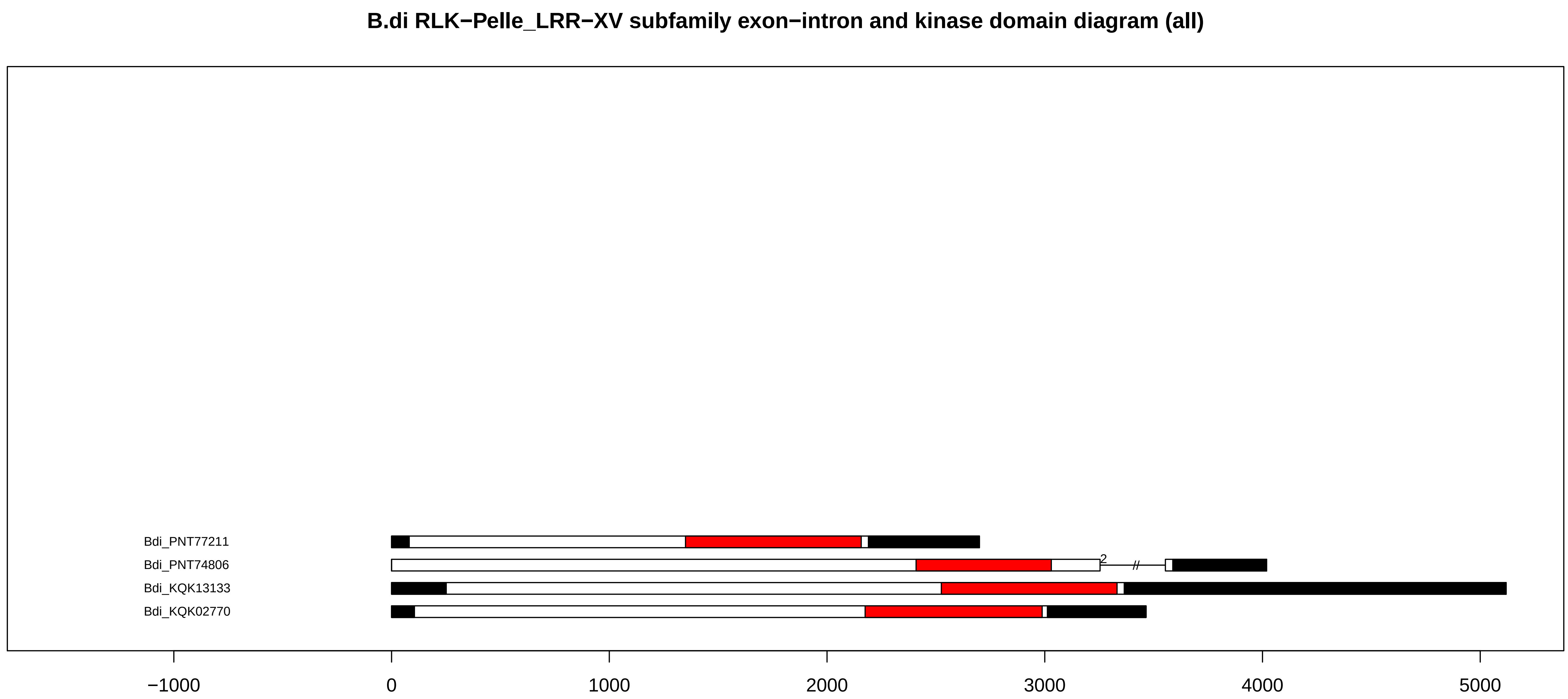

B.di RLK−Pelle_LRR−XV subfamily exon−intron and kinase domain diagram (all)
Bdi_PNT77211
Bdi_PNT74806
Bdi_KQK13133
Bdi_KQK02770
2
−1000
0
1000
2000
3000
4000
5000

B.di RLK−Pelle_LysM subfamily exon−intron and kinase domain diagram (all)

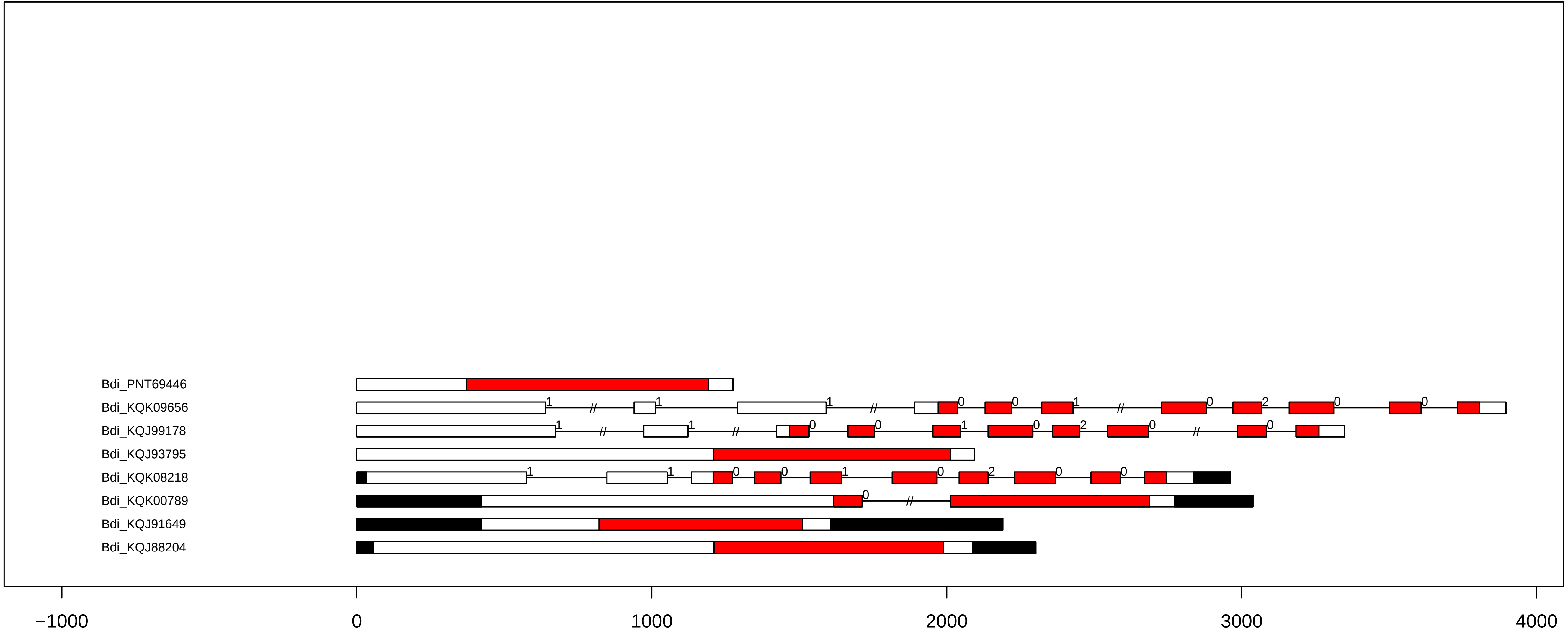

B.di RLK−Pelle_PERK−1 subfamily exon−intron and kinase domain diagram (all)

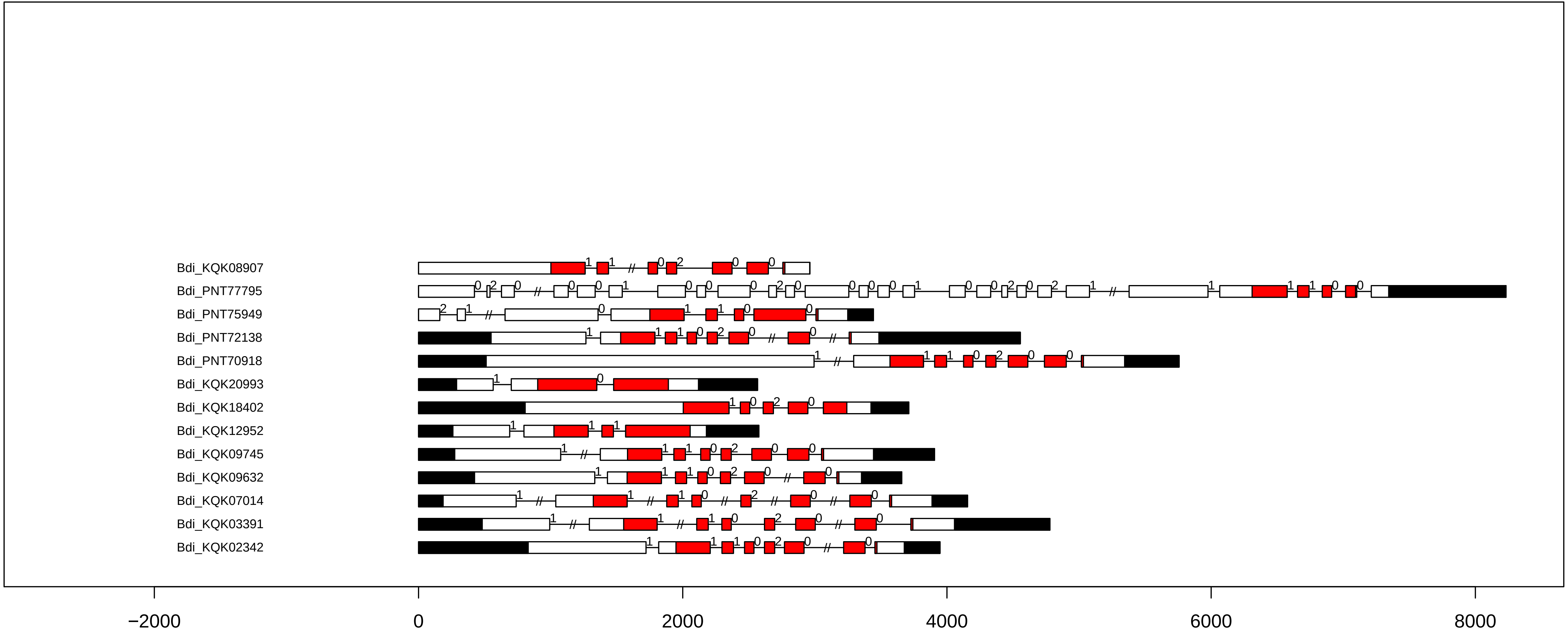

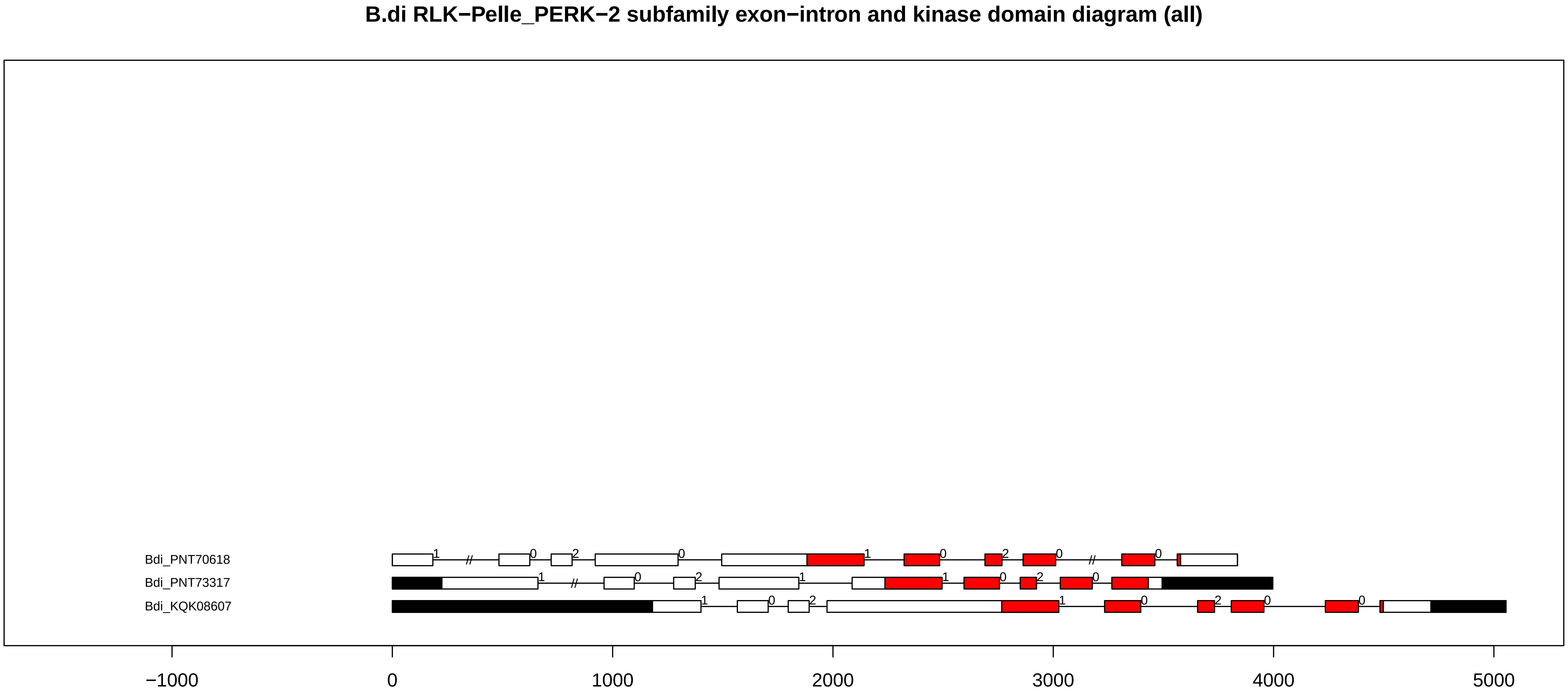

B.di RLK−Pelle_PERK−2 subfamily exon−intron and kinase domain diagram (all)
Bdi_PNT70618
Bdi_PNT73317
Bdi_KQK08607
−1000
0
1000
2000
3000
4000
5000

B.di RLK−Pelle_RKF3 subfamily exon−intron and kinase domain diagram (all)

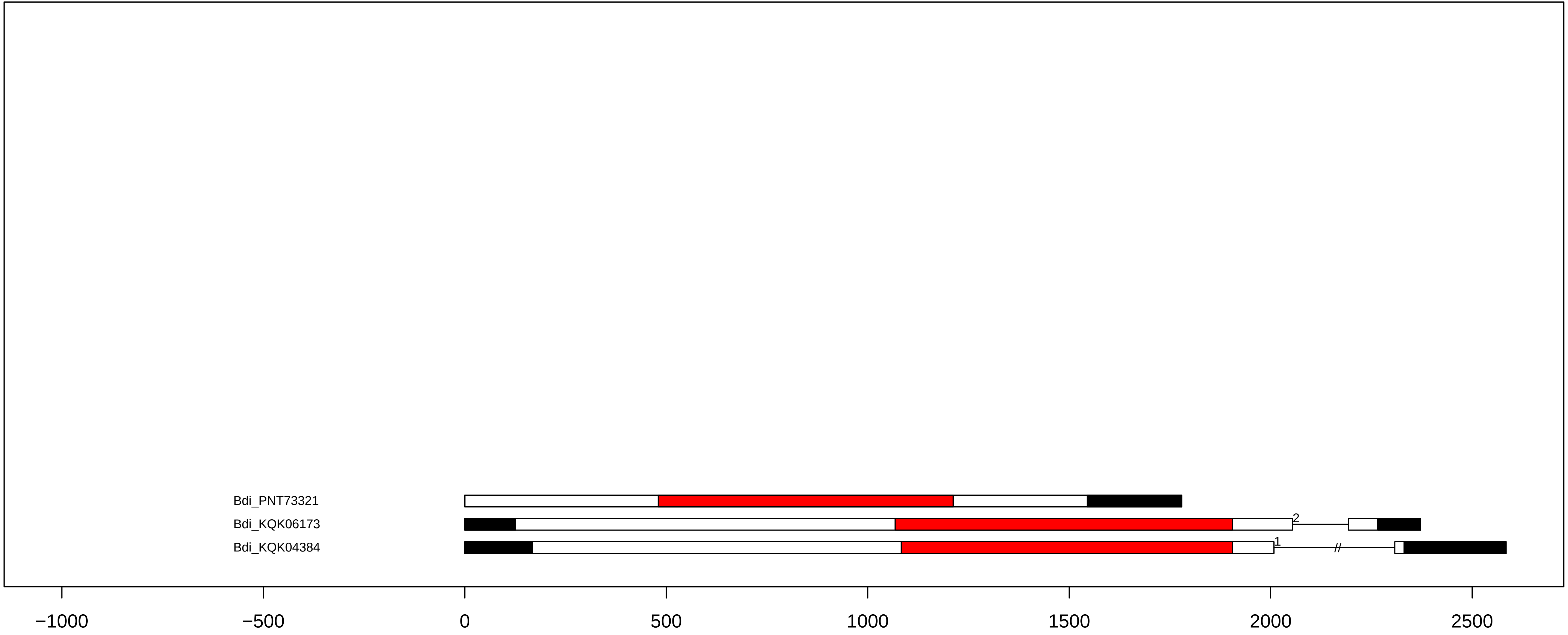

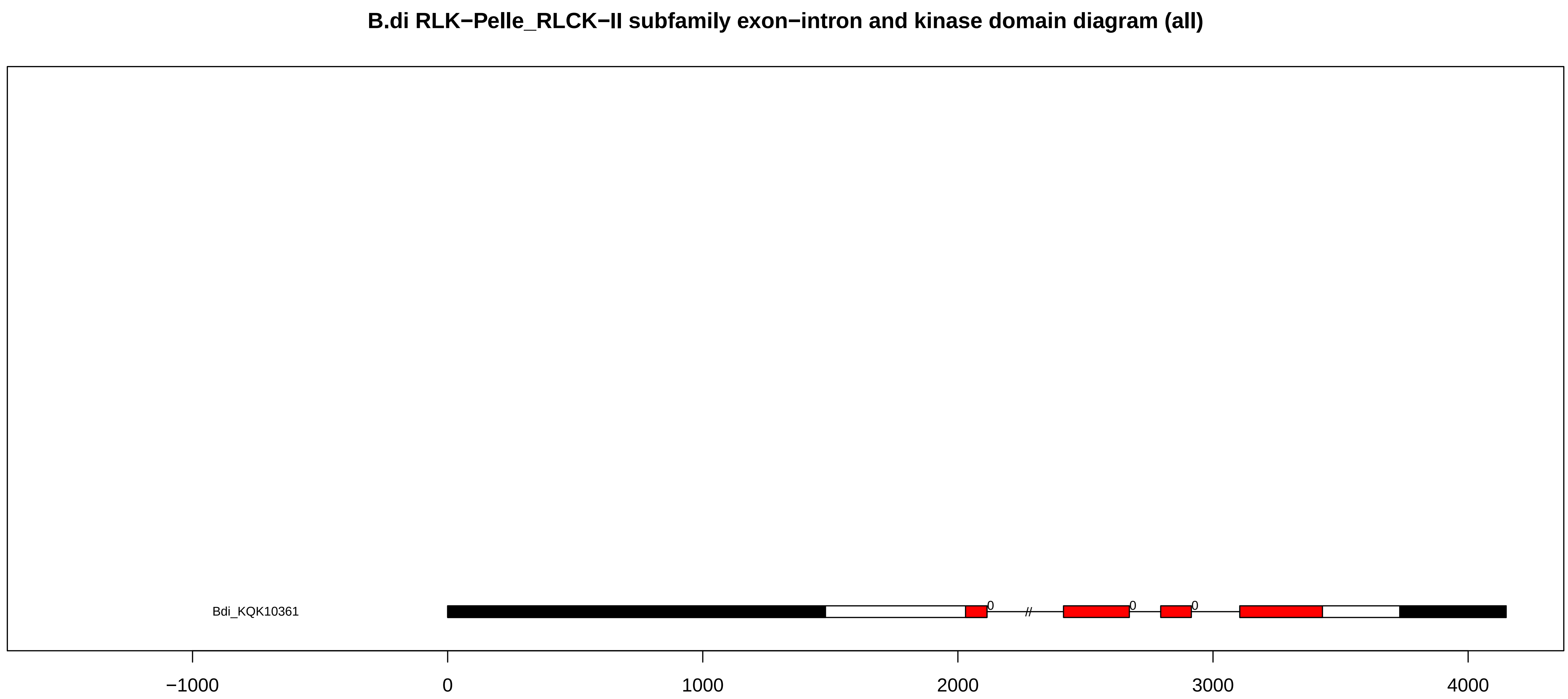

B.di RLK−Pelle_RLCK−II subfamily exon−intron and kinase domain diagram (all)
Bdi_KQK10361
0
0
0
−1000
0
1000
2000
3000
4000

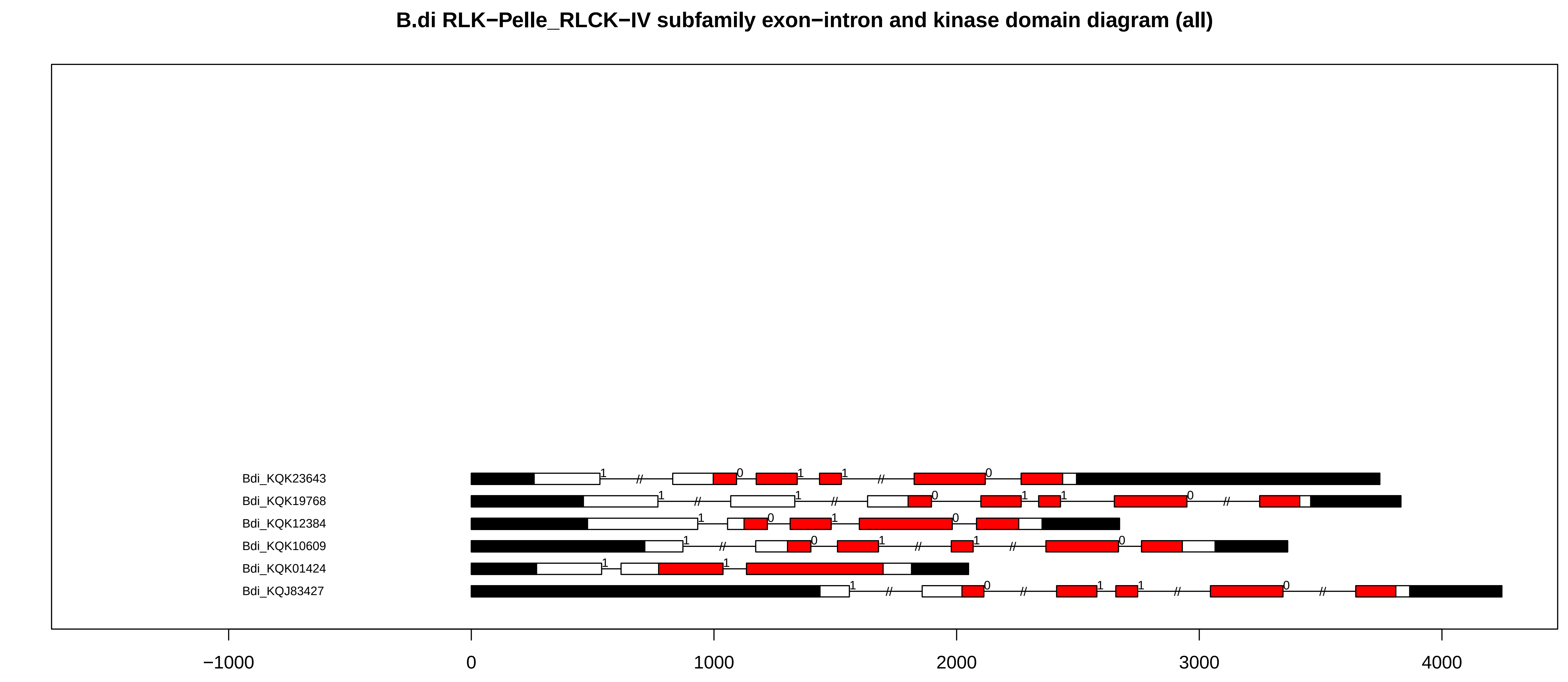

B.di RLK−Pelle_RLCK−IV subfamily exon−intron and kinase domain diagram (all)
Bdi_KQK23643
Bdi_KQK19768
Bdi_KQK12384
Bdi_KQK10609
Bdi_KQK01424
Bdi_KQJ83427
−1000
0
1000
2000
3000
4000

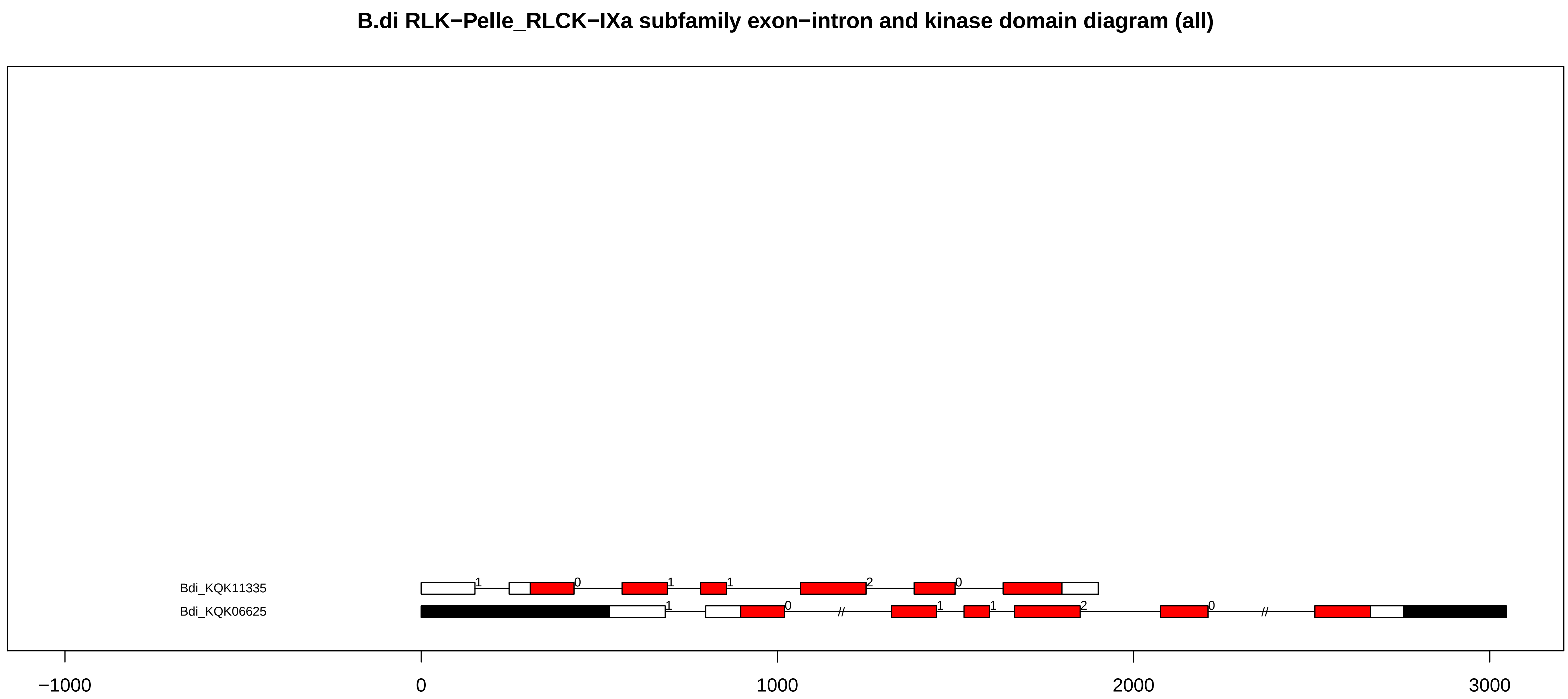
B.di RLK−Pelle_RLCK−IXa subfamily exon−intron and kinase domain diagram (all)
Bdi_KQK11335
Bdi_KQK06625
1
0
1
1
2
0
1
0
1
1
2
0
−1000
0
1000
2000
3000

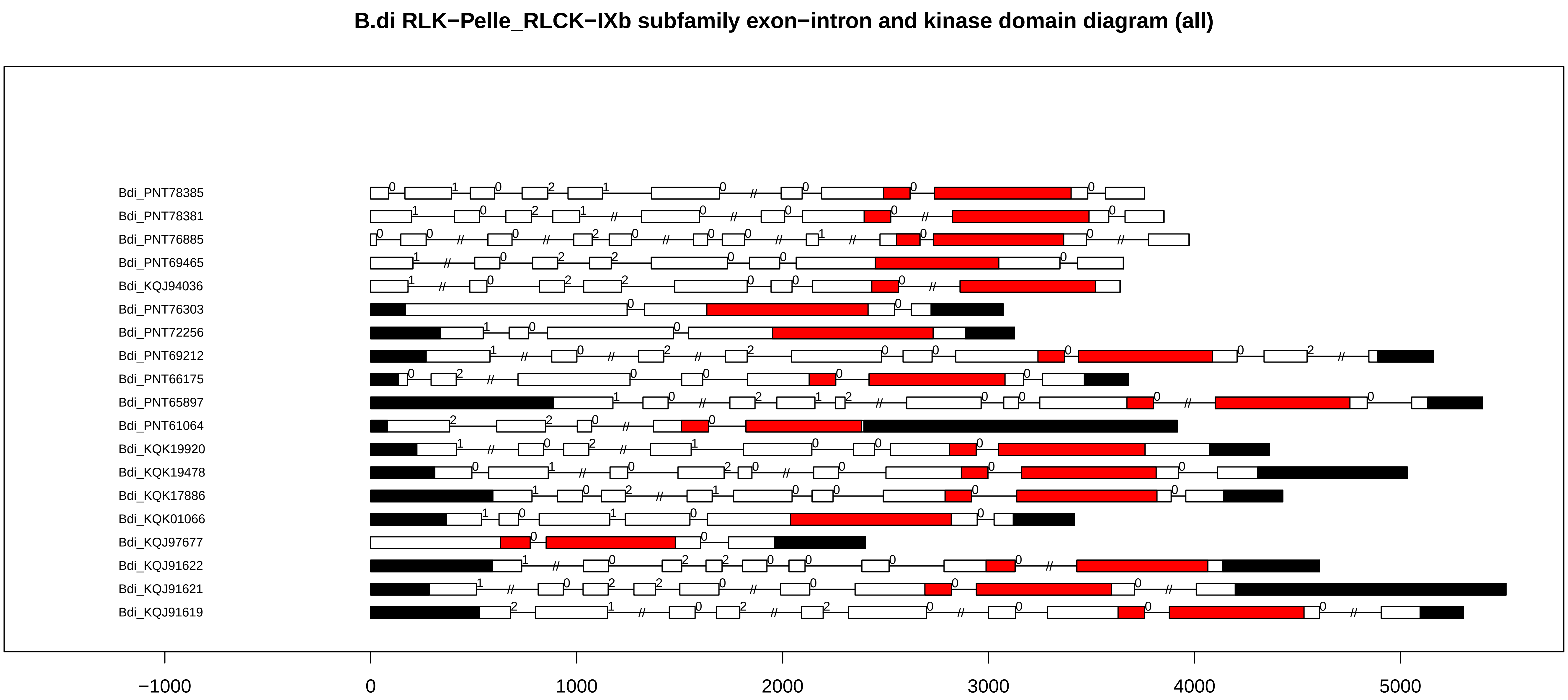
B.di RLK−Pelle_RLCK−IXb subfamily exon−intron and kinase domain diagram (all)
Bdi_PNT78385
Bdi_PNT78381
Bdi_PNT76885
Bdi_PNT69465
Bdi_KQJ94036
Bdi_PNT76303
Bdi_PNT72256
Bdi_PNT69212
Bdi_PNT66175
Bdi_PNT65897
Bdi_PNT61064
Bdi_KQK19920
Bdi_KQK19478
Bdi_KQK17886
Bdi_KQK01066
Bdi_KQJ97677
Bdi_KQJ91622
Bdi_KQJ91621
Bdi_KQJ91619
−1000
0
1000
2000
3000
4000
5000

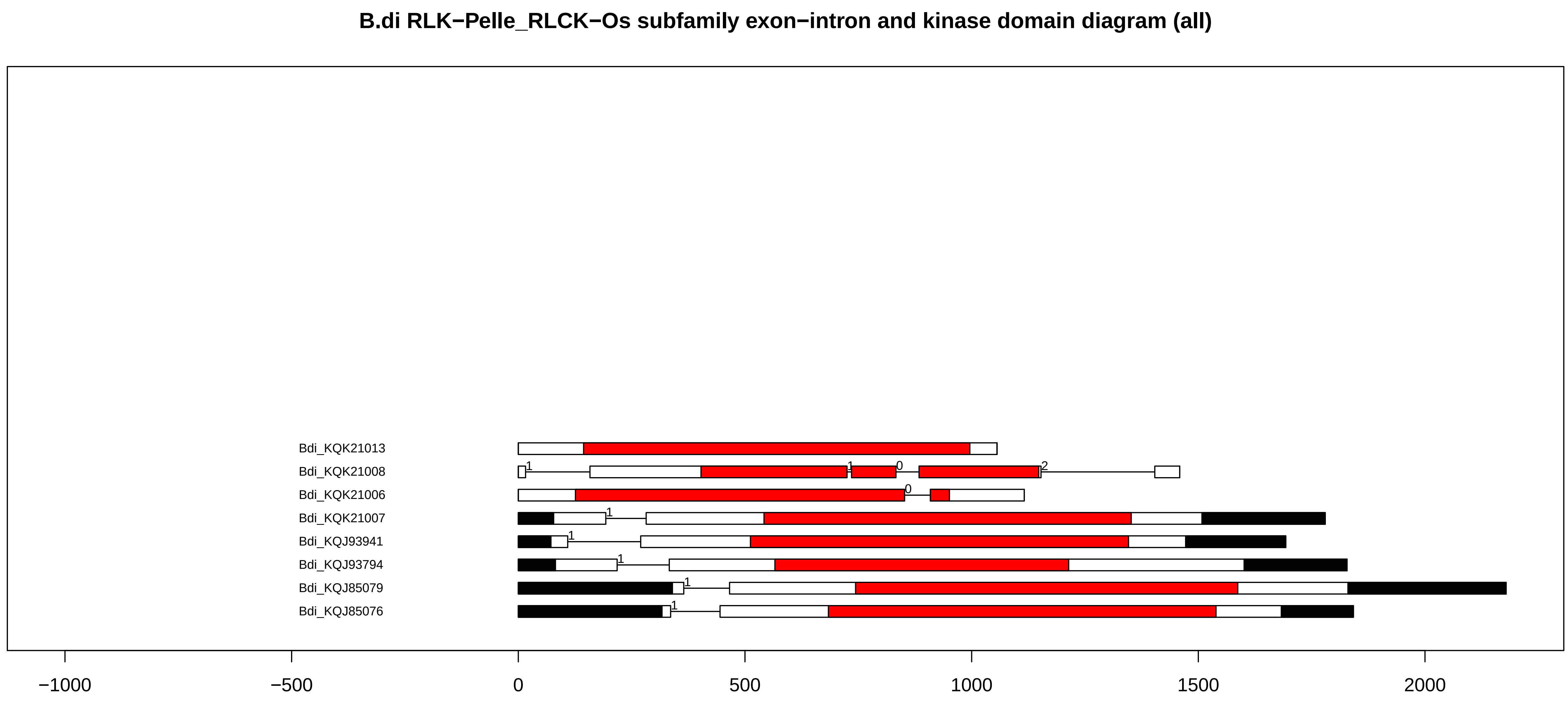
B.di RLK−Pelle_RLCK−Os subfamily exon−intron and kinase domain diagram (all)
Bdi_KQK21013
Bdi_KQK21008
Bdi_KQK21006
Bdi_KQK21007
Bdi_KQJ93941
Bdi_KQJ93794
Bdi_KQJ85079
Bdi_KQJ85076
−1000
−500
0
500
1000
1500
2000

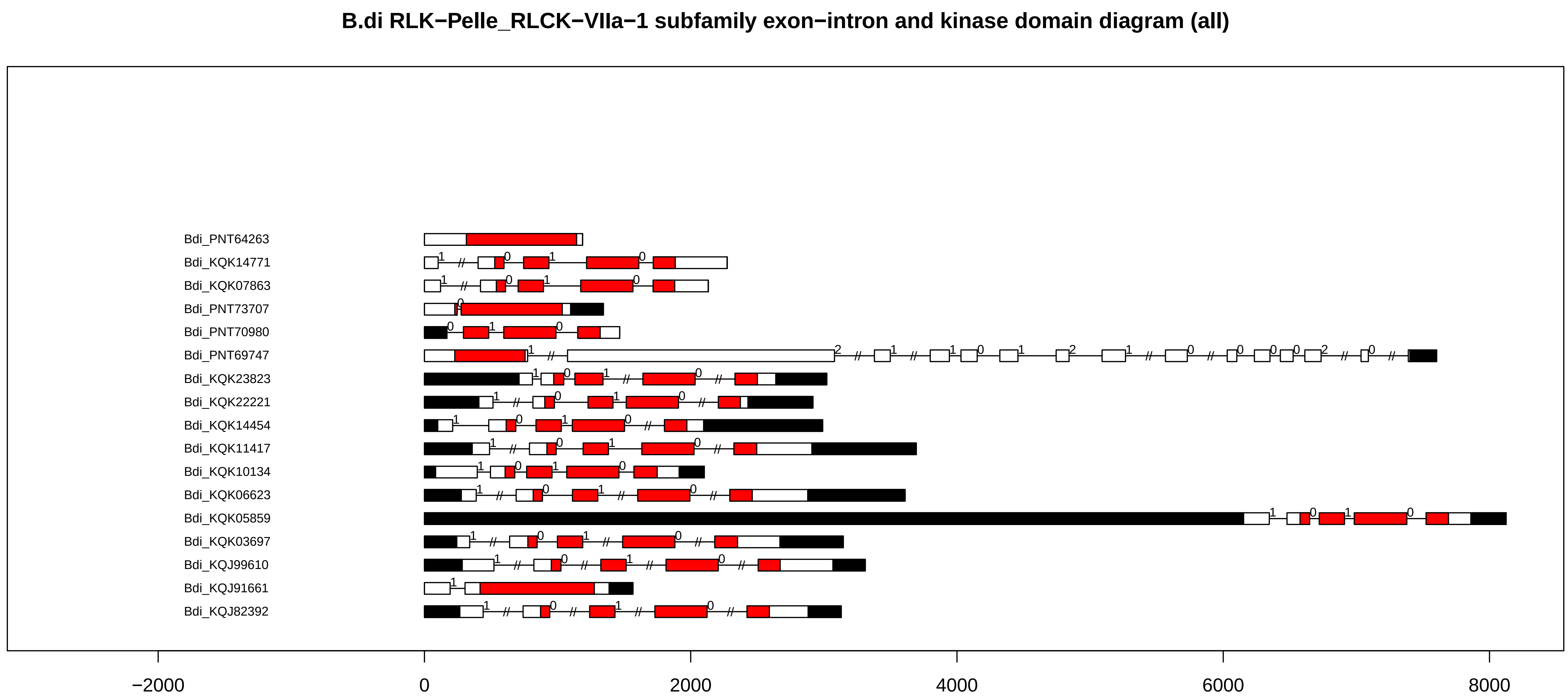
B.di RLK−Pelle_RLCK−VIIa−1 subfamily exon−intron and kinase domain diagram (all)
Bdi_PNT64263
Bdi_KQK14771
Bdi_KQK07863
Bdi_PNT73707
Bdi_PNT70980
Bdi_PNT69747
Bdi_KQK23823
Bdi_KQK22221
Bdi_KQK14454
Bdi_KQK11417
Bdi_KQK10134
Bdi_KQK06623
Bdi_KQK05859
Bdi_KQK03697
Bdi_KQJ99610
Bdi_KQJ91661
Bdi_KQJ82392
−2000
0
2000
4000
6000
8000

B.di RLK−Pelle_RLCK−VIIa−2 subfamily exon−intron and kinase domain diagram (part 1)

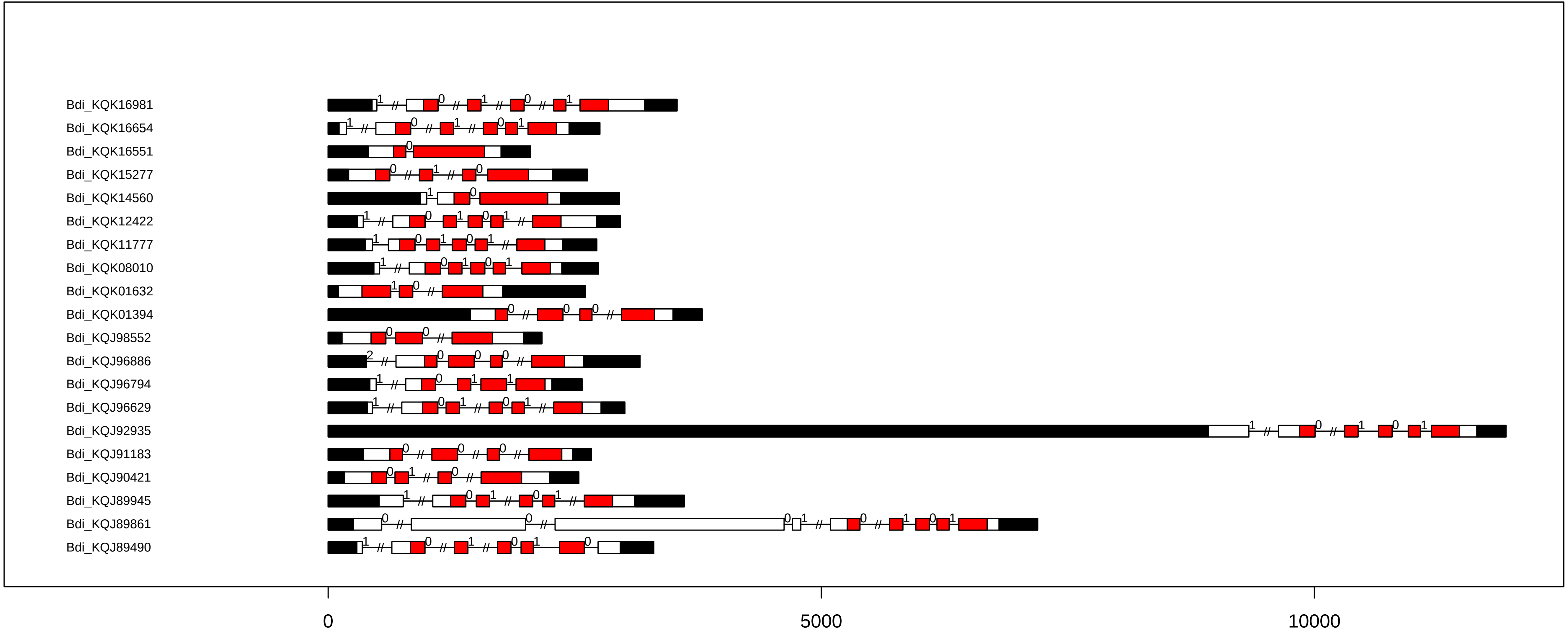

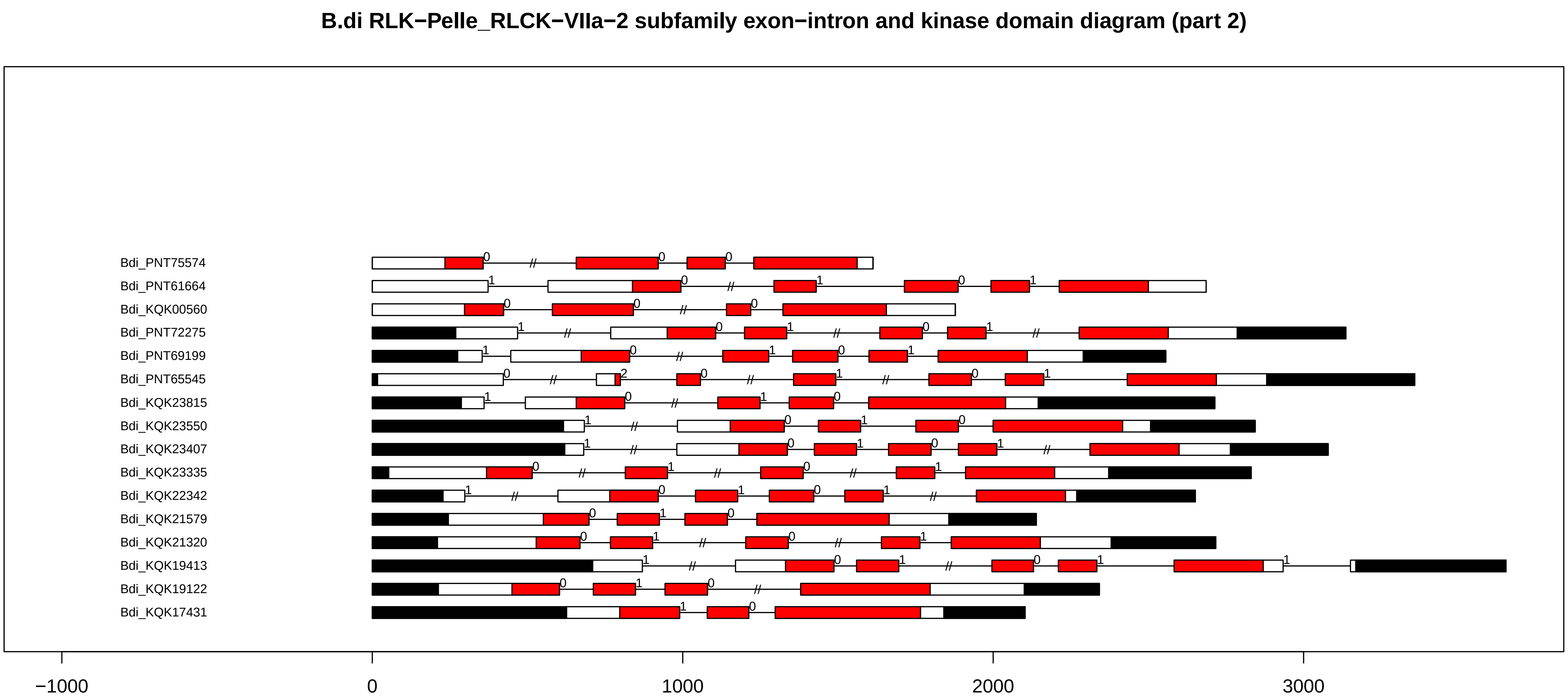

B.di RLK−Pelle_RLCK−VIIa−2 subfamily exon−intron and kinase domain diagram (part 2)
Bdi_PNT75574
Bdi_PNT61664
Bdi_KQK00560
Bdi_PNT72275
Bdi_PNT69199
Bdi_PNT65545
Bdi_KQK23815
Bdi_KQK23550
Bdi_KQK23407
Bdi_KQK23335
Bdi_KQK22342
Bdi_KQK21579
Bdi_KQK21320
Bdi_KQK19413
Bdi_KQK19122
Bdi_KQK17431
−1000
0
1000
2000
3000

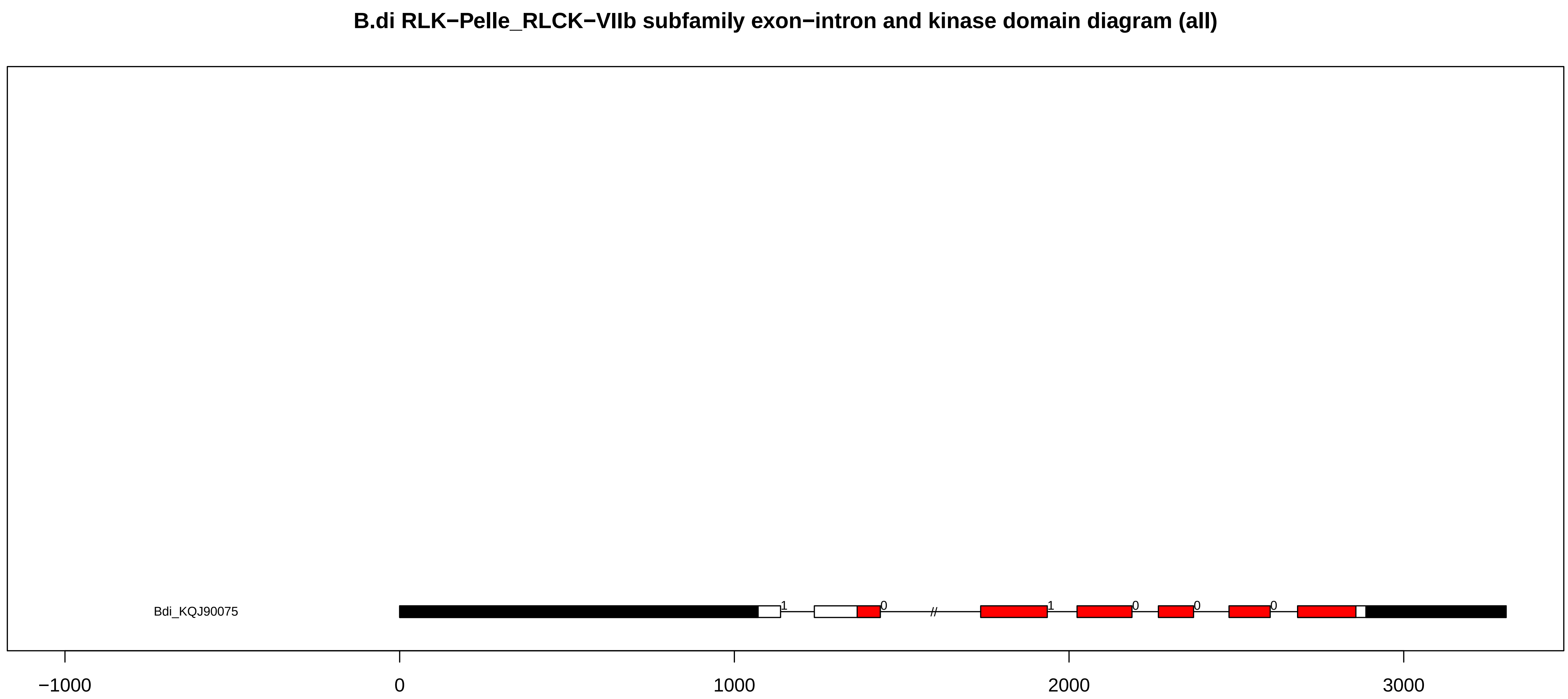
B.di RLK−Pelle_RLCK−VIIb subfamily exon−intron and kinase domain diagram (all)
Bdi_KQJ90075
1
0
//
1
0
0
0
−1000
0
1000
2000
3000

B.di RLK−Pelle_RLCK−VIII subfamily exon−intron and kinase domain diagram (all)

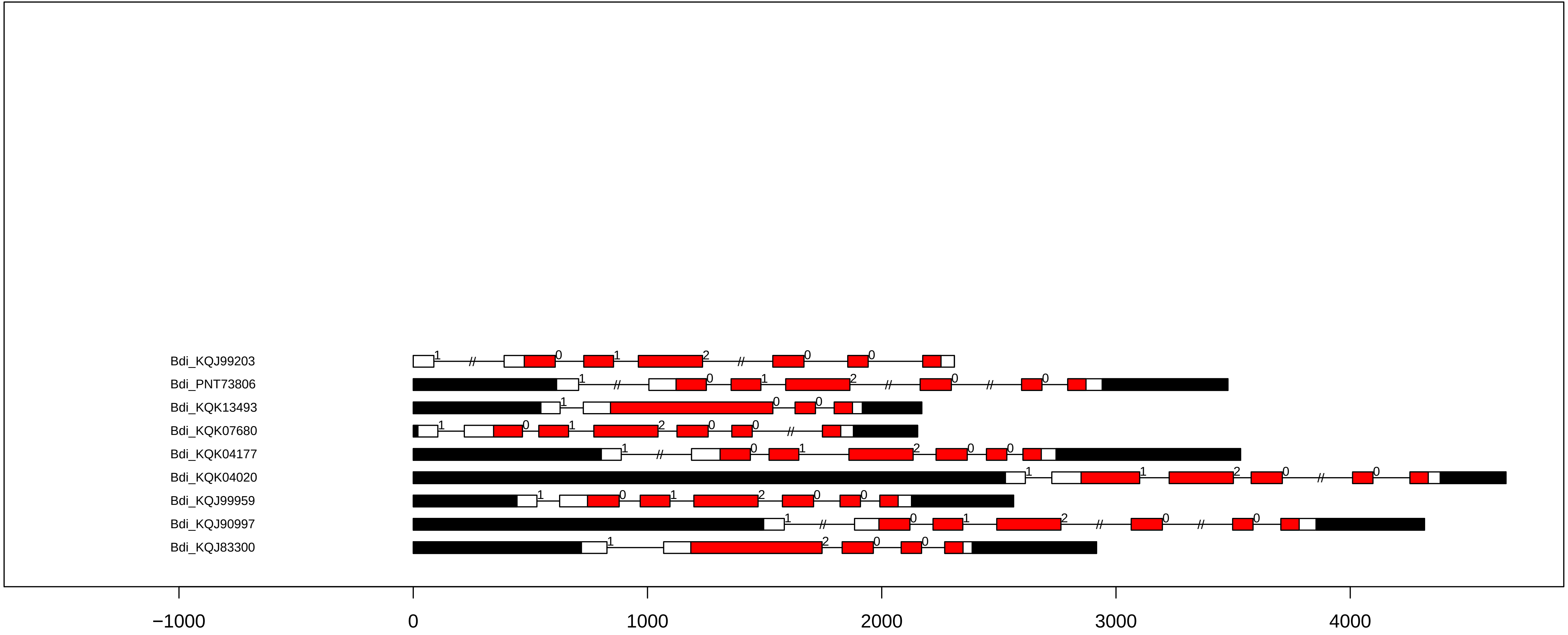

B.di RLK−Pelle_RLCK−VI subfamily exon−intron and kinase domain diagram (all)

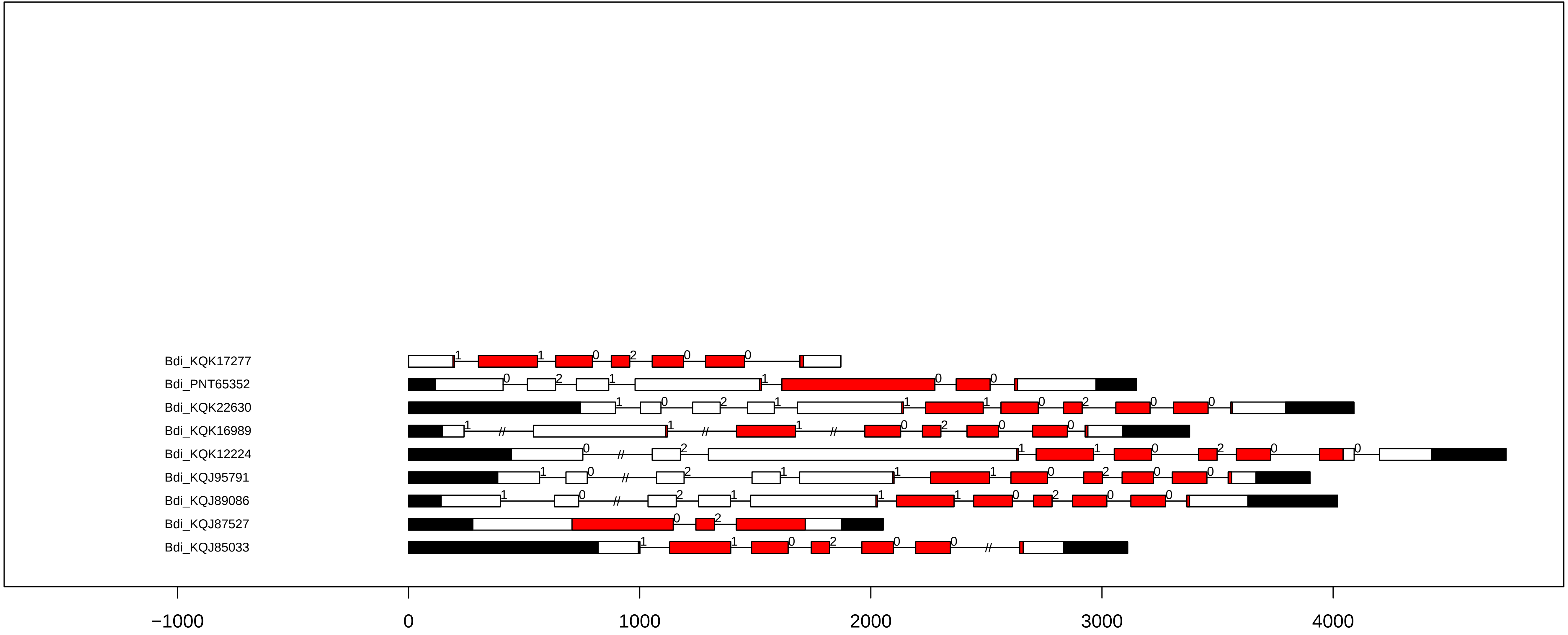

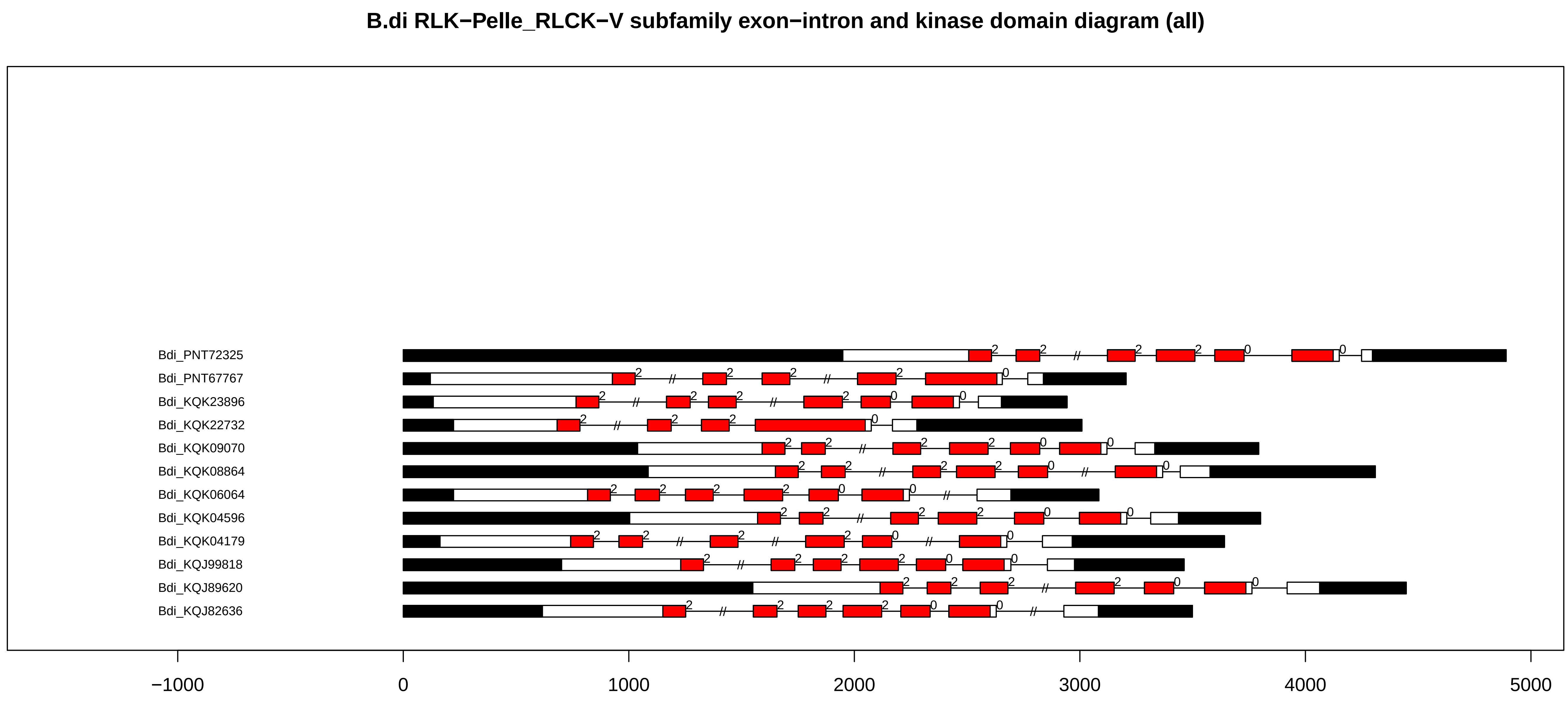
B.di RLK−Pelle_RLCK−V subfamily exon−intron and kinase domain diagram (all)
Bdi_PNT72325
Bdi_PNT67767
Bdi_KQK23896
Bdi_KQK22732
Bdi_KQK09070
Bdi_KQK08864
Bdi_KQK06064
Bdi_KQK04596
Bdi_KQK04179
Bdi_KQJ99818
Bdi_KQJ89620
Bdi_KQJ82636
−1000
0
1000
2000
3000
4000
5000

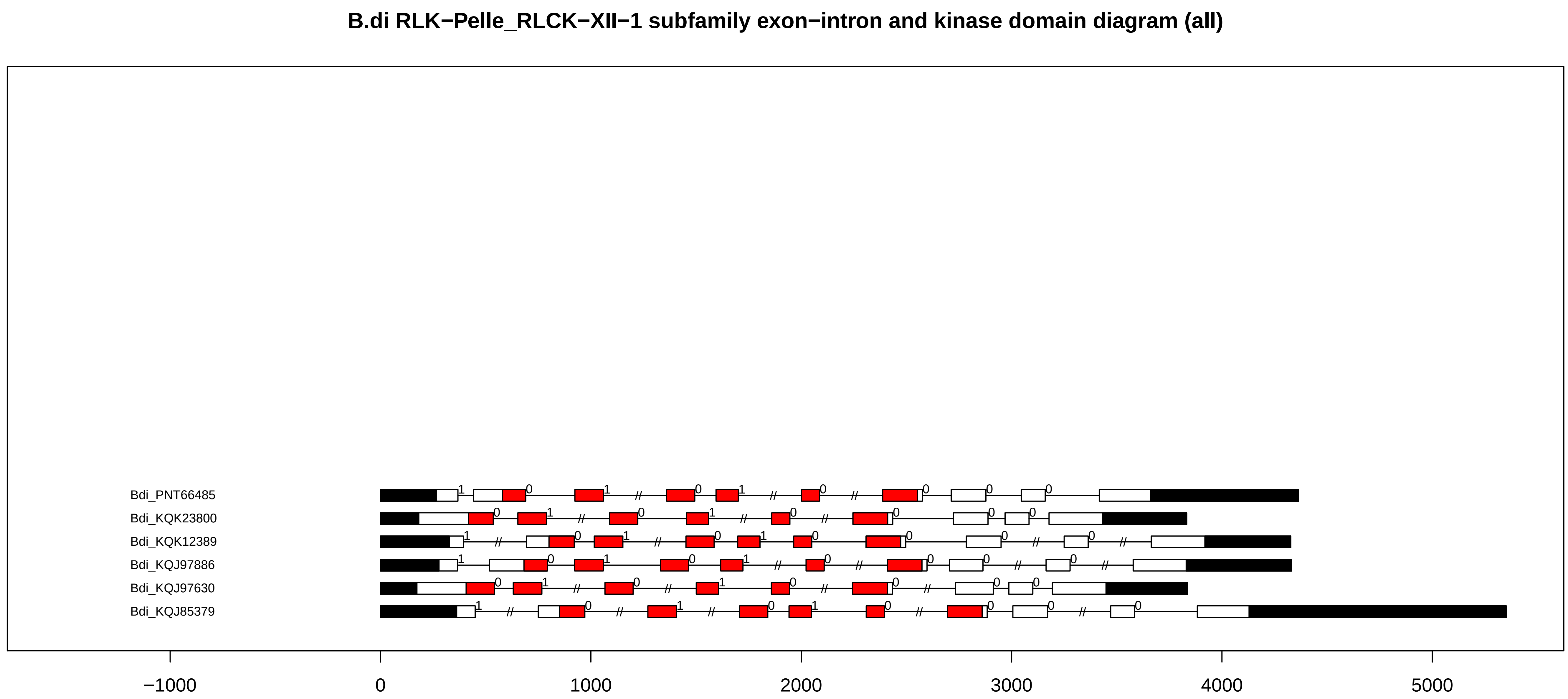

B.di RLK−Pelle_RLCK−XII−1 subfamily exon−intron and kinase domain diagram (all)
Bdi_PNT66485
Bdi_KQK23800
Bdi_KQK12389
Bdi_KQJ97886
Bdi_KQJ97630
Bdi_KQJ85379
−1000
0
1000
2000
3000
4000
5000

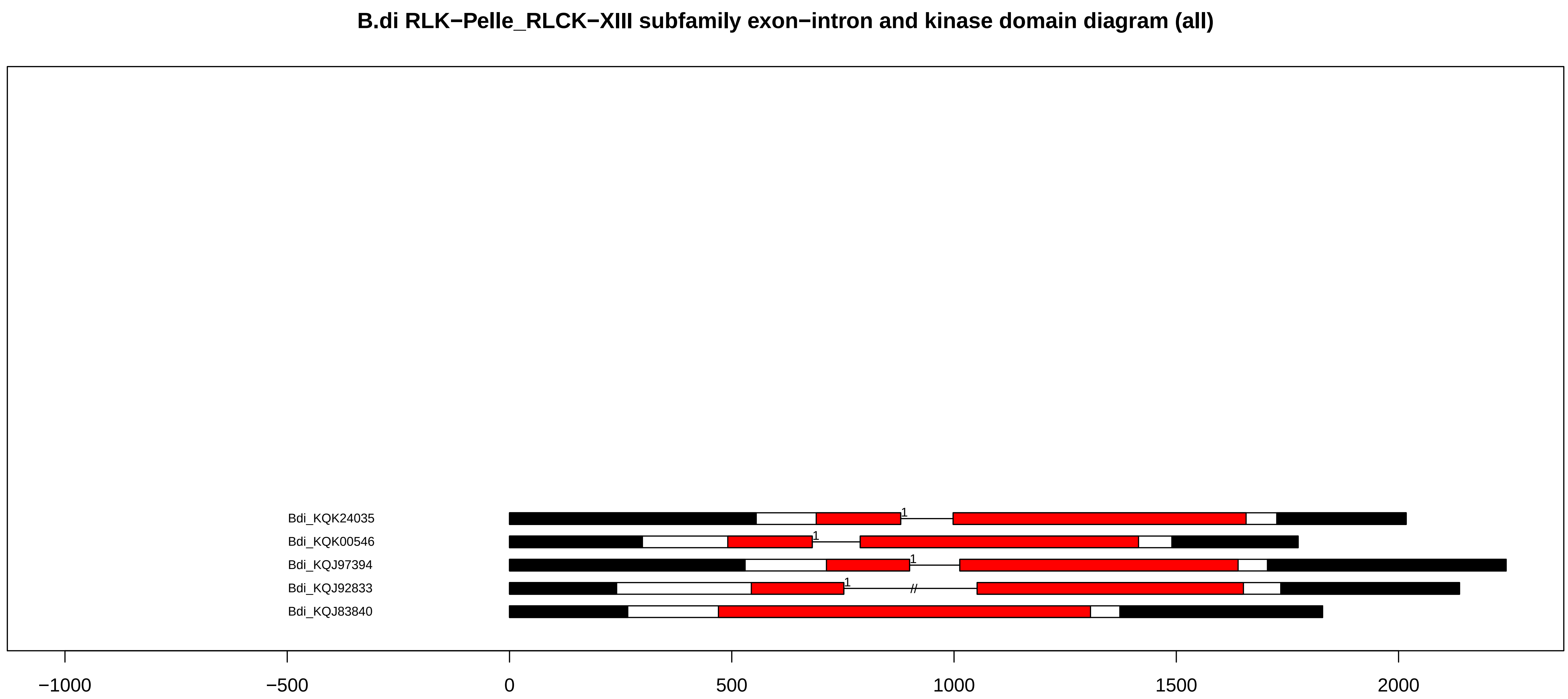
B.di RLK−Pelle_RLCK−XIII subfamily exon−intron and kinase domain diagram (all)
Bdi_KQK24035
Bdi_KQK00546
Bdi_KQJ97394
Bdi_KQJ92833
Bdi_KQJ83840
1
1
1
1
−1000
−500
0
500
1000
1500
2000

B.di RLK−Pelle_RLCK−XI subfamily exon−intron and kinase domain diagram (all)

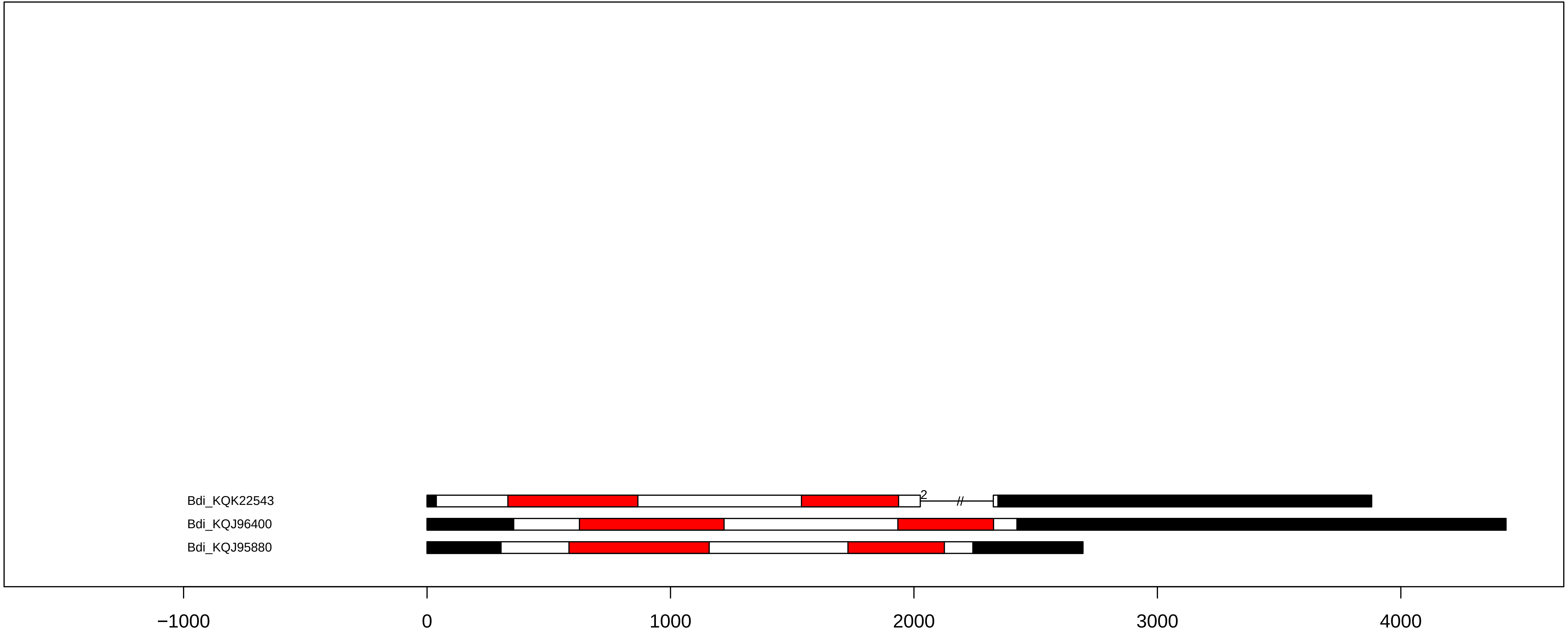

B.di RLK−Pelle_RLCK−X subfamily exon−intron and kinase domain diagram (all)

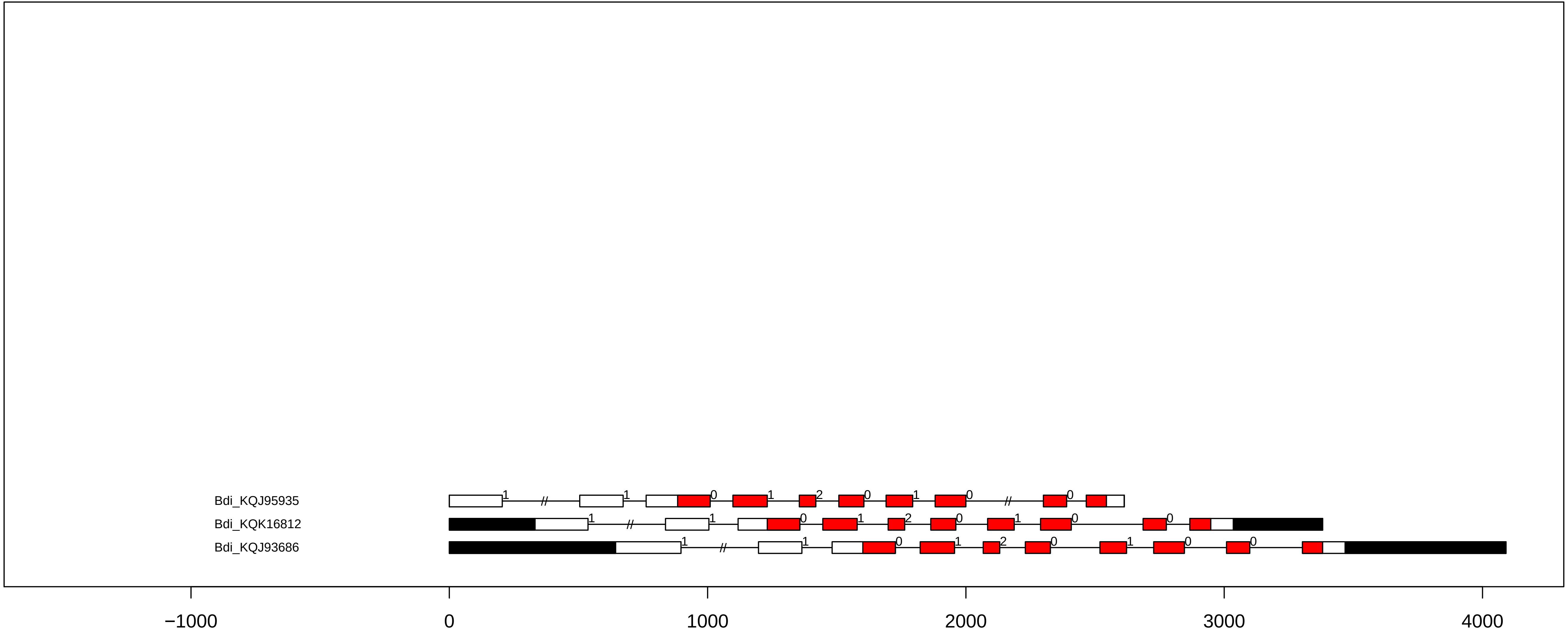

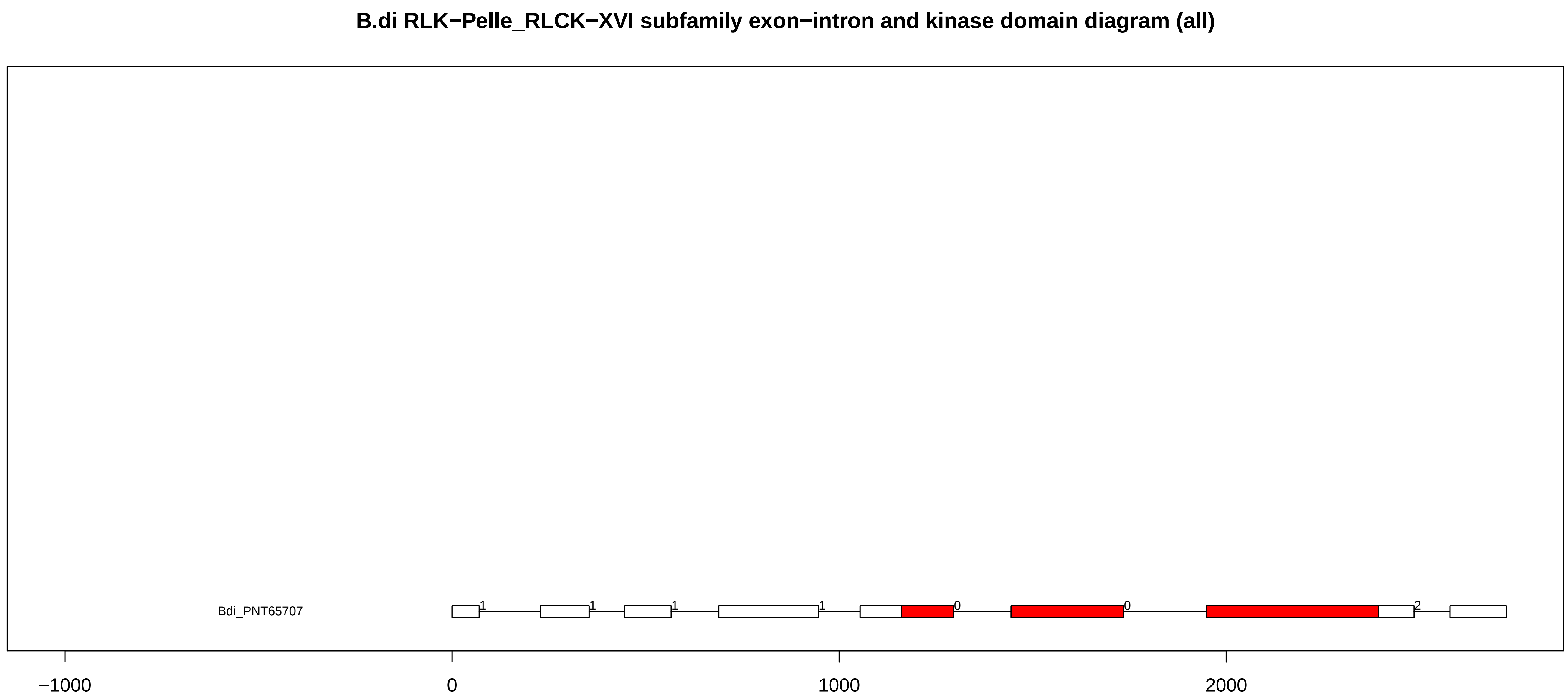
B.di RLK−Pelle_RLCK−XVI subfamily exon−intron and kinase domain diagram (all)
Bdi_PNT65707
1
1
1
1
0
0
2
−1000
0
1000
2000

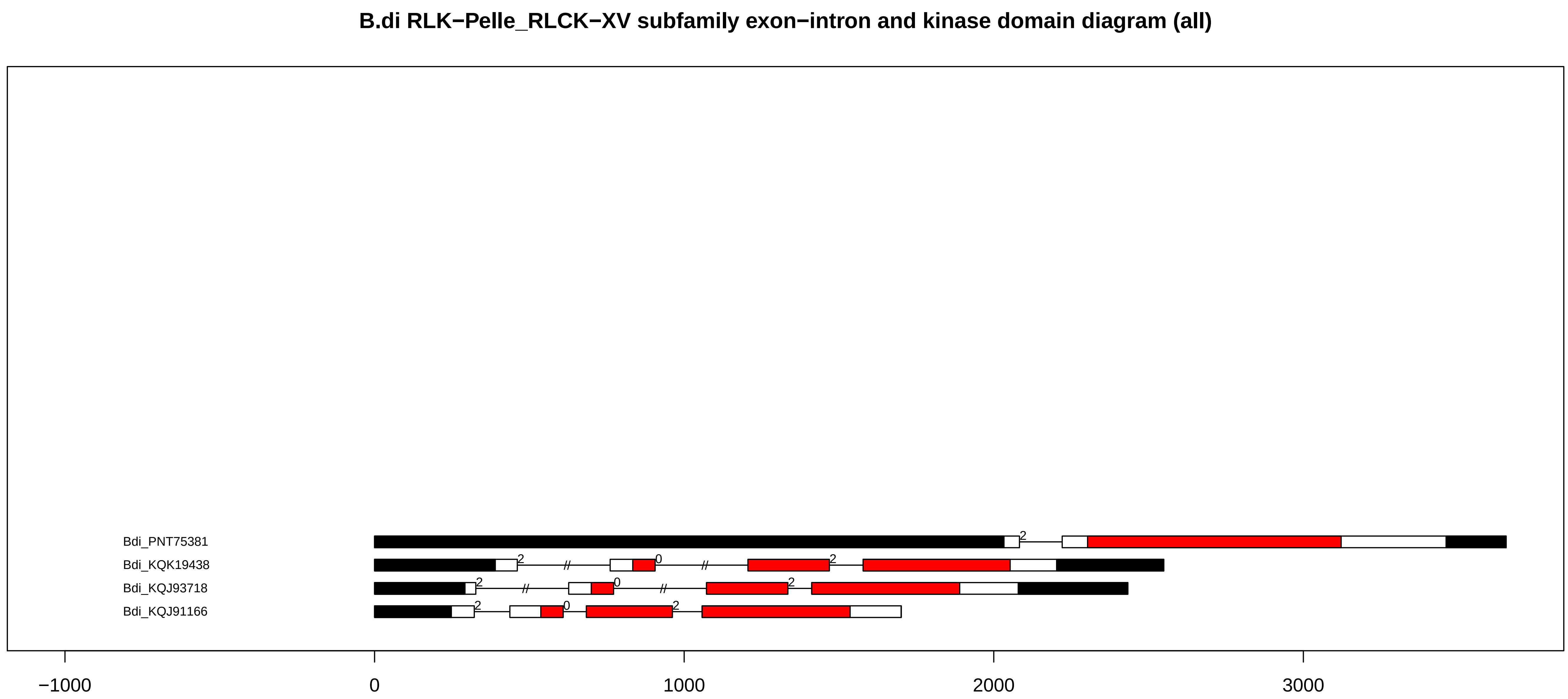

B.di RLK−Pelle_RLCK−XV subfamily exon−intron and kinase domain diagram (all)
Bdi_PNT75381
Bdi_KQK19438
Bdi_KQJ93718
Bdi_KQJ91166
2
2
0
2
2
0
2
2
0
2
−1000
0
1000
2000
3000

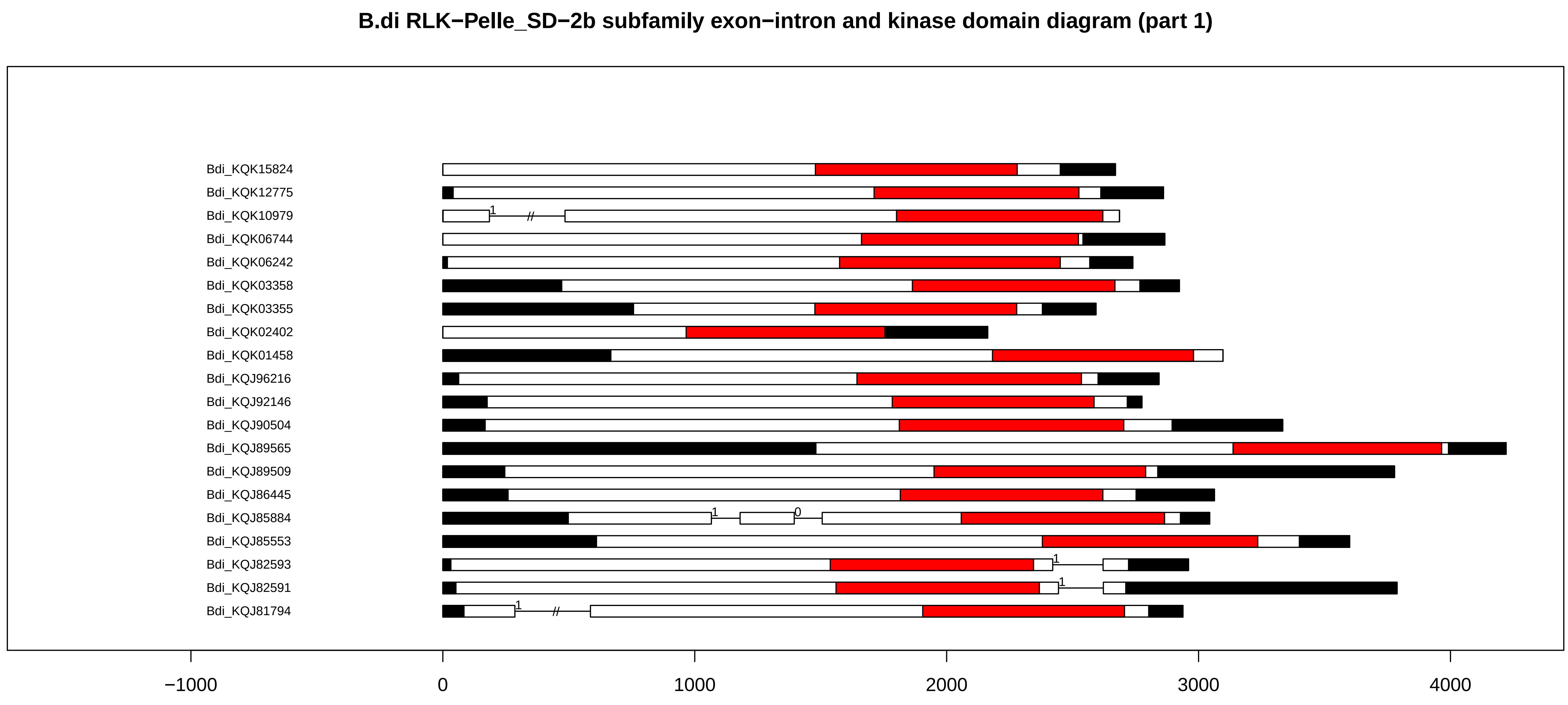

B.di RLK−Pelle_SD−2b subfamily exon−intron and kinase domain diagram (part 1)
Bdi_KQK15824
Bdi_KQK12775
Bdi_KQK10979
Bdi_KQK06744
Bdi_KQK06242
Bdi_KQK03358
Bdi_KQK03355
Bdi_KQK02402
Bdi_KQK01458
Bdi_KQJ96216
Bdi_KQJ92146
Bdi_KQJ90504
Bdi_KQJ89565
Bdi_KQJ89509
Bdi_KQJ86445
Bdi_KQJ85884
Bdi_KQJ85553
Bdi_KQJ82593
Bdi_KQJ82591
Bdi_KQJ81794
−1000
0
1000
2000
3000
4000

B.di RLK−Pelle_SD−2b subfamily exon−intron and kinase domain diagram (part 2)

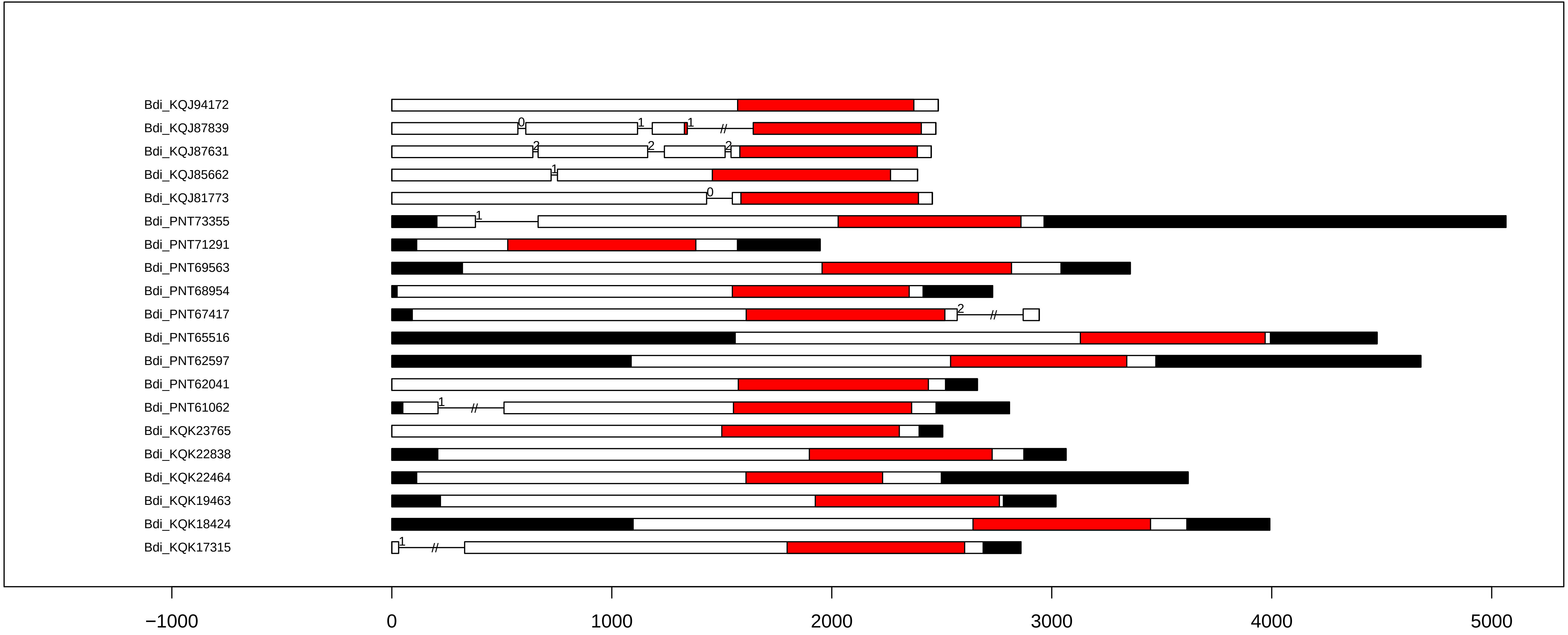

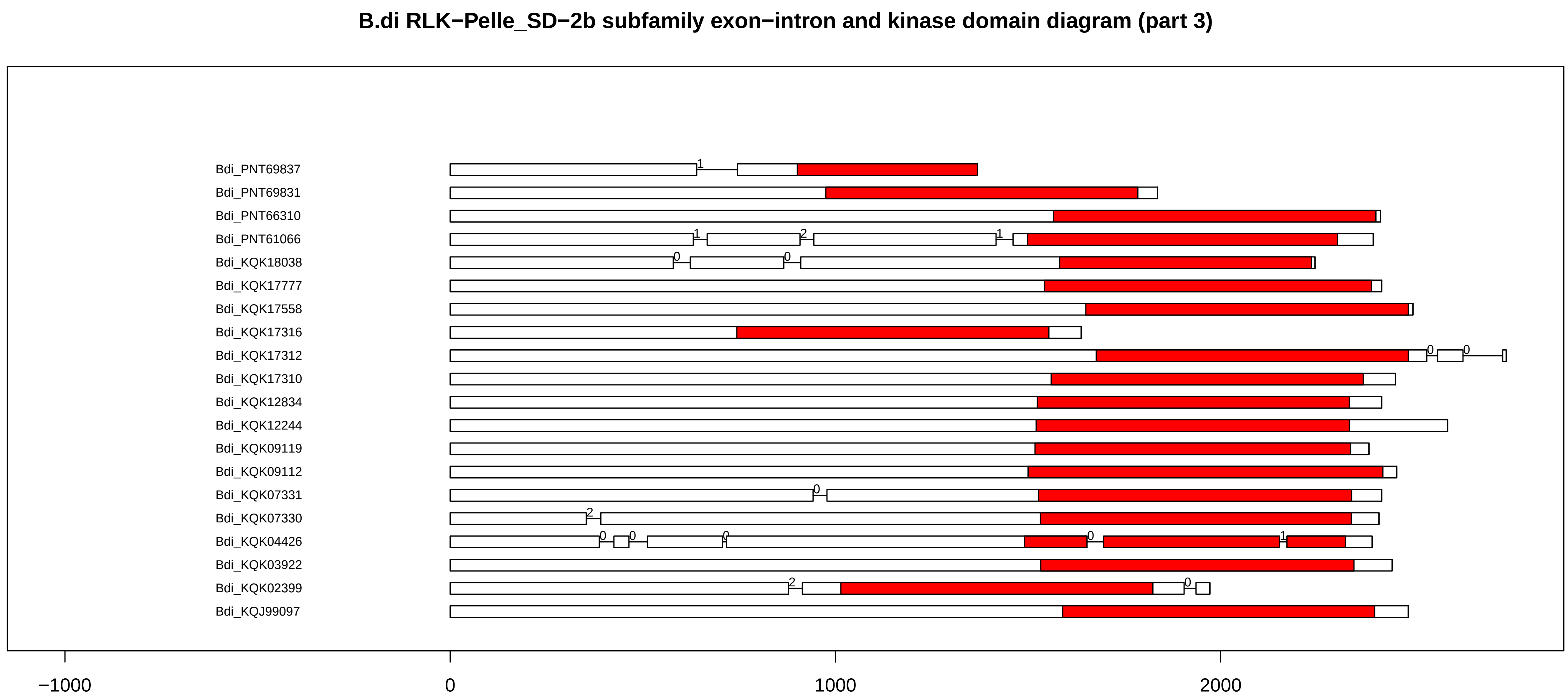
B.di RLK−Pelle_SD−2b subfamily exon−intron and kinase domain diagram (part 3)
Bdi_PNT69837
Bdi_PNT69831
Bdi_PNT66310
Bdi_PNT61066
Bdi_KQK18038
Bdi_KQK17777
Bdi_KQK17558
Bdi_KQK17316
Bdi_KQK17312
Bdi_KQK17310
Bdi_KQK12834
Bdi_KQK12244
Bdi_KQK09119
Bdi_KQK09112
Bdi_KQK07331
Bdi_KQK07330
Bdi_KQK04426
Bdi_KQK03922
Bdi_KQK02399
Bdi_KQJ99097
−1000
0
1000
2000

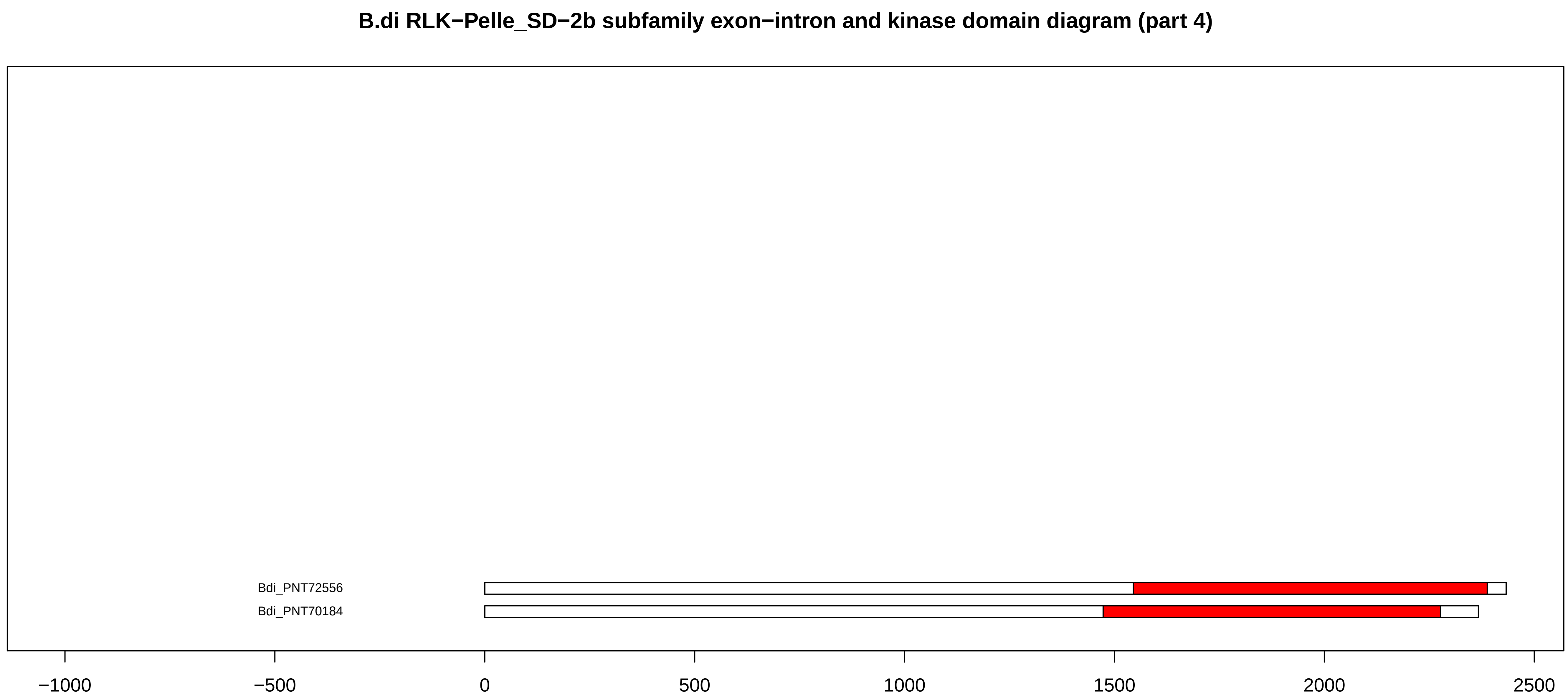
B.di RLK−Pelle_SD−2b subfamily exon−intron and kinase domain diagram (part 4)
Bdi_PNT72556
Bdi_PNT70184
−1000
−500
0
500
1000
1500
2000
2500

B.di RLK−Pelle_URK−1 subfamily exon−intron and kinase domain diagram (all)

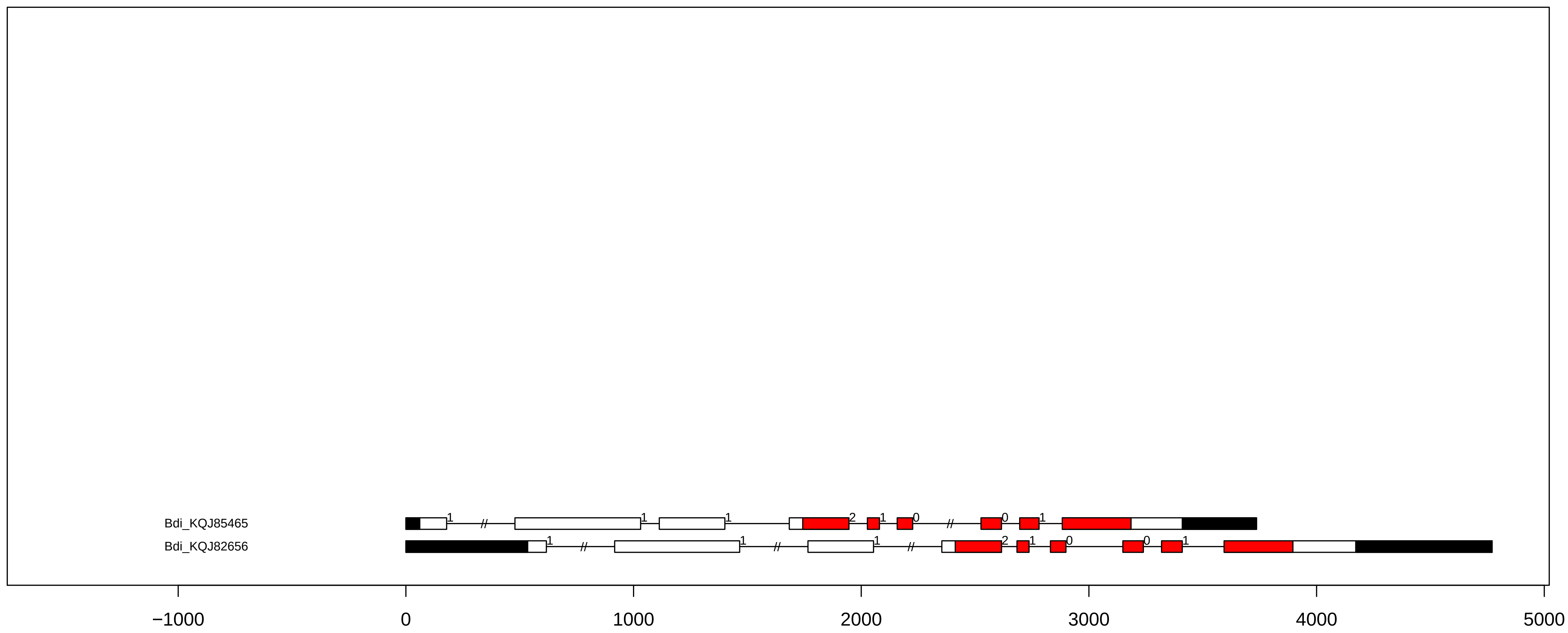

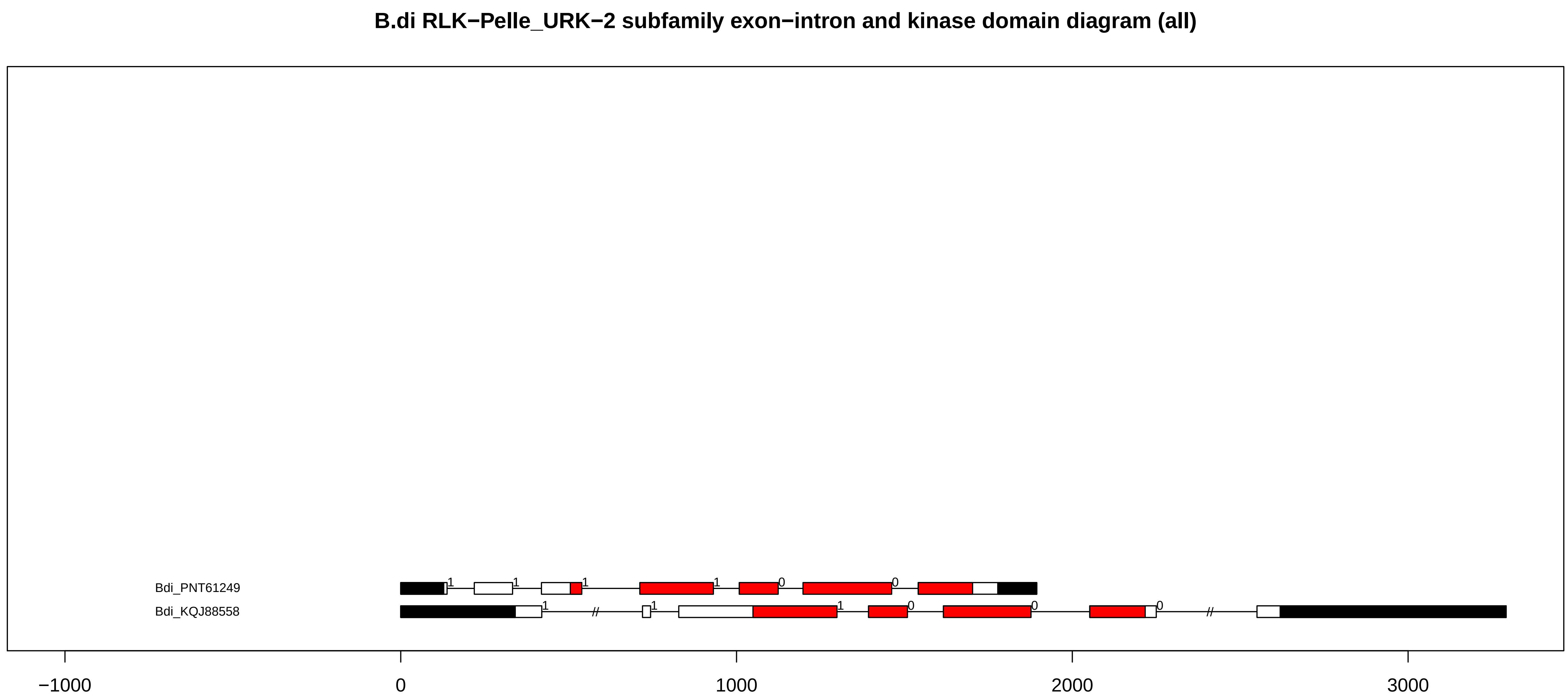
B.di RLK−Pelle_URK−2 subfamily exon−intron and kinase domain diagram (all)
Bdi_PNT61249
Bdi_KQJ88558
1
1
1
1
0
0
1
1
1
0
0
0
−1000
0
1000
2000
3000

B.di RLK−Pelle_WAK_LRK10L−1 subfamily exon−intron and kinase domain diagram (all)

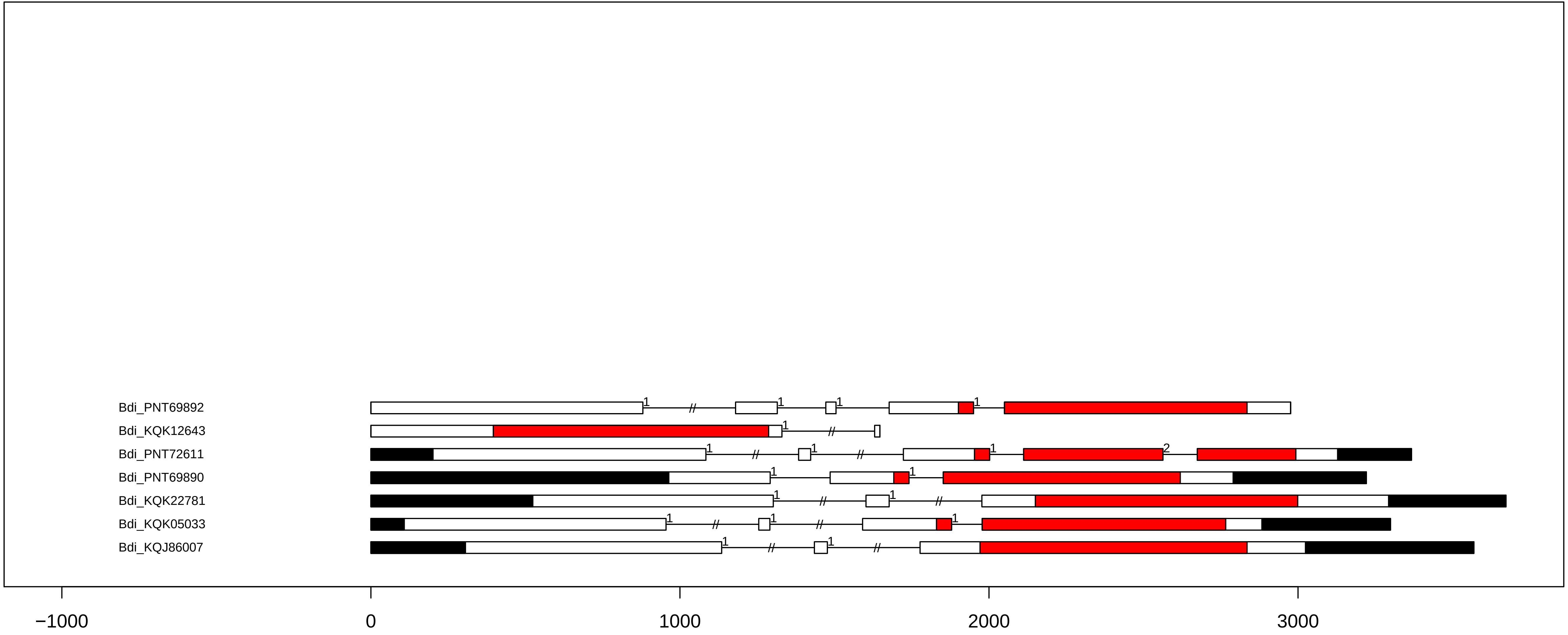

B.di RLK−Pelle_WAK subfamily exon−intron and kinase domain diagram (part 1)

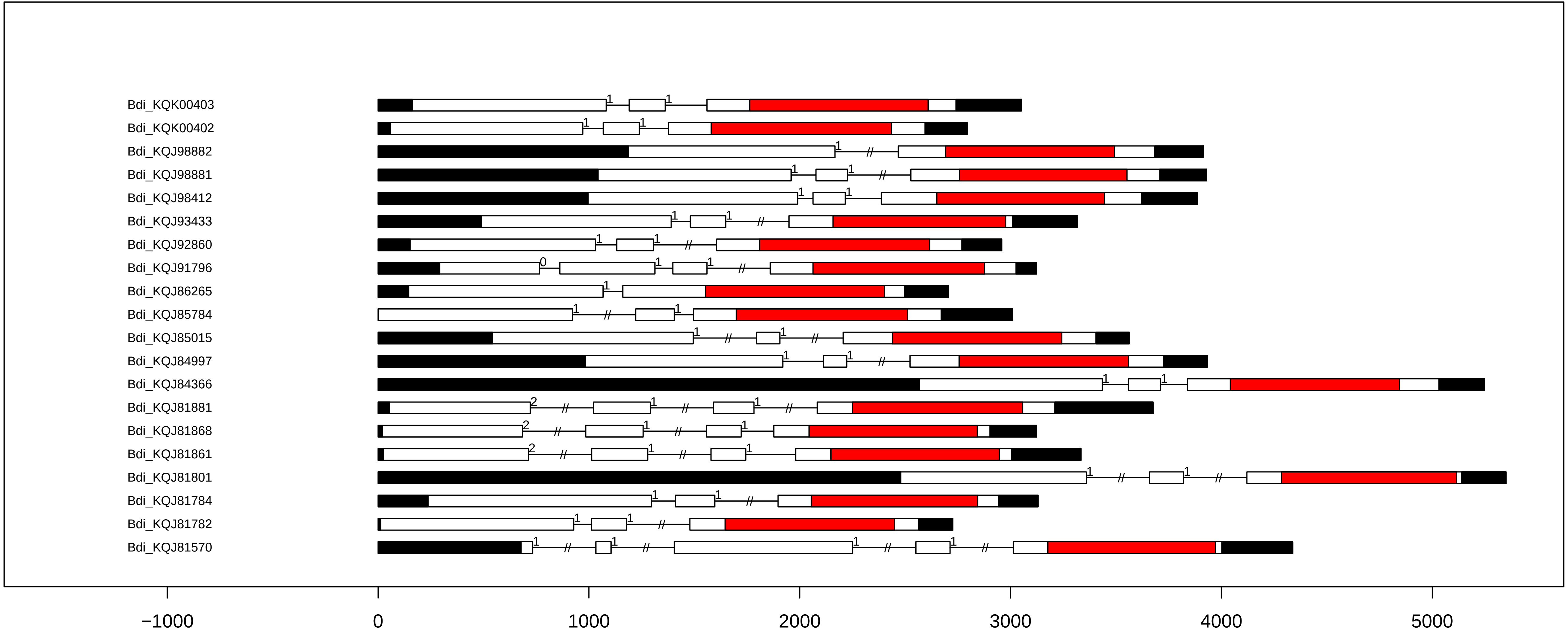

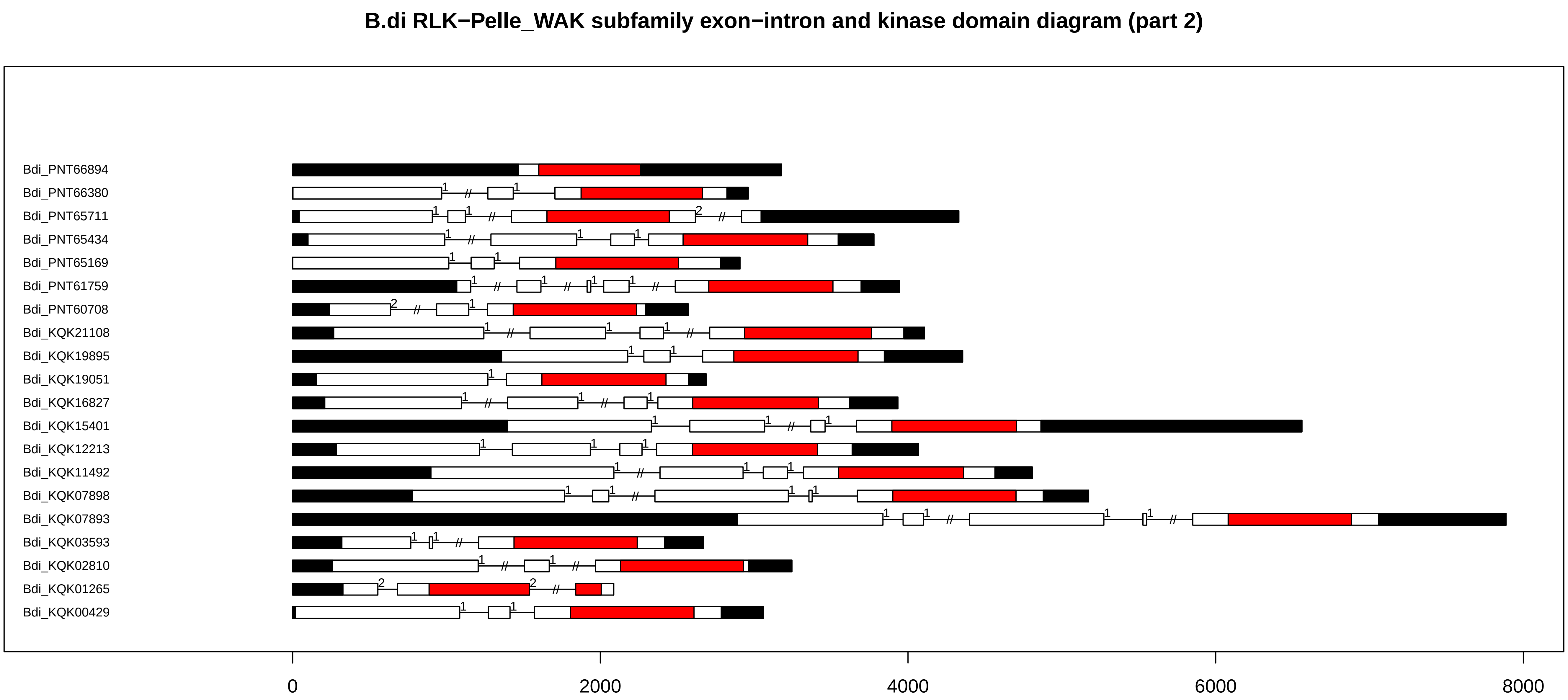
B.di RLK−Pelle_WAK subfamily exon−intron and kinase domain diagram (part 2)
Bdi_PNT66894
Bdi_PNT66380
Bdi_PNT65711
Bdi_PNT65434
Bdi_PNT65169
Bdi_PNT61759
Bdi_PNT60708
Bdi_KQK21108
Bdi_KQK19895
Bdi_KQK19051
Bdi_KQK16827
Bdi_KQK15401
Bdi_KQK12213
Bdi_KQK11492
Bdi_KQK07898
Bdi_KQK07893
Bdi_KQK03593
Bdi_KQK02810
Bdi_KQK01265
Bdi_KQK00429
0
2000
4000
6000
8000

B.di RLK−Pelle_WAK subfamily exon−intron and kinase domain diagram (part 3)

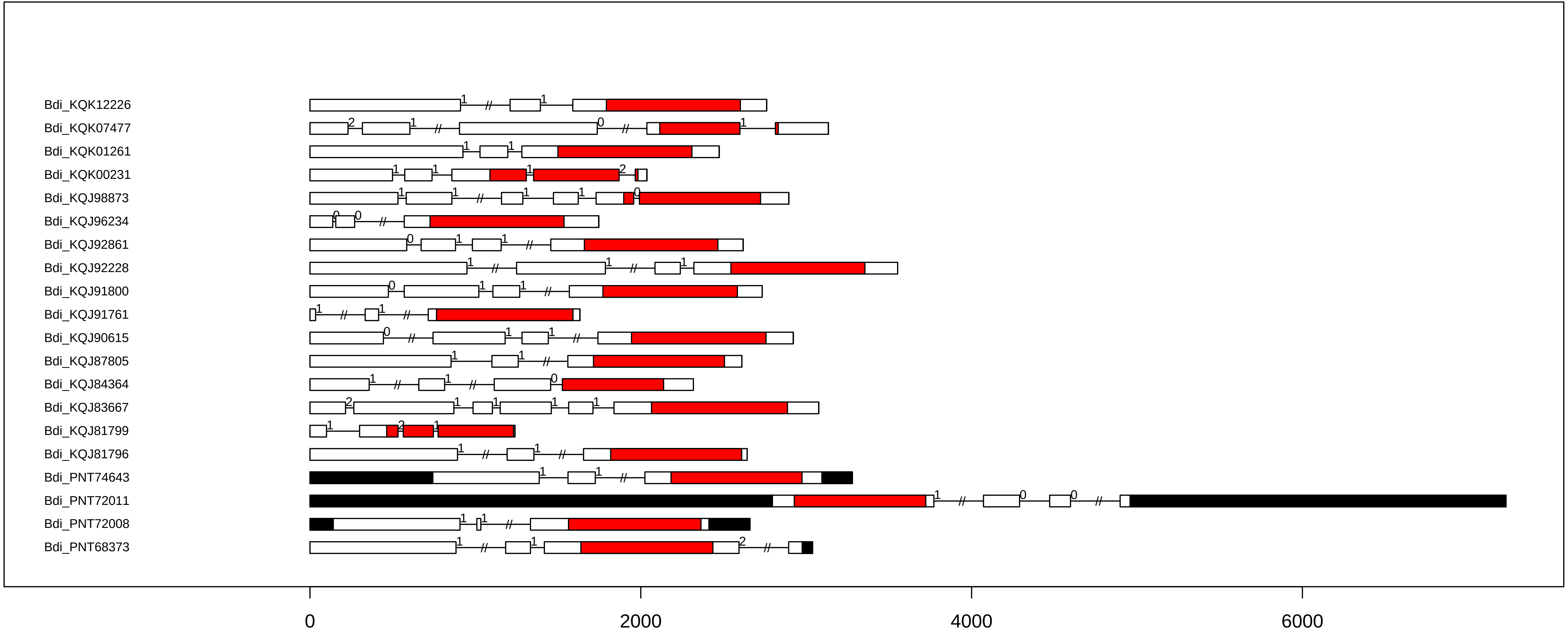

B.di RLK−Pelle_WAK subfamily exon−intron and kinase domain diagram (part 4)

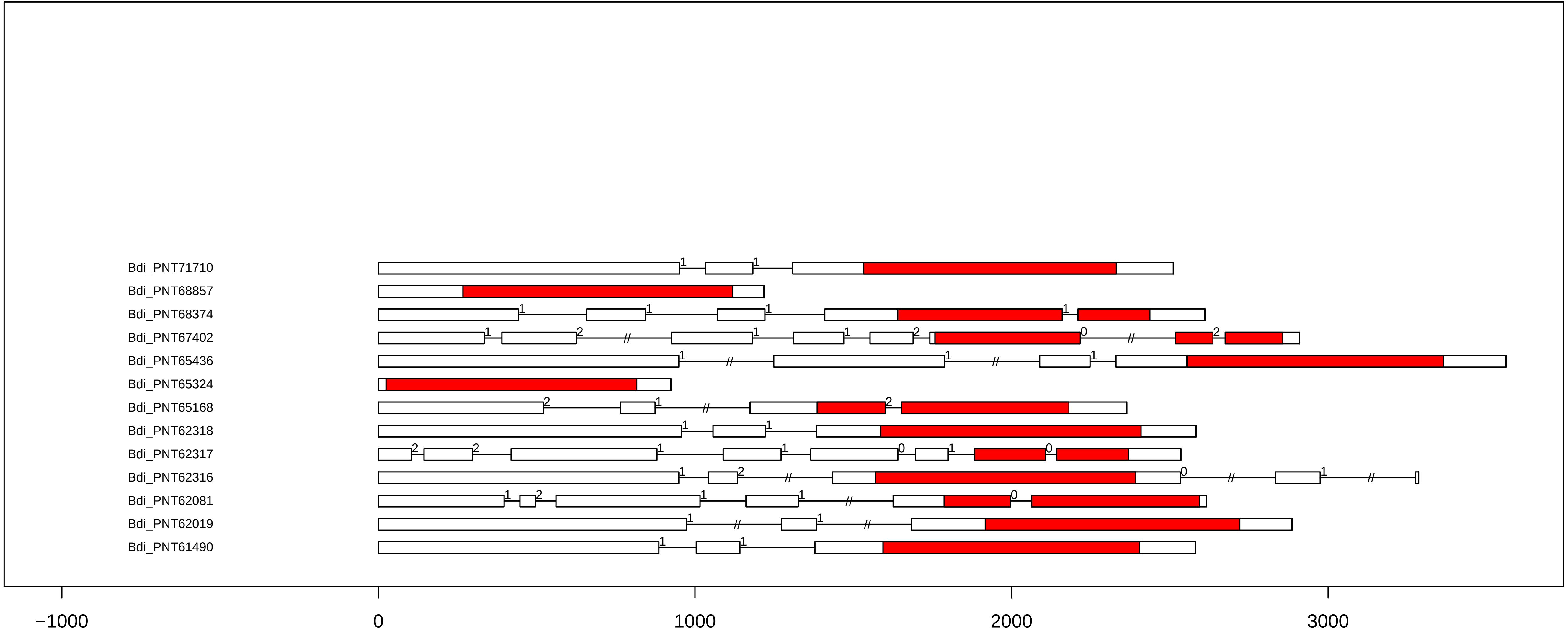

O.sa RLK−Pelle_C−LEC subfamily exon−intron and kinase domain diagram (all)

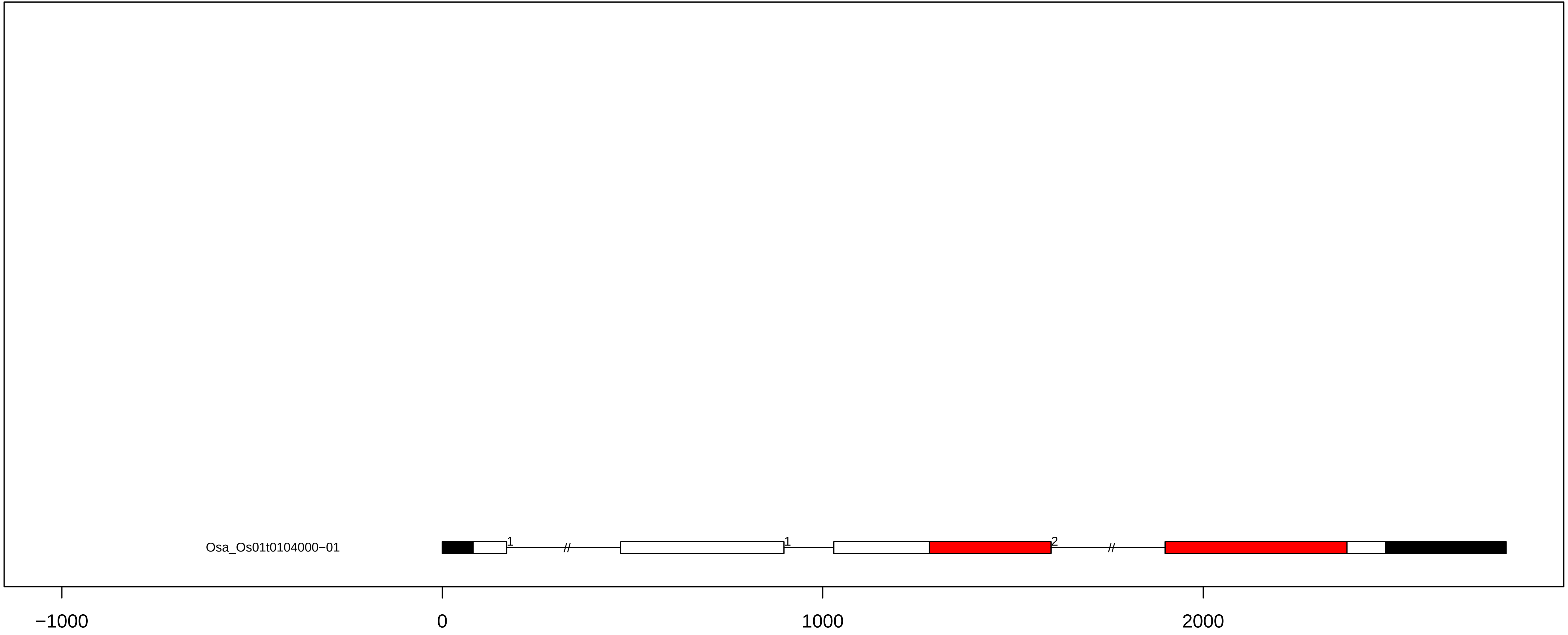

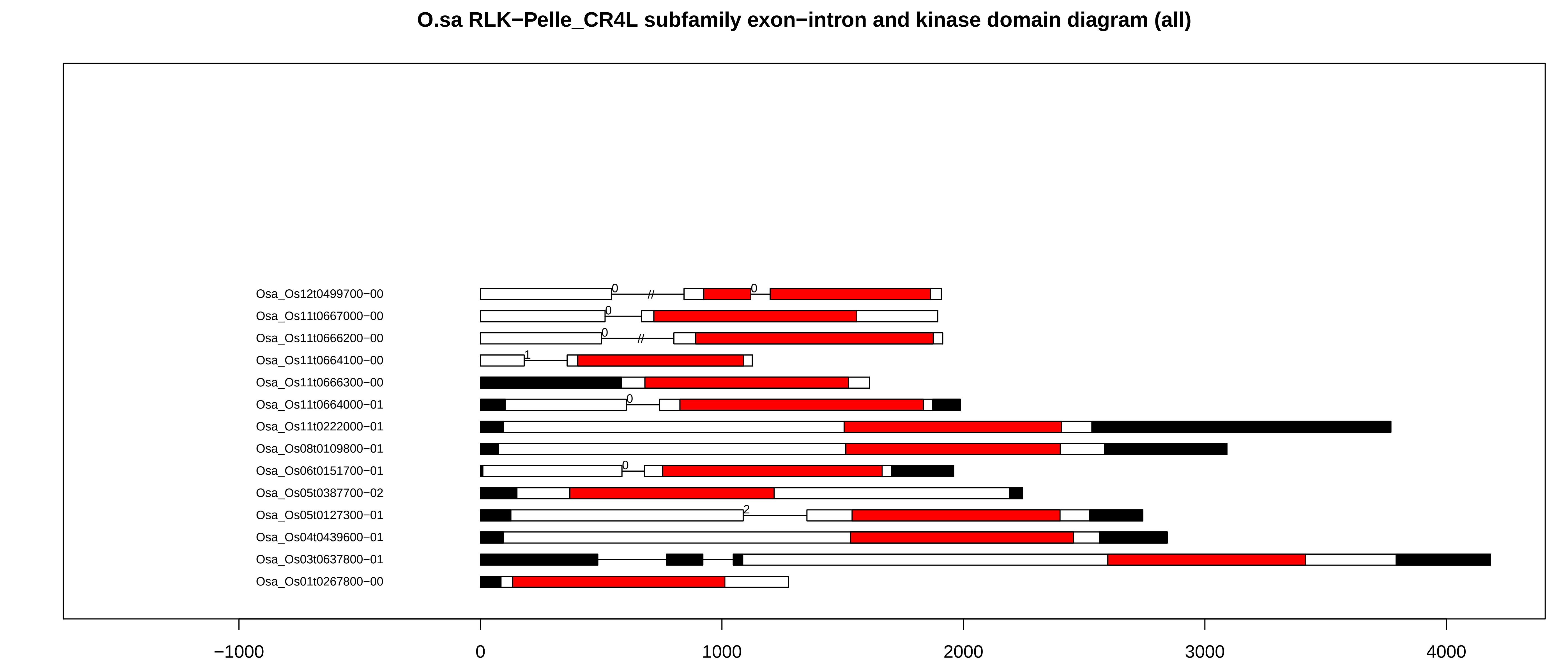

O.sa RLK−Pelle_CR4L subfamily exon−intron and kinase domain diagram (all)
Osa_Os12t0499700−00
Osa_Os11t0667000−00
Osa_Os11t0666200−00
Osa_Os11t0664100−00
Osa_Os11t0666300−00
Osa_Os11t0664000−01
Osa_Os11t0222000−01
Osa_Os08t0109800−01
Osa_Os06t0151700−01
Osa_Os05t0387700−02
Osa_Os05t0127300−01
Osa_Os04t0439600−01
Osa_Os03t0637800−01
Osa_Os01t0267800−00
−1000
0
1000
2000
3000
4000

O.sa RLK−Pelle_CrRLK1L−1 subfamily exon−intron and kinase domain diagram (all)

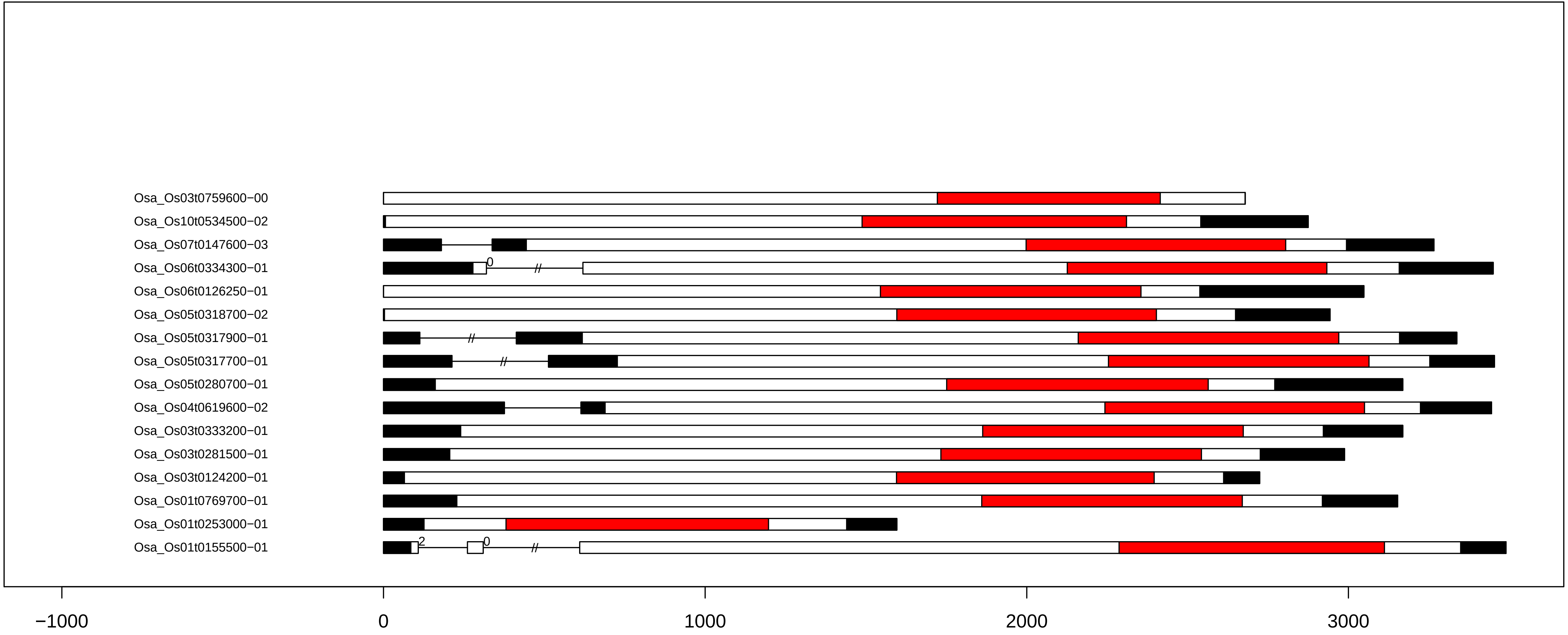

O.sa RLK−Pelle_DLSV subfamily exon−intron and kinase domain diagram (part 1)

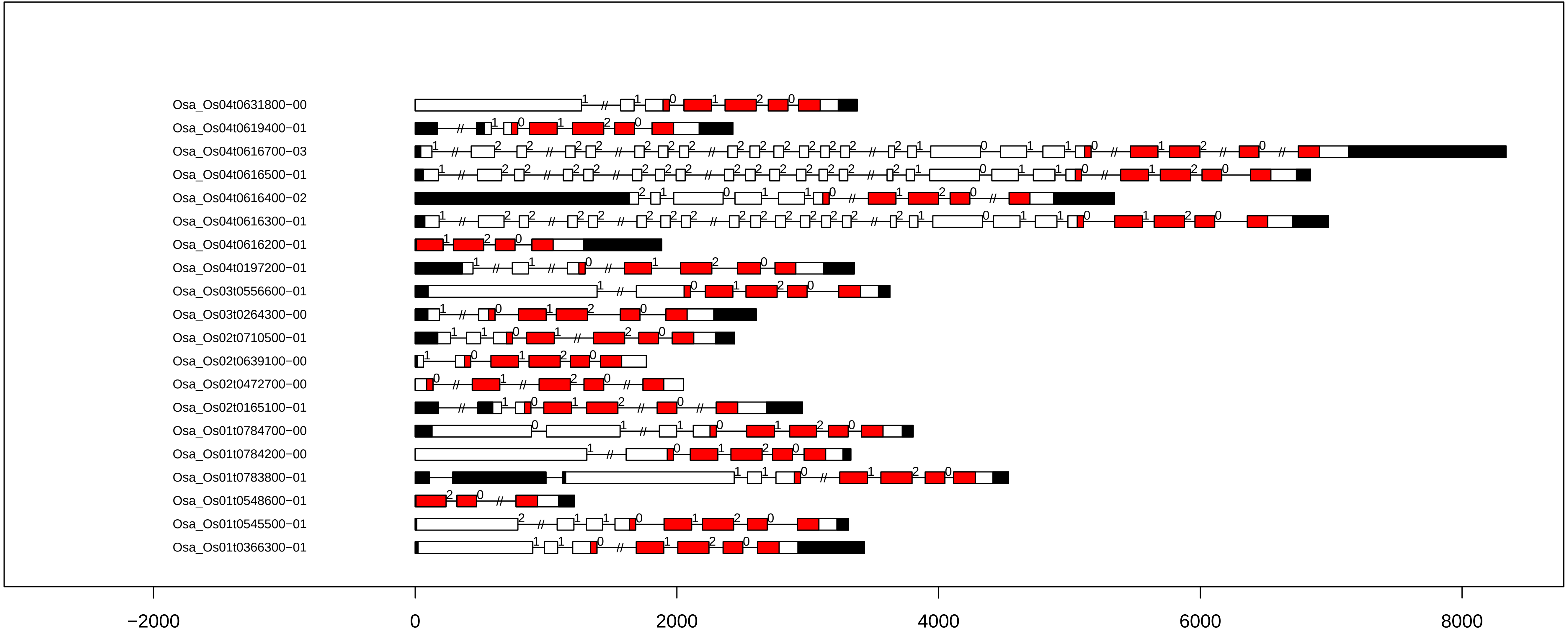

O.sa RLK−Pelle_DLSV subfamily exon−intron and kinase domain diagram (part 2)

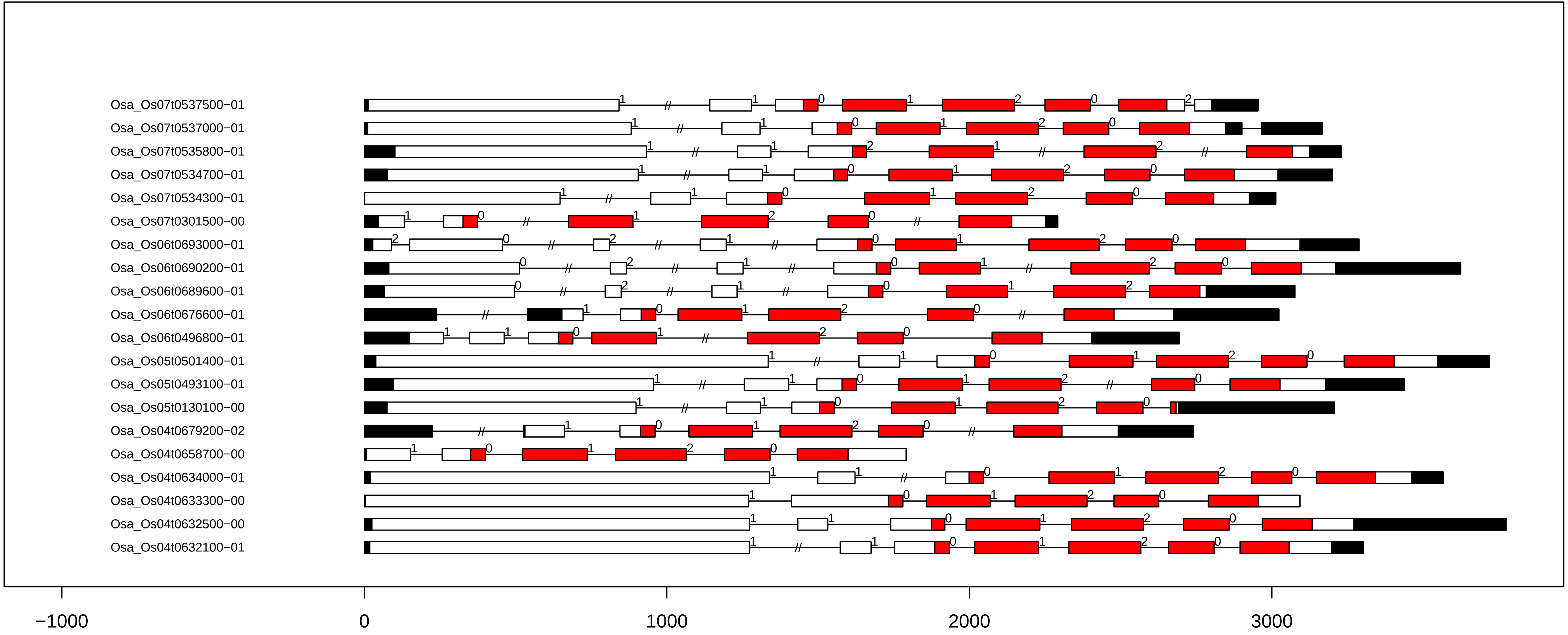

O.sa RLK−Pelle_DLSV subfamily exon−intron and kinase domain diagram (part 3)

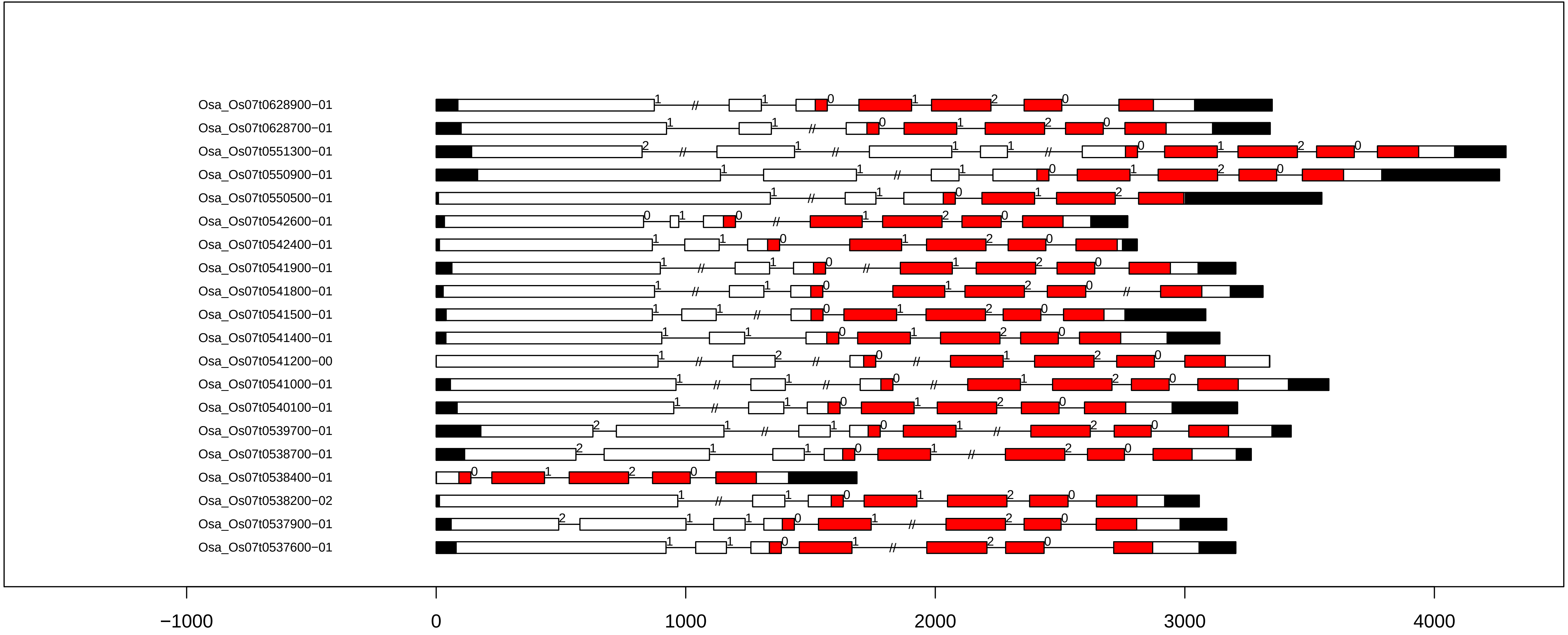

O.sa RLK−Pelle_DLSV subfamily exon−intron and kinase domain diagram (part 4)

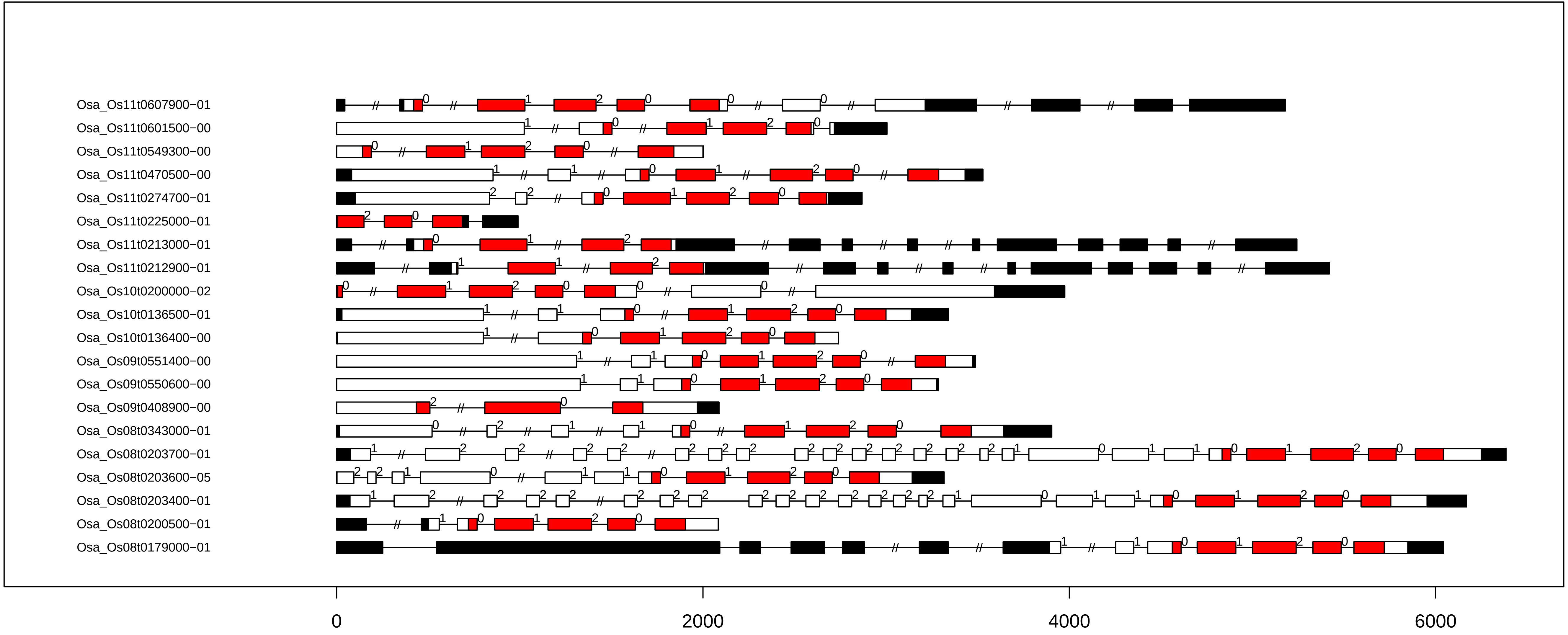

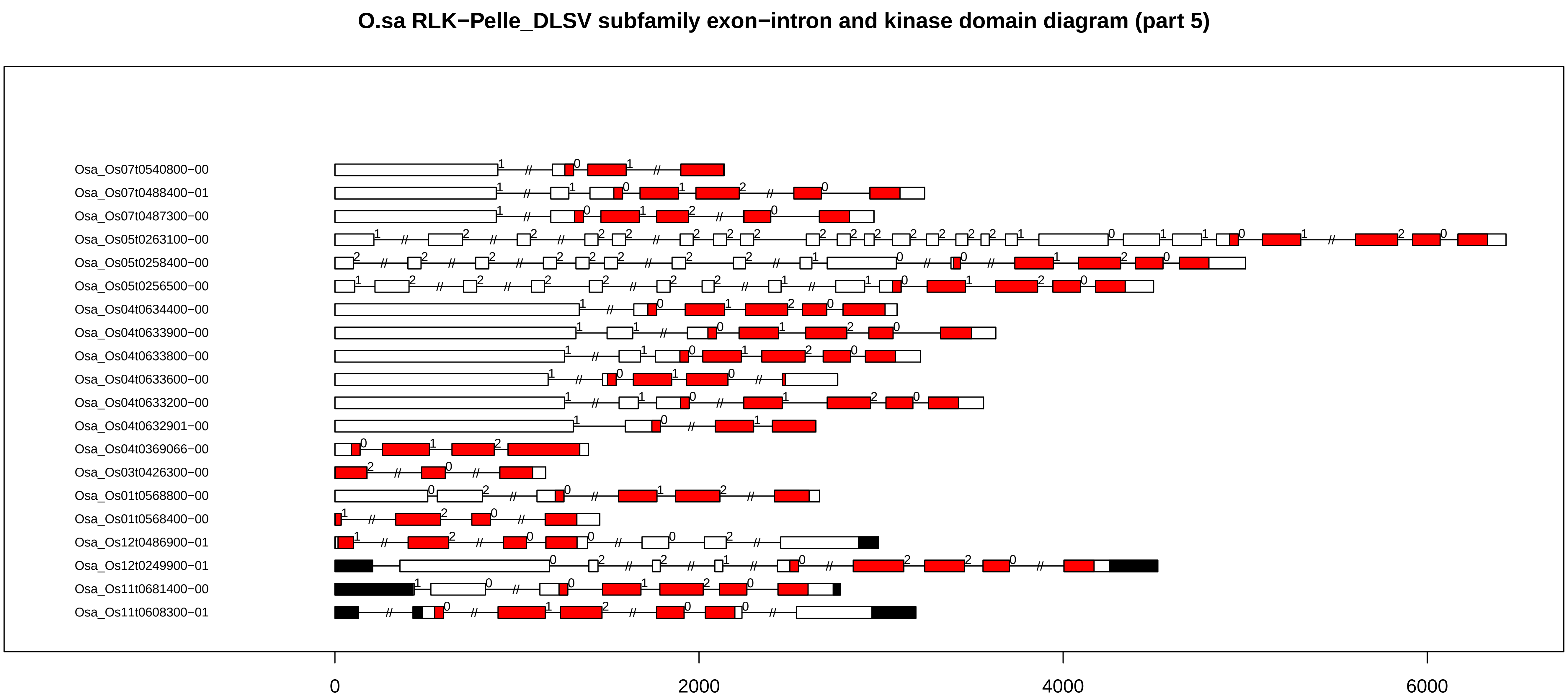

O.sa RLK−Pelle_DLSV subfamily exon−intron and kinase domain diagram (part 5)
Osa_Os07t0540800−00
Osa_Os07t0488400−01
Osa_Os07t0487300−00
Osa_Os05t0263100−00
Osa_Os05t0258400−00
Osa_Os05t0256500−00
Osa_Os04t0634400−00
Osa_Os04t0633900−00
Osa_Os04t0633800−00
Osa_Os04t0633600−00
Osa_Os04t0633200−00
Osa_Os04t0632901−00
Osa_Os04t0369066−00
Osa_Os03t0426300−00
Osa_Os01t0568800−00
Osa_Os01t0568400−00
Osa_Os12t0486900−01
Osa_Os12t0249900−01
Osa_Os11t0681400−00
Osa_Os11t0608300−01
0
2000
4000
6000

O.sa RLK−Pelle_DLSV subfamily exon−intron and kinase domain diagram (part 6)

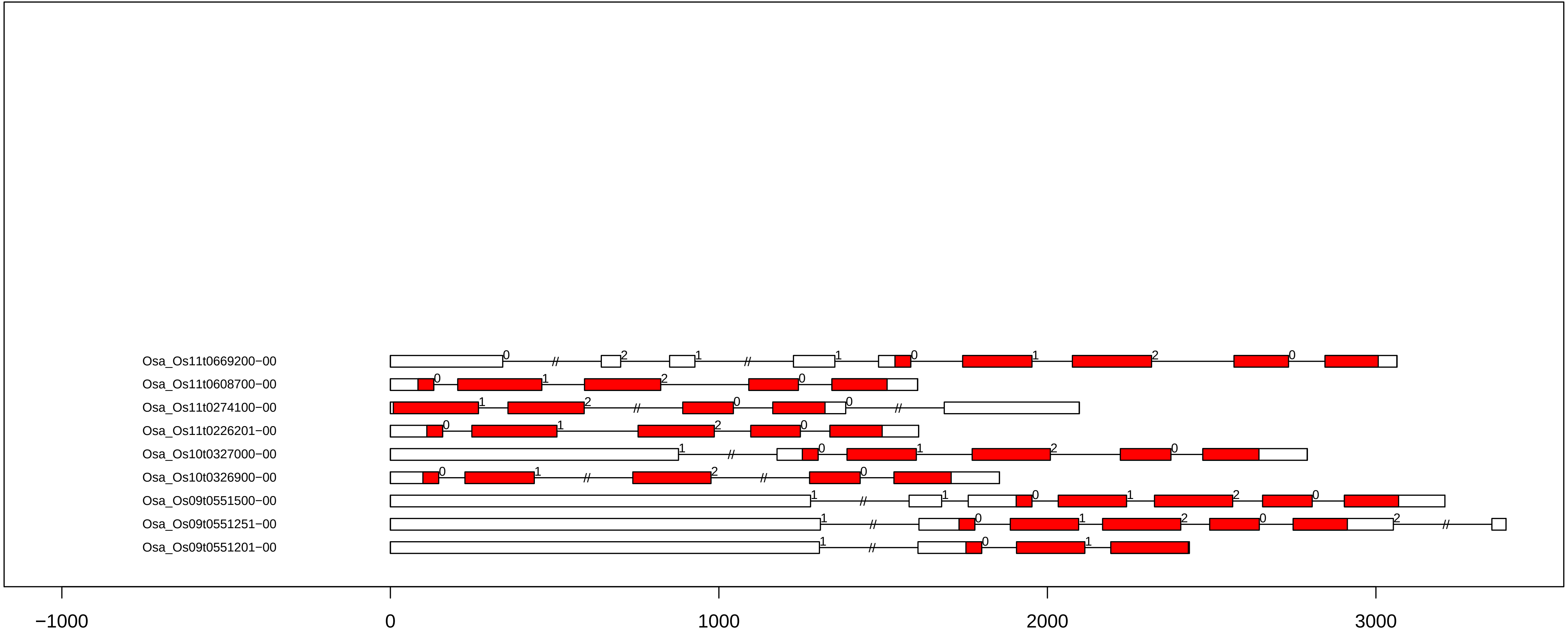

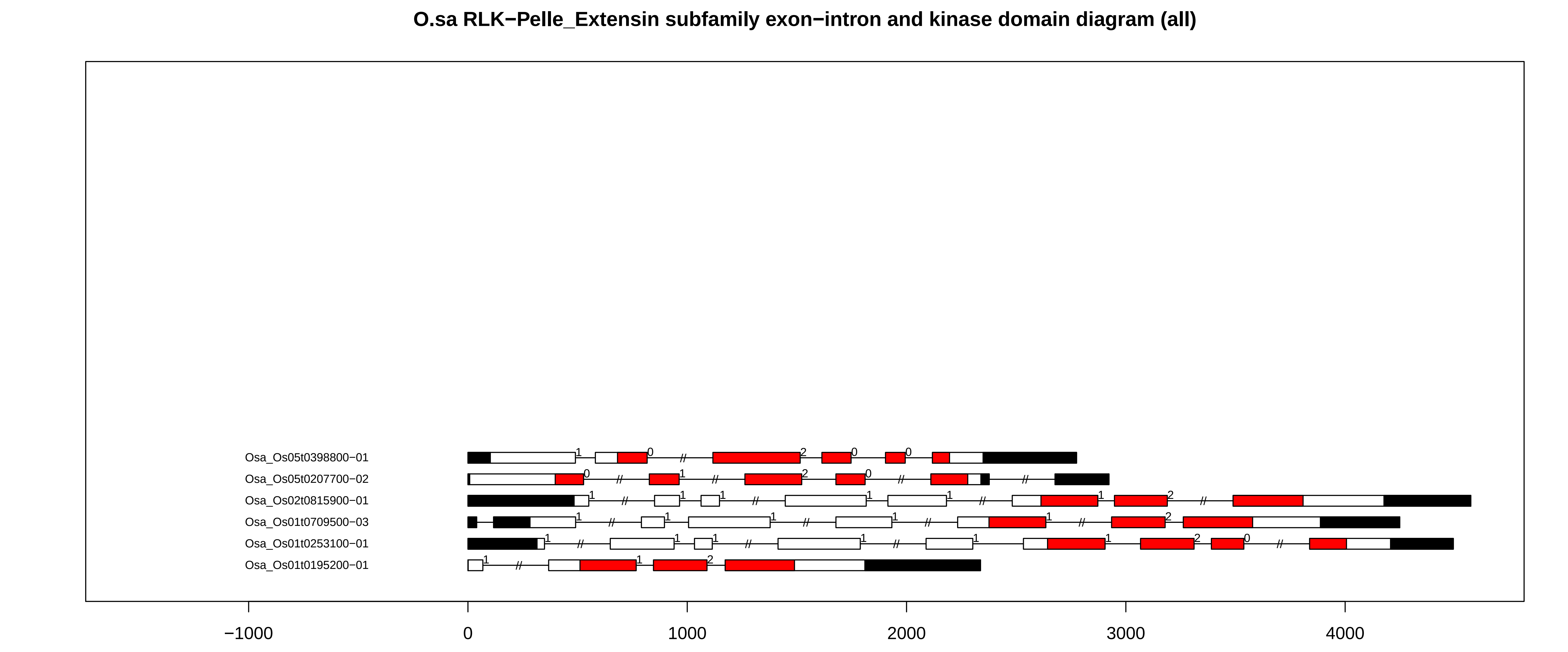
O.sa RLK−Pelle_Extensin subfamily exon−intron and kinase domain diagram (all)
Osa_Os05t0398800−01
Osa_Os05t0207700−02
Osa_Os02t0815900−01
Osa_Os01t0709500−03
Osa_Os01t0253100−01
Osa_Os01t0195200−01
−1000
0
1000
2000
3000
4000

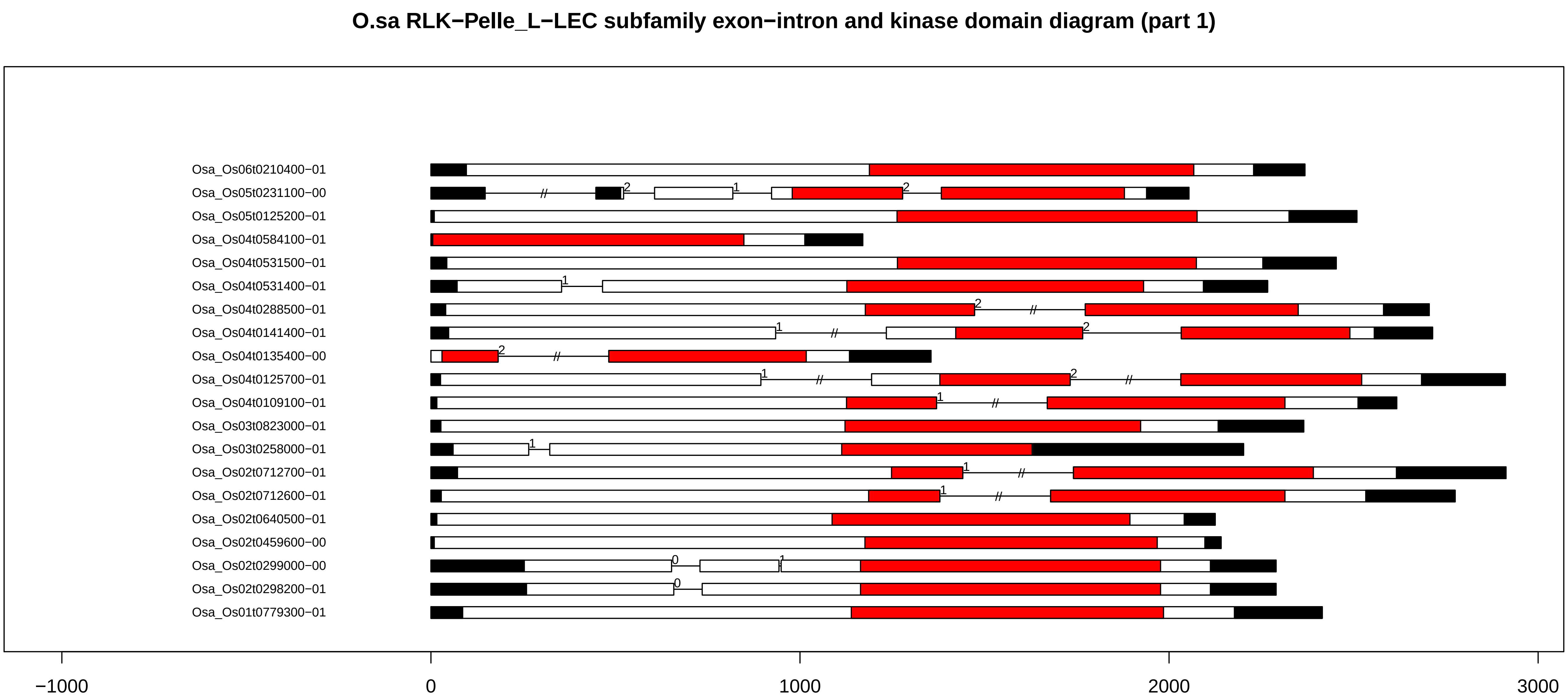
O.sa RLK−Pelle_L−LEC subfamily exon−intron and kinase domain diagram (part 1)
Osa_Os06t0210400−01
Osa_Os05t0231100−00
Osa_Os05t0125200−01
Osa_Os04t0584100−01
Osa_Os04t0531500−01
Osa_Os04t0531400−01
Osa_Os04t0288500−01
Osa_Os04t0141400−01
Osa_Os04t0135400−00
Osa_Os04t0125700−01
Osa_Os04t0109100−01
Osa_Os03t0823000−01
Osa_Os03t0258000−01
Osa_Os02t0712700−01
Osa_Os02t0712600−01
Osa_Os02t0640500−01
Osa_Os02t0459600−00
Osa_Os02t0299000−00
Osa_Os02t0298200−01
Osa_Os01t0779300−01
−1000
0
1000
2000
3000

O.sa RLK−Pelle_L−LEC subfamily exon−intron and kinase domain diagram (part 2)

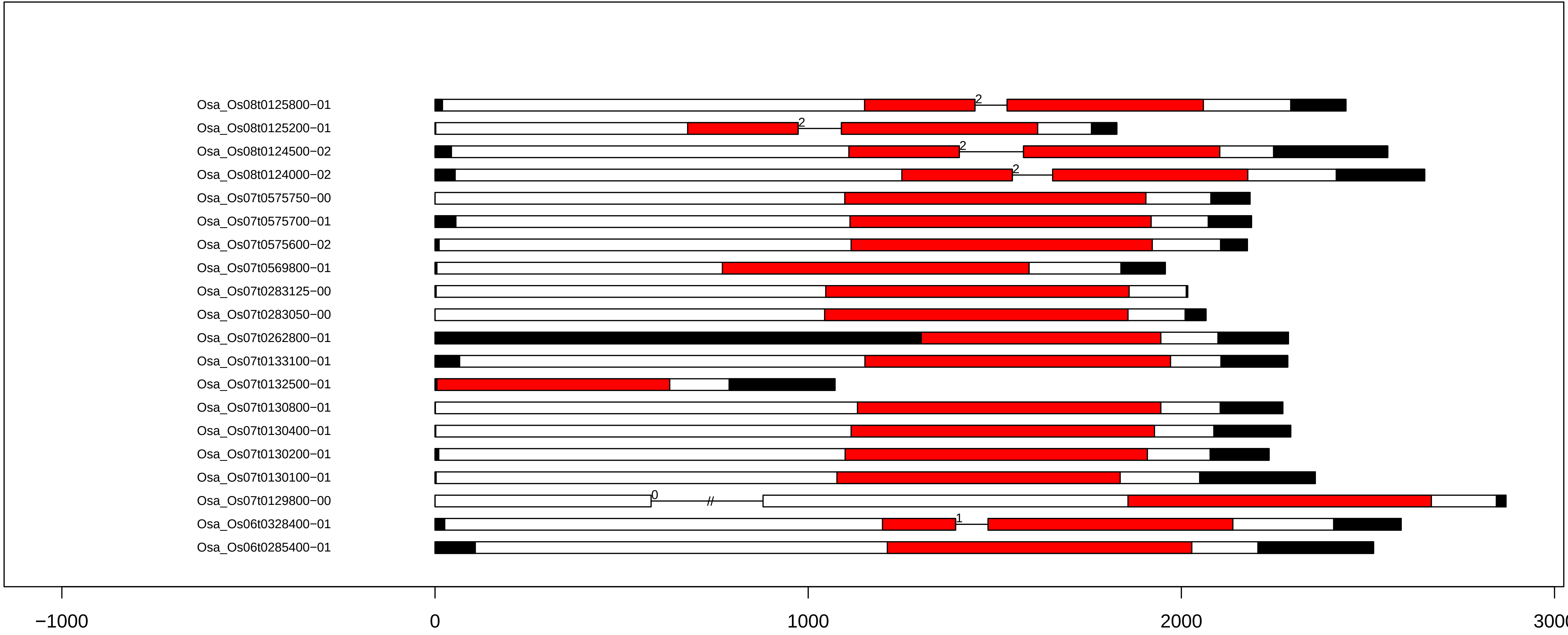

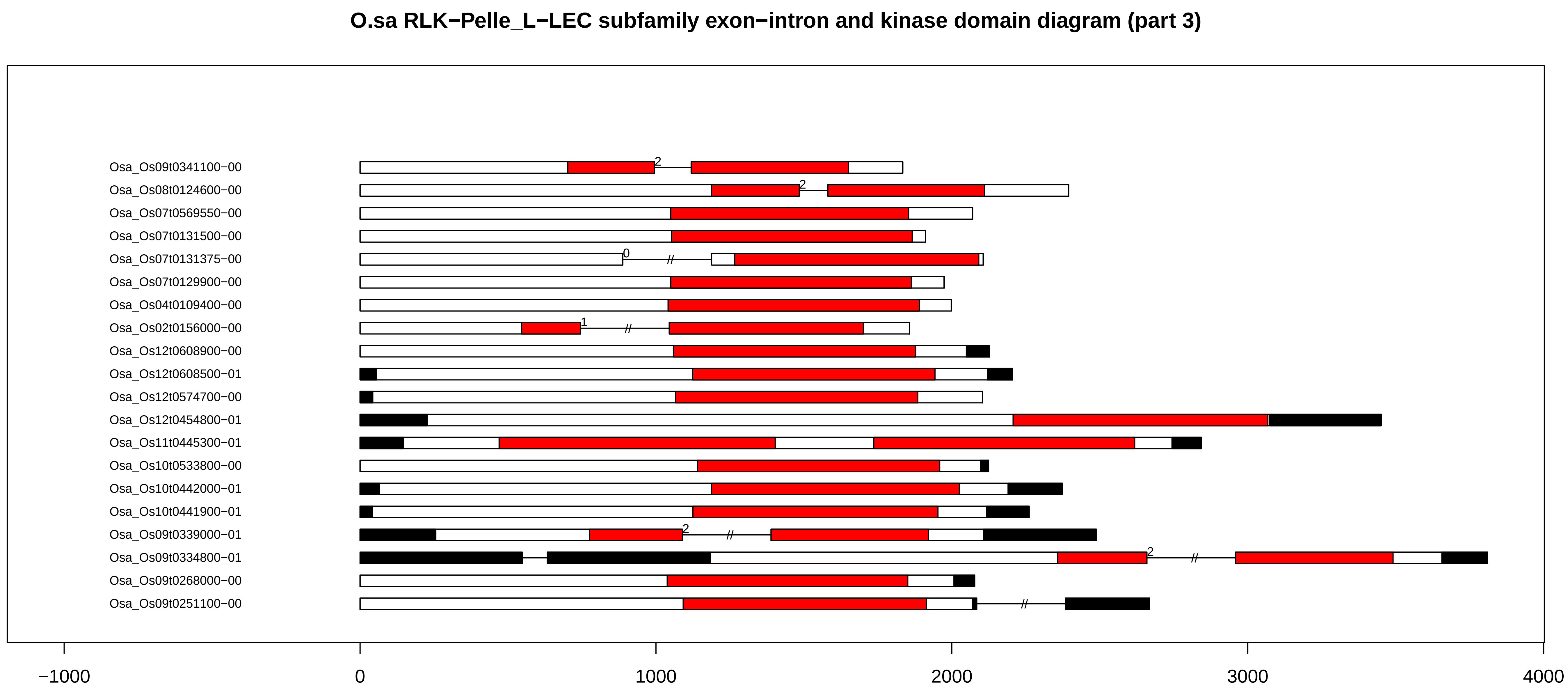
O.sa RLK−Pelle_L−LEC subfamily exon−intron and kinase domain diagram (part 3)
Osa_Os09t0341100−00
Osa_Os08t0124600−00
Osa_Os07t0569550−00
Osa_Os07t0131500−00
Osa_Os07t0131375−00
Osa_Os07t0129900−00
Osa_Os04t0109400−00
Osa_Os02t0156000−00
Osa_Os12t0608900−00
Osa_Os12t0608500−01
Osa_Os12t0574700−00
Osa_Os12t0454800−01
Osa_Os11t0445300−01
Osa_Os10t0533800−00
Osa_Os10t0442000−01
Osa_Os10t0441900−01
Osa_Os09t0339000−01
Osa_Os09t0334800−01
Osa_Os09t0268000−00
Osa_Os09t0251100−00
−1000
0
1000
2000
3000
4000

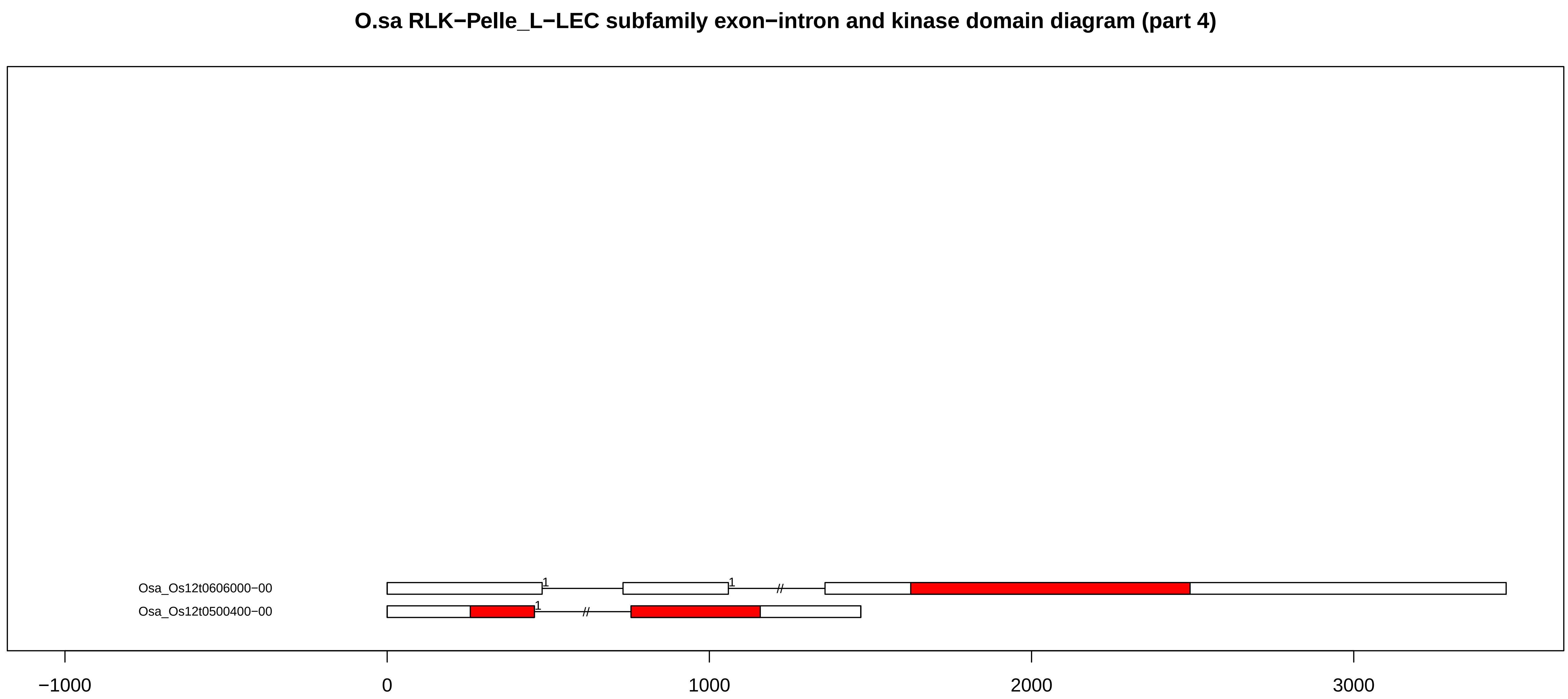

O.sa RLK−Pelle_L−LEC subfamily exon−intron and kinase domain diagram (part 4)
Osa_Os12t0606000−00
Osa_Os12t0500400−00
1
1
1
−1000
0
1000
2000
3000

O.sa RLK−Pelle_LRK10L−2 subfamily exon−intron and kinase domain diagram (part 1)

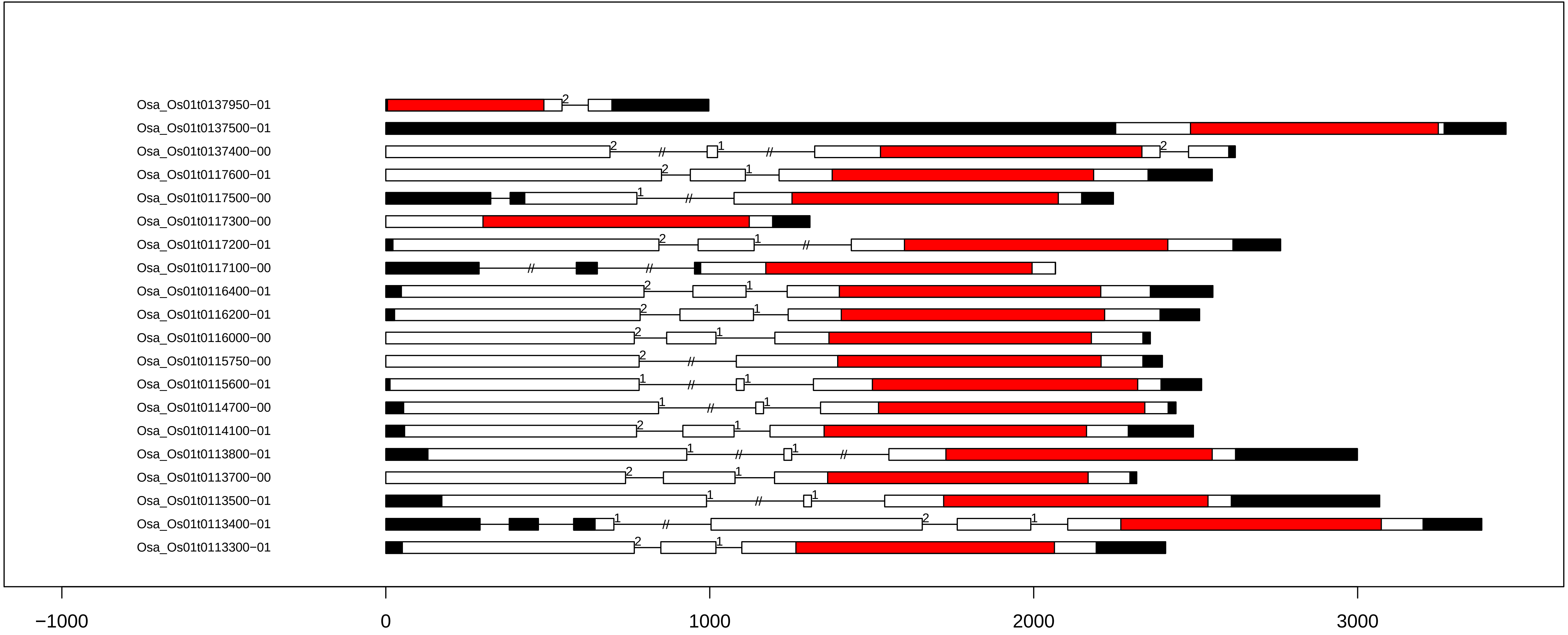

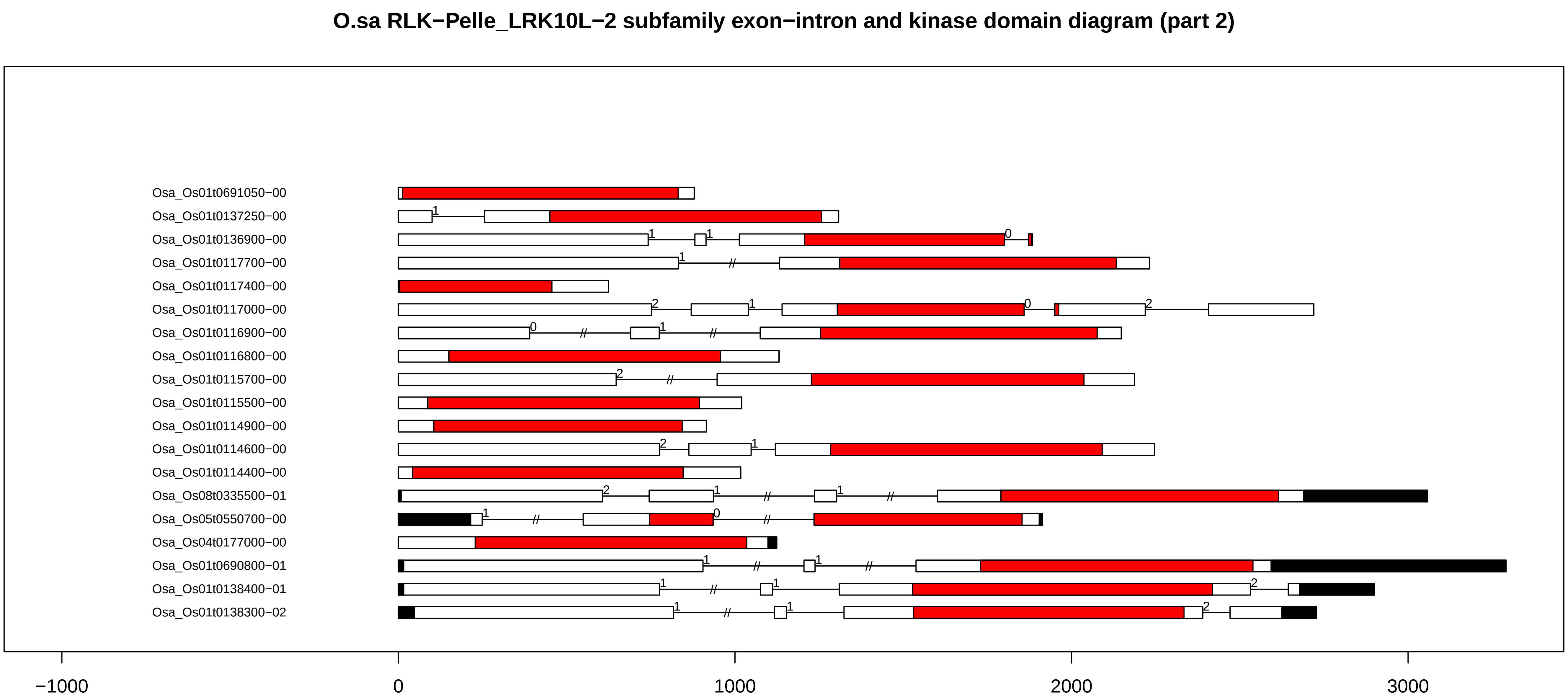

O.sa RLK−Pelle_LRK10L−2 subfamily exon−intron and kinase domain diagram (part 2)
Osa_Os01t0691050−00
Osa_Os01t0137250−00
Osa_Os01t0136900−00
Osa_Os01t0117700−00
Osa_Os01t0117400−00
Osa_Os01t0117000−00
Osa_Os01t0116900−00
Osa_Os01t0116800−00
Osa_Os01t0115700−00
Osa_Os01t0115500−00
Osa_Os01t0114900−00
Osa_Os01t0114600−00
Osa_Os01t0114400−00
Osa_Os08t0335500−01
Osa_Os05t0550700−00
Osa_Os04t0177000−00
Osa_Os01t0690800−01
Osa_Os01t0138400−01
Osa_Os01t0138300−02
−1000
0
1000
2000
3000

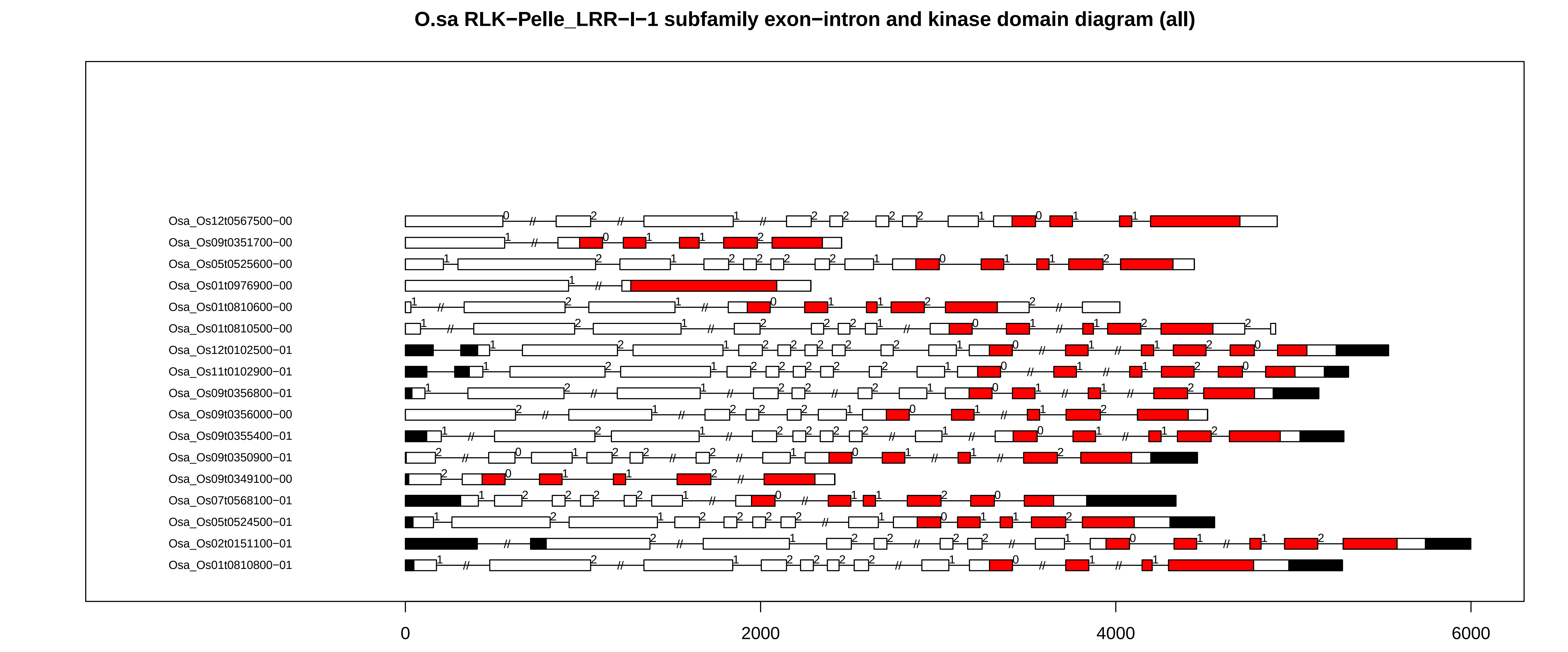
O.sa RLK−Pelle_LRR−I−1 subfamily exon−intron and kinase domain diagram (all)
Osa_Os12t0567500−00
Osa_Os09t0351700−00
Osa_Os05t0525600−00
Osa_Os01t0976900−00
Osa_Os01t0810600−00
Osa_Os01t0810500−00
Osa_Os12t0102500−01
Osa_Os11t0102900−01
Osa_Os09t0356800−01
Osa_Os09t0356000−00
Osa_Os09t0355400−01
Osa_Os09t0350900−01
Osa_Os09t0349100−00
Osa_Os07t0568100−01
Osa_Os05t0524500−01
Osa_Os02t0151100−01
Osa_Os01t0810800−01
0
2000
4000
6000

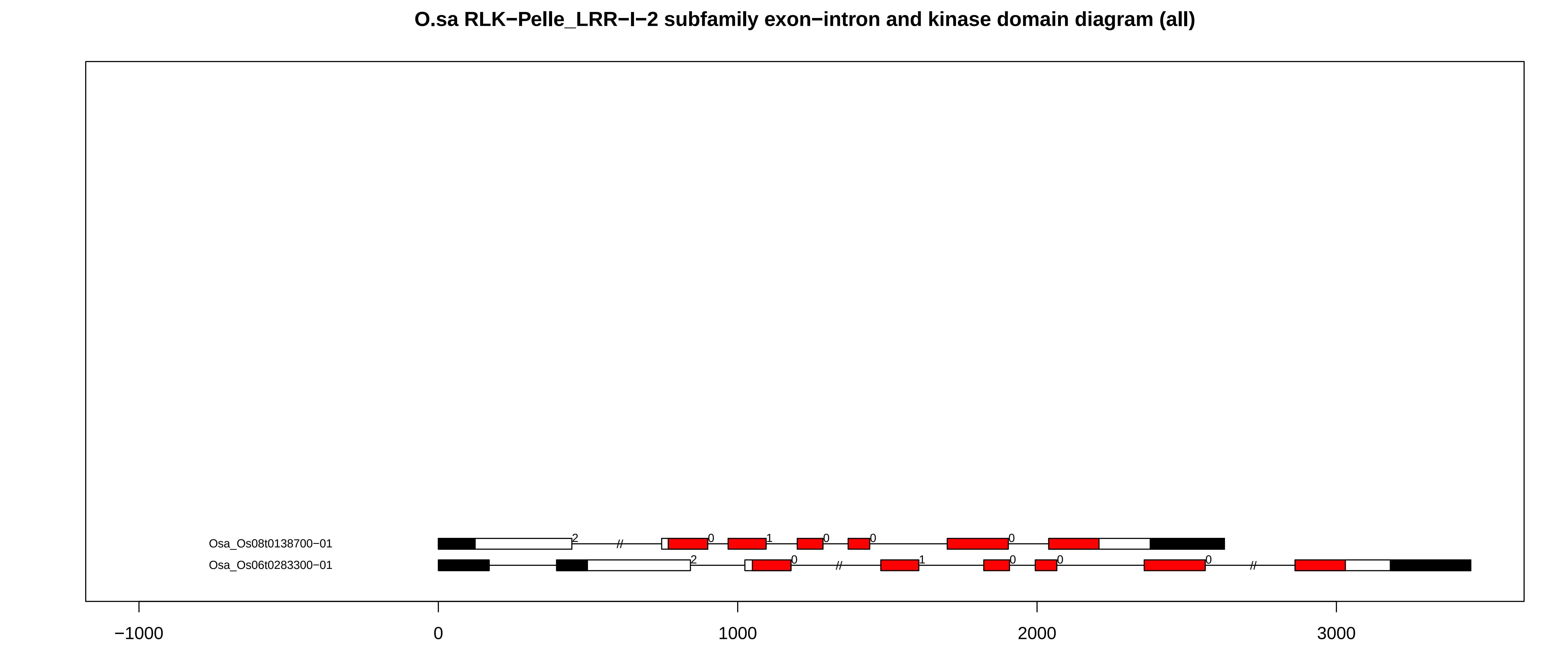
O.sa RLK−Pelle_LRR−I−2 subfamily exon−intron and kinase domain diagram (all)
Osa_Os08t0138700−01
Osa_Os06t0283300−01
2
0
1
0
0
0
2
0
1
0
0
0
−1000
0
1000
2000
3000

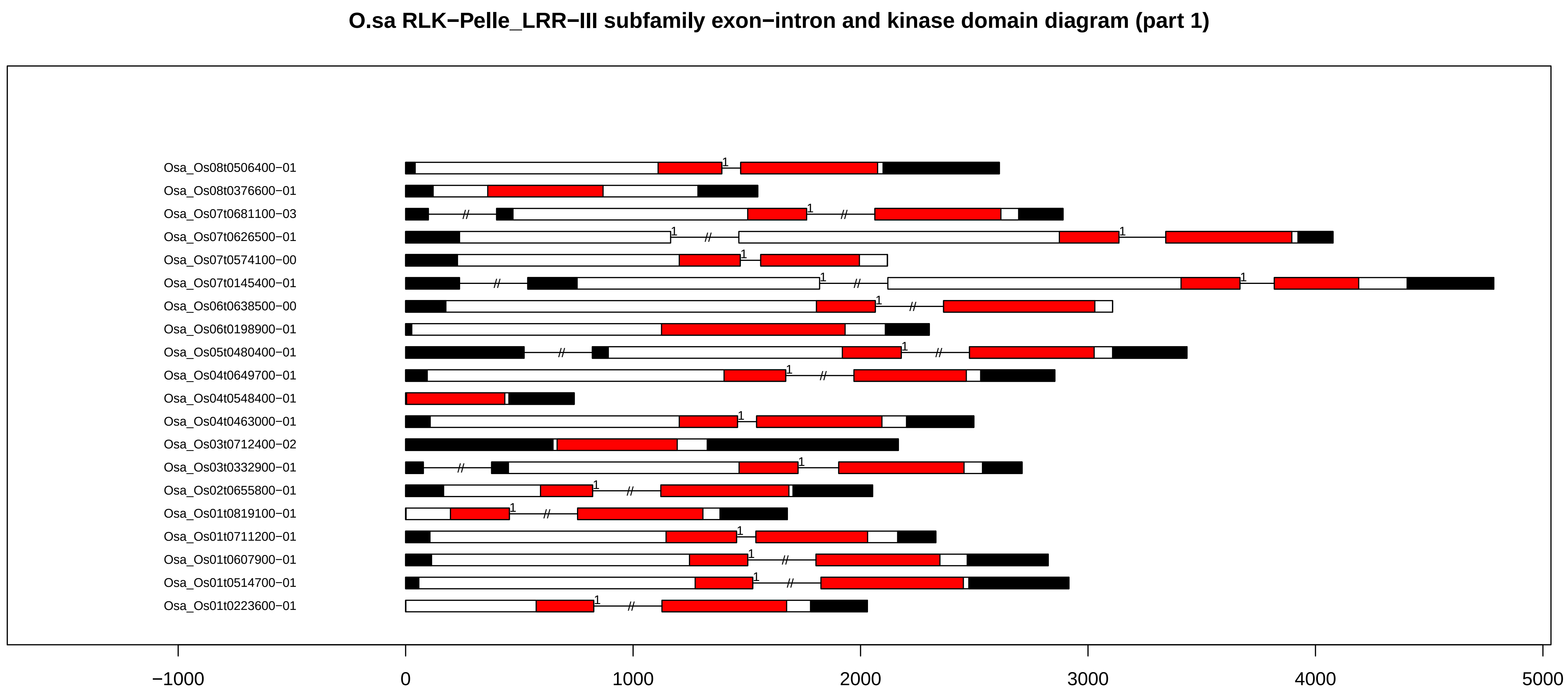
O.sa RLK−Pelle_LRR−III subfamily exon−intron and kinase domain diagram (part 1)
Osa_Os08t0506400−01
Osa_Os08t0376600−01
Osa_Os07t0681100−03
Osa_Os07t0626500−01
Osa_Os07t0574100−00
Osa_Os07t0145400−01
Osa_Os06t0638500−00
Osa_Os06t0198900−01
Osa_Os05t0480400−01
Osa_Os04t0649700−01
Osa_Os04t0548400−01
Osa_Os04t0463000−01
Osa_Os03t0712400−02
Osa_Os03t0332900−01
Osa_Os02t0655800−01
Osa_Os01t0819100−01
Osa_Os01t0711200−01
Osa_Os01t0607900−01
Osa_Os01t0514700−01
Osa_Os01t0223600−01
−1000
0
1000
2000
3000
4000
5000

O.sa RLK−Pelle_LRR−III subfamily exon−intron and kinase domain diagram (part 2)

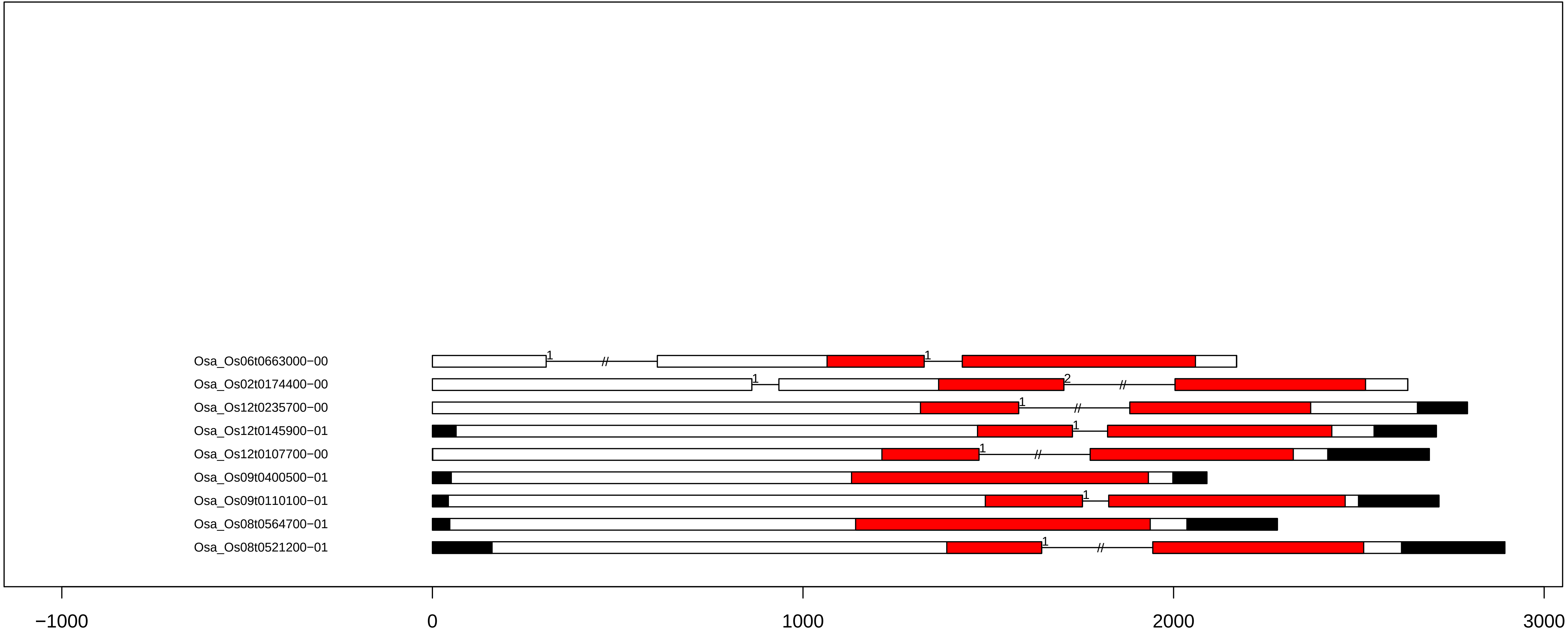

O.sa RLK−Pelle_LRR−II subfamily exon−intron and kinase domain diagram (all)

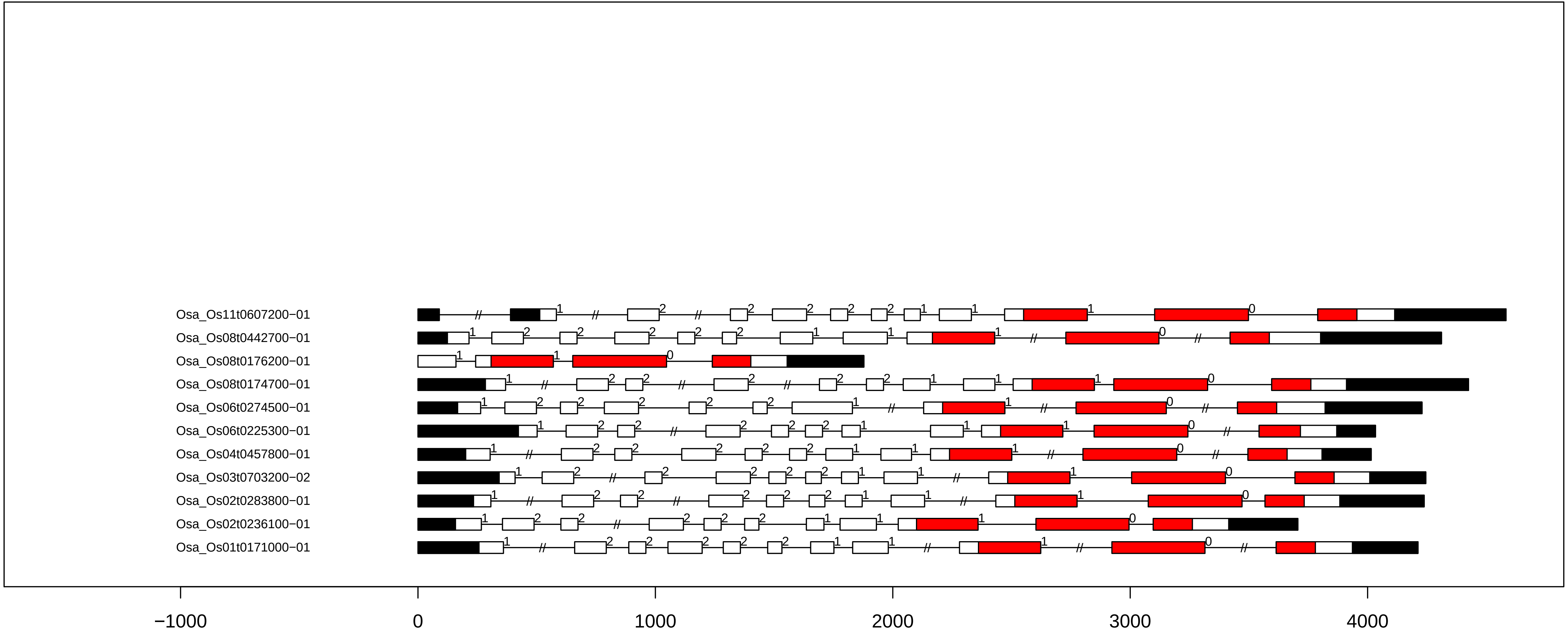

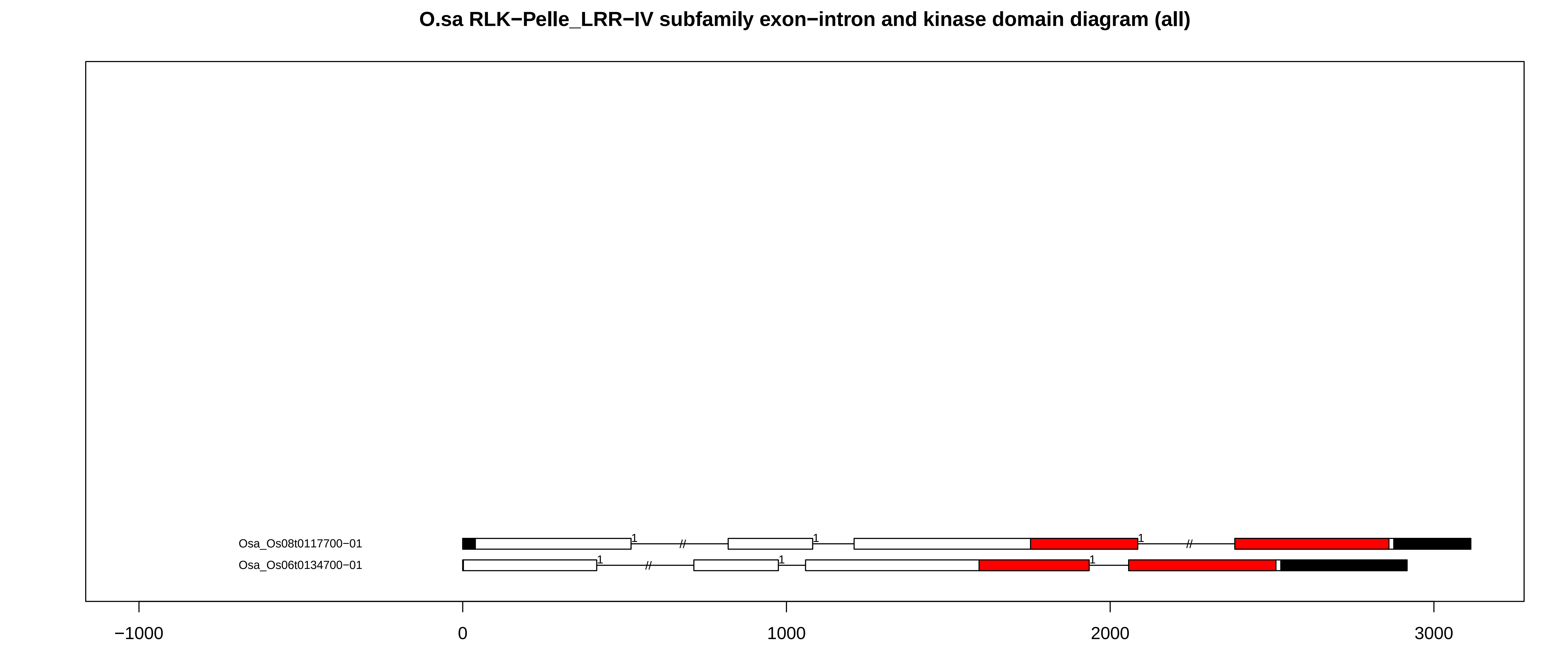
O.sa RLK−Pelle_LRR−IV subfamily exon−intron and kinase domain diagram (all)
Osa_Os08t0117700−01
Osa_Os06t0134700−01
1
1
1
1
1
1
−1000
0
1000
2000
3000

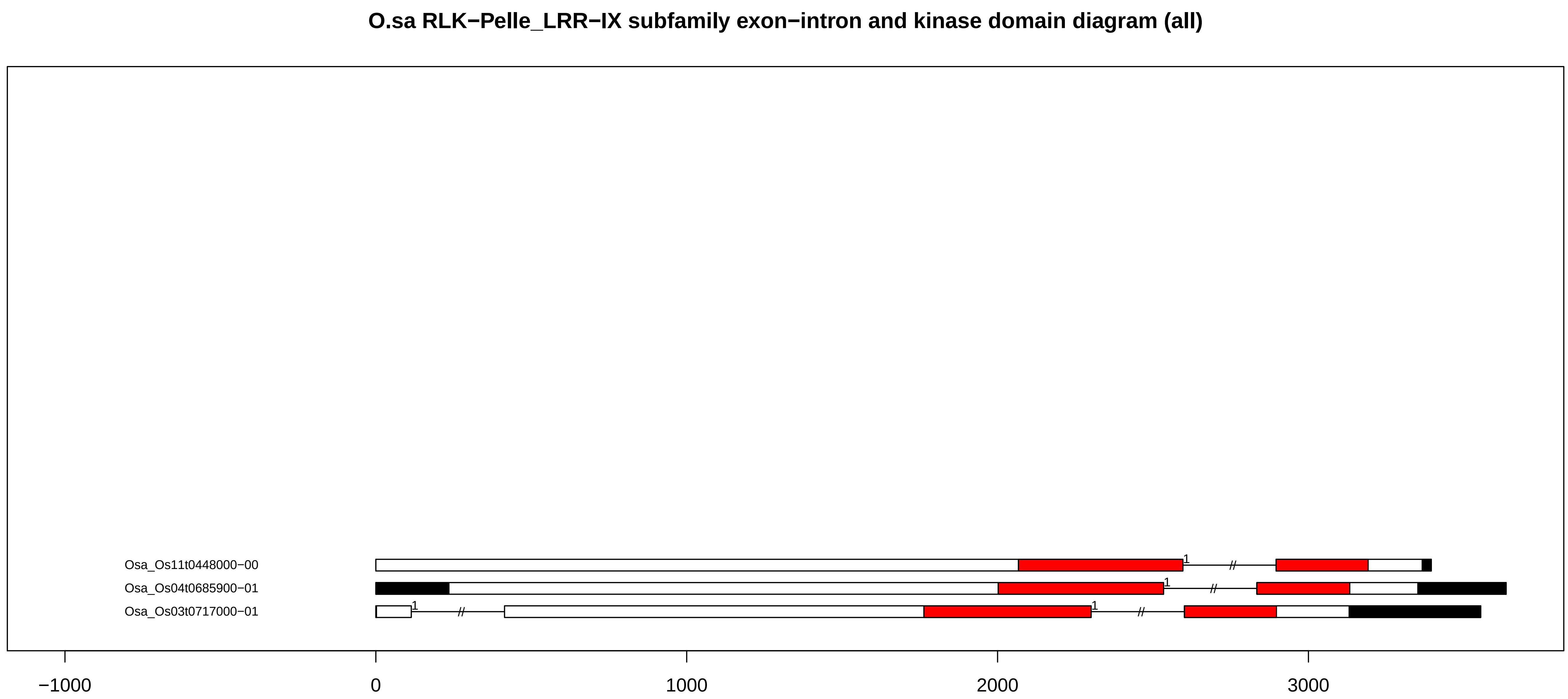
O.sa RLK−Pelle_LRR−IX subfamily exon−intron and kinase domain diagram (all)
Osa_Os11t0448000−00
Osa_Os04t0685900−01
Osa_Os03t0717000−01
1
1
1
1
−1000
0
1000
2000
3000

O.sa RLK−Pelle_LRR−VI−1 subfamily exon−intron and kinase domain diagram (all)

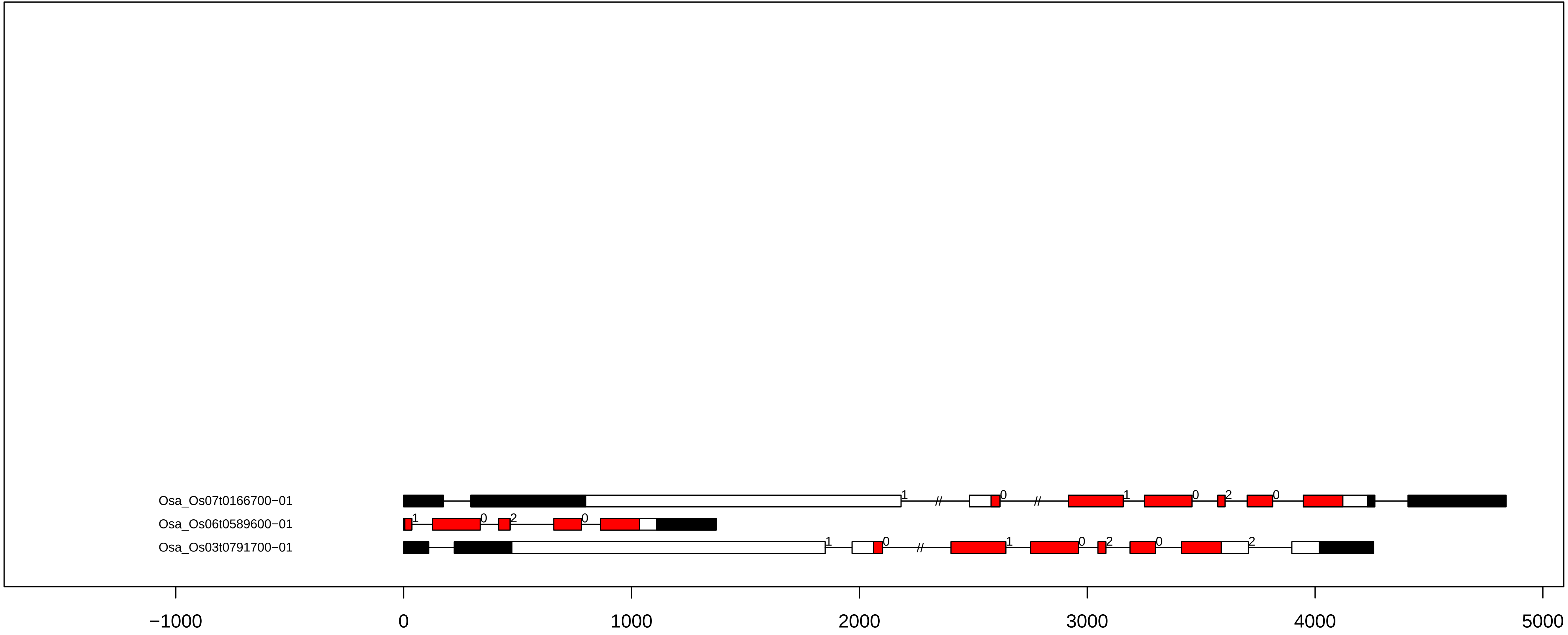

O.sa RLK−Pelle_LRR−VI−2 subfamily exon−intron and kinase domain diagram (all)

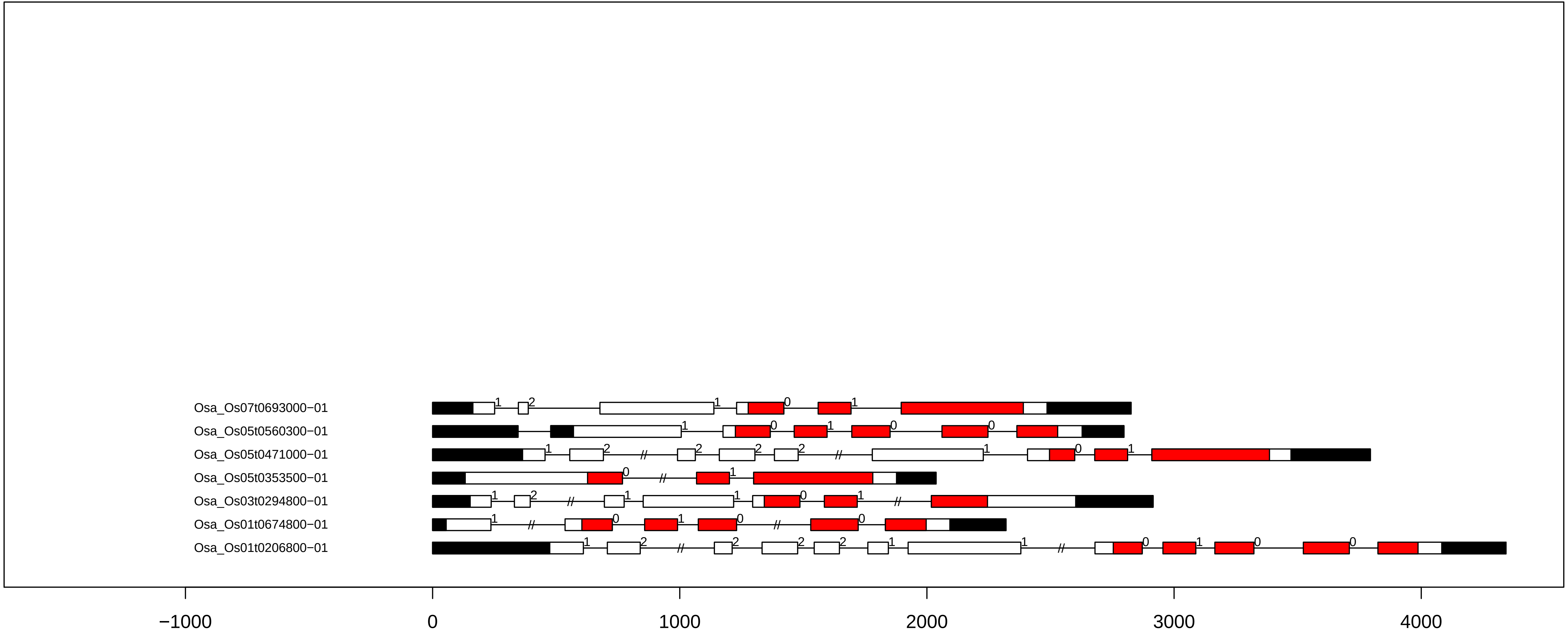

O.sa RLK−Pelle_LRR−VII−1 subfamily exon−intron and kinase domain diagram (all)

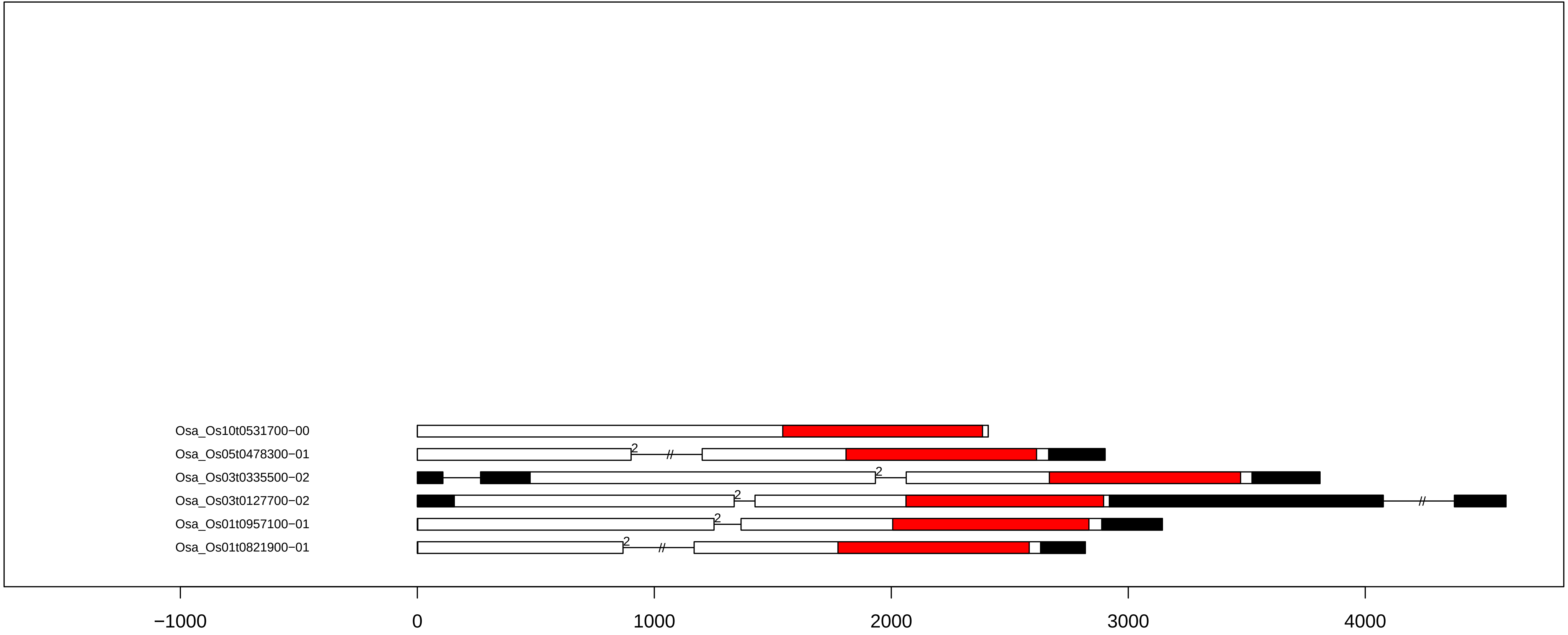

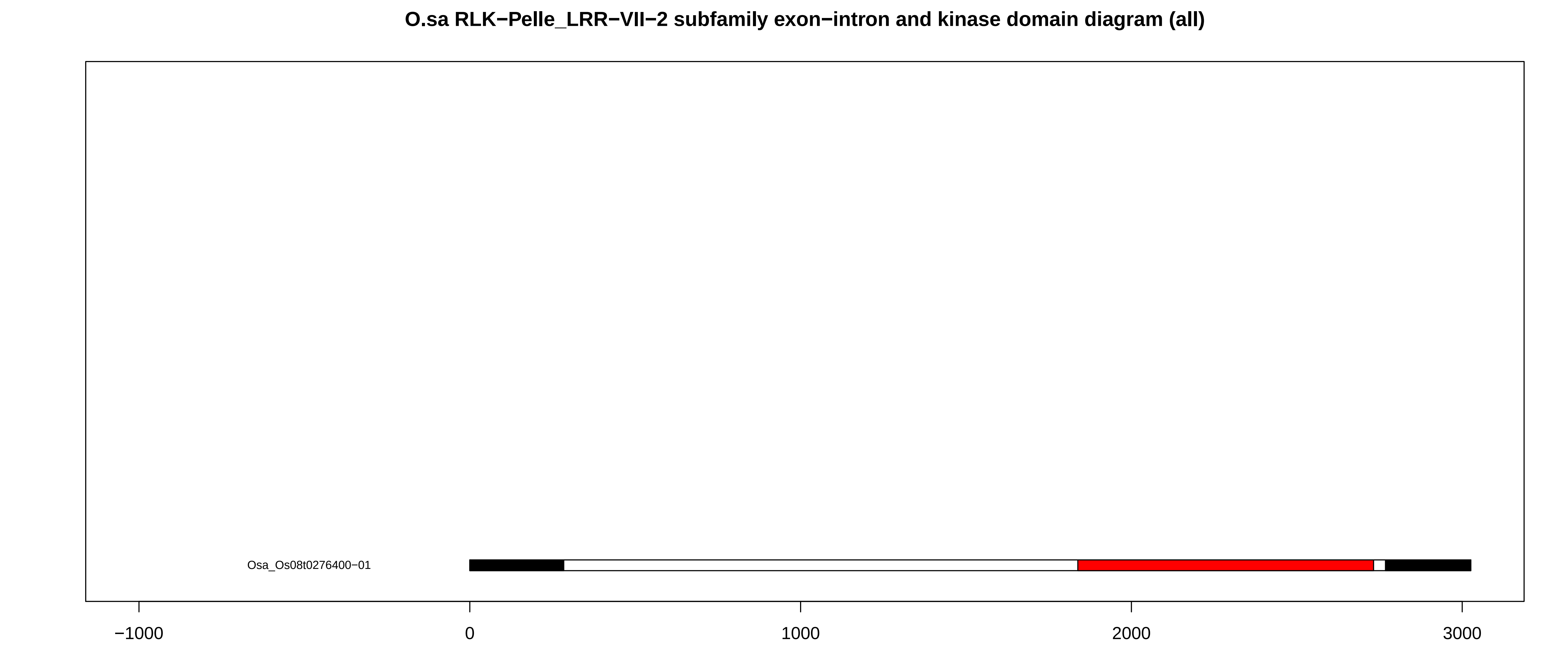
O.sa RLK−Pelle_LRR−VII−2 subfamily exon−intron and kinase domain diagram (all)
Osa_Os08t0276400−01
−1000
0
1000
2000
3000

O.sa RLK−Pelle_LRR−VII−3 subfamily exon−intron and kinase domain diagram (all)

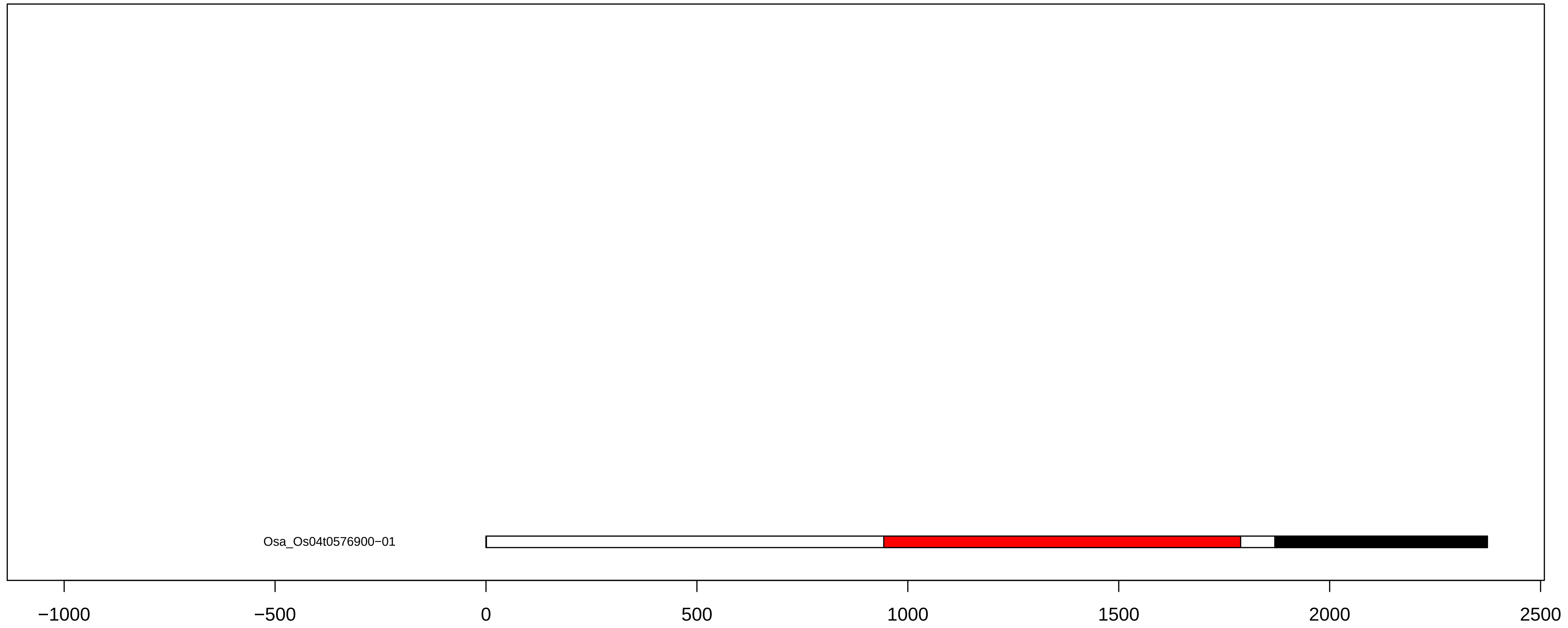

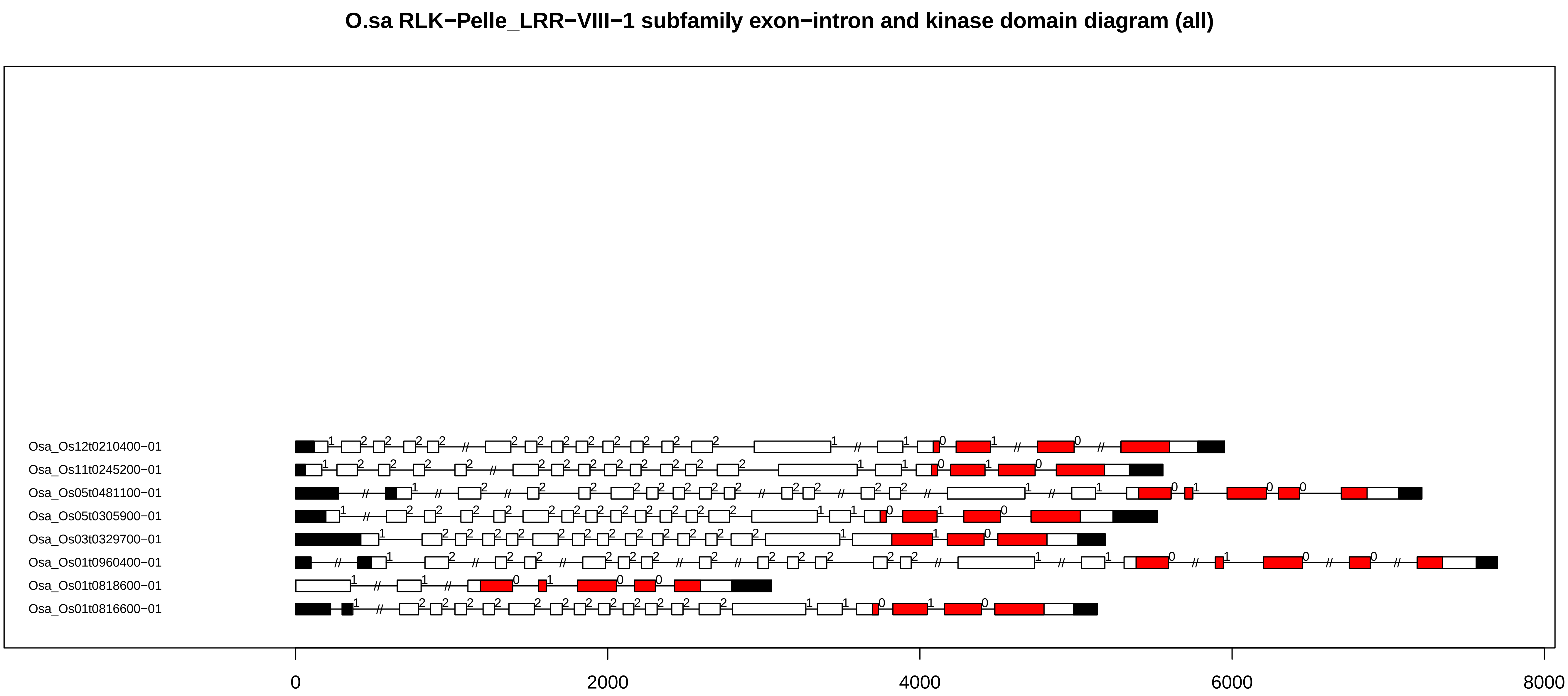
O.sa RLK−Pelle_LRR−VIII−1 subfamily exon−intron and kinase domain diagram (all)
Osa_Os12t0210400−01
Osa_Os11t0245200−01
Osa_Os05t0481100−01
Osa_Os05t0305900−01
Osa_Os03t0329700−01
Osa_Os01t0960400−01
Osa_Os01t0818600−01
Osa_Os01t0816600−01
0
2000
4000
6000
8000

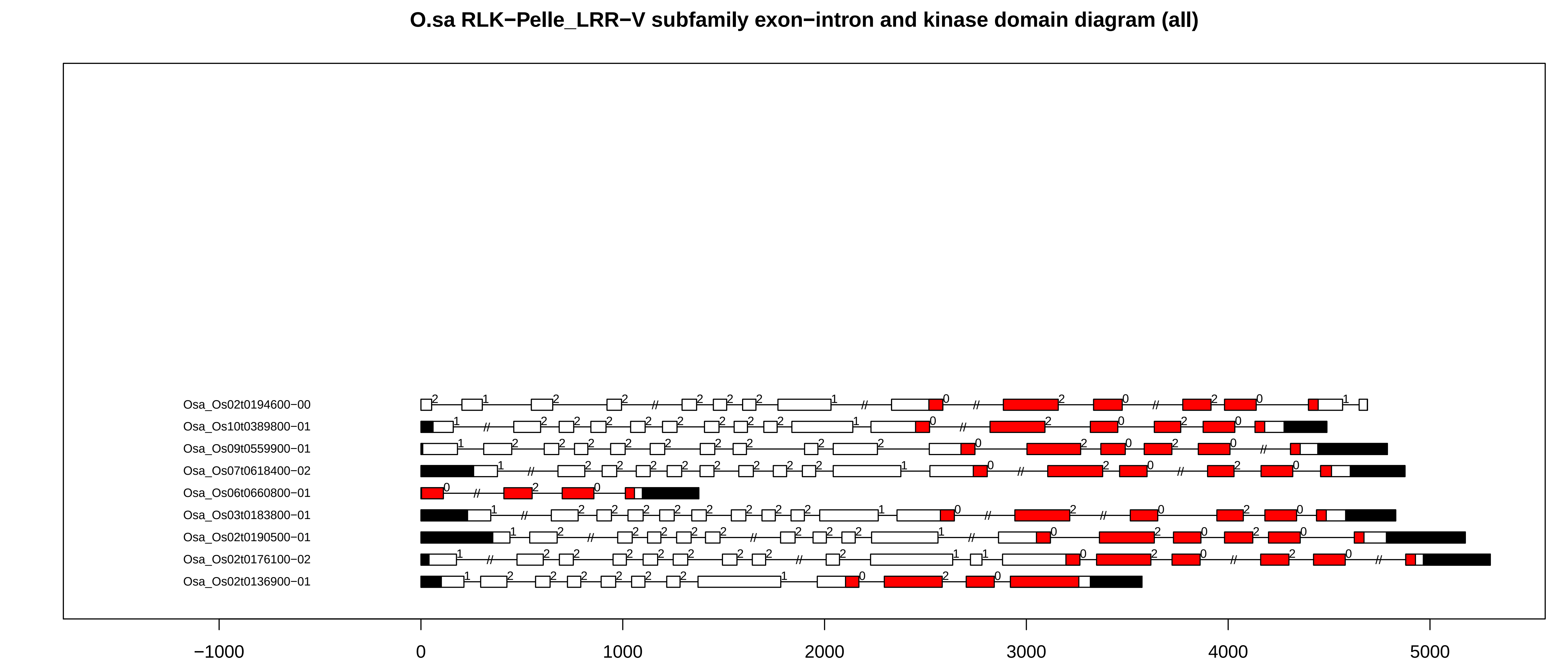
O.sa RLK−Pelle_LRR−V subfamily exon−intron and kinase domain diagram (all)
Osa_Os02t0194600−00
Osa_Os10t0389800−01
Osa_Os09t0559900−01
Osa_Os07t0618400−02
Osa_Os06t0660800−01
Osa_Os03t0183800−01
Osa_Os02t0190500−01
Osa_Os02t0176100−02
Osa_Os02t0136900−01
−1000
0
1000
2000
3000
4000
5000

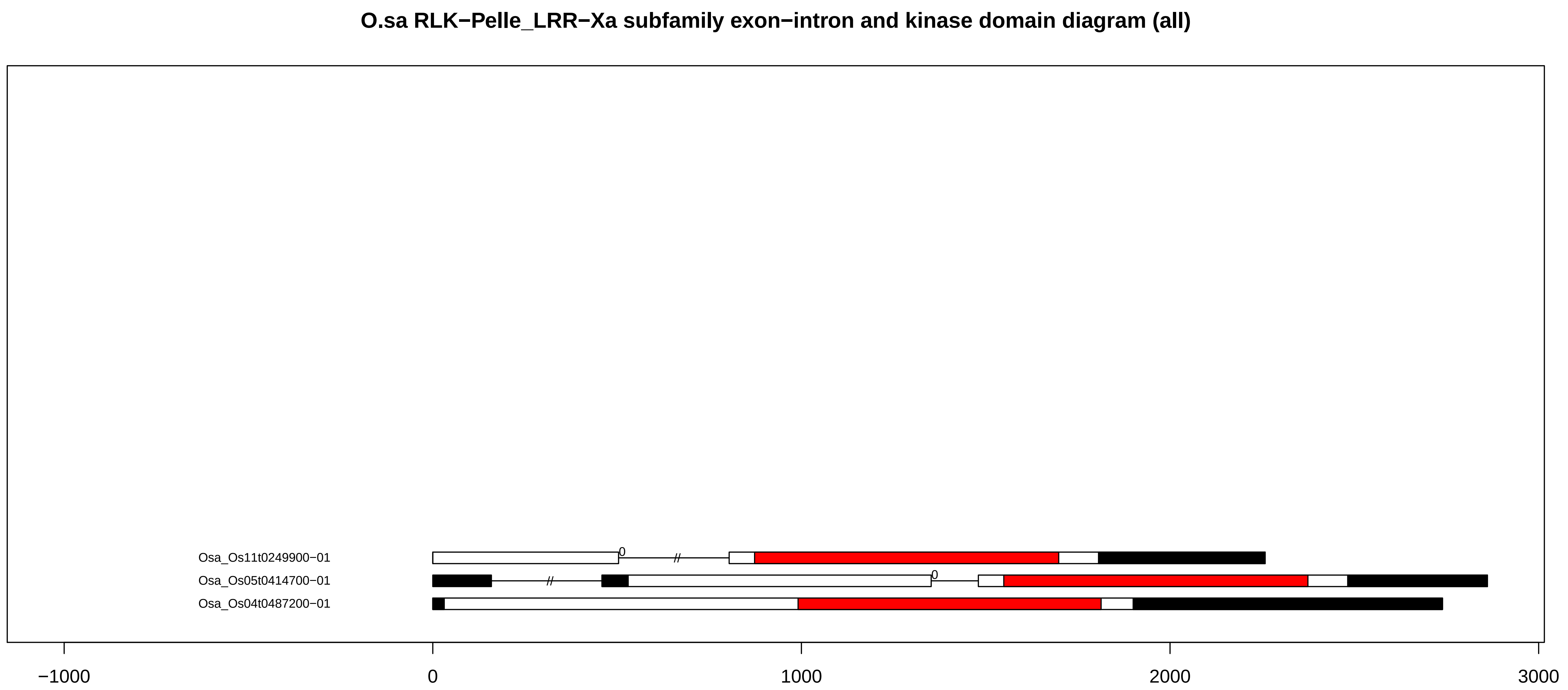
O.sa RLK−Pelle_LRR−Xa subfamily exon−intron and kinase domain diagram (all)
Osa_Os11t0249900−01
Osa_Os05t0414700−01
Osa_Os04t0487200−01
0
0
−1000
0
1000
2000
3000

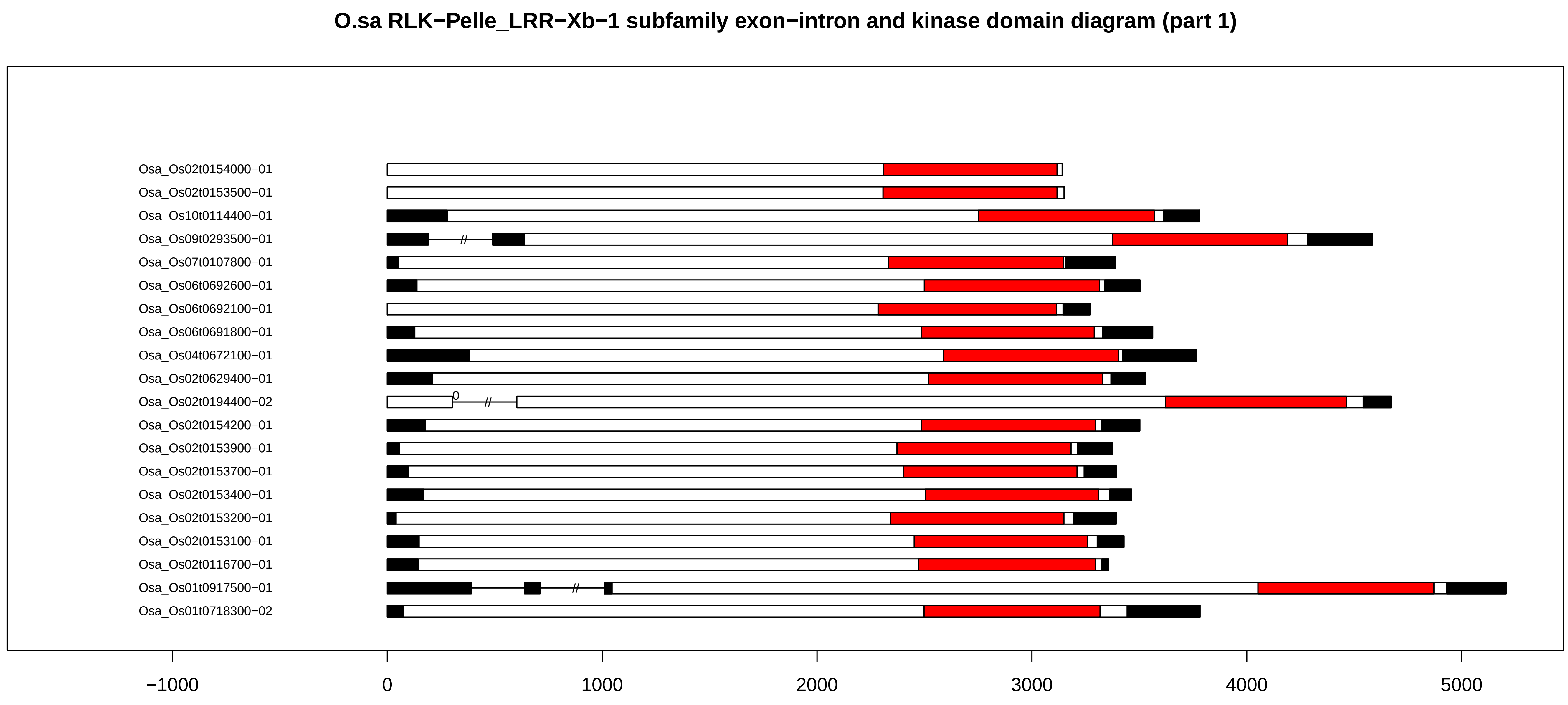

O.sa RLK−Pelle_LRR−Xb−1 subfamily exon−intron and kinase domain diagram (part 1)
Osa_Os02t0154000−01
Osa_Os02t0153500−01
Osa_Os10t0114400−01
Osa_Os09t0293500−01
Osa_Os07t0107800−01
Osa_Os06t0692600−01
Osa_Os06t0692100−01
Osa_Os06t0691800−01
Osa_Os04t0672100−01
Osa_Os02t0629400−01
Osa_Os02t0194400−02
Osa_Os02t0154200−01
Osa_Os02t0153900−01
Osa_Os02t0153700−01
Osa_Os02t0153400−01
Osa_Os02t0153200−01
Osa_Os02t0153100−01
Osa_Os02t0116700−01
Osa_Os01t0917500−01
Osa_Os01t0718300−02
0
−1000
0
1000
2000
3000
4000
5000

O.sa RLK−Pelle_LRR−Xb−1 subfamily exon−intron and kinase domain diagram (part 2)

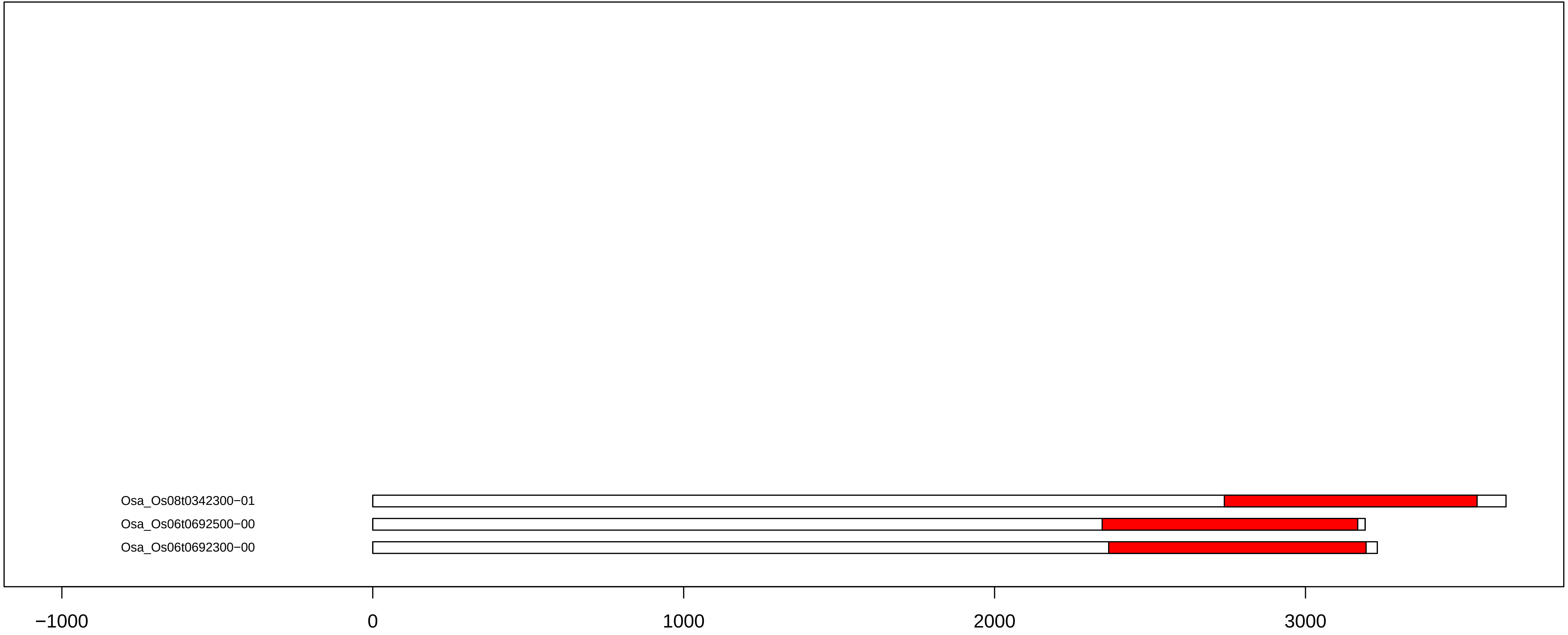

O.sa RLK−Pelle_LRR−Xb−2 subfamily exon−intron and kinase domain diagram (all)

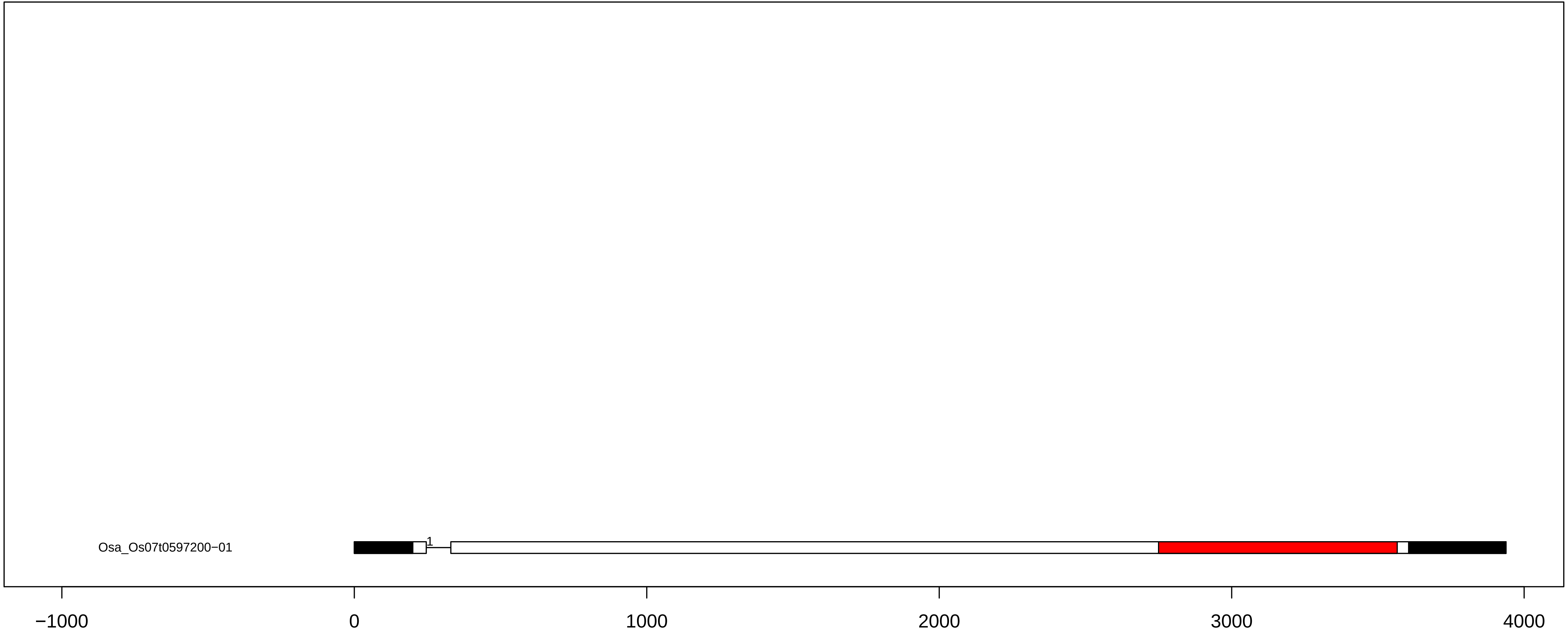

O.sa RLK−Pelle_LRR−XI−1 subfamily exon−intron and kinase domain diagram (part 1)

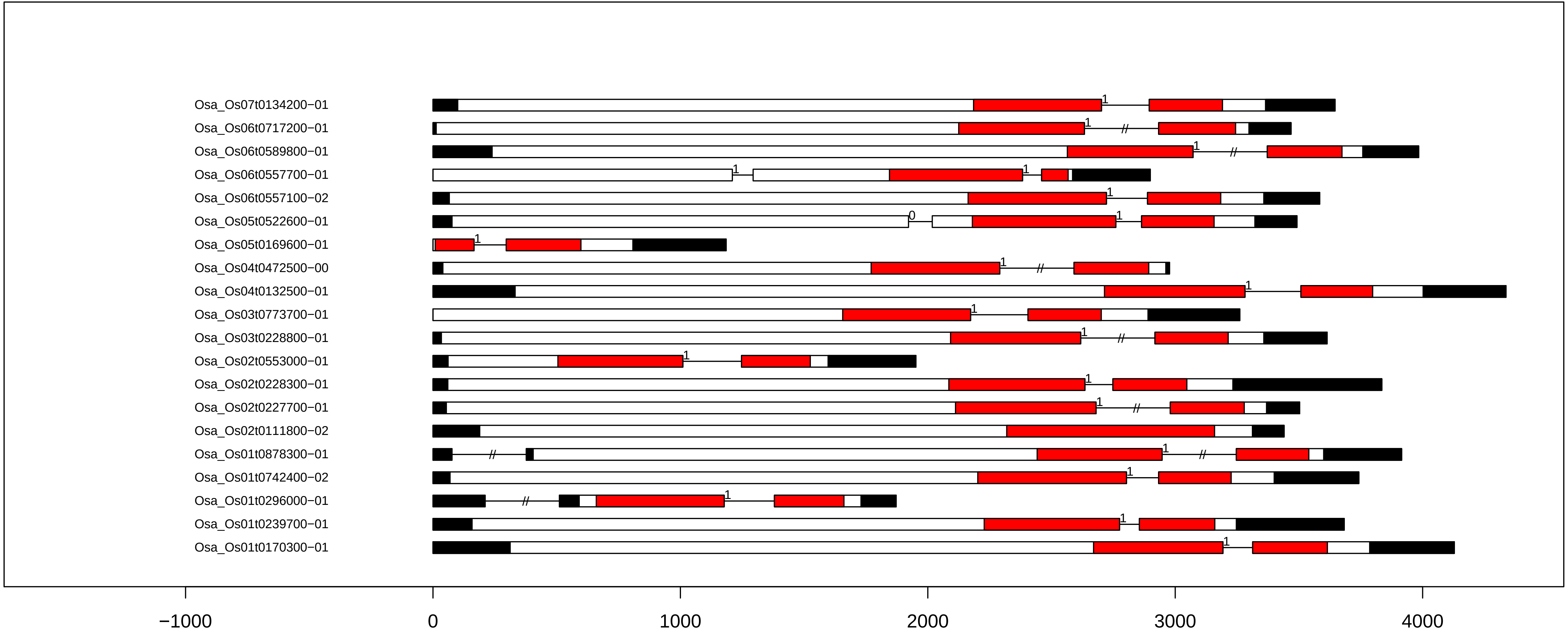

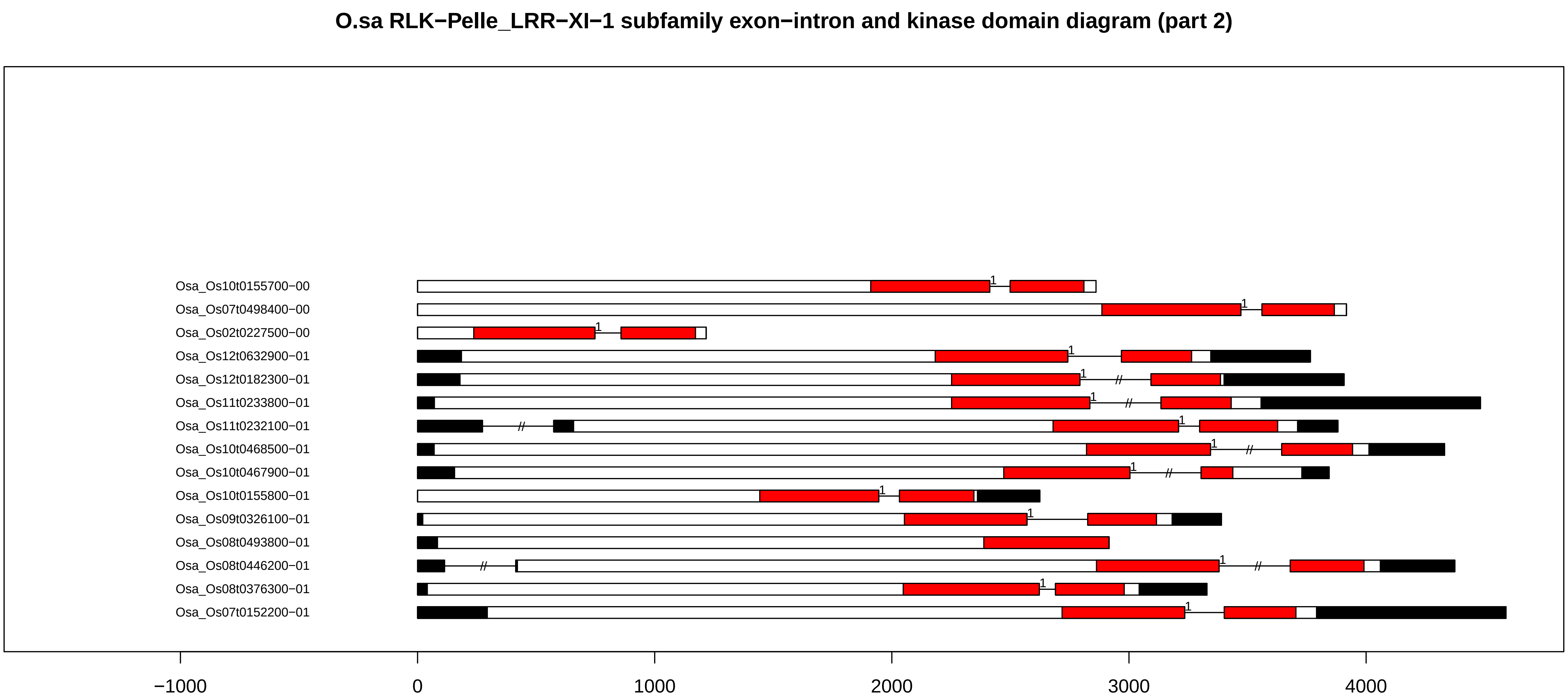
O.sa RLK−Pelle_LRR−XI−1 subfamily exon−intron and kinase domain diagram (part 2)
Osa_Os10t0155700−00
Osa_Os07t0498400−00
Osa_Os02t0227500−00
Osa_Os12t0632900−01
Osa_Os12t0182300−01
Osa_Os11t0233800−01
Osa_Os11t0232100−01
Osa_Os10t0468500−01
Osa_Os10t0467900−01
Osa_Os10t0155800−01
Osa_Os09t0326100−01
Osa_Os08t0493800−01
Osa_Os08t0446200−01
Osa_Os08t0376300−01
Osa_Os07t0152200−01
−1000
0
1000
2000
3000
4000

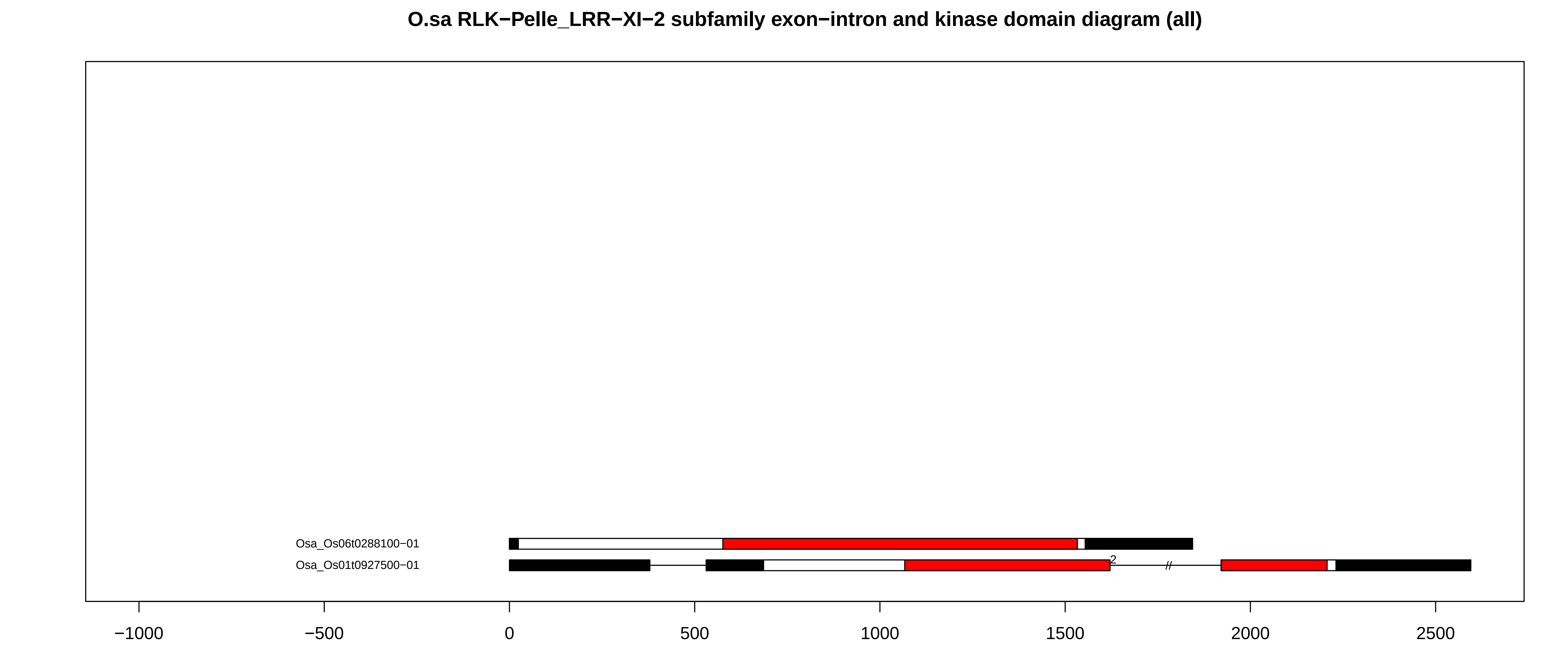

O.sa RLK−Pelle_LRR−XI−2 subfamily exon−intron and kinase domain diagram (all)
Osa_Os06t0288100−01
Osa_Os01t0927500−01
2
−1000
−500
0
500
1000
1500
2000
2500

O.sa RLK−Pelle_LRR−XII−1 subfamily exon−intron and kinase domain diagram (part 1)

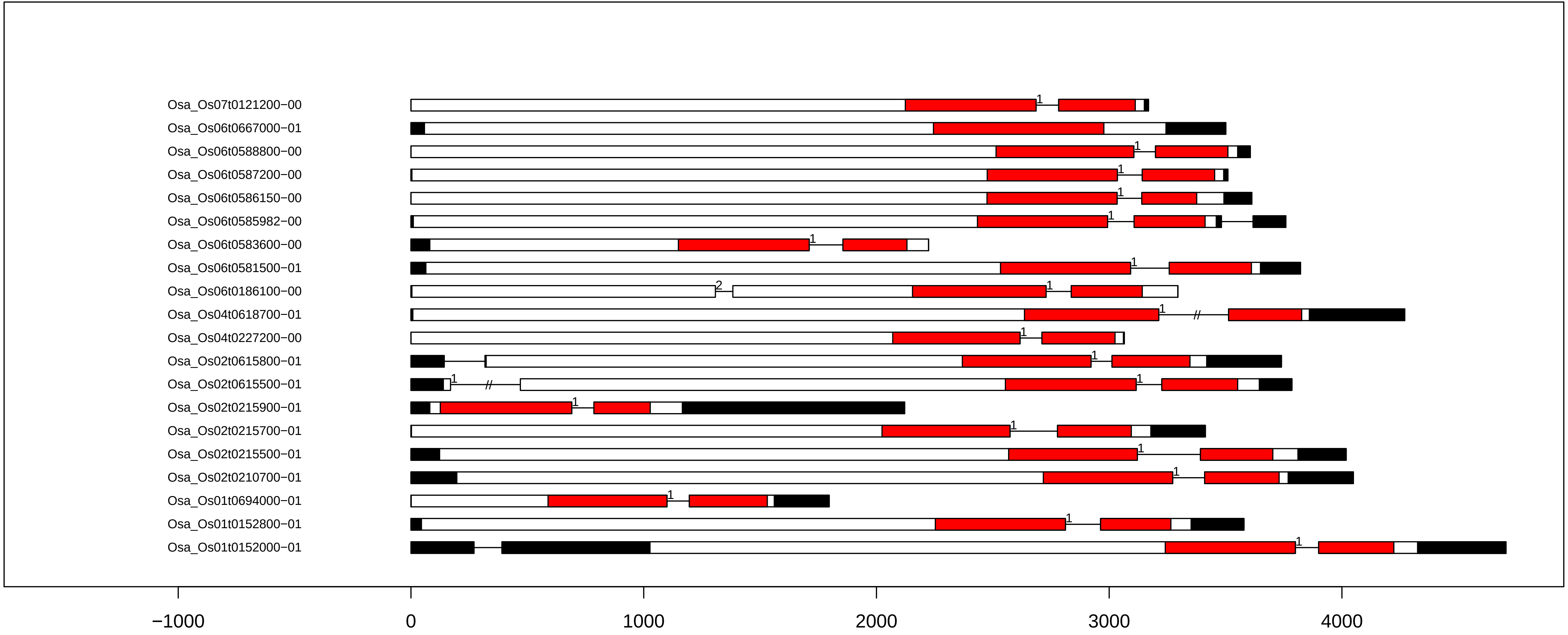

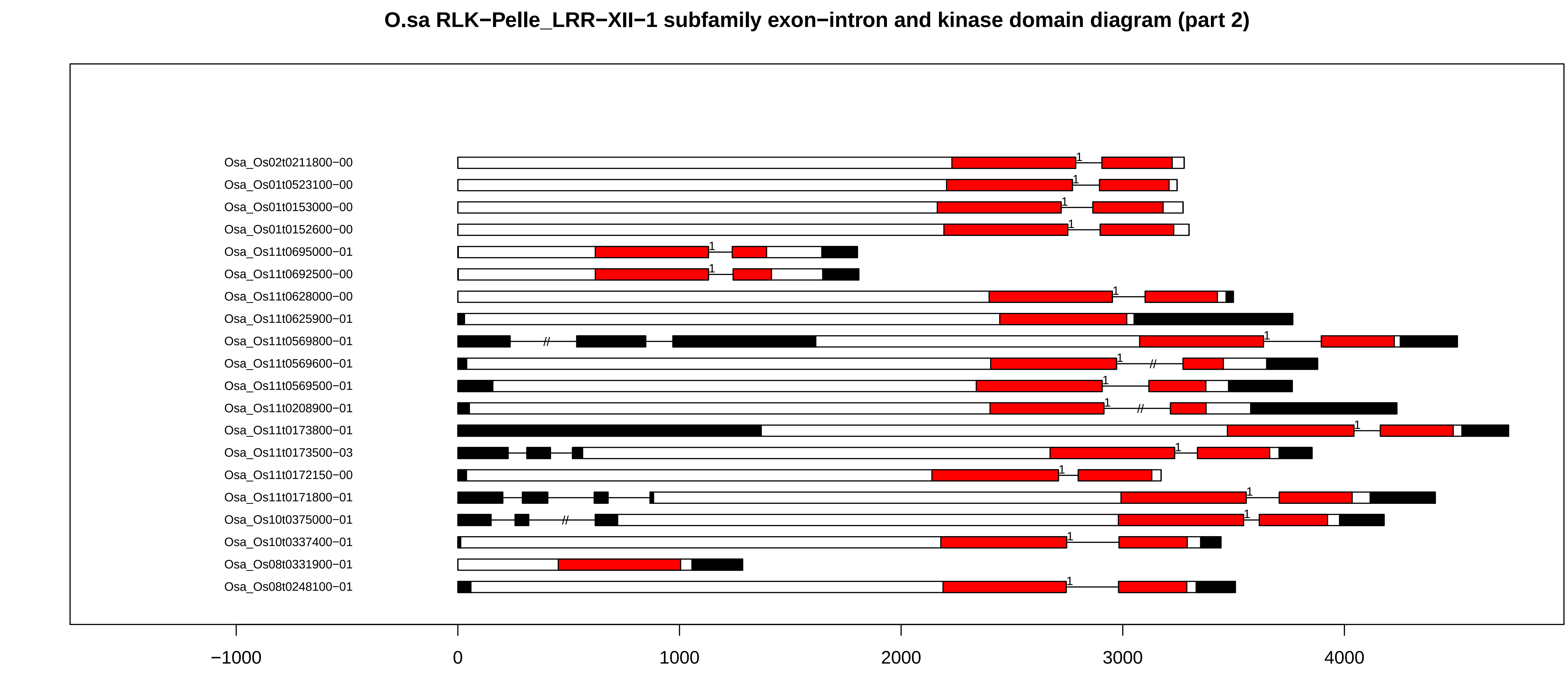
O.sa RLK−Pelle_LRR−XII−1 subfamily exon−intron and kinase domain diagram (part 2)
Osa_Os02t0211800−00
Osa_Os01t0523100−00
Osa_Os01t0153000−00
Osa_Os01t0152600−00
Osa_Os11t0695000−01
Osa_Os11t0692500−00
Osa_Os11t0628000−00
Osa_Os11t0625900−01
Osa_Os11t0569800−01
Osa_Os11t0569600−01
Osa_Os11t0569500−01
Osa_Os11t0208900−01
Osa_Os11t0173800−01
Osa_Os11t0173500−03
Osa_Os11t0172150−00
Osa_Os11t0171800−01
Osa_Os10t0375000−01
Osa_Os10t0337400−01
Osa_Os08t0331900−01
Osa_Os08t0248100−01
−1000
0
1000
2000
3000
4000

O.sa RLK−Pelle_LRR−XII−1 subfamily exon−intron and kinase domain diagram (part 3)

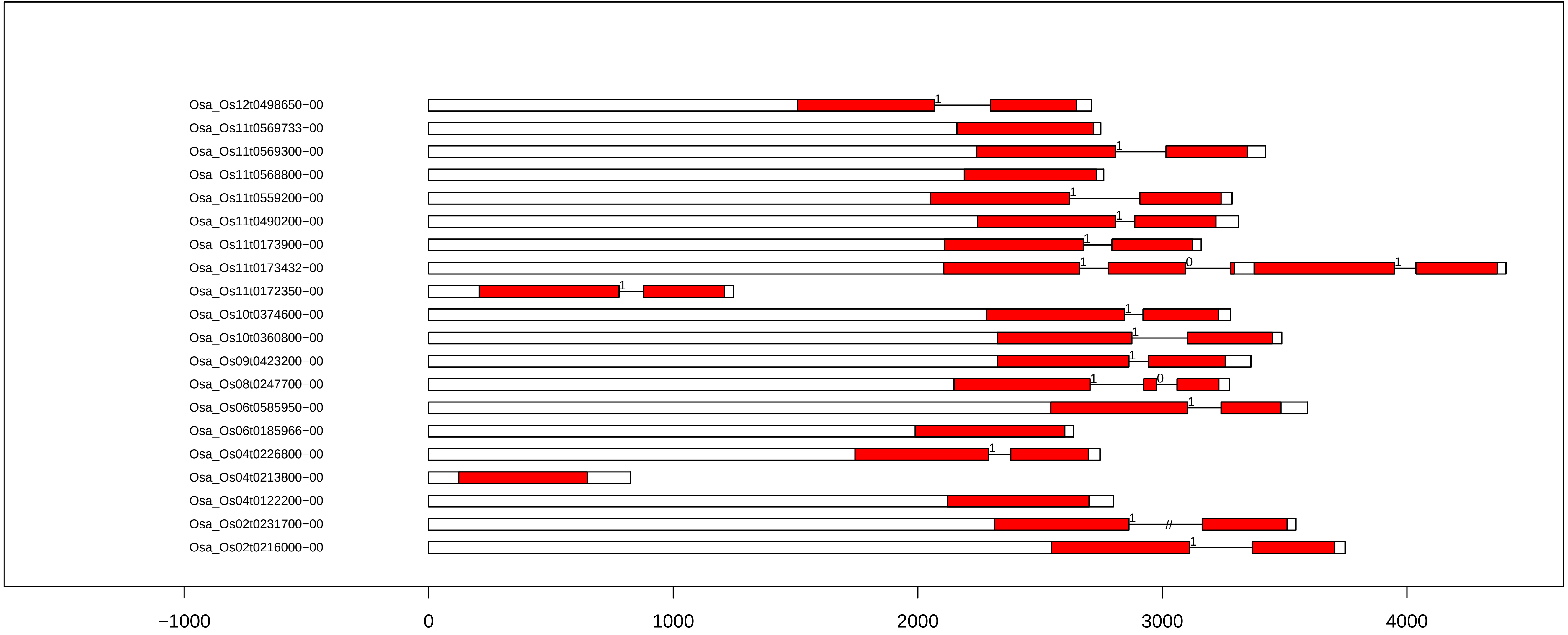

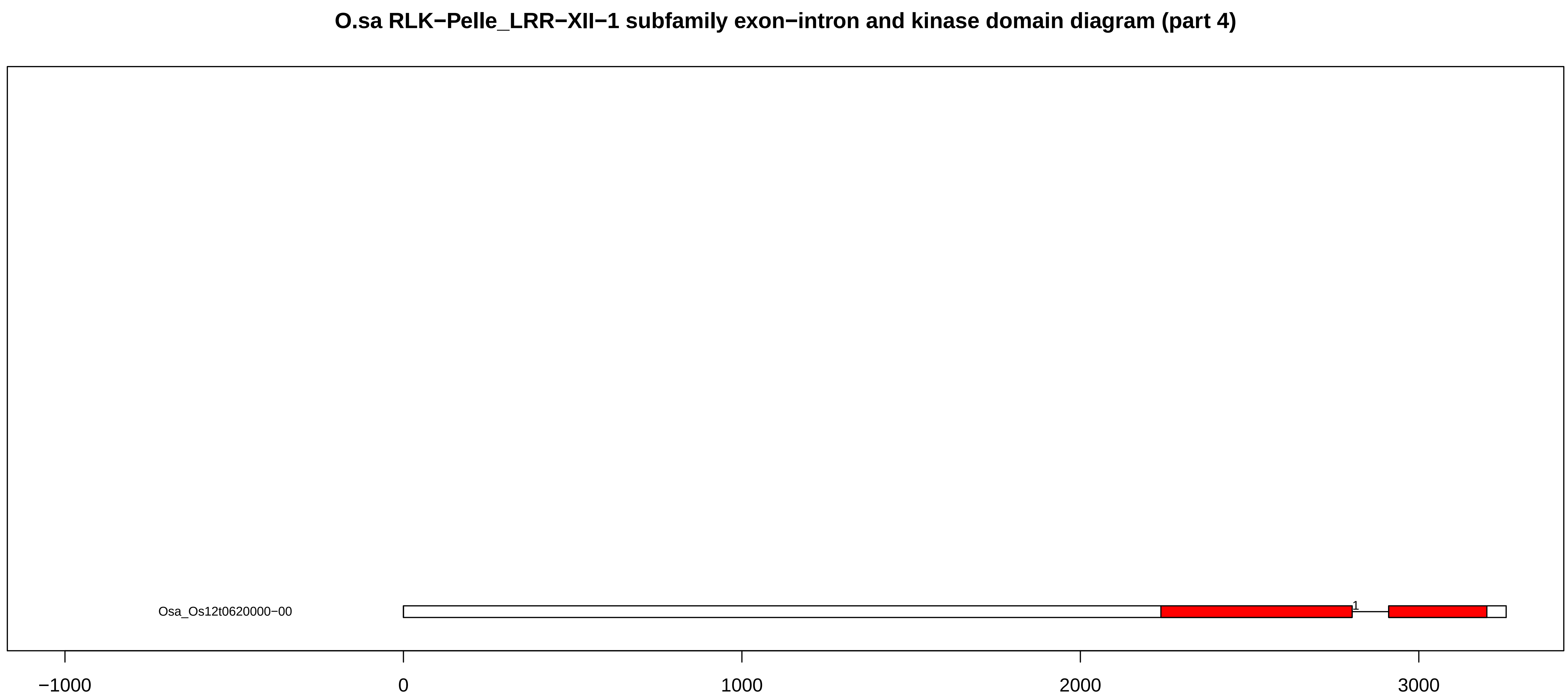
O.sa RLK−Pelle_LRR−XII−1 subfamily exon−intron and kinase domain diagram (part 4)
Osa_Os12t0620000−00
1
−1000
0
1000
2000
3000

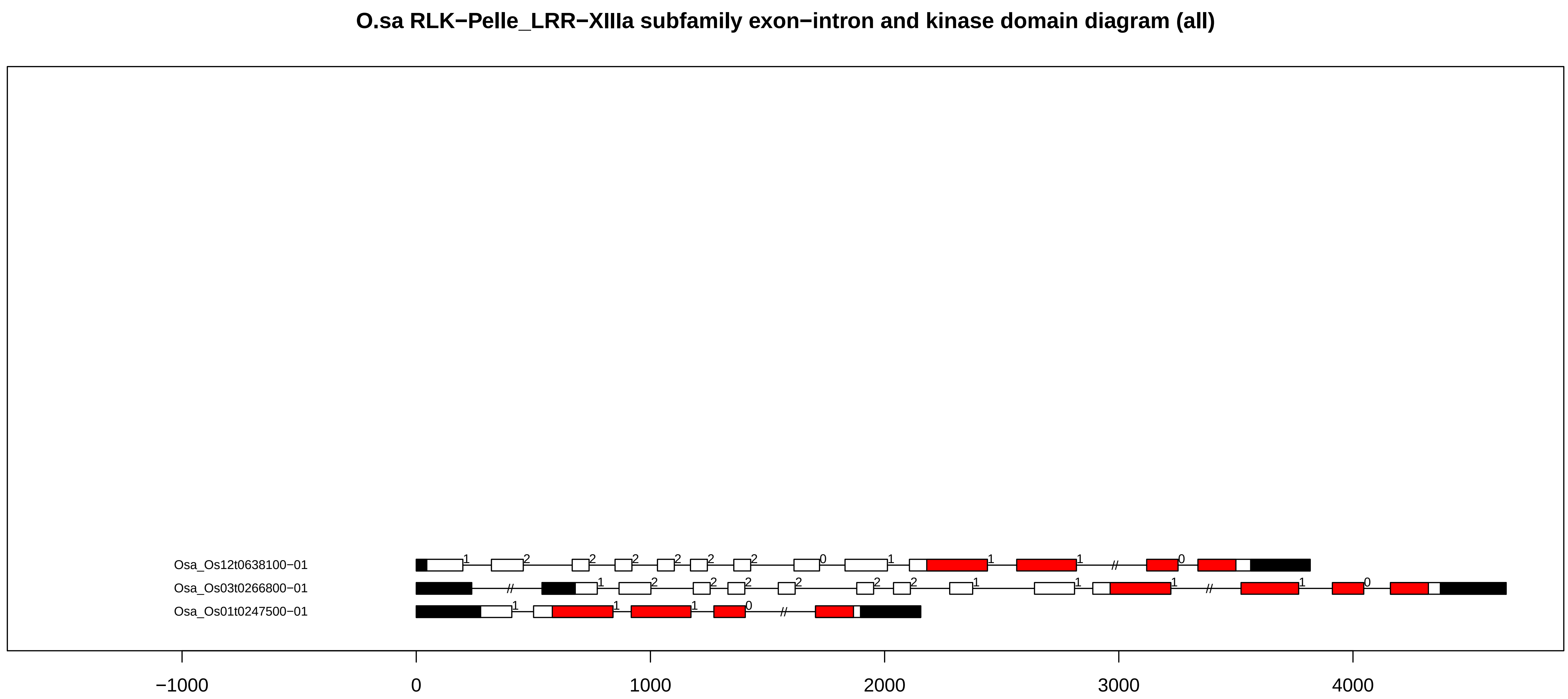

O.sa RLK−Pelle_LRR−XIIIa subfamily exon−intron and kinase domain diagram (all)
Osa_Os12t0638100−01
Osa_Os03t0266800−01
Osa_Os01t0247500−01
−1000
0
1000
2000
3000
4000

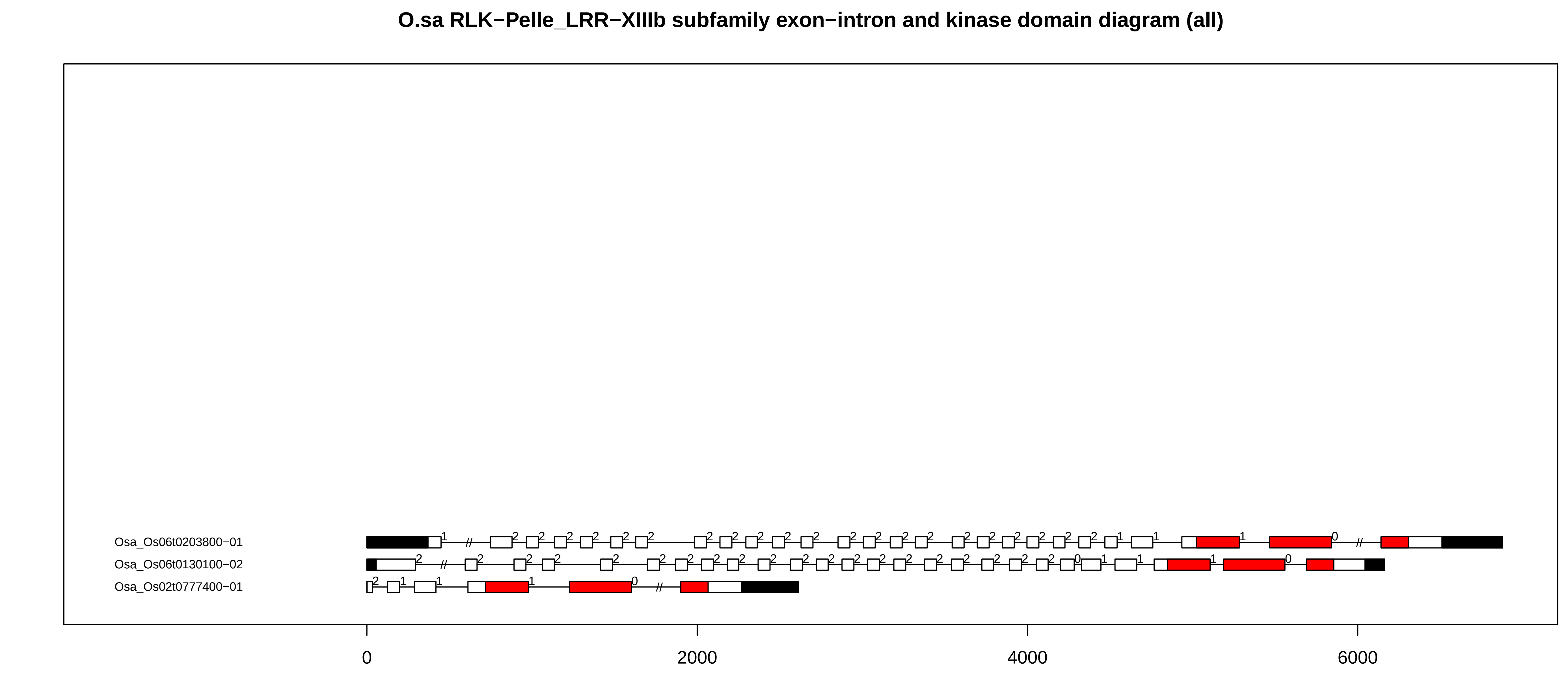
O.sa RLK−Pelle_LRR−XIIIb subfamily exon−intron and kinase domain diagram (all)
Osa_Os06t0203800−01
Osa_Os06t0130100−02
Osa_Os02t0777400−01
0
2000
4000
6000

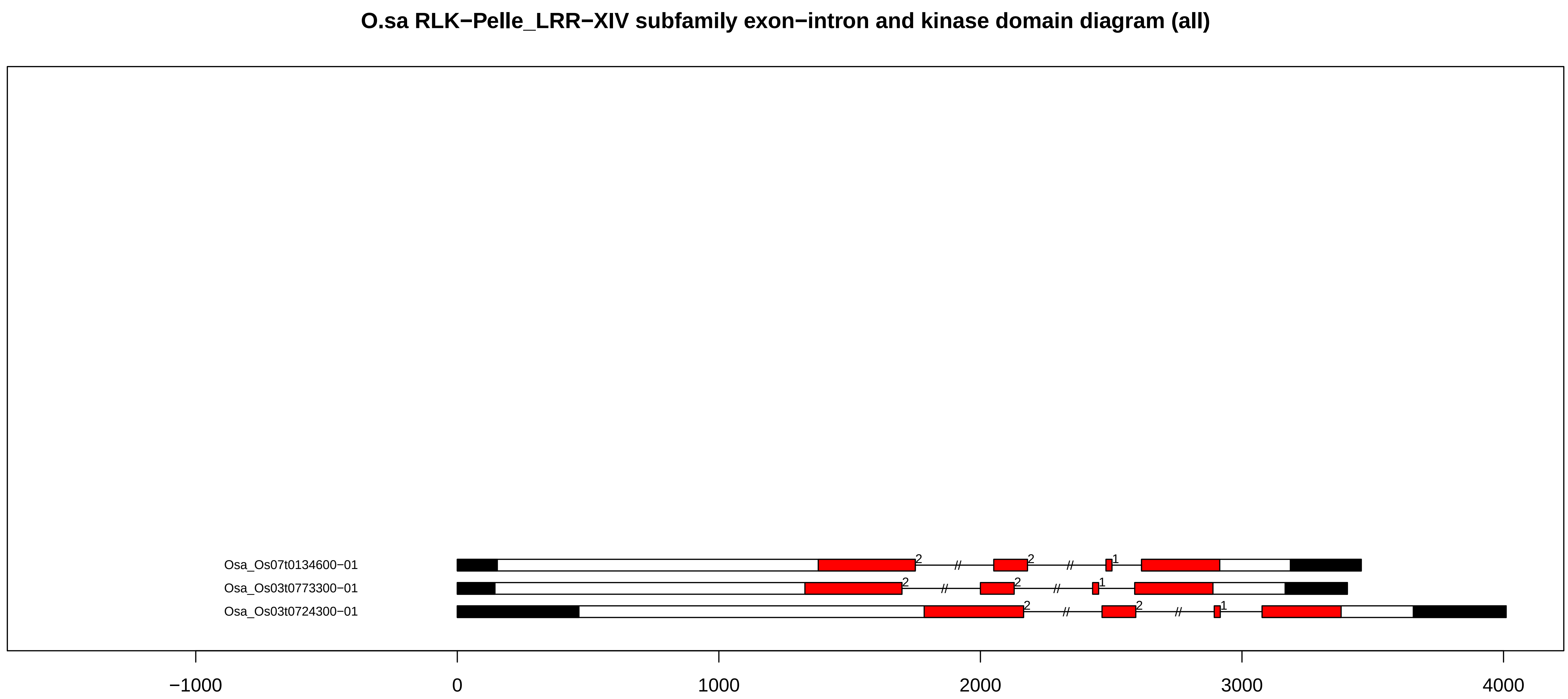

O.sa RLK−Pelle_LRR−XIV subfamily exon−intron and kinase domain diagram (all)
Osa_Os07t0134600−01
Osa_Os03t0773300−01
Osa_Os03t0724300−01
−1000
0
1000
2000
3000
4000

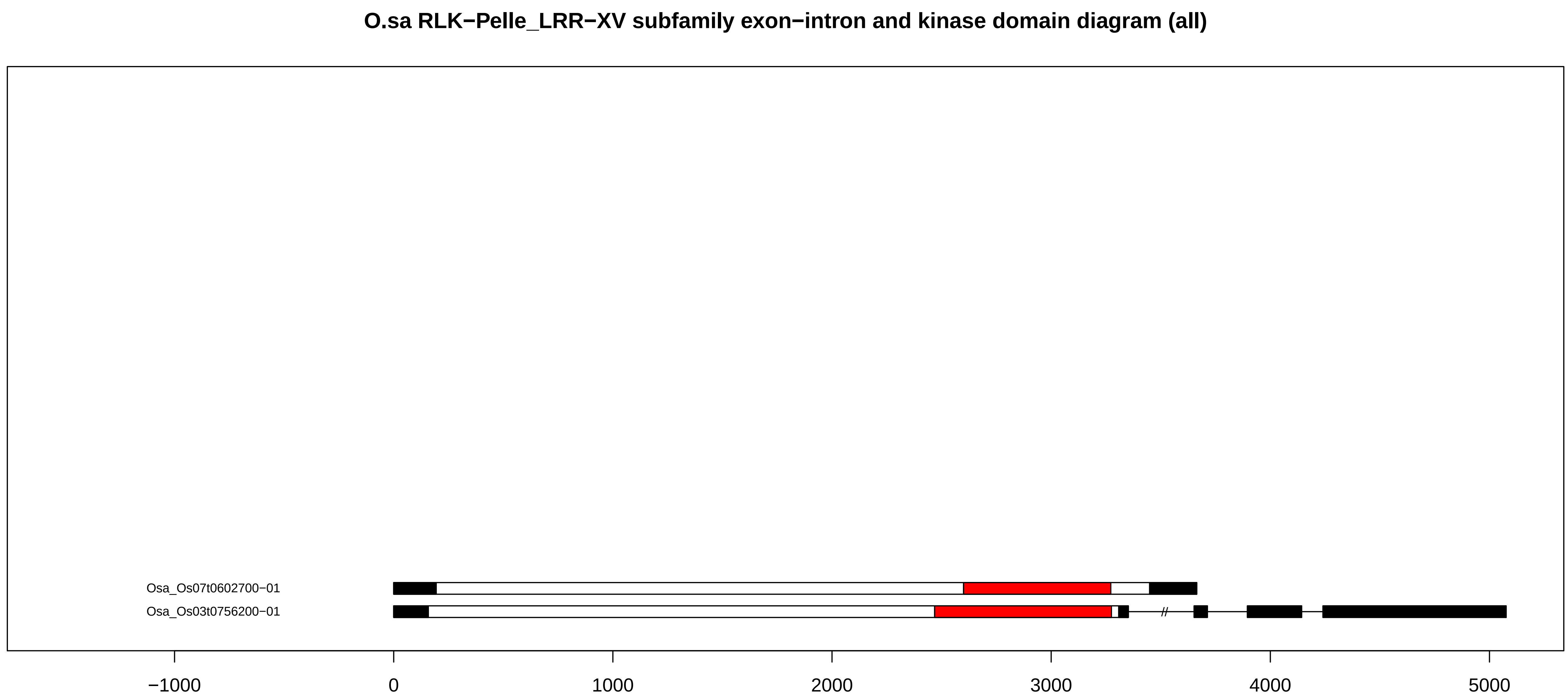
O.sa RLK−Pelle_LRR−XV subfamily exon−intron and kinase domain diagram (all)
Osa_Os07t0602700−01
Osa_Os03t0756200−01
−1000
0
1000
2000
3000
4000
5000

O.sa RLK−Pelle_LysM subfamily exon−intron and kinase domain diagram (all)

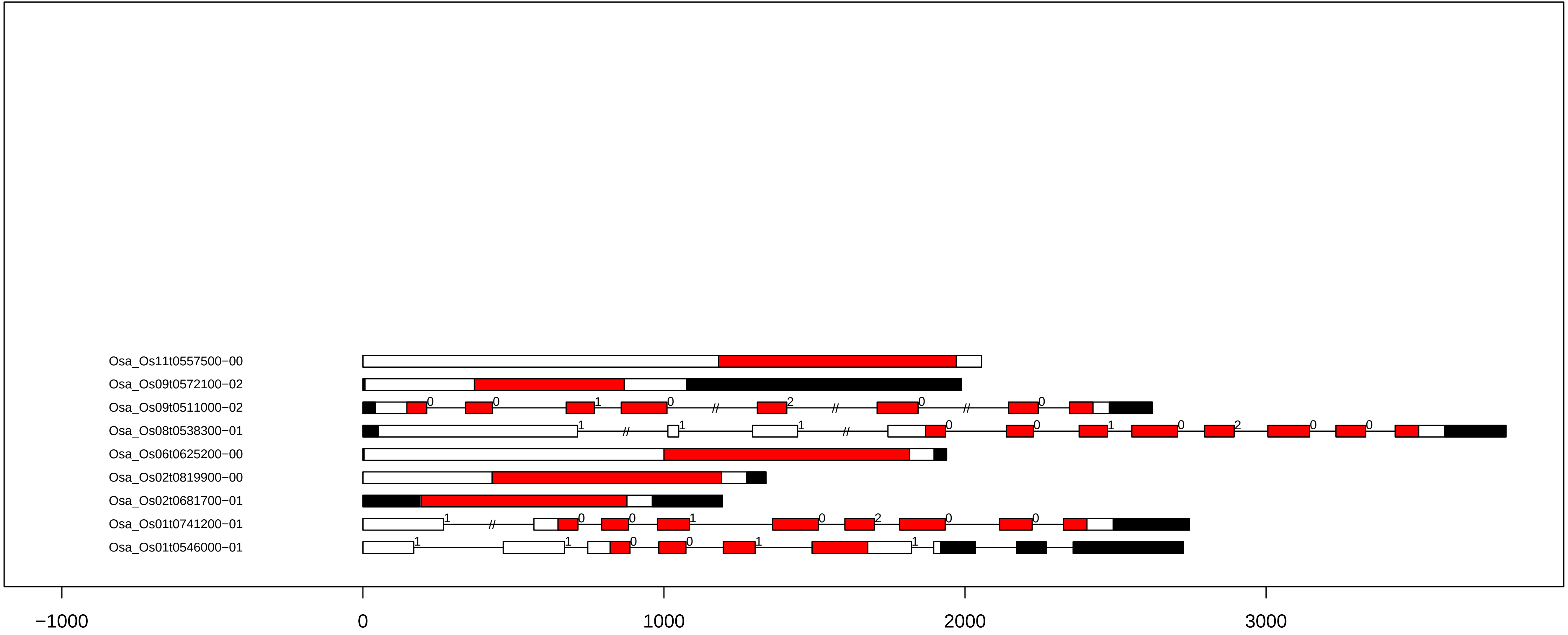

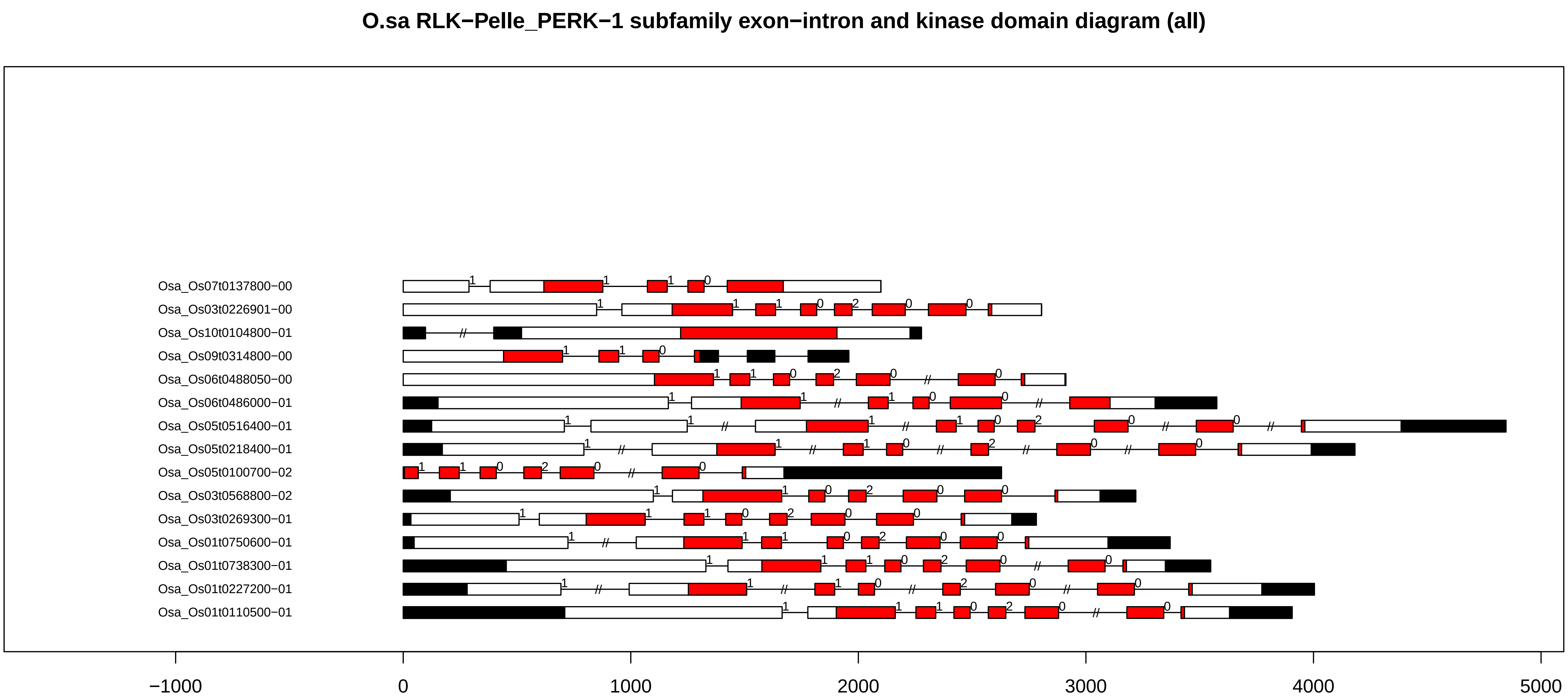
O.sa RLK−Pelle_PERK−1 subfamily exon−intron and kinase domain diagram (all)
Osa_Os07t0137800−00
Osa_Os03t0226901−00
Osa_Os10t0104800−01
Osa_Os09t0314800−00
Osa_Os06t0488050−00
Osa_Os06t0486000−01
Osa_Os05t0516400−01
Osa_Os05t0218400−01
Osa_Os05t0100700−02
Osa_Os03t0568800−02
Osa_Os03t0269300−01
Osa_Os01t0750600−01
Osa_Os01t0738300−01
Osa_Os01t0227200−01
Osa_Os01t0110500−01
−1000
0
1000
2000
3000
4000
5000

O.sa RLK−Pelle_PERK−2 subfamily exon−intron and kinase domain diagram (all)

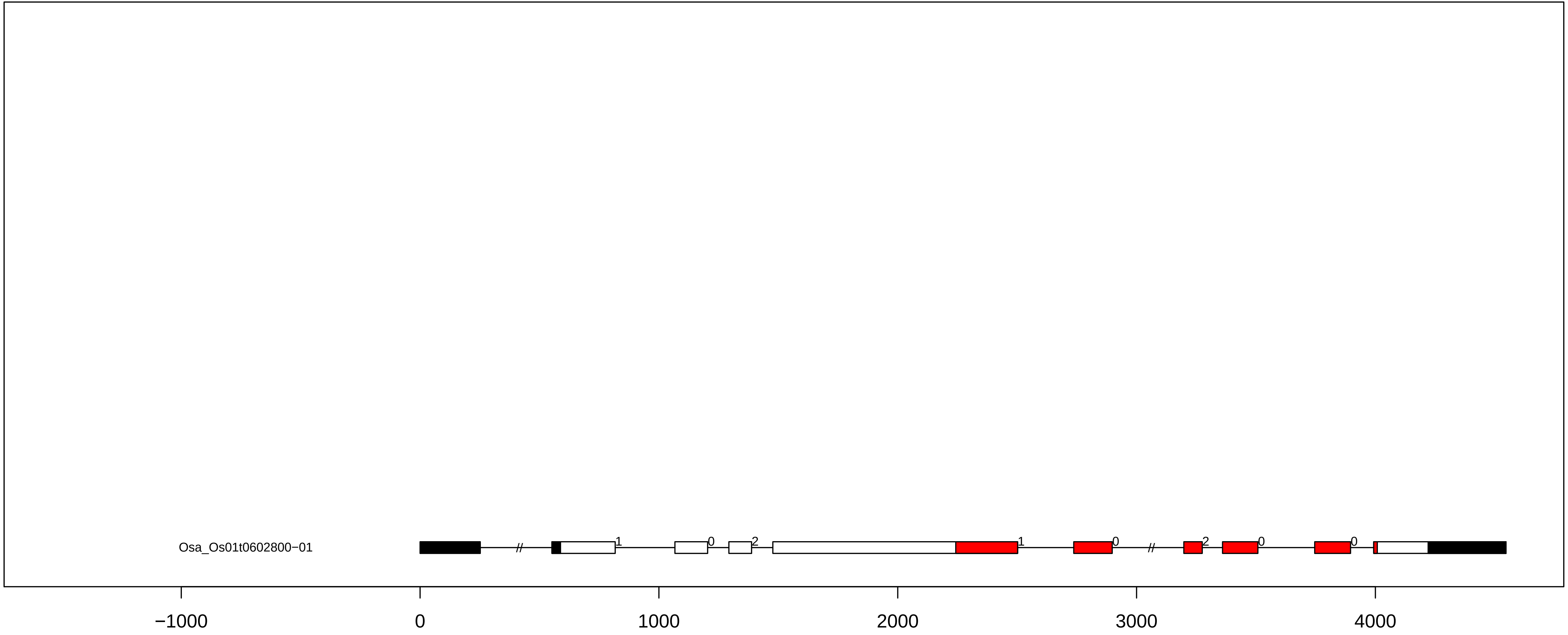

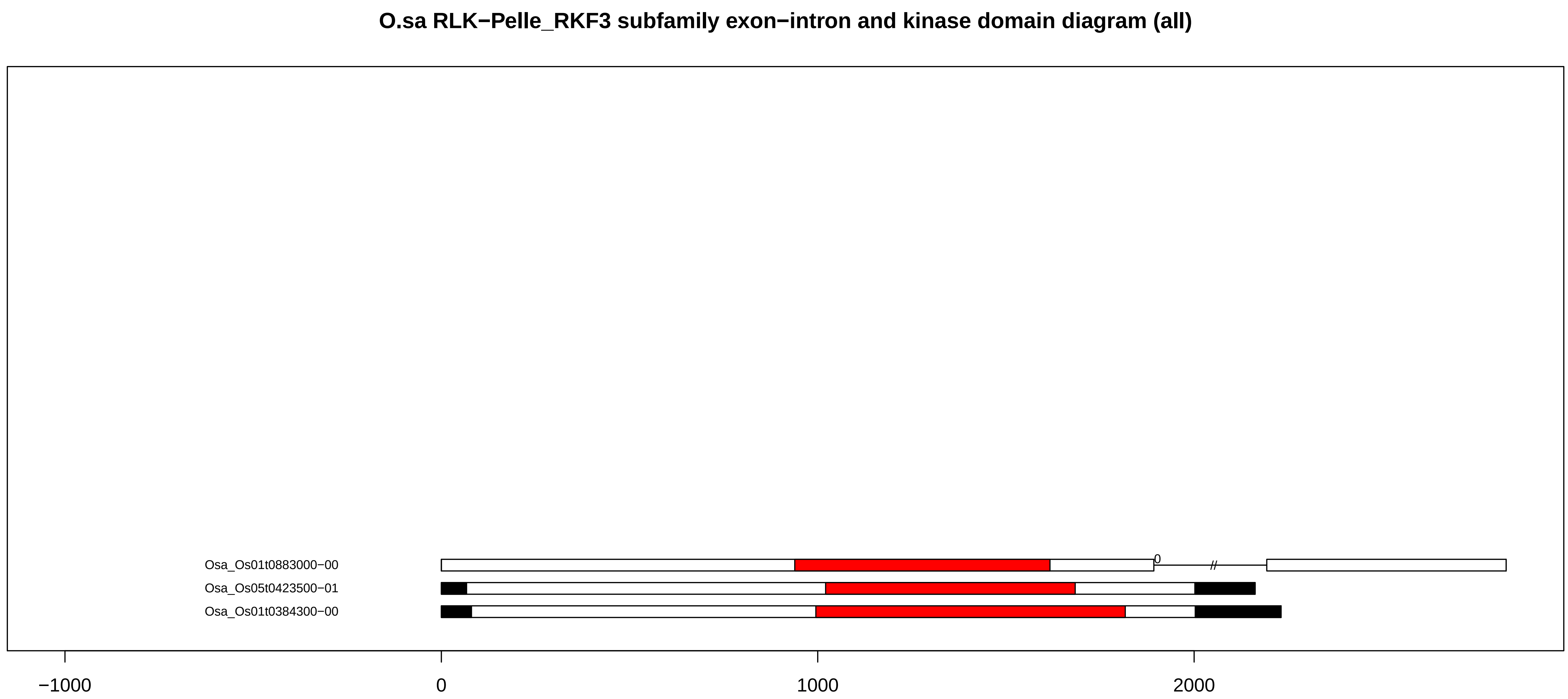
O.sa RLK−Pelle_RKF3 subfamily exon−intron and kinase domain diagram (all)
Osa_Os01t0883000−00
Osa_Os05t0423500−01
Osa_Os01t0384300−00
0
−1000
0
1000
2000

O.sa RLK−Pelle_RLCK−II subfamily exon−intron and kinase domain diagram (all)

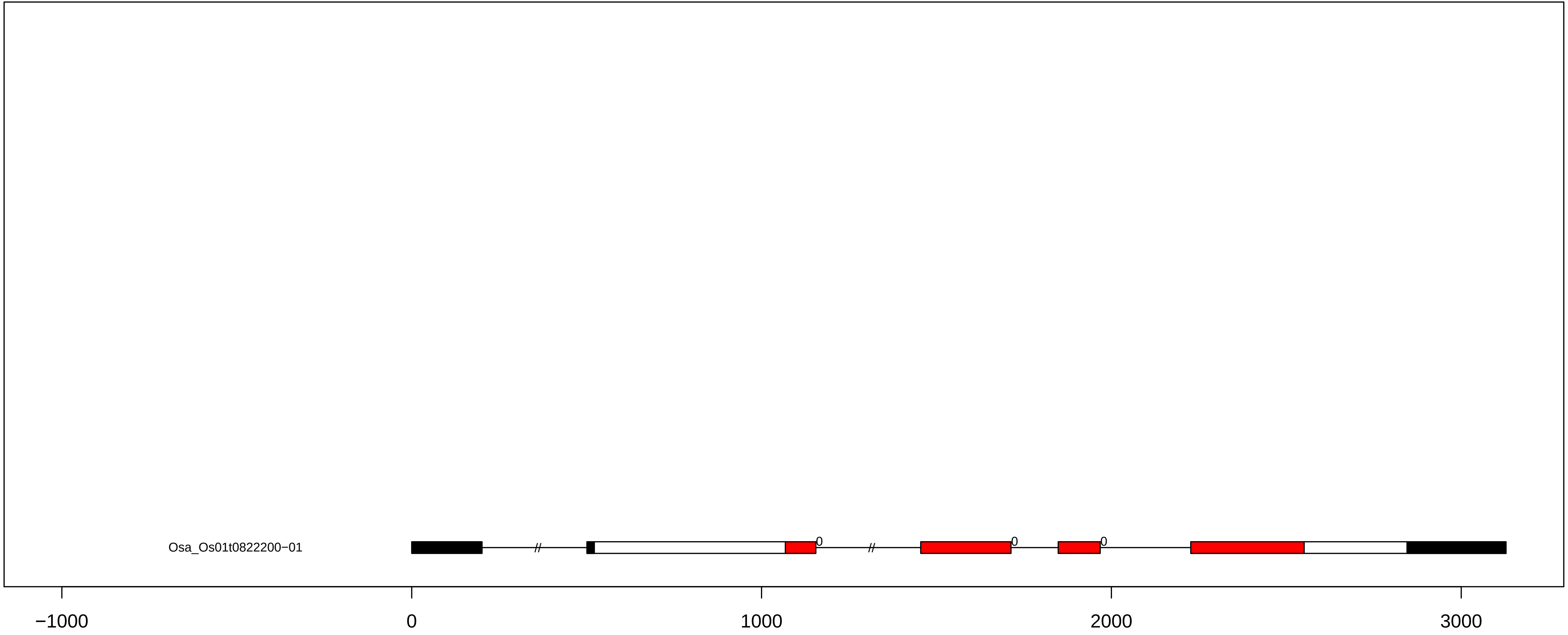

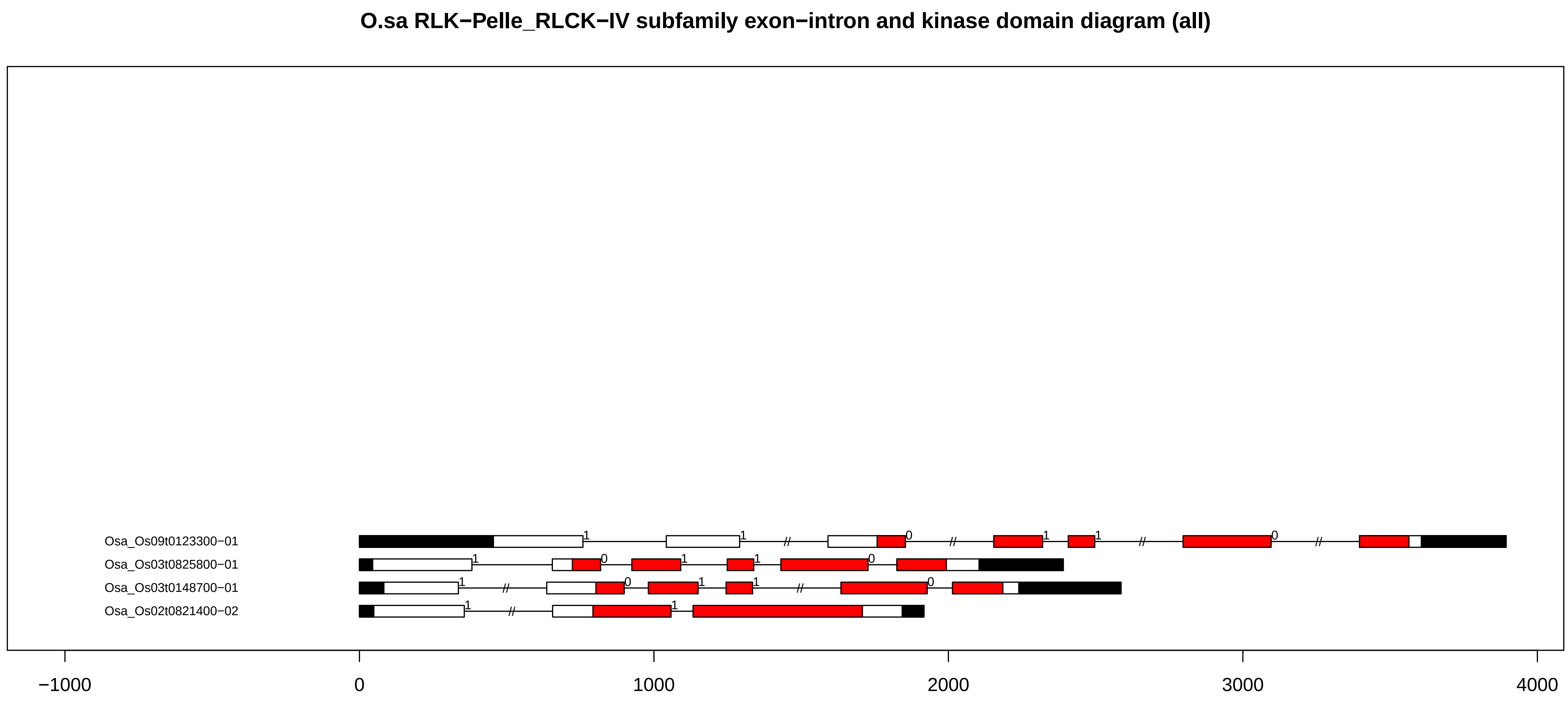
O.sa RLK−Pelle_RLCK−IV subfamily exon−intron and kinase domain diagram (all)
Osa_Os09t0123300−01
Osa_Os03t0825800−01
Osa_Os03t0148700−01
Osa_Os02t0821400−02
−1000
0
1000
2000
3000
4000

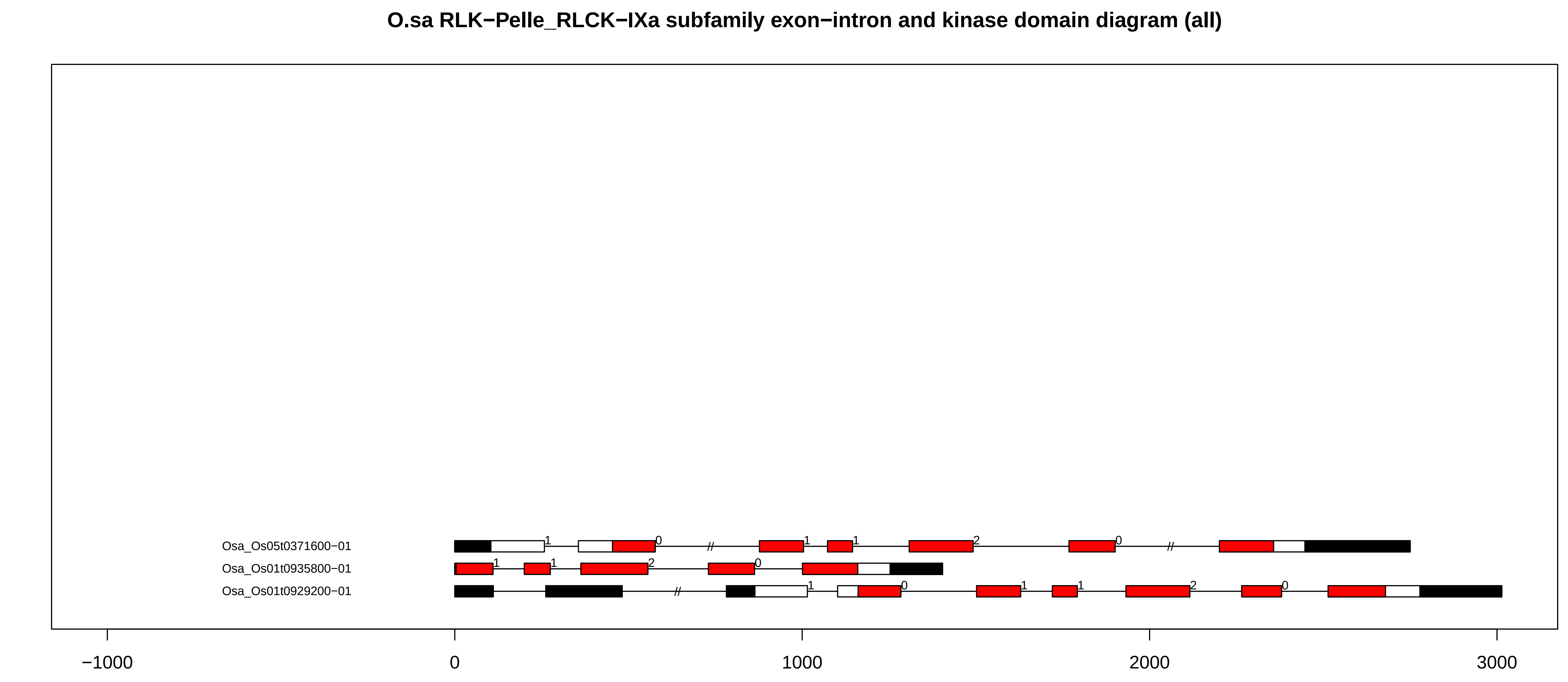

O.sa RLK−Pelle_RLCK−IXa subfamily exon−intron and kinase domain diagram (all)
Osa_Os05t0371600−01
Osa_Os01t0935800−01
Osa_Os01t0929200−01
−1000
0
1000
2000
3000

O.sa RLK−Pelle_RLCK−IXb subfamily exon−intron and kinase domain diagram (all)

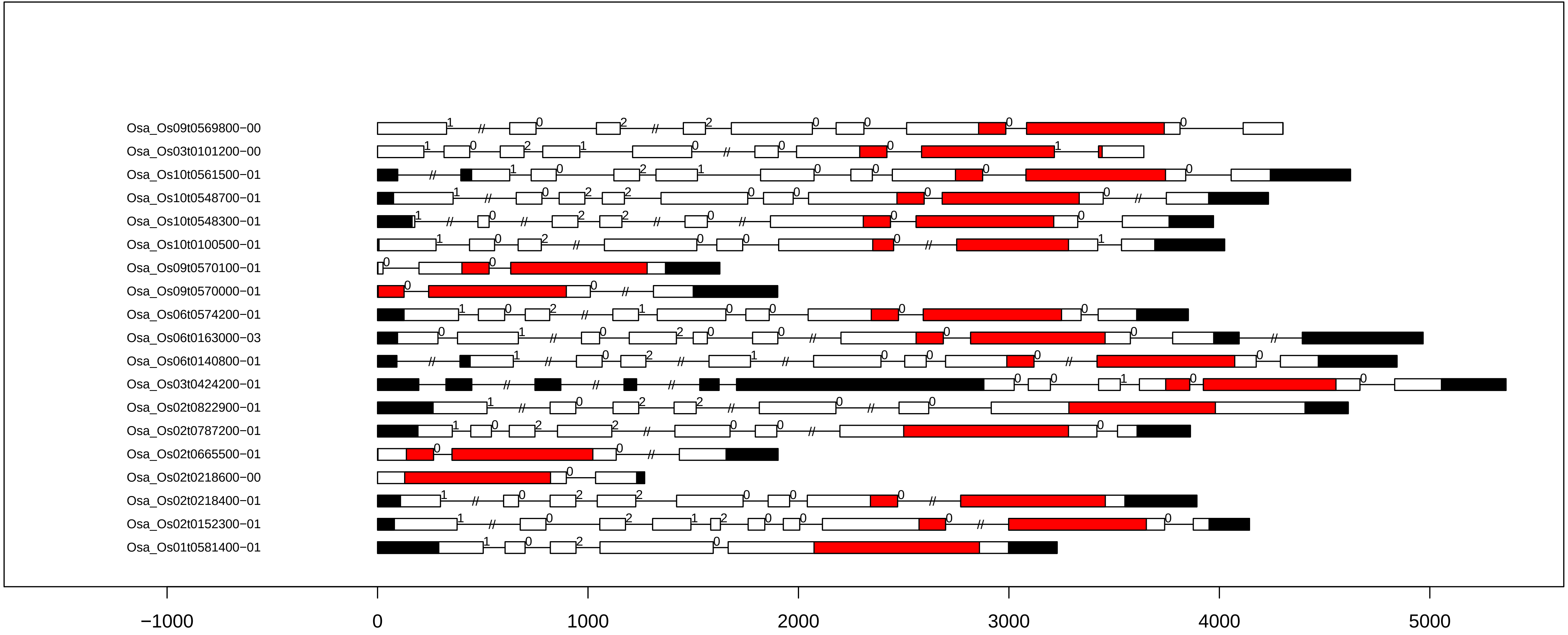

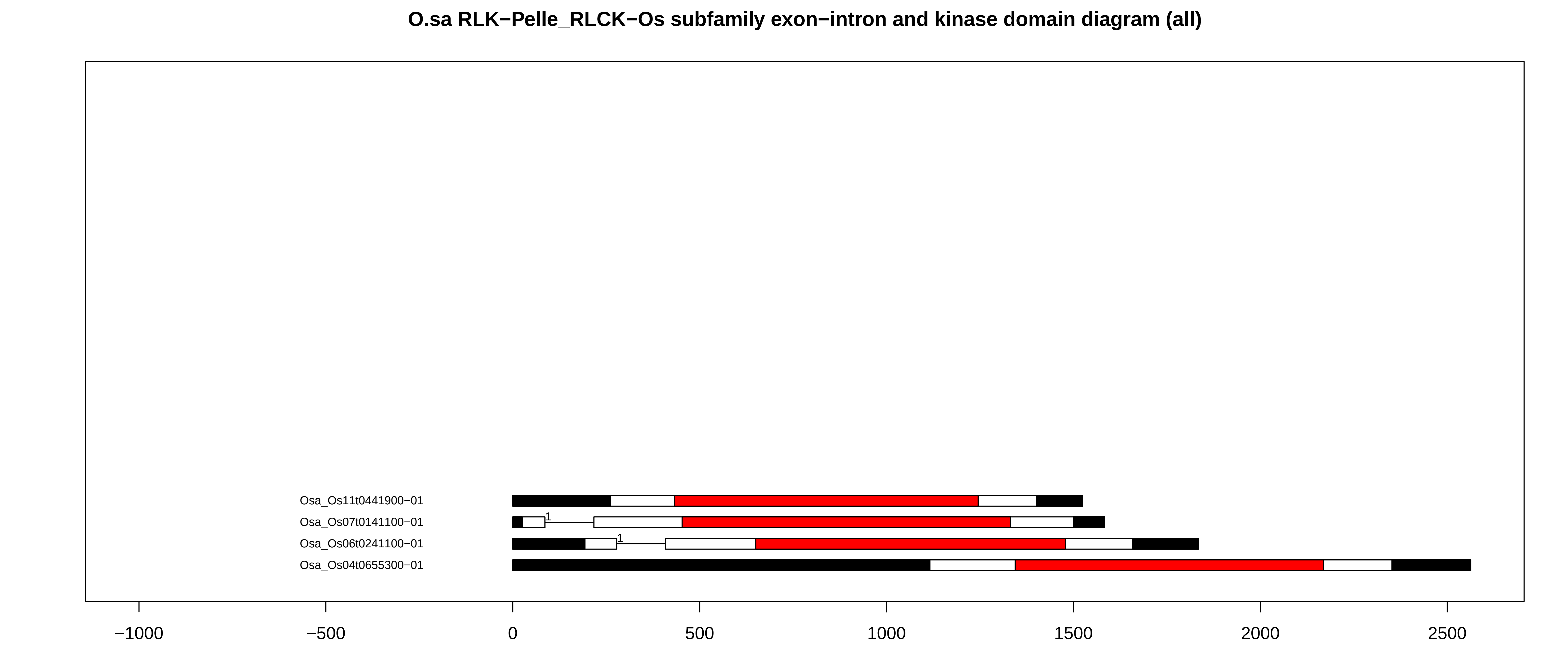
O.sa RLK−Pelle_RLCK−Os subfamily exon−intron and kinase domain diagram (all)
Osa_Os11t0441900−01
Osa_Os07t0141100−01
Osa_Os06t0241100−01
Osa_Os04t0655300−01
1
1
−1000
−500
0
500
1000
1500
2000
2500

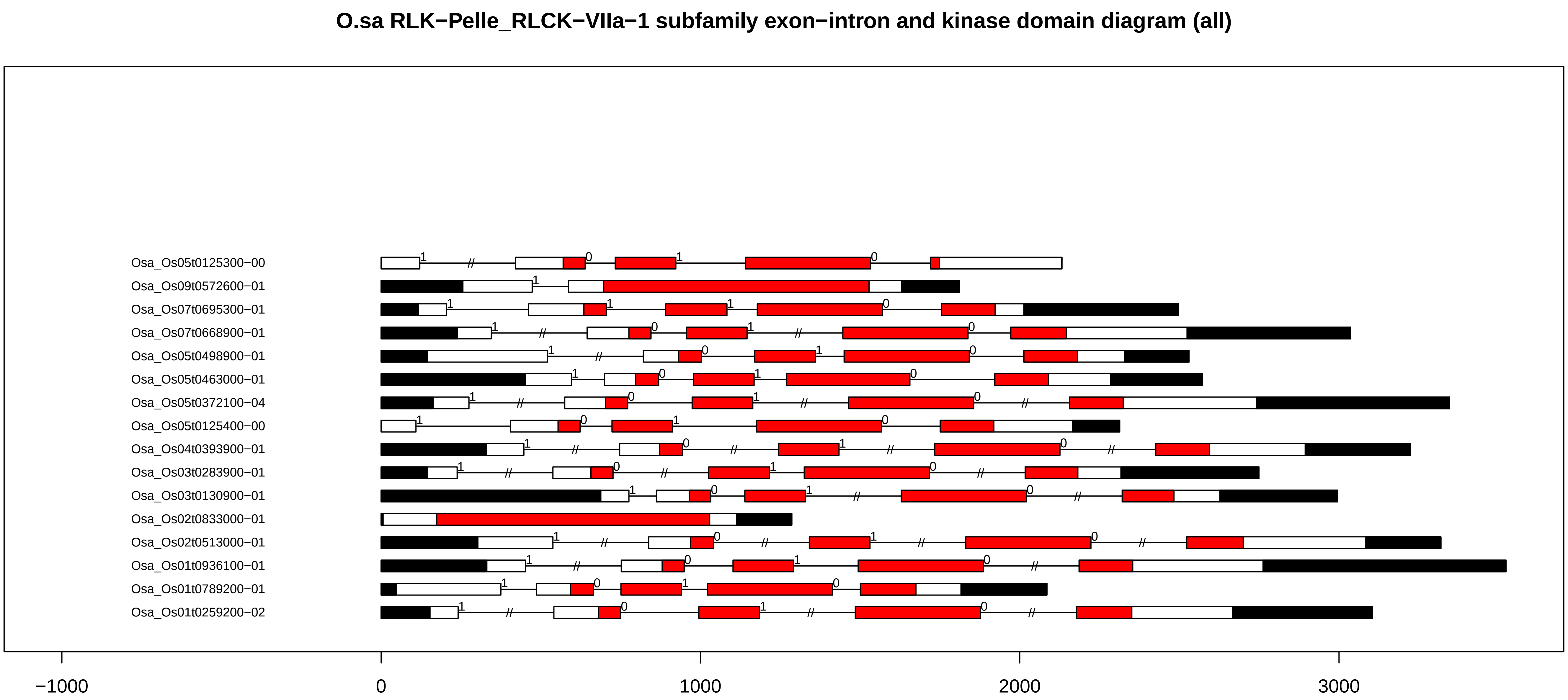

O.sa RLK−Pelle_RLCK−VIIa−1 subfamily exon−intron and kinase domain diagram (all)
Osa_Os05t0125300−00
Osa_Os09t0572600−01
Osa_Os07t0695300−01
Osa_Os07t0668900−01
Osa_Os05t0498900−01
Osa_Os05t0463000−01
Osa_Os05t0372100−04
Osa_Os05t0125400−00
Osa_Os04t0393900−01
Osa_Os03t0283900−01
Osa_Os03t0130900−01
Osa_Os02t0833000−01
Osa_Os02t0513000−01
Osa_Os01t0936100−01
Osa_Os01t0789200−01
Osa_Os01t0259200−02
−1000
0
1000
2000
3000

O.sa RLK−Pelle_RLCK−VIIa−2 subfamily exon−intron and kinase domain diagram (part 1)

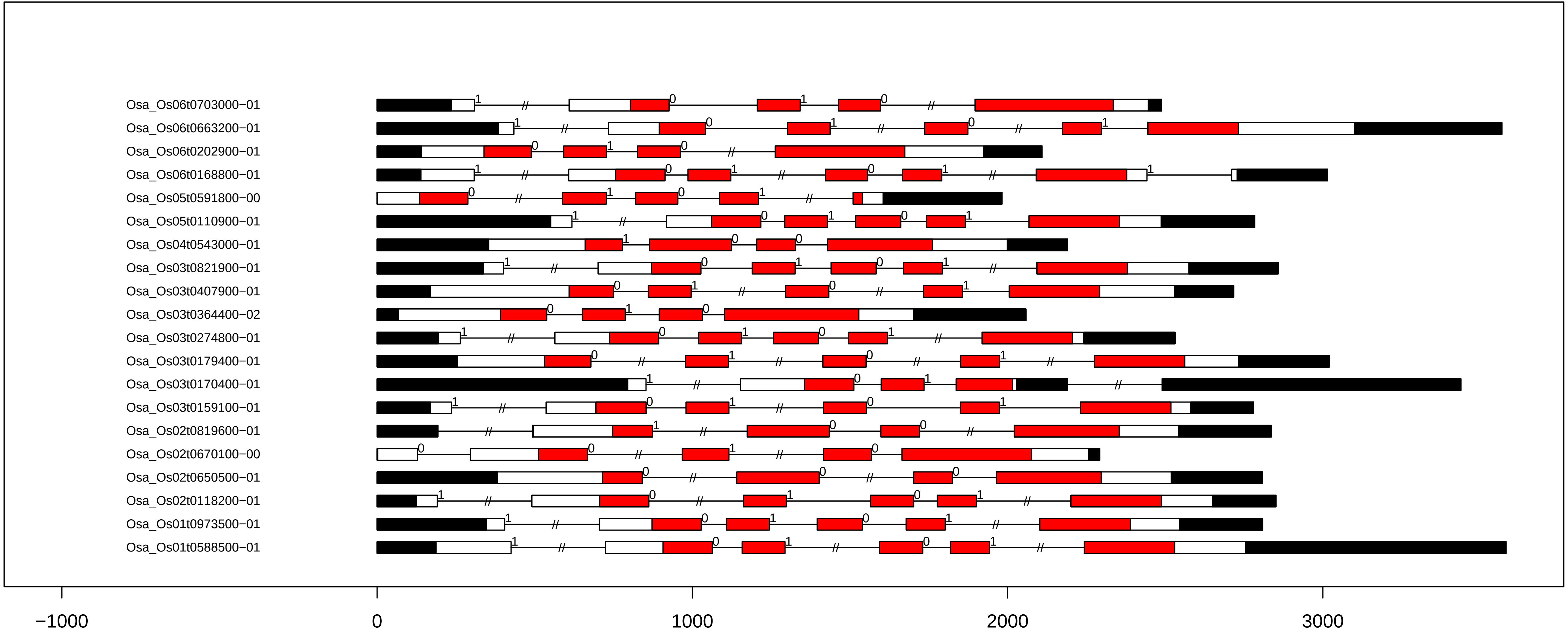

O.sa RLK−Pelle_RLCK−VIIa−2 subfamily exon−intron and kinase domain diagram (part 2)

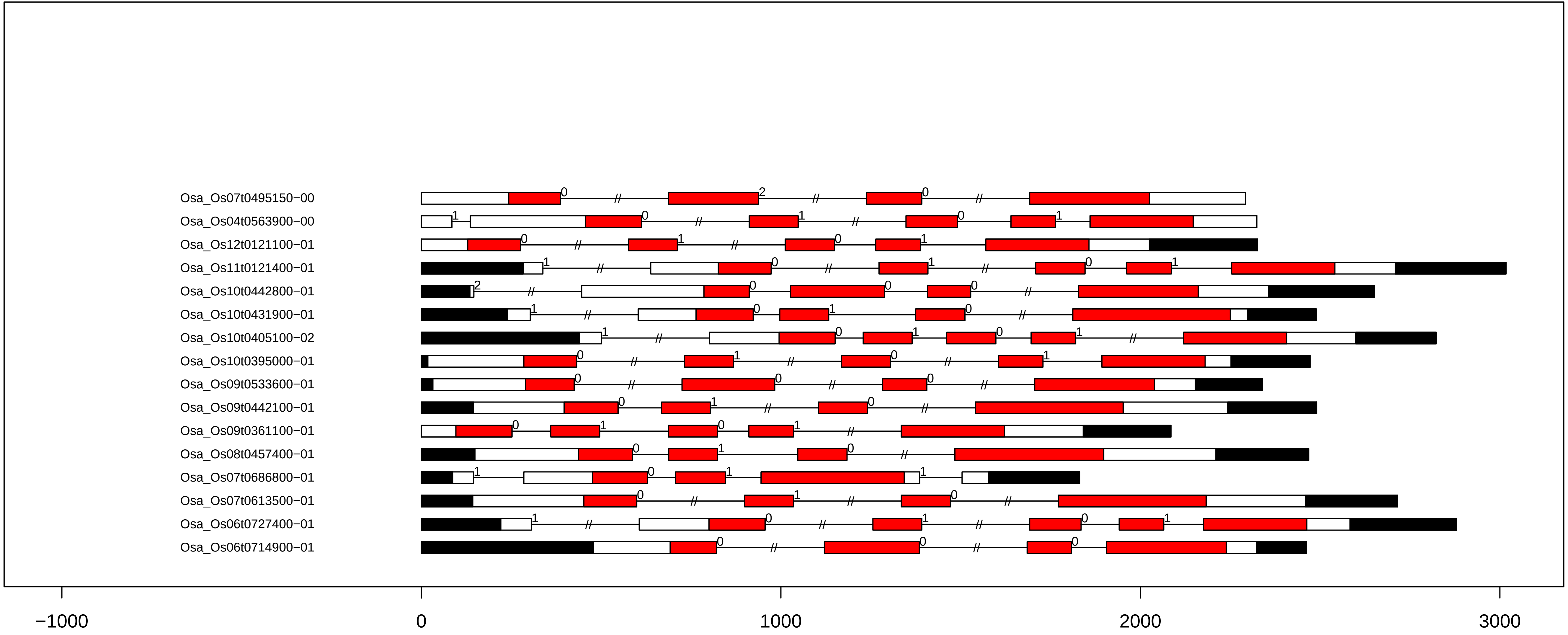

O.sa RLK−Pelle_RLCK−VIIb subfamily exon−intron and kinase domain diagram (all)

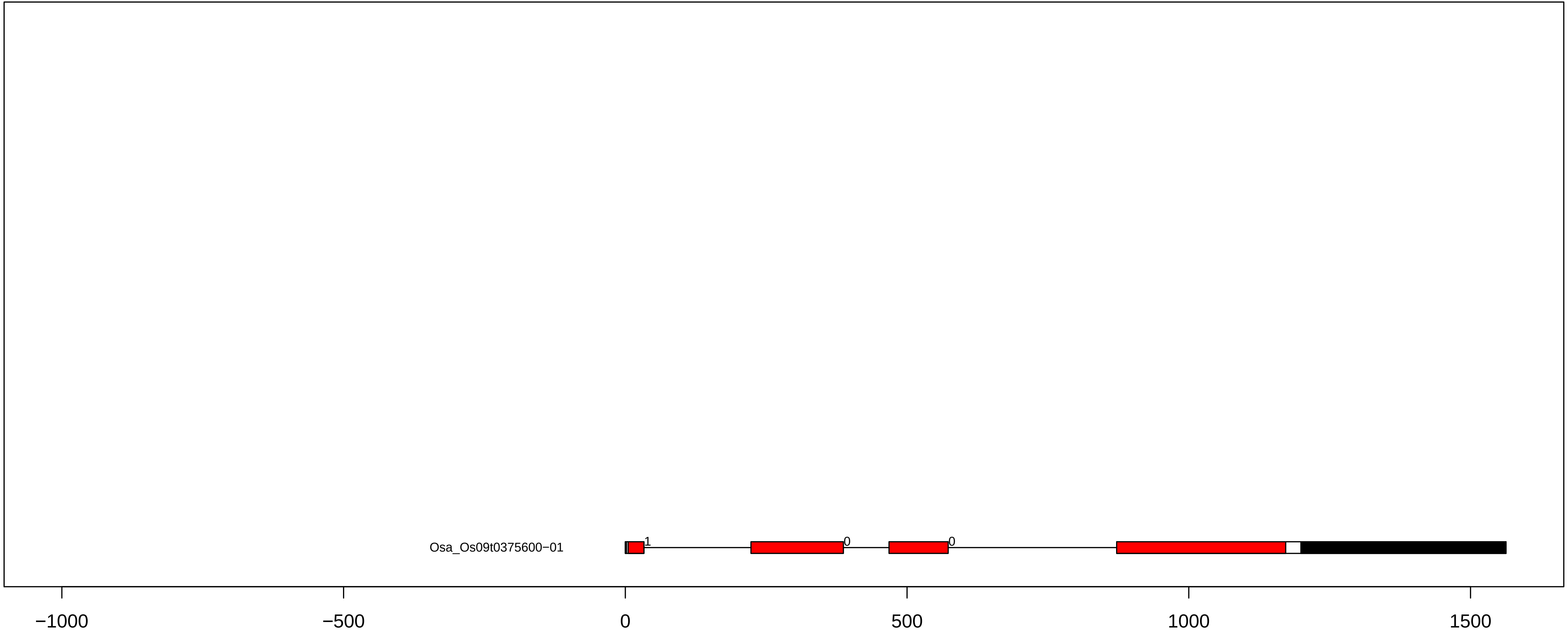

O.sa RLK−Pelle_RLCK−VIII subfamily exon−intron and kinase domain diagram (all)

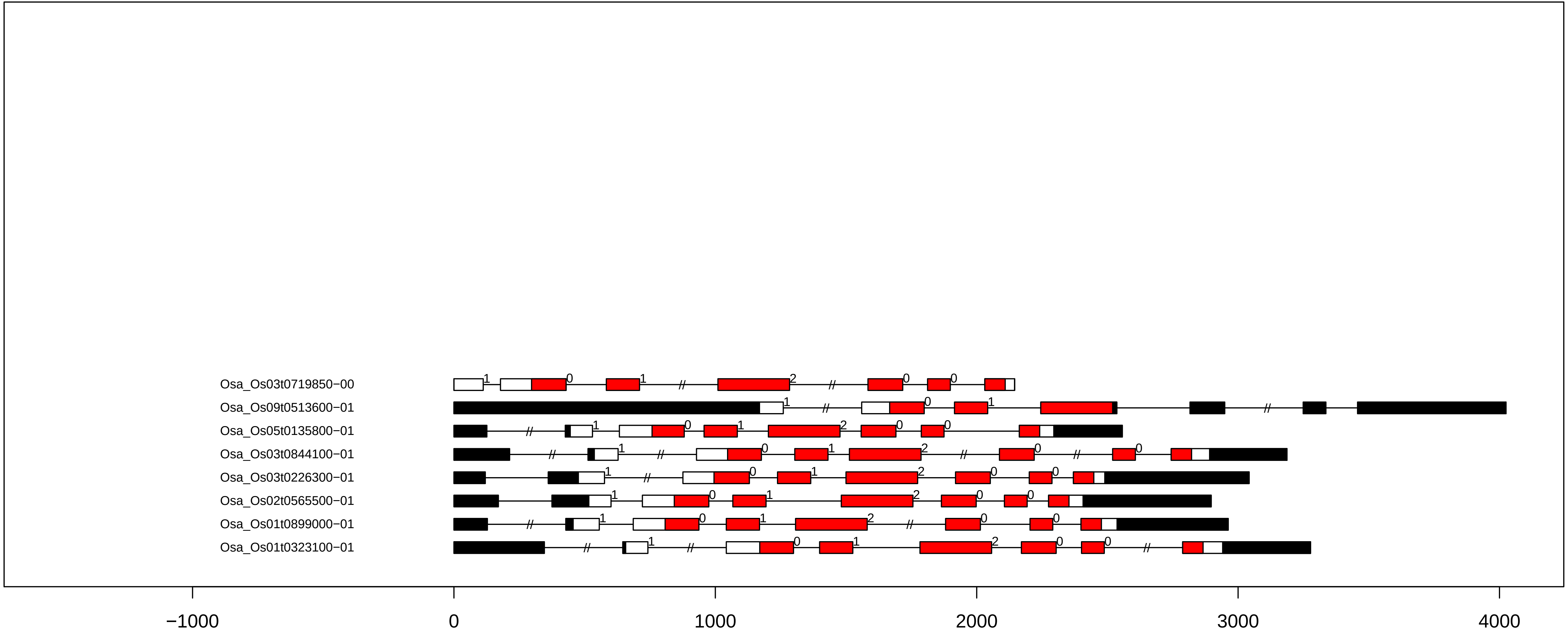

O.sa RLK−Pelle_RLCK−VI subfamily exon−intron and kinase domain diagram (all)

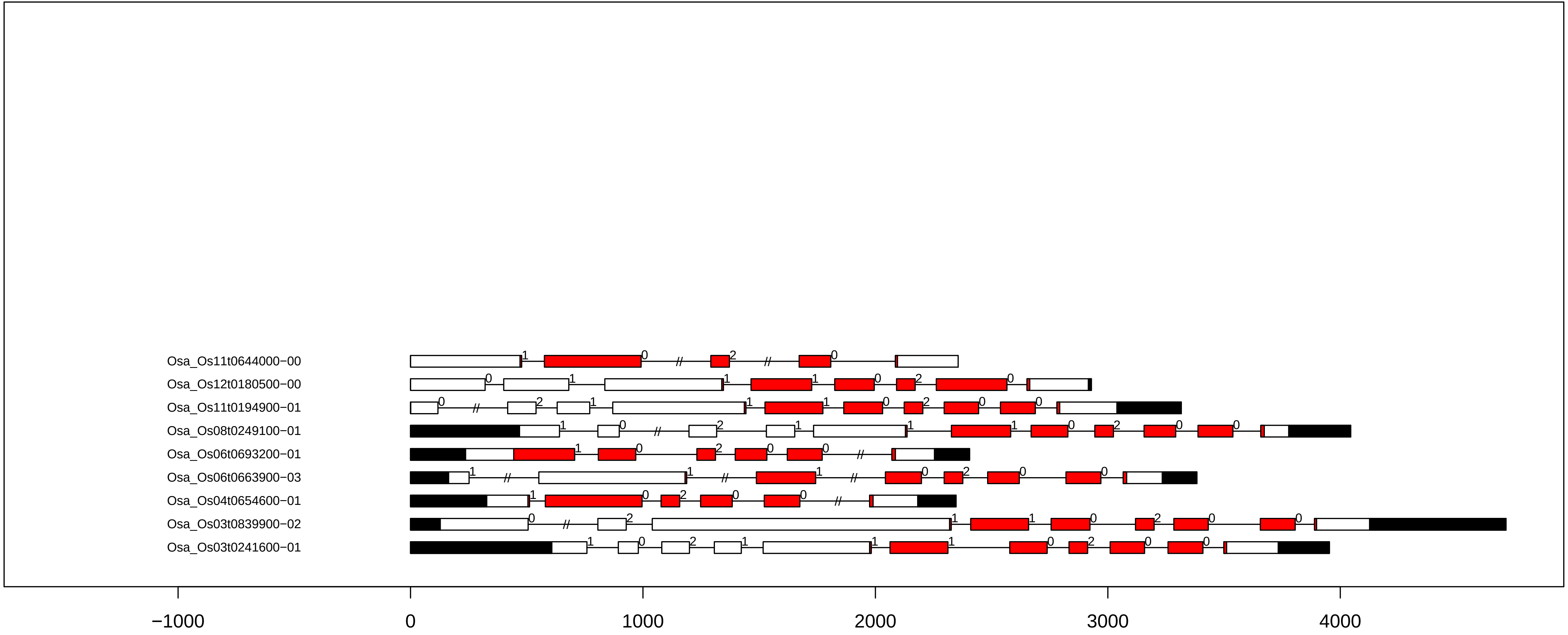

O.sa RLK−Pelle_RLCK−V subfamily exon−intron and kinase domain diagram (all)

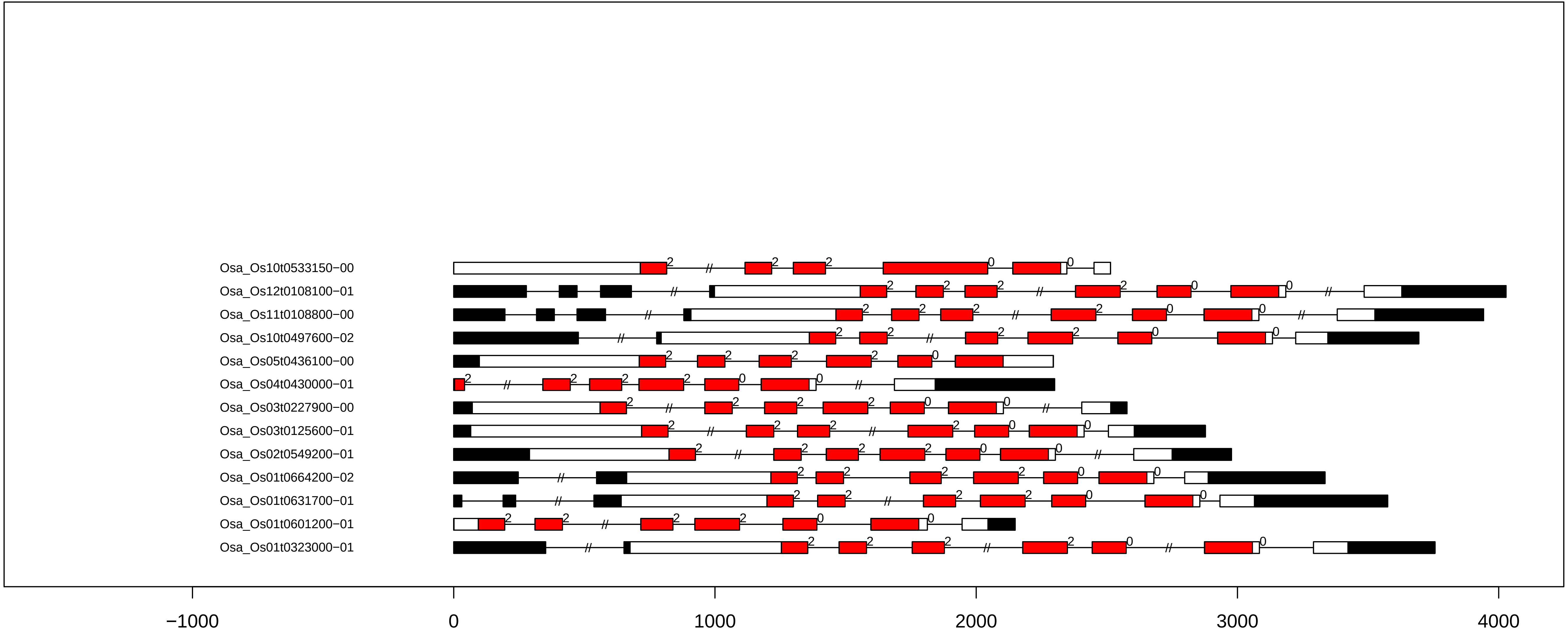

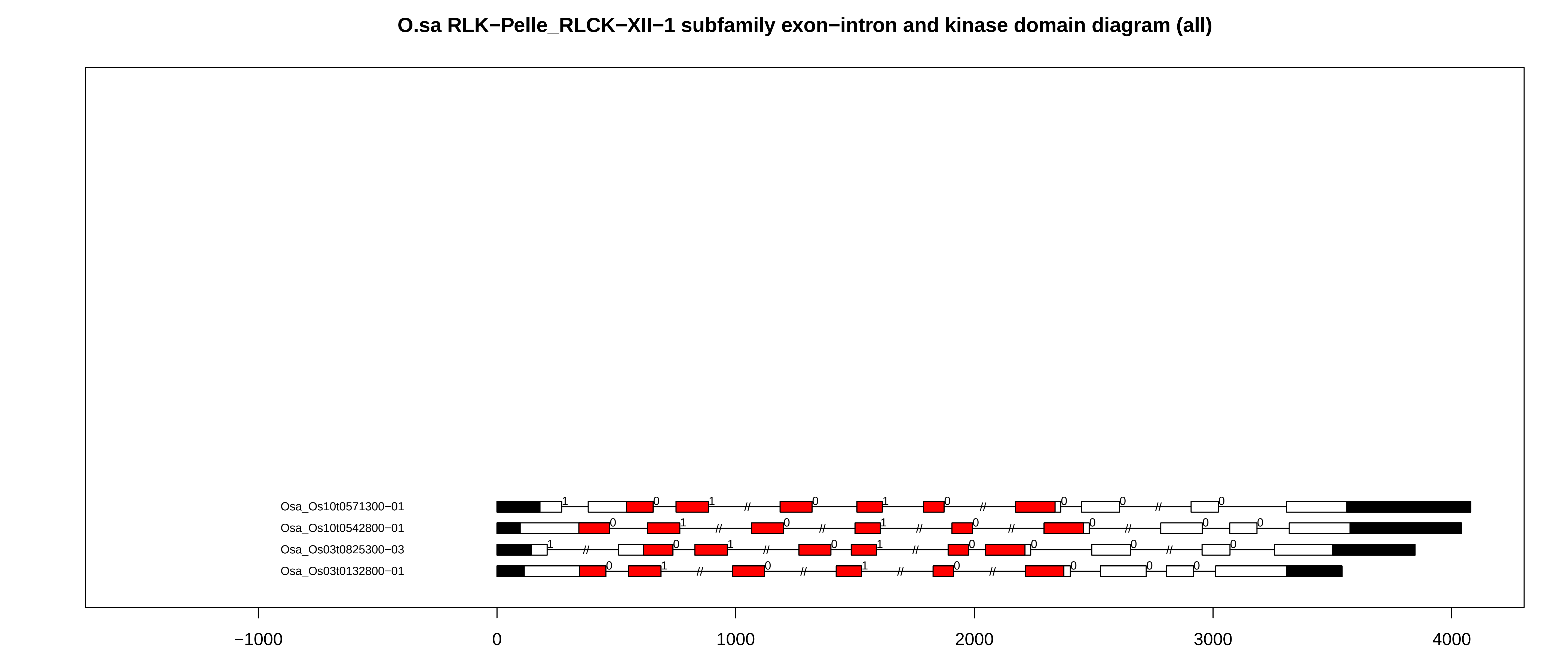

O.sa RLK−Pelle_RLCK−XII−1 subfamily exon−intron and kinase domain diagram (all)
Osa_Os10t0571300−01
Osa_Os10t0542800−01
Osa_Os03t0825300−03
Osa_Os03t0132800−01
−1000
0
1000
2000
3000
4000

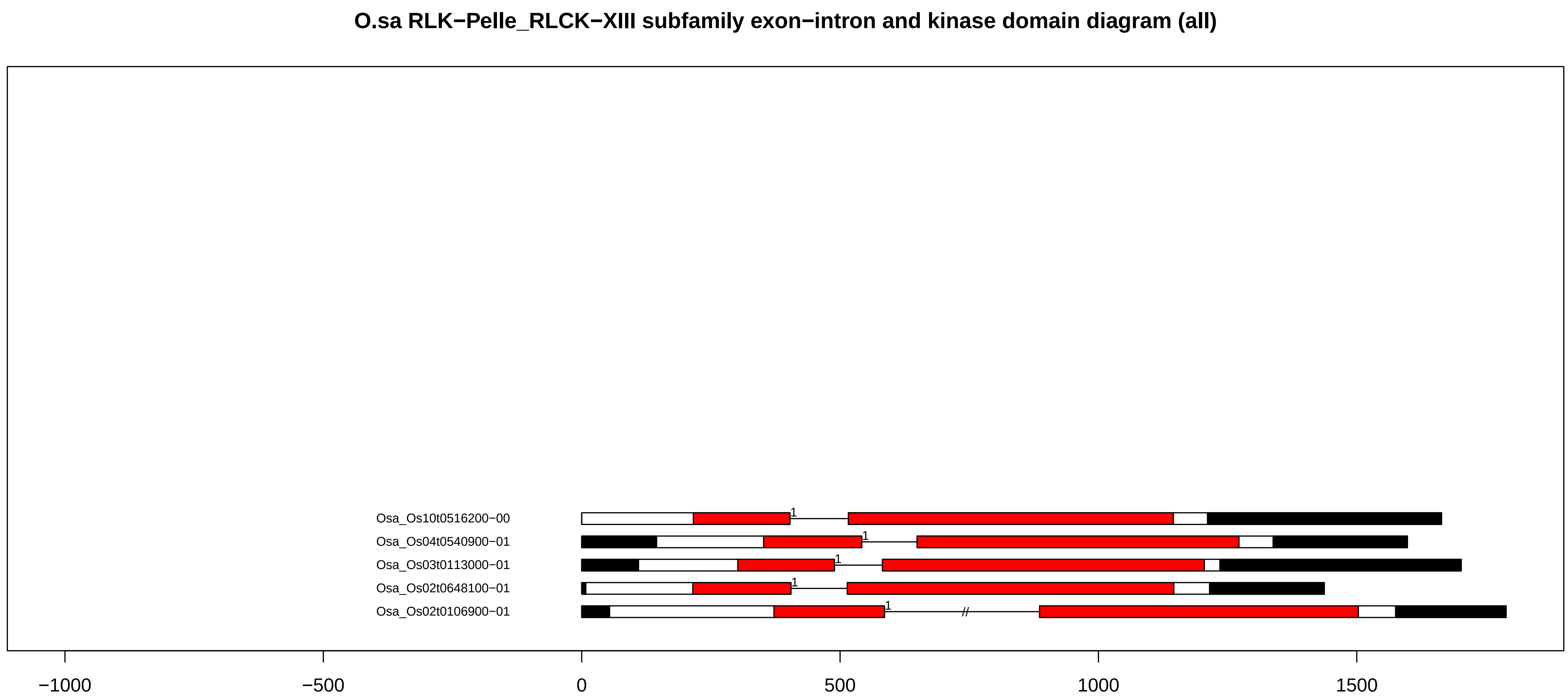
O.sa RLK−Pelle_RLCK−XIII subfamily exon−intron and kinase domain diagram (all)
Osa_Os10t0516200−00
Osa_Os04t0540900−01
Osa_Os03t0113000−01
Osa_Os02t0648100−01
Osa_Os02t0106900−01
1
1
1
1
1
−1000
−500
0
500
1000
1500

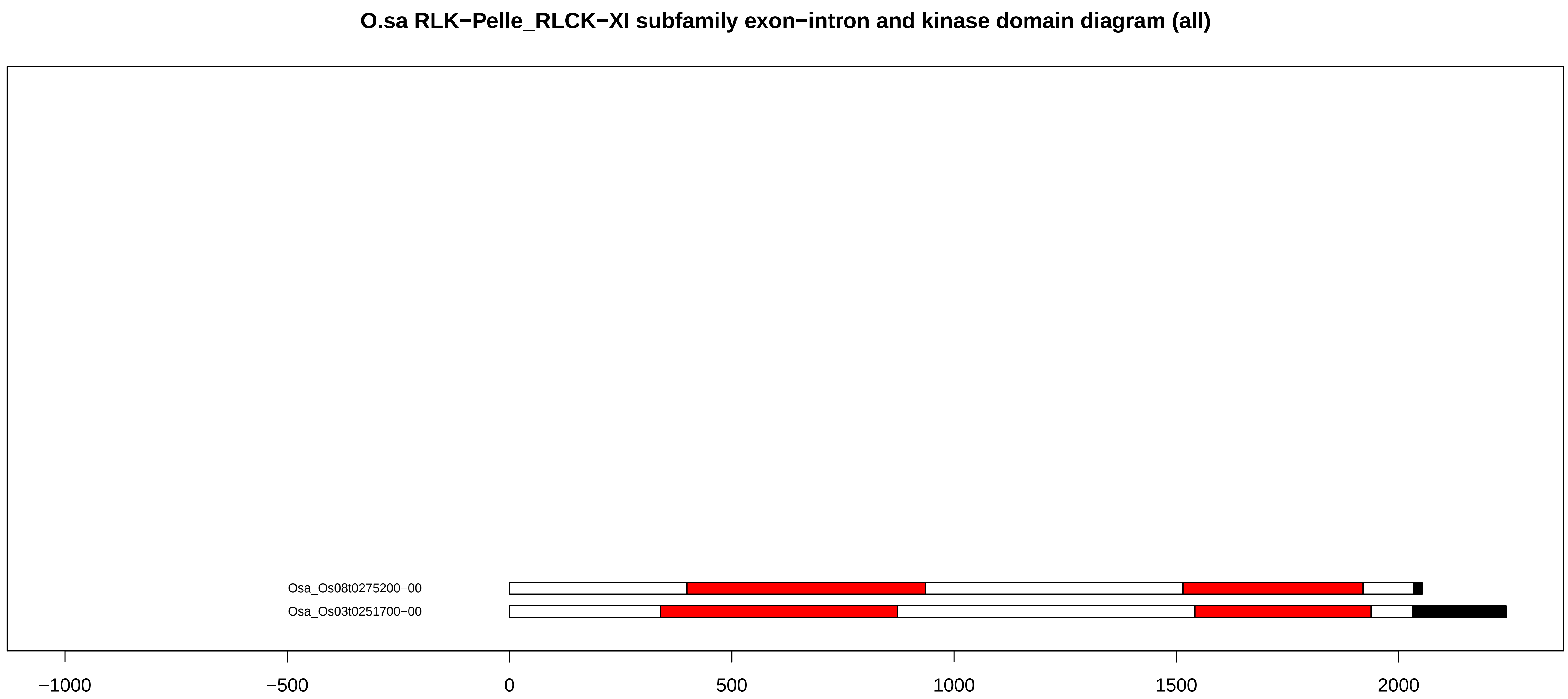
O.sa RLK−Pelle_RLCK−XI subfamily exon−intron and kinase domain diagram (all)
Osa_Os08t0275200−00
Osa_Os03t0251700−00
−1000
−500
0
500
1000
1500
2000

O.sa RLK−Pelle_RLCK−X subfamily exon−intron and kinase domain diagram (all)

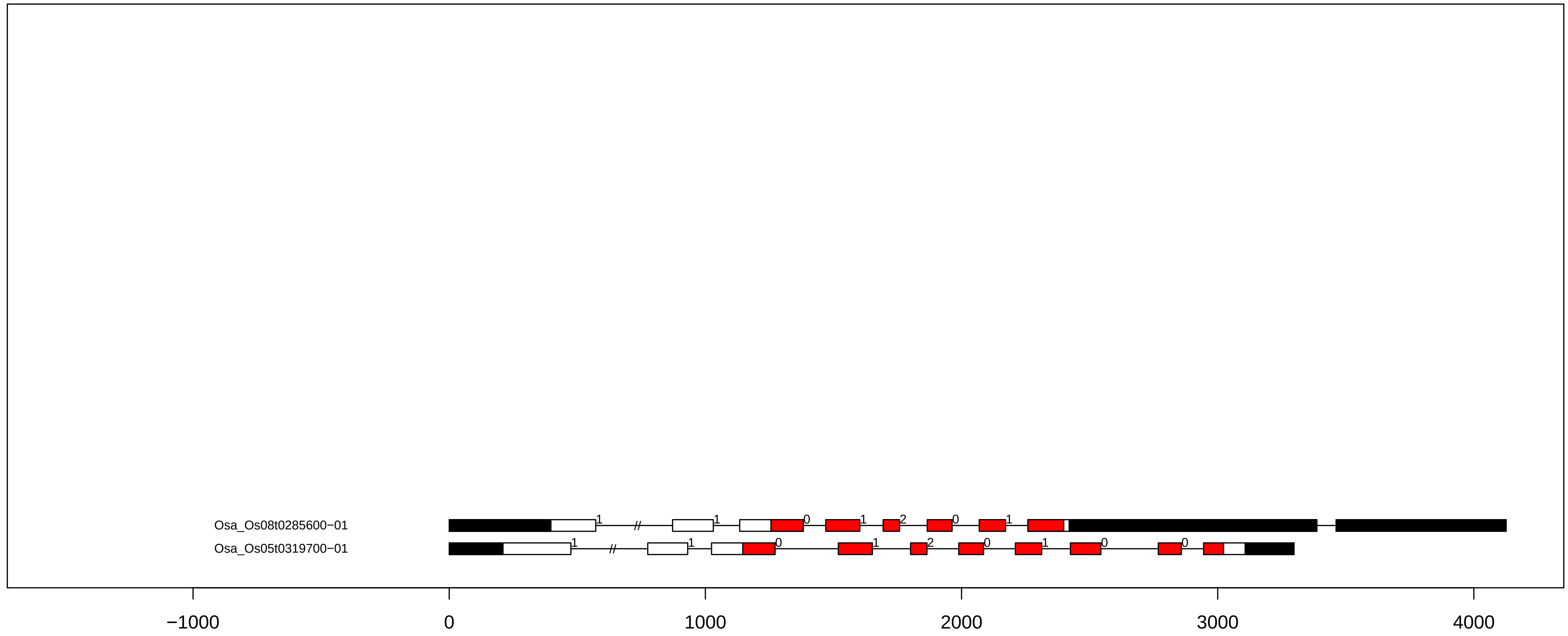

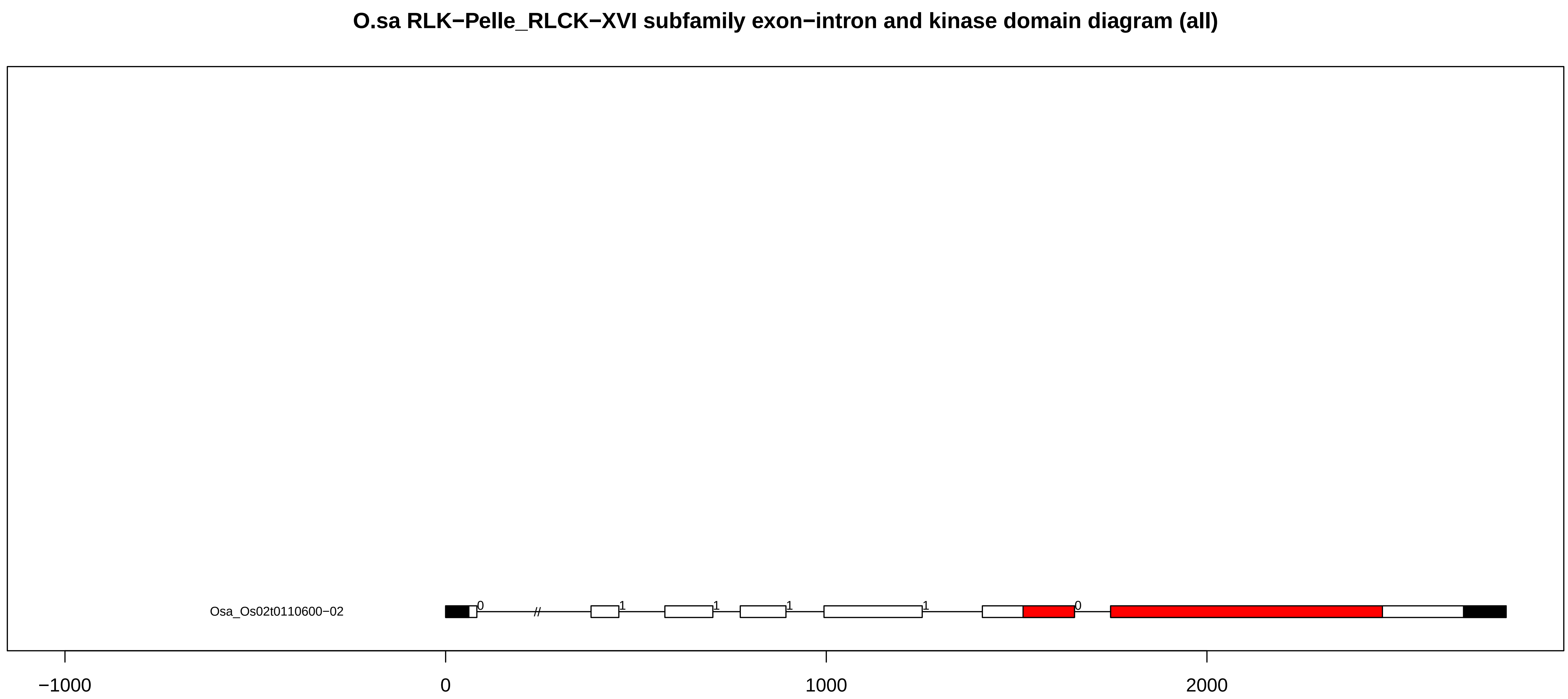

O.sa RLK−Pelle_RLCK−XVI subfamily exon−intron and kinase domain diagram (all)
Osa_Os02t0110600−02
0
1
1
1
1
0
−1000
0
1000
2000

O.sa RLK−Pelle_RLCK−XV subfamily exon−intron and kinase domain diagram (all)

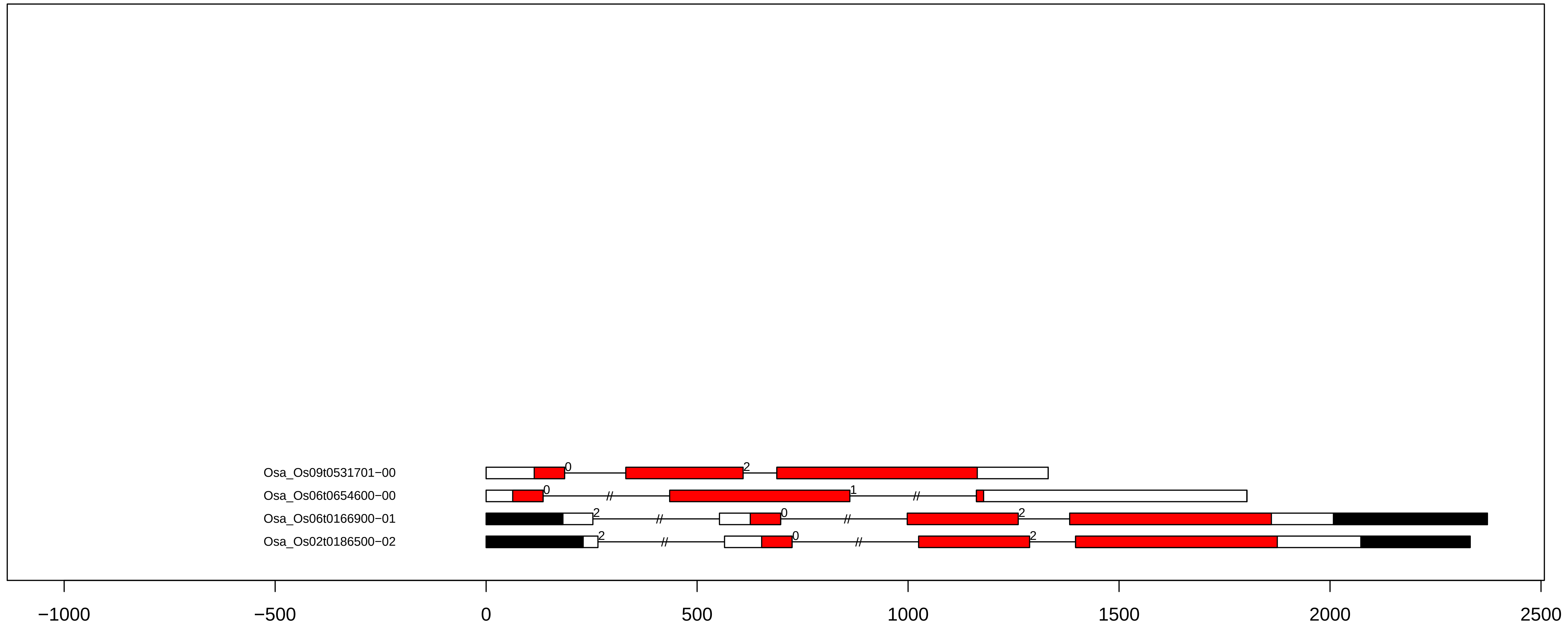

O.sa RLK−Pelle_SD−2b subfamily exon−intron and kinase domain diagram (part 1)

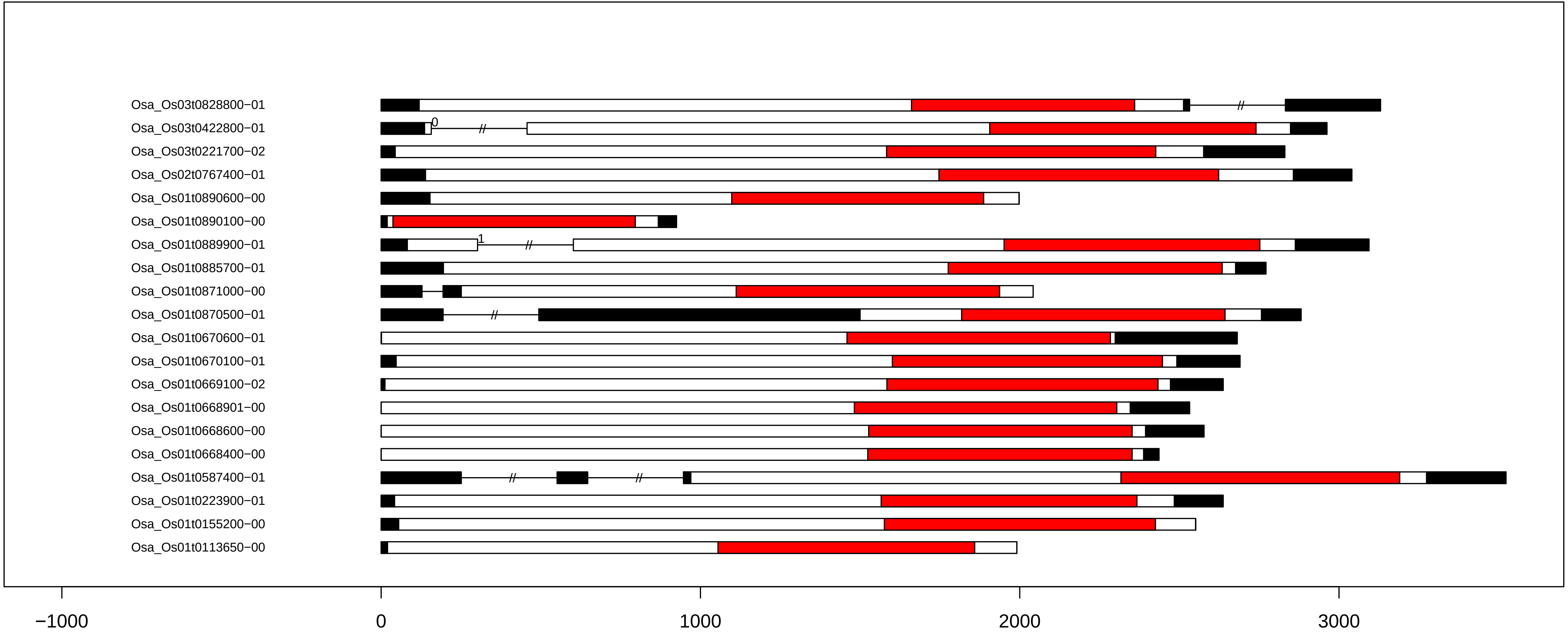

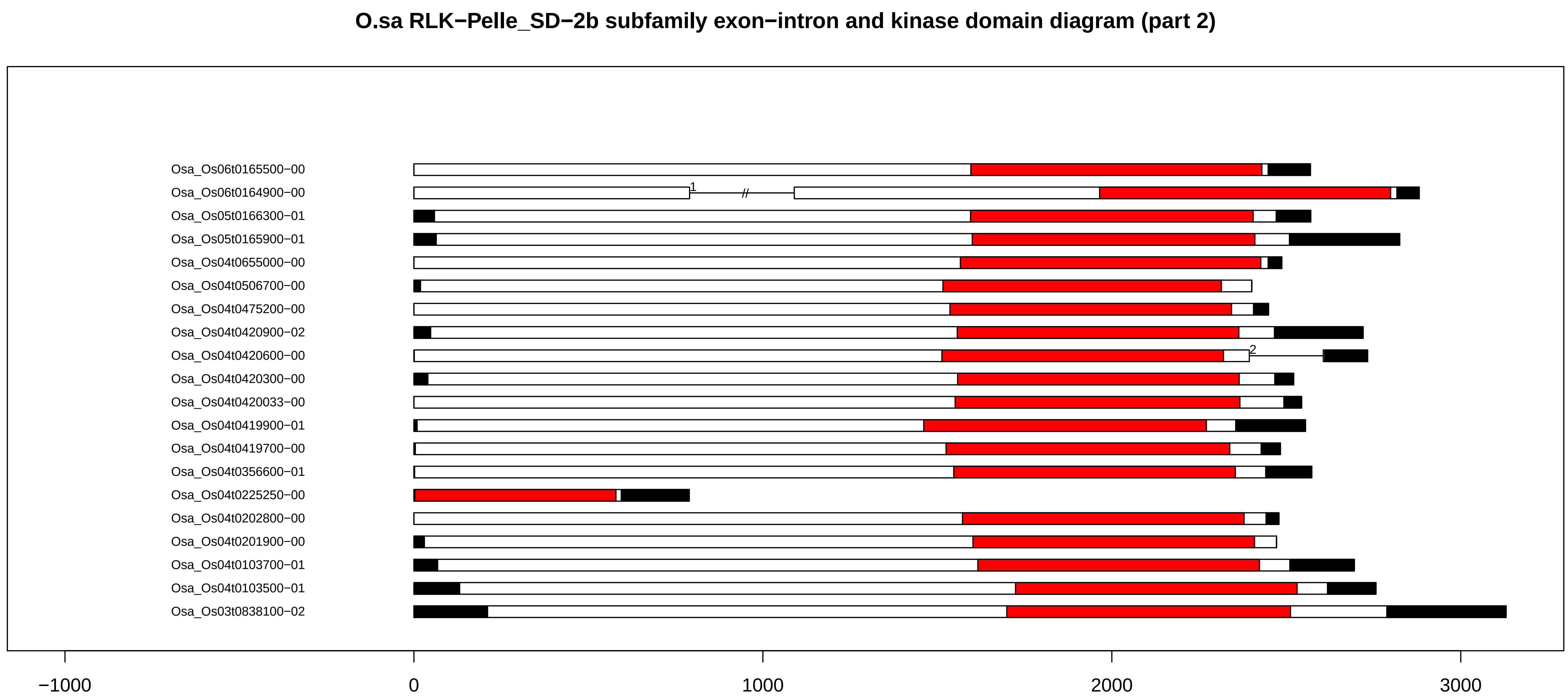

O.sa RLK−Pelle_SD−2b subfamily exon−intron and kinase domain diagram (part 2)
Osa_Os06t0165500−00
Osa_Os06t0164900−00
Osa_Os05t0166300−01
Osa_Os05t0165900−01
Osa_Os04t0655000−00
Osa_Os04t0506700−00
Osa_Os04t0475200−00
Osa_Os04t0420900−02
Osa_Os04t0420600−00
Osa_Os04t0420300−00
Osa_Os04t0420033−00
Osa_Os04t0419900−01
Osa_Os04t0419700−00
Osa_Os04t0356600−01
Osa_Os04t0225250−00
Osa_Os04t0202800−00
Osa_Os04t0201900−00
Osa_Os04t0103700−01
Osa_Os04t0103500−01
Osa_Os03t0838100−02
1
2
−1000
0
1000
2000
3000

O.sa RLK−Pelle_SD−2b subfamily exon−intron and kinase domain diagram (part 3)

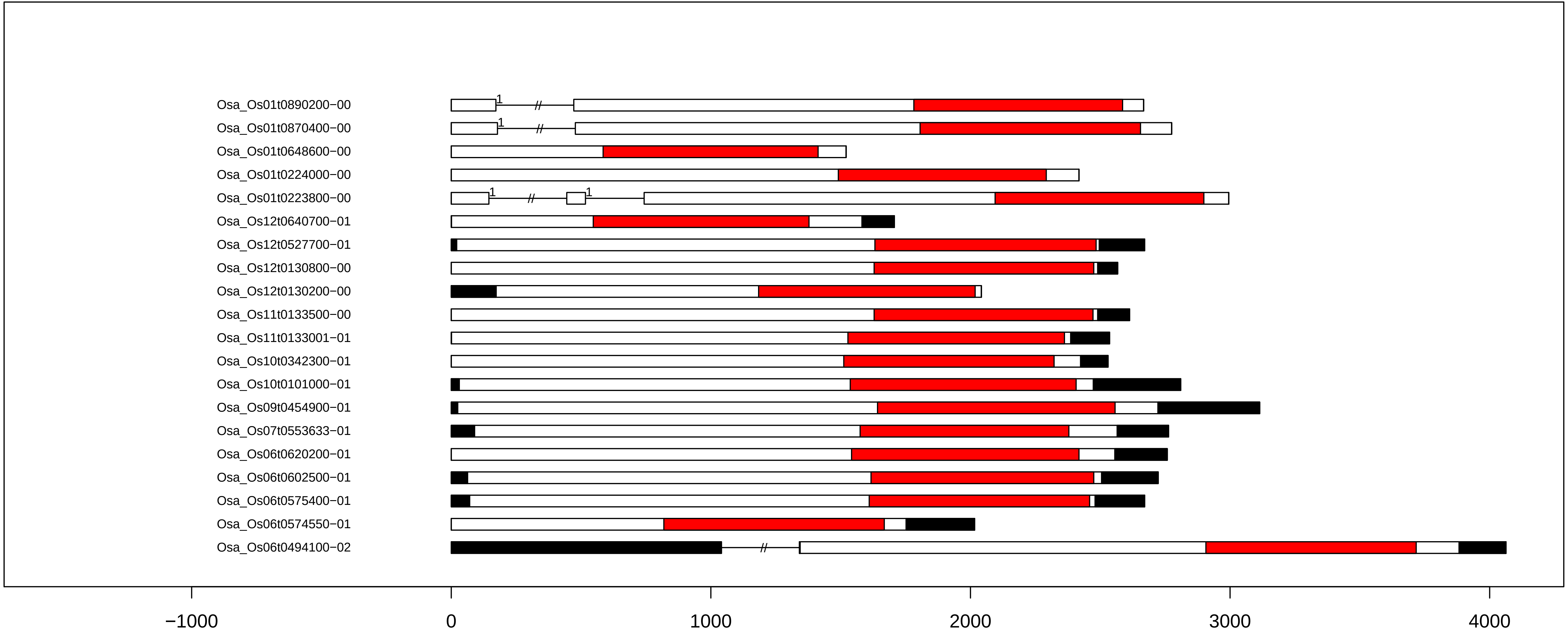

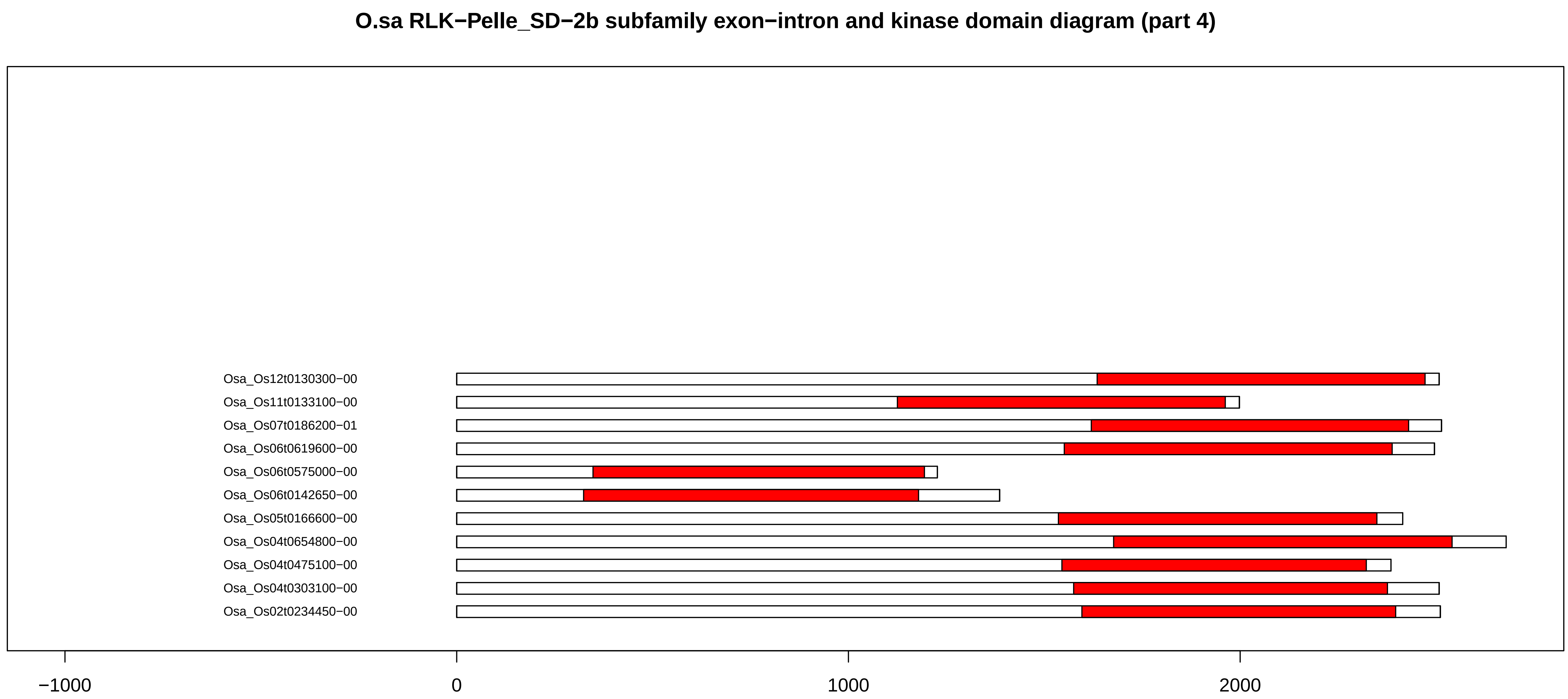
O.sa RLK−Pelle_SD−2b subfamily exon−intron and kinase domain diagram (part 4)
Osa_Os12t0130300−00
Osa_Os11t0133100−00
Osa_Os07t0186200−01
Osa_Os06t0619600−00
Osa_Os06t0575000−00
Osa_Os06t0142650−00
Osa_Os05t0166600−00
Osa_Os04t0654800−00
Osa_Os04t0475100−00
Osa_Os04t0303100−00
Osa_Os02t0234450−00
−1000
0
1000
2000

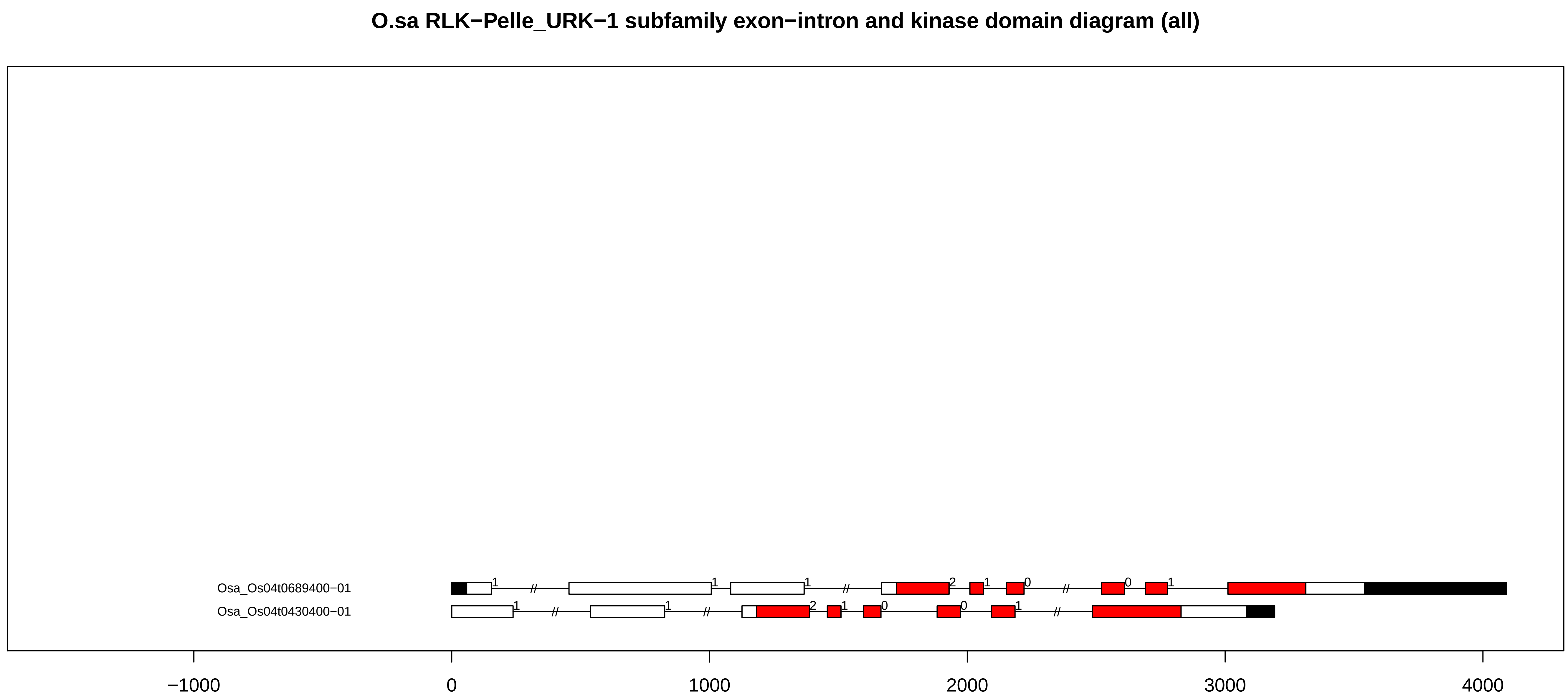
O.sa RLK−Pelle_URK−1 subfamily exon−intron and kinase domain diagram (all)
Osa_Os04t0689400−01
Osa_Os04t0430400−01
−1000
0
1000
2000
3000
4000

O.sa RLK−Pelle_URK−2 subfamily exon−intron and kinase domain diagram (all)

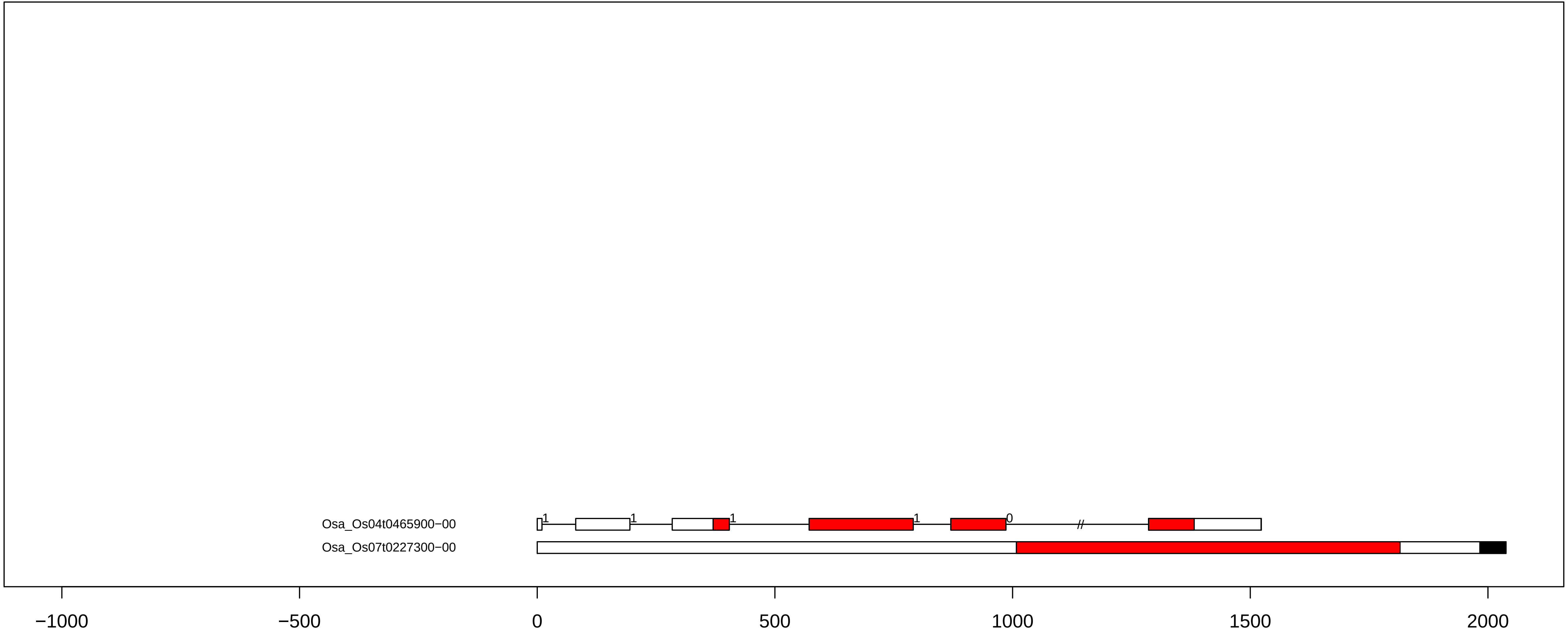

O.sa RLK−Pelle_URK−3 subfamily exon−intron and kinase domain diagram (all)

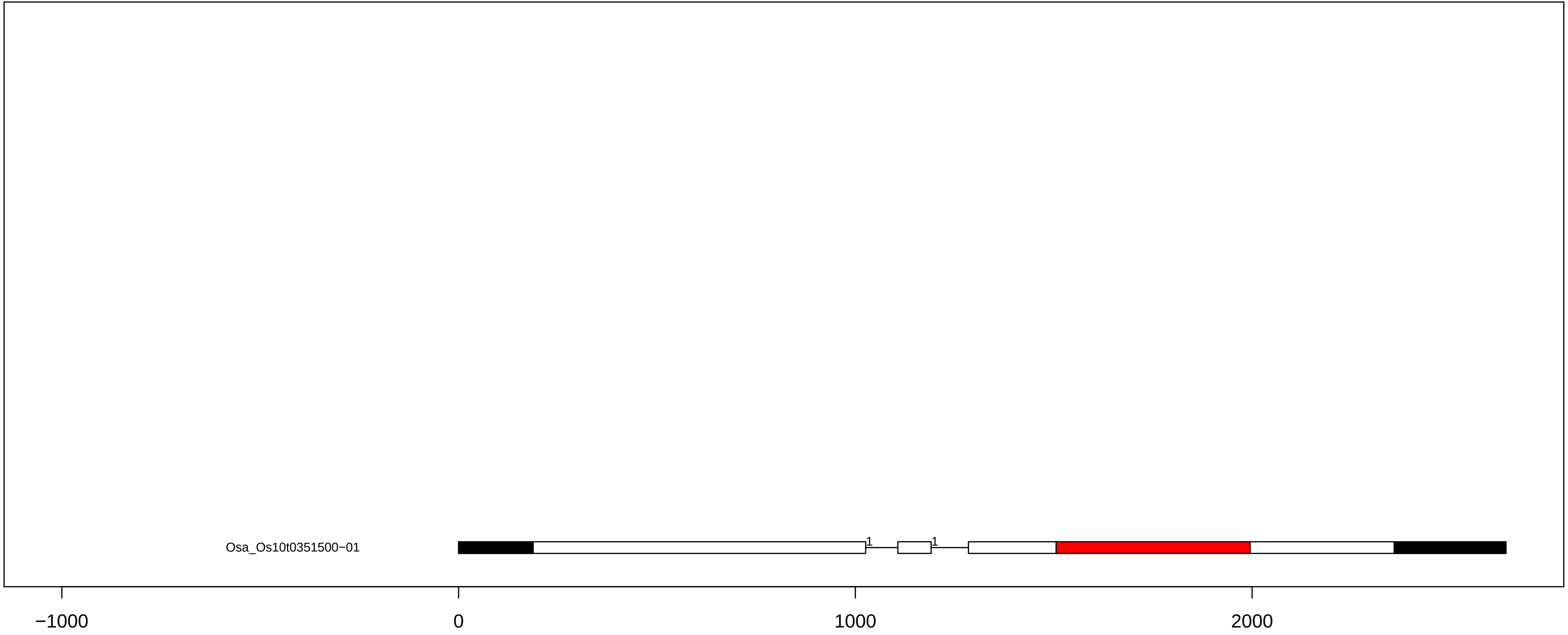

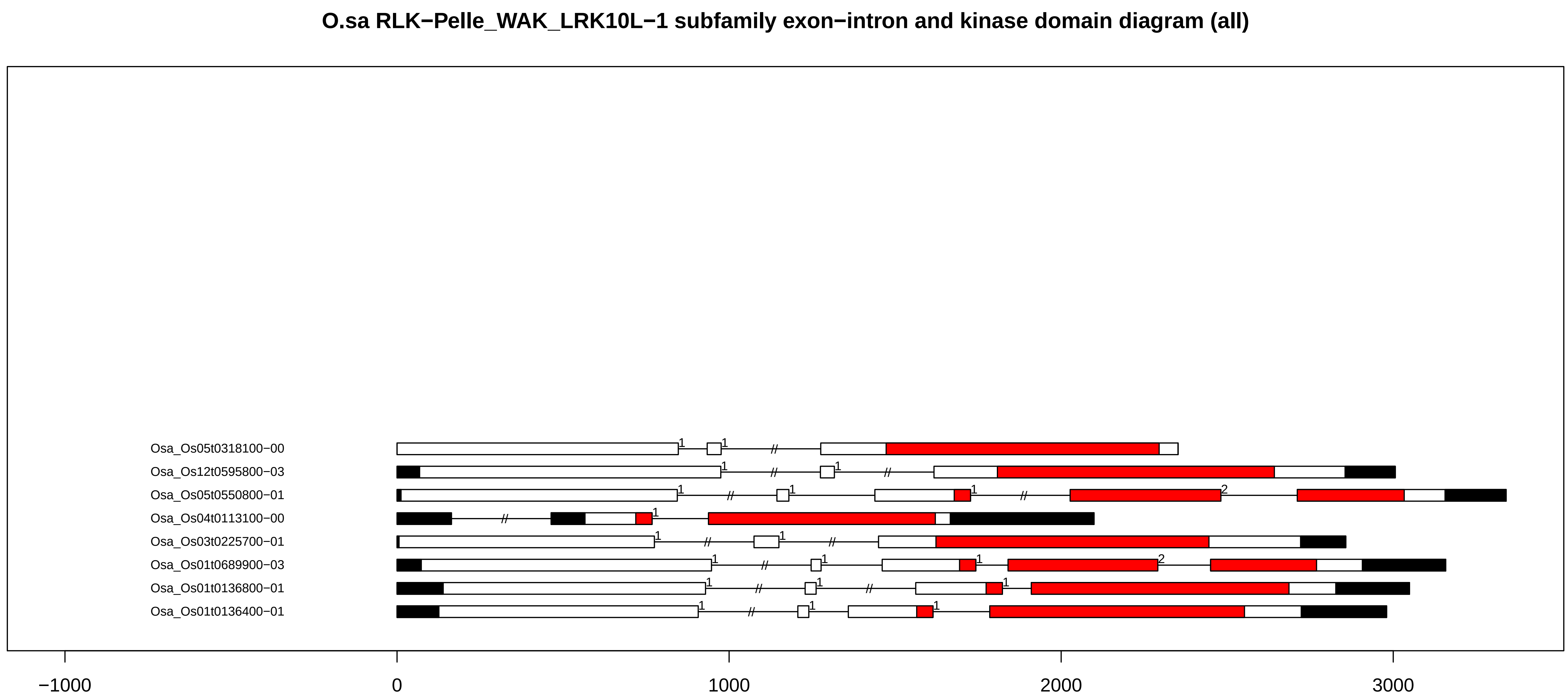
O.sa RLK−Pelle_WAK_LRK10L−1 subfamily exon−intron and kinase domain diagram (all)
Osa_Os05t0318100−00
Osa_Os12t0595800−03
Osa_Os05t0550800−01
Osa_Os04t0113100−00
Osa_Os03t0225700−01
Osa_Os01t0689900−03
Osa_Os01t0136800−01
Osa_Os01t0136400−01
−1000
0
1000
2000
3000

O.sa RLK−Pelle_WAK subfamily exon−intron and kinase domain diagram (part 1)

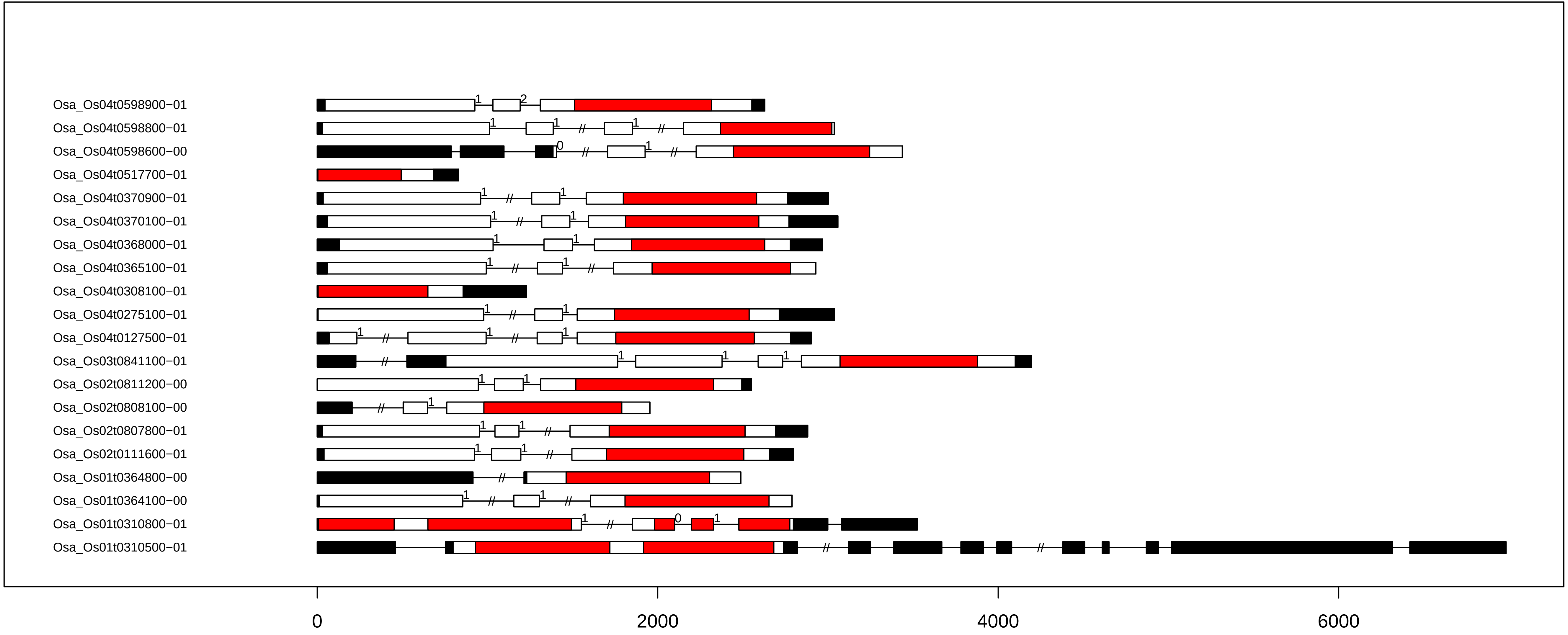

O.sa RLK−Pelle_WAK subfamily exon−intron and kinase domain diagram (part 2)

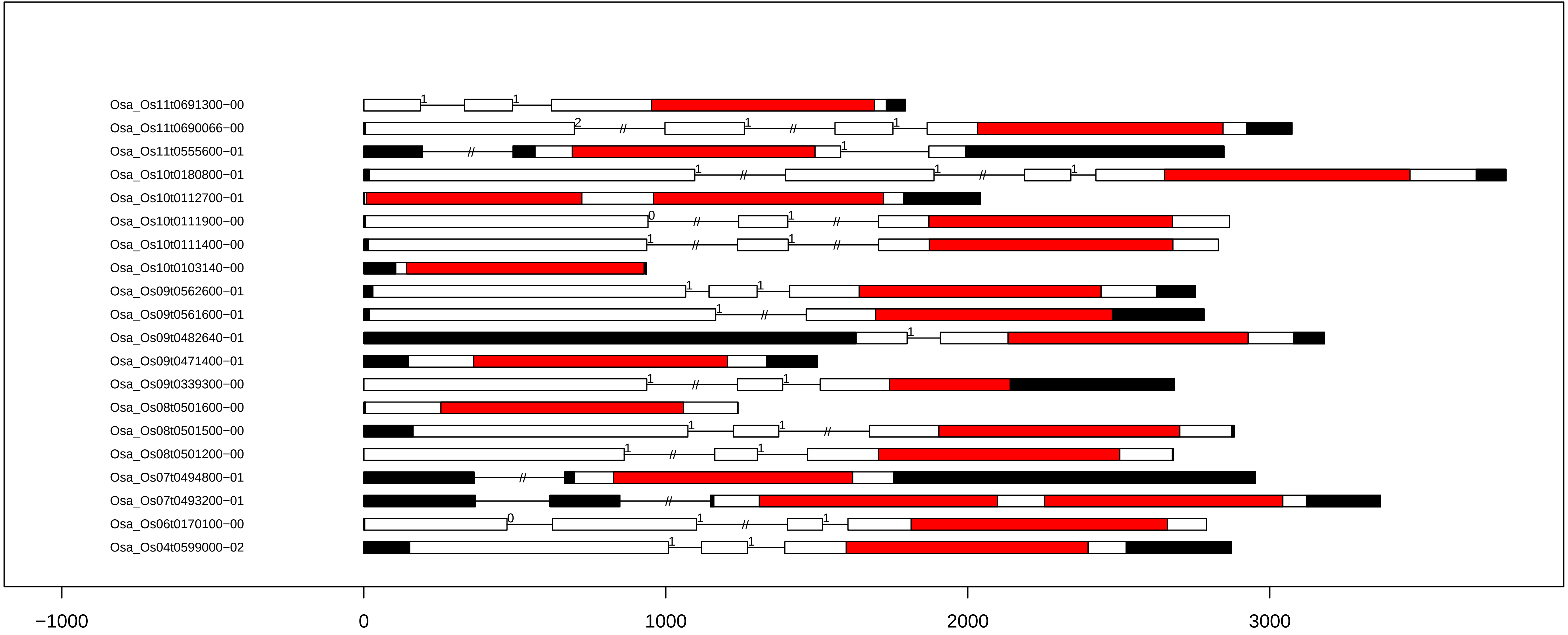

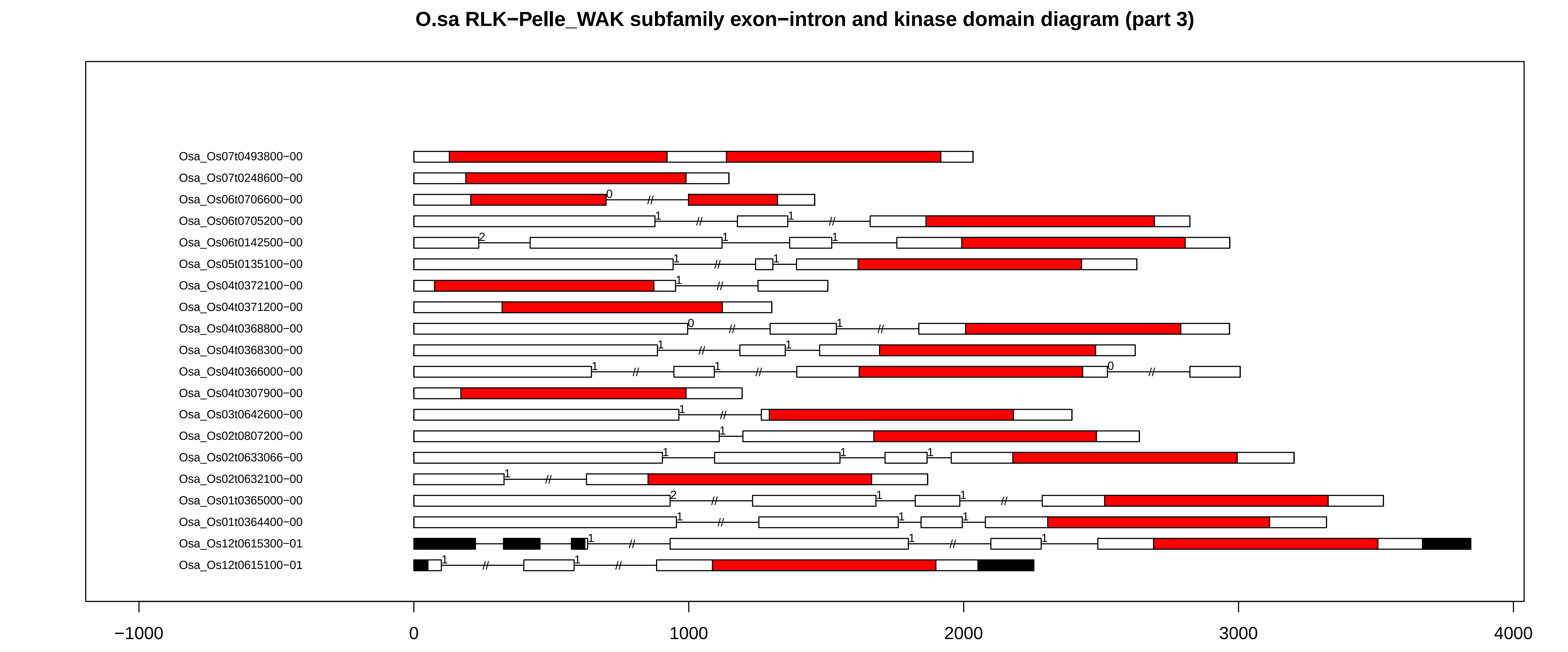

O.sa RLK−Pelle_WAK subfamily exon−intron and kinase domain diagram (part 3)
Osa_Os07t0493800−00
Osa_Os07t0248600−00
Osa_Os06t0706600−00
Osa_Os06t0705200−00
Osa_Os06t0142500−00
Osa_Os05t0135100−00
Osa_Os04t0372100−00
Osa_Os04t0371200−00
Osa_Os04t0368800−00
Osa_Os04t0368300−00
Osa_Os04t0366000−00
Osa_Os04t0307900−00
Osa_Os03t0642600−00
Osa_Os02t0807200−00
Osa_Os02t0633066−00
Osa_Os02t0632100−00
Osa_Os01t0365000−00
Osa_Os01t0364400−00
Osa_Os12t0615300−01
Osa_Os12t0615100−01
−1000
0
1000
2000
3000
4000

O.sa RLK−Pelle_WAK subfamily exon−intron and kinase domain diagram (part 4)

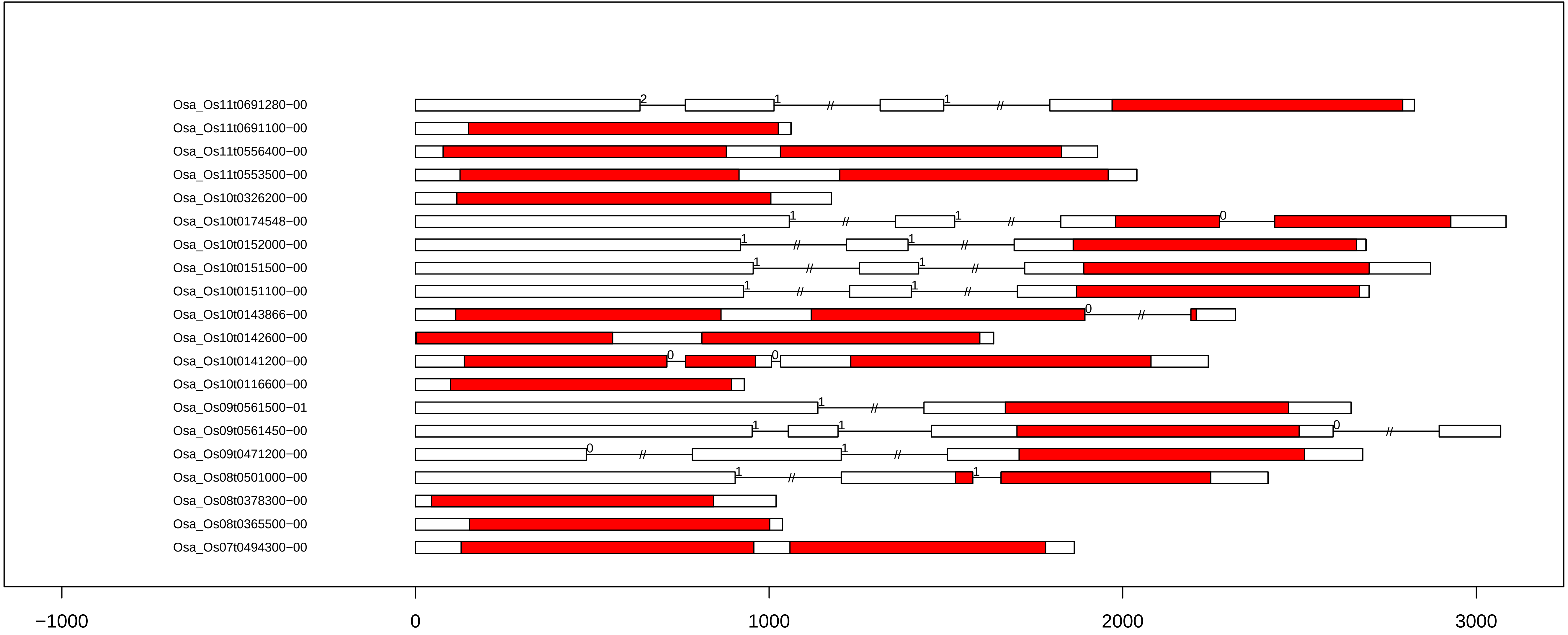

O.sa RLK−Pelle_WAK subfamily exon−intron and kinase domain diagram (part 5)

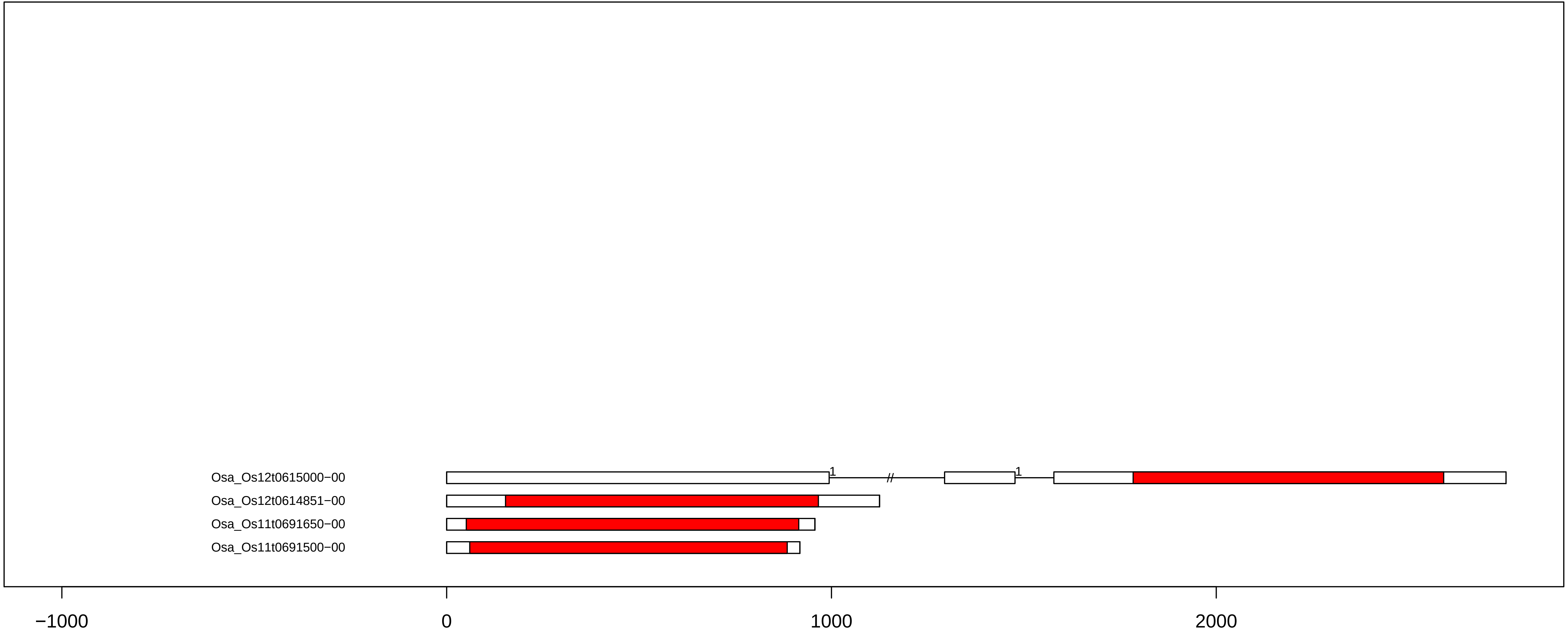

Z.ma RLK−Pelle_C−LEC subfamily exon−intron and kinase domain diagram (all)

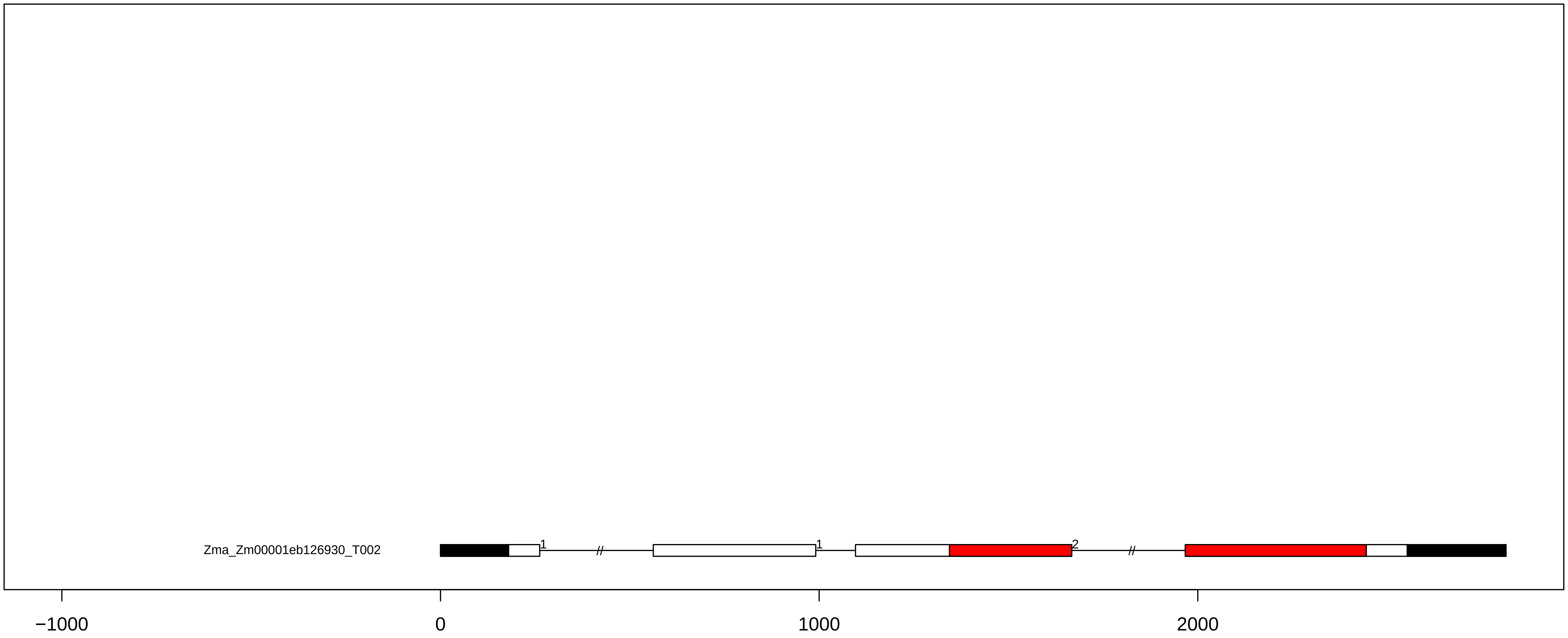

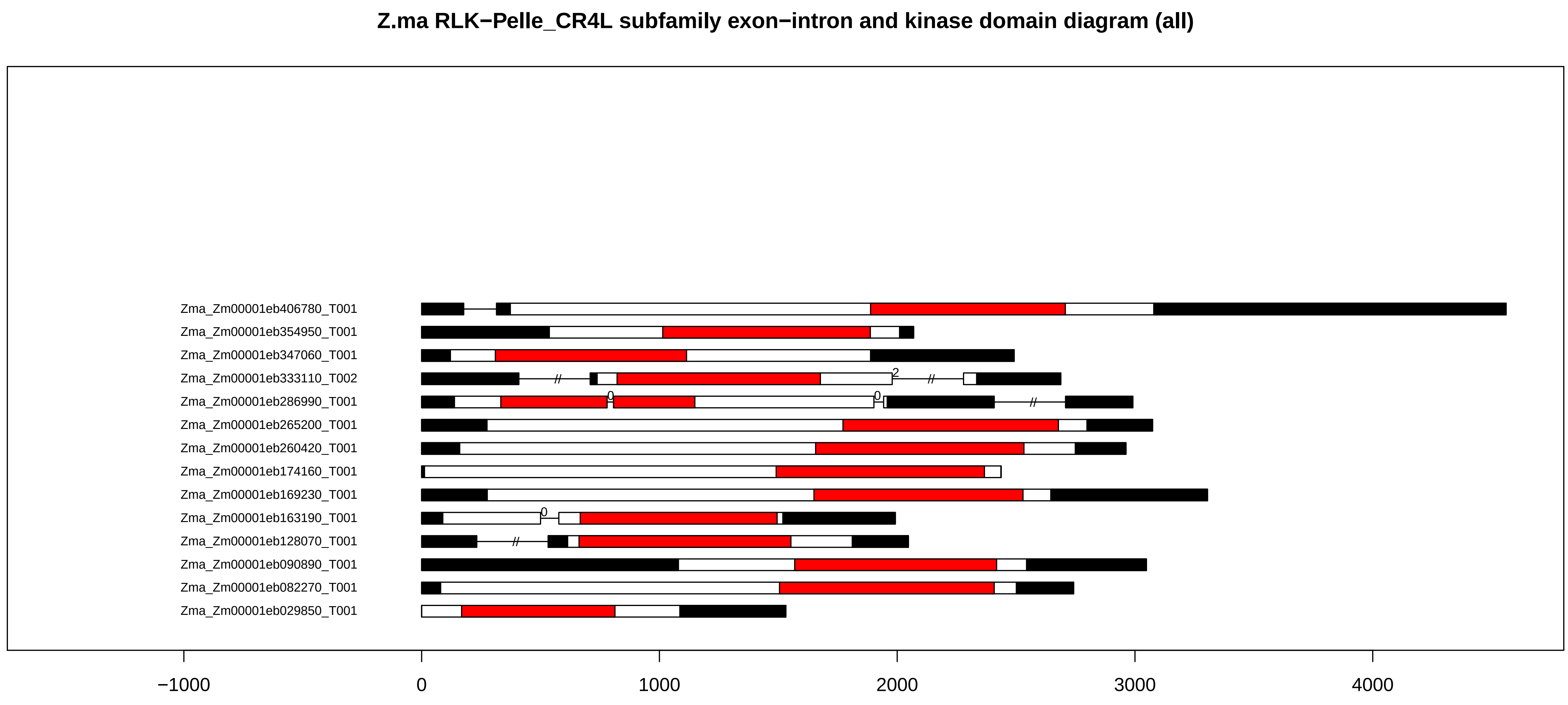
Z.ma RLK−Pelle_CR4L subfamily exon−intron and kinase domain diagram (all)
Zma_Zm00001eb406780_T001
Zma_Zm00001eb354950_T001
Zma_Zm00001eb347060_T001
Zma_Zm00001eb333110_T002
Zma_Zm00001eb286990_T001
Zma_Zm00001eb265200_T001
Zma_Zm00001eb260420_T001
Zma_Zm00001eb174160_T001
Zma_Zm00001eb169230_T001
Zma_Zm00001eb163190_T001
Zma_Zm00001eb128070_T001
Zma_Zm00001eb090890_T001
Zma_Zm00001eb082270_T001
Zma_Zm00001eb029850_T001
2
0
0
0
−1000
0
1000
2000
3000
4000

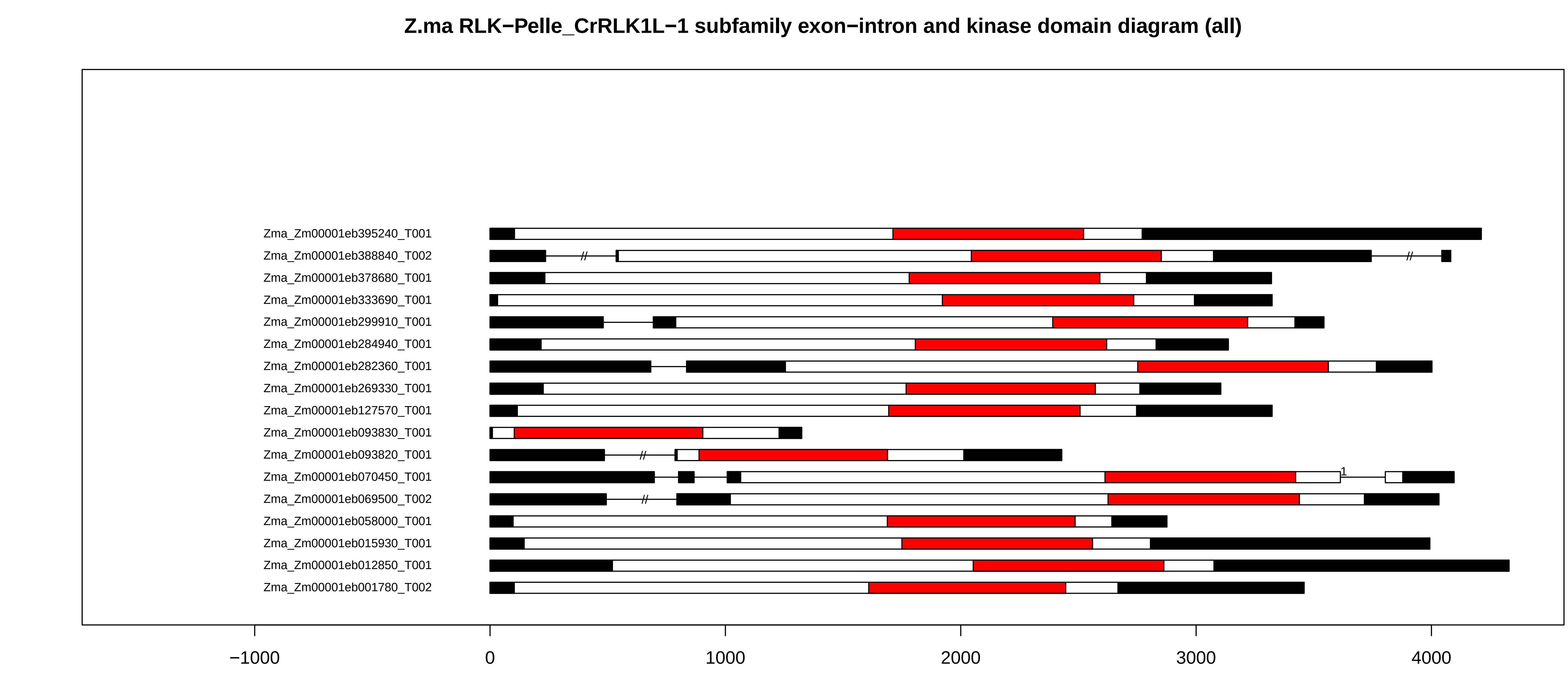
Z.ma RLK−Pelle_CrRLK1L−1 subfamily exon−intron and kinase domain diagram (all)
Zma_Zm00001eb395240_T001
Zma_Zm00001eb388840_T002
Zma_Zm00001eb378680_T001
Zma_Zm00001eb333690_T001
Zma_Zm00001eb299910_T001
Zma_Zm00001eb284940_T001
Zma_Zm00001eb282360_T001
Zma_Zm00001eb269330_T001
Zma_Zm00001eb127570_T001
Zma_Zm00001eb093830_T001
Zma_Zm00001eb093820_T001
Zma_Zm00001eb070450_T001
Zma_Zm00001eb069500_T002
Zma_Zm00001eb058000_T001
Zma_Zm00001eb015930_T001
Zma_Zm00001eb012850_T001
Zma_Zm00001eb001780_T002
1
−1000
0
1000
2000
3000
4000

Z.ma RLK−Pelle_DLSV subfamily exon−intron and kinase domain diagram (part 1)

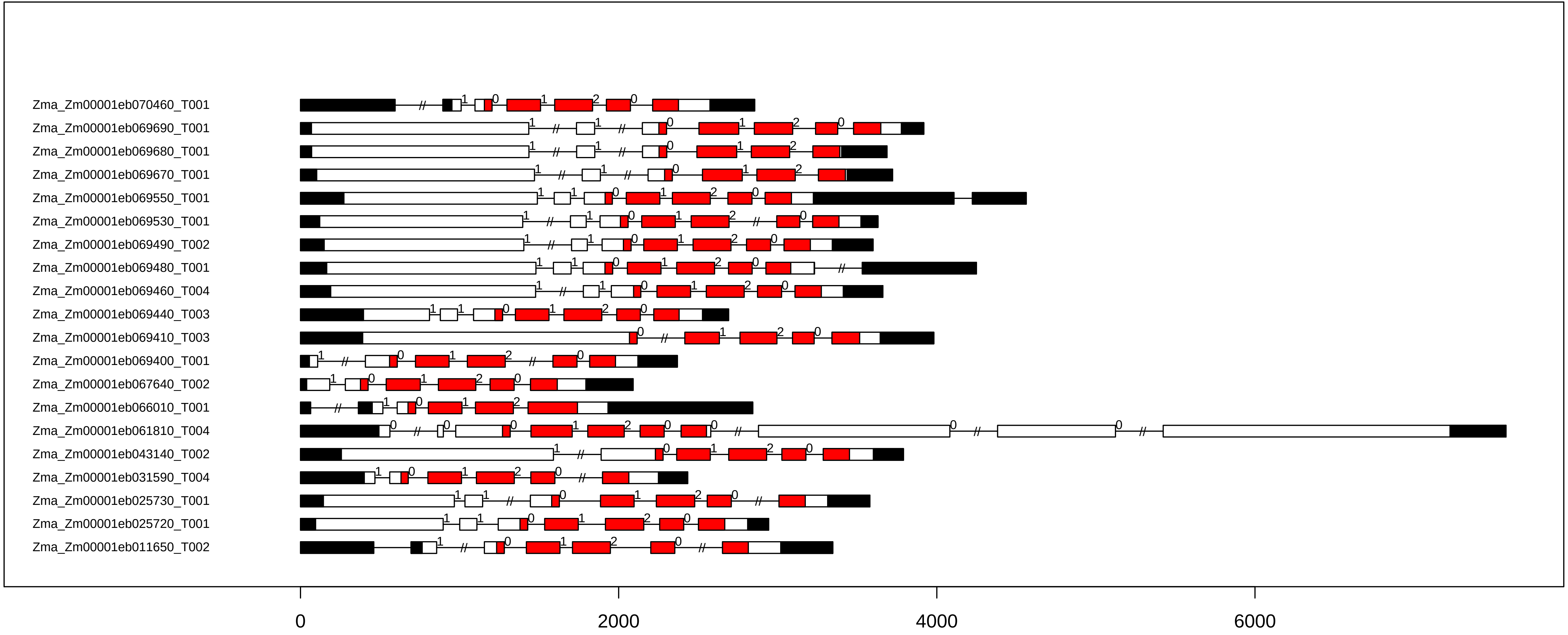

Z.ma RLK−Pelle_DLSV subfamily exon−intron and kinase domain diagram (part 2)

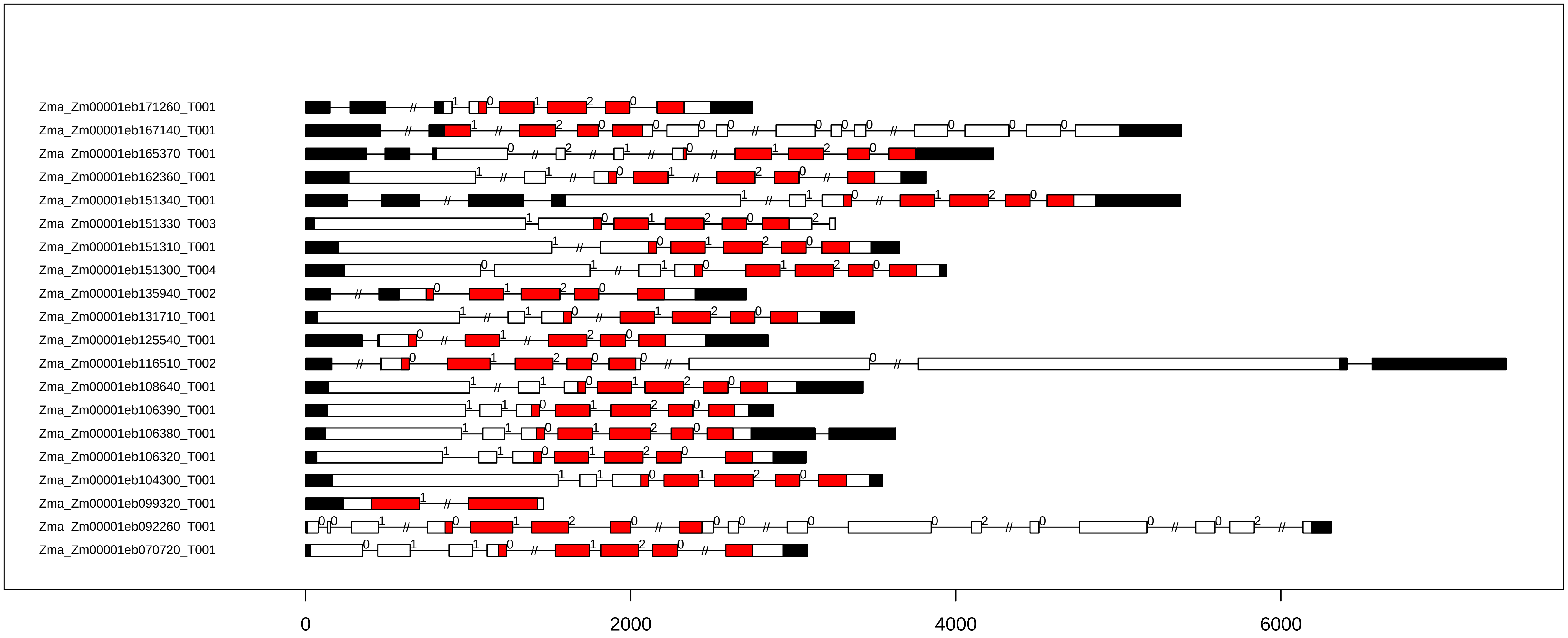

Z.ma RLK−Pelle_DLSV subfamily exon−intron and kinase domain diagram (part 3)

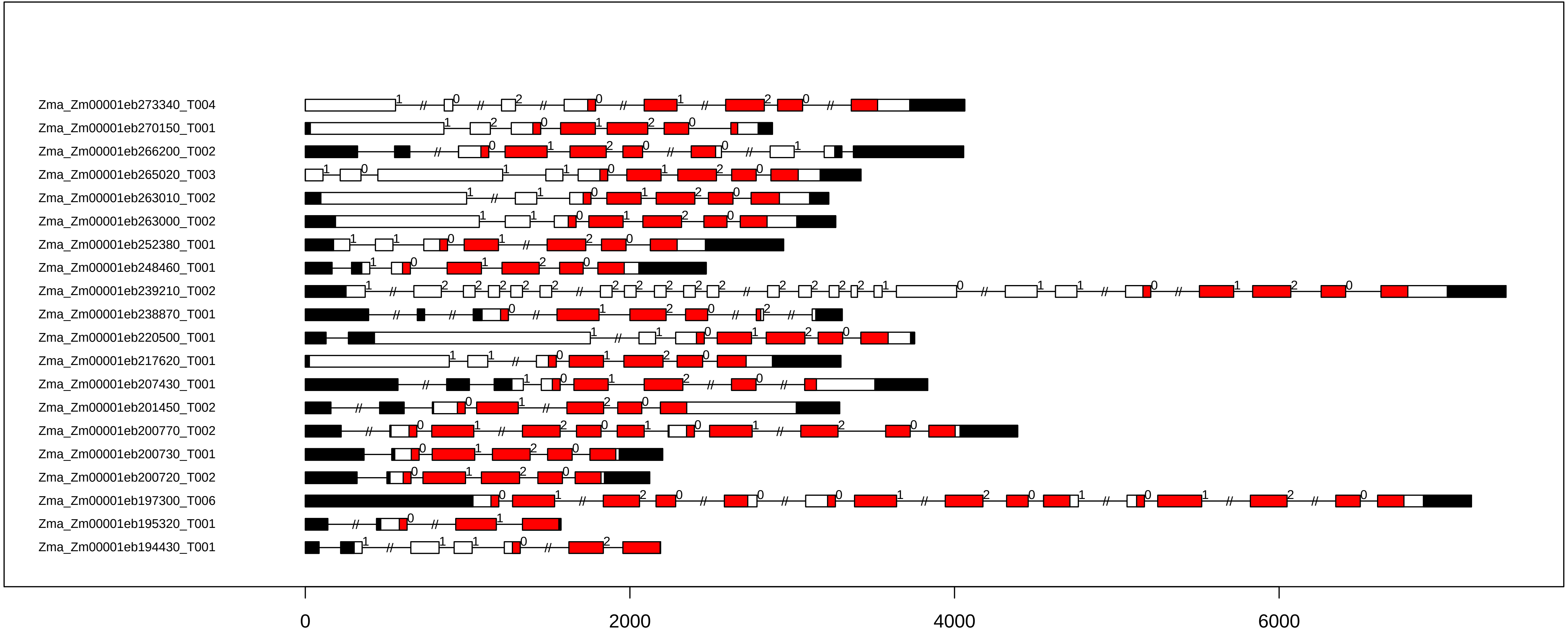

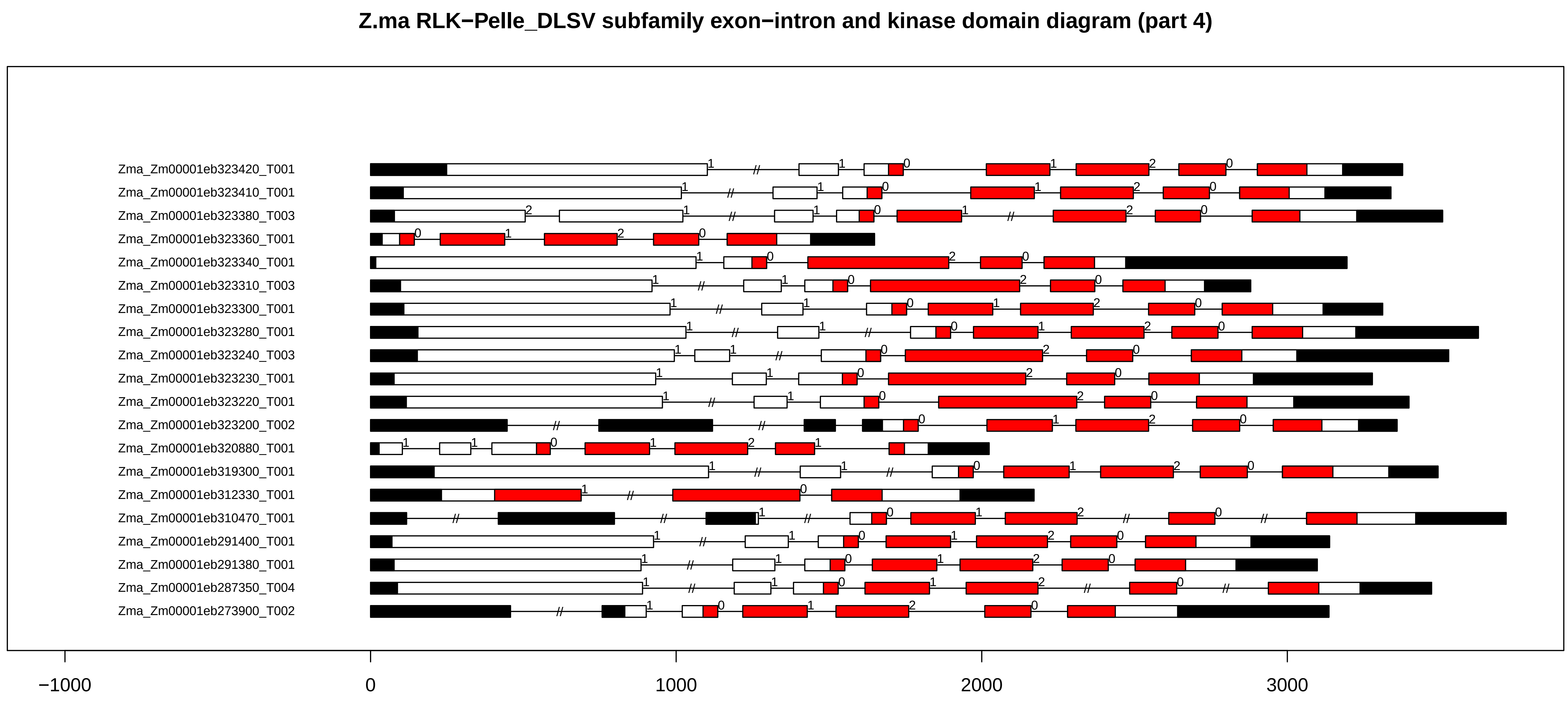

Z.ma RLK−Pelle_DLSV subfamily exon−intron and kinase domain diagram (part 4)
Zma_Zm00001eb323420_T001
Zma_Zm00001eb323410_T001
Zma_Zm00001eb323380_T003
Zma_Zm00001eb323360_T001
Zma_Zm00001eb323340_T001
Zma_Zm00001eb323310_T003
Zma_Zm00001eb323300_T001
Zma_Zm00001eb323280_T001
Zma_Zm00001eb323240_T003
Zma_Zm00001eb323230_T001
Zma_Zm00001eb323220_T001
Zma_Zm00001eb323200_T002
Zma_Zm00001eb320880_T001
Zma_Zm00001eb319300_T001
Zma_Zm00001eb312330_T001
Zma_Zm00001eb310470_T001
Zma_Zm00001eb291400_T001
Zma_Zm00001eb291380_T001
Zma_Zm00001eb287350_T004
Zma_Zm00001eb273900_T002
−1000
0
1000
2000
3000

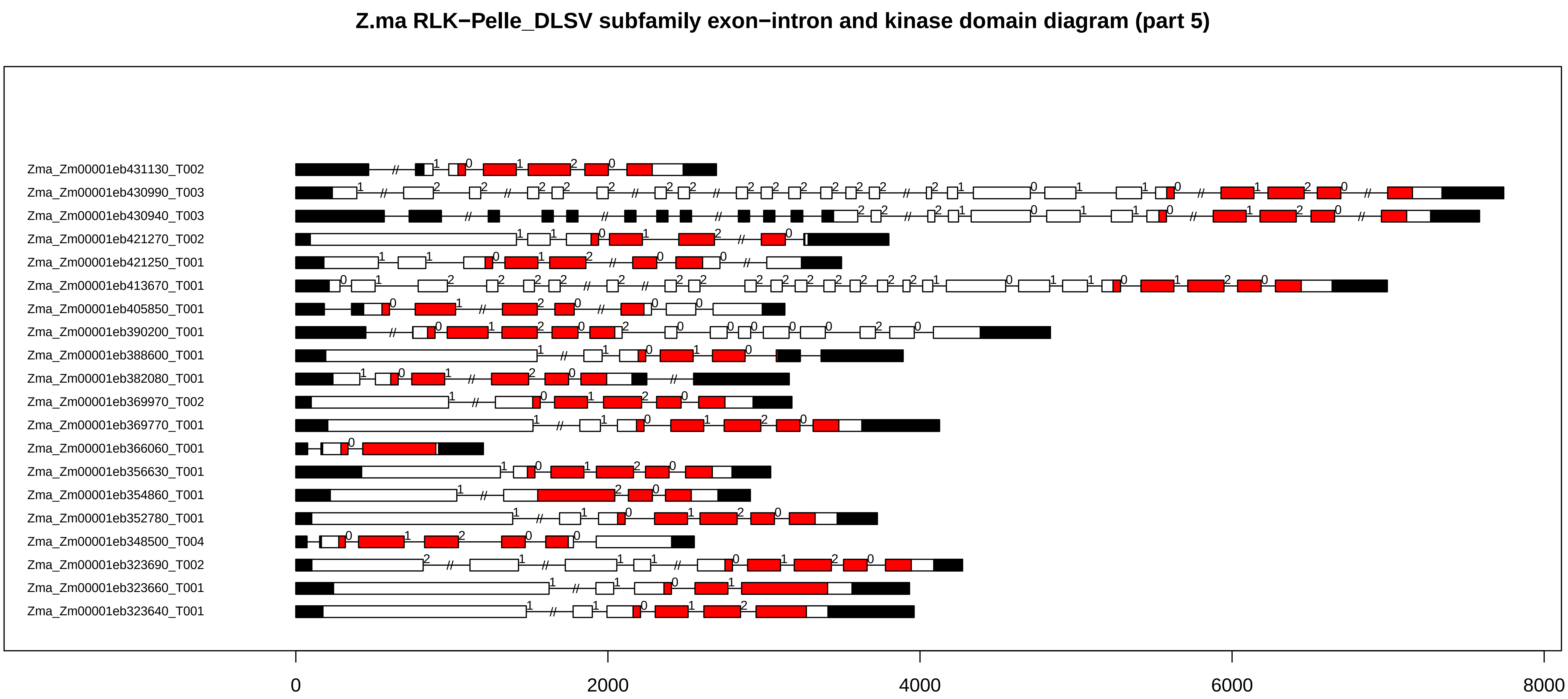
Z.ma RLK−Pelle_DLSV subfamily exon−intron and kinase domain diagram (part 5)
Zma_Zm00001eb431130_T002
Zma_Zm00001eb430990_T003
Zma_Zm00001eb430940_T003
Zma_Zm00001eb421270_T002
Zma_Zm00001eb421250_T001
Zma_Zm00001eb413670_T001
Zma_Zm00001eb405850_T001
Zma_Zm00001eb390200_T001
Zma_Zm00001eb388600_T001
Zma_Zm00001eb382080_T001
Zma_Zm00001eb369970_T002
Zma_Zm00001eb369770_T001
Zma_Zm00001eb366060_T001
Zma_Zm00001eb356630_T001
Zma_Zm00001eb354860_T001
Zma_Zm00001eb352780_T001
Zma_Zm00001eb348500_T004
Zma_Zm00001eb323690_T002
Zma_Zm00001eb323660_T001
Zma_Zm00001eb323640_T001
0
2000
4000
6000
8000

Z.ma RLK−Pelle_DLSV subfamily exon−intron and kinase domain diagram (part 6)

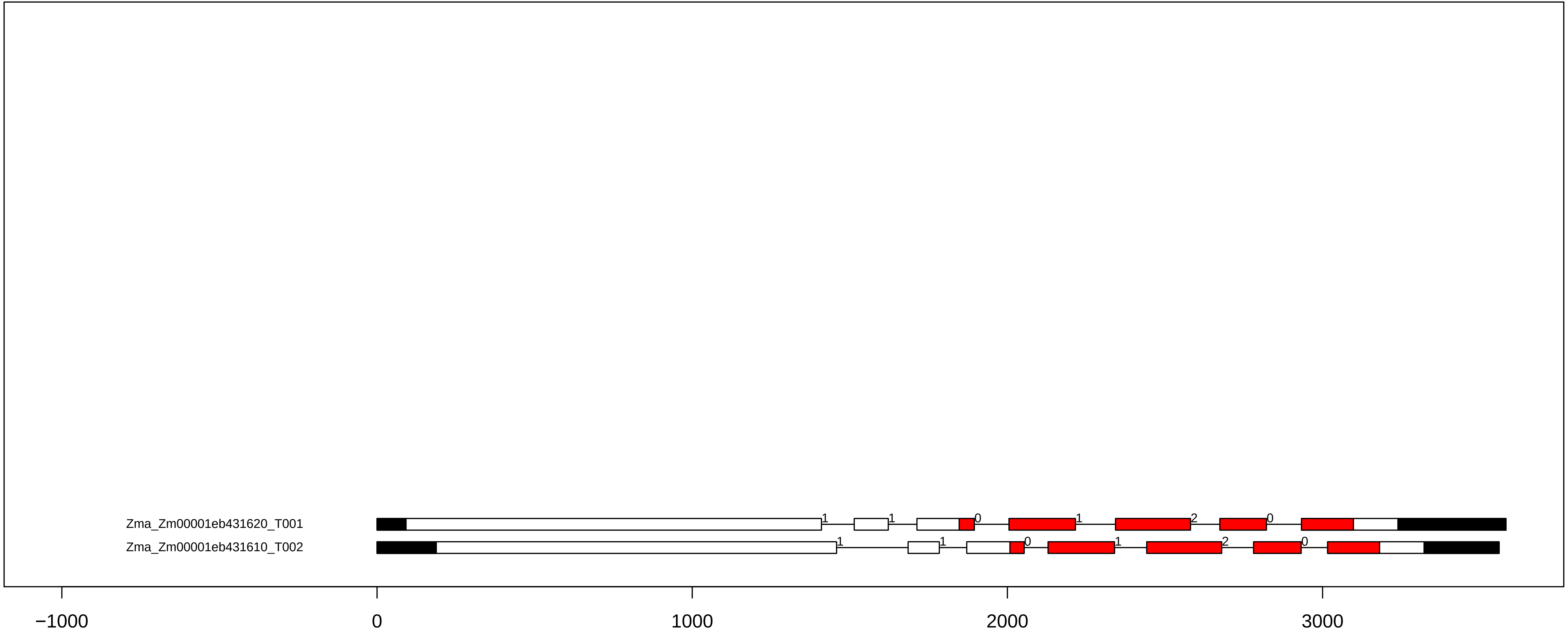

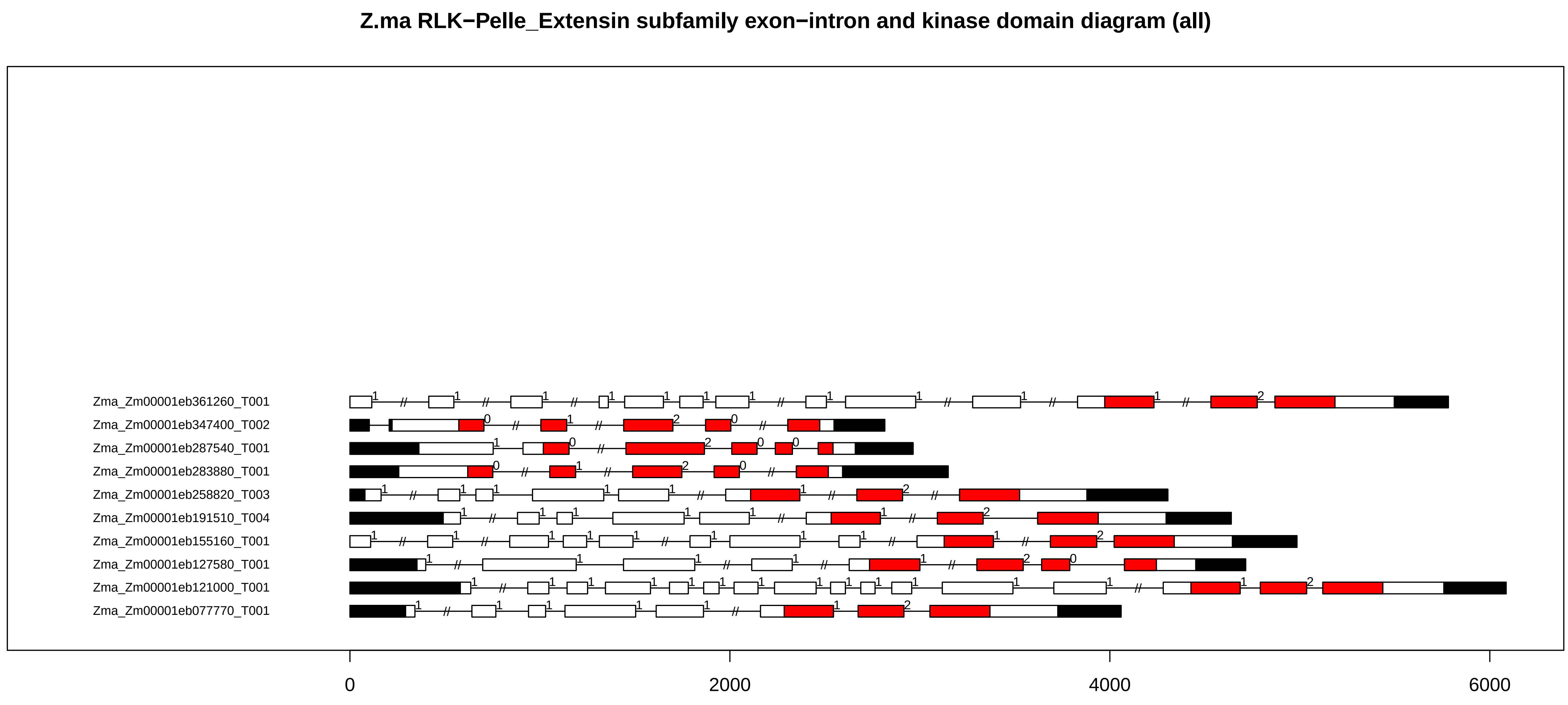

Z.ma RLK−Pelle_Extensin subfamily exon−intron and kinase domain diagram (all)
Zma_Zm00001eb361260_T001
Zma_Zm00001eb347400_T002
Zma_Zm00001eb287540_T001
Zma_Zm00001eb283880_T001
Zma_Zm00001eb258820_T003
Zma_Zm00001eb191510_T004
Zma_Zm00001eb155160_T001
Zma_Zm00001eb127580_T001
Zma_Zm00001eb121000_T001
Zma_Zm00001eb077770_T001
0
2000
4000
6000

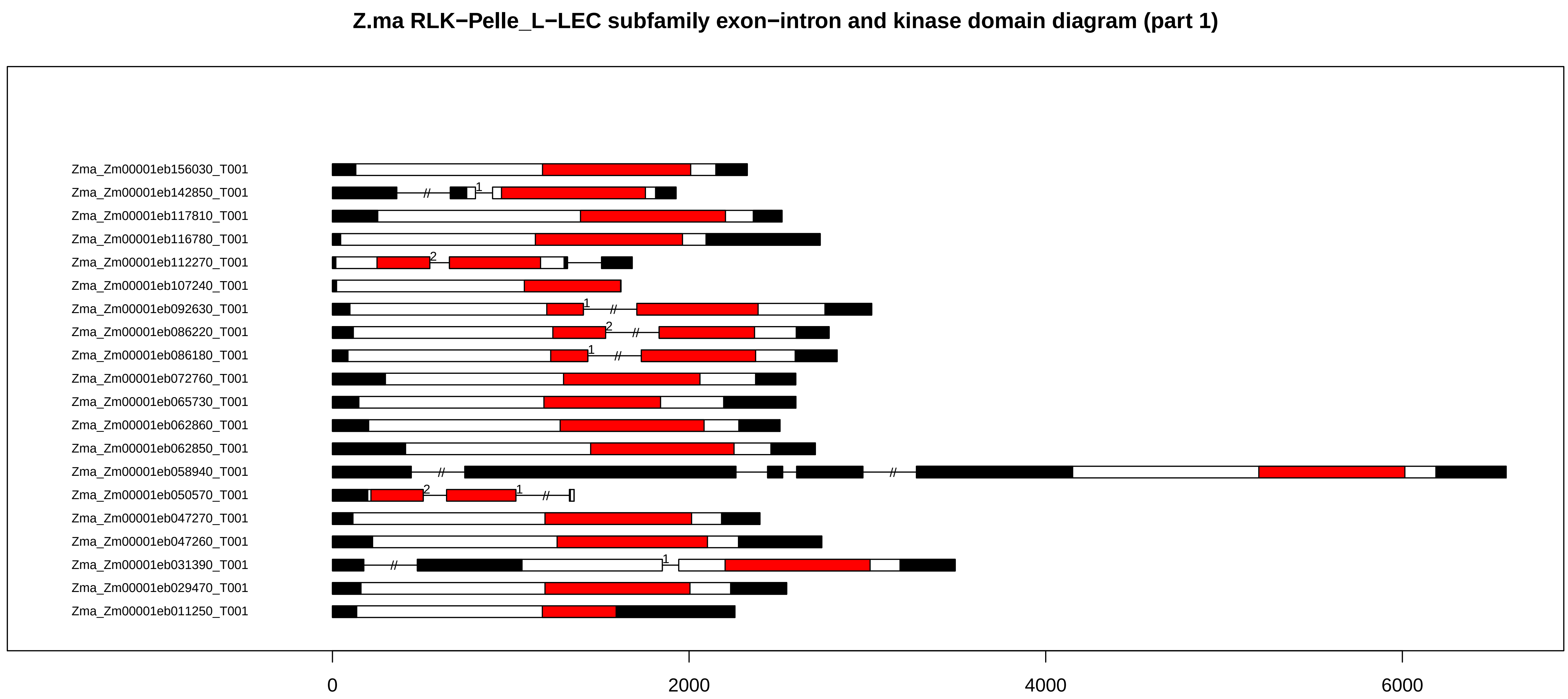

Z.ma RLK−Pelle_L−LEC subfamily exon−intron and kinase domain diagram (part 1)
Zma_Zm00001eb156030_T001
Zma_Zm00001eb142850_T001
Zma_Zm00001eb117810_T001
Zma_Zm00001eb116780_T001
Zma_Zm00001eb112270_T001
Zma_Zm00001eb107240_T001
Zma_Zm00001eb092630_T001
Zma_Zm00001eb086220_T001
Zma_Zm00001eb086180_T001
Zma_Zm00001eb072760_T001
Zma_Zm00001eb065730_T001
Zma_Zm00001eb062860_T001
Zma_Zm00001eb062850_T001
Zma_Zm00001eb058940_T001
Zma_Zm00001eb050570_T001
Zma_Zm00001eb047270_T001
Zma_Zm00001eb047260_T001
Zma_Zm00001eb031390_T001
Zma_Zm00001eb029470_T001
Zma_Zm00001eb011250_T001
0
2000
4000
6000

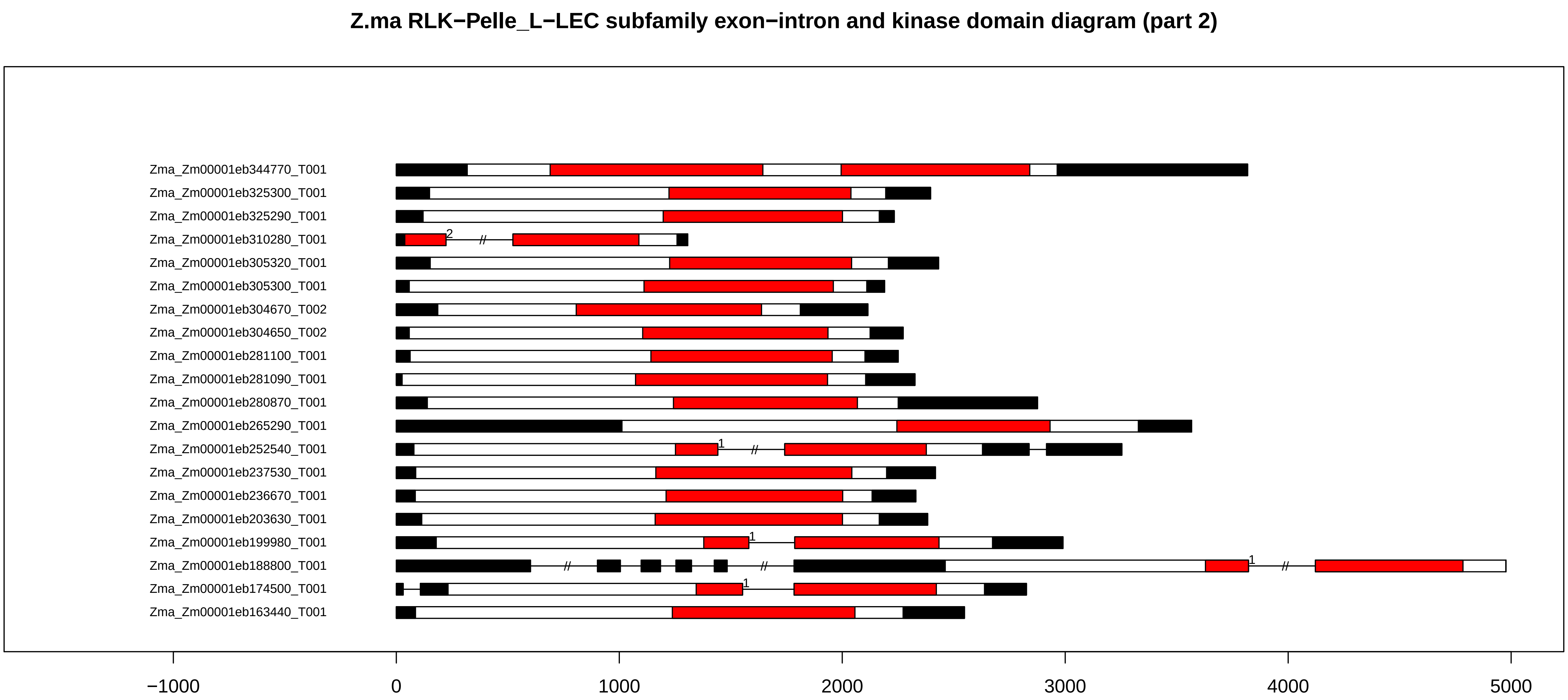
Z.ma RLK−Pelle_L−LEC subfamily exon−intron and kinase domain diagram (part 2)
Zma_Zm00001eb344770_T001
Zma_Zm00001eb325300_T001
Zma_Zm00001eb325290_T001
Zma_Zm00001eb310280_T001
Zma_Zm00001eb305320_T001
Zma_Zm00001eb305300_T001
Zma_Zm00001eb304670_T002
Zma_Zm00001eb304650_T002
Zma_Zm00001eb281100_T001
Zma_Zm00001eb281090_T001
Zma_Zm00001eb280870_T001
Zma_Zm00001eb265290_T001
Zma_Zm00001eb252540_T001
Zma_Zm00001eb237530_T001
Zma_Zm00001eb236670_T001
Zma_Zm00001eb203630_T001
Zma_Zm00001eb199980_T001
Zma_Zm00001eb188800_T001
Zma_Zm00001eb174500_T001
Zma_Zm00001eb163440_T001
2
1
1
1
1
−1000
0
1000
2000
3000
4000
5000

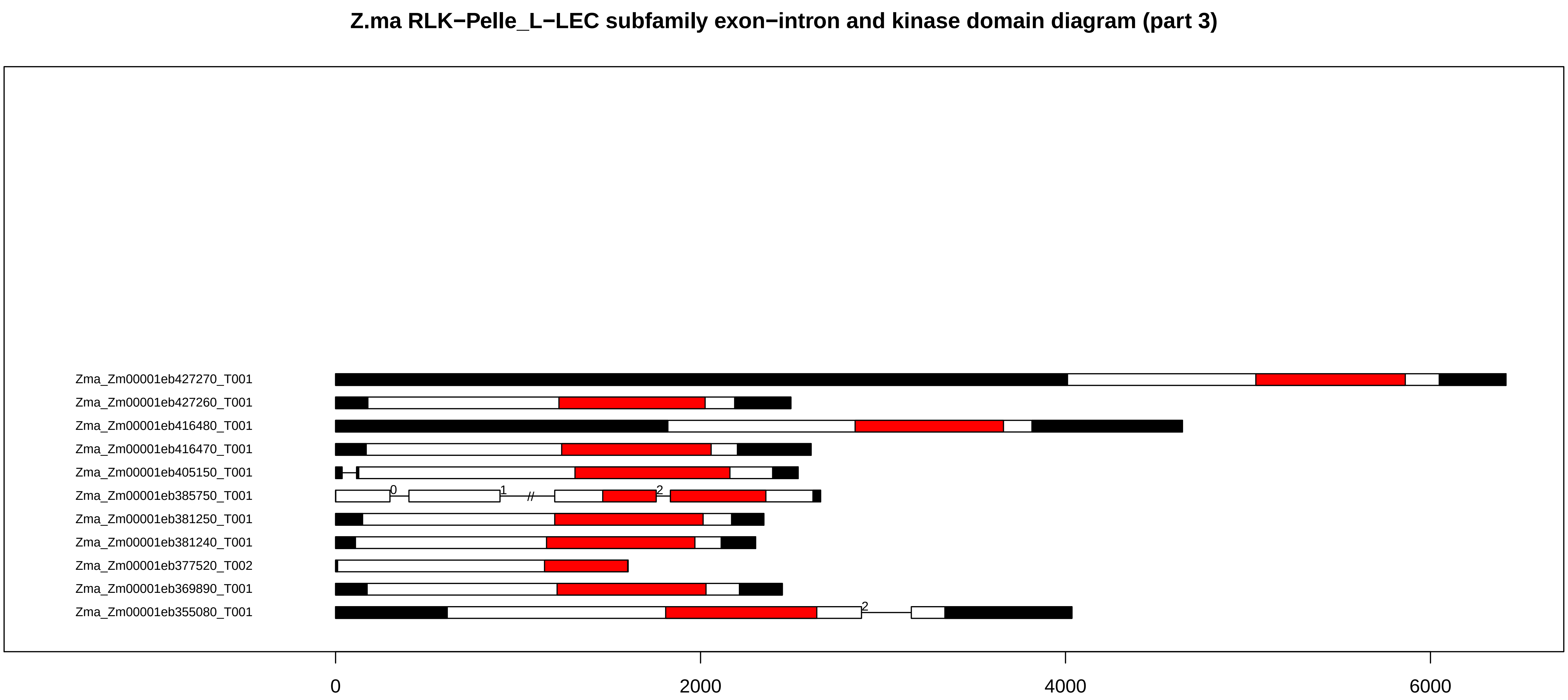

Z.ma RLK−Pelle_L−LEC subfamily exon−intron and kinase domain diagram (part 3)
Zma_Zm00001eb427270_T001
Zma_Zm00001eb427260_T001
Zma_Zm00001eb416480_T001
Zma_Zm00001eb416470_T001
Zma_Zm00001eb405150_T001
Zma_Zm00001eb385750_T001
Zma_Zm00001eb381250_T001
Zma_Zm00001eb381240_T001
Zma_Zm00001eb377520_T002
Zma_Zm00001eb369890_T001
Zma_Zm00001eb355080_T001
0
1
2
2
0
2000
4000
6000

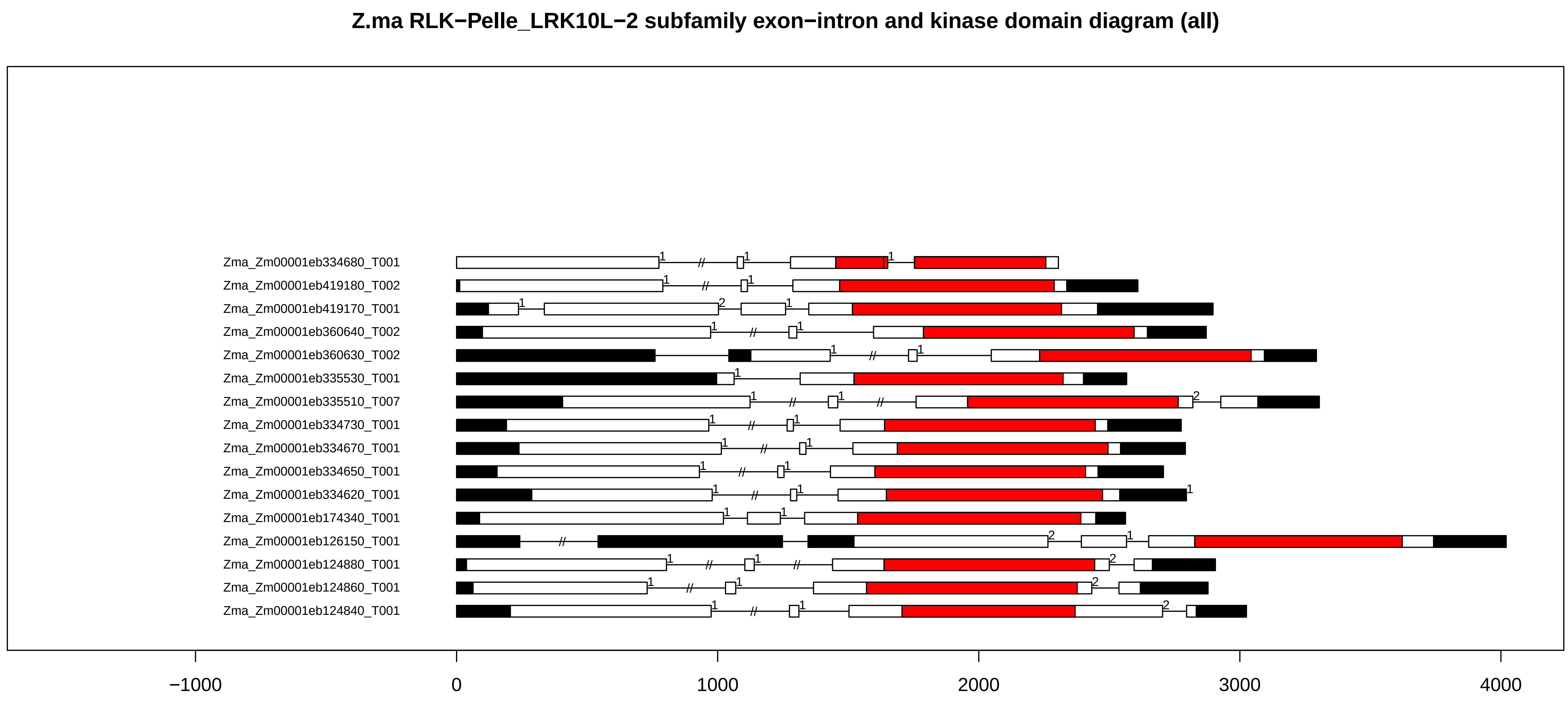

Z.ma RLK−Pelle_LRK10L−2 subfamily exon−intron and kinase domain diagram (all)
Zma_Zm00001eb334680_T001
Zma_Zm00001eb419180_T002
Zma_Zm00001eb419170_T001
Zma_Zm00001eb360640_T002
Zma_Zm00001eb360630_T002
Zma_Zm00001eb335530_T001
Zma_Zm00001eb335510_T007
Zma_Zm00001eb334730_T001
Zma_Zm00001eb334670_T001
Zma_Zm00001eb334650_T001
Zma_Zm00001eb334620_T001
Zma_Zm00001eb174340_T001
Zma_Zm00001eb126150_T001
Zma_Zm00001eb124880_T001
Zma_Zm00001eb124860_T001
Zma_Zm00001eb124840_T001
−1000
0
1000
2000
3000
4000

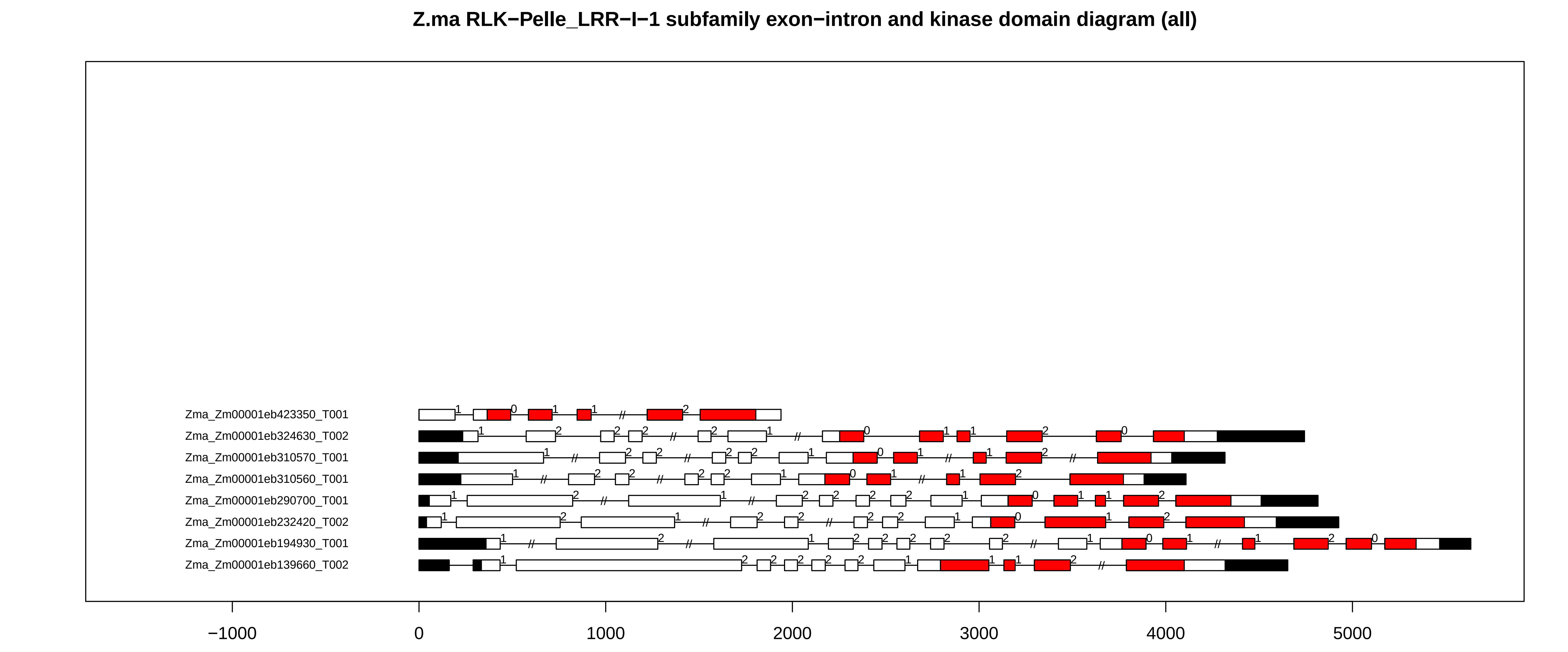

Z.ma RLK−Pelle_LRR−I−1 subfamily exon−intron and kinase domain diagram (all)
Zma_Zm00001eb423350_T001
Zma_Zm00001eb324630_T002
Zma_Zm00001eb310570_T001
Zma_Zm00001eb310560_T001
Zma_Zm00001eb290700_T001
Zma_Zm00001eb232420_T002
Zma_Zm00001eb194930_T001
Zma_Zm00001eb139660_T002
−1000
0
1000
2000
3000
4000
5000

Z.ma RLK−Pelle_LRR−I−2 subfamily exon−intron and kinase domain diagram (all)

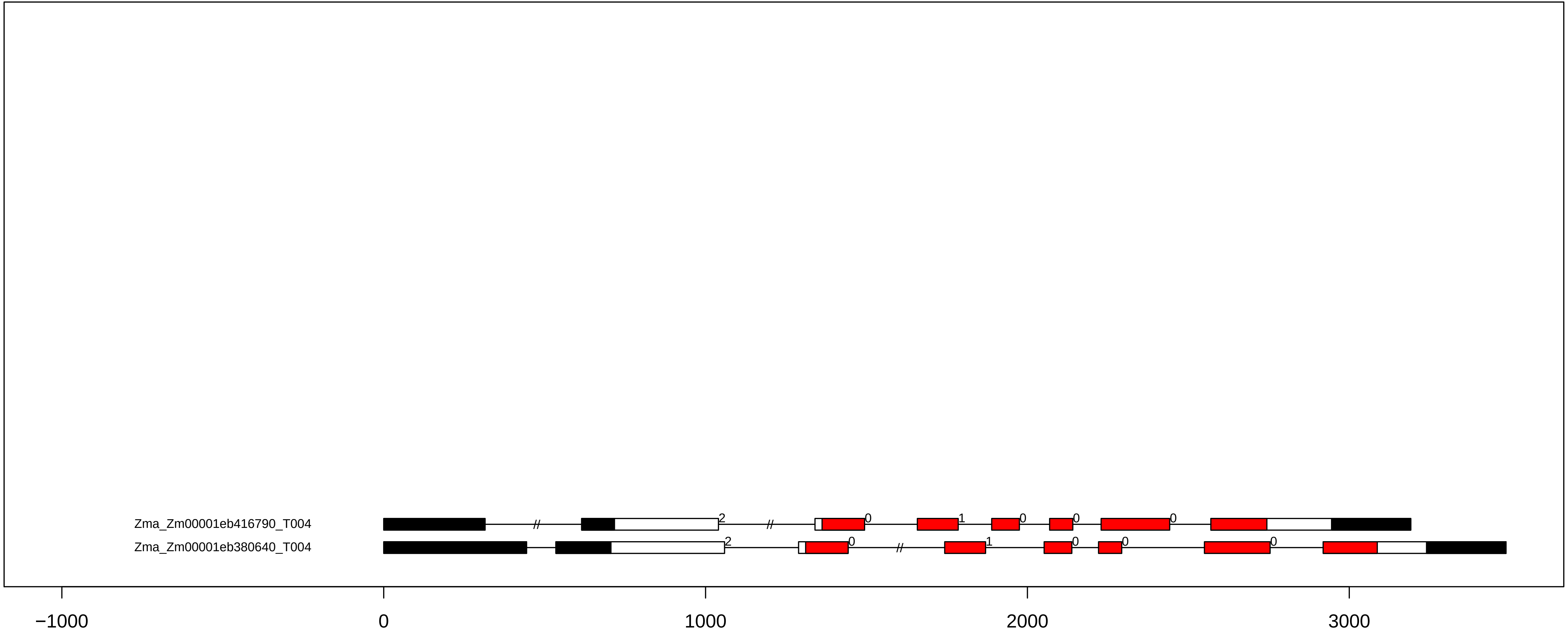

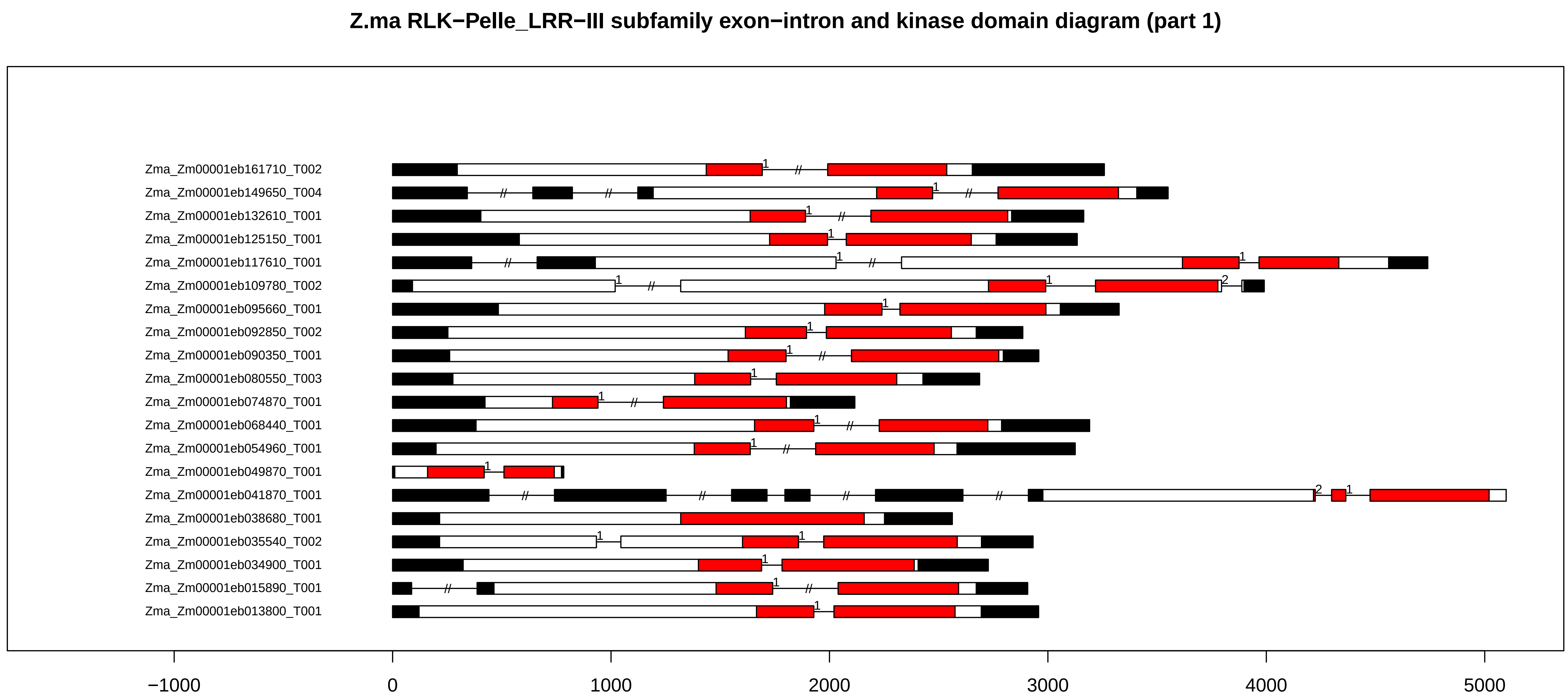
Z.ma RLK−Pelle_LRR−III subfamily exon−intron and kinase domain diagram (part 1)
Zma_Zm00001eb161710_T002
Zma_Zm00001eb149650_T004
Zma_Zm00001eb132610_T001
Zma_Zm00001eb125150_T001
Zma_Zm00001eb117610_T001
Zma_Zm00001eb109780_T002
Zma_Zm00001eb095660_T001
Zma_Zm00001eb092850_T002
Zma_Zm00001eb090350_T001
Zma_Zm00001eb080550_T003
Zma_Zm00001eb074870_T001
Zma_Zm00001eb068440_T001
Zma_Zm00001eb054960_T001
Zma_Zm00001eb049870_T001
Zma_Zm00001eb041870_T001
Zma_Zm00001eb038680_T001
Zma_Zm00001eb035540_T002
Zma_Zm00001eb034900_T001
Zma_Zm00001eb015890_T001
Zma_Zm00001eb013800_T001
−1000
0
1000
2000
3000
4000
5000

Z.ma RLK−Pelle_LRR−III subfamily exon−intron and kinase domain diagram (part 2)

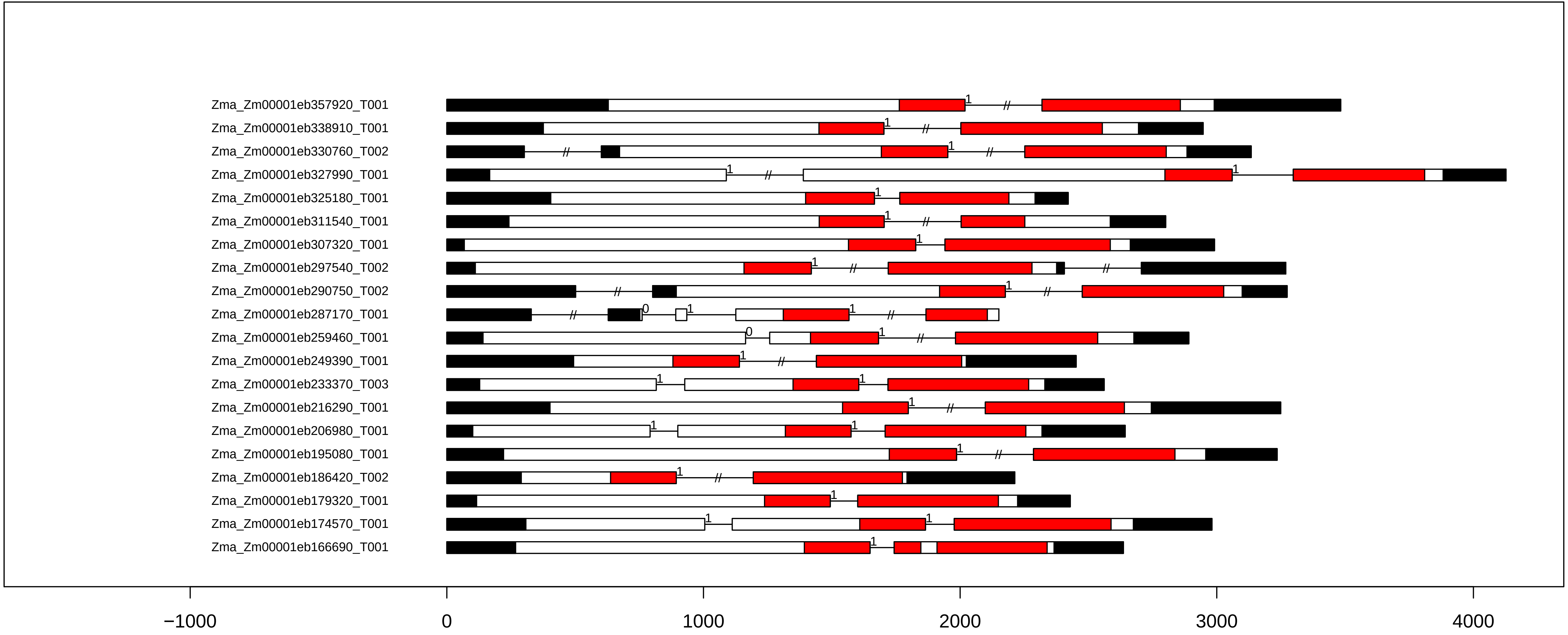

Z.ma RLK−Pelle_LRR−III subfamily exon−intron and kinase domain diagram (part 3)

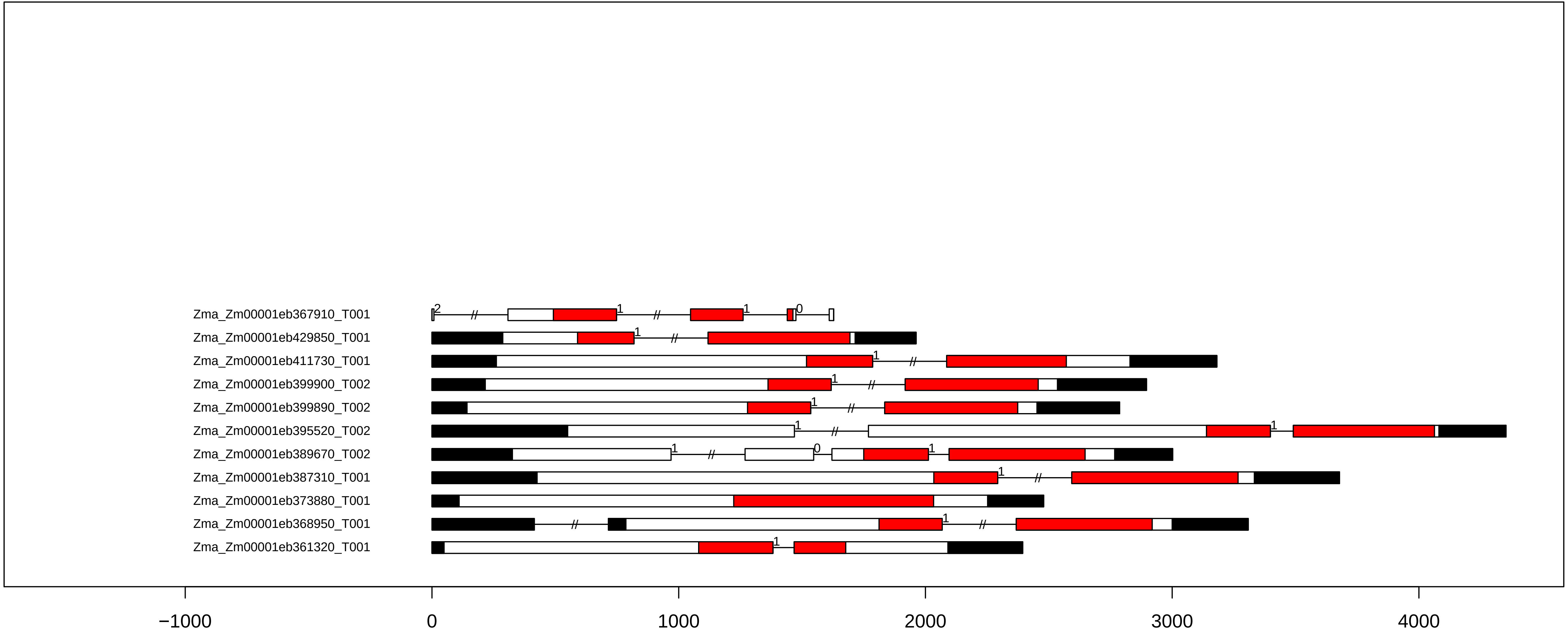

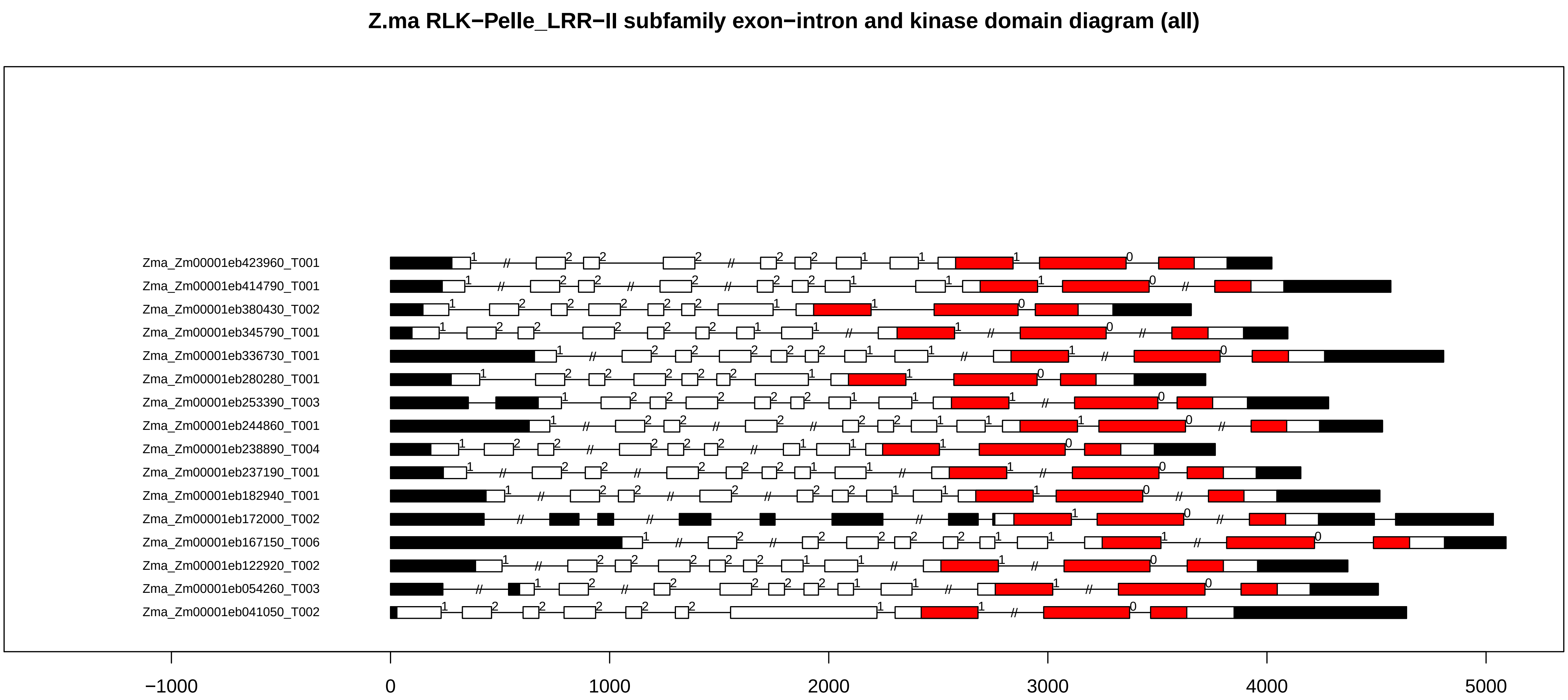

Z.ma RLK−Pelle_LRR−II subfamily exon−intron and kinase domain diagram (all)
Zma_Zm00001eb423960_T001
Zma_Zm00001eb414790_T001
Zma_Zm00001eb380430_T002
Zma_Zm00001eb345790_T001
Zma_Zm00001eb336730_T001
Zma_Zm00001eb280280_T001
Zma_Zm00001eb253390_T003
Zma_Zm00001eb244860_T001
Zma_Zm00001eb238890_T004
Zma_Zm00001eb237190_T001
Zma_Zm00001eb182940_T001
Zma_Zm00001eb172000_T002
Zma_Zm00001eb167150_T006
Zma_Zm00001eb122920_T002
Zma_Zm00001eb054260_T003
Zma_Zm00001eb041050_T002
−1000
0
1000
2000
3000
4000
5000

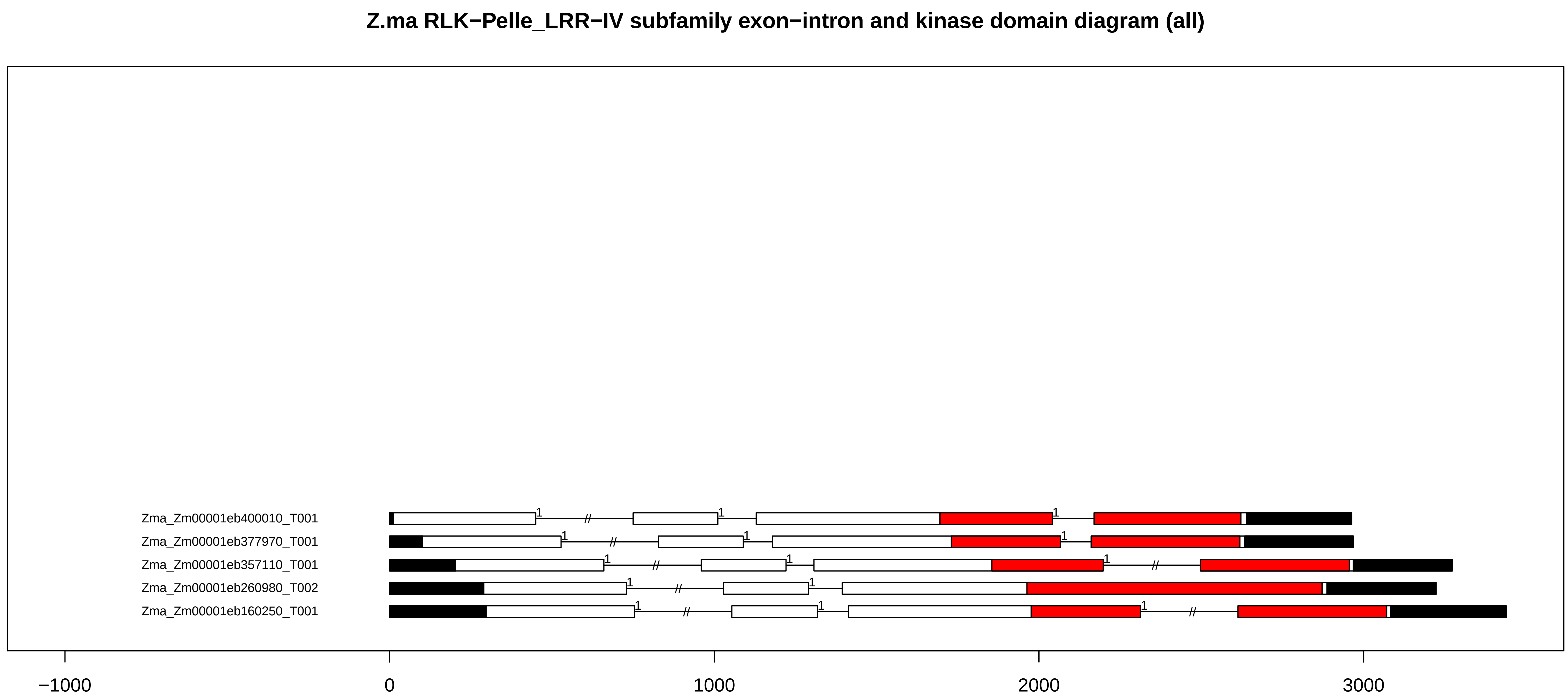

Z.ma RLK−Pelle_LRR−IV subfamily exon−intron and kinase domain diagram (all)
Zma_Zm00001eb400010_T001
Zma_Zm00001eb377970_T001
Zma_Zm00001eb357110_T001
Zma_Zm00001eb260980_T002
Zma_Zm00001eb160250_T001
−1000
0
1000
2000
3000

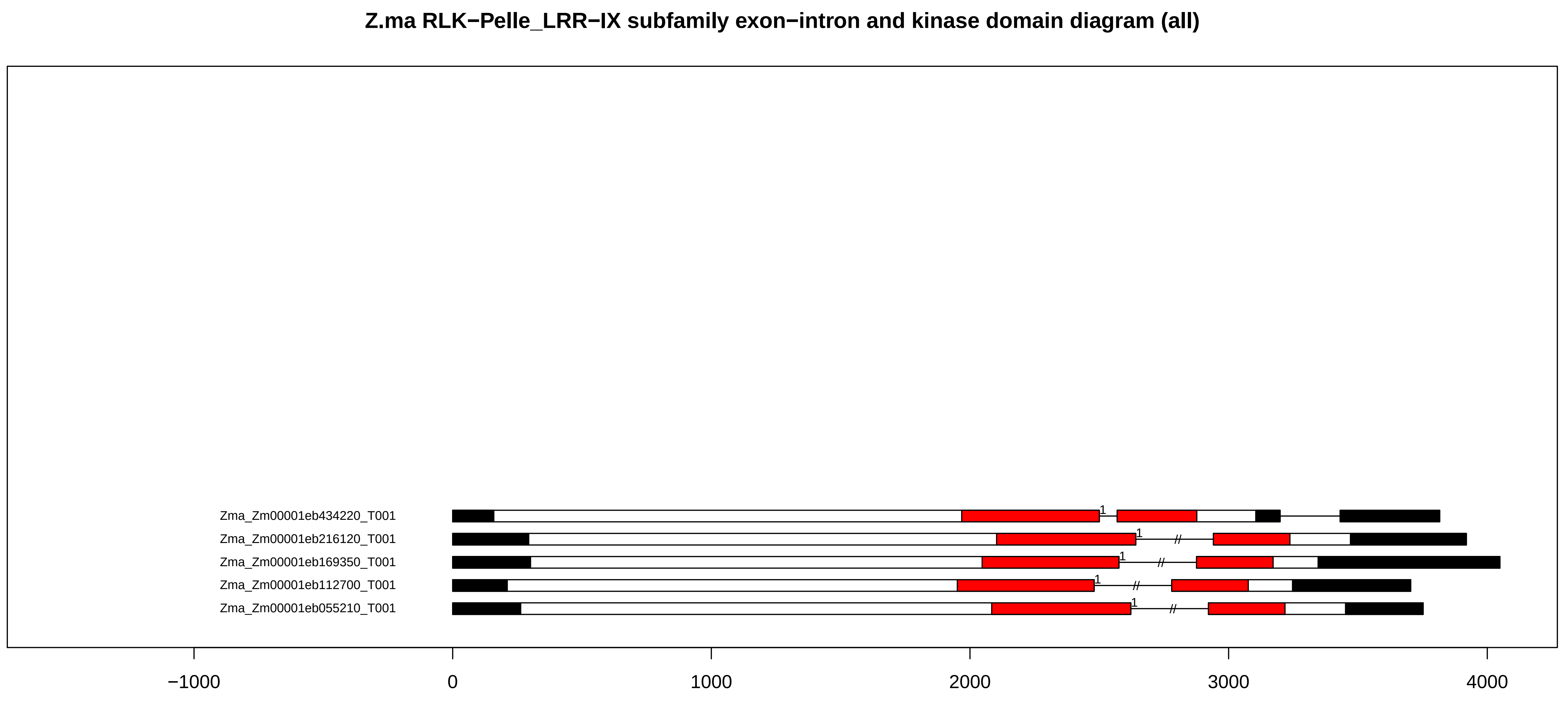
Z.ma RLK−Pelle_LRR−IX subfamily exon−intron and kinase domain diagram (all)
Zma_Zm00001eb434220_T001
Zma_Zm00001eb216120_T001
Zma_Zm00001eb169350_T001
Zma_Zm00001eb112700_T001
Zma_Zm00001eb055210_T001
1
1
1
1
1
−1000
0
1000
2000
3000
4000

Z.ma RLK−Pelle_LRR−VI−1 subfamily exon−intron and kinase domain diagram (all)

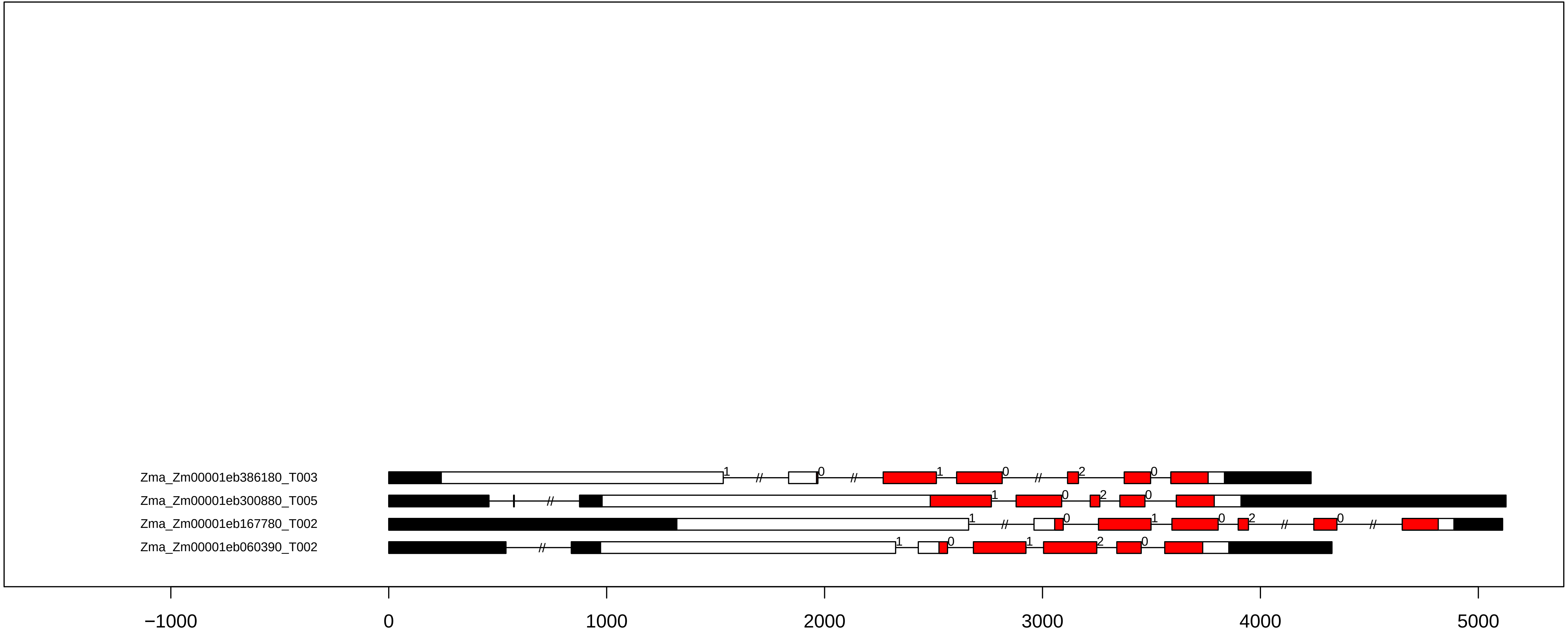

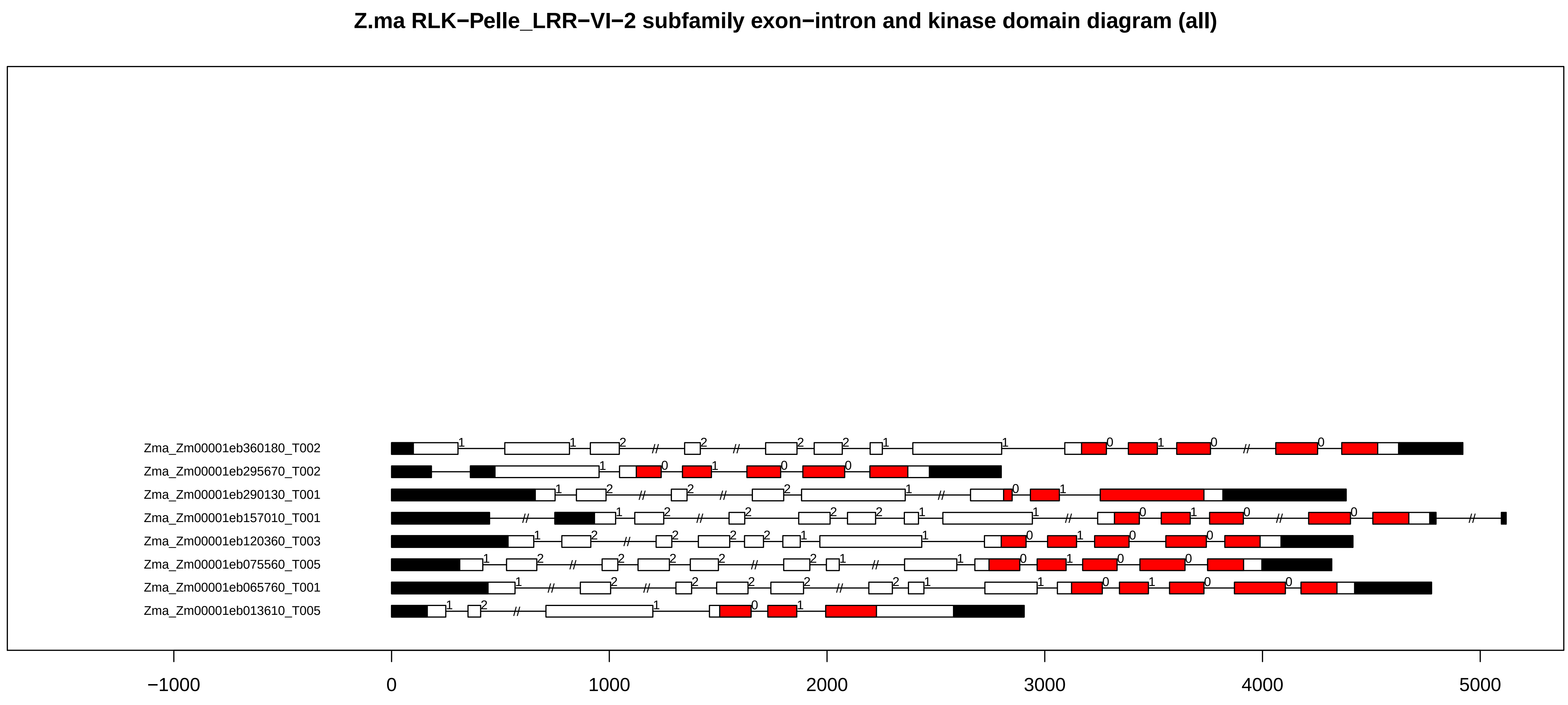

Z.ma RLK−Pelle_LRR−VI−2 subfamily exon−intron and kinase domain diagram (all)
Zma_Zm00001eb360180_T002
Zma_Zm00001eb295670_T002
Zma_Zm00001eb290130_T001
Zma_Zm00001eb157010_T001
Zma_Zm00001eb120360_T003
Zma_Zm00001eb075560_T005
Zma_Zm00001eb065760_T001
Zma_Zm00001eb013610_T005
−1000
0
1000
2000
3000
4000
5000

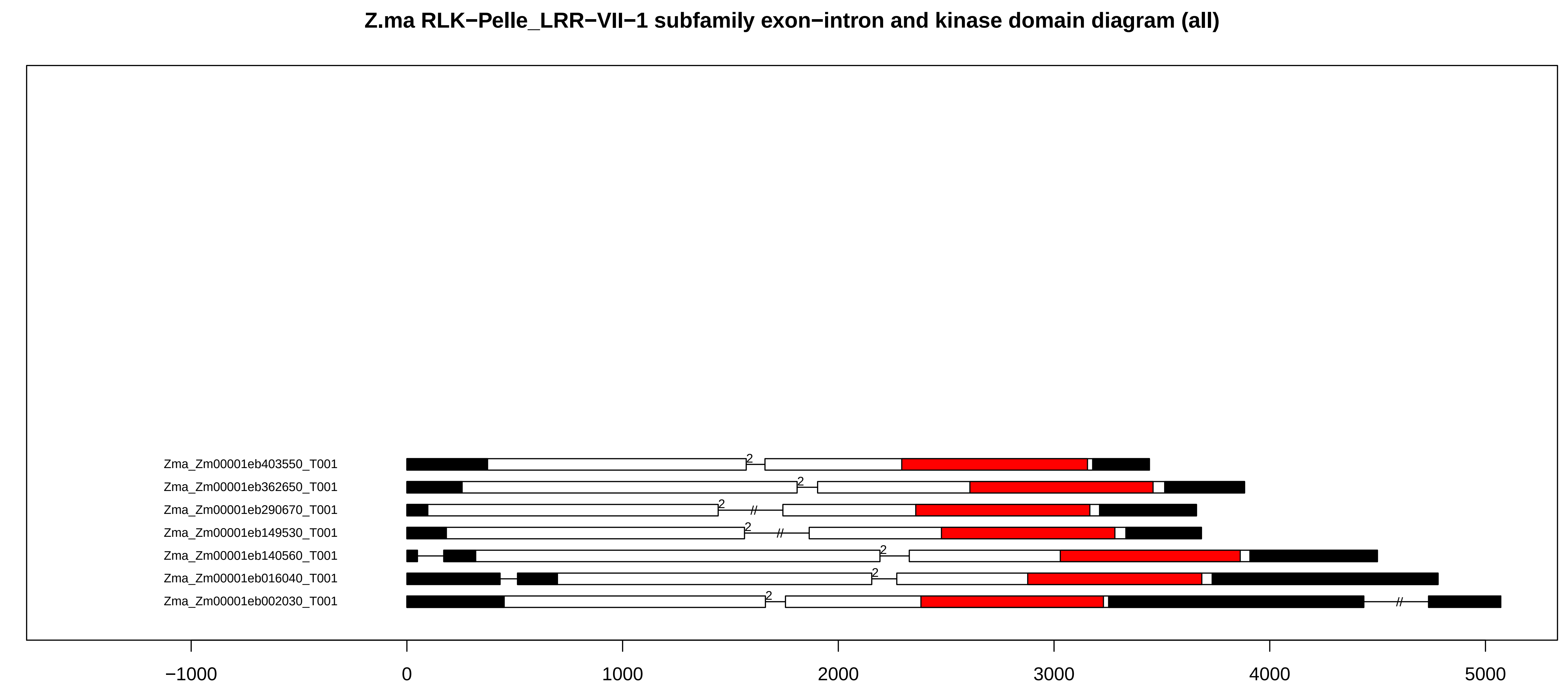

Z.ma RLK−Pelle_LRR−VII−1 subfamily exon−intron and kinase domain diagram (all)
Zma_Zm00001eb403550_T001
Zma_Zm00001eb362650_T001
Zma_Zm00001eb290670_T001
Zma_Zm00001eb149530_T001
Zma_Zm00001eb140560_T001
Zma_Zm00001eb016040_T001
Zma_Zm00001eb002030_T001
2
2
2
2
2
2
2
−1000
0
1000
2000
3000
4000
5000

Z.ma RLK−Pelle_LRR−VII−2 subfamily exon−intron and kinase domain diagram (all)

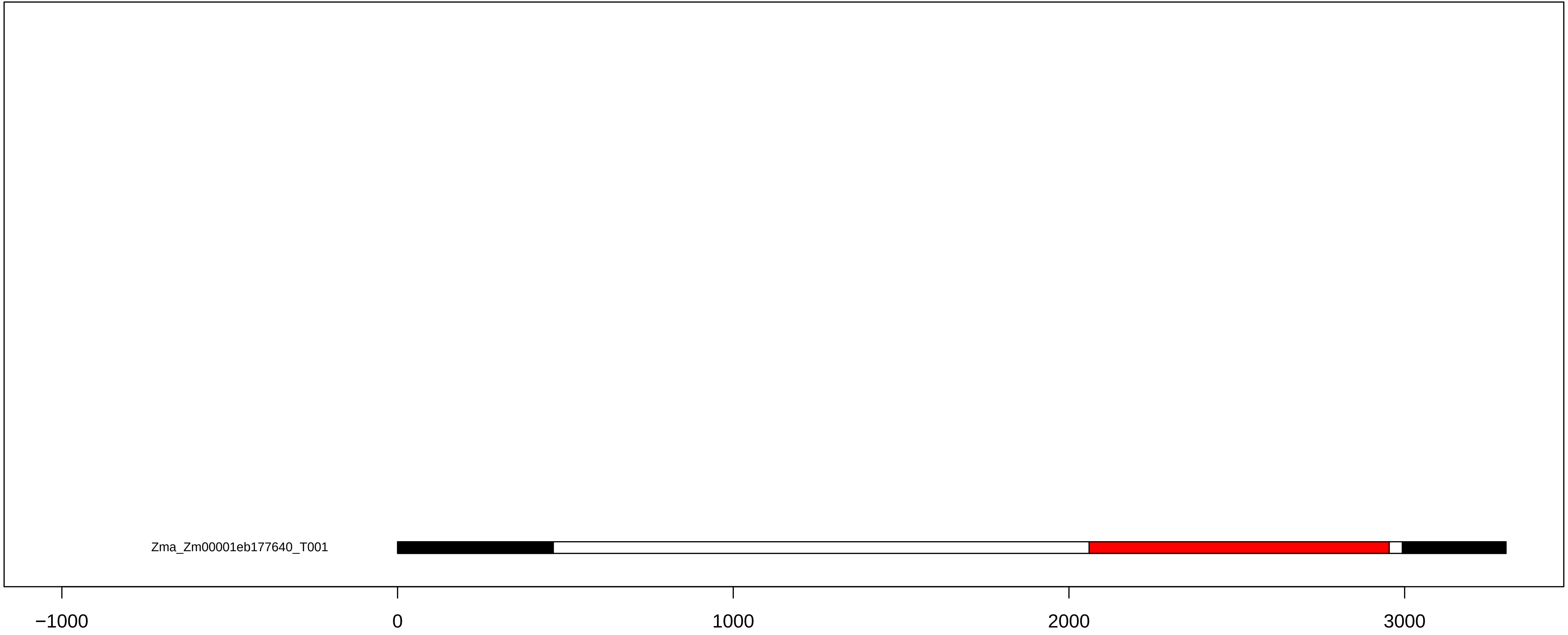

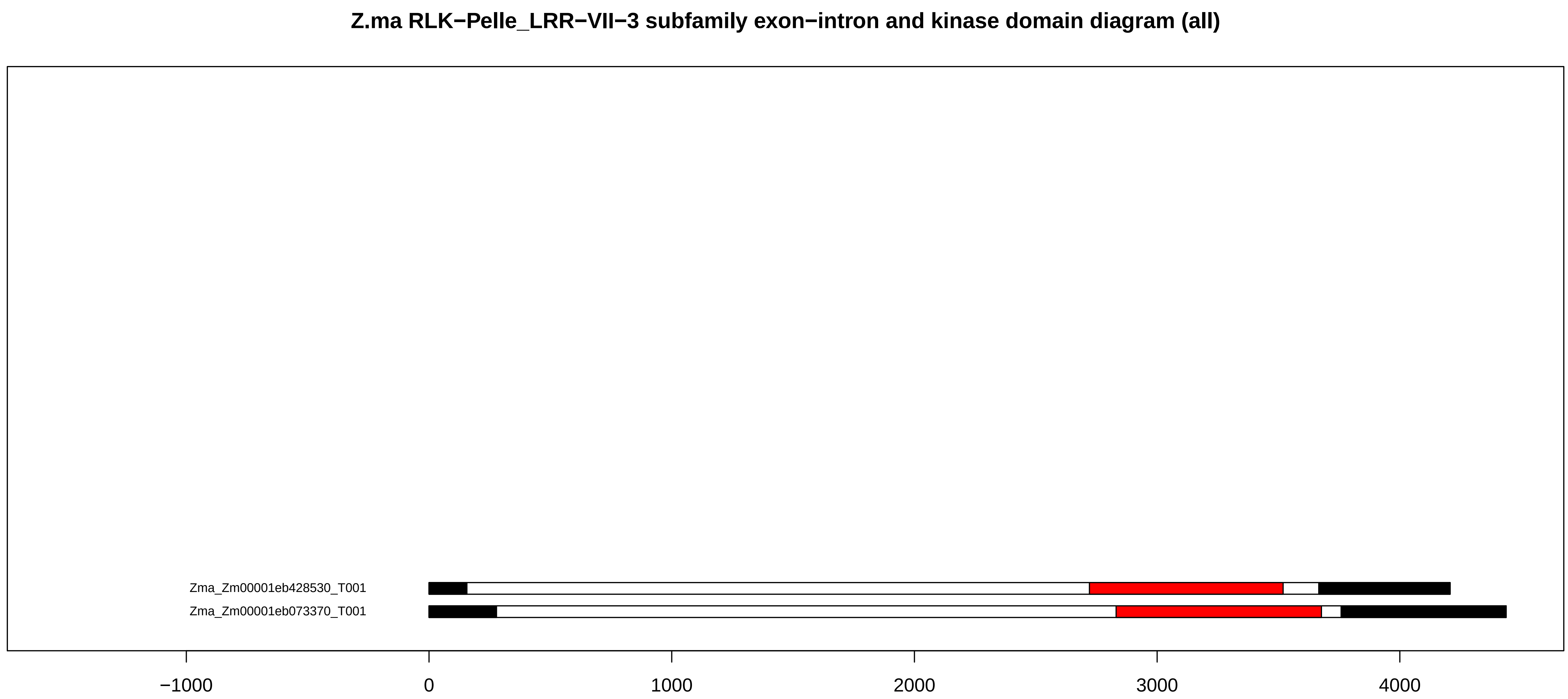
Z.ma RLK−Pelle_LRR−VII−3 subfamily exon−intron and kinase domain diagram (all)
Zma_Zm00001eb428530_T001
Zma_Zm00001eb073370_T001
−1000
0
1000
2000
3000
4000

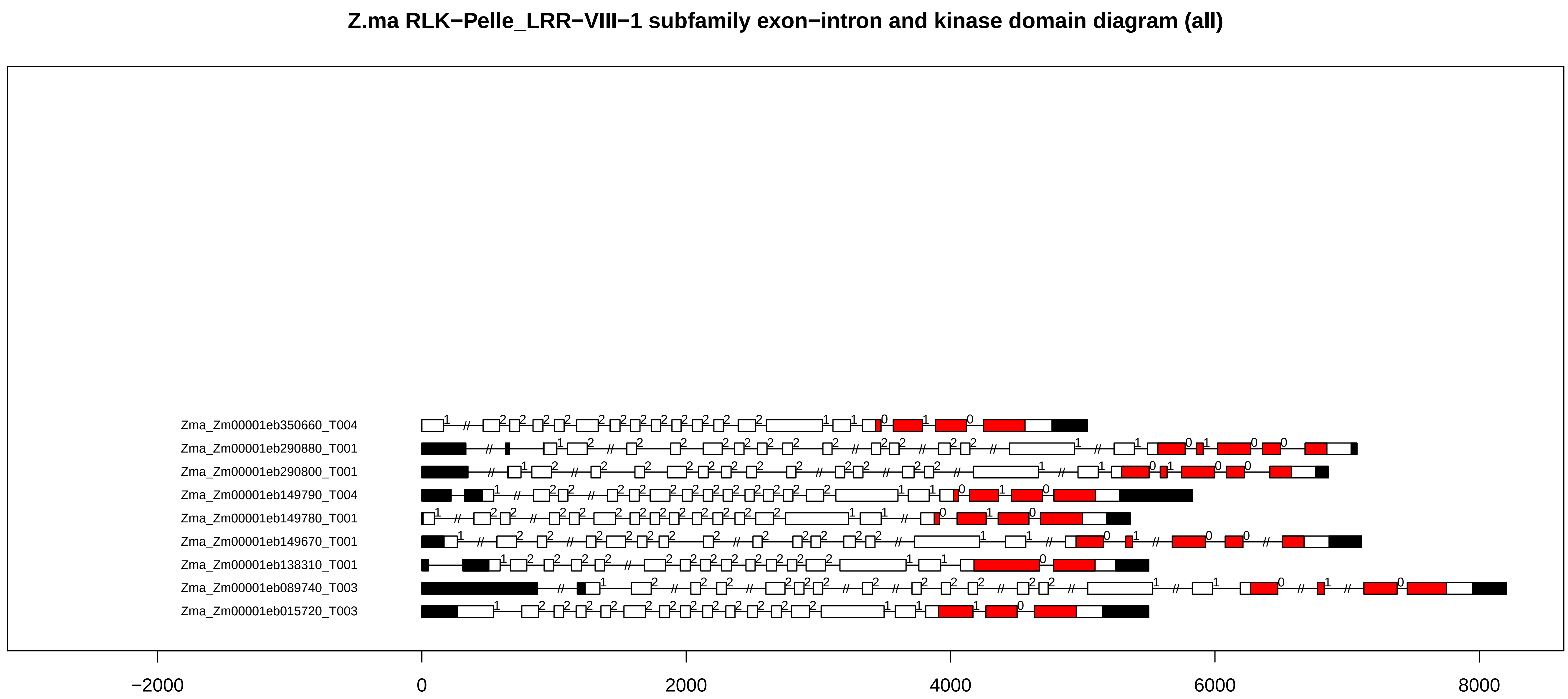

Z.ma RLK−Pelle_LRR−VIII−1 subfamily exon−intron and kinase domain diagram (all)
Zma_Zm00001eb350660_T004
Zma_Zm00001eb290880_T001
Zma_Zm00001eb290800_T001
Zma_Zm00001eb149790_T004
Zma_Zm00001eb149780_T001
Zma_Zm00001eb149670_T001
Zma_Zm00001eb138310_T001
Zma_Zm00001eb089740_T003
Zma_Zm00001eb015720_T003
−2000
0
2000
4000
6000
8000

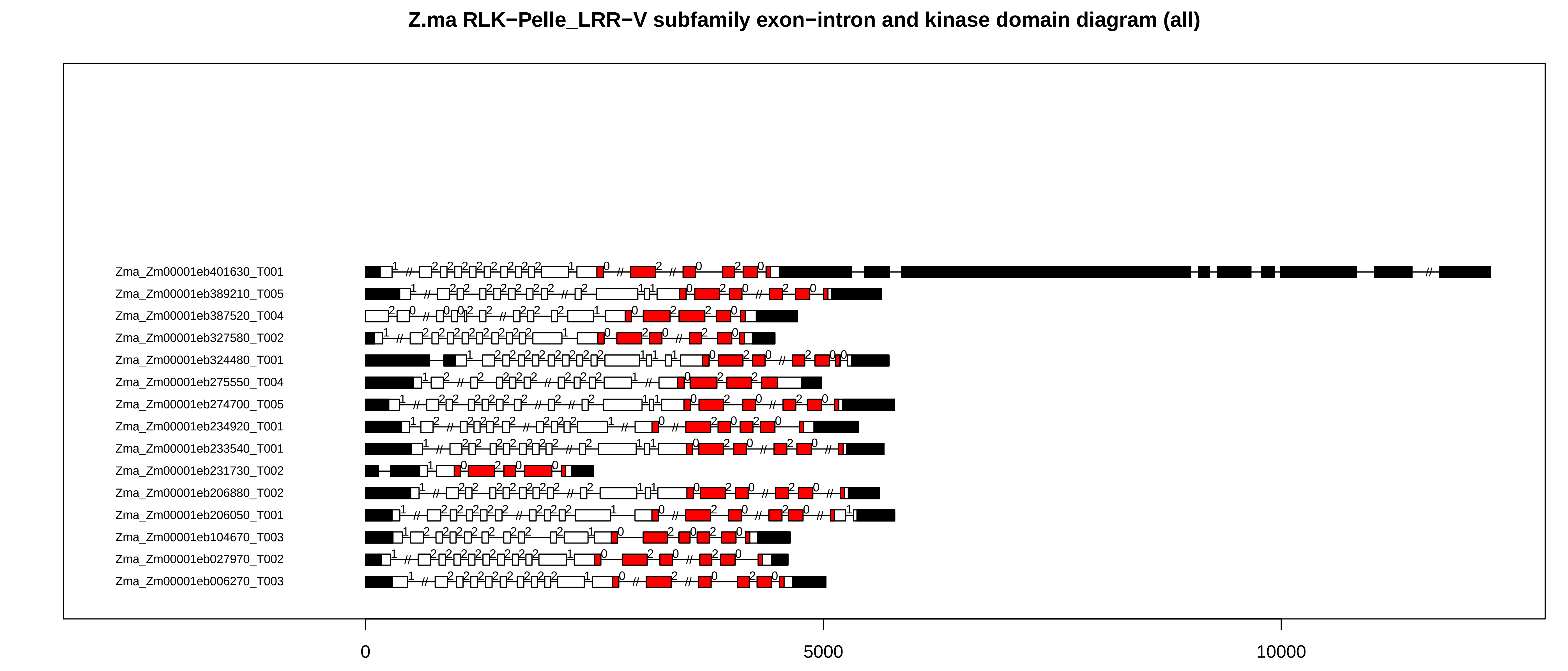
Z.ma RLK−Pelle_LRR−V subfamily exon−intron and kinase domain diagram (all)
Zma_Zm00001eb401630_T001
Zma_Zm00001eb389210_T005
Zma_Zm00001eb387520_T004
Zma_Zm00001eb327580_T002
Zma_Zm00001eb324480_T001
Zma_Zm00001eb275550_T004
Zma_Zm00001eb274700_T005
Zma_Zm00001eb234920_T001
Zma_Zm00001eb233540_T001
Zma_Zm00001eb231730_T002
Zma_Zm00001eb206880_T002
Zma_Zm00001eb206050_T001
Zma_Zm00001eb104670_T003
Zma_Zm00001eb027970_T002
Zma_Zm00001eb006270_T003
0
5000
10000

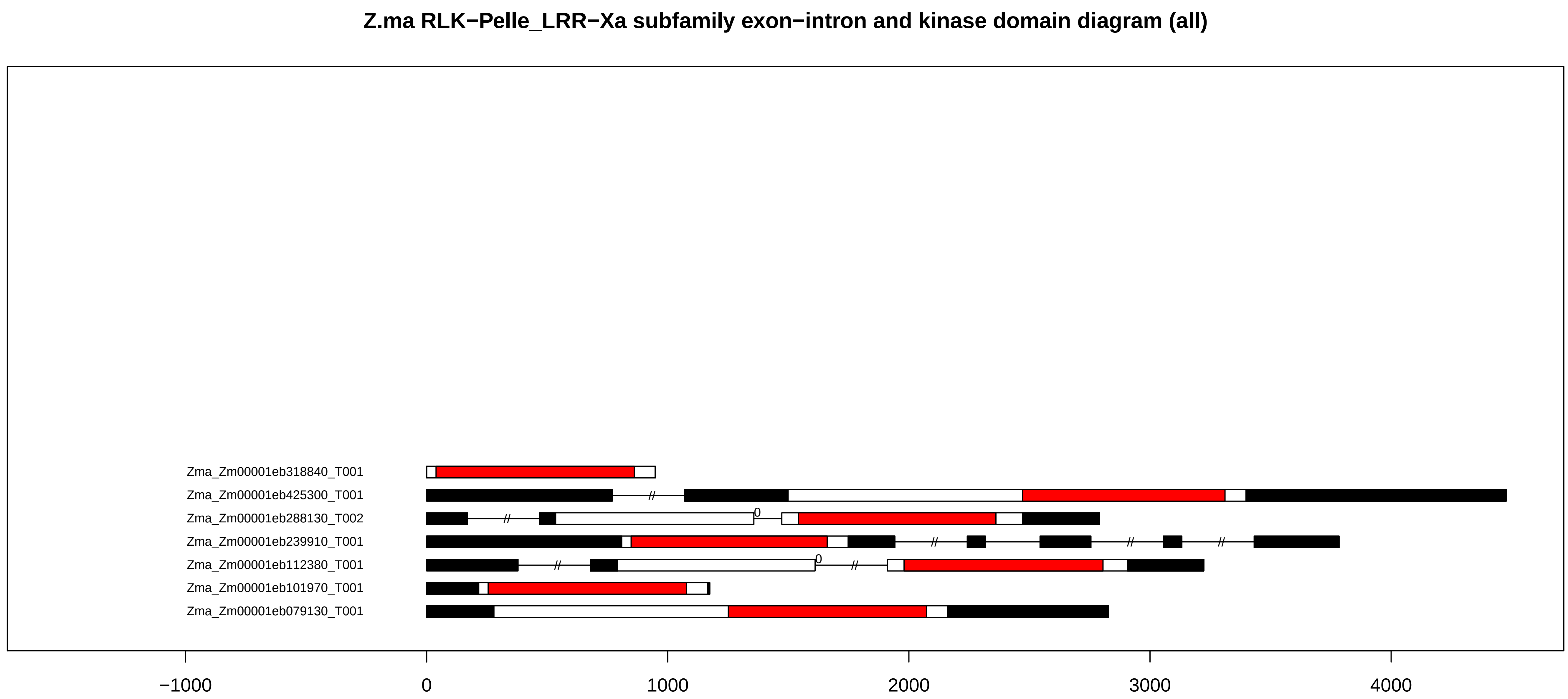
Z.ma RLK−Pelle_LRR−Xa subfamily exon−intron and kinase domain diagram (all)
Zma_Zm00001eb318840_T001
Zma_Zm00001eb425300_T001
Zma_Zm00001eb288130_T002
Zma_Zm00001eb239910_T001
Zma_Zm00001eb112380_T001
Zma_Zm00001eb101970_T001
Zma_Zm00001eb079130_T001
0
0
−1000
0
1000
2000
3000
4000

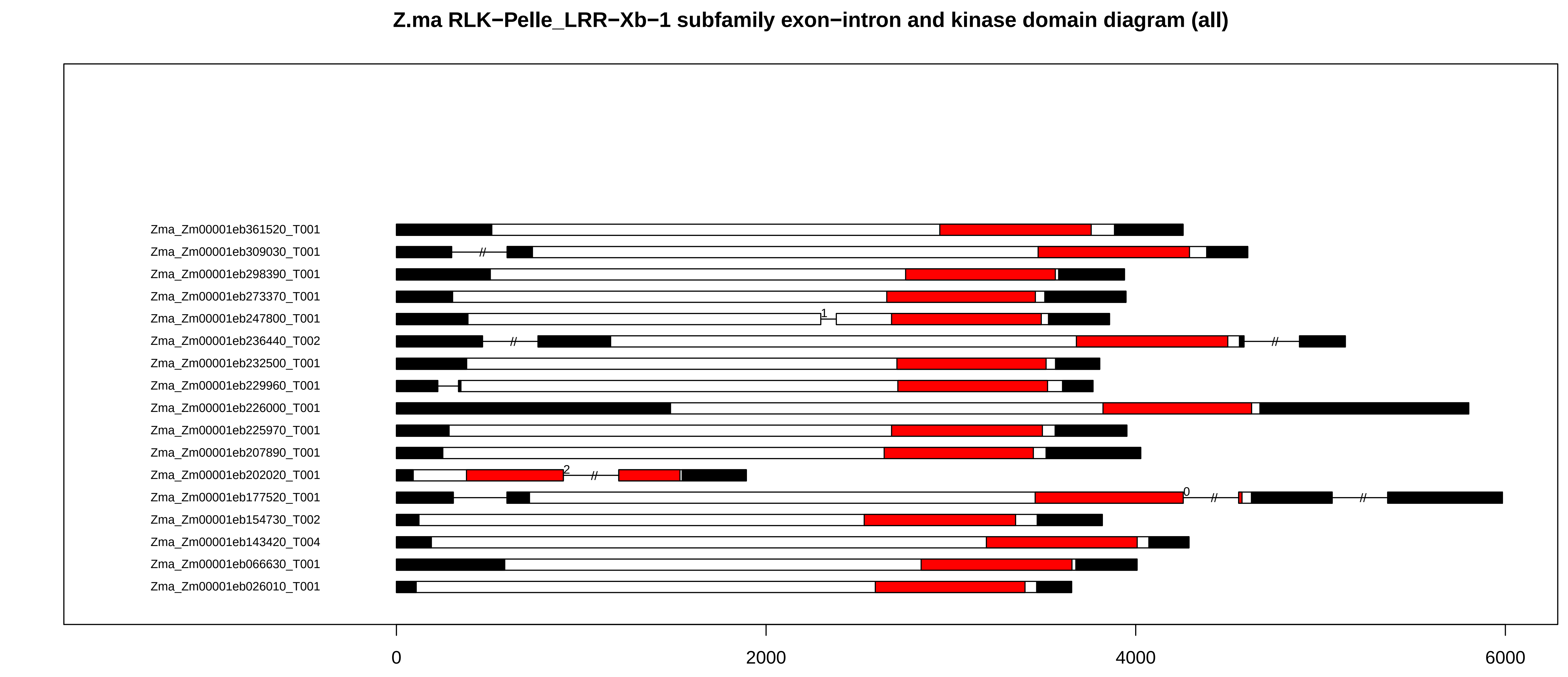

Z.ma RLK−Pelle_LRR−Xb−1 subfamily exon−intron and kinase domain diagram (all)
Zma_Zm00001eb361520_T001
Zma_Zm00001eb309030_T001
Zma_Zm00001eb298390_T001
Zma_Zm00001eb273370_T001
Zma_Zm00001eb247800_T001
Zma_Zm00001eb236440_T002
Zma_Zm00001eb232500_T001
Zma_Zm00001eb229960_T001
Zma_Zm00001eb226000_T001
Zma_Zm00001eb225970_T001
Zma_Zm00001eb207890_T001
Zma_Zm00001eb202020_T001
Zma_Zm00001eb177520_T001
Zma_Zm00001eb154730_T002
Zma_Zm00001eb143420_T004
Zma_Zm00001eb066630_T001
Zma_Zm00001eb026010_T001
1
2
0
0
2000
4000
6000

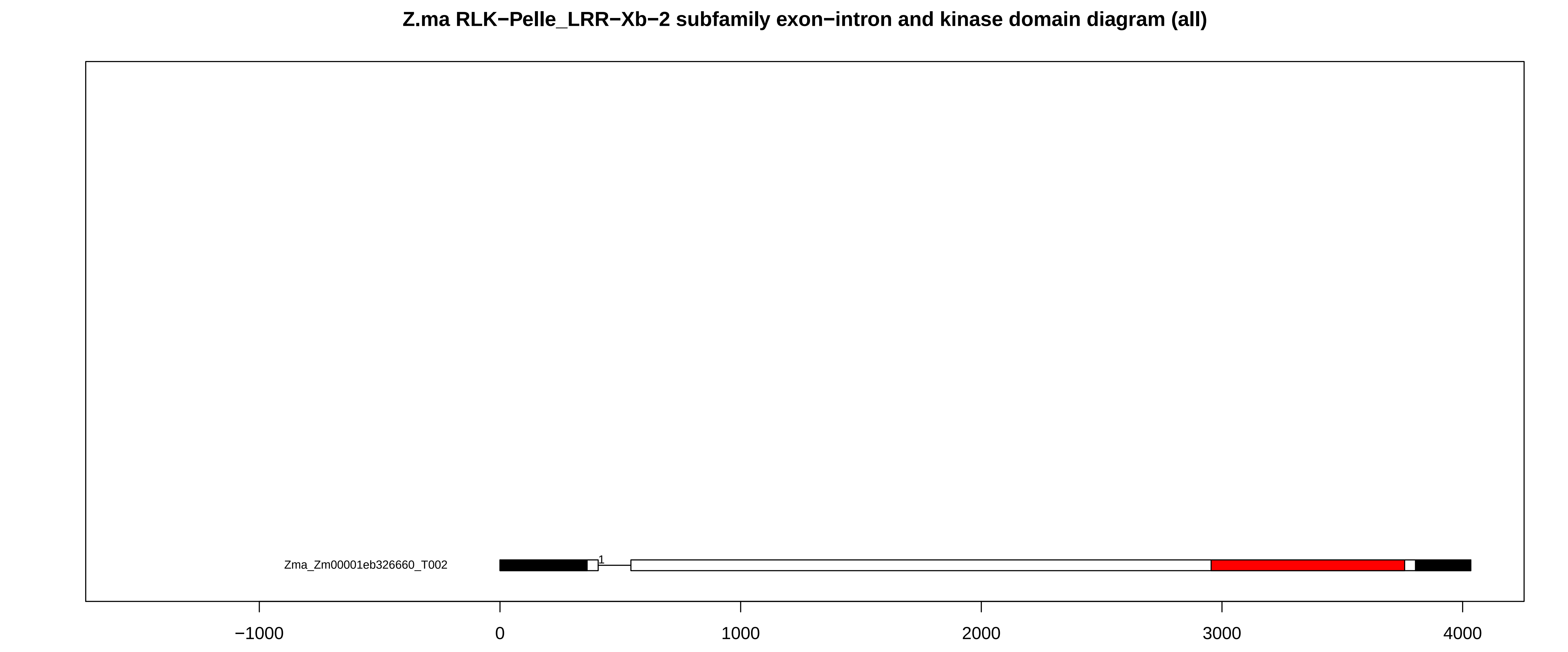

Z.ma RLK−Pelle_LRR−Xb−2 subfamily exon−intron and kinase domain diagram (all)
Zma_Zm00001eb326660_T002
1
−1000
0
1000
2000
3000
4000

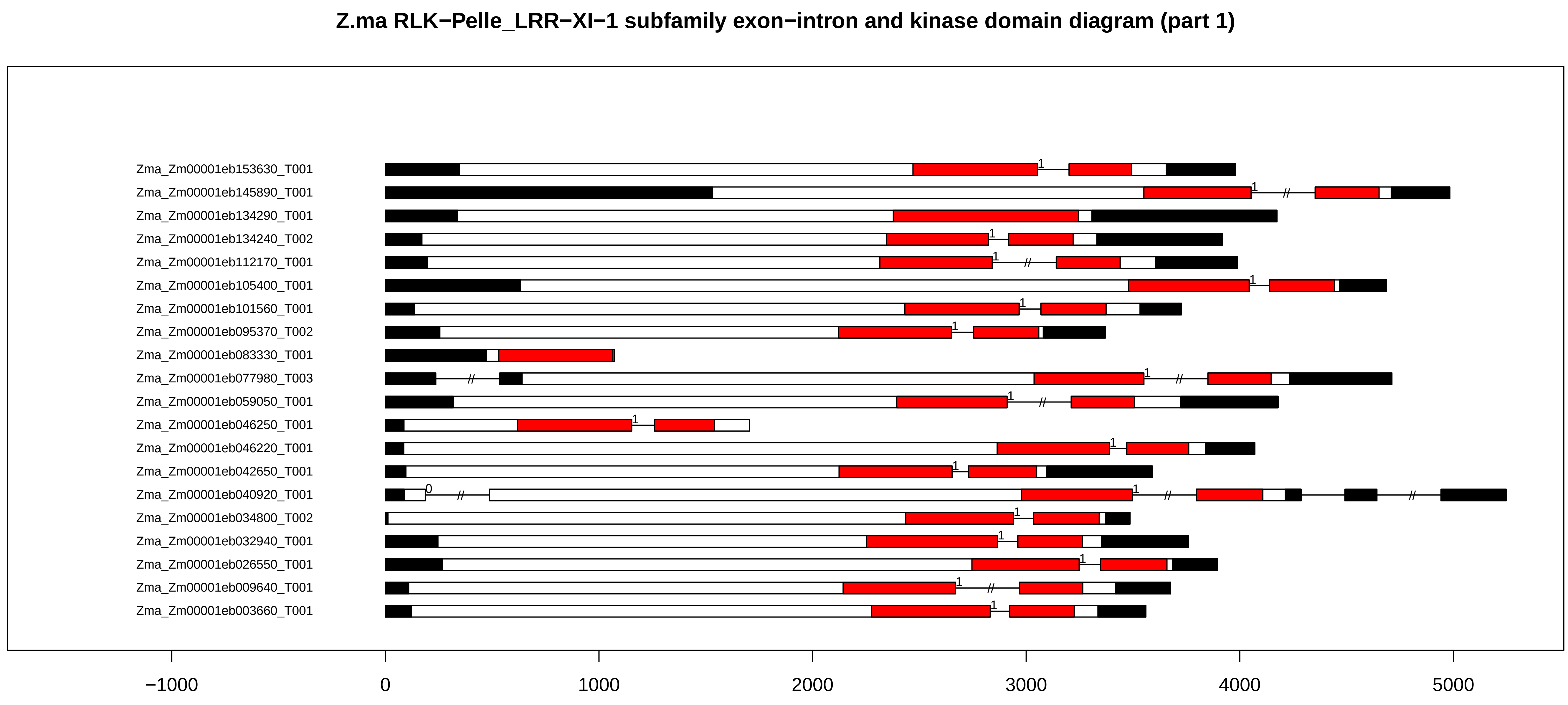
Z.ma RLK−Pelle_LRR−XI−1 subfamily exon−intron and kinase domain diagram (part 1)
Zma_Zm00001eb153630_T001
Zma_Zm00001eb145890_T001
Zma_Zm00001eb134290_T001
Zma_Zm00001eb134240_T002
Zma_Zm00001eb112170_T001
Zma_Zm00001eb105400_T001
Zma_Zm00001eb101560_T001
Zma_Zm00001eb095370_T002
Zma_Zm00001eb083330_T001
Zma_Zm00001eb077980_T003
Zma_Zm00001eb059050_T001
Zma_Zm00001eb046250_T001
Zma_Zm00001eb046220_T001
Zma_Zm00001eb042650_T001
Zma_Zm00001eb040920_T001
Zma_Zm00001eb034800_T002
Zma_Zm00001eb032940_T001
Zma_Zm00001eb026550_T001
Zma_Zm00001eb009640_T001
Zma_Zm00001eb003660_T001
−1000
0
1000
2000
3000
4000
5000

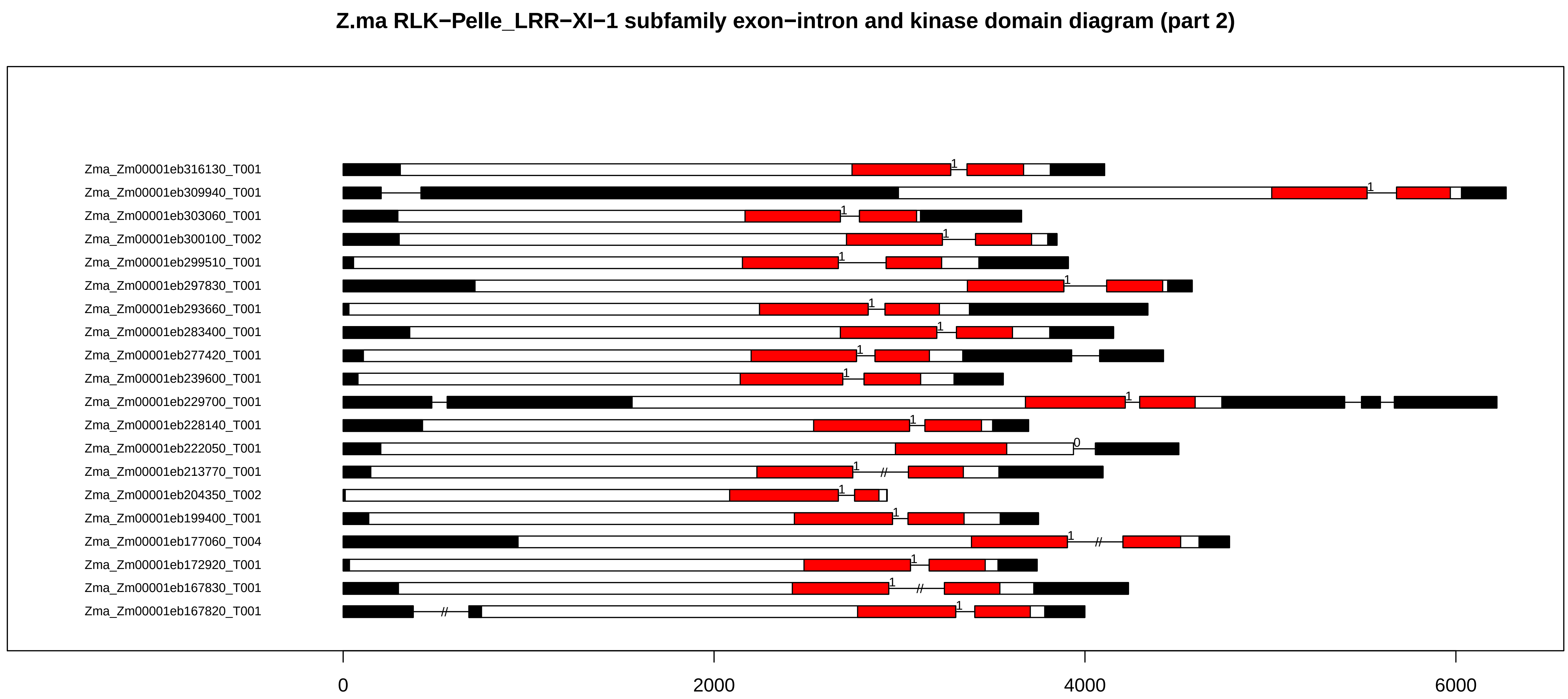
Z.ma RLK−Pelle_LRR−XI−1 subfamily exon−intron and kinase domain diagram (part 2)
Zma_Zm00001eb316130_T001
Zma_Zm00001eb309940_T001
Zma_Zm00001eb303060_T001
Zma_Zm00001eb300100_T002
Zma_Zm00001eb299510_T001
Zma_Zm00001eb297830_T001
Zma_Zm00001eb293660_T001
Zma_Zm00001eb283400_T001
Zma_Zm00001eb277420_T001
Zma_Zm00001eb239600_T001
Zma_Zm00001eb229700_T001
Zma_Zm00001eb228140_T001
Zma_Zm00001eb222050_T001
Zma_Zm00001eb213770_T001
Zma_Zm00001eb204350_T002
Zma_Zm00001eb199400_T001
Zma_Zm00001eb177060_T004
Zma_Zm00001eb172920_T001
Zma_Zm00001eb167830_T001
Zma_Zm00001eb167820_T001
0
2000
4000
6000

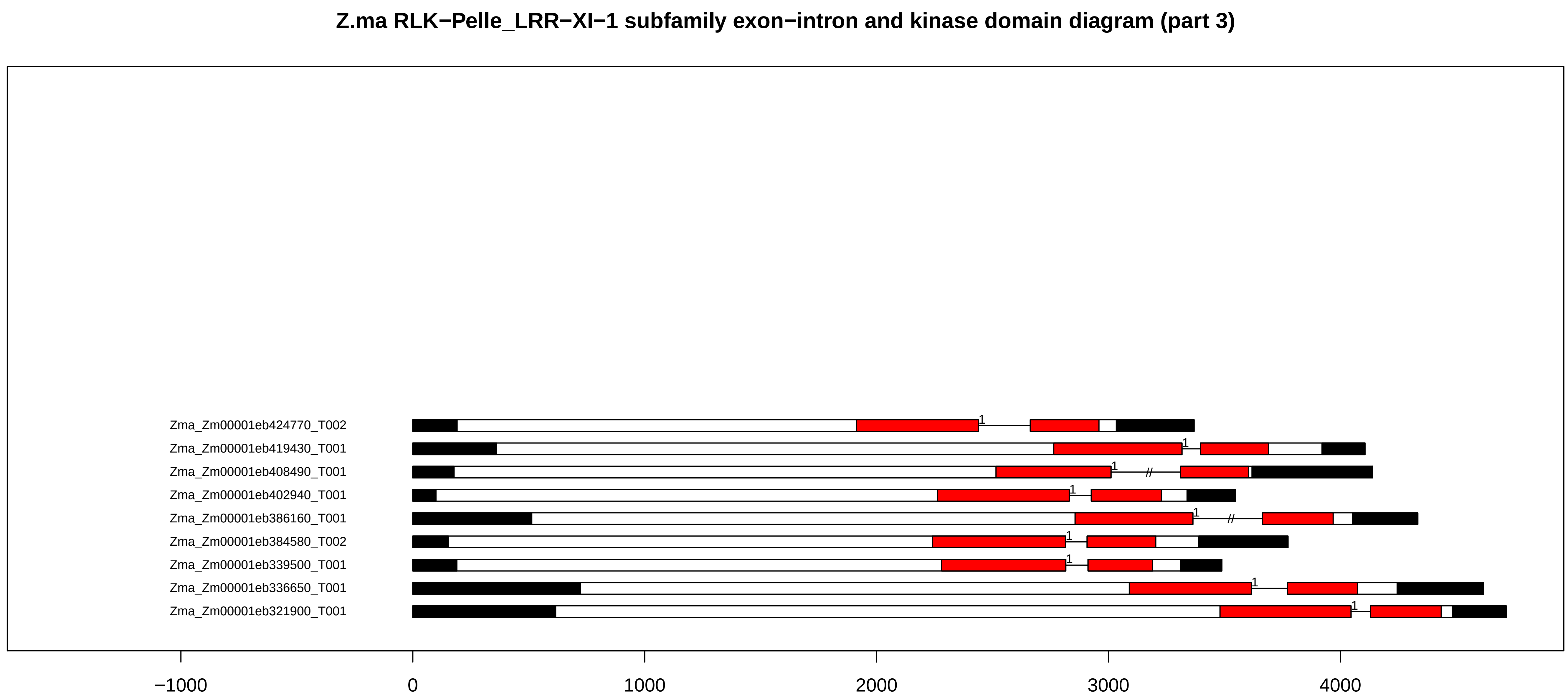

Z.ma RLK−Pelle_LRR−XI−1 subfamily exon−intron and kinase domain diagram (part 3)
Zma_Zm00001eb424770_T002
Zma_Zm00001eb419430_T001
Zma_Zm00001eb408490_T001
Zma_Zm00001eb402940_T001
Zma_Zm00001eb386160_T001
Zma_Zm00001eb384580_T002
Zma_Zm00001eb339500_T001
Zma_Zm00001eb336650_T001
Zma_Zm00001eb321900_T001
−1000
0
1000
2000
3000
4000

Z.ma RLK−Pelle_LRR−XI−2 subfamily exon−intron and kinase domain diagram (all)

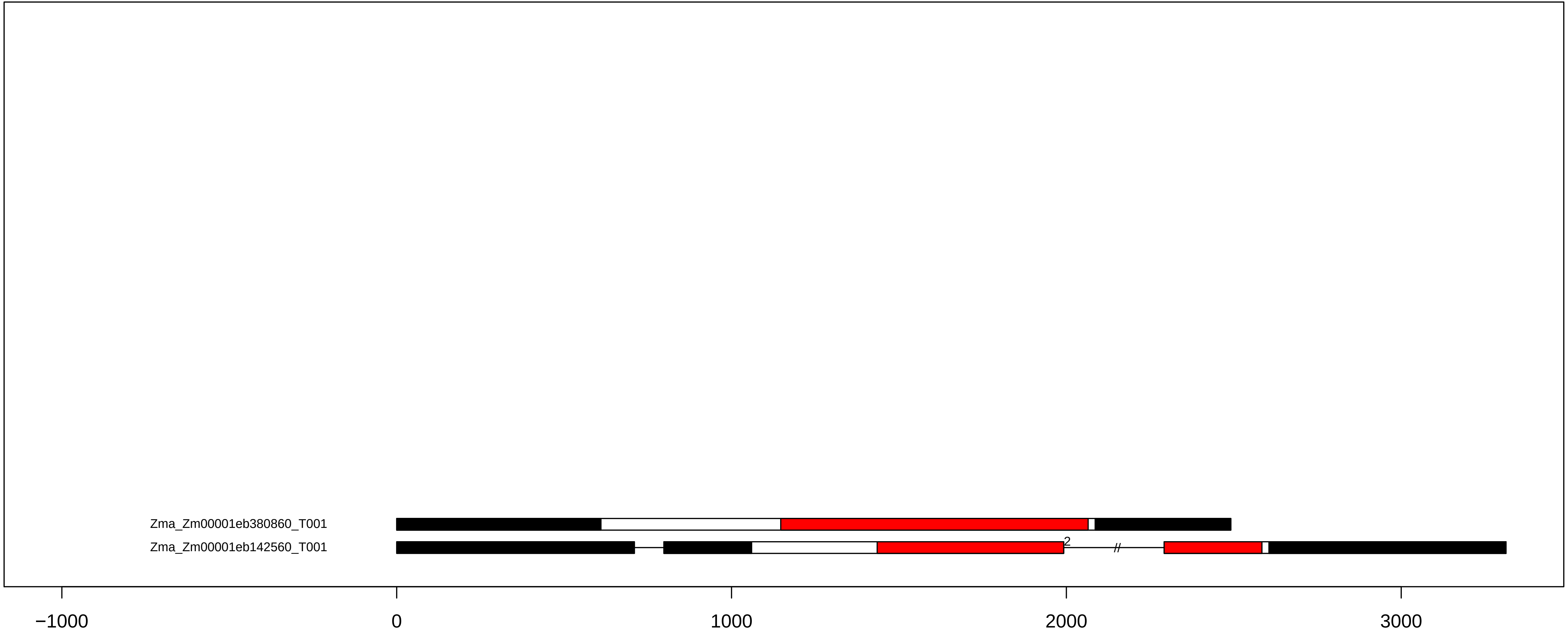

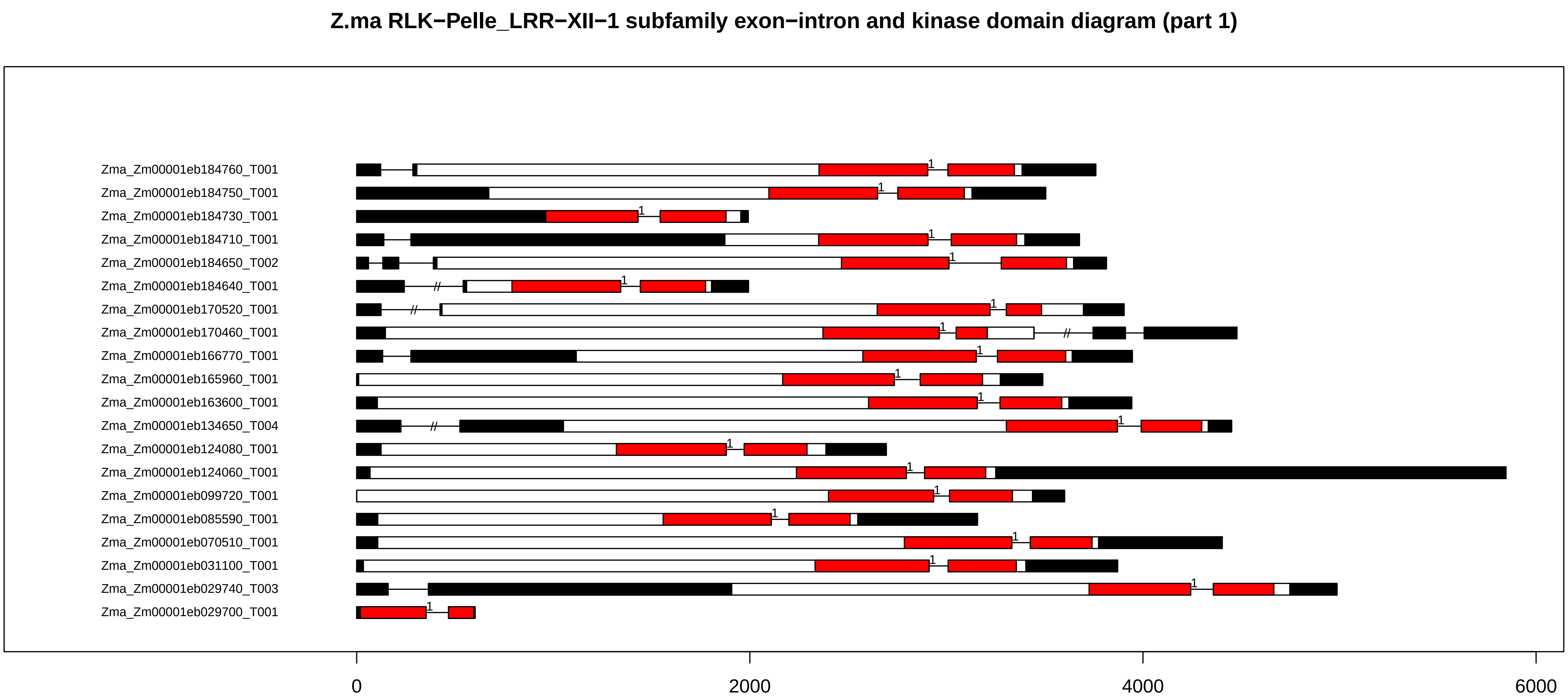
Z.ma RLK−Pelle_LRR−XII−1 subfamily exon−intron and kinase domain diagram (part 1)
Zma_Zm00001eb184760_T001
Zma_Zm00001eb184750_T001
Zma_Zm00001eb184730_T001
Zma_Zm00001eb184710_T001
Zma_Zm00001eb184650_T002
Zma_Zm00001eb184640_T001
Zma_Zm00001eb170520_T001
Zma_Zm00001eb170460_T001
Zma_Zm00001eb166770_T001
Zma_Zm00001eb165960_T001
Zma_Zm00001eb163600_T001
Zma_Zm00001eb134650_T004
Zma_Zm00001eb124080_T001
Zma_Zm00001eb124060_T001
Zma_Zm00001eb099720_T001
Zma_Zm00001eb085590_T001
Zma_Zm00001eb070510_T001
Zma_Zm00001eb031100_T001
Zma_Zm00001eb029740_T003
Zma_Zm00001eb029700_T001
0
2000
4000
6000

Z.ma RLK−Pelle_LRR−XII−1 subfamily exon−intron and kinase domain diagram (part 2)

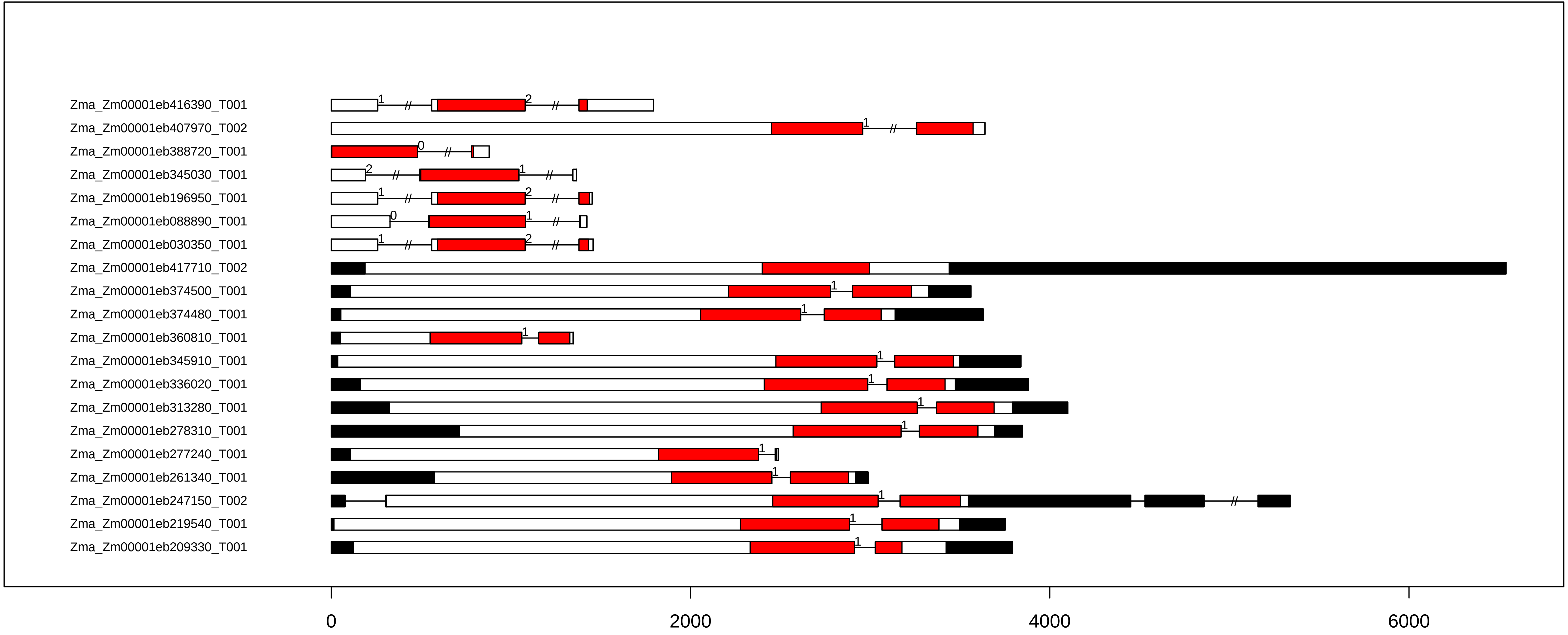

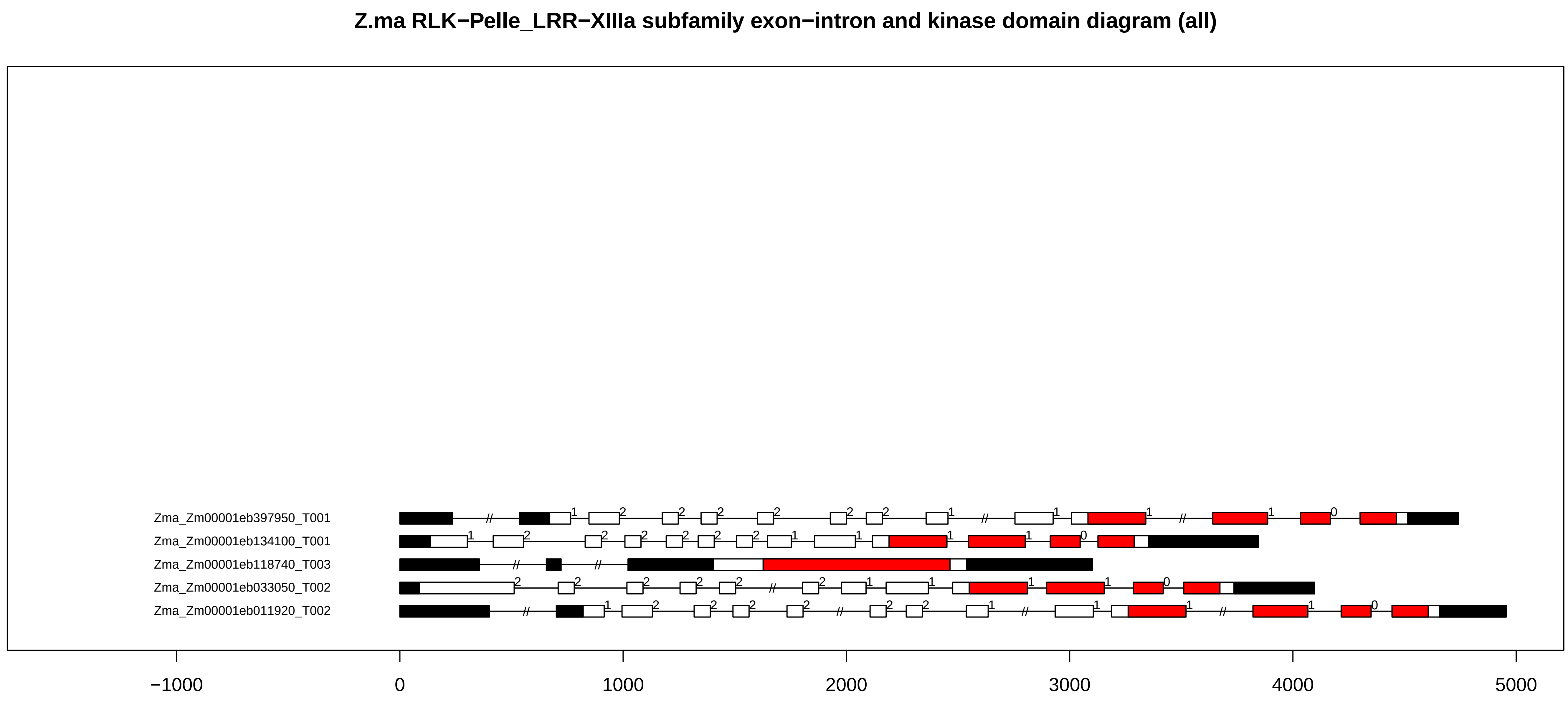

Z.ma RLK−Pelle_LRR−XIIIa subfamily exon−intron and kinase domain diagram (all)
Zma_Zm00001eb397950_T001
Zma_Zm00001eb134100_T001
Zma_Zm00001eb118740_T003
Zma_Zm00001eb033050_T002
Zma_Zm00001eb011920_T002
−1000
0
1000
2000
3000
4000
5000

Z.ma RLK−Pelle_LRR−XIIIb subfamily exon−intron and kinase domain diagram (all)

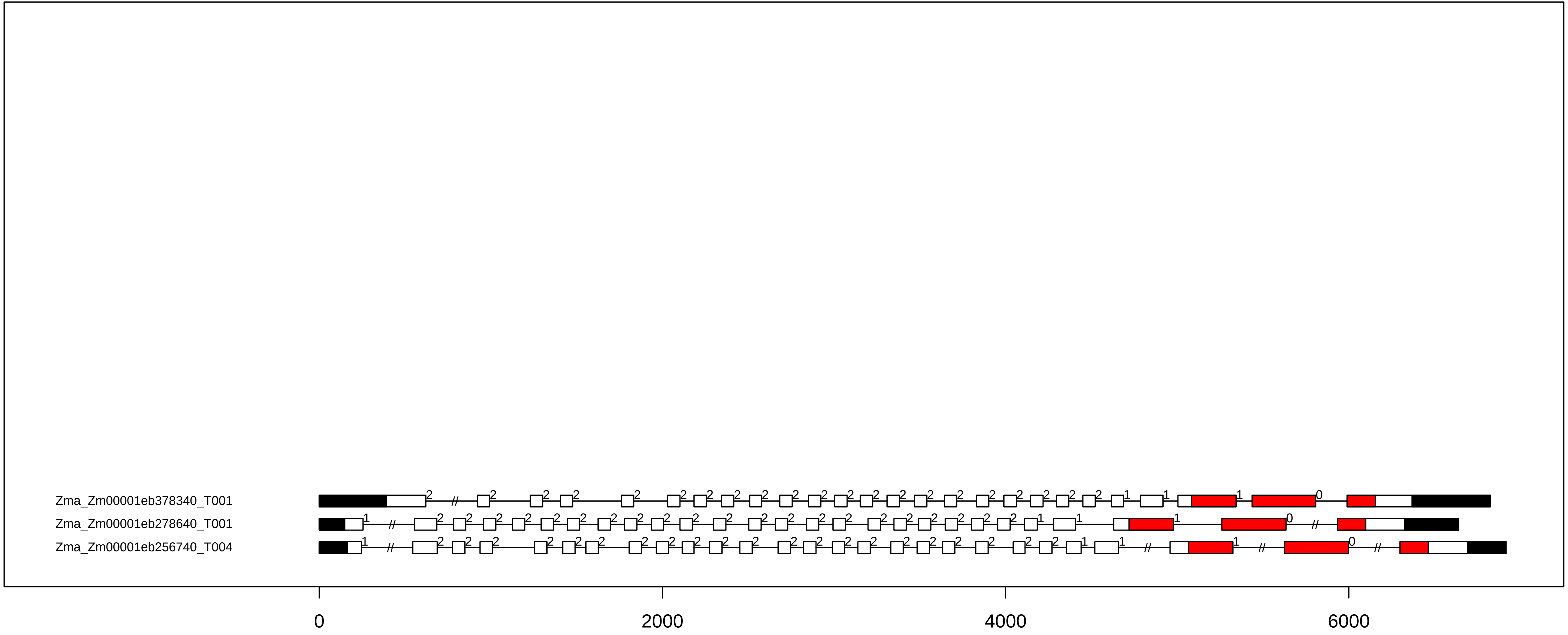

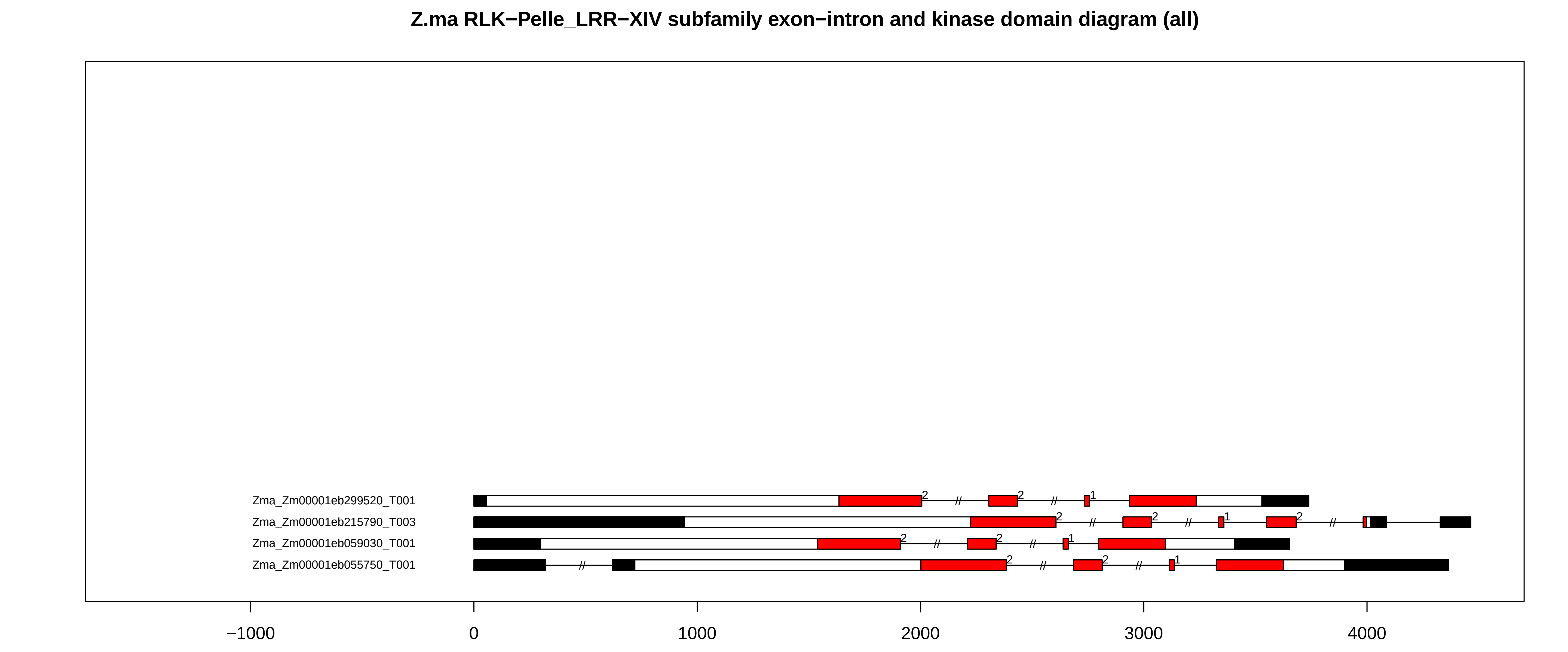

Z.ma RLK−Pelle_LRR−XIV subfamily exon−intron and kinase domain diagram (all)
Zma_Zm00001eb299520_T001
Zma_Zm00001eb215790_T003
Zma_Zm00001eb059030_T001
Zma_Zm00001eb055750_T001
−1000
0
1000
2000
3000
4000

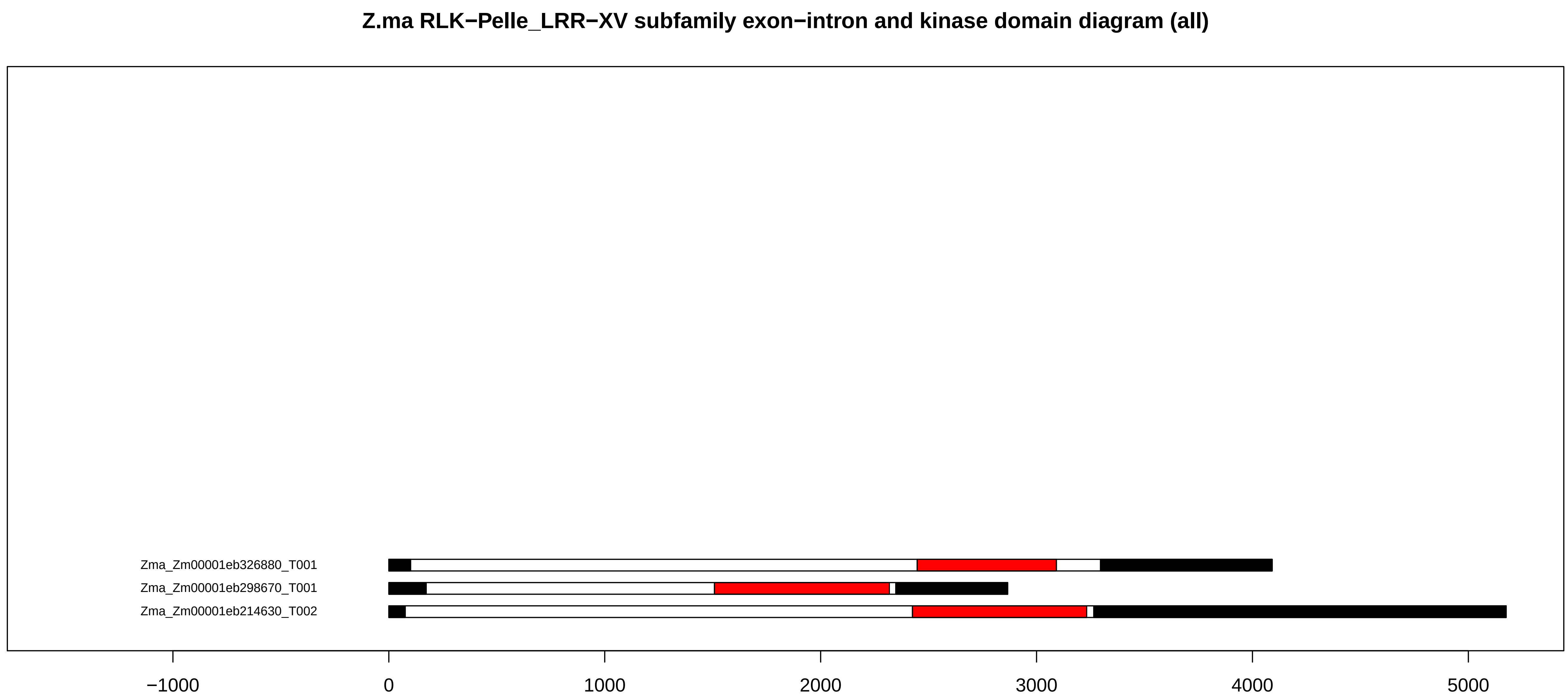

Z.ma RLK−Pelle_LRR−XV subfamily exon−intron and kinase domain diagram (all)
Zma_Zm00001eb326880_T001
Zma_Zm00001eb298670_T001
Zma_Zm00001eb214630_T002
−1000
0
1000
2000
3000
4000
5000

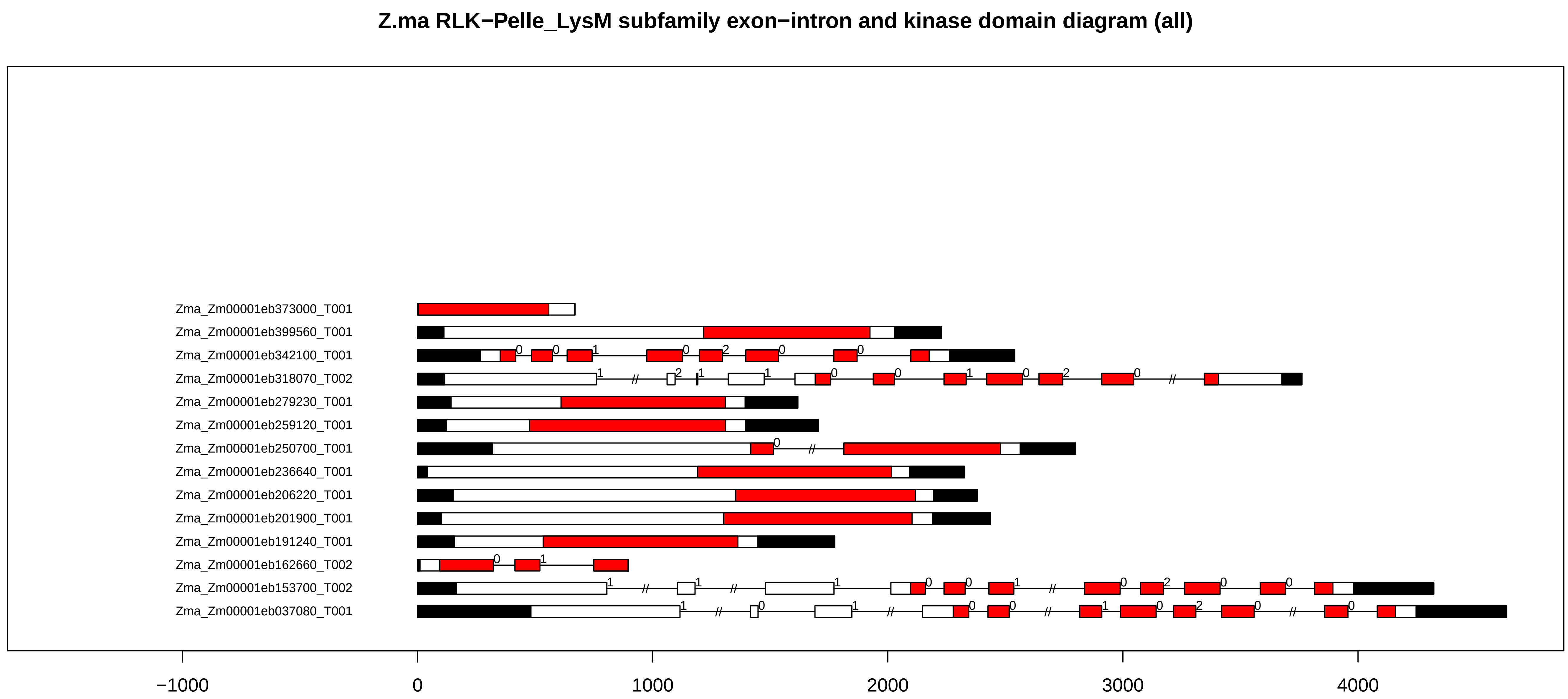
Z.ma RLK−Pelle_LysM subfamily exon−intron and kinase domain diagram (all)
Zma_Zm00001eb373000_T001
Zma_Zm00001eb399560_T001
Zma_Zm00001eb342100_T001
Zma_Zm00001eb318070_T002
Zma_Zm00001eb279230_T001
Zma_Zm00001eb259120_T001
Zma_Zm00001eb250700_T001
Zma_Zm00001eb236640_T001
Zma_Zm00001eb206220_T001
Zma_Zm00001eb201900_T001
Zma_Zm00001eb191240_T001
Zma_Zm00001eb162660_T002
Zma_Zm00001eb153700_T002
Zma_Zm00001eb037080_T001
−1000
0
1000
2000
3000
4000

Z.ma RLK−Pelle_PERK−1 subfamily exon−intron and kinase domain diagram (all)

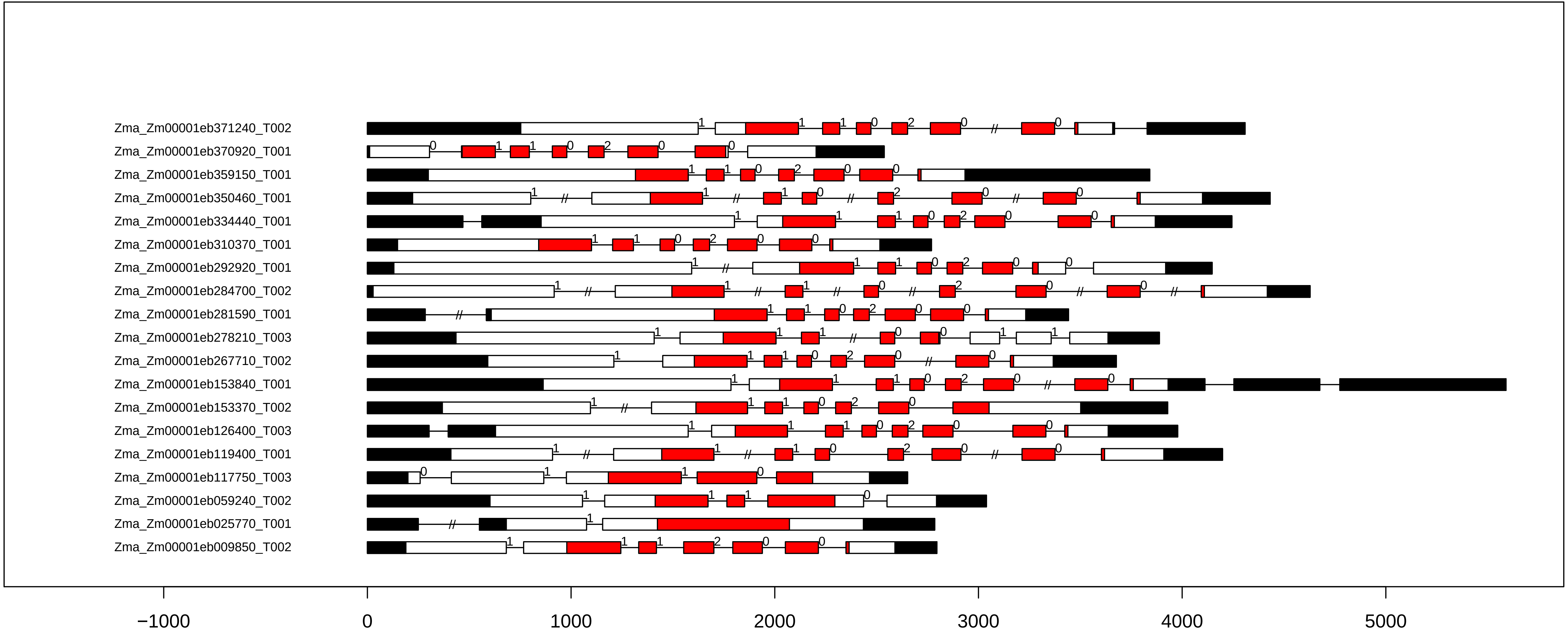

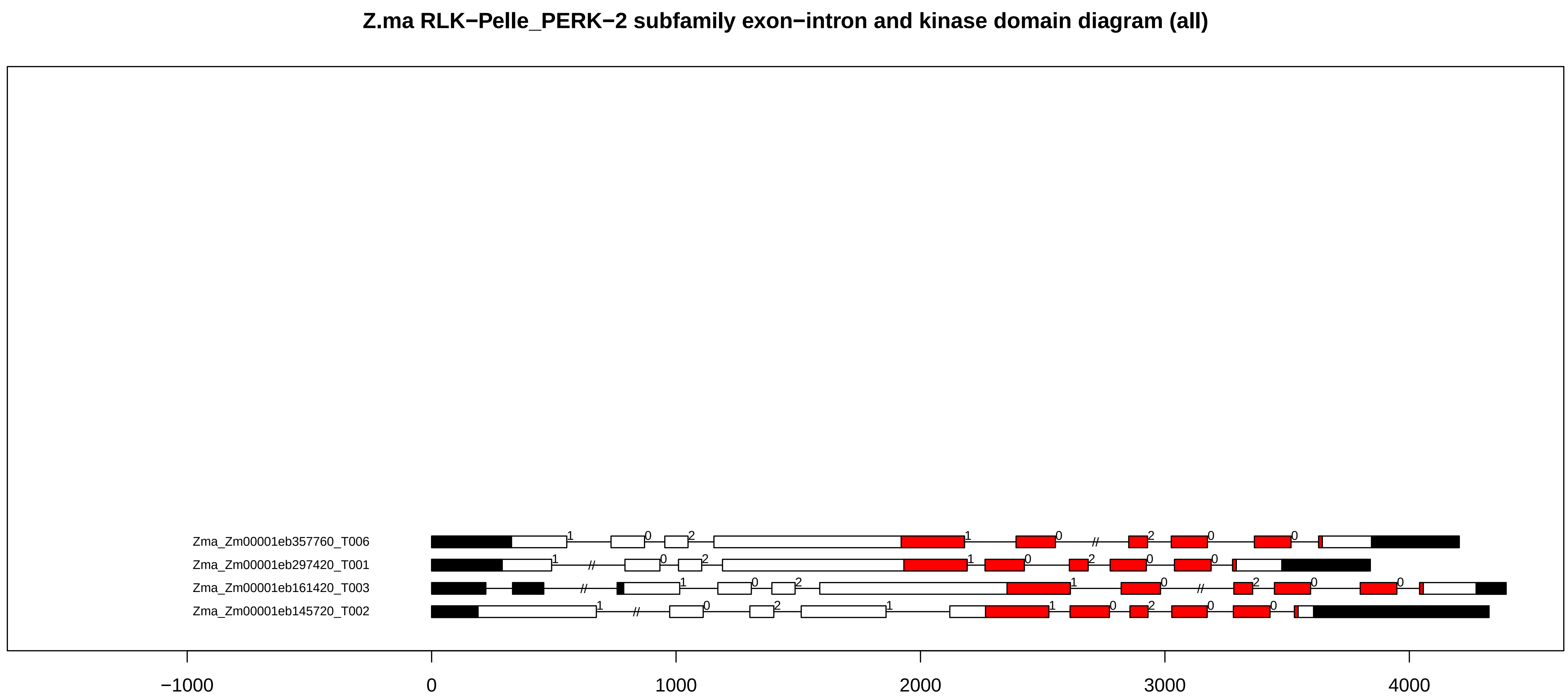
Z.ma RLK−Pelle_PERK−2 subfamily exon−intron and kinase domain diagram (all)
Zma_Zm00001eb357760_T006
Zma_Zm00001eb297420_T001
Zma_Zm00001eb161420_T003
Zma_Zm00001eb145720_T002
−1000
0
1000
2000
3000
4000

Z.ma RLK−Pelle_RKF3 subfamily exon−intron and kinase domain diagram (all)

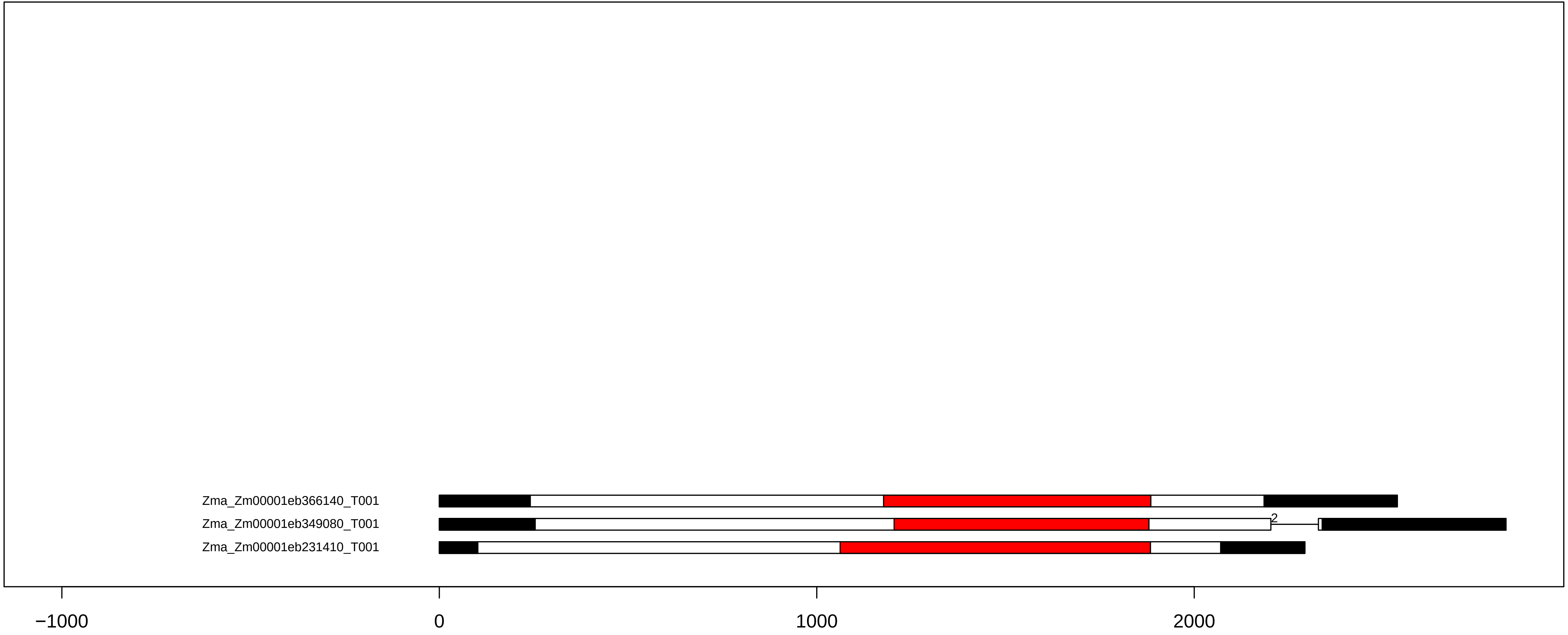

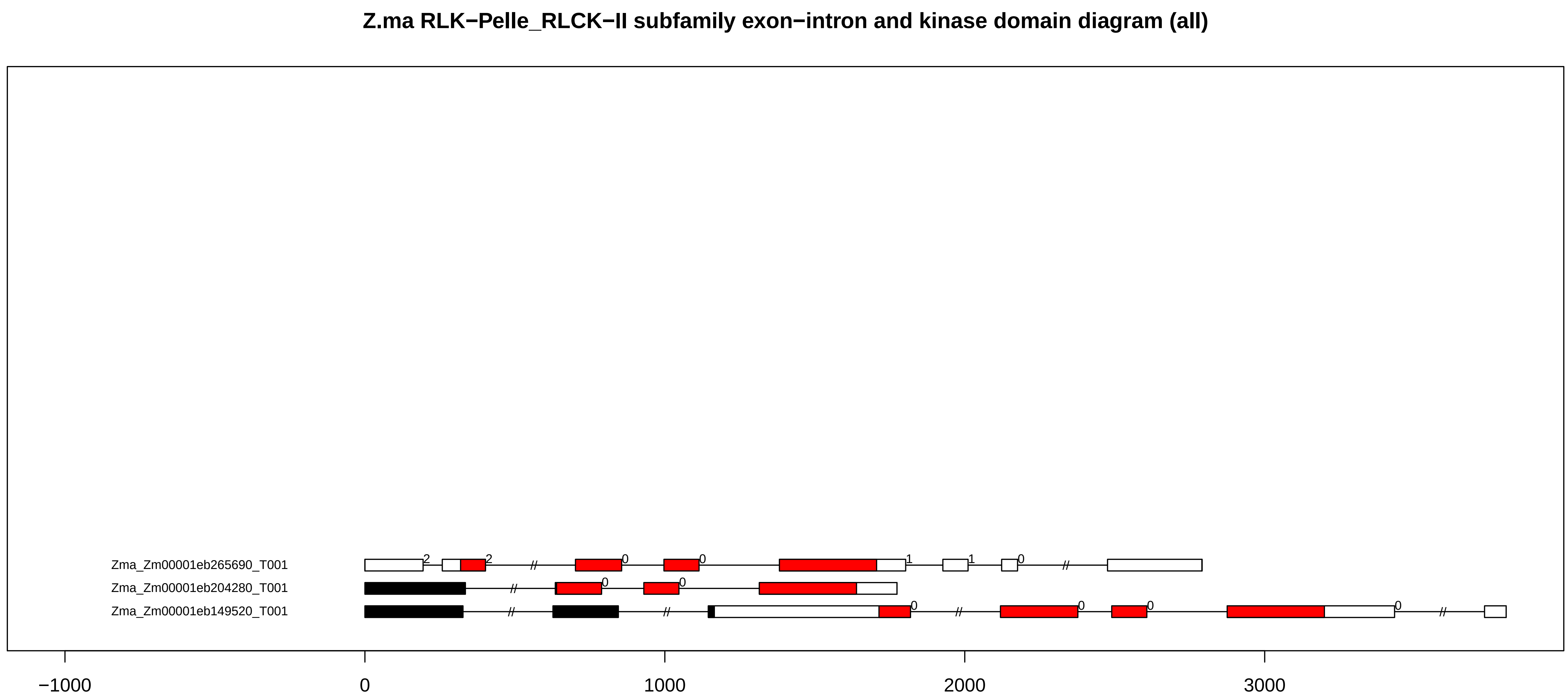

Z.ma RLK−Pelle_RLCK−II subfamily exon−intron and kinase domain diagram (all)
Zma_Zm00001eb265690_T001
Zma_Zm00001eb204280_T001
Zma_Zm00001eb149520_T001
2
2
0
0
1
1
0
0
0
0
0
0
0
−1000
0
1000
2000
3000

Z.ma RLK−Pelle_RLCK−IV subfamily exon−intron and kinase domain diagram (all)

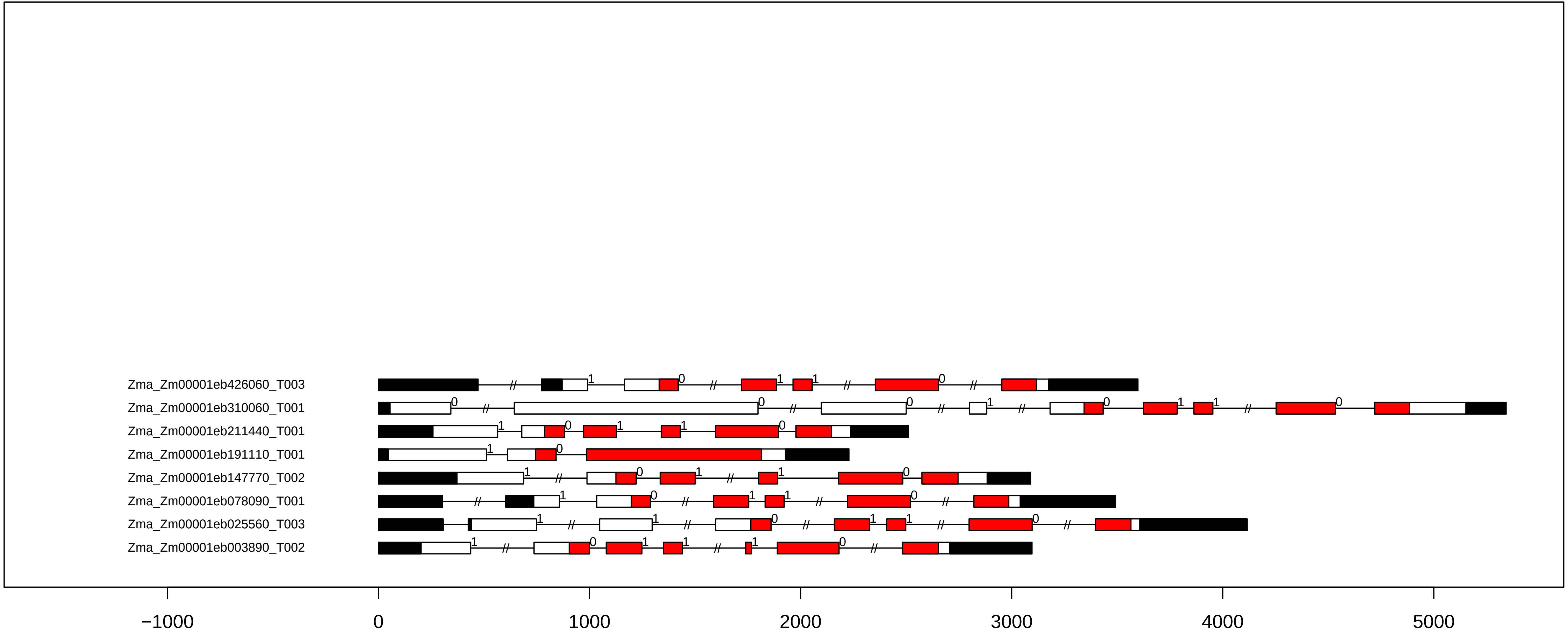

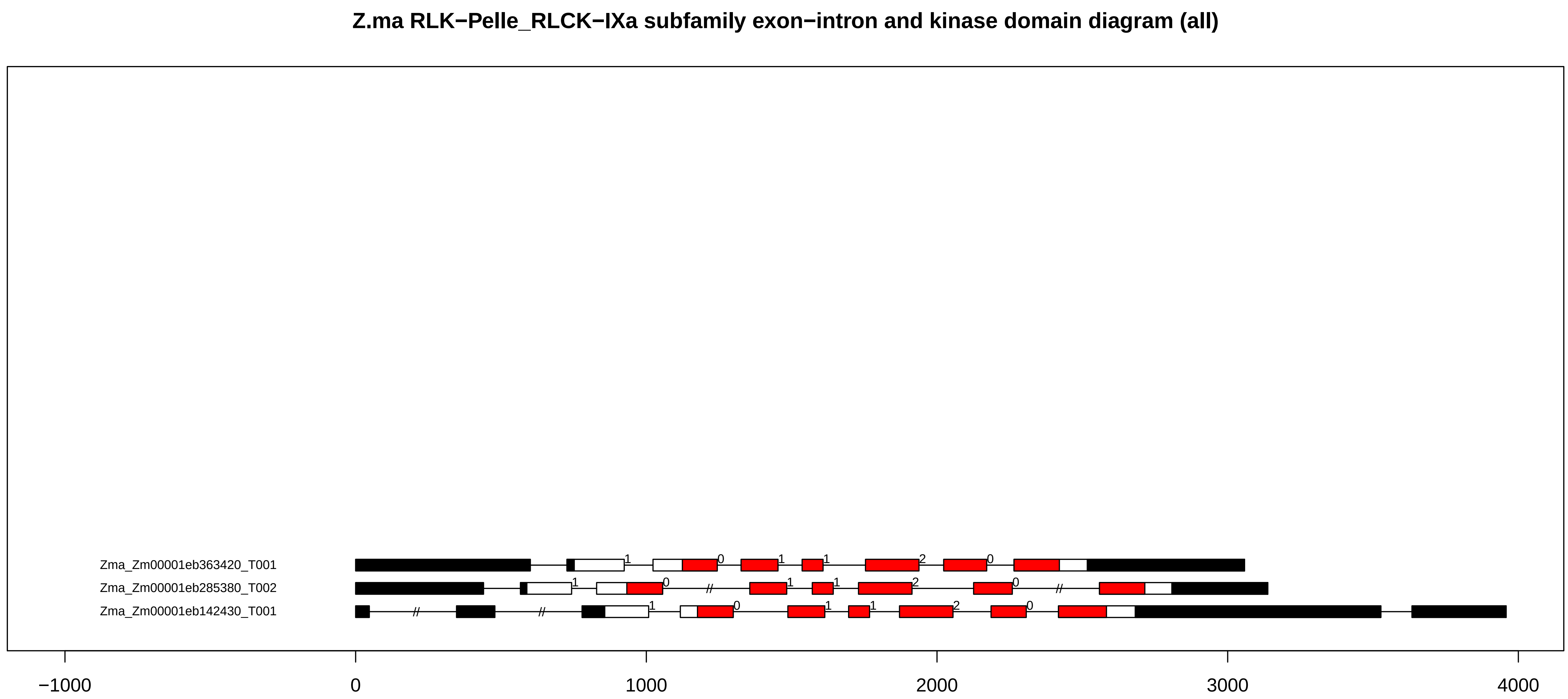
Z.ma RLK−Pelle_RLCK−IXa subfamily exon−intron and kinase domain diagram (all)
Zma_Zm00001eb363420_T001
Zma_Zm00001eb285380_T002
Zma_Zm00001eb142430_T001
−1000
0
1000
2000
3000
4000

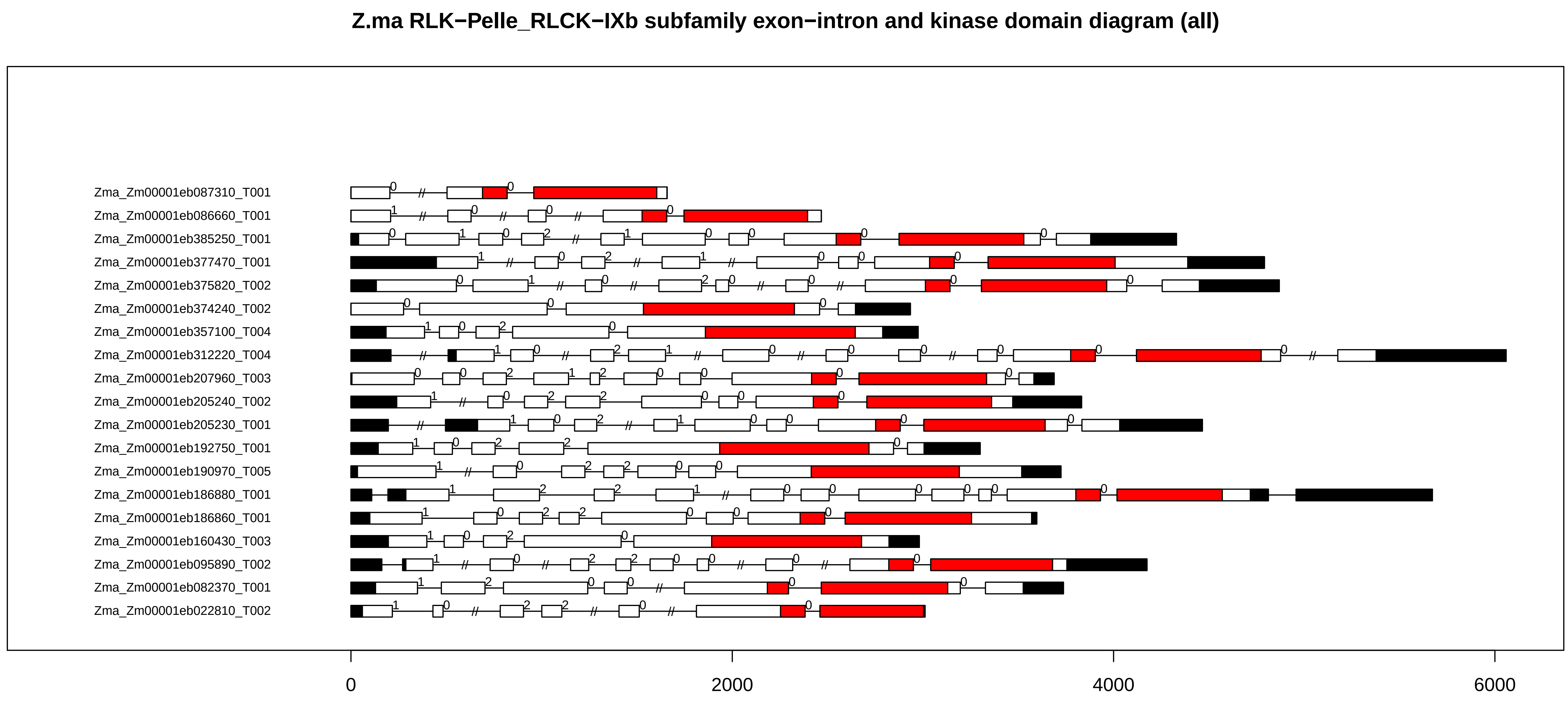

Z.ma RLK−Pelle_RLCK−IXb subfamily exon−intron and kinase domain diagram (all)
Zma_Zm00001eb087310_T001
Zma_Zm00001eb086660_T001
Zma_Zm00001eb385250_T001
Zma_Zm00001eb377470_T001
Zma_Zm00001eb375820_T002
Zma_Zm00001eb374240_T002
Zma_Zm00001eb357100_T004
Zma_Zm00001eb312220_T004
Zma_Zm00001eb207960_T003
Zma_Zm00001eb205240_T002
Zma_Zm00001eb205230_T001
Zma_Zm00001eb192750_T001
Zma_Zm00001eb190970_T005
Zma_Zm00001eb186880_T001
Zma_Zm00001eb186860_T001
Zma_Zm00001eb160430_T003
Zma_Zm00001eb095890_T002
Zma_Zm00001eb082370_T001
Zma_Zm00001eb022810_T002
0
2000
4000
6000

Z.ma RLK−Pelle_RLCK−Os subfamily exon−intron and kinase domain diagram (all)

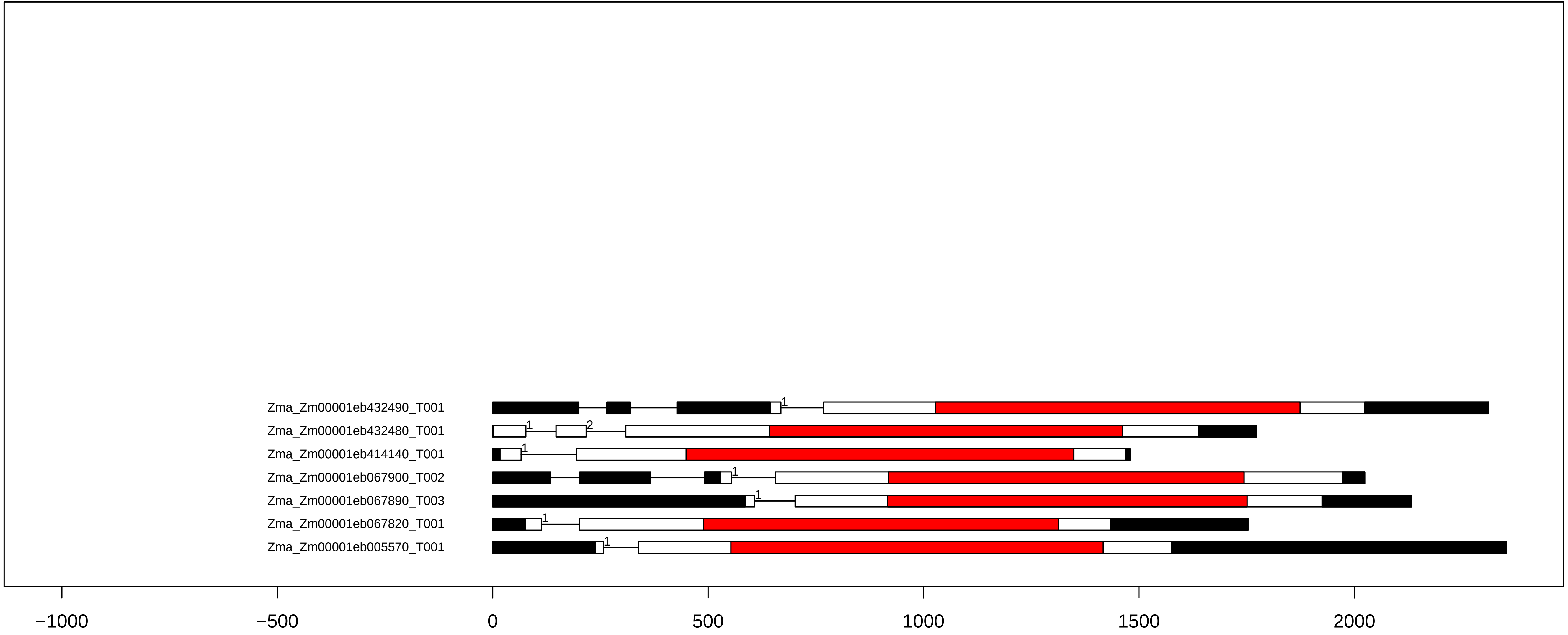

Z.ma RLK−Pelle_RLCK−VIIa−1 subfamily exon−intron and kinase domain diagram (part 1)

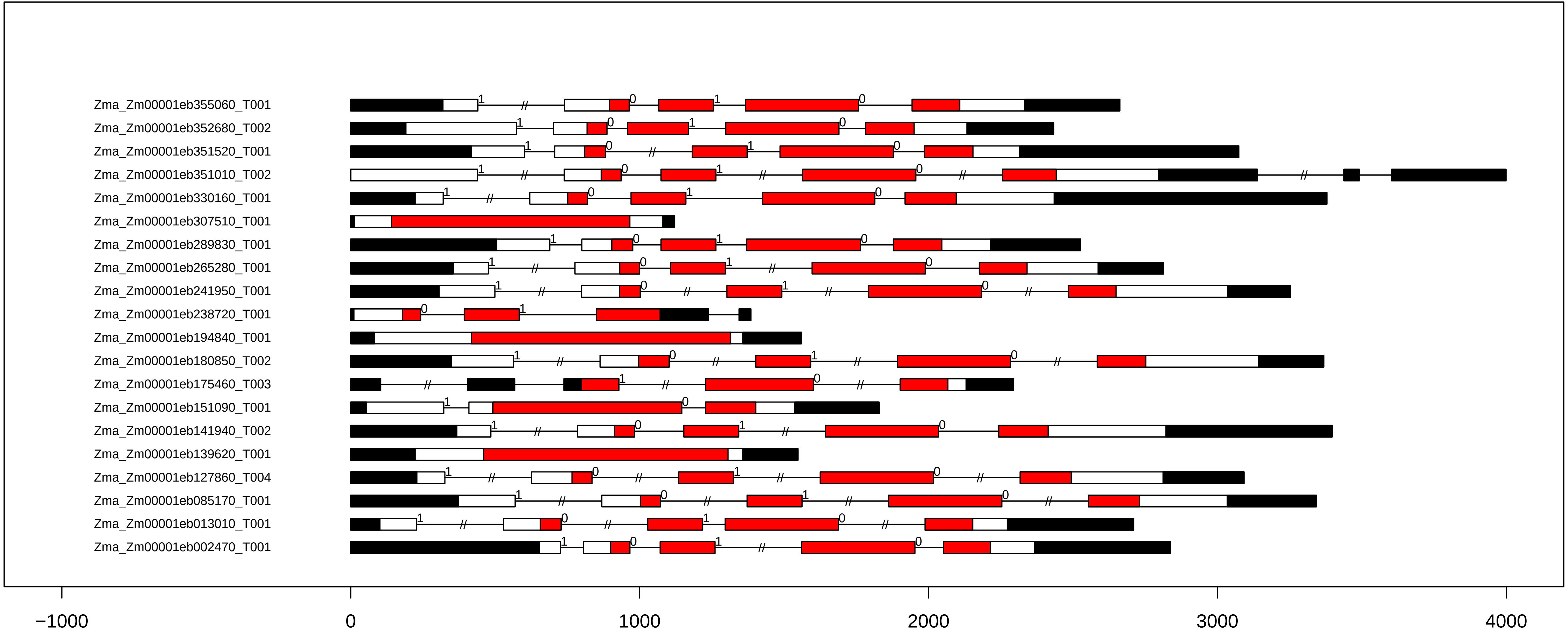

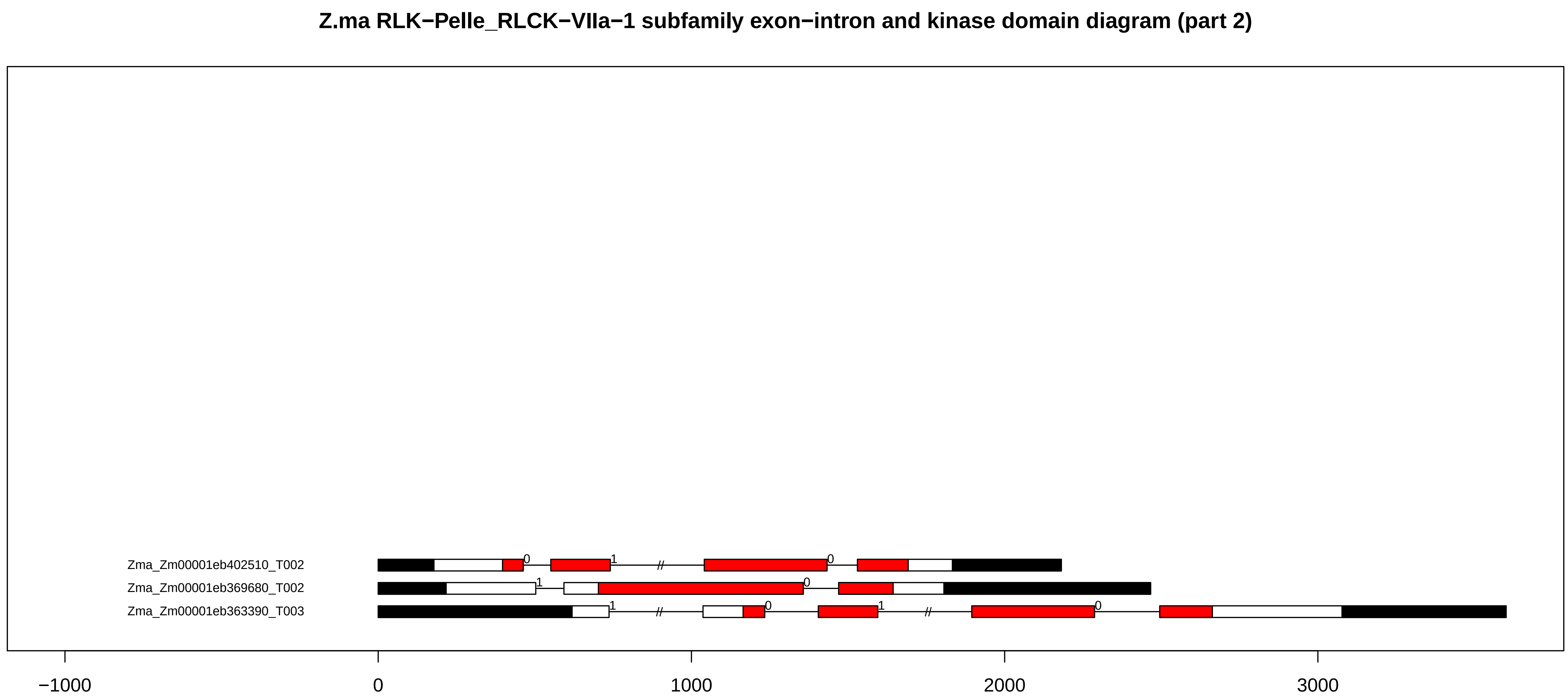
Z.ma RLK−Pelle_RLCK−VIIa−1 subfamily exon−intron and kinase domain diagram (part 2)
Zma_Zm00001eb402510_T002
Zma_Zm00001eb369680_T002
Zma_Zm00001eb363390_T003
0
1
0
1
0
1
0
1
0
−1000
0
1000
2000
3000

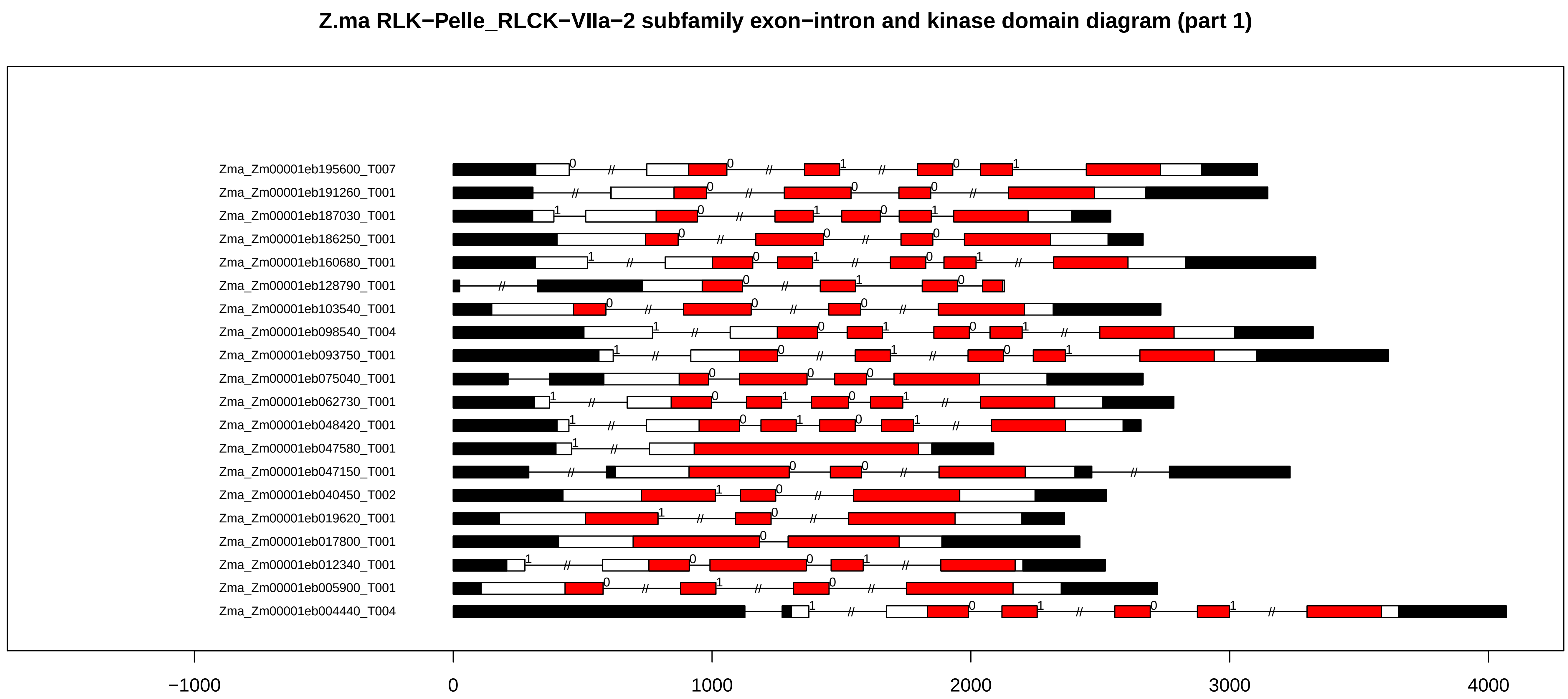

Z.ma RLK−Pelle_RLCK−VIIa−2 subfamily exon−intron and kinase domain diagram (part 1)
Zma_Zm00001eb195600_T007
Zma_Zm00001eb191260_T001
Zma_Zm00001eb187030_T001
Zma_Zm00001eb186250_T001
Zma_Zm00001eb160680_T001
Zma_Zm00001eb128790_T001
Zma_Zm00001eb103540_T001
Zma_Zm00001eb098540_T004
Zma_Zm00001eb093750_T001
Zma_Zm00001eb075040_T001
Zma_Zm00001eb062730_T001
Zma_Zm00001eb048420_T001
Zma_Zm00001eb047580_T001
Zma_Zm00001eb047150_T001
Zma_Zm00001eb040450_T002
Zma_Zm00001eb019620_T001
Zma_Zm00001eb017800_T001
Zma_Zm00001eb012340_T001
Zma_Zm00001eb005900_T001
Zma_Zm00001eb004440_T004
−1000
0
1000
2000
3000
4000

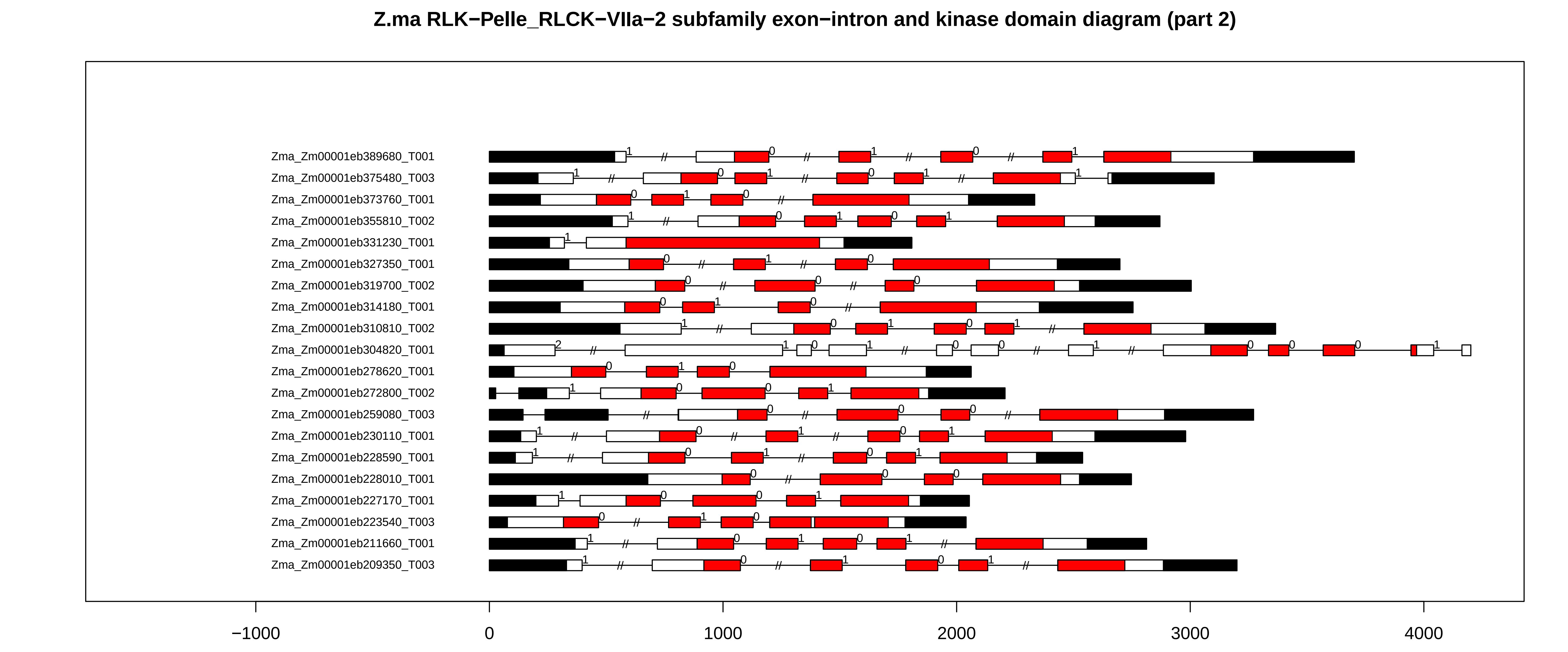

Z.ma RLK−Pelle_RLCK−VIIa−2 subfamily exon−intron and kinase domain diagram (part 2)
Zma_Zm00001eb389680_T001
Zma_Zm00001eb375480_T003
Zma_Zm00001eb373760_T001
Zma_Zm00001eb355810_T002
Zma_Zm00001eb331230_T001
Zma_Zm00001eb327350_T001
Zma_Zm00001eb319700_T002
Zma_Zm00001eb314180_T001
Zma_Zm00001eb310810_T002
Zma_Zm00001eb304820_T001
Zma_Zm00001eb278620_T001
Zma_Zm00001eb272800_T002
Zma_Zm00001eb259080_T003
Zma_Zm00001eb230110_T001
Zma_Zm00001eb228590_T001
Zma_Zm00001eb228010_T001
Zma_Zm00001eb227170_T001
Zma_Zm00001eb223540_T003
Zma_Zm00001eb211660_T001
Zma_Zm00001eb209350_T003
−1000
0
1000
2000
3000
4000

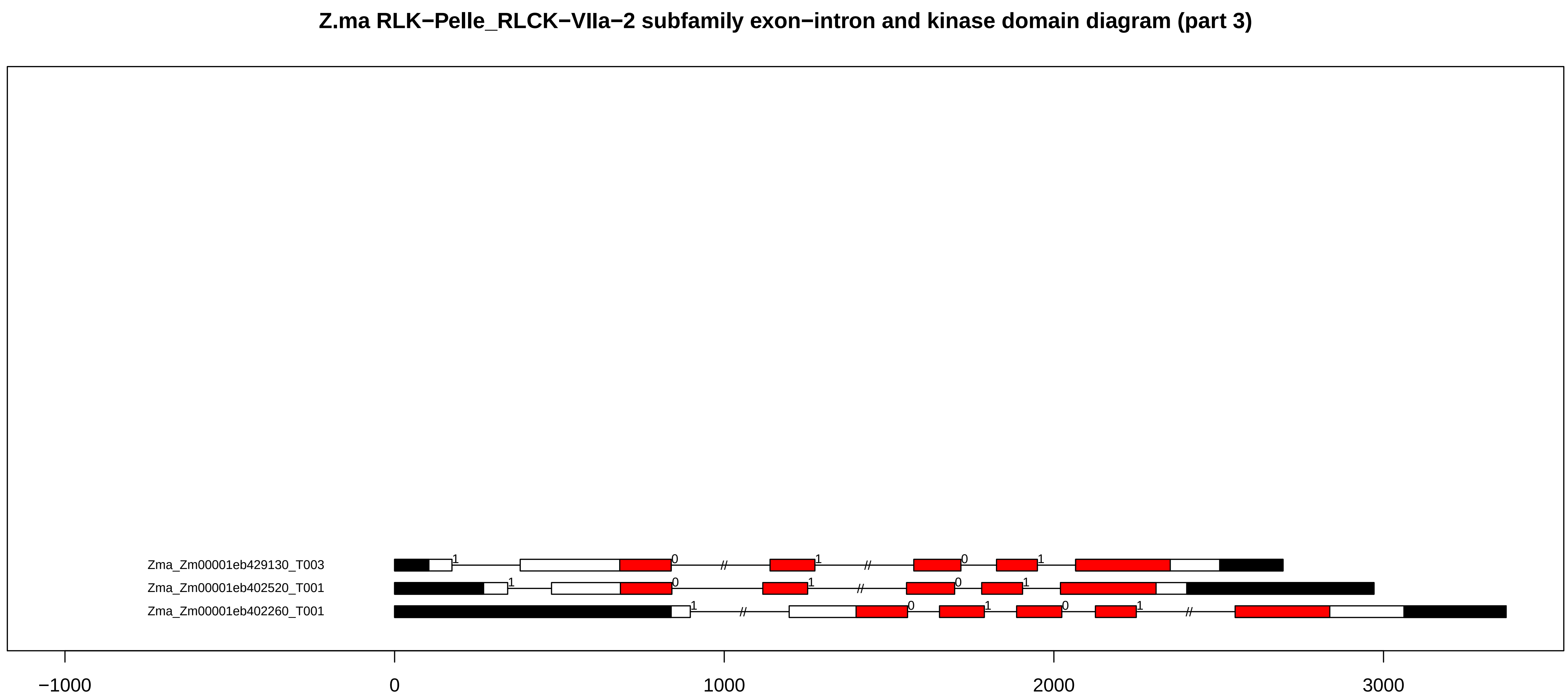

Z.ma RLK−Pelle_RLCK−VIIa−2 subfamily exon−intron and kinase domain diagram (part 3)
Zma_Zm00001eb429130_T003
Zma_Zm00001eb402520_T001
Zma_Zm00001eb402260_T001
−1000
0
1000
2000
3000

Z.ma RLK−Pelle_RLCK−VIIb subfamily exon−intron and kinase domain diagram (all)

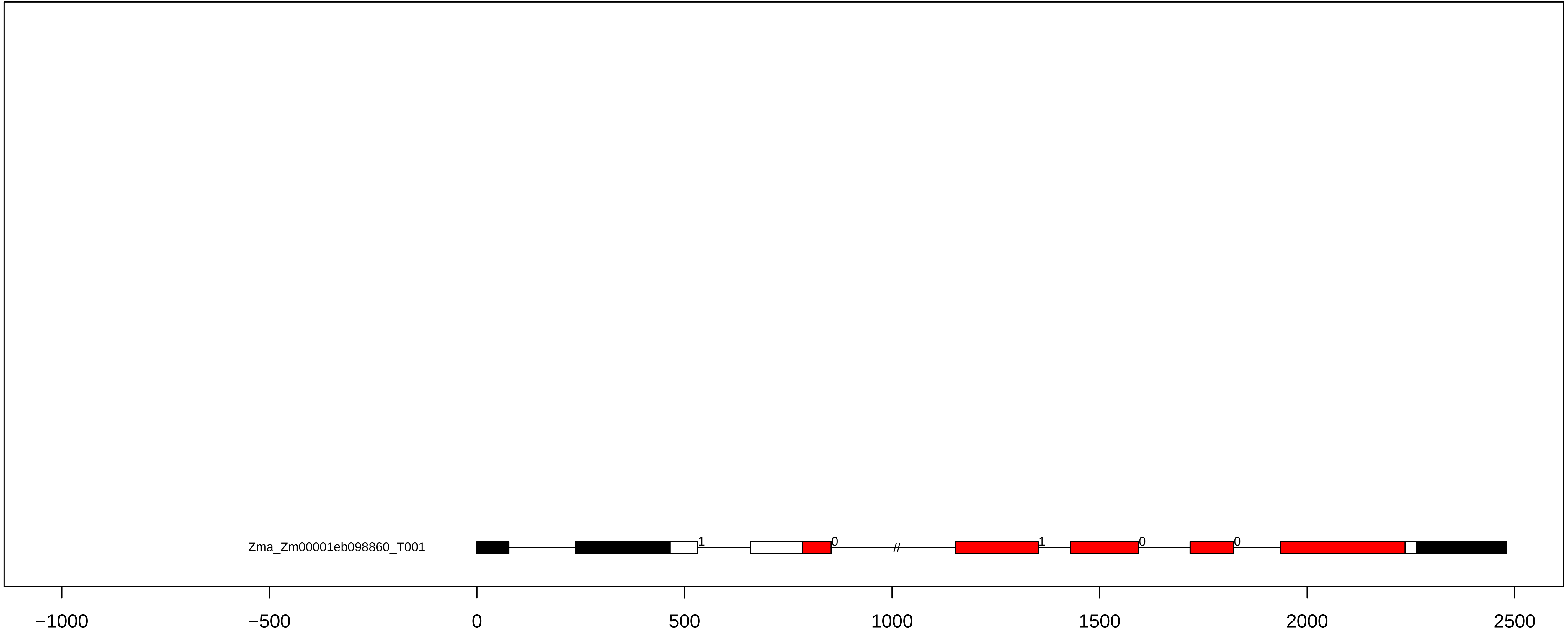

Z.ma RLK−Pelle_RLCK−VIII subfamily exon−intron and kinase domain diagram (all)

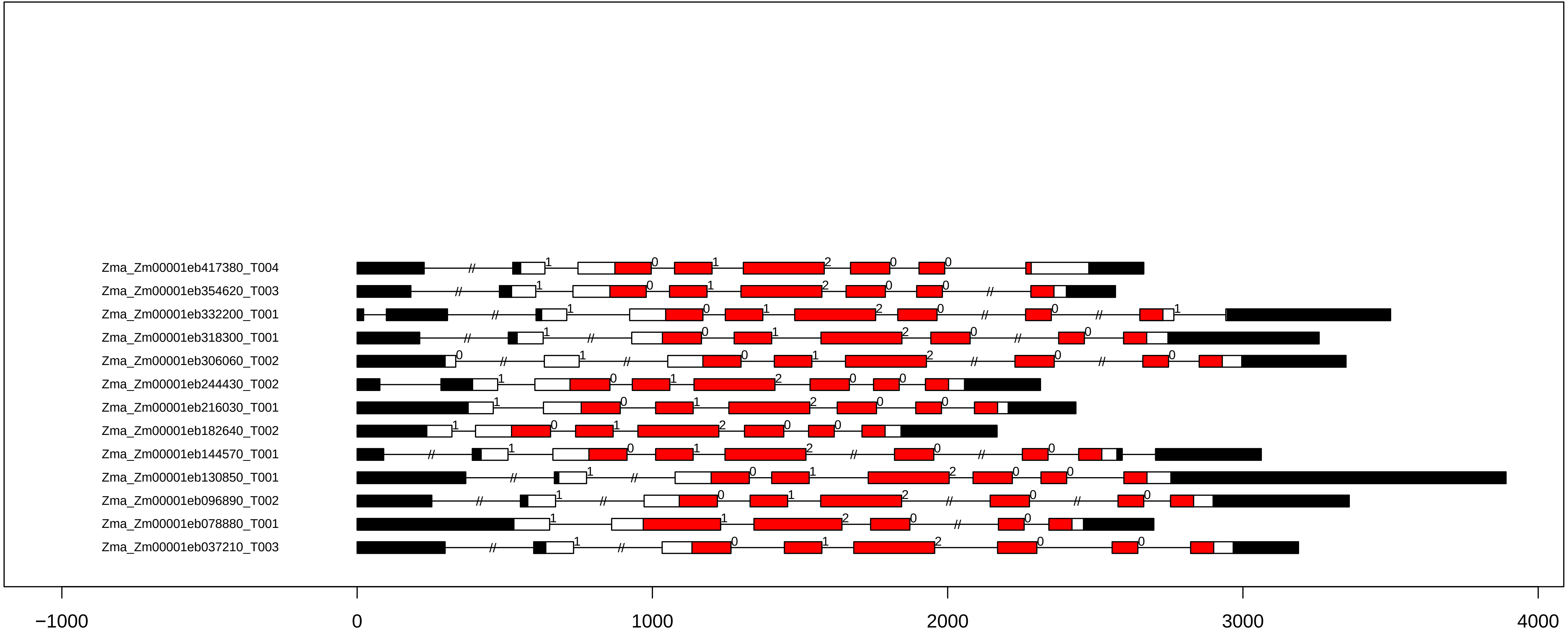

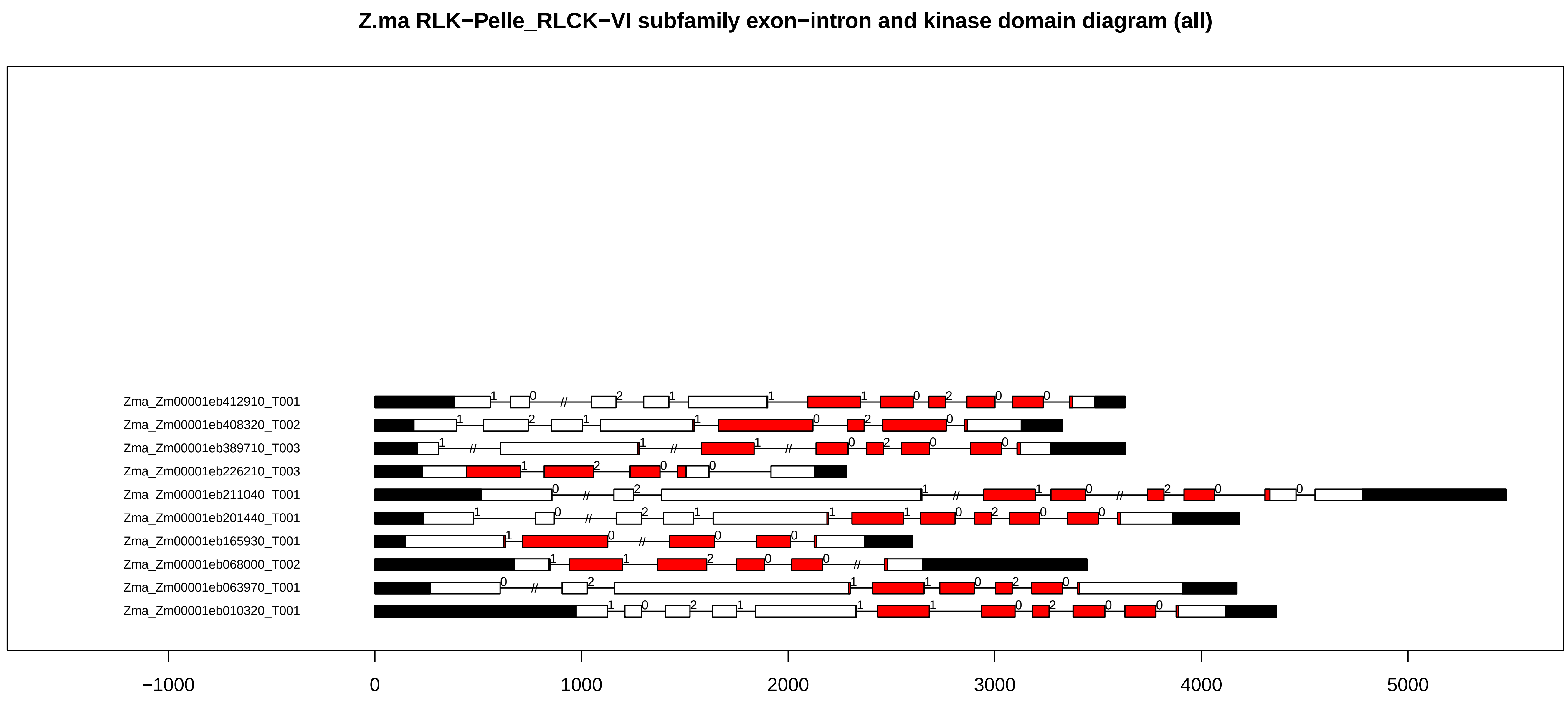

Z.ma RLK−Pelle_RLCK−VI subfamily exon−intron and kinase domain diagram (all)
Zma_Zm00001eb412910_T001
Zma_Zm00001eb408320_T002
Zma_Zm00001eb389710_T003
Zma_Zm00001eb226210_T003
Zma_Zm00001eb211040_T001
Zma_Zm00001eb201440_T001
Zma_Zm00001eb165930_T001
Zma_Zm00001eb068000_T002
Zma_Zm00001eb063970_T001
Zma_Zm00001eb010320_T001
−1000
0
1000
2000
3000
4000
5000

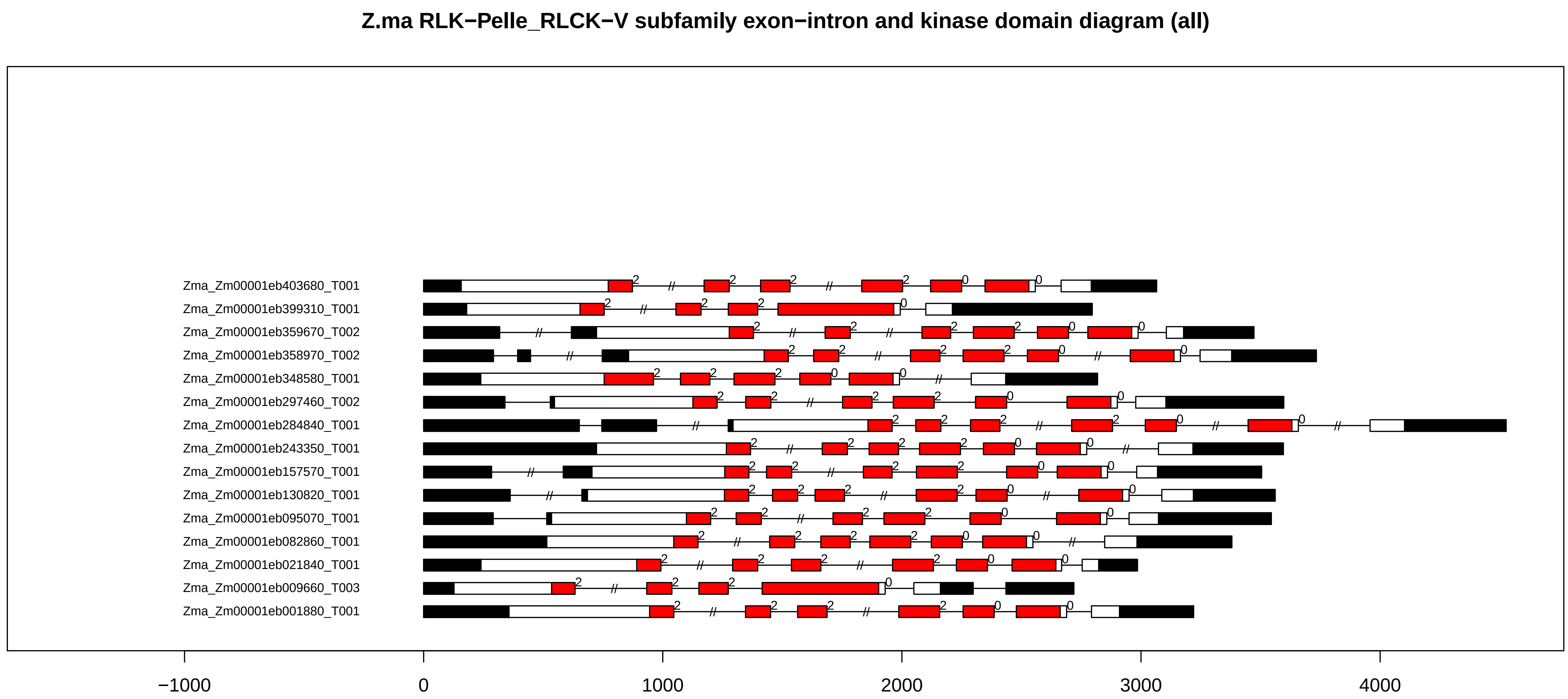
Z.ma RLK−Pelle_RLCK−V subfamily exon−intron and kinase domain diagram (all)
Zma_Zm00001eb403680_T001
Zma_Zm00001eb399310_T001
Zma_Zm00001eb359670_T002
Zma_Zm00001eb358970_T002
Zma_Zm00001eb348580_T001
Zma_Zm00001eb297460_T002
Zma_Zm00001eb284840_T001
Zma_Zm00001eb243350_T001
Zma_Zm00001eb157570_T001
Zma_Zm00001eb130820_T001
Zma_Zm00001eb095070_T001
Zma_Zm00001eb082860_T001
Zma_Zm00001eb021840_T001
Zma_Zm00001eb009660_T003
Zma_Zm00001eb001880_T001
−1000
0
1000
2000
3000
4000

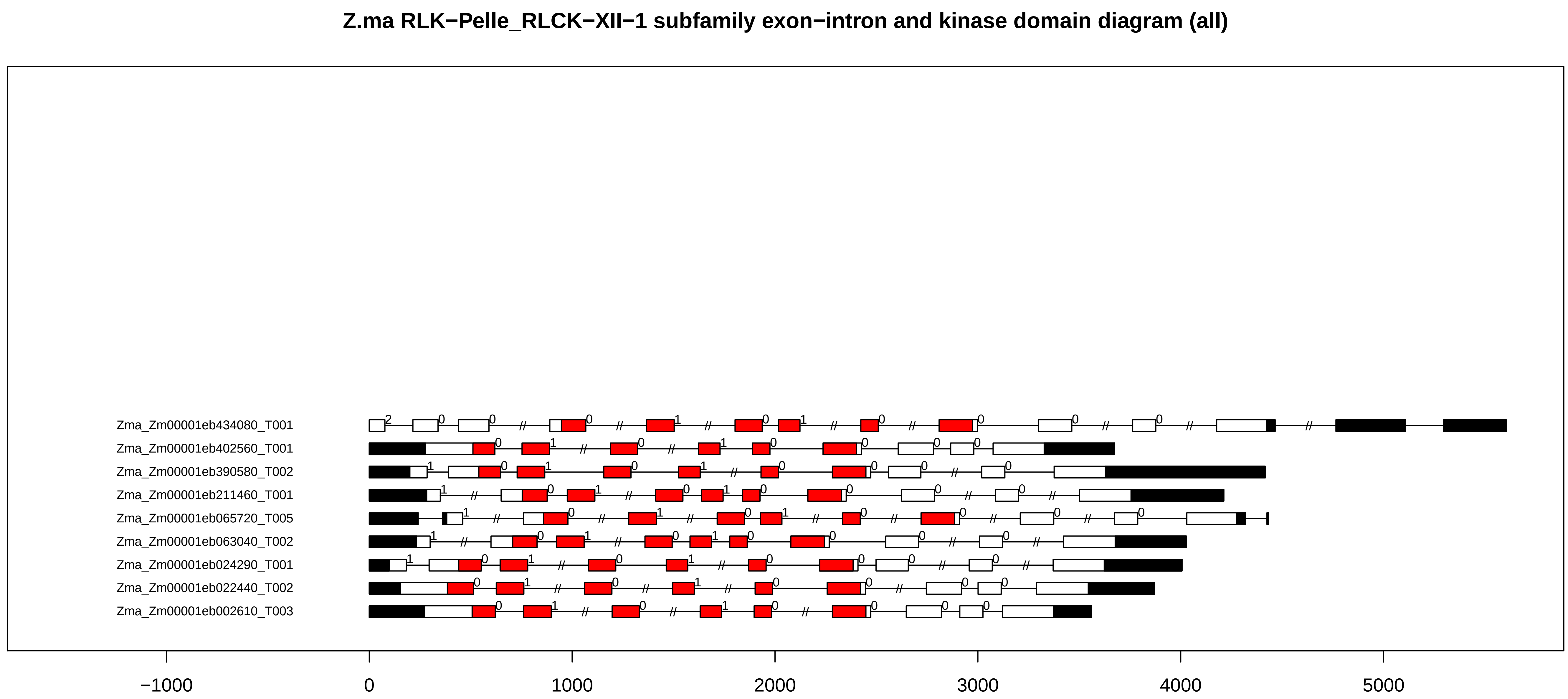
Z.ma RLK−Pelle_RLCK−XII−1 subfamily exon−intron and kinase domain diagram (all)
Zma_Zm00001eb434080_T001
Zma_Zm00001eb402560_T001
Zma_Zm00001eb390580_T002
Zma_Zm00001eb211460_T001
Zma_Zm00001eb065720_T005
Zma_Zm00001eb063040_T002
Zma_Zm00001eb024290_T001
Zma_Zm00001eb022440_T002
Zma_Zm00001eb002610_T003
−1000
0
1000
2000
3000
4000
5000

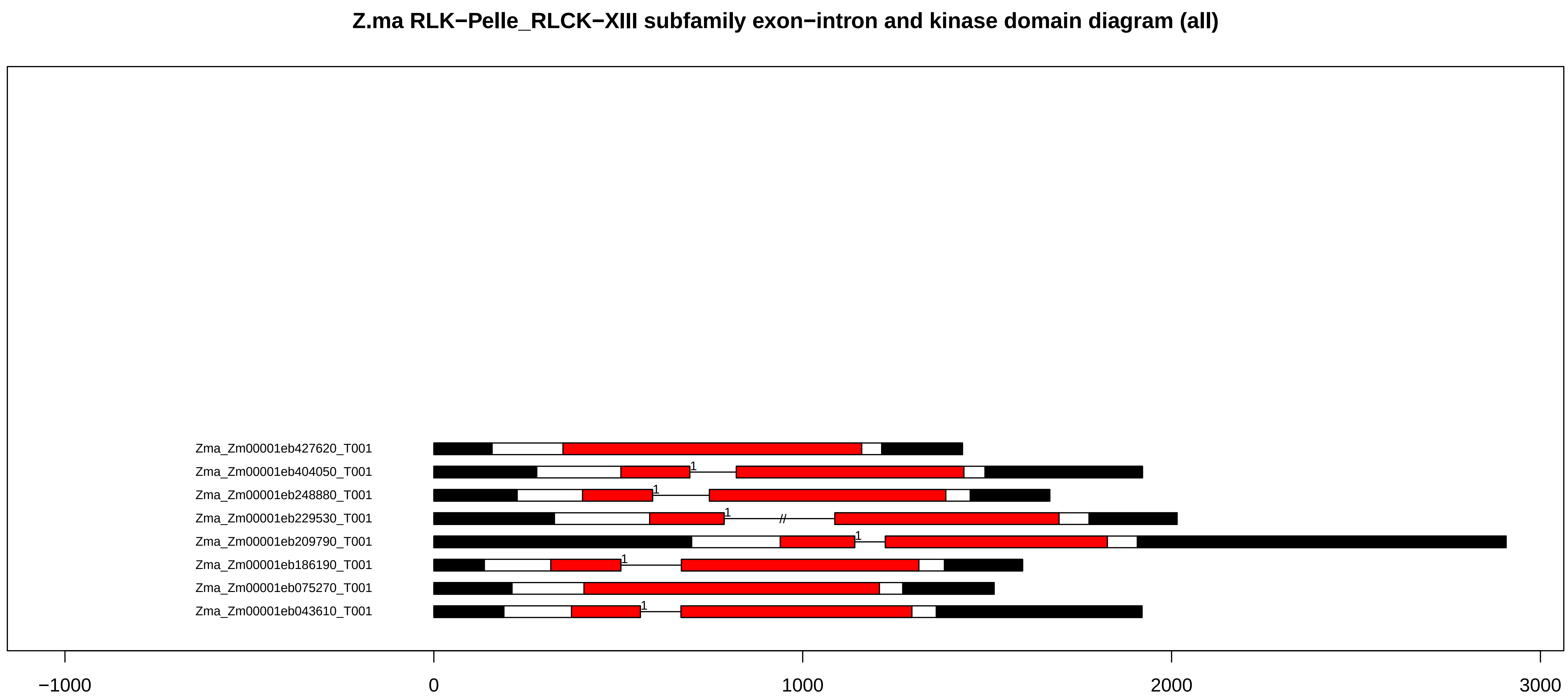
Z.ma RLK−Pelle_RLCK−XIII subfamily exon−intron and kinase domain diagram (all)
Zma_Zm00001eb427620_T001
Zma_Zm00001eb404050_T001
Zma_Zm00001eb248880_T001
Zma_Zm00001eb229530_T001
Zma_Zm00001eb209790_T001
Zma_Zm00001eb186190_T001
Zma_Zm00001eb075270_T001
Zma_Zm00001eb043610_T001
1
1
1
1
1
1
−1000
0
1000
2000
3000

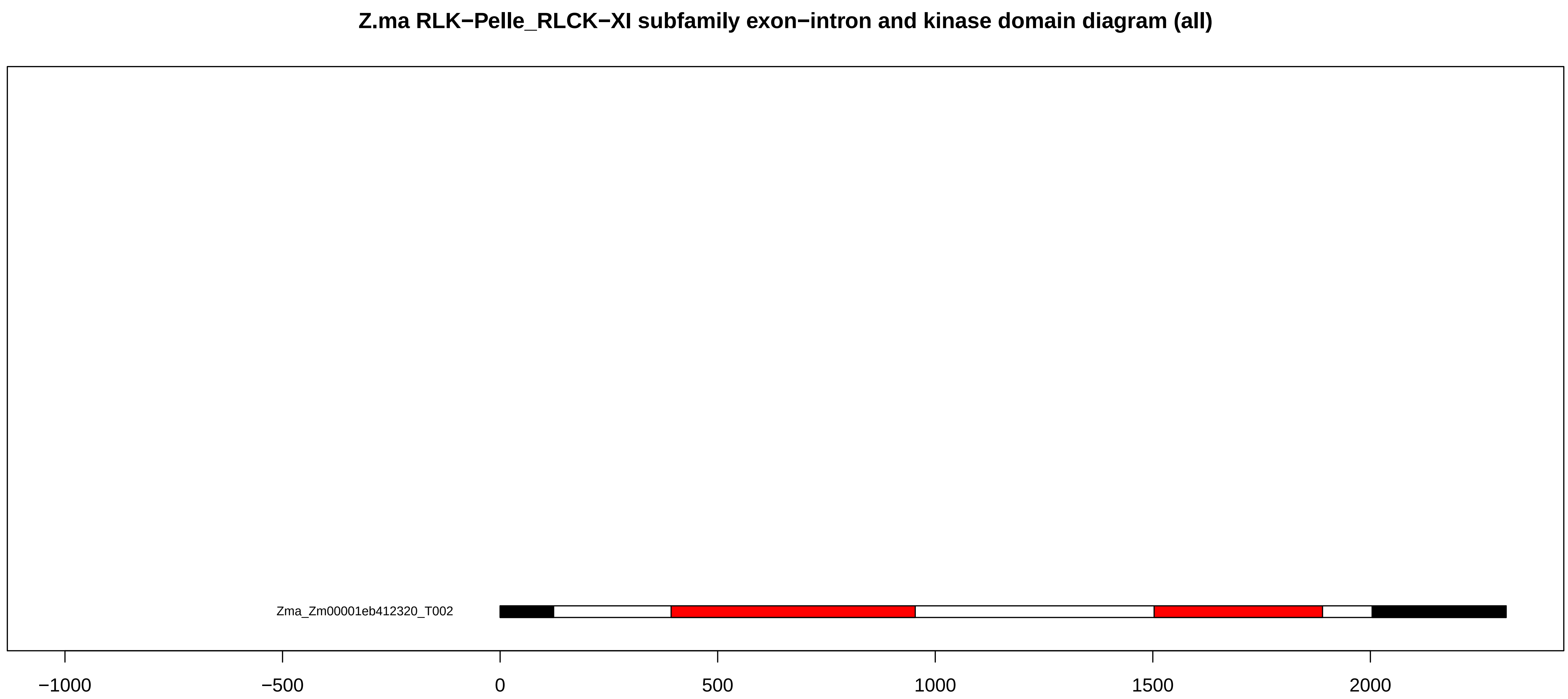
Z.ma RLK−Pelle_RLCK−XI subfamily exon−intron and kinase domain diagram (all)
Zma_Zm00001eb412320_T002
−1000
−500
0
500
1000
1500
2000

Z.ma RLK−Pelle_RLCK−X subfamily exon−intron and kinase domain diagram (all)

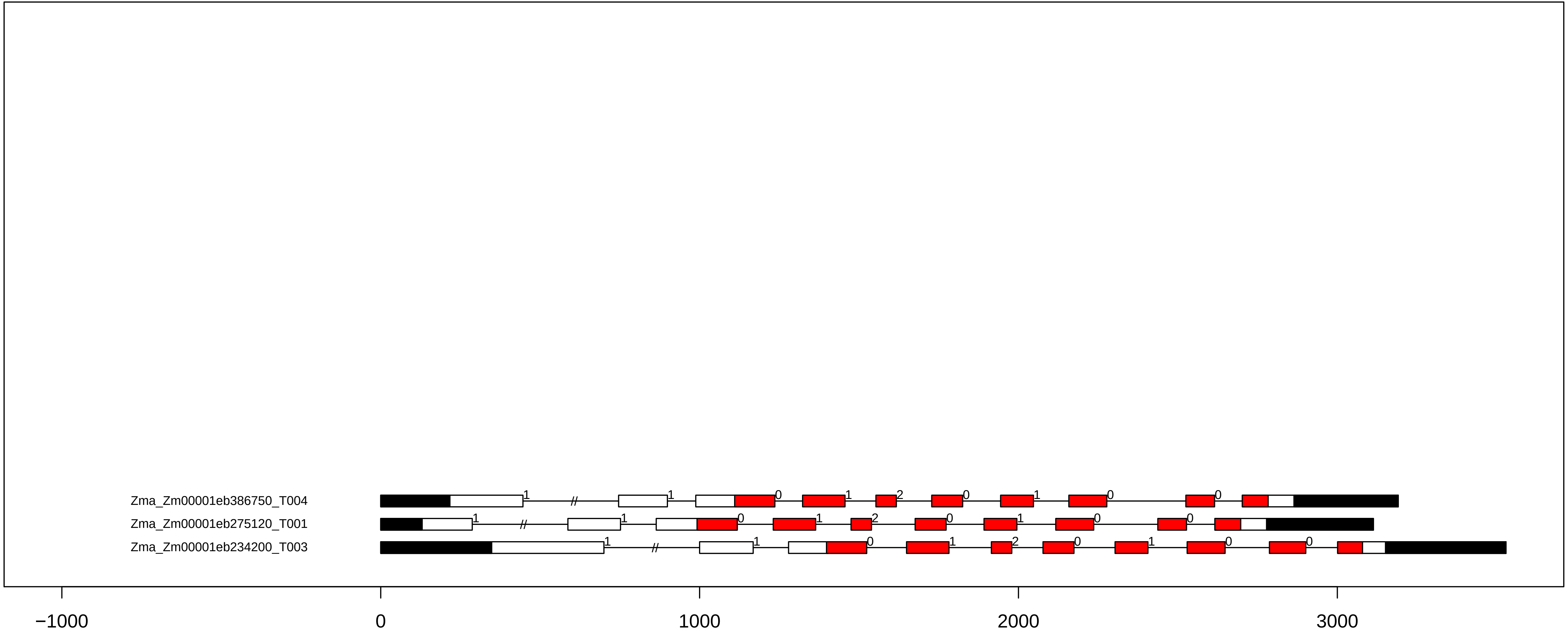

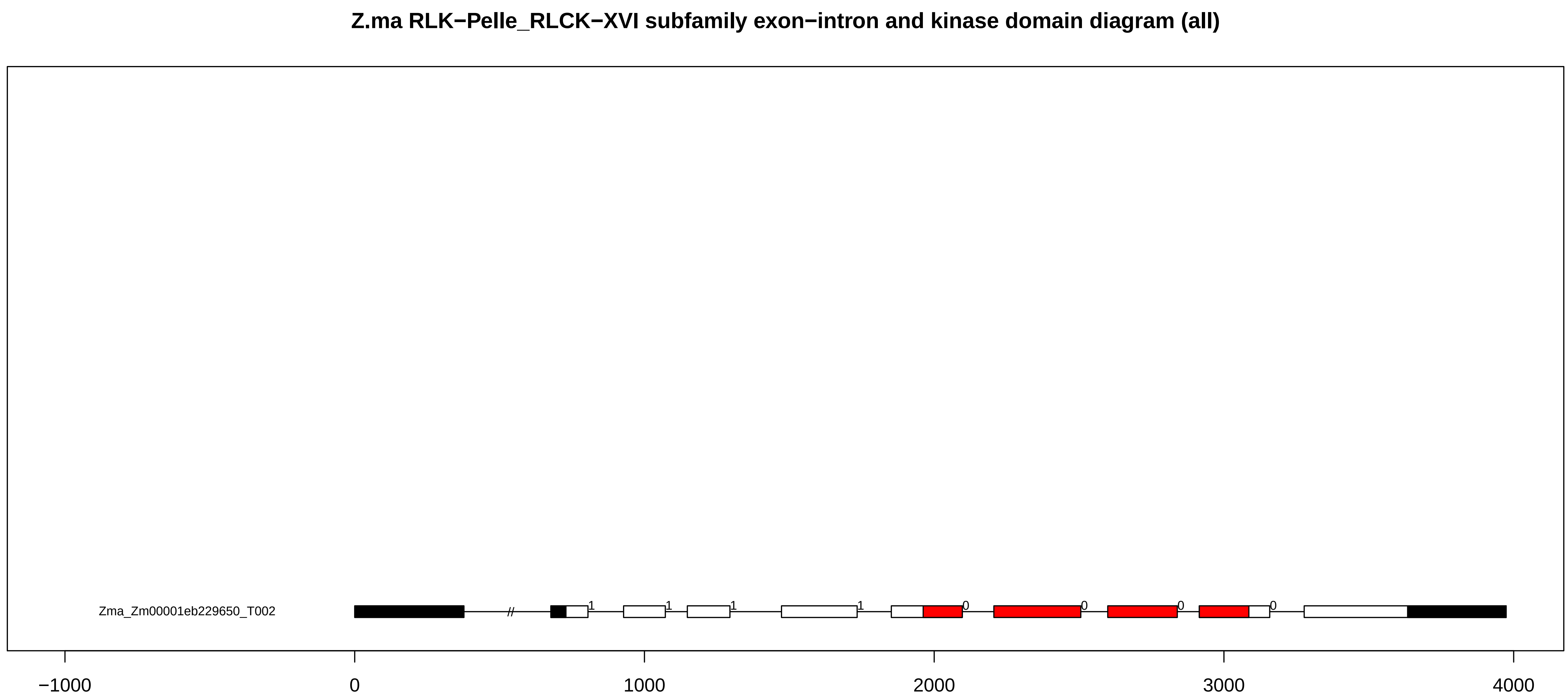

Z.ma RLK−Pelle_RLCK−XVI subfamily exon−intron and kinase domain diagram (all)
Zma_Zm00001eb229650_T002
1
1
1
1
0
0
0
0
−1000
0
1000
2000
3000
4000

Z.ma RLK−Pelle_RLCK−XV subfamily exon−intron and kinase domain diagram (all)

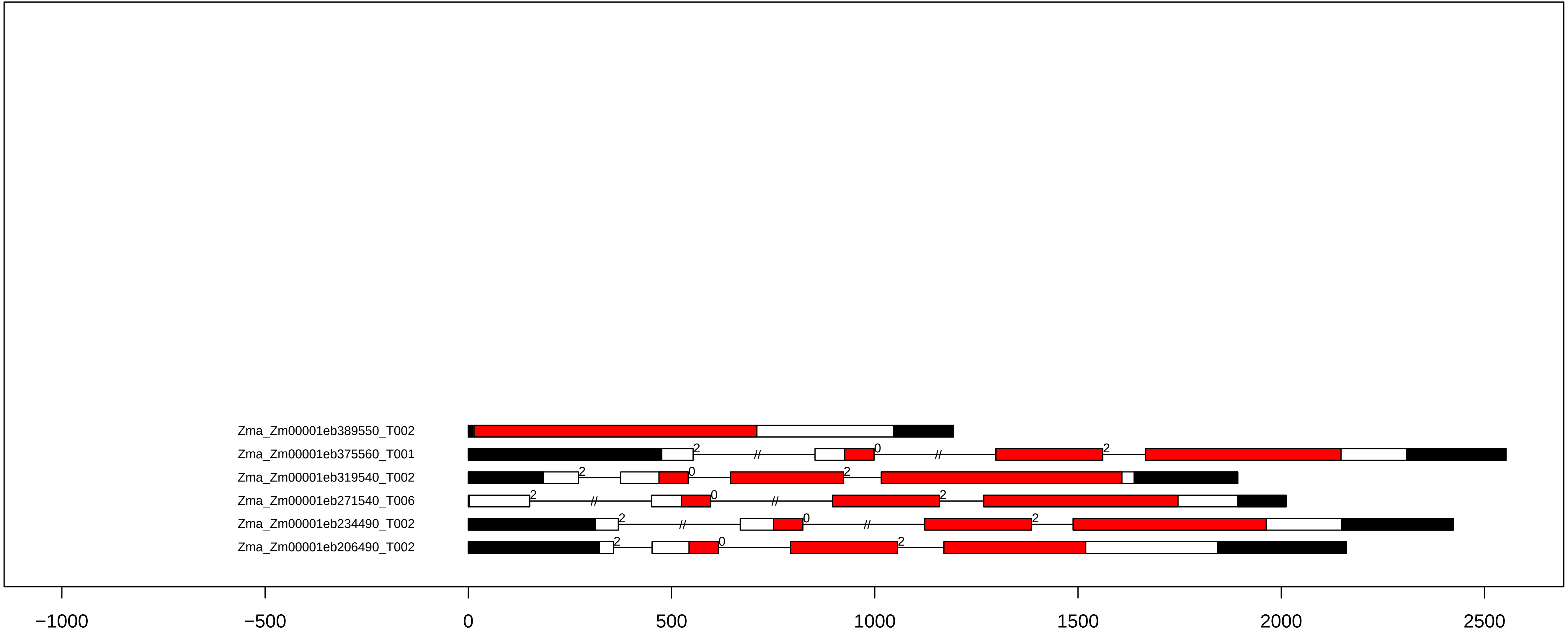

Z.ma RLK−Pelle_SD−2b subfamily exon−intron and kinase domain diagram (part 1)

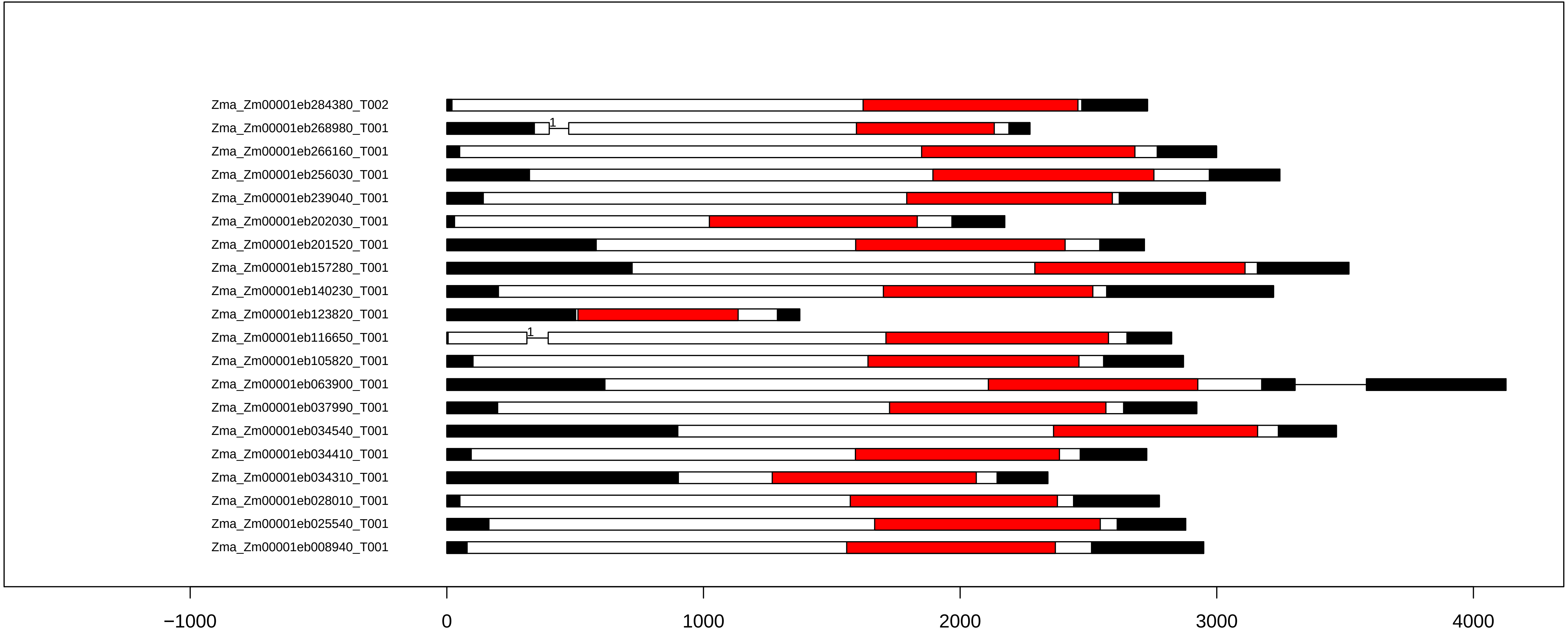

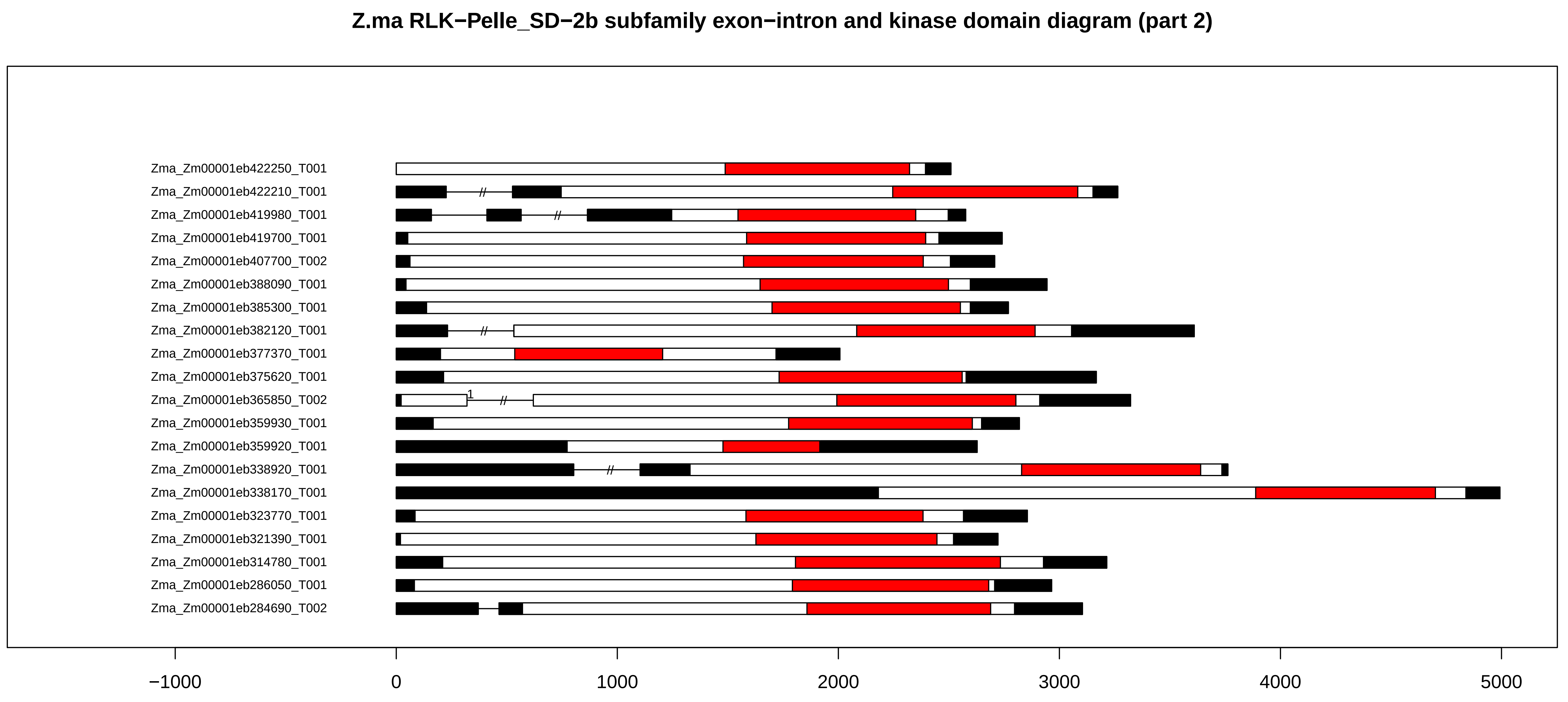

Z.ma RLK−Pelle_SD−2b subfamily exon−intron and kinase domain diagram (part 2)
Zma_Zm00001eb422250_T001
Zma_Zm00001eb422210_T001
Zma_Zm00001eb419980_T001
Zma_Zm00001eb419700_T001
Zma_Zm00001eb407700_T002
Zma_Zm00001eb388090_T001
Zma_Zm00001eb385300_T001
Zma_Zm00001eb382120_T001
Zma_Zm00001eb377370_T001
Zma_Zm00001eb375620_T001
Zma_Zm00001eb365850_T002
Zma_Zm00001eb359930_T001
Zma_Zm00001eb359920_T001
Zma_Zm00001eb338920_T001
Zma_Zm00001eb338170_T001
Zma_Zm00001eb323770_T001
Zma_Zm00001eb321390_T001
Zma_Zm00001eb314780_T001
Zma_Zm00001eb286050_T001
Zma_Zm00001eb284690_T002
1
−1000
0
1000
2000
3000
4000
5000

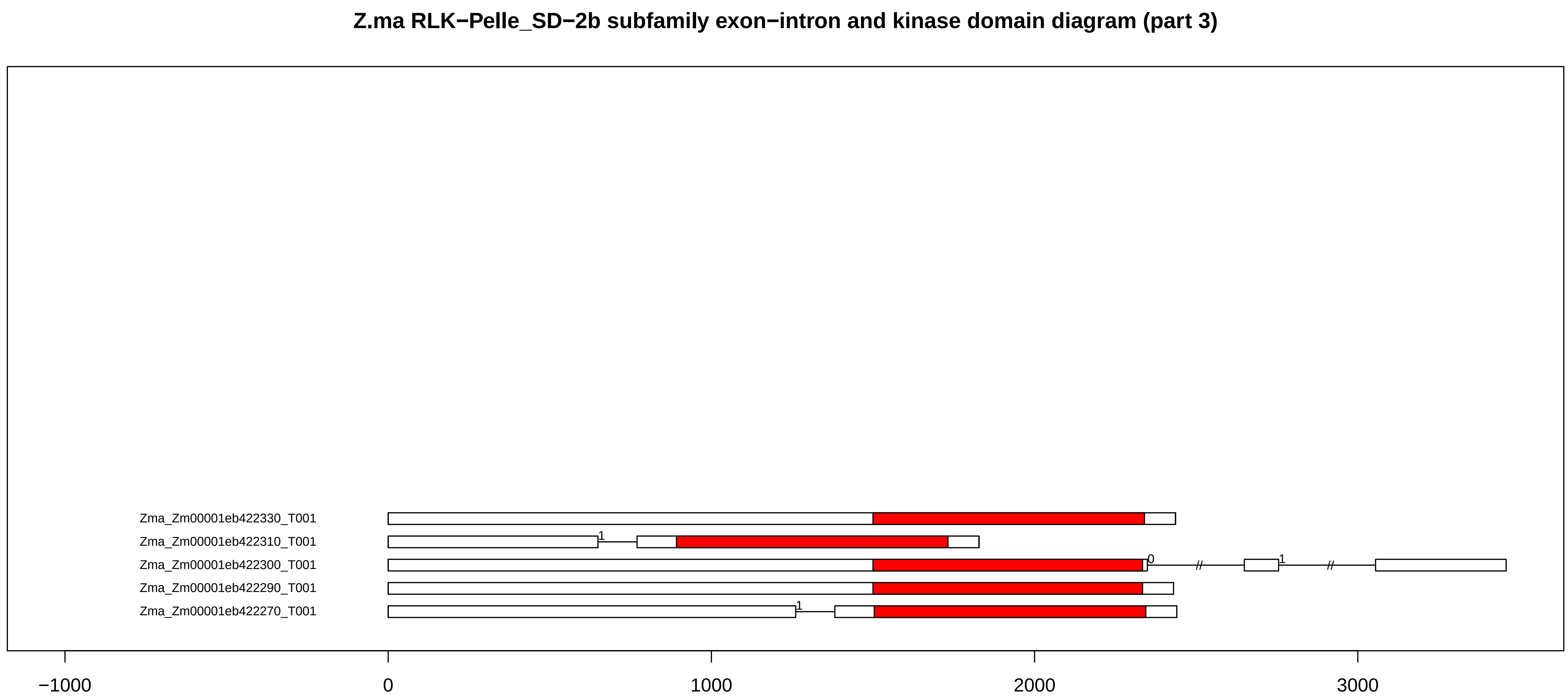

Z.ma RLK−Pelle_SD−2b subfamily exon−intron and kinase domain diagram (part 3)
Zma_Zm00001eb422330_T001
Zma_Zm00001eb422310_T001
Zma_Zm00001eb422300_T001
Zma_Zm00001eb422290_T001
Zma_Zm00001eb422270_T001
1
0
1
1
−1000
0
1000
2000
3000

Z.ma RLK−Pelle_URK−1 subfamily exon−intron and kinase domain diagram (all)

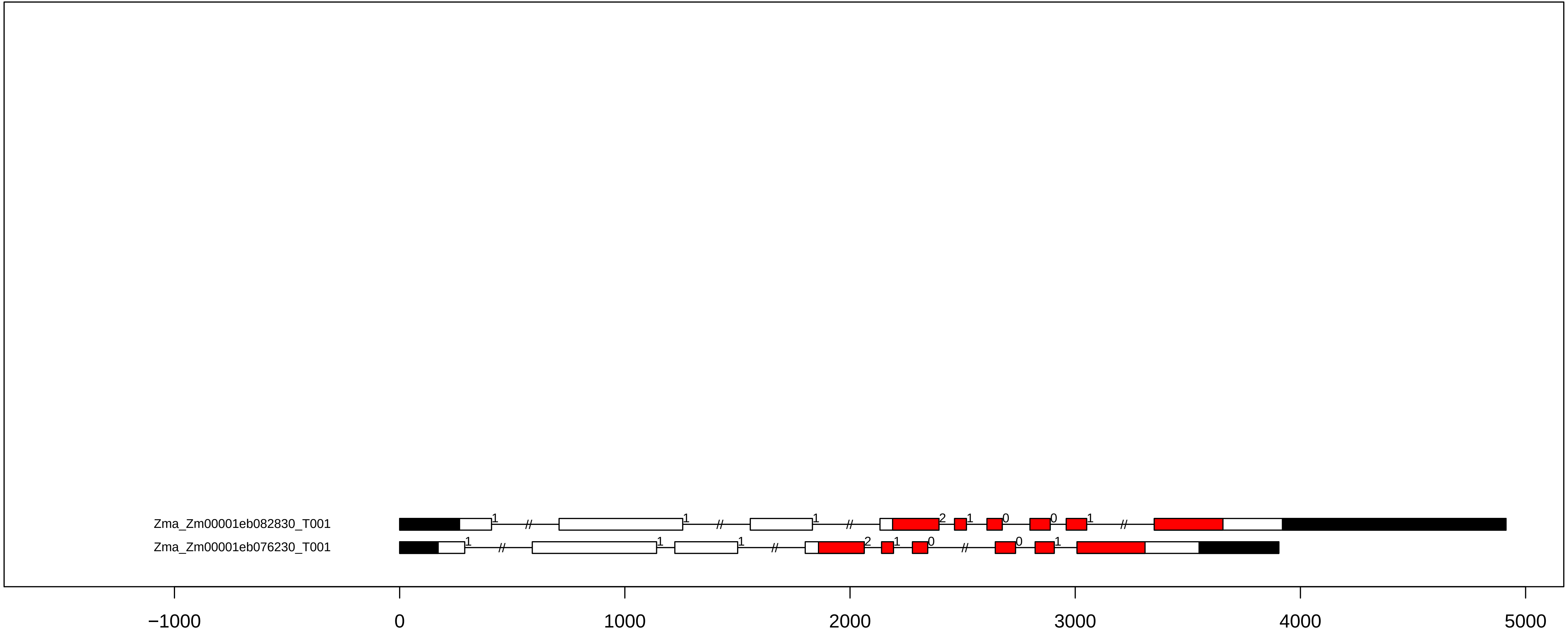

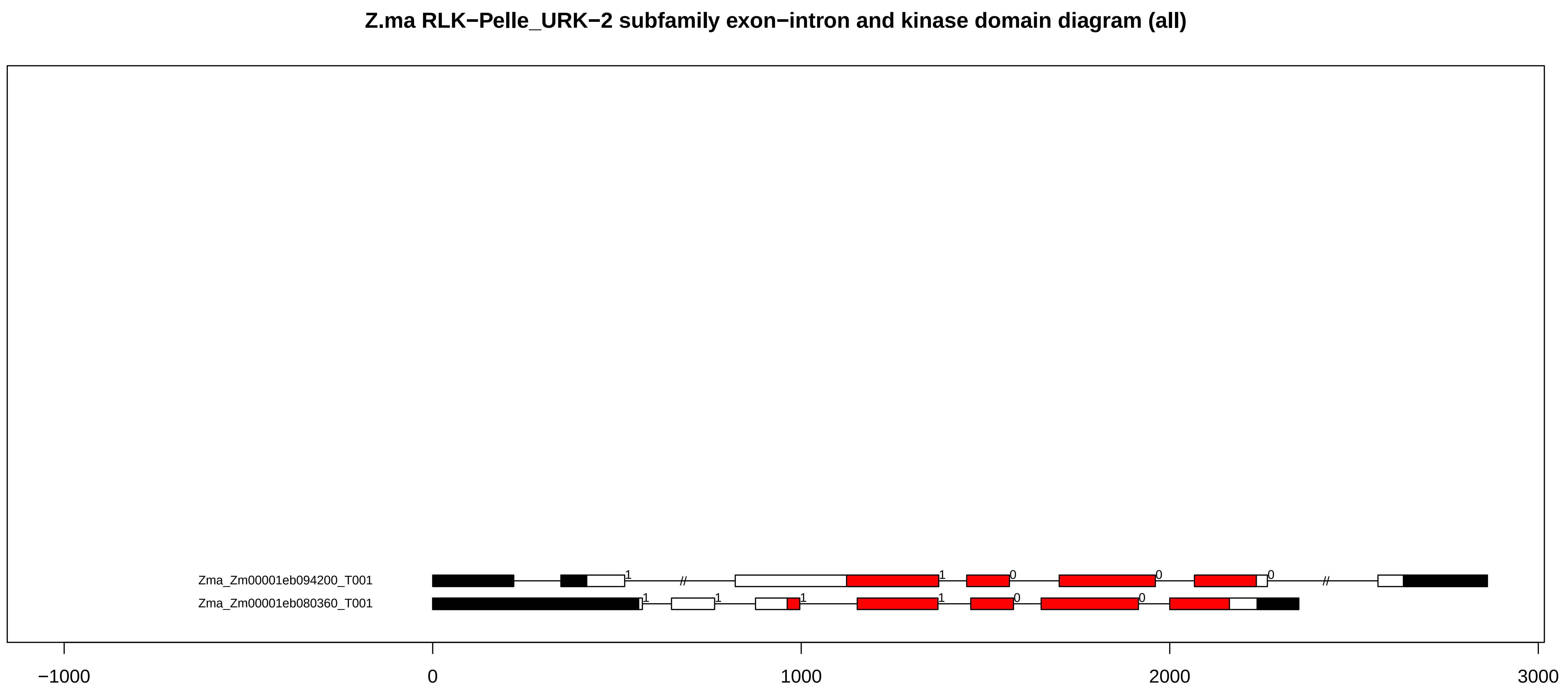
Z.ma RLK−Pelle_URK−2 subfamily exon−intron and kinase domain diagram (all)
Zma_Zm00001eb094200_T001
Zma_Zm00001eb080360_T001
−1000
0
1000
2000
3000

Z.ma RLK−Pelle_URK−3 subfamily exon−intron and kinase domain diagram (all)

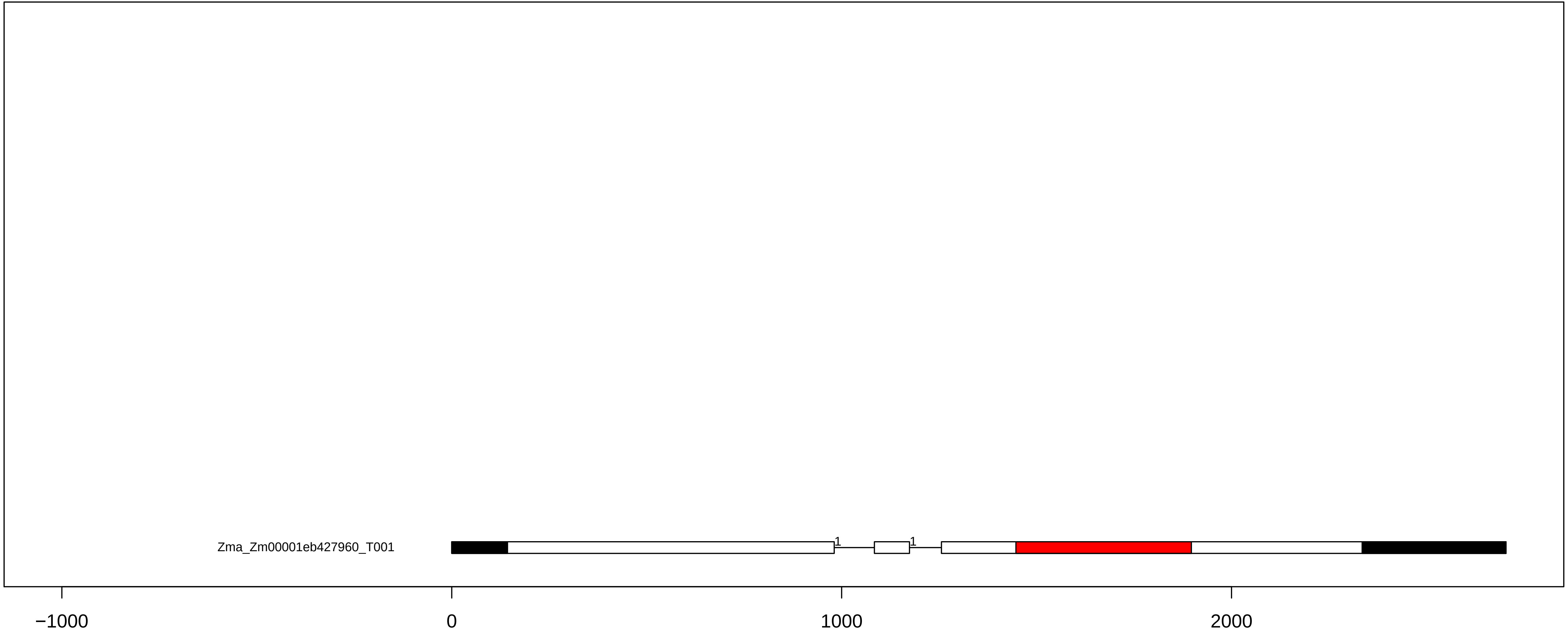

Z.ma RLK−Pelle_WAK_LRK10L−1 subfamily exon−intron and kinase domain diagram (all)

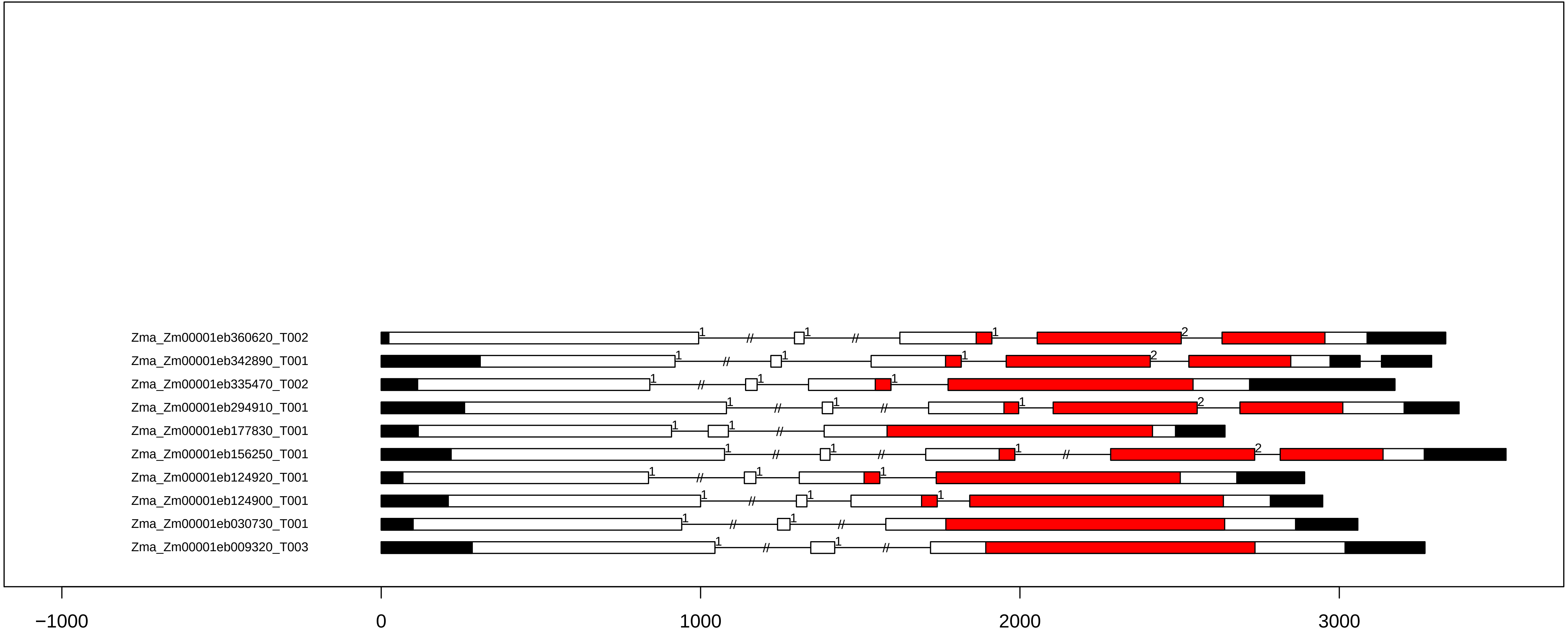

Z.ma RLK−Pelle_WAK subfamily exon−intron and kinase domain diagram (part 1)

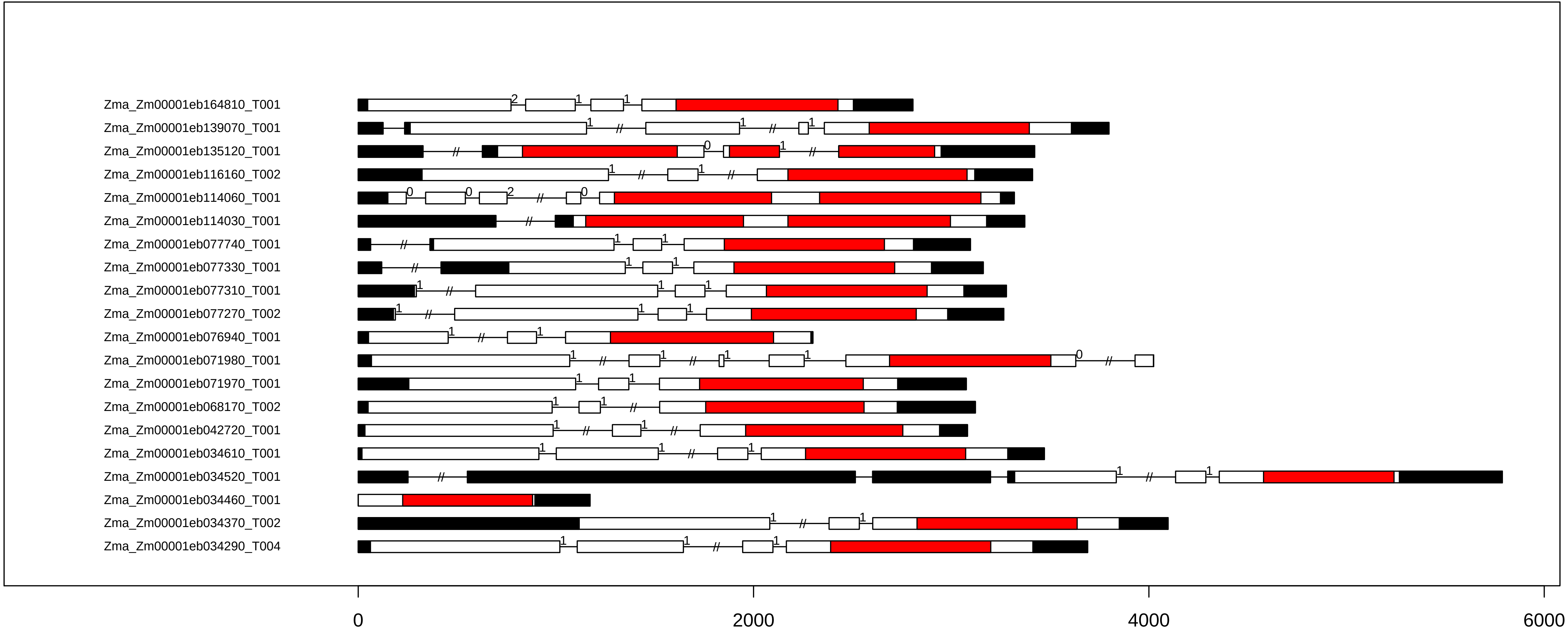

Z.ma RLK−Pelle_WAK subfamily exon−intron and kinase domain diagram (part 2)

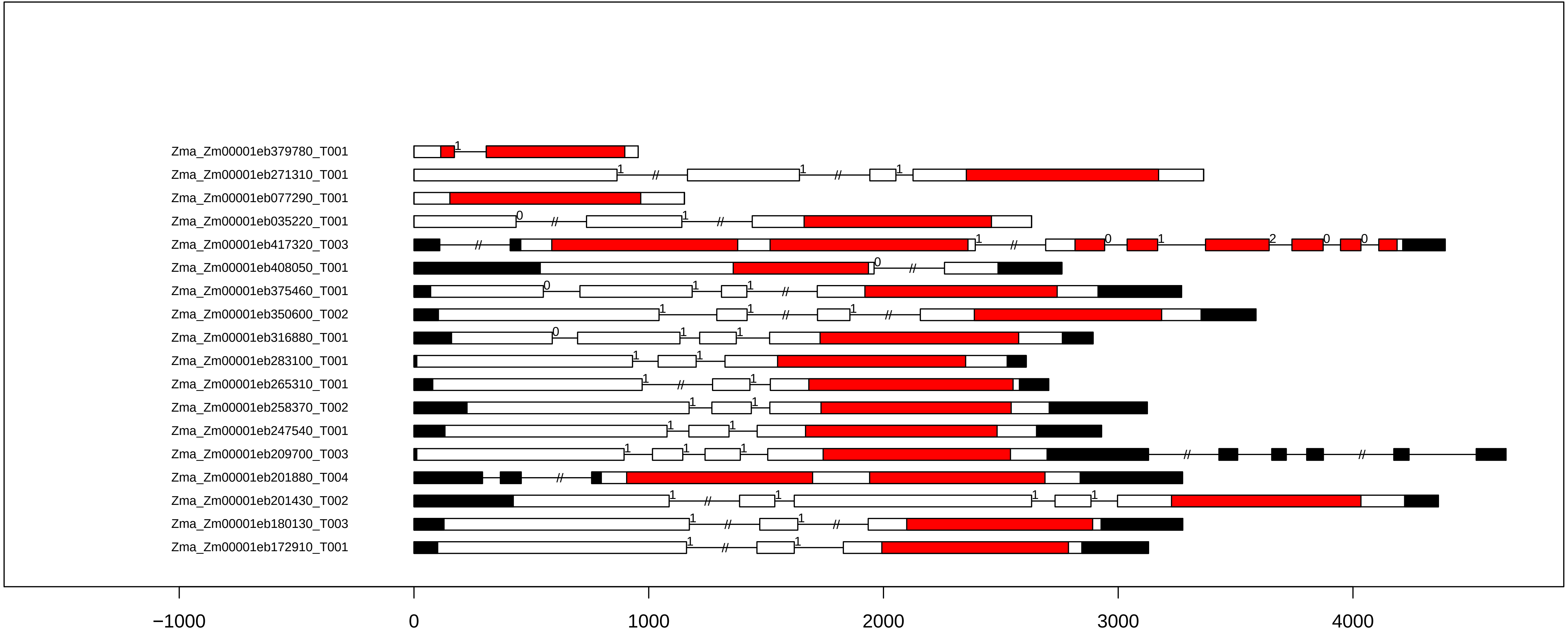

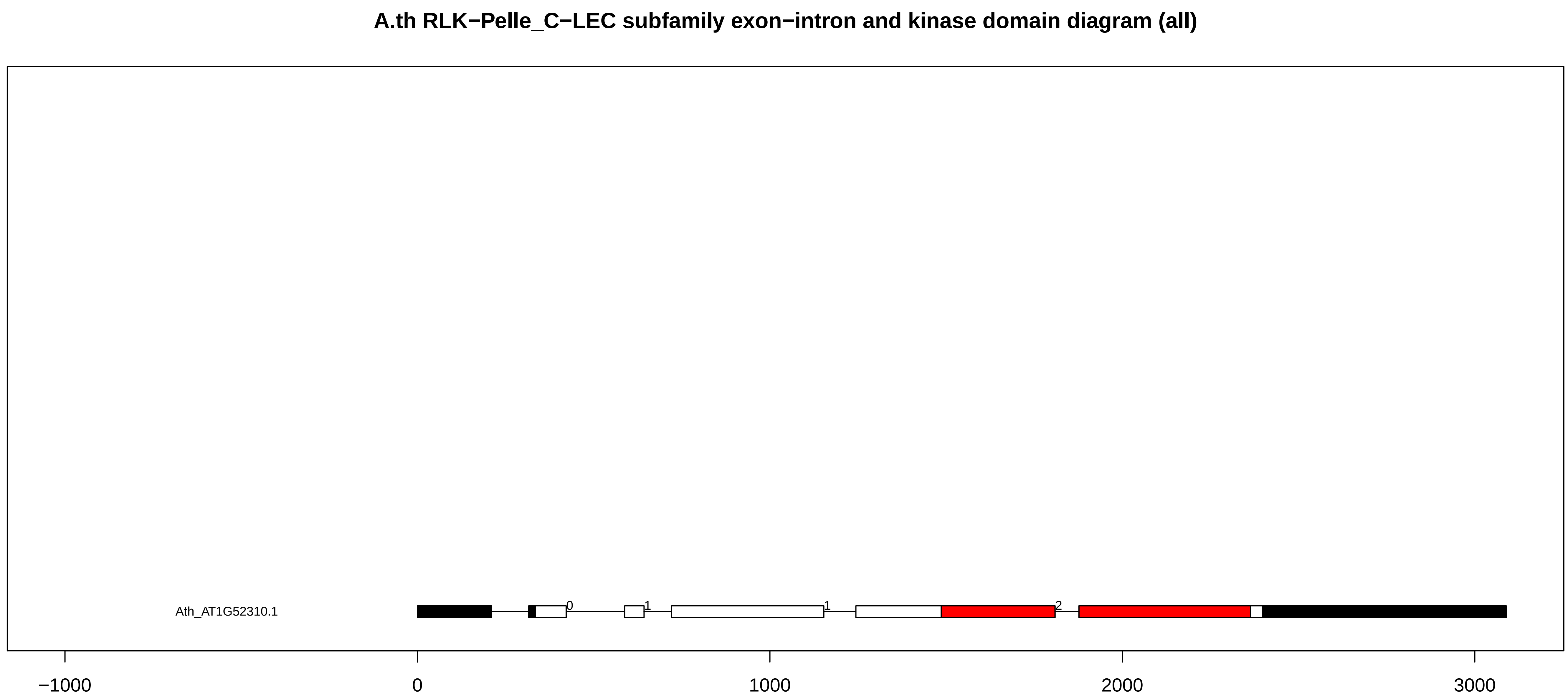

A.th RLK−Pelle_C−LEC subfamily exon−intron and kinase domain diagram (all)
Ath_AT1G52310.1
0
1
1
2
−1000
0
1000
2000
3000

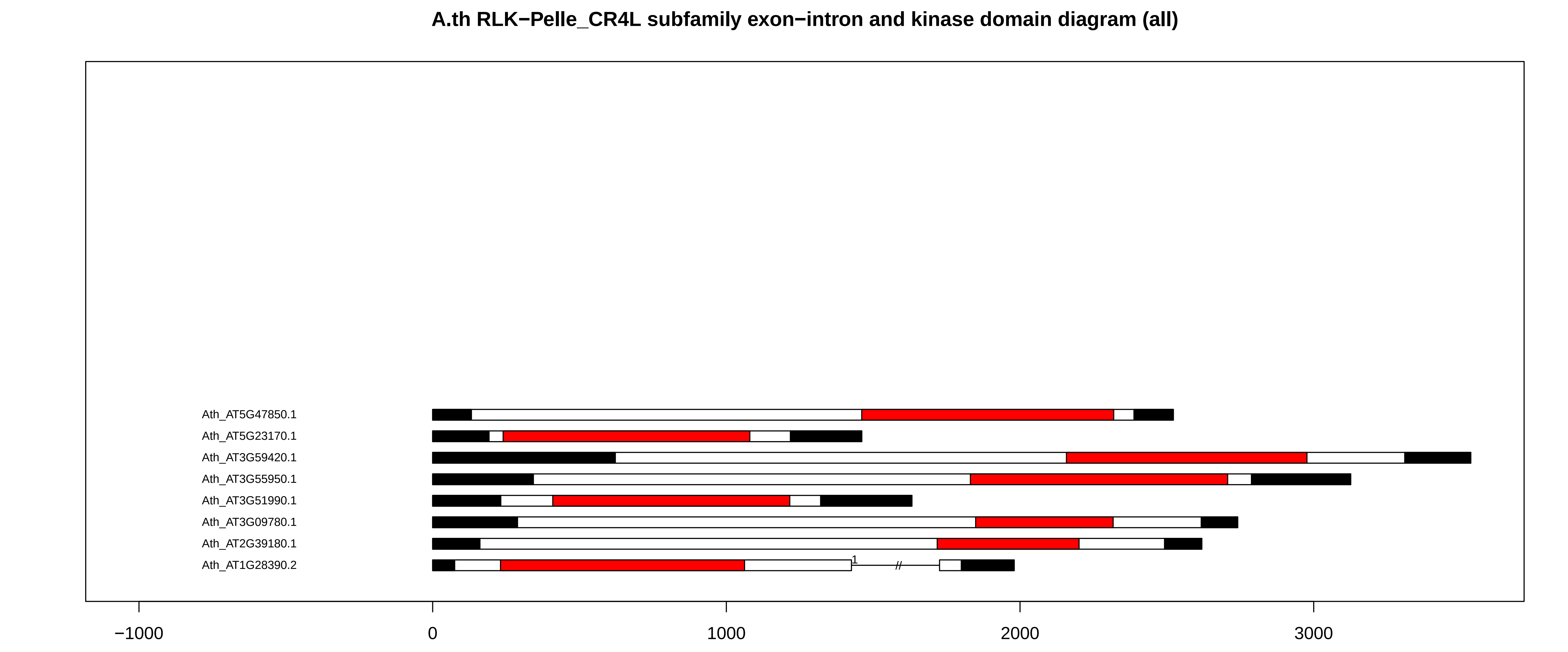
A.th RLK−Pelle_CR4L subfamily exon−intron and kinase domain diagram (all)
Ath_AT5G47850.1
Ath_AT5G23170.1
Ath_AT3G59420.1
Ath_AT3G55950.1
Ath_AT3G51990.1
Ath_AT3G09780.1
Ath_AT2G39180.1
Ath_AT1G28390.2
1
−1000
0
1000
2000
3000

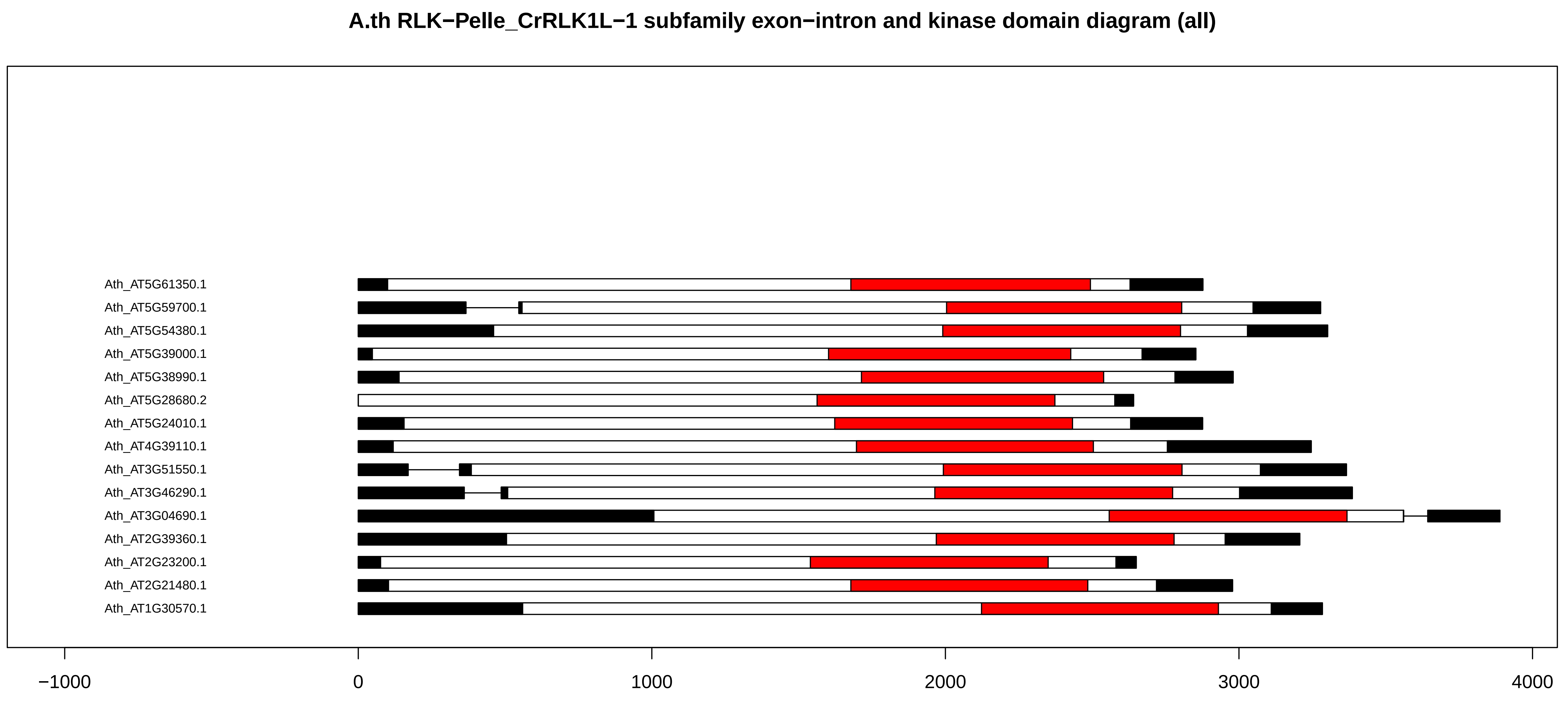
A.th RLK−Pelle_CrRLK1L−1 subfamily exon−intron and kinase domain diagram (all)
Ath_AT5G61350.1
Ath_AT5G59700.1
Ath_AT5G54380.1
Ath_AT5G39000.1
Ath_AT5G38990.1
Ath_AT5G28680.2
Ath_AT5G24010.1
Ath_AT4G39110.1
Ath_AT3G51550.1
Ath_AT3G46290.1
Ath_AT3G04690.1
Ath_AT2G39360.1
Ath_AT2G23200.1
Ath_AT2G21480.1
Ath_AT1G30570.1
−1000
0
1000
2000
3000
4000

A.th RLK−Pelle_DLSV subfamily exon−intron and kinase domain diagram (part 1)

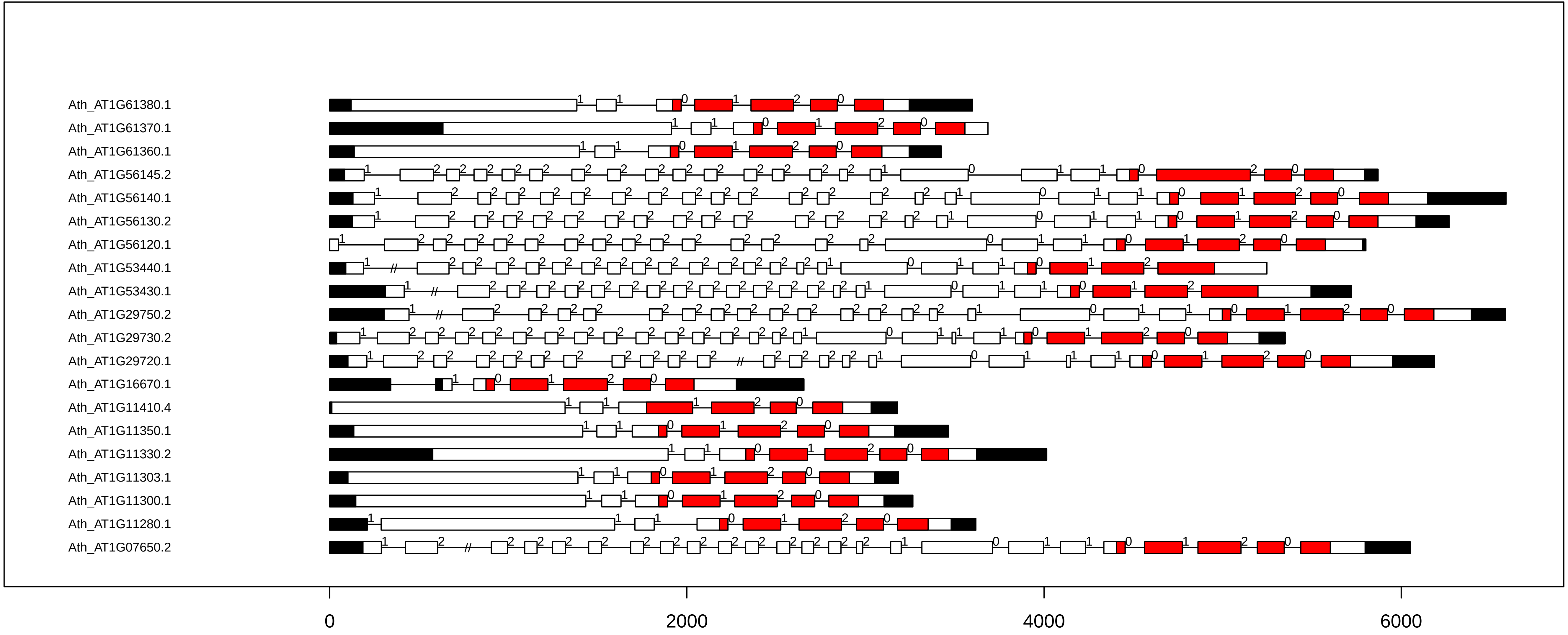

A.th RLK−Pelle_DLSV subfamily exon−intron and kinase domain diagram (part 2)

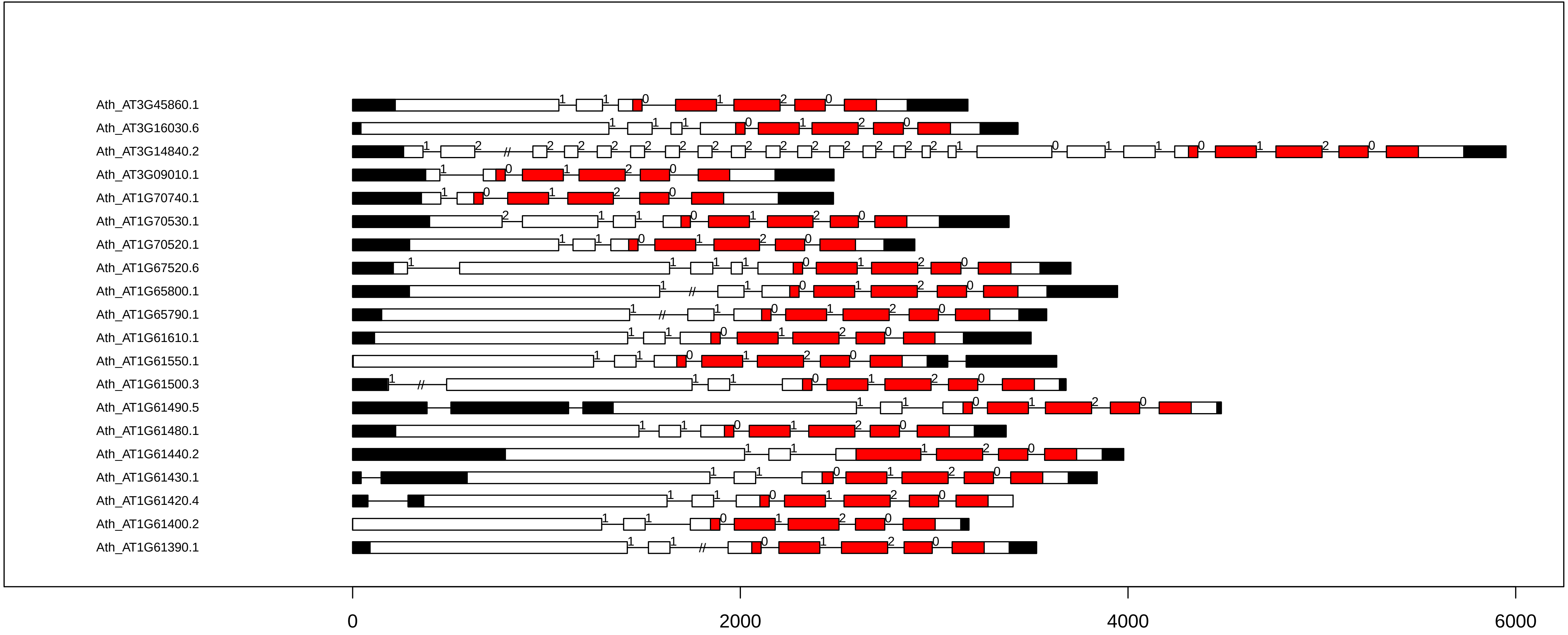

A.th RLK−Pelle_DLSV subfamily exon−intron and kinase domain diagram (part 3)

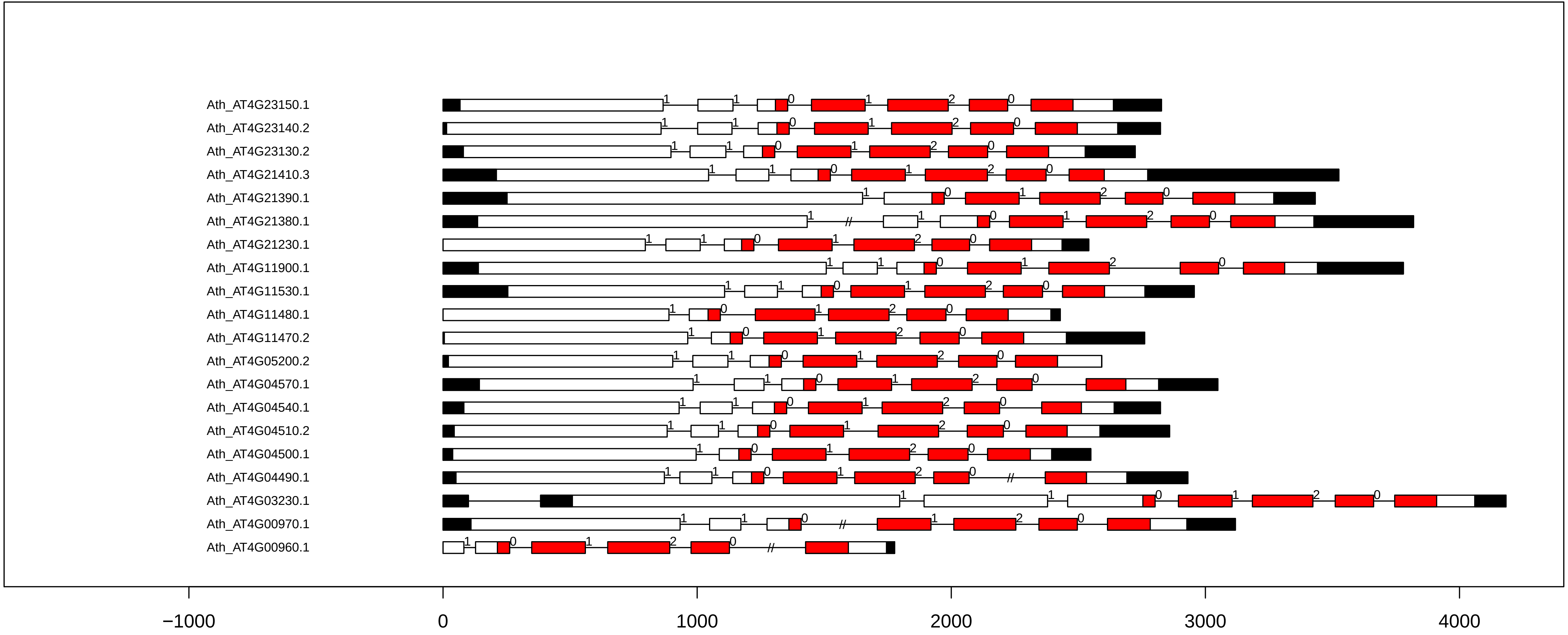

A.th RLK−Pelle_DLSV subfamily exon−intron and kinase domain diagram (part 4)

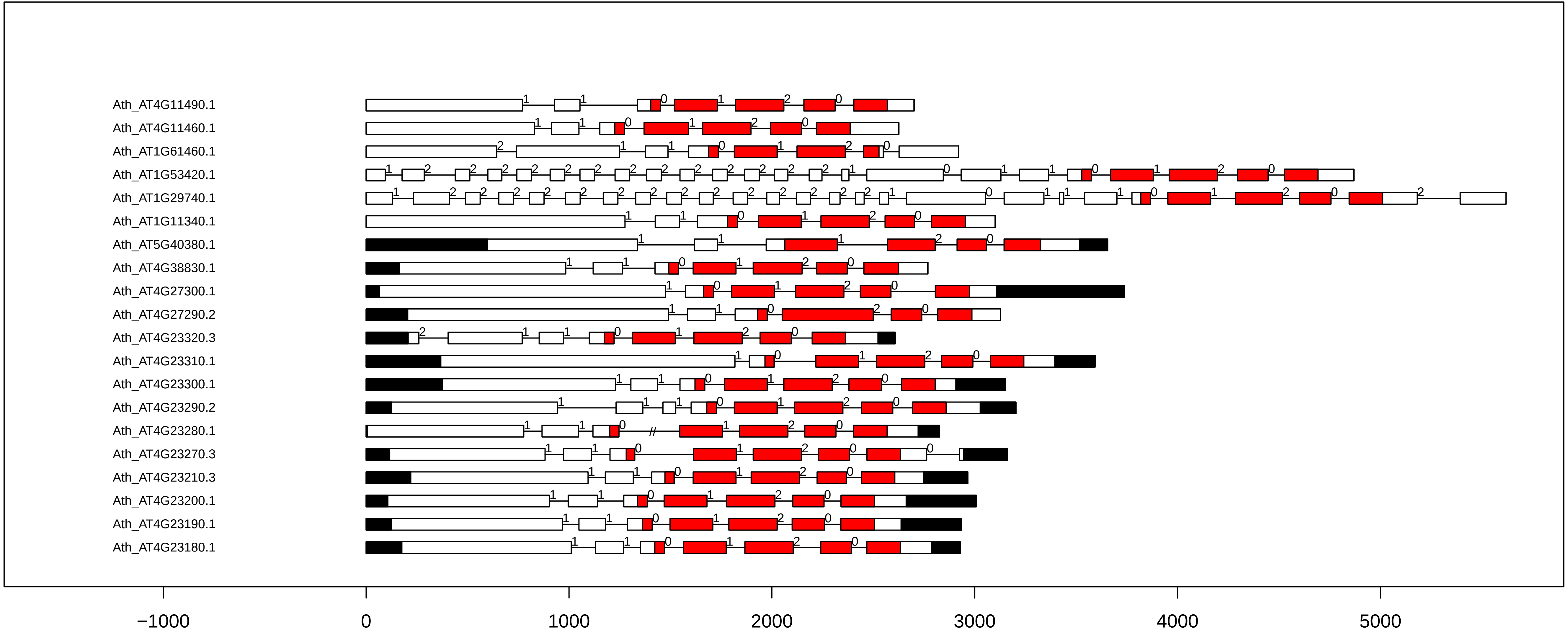

A.th RLK−Pelle_DLSV subfamily exon−intron and kinase domain diagram (part 5)

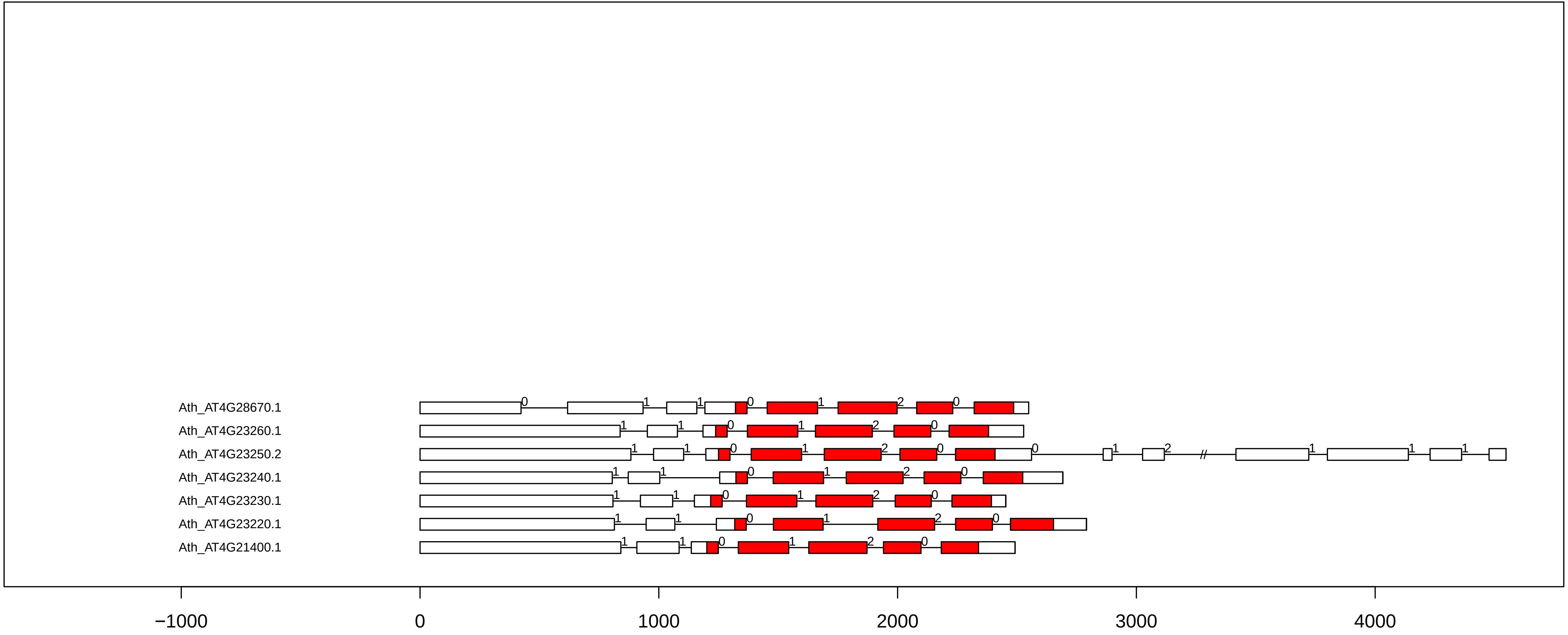

A.th RLK−Pelle_DSLV subfamily exon−intron and kinase domain diagram (all)

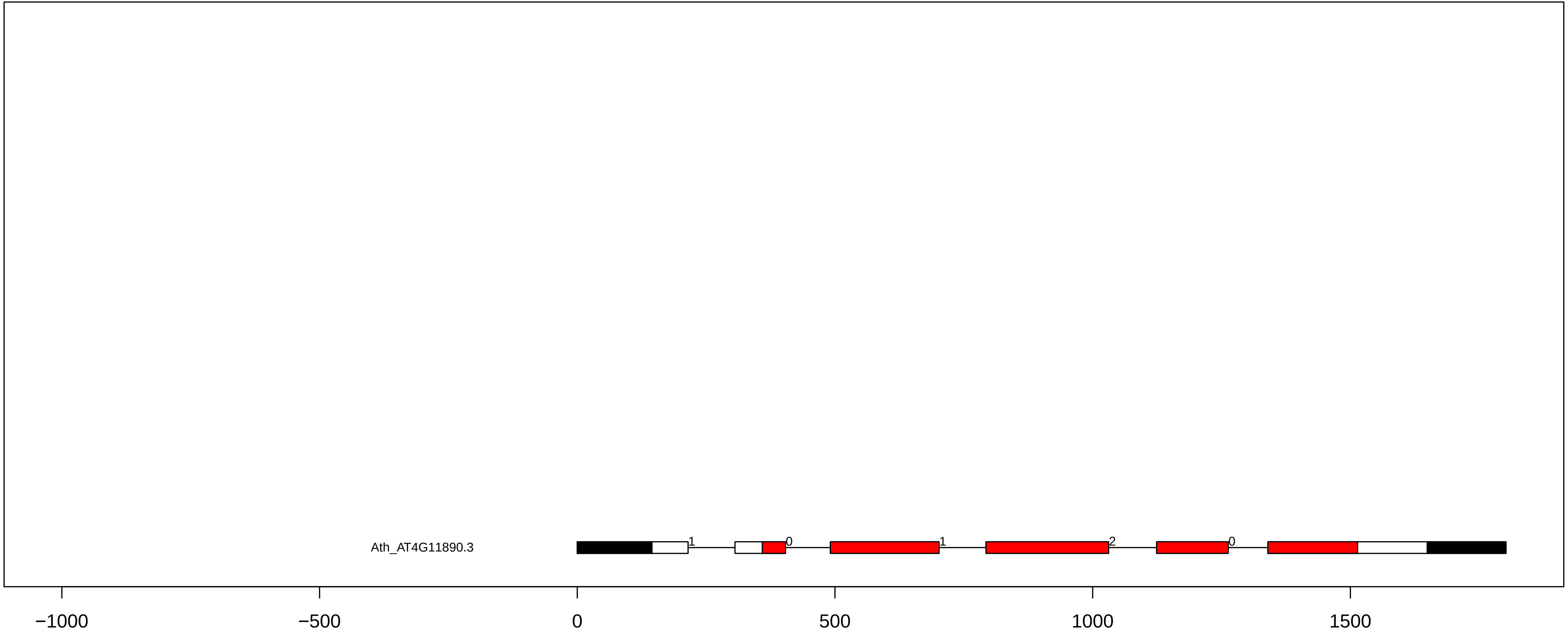

A.th RLK−Pelle_Extensin subfamily exon−intron and kinase domain diagram (all)

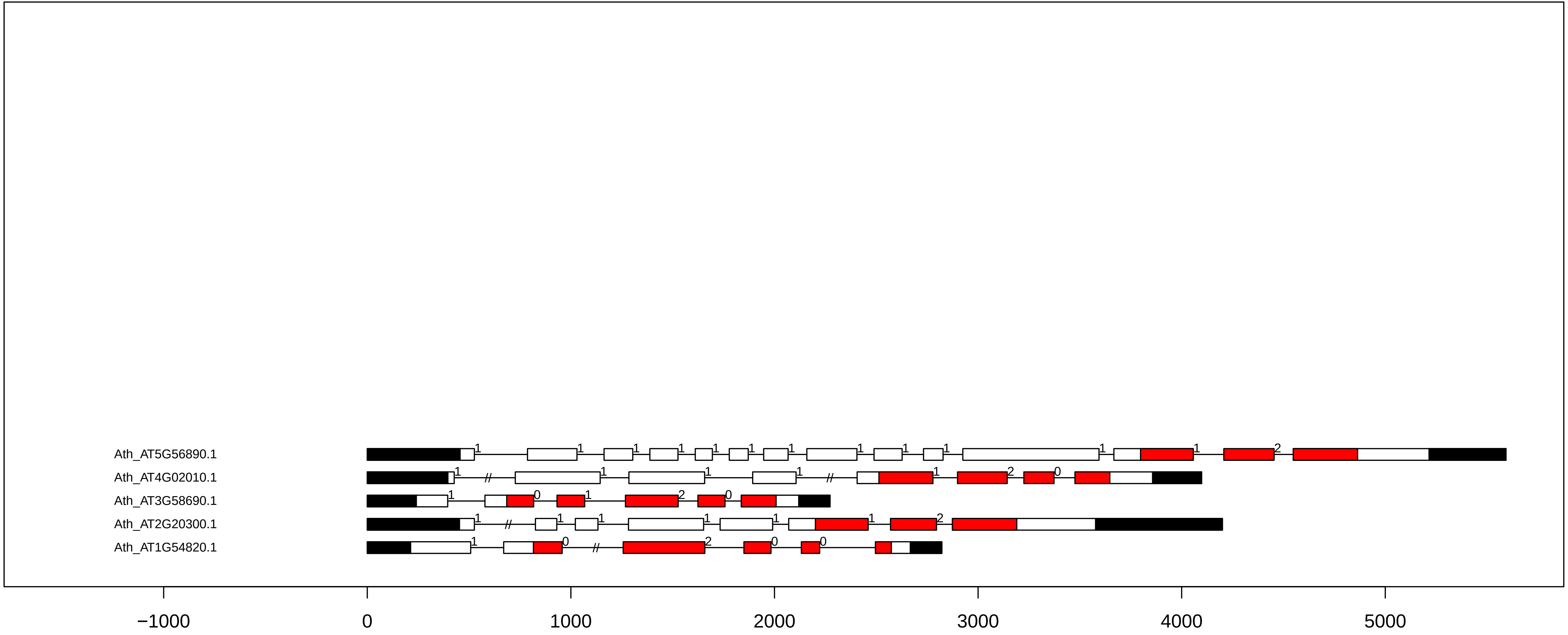

A.th RLK−Pelle_L−LEC subfamily exon−intron and kinase domain diagram (part 1)

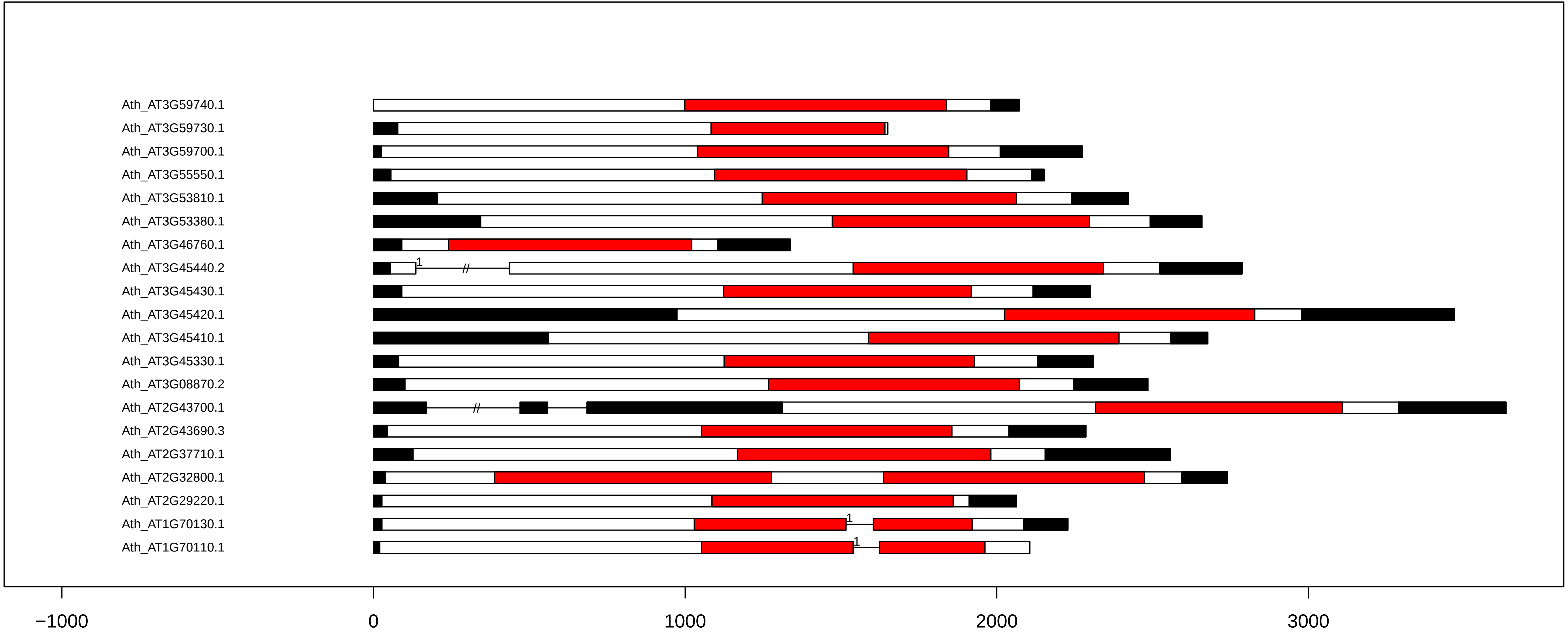

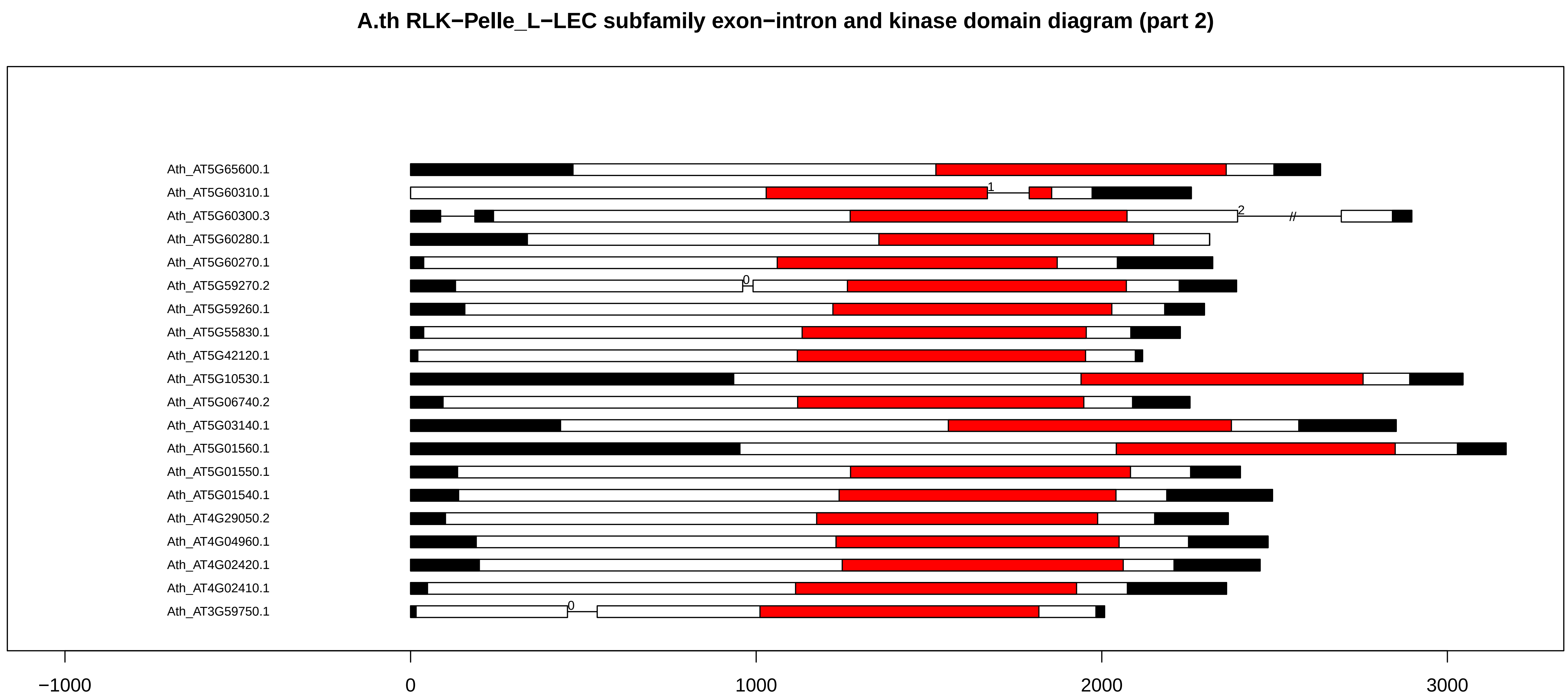
A.th RLK−Pelle_L−LEC subfamily exon−intron and kinase domain diagram (part 2)
Ath_AT5G65600.1
Ath_AT5G60310.1
Ath_AT5G60300.3
Ath_AT5G60280.1
Ath_AT5G60270.1
Ath_AT5G59270.2
Ath_AT5G59260.1
Ath_AT5G55830.1
Ath_AT5G42120.1
Ath_AT5G10530.1
Ath_AT5G06740.2
Ath_AT5G03140.1
Ath_AT5G01560.1
Ath_AT5G01550.1
Ath_AT5G01540.1
Ath_AT4G29050.2
Ath_AT4G04960.1
Ath_AT4G02420.1
Ath_AT4G02410.1
Ath_AT3G59750.1
1
2
0
0
−1000
0
1000
2000
3000

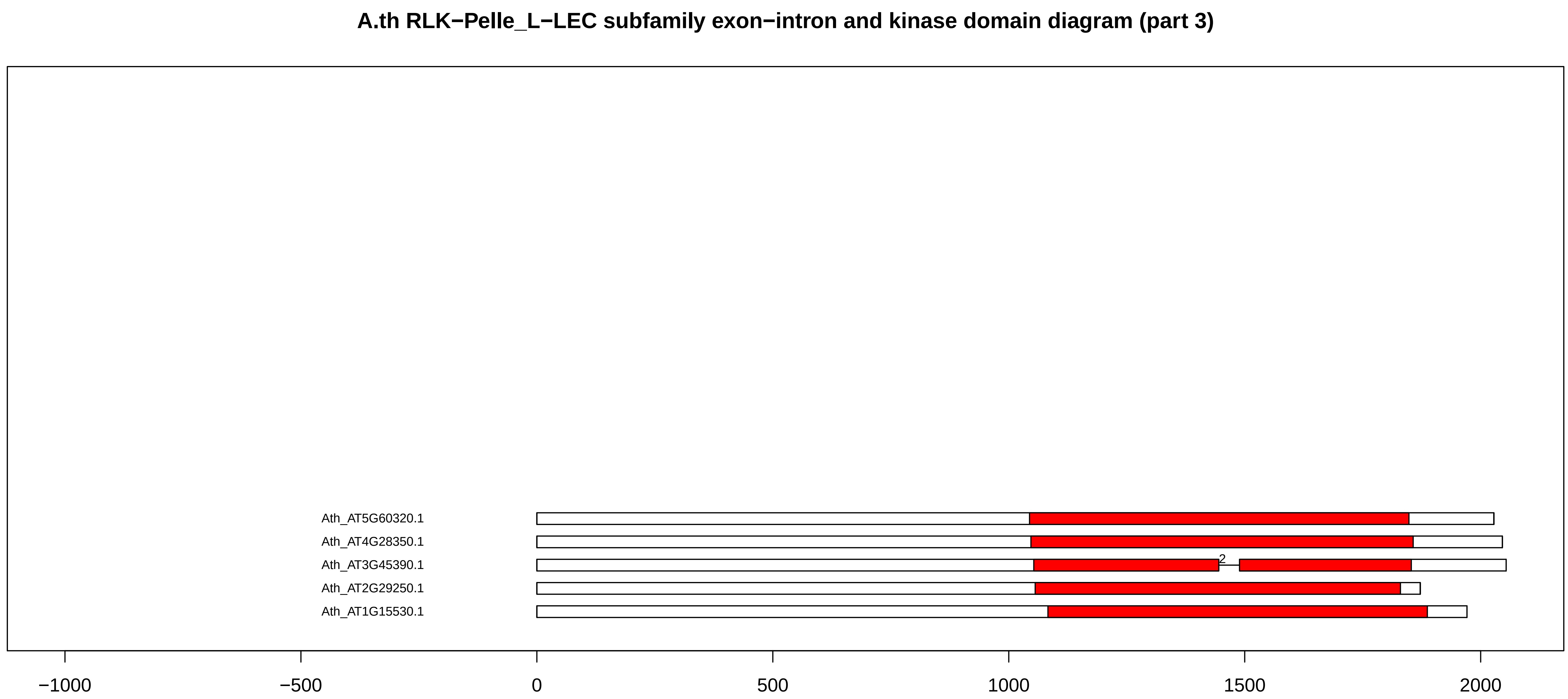
A.th RLK−Pelle_L−LEC subfamily exon−intron and kinase domain diagram (part 3)
Ath_AT5G60320.1
Ath_AT4G28350.1
Ath_AT3G45390.1
2
Ath_AT2G29250.1
Ath_AT1G15530.1
−1000
−500
0
500
1000
1500
2000

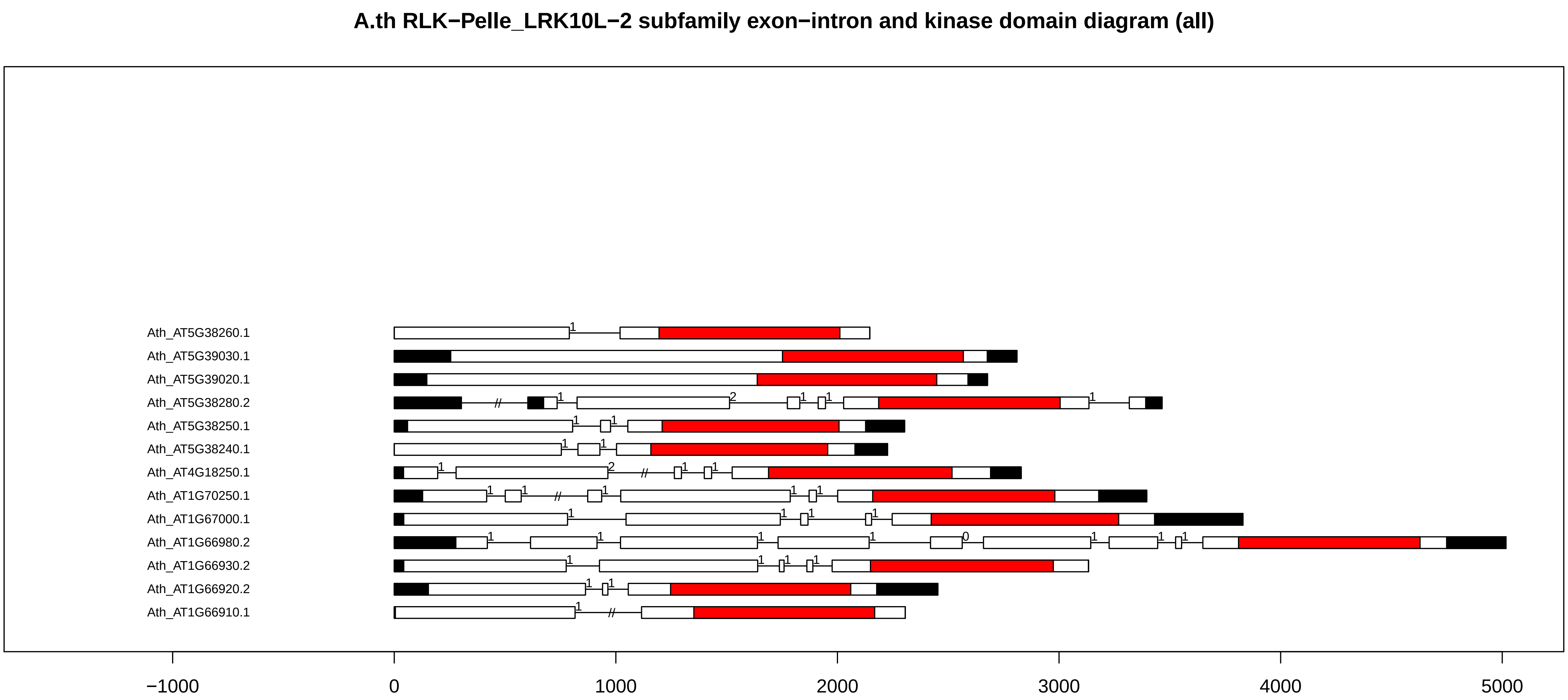
A.th RLK−Pelle_LRK10L−2 subfamily exon−intron and kinase domain diagram (all)
Ath_AT5G38260.1
Ath_AT5G39030.1
Ath_AT5G39020.1
Ath_AT5G38280.2
Ath_AT5G38250.1
Ath_AT5G38240.1
Ath_AT4G18250.1
Ath_AT1G70250.1
Ath_AT1G67000.1
Ath_AT1G66980.2
Ath_AT1G66930.2
Ath_AT1G66920.2
Ath_AT1G66910.1
−1000
0
1000
2000
3000
4000
5000

A.th RLK−Pelle_LRR−I−1 subfamily exon−intron and kinase domain diagram (part 1)

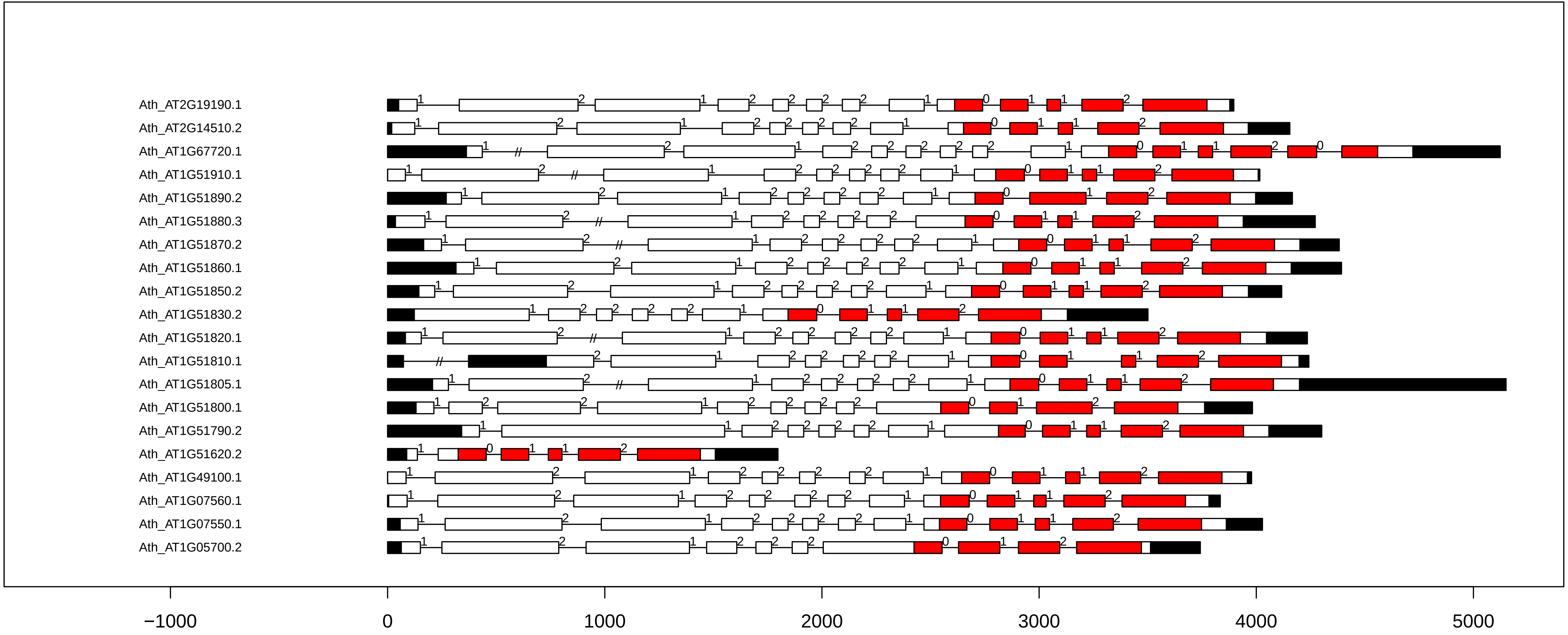

A.th RLK−Pelle_LRR−I−1 subfamily exon−intron and kinase domain diagram (part 2)

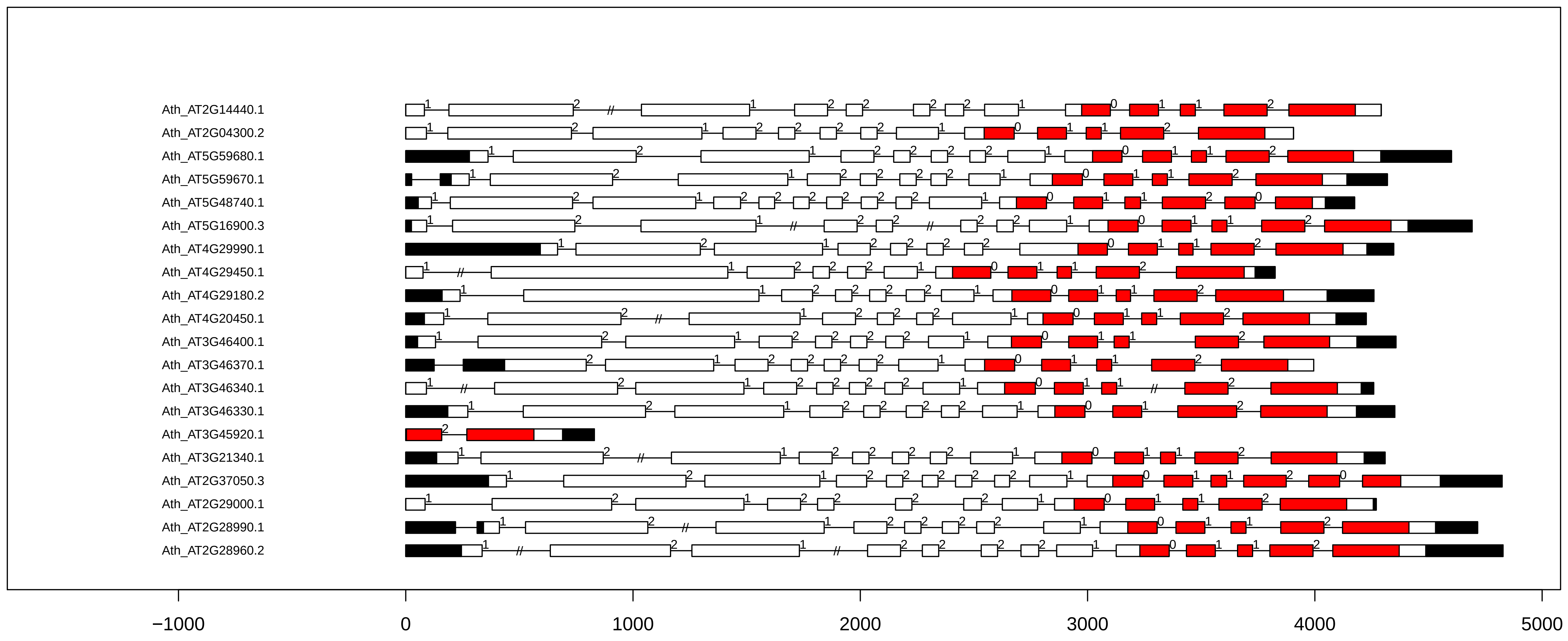

A.th RLK−Pelle_LRR−I−1 subfamily exon−intron and kinase domain diagram (part 3)

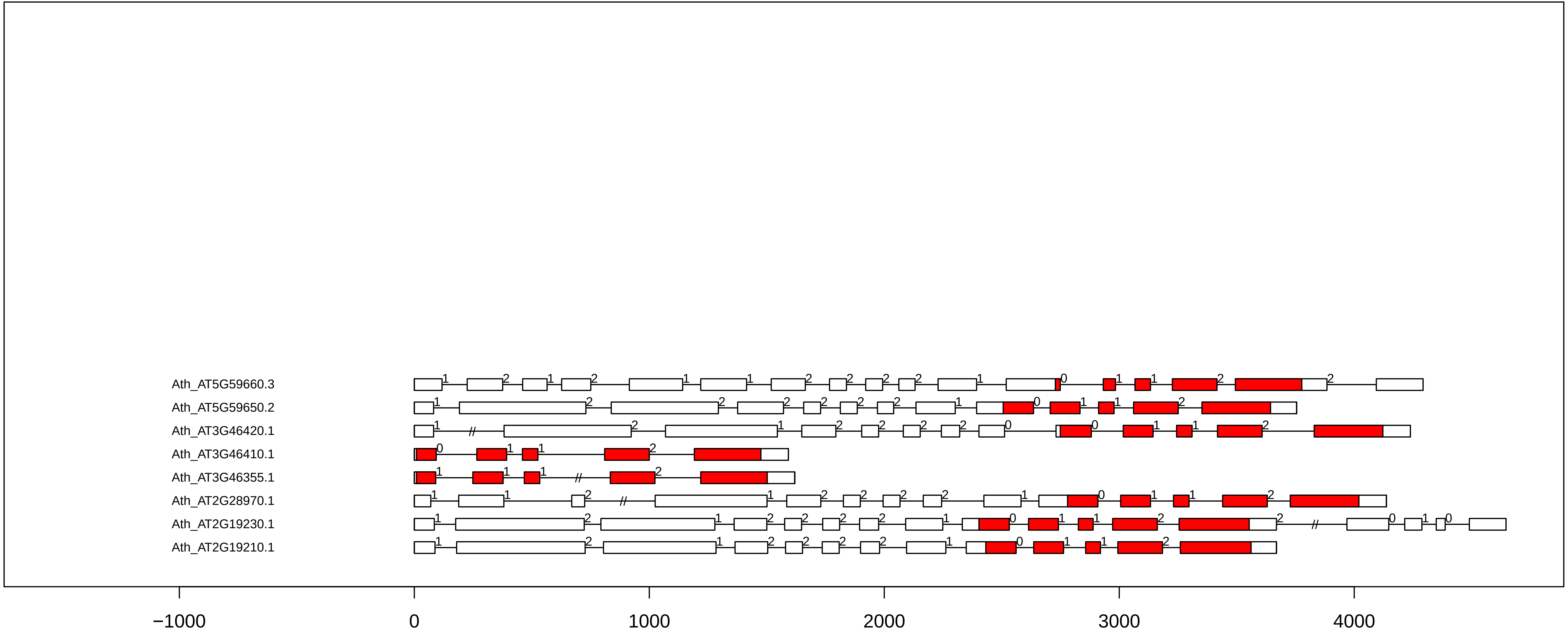

A.th RLK−Pelle_LRR−I−2 subfamily exon−intron and kinase domain diagram (all)

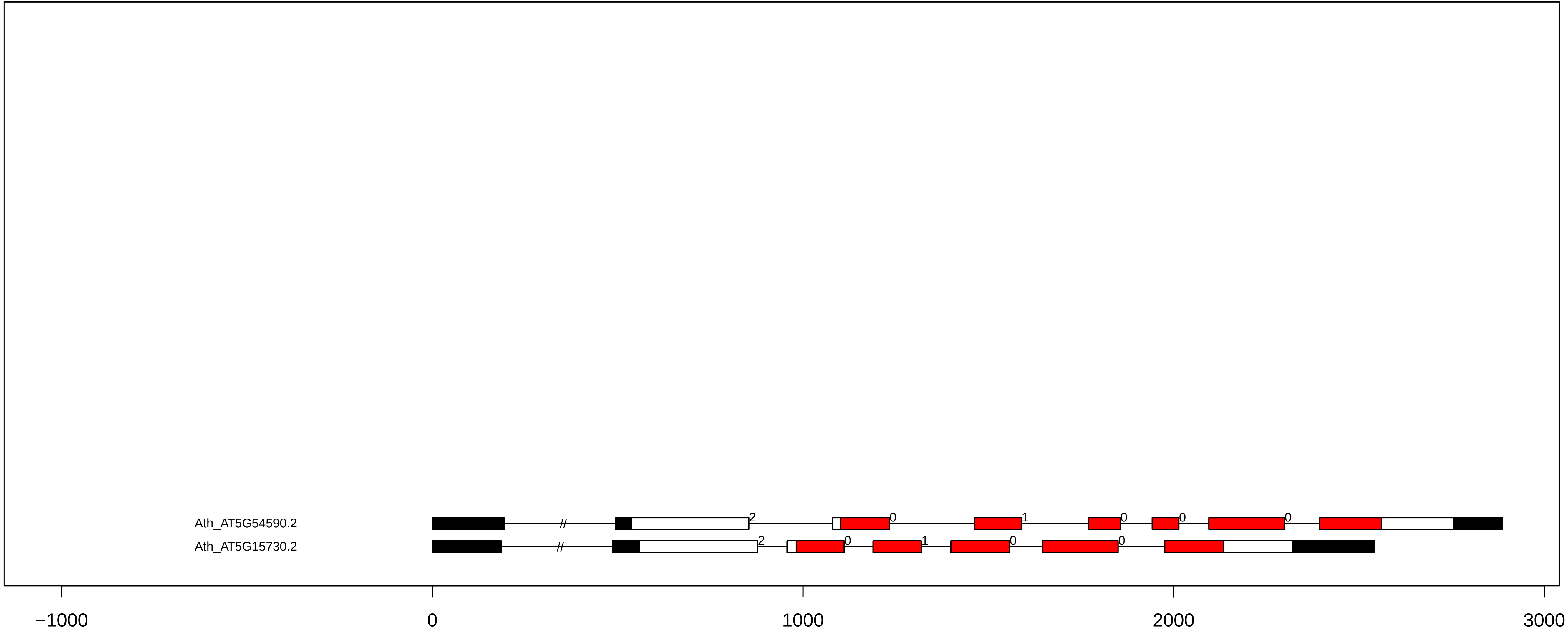

A.th RLK−Pelle_LRR−III subfamily exon−intron and kinase domain diagram (part 1)

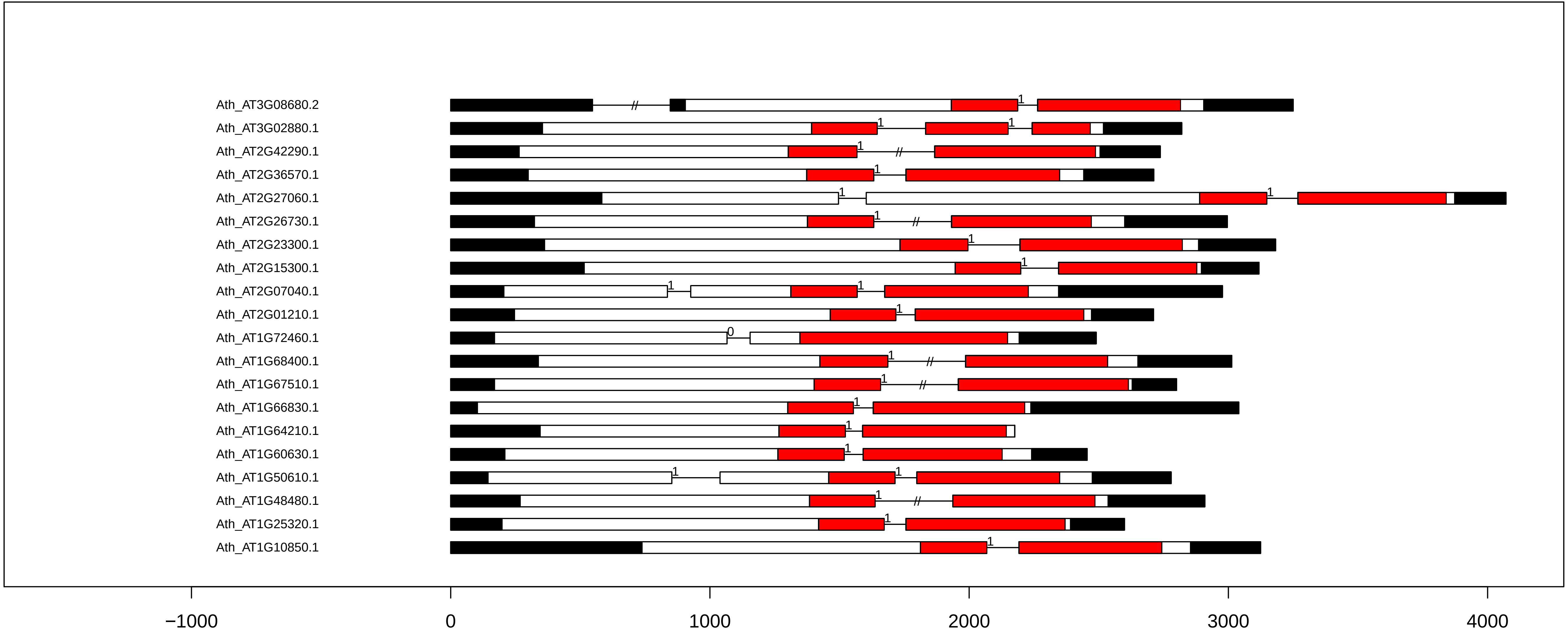

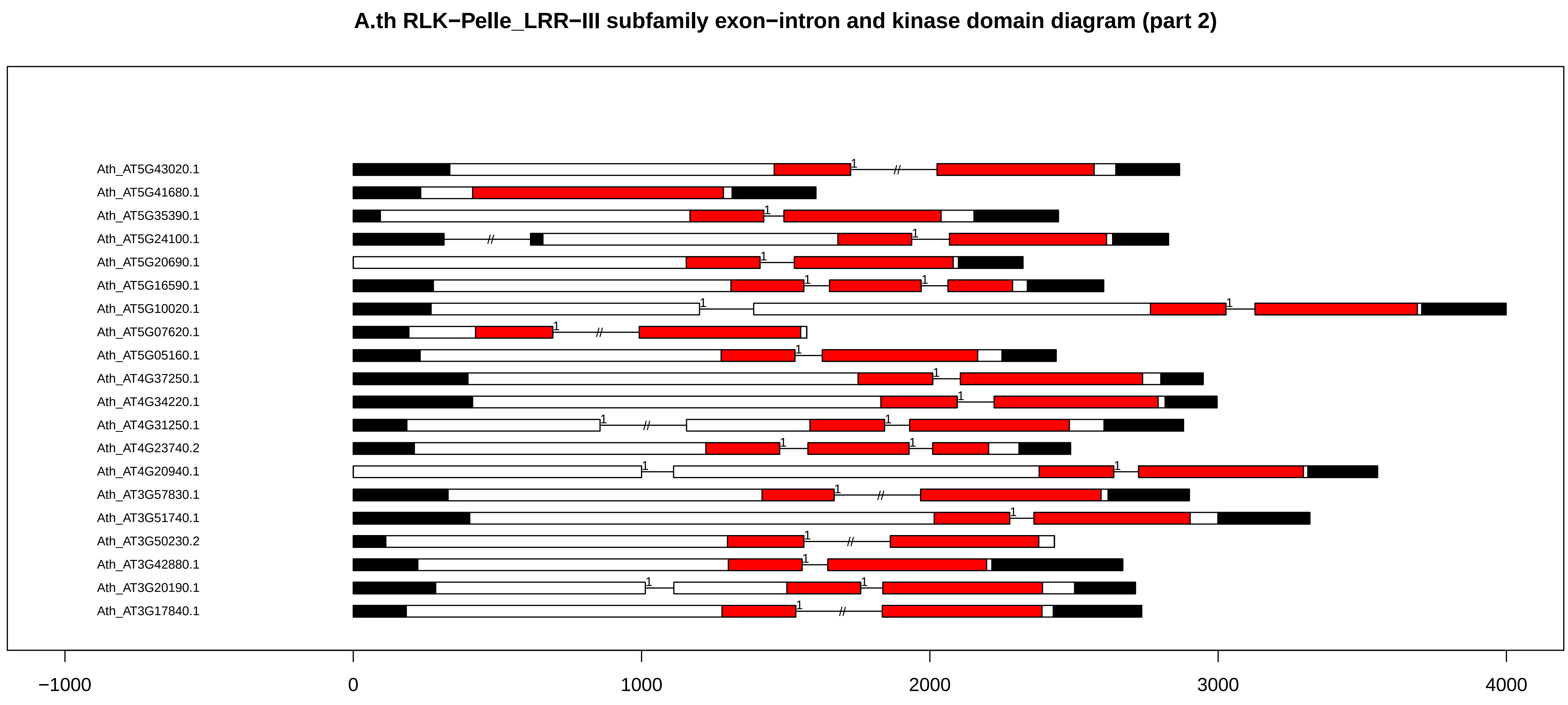

A.th RLK−Pelle_LRR−III subfamily exon−intron and kinase domain diagram (part 2)
Ath_AT5G43020.1
Ath_AT5G41680.1
Ath_AT5G35390.1
Ath_AT5G24100.1
Ath_AT5G20690.1
Ath_AT5G16590.1
Ath_AT5G10020.1
Ath_AT5G07620.1
Ath_AT5G05160.1
Ath_AT4G37250.1
Ath_AT4G34220.1
Ath_AT4G31250.1
Ath_AT4G23740.2
Ath_AT4G20940.1
Ath_AT3G57830.1
Ath_AT3G51740.1
Ath_AT3G50230.2
Ath_AT3G42880.1
Ath_AT3G20190.1
Ath_AT3G17840.1
−1000
0
1000
2000
3000
4000

A.th RLK−Pelle_LRR−III subfamily exon−intron and kinase domain diagram (part 3)

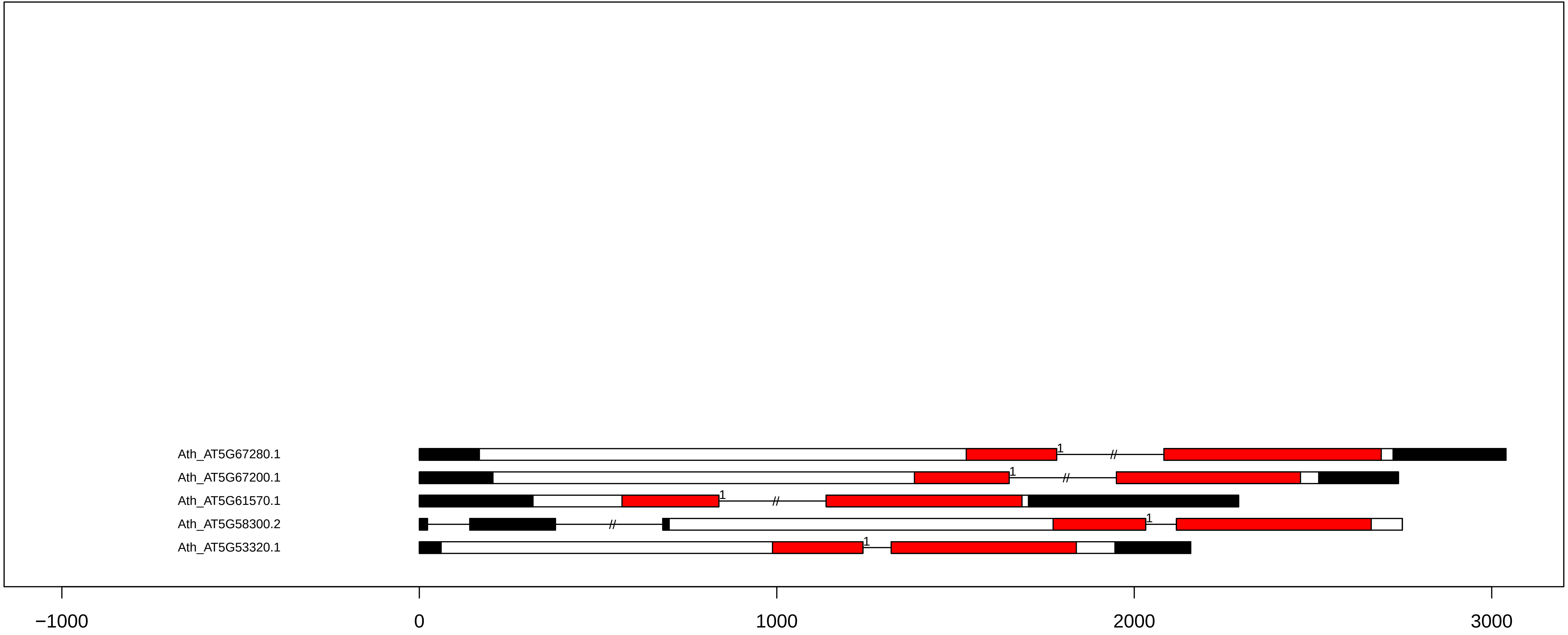

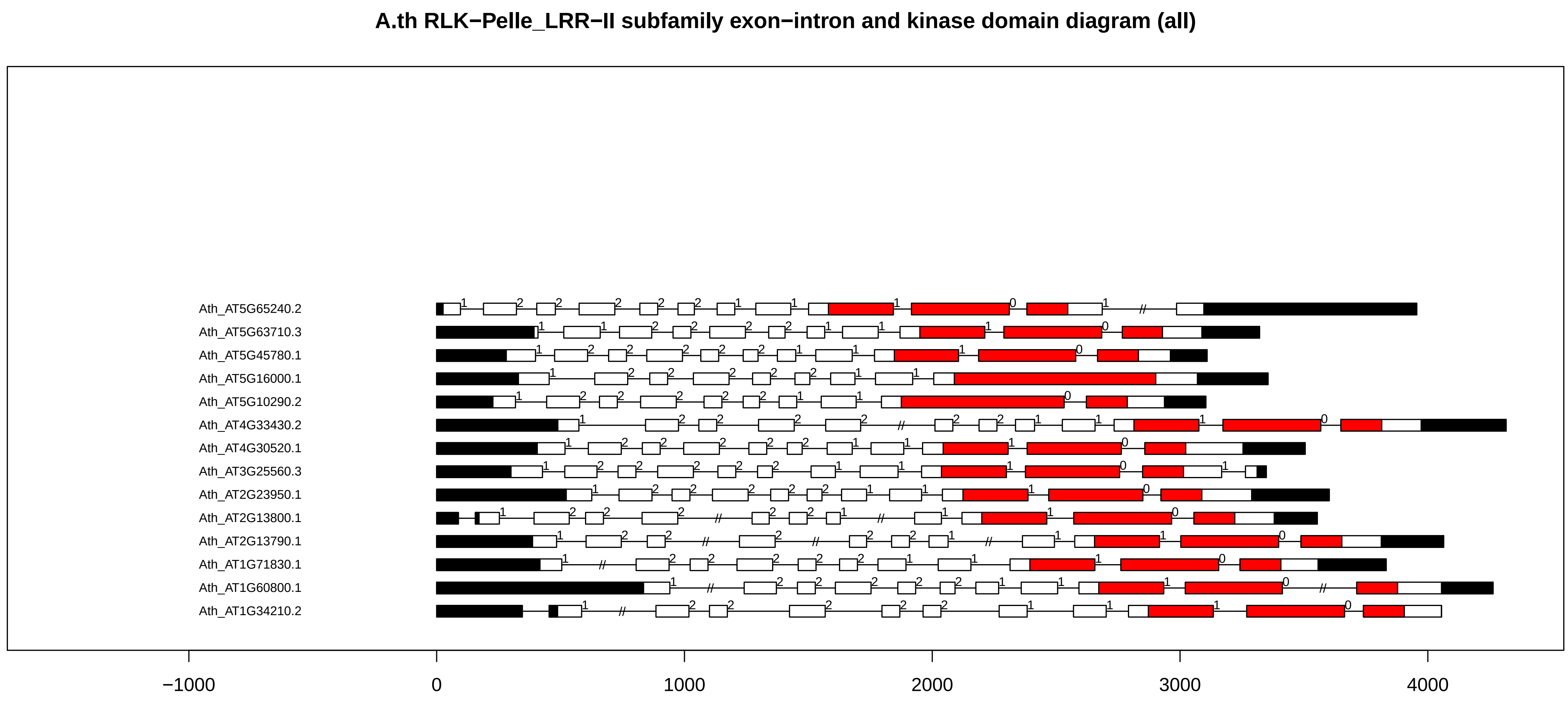
A.th RLK−Pelle_LRR−II subfamily exon−intron and kinase domain diagram (all)
Ath_AT5G65240.2
Ath_AT5G63710.3
Ath_AT5G45780.1
Ath_AT5G16000.1
Ath_AT5G10290.2
Ath_AT4G33430.2
Ath_AT4G30520.1
Ath_AT3G25560.3
Ath_AT2G23950.1
Ath_AT2G13800.1
Ath_AT2G13790.1
Ath_AT1G71830.1
Ath_AT1G60800.1
Ath_AT1G34210.2
−1000
0
1000
2000
3000
4000

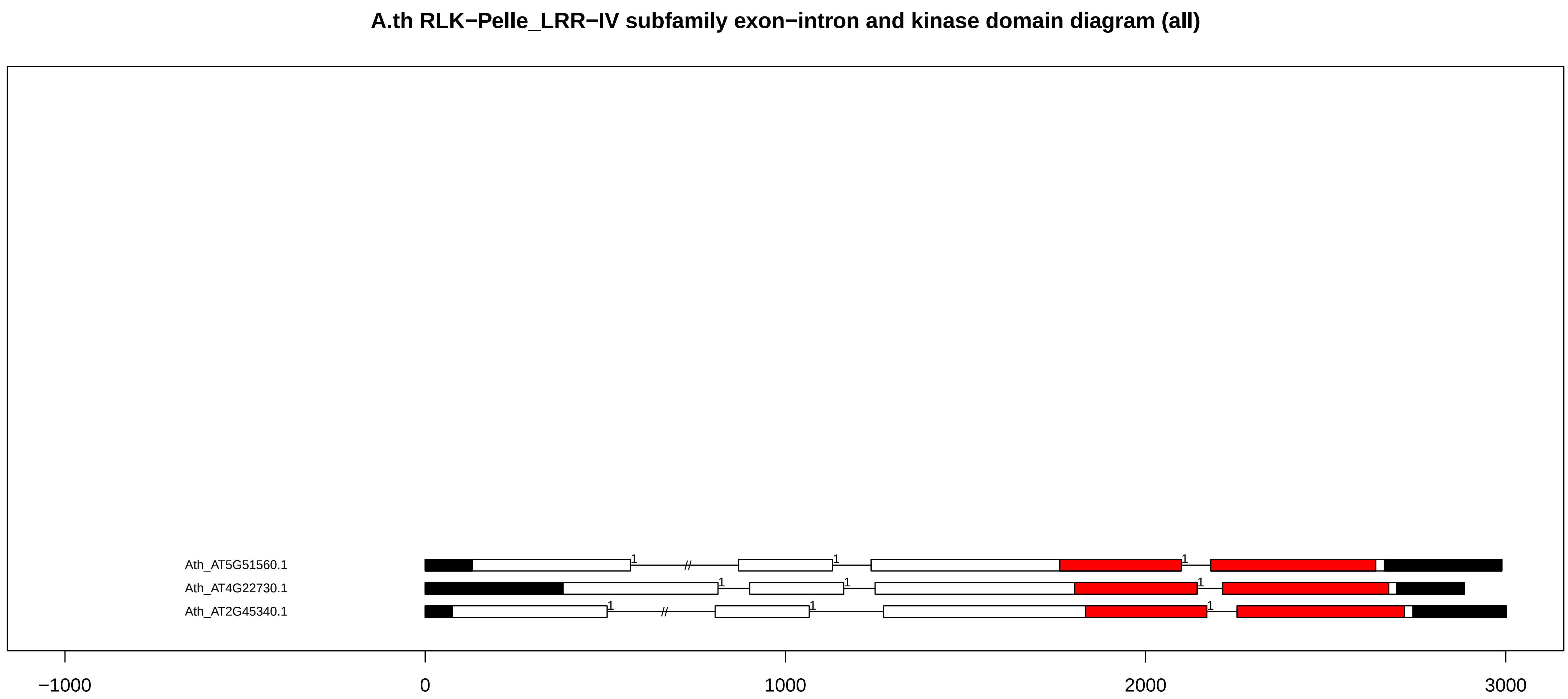
A.th RLK−Pelle_LRR−IV subfamily exon−intron and kinase domain diagram (all)
Ath_AT5G51560.1
Ath_AT4G22730.1
Ath_AT2G45340.1
−1000
0
1000
2000
3000

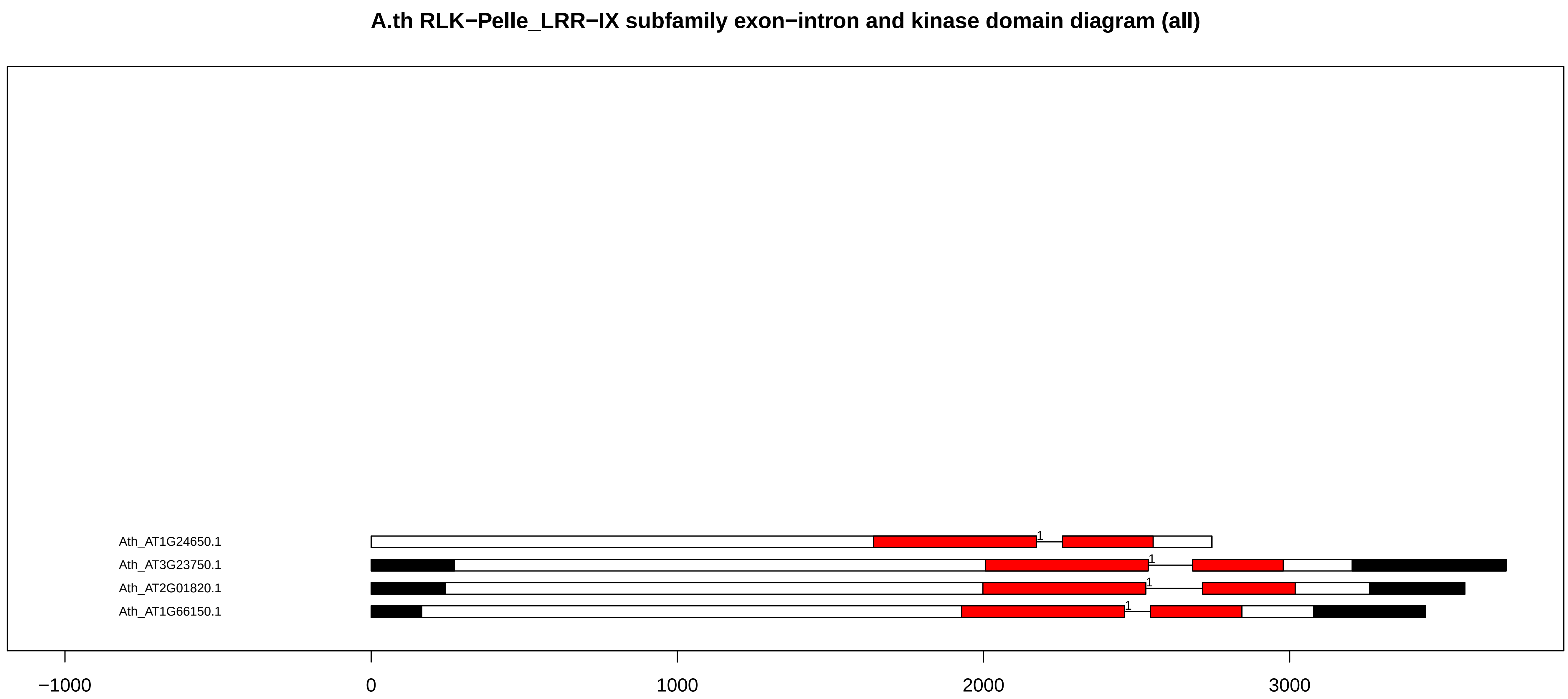
A.th RLK−Pelle_LRR−IX subfamily exon−intron and kinase domain diagram (all)
Ath_AT1G24650.1
Ath_AT3G23750.1
Ath_AT2G01820.1
Ath_AT1G66150.1
1
1
1
1
−1000
0
1000
2000
3000

A.th RLK−Pelle_LRR−VI−1 subfamily exon−intron and kinase domain diagram (all)

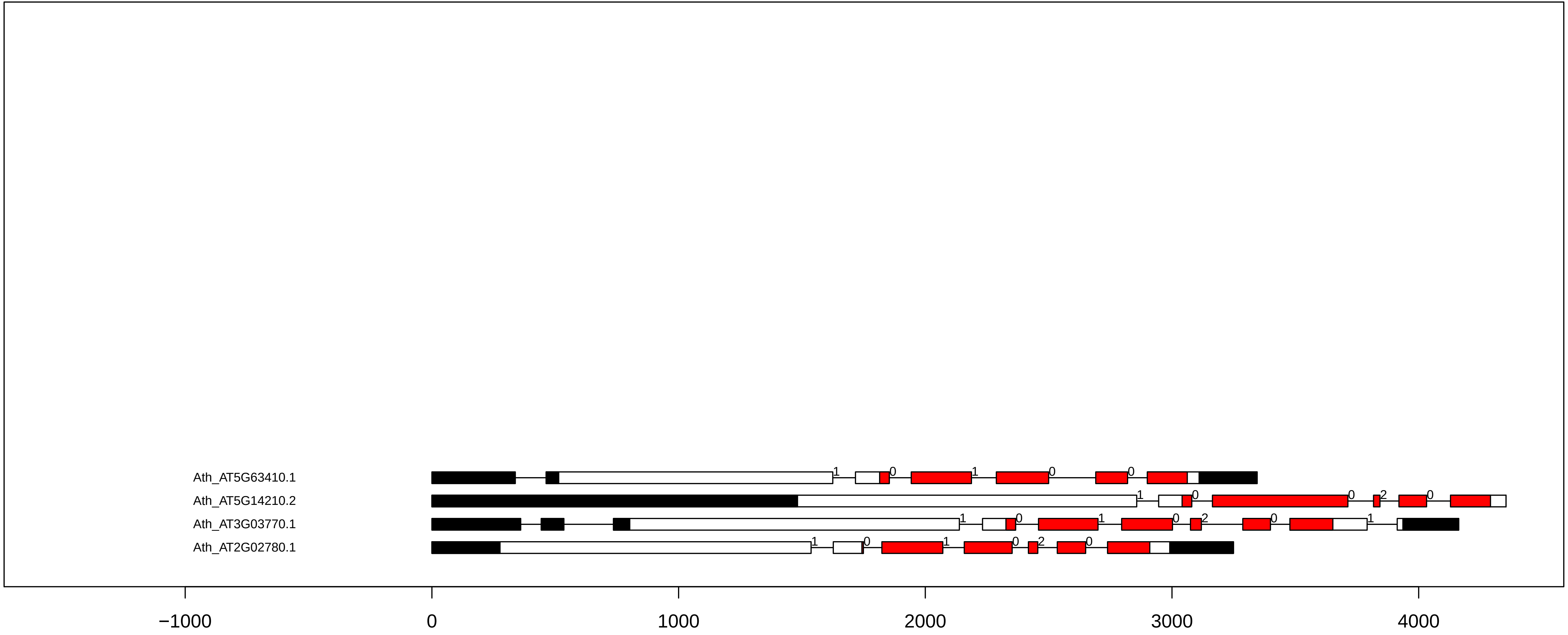

A.th RLK−Pelle_LRR−VI−2 subfamily exon−intron and kinase domain diagram (all)

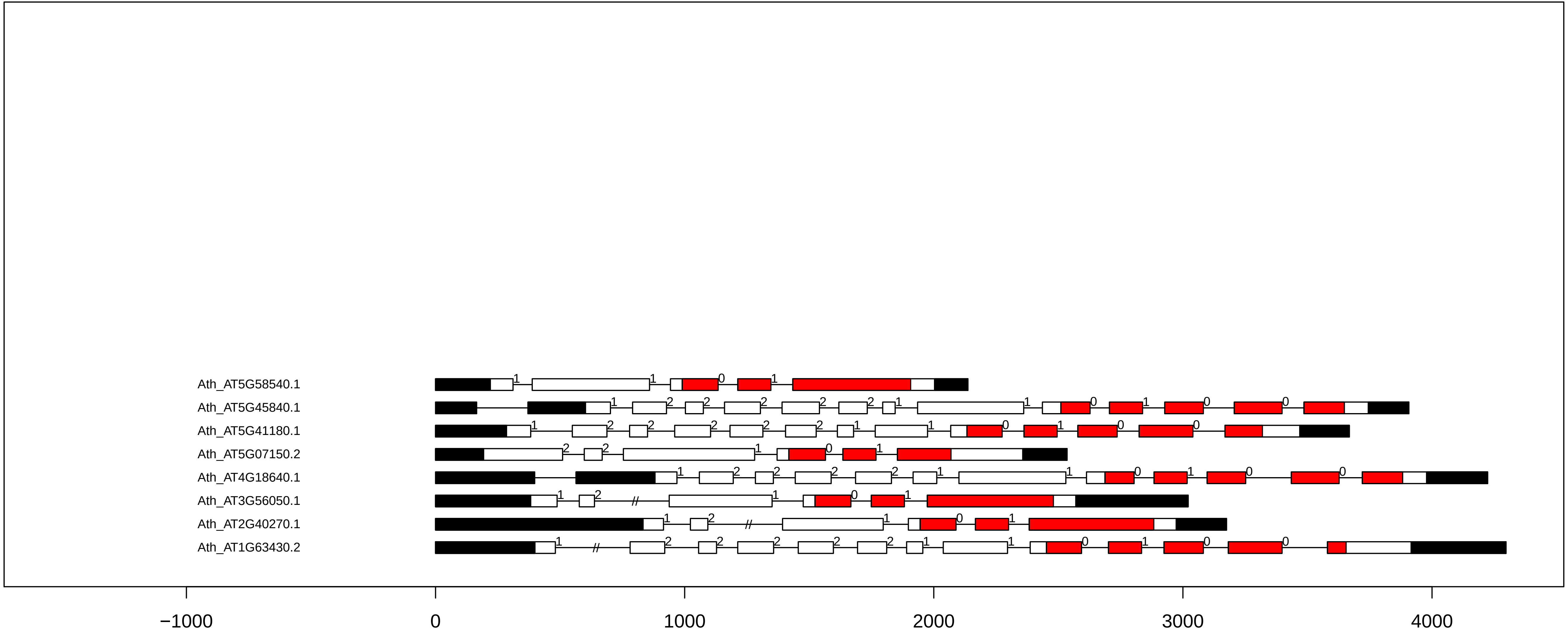

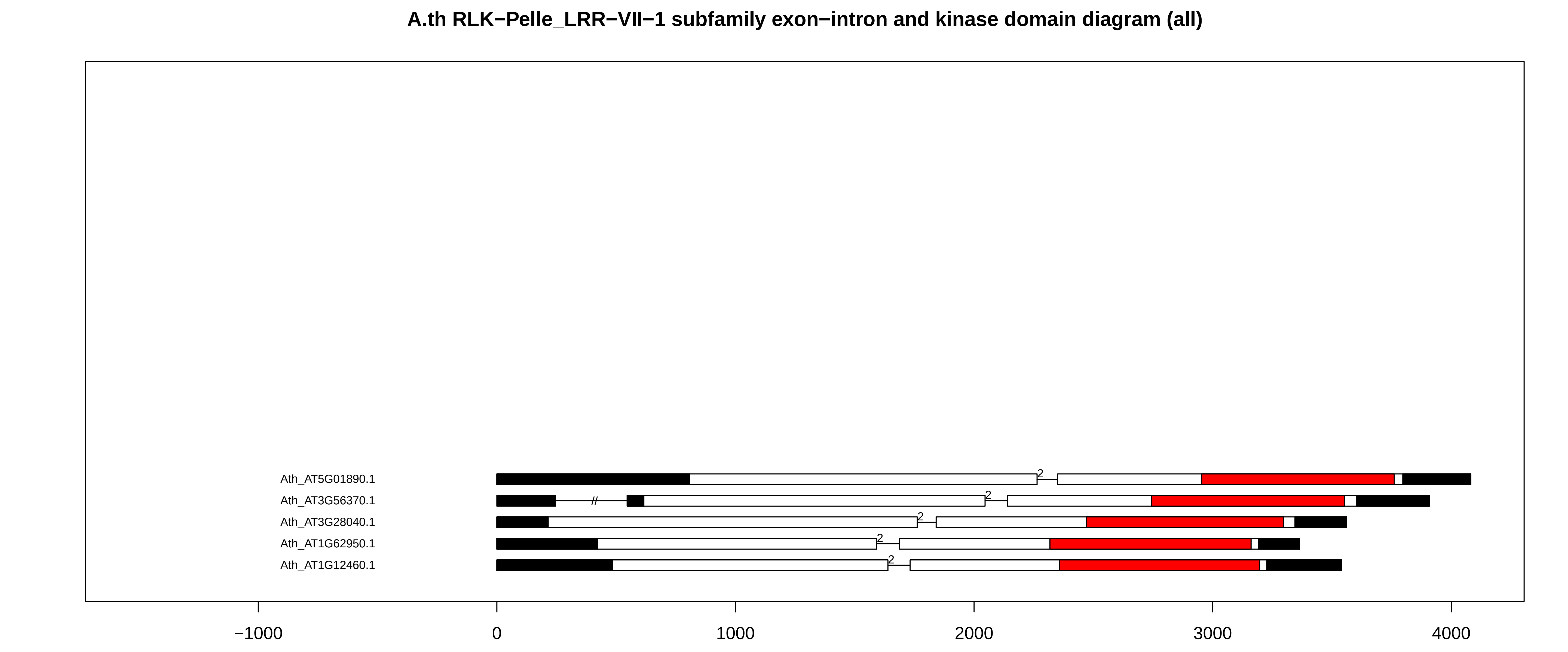
A.th RLK−Pelle_LRR−VII−1 subfamily exon−intron and kinase domain diagram (all)
Ath_AT5G01890.1
Ath_AT3G56370.1
Ath_AT3G28040.1
Ath_AT1G62950.1
Ath_AT1G12460.1
2
2
2
2
2
−1000
0
1000
2000
3000
4000

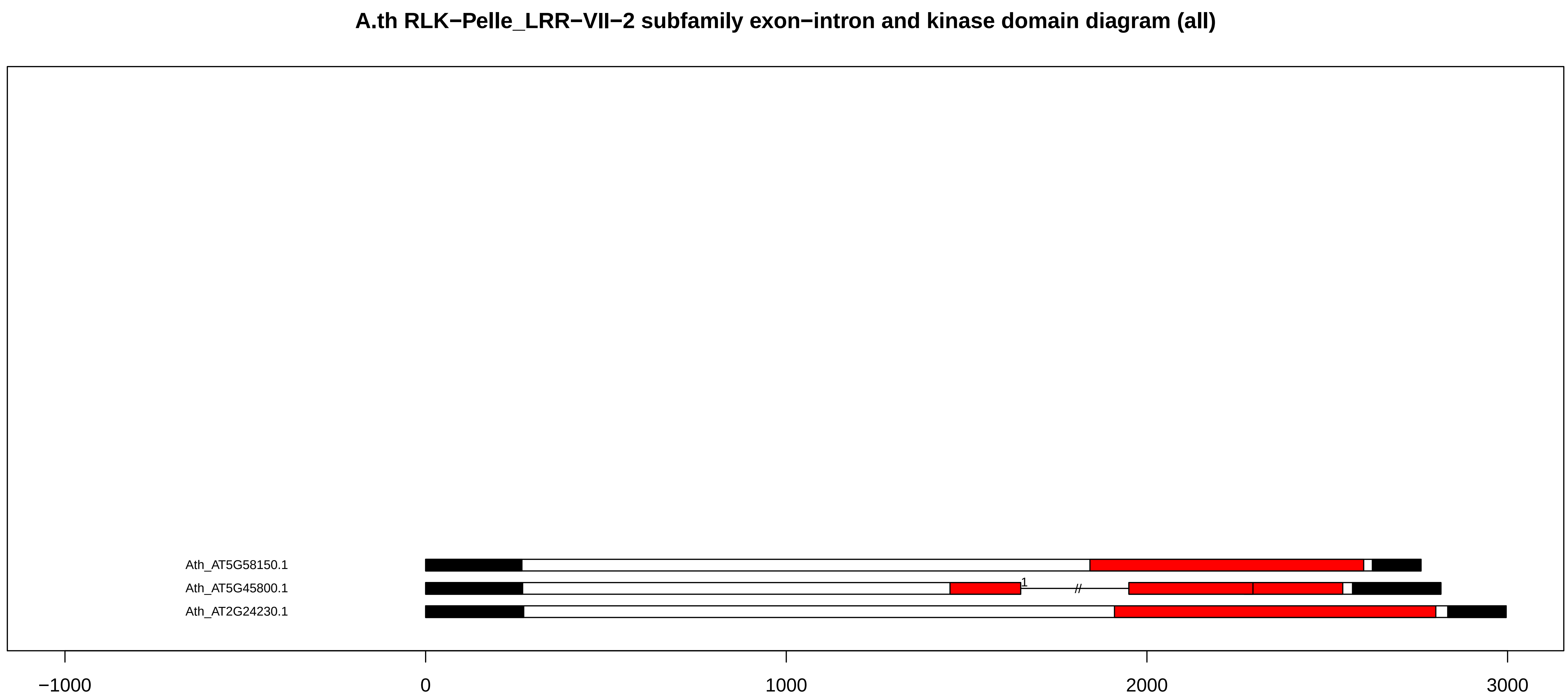
A.th RLK−Pelle_LRR−VII−2 subfamily exon−intron and kinase domain diagram (all)
Ath_AT5G58150.1
Ath_AT5G45800.1
Ath_AT2G24230.1
1
−1000
0
1000
2000
3000

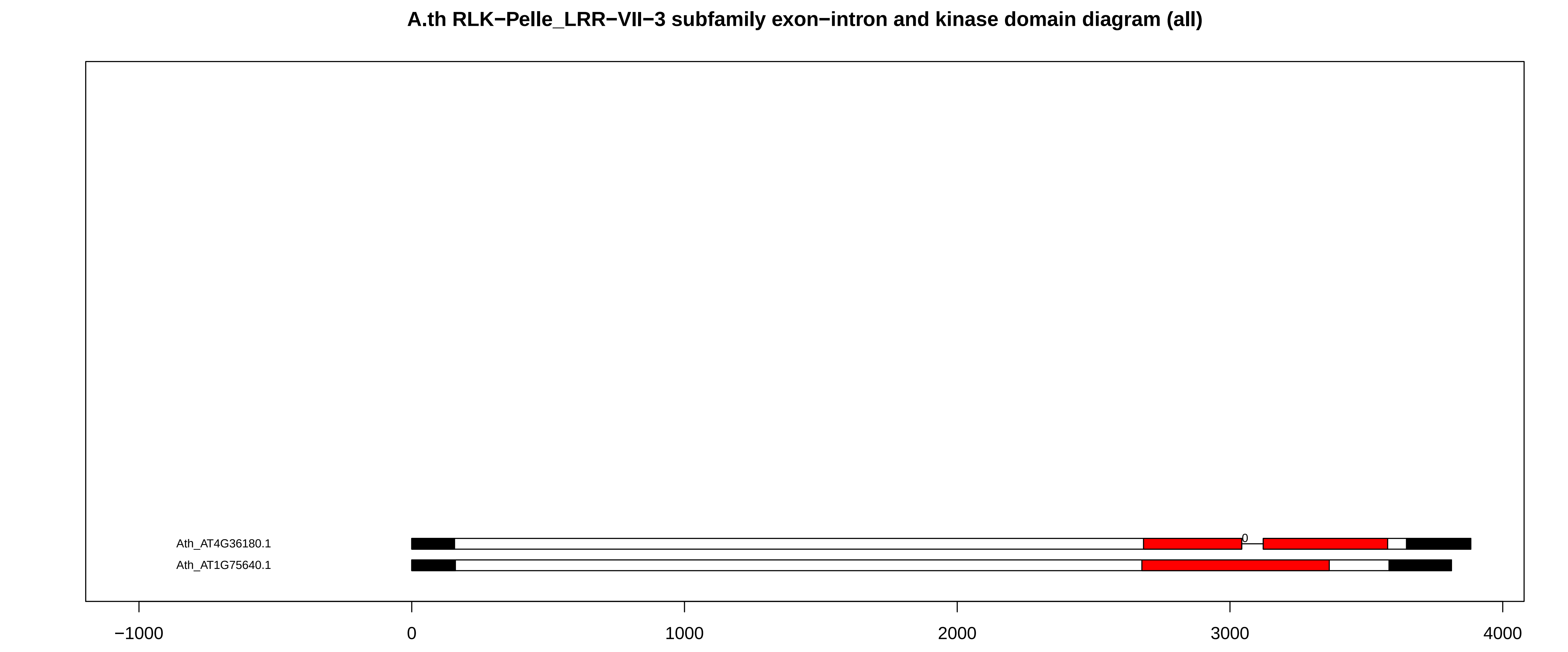

A.th RLK−Pelle_LRR−VII−3 subfamily exon−intron and kinase domain diagram (all)
Ath_AT4G36180.1
Ath_AT1G75640.1
0
−1000
0
1000
2000
3000
4000

A.th RLK−Pelle_LRR−VIII−1 subfamily exon−intron and kinase domain diagram (all)

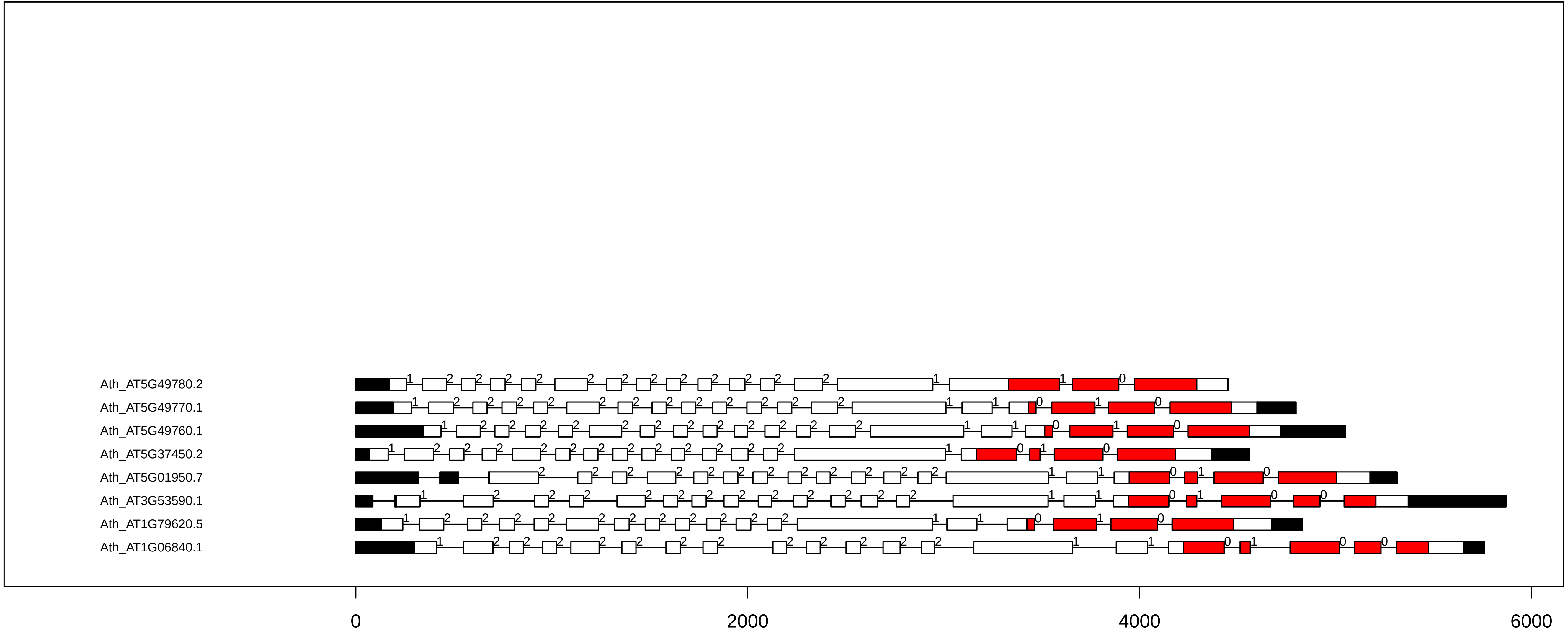

A.th RLK−Pelle_LRR−V subfamily exon−intron and kinase domain diagram (all)

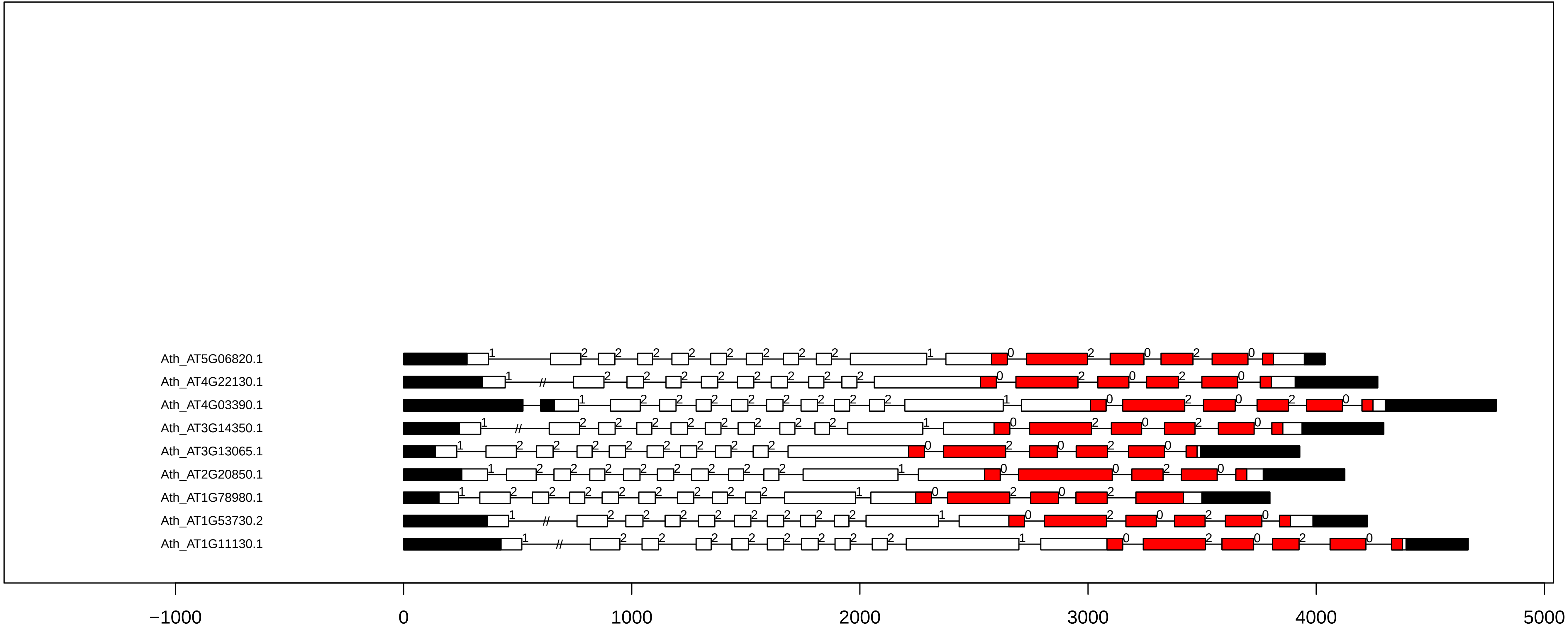

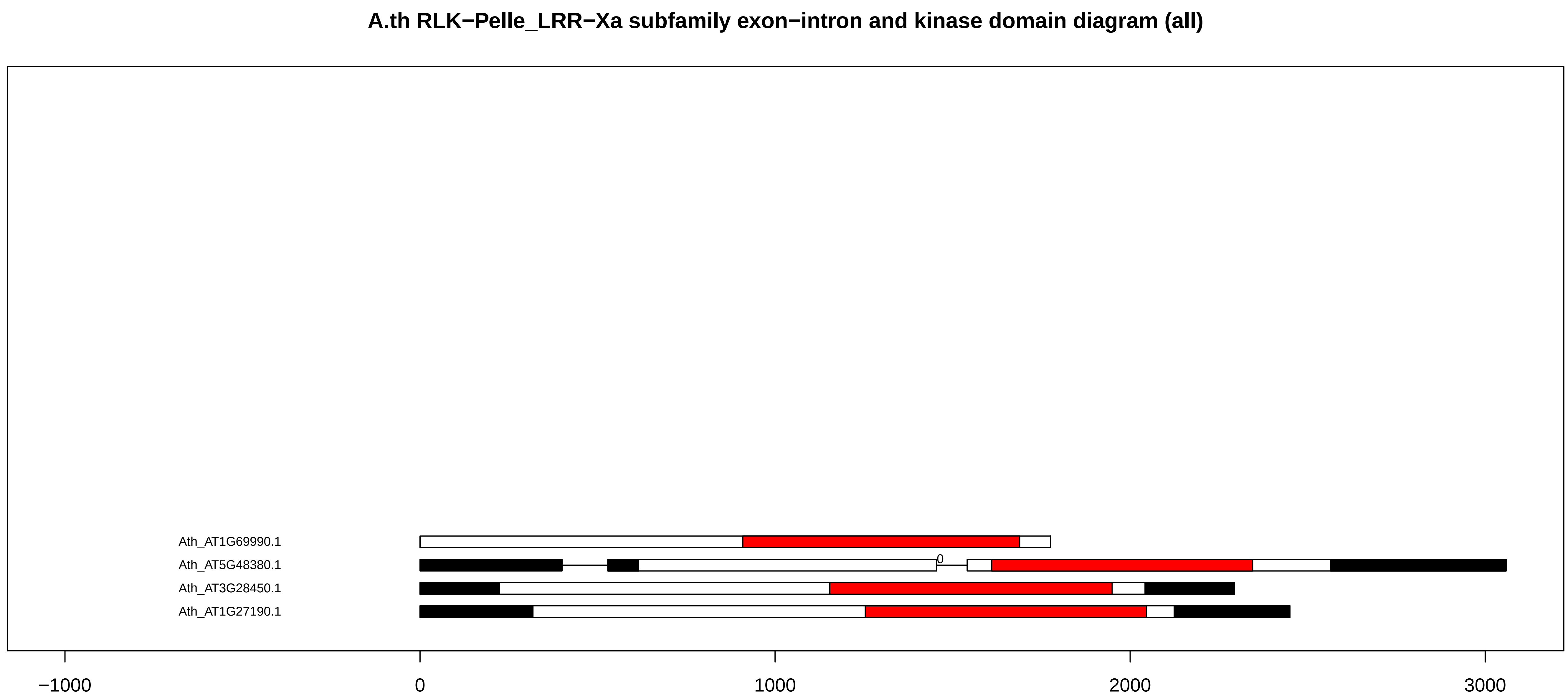
A.th RLK−Pelle_LRR−Xa subfamily exon−intron and kinase domain diagram (all)
Ath_AT1G69990.1
Ath_AT5G48380.1
Ath_AT3G28450.1
Ath_AT1G27190.1
0
−1000
0
1000
2000
3000

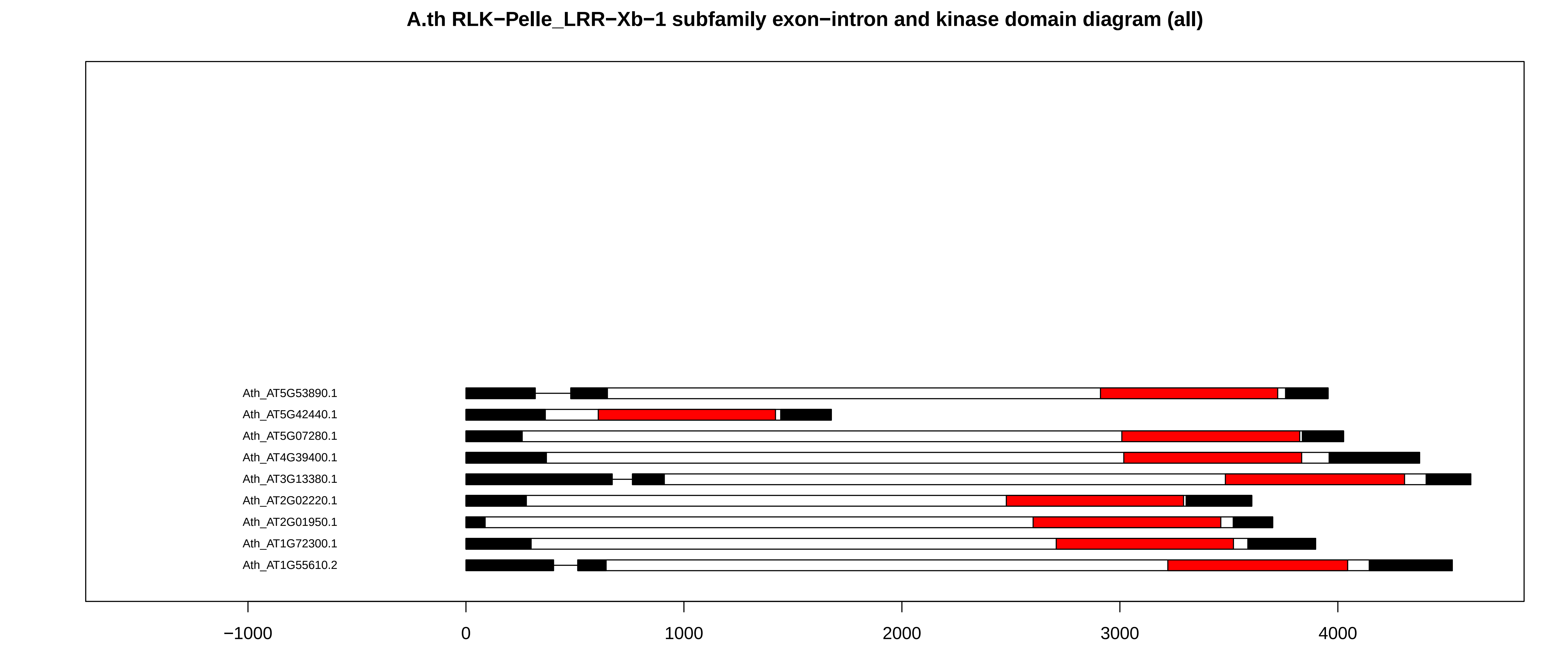

A.th RLK−Pelle_LRR−Xb−1 subfamily exon−intron and kinase domain diagram (all)
Ath_AT5G53890.1
Ath_AT5G42440.1
Ath_AT5G07280.1
Ath_AT4G39400.1
Ath_AT3G13380.1
Ath_AT2G02220.1
Ath_AT2G01950.1
Ath_AT1G72300.1
Ath_AT1G55610.2
−1000
0
1000
2000
3000
4000

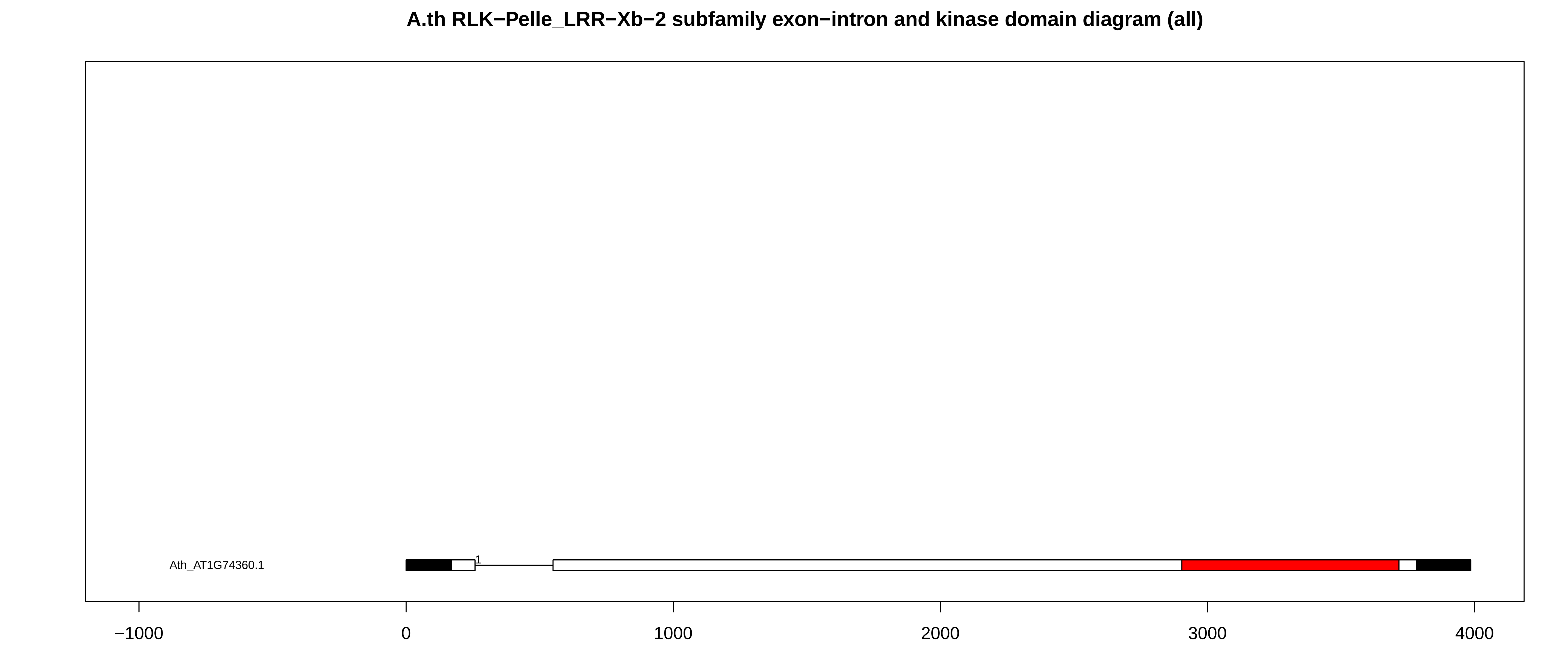

A.th RLK−Pelle_LRR−Xb−2 subfamily exon−intron and kinase domain diagram (all)
Ath_AT1G74360.1
1
−1000
0
1000
2000
3000
4000

A.th RLK−Pelle_LRR−XI−1 subfamily exon−intron and kinase domain diagram (part 1)

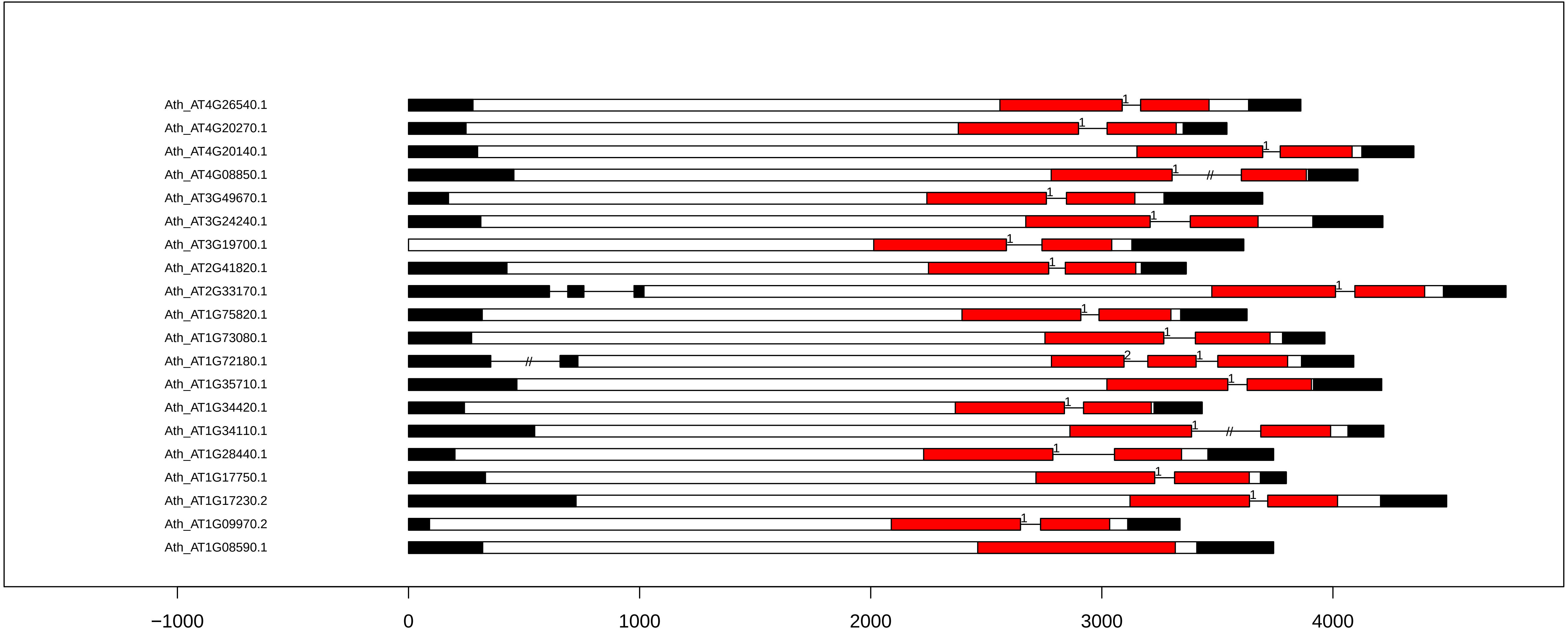

A.th RLK−Pelle_LRR−XI−1 subfamily exon−intron and kinase domain diagram (part 2)

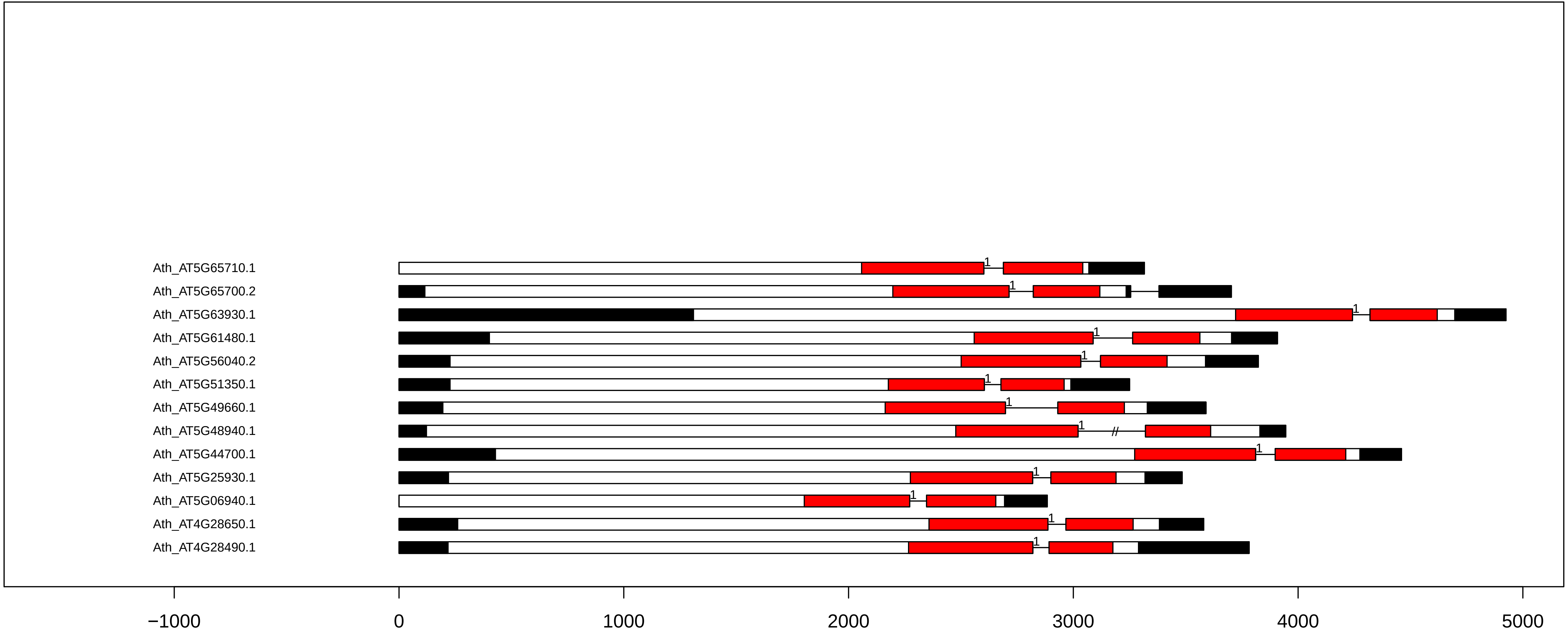

A.th RLK−Pelle_LRR−XI−2 subfamily exon−intron and kinase domain diagram (all)

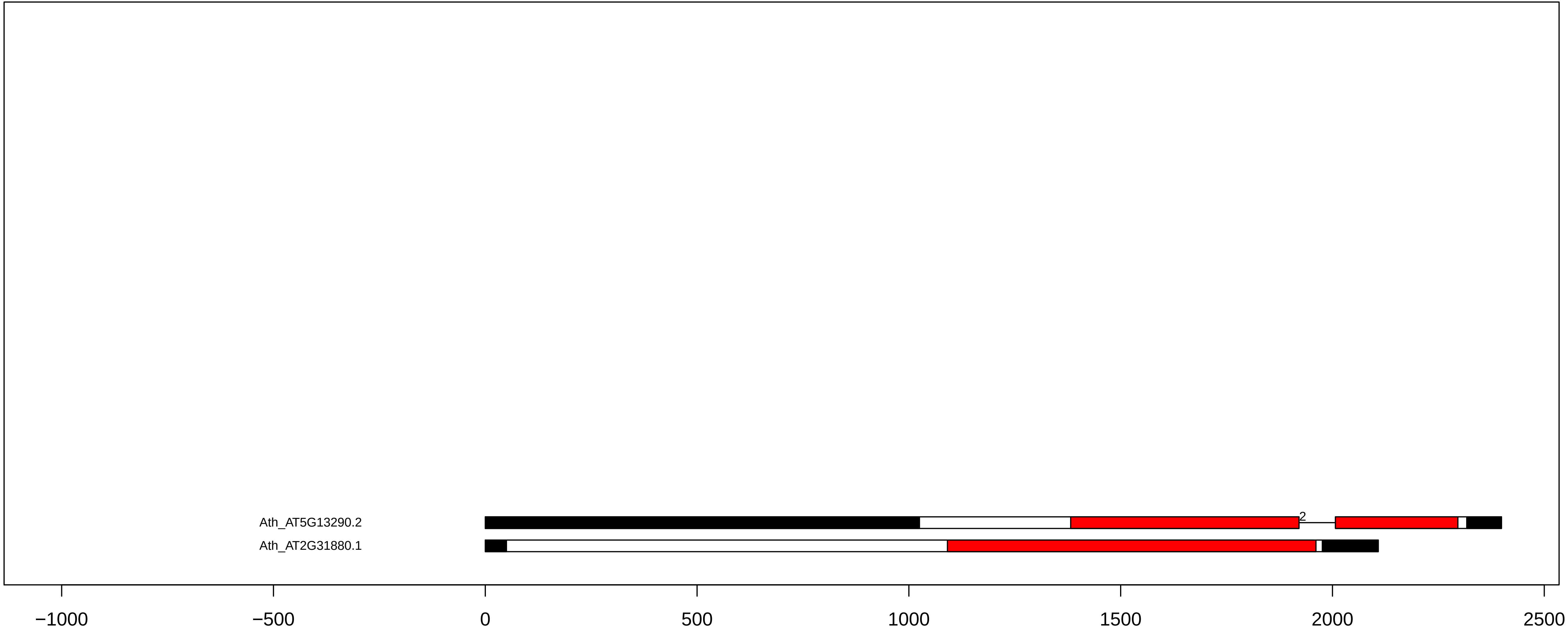

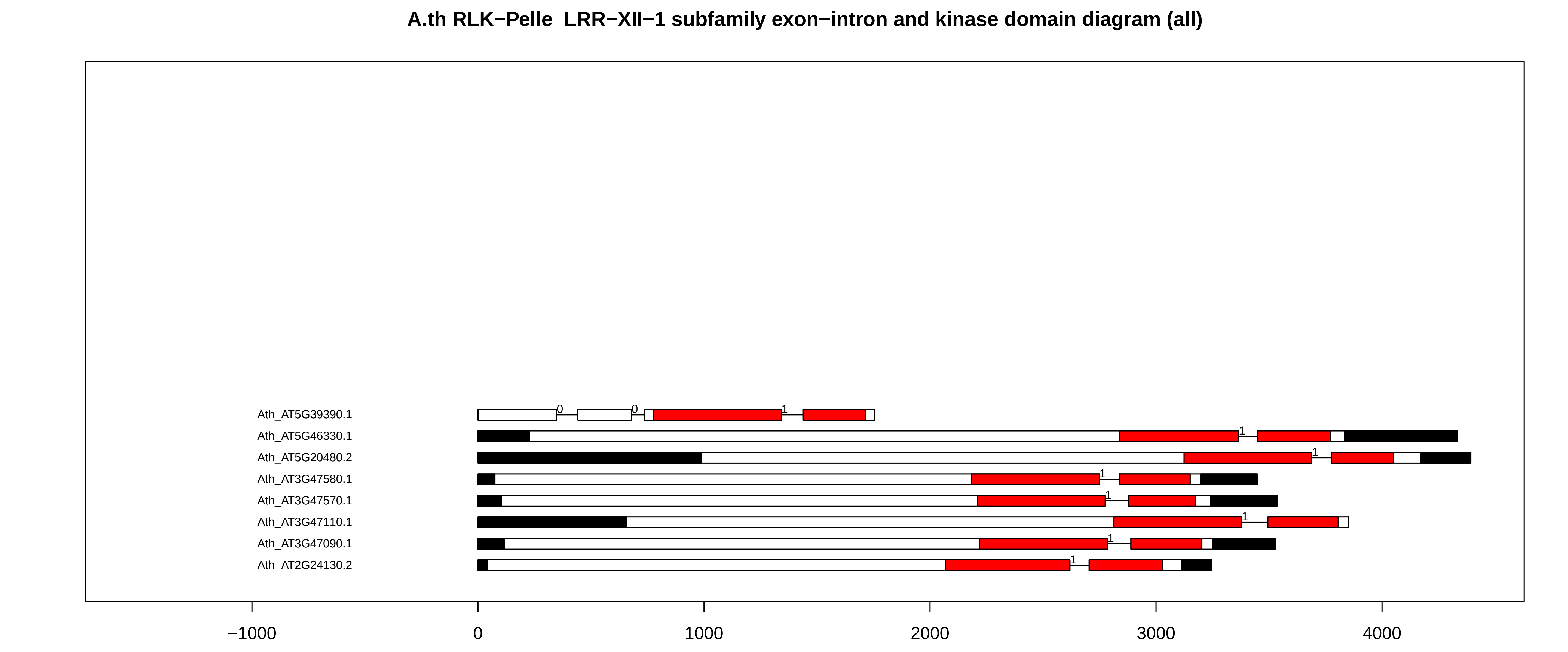
A.th RLK−Pelle_LRR−XII−1 subfamily exon−intron and kinase domain diagram (all)
Ath_AT5G39390.1
Ath_AT5G46330.1
Ath_AT5G20480.2
Ath_AT3G47580.1
Ath_AT3G47570.1
Ath_AT3G47110.1
Ath_AT3G47090.1
Ath_AT2G24130.2
0
0
1
−1000
0
1000
2000
3000
4000

A.th RLK−Pelle_LRR−XIIIa subfamily exon−intron and kinase domain diagram (all)

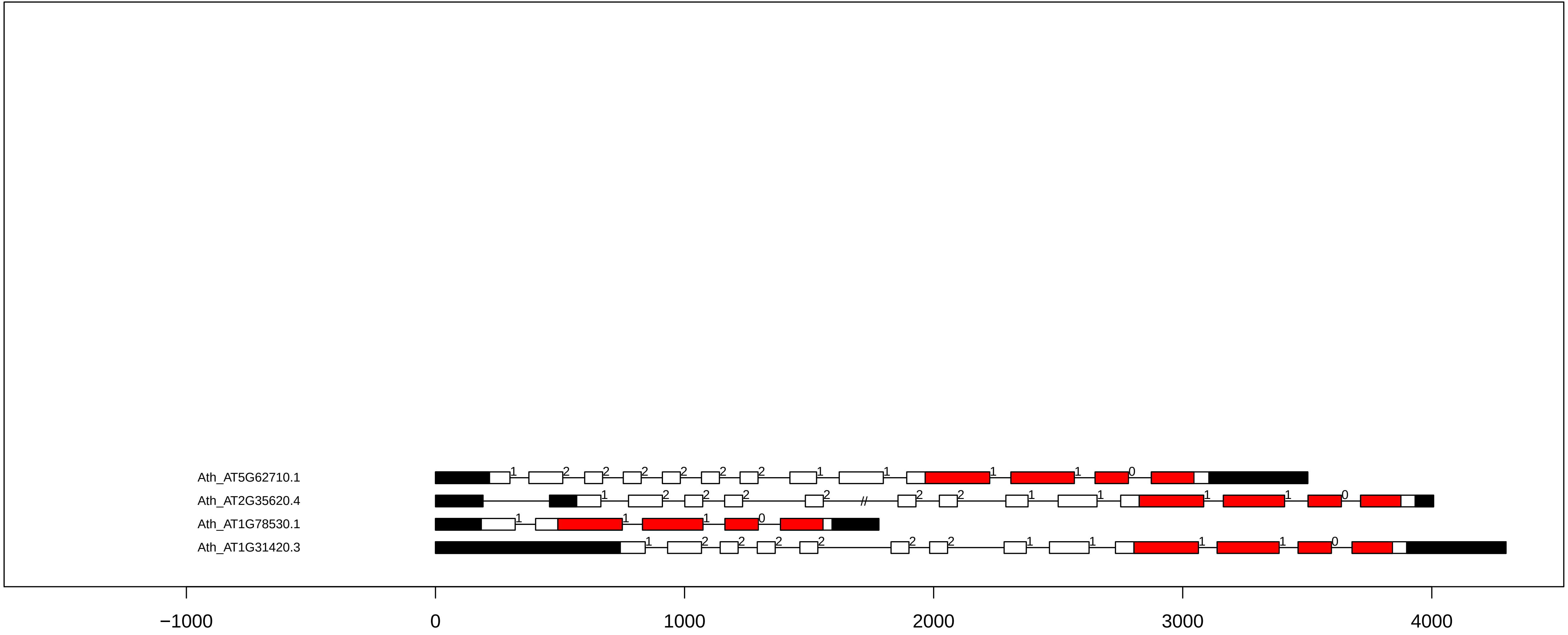

A.th RLK−Pelle_LRR−XIIIb subfamily exon−intron and kinase domain diagram (all)

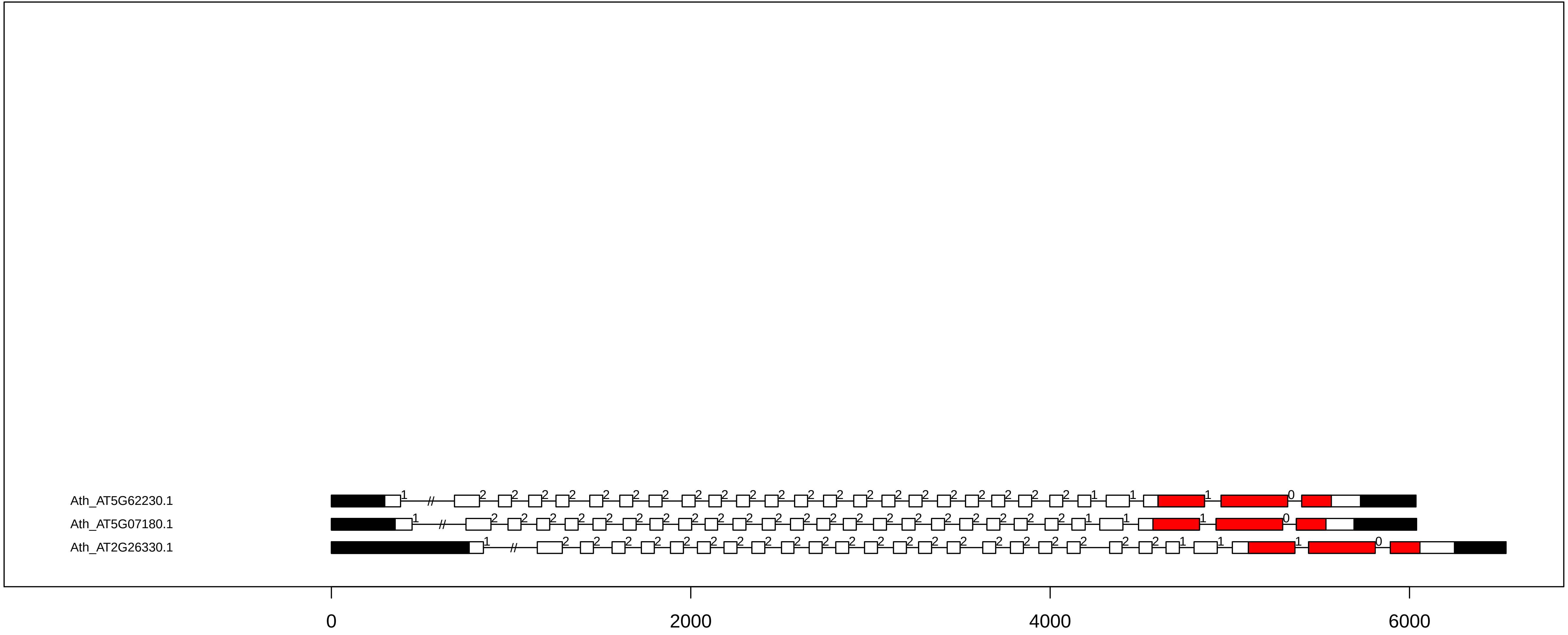

A.th RLK−Pelle_LRR−XIV subfamily exon−intron and kinase domain diagram (all)

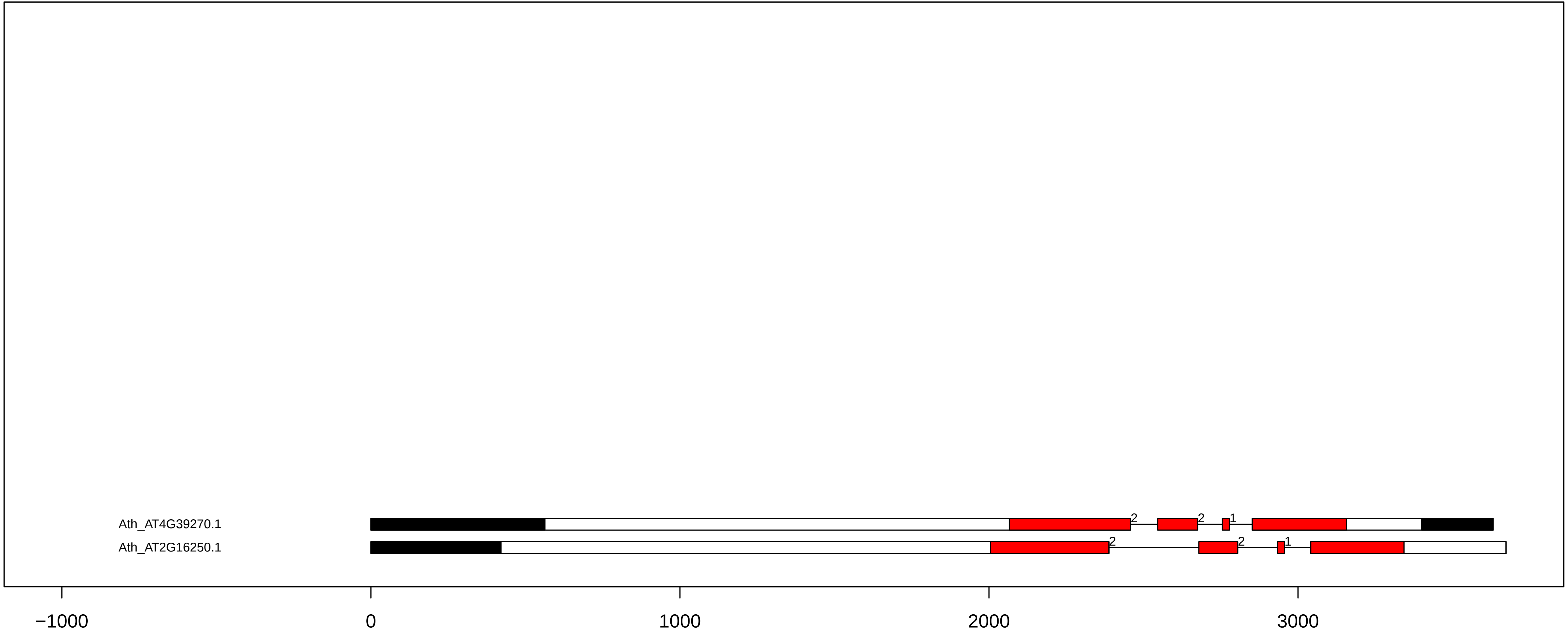

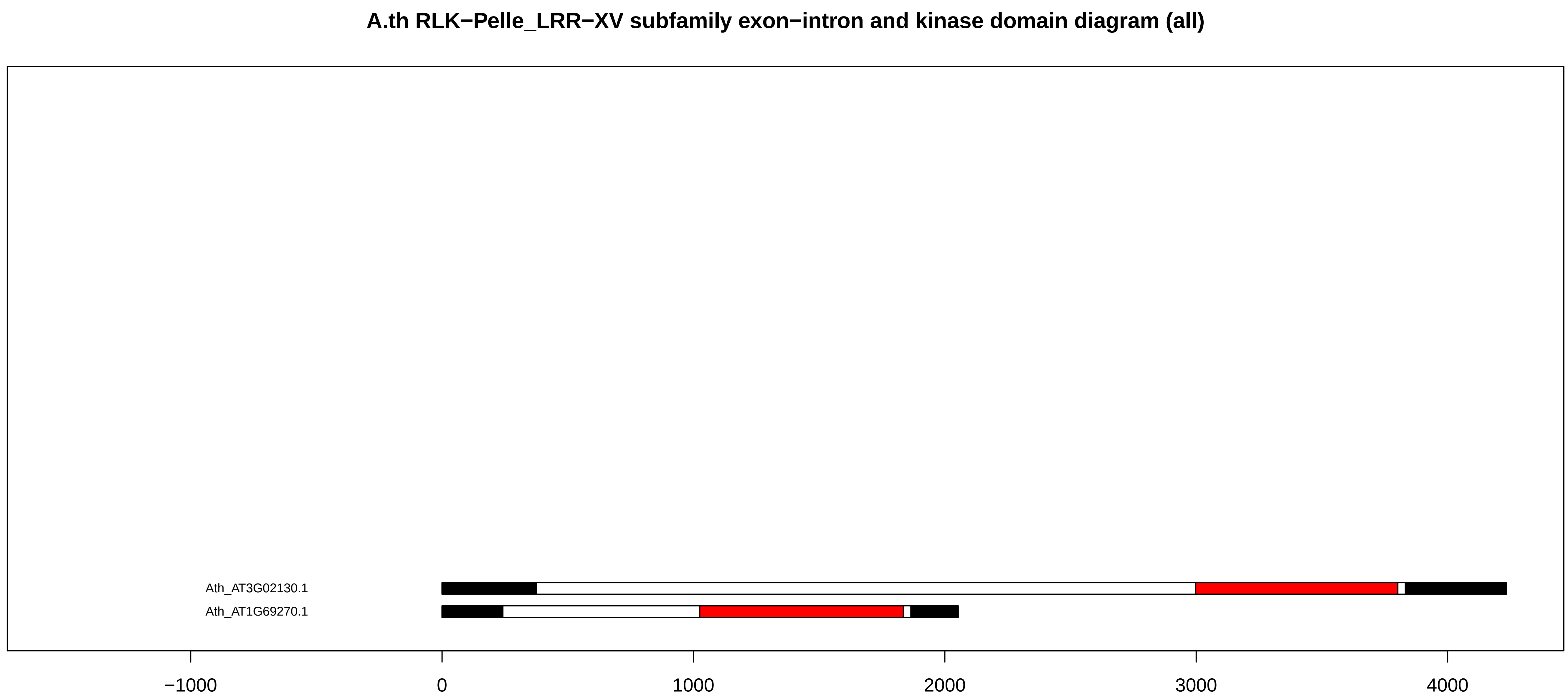
A.th RLK−Pelle_LRR−XV subfamily exon−intron and kinase domain diagram (all)
Ath_AT3G02130.1
Ath_AT1G69270.1
−1000
0
1000
2000
3000
4000

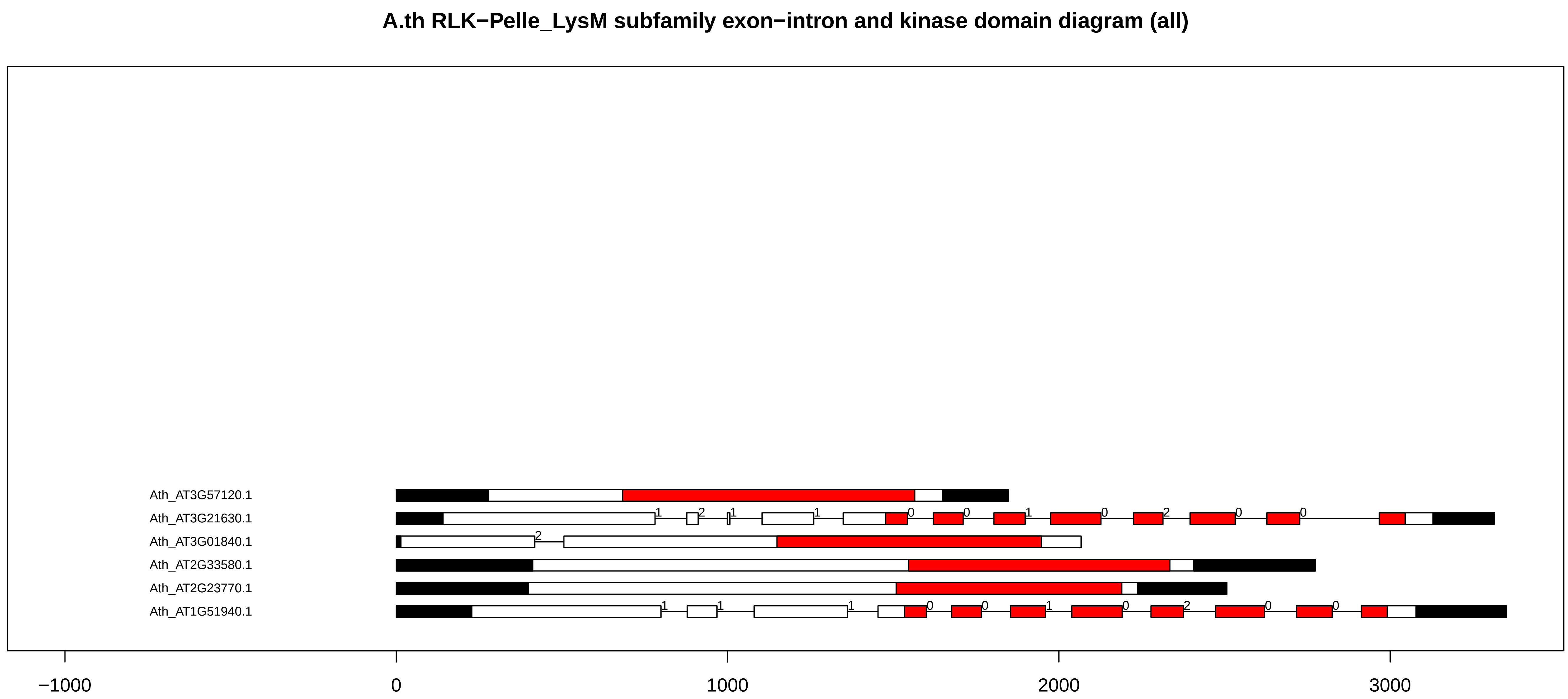

A.th RLK−Pelle_LysM subfamily exon−intron and kinase domain diagram (all)
Ath_AT3G57120.1
Ath_AT3G21630.1
Ath_AT3G01840.1
Ath_AT2G33580.1
Ath_AT2G23770.1
Ath_AT1G51940.1
−1000
0
1000
2000
3000

A.th RLK−Pelle_PERK−1 subfamily exon−intron and kinase domain diagram (all)

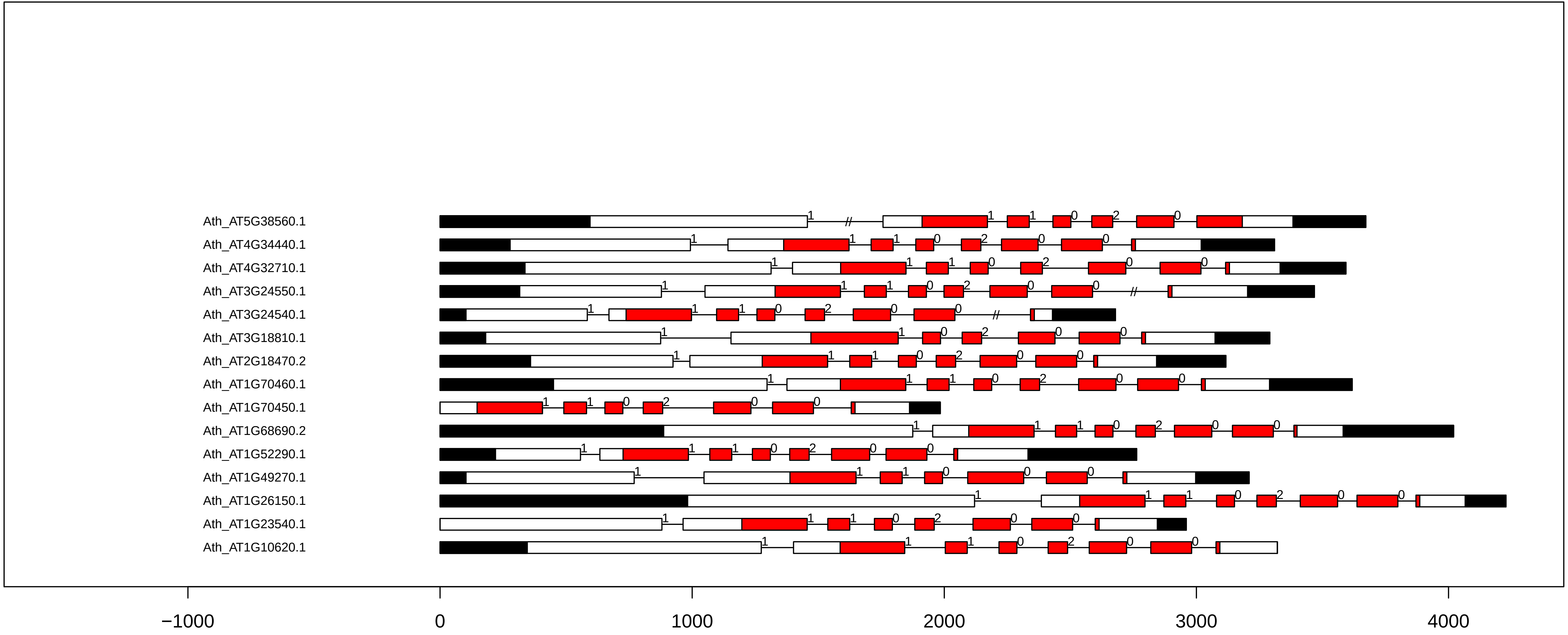

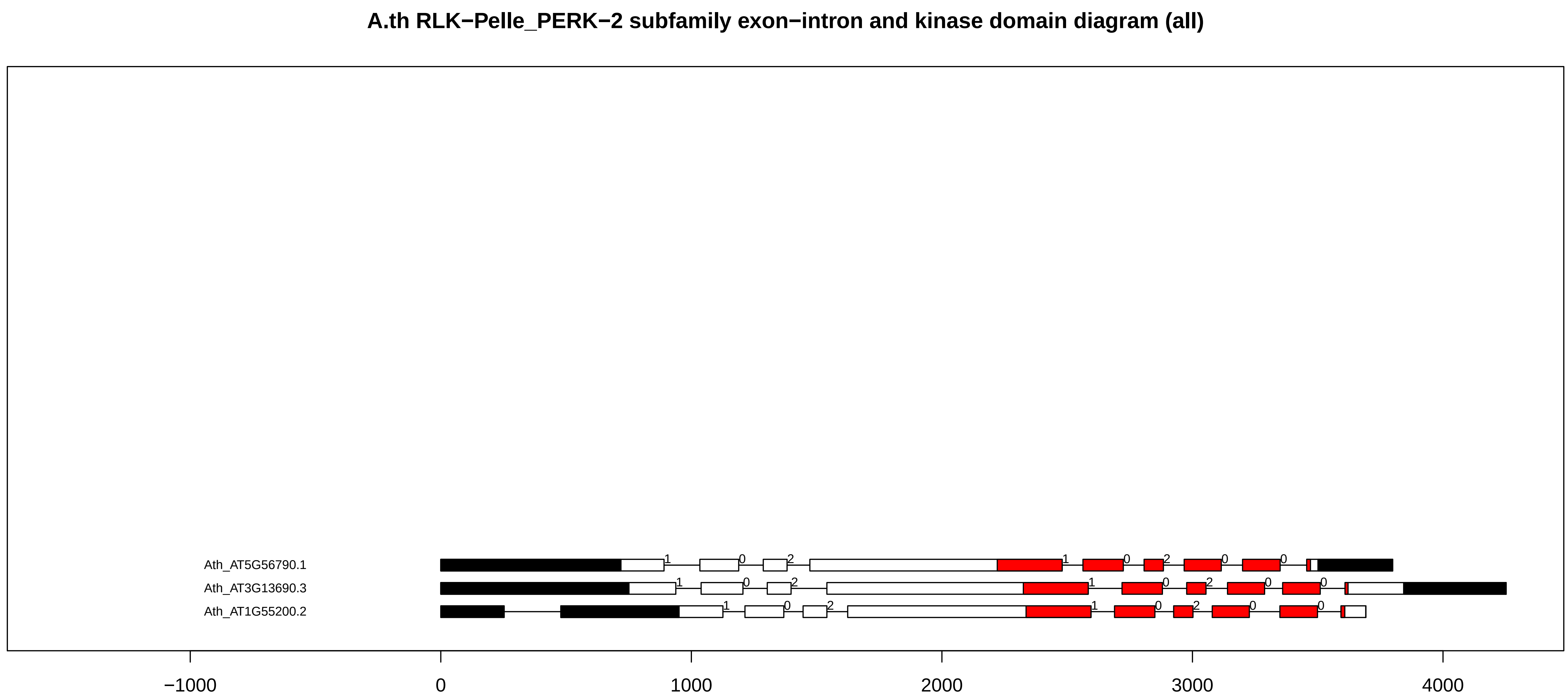
A.th RLK−Pelle_PERK−2 subfamily exon−intron and kinase domain diagram (all)
Ath_AT5G56790.1
Ath_AT3G13690.3
Ath_AT1G55200.2
1
0
2
1
0
2
0
0
1
0
2
1
0
2
0
0
1
0
2
1
0
2
0
0
−1000
0
1000
2000
3000
4000

A.th RLK−Pelle_RKF3 subfamily exon−intron and kinase domain diagram (all)

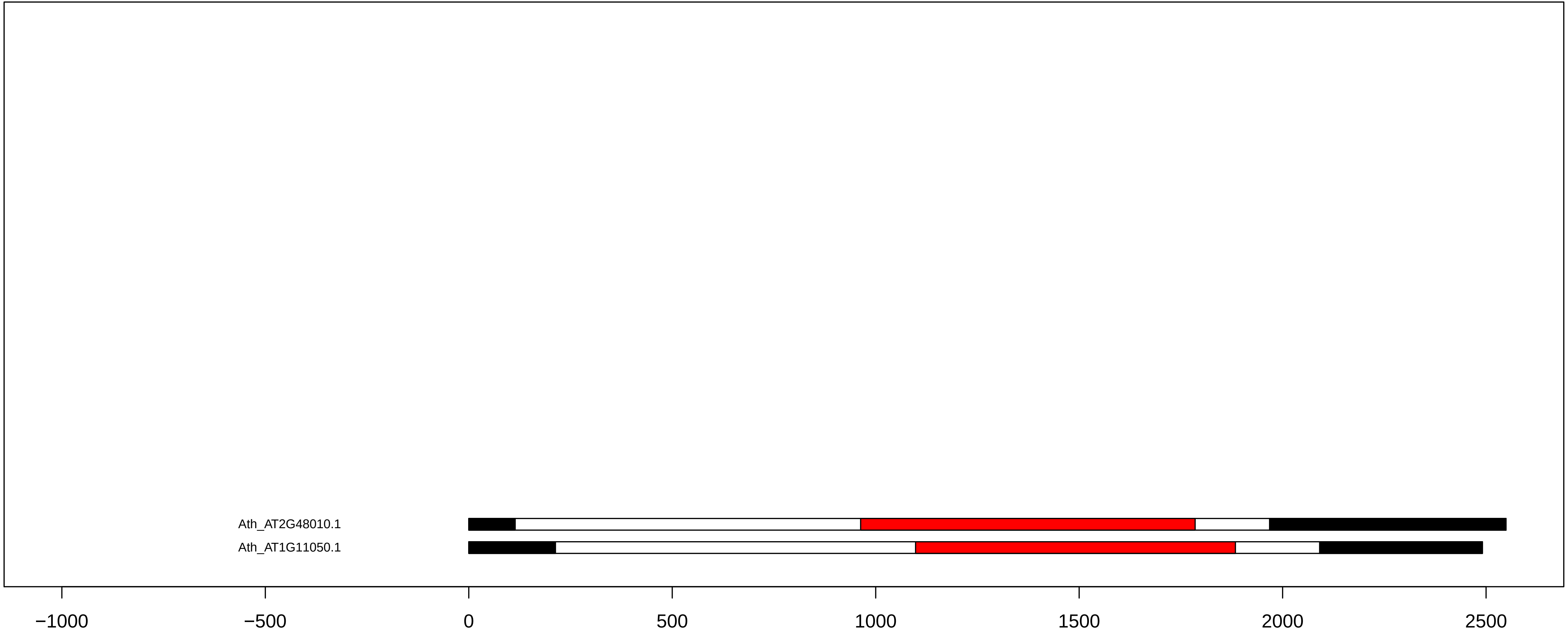

A.th RLK−Pelle_RLCK−II subfamily exon−intron and kinase domain diagram (all)

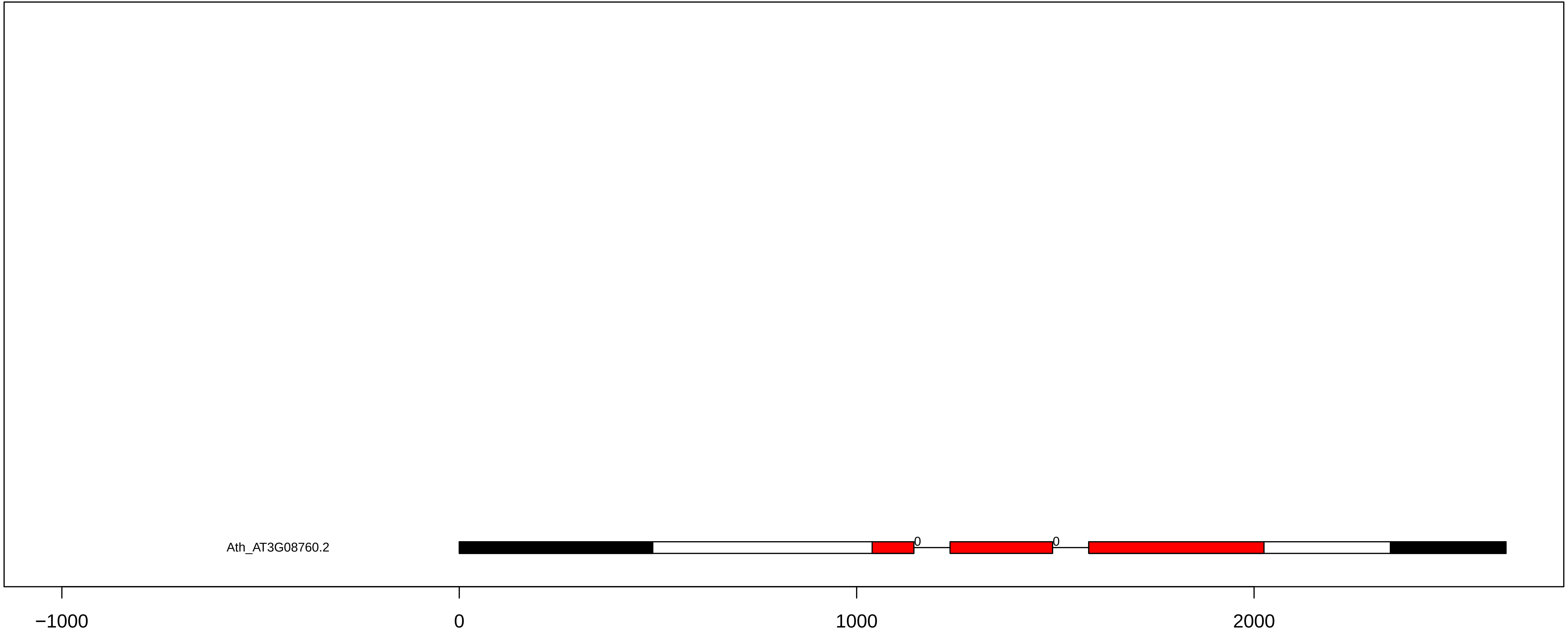

A.th RLK−Pelle_RLCK−IV subfamily exon−intron and kinase domain diagram (all)

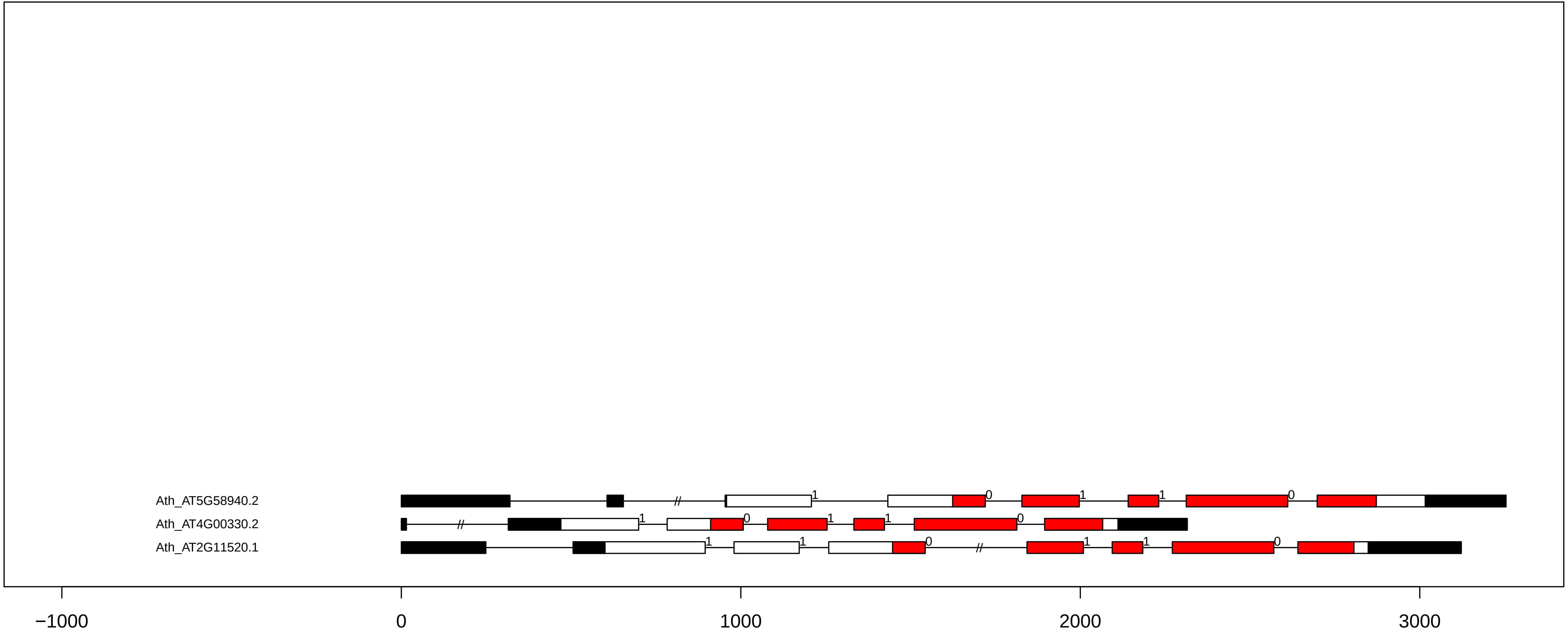

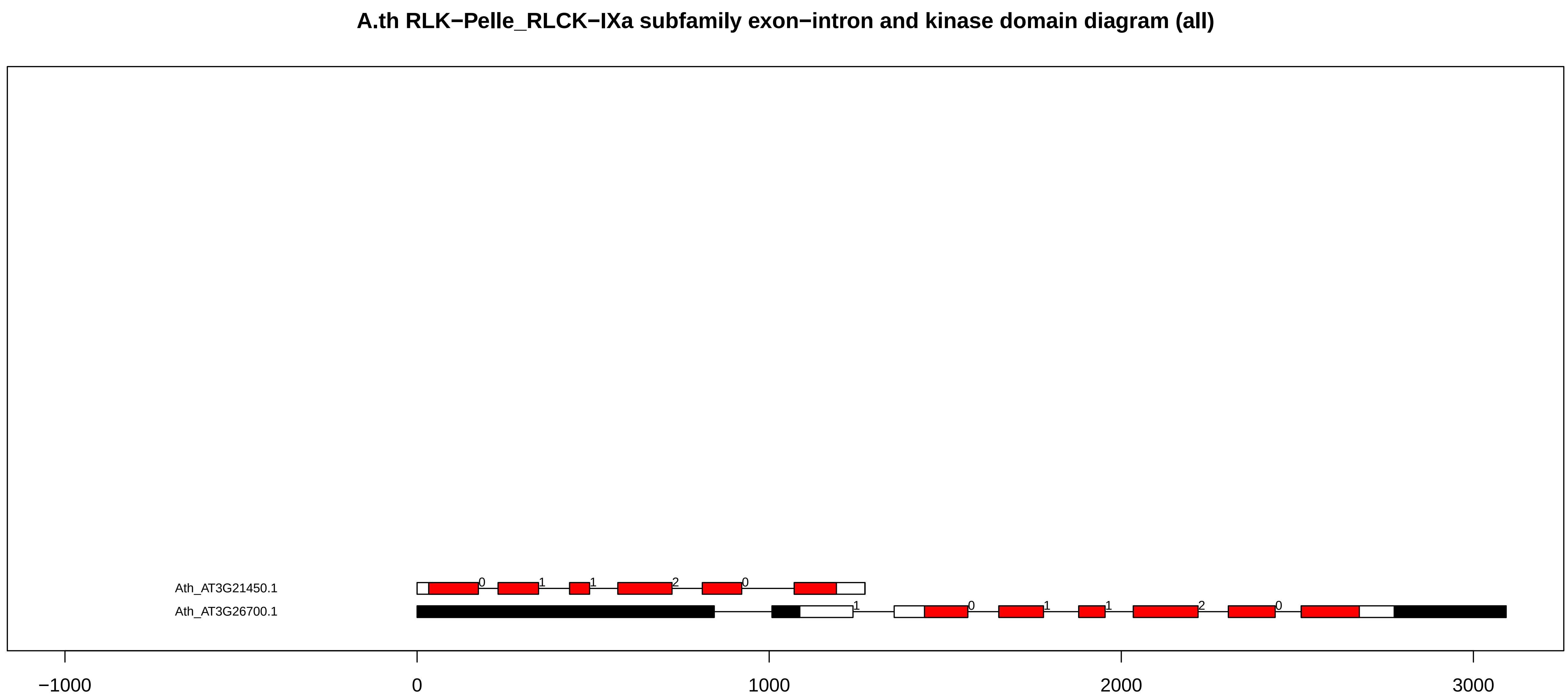

A.th RLK−Pelle_RLCK−IXa subfamily exon−intron and kinase domain diagram (all)
Ath_AT3G21450.1
Ath_AT3G26700.1
0
1
1
2
0
1
0
1
1
2
0
−1000
0
1000
2000
3000

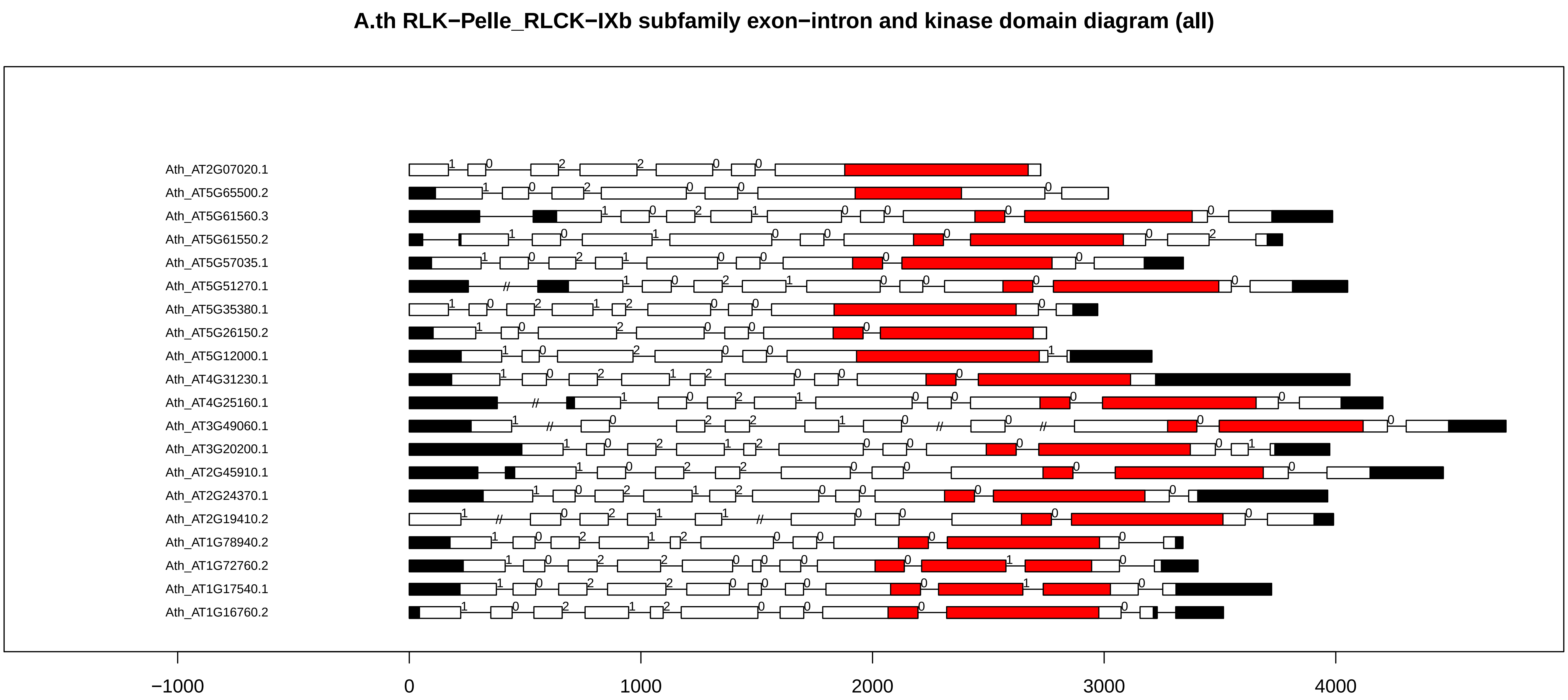

A.th RLK−Pelle_RLCK−IXb subfamily exon−intron and kinase domain diagram (all)
Ath_AT2G07020.1
Ath_AT5G65500.2
Ath_AT5G61560.3
Ath_AT5G61550.2
Ath_AT5G57035.1
Ath_AT5G51270.1
Ath_AT5G35380.1
Ath_AT5G26150.2
Ath_AT5G12000.1
Ath_AT4G31230.1
Ath_AT4G25160.1
Ath_AT3G49060.1
Ath_AT3G20200.1
Ath_AT2G45910.1
Ath_AT2G24370.1
Ath_AT2G19410.2
Ath_AT1G78940.2
Ath_AT1G72760.2
Ath_AT1G17540.1
Ath_AT1G16760.2
−1000
0
1000
2000
3000
4000

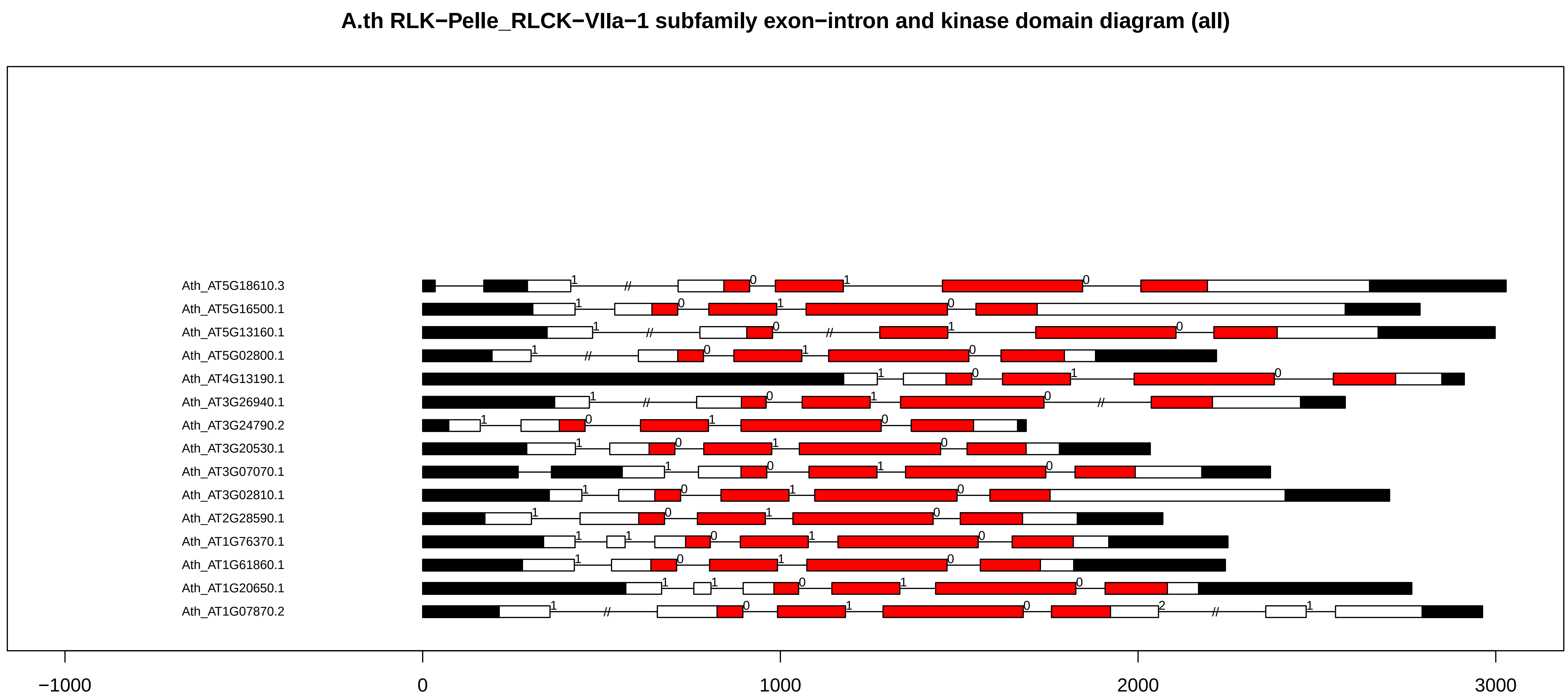
A.th RLK−Pelle_RLCK−VIIa−1 subfamily exon−intron and kinase domain diagram (all)
Ath_AT5G18610.3
Ath_AT5G16500.1
Ath_AT5G13160.1
Ath_AT5G02800.1
Ath_AT4G13190.1
Ath_AT3G26940.1
Ath_AT3G24790.2
Ath_AT3G20530.1
Ath_AT3G07070.1
Ath_AT3G02810.1
Ath_AT2G28590.1
Ath_AT1G76370.1
Ath_AT1G61860.1
Ath_AT1G20650.1
Ath_AT1G07870.2
−1000
0
1000
2000
3000

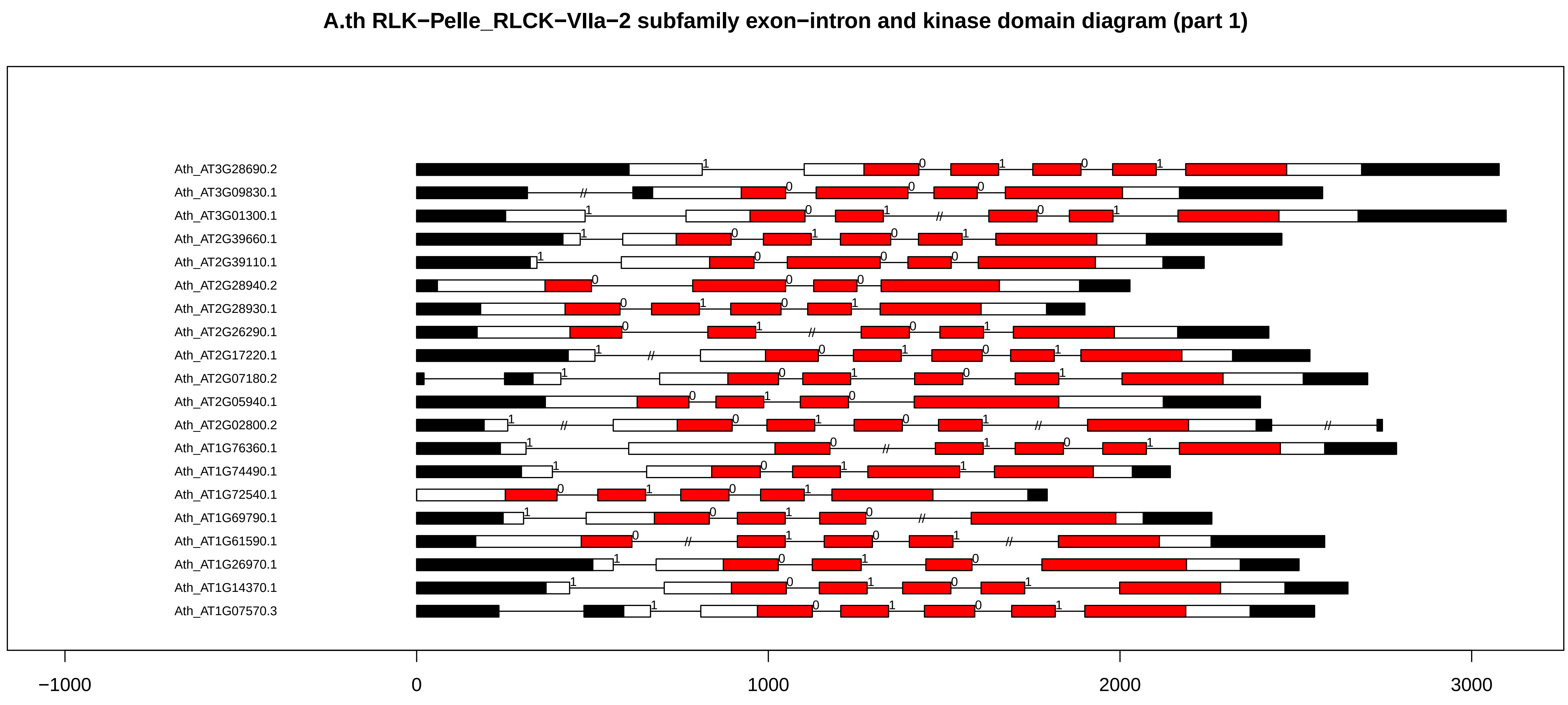
A.th RLK−Pelle_RLCK−VIIa−2 subfamily exon−intron and kinase domain diagram (part 1)
Ath_AT3G28690.2
Ath_AT3G09830.1
Ath_AT3G01300.1
Ath_AT2G39660.1
Ath_AT2G39110.1
Ath_AT2G28940.2
Ath_AT2G28930.1
Ath_AT2G26290.1
Ath_AT2G17220.1
Ath_AT2G07180.2
Ath_AT2G05940.1
Ath_AT2G02800.2
Ath_AT1G76360.1
Ath_AT1G74490.1
Ath_AT1G72540.1
Ath_AT1G69790.1
Ath_AT1G61590.1
Ath_AT1G26970.1
Ath_AT1G14370.1
Ath_AT1G07570.3
−1000
0
1000
2000
3000

A.th RLK−Pelle_RLCK−VIIa−2 subfamily exon−intron and kinase domain diagram (part 2)

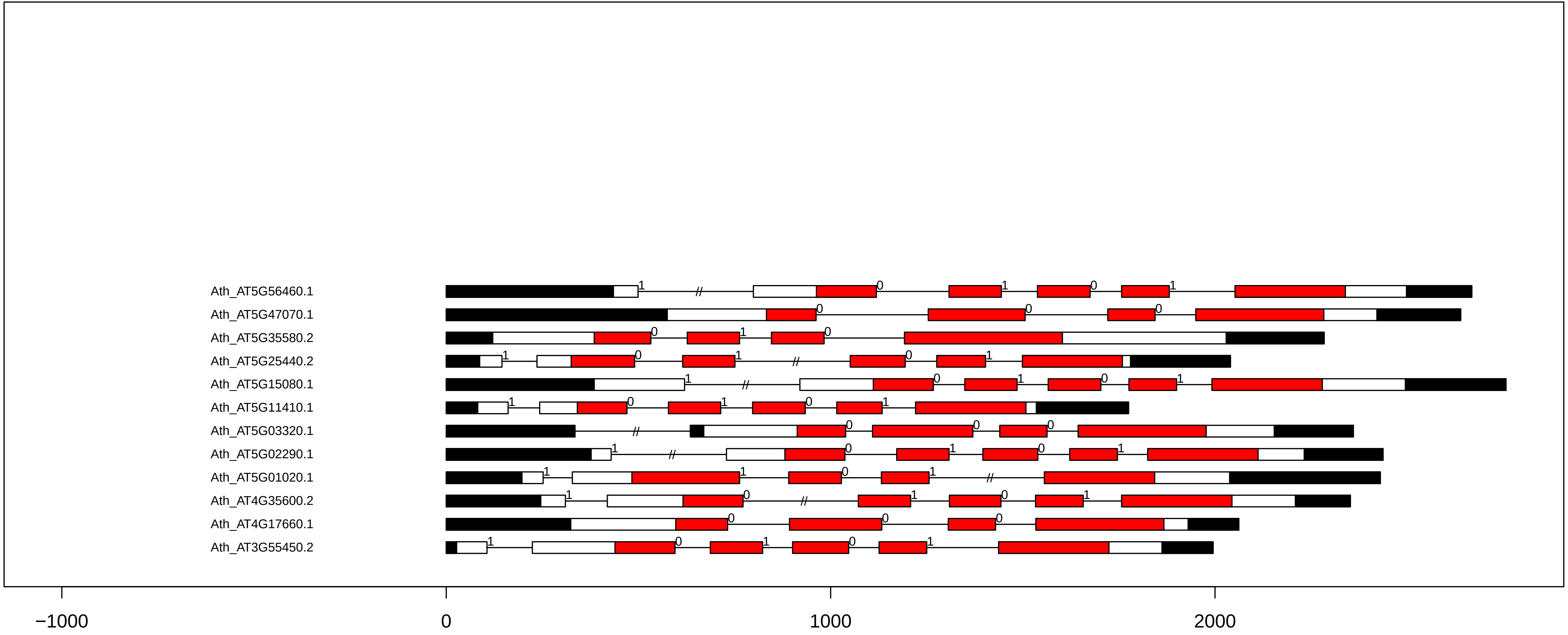

A.th RLK−Pelle_RLCK−VIIb subfamily exon−intron and kinase domain diagram (all)

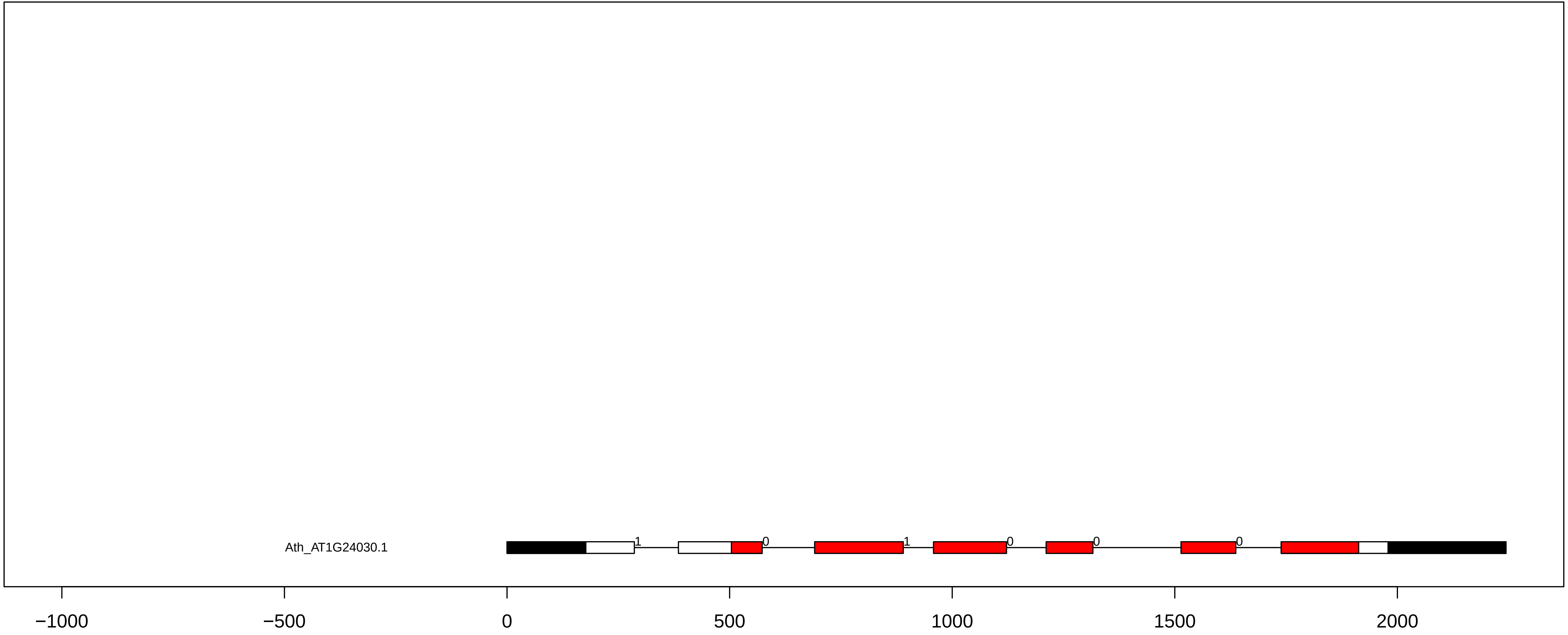

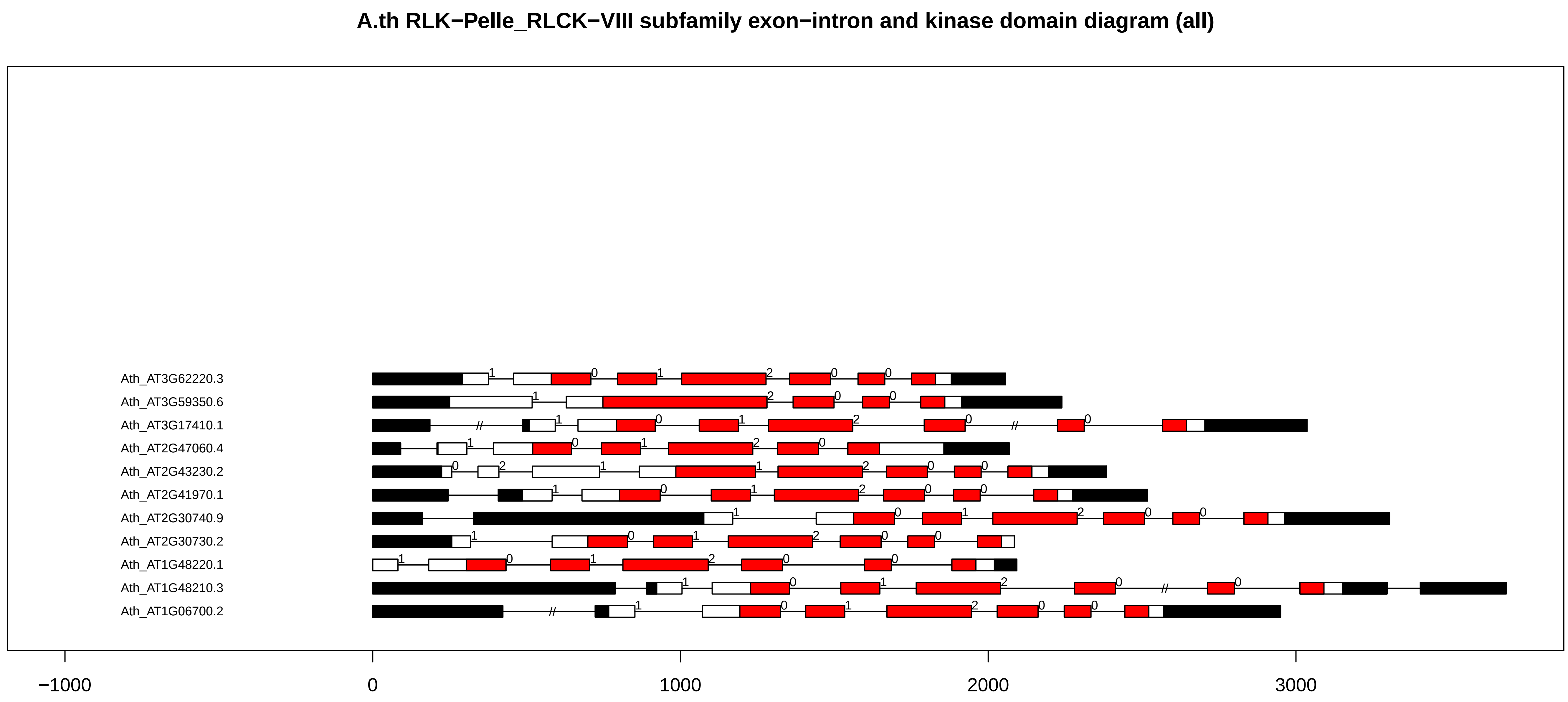

A.th RLK−Pelle_RLCK−VIII subfamily exon−intron and kinase domain diagram (all)
Ath_AT3G62220.3
Ath_AT3G59350.6
Ath_AT3G17410.1
Ath_AT2G47060.4
Ath_AT2G43230.2
Ath_AT2G41970.1
Ath_AT2G30740.9
Ath_AT2G30730.2
Ath_AT1G48220.1
Ath_AT1G48210.3
Ath_AT1G06700.2
−1000
0
1000
2000
3000

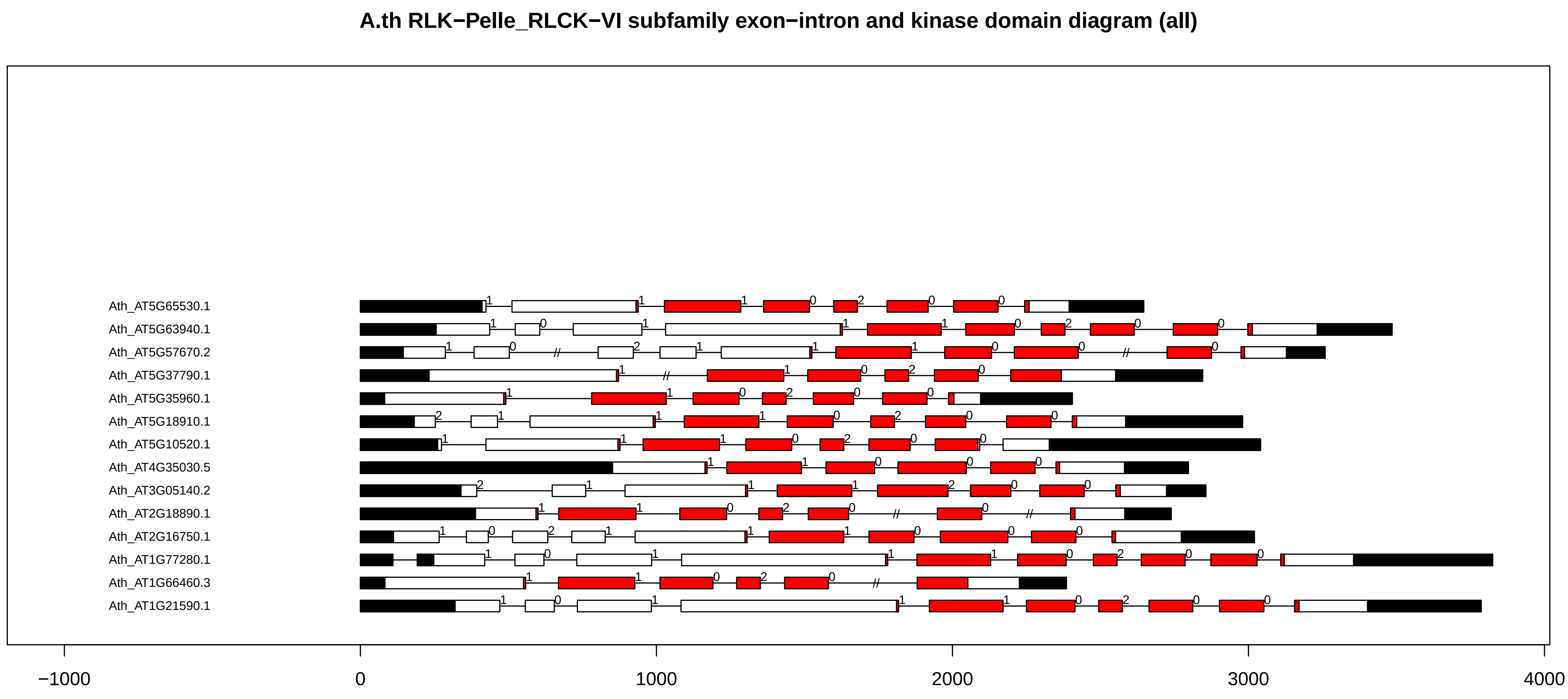
A.th RLK−Pelle_RLCK−VI subfamily exon−intron and kinase domain diagram (all)
Ath_AT5G65530.1
Ath_AT5G63940.1
Ath_AT5G57670.2
Ath_AT5G37790.1
Ath_AT5G35960.1
Ath_AT5G18910.1
Ath_AT5G10520.1
Ath_AT4G35030.5
Ath_AT3G05140.2
Ath_AT2G18890.1
Ath_AT2G16750.1
Ath_AT1G77280.1
Ath_AT1G66460.3
Ath_AT1G21590.1
−1000
0
1000
2000
3000
4000

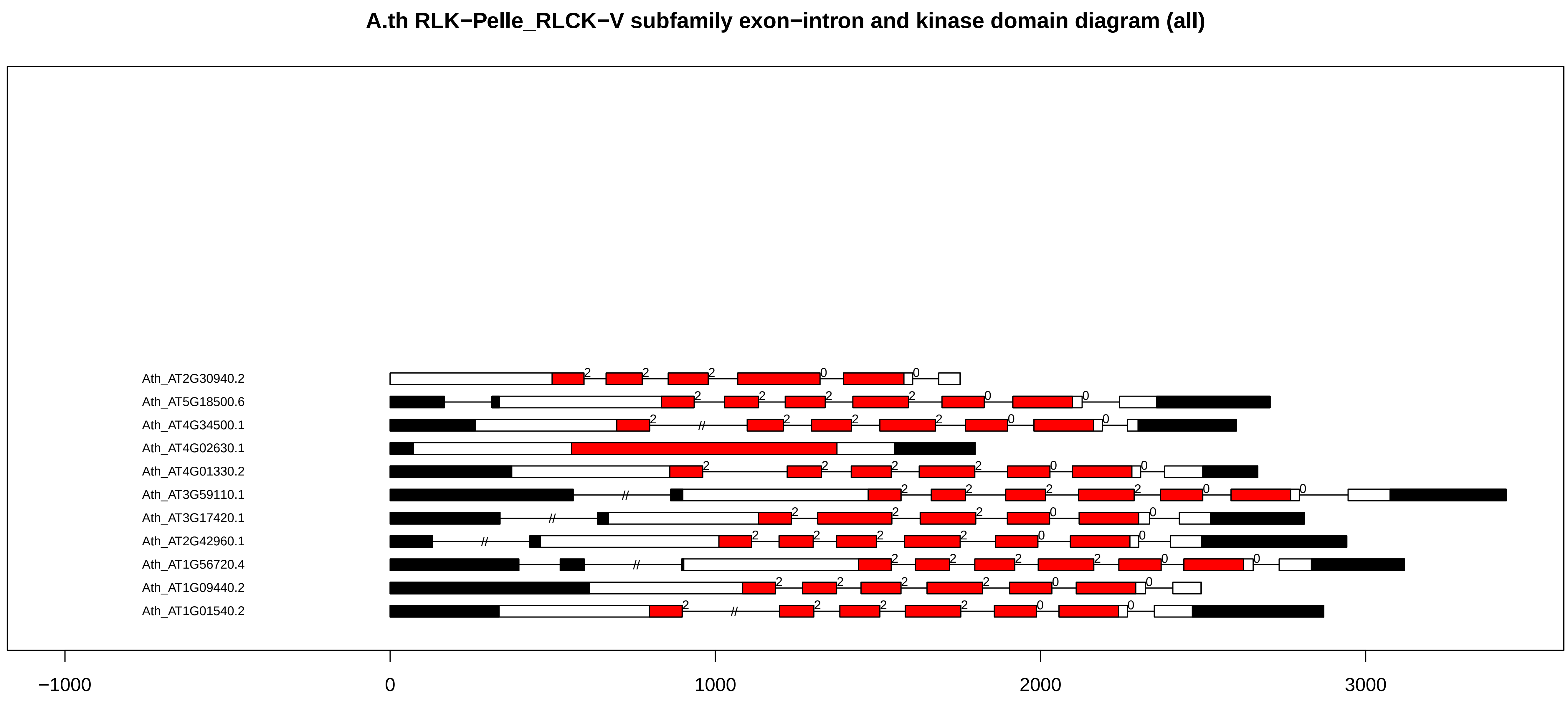

A.th RLK−Pelle_RLCK−V subfamily exon−intron and kinase domain diagram (all)
Ath_AT2G30940.2
Ath_AT5G18500.6
Ath_AT4G34500.1
Ath_AT4G02630.1
Ath_AT4G01330.2
Ath_AT3G59110.1
Ath_AT3G17420.1
Ath_AT2G42960.1
Ath_AT1G56720.4
Ath_AT1G09440.2
Ath_AT1G01540.2
−1000
0
1000
2000
3000

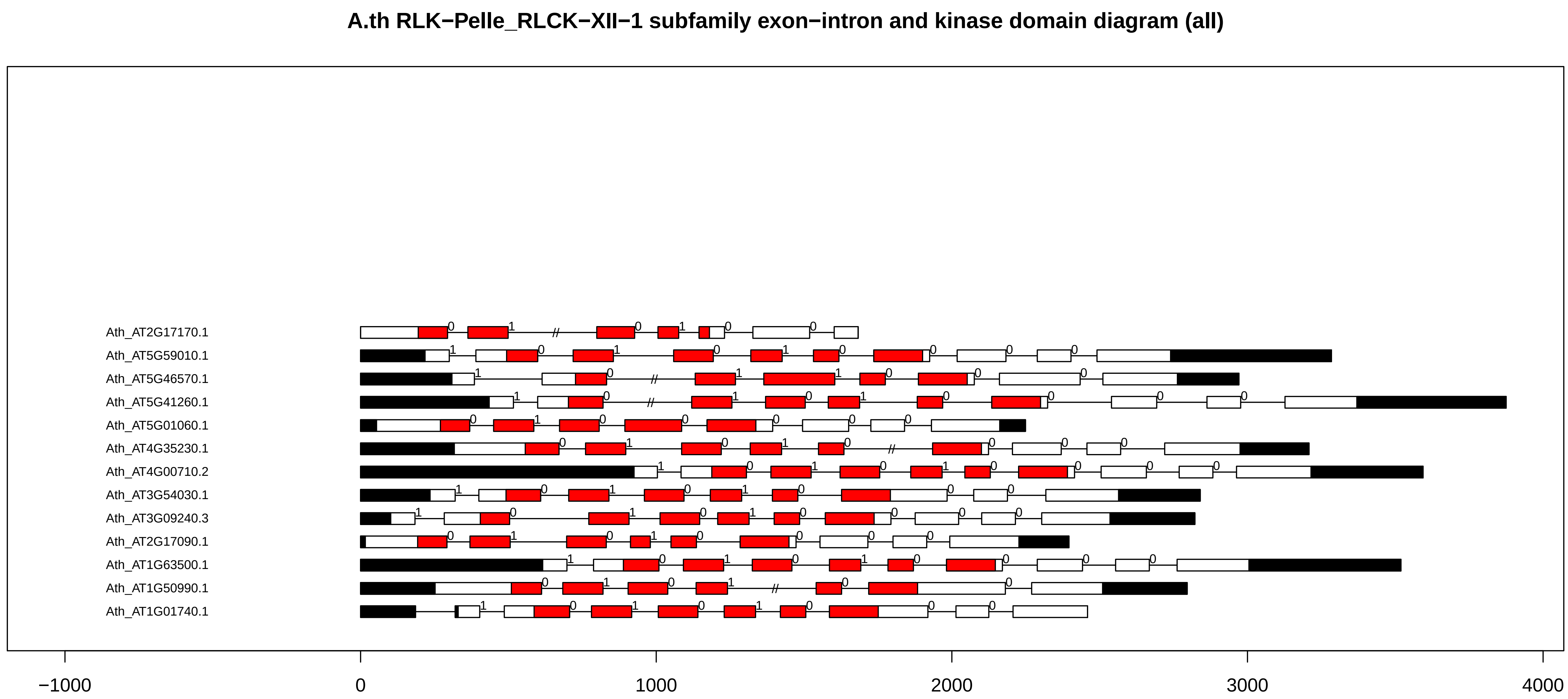
A.th RLK−Pelle_RLCK−XII−1 subfamily exon−intron and kinase domain diagram (all)
Ath_AT2G17170.1
Ath_AT5G59010.1
Ath_AT5G46570.1
Ath_AT5G41260.1
Ath_AT5G01060.1
Ath_AT4G35230.1
Ath_AT4G00710.2
Ath_AT3G54030.1
Ath_AT3G09240.3
Ath_AT2G17090.1
Ath_AT1G63500.1
Ath_AT1G50990.1
Ath_AT1G01740.1
−1000
0
1000
2000
3000
4000

A.th RLK−Pelle_RLCK−XII−2 subfamily exon−intron and kinase domain diagram (all)

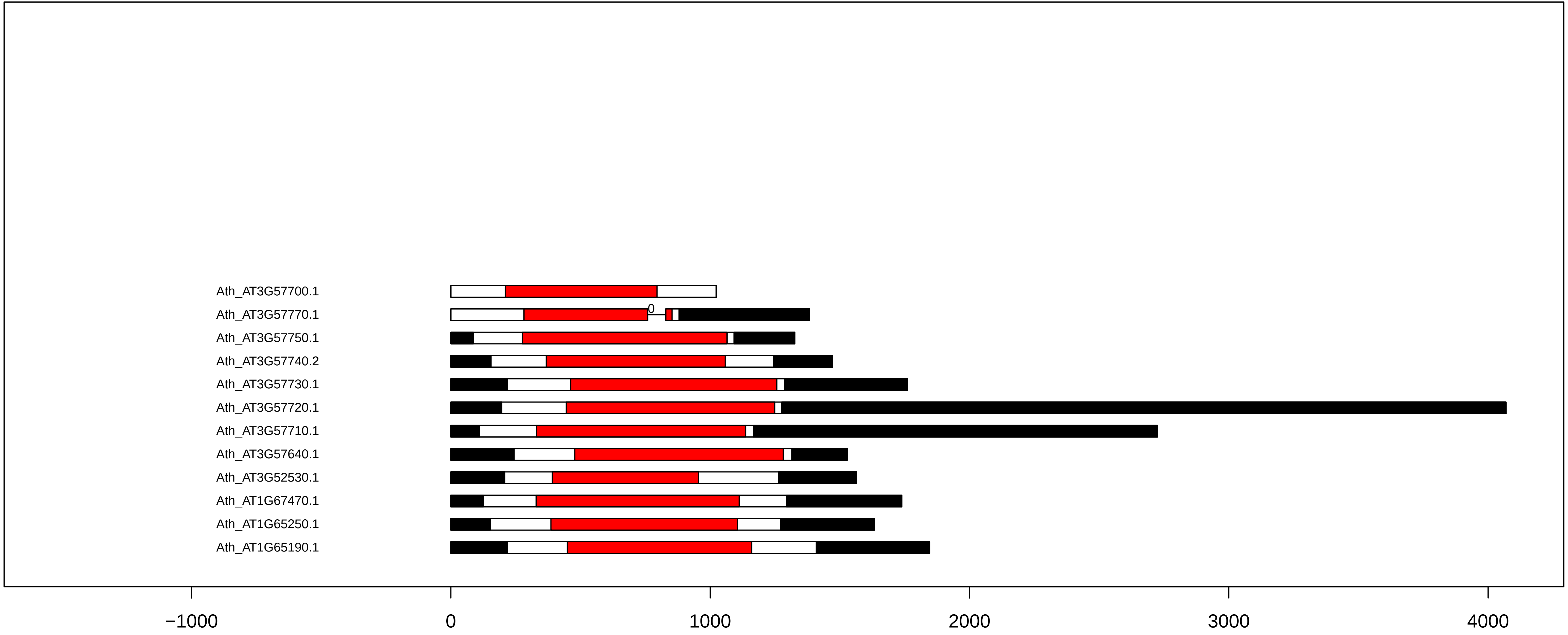

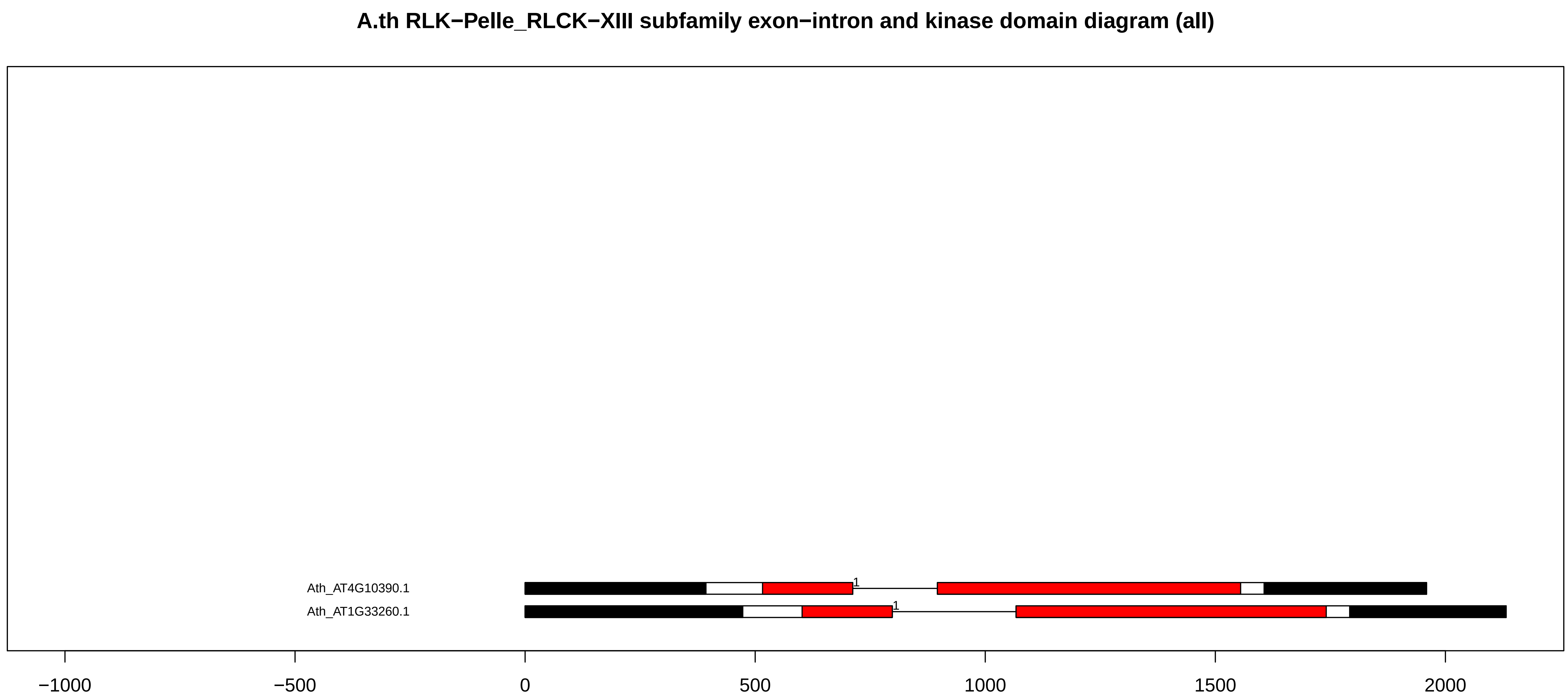
A.th RLK−Pelle_RLCK−XIII subfamily exon−intron and kinase domain diagram (all)
Ath_AT4G10390.1
Ath_AT1G33260.1
1
1
−1000
−500
0
500
1000
1500
2000

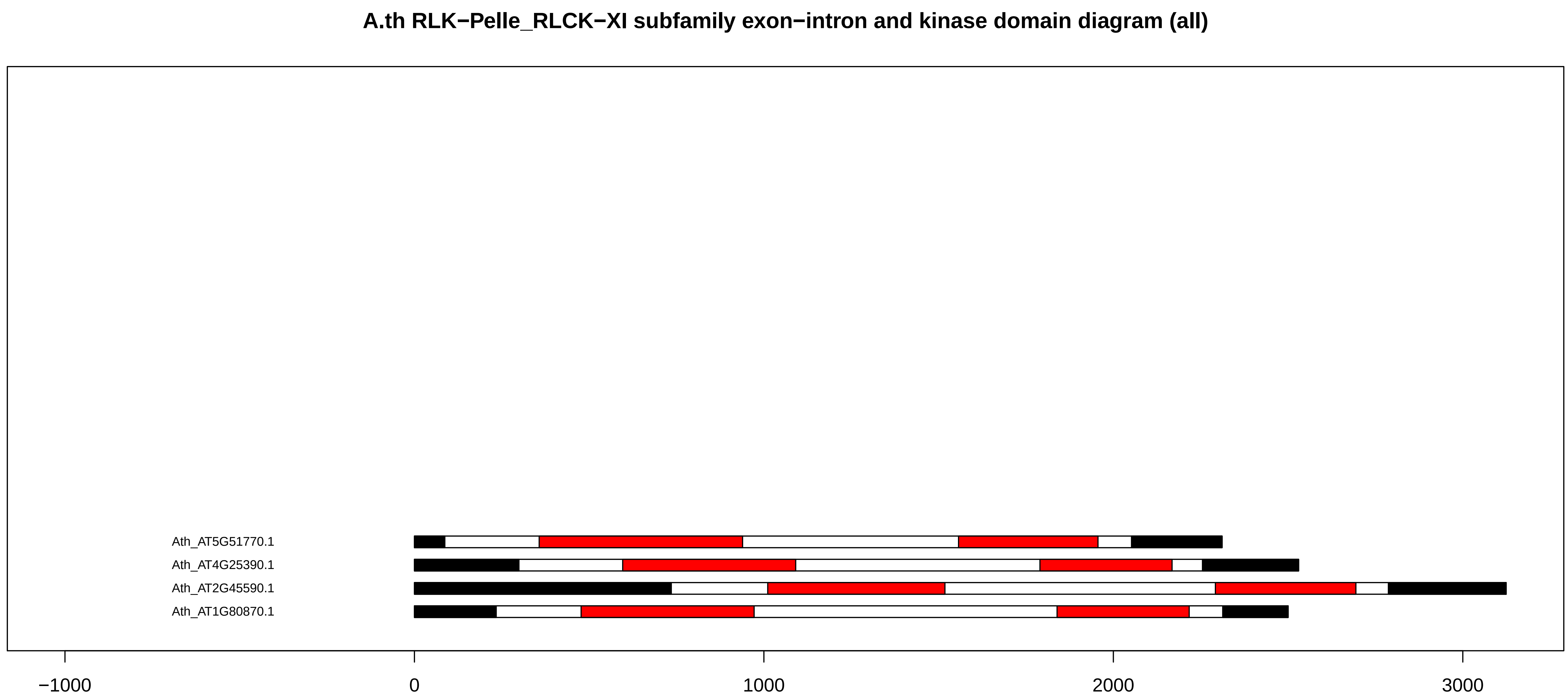

A.th RLK−Pelle_RLCK−XI subfamily exon−intron and kinase domain diagram (all)
Ath_AT5G51770.1
Ath_AT4G25390.1
Ath_AT2G45590.1
Ath_AT1G80870.1
−1000
0
1000
2000
3000

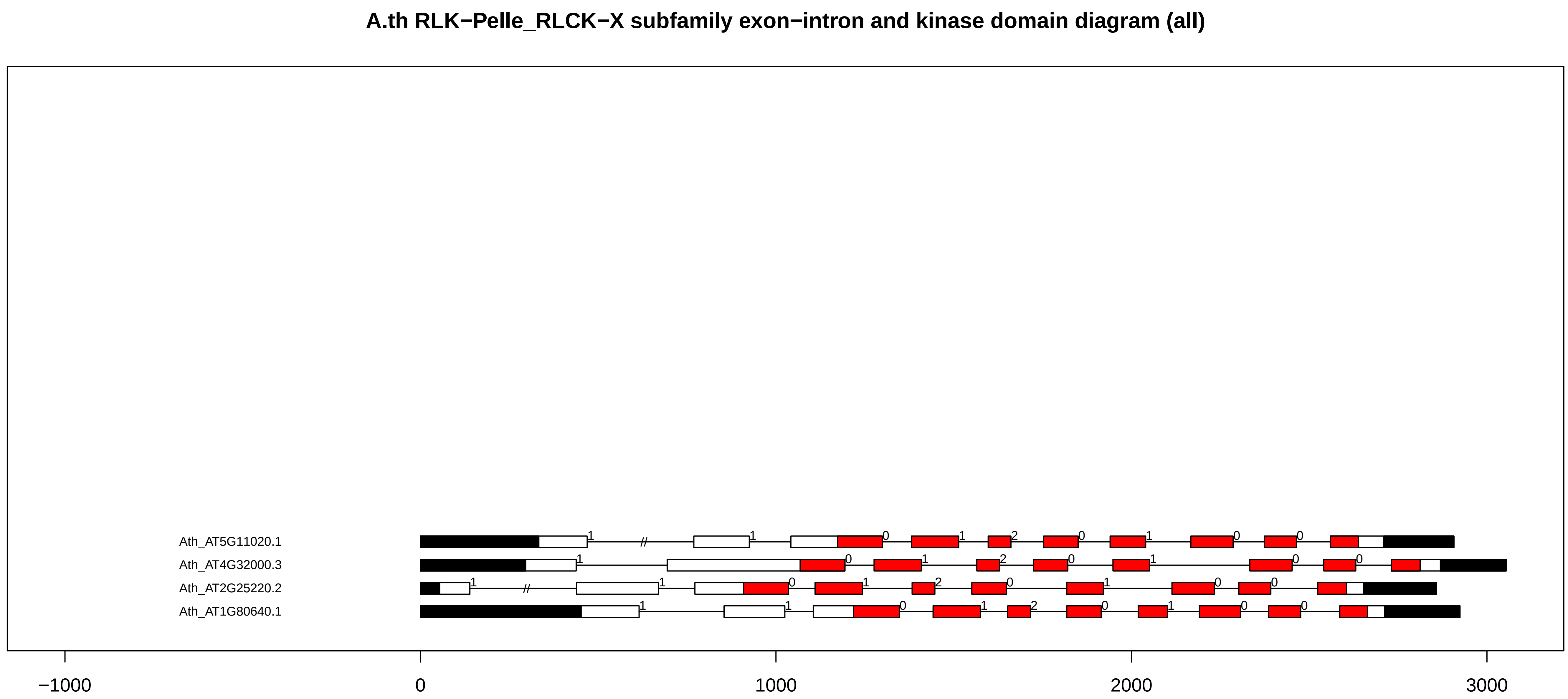
A.th RLK−Pelle_RLCK−X subfamily exon−intron and kinase domain diagram (all)
Ath_AT5G11020.1
Ath_AT4G32000.3
Ath_AT2G25220.2
Ath_AT1G80640.1
−1000
0
1000
2000
3000

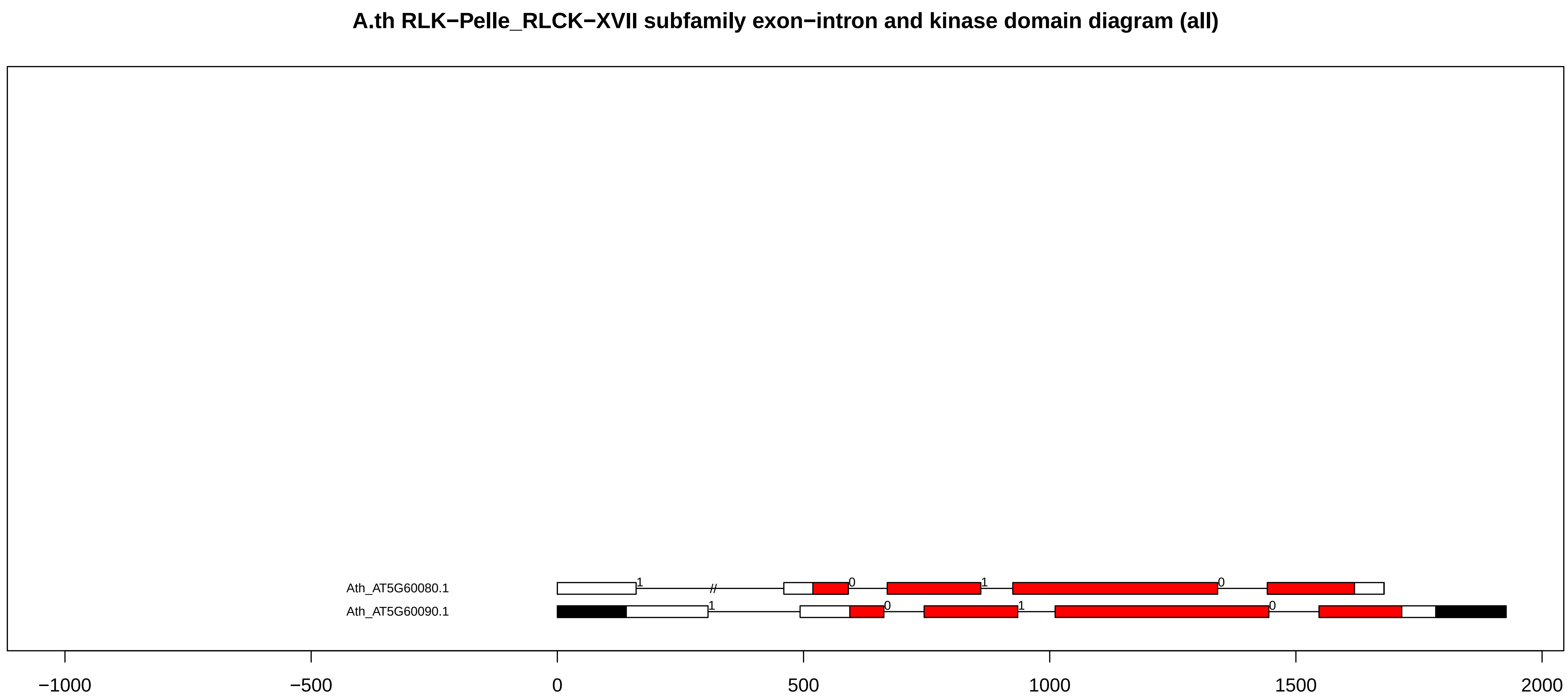
A.th RLK−Pelle_RLCK−XVII subfamily exon−intron and kinase domain diagram (all)
Ath_AT5G60080.1
Ath_AT5G60090.1
1
0
1
0
1
0
1
0
−1000
−500
0
500
1000
1500
2000

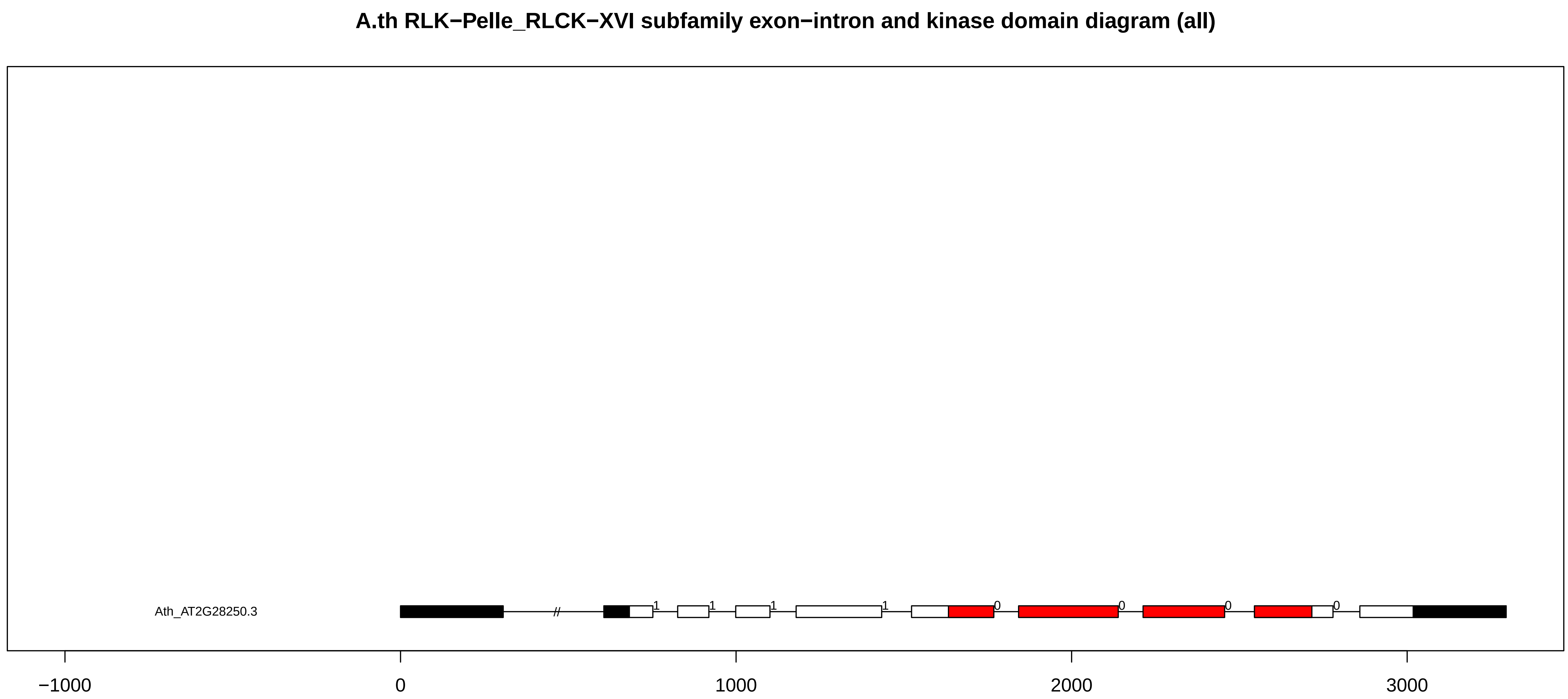
A.th RLK−Pelle_RLCK−XVI subfamily exon−intron and kinase domain diagram (all)
Ath_AT2G28250.3
//
1
1
1
1
0
0
0
0
−1000
0
1000
2000
3000

A.th RLK−Pelle_RLCK−XV subfamily exon−intron and kinase domain diagram (all)

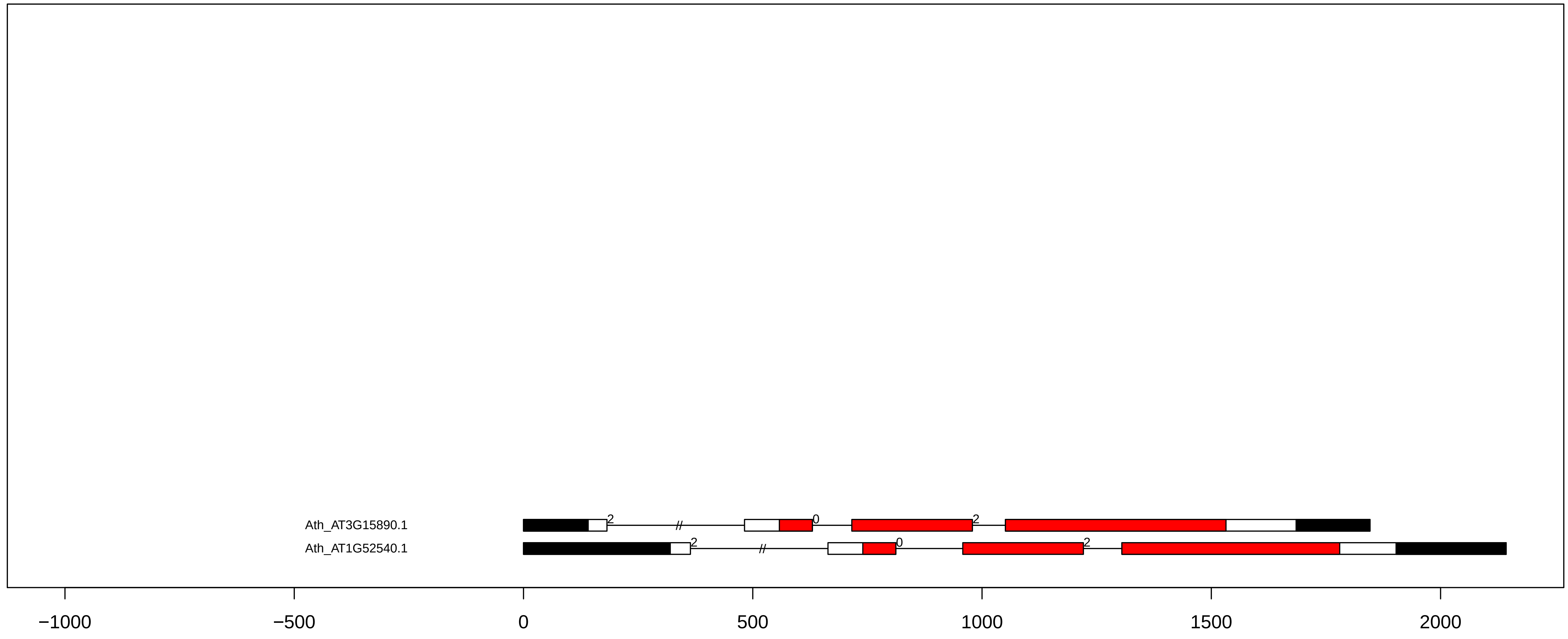

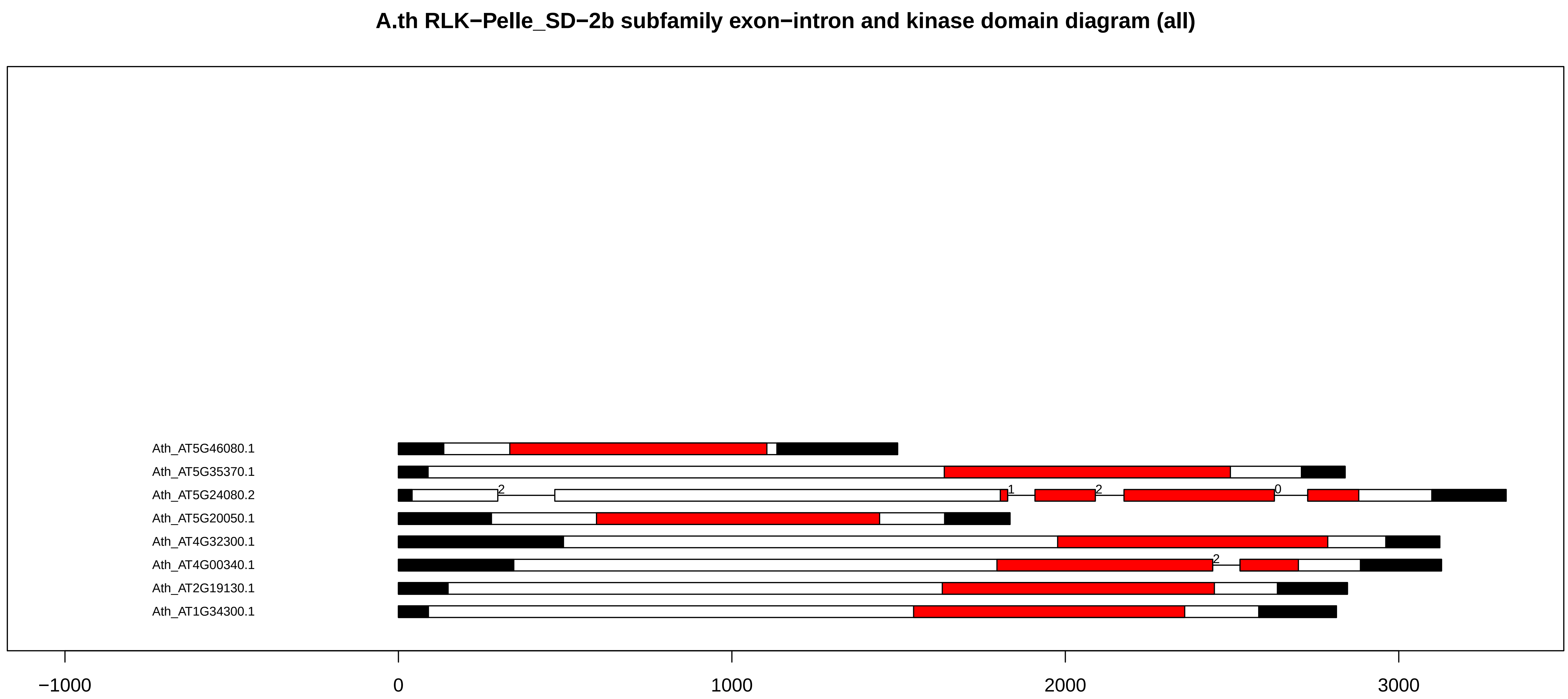
A.th RLK−Pelle_SD−2b subfamily exon−intron and kinase domain diagram (all)
Ath_AT5G46080.1
Ath_AT5G35370.1
Ath_AT5G24080.2
Ath_AT5G20050.1
Ath_AT4G32300.1
Ath_AT4G00340.1
Ath_AT2G19130.1
Ath_AT1G34300.1
2
1
2
0
2
−1000
0
1000
2000
3000

A.th RLK−Pelle_Singleton subfamily exon−intron and kinase domain diagram (all)

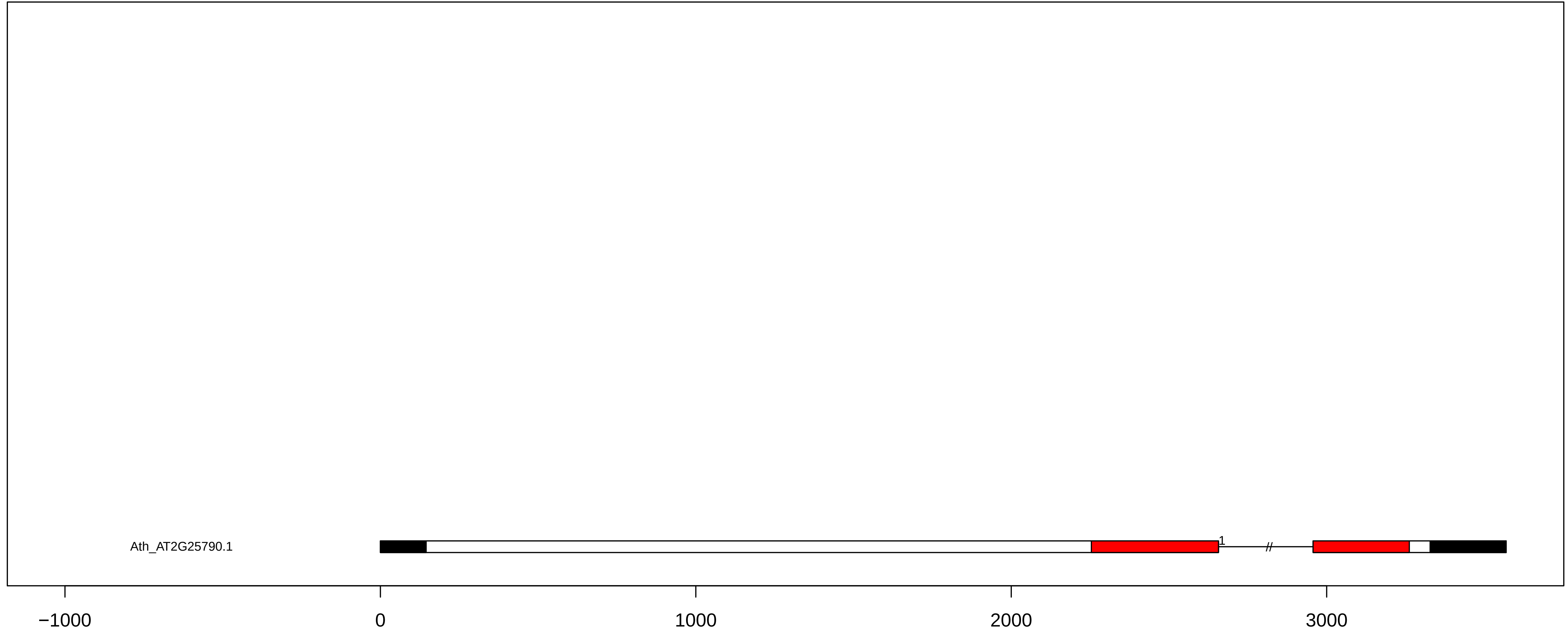

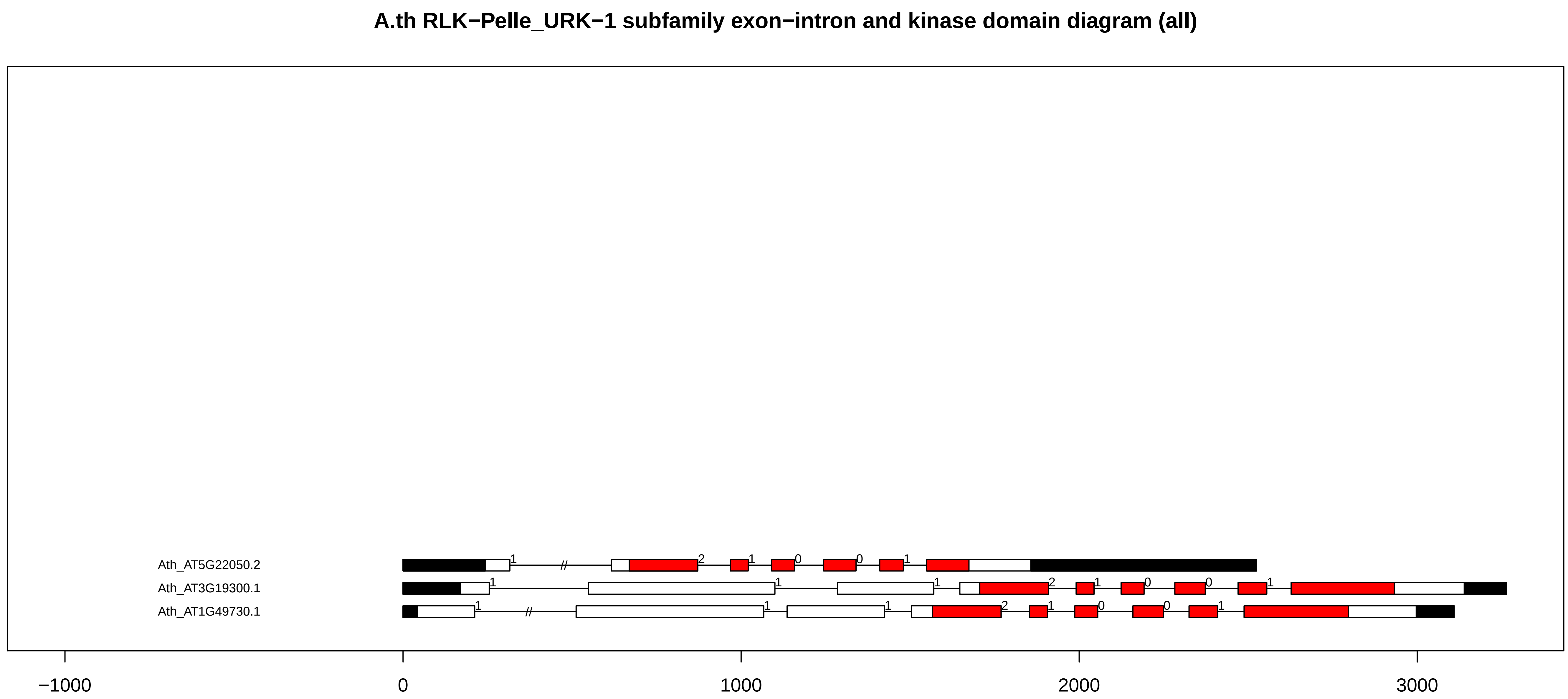
A.th RLK−Pelle_URK−1 subfamily exon−intron and kinase domain diagram (all)
Ath_AT5G22050.2
Ath_AT3G19300.1
Ath_AT1G49730.1
−1000
0
1000
2000
3000

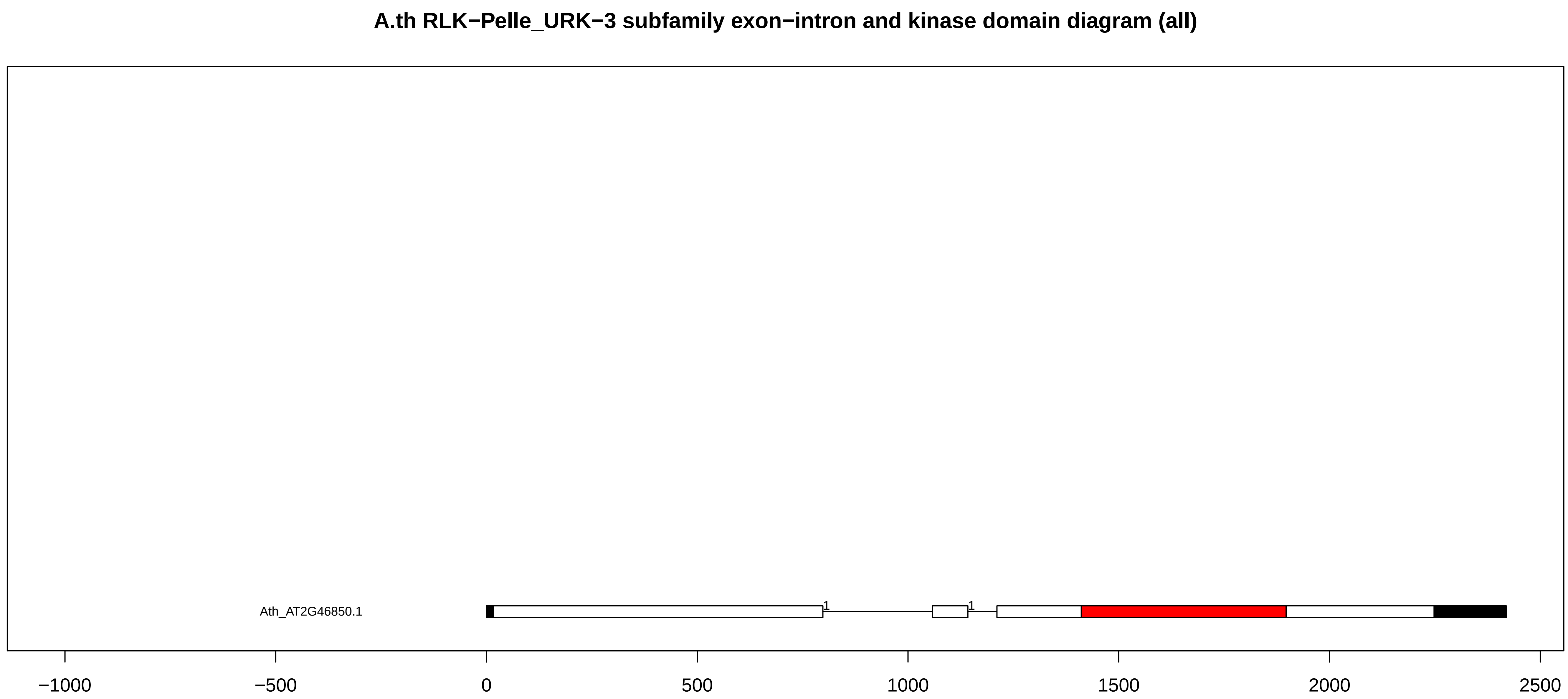

A.th RLK−Pelle_URK−3 subfamily exon−intron and kinase domain diagram (all)
Ath_AT2G46850.1
1
1
−1000
−500
0
500
1000
1500
2000
2500

A.th RLK−Pelle_WAK_LRK10L−1 subfamily exon−intron and kinase domain diagram (all)

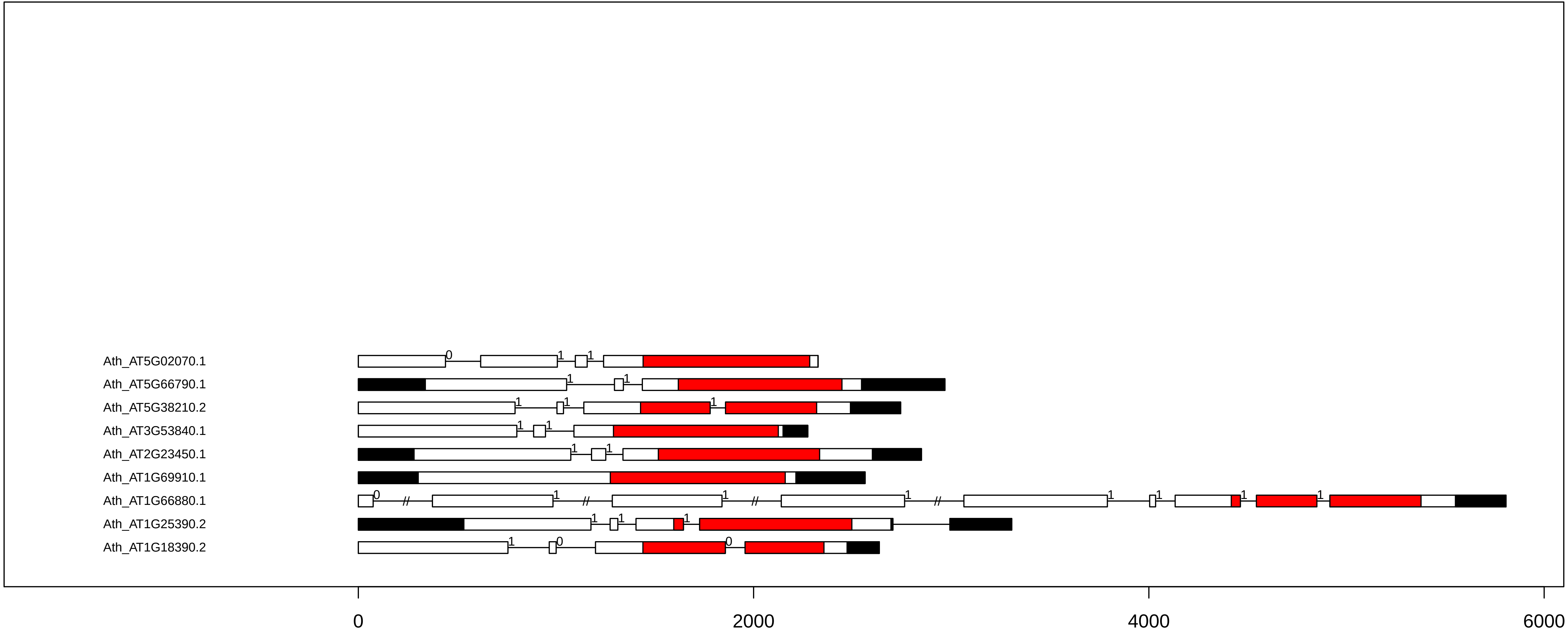

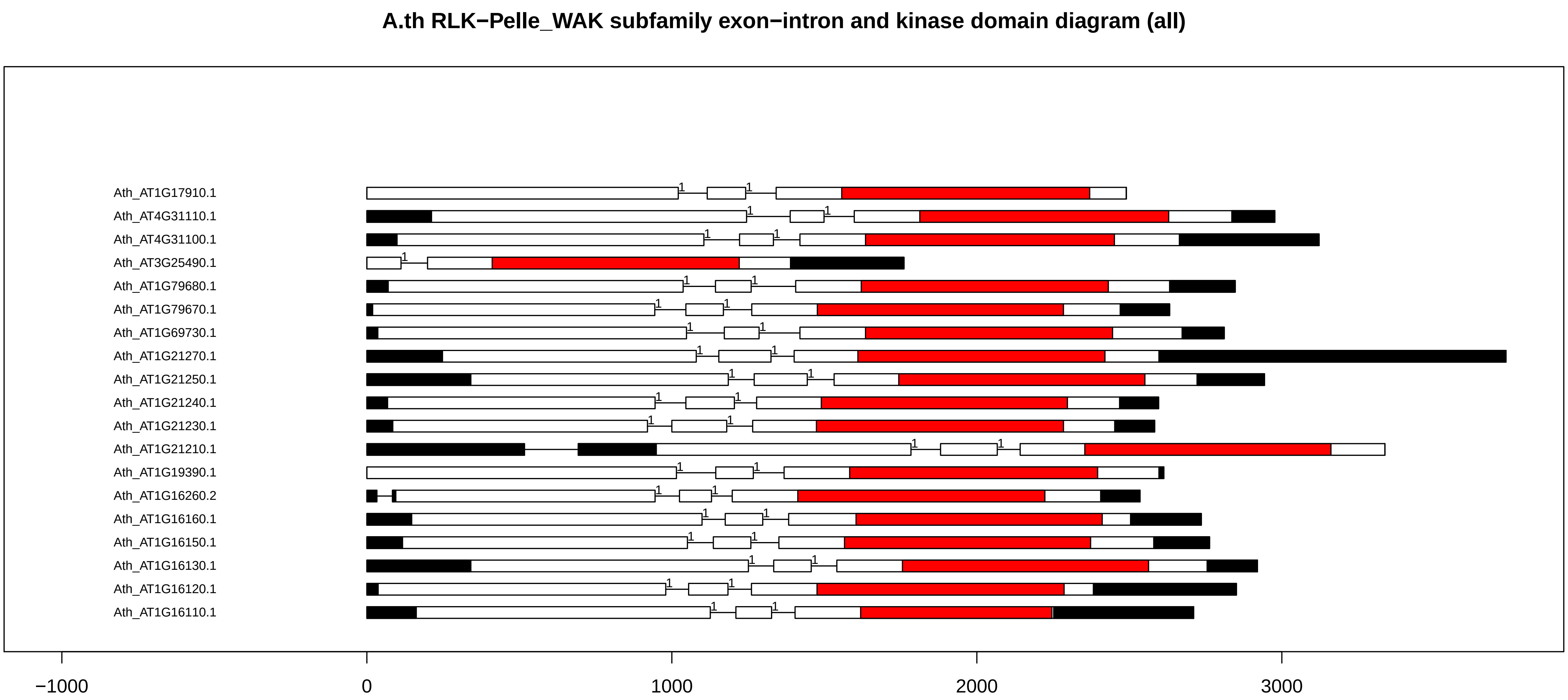

A.th RLK−Pelle_WAK subfamily exon−intron and kinase domain diagram (all)
Ath_AT1G17910.1
Ath_AT4G31110.1
Ath_AT4G31100.1
Ath_AT3G25490.1
Ath_AT1G79680.1
Ath_AT1G79670.1
Ath_AT1G69730.1
Ath_AT1G21270.1
Ath_AT1G21250.1
Ath_AT1G21240.1
Ath_AT1G21230.1
Ath_AT1G21210.1
Ath_AT1G19390.1
Ath_AT1G16260.2
Ath_AT1G16160.1
Ath_AT1G16150.1
Ath_AT1G16130.1
Ath_AT1G16120.1
Ath_AT1G16110.1
−1000
0
1000
2000
3000

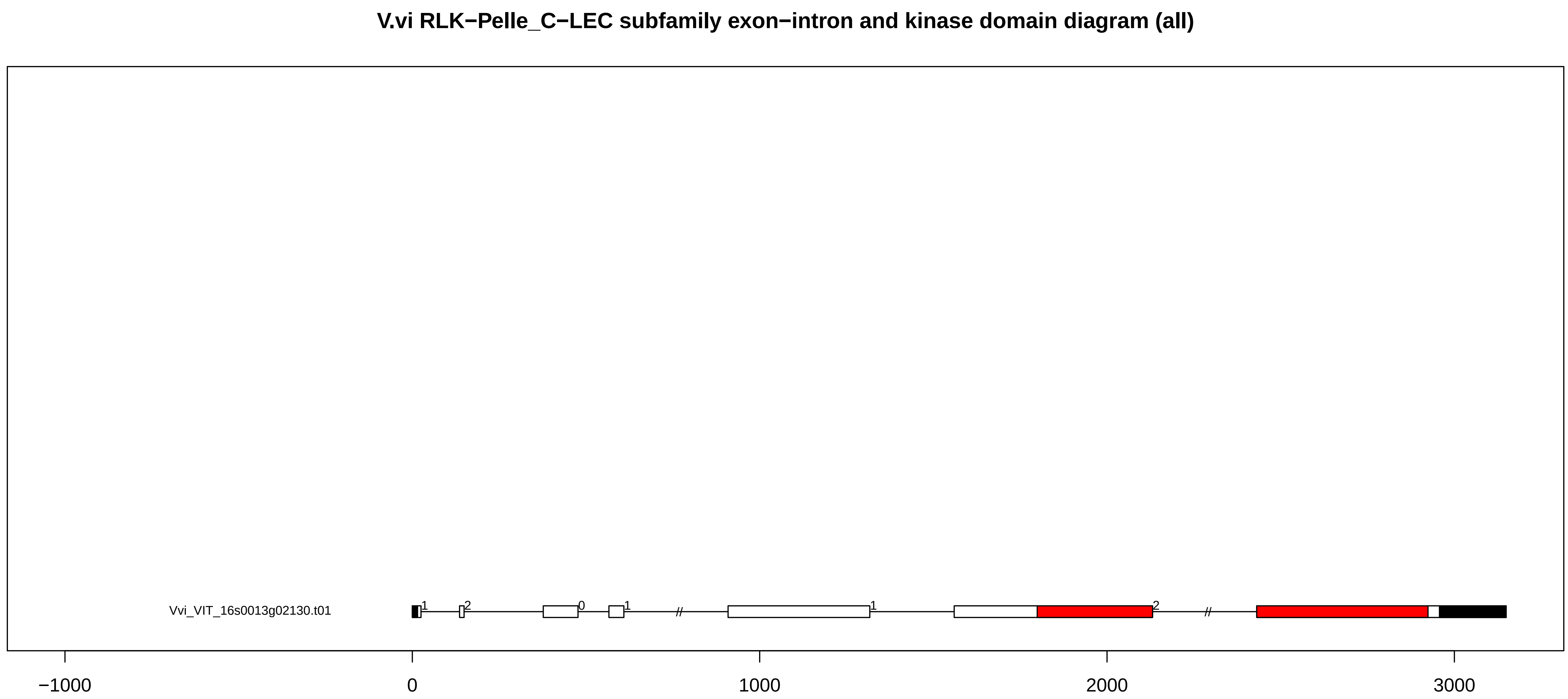

V.vi RLK−Pelle_C−LEC subfamily exon−intron and kinase domain diagram (all)
Vvi_VIT_16s0013g02130.t01
1
2
0
1
1
2
−1000
0
1000
2000
3000

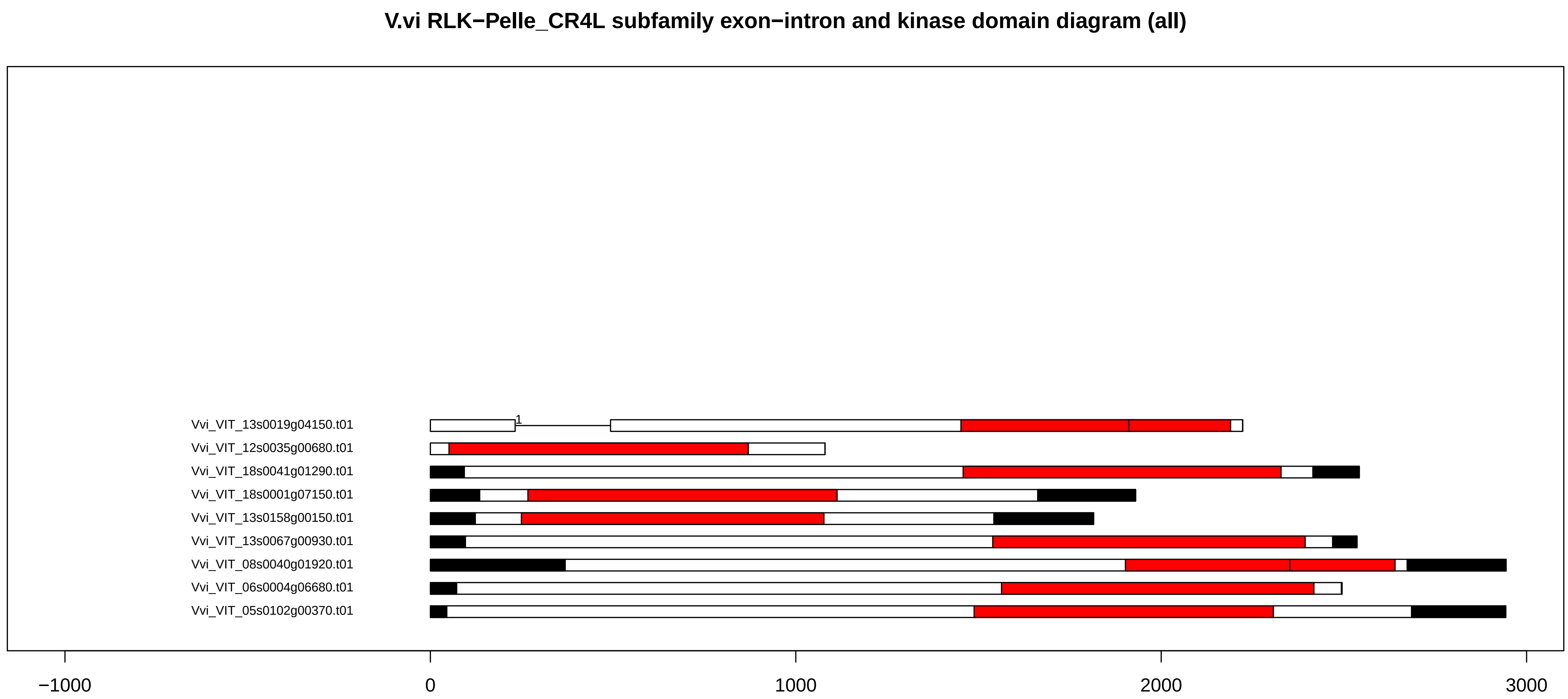

V.vi RLK−Pelle_CR4L subfamily exon−intron and kinase domain diagram (all)
Vvi_VIT_13s0019g04150.t01
1
Vvi_VIT_12s0035g00680.t01
Vvi_VIT_18s0041g01290.t01
Vvi_VIT_18s0001g07150.t01
Vvi_VIT_13s0158g00150.t01
Vvi_VIT_13s0067g00930.t01
Vvi_VIT_08s0040g01920.t01
Vvi_VIT_06s0004g06680.t01
Vvi_VIT_05s0102g00370.t01
−1000
0
1000
2000
3000

V.vi RLK−Pelle_CrRLK1L−1 subfamily exon−intron and kinase domain diagram (part 1)

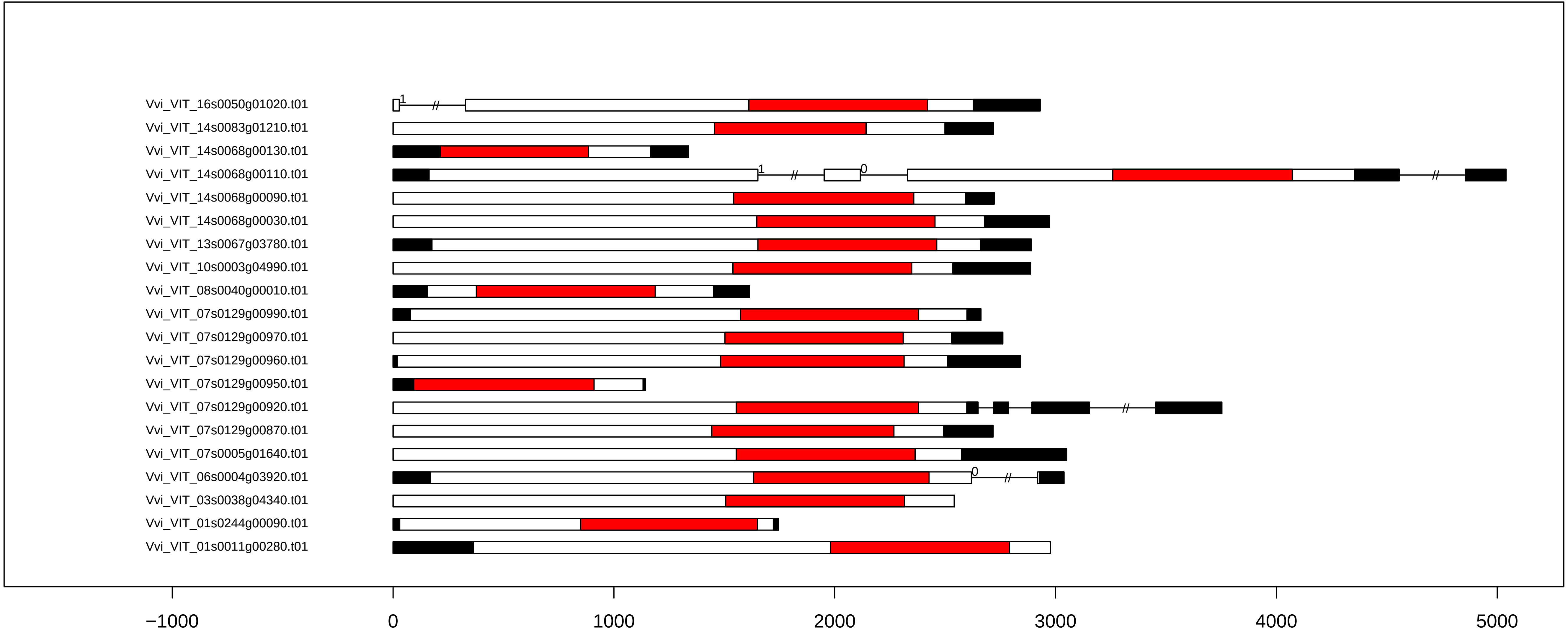

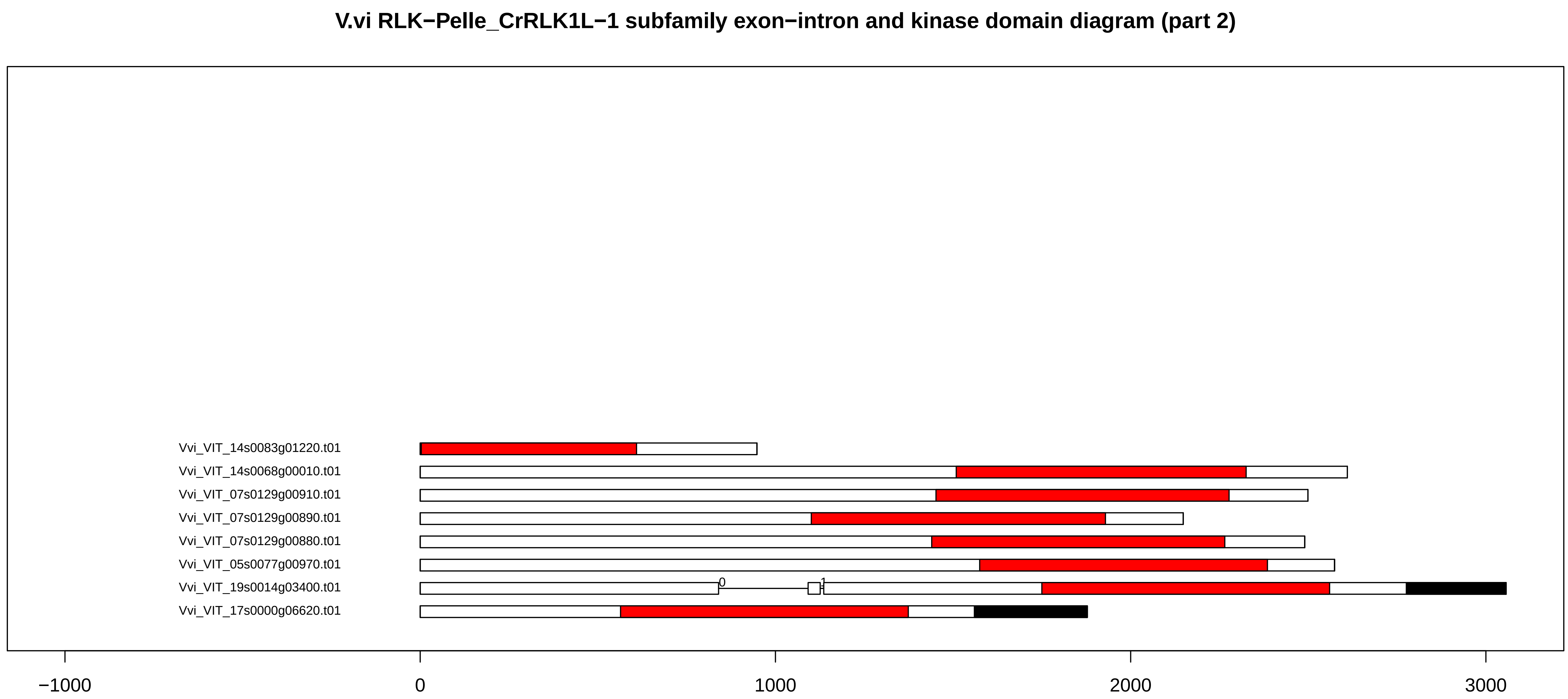
V.vi RLK−Pelle_CrRLK1L−1 subfamily exon−intron and kinase domain diagram (part 2)
Vvi_VIT_14s0083g01220.t01
Vvi_VIT_14s0068g00010.t01
Vvi_VIT_07s0129g00910.t01
Vvi_VIT_07s0129g00890.t01
Vvi_VIT_07s0129g00880.t01
Vvi_VIT_05s0077g00970.t01
Vvi_VIT_19s0014g03400.t01
Vvi_VIT_17s0000g06620.t01
0
1
−1000
0
1000
2000
3000

V.vi RLK−Pelle_DLSV subfamily exon−intron and kinase domain diagram (part 1)

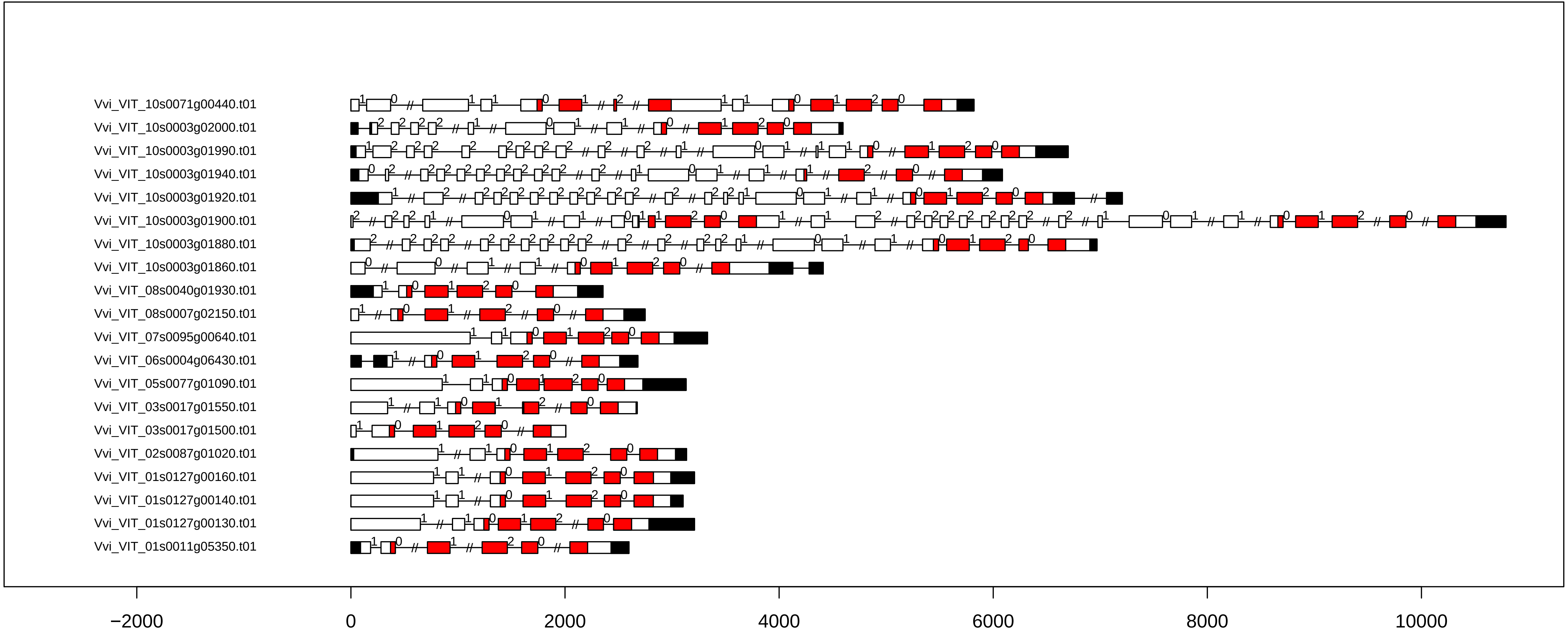

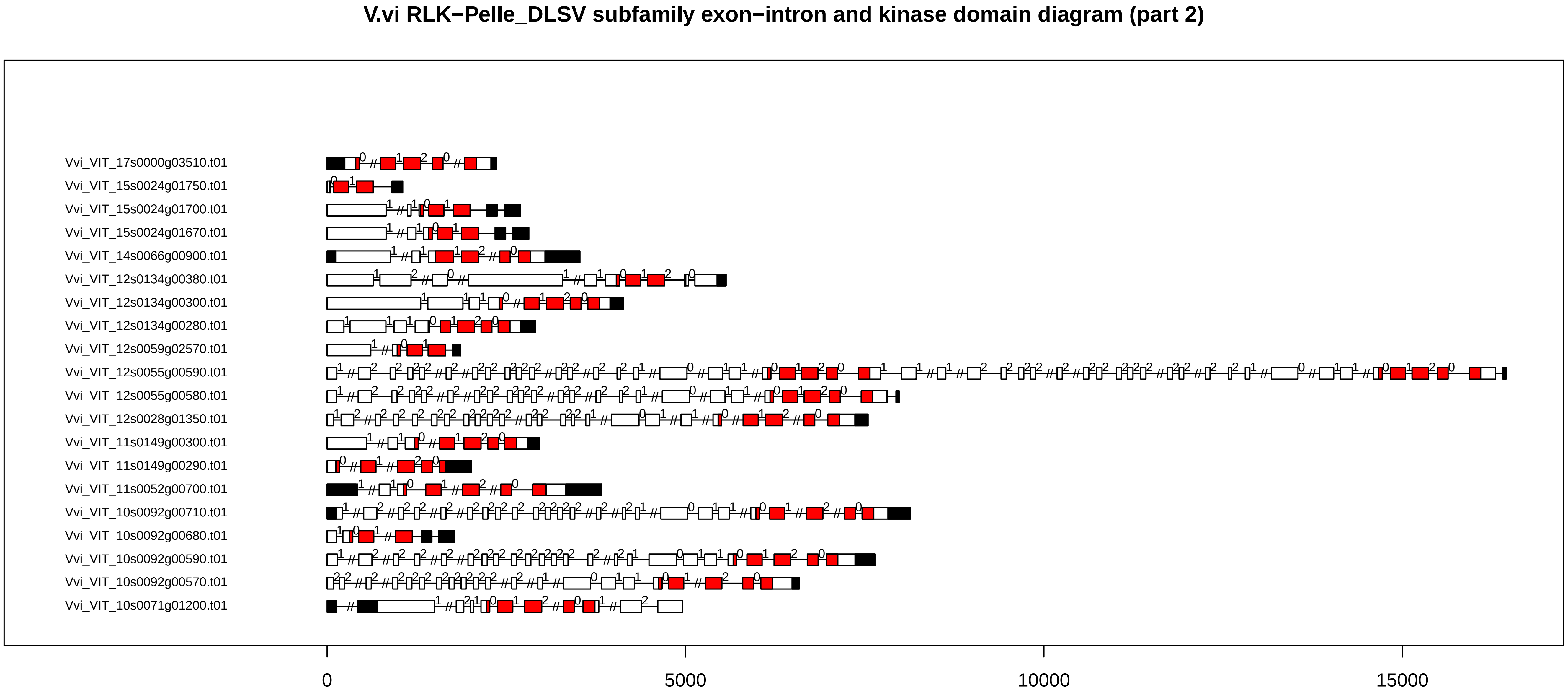
V.vi RLK−Pelle_DLSV subfamily exon−intron and kinase domain diagram (part 2)
Vvi_VIT_17s0000g03510.t01
Vvi_VIT_15s0024g01750.t01
Vvi_VIT_15s0024g01700.t01
Vvi_VIT_15s0024g01670.t01
Vvi_VIT_14s0066g00900.t01
Vvi_VIT_12s0134g00380.t01
Vvi_VIT_12s0134g00300.t01
Vvi_VIT_12s0134g00280.t01
Vvi_VIT_12s0059g02570.t01
Vvi_VIT_12s0055g00590.t01
Vvi_VIT_12s0055g00580.t01
Vvi_VIT_12s0028g01350.t01
Vvi_VIT_11s0149g00300.t01
Vvi_VIT_11s0149g00290.t01
Vvi_VIT_11s0052g00700.t01
Vvi_VIT_10s0092g00710.t01
Vvi_VIT_10s0092g00680.t01
Vvi_VIT_10s0092g00590.t01
Vvi_VIT_10s0092g00570.t01
Vvi_VIT_10s0071g01200.t01
0
5000
10000
15000

V.vi RLK−Pelle_DLSV subfamily exon−intron and kinase domain diagram (part 3)

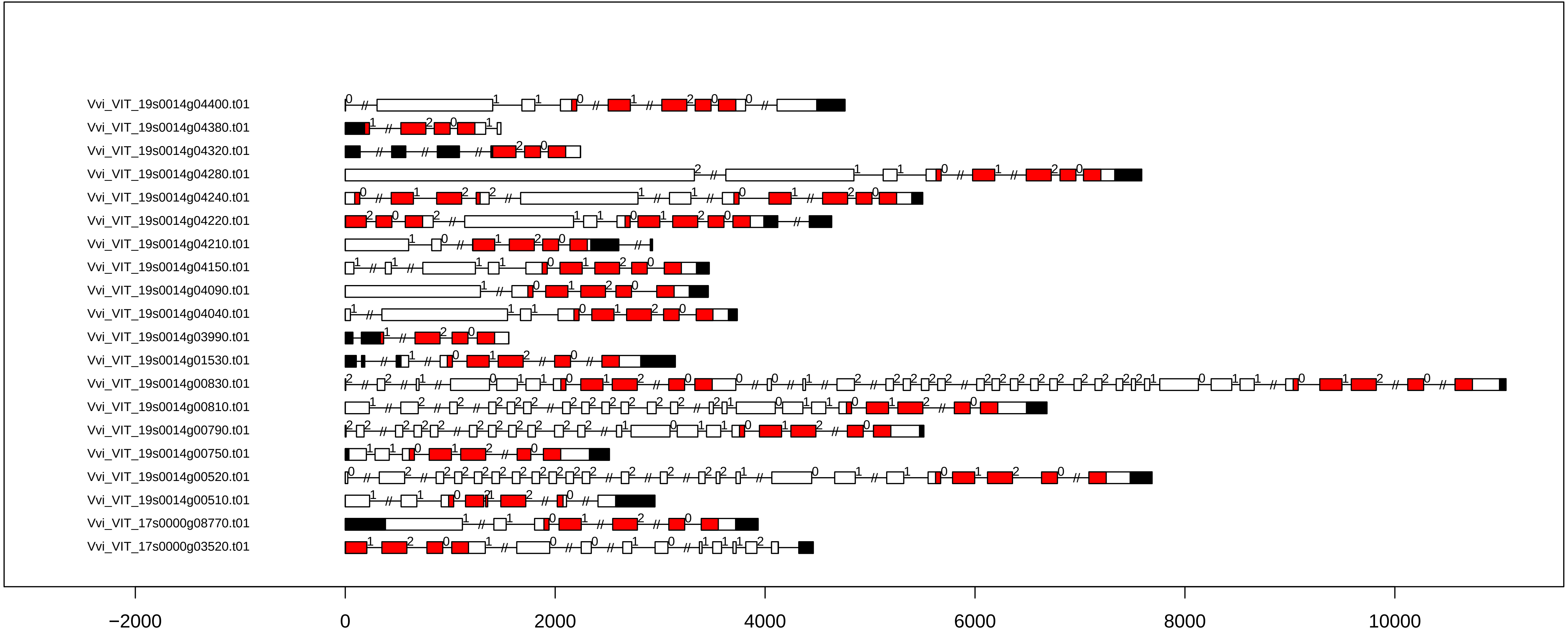

V.vi RLK−Pelle_DLSV subfamily exon−intron and kinase domain diagram (part 4)

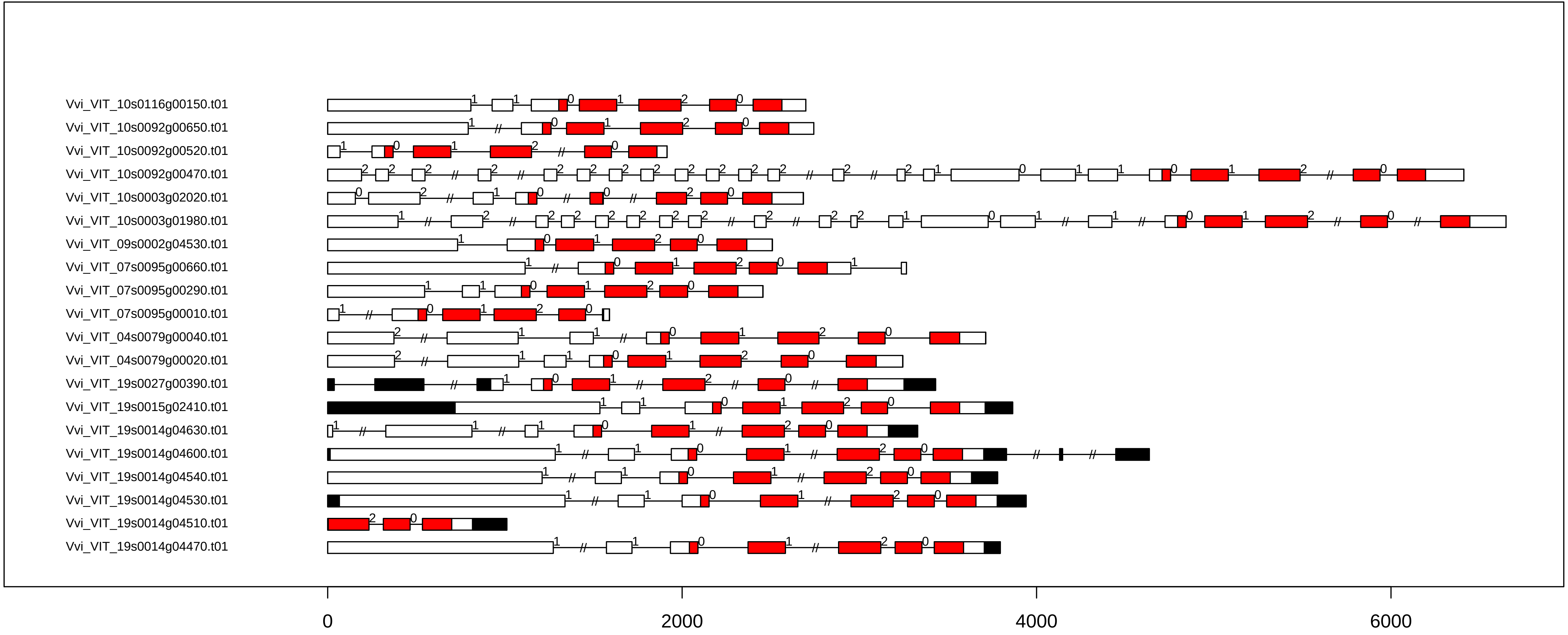

V.vi RLK−Pelle_DLSV subfamily exon−intron and kinase domain diagram (part 5)

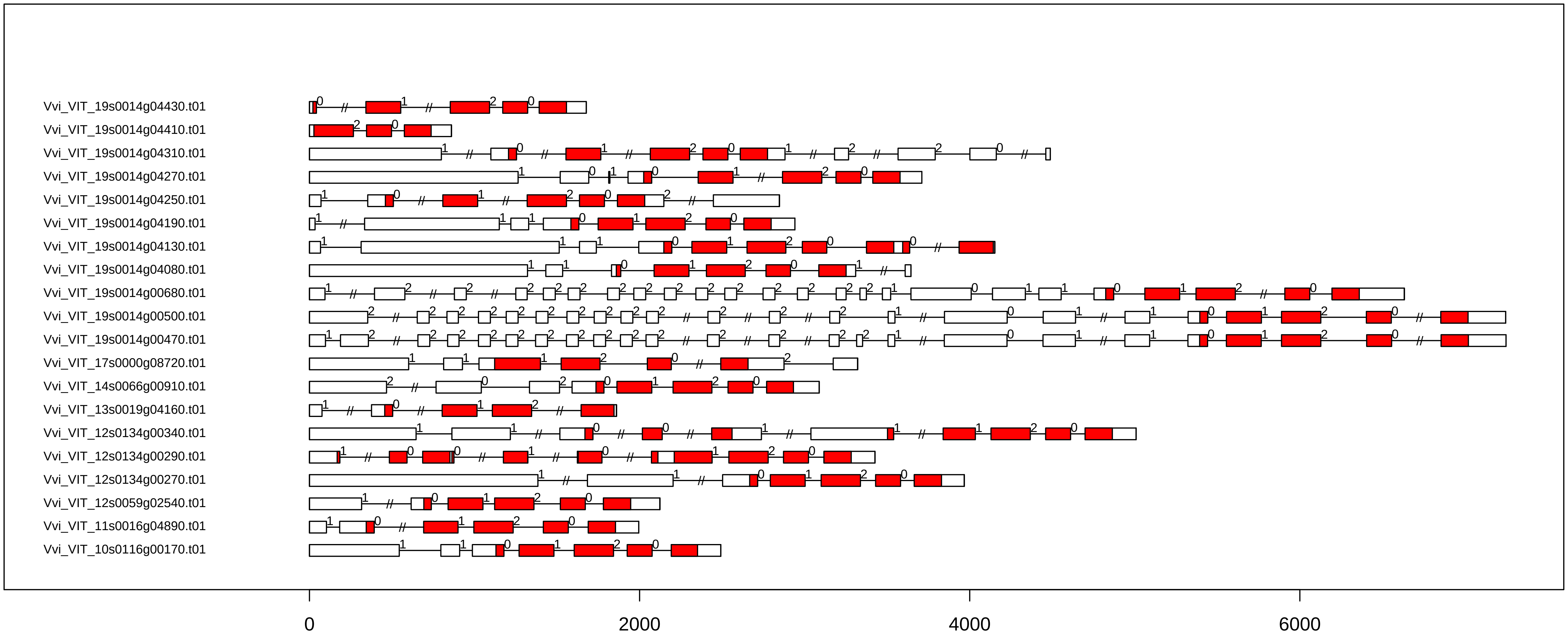

V.vi RLK−Pelle_DLSV subfamily exon−intron and kinase domain diagram (part 6)

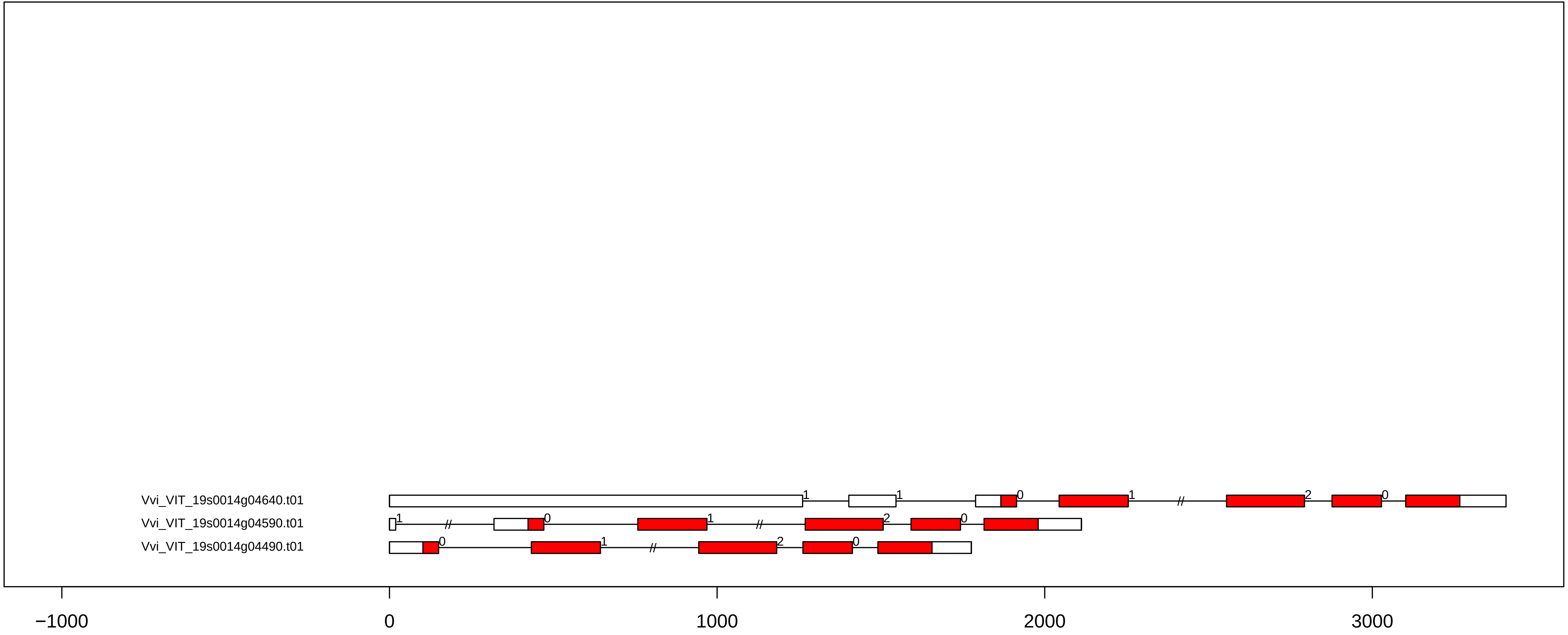

V.vi RLK−Pelle_Extensin subfamily exon−intron and kinase domain diagram (all)

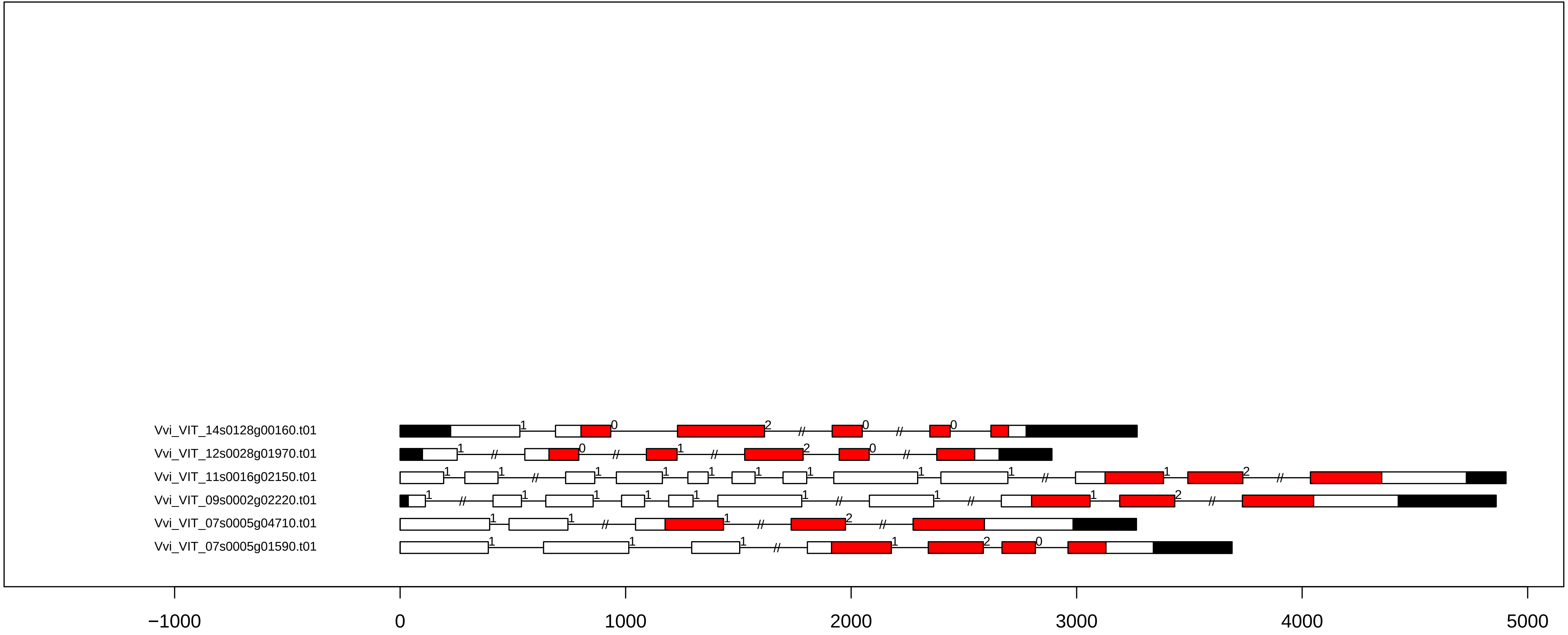

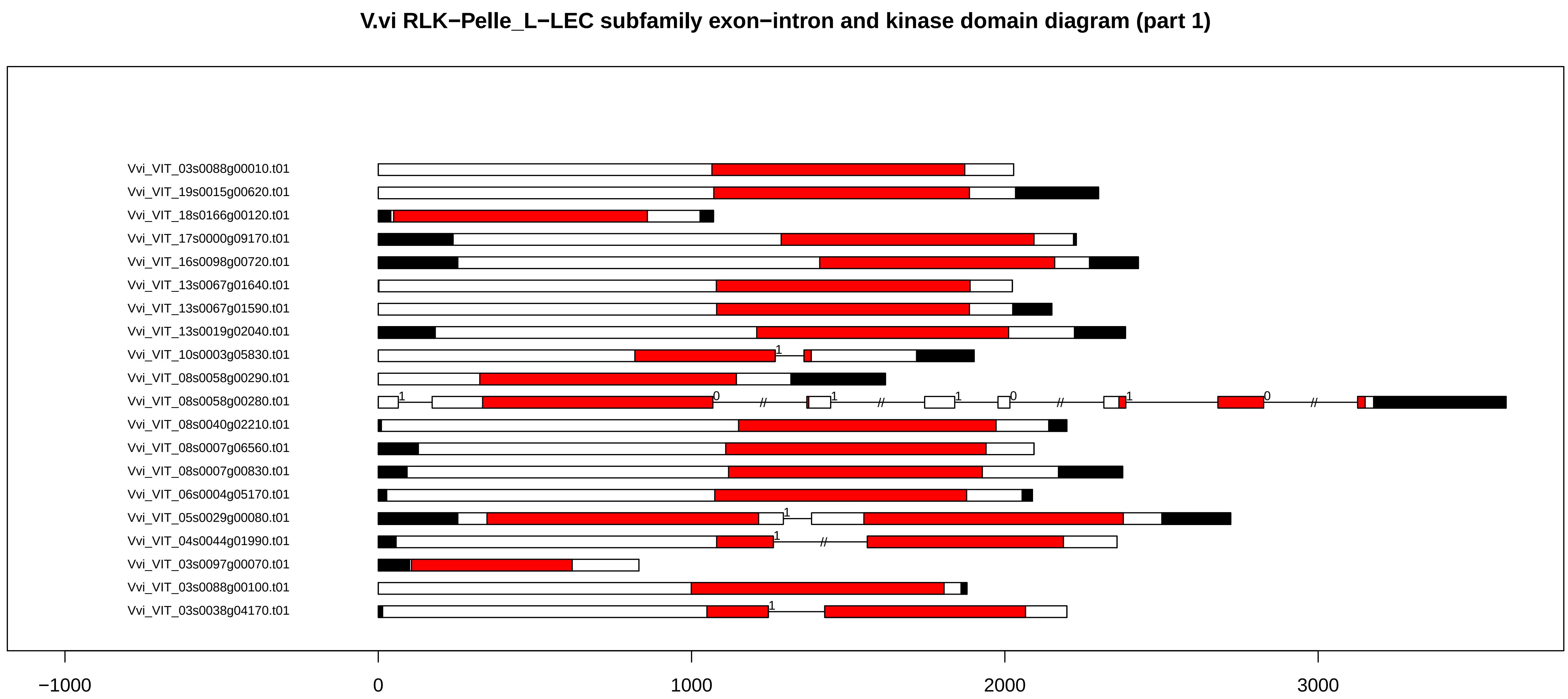
V.vi RLK−Pelle_L−LEC subfamily exon−intron and kinase domain diagram (part 1)
Vvi_VIT_03s0088g00010.t01
Vvi_VIT_19s0015g00620.t01
Vvi_VIT_18s0166g00120.t01
Vvi_VIT_17s0000g09170.t01
Vvi_VIT_16s0098g00720.t01
Vvi_VIT_13s0067g01640.t01
Vvi_VIT_13s0067g01590.t01
Vvi_VIT_13s0019g02040.t01
Vvi_VIT_10s0003g05830.t01
Vvi_VIT_08s0058g00290.t01
Vvi_VIT_08s0058g00280.t01
Vvi_VIT_08s0040g02210.t01
Vvi_VIT_08s0007g06560.t01
Vvi_VIT_08s0007g00830.t01
Vvi_VIT_06s0004g05170.t01
Vvi_VIT_05s0029g00080.t01
Vvi_VIT_04s0044g01990.t01
Vvi_VIT_03s0097g00070.t01
Vvi_VIT_03s0088g00100.t01
Vvi_VIT_03s0038g04170.t01
−1000
0
1000
2000
3000

V.vi RLK−Pelle_L−LEC subfamily exon−intron and kinase domain diagram (part 2)

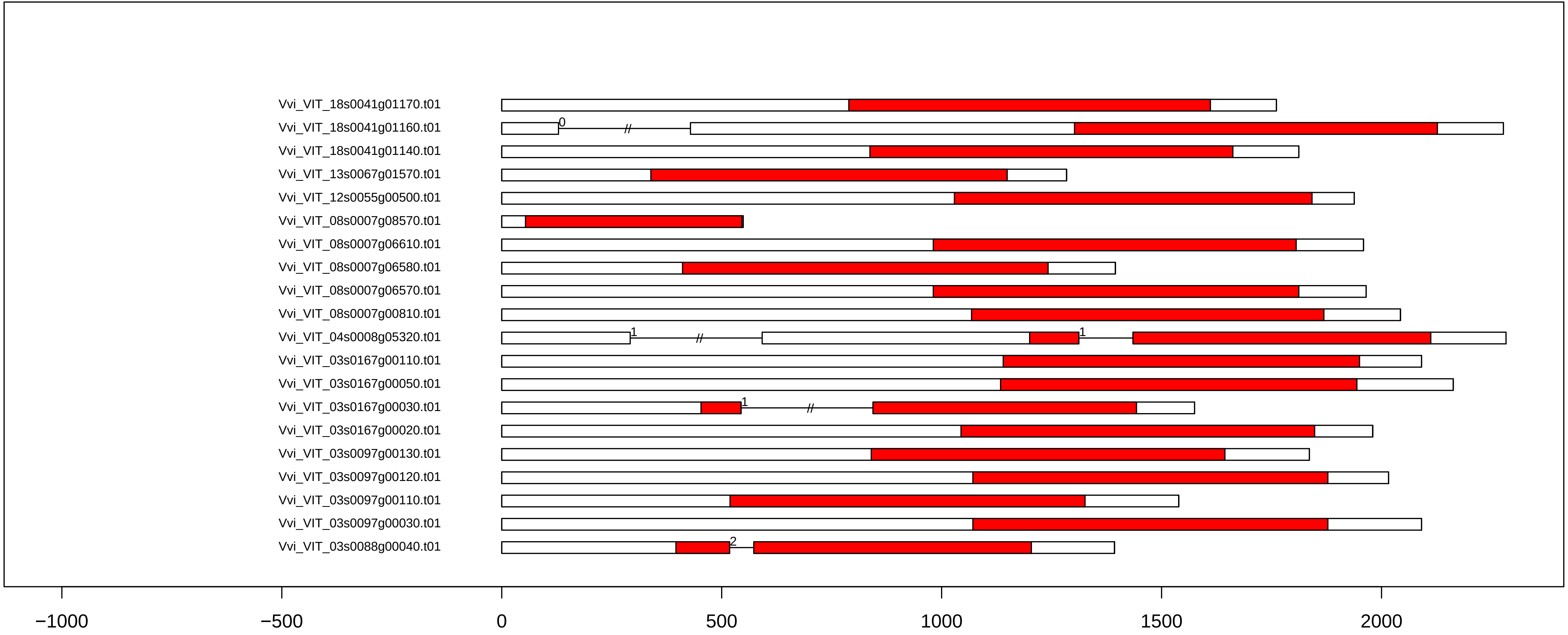

V.vi RLK−Pelle_L−LEC subfamily exon−intron and kinase domain diagram (part 3)

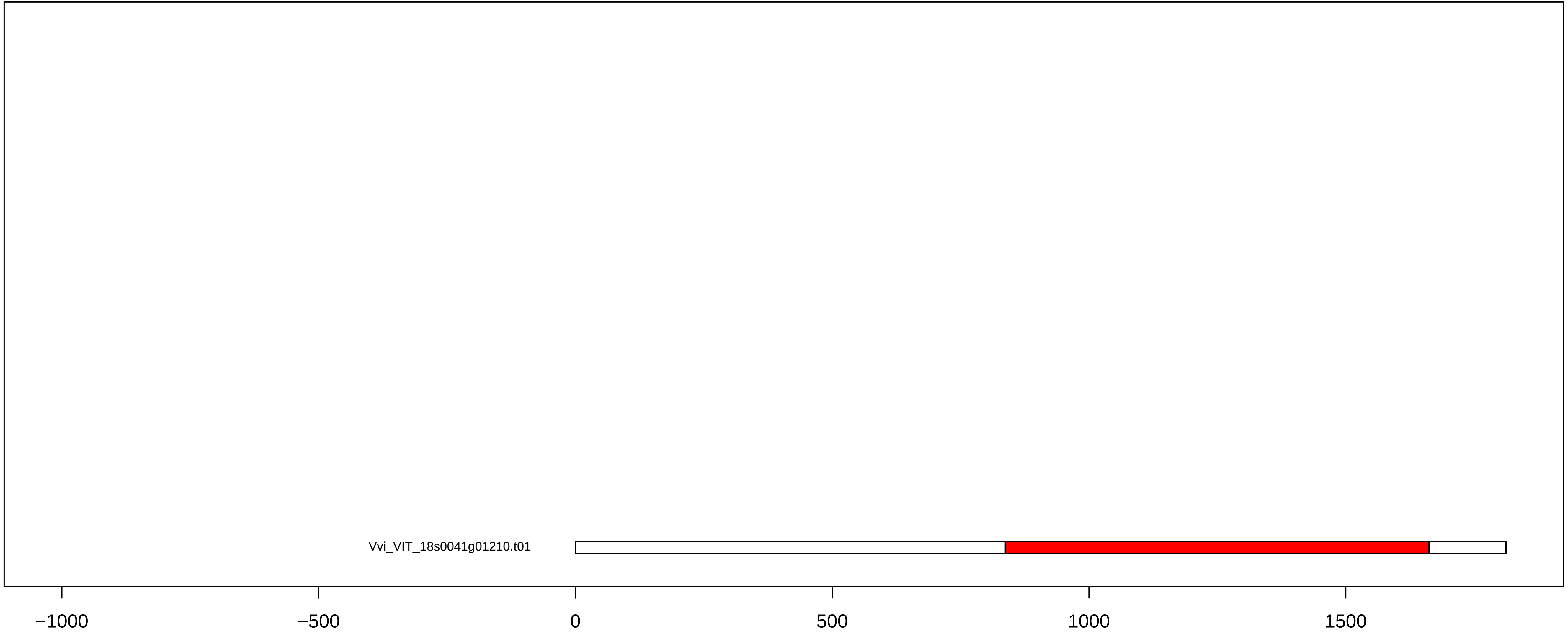

V.vi RLK−Pelle_LRK10L−2 subfamily exon−intron and kinase domain diagram (part 1)

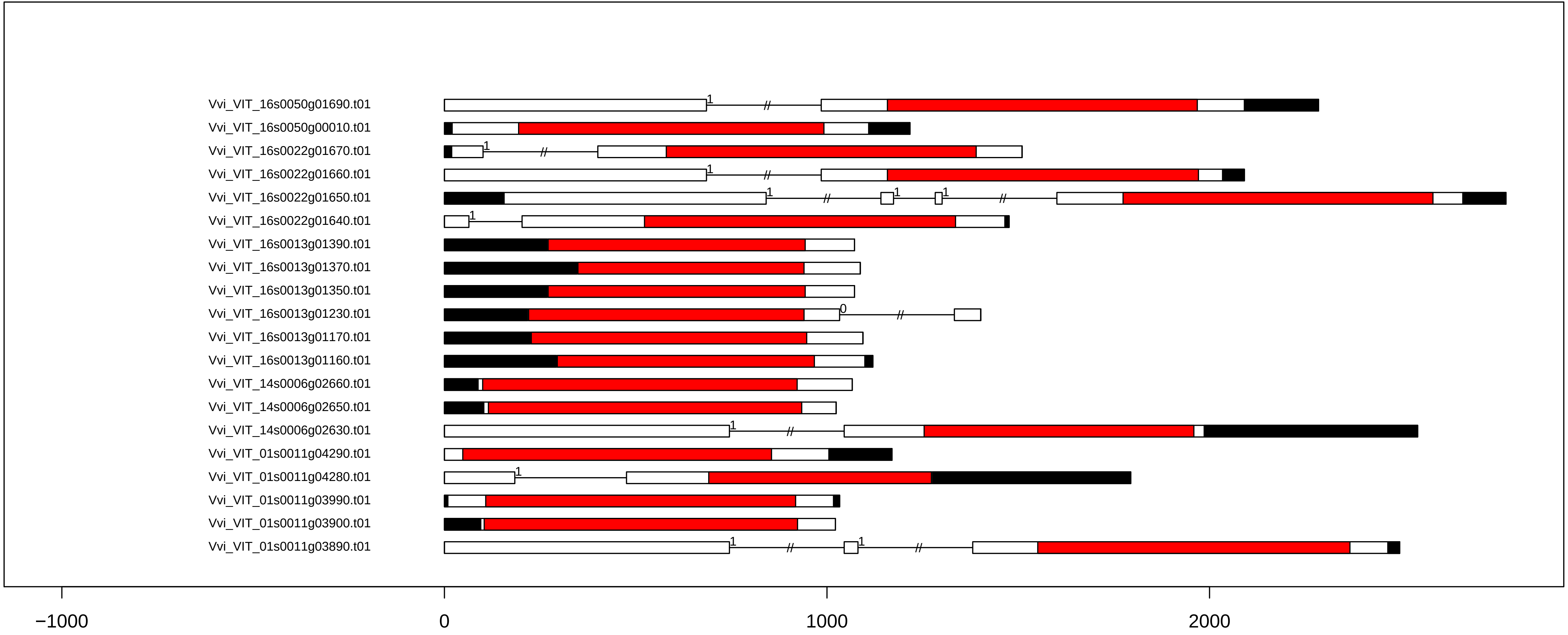

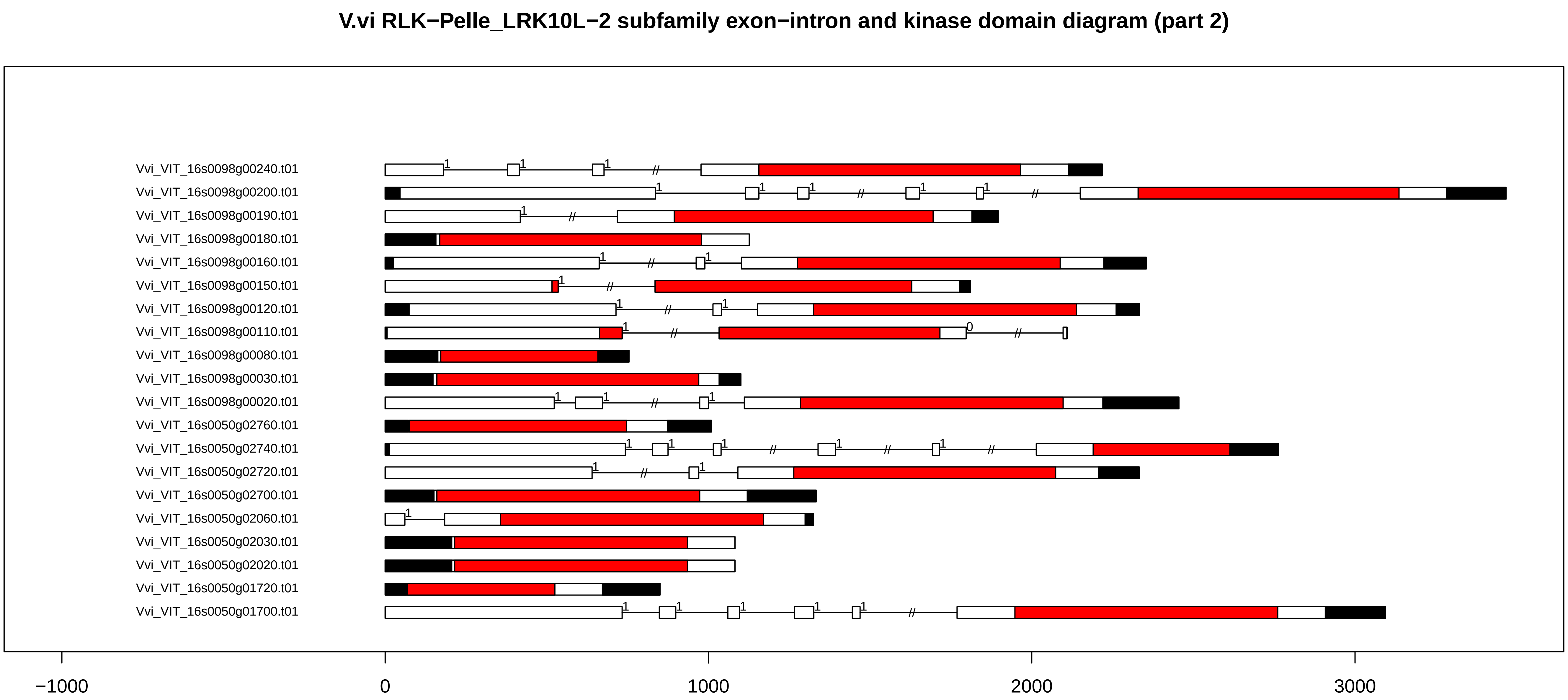
V.vi RLK−Pelle_LRK10L−2 subfamily exon−intron and kinase domain diagram (part 2)
Vvi_VIT_16s0098g00240.t01
Vvi_VIT_16s0098g00200.t01
Vvi_VIT_16s0098g00190.t01
Vvi_VIT_16s0098g00180.t01
Vvi_VIT_16s0098g00160.t01
Vvi_VIT_16s0098g00150.t01
Vvi_VIT_16s0098g00120.t01
Vvi_VIT_16s0098g00110.t01
Vvi_VIT_16s0098g00080.t01
Vvi_VIT_16s0098g00030.t01
Vvi_VIT_16s0098g00020.t01
Vvi_VIT_16s0050g02760.t01
Vvi_VIT_16s0050g02740.t01
Vvi_VIT_16s0050g02720.t01
Vvi_VIT_16s0050g02700.t01
Vvi_VIT_16s0050g02060.t01
Vvi_VIT_16s0050g02030.t01
Vvi_VIT_16s0050g02020.t01
Vvi_VIT_16s0050g01720.t01
Vvi_VIT_16s0050g01700.t01
−1000
0
1000
2000
3000

V.vi RLK−Pelle_LRK10L−2 subfamily exon−intron and kinase domain diagram (part 3)

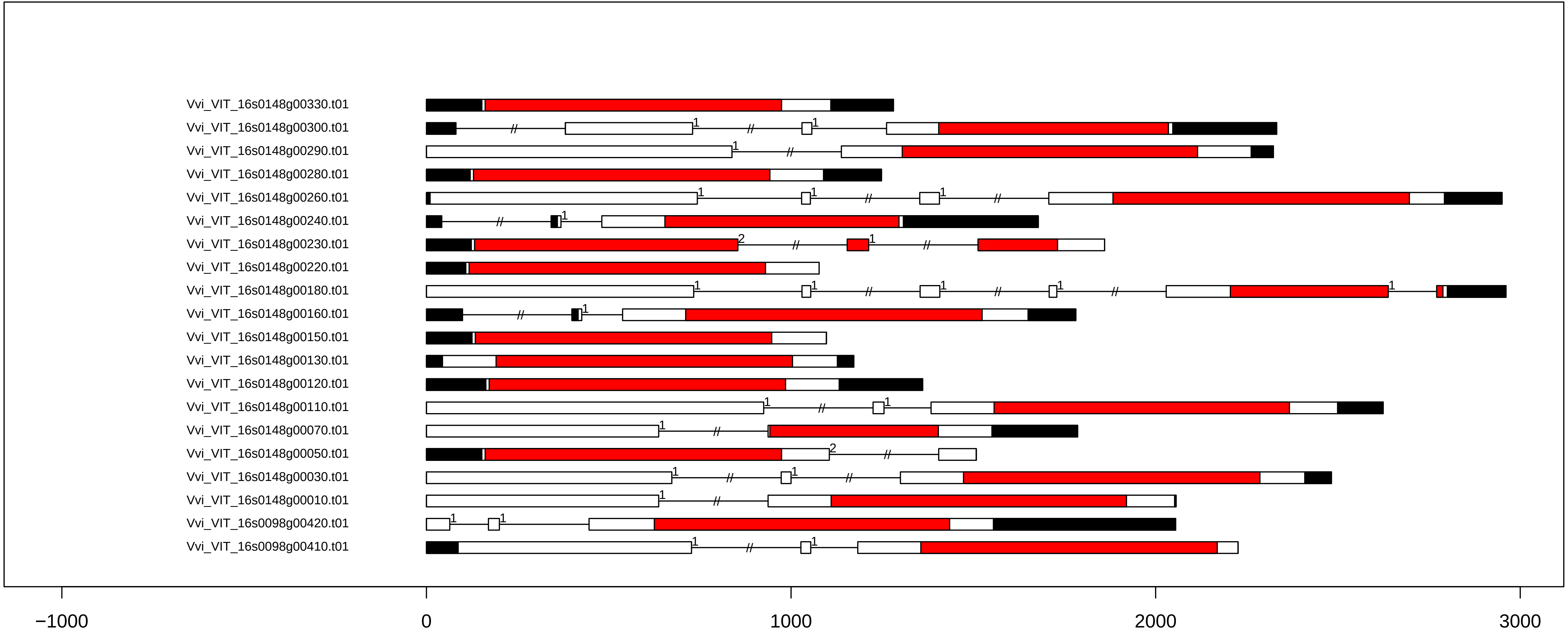

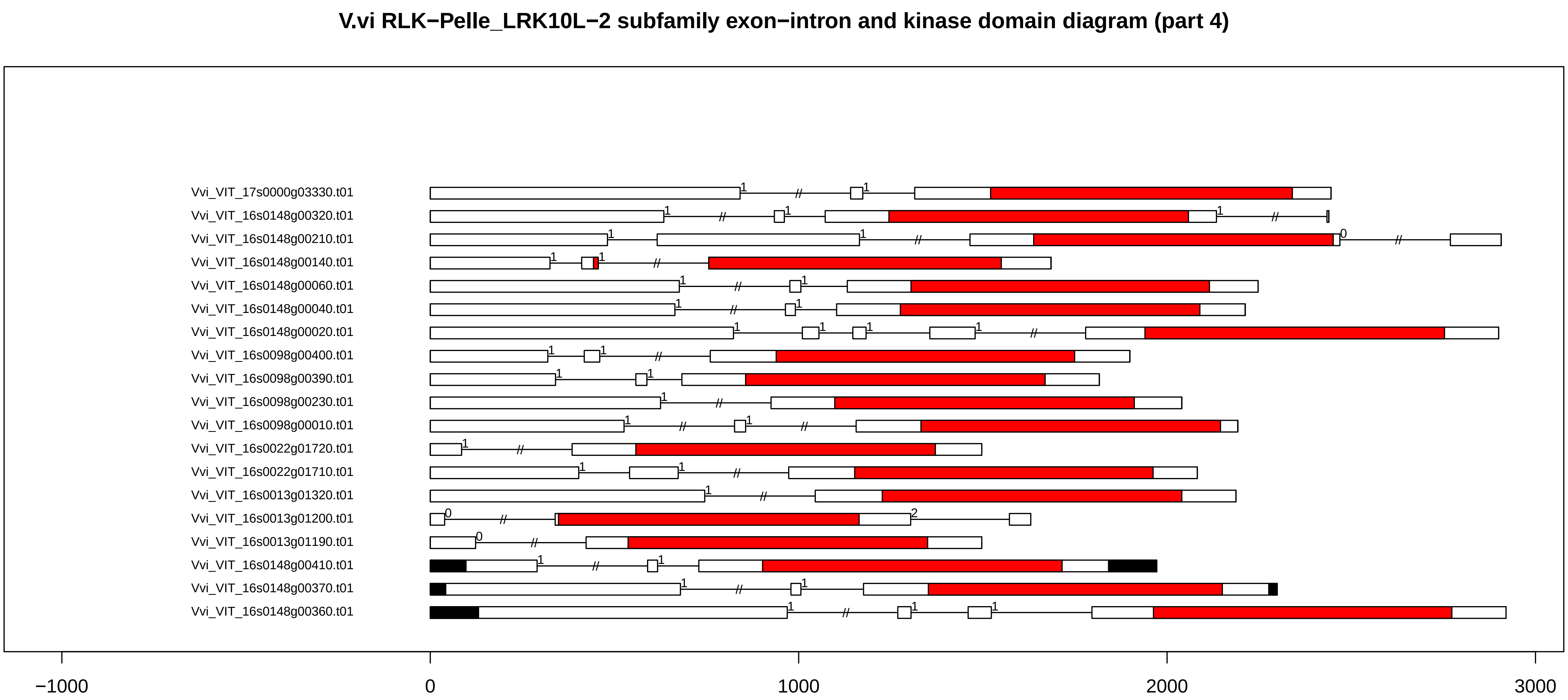
V.vi RLK−Pelle_LRK10L−2 subfamily exon−intron and kinase domain diagram (part 4)
Vvi_VIT_17s0000g03330.t01
Vvi_VIT_16s0148g00320.t01
Vvi_VIT_16s0148g00210.t01
Vvi_VIT_16s0148g00140.t01
Vvi_VIT_16s0148g00060.t01
Vvi_VIT_16s0148g00040.t01
Vvi_VIT_16s0148g00020.t01
Vvi_VIT_16s0098g00400.t01
Vvi_VIT_16s0098g00390.t01
Vvi_VIT_16s0098g00230.t01
Vvi_VIT_16s0098g00010.t01
Vvi_VIT_16s0022g01720.t01
Vvi_VIT_16s0022g01710.t01
Vvi_VIT_16s0013g01320.t01
Vvi_VIT_16s0013g01200.t01
Vvi_VIT_16s0013g01190.t01
Vvi_VIT_16s0148g00410.t01
Vvi_VIT_16s0148g00370.t01
Vvi_VIT_16s0148g00360.t01
−1000
0
1000
2000
3000

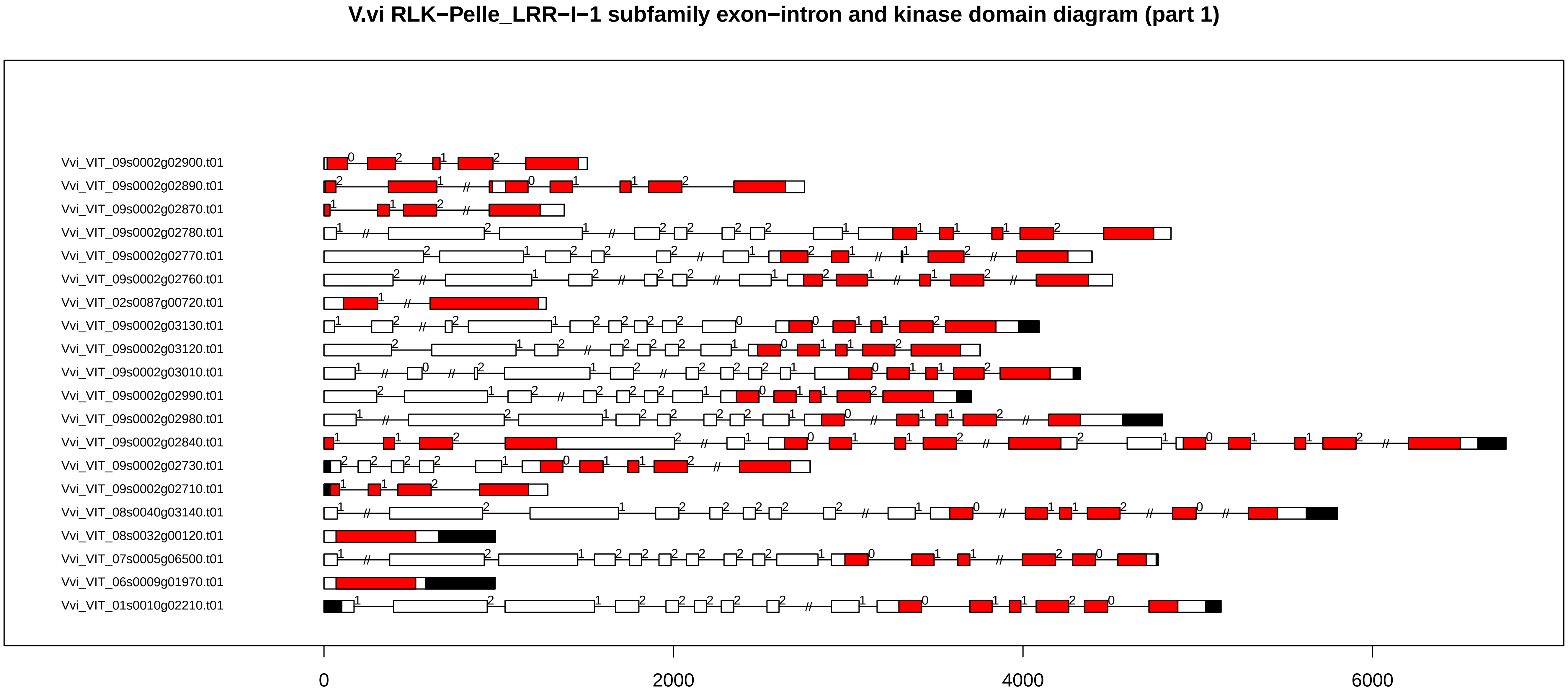
V.vi RLK−Pelle_LRR−I−1 subfamily exon−intron and kinase domain diagram (part 1)
Vvi_VIT_09s0002g02900.t01
Vvi_VIT_09s0002g02890.t01
Vvi_VIT_09s0002g02870.t01
Vvi_VIT_09s0002g02780.t01
Vvi_VIT_09s0002g02770.t01
Vvi_VIT_09s0002g02760.t01
Vvi_VIT_02s0087g00720.t01
Vvi_VIT_09s0002g03130.t01
Vvi_VIT_09s0002g03120.t01
Vvi_VIT_09s0002g03010.t01
Vvi_VIT_09s0002g02990.t01
Vvi_VIT_09s0002g02980.t01
Vvi_VIT_09s0002g02840.t01
Vvi_VIT_09s0002g02730.t01
Vvi_VIT_09s0002g02710.t01
Vvi_VIT_08s0040g03140.t01
Vvi_VIT_08s0032g00120.t01
Vvi_VIT_07s0005g06500.t01
Vvi_VIT_06s0009g01970.t01
Vvi_VIT_01s0010g02210.t01
0
2000
4000
6000

V.vi RLK−Pelle_LRR−I−1 subfamily exon−intron and kinase domain diagram (part 2)

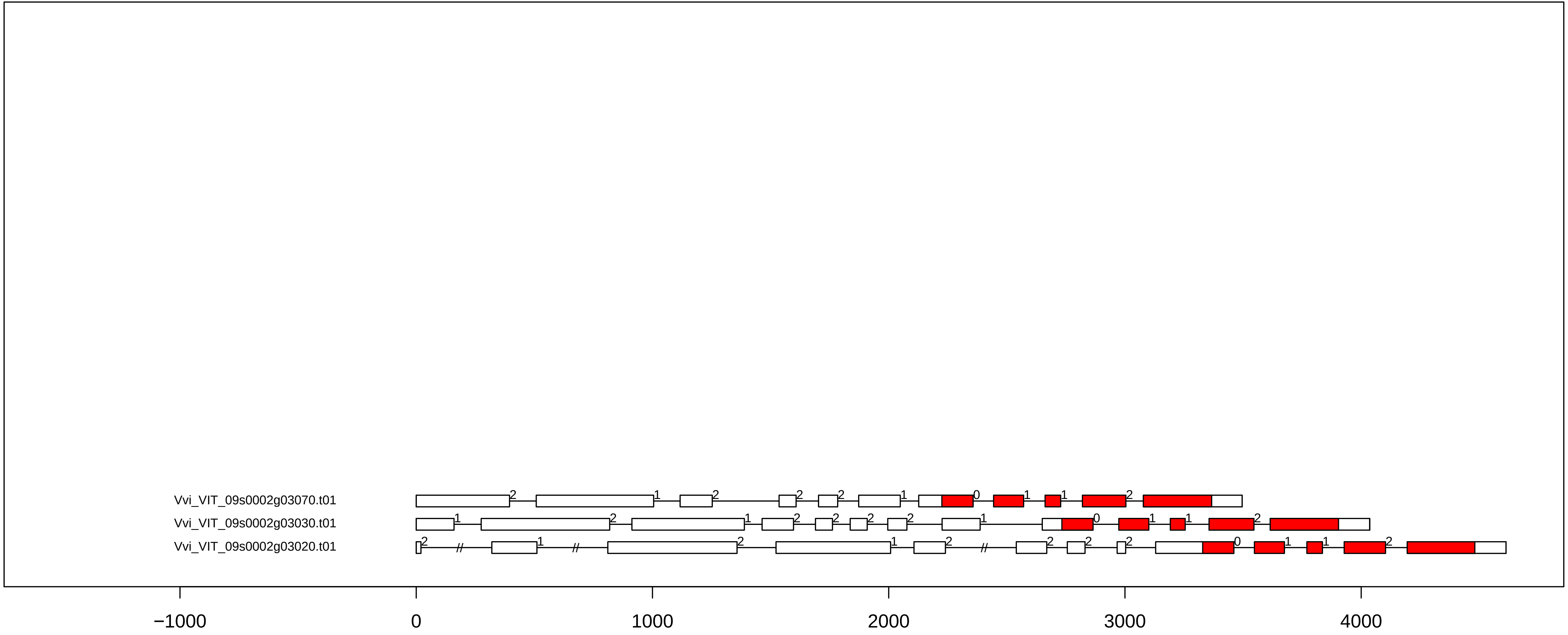

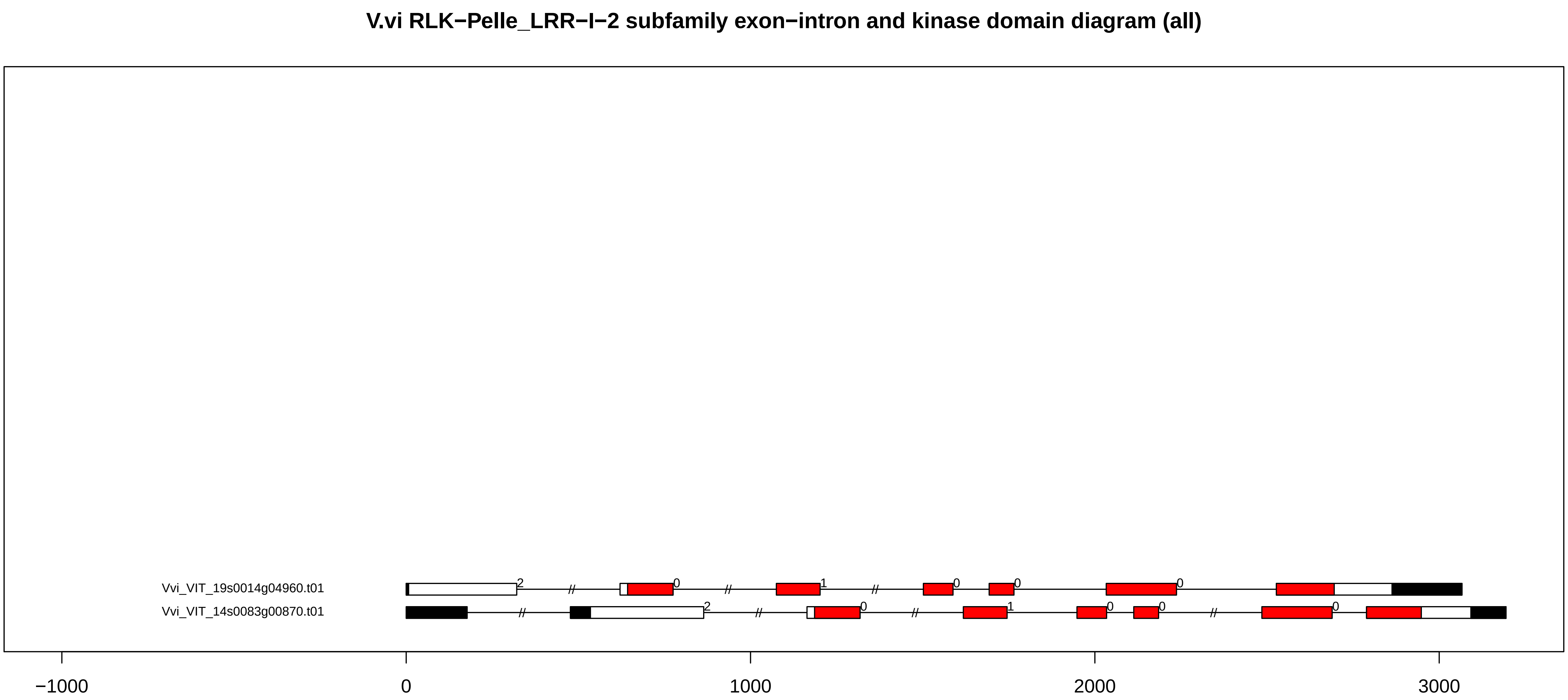

V.vi RLK−Pelle_LRR−I−2 subfamily exon−intron and kinase domain diagram (all)
Vvi_VIT_19s0014g04960.t01
Vvi_VIT_14s0083g00870.t01
2
0
1
0
0
0
2
0
1
0
0
0
−1000
0
1000
2000
3000

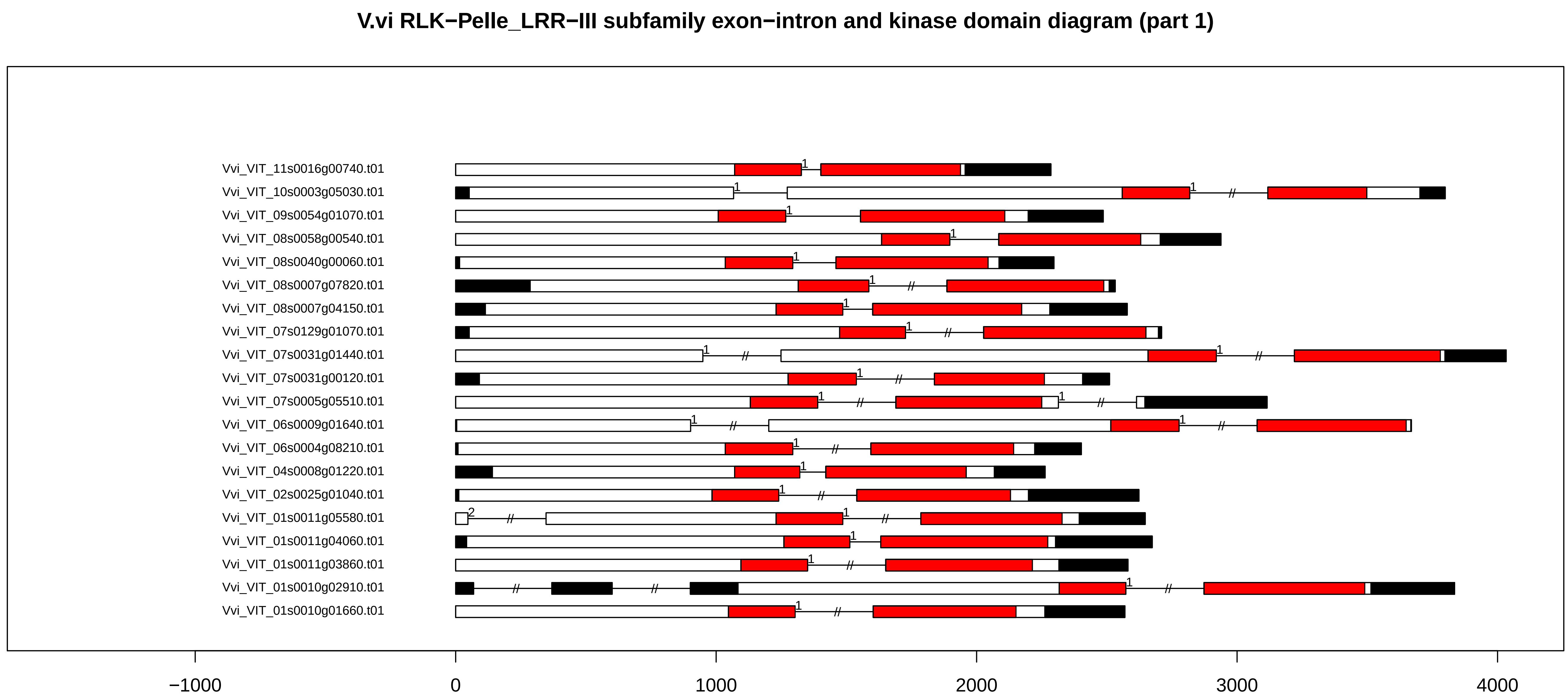
V.vi RLK−Pelle_LRR−III subfamily exon−intron and kinase domain diagram (part 1)
Vvi_VIT_11s0016g00740.t01
Vvi_VIT_10s0003g05030.t01
Vvi_VIT_09s0054g01070.t01
Vvi_VIT_08s0058g00540.t01
Vvi_VIT_08s0040g00060.t01
Vvi_VIT_08s0007g07820.t01
Vvi_VIT_08s0007g04150.t01
Vvi_VIT_07s0129g01070.t01
Vvi_VIT_07s0031g01440.t01
Vvi_VIT_07s0031g00120.t01
Vvi_VIT_07s0005g05510.t01
Vvi_VIT_06s0009g01640.t01
Vvi_VIT_06s0004g08210.t01
Vvi_VIT_04s0008g01220.t01
Vvi_VIT_02s0025g01040.t01
Vvi_VIT_01s0011g05580.t01
Vvi_VIT_01s0011g04060.t01
Vvi_VIT_01s0011g03860.t01
Vvi_VIT_01s0010g02910.t01
Vvi_VIT_01s0010g01660.t01
−1000
0
1000
2000
3000
4000

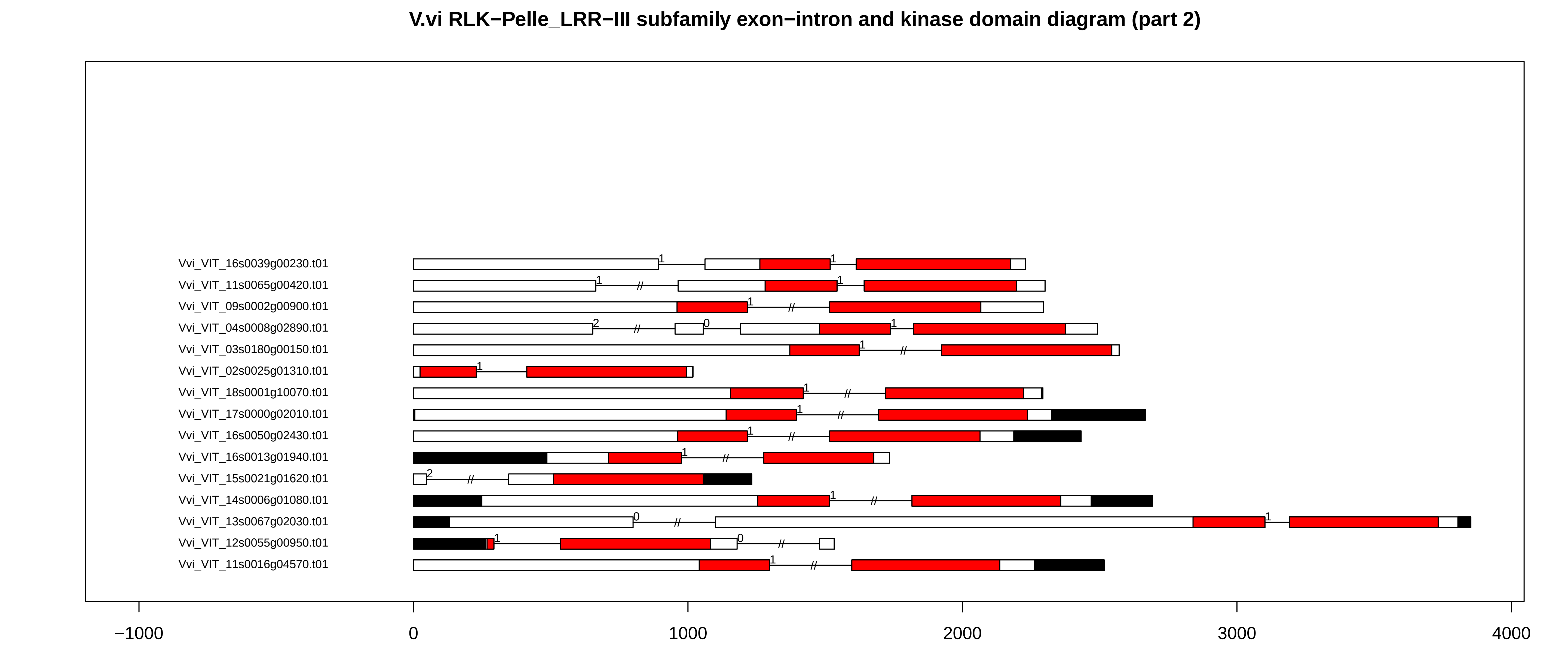
V.vi RLK−Pelle_LRR−III subfamily exon−intron and kinase domain diagram (part 2)
Vvi_VIT_16s0039g00230.t01
Vvi_VIT_11s0065g00420.t01
Vvi_VIT_09s0002g00900.t01
Vvi_VIT_04s0008g02890.t01
Vvi_VIT_03s0180g00150.t01
Vvi_VIT_02s0025g01310.t01
Vvi_VIT_18s0001g10070.t01
Vvi_VIT_17s0000g02010.t01
Vvi_VIT_16s0050g02430.t01
Vvi_VIT_16s0013g01940.t01
Vvi_VIT_15s0021g01620.t01
Vvi_VIT_14s0006g01080.t01
Vvi_VIT_13s0067g02030.t01
Vvi_VIT_12s0055g00950.t01
Vvi_VIT_11s0016g04570.t01
−1000
0
1000
2000
3000
4000

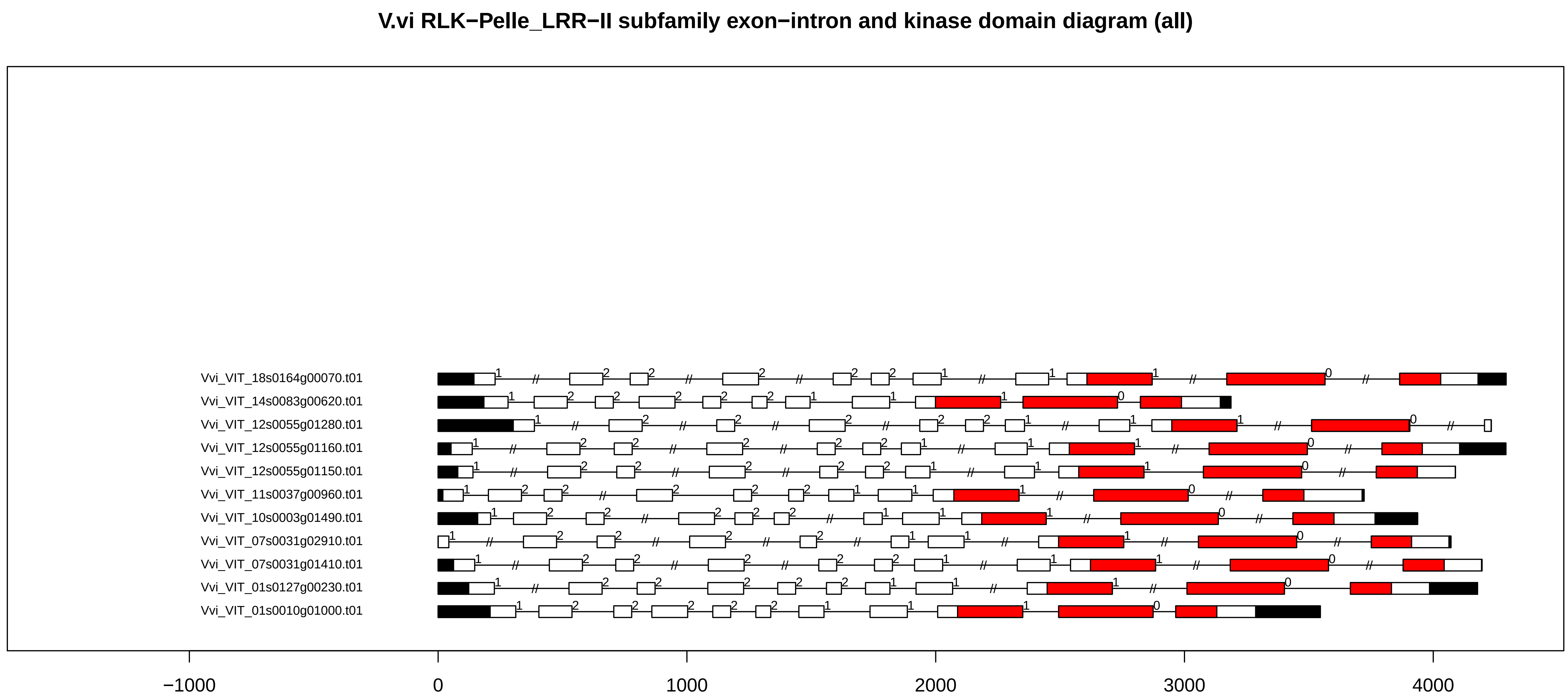
V.vi RLK−Pelle_LRR−II subfamily exon−intron and kinase domain diagram (all)
Vvi_VIT_18s0164g00070.t01
Vvi_VIT_14s0083g00620.t01
Vvi_VIT_12s0055g01280.t01
Vvi_VIT_12s0055g01160.t01
Vvi_VIT_12s0055g01150.t01
Vvi_VIT_11s0037g00960.t01
Vvi_VIT_10s0003g01490.t01
Vvi_VIT_07s0031g02910.t01
Vvi_VIT_07s0031g01410.t01
Vvi_VIT_01s0127g00230.t01
Vvi_VIT_01s0010g01000.t01
−1000
0
1000
2000
3000
4000

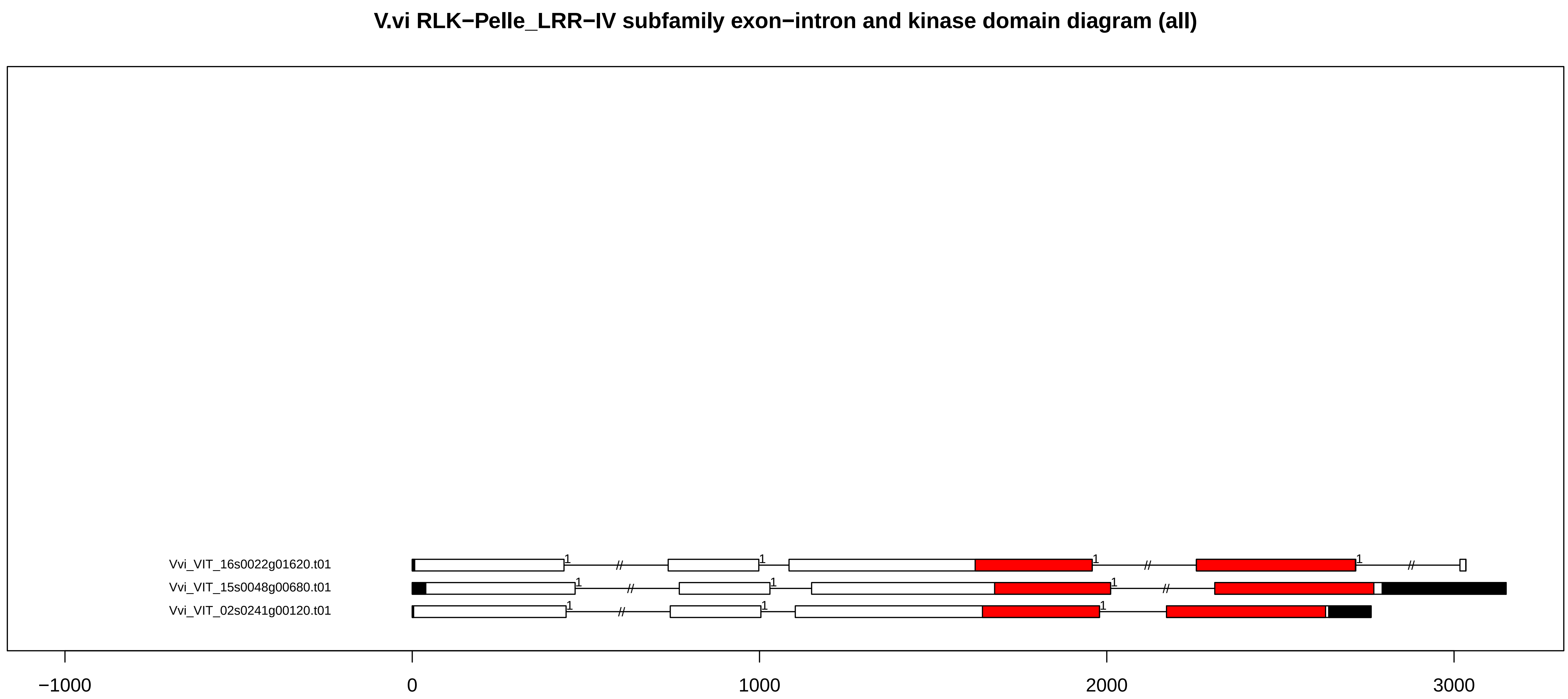
V.vi RLK−Pelle_LRR−IV subfamily exon−intron and kinase domain diagram (all)
Vvi_VIT_16s0022g01620.t01
Vvi_VIT_15s0048g00680.t01
Vvi_VIT_02s0241g00120.t01
−1000
0
1000
2000
3000

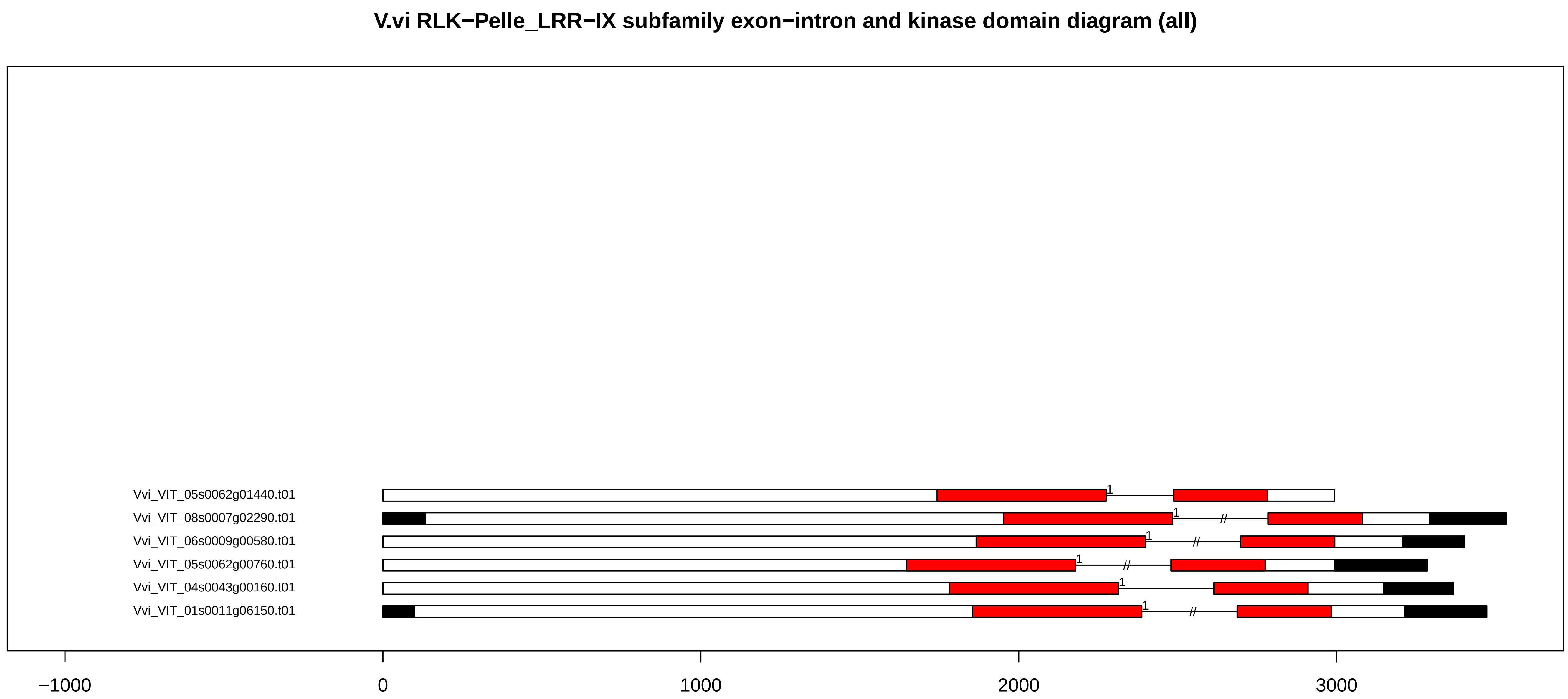
V.vi RLK−Pelle_LRR−IX subfamily exon−intron and kinase domain diagram (all)
Vvi_VIT_05s0062g01440.t01
Vvi_VIT_08s0007g02290.t01
Vvi_VIT_06s0009g00580.t01
Vvi_VIT_05s0062g00760.t01
Vvi_VIT_04s0043g00160.t01
Vvi_VIT_01s0011g06150.t01
−1000
0
1000
2000
3000

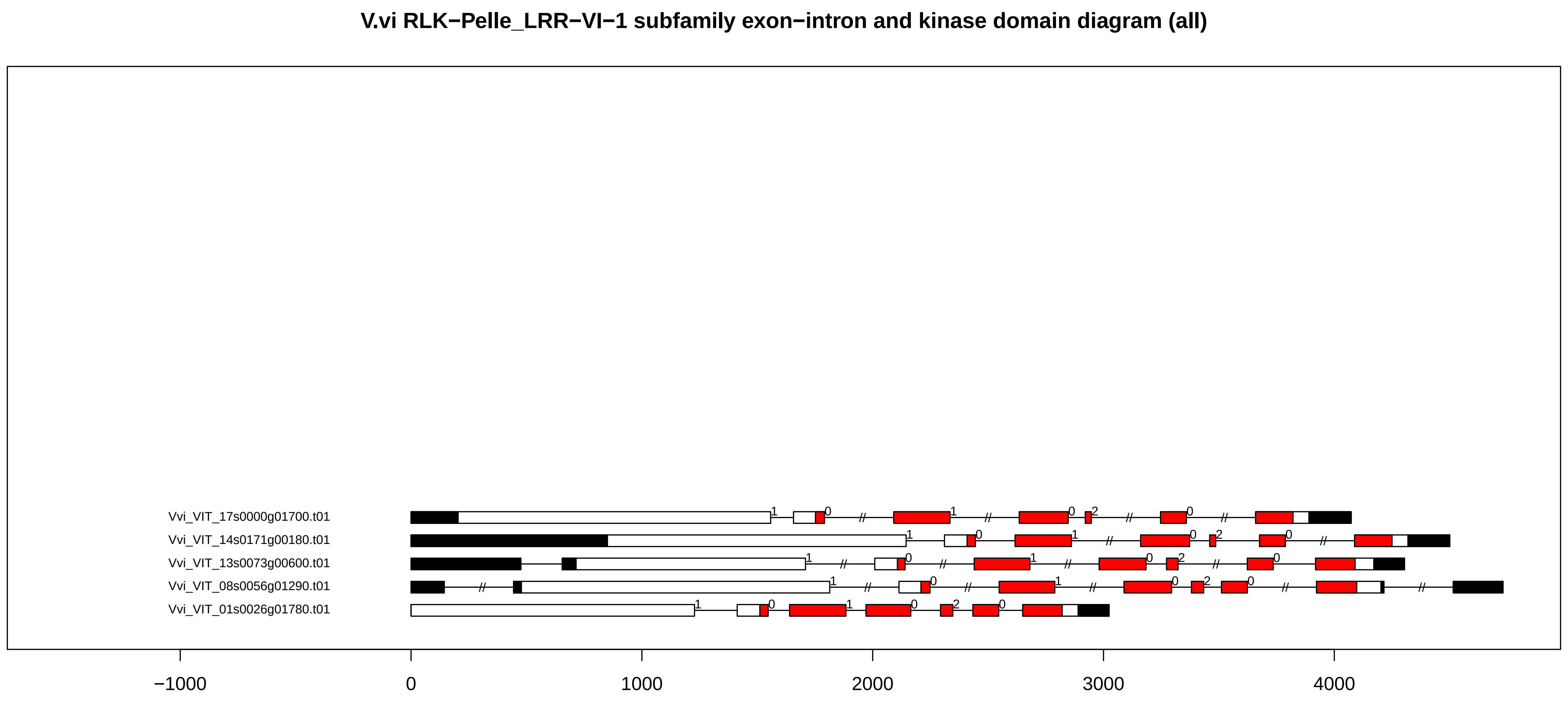
V.vi RLK−Pelle_LRR−VI−1 subfamily exon−intron and kinase domain diagram (all)
Vvi_VIT_17s0000g01700.t01
Vvi_VIT_14s0171g00180.t01
Vvi_VIT_13s0073g00600.t01
Vvi_VIT_08s0056g01290.t01
Vvi_VIT_01s0026g01780.t01
−1000
0
1000
2000
3000
4000

V.vi RLK−Pelle_LRR−VI−2 subfamily exon−intron and kinase domain diagram (all)

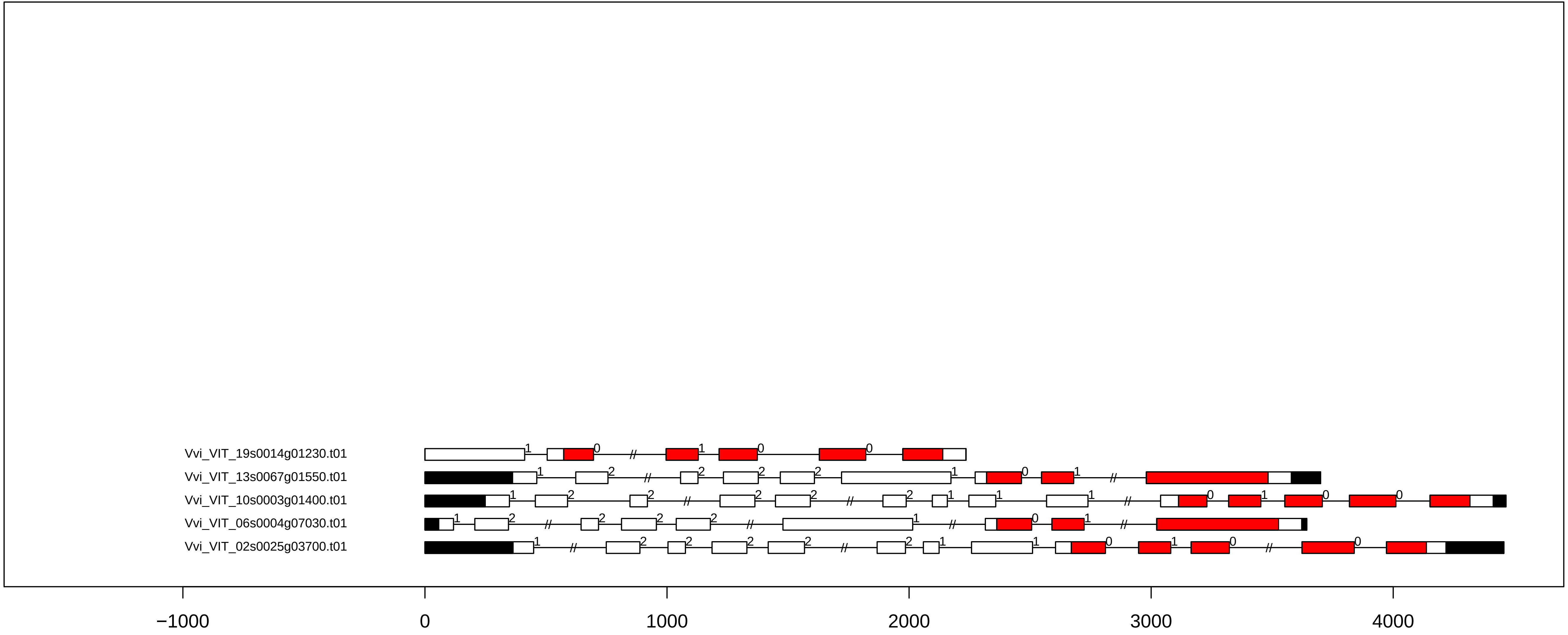

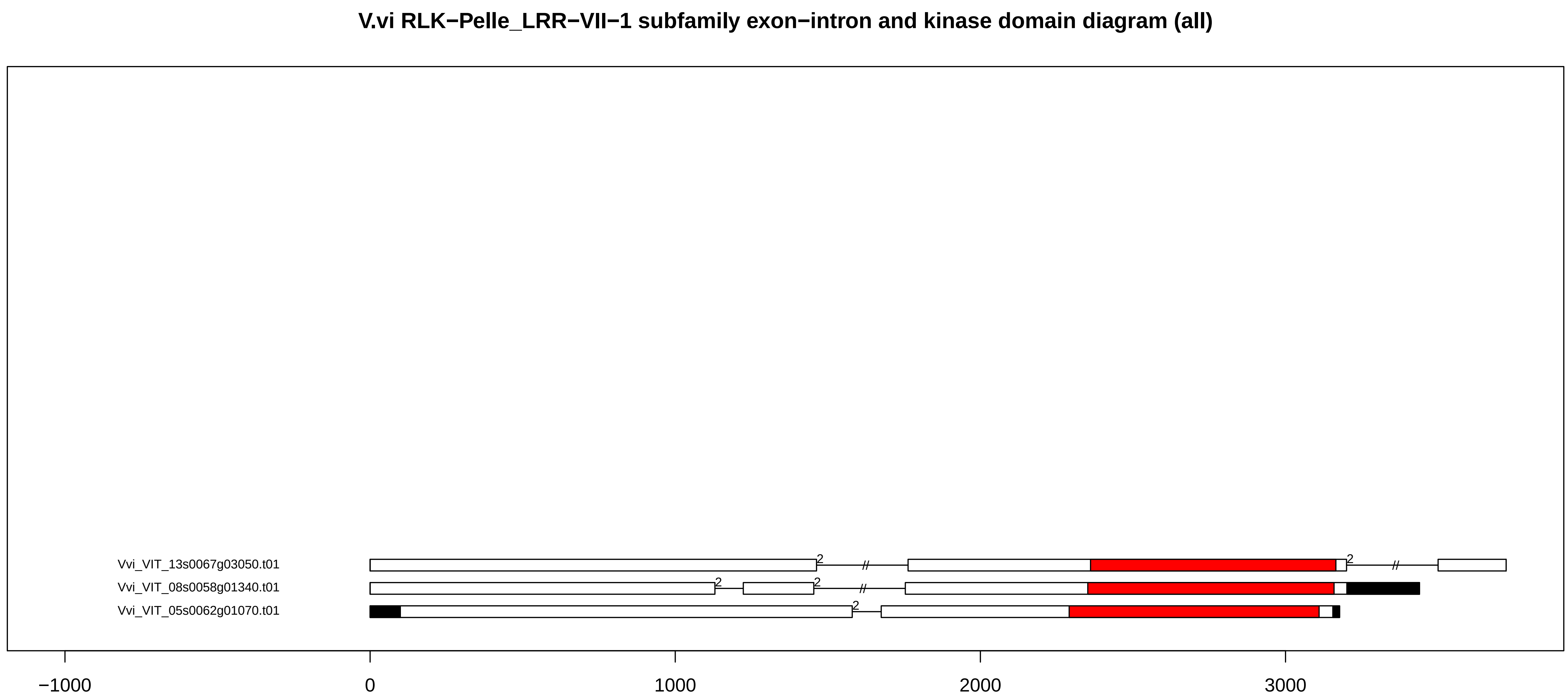

V.vi RLK−Pelle_LRR−VII−1 subfamily exon−intron and kinase domain diagram (all)
Vvi_VIT_13s0067g03050.t01
Vvi_VIT_08s0058g01340.t01
Vvi_VIT_05s0062g01070.t01
2
2
2
2
2
−1000
0
1000
2000
3000

V.vi RLK−Pelle_LRR−VII−2 subfamily exon−intron and kinase domain diagram (all)

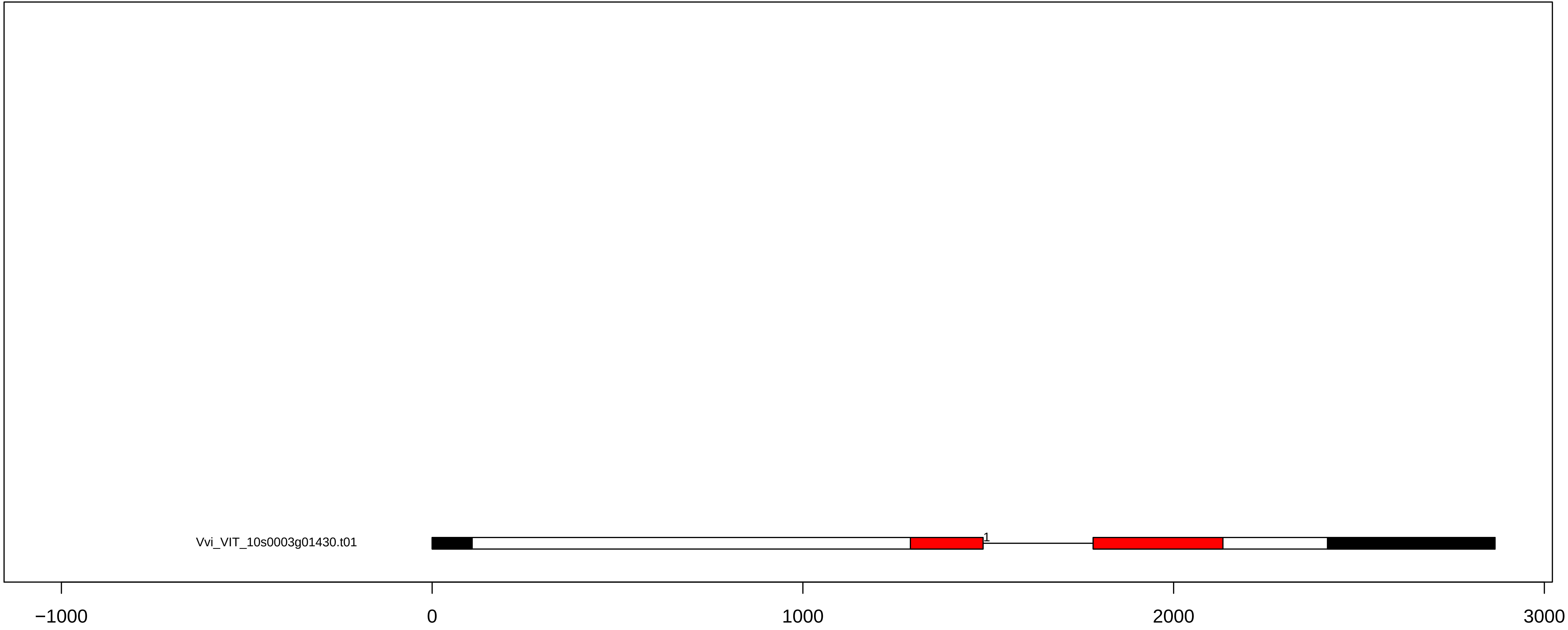

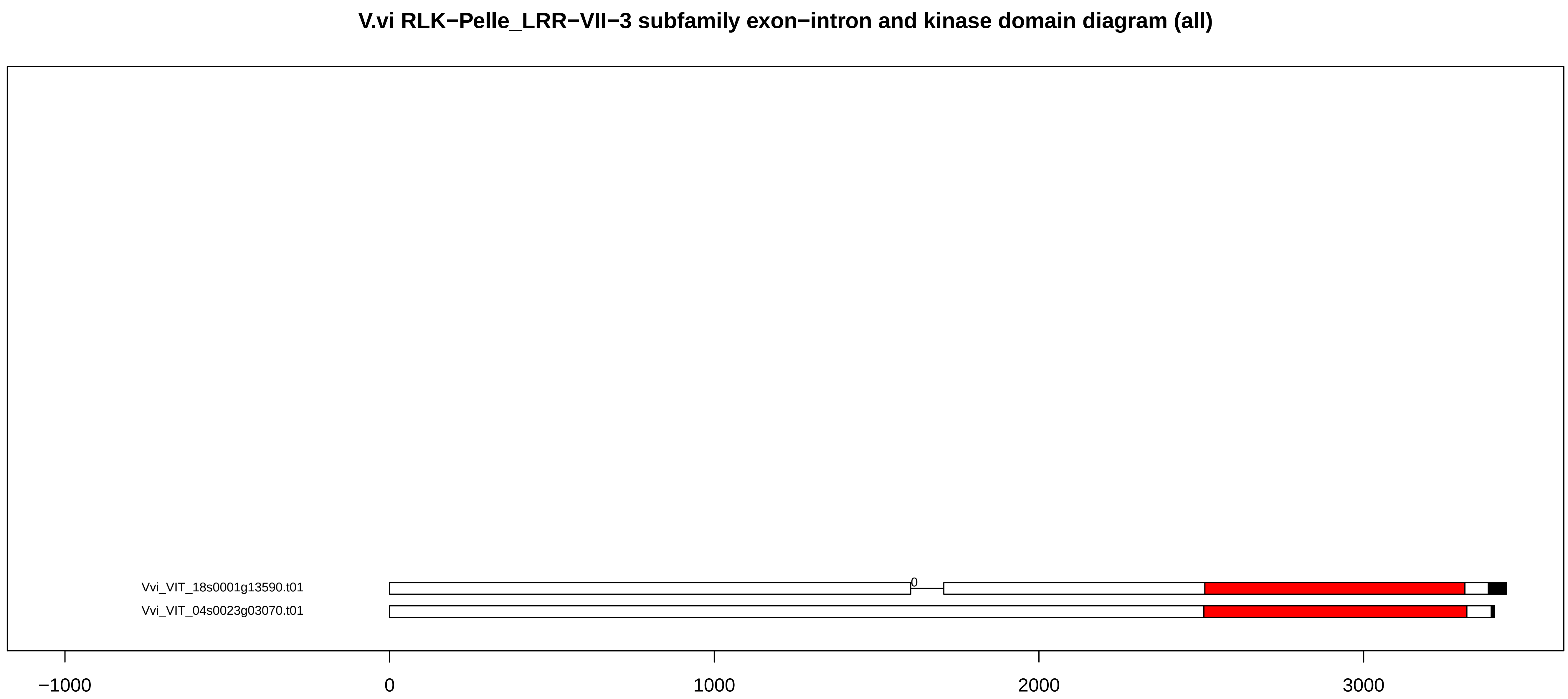
V.vi RLK−Pelle_LRR−VII−3 subfamily exon−intron and kinase domain diagram (all)
Vvi_VIT_18s0001g13590.t01
Vvi_VIT_04s0023g03070.t01
0
−1000
0
1000
2000
3000

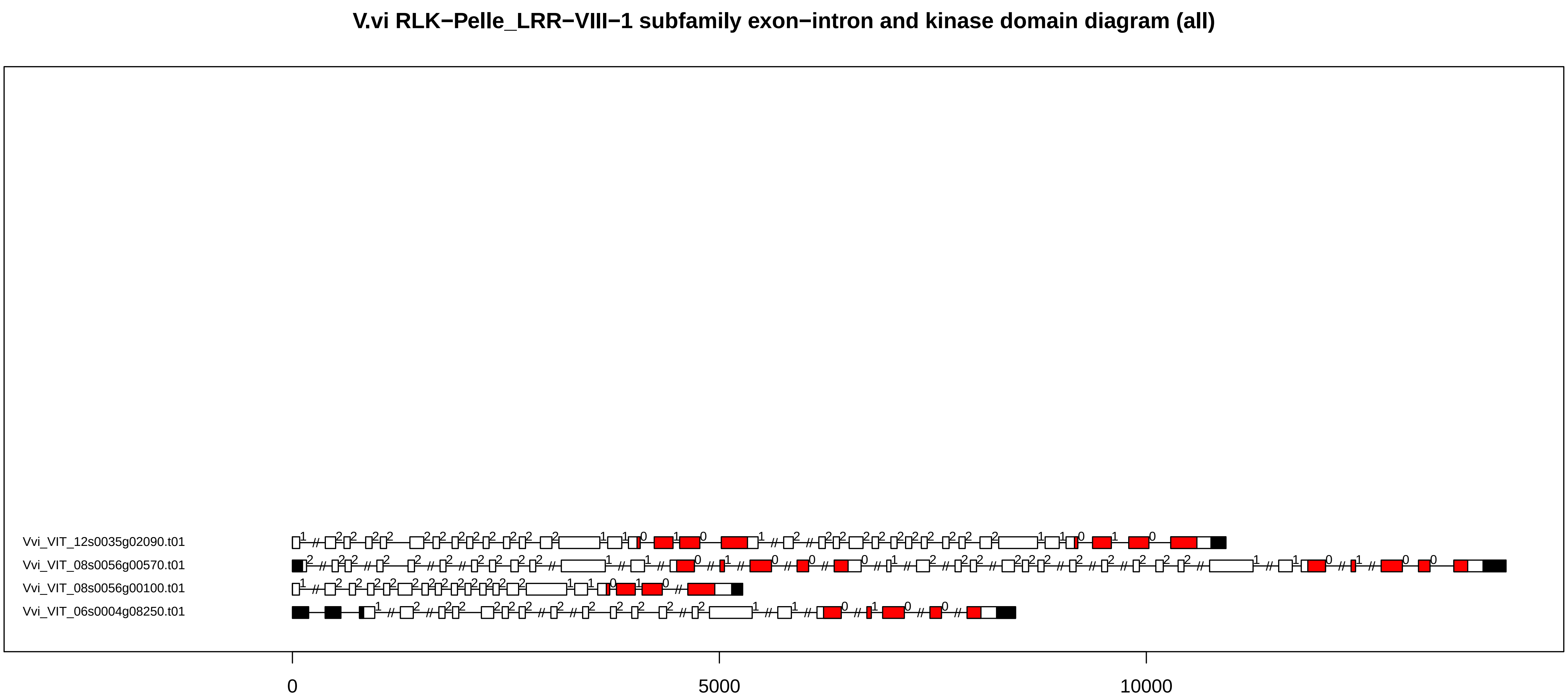

V.vi RLK−Pelle_LRR−VIII−1 subfamily exon−intron and kinase domain diagram (all)
Vvi_VIT_12s0035g02090.t01
Vvi_VIT_08s0056g00570.t01
Vvi_VIT_08s0056g00100.t01
Vvi_VIT_06s0004g08250.t01
0
5000
10000

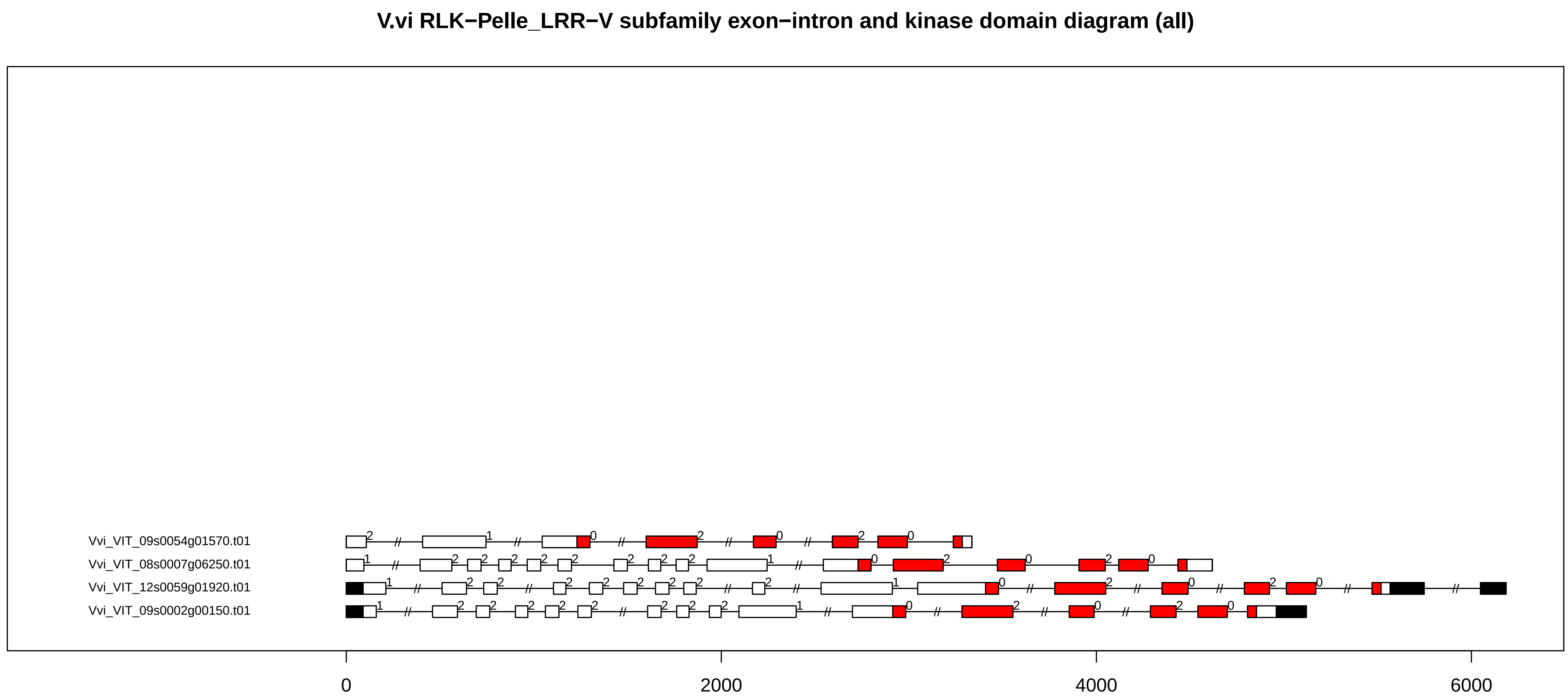
V.vi RLK−Pelle_LRR−V subfamily exon−intron and kinase domain diagram (all)
Vvi_VIT_09s0054g01570.t01
Vvi_VIT_08s0007g06250.t01
Vvi_VIT_12s0059g01920.t01
Vvi_VIT_09s0002g00150.t01
0
2000
4000
6000

V.vi RLK−Pelle_LRR−Xa subfamily exon−intron and kinase domain diagram (all)

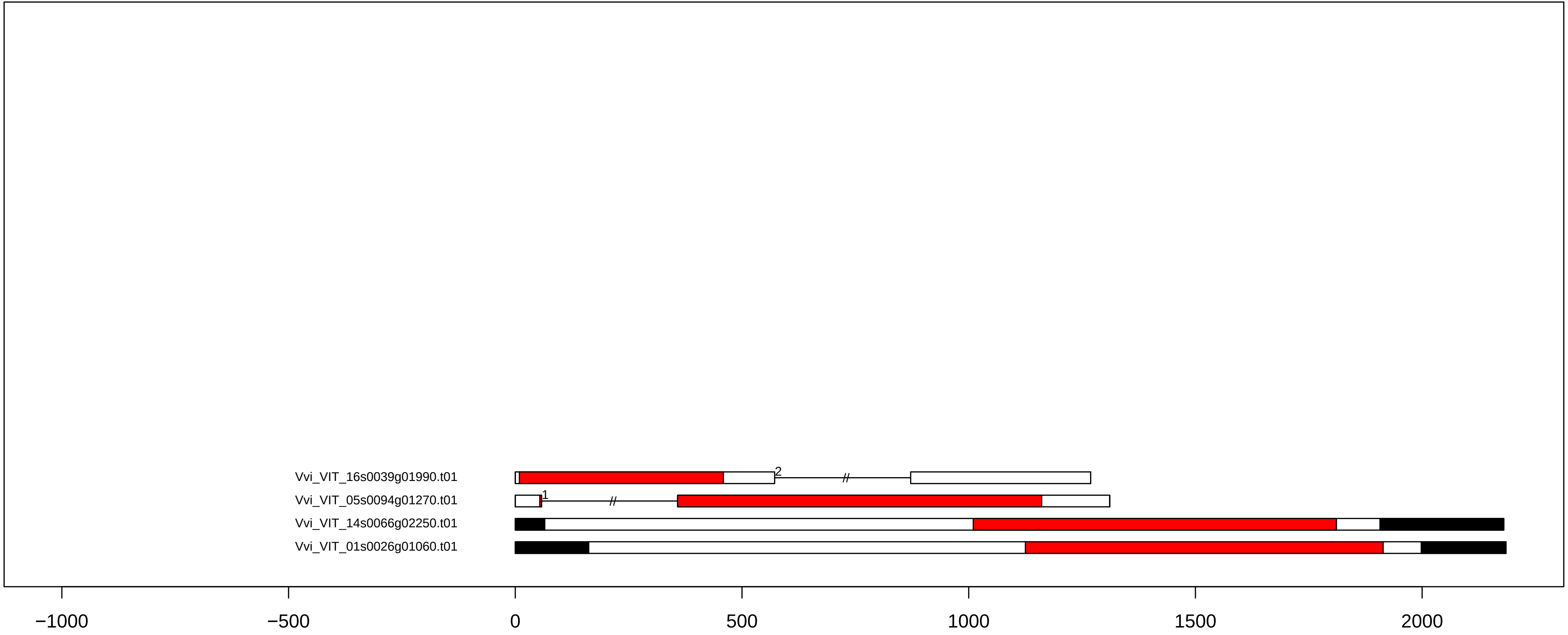

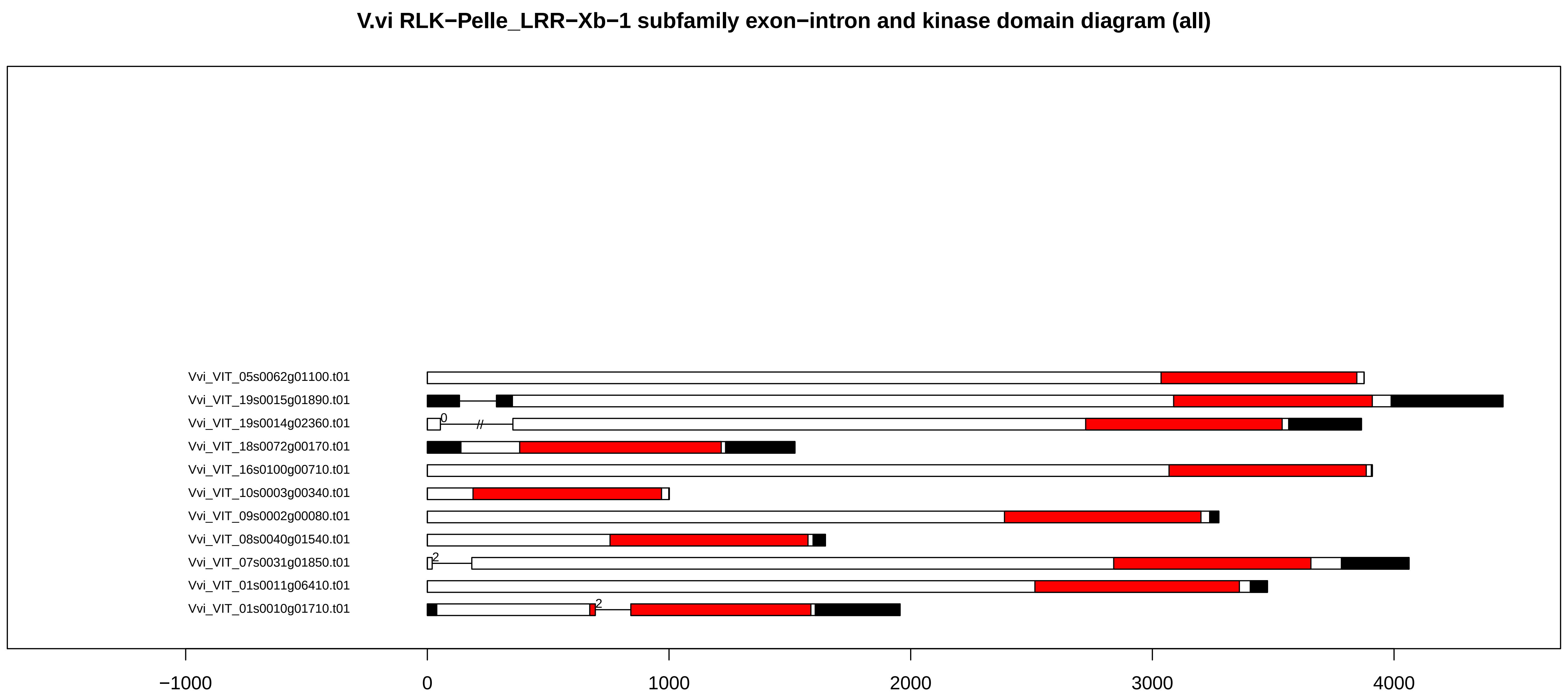
V.vi RLK−Pelle_LRR−Xb−1 subfamily exon−intron and kinase domain diagram (all)
Vvi_VIT_05s0062g01100.t01
Vvi_VIT_19s0015g01890.t01
Vvi_VIT_19s0014g02360.t01
Vvi_VIT_18s0072g00170.t01
Vvi_VIT_16s0100g00710.t01
Vvi_VIT_10s0003g00340.t01
Vvi_VIT_09s0002g00080.t01
Vvi_VIT_08s0040g01540.t01
Vvi_VIT_07s0031g01850.t01
Vvi_VIT_01s0011g06410.t01
Vvi_VIT_01s0010g01710.t01
0
2
2
−1000
0
1000
2000
3000
4000

V.vi RLK−Pelle_LRR−Xb−2 subfamily exon−intron and kinase domain diagram (all)

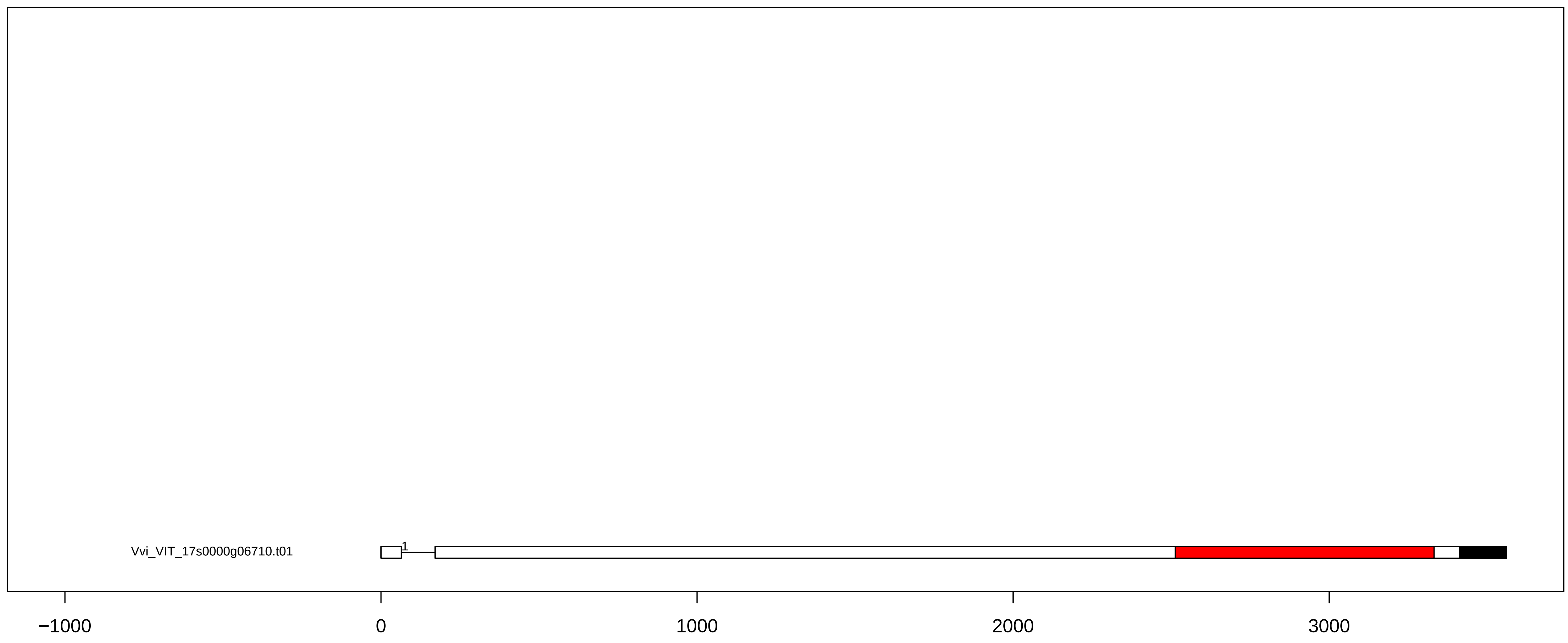

V.vi RLK−Pelle_LRR−XI−1 subfamily exon−intron and kinase domain diagram (part 1)

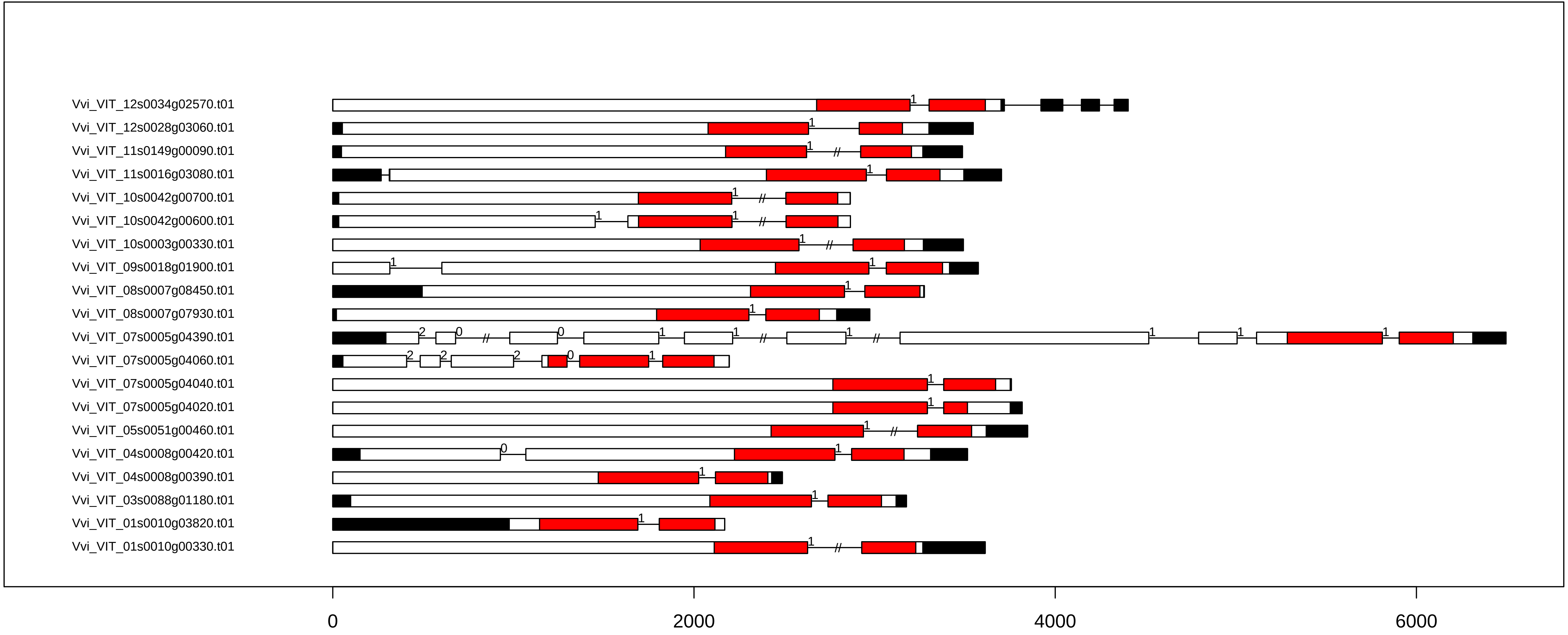

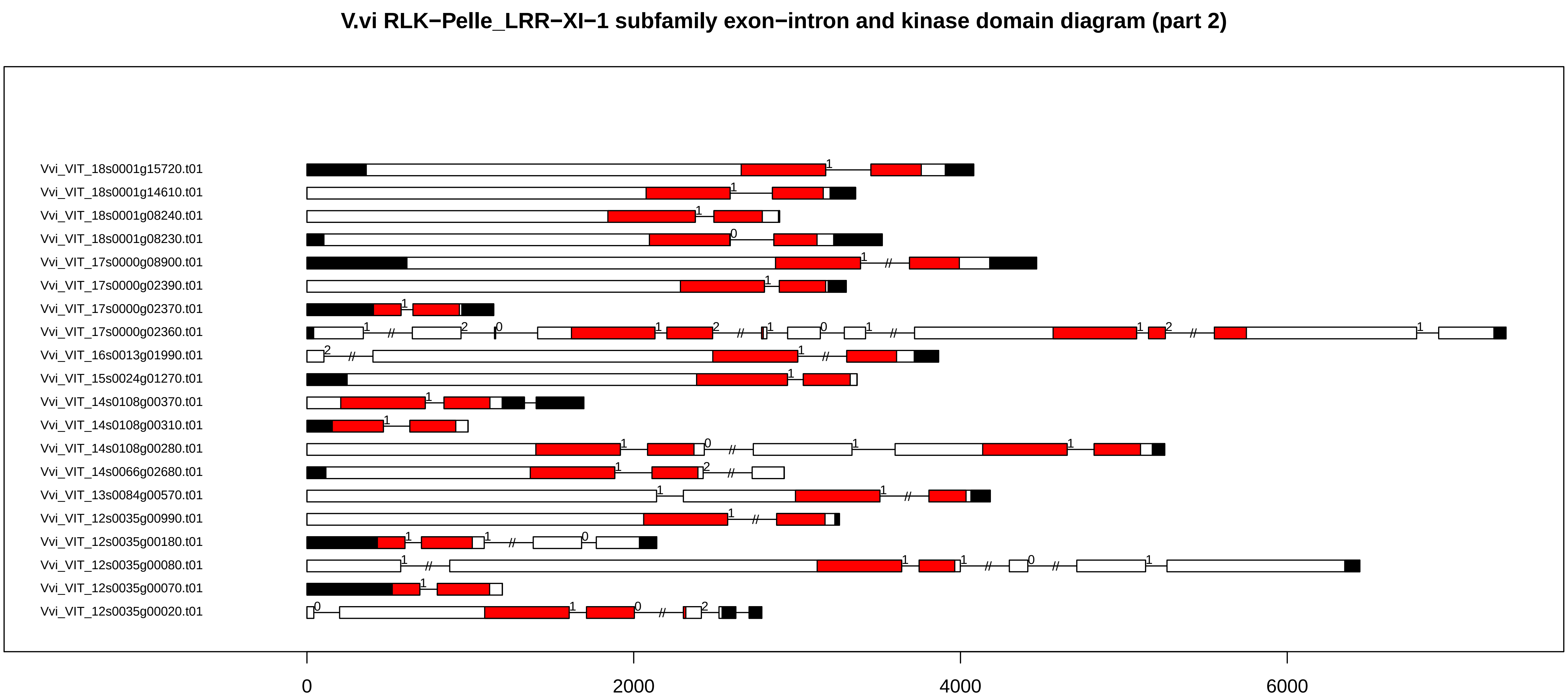
V.vi RLK−Pelle_LRR−XI−1 subfamily exon−intron and kinase domain diagram (part 2)
Vvi_VIT_18s0001g15720.t01
Vvi_VIT_18s0001g14610.t01
Vvi_VIT_18s0001g08240.t01
Vvi_VIT_18s0001g08230.t01
Vvi_VIT_17s0000g08900.t01
Vvi_VIT_17s0000g02390.t01
Vvi_VIT_17s0000g02370.t01
Vvi_VIT_17s0000g02360.t01
Vvi_VIT_16s0013g01990.t01
Vvi_VIT_15s0024g01270.t01
Vvi_VIT_14s0108g00370.t01
Vvi_VIT_14s0108g00310.t01
Vvi_VIT_14s0108g00280.t01
Vvi_VIT_14s0066g02680.t01
Vvi_VIT_13s0084g00570.t01
Vvi_VIT_12s0035g00990.t01
Vvi_VIT_12s0035g00180.t01
Vvi_VIT_12s0035g00080.t01
Vvi_VIT_12s0035g00070.t01
Vvi_VIT_12s0035g00020.t01
0
2000
4000
6000

V.vi RLK−Pelle_LRR−XI−1 subfamily exon−intron and kinase domain diagram (part 3)

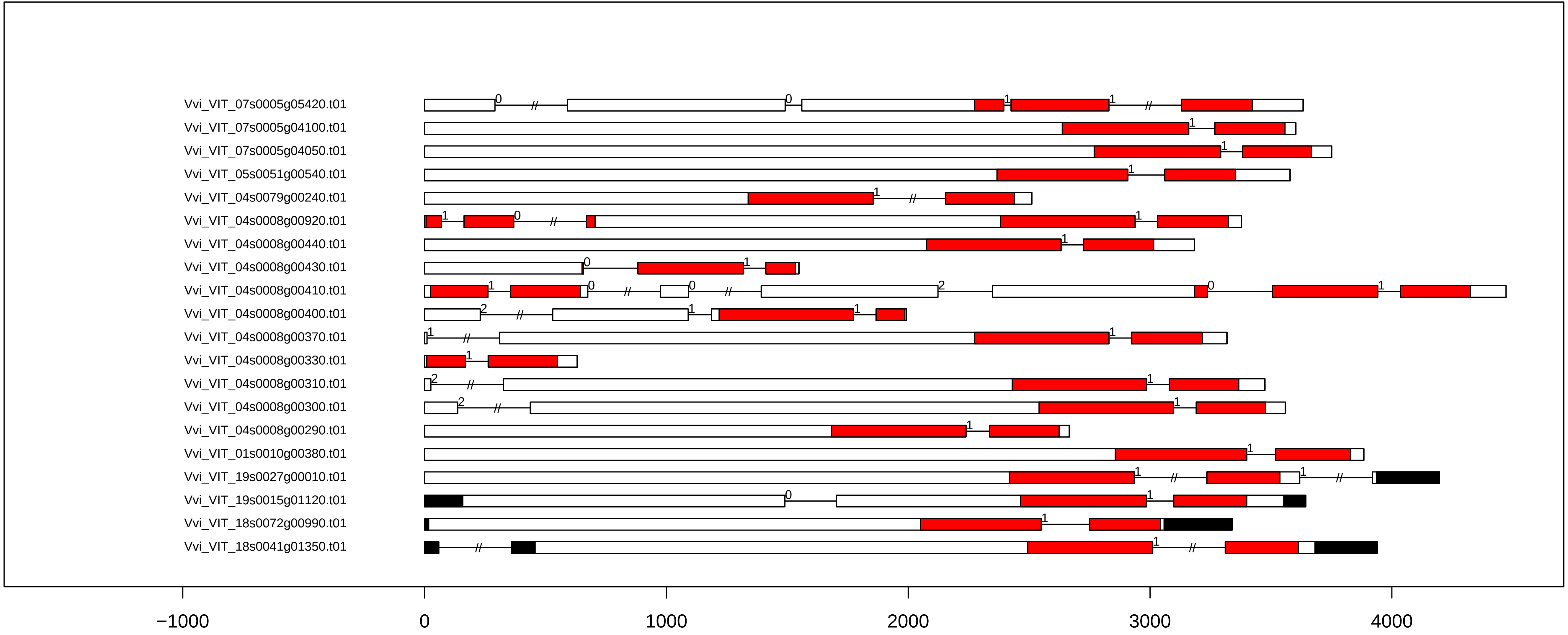

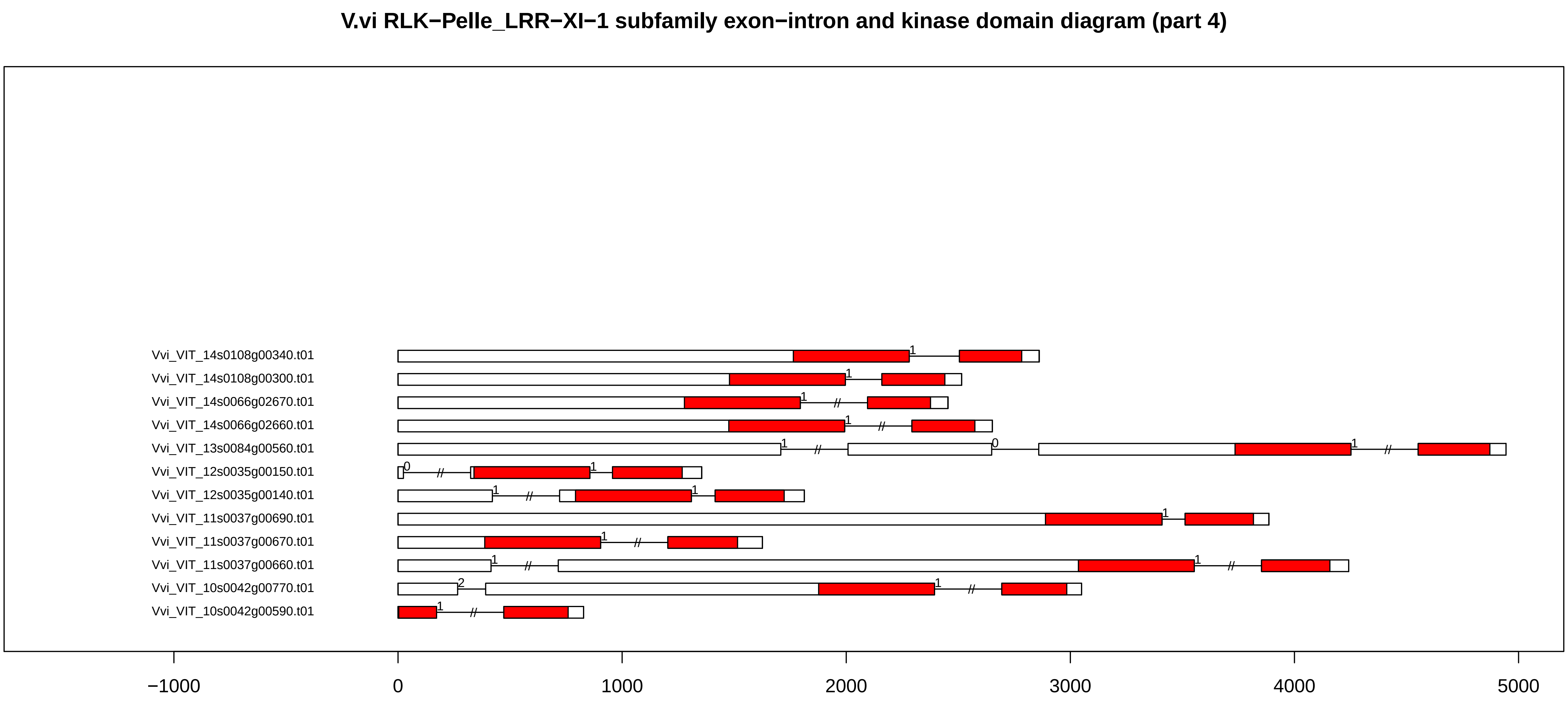
V.vi RLK−Pelle_LRR−XI−1 subfamily exon−intron and kinase domain diagram (part 4)
Vvi_VIT_14s0108g00340.t01
Vvi_VIT_14s0108g00300.t01
Vvi_VIT_14s0066g02670.t01
Vvi_VIT_14s0066g02660.t01
Vvi_VIT_13s0084g00560.t01
Vvi_VIT_12s0035g00150.t01
Vvi_VIT_12s0035g00140.t01
Vvi_VIT_11s0037g00690.t01
Vvi_VIT_11s0037g00670.t01
Vvi_VIT_11s0037g00660.t01
Vvi_VIT_10s0042g00770.t01
Vvi_VIT_10s0042g00590.t01
−1000
0
1000
2000
3000
4000
5000

V.vi RLK−Pelle_LRR−XI−2 subfamily exon−intron and kinase domain diagram (all)

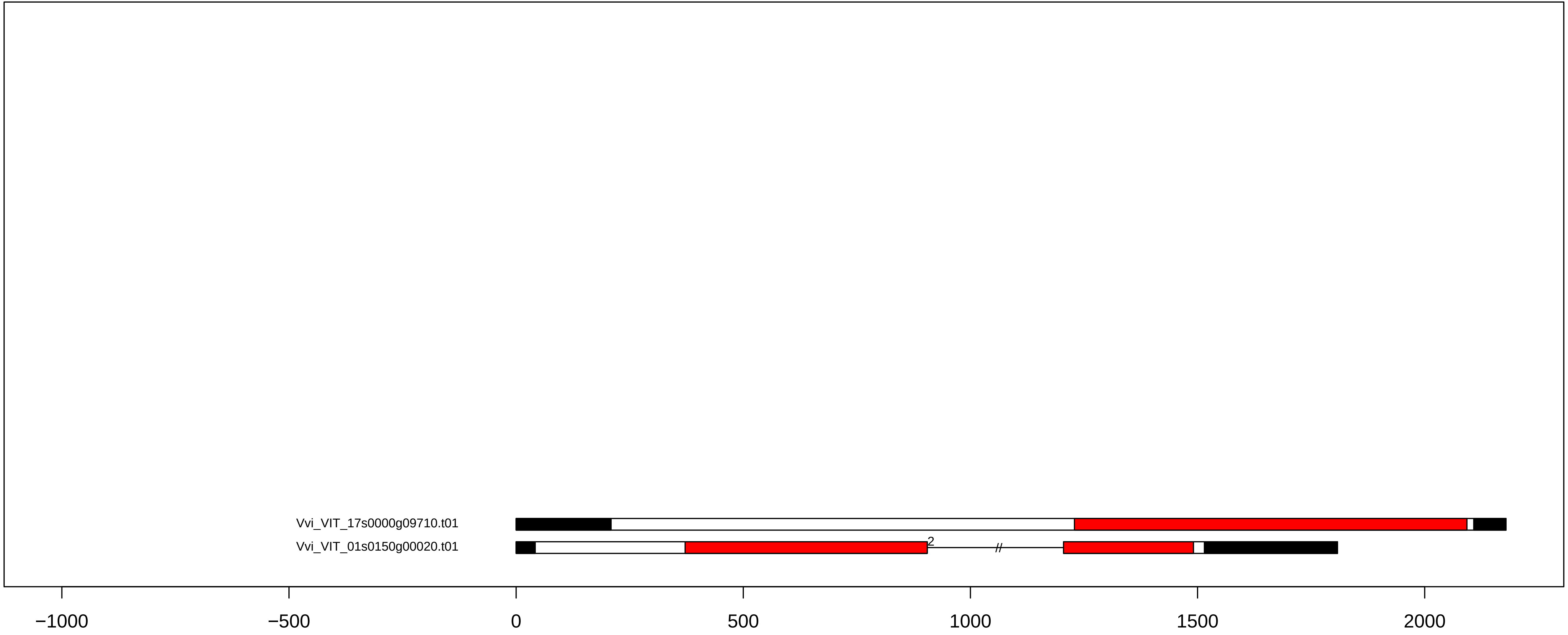

V.vi RLK−Pelle_LRR−XII−1 subfamily exon−intron and kinase domain diagram (part 1)

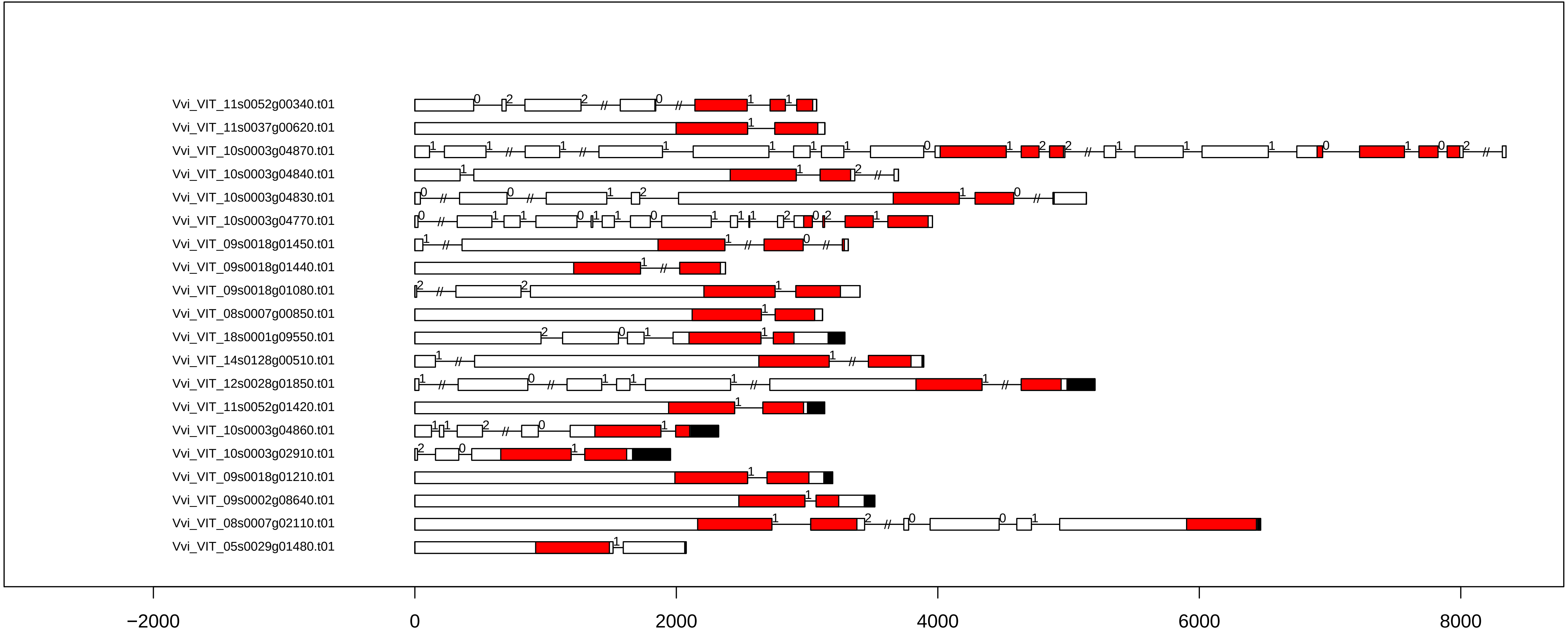

V.vi RLK−Pelle_LRR−XII−1 subfamily exon−intron and kinase domain diagram (part 2)

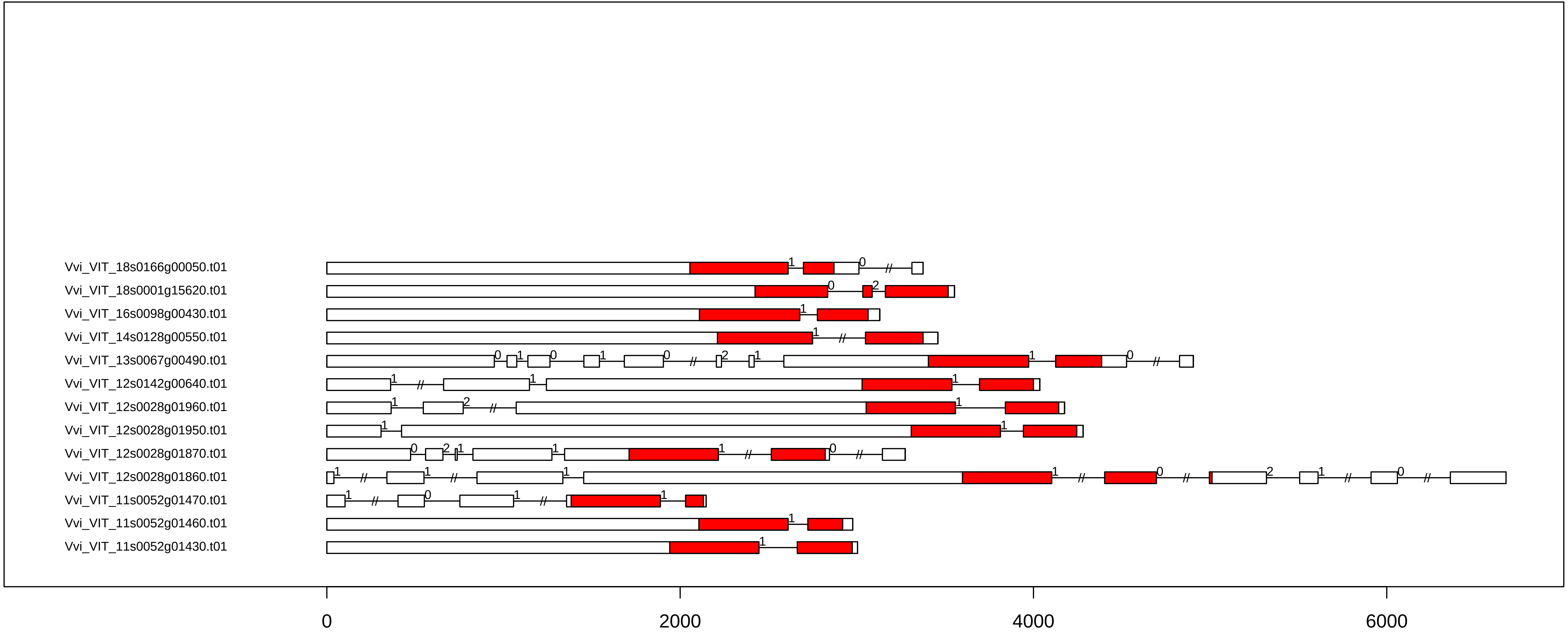

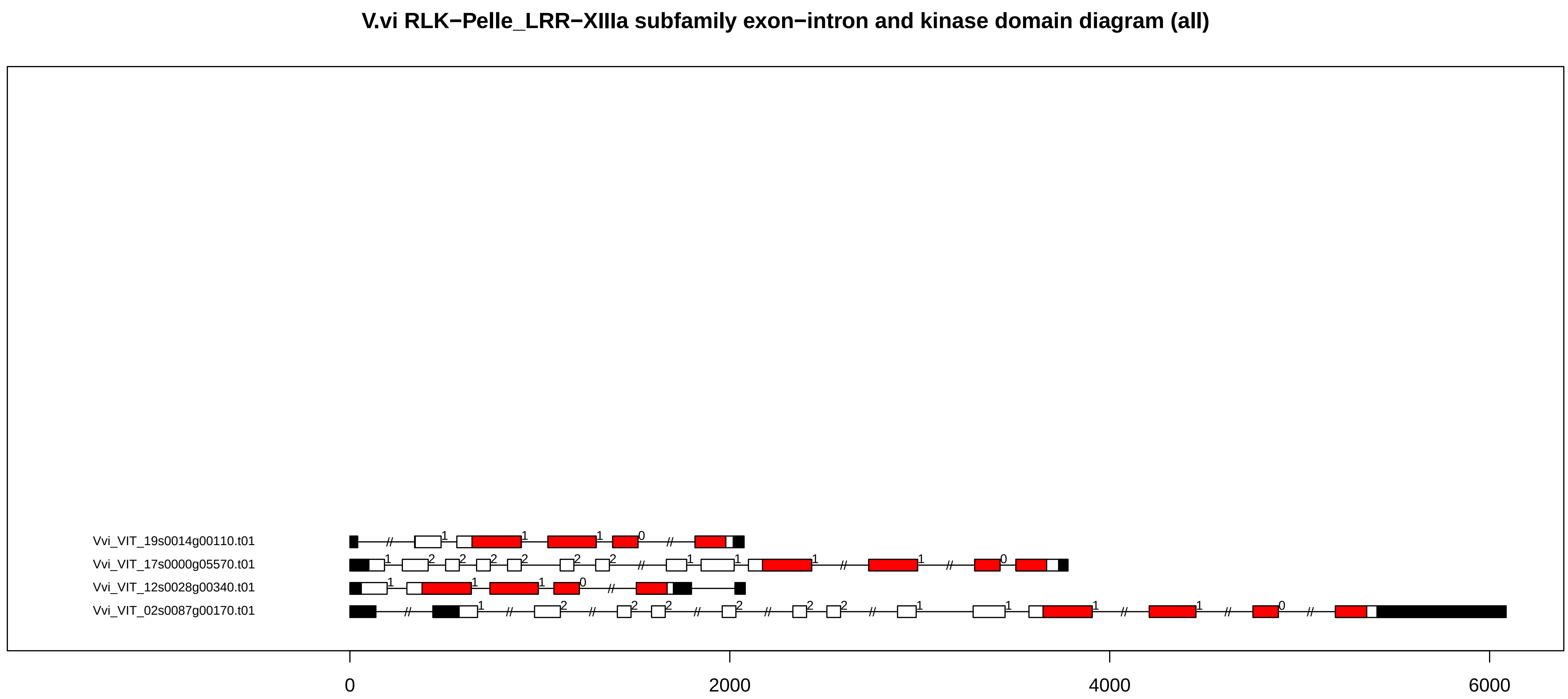
V.vi RLK−Pelle_LRR−XIIIa subfamily exon−intron and kinase domain diagram (all)
Vvi_VIT_19s0014g00110.t01
Vvi_VIT_17s0000g05570.t01
Vvi_VIT_12s0028g00340.t01
Vvi_VIT_02s0087g00170.t01
0
2000
4000
6000

V.vi RLK−Pelle_LRR−XIIIb subfamily exon−intron and kinase domain diagram (all)

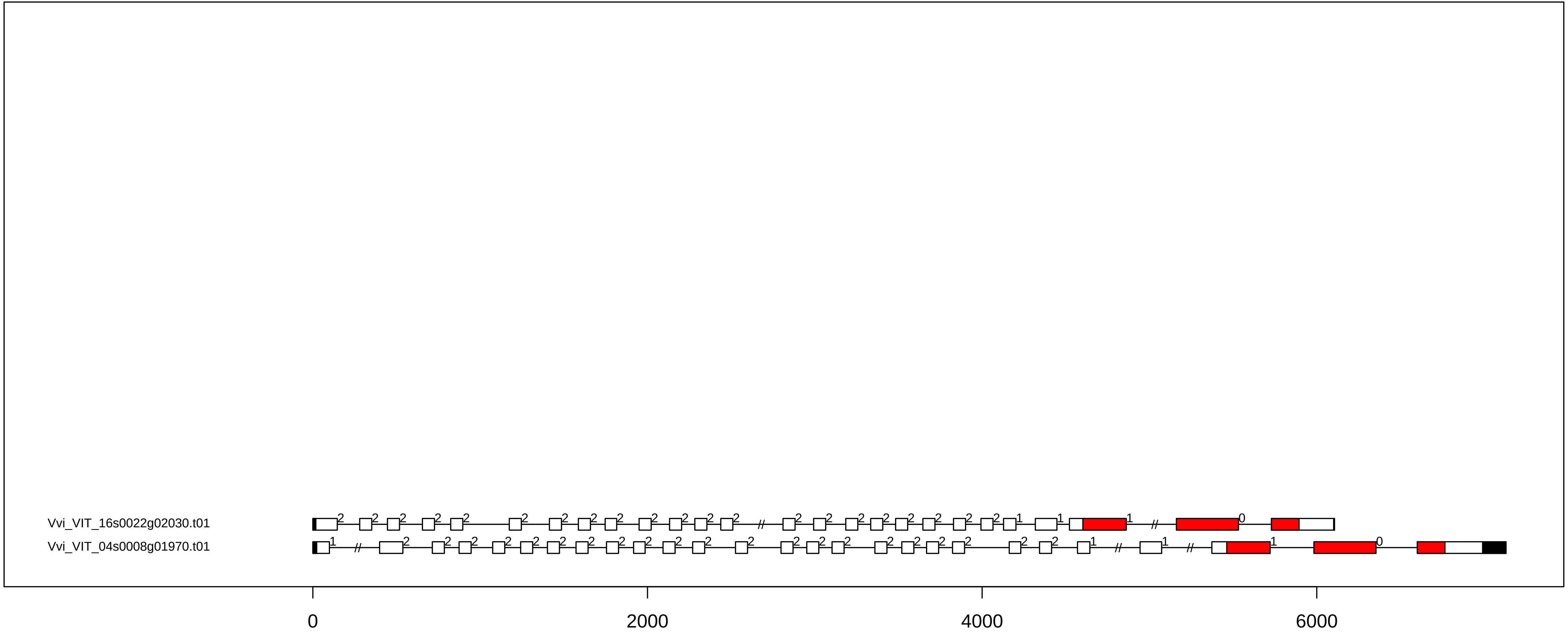

V.vi RLK−Pelle_LRR−XIV subfamily exon−intron and kinase domain diagram (all)

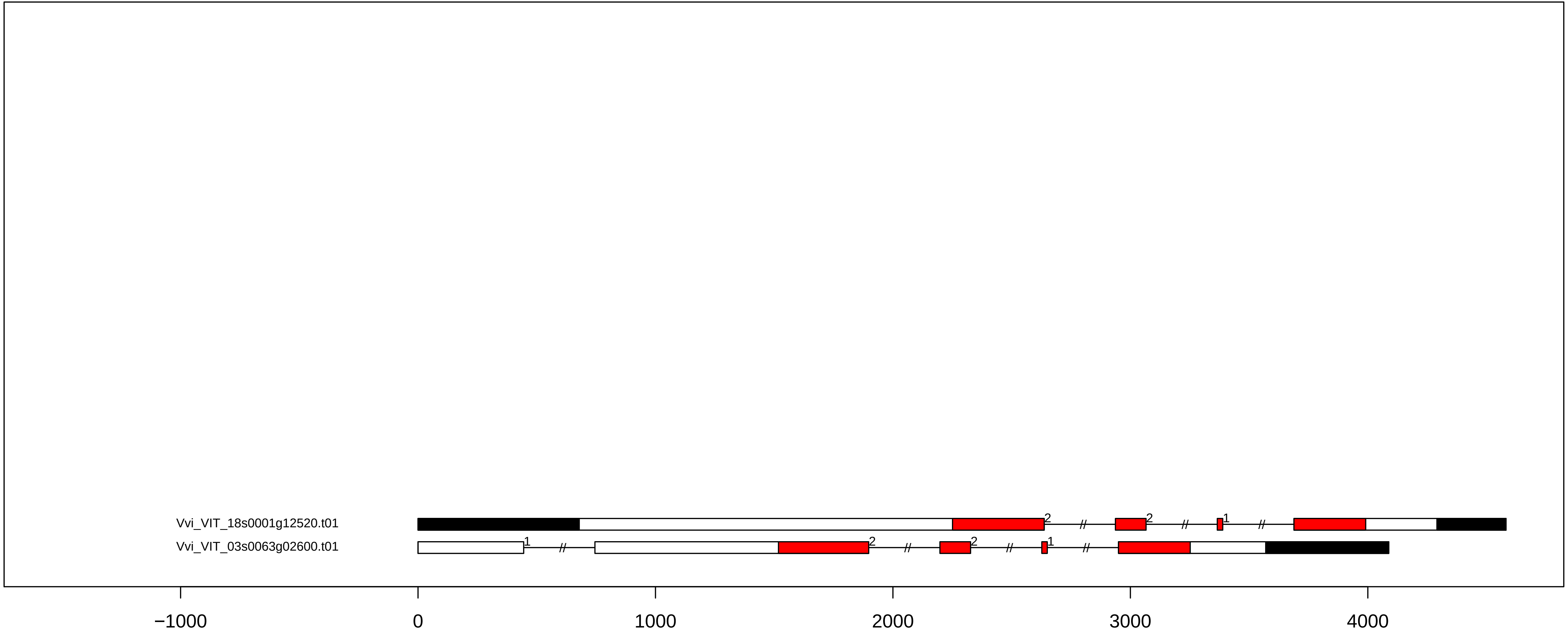

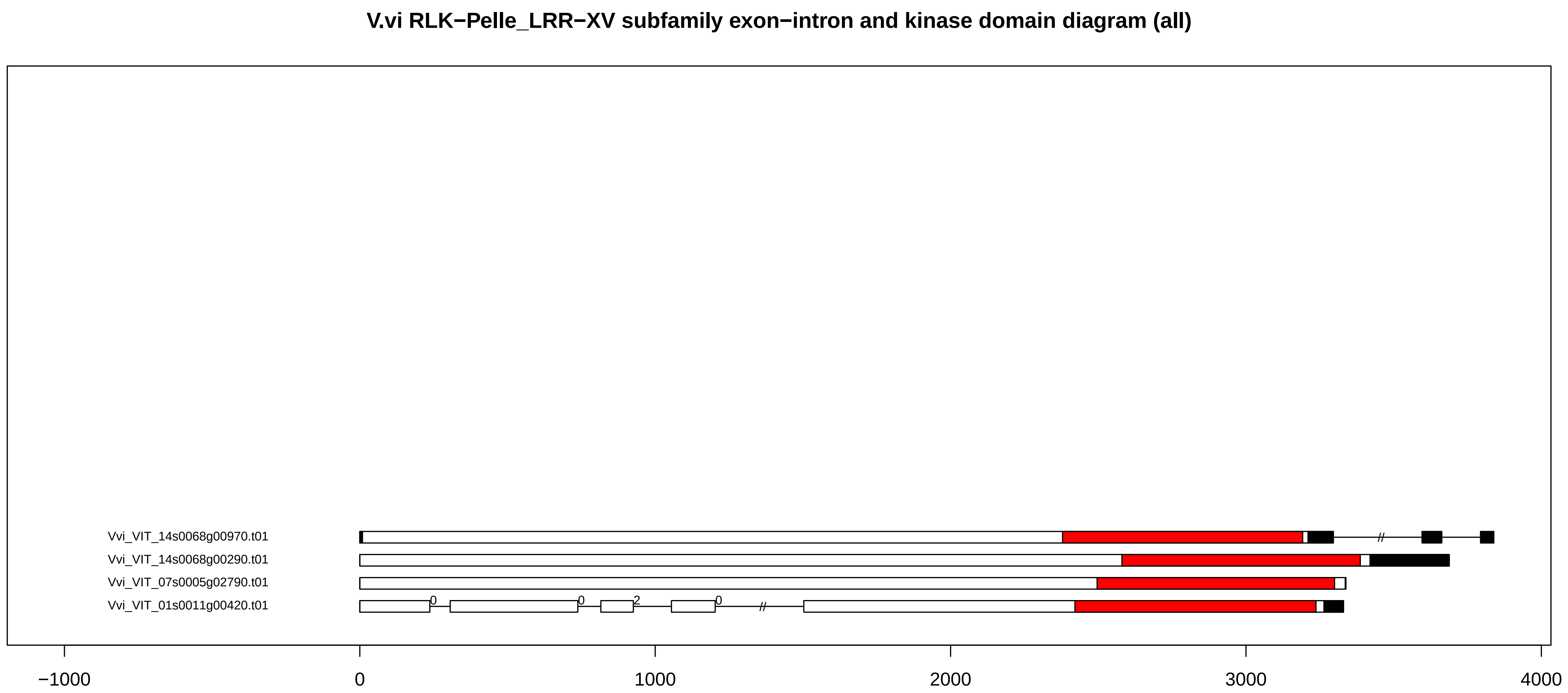

V.vi RLK−Pelle_LRR−XV subfamily exon−intron and kinase domain diagram (all)
Vvi_VIT_14s0068g00970.t01
Vvi_VIT_14s0068g00290.t01
Vvi_VIT_07s0005g02790.t01
Vvi_VIT_01s0011g00420.t01
0
0
2
0
−1000
0
1000
2000
3000
4000

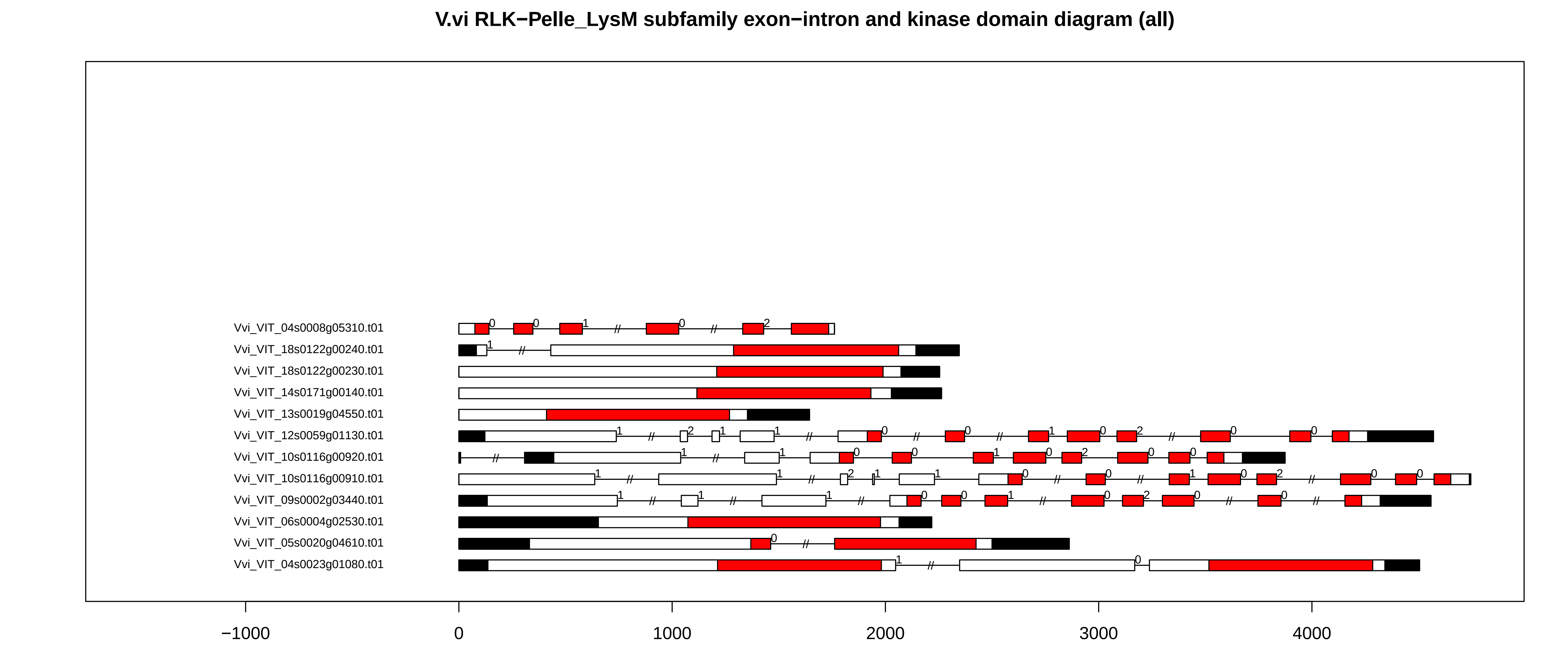

V.vi RLK−Pelle_LysM subfamily exon−intron and kinase domain diagram (all)
Vvi_VIT_04s0008g05310.t01
Vvi_VIT_18s0122g00240.t01
Vvi_VIT_18s0122g00230.t01
Vvi_VIT_14s0171g00140.t01
Vvi_VIT_13s0019g04550.t01
Vvi_VIT_12s0059g01130.t01
Vvi_VIT_10s0116g00920.t01
Vvi_VIT_10s0116g00910.t01
Vvi_VIT_09s0002g03440.t01
Vvi_VIT_06s0004g02530.t01
Vvi_VIT_05s0020g04610.t01
Vvi_VIT_04s0023g01080.t01
−1000
0
1000
2000
3000
4000

V.vi RLK−Pelle_PERK−1 subfamily exon−intron and kinase domain diagram (all)

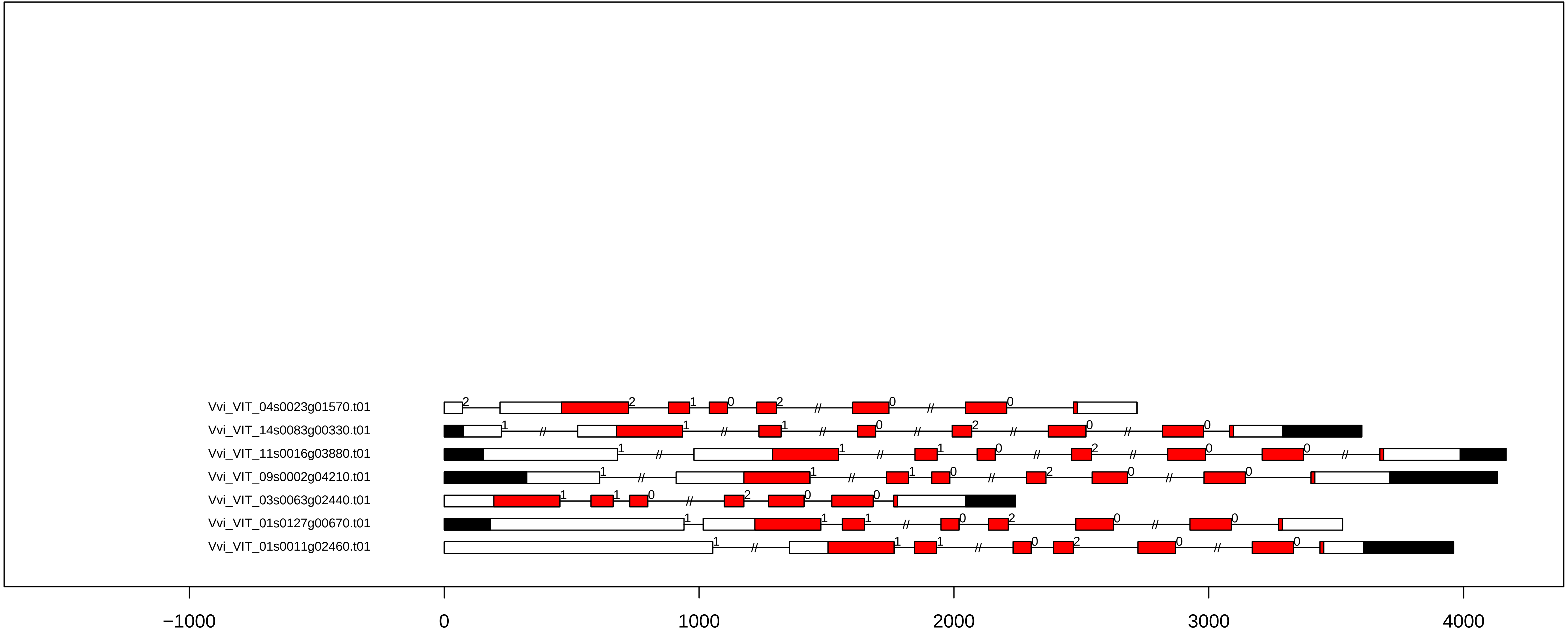

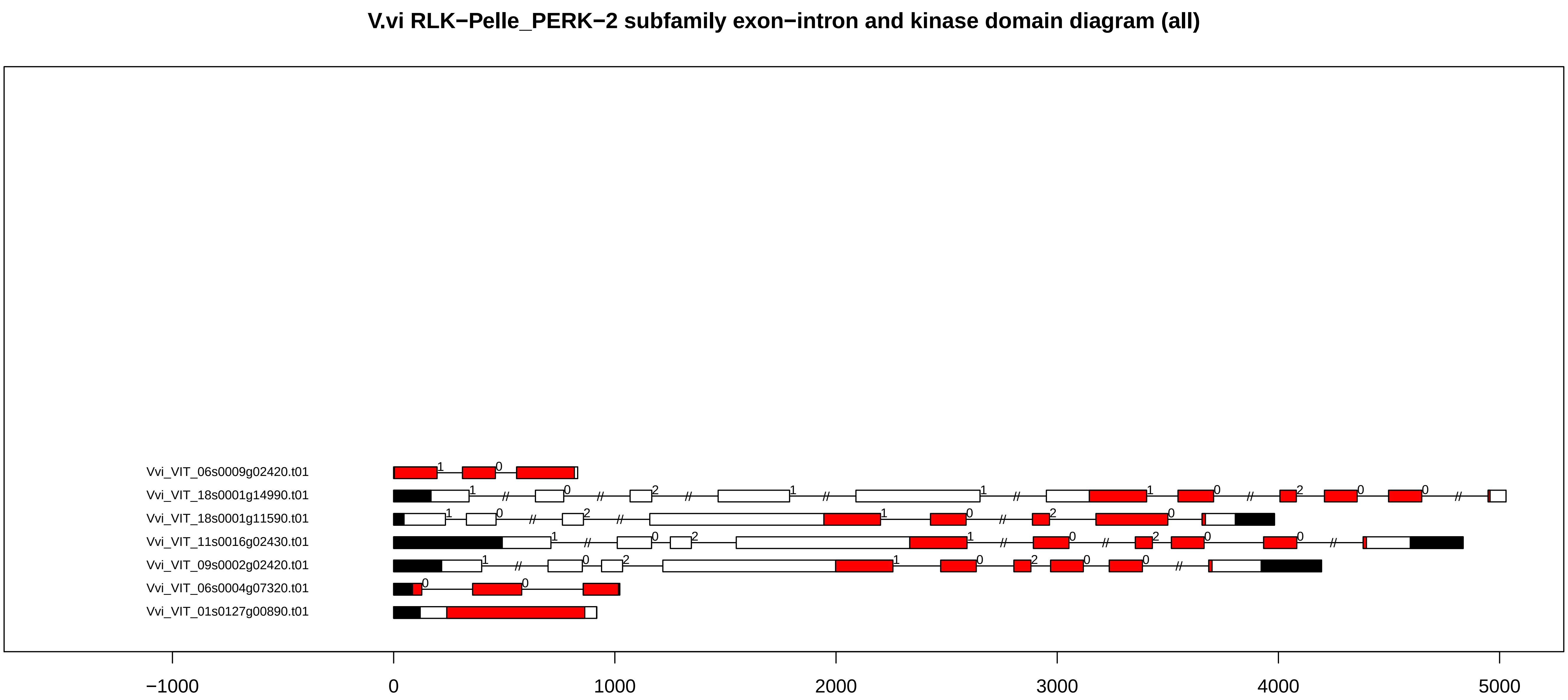

V.vi RLK−Pelle_PERK−2 subfamily exon−intron and kinase domain diagram (all)
Vvi_VIT_06s0009g02420.t01
Vvi_VIT_18s0001g14990.t01
Vvi_VIT_18s0001g11590.t01
Vvi_VIT_11s0016g02430.t01
Vvi_VIT_09s0002g02420.t01
Vvi_VIT_06s0004g07320.t01
Vvi_VIT_01s0127g00890.t01
−1000
0
1000
2000
3000
4000
5000

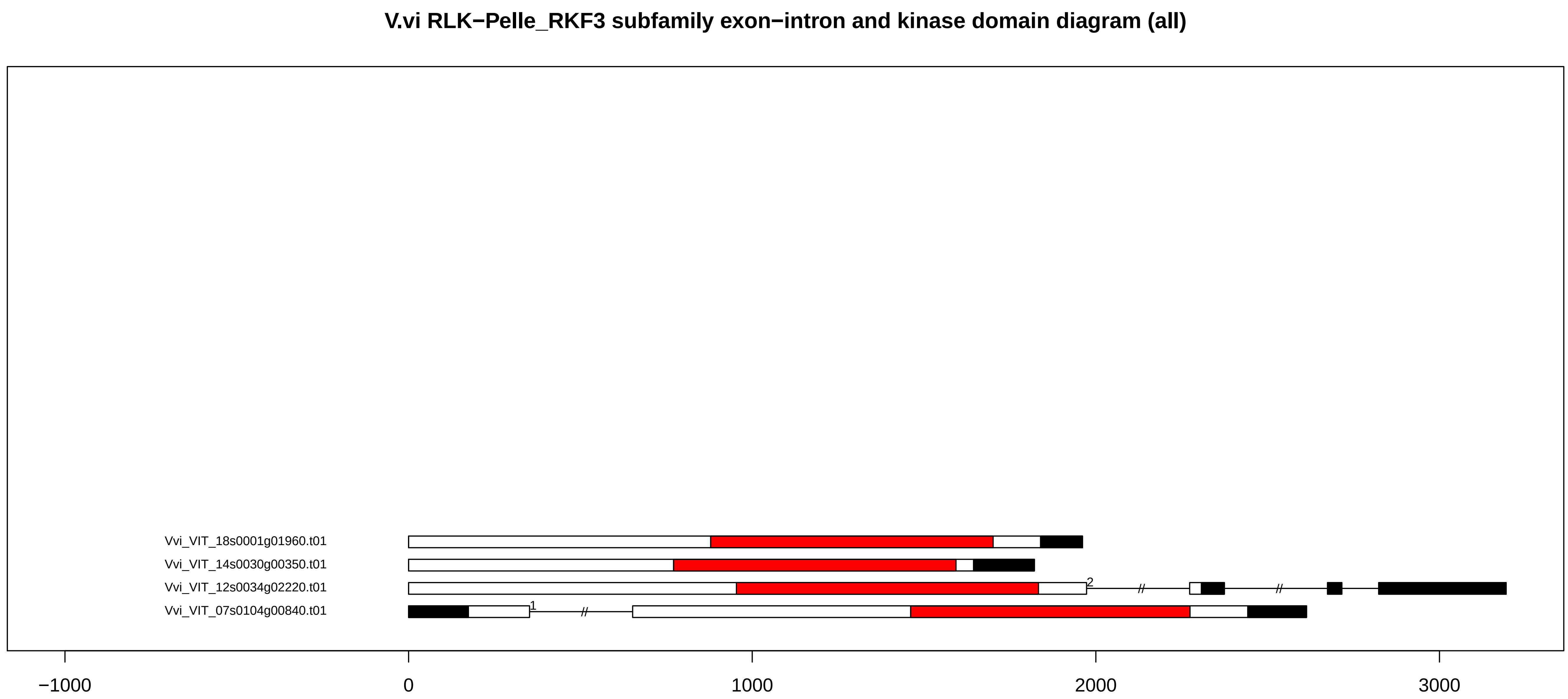
V.vi RLK−Pelle_RKF3 subfamily exon−intron and kinase domain diagram (all)
Vvi_VIT_18s0001g01960.t01
Vvi_VIT_14s0030g00350.t01
Vvi_VIT_12s0034g02220.t01
Vvi_VIT_07s0104g00840.t01
2
1
−1000
0
1000
2000
3000

V.vi RLK−Pelle_RLCK−II subfamily exon−intron and kinase domain diagram (all)

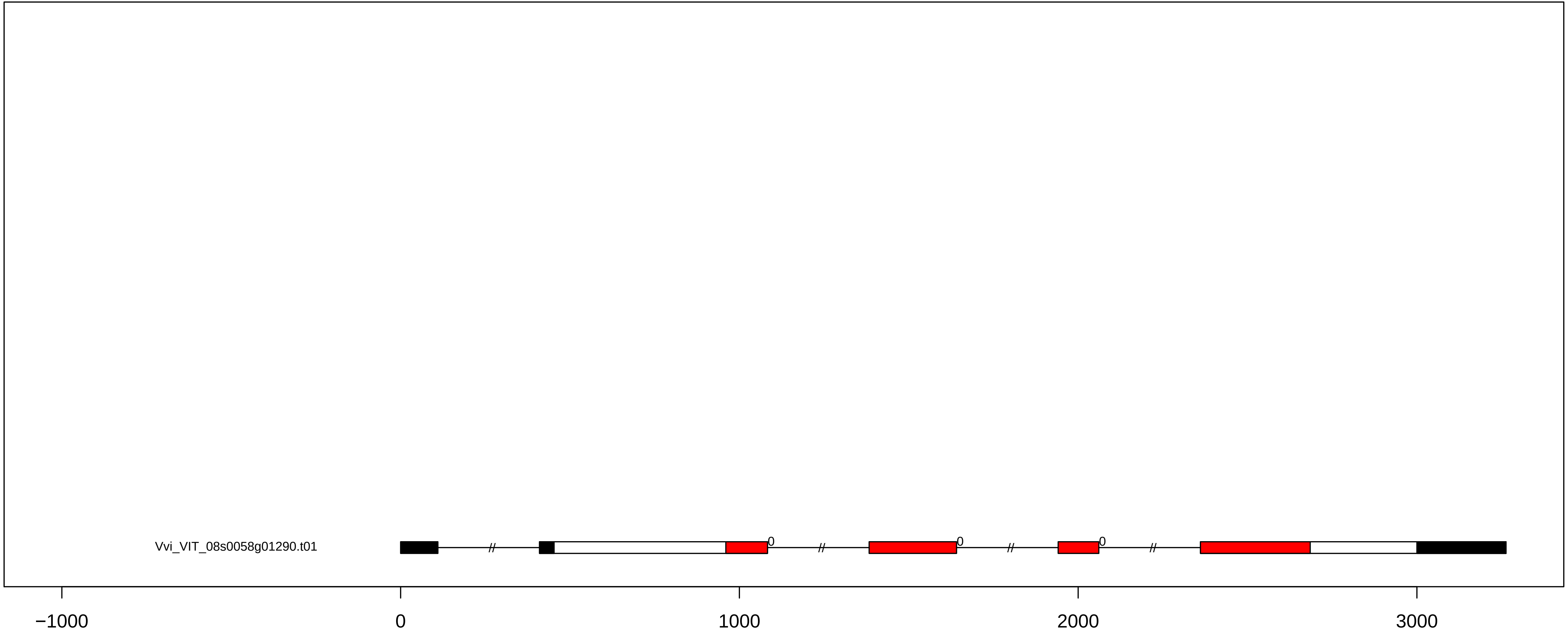

V.vi RLK−Pelle_RLCK−IV subfamily exon−intron and kinase domain diagram (all)

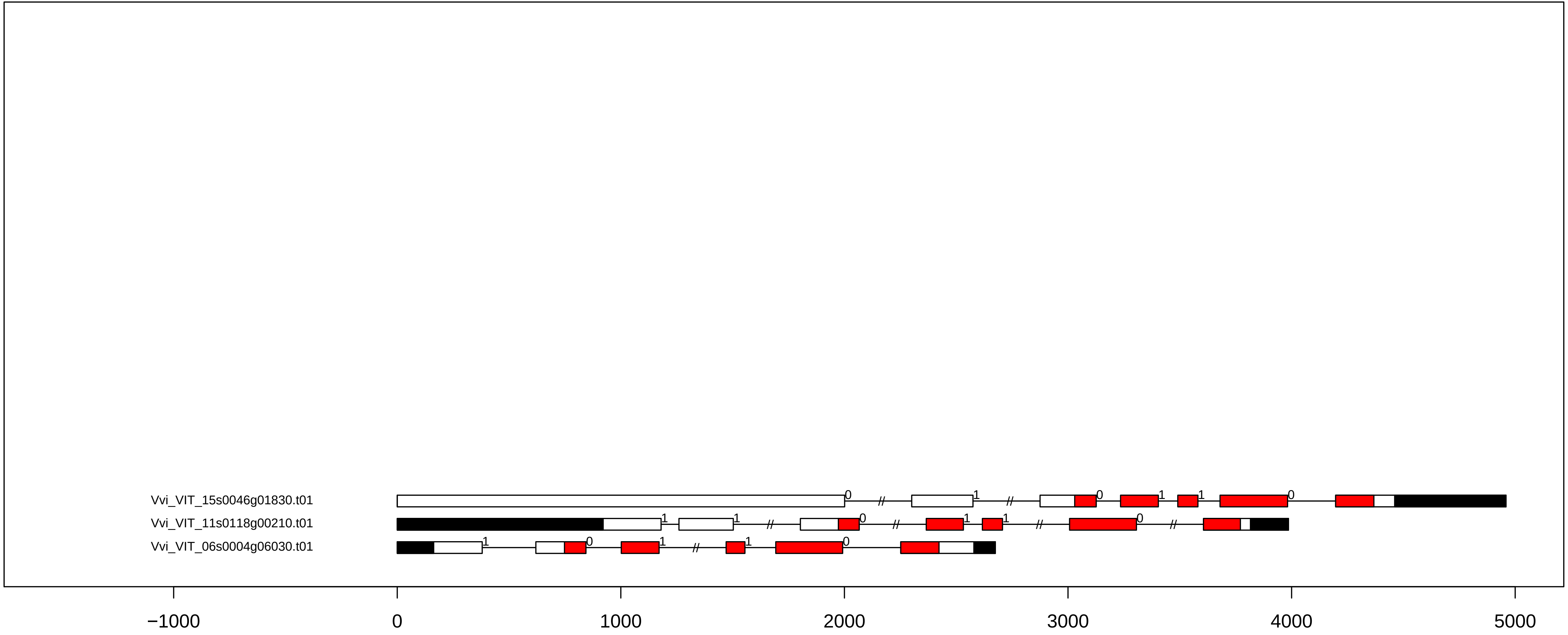

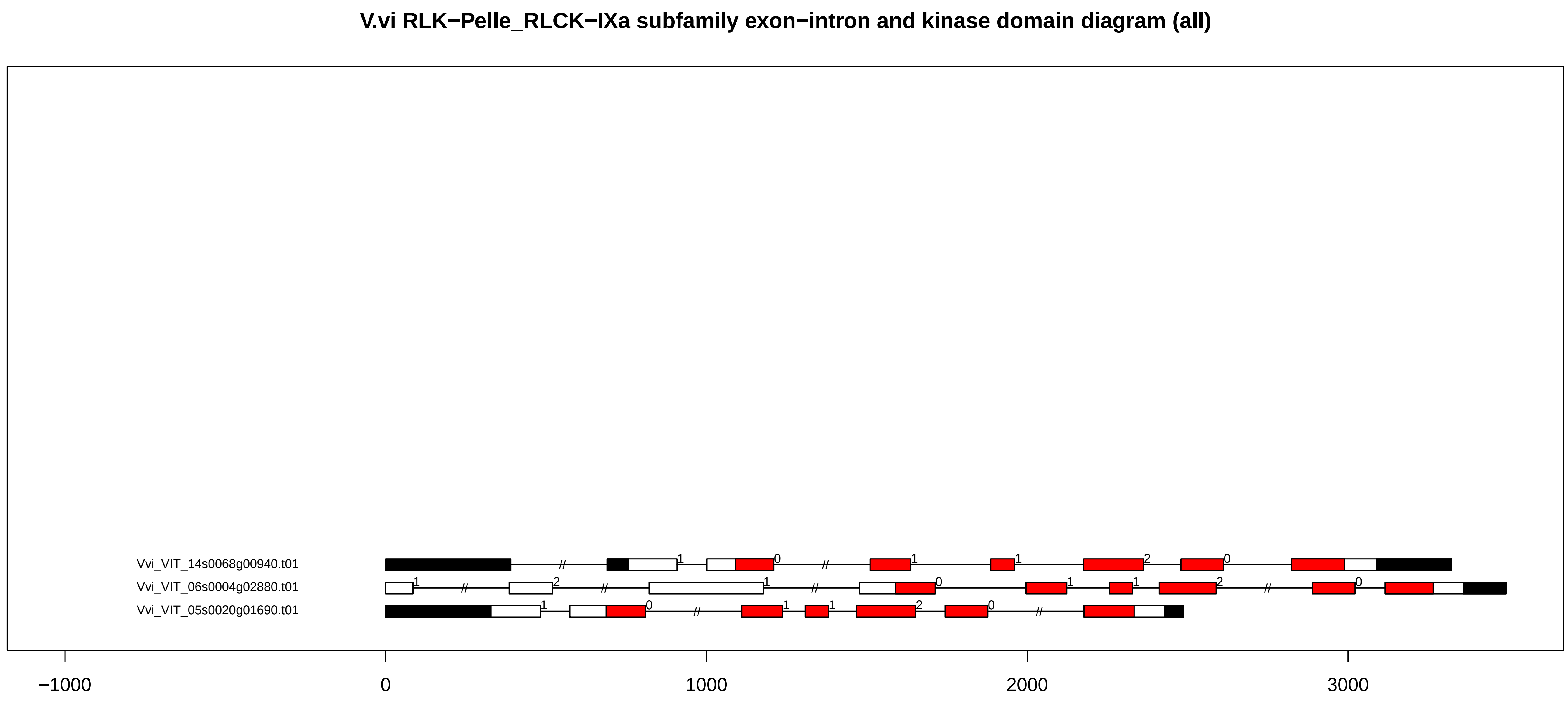
V.vi RLK−Pelle_RLCK−IXa subfamily exon−intron and kinase domain diagram (all)
Vvi_VIT_14s0068g00940.t01
Vvi_VIT_06s0004g02880.t01
Vvi_VIT_05s0020g01690.t01
−1000
0
1000
2000
3000

V.vi RLK−Pelle_RLCK−IXb subfamily exon−intron and kinase domain diagram (all)

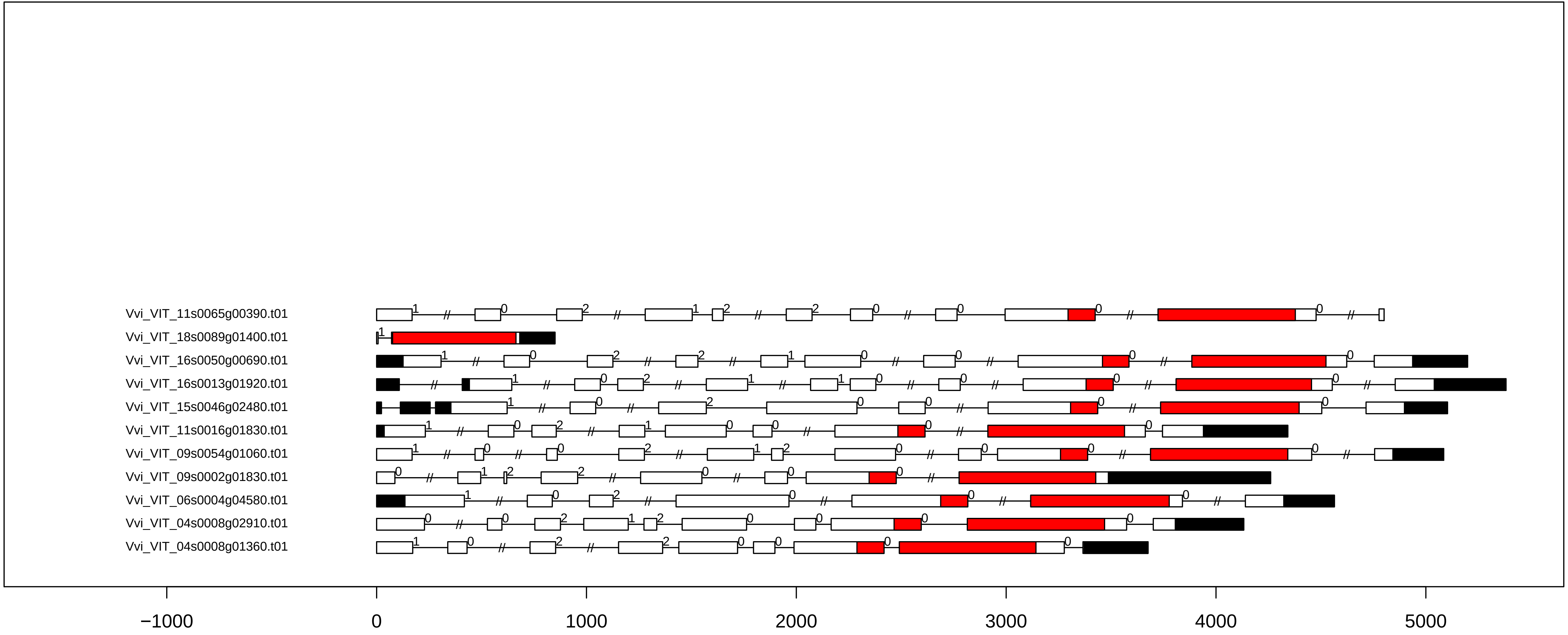

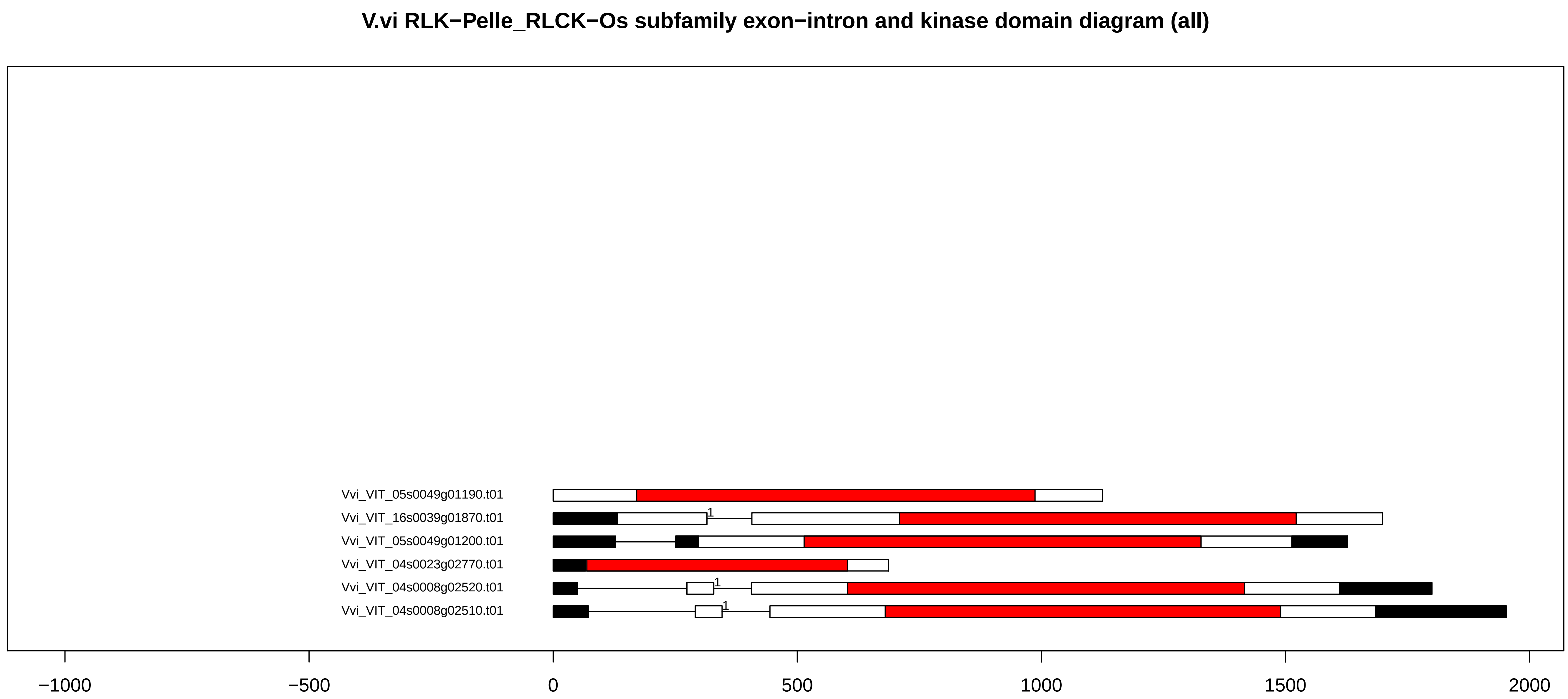
V.vi RLK−Pelle_RLCK−Os subfamily exon−intron and kinase domain diagram (all)
Vvi_VIT_05s0049g01190.t01
Vvi_VIT_16s0039g01870.t01
Vvi_VIT_05s0049g01200.t01
Vvi_VIT_04s0023g02770.t01
Vvi_VIT_04s0008g02520.t01
Vvi_VIT_04s0008g02510.t01
1
1
1
−1000
−500
0
500
1000
1500
2000

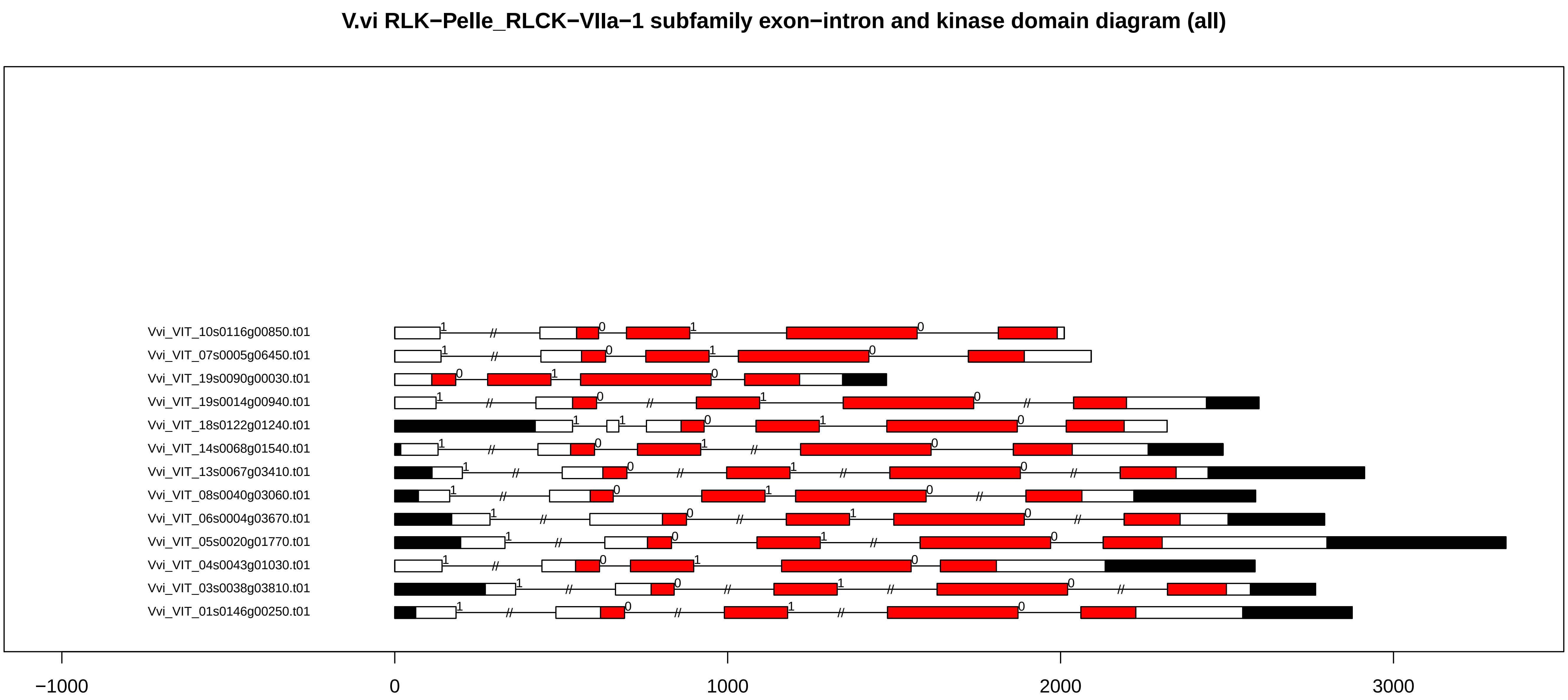

V.vi RLK−Pelle_RLCK−VIIa−1 subfamily exon−intron and kinase domain diagram (all)
Vvi_VIT_10s0116g00850.t01
Vvi_VIT_07s0005g06450.t01
Vvi_VIT_19s0090g00030.t01
Vvi_VIT_19s0014g00940.t01
Vvi_VIT_18s0122g01240.t01
Vvi_VIT_14s0068g01540.t01
Vvi_VIT_13s0067g03410.t01
Vvi_VIT_08s0040g03060.t01
Vvi_VIT_06s0004g03670.t01
Vvi_VIT_05s0020g01770.t01
Vvi_VIT_04s0043g01030.t01
Vvi_VIT_03s0038g03810.t01
Vvi_VIT_01s0146g00250.t01
−1000
0
1000
2000
3000

V.vi RLK−Pelle_RLCK−VIIa−2 subfamily exon−intron and kinase domain diagram (part 1)

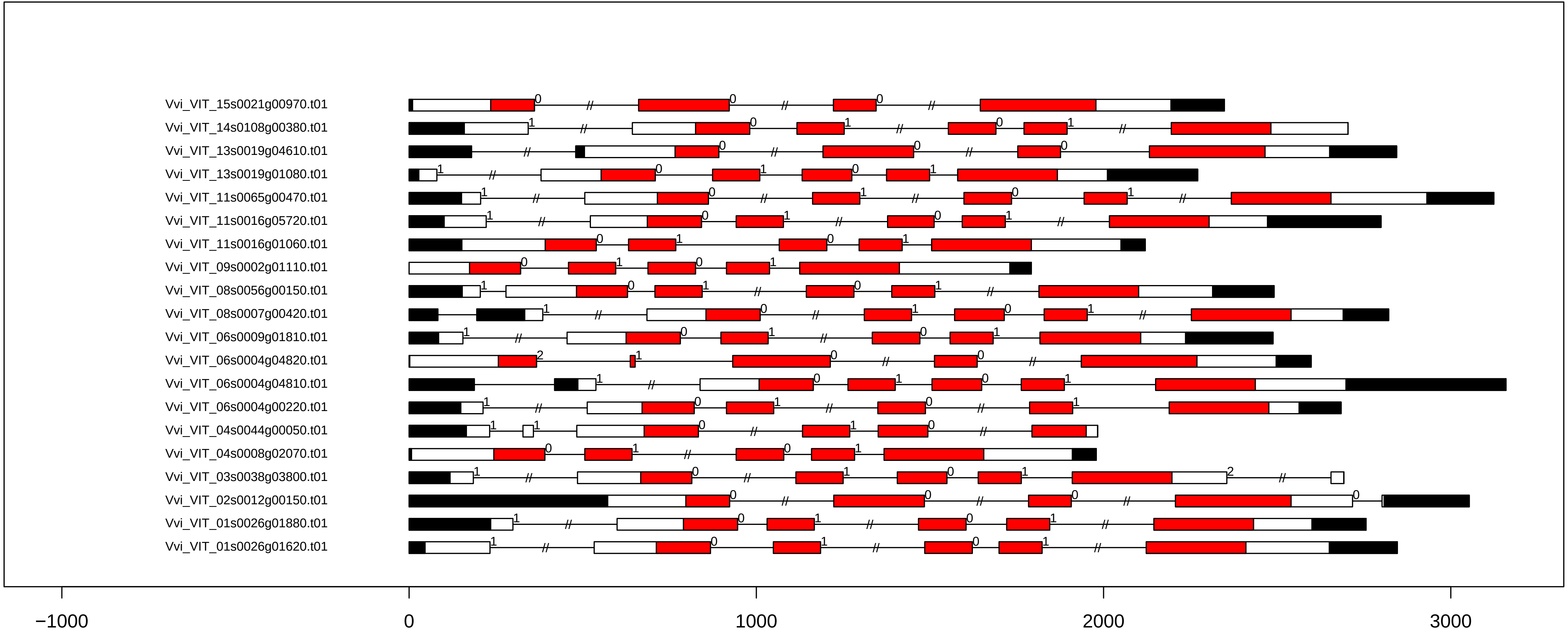

V.vi RLK−Pelle_RLCK−VIIa−2 subfamily exon−intron and kinase domain diagram (part 2)

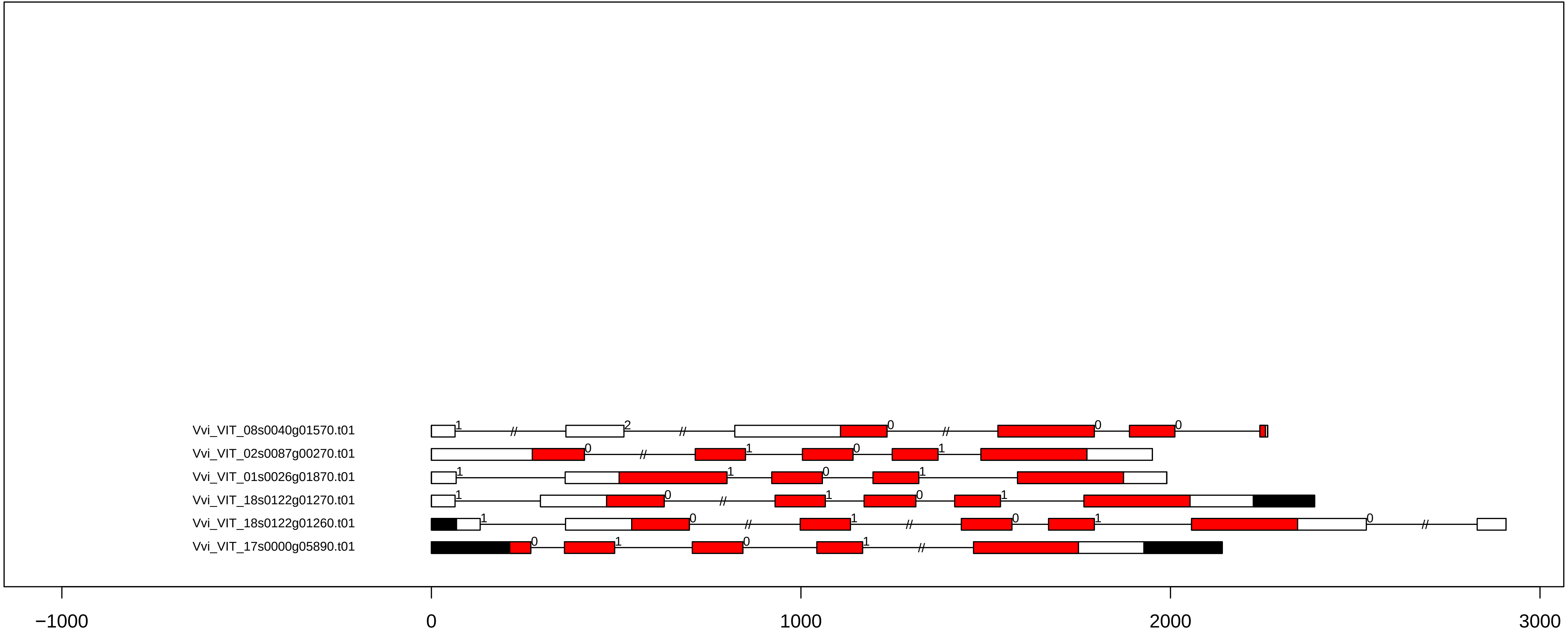

V.vi RLK−Pelle_RLCK−VIIb subfamily exon−intron and kinase domain diagram (all)

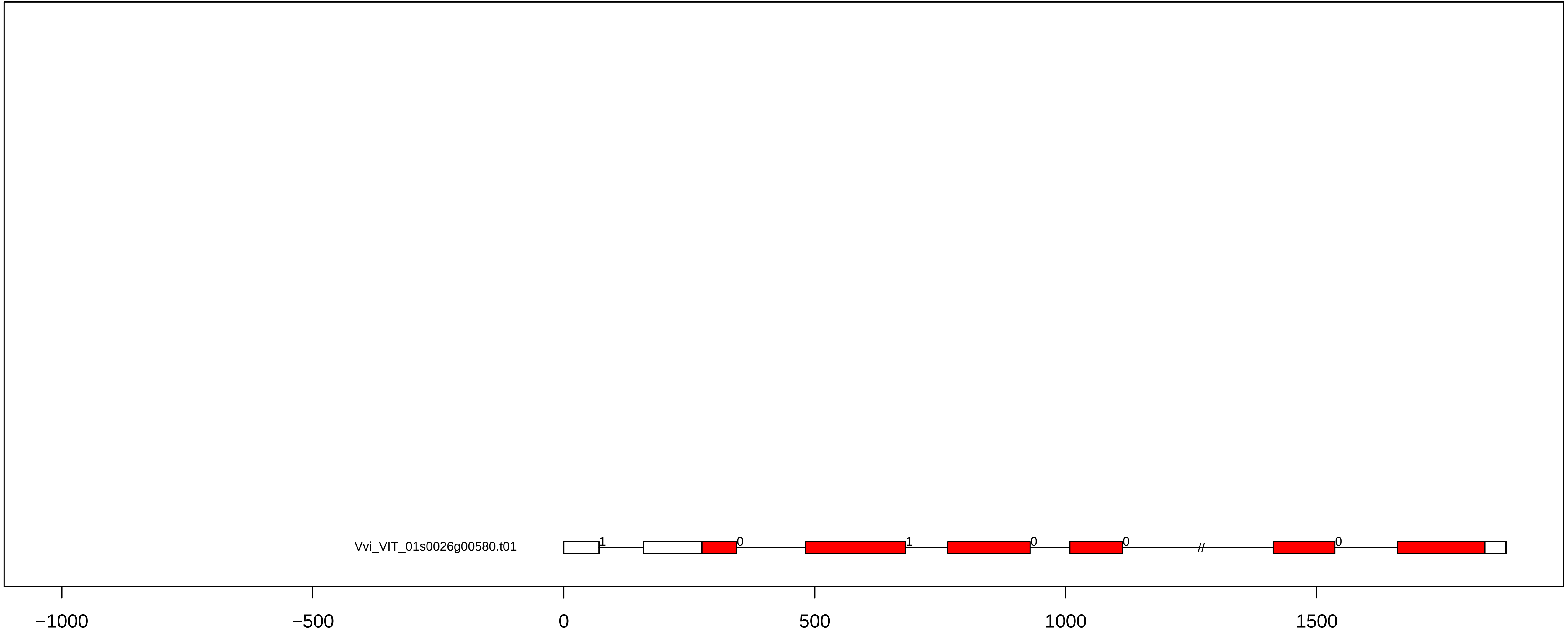

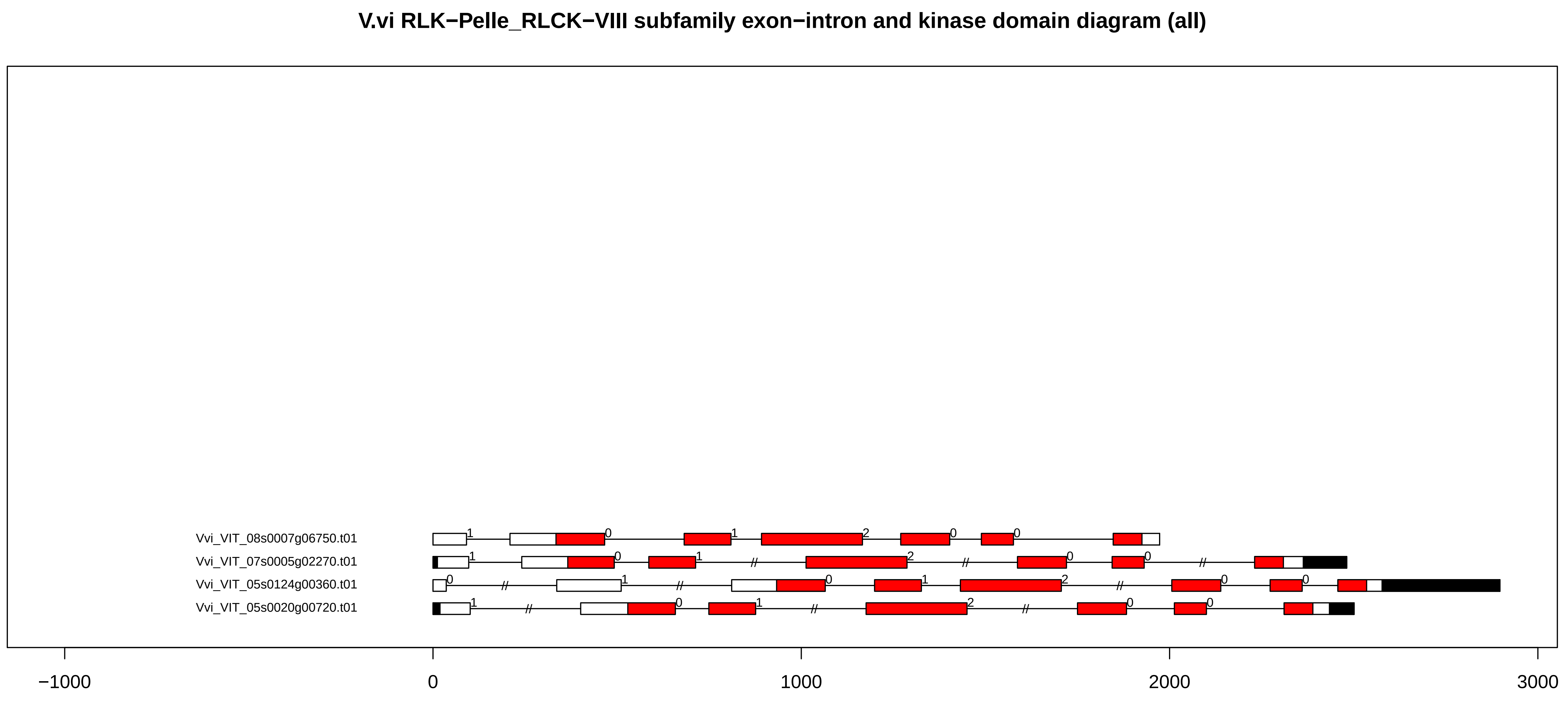

V.vi RLK−Pelle_RLCK−VIII subfamily exon−intron and kinase domain diagram (all)
Vvi_VIT_08s0007g06750.t01
Vvi_VIT_07s0005g02270.t01
Vvi_VIT_05s0124g00360.t01
Vvi_VIT_05s0020g00720.t01
−1000
0
1000
2000
3000

V.vi RLK−Pelle_RLCK−VI subfamily exon−intron and kinase domain diagram (all)

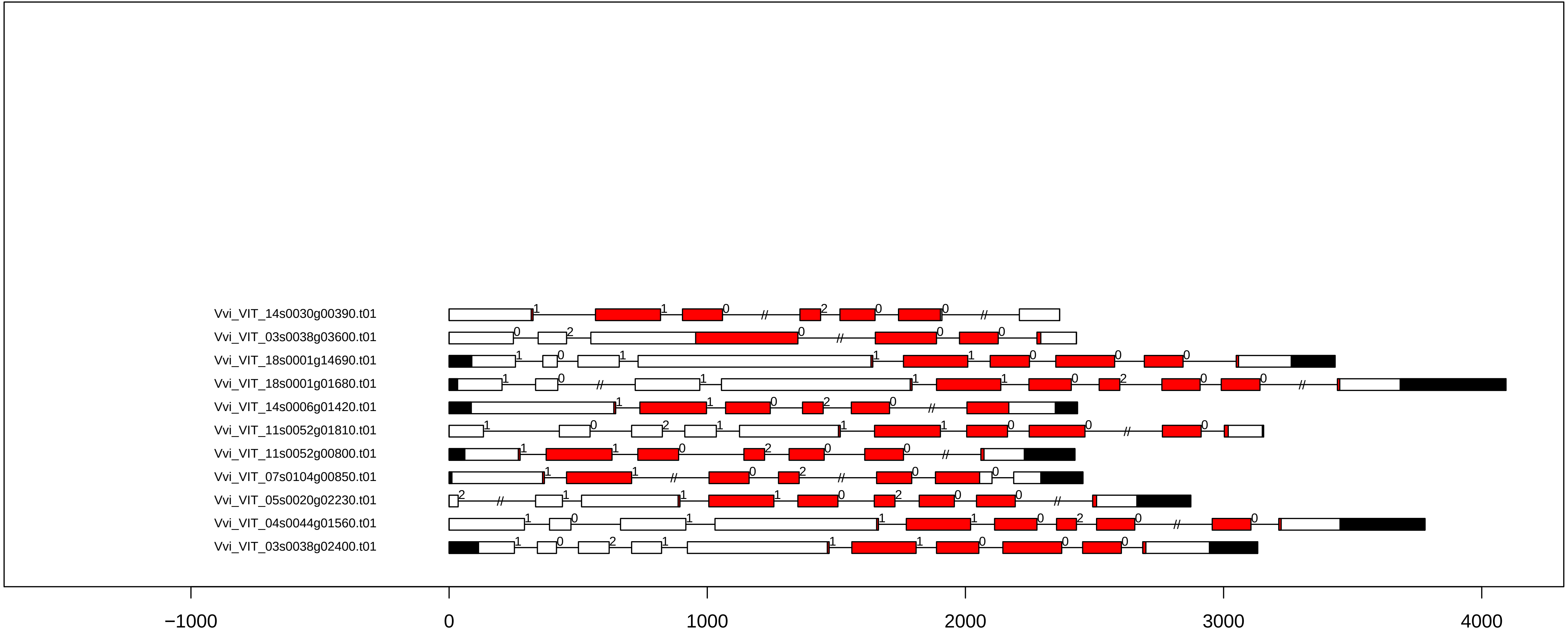

V.vi RLK−Pelle_RLCK−V subfamily exon−intron and kinase domain diagram (all)

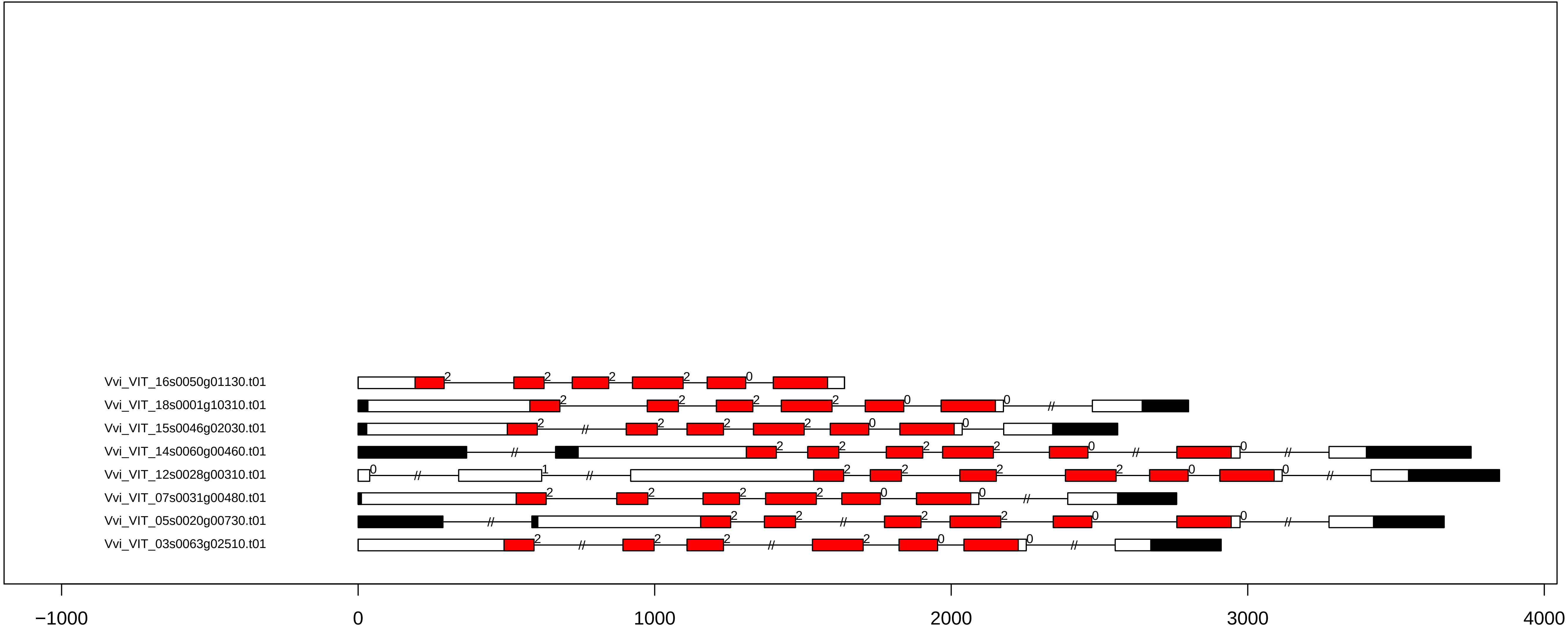

V.vi RLK−Pelle_RLCK−XII−1 subfamily exon−intron and kinase domain diagram (all)

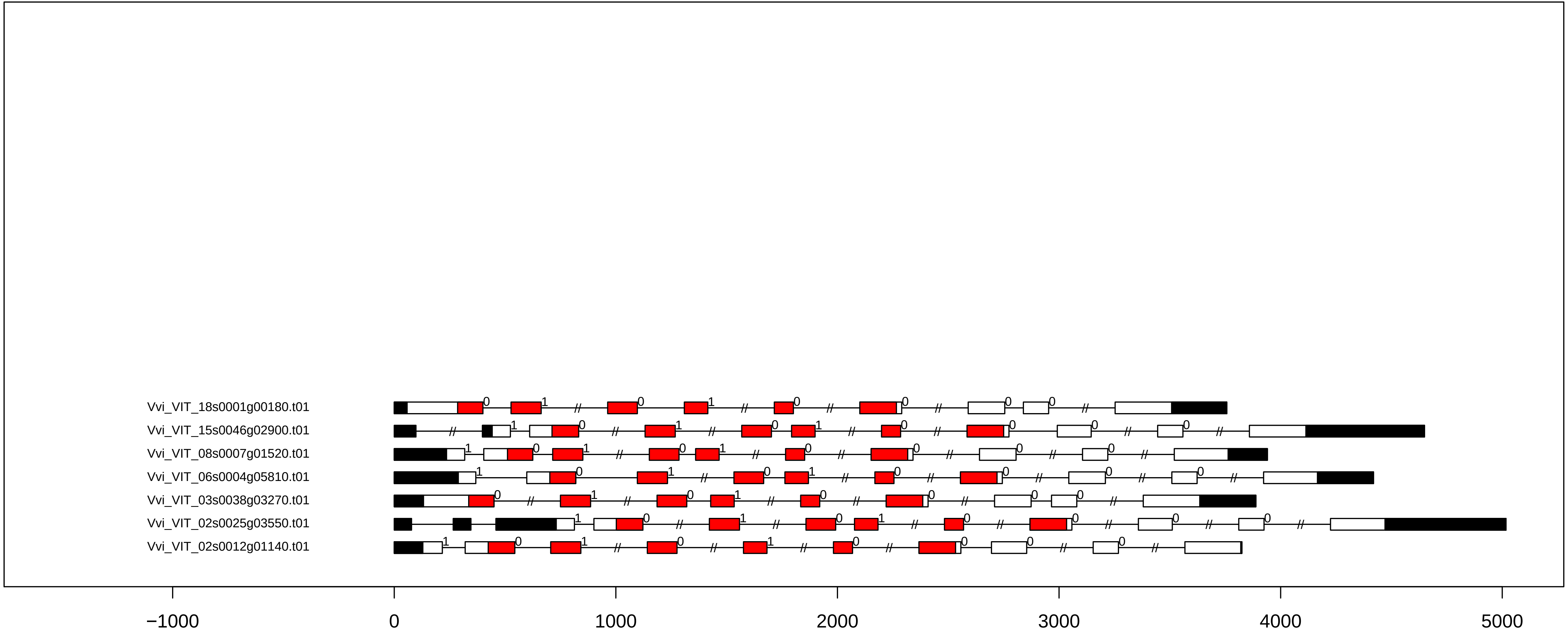

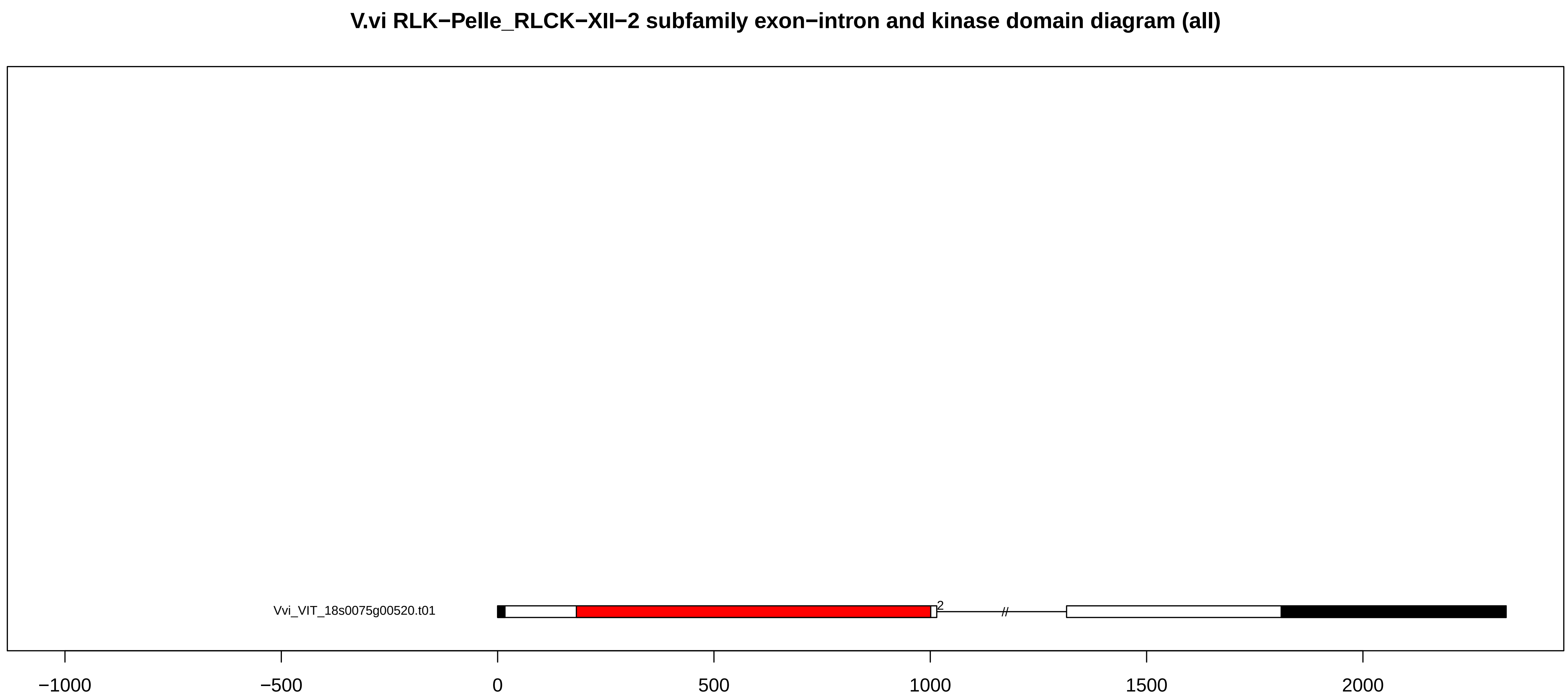
V.vi RLK−Pelle_RLCK−XII−2 subfamily exon−intron and kinase domain diagram (all)
Vvi_VIT_18s0075g00520.t01
2
−1000
−500
0
500
1000
1500
2000

V.vi RLK−Pelle_RLCK−XIII subfamily exon−intron and kinase domain diagram (all)

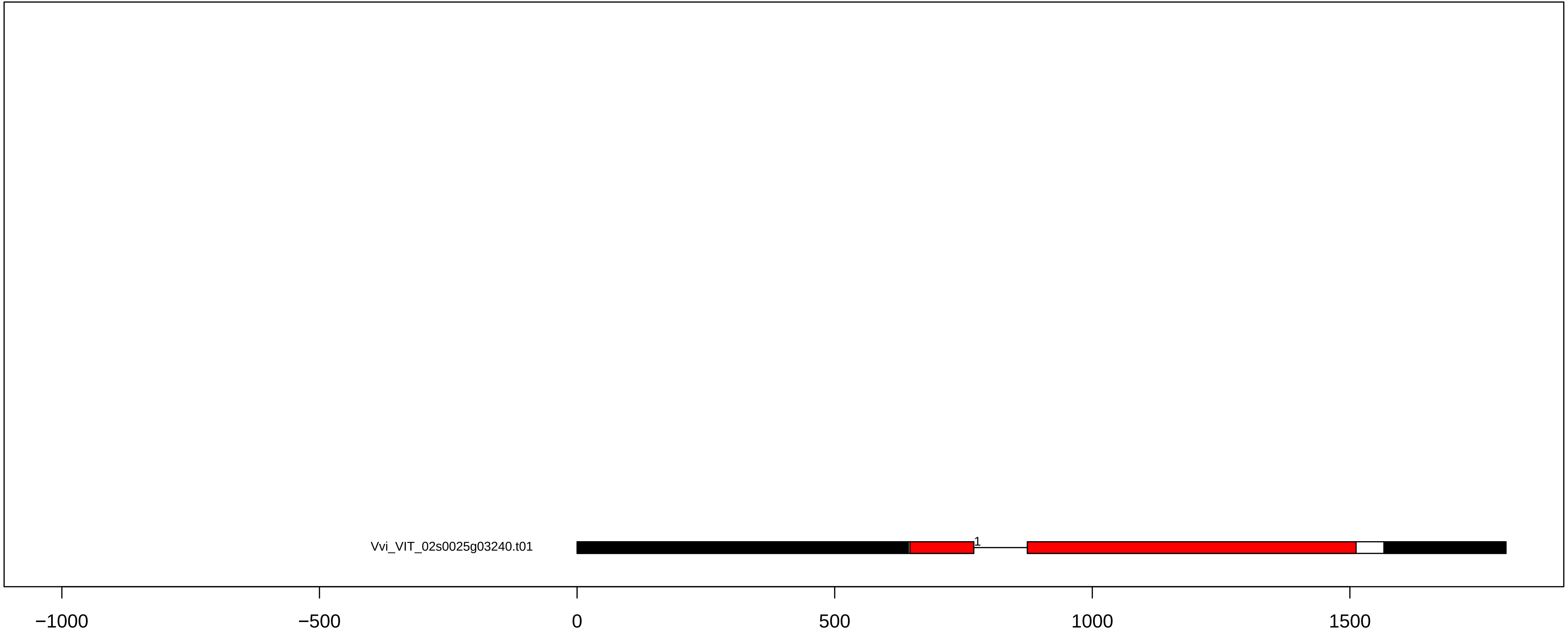

V.vi RLK−Pelle_RLCK−XI subfamily exon−intron and kinase domain diagram (all)

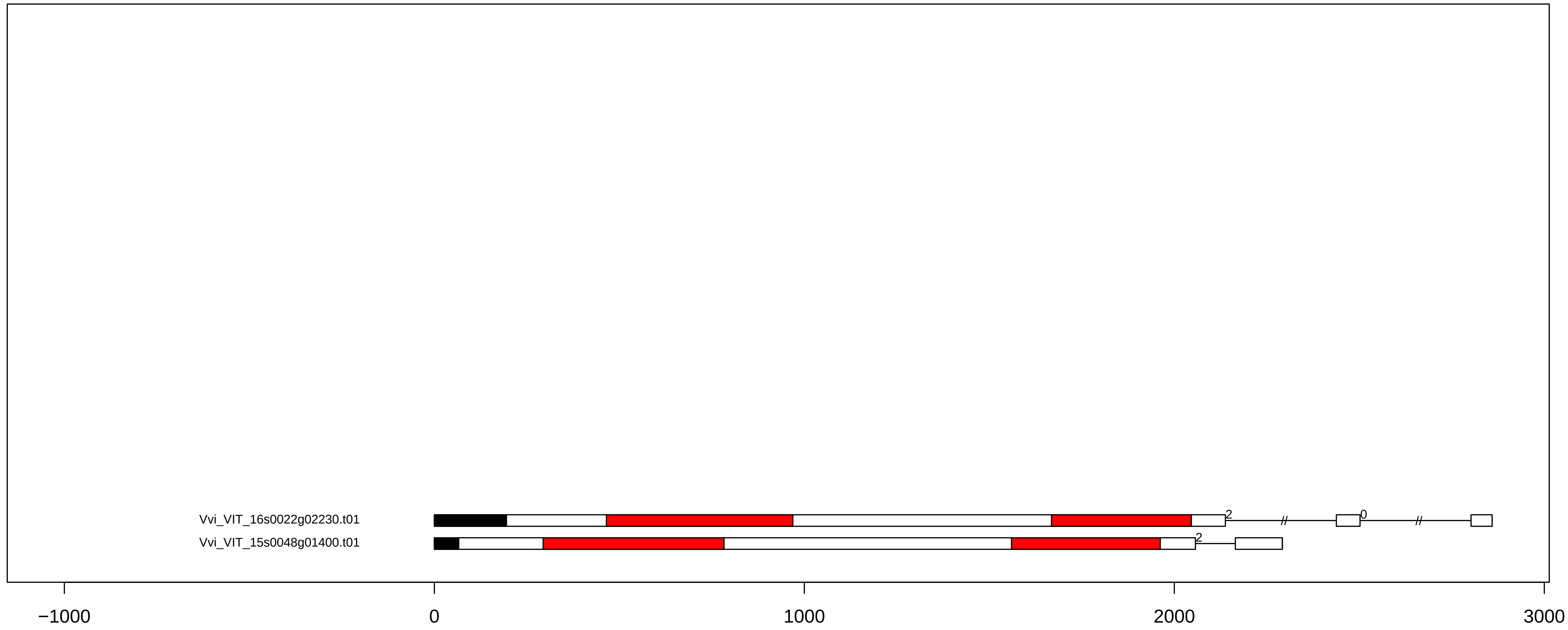

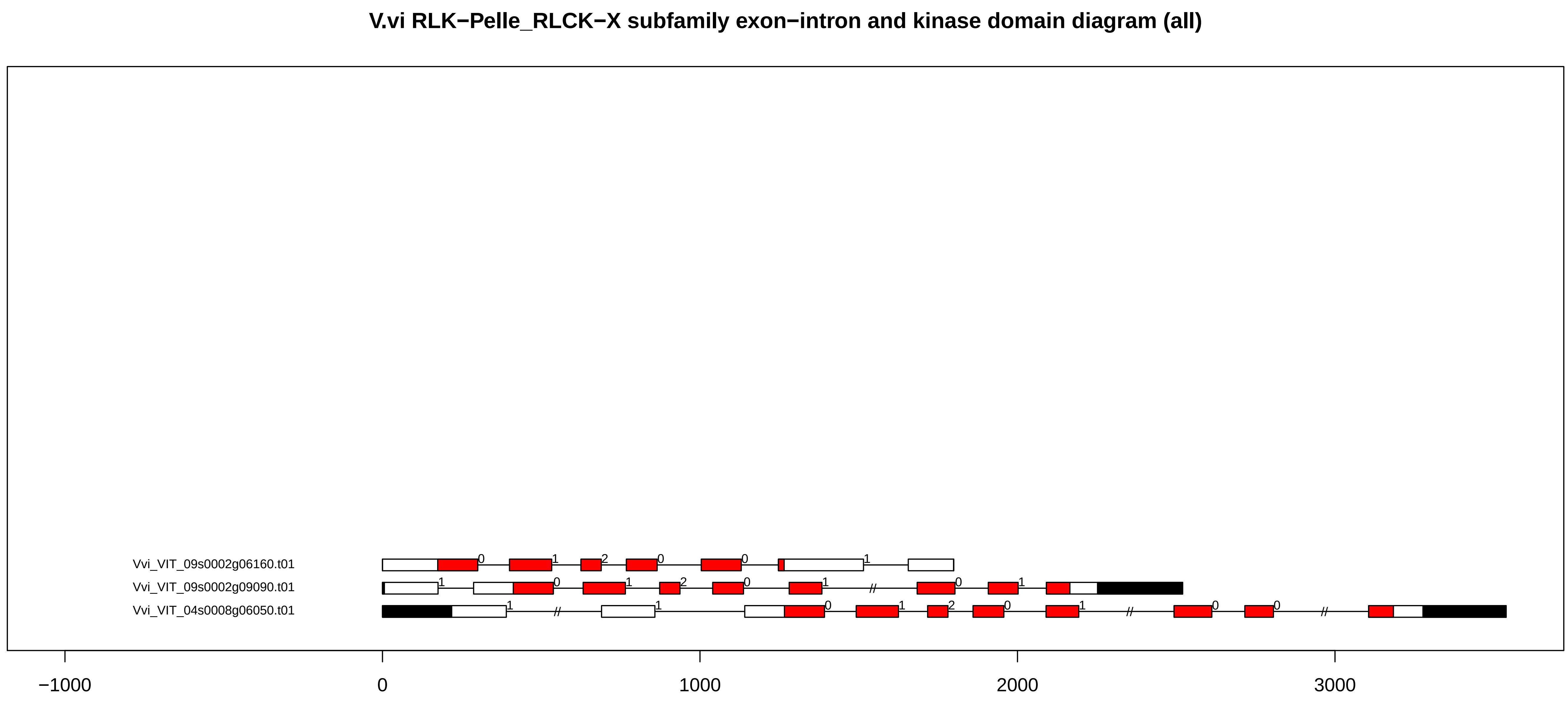

V.vi RLK−Pelle_RLCK−X subfamily exon−intron and kinase domain diagram (all)
Vvi_VIT_09s0002g06160.t01
Vvi_VIT_09s0002g09090.t01
Vvi_VIT_04s0008g06050.t01
−1000
0
1000
2000
3000

V.vi RLK−Pelle_RLCK−XVI subfamily exon−intron and kinase domain diagram (all)

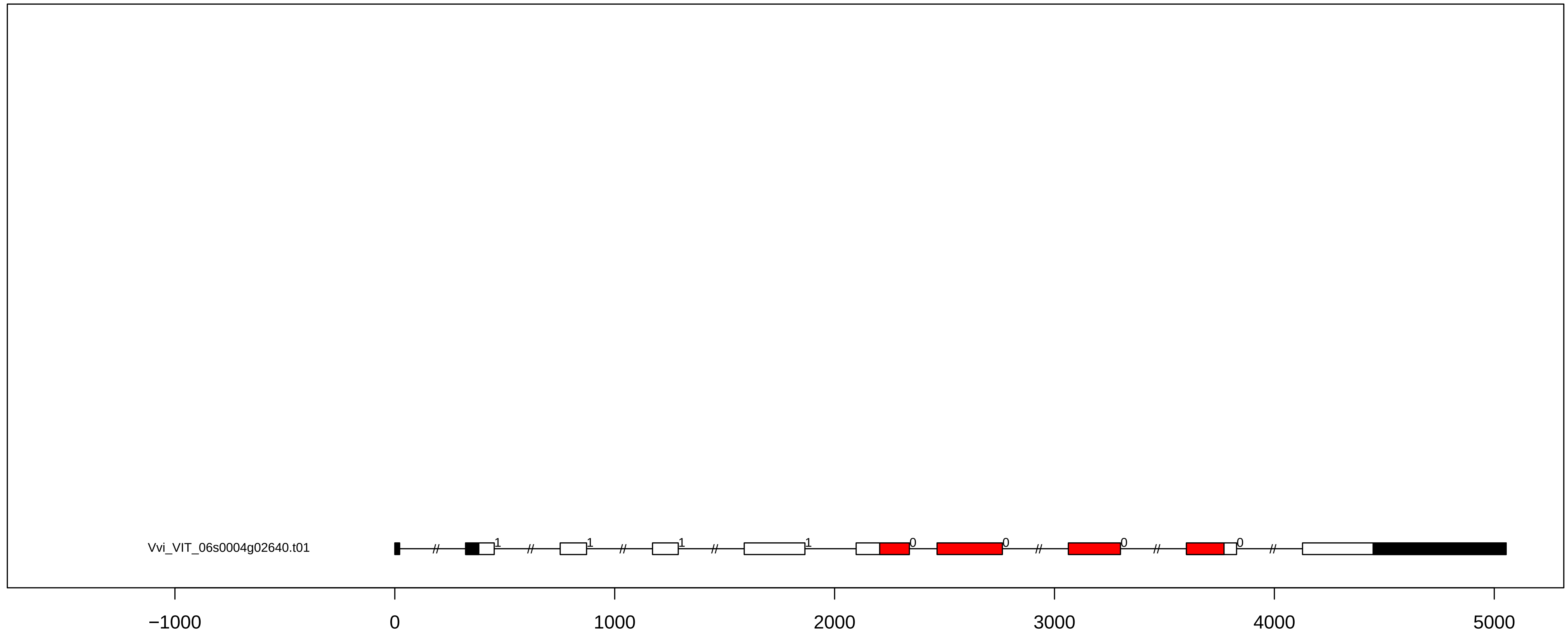

V.vi RLK−Pelle_RLCK−XV subfamily exon−intron and kinase domain diagram (all)

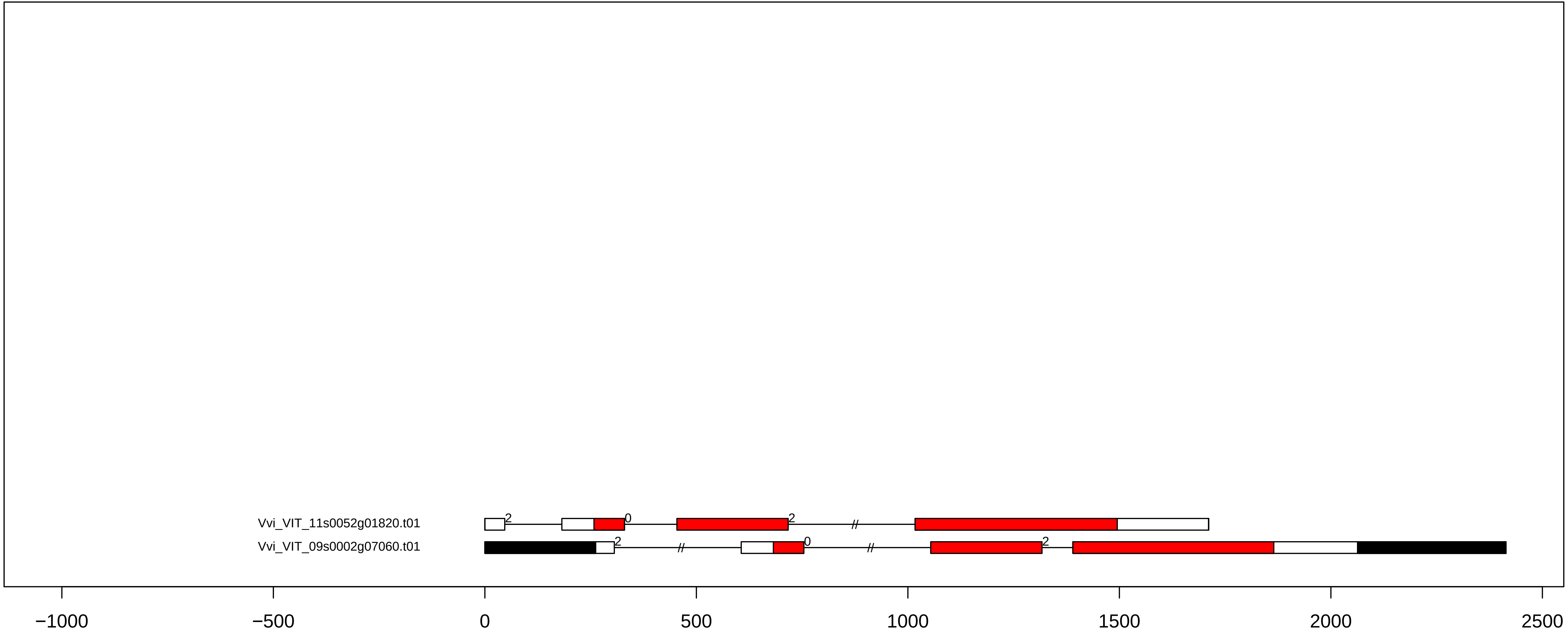

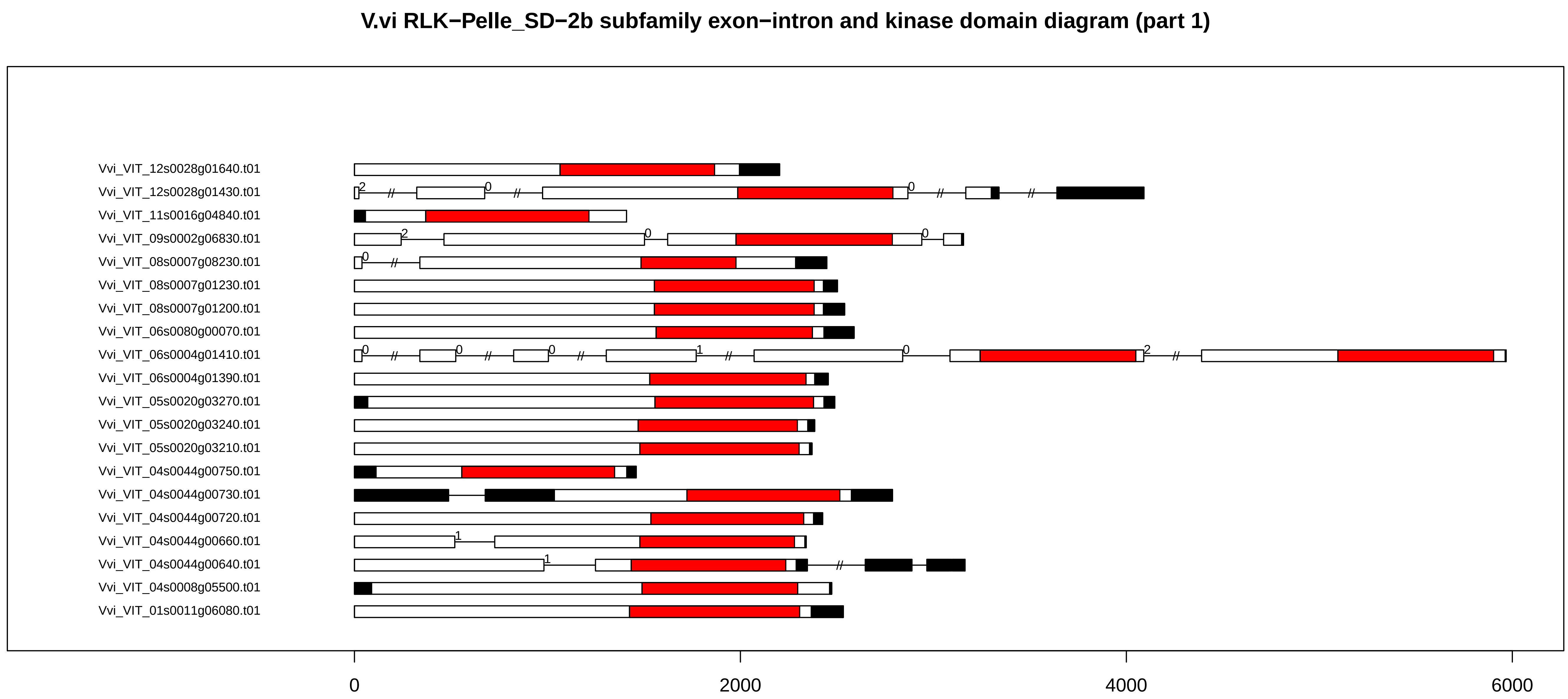
V.vi RLK−Pelle_SD−2b subfamily exon−intron and kinase domain diagram (part 1)
Vvi_VIT_12s0028g01640.t01
Vvi_VIT_12s0028g01430.t01
Vvi_VIT_11s0016g04840.t01
Vvi_VIT_09s0002g06830.t01
Vvi_VIT_08s0007g08230.t01
Vvi_VIT_08s0007g01230.t01
Vvi_VIT_08s0007g01200.t01
Vvi_VIT_06s0080g00070.t01
Vvi_VIT_06s0004g01410.t01
Vvi_VIT_06s0004g01390.t01
Vvi_VIT_05s0020g03270.t01
Vvi_VIT_05s0020g03240.t01
Vvi_VIT_05s0020g03210.t01
Vvi_VIT_04s0044g00750.t01
Vvi_VIT_04s0044g00730.t01
Vvi_VIT_04s0044g00720.t01
Vvi_VIT_04s0044g00660.t01
Vvi_VIT_04s0044g00640.t01
Vvi_VIT_04s0008g05500.t01
Vvi_VIT_01s0011g06080.t01
0
2000
4000
6000

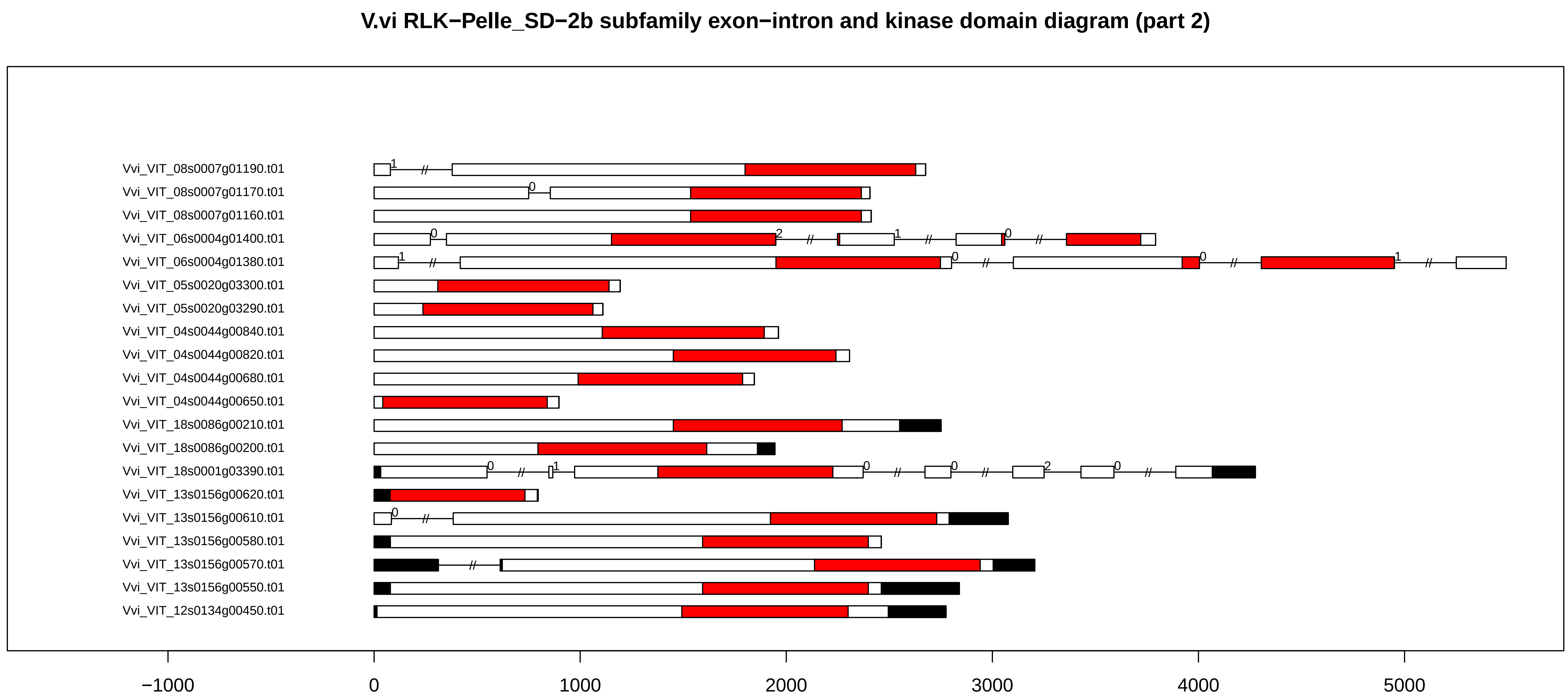
V.vi RLK−Pelle_SD−2b subfamily exon−intron and kinase domain diagram (part 2)
Vvi_VIT_08s0007g01190.t01
Vvi_VIT_08s0007g01170.t01
Vvi_VIT_08s0007g01160.t01
Vvi_VIT_06s0004g01400.t01
Vvi_VIT_06s0004g01380.t01
Vvi_VIT_05s0020g03300.t01
Vvi_VIT_05s0020g03290.t01
Vvi_VIT_04s0044g00840.t01
Vvi_VIT_04s0044g00820.t01
Vvi_VIT_04s0044g00680.t01
Vvi_VIT_04s0044g00650.t01
Vvi_VIT_18s0086g00210.t01
Vvi_VIT_18s0086g00200.t01
Vvi_VIT_18s0001g03390.t01
Vvi_VIT_13s0156g00620.t01
Vvi_VIT_13s0156g00610.t01
Vvi_VIT_13s0156g00580.t01
Vvi_VIT_13s0156g00570.t01
Vvi_VIT_13s0156g00550.t01
Vvi_VIT_12s0134g00450.t01
−1000
0
1000
2000
3000
4000
5000

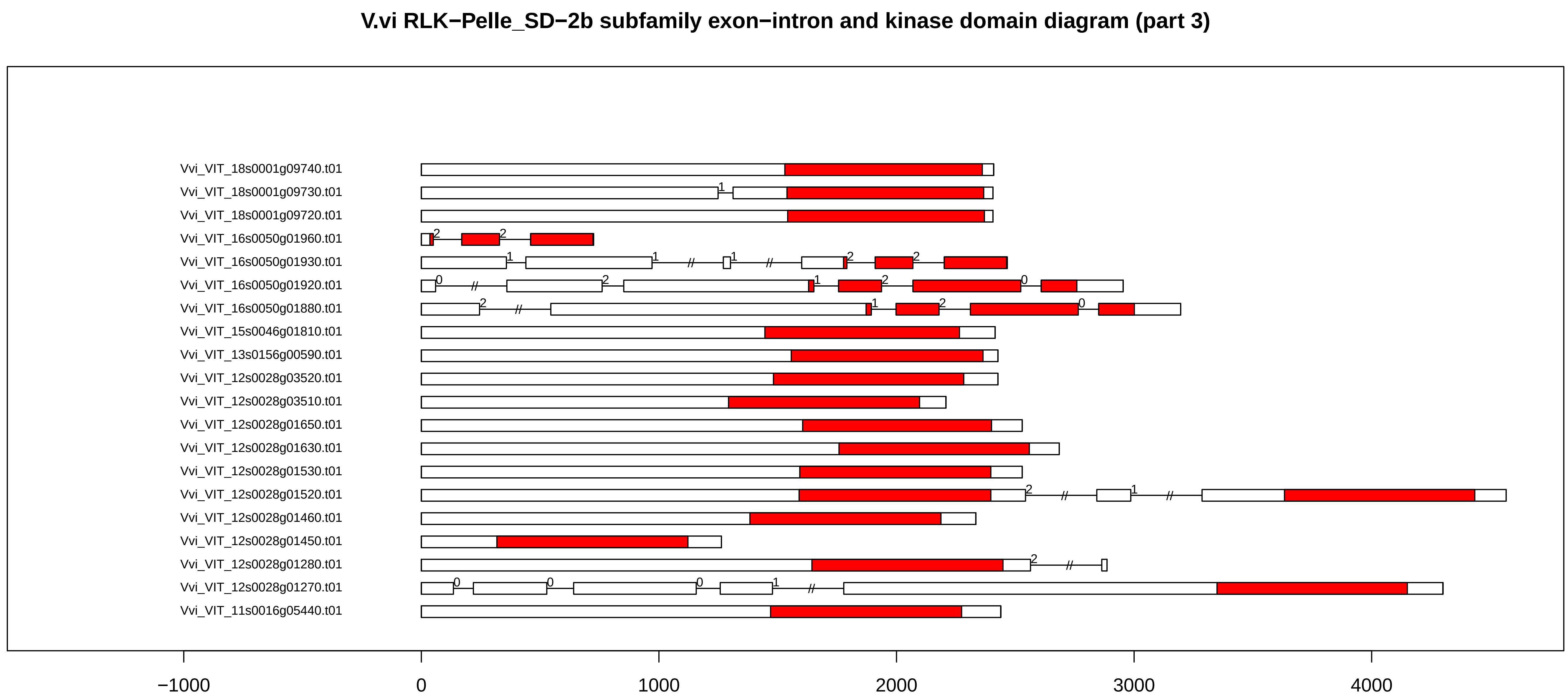
V.vi RLK−Pelle_SD−2b subfamily exon−intron and kinase domain diagram (part 3)
Vvi_VIT_18s0001g09740.t01
Vvi_VIT_18s0001g09730.t01
Vvi_VIT_18s0001g09720.t01
Vvi_VIT_16s0050g01960.t01
Vvi_VIT_16s0050g01930.t01
Vvi_VIT_16s0050g01920.t01
Vvi_VIT_16s0050g01880.t01
Vvi_VIT_15s0046g01810.t01
Vvi_VIT_13s0156g00590.t01
Vvi_VIT_12s0028g03520.t01
Vvi_VIT_12s0028g03510.t01
Vvi_VIT_12s0028g01650.t01
Vvi_VIT_12s0028g01630.t01
Vvi_VIT_12s0028g01530.t01
Vvi_VIT_12s0028g01520.t01
Vvi_VIT_12s0028g01460.t01
Vvi_VIT_12s0028g01450.t01
Vvi_VIT_12s0028g01280.t01
Vvi_VIT_12s0028g01270.t01
Vvi_VIT_11s0016g05440.t01
−1000
0
1000
2000
3000
4000

V.vi RLK−Pelle_SD−2b subfamily exon−intron and kinase domain diagram (part 4)

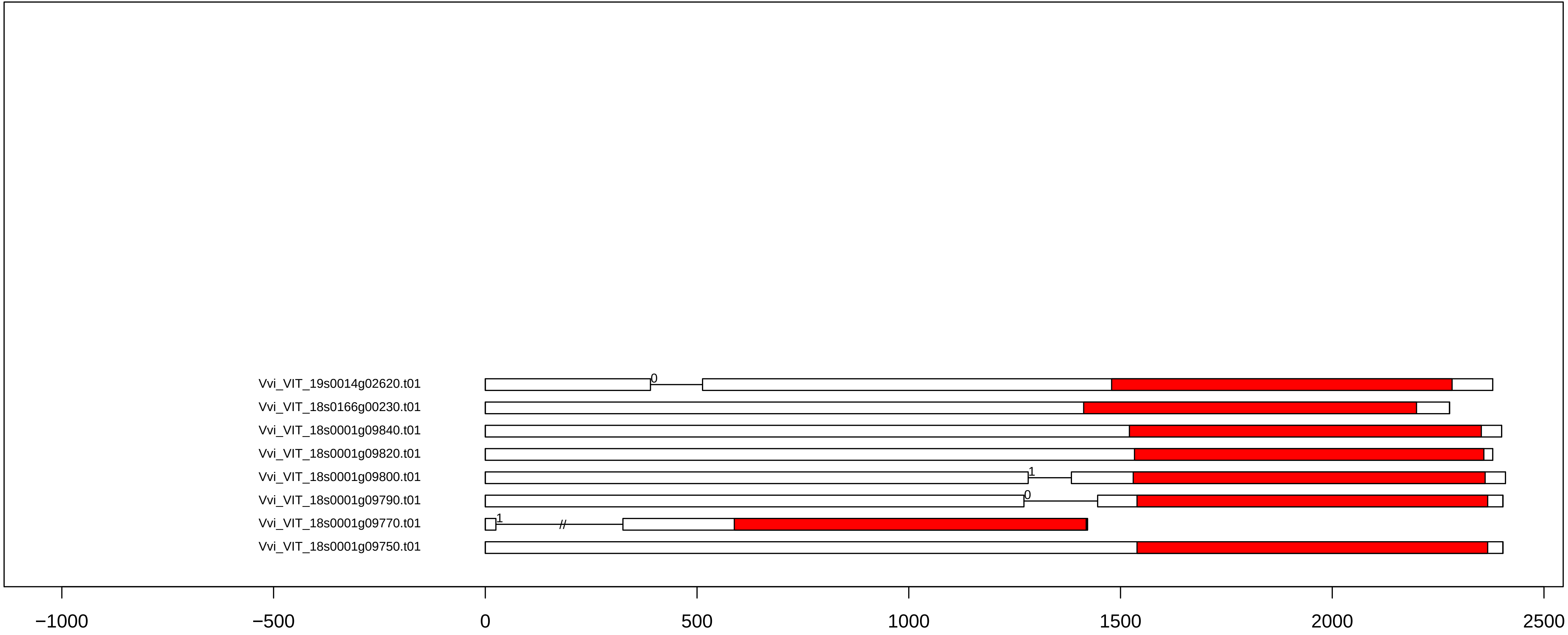

V.vi RLK−Pelle_Singleton subfamily exon−intron and kinase domain diagram (all)

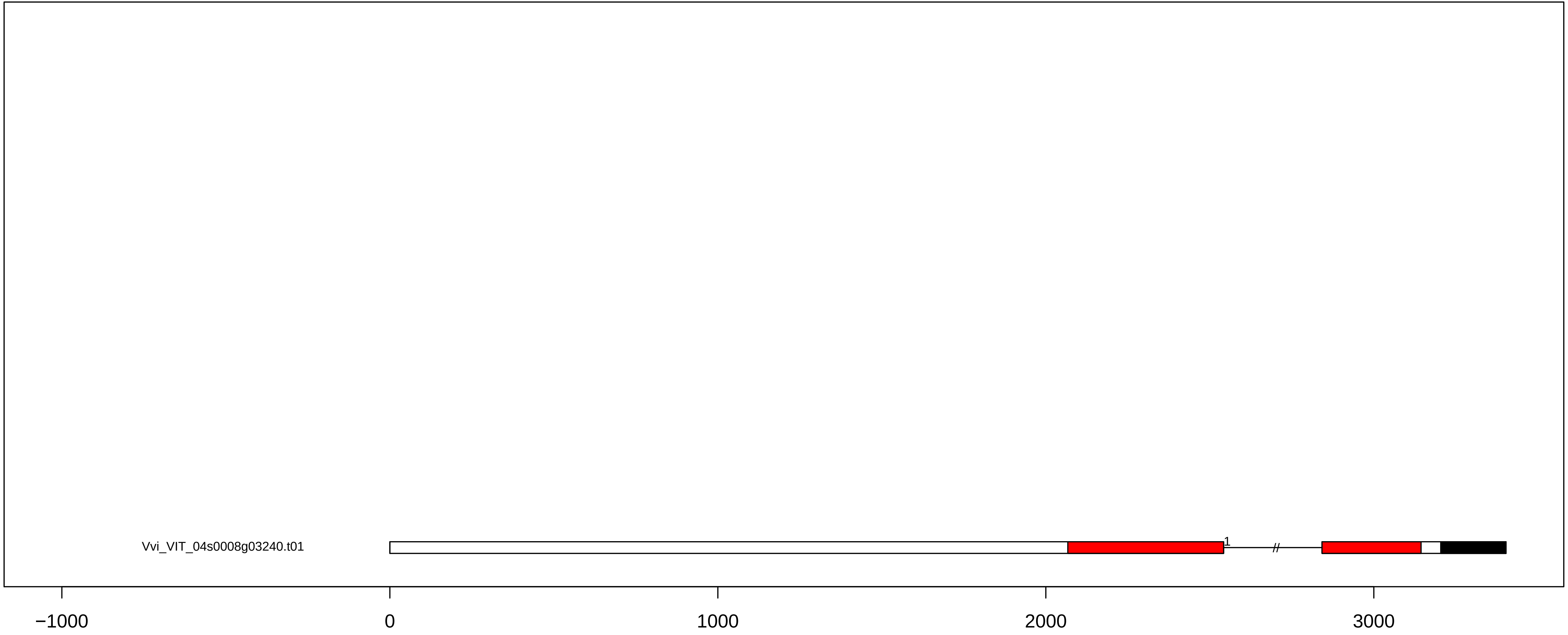

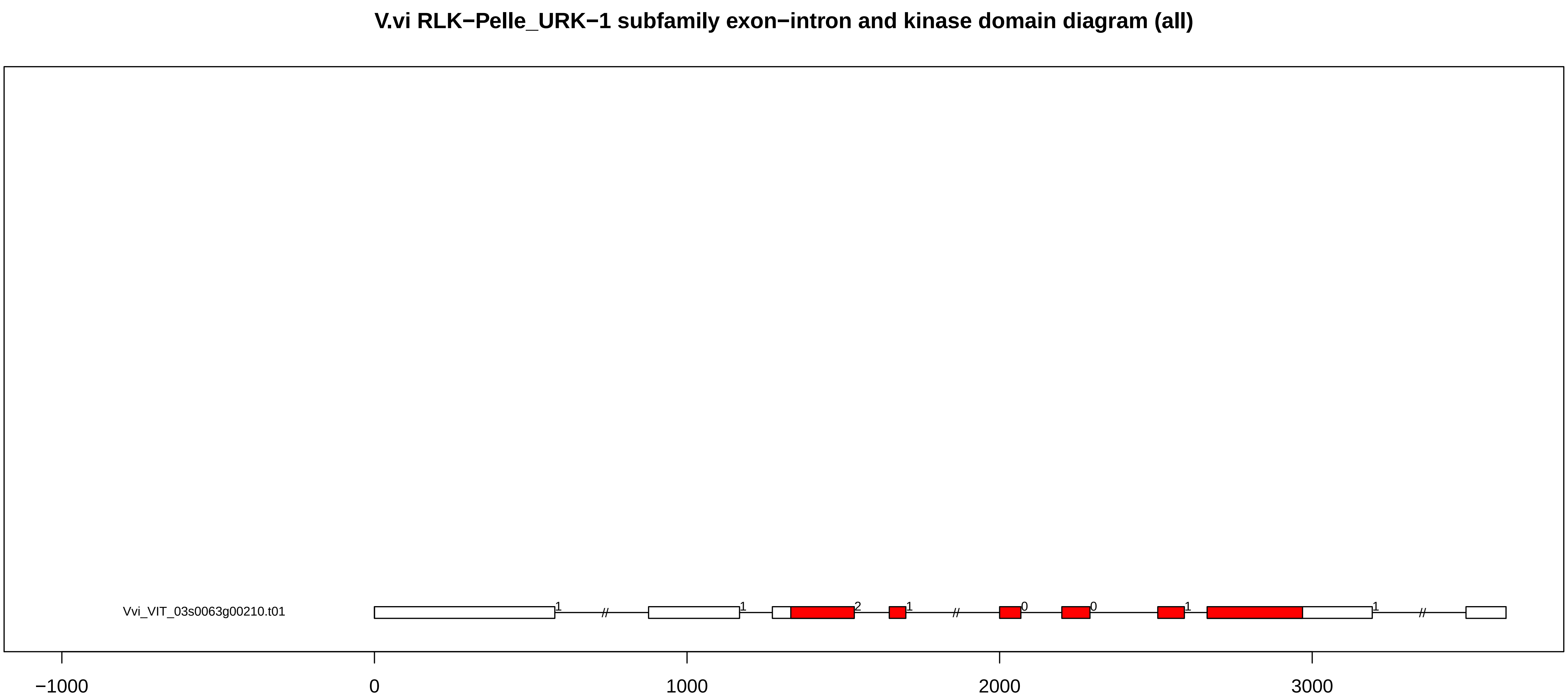

V.vi RLK−Pelle_URK−1 subfamily exon−intron and kinase domain diagram (all)
Vvi_VIT_03s0063g00210.t01
1
1
2
1
0
0
1
1
−1000
0
1000
2000
3000

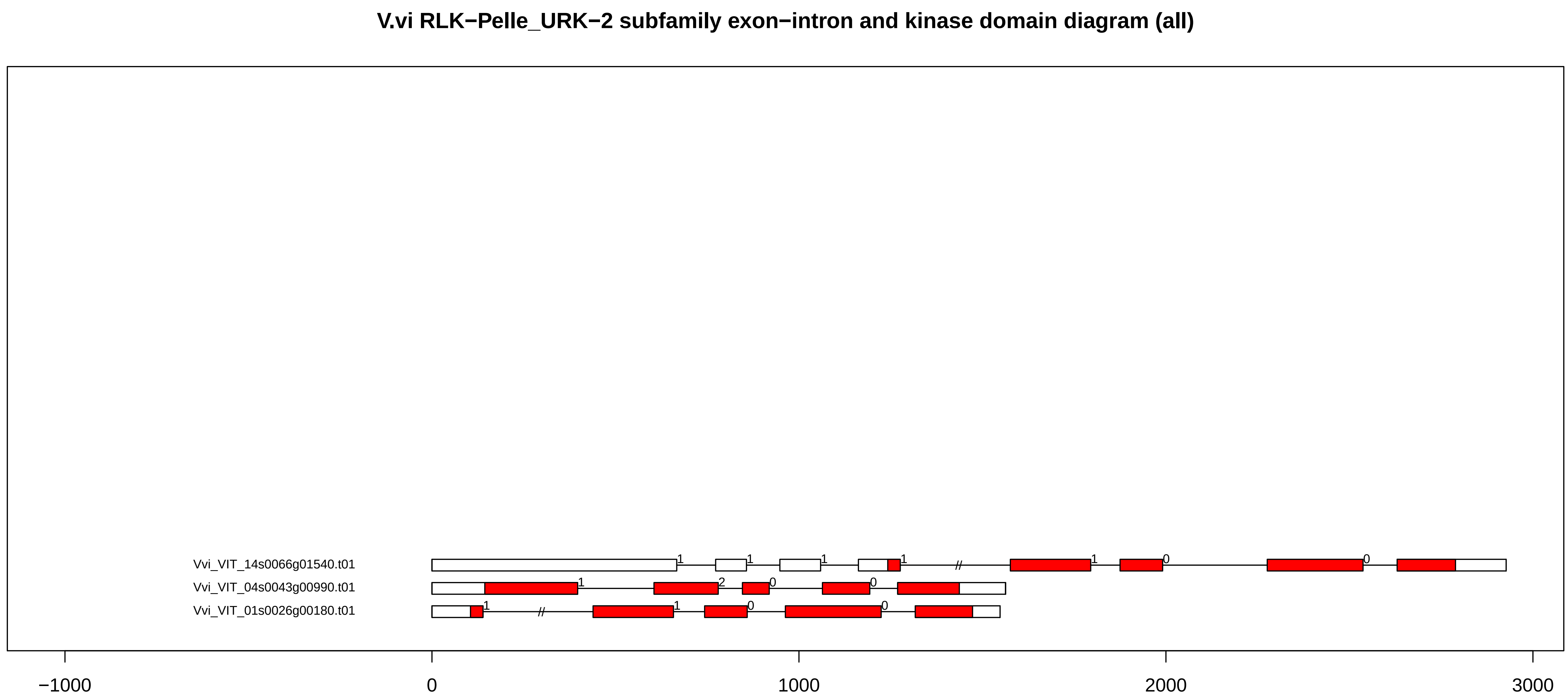
V.vi RLK−Pelle_URK−2 subfamily exon−intron and kinase domain diagram (all)
Vvi_VIT_14s0066g01540.t01
Vvi_VIT_04s0043g00990.t01
Vvi_VIT_01s0026g00180.t01
−1000
0
1000
2000
3000

V.vi RLK−Pelle_URK−3 subfamily exon−intron and kinase domain diagram (all)

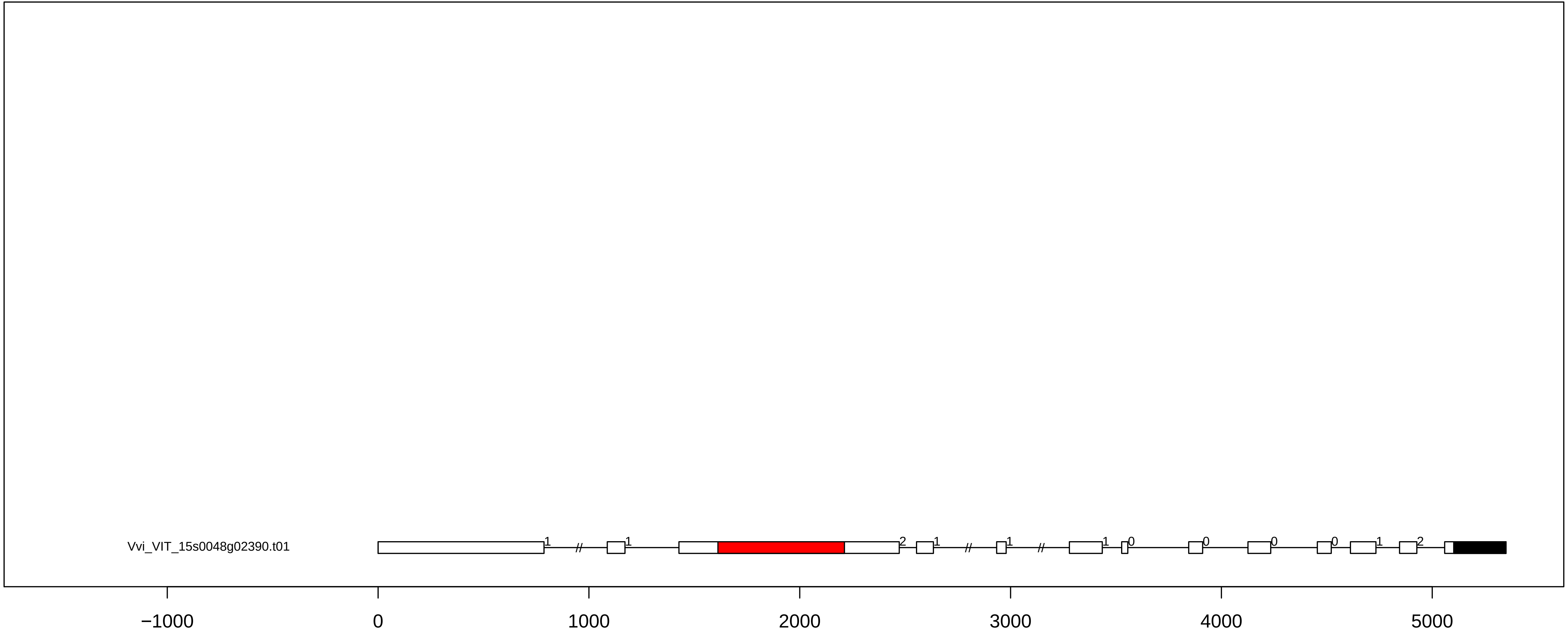

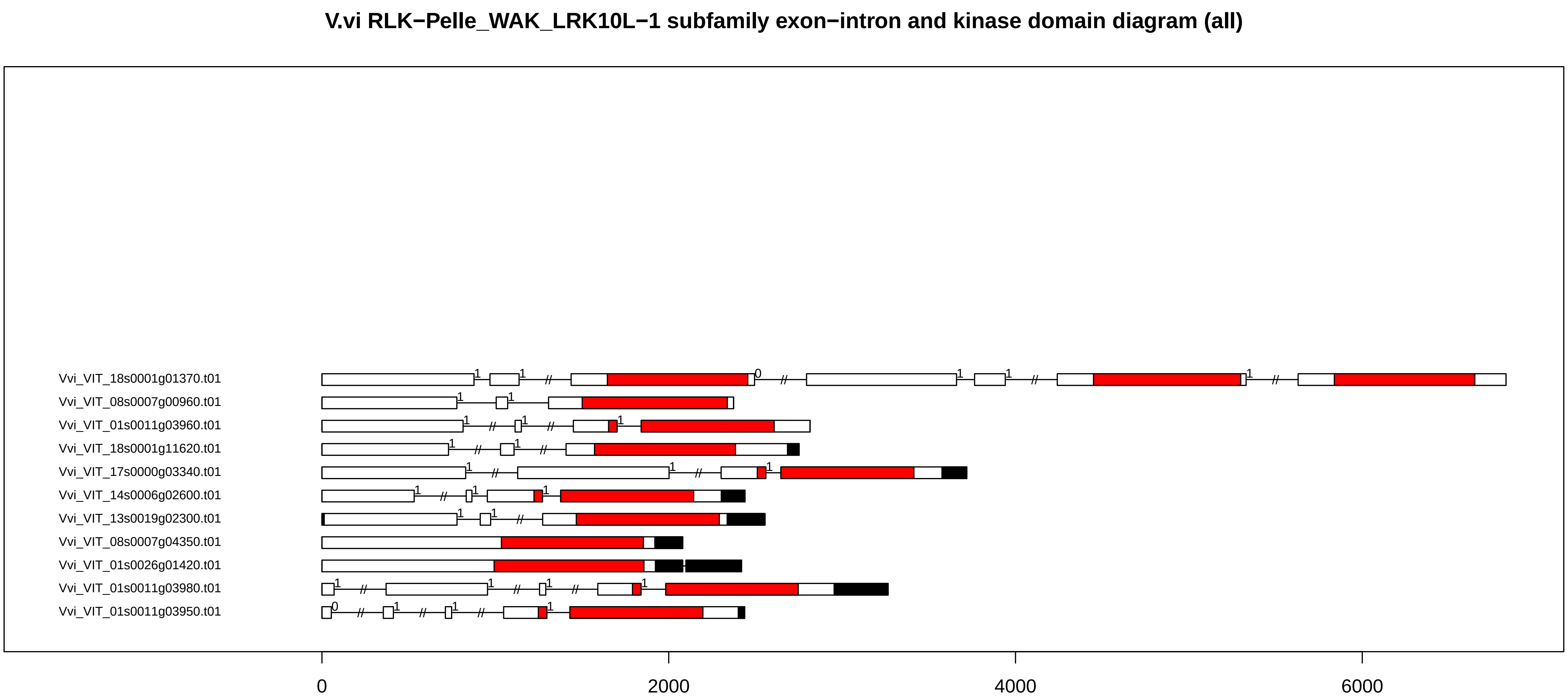
V.vi RLK−Pelle_WAK_LRK10L−1 subfamily exon−intron and kinase domain diagram (all)
Vvi_VIT_18s0001g01370.t01
Vvi_VIT_08s0007g00960.t01
Vvi_VIT_01s0011g03960.t01
Vvi_VIT_18s0001g11620.t01
Vvi_VIT_17s0000g03340.t01
Vvi_VIT_14s0006g02600.t01
Vvi_VIT_13s0019g02300.t01
Vvi_VIT_08s0007g04350.t01
Vvi_VIT_01s0026g01420.t01
Vvi_VIT_01s0011g03980.t01
Vvi_VIT_01s0011g03950.t01
0
2000
4000
6000

V.vi RLK−Pelle_WAK subfamily exon−intron and kinase domain diagram (part 1)

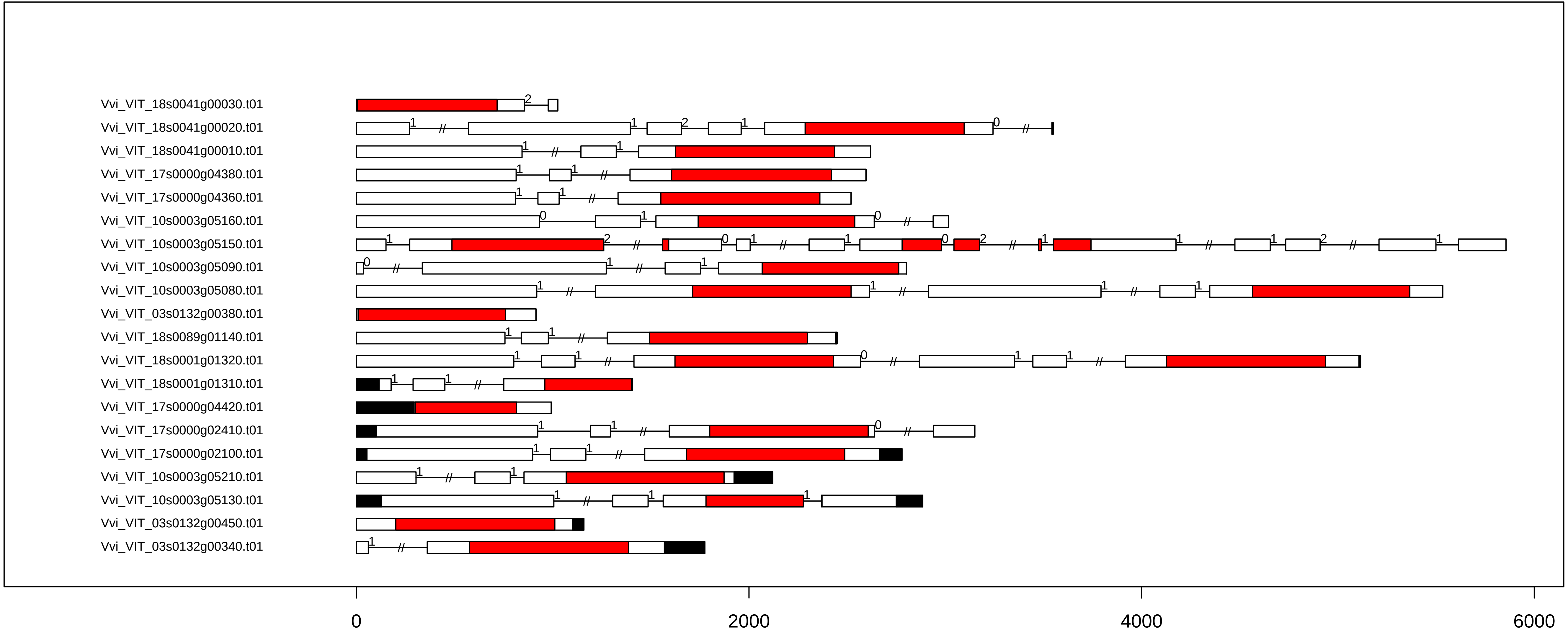

V.vi RLK−Pelle_WAK subfamily exon−intron and kinase domain diagram (part 2)

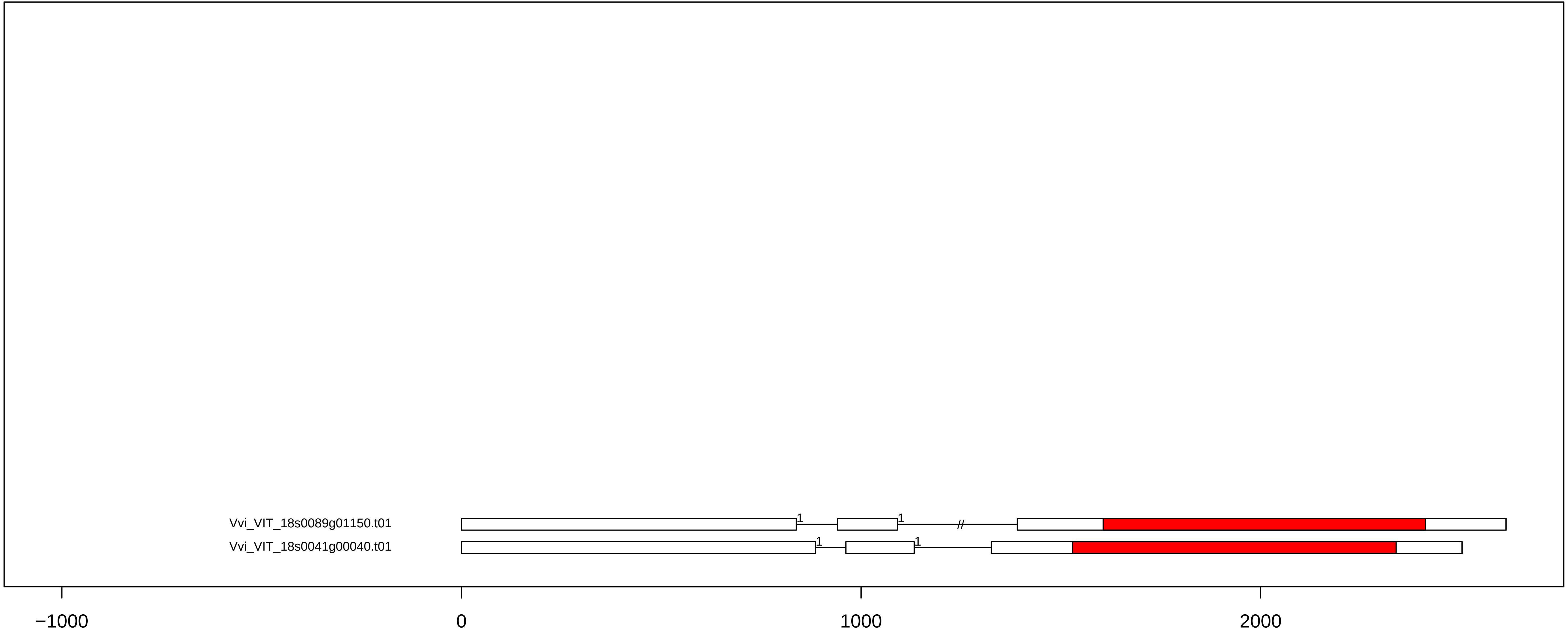

A.tr RLK−Pelle_C−LEC subfamily exon−intron and kinase domain diagram (all)

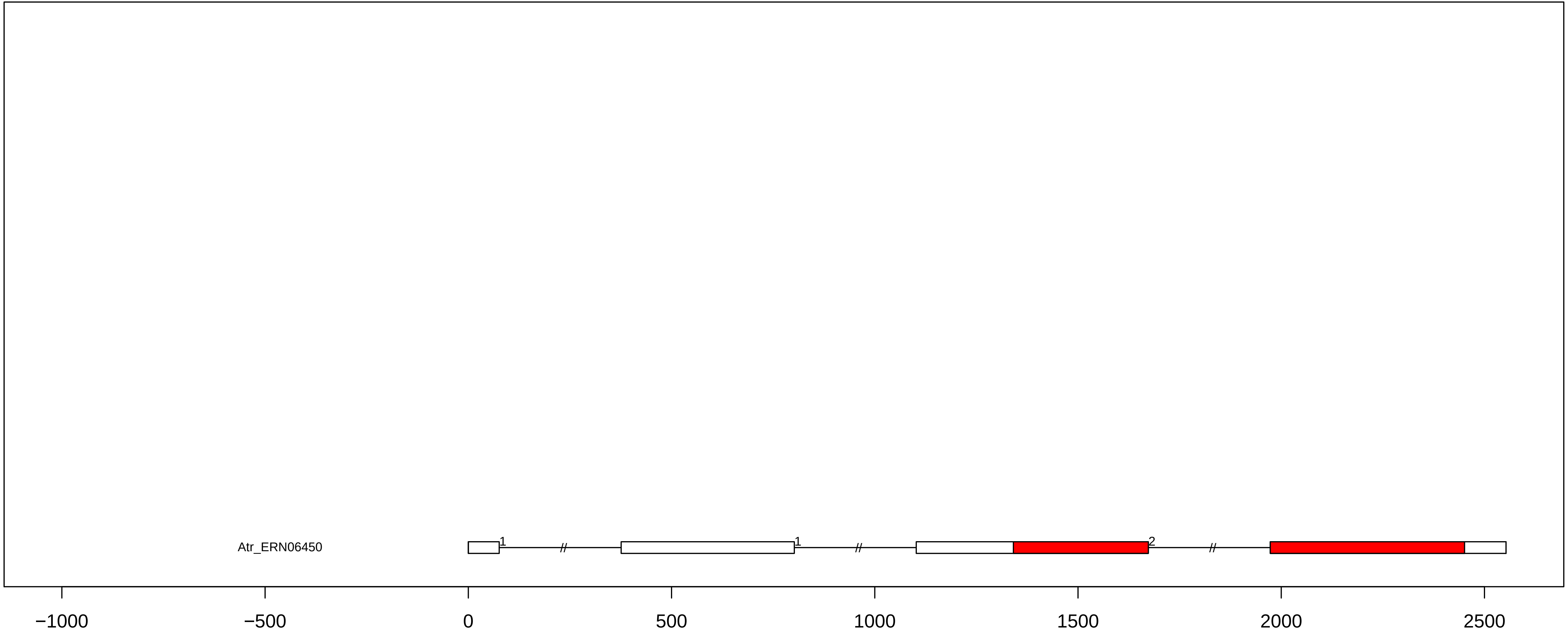

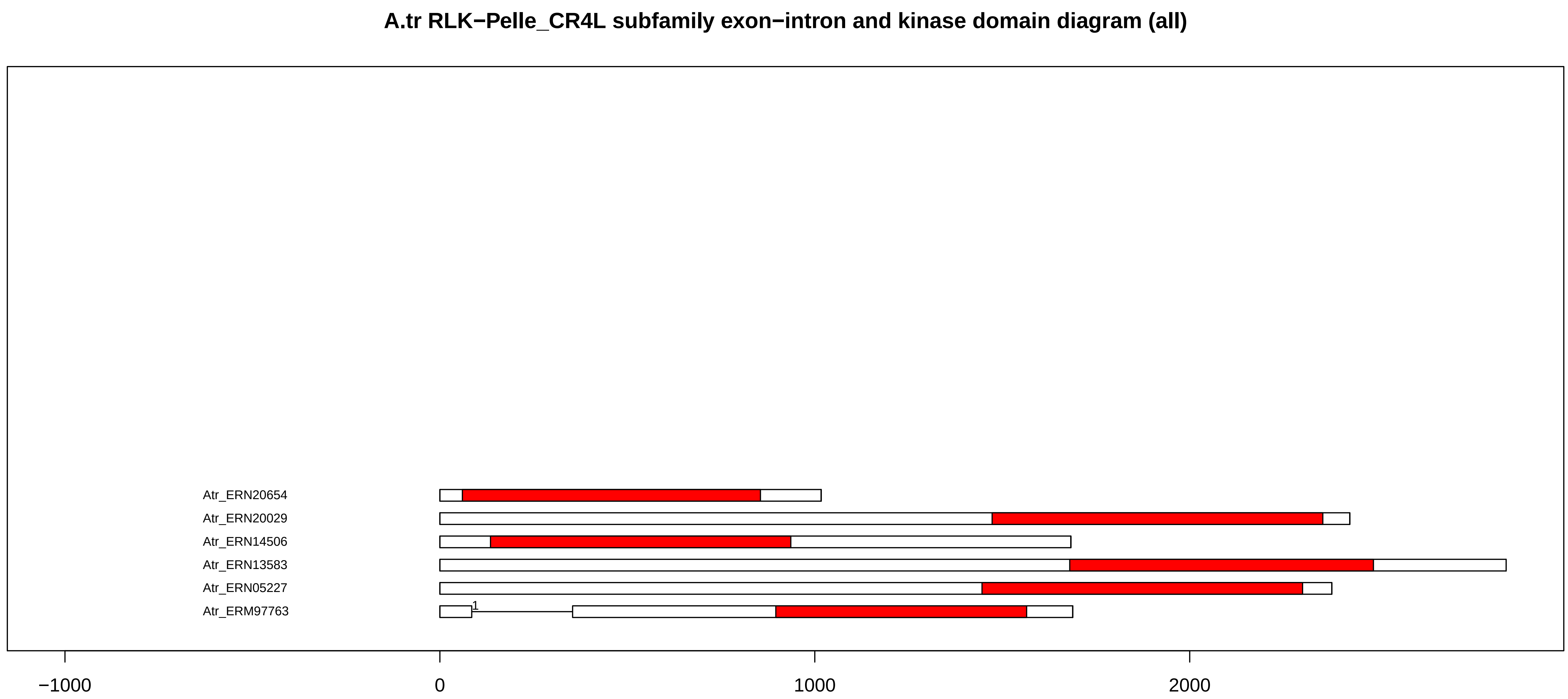

A.tr RLK−Pelle_CR4L subfamily exon−intron and kinase domain diagram (all)
Atr_ERN20654
Atr_ERN20029
Atr_ERN14506
Atr_ERN13583
Atr_ERN05227
Atr_ERM97763
1
−1000
0
1000
2000

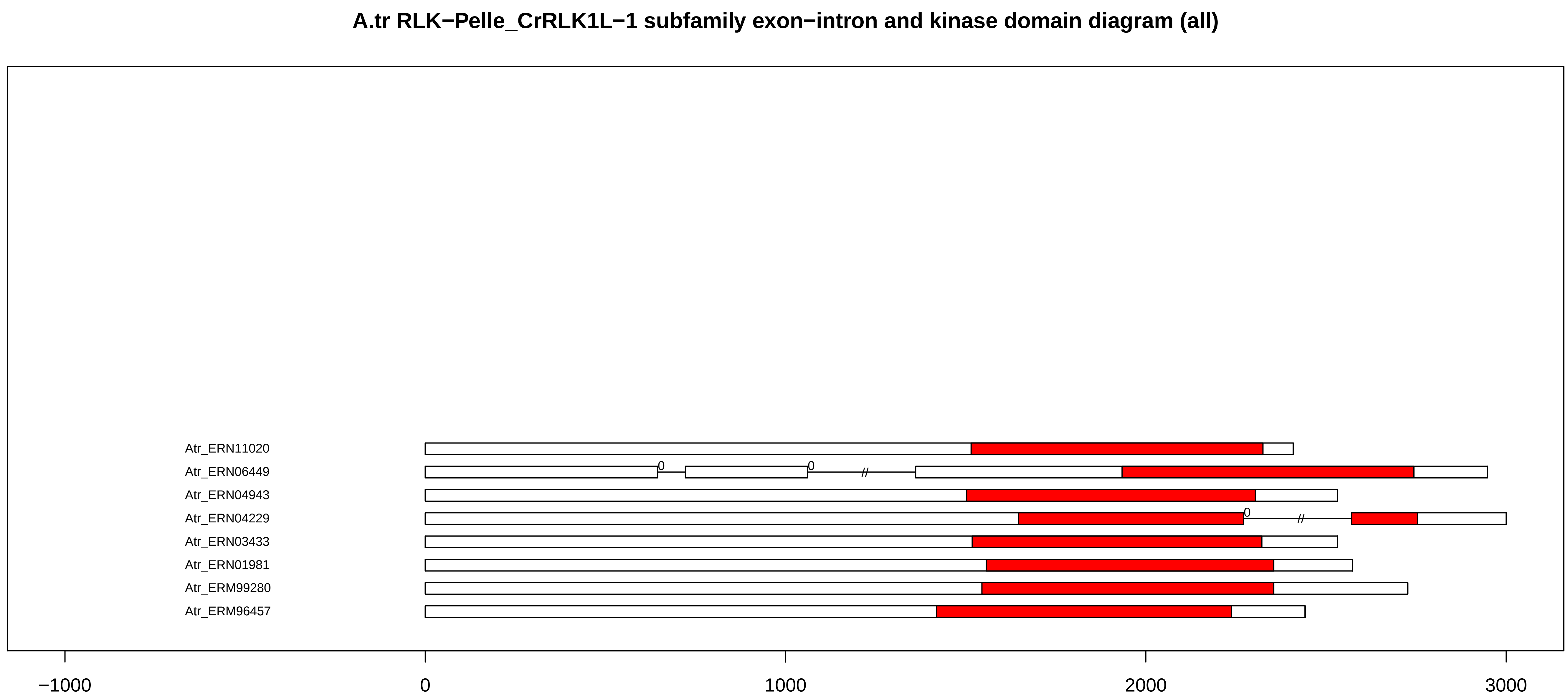
A.tr RLK−Pelle_CrRLK1L−1 subfamily exon−intron and kinase domain diagram (all)
Atr_ERN11020
Atr_ERN06449
Atr_ERN04943
Atr_ERN04229
Atr_ERN03433
Atr_ERN01981
Atr_ERM99280
Atr_ERM96457
0
0
0
−1000
0
1000
2000
3000

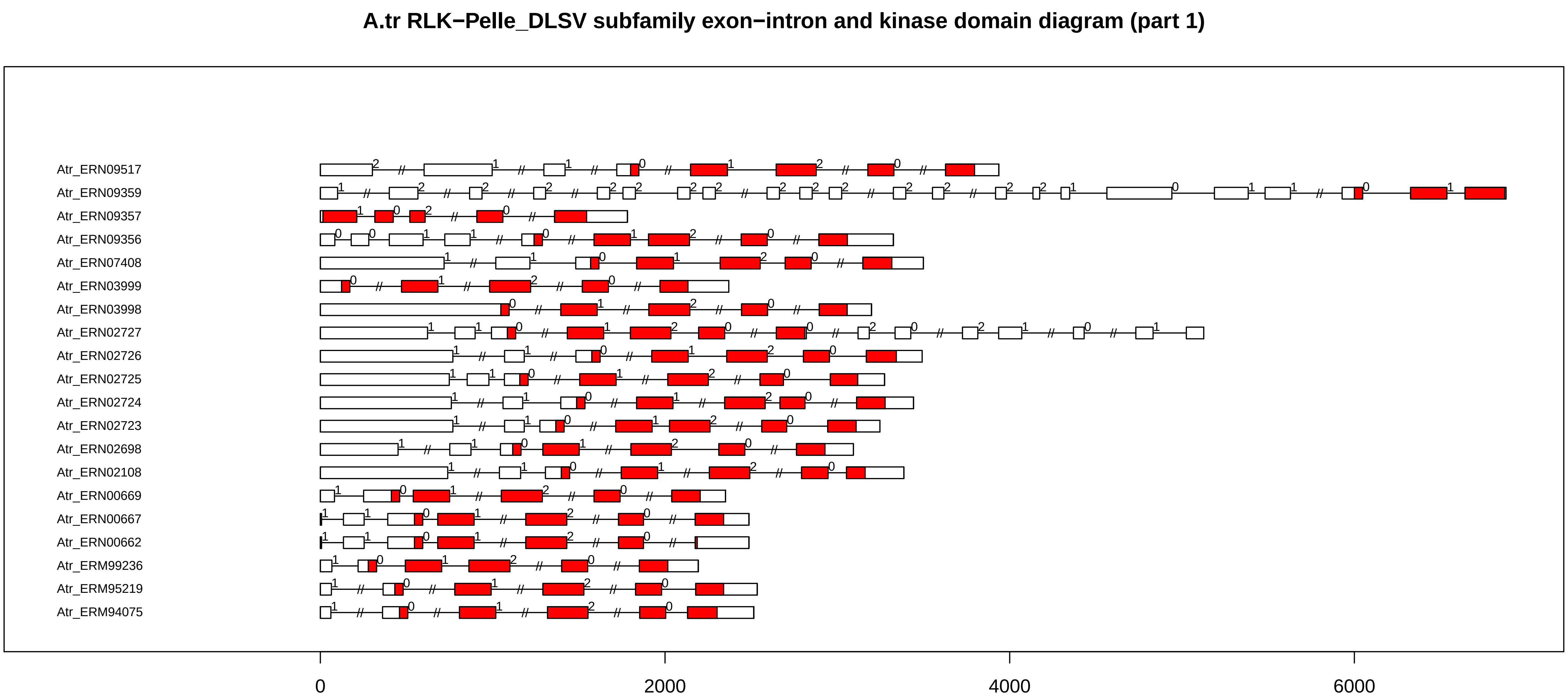
A.tr RLK−Pelle_DLSV subfamily exon−intron and kinase domain diagram (part 1)
Atr_ERN09517
Atr_ERN09359
Atr_ERN09357
Atr_ERN09356
Atr_ERN07408
Atr_ERN03999
Atr_ERN03998
Atr_ERN02727
Atr_ERN02726
Atr_ERN02725
Atr_ERN02724
Atr_ERN02723
Atr_ERN02698
Atr_ERN02108
Atr_ERN00669
Atr_ERN00667
Atr_ERN00662
Atr_ERM99236
Atr_ERM95219
Atr_ERM94075
0
2000
4000
6000

A.tr RLK−Pelle_DLSV subfamily exon−intron and kinase domain diagram (part 2)

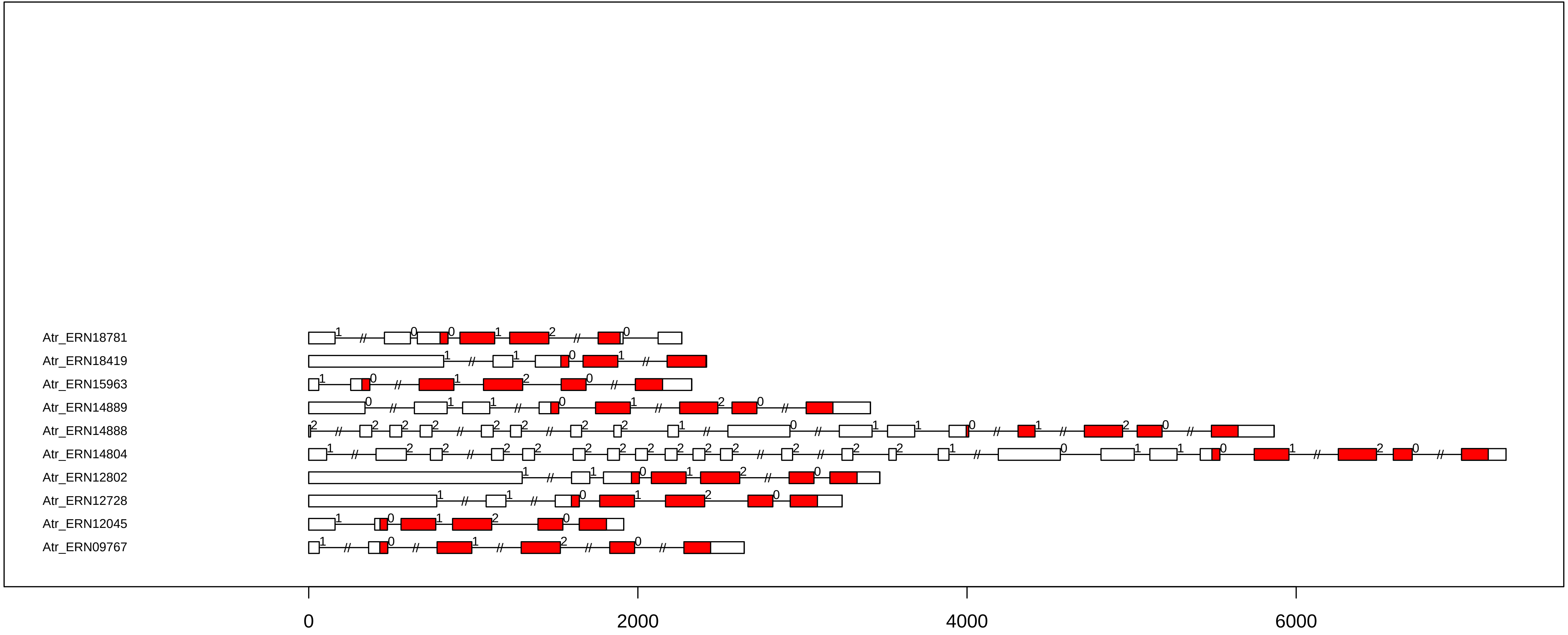

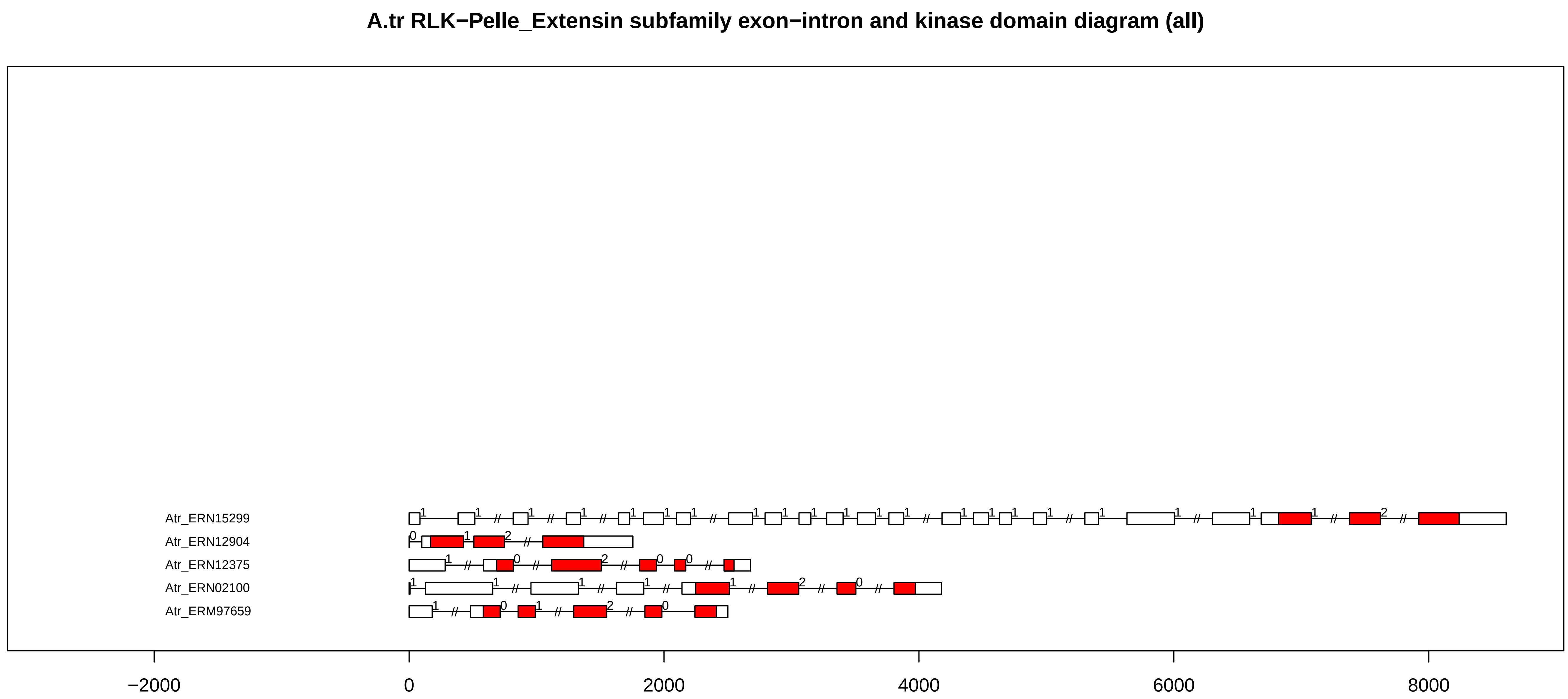

A.tr RLK−Pelle_Extensin subfamily exon−intron and kinase domain diagram (all)
Atr_ERN15299
Atr_ERN12904
Atr_ERN12375
Atr_ERN02100
Atr_ERM97659
−2000
0
2000
4000
6000
8000

A.tr RLK−Pelle_L−LEC subfamily exon−intron and kinase domain diagram (part 1)

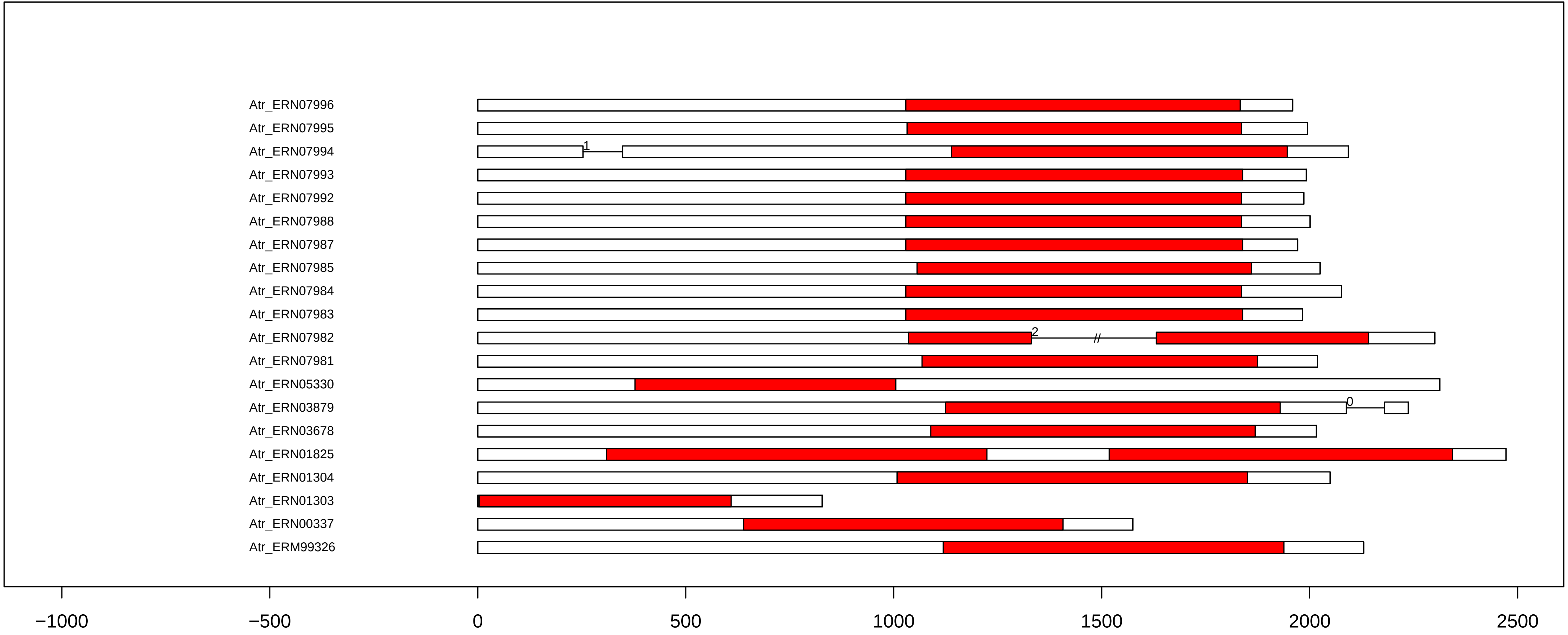

A.tr RLK−Pelle_L−LEC subfamily exon−intron and kinase domain diagram (part 2)

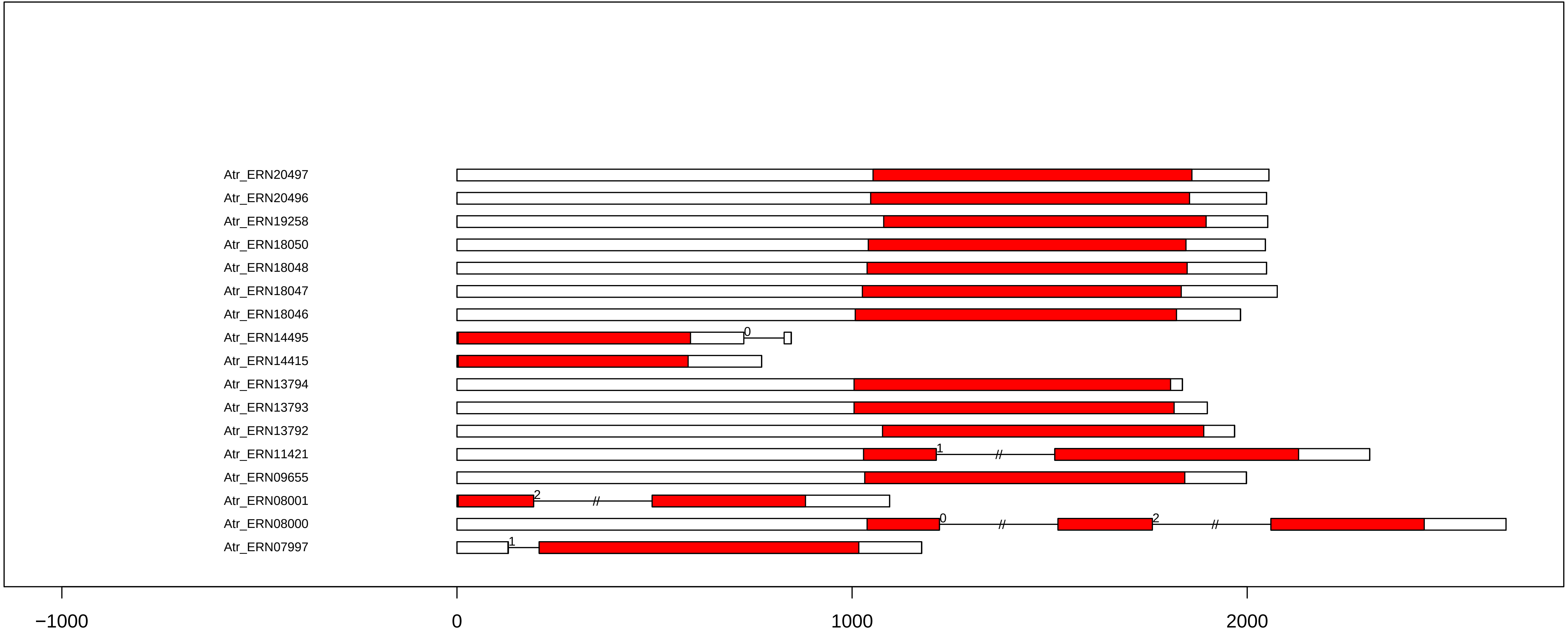

A.tr RLK−Pelle_LRK10L−2 subfamily exon−intron and kinase domain diagram (all)

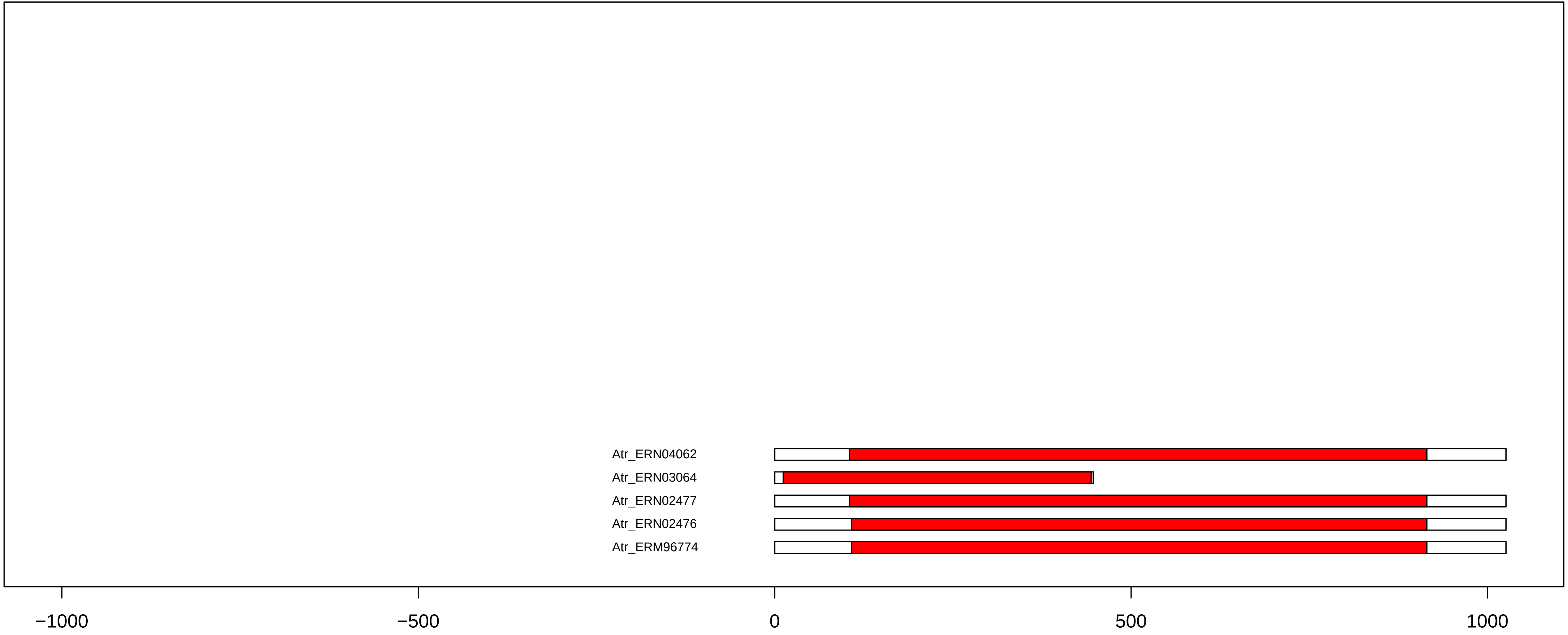

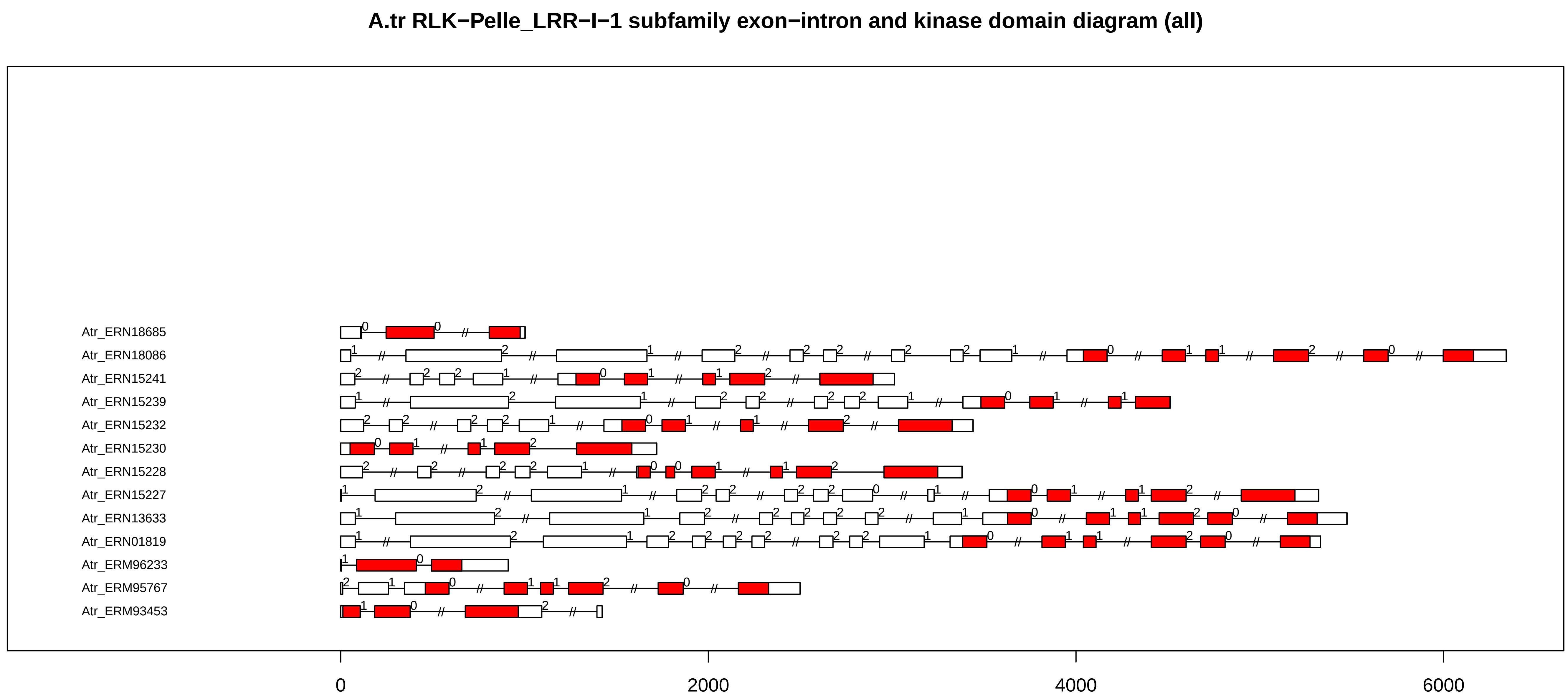
A.tr RLK−Pelle_LRR−I−1 subfamily exon−intron and kinase domain diagram (all)
Atr_ERN18685
Atr_ERN18086
Atr_ERN15241
Atr_ERN15239
Atr_ERN15232
Atr_ERN15230
Atr_ERN15228
Atr_ERN15227
Atr_ERN13633
Atr_ERN01819
Atr_ERM96233
Atr_ERM95767
Atr_ERM93453
0
2000
4000
6000

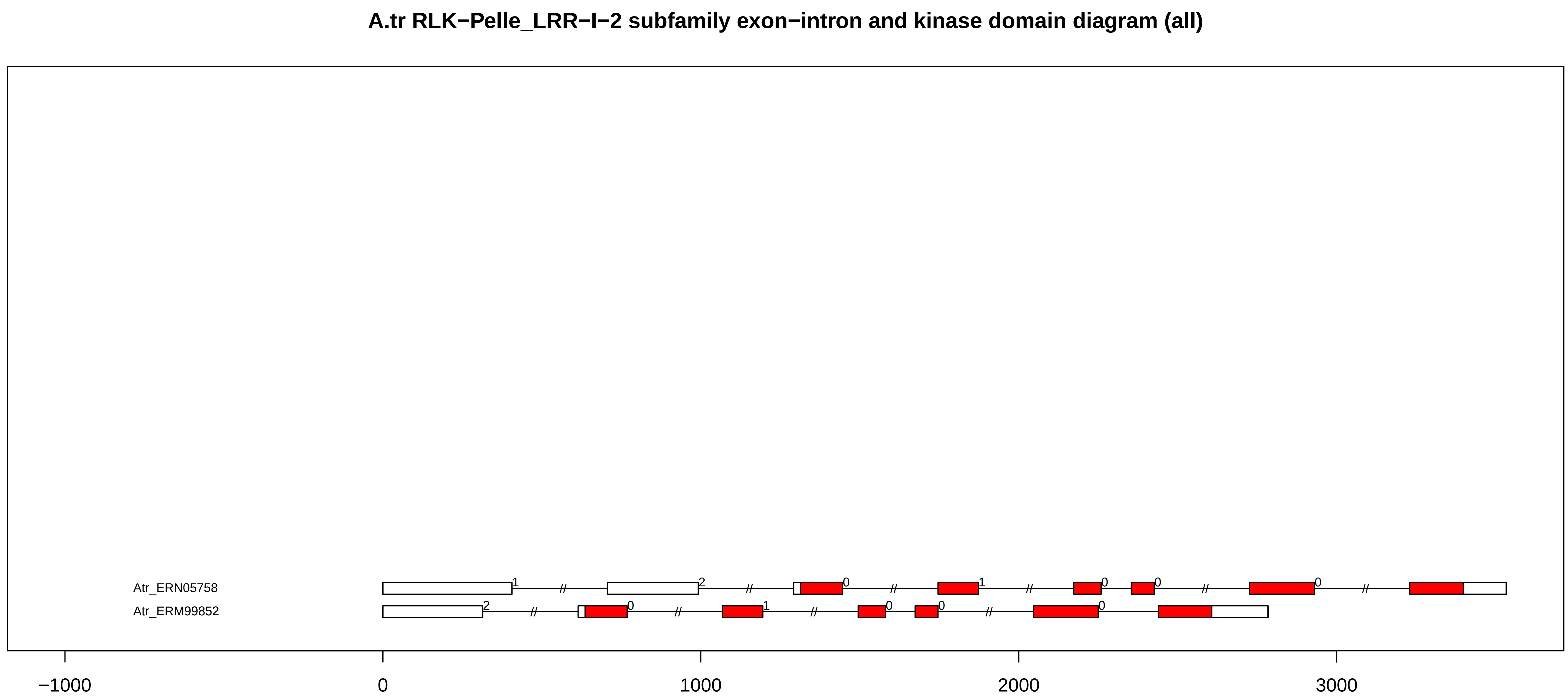

A.tr RLK−Pelle_LRR−I−2 subfamily exon−intron and kinase domain diagram (all)
Atr_ERN05758
Atr_ERM99852
1
2
0
1
0
0
0
2
0
1
0
0
0
−1000
0
1000
2000
3000

A.tr RLK−Pelle_LRR−III subfamily exon−intron and kinase domain diagram (part 1)

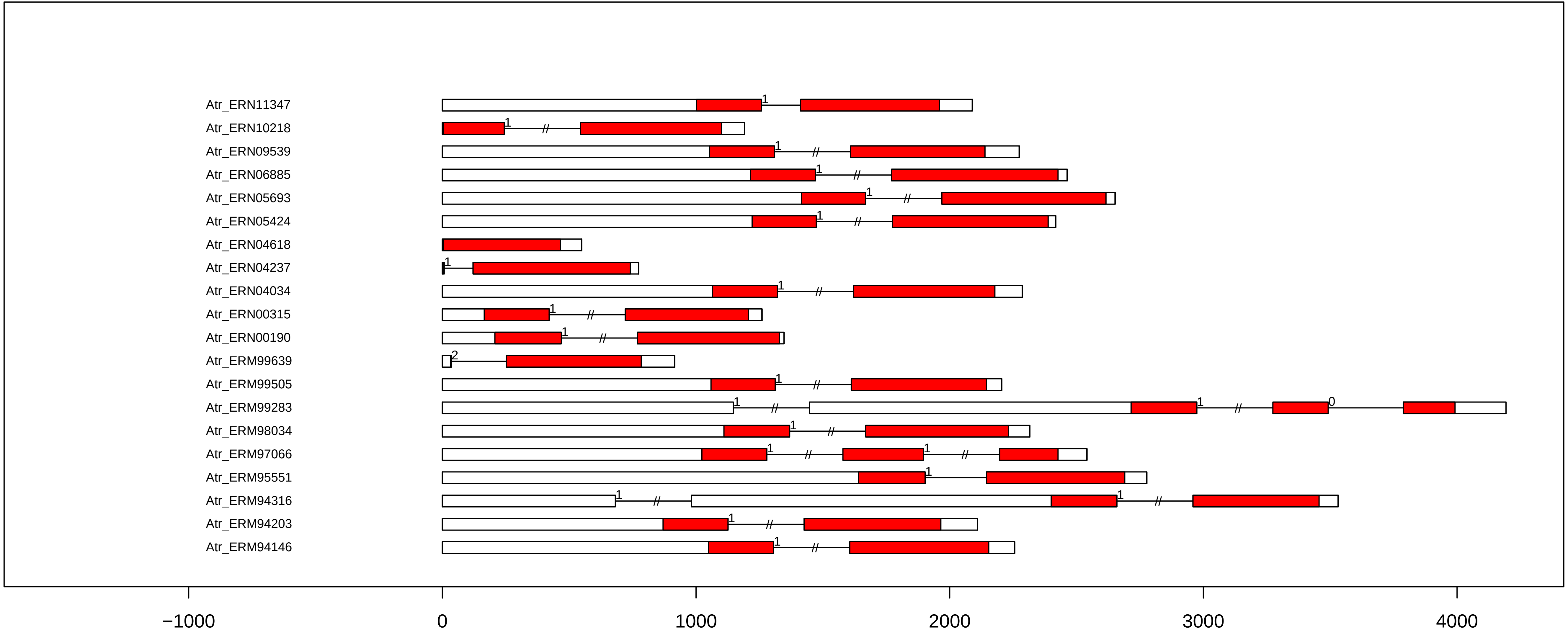

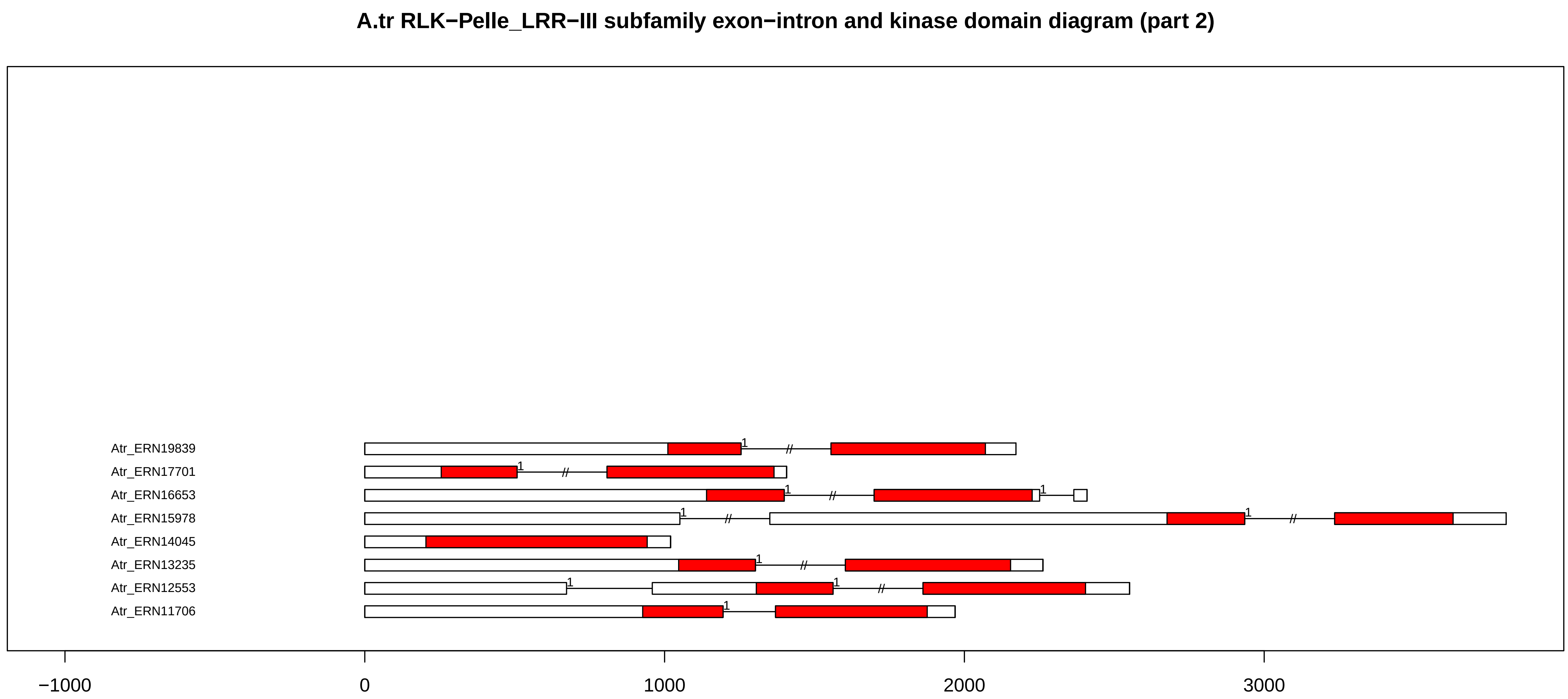
A.tr RLK−Pelle_LRR−III subfamily exon−intron and kinase domain diagram (part 2)
Atr_ERN19839
Atr_ERN17701
Atr_ERN16653
Atr_ERN15978
Atr_ERN14045
Atr_ERN13235
Atr_ERN12553
Atr_ERN11706
−1000
0
1000
2000
3000

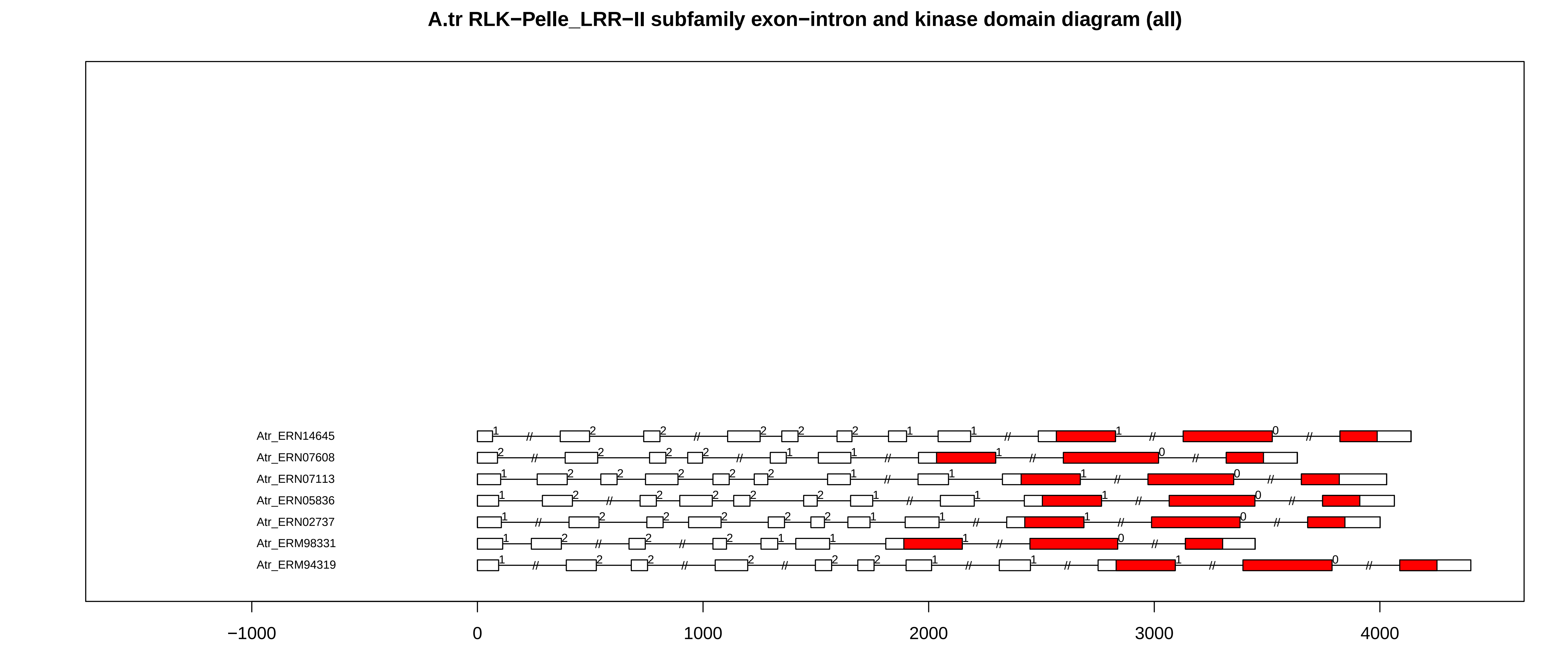

A.tr RLK−Pelle_LRR−II subfamily exon−intron and kinase domain diagram (all)
Atr_ERN14645
Atr_ERN07608
Atr_ERN07113
Atr_ERN05836
Atr_ERN02737
Atr_ERM98331
Atr_ERM94319
−1000
0
1000
2000
3000
4000

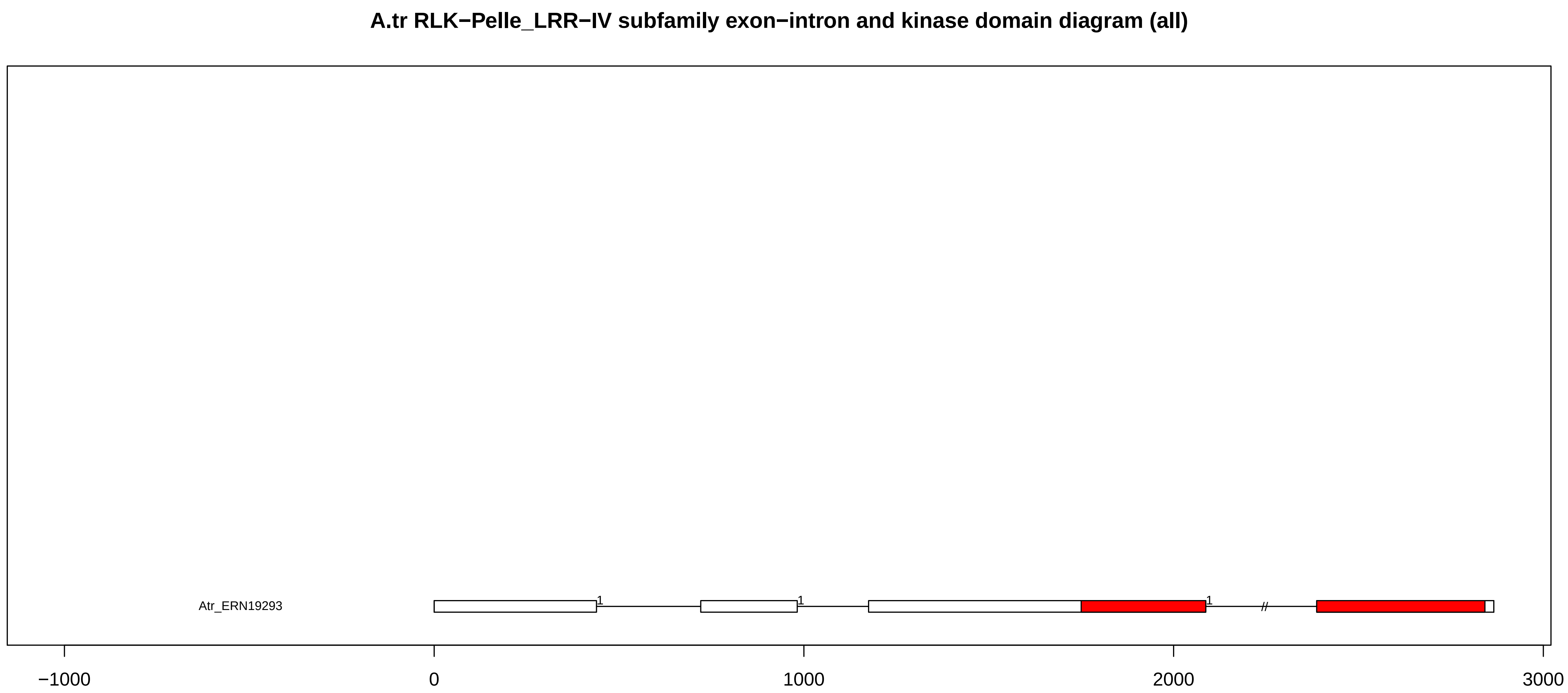
A.tr RLK−Pelle_LRR−IV subfamily exon−intron and kinase domain diagram (all)
Atr_ERN19293
1
1
1
−1000
0
1000
2000
3000

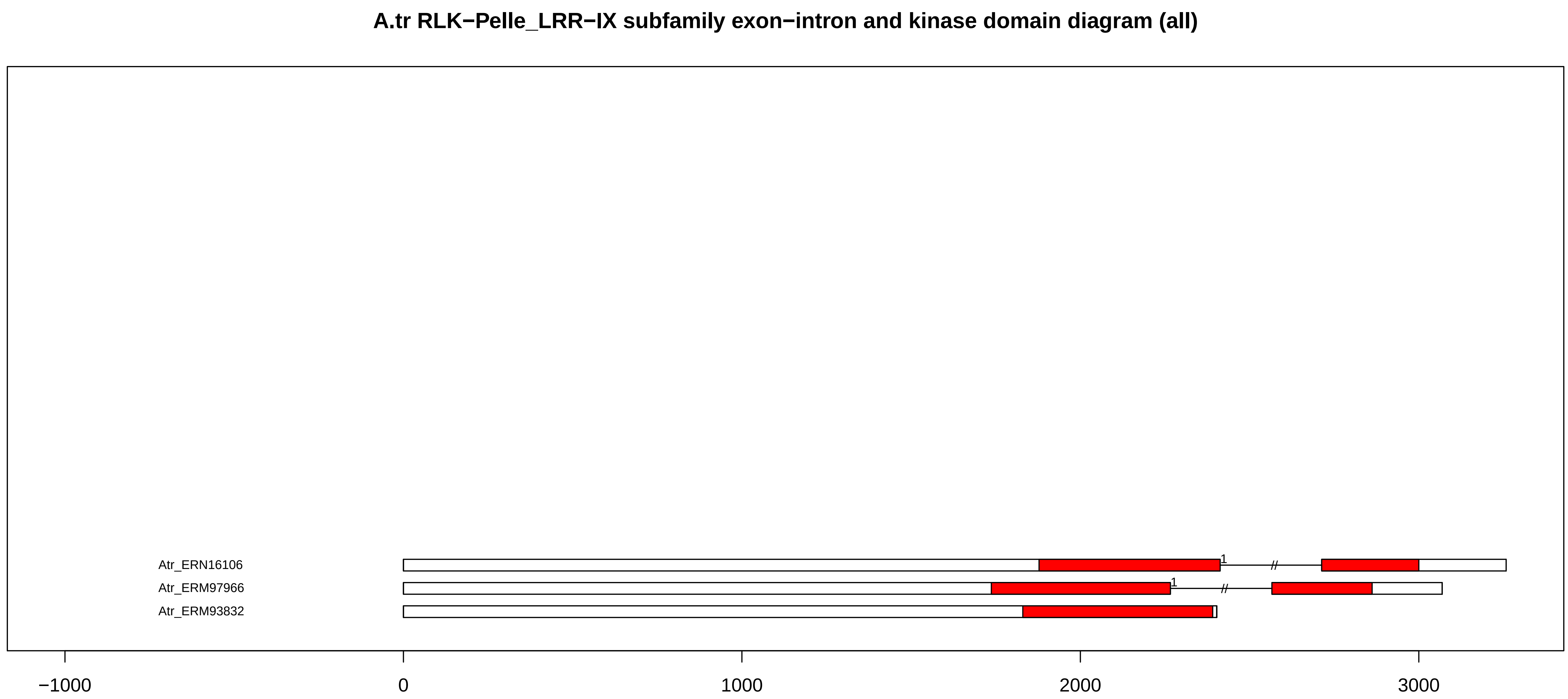
A.tr RLK−Pelle_LRR−IX subfamily exon−intron and kinase domain diagram (all)
Atr_ERN16106
Atr_ERM97966
Atr_ERM93832
1
1
−1000
0
1000
2000
3000

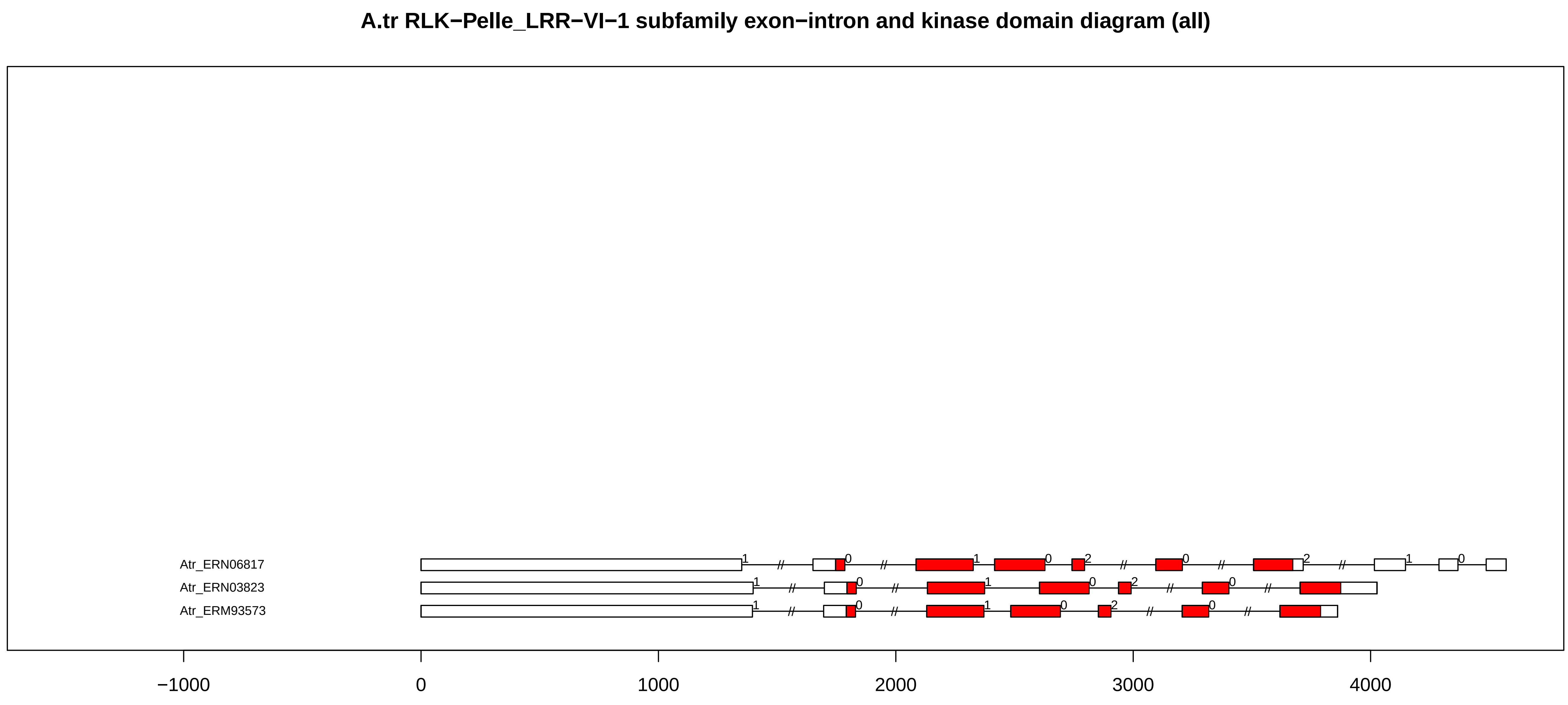

A.tr RLK−Pelle_LRR−VI−1 subfamily exon−intron and kinase domain diagram (all)
Atr_ERN06817
Atr_ERN03823
Atr_ERM93573
−1000
0
1000
2000
3000
4000

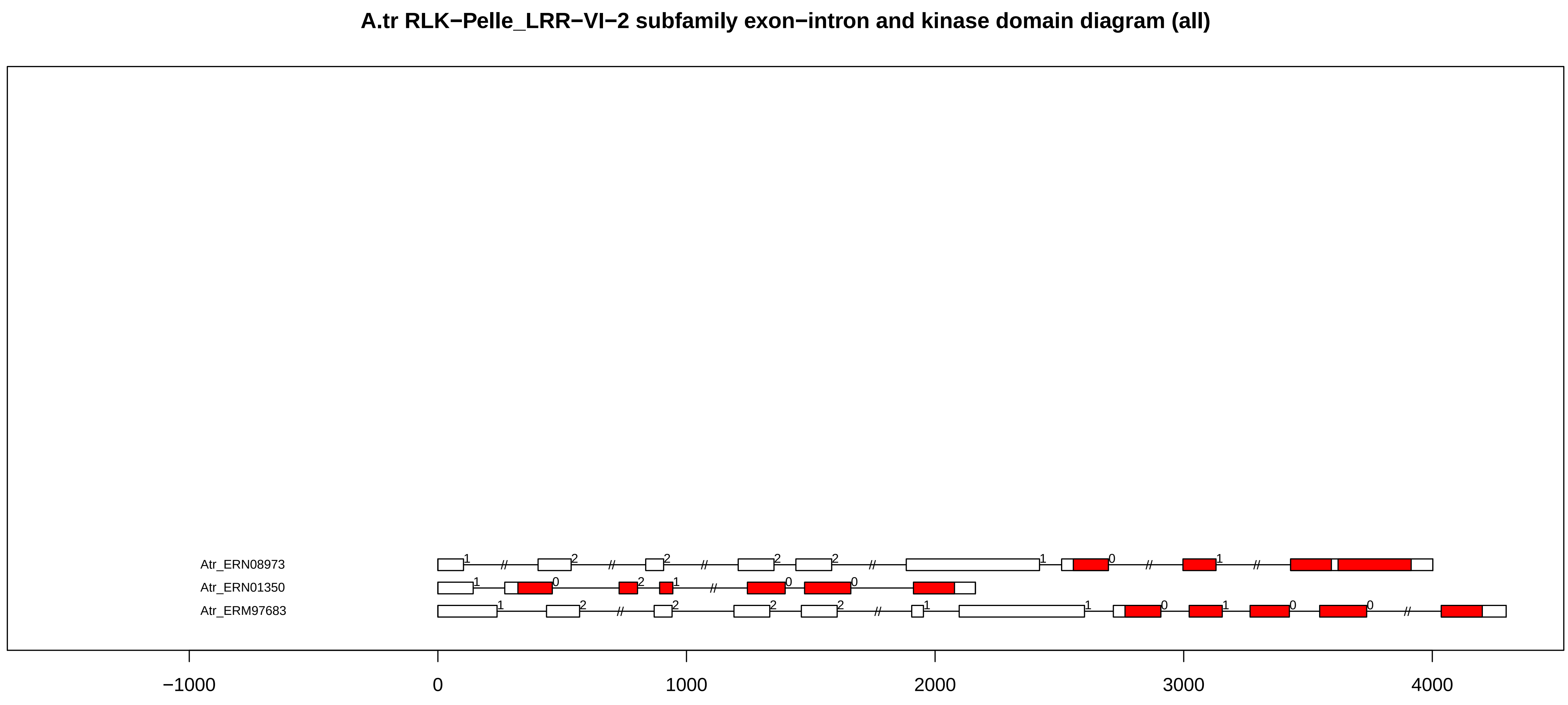
A.tr RLK−Pelle_LRR−VI−2 subfamily exon−intron and kinase domain diagram (all)
Atr_ERN08973
Atr_ERN01350
Atr_ERM97683
−1000
0
1000
2000
3000
4000

A.tr RLK−Pelle_LRR−VII−1 subfamily exon−intron and kinase domain diagram (all)

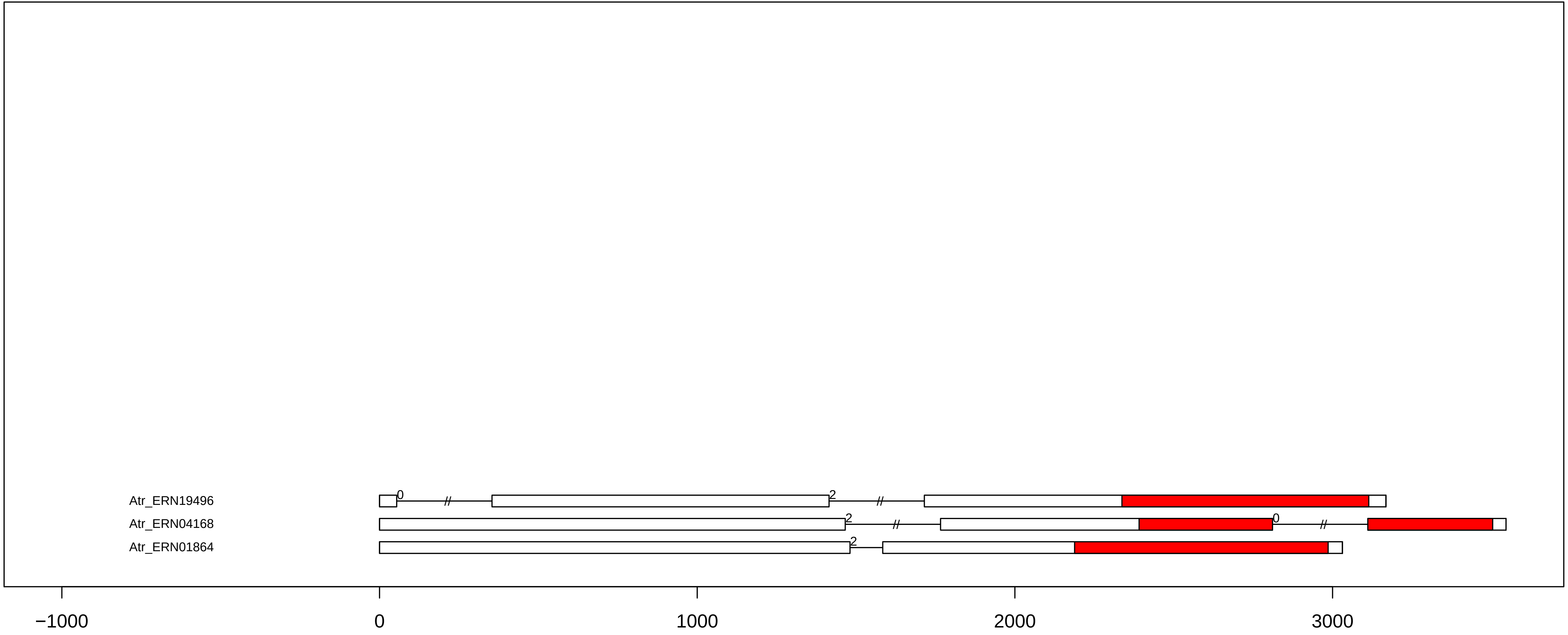

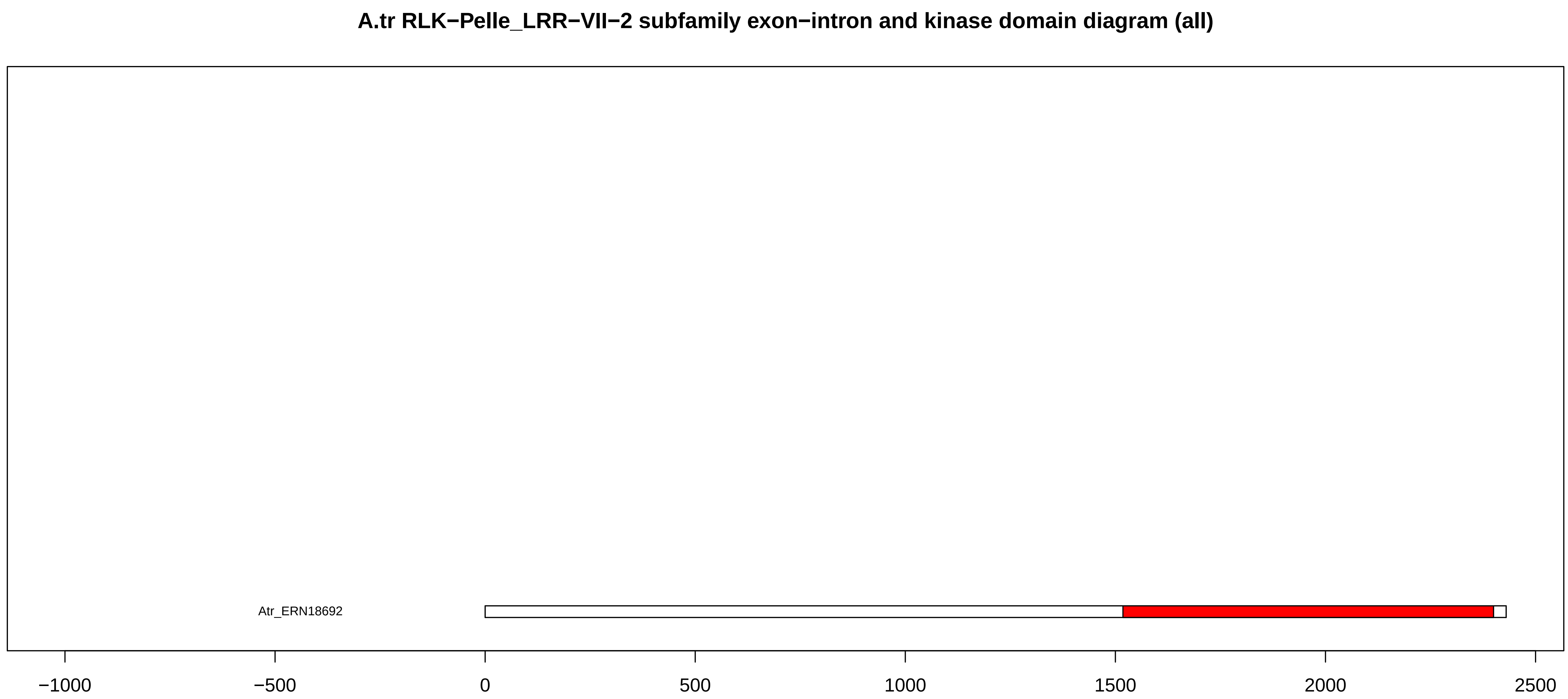
A.tr RLK−Pelle_LRR−VII−2 subfamily exon−intron and kinase domain diagram (all)
Atr_ERN18692
−1000
−500
0
500
1000
1500
2000
2500

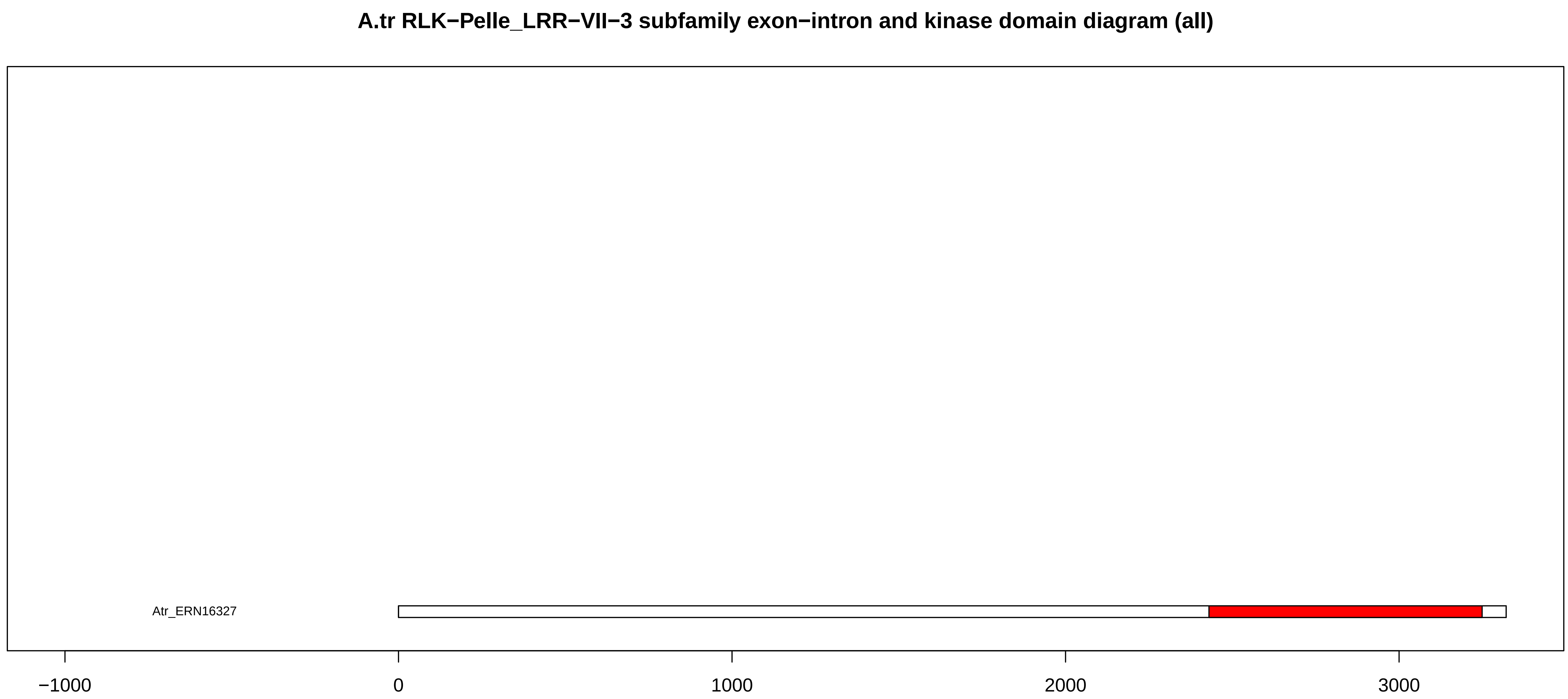

A.tr RLK−Pelle_LRR−VII−3 subfamily exon−intron and kinase domain diagram (all)
Atr_ERN16327
−1000
0
1000
2000
3000

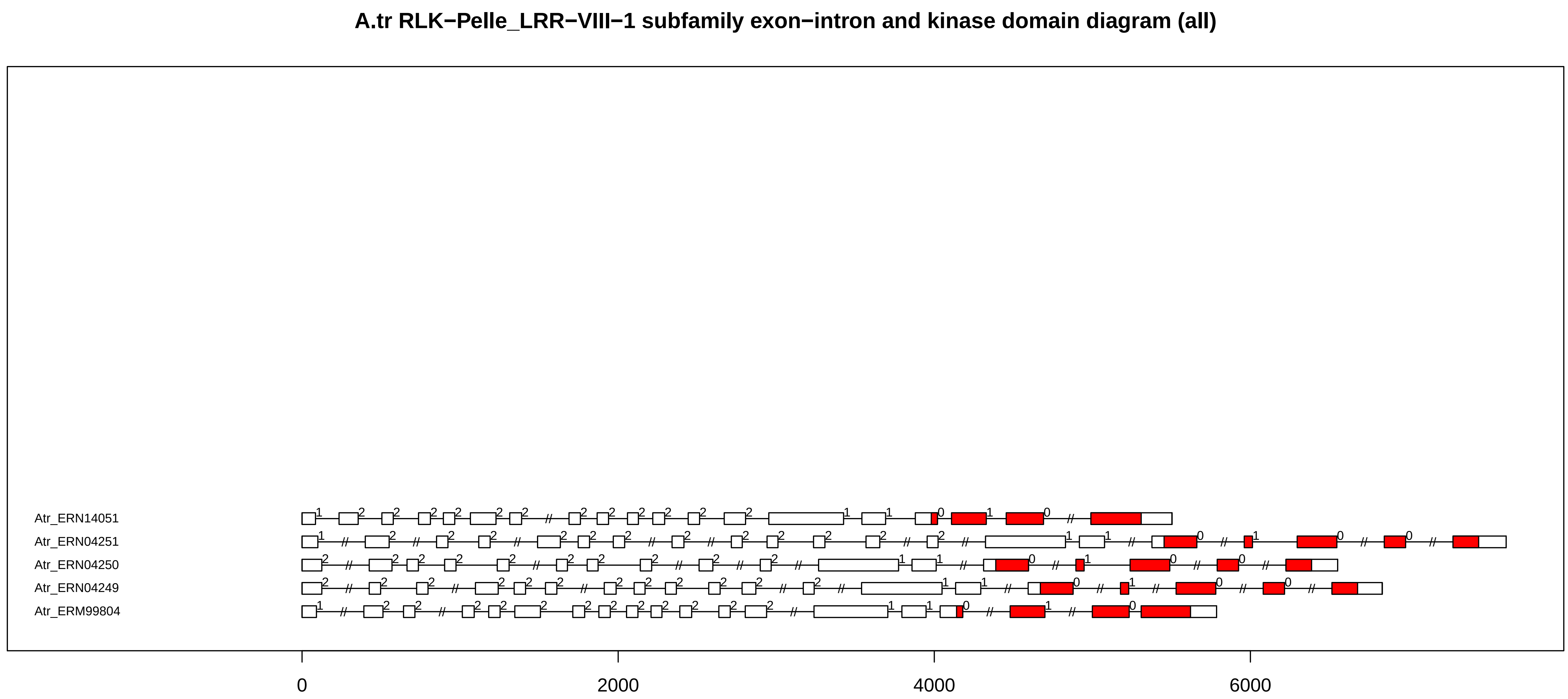
A.tr RLK−Pelle_LRR−VIII−1 subfamily exon−intron and kinase domain diagram (all)
Atr_ERN14051
Atr_ERN04251
Atr_ERN04250
Atr_ERN04249
Atr_ERM99804
0
2000
4000
6000

A.tr RLK−Pelle_LRR−V subfamily exon−intron and kinase domain diagram (all)

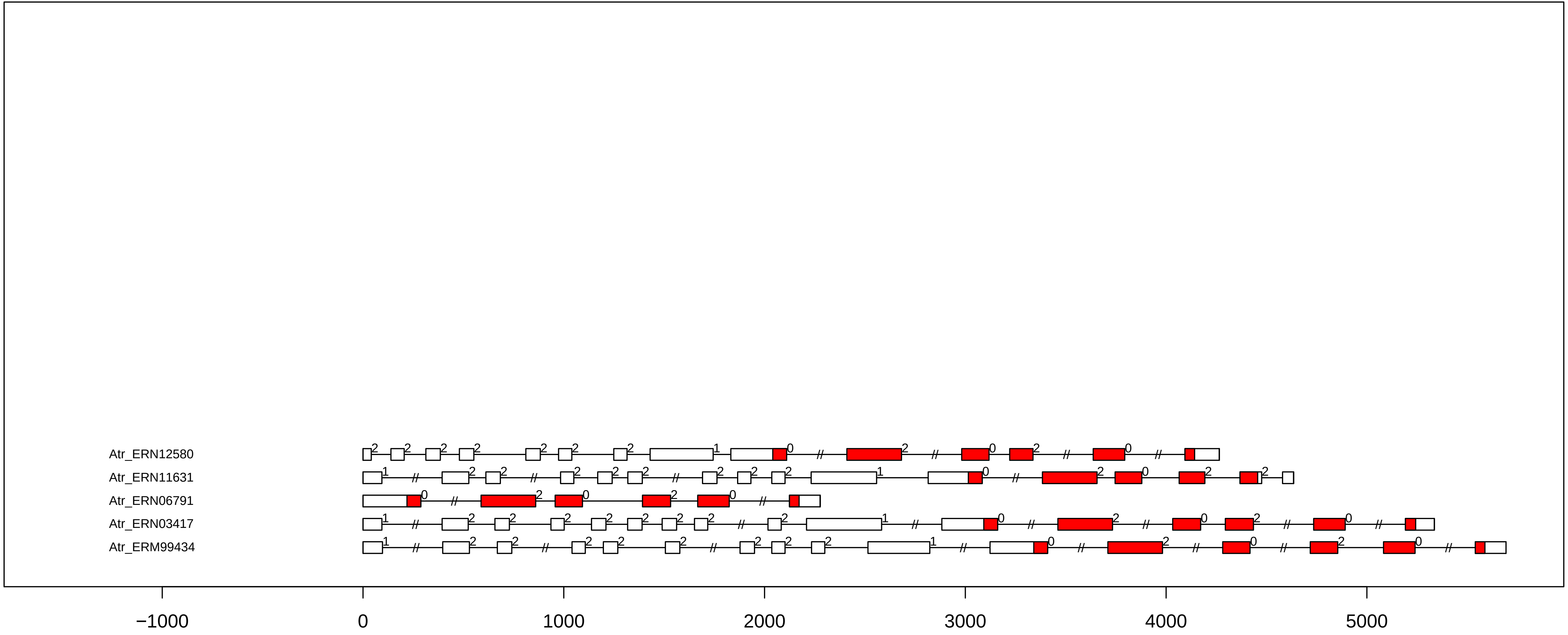

A.tr RLK−Pelle_LRR−Xa subfamily exon−intron and kinase domain diagram (all)

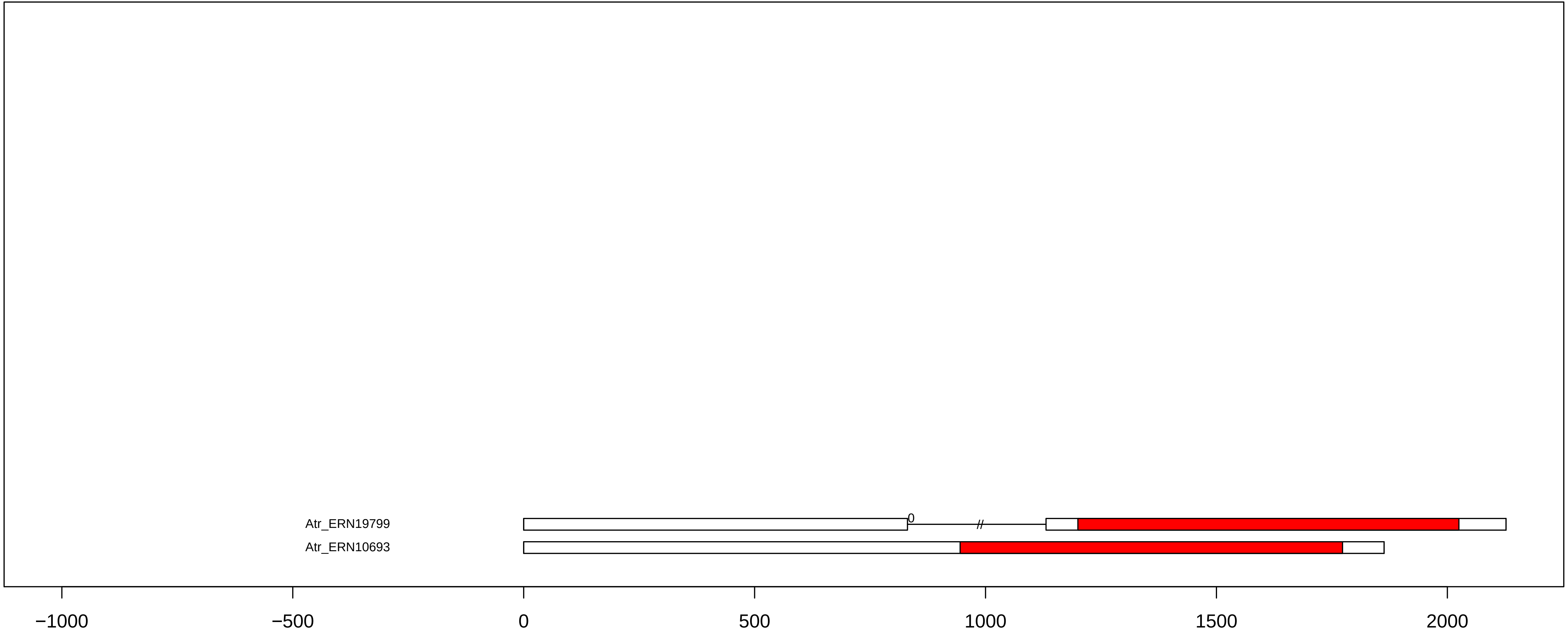

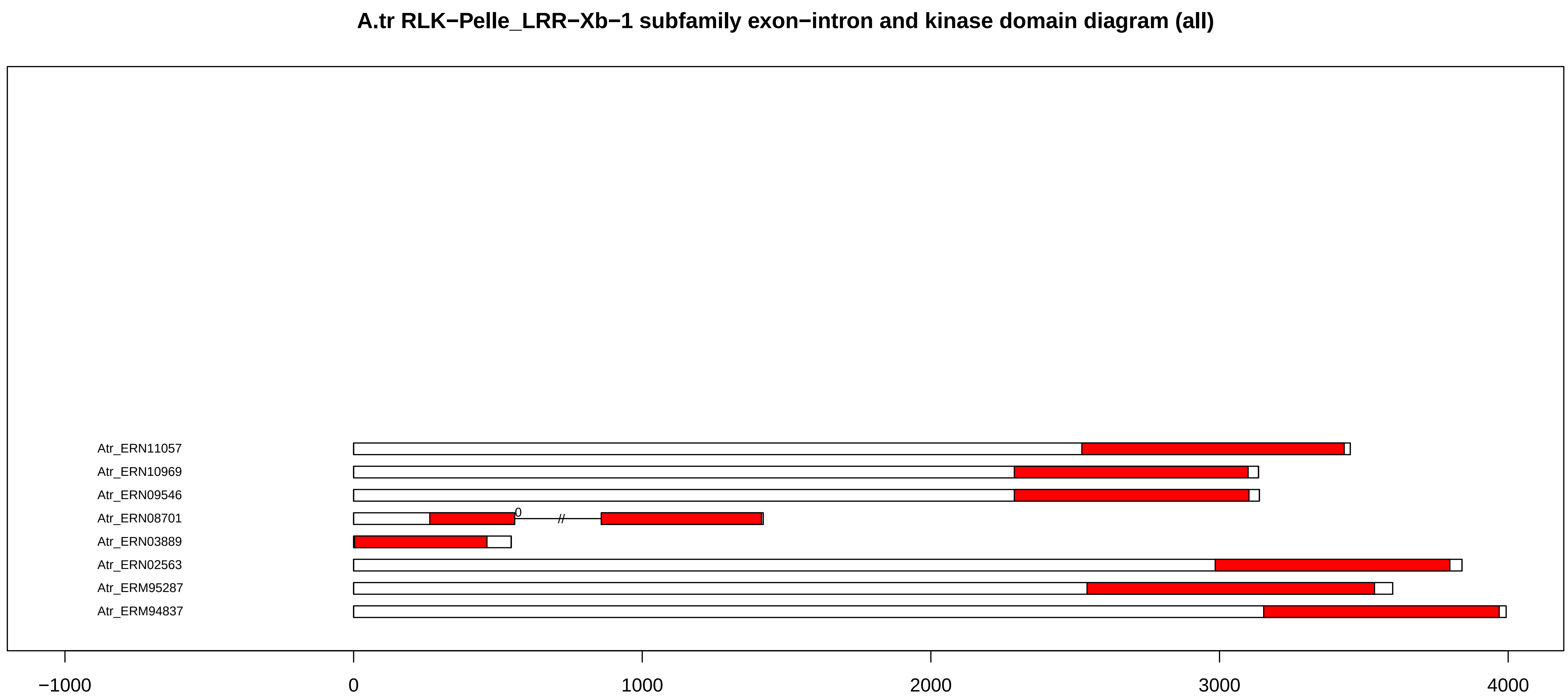
A.tr RLK−Pelle_LRR−Xb−1 subfamily exon−intron and kinase domain diagram (all)
Atr_ERN11057
Atr_ERN10969
Atr_ERN09546
Atr_ERN08701
Atr_ERN03889
Atr_ERN02563
Atr_ERM95287
Atr_ERM94837
0
//
−1000
0
1000
2000
3000
4000

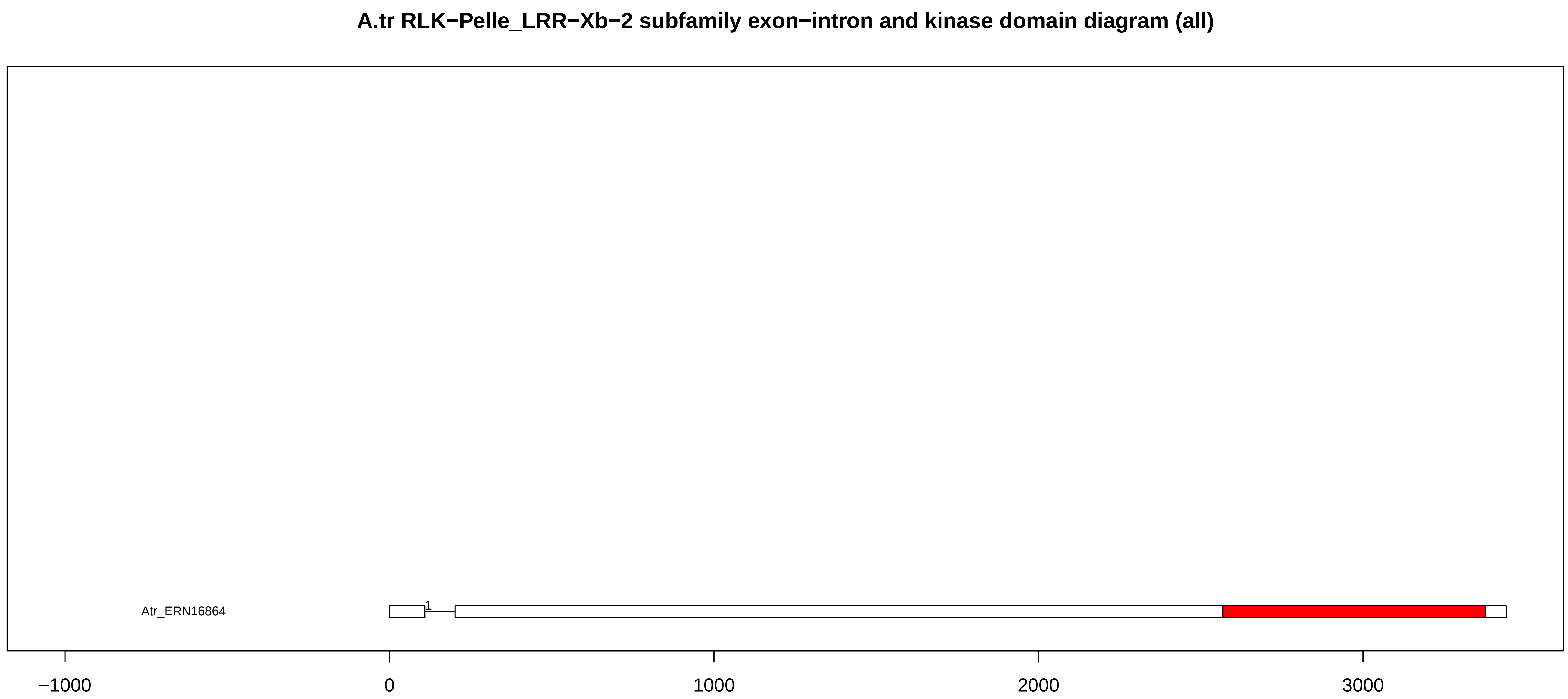

A.tr RLK−Pelle_LRR−Xb−2 subfamily exon−intron and kinase domain diagram (all)
Atr_ERN16864
1
−1000
0
1000
2000
3000

A.tr RLK−Pelle_LRR−XI−1 subfamily exon−intron and kinase domain diagram (part 1)

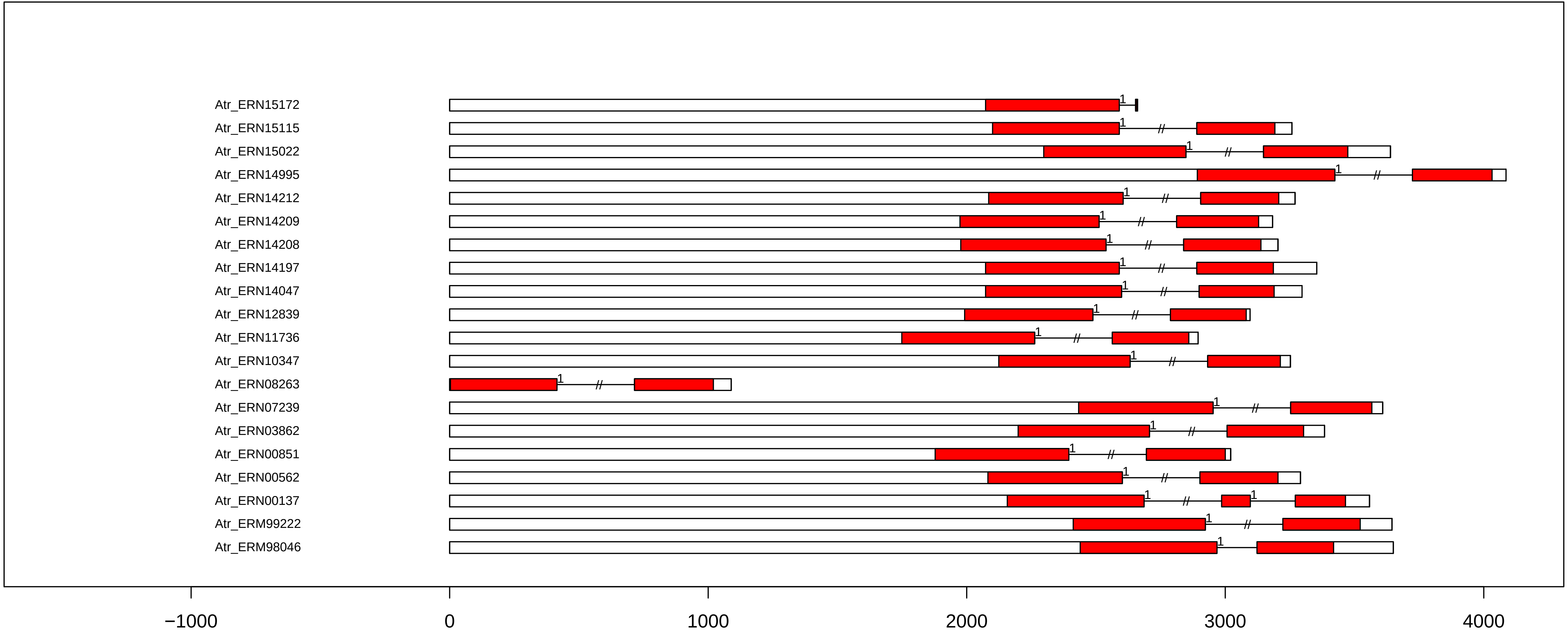

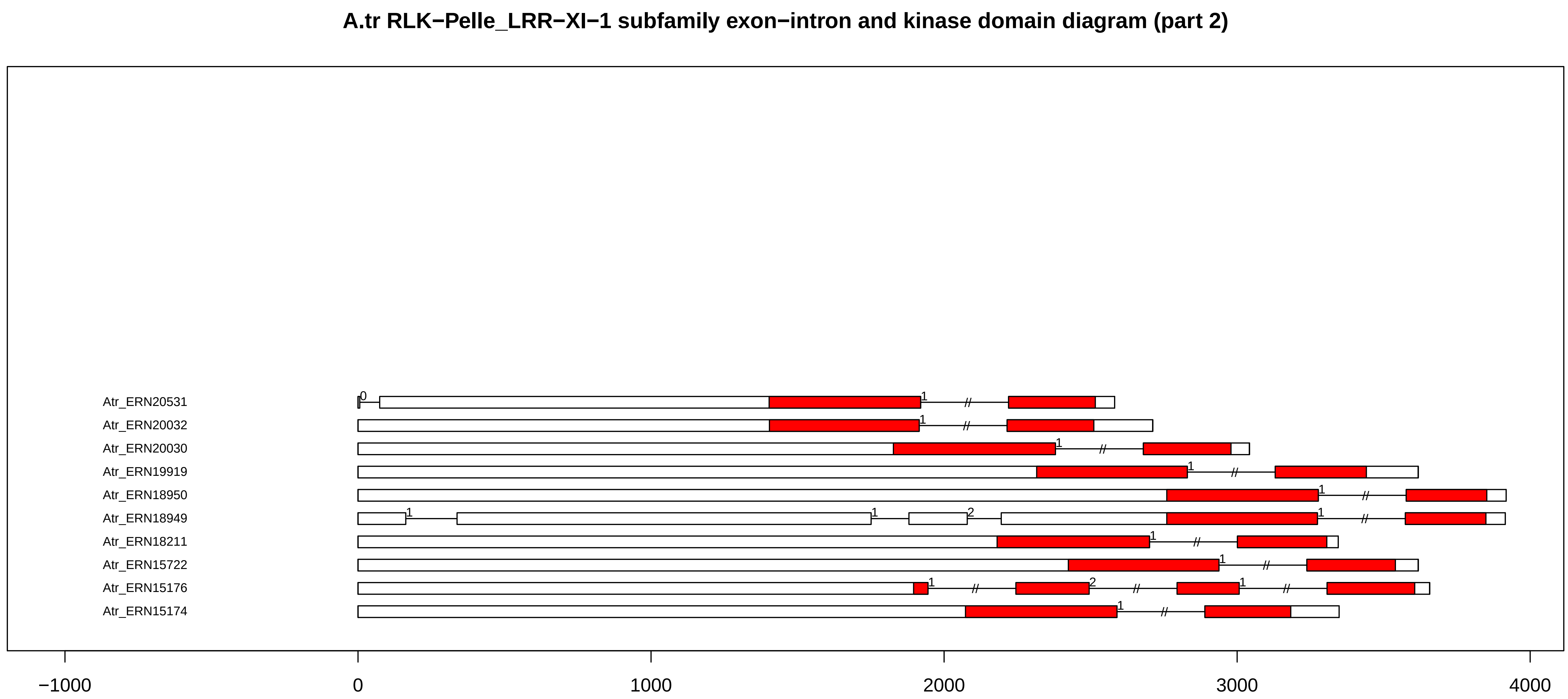

A.tr RLK−Pelle_LRR−XI−1 subfamily exon−intron and kinase domain diagram (part 2)
Atr_ERN20531
Atr_ERN20032
Atr_ERN20030
Atr_ERN19919
Atr_ERN18950
Atr_ERN18949
Atr_ERN18211
Atr_ERN15722
Atr_ERN15176
Atr_ERN15174
−1000
0
1000
2000
3000
4000

A.tr RLK−Pelle_LRR−XI−2 subfamily exon−intron and kinase domain diagram (all)

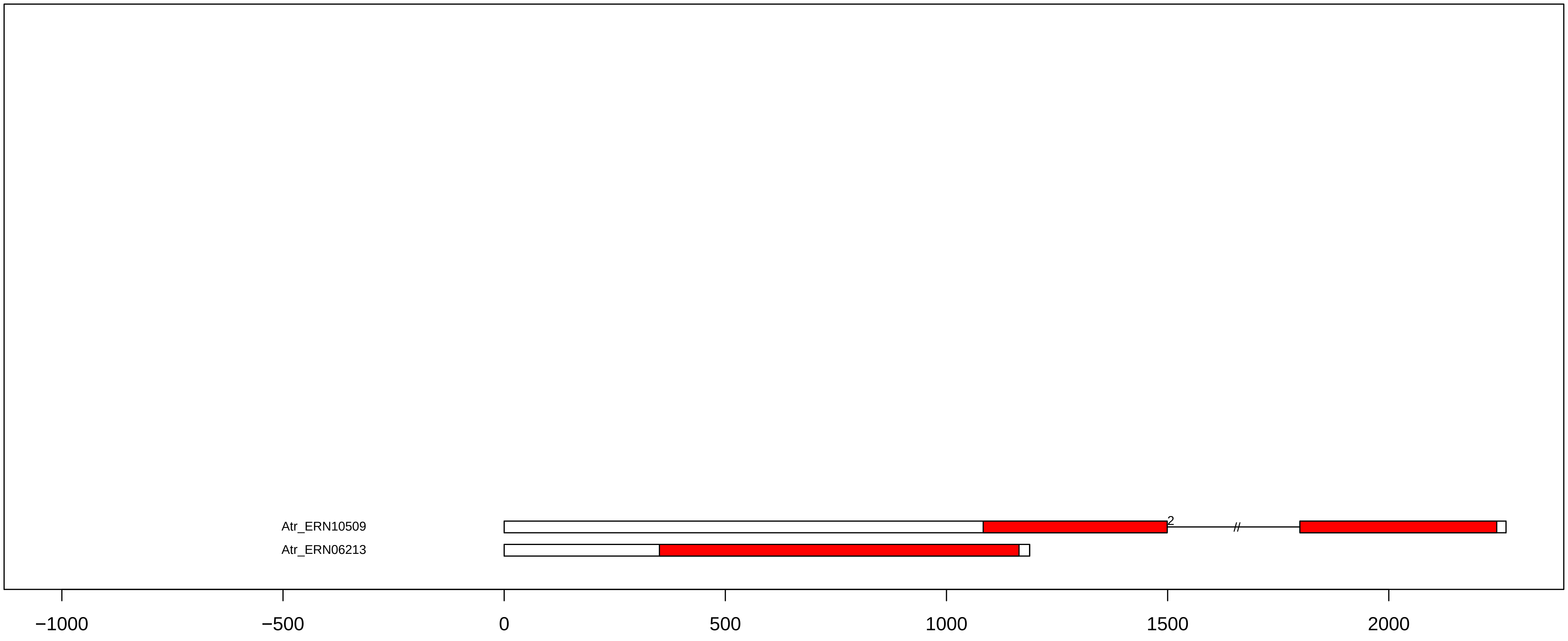

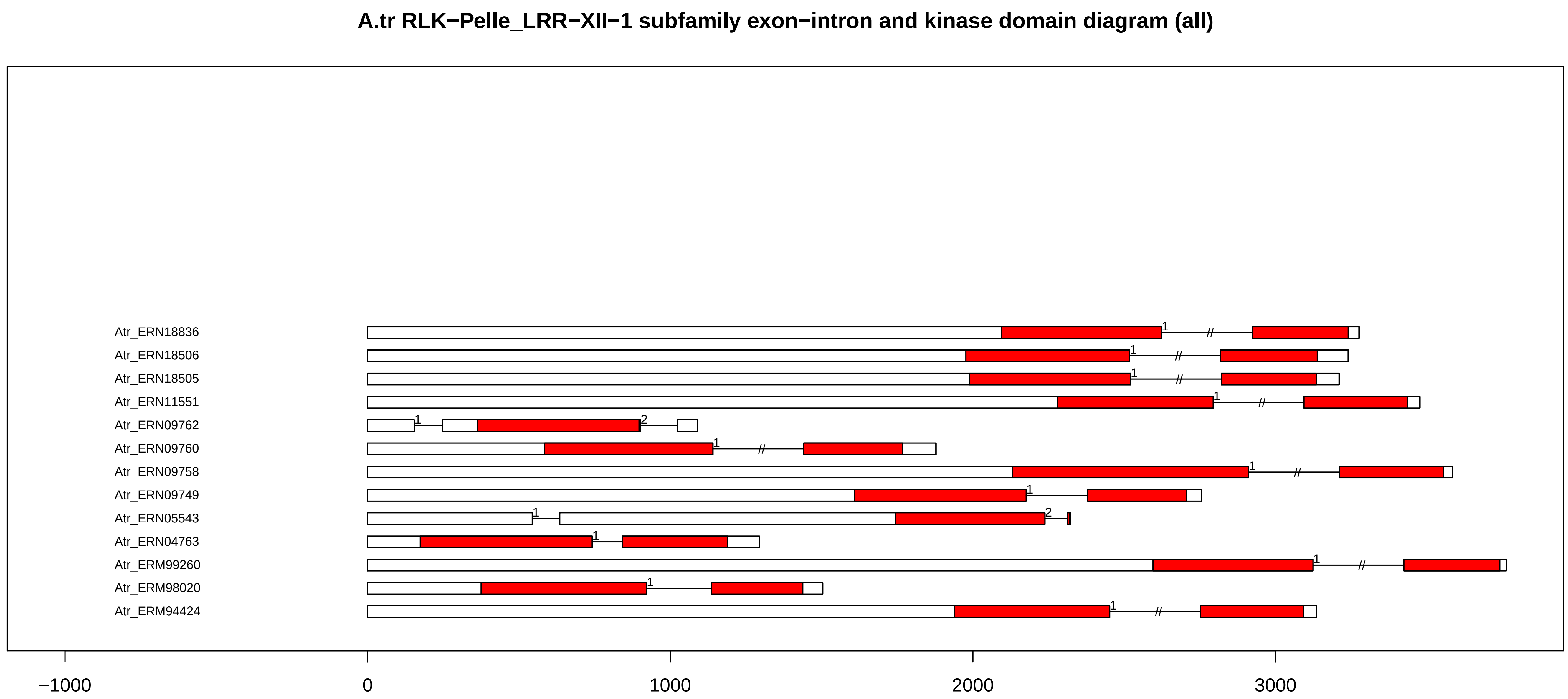

A.tr RLK−Pelle_LRR−XII−1 subfamily exon−intron and kinase domain diagram (all)
Atr_ERN18836
Atr_ERN18506
Atr_ERN18505
Atr_ERN11551
Atr_ERN09762
Atr_ERN09760
Atr_ERN09758
Atr_ERN09749
Atr_ERN05543
Atr_ERN04763
Atr_ERM99260
Atr_ERM98020
Atr_ERM94424
−1000
0
1000
2000
3000

A.tr RLK−Pelle_LRR−XIIIa subfamily exon−intron and kinase domain diagram (all)

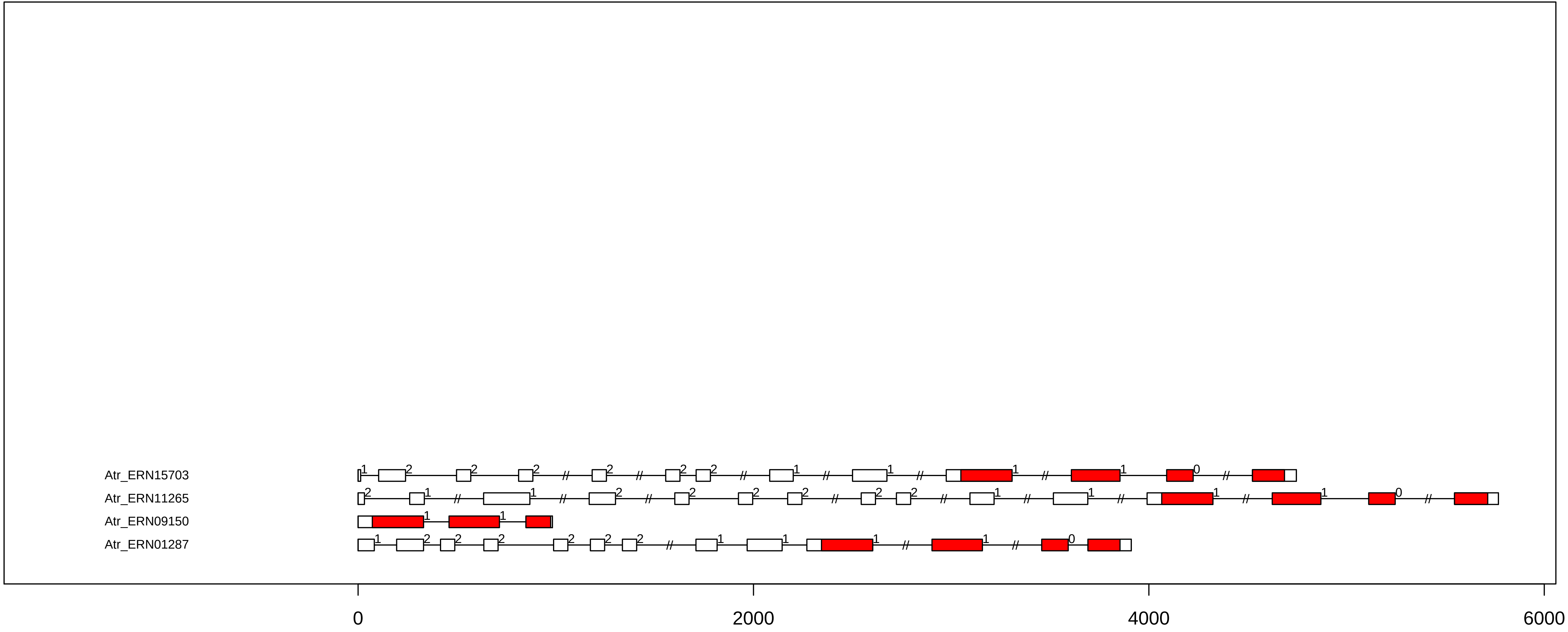

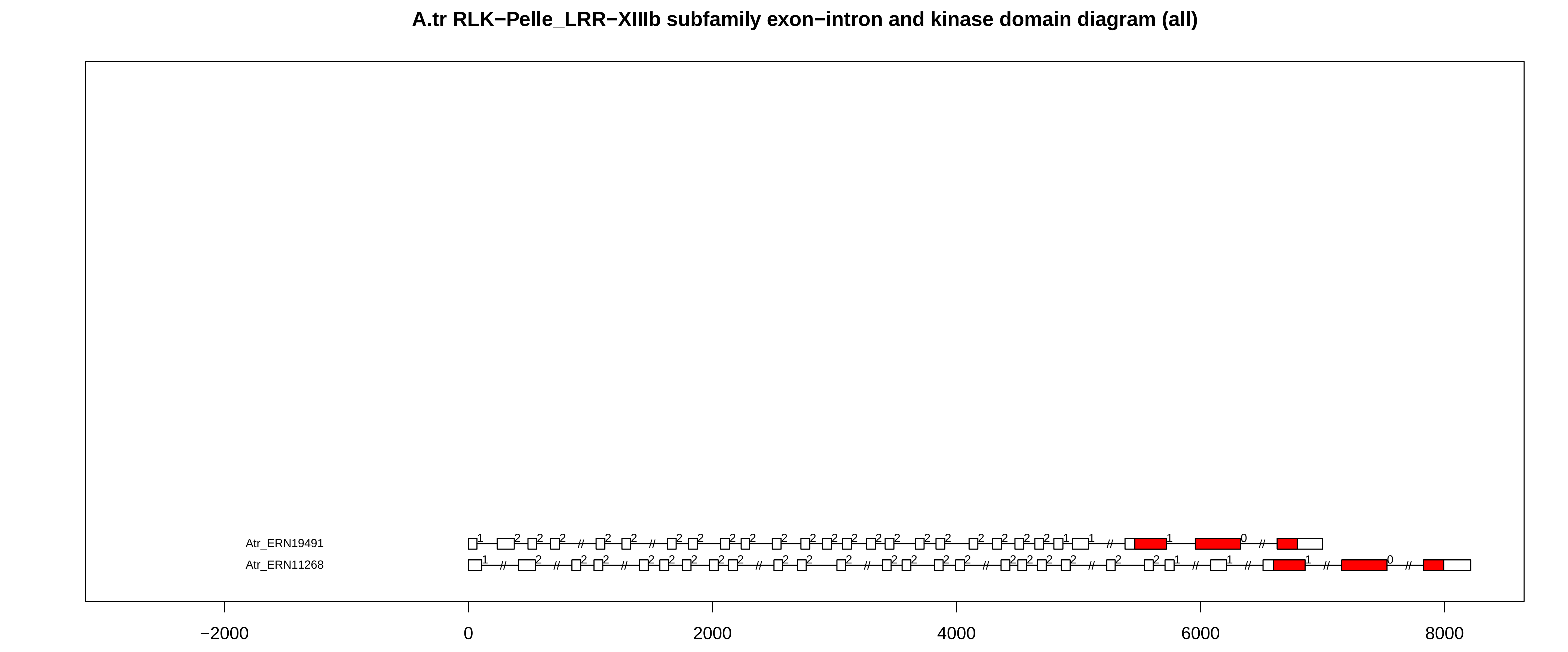
A.tr RLK−Pelle_LRR−XIIIb subfamily exon−intron and kinase domain diagram (all)
Atr_ERN19491
Atr_ERN11268
−2000
0
2000
4000
6000
8000

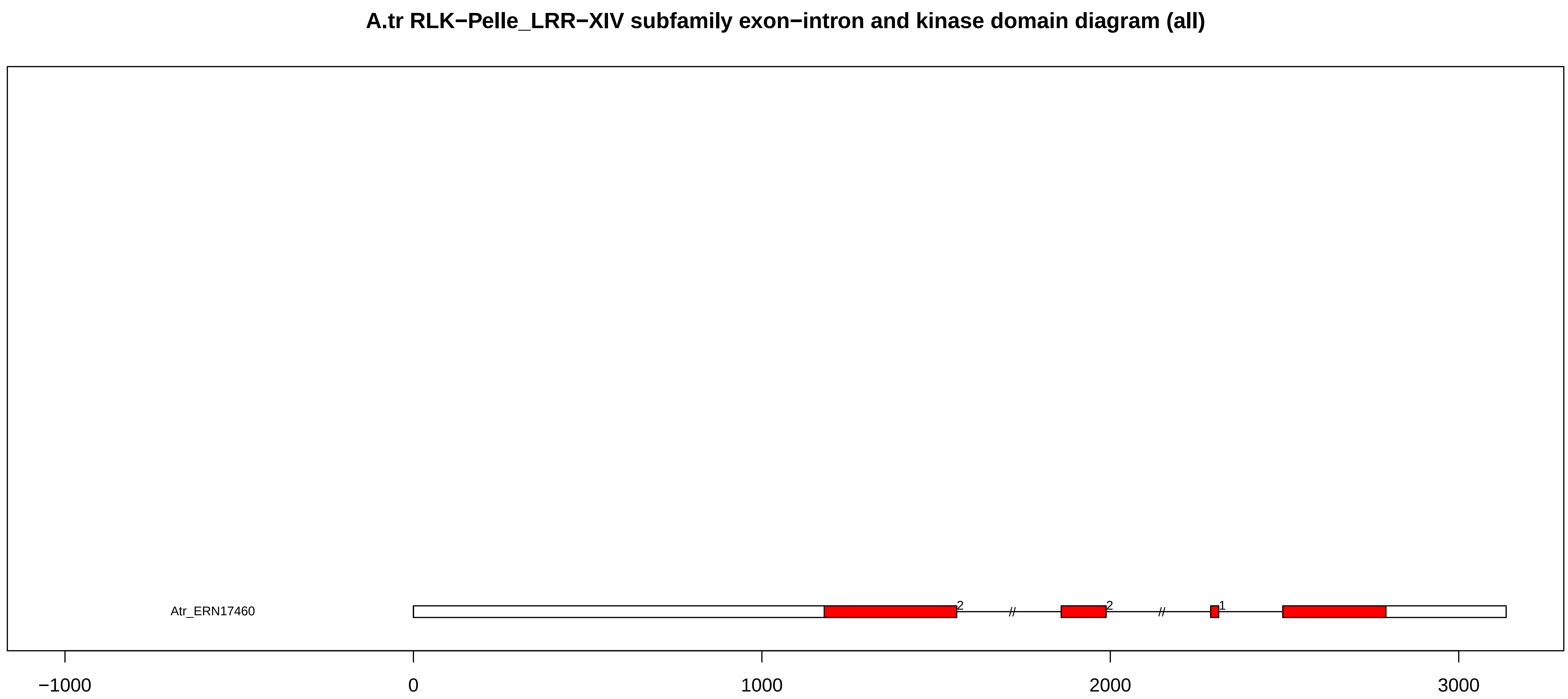

A.tr RLK−Pelle_LRR−XIV subfamily exon−intron and kinase domain diagram (all)
Atr_ERN17460
2
2
1
−1000
0
1000
2000
3000

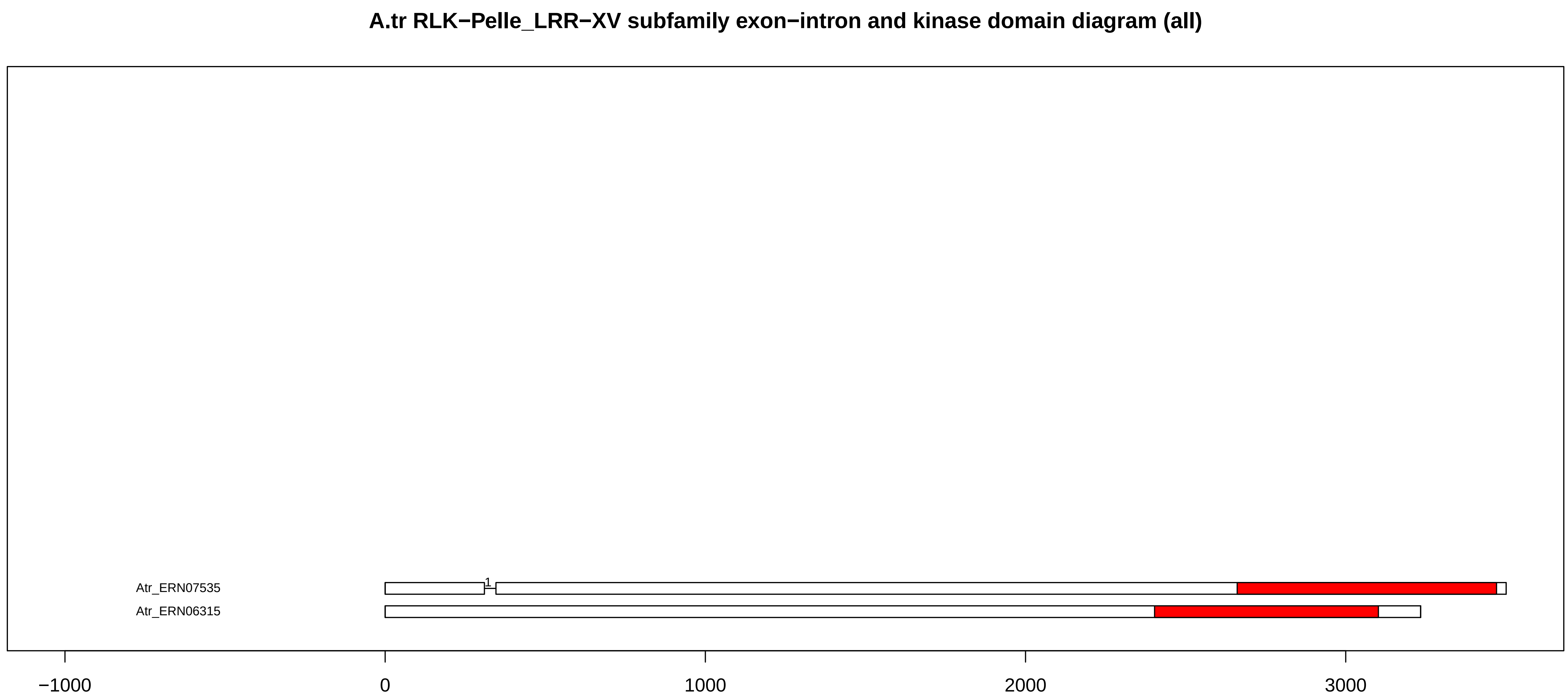
A.tr RLK−Pelle_LRR−XV subfamily exon−intron and kinase domain diagram (all)
Atr_ERN07535
1
Atr_ERN06315
−1000
0
1000
2000
3000

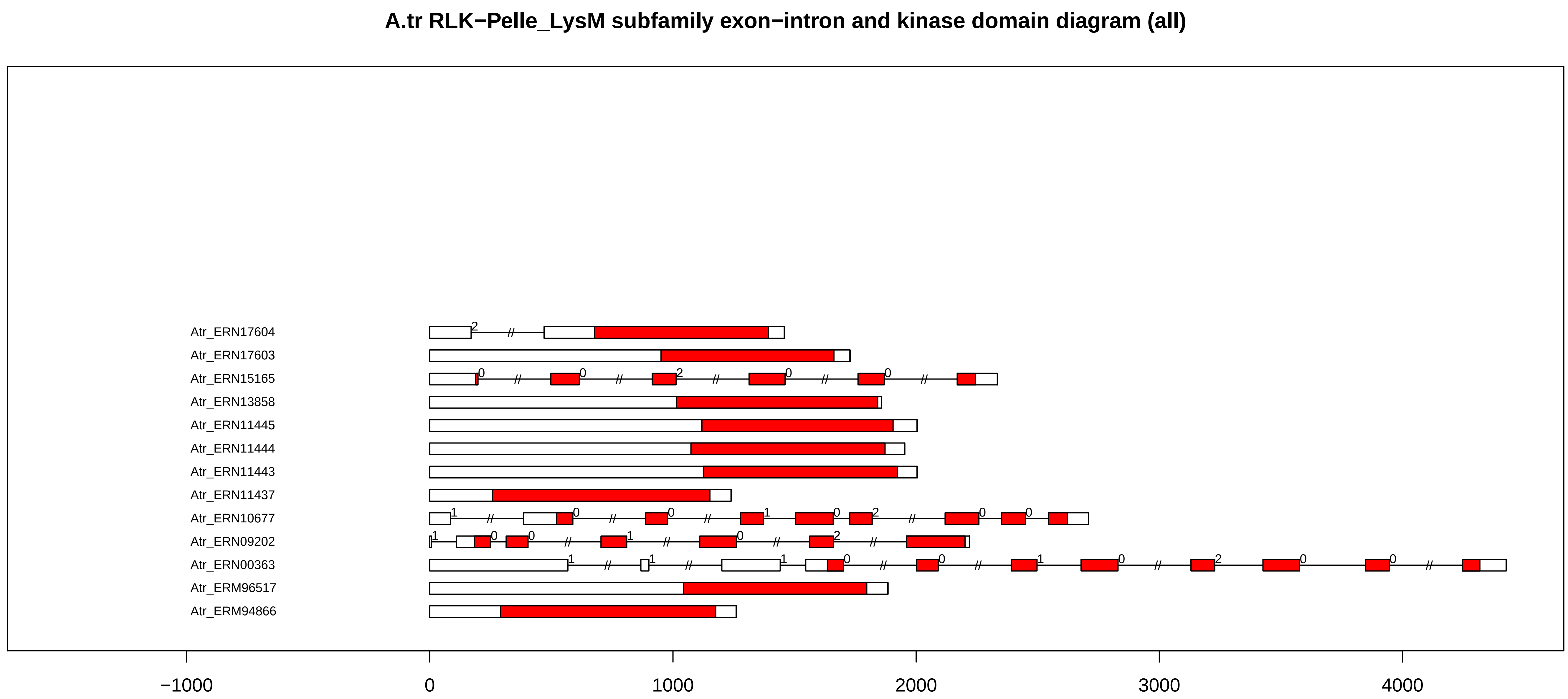

A.tr RLK−Pelle_LysM subfamily exon−intron and kinase domain diagram (all)
Atr_ERN17604
Atr_ERN17603
Atr_ERN15165
Atr_ERN13858
Atr_ERN11445
Atr_ERN11444
Atr_ERN11443
Atr_ERN11437
Atr_ERN10677
Atr_ERN09202
Atr_ERN00363
Atr_ERM96517
Atr_ERM94866
−1000
0
1000
2000
3000
4000

A.tr RLK−Pelle_PERK−1 subfamily exon−intron and kinase domain diagram (all)

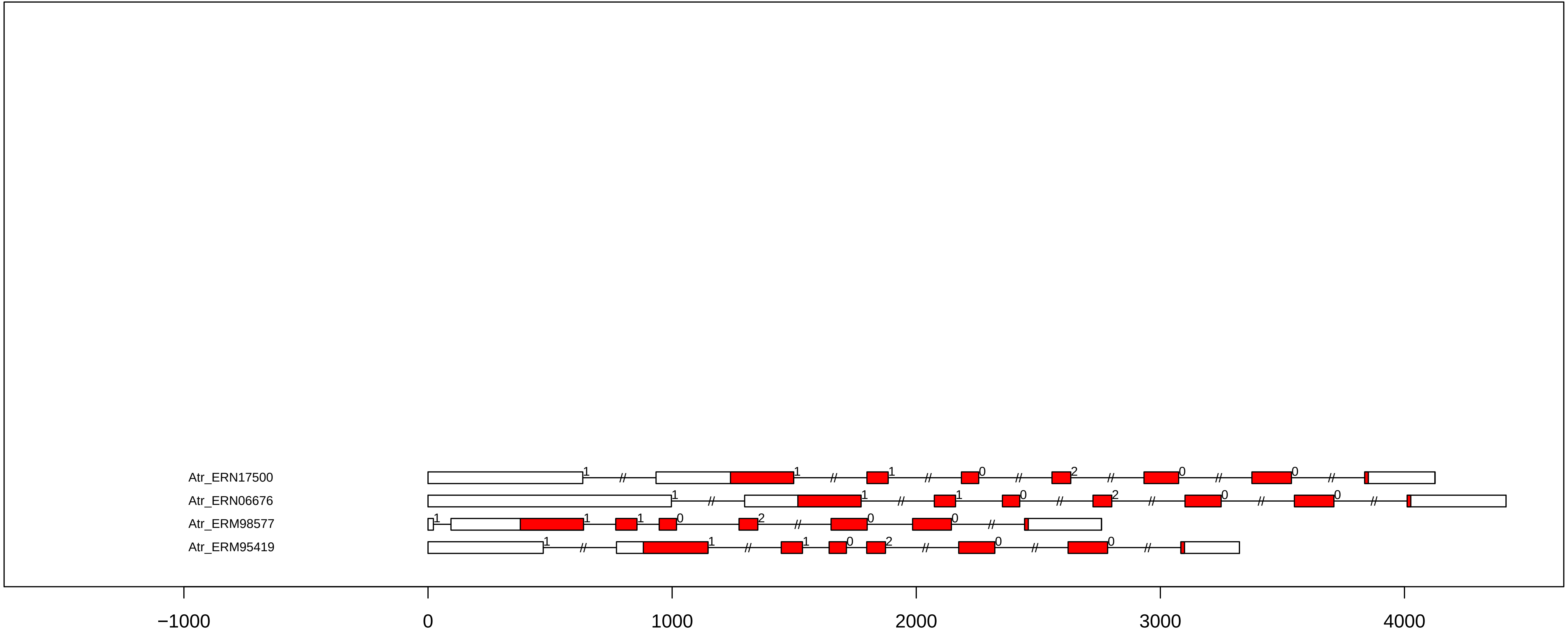

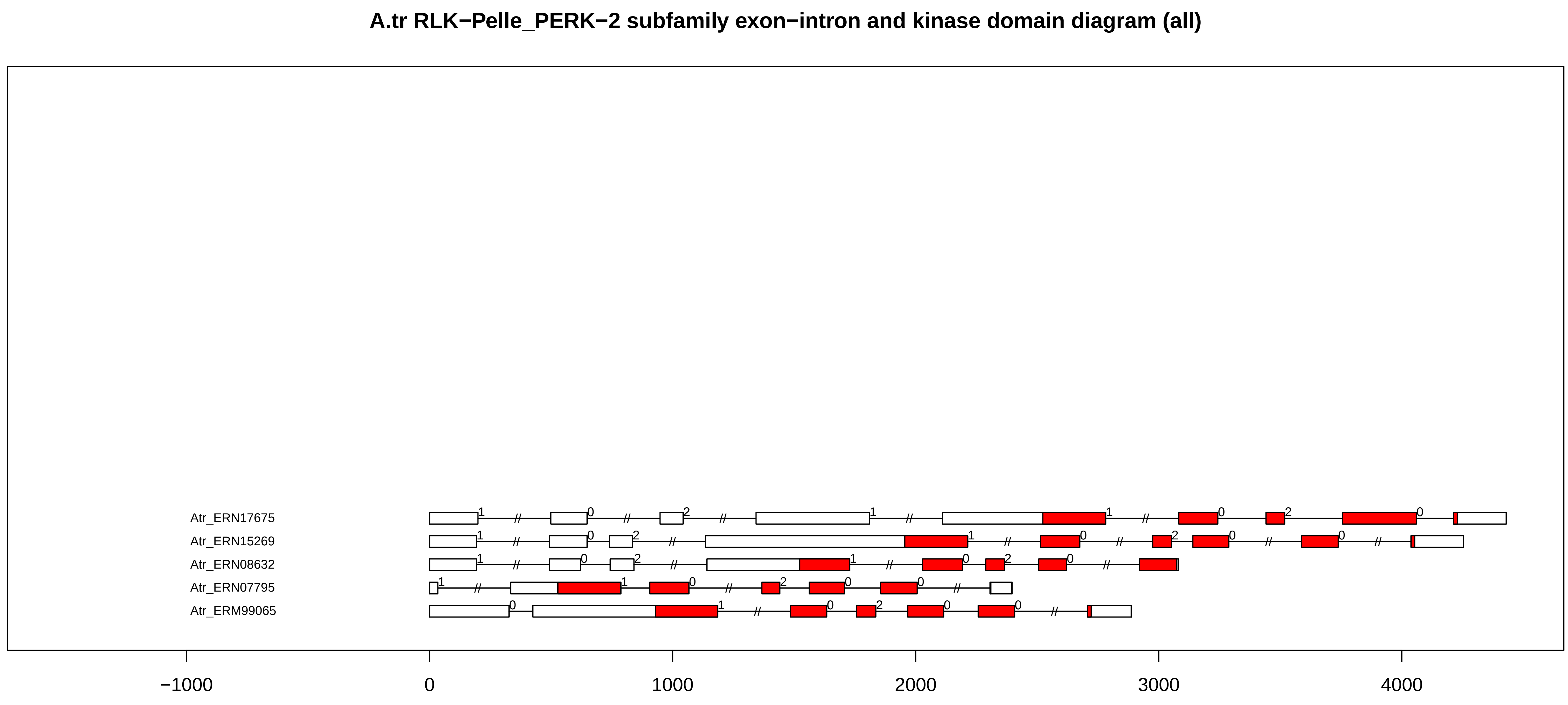

A.tr RLK−Pelle_PERK−2 subfamily exon−intron and kinase domain diagram (all)
Atr_ERN17675
Atr_ERN15269
Atr_ERN08632
Atr_ERN07795
Atr_ERM99065
−1000
0
1000
2000
3000
4000

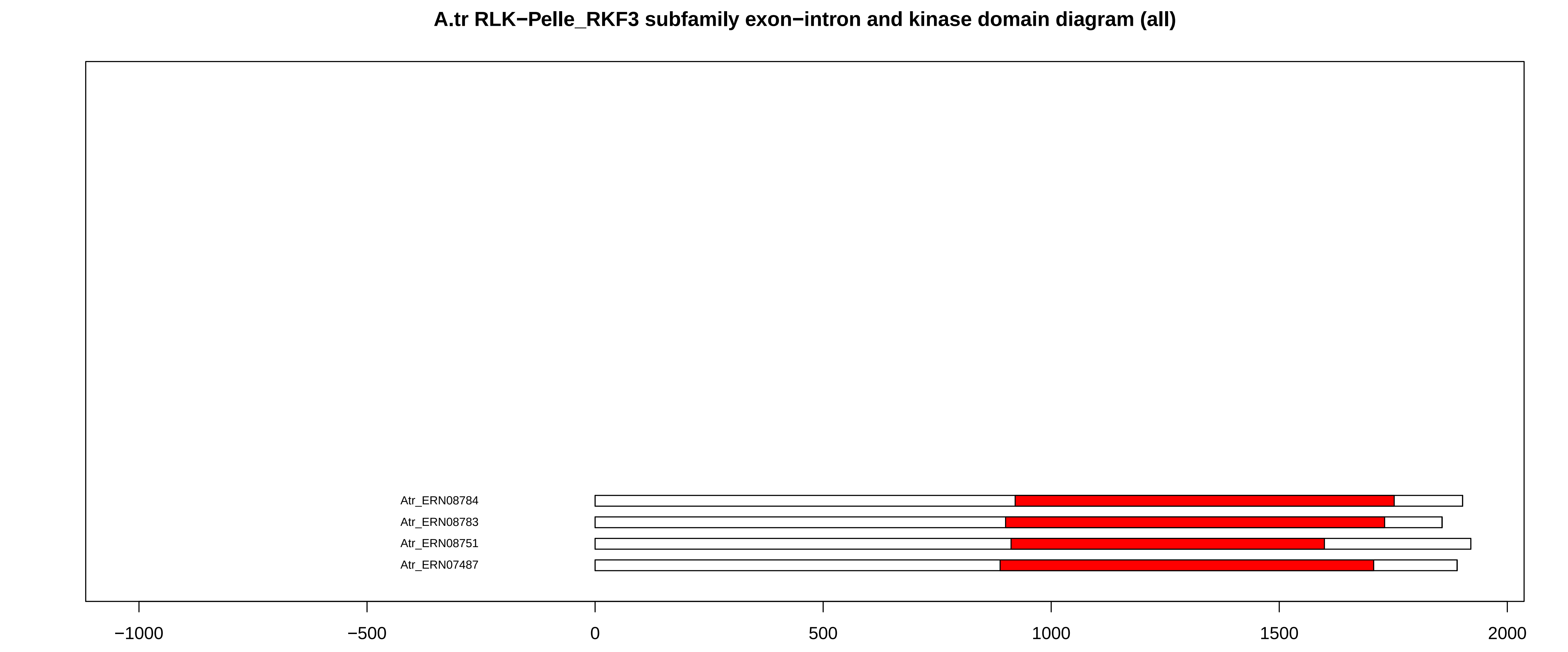
A.tr RLK−Pelle_RKF3 subfamily exon−intron and kinase domain diagram (all)
Atr_ERN08784
Atr_ERN08783
Atr_ERN08751
Atr_ERN07487
−1000
−500
0
500
1000
1500
2000

A.tr RLK−Pelle_RLCK−II subfamily exon−intron and kinase domain diagram (all)

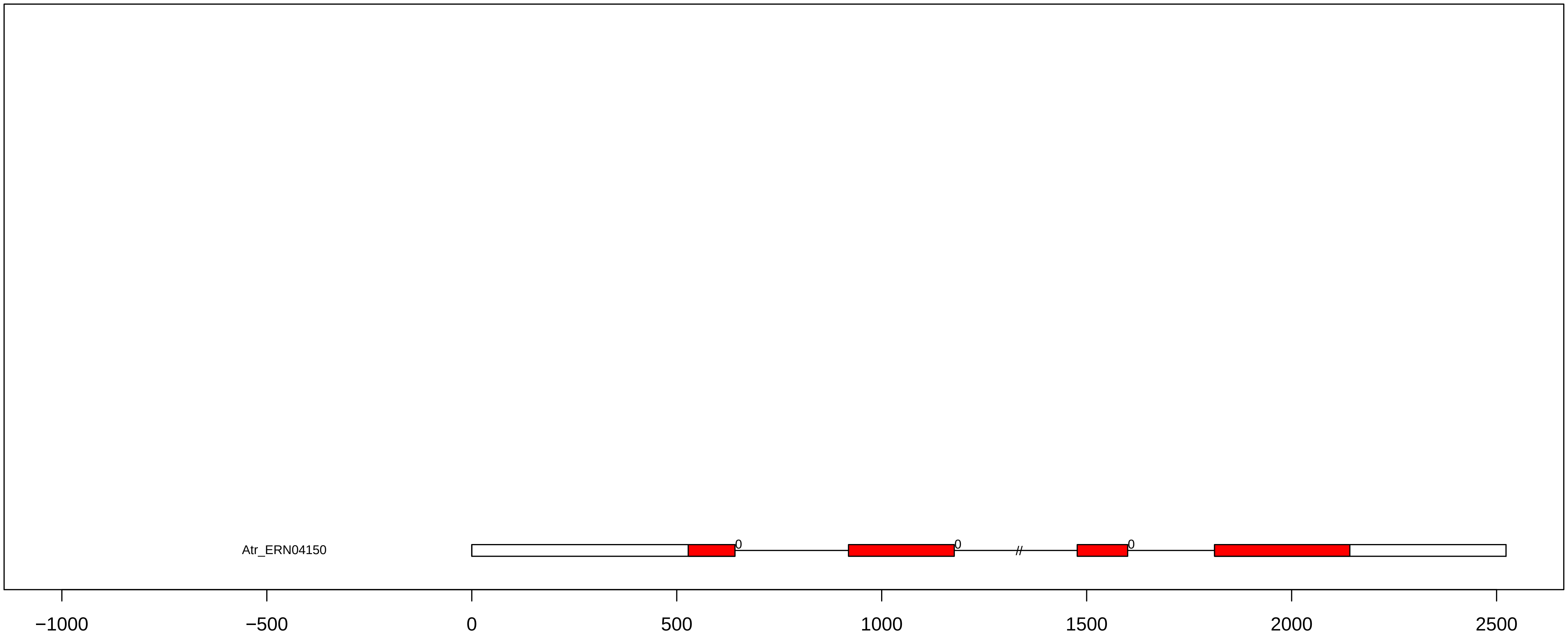

A.tr RLK−Pelle_RLCK−IV subfamily exon−intron and kinase domain diagram (all)

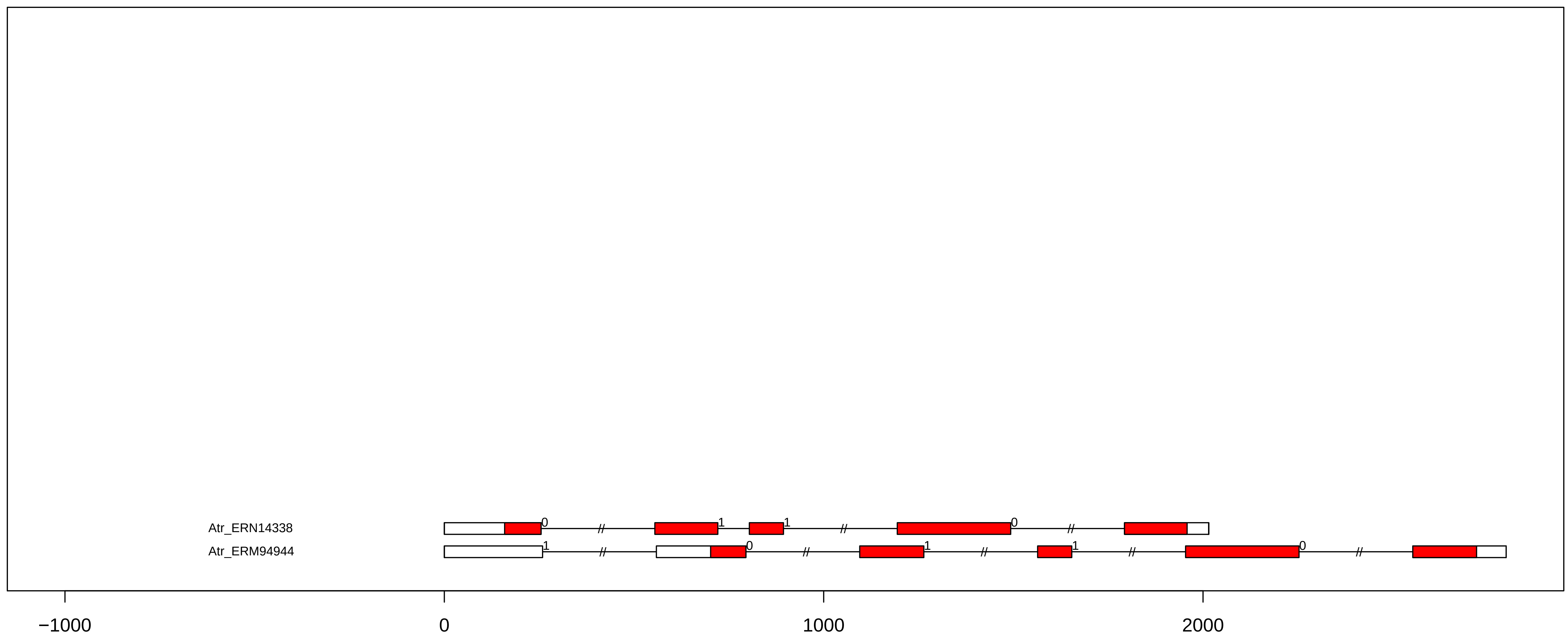

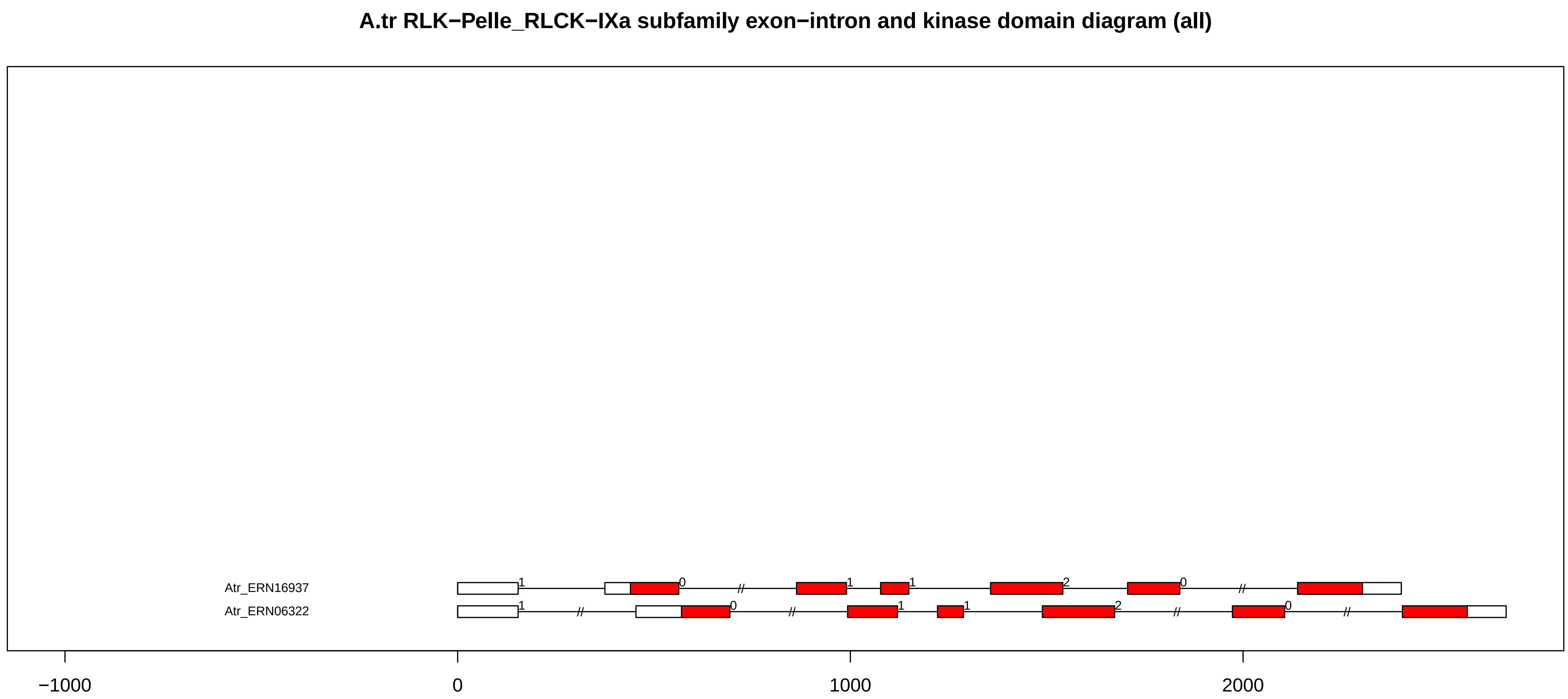
A.tr RLK−Pelle_RLCK−IXa subfamily exon−intron and kinase domain diagram (all)
Atr_ERN16937
Atr_ERN06322
−1000
0
1000
2000

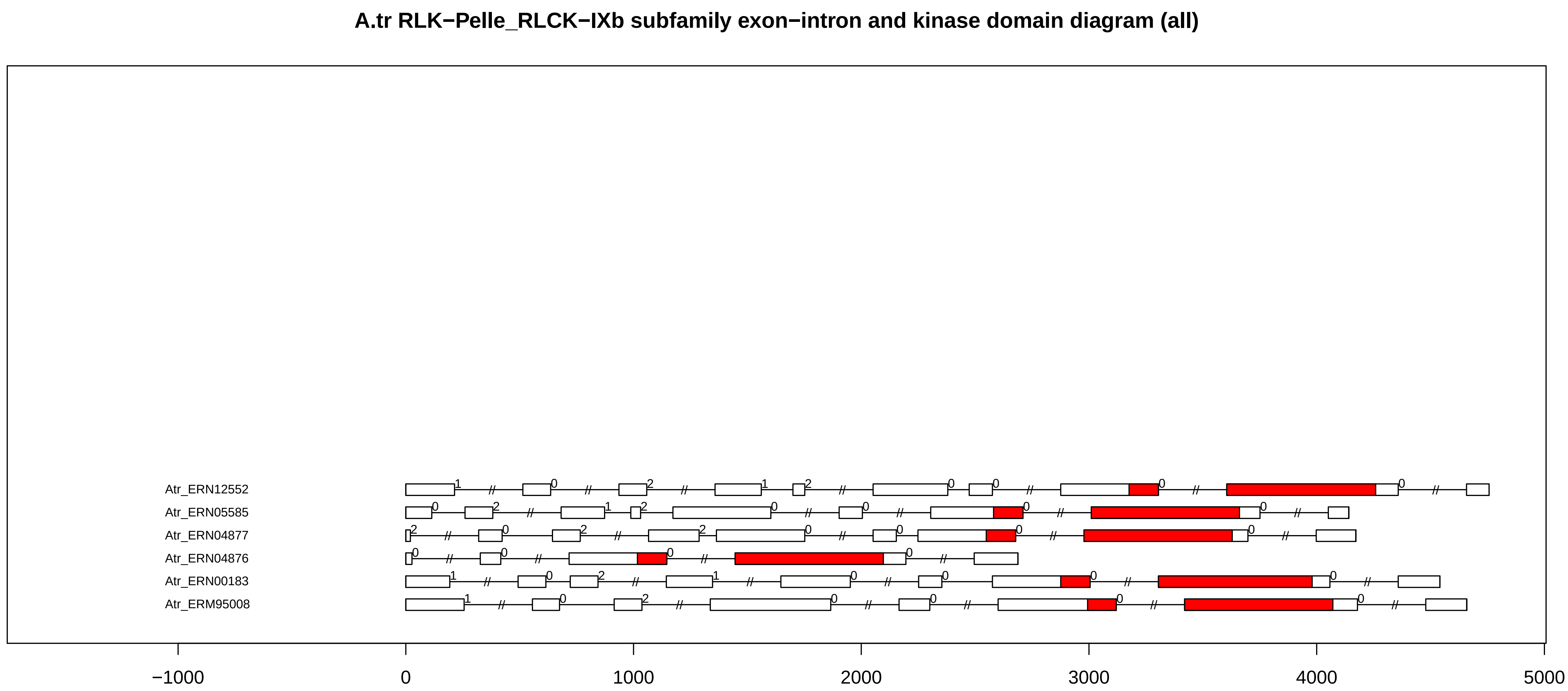
A.tr RLK−Pelle_RLCK−IXb subfamily exon−intron and kinase domain diagram (all)
Atr_ERN12552
Atr_ERN05585
Atr_ERN04877
Atr_ERN04876
Atr_ERN00183
Atr_ERM95008
−1000
0
1000
2000
3000
4000
5000

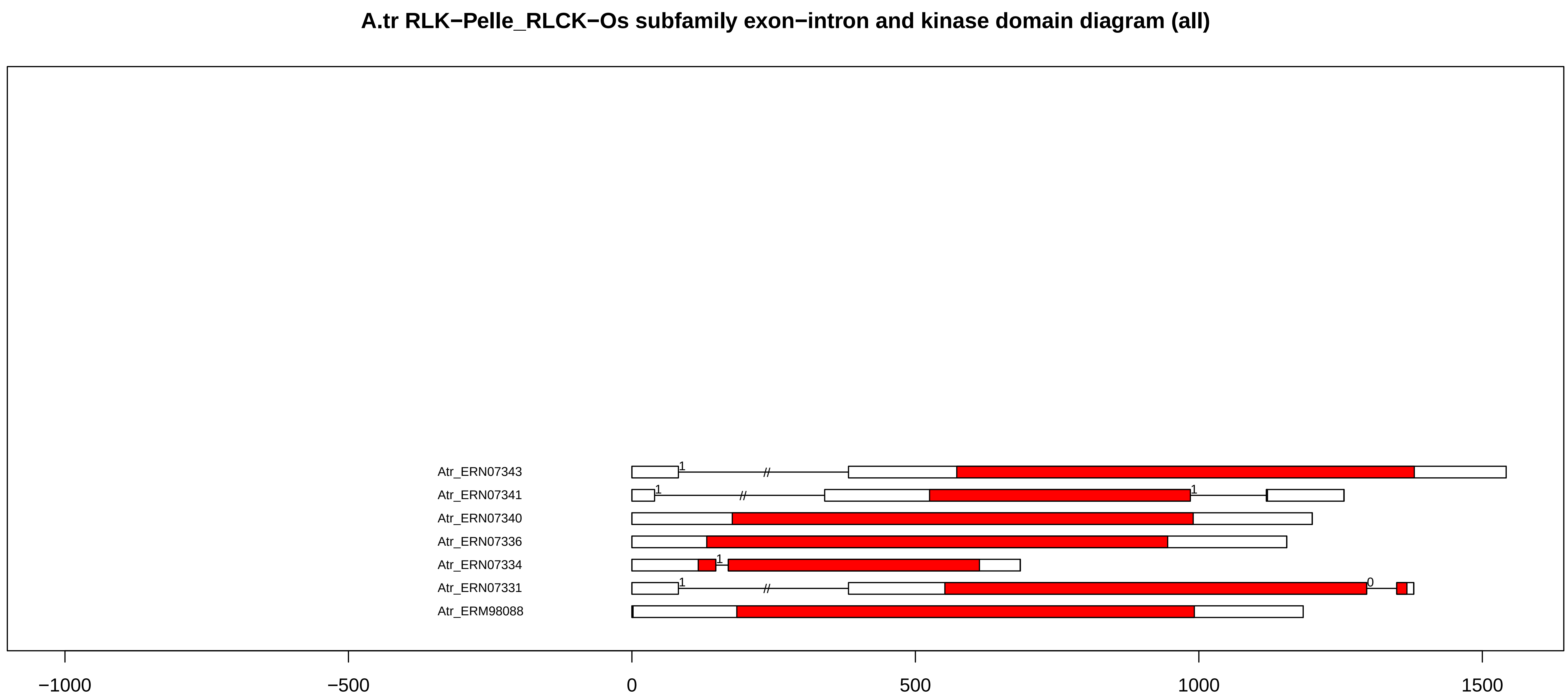
A.tr RLK−Pelle_RLCK−Os subfamily exon−intron and kinase domain diagram (all)
Atr_ERN07343
Atr_ERN07341
Atr_ERN07340
Atr_ERN07336
Atr_ERN07334
Atr_ERN07331
Atr_ERM98088
−1000
−500
0
500
1000
1500

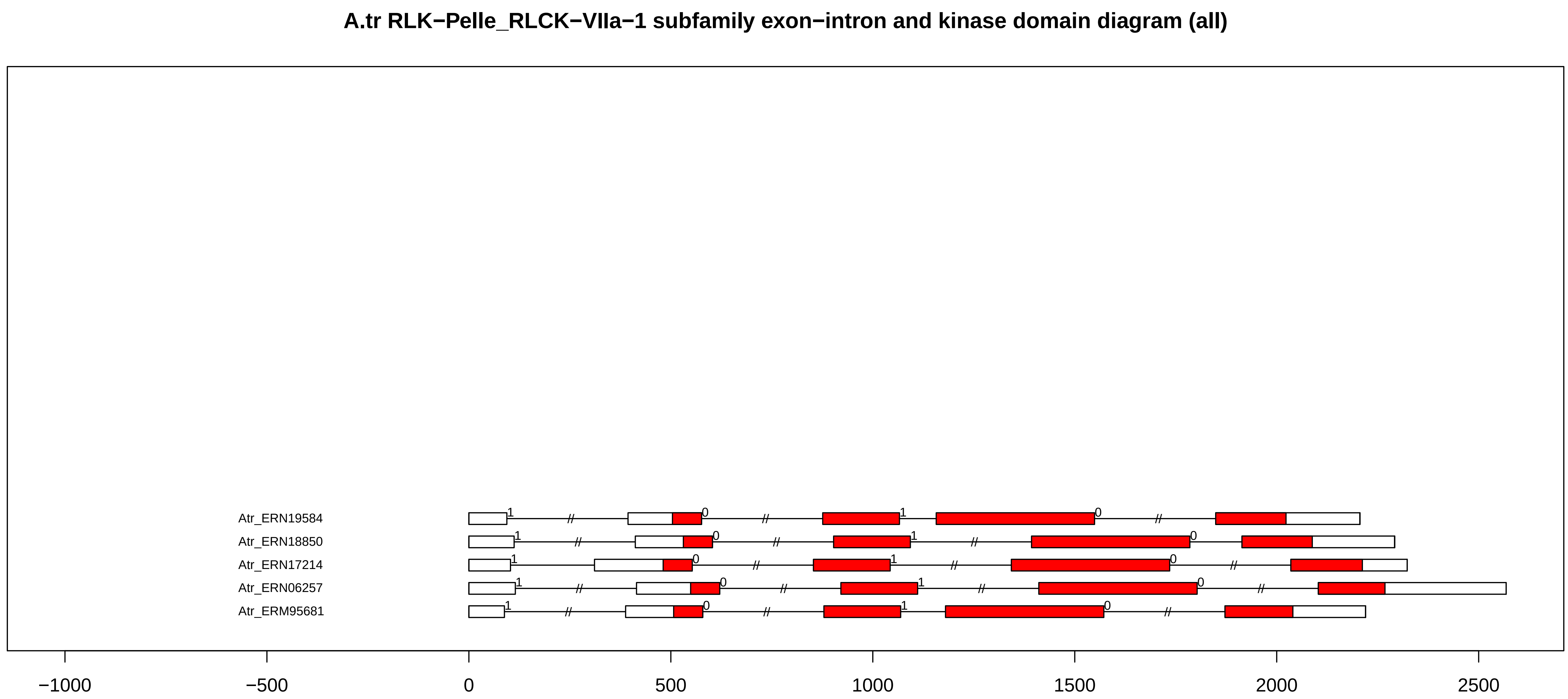
A.tr RLK−Pelle_RLCK−VIIa−1 subfamily exon−intron and kinase domain diagram (all)
Atr_ERN19584
Atr_ERN18850
Atr_ERN17214
Atr_ERN06257
Atr_ERM95681
−1000
−500
0
500
1000
1500
2000
2500

A.tr RLK−Pelle_RLCK−VIIa−2 subfamily exon−intron and kinase domain diagram (all)

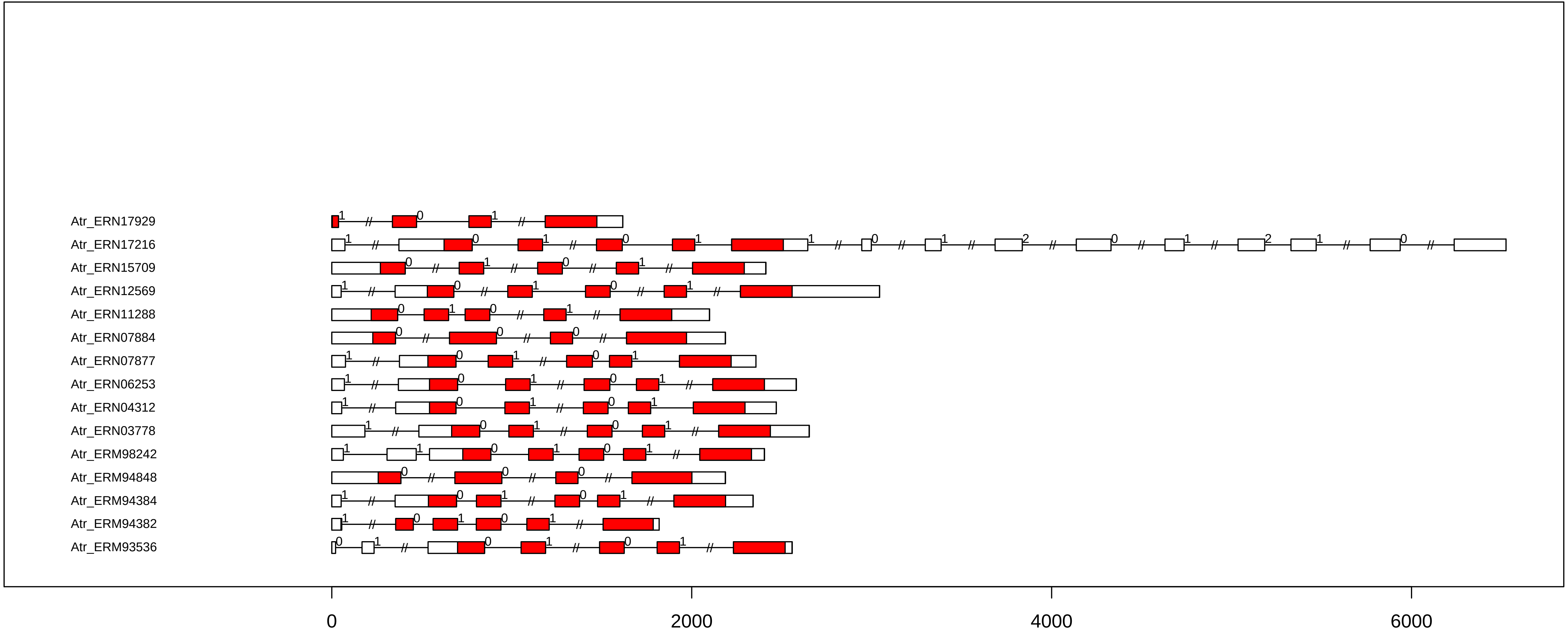

A.tr RLK−Pelle_RLCK−VIIb subfamily exon−intron and kinase domain diagram (all)

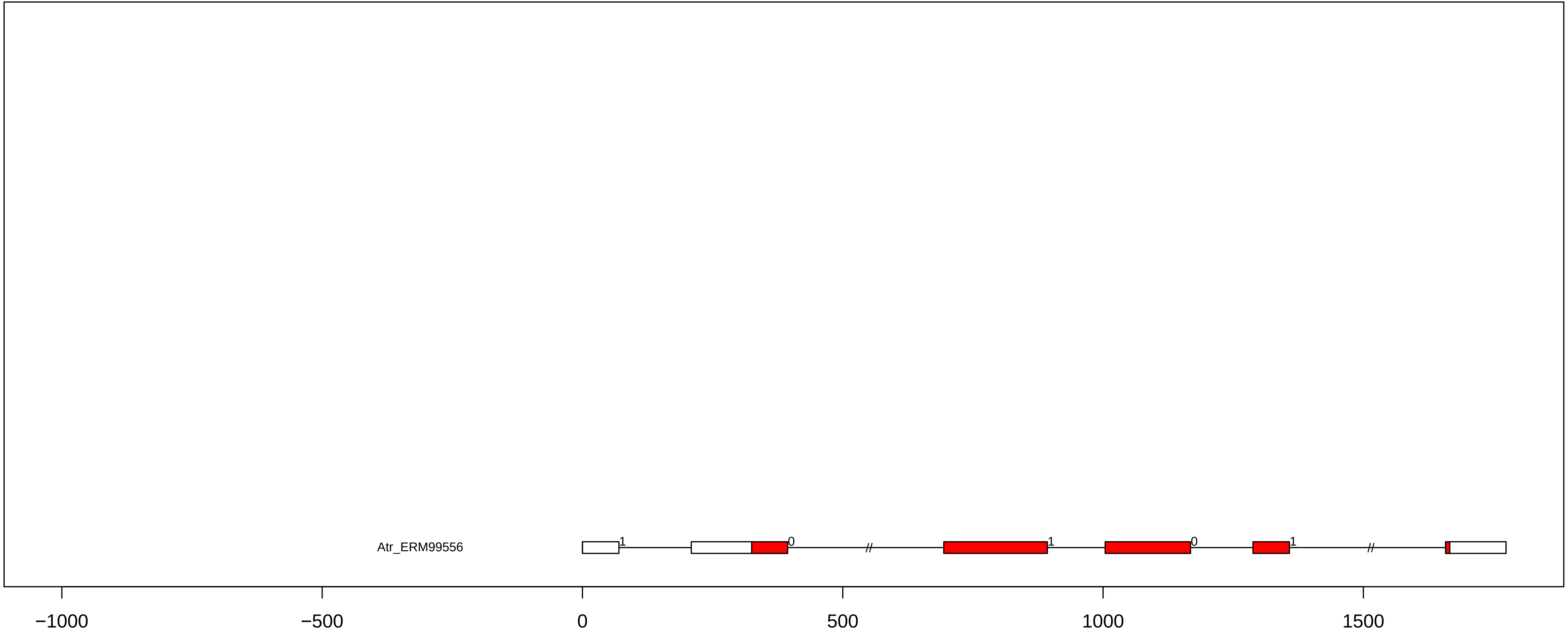

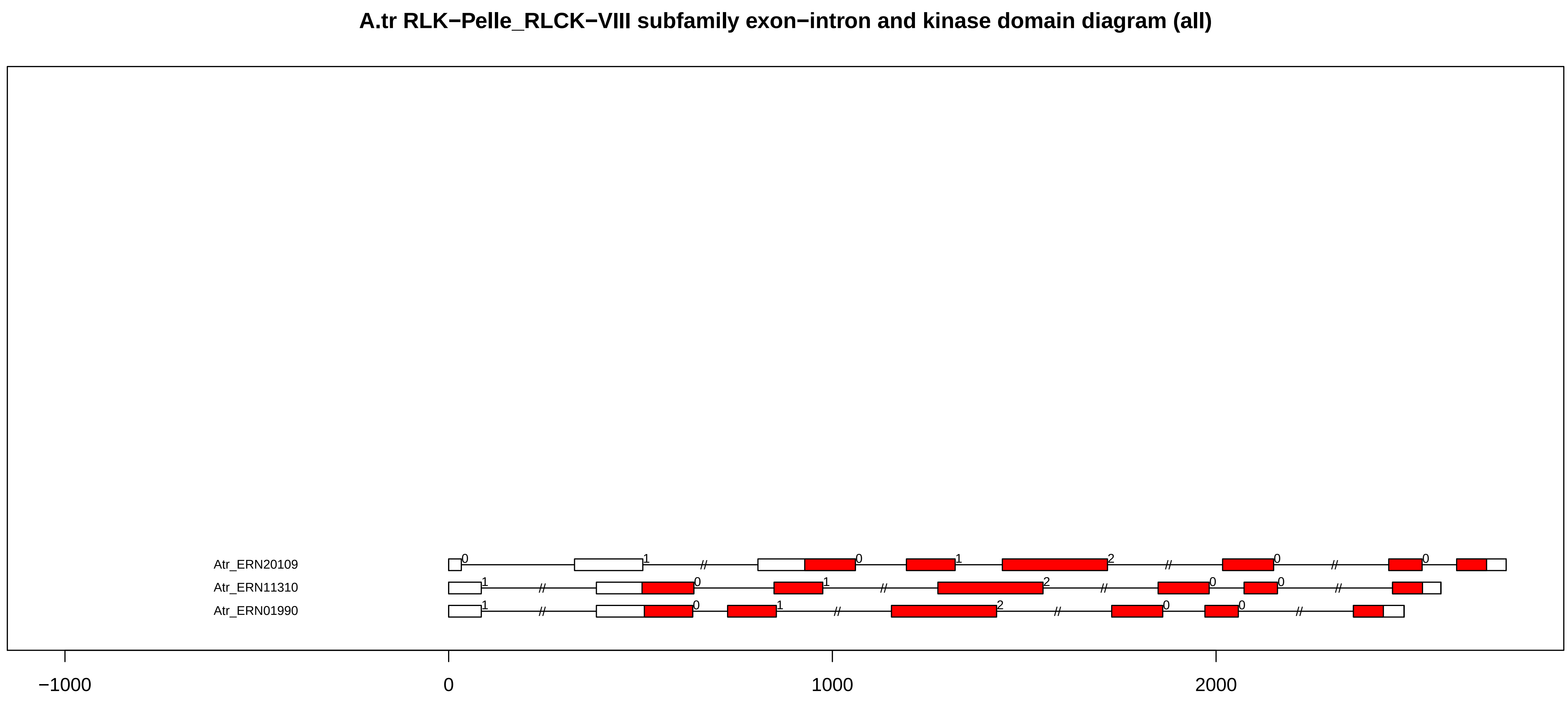
A.tr RLK−Pelle_RLCK−VIII subfamily exon−intron and kinase domain diagram (all)
Atr_ERN20109
Atr_ERN11310
Atr_ERN01990
−1000
0
1000
2000

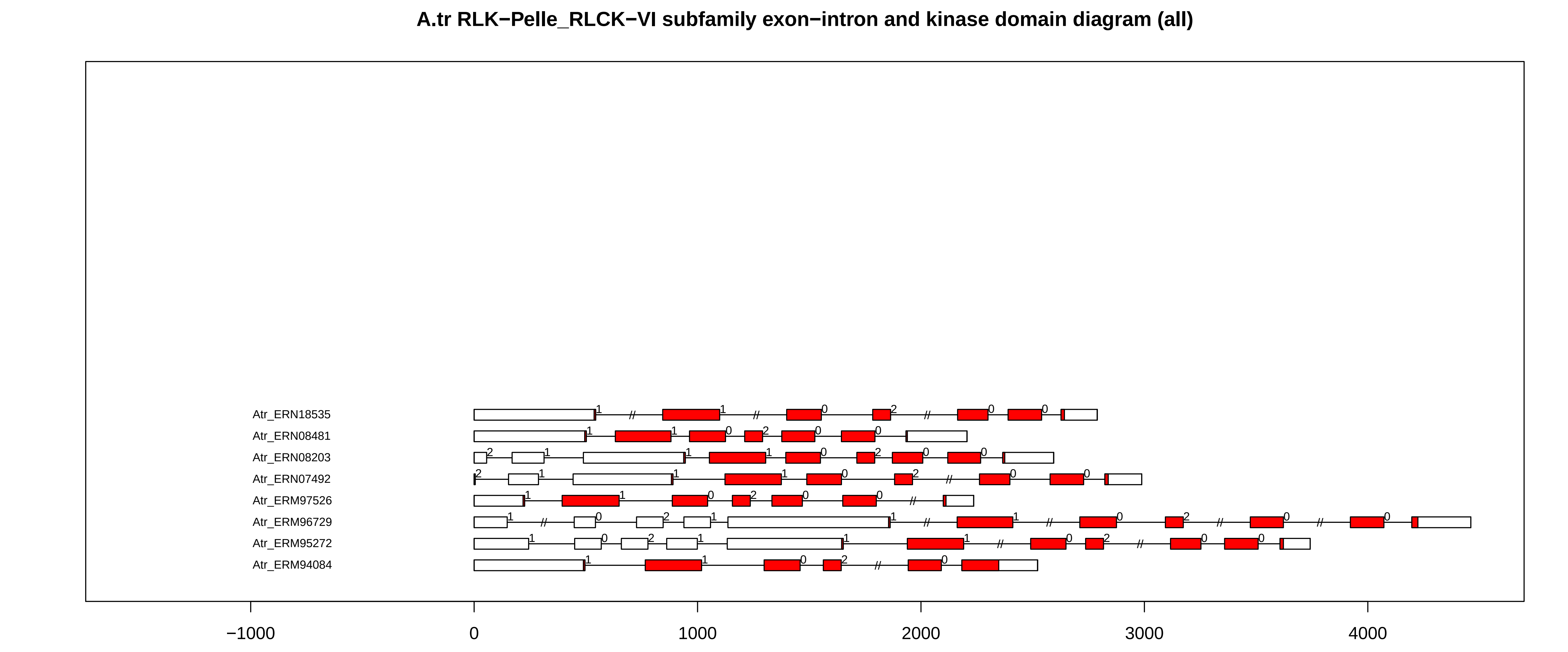

A.tr RLK−Pelle_RLCK−VI subfamily exon−intron and kinase domain diagram (all)
Atr_ERN18535
Atr_ERN08481
Atr_ERN08203
Atr_ERN07492
Atr_ERM97526
Atr_ERM96729
Atr_ERM95272
Atr_ERM94084
−1000
0
1000
2000
3000
4000

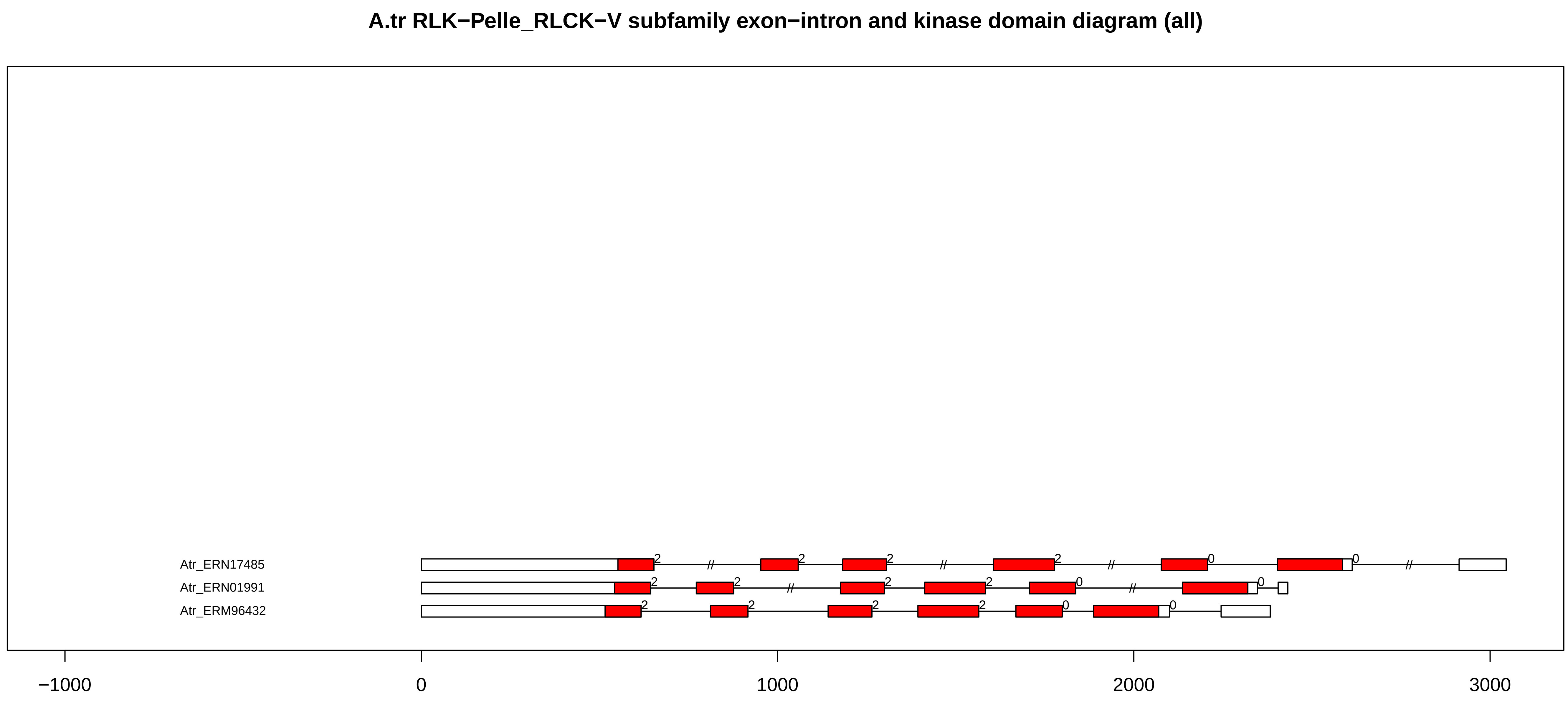

A.tr RLK−Pelle_RLCK−V subfamily exon−intron and kinase domain diagram (all)
Atr_ERN17485
Atr_ERN01991
Atr_ERM96432
2
0
−1000
0
1000
2000
3000

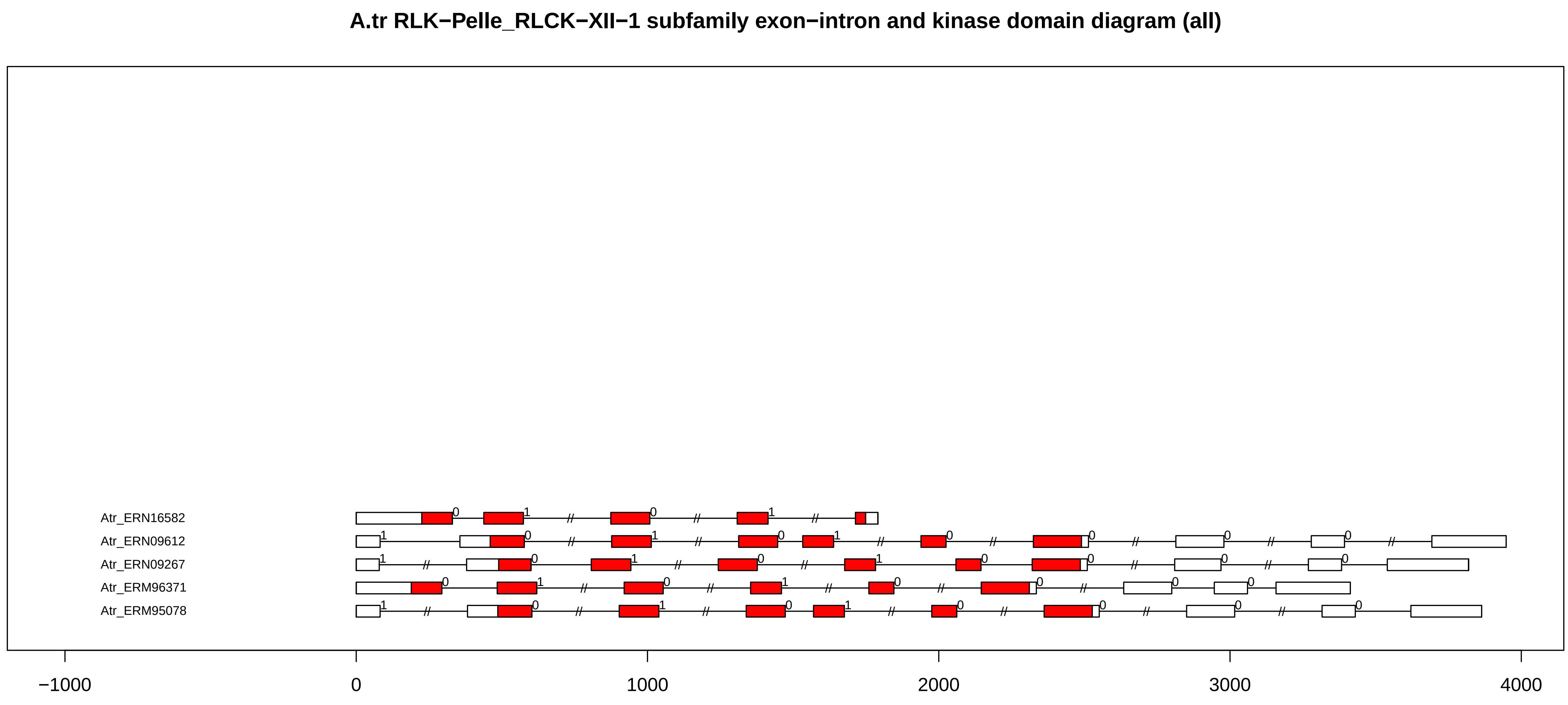
A.tr RLK−Pelle_RLCK−XII−1 subfamily exon−intron and kinase domain diagram (all)
Atr_ERN16582
Atr_ERN09612
Atr_ERN09267
Atr_ERM96371
Atr_ERM95078
−1000
0
1000
2000
3000
4000

A.tr RLK−Pelle_RLCK−XIII subfamily exon−intron and kinase domain diagram (all)

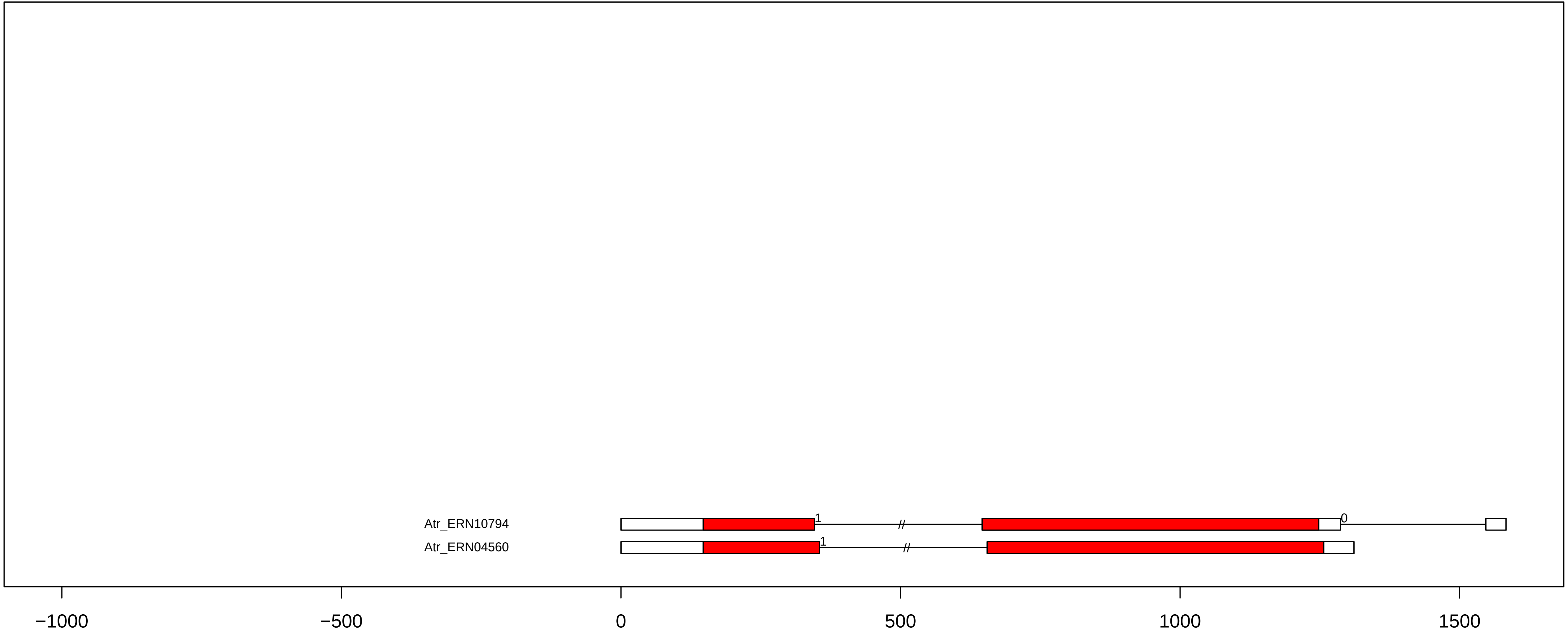

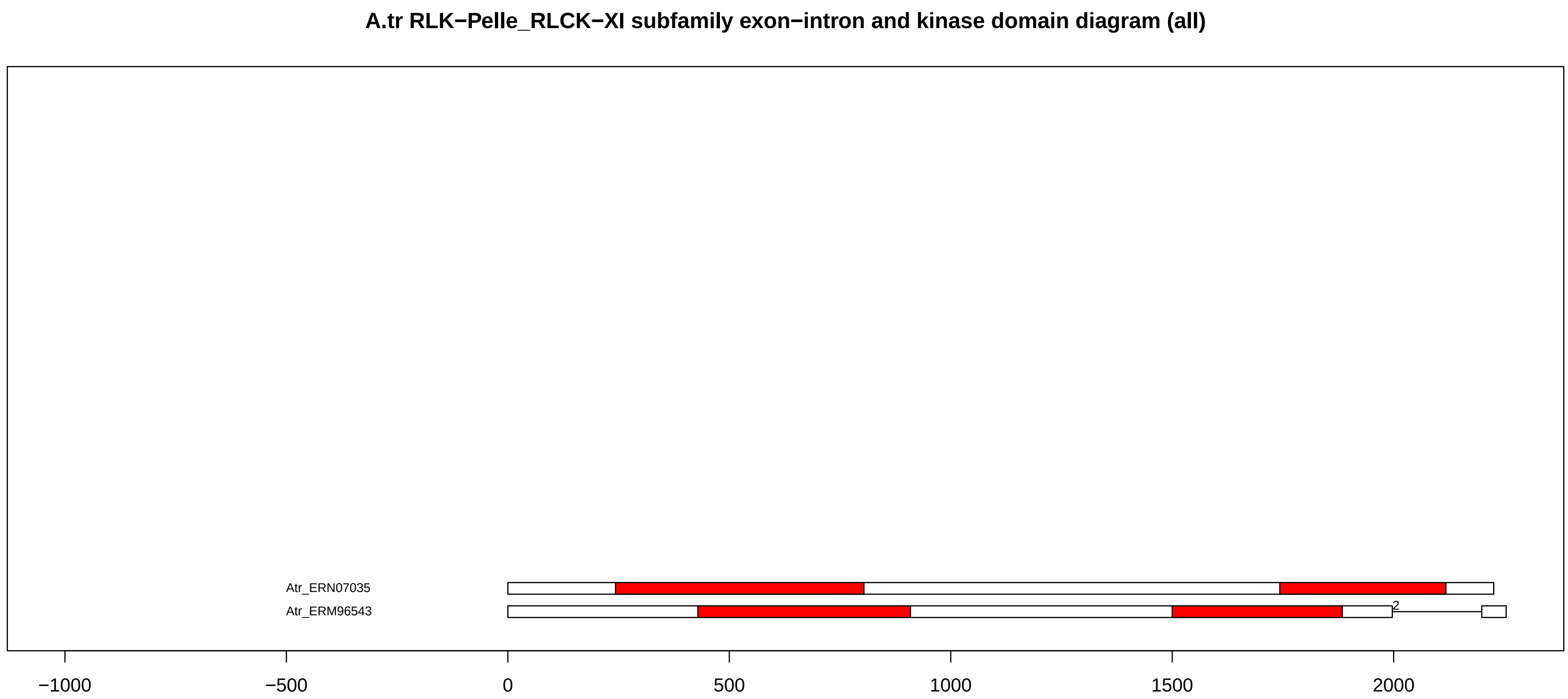
A.tr RLK−Pelle_RLCK−XI subfamily exon−intron and kinase domain diagram (all)
Atr_ERN07035
Atr_ERM96543
2
−1000
−500
0
500
1000
1500
2000

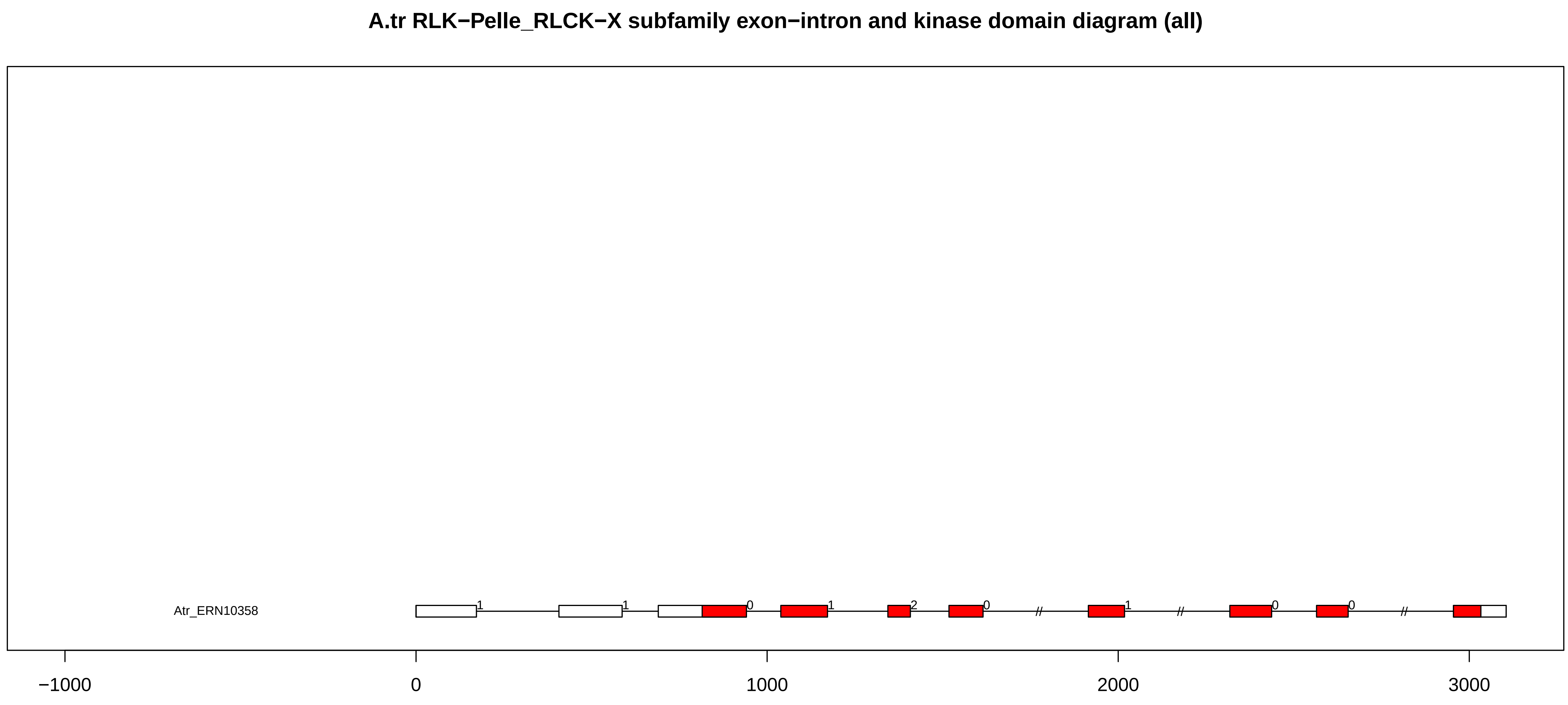
A.tr RLK−Pelle_RLCK−X subfamily exon−intron and kinase domain diagram (all)
Atr_ERN10358
1
1
0
1
2
0
1
0
0
−1000
0
1000
2000
3000

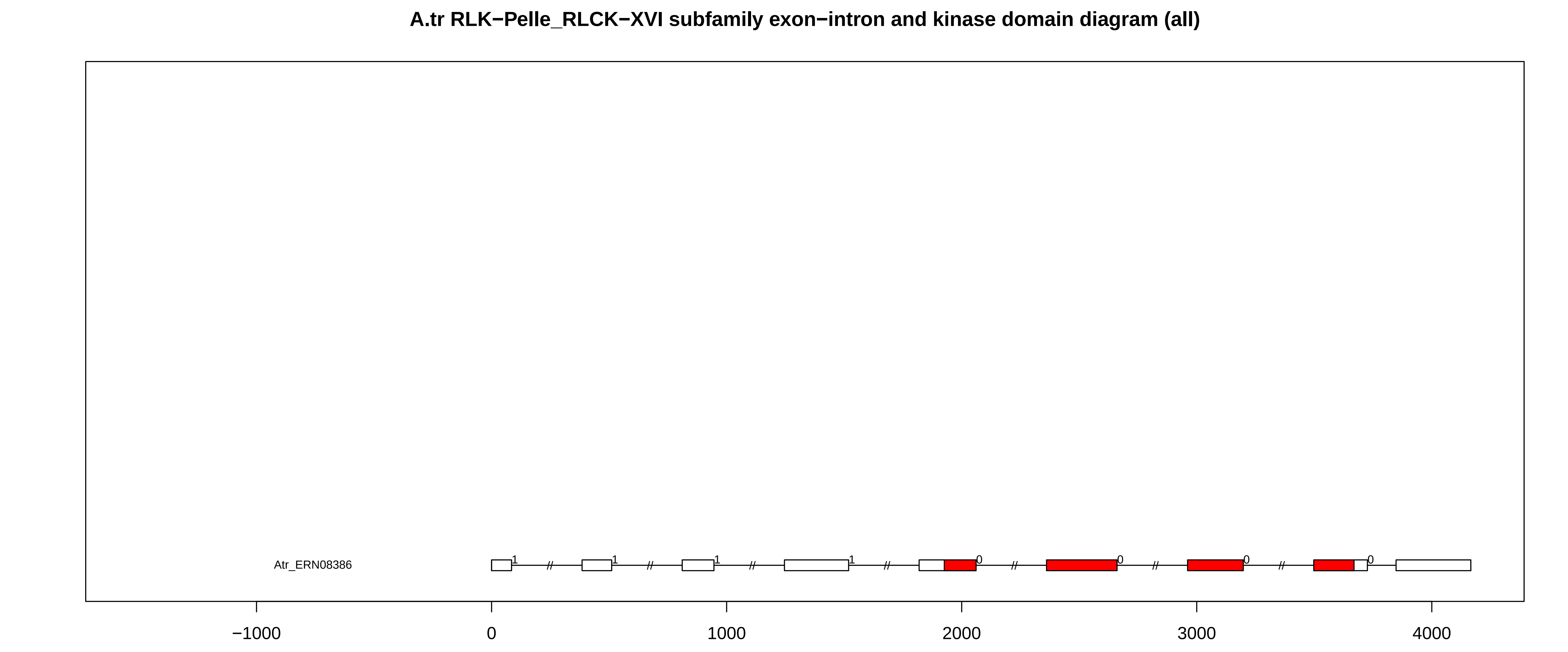
A.tr RLK−Pelle_RLCK−XVI subfamily exon−intron and kinase domain diagram (all)
Atr_ERN08386
1
1
1
1
0
0
0
0
−1000
0
1000
2000
3000
4000

A.tr RLK−Pelle_RLCK−XV subfamily exon−intron and kinase domain diagram (all)

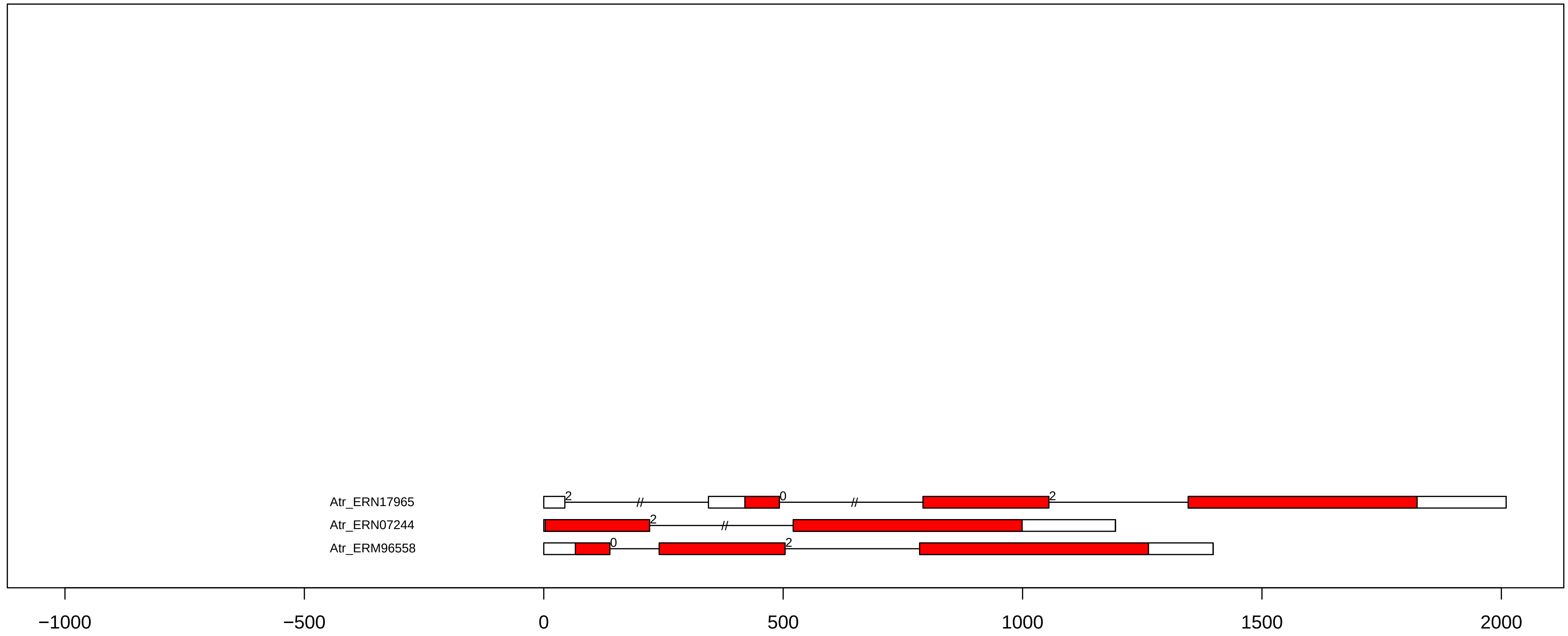

A.tr RLK−Pelle_SD−2b subfamily exon−intron and kinase domain diagram (part 1)

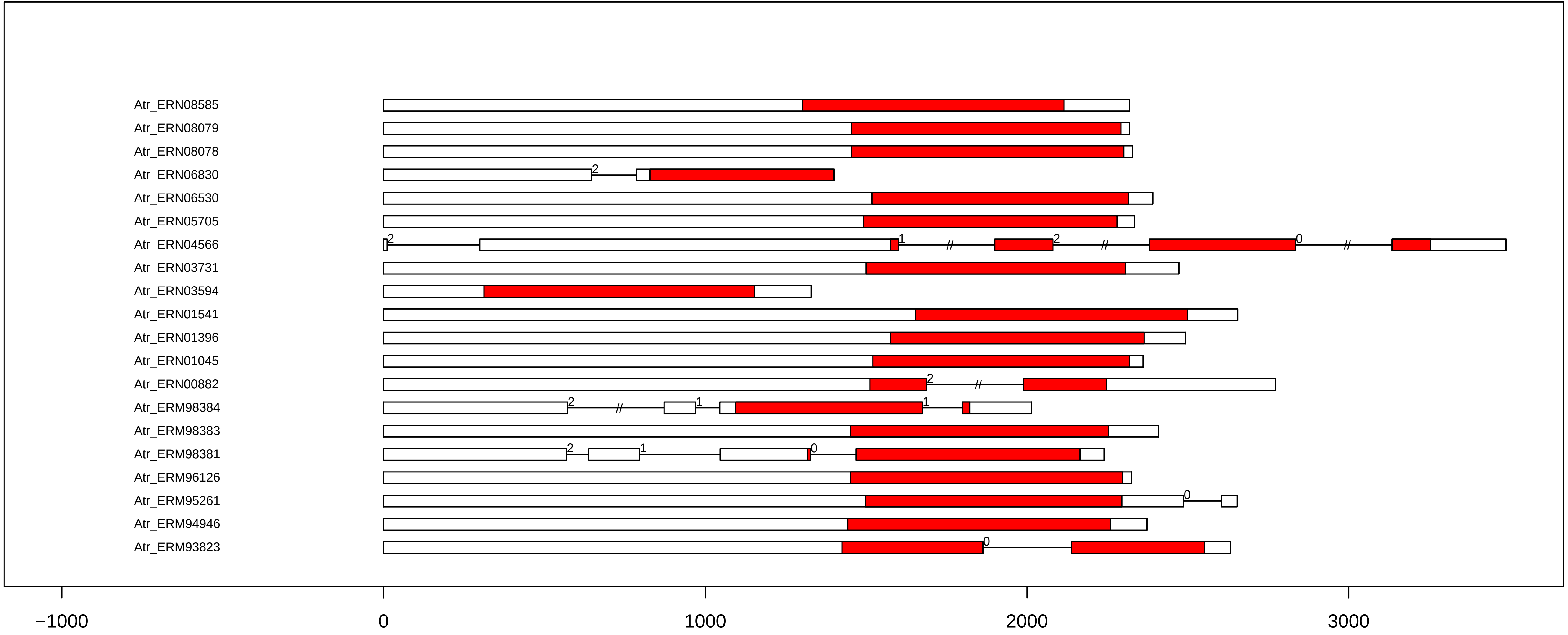

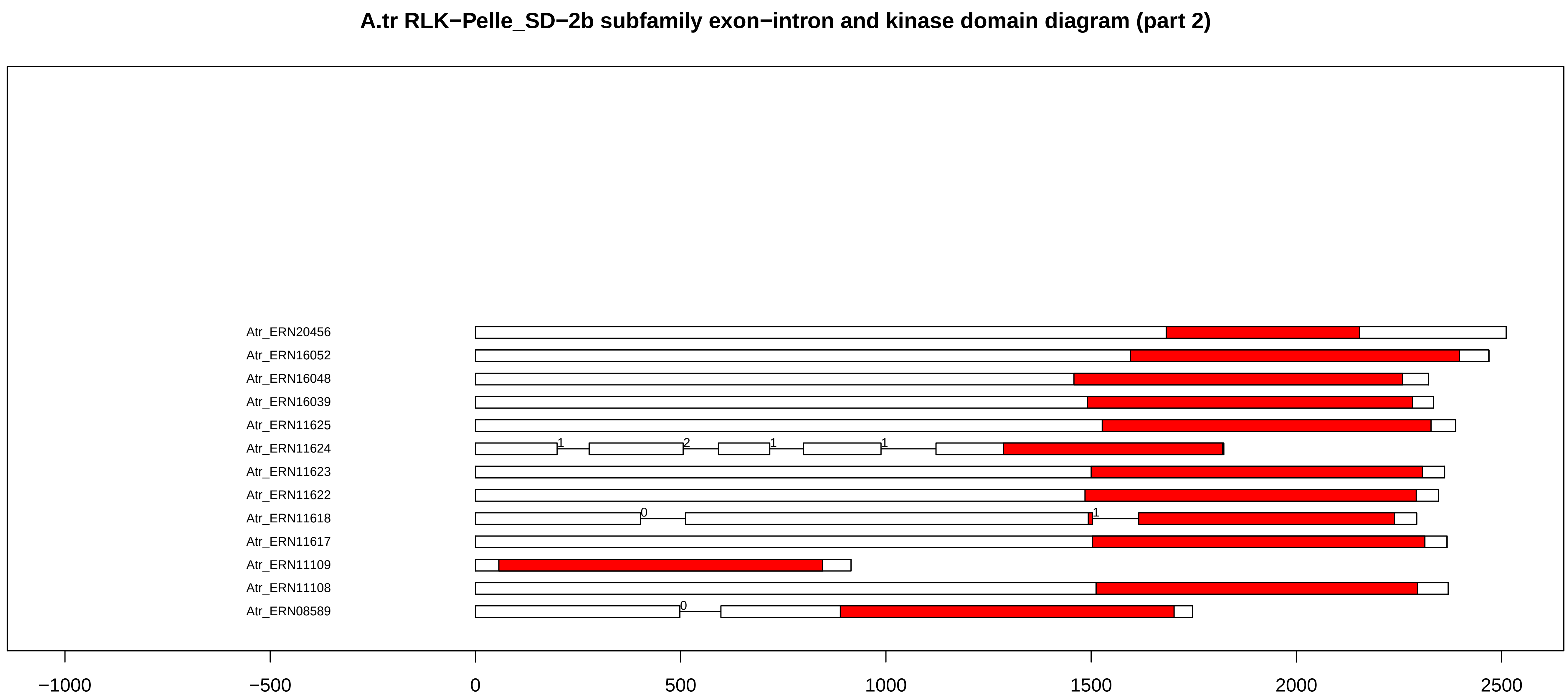
A.tr RLK−Pelle_SD−2b subfamily exon−intron and kinase domain diagram (part 2)
Atr_ERN20456
Atr_ERN16052
Atr_ERN16048
Atr_ERN16039
Atr_ERN11625
Atr_ERN11624
Atr_ERN11623
Atr_ERN11622
Atr_ERN11618
Atr_ERN11617
Atr_ERN11109
Atr_ERN11108
Atr_ERN08589
−1000
−500
0
500
1000
1500
2000
2500

A.tr RLK−Pelle_URK−1 subfamily exon−intron and kinase domain diagram (all)

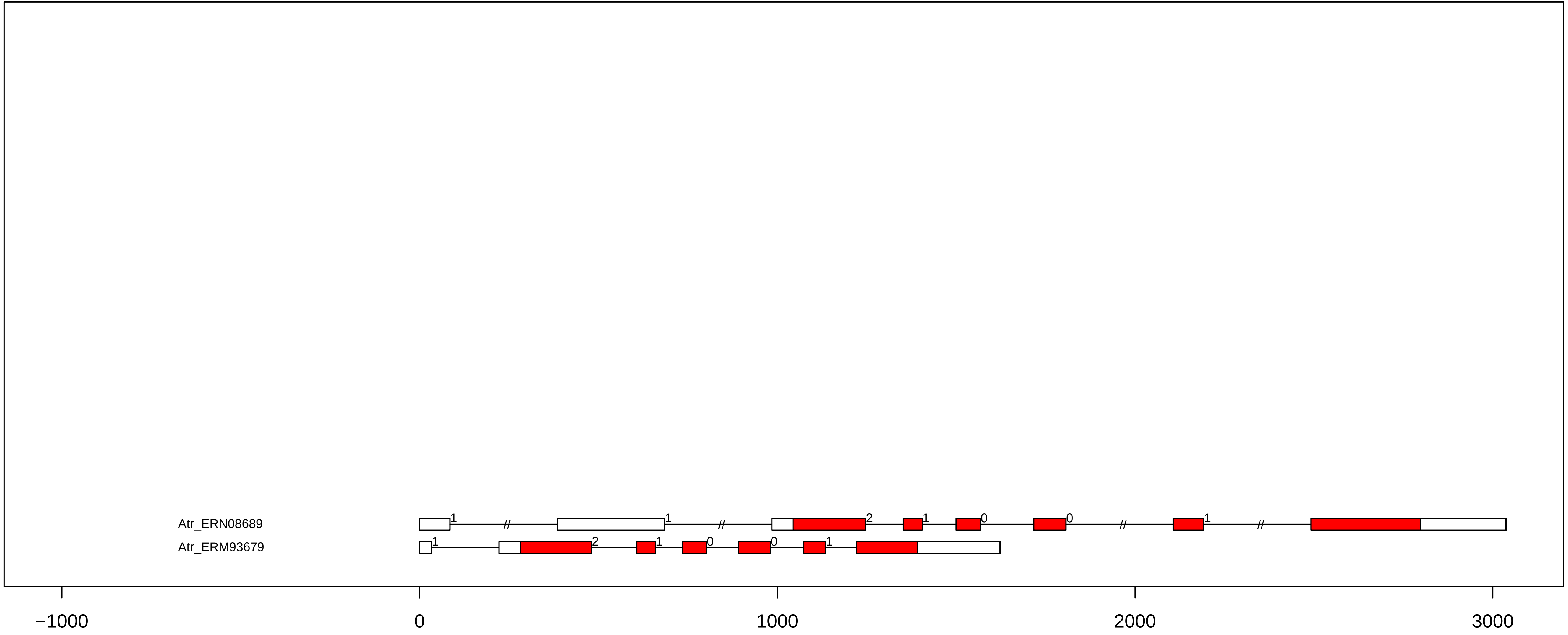

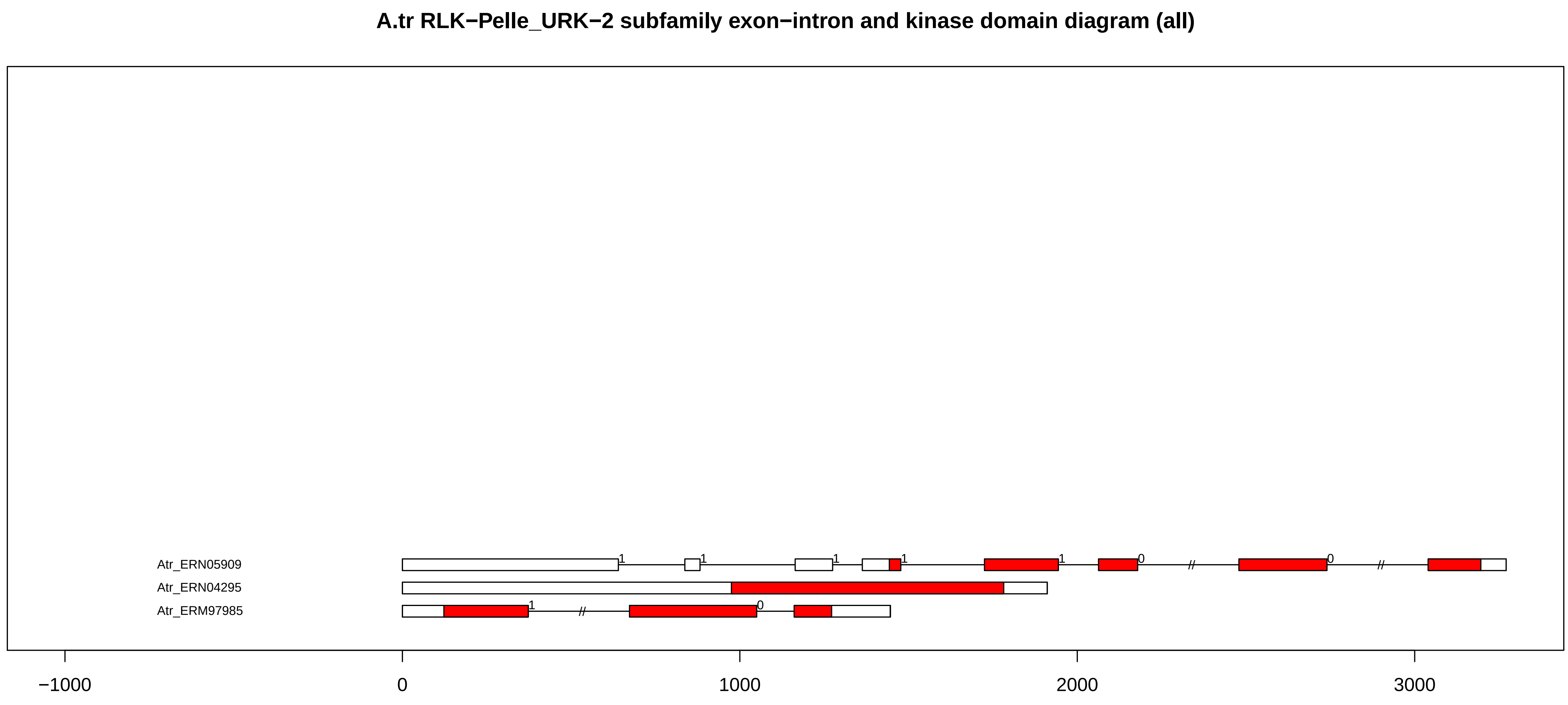

A.tr RLK−Pelle_URK−2 subfamily exon−intron and kinase domain diagram (all)
Atr_ERN05909
Atr_ERN04295
Atr_ERM97985
1
1
1
1
1
0
0
1
0
−1000
0
1000
2000
3000

A.tr RLK−Pelle_WAK_LRK10L−1 subfamily exon−intron and kinase domain diagram (all)

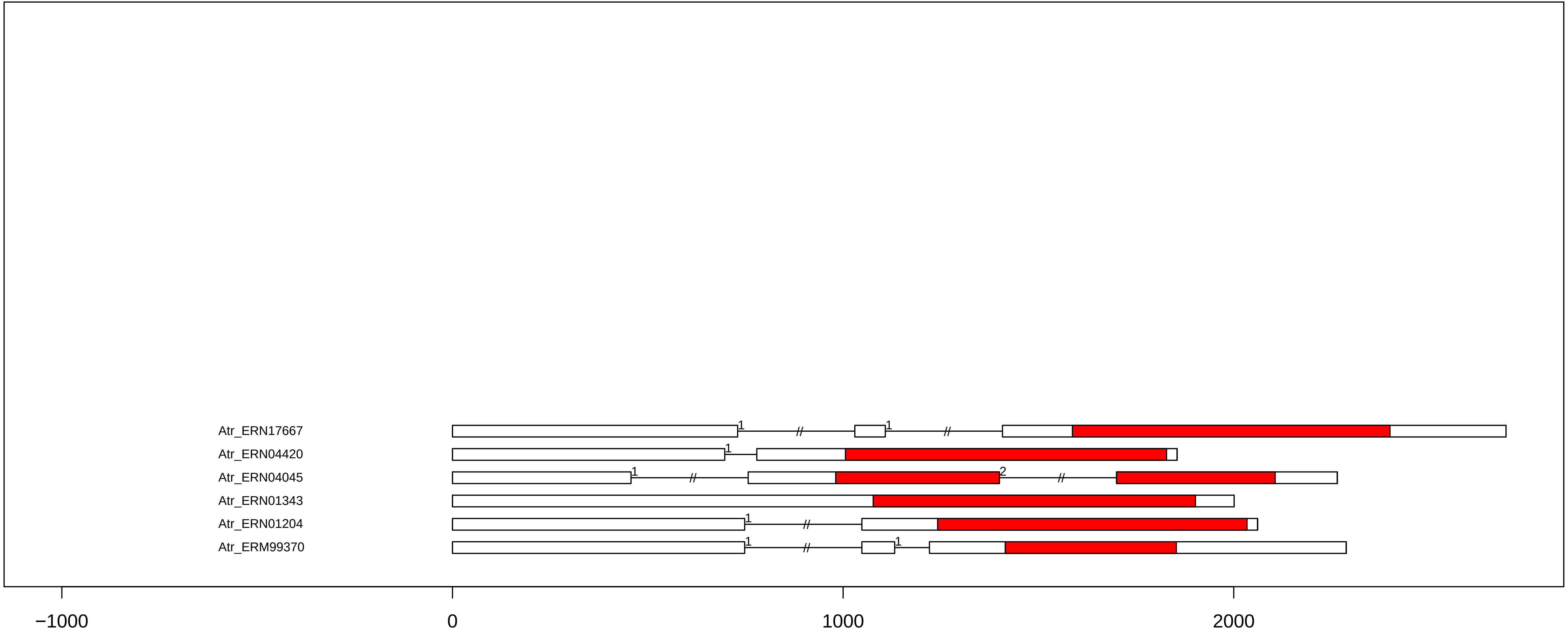

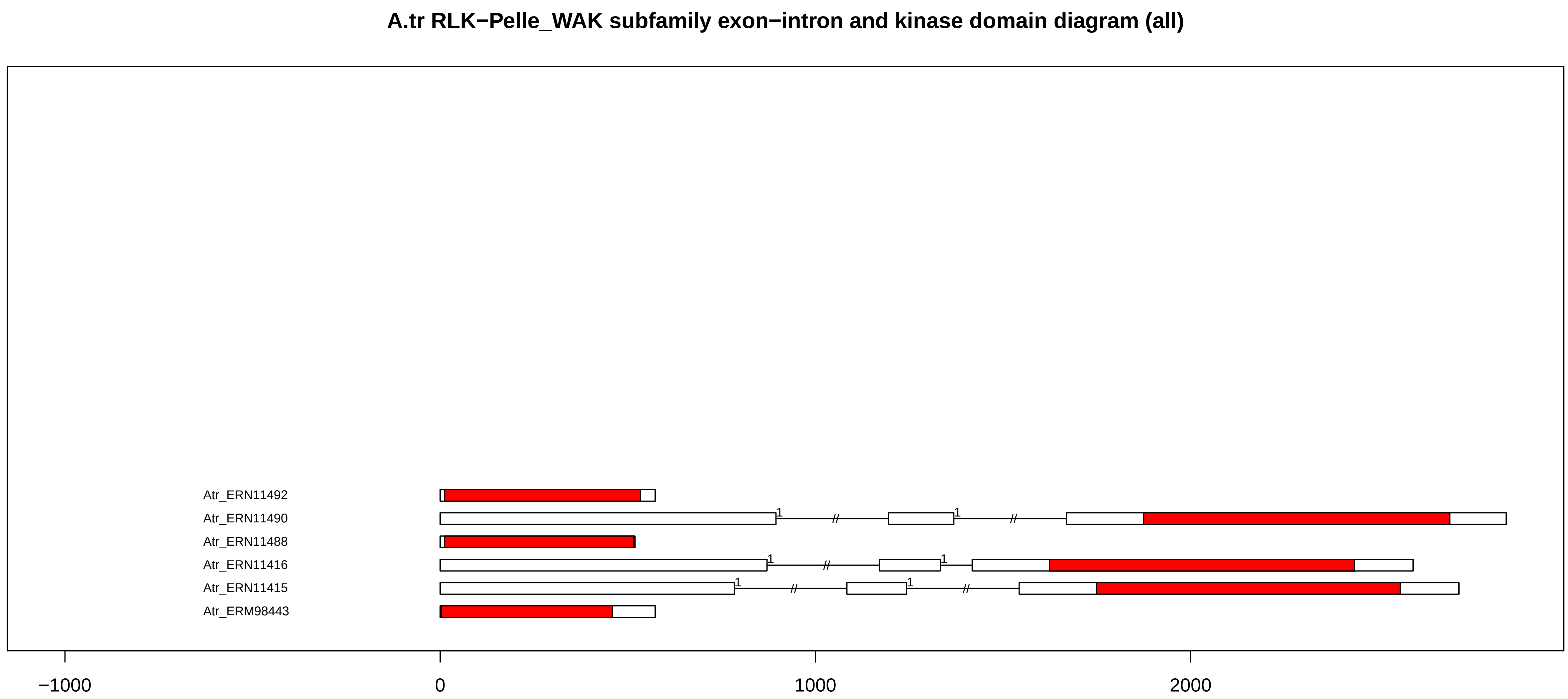

A.tr RLK−Pelle_WAK subfamily exon−intron and kinase domain diagram (all)
Atr_ERN11492
Atr_ERN11490
Atr_ERN11488
Atr_ERN11416
Atr_ERN11415
Atr_ERM98443
1
1
1
1
1
1
−1000
0
1000
2000

S.mo RLK−Pelle_C−LEC subfamily exon−intron and kinase domain diagram (all)

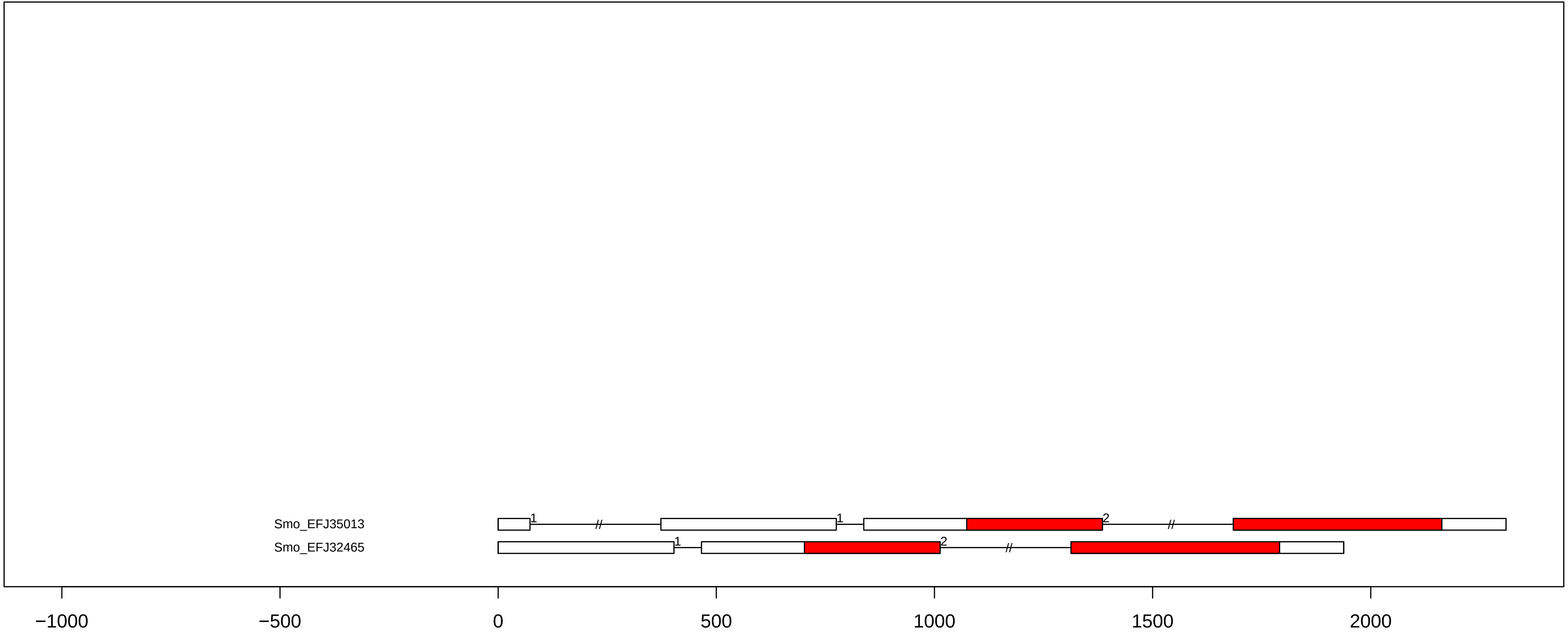

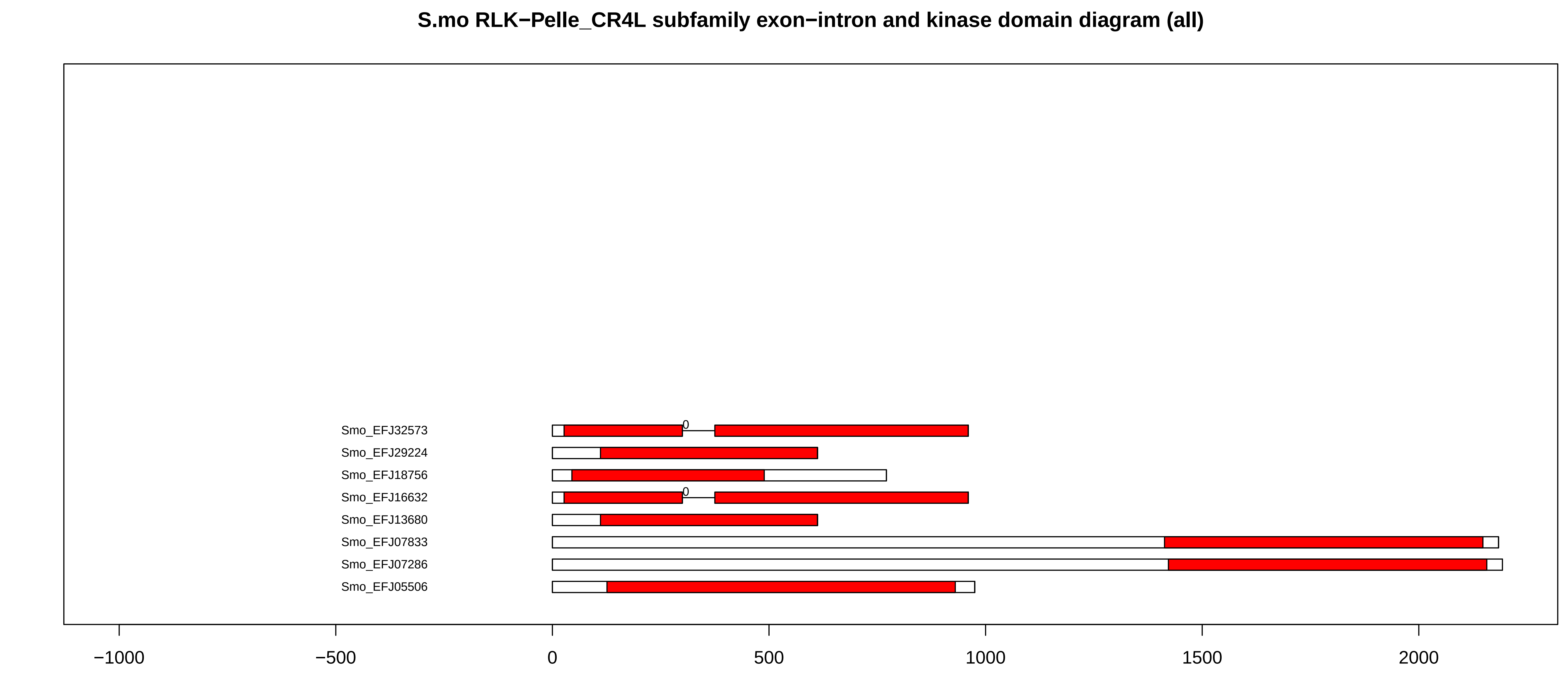
S.mo RLK−Pelle_CR4L subfamily exon−intron and kinase domain diagram (all)
Smo_EFJ32573
Smo_EFJ29224
Smo_EFJ18756
Smo_EFJ16632
Smo_EFJ13680
Smo_EFJ07833
Smo_EFJ07286
Smo_EFJ05506
0
0
−1000
−500
0
500
1000
1500
2000

S.mo RLK−Pelle_CrRLK1L−1 subfamily exon−intron and kinase domain diagram (all)

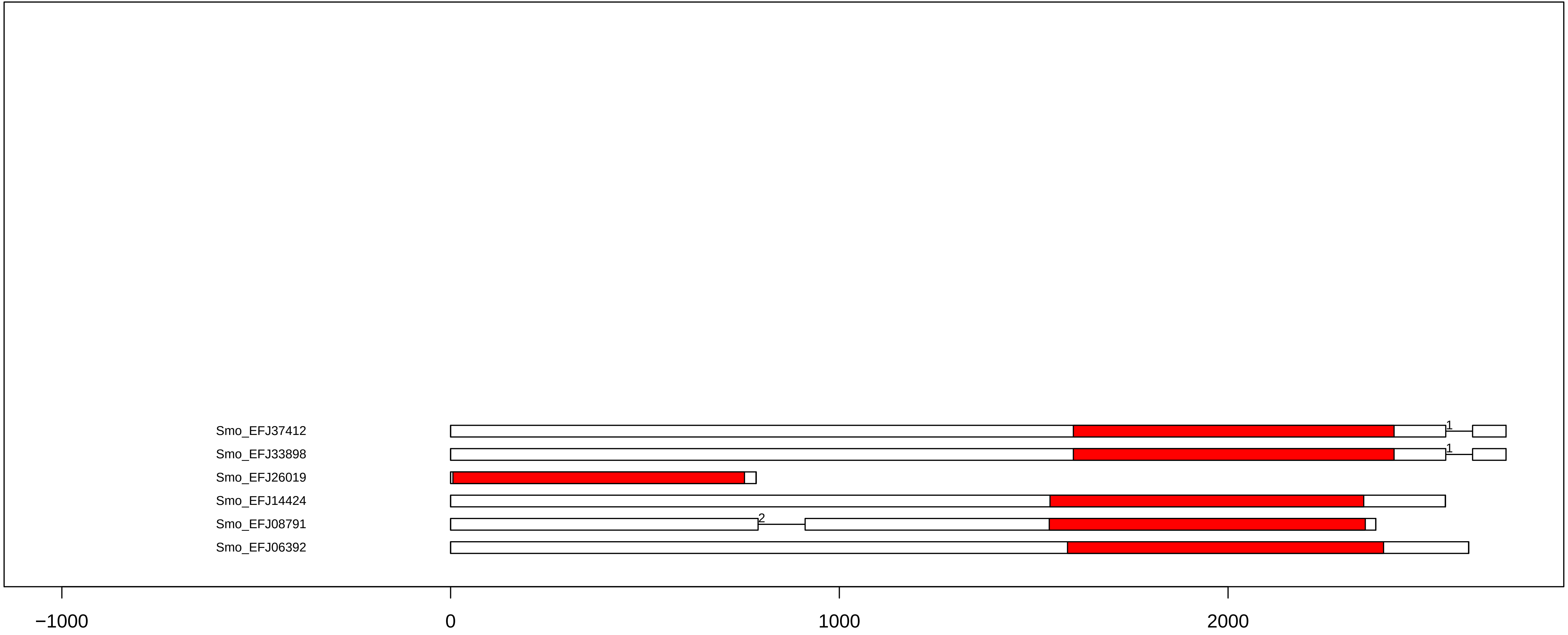

S.mo RLK−Pelle_DLSV subfamily exon−intron and kinase domain diagram (all)

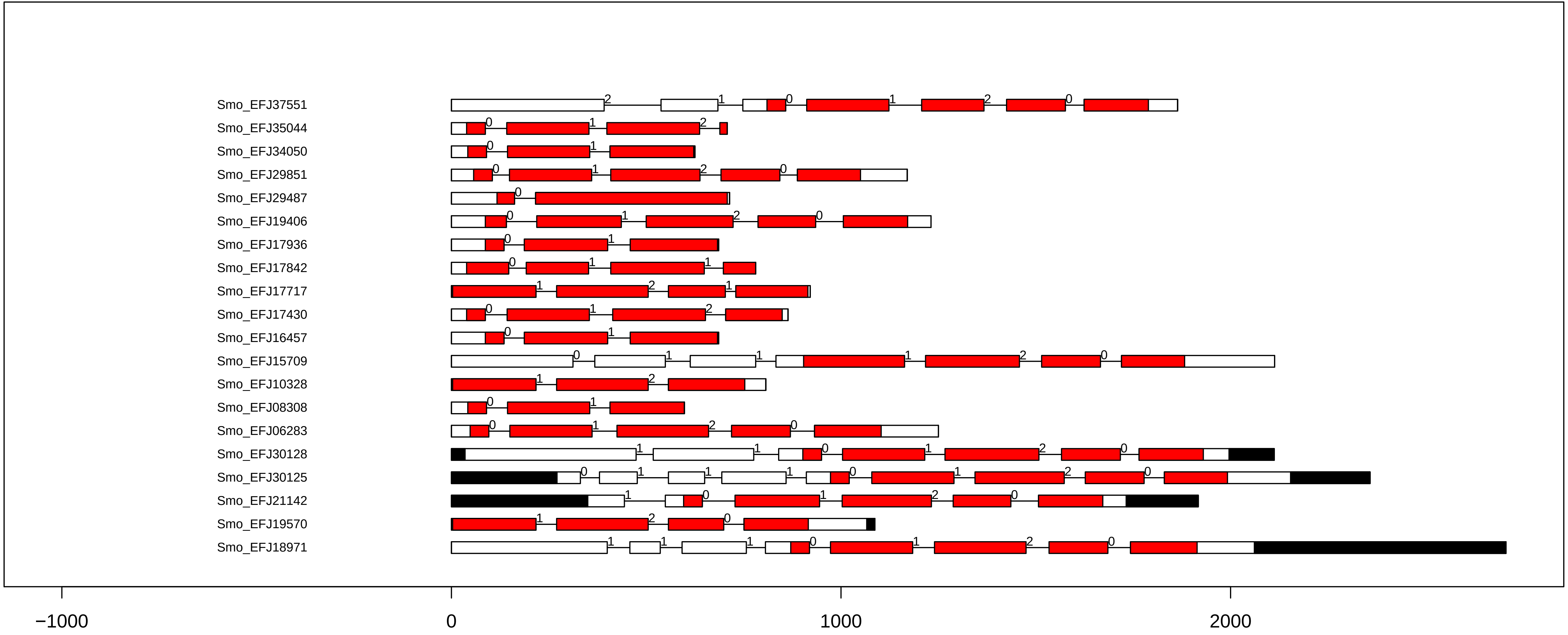

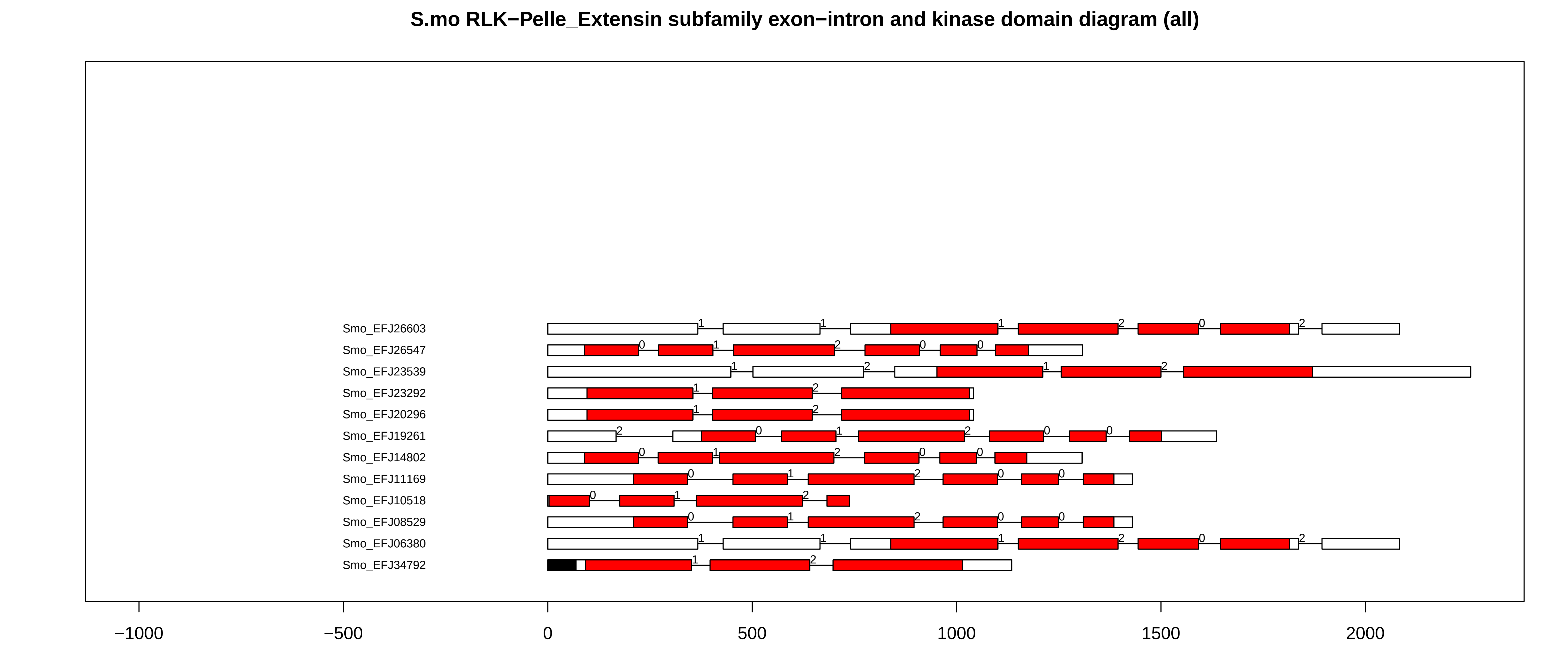
S.mo RLK−Pelle_Extensin subfamily exon−intron and kinase domain diagram (all)
Smo_EFJ26603
Smo_EFJ26547
Smo_EFJ23539
Smo_EFJ23292
Smo_EFJ20296
Smo_EFJ19261
Smo_EFJ14802
Smo_EFJ11169
Smo_EFJ10518
Smo_EFJ08529
Smo_EFJ06380
Smo_EFJ34792
−1000
−500
0
500
1000
1500
2000

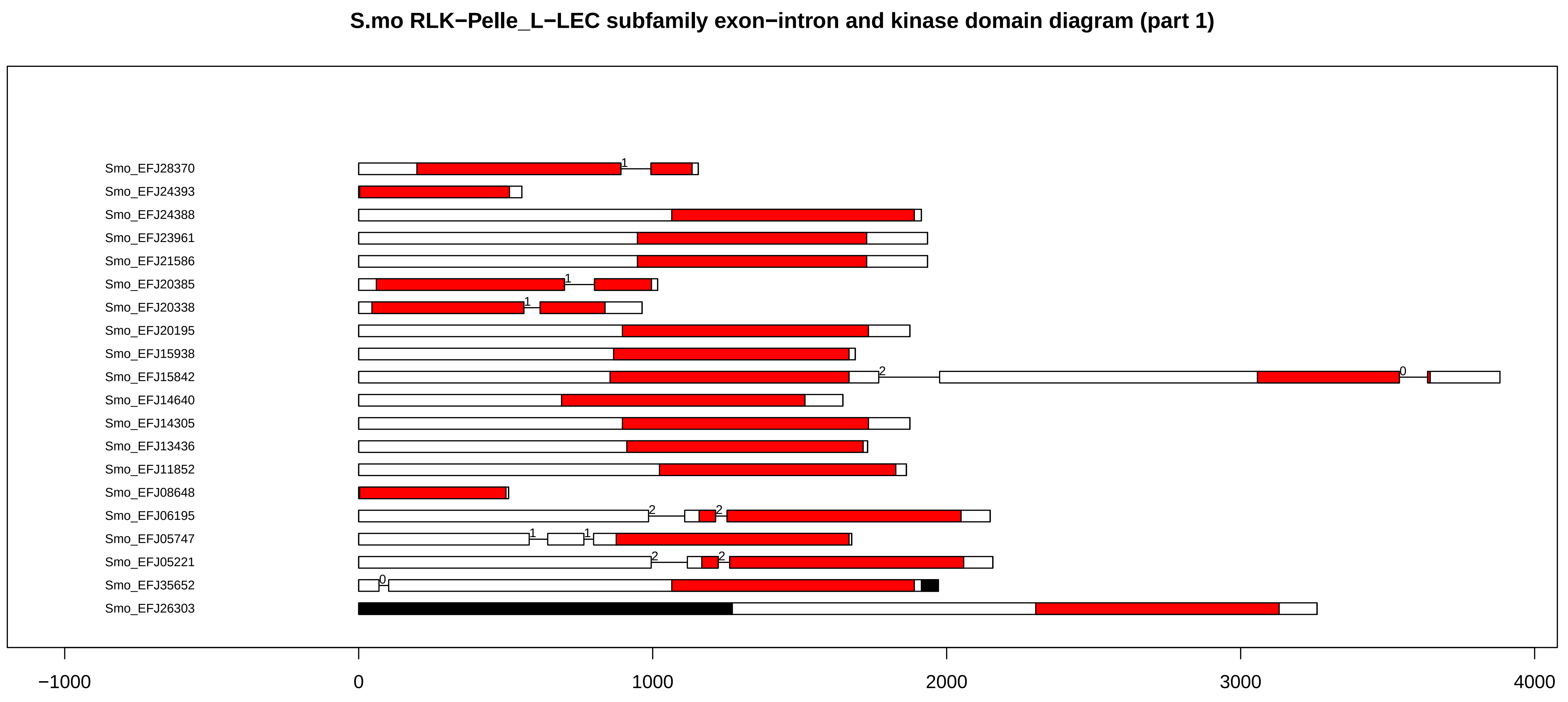
S.mo RLK−Pelle_L−LEC subfamily exon−intron and kinase domain diagram (part 1)
Smo_EFJ28370
Smo_EFJ24393
Smo_EFJ24388
Smo_EFJ23961
Smo_EFJ21586
Smo_EFJ20385
Smo_EFJ20338
Smo_EFJ20195
Smo_EFJ15938
Smo_EFJ15842
Smo_EFJ14640
Smo_EFJ14305
Smo_EFJ13436
Smo_EFJ11852
Smo_EFJ08648
Smo_EFJ06195
Smo_EFJ05747
Smo_EFJ05221
Smo_EFJ35652
Smo_EFJ26303
−1000
0
1000
2000
3000
4000

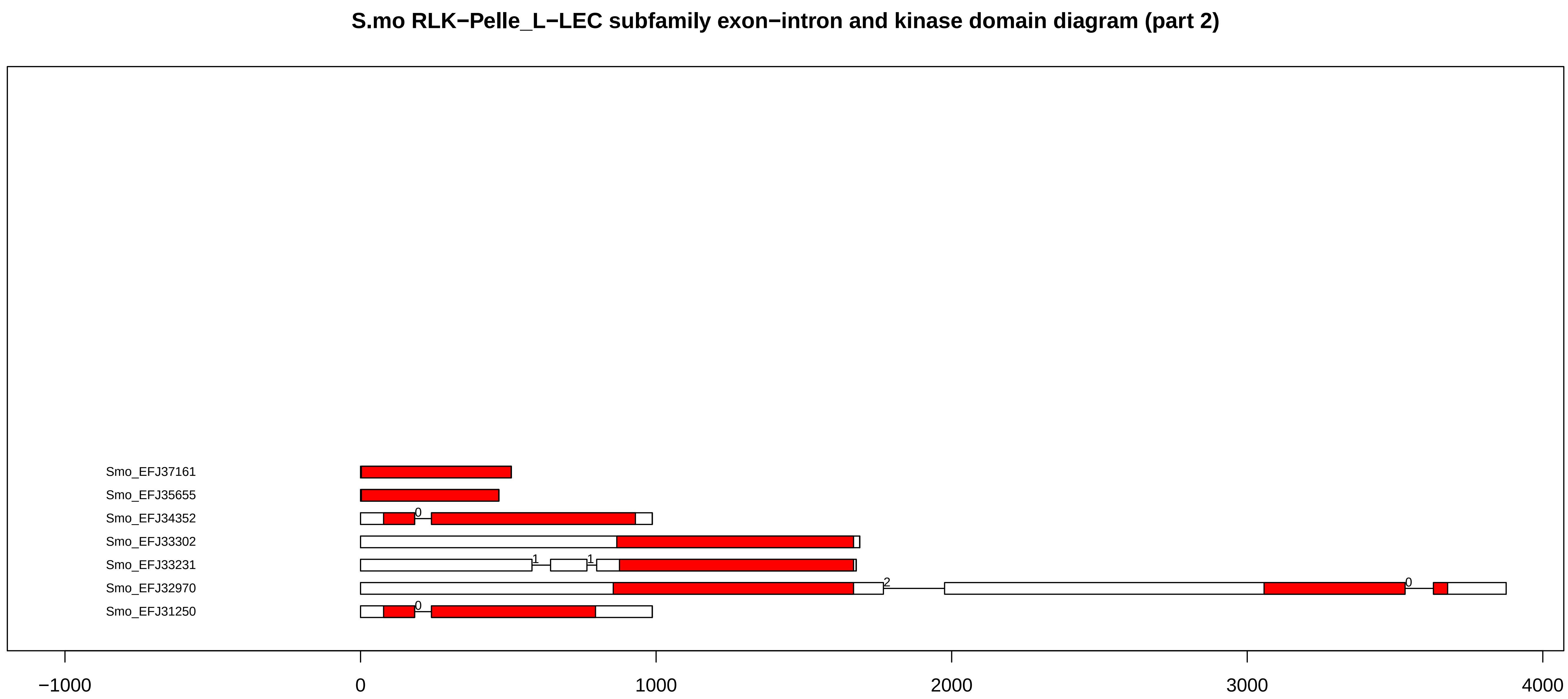
S.mo RLK−Pelle_L−LEC subfamily exon−intron and kinase domain diagram (part 2)
Smo_EFJ37161
Smo_EFJ35655
Smo_EFJ34352
Smo_EFJ33302
Smo_EFJ33231
Smo_EFJ32970
Smo_EFJ31250
0
1
1
2
0
0
−1000
0
1000
2000
3000
4000

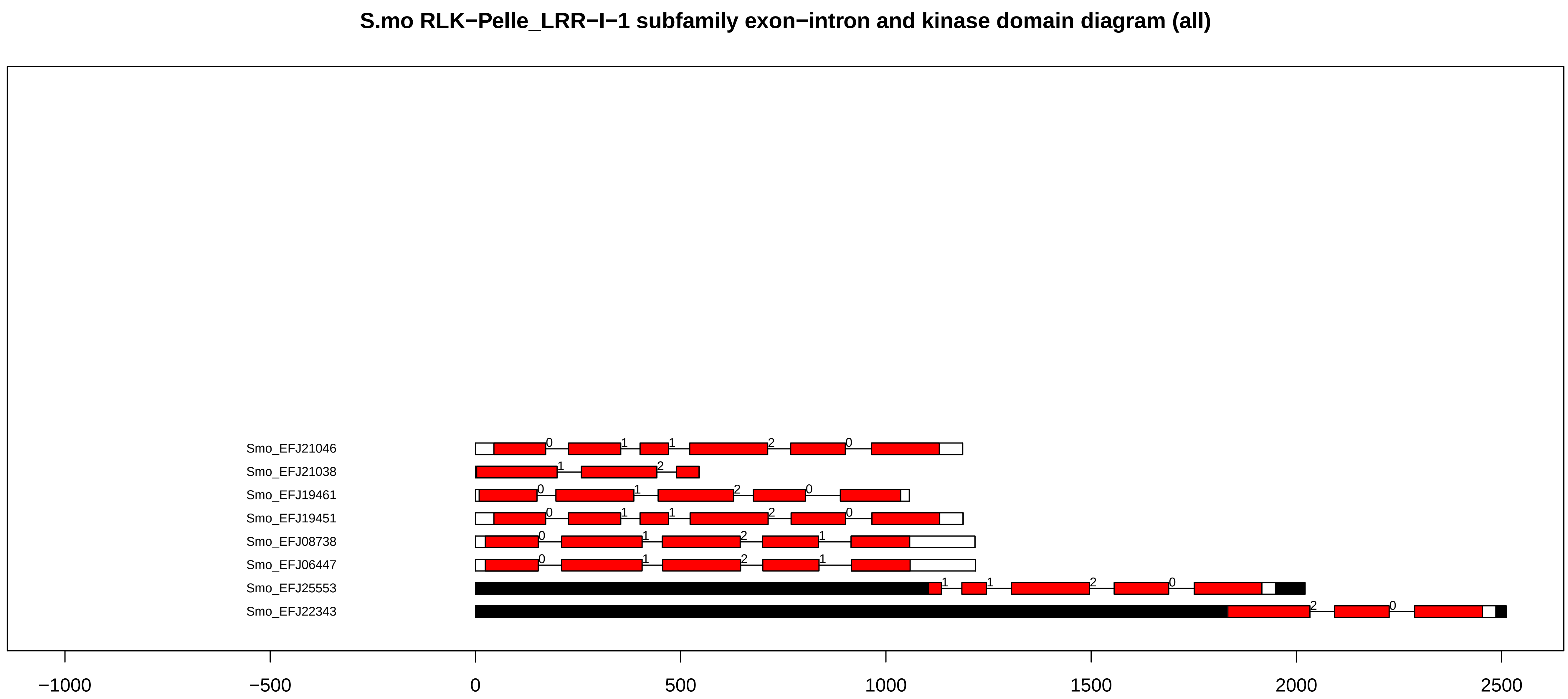
S.mo RLK−Pelle_LRR−I−1 subfamily exon−intron and kinase domain diagram (all)
Smo_EFJ21046
Smo_EFJ21038
Smo_EFJ19461
Smo_EFJ19451
Smo_EFJ08738
Smo_EFJ06447
Smo_EFJ25553
Smo_EFJ22343
−1000
−500
0
500
1000
1500
2000
2500

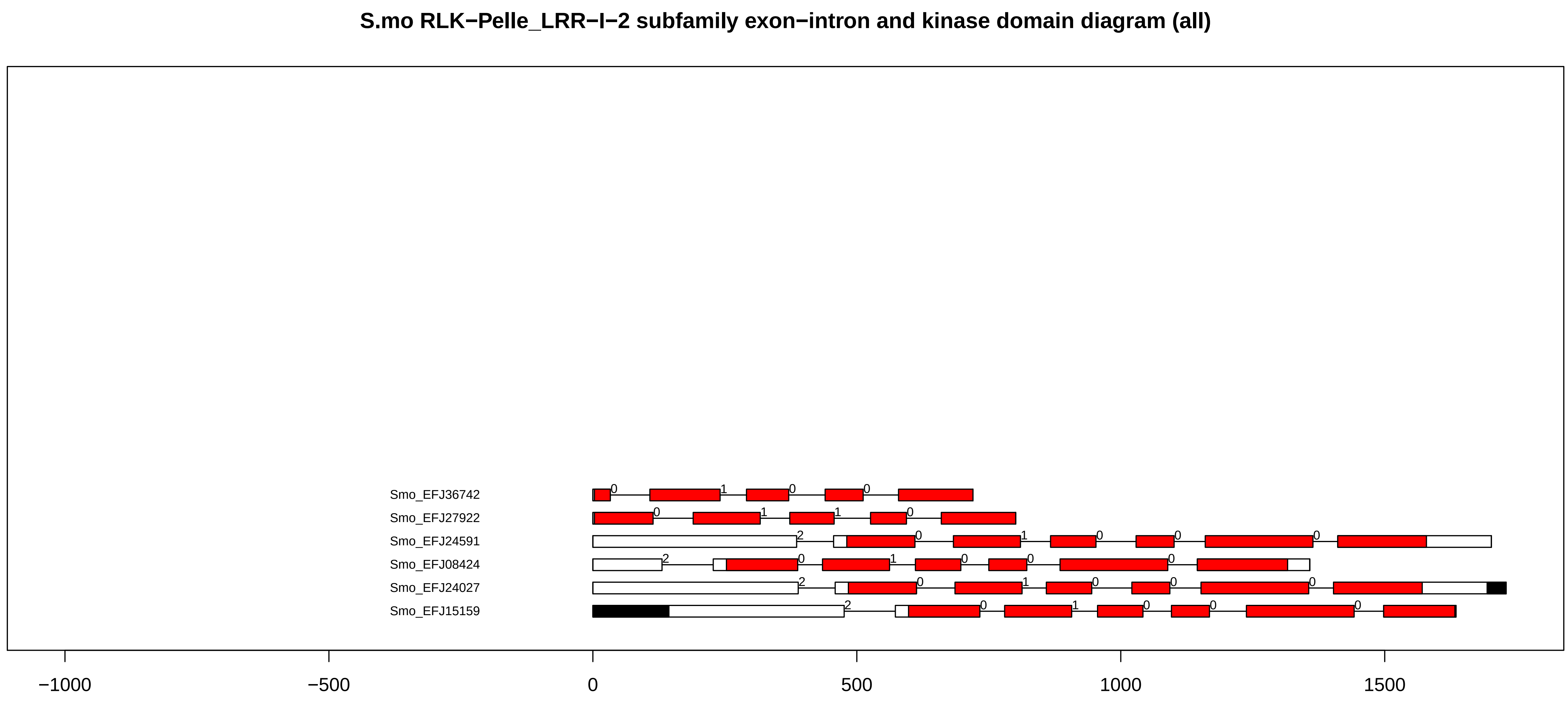
S.mo RLK−Pelle_LRR−I−2 subfamily exon−intron and kinase domain diagram (all)
Smo_EFJ36742
Smo_EFJ27922
Smo_EFJ24591
Smo_EFJ08424
Smo_EFJ24027
Smo_EFJ15159
−1000
−500
0
500
1000
1500

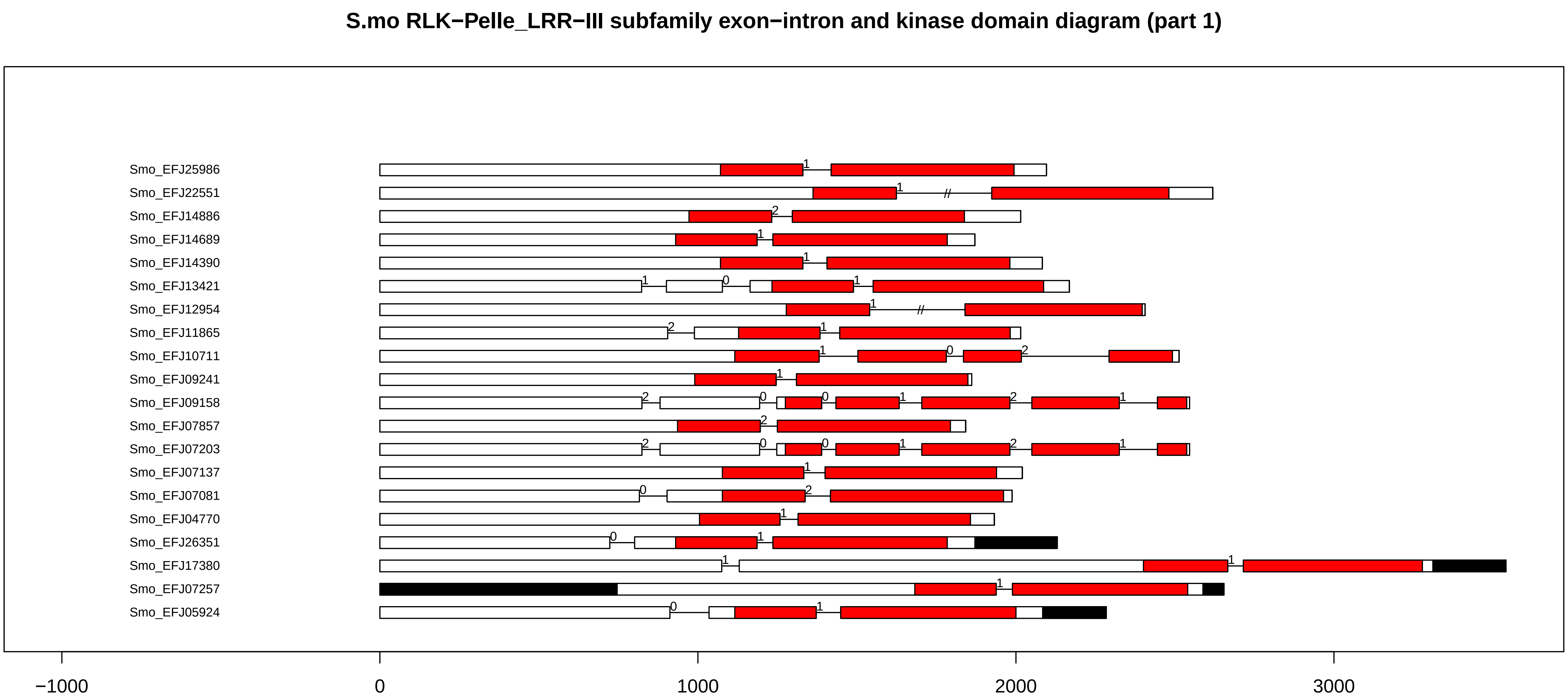
S.mo RLK−Pelle_LRR−III subfamily exon−intron and kinase domain diagram (part 1)
Smo_EFJ25986
Smo_EFJ22551
Smo_EFJ14886
Smo_EFJ14689
Smo_EFJ14390
Smo_EFJ13421
Smo_EFJ12954
Smo_EFJ11865
Smo_EFJ10711
Smo_EFJ09241
Smo_EFJ09158
Smo_EFJ07857
Smo_EFJ07203
Smo_EFJ07137
Smo_EFJ07081
Smo_EFJ04770
Smo_EFJ26351
Smo_EFJ17380
Smo_EFJ07257
Smo_EFJ05924
−1000
0
1000
2000
3000

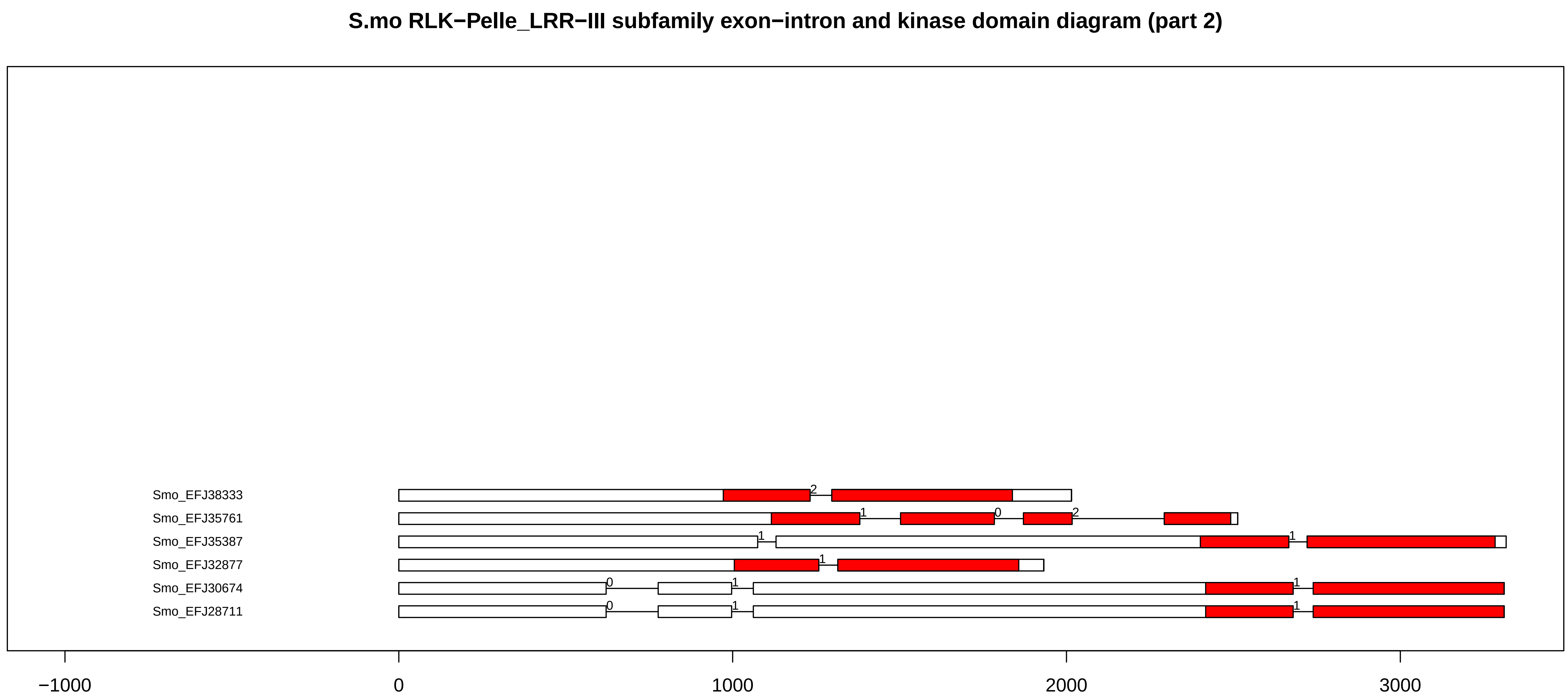
S.mo RLK−Pelle_LRR−III subfamily exon−intron and kinase domain diagram (part 2)
Smo_EFJ38333
Smo_EFJ35761
Smo_EFJ35387
Smo_EFJ32877
Smo_EFJ30674
Smo_EFJ28711
2
1
0
2
1
1
1
0
1
1
0
1
1
−1000
0
1000
2000
3000

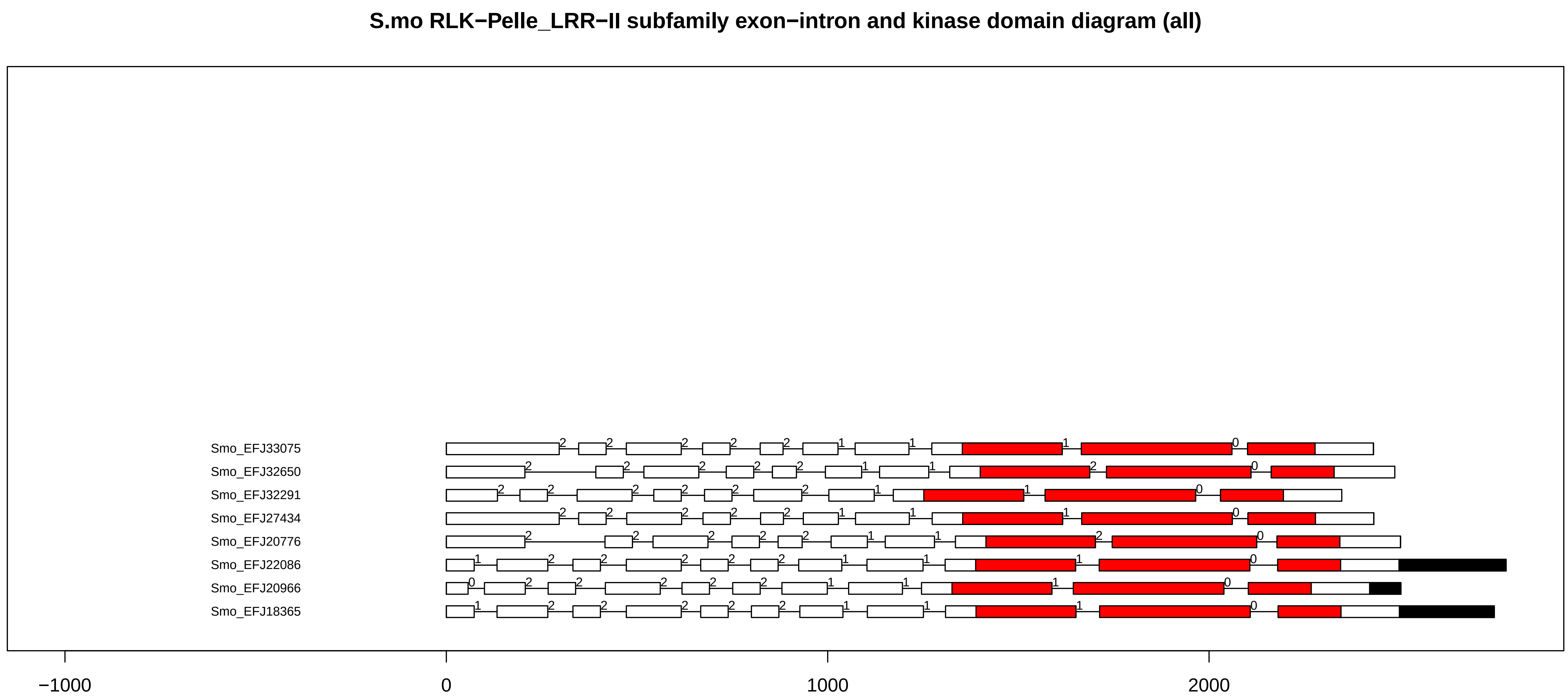

S.mo RLK−Pelle_LRR−II subfamily exon−intron and kinase domain diagram (all)
Smo_EFJ33075
Smo_EFJ32650
Smo_EFJ32291
Smo_EFJ27434
Smo_EFJ20776
Smo_EFJ22086
Smo_EFJ20966
Smo_EFJ18365
−1000
0
1000
2000

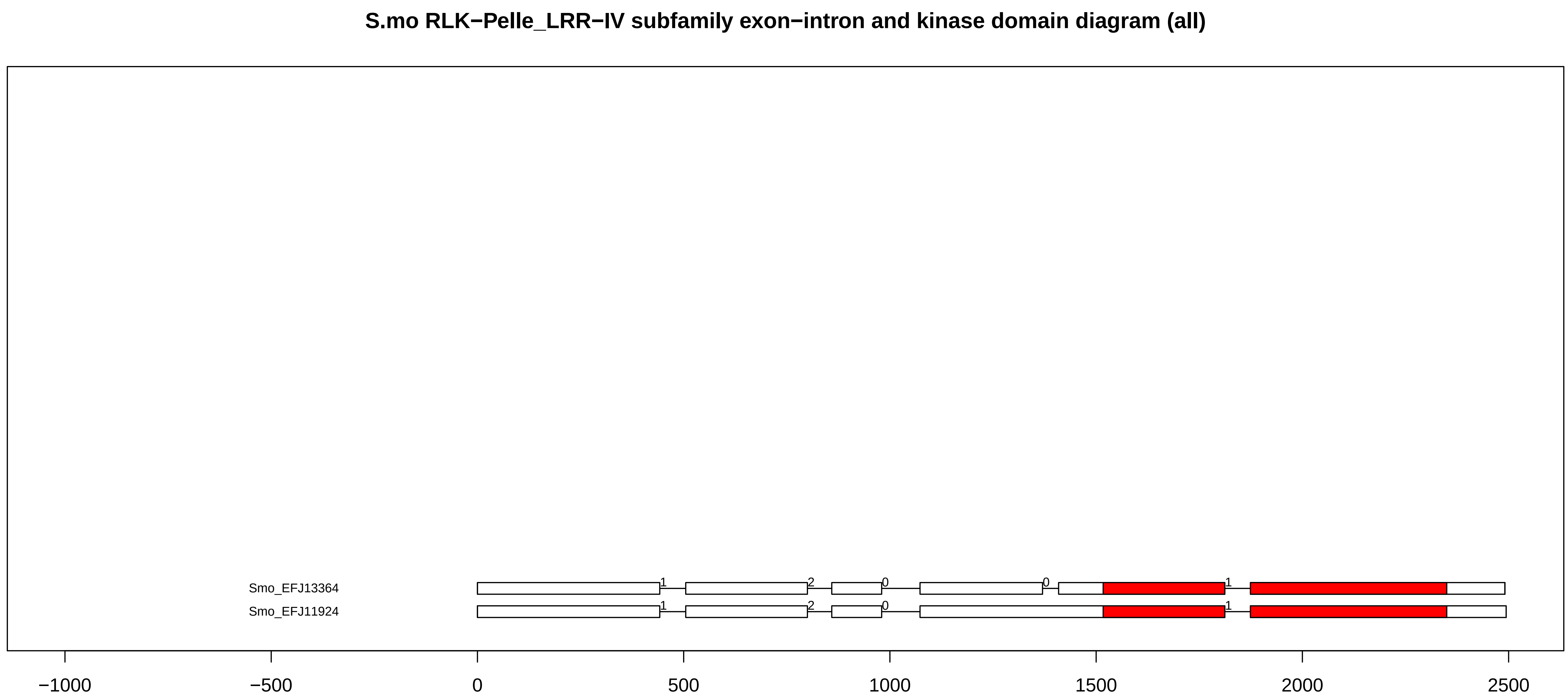
S.mo RLK−Pelle_LRR−IV subfamily exon−intron and kinase domain diagram (all)
Smo_EFJ13364
Smo_EFJ11924
1
2
0
0
1
1
2
0
1
−1000
−500
0
500
1000
1500
2000
2500

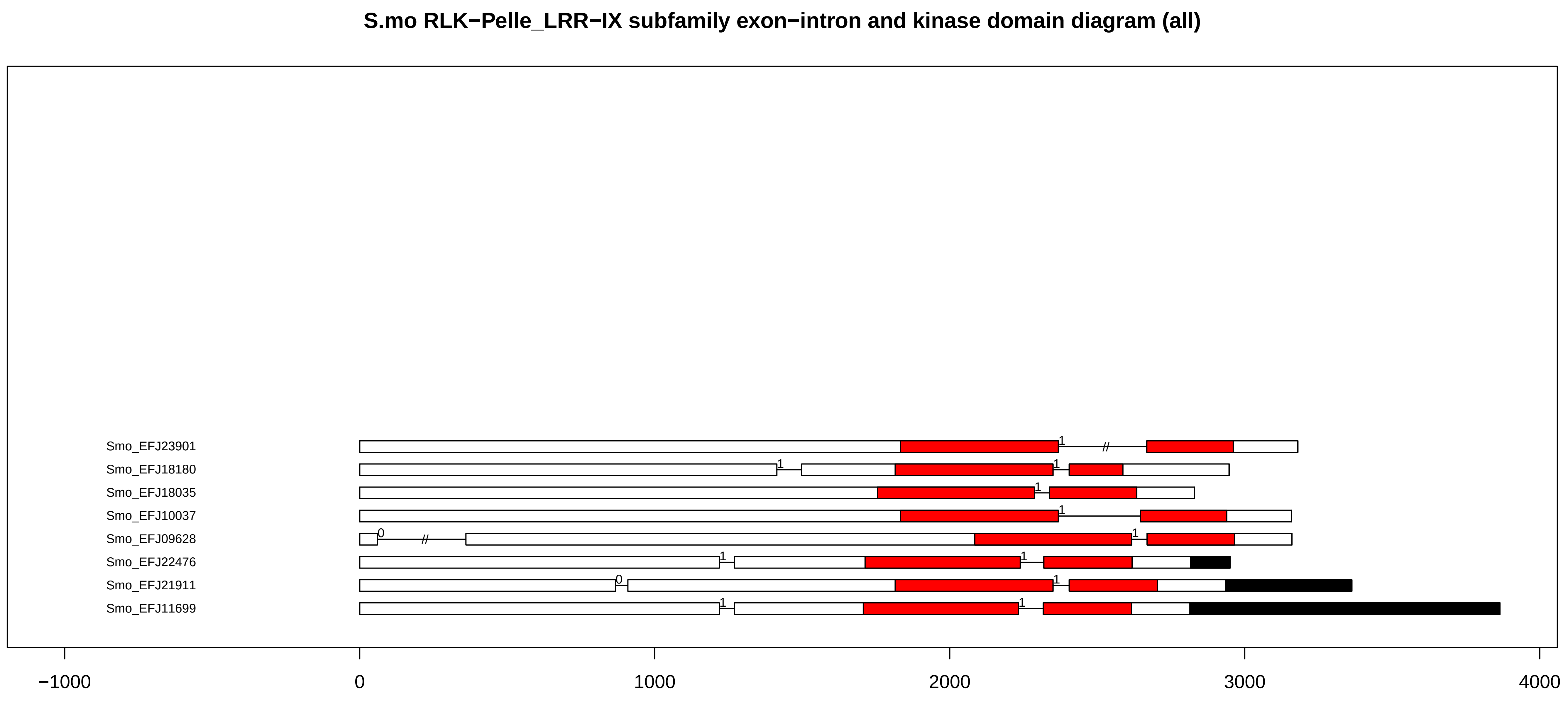
S.mo RLK−Pelle_LRR−IX subfamily exon−intron and kinase domain diagram (all)
Smo_EFJ23901
Smo_EFJ18180
Smo_EFJ18035
Smo_EFJ10037
Smo_EFJ09628
Smo_EFJ22476
Smo_EFJ21911
Smo_EFJ11699
−1000
0
1000
2000
3000
4000

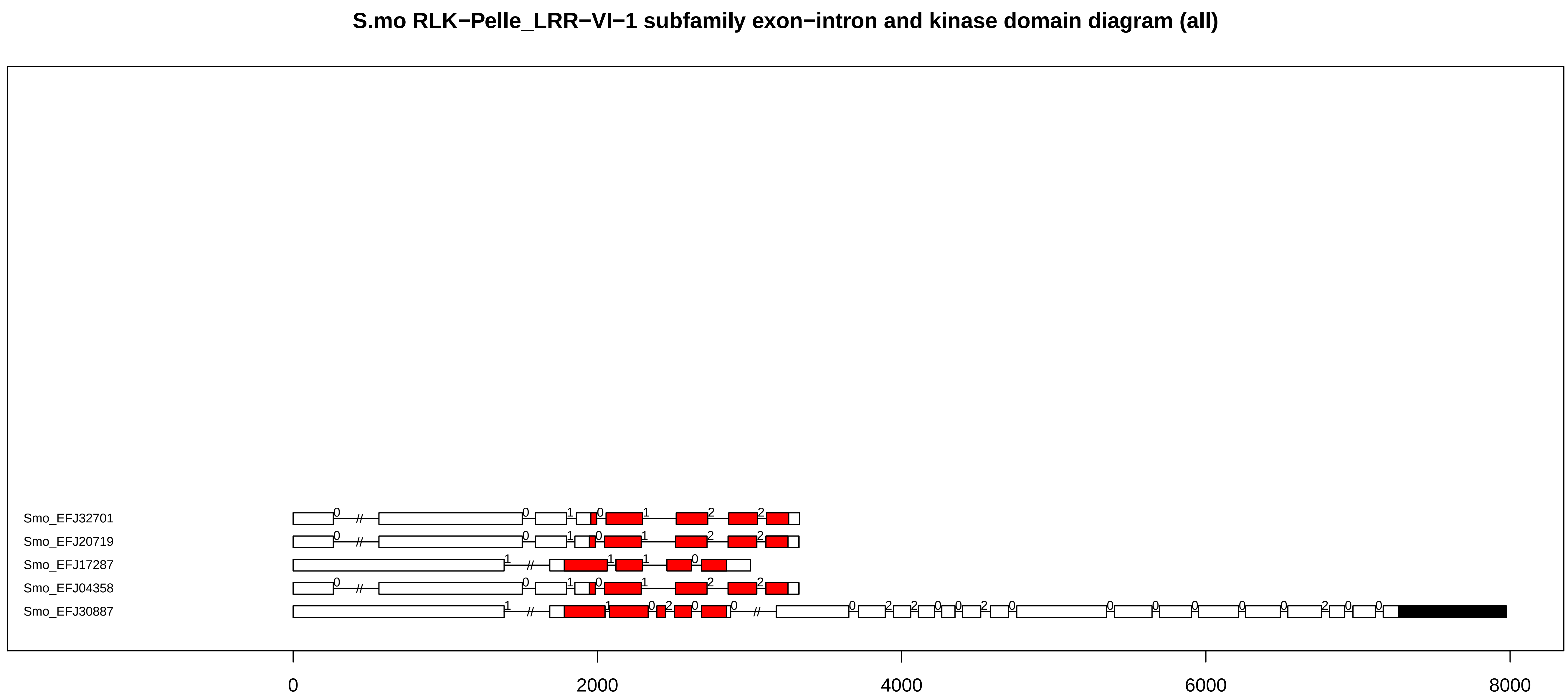
S.mo RLK−Pelle_LRR−VI−1 subfamily exon−intron and kinase domain diagram (all)
Smo_EFJ32701
Smo_EFJ20719
Smo_EFJ17287
Smo_EFJ04358
Smo_EFJ30887
0
2000
4000
6000
8000

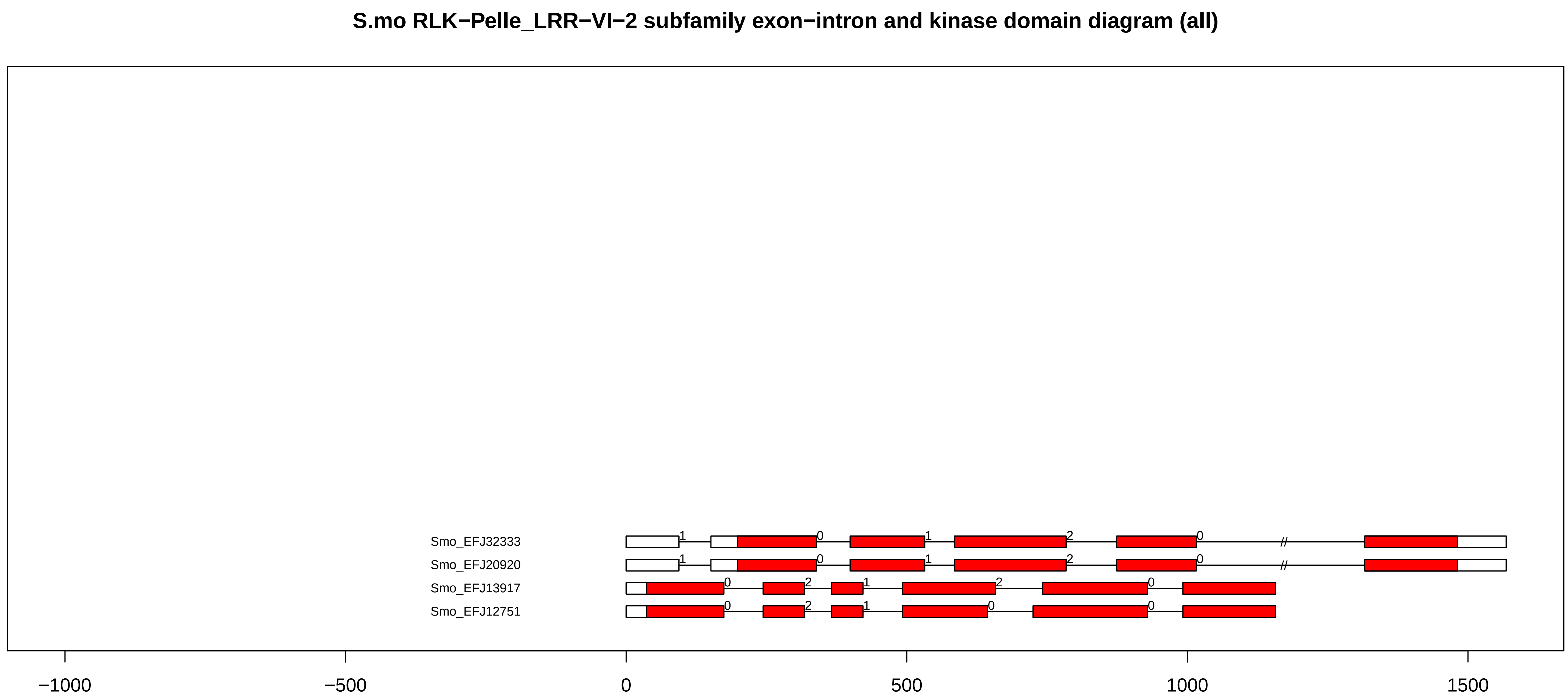

S.mo RLK−Pelle_LRR−VI−2 subfamily exon−intron and kinase domain diagram (all)
Smo_EFJ32333
Smo_EFJ20920
Smo_EFJ13917
Smo_EFJ12751
−1000
−500
0
500
1000
1500

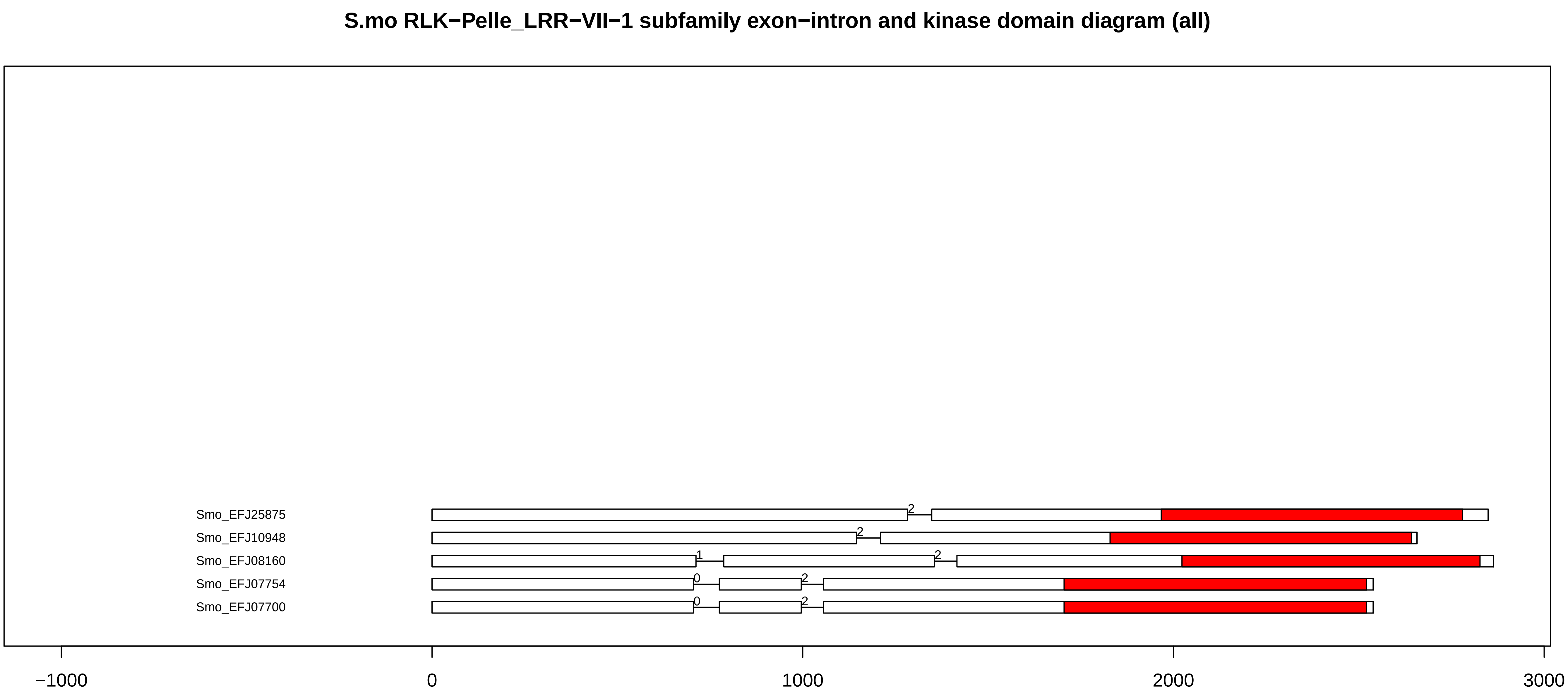

S.mo RLK−Pelle_LRR−VII−1 subfamily exon−intron and kinase domain diagram (all)
Smo_EFJ25875
Smo_EFJ10948
Smo_EFJ08160
Smo_EFJ07754
Smo_EFJ07700
2
2
1
2
0
2
0
2
−1000
0
1000
2000
3000

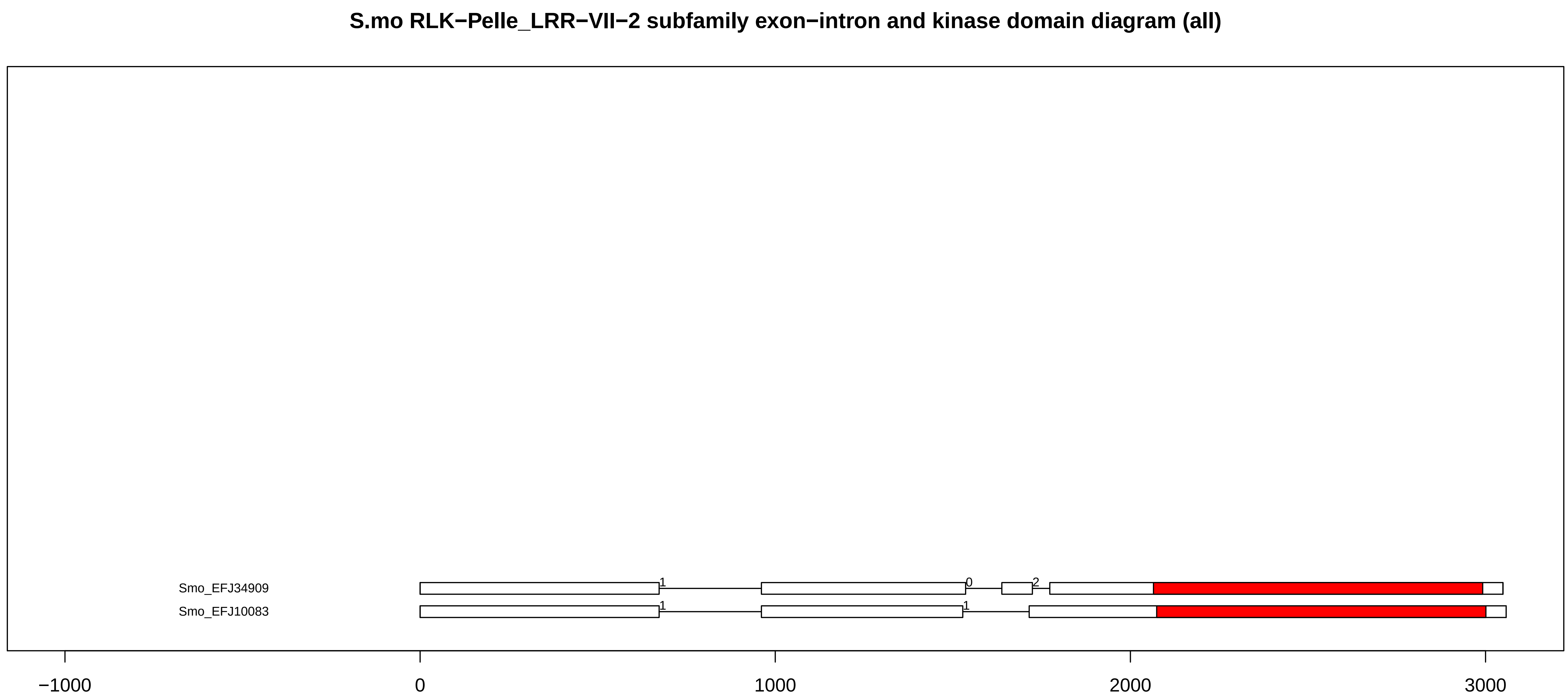
S.mo RLK−Pelle_LRR−VII−2 subfamily exon−intron and kinase domain diagram (all)
Smo_EFJ34909
Smo_EFJ10083
1
0
2
1
1
−1000
0
1000
2000
3000

S.mo RLK−Pelle_LRR−VII−3 subfamily exon−intron and kinase domain diagram (all)

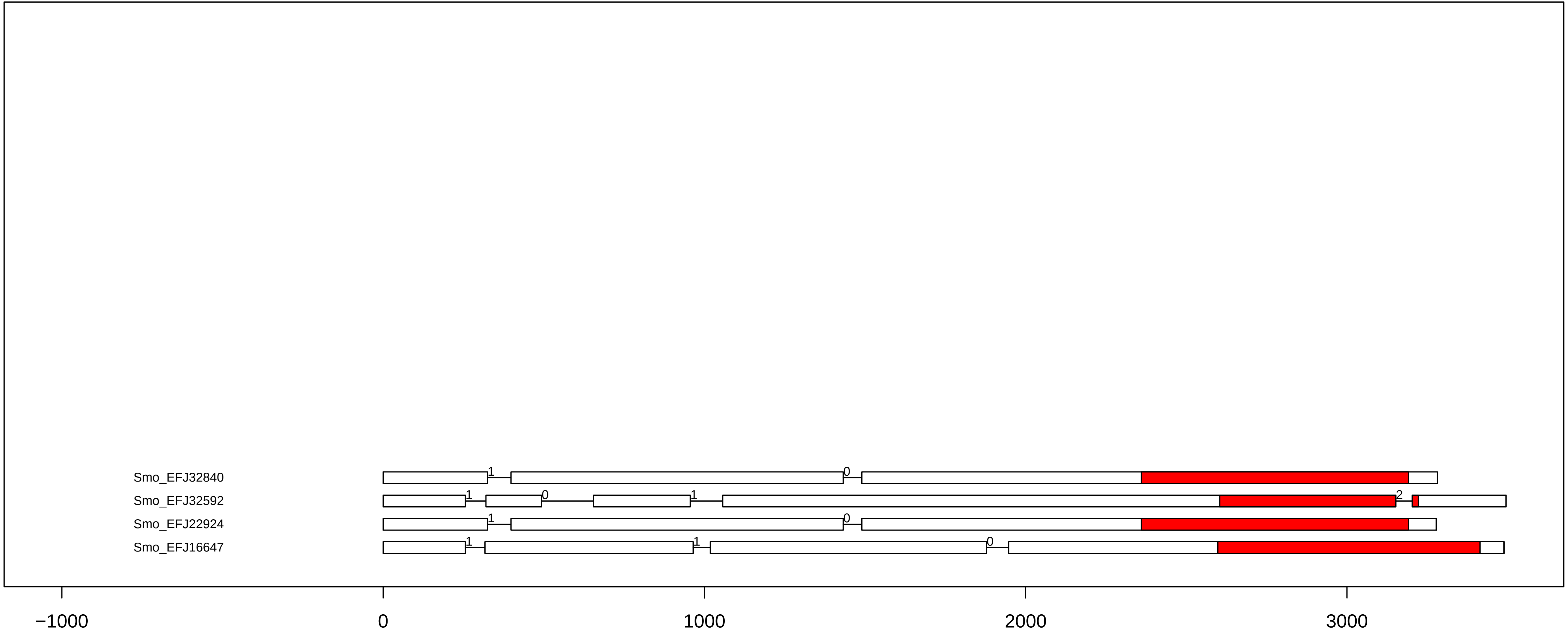

S.mo RLK−Pelle_LRR−VIII−1 subfamily exon−intron and kinase domain diagram (all)

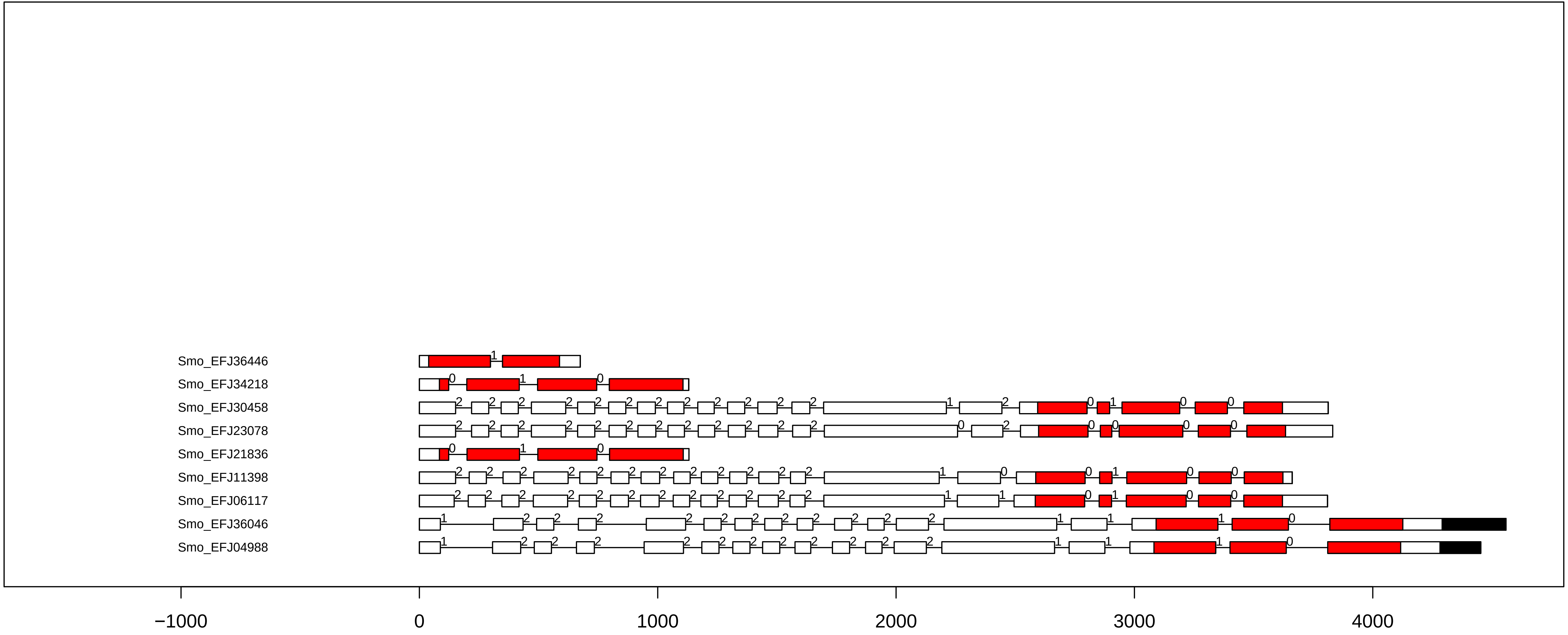

S.mo RLK−Pelle_LRR−V subfamily exon−intron and kinase domain diagram (all)

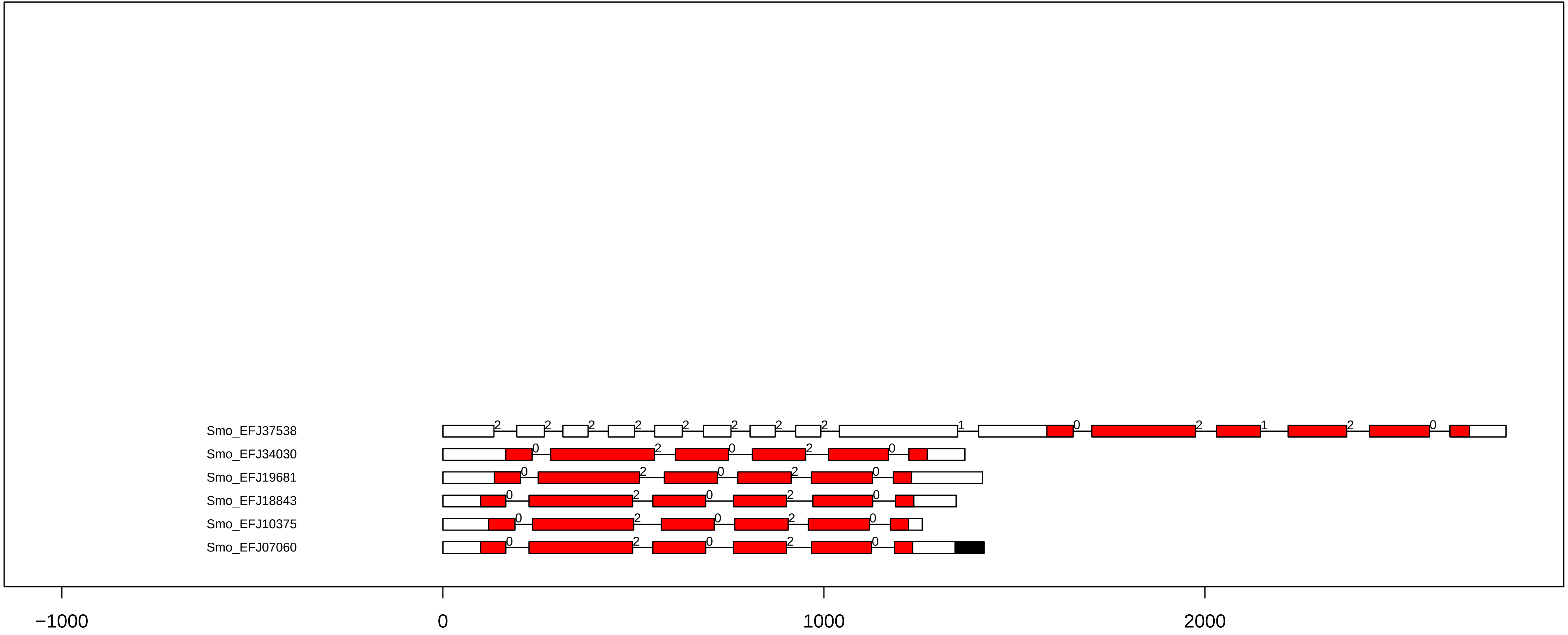

S.mo RLK−Pelle_LRR−Xa subfamily exon−intron and kinase domain diagram (all)

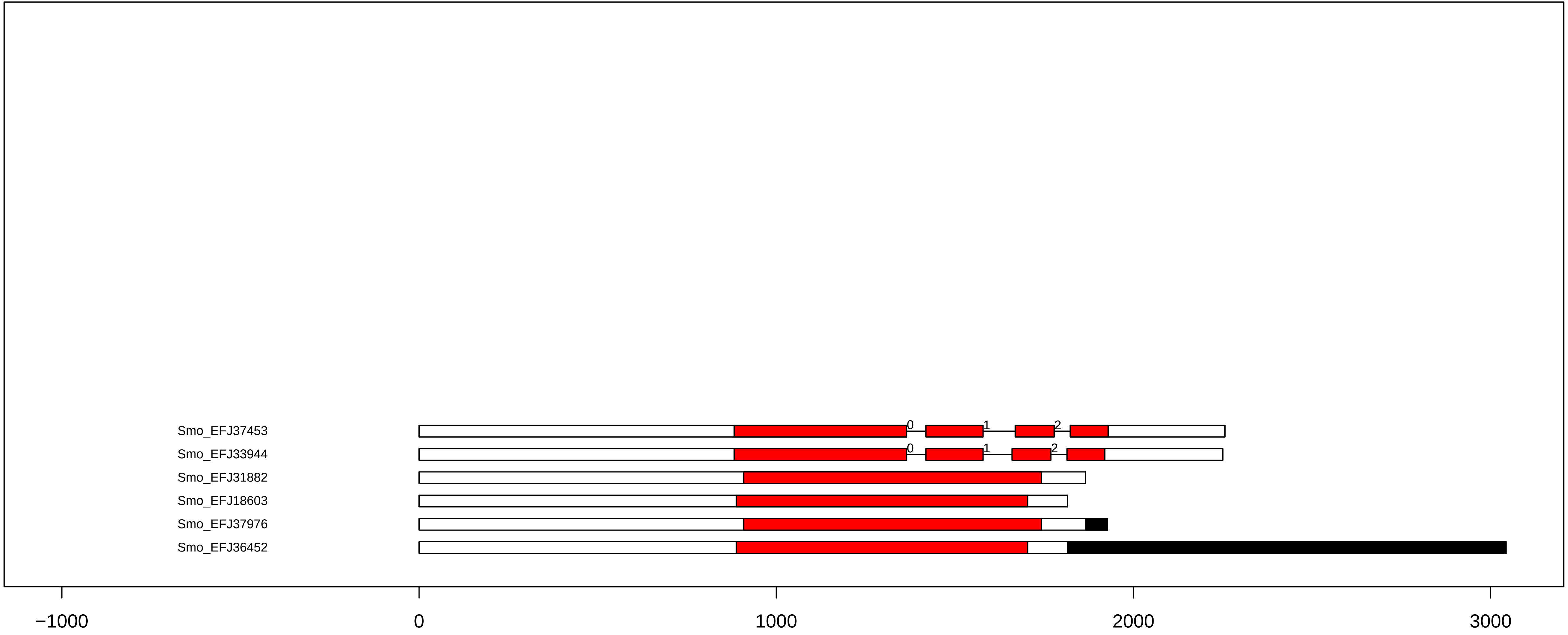

S.mo RLK−Pelle_LRR−Xb−1 subfamily exon−intron and kinase domain diagram (all)

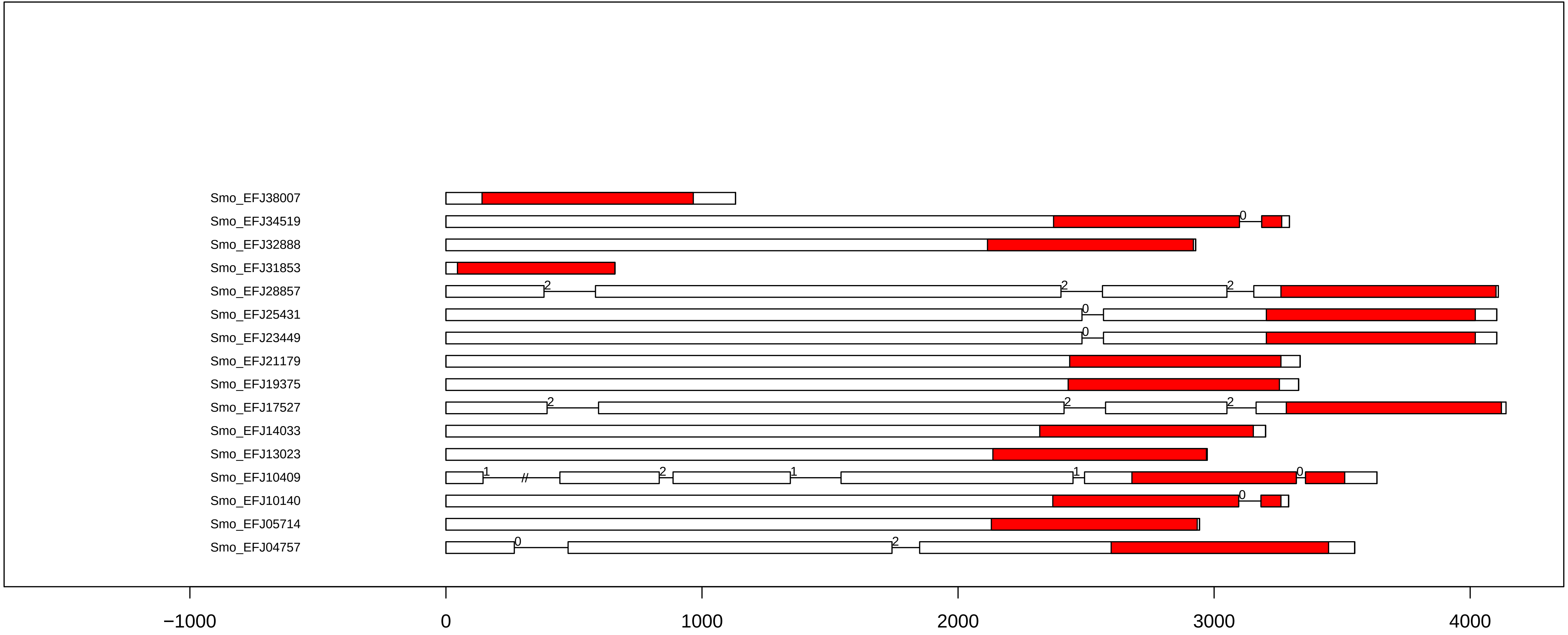

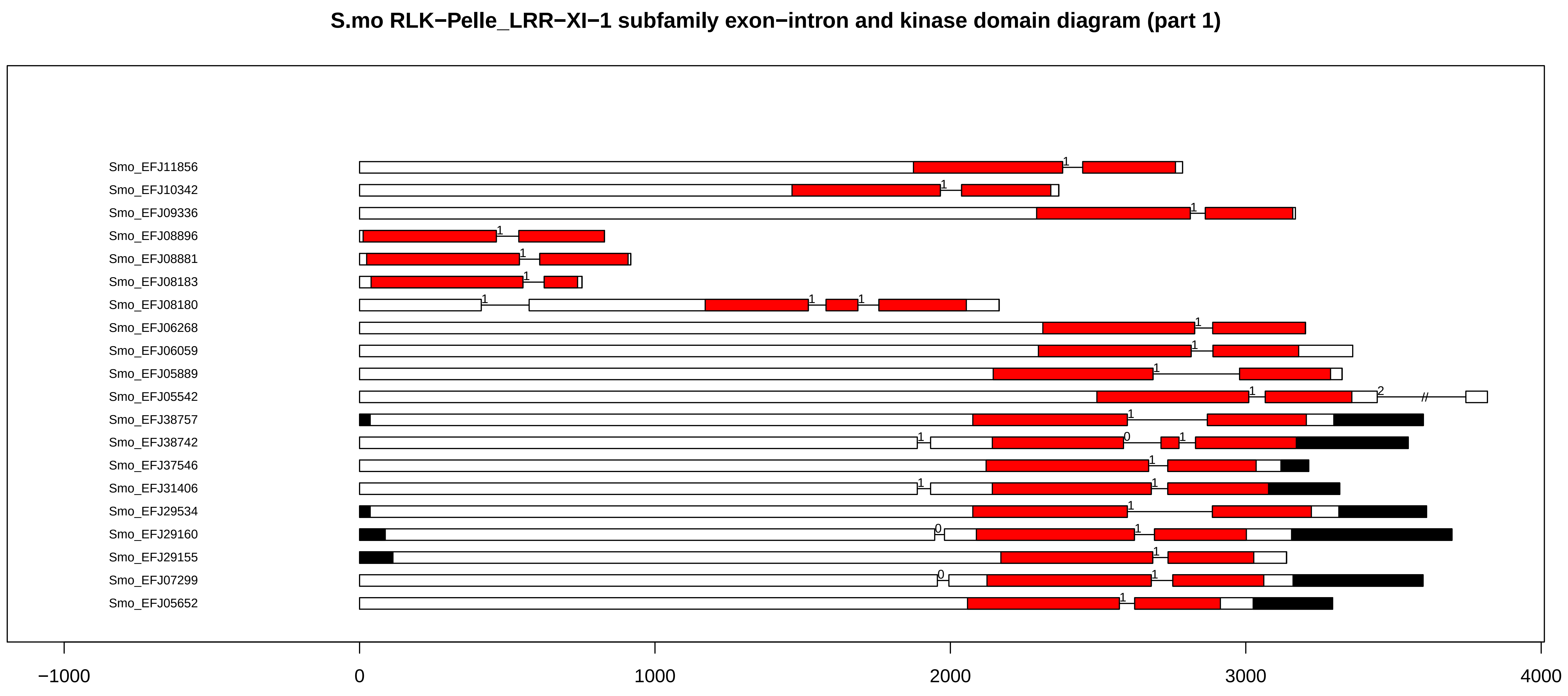
S.mo RLK−Pelle_LRR−XI−1 subfamily exon−intron and kinase domain diagram (part 1)
Smo_EFJ11856
Smo_EFJ10342
Smo_EFJ09336
Smo_EFJ08896
Smo_EFJ08881
Smo_EFJ08183
Smo_EFJ08180
Smo_EFJ06268
Smo_EFJ06059
Smo_EFJ05889
Smo_EFJ05542
Smo_EFJ38757
Smo_EFJ38742
Smo_EFJ37546
Smo_EFJ31406
Smo_EFJ29534
Smo_EFJ29160
Smo_EFJ29155
Smo_EFJ07299
Smo_EFJ05652
−1000
0
1000
2000
3000
4000

S.mo RLK−Pelle_LRR−XI−1 subfamily exon−intron and kinase domain diagram (part 2)

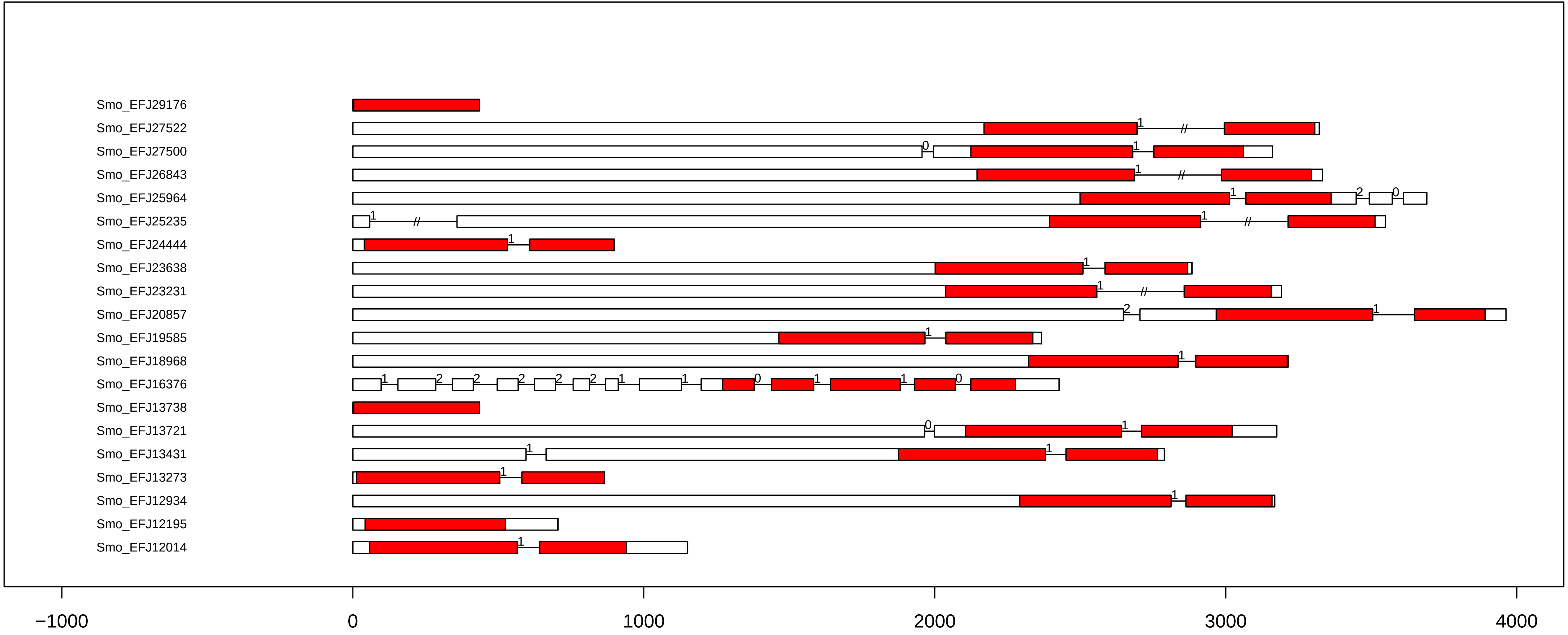

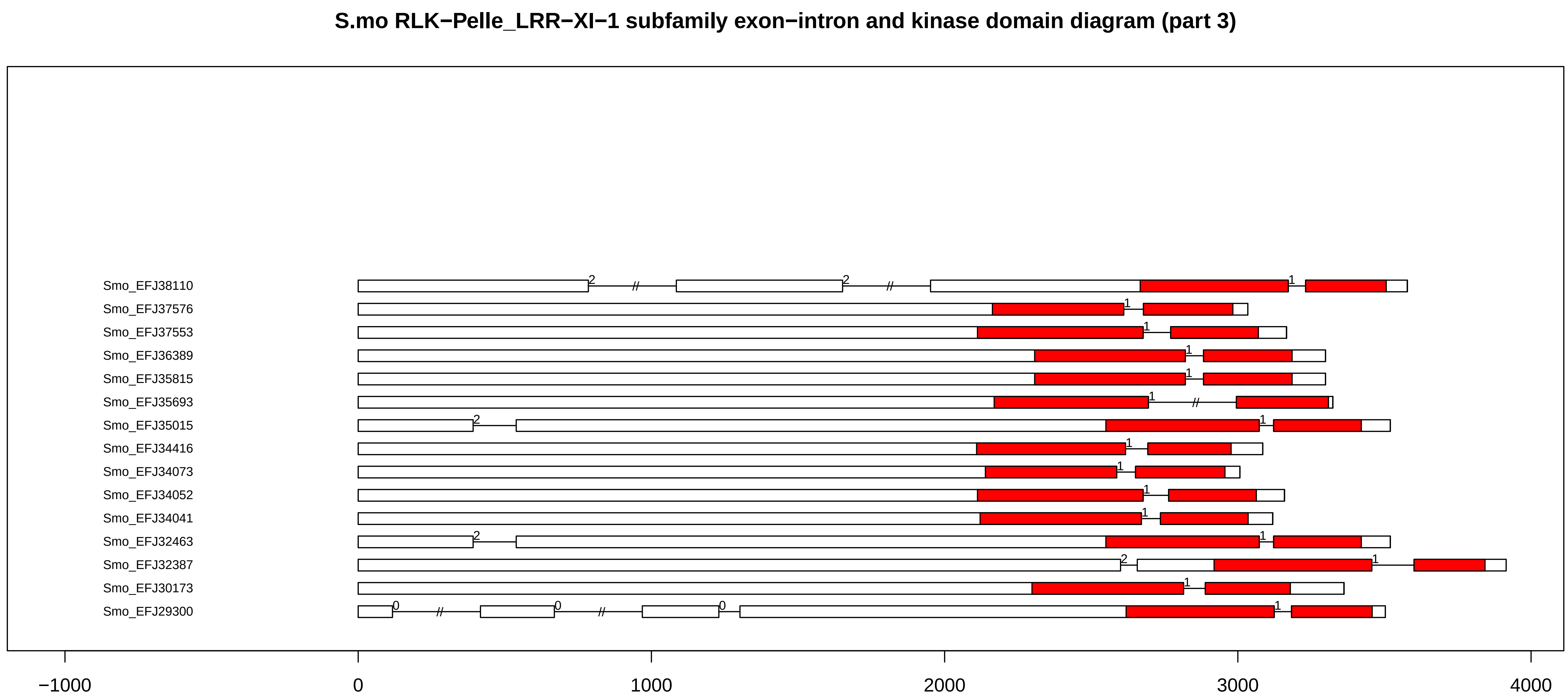
S.mo RLK−Pelle_LRR−XI−1 subfamily exon−intron and kinase domain diagram (part 3)
Smo_EFJ38110
Smo_EFJ37576
Smo_EFJ37553
Smo_EFJ36389
Smo_EFJ35815
Smo_EFJ35693
Smo_EFJ35015
Smo_EFJ34416
Smo_EFJ34073
Smo_EFJ34052
Smo_EFJ34041
Smo_EFJ32463
Smo_EFJ32387
Smo_EFJ30173
Smo_EFJ29300
−1000
0
1000
2000
3000
4000

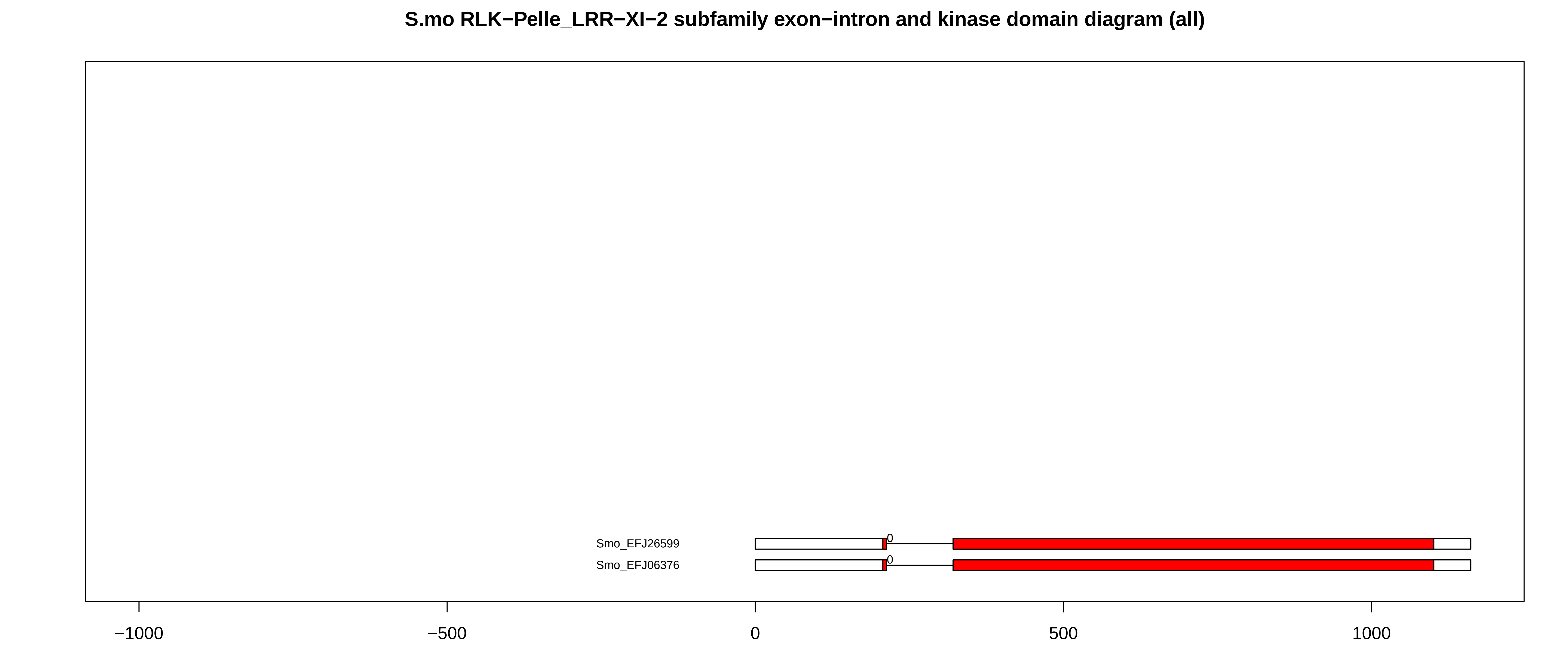
S.mo RLK−Pelle_LRR−XI−2 subfamily exon−intron and kinase domain diagram (all)
Smo_EFJ26599
Smo_EFJ06376
0
0
−1000
−500
0
500
1000

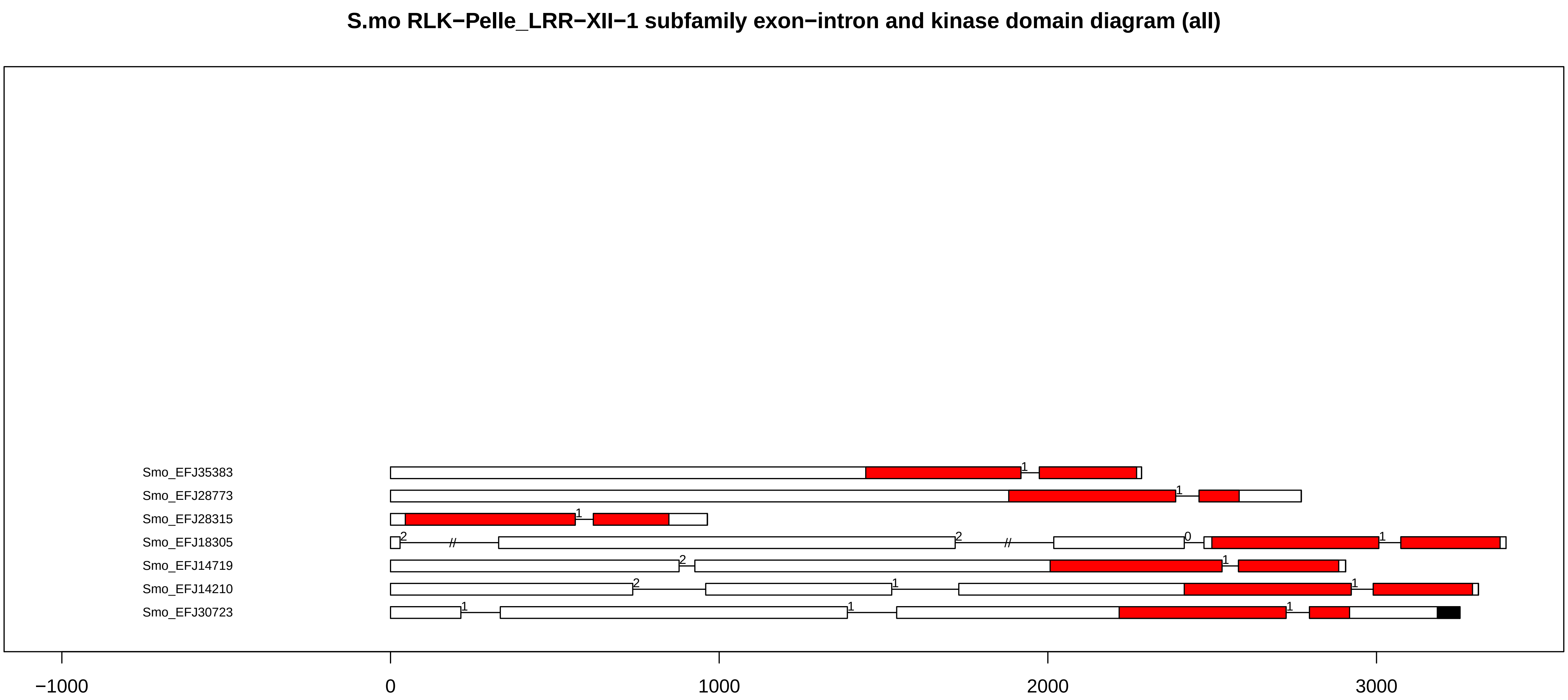
S.mo RLK−Pelle_LRR−XII−1 subfamily exon−intron and kinase domain diagram (all)
Smo_EFJ35383
Smo_EFJ28773
Smo_EFJ28315
Smo_EFJ18305
Smo_EFJ14719
Smo_EFJ14210
Smo_EFJ30723
1
1
1
2
2
0
1
2
1
2
1
1
1
1
1
−1000
0
1000
2000
3000

S.mo RLK−Pelle_LRR−XIIIa subfamily exon−intron and kinase domain diagram (all)

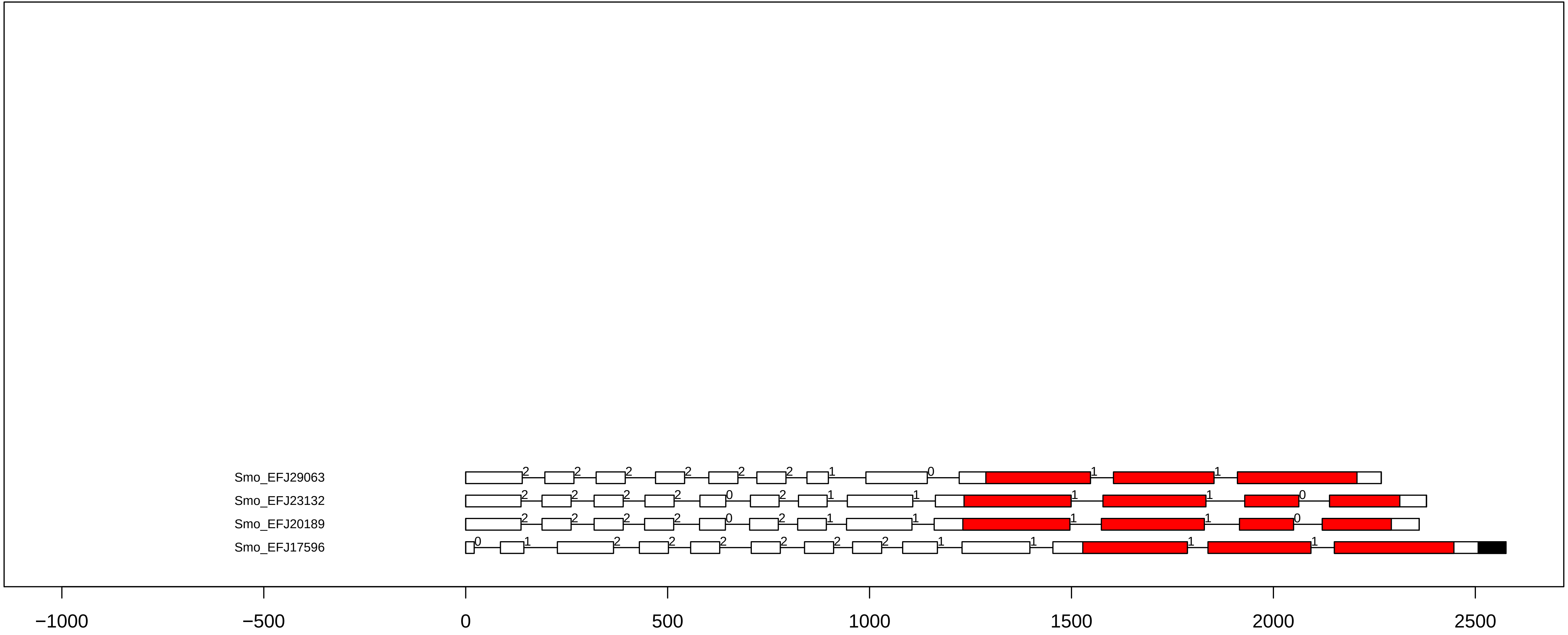

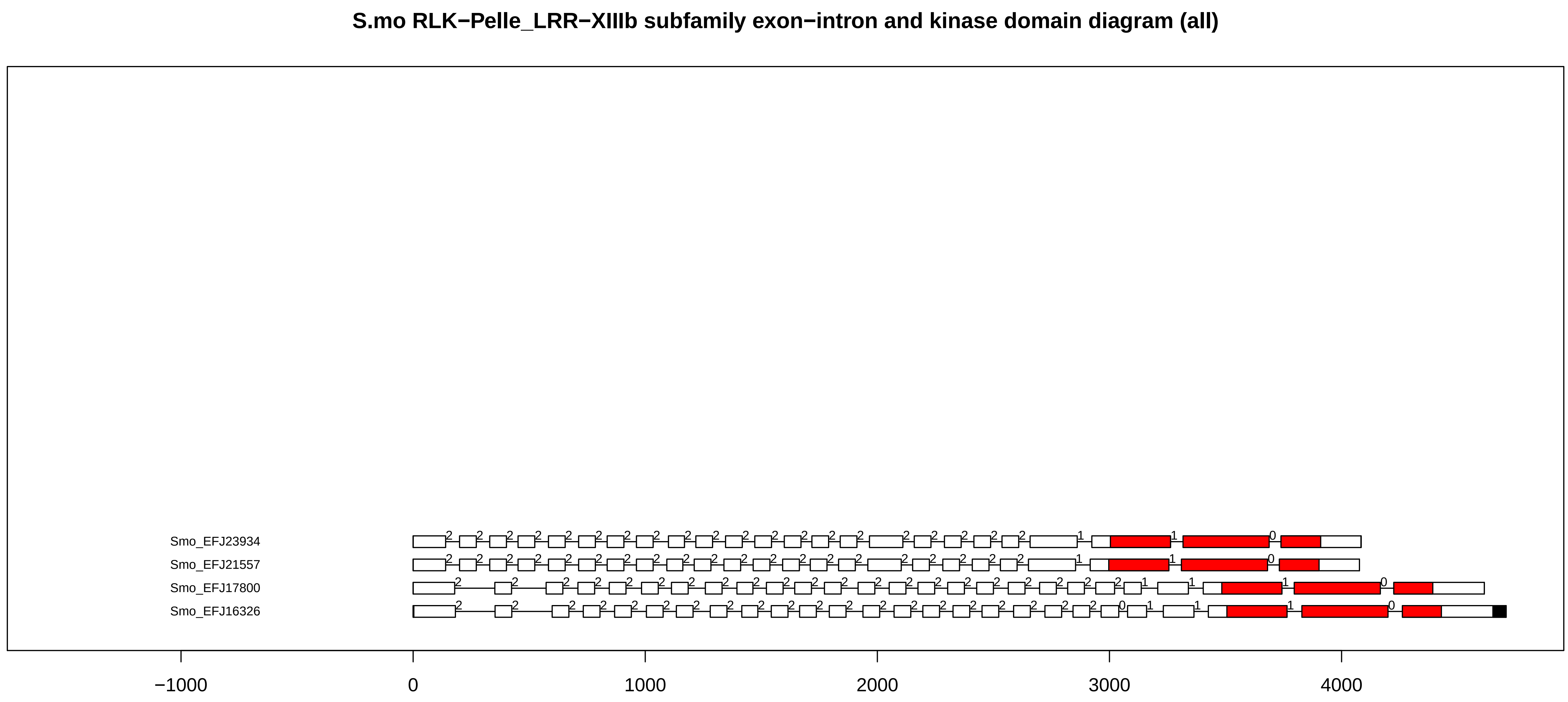
S.mo RLK−Pelle_LRR−XIIIb subfamily exon−intron and kinase domain diagram (all)
Smo_EFJ23934
Smo_EFJ21557
Smo_EFJ17800
Smo_EFJ16326
−1000
0
1000
2000
3000
4000

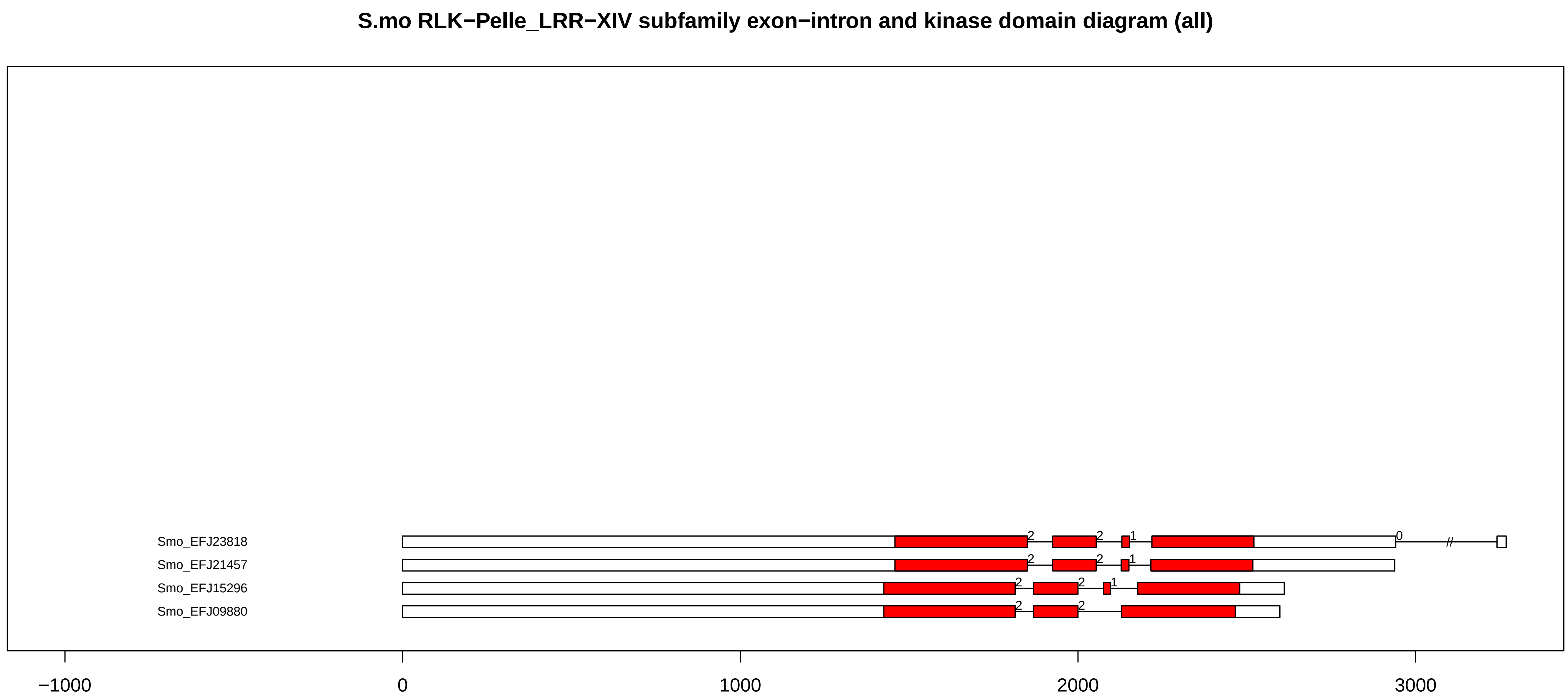
S.mo RLK−Pelle_LRR−XIV subfamily exon−intron and kinase domain diagram (all)
Smo_EFJ23818
Smo_EFJ21457
Smo_EFJ15296
Smo_EFJ09880
2
2
1
0
−1000
0
1000
2000
3000

S.mo RLK−Pelle_LRR−XV subfamily exon−intron and kinase domain diagram (all)

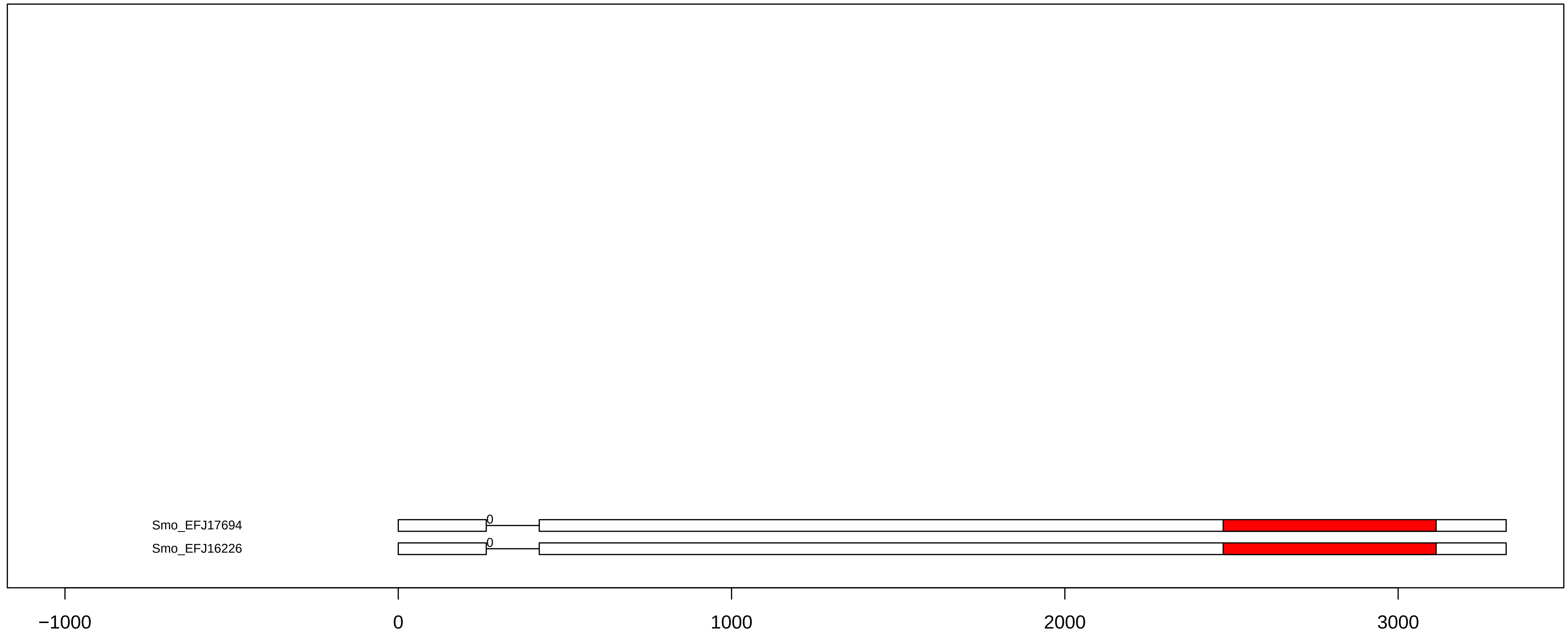

S.mo RLK−Pelle_LysM subfamily exon−intron and kinase domain diagram (all)

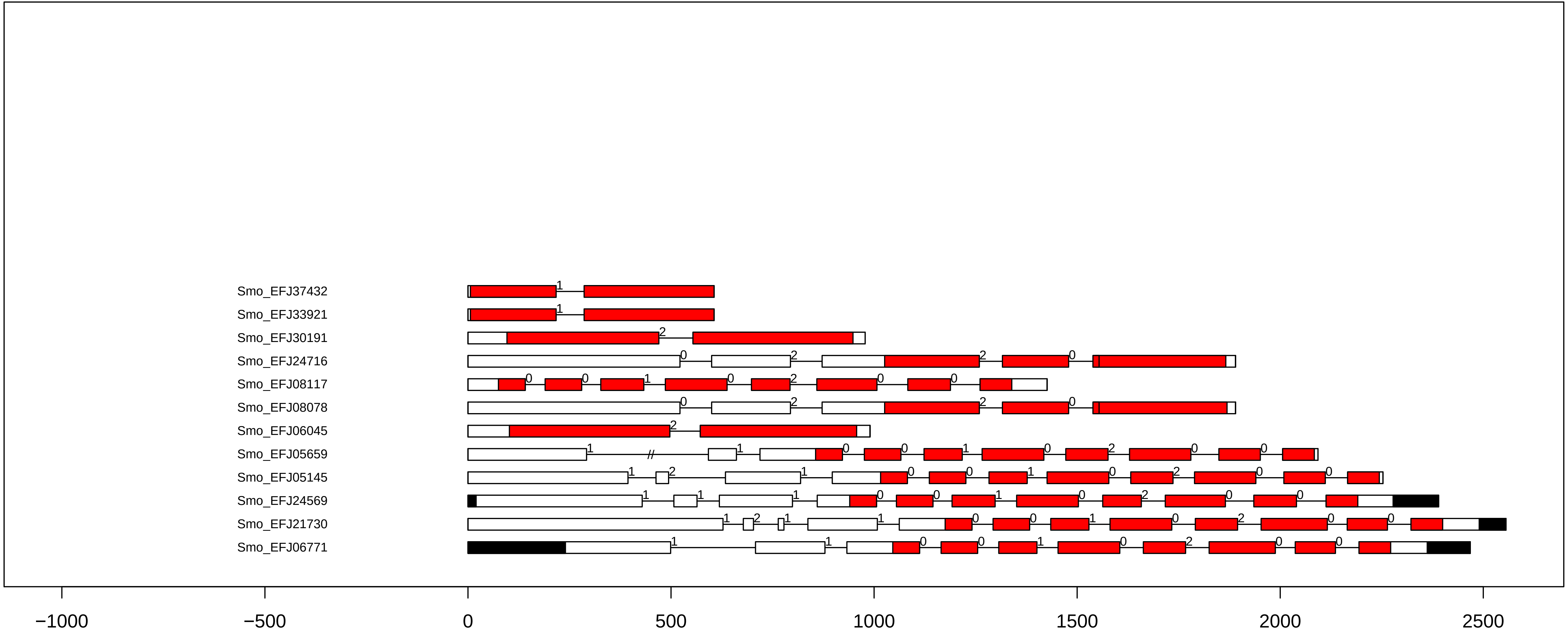

S.mo RLK−Pelle_PERK−1 subfamily exon−intron and kinase domain diagram (all)

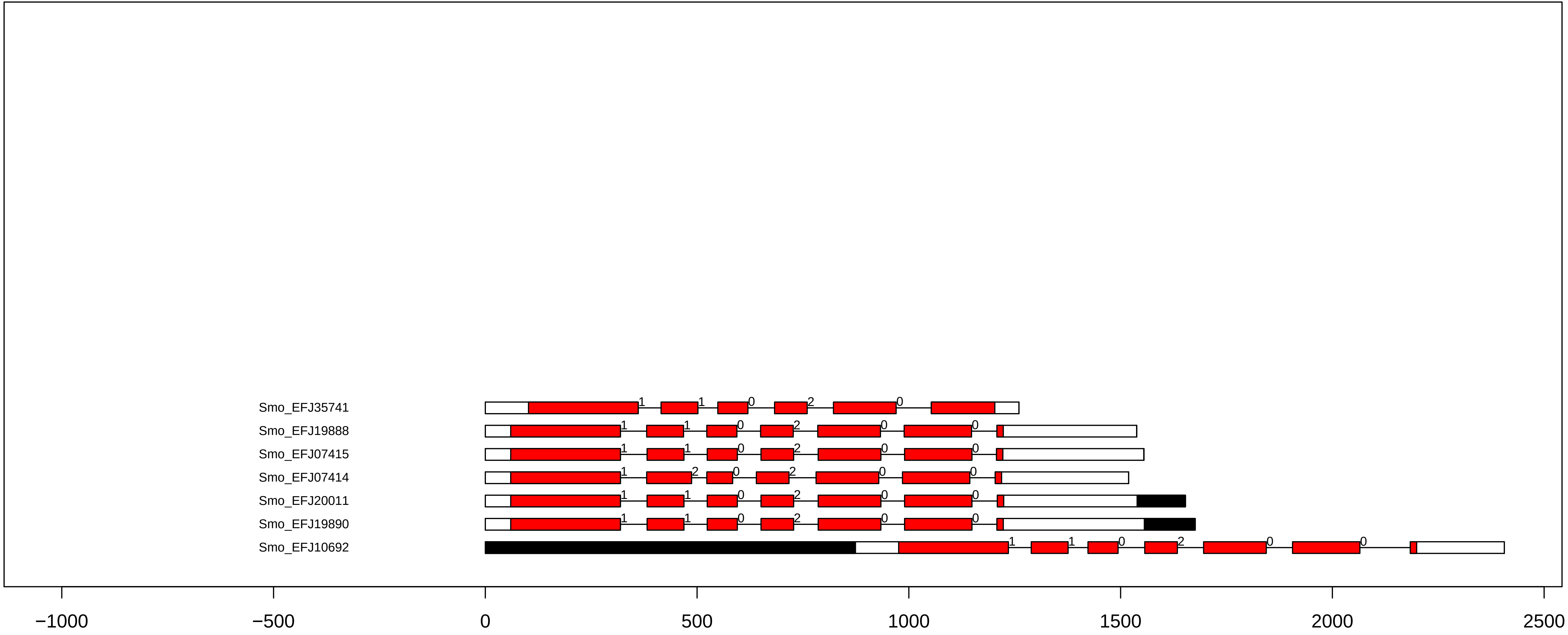

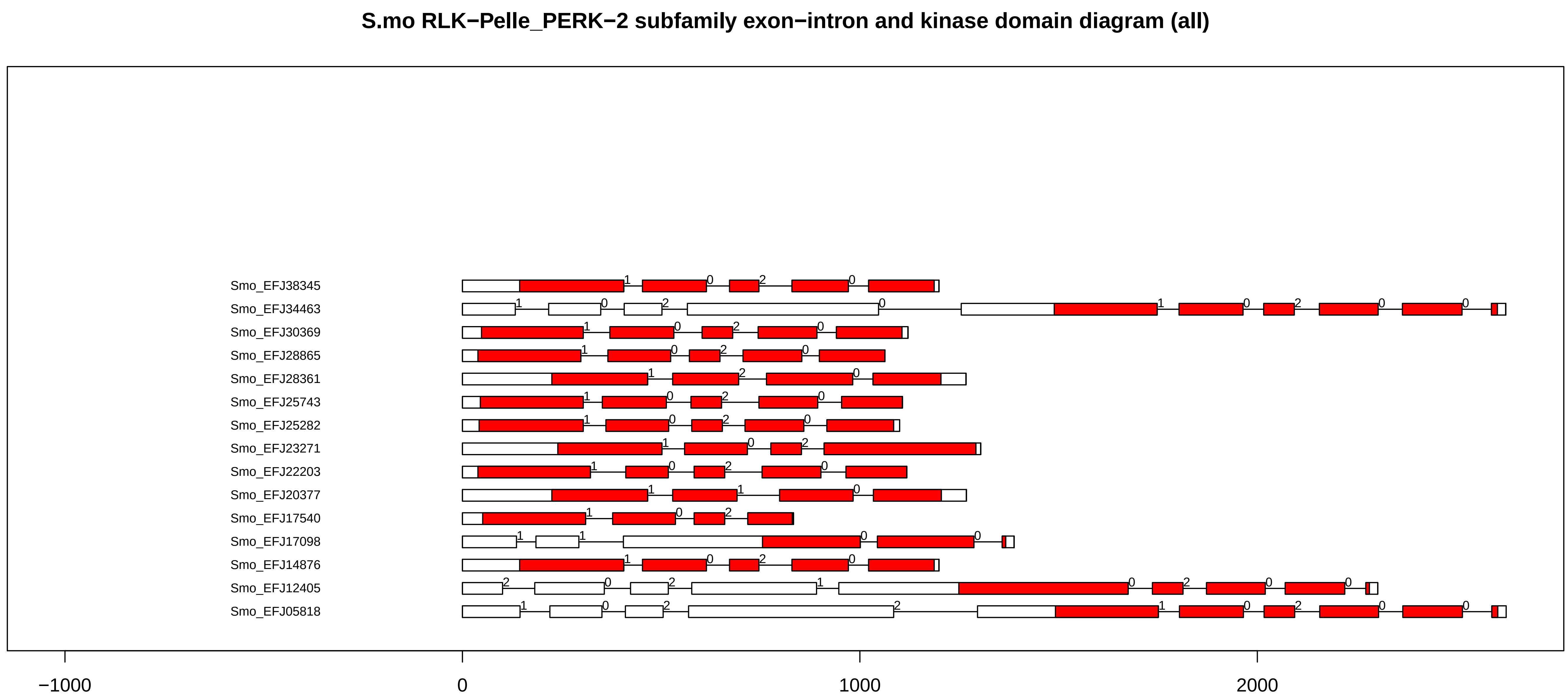
S.mo RLK−Pelle_PERK−2 subfamily exon−intron and kinase domain diagram (all)
Smo_EFJ38345
Smo_EFJ34463
Smo_EFJ30369
Smo_EFJ28865
Smo_EFJ28361
Smo_EFJ25743
Smo_EFJ25282
Smo_EFJ23271
Smo_EFJ22203
Smo_EFJ20377
Smo_EFJ17540
Smo_EFJ17098
Smo_EFJ14876
Smo_EFJ12405
Smo_EFJ05818
−1000
0
1000
2000

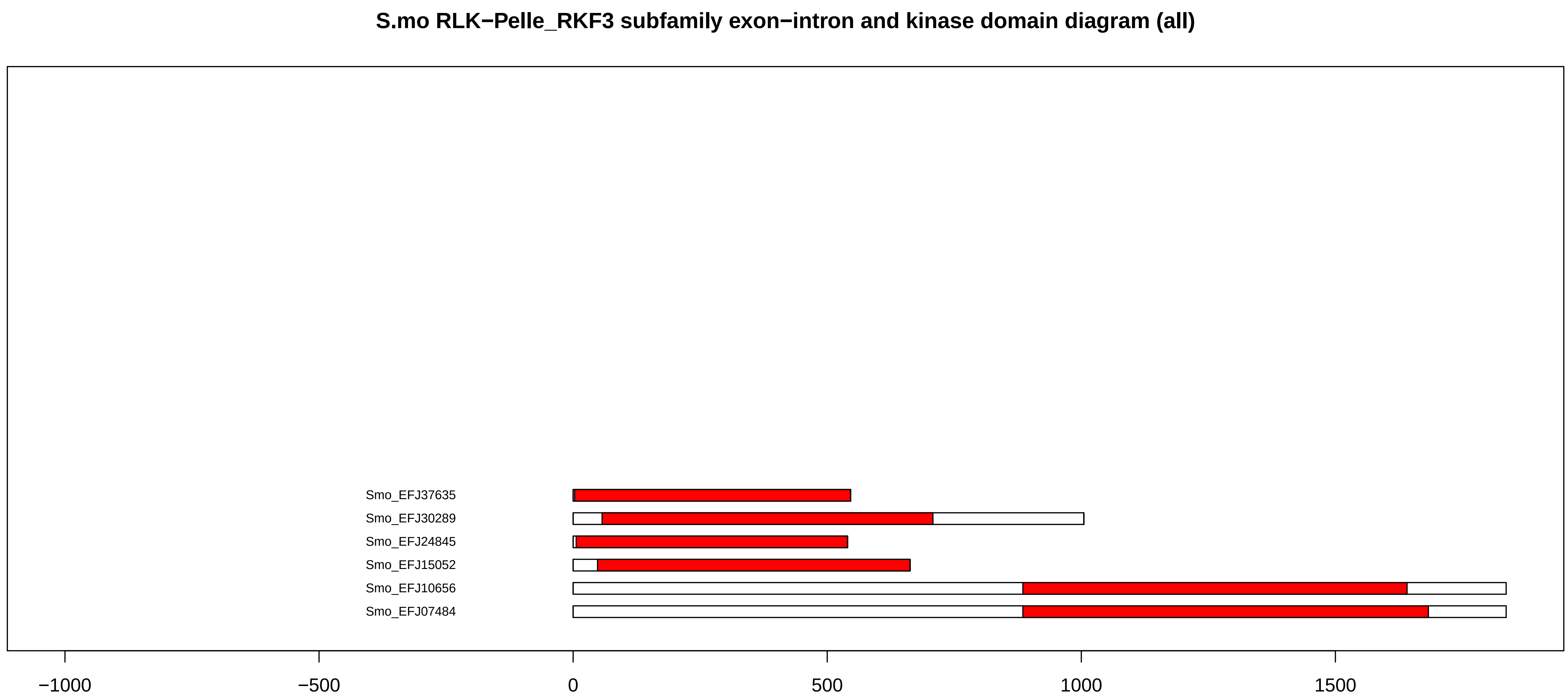
S.mo RLK−Pelle_RKF3 subfamily exon−intron and kinase domain diagram (all)
Smo_EFJ37635
Smo_EFJ30289
Smo_EFJ24845
Smo_EFJ15052
Smo_EFJ10656
Smo_EFJ07484
−1000
−500
0
500
1000
1500

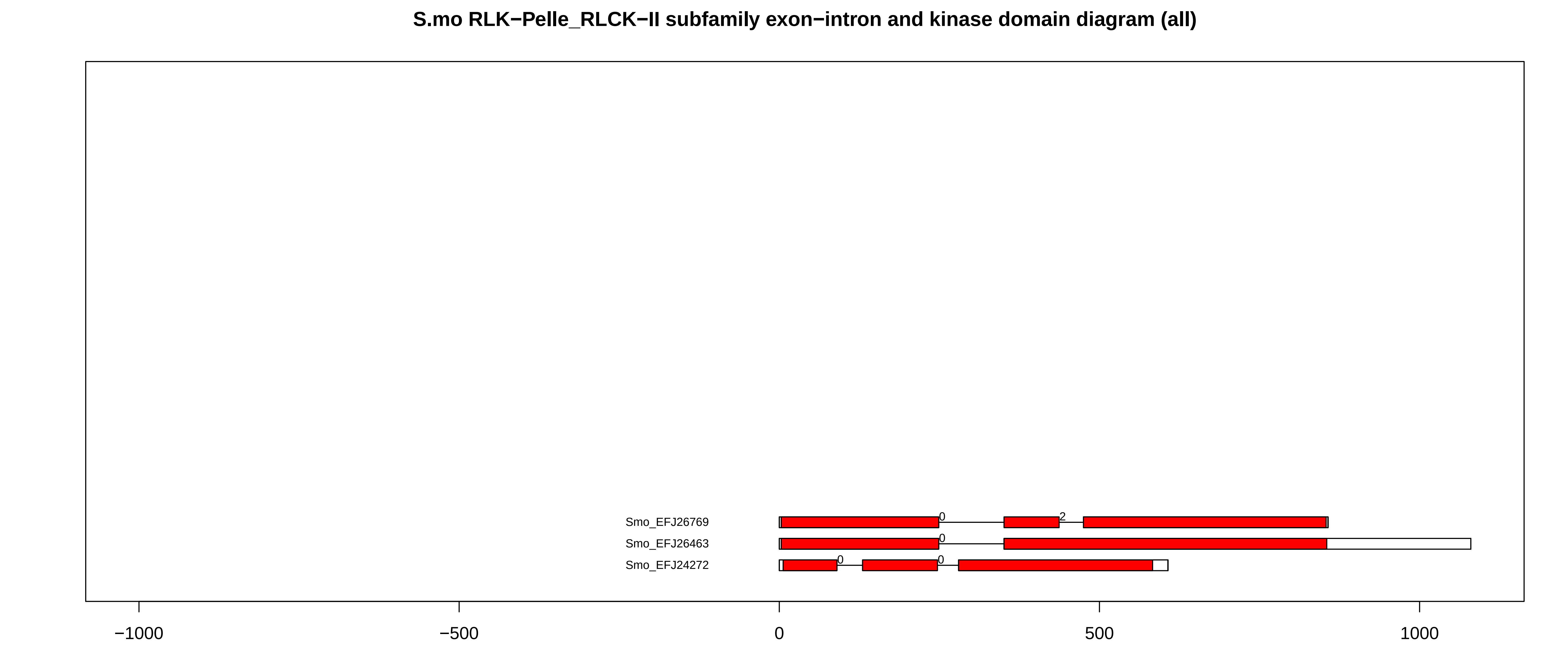
S.mo RLK−Pelle_RLCK−II subfamily exon−intron and kinase domain diagram (all)
Smo_EFJ26769
Smo_EFJ26463
Smo_EFJ24272
0
2
0
0
0
−1000
−500
0
500
1000

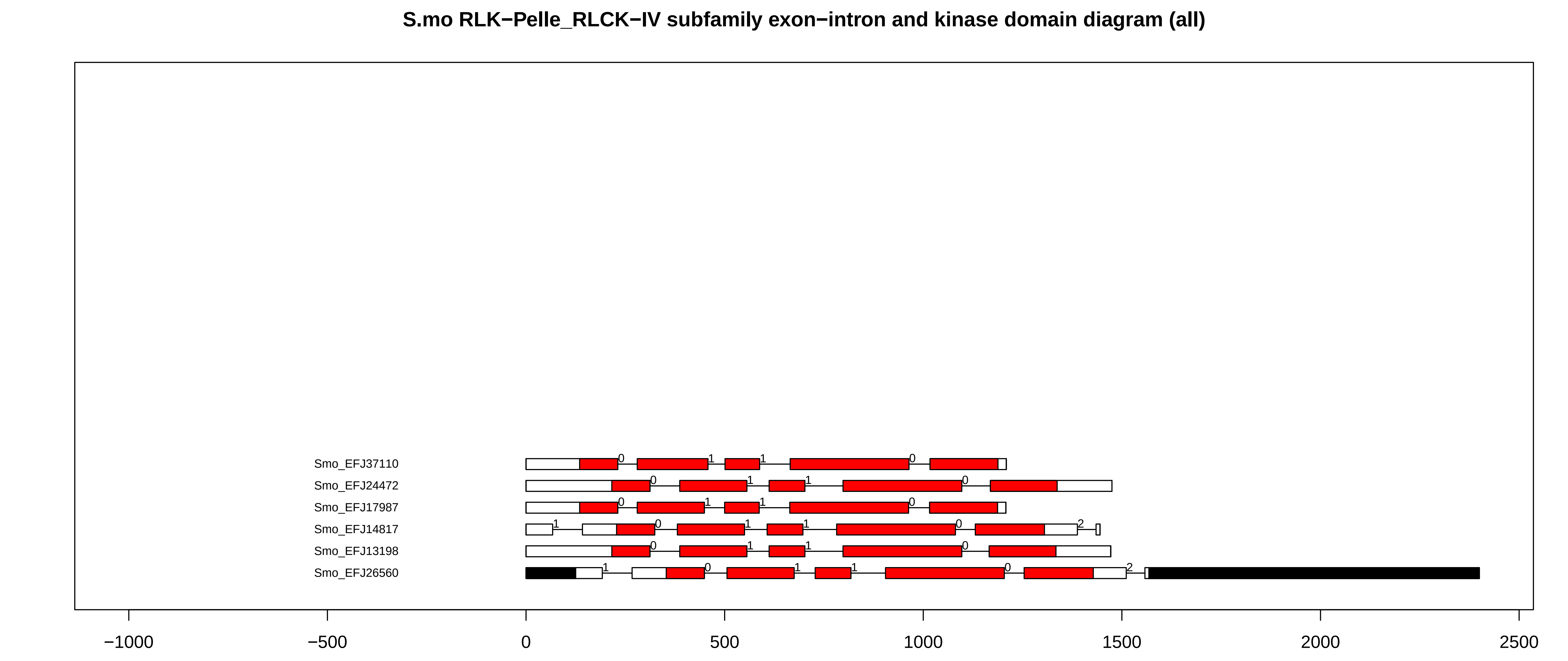

S.mo RLK−Pelle_RLCK−IV subfamily exon−intron and kinase domain diagram (all)
Smo_EFJ37110
Smo_EFJ24472
Smo_EFJ17987
Smo_EFJ14817
Smo_EFJ13198
Smo_EFJ26560
−1000
−500
0
500
1000
1500
2000
2500

S.mo RLK−Pelle_RLCK−IXa subfamily exon−intron and kinase domain diagram (all)

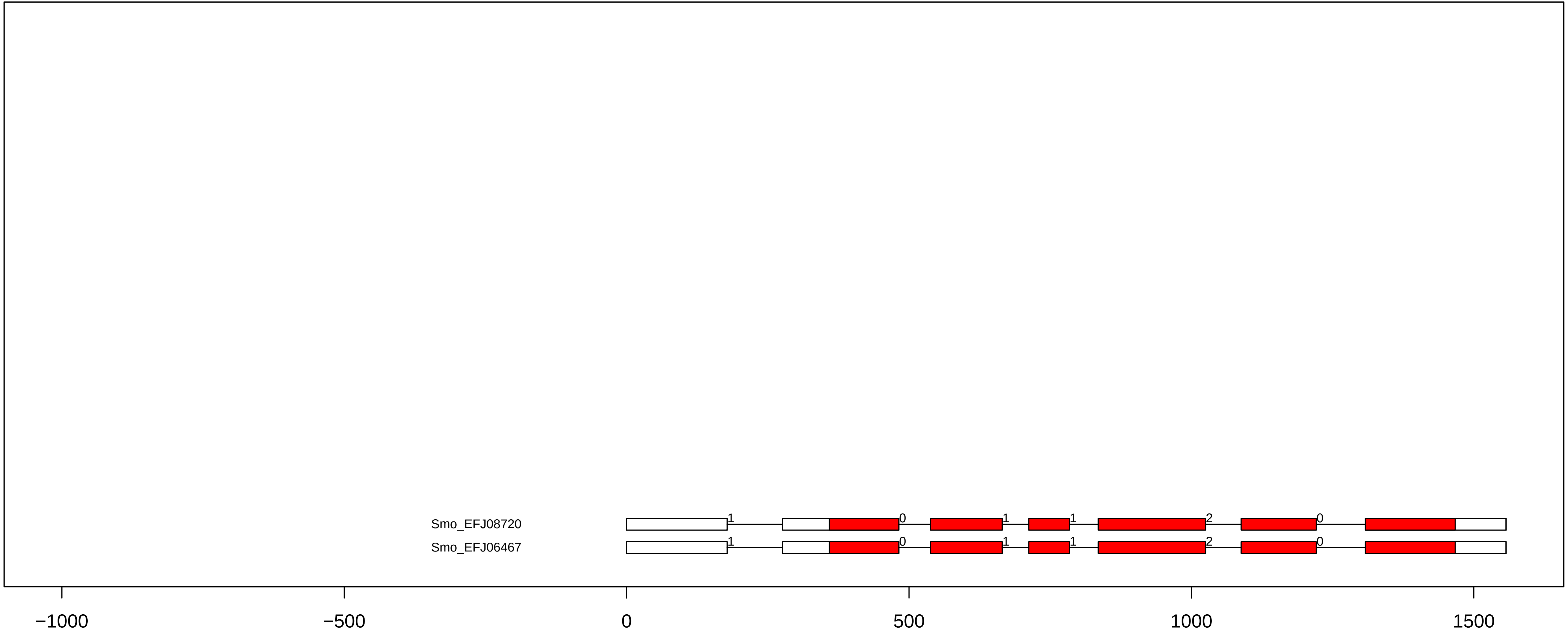

S.mo RLK−Pelle_RLCK−IXb subfamily exon−intron and kinase domain diagram (all)

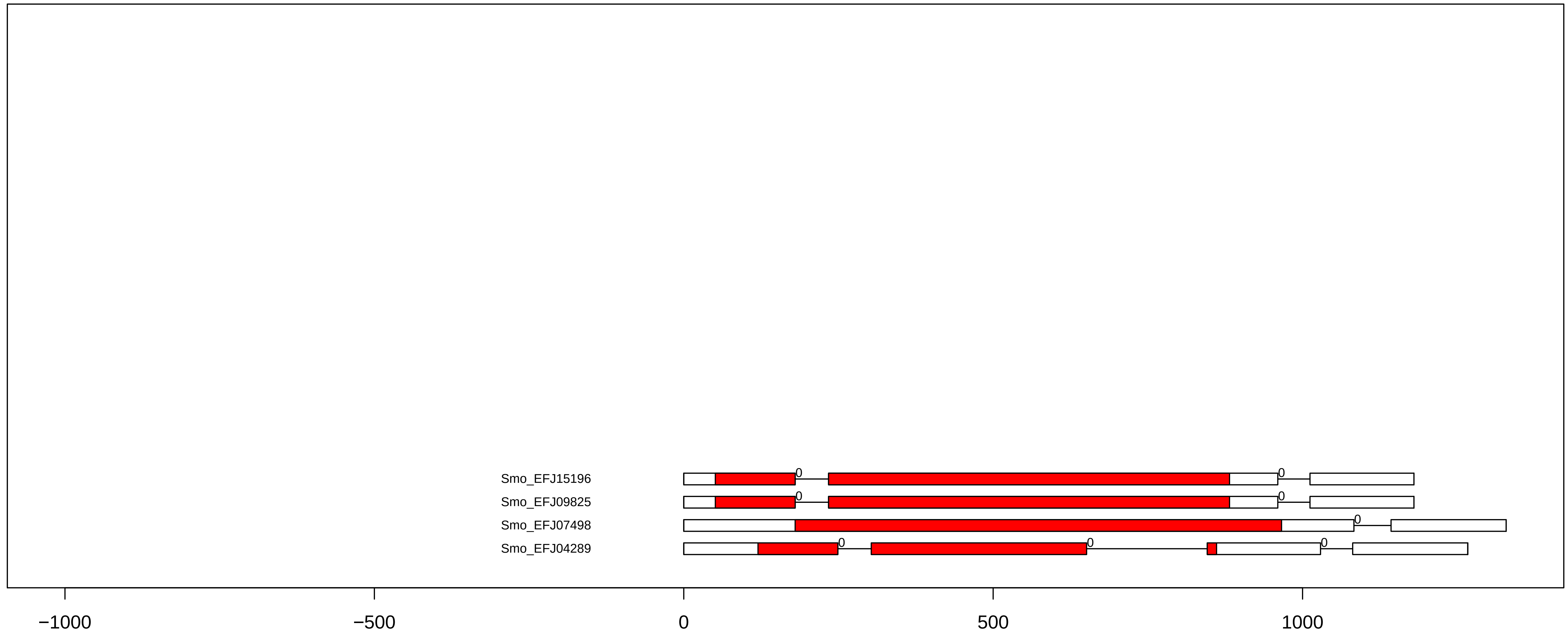

S.mo RLK−Pelle_RLCK−VIIa−1 subfamily exon−intron and kinase domain diagram (all)

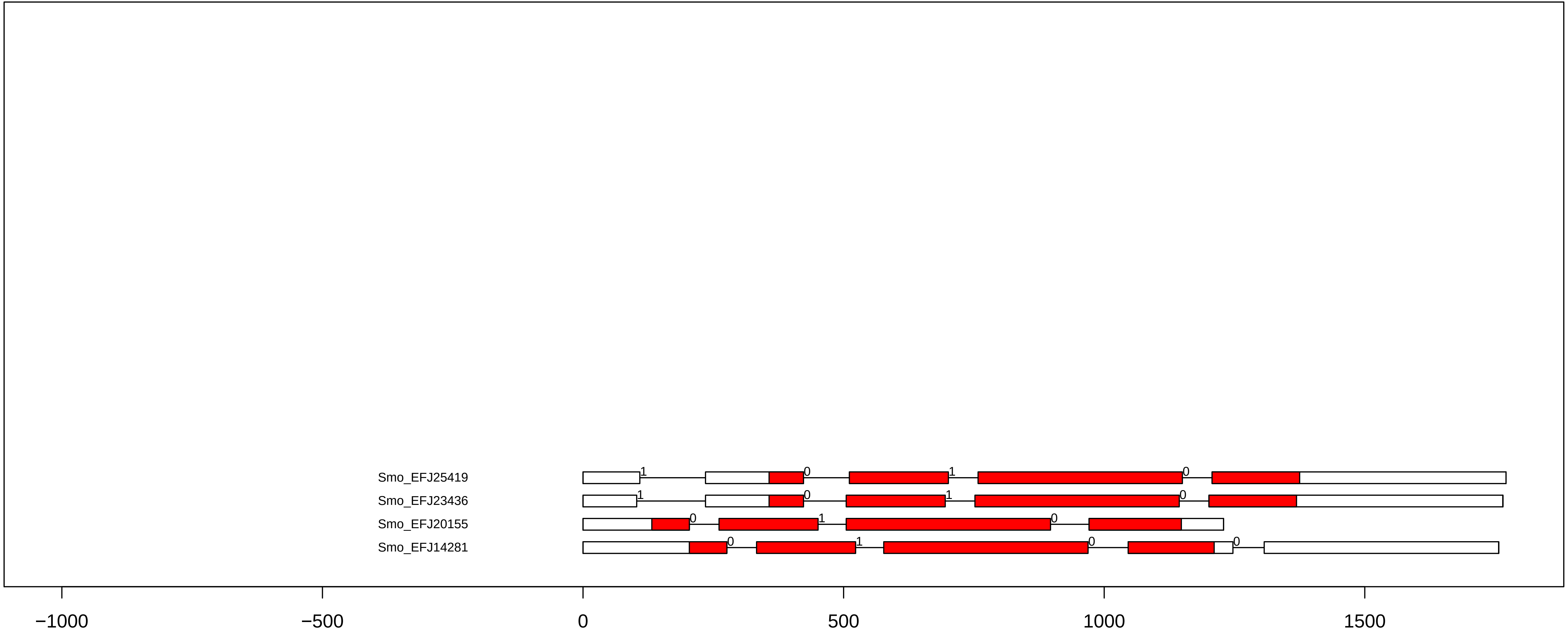

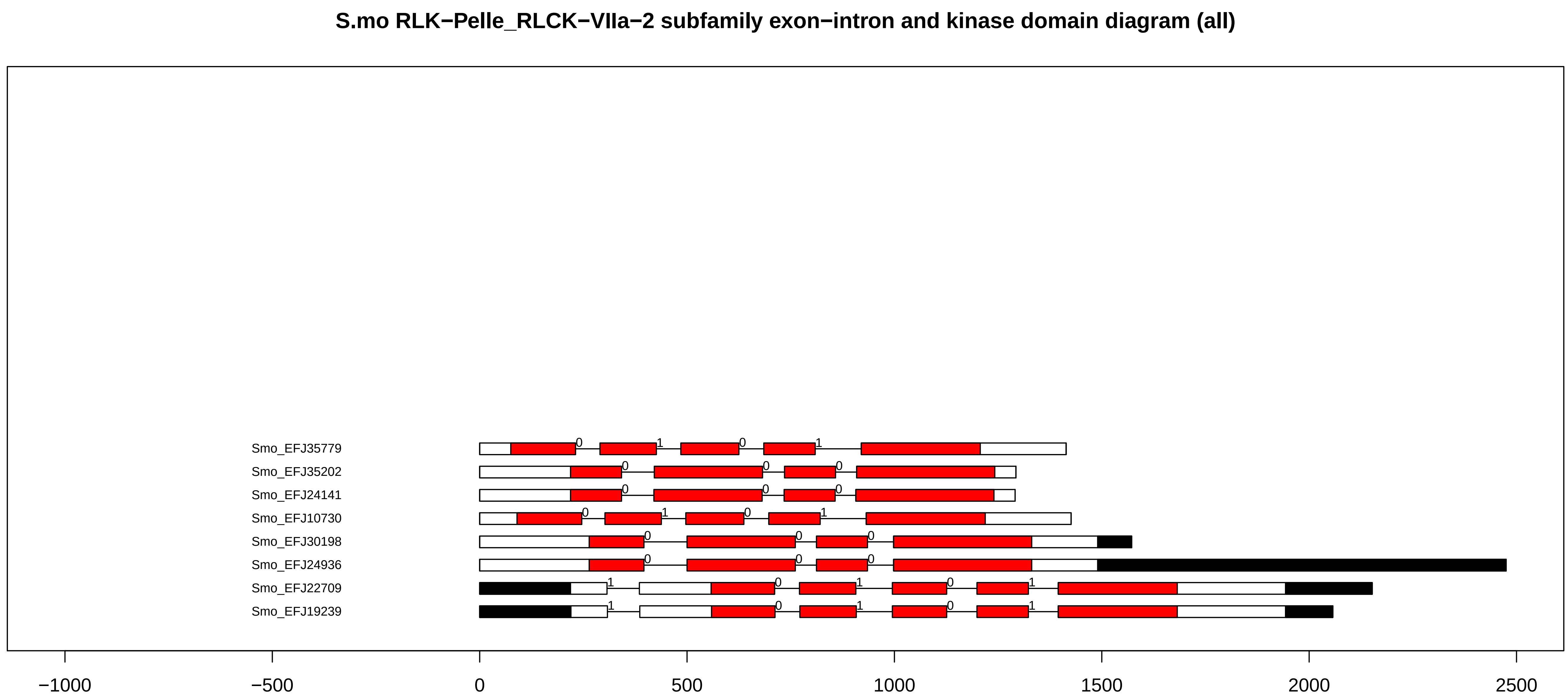

S.mo RLK−Pelle_RLCK−VIIa−2 subfamily exon−intron and kinase domain diagram (all)
Smo_EFJ35779
Smo_EFJ35202
Smo_EFJ24141
Smo_EFJ10730
Smo_EFJ30198
Smo_EFJ24936
Smo_EFJ22709
Smo_EFJ19239
−1000
−500
0
500
1000
1500
2000
2500

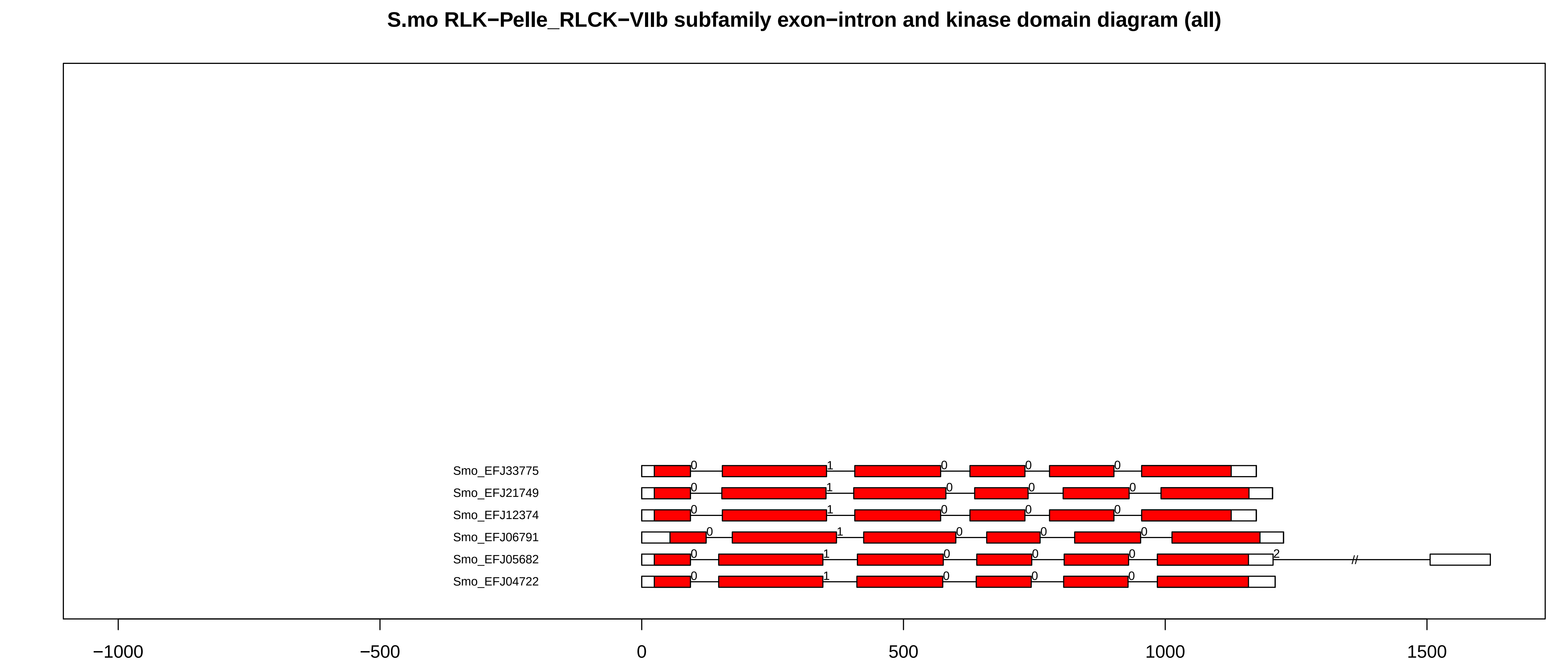

S.mo RLK−Pelle_RLCK−VIIb subfamily exon−intron and kinase domain diagram (all)
Smo_EFJ33775
Smo_EFJ21749
Smo_EFJ12374
Smo_EFJ06791
Smo_EFJ05682
Smo_EFJ04722
−1000
−500
0
500
1000
1500

S.mo RLK−Pelle_RLCK−VIII subfamily exon−intron and kinase domain diagram (all)

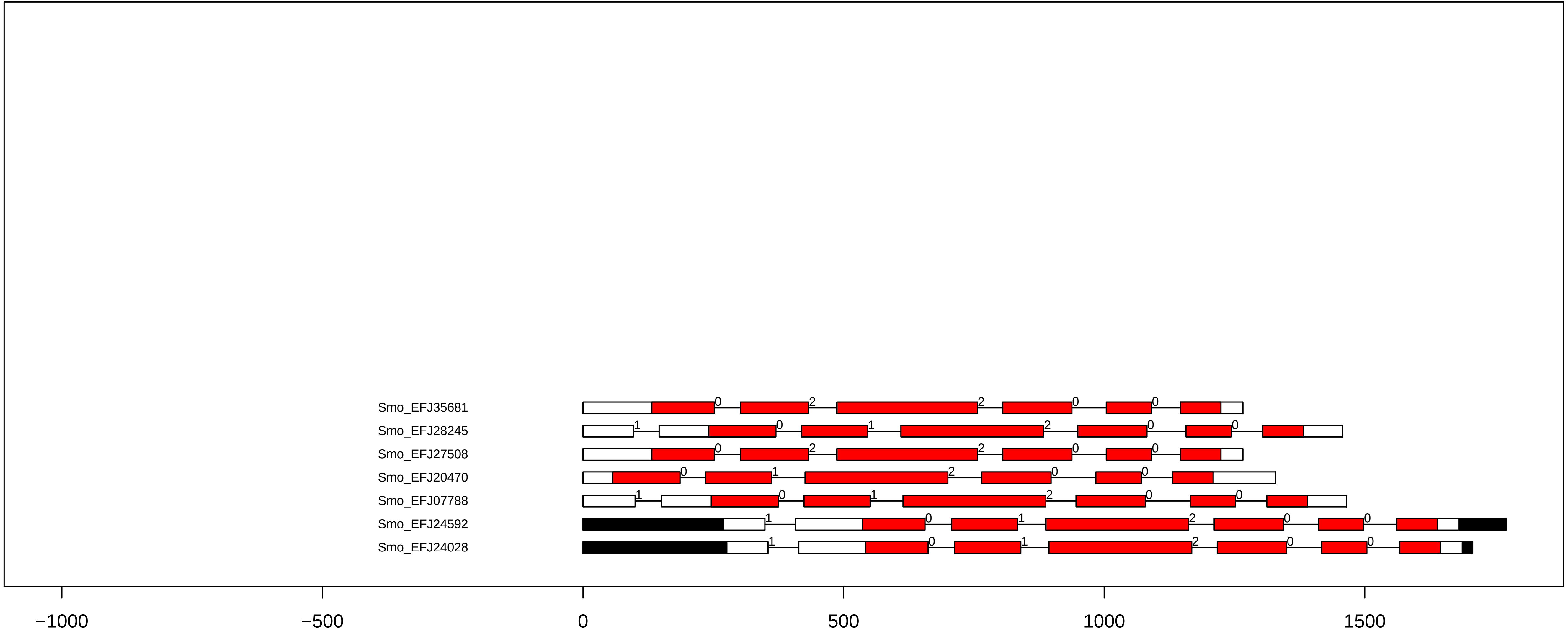

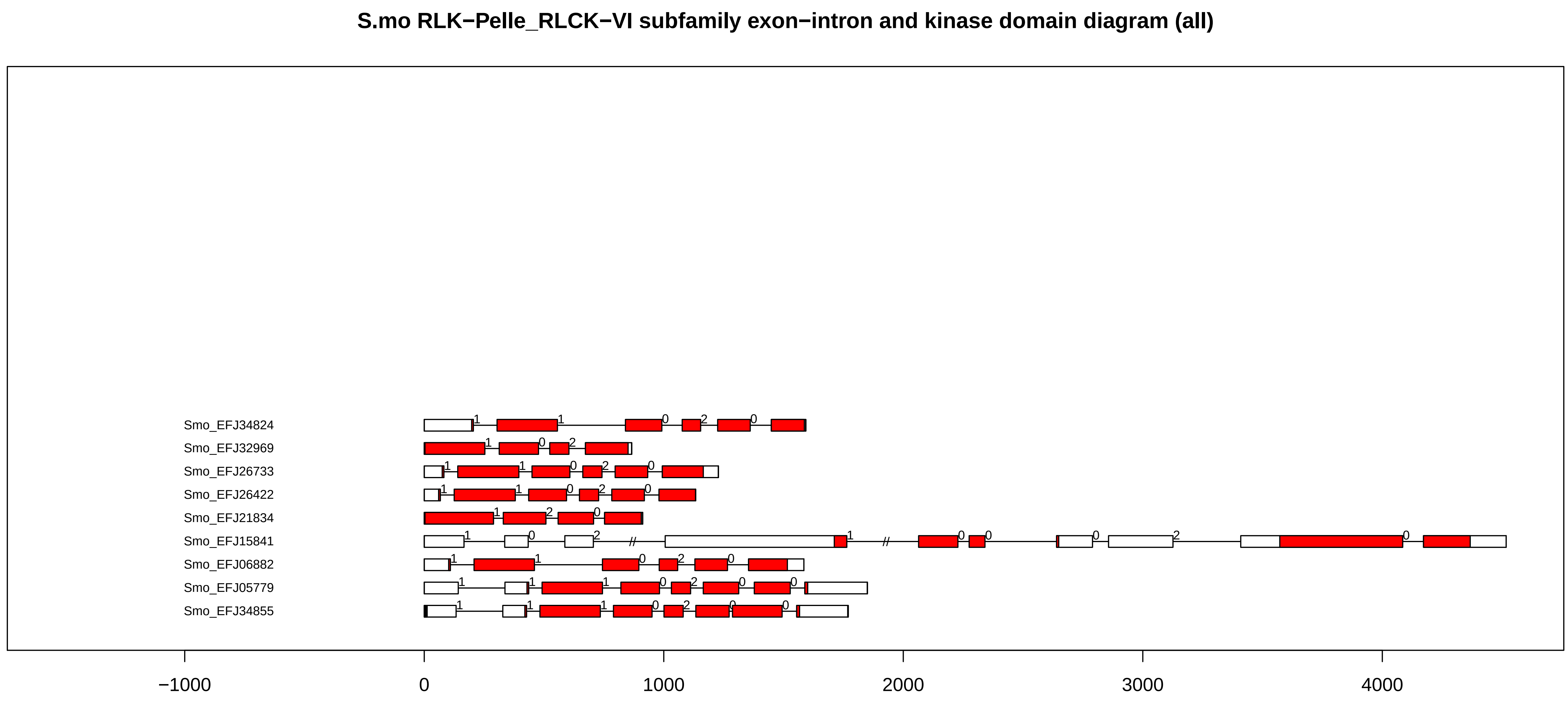
S.mo RLK−Pelle_RLCK−VI subfamily exon−intron and kinase domain diagram (all)
Smo_EFJ34824
Smo_EFJ32969
Smo_EFJ26733
Smo_EFJ26422
Smo_EFJ21834
Smo_EFJ15841
Smo_EFJ06882
Smo_EFJ05779
Smo_EFJ34855
−1000
0
1000
2000
3000
4000

S.mo RLK−Pelle_RLCK−V subfamily exon−intron and kinase domain diagram (all)

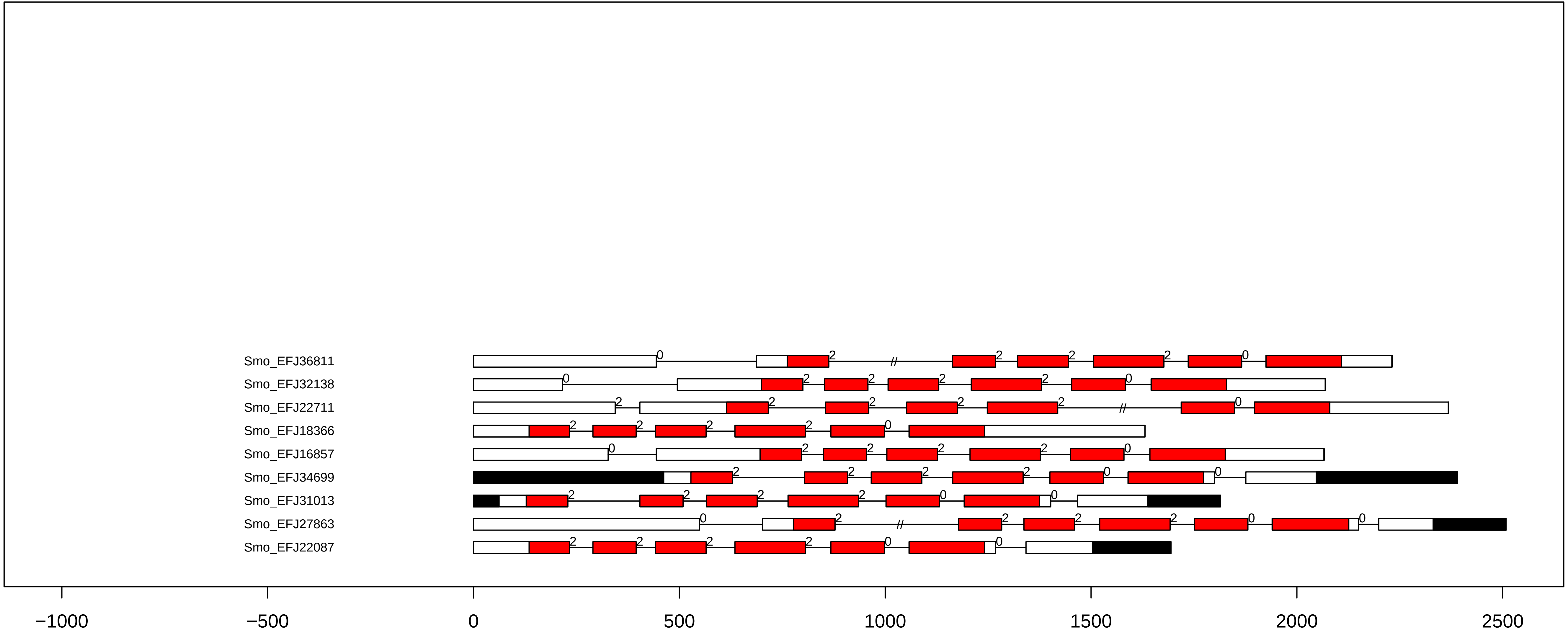

S.mo RLK−Pelle_RLCK−XII−1 subfamily exon−intron and kinase domain diagram (all)

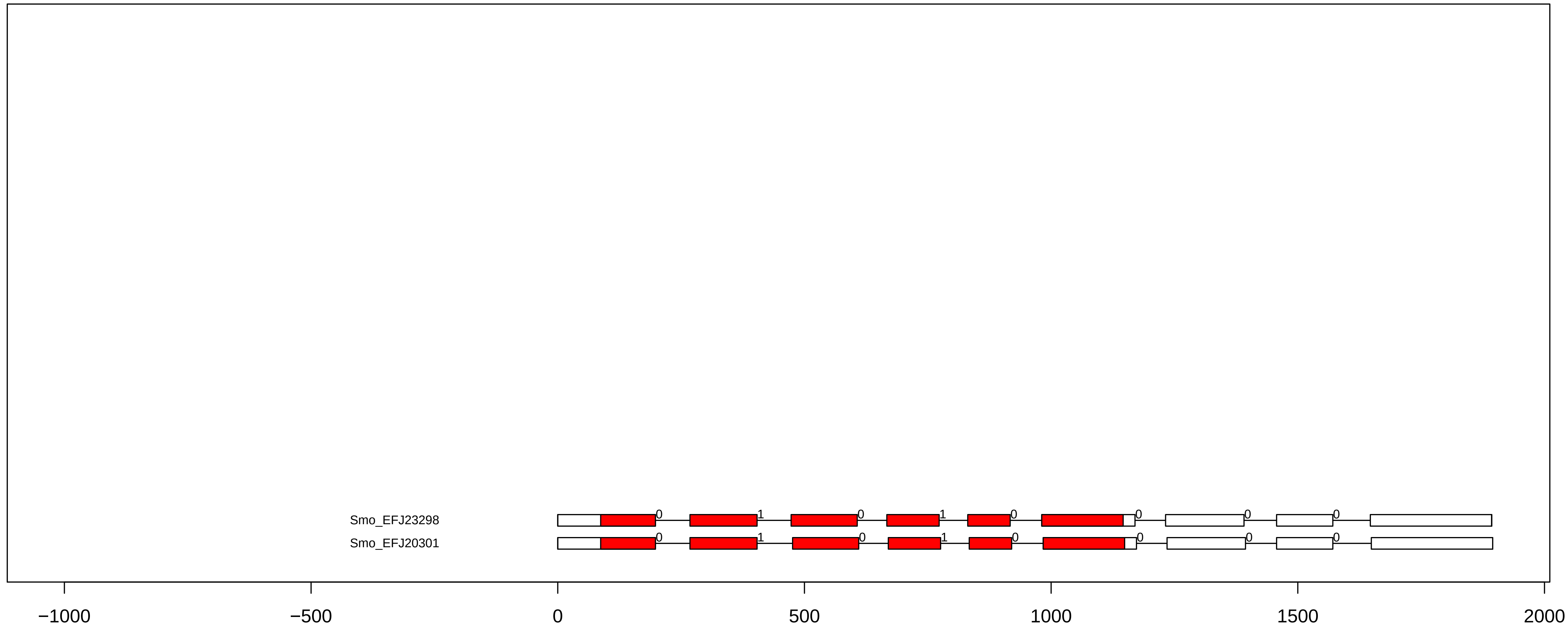

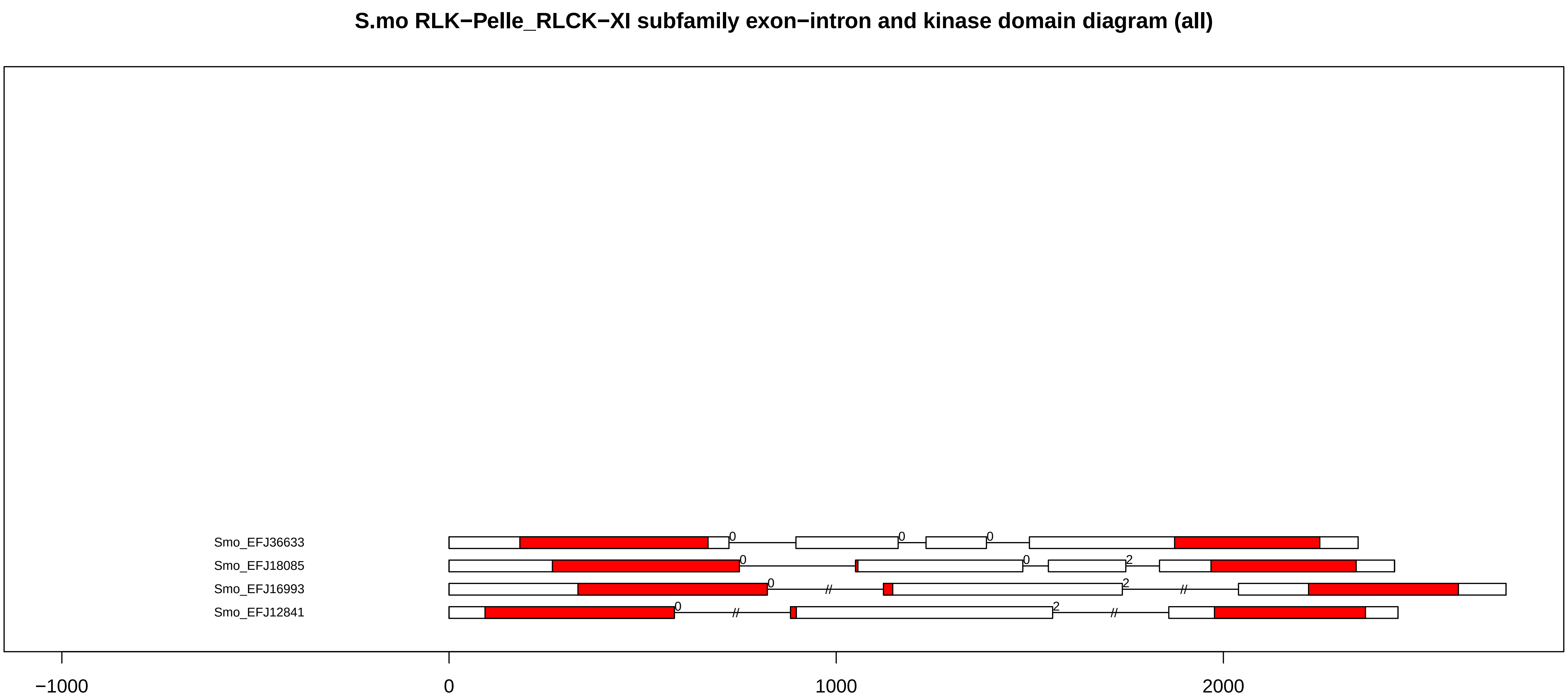

S.mo RLK−Pelle_RLCK−XI subfamily exon−intron and kinase domain diagram (all)
Smo_EFJ36633
Smo_EFJ18085
Smo_EFJ16993
Smo_EFJ12841
0
0
0
0
0
2
0
2
0
2
−1000
0
1000
2000

S.mo RLK−Pelle_RLCK−X subfamily exon−intron and kinase domain diagram (all)

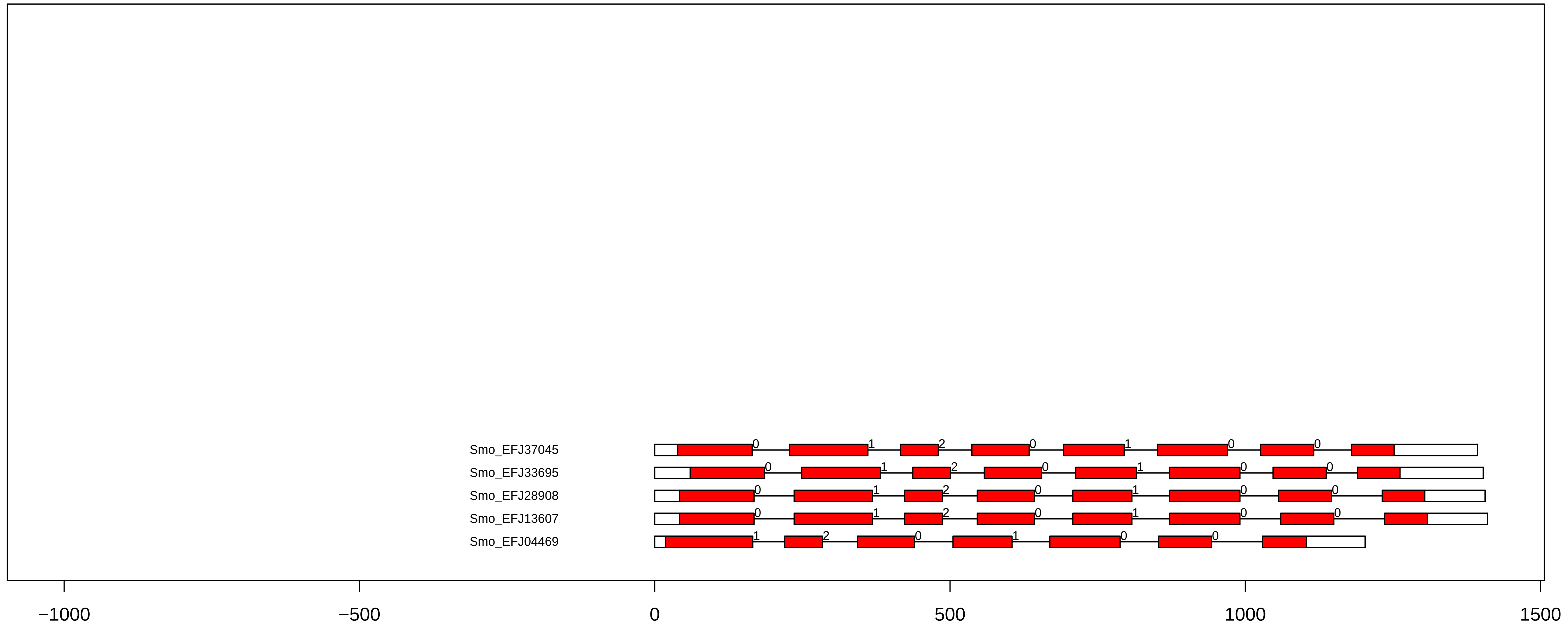

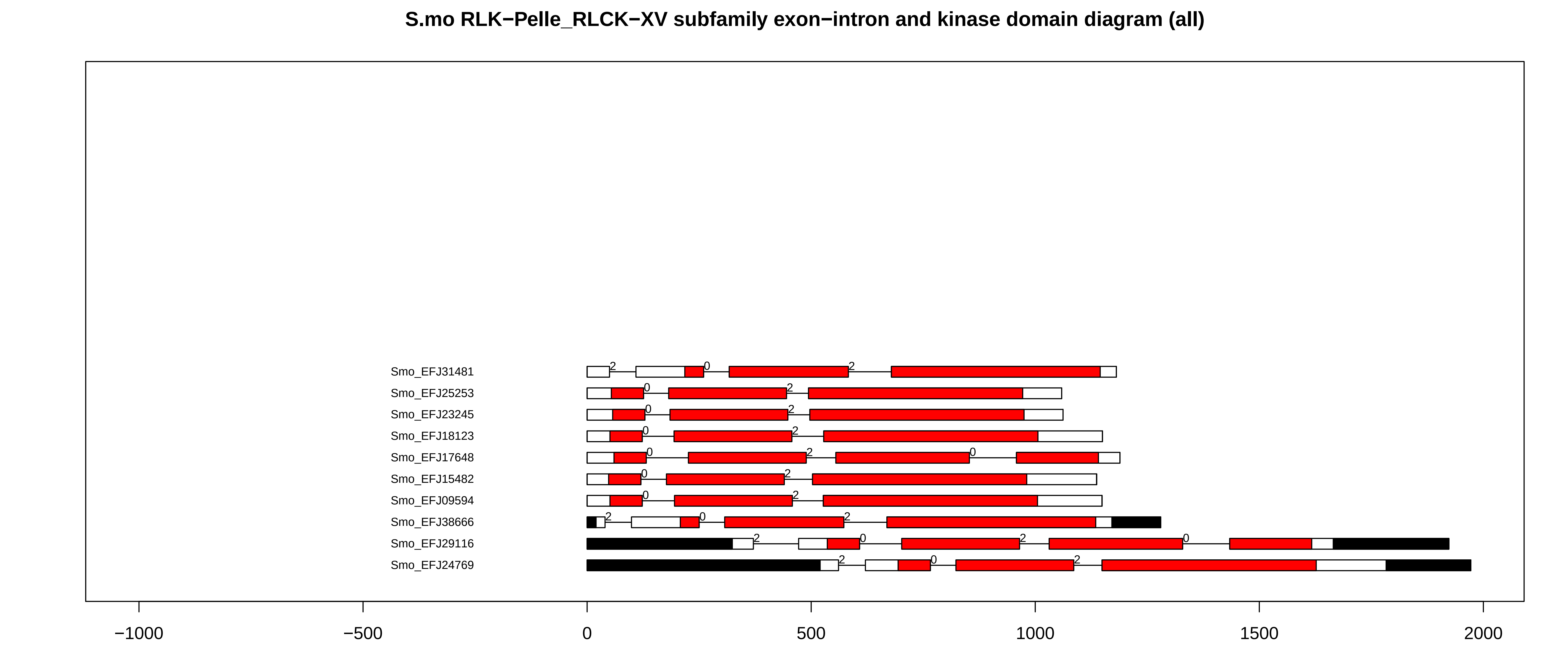

S.mo RLK−Pelle_RLCK−XV subfamily exon−intron and kinase domain diagram (all)
Smo_EFJ31481
Smo_EFJ25253
Smo_EFJ23245
Smo_EFJ18123
Smo_EFJ17648
Smo_EFJ15482
Smo_EFJ09594
Smo_EFJ38666
Smo_EFJ29116
Smo_EFJ24769
−1000
−500
0
500
1000
1500
2000

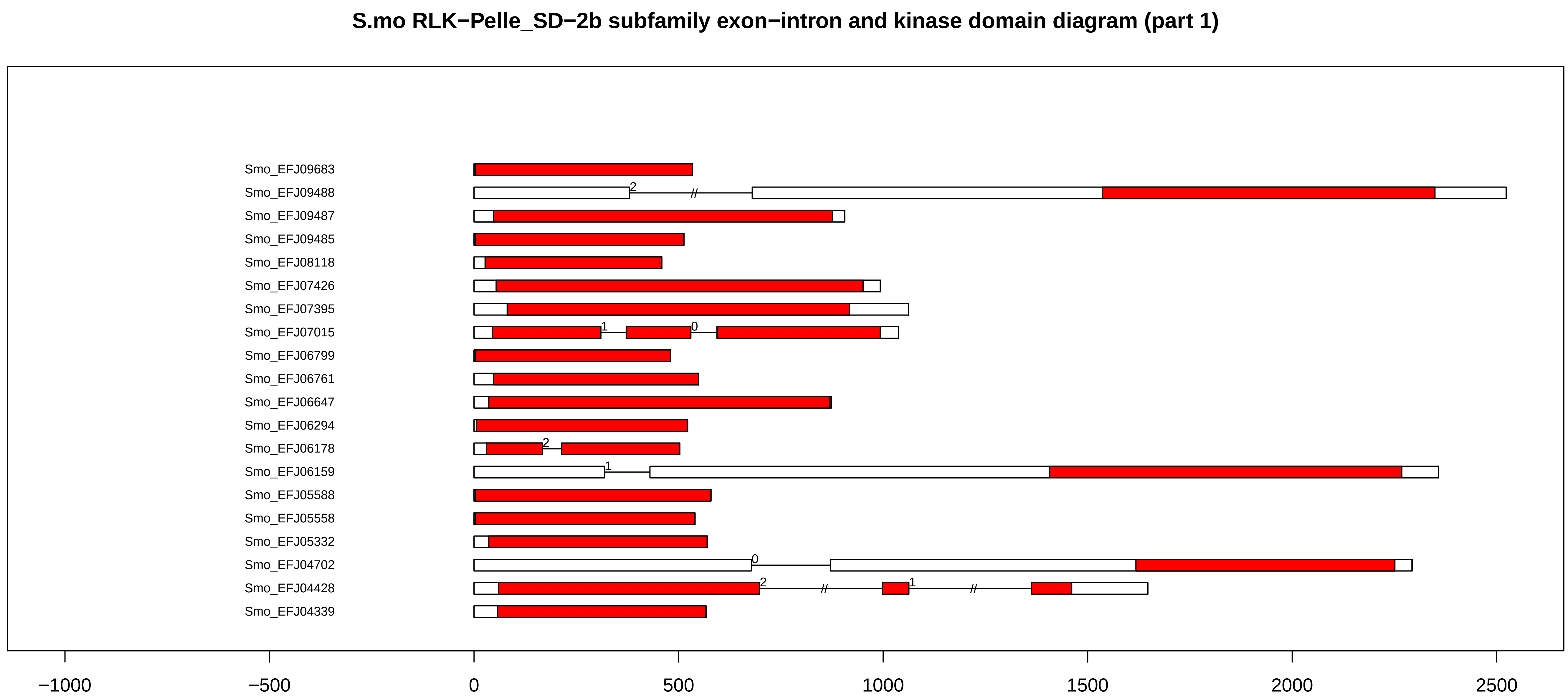

S.mo RLK−Pelle_SD−2b subfamily exon−intron and kinase domain diagram (part 1)
Smo_EFJ09683
Smo_EFJ09488
Smo_EFJ09487
Smo_EFJ09485
Smo_EFJ08118
Smo_EFJ07426
Smo_EFJ07395
Smo_EFJ07015
Smo_EFJ06799
Smo_EFJ06761
Smo_EFJ06647
Smo_EFJ06294
Smo_EFJ06178
Smo_EFJ06159
Smo_EFJ05588
Smo_EFJ05558
Smo_EFJ05332
Smo_EFJ04702
Smo_EFJ04428
Smo_EFJ04339
−1000
−500
0
500
1000
1500
2000
2500

S.mo RLK−Pelle_SD−2b subfamily exon−intron and kinase domain diagram (part 2)

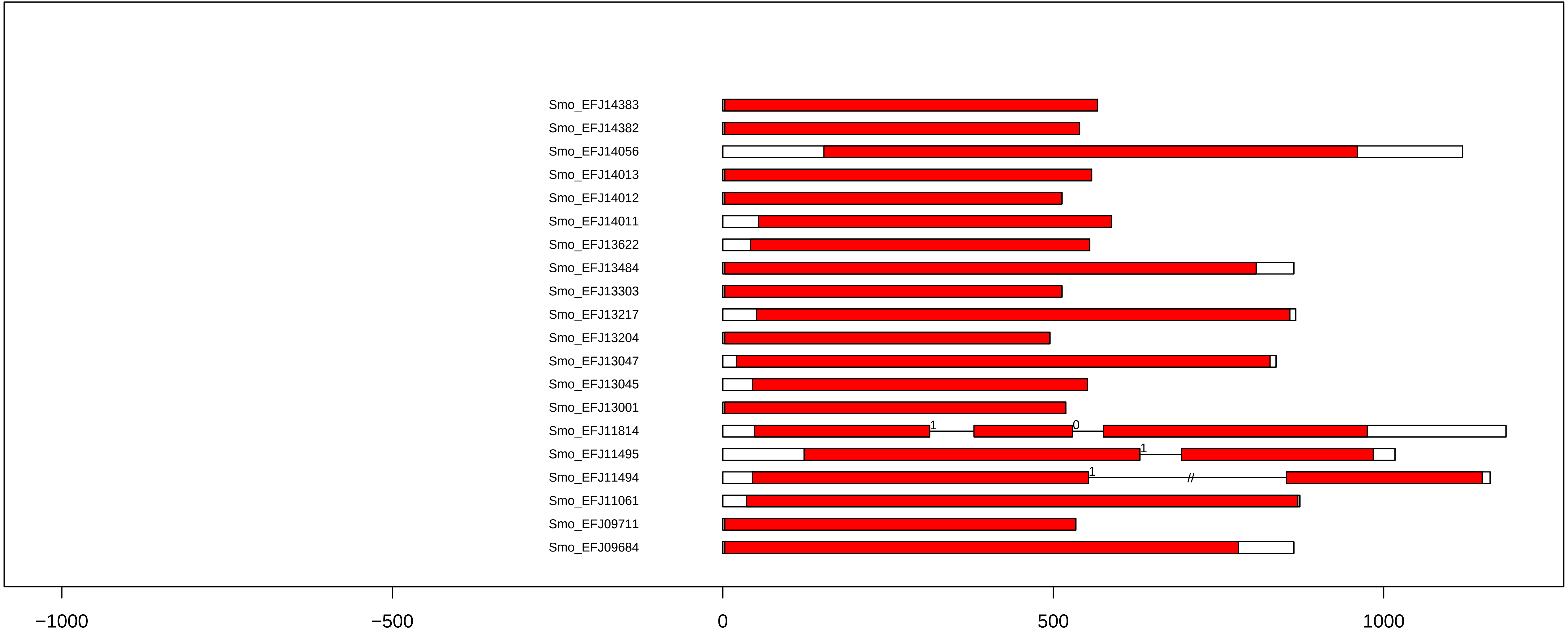

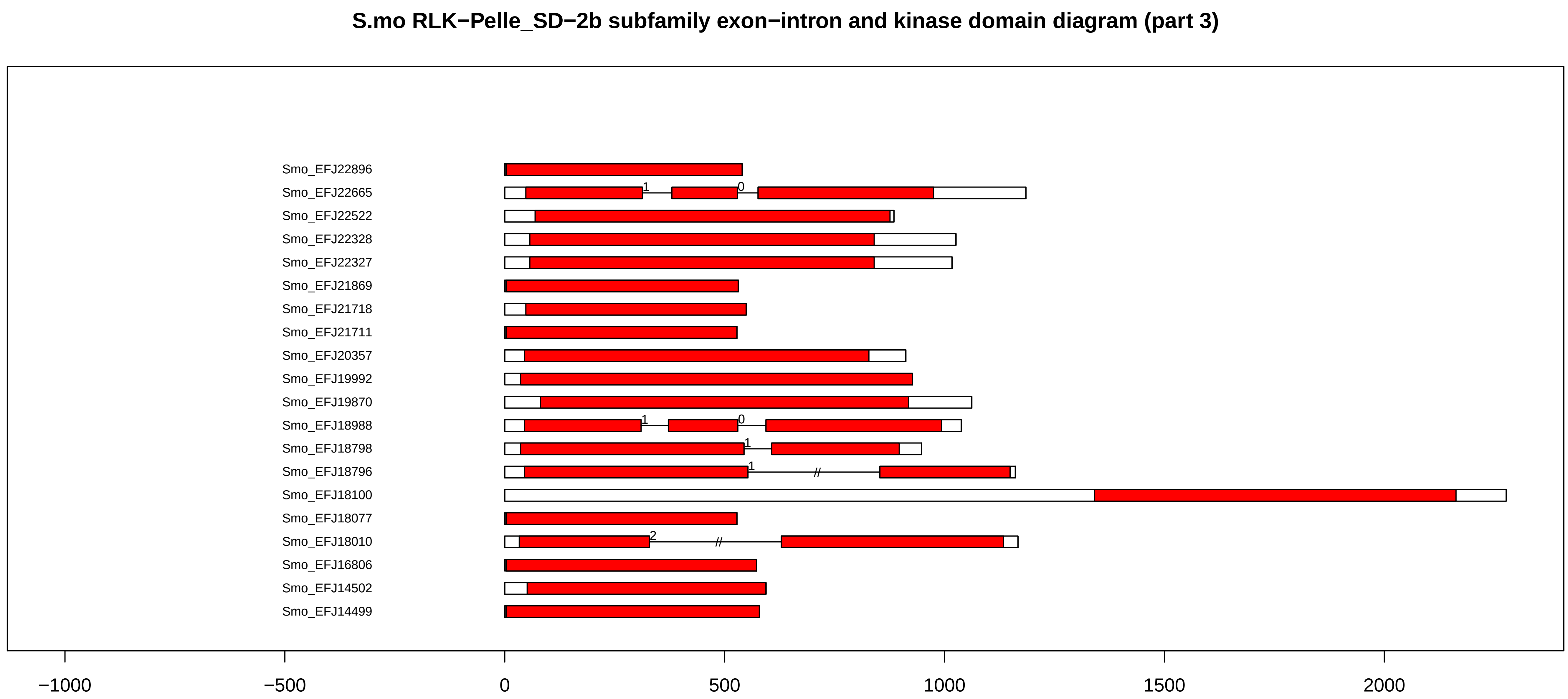
S.mo RLK−Pelle_SD−2b subfamily exon−intron and kinase domain diagram (part 3)
Smo_EFJ22896
Smo_EFJ22665
Smo_EFJ22522
Smo_EFJ22328
Smo_EFJ22327
Smo_EFJ21869
Smo_EFJ21718
Smo_EFJ21711
Smo_EFJ20357
Smo_EFJ19992
Smo_EFJ19870
Smo_EFJ18988
Smo_EFJ18798
Smo_EFJ18796
Smo_EFJ18100
Smo_EFJ18077
Smo_EFJ18010
Smo_EFJ16806
Smo_EFJ14502
Smo_EFJ14499
1
0
1
0
1
1
2
−1000
−500
0
500
1000
1500
2000

S.mo RLK−Pelle_SD−2b subfamily exon−intron and kinase domain diagram (part 4)

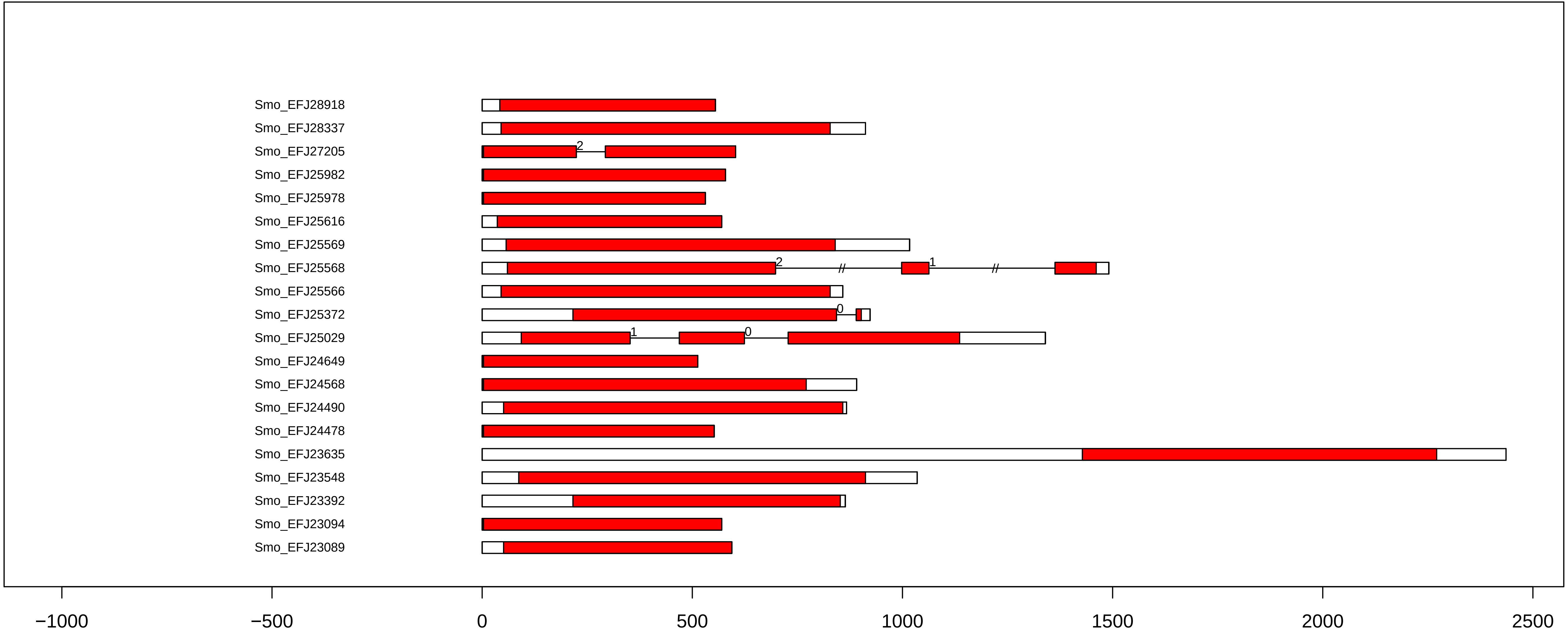

S.mo RLK−Pelle_SD−2b subfamily exon−intron and kinase domain diagram (part 5)

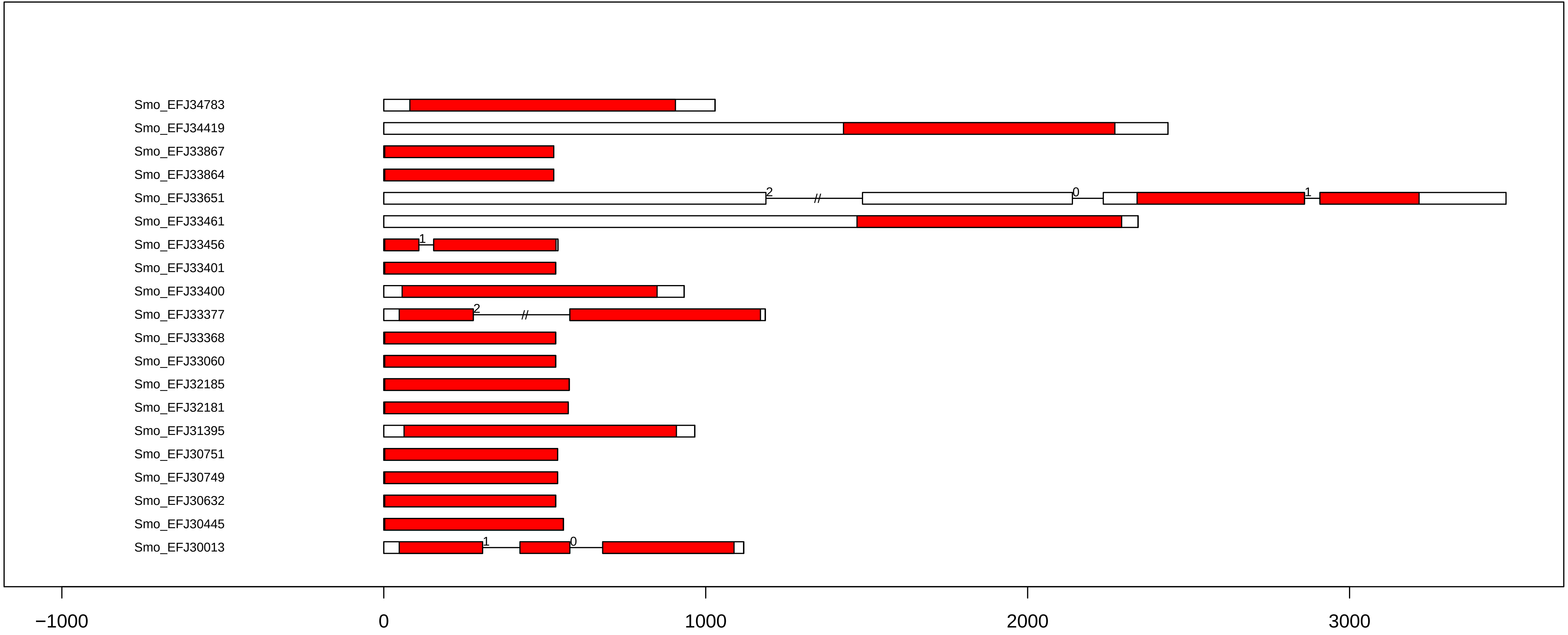

S.mo RLK−Pelle_SD−2b subfamily exon−intron and kinase domain diagram (part 6)

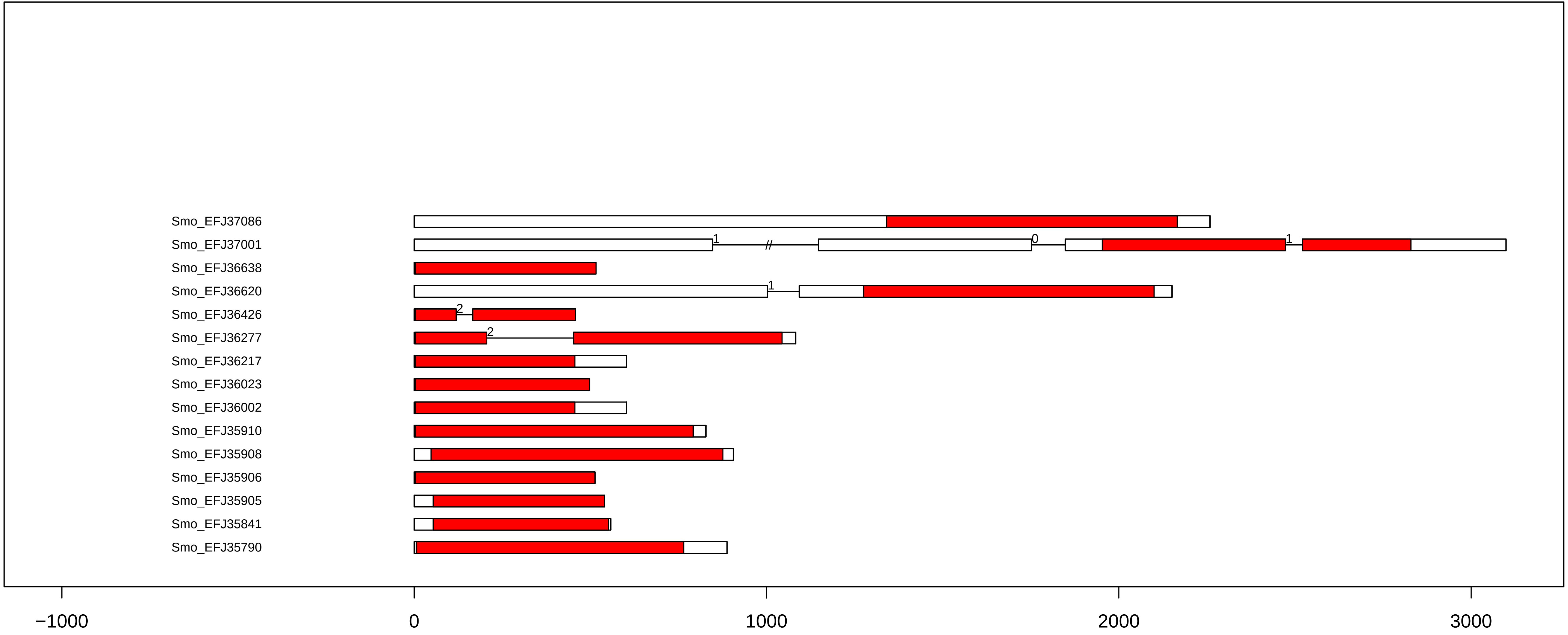

S.mo RLK−Pelle_URK−1 subfamily exon−intron and kinase domain diagram (all)

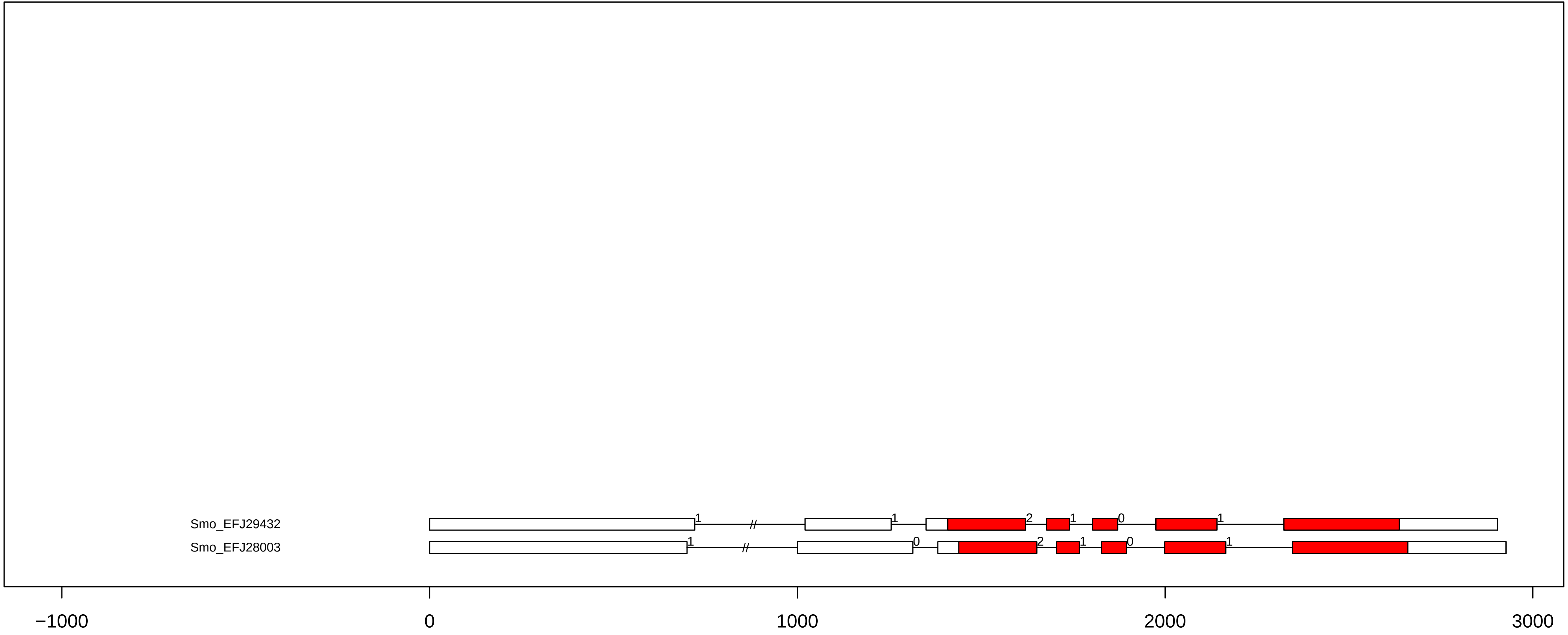

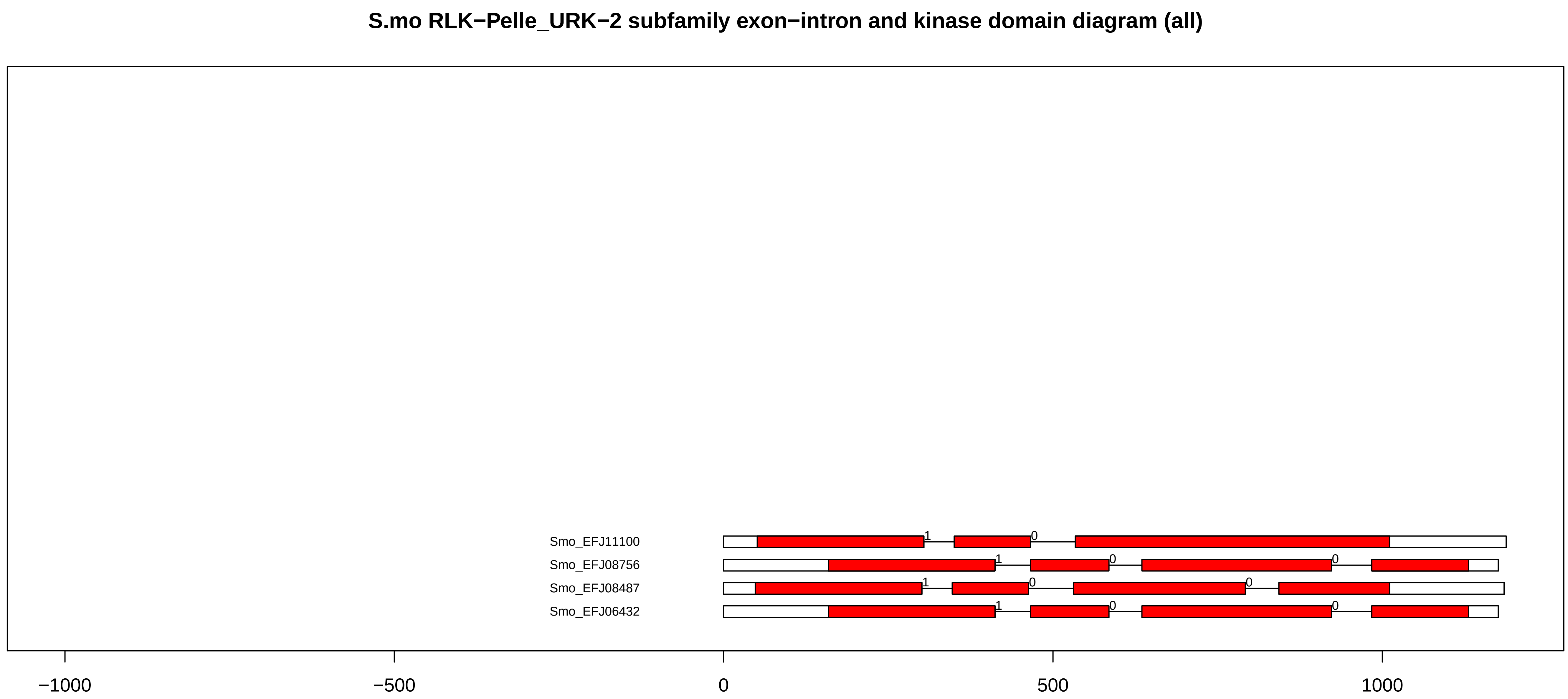
S.mo RLK−Pelle_URK−2 subfamily exon−intron and kinase domain diagram (all)
Smo_EFJ11100
Smo_EFJ08756
Smo_EFJ08487
Smo_EFJ06432
−1000
−500
0
500
1000

S.mo RLK−Pelle_URK−Pp−1 subfamily exon−intron and kinase domain diagram (all)

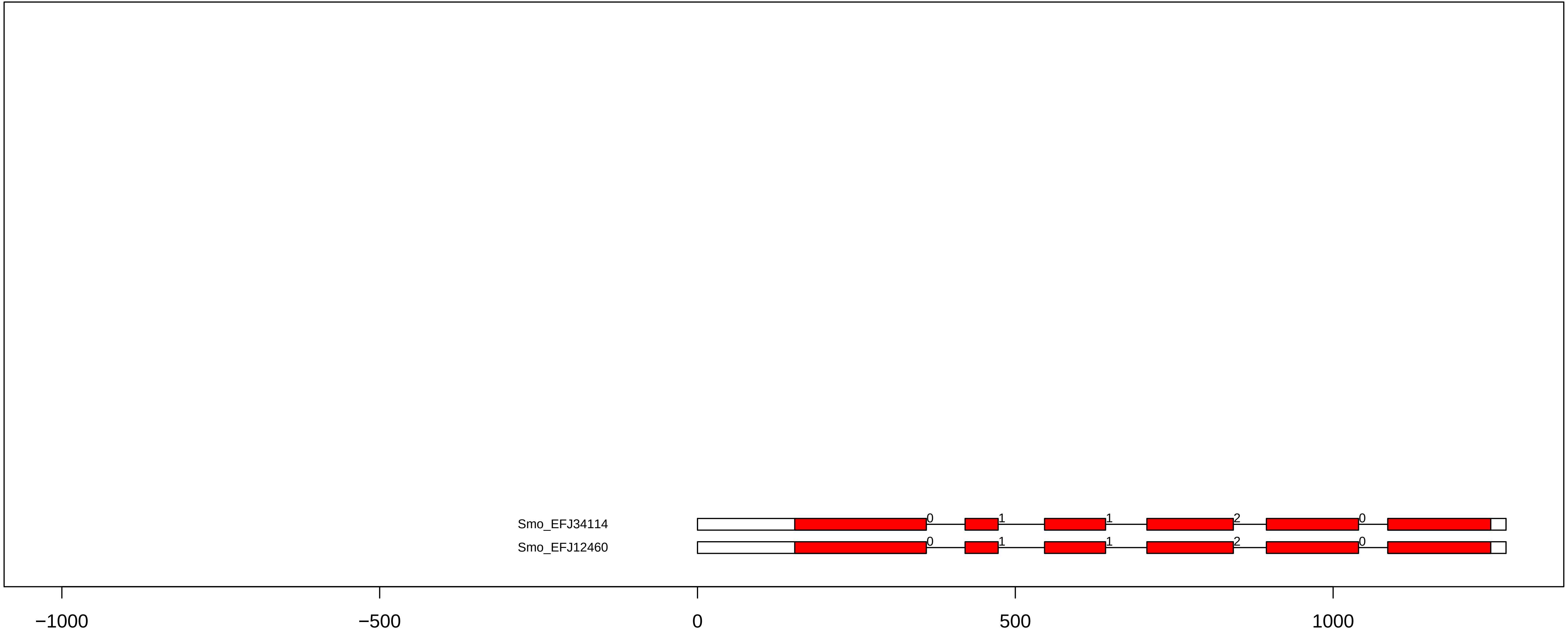

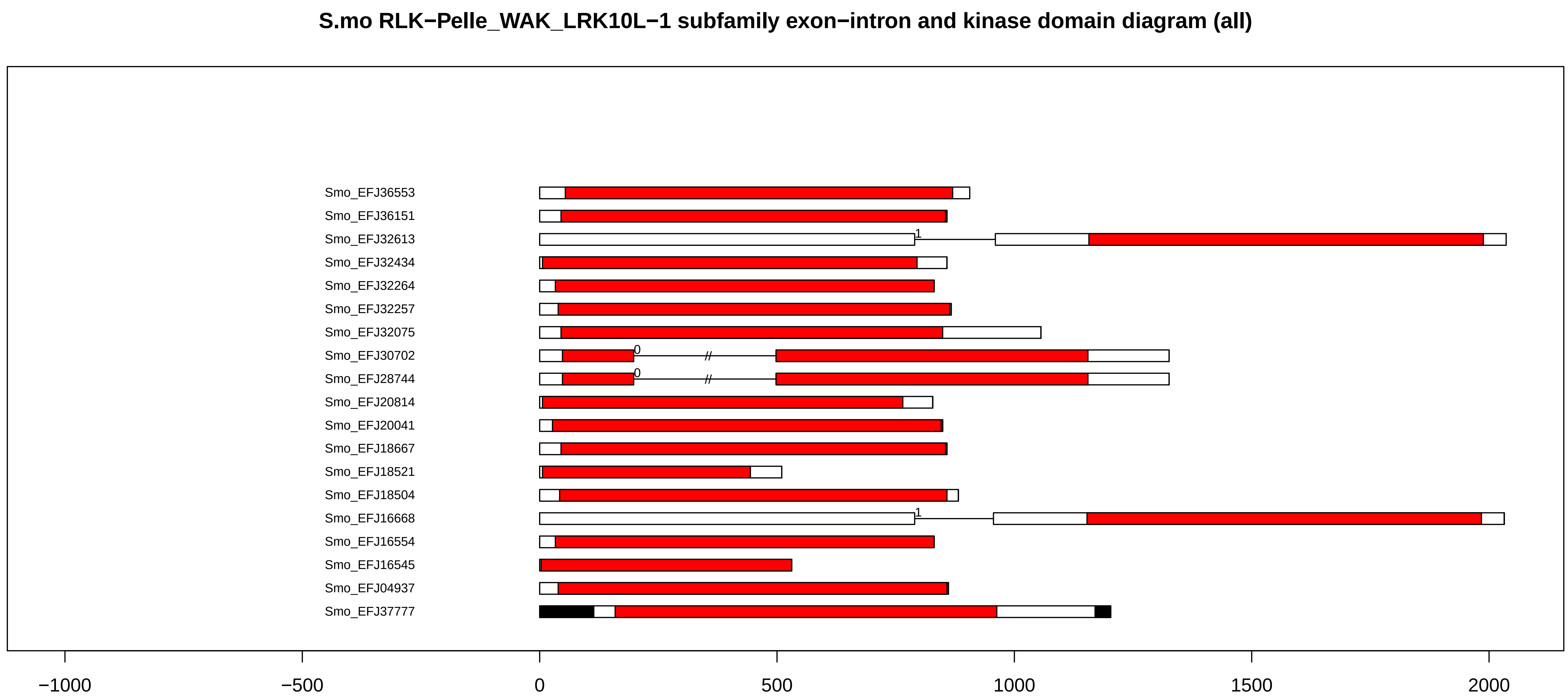
S.mo RLK−Pelle_WAK_LRK10L−1 subfamily exon−intron and kinase domain diagram (all)
Smo_EFJ36553
Smo_EFJ36151
Smo_EFJ32613
Smo_EFJ32434
Smo_EFJ32264
Smo_EFJ32257
Smo_EFJ32075
Smo_EFJ30702
Smo_EFJ28744
Smo_EFJ20814
Smo_EFJ20041
Smo_EFJ18667
Smo_EFJ18521
Smo_EFJ18504
Smo_EFJ16668
Smo_EFJ16554
Smo_EFJ16545
Smo_EFJ04937
Smo_EFJ37777
1
0
0
1
−1000
−500
0
500
1000
1500
2000

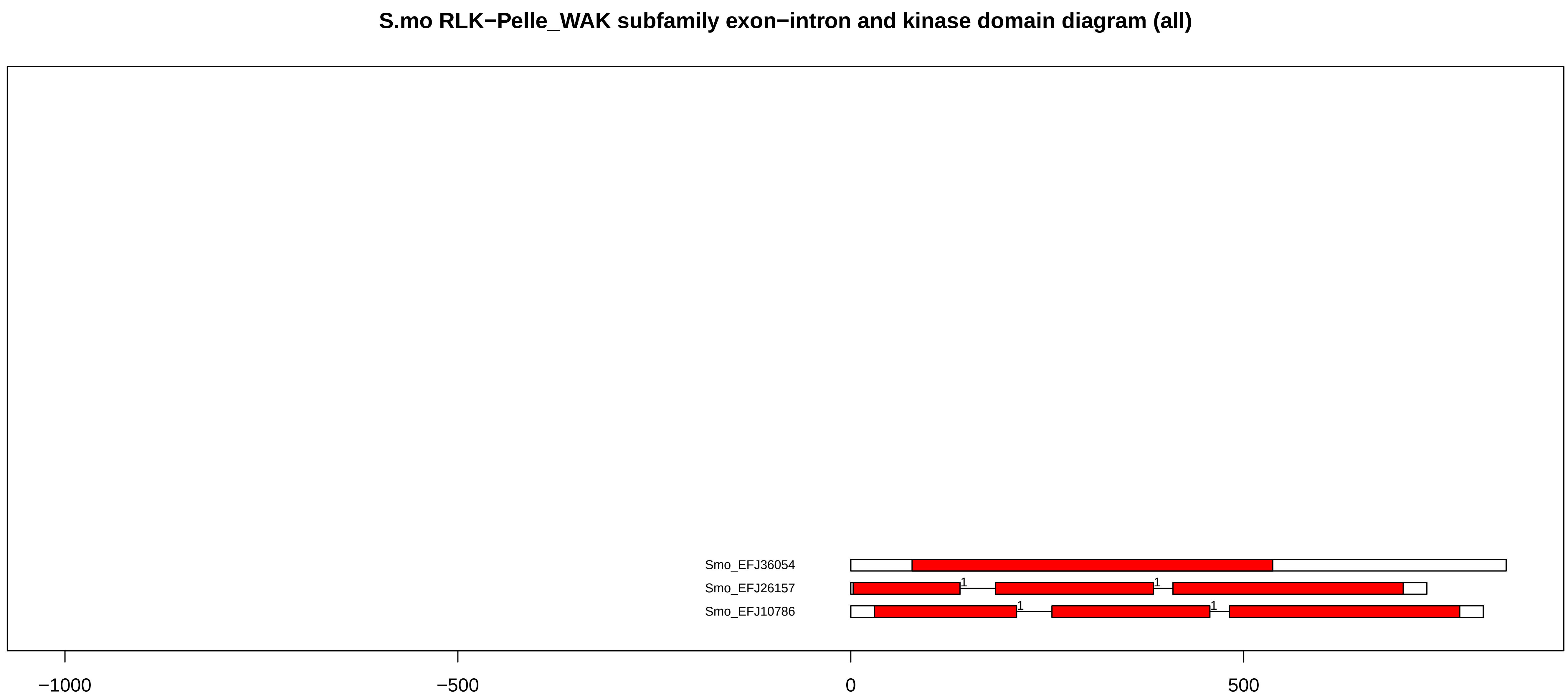
S.mo RLK−Pelle_WAK subfamily exon−intron and kinase domain diagram (all)
Smo_EFJ36054
Smo_EFJ26157
Smo_EFJ10786
1
1
1
1
−1000
−500
0
500

P.pa RLK−Pelle_C−LEC subfamily exon−intron and kinase domain diagram (all)

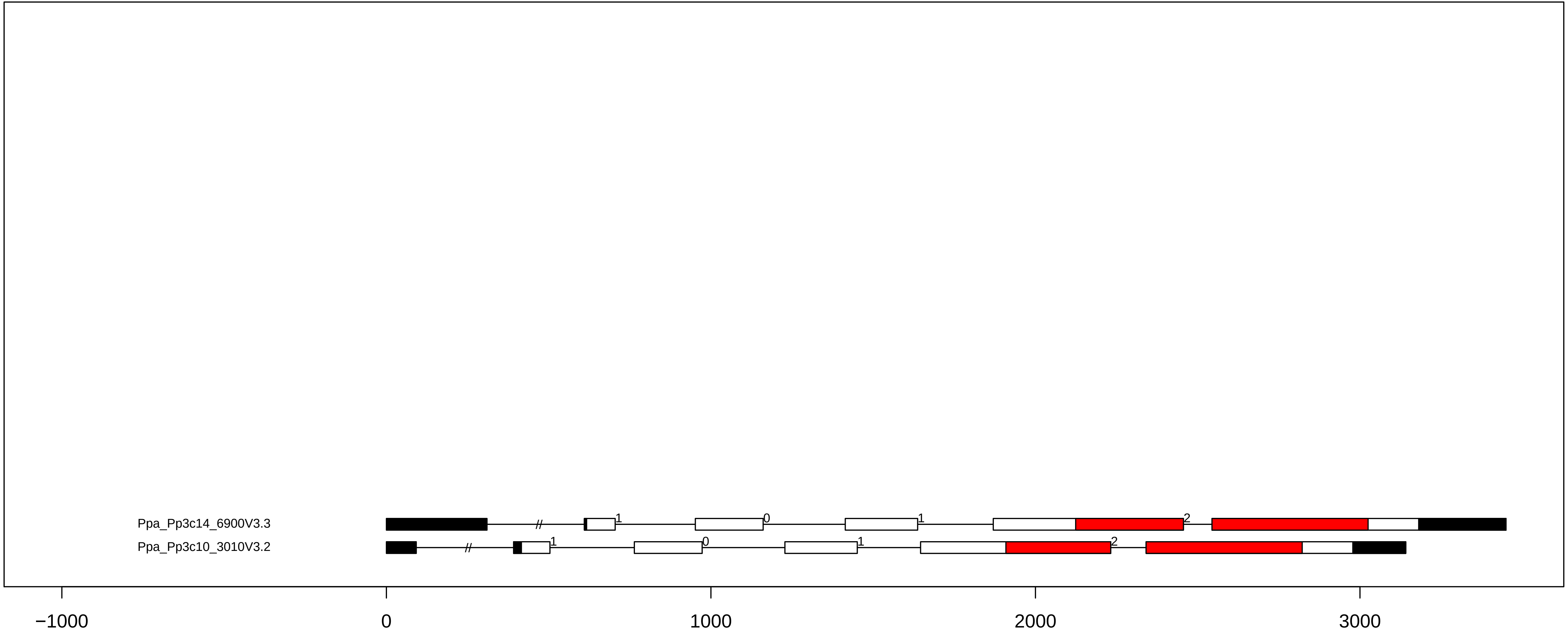

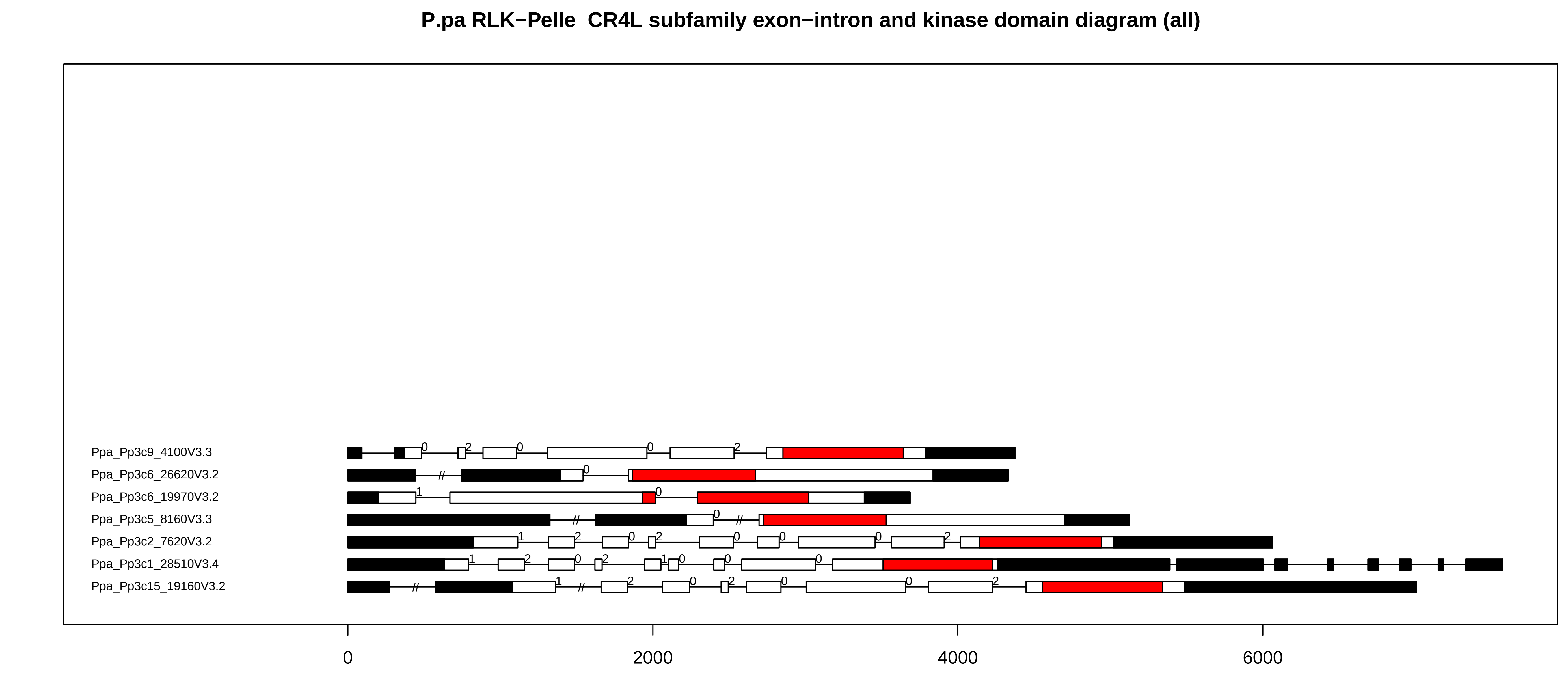
P.pa RLK−Pelle_CR4L subfamily exon−intron and kinase domain diagram (all)
Ppa_Pp3c9_4100V3.3
Ppa_Pp3c6_26620V3.2
Ppa_Pp3c6_19970V3.2
Ppa_Pp3c5_8160V3.3
Ppa_Pp3c2_7620V3.2
Ppa_Pp3c1_28510V3.4
Ppa_Pp3c15_19160V3.2
0
2000
4000
6000

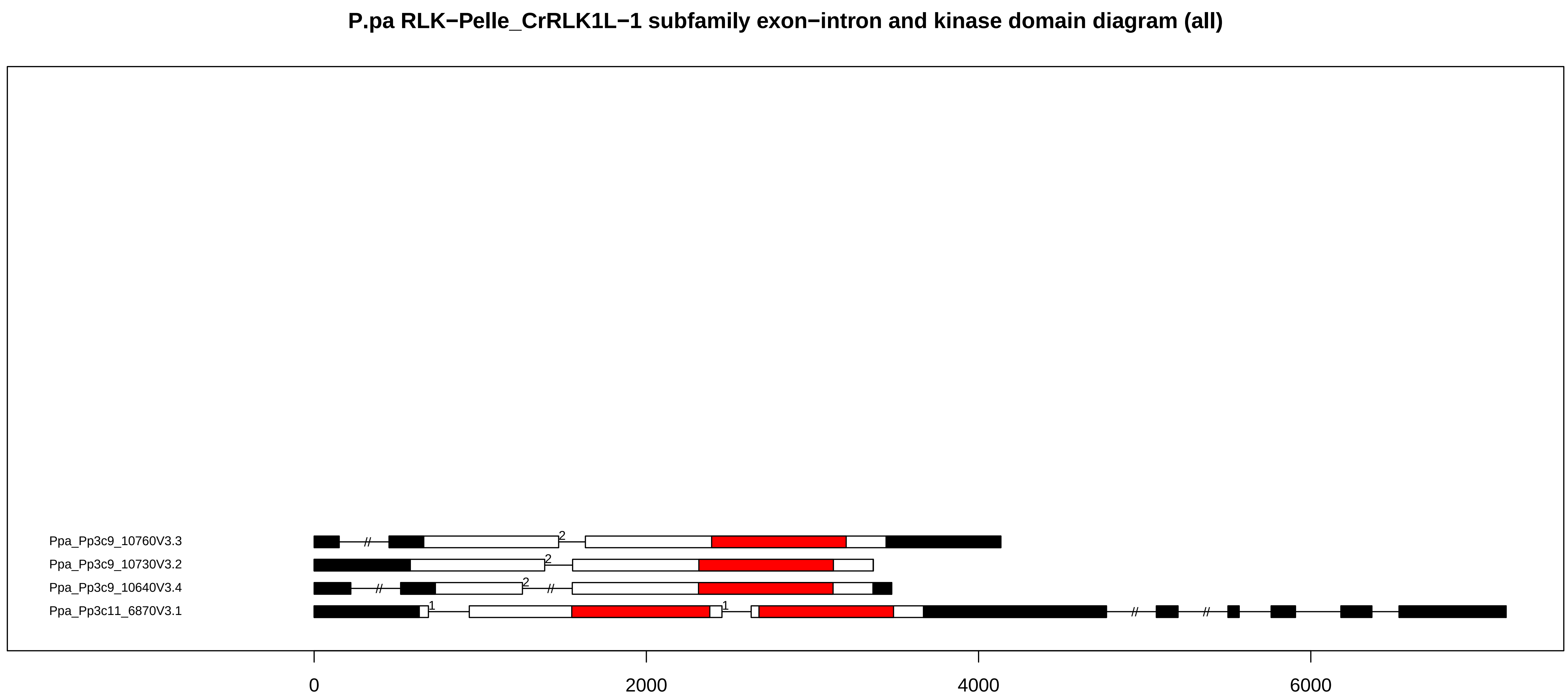

P.pa RLK−Pelle_CrRLK1L−1 subfamily exon−intron and kinase domain diagram (all)
Ppa_Pp3c9_10760V3.3
Ppa_Pp3c9_10730V3.2
Ppa_Pp3c9_10640V3.4
Ppa_Pp3c11_6870V3.1
2
2
2
1
1
0
2000
4000
6000

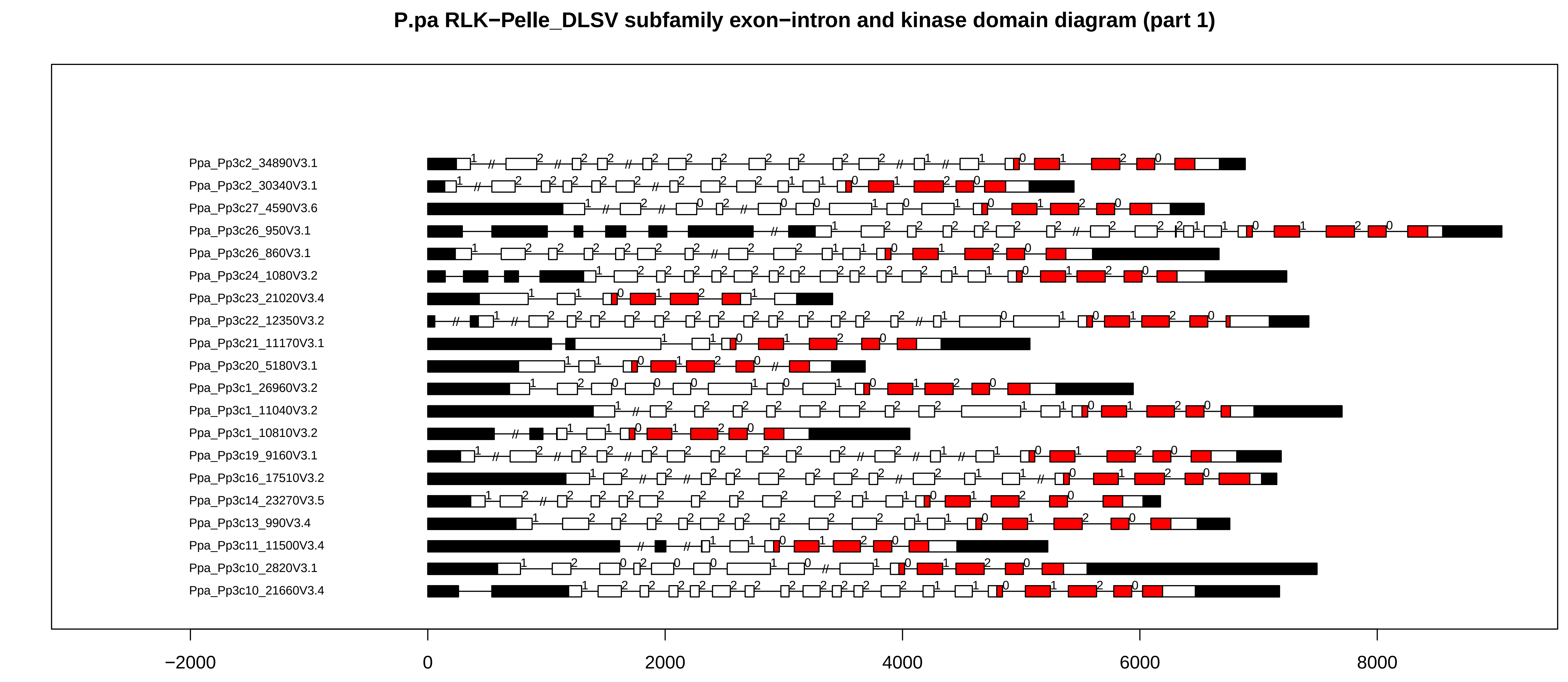

P.pa RLK−Pelle_DLSV subfamily exon−intron and kinase domain diagram (part 1)
Ppa_Pp3c2_34890V3.1
Ppa_Pp3c2_30340V3.1
Ppa_Pp3c27_4590V3.6
Ppa_Pp3c26_950V3.1
Ppa_Pp3c26_860V3.1
Ppa_Pp3c24_1080V3.2
Ppa_Pp3c23_21020V3.4
Ppa_Pp3c22_12350V3.2
Ppa_Pp3c21_11170V3.1
Ppa_Pp3c20_5180V3.1
Ppa_Pp3c1_26960V3.2
Ppa_Pp3c1_11040V3.2
Ppa_Pp3c1_10810V3.2
Ppa_Pp3c19_9160V3.1
Ppa_Pp3c16_17510V3.2
Ppa_Pp3c14_23270V3.5
Ppa_Pp3c13_990V3.4
Ppa_Pp3c11_11500V3.4
Ppa_Pp3c10_2820V3.1
Ppa_Pp3c10_21660V3.4
−2000
0
2000
4000
6000
8000

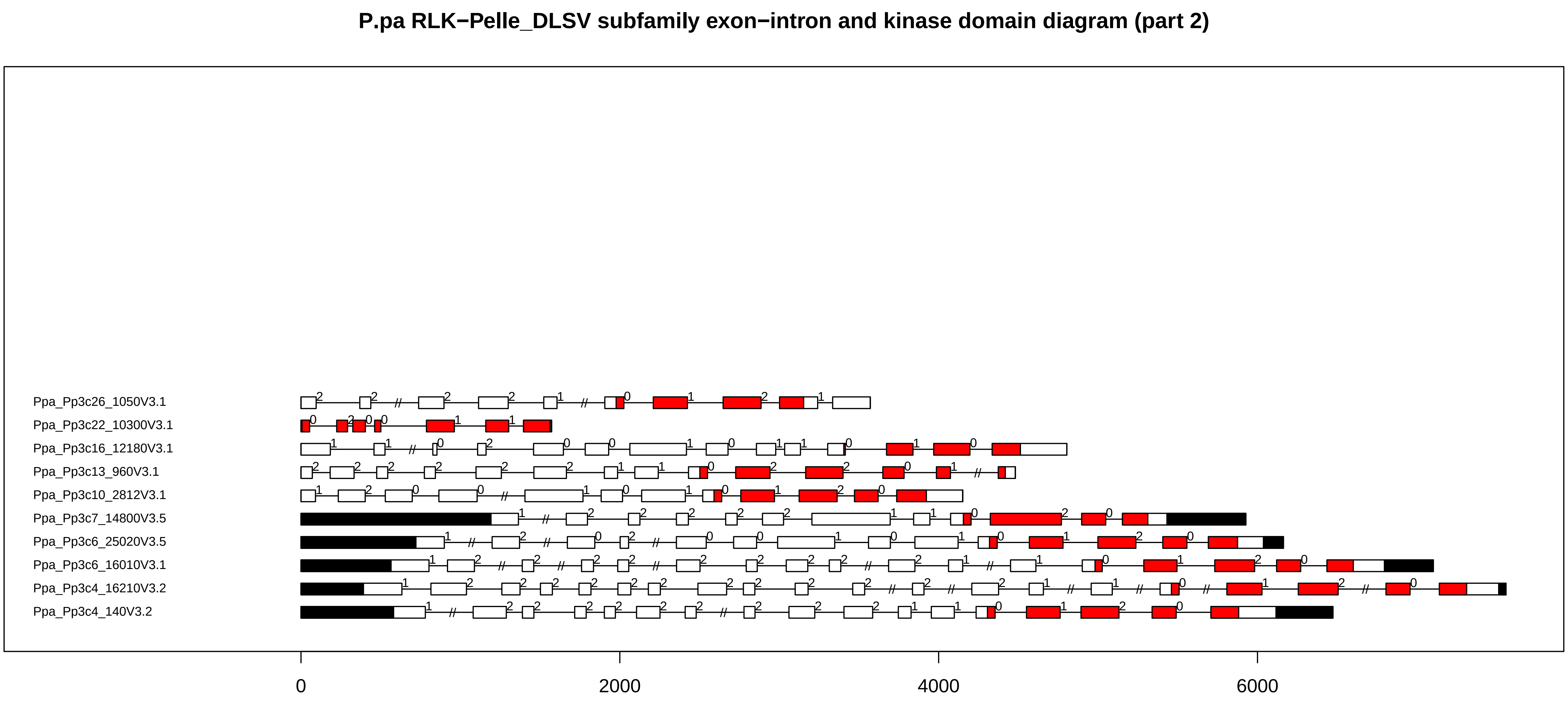

P.pa RLK−Pelle_DLSV subfamily exon−intron and kinase domain diagram (part 2)
Ppa_Pp3c26_1050V3.1
Ppa_Pp3c22_10300V3.1
Ppa_Pp3c16_12180V3.1
Ppa_Pp3c13_960V3.1
Ppa_Pp3c10_2812V3.1
Ppa_Pp3c7_14800V3.5
Ppa_Pp3c6_25020V3.5
Ppa_Pp3c6_16010V3.1
Ppa_Pp3c4_16210V3.2
Ppa_Pp3c4_140V3.2
0
2000
4000
6000

P.pa RLK−Pelle_DSLV subfamily exon−intron and kinase domain diagram (all)

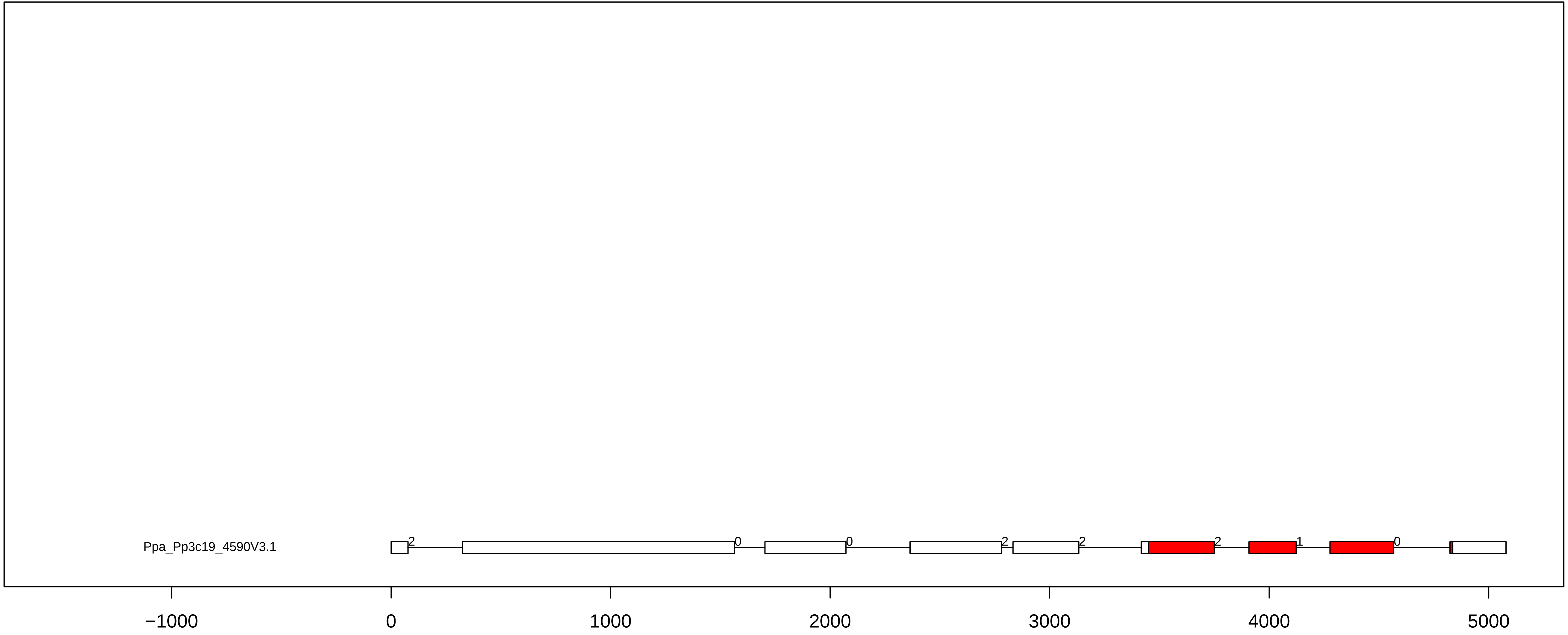

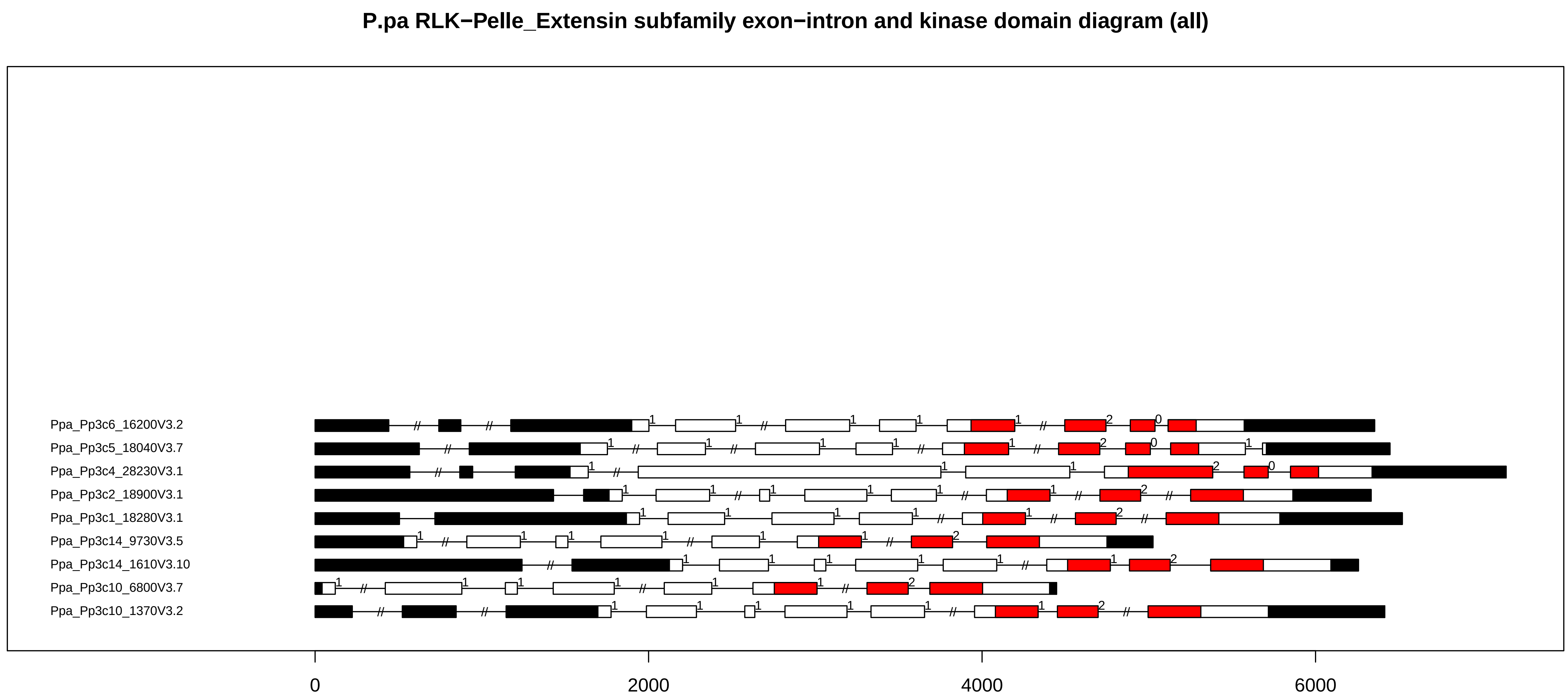

P.pa RLK−Pelle_Extensin subfamily exon−intron and kinase domain diagram (all)
Ppa_Pp3c6_16200V3.2
Ppa_Pp3c5_18040V3.7
Ppa_Pp3c4_28230V3.1
Ppa_Pp3c2_18900V3.1
Ppa_Pp3c1_18280V3.1
Ppa_Pp3c14_9730V3.5
Ppa_Pp3c14_1610V3.10
Ppa_Pp3c10_6800V3.7
Ppa_Pp3c10_1370V3.2
0
2000
4000
6000

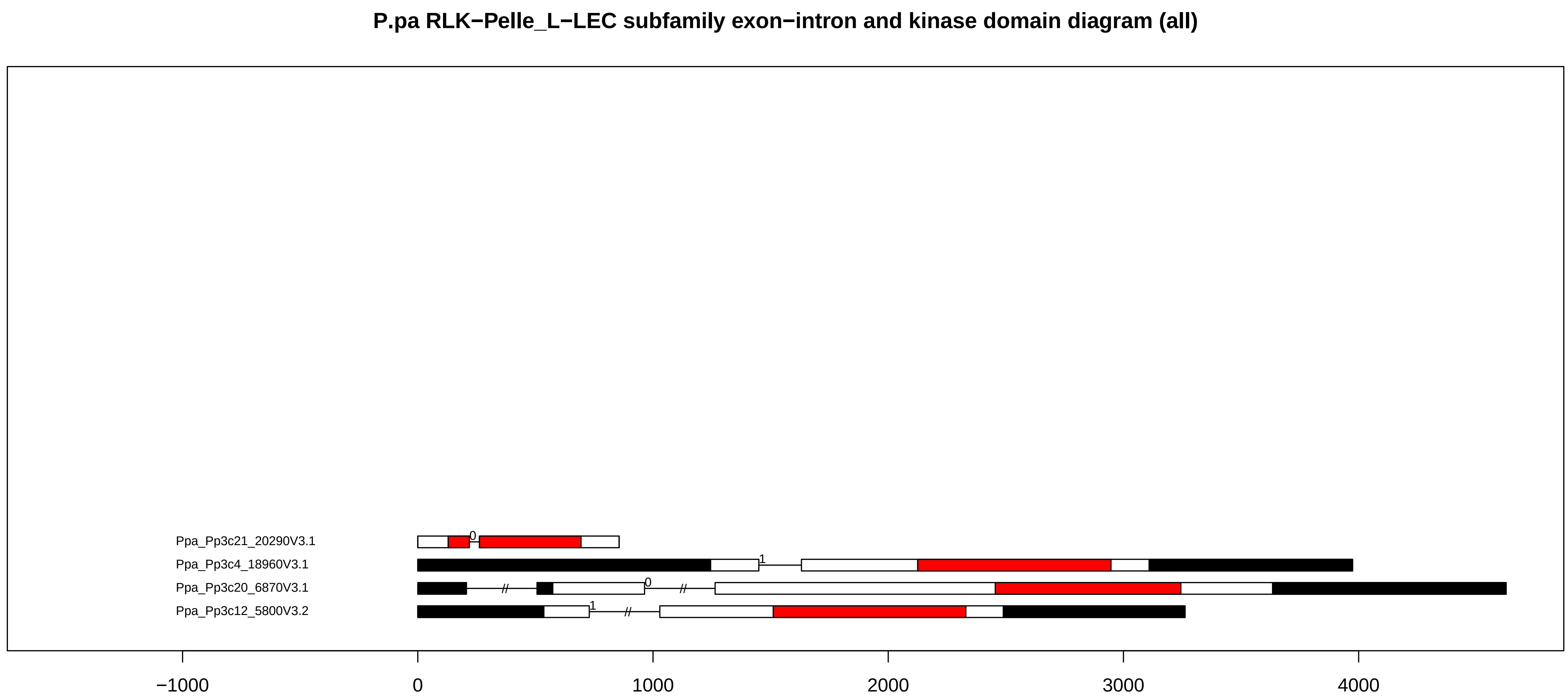
P.pa RLK−Pelle_L−LEC subfamily exon−intron and kinase domain diagram (all)
Ppa_Pp3c21_20290V3.1
Ppa_Pp3c4_18960V3.1
Ppa_Pp3c20_6870V3.1
Ppa_Pp3c12_5800V3.2
0
1
0
1
−1000
0
1000
2000
3000
4000

P.pa RLK−Pelle_LRR−I−1 subfamily exon−intron and kinase domain diagram (part 1)

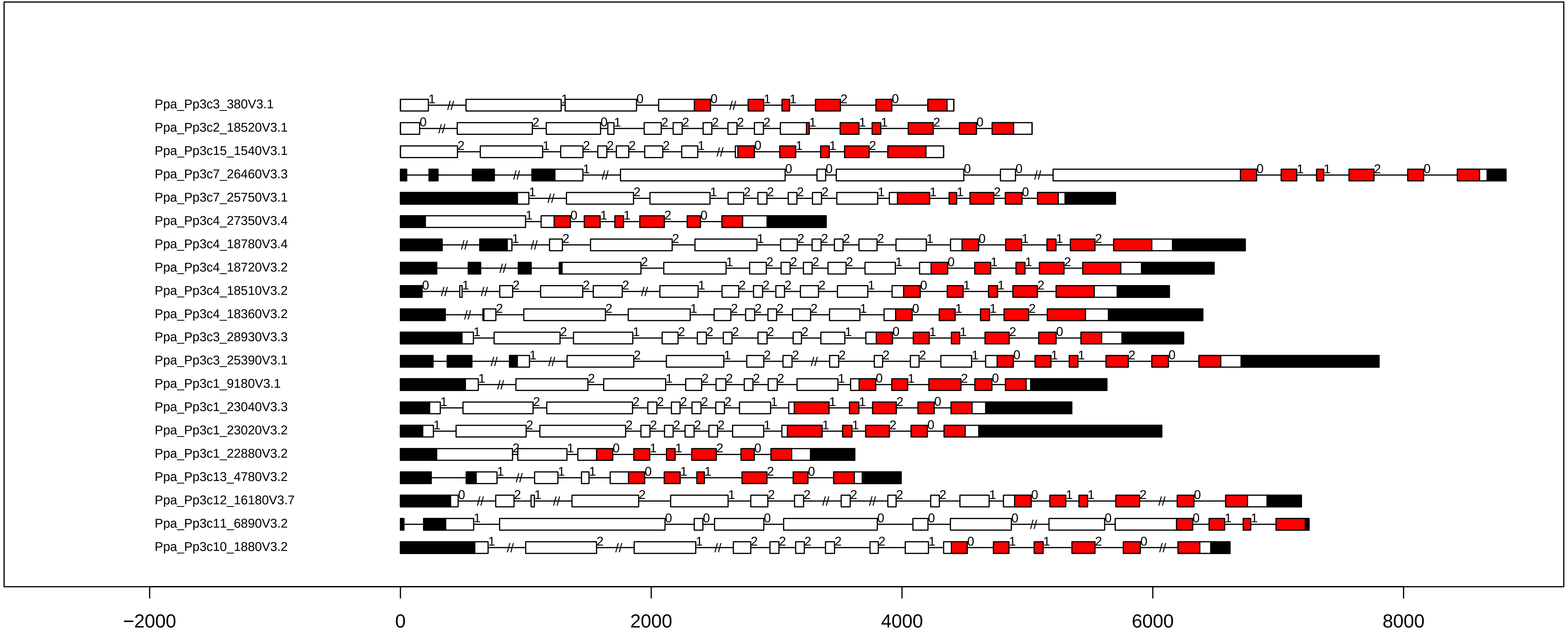

P.pa RLK−Pelle_LRR−I−1 subfamily exon−intron and kinase domain diagram (part 2)

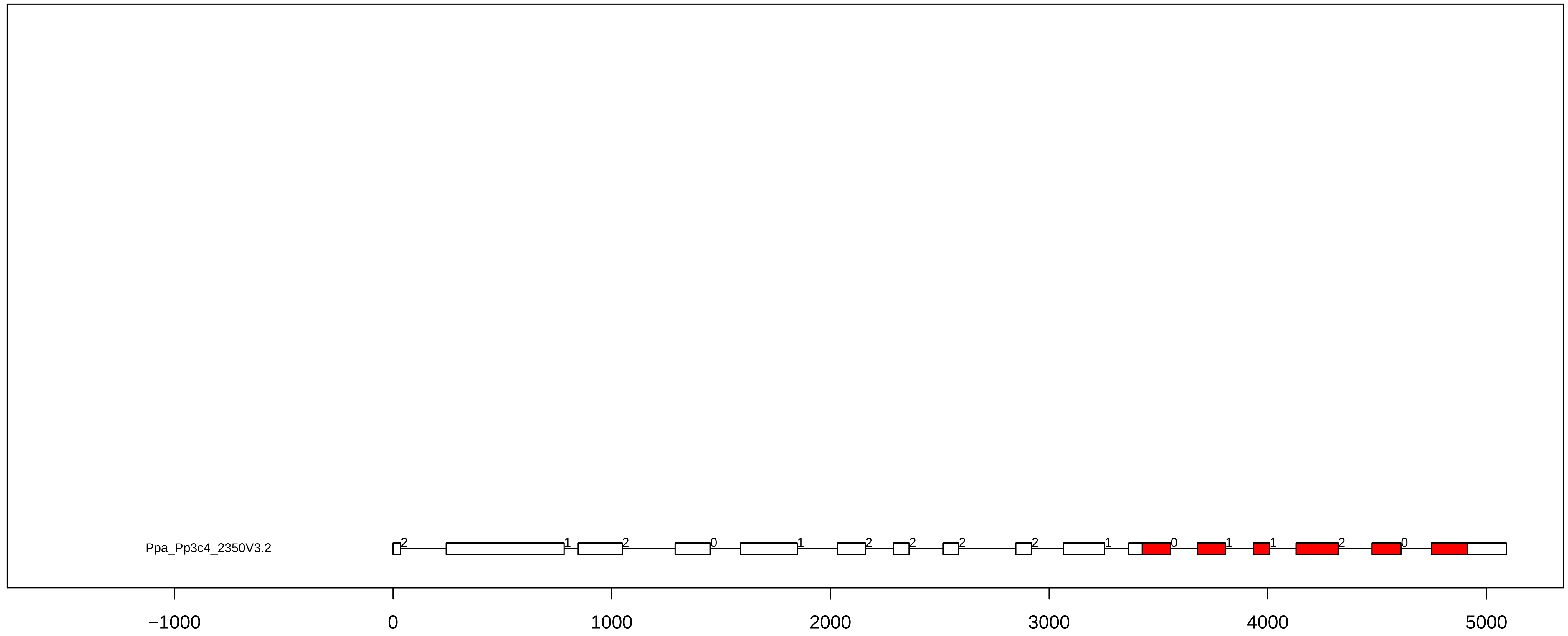

P.pa RLK−Pelle_LRR−I−2 subfamily exon−intron and kinase domain diagram (all)

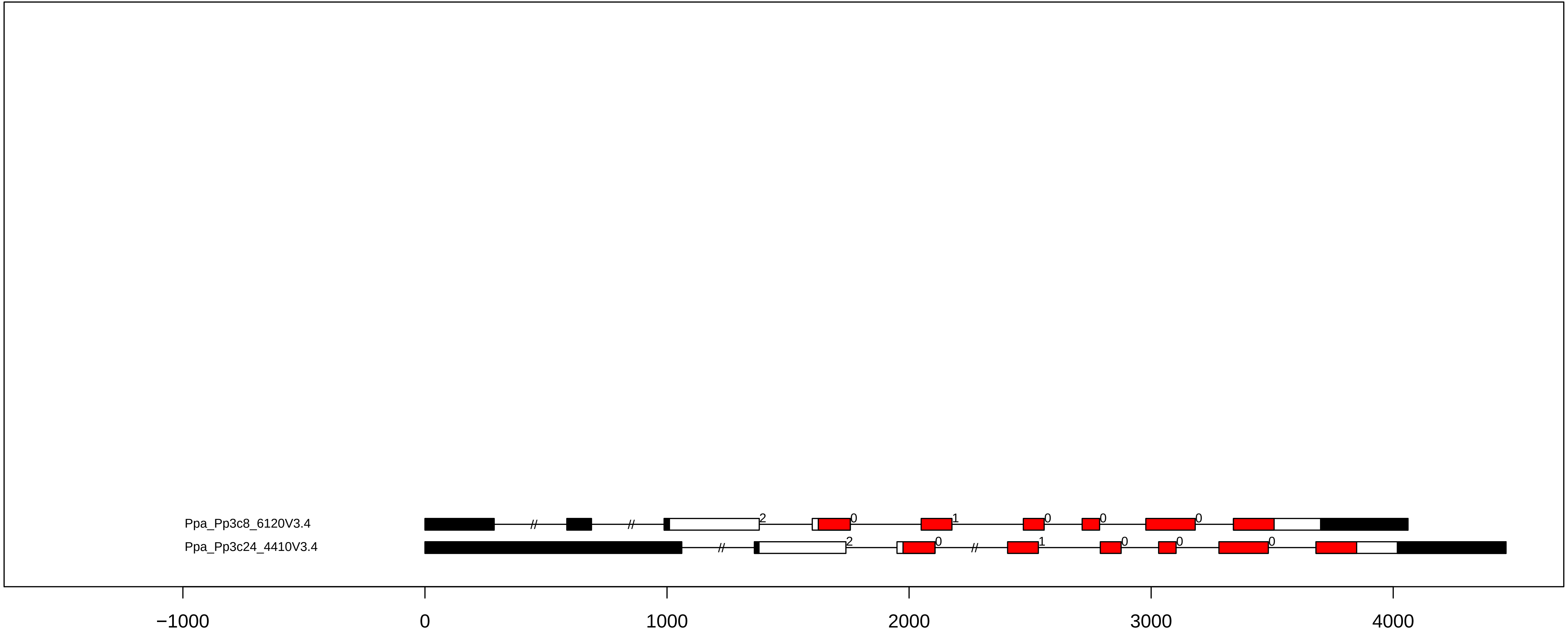

P.pa RLK−Pelle_LRR−III subfamily exon−intron and kinase domain diagram (all)

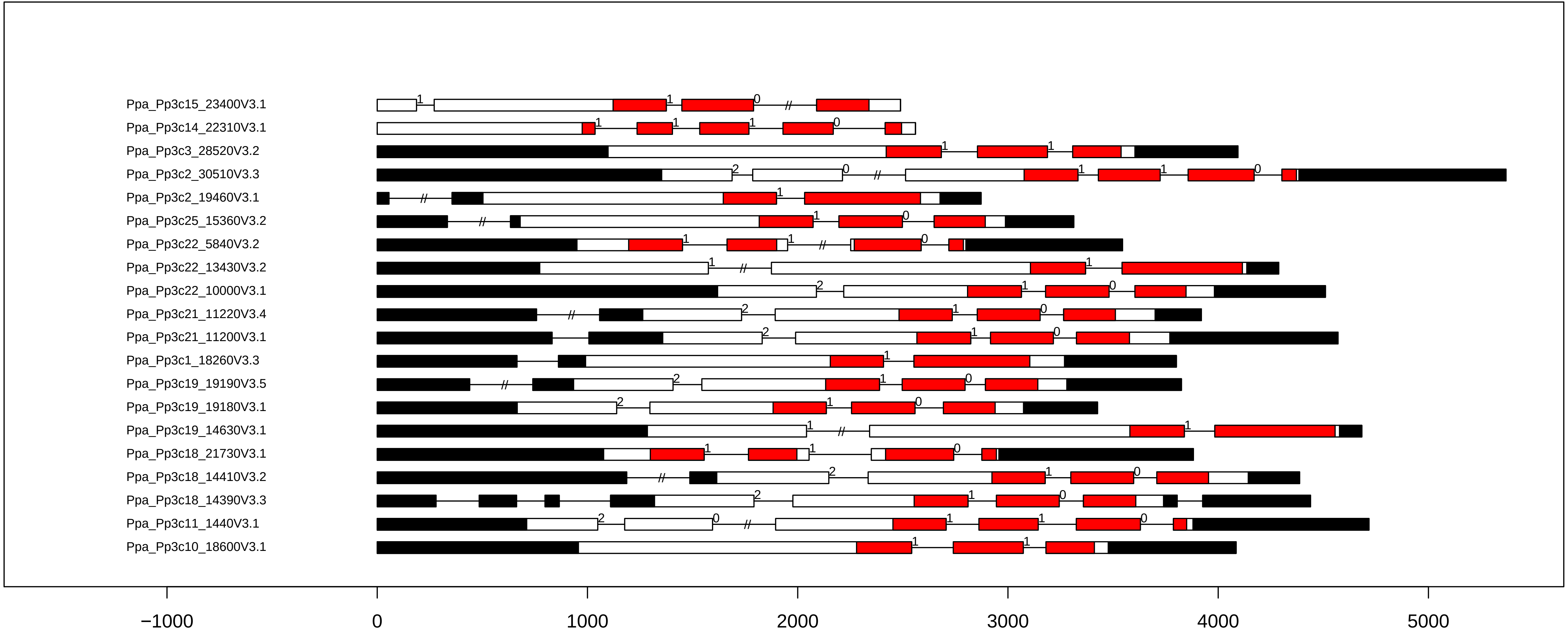

P.pa RLK−Pelle_LRR−II subfamily exon−intron and kinase domain diagram (all)

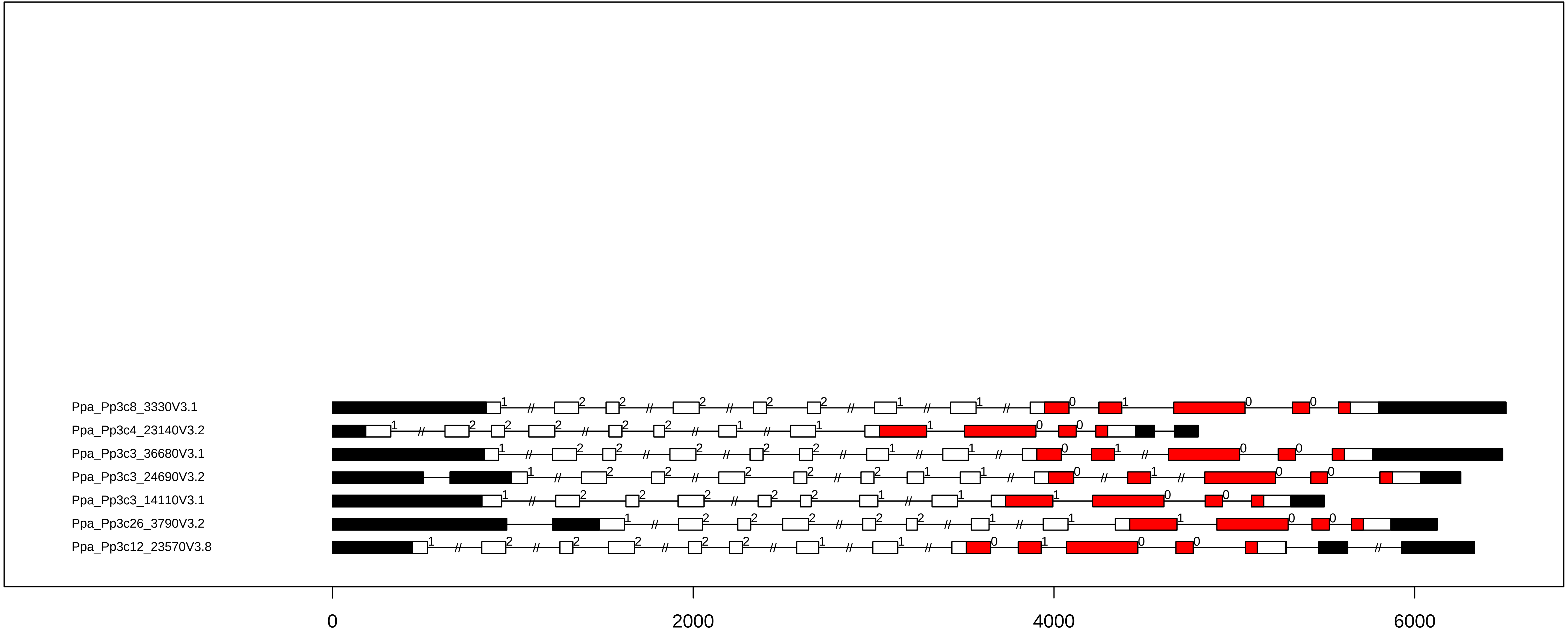

P.pa RLK−Pelle_LRR−IV subfamily exon−intron and kinase domain diagram (all)

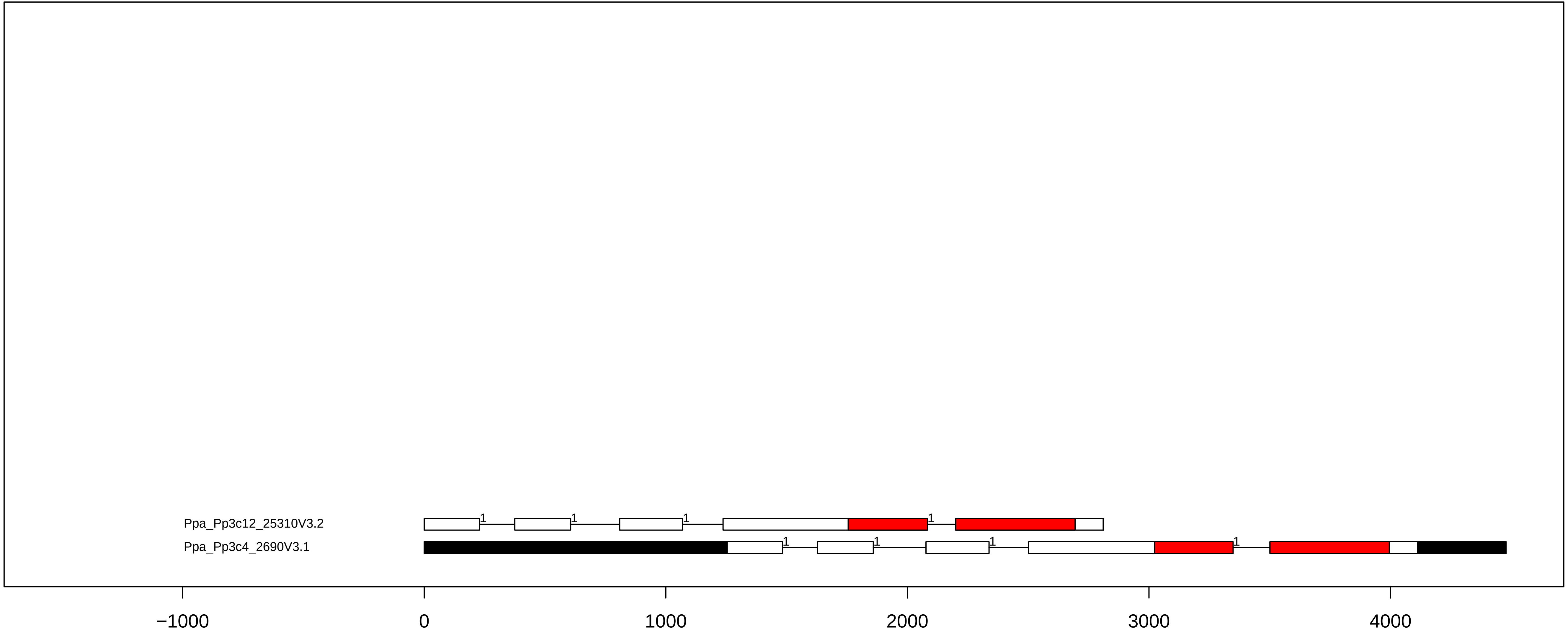

P.pa RLK−Pelle_LRR−IX subfamily exon−intron and kinase domain diagram (all)

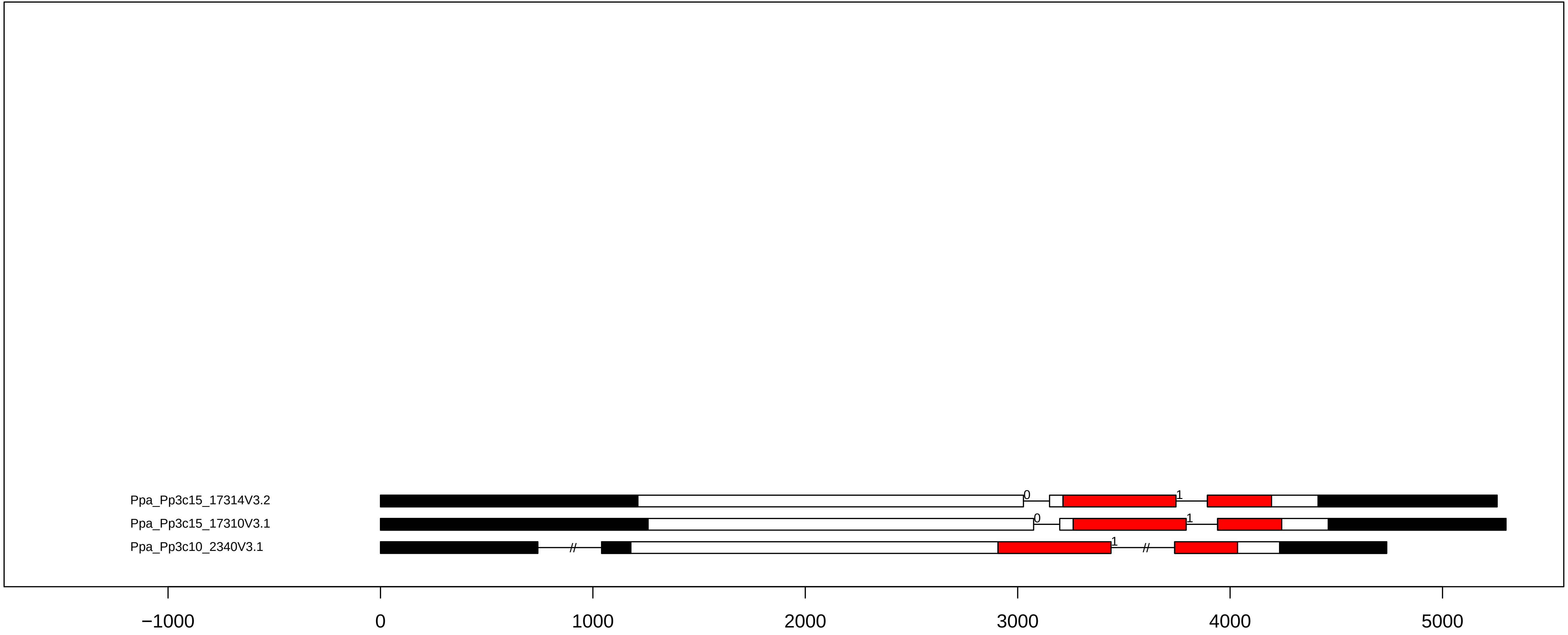

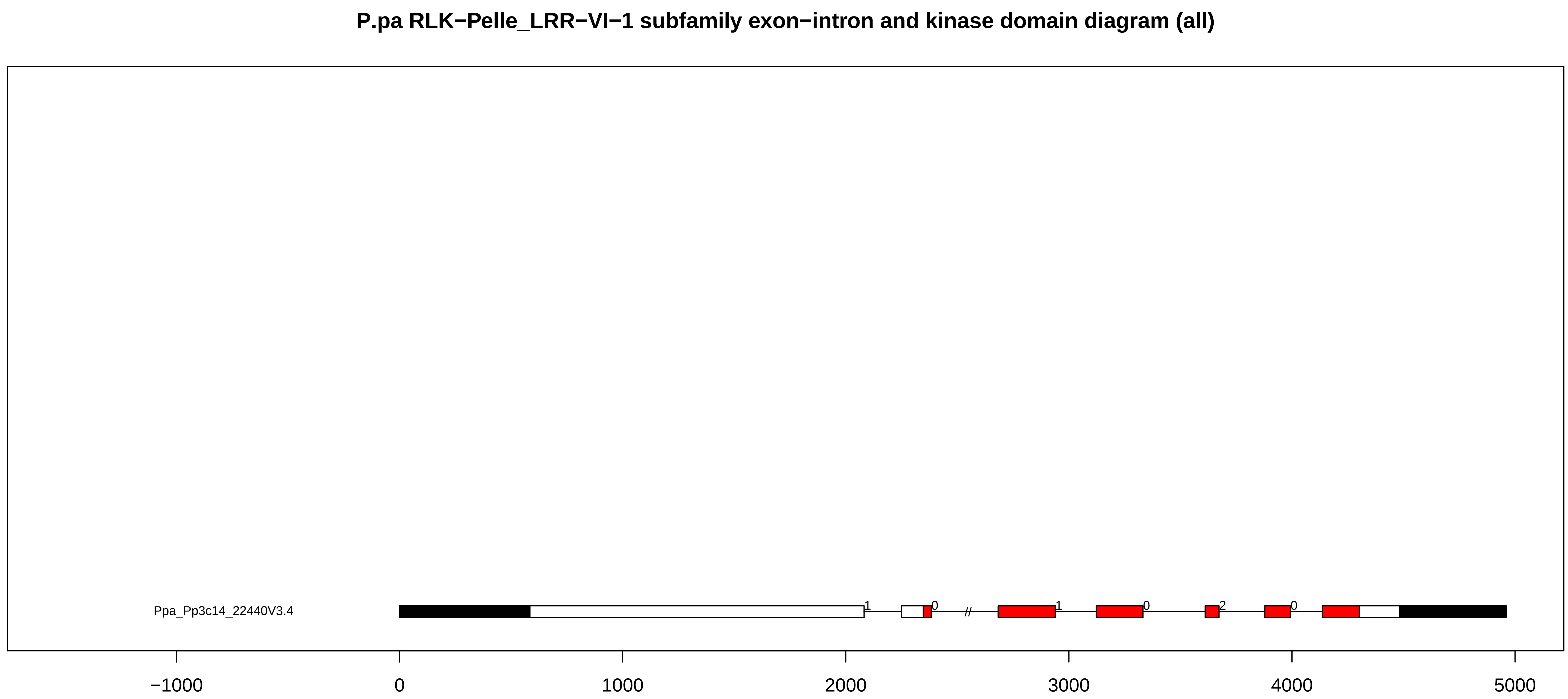

P.pa RLK−Pelle_LRR−VI−1 subfamily exon−intron and kinase domain diagram (all)
Ppa_Pp3c14_22440V3.4
1
0
//
1
0
2
0
−1000
0
1000
2000
3000
4000
5000

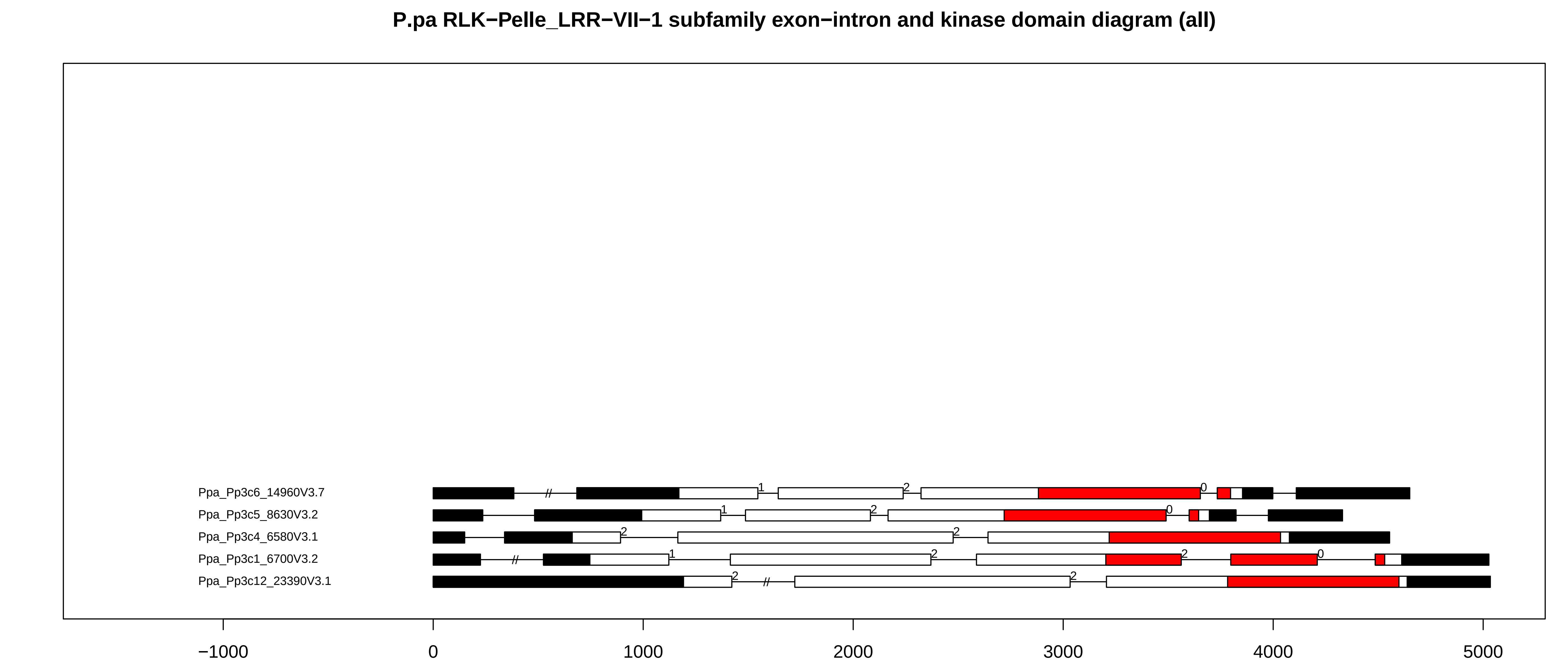
P.pa RLK−Pelle_LRR−VII−1 subfamily exon−intron and kinase domain diagram (all)
Ppa_Pp3c6_14960V3.7
Ppa_Pp3c5_8630V3.2
Ppa_Pp3c4_6580V3.1
Ppa_Pp3c1_6700V3.2
Ppa_Pp3c12_23390V3.1
−1000
0
1000
2000
3000
4000
5000

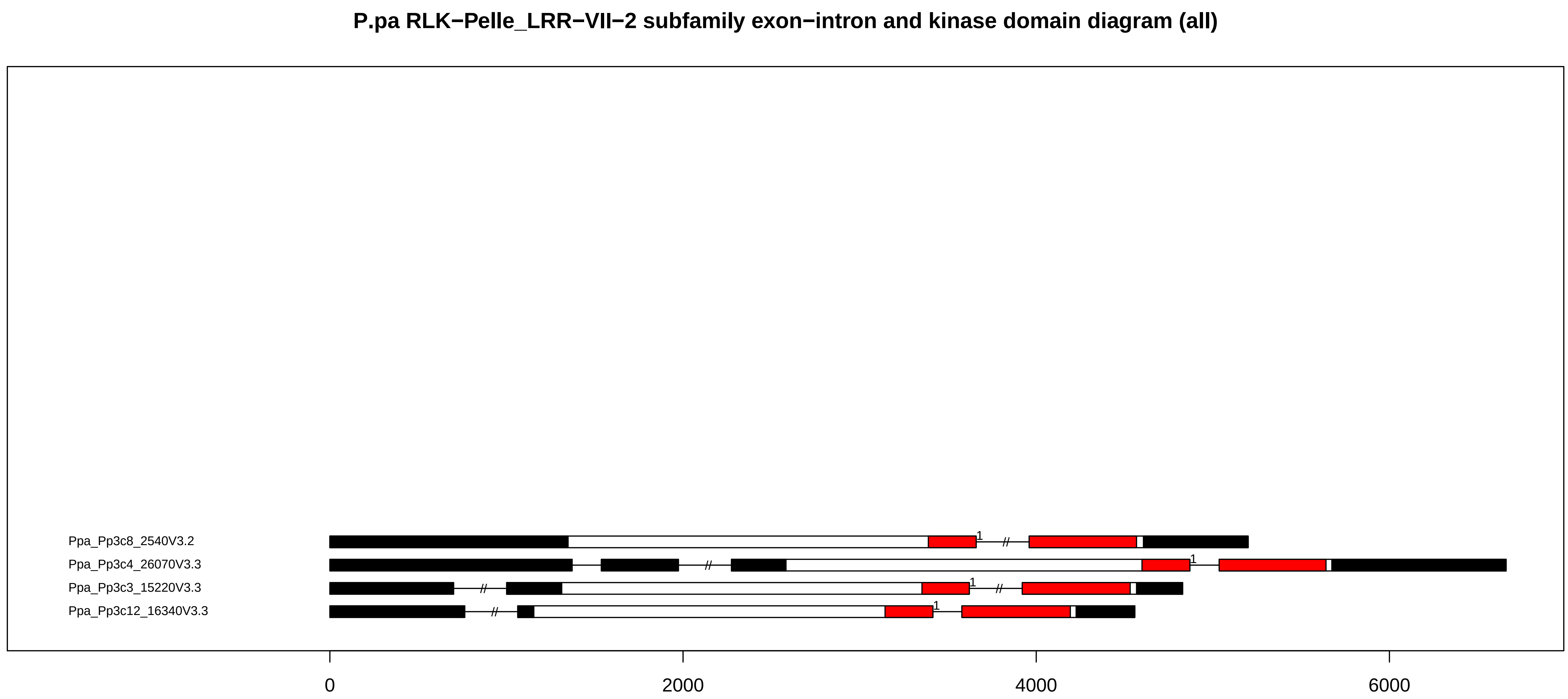
P.pa RLK−Pelle_LRR−VII−2 subfamily exon−intron and kinase domain diagram (all)
Ppa_Pp3c8_2540V3.2
Ppa_Pp3c4_26070V3.3
Ppa_Pp3c3_15220V3.3
Ppa_Pp3c12_16340V3.3
1
1
1
1
0
2000
4000
6000

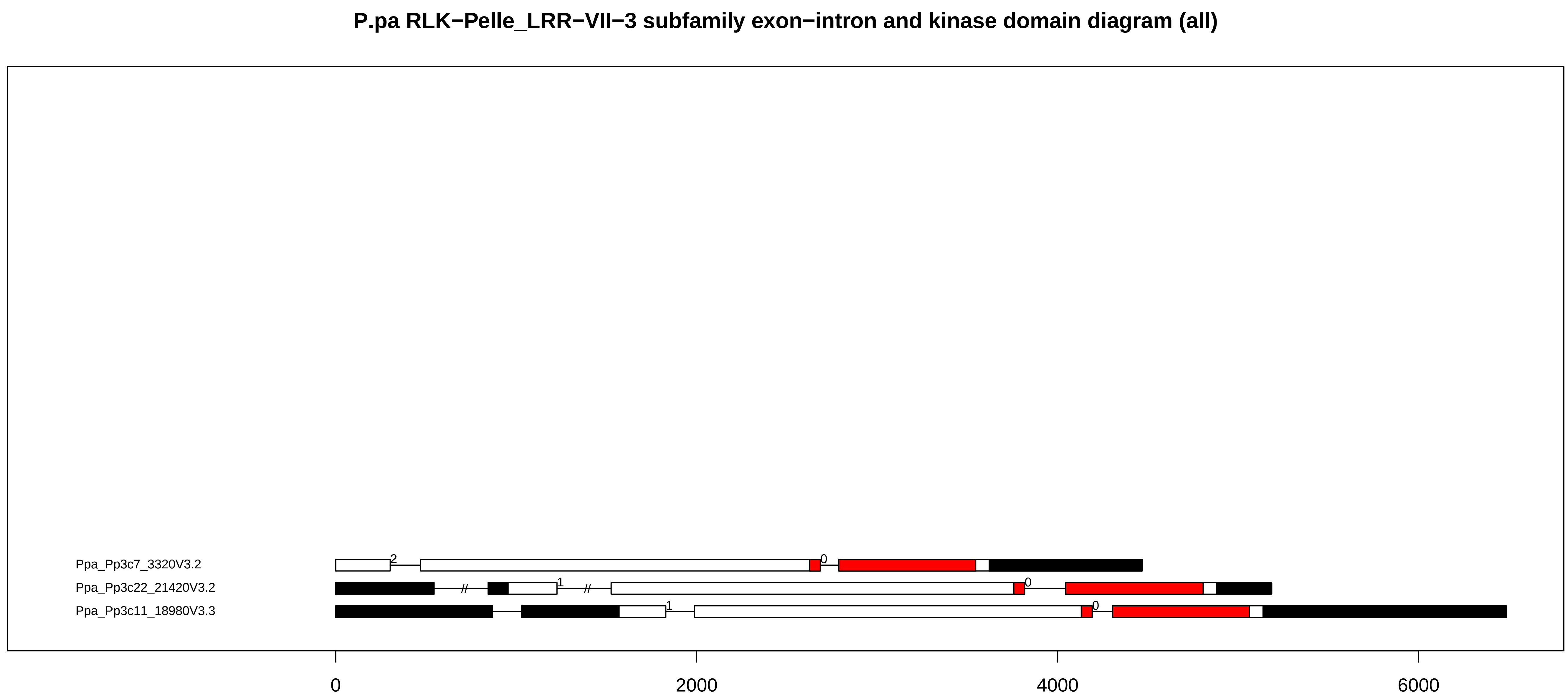
P.pa RLK−Pelle_LRR−VII−3 subfamily exon−intron and kinase domain diagram (all)
Ppa_Pp3c7_3320V3.2
Ppa_Pp3c22_21420V3.2
Ppa_Pp3c11_18980V3.3
2
0
1
0
1
0
0
2000
4000
6000

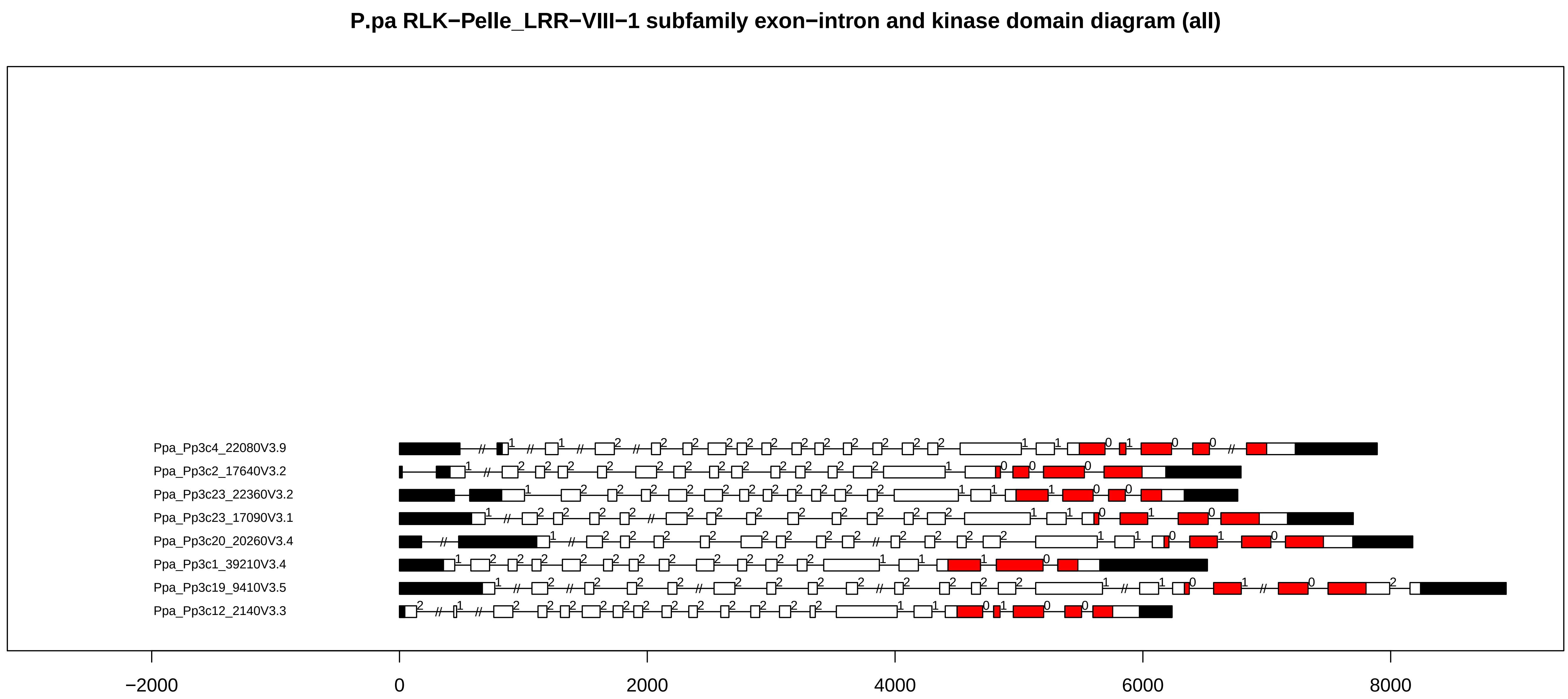

P.pa RLK−Pelle_LRR−VIII−1 subfamily exon−intron and kinase domain diagram (all)
Ppa_Pp3c4_22080V3.9
Ppa_Pp3c2_17640V3.2
Ppa_Pp3c23_22360V3.2
Ppa_Pp3c23_17090V3.1
Ppa_Pp3c20_20260V3.4
Ppa_Pp3c1_39210V3.4
Ppa_Pp3c19_9410V3.5
Ppa_Pp3c12_2140V3.3
−2000
0
2000
4000
6000
8000

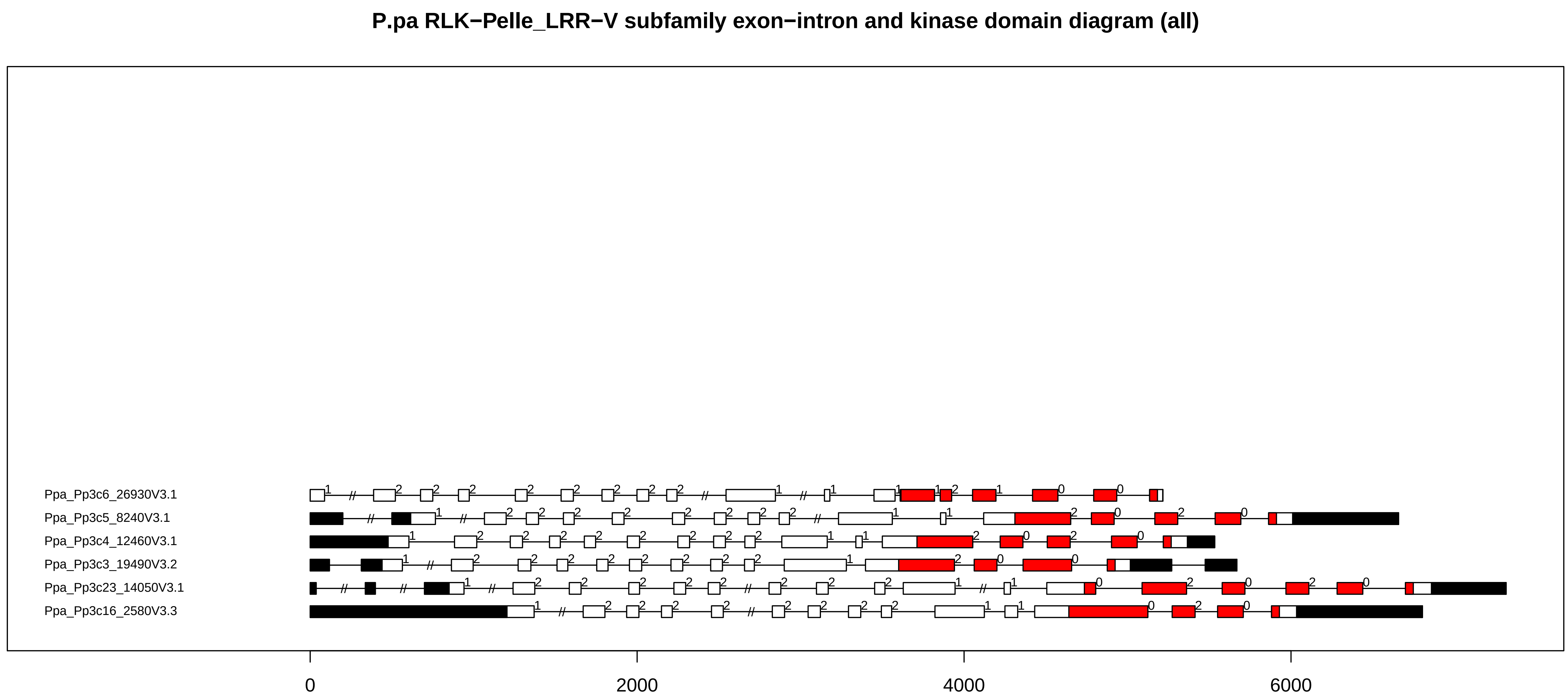
P.pa RLK−Pelle_LRR−V subfamily exon−intron and kinase domain diagram (all)
Ppa_Pp3c6_26930V3.1
Ppa_Pp3c5_8240V3.1
Ppa_Pp3c4_12460V3.1
Ppa_Pp3c3_19490V3.2
Ppa_Pp3c23_14050V3.1
Ppa_Pp3c16_2580V3.3
0
2000
4000
6000

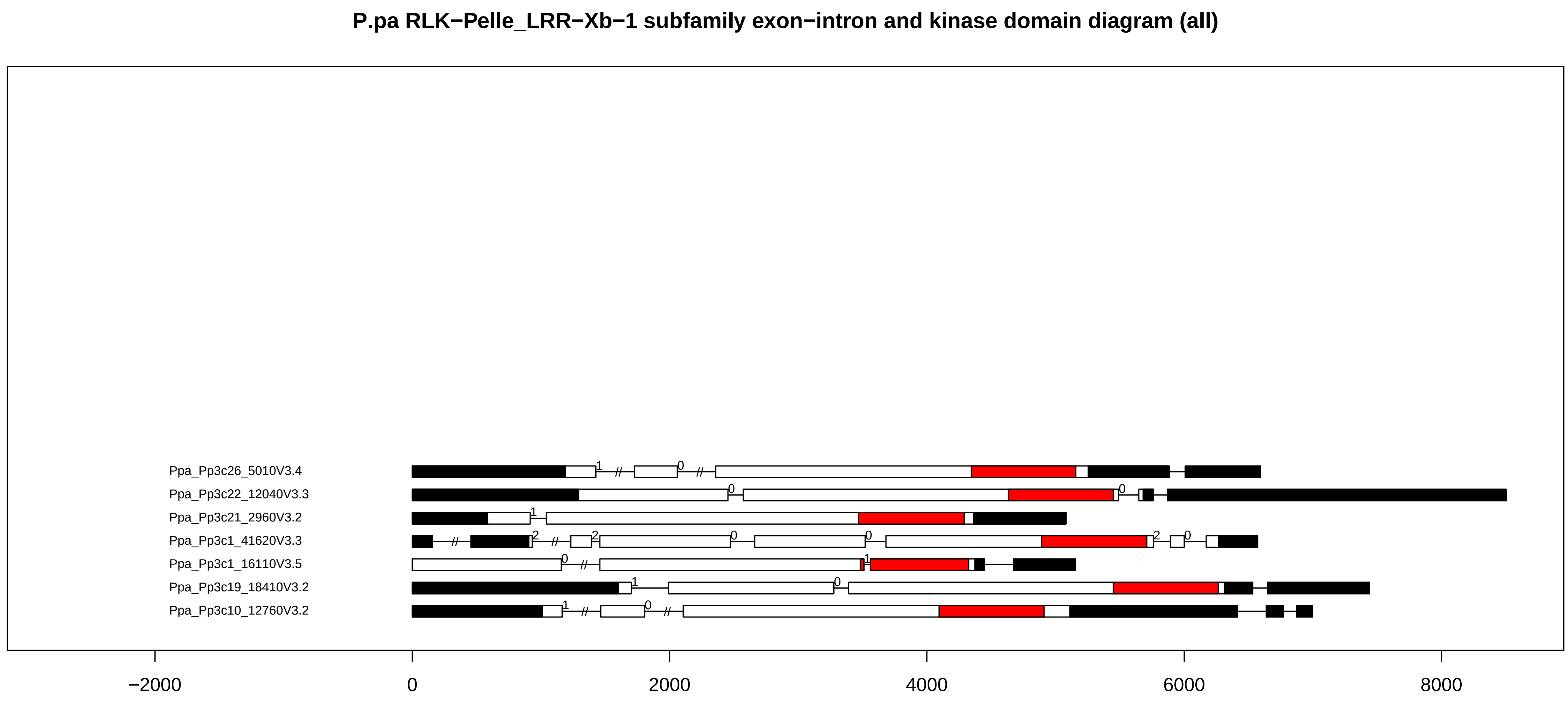
P.pa RLK−Pelle_LRR−Xb−1 subfamily exon−intron and kinase domain diagram (all)
Ppa_Pp3c26_5010V3.4
Ppa_Pp3c22_12040V3.3
Ppa_Pp3c21_2960V3.2
Ppa_Pp3c1_41620V3.3
Ppa_Pp3c1_16110V3.5
Ppa_Pp3c19_18410V3.2
Ppa_Pp3c10_12760V3.2
−2000
0
2000
4000
6000
8000

P.pa RLK−Pelle_LRR−XI−1 subfamily exon−intron and kinase domain diagram (all)

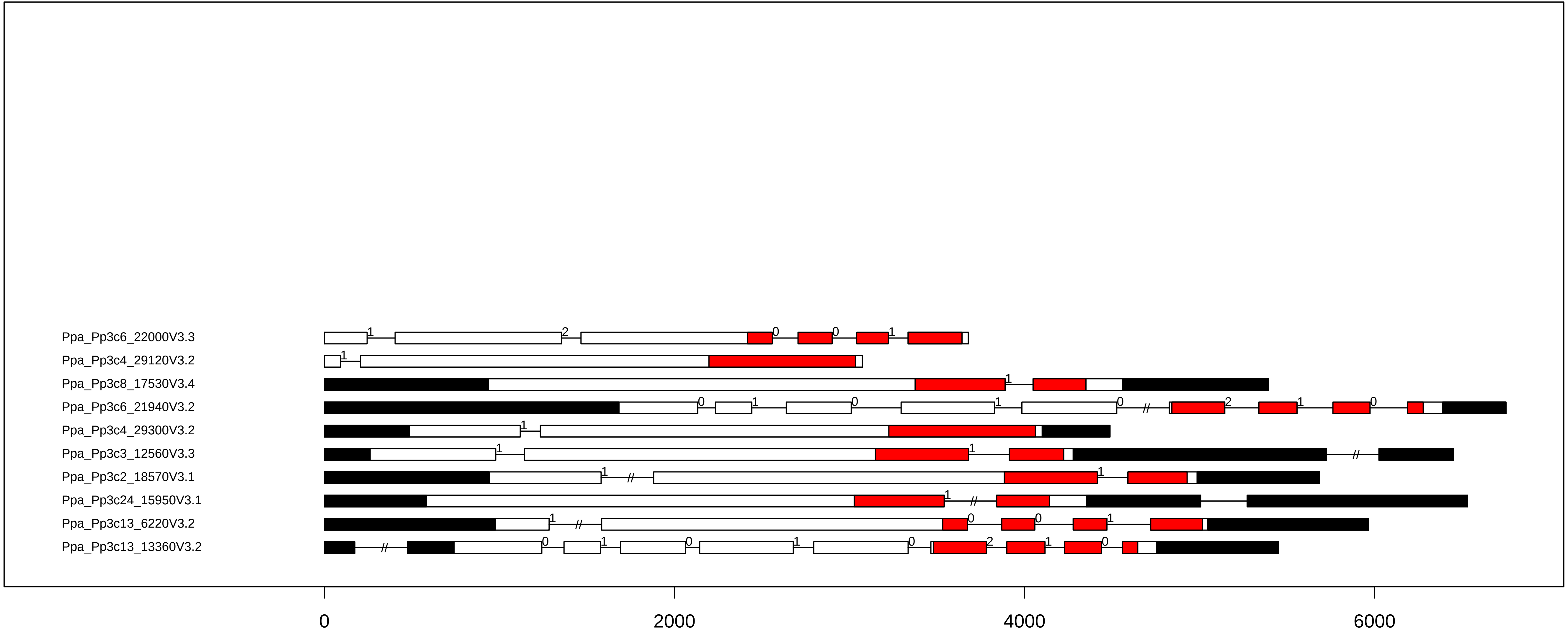

P.pa RLK−Pelle_LRR−XI−2 subfamily exon−intron and kinase domain diagram (all)

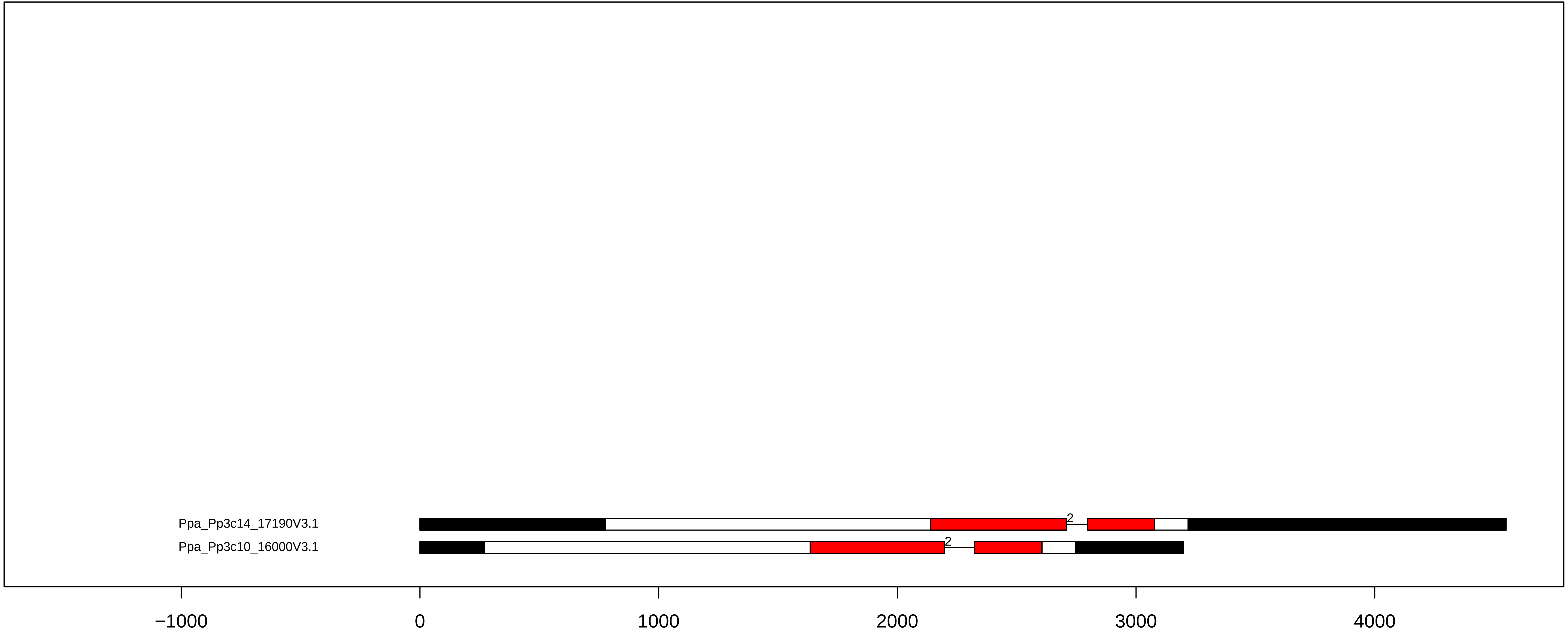

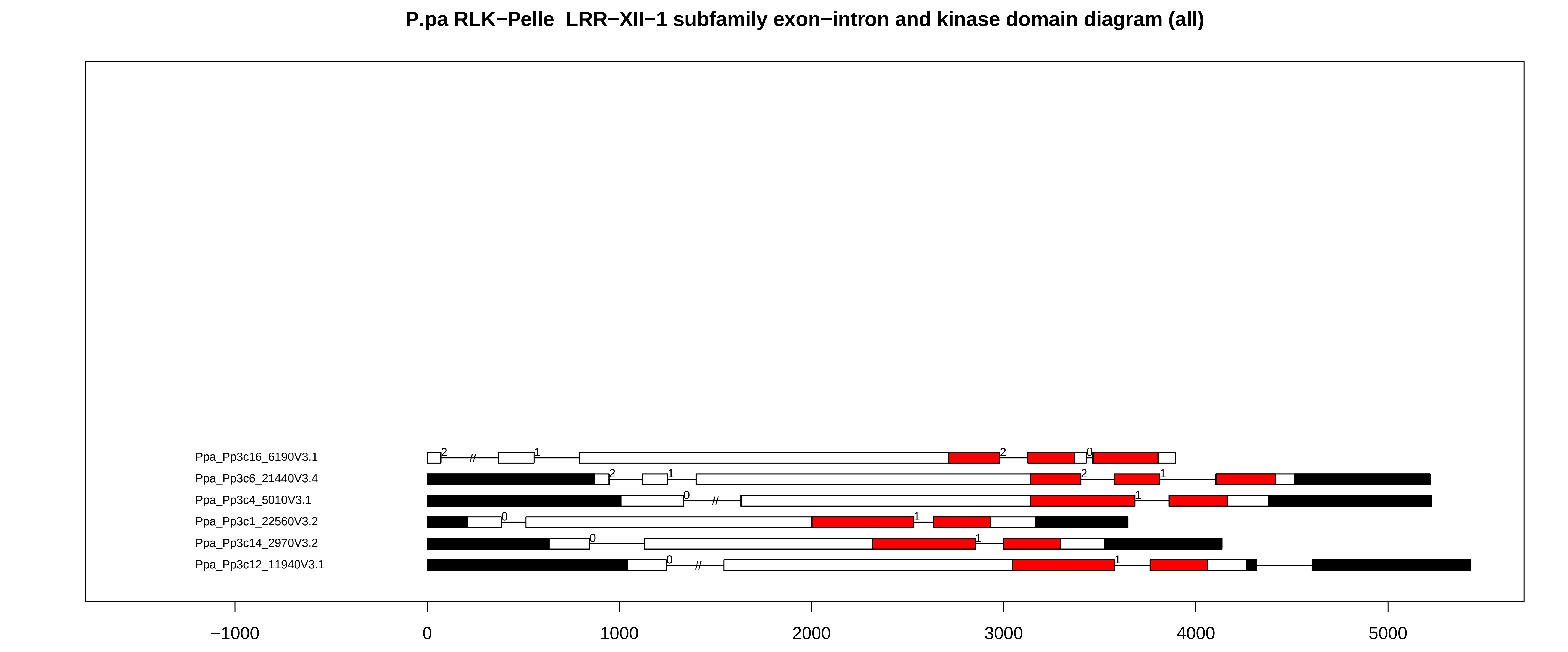

P.pa RLK−Pelle_LRR−XII−1 subfamily exon−intron and kinase domain diagram (all)
Ppa_Pp3c16_6190V3.1
Ppa_Pp3c6_21440V3.4
Ppa_Pp3c4_5010V3.1
Ppa_Pp3c1_22560V3.2
Ppa_Pp3c14_2970V3.2
Ppa_Pp3c12_11940V3.1
−1000
0
1000
2000
3000
4000
5000

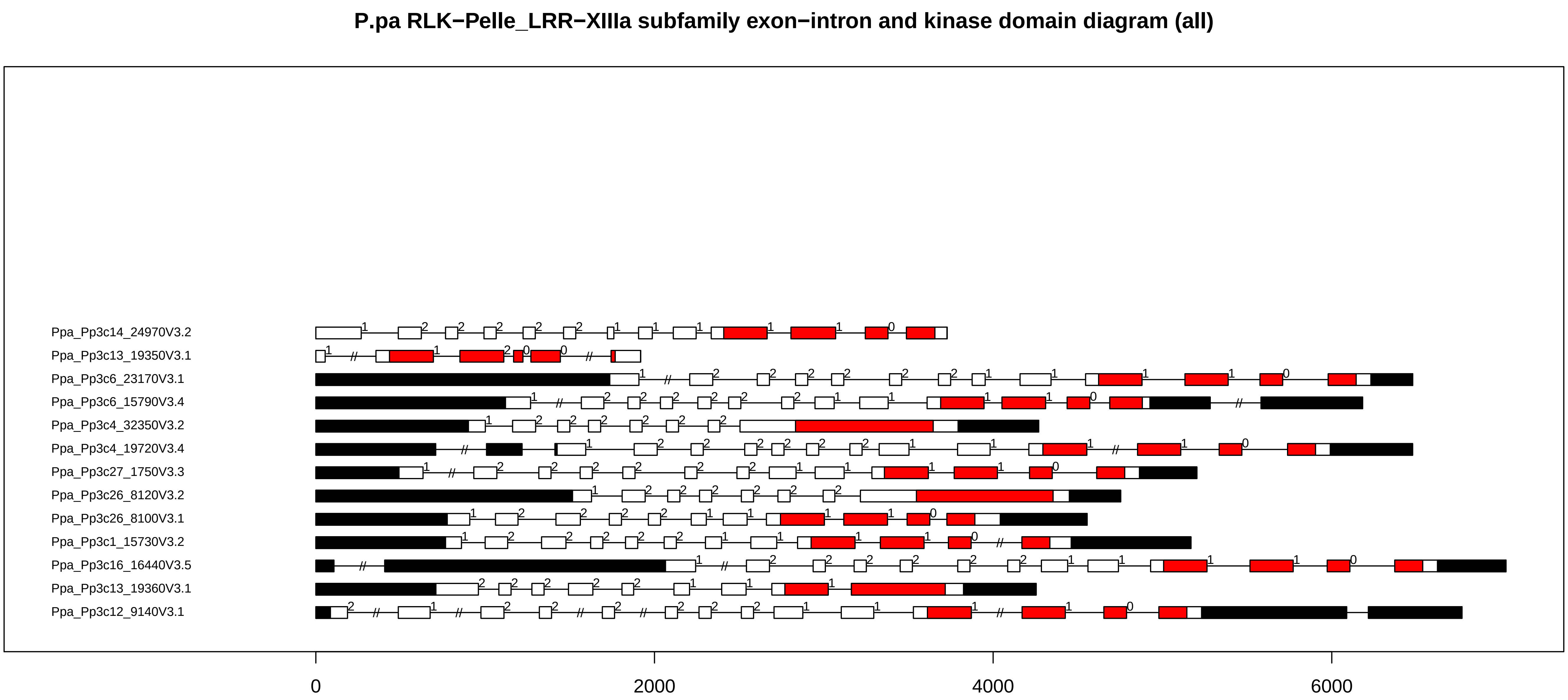

P.pa RLK−Pelle_LRR−XIIIa subfamily exon−intron and kinase domain diagram (all)
Ppa_Pp3c14_24970V3.2
Ppa_Pp3c13_19350V3.1
Ppa_Pp3c6_23170V3.1
Ppa_Pp3c6_15790V3.4
Ppa_Pp3c4_32350V3.2
Ppa_Pp3c4_19720V3.4
Ppa_Pp3c27_1750V3.3
Ppa_Pp3c26_8120V3.2
Ppa_Pp3c26_8100V3.1
Ppa_Pp3c1_15730V3.2
Ppa_Pp3c16_16440V3.5
Ppa_Pp3c13_19360V3.1
Ppa_Pp3c12_9140V3.1
0
2000
4000
6000

P.pa RLK−Pelle_LRR−XIIIb subfamily exon−intron and kinase domain diagram (all)

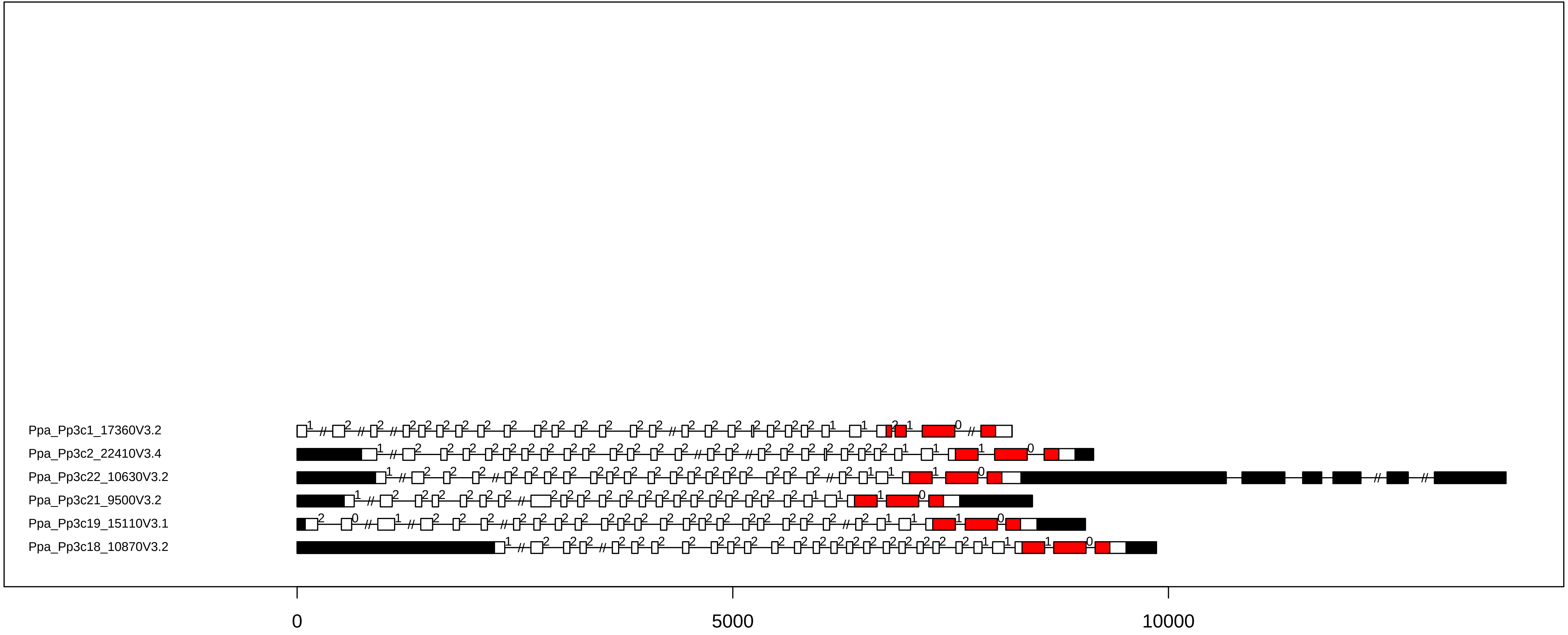

P.pa RLK−Pelle_LRR−XIV subfamily exon−intron and kinase domain diagram (all)

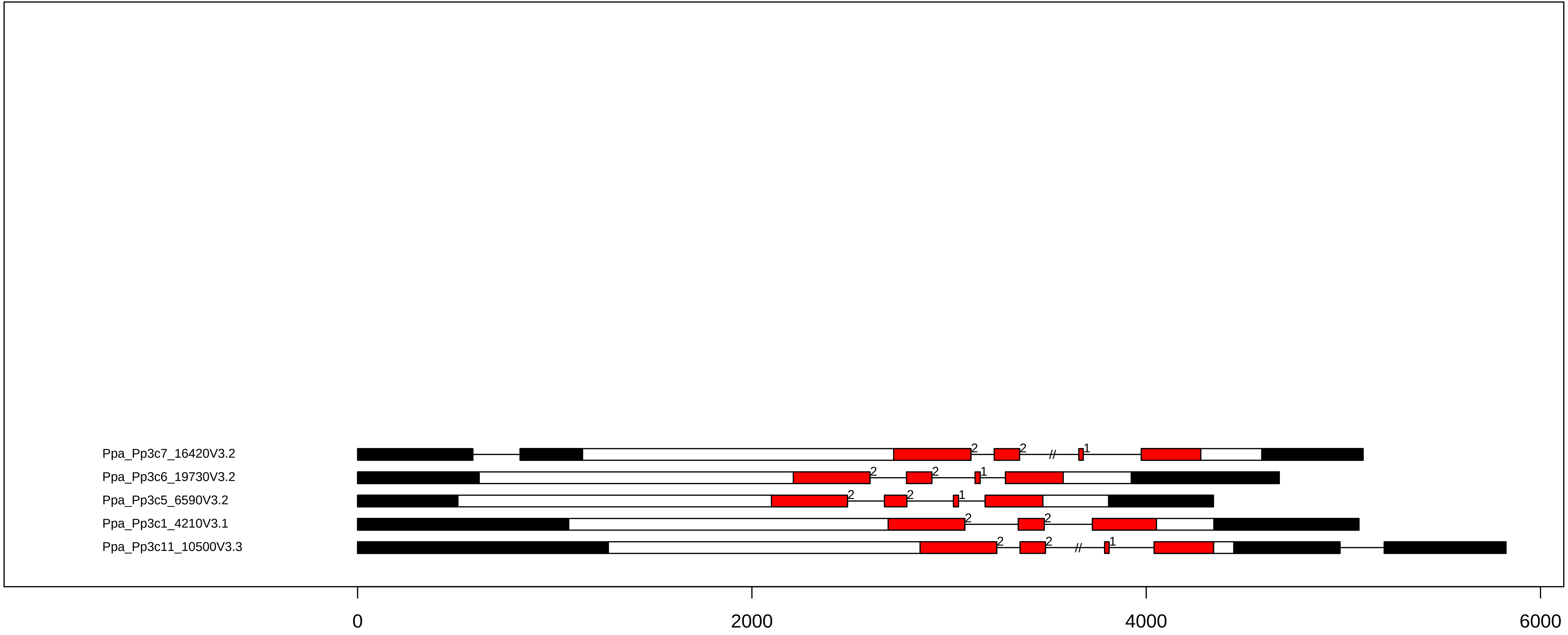

P.pa RLK−Pelle_LysM subfamily exon−intron and kinase domain diagram (all)

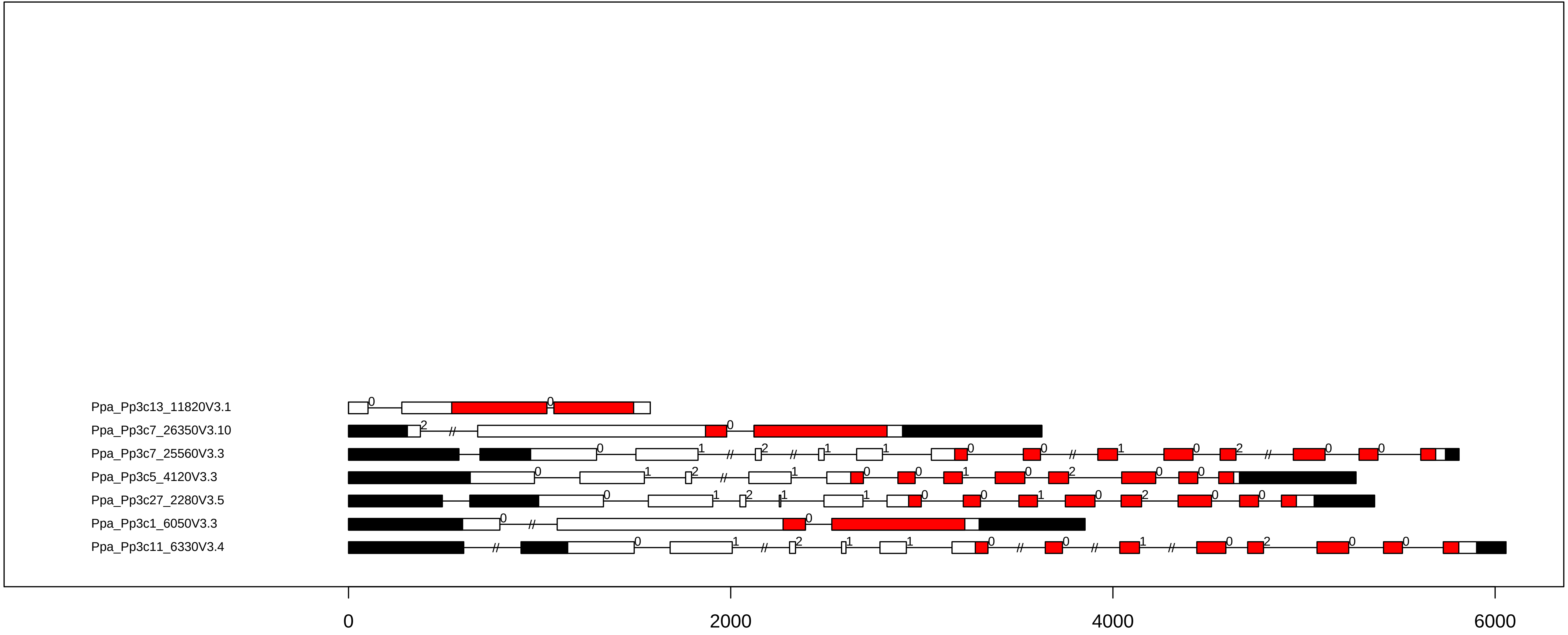

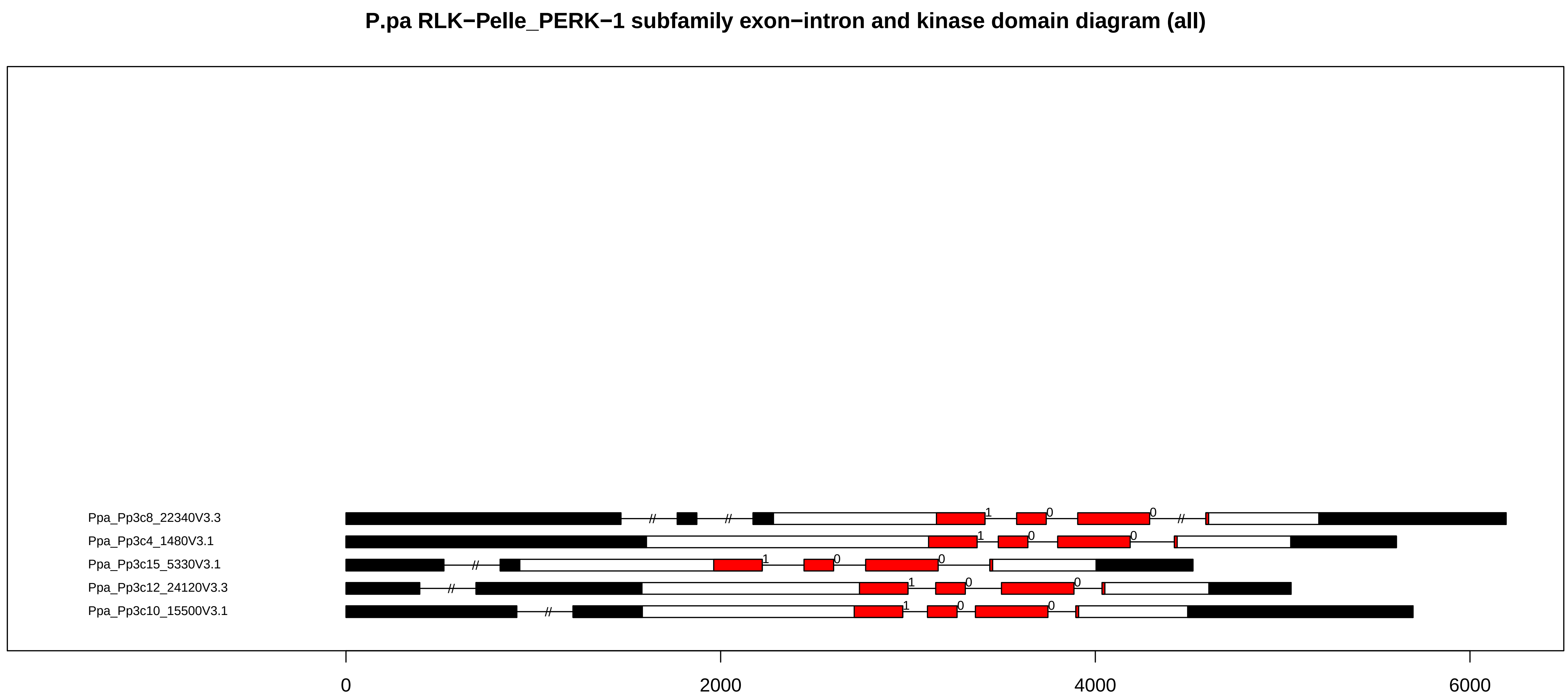
P.pa RLK−Pelle_PERK−1 subfamily exon−intron and kinase domain diagram (all)
Ppa_Pp3c8_22340V3.3
Ppa_Pp3c4_1480V3.1
Ppa_Pp3c15_5330V3.1
Ppa_Pp3c12_24120V3.3
Ppa_Pp3c10_15500V3.1
0
2000
4000
6000

P.pa RLK−Pelle_PERK−2 subfamily exon−intron and kinase domain diagram (all)

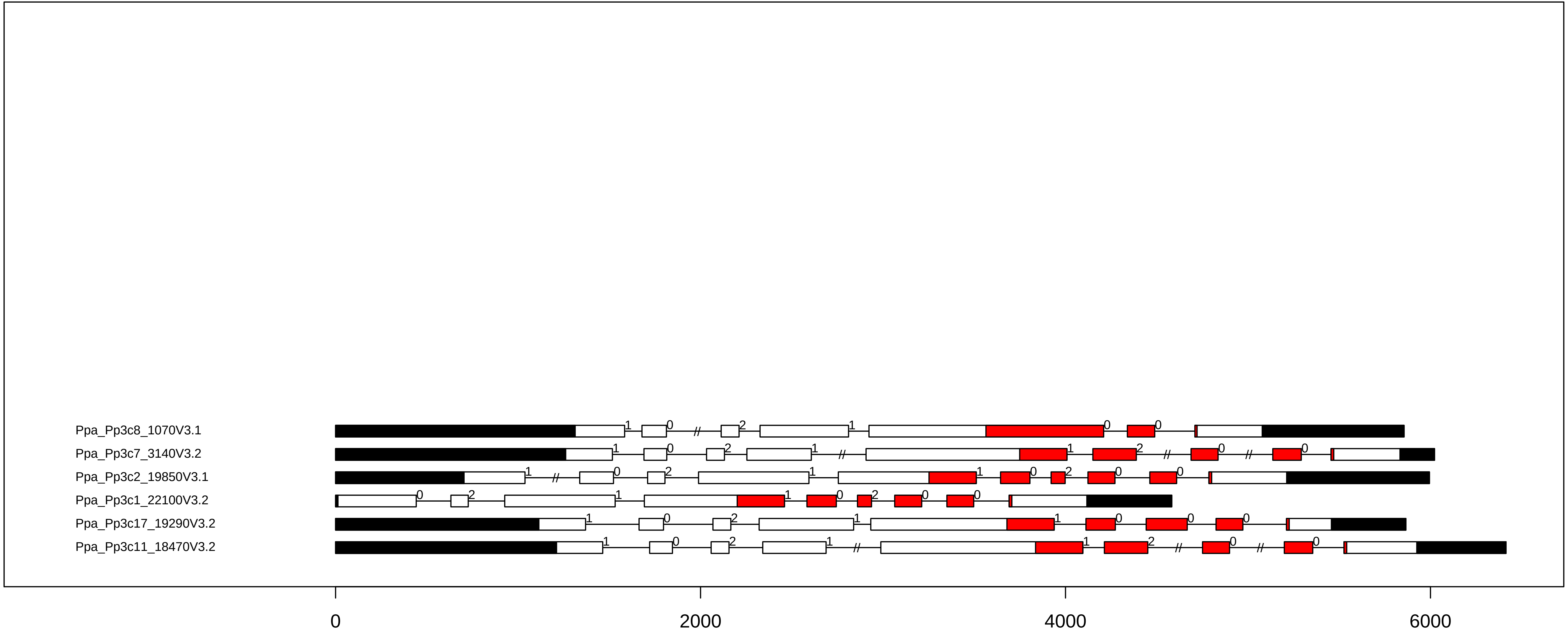

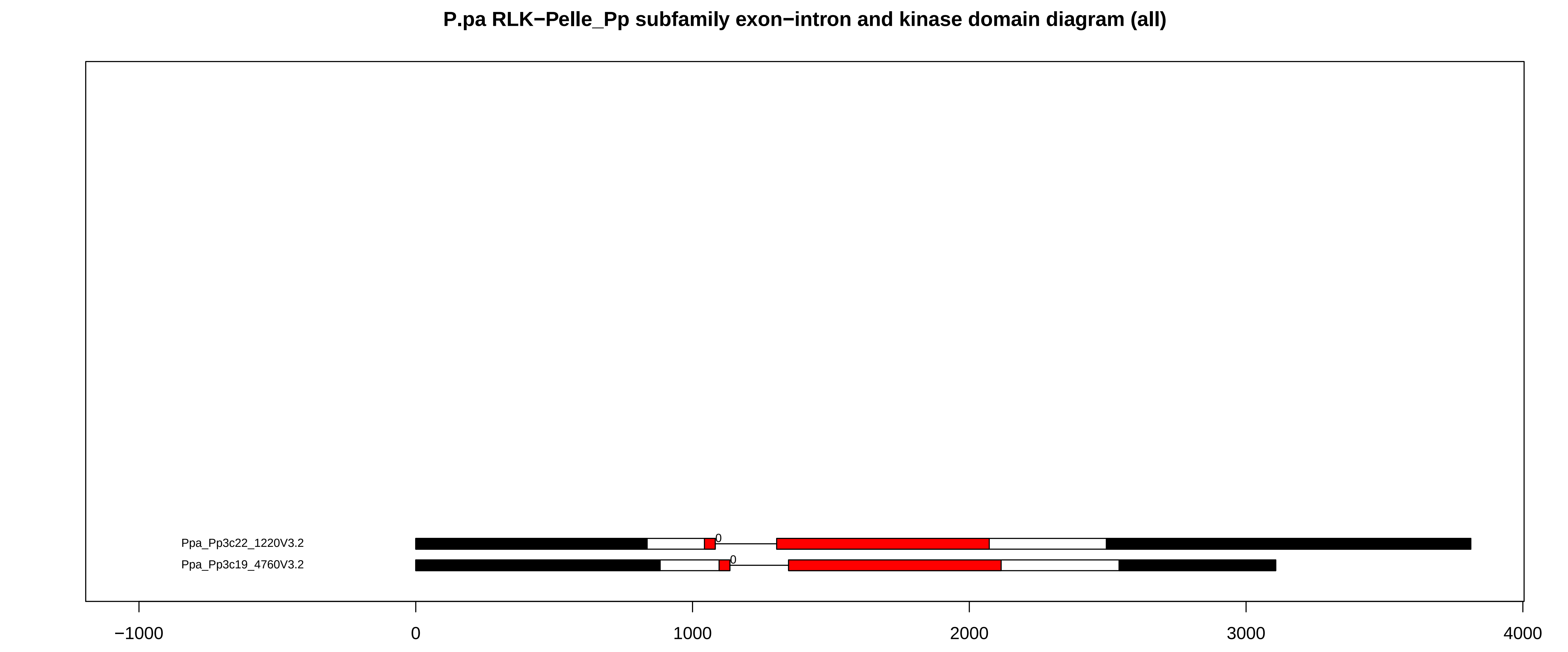

P.pa RLK−Pelle_Pp subfamily exon−intron and kinase domain diagram (all)
Ppa_Pp3c22_1220V3.2
Ppa_Pp3c19_4760V3.2
0
0
−1000
0
1000
2000
3000
4000

P.pa RLK−Pelle_RKF3 subfamily exon−intron and kinase domain diagram (all)

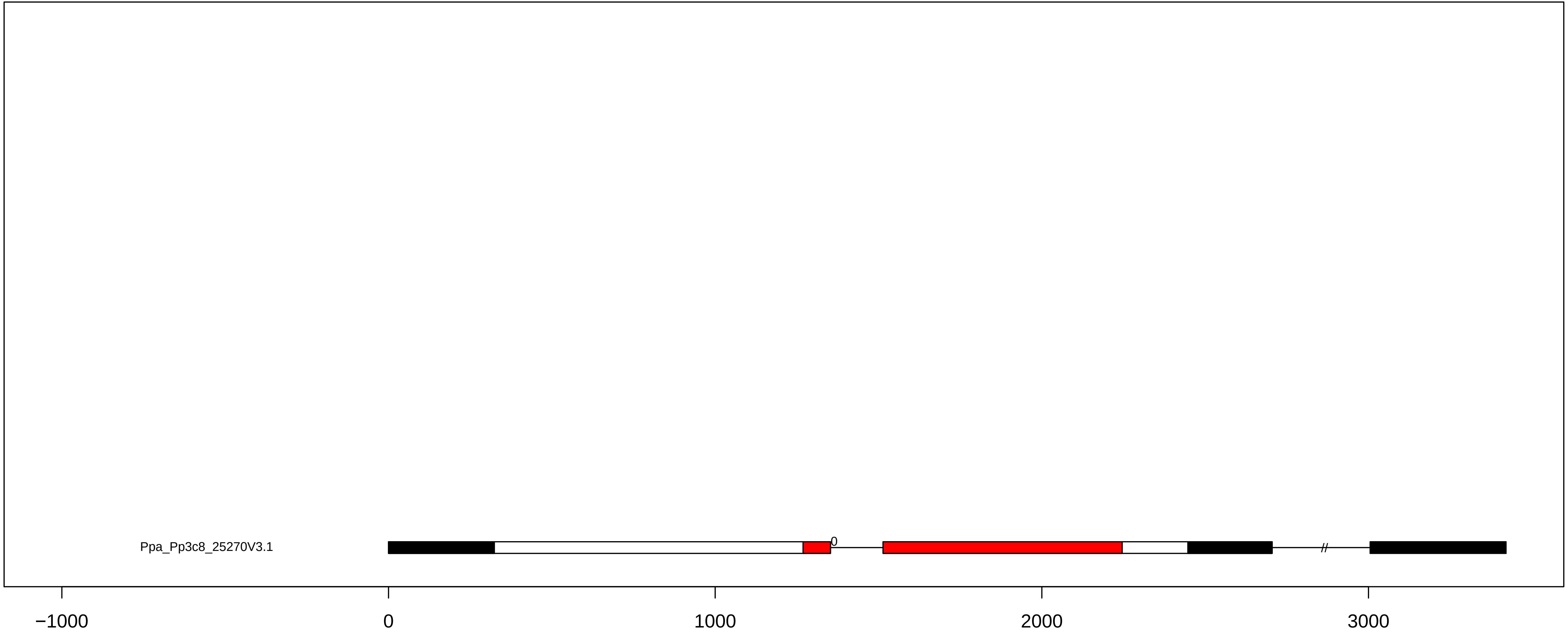

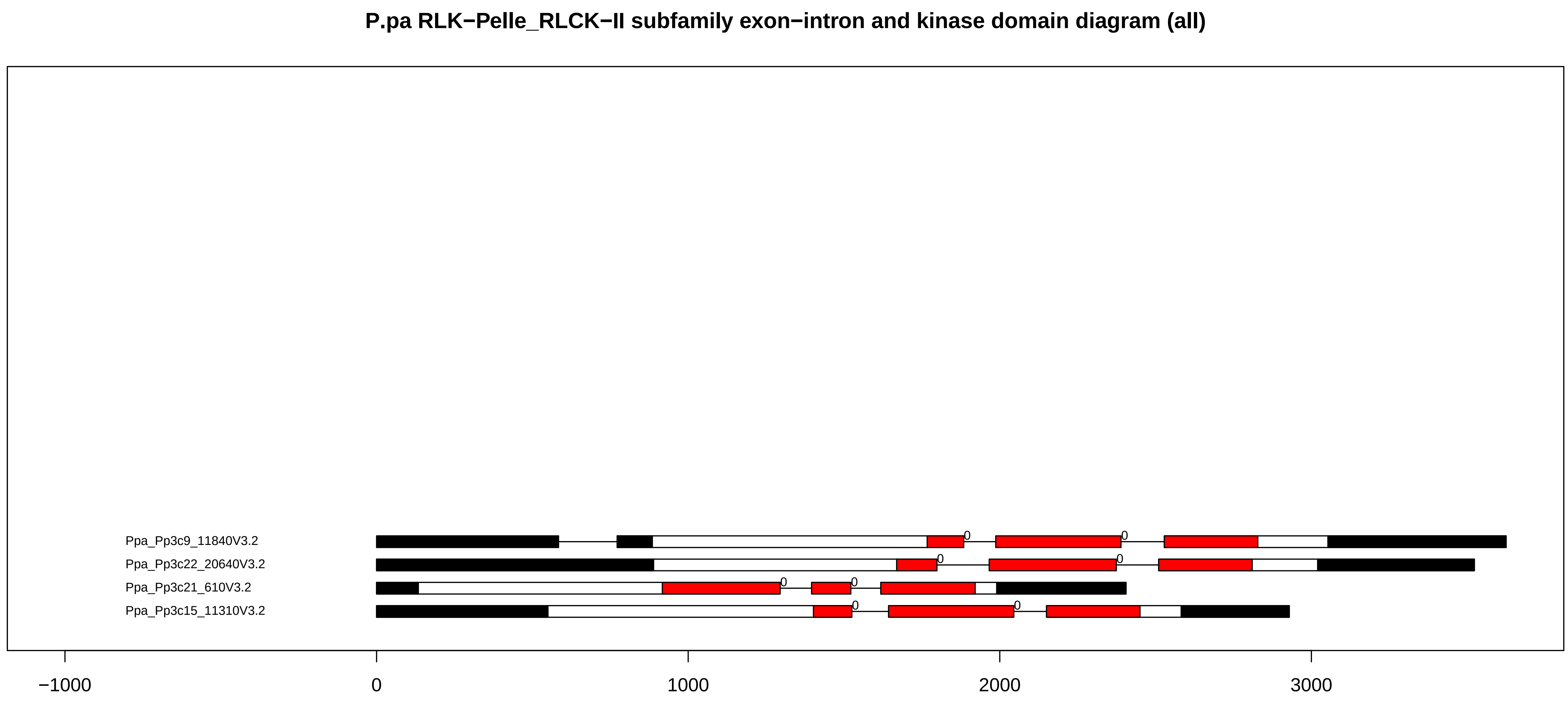

P.pa RLK−Pelle_RLCK−II subfamily exon−intron and kinase domain diagram (all)
Ppa_Pp3c9_11840V3.2
Ppa_Pp3c22_20640V3.2
Ppa_Pp3c21_610V3.2
Ppa_Pp3c15_11310V3.2
0
0
0
0
0
0
0
0
−1000
0
1000
2000
3000

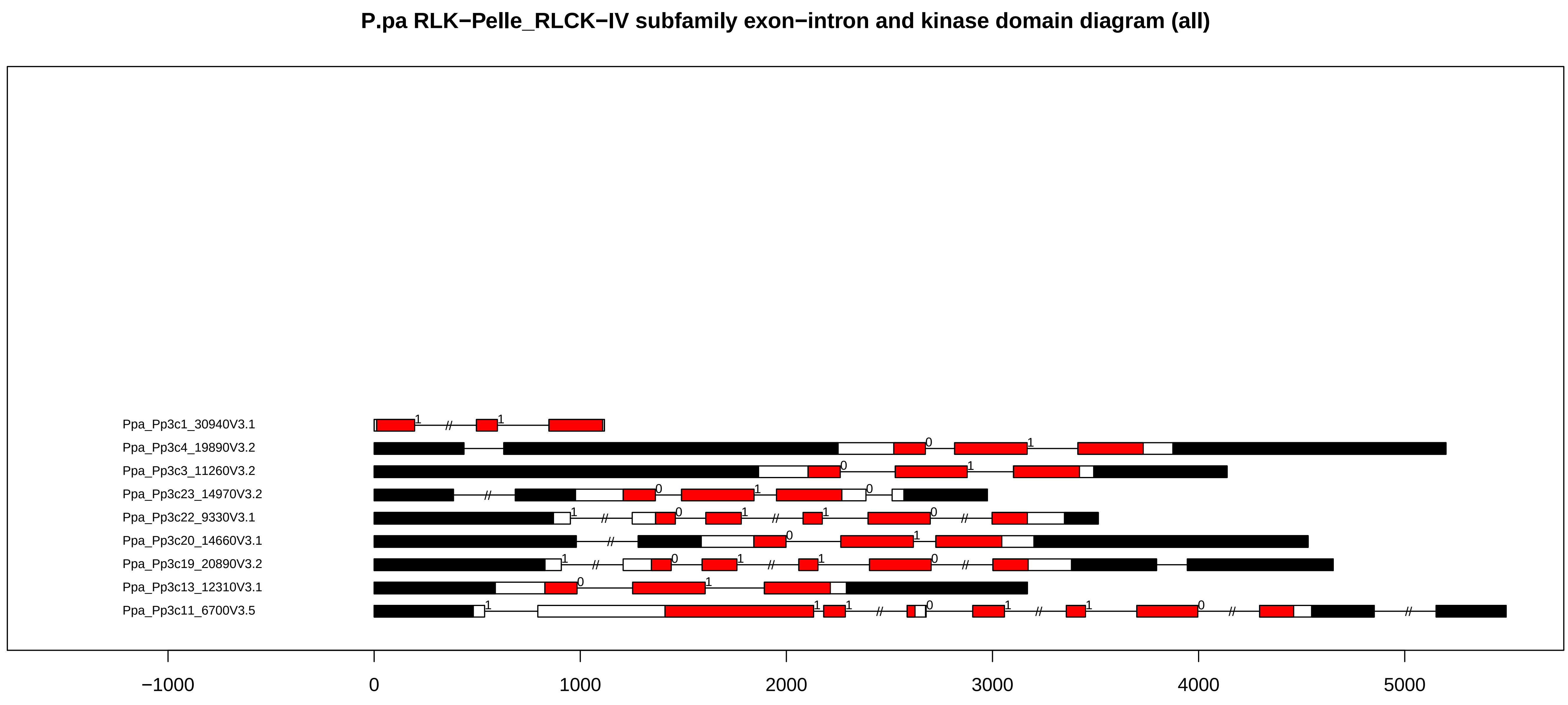
P.pa RLK−Pelle_RLCK−IV subfamily exon−intron and kinase domain diagram (all)
Ppa_Pp3c1_30940V3.1
Ppa_Pp3c4_19890V3.2
Ppa_Pp3c3_11260V3.2
Ppa_Pp3c23_14970V3.2
Ppa_Pp3c22_9330V3.1
Ppa_Pp3c20_14660V3.1
Ppa_Pp3c19_20890V3.2
Ppa_Pp3c13_12310V3.1
Ppa_Pp3c11_6700V3.5
−1000
0
1000
2000
3000
4000
5000

P.pa RLK−Pelle_RLCK−Pp subfamily exon−intron and kinase domain diagram (all)

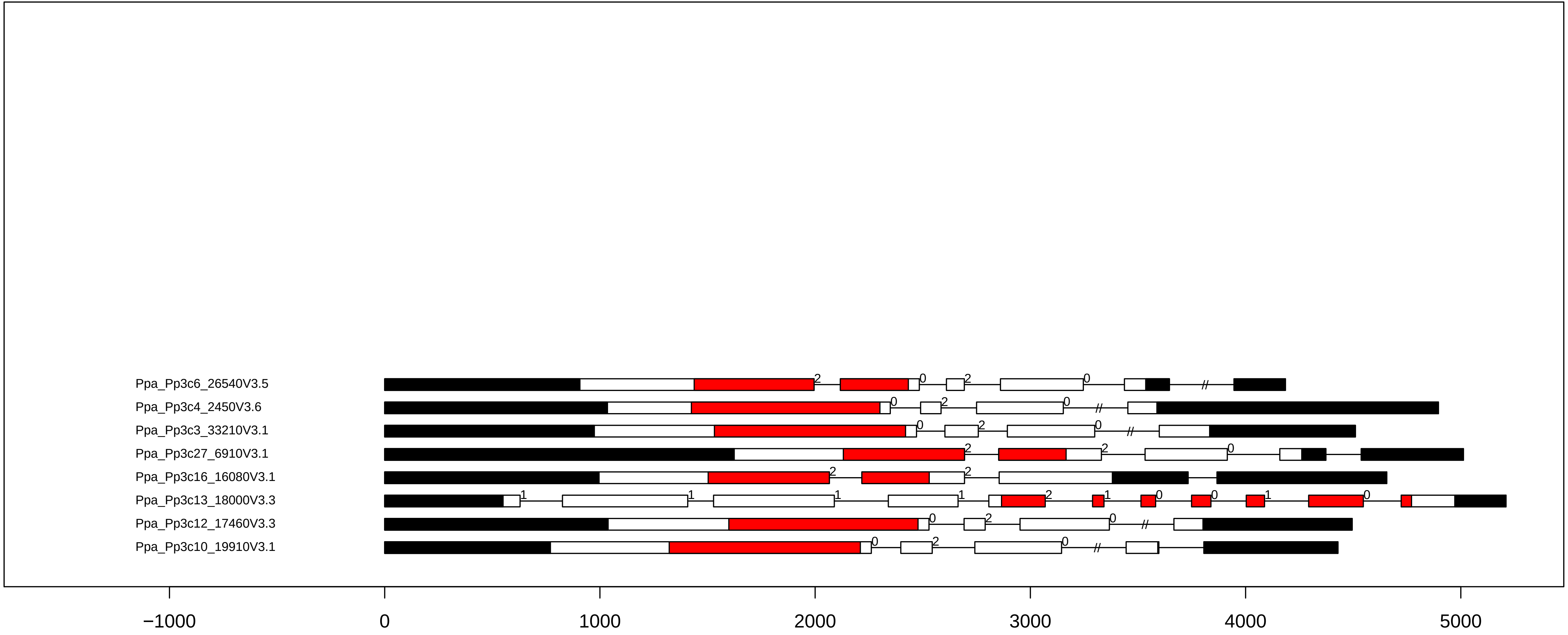

P.pa RLK−Pelle_RLCK−VIIa−1 subfamily exon−intron and kinase domain diagram (all)

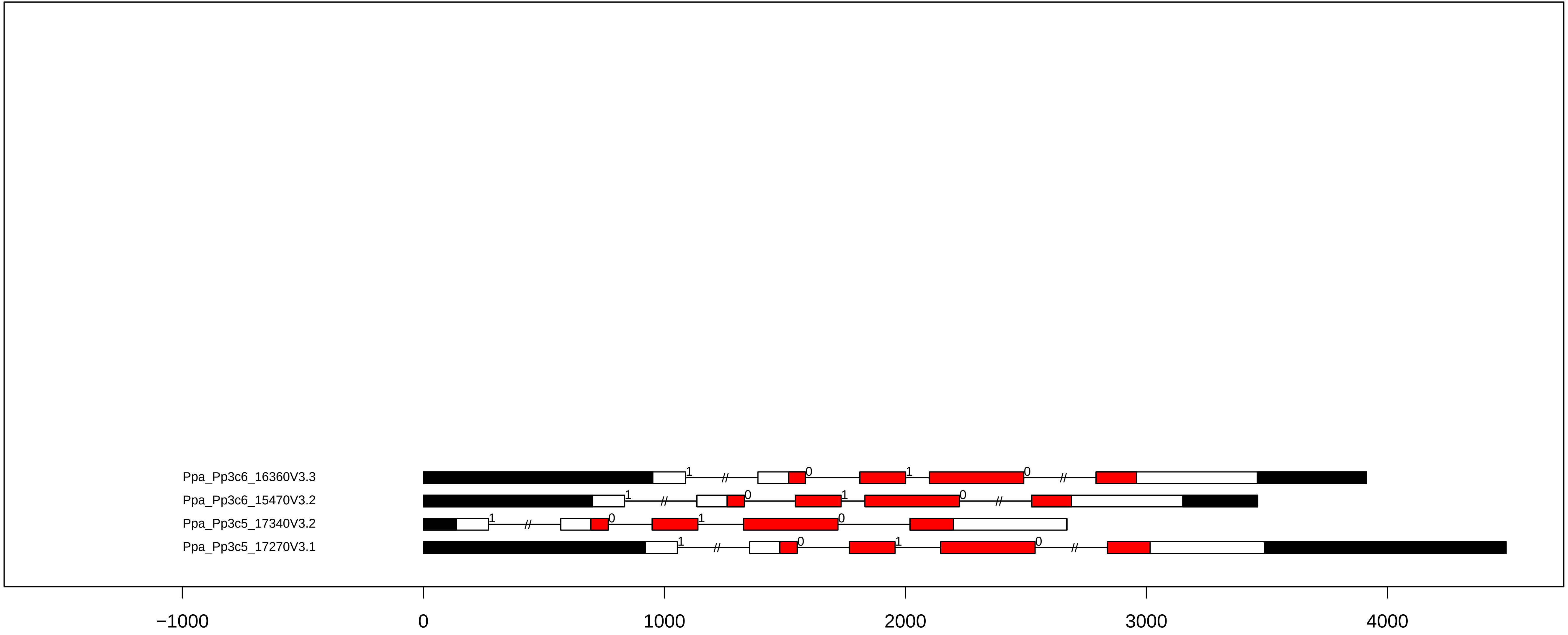

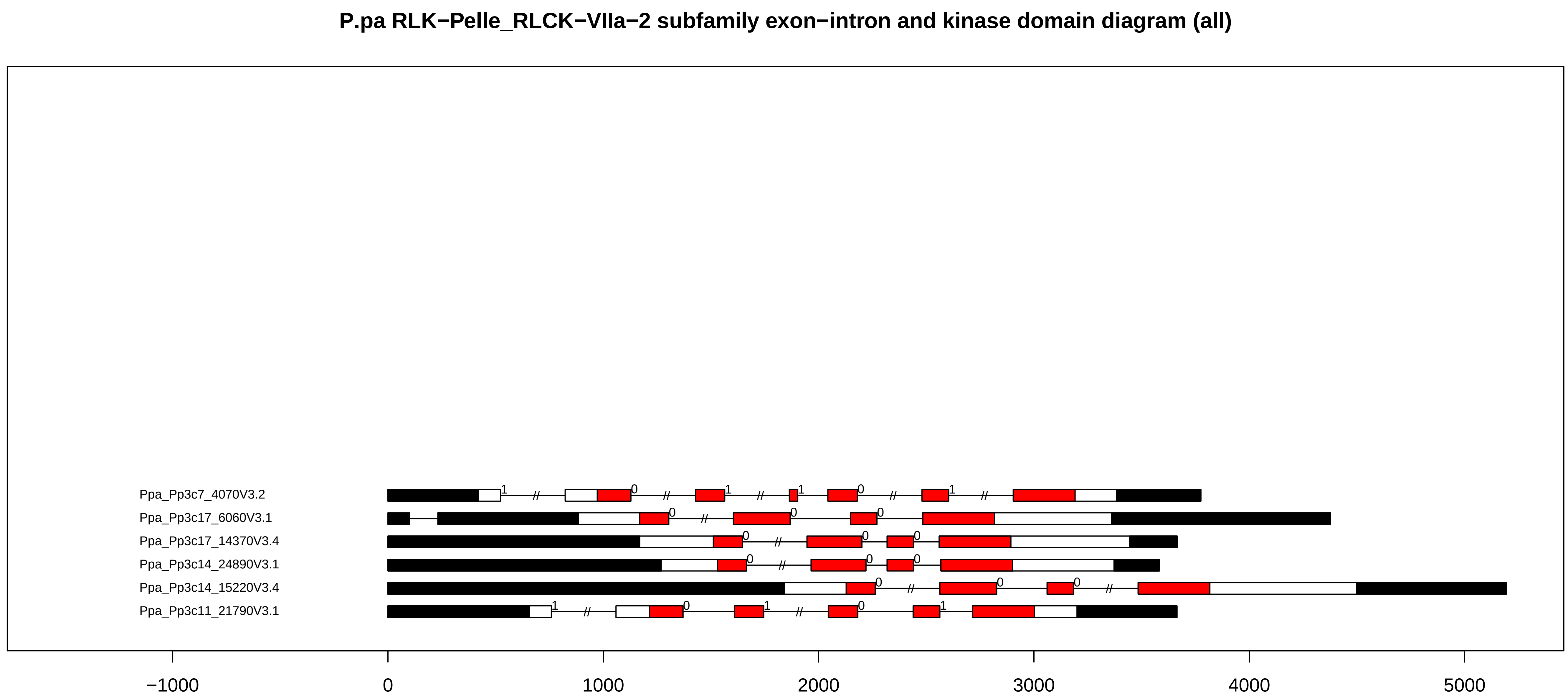
P.pa RLK−Pelle_RLCK−VIIa−2 subfamily exon−intron and kinase domain diagram (all)
Ppa_Pp3c7_4070V3.2
Ppa_Pp3c17_6060V3.1
Ppa_Pp3c17_14370V3.4
Ppa_Pp3c14_24890V3.1
Ppa_Pp3c14_15220V3.4
Ppa_Pp3c11_21790V3.1
−1000
0
1000
2000
3000
4000
5000

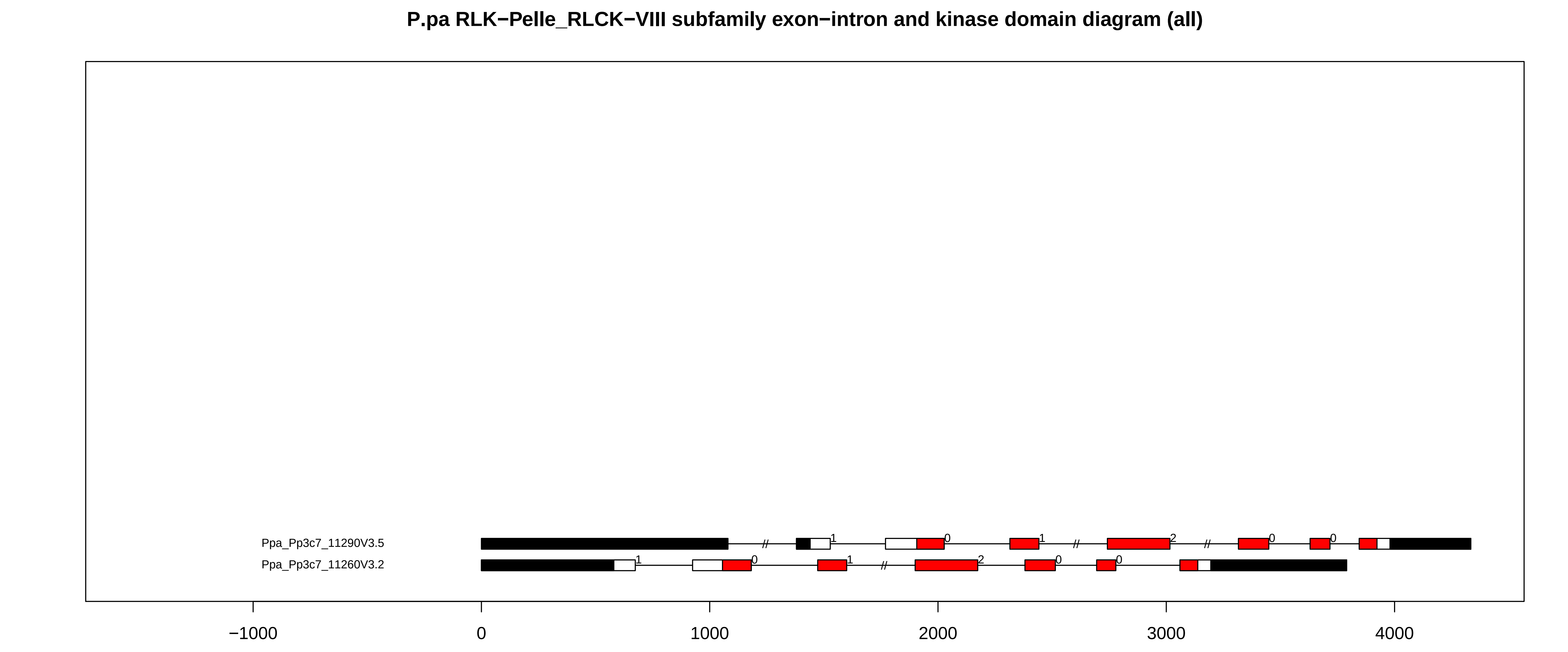
P.pa RLK−Pelle_RLCK−VIII subfamily exon−intron and kinase domain diagram (all)
Ppa_Pp3c7_11290V3.5
Ppa_Pp3c7_11260V3.2
1
0
1
2
0
0
1
0
1
2
0
0
−1000
0
1000
2000
3000
4000

P.pa RLK−Pelle_RLCK−VI subfamily exon−intron and kinase domain diagram (all)

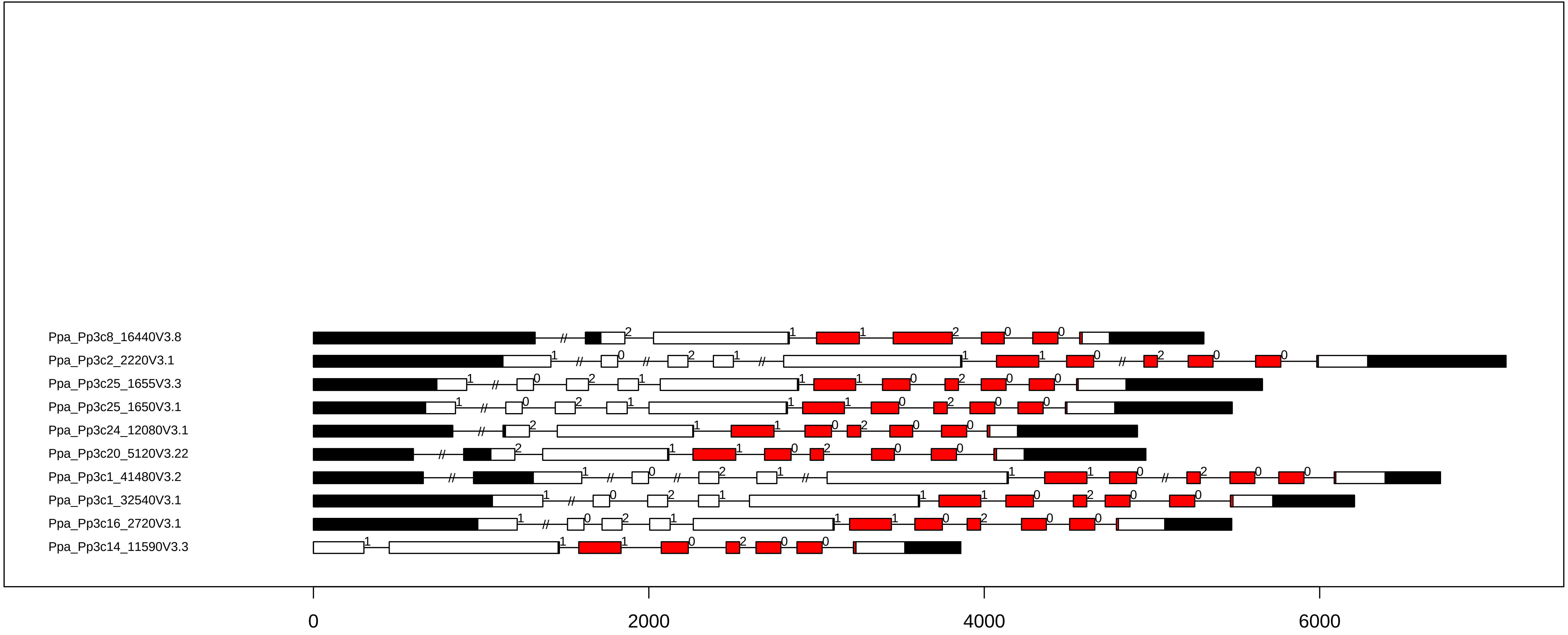

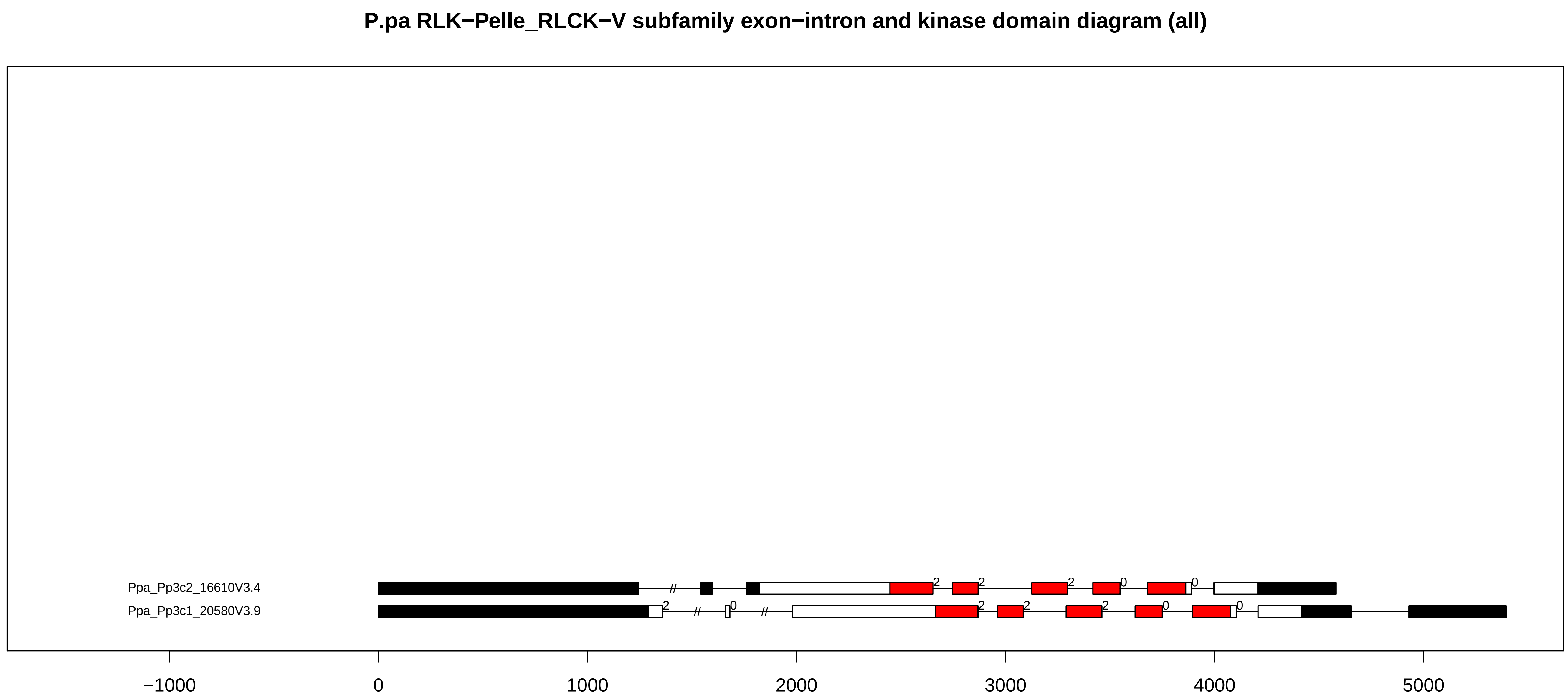
P.pa RLK−Pelle_RLCK−V subfamily exon−intron and kinase domain diagram (all)
Ppa_Pp3c2_16610V3.4
Ppa_Pp3c1_20580V3.9
2
2
2
0
0
2
0
2
2
2
0
0
−1000
0
1000
2000
3000
4000
5000

P.pa RLK−Pelle_RLCK−XII−1 subfamily exon−intron and kinase domain diagram (all)

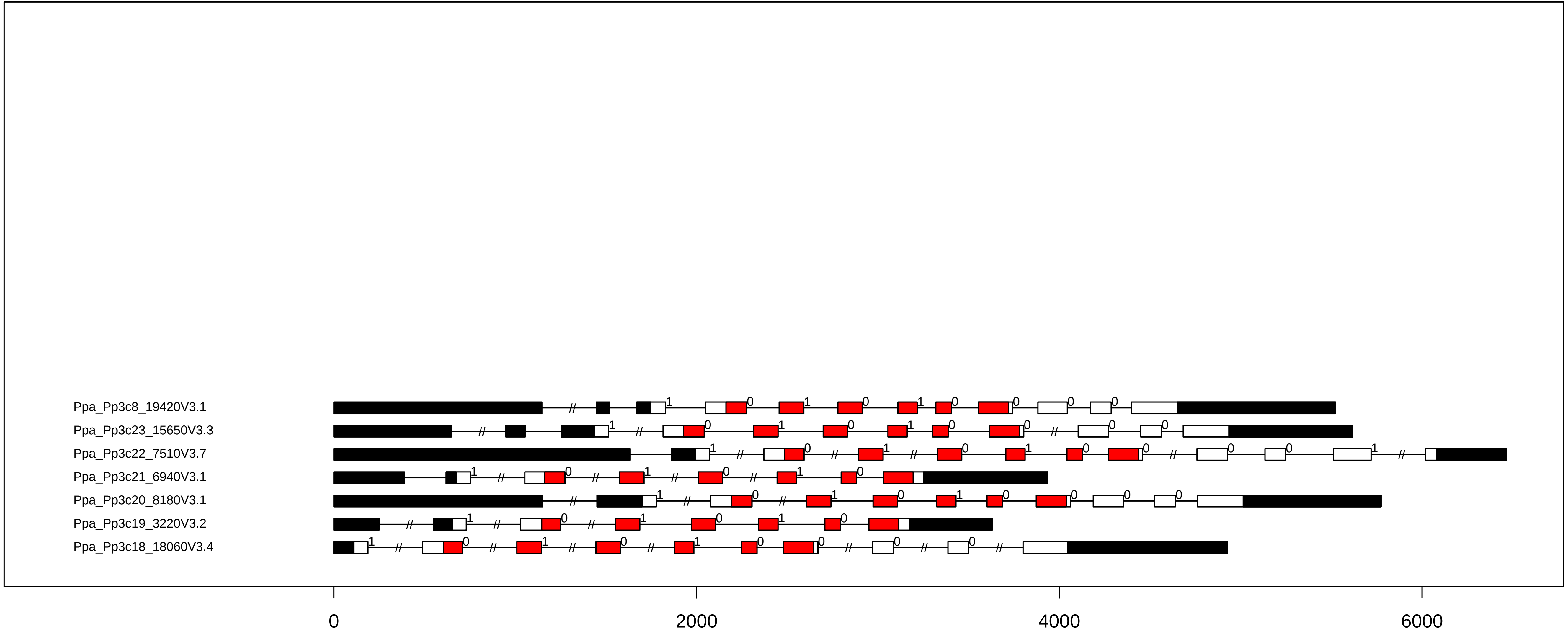

P.pa RLK−Pelle_RLCK−X subfamily exon−intron and kinase domain diagram (all)

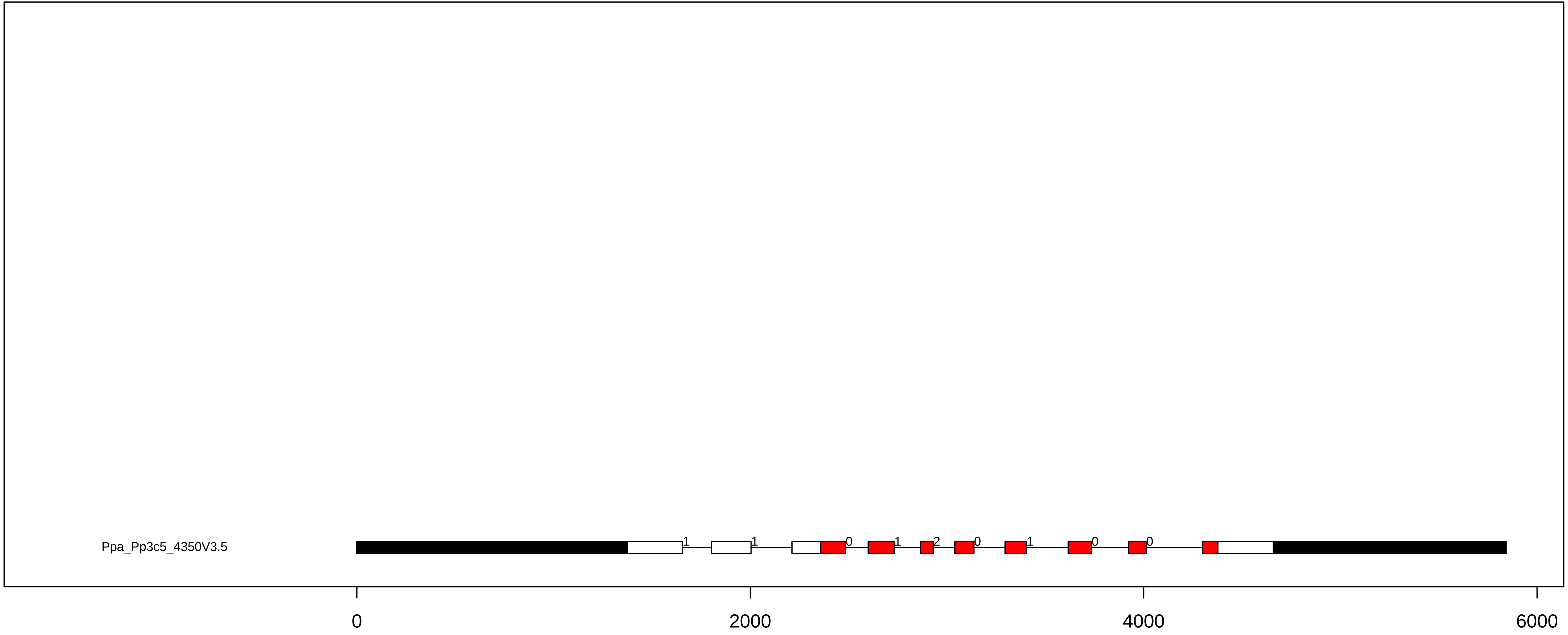

P.pa RLK−Pelle_RLCK−XV subfamily exon−intron and kinase domain diagram (all)

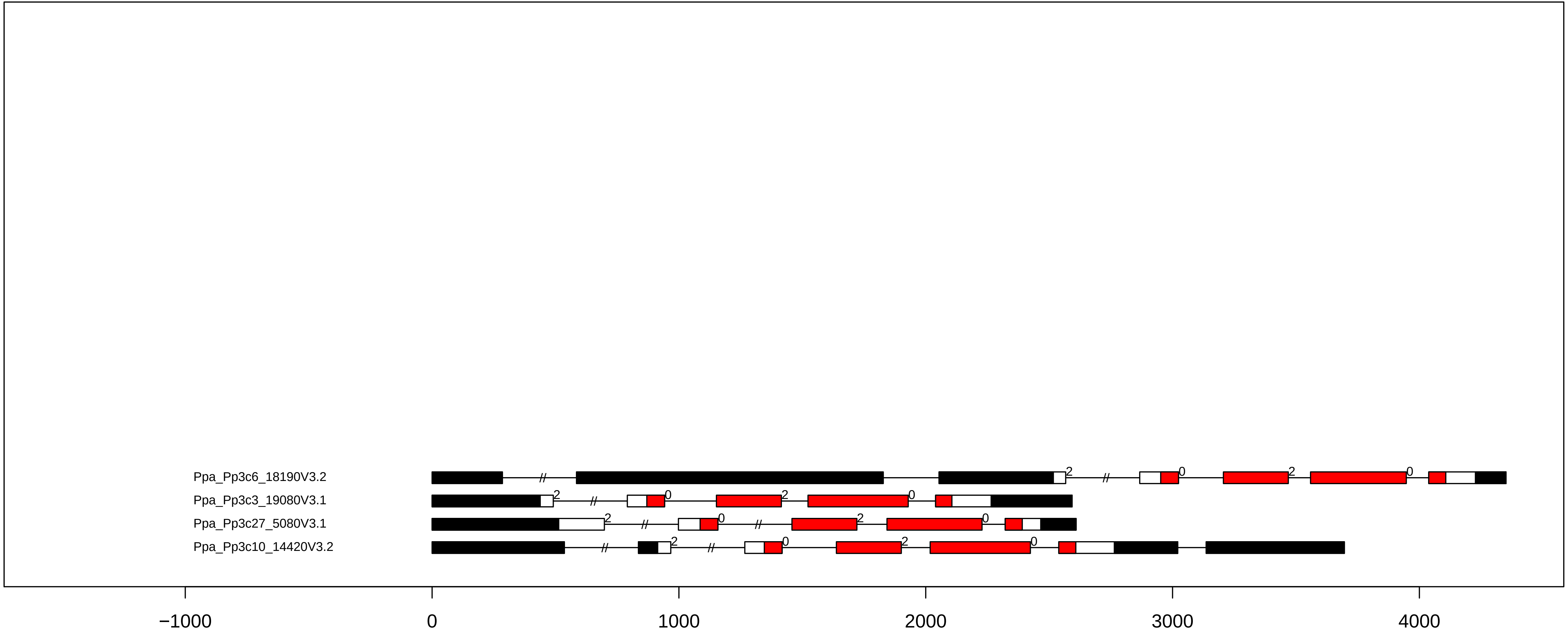

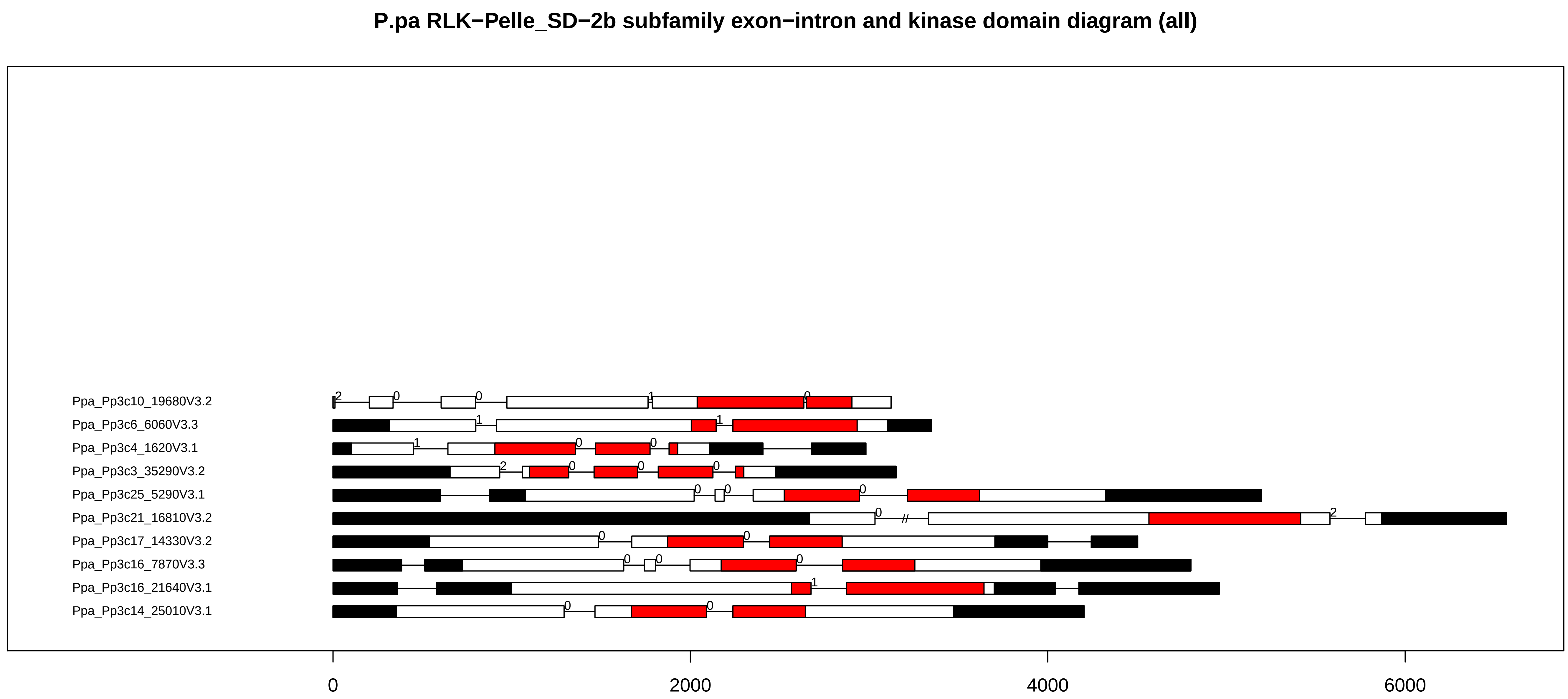
P.pa RLK−Pelle_SD−2b subfamily exon−intron and kinase domain diagram (all)
Ppa_Pp3c10_19680V3.2
Ppa_Pp3c6_6060V3.3
Ppa_Pp3c4_1620V3.1
Ppa_Pp3c3_35290V3.2
Ppa_Pp3c25_5290V3.1
Ppa_Pp3c21_16810V3.2
Ppa_Pp3c17_14330V3.2
Ppa_Pp3c16_7870V3.3
Ppa_Pp3c16_21640V3.1
Ppa_Pp3c14_25010V3.1
0
2000
4000
6000

P.pa RLK−Pelle_Singleton subfamily exon−intron and kinase domain diagram (all)

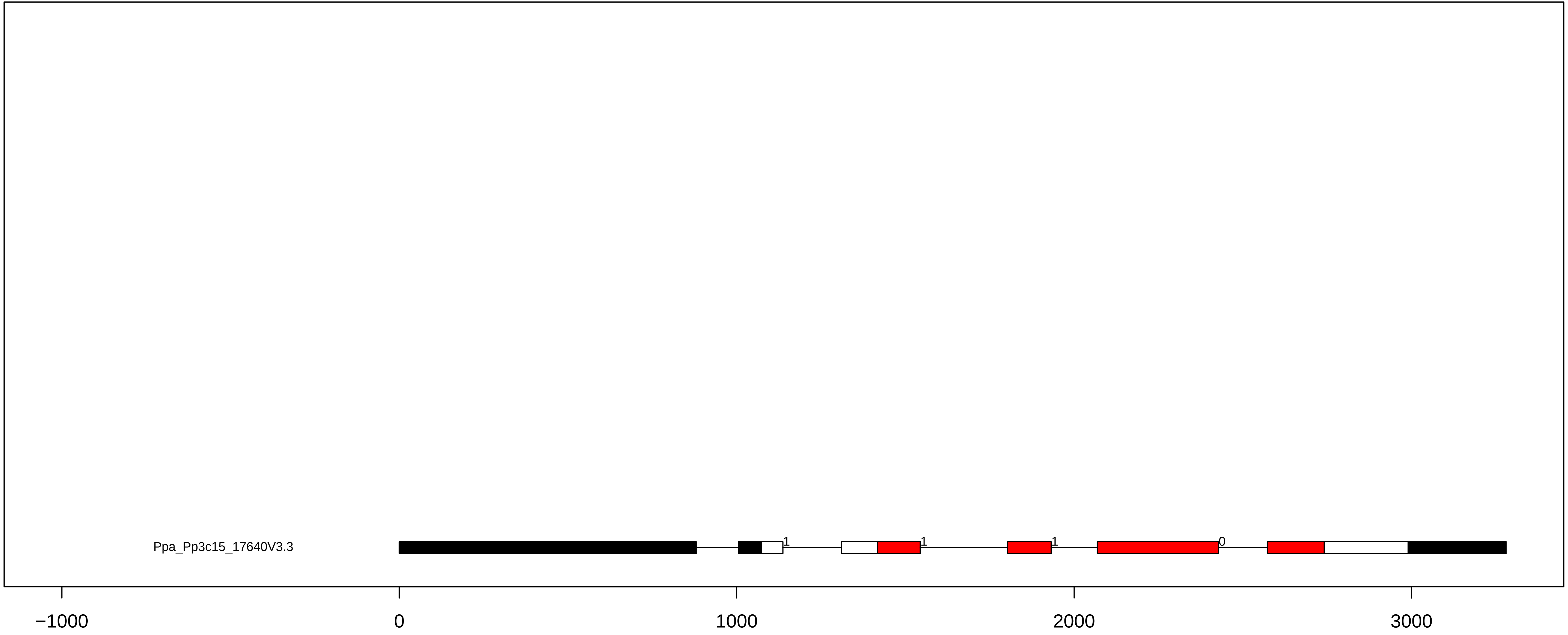

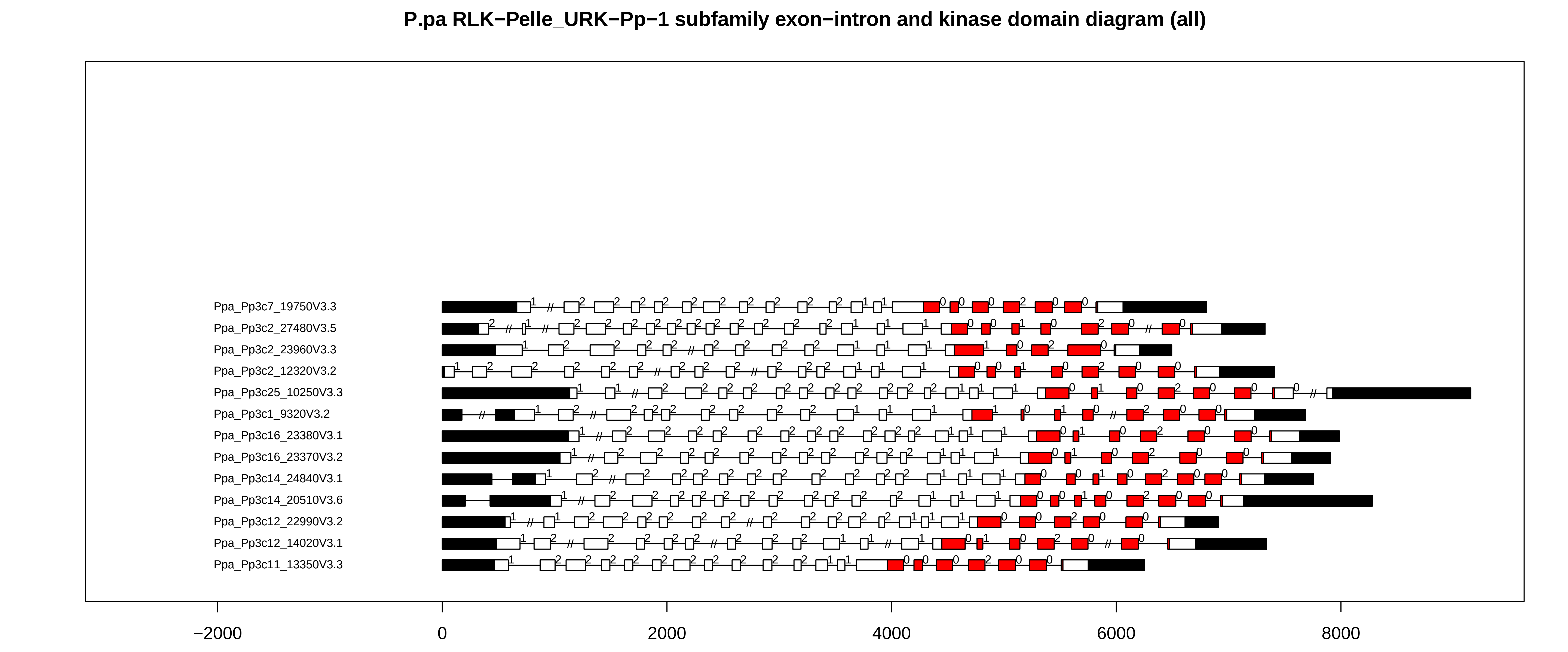
P.pa RLK−Pelle_URK−Pp−1 subfamily exon−intron and kinase domain diagram (all)
Ppa_Pp3c7_19750V3.3
Ppa_Pp3c2_27480V3.5
Ppa_Pp3c2_23960V3.3
Ppa_Pp3c2_12320V3.2
Ppa_Pp3c25_10250V3.3
Ppa_Pp3c1_9320V3.2
Ppa_Pp3c16_23380V3.1
Ppa_Pp3c16_23370V3.2
Ppa_Pp3c14_24840V3.1
Ppa_Pp3c14_20510V3.6
Ppa_Pp3c12_22990V3.2
Ppa_Pp3c12_14020V3.1
Ppa_Pp3c11_13350V3.3
−2000
0
2000
4000
6000
8000

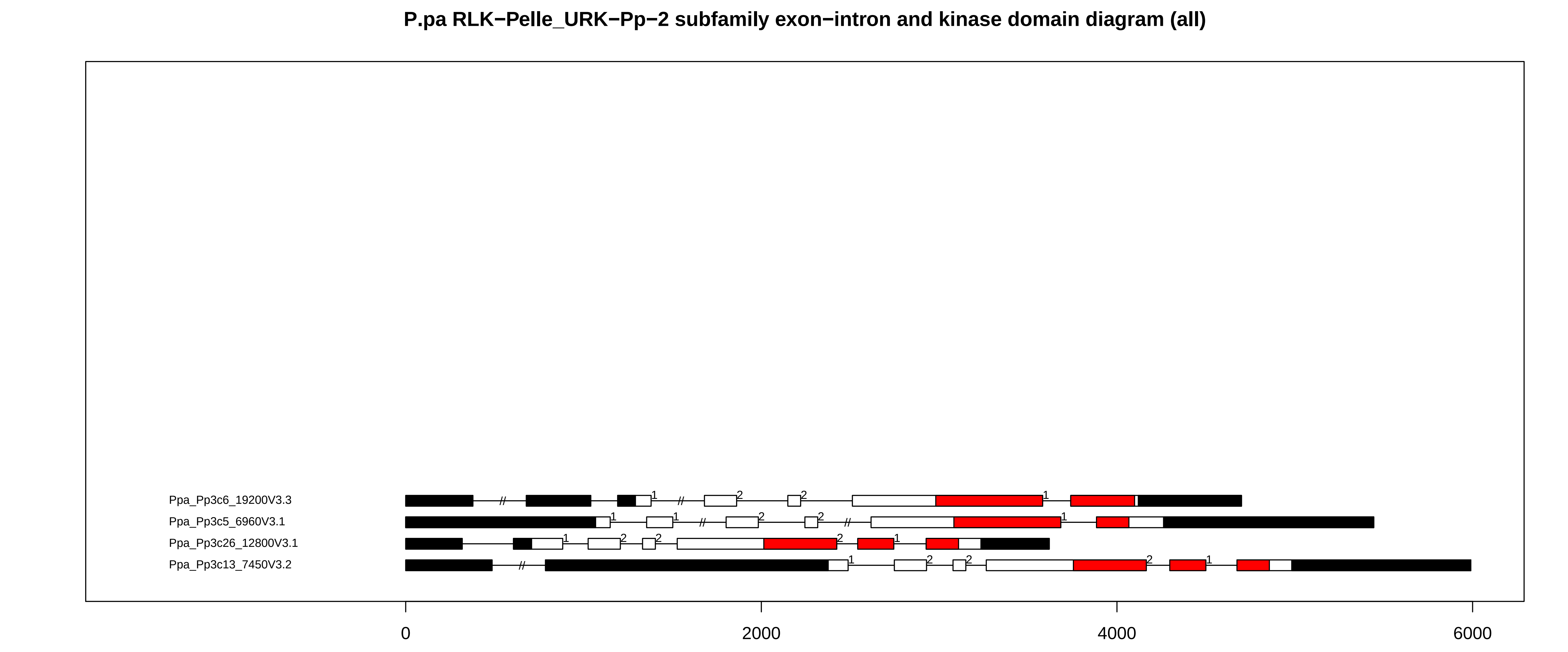
P.pa RLK−Pelle_URK−Pp−2 subfamily exon−intron and kinase domain diagram (all)
Ppa_Pp3c6_19200V3.3
Ppa_Pp3c5_6960V3.1
Ppa_Pp3c26_12800V3.1
Ppa_Pp3c13_7450V3.2
0
2000
4000
6000

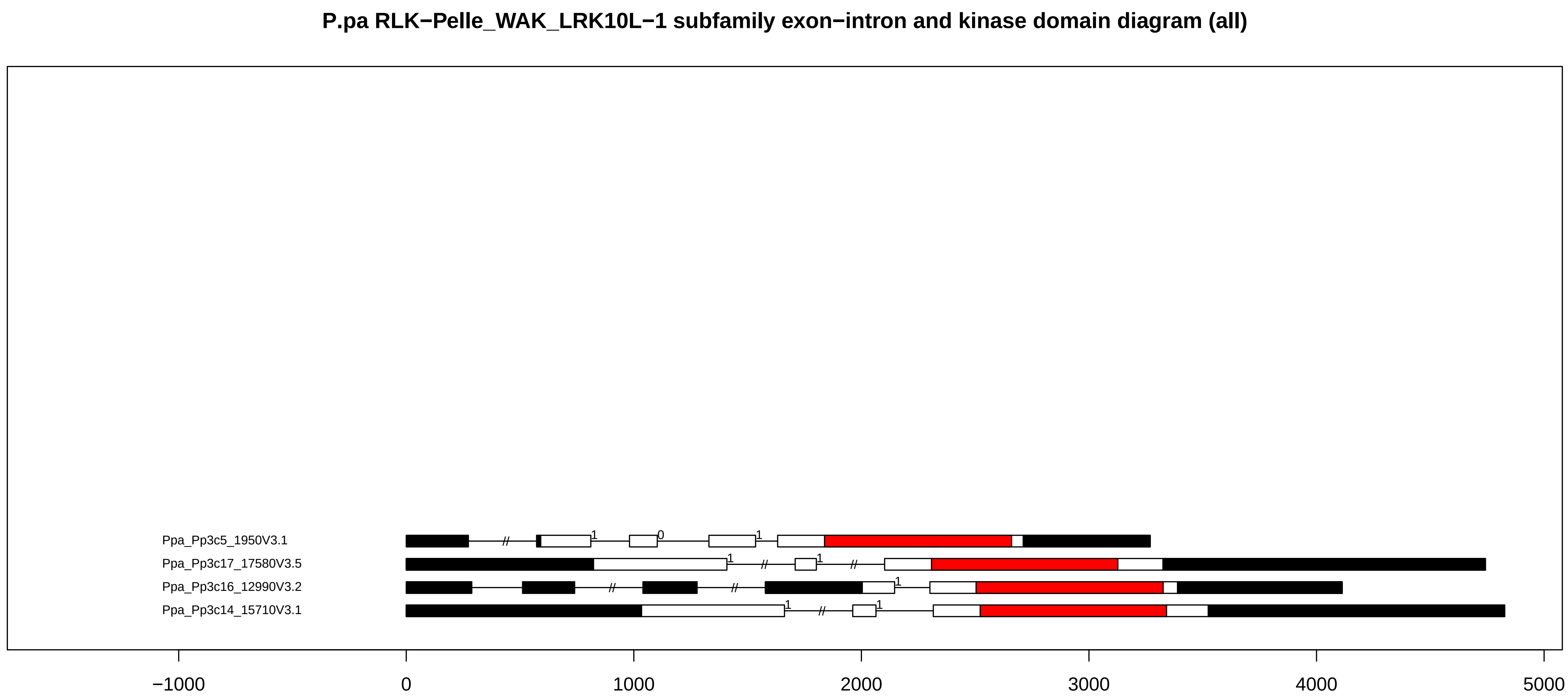
P.pa RLK−Pelle_WAK_LRK10L−1 subfamily exon−intron and kinase domain diagram (all)
Ppa_Pp3c5_1950V3.1
Ppa_Pp3c17_17580V3.5
Ppa_Pp3c16_12990V3.2
Ppa_Pp3c14_15710V3.1
−1000
0
1000
2000
3000
4000
5000

C.re RLK−Pelle_C−LEC subfamily exon−intron and kinase domain diagram (all)

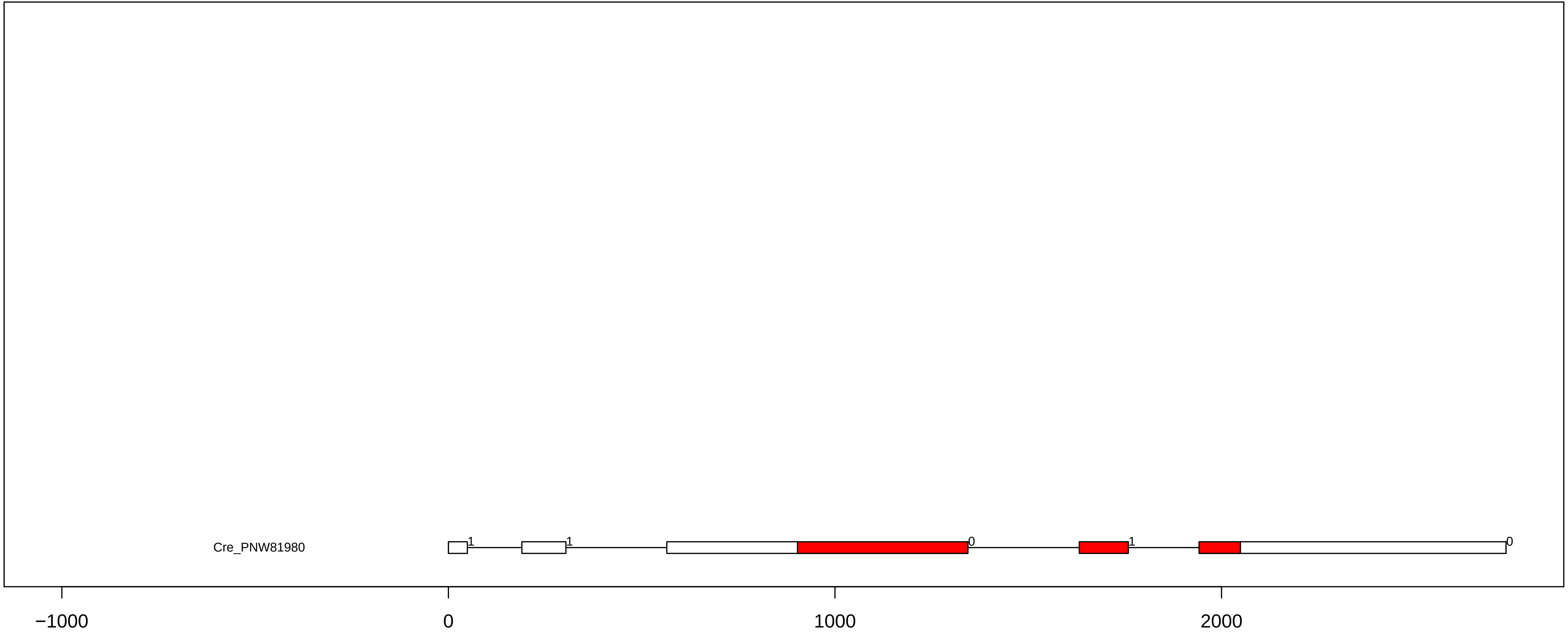

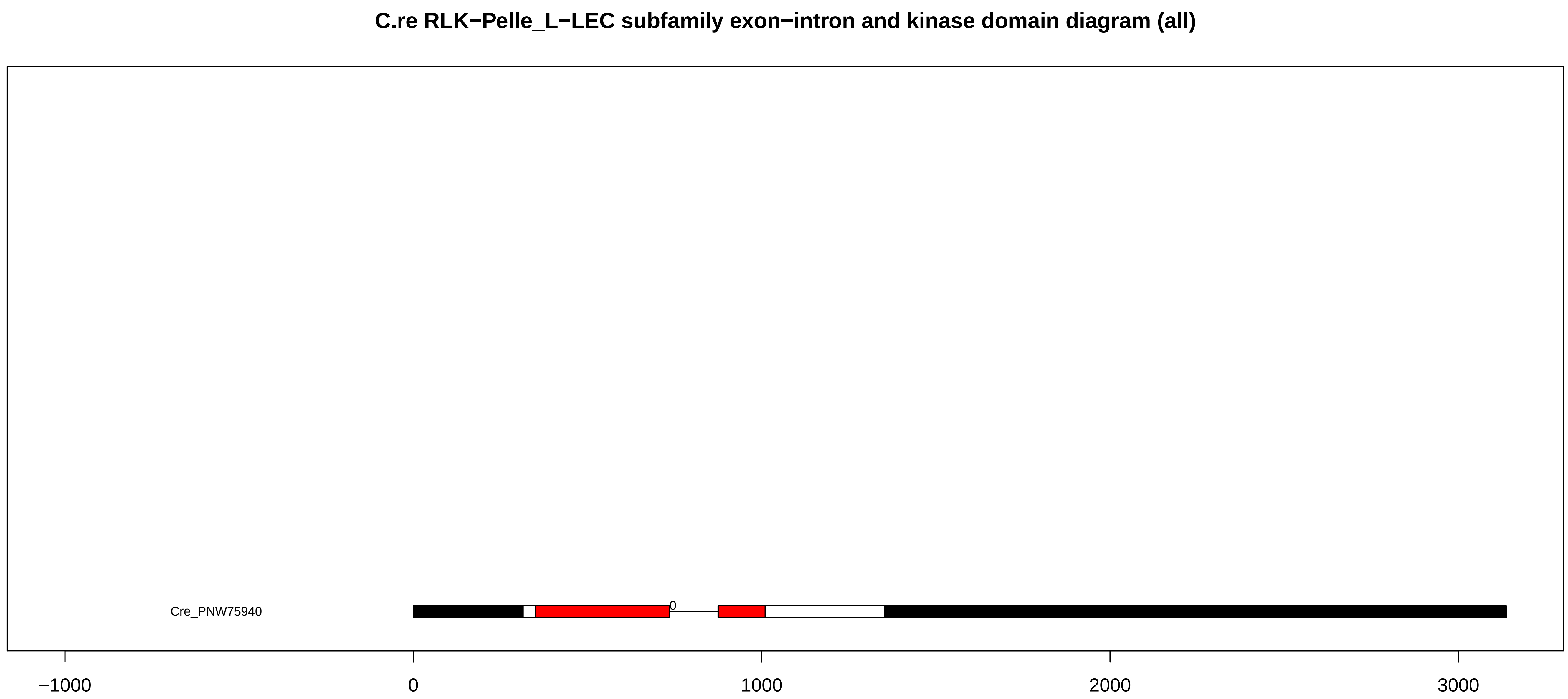
C.re RLK−Pelle_L−LEC subfamily exon−intron and kinase domain diagram (all)
Cre_PNW75940
0
−1000
0
1000
2000
3000

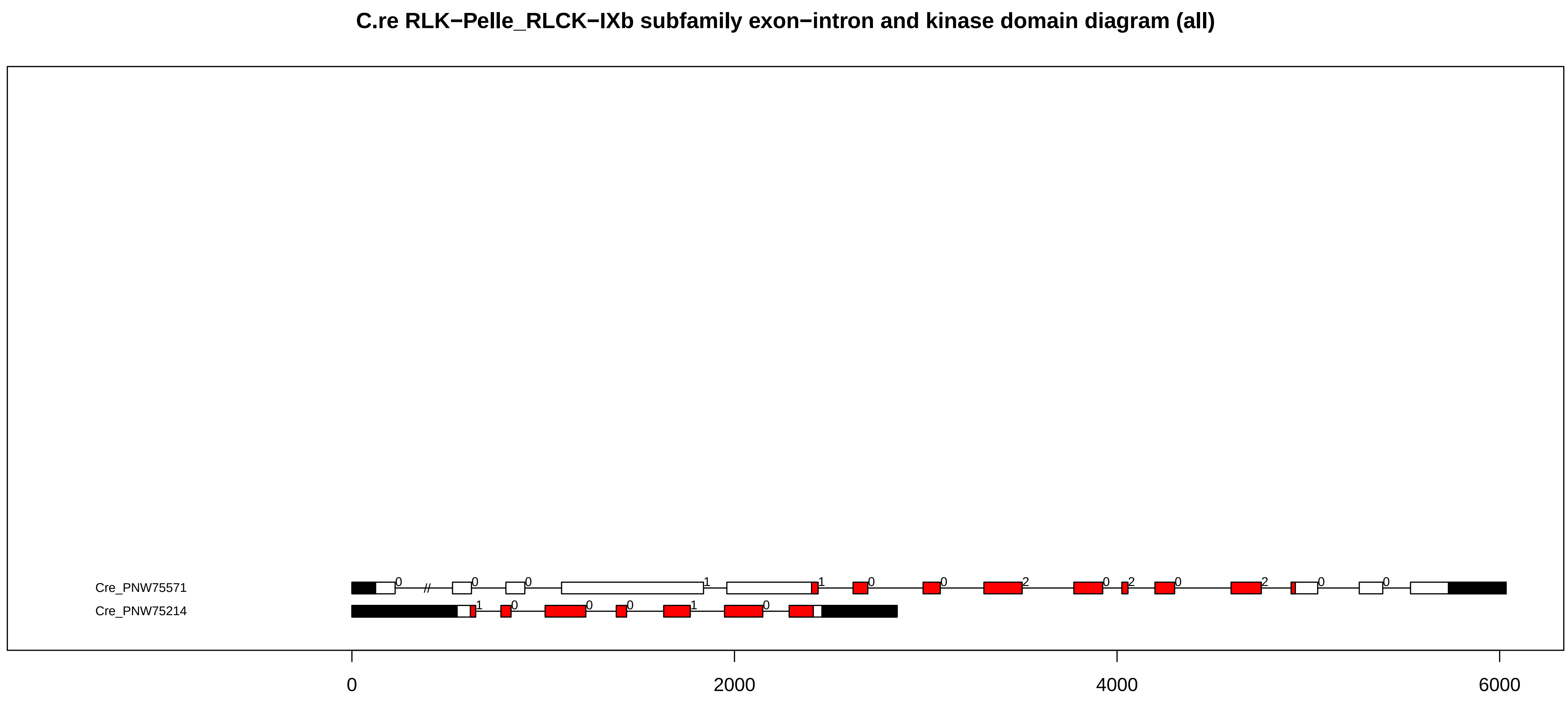
C.re RLK−Pelle_RLCK−IXb subfamily exon−intron and kinase domain diagram (all)
Cre_PNW75571
Cre_PNW75214
0
2000
4000
6000
