# Supplementary material for: Phylogeny of the plant receptor-like kinase (RLK) gene family and expression analysis of wheat RLK genes in response to biotic and abiotic stresses
Source: BMC Genomics. 2023 May 1;24:224. doi: 10.1186/s12864-023-09303-7 (PMC10152718; doi:10.1186/s12864-023-09303-7)

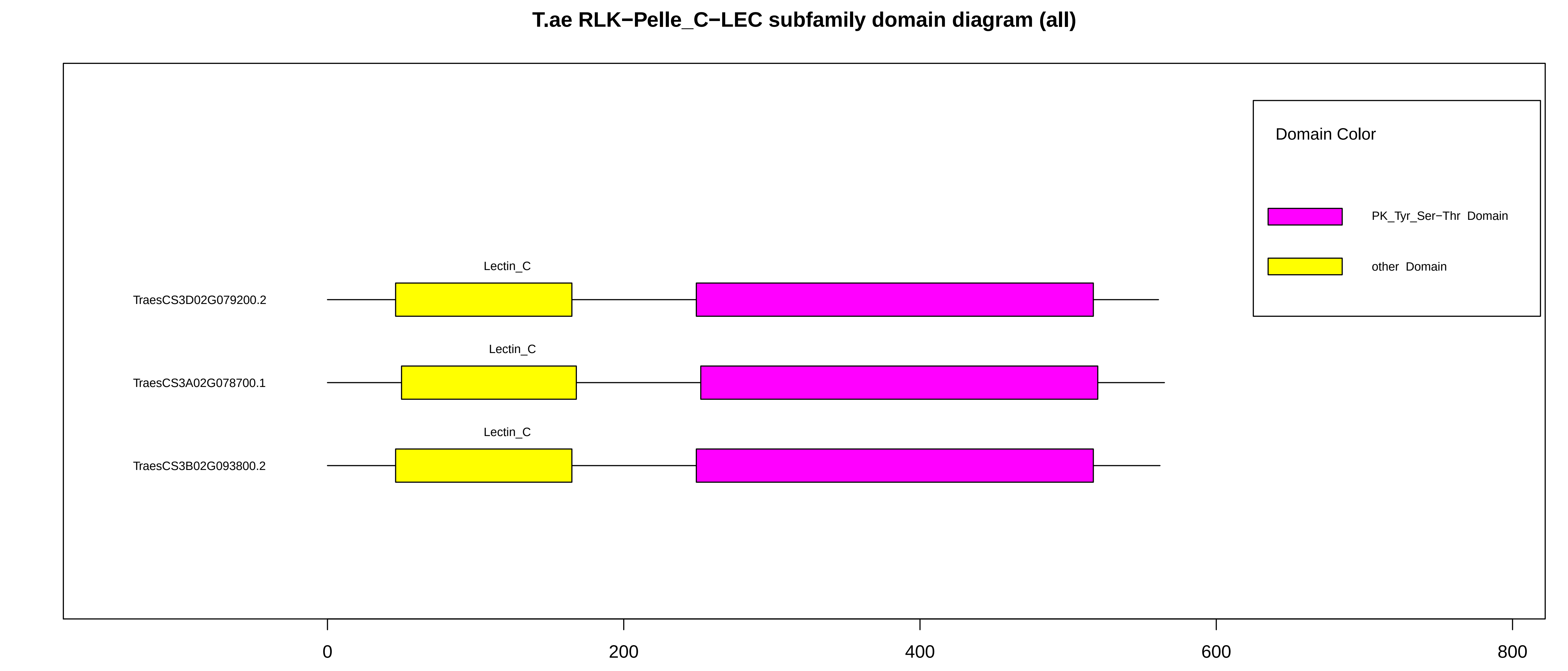
T.ae RLK−Pelle_C−LEC subfamily domain diagram (all)
Domain Color
PK_Tyr_Ser−Thr Domain
other Domain
Lectin_C
TraesCS3D02G079200.2
Lectin_C
TraesCS3A02G078700.1
Lectin_C
TraesCS3B02G093800.2
0
200
400
600
800

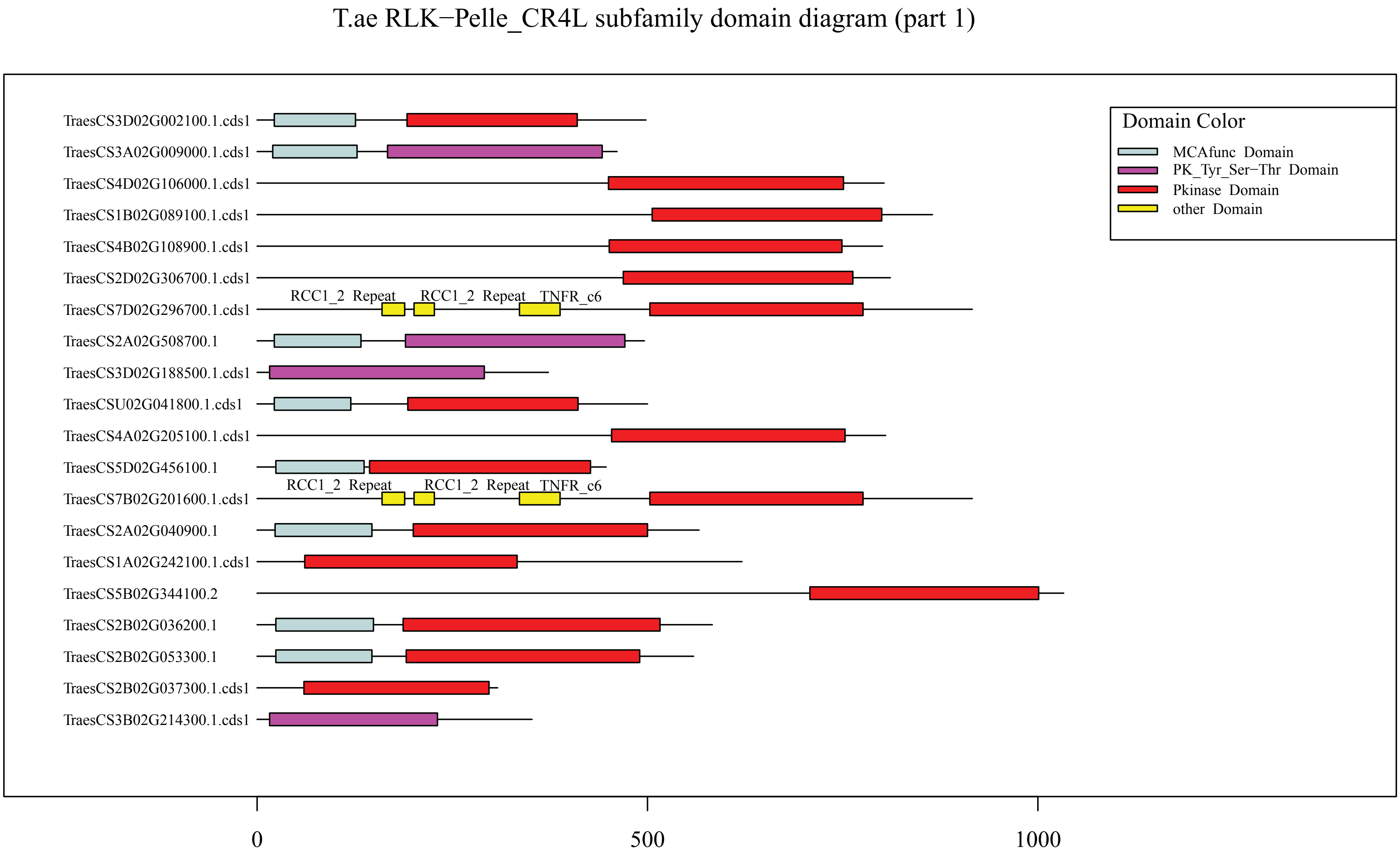

T.ae RLK−Pelle_CR4L subfamily domain diagram (part 1)
TraesCS3D02G002100.1.cds1
TraesCS3A02G009000.1.cds1
TraesCS4D02G106000.1.cds1
TraesCS1B02G089100.1.cds1
TraesCS4B02G108900.1.cds1
TraesCS2D02G306700.1.cds1
RCC1_2 Repeat
RCC1_2 Repeat
TNFR_c6
TraesCS7D02G296700.1.cds1
TraesCS2A02G508700.1
TraesCS3D02G188500.1.cds1
TraesCSU02G041800.1.cds1
TraesCS4A02G205100.1.cds1
TraesCS5D02G456100.1
RCC1_2 Repeat
RCC1_2 Repeat
TNFR_c6
TraesCS7B02G201600.1.cds1
TraesCS2A02G040900.1
TraesCS1A02G242100.1.cds1
TraesCS5B02G344100.2
TraesCS2B02G036200.1
TraesCS2B02G053300.1
TraesCS2B02G037300.1.cds1
TraesCS3B02G214300.1.cds1
Domain Color
MCAfunc Domain
PK_Tyr_Ser−Thr Domain
Pkinase Domain
other Domain
0
500
1000

T.ae RLK−Pelle_CR4L subfamily domain diagram (part 2)

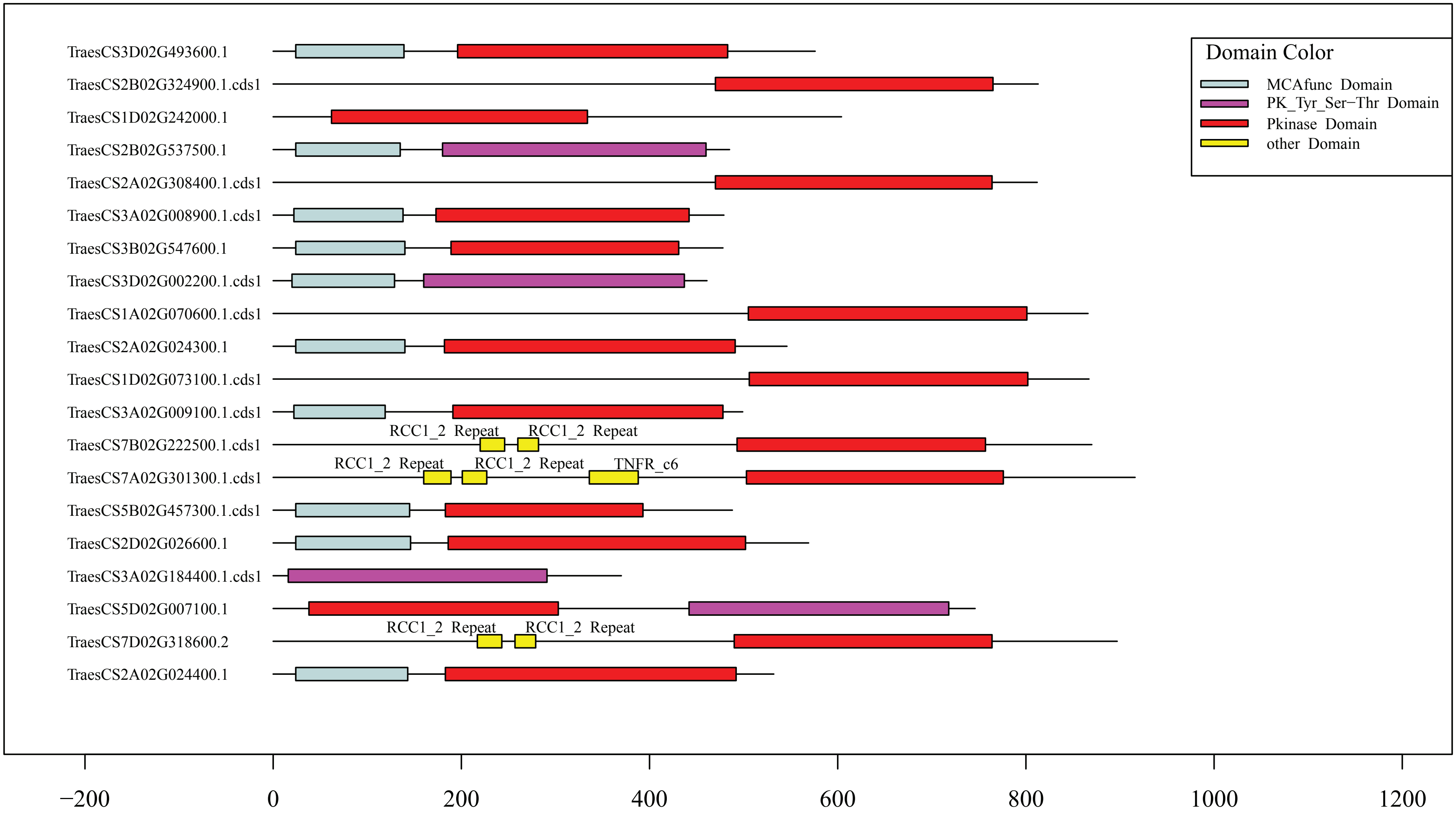

T.ae RLK−Pelle_CR4L subfamily domain diagram (part 3)

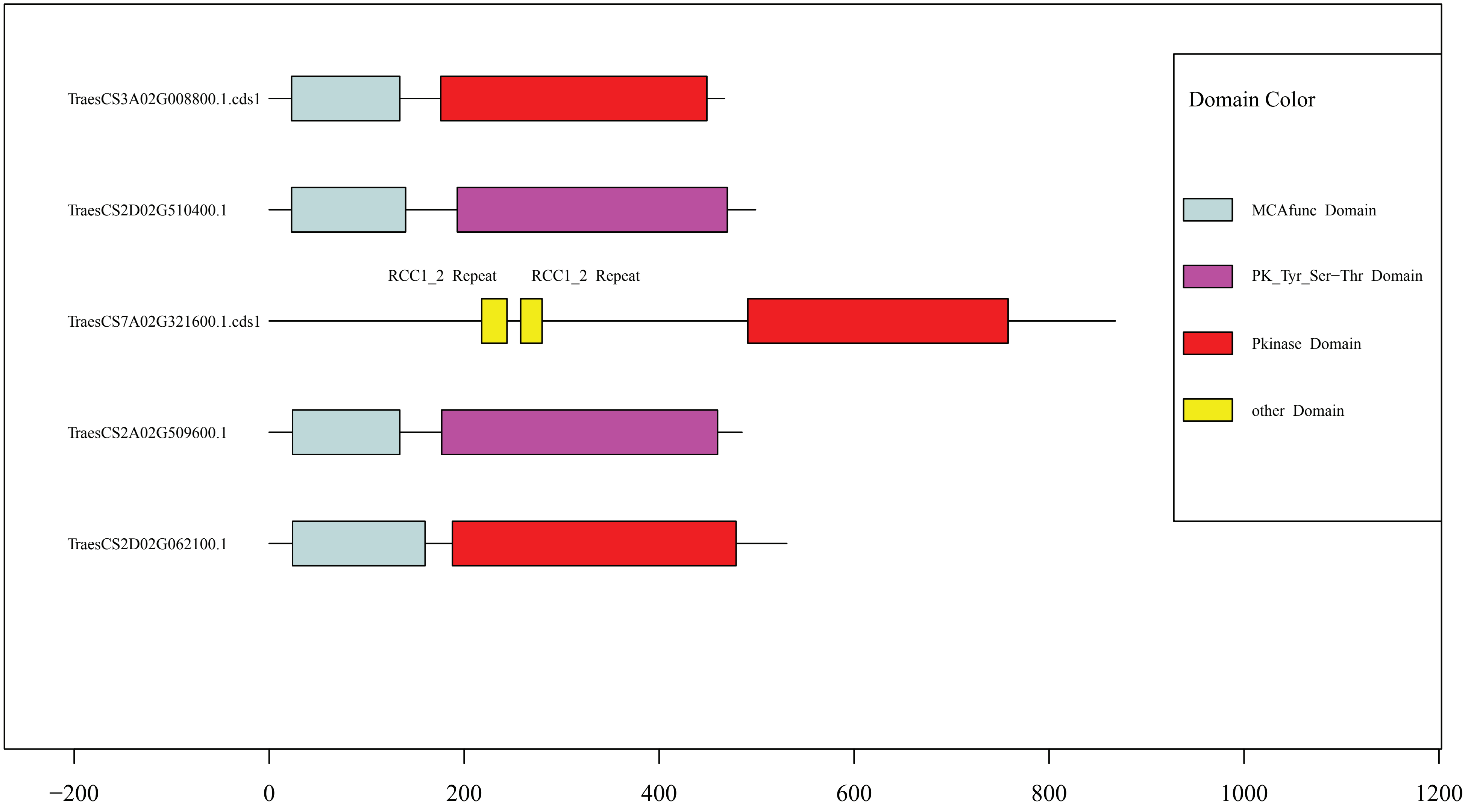

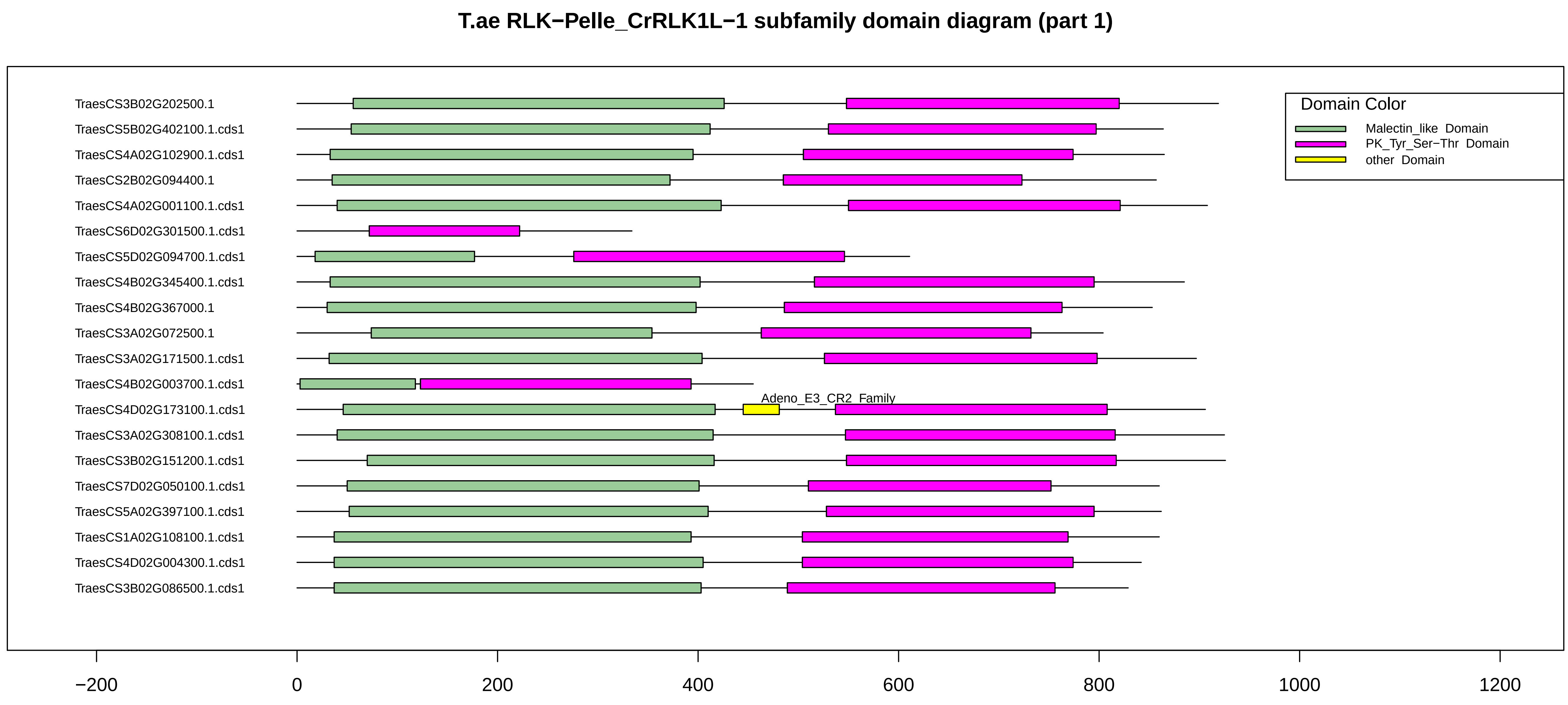
T.ae RLK−Pelle_CrRLK1L−1 subfamily domain diagram (part 1)
TraesCS3B02G202500.1
TraesCS5B02G402100.1.cds1
TraesCS4A02G102900.1.cds1
TraesCS2B02G094400.1
TraesCS4A02G001100.1.cds1
TraesCS6D02G301500.1.cds1
TraesCS5D02G094700.1.cds1
TraesCS4B02G345400.1.cds1
TraesCS4B02G367000.1
TraesCS3A02G072500.1
TraesCS3A02G171500.1.cds1
TraesCS4B02G003700.1.cds1
TraesCS4D02G173100.1.cds1
TraesCS3A02G308100.1.cds1
TraesCS3B02G151200.1.cds1
TraesCS7D02G050100.1.cds1
TraesCS5A02G397100.1.cds1
TraesCS1A02G108100.1.cds1
TraesCS4D02G004300.1.cds1
TraesCS3B02G086500.1.cds1
Adeno_E3_CR2_Family
Domain Color
Malectin_like Domain
PK_Tyr_Ser−Thr Domain
other Domain
−200
0
200
400
600
800
1000
1200

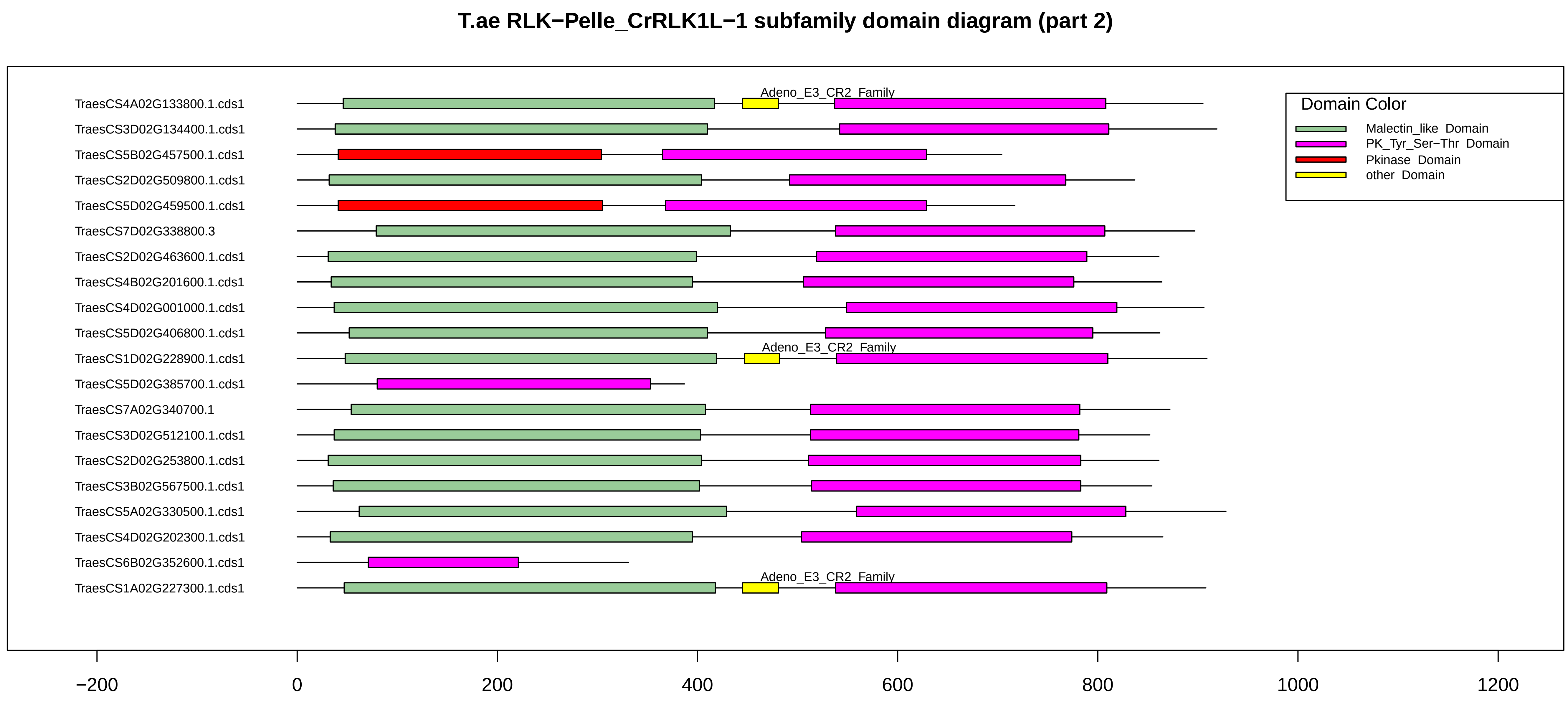
T.ae RLK−Pelle_CrRLK1L−1 subfamily domain diagram (part 2)
TraesCS4A02G133800.1.cds1
TraesCS3D02G134400.1.cds1
TraesCS5B02G457500.1.cds1
TraesCS2D02G509800.1.cds1
TraesCS5D02G459500.1.cds1
TraesCS7D02G338800.3
TraesCS2D02G463600.1.cds1
TraesCS4B02G201600.1.cds1
TraesCS4D02G001000.1.cds1
TraesCS5D02G406800.1.cds1
TraesCS1D02G228900.1.cds1
TraesCS5D02G385700.1.cds1
TraesCS7A02G340700.1
TraesCS3D02G512100.1.cds1
TraesCS2D02G253800.1.cds1
TraesCS3B02G567500.1.cds1
TraesCS5A02G330500.1.cds1
TraesCS4D02G202300.1.cds1
TraesCS6B02G352600.1.cds1
TraesCS1A02G227300.1.cds1
Adeno_E3_CR2_Family
Adeno_E3_CR2_Family
Adeno_E3_CR2_Family
Domain Color
Malectin_like Domain
PK_Tyr_Ser−Thr Domain
Pkinase Domain
other Domain
−200
0
200
400
600
800
1000
1200

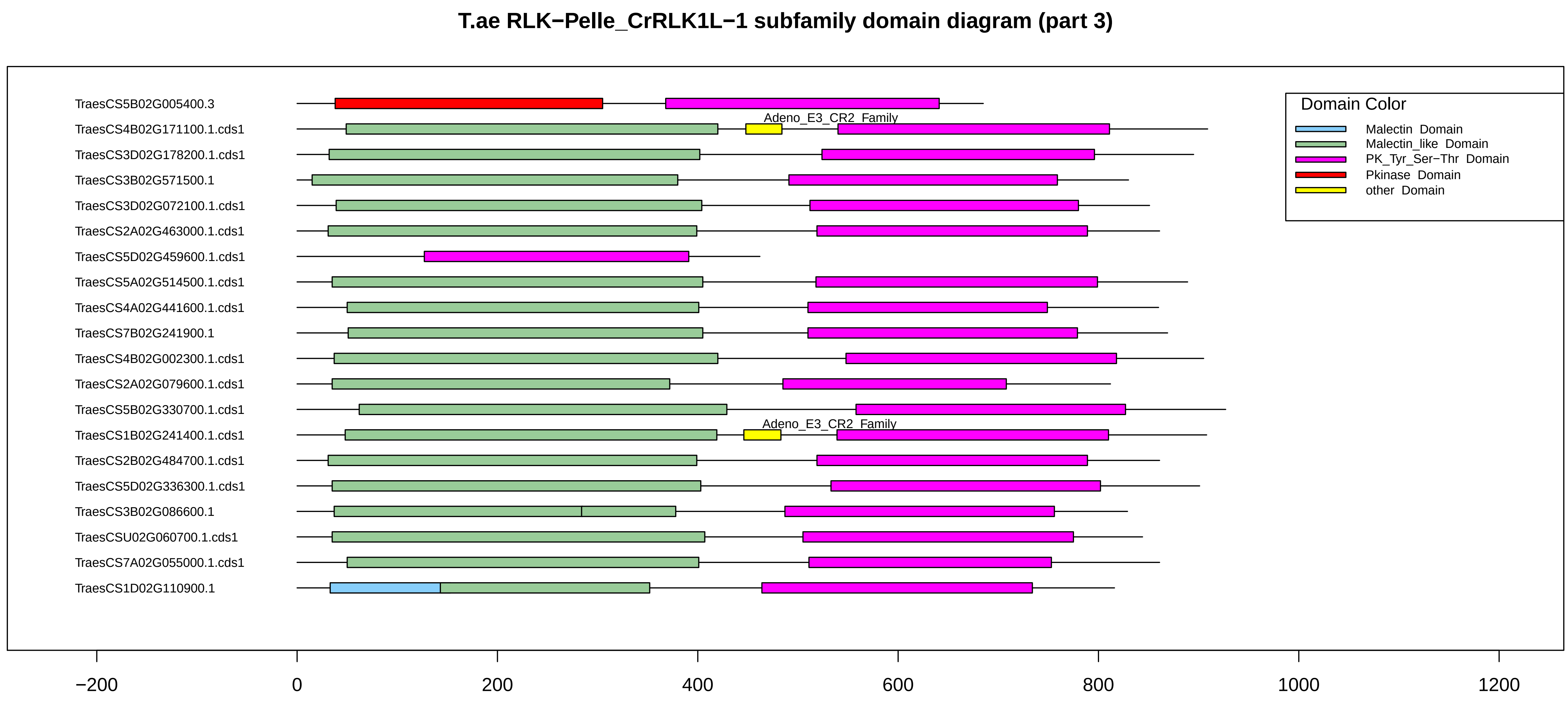

T.ae RLK−Pelle_CrRLK1L−1 subfamily domain diagram (part 3)
TraesCS5B02G005400.3
TraesCS4B02G171100.1.cds1
Adeno_E3_CR2_Family
TraesCS3D02G178200.1.cds1
TraesCS3B02G571500.1
TraesCS3D02G072100.1.cds1
TraesCS2A02G463000.1.cds1
TraesCS5D02G459600.1.cds1
TraesCS5A02G514500.1.cds1
TraesCS4A02G441600.1.cds1
TraesCS7B02G241900.1
TraesCS4B02G002300.1.cds1
TraesCS2A02G079600.1.cds1
TraesCS5B02G330700.1.cds1
TraesCS1B02G241400.1.cds1
Adeno_E3_CR2_Family
TraesCS2B02G484700.1.cds1
TraesCS5D02G336300.1.cds1
TraesCS3B02G086600.1
TraesCSU02G060700.1.cds1
TraesCS7A02G055000.1.cds1
TraesCS1D02G110900.1
Domain Color
Malectin Domain
Malectin_like Domain
PK_Tyr_Ser−Thr Domain
Pkinase Domain
other Domain
−200
0
200
400
600
800
1000
1200

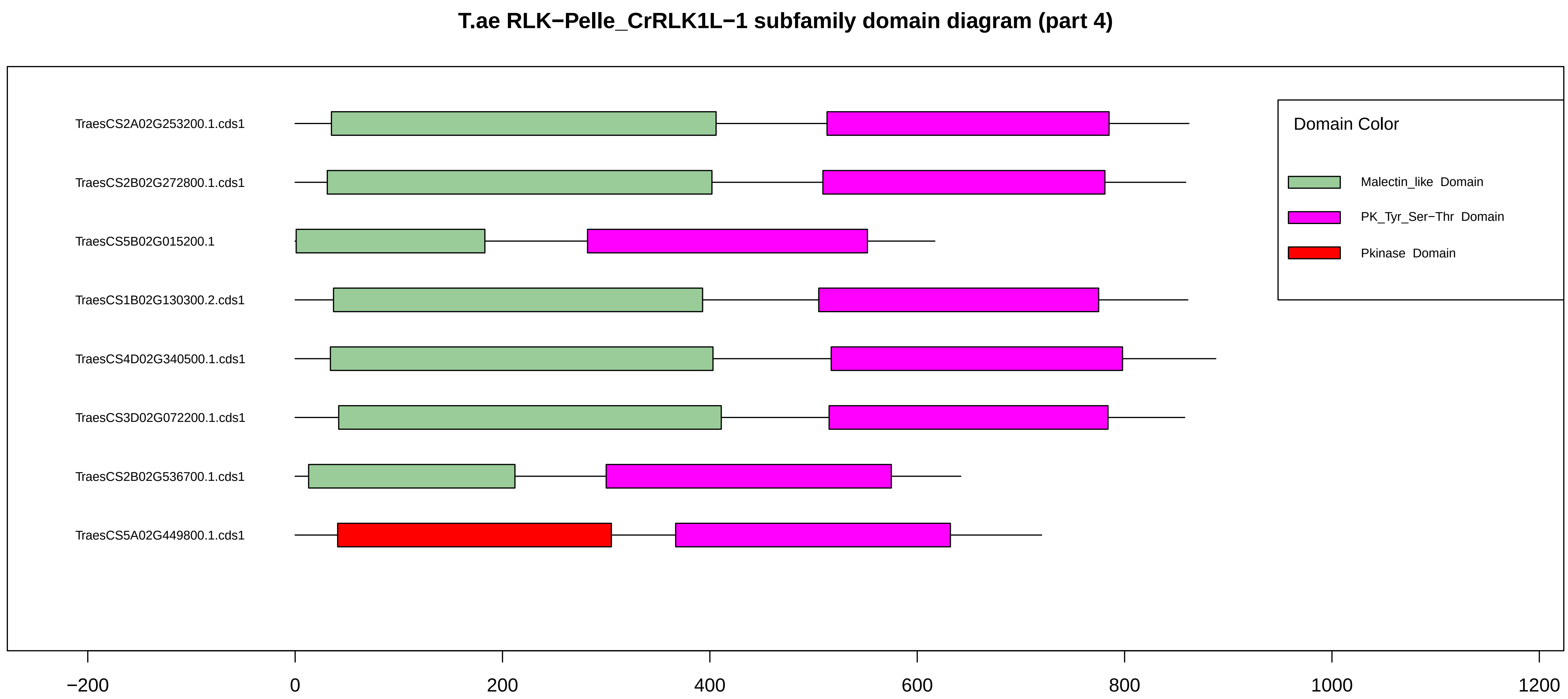
T.ae RLK−Pelle_CrRLK1L−1 subfamily domain diagram (part 4)
TraesCS2A02G253200.1.cds1
TraesCS2B02G272800.1.cds1
TraesCS5B02G015200.1
TraesCS1B02G130300.2.cds1
TraesCS4D02G340500.1.cds1
TraesCS3D02G072200.1.cds1
TraesCS2B02G536700.1.cds1
TraesCS5A02G449800.1.cds1
Domain Color
Malectin_like Domain
PK_Tyr_Ser−Thr Domain
Pkinase Domain
−200
0
200
400
600
800
1000
1200

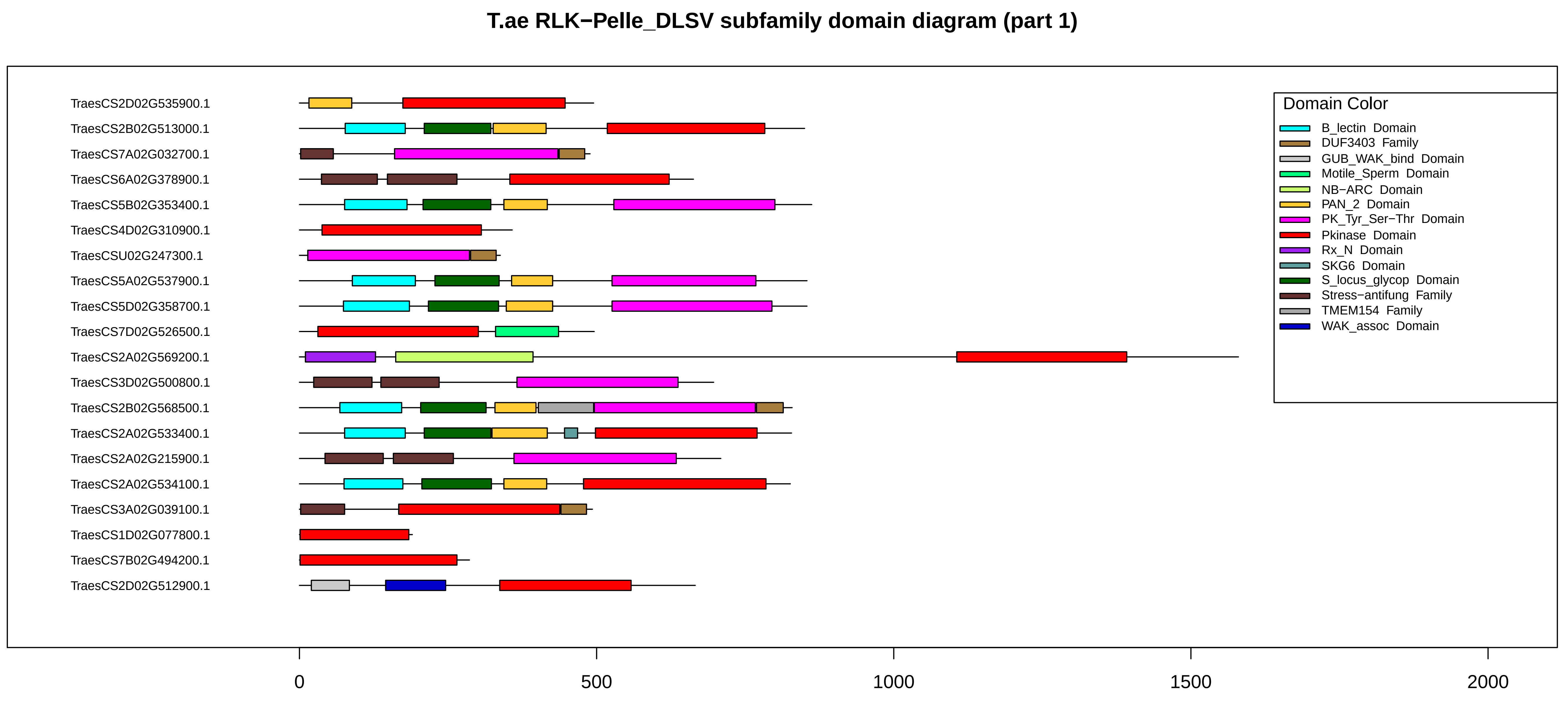
T.ae RLK−Pelle_DLSV subfamily domain diagram (part 1)
TraesCS2D02G535900.1
TraesCS2B02G513000.1
TraesCS7A02G032700.1
TraesCS6A02G378900.1
TraesCS5B02G353400.1
TraesCS4D02G310900.1
TraesCSU02G247300.1
TraesCS5A02G537900.1
TraesCS5D02G358700.1
TraesCS7D02G526500.1
TraesCS2A02G569200.1
TraesCS3D02G500800.1
TraesCS2B02G568500.1
TraesCS2A02G533400.1
TraesCS2A02G215900.1
TraesCS2A02G534100.1
TraesCS3A02G039100.1
TraesCS1D02G077800.1
TraesCS7B02G494200.1
TraesCS2D02G512900.1
Domain Color
B_lectin Domain
DUF3403 Family
GUB_WAK_bind Domain
Motile_Sperm Domain
NB−ARC Domain
PAN_2 Domain
PK_Tyr_Ser−Thr Domain
Pkinase Domain
Rx_N Domain
SKG6 Domain
S_locus_glycop Domain
Stress−antifung Family
TMEM154 Family
WAK_assoc Domain
0
500
1000
1500
2000

T.ae RLK−Pelle_DLSV subfamily domain diagram (part 2)

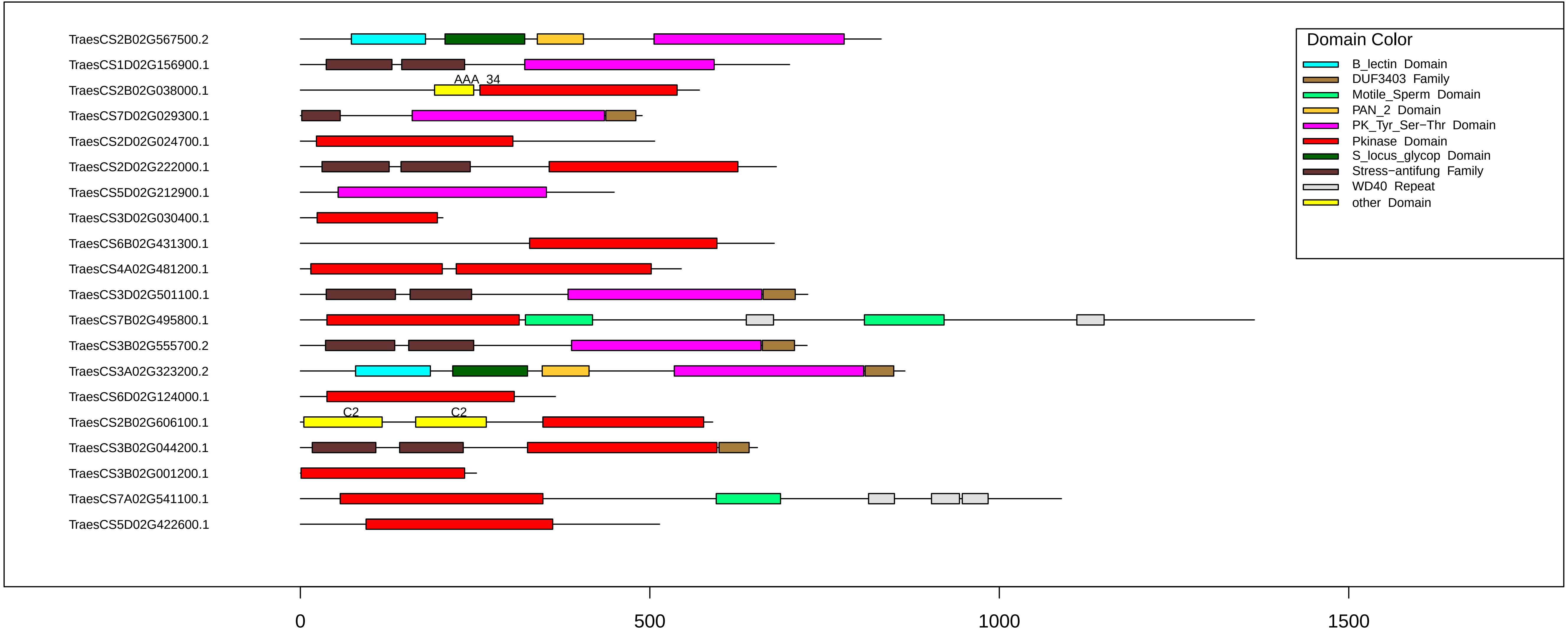

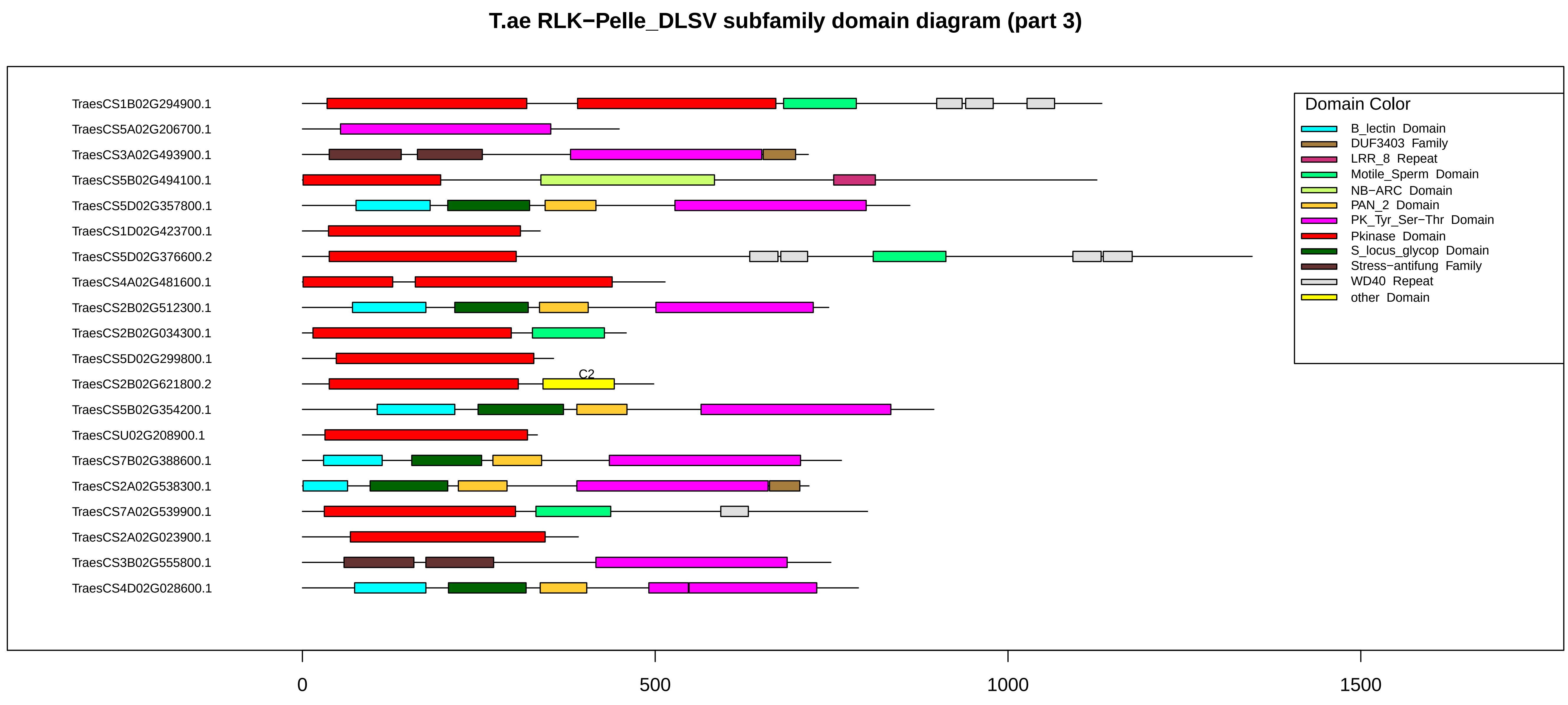
T.ae RLK−Pelle_DLSV subfamily domain diagram (part 3)
TraesCS1B02G294900.1
TraesCS5A02G206700.1
TraesCS3A02G493900.1
TraesCS5B02G494100.1
TraesCS5D02G357800.1
TraesCS1D02G423700.1
TraesCS5D02G376600.2
TraesCS4A02G481600.1
TraesCS2B02G512300.1
TraesCS2B02G034300.1
TraesCS5D02G299800.1
TraesCS2B02G621800.2
C2
TraesCS5B02G354200.1
TraesCSU02G208900.1
TraesCS7B02G388600.1
TraesCS2A02G538300.1
TraesCS7A02G539900.1
TraesCS2A02G023900.1
TraesCS3B02G555800.1
TraesCS4D02G028600.1
Domain Color
B_lectin Domain
DUF3403 Family
LRR_8 Repeat
Motile_Sperm Domain
NB−ARC Domain
PAN_2 Domain
PK_Tyr_Ser−Thr Domain
Pkinase Domain
S_locus_glycop Domain
Stress−antifung Family
WD40 Repeat
other Domain
0
500
1000
1500

T.ae RLK−Pelle_DLSV subfamily domain diagram (part 4)

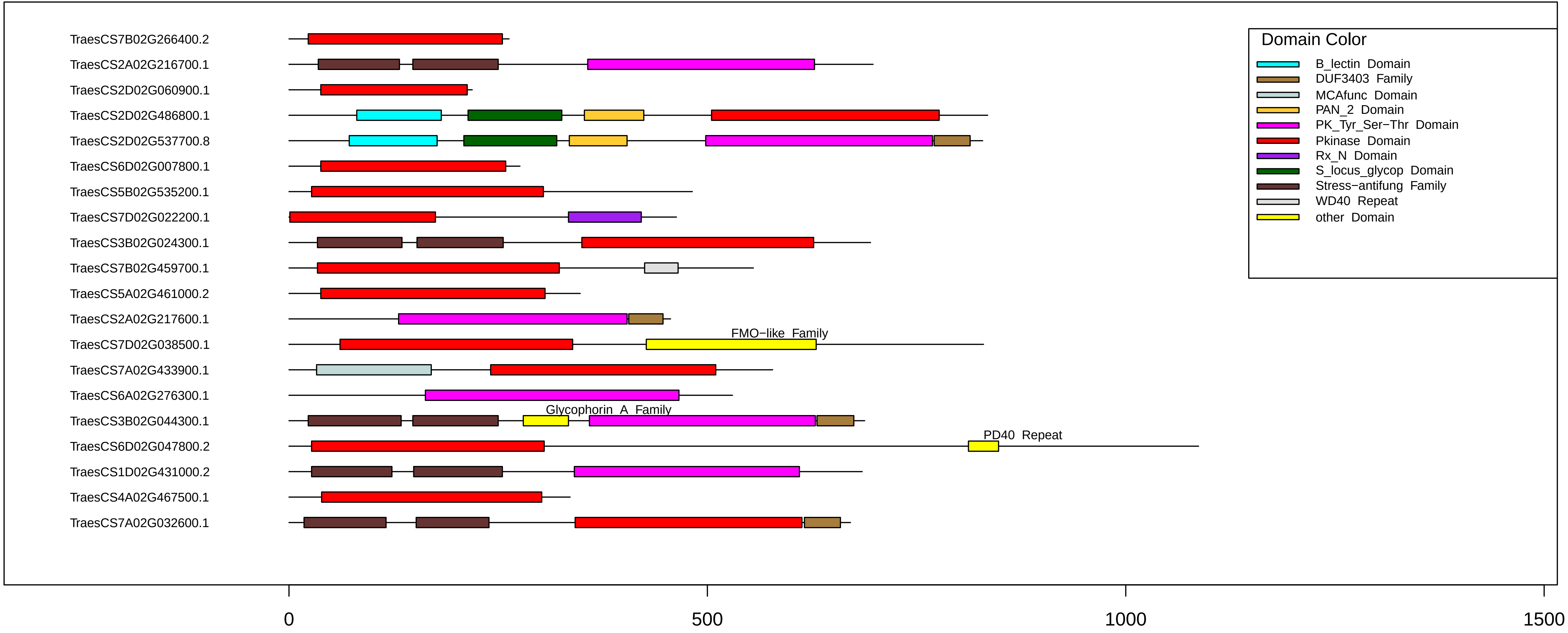

T.ae RLK−Pelle_DLSV subfamily domain diagram (part 5)

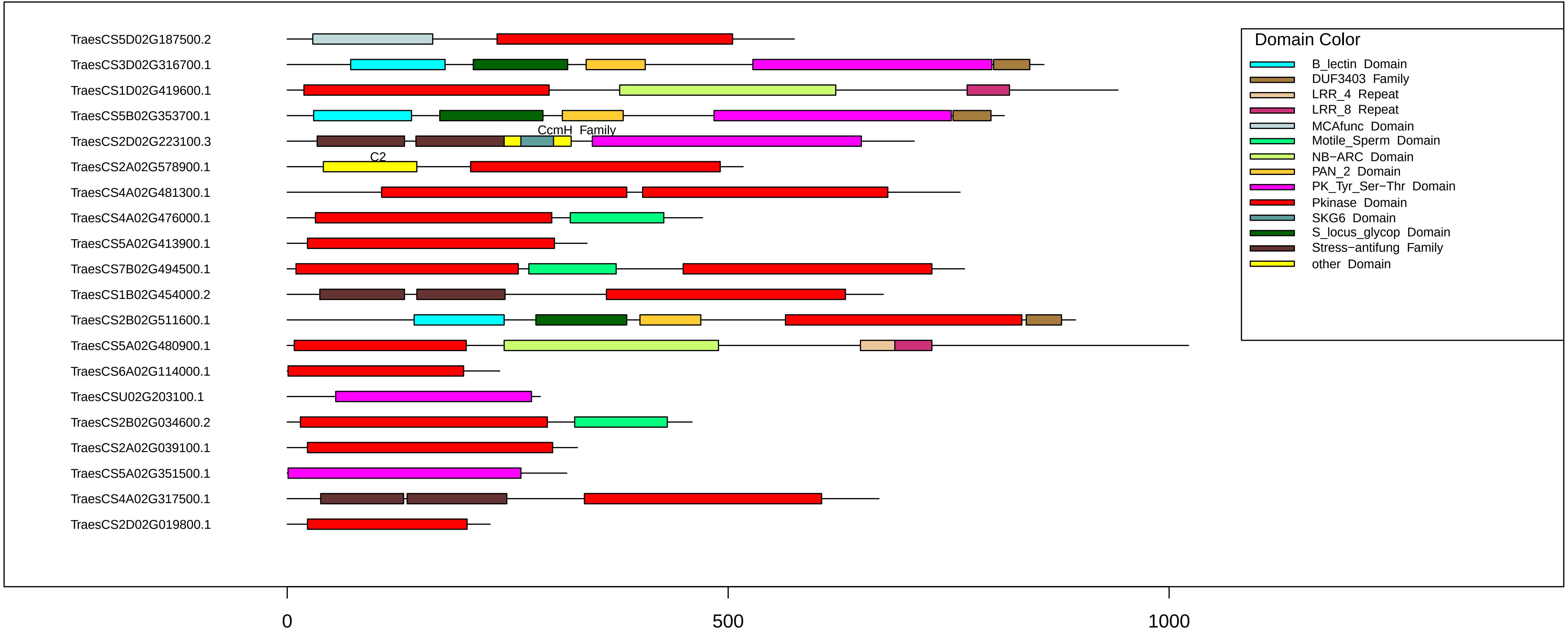

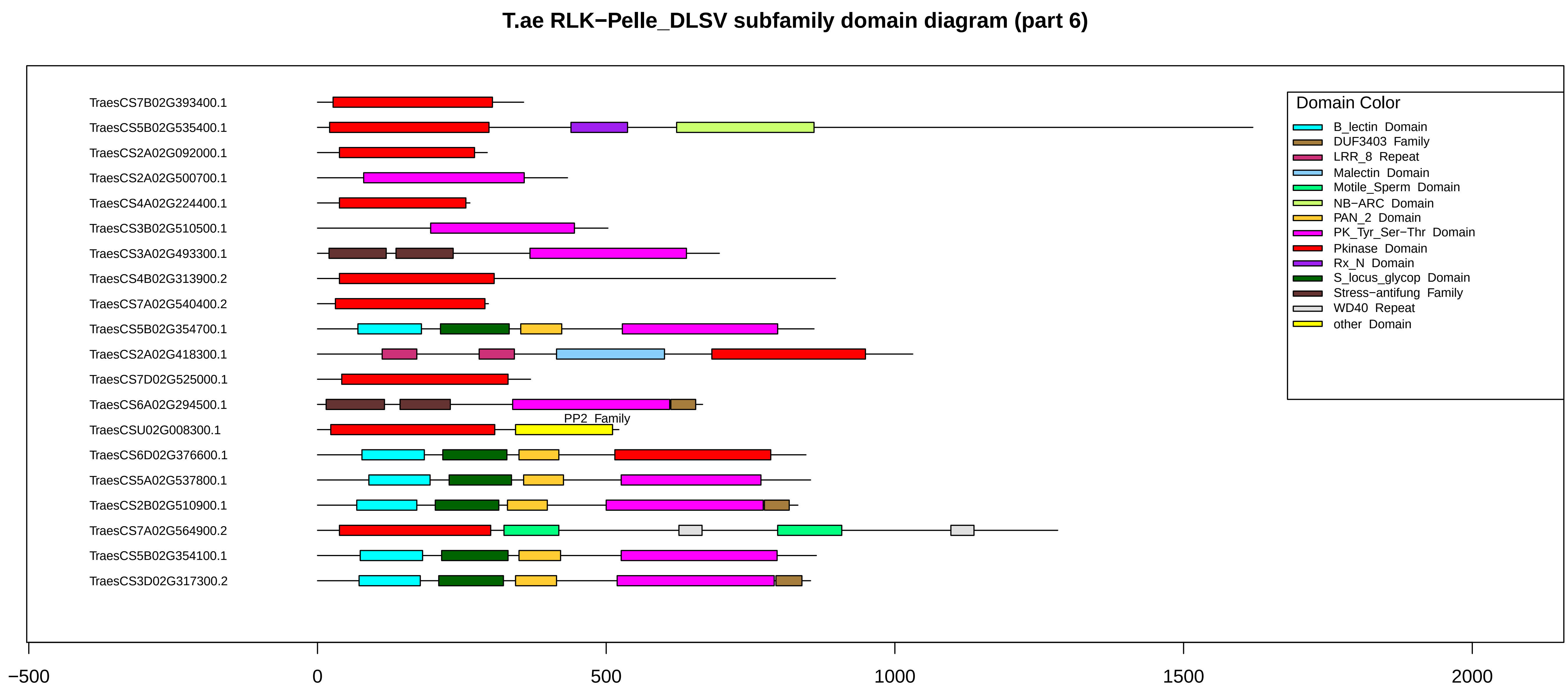
T.ae RLK−Pelle_DLSV subfamily domain diagram (part 6)
TraesCS7B02G393400.1
TraesCS5B02G535400.1
TraesCS2A02G092000.1
TraesCS2A02G500700.1
TraesCS4A02G224400.1
TraesCS3B02G510500.1
TraesCS3A02G493300.1
TraesCS4B02G313900.2
TraesCS7A02G540400.2
TraesCS5B02G354700.1
TraesCS2A02G418300.1
TraesCS7D02G525000.1
TraesCS6A02G294500.1
TraesCSU02G008300.1
PP2 Family
TraesCS6D02G376600.1
TraesCS5A02G537800.1
TraesCS2B02G510900.1
TraesCS7A02G564900.2
TraesCS5B02G354100.1
TraesCS3D02G317300.2
Domain Color
B_lectin Domain
DUF3403 Family
LRR_8 Repeat
Malectin Domain
Motile_Sperm Domain
NB−ARC Domain
PAN_2 Domain
PK_Tyr_Ser−Thr Domain
Pkinase Domain
Rx_N Domain
S_locus_glycop Domain
Stress−antifung Family
WD40 Repeat
other Domain
−500
0
500
1000
1500
2000

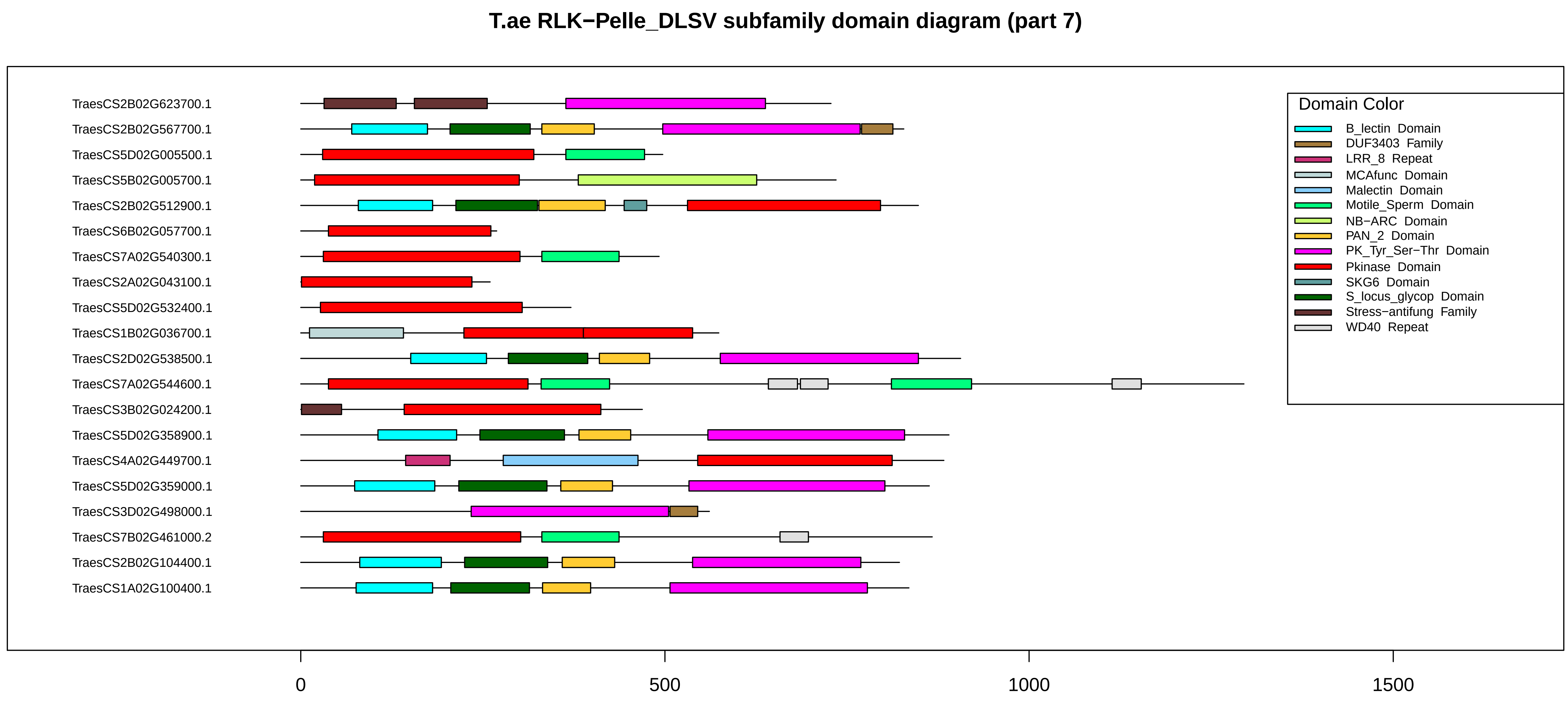
T.ae RLK−Pelle_DLSV subfamily domain diagram (part 7)
TraesCS2B02G623700.1
TraesCS2B02G567700.1
TraesCS5D02G005500.1
TraesCS5B02G005700.1
TraesCS2B02G512900.1
TraesCS6B02G057700.1
TraesCS7A02G540300.1
TraesCS2A02G043100.1
TraesCS5D02G532400.1
TraesCS1B02G036700.1
TraesCS2D02G538500.1
TraesCS7A02G544600.1
TraesCS3B02G024200.1
TraesCS5D02G358900.1
TraesCS4A02G449700.1
TraesCS5D02G359000.1
TraesCS3D02G498000.1
TraesCS7B02G461000.2
TraesCS2B02G104400.1
TraesCS1A02G100400.1
Domain Color
B_lectin Domain
DUF3403 Family
LRR_8 Repeat
MCAfunc Domain
Malectin Domain
Motile_Sperm Domain
NB−ARC Domain
PAN_2 Domain
PK_Tyr_Ser−Thr Domain
Pkinase Domain
SKG6 Domain
S_locus_glycop Domain
Stress−antifung Family
WD40 Repeat
0
500
1000
1500

T.ae RLK−Pelle_DLSV subfamily domain diagram (part 8)

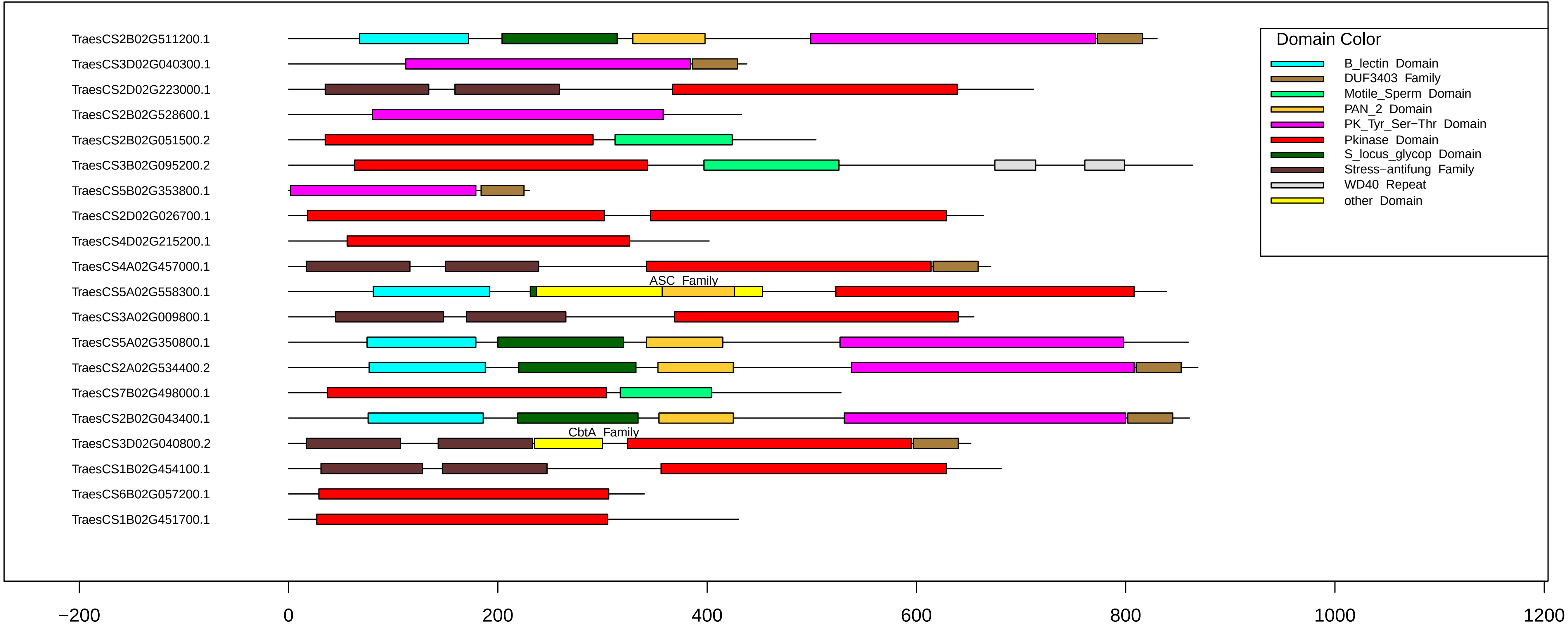

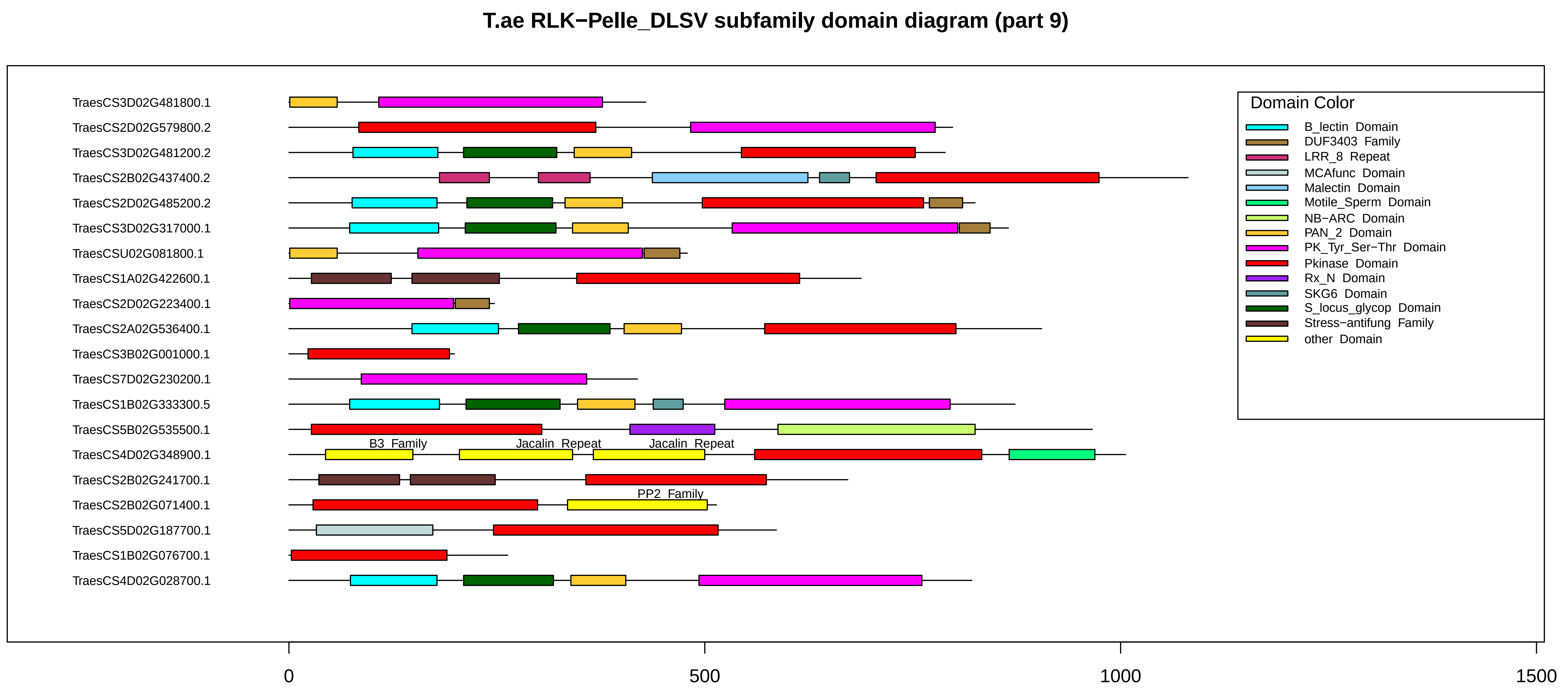
T.ae RLK−Pelle_DLSV subfamily domain diagram (part 9)
TraesCS3D02G481800.1
TraesCS2D02G579800.2
TraesCS3D02G481200.2
TraesCS2B02G437400.2
TraesCS2D02G485200.2
TraesCS3D02G317000.1
TraesCSU02G081800.1
TraesCS1A02G422600.1
TraesCS2D02G223400.1
TraesCS2A02G536400.1
TraesCS3B02G001000.1
TraesCS7D02G230200.1
TraesCS1B02G333300.5
TraesCS5B02G535500.1
TraesCS4D02G348900.1
TraesCS2B02G241700.1
TraesCS2B02G071400.1
TraesCS5D02G187700.1
TraesCS1B02G076700.1
TraesCS4D02G028700.1
B3 Family
Jacalin Repeat
Jacalin Repeat
PP2 Family
Domain Color
B_lectin Domain
DUF3403 Family
LRR_8 Repeat
MCAfunc Domain
Malectin Domain
Motile_Sperm Domain
NB−ARC Domain
PAN_2 Domain
PK_Tyr_Ser−Thr Domain
Pkinase Domain
Rx_N Domain
SKG6 Domain
S_locus_glycop Domain
Stress−antifung Family
other Domain
0
500
1000
1500

T.ae RLK−Pelle_DLSV subfamily domain diagram (part 10)

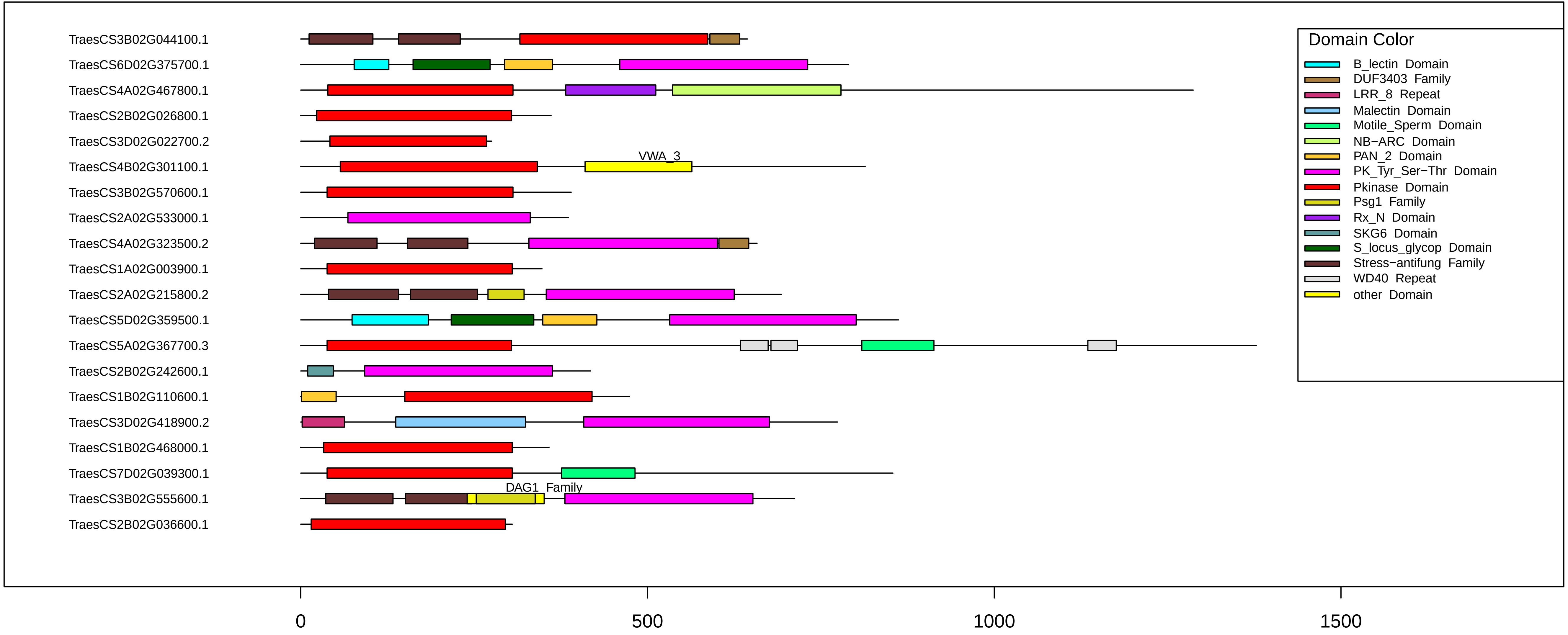

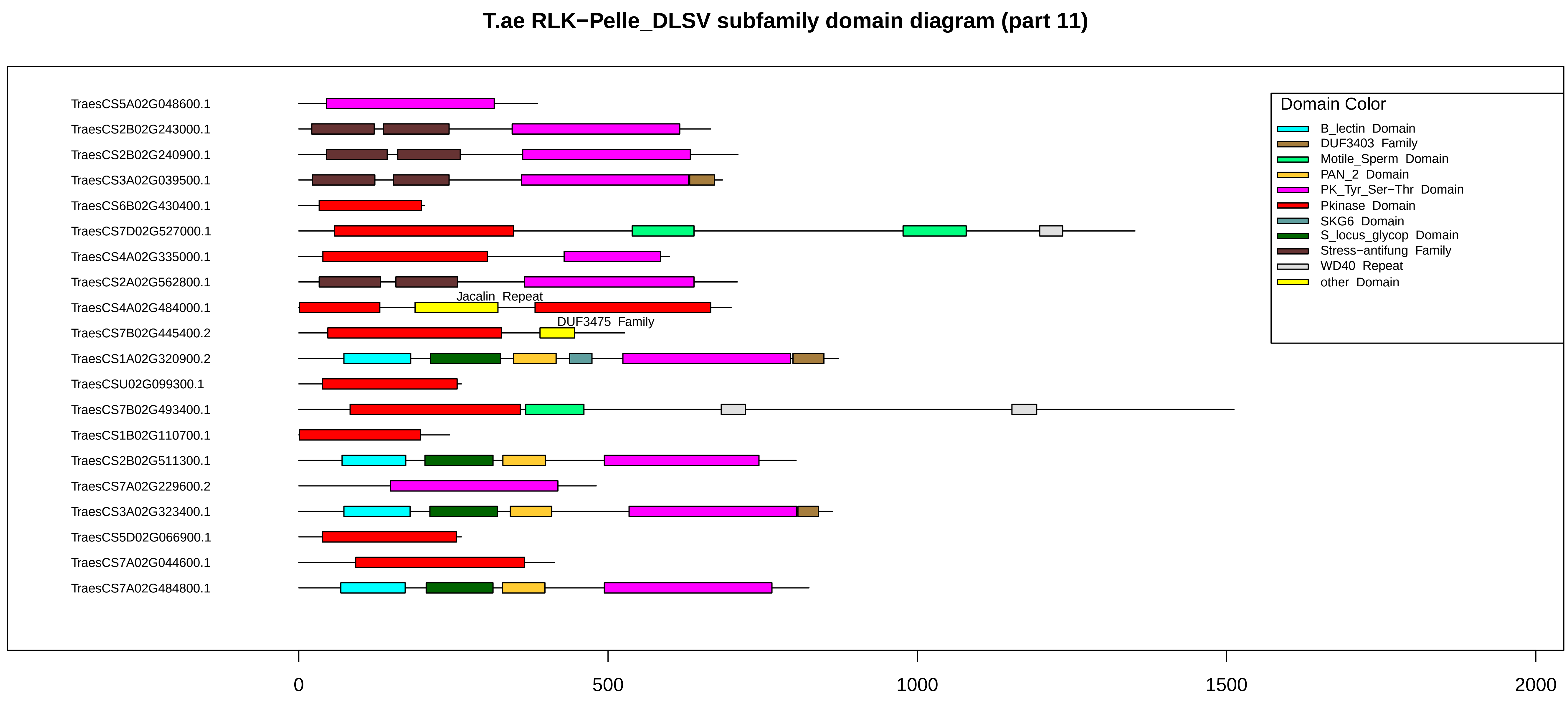

T.ae RLK−Pelle_DLSV subfamily domain diagram (part 11)
TraesCS5A02G048600.1
TraesCS2B02G243000.1
TraesCS2B02G240900.1
TraesCS3A02G039500.1
TraesCS6B02G430400.1
TraesCS7D02G527000.1
TraesCS4A02G335000.1
TraesCS2A02G562800.1
TraesCS4A02G484000.1
Jacalin Repeat
TraesCS7B02G445400.2
DUF3475 Family
TraesCS1A02G320900.2
TraesCSU02G099300.1
TraesCS7B02G493400.1
TraesCS1B02G110700.1
TraesCS2B02G511300.1
TraesCS7A02G229600.2
TraesCS3A02G323400.1
TraesCS5D02G066900.1
TraesCS7A02G044600.1
TraesCS7A02G484800.1
Domain Color
B_lectin Domain
DUF3403 Family
Motile_Sperm Domain
PAN_2 Domain
PK_Tyr_Ser−Thr Domain
Pkinase Domain
SKG6 Domain
S_locus_glycop Domain
Stress−antifung Family
WD40 Repeat
other Domain
0
500
1000
1500
2000

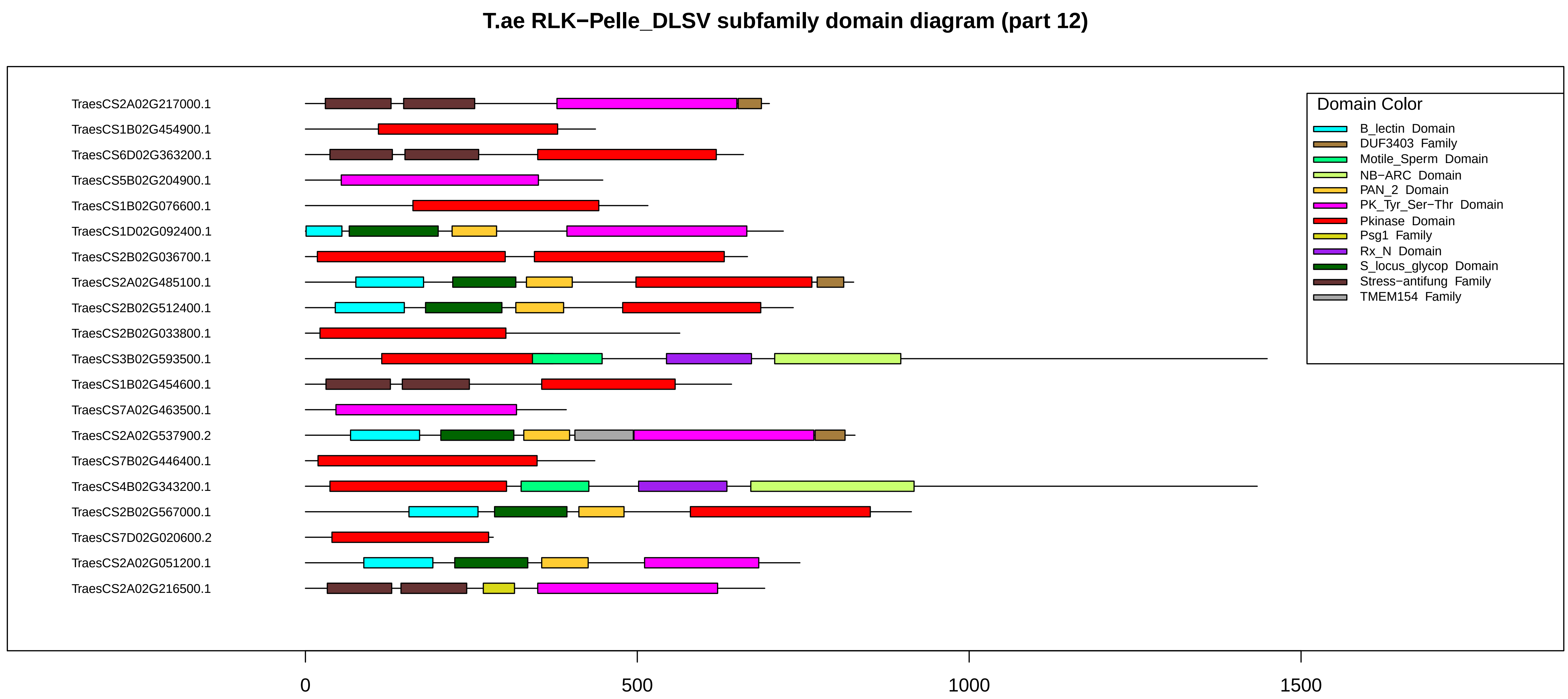
T.ae RLK−Pelle_DLSV subfamily domain diagram (part 12)
TraesCS2A02G217000.1
TraesCS1B02G454900.1
TraesCS6D02G363200.1
TraesCS5B02G204900.1
TraesCS1B02G076600.1
TraesCS1D02G092400.1
TraesCS2B02G036700.1
TraesCS2A02G485100.1
TraesCS2B02G512400.1
TraesCS2B02G033800.1
TraesCS3B02G593500.1
TraesCS1B02G454600.1
TraesCS7A02G463500.1
TraesCS2A02G537900.2
TraesCS7B02G446400.1
TraesCS4B02G343200.1
TraesCS2B02G567000.1
TraesCS7D02G020600.2
TraesCS2A02G051200.1
TraesCS2A02G216500.1
Domain Color
B_lectin Domain
DUF3403 Family
Motile_Sperm Domain
NB−ARC Domain
PAN_2 Domain
PK_Tyr_Ser−Thr Domain
Pkinase Domain
Psg1 Family
Rx_N Domain
S_locus_glycop Domain
Stress−antifung Family
TMEM154 Family
0
500
1000
1500

T.ae RLK−Pelle_DLSV subfamily domain diagram (part 13)

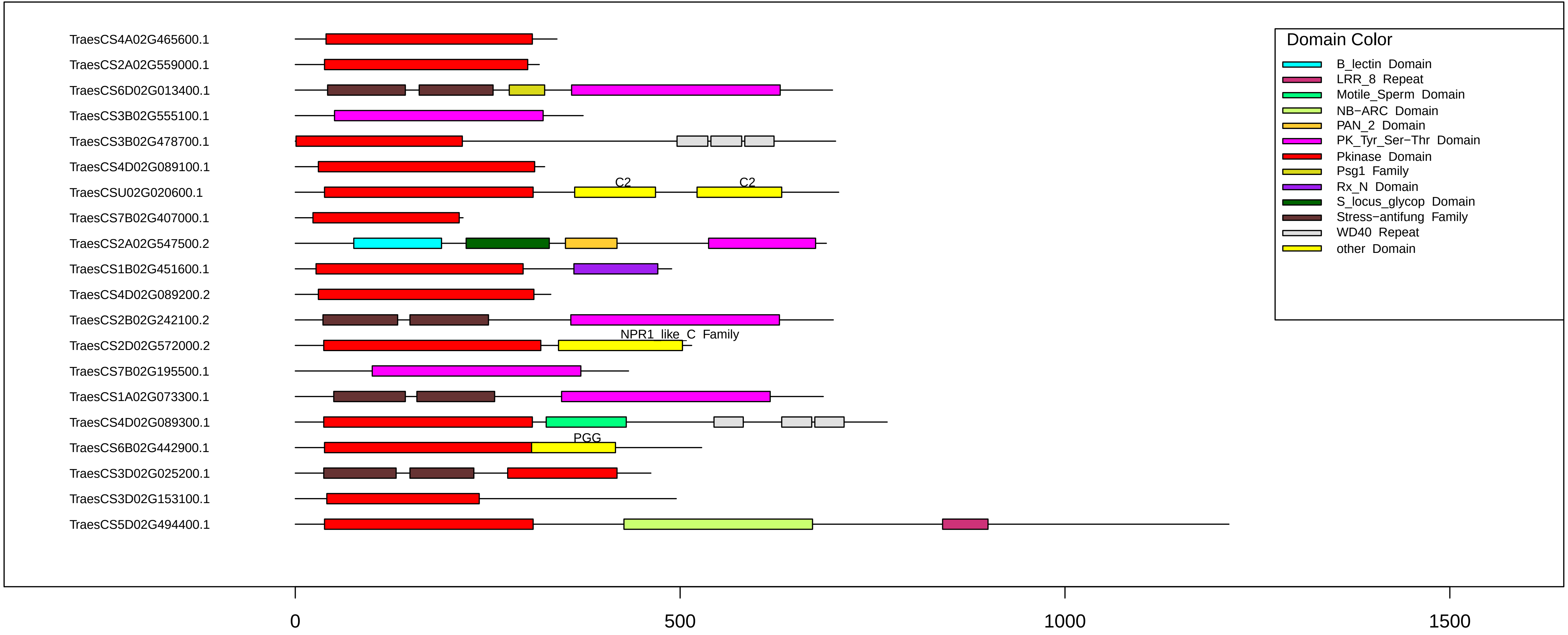

T.ae RLK−Pelle_DLSV subfamily domain diagram (part 14)

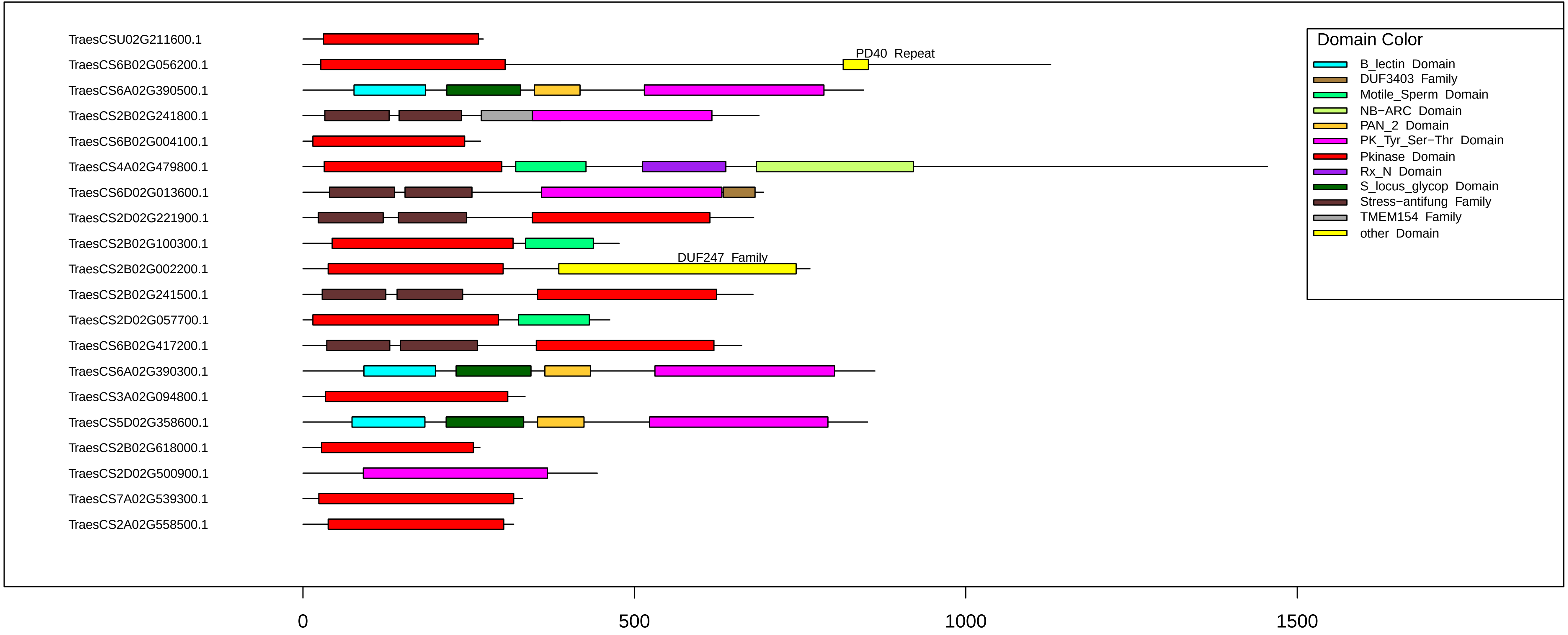

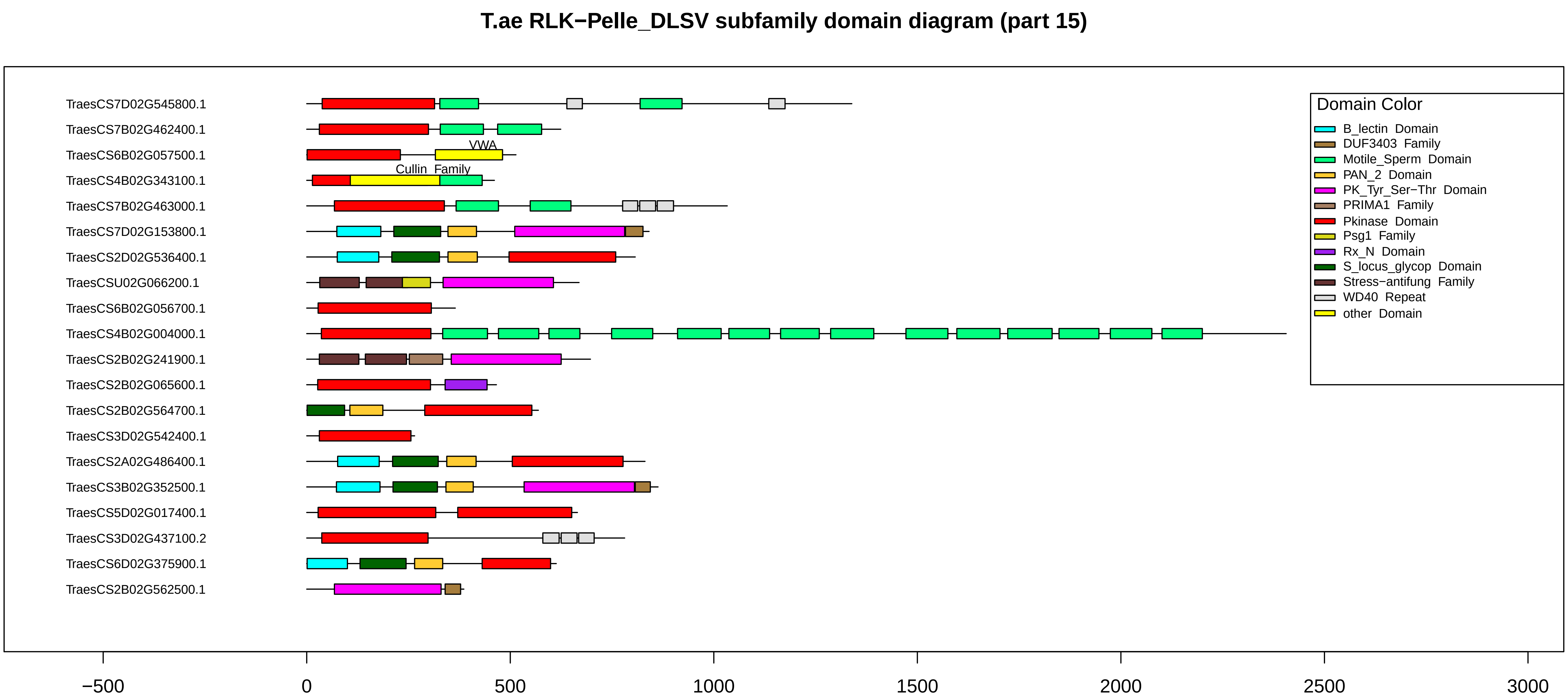
T.ae RLK−Pelle_DLSV subfamily domain diagram (part 15)
TraesCS7D02G545800.1
TraesCS7B02G462400.1
TraesCS6B02G057500.1
TraesCS4B02G343100.1
TraesCS7B02G463000.1
TraesCS7D02G153800.1
TraesCS2D02G536400.1
TraesCSU02G066200.1
TraesCS6B02G056700.1
TraesCS4B02G004000.1
TraesCS2B02G241900.1
TraesCS2B02G065600.1
TraesCS2B02G564700.1
TraesCS3D02G542400.1
TraesCS2A02G486400.1
TraesCS3B02G352500.1
TraesCS5D02G017400.1
TraesCS3D02G437100.2
TraesCS6D02G375900.1
TraesCS2B02G562500.1
VWA
Cullin Family
Domain Color
B_lectin Domain
DUF3403 Family
Motile_Sperm Domain
PAN_2 Domain
PK_Tyr_Ser−Thr Domain
PRIMA1 Family
Pkinase Domain
Psg1 Family
Rx_N Domain
S_locus_glycop Domain
Stress−antifung Family
WD40 Repeat
other Domain
−500
0
500
1000
1500
2000
2500
3000

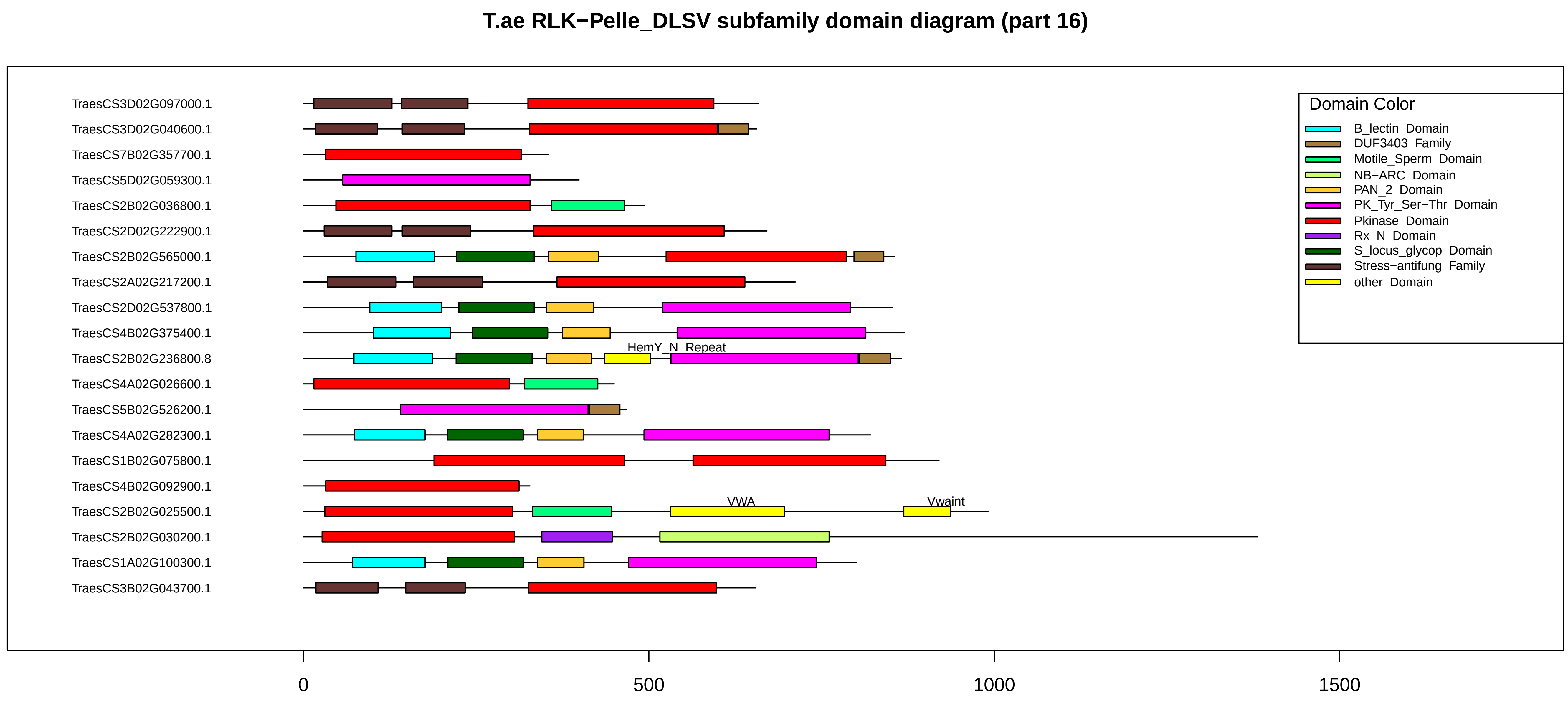
T.ae RLK−Pelle_DLSV subfamily domain diagram (part 16)
TraesCS3D02G097000.1
TraesCS3D02G040600.1
TraesCS7B02G357700.1
TraesCS5D02G059300.1
TraesCS2B02G036800.1
TraesCS2D02G222900.1
TraesCS2B02G565000.1
TraesCS2A02G217200.1
TraesCS2D02G537800.1
TraesCS4B02G375400.1
TraesCS2B02G236800.8
TraesCS4A02G026600.1
TraesCS5B02G526200.1
TraesCS4A02G282300.1
TraesCS1B02G075800.1
TraesCS4B02G092900.1
TraesCS2B02G025500.1
TraesCS2B02G030200.1
TraesCS1A02G100300.1
TraesCS3B02G043700.1
HemY_N_Repeat
VWA
Vwaint
Domain Color
B_lectin Domain
DUF3403 Family
Motile_Sperm Domain
NB−ARC Domain
PAN_2 Domain
PK_Tyr_Ser−Thr Domain
Pkinase Domain
Rx_N Domain
S_locus_glycop Domain
Stress−antifung Family
other Domain
0
500
1000
1500

T.ae RLK−Pelle_DLSV subfamily domain diagram (part 17)

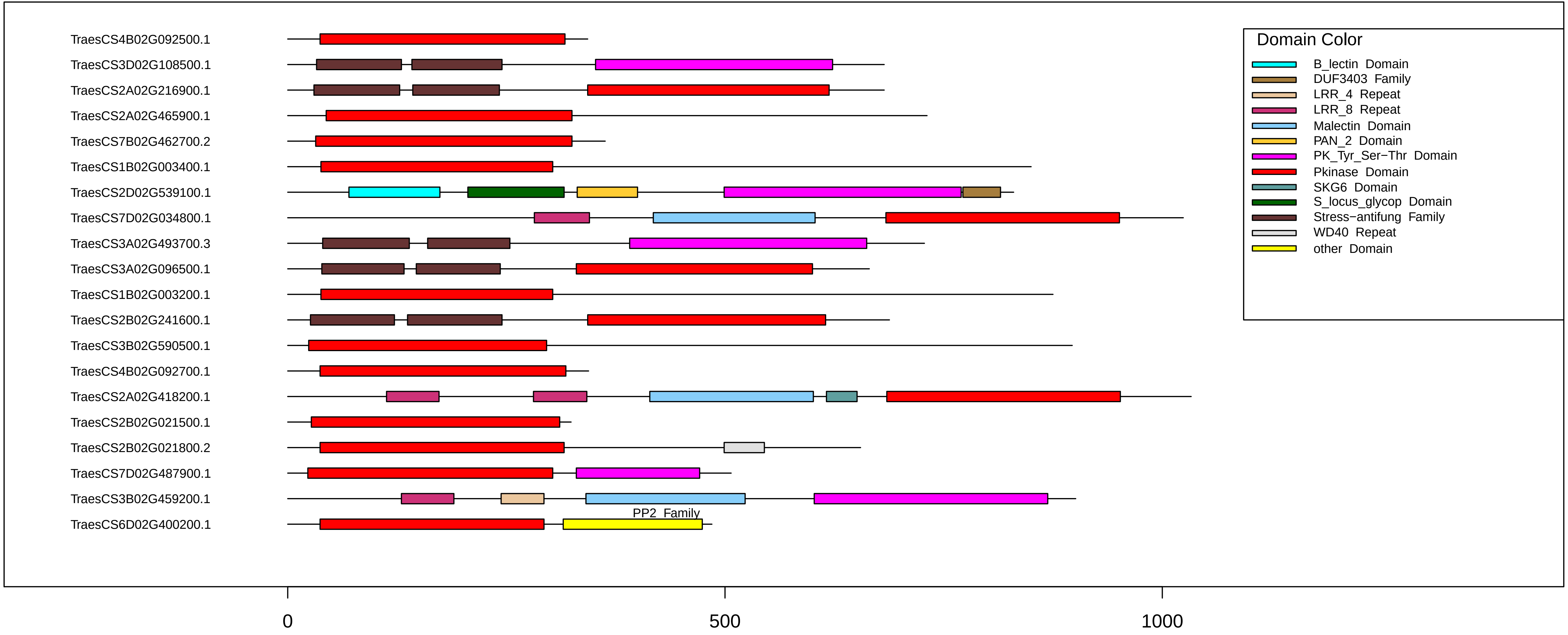

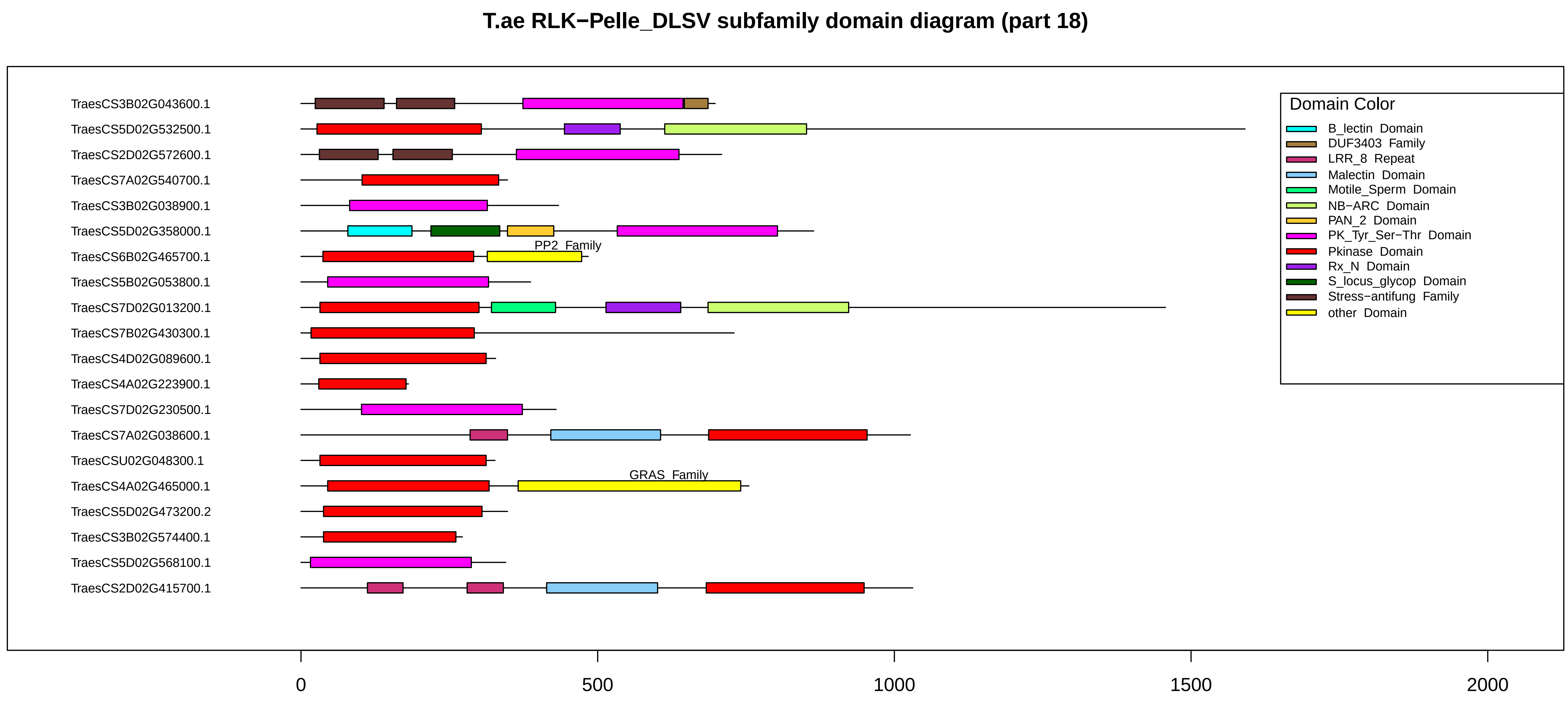
T.ae RLK−Pelle_DLSV subfamily domain diagram (part 18)
TraesCS3B02G043600.1
TraesCS5D02G532500.1
TraesCS2D02G572600.1
TraesCS7A02G540700.1
TraesCS3B02G038900.1
TraesCS5D02G358000.1
TraesCS6B02G465700.1
TraesCS5B02G053800.1
TraesCS7D02G013200.1
TraesCS7B02G430300.1
TraesCS4D02G089600.1
TraesCS4A02G223900.1
TraesCS7D02G230500.1
TraesCS7A02G038600.1
TraesCSU02G048300.1
TraesCS4A02G465000.1
TraesCS5D02G473200.2
TraesCS3B02G574400.1
TraesCS5D02G568100.1
TraesCS2D02G415700.1
PP2_Family
GRAS_Family
Domain Color
B_lectin Domain
DUF3403 Family
LRR_8 Repeat
Malectin Domain
Motile_Sperm Domain
NB−ARC Domain
PAN_2 Domain
PK_Tyr_Ser−Thr Domain
Pkinase Domain
Rx_N Domain
S_locus_glycop Domain
Stress−antifung Family
other Domain
0
500
1000
1500
2000

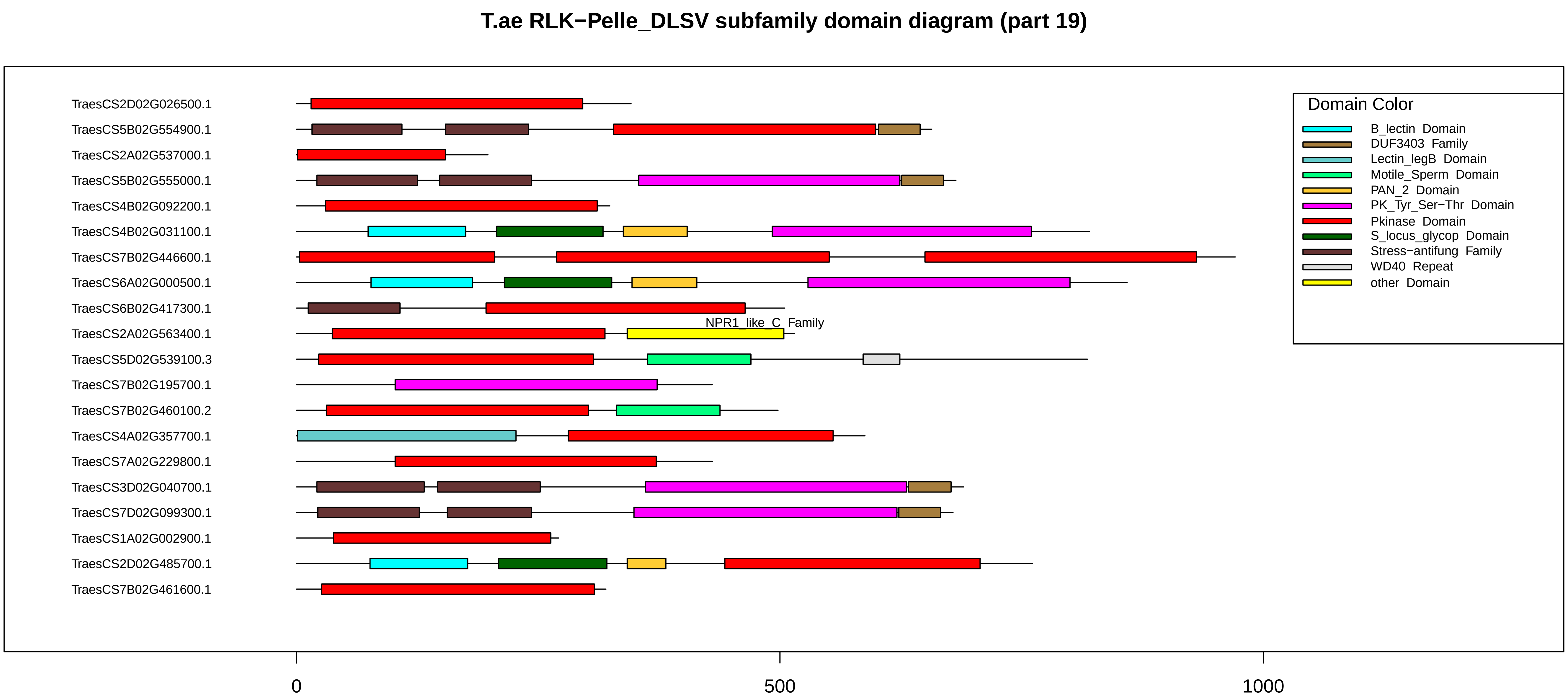
T.ae RLK−Pelle_DLSV subfamily domain diagram (part 19)
TraesCS2D02G026500.1
TraesCS5B02G554900.1
TraesCS2A02G537000.1
TraesCS5B02G555000.1
TraesCS4B02G092200.1
TraesCS4B02G031100.1
TraesCS7B02G446600.1
TraesCS6A02G000500.1
TraesCS6B02G417300.1
TraesCS2A02G563400.1
TraesCS5D02G539100.3
TraesCS7B02G195700.1
TraesCS7B02G460100.2
TraesCS4A02G357700.1
TraesCS7A02G229800.1
TraesCS3D02G040700.1
TraesCS7D02G099300.1
TraesCS1A02G002900.1
TraesCS2D02G485700.1
TraesCS7B02G461600.1
NPR1_like_C Family
Domain Color
B_lectin Domain
DUF3403 Family
Lectin_legB Domain
Motile_Sperm Domain
PAN_2 Domain
PK_Tyr_Ser−Thr Domain
Pkinase Domain
S_locus_glycop Domain
Stress−antifung Family
WD40 Repeat
other Domain
0
500
1000

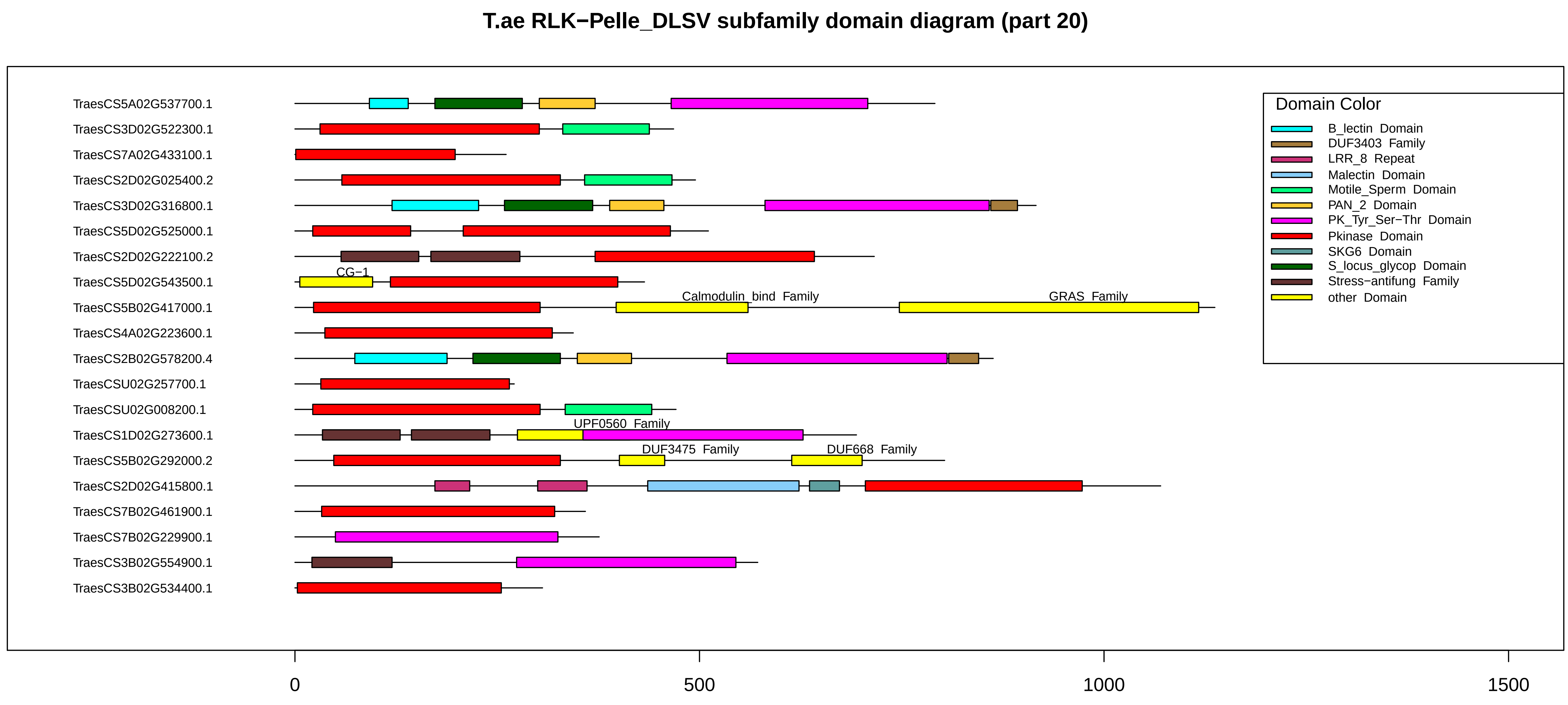
T.ae RLK−Pelle_DLSV subfamily domain diagram (part 20)
TraesCS5A02G537700.1
TraesCS3D02G522300.1
TraesCS7A02G433100.1
TraesCS2D02G025400.2
TraesCS3D02G316800.1
TraesCS5D02G525000.1
TraesCS2D02G222100.2
TraesCS5D02G543500.1
TraesCS5B02G417000.1
TraesCS4A02G223600.1
TraesCS2B02G578200.4
TraesCSU02G257700.1
TraesCSU02G008200.1
TraesCS1D02G273600.1
TraesCS5B02G292000.2
TraesCS2D02G415800.1
TraesCS7B02G461900.1
TraesCS7B02G229900.1
TraesCS3B02G554900.1
TraesCS3B02G534400.1
CG−1
Calmodulin_bind Family
GRAS Family
UPF0560 Family
DUF3475 Family
DUF668 Family
Domain Color
B_lectin Domain
DUF3403 Family
LRR_8 Repeat
Malectin Domain
Motile_Sperm Domain
PAN_2 Domain
PK_Tyr_Ser−Thr Domain
Pkinase Domain
SKG6 Domain
S_locus_glycop Domain
Stress−antifung Family
other Domain
0
500
1000
1500

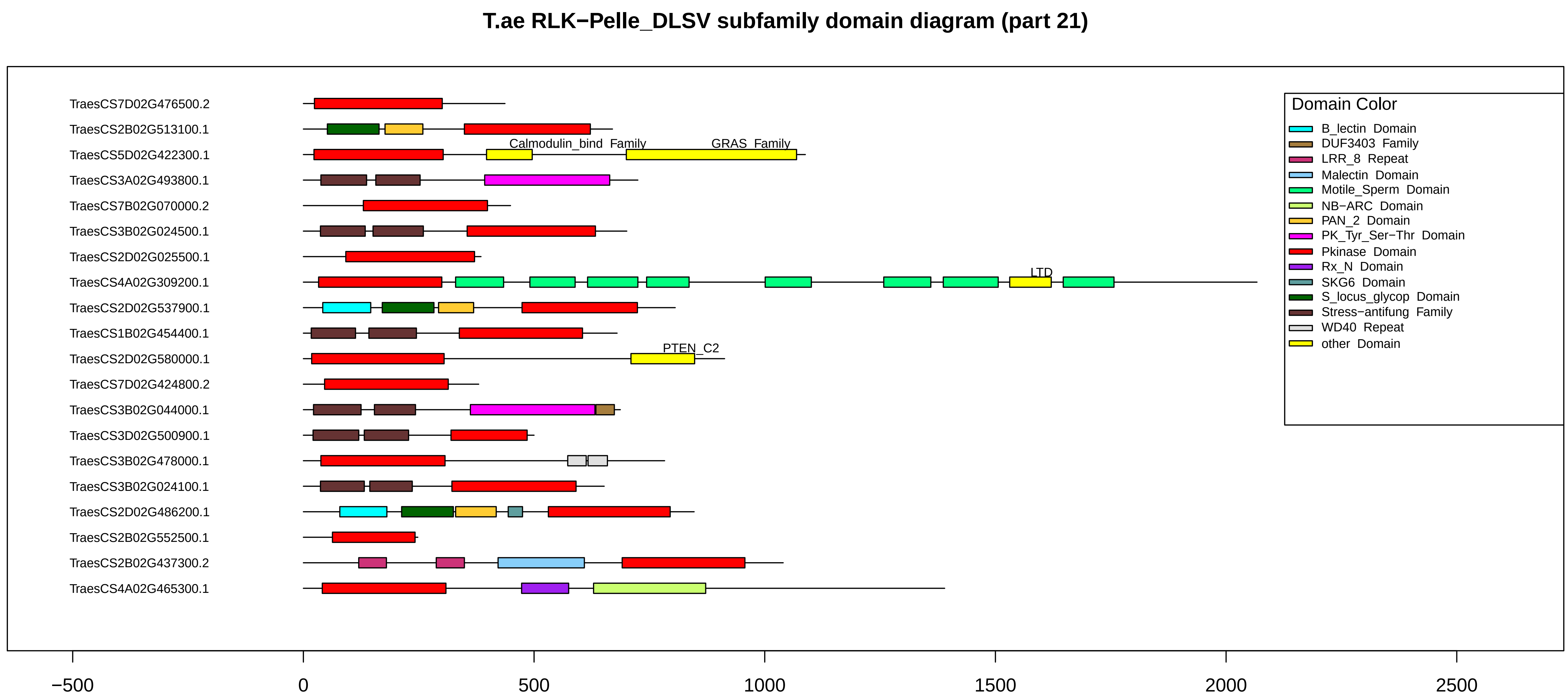
T.ae RLK−Pelle_DLSV subfamily domain diagram (part 21)
TraesCS7D02G476500.2
TraesCS2B02G513100.1
TraesCS5D02G422300.1
Calmodulin_bind Family
GRAS Family
TraesCS3A02G493800.1
TraesCS7B02G070000.2
TraesCS3B02G024500.1
TraesCS2D02G025500.1
TraesCS4A02G309200.1
LTD
TraesCS2D02G537900.1
TraesCS1B02G454400.1
TraesCS2D02G580000.1
PTEN_C2
TraesCS7D02G424800.2
TraesCS3B02G044000.1
TraesCS3D02G500900.1
TraesCS3B02G478000.1
TraesCS3B02G024100.1
TraesCS2D02G486200.1
TraesCS2B02G552500.1
TraesCS2B02G437300.2
TraesCS4A02G465300.1
Domain Color
B_lectin Domain
DUF3403 Family
LRR_8 Repeat
Malectin Domain
Motile_Sperm Domain
NB−ARC Domain
PAN_2 Domain
PK_Tyr_Ser−Thr Domain
Pkinase Domain
Rx_N Domain
SKG6 Domain
S_locus_glycop Domain
Stress−antifung Family
WD40 Repeat
other Domain
−500
0
500
1000
1500
2000
2500

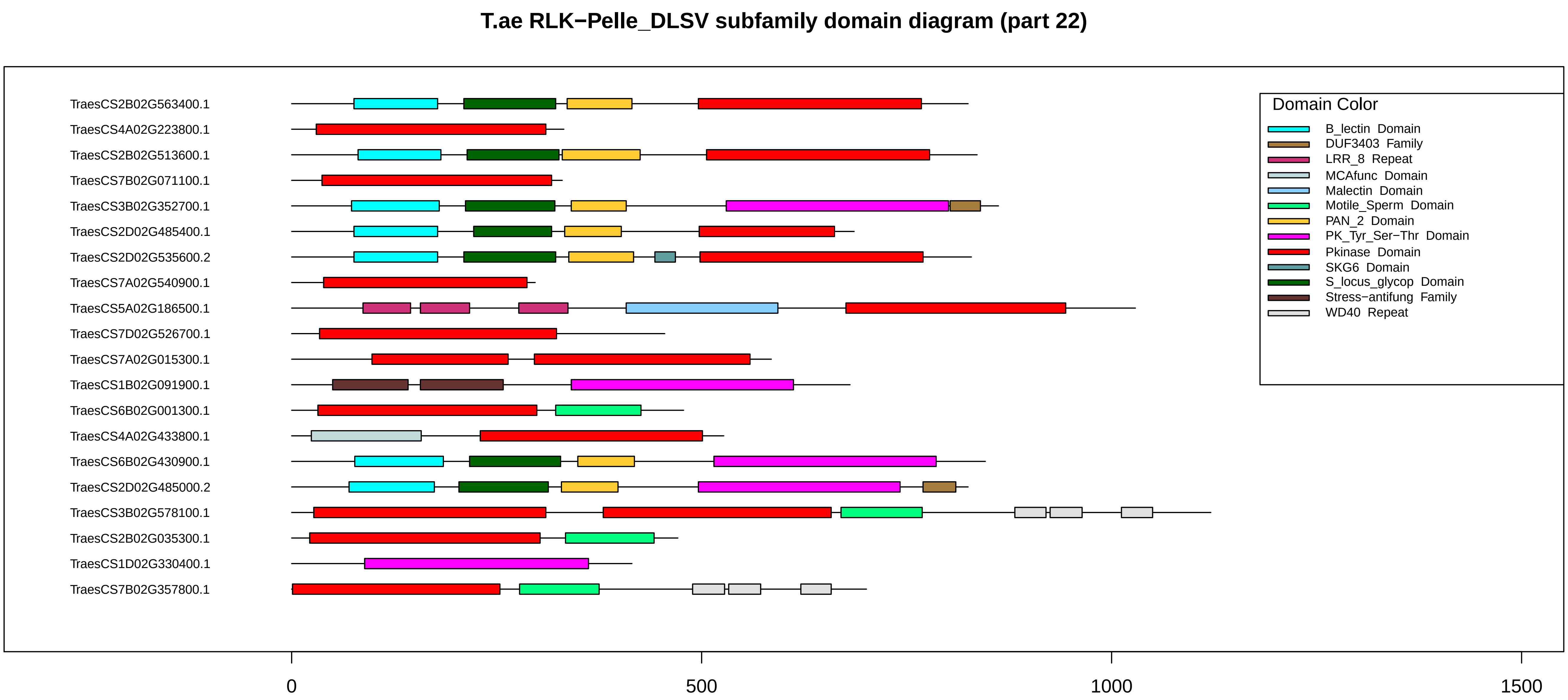
T.ae RLK−Pelle_DLSV subfamily domain diagram (part 22)
TraesCS2B02G563400.1
TraesCS4A02G223800.1
TraesCS2B02G513600.1
TraesCS7B02G071100.1
TraesCS3B02G352700.1
TraesCS2D02G485400.1
TraesCS2D02G535600.2
TraesCS7A02G540900.1
TraesCS5A02G186500.1
TraesCS7D02G526700.1
TraesCS7A02G015300.1
TraesCS1B02G091900.1
TraesCS6B02G001300.1
TraesCS4A02G433800.1
TraesCS6B02G430900.1
TraesCS2D02G485000.2
TraesCS3B02G578100.1
TraesCS2B02G035300.1
TraesCS1D02G330400.1
TraesCS7B02G357800.1
Domain Color
B_lectin Domain
DUF3403 Family
LRR_8 Repeat
MCAfunc Domain
Malectin Domain
Motile_Sperm Domain
PAN_2 Domain
PK_Tyr_Ser−Thr Domain
Pkinase Domain
SKG6 Domain
S_locus_glycop Domain
Stress−antifung Family
WD40 Repeat
0
500
1000
1500

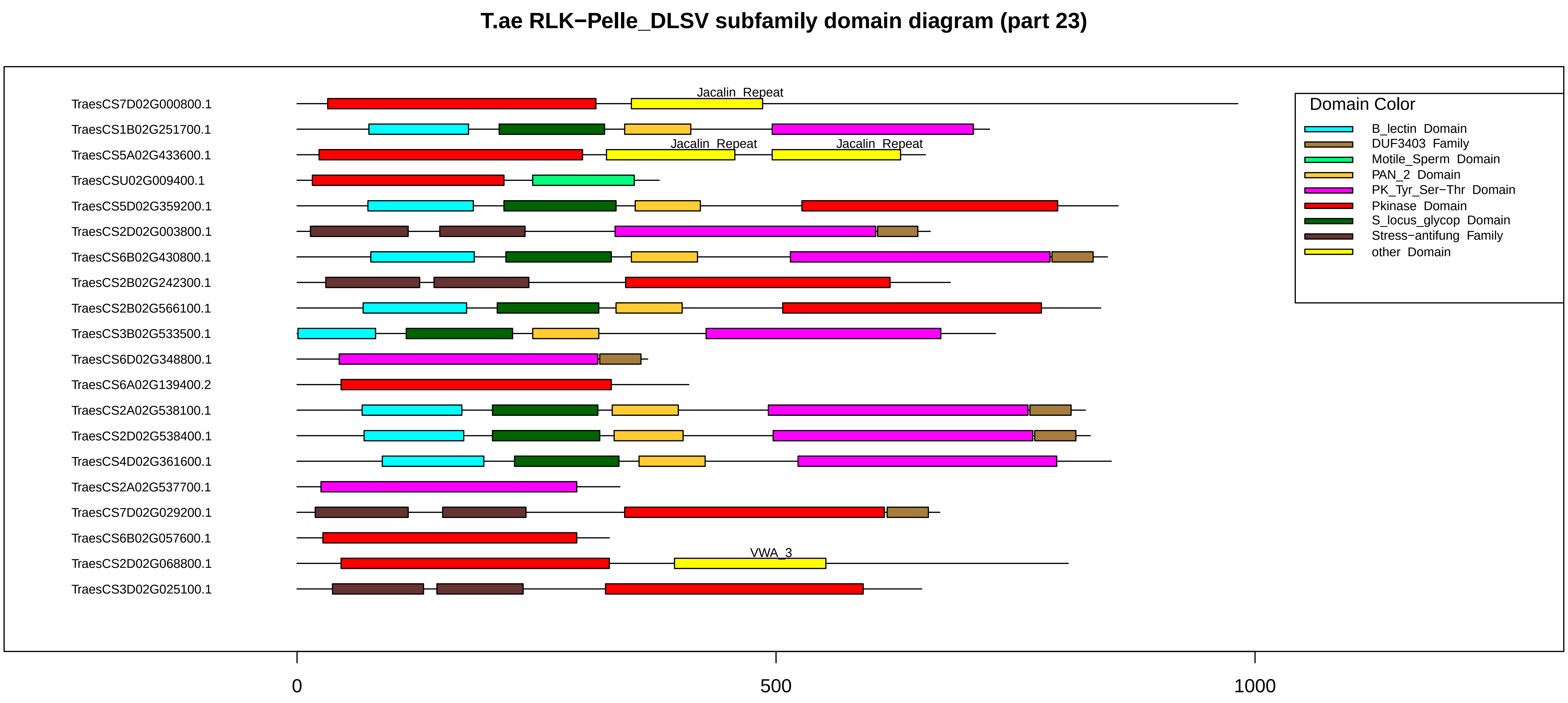

T.ae RLK−Pelle_DLSV subfamily domain diagram (part 23)
TraesCS7D02G000800.1
TraesCS1B02G251700.1
TraesCS5A02G433600.1
TraesCSU02G009400.1
TraesCS5D02G359200.1
TraesCS2D02G003800.1
TraesCS6B02G430800.1
TraesCS2B02G242300.1
TraesCS2B02G566100.1
TraesCS3B02G533500.1
TraesCS6D02G348800.1
TraesCS6A02G139400.2
TraesCS2A02G538100.1
TraesCS2D02G538400.1
TraesCS4D02G361600.1
TraesCS2A02G537700.1
TraesCS7D02G029200.1
TraesCS6B02G057600.1
TraesCS2D02G068800.1
TraesCS3D02G025100.1
Jacalin_Repeat
Jacalin_Repeat
Jacalin_Repeat
VWA_3
Domain Color
B_lectin Domain
DUF3403 Family
Motile_Sperm Domain
PAN_2 Domain
PK_Tyr_Ser−Thr Domain
Pkinase Domain
S_locus_glycop Domain
Stress−antifung Family
other Domain
0
500
1000

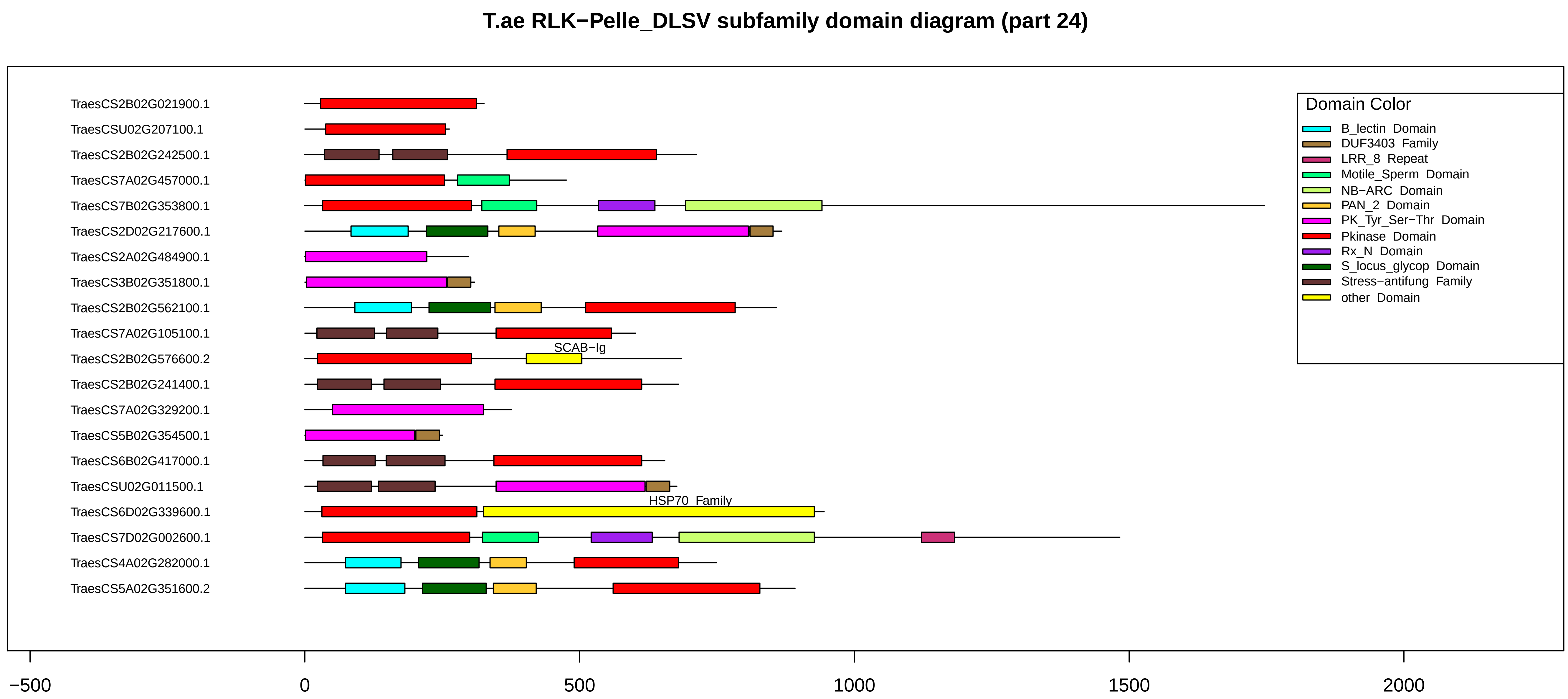

T.ae RLK−Pelle_DLSV subfamily domain diagram (part 24)
TraesCS2B02G021900.1
TraesCSU02G207100.1
TraesCS2B02G242500.1
TraesCS7A02G457000.1
TraesCS7B02G353800.1
TraesCS2D02G217600.1
TraesCS2A02G484900.1
TraesCS3B02G351800.1
TraesCS2B02G562100.1
TraesCS7A02G105100.1
TraesCS2B02G576600.2
TraesCS2B02G241400.1
TraesCS7A02G329200.1
TraesCS5B02G354500.1
TraesCS6B02G417000.1
TraesCSU02G011500.1
TraesCS6D02G339600.1
TraesCS7D02G002600.1
TraesCS4A02G282000.1
TraesCS5A02G351600.2
SCAB−Ig
HSP70_Family
Domain Color
B_lectin Domain
DUF3403 Family
LRR_8 Repeat
Motile_Sperm Domain
NB−ARC Domain
PAN_2 Domain
PK_Tyr_Ser−Thr Domain
Pkinase Domain
Rx_N Domain
S_locus_glycop Domain
Stress−antifung Family
other Domain
−500
0
500
1000
1500
2000

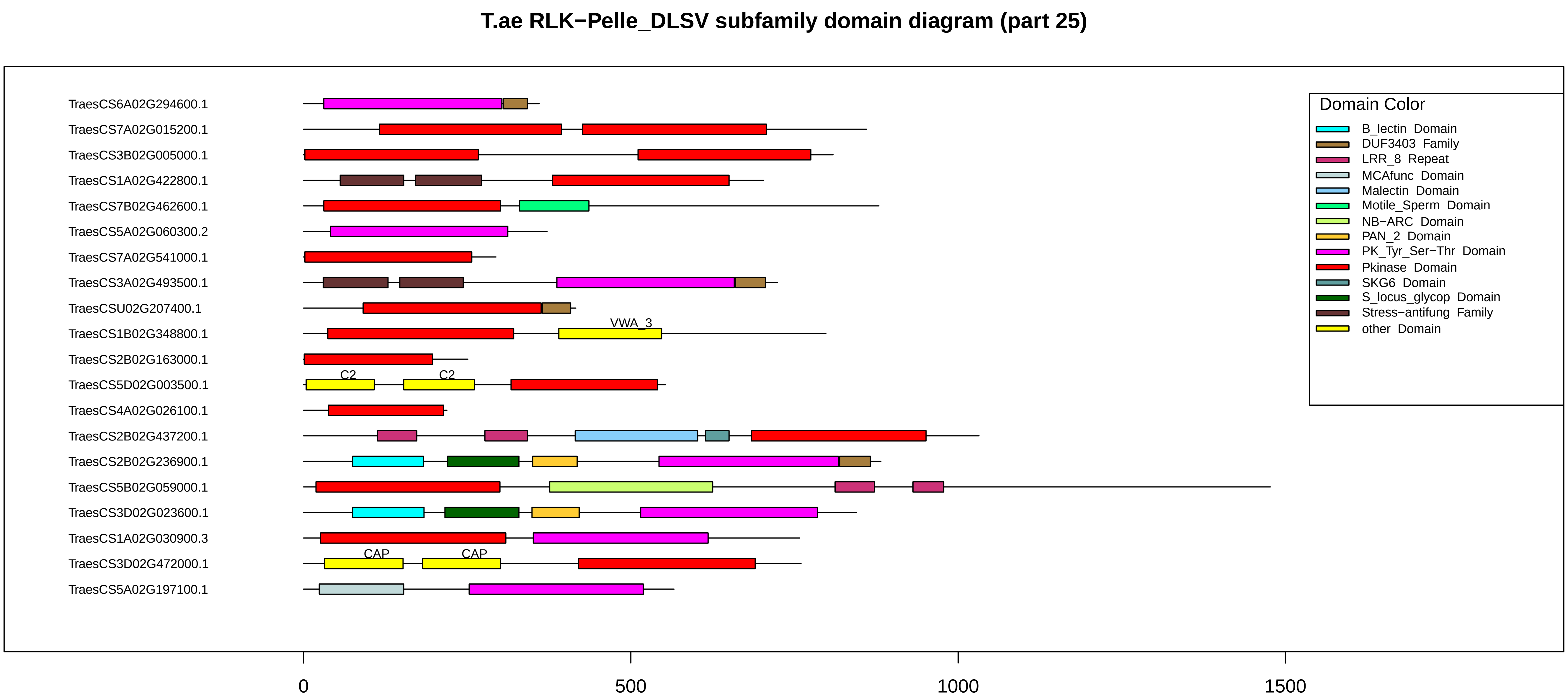
T.ae RLK−Pelle_DLSV subfamily domain diagram (part 25)
TraesCS6A02G294600.1
TraesCS7A02G015200.1
TraesCS3B02G005000.1
TraesCS1A02G422800.1
TraesCS7B02G462600.1
TraesCS5A02G060300.2
TraesCS7A02G541000.1
TraesCS3A02G493500.1
TraesCSU02G207400.1
TraesCS1B02G348800.1
VWA_3
TraesCS2B02G163000.1
TraesCS5D02G003500.1
C2
C2
TraesCS4A02G026100.1
TraesCS2B02G437200.1
TraesCS2B02G236900.1
TraesCS5B02G059000.1
TraesCS3D02G023600.1
TraesCS1A02G030900.3
TraesCS3D02G472000.1
CAP
CAP
TraesCS5A02G197100.1
Domain Color
B_lectin Domain
DUF3403 Family
LRR_8 Repeat
MCAfunc Domain
Malectin Domain
Motile_Sperm Domain
NB−ARC Domain
PAN_2 Domain
PK_Tyr_Ser−Thr Domain
Pkinase Domain
SKG6 Domain
S_locus_glycop Domain
Stress−antifung Family
other Domain
0
500
1000
1500

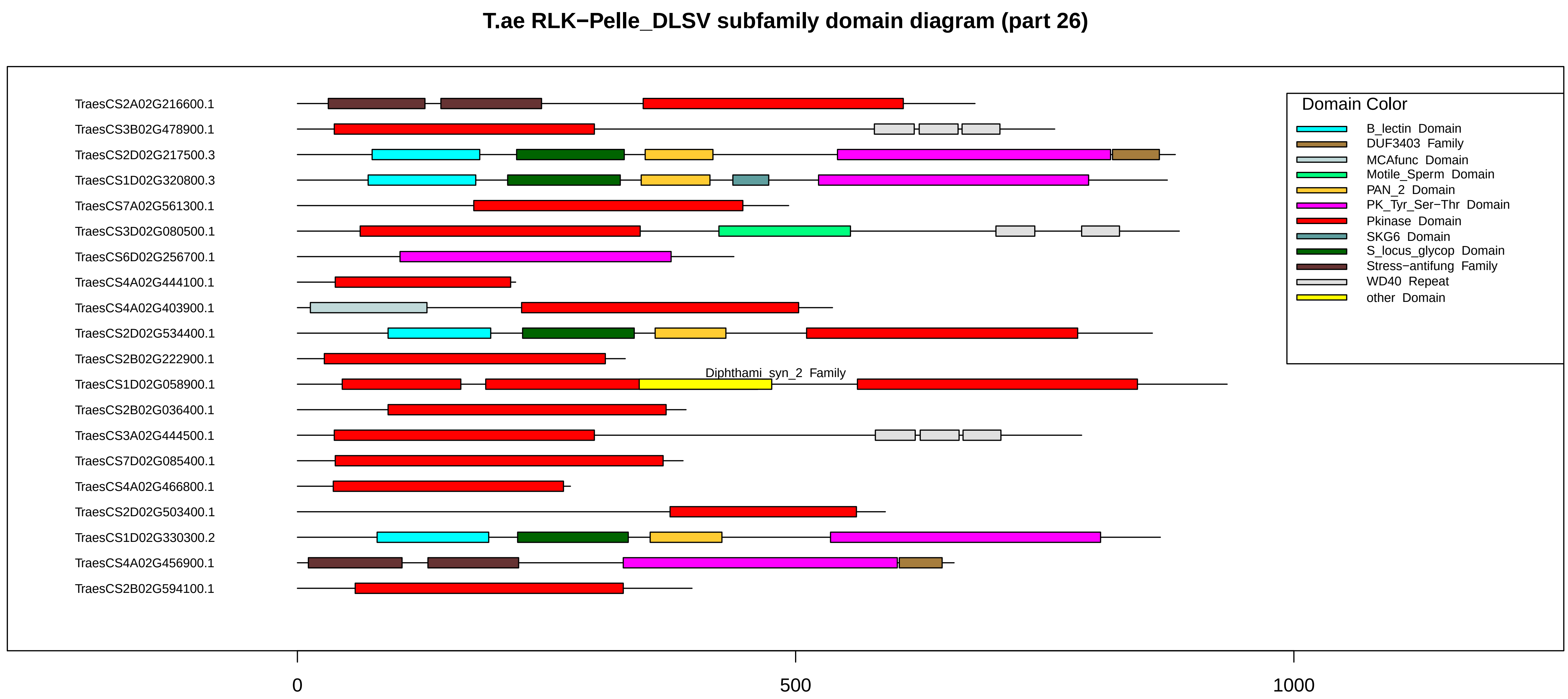
T.ae RLK−Pelle_DLSV subfamily domain diagram (part 26)
TraesCS2A02G216600.1
TraesCS3B02G478900.1
TraesCS2D02G217500.3
TraesCS1D02G320800.3
TraesCS7A02G561300.1
TraesCS3D02G080500.1
TraesCS6D02G256700.1
TraesCS4A02G444100.1
TraesCS4A02G403900.1
TraesCS2D02G534400.1
TraesCS2B02G222900.1
TraesCS1D02G058900.1
TraesCS2B02G036400.1
TraesCS3A02G444500.1
TraesCS7D02G085400.1
TraesCS4A02G466800.1
TraesCS2D02G503400.1
TraesCS1D02G330300.2
TraesCS4A02G456900.1
TraesCS2B02G594100.1
Diphthami_syn_2 Family
Domain Color
B_lectin Domain
DUF3403 Family
MCAfunc Domain
Motile_Sperm Domain
PAN_2 Domain
PK_Tyr_Ser−Thr Domain
Pkinase Domain
SKG6 Domain
S_locus_glycop Domain
Stress−antifung Family
WD40 Repeat
other Domain
0
500
1000

T.ae RLK−Pelle_DLSV subfamily domain diagram (part 27)

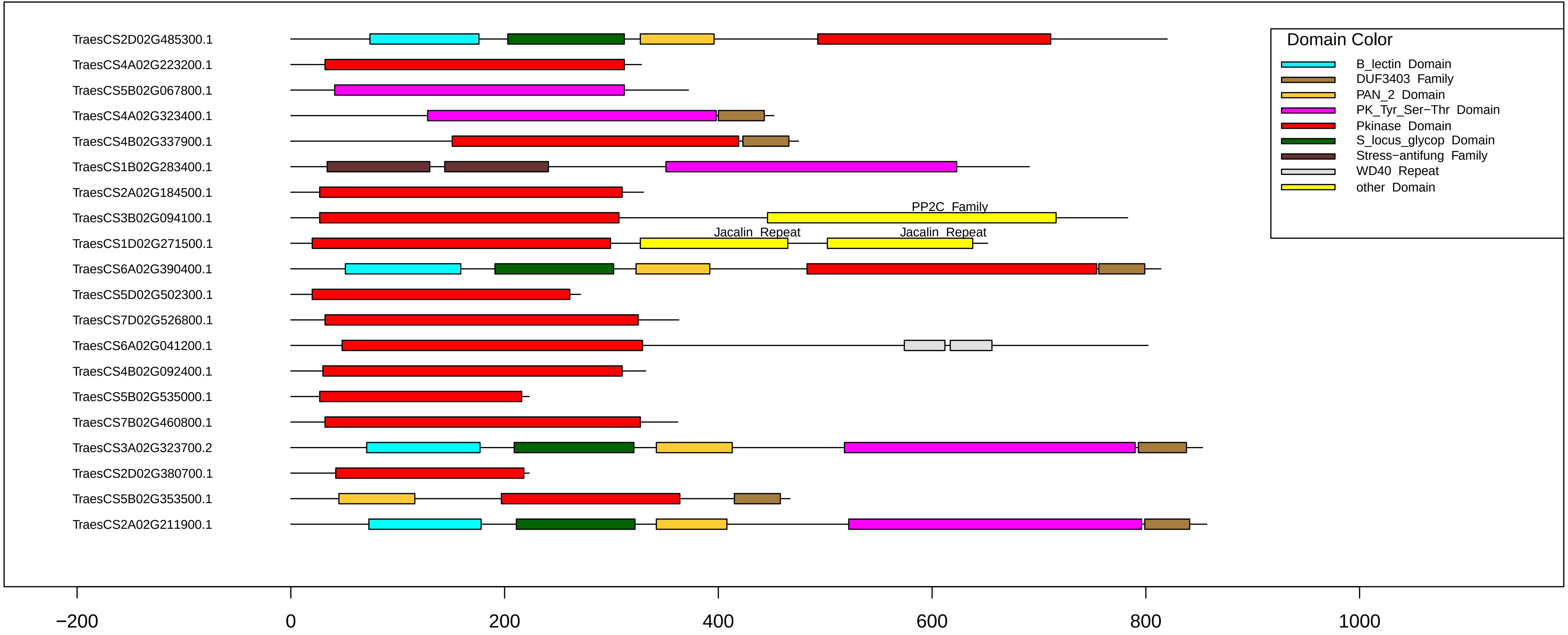

T.ae RLK−Pelle_DLSV subfamily domain diagram (part 28)

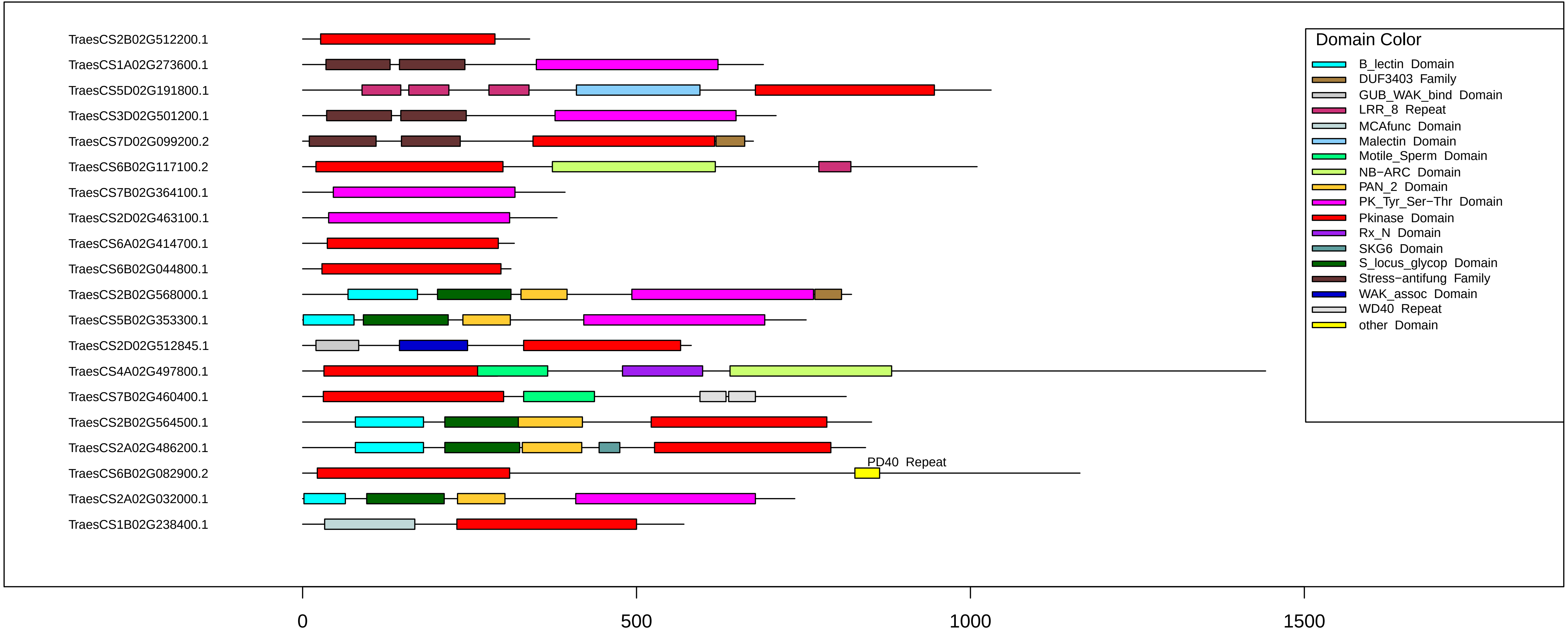

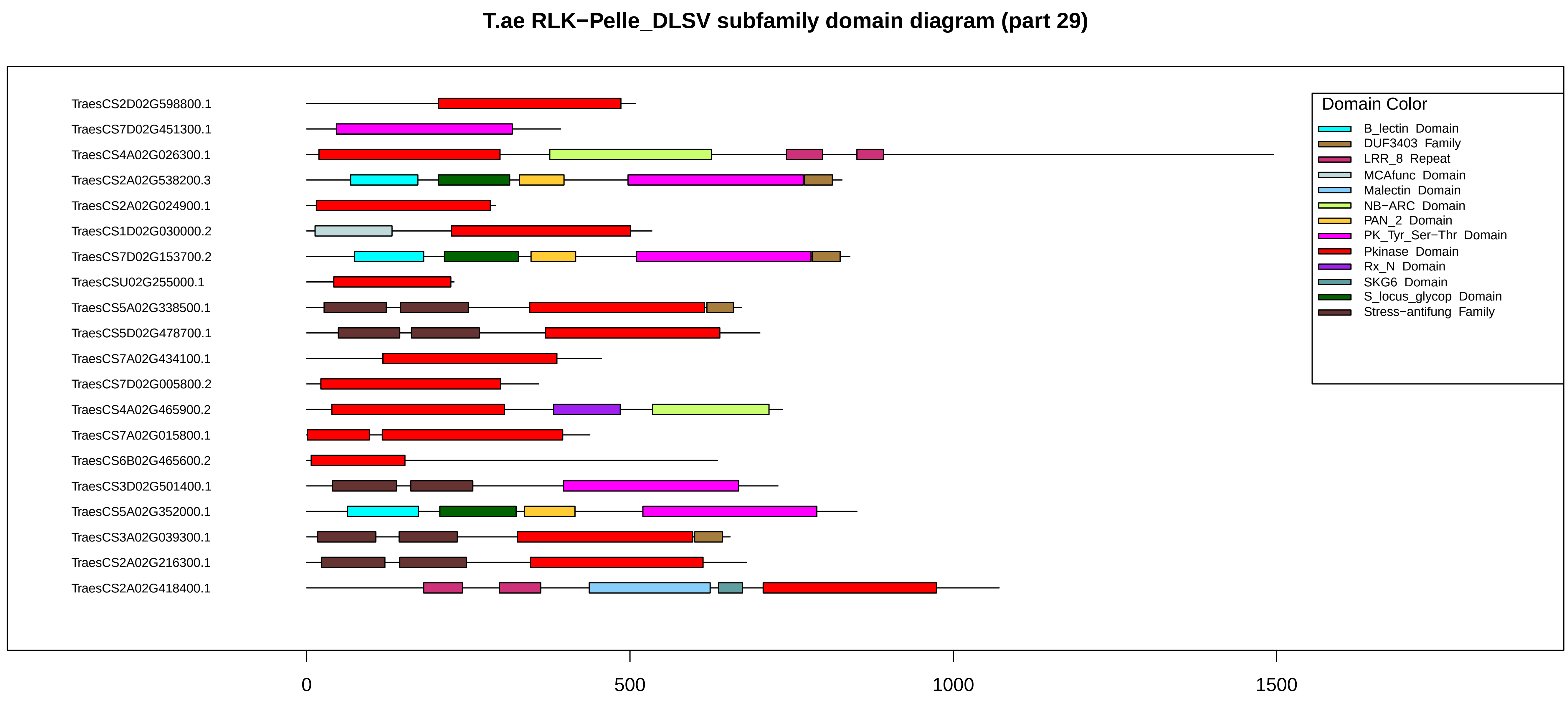
T.ae RLK−Pelle_DLSV subfamily domain diagram (part 29)
TraesCS2D02G598800.1
TraesCS7D02G451300.1
TraesCS4A02G026300.1
TraesCS2A02G538200.3
TraesCS2A02G024900.1
TraesCS1D02G030000.2
TraesCS7D02G153700.2
TraesCSU02G255000.1
TraesCS5A02G338500.1
TraesCS5D02G478700.1
TraesCS7A02G434100.1
TraesCS7D02G005800.2
TraesCS4A02G465900.2
TraesCS7A02G015800.1
TraesCS6B02G465600.2
TraesCS3D02G501400.1
TraesCS5A02G352000.1
TraesCS3A02G039300.1
TraesCS2A02G216300.1
TraesCS2A02G418400.1
Domain Color
B_lectin Domain
DUF3403 Family
LRR_8 Repeat
MCAfunc Domain
Malectin Domain
NB−ARC Domain
PAN_2 Domain
PK_Tyr_Ser−Thr Domain
Pkinase Domain
Rx_N Domain
SKG6 Domain
S_locus_glycop Domain
Stress−antifung Family
0
500
1000
1500

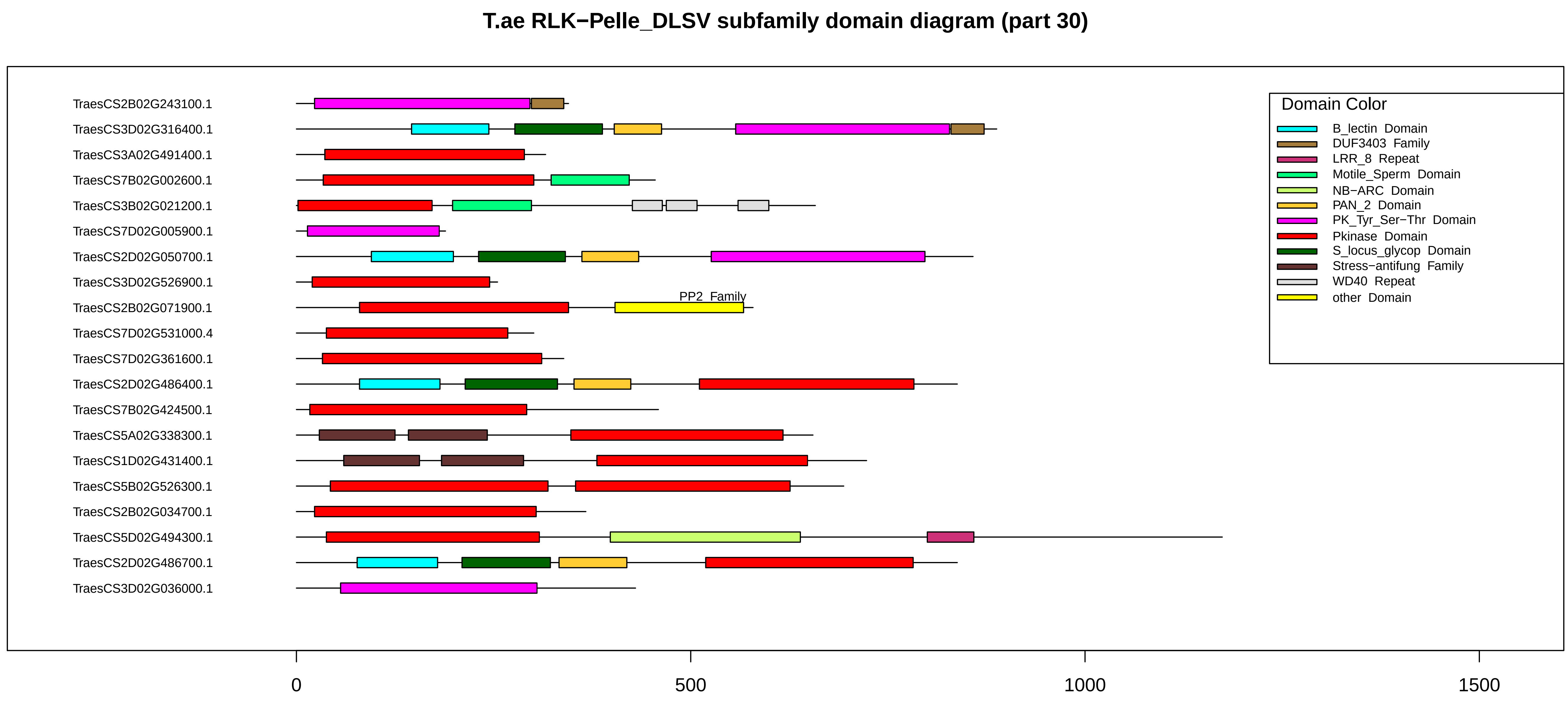
T.ae RLK−Pelle_DLSV subfamily domain diagram (part 30)
TraesCS2B02G243100.1
TraesCS3D02G316400.1
TraesCS3A02G491400.1
TraesCS7B02G002600.1
TraesCS3B02G021200.1
TraesCS7D02G005900.1
TraesCS2D02G050700.1
TraesCS3D02G526900.1
TraesCS2B02G071900.1
PP2_Family
TraesCS7D02G531000.4
TraesCS7D02G361600.1
TraesCS2D02G486400.1
TraesCS7B02G424500.1
TraesCS5A02G338300.1
TraesCS1D02G431400.1
TraesCS5B02G526300.1
TraesCS2B02G034700.1
TraesCS5D02G494300.1
TraesCS2D02G486700.1
TraesCS3D02G036000.1
Domain Color
B_lectin Domain
DUF3403 Family
LRR_8 Repeat
Motile_Sperm Domain
NB−ARC Domain
PAN_2 Domain
PK_Tyr_Ser−Thr Domain
Pkinase Domain
S_locus_glycop Domain
Stress−antifung Family
WD40 Repeat
other Domain
0
500
1000
1500

T.ae RLK−Pelle_DLSV subfamily domain diagram (part 31)

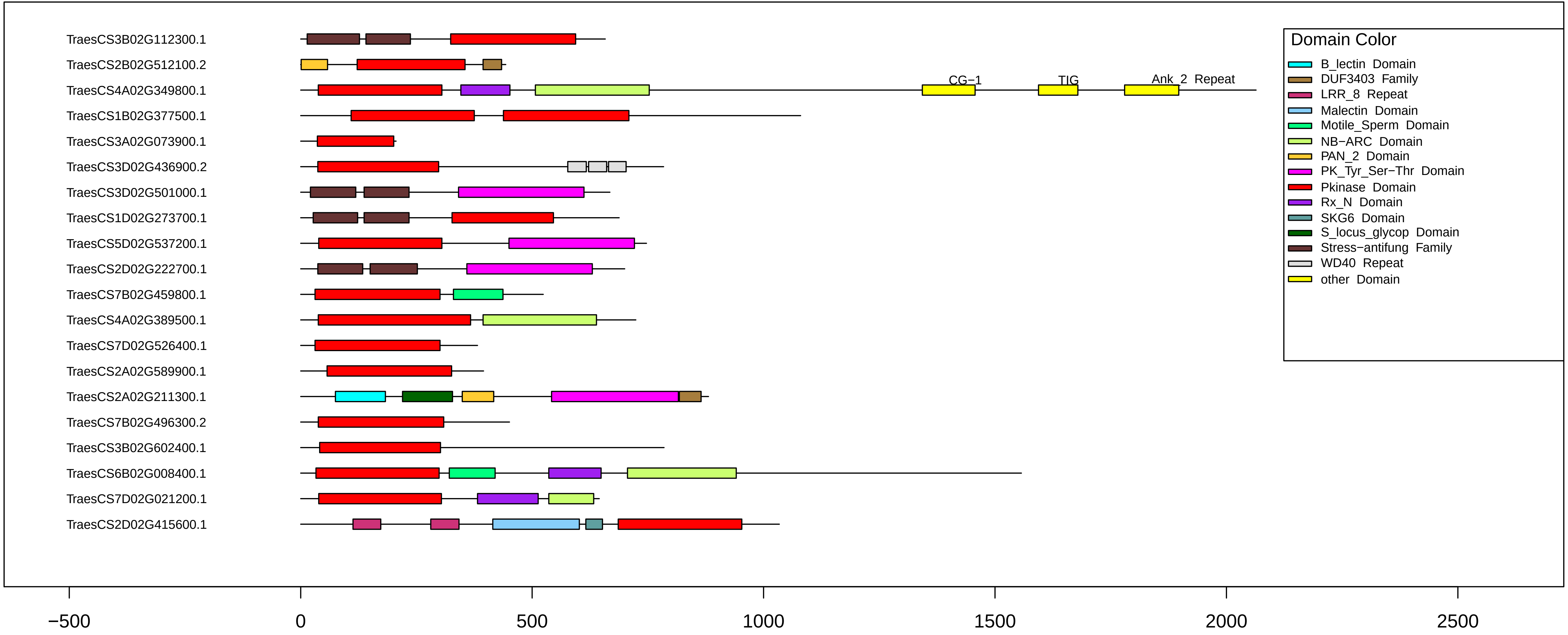

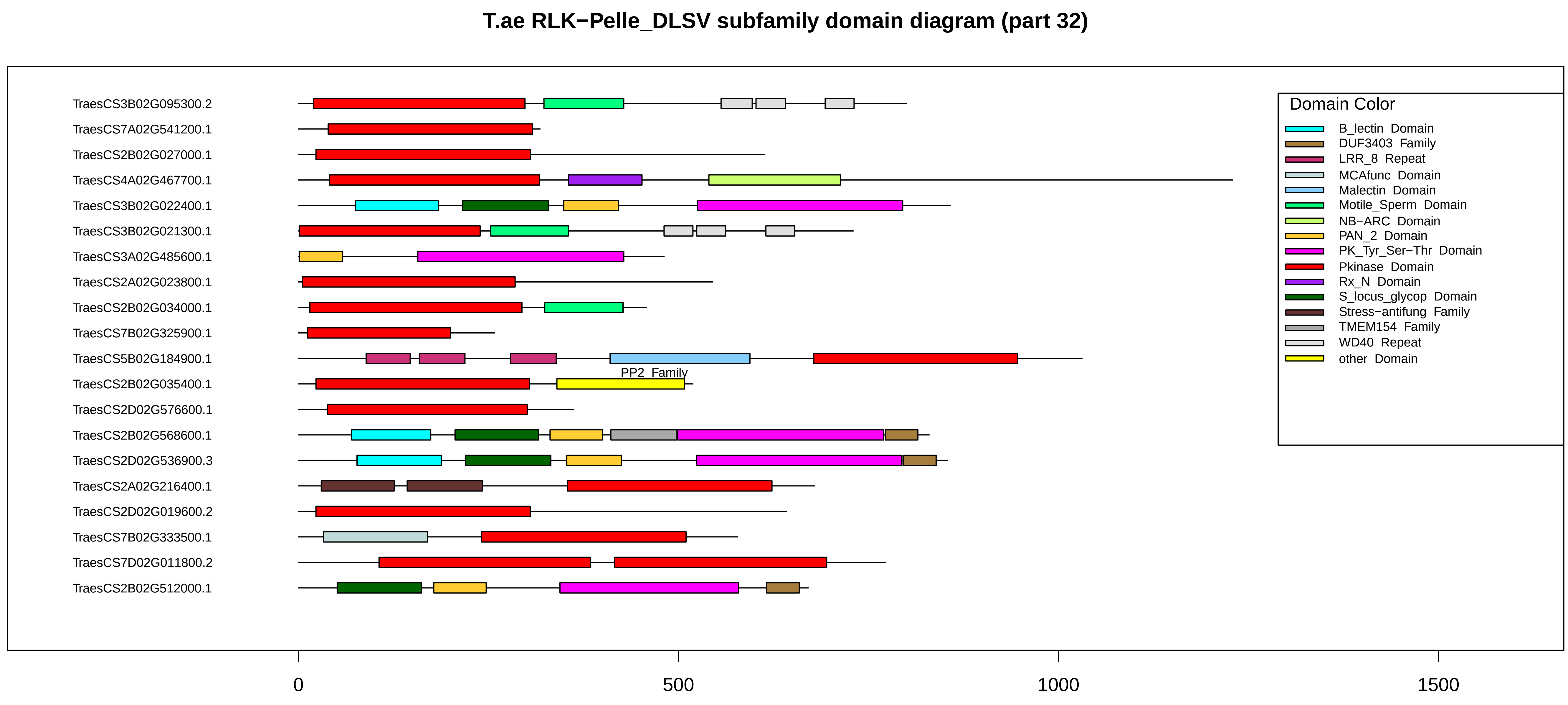

T.ae RLK−Pelle_DLSV subfamily domain diagram (part 32)
TraesCS3B02G095300.2
TraesCS7A02G541200.1
TraesCS2B02G027000.1
TraesCS4A02G467700.1
TraesCS3B02G022400.1
TraesCS3B02G021300.1
TraesCS3A02G485600.1
TraesCS2A02G023800.1
TraesCS2B02G034000.1
TraesCS7B02G325900.1
TraesCS5B02G184900.1
PP2_Family
TraesCS2B02G035400.1
TraesCS2D02G576600.1
TraesCS2B02G568600.1
TraesCS2D02G536900.3
TraesCS2A02G216400.1
TraesCS2D02G019600.2
TraesCS7B02G333500.1
TraesCS7D02G011800.2
TraesCS2B02G512000.1
Domain Color
B_lectin Domain
DUF3403 Family
LRR_8 Repeat
MCAfunc Domain
Malectin Domain
Motile_Sperm Domain
NB−ARC Domain
PAN_2 Domain
PK_Tyr_Ser−Thr Domain
Pkinase Domain
Rx_N Domain
S_locus_glycop Domain
Stress−antifung Family
TMEM154 Family
WD40 Repeat
other Domain
0
500
1000
1500

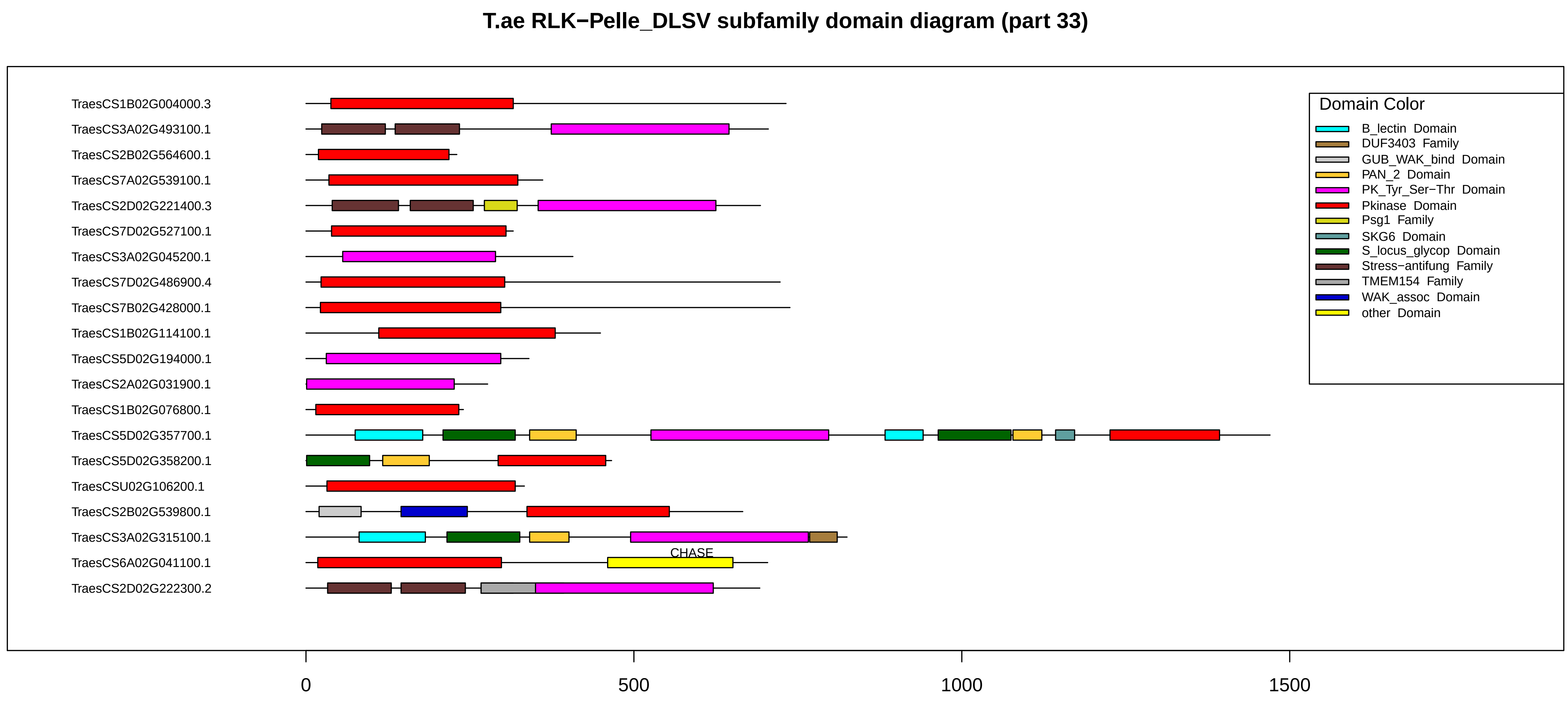

T.ae RLK−Pelle_DLSV subfamily domain diagram (part 33)
TraesCS1B02G004000.3
TraesCS3A02G493100.1
TraesCS2B02G564600.1
TraesCS7A02G539100.1
TraesCS2D02G221400.3
TraesCS7D02G527100.1
TraesCS3A02G045200.1
TraesCS7D02G486900.4
TraesCS7B02G428000.1
TraesCS1B02G114100.1
TraesCS5D02G194000.1
TraesCS2A02G031900.1
TraesCS1B02G076800.1
TraesCS5D02G357700.1
TraesCS5D02G358200.1
TraesCSU02G106200.1
TraesCS2B02G539800.1
TraesCS3A02G315100.1
TraesCS6A02G041100.1
TraesCS2D02G222300.2
CHASE
Domain Color
B_lectin Domain
DUF3403 Family
GUB_WAK_bind Domain
PAN_2 Domain
PK_Tyr_Ser−Thr Domain
Pkinase Domain
Psg1 Family
SKG6 Domain
S_locus_glycop Domain
Stress−antifung Family
TMEM154 Family
WAK_assoc Domain
other Domain
0
500
1000
1500

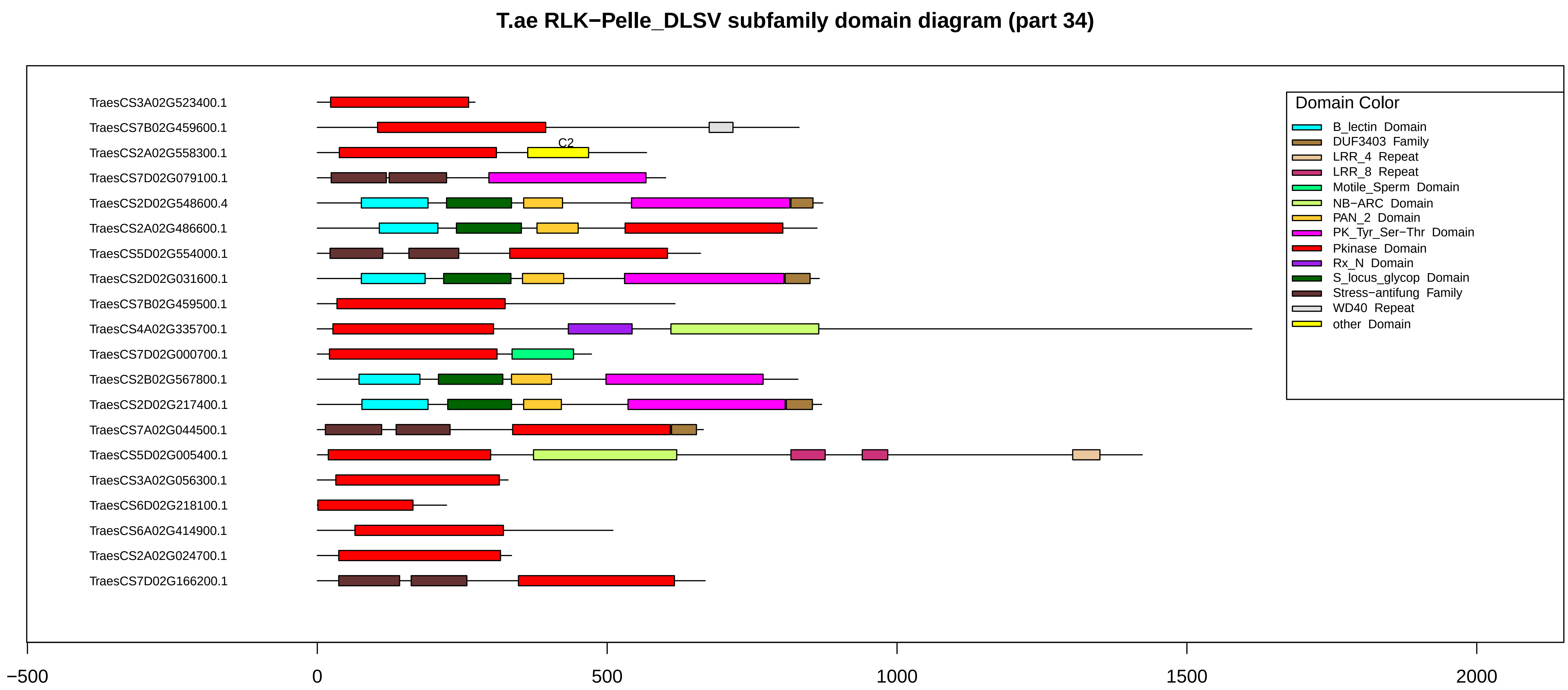
T.ae RLK−Pelle_DLSV subfamily domain diagram (part 34)
TraesCS3A02G523400.1
TraesCS7B02G459600.1
TraesCS2A02G558300.1
TraesCS7D02G079100.1
TraesCS2D02G548600.4
TraesCS2A02G486600.1
TraesCS5D02G554000.1
TraesCS2D02G031600.1
TraesCS7B02G459500.1
TraesCS4A02G335700.1
TraesCS7D02G000700.1
TraesCS2B02G567800.1
TraesCS2D02G217400.1
TraesCS7A02G044500.1
TraesCS5D02G005400.1
TraesCS3A02G056300.1
TraesCS6D02G218100.1
TraesCS6A02G414900.1
TraesCS2A02G024700.1
TraesCS7D02G166200.1
C2
Domain Color
B_lectin Domain
DUF3403 Family
LRR_4 Repeat
LRR_8 Repeat
Motile_Sperm Domain
NB−ARC Domain
PAN_2 Domain
PK_Tyr_Ser−Thr Domain
Pkinase Domain
Rx_N Domain
S_locus_glycop Domain
Stress−antifung Family
WD40 Repeat
other Domain
−500
0
500
1000
1500
2000

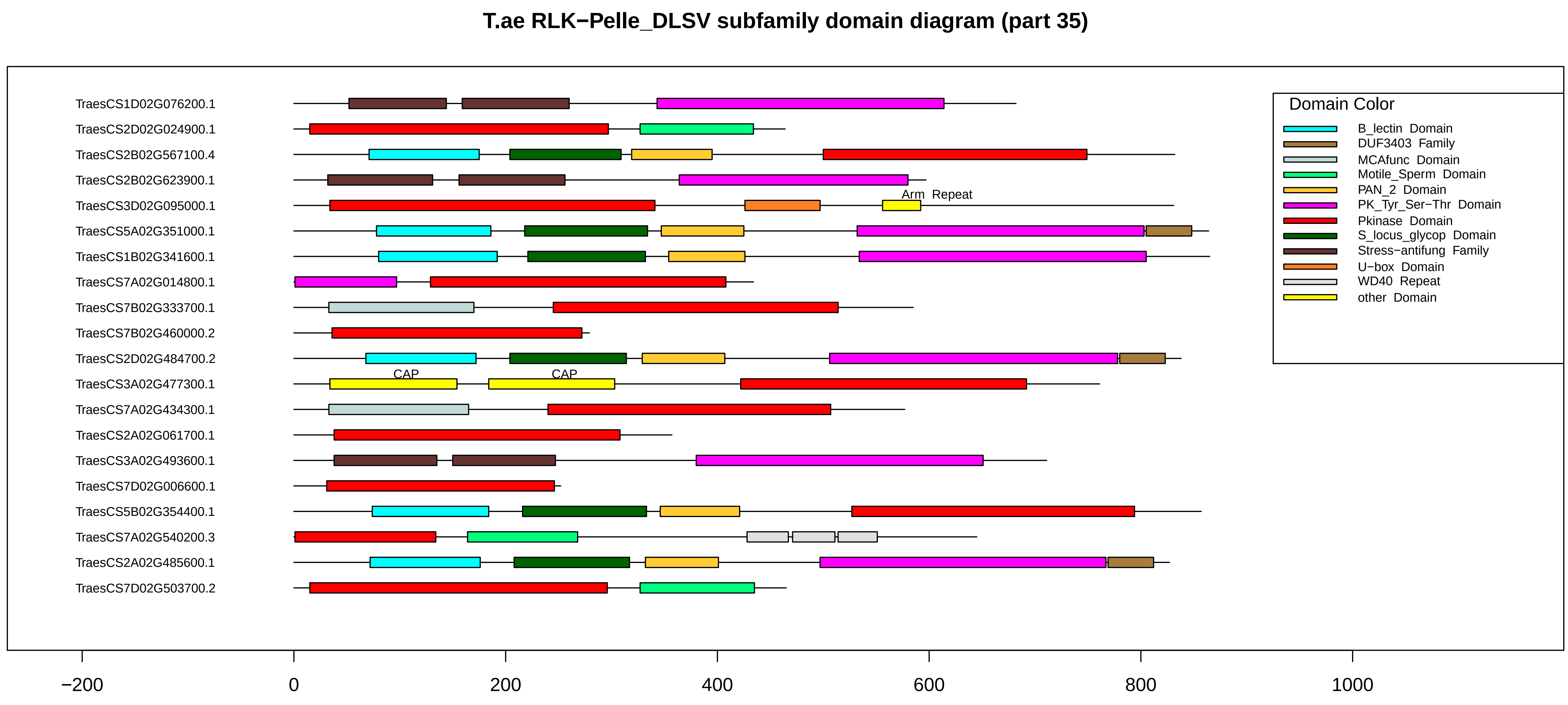
T.ae RLK−Pelle_DLSV subfamily domain diagram (part 35)
TraesCS1D02G076200.1
TraesCS2D02G024900.1
TraesCS2B02G567100.4
TraesCS2B02G623900.1
TraesCS3D02G095000.1
TraesCS5A02G351000.1
TraesCS1B02G341600.1
TraesCS7A02G014800.1
TraesCS7B02G333700.1
TraesCS7B02G460000.2
TraesCS2D02G484700.2
TraesCS3A02G477300.1
TraesCS7A02G434300.1
TraesCS2A02G061700.1
TraesCS3A02G493600.1
TraesCS7D02G006600.1
TraesCS5B02G354400.1
TraesCS7A02G540200.3
TraesCS2A02G485600.1
TraesCS7D02G503700.2
Arm Repeat
CAP
CAP
Domain Color
B_lectin Domain
DUF3403 Family
MCAfunc Domain
Motile_Sperm Domain
PAN_2 Domain
PK_Tyr_Ser−Thr Domain
Pkinase Domain
S_locus_glycop Domain
Stress−antifung Family
U−box Domain
WD40 Repeat
other Domain
−200
0
200
400
600
800
1000

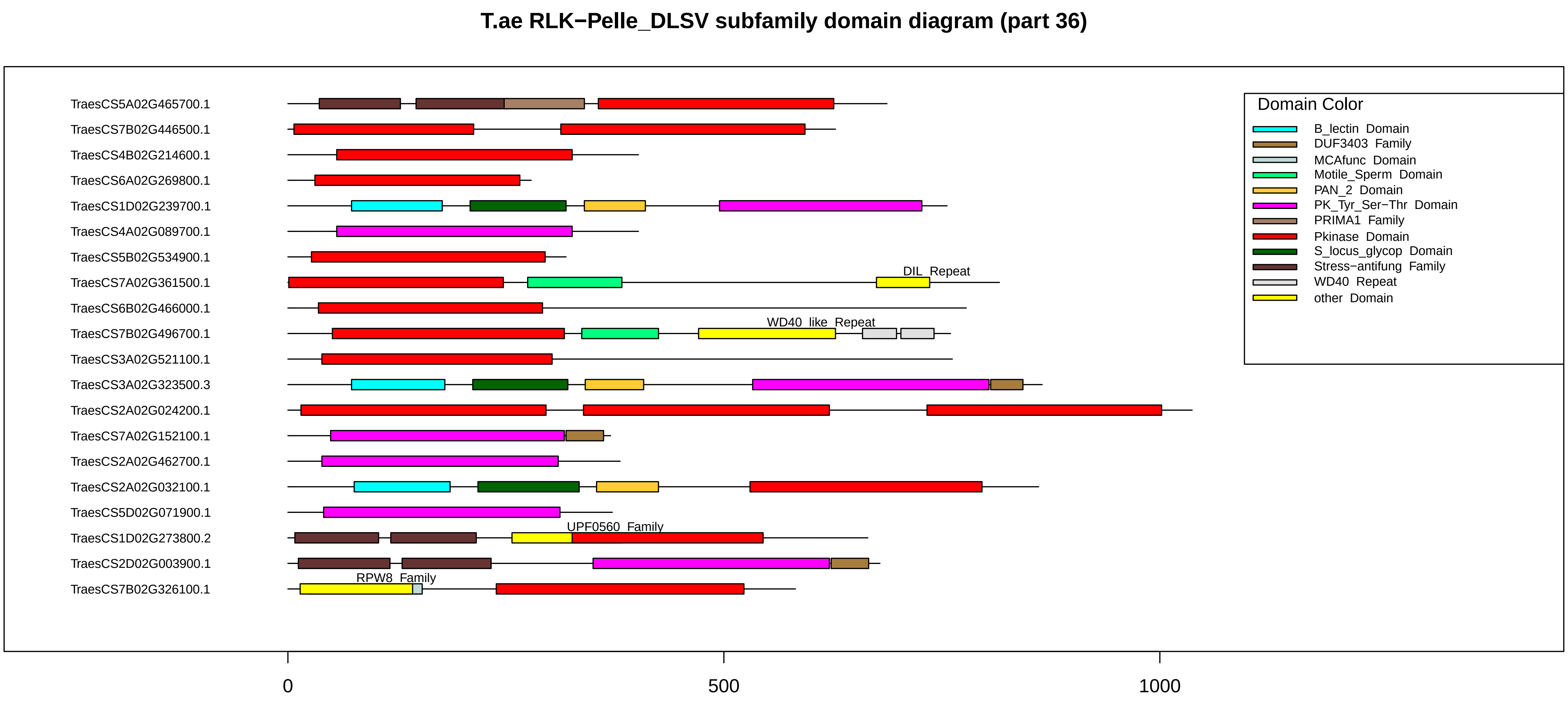
T.ae RLK−Pelle_DLSV subfamily domain diagram (part 36)
TraesCS5A02G465700.1
TraesCS7B02G446500.1
TraesCS4B02G214600.1
TraesCS6A02G269800.1
TraesCS1D02G239700.1
TraesCS4A02G089700.1
TraesCS5B02G534900.1
TraesCS7A02G361500.1
TraesCS6B02G466000.1
TraesCS7B02G496700.1
TraesCS3A02G521100.1
TraesCS3A02G323500.3
TraesCS2A02G024200.1
TraesCS7A02G152100.1
TraesCS2A02G462700.1
TraesCS2A02G032100.1
TraesCS5D02G071900.1
TraesCS1D02G273800.2
TraesCS2D02G003900.1
TraesCS7B02G326100.1
DIL_Repeat
WD40_like_Repeat
UPF0560_Family
RPW8_Family
Domain Color
B_lectin Domain
DUF3403 Family
MCAfunc Domain
Motile_Sperm Domain
PAN_2 Domain
PK_Tyr_Ser−Thr Domain
PRIMA1 Family
Pkinase Domain
S_locus_glycop Domain
Stress−antifung Family
WD40 Repeat
other Domain
0
500
1000

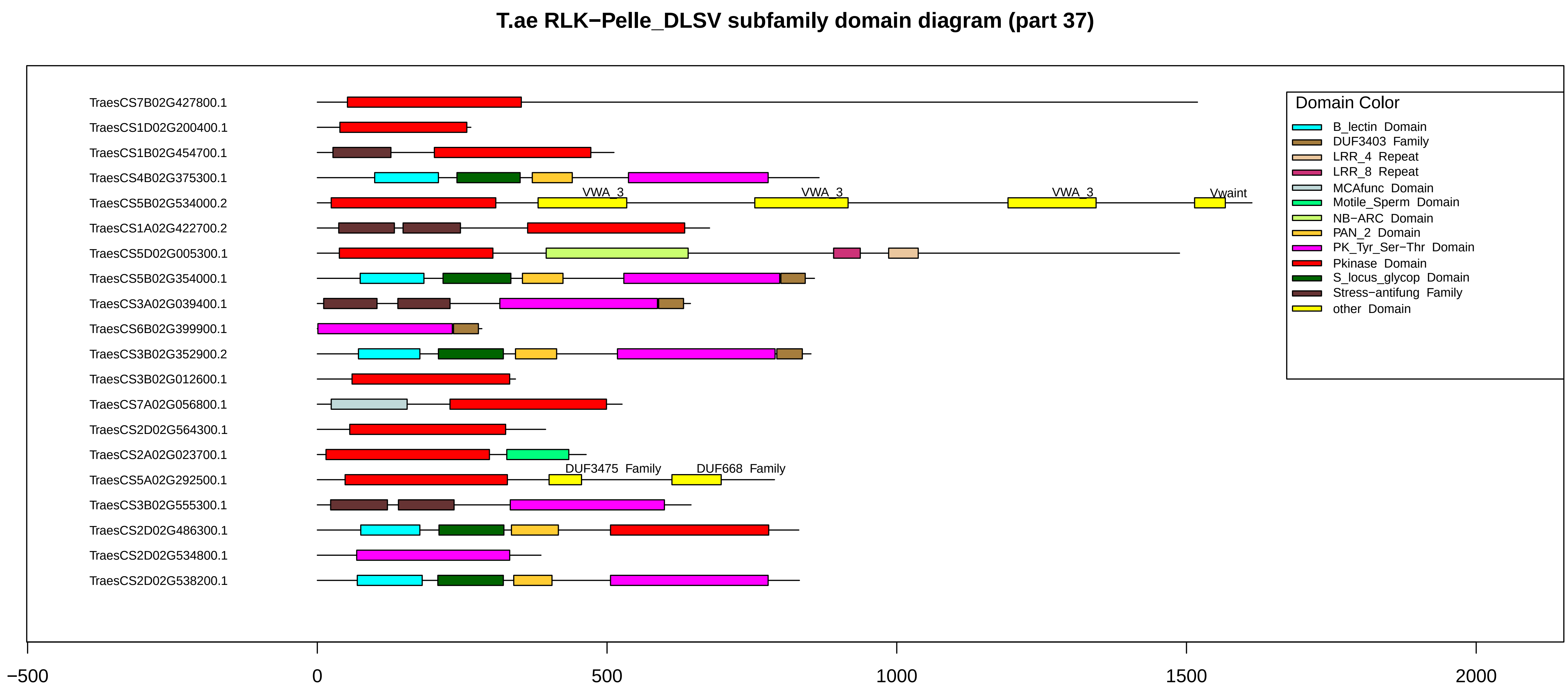

T.ae RLK−Pelle_DLSV subfamily domain diagram (part 37)
TraesCS7B02G427800.1
TraesCS1D02G200400.1
TraesCS1B02G454700.1
TraesCS4B02G375300.1
TraesCS5B02G534000.2
TraesCS1A02G422700.2
TraesCS5D02G005300.1
TraesCS5B02G354000.1
TraesCS3A02G039400.1
TraesCS6B02G399900.1
TraesCS3B02G352900.2
TraesCS3B02G012600.1
TraesCS7A02G056800.1
TraesCS2D02G564300.1
TraesCS2A02G023700.1
TraesCS5A02G292500.1
TraesCS3B02G555300.1
TraesCS2D02G486300.1
TraesCS2D02G534800.1
TraesCS2D02G538200.1
VWA_3
VWA_3
VWA_3
Vwaint
DUF3475 Family
DUF668 Family
Domain Color
B_lectin Domain
DUF3403 Family
LRR_4 Repeat
LRR_8 Repeat
MCAfunc Domain
Motile_Sperm Domain
NB−ARC Domain
PAN_2 Domain
PK_Tyr_Ser−Thr Domain
Pkinase Domain
S_locus_glycop Domain
Stress−antifung Family
other Domain
−500
0
500
1000
1500
2000

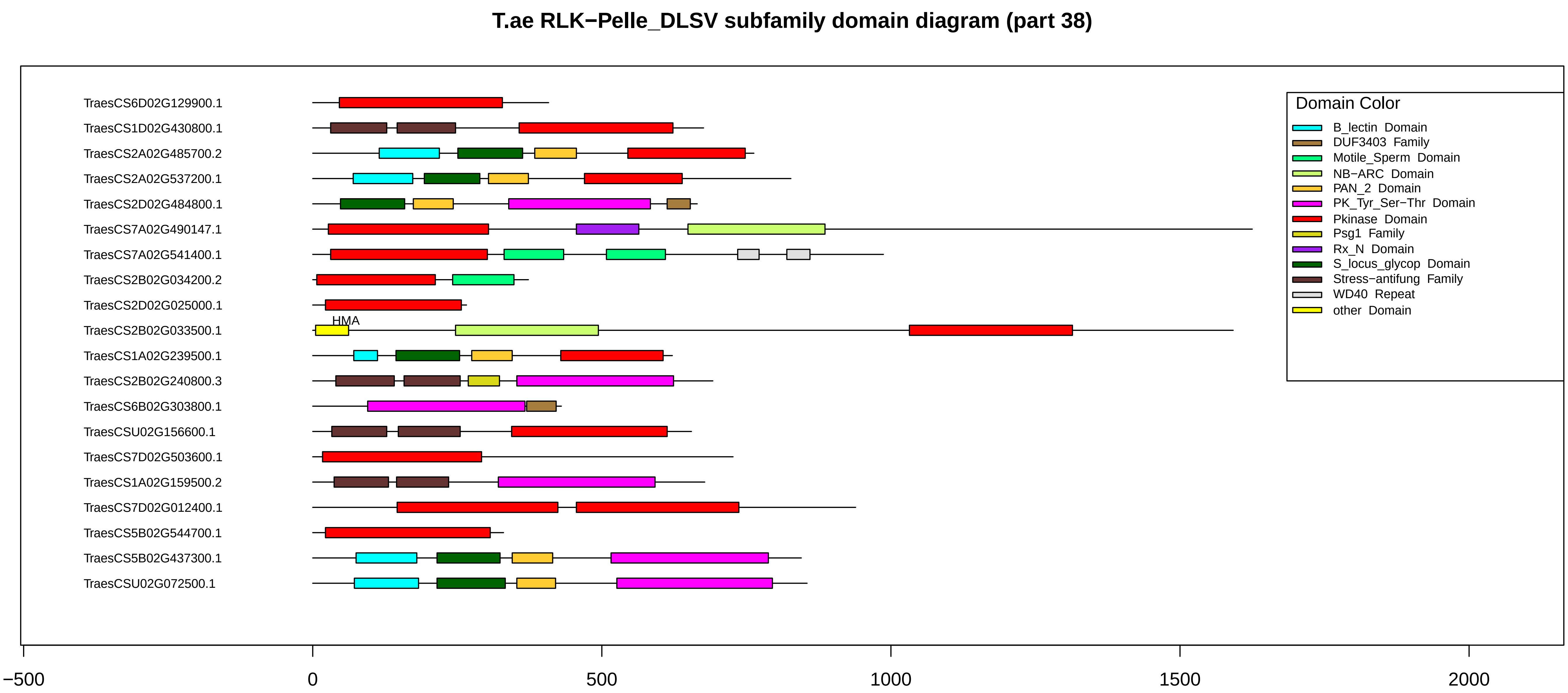

T.ae RLK−Pelle_DLSV subfamily domain diagram (part 38)
TraesCS6D02G129900.1
TraesCS1D02G430800.1
TraesCS2A02G485700.2
TraesCS2A02G537200.1
TraesCS2D02G484800.1
TraesCS7A02G490147.1
TraesCS7A02G541400.1
TraesCS2B02G034200.2
TraesCS2D02G025000.1
HMA
TraesCS2B02G033500.1
TraesCS1A02G239500.1
TraesCS2B02G240800.3
TraesCS6B02G303800.1
TraesCSU02G156600.1
TraesCS7D02G503600.1
TraesCS1A02G159500.2
TraesCS7D02G012400.1
TraesCS5B02G544700.1
TraesCS5B02G437300.1
TraesCSU02G072500.1
Domain Color
B_lectin Domain
DUF3403 Family
Motile_Sperm Domain
NB−ARC Domain
PAN_2 Domain
PK_Tyr_Ser−Thr Domain
Pkinase Domain
Psg1 Family
Rx_N Domain
S_locus_glycop Domain
Stress−antifung Family
WD40 Repeat
other Domain
−500
0
500
1000
1500
2000

T.ae RLK−Pelle_DLSV subfamily domain diagram (part 39)

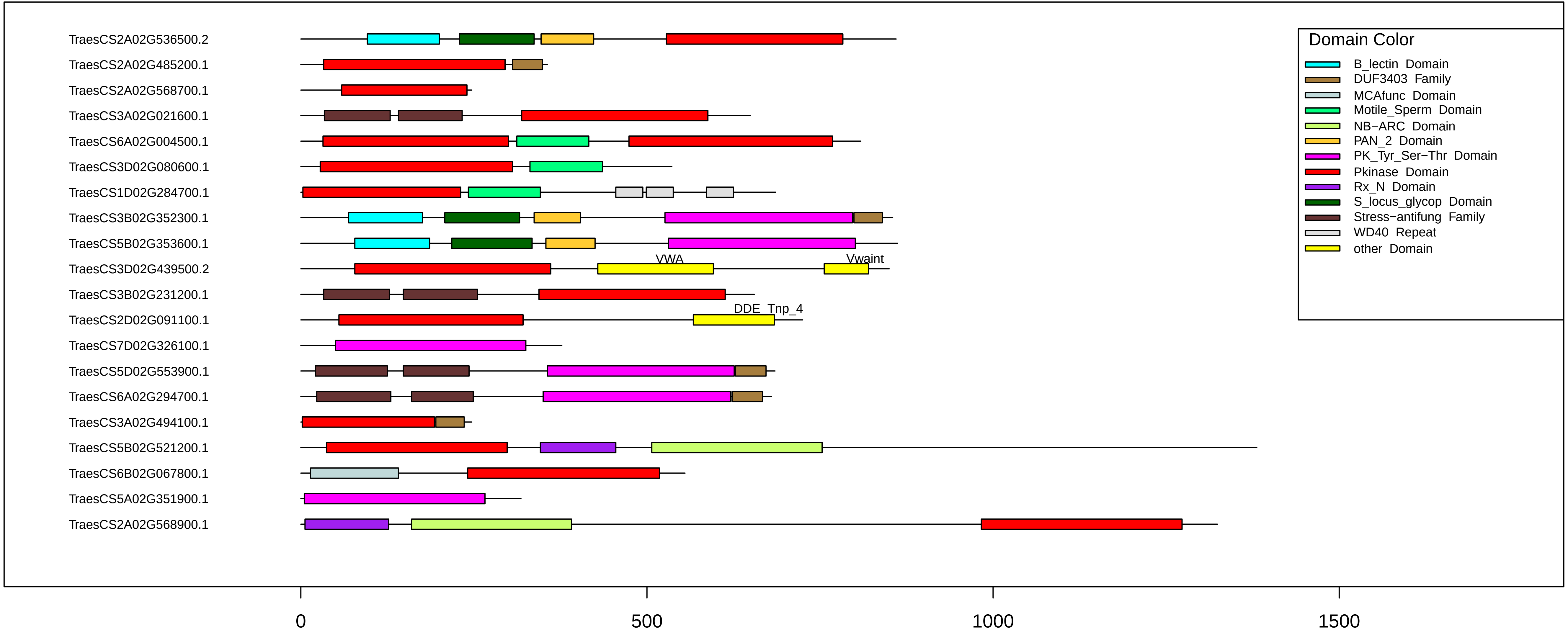

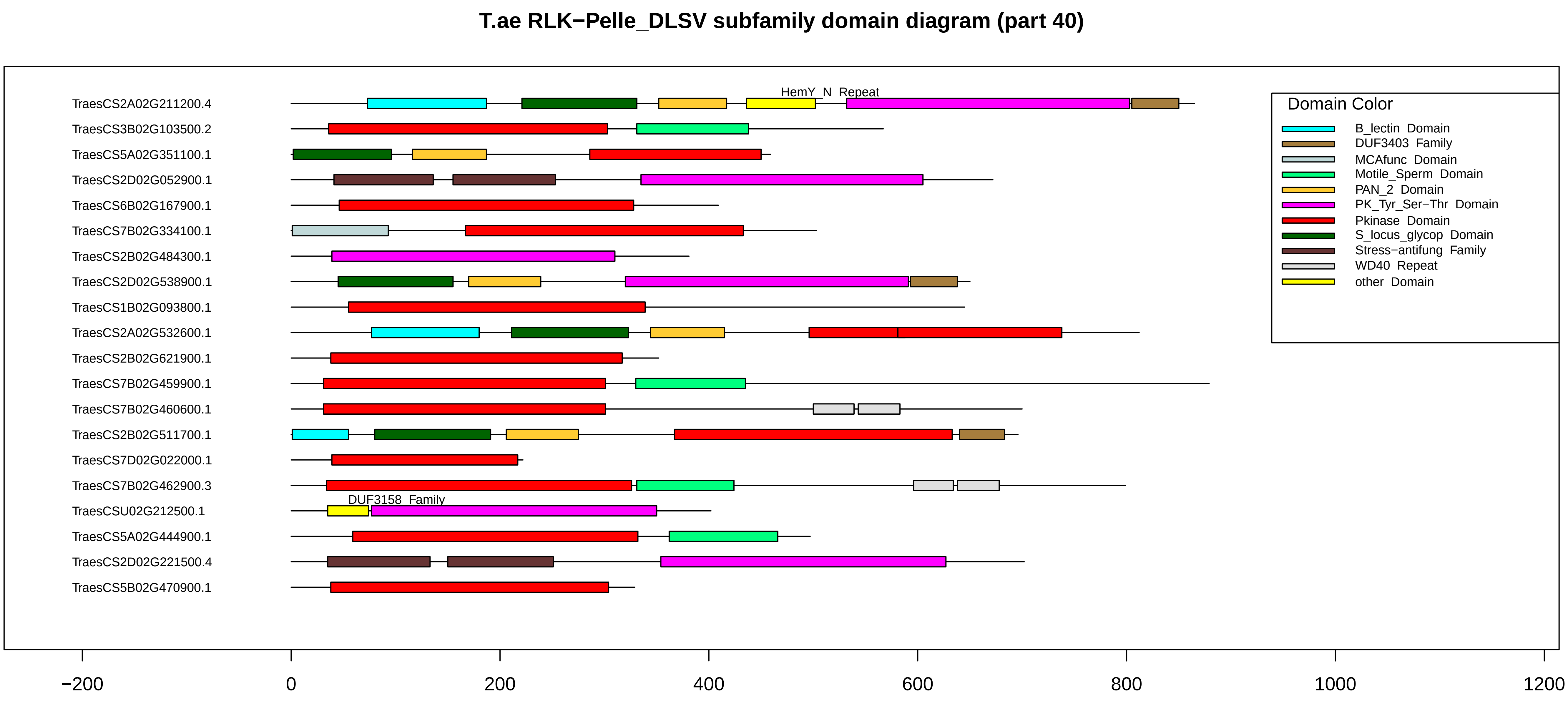
T.ae RLK−Pelle_DLSV subfamily domain diagram (part 40)
TraesCS2A02G211200.4
TraesCS3B02G103500.2
TraesCS5A02G351100.1
TraesCS2D02G052900.1
TraesCS6B02G167900.1
TraesCS7B02G334100.1
TraesCS2B02G484300.1
TraesCS2D02G538900.1
TraesCS1B02G093800.1
TraesCS2A02G532600.1
TraesCS2B02G621900.1
TraesCS7B02G459900.1
TraesCS7B02G460600.1
TraesCS2B02G511700.1
TraesCS7D02G022000.1
TraesCS7B02G462900.3
TraesCSU02G212500.1
TraesCS5A02G444900.1
TraesCS2D02G221500.4
TraesCS5B02G470900.1
HemY_N Repeat
DUF3158 Family
Domain Color
B_lectin Domain
DUF3403 Family
MCAfunc Domain
Motile_Sperm Domain
PAN_2 Domain
PK_Tyr_Ser−Thr Domain
Pkinase Domain
S_locus_glycop Domain
Stress−antifung Family
WD40 Repeat
other Domain
−200
0
200
400
600
800
1000
1200

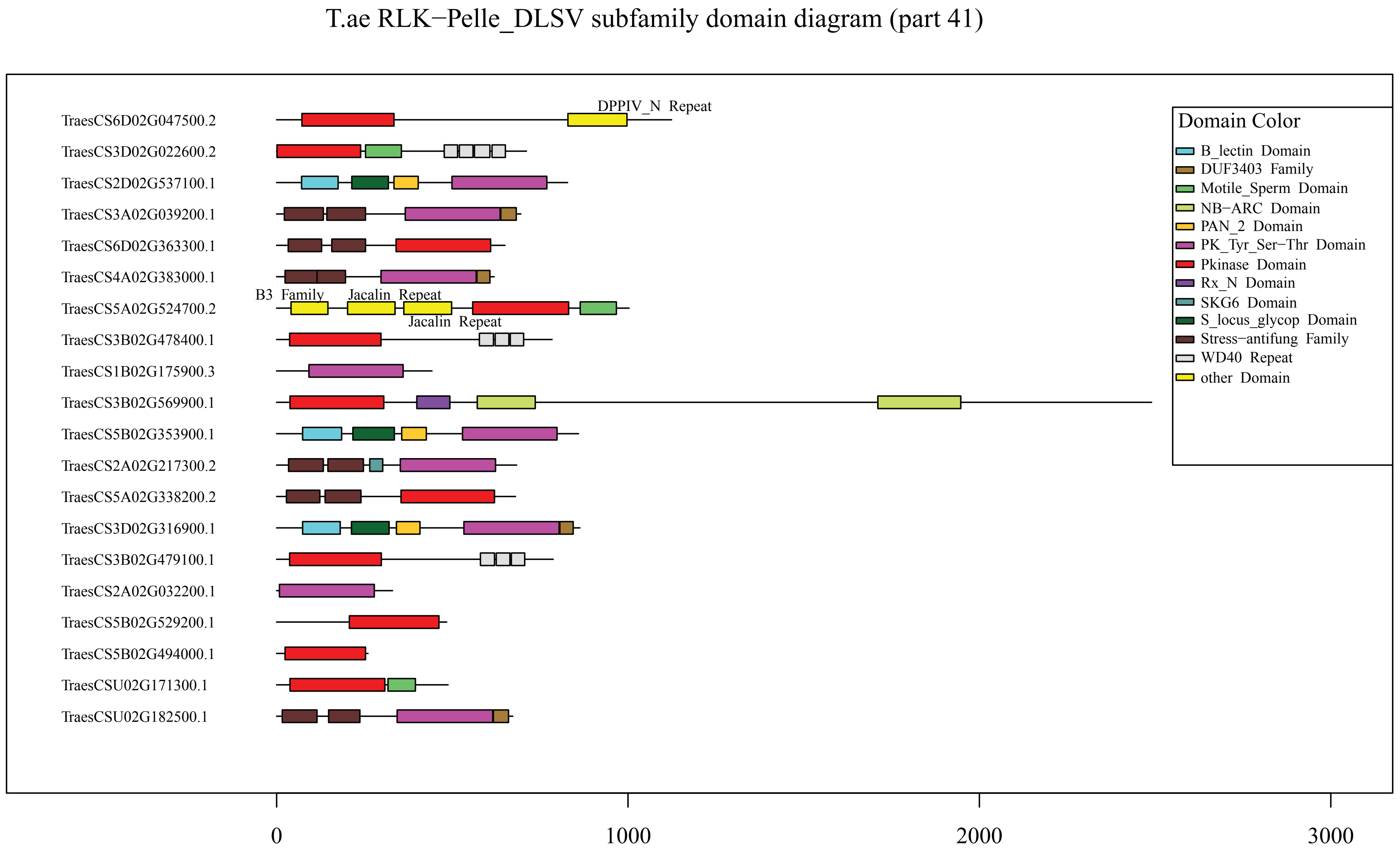

T.ae RLK−Pelle_DLSV subfamily domain diagram (part 41)
DPPIV_N Repeat
B3 Family
Jacalin Repeat
Jacalin Repeat
TraesCS6D02G047500.2
TraesCS3D02G022600.2
TraesCS2D02G537100.1
TraesCS3A02G039200.1
TraesCS6D02G363300.1
TraesCS4A02G383000.1
TraesCS5A02G524700.2
TraesCS3B02G478400.1
TraesCS1B02G175900.3
TraesCS3B02G569900.1
TraesCS5B02G353900.1
TraesCS2A02G217300.2
TraesCS5A02G338200.2
TraesCS3D02G316900.1
TraesCS3B02G479100.1
TraesCS2A02G032200.1
TraesCS5B02G529200.1
TraesCS5B02G494000.1
TraesCSU02G171300.1
TraesCSU02G182500.1
Domain Color
B_lectin Domain
DUF3403 Family
Motile_Sperm Domain
NB−ARC Domain
PAN_2 Domain
PK_Tyr_Ser−Thr Domain
Pkinase Domain
Rx_N Domain
SKG6 Domain
S_locus_glycop Domain
Stress−antifung Family
WD40 Repeat
other Domain
0
1000
2000
3000

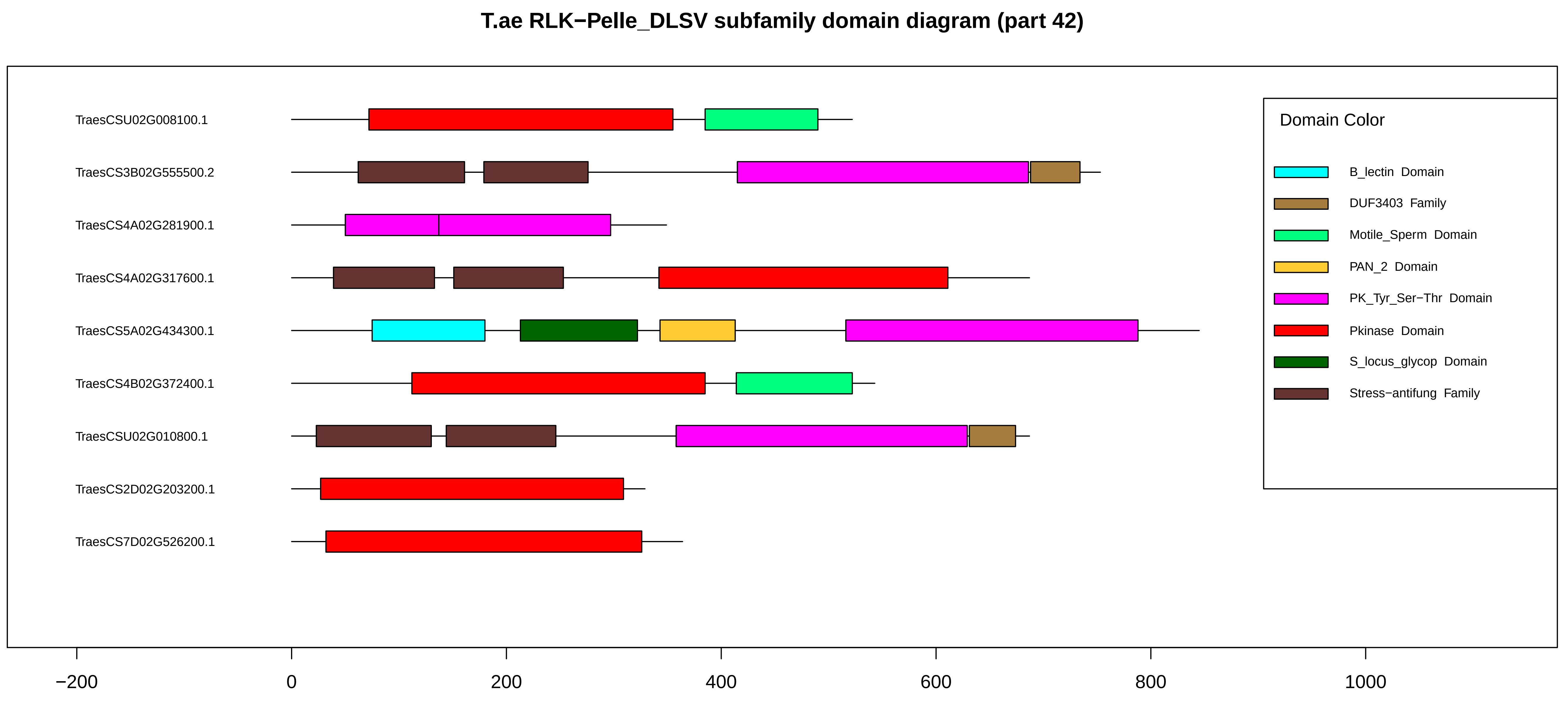
T.ae RLK−Pelle_DLSV subfamily domain diagram (part 42)
TraesCSU02G008100.1
TraesCS3B02G555500.2
TraesCS4A02G281900.1
TraesCS4A02G317600.1
TraesCS5A02G434300.1
TraesCS4B02G372400.1
TraesCSU02G010800.1
TraesCS2D02G203200.1
TraesCS7D02G526200.1
Domain Color
B_lectin Domain
DUF3403 Family
Motile_Sperm Domain
PAN_2 Domain
PK_Tyr_Ser−Thr Domain
Pkinase Domain
S_locus_glycop Domain
Stress−antifung Family
−200
0
200
400
600
800
1000

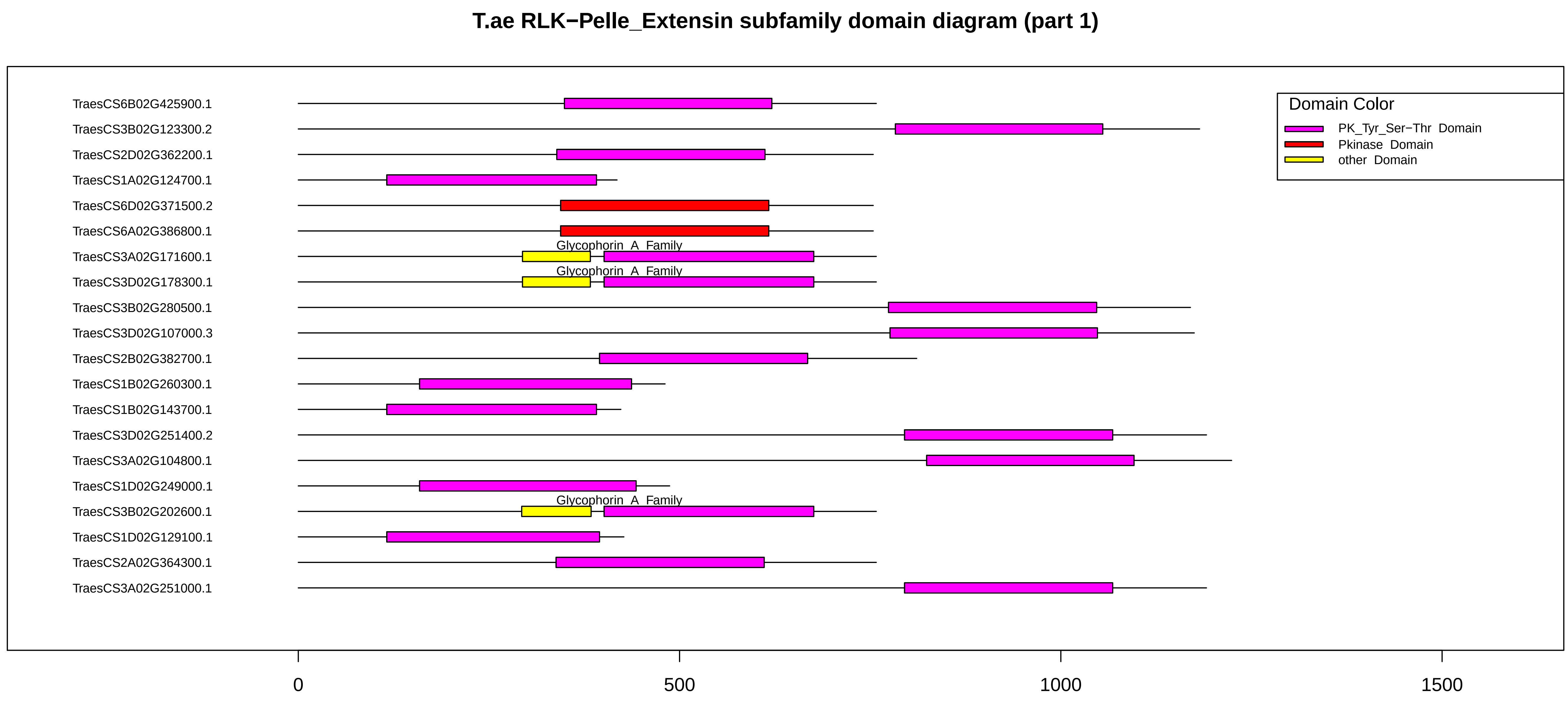
T.ae RLK−Pelle_Extensin subfamily domain diagram (part 1)
Domain Color
PK_Tyr_Ser−Thr Domain
Pkinase Domain
other Domain
TraesCS6B02G425900.1
TraesCS3B02G123300.2
TraesCS2D02G362200.1
TraesCS1A02G124700.1
TraesCS6D02G371500.2
TraesCS6A02G386800.1
TraesCS3A02G171600.1
Glycophorin_A_Family
TraesCS3D02G178300.1
Glycophorin_A_Family
TraesCS3B02G280500.1
TraesCS3D02G107000.3
TraesCS2B02G382700.1
TraesCS1B02G260300.1
TraesCS1B02G143700.1
TraesCS3D02G251400.2
TraesCS3A02G104800.1
TraesCS1D02G249000.1
TraesCS3B02G202600.1
Glycophorin_A_Family
TraesCS1D02G129100.1
TraesCS2A02G364300.1
TraesCS3A02G251000.1
0
500
1000
1500

T.ae RLK−Pelle_Extensin subfamily domain diagram (part 2)

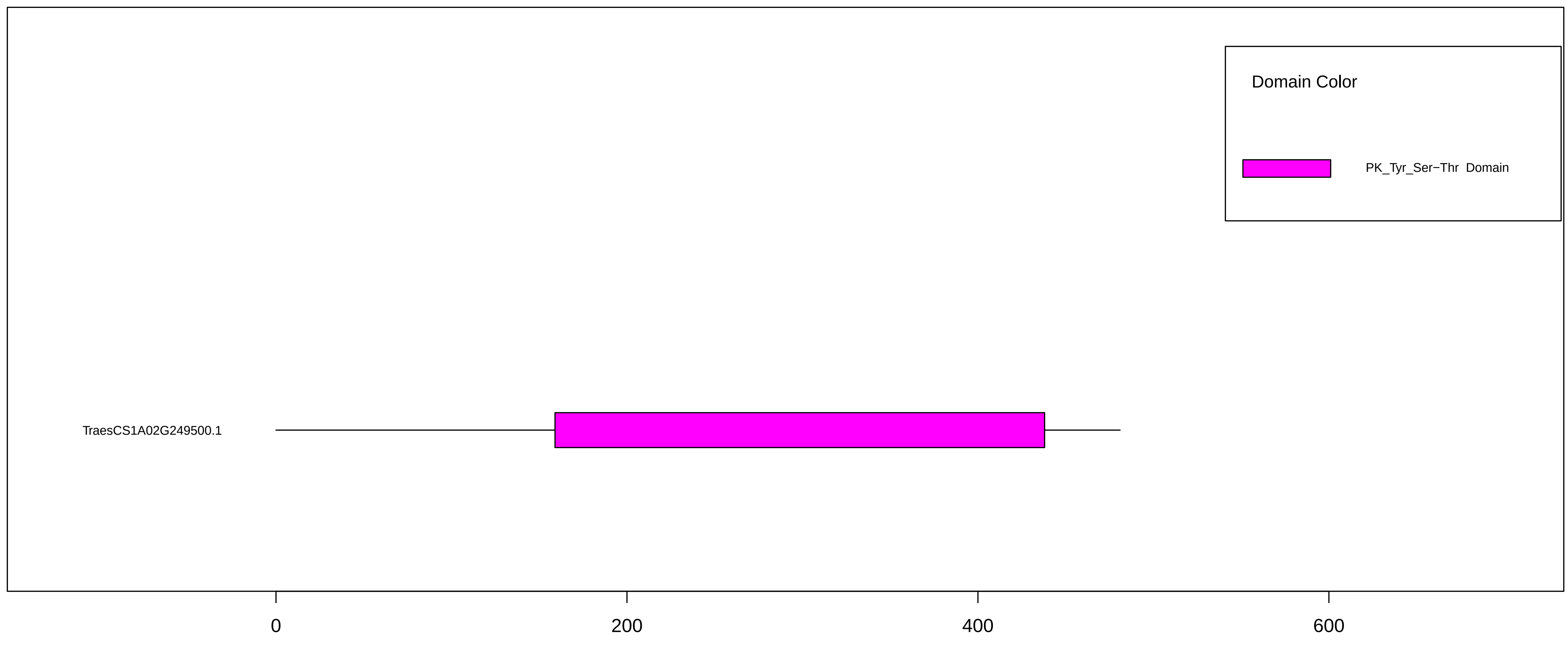

T.ae RLK−Pelle_L−LEC subfamily domain diagram (part 1)

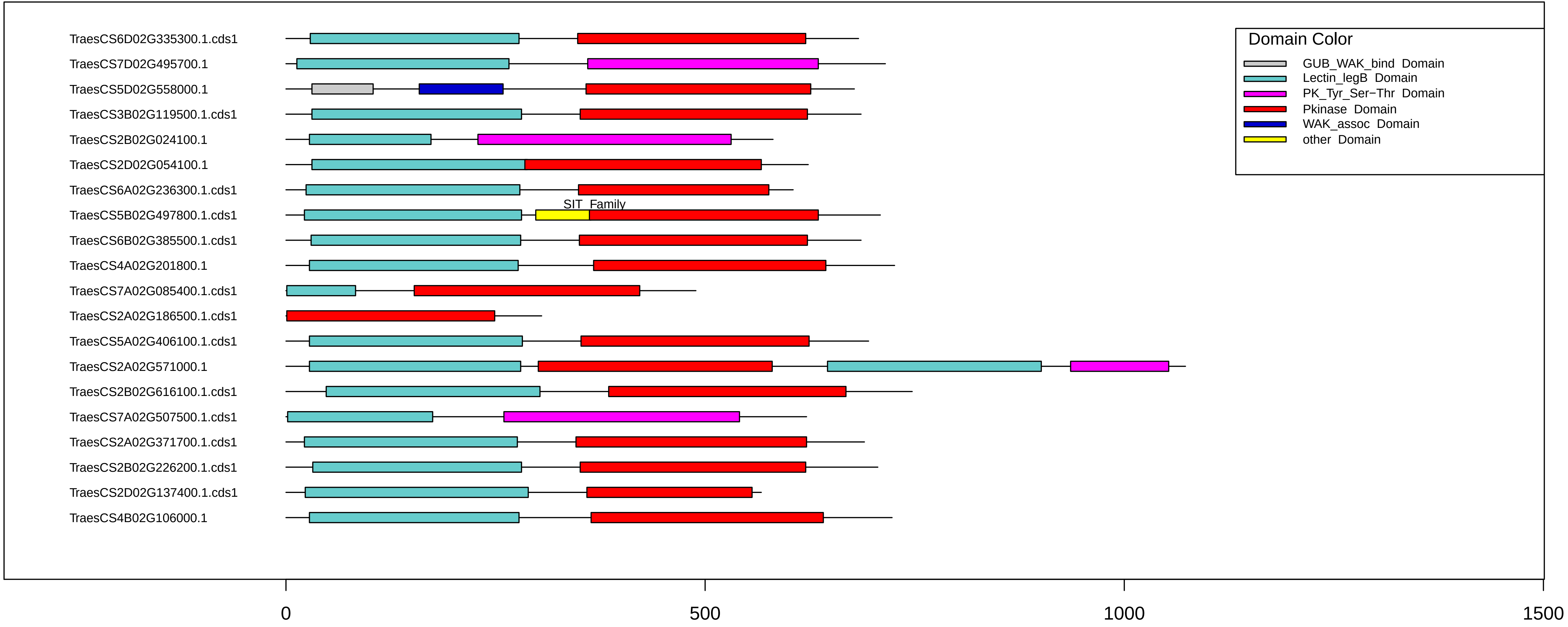

T.ae RLK−Pelle_L−LEC subfamily domain diagram (part 2)

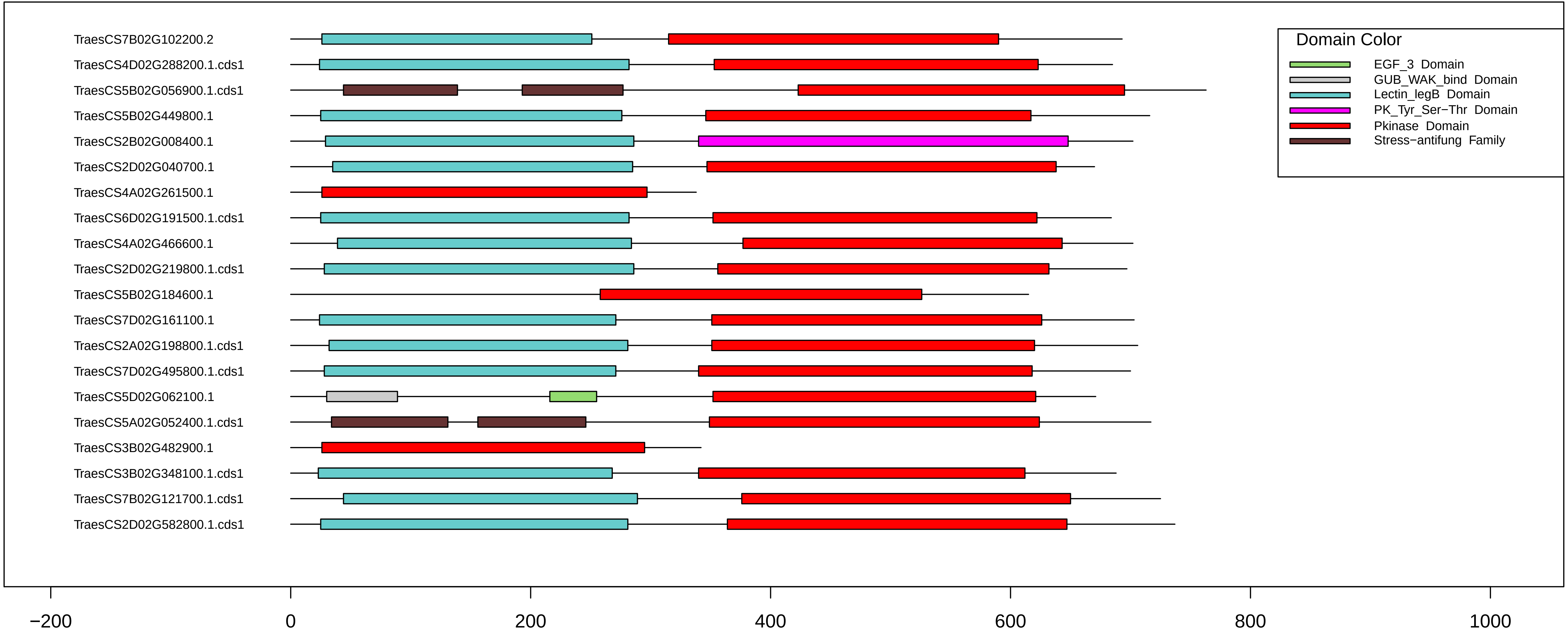

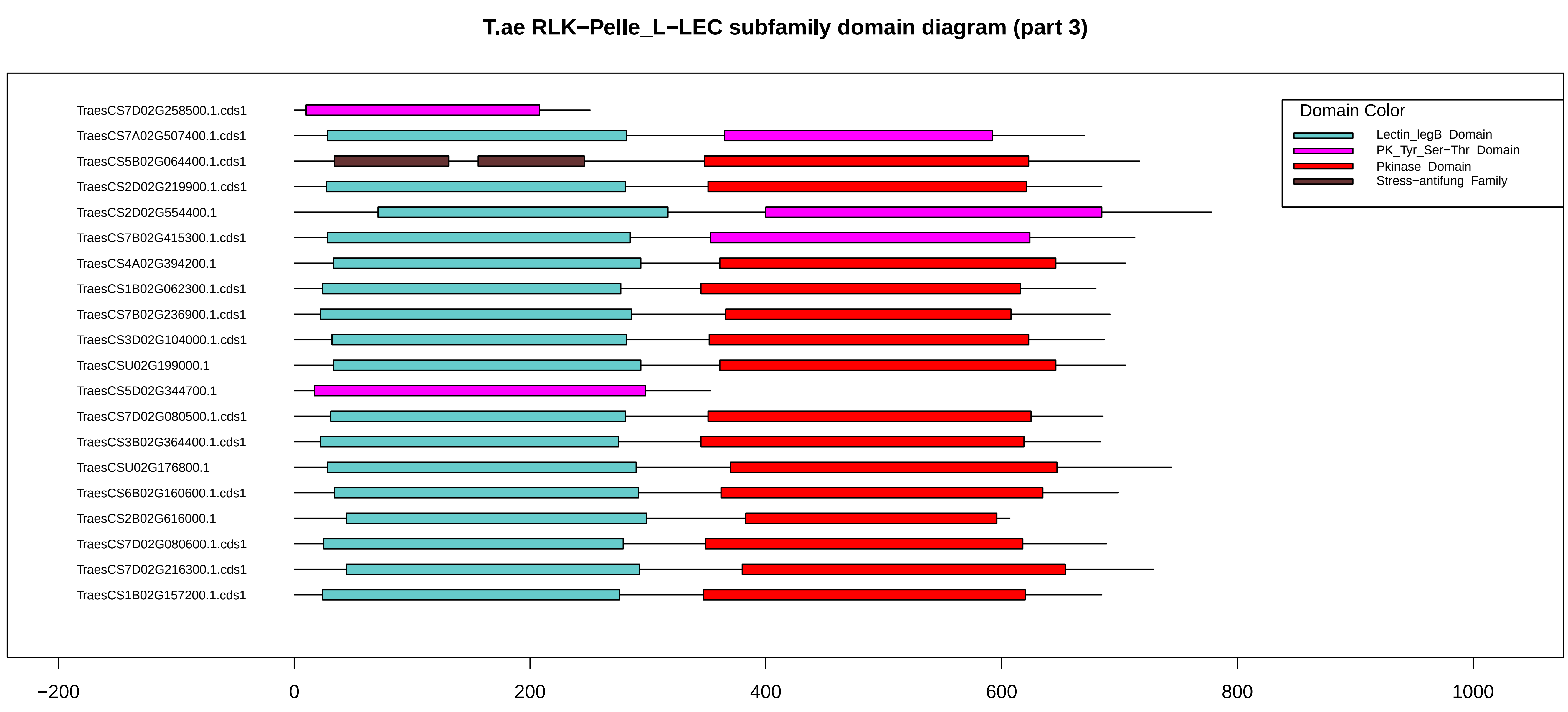
T.ae RLK−Pelle_L−LEC subfamily domain diagram (part 3)
TraesCS7D02G258500.1.cds1
TraesCS7A02G507400.1.cds1
TraesCS5B02G064400.1.cds1
TraesCS2D02G219900.1.cds1
TraesCS2D02G554400.1
TraesCS7B02G415300.1.cds1
TraesCS4A02G394200.1
TraesCS1B02G062300.1.cds1
TraesCS7B02G236900.1.cds1
TraesCS3D02G104000.1.cds1
TraesCSU02G199000.1
TraesCS5D02G344700.1
TraesCS7D02G080500.1.cds1
TraesCS3B02G364400.1.cds1
TraesCSU02G176800.1
TraesCS6B02G160600.1.cds1
TraesCS2B02G616000.1
TraesCS7D02G080600.1.cds1
TraesCS7D02G216300.1.cds1
TraesCS1B02G157200.1.cds1
Domain Color
Lectin_legB Domain
PK_Tyr_Ser−Thr Domain
Pkinase Domain
Stress−antifung Family
−200
0
200
400
600
800
1000

T.ae RLK−Pelle_L−LEC subfamily domain diagram (part 4)

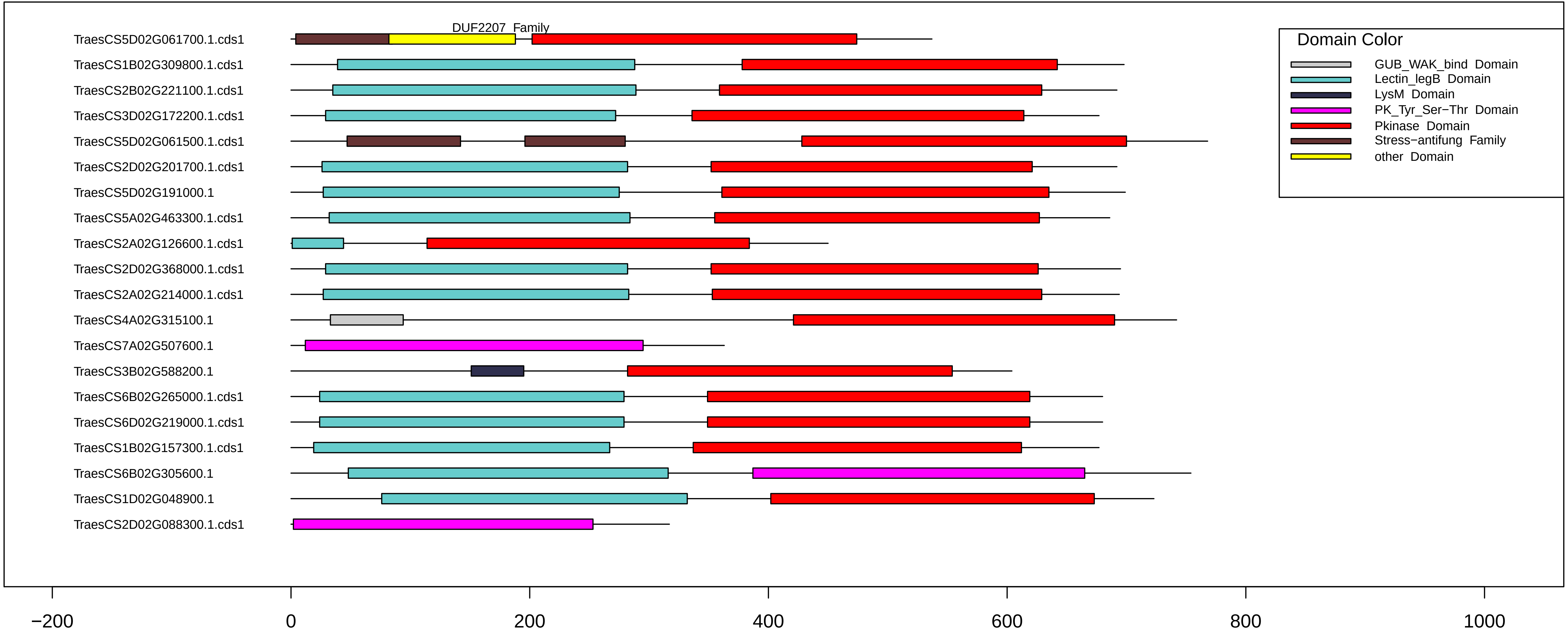

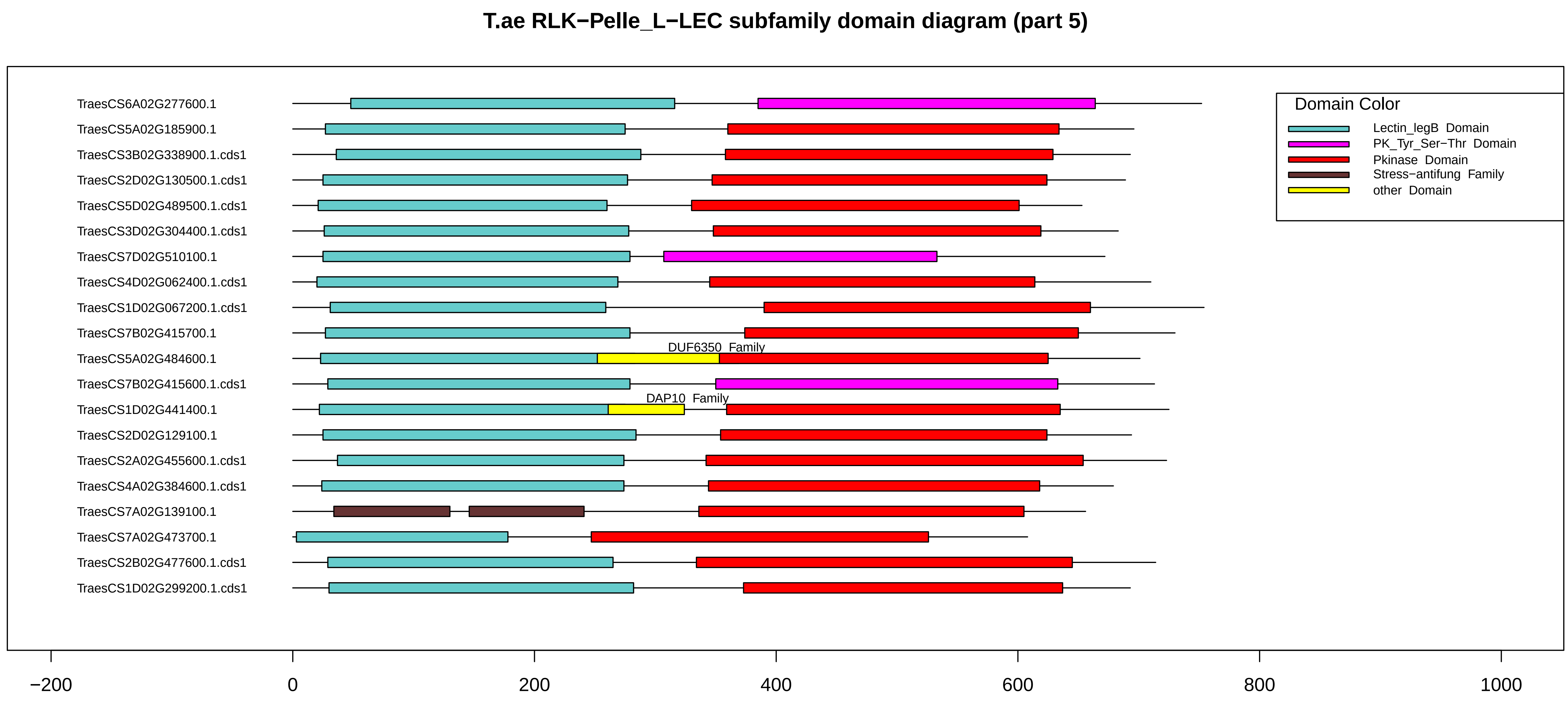

T.ae RLK−Pelle_L−LEC subfamily domain diagram (part 5)
TraesCS6A02G277600.1
TraesCS5A02G185900.1
TraesCS3B02G338900.1.cds1
TraesCS2D02G130500.1.cds1
TraesCS5D02G489500.1.cds1
TraesCS3D02G304400.1.cds1
TraesCS7D02G510100.1
TraesCS4D02G062400.1.cds1
TraesCS1D02G067200.1.cds1
TraesCS7B02G415700.1
TraesCS5A02G484600.1
DUF6350 Family
TraesCS7B02G415600.1.cds1
TraesCS1D02G441400.1
DAP10 Family
TraesCS2D02G129100.1
TraesCS2A02G455600.1.cds1
TraesCS4A02G384600.1.cds1
TraesCS7A02G139100.1
TraesCS7A02G473700.1
TraesCS2B02G477600.1.cds1
TraesCS1D02G299200.1.cds1
Domain Color
Lectin_legB Domain
PK_Tyr_Ser−Thr Domain
Pkinase Domain
Stress−antifung Family
other Domain
−200
0
200
400
600
800
1000

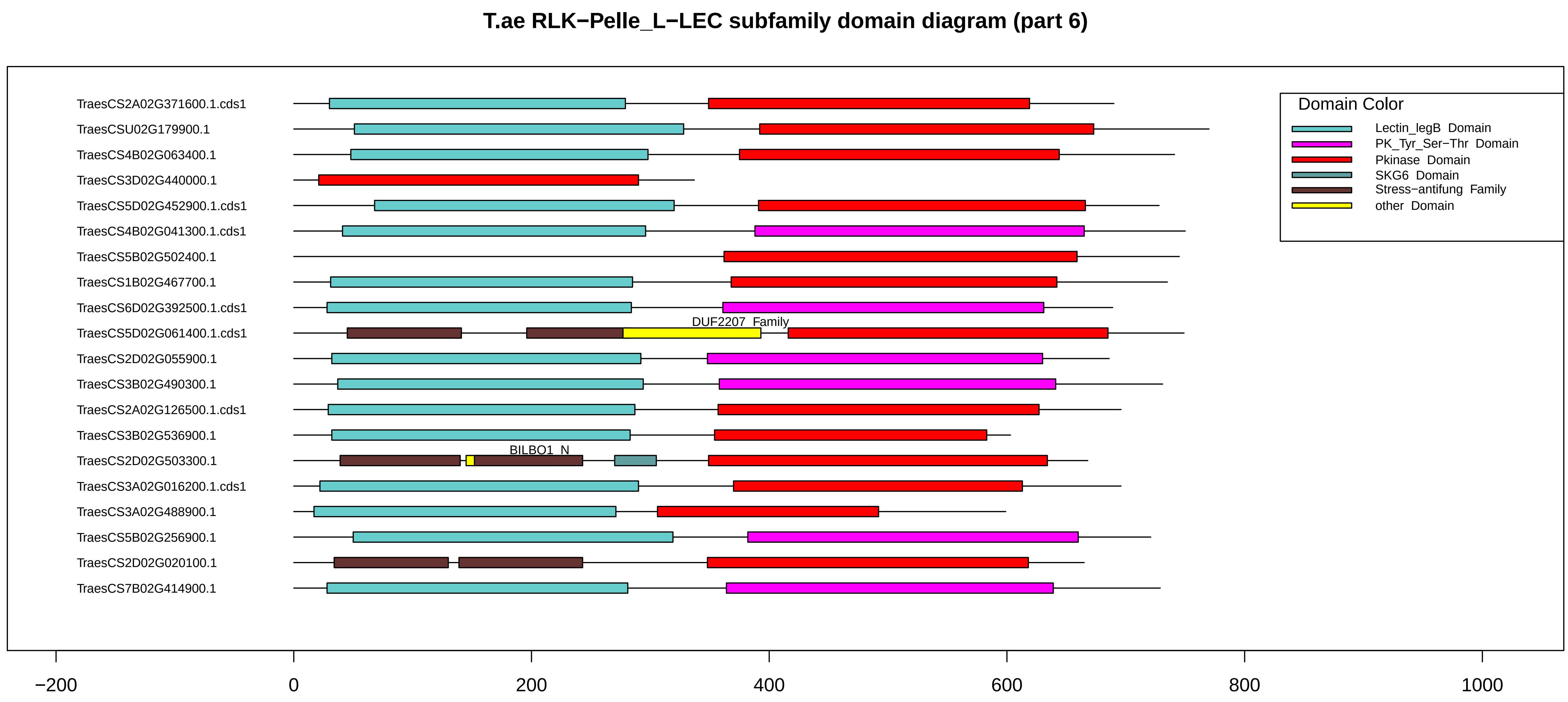
T.ae RLK−Pelle_L−LEC subfamily domain diagram (part 6)
TraesCS2A02G371600.1.cds1
TraesCSU02G179900.1
TraesCS4B02G063400.1
TraesCS3D02G440000.1
TraesCS5D02G452900.1.cds1
TraesCS4B02G041300.1.cds1
TraesCS5B02G502400.1
TraesCS1B02G467700.1
TraesCS6D02G392500.1.cds1
TraesCS5D02G061400.1.cds1
TraesCS2D02G055900.1
TraesCS3B02G490300.1
TraesCS2A02G126500.1.cds1
TraesCS3B02G536900.1
TraesCS2D02G503300.1
TraesCS3A02G016200.1.cds1
TraesCS3A02G488900.1
TraesCS5B02G256900.1
TraesCS2D02G020100.1
TraesCS7B02G414900.1
DUF2207_Family
BILBO1_N
Domain Color
Lectin_legB Domain
PK_Tyr_Ser−Thr Domain
Pkinase Domain
SKG6 Domain
Stress−antifung Family
other Domain
−200
0
200
400
600
800
1000

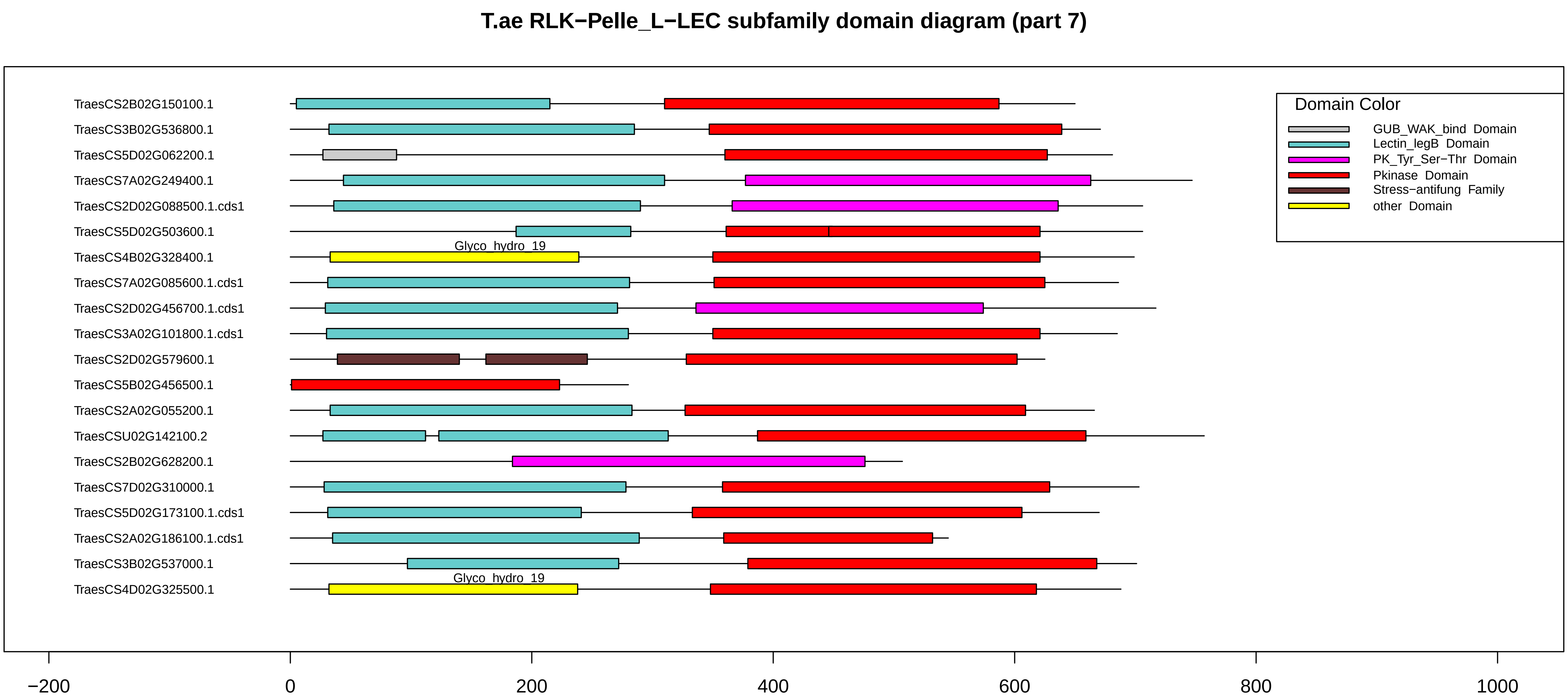
T.ae RLK−Pelle_L−LEC subfamily domain diagram (part 7)
TraesCS2B02G150100.1
TraesCS3B02G536800.1
TraesCS5D02G062200.1
TraesCS7A02G249400.1
TraesCS2D02G088500.1.cds1
TraesCS5D02G503600.1
Glyco_hydro_19
TraesCS4B02G328400.1
TraesCS7A02G085600.1.cds1
TraesCS2D02G456700.1.cds1
TraesCS3A02G101800.1.cds1
TraesCS2D02G579600.1
TraesCS5B02G456500.1
TraesCS2A02G055200.1
TraesCSU02G142100.2
TraesCS2B02G628200.1
TraesCS7D02G310000.1
TraesCS5D02G173100.1.cds1
TraesCS2A02G186100.1.cds1
TraesCS3B02G537000.1
Glyco_hydro_19
TraesCS4D02G325500.1
Domain Color
GUB_WAK_bind Domain
Lectin_legB Domain
PK_Tyr_Ser−Thr Domain
Pkinase Domain
Stress−antifung Family
other Domain
−200
0
200
400
600
800
1000

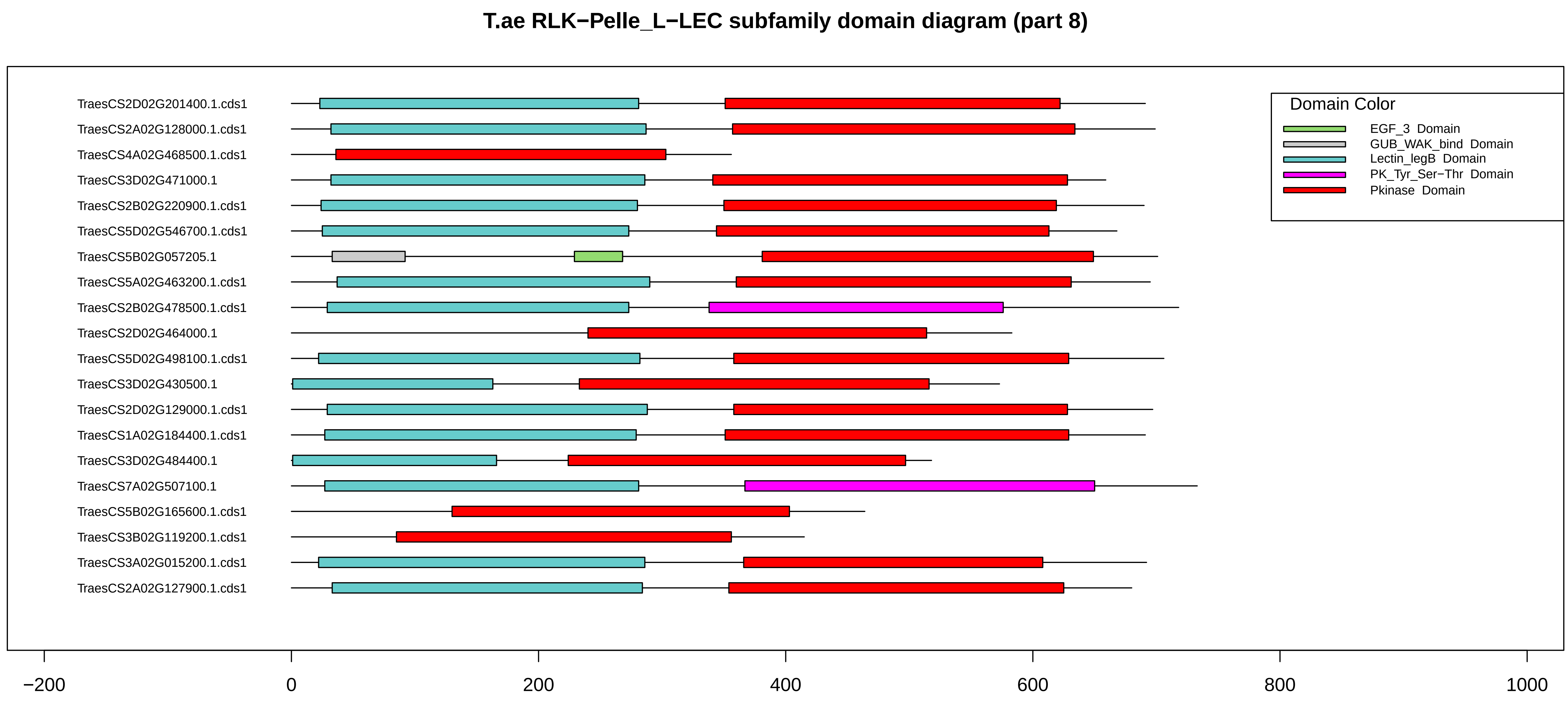
T.ae RLK−Pelle_L−LEC subfamily domain diagram (part 8)
TraesCS2D02G201400.1.cds1
TraesCS2A02G128000.1.cds1
TraesCS4A02G468500.1.cds1
TraesCS3D02G471000.1
TraesCS2B02G220900.1.cds1
TraesCS5D02G546700.1.cds1
TraesCS5B02G057205.1
TraesCS5A02G463200.1.cds1
TraesCS2B02G478500.1.cds1
TraesCS2D02G464000.1
TraesCS5D02G498100.1.cds1
TraesCS3D02G430500.1
TraesCS2D02G129000.1.cds1
TraesCS1A02G184400.1.cds1
TraesCS3D02G484400.1
TraesCS7A02G507100.1
TraesCS5B02G165600.1.cds1
TraesCS3B02G119200.1.cds1
TraesCS3A02G015200.1.cds1
TraesCS2A02G127900.1.cds1
Domain Color
EGF_3 Domain
GUB_WAK_bind Domain
Lectin_legB Domain
PK_Tyr_Ser−Thr Domain
Pkinase Domain
−200
0
200
400
600
800
1000

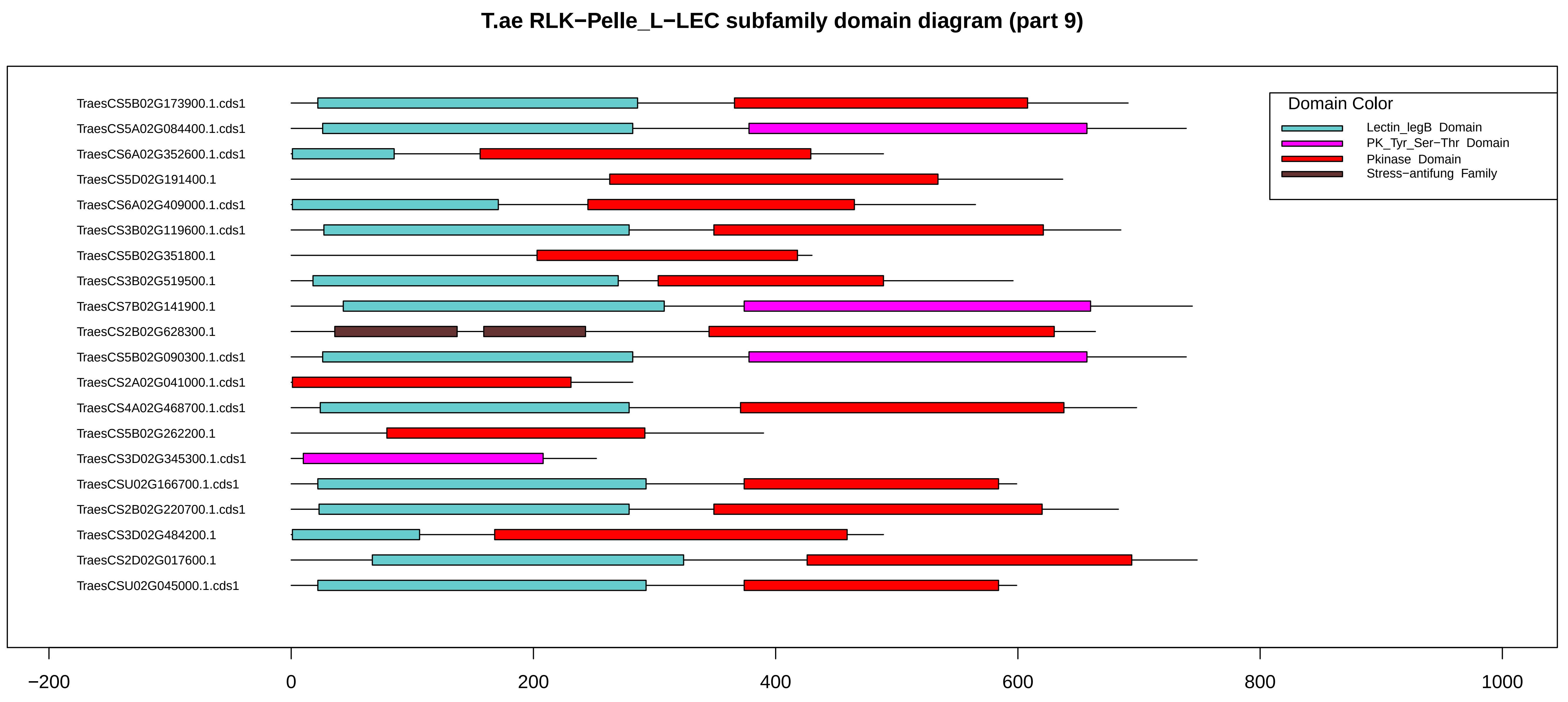
T.ae RLK−Pelle_L−LEC subfamily domain diagram (part 9)
TraesCS5B02G173900.1.cds1
TraesCS5A02G084400.1.cds1
TraesCS6A02G352600.1.cds1
TraesCS5D02G191400.1
TraesCS6A02G409000.1.cds1
TraesCS3B02G119600.1.cds1
TraesCS5B02G351800.1
TraesCS3B02G519500.1
TraesCS7B02G141900.1
TraesCS2B02G628300.1
TraesCS5B02G090300.1.cds1
TraesCS2A02G041000.1.cds1
TraesCS4A02G468700.1.cds1
TraesCS5B02G262200.1
TraesCS3D02G345300.1.cds1
TraesCSU02G166700.1.cds1
TraesCS2B02G220700.1.cds1
TraesCS3D02G484200.1
TraesCS2D02G017600.1
TraesCSU02G045000.1.cds1
Domain Color
Lectin_legB Domain
PK_Tyr_Ser−Thr Domain
Pkinase Domain
Stress−antifung Family
−200
0
200
400
600
800
1000

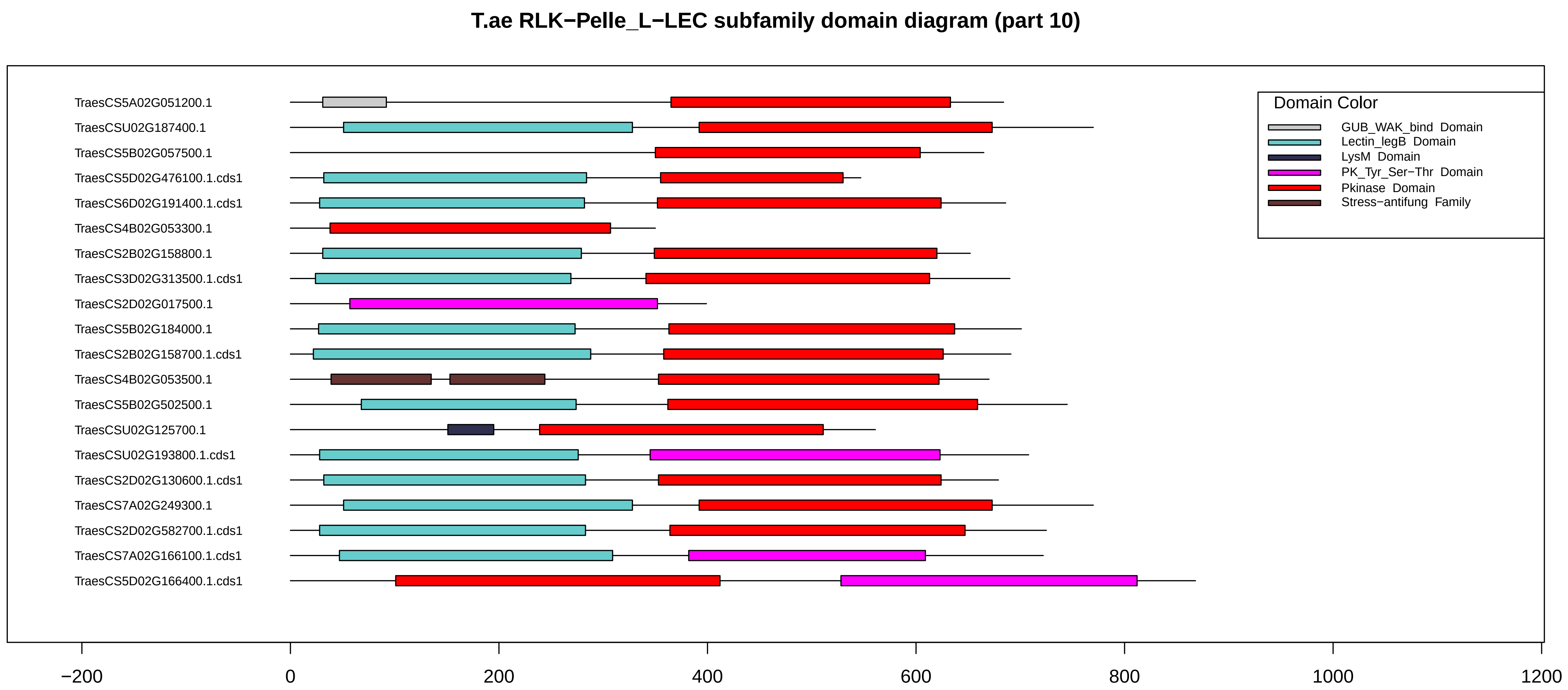
T.ae RLK−Pelle_L−LEC subfamily domain diagram (part 10)
TraesCS5A02G051200.1
TraesCSU02G187400.1
TraesCS5B02G057500.1
TraesCS5D02G476100.1.cds1
TraesCS6D02G191400.1.cds1
TraesCS4B02G053300.1
TraesCS2B02G158800.1
TraesCS3D02G313500.1.cds1
TraesCS2D02G017500.1
TraesCS5B02G184000.1
TraesCS2B02G158700.1.cds1
TraesCS4B02G053500.1
TraesCS5B02G502500.1
TraesCSU02G125700.1
TraesCSU02G193800.1.cds1
TraesCS2D02G130600.1.cds1
TraesCS7A02G249300.1
TraesCS2D02G582700.1.cds1
TraesCS7A02G166100.1.cds1
TraesCS5D02G166400.1.cds1
Domain Color
GUB_WAK_bind Domain
Lectin_legB Domain
LysM Domain
PK_Tyr_Ser−Thr Domain
Pkinase Domain
Stress−antifung Family
−200
0
200
400
600
800
1000
1200

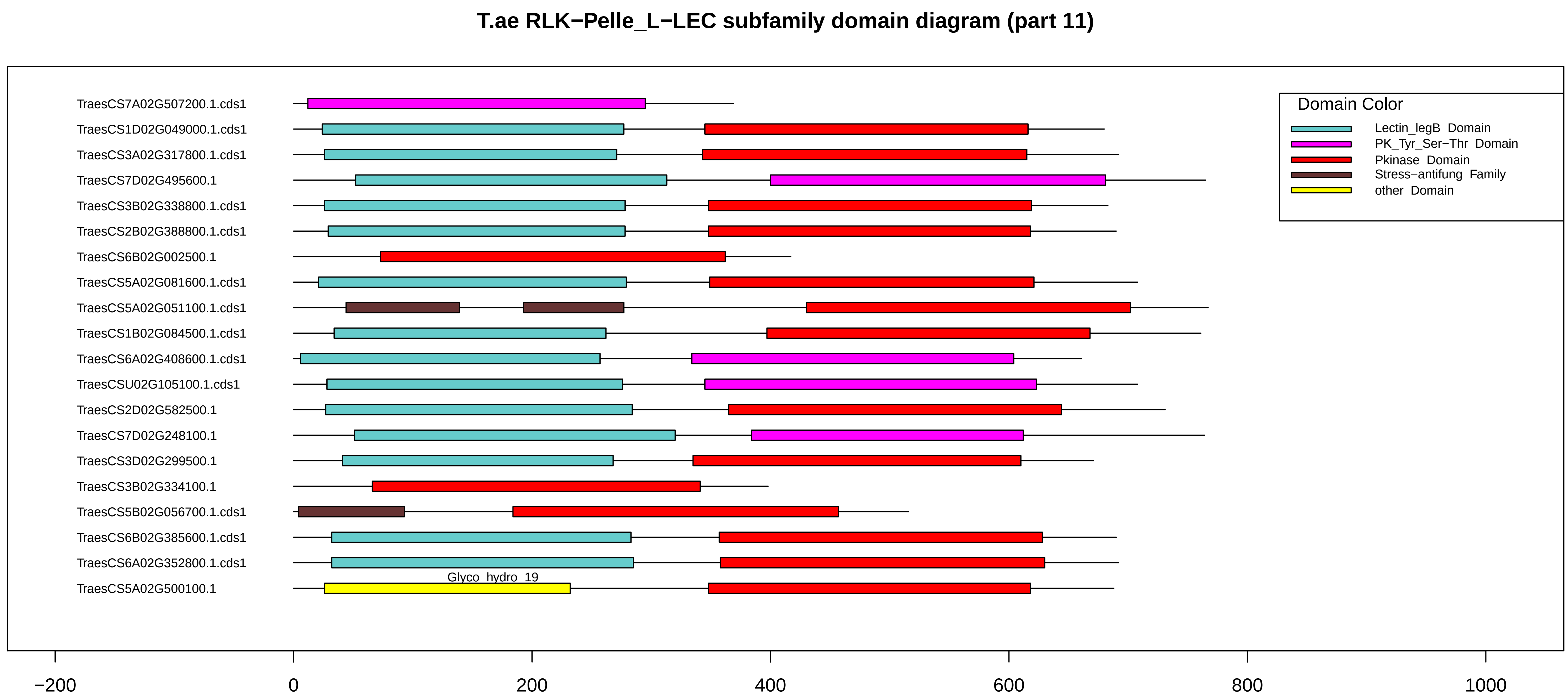
T.ae RLK−Pelle_L−LEC subfamily domain diagram (part 11)
TraesCS7A02G507200.1.cds1
TraesCS1D02G049000.1.cds1
TraesCS3A02G317800.1.cds1
TraesCS7D02G495600.1
TraesCS3B02G338800.1.cds1
TraesCS2B02G388800.1.cds1
TraesCS6B02G002500.1
TraesCS5A02G081600.1.cds1
TraesCS5A02G051100.1.cds1
TraesCS1B02G084500.1.cds1
TraesCS6A02G408600.1.cds1
TraesCSU02G105100.1.cds1
TraesCS2D02G582500.1
TraesCS7D02G248100.1
TraesCS3D02G299500.1
TraesCS3B02G334100.1
TraesCS5B02G056700.1.cds1
TraesCS6B02G385600.1.cds1
TraesCS6A02G352800.1.cds1
TraesCS5A02G500100.1
Glyco_hydro_19
Domain Color
Lectin_legB Domain
PK_Tyr_Ser−Thr Domain
Pkinase Domain
Stress−antifung Family
other Domain
−200
0
200
400
600
800
1000

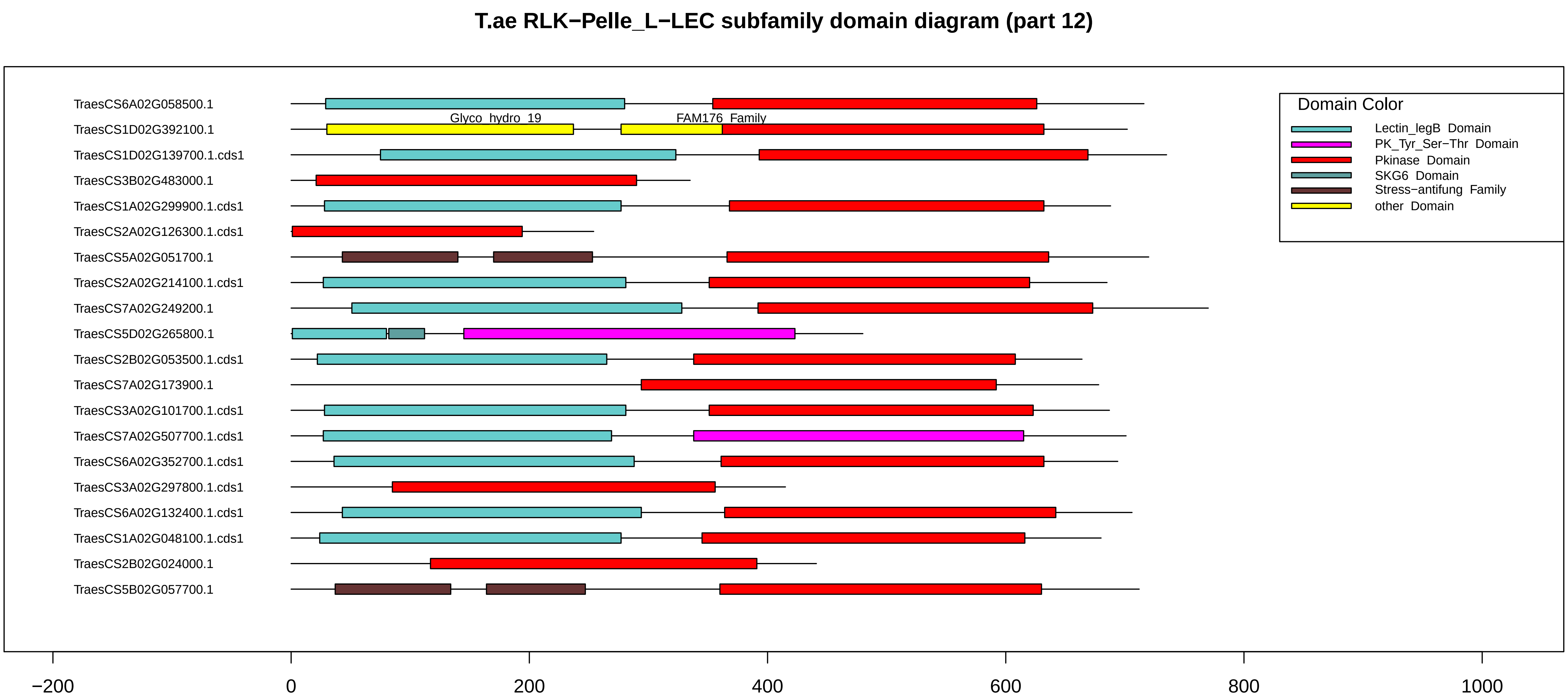

T.ae RLK−Pelle_L−LEC subfamily domain diagram (part 12)
TraesCS6A02G058500.1
TraesCS1D02G392100.1
TraesCS1D02G139700.1.cds1
TraesCS3B02G483000.1
TraesCS1A02G299900.1.cds1
TraesCS2A02G126300.1.cds1
TraesCS5A02G051700.1
TraesCS2A02G214100.1.cds1
TraesCS7A02G249200.1
TraesCS5D02G265800.1
TraesCS2B02G053500.1.cds1
TraesCS7A02G173900.1
TraesCS3A02G101700.1.cds1
TraesCS7A02G507700.1.cds1
TraesCS6A02G352700.1.cds1
TraesCS3A02G297800.1.cds1
TraesCS6A02G132400.1.cds1
TraesCS1A02G048100.1.cds1
TraesCS2B02G024000.1
TraesCS5B02G057700.1
Glyco_hydro_19
FAM176_Family
Domain Color
Lectin_legB Domain
PK_Tyr_Ser−Thr Domain
Pkinase Domain
SKG6 Domain
Stress−antifung Family
other Domain
−200
0
200
400
600
800
1000

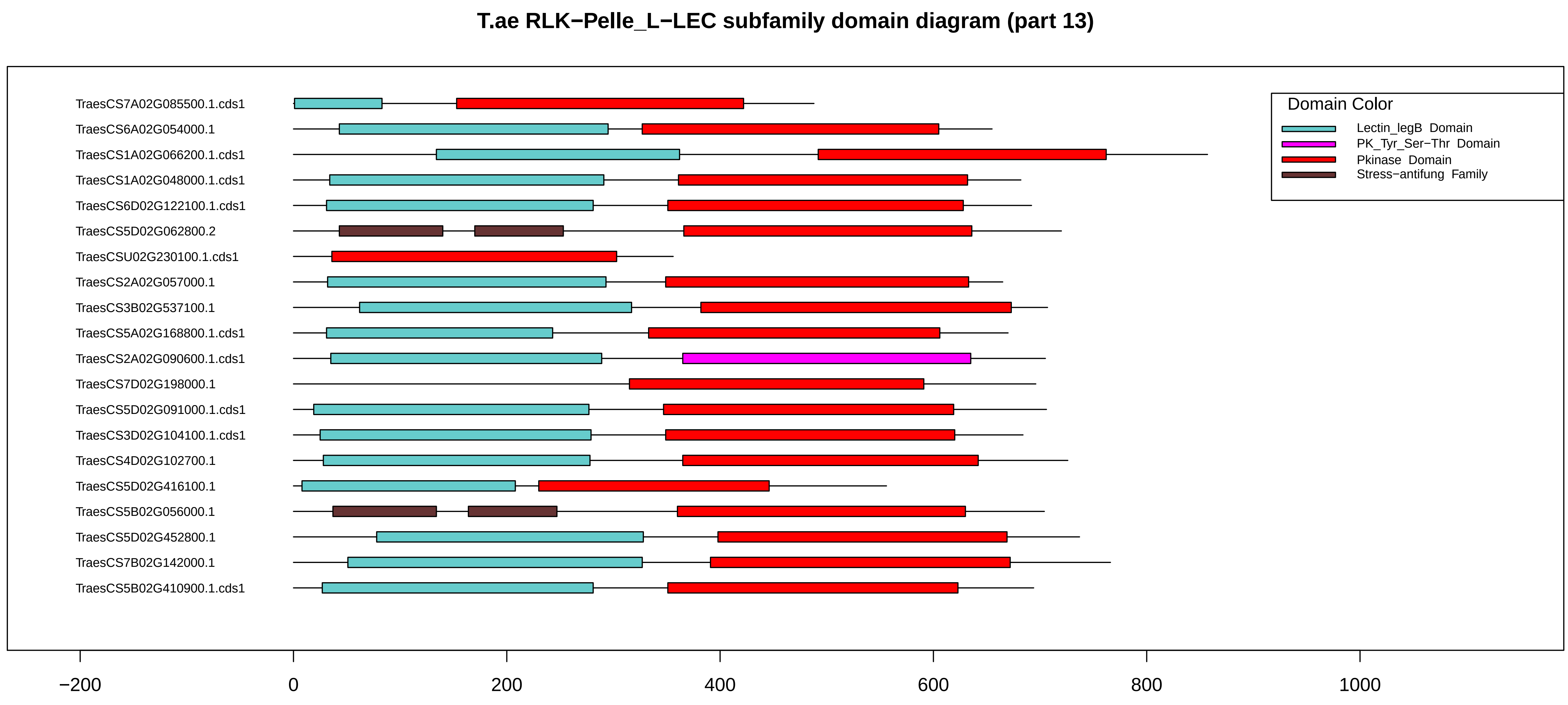
T.ae RLK−Pelle_L−LEC subfamily domain diagram (part 13)
TraesCS7A02G085500.1.cds1
TraesCS6A02G054000.1
TraesCS1A02G066200.1.cds1
TraesCS1A02G048000.1.cds1
TraesCS6D02G122100.1.cds1
TraesCS5D02G062800.2
TraesCSU02G230100.1.cds1
TraesCS2A02G057000.1
TraesCS3B02G537100.1
TraesCS5A02G168800.1.cds1
TraesCS2A02G090600.1.cds1
TraesCS7D02G198000.1
TraesCS5D02G091000.1.cds1
TraesCS3D02G104100.1.cds1
TraesCS4D02G102700.1
TraesCS5D02G416100.1
TraesCS5B02G056000.1
TraesCS5D02G452800.1
TraesCS7B02G142000.1
TraesCS5B02G410900.1.cds1
Domain Color
Lectin_legB Domain
PK_Tyr_Ser−Thr Domain
Pkinase Domain
Stress−antifung Family
−200
0
200
400
600
800
1000

T.ae RLK−Pelle_L−LEC subfamily domain diagram (part 14)

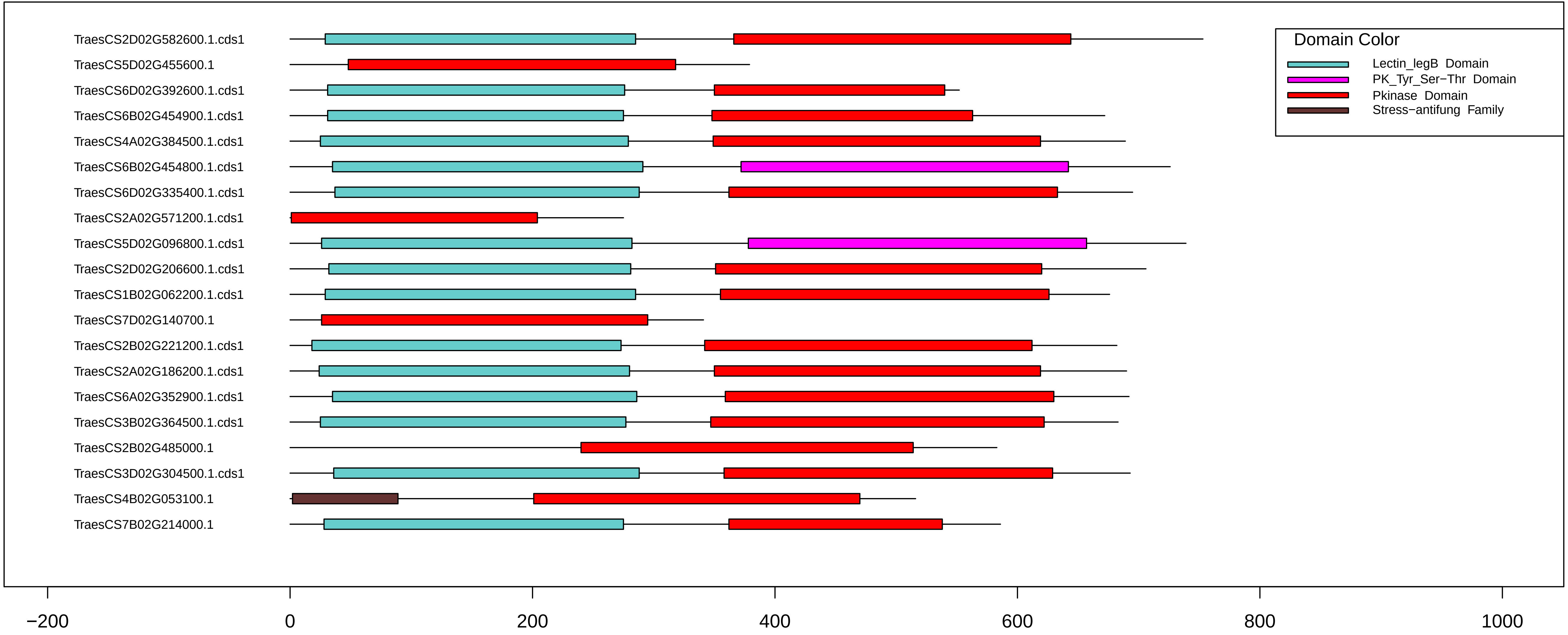

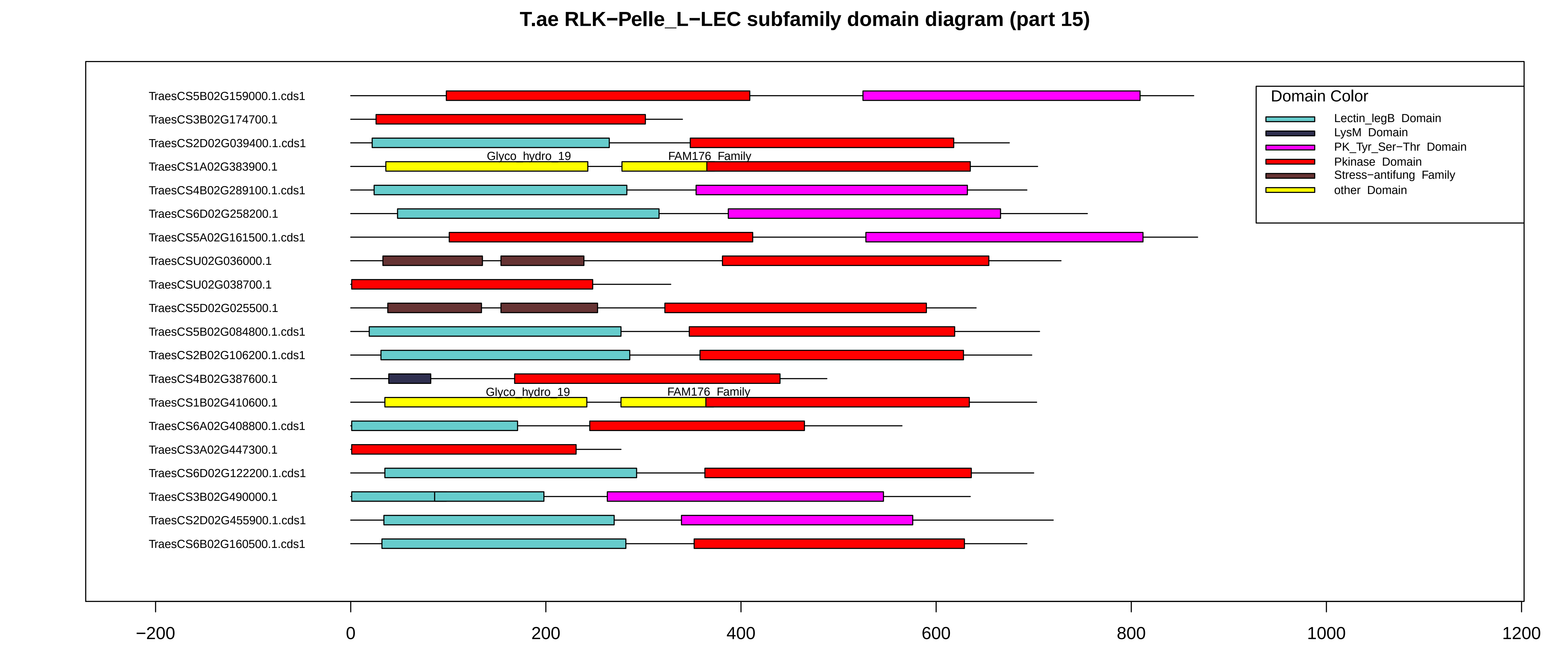

T.ae RLK−Pelle_L−LEC subfamily domain diagram (part 15)
TraesCS5B02G159000.1.cds1
TraesCS3B02G174700.1
TraesCS2D02G039400.1.cds1
TraesCS1A02G383900.1
TraesCS4B02G289100.1.cds1
TraesCS6D02G258200.1
TraesCS5A02G161500.1.cds1
TraesCSU02G036000.1
TraesCSU02G038700.1
TraesCS5D02G025500.1
TraesCS5B02G084800.1.cds1
TraesCS2B02G106200.1.cds1
TraesCS4B02G387600.1
TraesCS1B02G410600.1
TraesCS6A02G408800.1.cds1
TraesCS3A02G447300.1
TraesCS6D02G122200.1.cds1
TraesCS3B02G490000.1
TraesCS2D02G455900.1.cds1
TraesCS6B02G160500.1.cds1
Glyco_hydro_19
FAM176_Family
Glyco_hydro_19
FAM176_Family
Domain Color
Lectin_legB Domain
LysM Domain
PK_Tyr_Ser−Thr Domain
Pkinase Domain
Stress−antifung Family
other Domain
−200
0
200
400
600
800
1000
1200

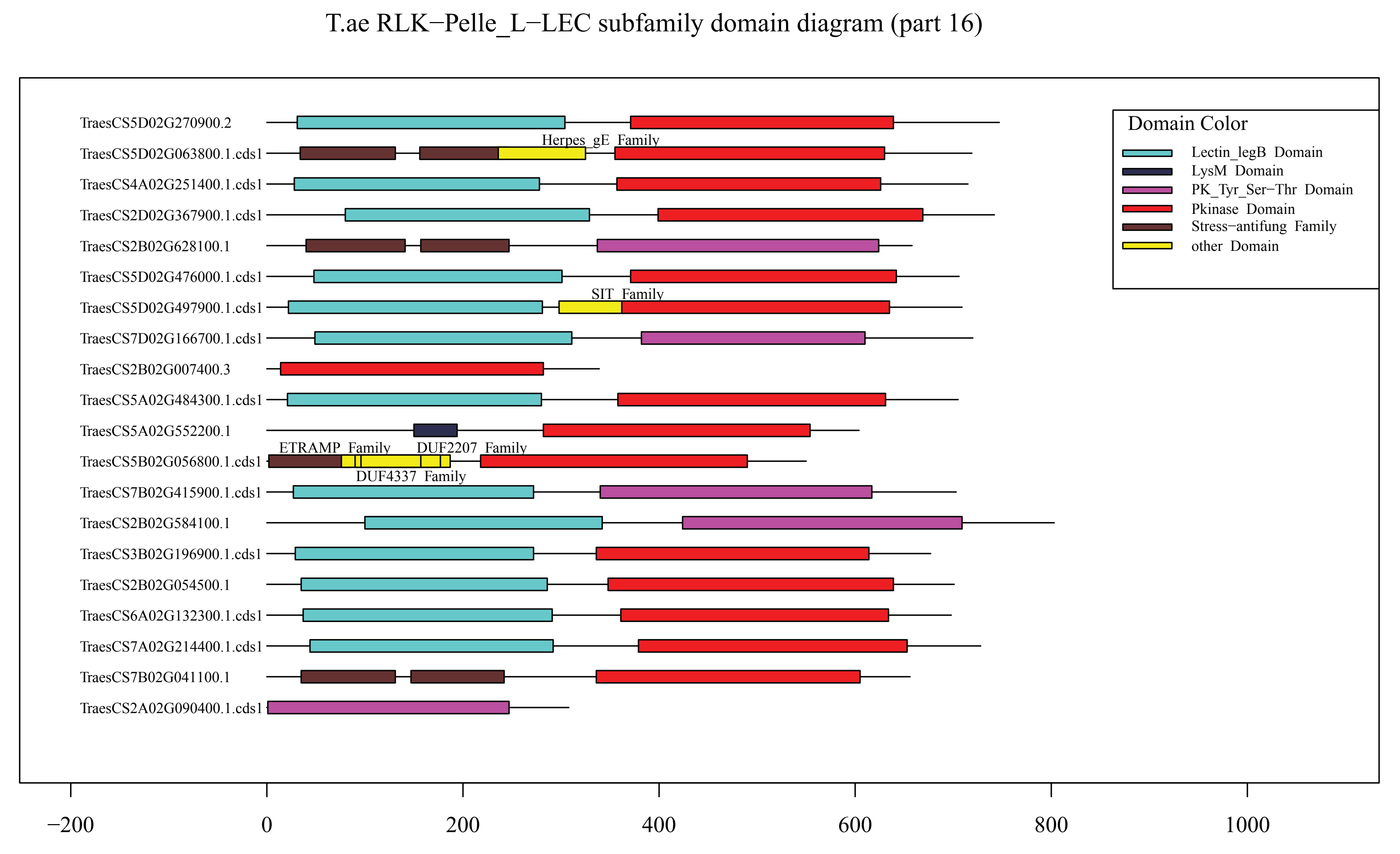
T.ae RLK−Pelle_L−LEC subfamily domain diagram (part 16)
TraesCS5D02G270900.2
TraesCS5D02G063800.1.cds1
Herpes_gE_Family
TraesCS4A02G251400.1.cds1
TraesCS2D02G367900.1.cds1
TraesCS2B02G628100.1
TraesCS5D02G476000.1.cds1
TraesCS5D02G497900.1.cds1
SIT_Family
TraesCS7D02G166700.1.cds1
TraesCS2B02G007400.3
TraesCS5A02G484300.1.cds1
TraesCS5A02G552200.1
TraesCS5B02G056800.1.cds1
ETRAMP_Family
DUF2207_Family
DUF4337_Family
TraesCS7B02G415900.1.cds1
TraesCS2B02G584100.1
TraesCS3B02G196900.1.cds1
TraesCS2B02G054500.1
TraesCS6A02G132300.1.cds1
TraesCS7A02G214400.1.cds1
TraesCS7B02G041100.1
TraesCS2A02G090400.1.cds1
Domain Color
Lectin_legB_Domain
LysM_Domain
PK_Tyr_Ser−Thr_Domain
Pkinase_Domain
Stress−antifung_Family
other_Domain
−200
0
200
400
600
800
1000

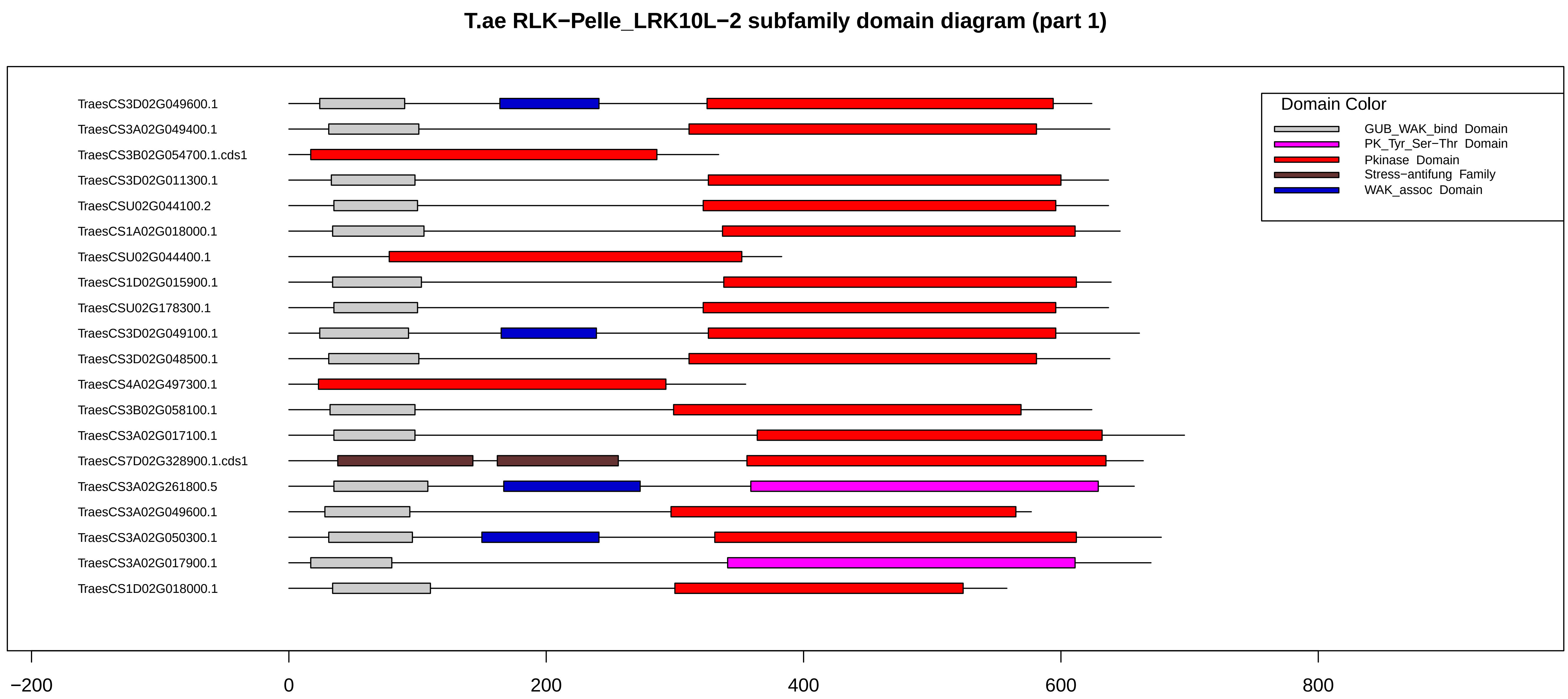

T.ae RLK−Pelle_LRK10L−2 subfamily domain diagram (part 1)
TraesCS3D02G049600.1
TraesCS3A02G049400.1
TraesCS3B02G054700.1.cds1
TraesCS3D02G011300.1
TraesCSU02G044100.2
TraesCS1A02G018000.1
TraesCSU02G044400.1
TraesCS1D02G015900.1
TraesCSU02G178300.1
TraesCS3D02G049100.1
TraesCS3D02G048500.1
TraesCS4A02G497300.1
TraesCS3B02G058100.1
TraesCS3A02G017100.1
TraesCS7D02G328900.1.cds1
TraesCS3A02G261800.5
TraesCS3A02G049600.1
TraesCS3A02G050300.1
TraesCS3A02G017900.1
TraesCS1D02G018000.1
Domain Color
GUB_WAK_bind Domain
PK_Tyr_Ser−Thr Domain
Pkinase Domain
Stress−antifung Family
WAK_assoc Domain
−200
0
200
400
600
800

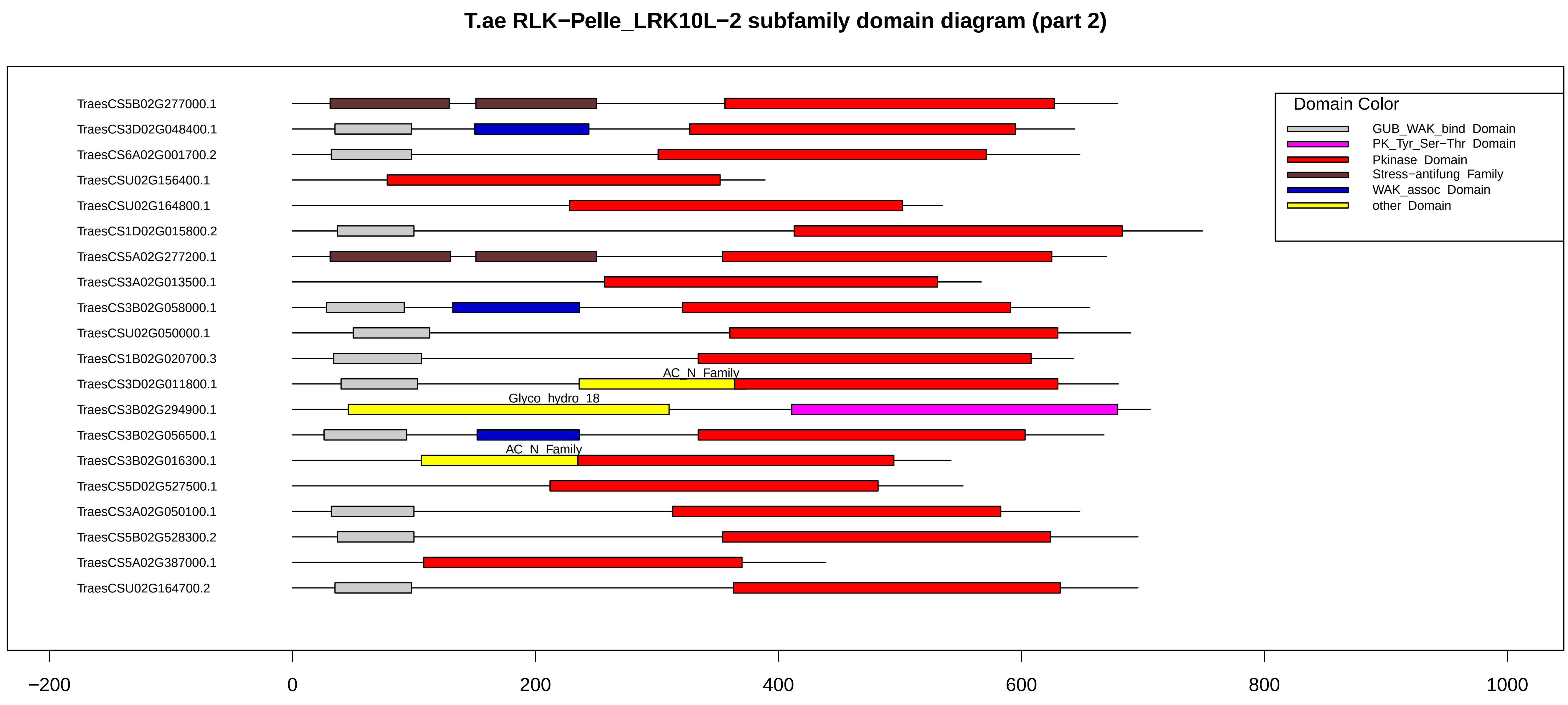
T.ae RLK−Pelle_LRK10L−2 subfamily domain diagram (part 2)
TraesCS5B02G277000.1
TraesCS3D02G048400.1
TraesCS6A02G001700.2
TraesCSU02G156400.1
TraesCSU02G164800.1
TraesCS1D02G015800.2
TraesCS5A02G277200.1
TraesCS3A02G013500.1
TraesCS3B02G058000.1
TraesCSU02G050000.1
TraesCS1B02G020700.3
TraesCS3D02G011800.1
AC_N_Family
TraesCS3B02G294900.1
Glyco_hydro_18
TraesCS3B02G056500.1
TraesCS3B02G016300.1
AC_N_Family
TraesCS5D02G527500.1
TraesCS3A02G050100.1
TraesCS5B02G528300.2
TraesCS5A02G387000.1
TraesCSU02G164700.2
Domain Color
GUB_WAK_bind Domain
PK_Tyr_Ser−Thr Domain
Pkinase Domain
Stress−antifung Family
WAK_assoc Domain
other Domain
−200
0
200
400
600
800
1000

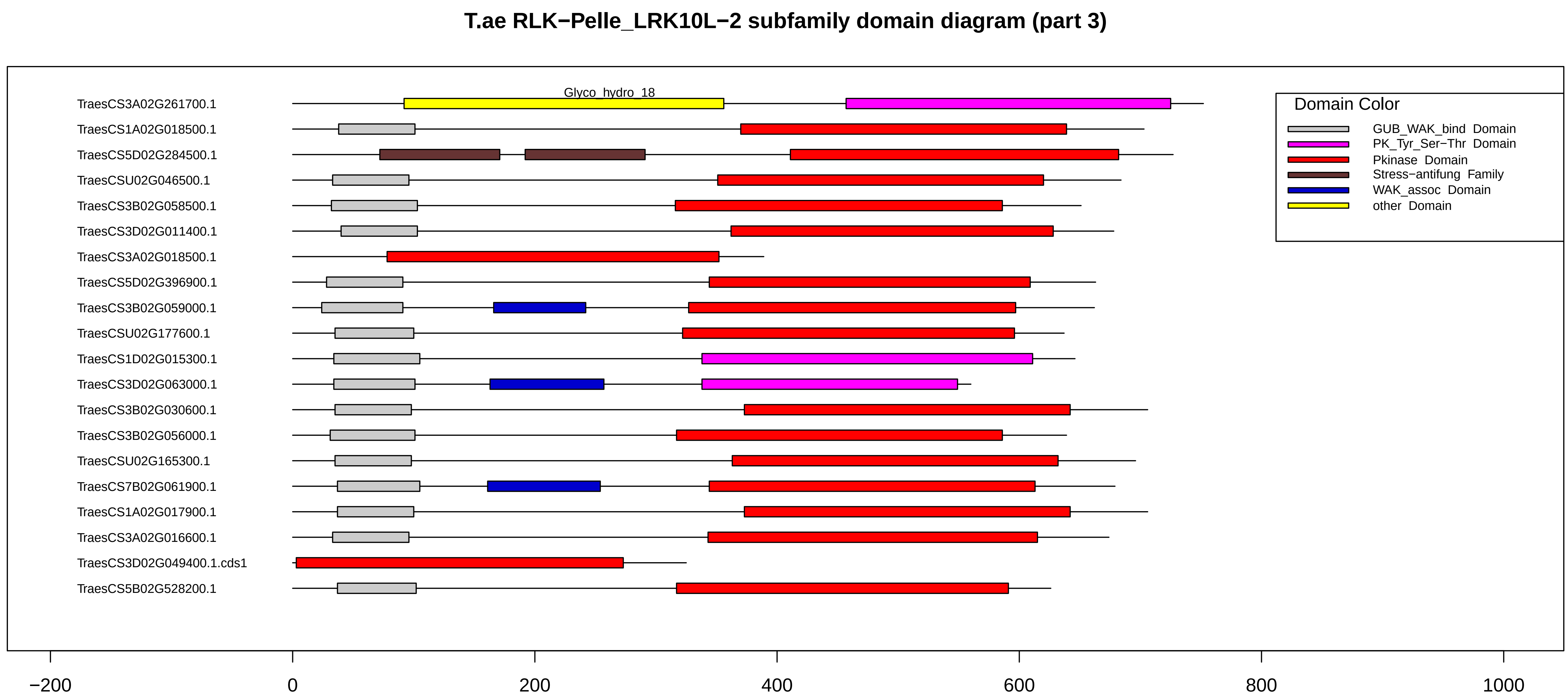
T.ae RLK−Pelle_LRK10L−2 subfamily domain diagram (part 3)
TraesCS3A02G261700.1
TraesCS1A02G018500.1
TraesCS5D02G284500.1
TraesCSU02G046500.1
TraesCS3B02G058500.1
TraesCS3D02G011400.1
TraesCS3A02G018500.1
TraesCS5D02G396900.1
TraesCS3B02G059000.1
TraesCSU02G177600.1
TraesCS1D02G015300.1
TraesCS3D02G063000.1
TraesCS3B02G030600.1
TraesCS3B02G056000.1
TraesCSU02G165300.1
TraesCS7B02G061900.1
TraesCS1A02G017900.1
TraesCS3A02G016600.1
TraesCS3D02G049400.1.cds1
TraesCS5B02G528200.1
Glyco_hydro_18
Domain Color
GUB_WAK_bind Domain
PK_Tyr_Ser−Thr Domain
Pkinase Domain
Stress−antifung Family
WAK_assoc Domain
other Domain
−200
0
200
400
600
800
1000

T.ae RLK−Pelle_LRK10L−2 subfamily domain diagram (part 4)

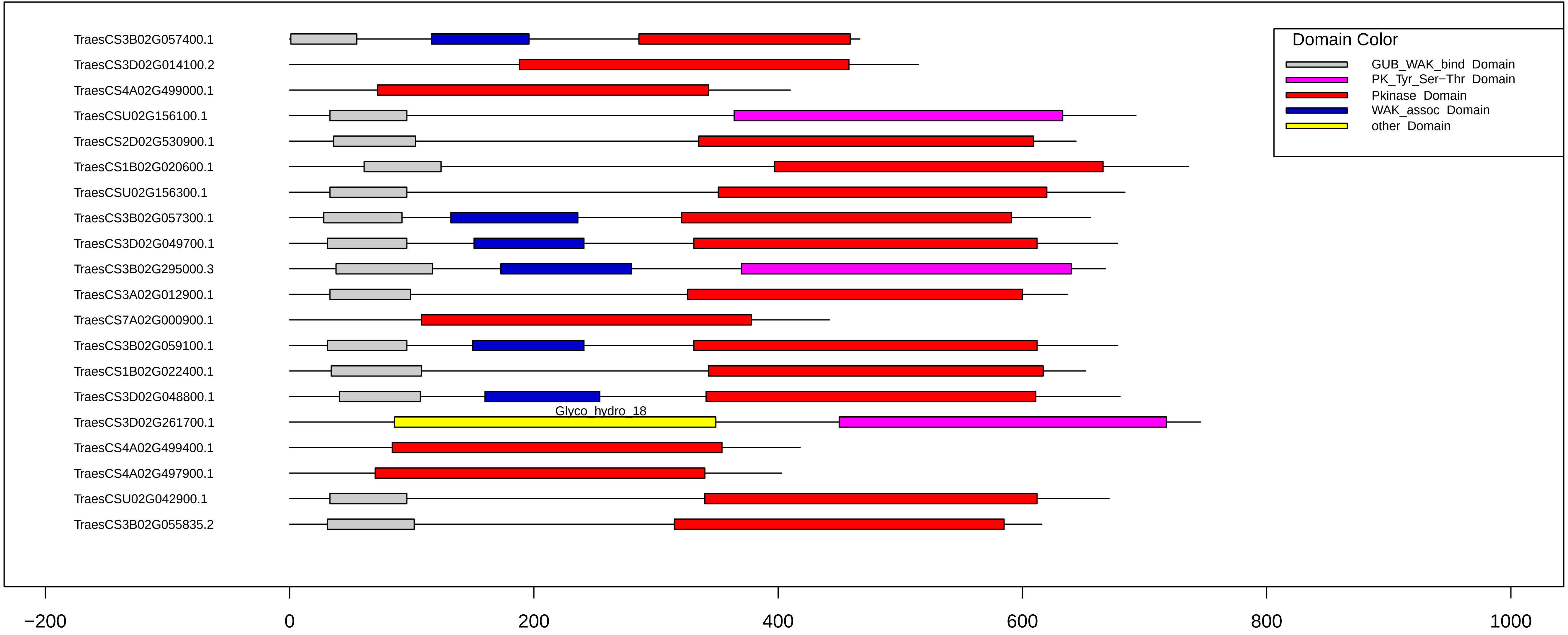

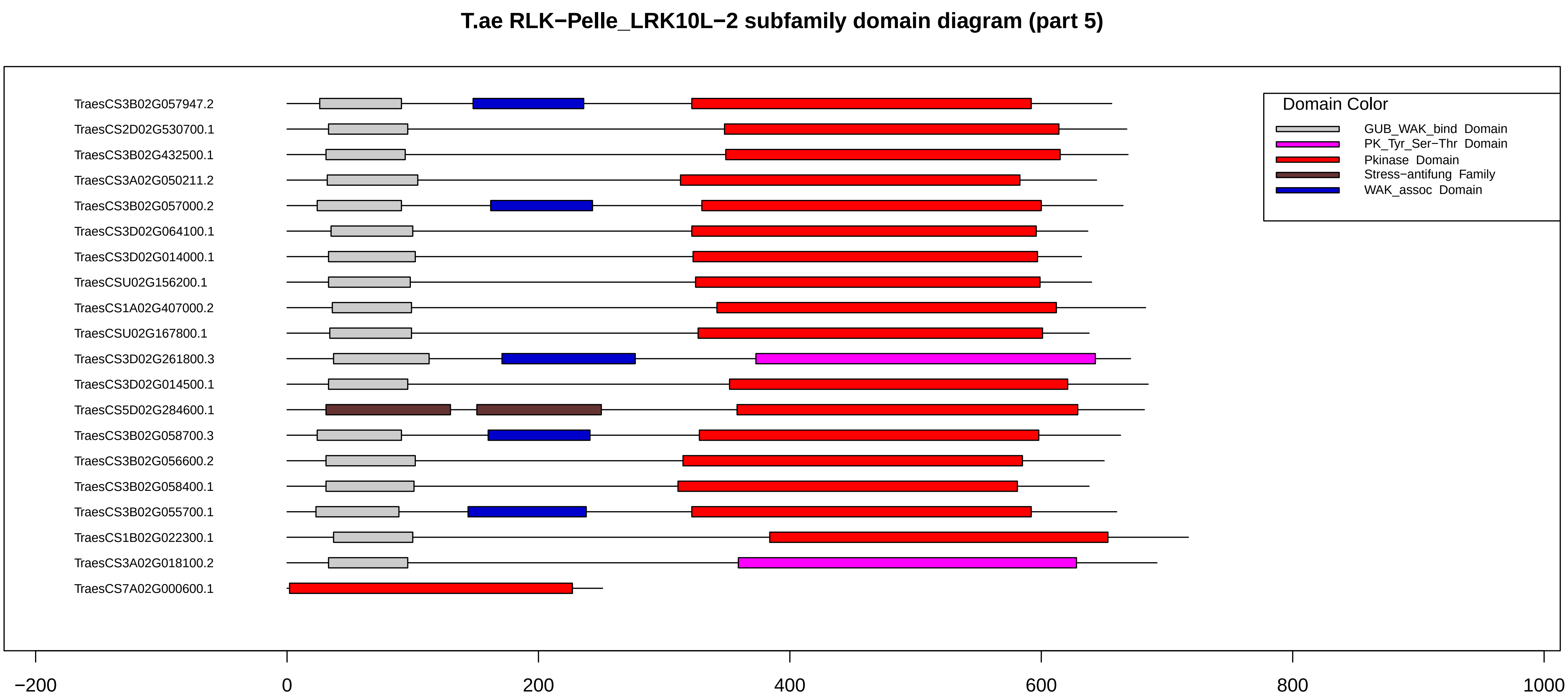
T.ae RLK−Pelle_LRK10L−2 subfamily domain diagram (part 5)
TraesCS3B02G057947.2
TraesCS2D02G530700.1
TraesCS3B02G432500.1
TraesCS3A02G050211.2
TraesCS3B02G057000.2
TraesCS3D02G064100.1
TraesCS3D02G014000.1
TraesCSU02G156200.1
TraesCS1A02G407000.2
TraesCSU02G167800.1
TraesCS3D02G261800.3
TraesCS3D02G014500.1
TraesCS5D02G284600.1
TraesCS3B02G058700.3
TraesCS3B02G056600.2
TraesCS3B02G058400.1
TraesCS3B02G055700.1
TraesCS1B02G022300.1
TraesCS3A02G018100.2
TraesCS7A02G000600.1
Domain Color
GUB_WAK_bind Domain
PK_Tyr_Ser−Thr Domain
Pkinase Domain
Stress−antifung Family
WAK_assoc Domain
−200
0
200
400
600
800
1000

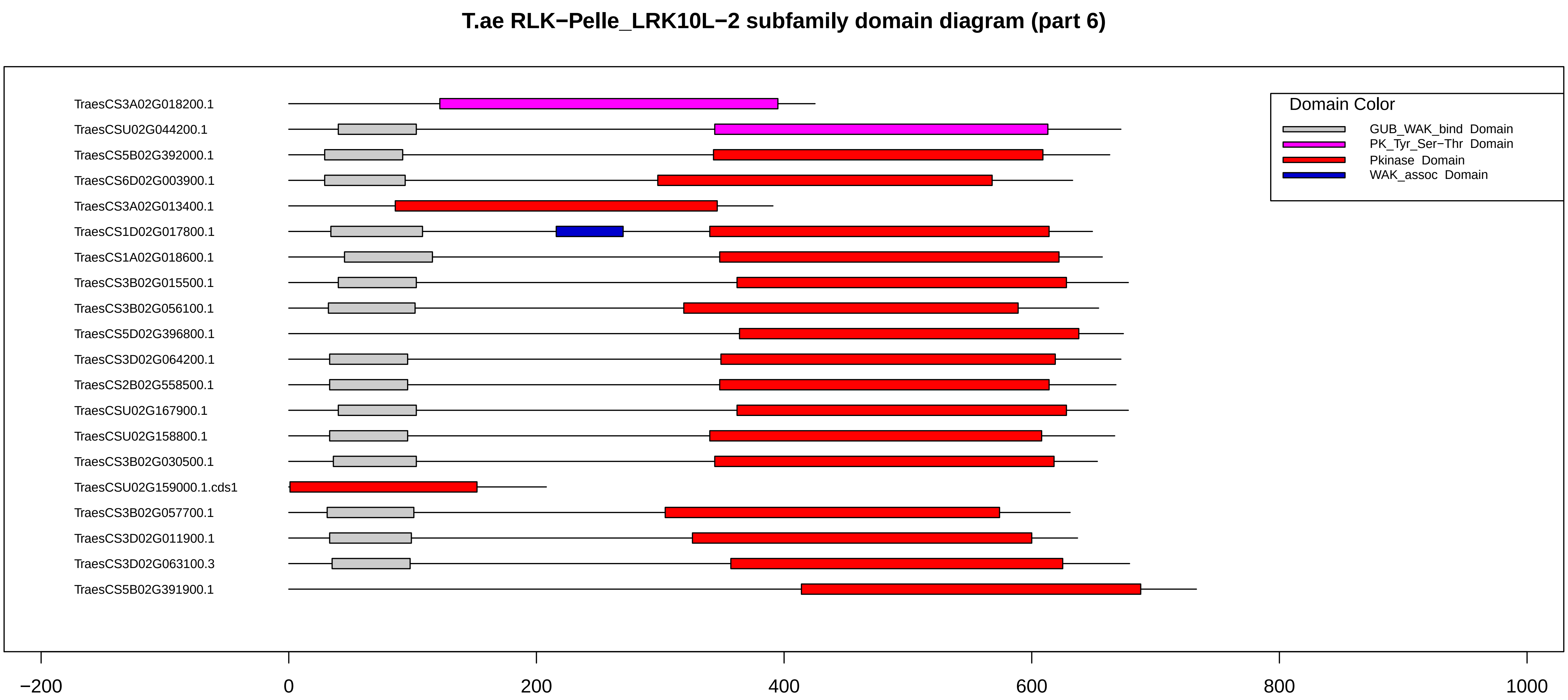
T.ae RLK−Pelle_LRK10L−2 subfamily domain diagram (part 6)
TraesCS3A02G018200.1
TraesCSU02G044200.1
TraesCS5B02G392000.1
TraesCS6D02G003900.1
TraesCS3A02G013400.1
TraesCS1D02G017800.1
TraesCS1A02G018600.1
TraesCS3B02G015500.1
TraesCS3B02G056100.1
TraesCS5D02G396800.1
TraesCS3D02G064200.1
TraesCS2B02G558500.1
TraesCSU02G167900.1
TraesCSU02G158800.1
TraesCS3B02G030500.1
TraesCSU02G159000.1.cds1
TraesCS3B02G057700.1
TraesCS3D02G011900.1
TraesCS3D02G063100.3
TraesCS5B02G391900.1
Domain Color
GUB_WAK_bind Domain
PK_Tyr_Ser−Thr Domain
Pkinase Domain
WAK_assoc Domain
−200
0
200
400
600
800
1000

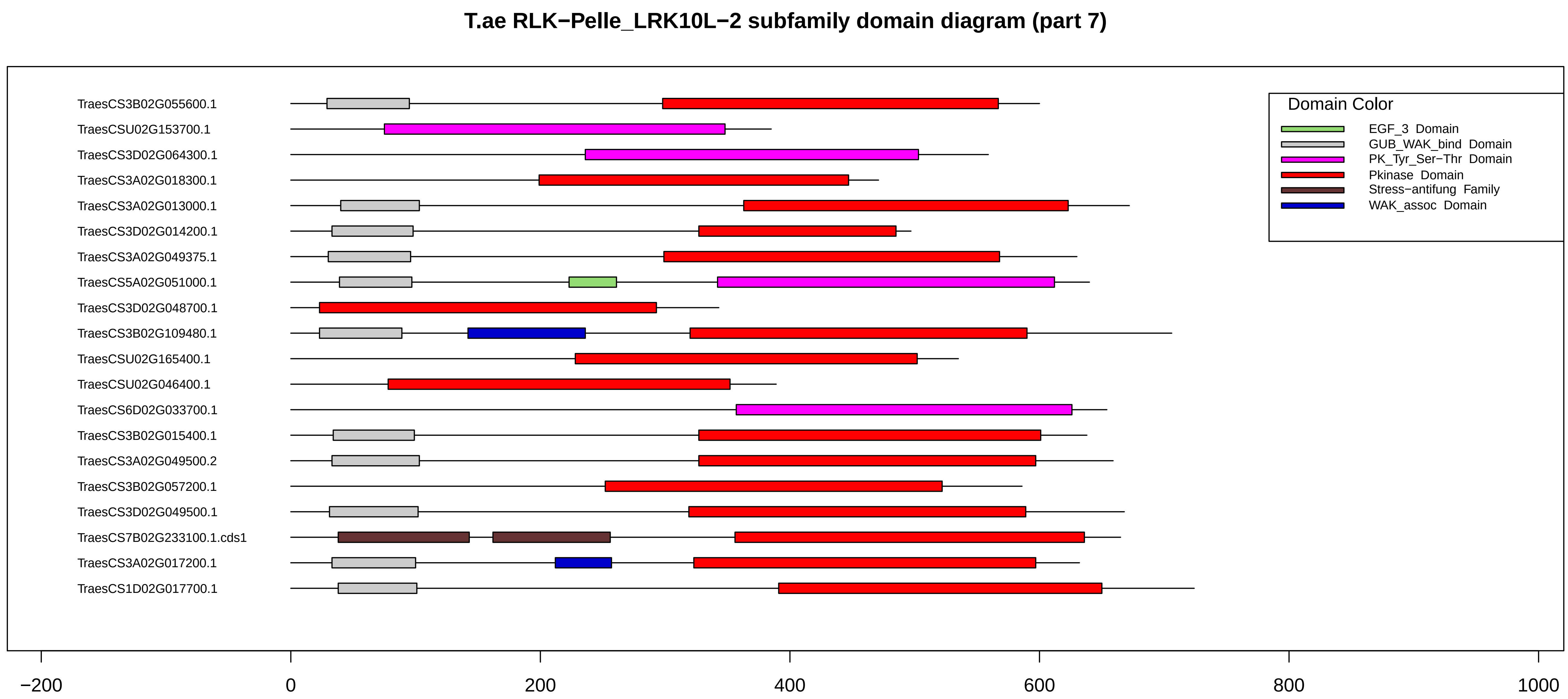
T.ae RLK−Pelle_LRK10L−2 subfamily domain diagram (part 7)
TraesCS3B02G055600.1
TraesCSU02G153700.1
TraesCS3D02G064300.1
TraesCS3A02G018300.1
TraesCS3A02G013000.1
TraesCS3D02G014200.1
TraesCS3A02G049375.1
TraesCS5A02G051000.1
TraesCS3D02G048700.1
TraesCS3B02G109480.1
TraesCSU02G165400.1
TraesCSU02G046400.1
TraesCS6D02G033700.1
TraesCS3B02G015400.1
TraesCS3A02G049500.2
TraesCS3B02G057200.1
TraesCS3D02G049500.1
TraesCS7B02G233100.1.cds1
TraesCS3A02G017200.1
TraesCS1D02G017700.1
Domain Color
EGF_3 Domain
GUB_WAK_bind Domain
PK_Tyr_Ser−Thr Domain
Pkinase Domain
Stress−antifung Family
WAK_assoc Domain
−200
0
200
400
600
800
1000

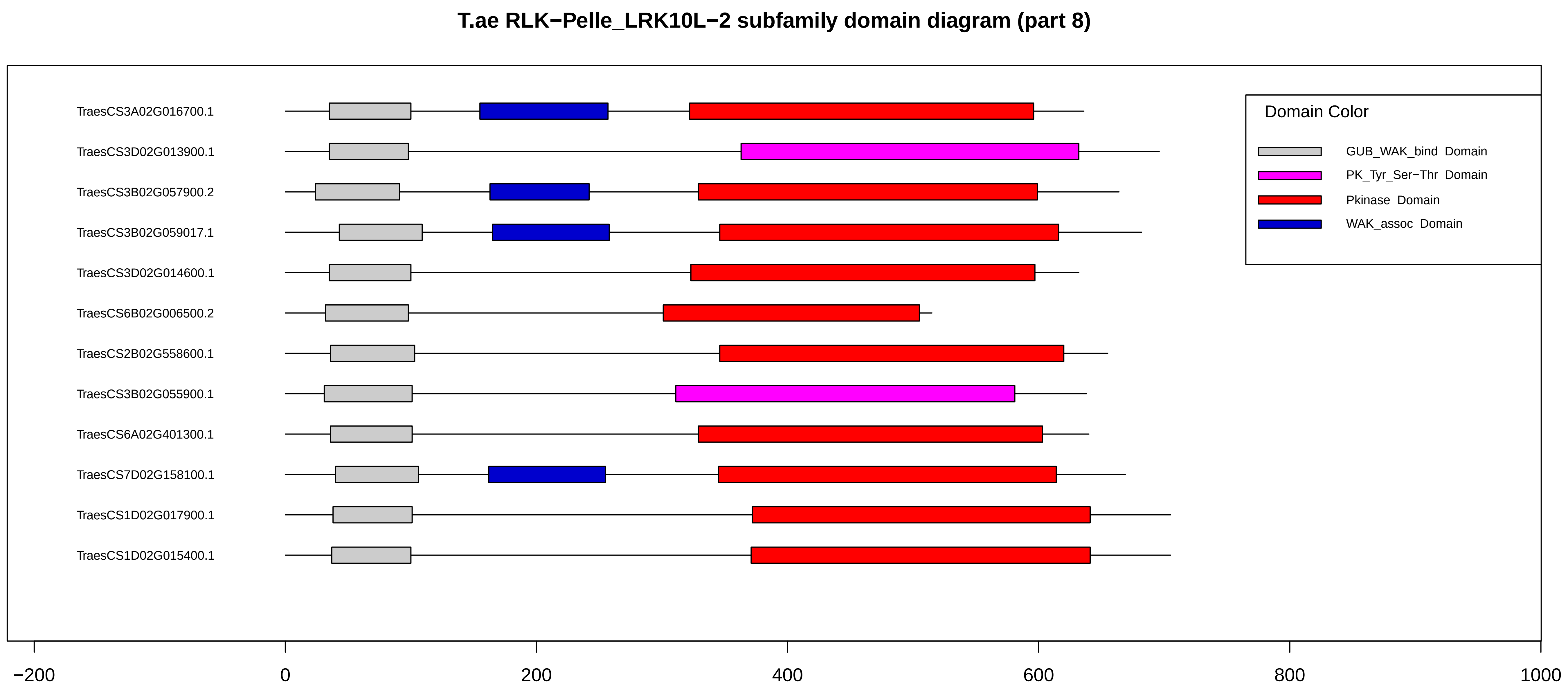
T.ae RLK−Pelle_LRK10L−2 subfamily domain diagram (part 8)
TraesCS3A02G016700.1
TraesCS3D02G013900.1
TraesCS3B02G057900.2
TraesCS3B02G059017.1
TraesCS3D02G014600.1
TraesCS6B02G006500.2
TraesCS2B02G558600.1
TraesCS3B02G055900.1
TraesCS6A02G401300.1
TraesCS7D02G158100.1
TraesCS1D02G017900.1
TraesCS1D02G015400.1
Domain Color
GUB_WAK_bind Domain
PK_Tyr_Ser−Thr Domain
Pkinase Domain
WAK_assoc Domain
−200
0
200
400
600
800
1000

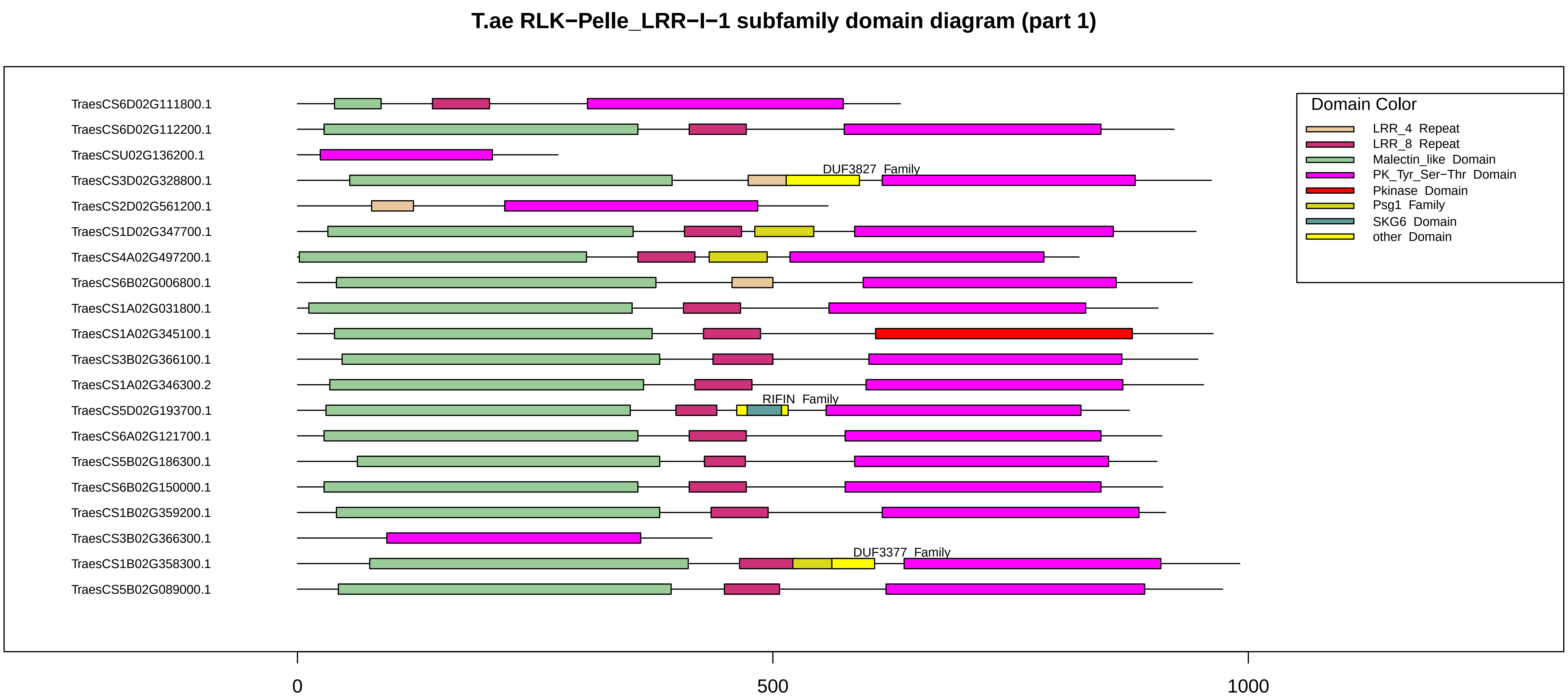
T.ae RLK−Pelle_LRR−I−1 subfamily domain diagram (part 1)
TraesCS6D02G111800.1
TraesCS6D02G112200.1
TraesCSU02G136200.1
TraesCS3D02G328800.1
TraesCS2D02G561200.1
TraesCS1D02G347700.1
TraesCS4A02G497200.1
TraesCS6B02G006800.1
TraesCS1A02G031800.1
TraesCS1A02G345100.1
TraesCS3B02G366100.1
TraesCS1A02G346300.2
TraesCS5D02G193700.1
TraesCS6A02G121700.1
TraesCS5B02G186300.1
TraesCS6B02G150000.1
TraesCS1B02G359200.1
TraesCS3B02G366300.1
TraesCS1B02G358300.1
TraesCS5B02G089000.1
DUF3827 Family
RIFIN Family
DUF3377 Family
Domain Color
LRR_4 Repeat
LRR_8 Repeat
Malectin_like Domain
PK_Tyr_Ser−Thr Domain
Pkinase Domain
Psg1 Family
SKG6 Domain
other Domain
0
500
1000

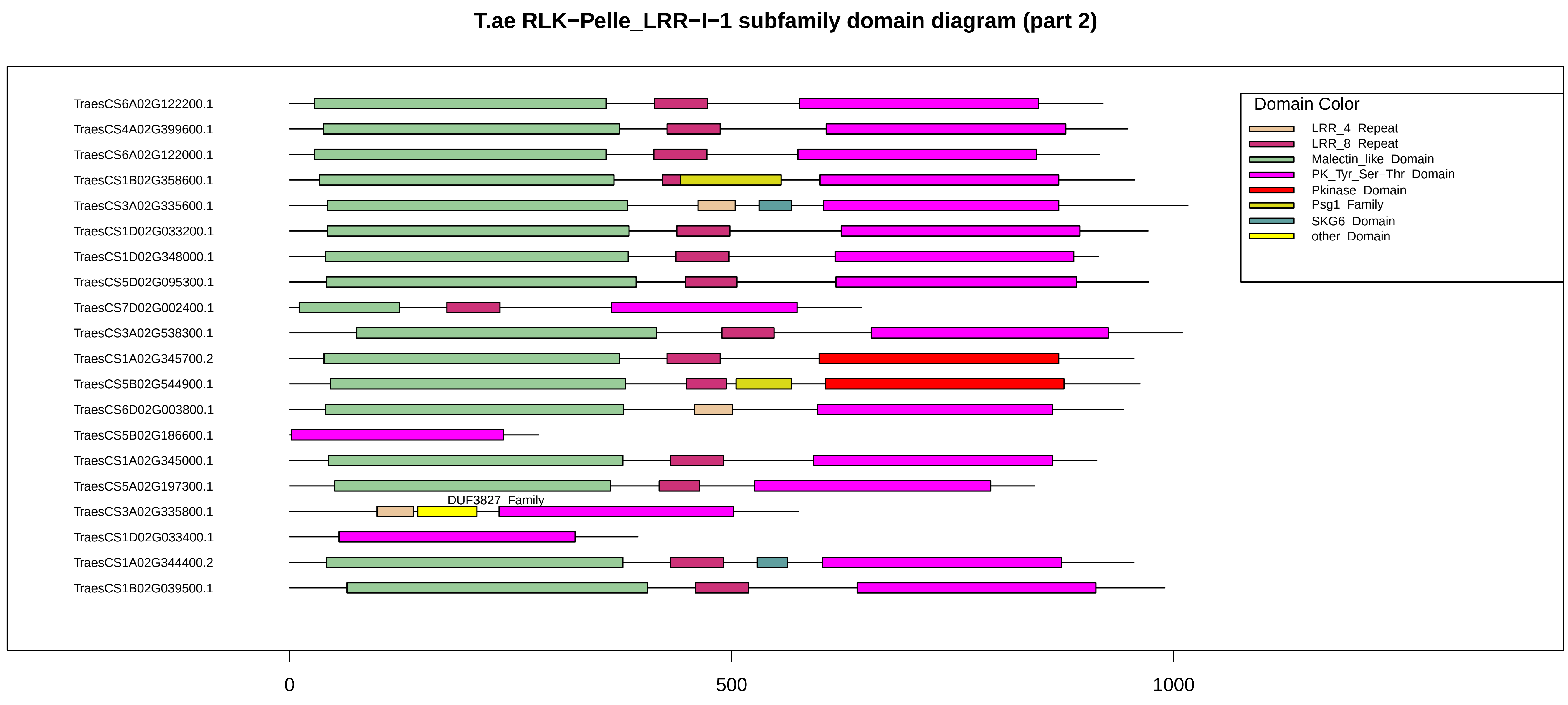
T.ae RLK−Pelle_LRR−I−1 subfamily domain diagram (part 2)
TraesCS6A02G122200.1
TraesCS4A02G399600.1
TraesCS6A02G122000.1
TraesCS1B02G358600.1
TraesCS3A02G335600.1
TraesCS1D02G033200.1
TraesCS1D02G348000.1
TraesCS5D02G095300.1
TraesCS7D02G002400.1
TraesCS3A02G538300.1
TraesCS1A02G345700.2
TraesCS5B02G544900.1
TraesCS6D02G003800.1
TraesCS5B02G186600.1
TraesCS1A02G345000.1
TraesCS5A02G197300.1
TraesCS3A02G335800.1
TraesCS1D02G033400.1
TraesCS1A02G344400.2
TraesCS1B02G039500.1
DUF3827_Family
Domain Color
LRR_4 Repeat
LRR_8 Repeat
Malectin_like Domain
PK_Tyr_Ser−Thr Domain
Pkinase Domain
Psg1 Family
SKG6 Domain
other Domain
0
500
1000

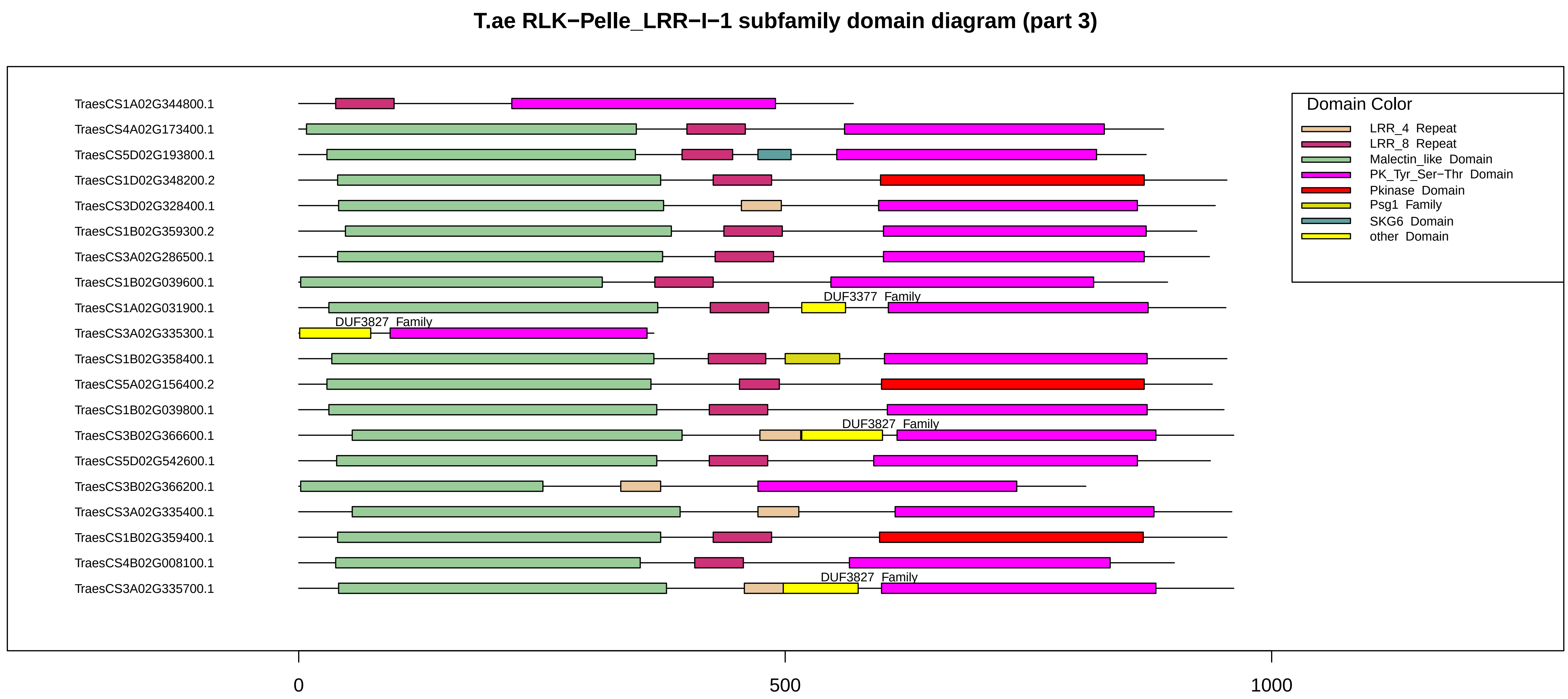

T.ae RLK−Pelle_LRR−I−1 subfamily domain diagram (part 3)
TraesCS1A02G344800.1
TraesCS4A02G173400.1
TraesCS5D02G193800.1
TraesCS1D02G348200.2
TraesCS3D02G328400.1
TraesCS1B02G359300.2
TraesCS3A02G286500.1
TraesCS1B02G039600.1
TraesCS1A02G031900.1
TraesCS3A02G335300.1
TraesCS1B02G358400.1
TraesCS5A02G156400.2
TraesCS1B02G039800.1
TraesCS3B02G366600.1
TraesCS5D02G542600.1
TraesCS3B02G366200.1
TraesCS3A02G335400.1
TraesCS1B02G359400.1
TraesCS4B02G008100.1
TraesCS3A02G335700.1
DUF3377 Family
DUF3827 Family
DUF3827 Family
DUF3827 Family
Domain Color
LRR_4 Repeat
LRR_8 Repeat
Malectin_like Domain
PK_Tyr_Ser−Thr Domain
Pkinase Domain
Psg1 Family
SKG6 Domain
other Domain
0
500
1000

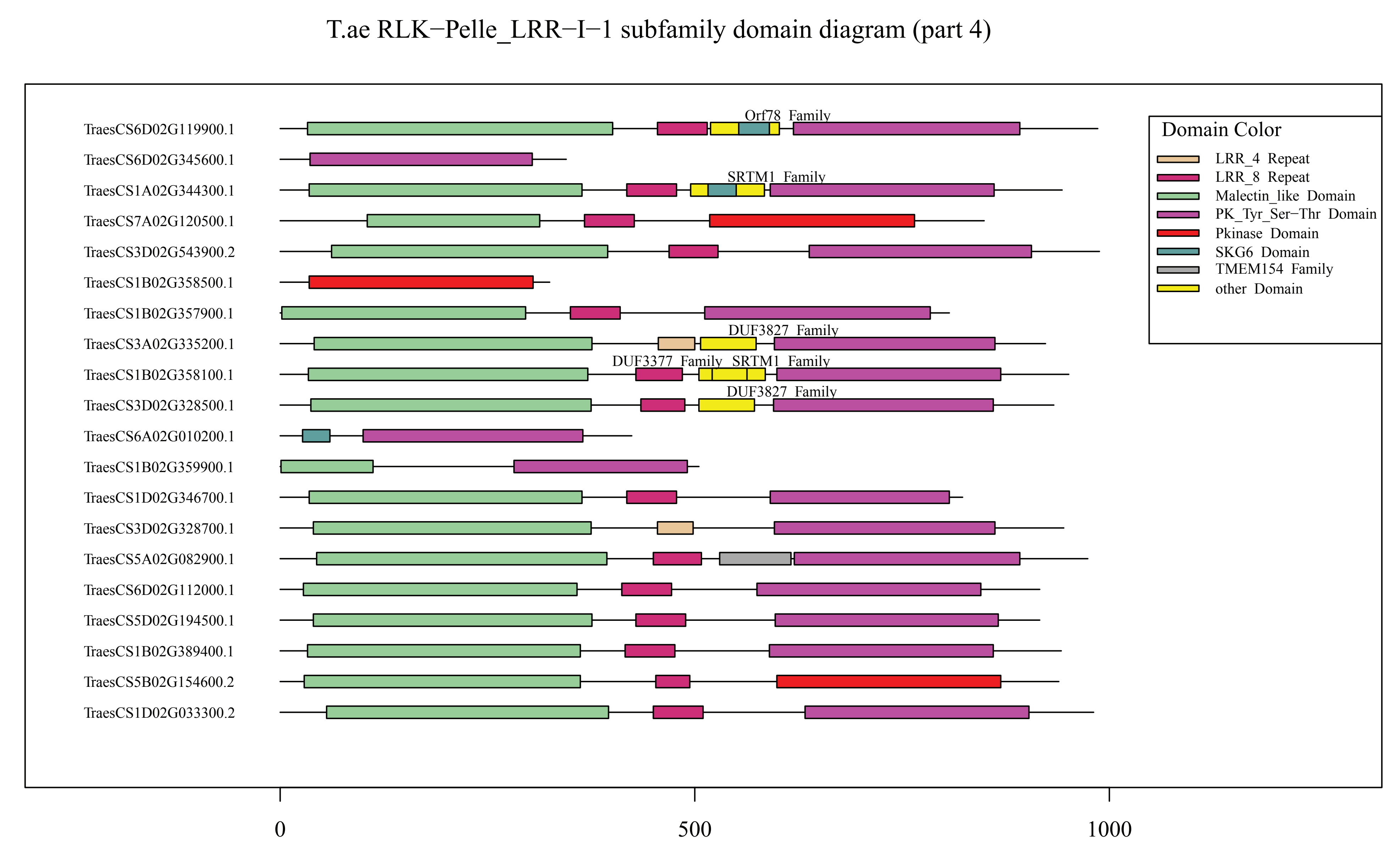
T.ae RLK−Pelle_LRR−I−1 subfamily domain diagram (part 4)
TraesCS6D02G119900.1
TraesCS6D02G345600.1
TraesCS1A02G344300.1
TraesCS7A02G120500.1
TraesCS3D02G543900.2
TraesCS1B02G358500.1
TraesCS1B02G357900.1
TraesCS3A02G335200.1
TraesCS1B02G358100.1
TraesCS3D02G328500.1
TraesCS6A02G010200.1
TraesCS1B02G359900.1
TraesCS1D02G346700.1
TraesCS3D02G328700.1
TraesCS5A02G082900.1
TraesCS6D02G112000.1
TraesCS5D02G194500.1
TraesCS1B02G389400.1
TraesCS5B02G154600.2
TraesCS1D02G033300.2
Orf78 Family
SRTM1 Family
DUF3827 Family
DUF3377 Family
SRTM1 Family
DUF3827 Family
Domain Color
LRR_4 Repeat
LRR_8 Repeat
Malectin_like Domain
PK_Tyr_Ser−Thr Domain
Pkinase Domain
SKG6 Domain
TMEM154 Family
other Domain
0
500
1000

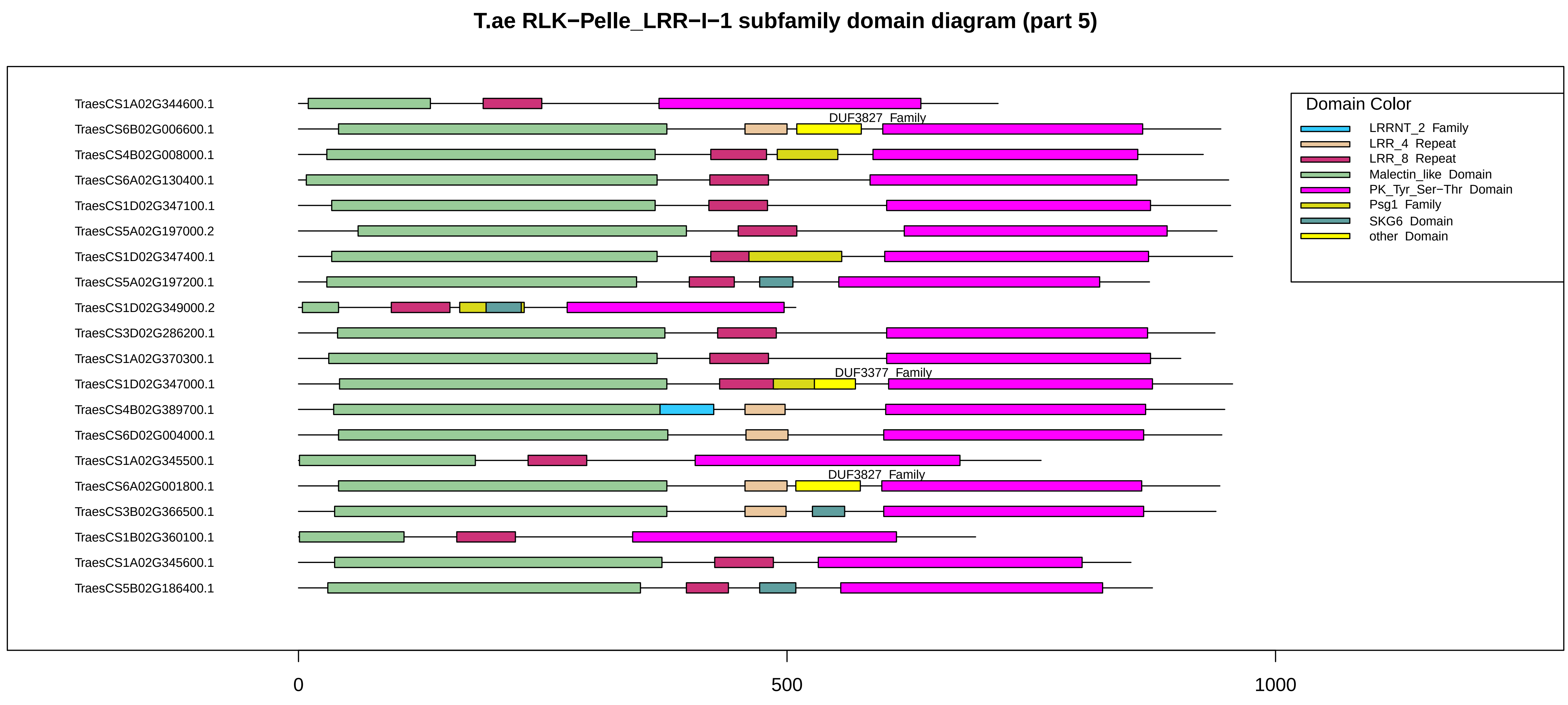
T.ae RLK−Pelle_LRR−I−1 subfamily domain diagram (part 5)
TraesCS1A02G344600.1
TraesCS6B02G006600.1
TraesCS4B02G008000.1
TraesCS6A02G130400.1
TraesCS1D02G347100.1
TraesCS5A02G197000.2
TraesCS1D02G347400.1
TraesCS5A02G197200.1
TraesCS1D02G349000.2
TraesCS3D02G286200.1
TraesCS1A02G370300.1
TraesCS1D02G347000.1
TraesCS4B02G389700.1
TraesCS6D02G004000.1
TraesCS1A02G345500.1
TraesCS6A02G001800.1
TraesCS3B02G366500.1
TraesCS1B02G360100.1
TraesCS1A02G345600.1
TraesCS5B02G186400.1
DUF3827_Family
DUF3377_Family
DUF3827_Family
Domain Color
LRRNT_2 Family
LRR_4 Repeat
LRR_8 Repeat
Malectin_like Domain
PK_Tyr_Ser−Thr Domain
Psg1 Family
SKG6 Domain
other Domain
0
500
1000

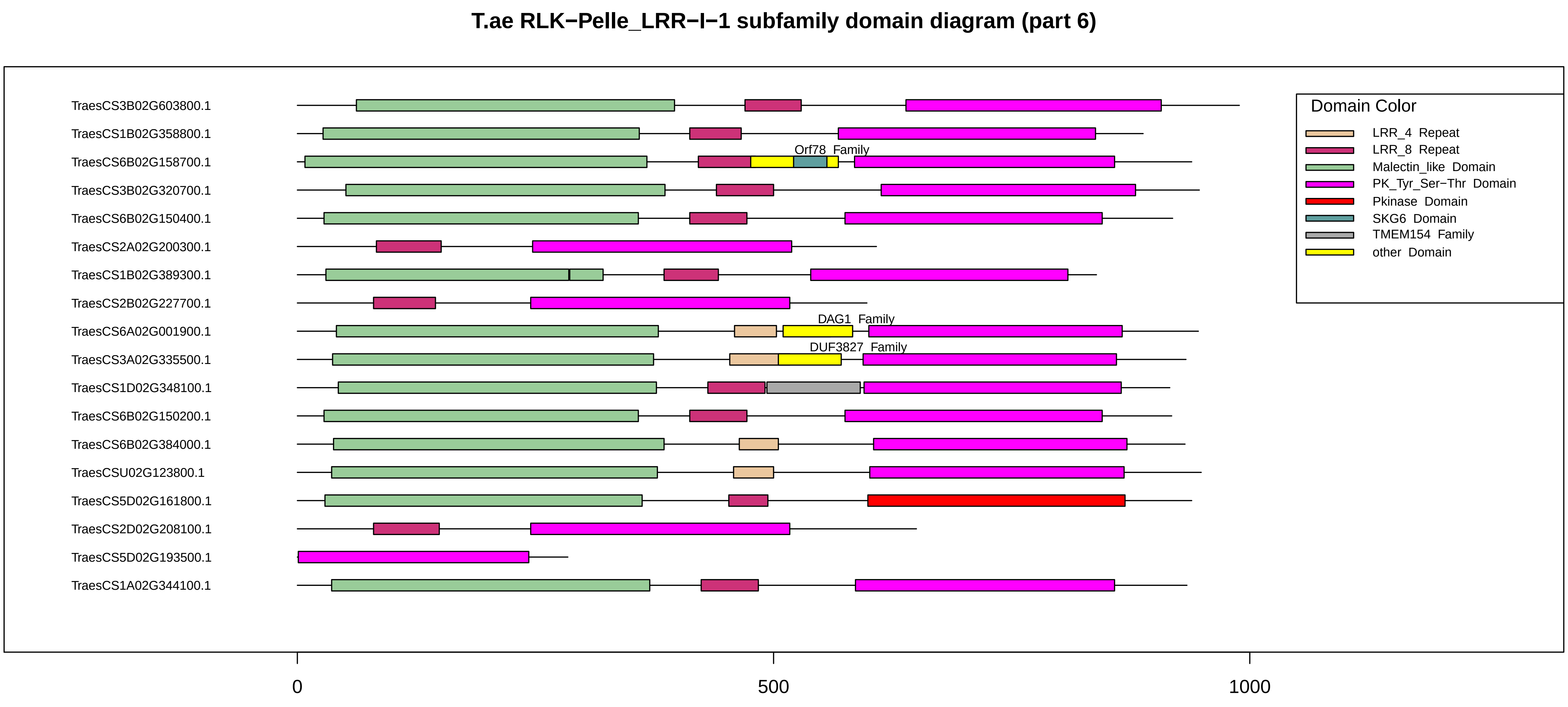

T.ae RLK−Pelle_LRR−I−1 subfamily domain diagram (part 6)
TraesCS3B02G603800.1
TraesCS1B02G358800.1
TraesCS6B02G158700.1
TraesCS3B02G320700.1
TraesCS6B02G150400.1
TraesCS2A02G200300.1
TraesCS1B02G389300.1
TraesCS2B02G227700.1
TraesCS6A02G001900.1
TraesCS3A02G335500.1
TraesCS1D02G348100.1
TraesCS6B02G150200.1
TraesCS6B02G384000.1
TraesCSU02G123800.1
TraesCS5D02G161800.1
TraesCS2D02G208100.1
TraesCS5D02G193500.1
TraesCS1A02G344100.1
Orf78 Family
DAG1 Family
DUF3827 Family
Domain Color
LRR_4 Repeat
LRR_8 Repeat
Malectin_like Domain
PK_Tyr_Ser−Thr Domain
Pkinase Domain
SKG6 Domain
TMEM154 Family
other Domain
0
500
1000

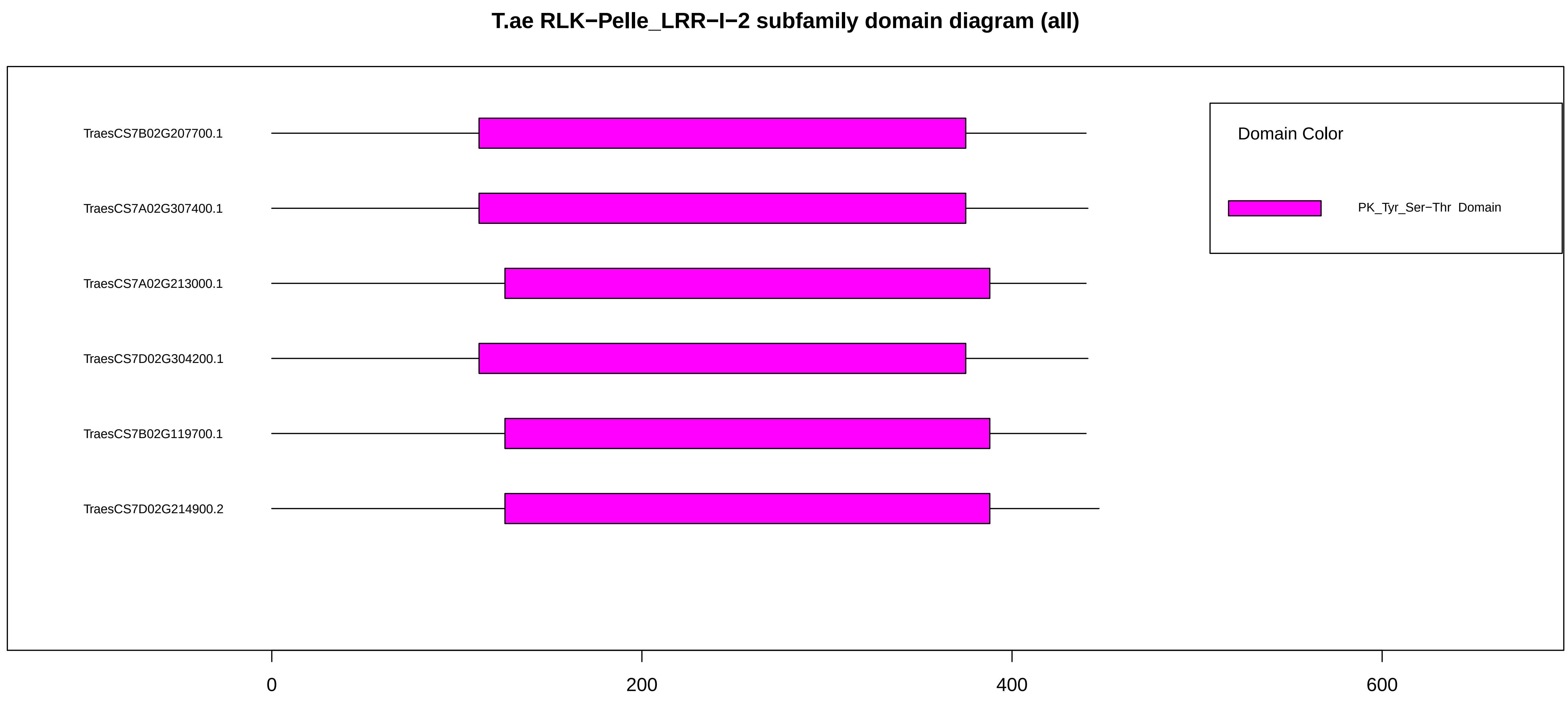
T.ae RLK−Pelle_LRR−I−2 subfamily domain diagram (all)
TraesCS7B02G207700.1
TraesCS7A02G307400.1
TraesCS7A02G213000.1
TraesCS7D02G304200.1
TraesCS7B02G119700.1
TraesCS7D02G214900.2
Domain Color
PK_Tyr_Ser−Thr Domain
0
200
400
600

T.ae RLK−Pelle_LRR−III subfamily domain diagram (part 1)

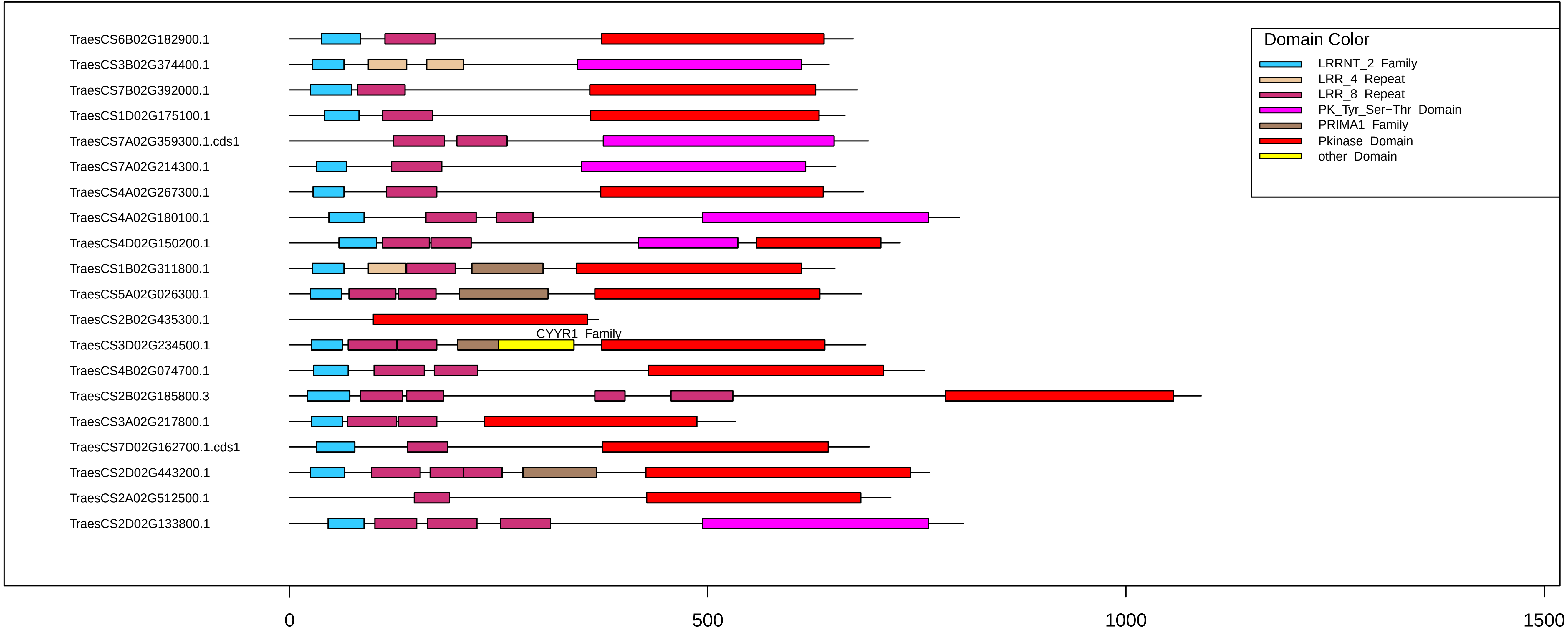

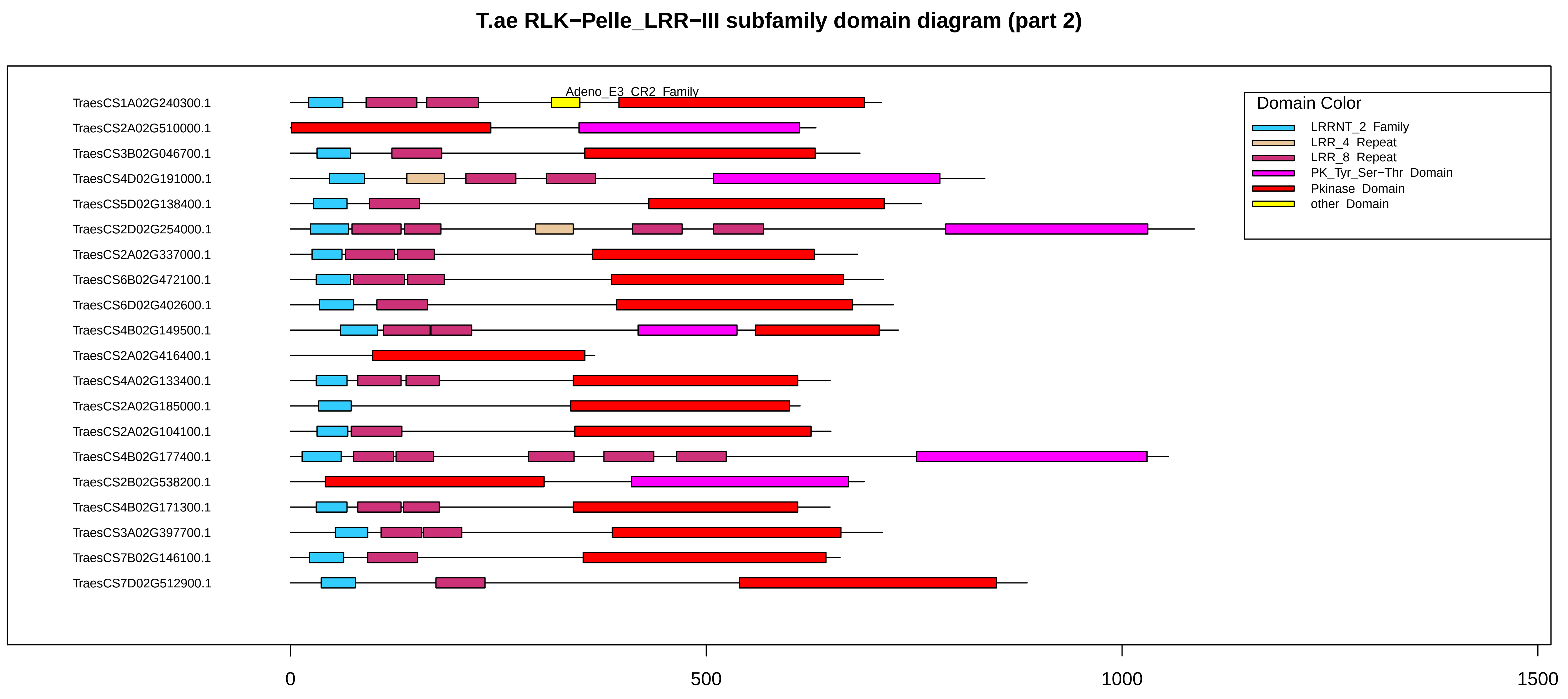
T.ae RLK−Pelle_LRR−III subfamily domain diagram (part 2)
Adeno_E3_CR2_Family
TraesCS1A02G240300.1
TraesCS2A02G510000.1
TraesCS3B02G046700.1
TraesCS4D02G191000.1
TraesCS5D02G138400.1
TraesCS2D02G254000.1
TraesCS2A02G337000.1
TraesCS6B02G472100.1
TraesCS6D02G402600.1
TraesCS4B02G149500.1
TraesCS2A02G416400.1
TraesCS4A02G133400.1
TraesCS2A02G185000.1
TraesCS2A02G104100.1
TraesCS4B02G177400.1
TraesCS2B02G538200.1
TraesCS4B02G171300.1
TraesCS3A02G397700.1
TraesCS7B02G146100.1
TraesCS7D02G512900.1
Domain Color
LRRNT_2 Family
LRR_4 Repeat
LRR_8 Repeat
PK_Tyr_Ser−Thr Domain
Pkinase Domain
other Domain
0
500
1000
1500

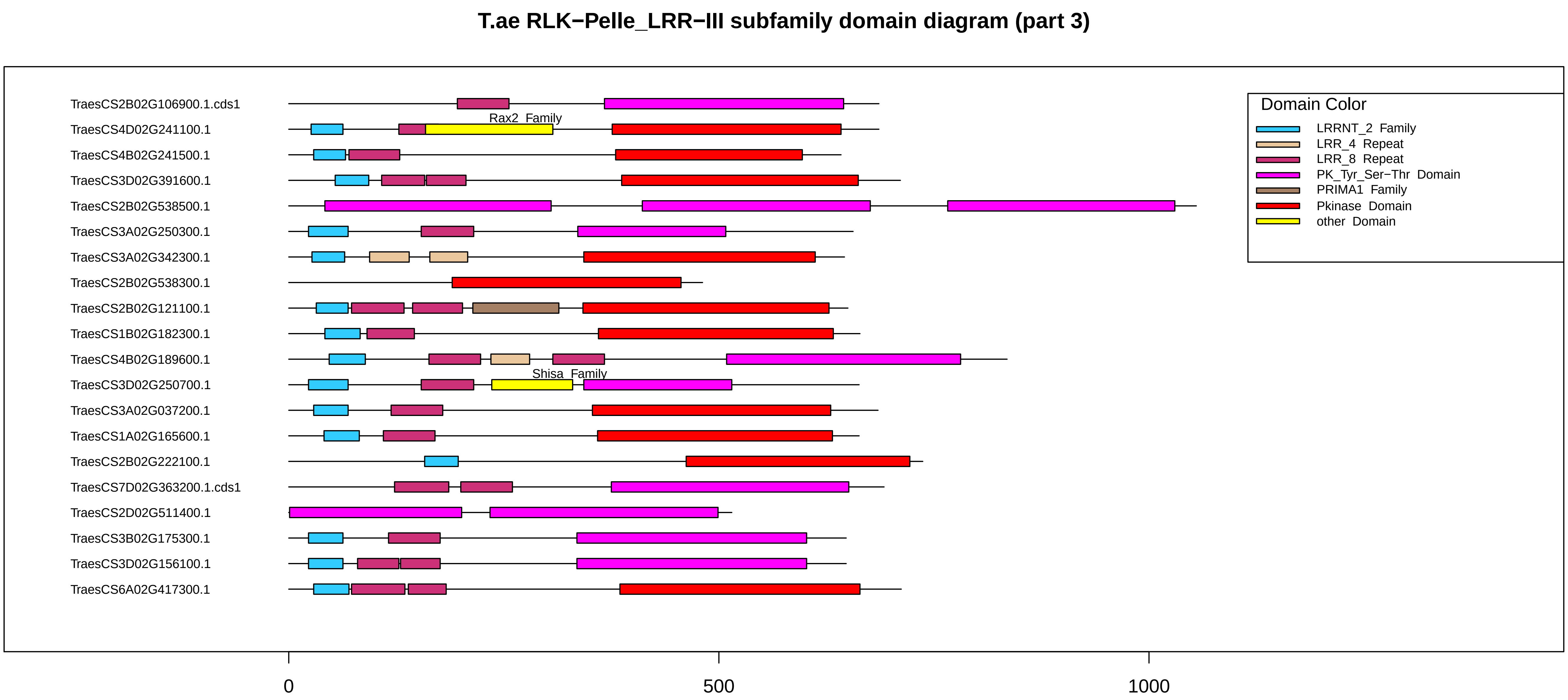
T.ae RLK−Pelle_LRR−III subfamily domain diagram (part 3)
TraesCS2B02G106900.1.cds1
TraesCS4D02G241100.1
Rax2 Family
TraesCS4B02G241500.1
TraesCS3D02G391600.1
TraesCS2B02G538500.1
TraesCS3A02G250300.1
TraesCS3A02G342300.1
TraesCS2B02G538300.1
TraesCS2B02G121100.1
TraesCS1B02G182300.1
TraesCS4B02G189600.1
TraesCS3D02G250700.1
Shisa Family
TraesCS3A02G037200.1
TraesCS1A02G165600.1
TraesCS2B02G222100.1
TraesCS7D02G363200.1.cds1
TraesCS2D02G511400.1
TraesCS3B02G175300.1
TraesCS3D02G156100.1
TraesCS6A02G417300.1
Domain Color
LRRNT_2 Family
LRR_4 Repeat
LRR_8 Repeat
PK_Tyr_Ser−Thr Domain
PRIMA1 Family
Pkinase Domain
other Domain
0
500
1000

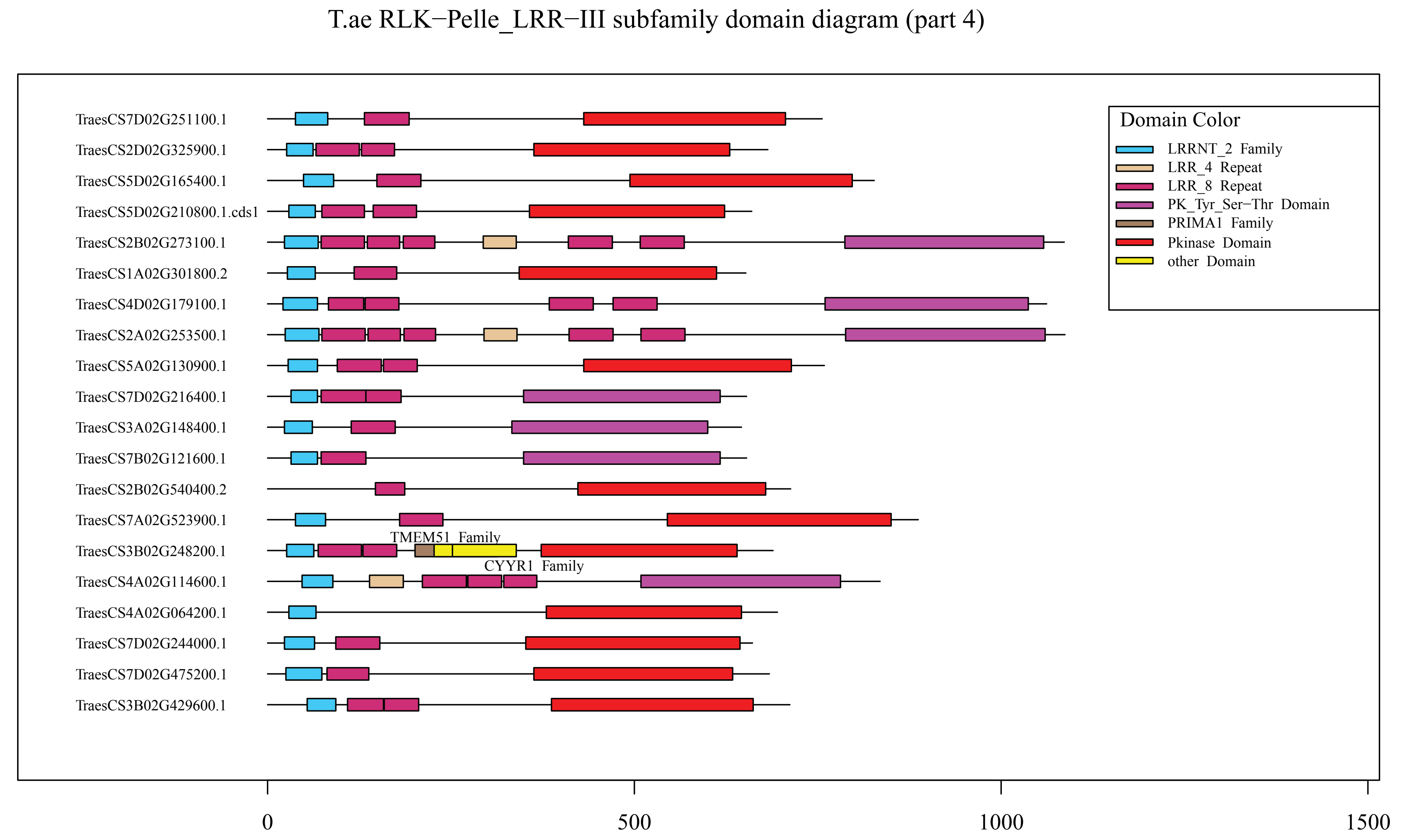

T.ae RLK−Pelle_LRR−III subfamily domain diagram (part 4)
TraesCS7D02G251100.1
TraesCS2D02G325900.1
TraesCS5D02G165400.1
TraesCS5D02G210800.1.cds1
TraesCS2B02G273100.1
TraesCS1A02G301800.2
TraesCS4D02G179100.1
TraesCS2A02G253500.1
TraesCS5A02G130900.1
TraesCS7D02G216400.1
TraesCS3A02G148400.1
TraesCS7B02G121600.1
TraesCS2B02G540400.2
TraesCS7A02G523900.1
TraesCS3B02G248200.1
TMEM51 Family
CYYR1 Family
TraesCS4A02G114600.1
TraesCS4A02G064200.1
TraesCS7D02G244000.1
TraesCS7D02G475200.1
TraesCS3B02G429600.1
Domain Color
LRRNT_2 Family
LRR_4 Repeat
LRR_8 Repeat
PK_Tyr_Ser−Thr Domain
PRIMA1 Family
Pkinase Domain
other Domain
0
500
1000
1500

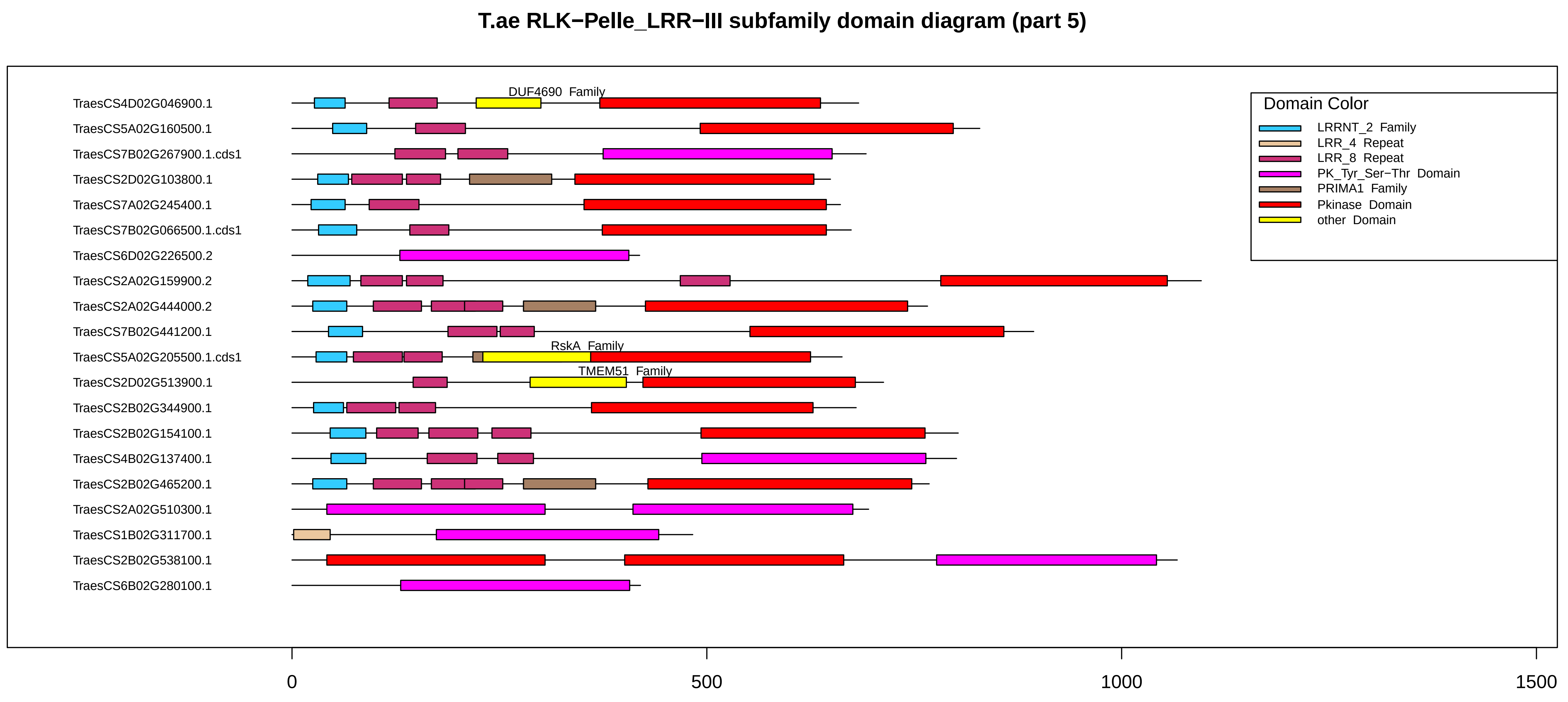
T.ae RLK−Pelle_LRR−III subfamily domain diagram (part 5)
TraesCS4D02G046900.1
TraesCS5A02G160500.1
TraesCS7B02G267900.1.cds1
TraesCS2D02G103800.1
TraesCS7A02G245400.1
TraesCS7B02G066500.1.cds1
TraesCS6D02G226500.2
TraesCS2A02G159900.2
TraesCS2A02G444000.2
TraesCS7B02G441200.1
TraesCS5A02G205500.1.cds1
TraesCS2D02G513900.1
TraesCS2B02G344900.1
TraesCS2B02G154100.1
TraesCS4B02G137400.1
TraesCS2B02G465200.1
TraesCS2A02G510300.1
TraesCS1B02G311700.1
TraesCS2B02G538100.1
TraesCS6B02G280100.1
DUF4690 Family
RskA Family
TMEM51 Family
Domain Color
LRRNT_2 Family
LRR_4 Repeat
LRR_8 Repeat
PK_Tyr_Ser−Thr Domain
PRIMA1 Family
Pkinase Domain
other Domain
0
500
1000
1500

T.ae RLK−Pelle_LRR−III subfamily domain diagram (part 6)

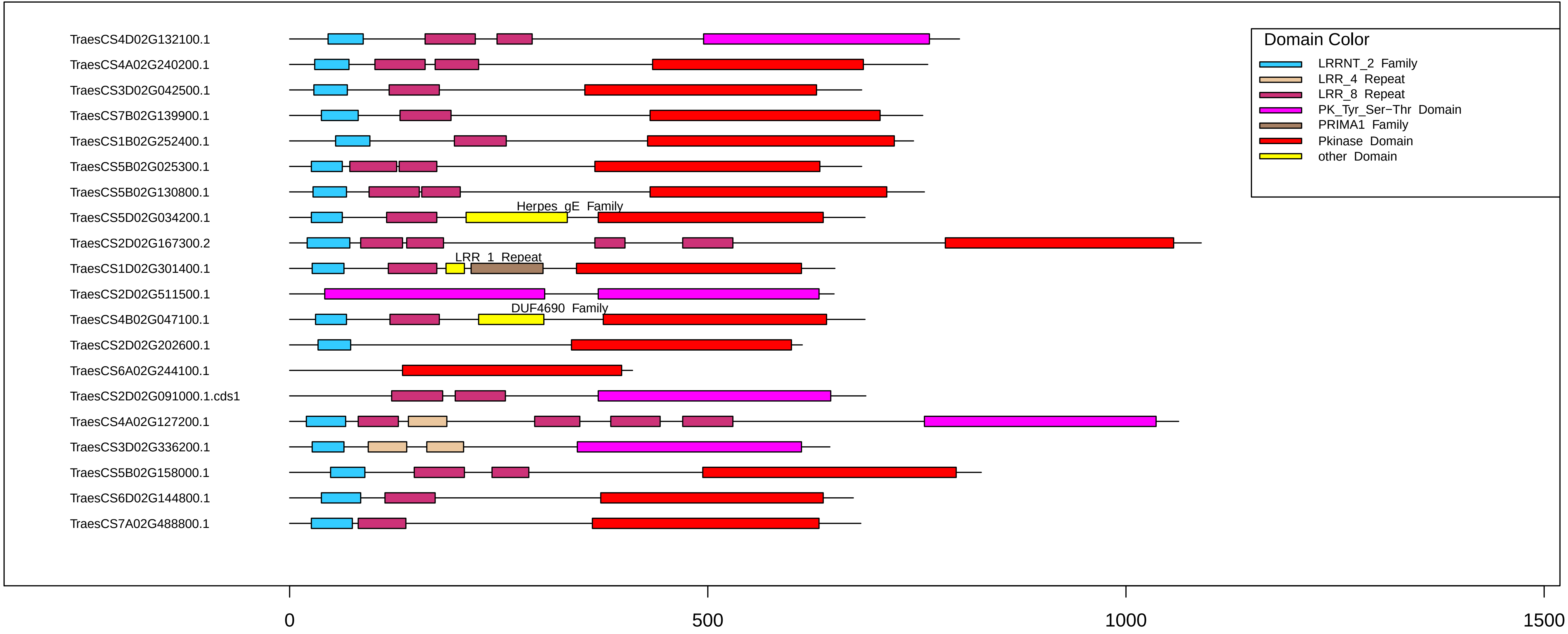

T.ae RLK−Pelle_LRR−III subfamily domain diagram (part 7)

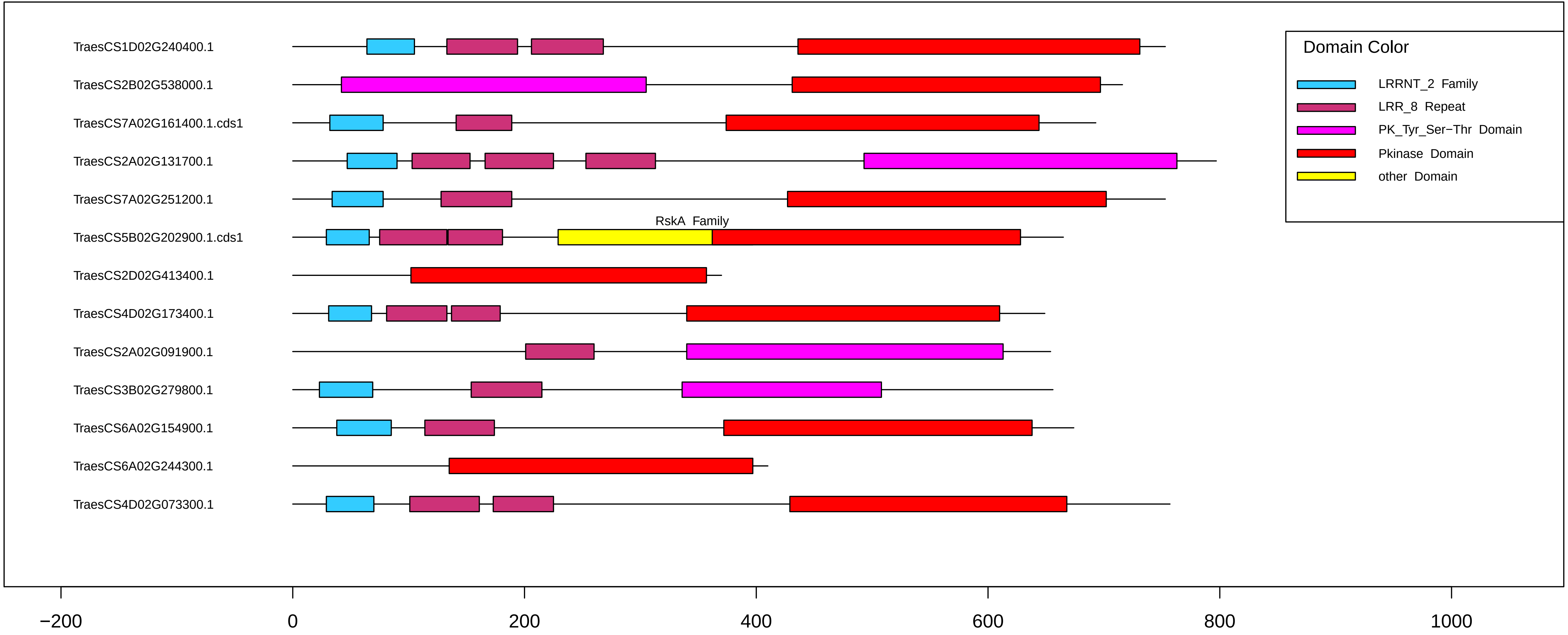

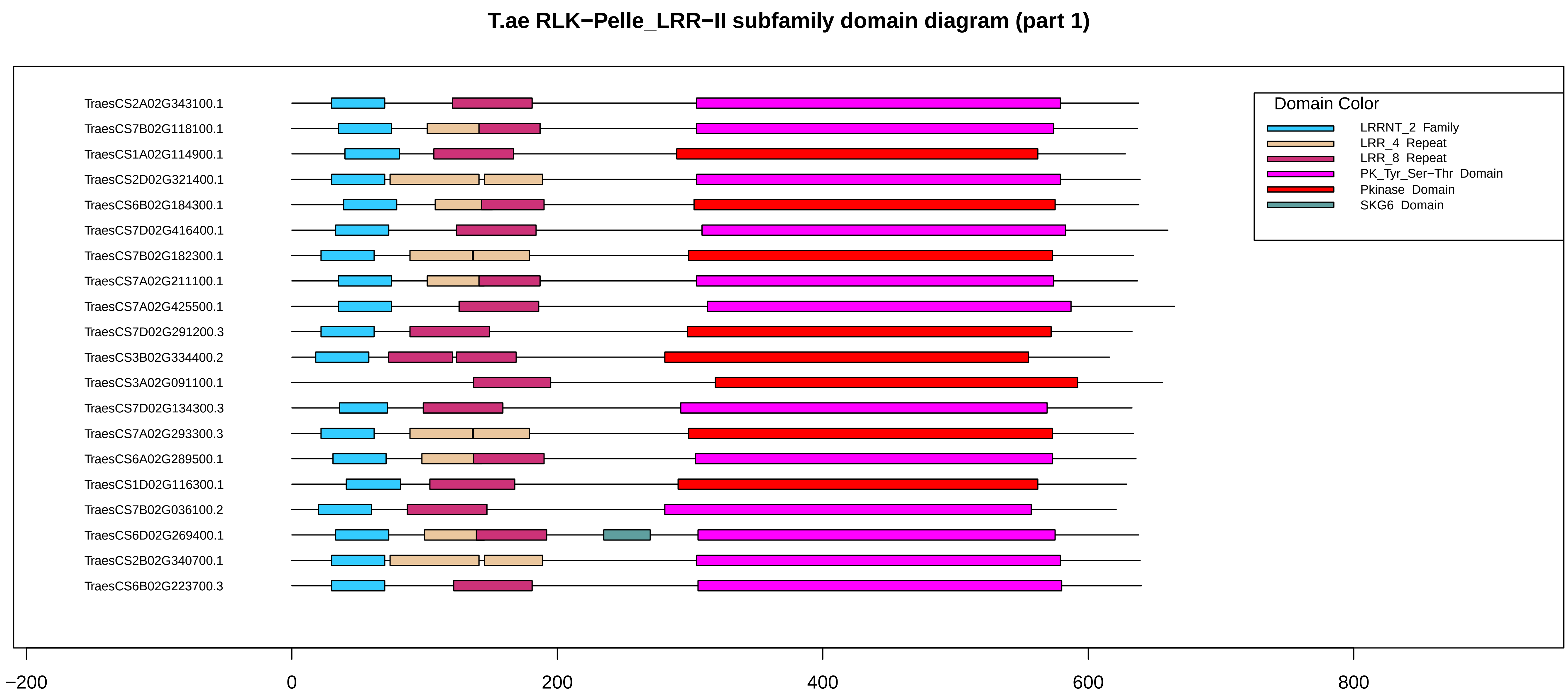
T.ae RLK−Pelle_LRR−II subfamily domain diagram (part 1)
TraesCS2A02G343100.1
TraesCS7B02G118100.1
TraesCS1A02G114900.1
TraesCS2D02G321400.1
TraesCS6B02G184300.1
TraesCS7D02G416400.1
TraesCS7B02G182300.1
TraesCS7A02G211100.1
TraesCS7A02G425500.1
TraesCS7D02G291200.3
TraesCS3B02G334400.2
TraesCS3A02G091100.1
TraesCS7D02G134300.3
TraesCS7A02G293300.3
TraesCS6A02G289500.1
TraesCS1D02G116300.1
TraesCS7B02G036100.2
TraesCS6D02G269400.1
TraesCS2B02G340700.1
TraesCS6B02G223700.3
Domain Color
LRRNT_2 Family
LRR_4 Repeat
LRR_8 Repeat
PK_Tyr_Ser−Thr Domain
Pkinase Domain
SKG6 Domain
−200
0
200
400
600
800

T.ae RLK−Pelle_LRR−II subfamily domain diagram (part 2)

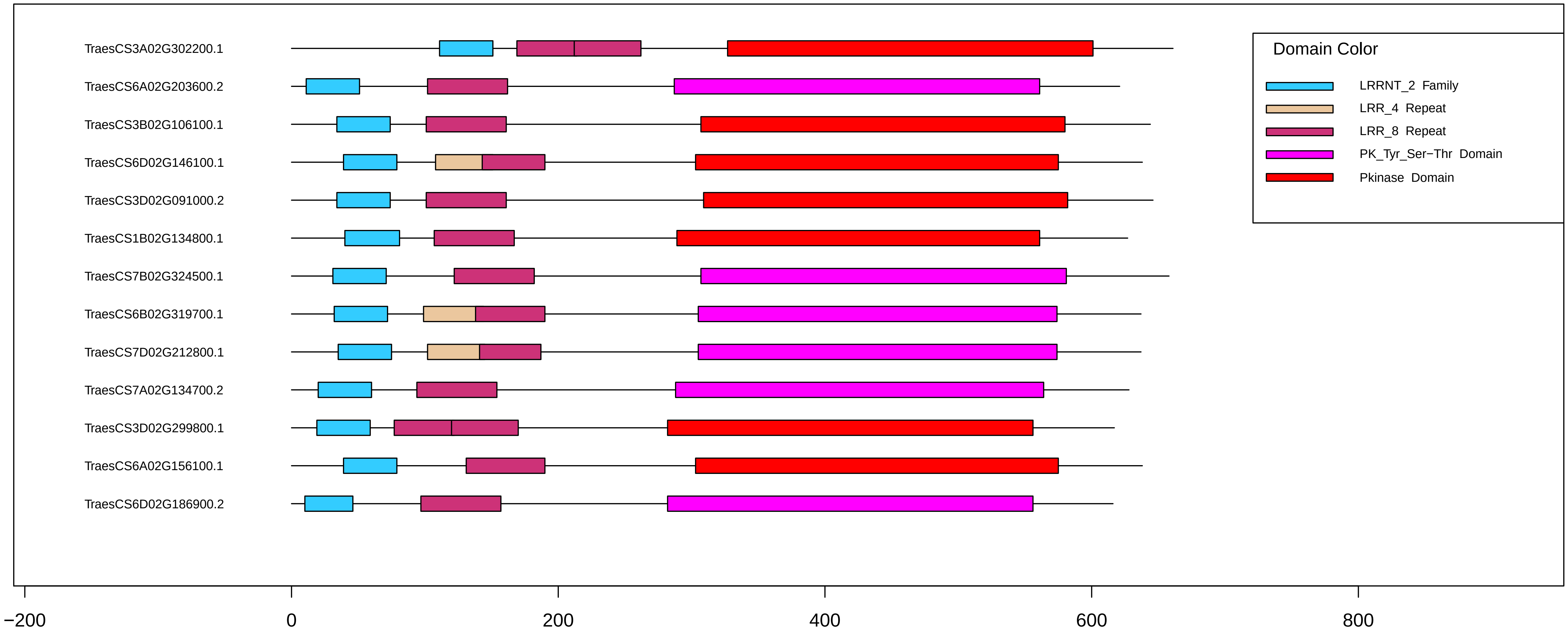

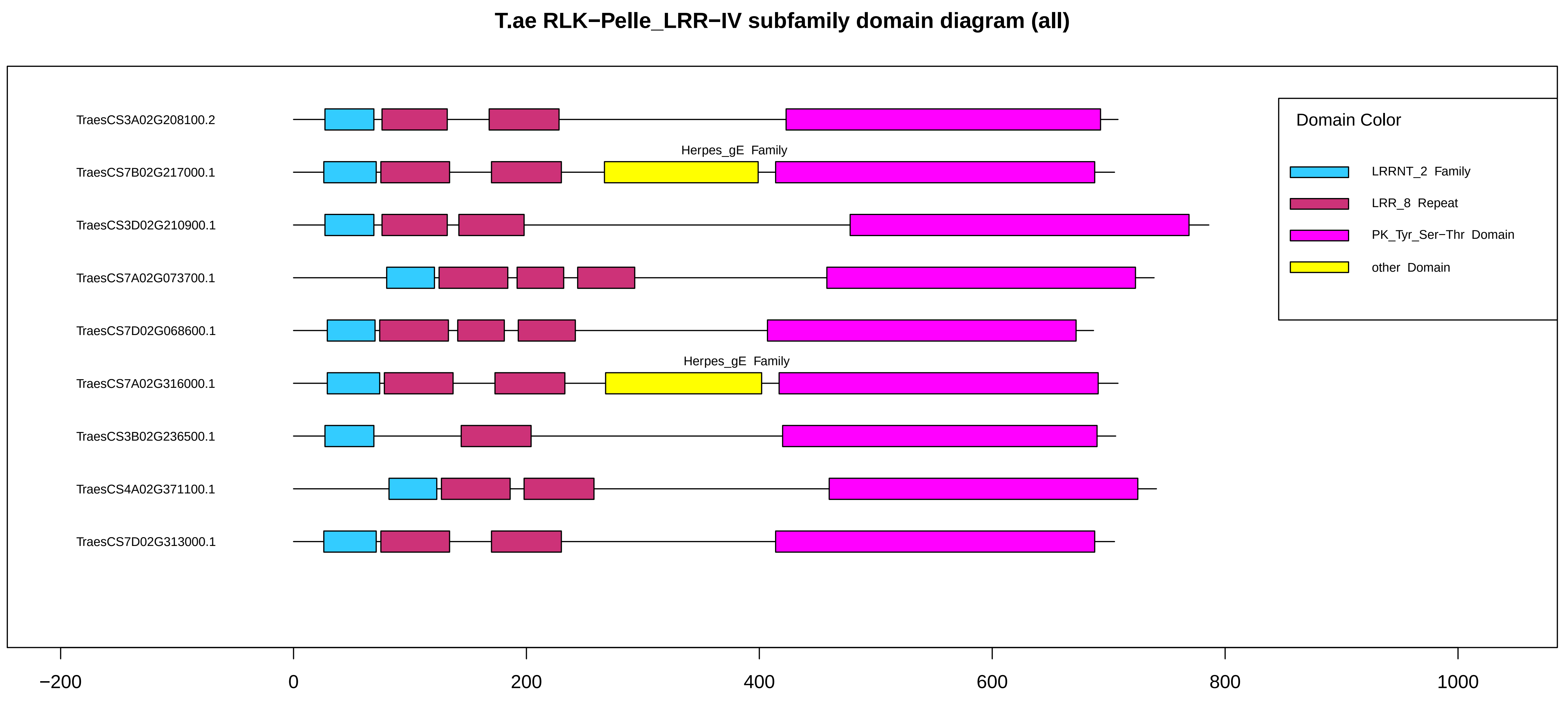

T.ae RLK−Pelle_LRR−IV subfamily domain diagram (all)
TraesCS3A02G208100.2
TraesCS7B02G217000.1
Herpes_gE Family
TraesCS3D02G210900.1
TraesCS7A02G073700.1
TraesCS7D02G068600.1
TraesCS7A02G316000.1
Herpes_gE Family
TraesCS3B02G236500.1
TraesCS4A02G371100.1
TraesCS7D02G313000.1
Domain Color
LRRNT_2 Family
LRR_8 Repeat
PK_Tyr_Ser−Thr Domain
other Domain
−200
0
200
400
600
800
1000

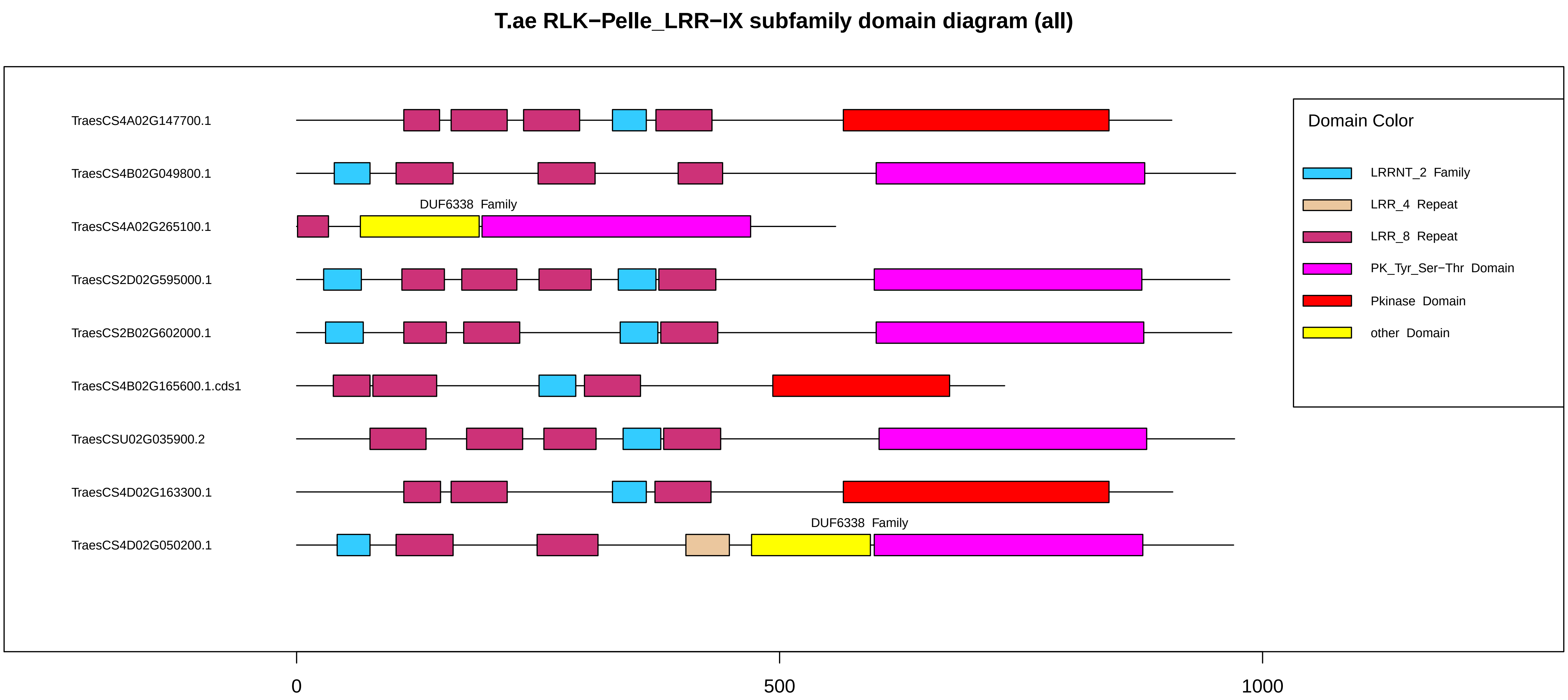
T.ae RLK−Pelle_LRR−IX subfamily domain diagram (all)
TraesCS4A02G147700.1
TraesCS4B02G049800.1
TraesCS4A02G265100.1
DUF6338 Family
TraesCS2D02G595000.1
TraesCS2B02G602000.1
TraesCS4B02G165600.1.cds1
TraesCSU02G035900.2
TraesCS4D02G163300.1
TraesCS4D02G050200.1
DUF6338 Family
Domain Color
LRRNT_2 Family
LRR_4 Repeat
LRR_8 Repeat
PK_Tyr_Ser−Thr Domain
Pkinase Domain
other Domain
0
500
1000

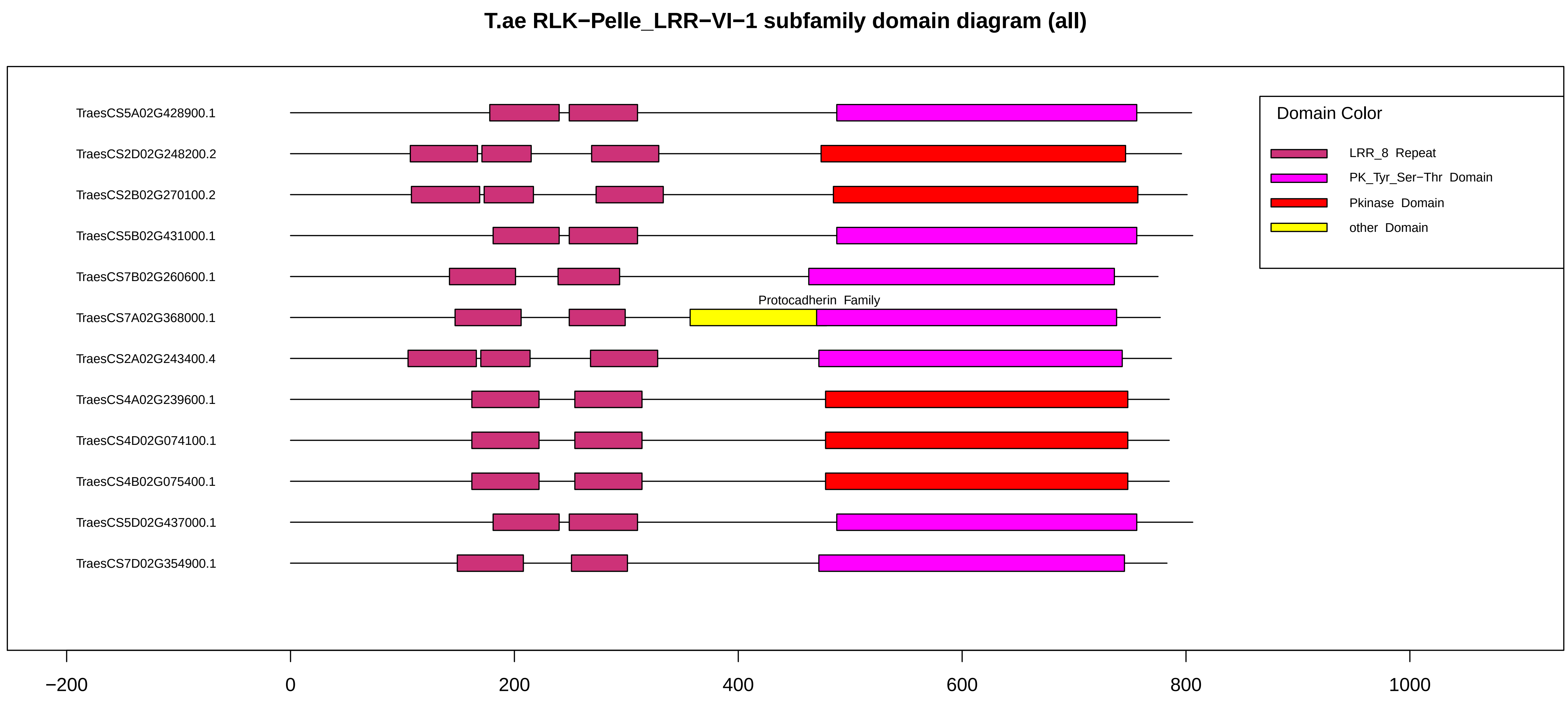

T.ae RLK−Pelle_LRR−VI−1 subfamily domain diagram (all)
TraesCS5A02G428900.1
TraesCS2D02G248200.2
TraesCS2B02G270100.2
TraesCS5B02G431000.1
TraesCS7B02G260600.1
TraesCS7A02G368000.1
TraesCS2A02G243400.4
TraesCS4A02G239600.1
TraesCS4D02G074100.1
TraesCS4B02G075400.1
TraesCS5D02G437000.1
TraesCS7D02G354900.1
Protocadherin Family
Domain Color
LRR_8 Repeat
PK_Tyr_Ser−Thr Domain
Pkinase Domain
other Domain
−200
0
200
400
600
800
1000

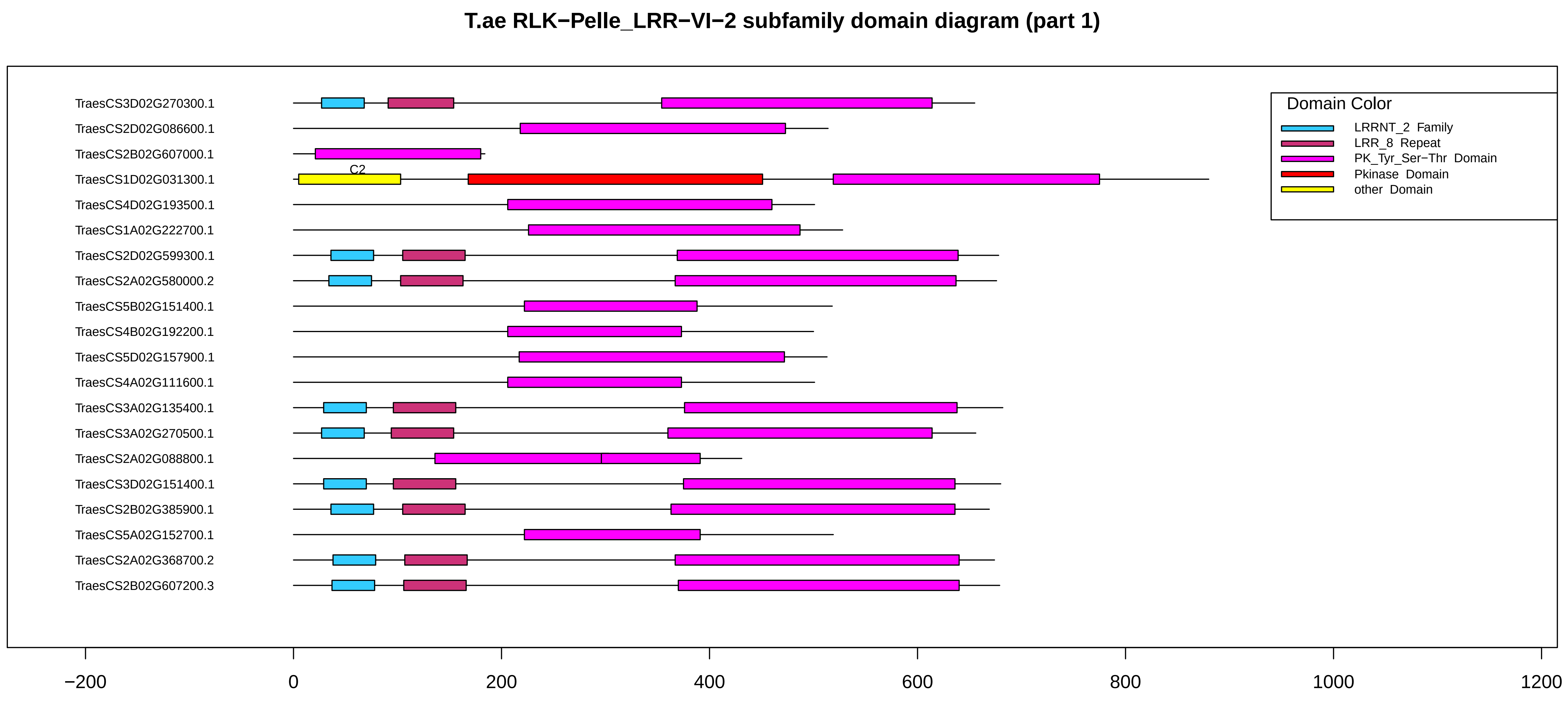
T.ae RLK−Pelle_LRR−VI−2 subfamily domain diagram (part 1)
TraesCS3D02G270300.1
TraesCS2D02G086600.1
TraesCS2B02G607000.1
TraesCS1D02G031300.1
C2
TraesCS4D02G193500.1
TraesCS1A02G222700.1
TraesCS2D02G599300.1
TraesCS2A02G580000.2
TraesCS5B02G151400.1
TraesCS4B02G192200.1
TraesCS5D02G157900.1
TraesCS4A02G111600.1
TraesCS3A02G135400.1
TraesCS3A02G270500.1
TraesCS2A02G088800.1
TraesCS3D02G151400.1
TraesCS2B02G385900.1
TraesCS5A02G152700.1
TraesCS2A02G368700.2
TraesCS2B02G607200.3
Domain Color
LRRNT_2 Family
LRR_8 Repeat
PK_Tyr_Ser−Thr Domain
Pkinase Domain
other Domain
−200
0
200
400
600
800
1000
1200

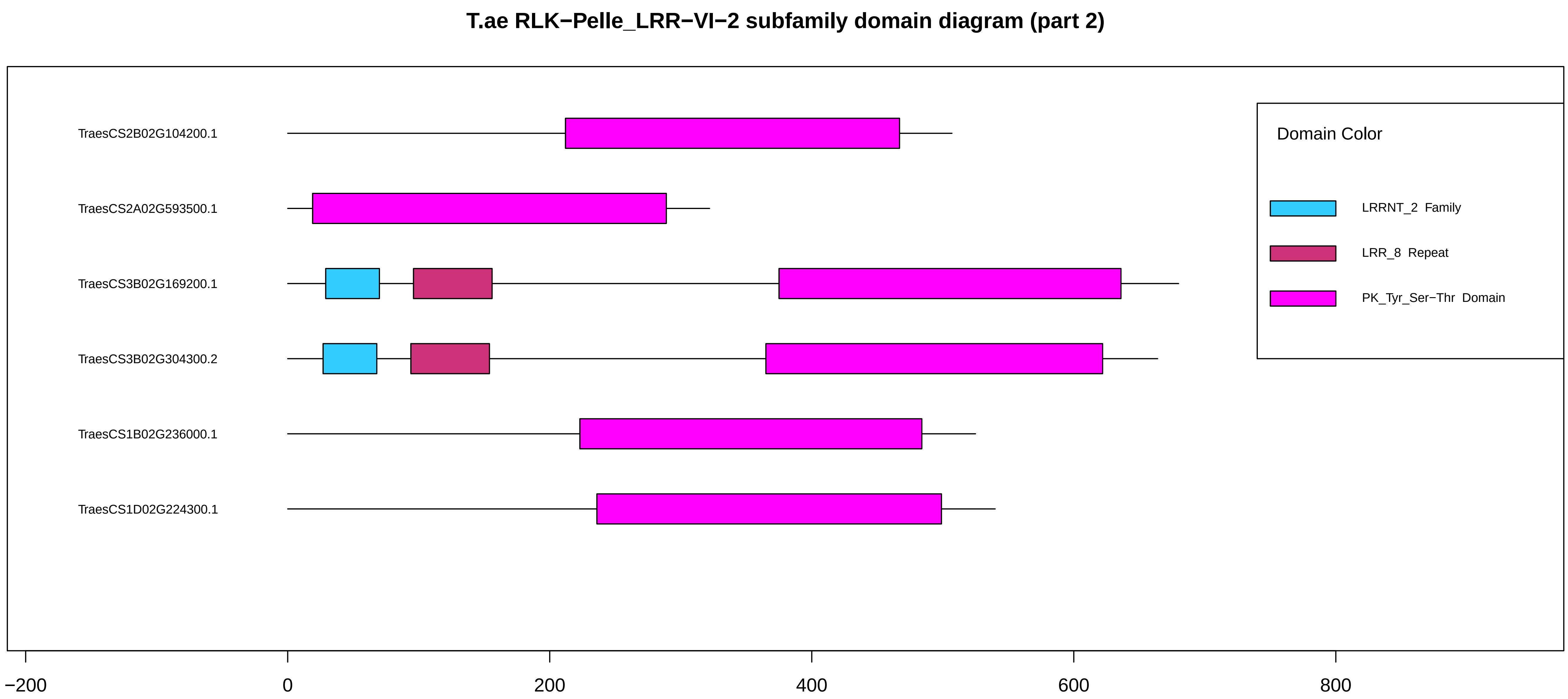
T.ae RLK−Pelle_LRR−VI−2 subfamily domain diagram (part 2)
TraesCS2B02G104200.1
TraesCS2A02G593500.1
TraesCS3B02G169200.1
TraesCS3B02G304300.2
TraesCS1B02G236000.1
TraesCS1D02G224300.1
Domain Color
LRRNT_2 Family
LRR_8 Repeat
PK_Tyr_Ser−Thr Domain
−200
0
200
400
600
800

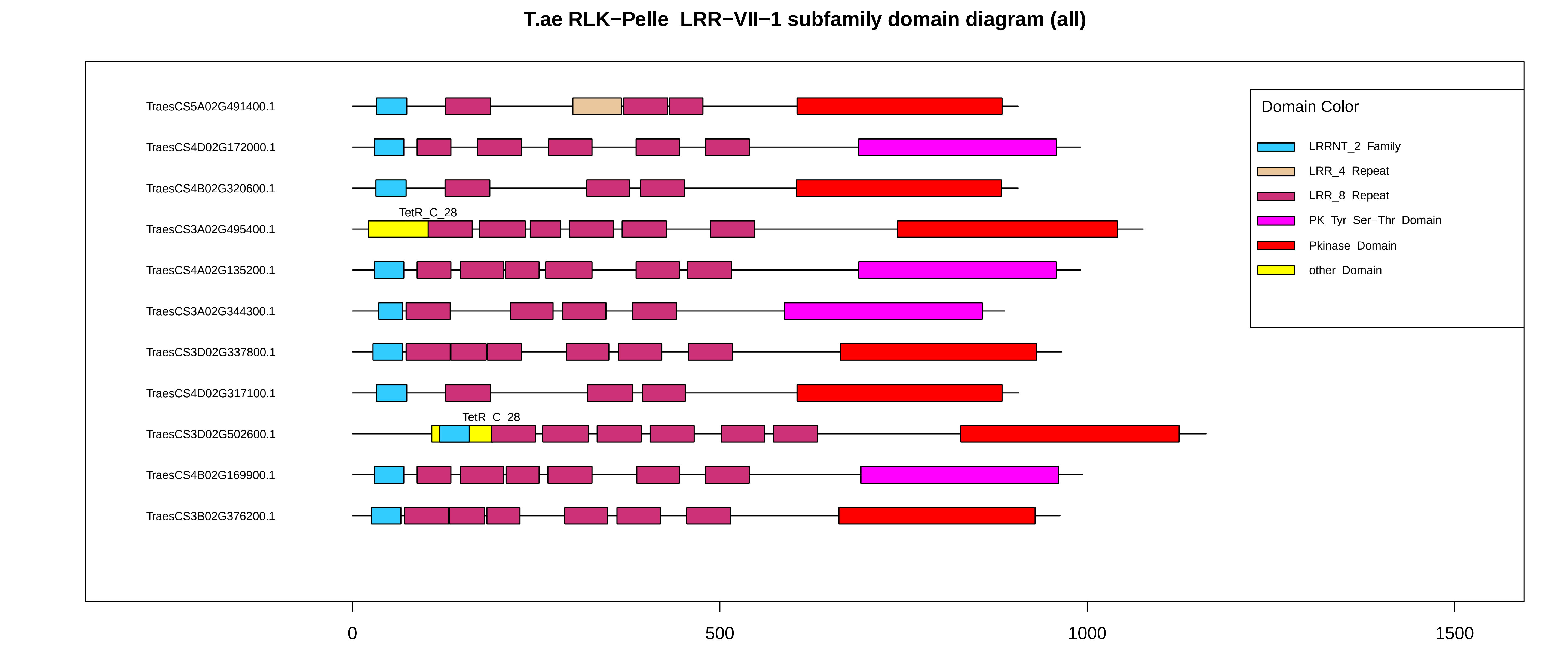

T.ae RLK−Pelle_LRR−VII−1 subfamily domain diagram (all)
TraesCS5A02G491400.1
TraesCS4D02G172000.1
TraesCS4B02G320600.1
TraesCS3A02G495400.1
TraesCS4A02G135200.1
TraesCS3A02G344300.1
TraesCS3D02G337800.1
TraesCS4D02G317100.1
TraesCS3D02G502600.1
TraesCS4B02G169900.1
TraesCS3B02G376200.1
TetR_C_28
TetR_C_28
Domain Color
LRRNT_2 Family
LRR_4 Repeat
LRR_8 Repeat
PK_Tyr_Ser−Thr Domain
Pkinase Domain
other Domain
0
500
1000
1500

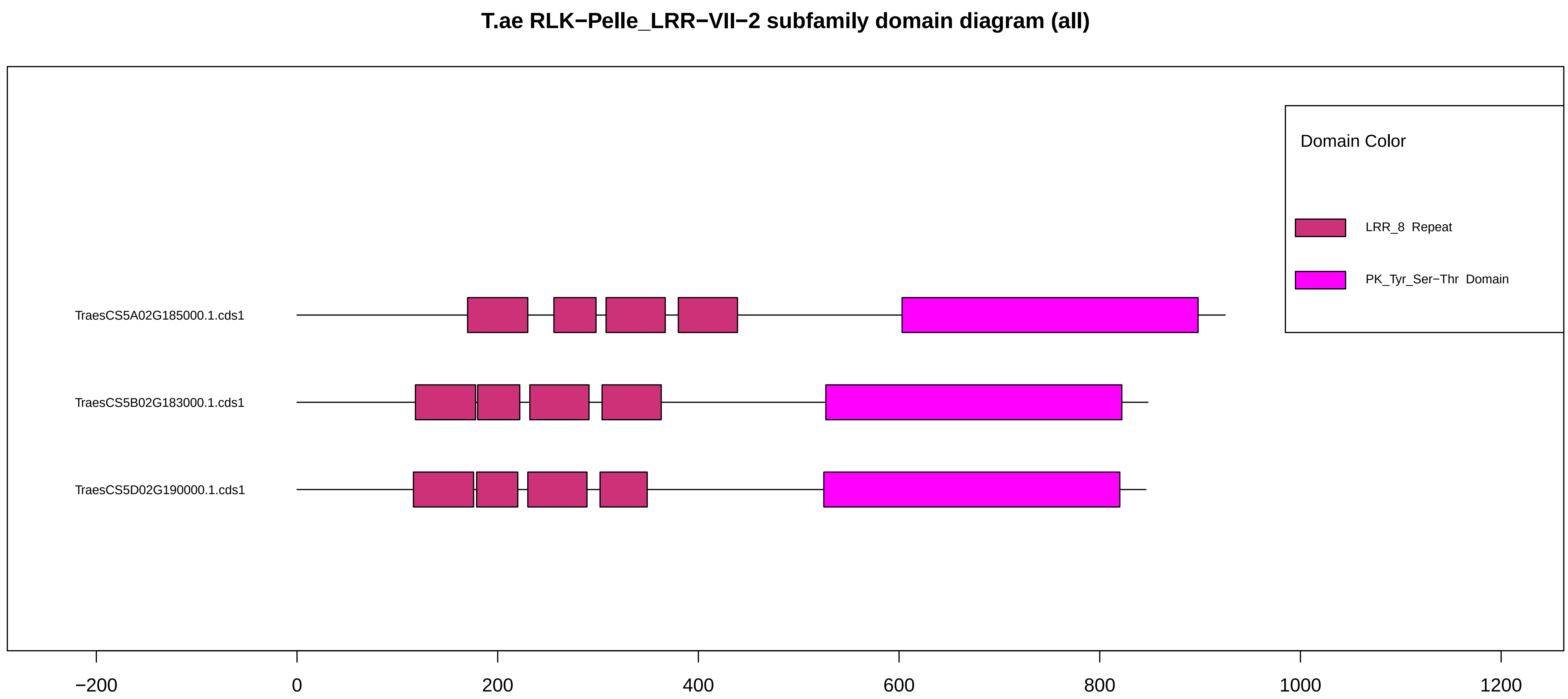
T.ae RLK−Pelle_LRR−VII−2 subfamily domain diagram (all)
Domain Color
LRR_8 Repeat
PK_Tyr_Ser−Thr Domain
TraesCS5A02G185000.1.cds1
TraesCS5B02G183000.1.cds1
TraesCS5D02G190000.1.cds1
−200
0
200
400
600
800
1000
1200

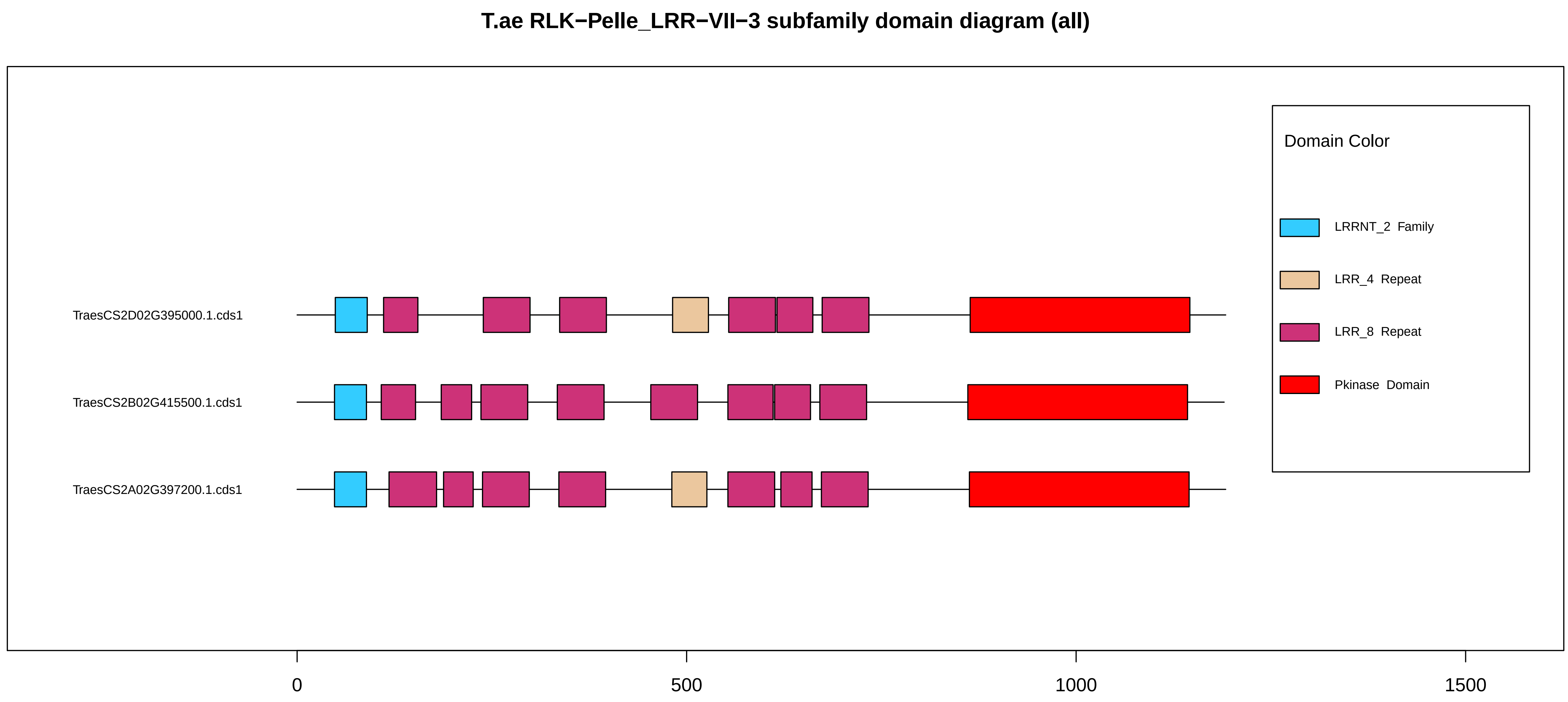
T.ae RLK−Pelle_LRR−VII−3 subfamily domain diagram (all)
TraesCS2D02G395000.1.cds1
TraesCS2B02G415500.1.cds1
TraesCS2A02G397200.1.cds1
Domain Color
LRRNT_2 Family
LRR_4 Repeat
LRR_8 Repeat
Pkinase Domain
0
500
1000
1500

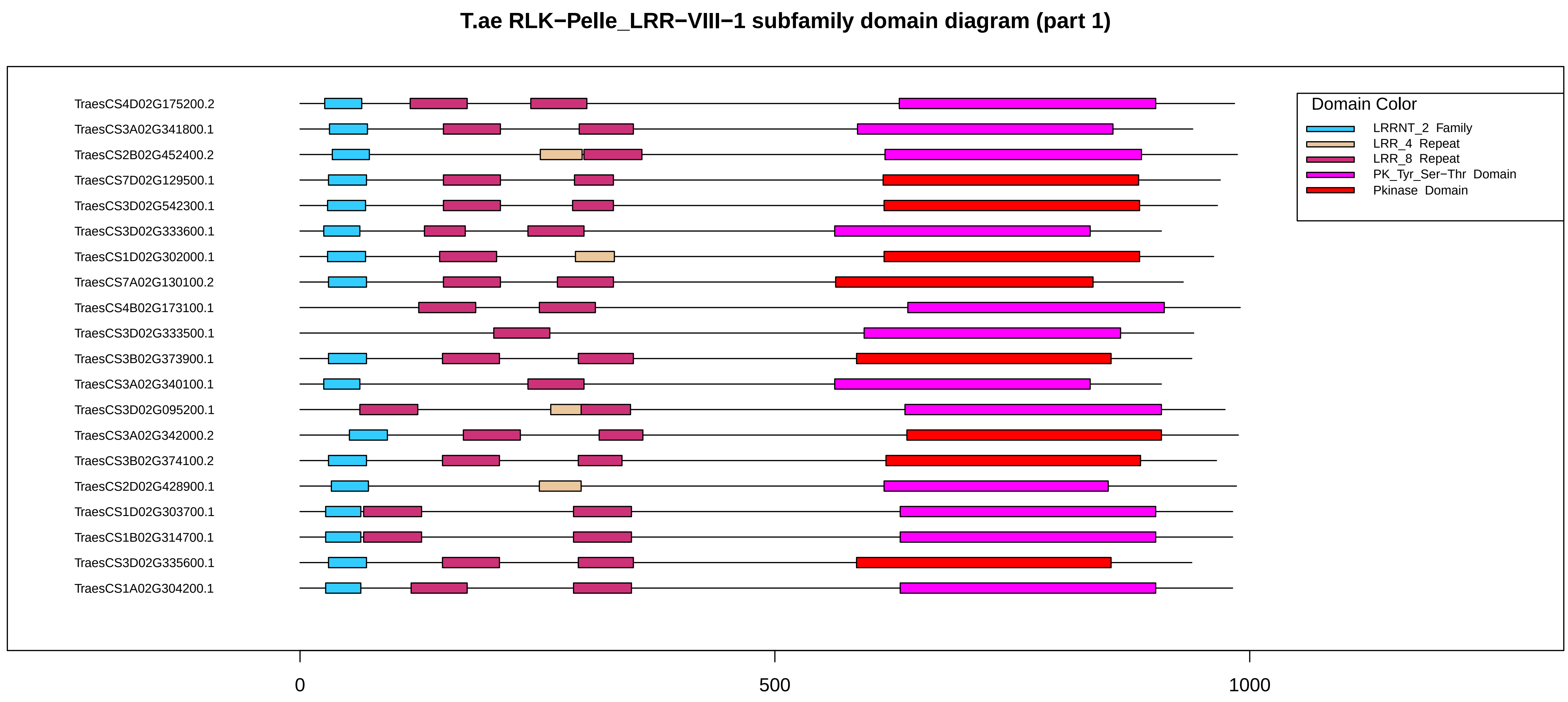
T.ae RLK−Pelle_LRR−VIII−1 subfamily domain diagram (part 1)
TraesCS4D02G175200.2
TraesCS3A02G341800.1
TraesCS2B02G452400.2
TraesCS7D02G129500.1
TraesCS3D02G542300.1
TraesCS3D02G333600.1
TraesCS1D02G302000.1
TraesCS7A02G130100.2
TraesCS4B02G173100.1
TraesCS3D02G333500.1
TraesCS3B02G373900.1
TraesCS3A02G340100.1
TraesCS3D02G095200.1
TraesCS3A02G342000.2
TraesCS3B02G374100.2
TraesCS2D02G428900.1
TraesCS1D02G303700.1
TraesCS1B02G314700.1
TraesCS3D02G335600.1
TraesCS1A02G304200.1
Domain Color
LRRNT_2 Family
LRR_4 Repeat
LRR_8 Repeat
PK_Tyr_Ser−Thr Domain
Pkinase Domain
0
500
1000

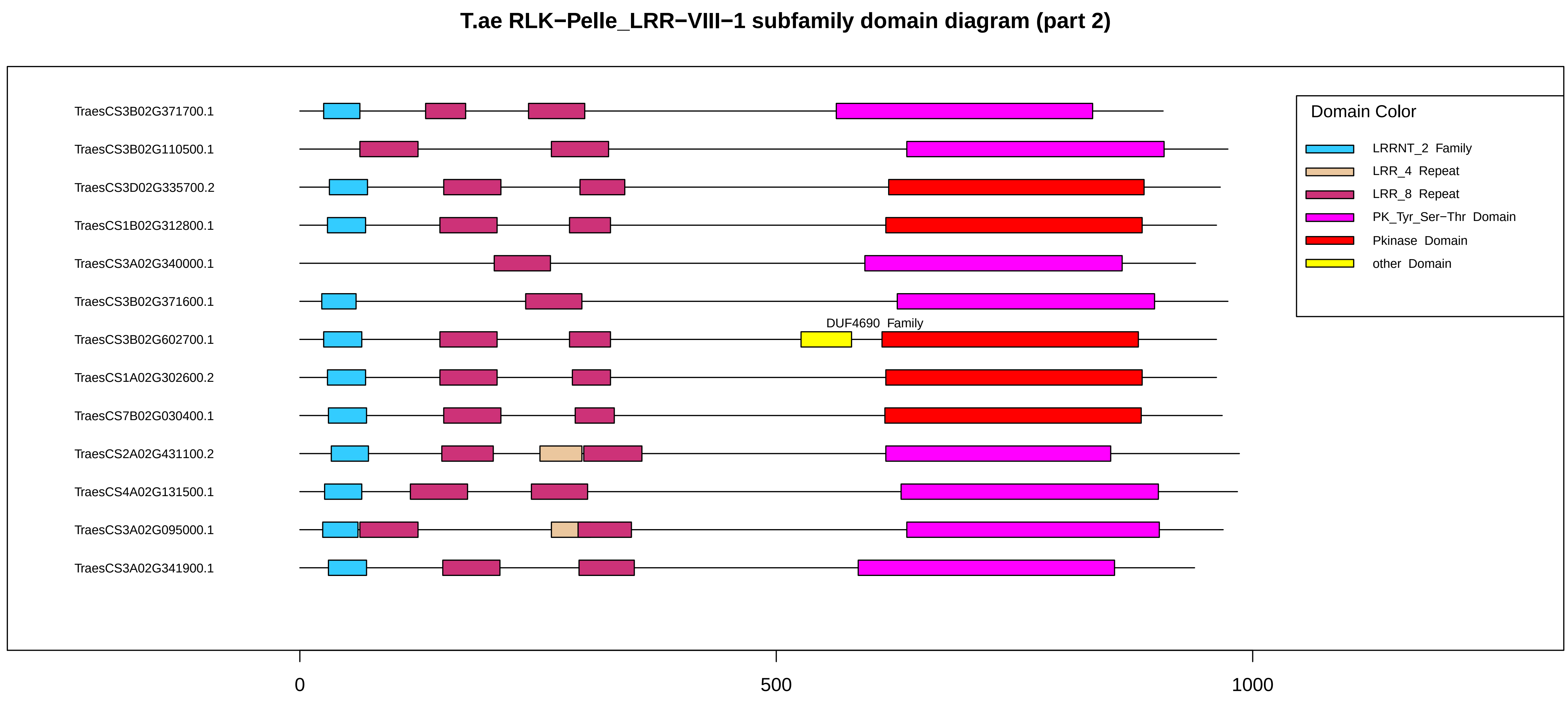
T.ae RLK−Pelle_LRR−VIII−1 subfamily domain diagram (part 2)
TraesCS3B02G371700.1
TraesCS3B02G110500.1
TraesCS3D02G335700.2
TraesCS1B02G312800.1
TraesCS3A02G340000.1
TraesCS3B02G371600.1
TraesCS3B02G602700.1
DUF4690 Family
TraesCS1A02G302600.2
TraesCS7B02G030400.1
TraesCS2A02G431100.2
TraesCS4A02G131500.1
TraesCS3A02G095000.1
TraesCS3A02G341900.1
Domain Color
LRRNT_2 Family
LRR_4 Repeat
LRR_8 Repeat
PK_Tyr_Ser−Thr Domain
Pkinase Domain
other Domain
0
500
1000

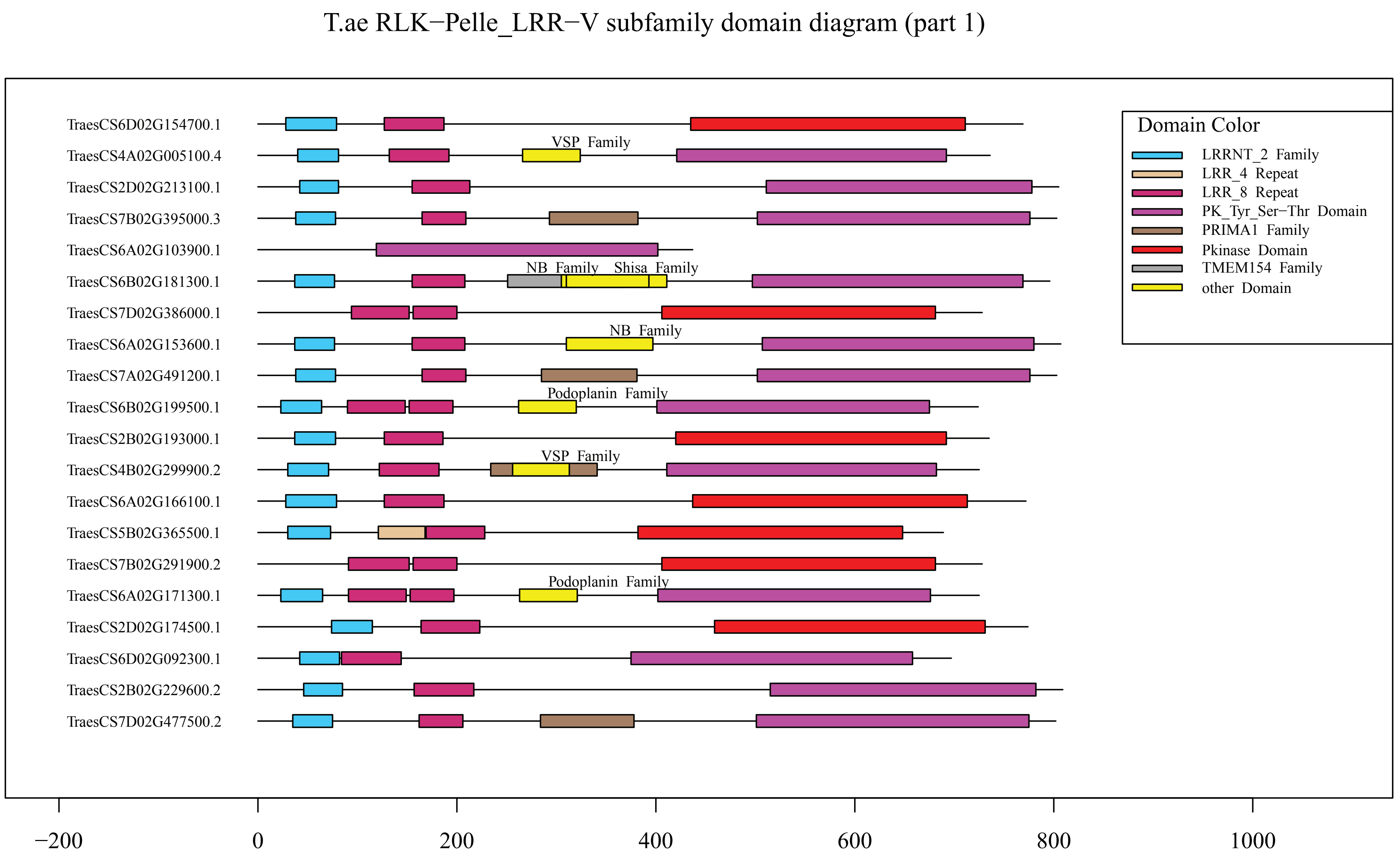
T.ae RLK−Pelle_LRR−V subfamily domain diagram (part 1)
TraesCS6D02G154700.1
TraesCS4A02G005100.4
VSP Family
TraesCS2D02G213100.1
TraesCS7B02G395000.3
TraesCS6A02G103900.1
TraesCS6B02G181300.1
NB Family
Shisa Family
TraesCS7D02G386000.1
TraesCS6A02G153600.1
NB Family
TraesCS7A02G491200.1
TraesCS6B02G199500.1
Podoplanin Family
TraesCS2B02G193000.1
TraesCS4B02G299900.2
VSP Family
TraesCS6A02G166100.1
TraesCS5B02G365500.1
TraesCS7B02G291900.2
TraesCS6A02G171300.1
Podoplanin Family
TraesCS2D02G174500.1
TraesCS6D02G092300.1
TraesCS2B02G229600.2
TraesCS7D02G477500.2
Domain Color
LRRNT_2 Family
LRR_4 Repeat
LRR_8 Repeat
PK_Tyr_Ser−Thr Domain
PRIMA1 Family
Pkinase Domain
TMEM154 Family
other Domain
−200
0
200
400
600
800
1000

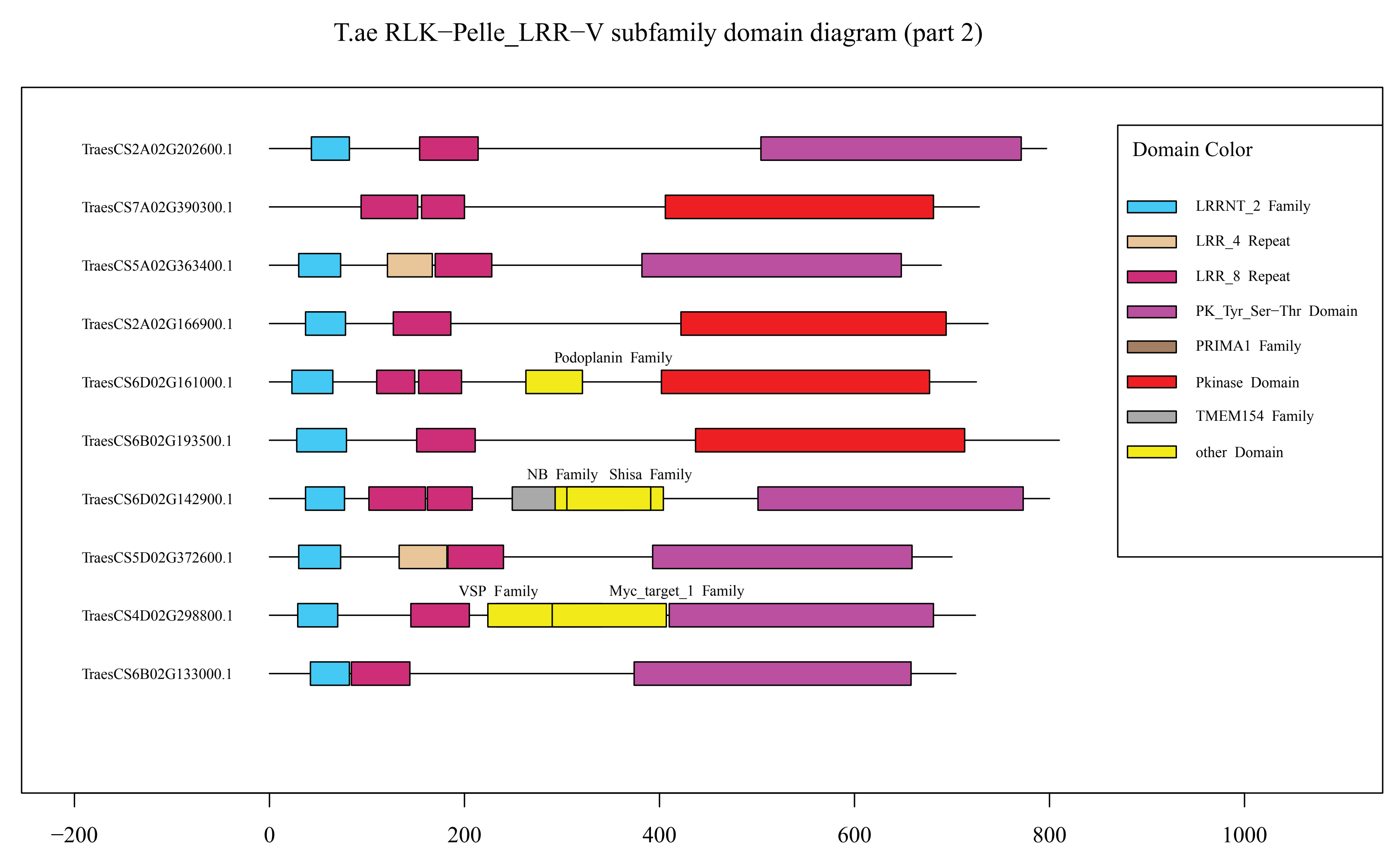

T.ae RLK−Pelle_LRR−V subfamily domain diagram (part 2)
TraesCS2A02G202600.1
TraesCS7A02G390300.1
TraesCS5A02G363400.1
TraesCS2A02G166900.1
TraesCS6D02G161000.1
TraesCS6B02G193500.1
TraesCS6D02G142900.1
TraesCS5D02G372600.1
TraesCS4D02G298800.1
TraesCS6B02G133000.1
Podoplanin Family
NB Family
Shisa Family
VSP Family
Myc_target_1 Family
Domain Color
LRRNT_2 Family
LRR_4 Repeat
LRR_8 Repeat
PK_Tyr_Ser−Thr Domain
PRIMA1 Family
Pkinase Domain
TMEM154 Family
other Domain
−200
0
200
400
600
800
1000

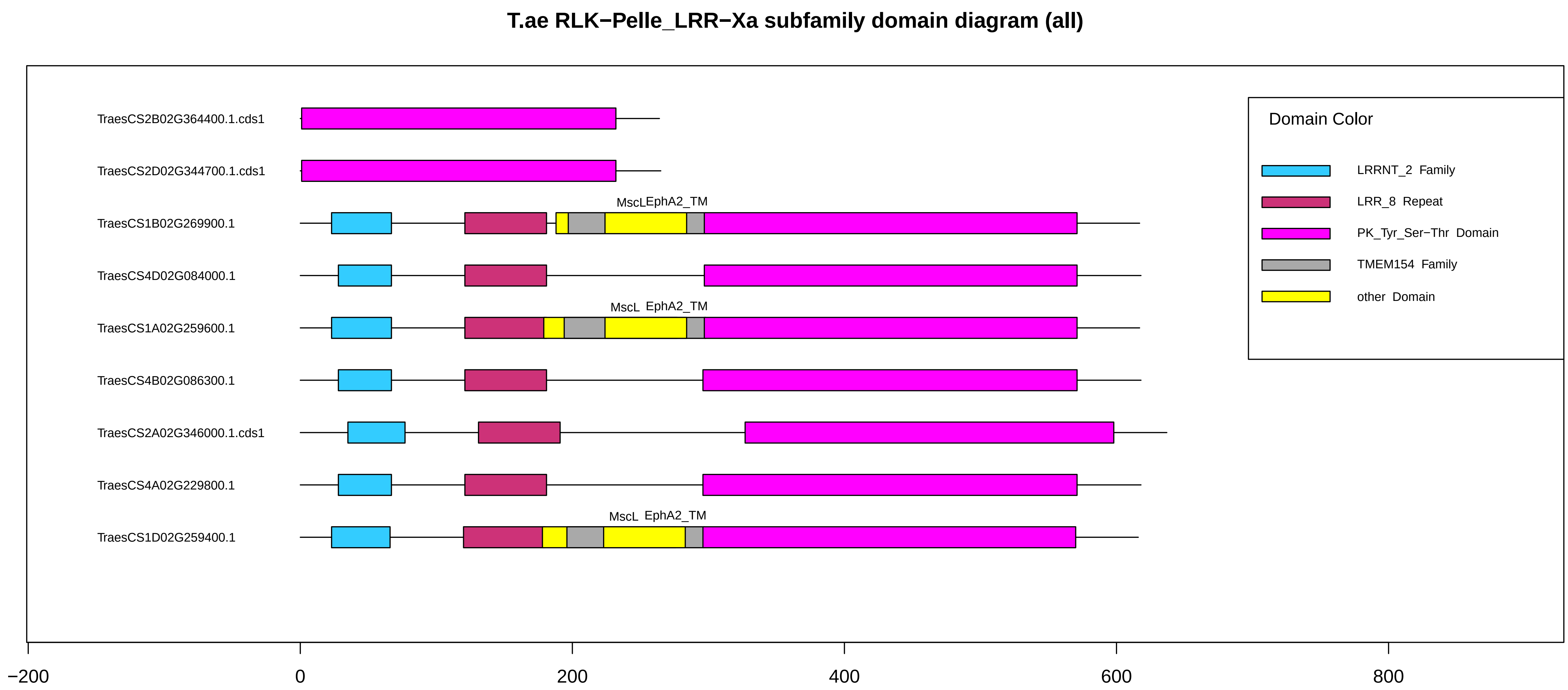
T.ae RLK−Pelle_LRR−Xa subfamily domain diagram (all)
TraesCS2B02G364400.1.cds1
TraesCS2D02G344700.1.cds1
TraesCS1B02G269900.1
TraesCS4D02G084000.1
TraesCS1A02G259600.1
TraesCS4B02G086300.1
TraesCS2A02G346000.1.cds1
TraesCS4A02G229800.1
TraesCS1D02G259400.1
MscL
EphA2_TM
Domain Color
LRRNT_2 Family
LRR_8 Repeat
PK_Tyr_Ser−Thr Domain
TMEM154 Family
other Domain
−200
0
200
400
600
800

T.ae RLK−Pelle_LRR−Xb−1 subfamily domain diagram (part 1)

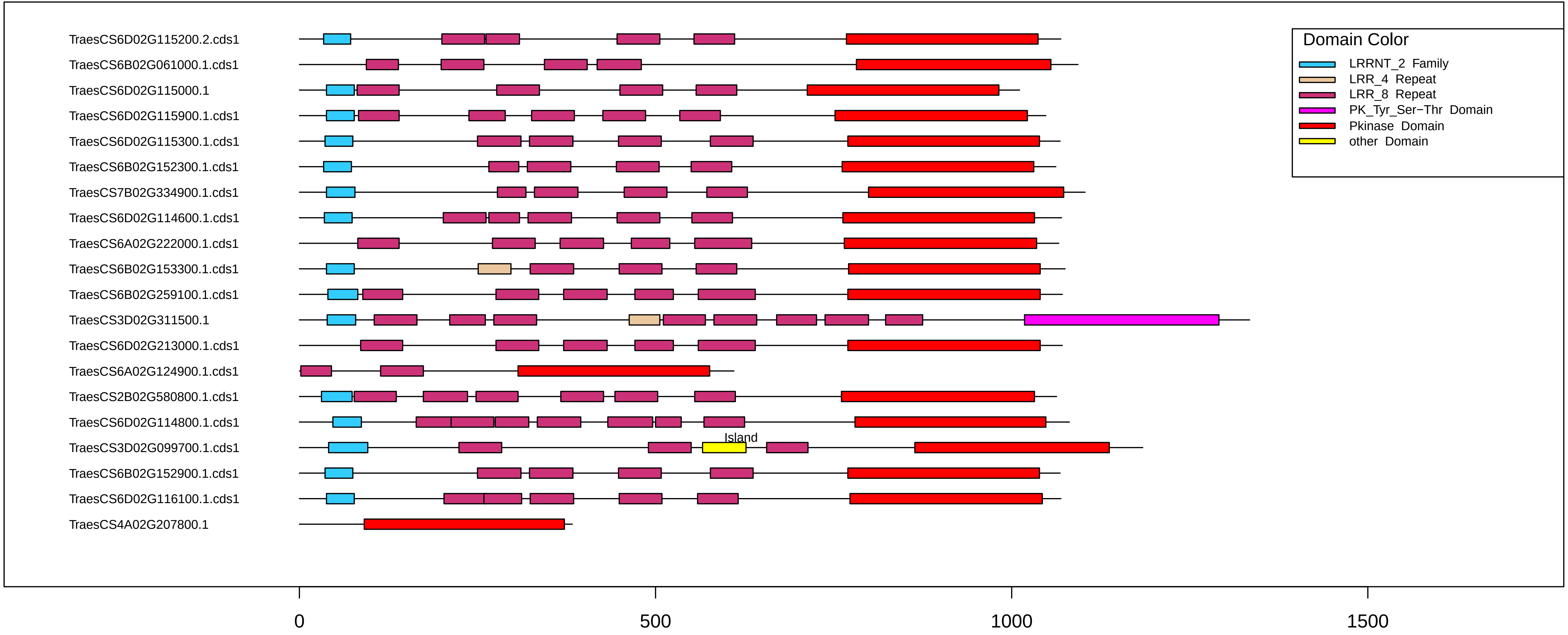

T.ae RLK−Pelle_LRR−Xb−1 subfamily domain diagram (part 2)

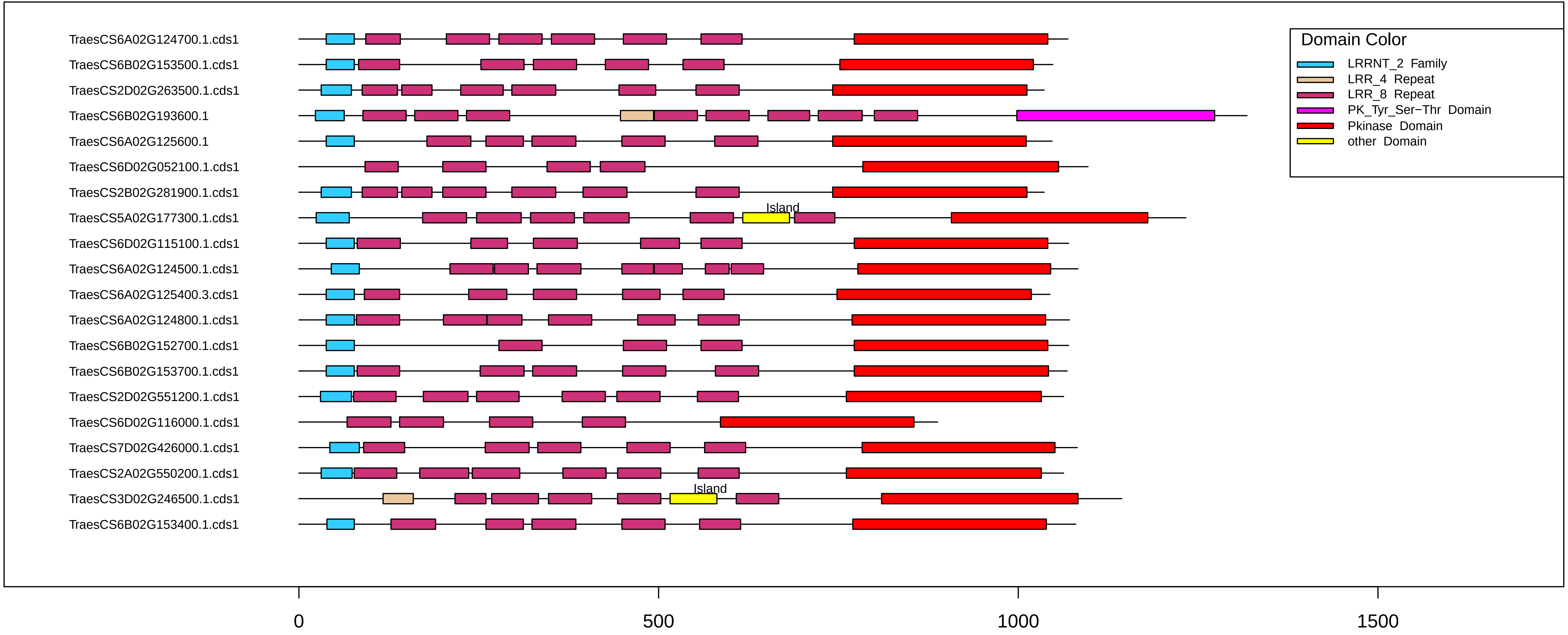

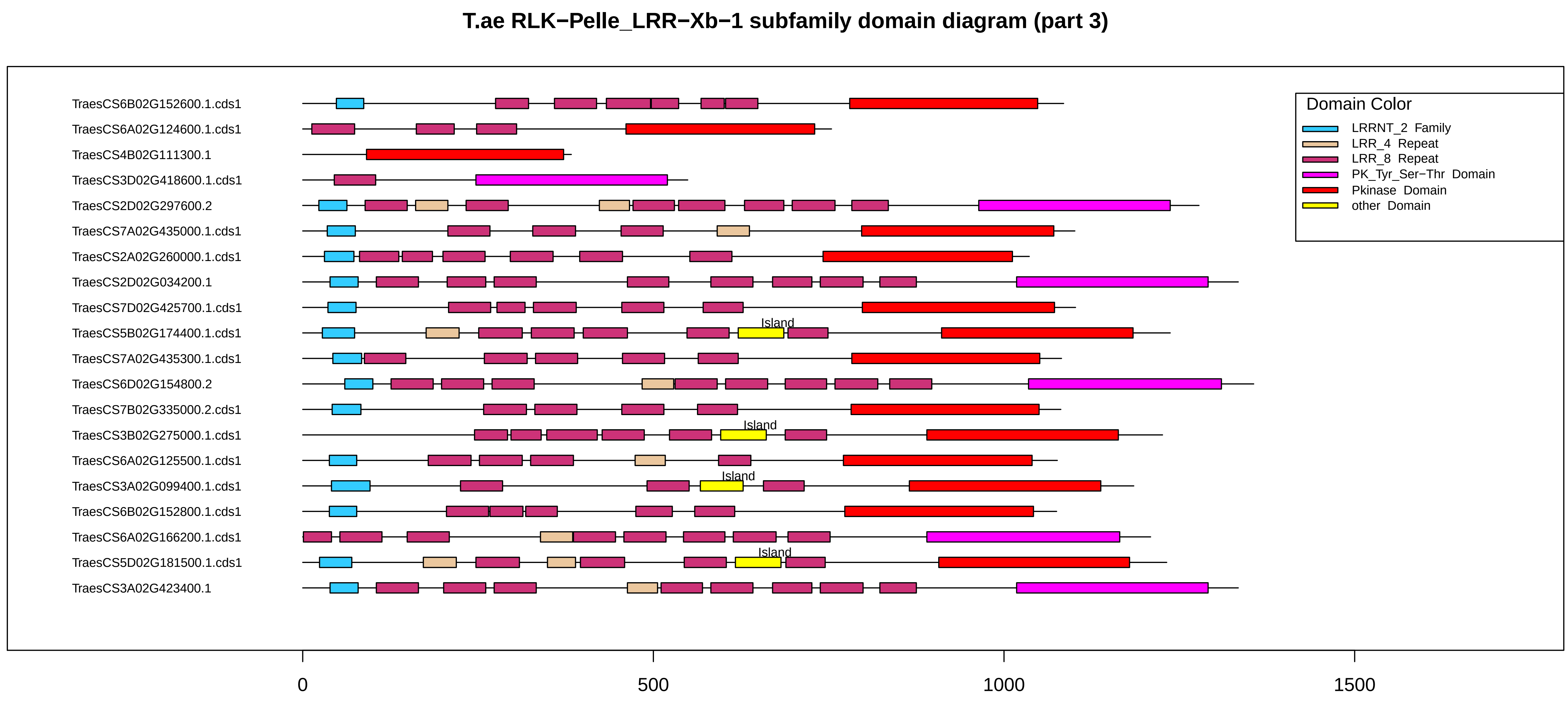

T.ae RLK−Pelle_LRR−Xb−1 subfamily domain diagram (part 3)
TraesCS6B02G152600.1.cds1
TraesCS6A02G124600.1.cds1
TraesCS4B02G111300.1
TraesCS3D02G418600.1.cds1
TraesCS2D02G297600.2
TraesCS7A02G435000.1.cds1
TraesCS2A02G260000.1.cds1
TraesCS2D02G034200.1
TraesCS7D02G425700.1.cds1
TraesCS5B02G174400.1.cds1
TraesCS7A02G435300.1.cds1
TraesCS6D02G154800.2
TraesCS7B02G335000.2.cds1
TraesCS3B02G275000.1.cds1
TraesCS6A02G125500.1.cds1
TraesCS3A02G099400.1.cds1
TraesCS6B02G152800.1.cds1
TraesCS6A02G166200.1.cds1
TraesCS5D02G181500.1.cds1
TraesCS3A02G423400.1
Island
Island
Island
Island
Domain Color
LRRNT_2 Family
LRR_4 Repeat
LRR_8 Repeat
PK_Tyr_Ser−Thr Domain
Pkinase Domain
other Domain
0
500
1000
1500

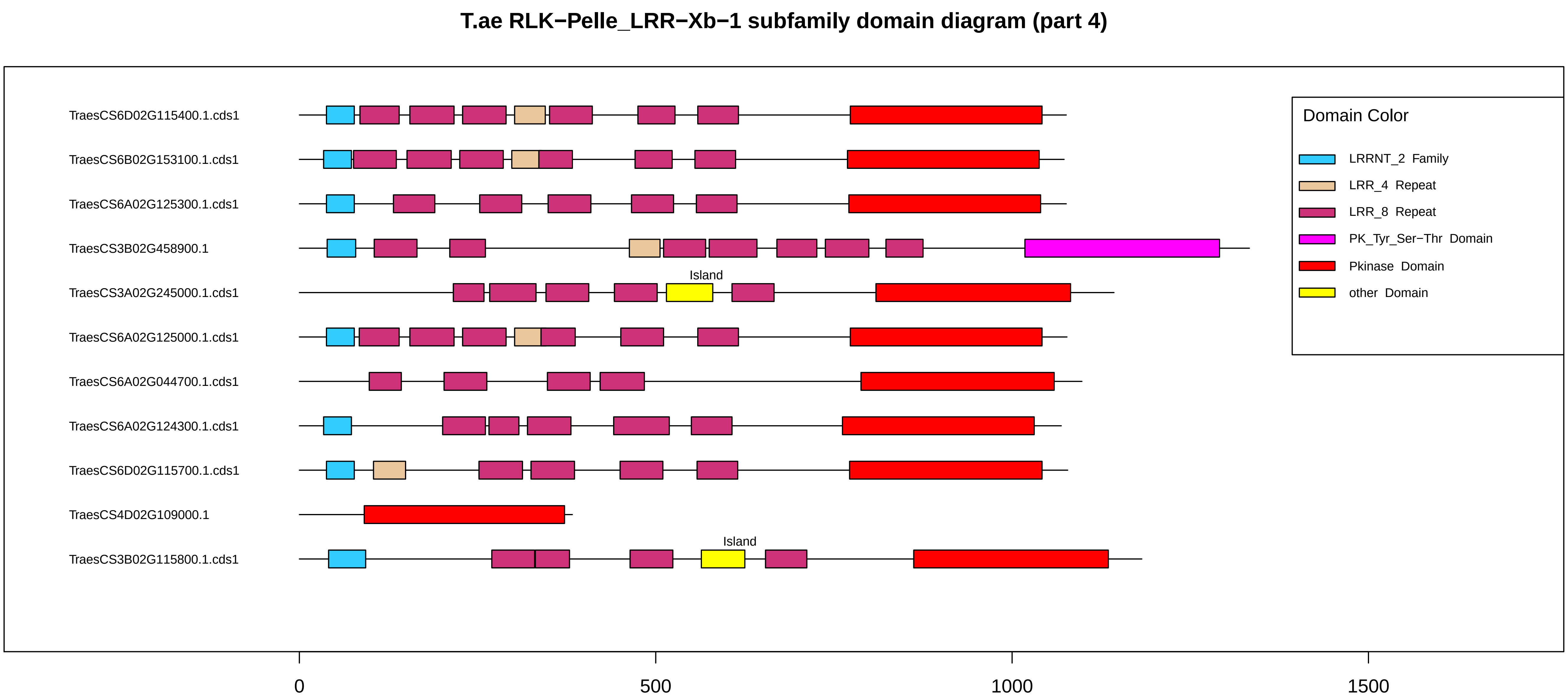
T.ae RLK−Pelle_LRR−Xb−1 subfamily domain diagram (part 4)
TraesCS6D02G115400.1.cds1
TraesCS6B02G153100.1.cds1
TraesCS6A02G125300.1.cds1
TraesCS3B02G458900.1
TraesCS3A02G245000.1.cds1
Island
TraesCS6A02G125000.1.cds1
TraesCS6A02G044700.1.cds1
TraesCS6A02G124300.1.cds1
TraesCS6D02G115700.1.cds1
TraesCS4D02G109000.1
TraesCS3B02G115800.1.cds1
Island
Domain Color
LRRNT_2 Family
LRR_4 Repeat
LRR_8 Repeat
PK_Tyr_Ser−Thr Domain
Pkinase Domain
other Domain
0
500
1000
1500

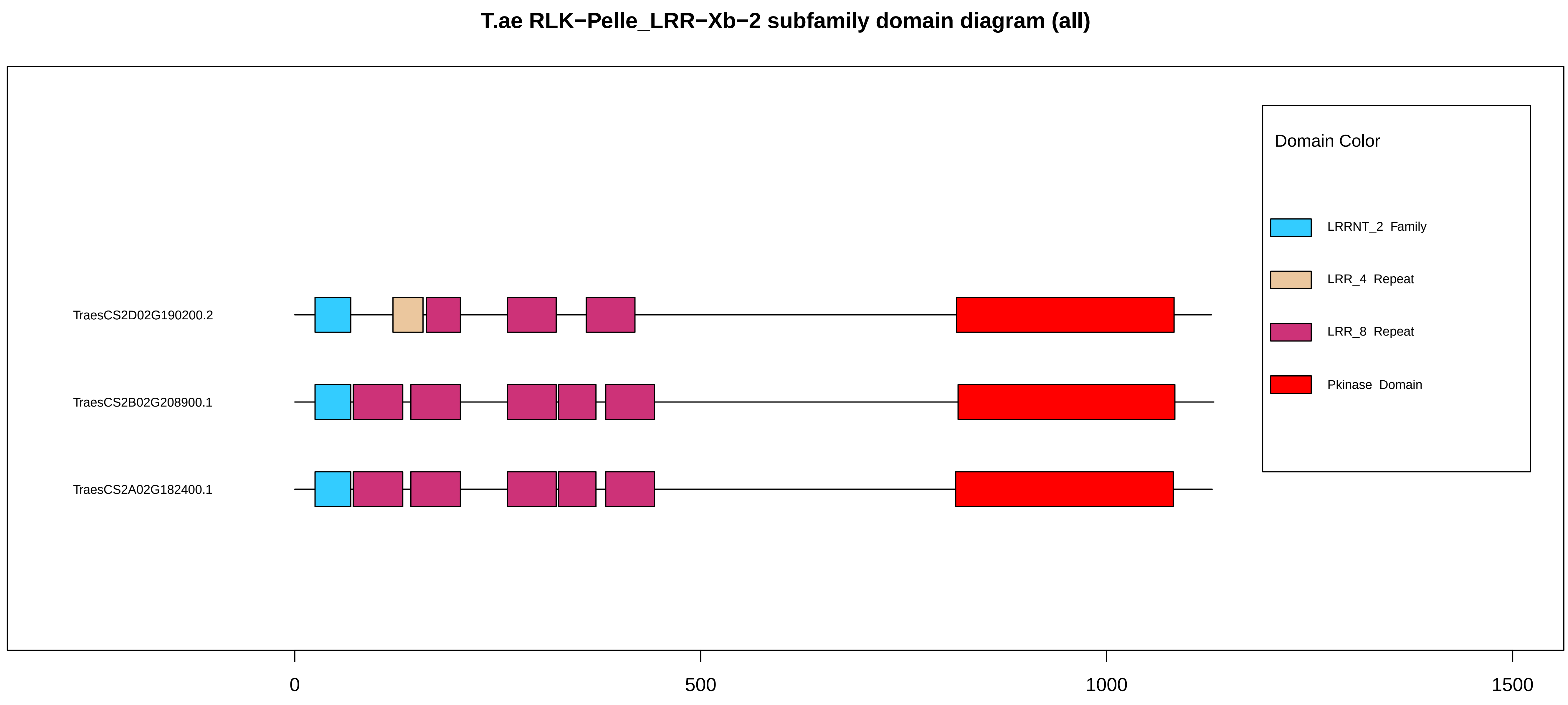

T.ae RLK−Pelle_LRR−Xb−2 subfamily domain diagram (all)
Domain Color
LRRNT_2 Family
LRR_4 Repeat
LRR_8 Repeat
Pkinase Domain
TraesCS2D02G190200.2
TraesCS2B02G208900.1
TraesCS2A02G182400.1
0
500
1000
1500

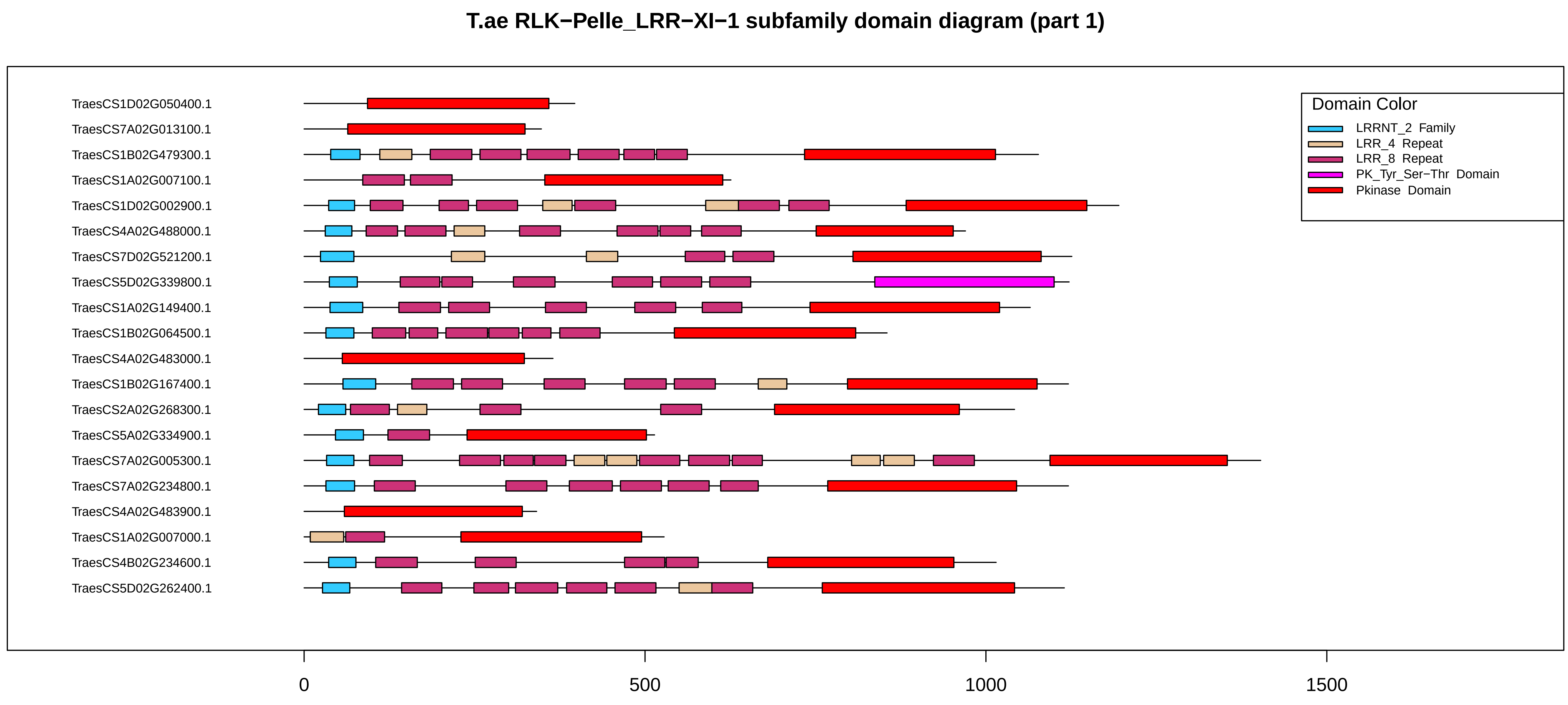

T.ae RLK−Pelle_LRR−XI−1 subfamily domain diagram (part 1)
Domain Color
LRRNT_2 Family
LRR_4 Repeat
LRR_8 Repeat
PK_Tyr_Ser−Thr Domain
Pkinase Domain
TraesCS1D02G050400.1
TraesCS7A02G013100.1
TraesCS1B02G479300.1
TraesCS1A02G007100.1
TraesCS1D02G002900.1
TraesCS4A02G488000.1
TraesCS7D02G521200.1
TraesCS5D02G339800.1
TraesCS1A02G149400.1
TraesCS1B02G064500.1
TraesCS4A02G483000.1
TraesCS1B02G167400.1
TraesCS2A02G268300.1
TraesCS5A02G334900.1
TraesCS7A02G005300.1
TraesCS7A02G234800.1
TraesCS4A02G483900.1
TraesCS1A02G007000.1
TraesCS4B02G234600.1
TraesCS5D02G262400.1
0
500
1000
1500

T.ae RLK−Pelle_LRR−XI−1 subfamily domain diagram (part 2)

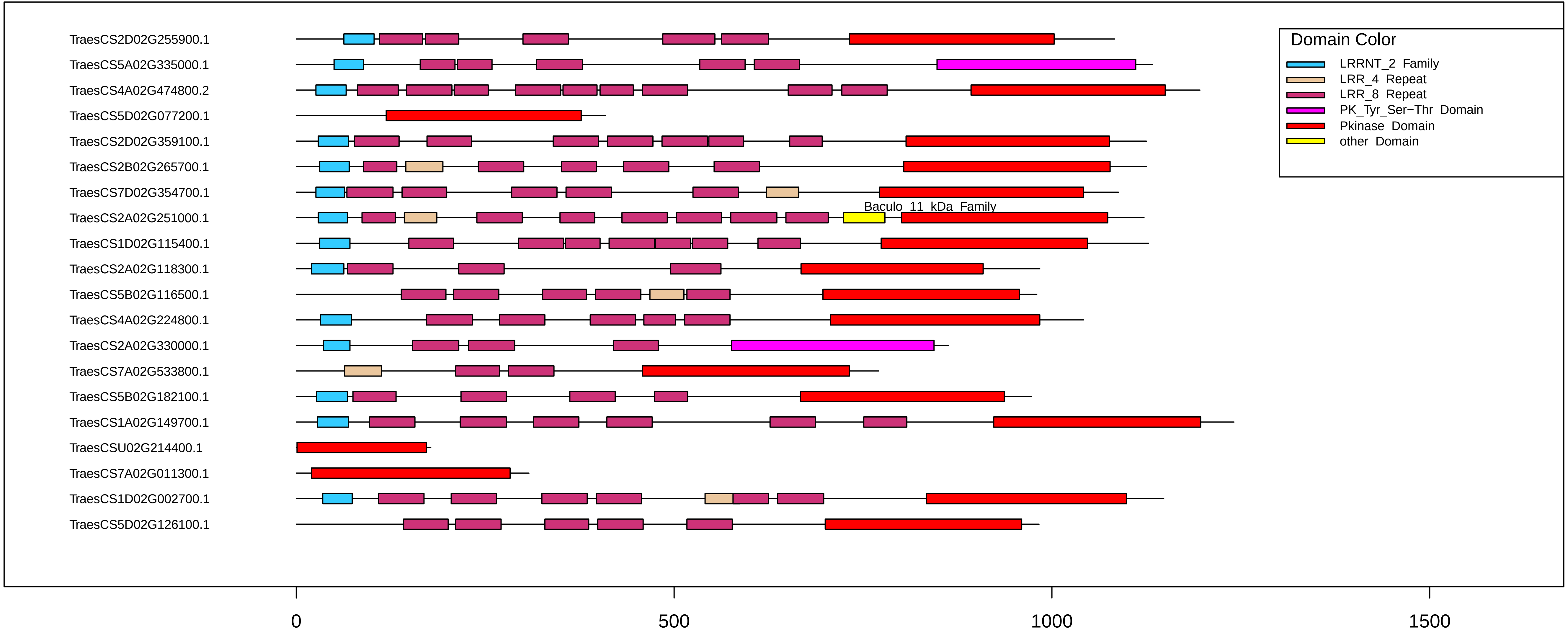

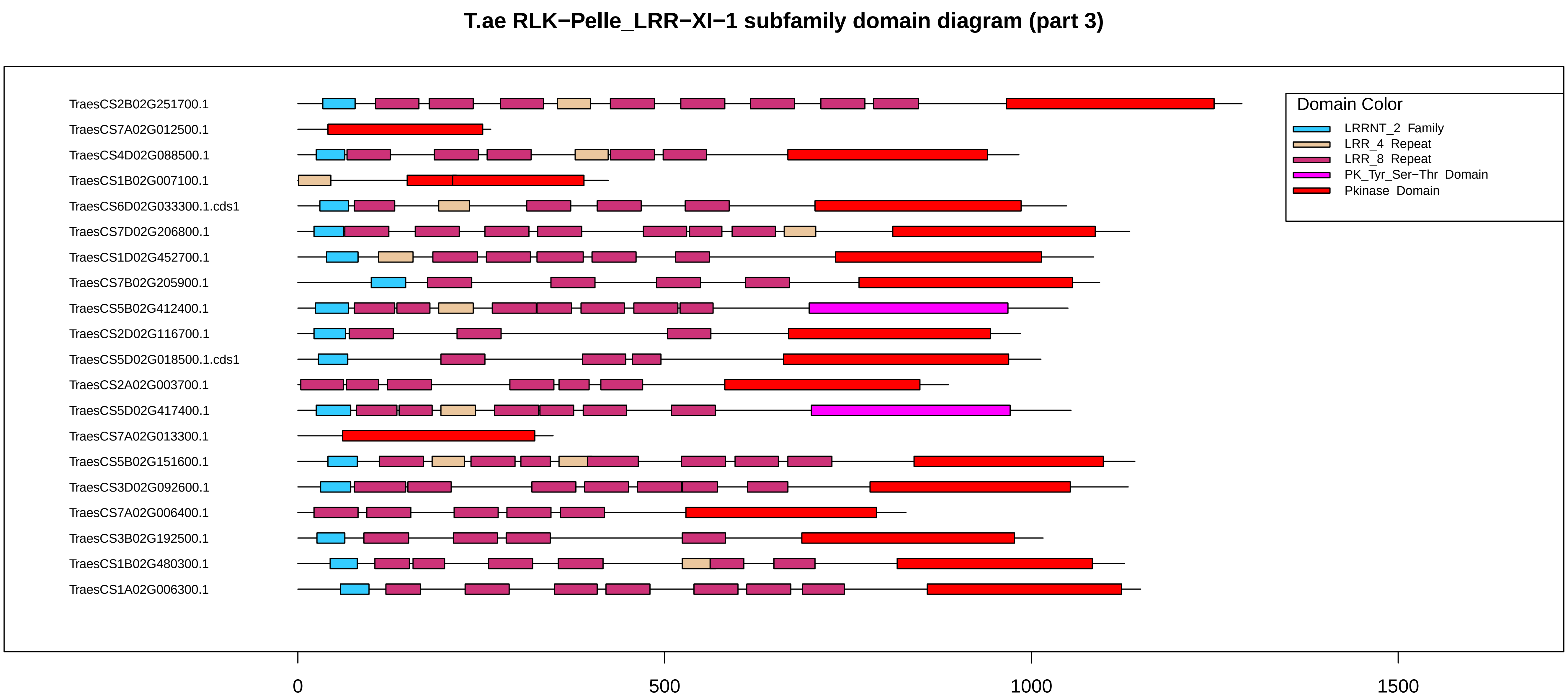
T.ae RLK−Pelle_LRR−XI−1 subfamily domain diagram (part 3)
TraesCS2B02G251700.1
TraesCS7A02G012500.1
TraesCS4D02G088500.1
TraesCS1B02G007100.1
TraesCS6D02G033300.1.cds1
TraesCS7D02G206800.1
TraesCS1D02G452700.1
TraesCS7B02G205900.1
TraesCS5B02G412400.1
TraesCS2D02G116700.1
TraesCS5D02G018500.1.cds1
TraesCS2A02G003700.1
TraesCS5D02G417400.1
TraesCS7A02G013300.1
TraesCS5B02G151600.1
TraesCS3D02G092600.1
TraesCS7A02G006400.1
TraesCS3B02G192500.1
TraesCS1B02G480300.1
TraesCS1A02G006300.1
Domain Color
LRRNT_2 Family
LRR_4 Repeat
LRR_8 Repeat
PK_Tyr_Ser−Thr Domain
Pkinase Domain
0
500
1000
1500

T.ae RLK−Pelle_LRR−XI−1 subfamily domain diagram (part 4)

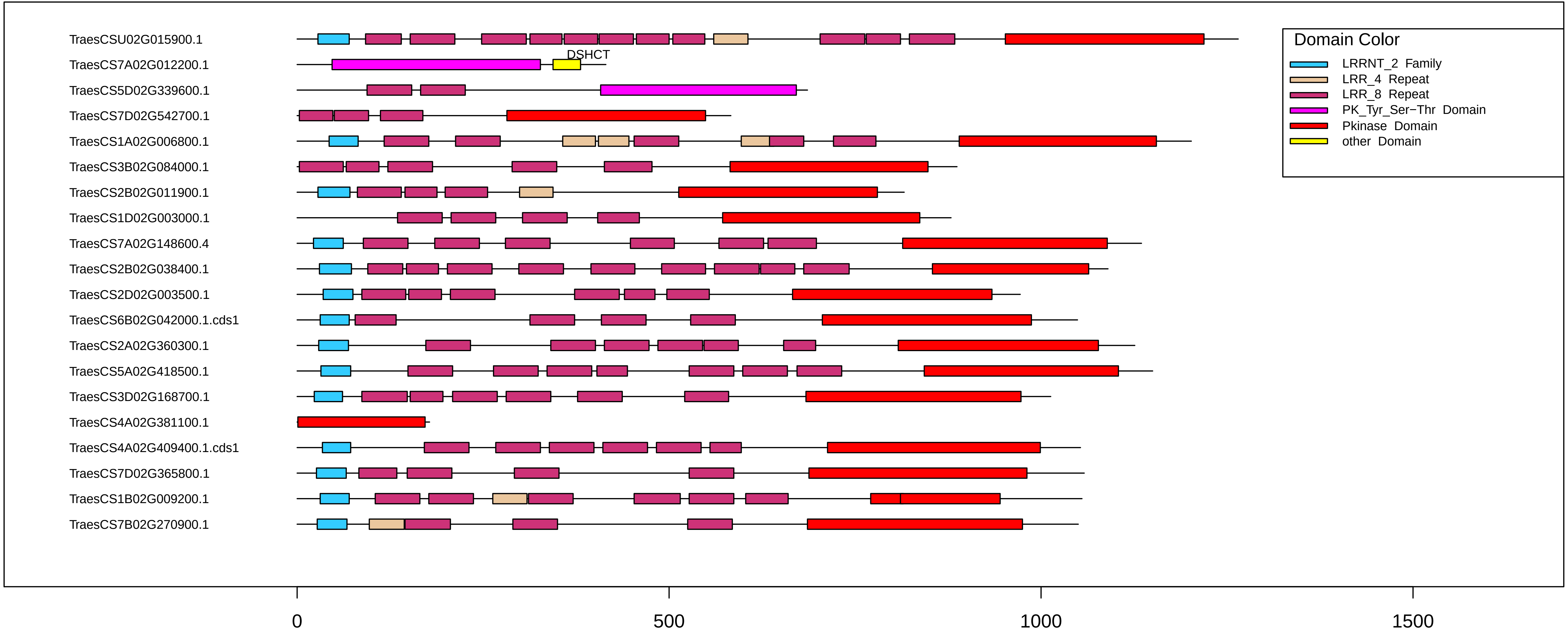

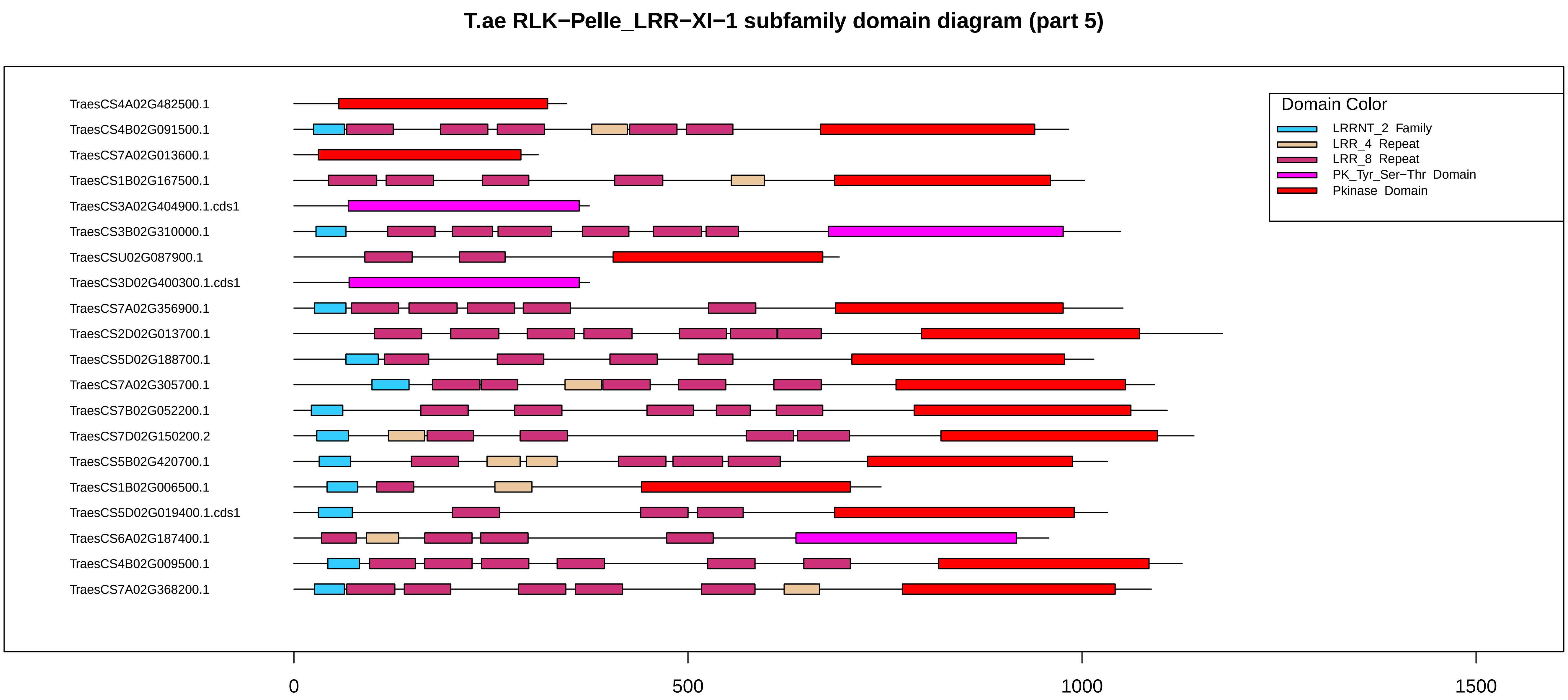
T.ae RLK−Pelle_LRR−XI−1 subfamily domain diagram (part 5)
TraesCS4A02G482500.1
TraesCS4B02G091500.1
TraesCS7A02G013600.1
TraesCS1B02G167500.1
TraesCS3A02G404900.1.cds1
TraesCS3B02G310000.1
TraesCSU02G087900.1
TraesCS3D02G400300.1.cds1
TraesCS7A02G356900.1
TraesCS2D02G013700.1
TraesCS5D02G188700.1
TraesCS7A02G305700.1
TraesCS7B02G052200.1
TraesCS7D02G150200.2
TraesCS5B02G420700.1
TraesCS1B02G006500.1
TraesCS5D02G019400.1.cds1
TraesCS6A02G187400.1
TraesCS4B02G009500.1
TraesCS7A02G368200.1
Domain Color
LRRNT_2 Family
LRR_4 Repeat
LRR_8 Repeat
PK_Tyr_Ser−Thr Domain
Pkinase Domain
0
500
1000
1500

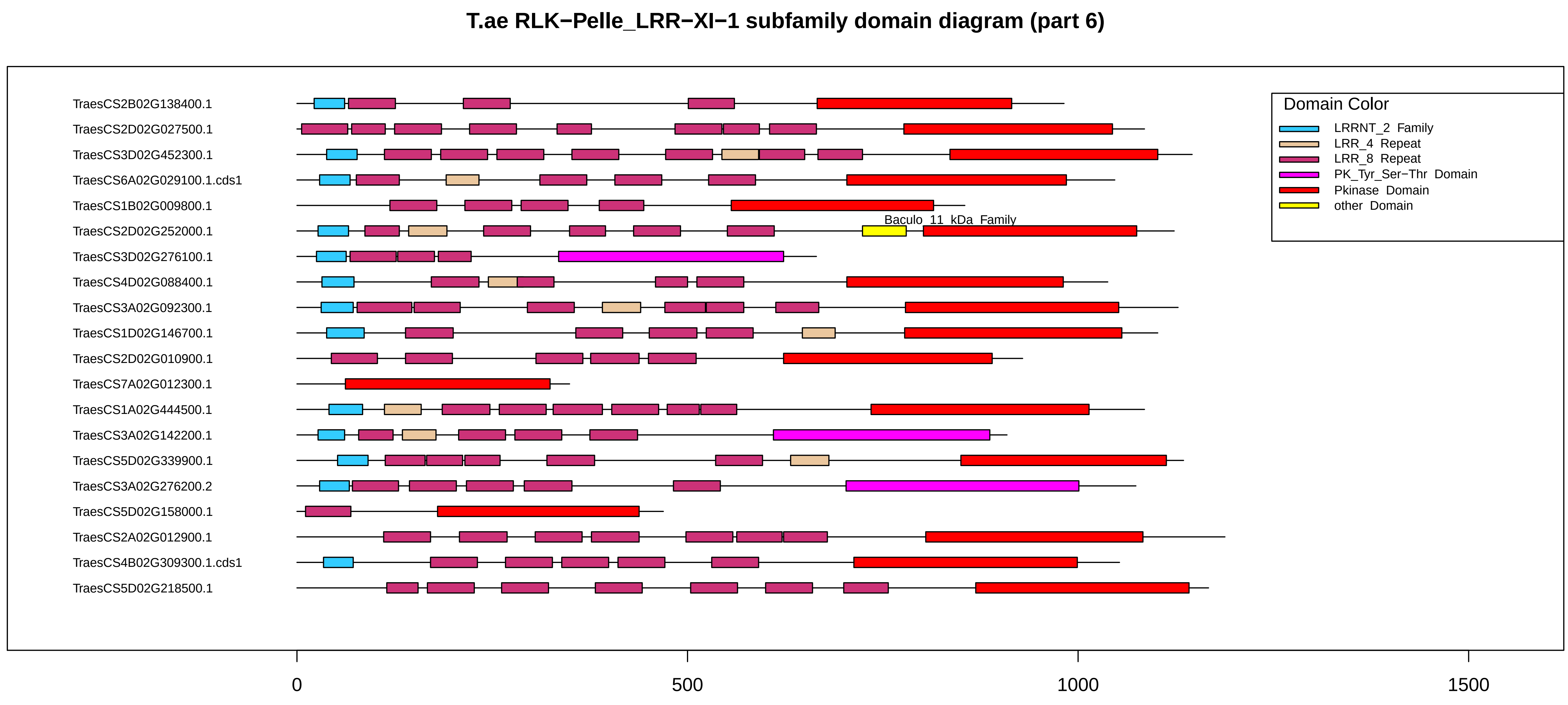

T.ae RLK−Pelle_LRR−XI−1 subfamily domain diagram (part 6)
TraesCS2B02G138400.1
TraesCS2D02G027500.1
TraesCS3D02G452300.1
TraesCS6A02G029100.1.cds1
TraesCS1B02G009800.1
TraesCS2D02G252000.1
TraesCS3D02G276100.1
TraesCS4D02G088400.1
TraesCS3A02G092300.1
TraesCS1D02G146700.1
TraesCS2D02G010900.1
TraesCS7A02G012300.1
TraesCS1A02G444500.1
TraesCS3A02G142200.1
TraesCS5D02G339900.1
TraesCS3A02G276200.2
TraesCS5D02G158000.1
TraesCS2A02G012900.1
TraesCS4B02G309300.1.cds1
TraesCS5D02G218500.1
Baculo_11_kDa_Family
Domain Color
LRRNT_2 Family
LRR_4 Repeat
LRR_8 Repeat
PK_Tyr_Ser−Thr Domain
Pkinase Domain
other Domain
0
500
1000
1500

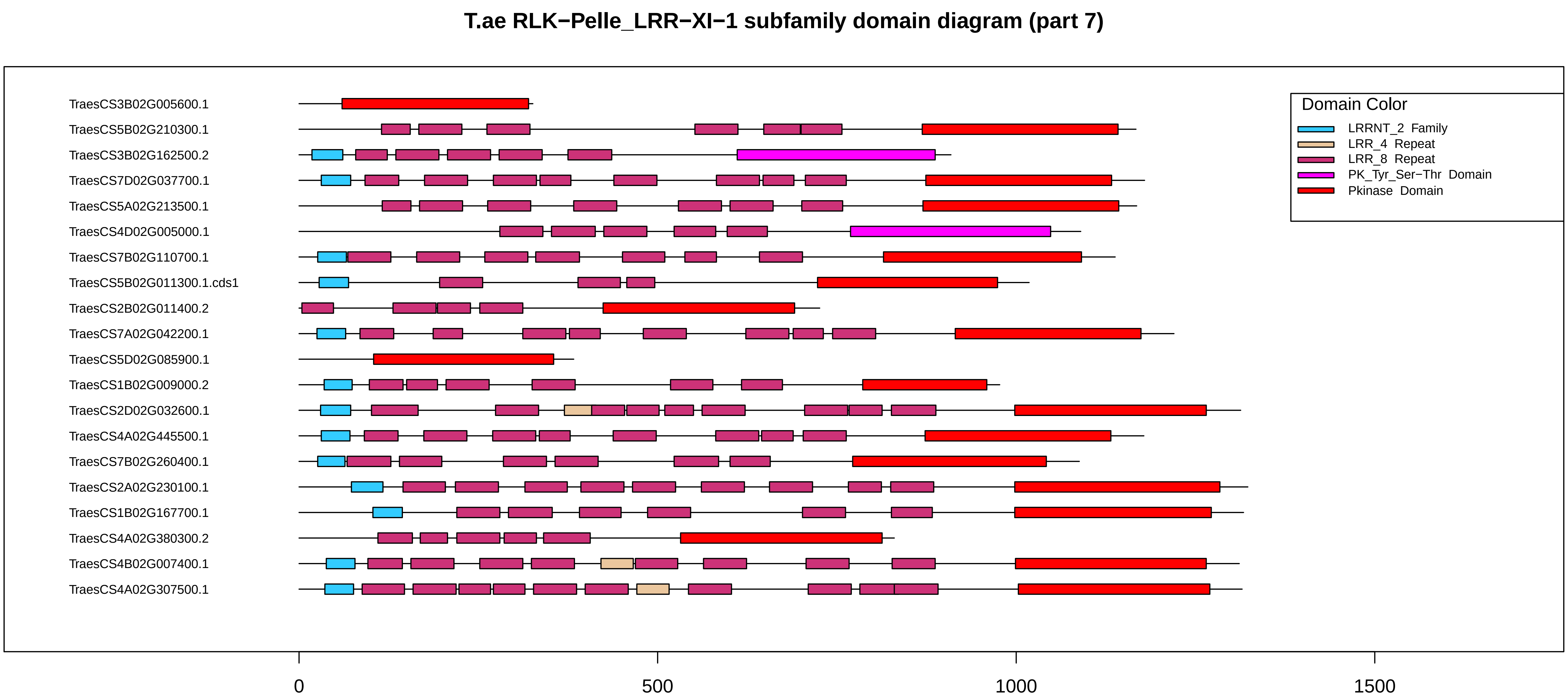
T.ae RLK−Pelle_LRR−XI−1 subfamily domain diagram (part 7)
TraesCS3B02G005600.1
TraesCS5B02G210300.1
TraesCS3B02G162500.2
TraesCS7D02G037700.1
TraesCS5A02G213500.1
TraesCS4D02G005000.1
TraesCS7B02G110700.1
TraesCS5B02G011300.1.cds1
TraesCS2B02G011400.2
TraesCS7A02G042200.1
TraesCS5D02G085900.1
TraesCS1B02G009000.2
TraesCS2D02G032600.1
TraesCS4A02G445500.1
TraesCS7B02G260400.1
TraesCS2A02G230100.1
TraesCS1B02G167700.1
TraesCS4A02G380300.2
TraesCS4B02G007400.1
TraesCS4A02G307500.1
Domain Color
LRRNT_2 Family
LRR_4 Repeat
LRR_8 Repeat
PK_Tyr_Ser−Thr Domain
Pkinase Domain
0
500
1000
1500

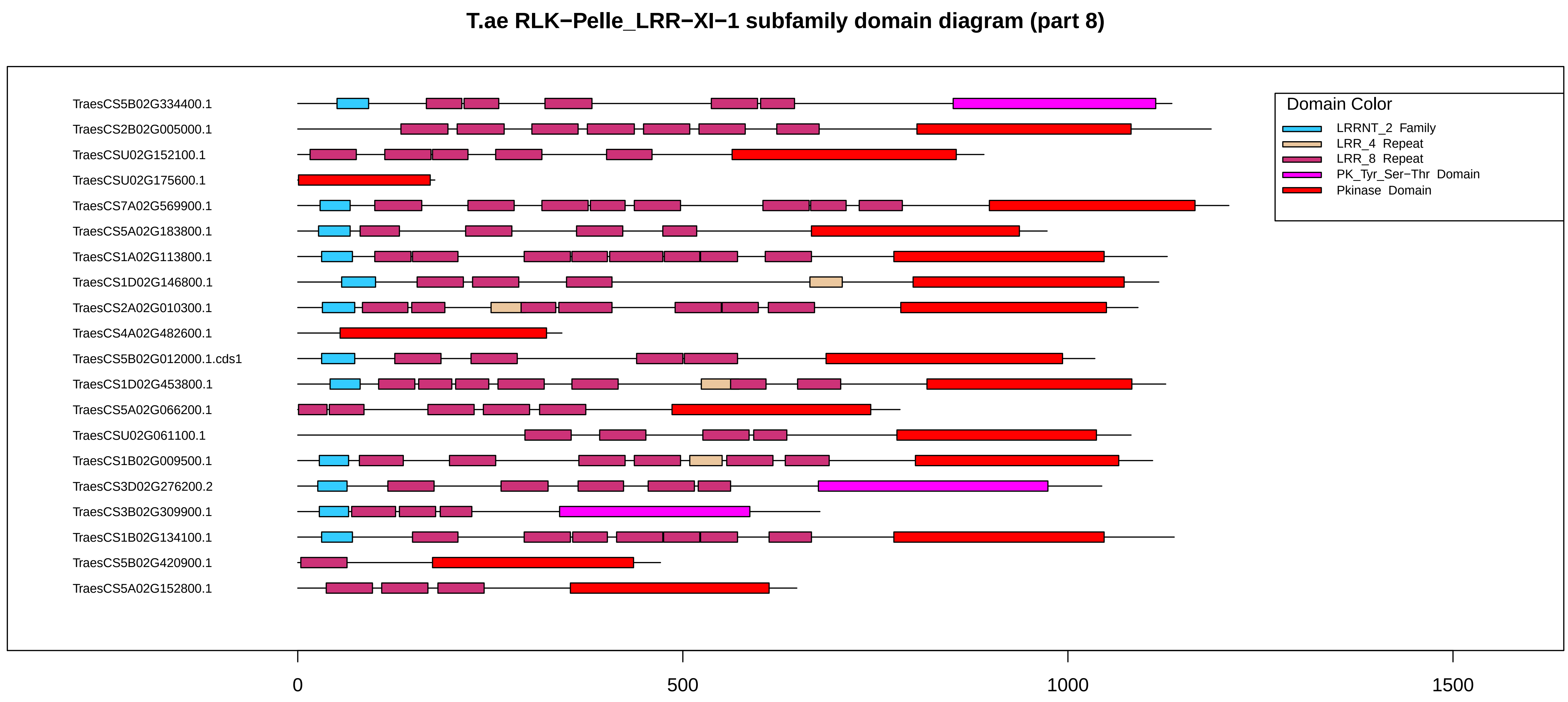

T.ae RLK−Pelle_LRR−XI−1 subfamily domain diagram (part 8)
TraesCS5B02G334400.1
TraesCS2B02G005000.1
TraesCSU02G152100.1
TraesCSU02G175600.1
TraesCS7A02G569900.1
TraesCS5A02G183800.1
TraesCS1A02G113800.1
TraesCS1D02G146800.1
TraesCS2A02G010300.1
TraesCS4A02G482600.1
TraesCS5B02G012000.1.cds1
TraesCS1D02G453800.1
TraesCS5A02G066200.1
TraesCSU02G061100.1
TraesCS1B02G009500.1
TraesCS3D02G276200.2
TraesCS3B02G309900.1
TraesCS1B02G134100.1
TraesCS5B02G420900.1
TraesCS5A02G152800.1
Domain Color
LRRNT_2 Family
LRR_4 Repeat
LRR_8 Repeat
PK_Tyr_Ser−Thr Domain
Pkinase Domain
0
500
1000
1500

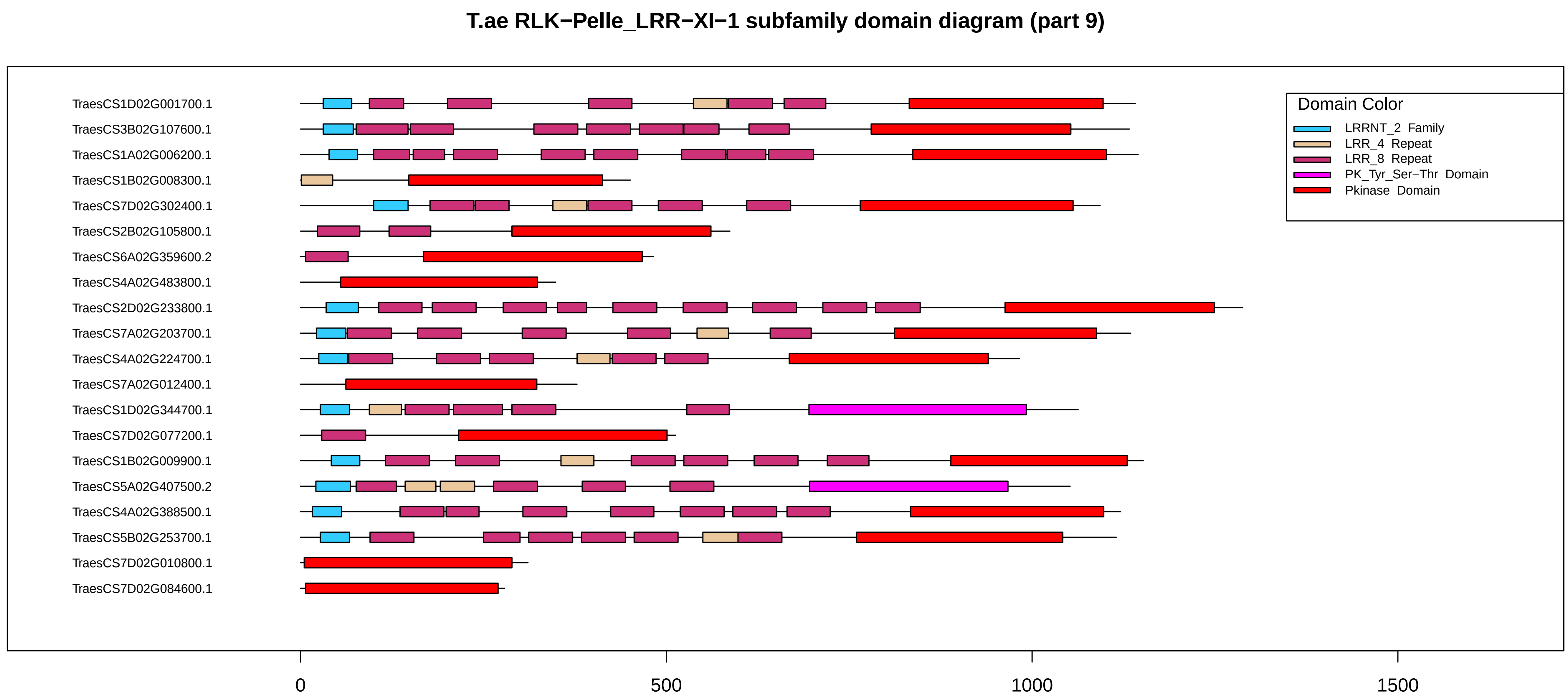
T.ae RLK−Pelle_LRR−XI−1 subfamily domain diagram (part 9)
TraesCS1D02G001700.1
TraesCS3B02G107600.1
TraesCS1A02G006200.1
TraesCS1B02G008300.1
TraesCS7D02G302400.1
TraesCS2B02G105800.1
TraesCS6A02G359600.2
TraesCS4A02G483800.1
TraesCS2D02G233800.1
TraesCS7A02G203700.1
TraesCS4A02G224700.1
TraesCS7A02G012400.1
TraesCS1D02G344700.1
TraesCS7D02G077200.1
TraesCS1B02G009900.1
TraesCS5A02G407500.2
TraesCS4A02G388500.1
TraesCS5B02G253700.1
TraesCS7D02G010800.1
TraesCS7D02G084600.1
Domain Color
LRRNT_2 Family
LRR_4 Repeat
LRR_8 Repeat
PK_Tyr_Ser−Thr Domain
Pkinase Domain
0
500
1000
1500

T.ae RLK−Pelle_LRR−XI−1 subfamily domain diagram (part 10)

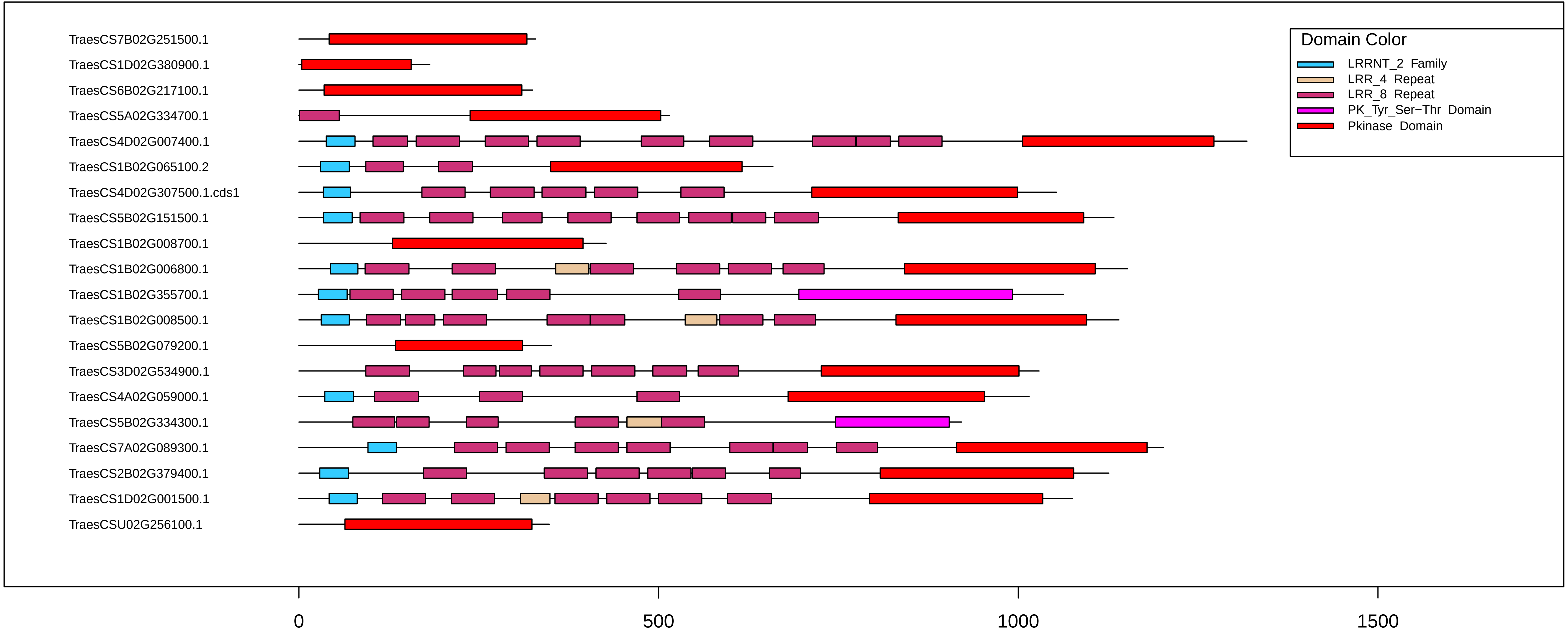

T.ae RLK−Pelle_LRR−XI−1 subfamily domain diagram (part 11)

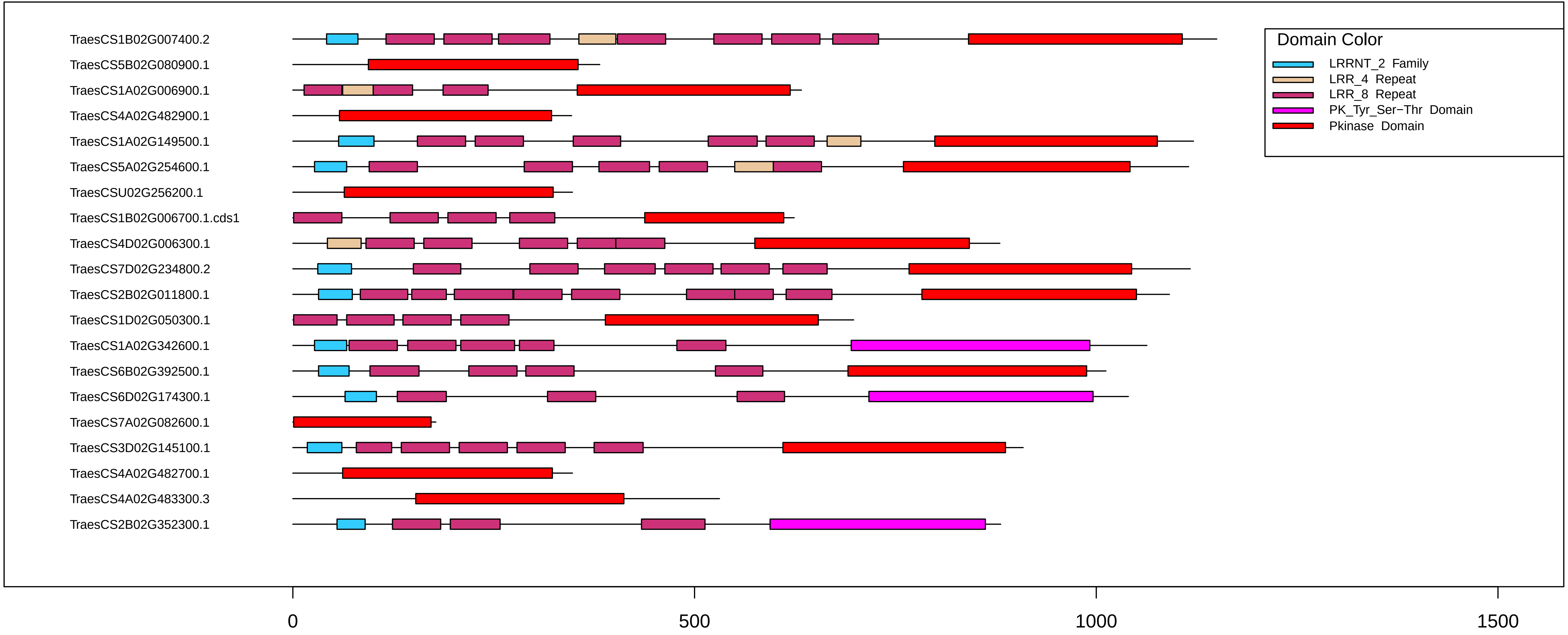

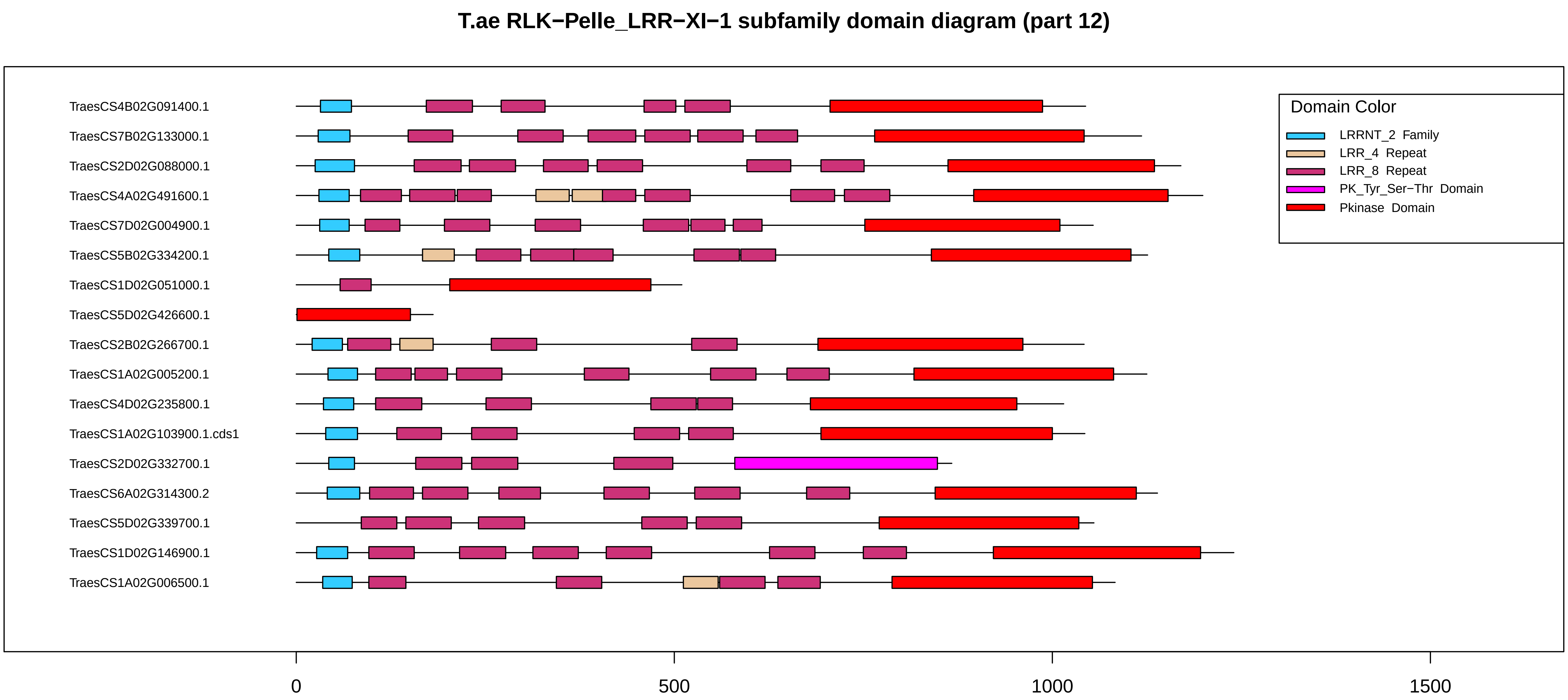

T.ae RLK−Pelle_LRR−XI−1 subfamily domain diagram (part 12)
TraesCS4B02G091400.1
TraesCS7B02G133000.1
TraesCS2D02G088000.1
TraesCS4A02G491600.1
TraesCS7D02G004900.1
TraesCS5B02G334200.1
TraesCS1D02G051000.1
TraesCS5D02G426600.1
TraesCS2B02G266700.1
TraesCS1A02G005200.1
TraesCS4D02G235800.1
TraesCS1A02G103900.1.cds1
TraesCS2D02G332700.1
TraesCS6A02G314300.2
TraesCS5D02G339700.1
TraesCS1D02G146900.1
TraesCS1A02G006500.1
Domain Color
LRRNT_2 Family
LRR_4 Repeat
LRR_8 Repeat
PK_Tyr_Ser−Thr Domain
Pkinase Domain
0
500
1000
1500

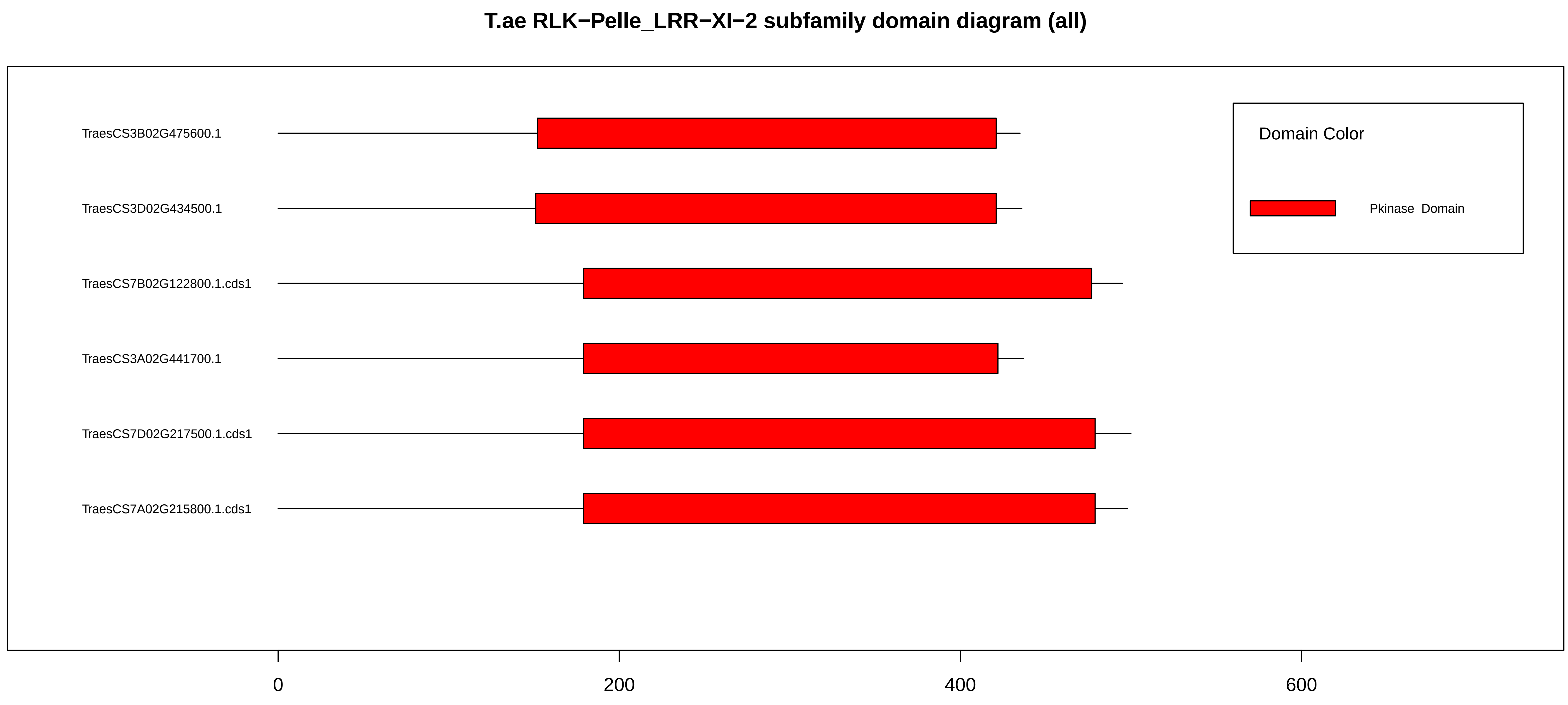
T.ae RLK−Pelle_LRR−XI−2 subfamily domain diagram (all)
TraesCS3B02G475600.1
TraesCS3D02G434500.1
TraesCS7B02G122800.1.cds1
TraesCS3A02G441700.1
TraesCS7D02G217500.1.cds1
TraesCS7A02G215800.1.cds1
Domain Color
Pkinase Domain
0
200
400
600

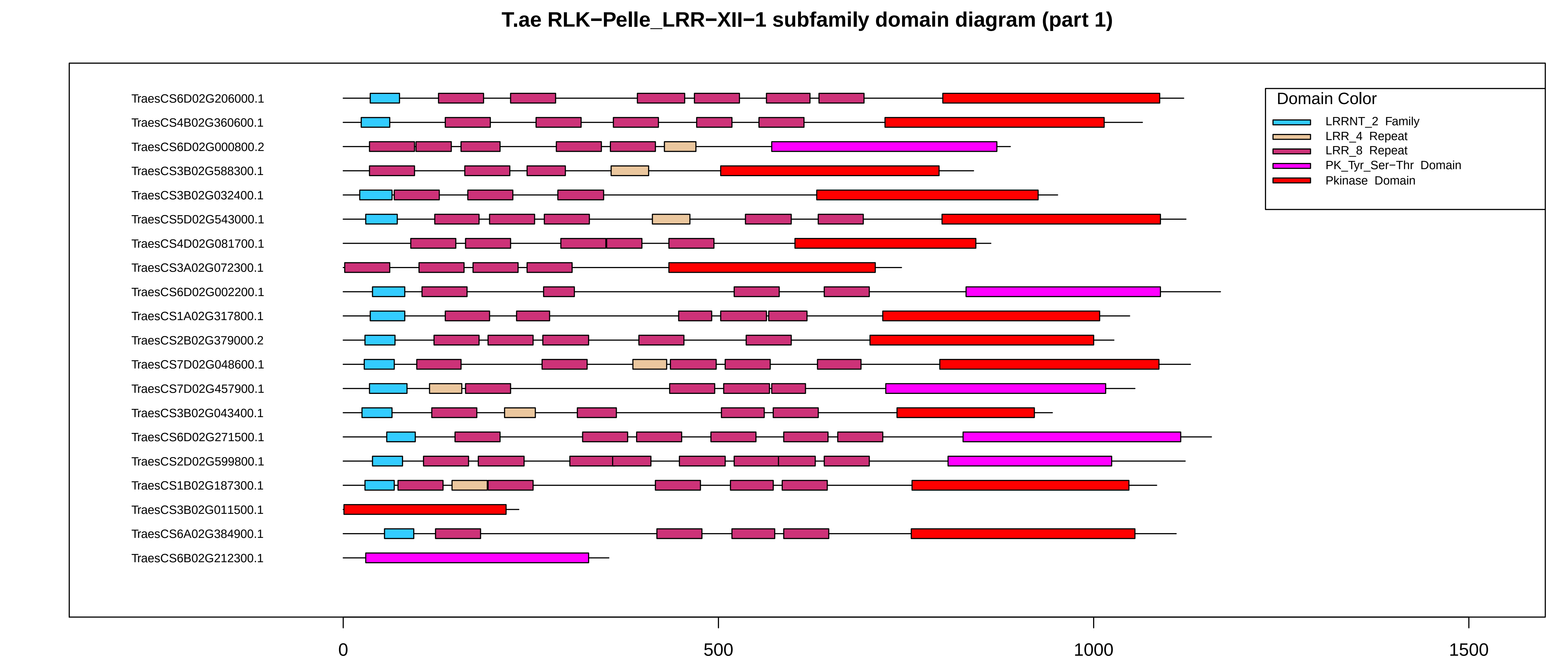
T.ae RLK−Pelle_LRR−XII−1 subfamily domain diagram (part 1)
TraesCS6D02G206000.1
TraesCS4B02G360600.1
TraesCS6D02G000800.2
TraesCS3B02G588300.1
TraesCS3B02G032400.1
TraesCS5D02G543000.1
TraesCS4D02G081700.1
TraesCS3A02G072300.1
TraesCS6D02G002200.1
TraesCS1A02G317800.1
TraesCS2B02G379000.2
TraesCS7D02G048600.1
TraesCS7D02G457900.1
TraesCS3B02G043400.1
TraesCS6D02G271500.1
TraesCS2D02G599800.1
TraesCS1B02G187300.1
TraesCS3B02G011500.1
TraesCS6A02G384900.1
TraesCS6B02G212300.1
Domain Color
LRRNT_2 Family
LRR_4 Repeat
LRR_8 Repeat
PK_Tyr_Ser−Thr Domain
Pkinase Domain
0
500
1000
1500

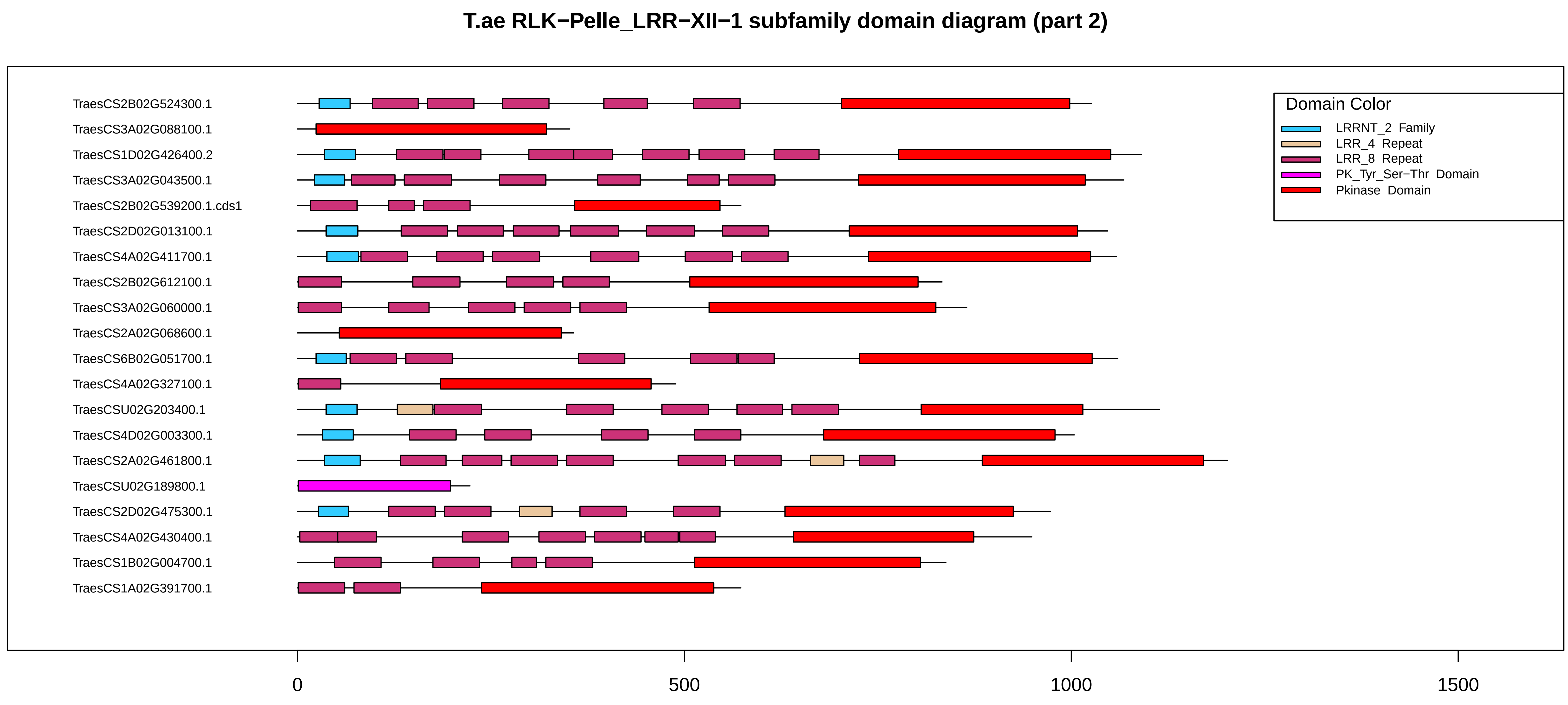

T.ae RLK−Pelle_LRR−XII−1 subfamily domain diagram (part 2)
TraesCS2B02G524300.1
TraesCS3A02G088100.1
TraesCS1D02G426400.2
TraesCS3A02G043500.1
TraesCS2B02G539200.1.cds1
TraesCS2D02G013100.1
TraesCS4A02G411700.1
TraesCS2B02G612100.1
TraesCS3A02G060000.1
TraesCS2A02G068600.1
TraesCS6B02G051700.1
TraesCS4A02G327100.1
TraesCSU02G203400.1
TraesCS4D02G003300.1
TraesCS2A02G461800.1
TraesCSU02G189800.1
TraesCS2D02G475300.1
TraesCS4A02G430400.1
TraesCS1B02G004700.1
TraesCS1A02G391700.1
Domain Color
LRRNT_2 Family
LRR_4 Repeat
LRR_8 Repeat
PK_Tyr_Ser−Thr Domain
Pkinase Domain
0
500
1000
1500

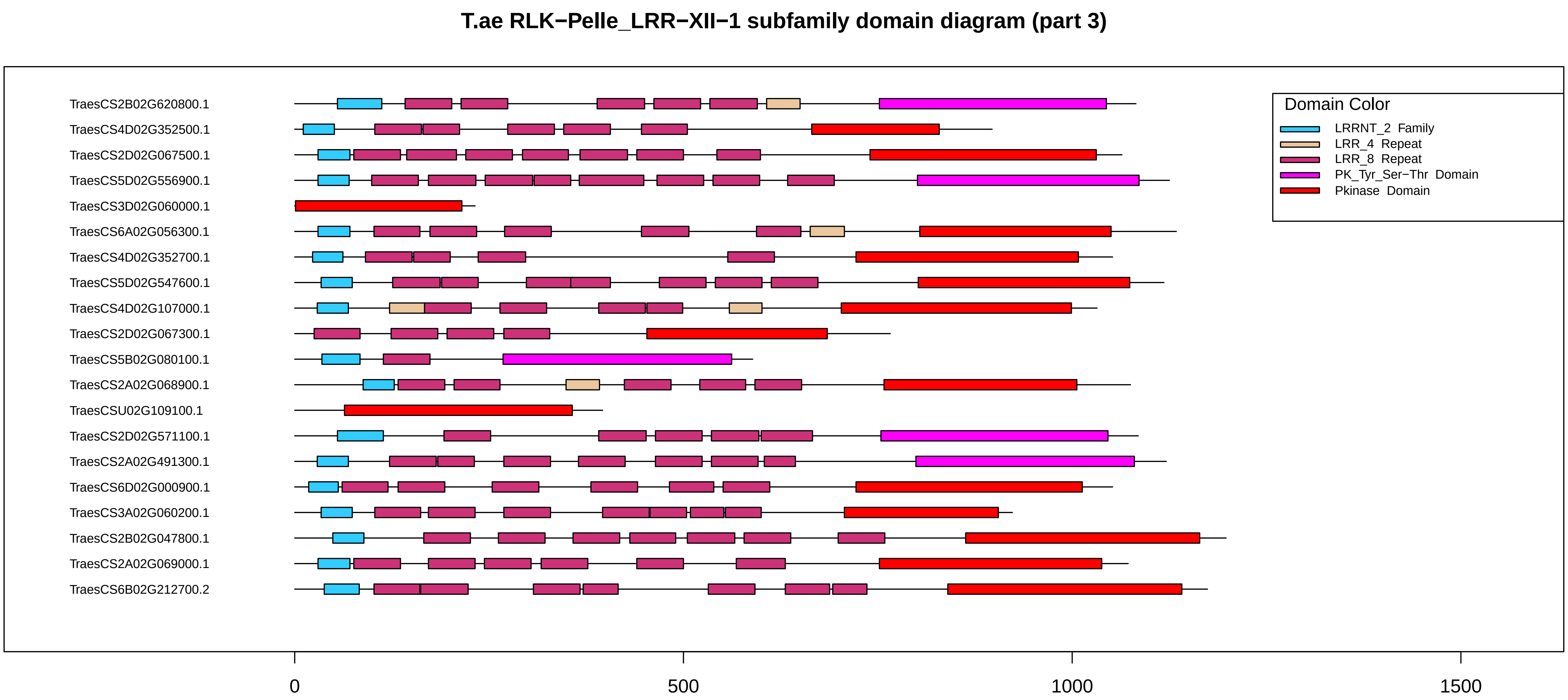
T.ae RLK−Pelle_LRR−XII−1 subfamily domain diagram (part 3)
TraesCS2B02G620800.1
TraesCS4D02G352500.1
TraesCS2D02G067500.1
TraesCS5D02G556900.1
TraesCS3D02G060000.1
TraesCS6A02G056300.1
TraesCS4D02G352700.1
TraesCS5D02G547600.1
TraesCS4D02G107000.1
TraesCS2D02G067300.1
TraesCS5B02G080100.1
TraesCS2A02G068900.1
TraesCSU02G109100.1
TraesCS2D02G571100.1
TraesCS2A02G491300.1
TraesCS6D02G000900.1
TraesCS3A02G060200.1
TraesCS2B02G047800.1
TraesCS2A02G069000.1
TraesCS6B02G212700.2
Domain Color
LRRNT_2 Family
LRR_4 Repeat
LRR_8 Repeat
PK_Tyr_Ser−Thr Domain
Pkinase Domain
0
500
1000
1500

T.ae RLK−Pelle_LRR−XII−1 subfamily domain diagram (part 4)

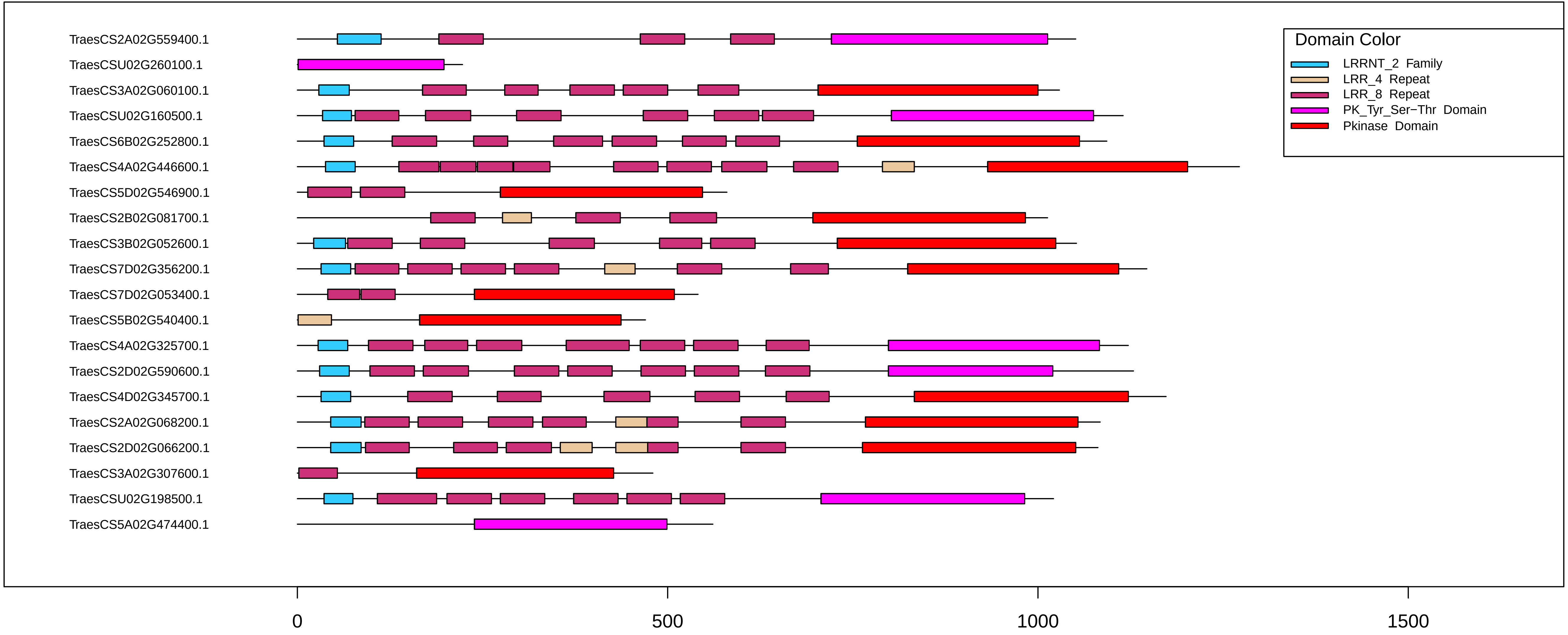

T.ae RLK−Pelle_LRR−XII−1 subfamily domain diagram (part 5)

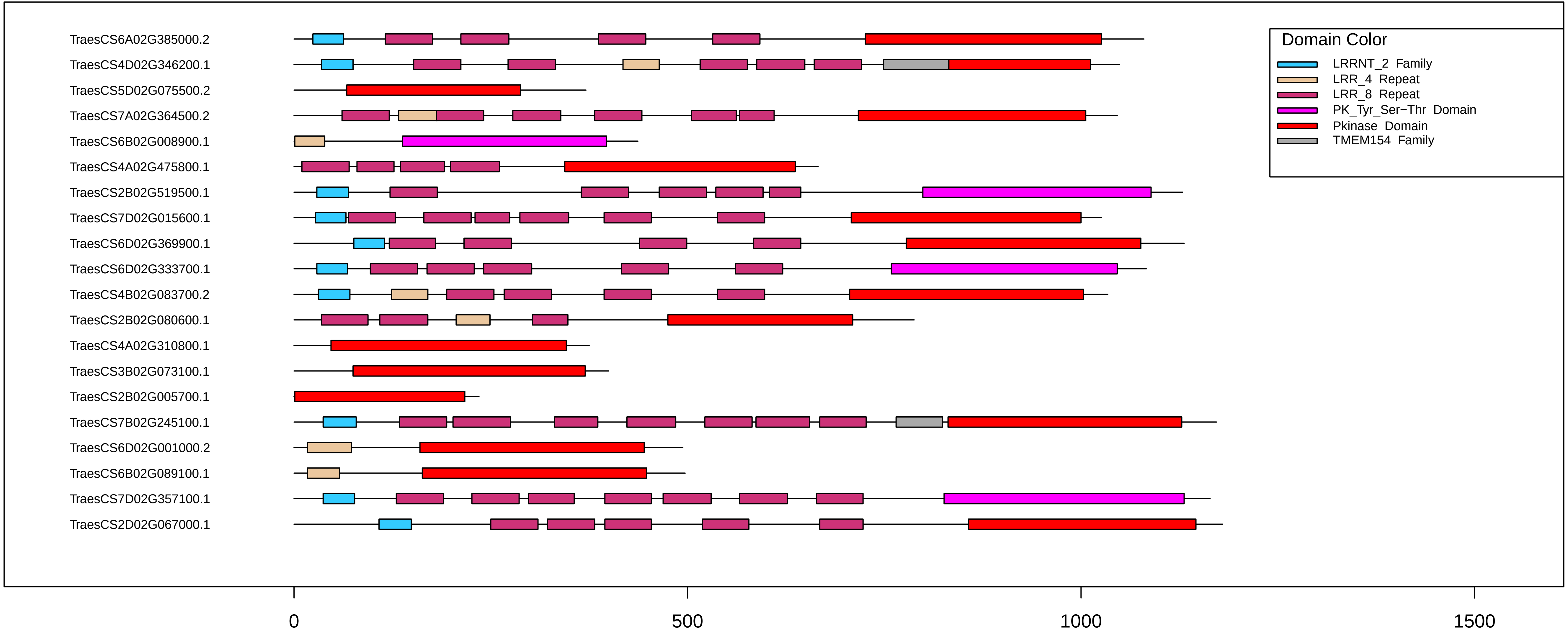

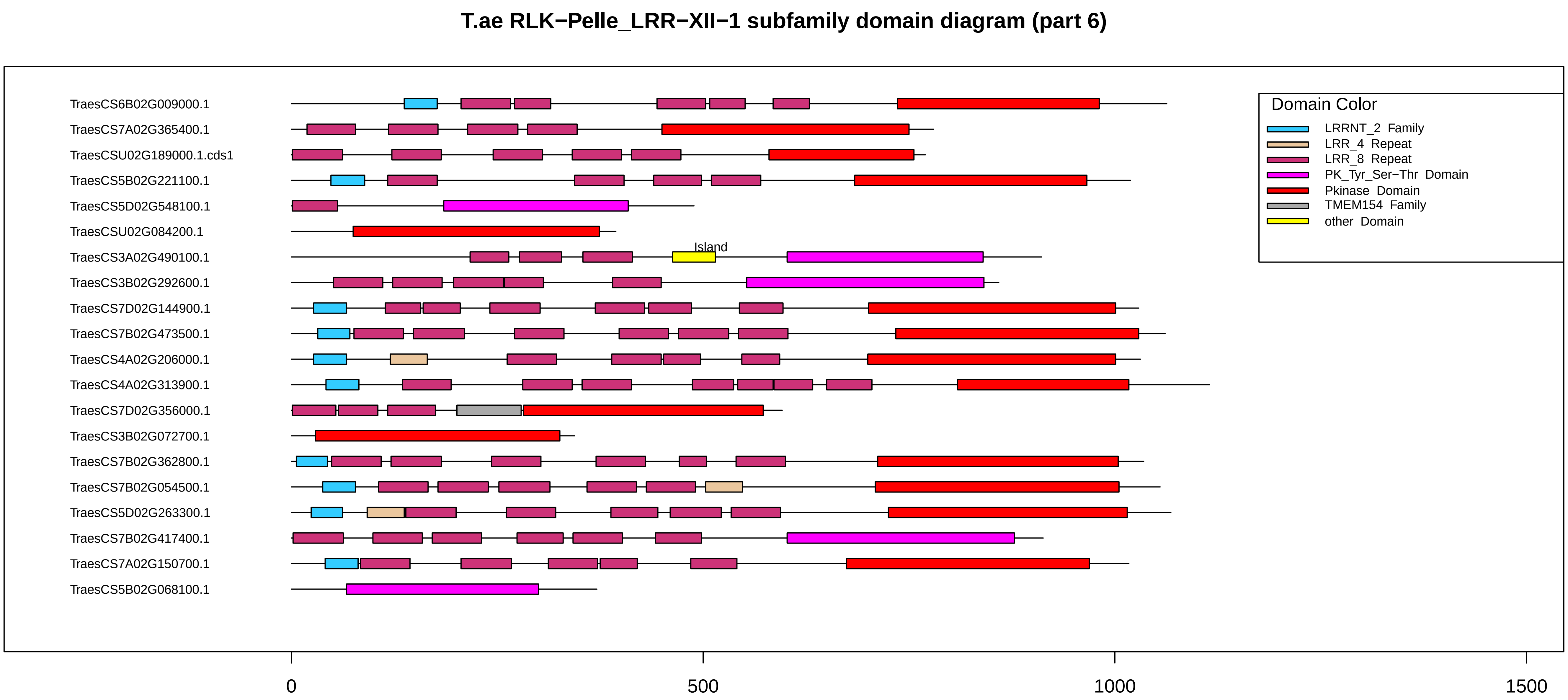
T.ae RLK−Pelle_LRR−XII−1 subfamily domain diagram (part 6)
TraesCS6B02G009000.1
TraesCS7A02G365400.1
TraesCSU02G189000.1.cds1
TraesCS5B02G221100.1
TraesCS5D02G548100.1
TraesCSU02G084200.1
TraesCS3A02G490100.1
Island
TraesCS3B02G292600.1
TraesCS7D02G144900.1
TraesCS7B02G473500.1
TraesCS4A02G206000.1
TraesCS4A02G313900.1
TraesCS7D02G356000.1
TraesCS3B02G072700.1
TraesCS7B02G362800.1
TraesCS7B02G054500.1
TraesCS5D02G263300.1
TraesCS7B02G417400.1
TraesCS7A02G150700.1
TraesCS5B02G068100.1
Domain Color
LRRNT_2 Family
LRR_4 Repeat
LRR_8 Repeat
PK_Tyr_Ser−Thr Domain
Pkinase Domain
TMEM154 Family
other Domain
0
500
1000
1500

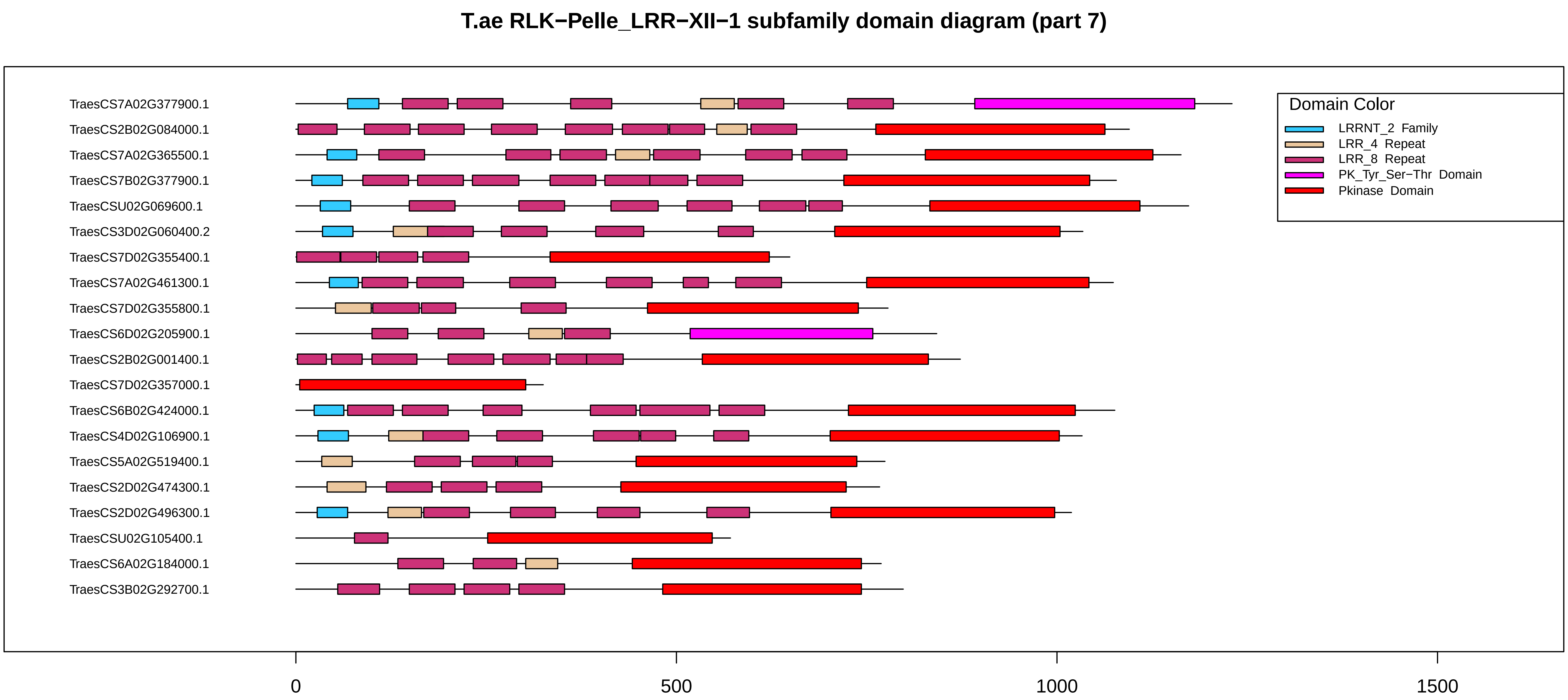
T.ae RLK−Pelle_LRR−XII−1 subfamily domain diagram (part 7)
TraesCS7A02G377900.1
TraesCS2B02G084000.1
TraesCS7A02G365500.1
TraesCS7B02G377900.1
TraesCSU02G069600.1
TraesCS3D02G060400.2
TraesCS7D02G355400.1
TraesCS7A02G461300.1
TraesCS7D02G355800.1
TraesCS6D02G205900.1
TraesCS2B02G001400.1
TraesCS7D02G357000.1
TraesCS6B02G424000.1
TraesCS4D02G106900.1
TraesCS5A02G519400.1
TraesCS2D02G474300.1
TraesCS2D02G496300.1
TraesCSU02G105400.1
TraesCS6A02G184000.1
TraesCS3B02G292700.1
Domain Color
LRRNT_2 Family
LRR_4 Repeat
LRR_8 Repeat
PK_Tyr_Ser−Thr Domain
Pkinase Domain
0
500
1000
1500

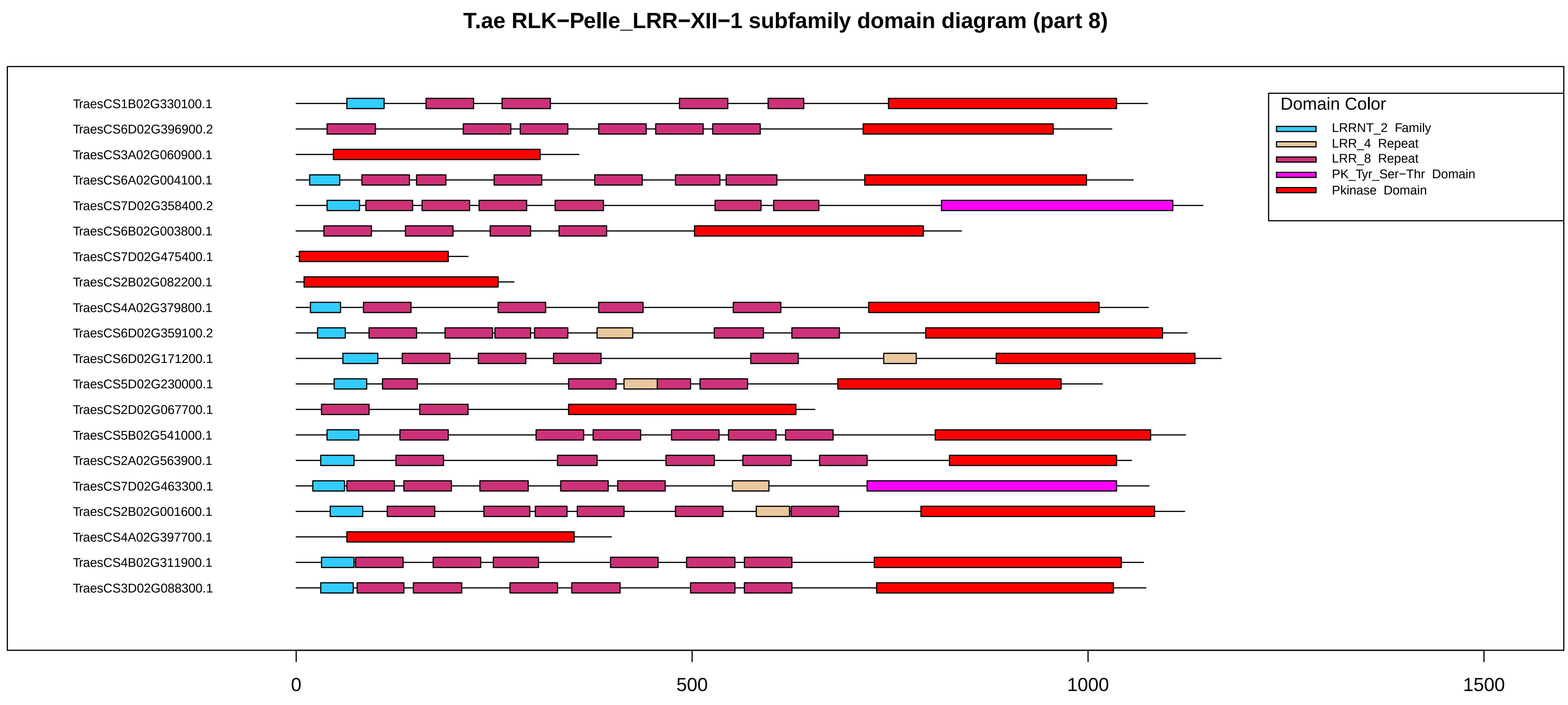

T.ae RLK−Pelle_LRR−XII−1 subfamily domain diagram (part 8)
TraesCS1B02G330100.1
TraesCS6D02G396900.2
TraesCS3A02G060900.1
TraesCS6A02G004100.1
TraesCS7D02G358400.2
TraesCS6B02G003800.1
TraesCS7D02G475400.1
TraesCS2B02G082200.1
TraesCS4A02G379800.1
TraesCS6D02G359100.2
TraesCS6D02G171200.1
TraesCS5D02G230000.1
TraesCS2D02G067700.1
TraesCS5B02G541000.1
TraesCS2A02G563900.1
TraesCS7D02G463300.1
TraesCS2B02G001600.1
TraesCS4A02G397700.1
TraesCS4B02G311900.1
TraesCS3D02G088300.1
Domain Color
LRRNT_2 Family
LRR_4 Repeat
LRR_8 Repeat
PK_Tyr_Ser−Thr Domain
Pkinase Domain
0
500
1000
1500

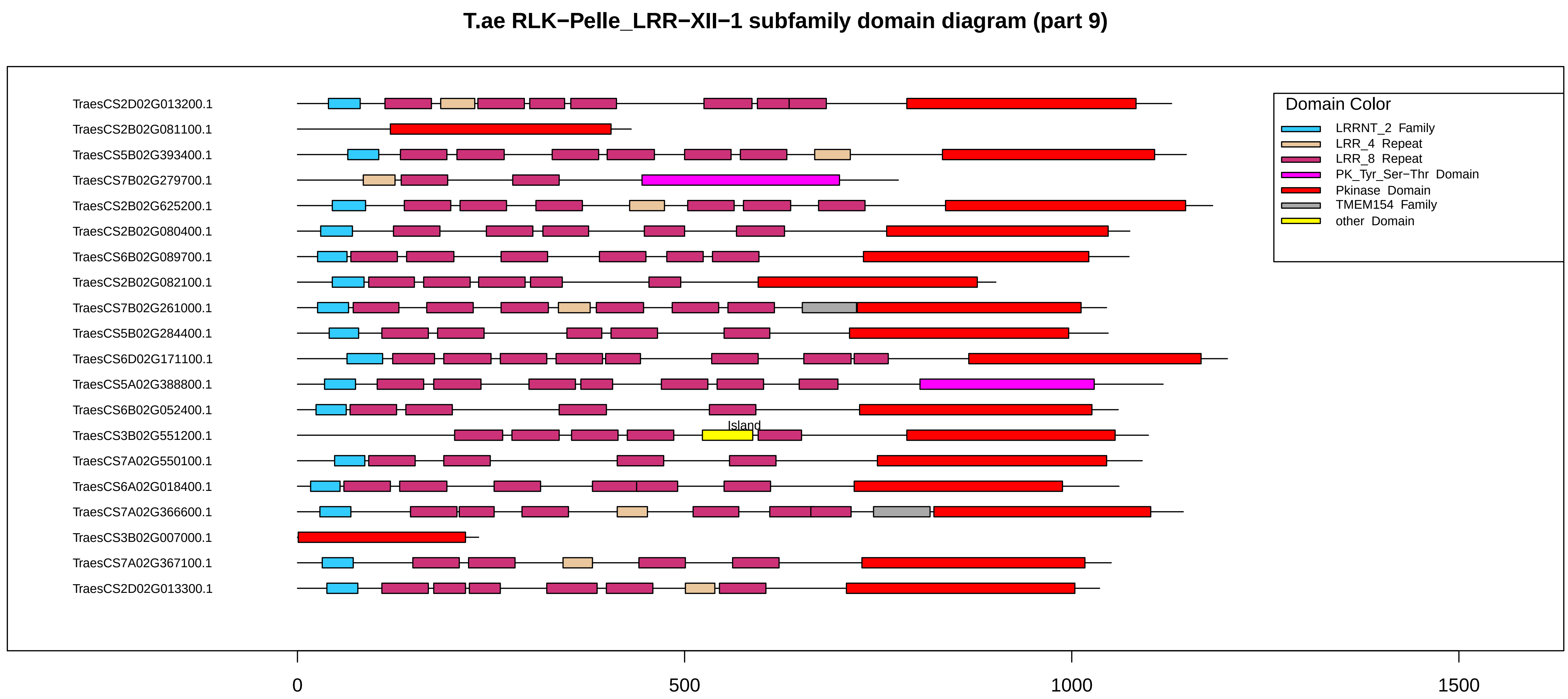
T.ae RLK−Pelle_LRR−XII−1 subfamily domain diagram (part 9)
TraesCS2D02G013200.1
TraesCS2B02G081100.1
TraesCS5B02G393400.1
TraesCS7B02G279700.1
TraesCS2B02G625200.1
TraesCS2B02G080400.1
TraesCS6B02G089700.1
TraesCS2B02G082100.1
TraesCS7B02G261000.1
TraesCS5B02G284400.1
TraesCS6D02G171100.1
TraesCS5A02G388800.1
TraesCS6B02G052400.1
TraesCS3B02G551200.1
TraesCS7A02G550100.1
TraesCS6A02G018400.1
TraesCS7A02G366600.1
TraesCS3B02G007000.1
TraesCS7A02G367100.1
TraesCS2D02G013300.1
Island
Domain Color
LRRNT_2 Family
LRR_4 Repeat
LRR_8 Repeat
PK_Tyr_Ser−Thr Domain
Pkinase Domain
TMEM154 Family
other Domain
0
500
1000
1500

T.ae RLK−Pelle_LRR−XII−1 subfamily domain diagram (part 10)

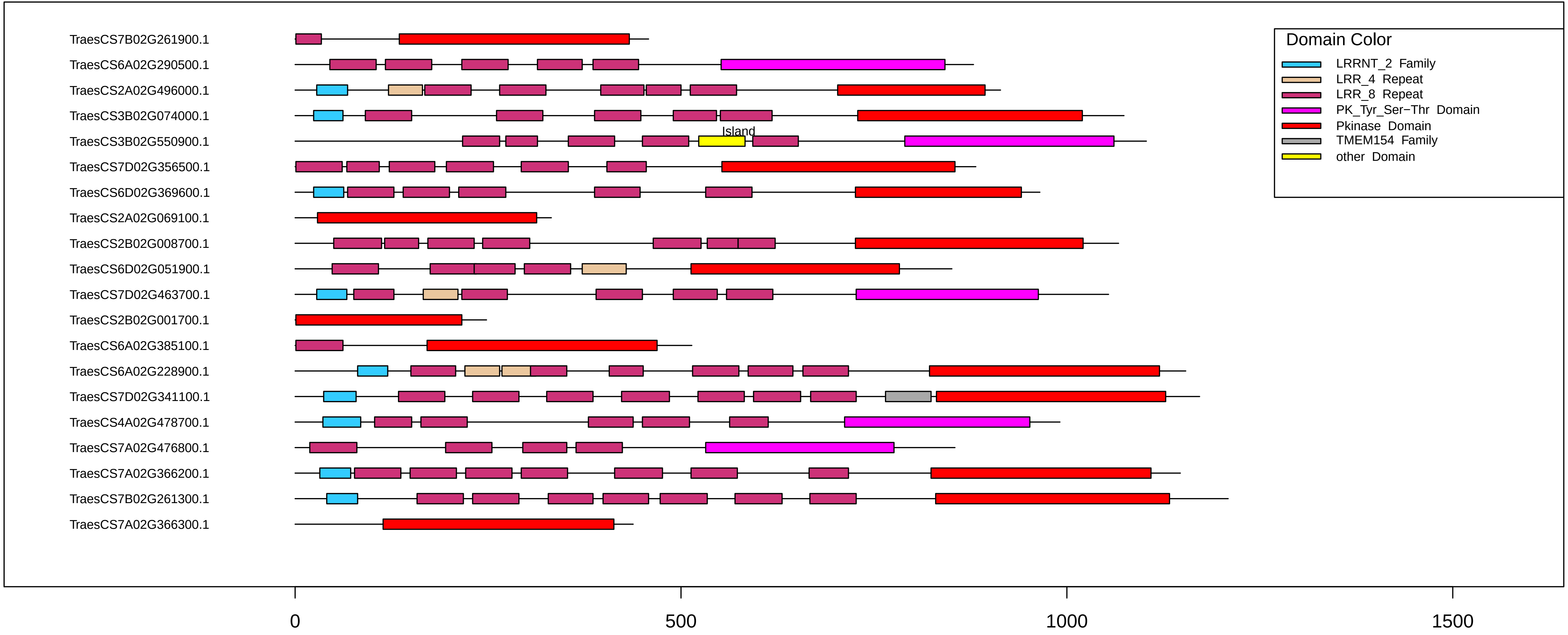

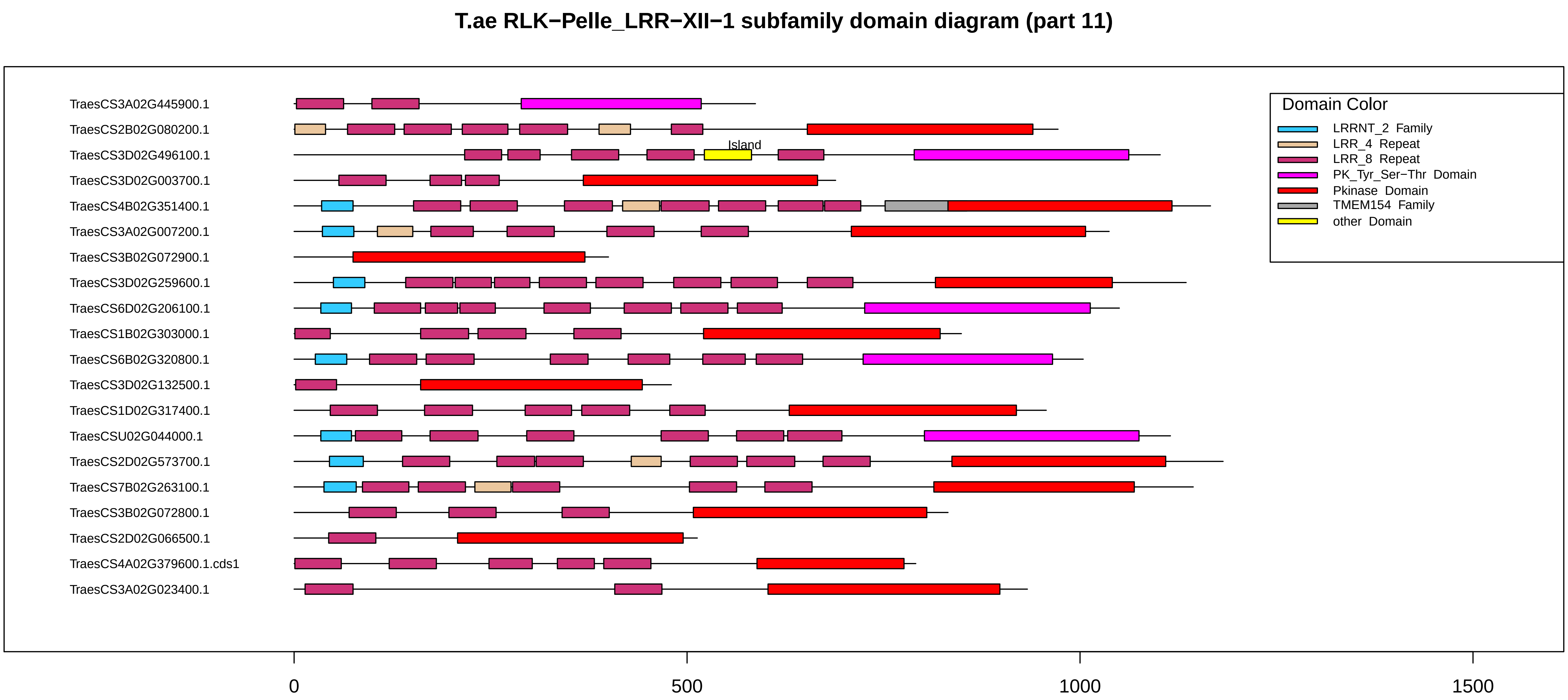
T.ae RLK−Pelle_LRR−XII−1 subfamily domain diagram (part 11)
TraesCS3A02G445900.1
TraesCS2B02G080200.1
TraesCS3D02G496100.1
Island
TraesCS3D02G003700.1
TraesCS4B02G351400.1
TraesCS3A02G007200.1
TraesCS3B02G072900.1
TraesCS3D02G259600.1
TraesCS6D02G206100.1
TraesCS1B02G303000.1
TraesCS6B02G320800.1
TraesCS3D02G132500.1
TraesCS1D02G317400.1
TraesCSU02G044000.1
TraesCS2D02G573700.1
TraesCS7B02G263100.1
TraesCS3B02G072800.1
TraesCS2D02G066500.1
TraesCS4A02G379600.1.cds1
TraesCS3A02G023400.1
Domain Color
LRRNT_2 Family
LRR_4 Repeat
LRR_8 Repeat
PK_Tyr_Ser−Thr Domain
Pkinase Domain
TMEM154 Family
other Domain
0
500
1000
1500

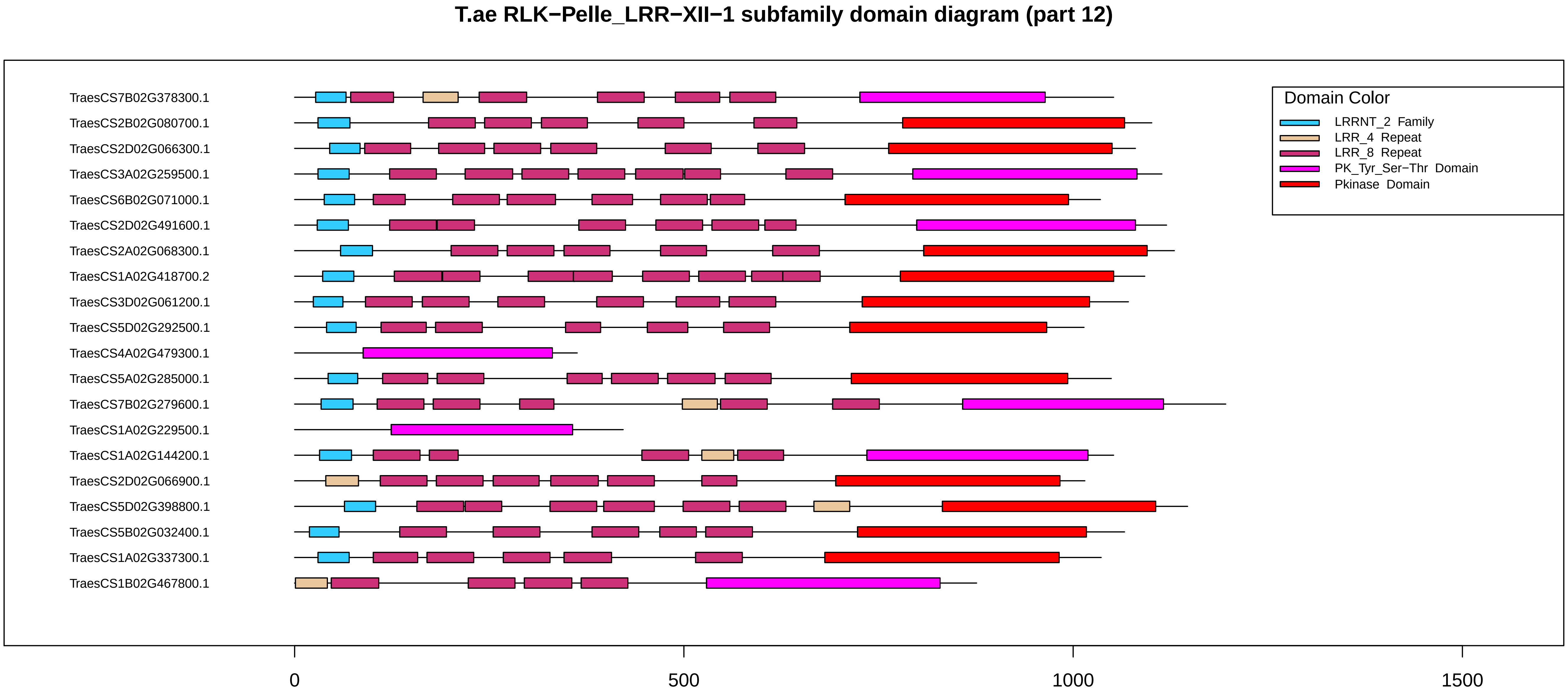
T.ae RLK−Pelle_LRR−XII−1 subfamily domain diagram (part 12)
TraesCS7B02G378300.1
TraesCS2B02G080700.1
TraesCS2D02G066300.1
TraesCS3A02G259500.1
TraesCS6B02G071000.1
TraesCS2D02G491600.1
TraesCS2A02G068300.1
TraesCS1A02G418700.2
TraesCS3D02G061200.1
TraesCS5D02G292500.1
TraesCS4A02G479300.1
TraesCS5A02G285000.1
TraesCS7B02G279600.1
TraesCS1A02G229500.1
TraesCS1A02G144200.1
TraesCS2D02G066900.1
TraesCS5D02G398800.1
TraesCS5B02G032400.1
TraesCS1A02G337300.1
TraesCS1B02G467800.1
Domain Color
LRRNT_2 Family
LRR_4 Repeat
LRR_8 Repeat
PK_Tyr_Ser−Thr Domain
Pkinase Domain
0
500
1000
1500

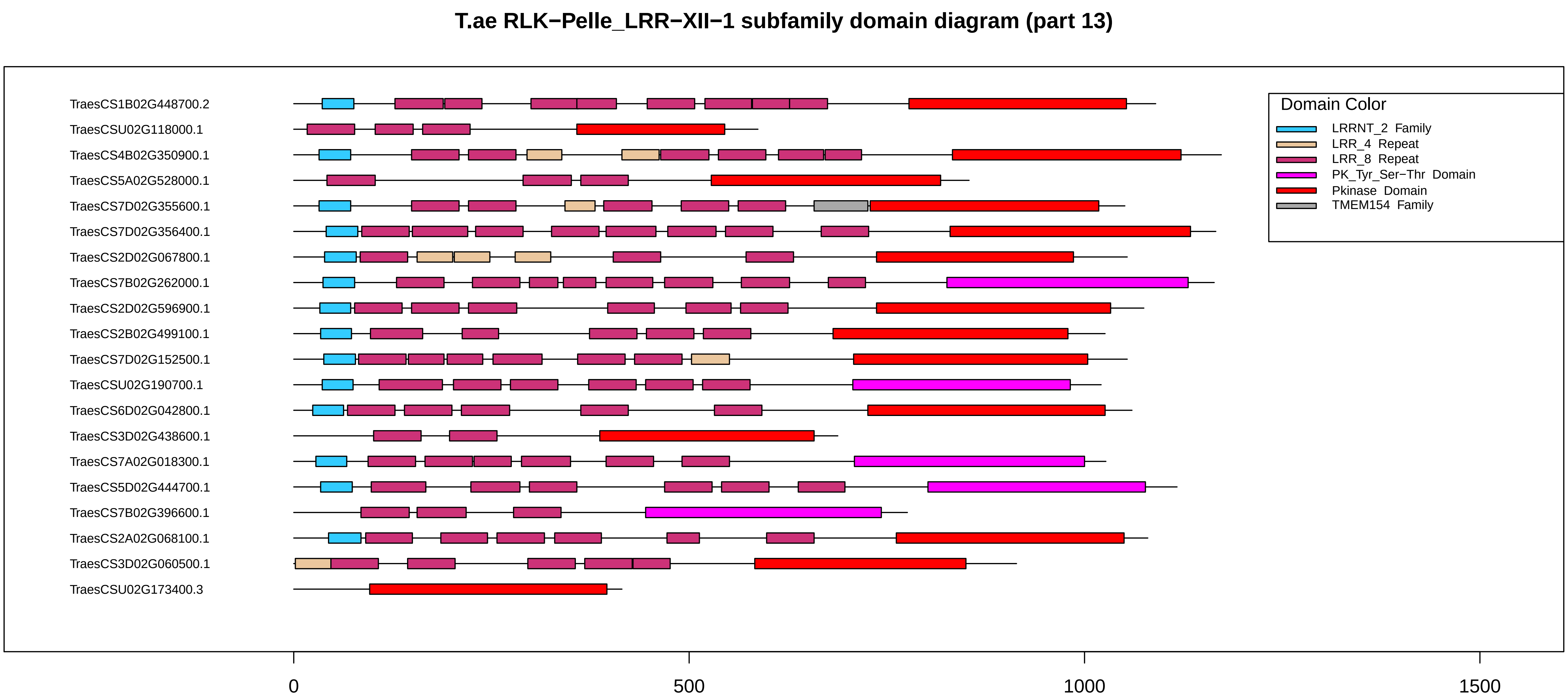

T.ae RLK−Pelle_LRR−XII−1 subfamily domain diagram (part 13)
TraesCS1B02G448700.2
TraesCSU02G118000.1
TraesCS4B02G350900.1
TraesCS5A02G528000.1
TraesCS7D02G355600.1
TraesCS7D02G356400.1
TraesCS2D02G067800.1
TraesCS7B02G262000.1
TraesCS2D02G596900.1
TraesCS2B02G499100.1
TraesCS7D02G152500.1
TraesCSU02G190700.1
TraesCS6D02G042800.1
TraesCS3D02G438600.1
TraesCS7A02G018300.1
TraesCS5D02G444700.1
TraesCS7B02G396600.1
TraesCS2A02G068100.1
TraesCS3D02G060500.1
TraesCSU02G173400.3
Domain Color
LRRNT_2 Family
LRR_4 Repeat
LRR_8 Repeat
PK_Tyr_Ser−Thr Domain
Pkinase Domain
TMEM154 Family
0
500
1000
1500

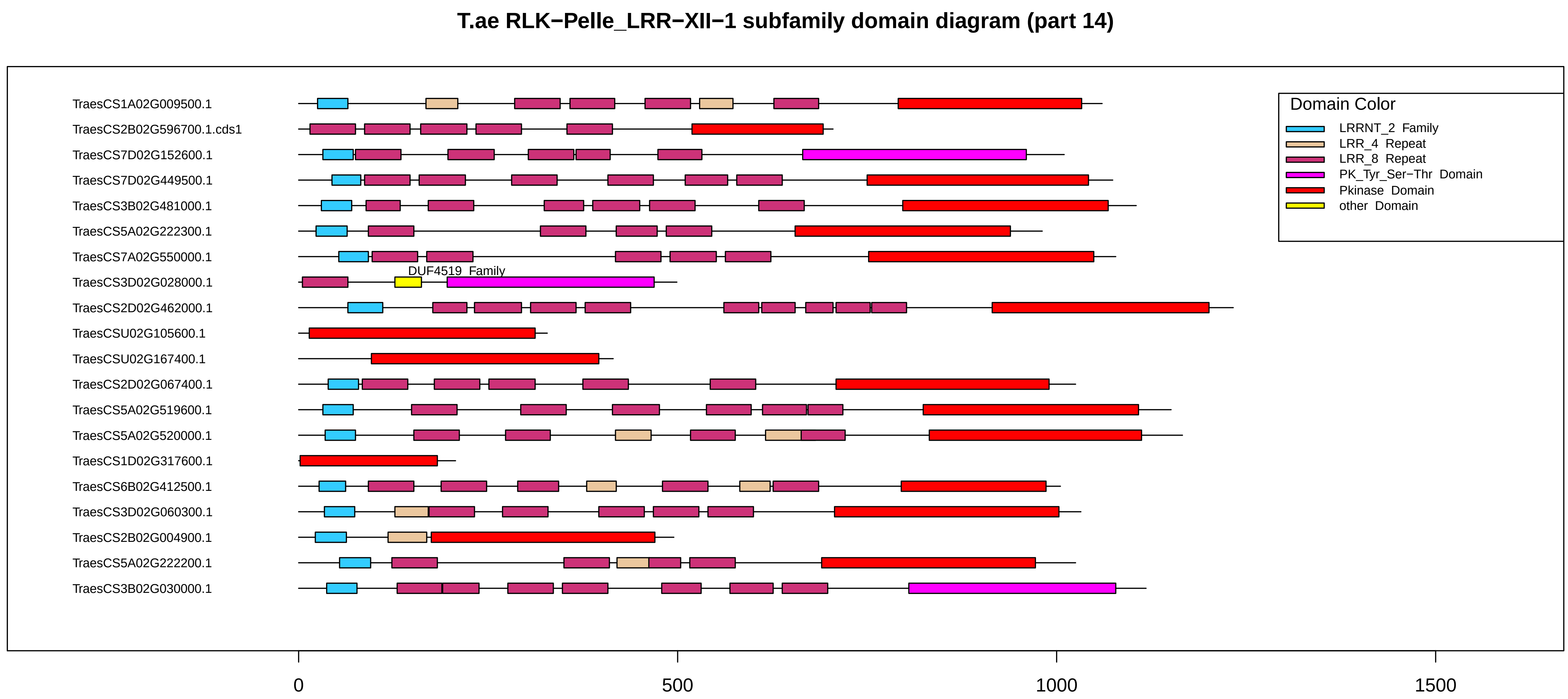
T.ae RLK−Pelle_LRR−XII−1 subfamily domain diagram (part 14)
TraesCS1A02G009500.1
TraesCS2B02G596700.1.cds1
TraesCS7D02G152600.1
TraesCS7D02G449500.1
TraesCS3B02G481000.1
TraesCS5A02G222300.1
TraesCS7A02G550000.1
TraesCS3D02G028000.1
DUF4519_Family
TraesCS2D02G462000.1
TraesCSU02G105600.1
TraesCSU02G167400.1
TraesCS2D02G067400.1
TraesCS5A02G519600.1
TraesCS5A02G520000.1
TraesCS1D02G317600.1
TraesCS6B02G412500.1
TraesCS3D02G060300.1
TraesCS2B02G004900.1
TraesCS5A02G222200.1
TraesCS3B02G030000.1
Domain Color
LRRNT_2 Family
LRR_4 Repeat
LRR_8 Repeat
PK_Tyr_Ser−Thr Domain
Pkinase Domain
other Domain
0
500
1000
1500

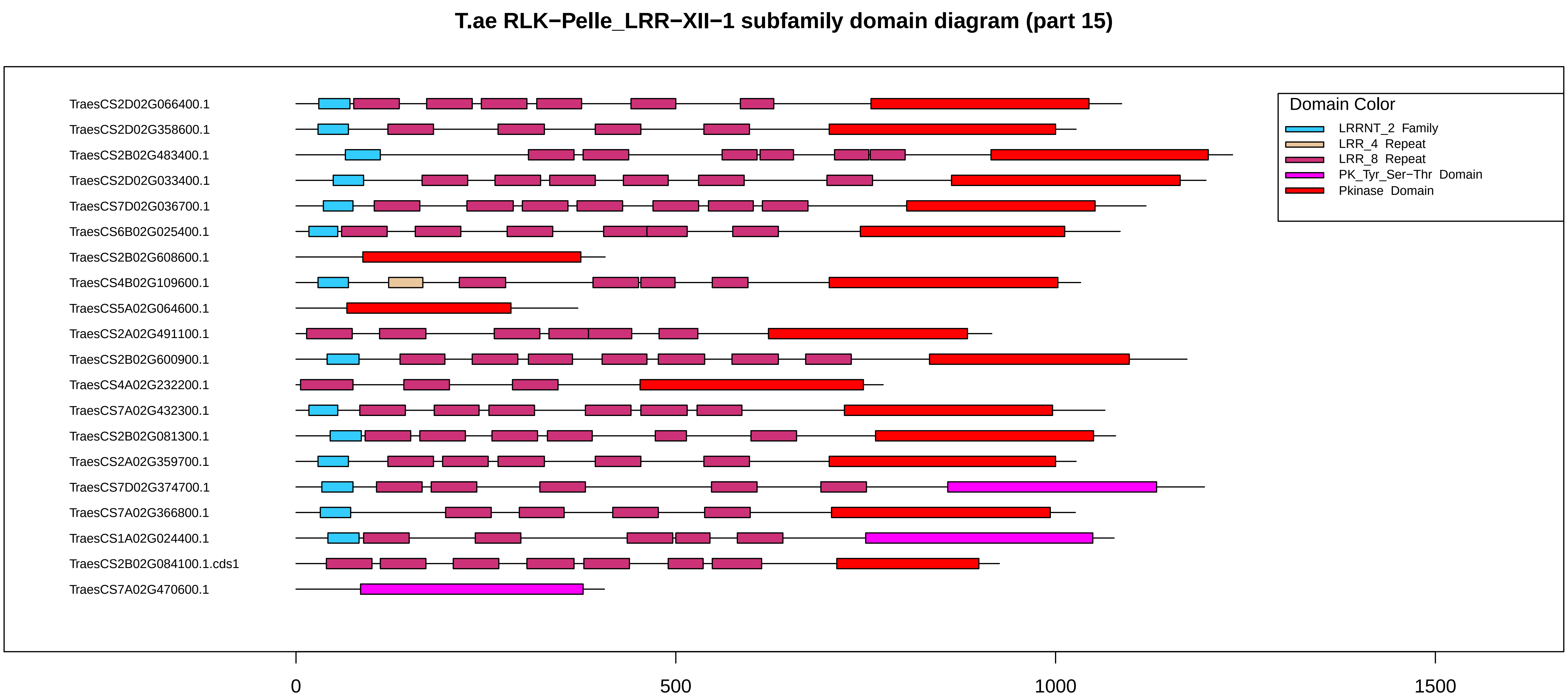
T.ae RLK−Pelle_LRR−XII−1 subfamily domain diagram (part 15)
TraesCS2D02G066400.1
TraesCS2D02G358600.1
TraesCS2B02G483400.1
TraesCS2D02G033400.1
TraesCS7D02G036700.1
TraesCS6B02G025400.1
TraesCS2B02G608600.1
TraesCS4B02G109600.1
TraesCS5A02G064600.1
TraesCS2A02G491100.1
TraesCS2B02G600900.1
TraesCS4A02G232200.1
TraesCS7A02G432300.1
TraesCS2B02G081300.1
TraesCS2A02G359700.1
TraesCS7D02G374700.1
TraesCS7A02G366800.1
TraesCS1A02G024400.1
TraesCS2B02G084100.1.cds1
TraesCS7A02G470600.1
Domain Color
LRRNT_2 Family
LRR_4 Repeat
LRR_8 Repeat
PK_Tyr_Ser−Thr Domain
Pkinase Domain
0
500
1000
1500

T.ae RLK−Pelle_LRR−XII−1 subfamily domain diagram (part 16)

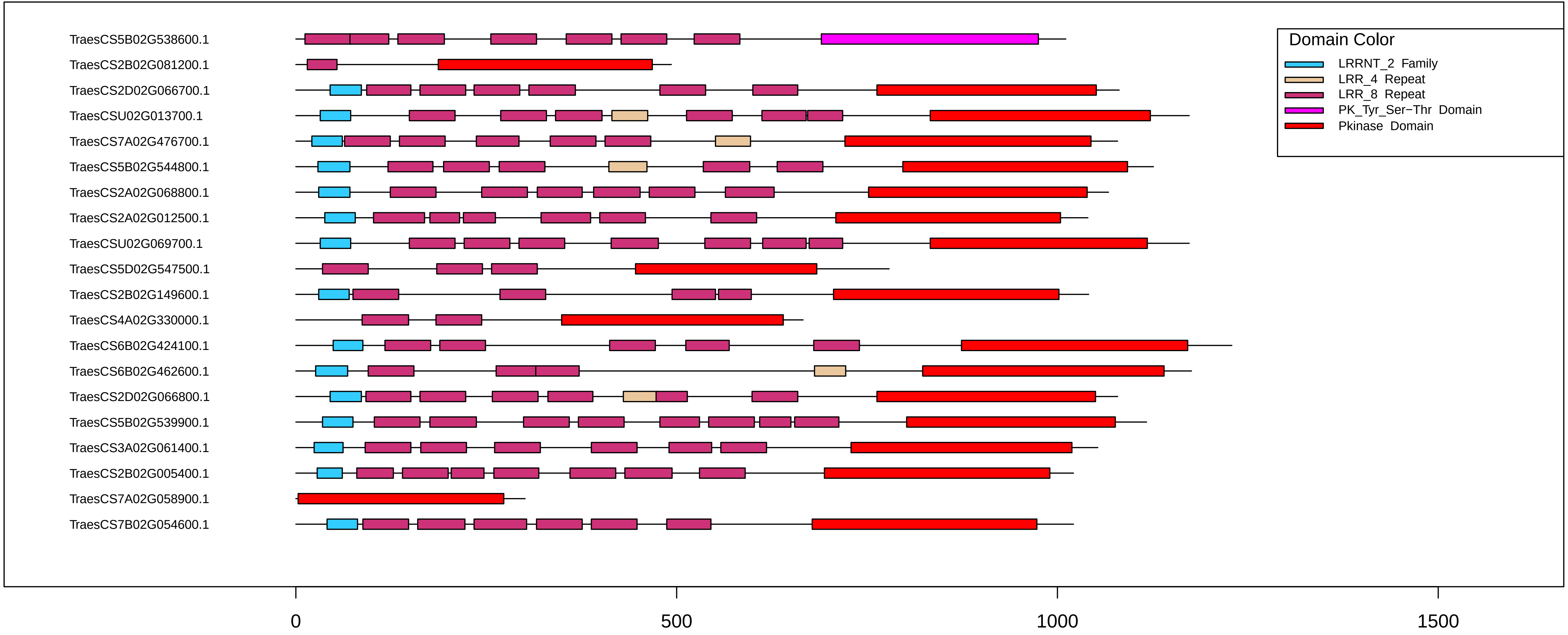

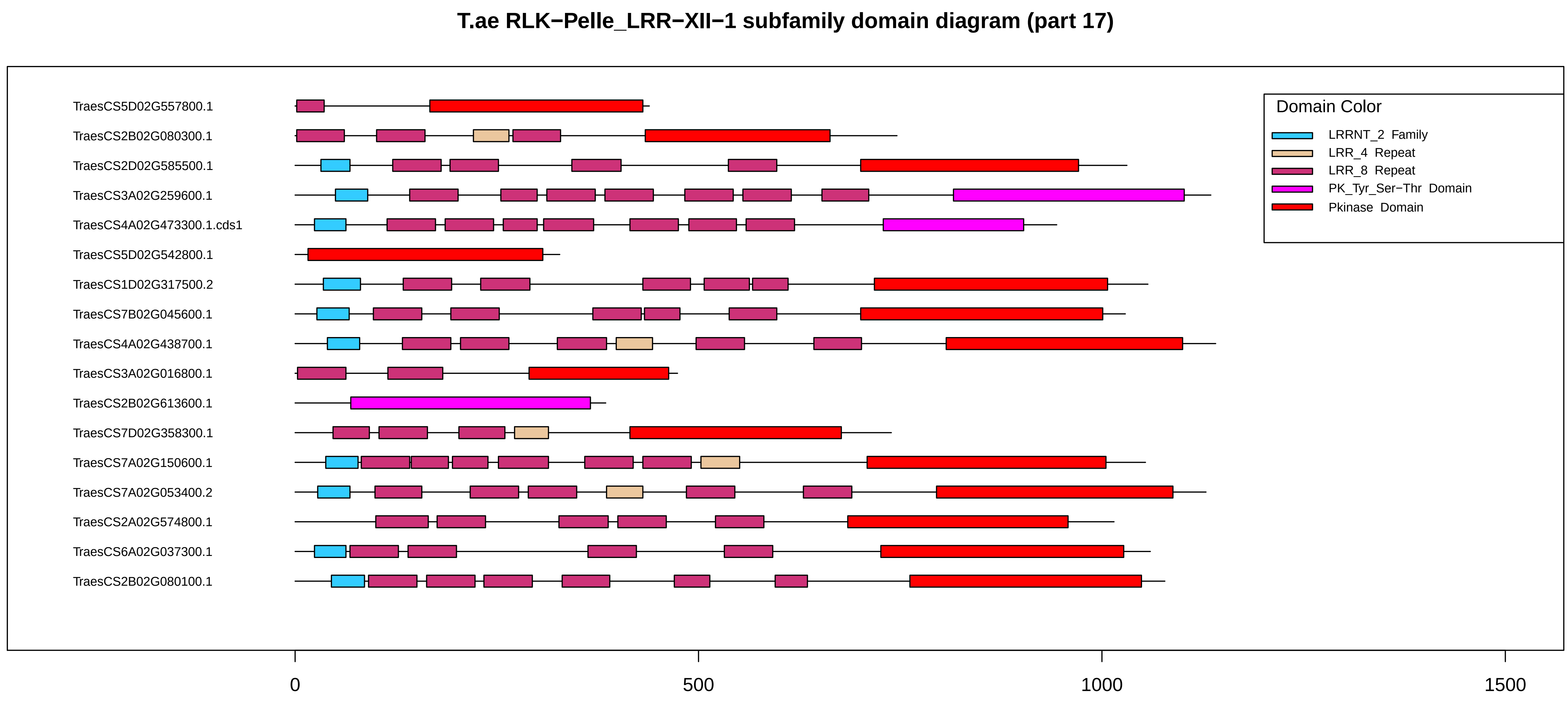
T.ae RLK−Pelle_LRR−XII−1 subfamily domain diagram (part 17)
TraesCS5D02G557800.1
TraesCS2B02G080300.1
TraesCS2D02G585500.1
TraesCS3A02G259600.1
TraesCS4A02G473300.1.cds1
TraesCS5D02G542800.1
TraesCS1D02G317500.2
TraesCS7B02G045600.1
TraesCS4A02G438700.1
TraesCS3A02G016800.1
TraesCS2B02G613600.1
TraesCS7D02G358300.1
TraesCS7A02G150600.1
TraesCS7A02G053400.2
TraesCS2A02G574800.1
TraesCS6A02G037300.1
TraesCS2B02G080100.1
Domain Color
LRRNT_2 Family
LRR_4 Repeat
LRR_8 Repeat
PK_Tyr_Ser−Thr Domain
Pkinase Domain
0
500
1000
1500

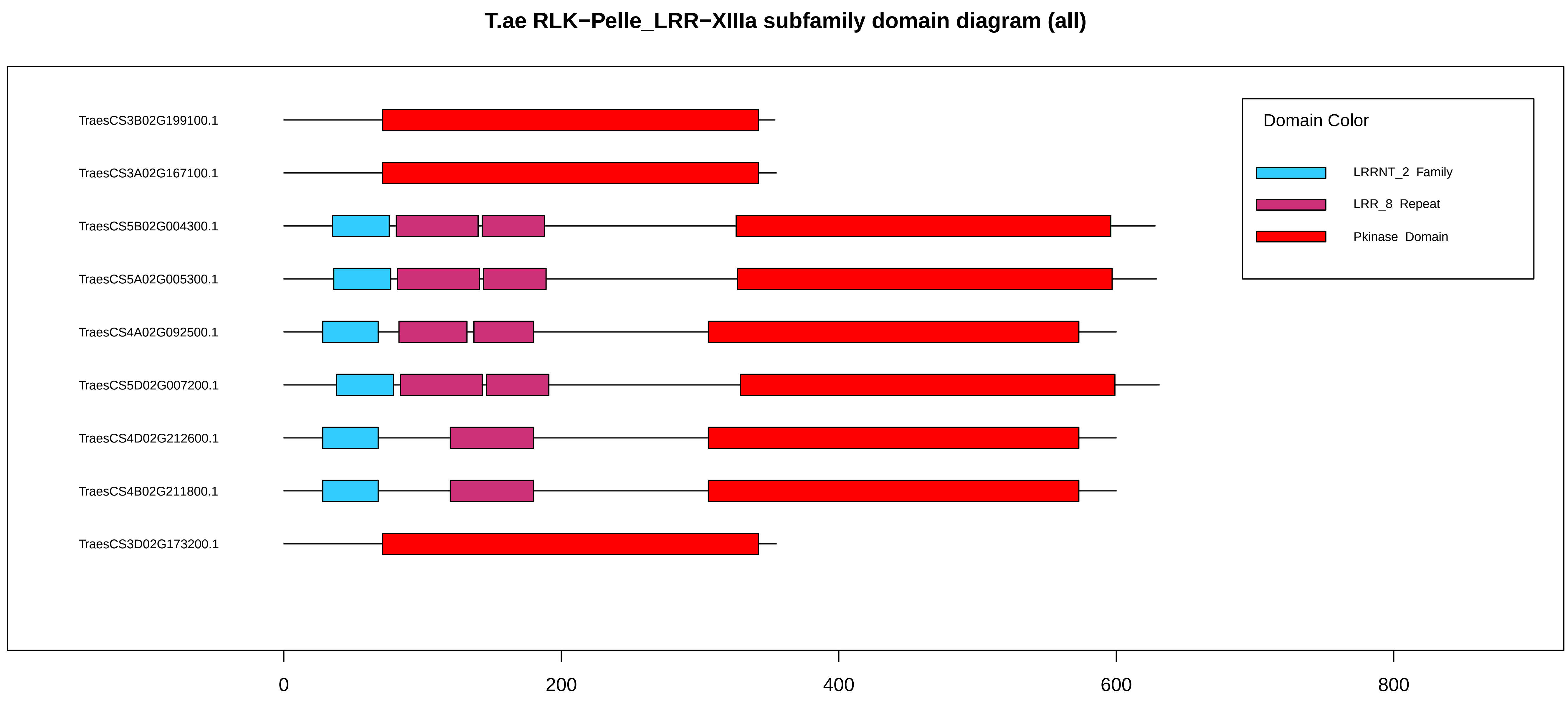
T.ae RLK−Pelle_LRR−XIIIa subfamily domain diagram (all)
TraesCS3B02G199100.1
TraesCS3A02G167100.1
TraesCS5B02G004300.1
TraesCS5A02G005300.1
TraesCS4A02G092500.1
TraesCS5D02G007200.1
TraesCS4D02G212600.1
TraesCS4B02G211800.1
TraesCS3D02G173200.1
Domain Color
LRRNT_2 Family
LRR_8 Repeat
Pkinase Domain
0
200
400
600
800

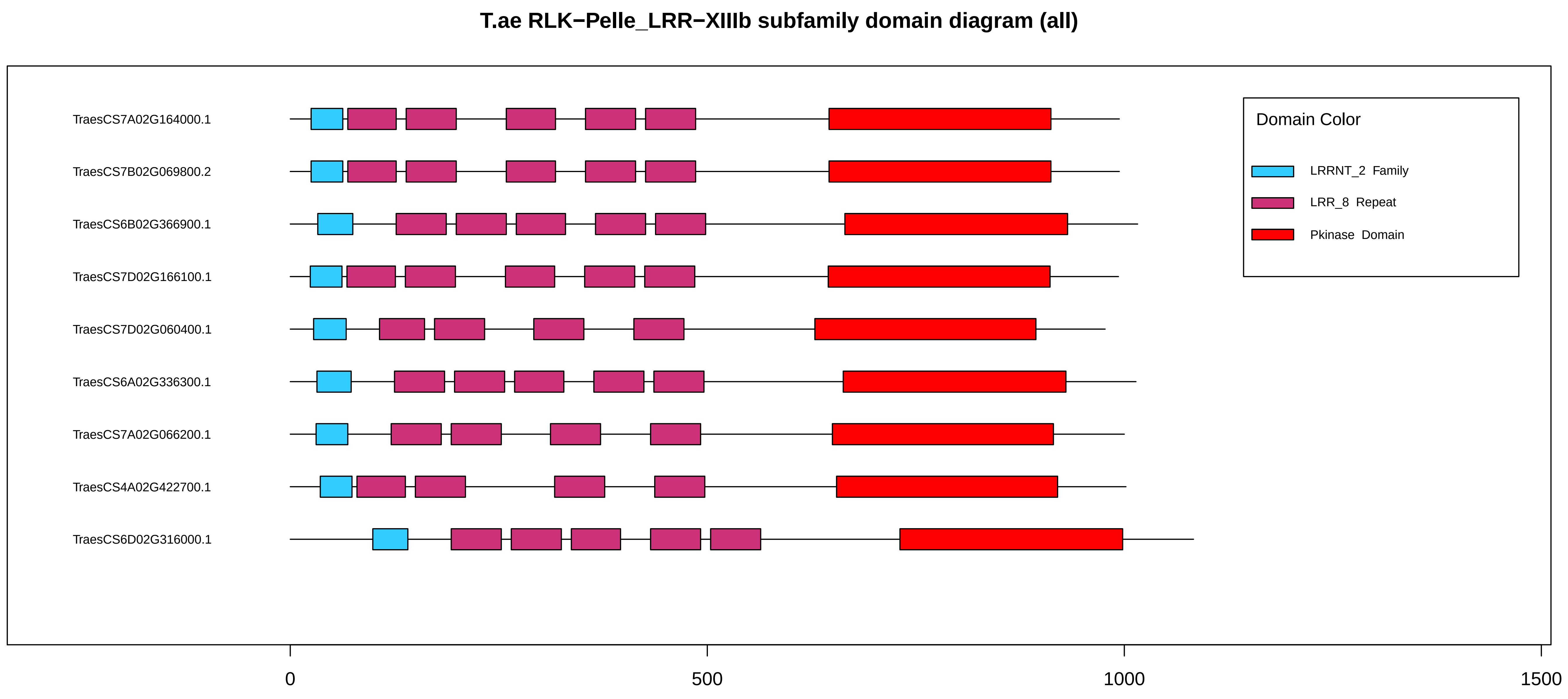

T.ae RLK−Pelle_LRR−XIIIb subfamily domain diagram (all)
TraesCS7A02G164000.1
TraesCS7B02G069800.2
TraesCS6B02G366900.1
TraesCS7D02G166100.1
TraesCS7D02G060400.1
TraesCS6A02G336300.1
TraesCS7A02G066200.1
TraesCS4A02G422700.1
TraesCS6D02G316000.1
Domain Color
LRRNT_2 Family
LRR_8 Repeat
Pkinase Domain
0
500
1000
1500

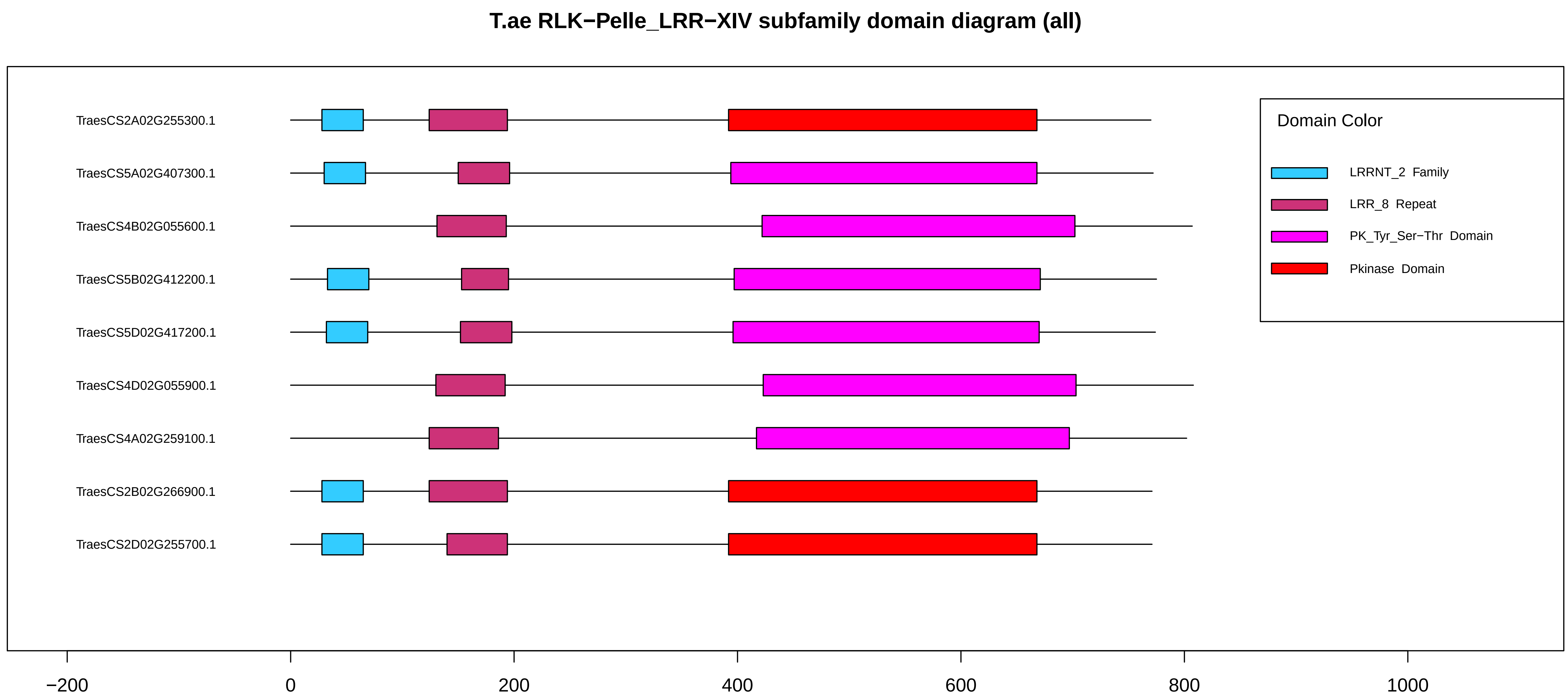
T.ae RLK−Pelle_LRR−XIV subfamily domain diagram (all)
TraesCS2A02G255300.1
TraesCS5A02G407300.1
TraesCS4B02G055600.1
TraesCS5B02G412200.1
TraesCS5D02G417200.1
TraesCS4D02G055900.1
TraesCS4A02G259100.1
TraesCS2B02G266900.1
TraesCS2D02G255700.1
Domain Color
LRRNT_2 Family
LRR_8 Repeat
PK_Tyr_Ser−Thr Domain
Pkinase Domain
−200
0
200
400
600
800
1000

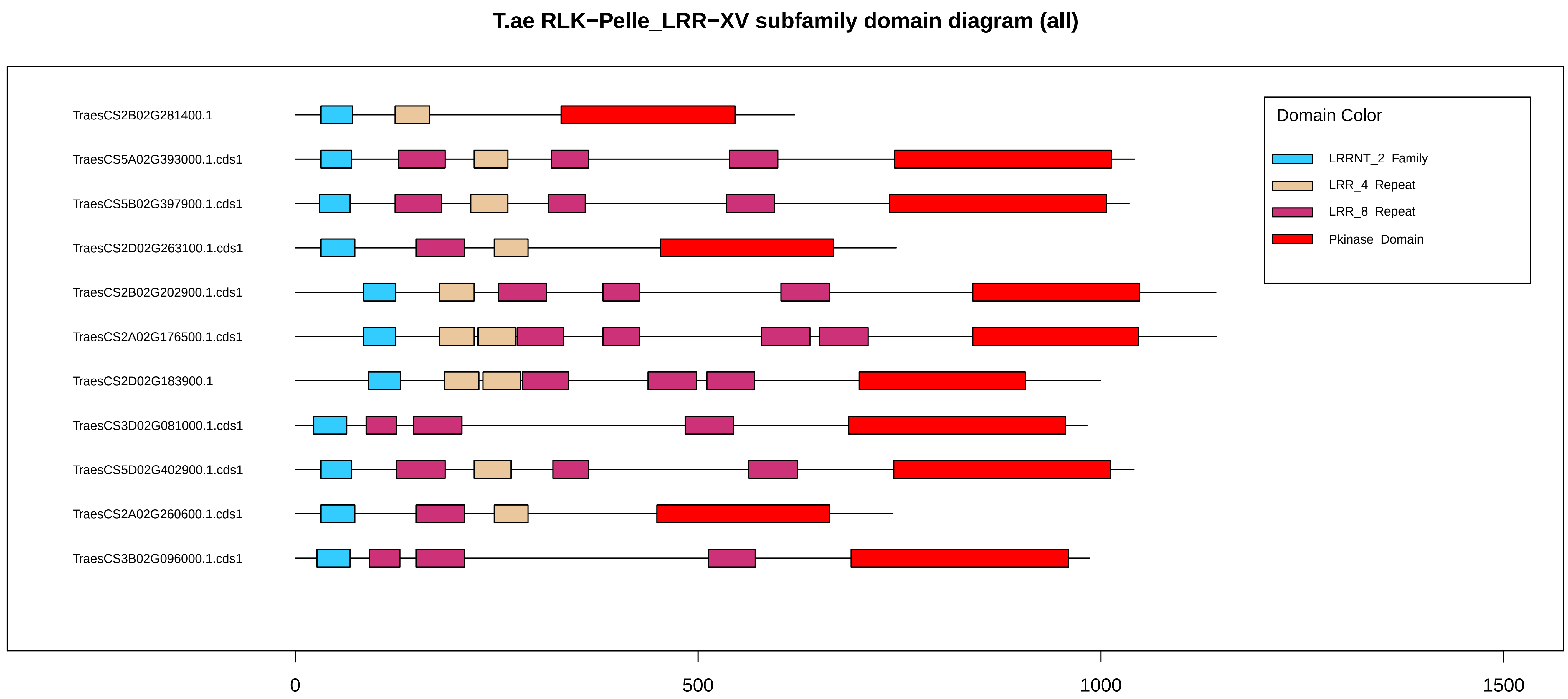

T.ae RLK−Pelle_LRR−XV subfamily domain diagram (all)
TraesCS2B02G281400.1
TraesCS5A02G393000.1.cds1
TraesCS5B02G397900.1.cds1
TraesCS2D02G263100.1.cds1
TraesCS2B02G202900.1.cds1
TraesCS2A02G176500.1.cds1
TraesCS2D02G183900.1
TraesCS3D02G081000.1.cds1
TraesCS5D02G402900.1.cds1
TraesCS2A02G260600.1.cds1
TraesCS3B02G096000.1.cds1
Domain Color
LRRNT_2 Family
LRR_4 Repeat
LRR_8 Repeat
Pkinase Domain
0
500
1000
1500

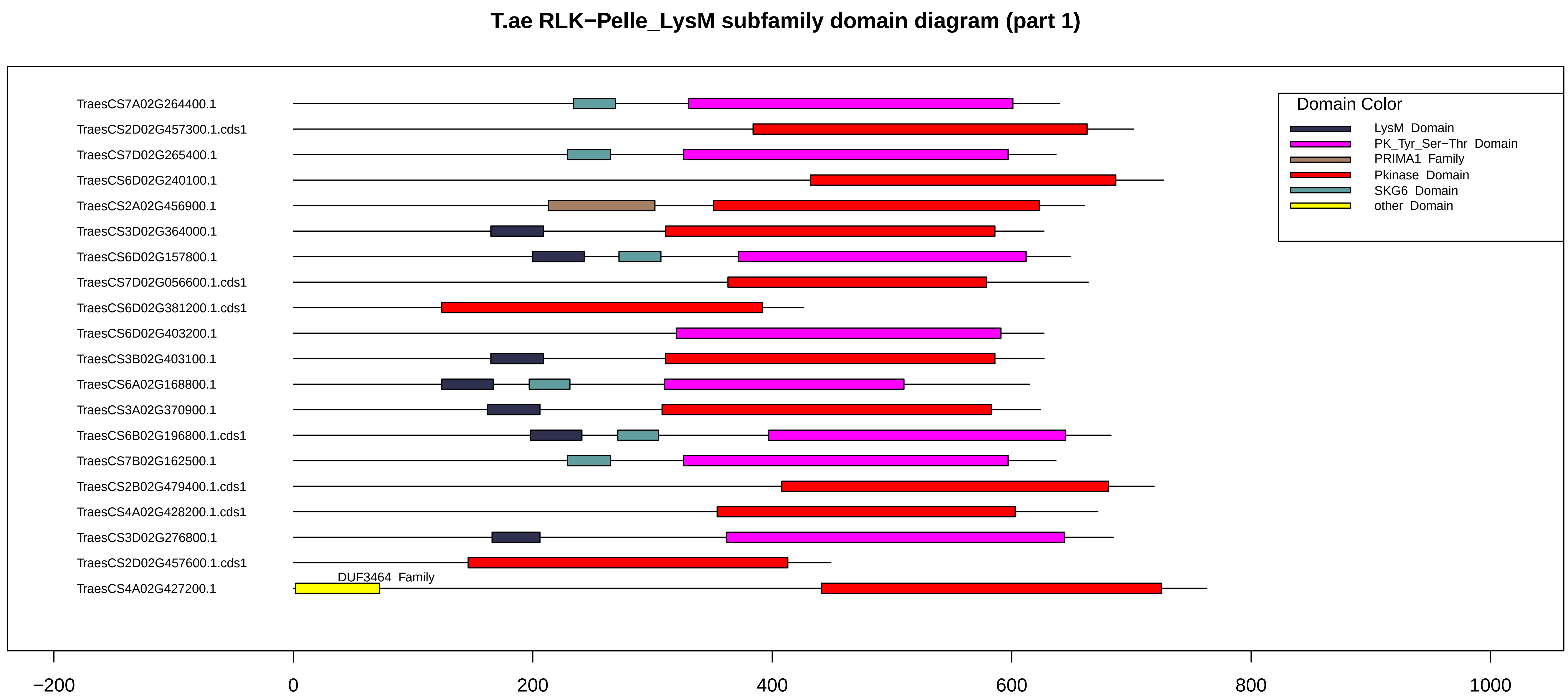
T.ae RLK−Pelle_LysM subfamily domain diagram (part 1)
TraesCS7A02G264400.1
TraesCS2D02G457300.1.cds1
TraesCS7D02G265400.1
TraesCS6D02G240100.1
TraesCS2A02G456900.1
TraesCS3D02G364000.1
TraesCS6D02G157800.1
TraesCS7D02G056600.1.cds1
TraesCS6D02G381200.1.cds1
TraesCS6D02G403200.1
TraesCS3B02G403100.1
TraesCS6A02G168800.1
TraesCS3A02G370900.1
TraesCS6B02G196800.1.cds1
TraesCS7B02G162500.1
TraesCS2B02G479400.1.cds1
TraesCS4A02G428200.1.cds1
TraesCS3D02G276800.1
TraesCS2D02G457600.1.cds1
TraesCS4A02G427200.1
DUF3464 Family
Domain Color
LysM Domain
PK_Tyr_Ser−Thr Domain
PRIMA1 Family
Pkinase Domain
SKG6 Domain
other Domain
−200
0
200
400
600
800
1000

T.ae RLK−Pelle_LysM subfamily domain diagram (part 2)

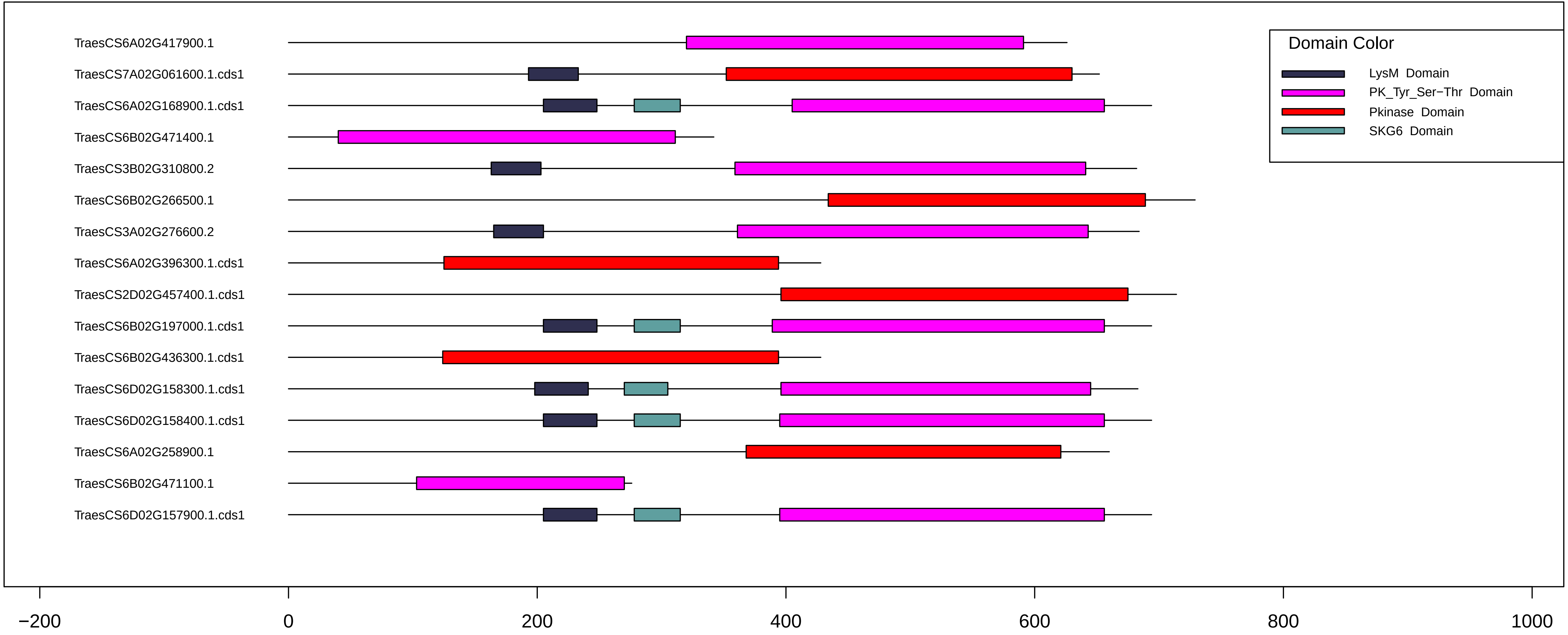

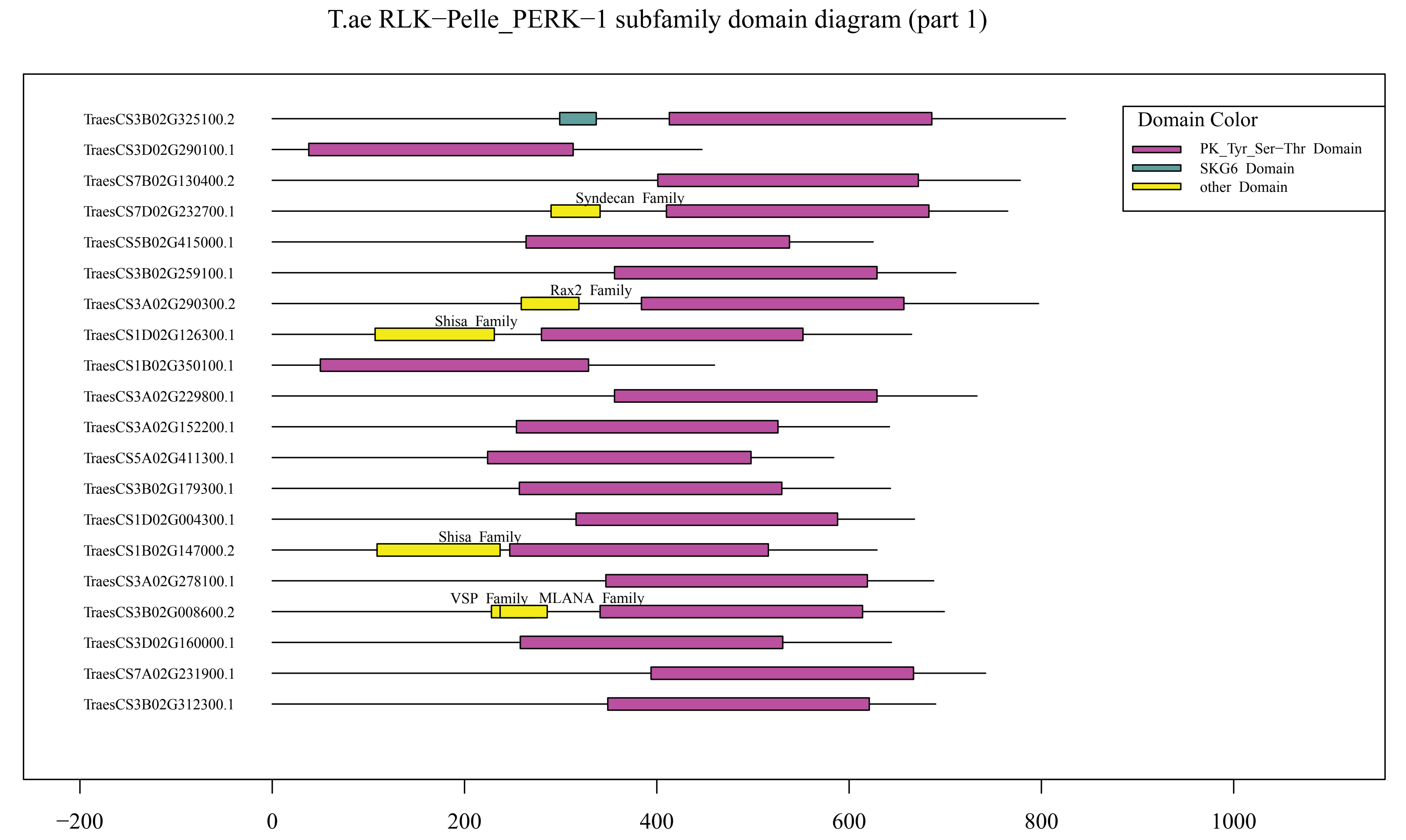
T.ae RLK−Pelle_PERK−1 subfamily domain diagram (part 1)
TraesCS3B02G325100.2
TraesCS3D02G290100.1
TraesCS7B02G130400.2
TraesCS7D02G232700.1
Syndecan Family
TraesCS5B02G415000.1
TraesCS3B02G259100.1
TraesCS3A02G290300.2
Rax2 Family
TraesCS1D02G126300.1
Shisa Family
TraesCS1B02G350100.1
TraesCS3A02G229800.1
TraesCS3A02G152200.1
TraesCS5A02G411300.1
TraesCS3B02G179300.1
TraesCS1D02G004300.1
TraesCS1B02G147000.2
Shisa Family
TraesCS3A02G278100.1
TraesCS3B02G008600.2
VSP Family
MLANA Family
TraesCS3D02G160000.1
TraesCS7A02G231900.1
TraesCS3B02G312300.1
Domain Color
PK_Tyr_Ser−Thr Domain
SKG6 Domain
other Domain
−200
0
200
400
600
800
1000

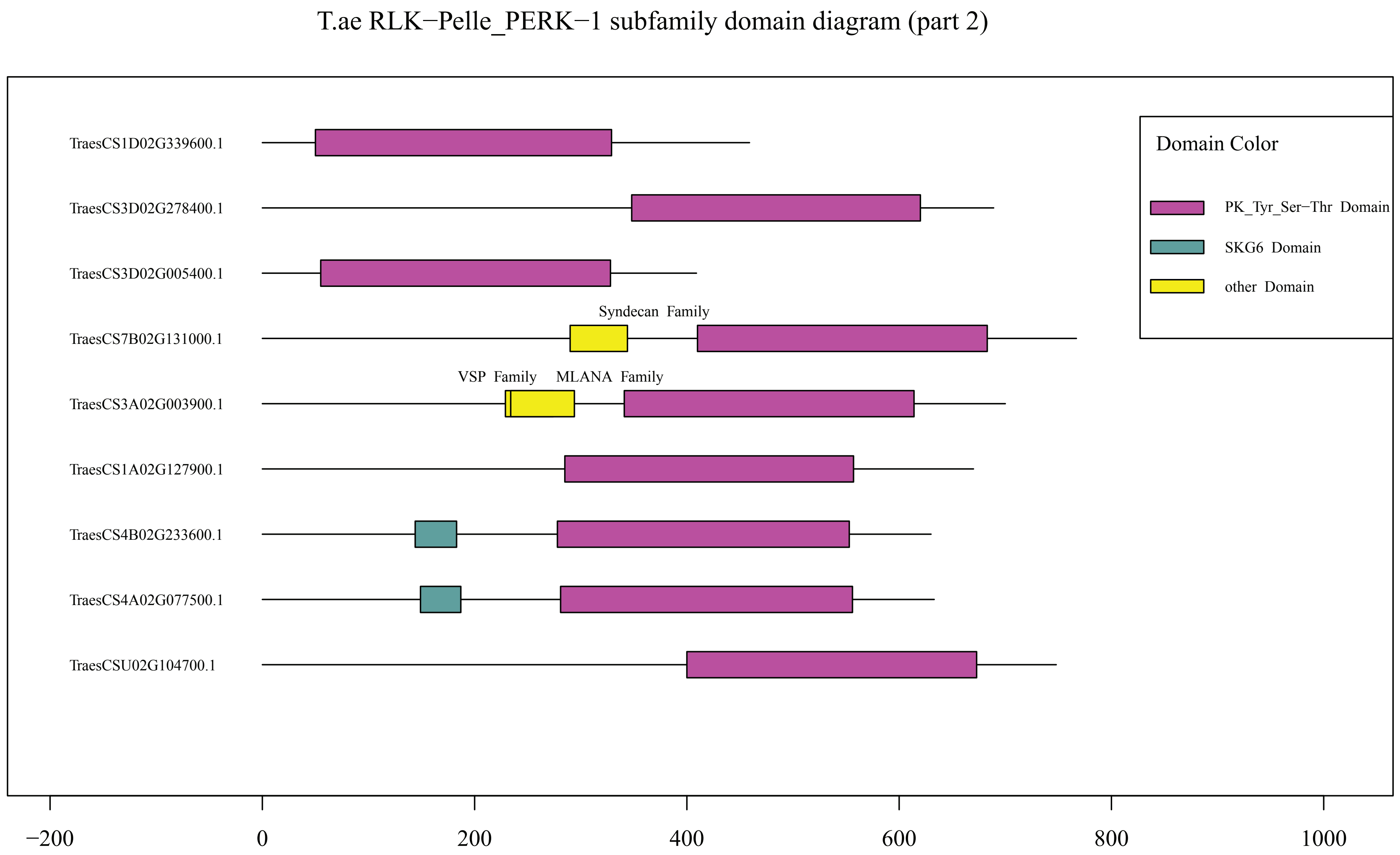
T.ae RLK−Pelle_PERK−1 subfamily domain diagram (part 2)
TraesCS1D02G339600.1
TraesCS3D02G278400.1
TraesCS3D02G005400.1
TraesCS7B02G131000.1
TraesCS3A02G003900.1
TraesCS1A02G127900.1
TraesCS4B02G233600.1
TraesCS4A02G077500.1
TraesCSU02G104700.1
Syndecan Family
VSP Family
MLANA Family
Domain Color
PK_Tyr_Ser−Thr Domain
SKG6 Domain
other Domain
−200
0
200
400
600
800
1000

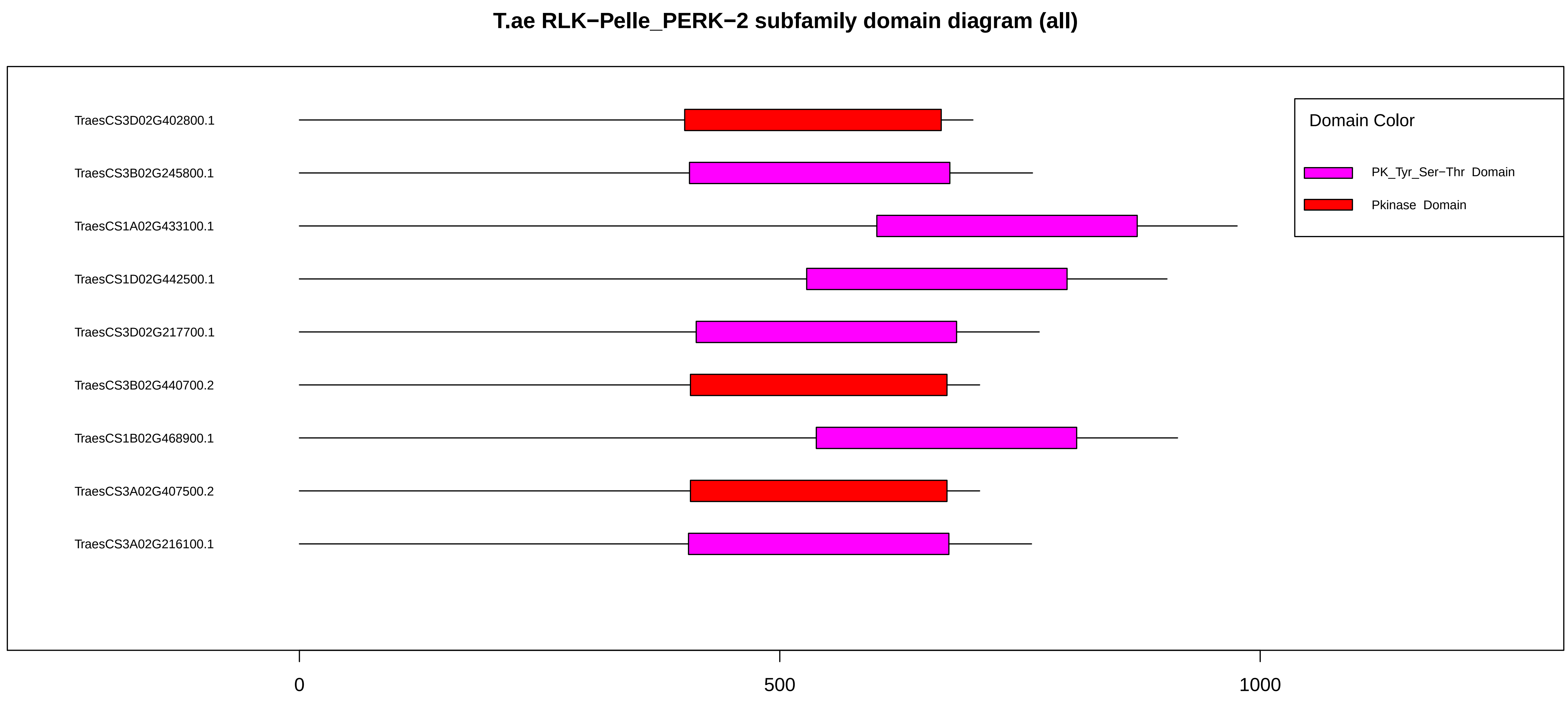
T.ae RLK−Pelle_PERK−2 subfamily domain diagram (all)
TraesCS3D02G402800.1
TraesCS3B02G245800.1
TraesCS1A02G433100.1
TraesCS1D02G442500.1
TraesCS3D02G217700.1
TraesCS3B02G440700.2
TraesCS1B02G468900.1
TraesCS3A02G407500.2
TraesCS3A02G216100.1
Domain Color
PK_Tyr_Ser−Thr Domain
Pkinase Domain
0
500
1000

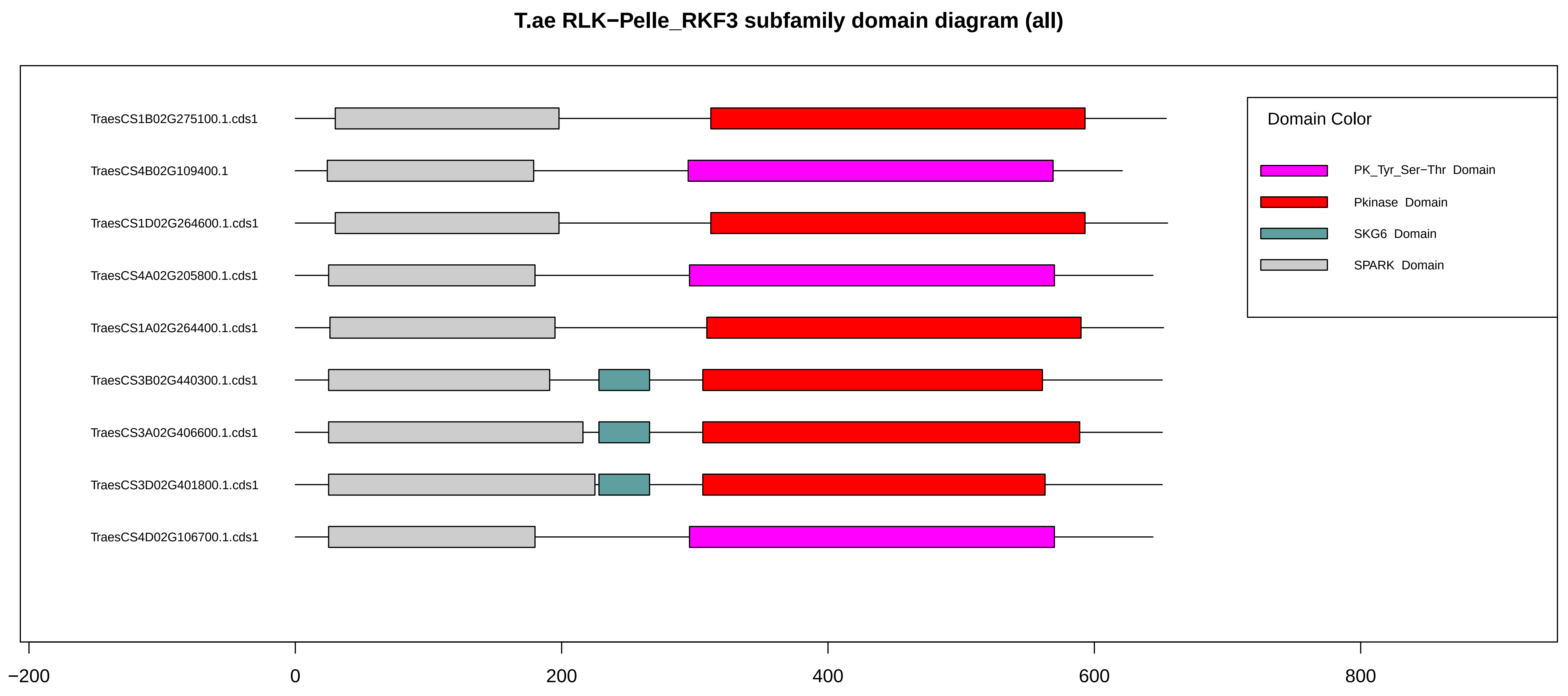

T.ae RLK−Pelle_RKF3 subfamily domain diagram (all)
TraesCS1B02G275100.1.cds1
TraesCS4B02G109400.1
TraesCS1D02G264600.1.cds1
TraesCS4A02G205800.1.cds1
TraesCS1A02G264400.1.cds1
TraesCS3B02G440300.1.cds1
TraesCS3A02G406600.1.cds1
TraesCS3D02G401800.1.cds1
TraesCS4D02G106700.1.cds1
Domain Color
PK_Tyr_Ser−Thr Domain
Pkinase Domain
SKG6 Domain
SPARK Domain
−200
0
200
400
600
800

T.ae RLK−Pelle_RLCK−II subfamily domain diagram (all)

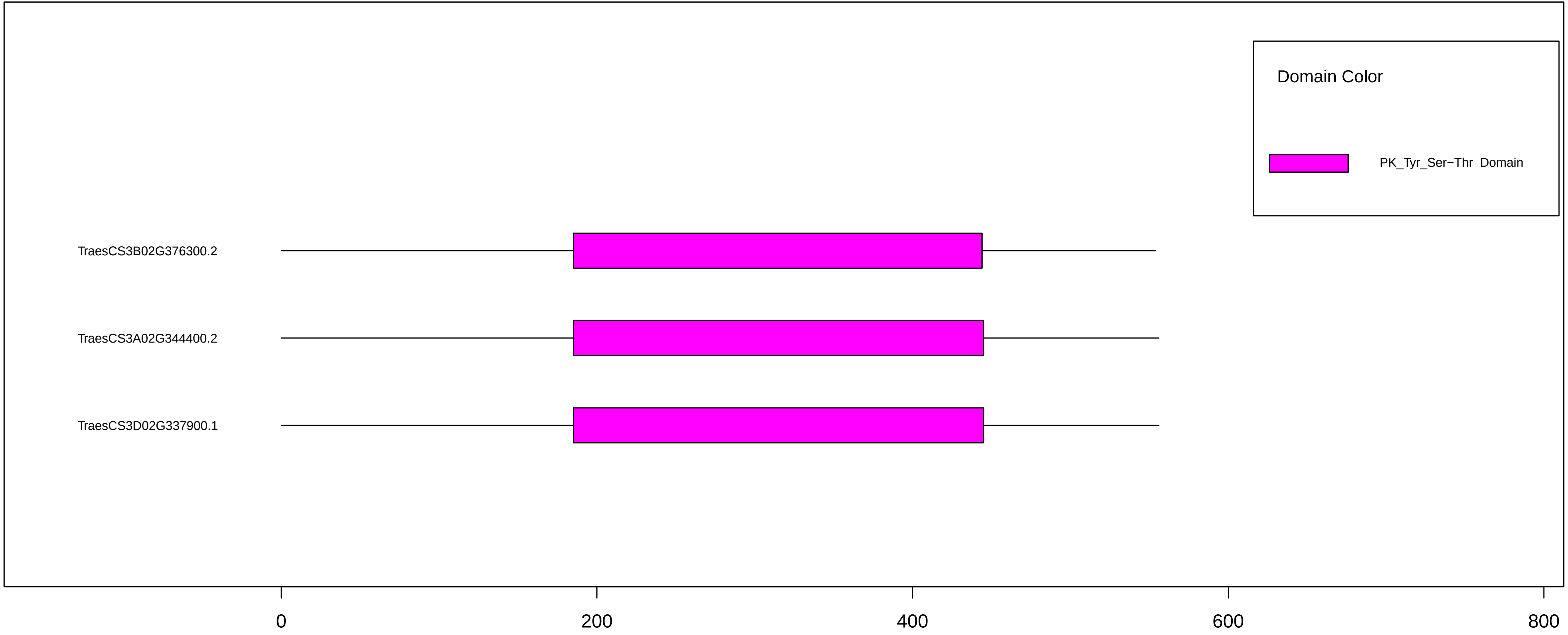

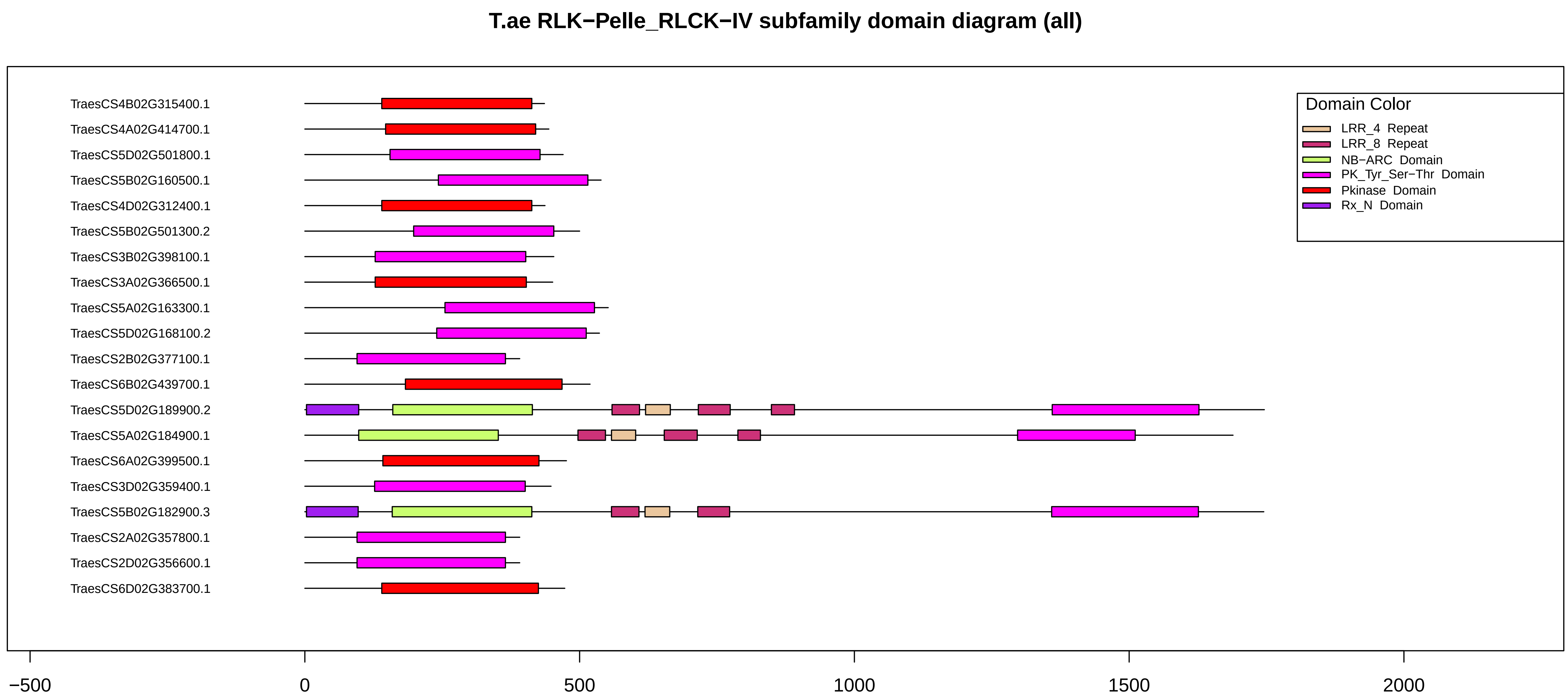
T.ae RLK−Pelle_RLCK−IV subfamily domain diagram (all)
TraesCS4B02G315400.1
TraesCS4A02G414700.1
TraesCS5D02G501800.1
TraesCS5B02G160500.1
TraesCS4D02G312400.1
TraesCS5B02G501300.2
TraesCS3B02G398100.1
TraesCS3A02G366500.1
TraesCS5A02G163300.1
TraesCS5D02G168100.2
TraesCS2B02G377100.1
TraesCS6B02G439700.1
TraesCS5D02G189900.2
TraesCS5A02G184900.1
TraesCS6A02G399500.1
TraesCS3D02G359400.1
TraesCS5B02G182900.3
TraesCS2A02G357800.1
TraesCS2D02G356600.1
TraesCS6D02G383700.1
Domain Color
LRR_4 Repeat
LRR_8 Repeat
NB−ARC Domain
PK_Tyr_Ser−Thr Domain
Pkinase Domain
Rx_N Domain
−500
0
500
1000
1500
2000

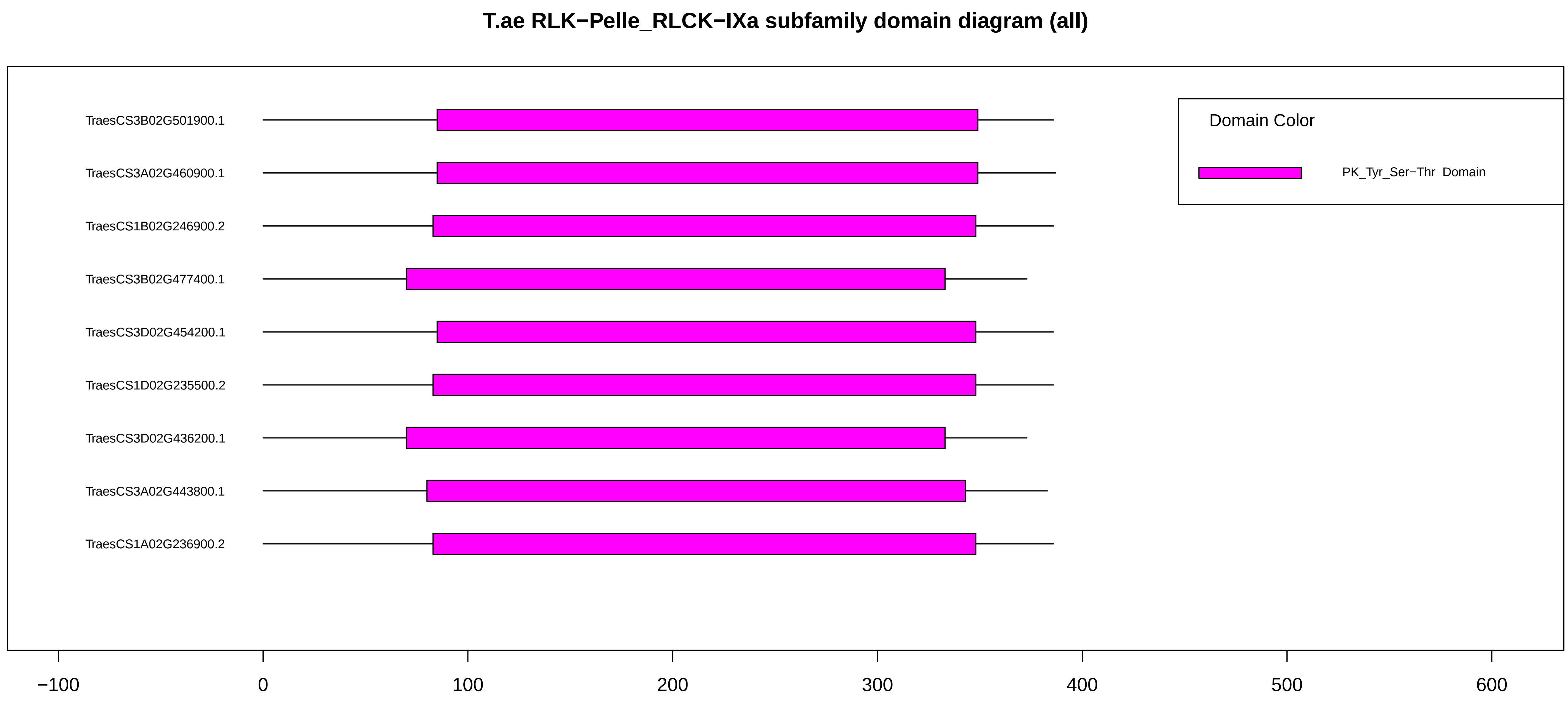
T.ae RLK−Pelle_RLCK−IXa subfamily domain diagram (all)
TraesCS3B02G501900.1
TraesCS3A02G460900.1
TraesCS1B02G246900.2
TraesCS3B02G477400.1
TraesCS3D02G454200.1
TraesCS1D02G235500.2
TraesCS3D02G436200.1
TraesCS3A02G443800.1
TraesCS1A02G236900.2
Domain Color
PK_Tyr_Ser−Thr Domain
−100
0
100
200
300
400
500
600

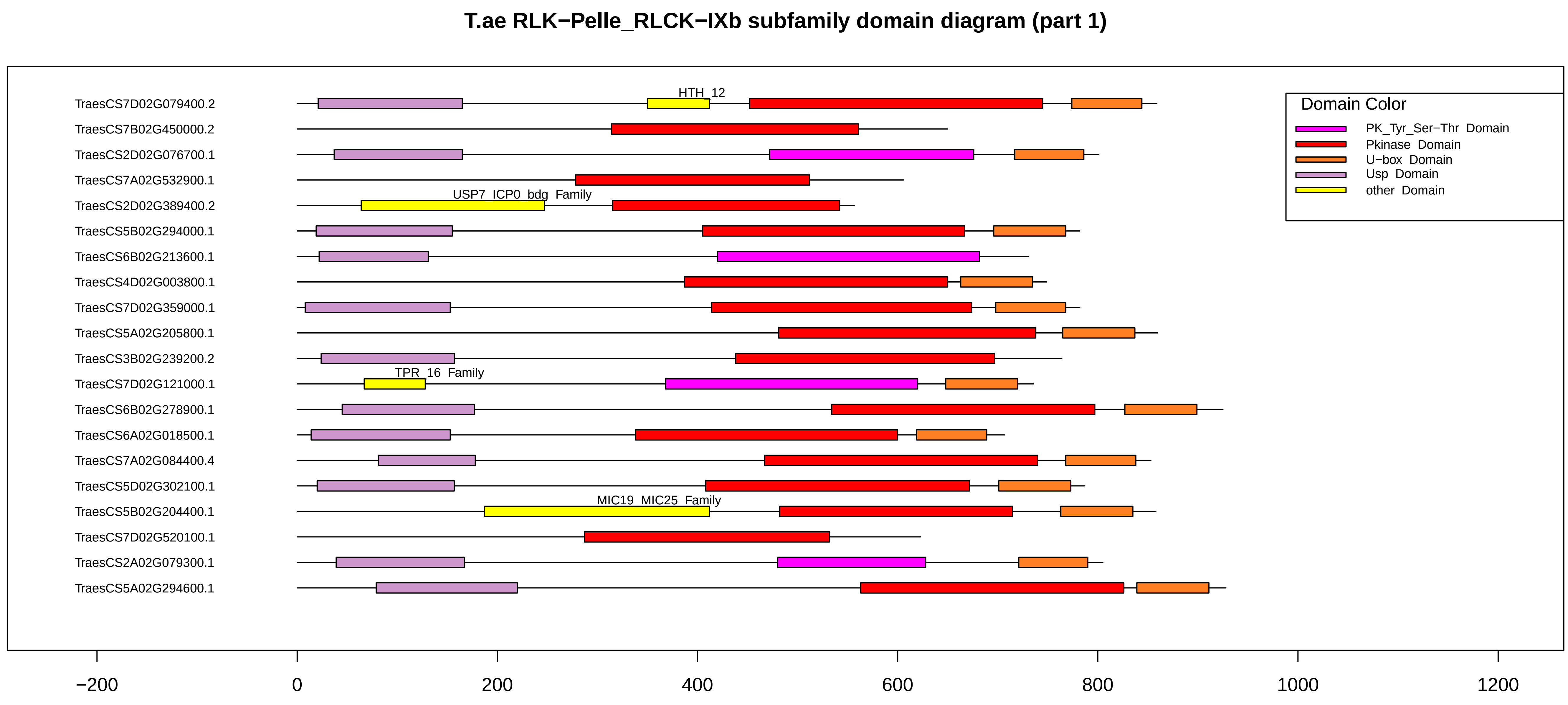
T.ae RLK−Pelle_RLCK−IXb subfamily domain diagram (part 1)
TraesCS7D02G079400.2
TraesCS7B02G450000.2
TraesCS2D02G076700.1
TraesCS7A02G532900.1
TraesCS2D02G389400.2
TraesCS5B02G294000.1
TraesCS6B02G213600.1
TraesCS4D02G003800.1
TraesCS7D02G359000.1
TraesCS5A02G205800.1
TraesCS3B02G239200.2
TraesCS7D02G121000.1
TraesCS6B02G278900.1
TraesCS6A02G018500.1
TraesCS7A02G084400.4
TraesCS5D02G302100.1
TraesCS5B02G204400.1
TraesCS7D02G520100.1
TraesCS2A02G079300.1
TraesCS5A02G294600.1
HTH_12
USP7_ICP0_bdg_Family
TPR_16_Family
MIC19_MIC25_Family
Domain Color
PK_Tyr_Ser−Thr Domain
Pkinase Domain
U−box Domain
Usp Domain
other Domain
−200
0
200
400
600
800
1000
1200

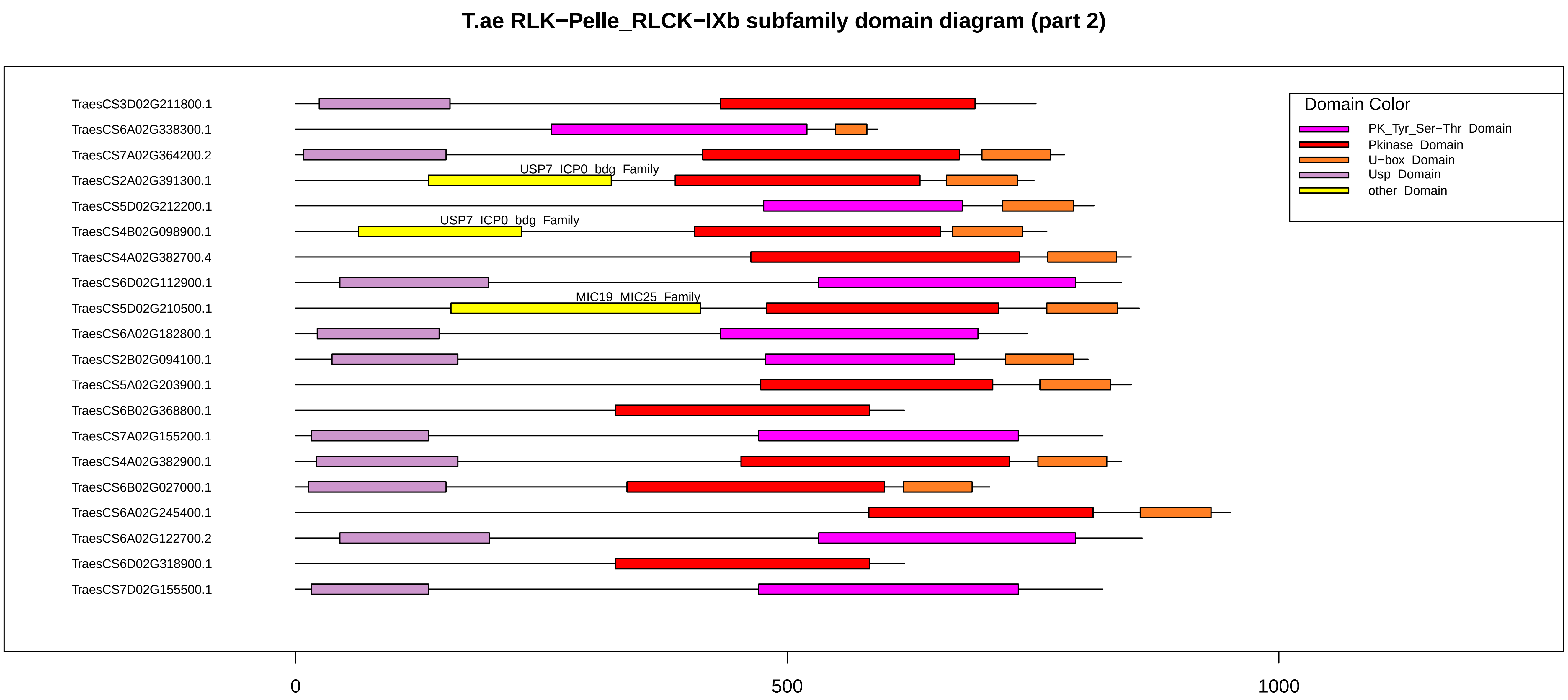
T.ae RLK−Pelle_RLCK−IXb subfamily domain diagram (part 2)
TraesCS3D02G211800.1
TraesCS6A02G338300.1
TraesCS7A02G364200.2
TraesCS2A02G391300.1
USP7_ICP0_bdg_Family
TraesCS5D02G212200.1
TraesCS4B02G098900.1
USP7_ICP0_bdg_Family
TraesCS4A02G382700.4
TraesCS6D02G112900.1
TraesCS5D02G210500.1
MIC19_MIC25_Family
TraesCS6A02G182800.1
TraesCS2B02G094100.1
TraesCS5A02G203900.1
TraesCS6B02G368800.1
TraesCS7A02G155200.1
TraesCS4A02G382900.1
TraesCS6B02G027000.1
TraesCS6A02G245400.1
TraesCS6A02G122700.2
TraesCS6D02G318900.1
TraesCS7D02G155500.1
Domain Color
PK_Tyr_Ser−Thr Domain
Pkinase Domain
U−box Domain
Usp Domain
other Domain
0
500
1000

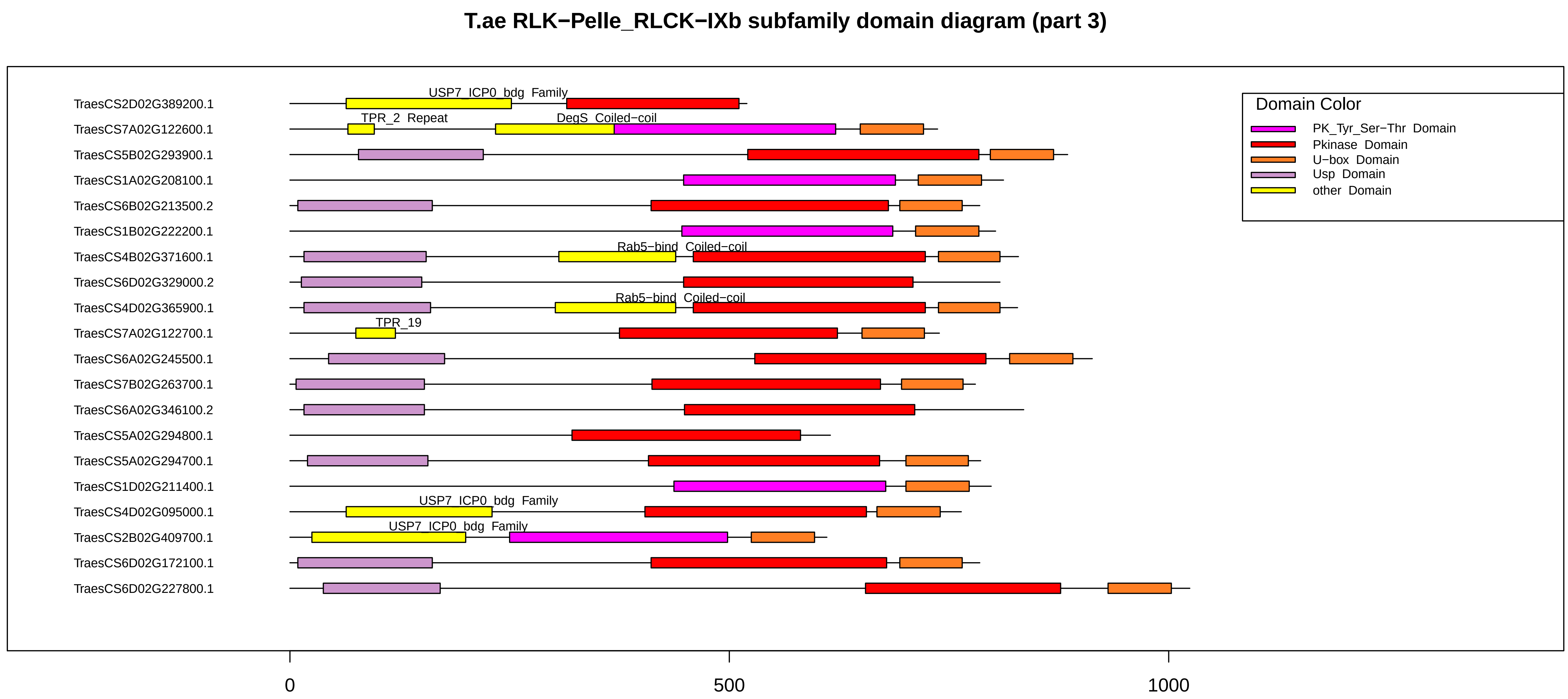
T.ae RLK−Pelle_RLCK−IXb subfamily domain diagram (part 3)
TraesCS2D02G389200.1
TraesCS7A02G122600.1
TraesCS5B02G293900.1
TraesCS1A02G208100.1
TraesCS6B02G213500.2
TraesCS1B02G222200.1
TraesCS4B02G371600.1
TraesCS6D02G329000.2
TraesCS4D02G365900.1
TraesCS7A02G122700.1
TraesCS6A02G245500.1
TraesCS7B02G263700.1
TraesCS6A02G346100.2
TraesCS5A02G294800.1
TraesCS5A02G294700.1
TraesCS1D02G211400.1
TraesCS4D02G095000.1
TraesCS2B02G409700.1
TraesCS6D02G172100.1
TraesCS6D02G227800.1
USP7_ICP0_bdg Family
TPR_2 Repeat
DegS Coiled−coil
Rab5−bind Coiled−coil
Rab5−bind Coiled−coil
TPR_19
USP7_ICP0_bdg Family
USP7_ICP0_bdg Family
Domain Color
PK_Tyr_Ser−Thr Domain
Pkinase Domain
U−box Domain
Usp Domain
other Domain
0
500
1000

T.ae RLK−Pelle_RLCK−IXb subfamily domain diagram (part 4)

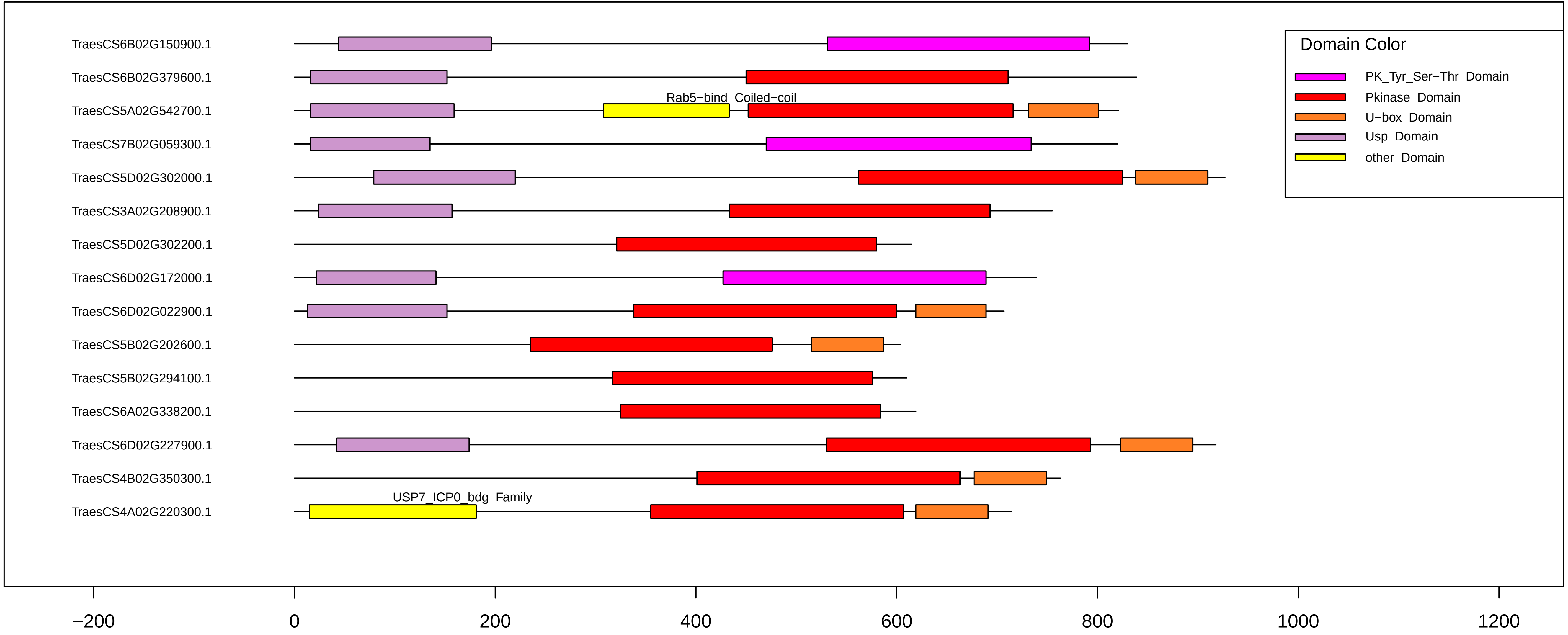

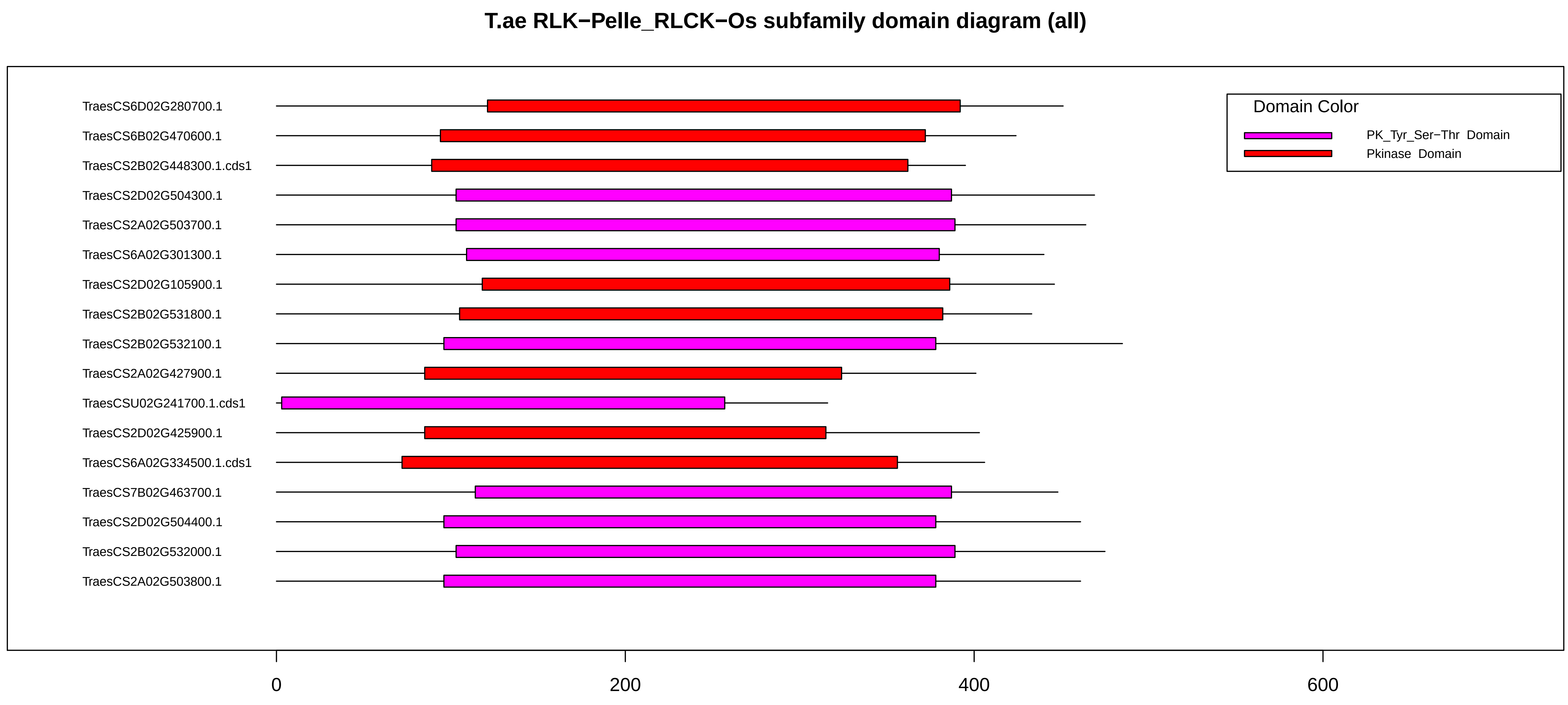

T.ae RLK−Pelle_RLCK−Os subfamily domain diagram (all)
TraesCS6D02G280700.1
TraesCS6B02G470600.1
TraesCS2B02G448300.1.cds1
TraesCS2D02G504300.1
TraesCS2A02G503700.1
TraesCS6A02G301300.1
TraesCS2D02G105900.1
TraesCS2B02G531800.1
TraesCS2B02G532100.1
TraesCS2A02G427900.1
TraesCSU02G241700.1.cds1
TraesCS2D02G425900.1
TraesCS6A02G334500.1.cds1
TraesCS7B02G463700.1
TraesCS2D02G504400.1
TraesCS2B02G532000.1
TraesCS2A02G503800.1
Domain Color
PK_Tyr_Ser−Thr Domain
Pkinase Domain
0
200
400
600

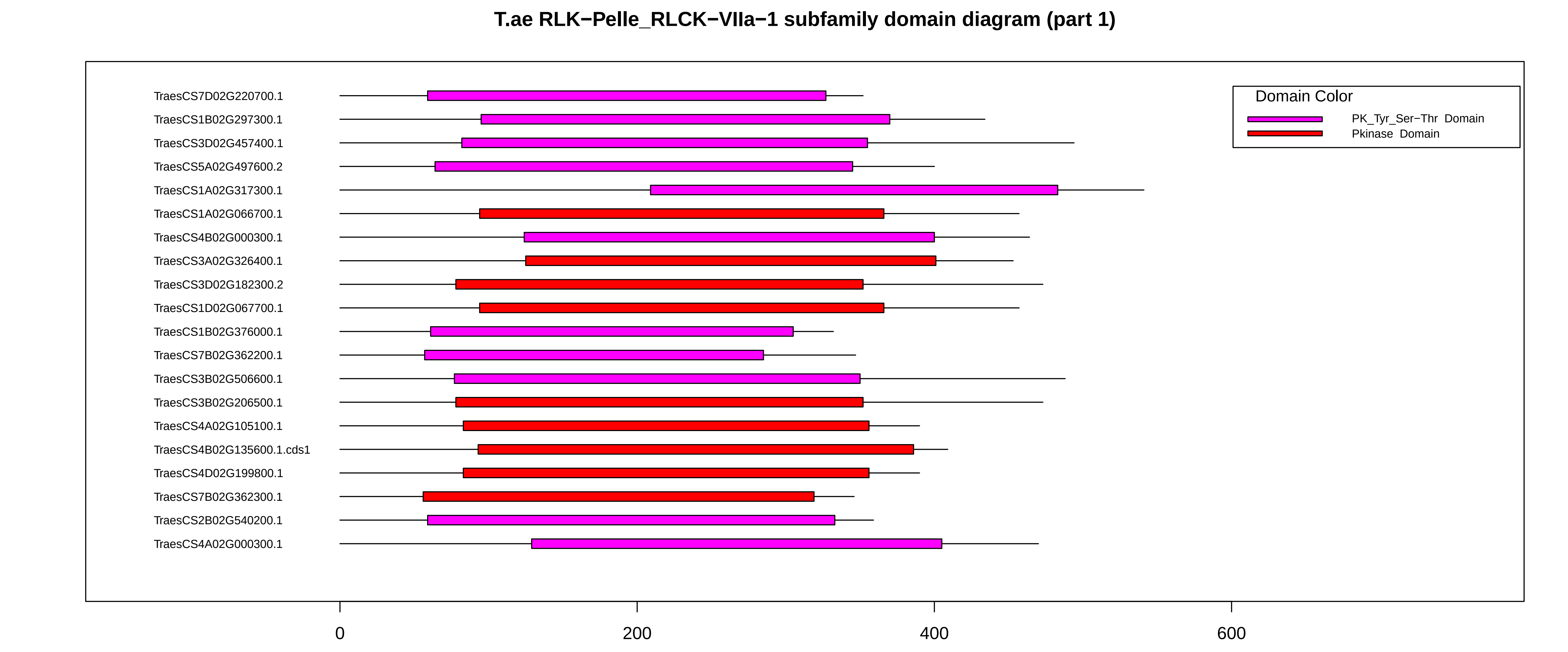
T.ae RLK−Pelle_RLCK−VIIa−1 subfamily domain diagram (part 1)
TraesCS7D02G220700.1
TraesCS1B02G297300.1
TraesCS3D02G457400.1
TraesCS5A02G497600.2
TraesCS1A02G317300.1
TraesCS1A02G066700.1
TraesCS4B02G000300.1
TraesCS3A02G326400.1
TraesCS3D02G182300.2
TraesCS1D02G067700.1
TraesCS1B02G376000.1
TraesCS7B02G362200.1
TraesCS3B02G506600.1
TraesCS3B02G206500.1
TraesCS4A02G105100.1
TraesCS4B02G135600.1.cds1
TraesCS4D02G199800.1
TraesCS7B02G362300.1
TraesCS2B02G540200.1
TraesCS4A02G000300.1
Domain Color
PK_Tyr_Ser−Thr Domain
Pkinase Domain
0
200
400
600

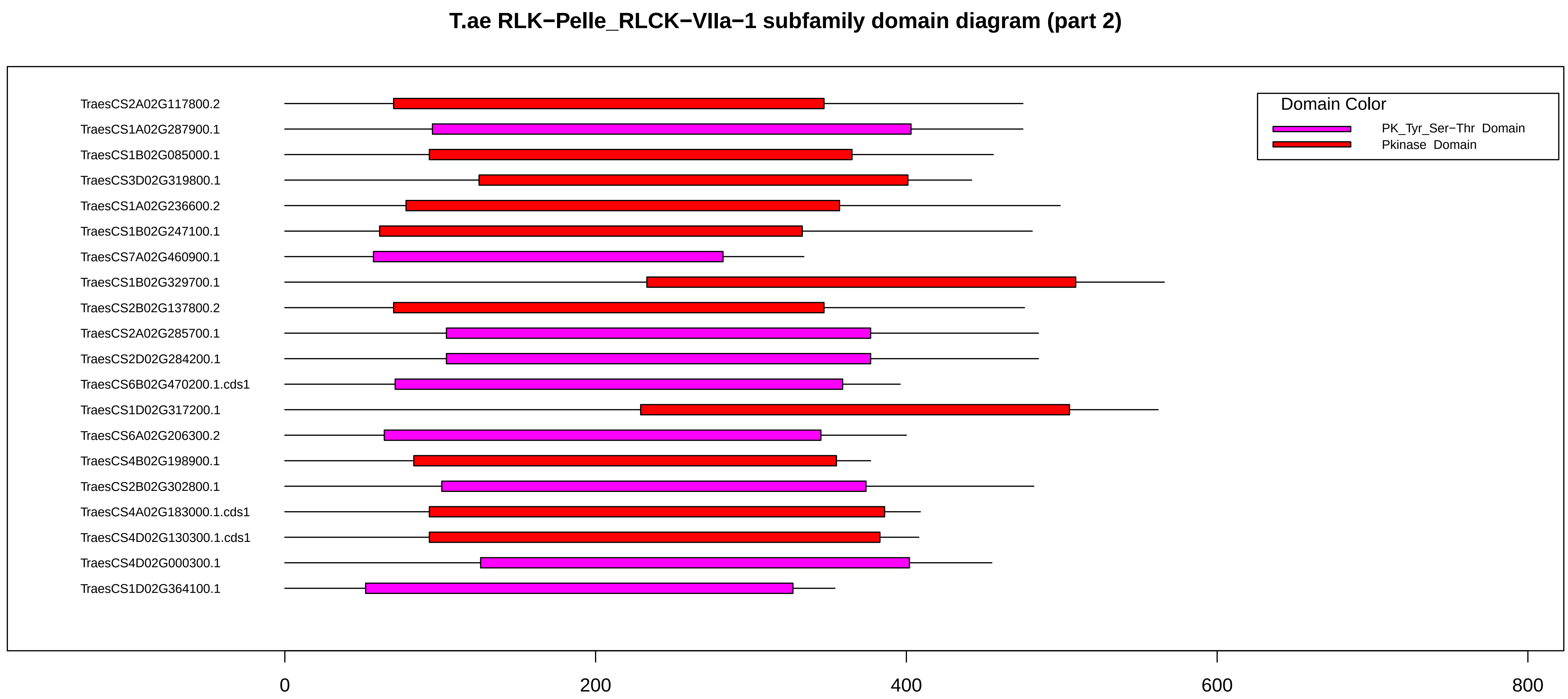

T.ae RLK−Pelle_RLCK−VIIa−1 subfamily domain diagram (part 2)
TraesCS2A02G117800.2
TraesCS1A02G287900.1
TraesCS1B02G085000.1
TraesCS3D02G319800.1
TraesCS1A02G236600.2
TraesCS1B02G247100.1
TraesCS7A02G460900.1
TraesCS1B02G329700.1
TraesCS2B02G137800.2
TraesCS2A02G285700.1
TraesCS2D02G284200.1
TraesCS6B02G470200.1.cds1
TraesCS1D02G317200.1
TraesCS6A02G206300.2
TraesCS4B02G198900.1
TraesCS2B02G302800.1
TraesCS4A02G183000.1.cds1
TraesCS4D02G130300.1.cds1
TraesCS4D02G000300.1
TraesCS1D02G364100.1
Domain Color
PK_Tyr_Ser−Thr Domain
Pkinase Domain
0
200
400
600
800

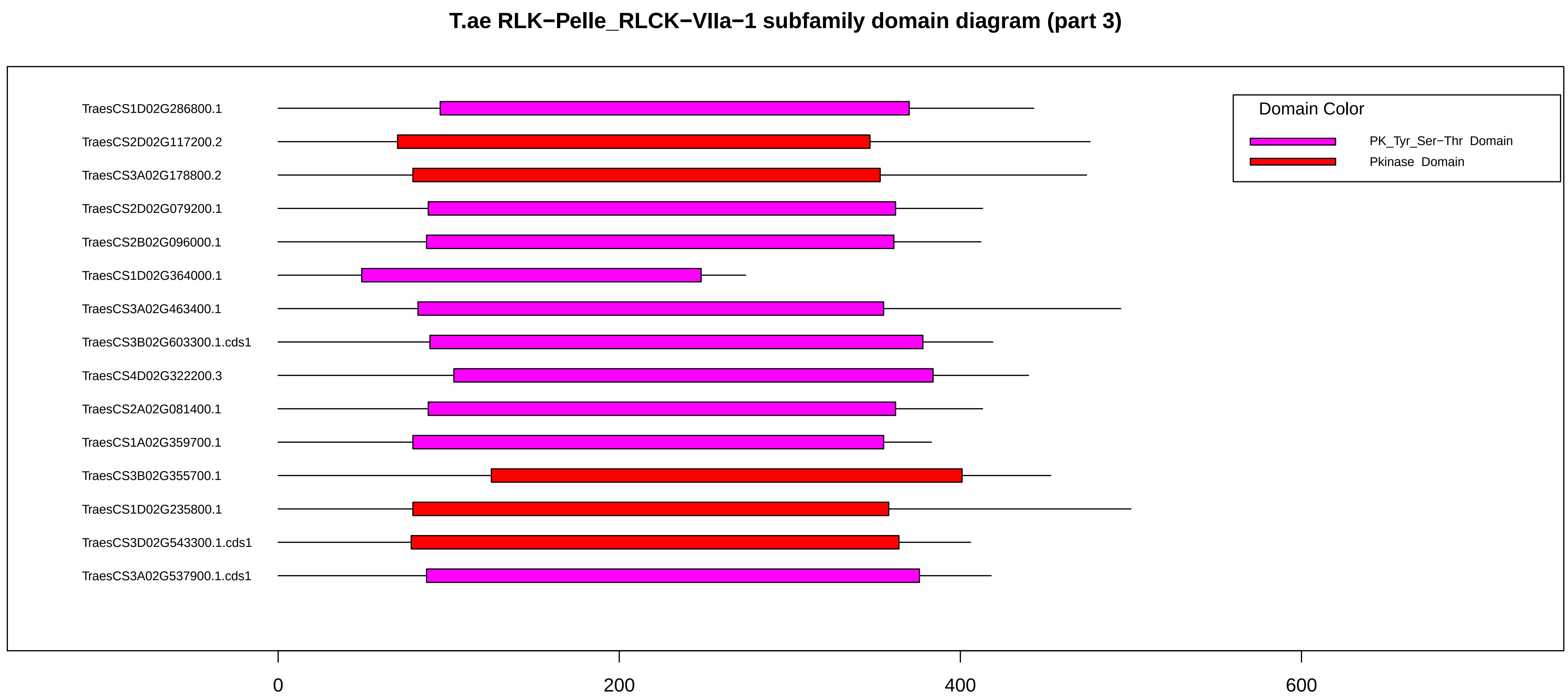

T.ae RLK−Pelle_RLCK−VIIa−1 subfamily domain diagram (part 3)
TraesCS1D02G286800.1
TraesCS2D02G117200.2
TraesCS3A02G178800.2
TraesCS2D02G079200.1
TraesCS2B02G096000.1
TraesCS1D02G364000.1
TraesCS3A02G463400.1
TraesCS3B02G603300.1.cds1
TraesCS4D02G322200.3
TraesCS2A02G081400.1
TraesCS1A02G359700.1
TraesCS3B02G355700.1
TraesCS1D02G235800.1
TraesCS3D02G543300.1.cds1
TraesCS3A02G537900.1.cds1
Domain Color
PK_Tyr_Ser−Thr Domain
Pkinase Domain
0
200
400
600

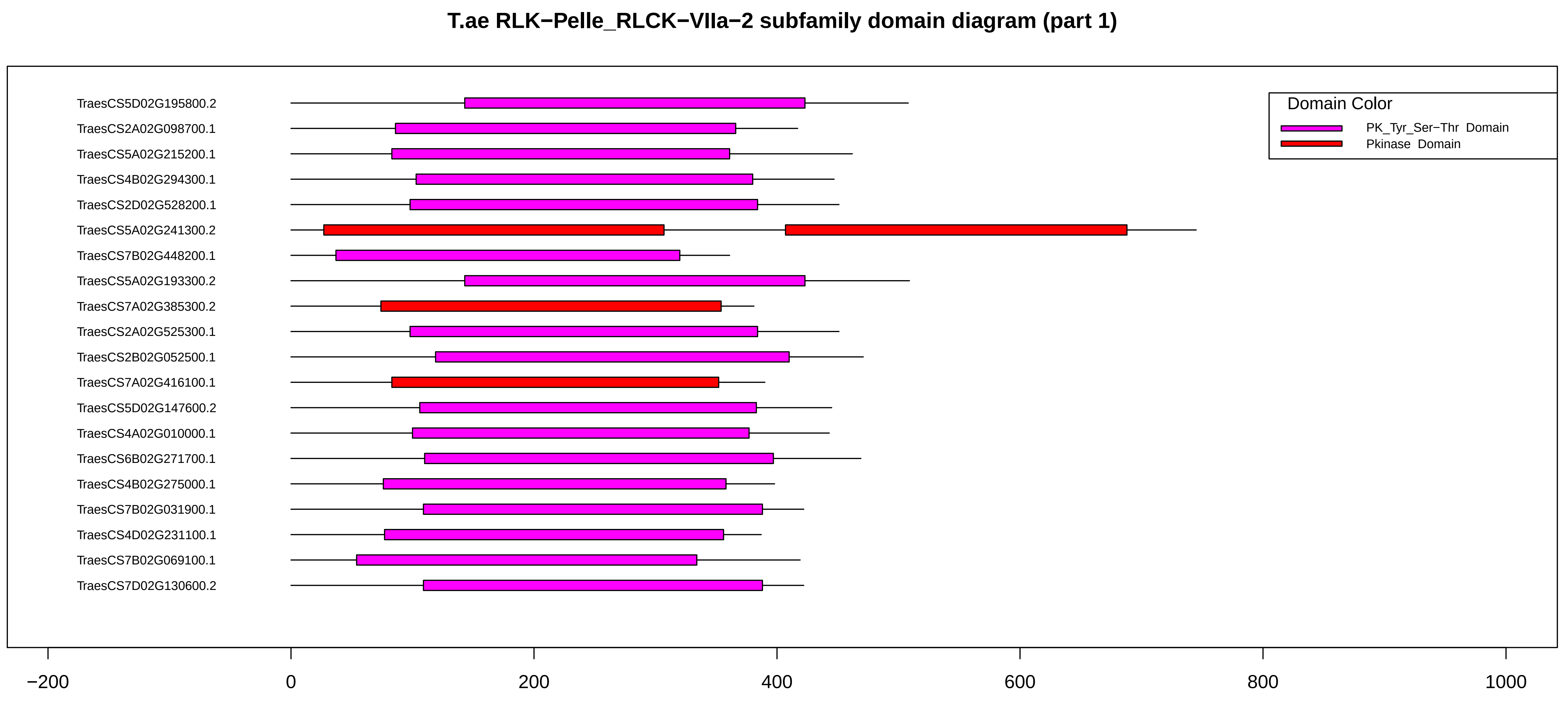

T.ae RLK−Pelle_RLCK−VIIa−2 subfamily domain diagram (part 1)
TraesCS5D02G195800.2
TraesCS2A02G098700.1
TraesCS5A02G215200.1
TraesCS4B02G294300.1
TraesCS2D02G528200.1
TraesCS5A02G241300.2
TraesCS7B02G448200.1
TraesCS5A02G193300.2
TraesCS7A02G385300.2
TraesCS2A02G525300.1
TraesCS2B02G052500.1
TraesCS7A02G416100.1
TraesCS5D02G147600.2
TraesCS4A02G010000.1
TraesCS6B02G271700.1
TraesCS4B02G275000.1
TraesCS7B02G031900.1
TraesCS4D02G231100.1
TraesCS7B02G069100.1
TraesCS7D02G130600.2
Domain Color
PK_Tyr_Ser−Thr Domain
Pkinase Domain
−200
0
200
400
600
800
1000

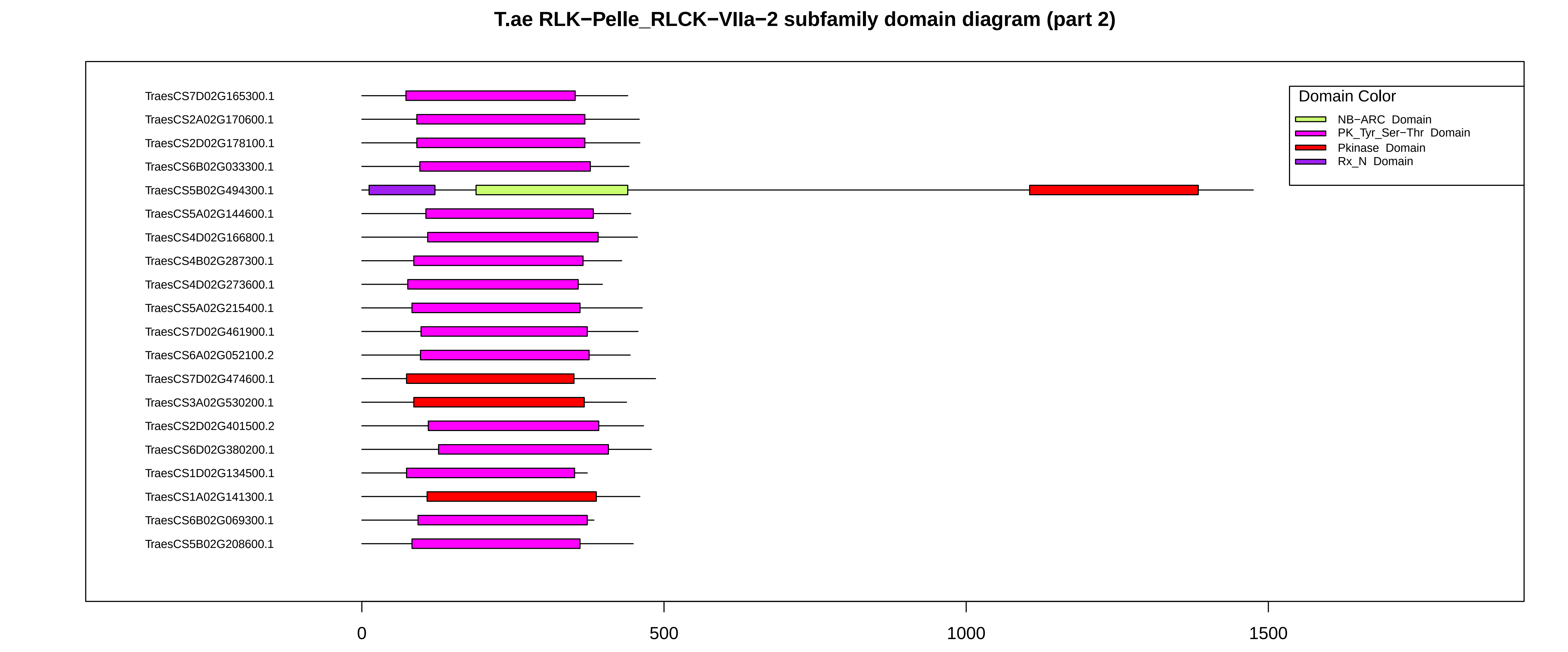
T.ae RLK−Pelle_RLCK−VIIa−2 subfamily domain diagram (part 2)
TraesCS7D02G165300.1
TraesCS2A02G170600.1
TraesCS2D02G178100.1
TraesCS6B02G033300.1
TraesCS5B02G494300.1
TraesCS5A02G144600.1
TraesCS4D02G166800.1
TraesCS4B02G287300.1
TraesCS4D02G273600.1
TraesCS5A02G215400.1
TraesCS7D02G461900.1
TraesCS6A02G052100.2
TraesCS7D02G474600.1
TraesCS3A02G530200.1
TraesCS2D02G401500.2
TraesCS6D02G380200.1
TraesCS1D02G134500.1
TraesCS1A02G141300.1
TraesCS6B02G069300.1
TraesCS5B02G208600.1
Domain Color
NB−ARC Domain
PK_Tyr_Ser−Thr Domain
Pkinase Domain
Rx_N Domain
0
500
1000
1500

T.ae RLK−Pelle_RLCK−VIIa−2 subfamily domain diagram (part 3)

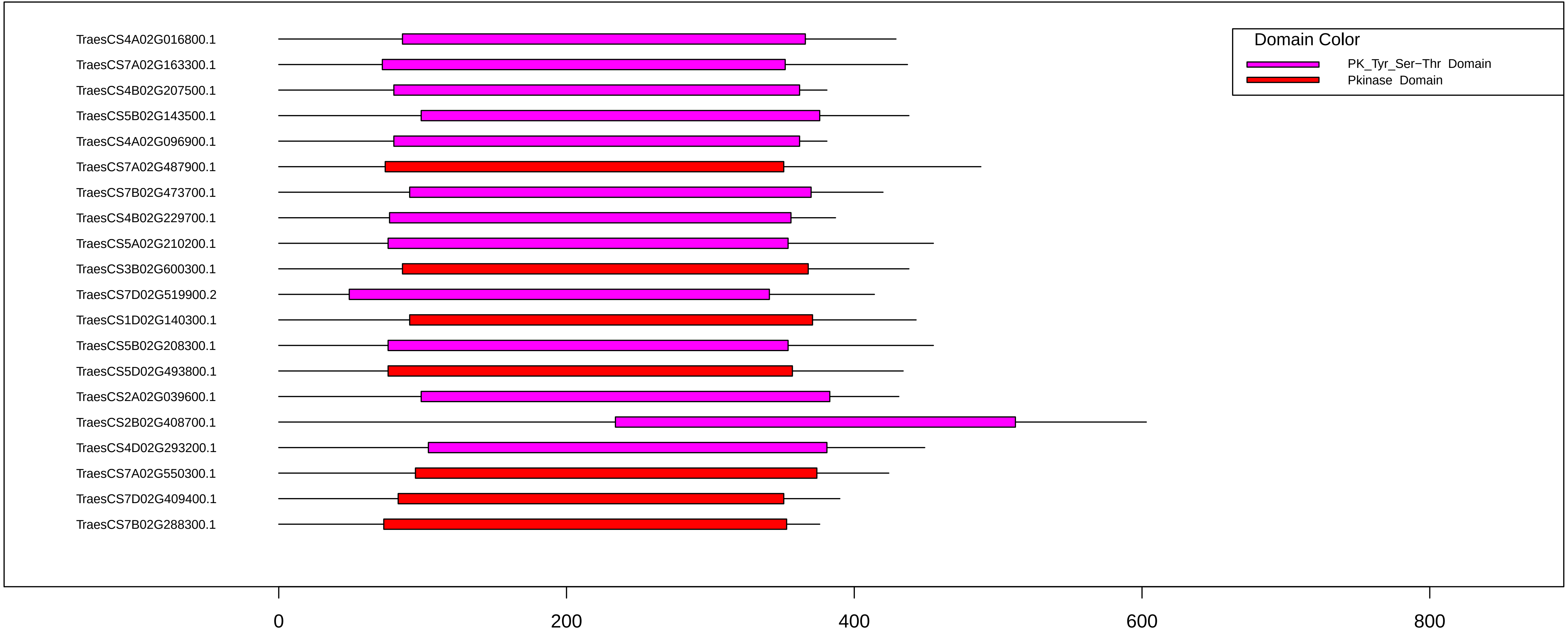

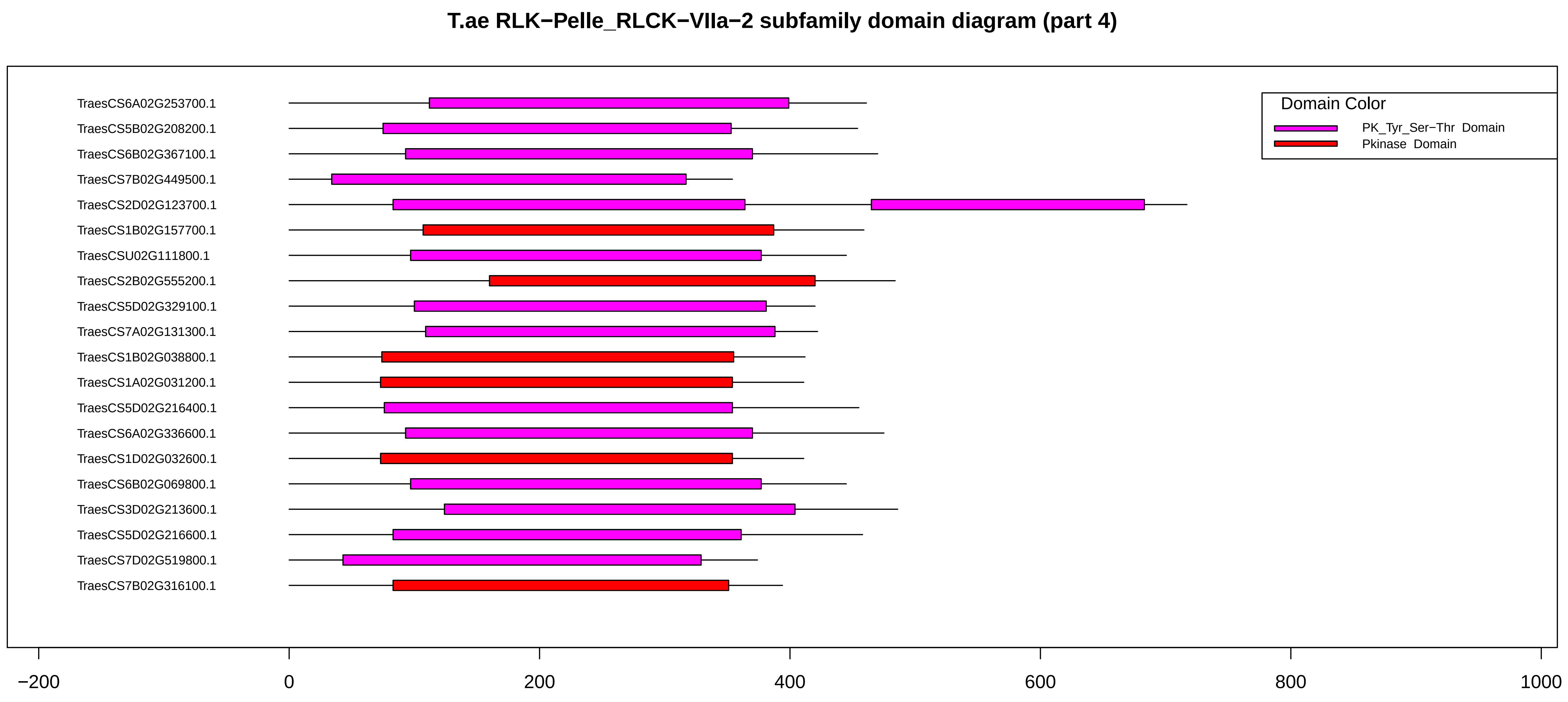
T.ae RLK−Pelle_RLCK−VIIa−2 subfamily domain diagram (part 4)
TraesCS6A02G253700.1
TraesCS5B02G208200.1
TraesCS6B02G367100.1
TraesCS7B02G449500.1
TraesCS2D02G123700.1
TraesCS1B02G157700.1
TraesCSU02G111800.1
TraesCS2B02G555200.1
TraesCS5D02G329100.1
TraesCS7A02G131300.1
TraesCS1B02G038800.1
TraesCS1A02G031200.1
TraesCS5D02G216400.1
TraesCS6A02G336600.1
TraesCS1D02G032600.1
TraesCS6B02G069800.1
TraesCS3D02G213600.1
TraesCS5D02G216600.1
TraesCS7D02G519800.1
TraesCS7B02G316100.1
Domain Color
PK_Tyr_Ser−Thr Domain
Pkinase Domain
−200
0
200
400
600
800
1000

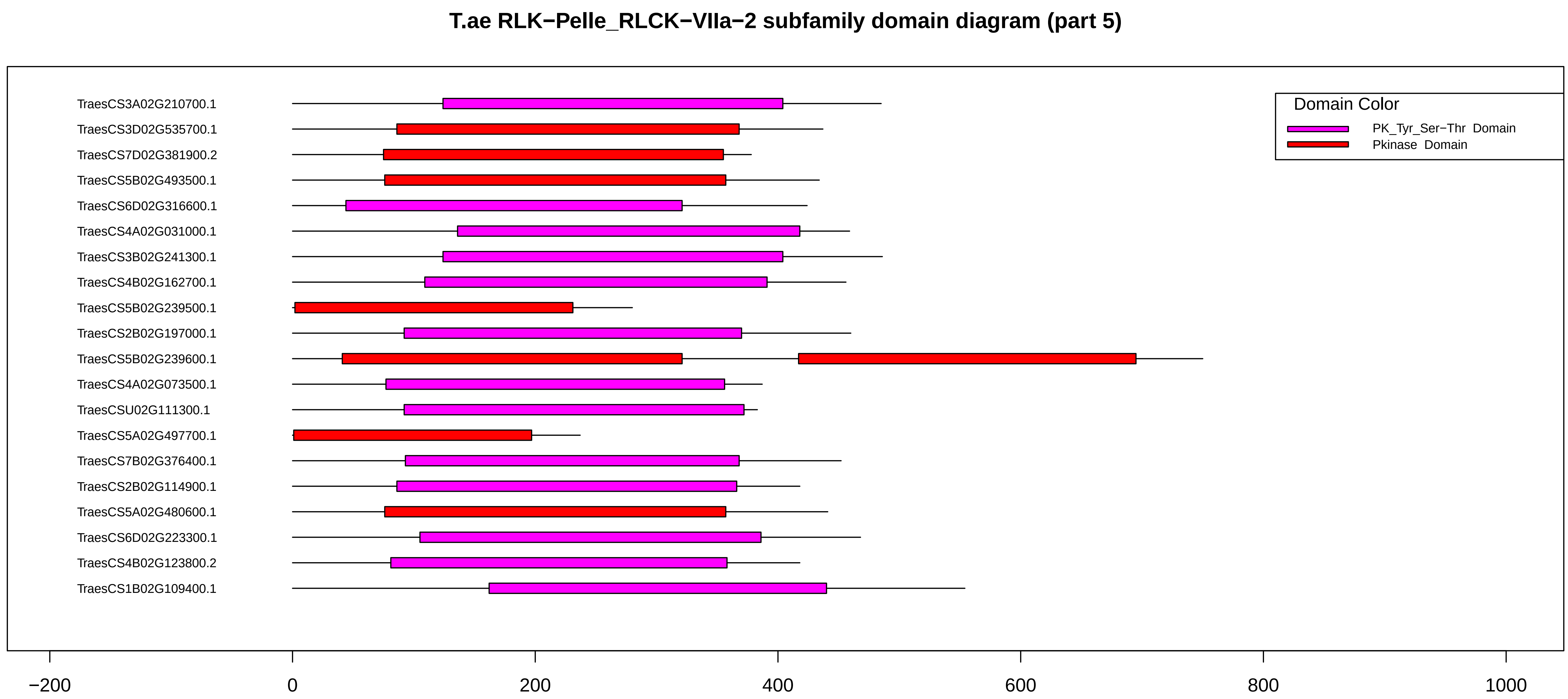
T.ae RLK−Pelle_RLCK−VIIa−2 subfamily domain diagram (part 5)
TraesCS3A02G210700.1
TraesCS3D02G535700.1
TraesCS7D02G381900.2
TraesCS5B02G493500.1
TraesCS6D02G316600.1
TraesCS4A02G031000.1
TraesCS3B02G241300.1
TraesCS4B02G162700.1
TraesCS5B02G239500.1
TraesCS2B02G197000.1
TraesCS5B02G239600.1
TraesCS4A02G073500.1
TraesCSU02G111300.1
TraesCS5A02G497700.1
TraesCS7B02G376400.1
TraesCS2B02G114900.1
TraesCS5A02G480600.1
TraesCS6D02G223300.1
TraesCS4B02G123800.2
TraesCS1B02G109400.1
Domain Color
PK_Tyr_Ser−Thr Domain
Pkinase Domain
−200
0
200
400
600
800
1000

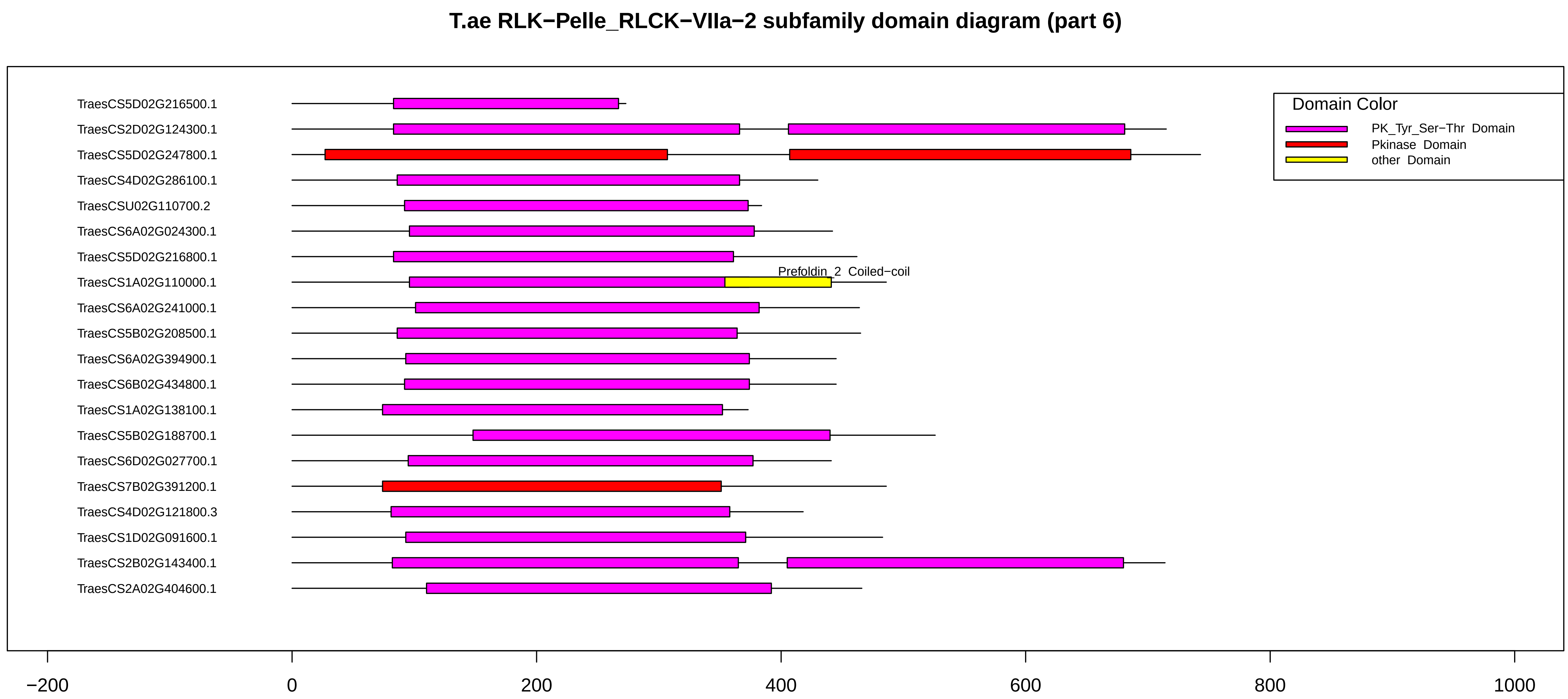
T.ae RLK−Pelle_RLCK−VIIa−2 subfamily domain diagram (part 6)
TraesCS5D02G216500.1
TraesCS2D02G124300.1
TraesCS5D02G247800.1
TraesCS4D02G286100.1
TraesCSU02G110700.2
TraesCS6A02G024300.1
TraesCS5D02G216800.1
TraesCS1A02G110000.1
TraesCS6A02G241000.1
TraesCS5B02G208500.1
TraesCS6A02G394900.1
TraesCS6B02G434800.1
TraesCS1A02G138100.1
TraesCS5B02G188700.1
TraesCS6D02G027700.1
TraesCS7B02G391200.1
TraesCS4D02G121800.3
TraesCS1D02G091600.1
TraesCS2B02G143400.1
TraesCS2A02G404600.1
Prefoldin_2 Coiled−coil
Domain Color
PK_Tyr_Ser−Thr Domain
Pkinase Domain
other Domain
−200
0
200
400
600
800
1000

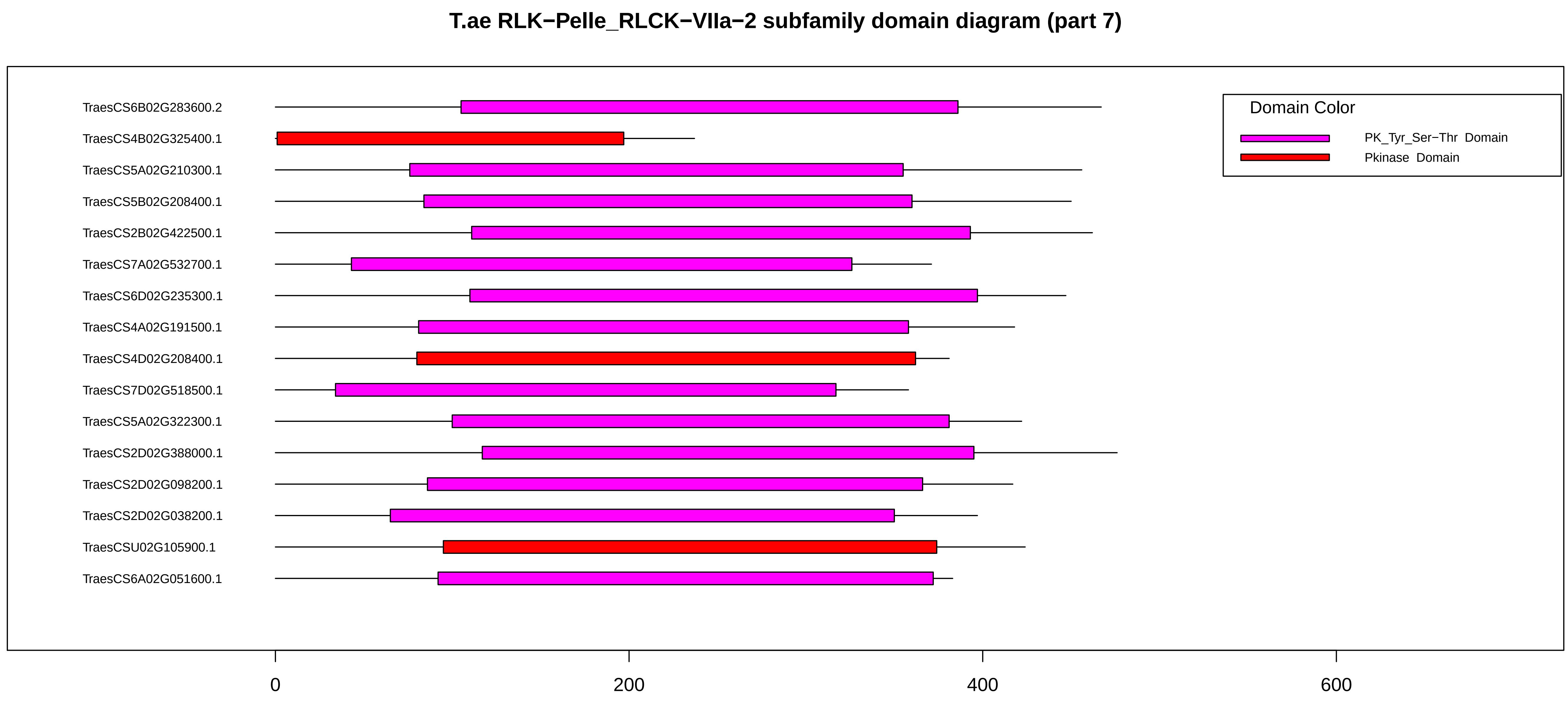
T.ae RLK−Pelle_RLCK−VIIa−2 subfamily domain diagram (part 7)
TraesCS6B02G283600.2
TraesCS4B02G325400.1
TraesCS5A02G210300.1
TraesCS5B02G208400.1
TraesCS2B02G422500.1
TraesCS7A02G532700.1
TraesCS6D02G235300.1
TraesCS4A02G191500.1
TraesCS4D02G208400.1
TraesCS7D02G518500.1
TraesCS5A02G322300.1
TraesCS2D02G388000.1
TraesCS2D02G098200.1
TraesCS2D02G038200.1
TraesCSU02G105900.1
TraesCS6A02G051600.1
Domain Color
PK_Tyr_Ser−Thr Domain
Pkinase Domain
0
200
400
600

T.ae RLK−Pelle_RLCK−VIIb subfamily domain diagram (all)

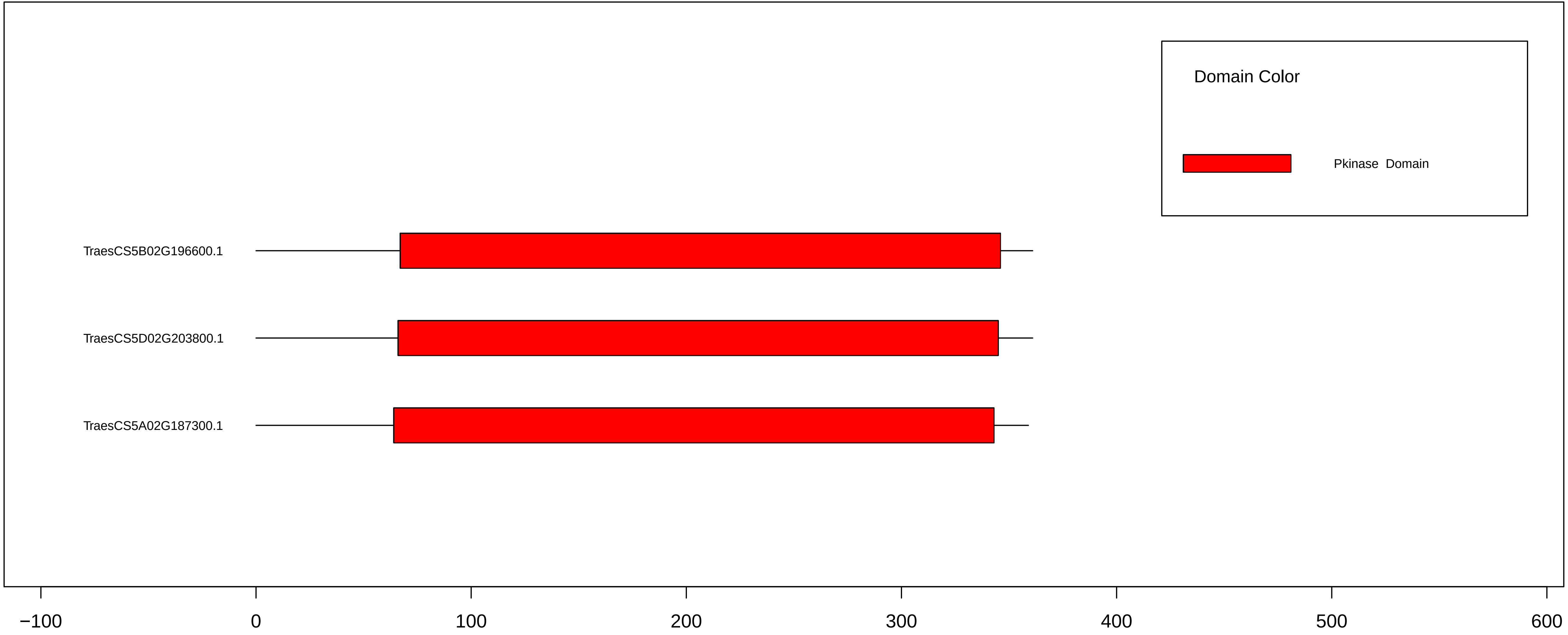

T.ae RLK−Pelle_RLCK−VIII subfamily domain diagram (part 1)

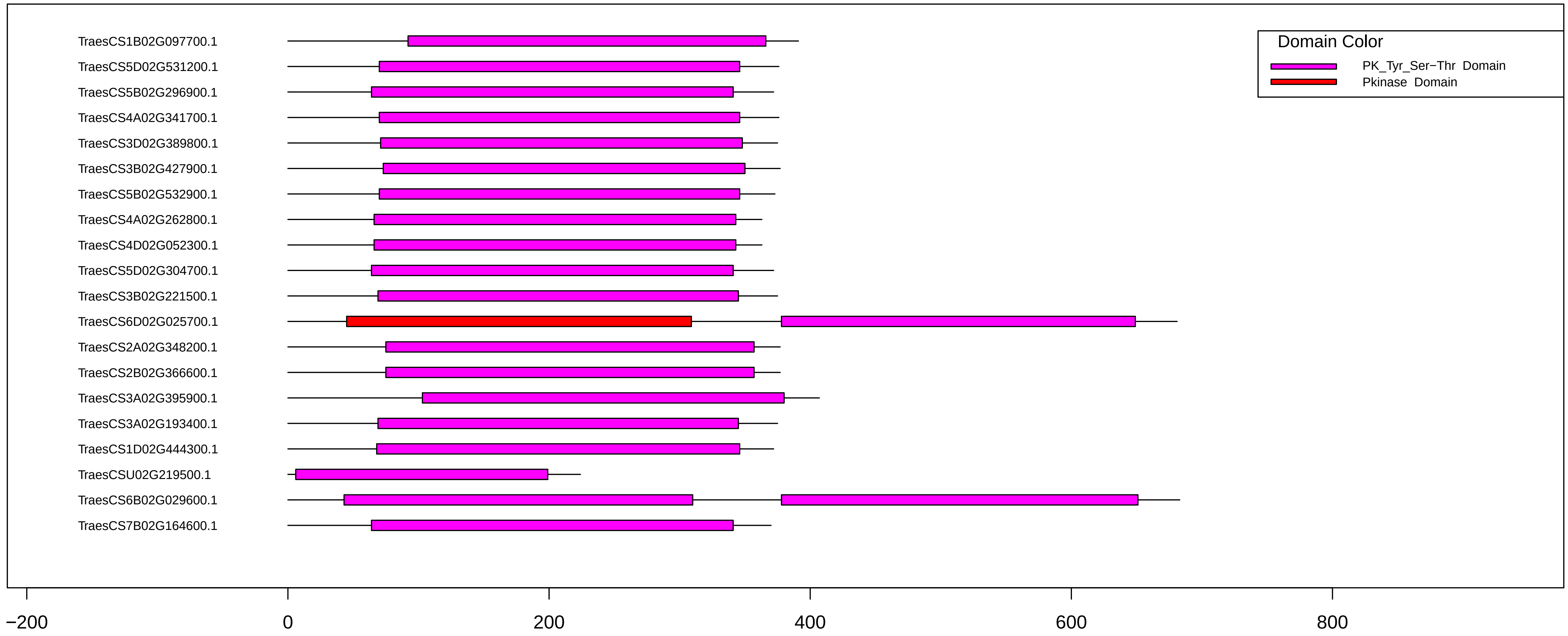

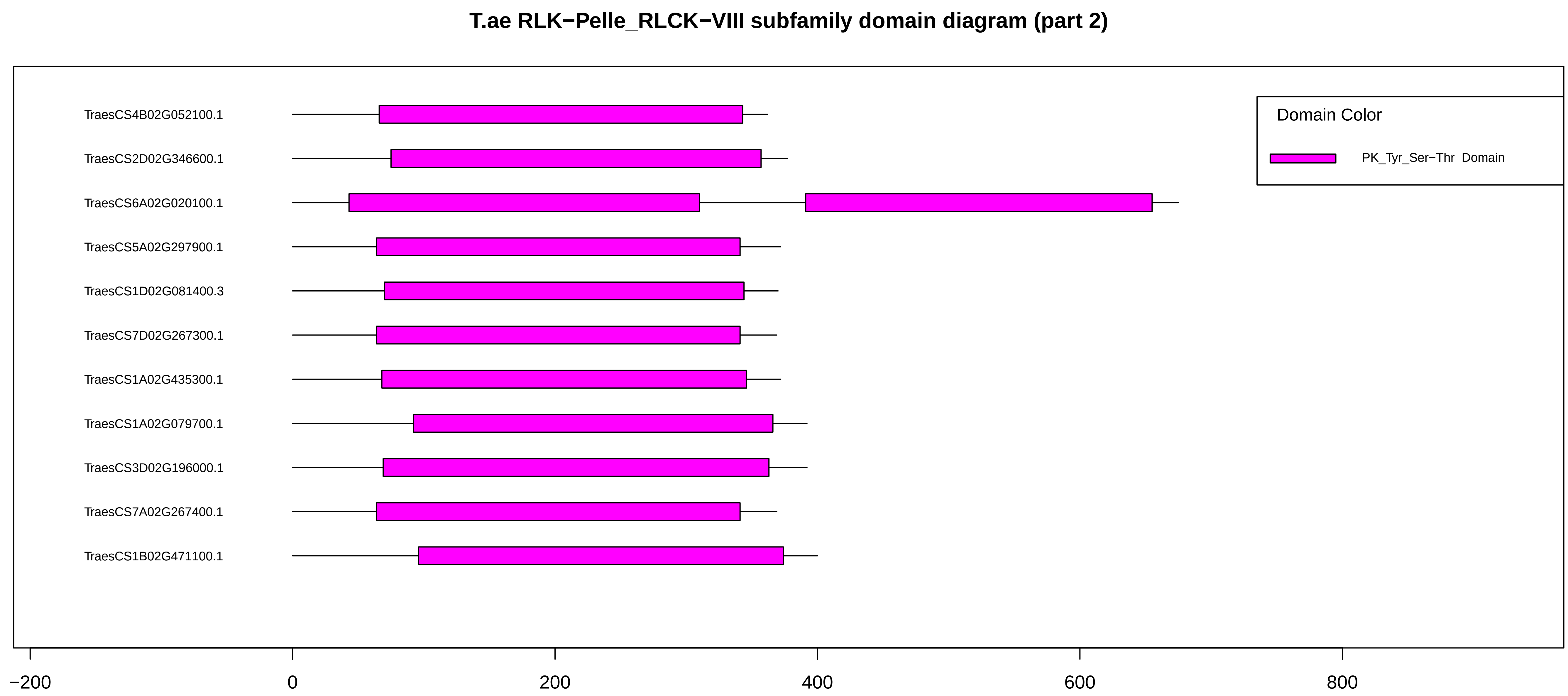

T.ae RLK−Pelle_RLCK−VIII subfamily domain diagram (part 2)
TraesCS4B02G052100.1
TraesCS2D02G346600.1
TraesCS6A02G020100.1
TraesCS5A02G297900.1
TraesCS1D02G081400.3
TraesCS7D02G267300.1
TraesCS1A02G435300.1
TraesCS1A02G079700.1
TraesCS3D02G196000.1
TraesCS7A02G267400.1
TraesCS1B02G471100.1
Domain Color
PK_Tyr_Ser−Thr Domain
−200
0
200
400
600
800

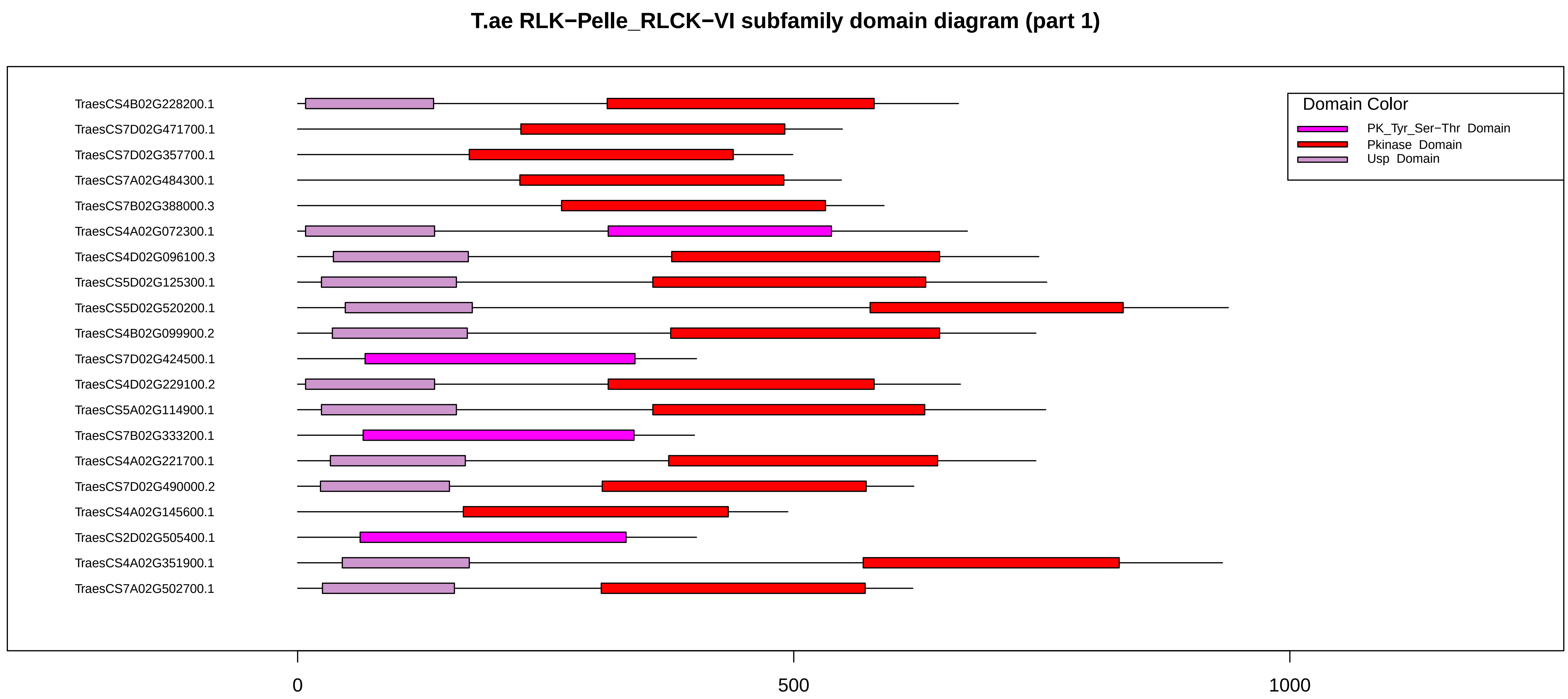
T.ae RLK−Pelle_RLCK−VI subfamily domain diagram (part 1)
TraesCS4B02G228200.1
TraesCS7D02G471700.1
TraesCS7D02G357700.1
TraesCS7A02G484300.1
TraesCS7B02G388000.3
TraesCS4A02G072300.1
TraesCS4D02G096100.3
TraesCS5D02G125300.1
TraesCS5D02G520200.1
TraesCS4B02G099900.2
TraesCS7D02G424500.1
TraesCS4D02G229100.2
TraesCS5A02G114900.1
TraesCS7B02G333200.1
TraesCS4A02G221700.1
TraesCS7D02G490000.2
TraesCS4A02G145600.1
TraesCS2D02G505400.1
TraesCS4A02G351900.1
TraesCS7A02G502700.1
Domain Color
PK_Tyr_Ser−Thr Domain
Pkinase Domain
Usp Domain
0
500
1000

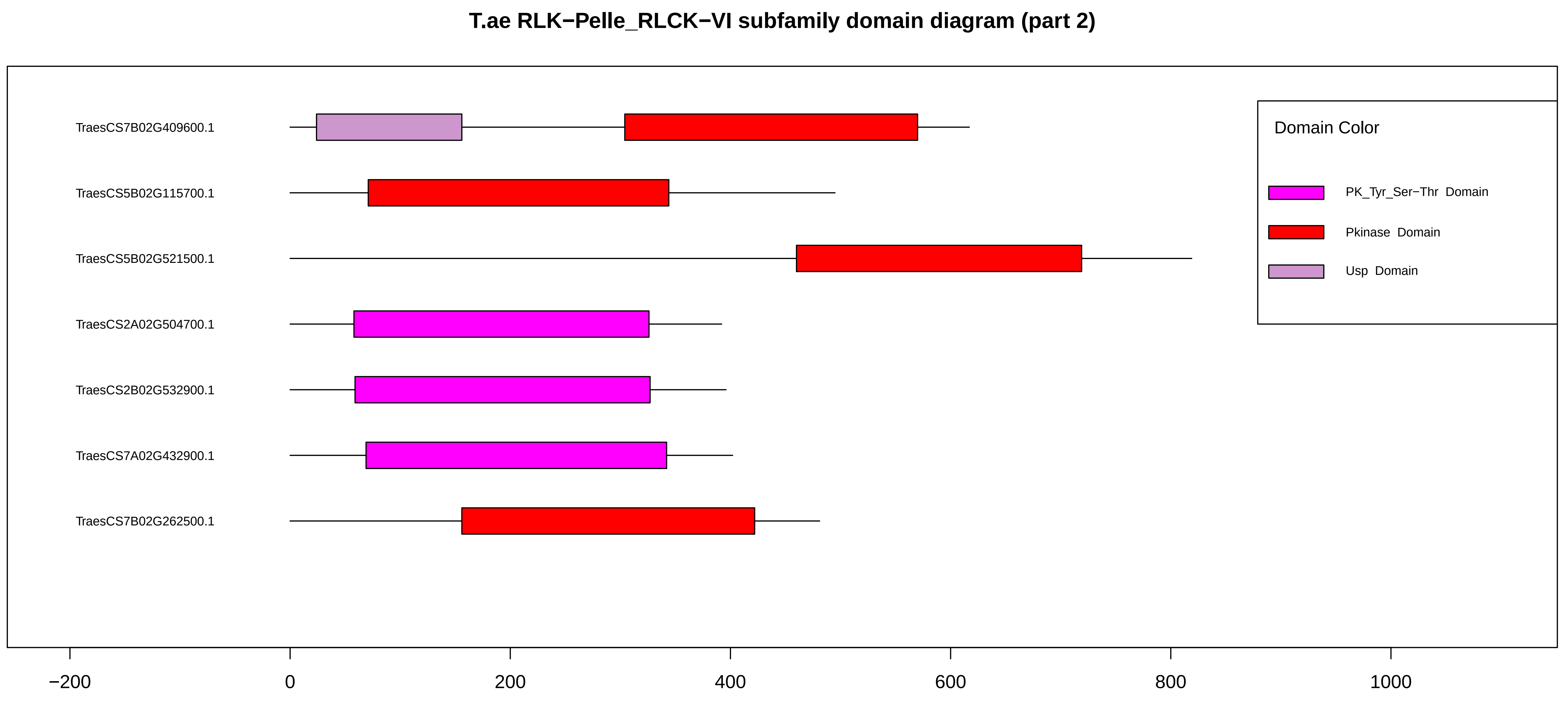

T.ae RLK−Pelle_RLCK−VI subfamily domain diagram (part 2)
TraesCS7B02G409600.1
TraesCS5B02G115700.1
TraesCS5B02G521500.1
TraesCS2A02G504700.1
TraesCS2B02G532900.1
TraesCS7A02G432900.1
TraesCS7B02G262500.1
Domain Color
PK_Tyr_Ser−Thr Domain
Pkinase Domain
Usp Domain
−200
0
200
400
600
800
1000

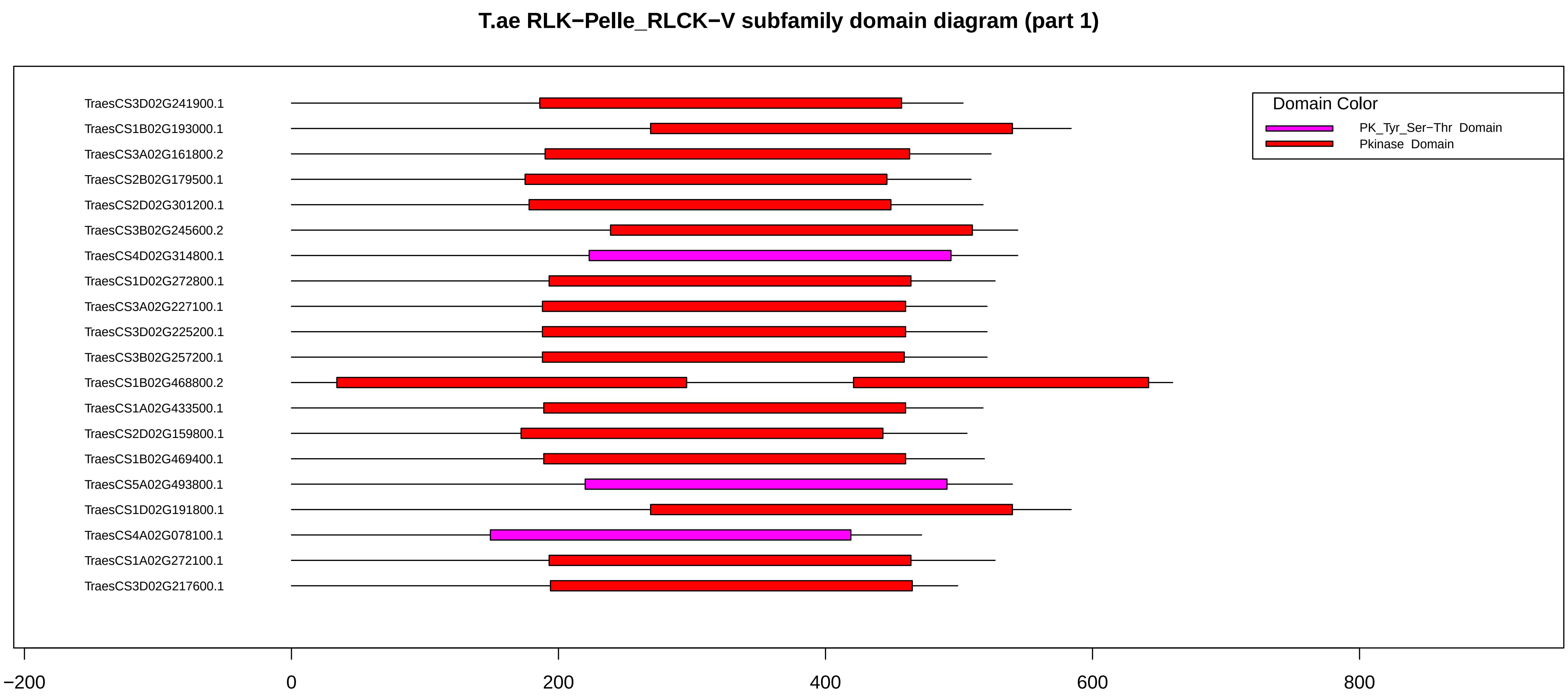
T.ae RLK−Pelle_RLCK−V subfamily domain diagram (part 1)
TraesCS3D02G241900.1
TraesCS1B02G193000.1
TraesCS3A02G161800.2
TraesCS2B02G179500.1
TraesCS2D02G301200.1
TraesCS3B02G245600.2
TraesCS4D02G314800.1
TraesCS1D02G272800.1
TraesCS3A02G227100.1
TraesCS3D02G225200.1
TraesCS3B02G257200.1
TraesCS1B02G468800.2
TraesCS1A02G433500.1
TraesCS2D02G159800.1
TraesCS1B02G469400.1
TraesCS5A02G493800.1
TraesCS1D02G191800.1
TraesCS4A02G078100.1
TraesCS1A02G272100.1
TraesCS3D02G217600.1
Domain Color
PK_Tyr_Ser−Thr Domain
Pkinase Domain
−200
0
200
400
600
800

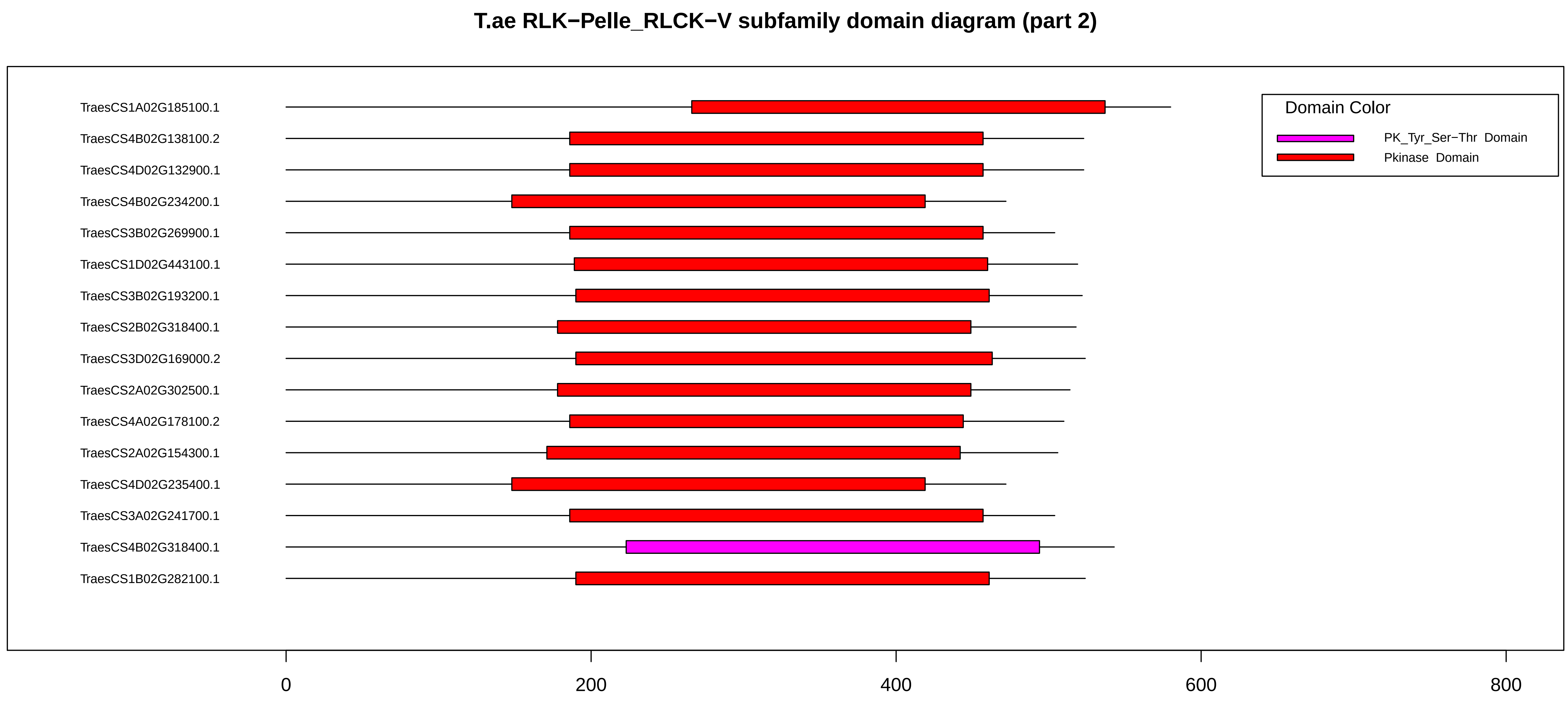
T.ae RLK−Pelle_RLCK−V subfamily domain diagram (part 2)
TraesCS1A02G185100.1
TraesCS4B02G138100.2
TraesCS4D02G132900.1
TraesCS4B02G234200.1
TraesCS3B02G269900.1
TraesCS1D02G443100.1
TraesCS3B02G193200.1
TraesCS2B02G318400.1
TraesCS3D02G169000.2
TraesCS2A02G302500.1
TraesCS4A02G178100.2
TraesCS2A02G154300.1
TraesCS4D02G235400.1
TraesCS3A02G241700.1
TraesCS4B02G318400.1
TraesCS1B02G282100.1
Domain Color
PK_Tyr_Ser−Thr Domain
Pkinase Domain
0
200
400
600
800

T.ae RLK−Pelle_RLCK−XII−1 subfamily domain diagram (all)

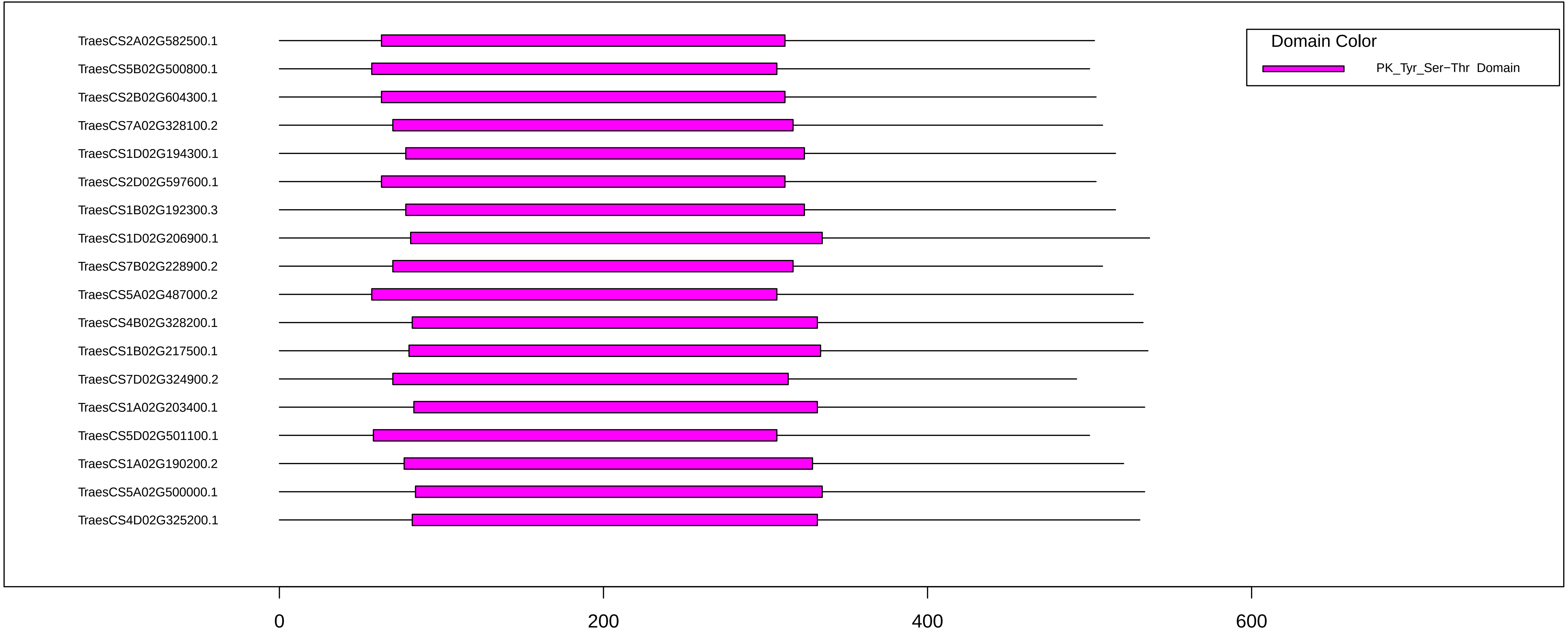

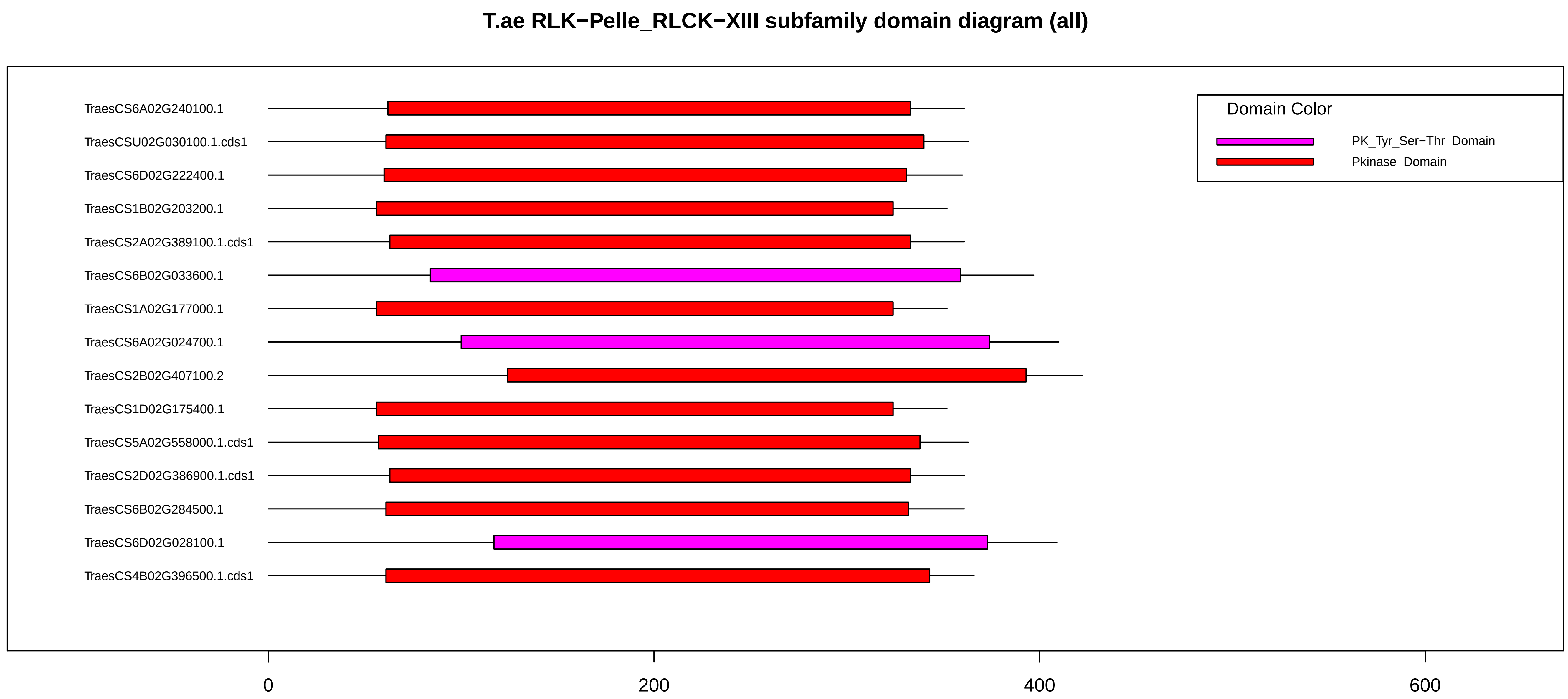
T.ae RLK−Pelle_RLCK−XIII subfamily domain diagram (all)
Domain Color
PK_Tyr_Ser−Thr Domain
Pkinase Domain
TraesCS6A02G240100.1
TraesCSU02G030100.1.cds1
TraesCS6D02G222400.1
TraesCS1B02G203200.1
TraesCS2A02G389100.1.cds1
TraesCS6B02G033600.1
TraesCS1A02G177000.1
TraesCS6A02G024700.1
TraesCS2B02G407100.2
TraesCS1D02G175400.1
TraesCS5A02G558000.1.cds1
TraesCS2D02G386900.1.cds1
TraesCS6B02G284500.1
TraesCS6D02G028100.1
TraesCS4B02G396500.1.cds1
0
200
400
600

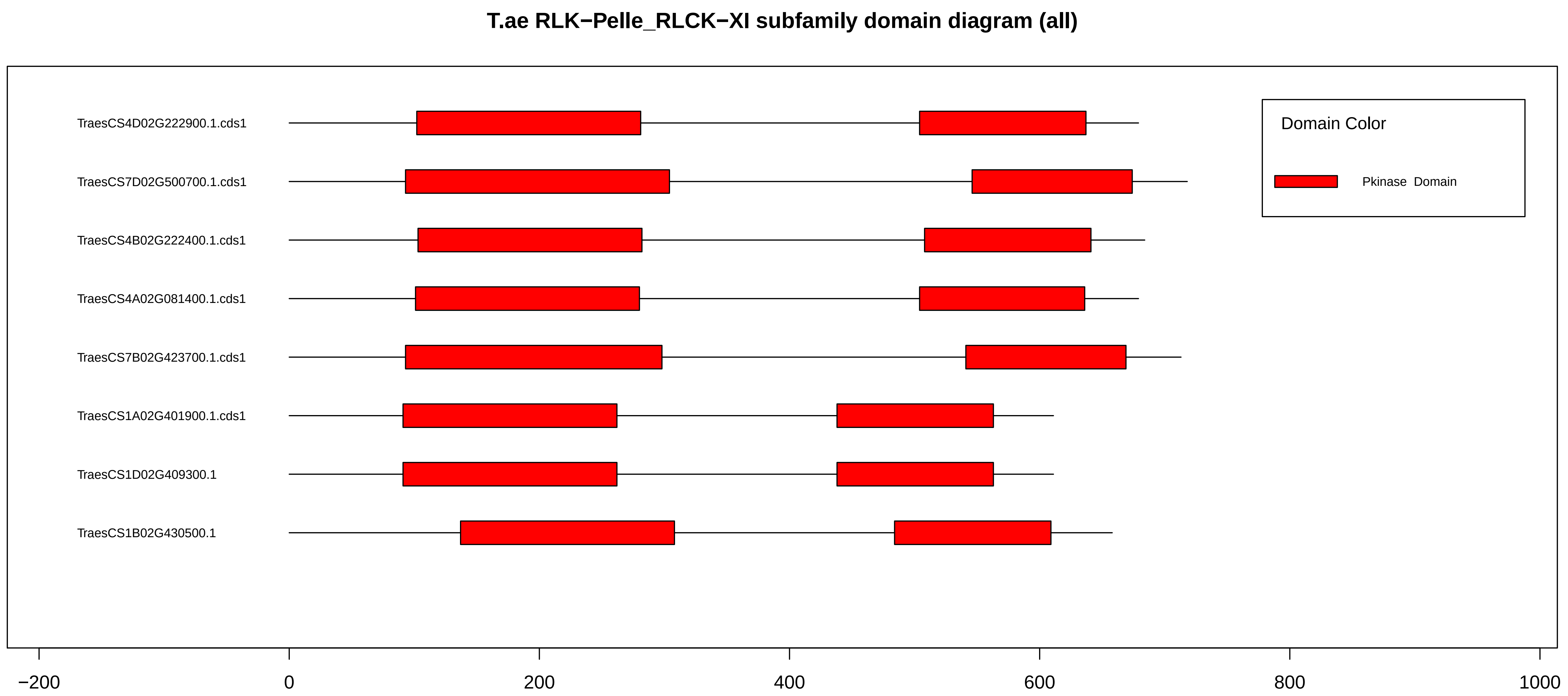
T.ae RLK−Pelle_RLCK−XI subfamily domain diagram (all)
TraesCS4D02G222900.1.cds1
TraesCS7D02G500700.1.cds1
TraesCS4B02G222400.1.cds1
TraesCS4A02G081400.1.cds1
TraesCS7B02G423700.1.cds1
TraesCS1A02G401900.1.cds1
TraesCS1D02G409300.1
TraesCS1B02G430500.1
Domain Color
Pkinase Domain
−200
0
200
400
600
800
1000

T.ae RLK−Pelle_RLCK−X subfamily domain diagram (all)

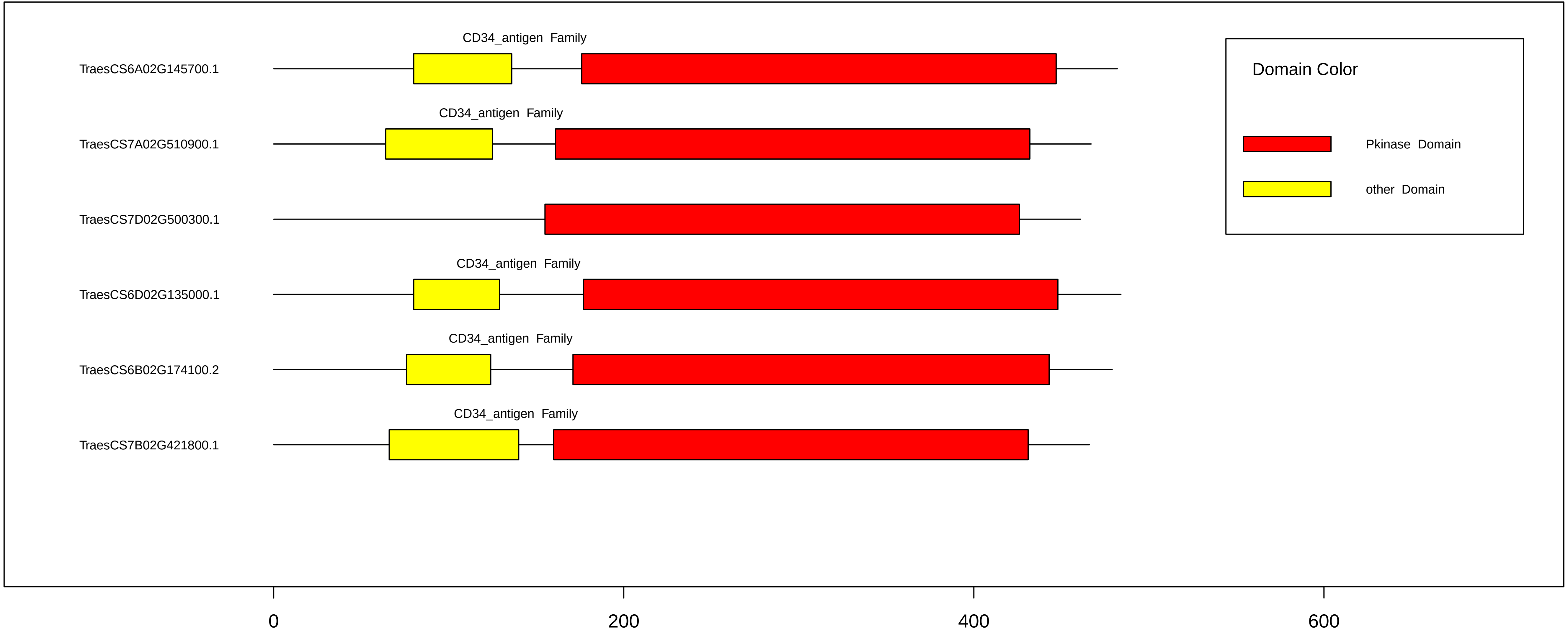

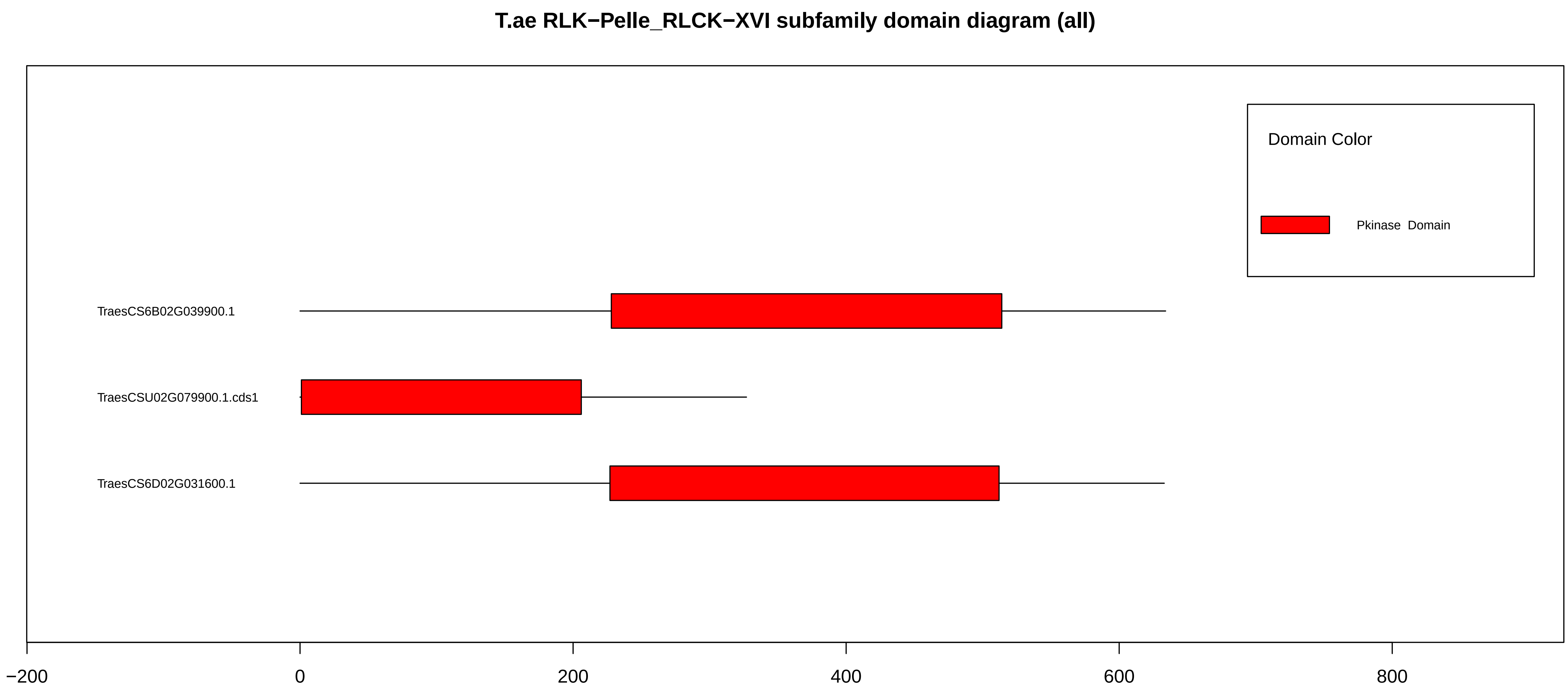
T.ae RLK−Pelle_RLCK−XVI subfamily domain diagram (all)
Domain Color
Pkinase Domain
TraesCS6B02G039900.1
TraesCSU02G079900.1.cds1
TraesCS6D02G031600.1
−200
0
200
400
600
800

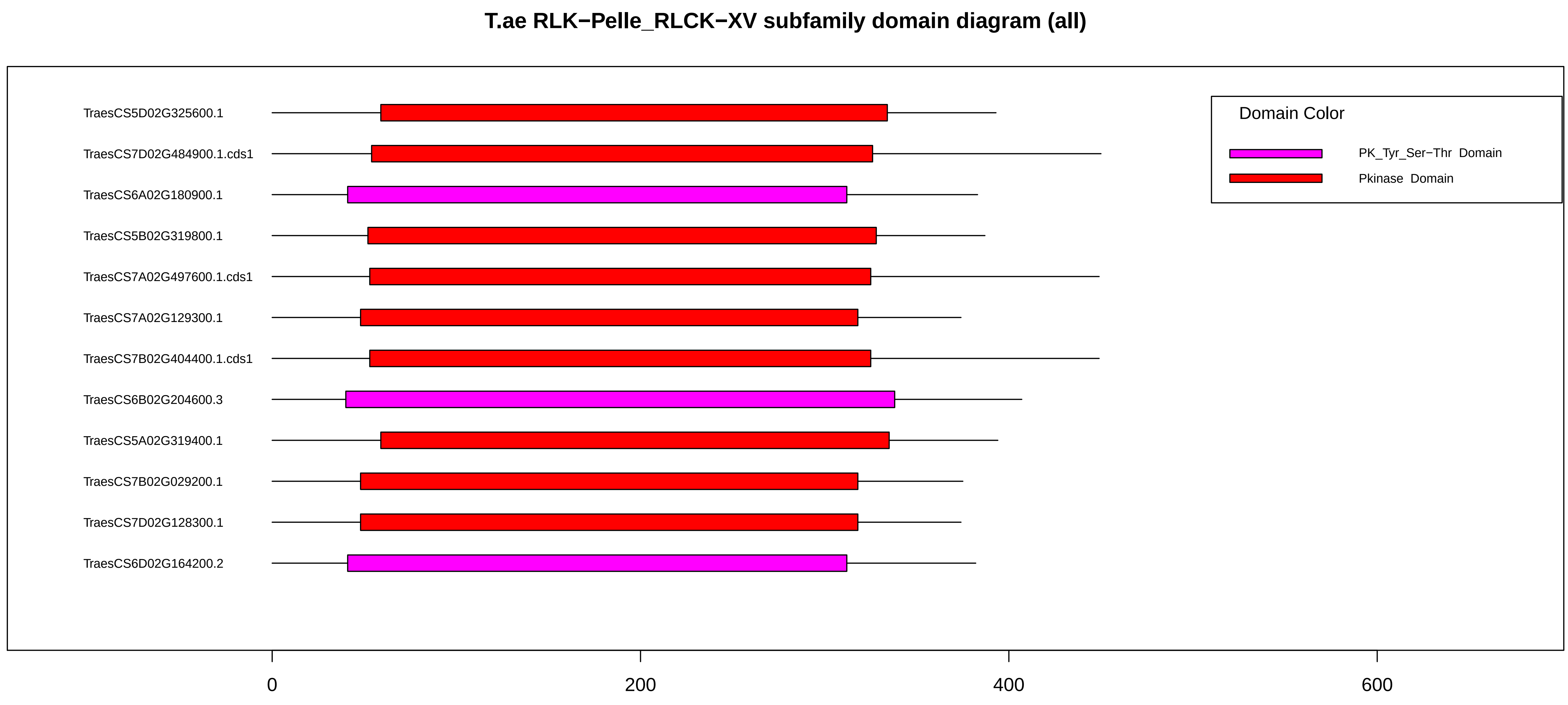
T.ae RLK−Pelle_RLCK−XV subfamily domain diagram (all)
TraesCS5D02G325600.1
TraesCS7D02G484900.1.cds1
TraesCS6A02G180900.1
TraesCS5B02G319800.1
TraesCS7A02G497600.1.cds1
TraesCS7A02G129300.1
TraesCS7B02G404400.1.cds1
TraesCS6B02G204600.3
TraesCS5A02G319400.1
TraesCS7B02G029200.1
TraesCS7D02G128300.1
TraesCS6D02G164200.2
Domain Color
PK_Tyr_Ser−Thr Domain
Pkinase Domain
0
200
400
600

T.ae RLK−Pelle_SD−2b subfamily domain diagram (part 1)

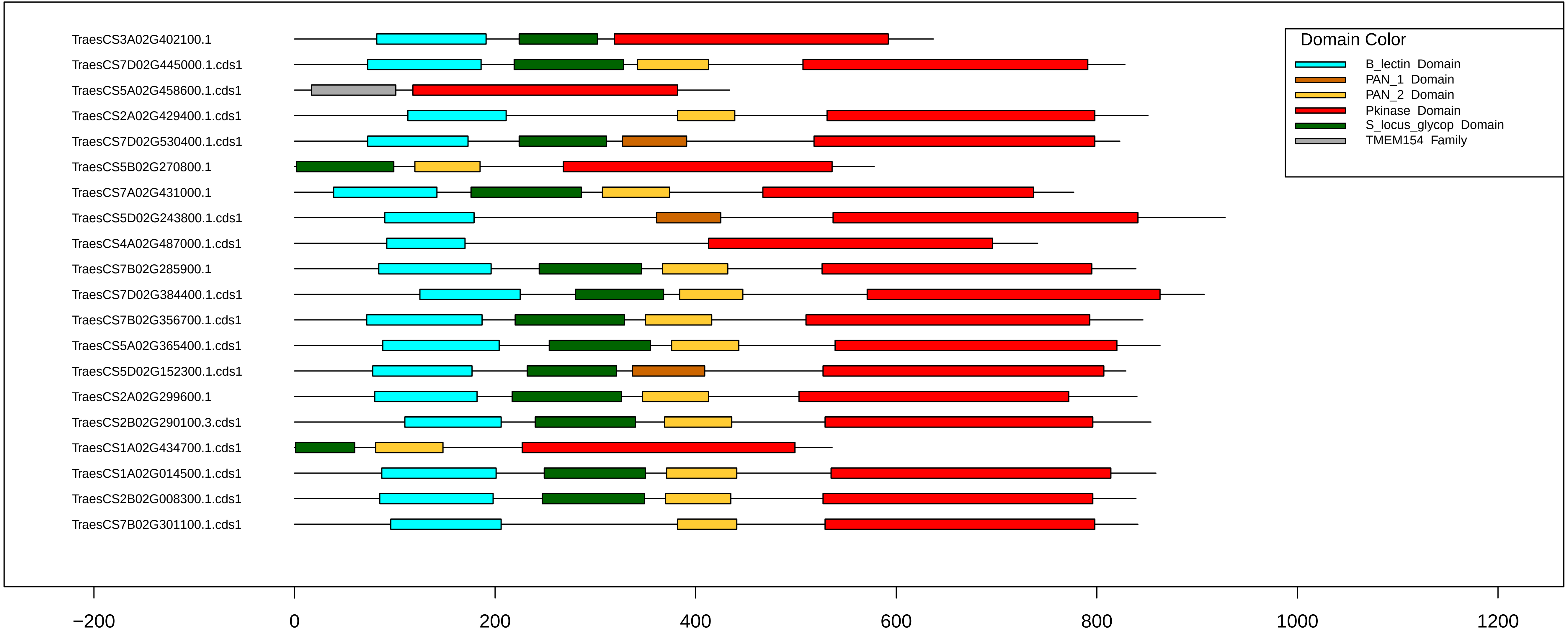

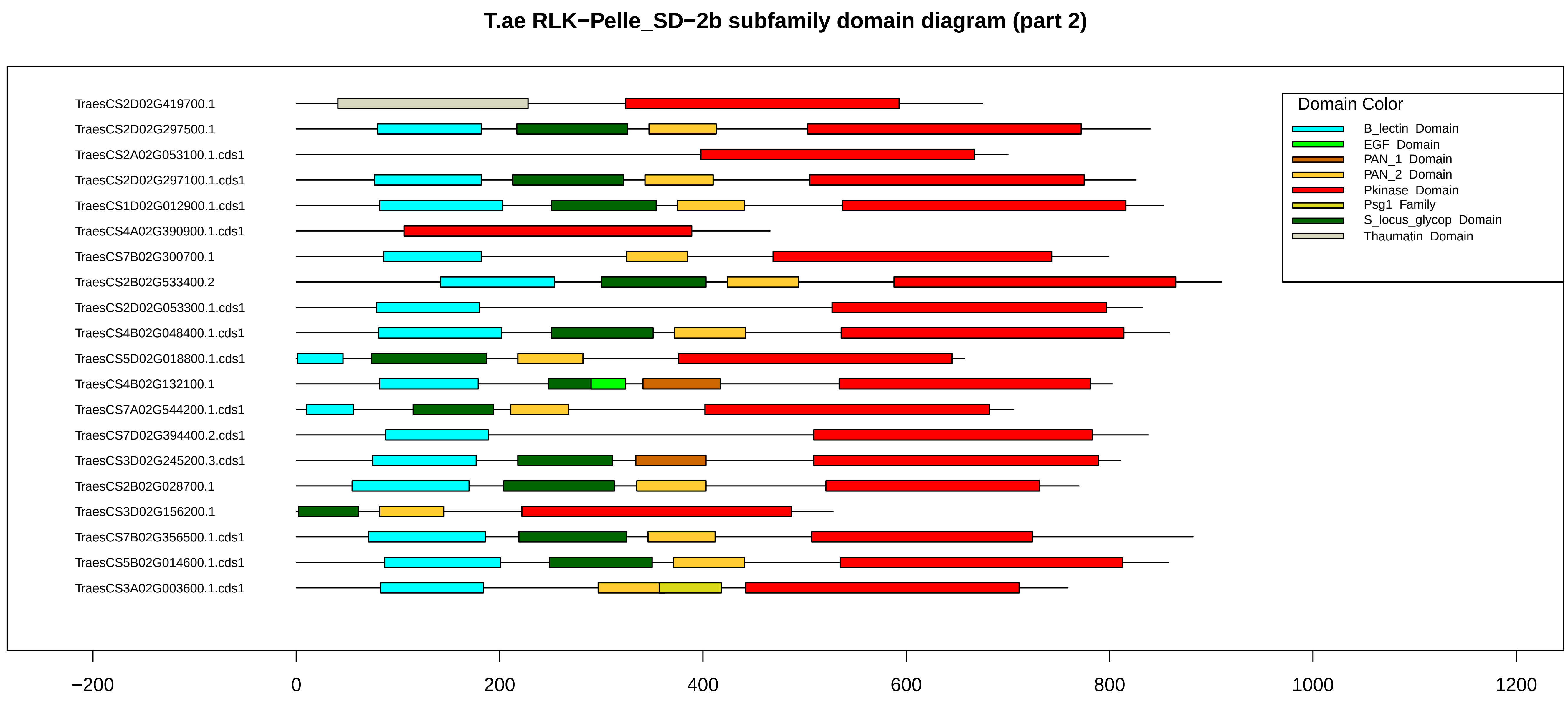
T.ae RLK−Pelle_SD−2b subfamily domain diagram (part 2)
TraesCS2D02G419700.1
TraesCS2D02G297500.1
TraesCS2A02G053100.1.cds1
TraesCS2D02G297100.1.cds1
TraesCS1D02G012900.1.cds1
TraesCS4A02G390900.1.cds1
TraesCS7B02G300700.1
TraesCS2B02G533400.2
TraesCS2D02G053300.1.cds1
TraesCS4B02G048400.1.cds1
TraesCS5D02G018800.1.cds1
TraesCS4B02G132100.1
TraesCS7A02G544200.1.cds1
TraesCS7D02G394400.2.cds1
TraesCS3D02G245200.3.cds1
TraesCS2B02G028700.1
TraesCS3D02G156200.1
TraesCS7B02G356500.1.cds1
TraesCS5B02G014600.1.cds1
TraesCS3A02G003600.1.cds1
Domain Color
B_lectin Domain
EGF Domain
PAN_1 Domain
PAN_2 Domain
Pkinase Domain
Psg1 Family
S_locus_glycop Domain
Thaumatin Domain
−200
0
200
400
600
800
1000
1200

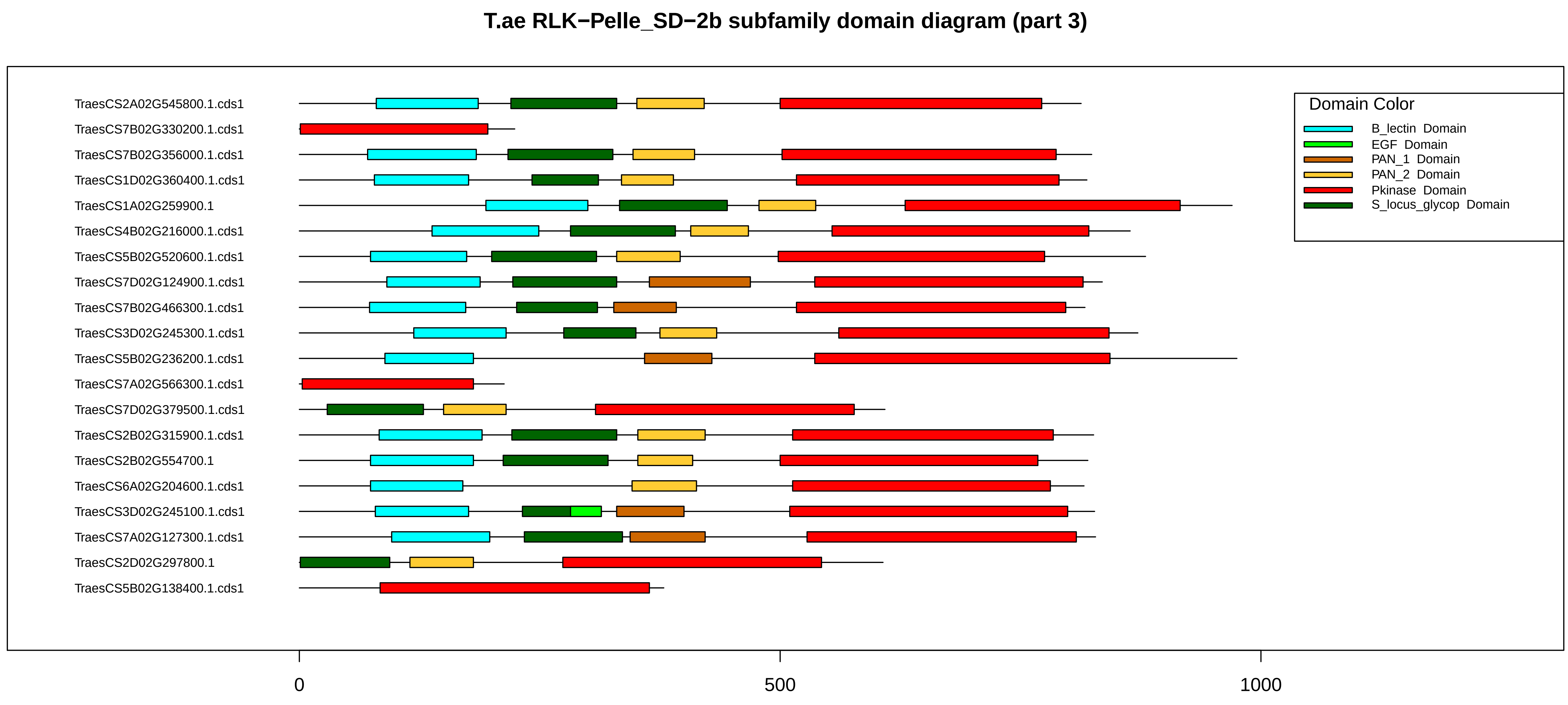
T.ae RLK−Pelle_SD−2b subfamily domain diagram (part 3)
TraesCS2A02G545800.1.cds1
TraesCS7B02G330200.1.cds1
TraesCS7B02G356000.1.cds1
TraesCS1D02G360400.1.cds1
TraesCS1A02G259900.1
TraesCS4B02G216000.1.cds1
TraesCS5B02G520600.1.cds1
TraesCS7D02G124900.1.cds1
TraesCS7B02G466300.1.cds1
TraesCS3D02G245300.1.cds1
TraesCS5B02G236200.1.cds1
TraesCS7A02G566300.1.cds1
TraesCS7D02G379500.1.cds1
TraesCS2B02G315900.1.cds1
TraesCS2B02G554700.1
TraesCS6A02G204600.1.cds1
TraesCS3D02G245100.1.cds1
TraesCS7A02G127300.1.cds1
TraesCS2D02G297800.1
TraesCS5B02G138400.1.cds1
Domain Color
B_lectin Domain
EGF Domain
PAN_1 Domain
PAN_2 Domain
Pkinase Domain
S_locus_glycop Domain
0
500
1000

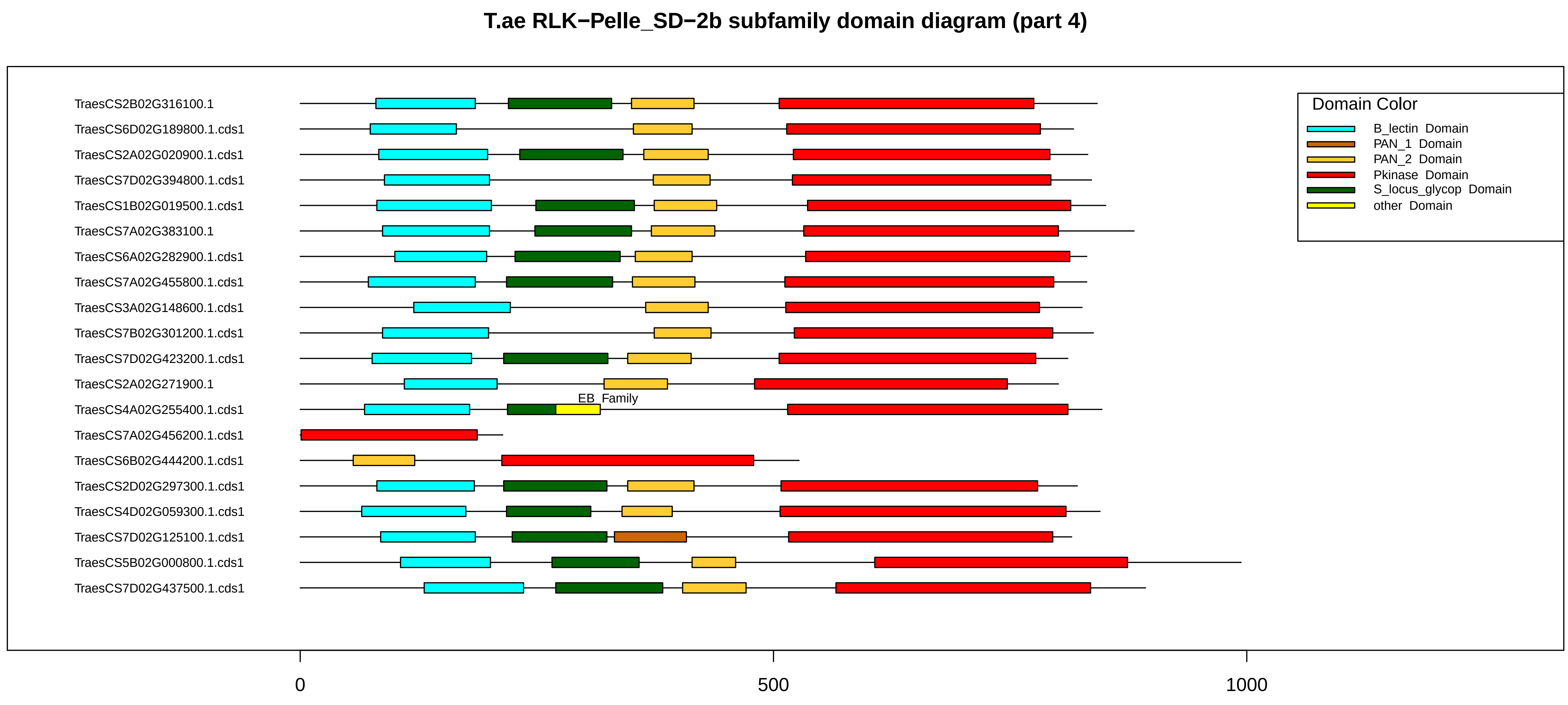
T.ae RLK−Pelle_SD−2b subfamily domain diagram (part 4)
TraesCS2B02G316100.1
TraesCS6D02G189800.1.cds1
TraesCS2A02G020900.1.cds1
TraesCS7D02G394800.1.cds1
TraesCS1B02G019500.1.cds1
TraesCS7A02G383100.1
TraesCS6A02G282900.1.cds1
TraesCS7A02G455800.1.cds1
TraesCS3A02G148600.1.cds1
TraesCS7B02G301200.1.cds1
TraesCS7D02G423200.1.cds1
TraesCS2A02G271900.1
TraesCS4A02G255400.1.cds1
EB_Family
TraesCS7A02G456200.1.cds1
TraesCS6B02G444200.1.cds1
TraesCS2D02G297300.1.cds1
TraesCS4D02G059300.1.cds1
TraesCS7D02G125100.1.cds1
TraesCS5B02G000800.1.cds1
TraesCS7D02G437500.1.cds1
Domain Color
B_lectin Domain
PAN_1 Domain
PAN_2 Domain
Pkinase Domain
S_locus_glycop Domain
other Domain
0
500
1000

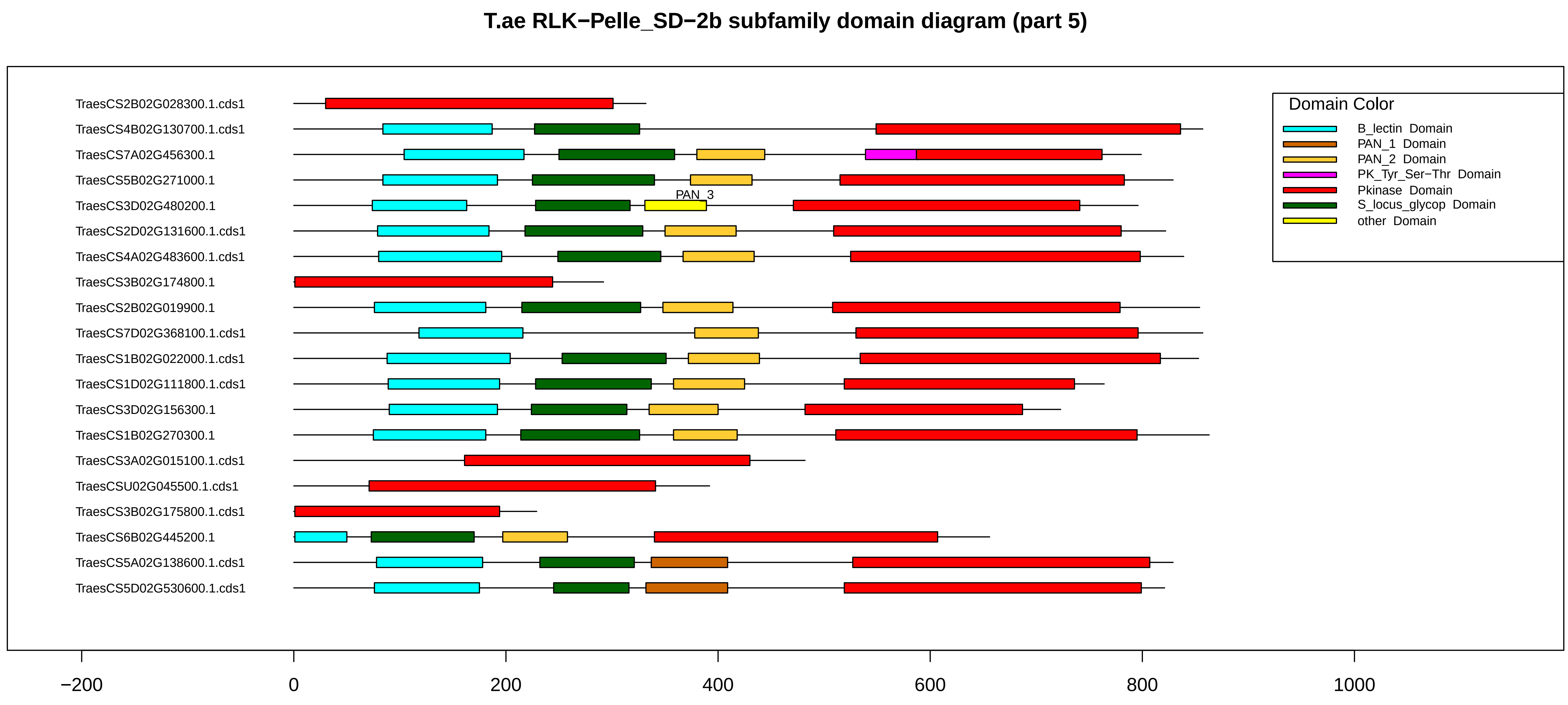
T.ae RLK−Pelle_SD−2b subfamily domain diagram (part 5)
TraesCS2B02G028300.1.cds1
TraesCS4B02G130700.1.cds1
TraesCS7A02G456300.1
TraesCS5B02G271000.1
TraesCS3D02G480200.1
PAN_3
TraesCS2D02G131600.1.cds1
TraesCS4A02G483600.1.cds1
TraesCS3B02G174800.1
TraesCS2B02G019900.1
TraesCS7D02G368100.1.cds1
TraesCS1B02G022000.1.cds1
TraesCS1D02G111800.1.cds1
TraesCS3D02G156300.1
TraesCS1B02G270300.1
TraesCS3A02G015100.1.cds1
TraesCSU02G045500.1.cds1
TraesCS3B02G175800.1.cds1
TraesCS6B02G445200.1
TraesCS5A02G138600.1.cds1
TraesCS5D02G530600.1.cds1
Domain Color
B_lectin Domain
PAN_1 Domain
PAN_2 Domain
PK_Tyr_Ser−Thr Domain
Pkinase Domain
S_locus_glycop Domain
other Domain
−200
0
200
400
600
800
1000

T.ae RLK−Pelle_SD−2b subfamily domain diagram (part 6)

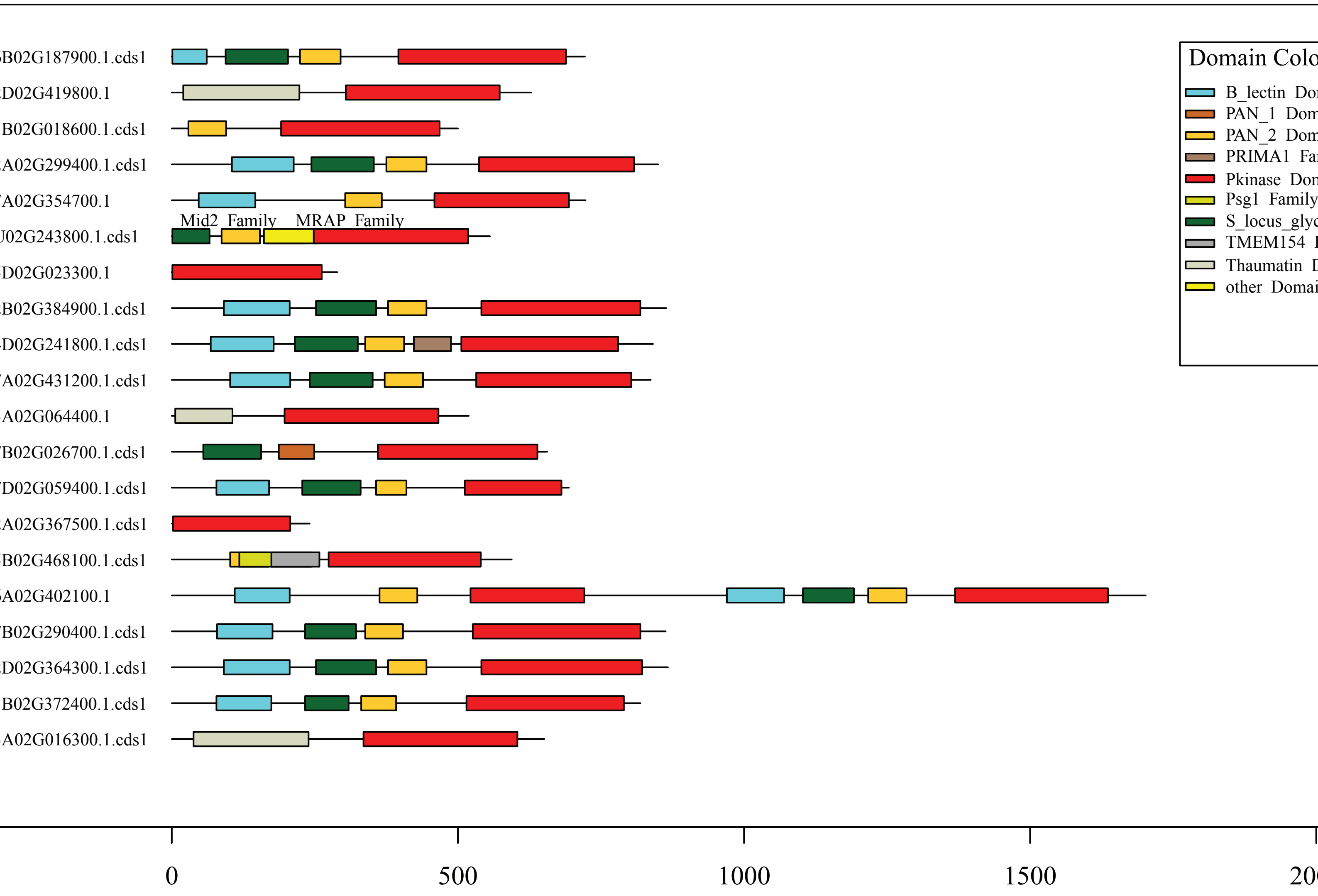

T.ae RLK−Pelle_SD−2b subfamily domain diagram (part 7)

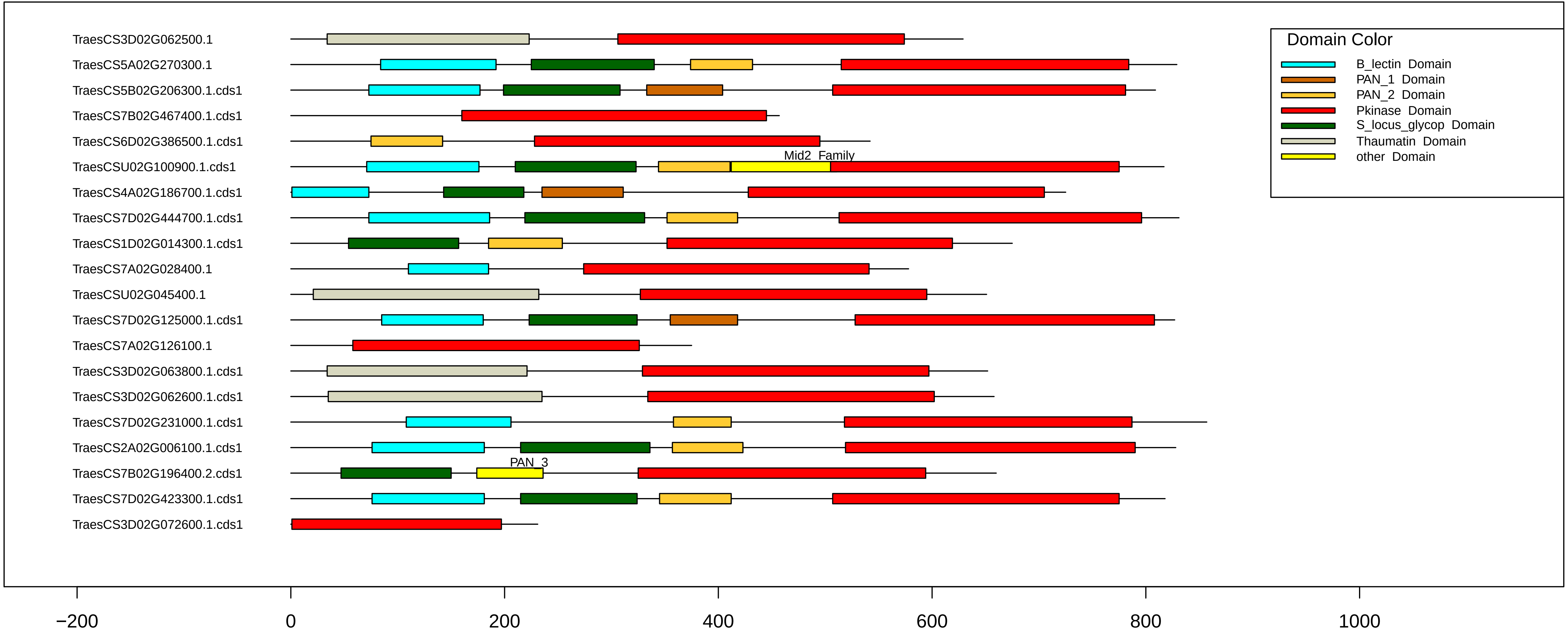

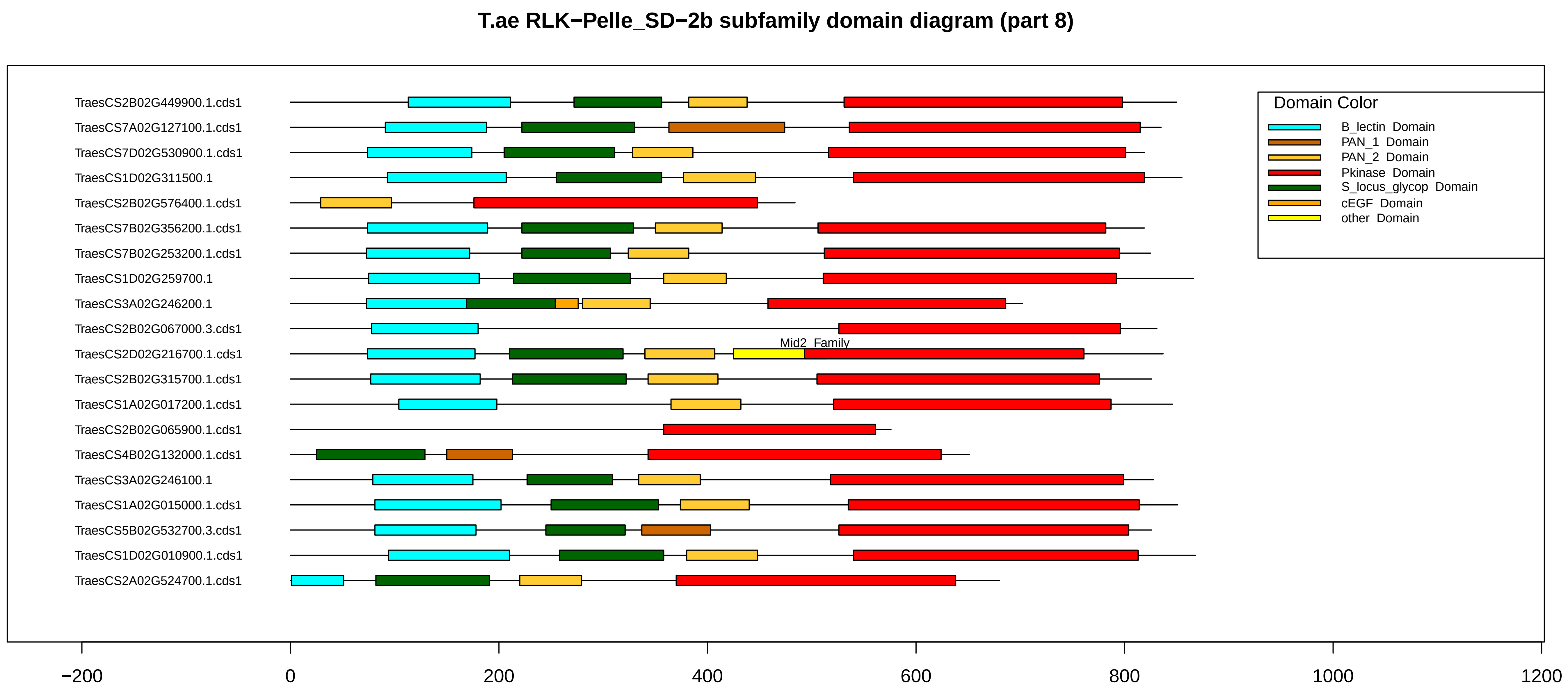
T.ae RLK−Pelle_SD−2b subfamily domain diagram (part 8)
TraesCS2B02G449900.1.cds1
TraesCS7A02G127100.1.cds1
TraesCS7D02G530900.1.cds1
TraesCS1D02G311500.1
TraesCS2B02G576400.1.cds1
TraesCS7B02G356200.1.cds1
TraesCS7B02G253200.1.cds1
TraesCS1D02G259700.1
TraesCS3A02G246200.1
TraesCS2B02G067000.3.cds1
TraesCS2D02G216700.1.cds1
Mid2_Family
TraesCS2B02G315700.1.cds1
TraesCS1A02G017200.1.cds1
TraesCS2B02G065900.1.cds1
TraesCS4B02G132000.1.cds1
TraesCS3A02G246100.1
TraesCS1A02G015000.1.cds1
TraesCS5B02G532700.3.cds1
TraesCS1D02G010900.1.cds1
TraesCS2A02G524700.1.cds1
Domain Color
B_lectin Domain
PAN_1 Domain
PAN_2 Domain
Pkinase Domain
S_locus_glycop Domain
cEGF Domain
other Domain
−200
0
200
400
600
800
1000
1200

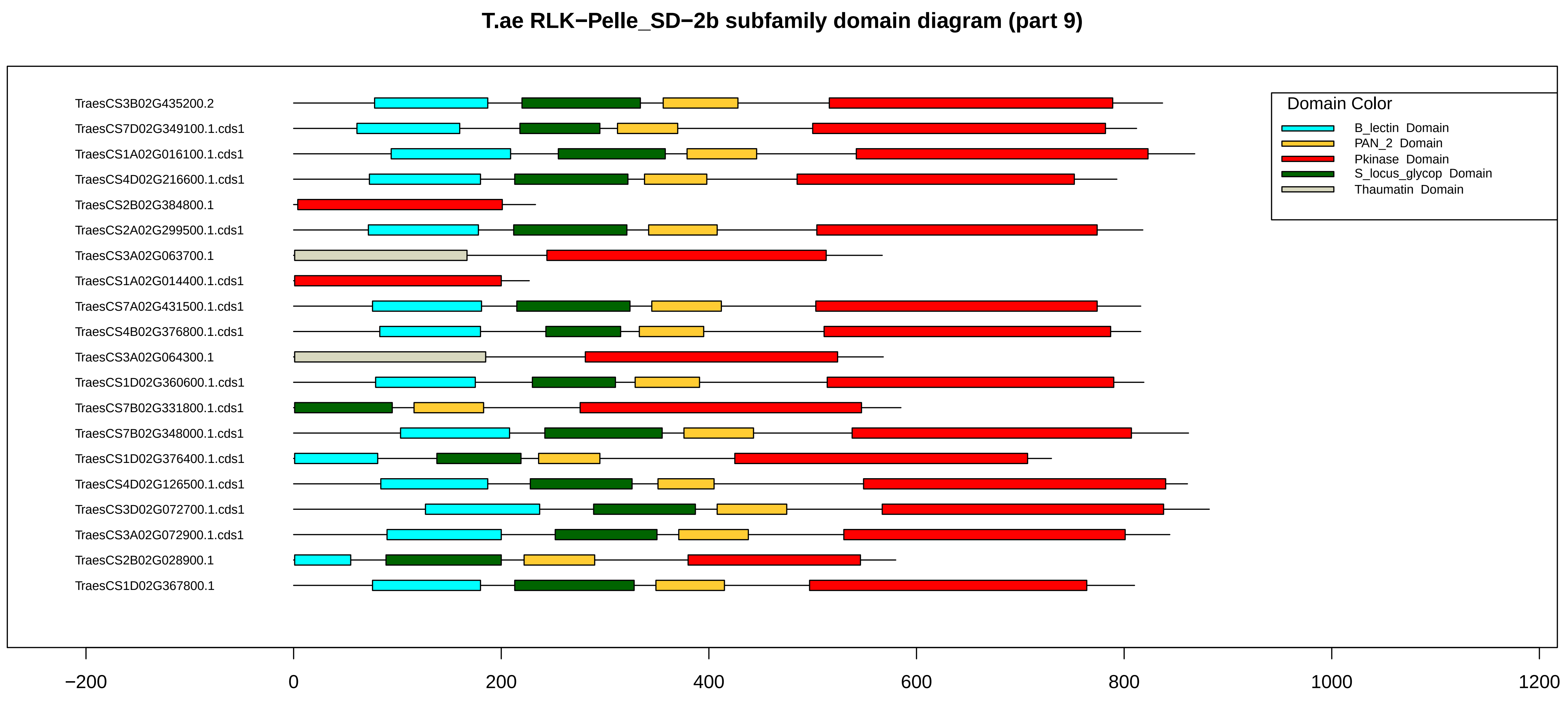
T.ae RLK−Pelle_SD−2b subfamily domain diagram (part 9)
TraesCS3B02G435200.2
TraesCS7D02G349100.1.cds1
TraesCS1A02G016100.1.cds1
TraesCS4D02G216600.1.cds1
TraesCS2B02G384800.1
TraesCS2A02G299500.1.cds1
TraesCS3A02G063700.1
TraesCS1A02G014400.1.cds1
TraesCS7A02G431500.1.cds1
TraesCS4B02G376800.1.cds1
TraesCS3A02G064300.1
TraesCS1D02G360600.1.cds1
TraesCS7B02G331800.1.cds1
TraesCS7B02G348000.1.cds1
TraesCS1D02G376400.1.cds1
TraesCS4D02G126500.1.cds1
TraesCS3D02G072700.1.cds1
TraesCS3A02G072900.1.cds1
TraesCS2B02G028900.1
TraesCS1D02G367800.1
Domain Color
B_lectin Domain
PAN_2 Domain
Pkinase Domain
S_locus_glycop Domain
Thaumatin Domain
−200
0
200
400
600
800
1000
1200

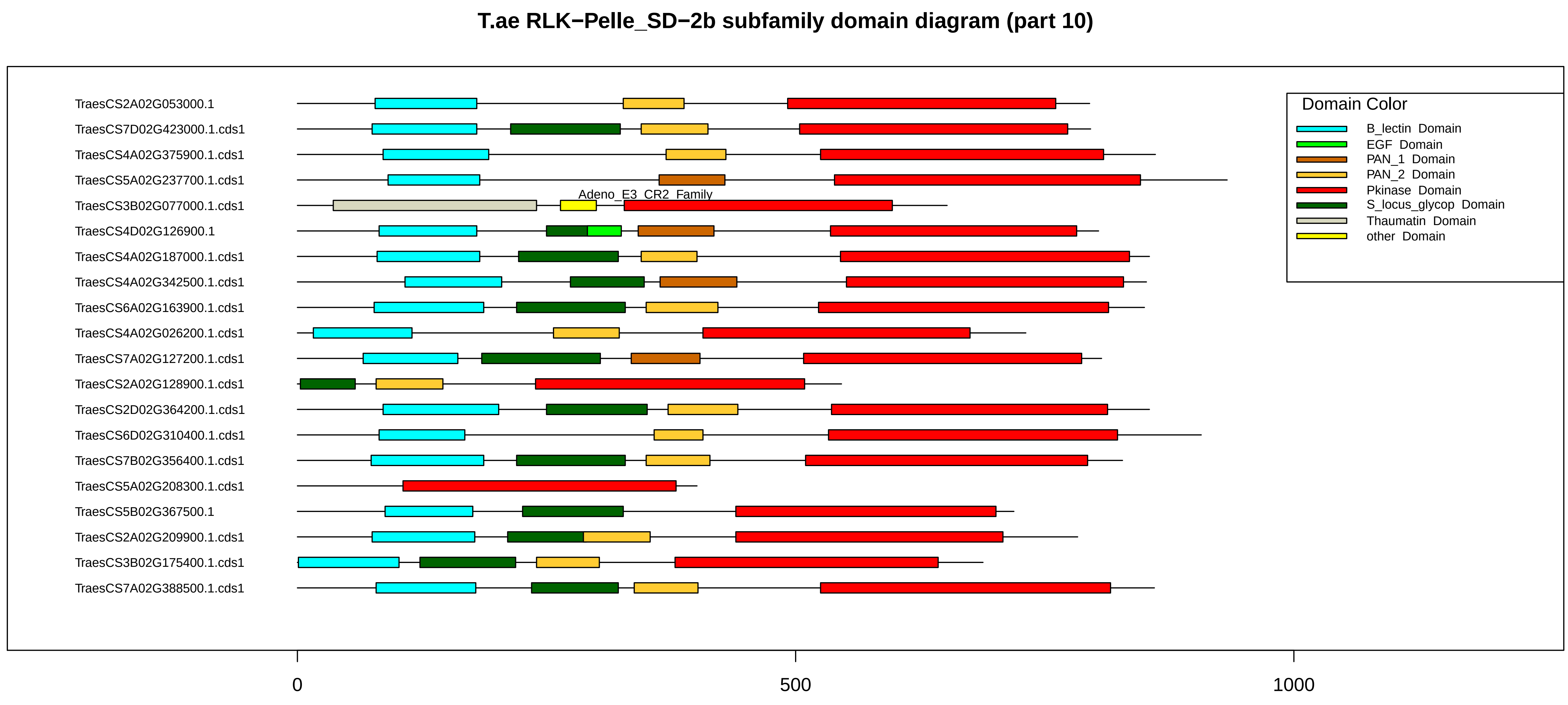
T.ae RLK−Pelle_SD−2b subfamily domain diagram (part 10)
TraesCS2A02G053000.1
TraesCS7D02G423000.1.cds1
TraesCS4A02G375900.1.cds1
TraesCS5A02G237700.1.cds1
TraesCS3B02G077000.1.cds1
Adeno_E3_CR2_Family
TraesCS4D02G126900.1
TraesCS4A02G187000.1.cds1
TraesCS4A02G342500.1.cds1
TraesCS6A02G163900.1.cds1
TraesCS4A02G026200.1.cds1
TraesCS7A02G127200.1.cds1
TraesCS2A02G128900.1.cds1
TraesCS2D02G364200.1.cds1
TraesCS6D02G310400.1.cds1
TraesCS7B02G356400.1.cds1
TraesCS5A02G208300.1.cds1
TraesCS5B02G367500.1
TraesCS2A02G209900.1.cds1
TraesCS3B02G175400.1.cds1
TraesCS7A02G388500.1.cds1
Domain Color
B_lectin Domain
EGF Domain
PAN_1 Domain
PAN_2 Domain
Pkinase Domain
S_locus_glycop Domain
Thaumatin Domain
other Domain
0
500
1000

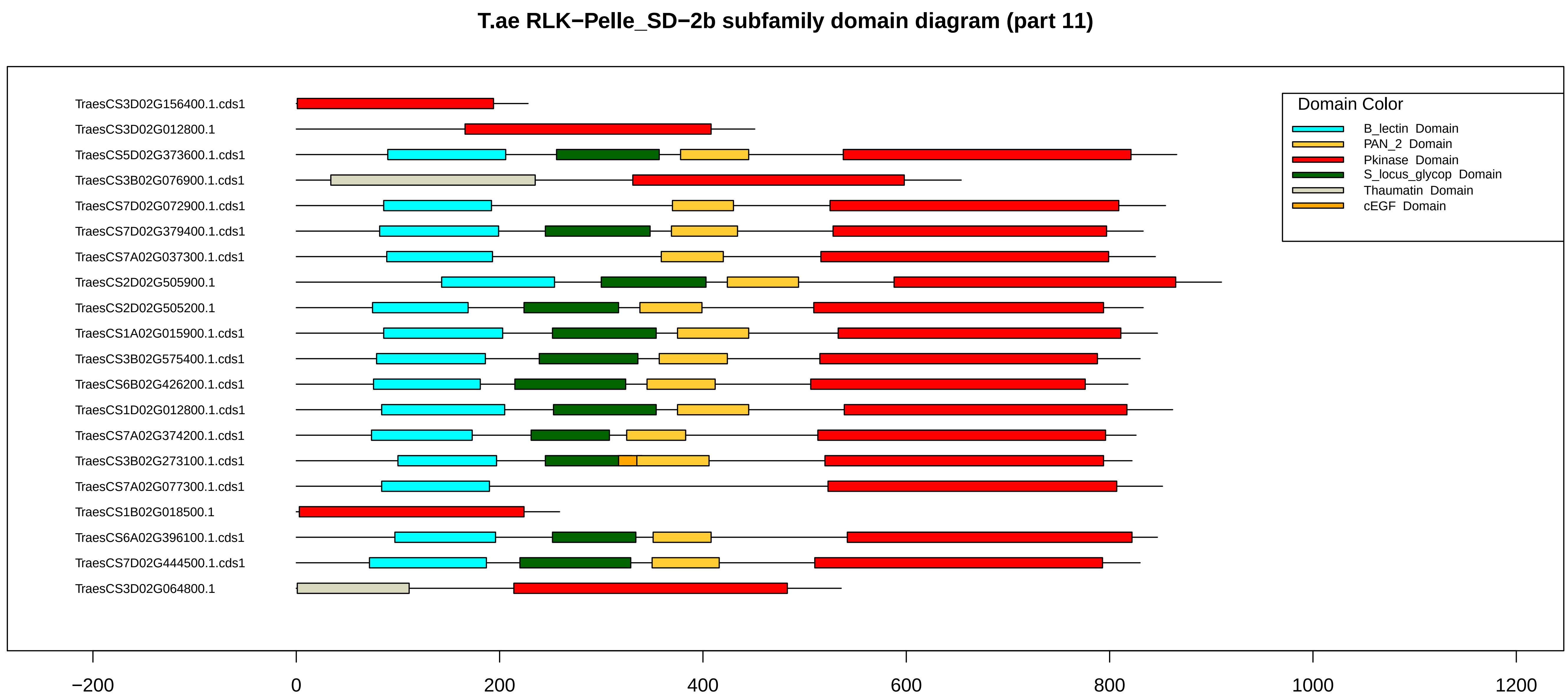
T.ae RLK−Pelle_SD−2b subfamily domain diagram (part 11)
TraesCS3D02G156400.1.cds1
TraesCS3D02G012800.1
TraesCS5D02G373600.1.cds1
TraesCS3B02G076900.1.cds1
TraesCS7D02G072900.1.cds1
TraesCS7D02G379400.1.cds1
TraesCS7A02G037300.1.cds1
TraesCS2D02G505900.1
TraesCS2D02G505200.1
TraesCS1A02G015900.1.cds1
TraesCS3B02G575400.1.cds1
TraesCS6B02G426200.1.cds1
TraesCS1D02G012800.1.cds1
TraesCS7A02G374200.1.cds1
TraesCS3B02G273100.1.cds1
TraesCS7A02G077300.1.cds1
TraesCS1B02G018500.1
TraesCS6A02G396100.1.cds1
TraesCS7D02G444500.1.cds1
TraesCS3D02G064800.1
Domain Color
B_lectin Domain
PAN_2 Domain
Pkinase Domain
S_locus_glycop Domain
Thaumatin Domain
cEGF Domain
−200
0
200
400
600
800
1000
1200

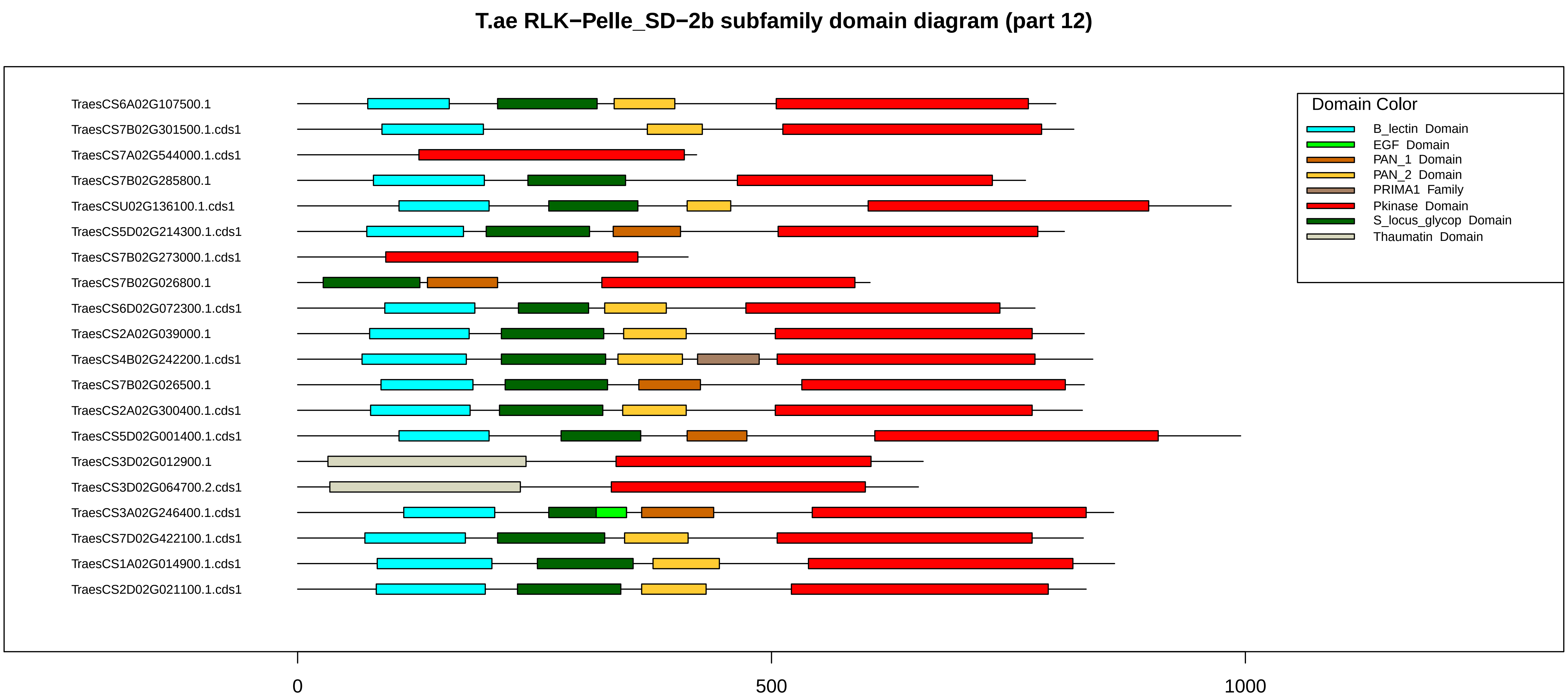
T.ae RLK−Pelle_SD−2b subfamily domain diagram (part 12)
TraesCS6A02G107500.1
TraesCS7B02G301500.1.cds1
TraesCS7A02G544000.1.cds1
TraesCS7B02G285800.1
TraesCSU02G136100.1.cds1
TraesCS5D02G214300.1.cds1
TraesCS7B02G273000.1.cds1
TraesCS7B02G026800.1
TraesCS6D02G072300.1.cds1
TraesCS2A02G039000.1
TraesCS4B02G242200.1.cds1
TraesCS7B02G026500.1
TraesCS2A02G300400.1.cds1
TraesCS5D02G001400.1.cds1
TraesCS3D02G012900.1
TraesCS3D02G064700.2.cds1
TraesCS3A02G246400.1.cds1
TraesCS7D02G422100.1.cds1
TraesCS1A02G014900.1.cds1
TraesCS2D02G021100.1.cds1
Domain Color
B_lectin Domain
EGF Domain
PAN_1 Domain
PAN_2 Domain
PRIMA1 Family
Pkinase Domain
S_locus_glycop Domain
Thaumatin Domain
0
500
1000

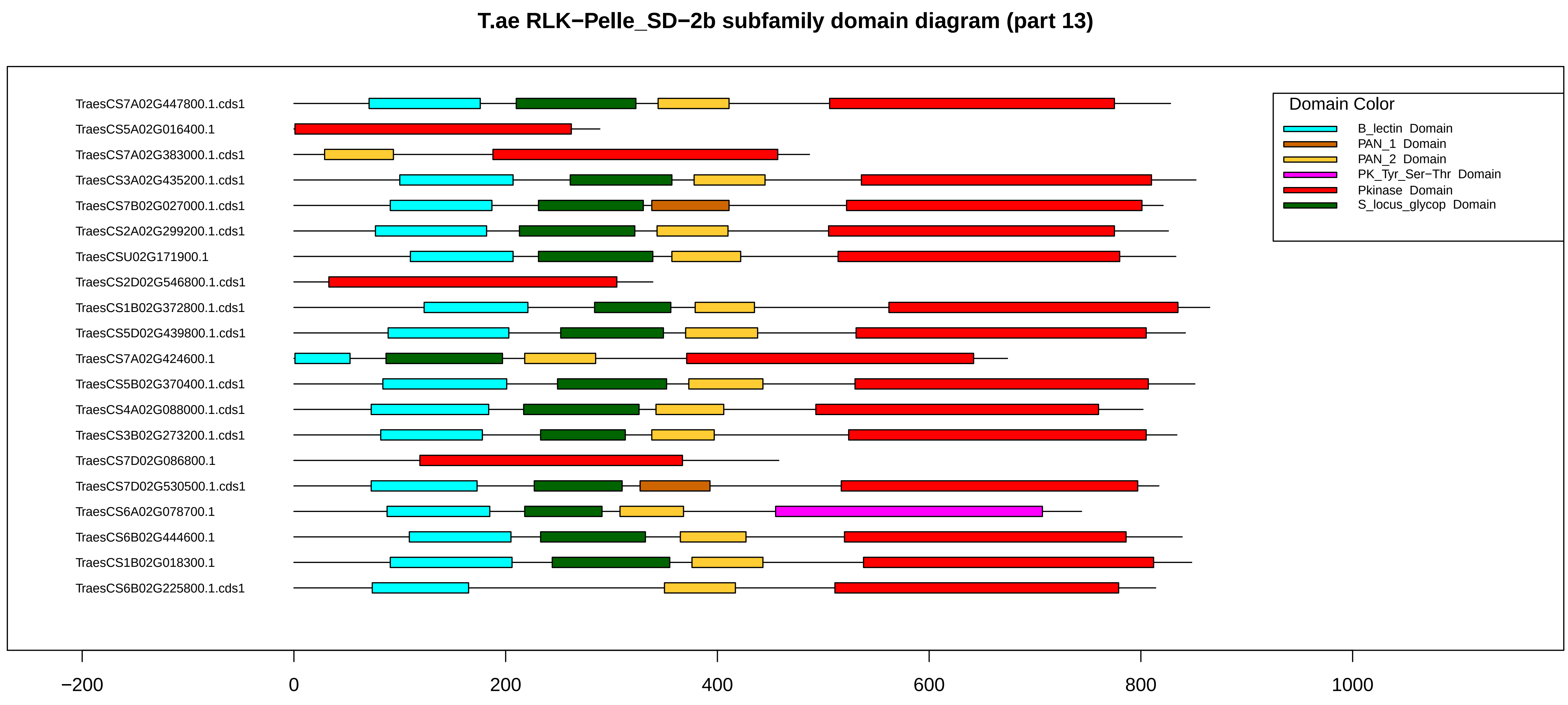
T.ae RLK−Pelle_SD−2b subfamily domain diagram (part 13)
TraesCS7A02G447800.1.cds1
TraesCS5A02G016400.1
TraesCS7A02G383000.1.cds1
TraesCS3A02G435200.1.cds1
TraesCS7B02G027000.1.cds1
TraesCS2A02G299200.1.cds1
TraesCSU02G171900.1
TraesCS2D02G546800.1.cds1
TraesCS1B02G372800.1.cds1
TraesCS5D02G439800.1.cds1
TraesCS7A02G424600.1
TraesCS5B02G370400.1.cds1
TraesCS4A02G088000.1.cds1
TraesCS3B02G273200.1.cds1
TraesCS7D02G086800.1
TraesCS7D02G530500.1.cds1
TraesCS6A02G078700.1
TraesCS6B02G444600.1
TraesCS1B02G018300.1
TraesCS6B02G225800.1.cds1
Domain Color
B_lectin Domain
PAN_1 Domain
PAN_2 Domain
PK_Tyr_Ser−Thr Domain
Pkinase Domain
S_locus_glycop Domain
−200
0
200
400
600
800
1000

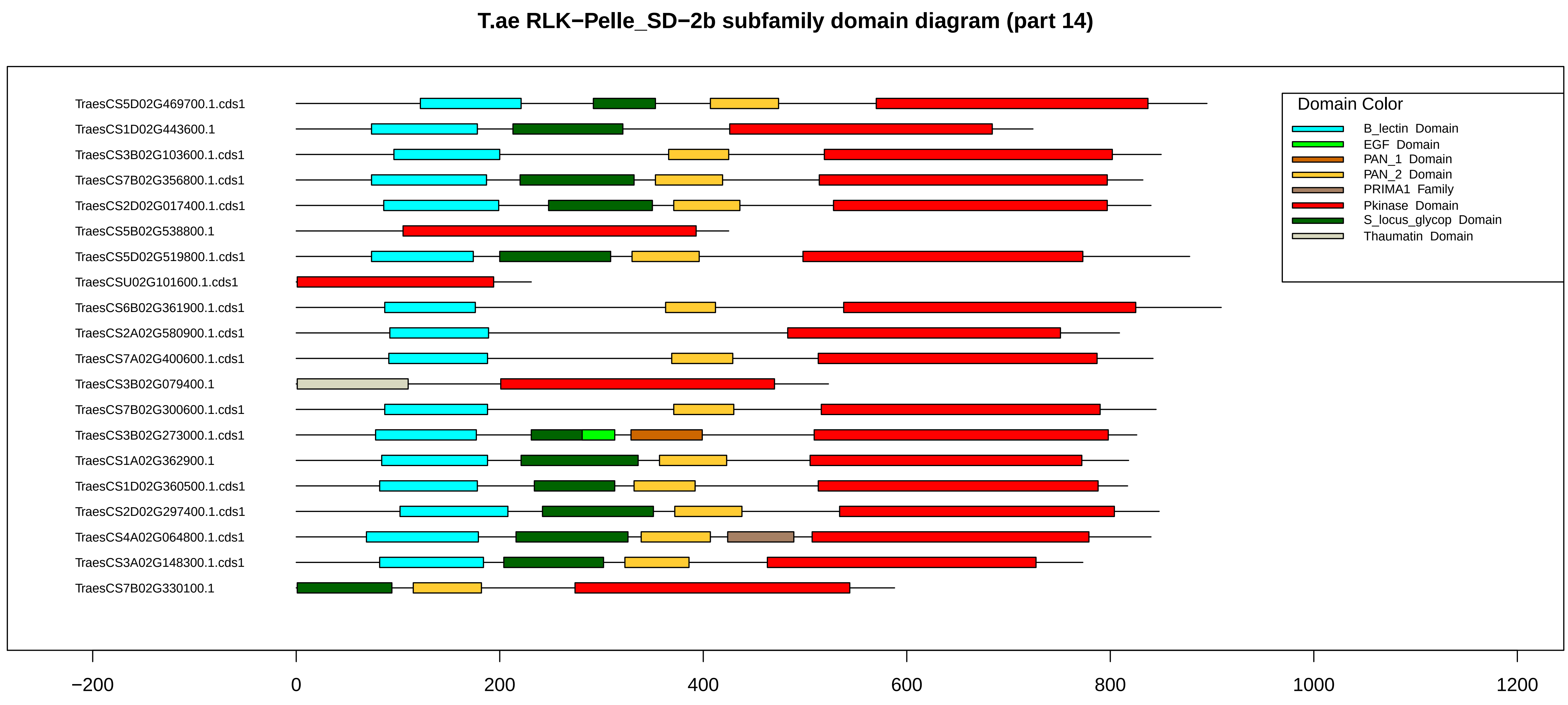
T.ae RLK−Pelle_SD−2b subfamily domain diagram (part 14)
TraesCS5D02G469700.1.cds1
TraesCS1D02G443600.1
TraesCS3B02G103600.1.cds1
TraesCS7B02G356800.1.cds1
TraesCS2D02G017400.1.cds1
TraesCS5B02G538800.1
TraesCS5D02G519800.1.cds1
TraesCSU02G101600.1.cds1
TraesCS6B02G361900.1.cds1
TraesCS2A02G580900.1.cds1
TraesCS7A02G400600.1.cds1
TraesCS3B02G079400.1
TraesCS7B02G300600.1.cds1
TraesCS3B02G273000.1.cds1
TraesCS1A02G362900.1
TraesCS1D02G360500.1.cds1
TraesCS2D02G297400.1.cds1
TraesCS4A02G064800.1.cds1
TraesCS3A02G148300.1.cds1
TraesCS7B02G330100.1
Domain Color
B_lectin Domain
EGF Domain
PAN_1 Domain
PAN_2 Domain
PRIMA1 Family
Pkinase Domain
S_locus_glycop Domain
Thaumatin Domain
−200
0
200
400
600
800
1000
1200

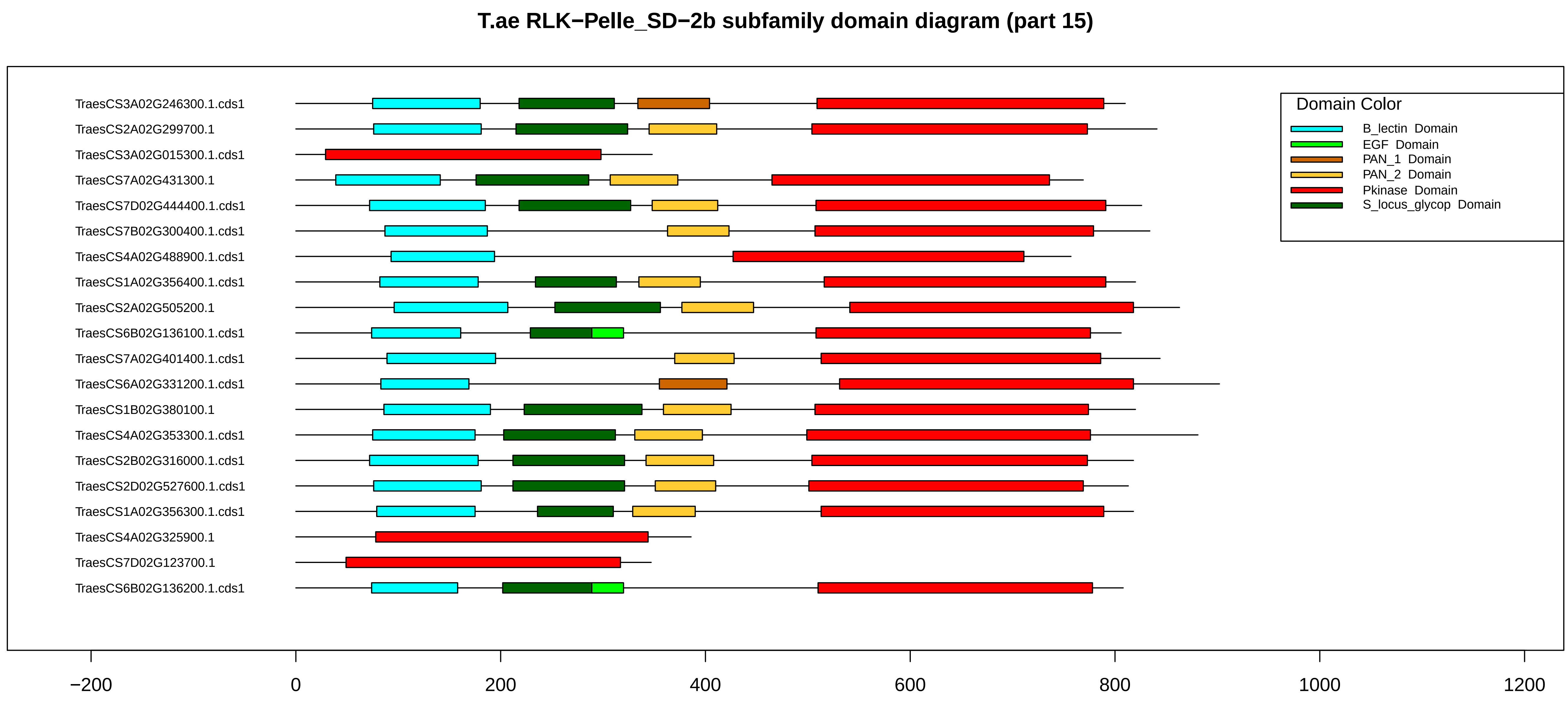

T.ae RLK−Pelle_SD−2b subfamily domain diagram (part 15)
TraesCS3A02G246300.1.cds1
TraesCS2A02G299700.1
TraesCS3A02G015300.1.cds1
TraesCS7A02G431300.1
TraesCS7D02G444400.1.cds1
TraesCS7B02G300400.1.cds1
TraesCS4A02G488900.1.cds1
TraesCS1A02G356400.1.cds1
TraesCS2A02G505200.1
TraesCS6B02G136100.1.cds1
TraesCS7A02G401400.1.cds1
TraesCS6A02G331200.1.cds1
TraesCS1B02G380100.1
TraesCS4A02G353300.1.cds1
TraesCS2B02G316000.1.cds1
TraesCS2D02G527600.1.cds1
TraesCS1A02G356300.1.cds1
TraesCS4A02G325900.1
TraesCS7D02G123700.1
TraesCS6B02G136200.1.cds1
Domain Color
B_lectin Domain
EGF Domain
PAN_1 Domain
PAN_2 Domain
Pkinase Domain
S_locus_glycop Domain
−200
0
200
400
600
800
1000
1200

T.ae RLK−Pelle_SD−2b subfamily domain diagram (part 16)

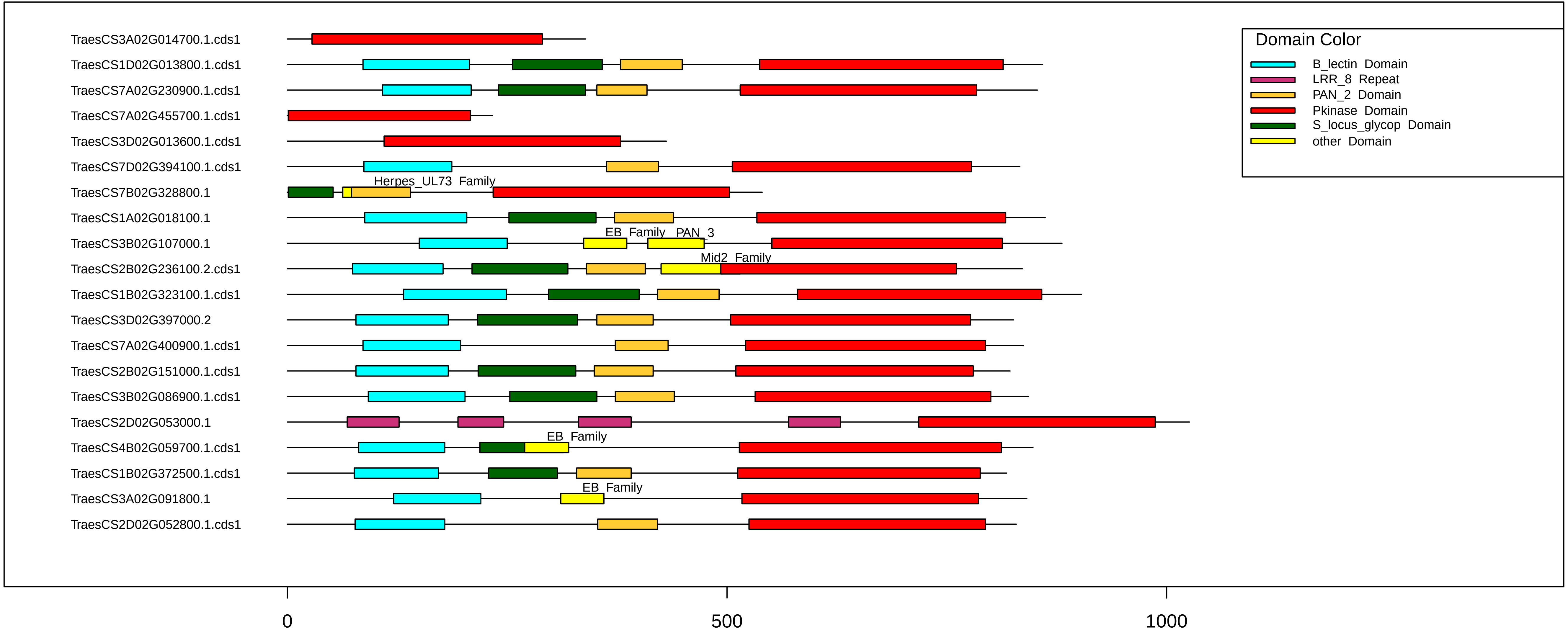

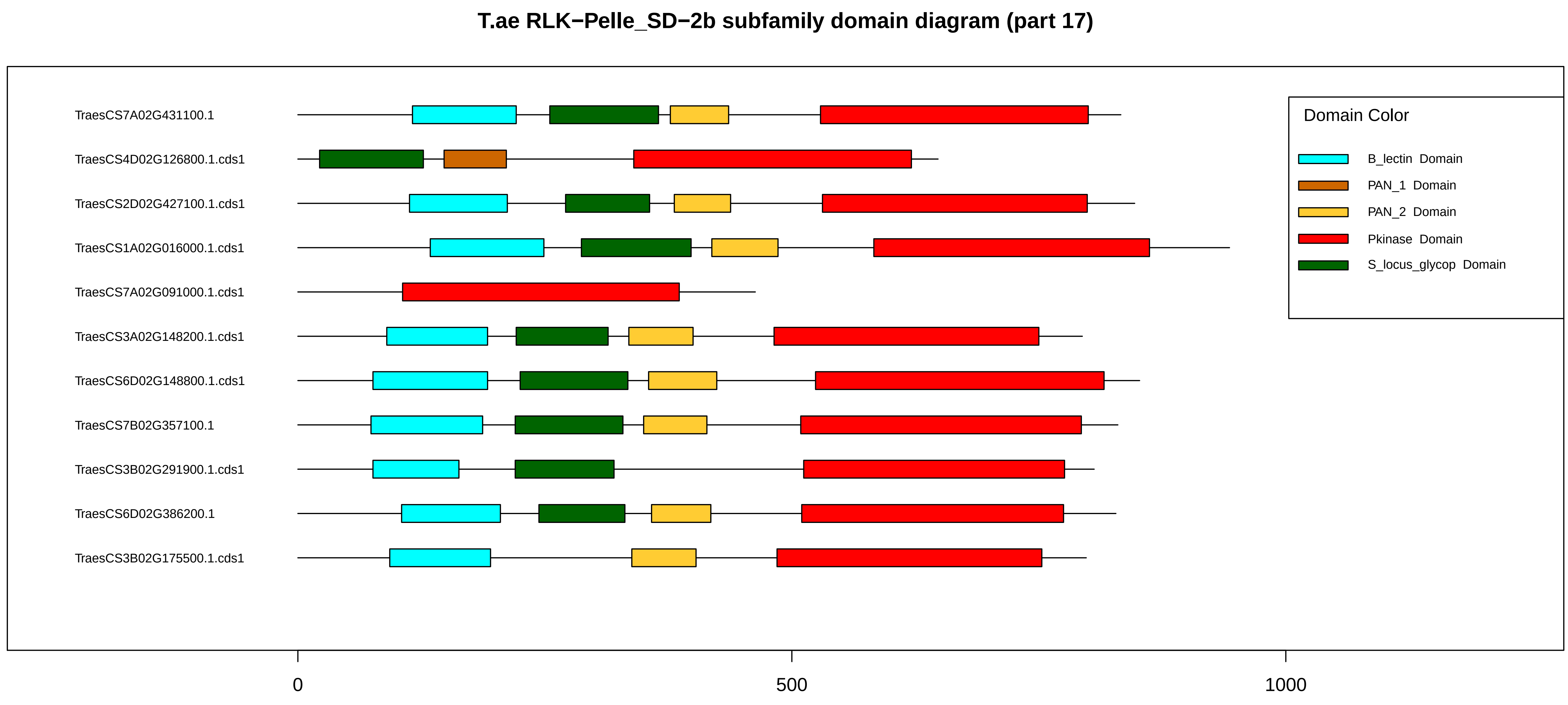
T.ae RLK−Pelle_SD−2b subfamily domain diagram (part 17)
TraesCS7A02G431100.1
TraesCS4D02G126800.1.cds1
TraesCS2D02G427100.1.cds1
TraesCS1A02G016000.1.cds1
TraesCS7A02G091000.1.cds1
TraesCS3A02G148200.1.cds1
TraesCS6D02G148800.1.cds1
TraesCS7B02G357100.1
TraesCS3B02G291900.1.cds1
TraesCS6D02G386200.1
TraesCS3B02G175500.1.cds1
Domain Color
B_lectin Domain
PAN_1 Domain
PAN_2 Domain
Pkinase Domain
S_locus_glycop Domain
0
500
1000

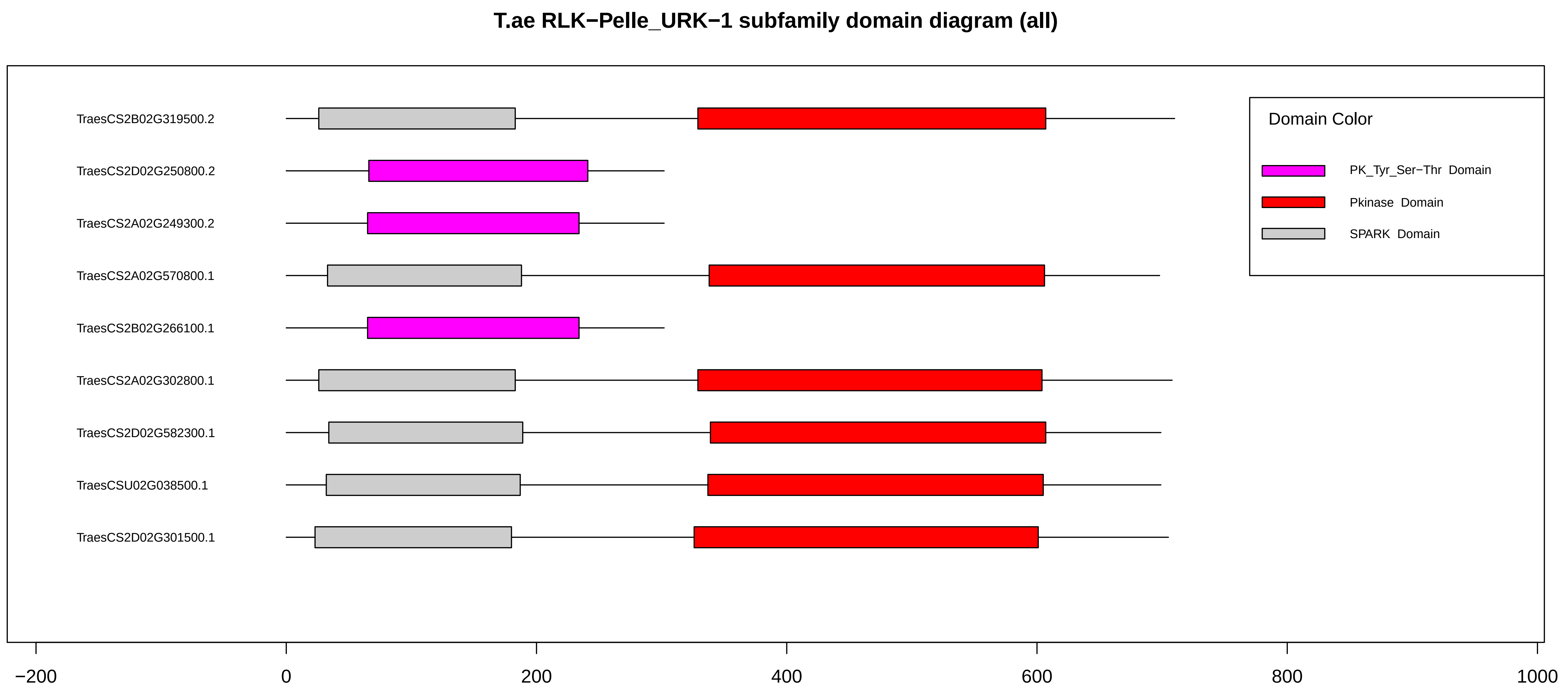
T.ae RLK−Pelle_URK−1 subfamily domain diagram (all)
TraesCS2B02G319500.2
TraesCS2D02G250800.2
TraesCS2A02G249300.2
TraesCS2A02G570800.1
TraesCS2B02G266100.1
TraesCS2A02G302800.1
TraesCS2D02G582300.1
TraesCSU02G038500.1
TraesCS2D02G301500.1
Domain Color
PK_Tyr_Ser−Thr Domain
Pkinase Domain
SPARK Domain
−200
0
200
400
600
800
1000

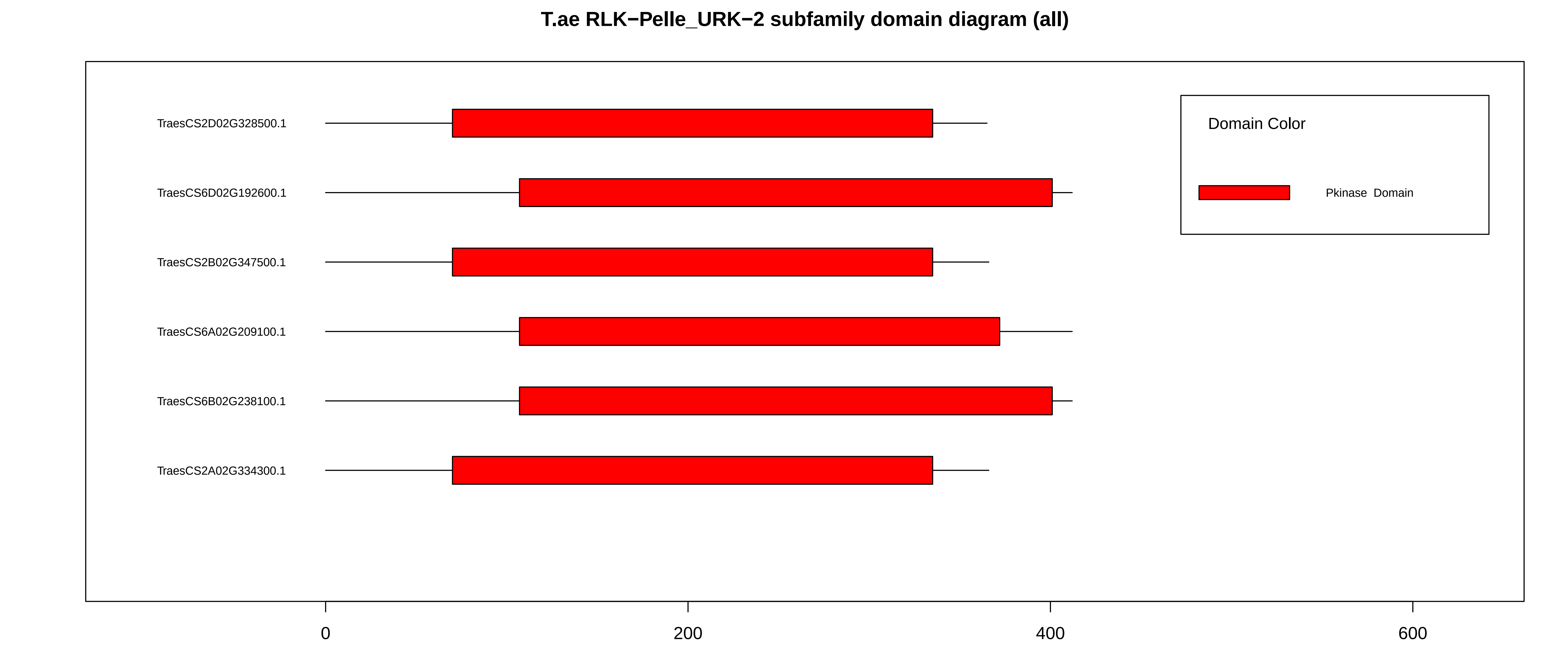

T.ae RLK−Pelle_URK−2 subfamily domain diagram (all)
TraesCS2D02G328500.1
TraesCS6D02G192600.1
TraesCS2B02G347500.1
TraesCS6A02G209100.1
TraesCS6B02G238100.1
TraesCS2A02G334300.1
Domain Color
Pkinase Domain
0
200
400
600

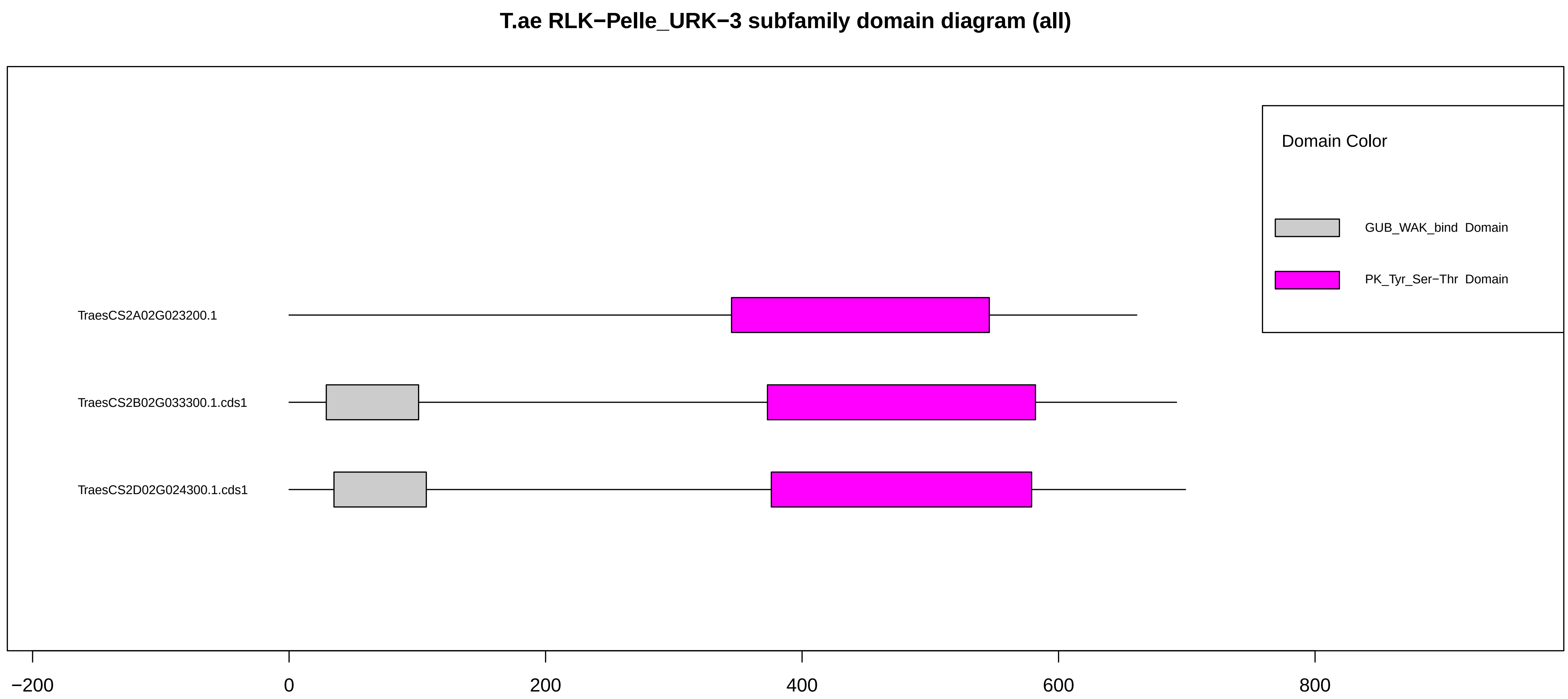
T.ae RLK−Pelle_URK−3 subfamily domain diagram (all)
Domain Color
GUB_WAK_bind Domain
PK_Tyr_Ser−Thr Domain
TraesCS2A02G023200.1
TraesCS2B02G033300.1.cds1
TraesCS2D02G024300.1.cds1
−200
0
200
400
600
800

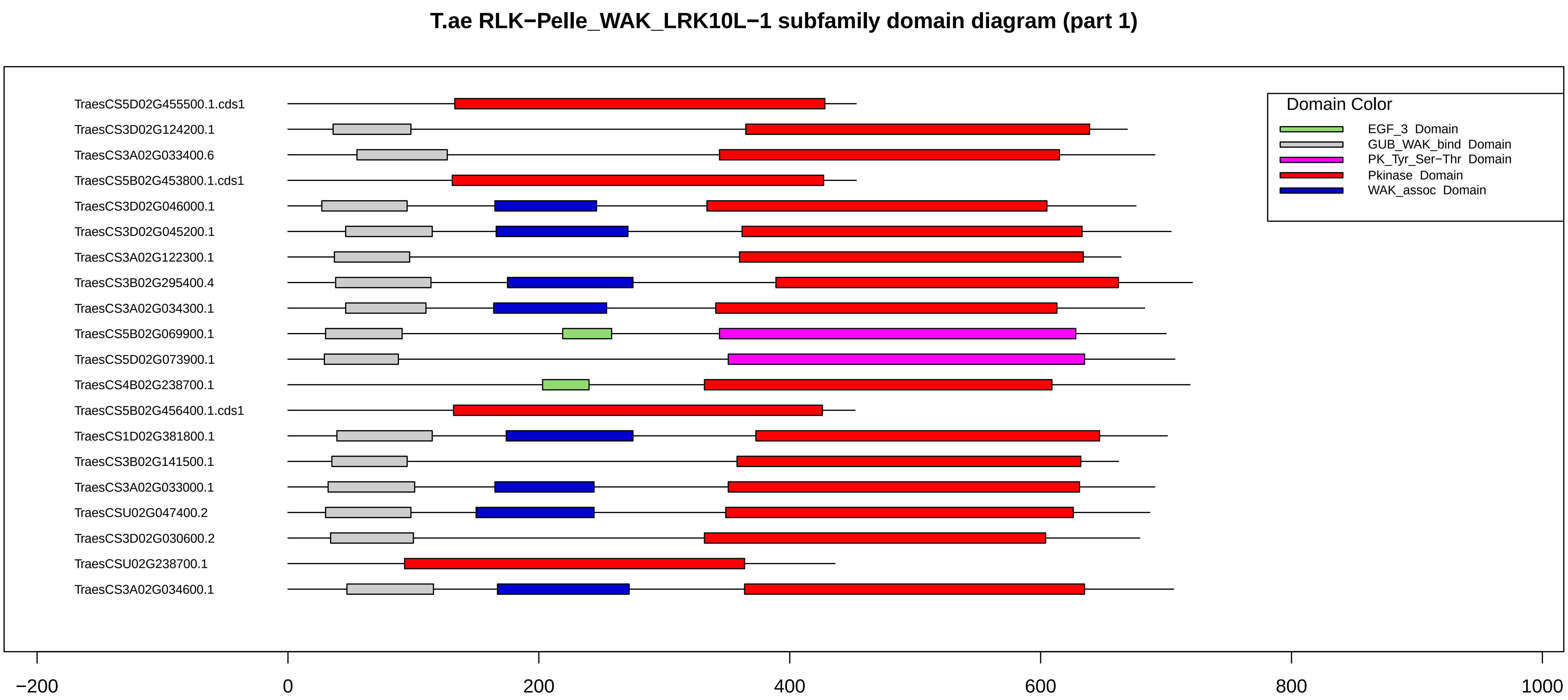
T.ae RLK−Pelle_WAK_LRK10L−1 subfamily domain diagram (part 1)
TraesCS5D02G455500.1.cds1
TraesCS3D02G124200.1
TraesCS3A02G033400.6
TraesCS5B02G453800.1.cds1
TraesCS3D02G046000.1
TraesCS3D02G045200.1
TraesCS3A02G122300.1
TraesCS3B02G295400.4
TraesCS3A02G034300.1
TraesCS5B02G069900.1
TraesCS5D02G073900.1
TraesCS4B02G238700.1
TraesCS5B02G456400.1.cds1
TraesCS1D02G381800.1
TraesCS3B02G141500.1
TraesCS3A02G033000.1
TraesCSU02G047400.2
TraesCS3D02G030600.2
TraesCSU02G238700.1
TraesCS3A02G034600.1
Domain Color
EGF_3 Domain
GUB_WAK_bind Domain
PK_Tyr_Ser−Thr Domain
Pkinase Domain
WAK_assoc Domain
−200
0
200
400
600
800
1000

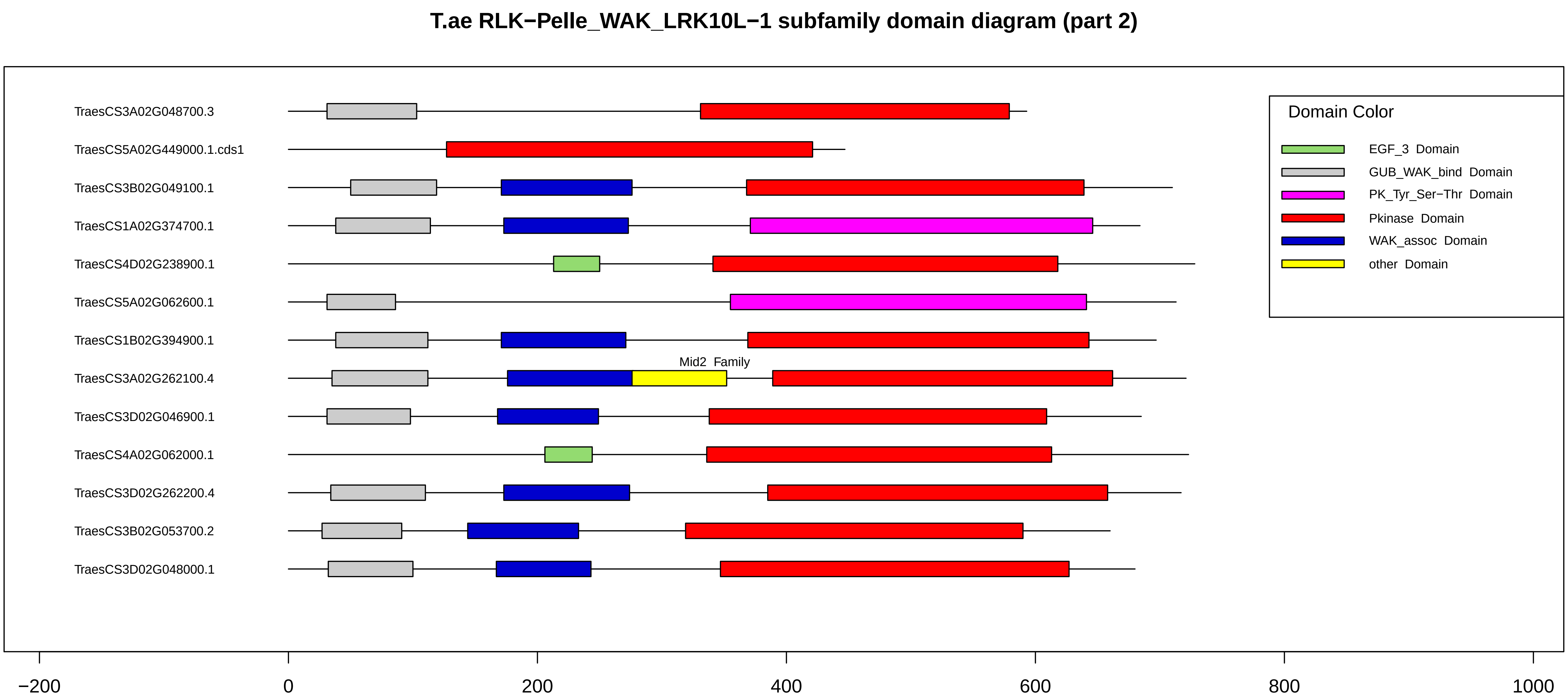
T.ae RLK−Pelle_WAK_LRK10L−1 subfamily domain diagram (part 2)
TraesCS3A02G048700.3
TraesCS5A02G449000.1.cds1
TraesCS3B02G049100.1
TraesCS1A02G374700.1
TraesCS4D02G238900.1
TraesCS5A02G062600.1
TraesCS1B02G394900.1
TraesCS3A02G262100.4
TraesCS3D02G046900.1
TraesCS4A02G062000.1
TraesCS3D02G262200.4
TraesCS3B02G053700.2
TraesCS3D02G048000.1
Mid2 Family
Domain Color
EGF_3 Domain
GUB_WAK_bind Domain
PK_Tyr_Ser−Thr Domain
Pkinase Domain
WAK_assoc Domain
other Domain
−200
0
200
400
600
800
1000

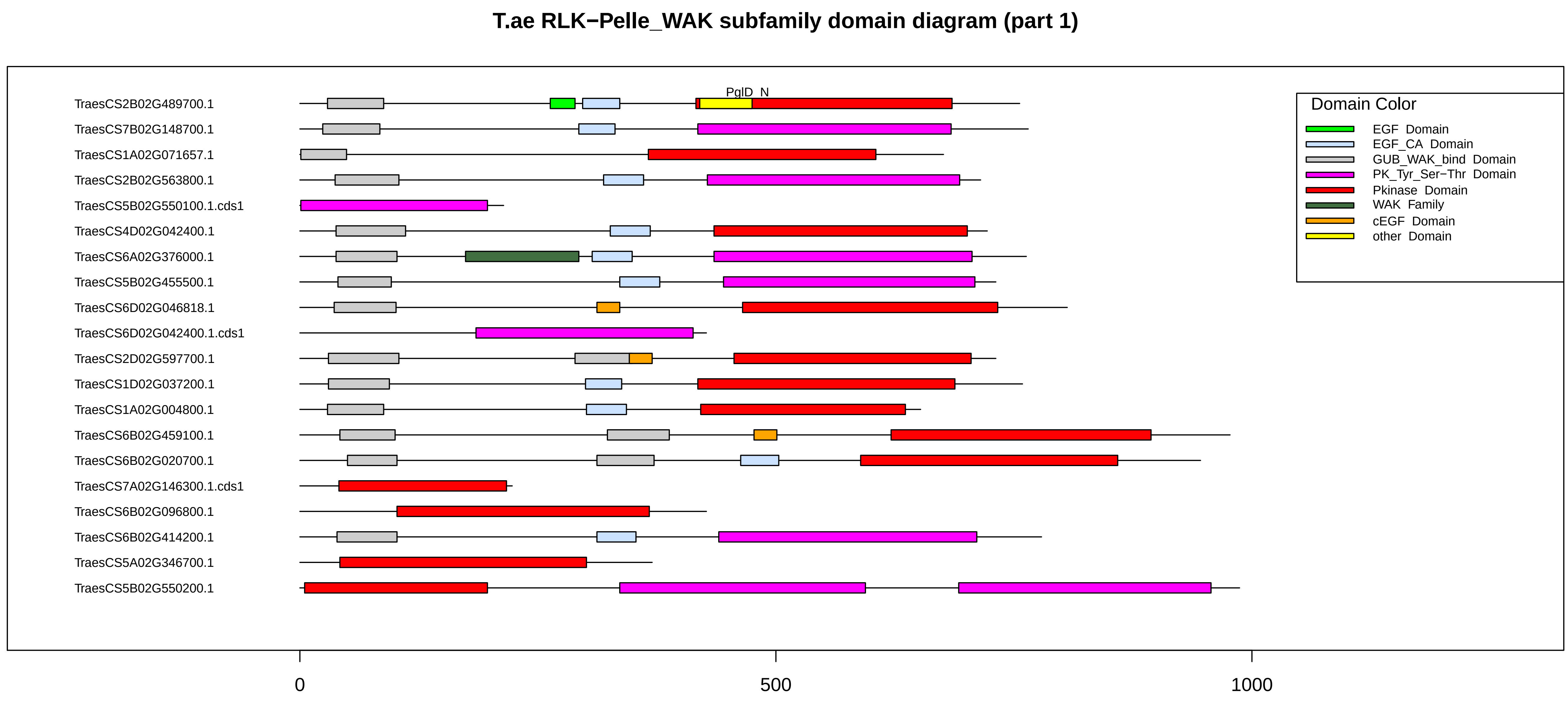
T.ae RLK−Pelle_WAK subfamily domain diagram (part 1)
TraesCS2B02G489700.1
TraesCS7B02G148700.1
TraesCS1A02G071657.1
TraesCS2B02G563800.1
TraesCS5B02G550100.1.cds1
TraesCS4D02G042400.1
TraesCS6A02G376000.1
TraesCS5B02G455500.1
TraesCS6D02G046818.1
TraesCS6D02G042400.1.cds1
TraesCS2D02G597700.1
TraesCS1D02G037200.1
TraesCS1A02G004800.1
TraesCS6B02G459100.1
TraesCS6B02G020700.1
TraesCS7A02G146300.1.cds1
TraesCS6B02G096800.1
TraesCS6B02G414200.1
TraesCS5A02G346700.1
TraesCS5B02G550200.1
PglD_N
Domain Color
EGF Domain
EGF_CA Domain
GUB_WAK_bind Domain
PK_Tyr_Ser−Thr Domain
Pkinase Domain
WAK Family
cEGF Domain
other Domain
0
500
1000

T.ae RLK−Pelle_WAK subfamily domain diagram (part 2)

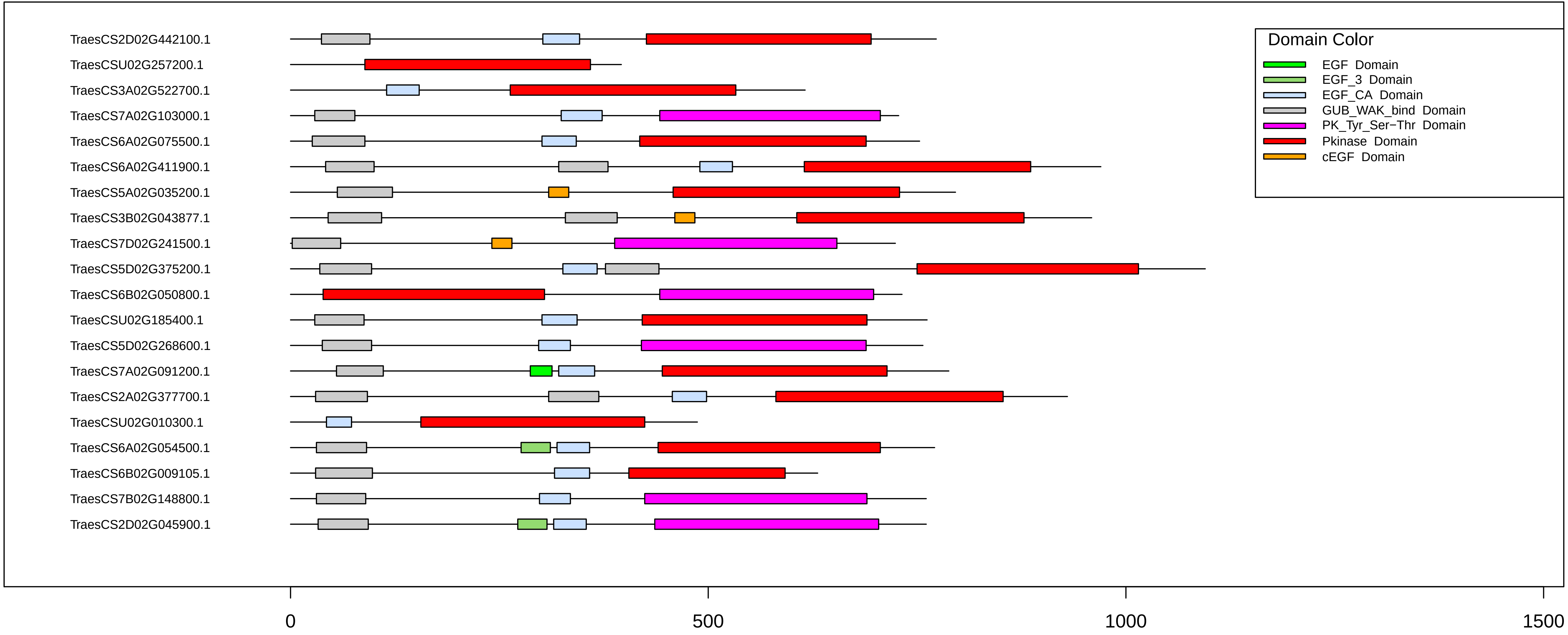

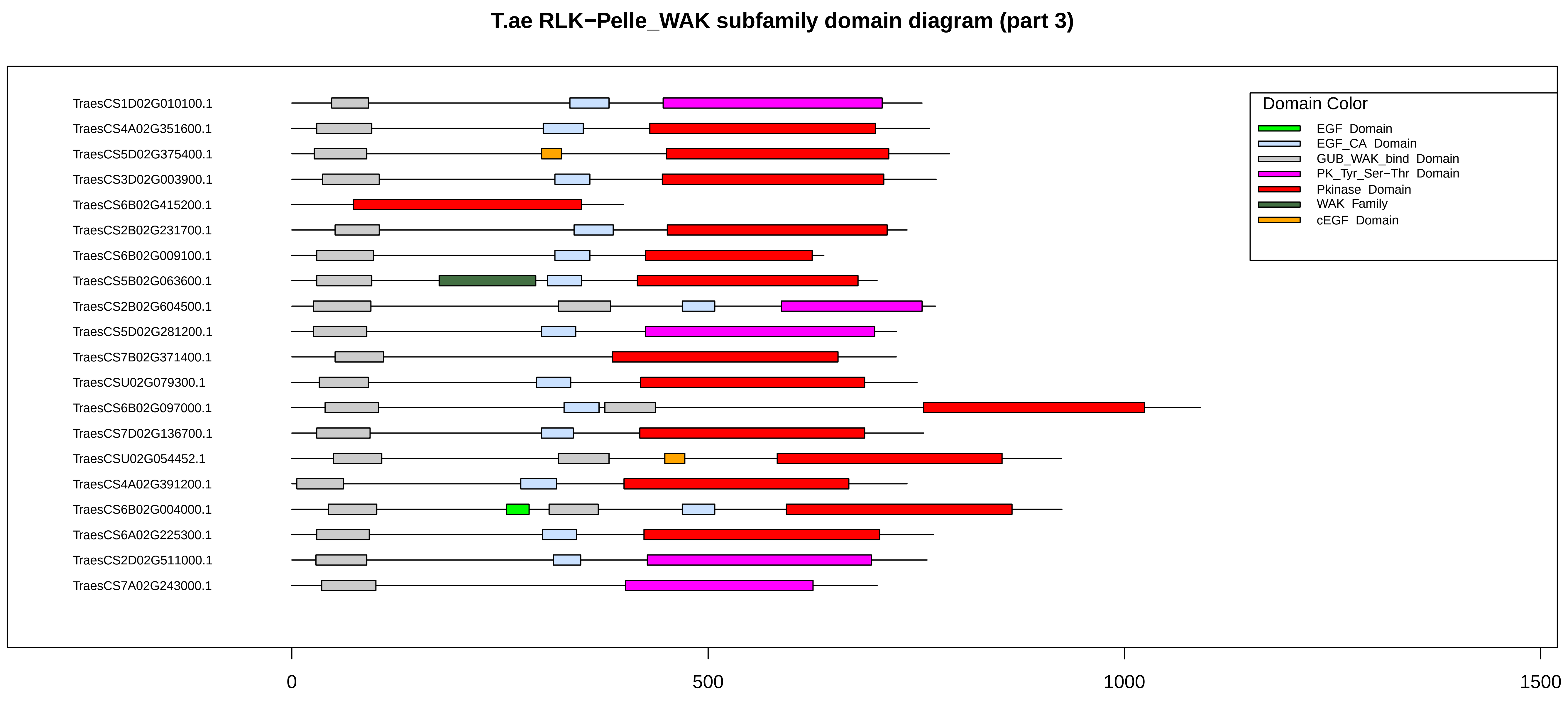
T.ae RLK−Pelle_WAK subfamily domain diagram (part 3)
TraesCS1D02G010100.1
TraesCS4A02G351600.1
TraesCS5D02G375400.1
TraesCS3D02G003900.1
TraesCS6B02G415200.1
TraesCS2B02G231700.1
TraesCS6B02G009100.1
TraesCS5B02G063600.1
TraesCS2B02G604500.1
TraesCS5D02G281200.1
TraesCS7B02G371400.1
TraesCSU02G079300.1
TraesCS6B02G097000.1
TraesCS7D02G136700.1
TraesCSU02G054452.1
TraesCS4A02G391200.1
TraesCS6B02G004000.1
TraesCS6A02G225300.1
TraesCS2D02G511000.1
TraesCS7A02G243000.1
Domain Color
EGF Domain
EGF_CA Domain
GUB_WAK_bind Domain
PK_Tyr_Ser−Thr Domain
Pkinase Domain
WAK Family
cEGF Domain
0
500
1000
1500

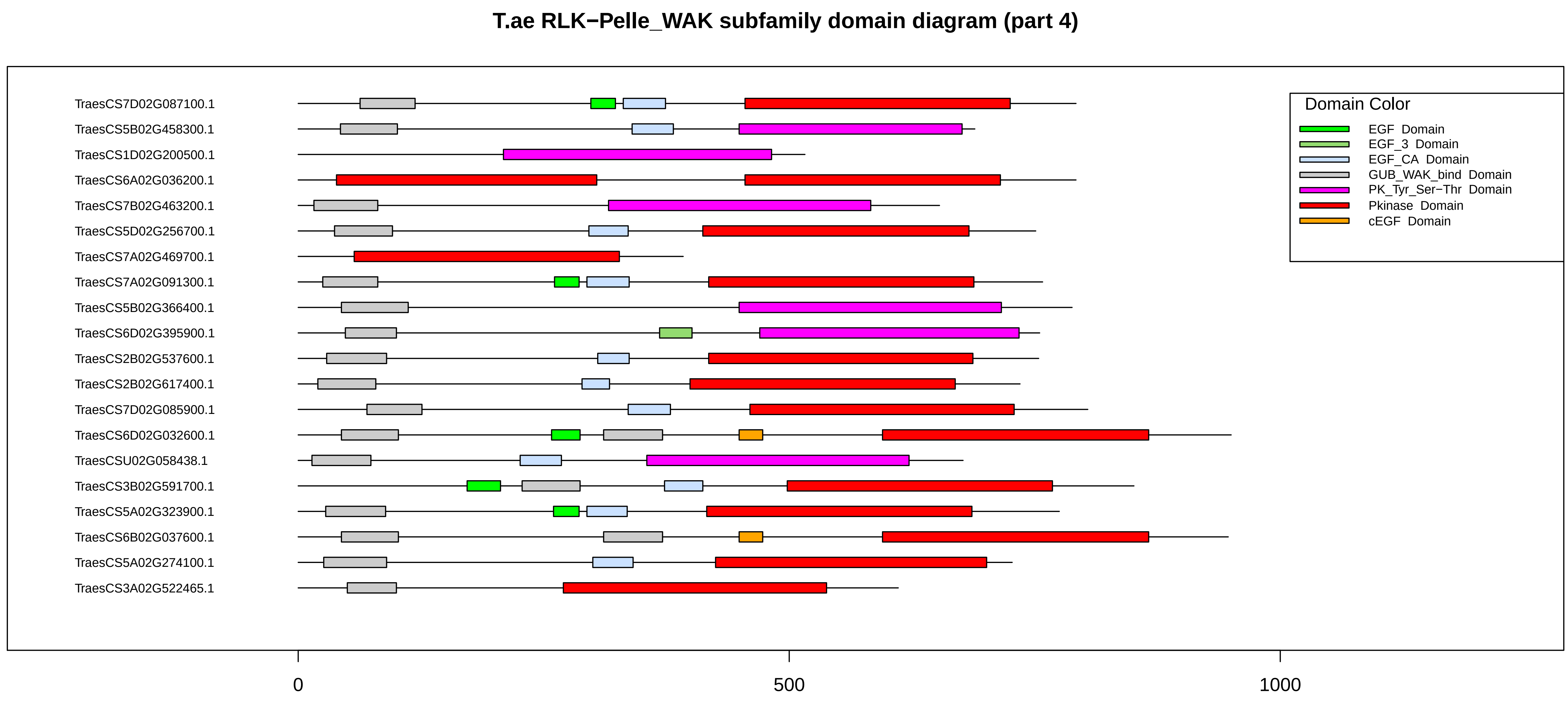
T.ae RLK−Pelle_WAK subfamily domain diagram (part 4)
TraesCS7D02G087100.1
TraesCS5B02G458300.1
TraesCS1D02G200500.1
TraesCS6A02G036200.1
TraesCS7B02G463200.1
TraesCS5D02G256700.1
TraesCS7A02G469700.1
TraesCS7A02G091300.1
TraesCS5B02G366400.1
TraesCS6D02G395900.1
TraesCS2B02G537600.1
TraesCS2B02G617400.1
TraesCS7D02G085900.1
TraesCS6D02G032600.1
TraesCSU02G058438.1
TraesCS3B02G591700.1
TraesCS5A02G323900.1
TraesCS6B02G037600.1
TraesCS5A02G274100.1
TraesCS3A02G522465.1
Domain Color
EGF Domain
EGF_3 Domain
EGF_CA Domain
GUB_WAK_bind Domain
PK_Tyr_Ser−Thr Domain
Pkinase Domain
cEGF Domain
0
500
1000

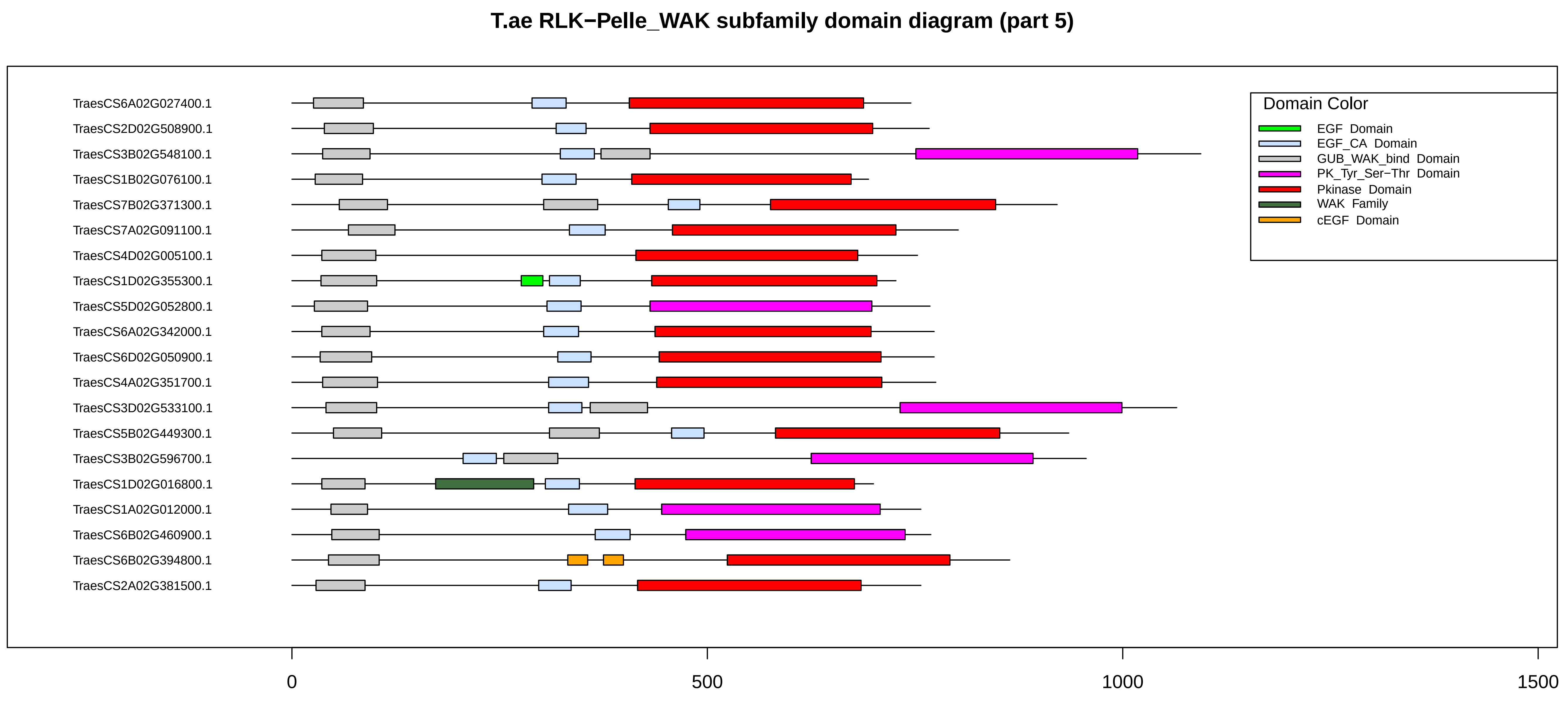
T.ae RLK−Pelle_WAK subfamily domain diagram (part 5)
TraesCS6A02G027400.1
TraesCS2D02G508900.1
TraesCS3B02G548100.1
TraesCS1B02G076100.1
TraesCS7B02G371300.1
TraesCS7A02G091100.1
TraesCS4D02G005100.1
TraesCS1D02G355300.1
TraesCS5D02G052800.1
TraesCS6A02G342000.1
TraesCS6D02G050900.1
TraesCS4A02G351700.1
TraesCS3D02G533100.1
TraesCS5B02G449300.1
TraesCS3B02G596700.1
TraesCS1D02G016800.1
TraesCS1A02G012000.1
TraesCS6B02G460900.1
TraesCS6B02G394800.1
TraesCS2A02G381500.1
Domain Color
EGF Domain
EGF_CA Domain
GUB_WAK_bind Domain
PK_Tyr_Ser−Thr Domain
Pkinase Domain
WAK Family
cEGF Domain
0
500
1000
1500

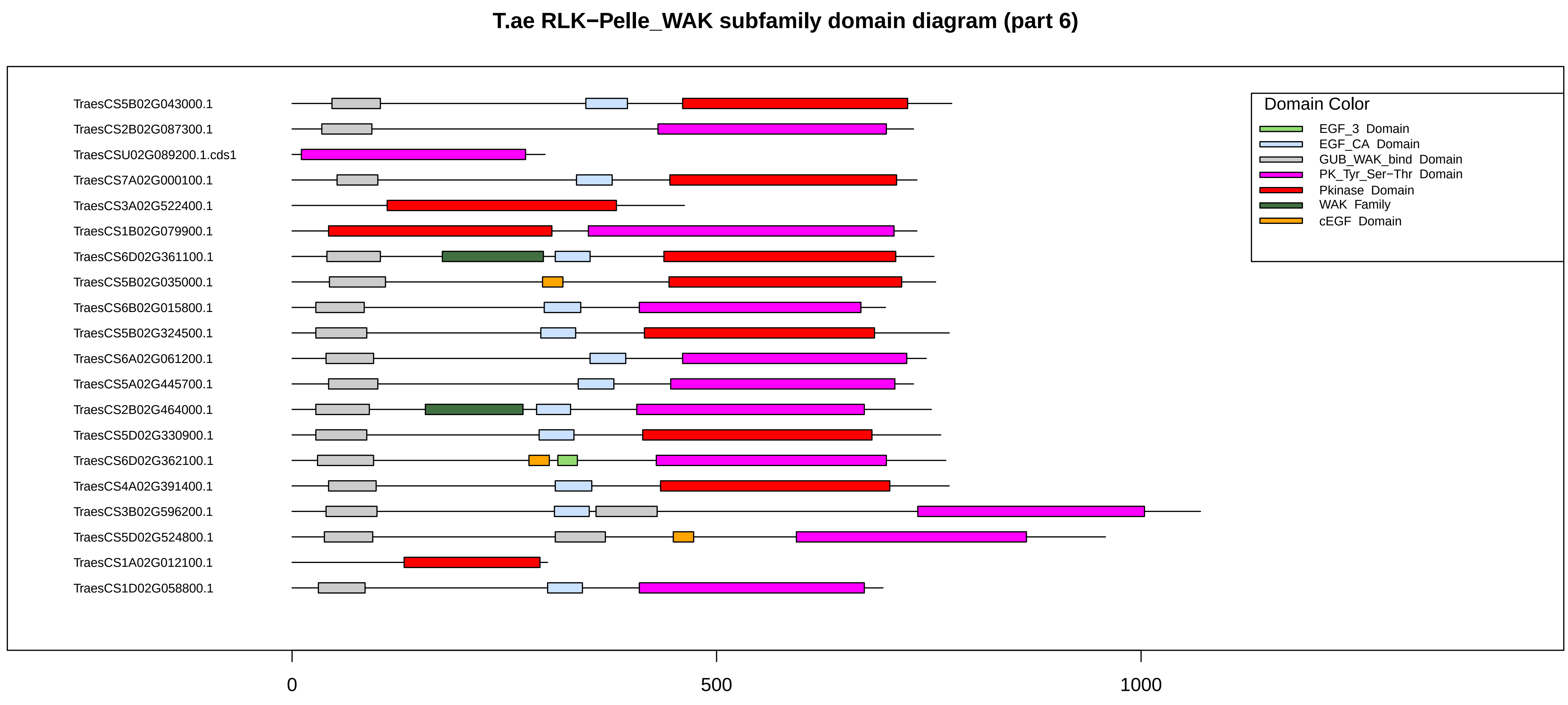
T.ae RLK−Pelle_WAK subfamily domain diagram (part 6)
TraesCS5B02G043000.1
TraesCS2B02G087300.1
TraesCSU02G089200.1.cds1
TraesCS7A02G000100.1
TraesCS3A02G522400.1
TraesCS1B02G079900.1
TraesCS6D02G361100.1
TraesCS5B02G035000.1
TraesCS6B02G015800.1
TraesCS5B02G324500.1
TraesCS6A02G061200.1
TraesCS5A02G445700.1
TraesCS2B02G464000.1
TraesCS5D02G330900.1
TraesCS6D02G362100.1
TraesCS4A02G391400.1
TraesCS3B02G596200.1
TraesCS5D02G524800.1
TraesCS1A02G012100.1
TraesCS1D02G058800.1
Domain Color
EGF_3 Domain
EGF_CA Domain
GUB_WAK_bind Domain
PK_Tyr_Ser−Thr Domain
Pkinase Domain
WAK Family
cEGF Domain
0
500
1000

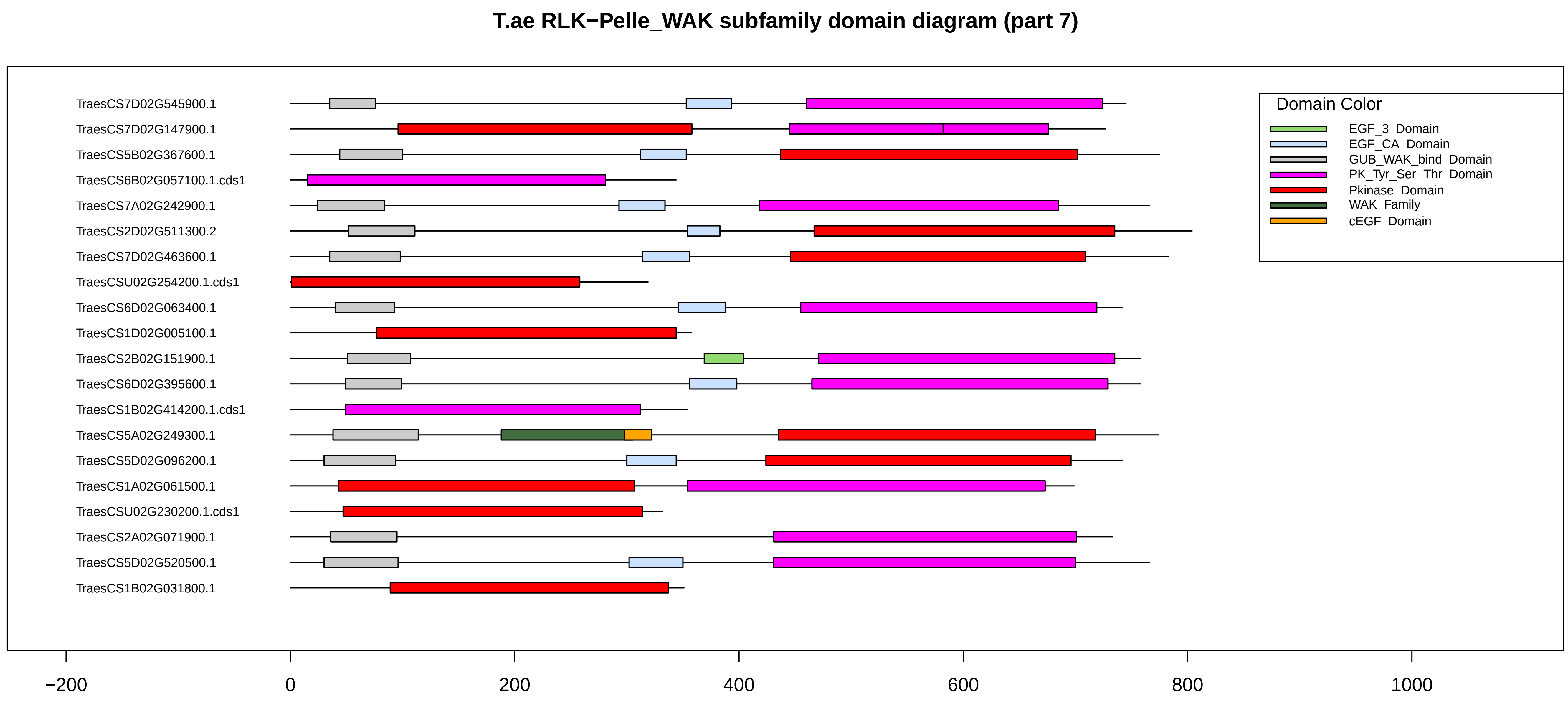
T.ae RLK−Pelle_WAK subfamily domain diagram (part 7)
TraesCS7D02G545900.1
TraesCS7D02G147900.1
TraesCS5B02G367600.1
TraesCS6B02G057100.1.cds1
TraesCS7A02G242900.1
TraesCS2D02G511300.2
TraesCS7D02G463600.1
TraesCSU02G254200.1.cds1
TraesCS6D02G063400.1
TraesCS1D02G005100.1
TraesCS2B02G151900.1
TraesCS6D02G395600.1
TraesCS1B02G414200.1.cds1
TraesCS5A02G249300.1
TraesCS5D02G096200.1
TraesCS1A02G061500.1
TraesCSU02G230200.1.cds1
TraesCS2A02G071900.1
TraesCS5D02G520500.1
TraesCS1B02G031800.1
Domain Color
EGF_3 Domain
EGF_CA Domain
GUB_WAK_bind Domain
PK_Tyr_Ser−Thr Domain
Pkinase Domain
WAK Family
cEGF Domain
−200
0
200
400
600
800
1000

T.ae RLK−Pelle_WAK subfamily domain diagram (part 8)

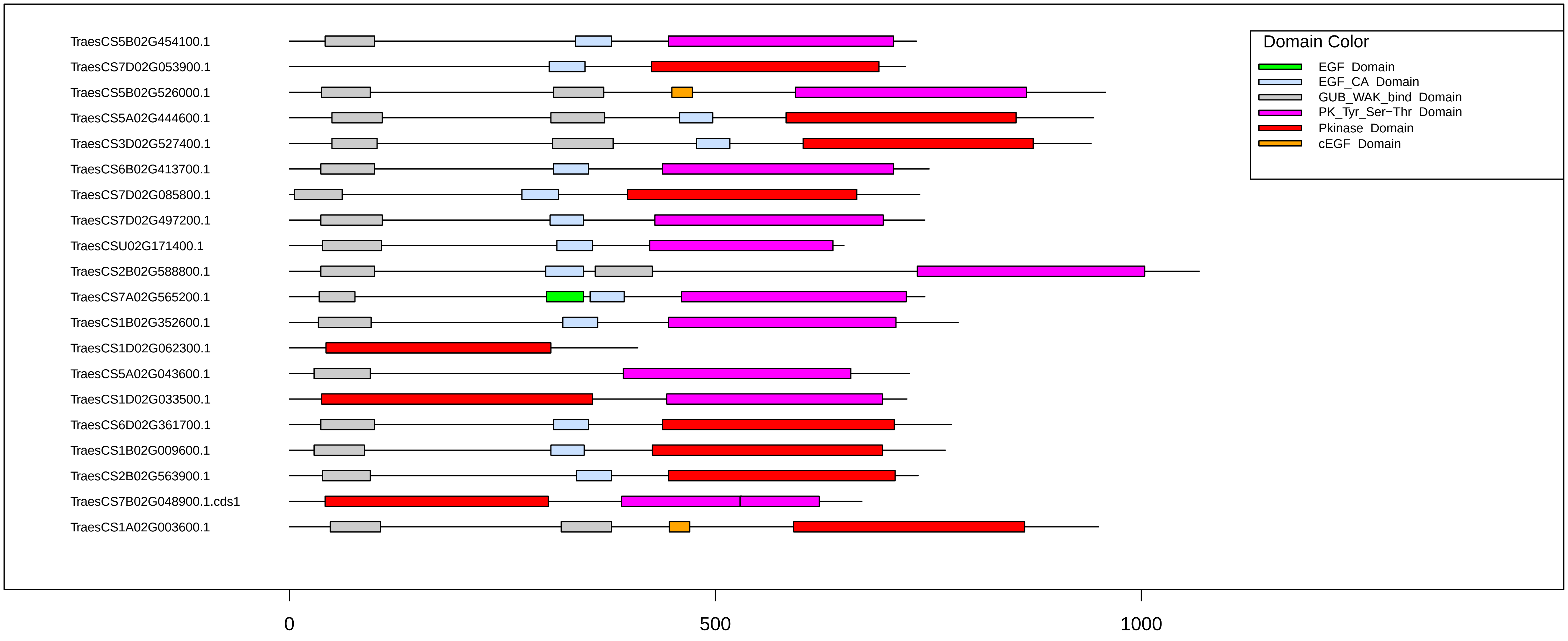

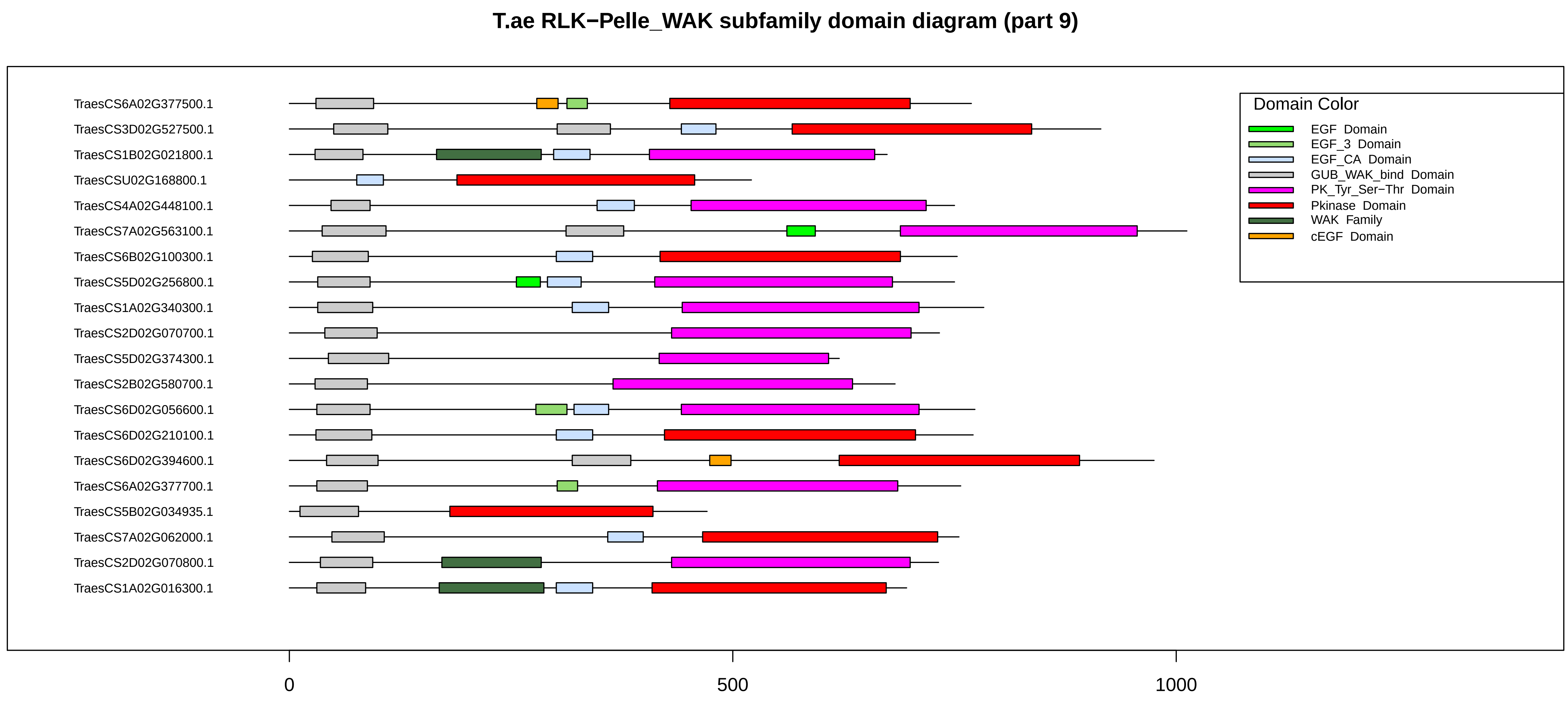

T.ae RLK−Pelle_WAK subfamily domain diagram (part 9)
TraesCS6A02G377500.1
TraesCS3D02G527500.1
TraesCS1B02G021800.1
TraesCSU02G168800.1
TraesCS4A02G448100.1
TraesCS7A02G563100.1
TraesCS6B02G100300.1
TraesCS5D02G256800.1
TraesCS1A02G340300.1
TraesCS2D02G070700.1
TraesCS5D02G374300.1
TraesCS2B02G580700.1
TraesCS6D02G056600.1
TraesCS6D02G210100.1
TraesCS6D02G394600.1
TraesCS6A02G377700.1
TraesCS5B02G034935.1
TraesCS7A02G062000.1
TraesCS2D02G070800.1
TraesCS1A02G016300.1
Domain Color
EGF Domain
EGF_3 Domain
EGF_CA Domain
GUB_WAK_bind Domain
PK_Tyr_Ser−Thr Domain
Pkinase Domain
WAK Family
cEGF Domain
0
500
1000

T.ae RLK−Pelle_WAK subfamily domain diagram (part 10)

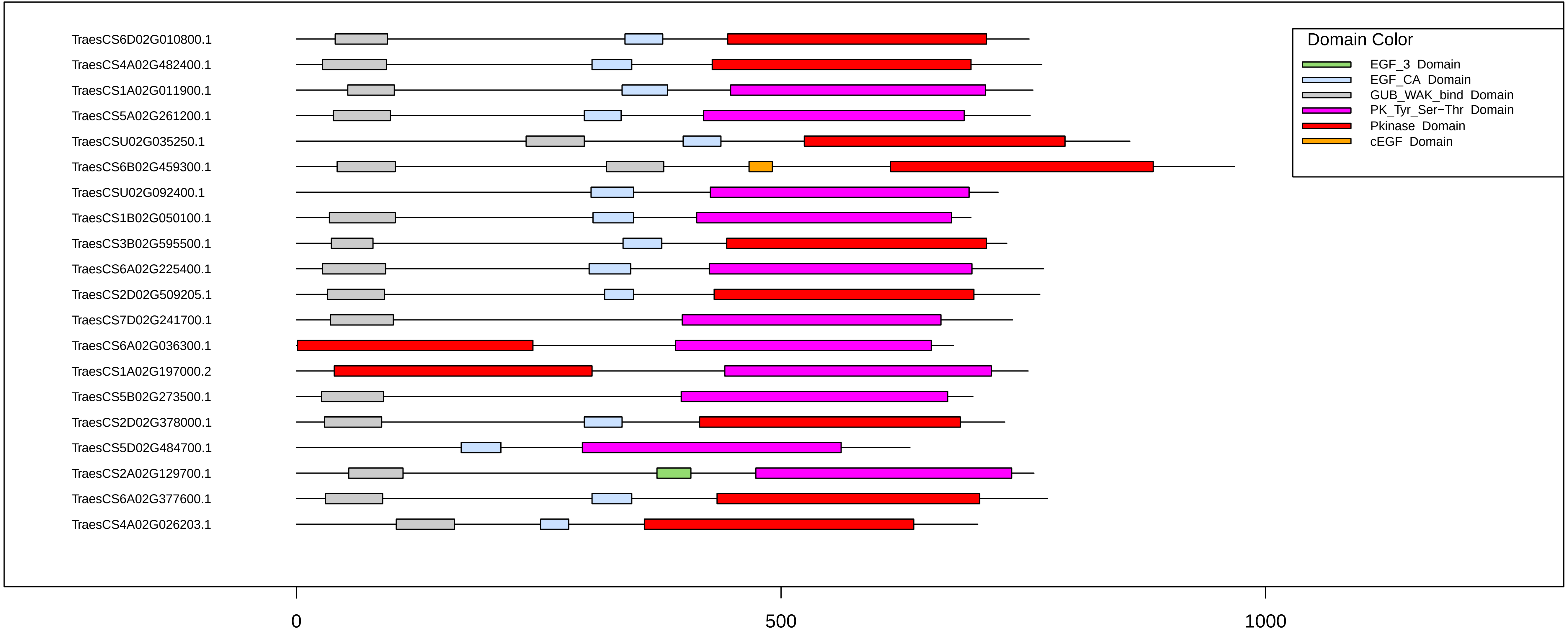

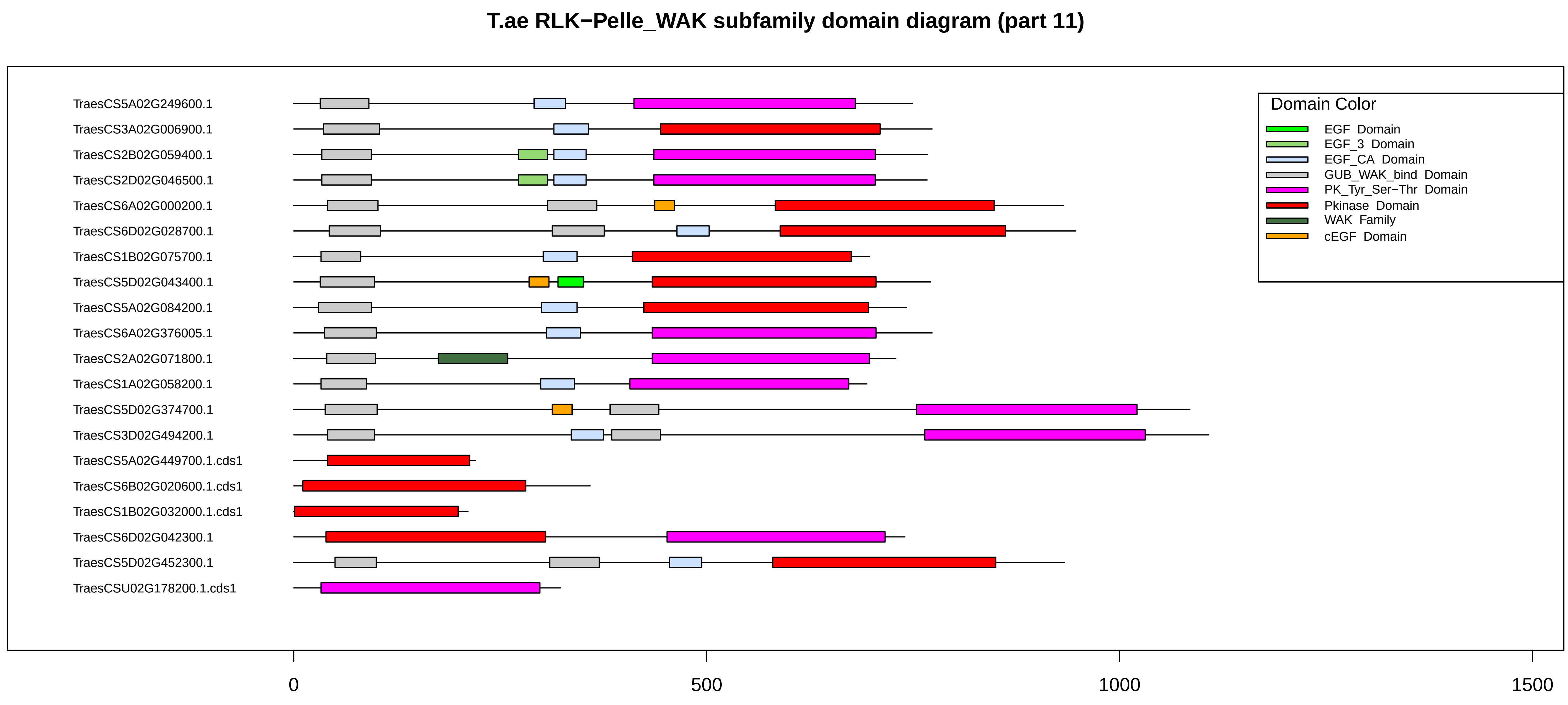

T.ae RLK−Pelle_WAK subfamily domain diagram (part 11)
TraesCS5A02G249600.1
TraesCS3A02G006900.1
TraesCS2B02G059400.1
TraesCS2D02G046500.1
TraesCS6A02G000200.1
TraesCS6D02G028700.1
TraesCS1B02G075700.1
TraesCS5D02G043400.1
TraesCS5A02G084200.1
TraesCS6A02G376005.1
TraesCS2A02G071800.1
TraesCS1A02G058200.1
TraesCS5D02G374700.1
TraesCS3D02G494200.1
TraesCS5A02G449700.1.cds1
TraesCS6B02G020600.1.cds1
TraesCS1B02G032000.1.cds1
TraesCS6D02G042300.1
TraesCS5D02G452300.1
TraesCSU02G178200.1.cds1
Domain Color
EGF Domain
EGF_3 Domain
EGF_CA Domain
GUB_WAK_bind Domain
PK_Tyr_Ser−Thr Domain
Pkinase Domain
WAK Family
cEGF Domain
0
500
1000
1500

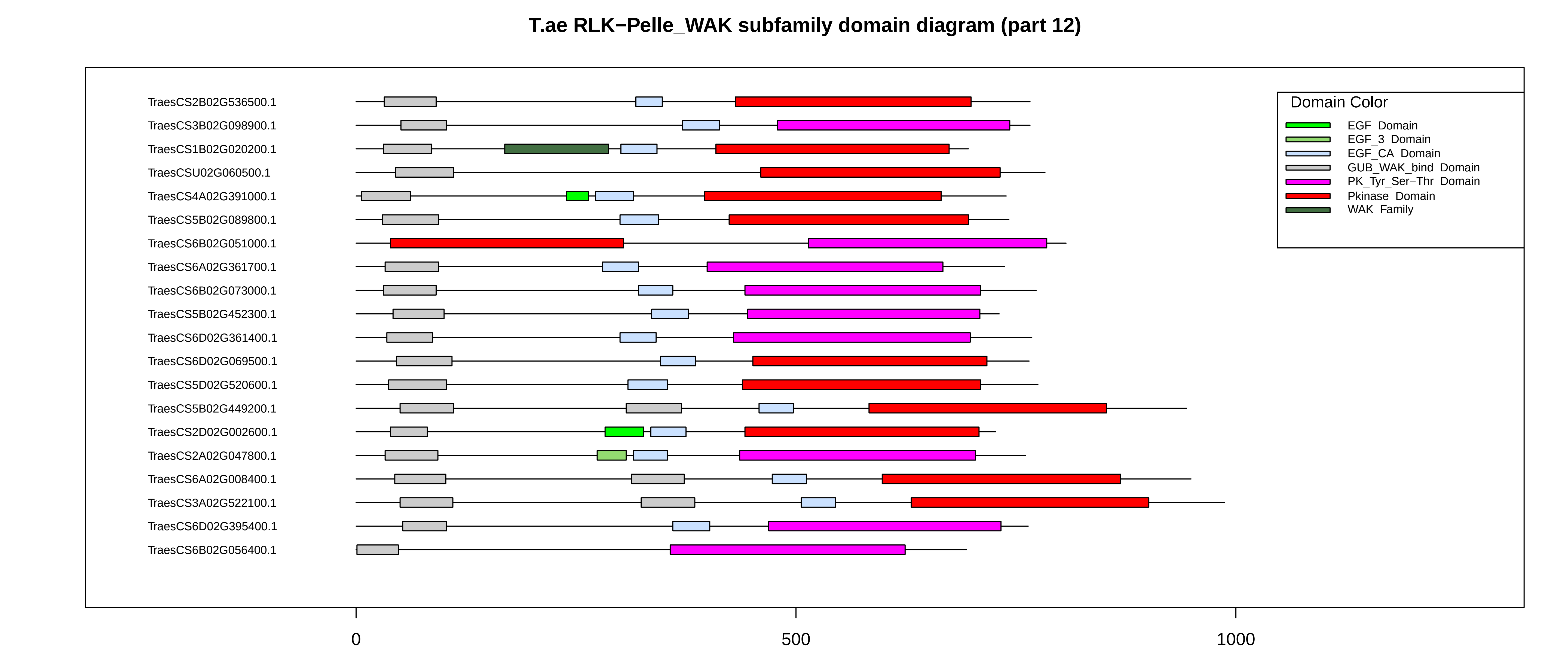
T.ae RLK−Pelle_WAK subfamily domain diagram (part 12)
TraesCS2B02G536500.1
TraesCS3B02G098900.1
TraesCS1B02G020200.1
TraesCSU02G060500.1
TraesCS4A02G391000.1
TraesCS5B02G089800.1
TraesCS6B02G051000.1
TraesCS6A02G361700.1
TraesCS6B02G073000.1
TraesCS5B02G452300.1
TraesCS6D02G361400.1
TraesCS6D02G069500.1
TraesCS5D02G520600.1
TraesCS5B02G449200.1
TraesCS2D02G002600.1
TraesCS2A02G047800.1
TraesCS6A02G008400.1
TraesCS3A02G522100.1
TraesCS6D02G395400.1
TraesCS6B02G056400.1
Domain Color
EGF Domain
EGF_3 Domain
EGF_CA Domain
GUB_WAK_bind Domain
PK_Tyr_Ser−Thr Domain
Pkinase Domain
WAK Family
0
500
1000

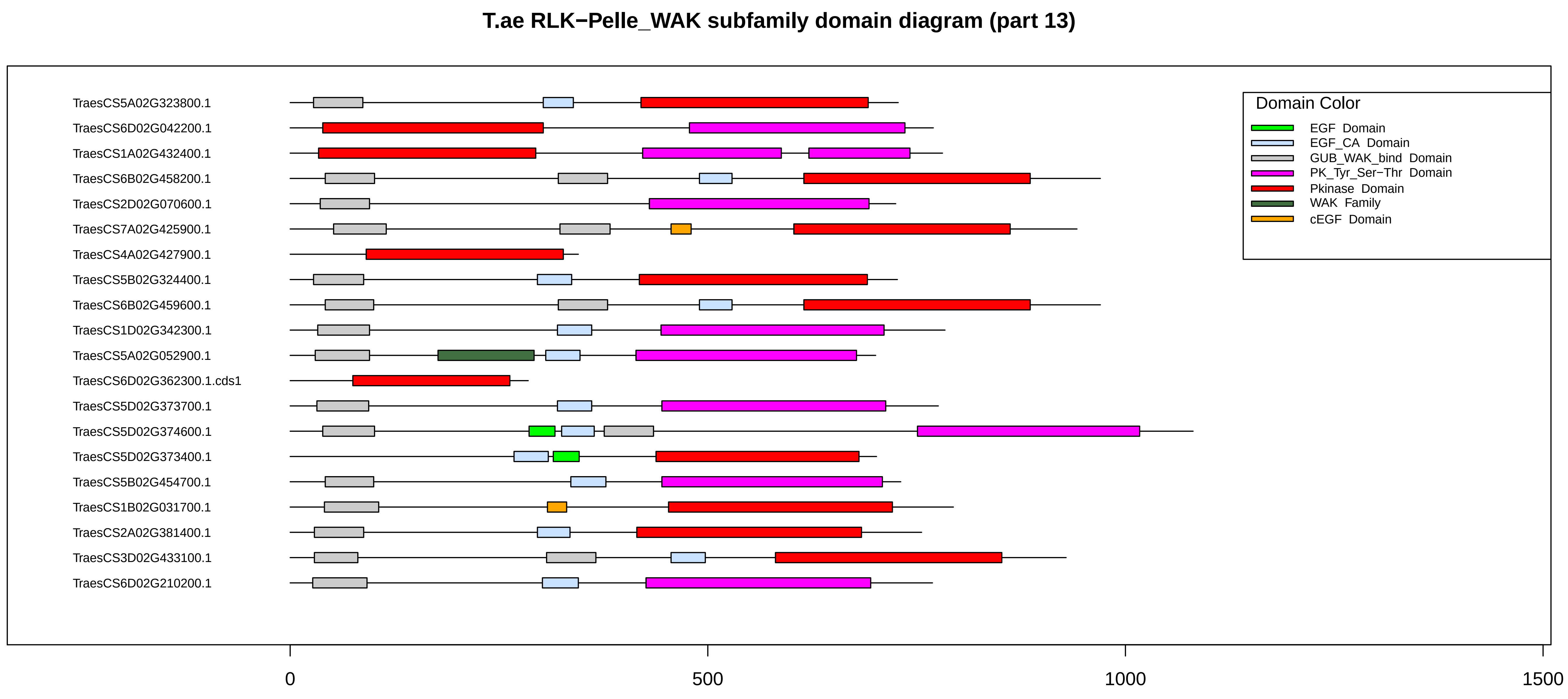

T.ae RLK−Pelle_WAK subfamily domain diagram (part 13)
TraesCS5A02G323800.1
TraesCS6D02G042200.1
TraesCS1A02G432400.1
TraesCS6B02G458200.1
TraesCS2D02G070600.1
TraesCS7A02G425900.1
TraesCS4A02G427900.1
TraesCS5B02G324400.1
TraesCS6B02G459600.1
TraesCS1D02G342300.1
TraesCS5A02G052900.1
TraesCS6D02G362300.1.cds1
TraesCS5D02G373700.1
TraesCS5D02G374600.1
TraesCS5D02G373400.1
TraesCS5B02G454700.1
TraesCS1B02G031700.1
TraesCS2A02G381400.1
TraesCS3D02G433100.1
TraesCS6D02G210200.1
Domain Color
EGF Domain
EGF_CA Domain
GUB_WAK_bind Domain
PK_Tyr_Ser−Thr Domain
Pkinase Domain
WAK Family
cEGF Domain
0
500
1000
1500

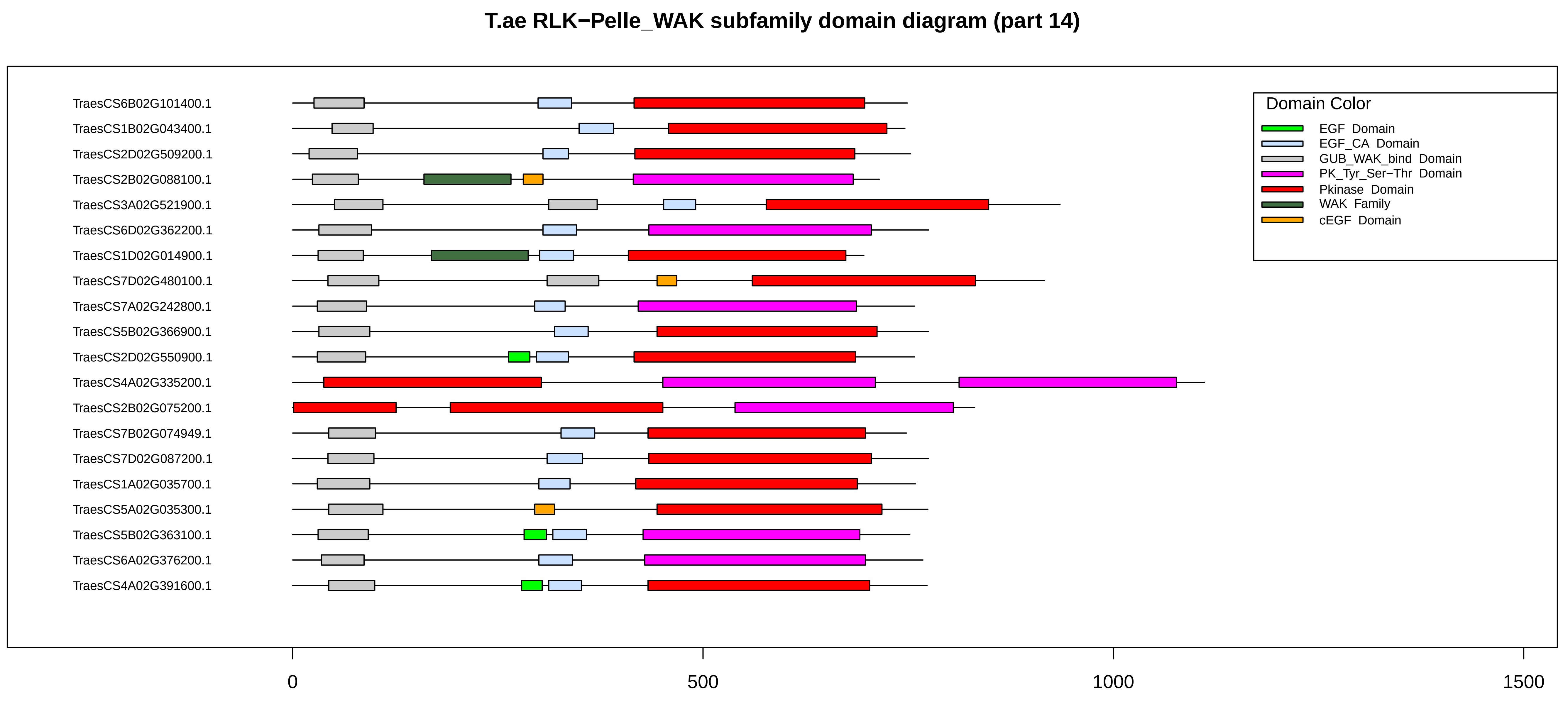

T.ae RLK−Pelle_WAK subfamily domain diagram (part 14)
TraesCS6B02G101400.1
TraesCS1B02G043400.1
TraesCS2D02G509200.1
TraesCS2B02G088100.1
TraesCS3A02G521900.1
TraesCS6D02G362200.1
TraesCS1D02G014900.1
TraesCS7D02G480100.1
TraesCS7A02G242800.1
TraesCS5B02G366900.1
TraesCS2D02G550900.1
TraesCS4A02G335200.1
TraesCS2B02G075200.1
TraesCS7B02G074949.1
TraesCS7D02G087200.1
TraesCS1A02G035700.1
TraesCS5A02G035300.1
TraesCS5B02G363100.1
TraesCS6A02G376200.1
TraesCS4A02G391600.1
Domain Color
EGF Domain
EGF_CA Domain
GUB_WAK_bind Domain
PK_Tyr_Ser−Thr Domain
Pkinase Domain
WAK Family
cEGF Domain
0
500
1000
1500

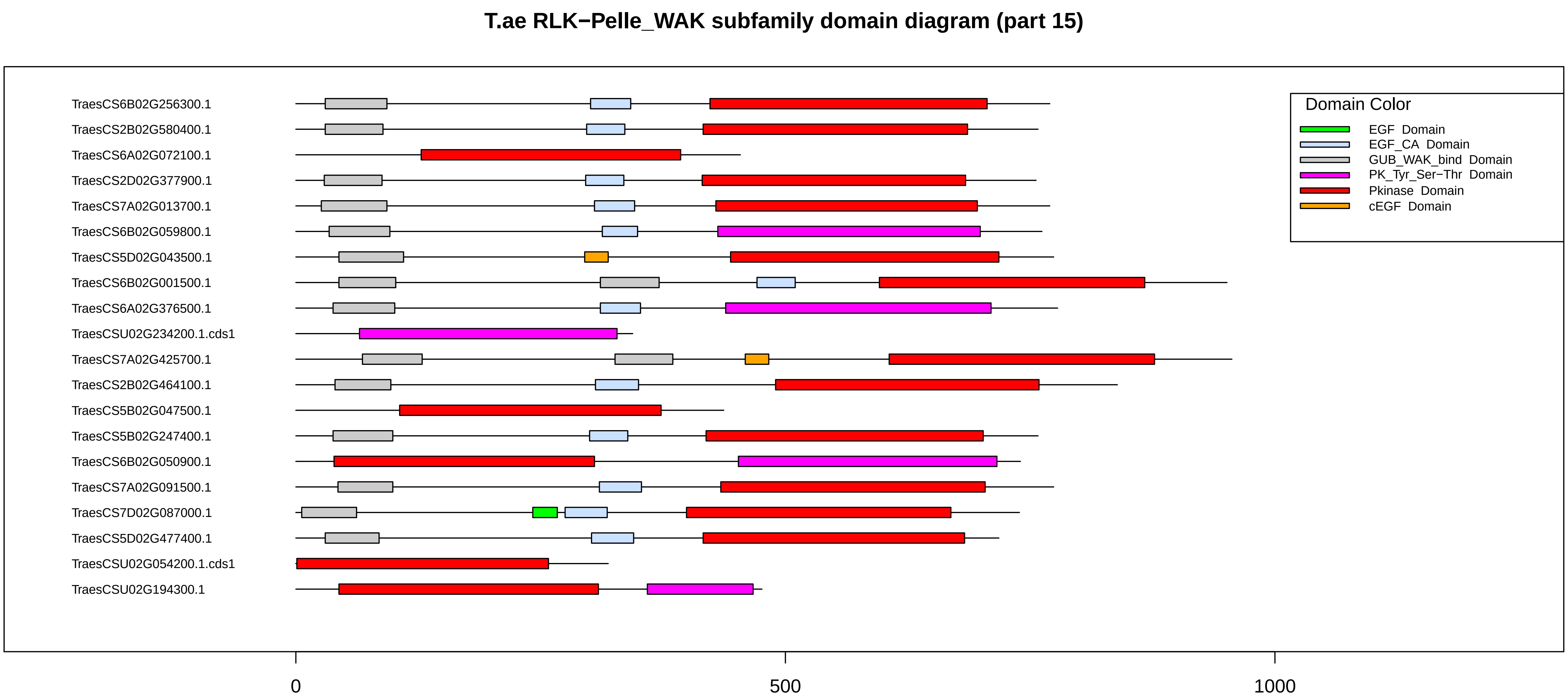
T.ae RLK−Pelle_WAK subfamily domain diagram (part 15)
TraesCS6B02G256300.1
TraesCS2B02G580400.1
TraesCS6A02G072100.1
TraesCS2D02G377900.1
TraesCS7A02G013700.1
TraesCS6B02G059800.1
TraesCS5D02G043500.1
TraesCS6B02G001500.1
TraesCS6A02G376500.1
TraesCSU02G234200.1.cds1
TraesCS7A02G425700.1
TraesCS2B02G464100.1
TraesCS5B02G047500.1
TraesCS5B02G247400.1
TraesCS6B02G050900.1
TraesCS7A02G091500.1
TraesCS7D02G087000.1
TraesCS5D02G477400.1
TraesCSU02G054200.1.cds1
TraesCSU02G194300.1
Domain Color
EGF Domain
EGF_CA Domain
GUB_WAK_bind Domain
PK_Tyr_Ser−Thr Domain
Pkinase Domain
cEGF Domain
0
500
1000

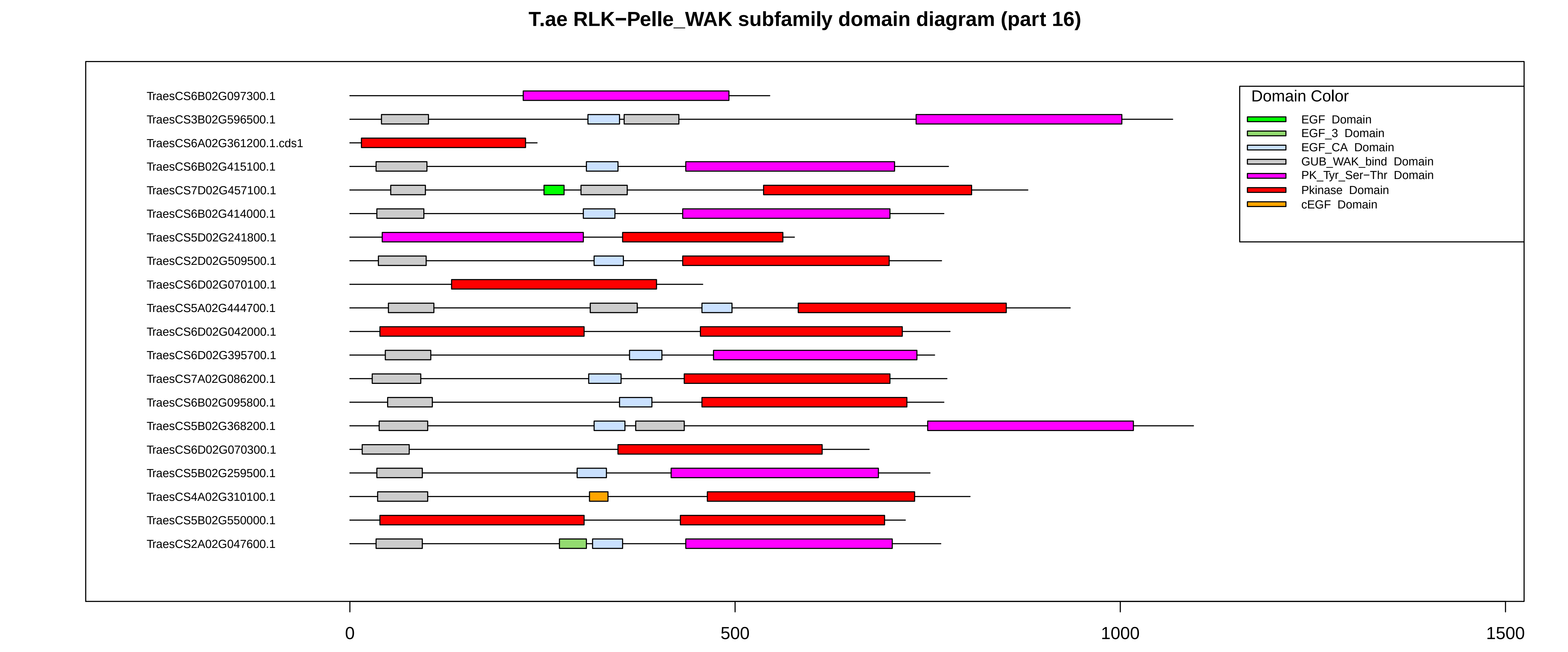

T.ae RLK−Pelle_WAK subfamily domain diagram (part 16)
TraesCS6B02G097300.1
TraesCS3B02G596500.1
TraesCS6A02G361200.1.cds1
TraesCS6B02G415100.1
TraesCS7D02G457100.1
TraesCS6B02G414000.1
TraesCS5D02G241800.1
TraesCS2D02G509500.1
TraesCS6D02G070100.1
TraesCS5A02G444700.1
TraesCS6D02G042000.1
TraesCS6D02G395700.1
TraesCS7A02G086200.1
TraesCS6B02G095800.1
TraesCS5B02G368200.1
TraesCS6D02G070300.1
TraesCS5B02G259500.1
TraesCS4A02G310100.1
TraesCS5B02G550000.1
TraesCS2A02G047600.1
Domain Color
EGF Domain
EGF_3 Domain
EGF_CA Domain
GUB_WAK_bind Domain
PK_Tyr_Ser−Thr Domain
Pkinase Domain
cEGF Domain
0
500
1000
1500

T.ae RLK−Pelle_WAK subfamily domain diagram (part 17)

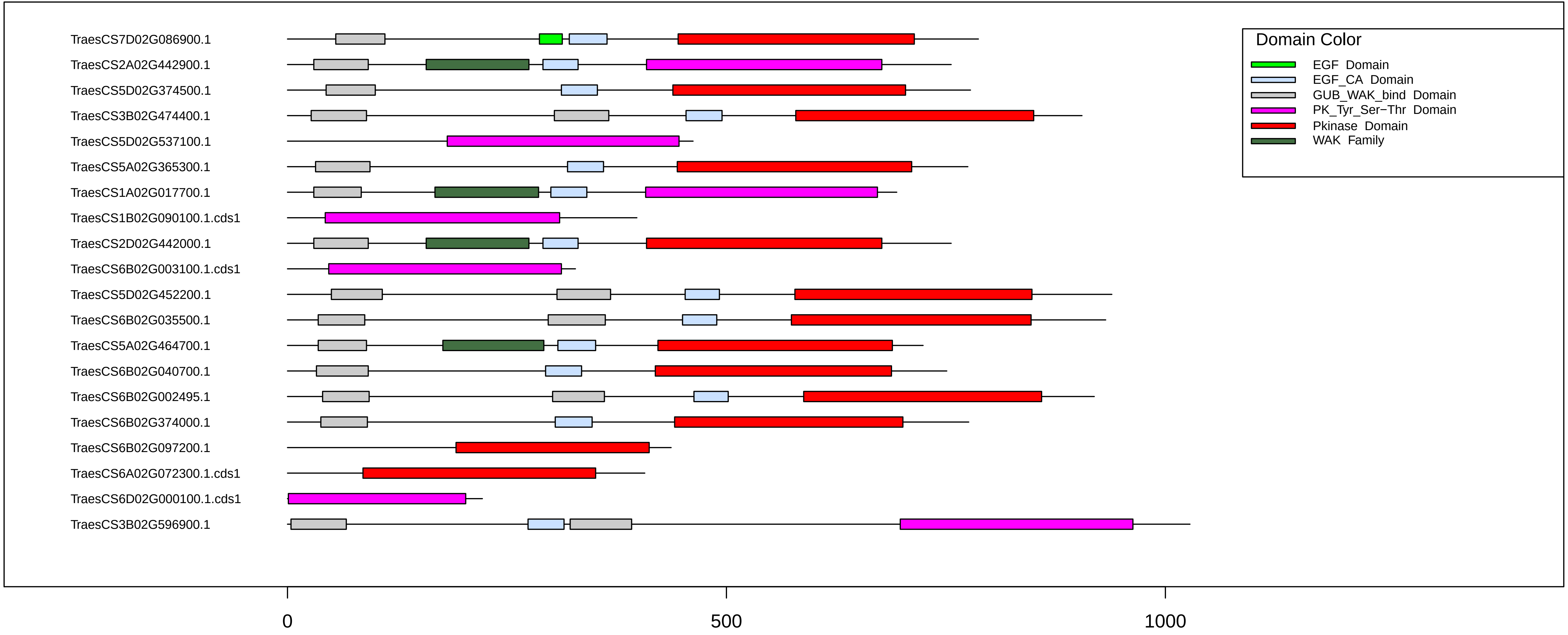

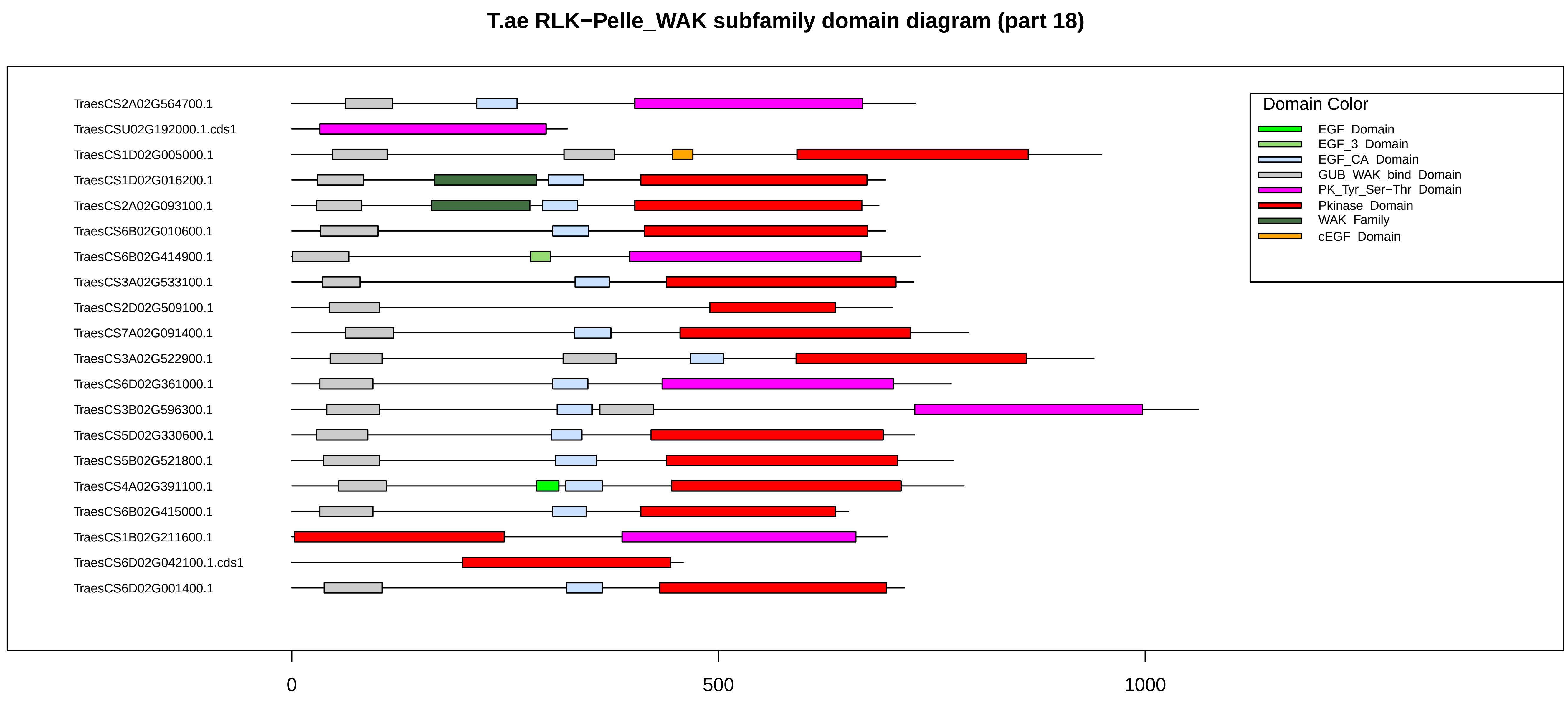

T.ae RLK−Pelle_WAK subfamily domain diagram (part 18)
TraesCS2A02G564700.1
TraesCSU02G192000.1.cds1
TraesCS1D02G005000.1
TraesCS1D02G016200.1
TraesCS2A02G093100.1
TraesCS6B02G010600.1
TraesCS6B02G414900.1
TraesCS3A02G533100.1
TraesCS2D02G509100.1
TraesCS7A02G091400.1
TraesCS3A02G522900.1
TraesCS6D02G361000.1
TraesCS3B02G596300.1
TraesCS5D02G330600.1
TraesCS5B02G521800.1
TraesCS4A02G391100.1
TraesCS6B02G415000.1
TraesCS1B02G211600.1
TraesCS6D02G042100.1.cds1
TraesCS6D02G001400.1
Domain Color
EGF Domain
EGF_3 Domain
EGF_CA Domain
GUB_WAK_bind Domain
PK_Tyr_Ser−Thr Domain
Pkinase Domain
WAK Family
cEGF Domain
0
500
1000

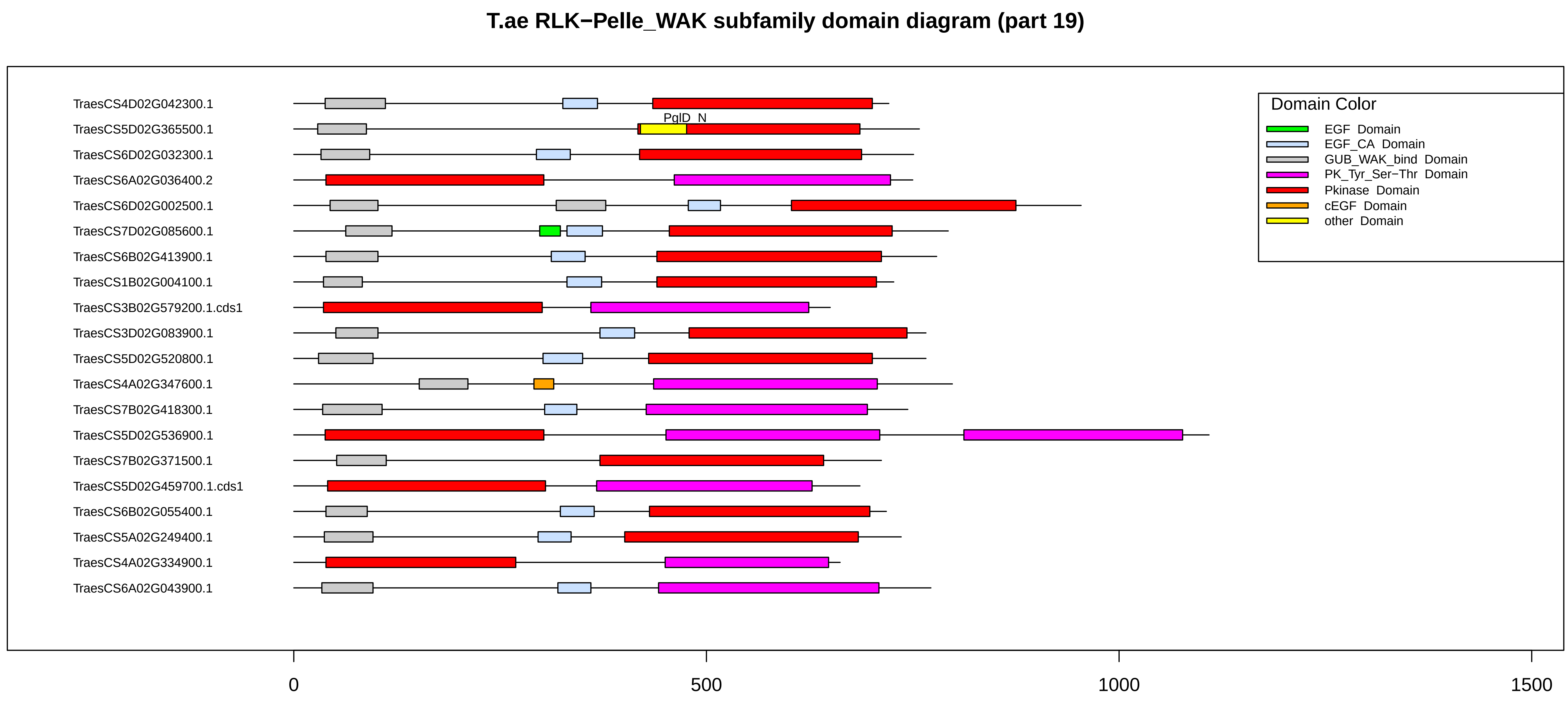

T.ae RLK−Pelle_WAK subfamily domain diagram (part 19)
TraesCS4D02G042300.1
TraesCS5D02G365500.1
TraesCS6D02G032300.1
TraesCS6A02G036400.2
TraesCS6D02G002500.1
TraesCS7D02G085600.1
TraesCS6B02G413900.1
TraesCS1B02G004100.1
TraesCS3B02G579200.1.cds1
TraesCS3D02G083900.1
TraesCS5D02G520800.1
TraesCS4A02G347600.1
TraesCS7B02G418300.1
TraesCS5D02G536900.1
TraesCS7B02G371500.1
TraesCS5D02G459700.1.cds1
TraesCS6B02G055400.1
TraesCS5A02G249400.1
TraesCS4A02G334900.1
TraesCS6A02G043900.1
PglD_N
Domain Color
EGF Domain
EGF_CA Domain
GUB_WAK_bind Domain
PK_Tyr_Ser−Thr Domain
Pkinase Domain
cEGF Domain
other Domain
0
500
1000
1500

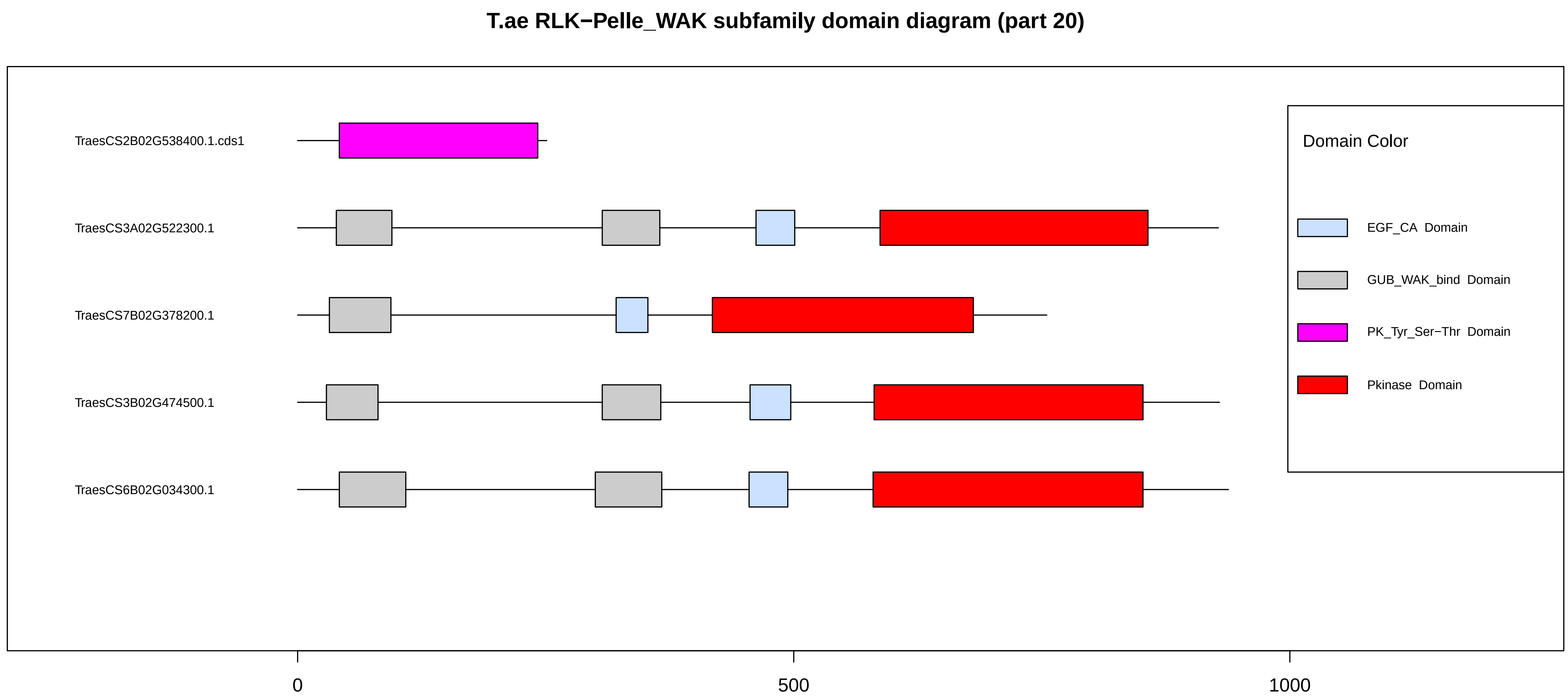

T.ae RLK−Pelle_WAK subfamily domain diagram (part 20)
TraesCS2B02G538400.1.cds1
TraesCS3A02G522300.1
TraesCS7B02G378200.1
TraesCS3B02G474500.1
TraesCS6B02G034300.1
Domain Color
EGF_CA Domain
GUB_WAK_bind Domain
PK_Tyr_Ser−Thr Domain
Pkinase Domain
0
500
1000

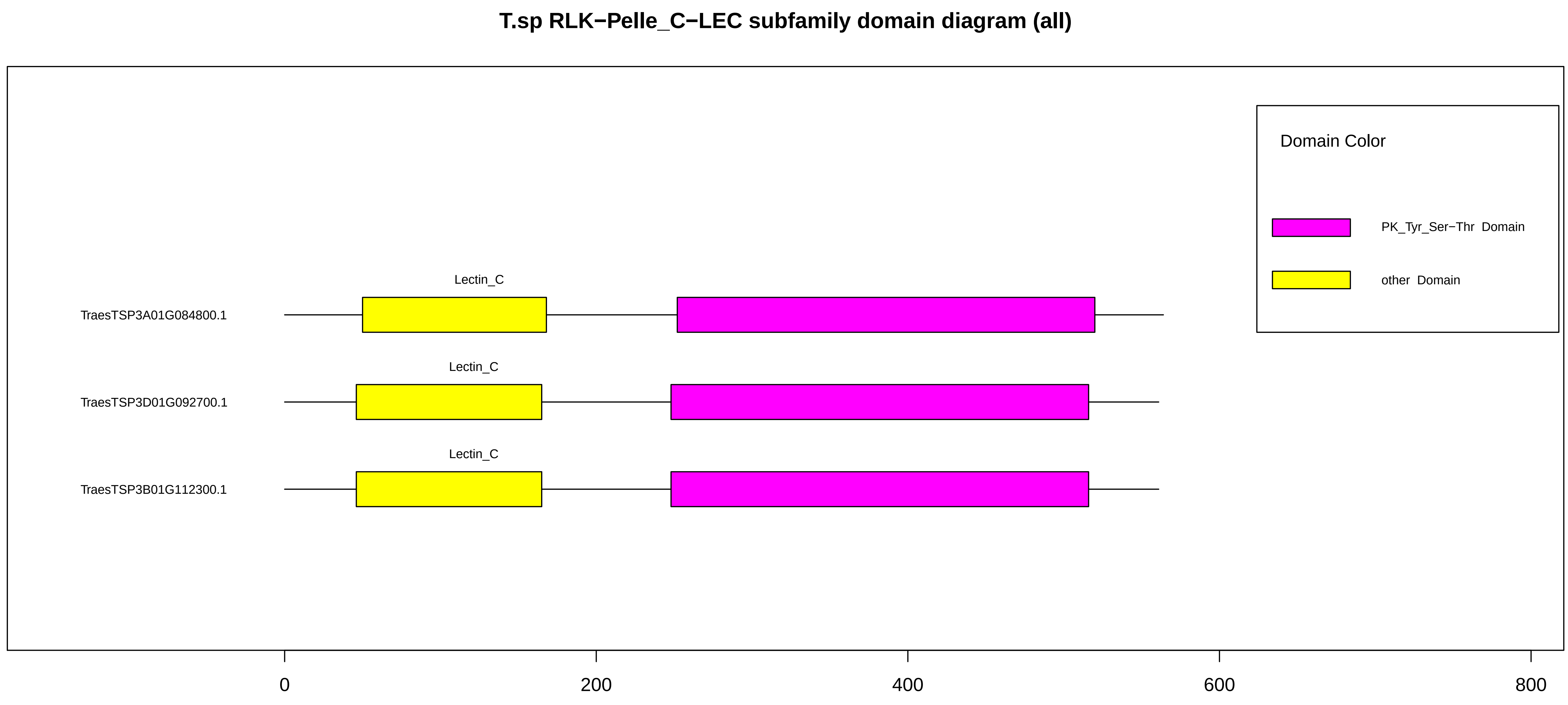
T.sp RLK−Pelle_C−LEC subfamily domain diagram (all)
Domain Color
PK_Tyr_Ser−Thr Domain
other Domain
Lectin_C
TraesTSP3A01G084800.1
Lectin_C
TraesTSP3D01G092700.1
Lectin_C
TraesTSP3B01G112300.1
0
200
400
600
800

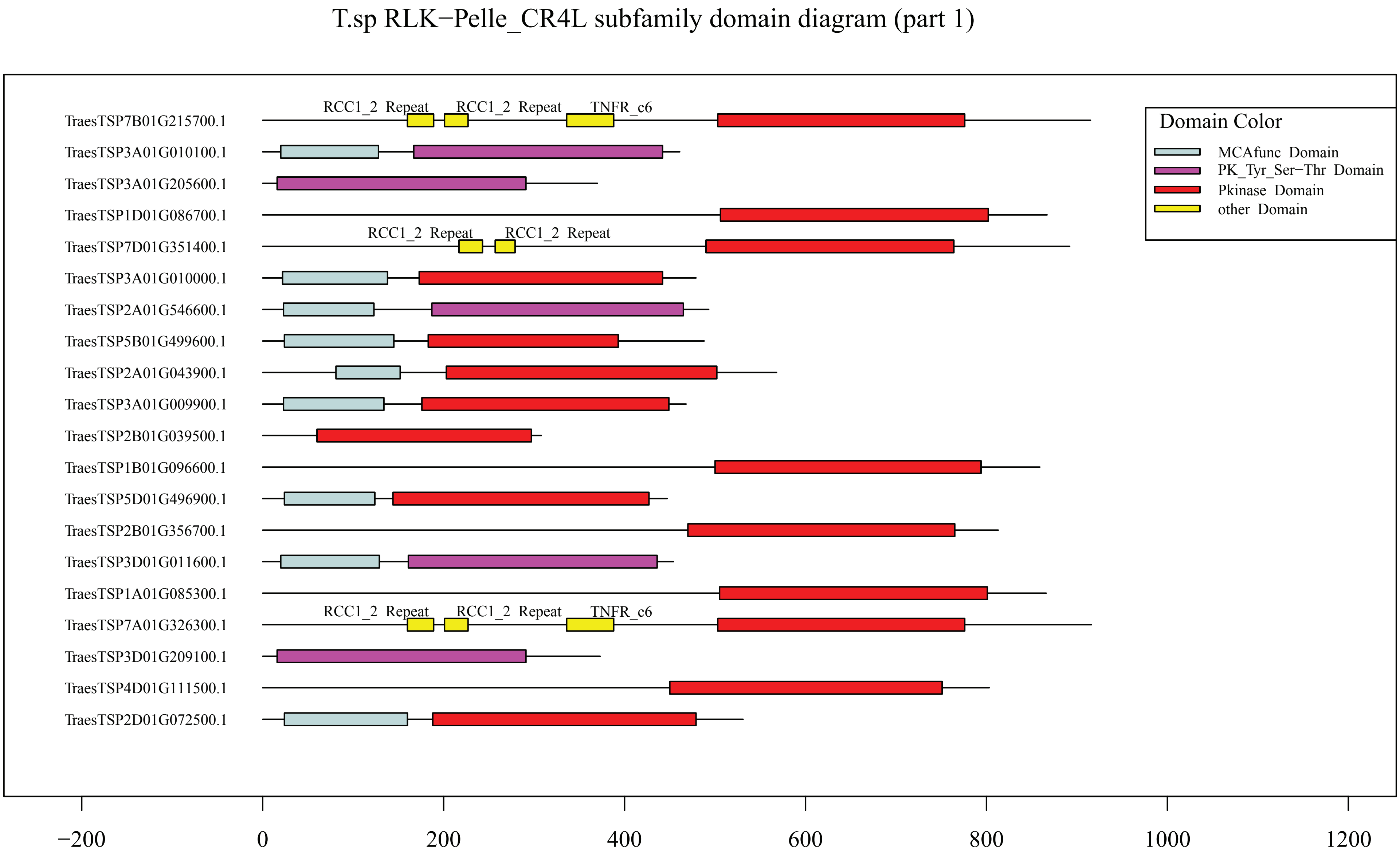

T.sp RLK−Pelle_CR4L subfamily domain diagram (part 1)
TraesTSP7B01G215700.1
TraesTSP3A01G010100.1
TraesTSP3A01G205600.1
TraesTSP1D01G086700.1
TraesTSP7D01G351400.1
TraesTSP3A01G010000.1
TraesTSP2A01G546600.1
TraesTSP5B01G499600.1
TraesTSP2A01G043900.1
TraesTSP3A01G009900.1
TraesTSP2B01G039500.1
TraesTSP1B01G096600.1
TraesTSP5D01G496900.1
TraesTSP2B01G356700.1
TraesTSP3D01G011600.1
TraesTSP1A01G085300.1
TraesTSP7A01G326300.1
TraesTSP3D01G209100.1
TraesTSP4D01G111500.1
TraesTSP2D01G072500.1
RCC1_2 Repeat
RCC1_2 Repeat
TNFR_c6
RCC1_2 Repeat
RCC1_2 Repeat
RCC1_2 Repeat
RCC1_2 Repeat
TNFR_c6
Domain Color
MCAfunc Domain
PK_Tyr_Ser−Thr Domain
Pkinase Domain
other Domain
−200
0
200
400
600
800
1000
1200

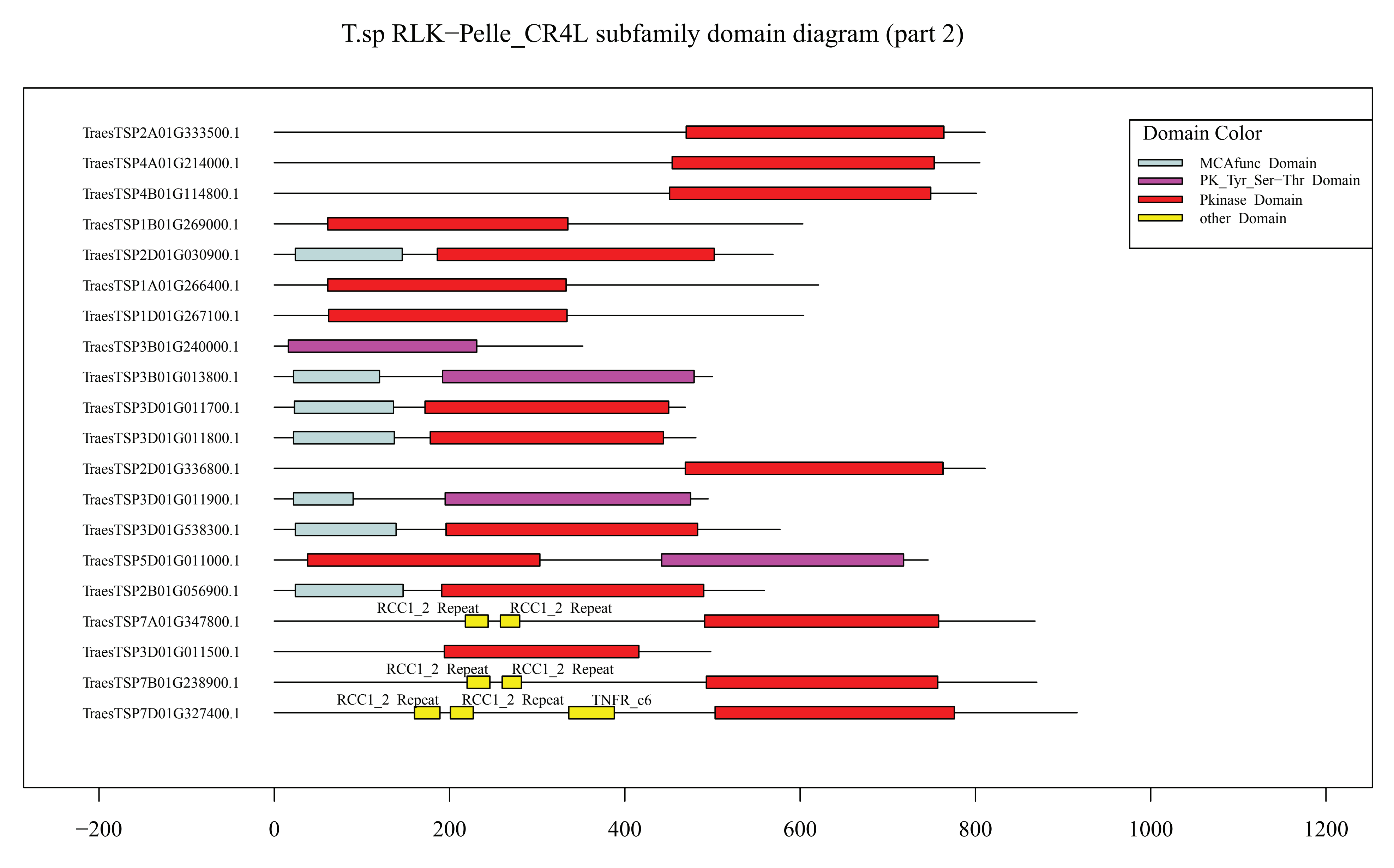

T.sp RLK−Pelle_CR4L subfamily domain diagram (part 2)
Domain Color
MCAfunc Domain
PK_Tyr_Ser−Thr Domain
Pkinase Domain
other Domain
TraesTSP2A01G333500.1
TraesTSP4A01G214000.1
TraesTSP4B01G114800.1
TraesTSP1B01G269000.1
TraesTSP2D01G030900.1
TraesTSP1A01G266400.1
TraesTSP1D01G267100.1
TraesTSP3B01G240000.1
TraesTSP3B01G013800.1
TraesTSP3D01G011700.1
TraesTSP3D01G011800.1
TraesTSP2D01G336800.1
TraesTSP3D01G011900.1
TraesTSP3D01G538300.1
TraesTSP5D01G011000.1
TraesTSP2B01G056900.1
RCC1_2 Repeat
RCC1_2 Repeat
TraesTSP7A01G347800.1
TraesTSP3D01G011500.1
RCC1_2 Repeat
RCC1_2 Repeat
TraesTSP7B01G238900.1
RCC1_2 Repeat
RCC1_2 Repeat
TNFR_c6
TraesTSP7D01G327400.1
−200
0
200
400
600
800
1000
1200

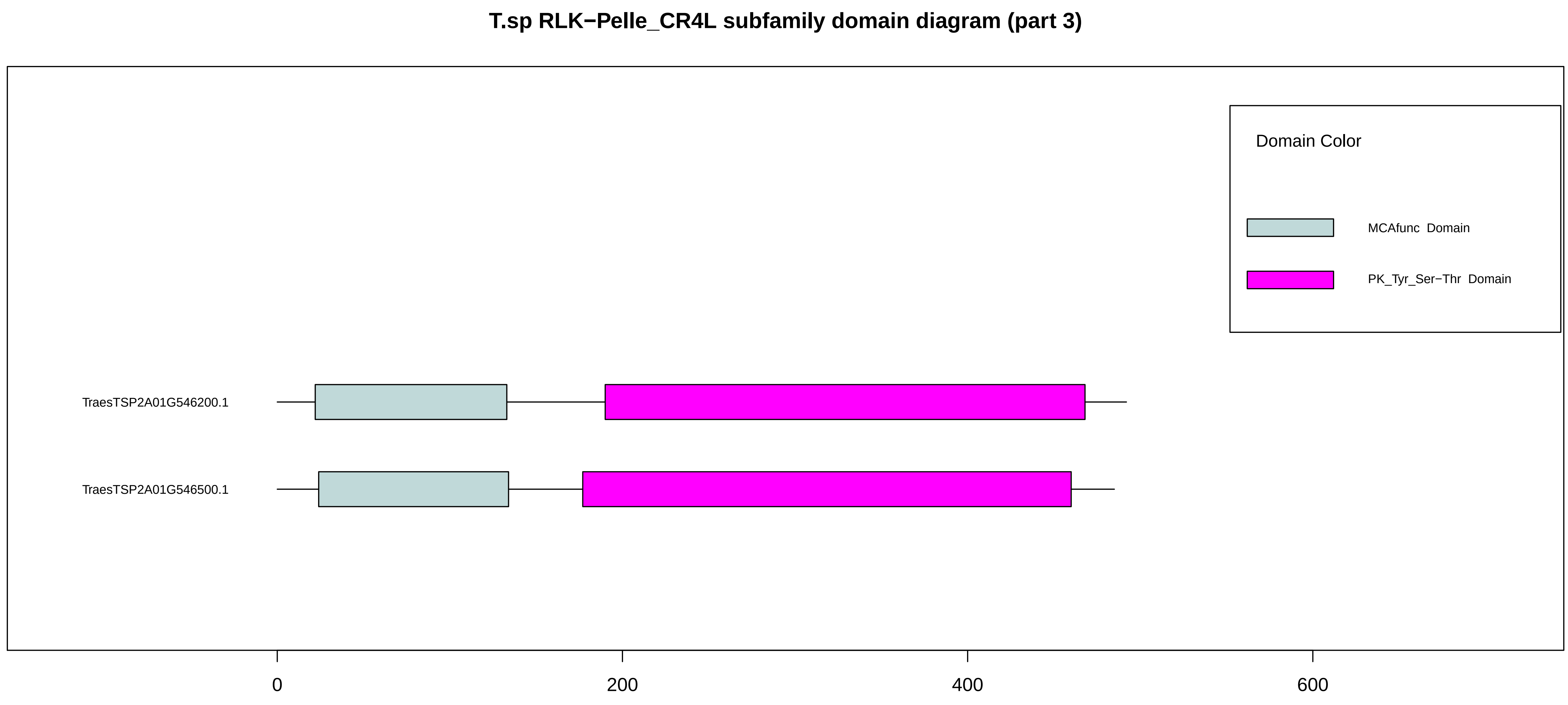
T.sp RLK−Pelle_CR4L subfamily domain diagram (part 3)
Domain Color
MCAfunc Domain
PK_Tyr_Ser−Thr Domain
TraesTSP2A01G546200.1
TraesTSP2A01G546500.1
0
200
400
600

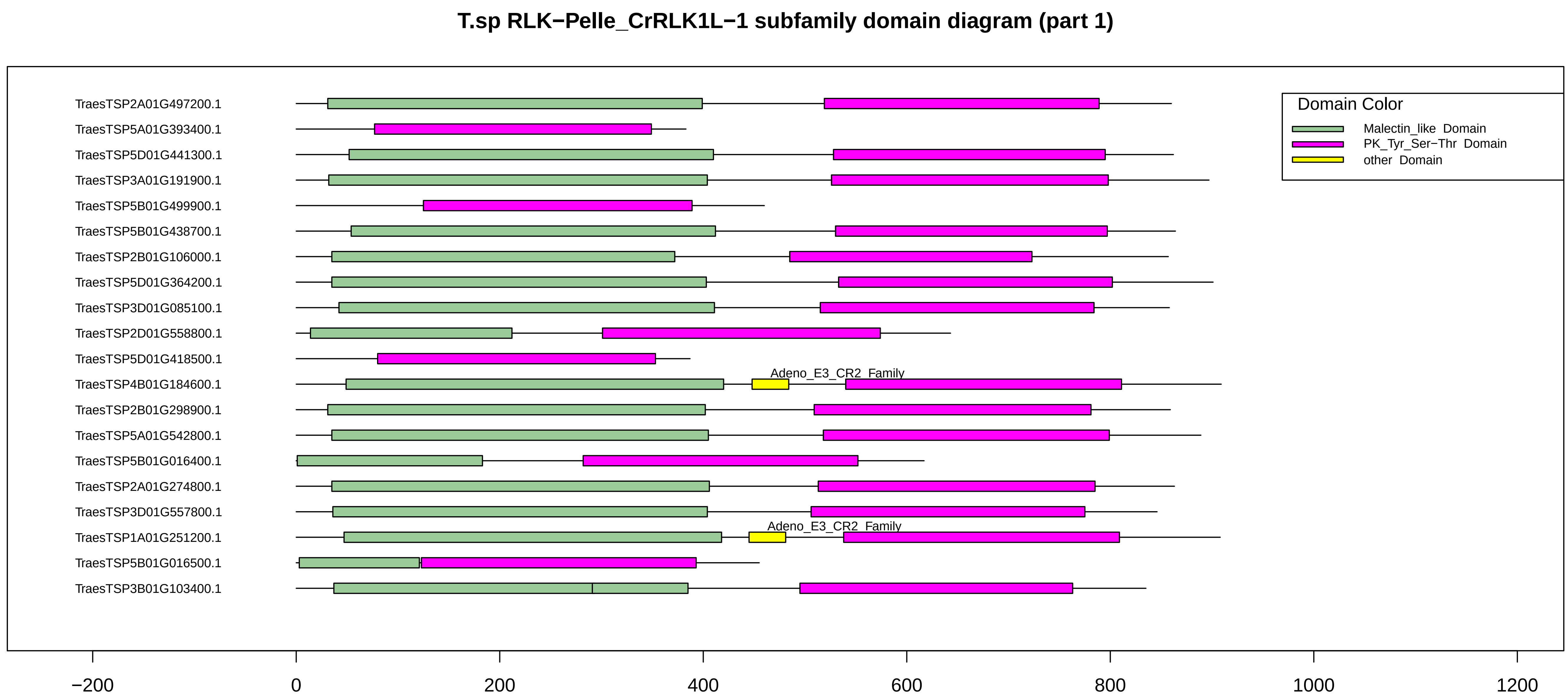

T.sp RLK−Pelle_CrRLK1L−1 subfamily domain diagram (part 1)
TraesTSP2A01G497200.1
TraesTSP5A01G393400.1
TraesTSP5D01G441300.1
TraesTSP3A01G191900.1
TraesTSP5B01G499900.1
TraesTSP5B01G438700.1
TraesTSP2B01G106000.1
TraesTSP5D01G364200.1
TraesTSP3D01G085100.1
TraesTSP2D01G558800.1
TraesTSP5D01G418500.1
TraesTSP4B01G184600.1
Adeno_E3_CR2_Family
TraesTSP2B01G298900.1
TraesTSP5A01G542800.1
TraesTSP5B01G016400.1
TraesTSP2A01G274800.1
TraesTSP3D01G557800.1
TraesTSP1A01G251200.1
Adeno_E3_CR2_Family
TraesTSP5B01G016500.1
TraesTSP3B01G103400.1
Domain Color
Malectin_like Domain
PK_Tyr_Ser−Thr Domain
other Domain
−200
0
200
400
600
800
1000
1200

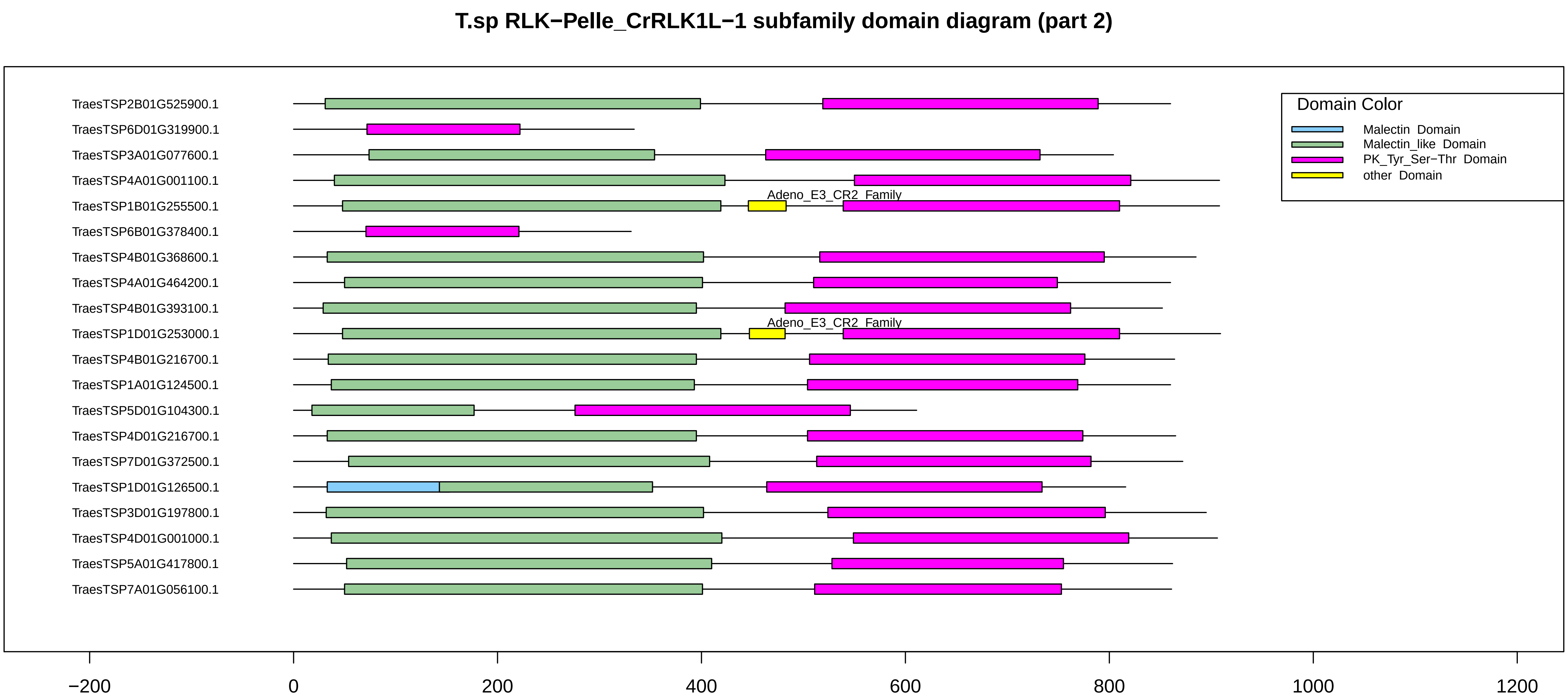
T.sp RLK−Pelle_CrRLK1L−1 subfamily domain diagram (part 2)
TraesTSP2B01G525900.1
TraesTSP6D01G319900.1
TraesTSP3A01G077600.1
TraesTSP4A01G001100.1
TraesTSP1B01G255500.1
Adeno_E3_CR2_Family
TraesTSP6B01G378400.1
TraesTSP4B01G368600.1
TraesTSP4A01G464200.1
TraesTSP4B01G393100.1
TraesTSP1D01G253000.1
Adeno_E3_CR2_Family
TraesTSP4B01G216700.1
TraesTSP1A01G124500.1
TraesTSP5D01G104300.1
TraesTSP4D01G216700.1
TraesTSP7D01G372500.1
TraesTSP1D01G126500.1
TraesTSP3D01G197800.1
TraesTSP4D01G001000.1
TraesTSP5A01G417800.1
TraesTSP7A01G056100.1
Domain Color
Malectin Domain
Malectin_like Domain
PK_Tyr_Ser−Thr Domain
other Domain
−200
0
200
400
600
800
1000
1200

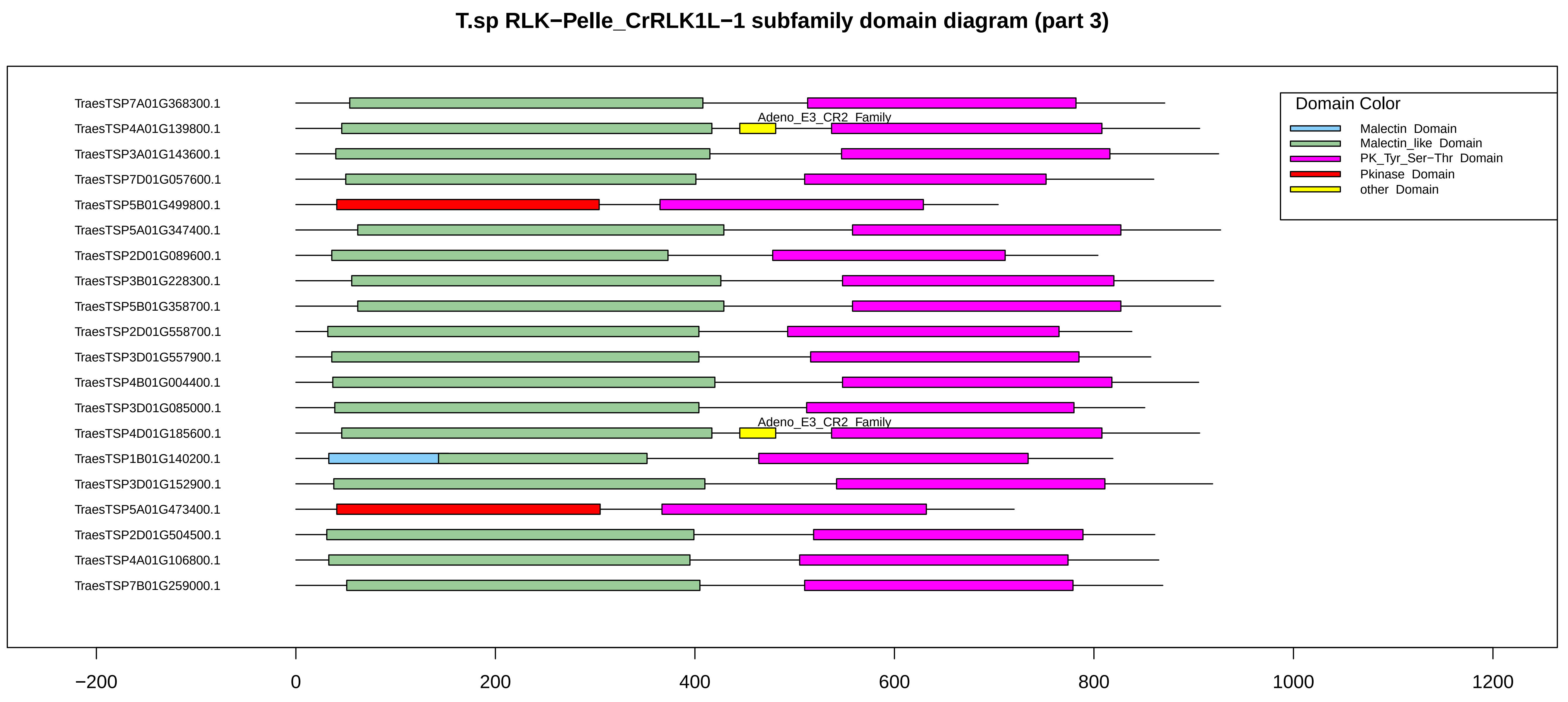

T.sp RLK−Pelle_CrRLK1L−1 subfamily domain diagram (part 3)
TraesTSP7A01G368300.1
TraesTSP4A01G139800.1
TraesTSP3A01G143600.1
TraesTSP7D01G057600.1
TraesTSP5B01G499800.1
TraesTSP5A01G347400.1
TraesTSP2D01G089600.1
TraesTSP3B01G228300.1
TraesTSP5B01G358700.1
TraesTSP2D01G558700.1
TraesTSP3D01G557900.1
TraesTSP4B01G004400.1
TraesTSP3D01G085000.1
TraesTSP4D01G185600.1
TraesTSP1B01G140200.1
TraesTSP3D01G152900.1
TraesTSP5A01G473400.1
TraesTSP2D01G504500.1
TraesTSP4A01G106800.1
TraesTSP7B01G259000.1
Adeno_E3_CR2_Family
Adeno_E3_CR2_Family
Domain Color
Malectin Domain
Malectin_like Domain
PK_Tyr_Ser−Thr Domain
Pkinase Domain
other Domain
−200
0
200
400
600
800
1000
1200

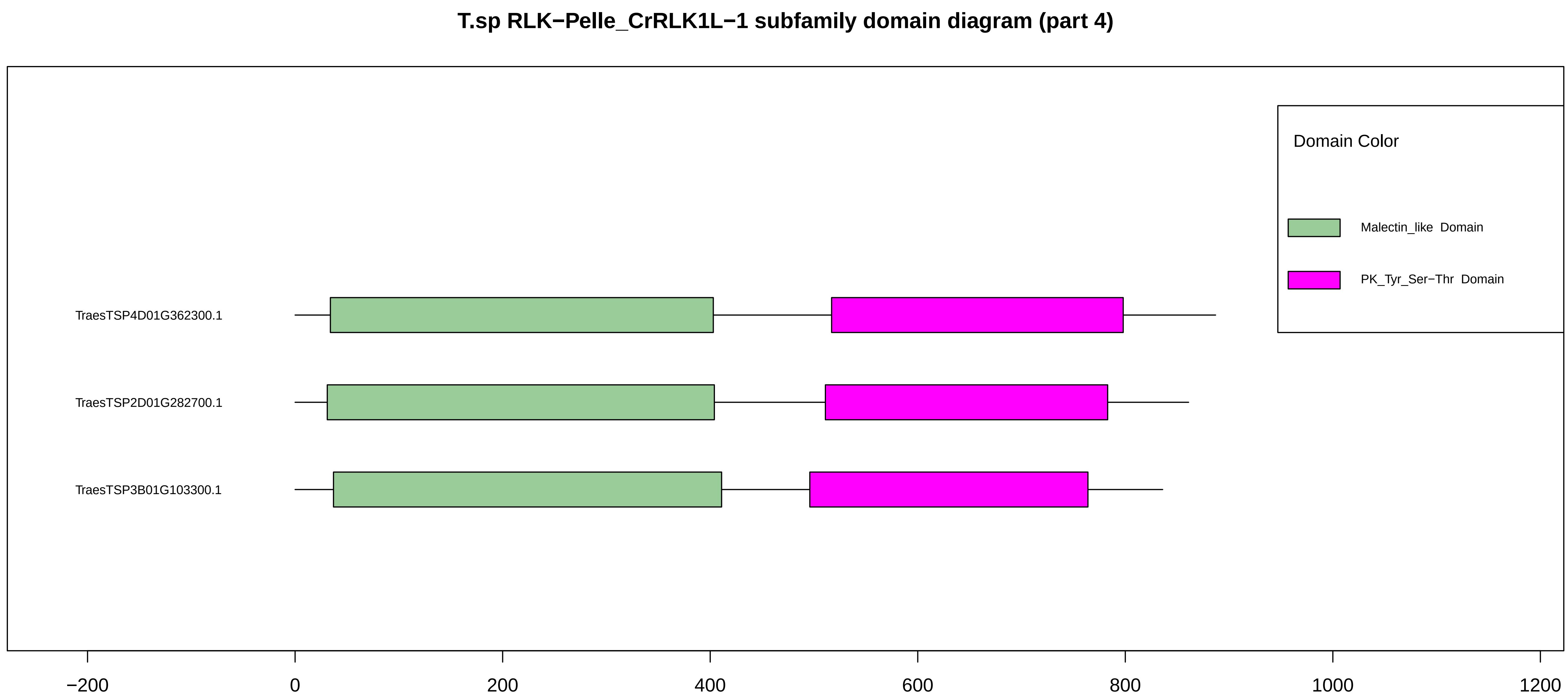
T.sp RLK−Pelle_CrRLK1L−1 subfamily domain diagram (part 4)
Domain Color
Malectin_like Domain
PK_Tyr_Ser−Thr Domain
TraesTSP4D01G362300.1
TraesTSP2D01G282700.1
TraesTSP3B01G103300.1
−200
0
200
400
600
800
1000
1200

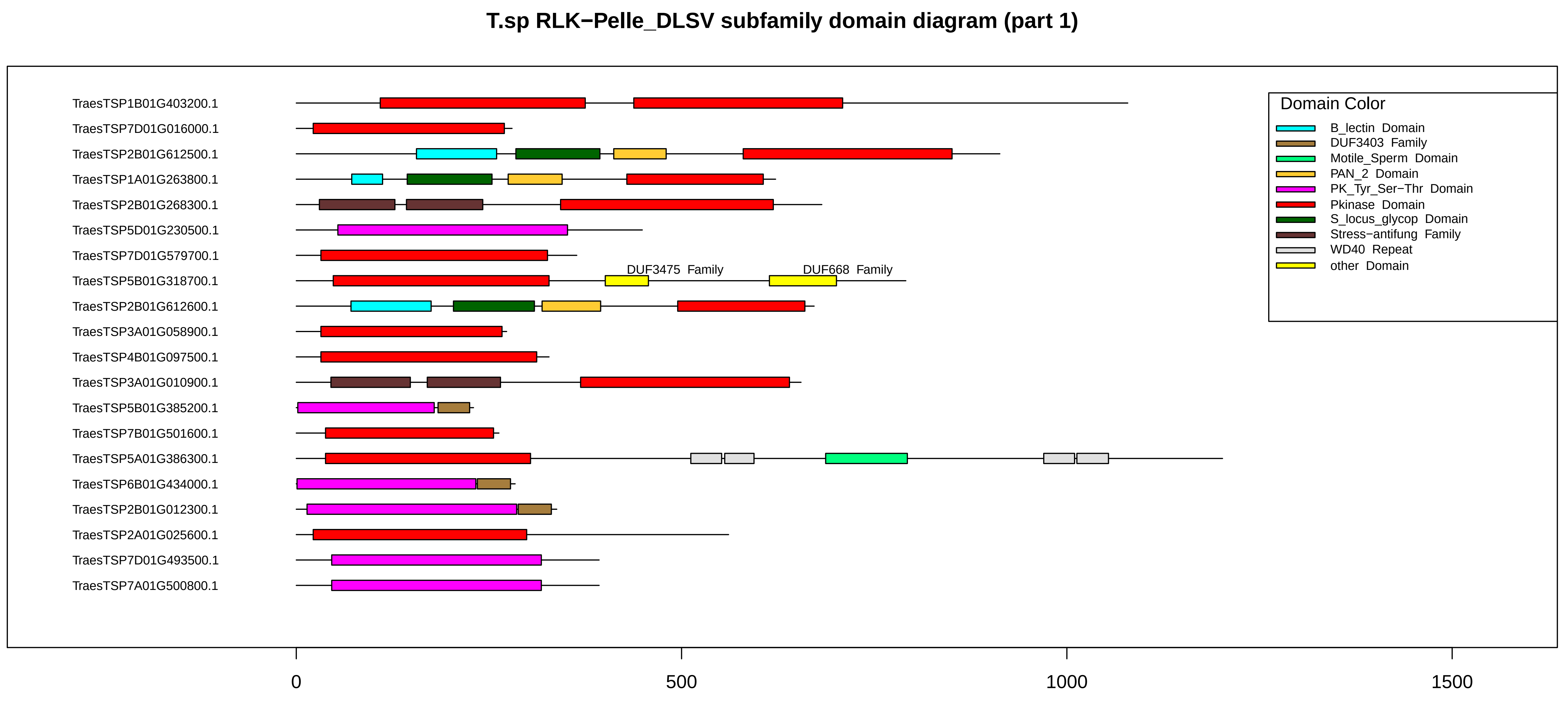

T.sp RLK−Pelle_DLSV subfamily domain diagram (part 1)
TraesTSP1B01G403200.1
TraesTSP7D01G016000.1
TraesTSP2B01G612500.1
TraesTSP1A01G263800.1
TraesTSP2B01G268300.1
TraesTSP5D01G230500.1
TraesTSP7D01G579700.1
TraesTSP5B01G318700.1
TraesTSP2B01G612600.1
TraesTSP3A01G058900.1
TraesTSP4B01G097500.1
TraesTSP3A01G010900.1
TraesTSP5B01G385200.1
TraesTSP7B01G501600.1
TraesTSP5A01G386300.1
TraesTSP6B01G434000.1
TraesTSP2B01G012300.1
TraesTSP2A01G025600.1
TraesTSP7D01G493500.1
TraesTSP7A01G500800.1
DUF3475 Family
DUF668 Family
Domain Color
B_lectin Domain
DUF3403 Family
Motile_Sperm Domain
PAN_2 Domain
PK_Tyr_Ser−Thr Domain
Pkinase Domain
S_locus_glycop Domain
Stress−antifung Family
WD40 Repeat
other Domain
0
500
1000
1500

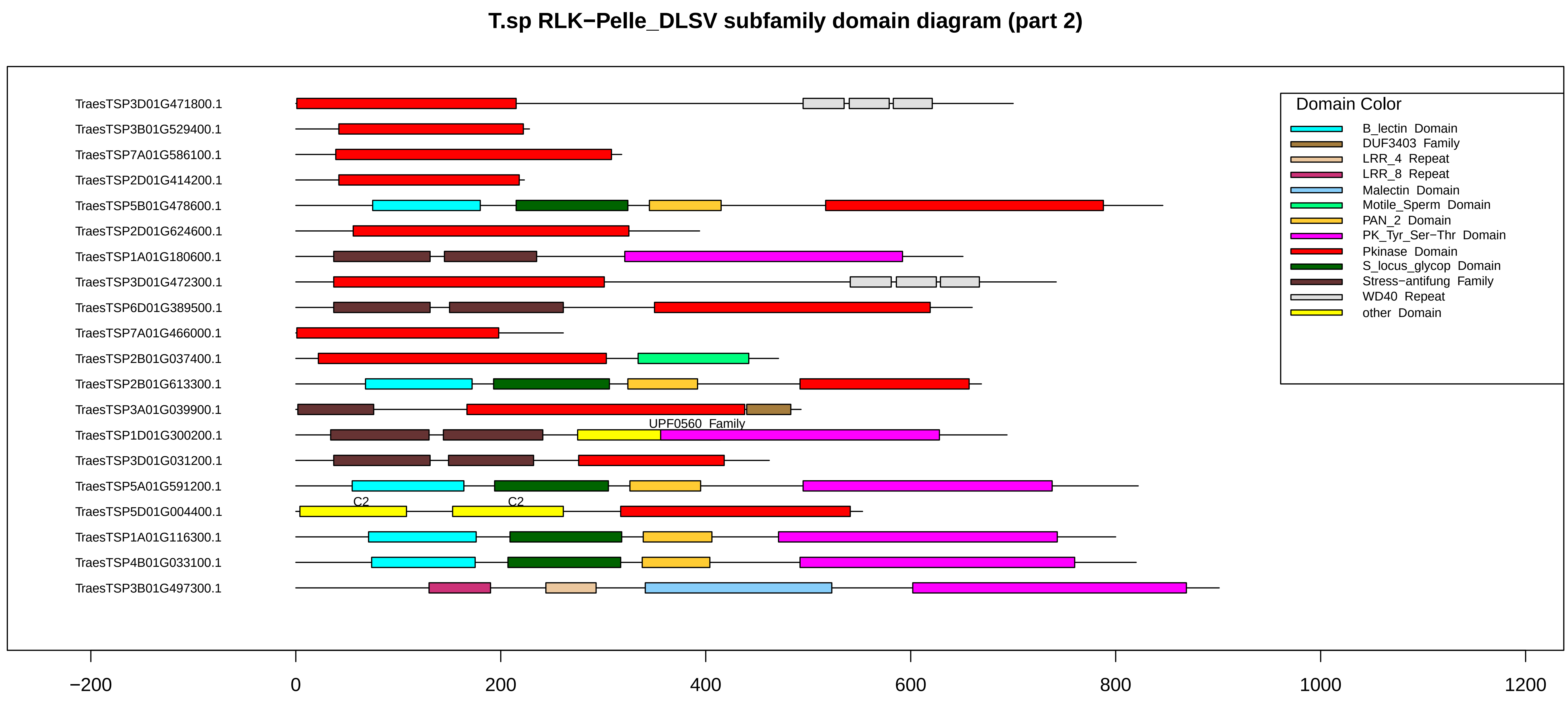
T.sp RLK−Pelle_DLSV subfamily domain diagram (part 2)
TraesTSP3D01G471800.1
TraesTSP3B01G529400.1
TraesTSP7A01G586100.1
TraesTSP2D01G414200.1
TraesTSP5B01G478600.1
TraesTSP2D01G624600.1
TraesTSP1A01G180600.1
TraesTSP3D01G472300.1
TraesTSP6D01G389500.1
TraesTSP7A01G466000.1
TraesTSP2B01G037400.1
TraesTSP2B01G613300.1
TraesTSP3A01G039900.1
UPF0560_Family
TraesTSP1D01G300200.1
TraesTSP3D01G031200.1
TraesTSP5A01G591200.1
C2
C2
TraesTSP5D01G004400.1
TraesTSP1A01G116300.1
TraesTSP4B01G033100.1
TraesTSP3B01G497300.1
Domain Color
B_lectin Domain
DUF3403 Family
LRR_4 Repeat
LRR_8 Repeat
Malectin Domain
Motile_Sperm Domain
PAN_2 Domain
PK_Tyr_Ser−Thr Domain
Pkinase Domain
S_locus_glycop Domain
Stress−antifung Family
WD40 Repeat
other Domain
−200
0
200
400
600
800
1000
1200

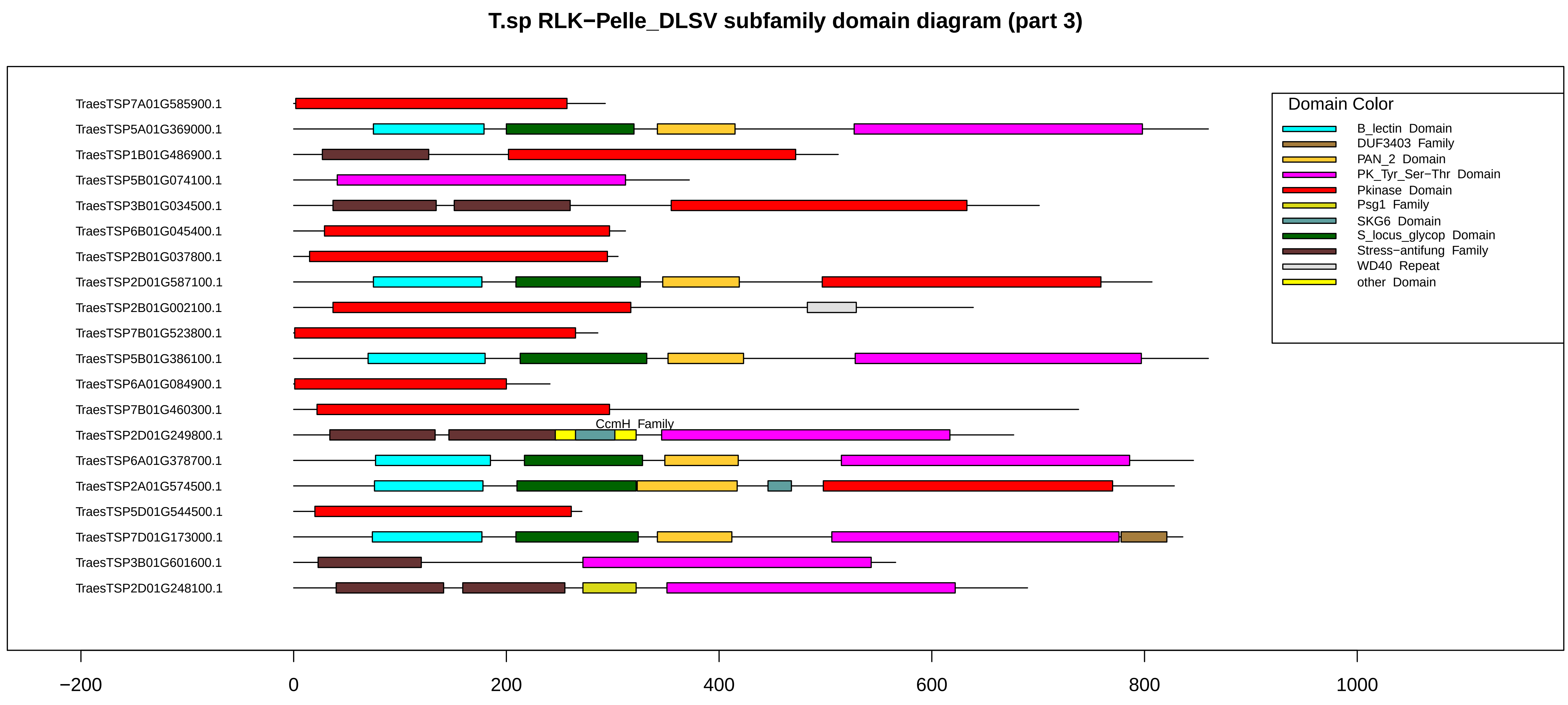

T.sp RLK−Pelle_DLSV subfamily domain diagram (part 3)
TraesTSP7A01G585900.1
TraesTSP5A01G369000.1
TraesTSP1B01G486900.1
TraesTSP5B01G074100.1
TraesTSP3B01G034500.1
TraesTSP6B01G045400.1
TraesTSP2B01G037800.1
TraesTSP2D01G587100.1
TraesTSP2B01G002100.1
TraesTSP7B01G523800.1
TraesTSP5B01G386100.1
TraesTSP6A01G084900.1
TraesTSP7B01G460300.1
TraesTSP2D01G249800.1
TraesTSP6A01G378700.1
TraesTSP2A01G574500.1
TraesTSP5D01G544500.1
TraesTSP7D01G173000.1
TraesTSP3B01G601600.1
TraesTSP2D01G248100.1
CcmH_Family
Domain Color
B_lectin Domain
DUF3403 Family
PAN_2 Domain
PK_Tyr_Ser−Thr Domain
Pkinase Domain
Psg1 Family
SKG6 Domain
S_locus_glycop Domain
Stress−antifung Family
WD40 Repeat
other Domain
−200
0
200
400
600
800
1000

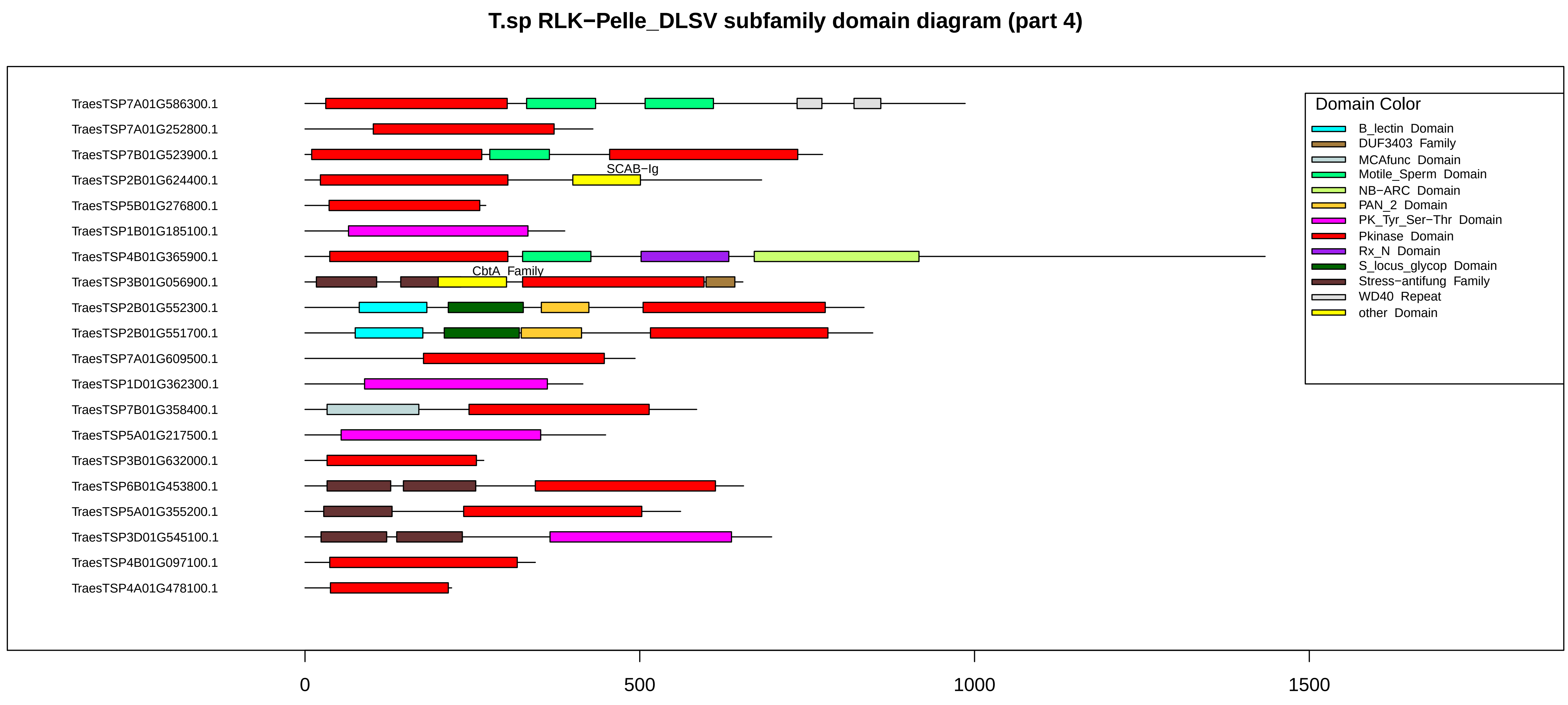

T.sp RLK−Pelle_DLSV subfamily domain diagram (part 4)
TraesTSP7A01G586300.1
TraesTSP7A01G252800.1
TraesTSP7B01G523900.1
TraesTSP2B01G624400.1
SCAB−Ig
TraesTSP5B01G276800.1
TraesTSP1B01G185100.1
TraesTSP4B01G365900.1
TraesTSP3B01G056900.1
CbtA_Family
TraesTSP2B01G552300.1
TraesTSP2B01G551700.1
TraesTSP7A01G609500.1
TraesTSP1D01G362300.1
TraesTSP7B01G358400.1
TraesTSP5A01G217500.1
TraesTSP3B01G632000.1
TraesTSP6B01G453800.1
TraesTSP5A01G355200.1
TraesTSP3D01G545100.1
TraesTSP4B01G097100.1
TraesTSP4A01G478100.1
0
500
1000
1500
Domain Color
B_lectin Domain
DUF3403 Family
MCAfunc Domain
Motile_Sperm Domain
NB−ARC Domain
PAN_2 Domain
PK_Tyr_Ser−Thr Domain
Pkinase Domain
Rx_N Domain
S_locus_glycop Domain
Stress−antifung Family
WD40 Repeat
other Domain

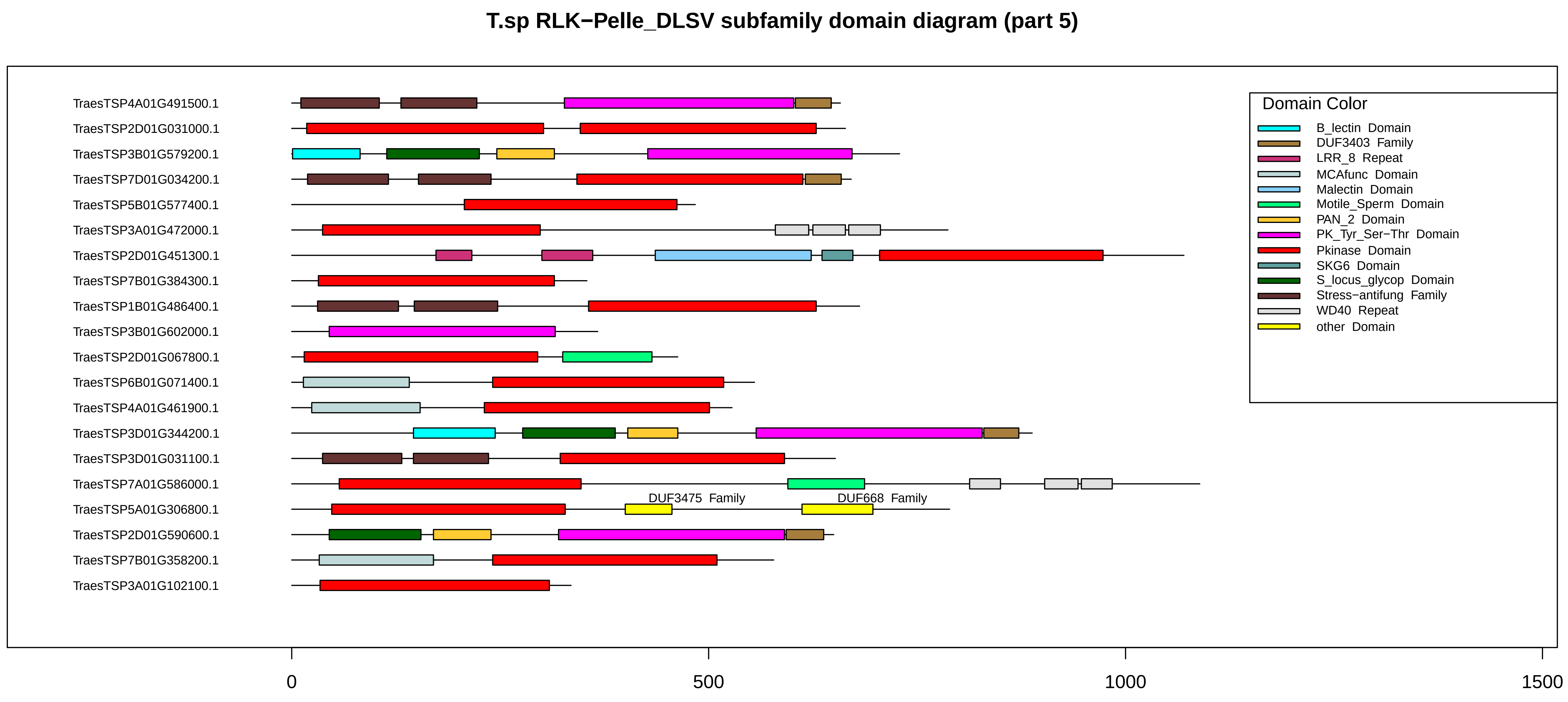
T.sp RLK−Pelle_DLSV subfamily domain diagram (part 5)
TraesTSP4A01G491500.1
TraesTSP2D01G031000.1
TraesTSP3B01G579200.1
TraesTSP7D01G034200.1
TraesTSP5B01G577400.1
TraesTSP3A01G472000.1
TraesTSP2D01G451300.1
TraesTSP7B01G384300.1
TraesTSP1B01G486400.1
TraesTSP3B01G602000.1
TraesTSP2D01G067800.1
TraesTSP6B01G071400.1
TraesTSP4A01G461900.1
TraesTSP3D01G344200.1
TraesTSP3D01G031100.1
TraesTSP7A01G586000.1
TraesTSP5A01G306800.1
TraesTSP2D01G590600.1
TraesTSP7B01G358200.1
TraesTSP3A01G102100.1
DUF3475 Family
DUF668 Family
Domain Color
B_lectin Domain
DUF3403 Family
LRR_8 Repeat
MCAfunc Domain
Malectin Domain
Motile_Sperm Domain
PAN_2 Domain
PK_Tyr_Ser−Thr Domain
Pkinase Domain
SKG6 Domain
S_locus_glycop Domain
Stress−antifung Family
WD40 Repeat
other Domain
0
500
1000
1500

T.sp RLK−Pelle_DLSV subfamily domain diagram (part 6)

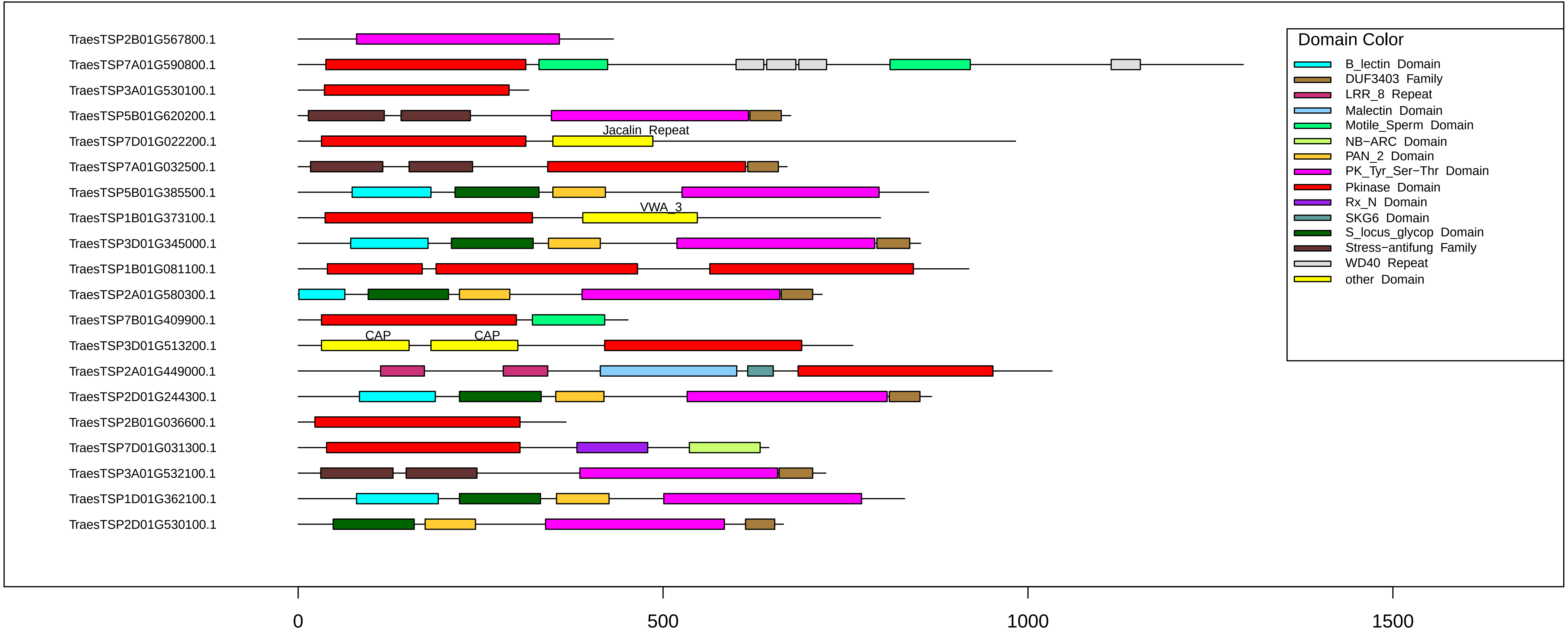

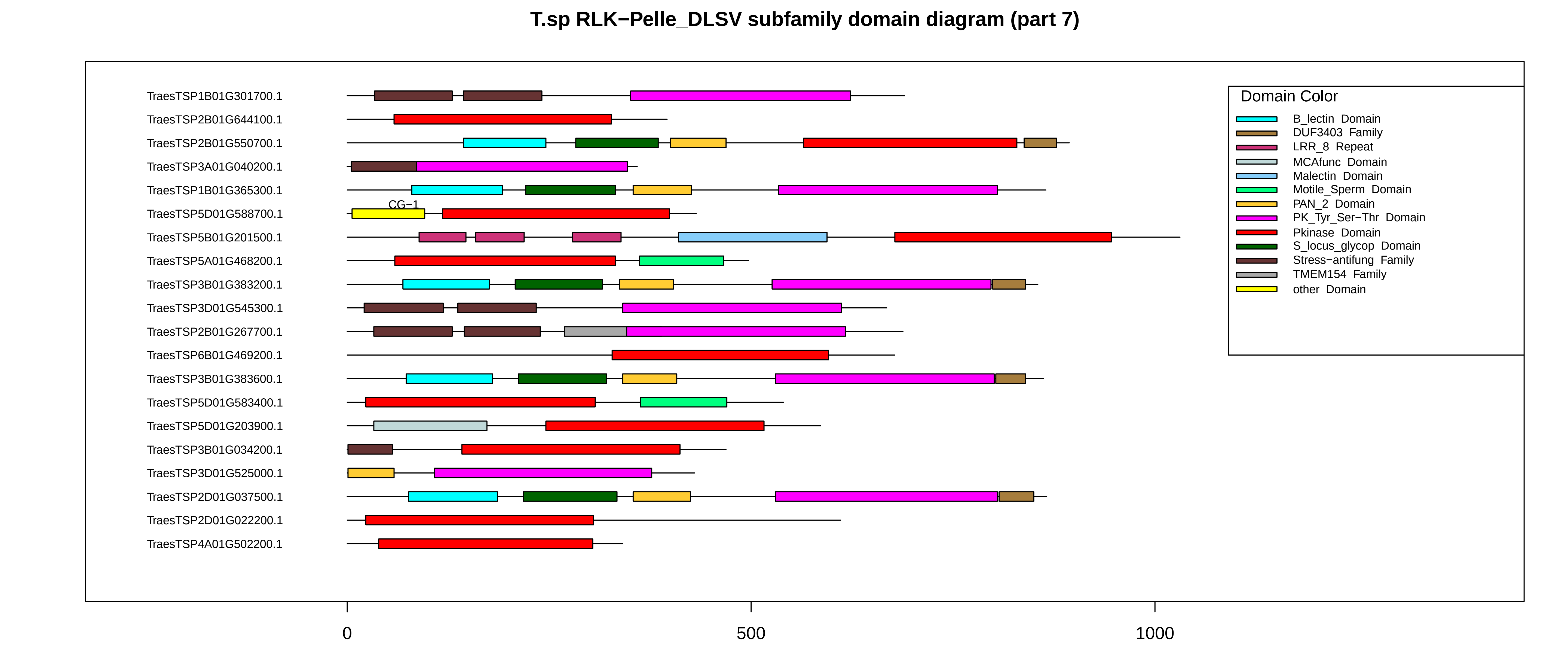
T.sp RLK−Pelle_DLSV subfamily domain diagram (part 7)
TraesTSP1B01G301700.1
TraesTSP2B01G644100.1
TraesTSP2B01G550700.1
TraesTSP3A01G040200.1
TraesTSP1B01G365300.1
TraesTSP5D01G588700.1
CG−1
TraesTSP5B01G201500.1
TraesTSP5A01G468200.1
TraesTSP3B01G383200.1
TraesTSP3D01G545300.1
TraesTSP2B01G267700.1
TraesTSP6B01G469200.1
TraesTSP3B01G383600.1
TraesTSP5D01G583400.1
TraesTSP5D01G203900.1
TraesTSP3B01G034200.1
TraesTSP3D01G525000.1
TraesTSP2D01G037500.1
TraesTSP2D01G022200.1
TraesTSP4A01G502200.1
Domain Color
B_lectin Domain
DUF3403 Family
LRR_8 Repeat
MCAfunc Domain
Malectin Domain
Motile_Sperm Domain
PAN_2 Domain
PK_Tyr_Ser−Thr Domain
Pkinase Domain
S_locus_glycop Domain
Stress−antifung Family
TMEM154 Family
other Domain
0
500
1000

T.sp RLK−Pelle_DLSV subfamily domain diagram (part 8)

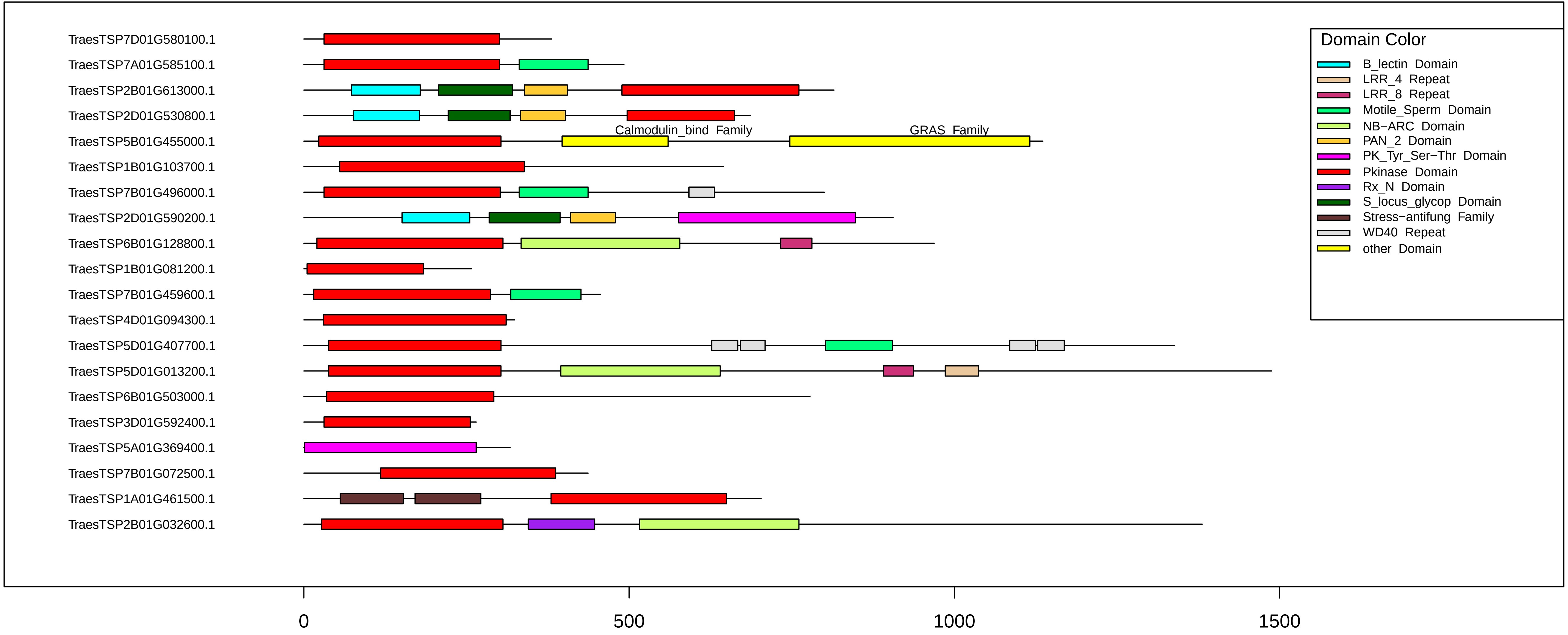

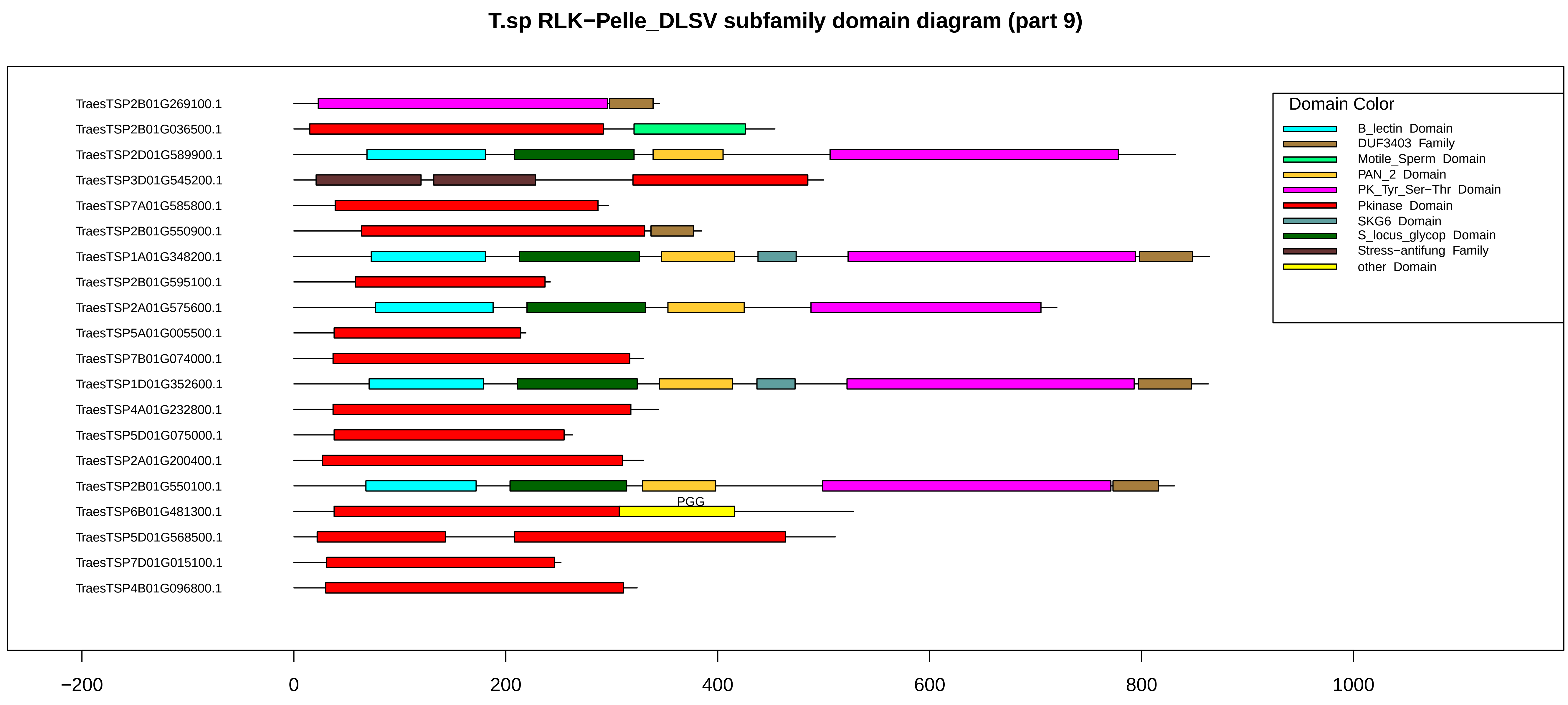
T.sp RLK−Pelle_DLSV subfamily domain diagram (part 9)
TraesTSP2B01G269100.1
TraesTSP2B01G036500.1
TraesTSP2D01G589900.1
TraesTSP3D01G545200.1
TraesTSP7A01G585800.1
TraesTSP2B01G550900.1
TraesTSP1A01G348200.1
TraesTSP2B01G595100.1
TraesTSP2A01G575600.1
TraesTSP5A01G005500.1
TraesTSP7B01G074000.1
TraesTSP1D01G352600.1
TraesTSP4A01G232800.1
TraesTSP5D01G075000.1
TraesTSP2A01G200400.1
TraesTSP2B01G550100.1
PGG
TraesTSP6B01G481300.1
TraesTSP5D01G568500.1
TraesTSP7D01G015100.1
TraesTSP4B01G096800.1
Domain Color
B_lectin Domain
DUF3403 Family
Motile_Sperm Domain
PAN_2 Domain
PK_Tyr_Ser−Thr Domain
Pkinase Domain
SKG6 Domain
S_locus_glycop Domain
Stress−antifung Family
other Domain
−200
0
200
400
600
800
1000

T.sp RLK−Pelle_DLSV subfamily domain diagram (part 10)

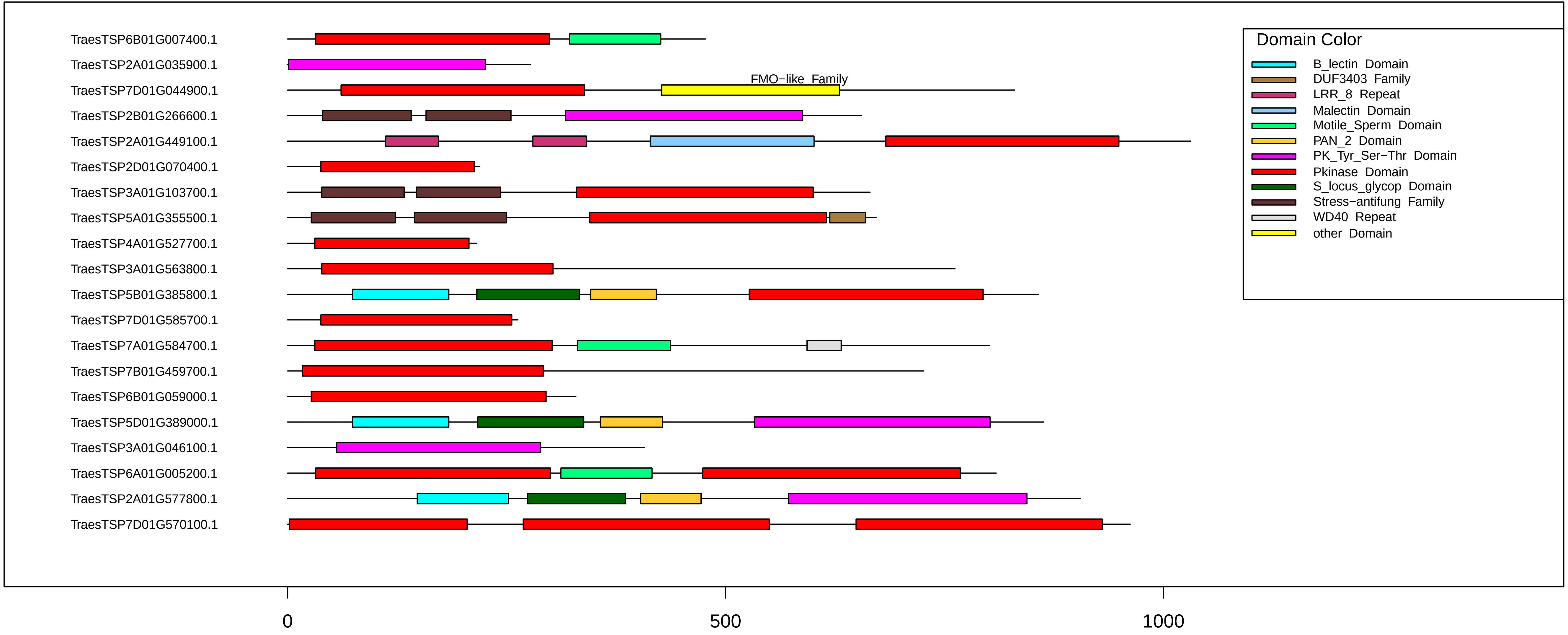

T.sp RLK−Pelle_DLSV subfamily domain diagram (part 11)

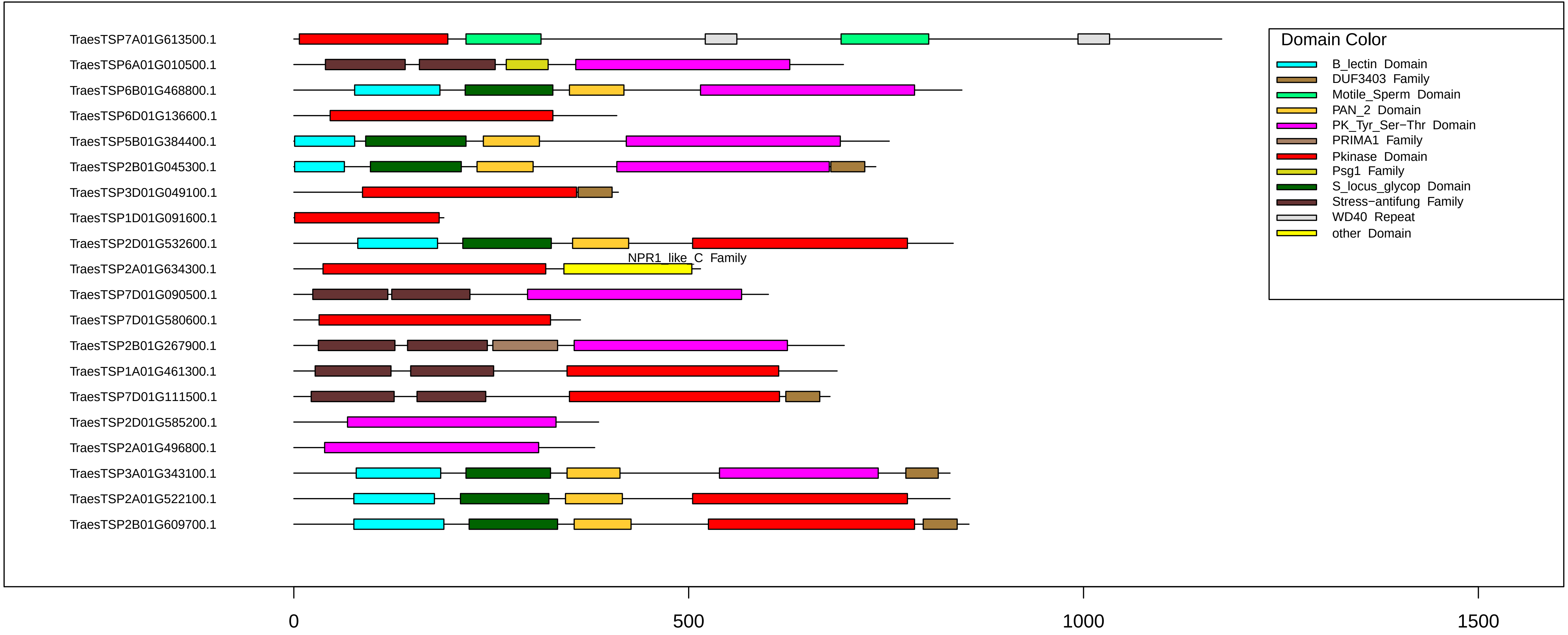

T.sp RLK−Pelle_DLSV subfamily domain diagram (part 12)

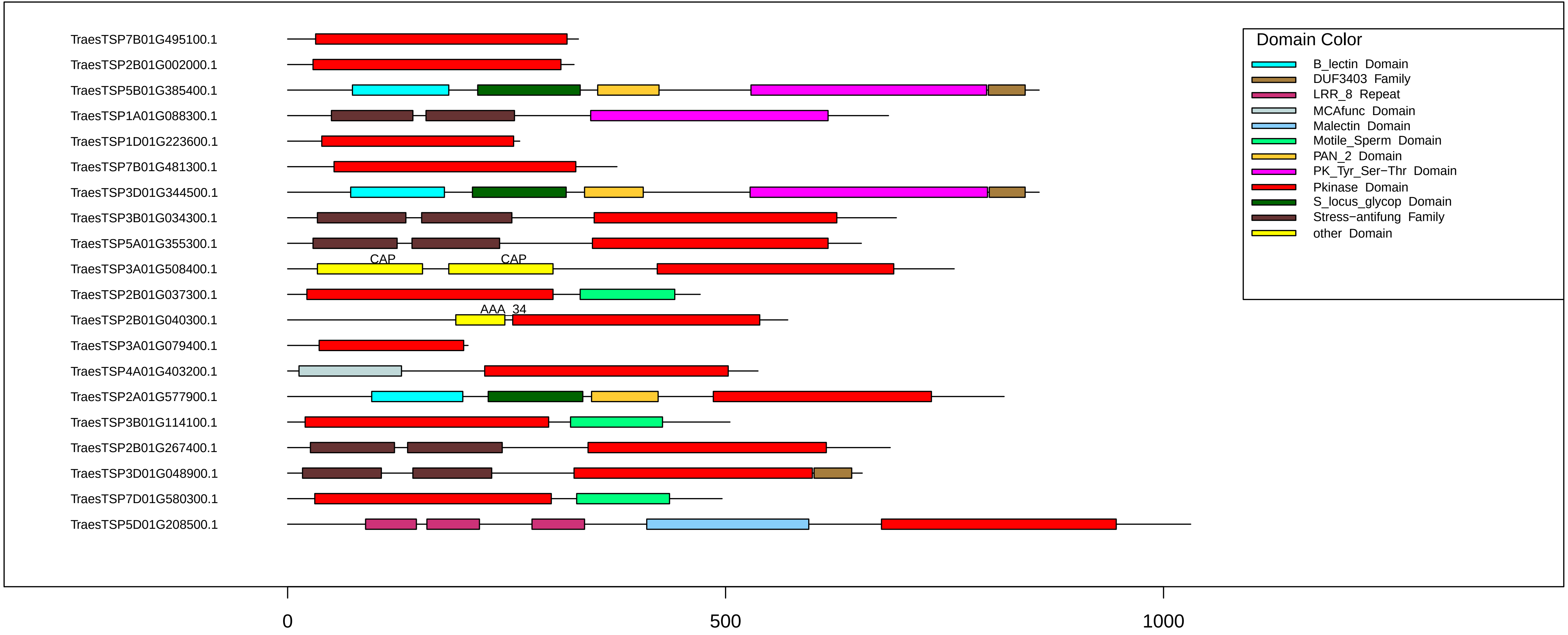

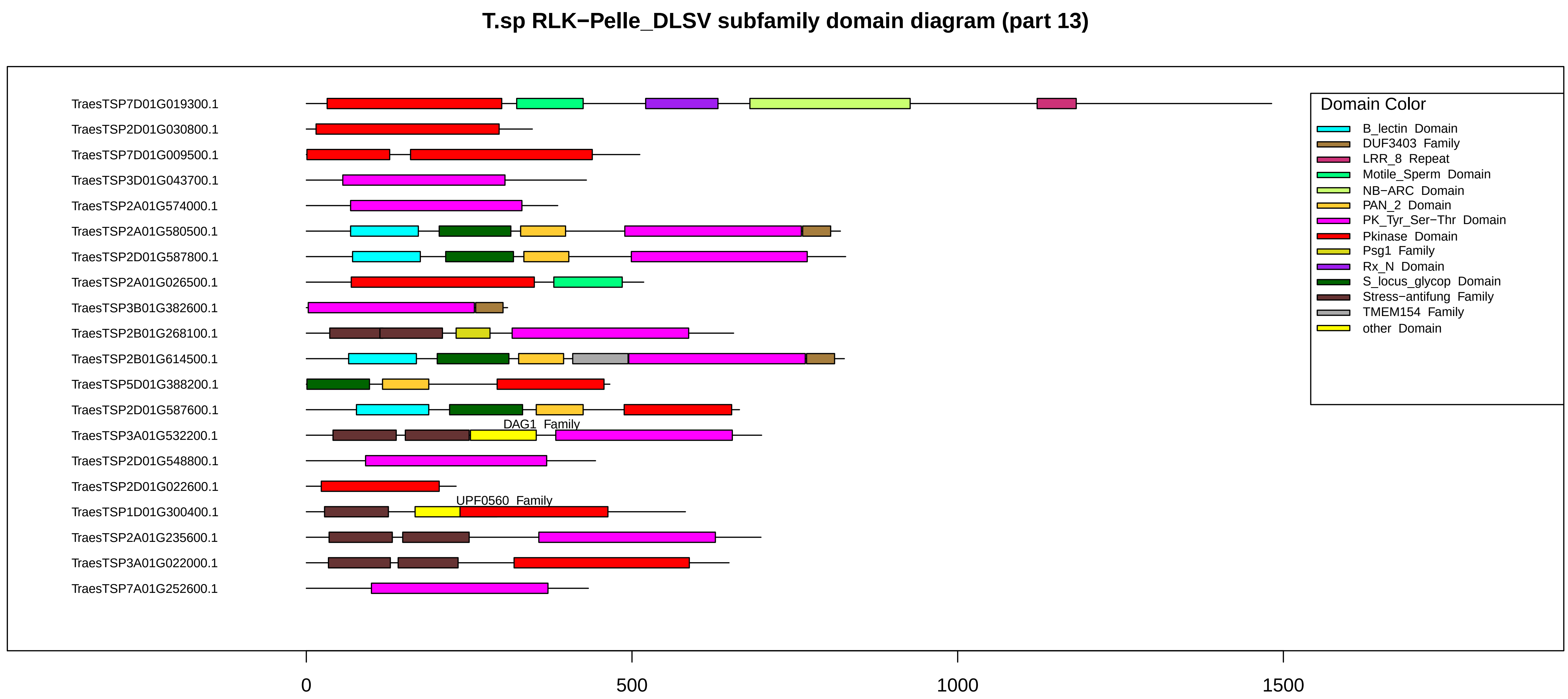
T.sp RLK−Pelle_DLSV subfamily domain diagram (part 13)
TraesTSP7D01G019300.1
TraesTSP2D01G030800.1
TraesTSP7D01G009500.1
TraesTSP3D01G043700.1
TraesTSP2A01G574000.1
TraesTSP2A01G580500.1
TraesTSP2D01G587800.1
TraesTSP2A01G026500.1
TraesTSP3B01G382600.1
TraesTSP2B01G268100.1
TraesTSP2B01G614500.1
TraesTSP5D01G388200.1
TraesTSP2D01G587600.1
TraesTSP3A01G532200.1
TraesTSP2D01G548800.1
TraesTSP2D01G022600.1
TraesTSP1D01G300400.1
TraesTSP2A01G235600.1
TraesTSP3A01G022000.1
TraesTSP7A01G252600.1
DAG1 Family
UPF0560 Family
Domain Color
B_lectin Domain
DUF3403 Family
LRR_8 Repeat
Motile_Sperm Domain
NB−ARC Domain
PAN_2 Domain
PK_Tyr_Ser−Thr Domain
Pkinase Domain
Psg1 Family
Rx_N Domain
S_locus_glycop Domain
Stress−antifung Family
TMEM154 Family
other Domain
0
500
1000
1500

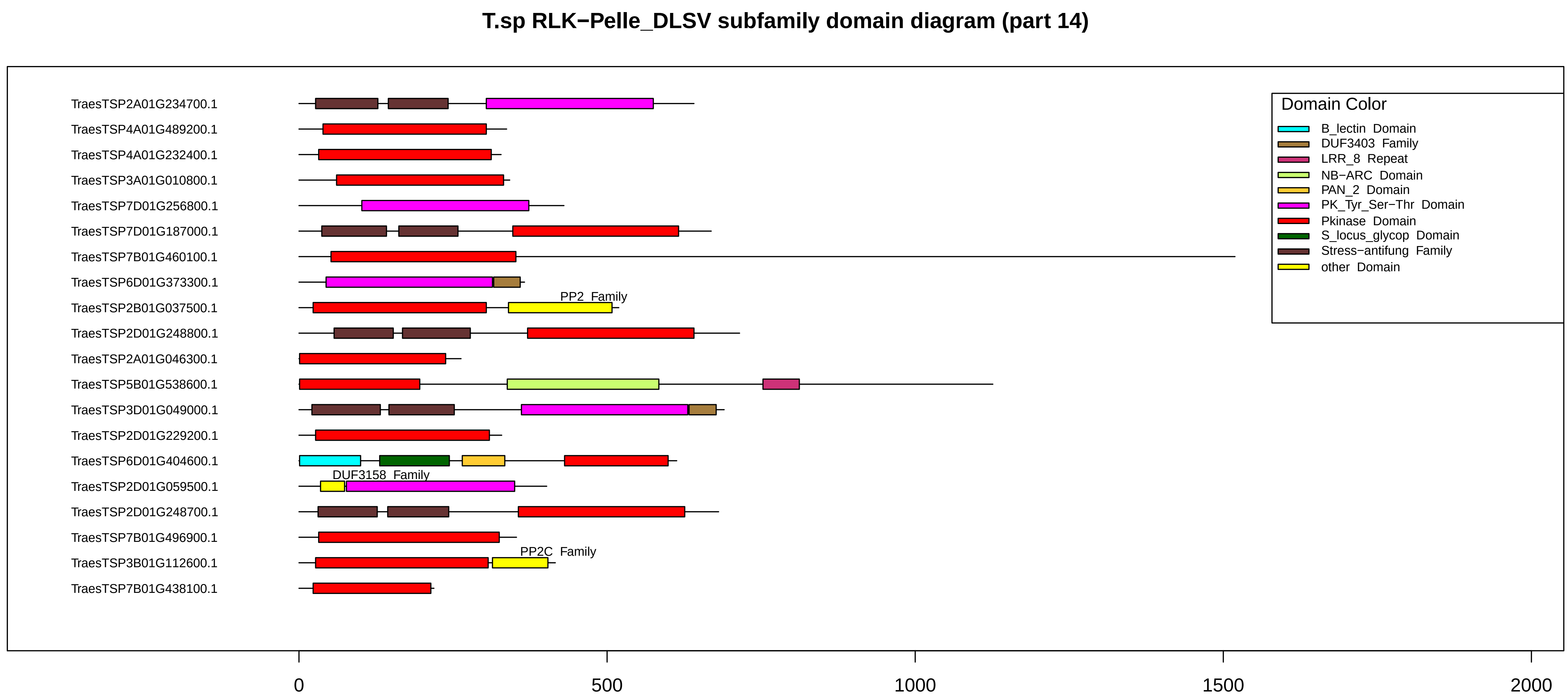
T.sp RLK−Pelle_DLSV subfamily domain diagram (part 14)
TraesTSP2A01G234700.1
TraesTSP4A01G489200.1
TraesTSP4A01G232400.1
TraesTSP3A01G010800.1
TraesTSP7D01G256800.1
TraesTSP7D01G187000.1
TraesTSP7B01G460100.1
TraesTSP6D01G373300.1
TraesTSP2B01G037500.1
PP2 Family
TraesTSP2D01G248800.1
TraesTSP2A01G046300.1
TraesTSP5B01G538600.1
TraesTSP3D01G049000.1
TraesTSP2D01G229200.1
TraesTSP6D01G404600.1
TraesTSP2D01G059500.1
DUF3158 Family
TraesTSP2D01G248700.1
TraesTSP7B01G496900.1
TraesTSP3B01G112600.1
PP2C Family
TraesTSP7B01G438100.1
Domain Color
B_lectin Domain
DUF3403 Family
LRR_8 Repeat
NB−ARC Domain
PAN_2 Domain
PK_Tyr_Ser−Thr Domain
Pkinase Domain
S_locus_glycop Domain
Stress−antifung Family
other Domain
0
500
1000
1500
2000

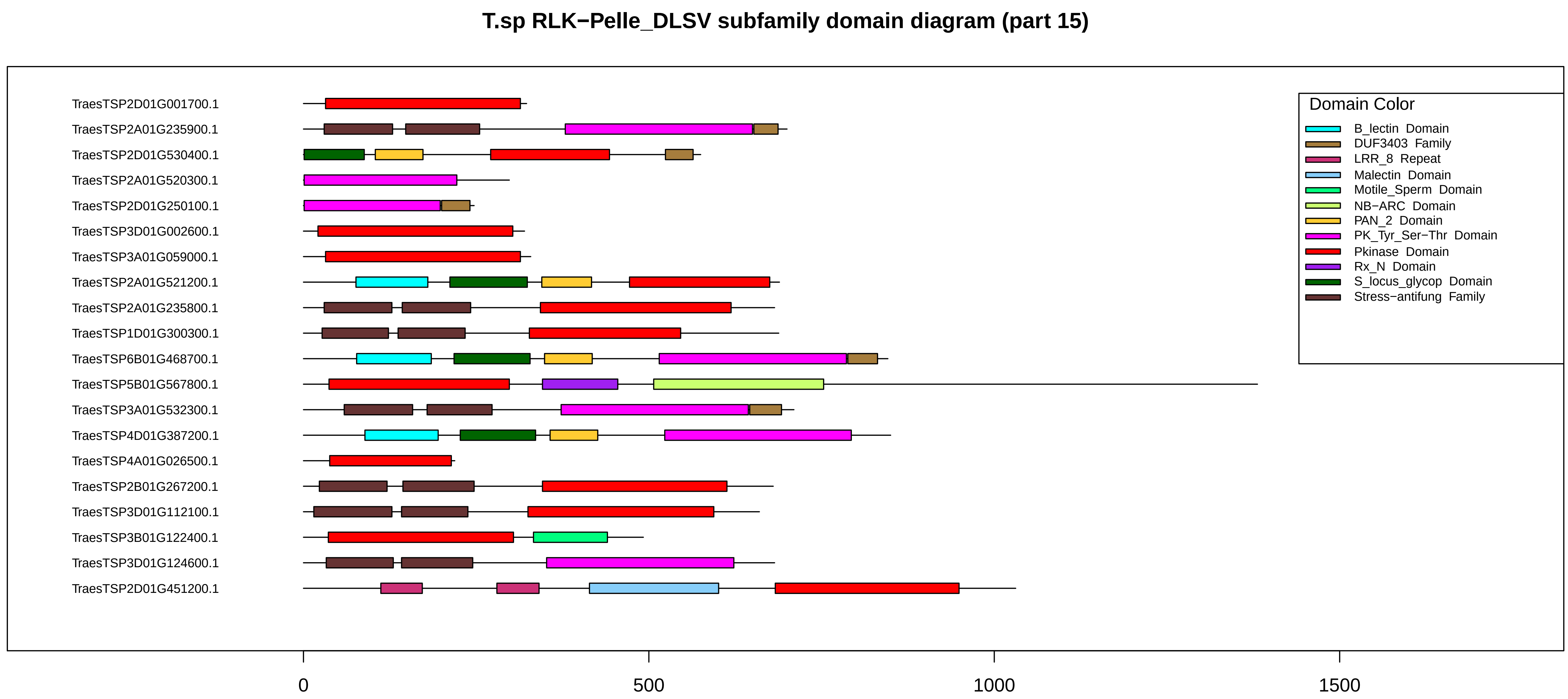
T.sp RLK−Pelle_DLSV subfamily domain diagram (part 15)
TraesTSP2D01G001700.1
TraesTSP2A01G235900.1
TraesTSP2D01G530400.1
TraesTSP2A01G520300.1
TraesTSP2D01G250100.1
TraesTSP3D01G002600.1
TraesTSP3A01G059000.1
TraesTSP2A01G521200.1
TraesTSP2A01G235800.1
TraesTSP1D01G300300.1
TraesTSP6B01G468700.1
TraesTSP5B01G567800.1
TraesTSP3A01G532300.1
TraesTSP4D01G387200.1
TraesTSP4A01G026500.1
TraesTSP2B01G267200.1
TraesTSP3D01G112100.1
TraesTSP3B01G122400.1
TraesTSP3D01G124600.1
TraesTSP2D01G451200.1
Domain Color
B_lectin Domain
DUF3403 Family
LRR_8 Repeat
Malectin Domain
Motile_Sperm Domain
NB−ARC Domain
PAN_2 Domain
PK_Tyr_Ser−Thr Domain
Pkinase Domain
Rx_N Domain
S_locus_glycop Domain
Stress−antifung Family
0
500
1000
1500

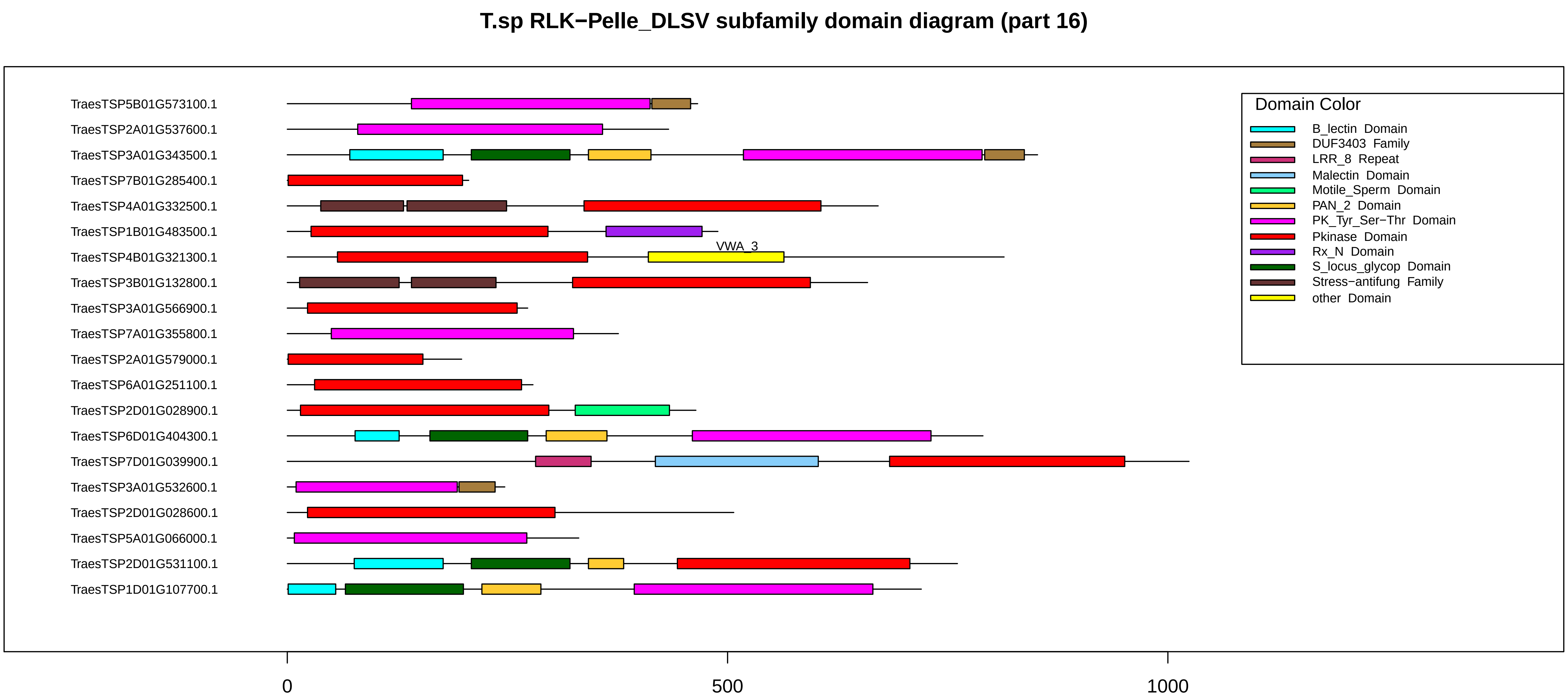
T.sp RLK−Pelle_DLSV subfamily domain diagram (part 16)
TraesTSP5B01G573100.1
TraesTSP2A01G537600.1
TraesTSP3A01G343500.1
TraesTSP7B01G285400.1
TraesTSP4A01G332500.1
TraesTSP1B01G483500.1
TraesTSP4B01G321300.1
TraesTSP3B01G132800.1
TraesTSP3A01G566900.1
TraesTSP7A01G355800.1
TraesTSP2A01G579000.1
TraesTSP6A01G251100.1
TraesTSP2D01G028900.1
TraesTSP6D01G404300.1
TraesTSP7D01G039900.1
TraesTSP3A01G532600.1
TraesTSP2D01G028600.1
TraesTSP5A01G066000.1
TraesTSP2D01G531100.1
TraesTSP1D01G107700.1
VWA_3
Domain Color
B_lectin Domain
DUF3403 Family
LRR_8 Repeat
Malectin Domain
Motile_Sperm Domain
PAN_2 Domain
PK_Tyr_Ser−Thr Domain
Pkinase Domain
Rx_N Domain
S_locus_glycop Domain
Stress−antifung Family
other Domain
0
500
1000

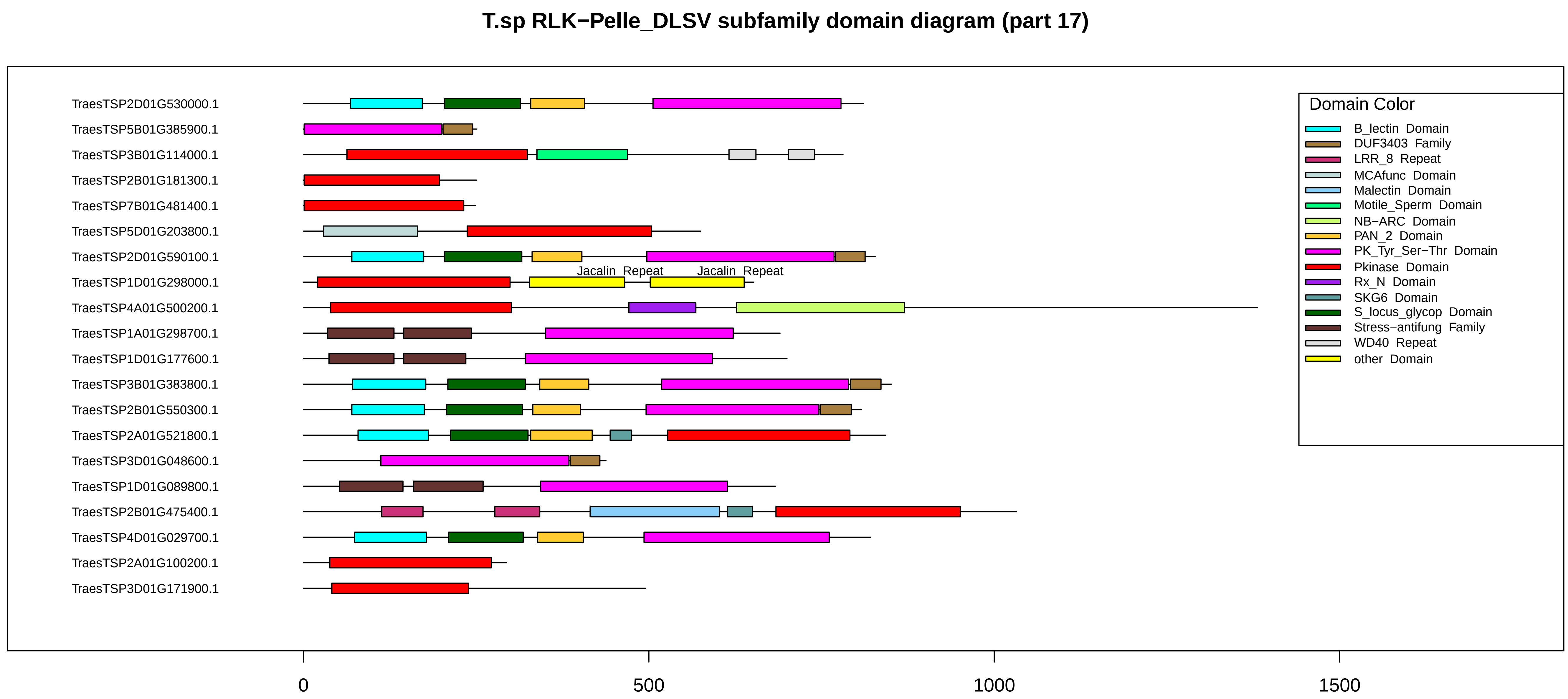
T.sp RLK−Pelle_DLSV subfamily domain diagram (part 17)
TraesTSP2D01G530000.1
TraesTSP5B01G385900.1
TraesTSP3B01G114000.1
TraesTSP2B01G181300.1
TraesTSP7B01G481400.1
TraesTSP5D01G203800.1
TraesTSP2D01G590100.1
TraesTSP1D01G298000.1
Jacalin Repeat
Jacalin Repeat
TraesTSP4A01G500200.1
TraesTSP1A01G298700.1
TraesTSP1D01G177600.1
TraesTSP3B01G383800.1
TraesTSP2B01G550300.1
TraesTSP2A01G521800.1
TraesTSP3D01G048600.1
TraesTSP1D01G089800.1
TraesTSP2B01G475400.1
TraesTSP4D01G029700.1
TraesTSP2A01G100200.1
TraesTSP3D01G171900.1
Domain Color
B_lectin Domain
DUF3403 Family
LRR_8 Repeat
MCAfunc Domain
Malectin Domain
Motile_Sperm Domain
NB−ARC Domain
PAN_2 Domain
PK_Tyr_Ser−Thr Domain
Pkinase Domain
Rx_N Domain
SKG6 Domain
S_locus_glycop Domain
Stress−antifung Family
WD40 Repeat
other Domain
0
500
1000
1500

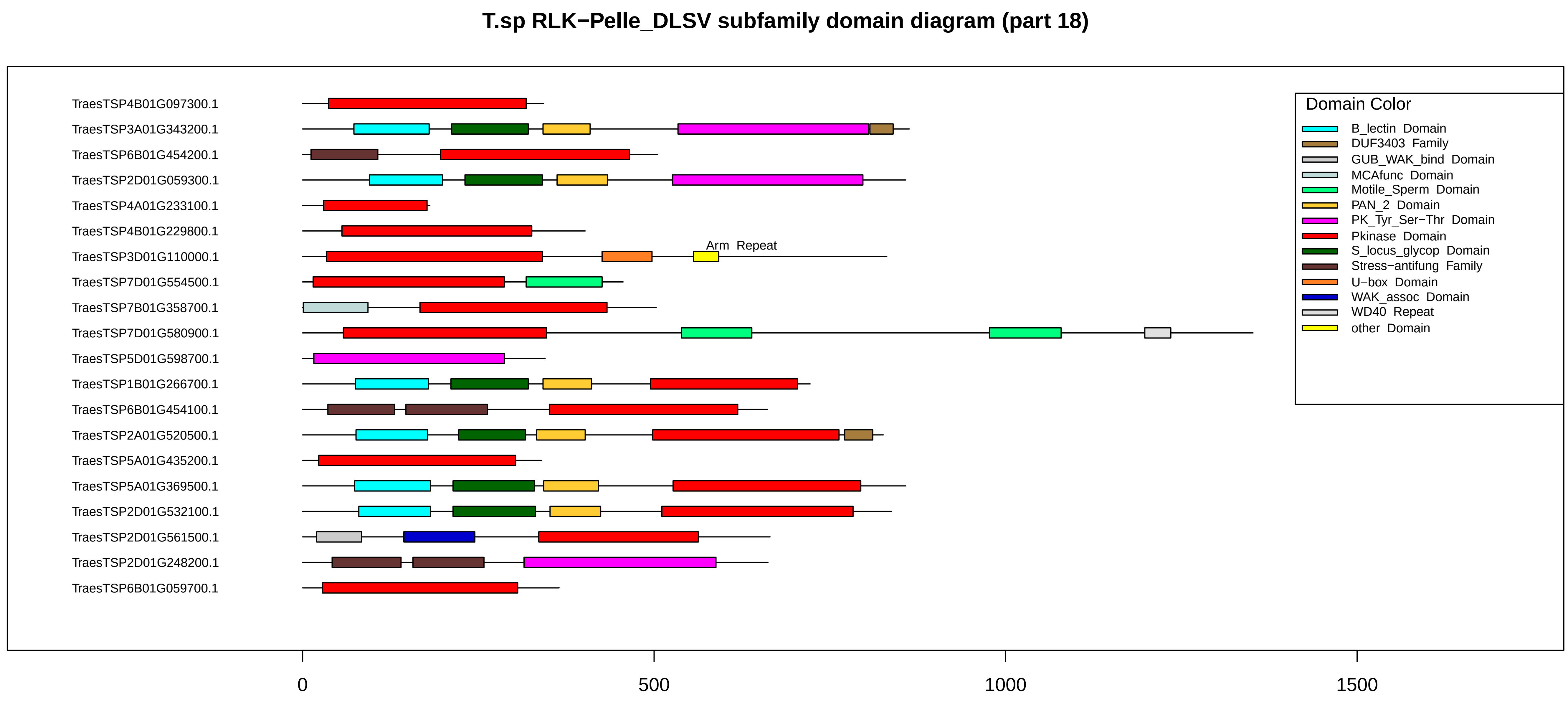
T.sp RLK−Pelle_DLSV subfamily domain diagram (part 18)
TraesTSP4B01G097300.1
TraesTSP3A01G343200.1
TraesTSP6B01G454200.1
TraesTSP2D01G059300.1
TraesTSP4A01G233100.1
TraesTSP4B01G229800.1
TraesTSP3D01G110000.1
TraesTSP7D01G554500.1
TraesTSP7B01G358700.1
TraesTSP7D01G580900.1
TraesTSP5D01G598700.1
TraesTSP1B01G266700.1
TraesTSP6B01G454100.1
TraesTSP2A01G520500.1
TraesTSP5A01G435200.1
TraesTSP5A01G369500.1
TraesTSP2D01G532100.1
TraesTSP2D01G561500.1
TraesTSP2D01G248200.1
TraesTSP6B01G059700.1
Arm Repeat
Domain Color
B_lectin Domain
DUF3403 Family
GUB_WAK_bind Domain
MCAfunc Domain
Motile_Sperm Domain
PAN_2 Domain
PK_Tyr_Ser−Thr Domain
Pkinase Domain
S_locus_glycop Domain
Stress−antifung Family
U−box Domain
WAK_assoc Domain
WD40 Repeat
other Domain
0
500
1000
1500

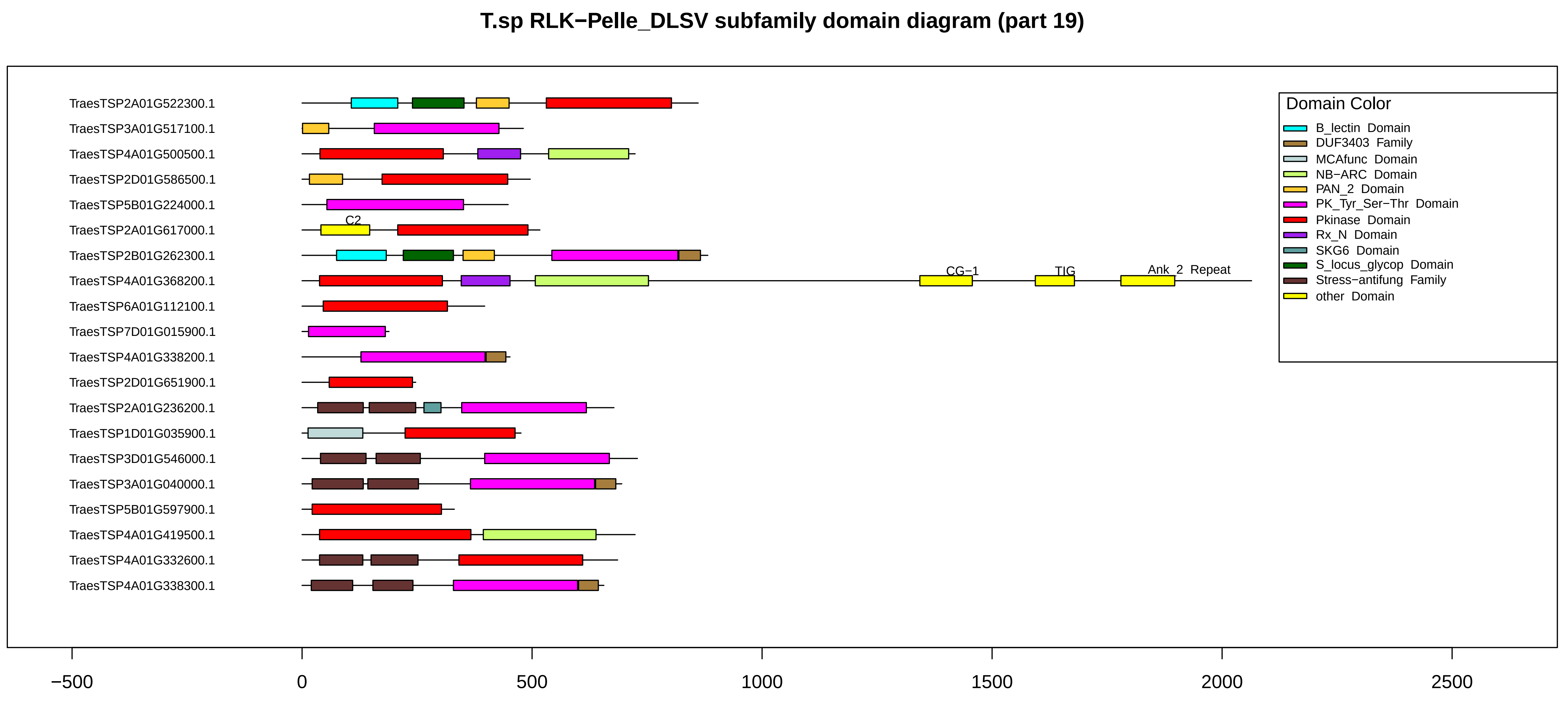

T.sp RLK−Pelle_DLSV subfamily domain diagram (part 19)
TraesTSP2A01G522300.1
TraesTSP3A01G517100.1
TraesTSP4A01G500500.1
TraesTSP2D01G586500.1
TraesTSP5B01G224000.1
TraesTSP2A01G617000.1
C2
TraesTSP2B01G262300.1
TraesTSP4A01G368200.1
CG−1
TIG
Ank_2 Repeat
TraesTSP6A01G112100.1
TraesTSP7D01G015900.1
TraesTSP4A01G338200.1
TraesTSP2D01G651900.1
TraesTSP2A01G236200.1
TraesTSP1D01G035900.1
TraesTSP3D01G546000.1
TraesTSP3A01G040000.1
TraesTSP5B01G597900.1
TraesTSP4A01G419500.1
TraesTSP4A01G332600.1
TraesTSP4A01G338300.1
Domain Color
B_lectin Domain
DUF3403 Family
MCAfunc Domain
NB−ARC Domain
PAN_2 Domain
PK_Tyr_Ser−Thr Domain
Pkinase Domain
Rx_N Domain
SKG6 Domain
S_locus_glycop Domain
Stress−antifung Family
other Domain
−500
0
500
1000
1500
2000
2500

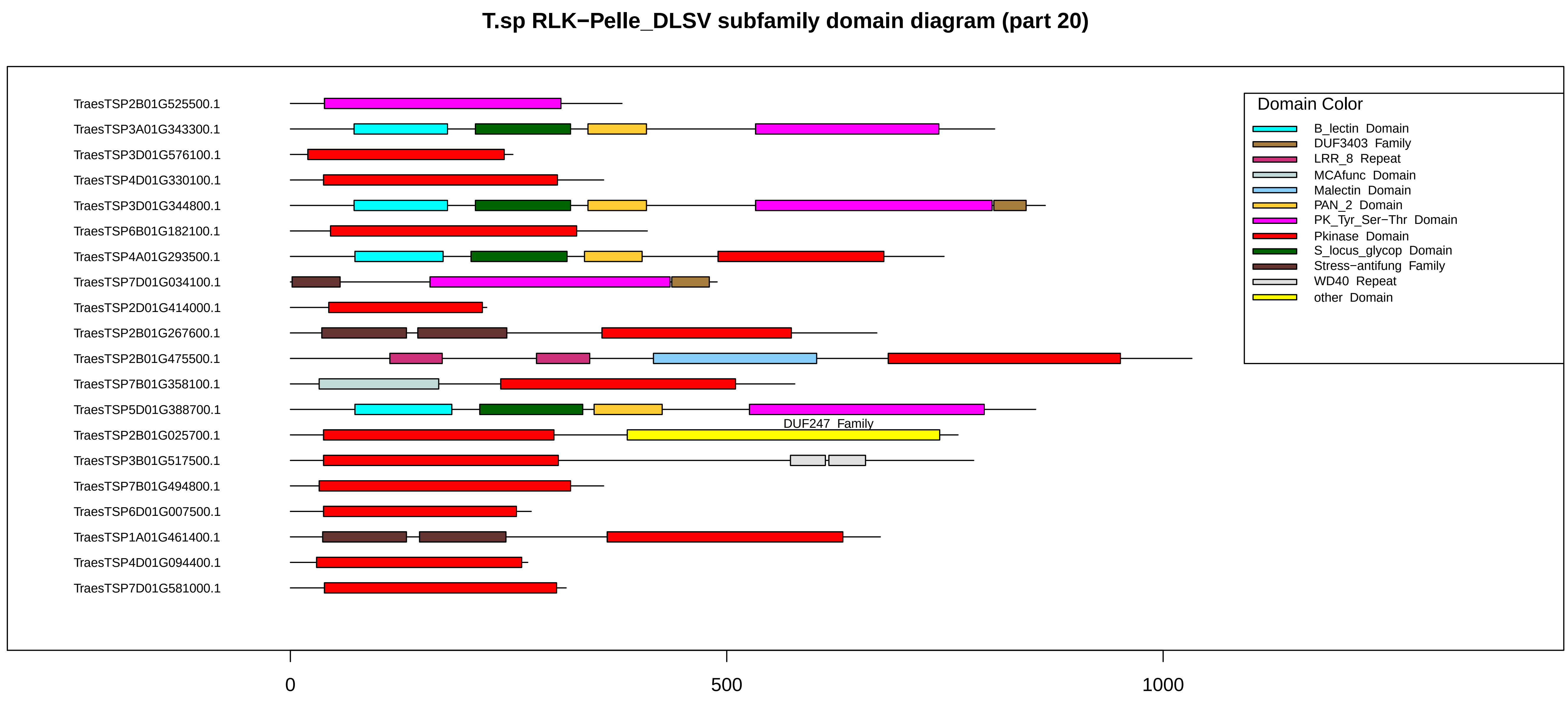

T.sp RLK−Pelle_DLSV subfamily domain diagram (part 20)
TraesTSP2B01G525500.1
TraesTSP3A01G343300.1
TraesTSP3D01G576100.1
TraesTSP4D01G330100.1
TraesTSP3D01G344800.1
TraesTSP6B01G182100.1
TraesTSP4A01G293500.1
TraesTSP7D01G034100.1
TraesTSP2D01G414000.1
TraesTSP2B01G267600.1
TraesTSP2B01G475500.1
TraesTSP7B01G358100.1
TraesTSP5D01G388700.1
TraesTSP2B01G025700.1
DUF247 Family
TraesTSP3B01G517500.1
TraesTSP7B01G494800.1
TraesTSP6D01G007500.1
TraesTSP1A01G461400.1
TraesTSP4D01G094400.1
TraesTSP7D01G581000.1
Domain Color
B_lectin Domain
DUF3403 Family
LRR_8 Repeat
MCAfunc Domain
Malectin Domain
PAN_2 Domain
PK_Tyr_Ser−Thr Domain
Pkinase Domain
S_locus_glycop Domain
Stress−antifung Family
WD40 Repeat
other Domain
0
500
1000

T.sp RLK−Pelle_DLSV subfamily domain diagram (part 21)

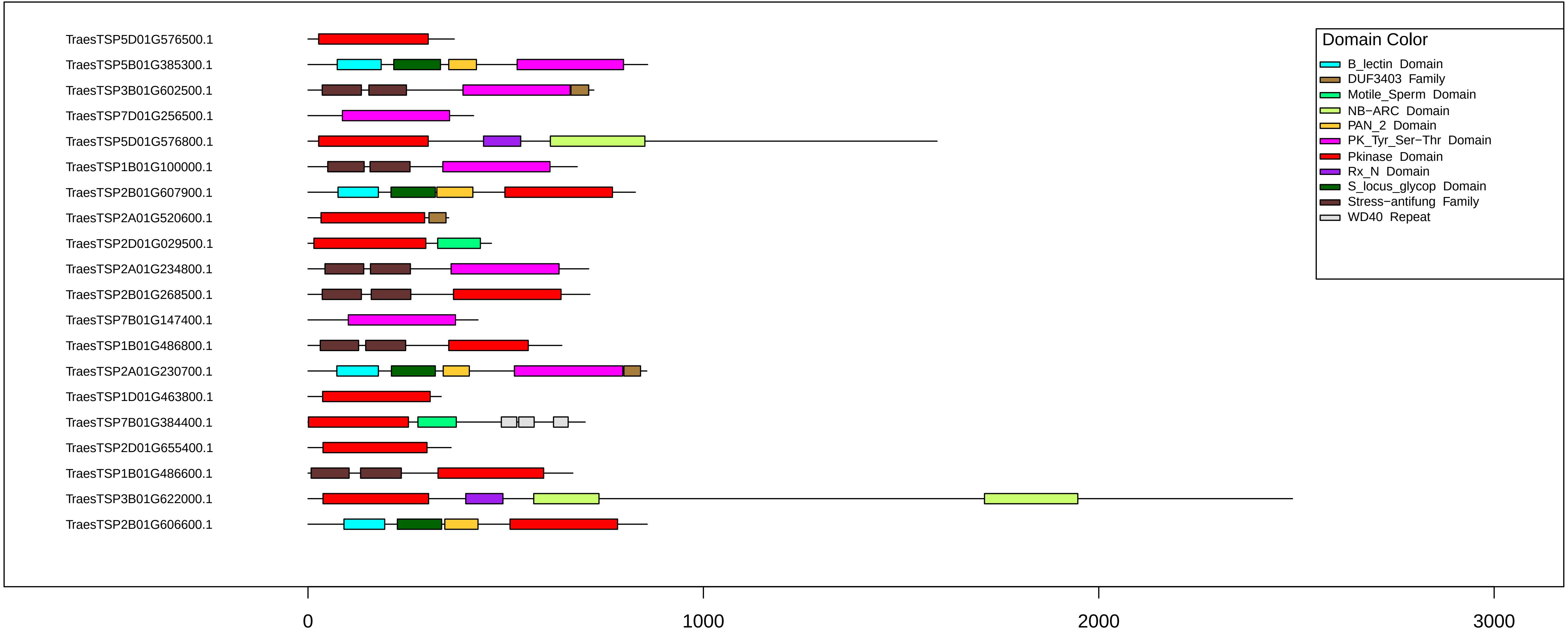

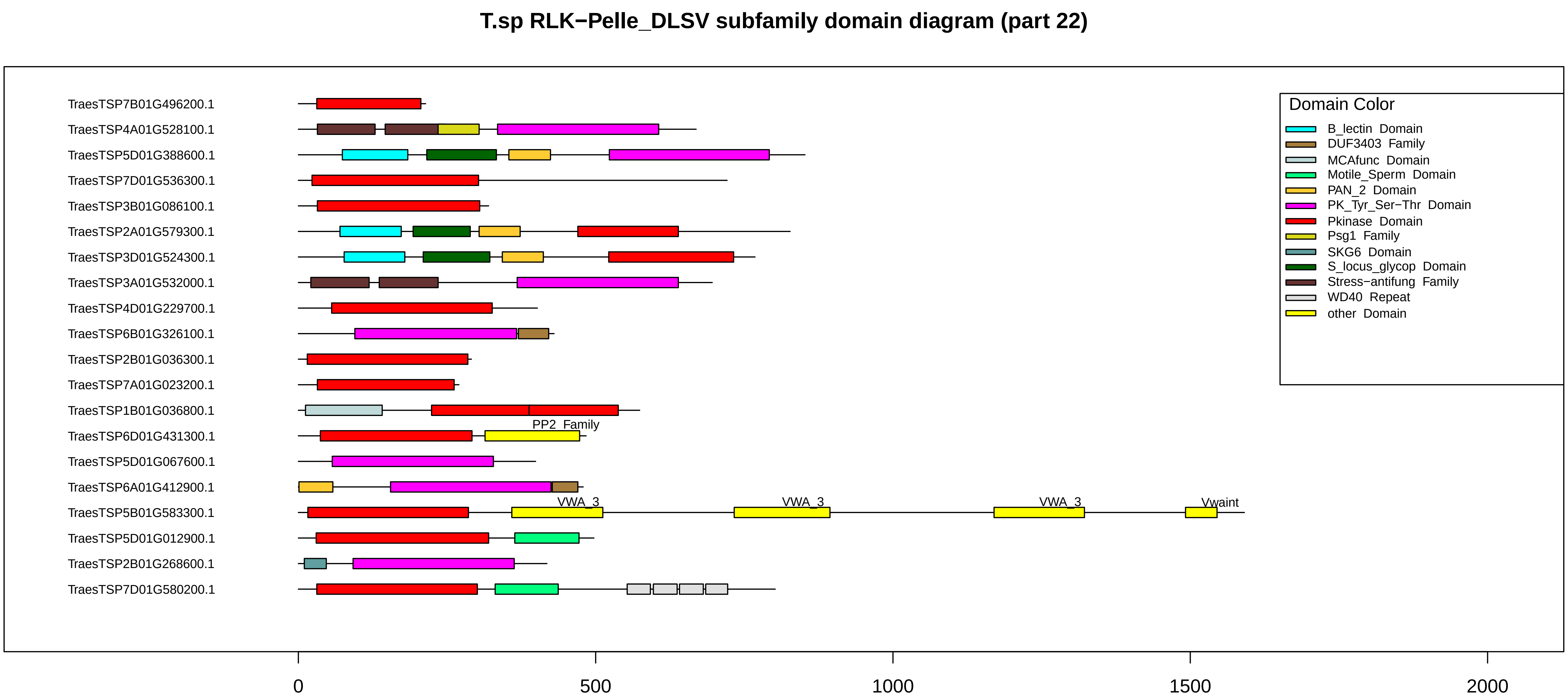
T.sp RLK−Pelle_DLSV subfamily domain diagram (part 22)
TraesTSP7B01G496200.1
TraesTSP4A01G528100.1
TraesTSP5D01G388600.1
TraesTSP7D01G536300.1
TraesTSP3B01G086100.1
TraesTSP2A01G579300.1
TraesTSP3D01G524300.1
TraesTSP3A01G532000.1
TraesTSP4D01G229700.1
TraesTSP6B01G326100.1
TraesTSP2B01G036300.1
TraesTSP7A01G023200.1
TraesTSP1B01G036800.1
TraesTSP6D01G431300.1
PP2_Family
TraesTSP5D01G067600.1
TraesTSP6A01G412900.1
TraesTSP5B01G583300.1
VWA_3
VWA_3
VWA_3
Vwaint
TraesTSP5D01G012900.1
TraesTSP2B01G268600.1
TraesTSP7D01G580200.1
Domain Color
B_lectin Domain
DUF3403 Family
MCAfunc Domain
Motile_Sperm Domain
PAN_2 Domain
PK_Tyr_Ser−Thr Domain
Pkinase Domain
Psg1 Family
SKG6 Domain
S_locus_glycop Domain
Stress−antifung Family
WD40 Repeat
other Domain
0
500
1000
1500
2000

T.sp RLK−Pelle_DLSV subfamily domain diagram (part 23)

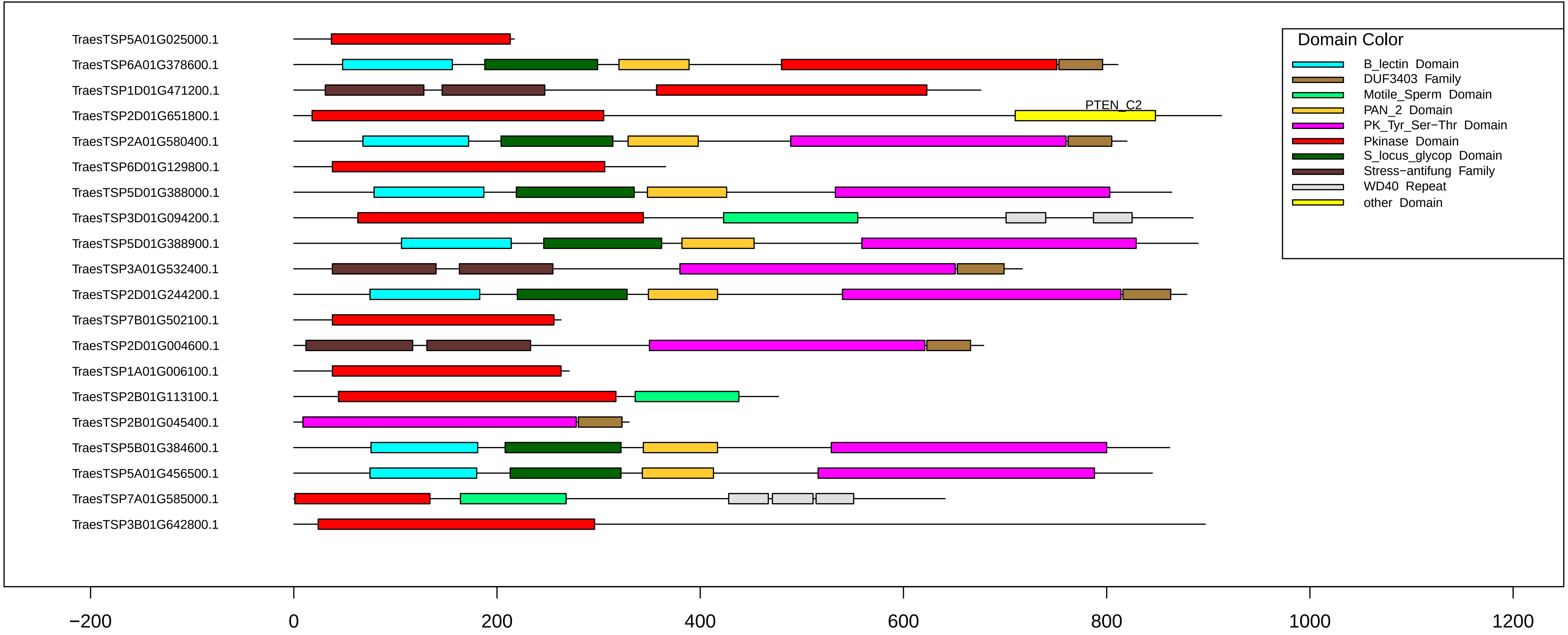

T.sp RLK−Pelle_DLSV subfamily domain diagram (part 24)

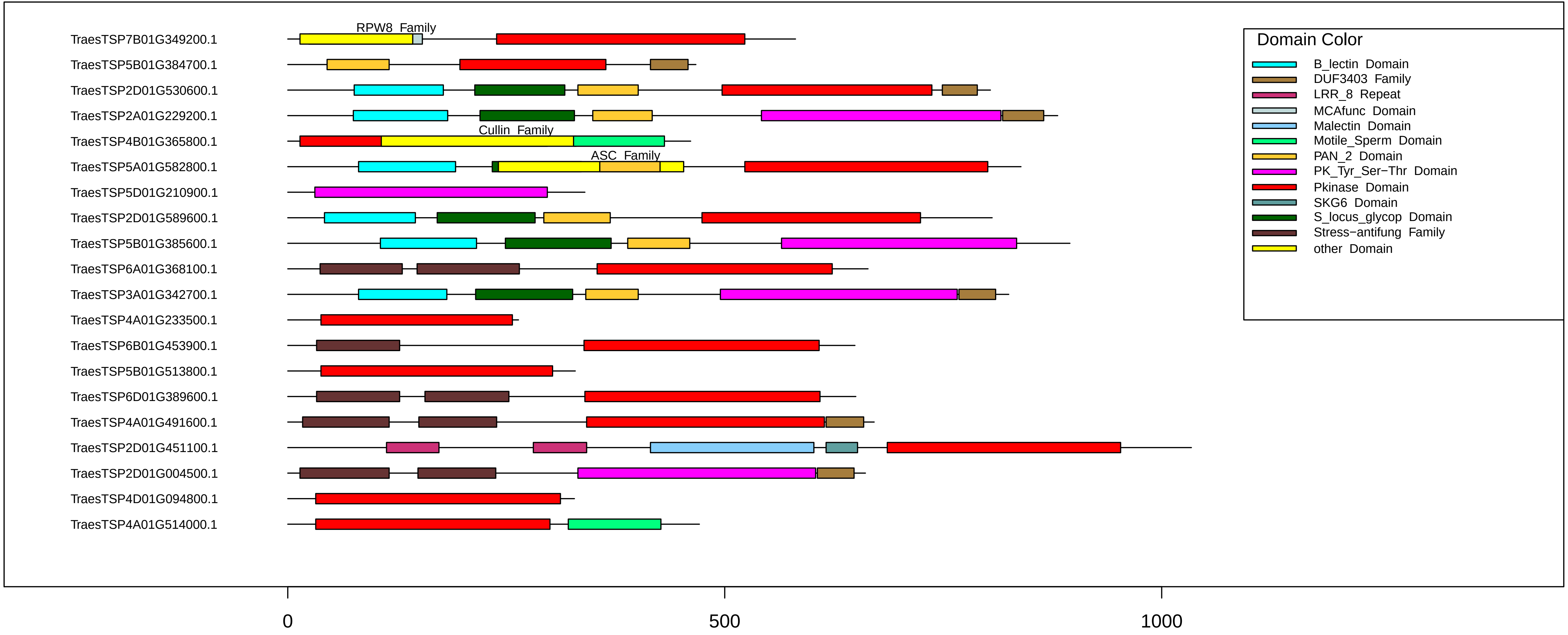

T.sp RLK−Pelle_DLSV subfamily domain diagram (part 25)

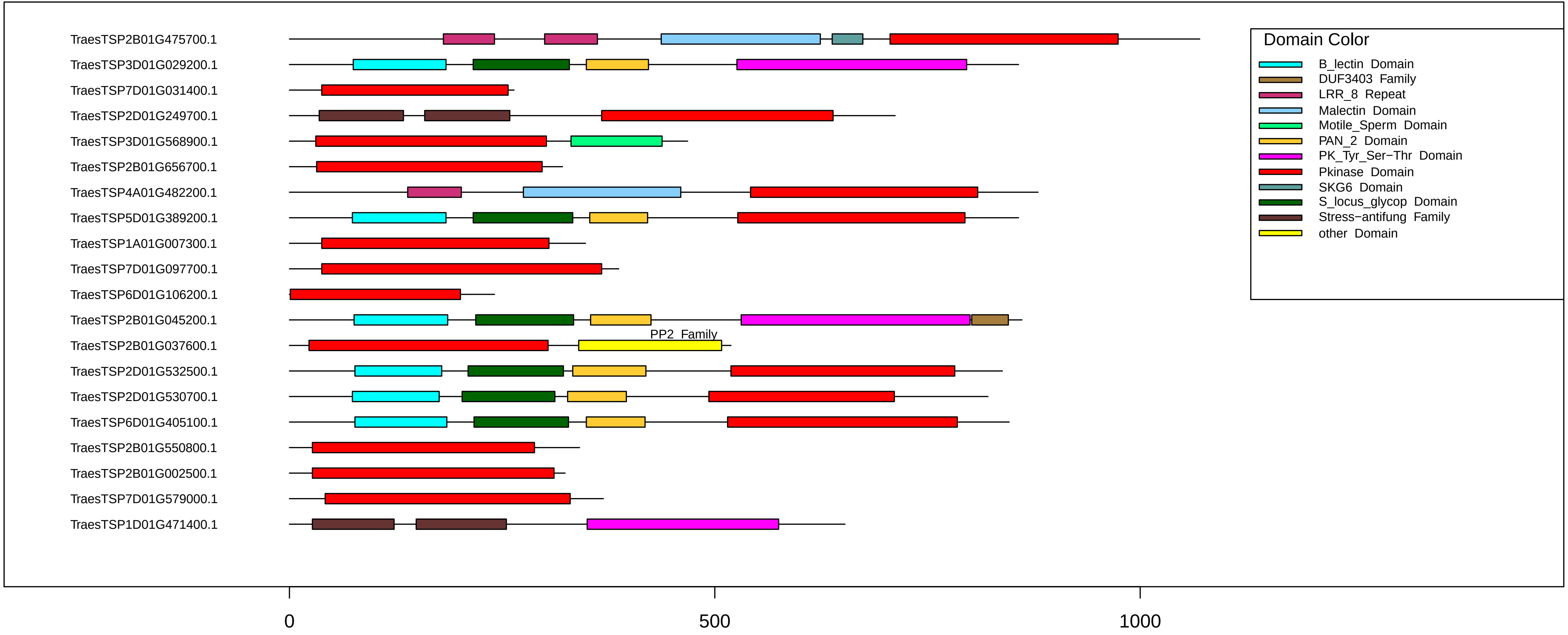

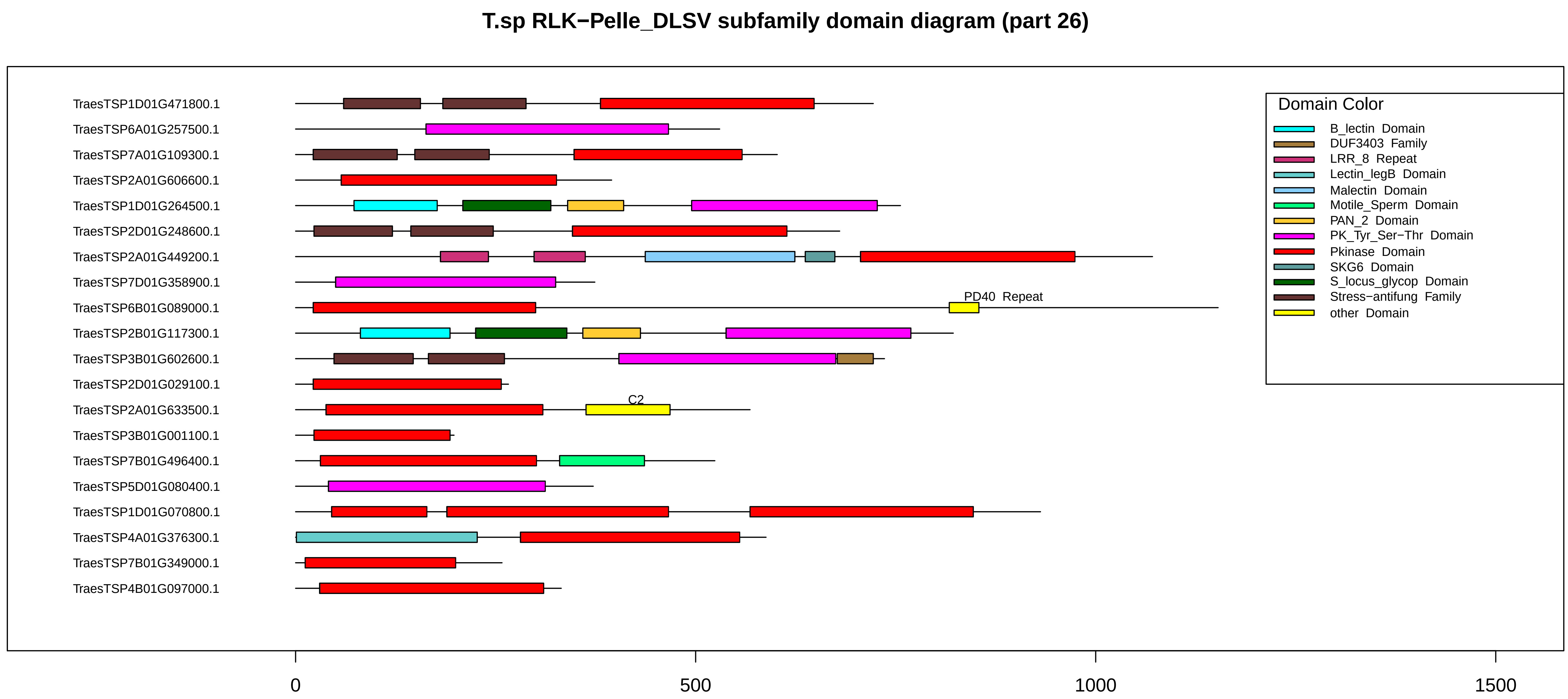

T.sp RLK−Pelle_DLSV subfamily domain diagram (part 26)
TraesTSP1D01G471800.1
TraesTSP6A01G257500.1
TraesTSP7A01G109300.1
TraesTSP2A01G606600.1
TraesTSP1D01G264500.1
TraesTSP2D01G248600.1
TraesTSP2A01G449200.1
TraesTSP7D01G358900.1
TraesTSP6B01G089000.1
TraesTSP2B01G117300.1
TraesTSP3B01G602600.1
TraesTSP2D01G029100.1
TraesTSP2A01G633500.1
TraesTSP3B01G001100.1
TraesTSP7B01G496400.1
TraesTSP5D01G080400.1
TraesTSP1D01G070800.1
TraesTSP4A01G376300.1
TraesTSP7B01G349000.1
TraesTSP4B01G097000.1
PD40 Repeat
C2
Domain Color
B_lectin Domain
DUF3403 Family
LRR_8 Repeat
Lectin_legB Domain
Malectin Domain
Motile_Sperm Domain
PAN_2 Domain
PK_Tyr_Ser−Thr Domain
Pkinase Domain
SKG6 Domain
S_locus_glycop Domain
Stress−antifung Family
other Domain
0
500
1000
1500

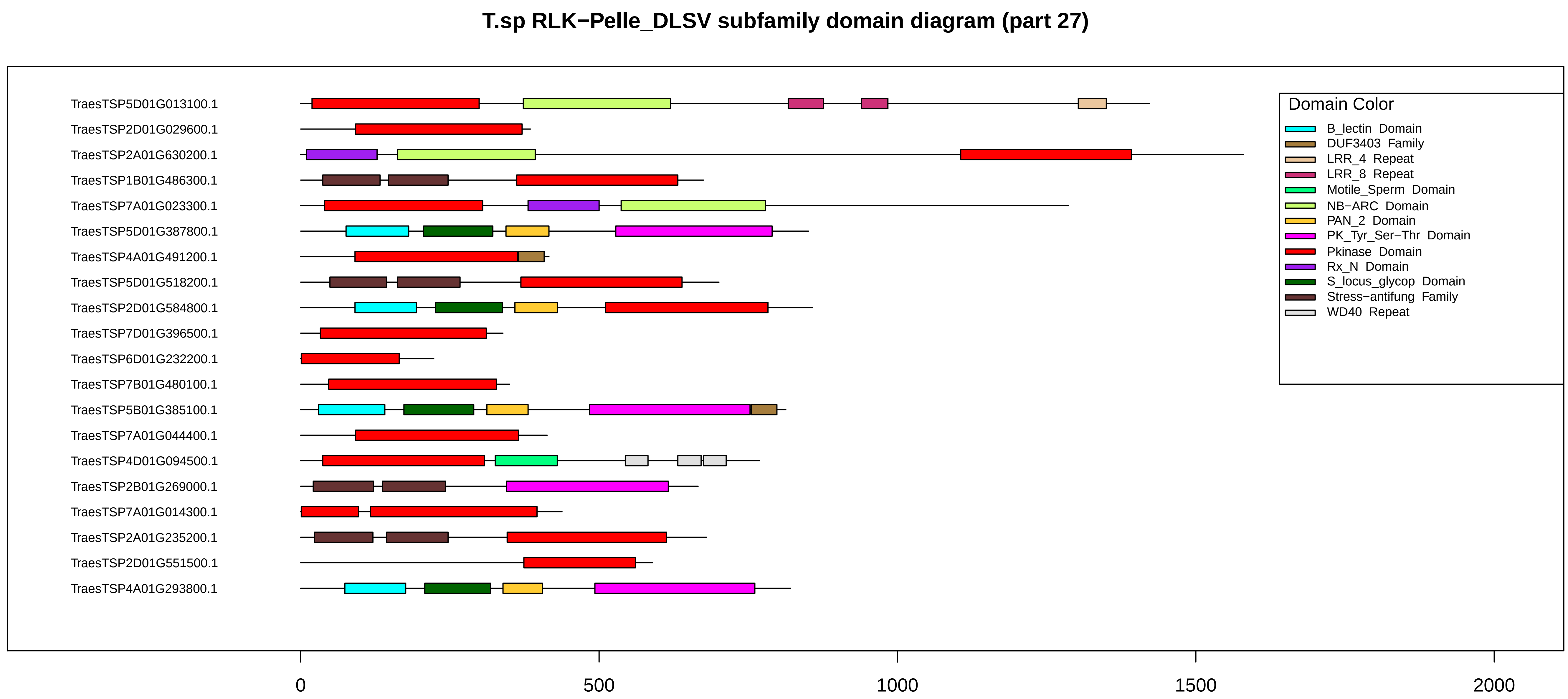
T.sp RLK−Pelle_DLSV subfamily domain diagram (part 27)
TraesTSP5D01G013100.1
TraesTSP2D01G029600.1
TraesTSP2A01G630200.1
TraesTSP1B01G486300.1
TraesTSP7A01G023300.1
TraesTSP5D01G387800.1
TraesTSP4A01G491200.1
TraesTSP5D01G518200.1
TraesTSP2D01G584800.1
TraesTSP7D01G396500.1
TraesTSP6D01G232200.1
TraesTSP7B01G480100.1
TraesTSP5B01G385100.1
TraesTSP7A01G044400.1
TraesTSP4D01G094500.1
TraesTSP2B01G269000.1
TraesTSP7A01G014300.1
TraesTSP2A01G235200.1
TraesTSP2D01G551500.1
TraesTSP4A01G293800.1
Domain Color
B_lectin Domain
DUF3403 Family
LRR_4 Repeat
LRR_8 Repeat
Motile_Sperm Domain
NB−ARC Domain
PAN_2 Domain
PK_Tyr_Ser−Thr Domain
Pkinase Domain
Rx_N Domain
S_locus_glycop Domain
Stress−antifung Family
WD40 Repeat
0
500
1000
1500
2000

T.sp RLK−Pelle_DLSV subfamily domain diagram (part 28)

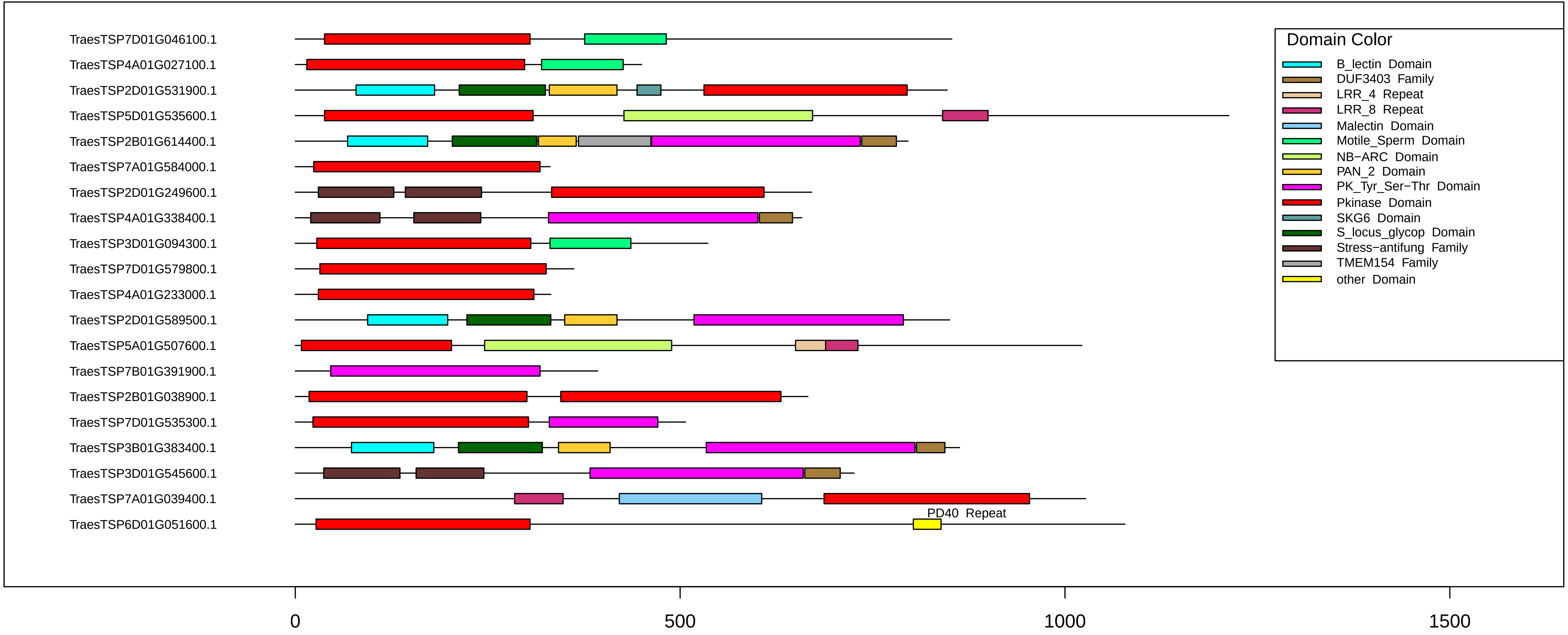

T.sp RLK−Pelle_DLSV subfamily domain diagram (part 29)

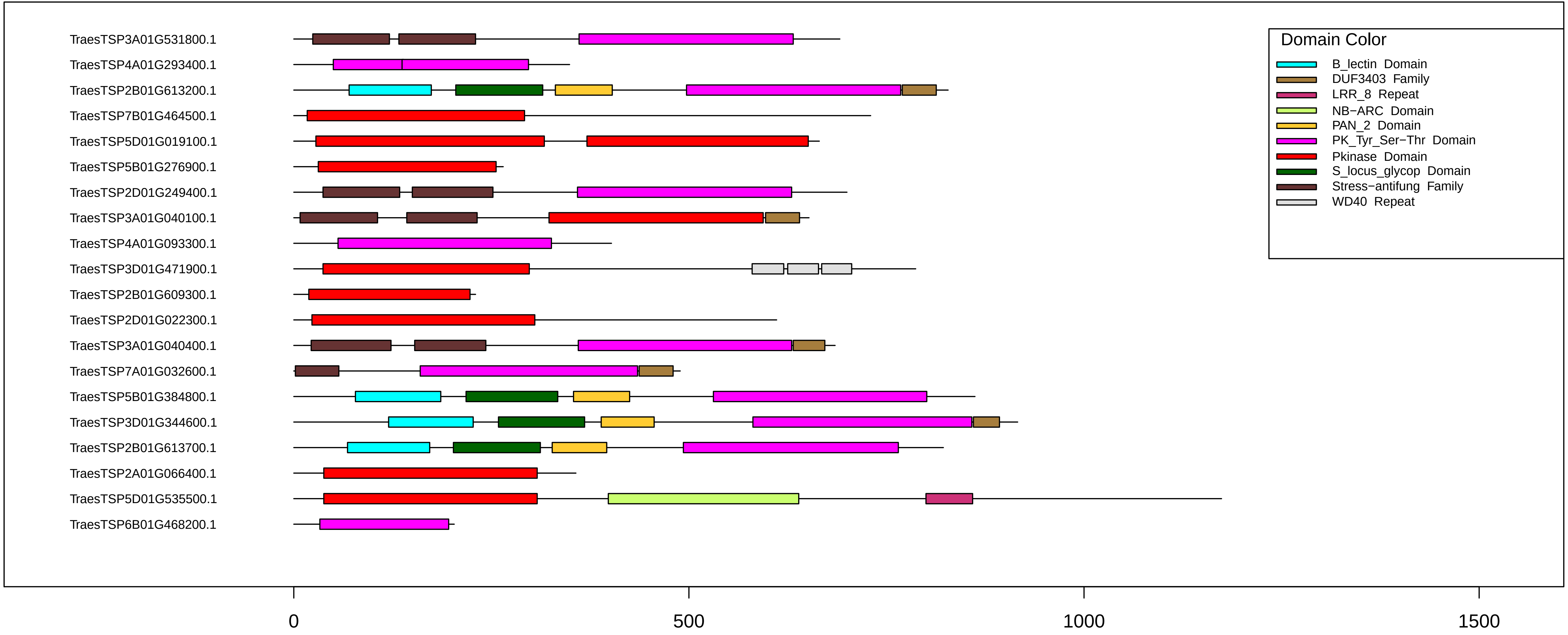

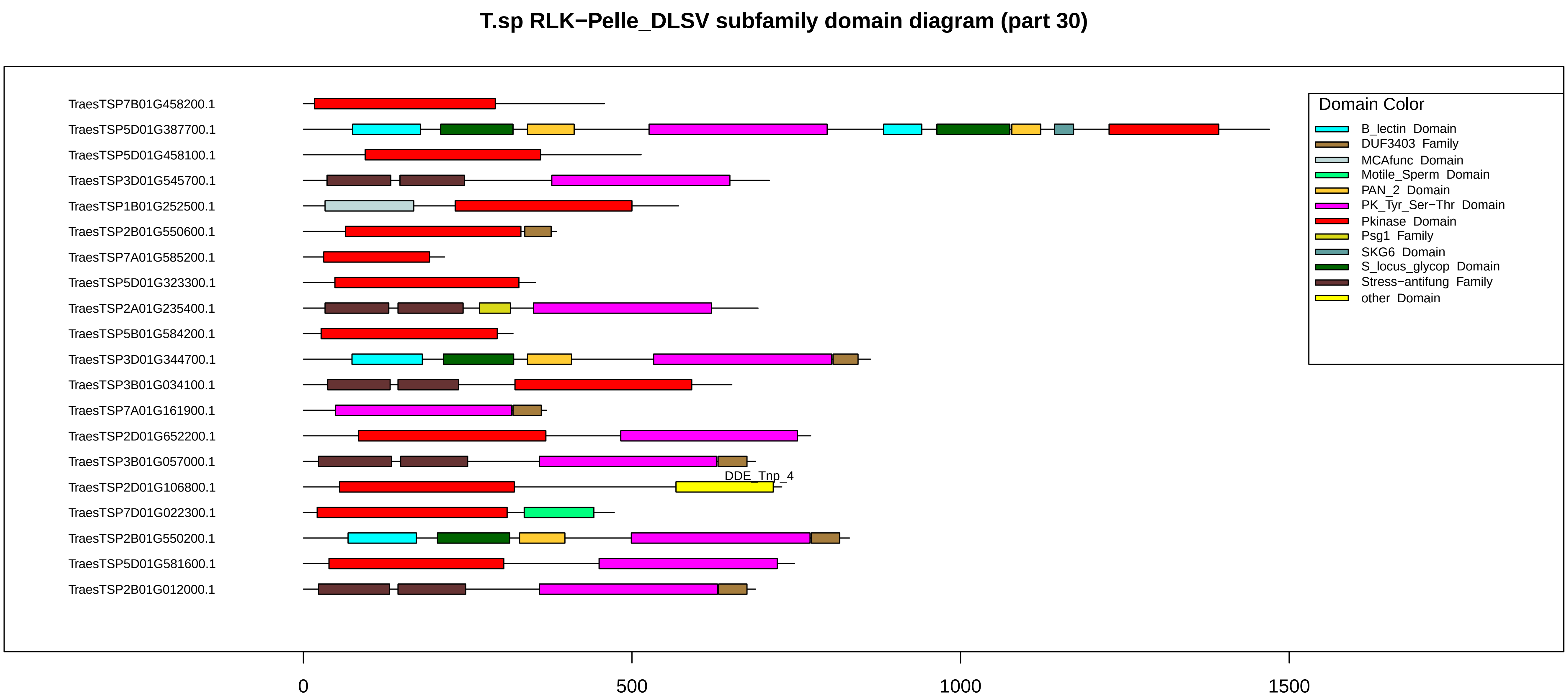

T.sp RLK−Pelle_DLSV subfamily domain diagram (part 30)
TraesTSP7B01G458200.1
TraesTSP5D01G387700.1
TraesTSP5D01G458100.1
TraesTSP3D01G545700.1
TraesTSP1B01G252500.1
TraesTSP2B01G550600.1
TraesTSP7A01G585200.1
TraesTSP5D01G323300.1
TraesTSP2A01G235400.1
TraesTSP5B01G584200.1
TraesTSP3D01G344700.1
TraesTSP3B01G034100.1
TraesTSP7A01G161900.1
TraesTSP2D01G652200.1
TraesTSP3B01G057000.1
TraesTSP2D01G106800.1
TraesTSP7D01G022300.1
TraesTSP2B01G550200.1
TraesTSP5D01G581600.1
TraesTSP2B01G012000.1
DDE_Tnp_4
Domain Color
B_lectin Domain
DUF3403 Family
MCAfunc Domain
Motile_Sperm Domain
PAN_2 Domain
PK_Tyr_Ser−Thr Domain
Pkinase Domain
Psg1 Family
SKG6 Domain
S_locus_glycop Domain
Stress−antifung Family
other Domain
0
500
1000
1500

T.sp RLK−Pelle_DLSV subfamily domain diagram (part 31)

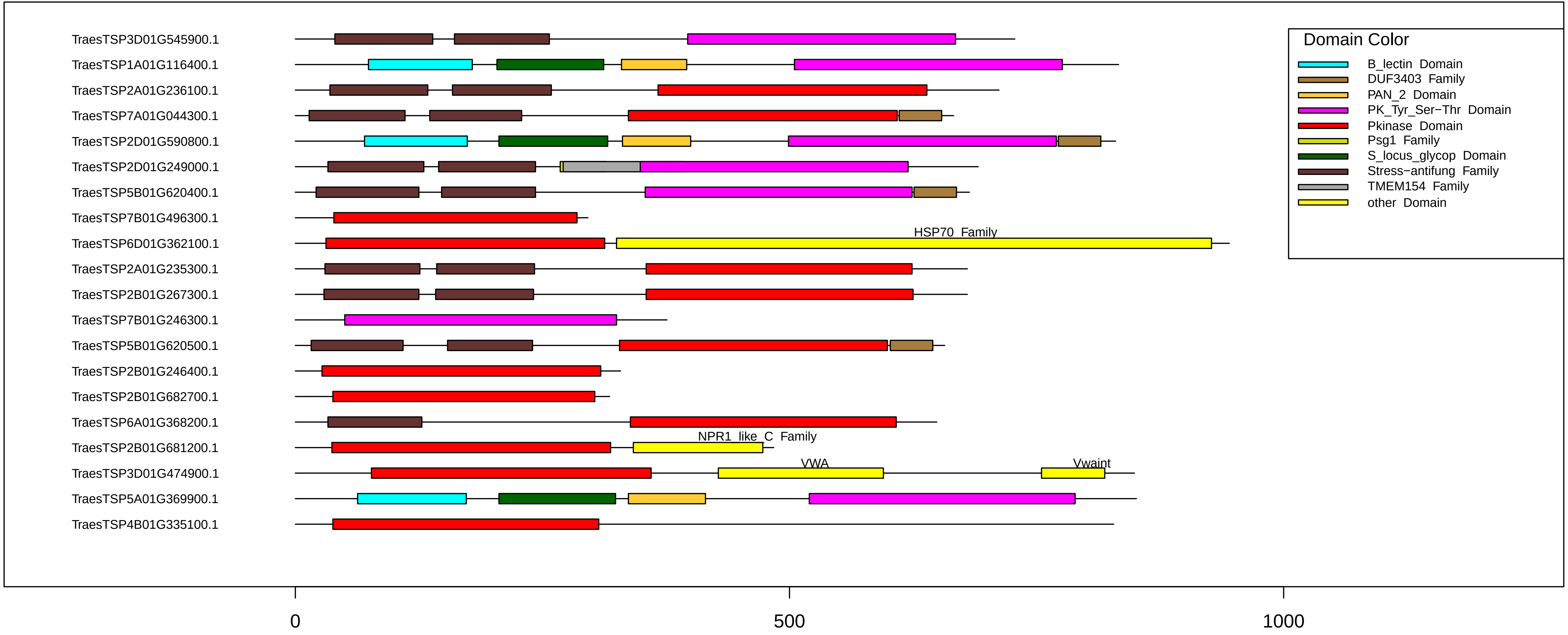

T.sp RLK−Pelle_DLSV subfamily domain diagram (part 32)

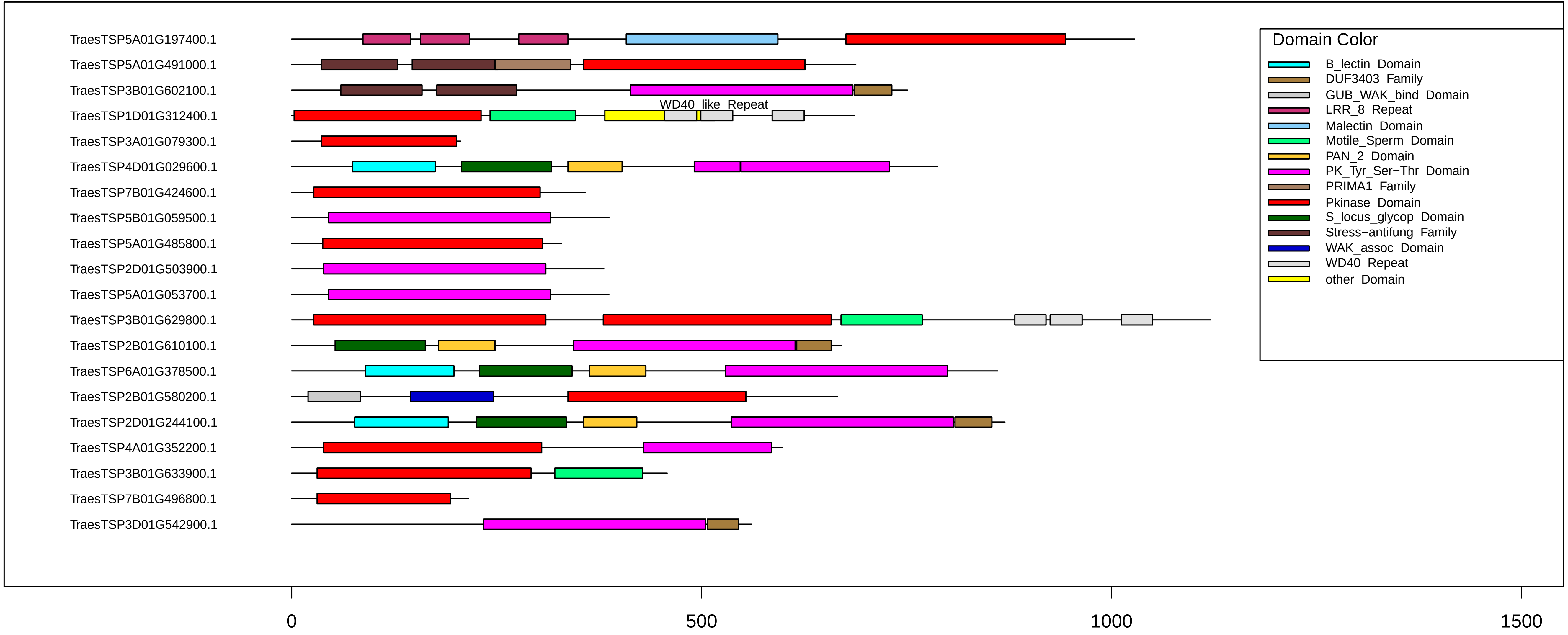

T.sp RLK−Pelle_DLSV subfamily domain diagram (part 33)

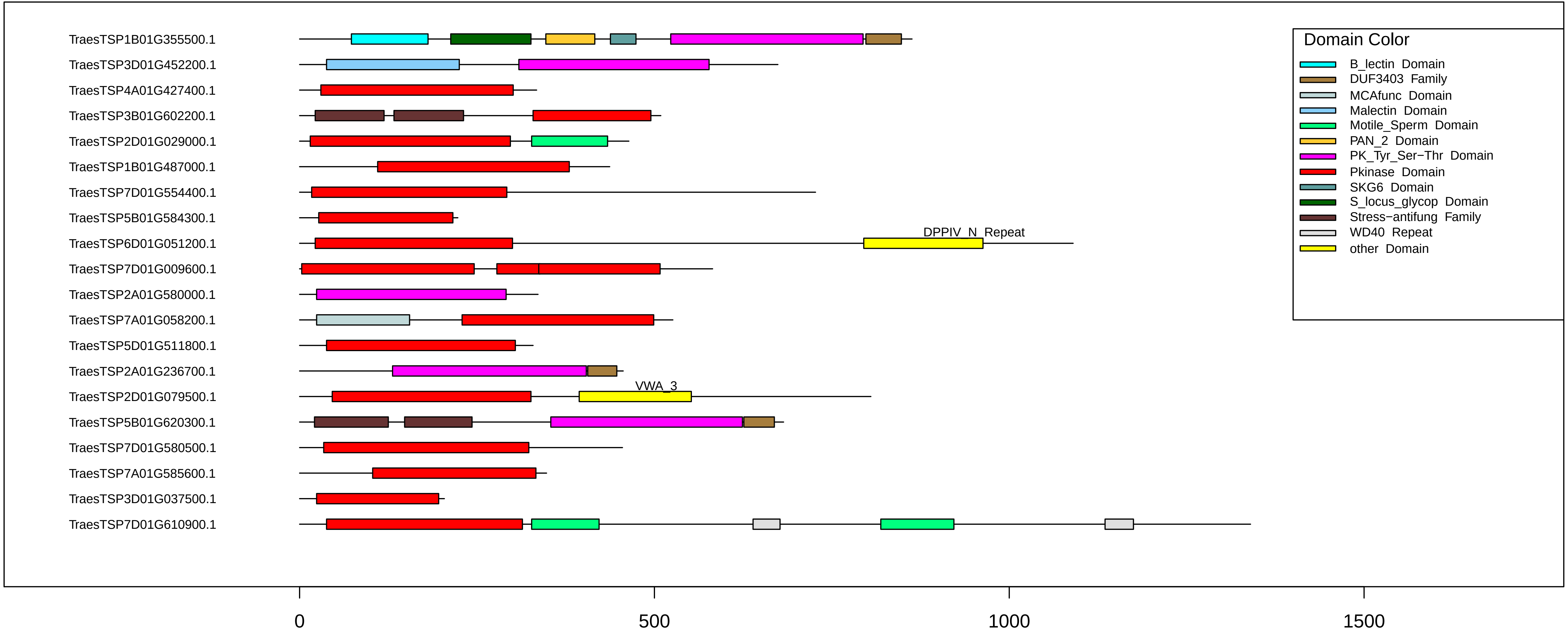

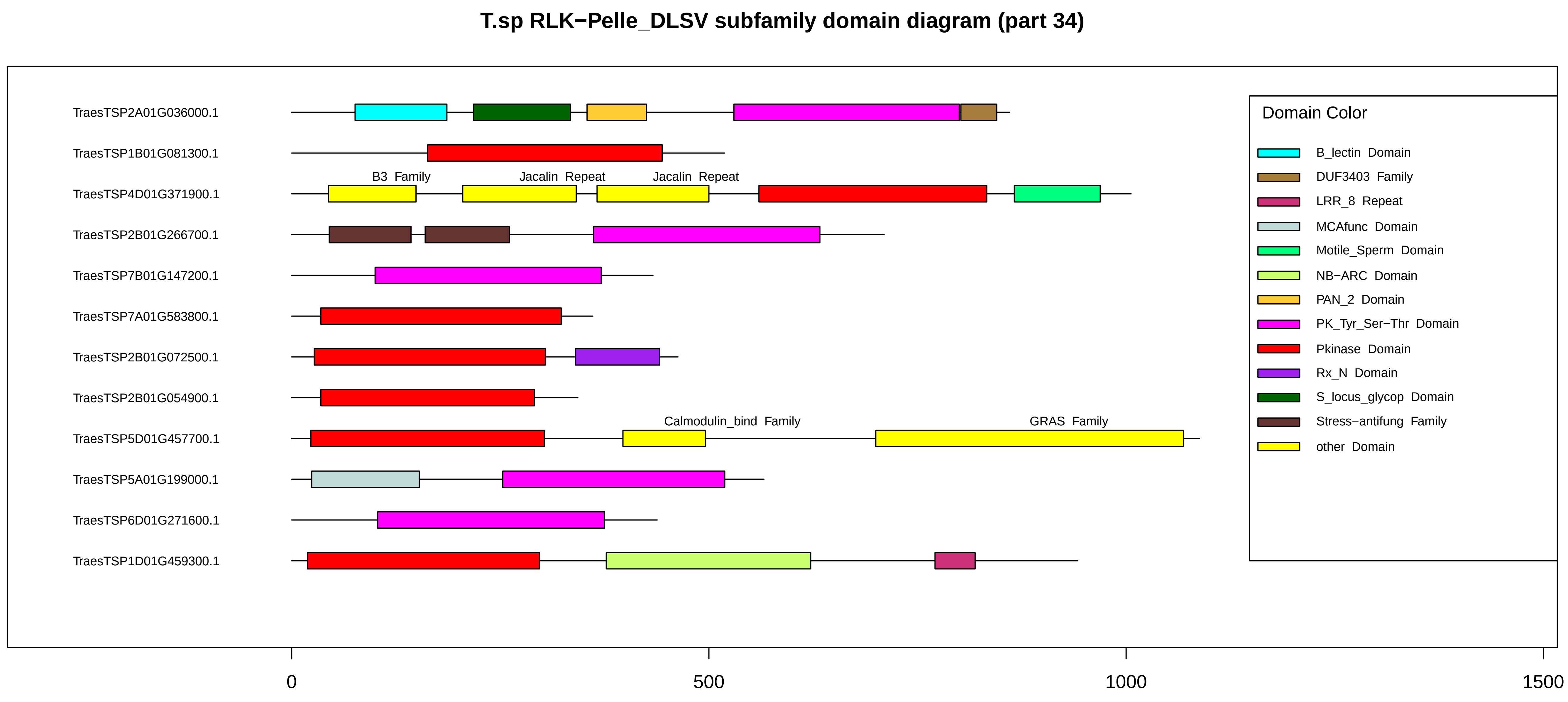
T.sp RLK−Pelle_DLSV subfamily domain diagram (part 34)
TraesTSP2A01G036000.1
TraesTSP1B01G081300.1
TraesTSP4D01G371900.1
TraesTSP2B01G266700.1
TraesTSP7B01G147200.1
TraesTSP7A01G583800.1
TraesTSP2B01G072500.1
TraesTSP2B01G054900.1
TraesTSP5D01G457700.1
TraesTSP5A01G199000.1
TraesTSP6D01G271600.1
TraesTSP1D01G459300.1
B3 Family
Jacalin Repeat
Jacalin Repeat
Calmodulin_bind Family
GRAS Family
Domain Color
B_lectin Domain
DUF3403 Family
LRR_8 Repeat
MCAfunc Domain
Motile_Sperm Domain
NB−ARC Domain
PAN_2 Domain
PK_Tyr_Ser−Thr Domain
Pkinase Domain
Rx_N Domain
S_locus_glycop Domain
Stress−antifung Family
other Domain
0
500
1000
1500

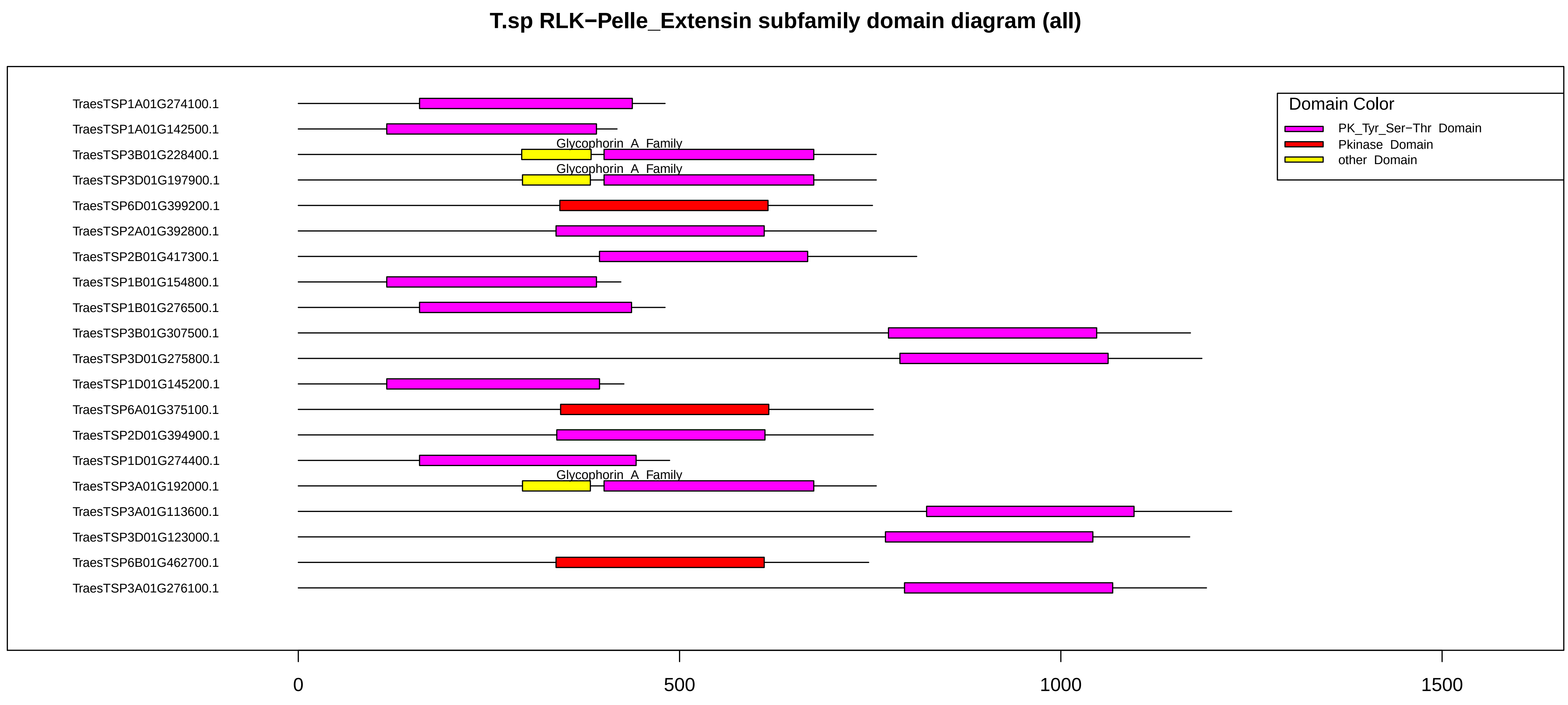
T.sp RLK−Pelle_Extensin subfamily domain diagram (all)
TraesTSP1A01G274100.1
TraesTSP1A01G142500.1
TraesTSP3B01G228400.1
TraesTSP3D01G197900.1
TraesTSP6D01G399200.1
TraesTSP2A01G392800.1
TraesTSP2B01G417300.1
TraesTSP1B01G154800.1
TraesTSP1B01G276500.1
TraesTSP3B01G307500.1
TraesTSP3D01G275800.1
TraesTSP1D01G145200.1
TraesTSP6A01G375100.1
TraesTSP2D01G394900.1
TraesTSP1D01G274400.1
TraesTSP3A01G192000.1
TraesTSP3A01G113600.1
TraesTSP3D01G123000.1
TraesTSP6B01G462700.1
TraesTSP3A01G276100.1
Glycophorin_A_Family
Glycophorin_A_Family
Glycophorin_A_Family
Domain Color
PK_Tyr_Ser−Thr Domain
Pkinase Domain
other Domain
0
500
1000
1500

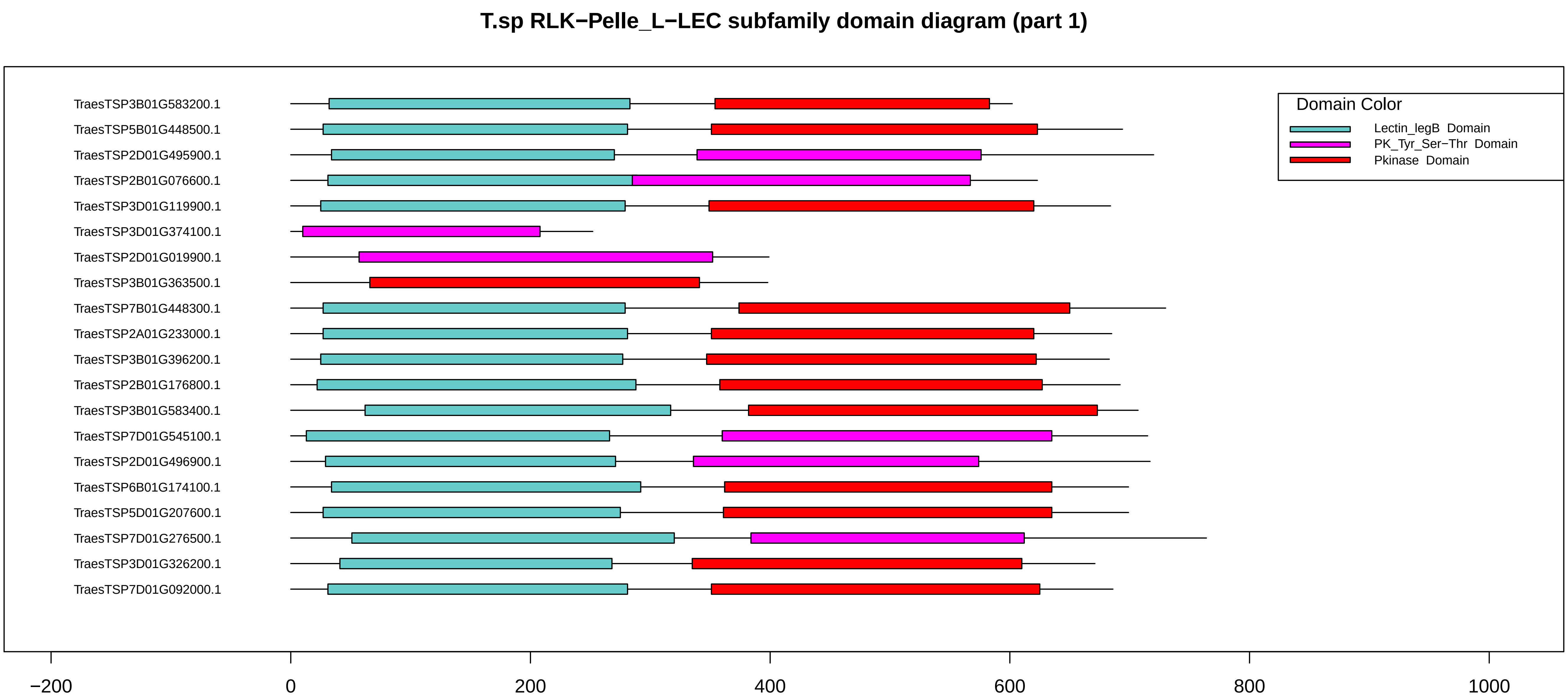

T.sp RLK−Pelle_L−LEC subfamily domain diagram (part 1)
TraesTSP3B01G583200.1
TraesTSP5B01G448500.1
TraesTSP2D01G495900.1
TraesTSP2B01G076600.1
TraesTSP3D01G119900.1
TraesTSP3D01G374100.1
TraesTSP2D01G019900.1
TraesTSP3B01G363500.1
TraesTSP7B01G448300.1
TraesTSP2A01G233000.1
TraesTSP3B01G396200.1
TraesTSP2B01G176800.1
TraesTSP3B01G583400.1
TraesTSP7D01G545100.1
TraesTSP2D01G496900.1
TraesTSP6B01G174100.1
TraesTSP5D01G207600.1
TraesTSP7D01G276500.1
TraesTSP3D01G326200.1
TraesTSP7D01G092000.1
Domain Color
Lectin_legB Domain
PK_Tyr_Ser−Thr Domain
Pkinase Domain
−200
0
200
400
600
800
1000

T.sp RLK−Pelle_L−LEC subfamily domain diagram (part 2)

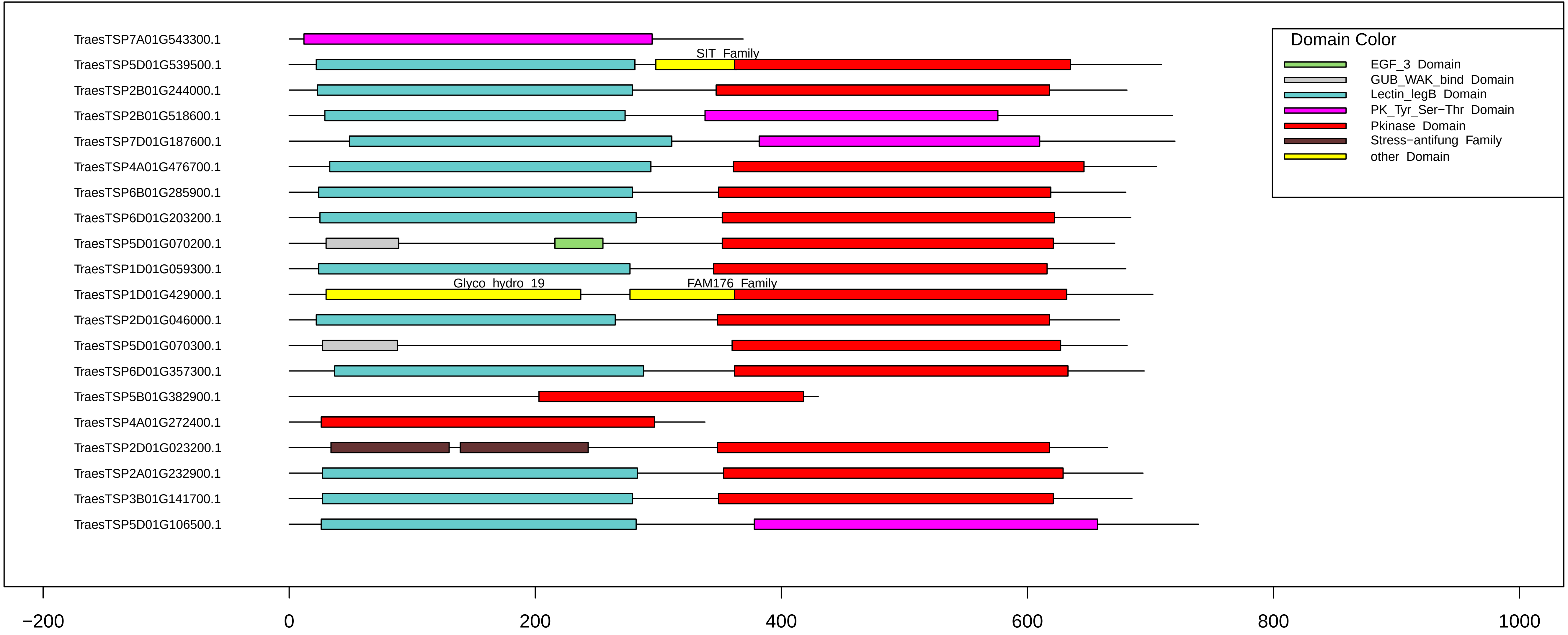

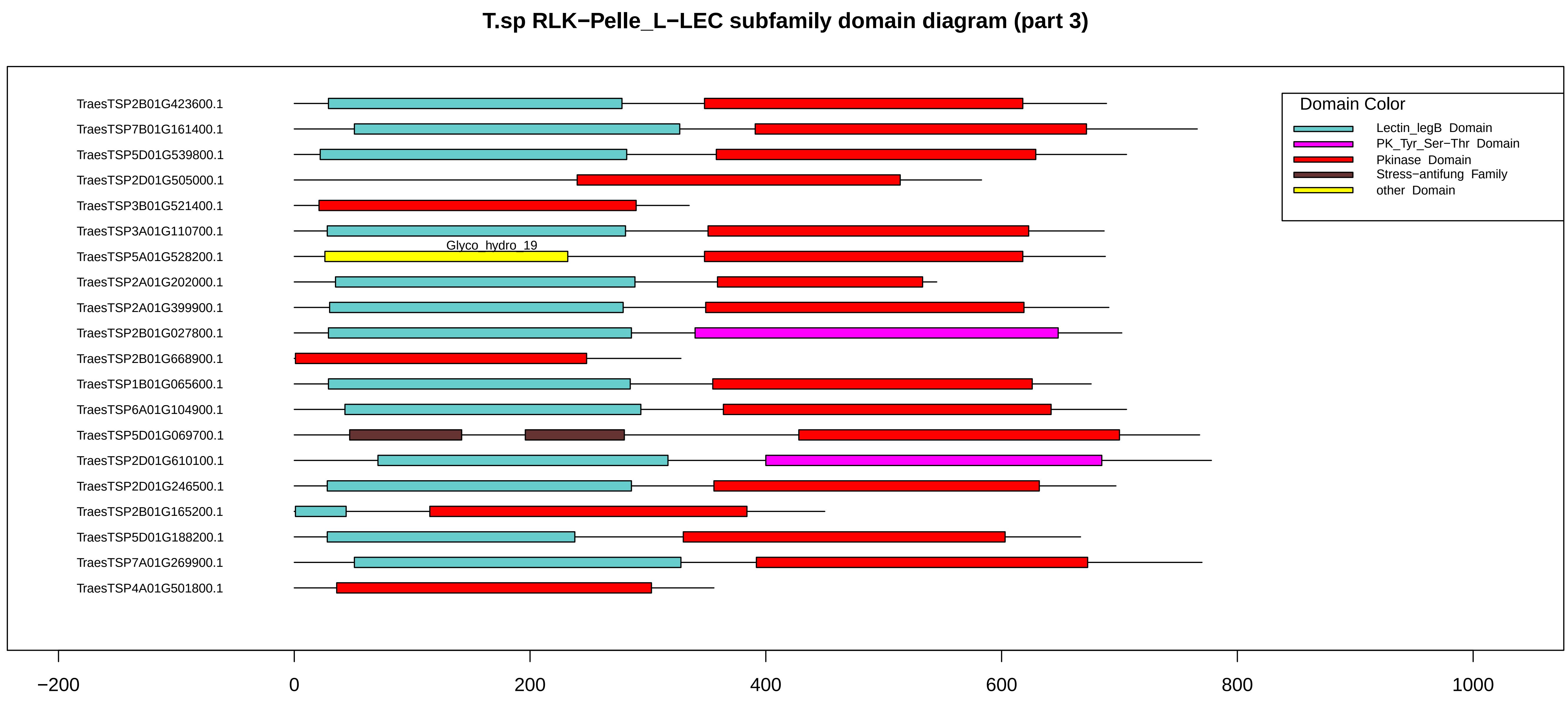
T.sp RLK−Pelle_L−LEC subfamily domain diagram (part 3)
TraesTSP2B01G423600.1
TraesTSP7B01G161400.1
TraesTSP5D01G539800.1
TraesTSP2D01G505000.1
TraesTSP3B01G521400.1
TraesTSP3A01G110700.1
TraesTSP5A01G528200.1
Glyco_hydro_19
TraesTSP2A01G202000.1
TraesTSP2A01G399900.1
TraesTSP2B01G027800.1
TraesTSP2B01G668900.1
TraesTSP1B01G065600.1
TraesTSP6A01G104900.1
TraesTSP5D01G069700.1
TraesTSP2D01G610100.1
TraesTSP2D01G246500.1
TraesTSP2B01G165200.1
TraesTSP5D01G188200.1
TraesTSP7A01G269900.1
TraesTSP4A01G501800.1
Domain Color
Lectin_legB Domain
PK_Tyr_Ser−Thr Domain
Pkinase Domain
Stress−antifung Family
other Domain
−200
0
200
400
600
800
1000

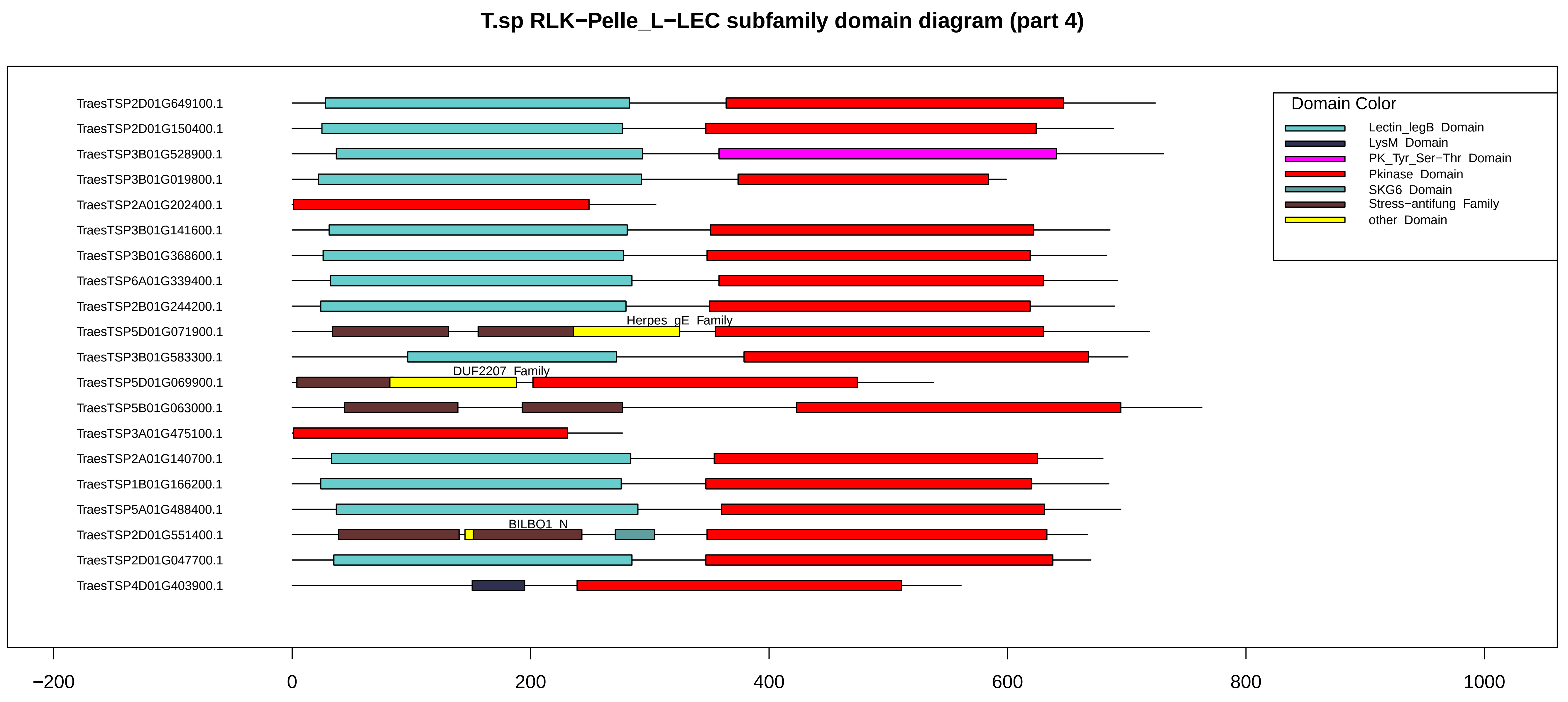
T.sp RLK−Pelle_L−LEC subfamily domain diagram (part 4)
TraesTSP2D01G649100.1
TraesTSP2D01G150400.1
TraesTSP3B01G528900.1
TraesTSP3B01G019800.1
TraesTSP2A01G202400.1
TraesTSP3B01G141600.1
TraesTSP3B01G368600.1
TraesTSP6A01G339400.1
TraesTSP2B01G244200.1
TraesTSP5D01G071900.1
Herpes_gE_Family
TraesTSP3B01G583300.1
TraesTSP5D01G069900.1
DUF2207_Family
TraesTSP5B01G063000.1
TraesTSP3A01G475100.1
TraesTSP2A01G140700.1
TraesTSP1B01G166200.1
TraesTSP5A01G488400.1
TraesTSP2D01G551400.1
BILBO1_N
TraesTSP2D01G047700.1
TraesTSP4D01G403900.1
Domain Color
Lectin_legB Domain
LysM Domain
PK_Tyr_Ser−Thr Domain
Pkinase Domain
SKG6 Domain
Stress−antifung Family
other Domain
−200
0
200
400
600
800
1000

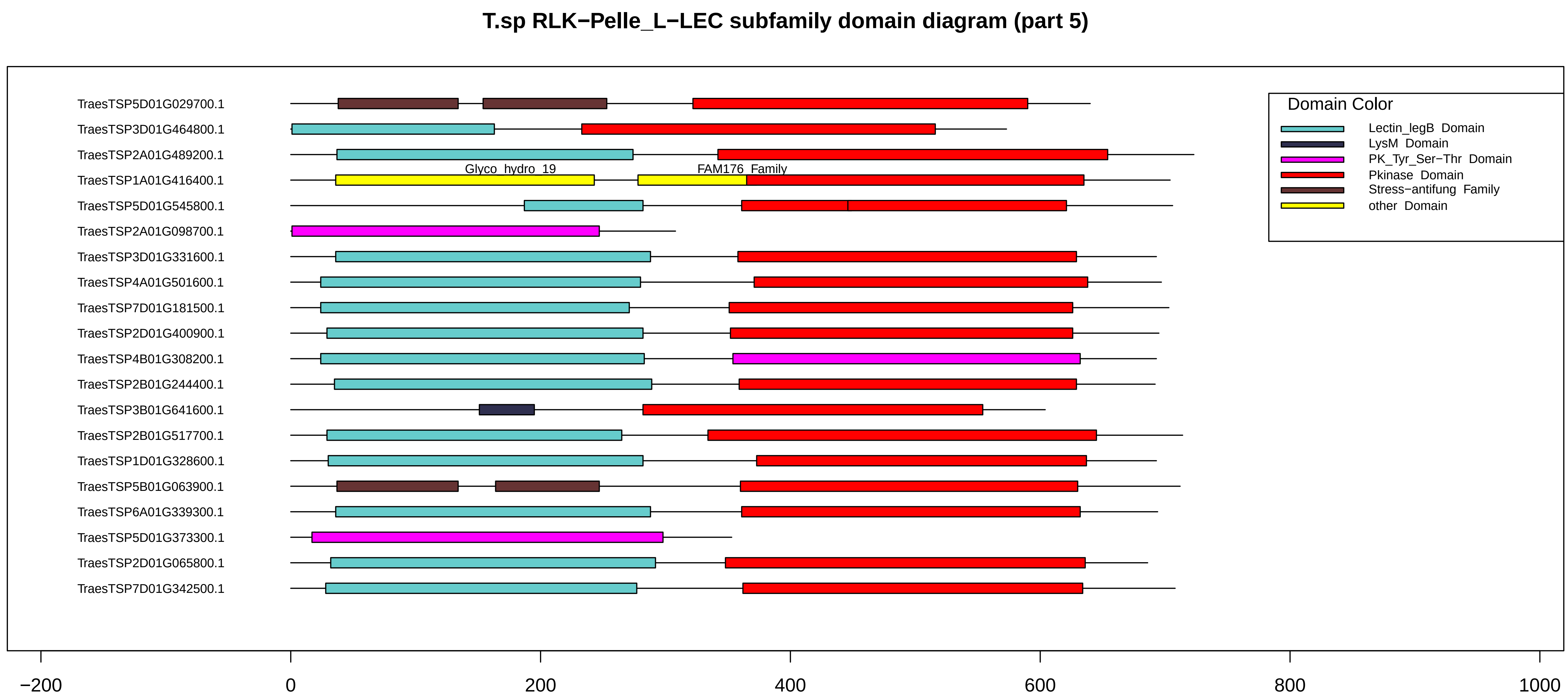

T.sp RLK−Pelle_L−LEC subfamily domain diagram (part 5)
TraesTSP5D01G029700.1
TraesTSP3D01G464800.1
TraesTSP2A01G489200.1
TraesTSP1A01G416400.1
TraesTSP5D01G545800.1
TraesTSP2A01G098700.1
TraesTSP3D01G331600.1
TraesTSP4A01G501600.1
TraesTSP7D01G181500.1
TraesTSP2D01G400900.1
TraesTSP4B01G308200.1
TraesTSP2B01G244400.1
TraesTSP3B01G641600.1
TraesTSP2B01G517700.1
TraesTSP1D01G328600.1
TraesTSP5B01G063900.1
TraesTSP6A01G339300.1
TraesTSP5D01G373300.1
TraesTSP2D01G065800.1
TraesTSP7D01G342500.1
Glyco_hydro_19
FAM176_Family
Domain Color
Lectin_legB Domain
LysM Domain
PK_Tyr_Ser−Thr Domain
Pkinase Domain
Stress−antifung Family
other Domain
−200
0
200
400
600
800
1000

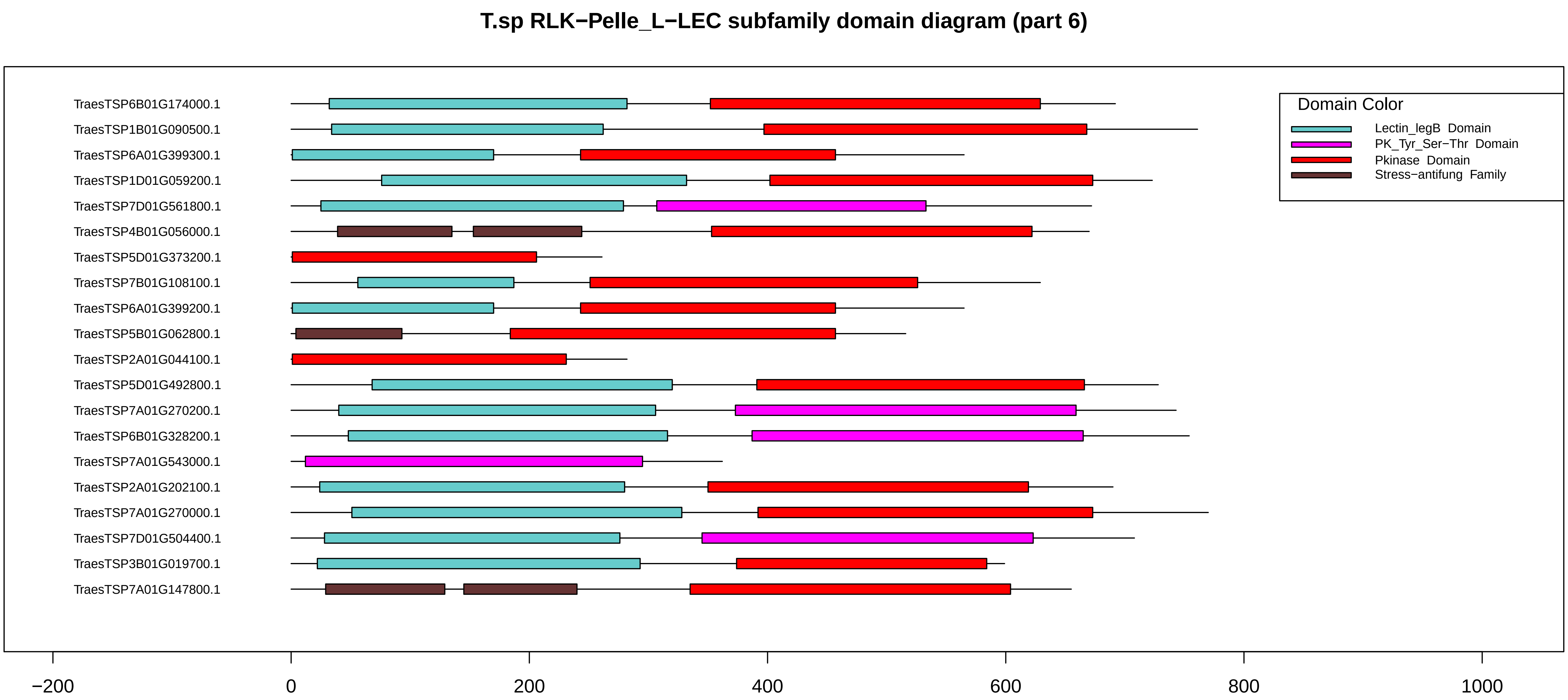
T.sp RLK−Pelle_L−LEC subfamily domain diagram (part 6)
TraesTSP6B01G174000.1
TraesTSP1B01G090500.1
TraesTSP6A01G399300.1
TraesTSP1D01G059200.1
TraesTSP7D01G561800.1
TraesTSP4B01G056000.1
TraesTSP5D01G373200.1
TraesTSP7B01G108100.1
TraesTSP6A01G399200.1
TraesTSP5B01G062800.1
TraesTSP2A01G044100.1
TraesTSP5D01G492800.1
TraesTSP7A01G270200.1
TraesTSP6B01G328200.1
TraesTSP7A01G543000.1
TraesTSP2A01G202100.1
TraesTSP7A01G270000.1
TraesTSP7D01G504400.1
TraesTSP3B01G019700.1
TraesTSP7A01G147800.1
Domain Color
Lectin_legB Domain
PK_Tyr_Ser−Thr Domain
Pkinase Domain
Stress−antifung Family
−200
0
200
400
600
800
1000

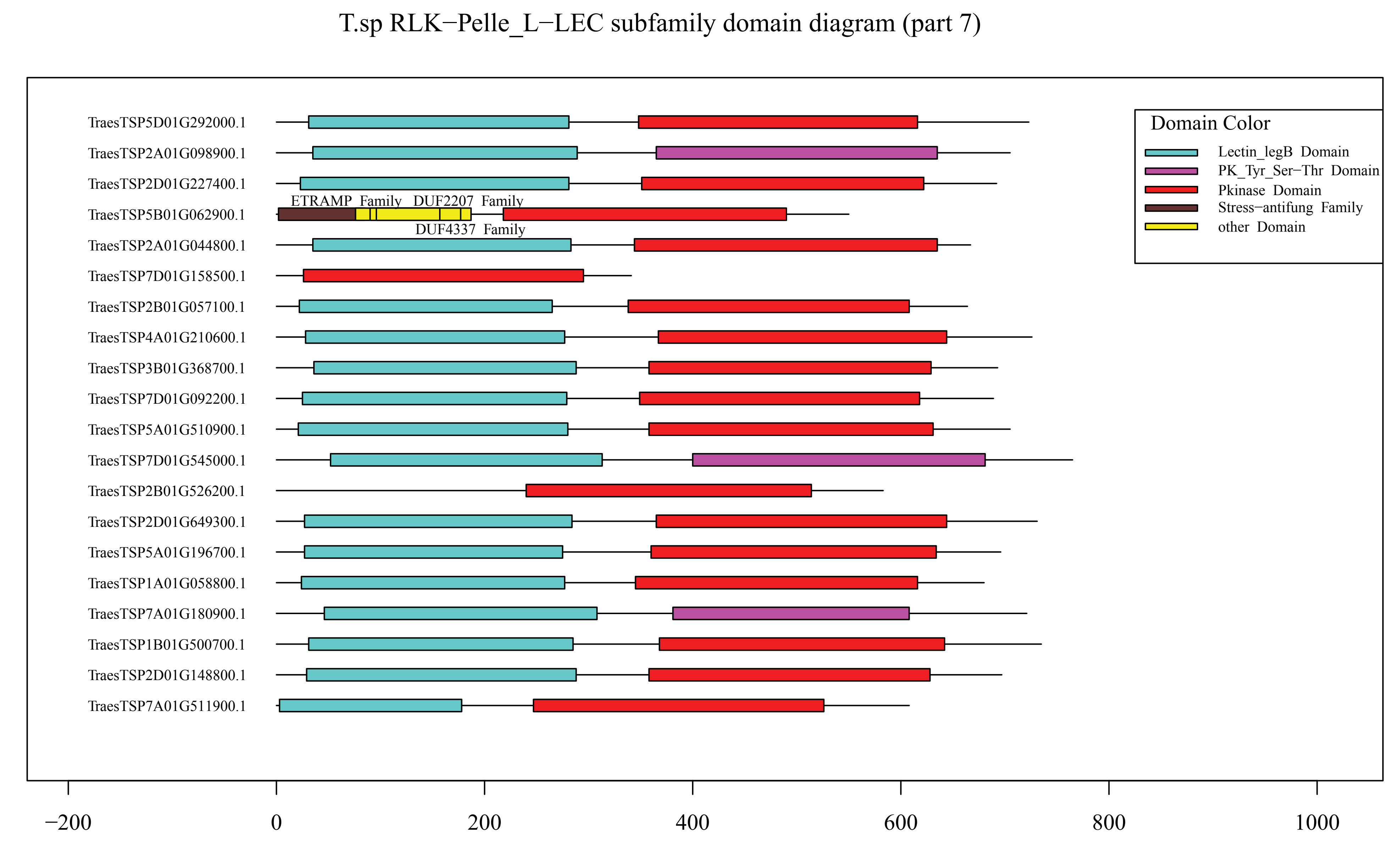
T.sp RLK−Pelle_L−LEC subfamily domain diagram (part 7)
TraesTSP5D01G292000.1
TraesTSP2A01G098900.1
TraesTSP2D01G227400.1
TraesTSP5B01G062900.1
ETRAMP Family
DUF2207 Family
DUF4337 Family
TraesTSP2A01G044800.1
TraesTSP7D01G158500.1
TraesTSP2B01G057100.1
TraesTSP4A01G210600.1
TraesTSP3B01G368700.1
TraesTSP7D01G092200.1
TraesTSP5A01G510900.1
TraesTSP7D01G545000.1
TraesTSP2B01G526200.1
TraesTSP2D01G649300.1
TraesTSP5A01G196700.1
TraesTSP1A01G058800.1
TraesTSP7A01G180900.1
TraesTSP1B01G500700.1
TraesTSP2D01G148800.1
TraesTSP7A01G511900.1
Domain Color
Lectin_legB Domain
PK_Tyr_Ser−Thr Domain
Pkinase Domain
Stress−antifung Family
other Domain
−200
0
200
400
600
800
1000

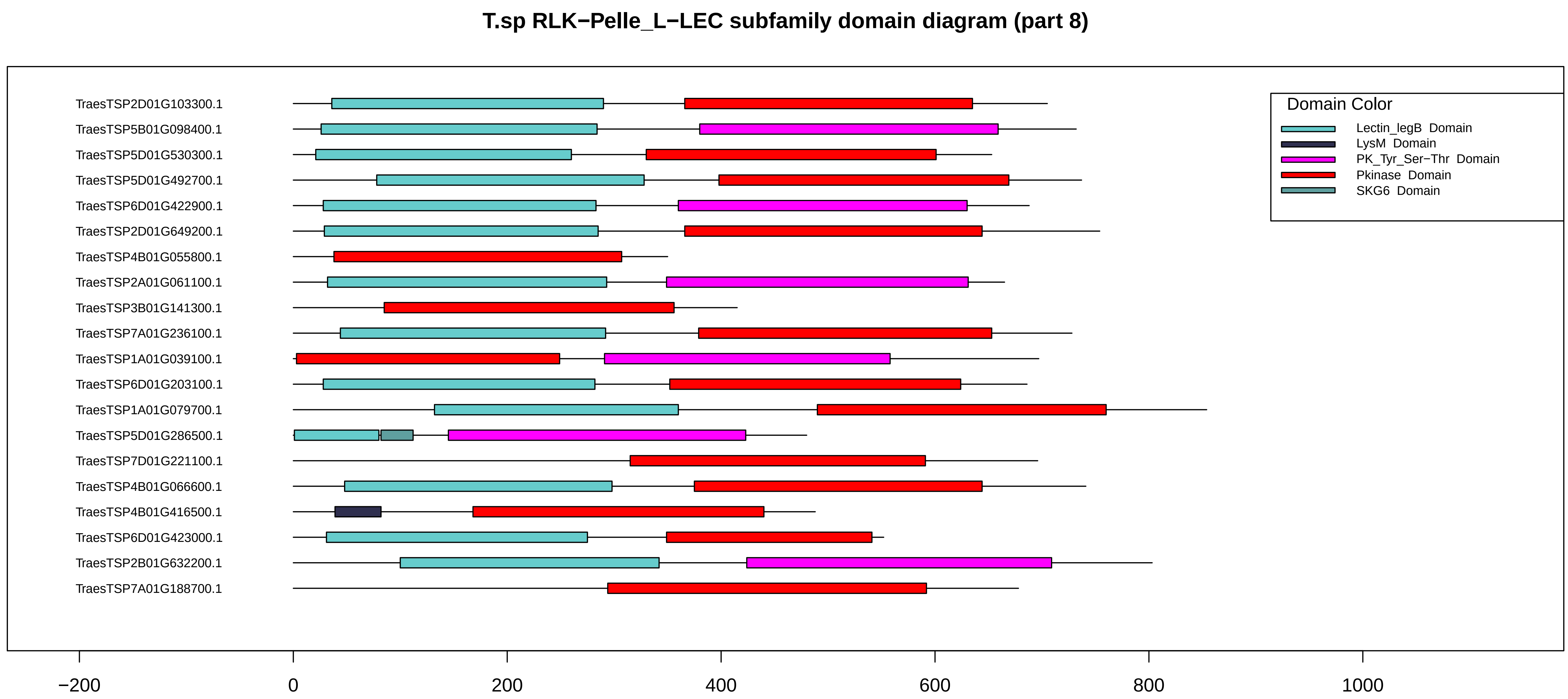
T.sp RLK−Pelle_L−LEC subfamily domain diagram (part 8)
TraesTSP2D01G103300.1
TraesTSP5B01G098400.1
TraesTSP5D01G530300.1
TraesTSP5D01G492700.1
TraesTSP6D01G422900.1
TraesTSP2D01G649200.1
TraesTSP4B01G055800.1
TraesTSP2A01G061100.1
TraesTSP3B01G141300.1
TraesTSP7A01G236100.1
TraesTSP1A01G039100.1
TraesTSP6D01G203100.1
TraesTSP1A01G079700.1
TraesTSP5D01G286500.1
TraesTSP7D01G221100.1
TraesTSP4B01G066600.1
TraesTSP4B01G416500.1
TraesTSP6D01G423000.1
TraesTSP2B01G632200.1
TraesTSP7A01G188700.1
Domain Color
Lectin_legB Domain
LysM Domain
PK_Tyr_Ser−Thr Domain
Pkinase Domain
SKG6 Domain
−200
0
200
400
600
800
1000

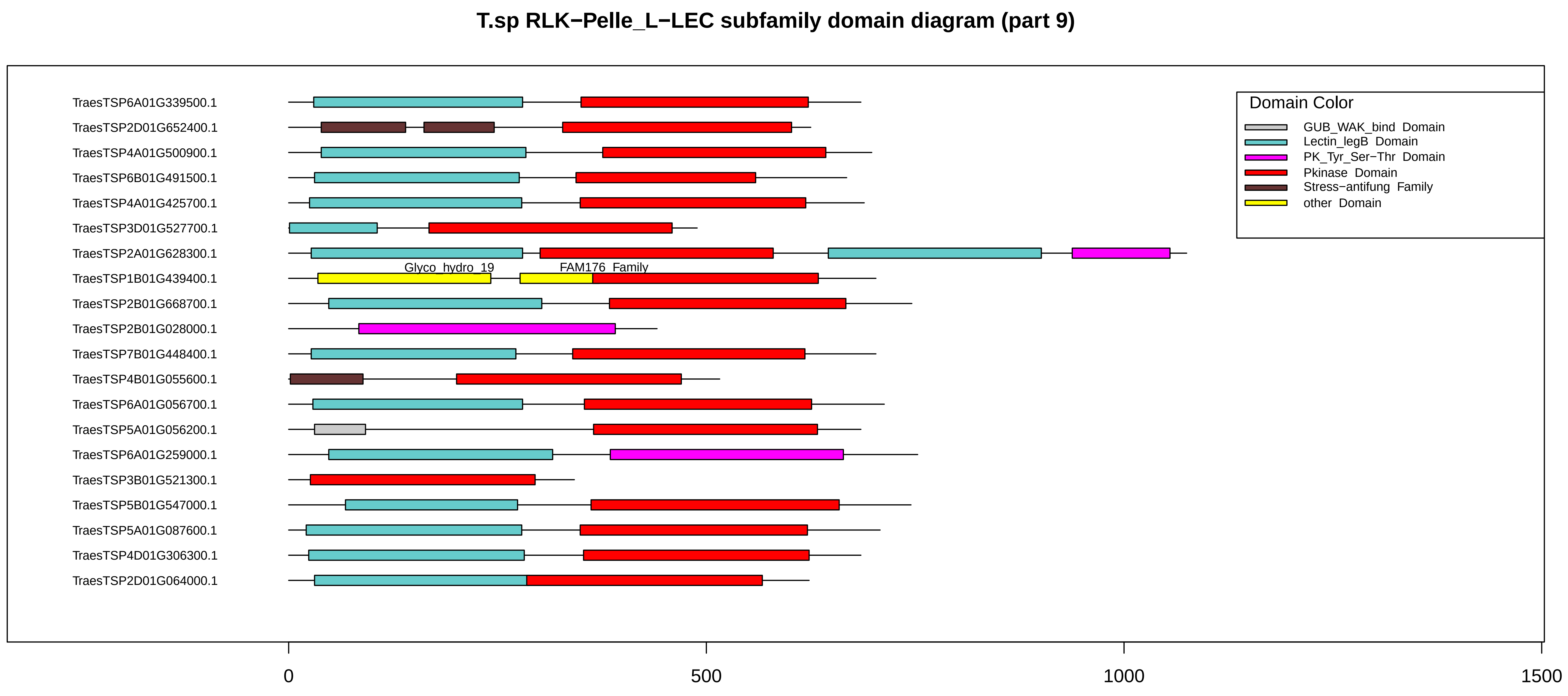
T.sp RLK−Pelle_L−LEC subfamily domain diagram (part 9)
TraesTSP6A01G339500.1
TraesTSP2D01G652400.1
TraesTSP4A01G500900.1
TraesTSP6B01G491500.1
TraesTSP4A01G425700.1
TraesTSP3D01G527700.1
TraesTSP2A01G628300.1
TraesTSP1B01G439400.1
TraesTSP2B01G668700.1
TraesTSP2B01G028000.1
TraesTSP7B01G448400.1
TraesTSP4B01G055600.1
TraesTSP6A01G056700.1
TraesTSP5A01G056200.1
TraesTSP6A01G259000.1
TraesTSP3B01G521300.1
TraesTSP5B01G547000.1
TraesTSP5A01G087600.1
TraesTSP4D01G306300.1
TraesTSP2D01G064000.1
Glyco_hydro_19
FAM176_Family
Domain Color
GUB_WAK_bind Domain
Lectin_legB Domain
PK_Tyr_Ser−Thr Domain
Pkinase Domain
Stress−antifung Family
other Domain
0
500
1000
1500

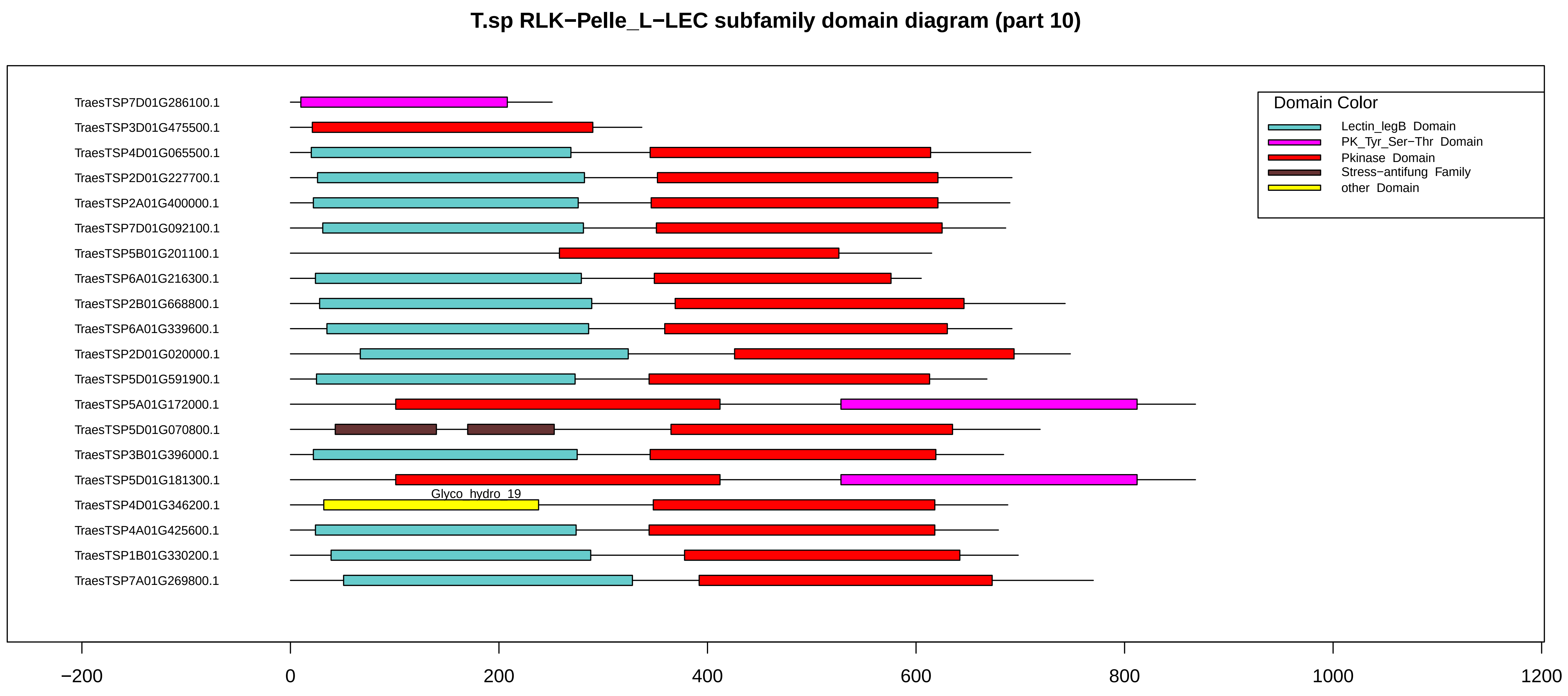

T.sp RLK−Pelle_L−LEC subfamily domain diagram (part 10)
TraesTSP7D01G286100.1
TraesTSP3D01G475500.1
TraesTSP4D01G065500.1
TraesTSP2D01G227700.1
TraesTSP2A01G400000.1
TraesTSP7D01G092100.1
TraesTSP5B01G201100.1
TraesTSP6A01G216300.1
TraesTSP2B01G668800.1
TraesTSP6A01G339600.1
TraesTSP2D01G020000.1
TraesTSP5D01G591900.1
TraesTSP5A01G172000.1
TraesTSP5D01G070800.1
TraesTSP3B01G396000.1
TraesTSP5D01G181300.1
TraesTSP4D01G346200.1
TraesTSP4A01G425600.1
TraesTSP1B01G330200.1
TraesTSP7A01G269800.1
Glyco_hydro_19
Domain Color
Lectin_legB Domain
PK_Tyr_Ser−Thr Domain
Pkinase Domain
Stress−antifung Family
other Domain
−200
0
200
400
600
800
1000
1200

T.sp RLK−Pelle_L−LEC subfamily domain diagram (part 11)

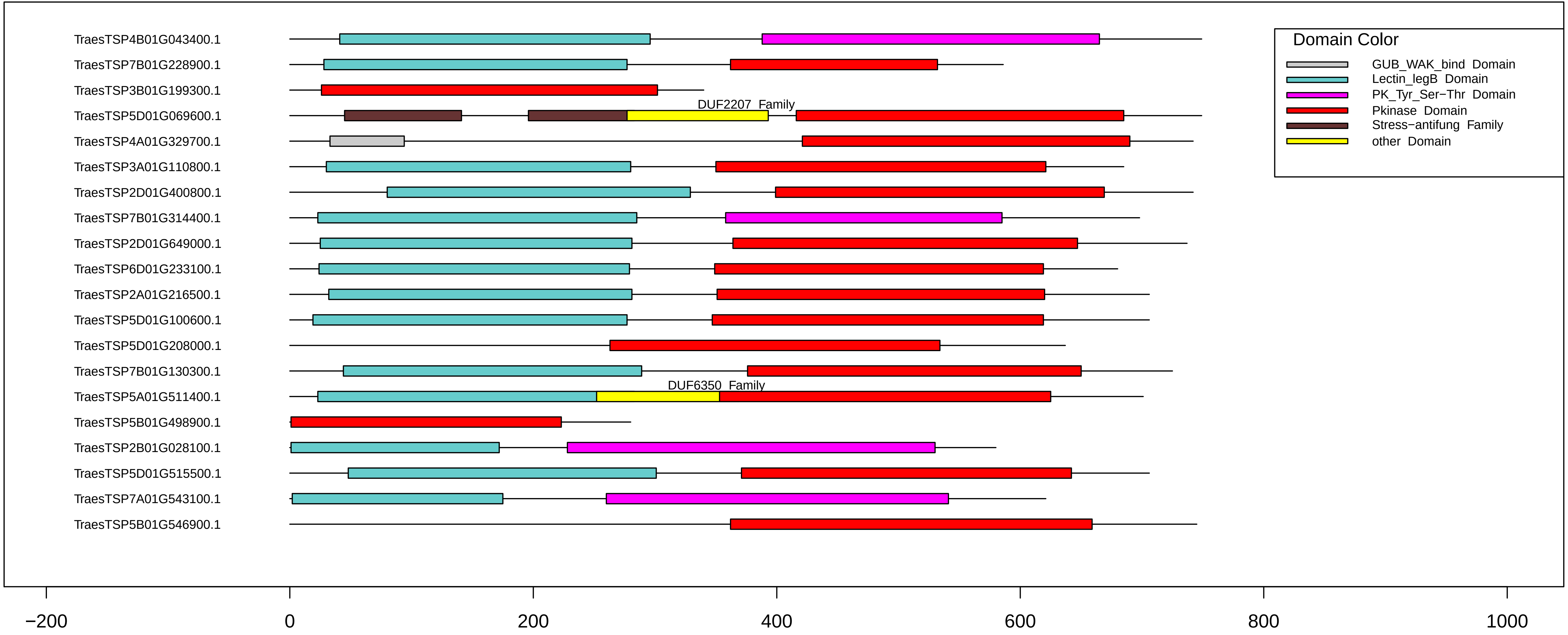

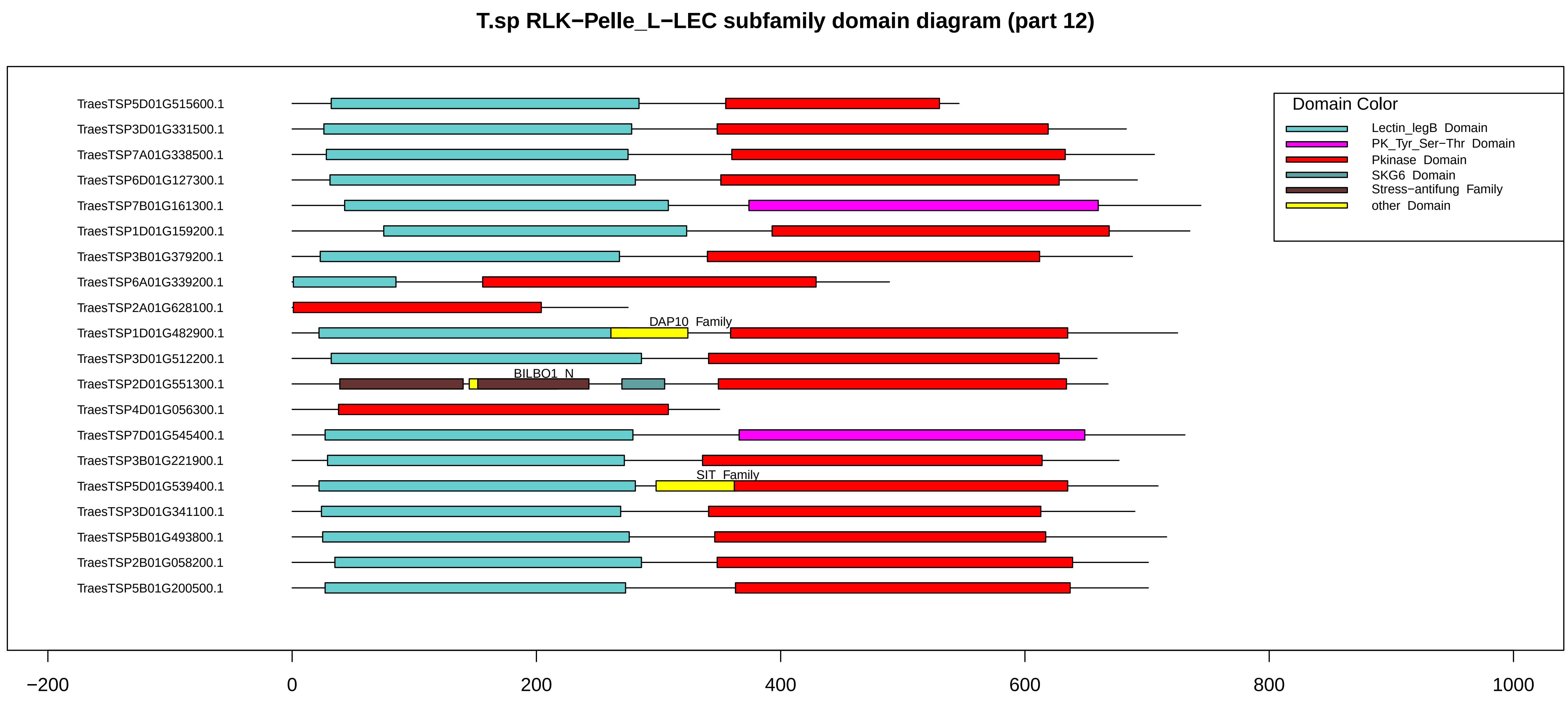

T.sp RLK−Pelle_L−LEC subfamily domain diagram (part 12)
TraesTSP5D01G515600.1
TraesTSP3D01G331500.1
TraesTSP7A01G338500.1
TraesTSP6D01G127300.1
TraesTSP7B01G161300.1
TraesTSP1D01G159200.1
TraesTSP3B01G379200.1
TraesTSP6A01G339200.1
TraesTSP2A01G628100.1
TraesTSP1D01G482900.1
DAP10 Family
TraesTSP3D01G512200.1
TraesTSP2D01G551300.1
BILBO1_N
TraesTSP4D01G056300.1
TraesTSP7D01G545400.1
TraesTSP3B01G221900.1
TraesTSP5D01G539400.1
SIT Family
TraesTSP3D01G341100.1
TraesTSP5B01G493800.1
TraesTSP2B01G058200.1
TraesTSP5B01G200500.1
Domain Color
Lectin_legB Domain
PK_Tyr_Ser−Thr Domain
Pkinase Domain
SKG6 Domain
Stress−antifung Family
other Domain
−200
0
200
400
600
800
1000

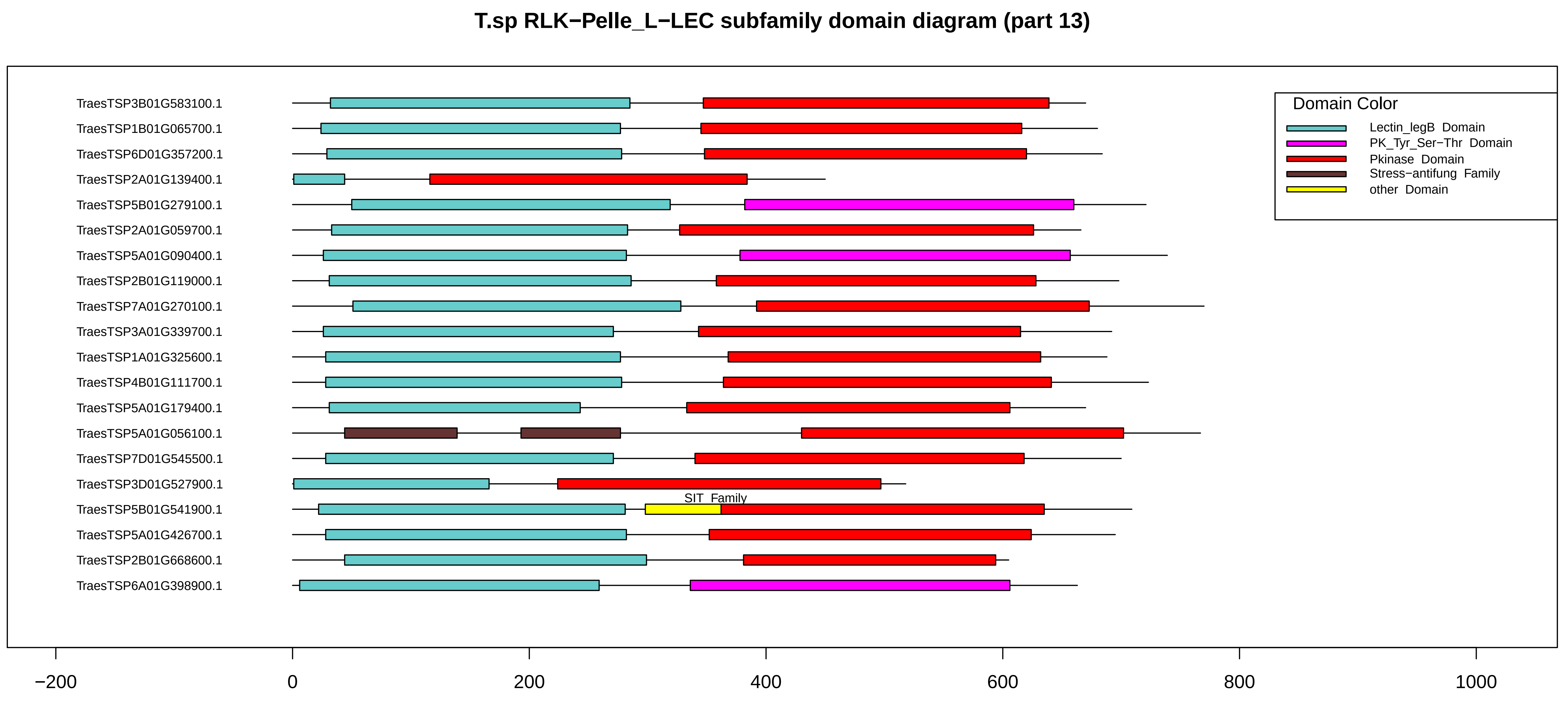
T.sp RLK−Pelle_L−LEC subfamily domain diagram (part 13)
TraesTSP3B01G583100.1
TraesTSP1B01G065700.1
TraesTSP6D01G357200.1
TraesTSP2A01G139400.1
TraesTSP5B01G279100.1
TraesTSP2A01G059700.1
TraesTSP5A01G090400.1
TraesTSP2B01G119000.1
TraesTSP7A01G270100.1
TraesTSP3A01G339700.1
TraesTSP1A01G325600.1
TraesTSP4B01G111700.1
TraesTSP5A01G179400.1
TraesTSP5A01G056100.1
TraesTSP7D01G545500.1
TraesTSP3D01G527900.1
TraesTSP5B01G541900.1
TraesTSP5A01G426700.1
TraesTSP2B01G668600.1
TraesTSP6A01G398900.1
SIT_Family
Domain Color
Lectin_legB Domain
PK_Tyr_Ser−Thr Domain
Pkinase Domain
Stress−antifung Family
other Domain
−200
0
200
400
600
800
1000

T.sp RLK−Pelle_L−LEC subfamily domain diagram (part 14)

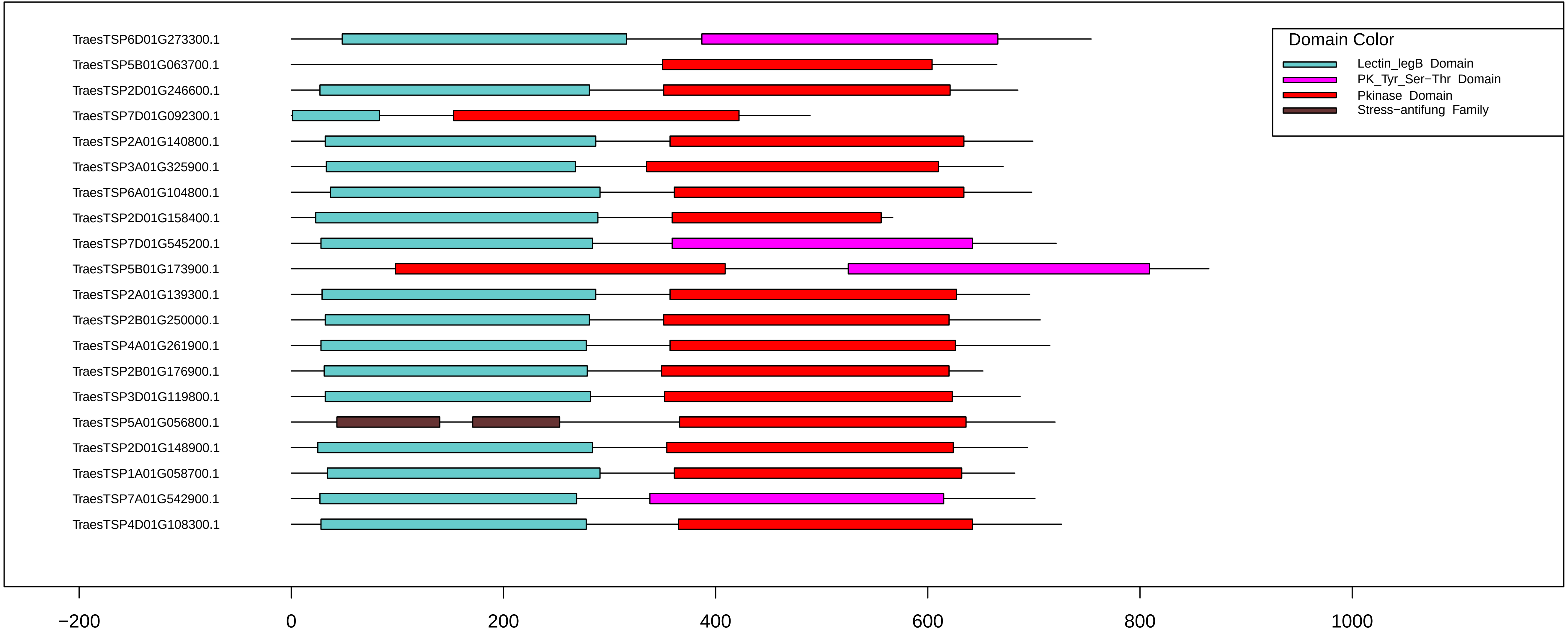

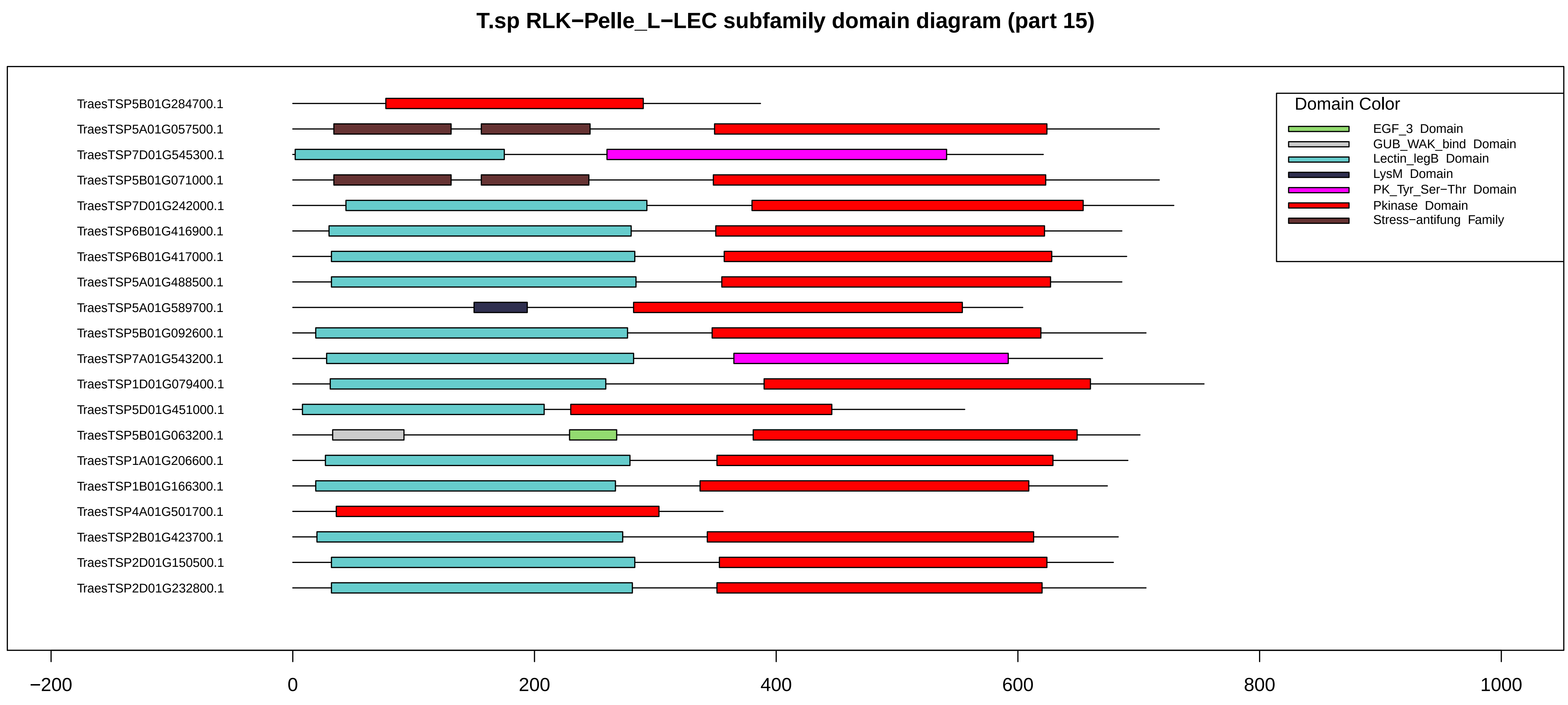
T.sp RLK−Pelle_L−LEC subfamily domain diagram (part 15)
TraesTSP5B01G284700.1
TraesTSP5A01G057500.1
TraesTSP7D01G545300.1
TraesTSP5B01G071000.1
TraesTSP7D01G242000.1
TraesTSP6B01G416900.1
TraesTSP6B01G417000.1
TraesTSP5A01G488500.1
TraesTSP5A01G589700.1
TraesTSP5B01G092600.1
TraesTSP7A01G543200.1
TraesTSP1D01G079400.1
TraesTSP5D01G451000.1
TraesTSP5B01G063200.1
TraesTSP1A01G206600.1
TraesTSP1B01G166300.1
TraesTSP4A01G501700.1
TraesTSP2B01G423700.1
TraesTSP2D01G150500.1
TraesTSP2D01G232800.1
Domain Color
EGF_3 Domain
GUB_WAK_bind Domain
Lectin_legB Domain
LysM Domain
PK_Tyr_Ser−Thr Domain
Pkinase Domain
Stress−antifung Family
−200
0
200
400
600
800
1000

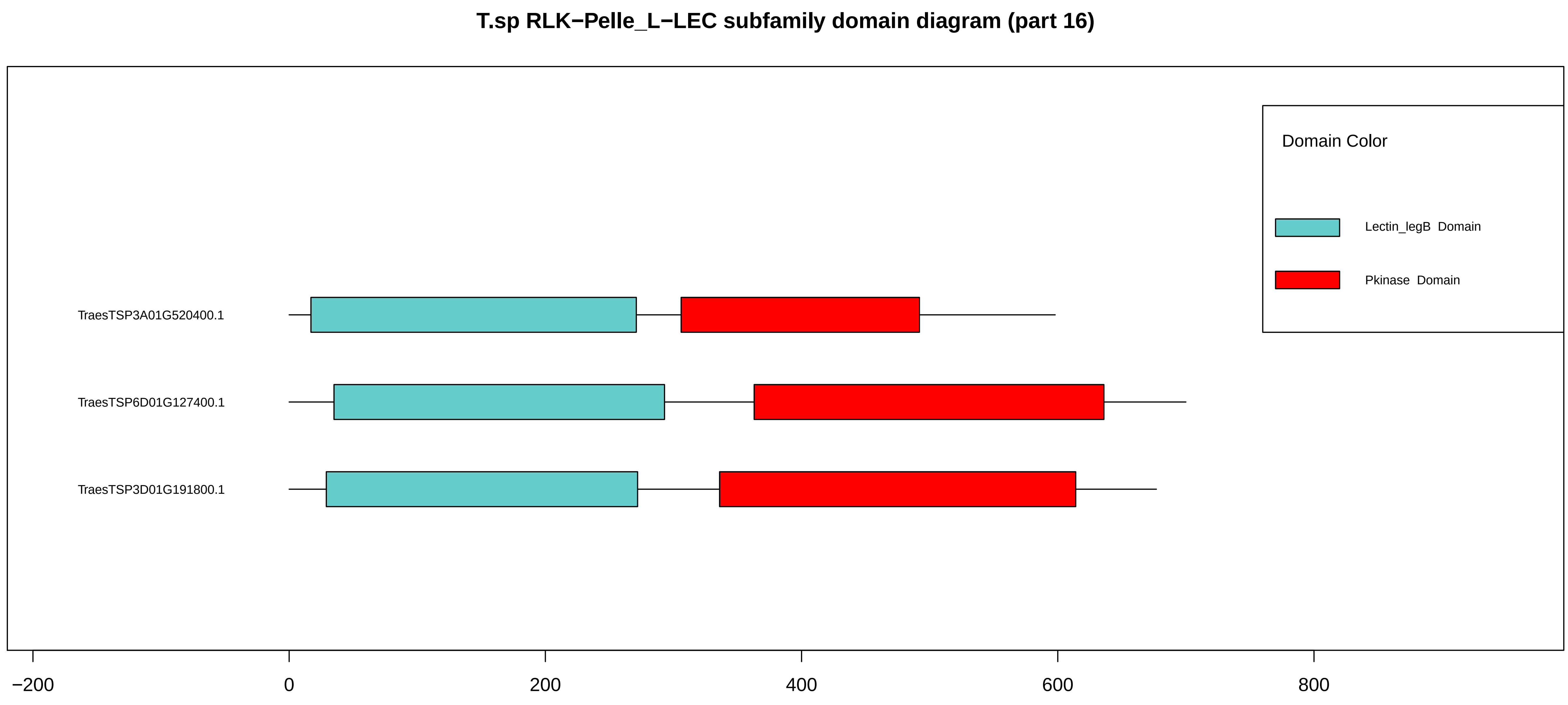

T.sp RLK−Pelle_L−LEC subfamily domain diagram (part 16)
Domain Color
Lectin_legB Domain
Pkinase Domain
TraesTSP3A01G520400.1
TraesTSP6D01G127400.1
TraesTSP3D01G191800.1
−200
0
200
400
600
800

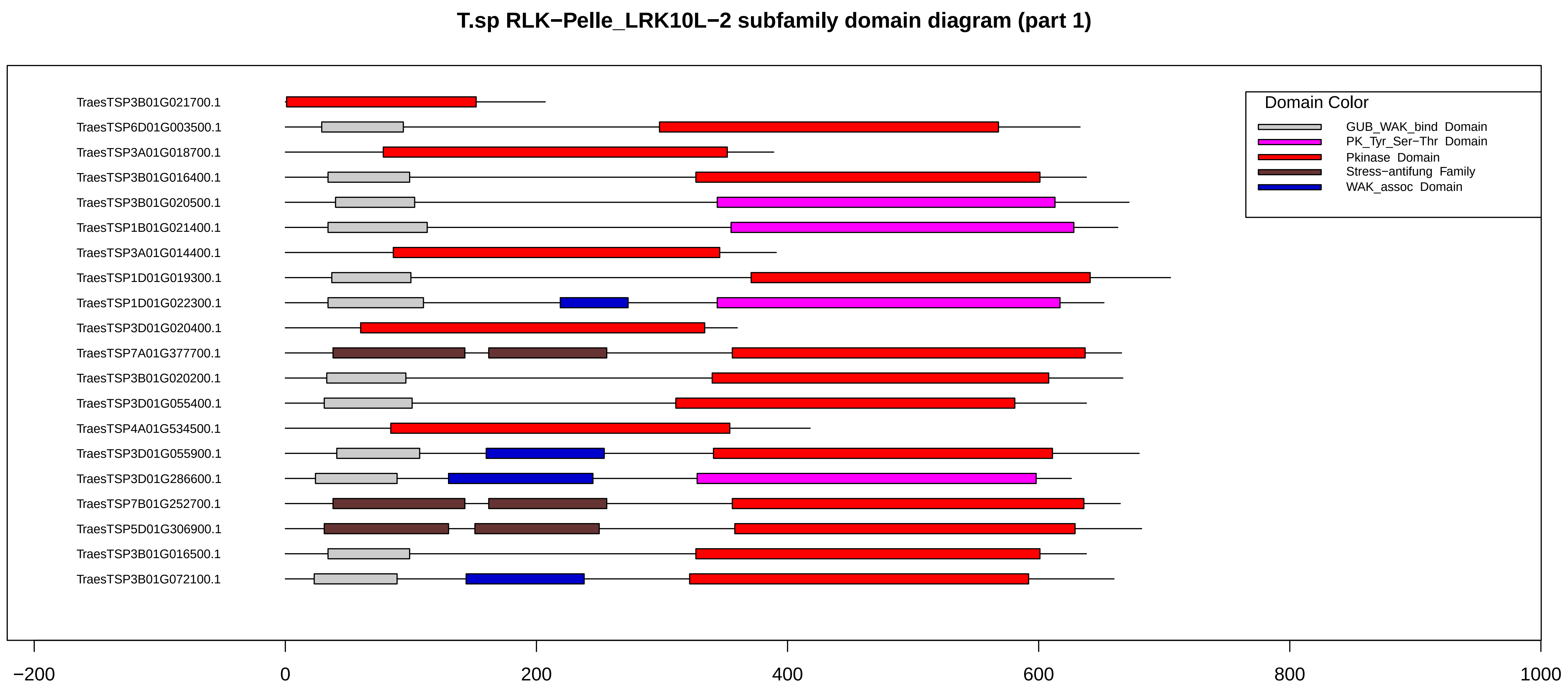
T.sp RLK−Pelle_LRK10L−2 subfamily domain diagram (part 1)
TraesTSP3B01G021700.1
TraesTSP6D01G003500.1
TraesTSP3A01G018700.1
TraesTSP3B01G016400.1
TraesTSP3B01G020500.1
TraesTSP1B01G021400.1
TraesTSP3A01G014400.1
TraesTSP1D01G019300.1
TraesTSP1D01G022300.1
TraesTSP3D01G020400.1
TraesTSP7A01G377700.1
TraesTSP3B01G020200.1
TraesTSP3D01G055400.1
TraesTSP4A01G534500.1
TraesTSP3D01G055900.1
TraesTSP3D01G286600.1
TraesTSP7B01G252700.1
TraesTSP5D01G306900.1
TraesTSP3B01G016500.1
TraesTSP3B01G072100.1
Domain Color
GUB_WAK_bind Domain
PK_Tyr_Ser−Thr Domain
Pkinase Domain
Stress−antifung Family
WAK_assoc Domain
−200
0
200
400
600
800
1000

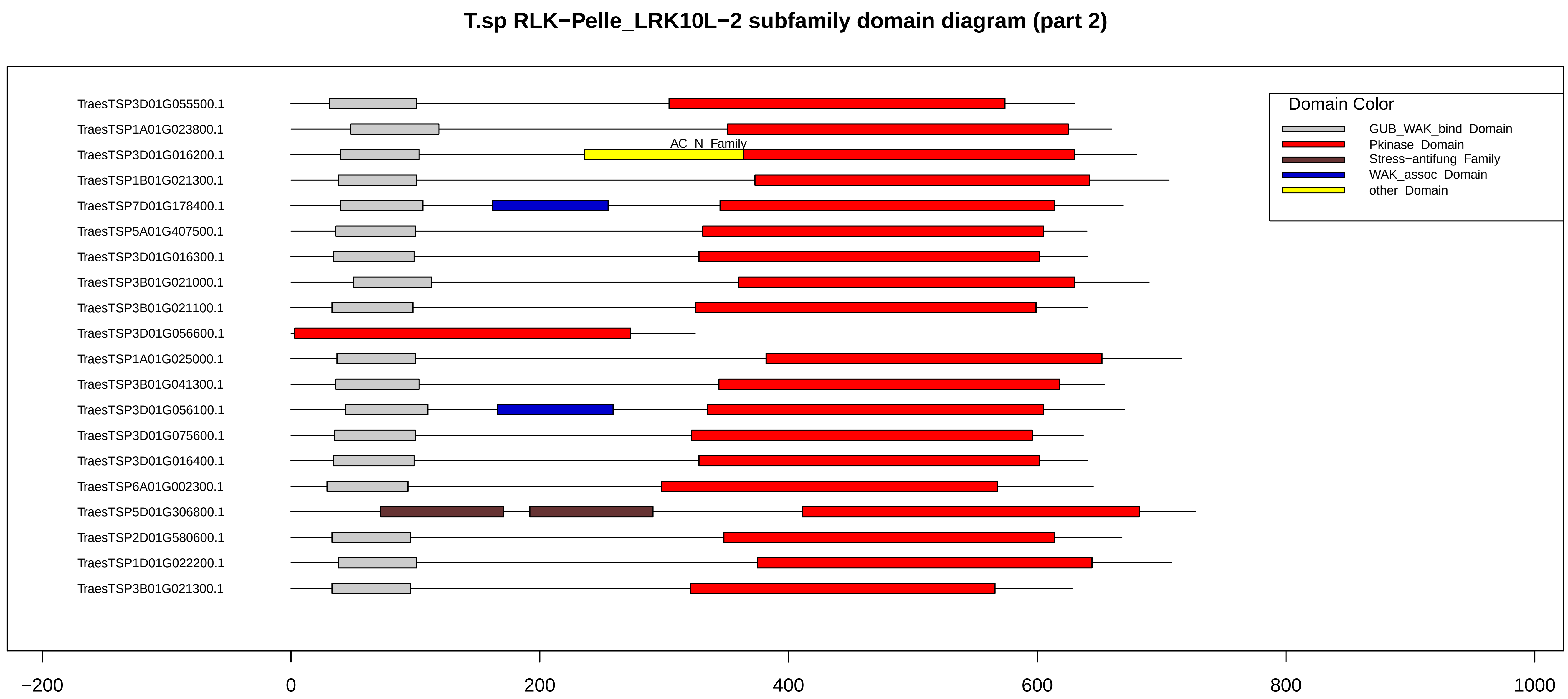
T.sp RLK−Pelle_LRK10L−2 subfamily domain diagram (part 2)
TraesTSP3D01G055500.1
TraesTSP1A01G023800.1
TraesTSP3D01G016200.1
AC_N_Family
TraesTSP1B01G021300.1
TraesTSP7D01G178400.1
TraesTSP5A01G407500.1
TraesTSP3D01G016300.1
TraesTSP3B01G021000.1
TraesTSP3B01G021100.1
TraesTSP3D01G056600.1
TraesTSP1A01G025000.1
TraesTSP3B01G041300.1
TraesTSP3D01G056100.1
TraesTSP3D01G075600.1
TraesTSP3D01G016400.1
TraesTSP6A01G002300.1
TraesTSP5D01G306800.1
TraesTSP2D01G580600.1
TraesTSP1D01G022200.1
TraesTSP3B01G021300.1
Domain Color
GUB_WAK_bind Domain
Pkinase Domain
Stress−antifung Family
WAK_assoc Domain
other Domain
−200
0
200
400
600
800
1000

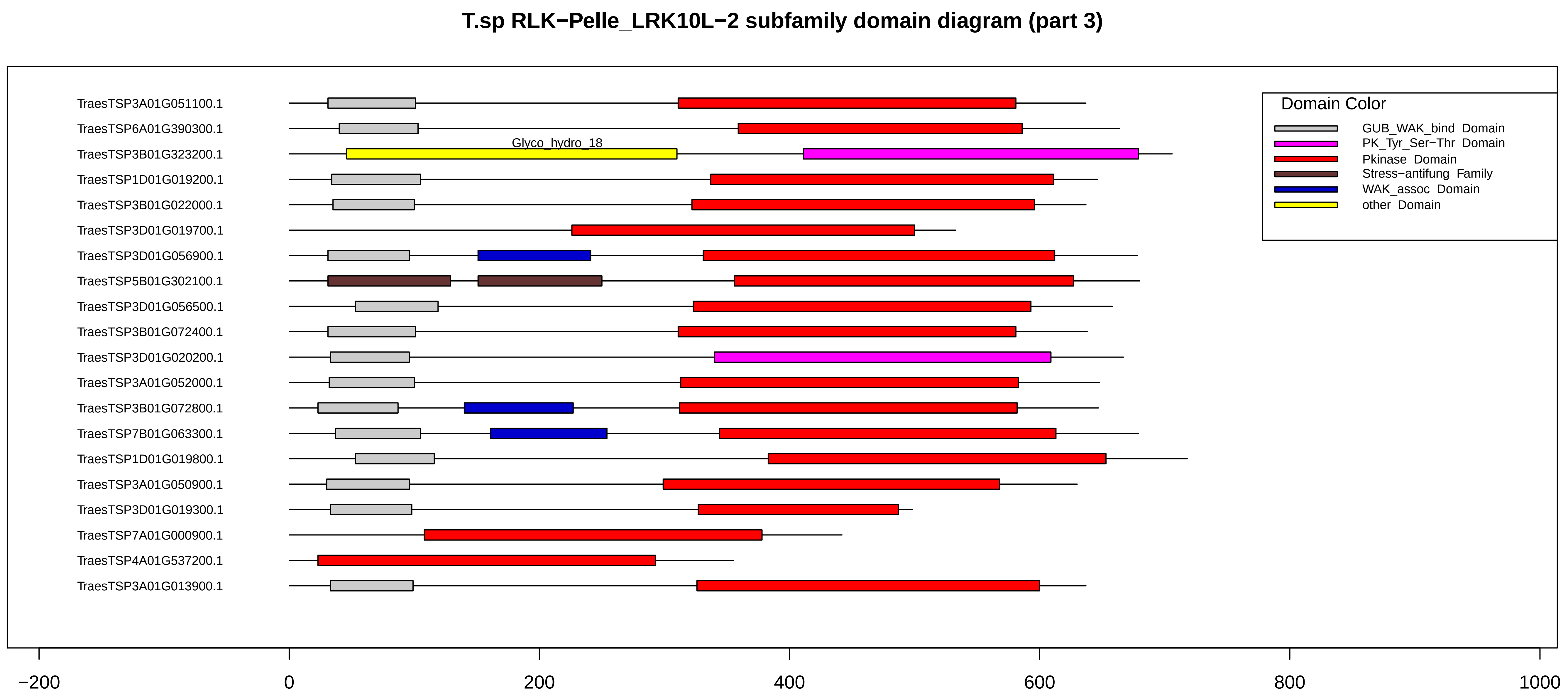
T.sp RLK−Pelle_LRK10L−2 subfamily domain diagram (part 3)
TraesTSP3A01G051100.1
TraesTSP6A01G390300.1
TraesTSP3B01G323200.1
Glyco_hydro_18
TraesTSP1D01G019200.1
TraesTSP3B01G022000.1
TraesTSP3D01G019700.1
TraesTSP3D01G056900.1
TraesTSP5B01G302100.1
TraesTSP3D01G056500.1
TraesTSP3B01G072400.1
TraesTSP3D01G020200.1
TraesTSP3A01G052000.1
TraesTSP3B01G072800.1
TraesTSP7B01G063300.1
TraesTSP1D01G019800.1
TraesTSP3A01G050900.1
TraesTSP3D01G019300.1
TraesTSP7A01G000900.1
TraesTSP4A01G537200.1
TraesTSP3A01G013900.1
Domain Color
GUB_WAK_bind Domain
PK_Tyr_Ser−Thr Domain
Pkinase Domain
Stress−antifung Family
WAK_assoc Domain
other Domain
−200
0
200
400
600
800
1000

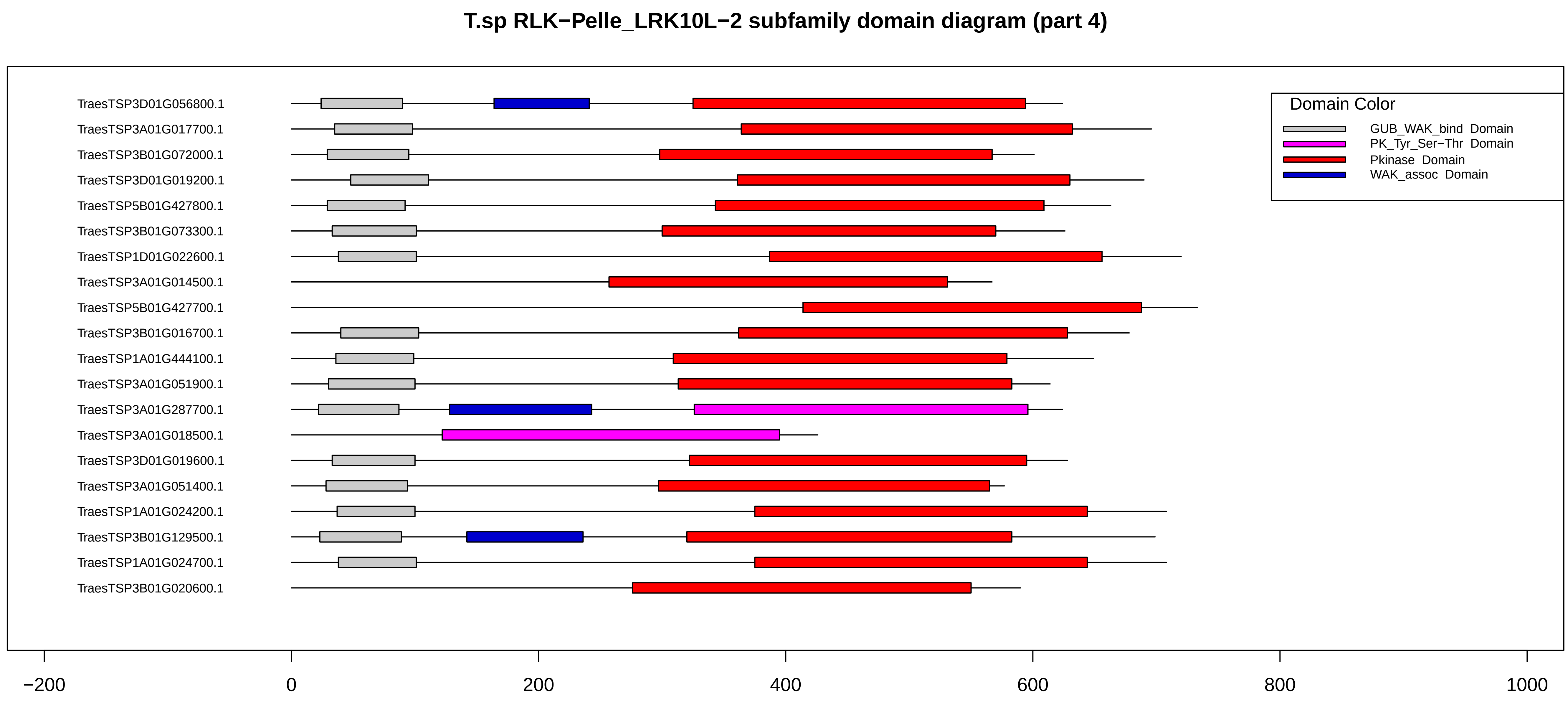

T.sp RLK−Pelle_LRK10L−2 subfamily domain diagram (part 4)
TraesTSP3D01G056800.1
TraesTSP3A01G017700.1
TraesTSP3B01G072000.1
TraesTSP3D01G019200.1
TraesTSP5B01G427800.1
TraesTSP3B01G073300.1
TraesTSP1D01G022600.1
TraesTSP3A01G014500.1
TraesTSP5B01G427700.1
TraesTSP3B01G016700.1
TraesTSP1A01G444100.1
TraesTSP3A01G051900.1
TraesTSP3A01G287700.1
TraesTSP3A01G018500.1
TraesTSP3D01G019600.1
TraesTSP3A01G051400.1
TraesTSP1A01G024200.1
TraesTSP3B01G129500.1
TraesTSP1A01G024700.1
TraesTSP3B01G020600.1
Domain Color
GUB_WAK_bind Domain
PK_Tyr_Ser−Thr Domain
Pkinase Domain
WAK_assoc Domain
−200
0
200
400
600
800
1000

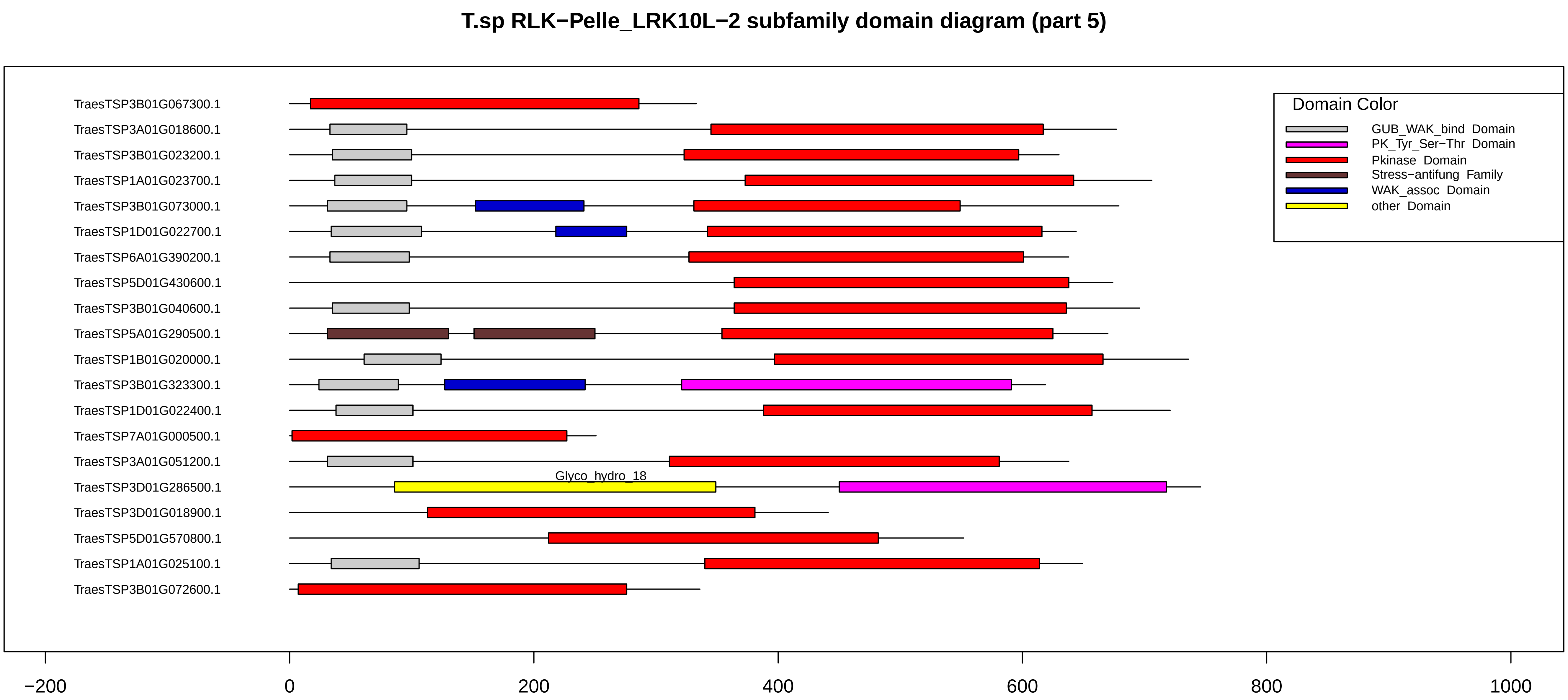

T.sp RLK−Pelle_LRK10L−2 subfamily domain diagram (part 5)
TraesTSP3B01G067300.1
TraesTSP3A01G018600.1
TraesTSP3B01G023200.1
TraesTSP1A01G023700.1
TraesTSP3B01G073000.1
TraesTSP1D01G022700.1
TraesTSP6A01G390200.1
TraesTSP5D01G430600.1
TraesTSP3B01G040600.1
TraesTSP5A01G290500.1
TraesTSP1B01G020000.1
TraesTSP3B01G323300.1
TraesTSP1D01G022400.1
TraesTSP7A01G000500.1
TraesTSP3A01G051200.1
TraesTSP3D01G286500.1
Glyco_hydro_18
TraesTSP3D01G018900.1
TraesTSP5D01G570800.1
TraesTSP1A01G025100.1
TraesTSP3B01G072600.1
Domain Color
GUB_WAK_bind Domain
PK_Tyr_Ser−Thr Domain
Pkinase Domain
Stress−antifung Family
WAK_assoc Domain
other Domain
−200
0
200
400
600
800
1000

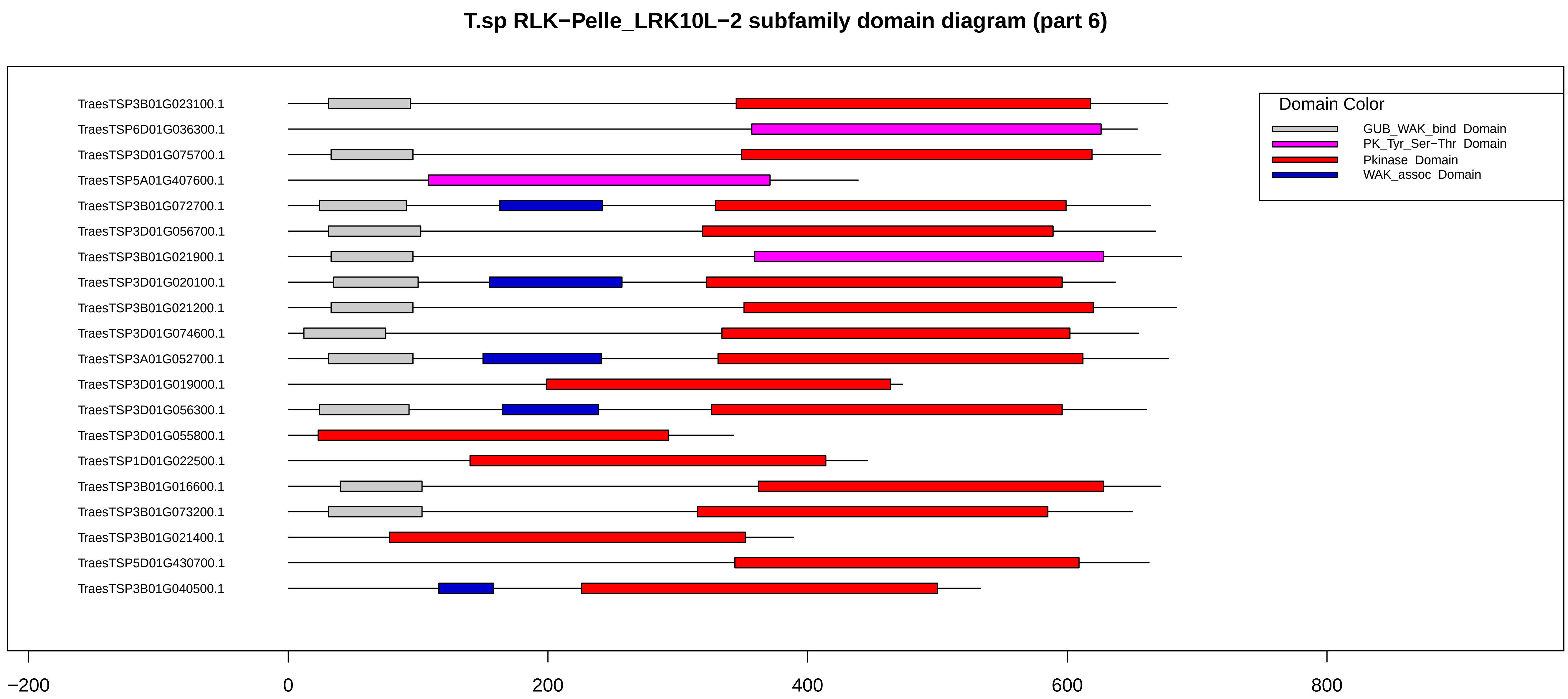
T.sp RLK−Pelle_LRK10L−2 subfamily domain diagram (part 6)
TraesTSP3B01G023100.1
TraesTSP6D01G036300.1
TraesTSP3D01G075700.1
TraesTSP5A01G407600.1
TraesTSP3B01G072700.1
TraesTSP3D01G056700.1
TraesTSP3B01G021900.1
TraesTSP3D01G020100.1
TraesTSP3B01G021200.1
TraesTSP3D01G074600.1
TraesTSP3A01G052700.1
TraesTSP3D01G019000.1
TraesTSP3D01G056300.1
TraesTSP3D01G055800.1
TraesTSP1D01G022500.1
TraesTSP3B01G016600.1
TraesTSP3B01G073200.1
TraesTSP3B01G021400.1
TraesTSP5D01G430700.1
TraesTSP3B01G040500.1
Domain Color
GUB_WAK_bind Domain
PK_Tyr_Ser−Thr Domain
Pkinase Domain
WAK_assoc Domain
−200
0
200
400
600
800

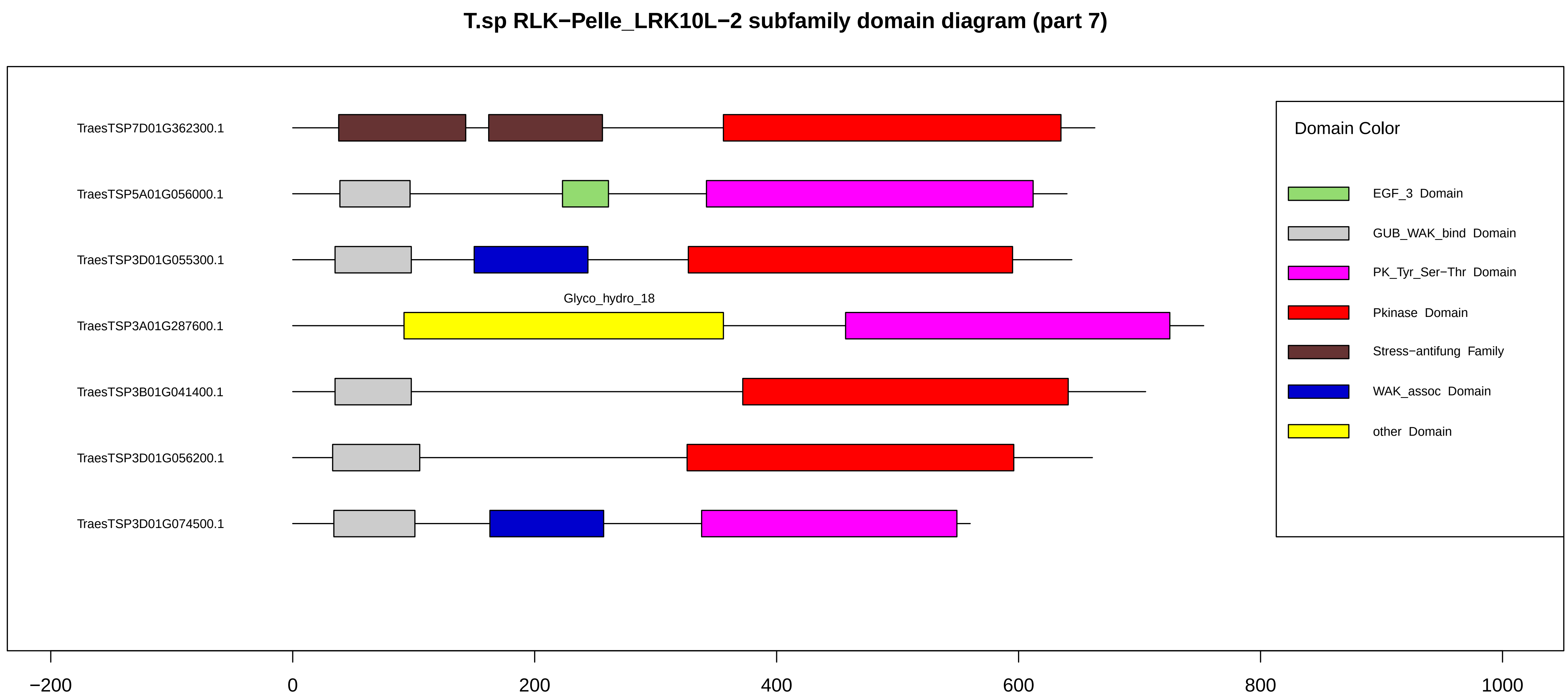
T.sp RLK−Pelle_LRK10L−2 subfamily domain diagram (part 7)
TraesTSP7D01G362300.1
TraesTSP5A01G056000.1
TraesTSP3D01G055300.1
Glyco_hydro_18
TraesTSP3A01G287600.1
TraesTSP3B01G041400.1
TraesTSP3D01G056200.1
TraesTSP3D01G074500.1
Domain Color
EGF_3 Domain
GUB_WAK_bind Domain
PK_Tyr_Ser−Thr Domain
Pkinase Domain
Stress−antifung Family
WAK_assoc Domain
other Domain
−200
0
200
400
600
800
1000

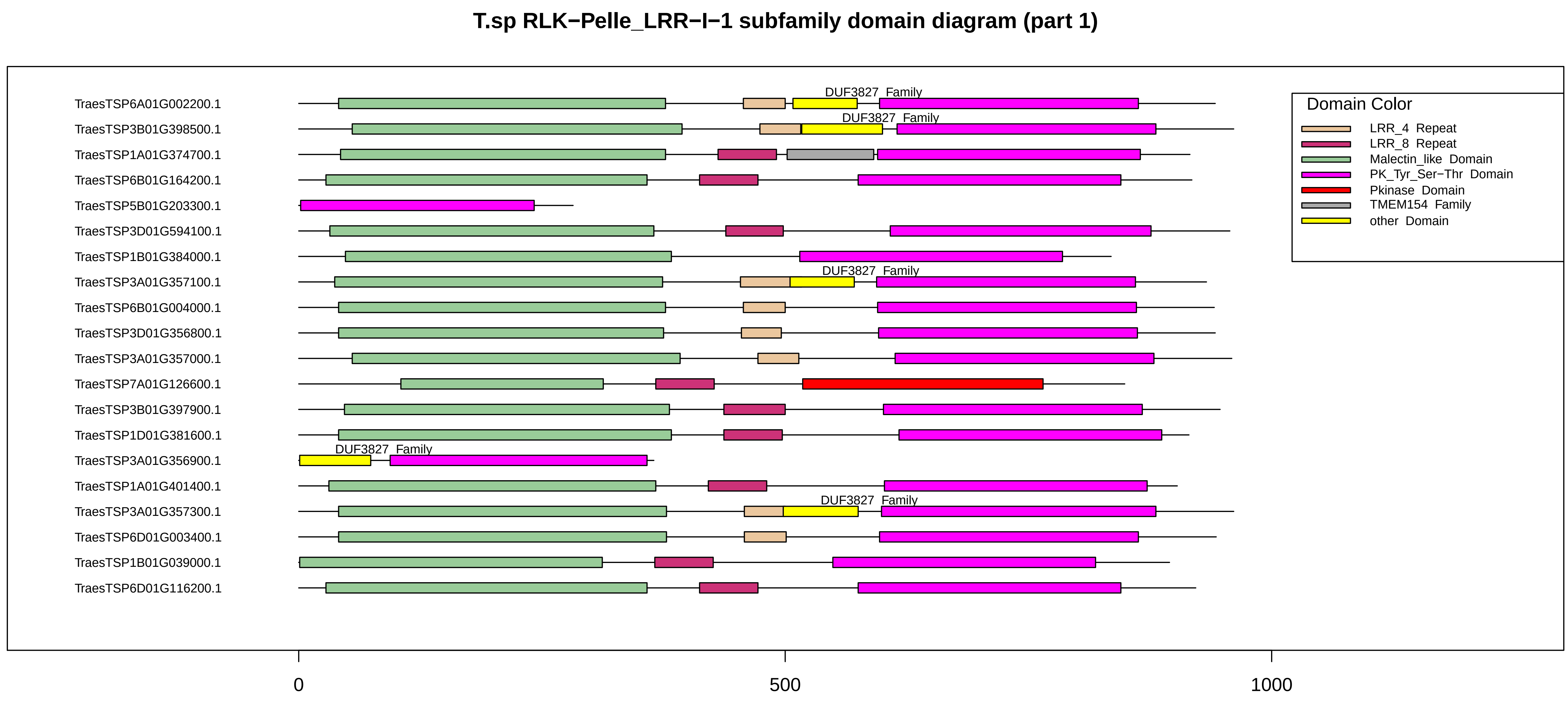
T.sp RLK−Pelle_LRR−I−1 subfamily domain diagram (part 1)
TraesTSP6A01G002200.1
TraesTSP3B01G398500.1
TraesTSP1A01G374700.1
TraesTSP6B01G164200.1
TraesTSP5B01G203300.1
TraesTSP3D01G594100.1
TraesTSP1B01G384000.1
TraesTSP3A01G357100.1
TraesTSP6B01G004000.1
TraesTSP3D01G356800.1
TraesTSP3A01G357000.1
TraesTSP7A01G126600.1
TraesTSP3B01G397900.1
TraesTSP1D01G381600.1
TraesTSP3A01G356900.1
TraesTSP1A01G401400.1
TraesTSP3A01G357300.1
TraesTSP6D01G003400.1
TraesTSP1B01G039000.1
TraesTSP6D01G116200.1
DUF3827 Family
Domain Color
LRR_4 Repeat
LRR_8 Repeat
Malectin_like Domain
PK_Tyr_Ser−Thr Domain
Pkinase Domain
TMEM154 Family
other Domain
0
500
1000

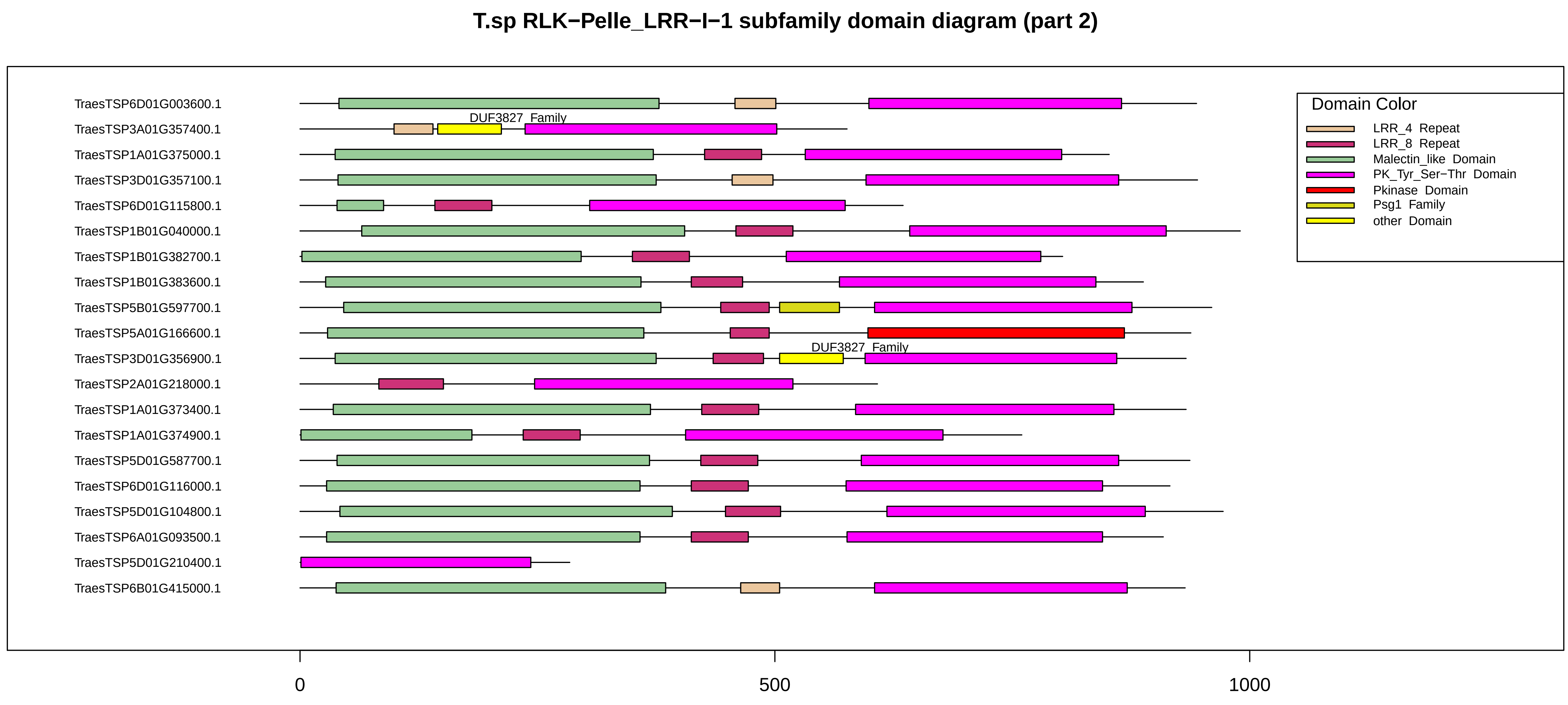
T.sp RLK−Pelle_LRR−I−1 subfamily domain diagram (part 2)
TraesTSP6D01G003600.1
TraesTSP3A01G357400.1
DUF3827_Family
TraesTSP1A01G375000.1
TraesTSP3D01G357100.1
TraesTSP6D01G115800.1
TraesTSP1B01G040000.1
TraesTSP1B01G382700.1
TraesTSP1B01G383600.1
TraesTSP5B01G597700.1
TraesTSP5A01G166600.1
TraesTSP3D01G356900.1
DUF3827_Family
TraesTSP2A01G218000.1
TraesTSP1A01G373400.1
TraesTSP1A01G374900.1
TraesTSP5D01G587700.1
TraesTSP6D01G116000.1
TraesTSP5D01G104800.1
TraesTSP6A01G093500.1
TraesTSP5D01G210400.1
TraesTSP6B01G415000.1
Domain Color
LRR_4 Repeat
LRR_8 Repeat
Malectin_like Domain
PK_Tyr_Ser−Thr Domain
Pkinase Domain
Psg1 Family
other Domain
0
500
1000

T.sp RLK−Pelle_LRR−I−1 subfamily domain diagram (part 3)

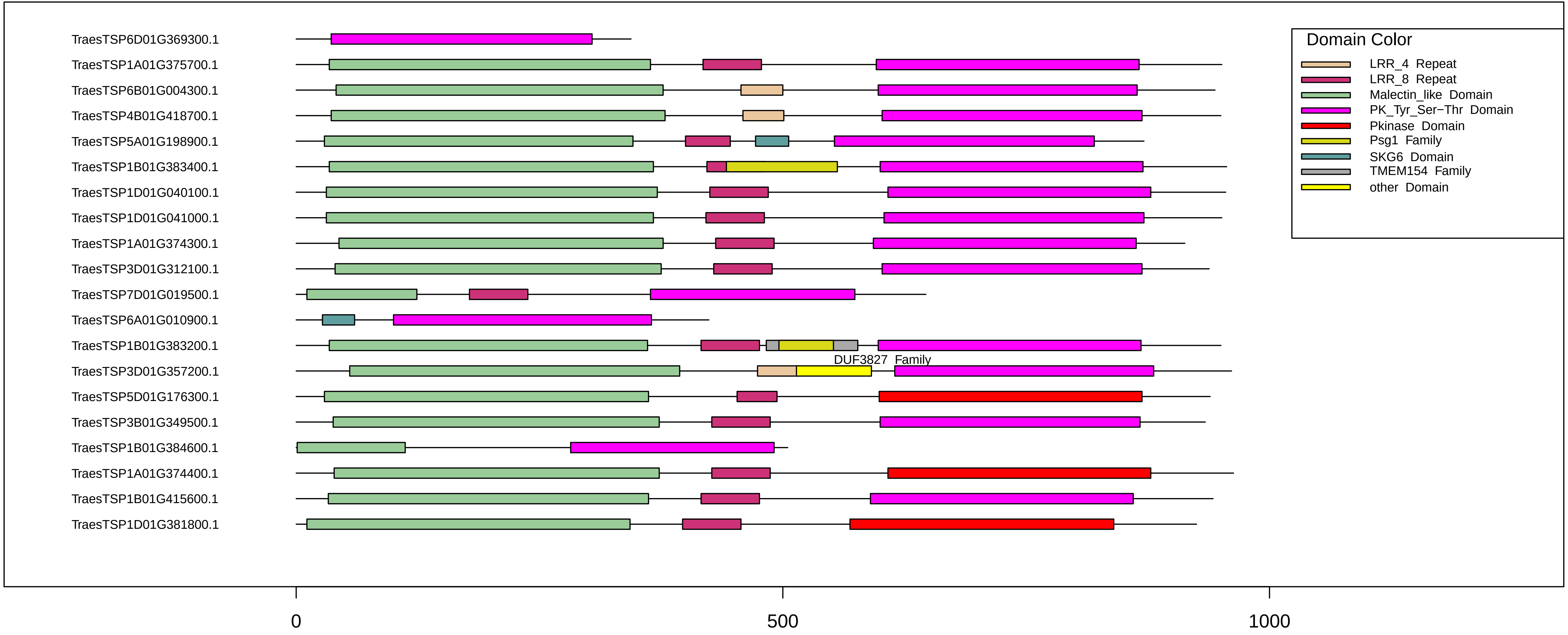

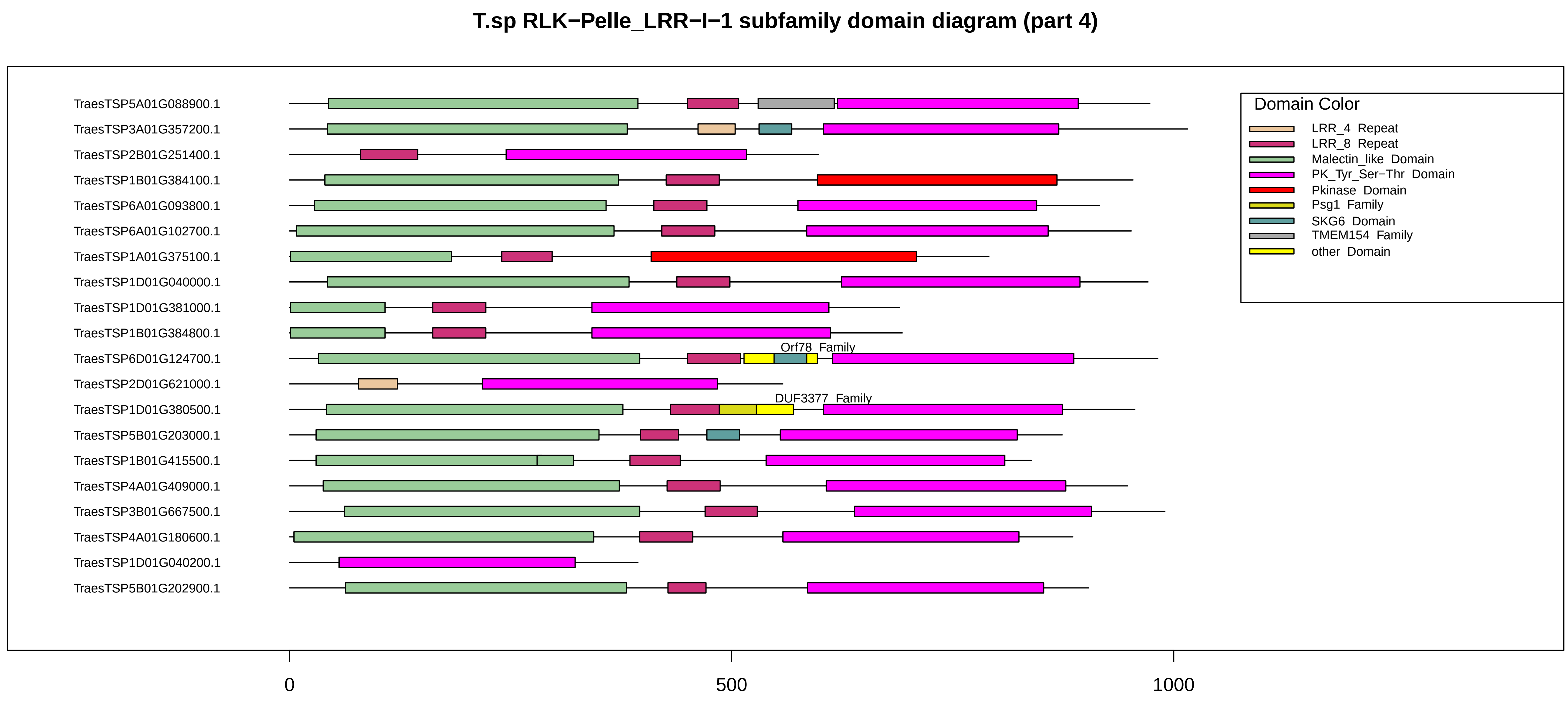
T.sp RLK−Pelle_LRR−I−1 subfamily domain diagram (part 4)
TraesTSP5A01G088900.1
TraesTSP3A01G357200.1
TraesTSP2B01G251400.1
TraesTSP1B01G384100.1
TraesTSP6A01G093800.1
TraesTSP6A01G102700.1
TraesTSP1A01G375100.1
TraesTSP1D01G040000.1
TraesTSP1D01G381000.1
TraesTSP1B01G384800.1
TraesTSP6D01G124700.1
TraesTSP2D01G621000.1
TraesTSP1D01G380500.1
TraesTSP5B01G203000.1
TraesTSP1B01G415500.1
TraesTSP4A01G409000.1
TraesTSP3B01G667500.1
TraesTSP4A01G180600.1
TraesTSP1D01G040200.1
TraesTSP5B01G202900.1
Orf78_Family
DUF3377_Family
Domain Color
LRR_4 Repeat
LRR_8 Repeat
Malectin_like Domain
PK_Tyr_Ser−Thr Domain
Pkinase Domain
Psg1 Family
SKG6 Domain
TMEM154 Family
other Domain
0
500
1000

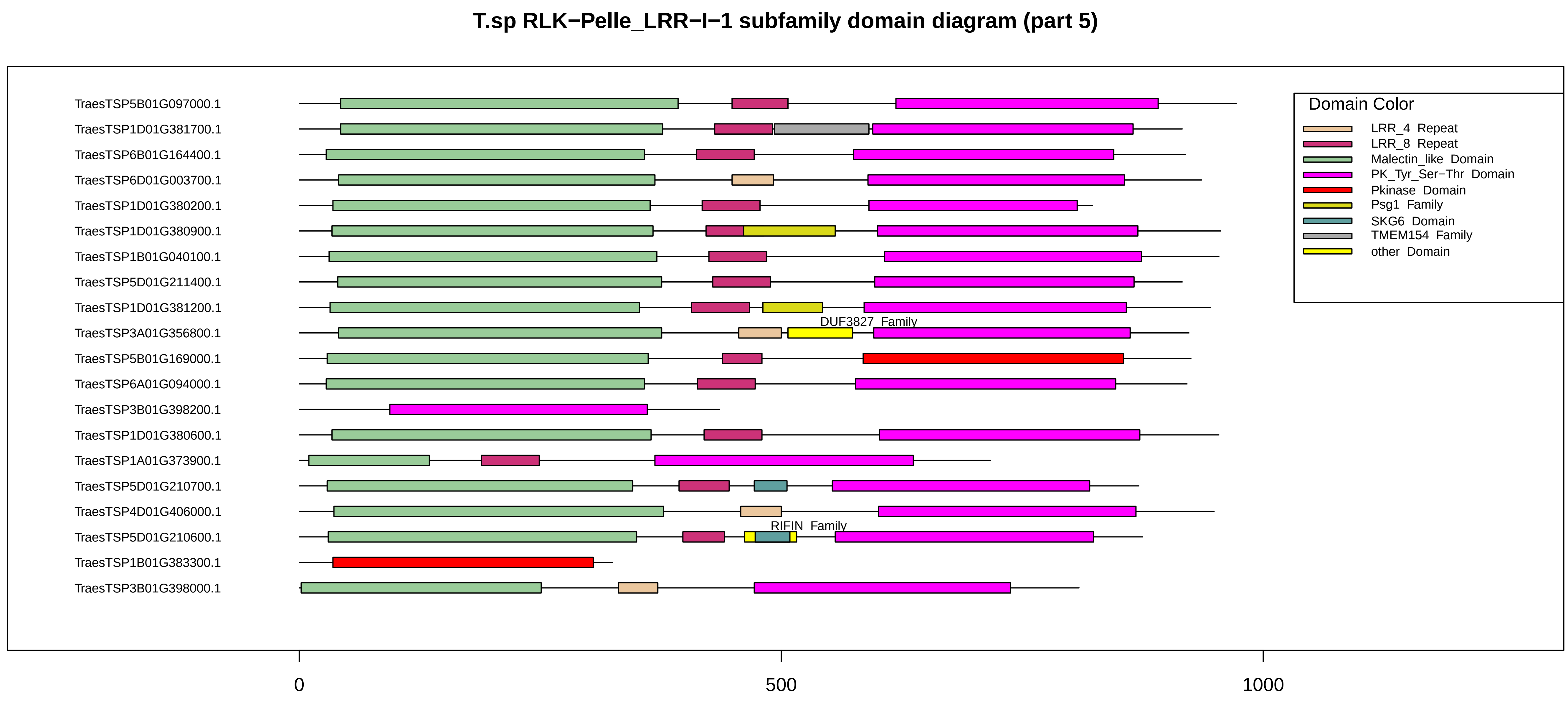
T.sp RLK−Pelle_LRR−I−1 subfamily domain diagram (part 5)
TraesTSP5B01G097000.1
TraesTSP1D01G381700.1
TraesTSP6B01G164400.1
TraesTSP6D01G003700.1
TraesTSP1D01G380200.1
TraesTSP1D01G380900.1
TraesTSP1B01G040100.1
TraesTSP5D01G211400.1
TraesTSP1D01G381200.1
TraesTSP3A01G356800.1
TraesTSP5B01G169000.1
TraesTSP6A01G094000.1
TraesTSP3B01G398200.1
TraesTSP1D01G380600.1
TraesTSP1A01G373900.1
TraesTSP5D01G210700.1
TraesTSP4D01G406000.1
TraesTSP5D01G210600.1
TraesTSP1B01G383300.1
TraesTSP3B01G398000.1
DUF3827 Family
RIFIN Family
Domain Color
LRR_4 Repeat
LRR_8 Repeat
Malectin_like Domain
PK_Tyr_Ser−Thr Domain
Pkinase Domain
Psg1 Family
SKG6 Domain
TMEM154 Family
other Domain
0
500
1000

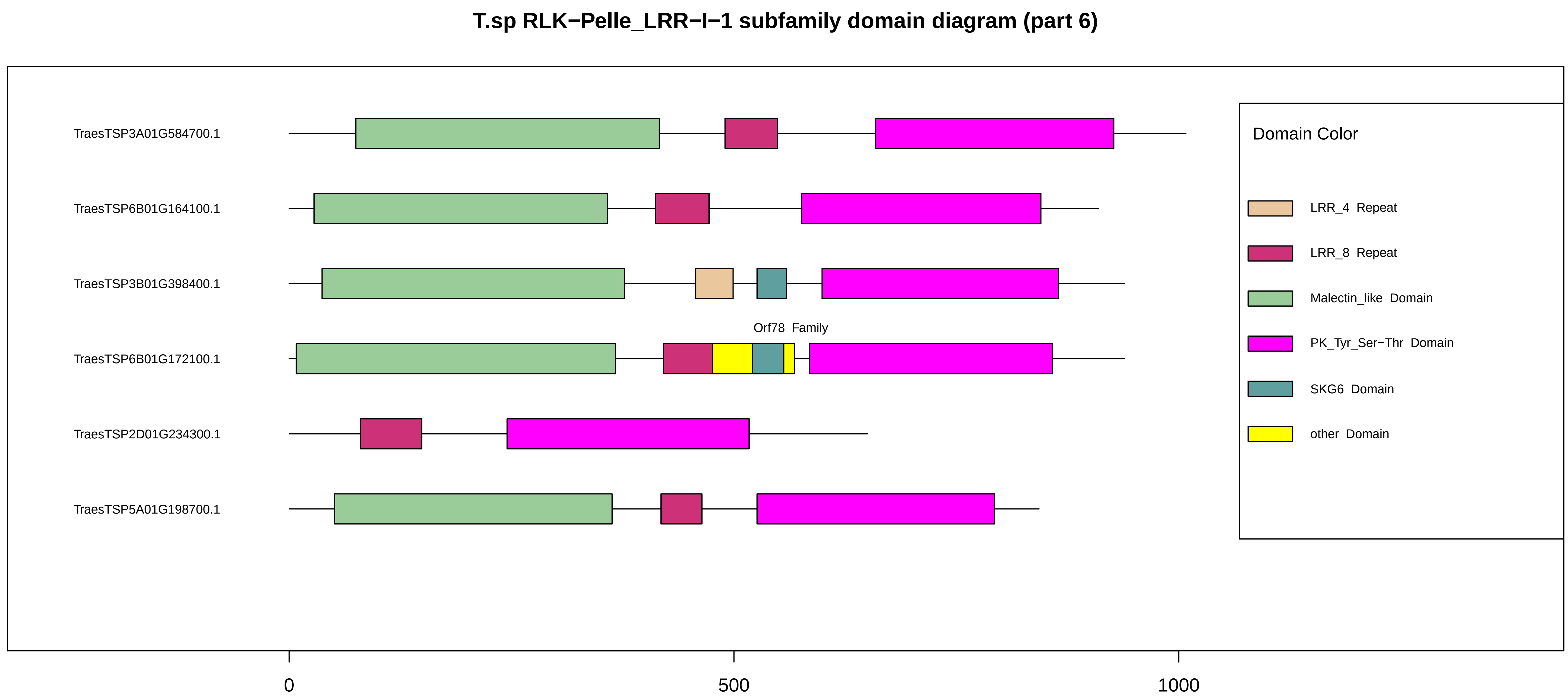

T.sp RLK−Pelle_LRR−I−1 subfamily domain diagram (part 6)
TraesTSP3A01G584700.1
TraesTSP6B01G164100.1
TraesTSP3B01G398400.1
TraesTSP6B01G172100.1
TraesTSP2D01G234300.1
TraesTSP5A01G198700.1
Orf78 Family
Domain Color
LRR_4 Repeat
LRR_8 Repeat
Malectin_like Domain
PK_Tyr_Ser−Thr Domain
SKG6 Domain
other Domain
0
500
1000

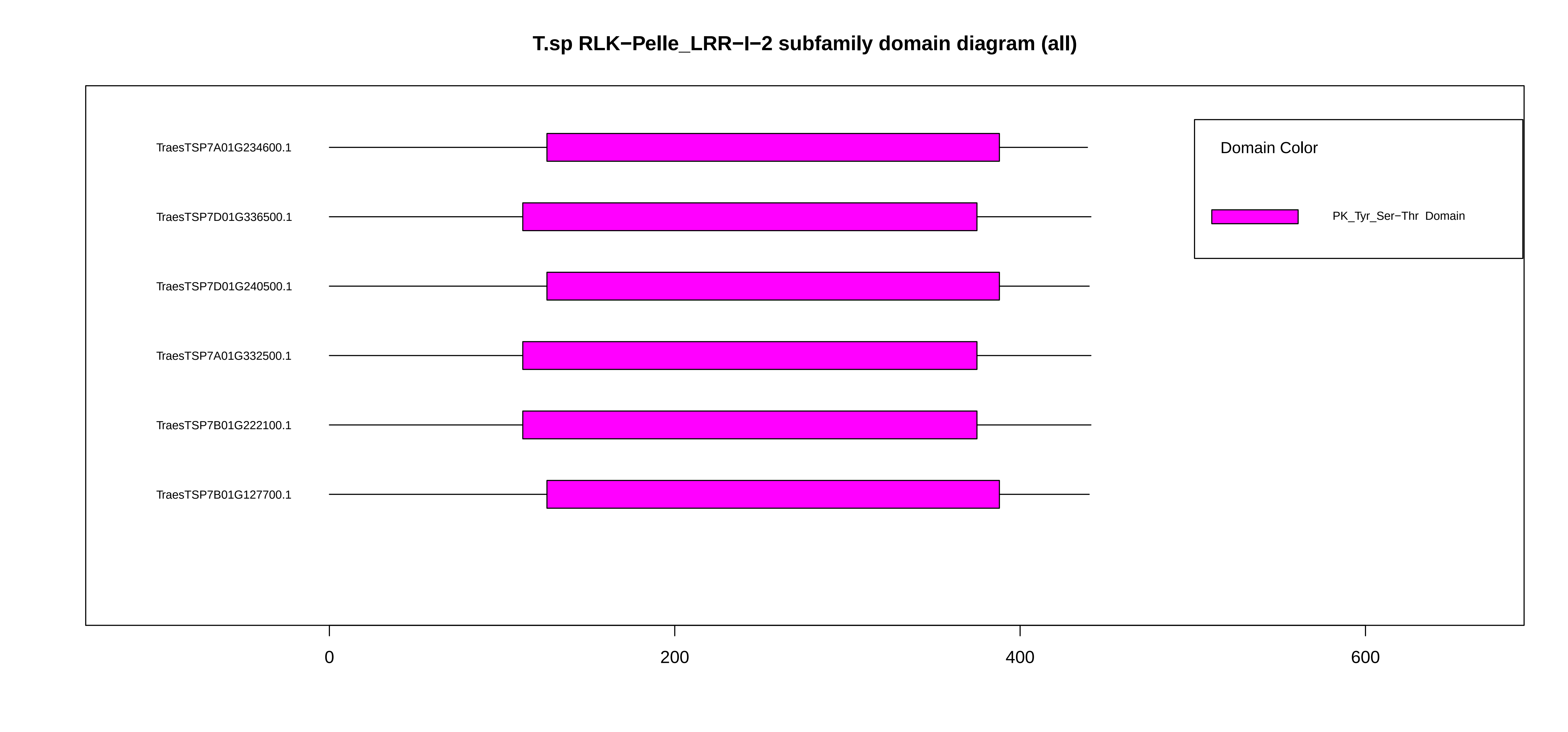
T.sp RLK−Pelle_LRR−I−2 subfamily domain diagram (all)
TraesTSP7A01G234600.1
TraesTSP7D01G336500.1
TraesTSP7D01G240500.1
TraesTSP7A01G332500.1
TraesTSP7B01G222100.1
TraesTSP7B01G127700.1
Domain Color
PK_Tyr_Ser−Thr Domain
0
200
400
600

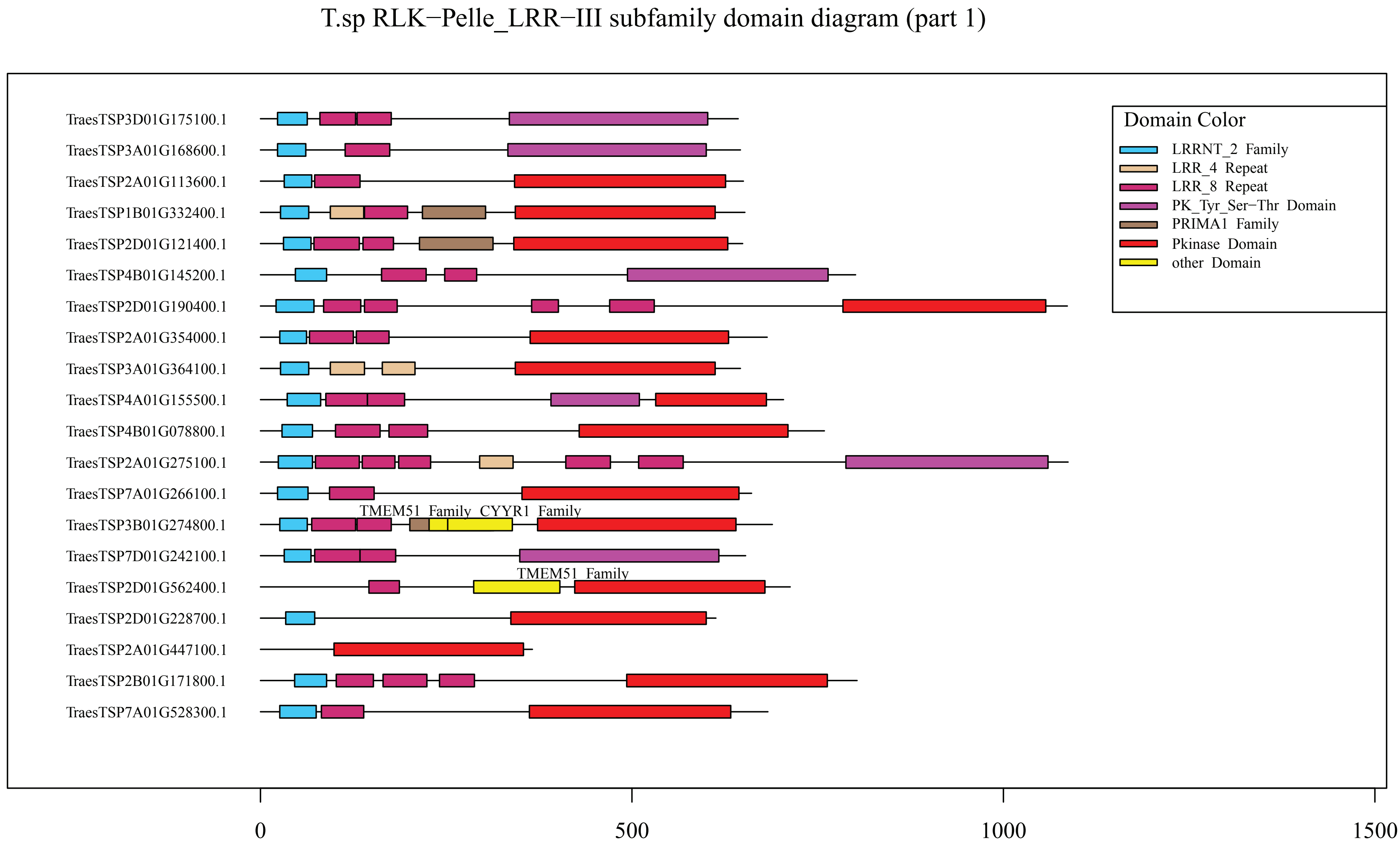

T.sp RLK−Pelle_LRR−III subfamily domain diagram (part 1)
TraesTSP3D01G175100.1
TraesTSP3A01G168600.1
TraesTSP2A01G113600.1
TraesTSP1B01G332400.1
TraesTSP2D01G121400.1
TraesTSP4B01G145200.1
TraesTSP2D01G190400.1
TraesTSP2A01G354000.1
TraesTSP3A01G364100.1
TraesTSP4A01G155500.1
TraesTSP4B01G078800.1
TraesTSP2A01G275100.1
TraesTSP7A01G266100.1
TMEM51_Family CYYR1_Family
TraesTSP3B01G274800.1
TraesTSP7D01G242100.1
TMEM51_Family
TraesTSP2D01G562400.1
TraesTSP2D01G228700.1
TraesTSP2A01G447100.1
TraesTSP2B01G171800.1
TraesTSP7A01G528300.1
Domain Color
LRRNT_2 Family
LRR_4 Repeat
LRR_8 Repeat
PK_Tyr_Ser−Thr Domain
PRIMA1 Family
Pkinase Domain
other Domain
0
500
1000
1500

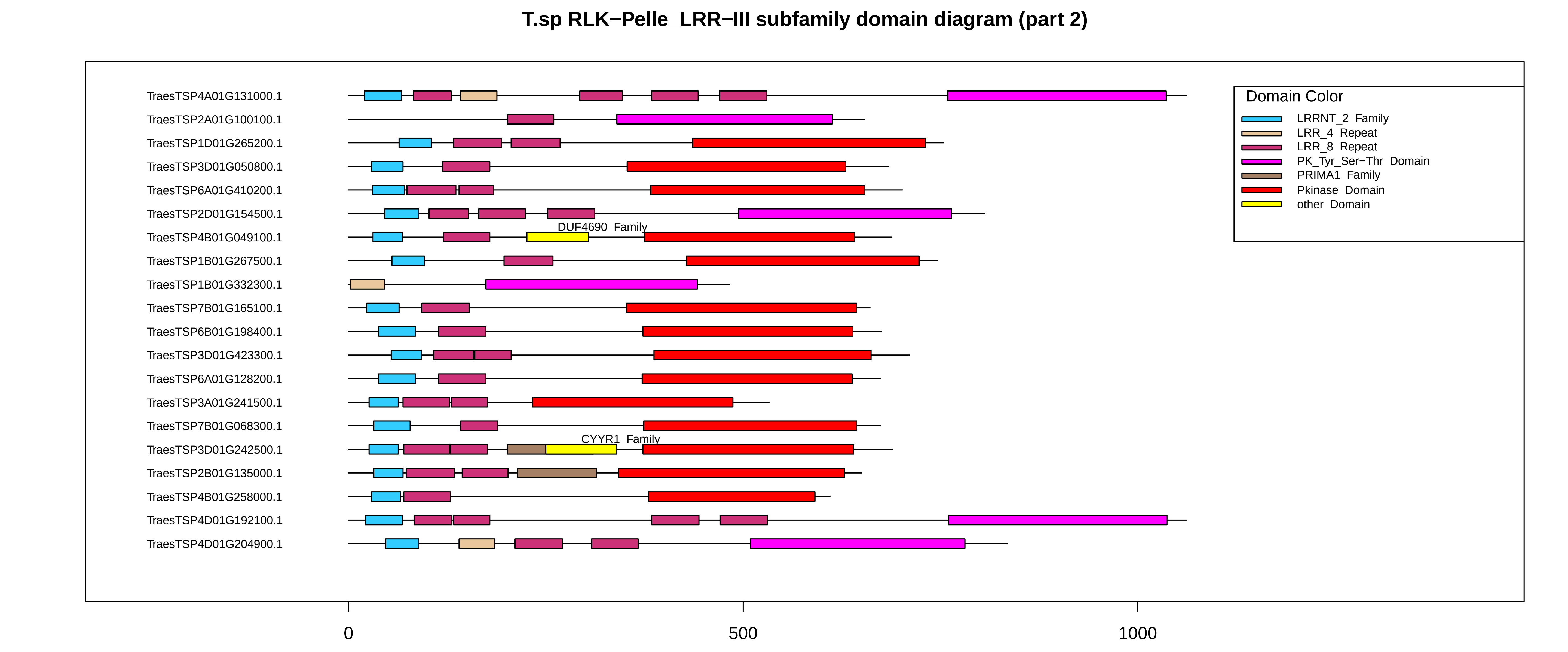
T.sp RLK−Pelle_LRR−III subfamily domain diagram (part 2)
TraesTSP4A01G131000.1
TraesTSP2A01G100100.1
TraesTSP1D01G265200.1
TraesTSP3D01G050800.1
TraesTSP6A01G410200.1
TraesTSP2D01G154500.1
TraesTSP4B01G049100.1
TraesTSP1B01G267500.1
TraesTSP1B01G332300.1
TraesTSP7B01G165100.1
TraesTSP6B01G198400.1
TraesTSP3D01G423300.1
TraesTSP6A01G128200.1
TraesTSP3A01G241500.1
TraesTSP7B01G068300.1
TraesTSP3D01G242500.1
TraesTSP2B01G135000.1
TraesTSP4B01G258000.1
TraesTSP4D01G192100.1
TraesTSP4D01G204900.1
DUF4690 Family
CYYR1 Family
Domain Color
LRRNT_2 Family
LRR_4 Repeat
LRR_8 Repeat
PK_Tyr_Ser−Thr Domain
PRIMA1 Family
Pkinase Domain
other Domain
0
500
1000

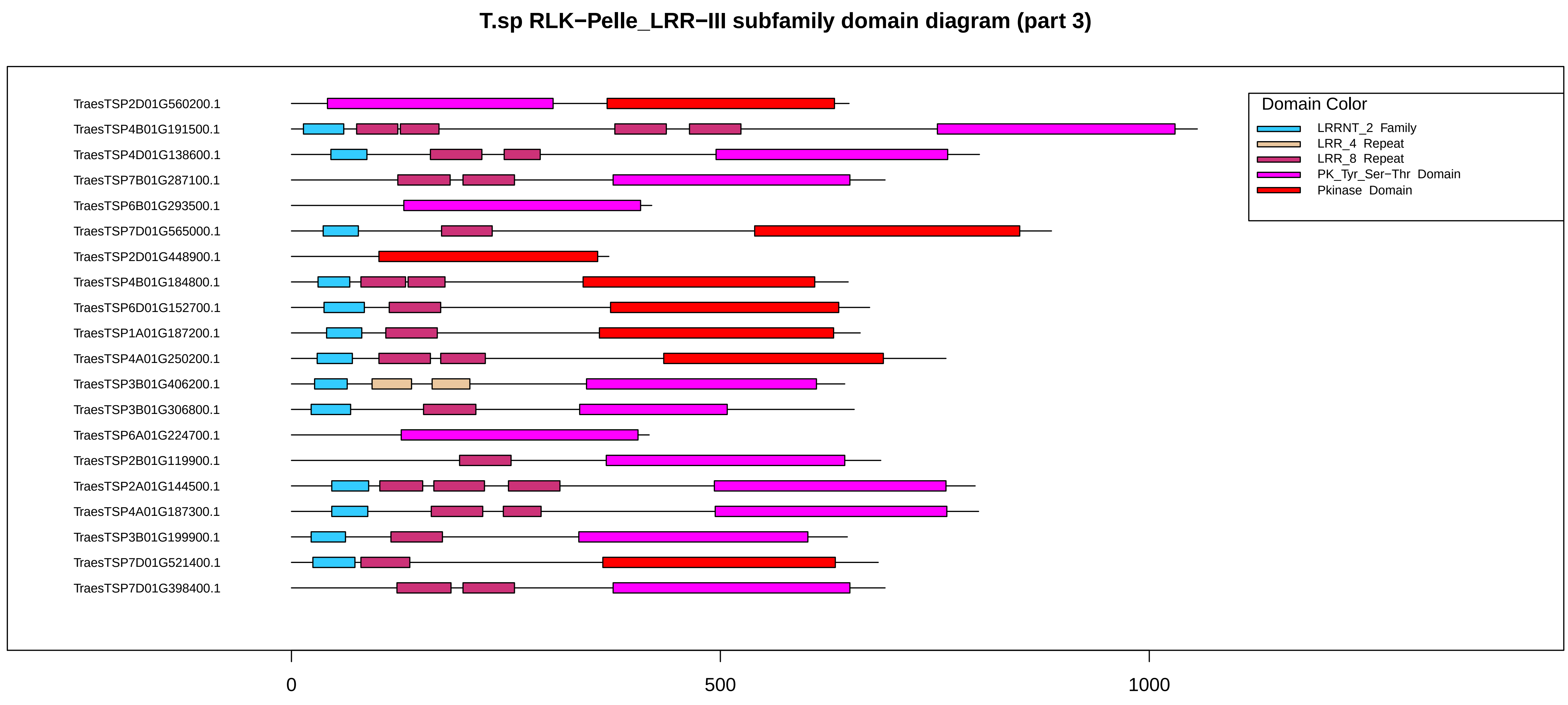
T.sp RLK−Pelle_LRR−III subfamily domain diagram (part 3)
TraesTSP2D01G560200.1
TraesTSP4B01G191500.1
TraesTSP4D01G138600.1
TraesTSP7B01G287100.1
TraesTSP6B01G293500.1
TraesTSP7D01G565000.1
TraesTSP2D01G448900.1
TraesTSP4B01G184800.1
TraesTSP6D01G152700.1
TraesTSP1A01G187200.1
TraesTSP4A01G250200.1
TraesTSP3B01G406200.1
TraesTSP3B01G306800.1
TraesTSP6A01G224700.1
TraesTSP2B01G119900.1
TraesTSP2A01G144500.1
TraesTSP4A01G187300.1
TraesTSP3B01G199900.1
TraesTSP7D01G521400.1
TraesTSP7D01G398400.1
Domain Color
LRRNT_2 Family
LRR_4 Repeat
LRR_8 Repeat
PK_Tyr_Ser−Thr Domain
Pkinase Domain
0
500
1000

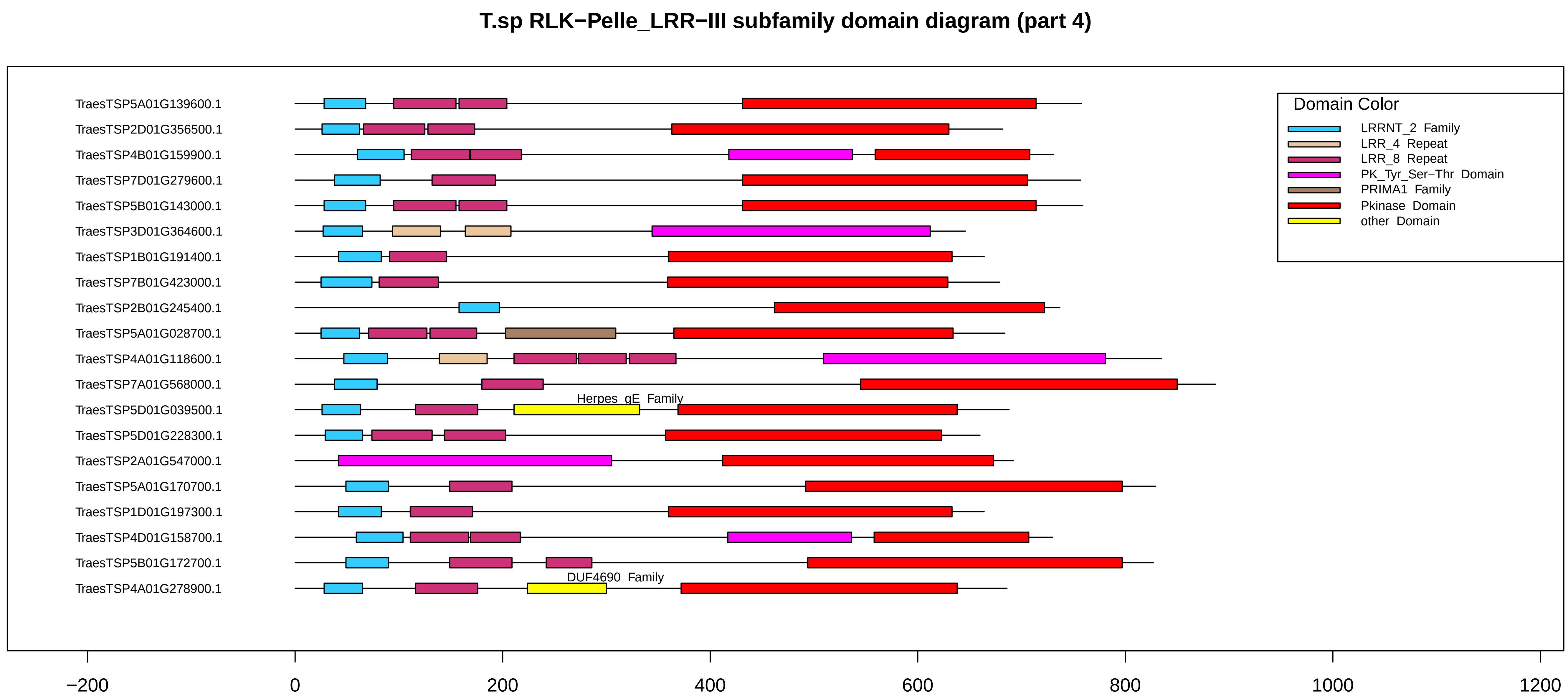

T.sp RLK−Pelle_LRR−III subfamily domain diagram (part 4)
TraesTSP5A01G139600.1
TraesTSP2D01G356500.1
TraesTSP4B01G159900.1
TraesTSP7D01G279600.1
TraesTSP5B01G143000.1
TraesTSP3D01G364600.1
TraesTSP1B01G191400.1
TraesTSP7B01G423000.1
TraesTSP2B01G245400.1
TraesTSP5A01G028700.1
TraesTSP4A01G118600.1
TraesTSP7A01G568000.1
TraesTSP5D01G039500.1
TraesTSP5D01G228300.1
TraesTSP2A01G547000.1
TraesTSP5A01G170700.1
TraesTSP1D01G197300.1
TraesTSP4D01G158700.1
TraesTSP5B01G172700.1
TraesTSP4A01G278900.1
Herpes_gE Family
DUF4690 Family
Domain Color
LRRNT_2 Family
LRR_4 Repeat
LRR_8 Repeat
PK_Tyr_Ser−Thr Domain
PRIMA1 Family
Pkinase Domain
other Domain
−200
0
200
400
600
800
1000
1200

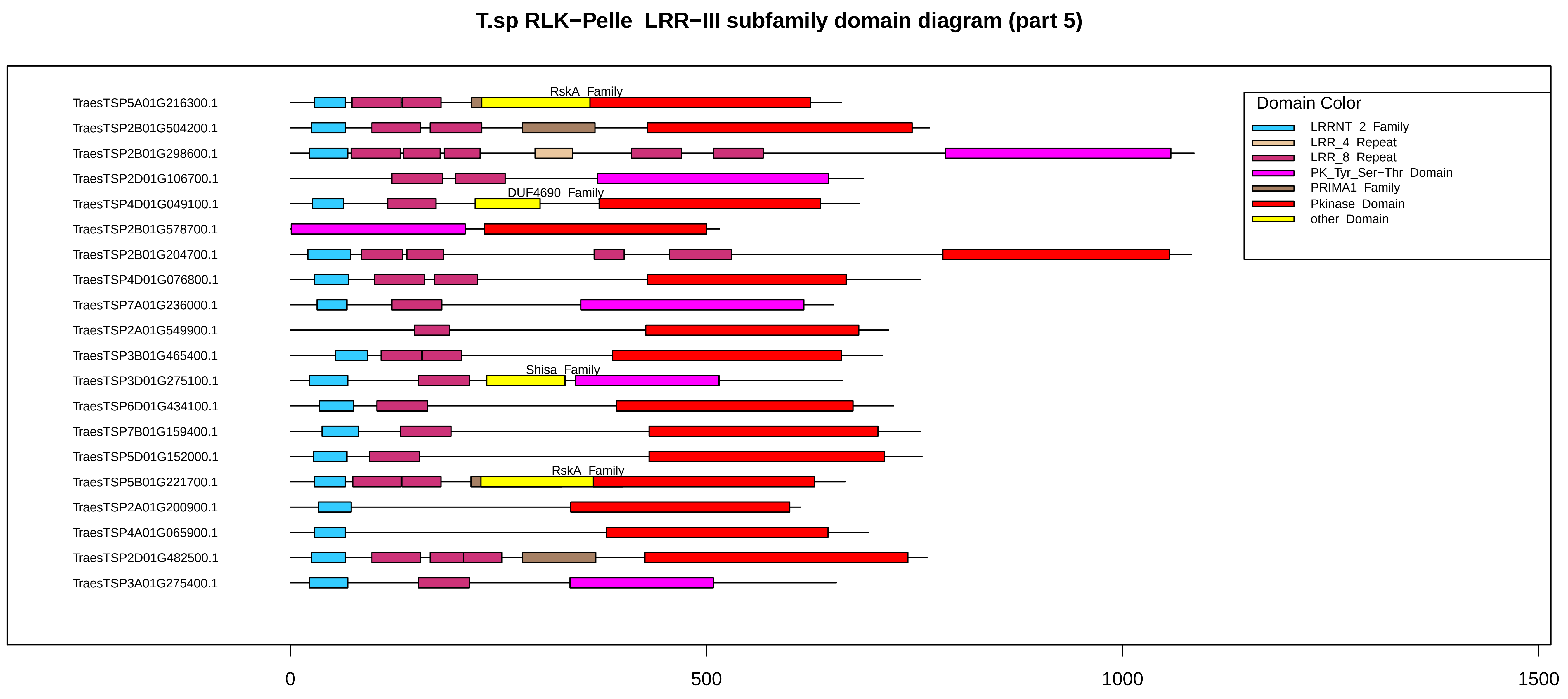

T.sp RLK−Pelle_LRR−III subfamily domain diagram (part 5)
TraesTSP5A01G216300.1
TraesTSP2B01G504200.1
TraesTSP2B01G298600.1
TraesTSP2D01G106700.1
TraesTSP4D01G049100.1
TraesTSP2B01G578700.1
TraesTSP2B01G204700.1
TraesTSP4D01G076800.1
TraesTSP7A01G236000.1
TraesTSP2A01G549900.1
TraesTSP3B01G465400.1
TraesTSP3D01G275100.1
TraesTSP6D01G434100.1
TraesTSP7B01G159400.1
TraesTSP5D01G152000.1
TraesTSP5B01G221700.1
TraesTSP2A01G200900.1
TraesTSP4A01G065900.1
TraesTSP2D01G482500.1
TraesTSP3A01G275400.1
RskA Family
DUF4690 Family
Shisa Family
RskA Family
Domain Color
LRRNT_2 Family
LRR_4 Repeat
LRR_8 Repeat
PK_Tyr_Ser−Thr Domain
PRIMA1 Family
Pkinase Domain
other Domain
0
500
1000
1500

T.sp RLK−Pelle_LRR−III subfamily domain diagram (part 6)

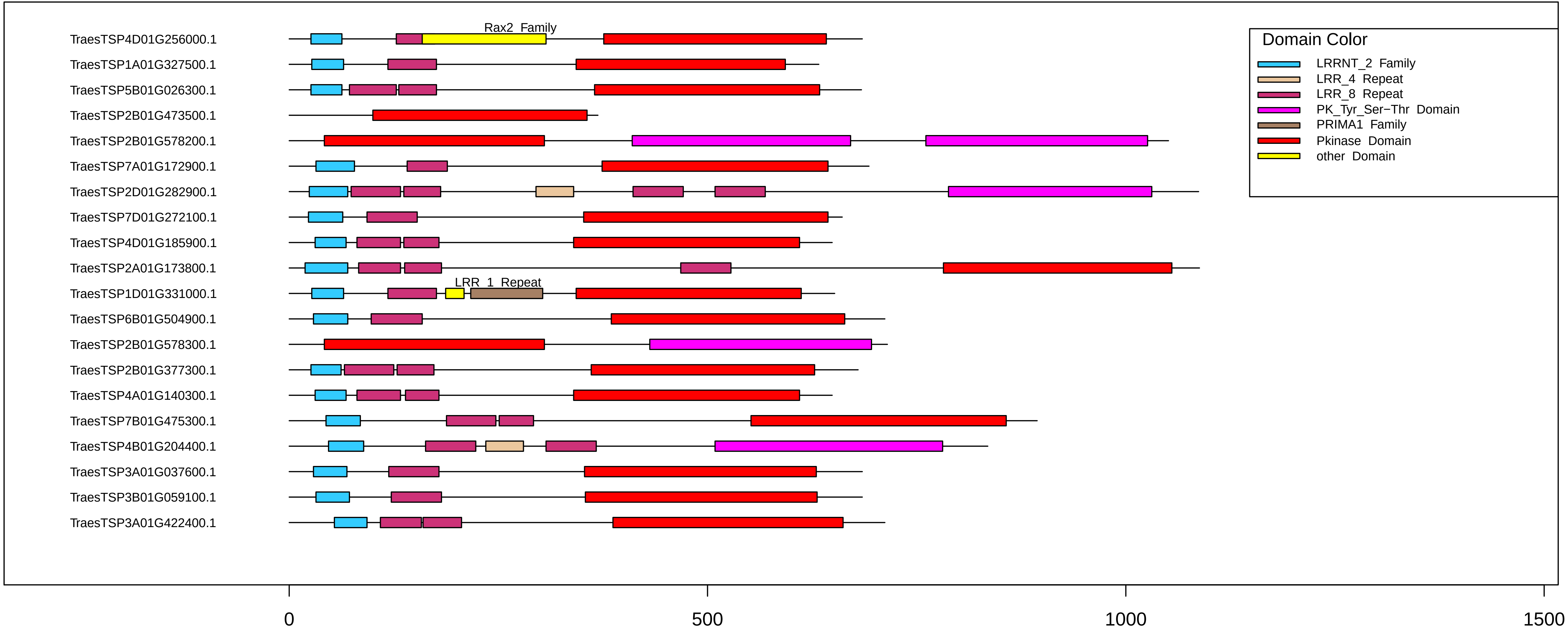

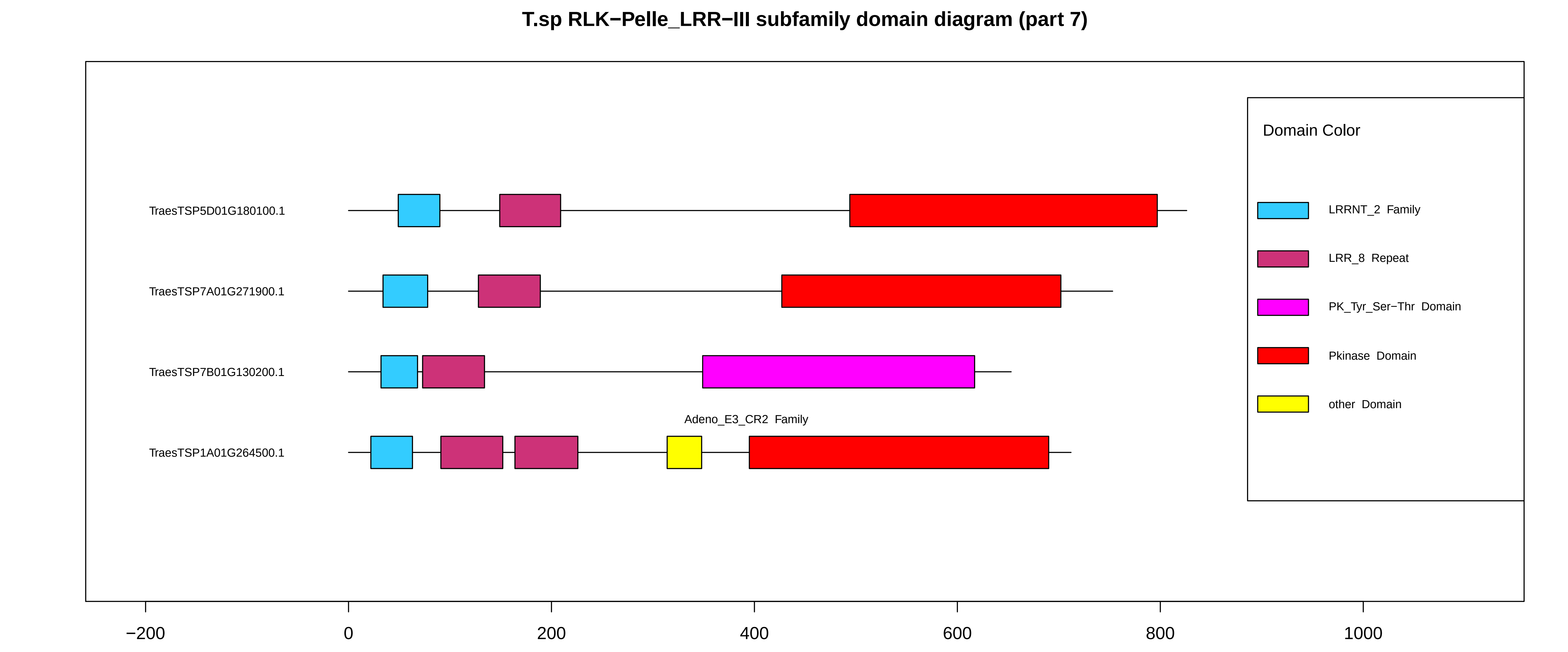
T.sp RLK−Pelle_LRR−III subfamily domain diagram (part 7)
TraesTSP5D01G180100.1
TraesTSP7A01G271900.1
TraesTSP7B01G130200.1
TraesTSP1A01G264500.1
Adeno_E3_CR2 Family
Domain Color
LRRNT_2 Family
LRR_8 Repeat
PK_Tyr_Ser−Thr Domain
Pkinase Domain
other Domain
−200
0
200
400
600
800
1000

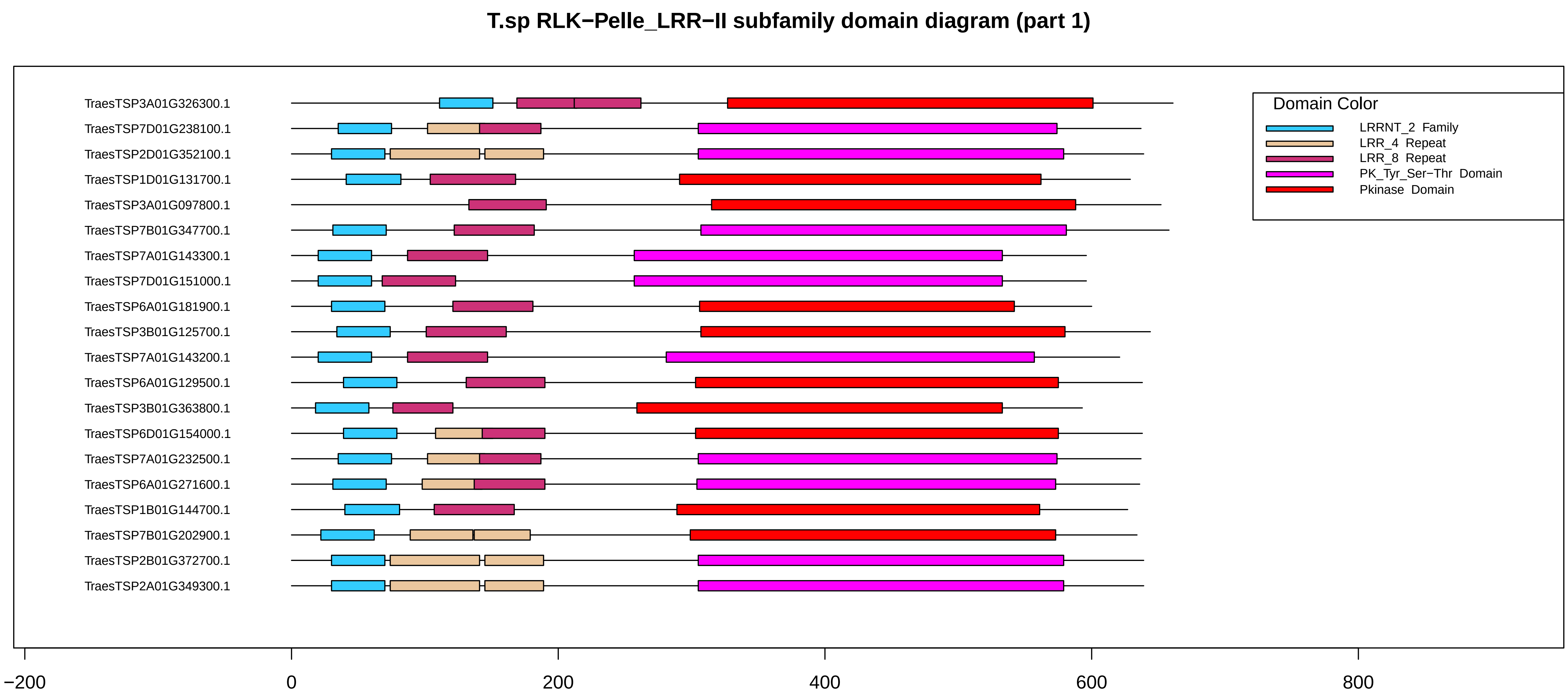
T.sp RLK−Pelle_LRR−II subfamily domain diagram (part 1)
TraesTSP3A01G326300.1
TraesTSP7D01G238100.1
TraesTSP2D01G352100.1
TraesTSP1D01G131700.1
TraesTSP3A01G097800.1
TraesTSP7B01G347700.1
TraesTSP7A01G143300.1
TraesTSP7D01G151000.1
TraesTSP6A01G181900.1
TraesTSP3B01G125700.1
TraesTSP7A01G143200.1
TraesTSP6A01G129500.1
TraesTSP3B01G363800.1
TraesTSP6D01G154000.1
TraesTSP7A01G232500.1
TraesTSP6A01G271600.1
TraesTSP1B01G144700.1
TraesTSP7B01G202900.1
TraesTSP2B01G372700.1
TraesTSP2A01G349300.1
Domain Color
LRRNT_2 Family
LRR_4 Repeat
LRR_8 Repeat
PK_Tyr_Ser−Thr Domain
Pkinase Domain
−200
0
200
400
600
800

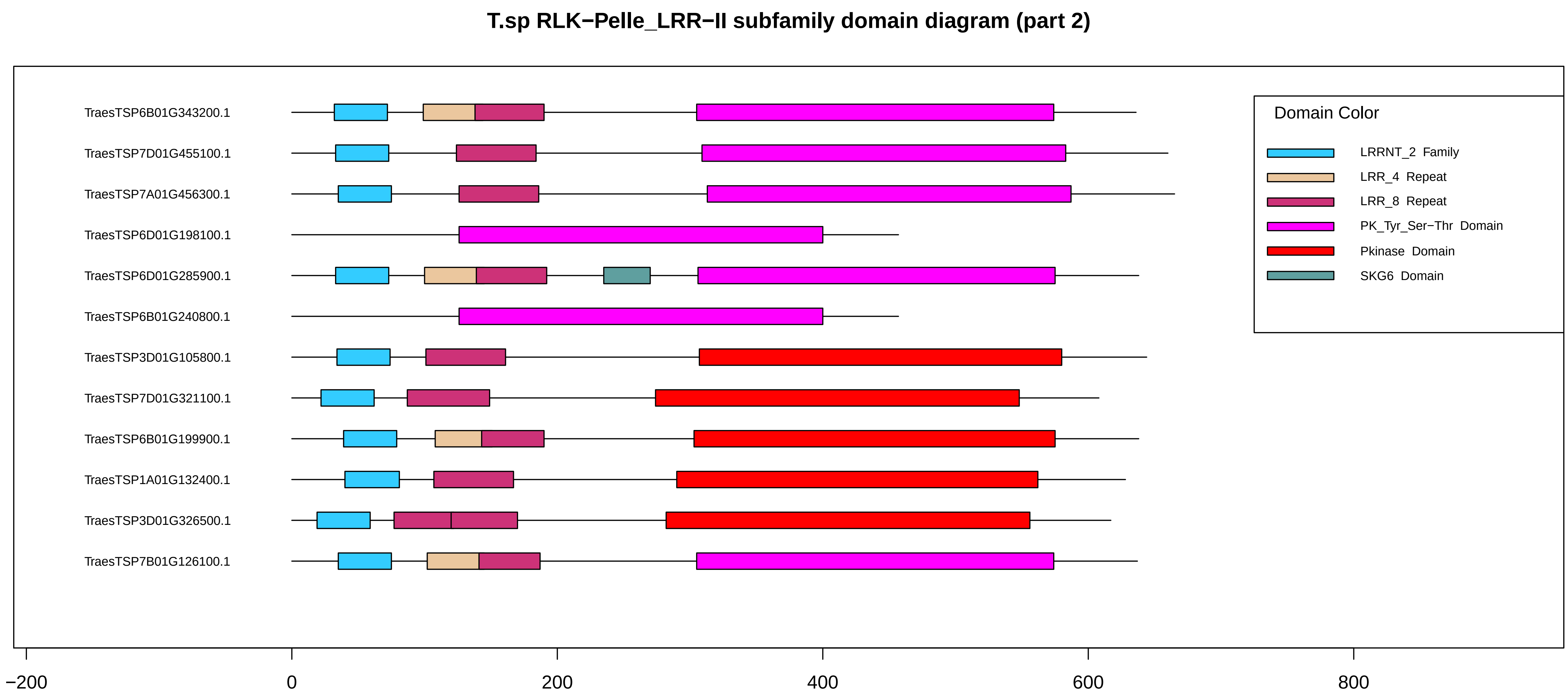

T.sp RLK−Pelle_LRR−II subfamily domain diagram (part 2)
TraesTSP6B01G343200.1
TraesTSP7D01G455100.1
TraesTSP7A01G456300.1
TraesTSP6D01G198100.1
TraesTSP6D01G285900.1
TraesTSP6B01G240800.1
TraesTSP3D01G105800.1
TraesTSP7D01G321100.1
TraesTSP6B01G199900.1
TraesTSP1A01G132400.1
TraesTSP3D01G326500.1
TraesTSP7B01G126100.1
Domain Color
LRRNT_2 Family
LRR_4 Repeat
LRR_8 Repeat
PK_Tyr_Ser−Thr Domain
Pkinase Domain
SKG6 Domain
−200
0
200
400
600
800

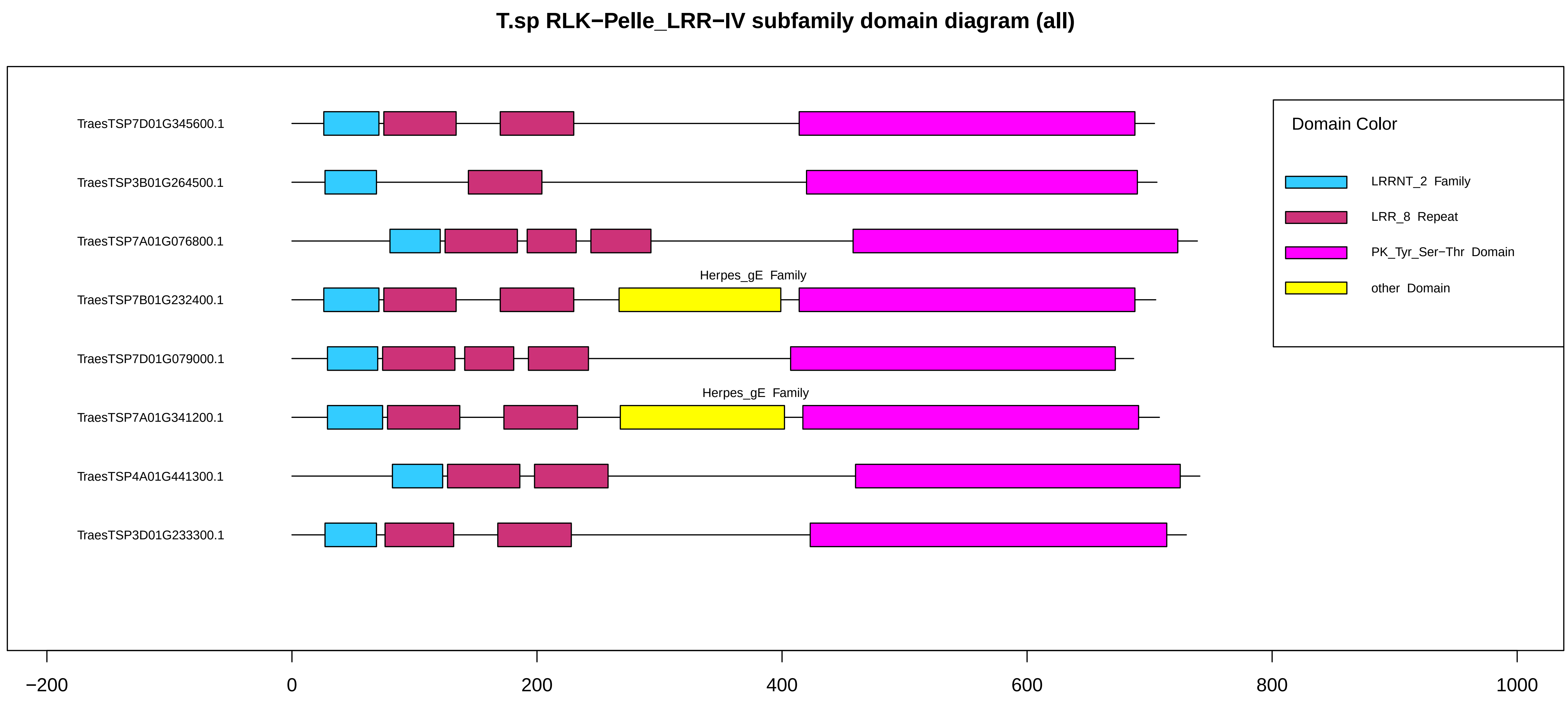

T.sp RLK−Pelle_LRR−IV subfamily domain diagram (all)
TraesTSP7D01G345600.1
TraesTSP3B01G264500.1
TraesTSP7A01G076800.1
TraesTSP7B01G232400.1
TraesTSP7D01G079000.1
TraesTSP7A01G341200.1
TraesTSP4A01G441300.1
TraesTSP3D01G233300.1
Herpes_gE Family
Herpes_gE Family
Domain Color
LRRNT_2 Family
LRR_8 Repeat
PK_Tyr_Ser−Thr Domain
other Domain
−200
0
200
400
600
800
1000

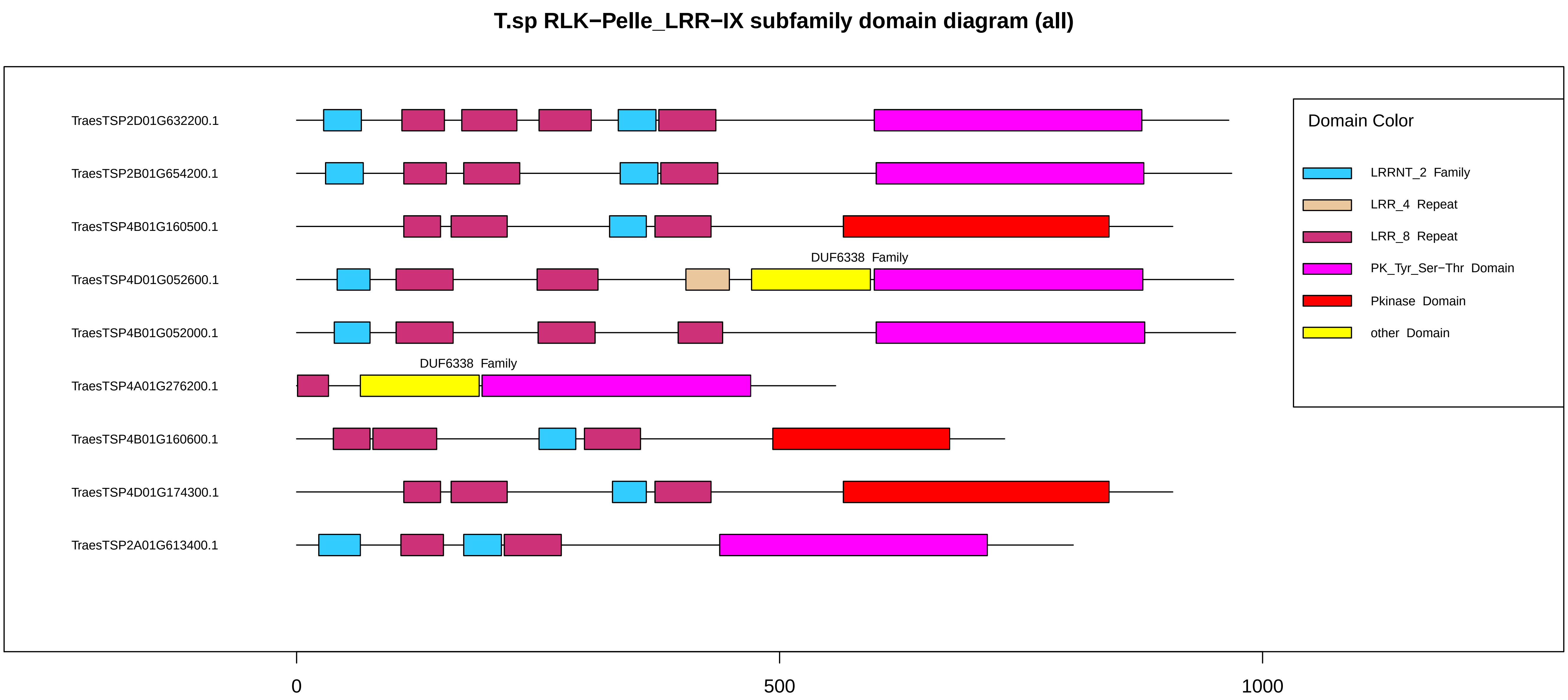

T.sp RLK−Pelle_LRR−IX subfamily domain diagram (all)
TraesTSP2D01G632200.1
TraesTSP2B01G654200.1
TraesTSP4B01G160500.1
TraesTSP4D01G052600.1
TraesTSP4B01G052000.1
TraesTSP4A01G276200.1
TraesTSP4B01G160600.1
TraesTSP4D01G174300.1
TraesTSP2A01G613400.1
DUF6338 Family
DUF6338 Family
Domain Color
LRRNT_2 Family
LRR_4 Repeat
LRR_8 Repeat
PK_Tyr_Ser−Thr Domain
Pkinase Domain
other Domain
0
500
1000

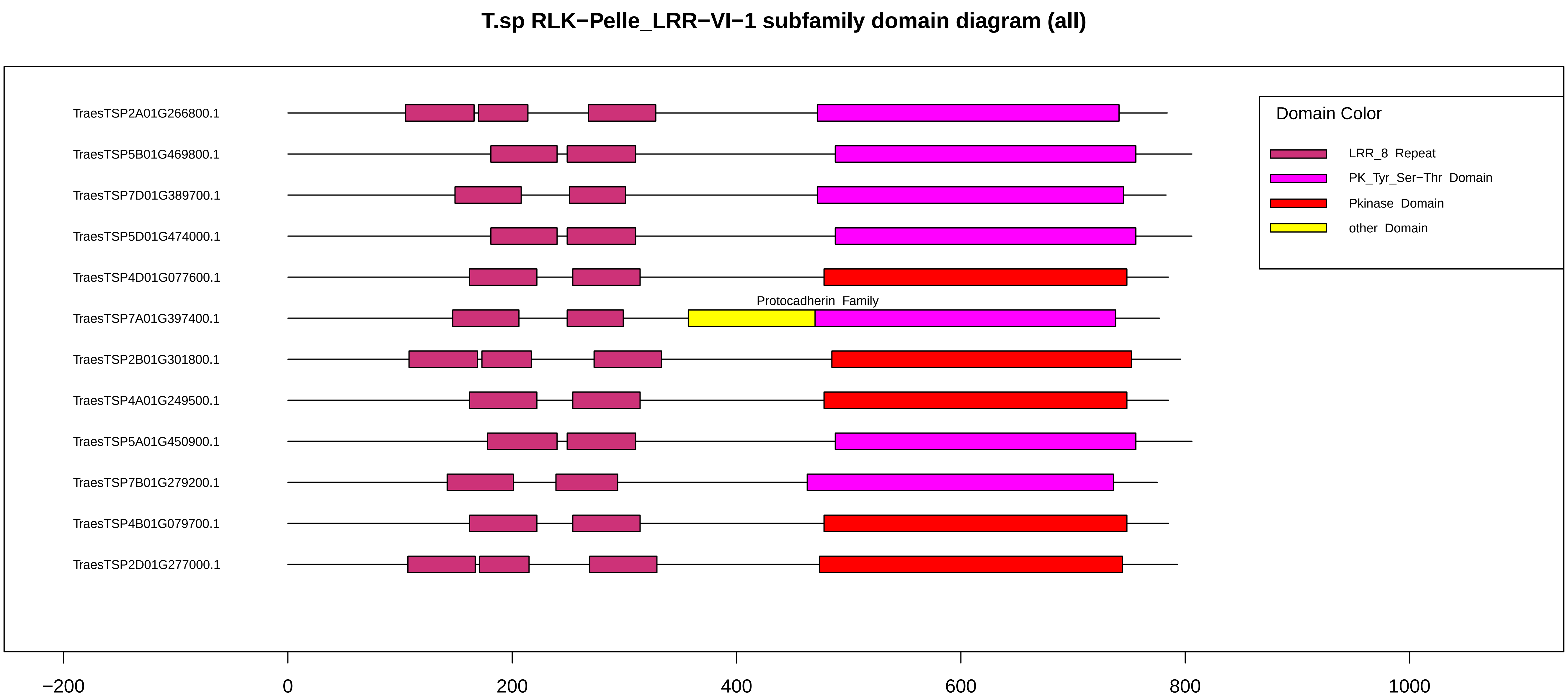
T.sp RLK−Pelle_LRR−VI−1 subfamily domain diagram (all)
TraesTSP2A01G266800.1
TraesTSP5B01G469800.1
TraesTSP7D01G389700.1
TraesTSP5D01G474000.1
TraesTSP4D01G077600.1
TraesTSP7A01G397400.1
TraesTSP2B01G301800.1
TraesTSP4A01G249500.1
TraesTSP5A01G450900.1
TraesTSP7B01G279200.1
TraesTSP4B01G079700.1
TraesTSP2D01G277000.1
Protocadherin Family
Domain Color
LRR_8 Repeat
PK_Tyr_Ser−Thr Domain
Pkinase Domain
other Domain
−200
0
200
400
600
800
1000

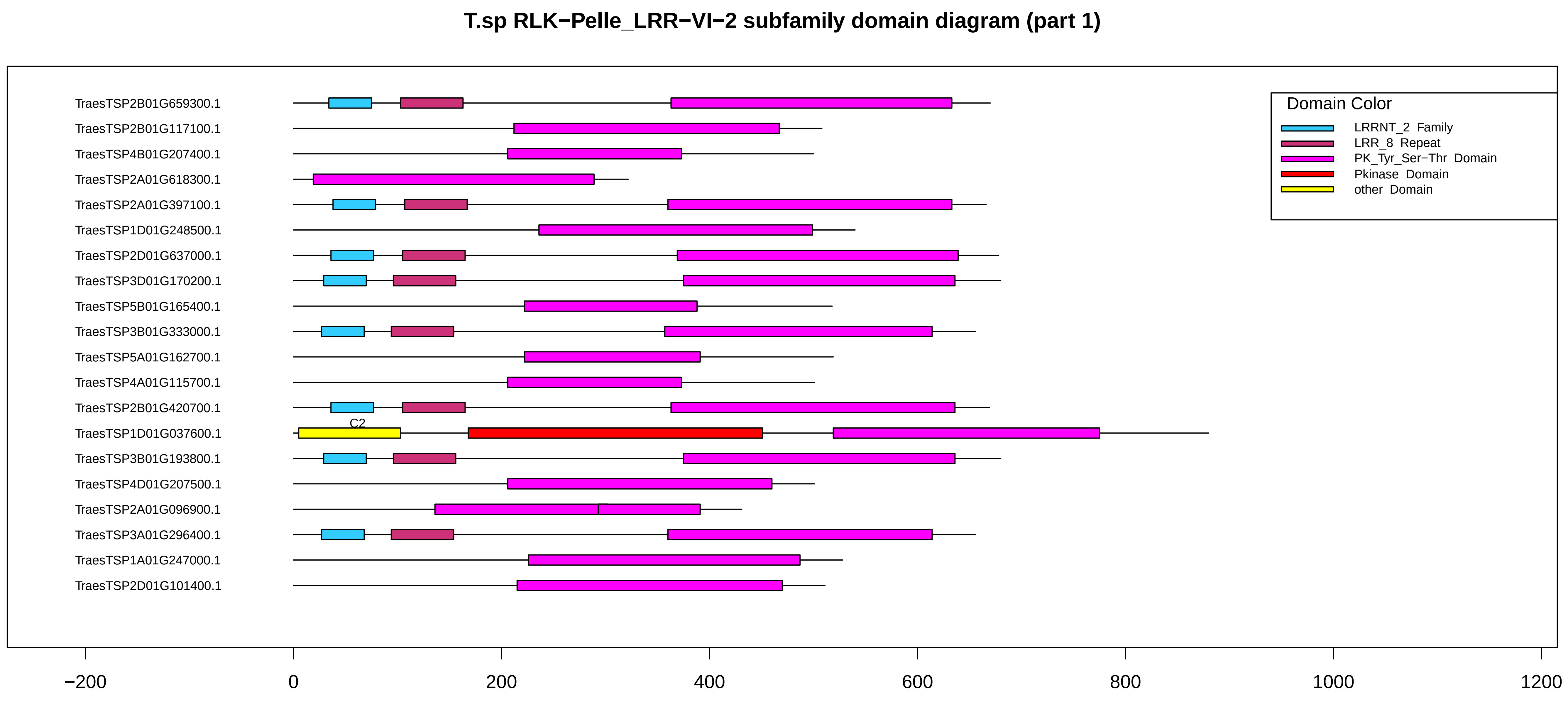
T.sp RLK−Pelle_LRR−VI−2 subfamily domain diagram (part 1)
TraesTSP2B01G659300.1
TraesTSP2B01G117100.1
TraesTSP4B01G207400.1
TraesTSP2A01G618300.1
TraesTSP2A01G397100.1
TraesTSP1D01G248500.1
TraesTSP2D01G637000.1
TraesTSP3D01G170200.1
TraesTSP5B01G165400.1
TraesTSP3B01G333000.1
TraesTSP5A01G162700.1
TraesTSP4A01G115700.1
TraesTSP2B01G420700.1
TraesTSP1D01G037600.1
C2
TraesTSP3B01G193800.1
TraesTSP4D01G207500.1
TraesTSP2A01G096900.1
TraesTSP3A01G296400.1
TraesTSP1A01G247000.1
TraesTSP2D01G101400.1
Domain Color
LRRNT_2 Family
LRR_8 Repeat
PK_Tyr_Ser−Thr Domain
Pkinase Domain
other Domain
−200
0
200
400
600
800
1000
1200

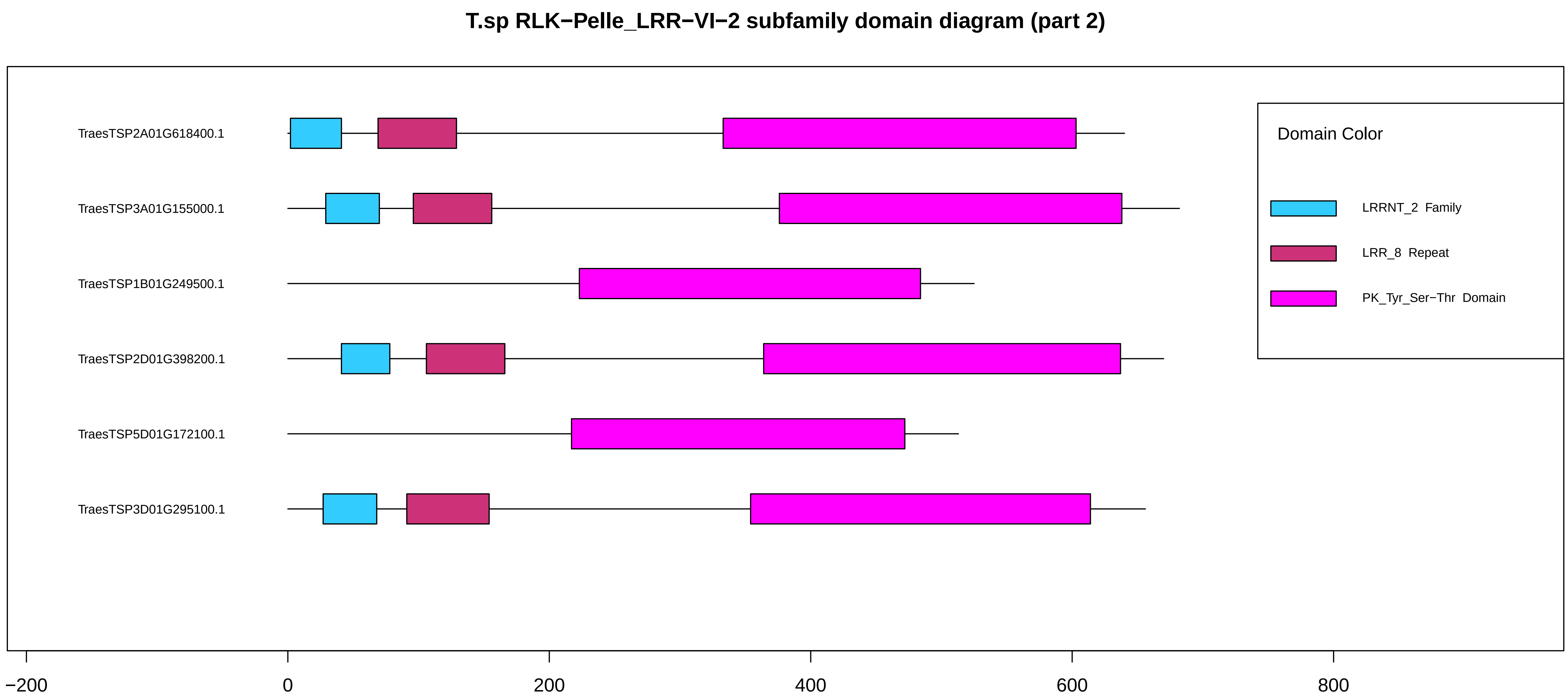

T.sp RLK−Pelle_LRR−VI−2 subfamily domain diagram (part 2)
TraesTSP2A01G618400.1
TraesTSP3A01G155000.1
TraesTSP1B01G249500.1
TraesTSP2D01G398200.1
TraesTSP5D01G172100.1
TraesTSP3D01G295100.1
Domain Color
LRRNT_2 Family
LRR_8 Repeat
PK_Tyr_Ser−Thr Domain
−200
0
200
400
600
800

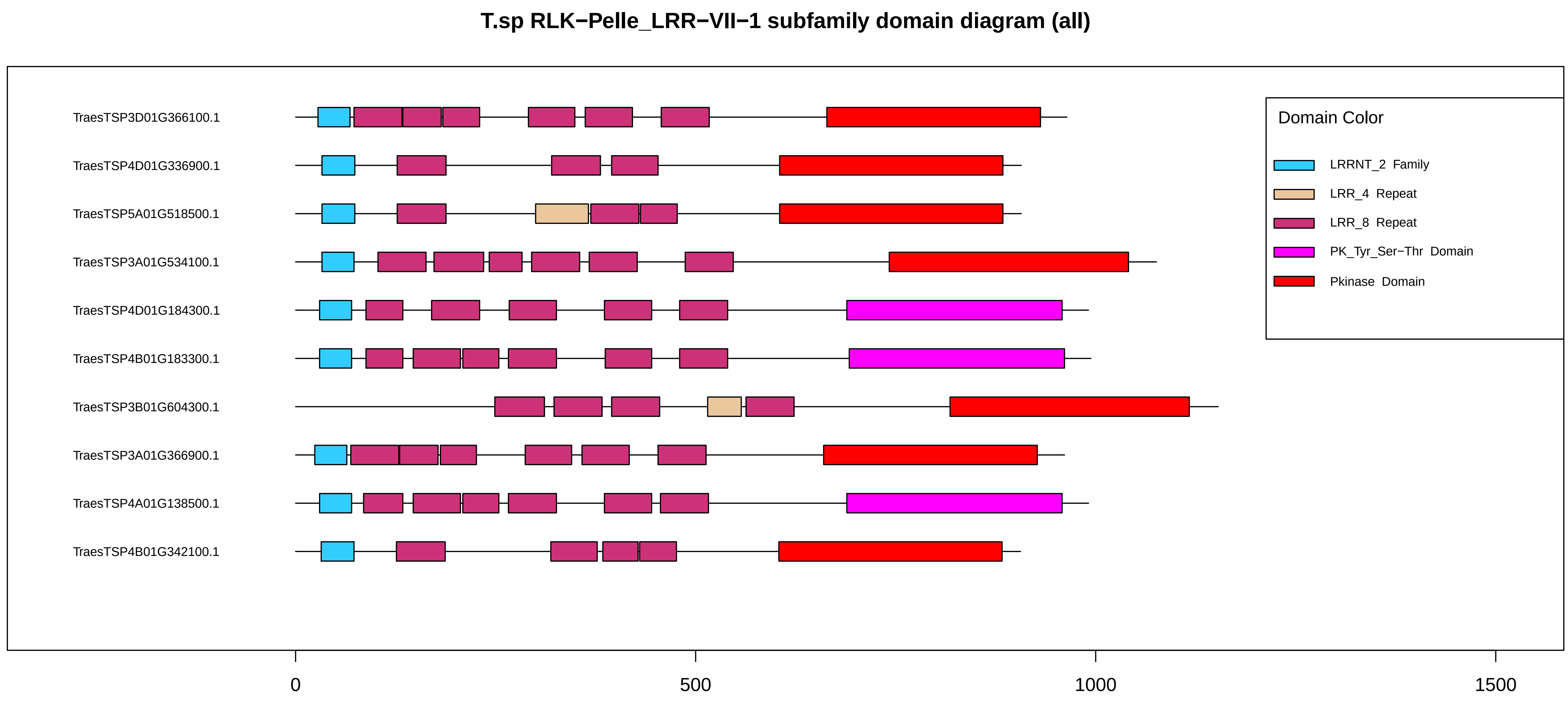
T.sp RLK−Pelle_LRR−VII−1 subfamily domain diagram (all)
TraesTSP3D01G366100.1
TraesTSP4D01G336900.1
TraesTSP5A01G518500.1
TraesTSP3A01G534100.1
TraesTSP4D01G184300.1
TraesTSP4B01G183300.1
TraesTSP3B01G604300.1
TraesTSP3A01G366900.1
TraesTSP4A01G138500.1
TraesTSP4B01G342100.1
Domain Color
LRRNT_2 Family
LRR_4 Repeat
LRR_8 Repeat
PK_Tyr_Ser−Thr Domain
Pkinase Domain
0
500
1000
1500

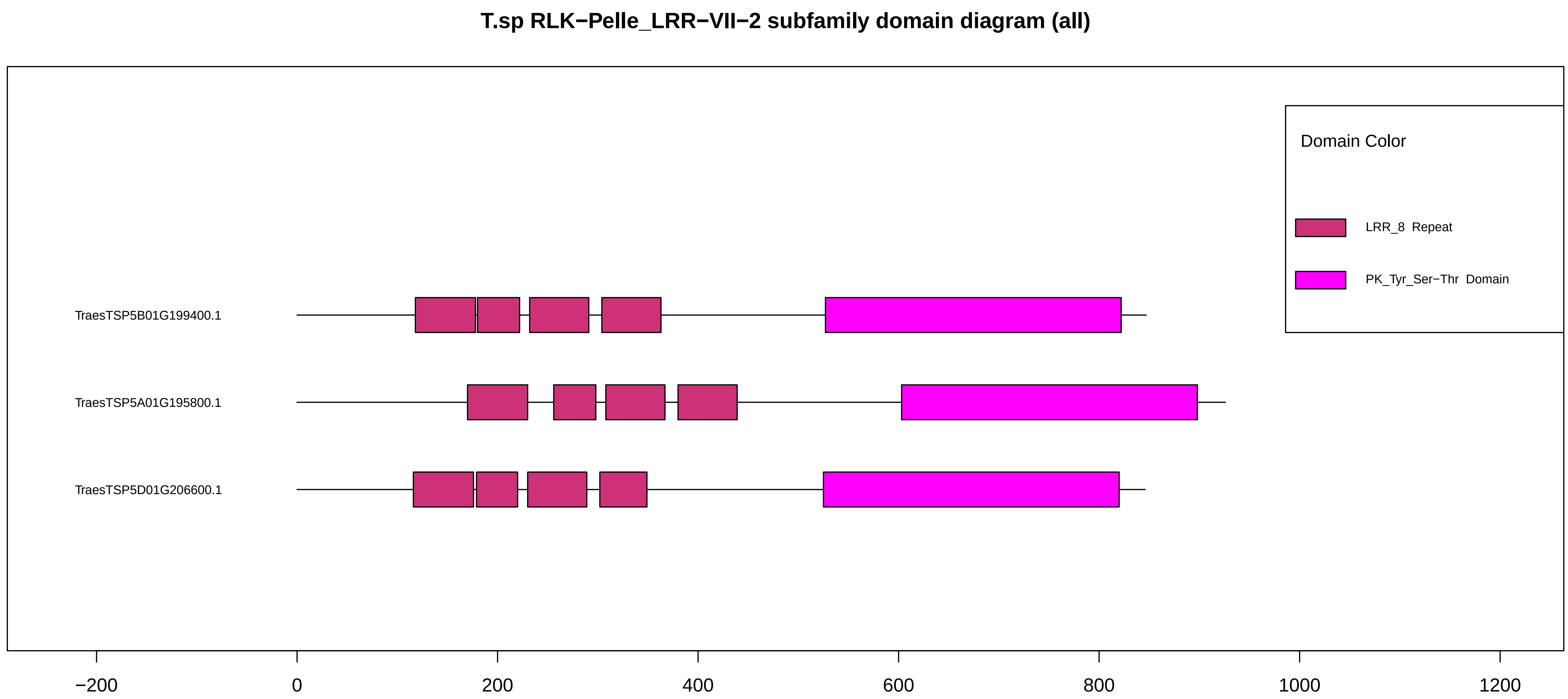

T.sp RLK−Pelle_LRR−VII−2 subfamily domain diagram (all)
Domain Color
LRR_8 Repeat
PK_Tyr_Ser−Thr Domain
TraesTSP5B01G199400.1
TraesTSP5A01G195800.1
TraesTSP5D01G206600.1
−200
0
200
400
600
800
1000
1200

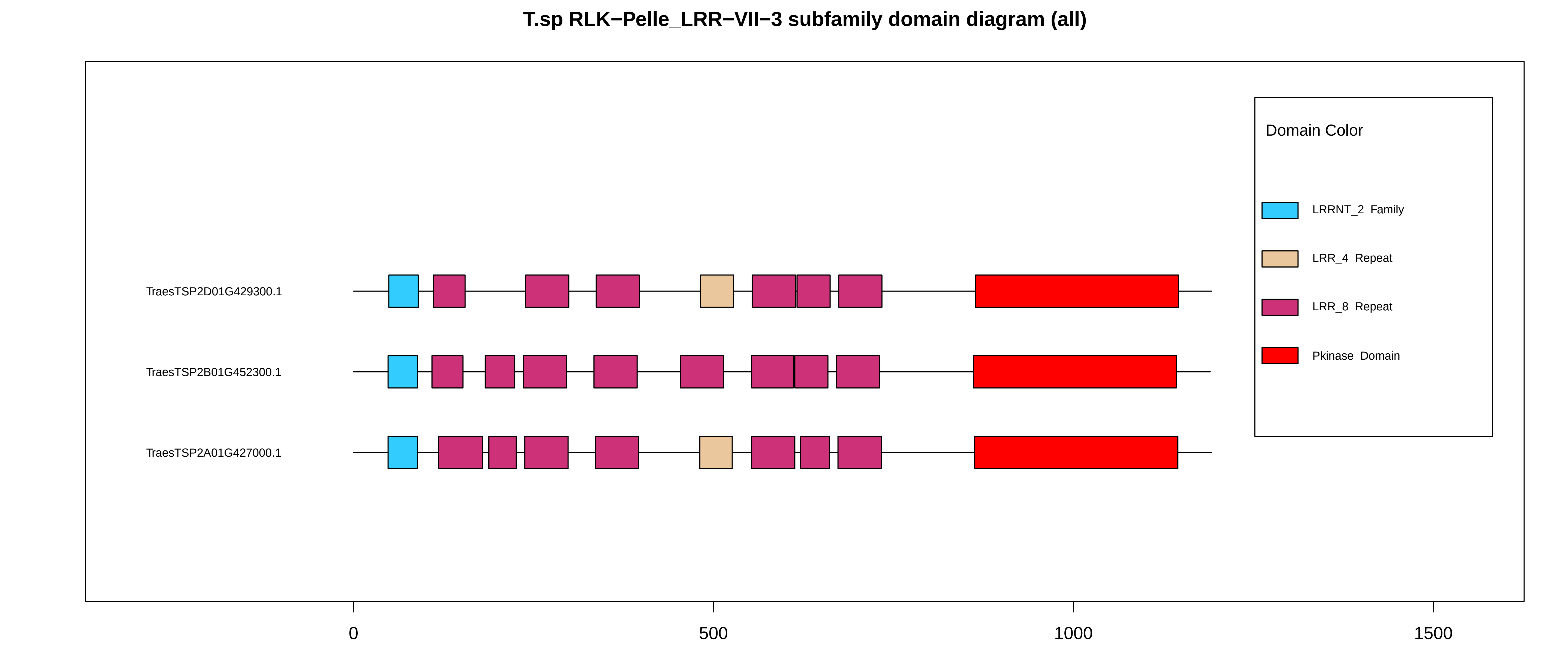

T.sp RLK−Pelle_LRR−VII−3 subfamily domain diagram (all)
TraesTSP2D01G429300.1
TraesTSP2B01G452300.1
TraesTSP2A01G427000.1
Domain Color
LRRNT_2 Family
LRR_4 Repeat
LRR_8 Repeat
Pkinase Domain
0
500
1000
1500

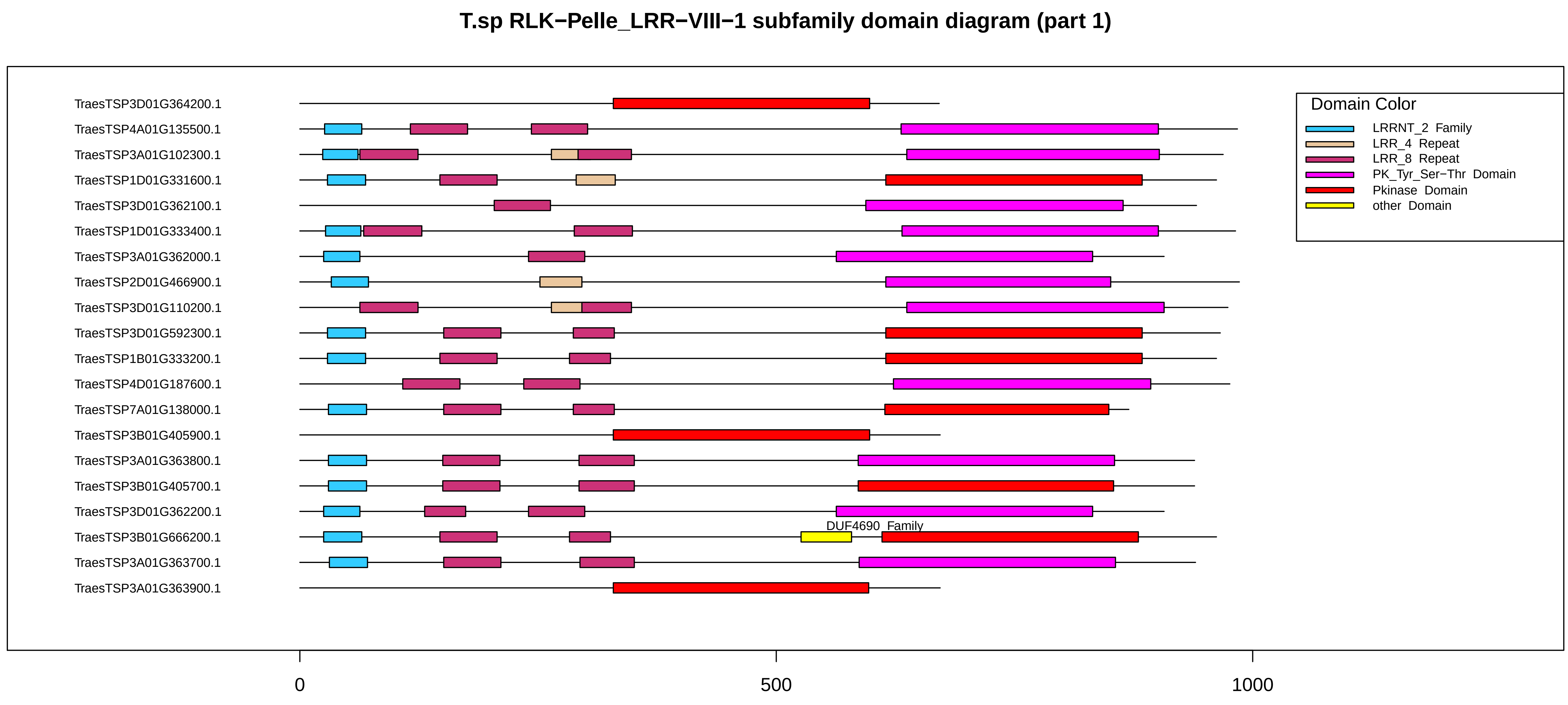

T.sp RLK−Pelle_LRR−VIII−1 subfamily domain diagram (part 1)
TraesTSP3D01G364200.1
TraesTSP4A01G135500.1
TraesTSP3A01G102300.1
TraesTSP1D01G331600.1
TraesTSP3D01G362100.1
TraesTSP1D01G333400.1
TraesTSP3A01G362000.1
TraesTSP2D01G466900.1
TraesTSP3D01G110200.1
TraesTSP3D01G592300.1
TraesTSP1B01G333200.1
TraesTSP4D01G187600.1
TraesTSP7A01G138000.1
TraesTSP3B01G405900.1
TraesTSP3A01G363800.1
TraesTSP3B01G405700.1
TraesTSP3D01G362200.1
TraesTSP3B01G666200.1
TraesTSP3A01G363700.1
TraesTSP3A01G363900.1
DUF4690 Family
Domain Color
LRRNT_2 Family
LRR_4 Repeat
LRR_8 Repeat
PK_Tyr_Ser−Thr Domain
Pkinase Domain
other Domain
0
500
1000

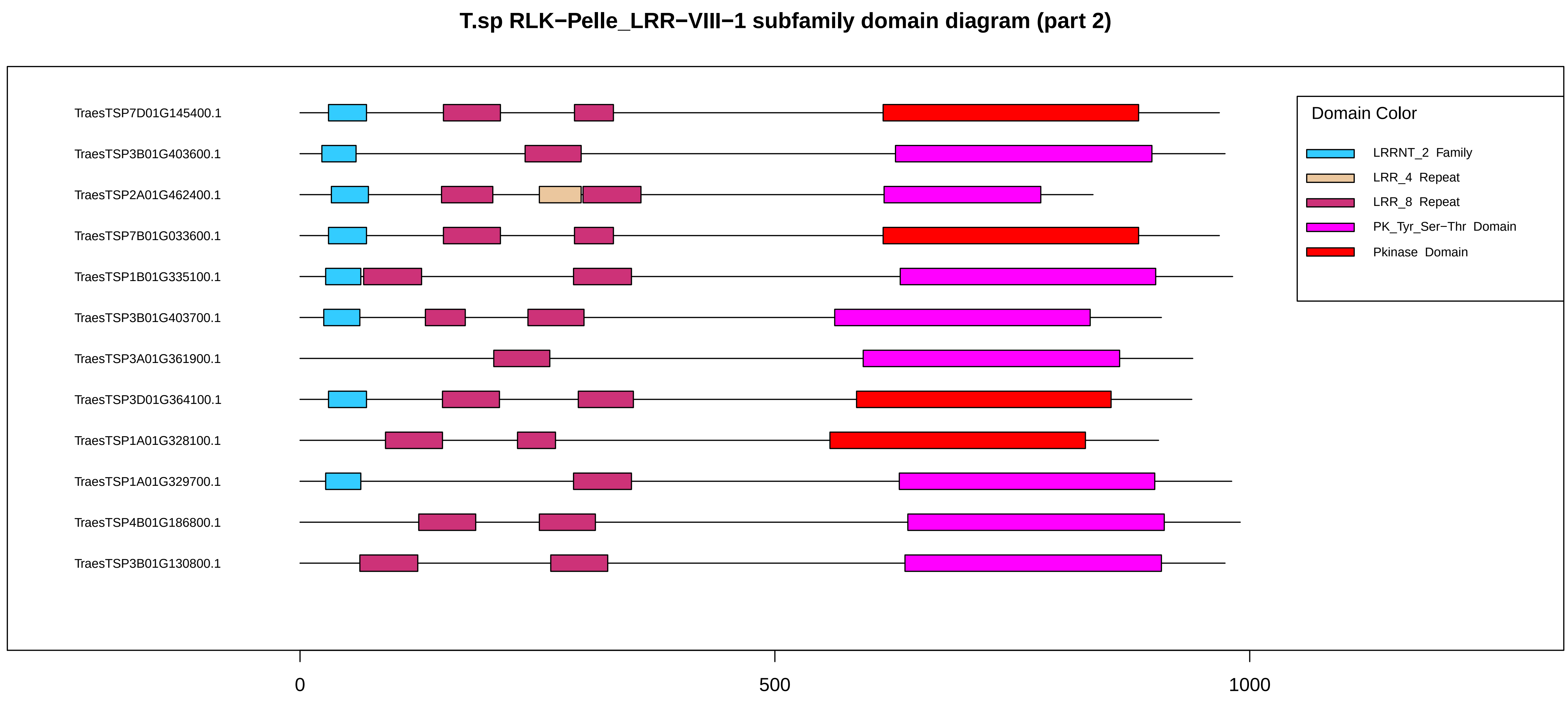
T.sp RLK−Pelle_LRR−VIII−1 subfamily domain diagram (part 2)
TraesTSP7D01G145400.1
TraesTSP3B01G403600.1
TraesTSP2A01G462400.1
TraesTSP7B01G033600.1
TraesTSP1B01G335100.1
TraesTSP3B01G403700.1
TraesTSP3A01G361900.1
TraesTSP3D01G364100.1
TraesTSP1A01G328100.1
TraesTSP1A01G329700.1
TraesTSP4B01G186800.1
TraesTSP3B01G130800.1
Domain Color
LRRNT_2 Family
LRR_4 Repeat
LRR_8 Repeat
PK_Tyr_Ser−Thr Domain
Pkinase Domain
0
500
1000

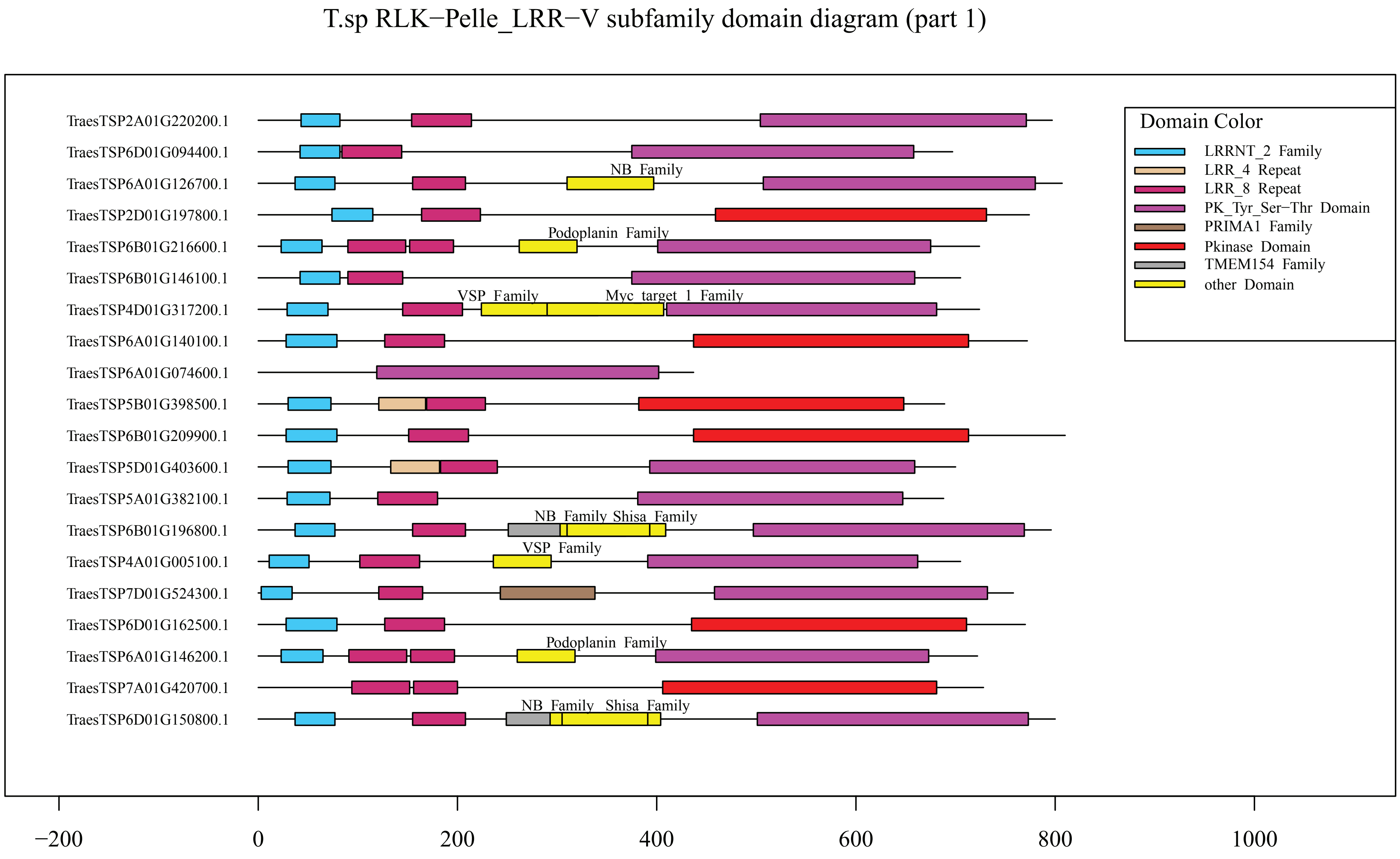
T.sp RLK−Pelle_LRR−V subfamily domain diagram (part 1)
TraesTSP2A01G220200.1
TraesTSP6D01G094400.1
TraesTSP6A01G126700.1
TraesTSP2D01G197800.1
TraesTSP6B01G216600.1
TraesTSP6B01G146100.1
TraesTSP4D01G317200.1
TraesTSP6A01G140100.1
TraesTSP6A01G074600.1
TraesTSP5B01G398500.1
TraesTSP6B01G209900.1
TraesTSP5D01G403600.1
TraesTSP5A01G382100.1
TraesTSP6B01G196800.1
TraesTSP4A01G005100.1
TraesTSP7D01G524300.1
TraesTSP6D01G162500.1
TraesTSP6A01G146200.1
TraesTSP7A01G420700.1
TraesTSP6D01G150800.1
NB_Family
Podoplanin_Family
VSP_Family
Myc_target_1_Family
NB_Family Shisa_Family
VSP_Family
Podoplanin_Family
NB_Family Shisa_Family
Domain Color
LRRNT_2_Family
LRR_4_Repeat
LRR_8_Repeat
PK_Tyr_Ser−Thr_Domain
PRIMA1_Family
Pkinase_Domain
TMEM154_Family
other_Domain
−200
0
200
400
600
800
1000

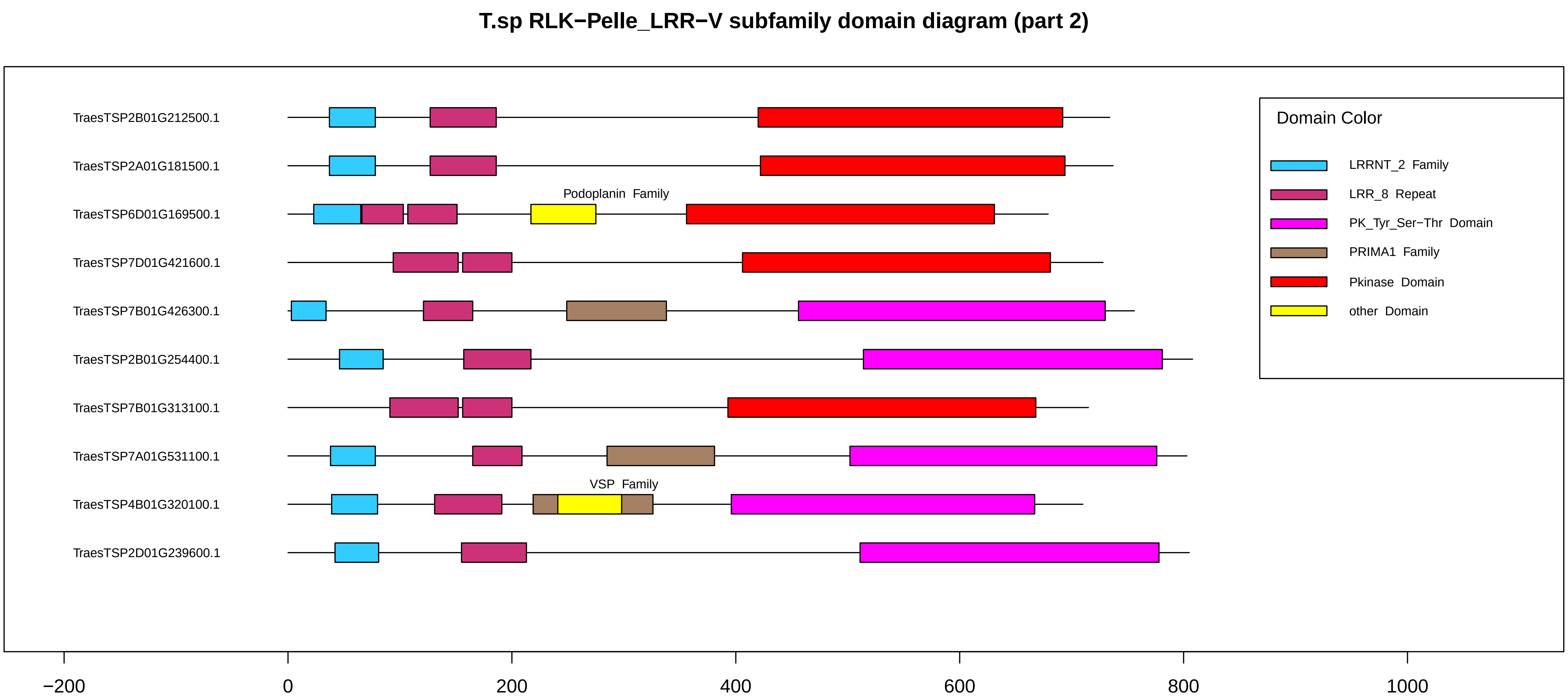
T.sp RLK−Pelle_LRR−V subfamily domain diagram (part 2)
TraesTSP2B01G212500.1
TraesTSP2A01G181500.1
TraesTSP6D01G169500.1
TraesTSP7D01G421600.1
TraesTSP7B01G426300.1
TraesTSP2B01G254400.1
TraesTSP7B01G313100.1
TraesTSP7A01G531100.1
TraesTSP4B01G320100.1
TraesTSP2D01G239600.1
Podoplanin Family
VSP Family
Domain Color
LRRNT_2 Family
LRR_8 Repeat
PK_Tyr_Ser−Thr Domain
PRIMA1 Family
Pkinase Domain
other Domain
−200
0
200
400
600
800
1000

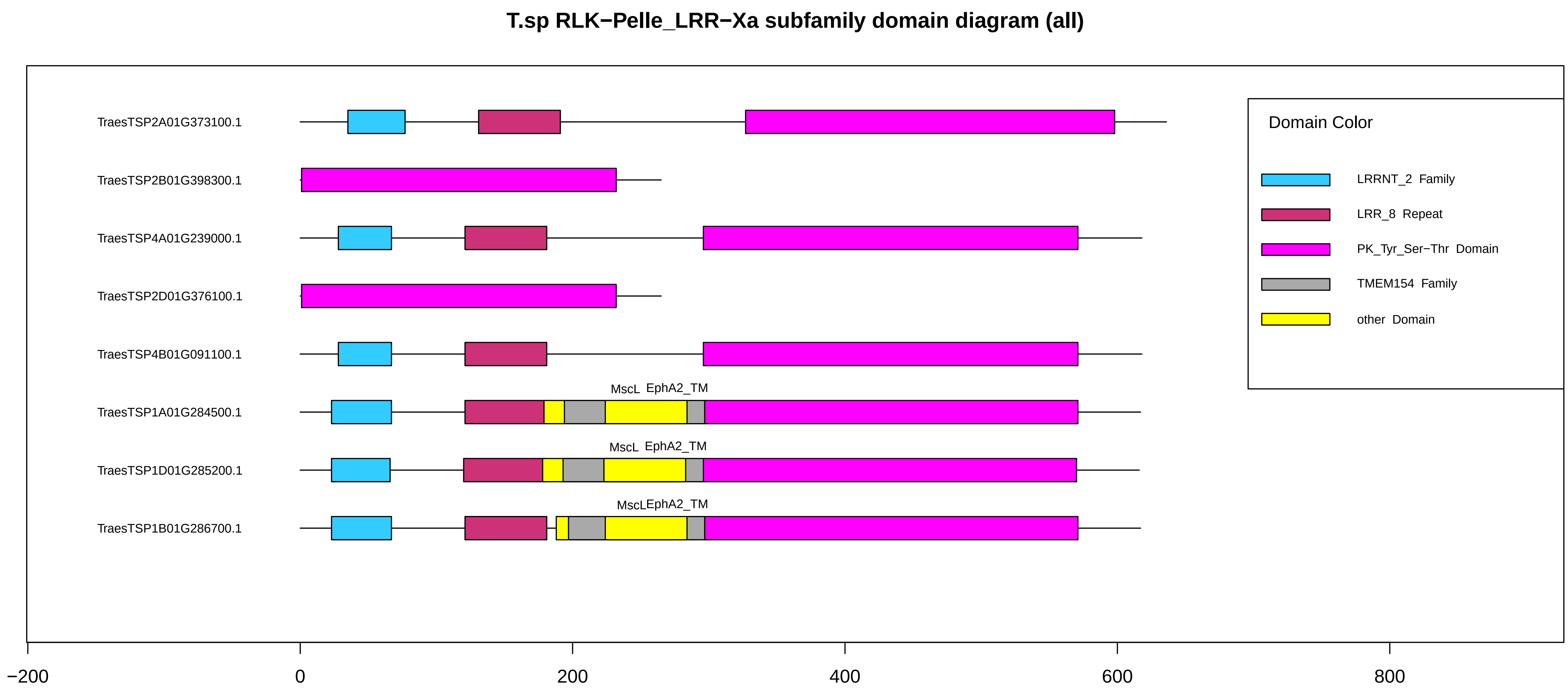
T.sp RLK−Pelle_LRR−Xa subfamily domain diagram (all)
TraesTSP2A01G373100.1
TraesTSP2B01G398300.1
TraesTSP4A01G239000.1
TraesTSP2D01G376100.1
TraesTSP4B01G091100.1
TraesTSP1A01G284500.1
TraesTSP1D01G285200.1
TraesTSP1B01G286700.1
MscL
EphA2_TM
Domain Color
LRRNT_2 Family
LRR_8 Repeat
PK_Tyr_Ser−Thr Domain
TMEM154 Family
other Domain
−200
0
200
400
600
800

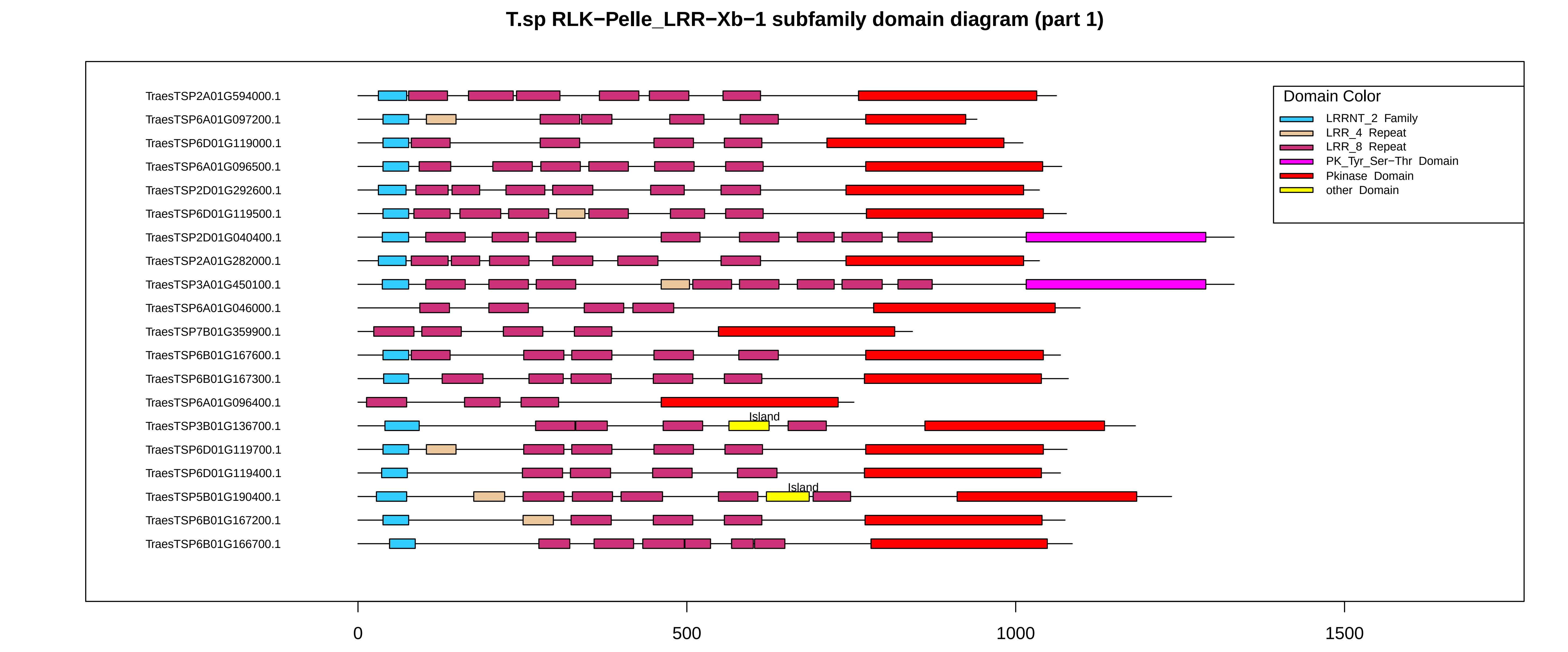
T.sp RLK−Pelle_LRR−Xb−1 subfamily domain diagram (part 1)
TraesTSP2A01G594000.1
TraesTSP6A01G097200.1
TraesTSP6D01G119000.1
TraesTSP6A01G096500.1
TraesTSP2D01G292600.1
TraesTSP6D01G119500.1
TraesTSP2D01G040400.1
TraesTSP2A01G282000.1
TraesTSP3A01G450100.1
TraesTSP6A01G046000.1
TraesTSP7B01G359900.1
TraesTSP6B01G167600.1
TraesTSP6B01G167300.1
TraesTSP6A01G096400.1
TraesTSP3B01G136700.1
TraesTSP6D01G119700.1
TraesTSP6D01G119400.1
TraesTSP5B01G190400.1
TraesTSP6B01G167200.1
TraesTSP6B01G166700.1
Island
Island
Domain Color
LRRNT_2 Family
LRR_4 Repeat
LRR_8 Repeat
PK_Tyr_Ser−Thr Domain
Pkinase Domain
other Domain
0
500
1000
1500

T.sp RLK−Pelle_LRR−Xb−1 subfamily domain diagram (part 2)

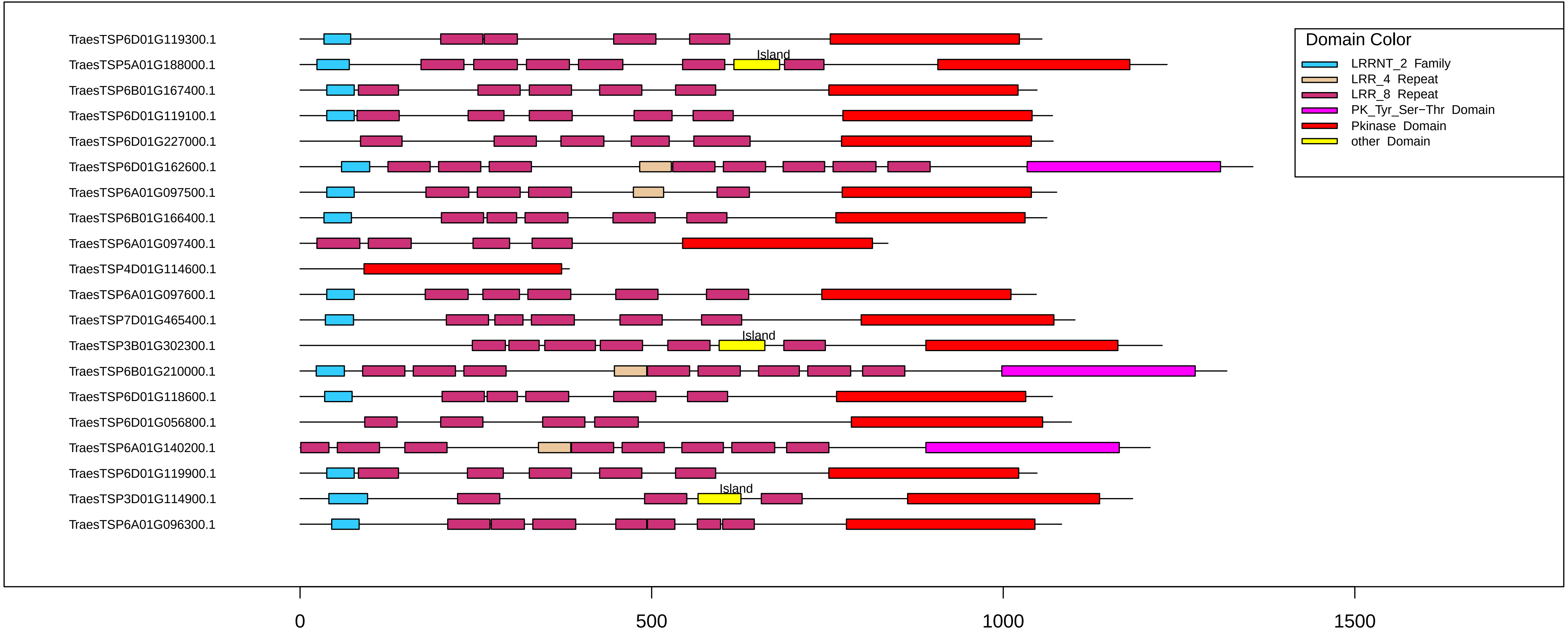

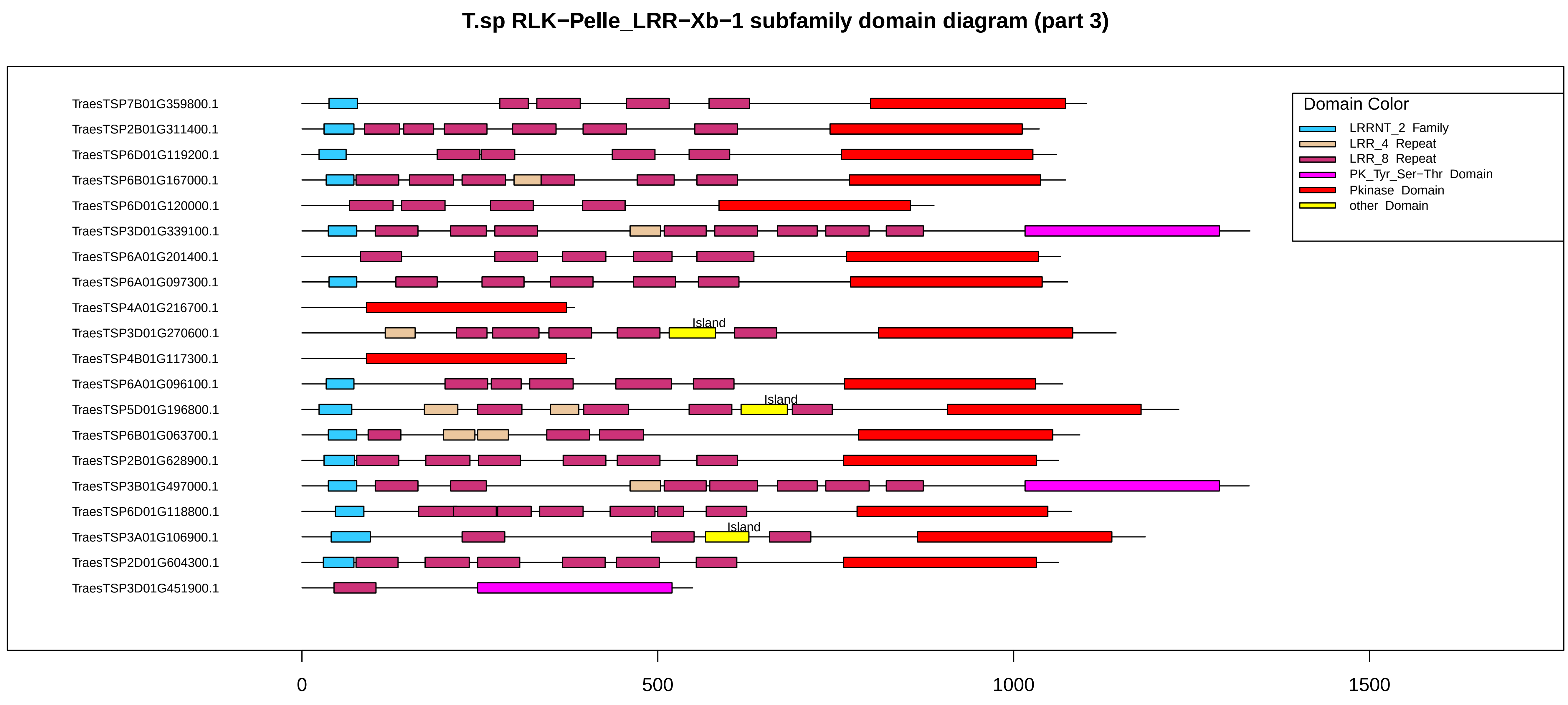
T.sp RLK−Pelle_LRR−Xb−1 subfamily domain diagram (part 3)
TraesTSP7B01G359800.1
TraesTSP2B01G311400.1
TraesTSP6D01G119200.1
TraesTSP6B01G167000.1
TraesTSP6D01G120000.1
TraesTSP3D01G339100.1
TraesTSP6A01G201400.1
TraesTSP6A01G097300.1
TraesTSP4A01G216700.1
TraesTSP3D01G270600.1
Island
TraesTSP4B01G117300.1
TraesTSP6A01G096100.1
TraesTSP5D01G196800.1
Island
TraesTSP6B01G063700.1
TraesTSP2B01G628900.1
TraesTSP3B01G497000.1
TraesTSP6D01G118800.1
TraesTSP3A01G106900.1
Island
TraesTSP2D01G604300.1
TraesTSP3D01G451900.1
Domain Color
LRRNT_2 Family
LRR_4 Repeat
LRR_8 Repeat
PK_Tyr_Ser−Thr Domain
Pkinase Domain
other Domain
0
500
1000
1500

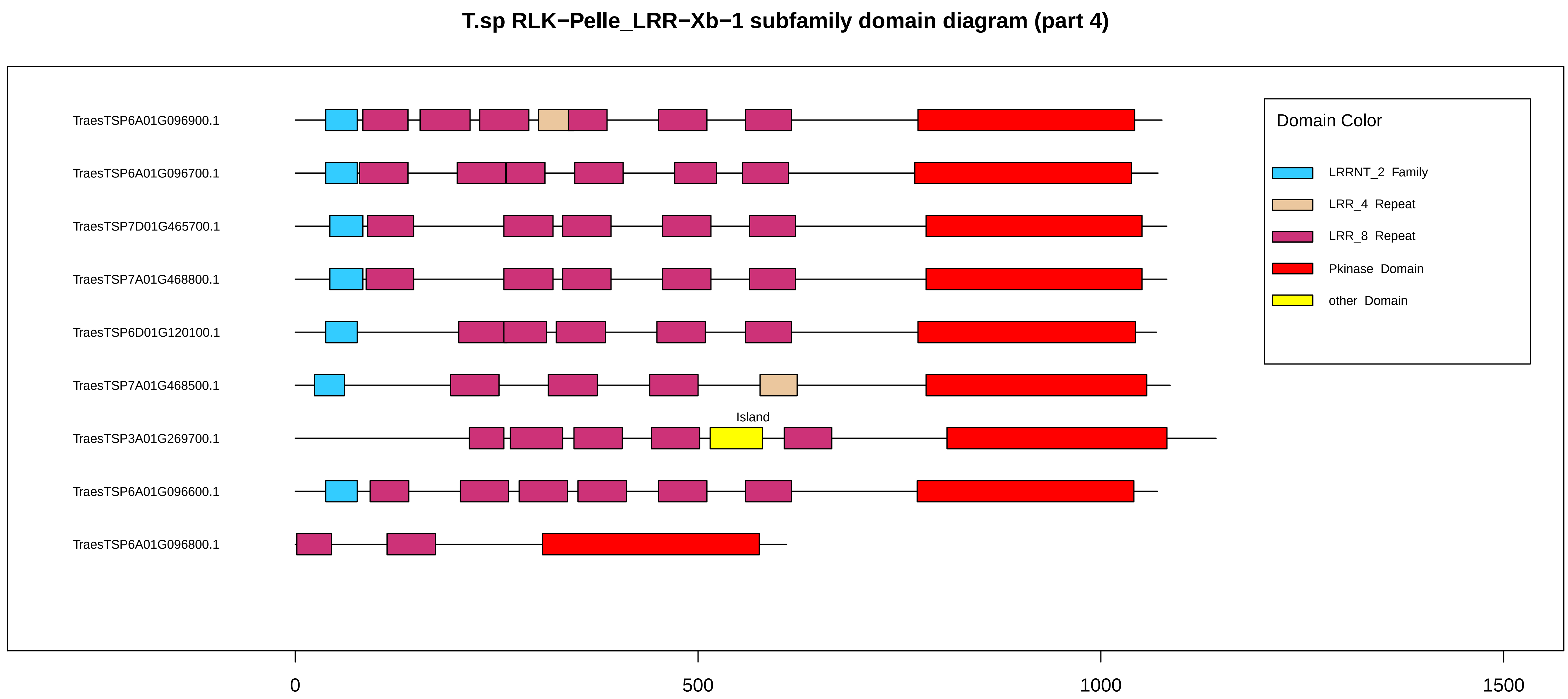
T.sp RLK−Pelle_LRR−Xb−1 subfamily domain diagram (part 4)
TraesTSP6A01G096900.1
TraesTSP6A01G096700.1
TraesTSP7D01G465700.1
TraesTSP7A01G468800.1
TraesTSP6D01G120100.1
TraesTSP7A01G468500.1
TraesTSP3A01G269700.1
TraesTSP6A01G096600.1
TraesTSP6A01G096800.1
Island
Domain Color
LRRNT_2 Family
LRR_4 Repeat
LRR_8 Repeat
Pkinase Domain
other Domain
0
500
1000
1500

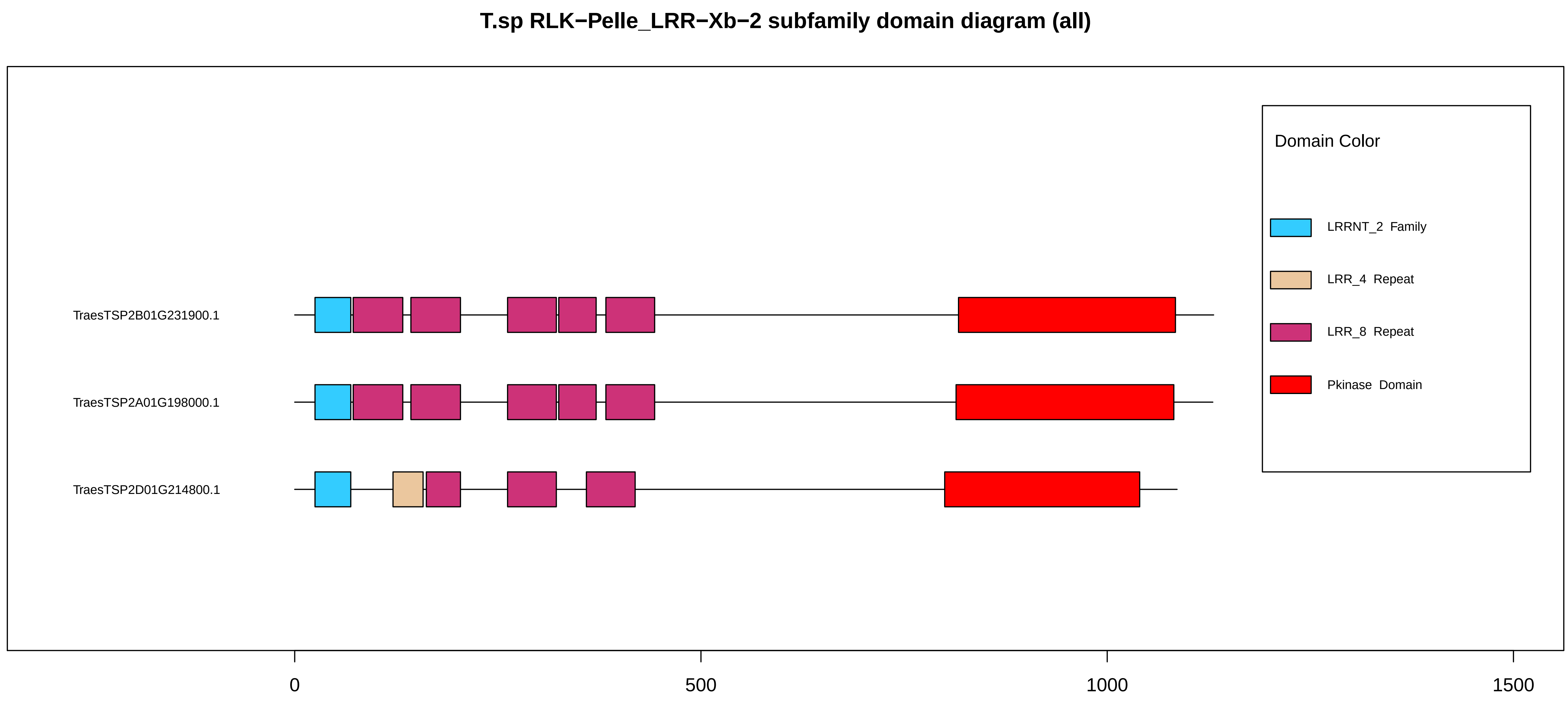
T.sp RLK−Pelle_LRR−Xb−2 subfamily domain diagram (all)
TraesTSP2B01G231900.1
TraesTSP2A01G198000.1
TraesTSP2D01G214800.1
Domain Color
LRRNT_2 Family
LRR_4 Repeat
LRR_8 Repeat
Pkinase Domain
0
500
1000
1500

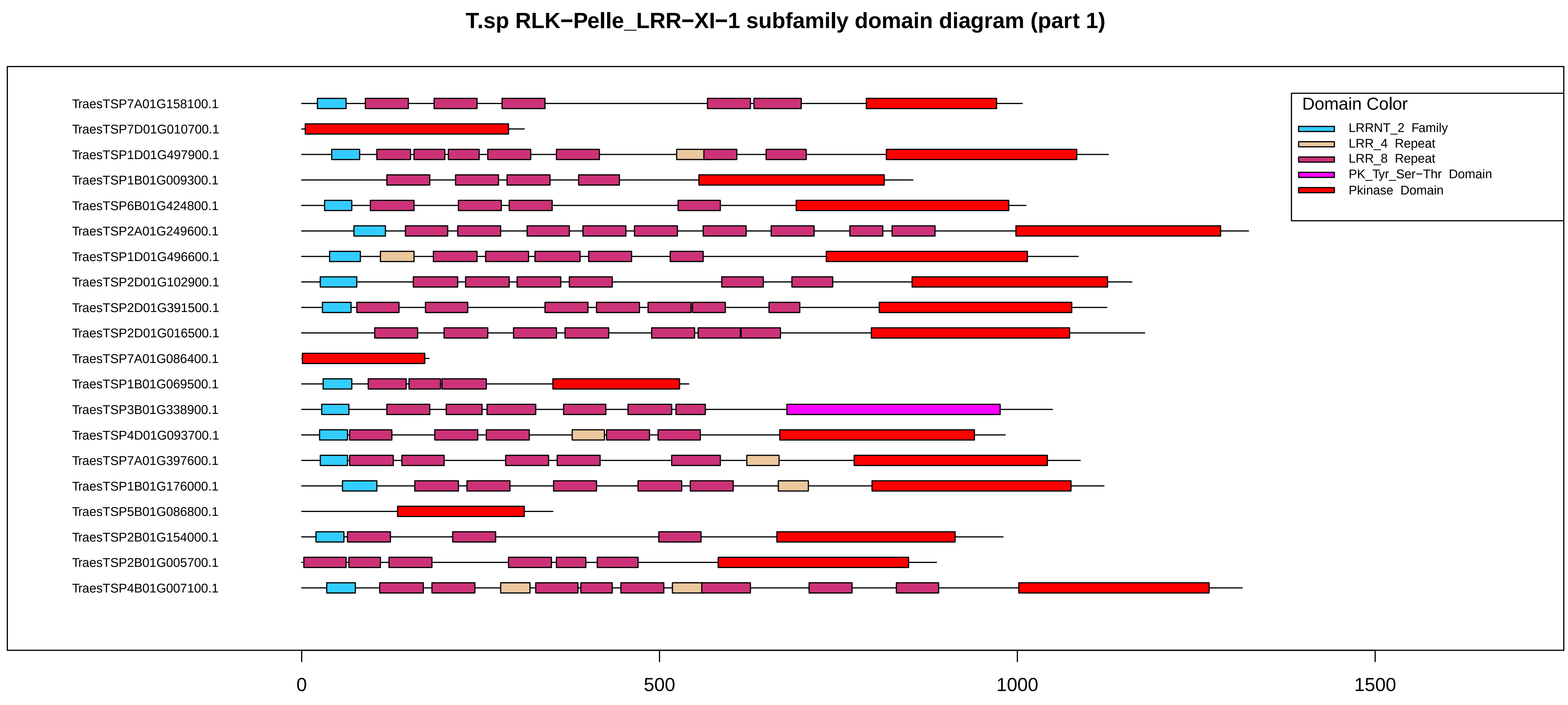

T.sp RLK−Pelle_LRR−XI−1 subfamily domain diagram (part 1)
TraesTSP7A01G158100.1
TraesTSP7D01G010700.1
TraesTSP1D01G497900.1
TraesTSP1B01G009300.1
TraesTSP6B01G424800.1
TraesTSP2A01G249600.1
TraesTSP1D01G496600.1
TraesTSP2D01G102900.1
TraesTSP2D01G391500.1
TraesTSP2D01G016500.1
TraesTSP7A01G086400.1
TraesTSP1B01G069500.1
TraesTSP3B01G338900.1
TraesTSP4D01G093700.1
TraesTSP7A01G397600.1
TraesTSP1B01G176000.1
TraesTSP5B01G086800.1
TraesTSP2B01G154000.1
TraesTSP2B01G005700.1
TraesTSP4B01G007100.1
Domain Color
LRRNT_2 Family
LRR_4 Repeat
LRR_8 Repeat
PK_Tyr_Ser−Thr Domain
Pkinase Domain
0
500
1000
1500

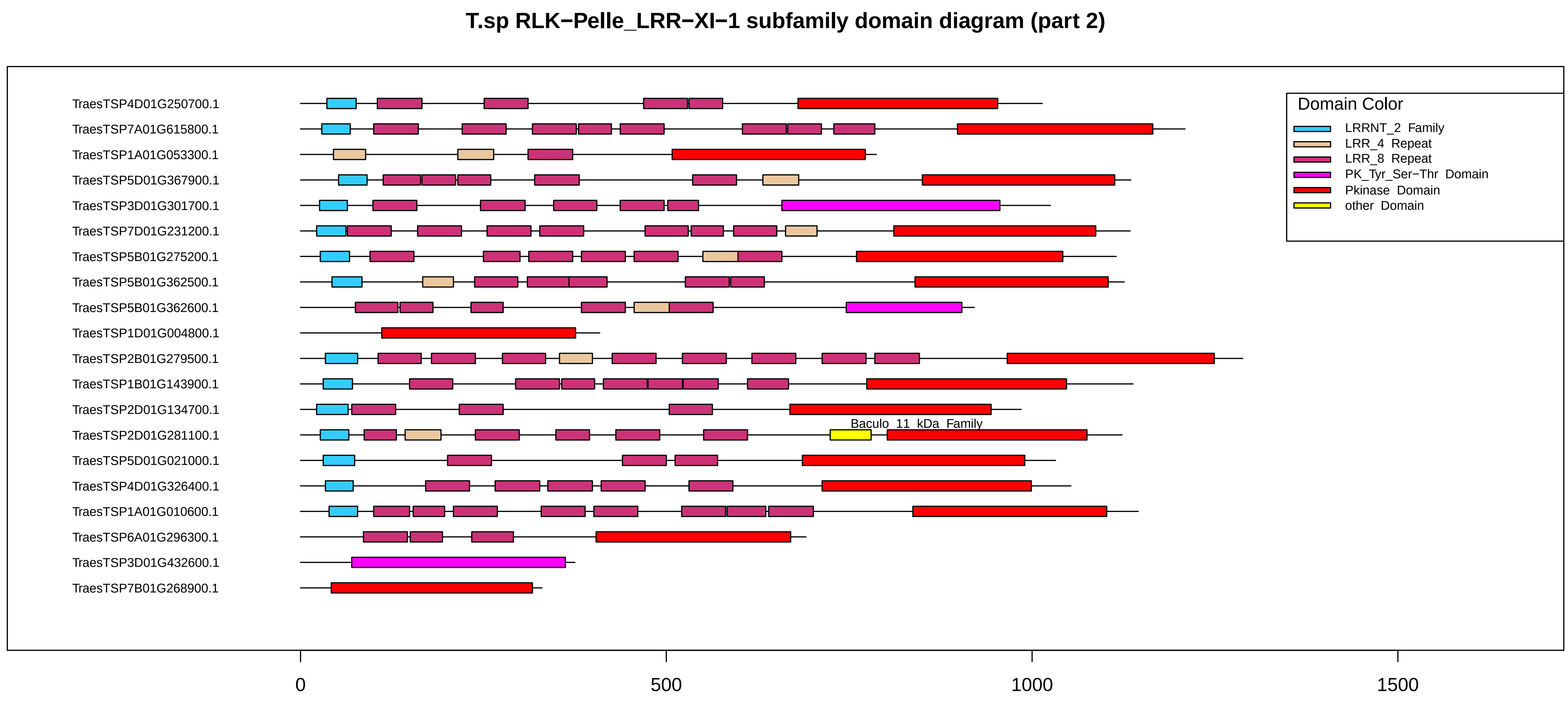
T.sp RLK−Pelle_LRR−XI−1 subfamily domain diagram (part 2)
TraesTSP4D01G250700.1
TraesTSP7A01G615800.1
TraesTSP1A01G053300.1
TraesTSP5D01G367900.1
TraesTSP3D01G301700.1
TraesTSP7D01G231200.1
TraesTSP5B01G275200.1
TraesTSP5B01G362500.1
TraesTSP5B01G362600.1
TraesTSP1D01G004800.1
TraesTSP2B01G279500.1
TraesTSP1B01G143900.1
TraesTSP2D01G134700.1
TraesTSP2D01G281100.1
Baculo_11_kDa_Family
TraesTSP5D01G021000.1
TraesTSP4D01G326400.1
TraesTSP1A01G010600.1
TraesTSP6A01G296300.1
TraesTSP3D01G432600.1
TraesTSP7B01G268900.1
Domain Color
LRRNT_2 Family
LRR_4 Repeat
LRR_8 Repeat
PK_Tyr_Ser−Thr Domain
Pkinase Domain
other Domain
0
500
1000
1500

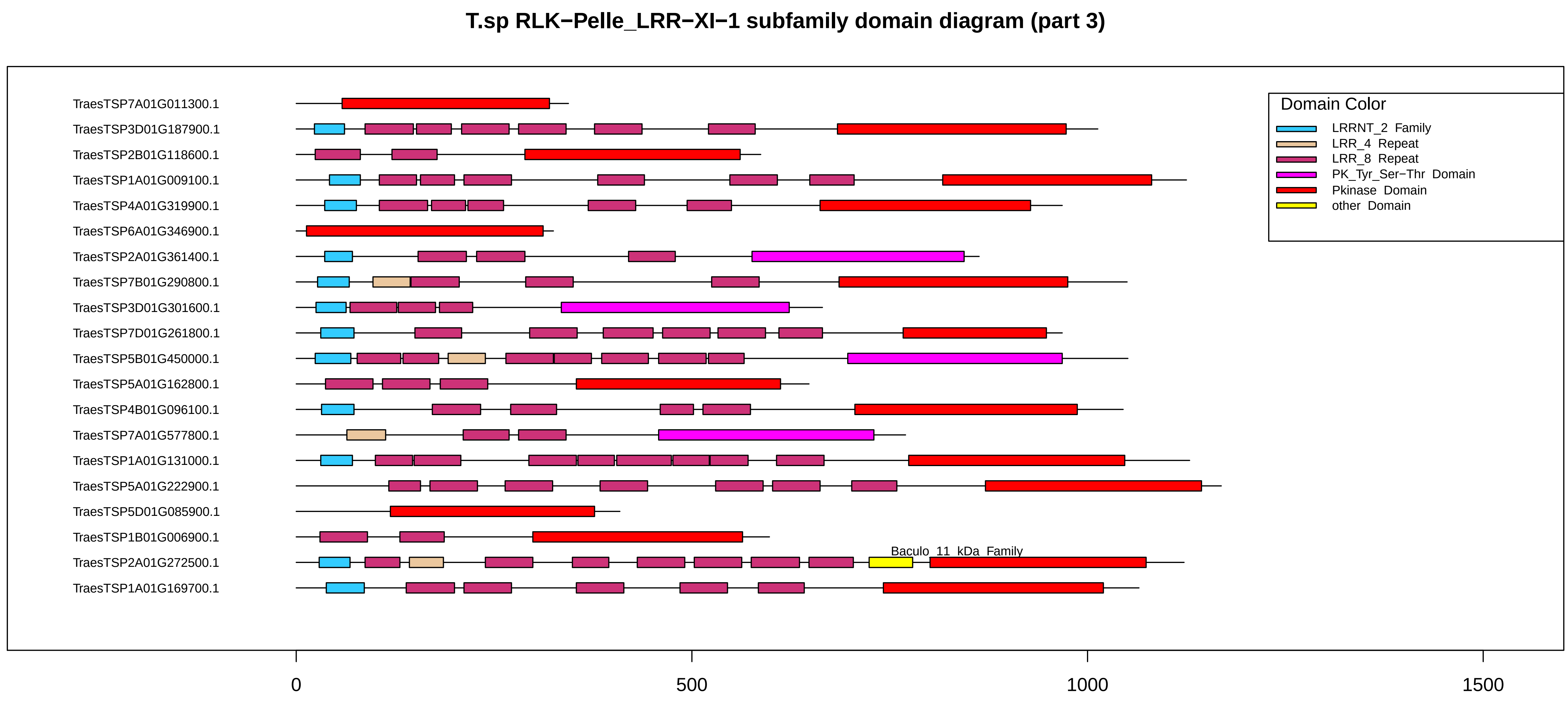
T.sp RLK−Pelle_LRR−XI−1 subfamily domain diagram (part 3)
TraesTSP7A01G011300.1
TraesTSP3D01G187900.1
TraesTSP2B01G118600.1
TraesTSP1A01G009100.1
TraesTSP4A01G319900.1
TraesTSP6A01G346900.1
TraesTSP2A01G361400.1
TraesTSP7B01G290800.1
TraesTSP3D01G301600.1
TraesTSP7D01G261800.1
TraesTSP5B01G450000.1
TraesTSP5A01G162800.1
TraesTSP4B01G096100.1
TraesTSP7A01G577800.1
TraesTSP1A01G131000.1
TraesTSP5A01G222900.1
TraesTSP5D01G085900.1
TraesTSP1B01G006900.1
TraesTSP2A01G272500.1
TraesTSP1A01G169700.1
Baculo_11_kDa_Family
Domain Color
LRRNT_2 Family
LRR_4 Repeat
LRR_8 Repeat
PK_Tyr_Ser−Thr Domain
Pkinase Domain
other Domain
0
500
1000
1500

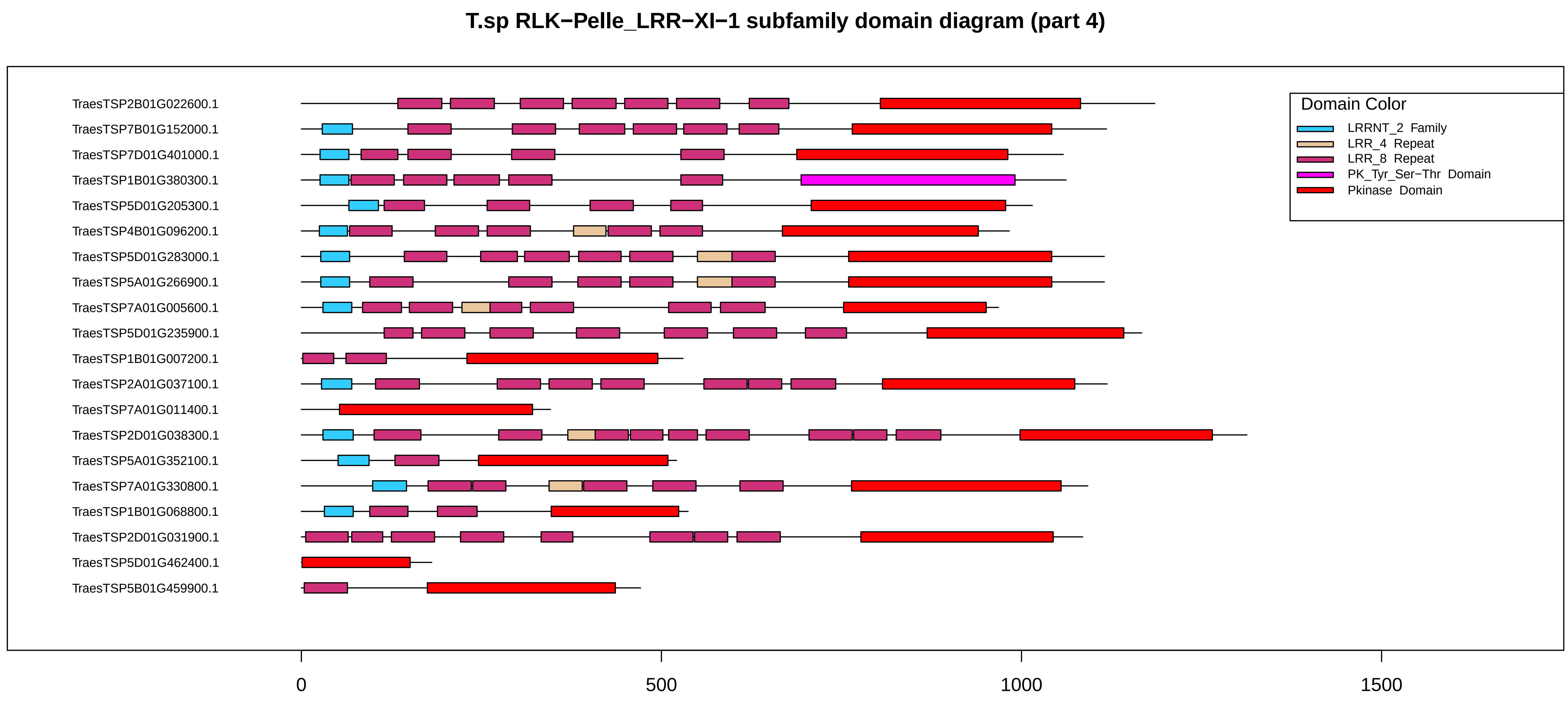
T.sp RLK−Pelle_LRR−XI−1 subfamily domain diagram (part 4)
TraesTSP2B01G022600.1
TraesTSP7B01G152000.1
TraesTSP7D01G401000.1
TraesTSP1B01G380300.1
TraesTSP5D01G205300.1
TraesTSP4B01G096200.1
TraesTSP5D01G283000.1
TraesTSP5A01G266900.1
TraesTSP7A01G005600.1
TraesTSP5D01G235900.1
TraesTSP1B01G007200.1
TraesTSP2A01G037100.1
TraesTSP7A01G011400.1
TraesTSP2D01G038300.1
TraesTSP5A01G352100.1
TraesTSP7A01G330800.1
TraesTSP1B01G068800.1
TraesTSP2D01G031900.1
TraesTSP5D01G462400.1
TraesTSP5B01G459900.1
Domain Color
LRRNT_2 Family
LRR_4 Repeat
LRR_8 Repeat
PK_Tyr_Ser−Thr Domain
Pkinase Domain
0
500
1000
1500

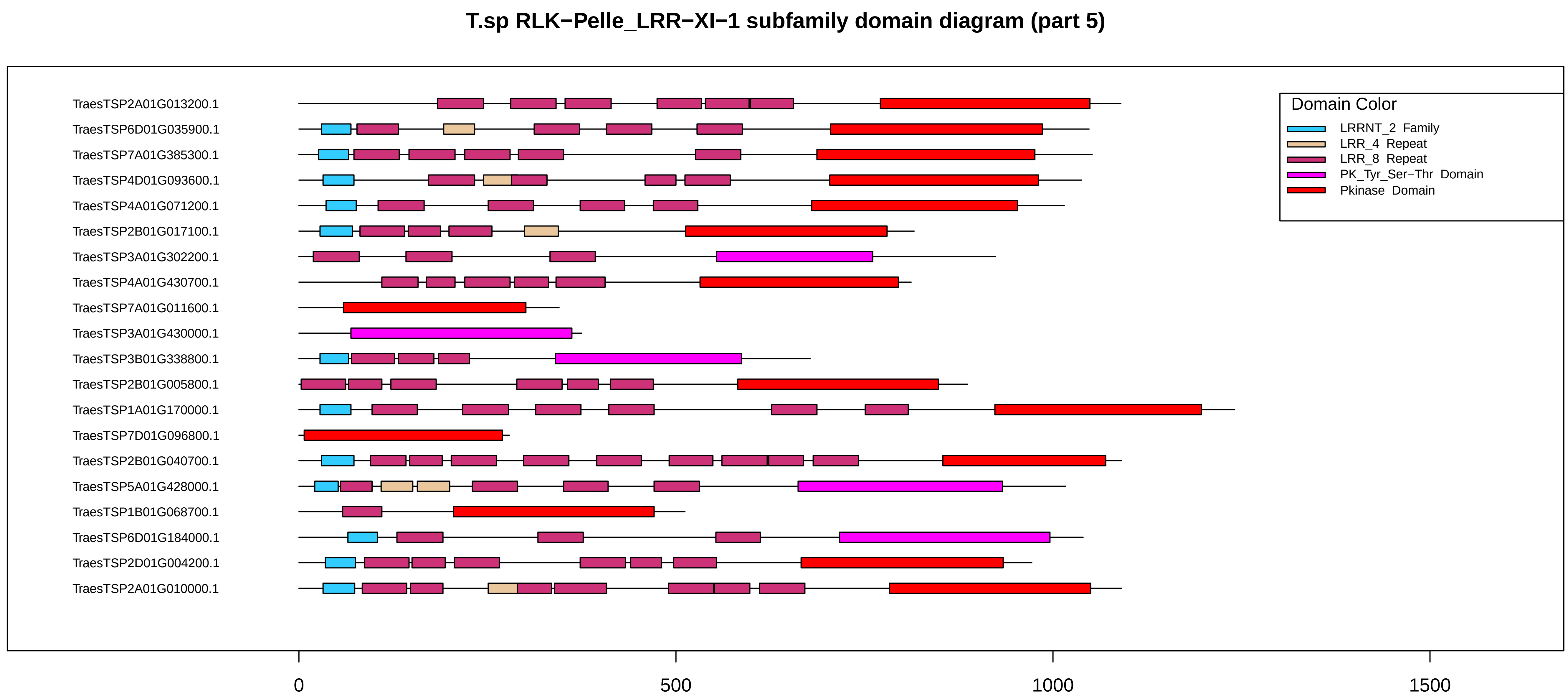
T.sp RLK−Pelle_LRR−XI−1 subfamily domain diagram (part 5)
TraesTSP2A01G013200.1
TraesTSP6D01G035900.1
TraesTSP7A01G385300.1
TraesTSP4D01G093600.1
TraesTSP4A01G071200.1
TraesTSP2B01G017100.1
TraesTSP3A01G302200.1
TraesTSP4A01G430700.1
TraesTSP7A01G011600.1
TraesTSP3A01G430000.1
TraesTSP3B01G338800.1
TraesTSP2B01G005800.1
TraesTSP1A01G170000.1
TraesTSP7D01G096800.1
TraesTSP2B01G040700.1
TraesTSP5A01G428000.1
TraesTSP1B01G068700.1
TraesTSP6D01G184000.1
TraesTSP2D01G004200.1
TraesTSP2A01G010000.1
Domain Color
LRRNT_2 Family
LRR_4 Repeat
LRR_8 Repeat
PK_Tyr_Ser−Thr Domain
Pkinase Domain
0
500
1000
1500

T.sp RLK−Pelle_LRR−XI−1 subfamily domain diagram (part 6)

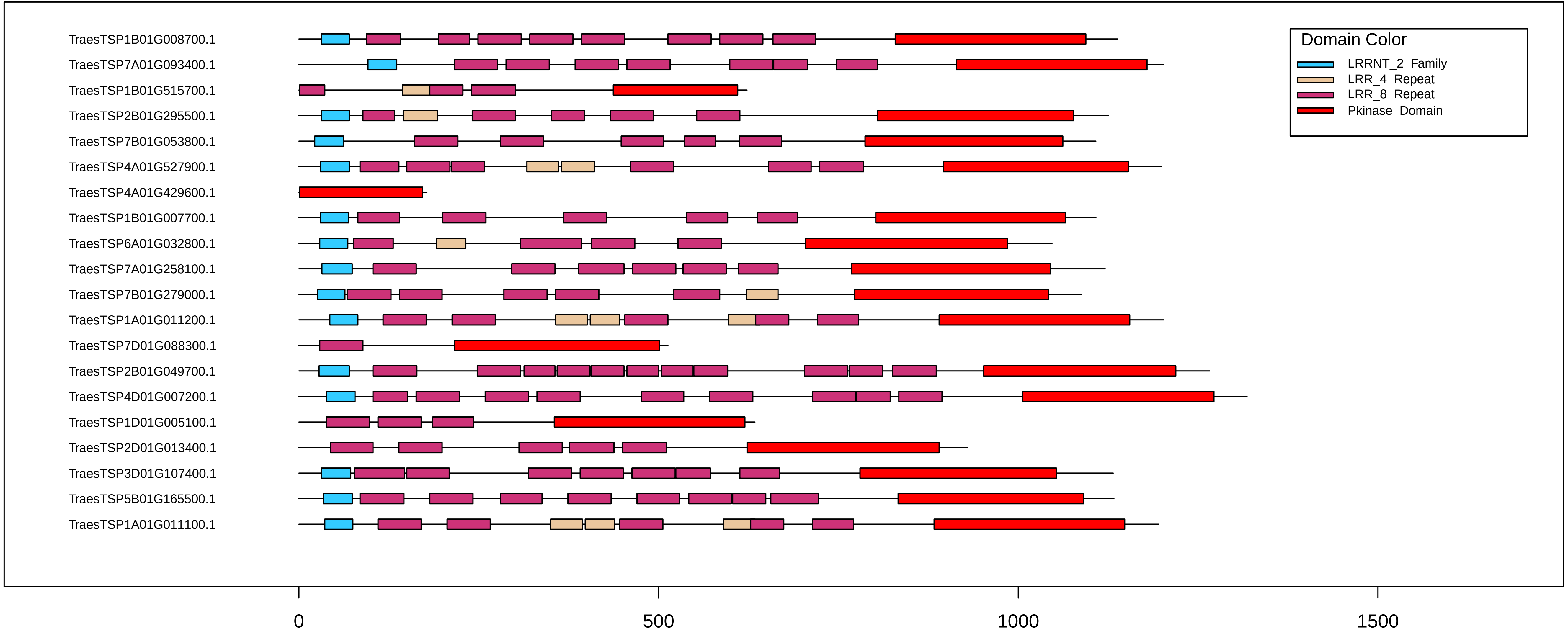

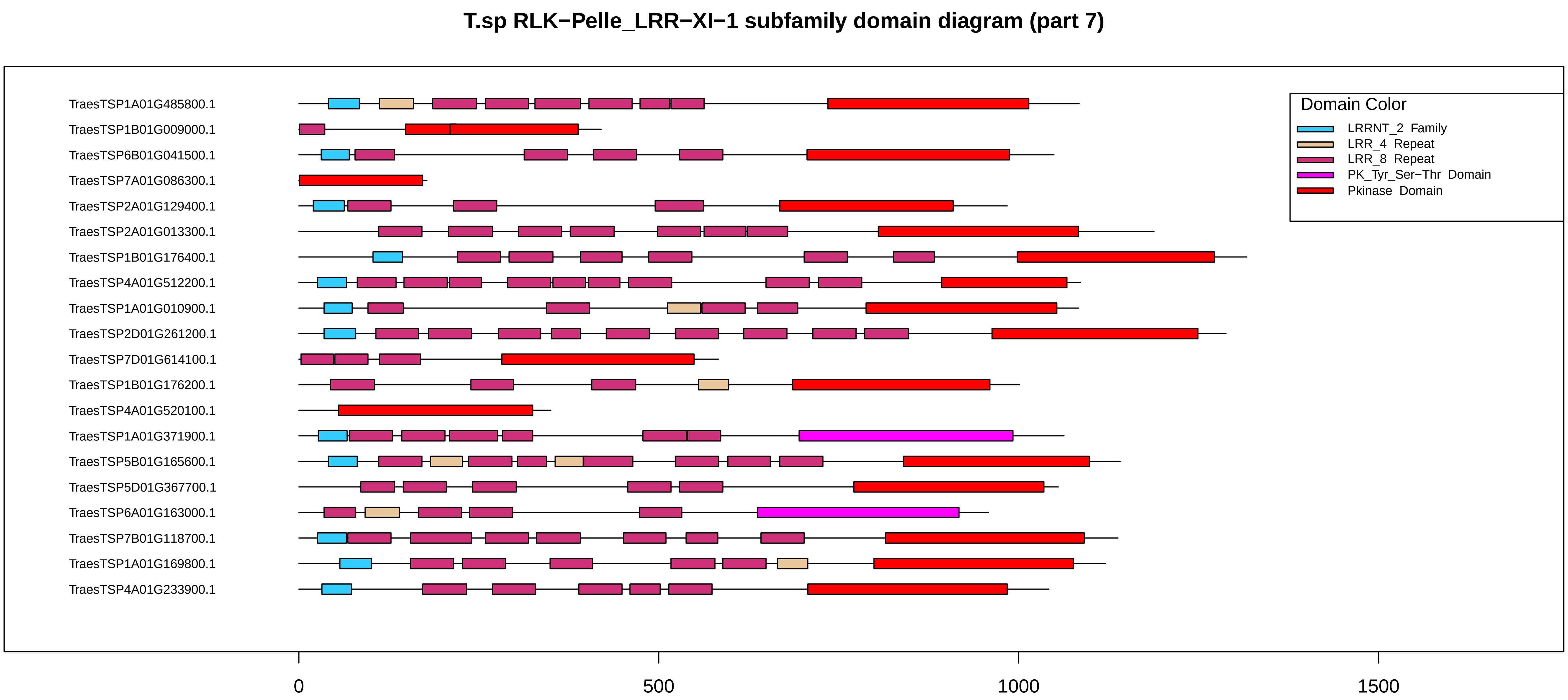
T.sp RLK−Pelle_LRR−XI−1 subfamily domain diagram (part 7)
TraesTSP1A01G485800.1
TraesTSP1B01G009000.1
TraesTSP6B01G041500.1
TraesTSP7A01G086300.1
TraesTSP2A01G129400.1
TraesTSP2A01G013300.1
TraesTSP1B01G176400.1
TraesTSP4A01G512200.1
TraesTSP1A01G010900.1
TraesTSP2D01G261200.1
TraesTSP7D01G614100.1
TraesTSP1B01G176200.1
TraesTSP4A01G520100.1
TraesTSP1A01G371900.1
TraesTSP5B01G165600.1
TraesTSP5D01G367700.1
TraesTSP6A01G163000.1
TraesTSP7B01G118700.1
TraesTSP1A01G169800.1
TraesTSP4A01G233900.1
Domain Color
LRRNT_2 Family
LRR_4 Repeat
LRR_8 Repeat
PK_Tyr_Ser−Thr Domain
Pkinase Domain
0
500
1000
1500

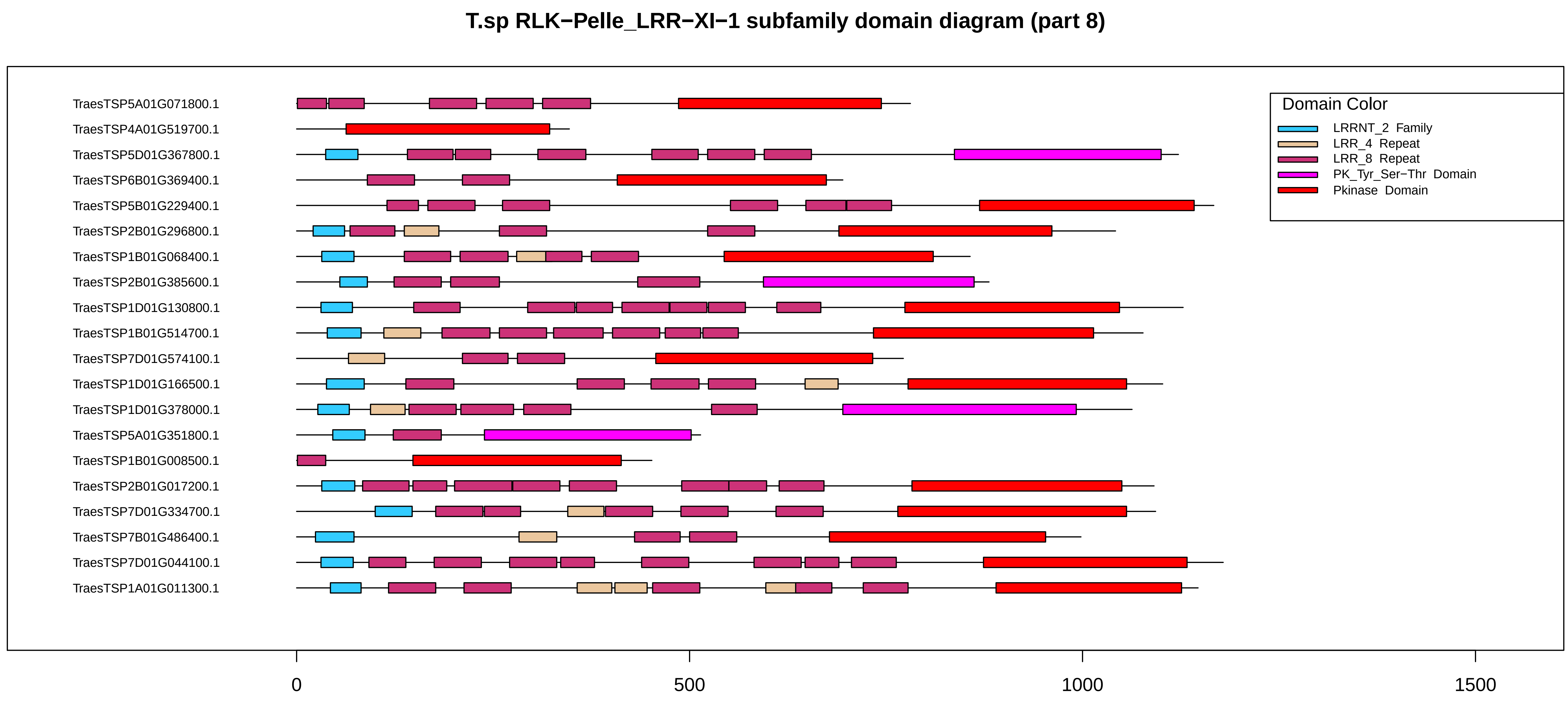
T.sp RLK−Pelle_LRR−XI−1 subfamily domain diagram (part 8)
TraesTSP5A01G071800.1
TraesTSP4A01G519700.1
TraesTSP5D01G367800.1
TraesTSP6B01G369400.1
TraesTSP5B01G229400.1
TraesTSP2B01G296800.1
TraesTSP1B01G068400.1
TraesTSP2B01G385600.1
TraesTSP1D01G130800.1
TraesTSP1B01G514700.1
TraesTSP7D01G574100.1
TraesTSP1D01G166500.1
TraesTSP1D01G378000.1
TraesTSP5A01G351800.1
TraesTSP1B01G008500.1
TraesTSP2B01G017200.1
TraesTSP7D01G334700.1
TraesTSP7B01G486400.1
TraesTSP7D01G044100.1
TraesTSP1A01G011300.1
Domain Color
LRRNT_2 Family
LRR_4 Repeat
LRR_8 Repeat
PK_Tyr_Ser−Thr Domain
Pkinase Domain
0
500
1000
1500

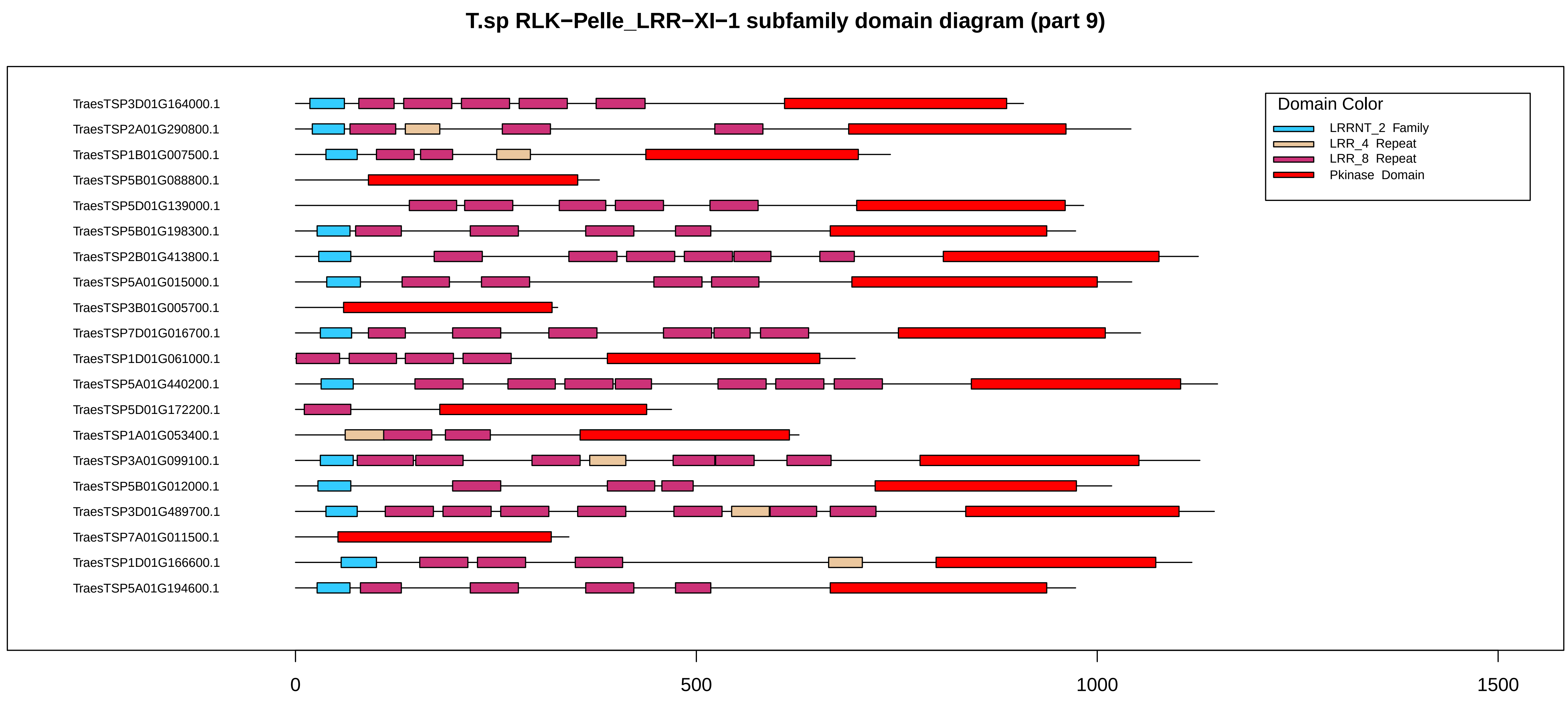
T.sp RLK−Pelle_LRR−XI−1 subfamily domain diagram (part 9)
TraesTSP3D01G164000.1
TraesTSP2A01G290800.1
TraesTSP1B01G007500.1
TraesTSP5B01G088800.1
TraesTSP5D01G139000.1
TraesTSP5B01G198300.1
TraesTSP2B01G413800.1
TraesTSP5A01G015000.1
TraesTSP3B01G005700.1
TraesTSP7D01G016700.1
TraesTSP1D01G061000.1
TraesTSP5A01G440200.1
TraesTSP5D01G172200.1
TraesTSP1A01G053400.1
TraesTSP3A01G099100.1
TraesTSP5B01G012000.1
TraesTSP3D01G489700.1
TraesTSP7A01G011500.1
TraesTSP1D01G166600.1
TraesTSP5A01G194600.1
Domain Color
LRRNT_2 Family
LRR_4 Repeat
LRR_8 Repeat
Pkinase Domain
0
500
1000
1500

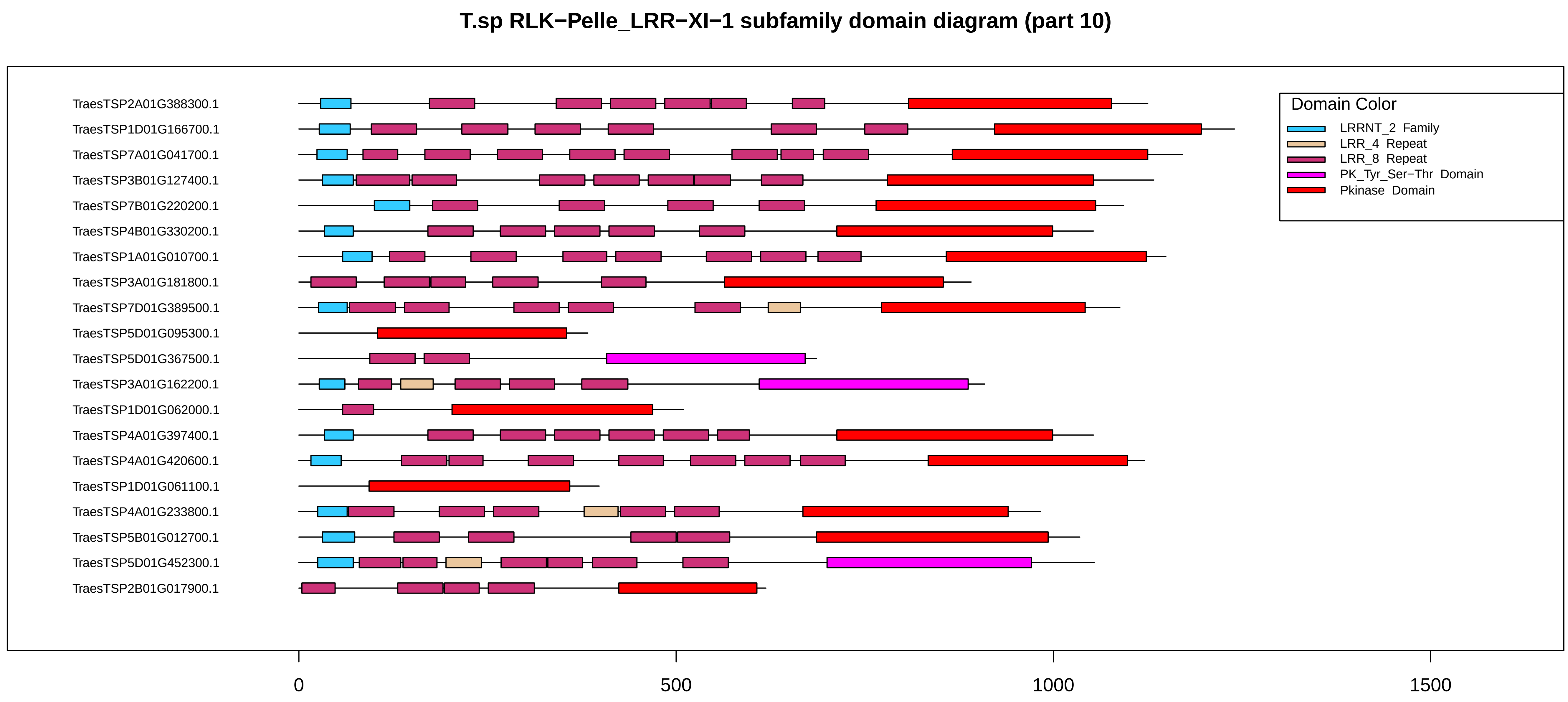

T.sp RLK−Pelle_LRR−XI−1 subfamily domain diagram (part 10)
TraesTSP2A01G388300.1
TraesTSP1D01G166700.1
TraesTSP7A01G041700.1
TraesTSP3B01G127400.1
TraesTSP7B01G220200.1
TraesTSP4B01G330200.1
TraesTSP1A01G010700.1
TraesTSP3A01G181800.1
TraesTSP7D01G389500.1
TraesTSP5D01G095300.1
TraesTSP5D01G367500.1
TraesTSP3A01G162200.1
TraesTSP1D01G062000.1
TraesTSP4A01G397400.1
TraesTSP4A01G420600.1
TraesTSP1D01G061100.1
TraesTSP4A01G233800.1
TraesTSP5B01G012700.1
TraesTSP5D01G452300.1
TraesTSP2B01G017900.1
Domain Color
LRRNT_2 Family
LRR_4 Repeat
LRR_8 Repeat
PK_Tyr_Ser−Thr Domain
Pkinase Domain
0
500
1000
1500

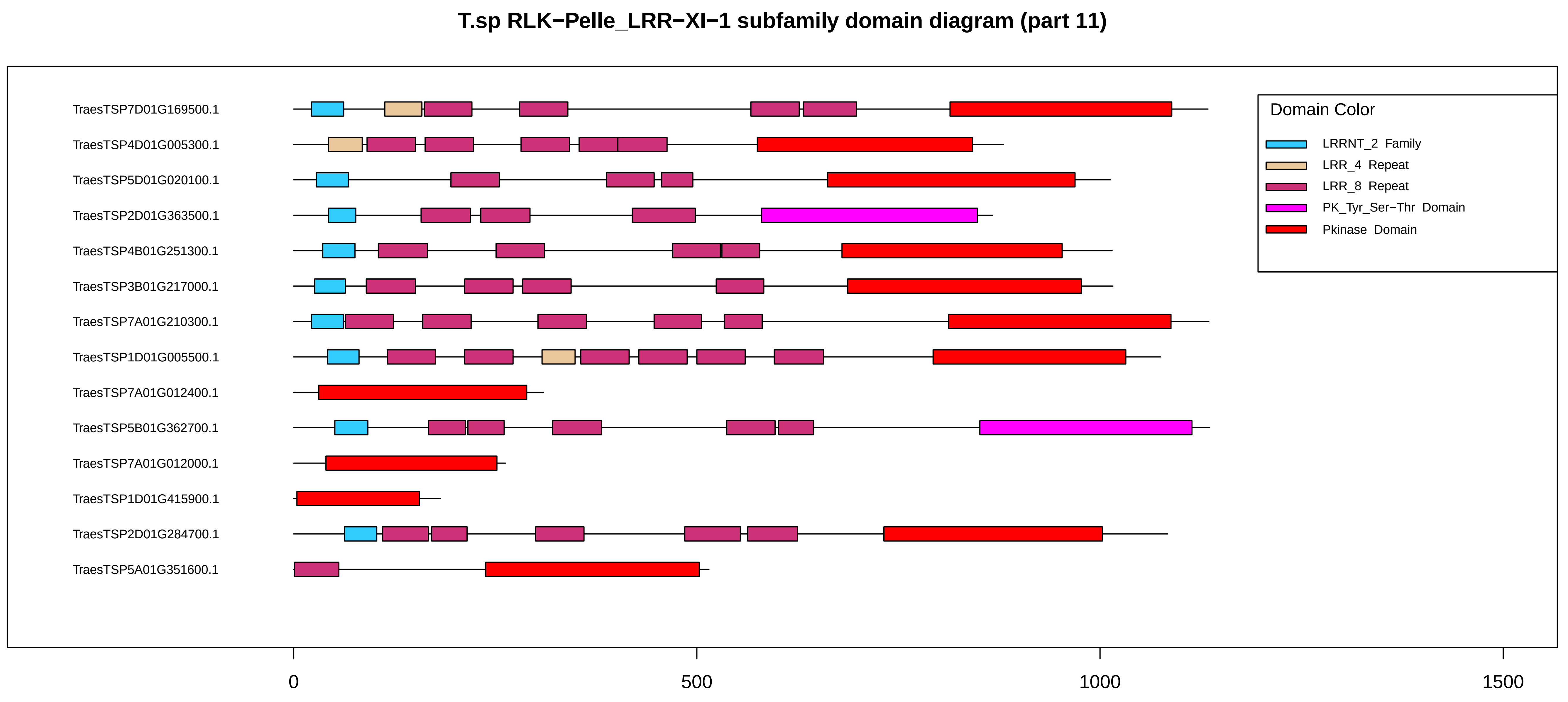
T.sp RLK−Pelle_LRR−XI−1 subfamily domain diagram (part 11)
TraesTSP7D01G169500.1
TraesTSP4D01G005300.1
TraesTSP5D01G020100.1
TraesTSP2D01G363500.1
TraesTSP4B01G251300.1
TraesTSP3B01G217000.1
TraesTSP7A01G210300.1
TraesTSP1D01G005500.1
TraesTSP7A01G012400.1
TraesTSP5B01G362700.1
TraesTSP7A01G012000.1
TraesTSP1D01G415900.1
TraesTSP2D01G284700.1
TraesTSP5A01G351600.1
Domain Color
LRRNT_2 Family
LRR_4 Repeat
LRR_8 Repeat
PK_Tyr_Ser−Thr Domain
Pkinase Domain
0
500
1000
1500

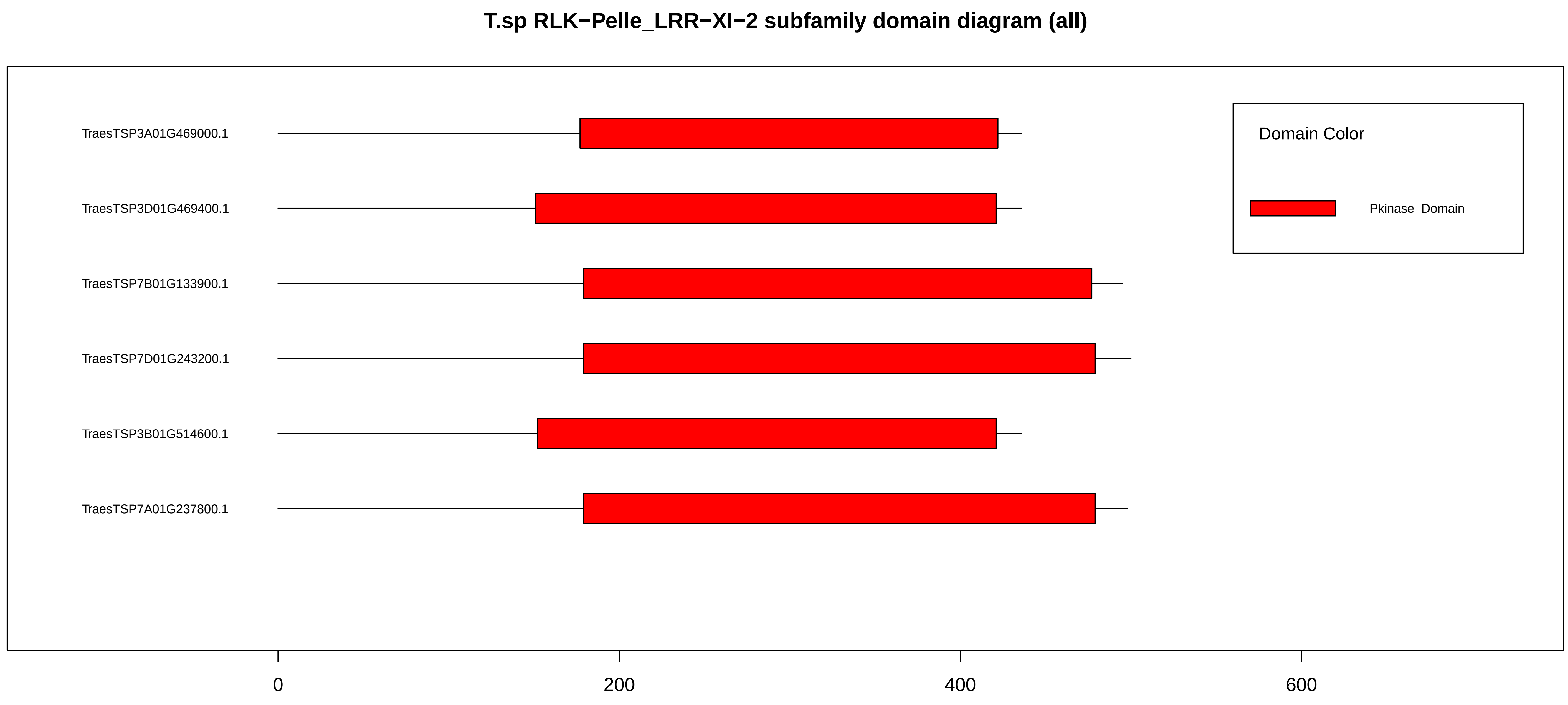
T.sp RLK−Pelle_LRR−XI−2 subfamily domain diagram (all)
TraesTSP3A01G469000.1
TraesTSP3D01G469400.1
TraesTSP7B01G133900.1
TraesTSP7D01G243200.1
TraesTSP3B01G514600.1
TraesTSP7A01G237800.1
Domain Color
Pkinase Domain
0
200
400
600

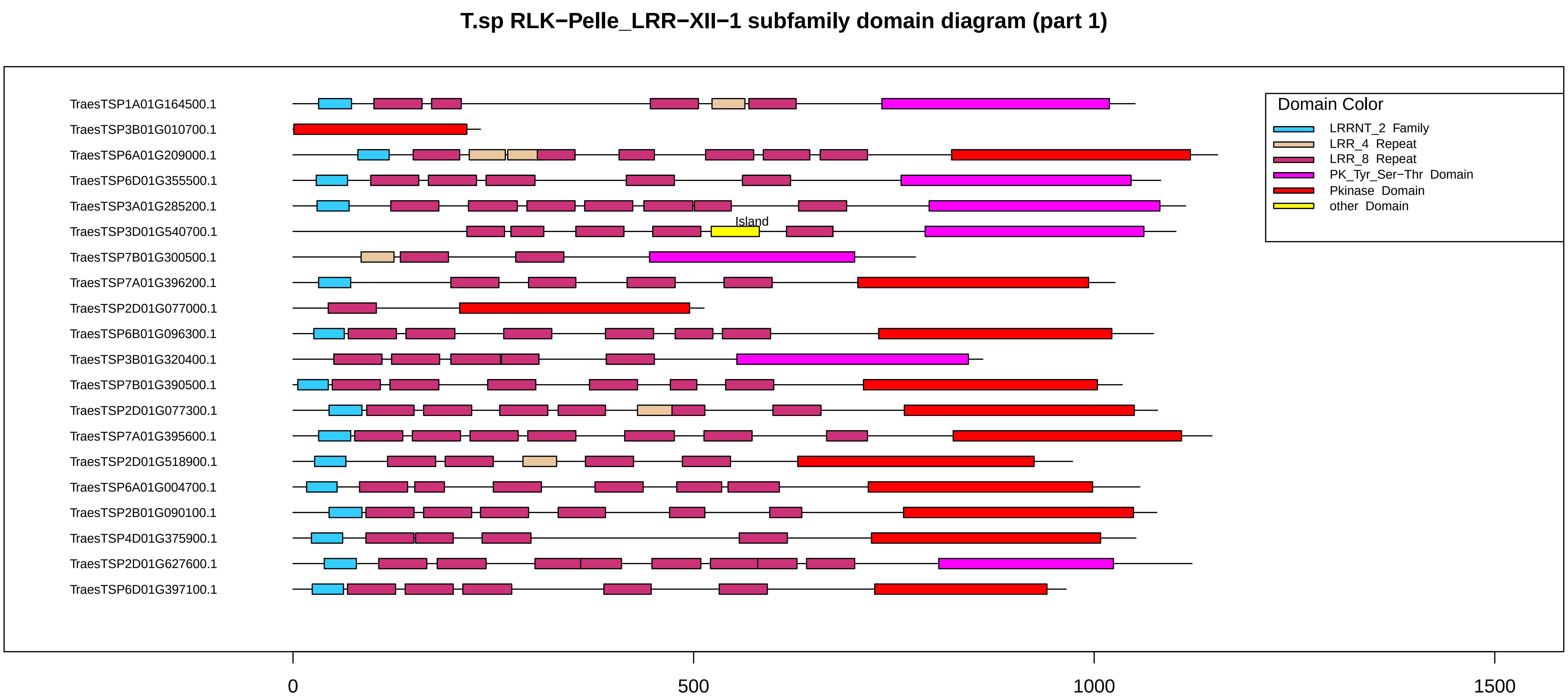
T.sp RLK−Pelle_LRR−XII−1 subfamily domain diagram (part 1)
TraesTSP1A01G164500.1
TraesTSP3B01G010700.1
TraesTSP6A01G209000.1
TraesTSP6D01G355500.1
TraesTSP3A01G285200.1
TraesTSP3D01G540700.1
Island
TraesTSP7B01G300500.1
TraesTSP7A01G396200.1
TraesTSP2D01G077000.1
TraesTSP6B01G096300.1
TraesTSP3B01G320400.1
TraesTSP7B01G390500.1
TraesTSP2D01G077300.1
TraesTSP7A01G395600.1
TraesTSP2D01G518900.1
TraesTSP6A01G004700.1
TraesTSP2B01G090100.1
TraesTSP4D01G375900.1
TraesTSP2D01G627600.1
TraesTSP6D01G397100.1
Domain Color
LRRNT_2 Family
LRR_4 Repeat
LRR_8 Repeat
PK_Tyr_Ser−Thr Domain
Pkinase Domain
other Domain
0
500
1000
1500

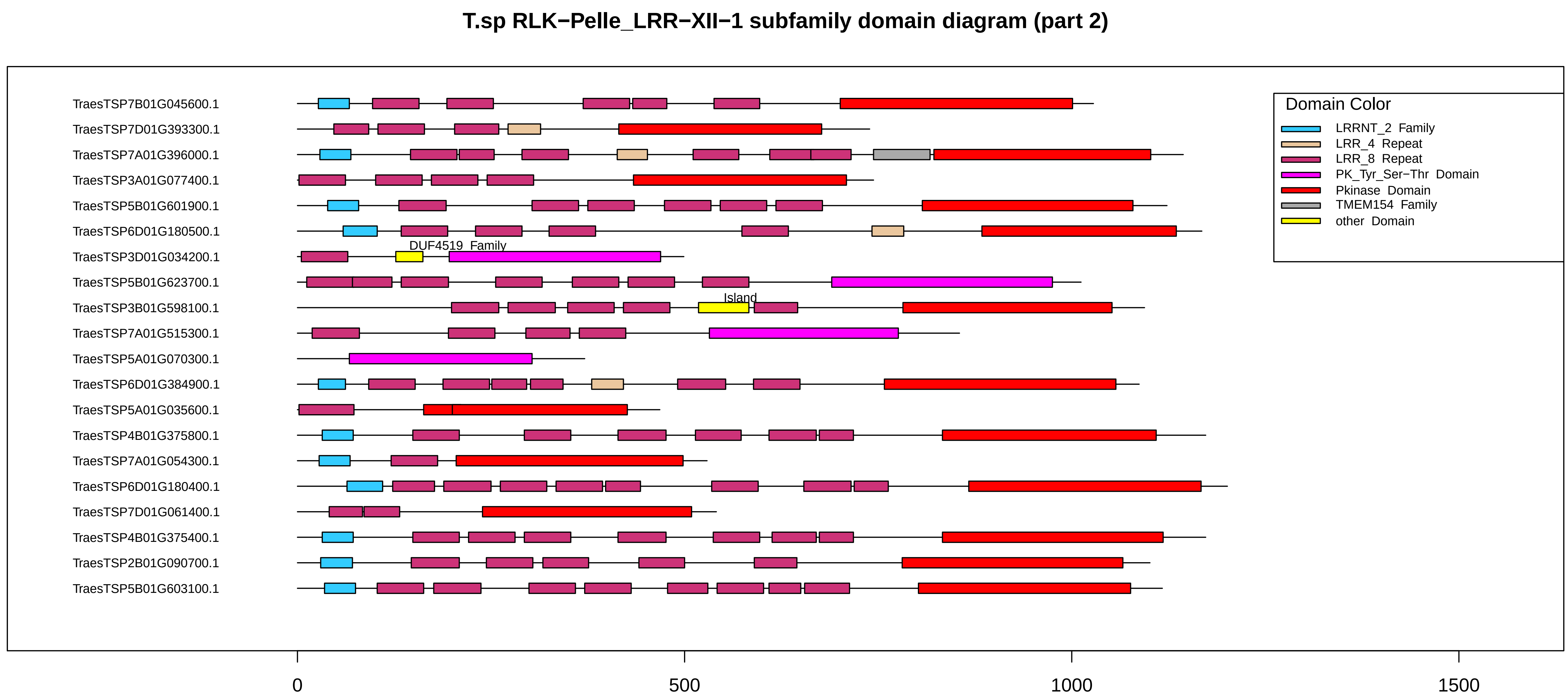
T.sp RLK−Pelle_LRR−XII−1 subfamily domain diagram (part 2)
TraesTSP7B01G045600.1
TraesTSP7D01G393300.1
TraesTSP7A01G396000.1
TraesTSP3A01G077400.1
TraesTSP5B01G601900.1
TraesTSP6D01G180500.1
TraesTSP3D01G034200.1
TraesTSP5B01G623700.1
TraesTSP3B01G598100.1
TraesTSP7A01G515300.1
TraesTSP5A01G070300.1
TraesTSP6D01G384900.1
TraesTSP5A01G035600.1
TraesTSP4B01G375800.1
TraesTSP7A01G054300.1
TraesTSP6D01G180400.1
TraesTSP7D01G061400.1
TraesTSP4B01G375400.1
TraesTSP2B01G090700.1
TraesTSP5B01G603100.1
DUF4519 Family
Island
Domain Color
LRRNT_2 Family
LRR_4 Repeat
LRR_8 Repeat
PK_Tyr_Ser−Thr Domain
Pkinase Domain
TMEM154 Family
other Domain
0
500
1000
1500

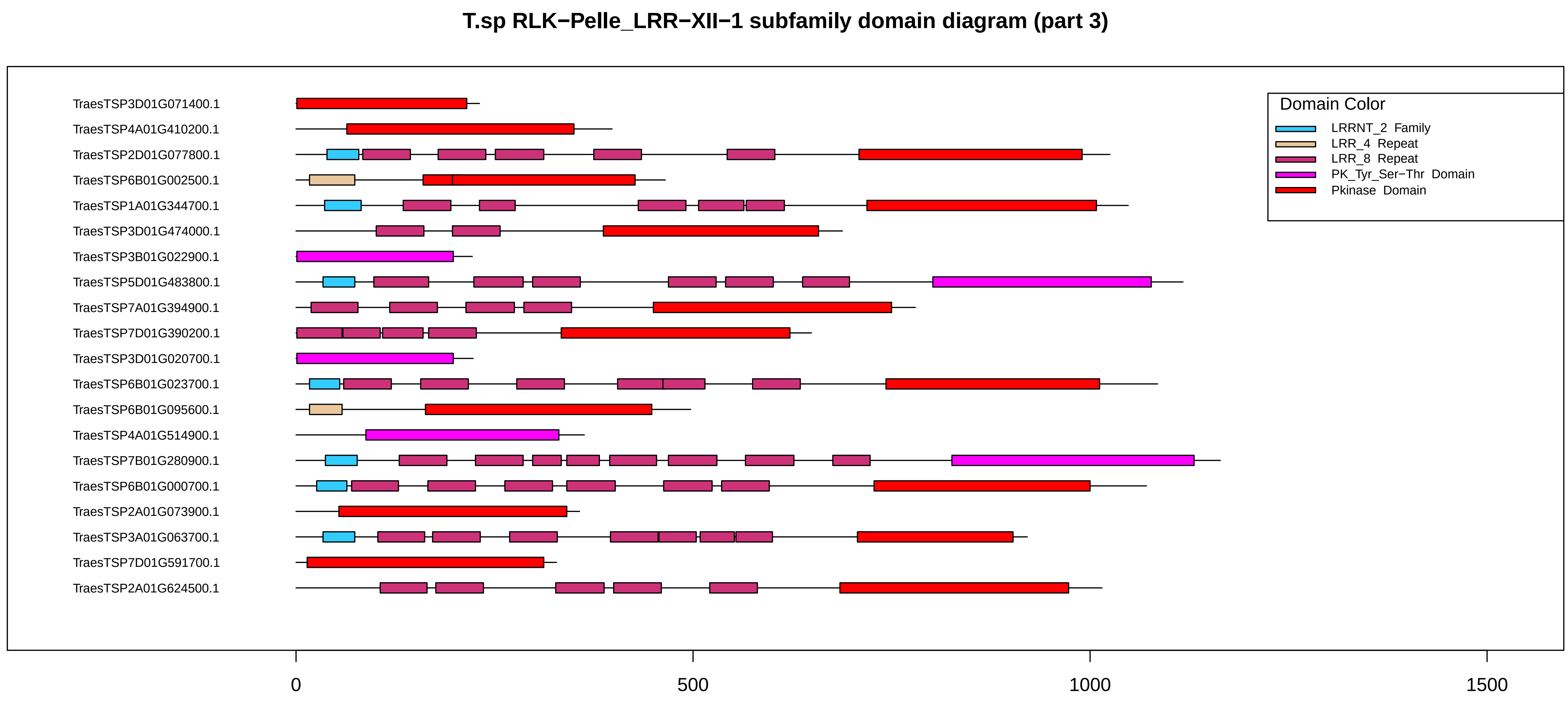
T.sp RLK−Pelle_LRR−XII−1 subfamily domain diagram (part 3)
TraesTSP3D01G071400.1
TraesTSP4A01G410200.1
TraesTSP2D01G077800.1
TraesTSP6B01G002500.1
TraesTSP1A01G344700.1
TraesTSP3D01G474000.1
TraesTSP3B01G022900.1
TraesTSP5D01G483800.1
TraesTSP7A01G394900.1
TraesTSP7D01G390200.1
TraesTSP3D01G020700.1
TraesTSP6B01G023700.1
TraesTSP6B01G095600.1
TraesTSP4A01G514900.1
TraesTSP7B01G280900.1
TraesTSP6B01G000700.1
TraesTSP2A01G073900.1
TraesTSP3A01G063700.1
TraesTSP7D01G591700.1
TraesTSP2A01G624500.1
Domain Color
LRRNT_2 Family
LRR_4 Repeat
LRR_8 Repeat
PK_Tyr_Ser−Thr Domain
Pkinase Domain
0
500
1000
1500

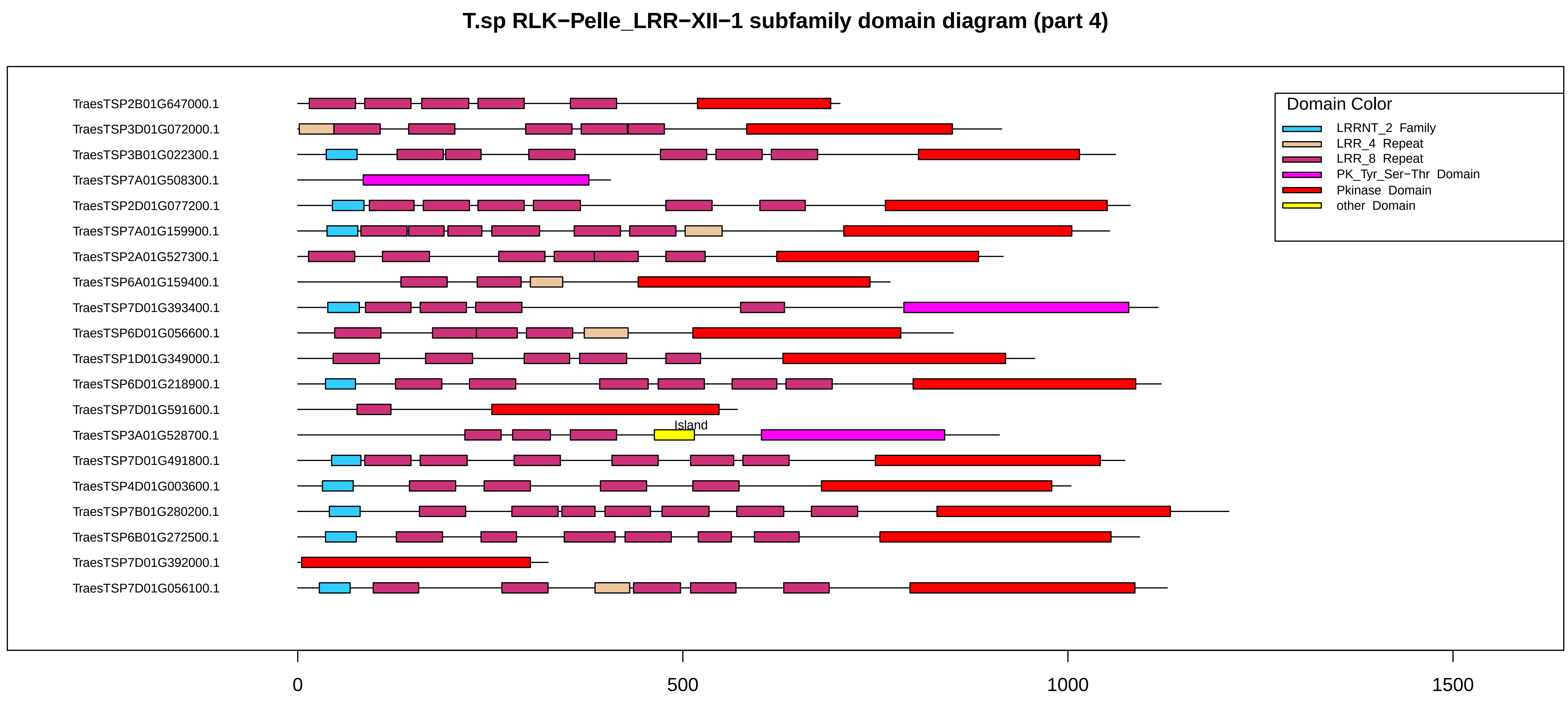

T.sp RLK−Pelle_LRR−XII−1 subfamily domain diagram (part 4)
TraesTSP2B01G647000.1
TraesTSP3D01G072000.1
TraesTSP3B01G022300.1
TraesTSP7A01G508300.1
TraesTSP2D01G077200.1
TraesTSP7A01G159900.1
TraesTSP2A01G527300.1
TraesTSP6A01G159400.1
TraesTSP7D01G393400.1
TraesTSP6D01G056600.1
TraesTSP1D01G349000.1
TraesTSP6D01G218900.1
TraesTSP7D01G591600.1
TraesTSP3A01G528700.1
TraesTSP7D01G491800.1
TraesTSP4D01G003600.1
TraesTSP7B01G280200.1
TraesTSP6B01G272500.1
TraesTSP7D01G392000.1
TraesTSP7D01G056100.1
Island
Domain Color
LRRNT_2 Family
LRR_4 Repeat
LRR_8 Repeat
PK_Tyr_Ser−Thr Domain
Pkinase Domain
other Domain
0
500
1000
1500

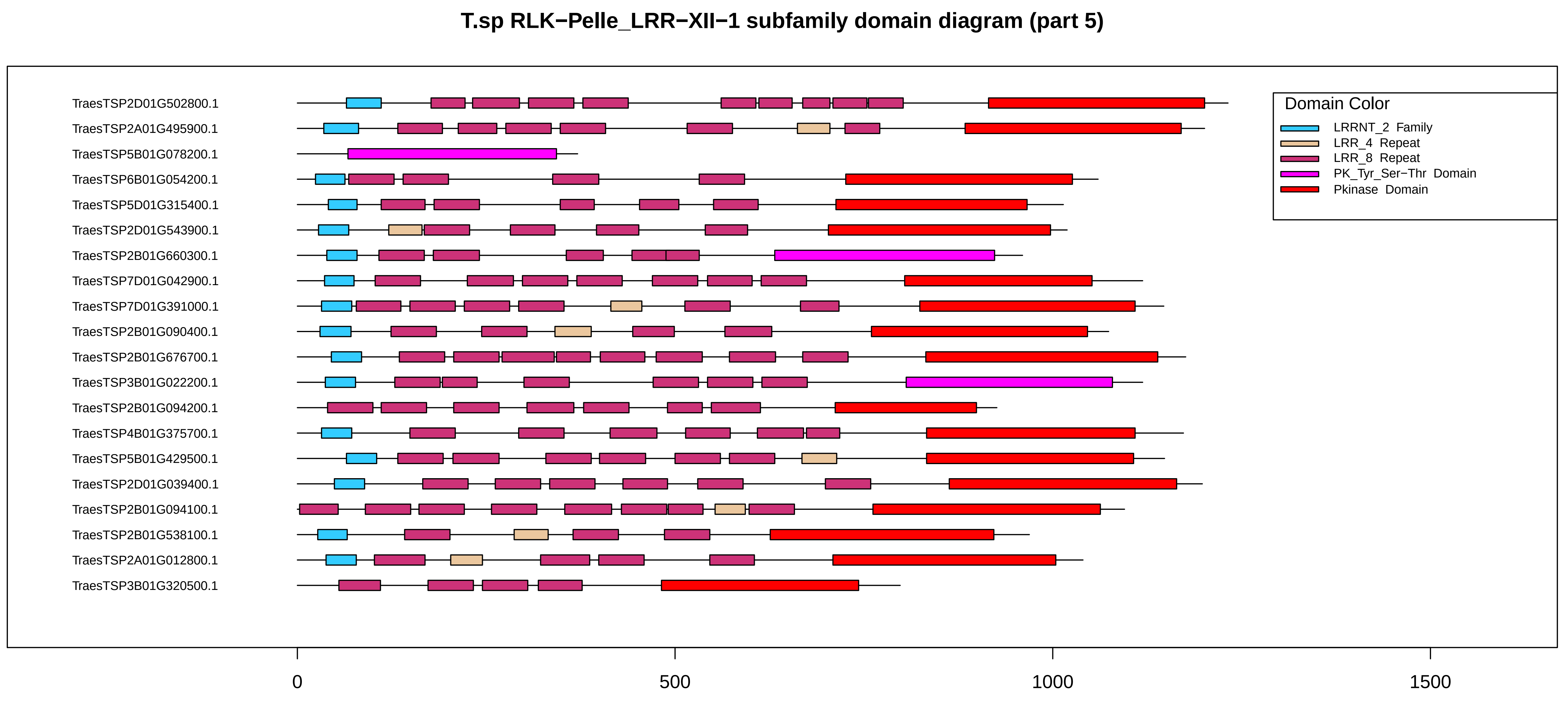
T.sp RLK−Pelle_LRR−XII−1 subfamily domain diagram (part 5)
TraesTSP2D01G502800.1
TraesTSP2A01G495900.1
TraesTSP5B01G078200.1
TraesTSP6B01G054200.1
TraesTSP5D01G315400.1
TraesTSP2D01G543900.1
TraesTSP2B01G660300.1
TraesTSP7D01G042900.1
TraesTSP7D01G391000.1
TraesTSP2B01G090400.1
TraesTSP2B01G676700.1
TraesTSP3B01G022200.1
TraesTSP2B01G094200.1
TraesTSP4B01G375700.1
TraesTSP5B01G429500.1
TraesTSP2D01G039400.1
TraesTSP2B01G094100.1
TraesTSP2B01G538100.1
TraesTSP2A01G012800.1
TraesTSP3B01G320500.1
Domain Color
LRRNT_2 Family
LRR_4 Repeat
LRR_8 Repeat
PK_Tyr_Ser−Thr Domain
Pkinase Domain
0
500
1000
1500

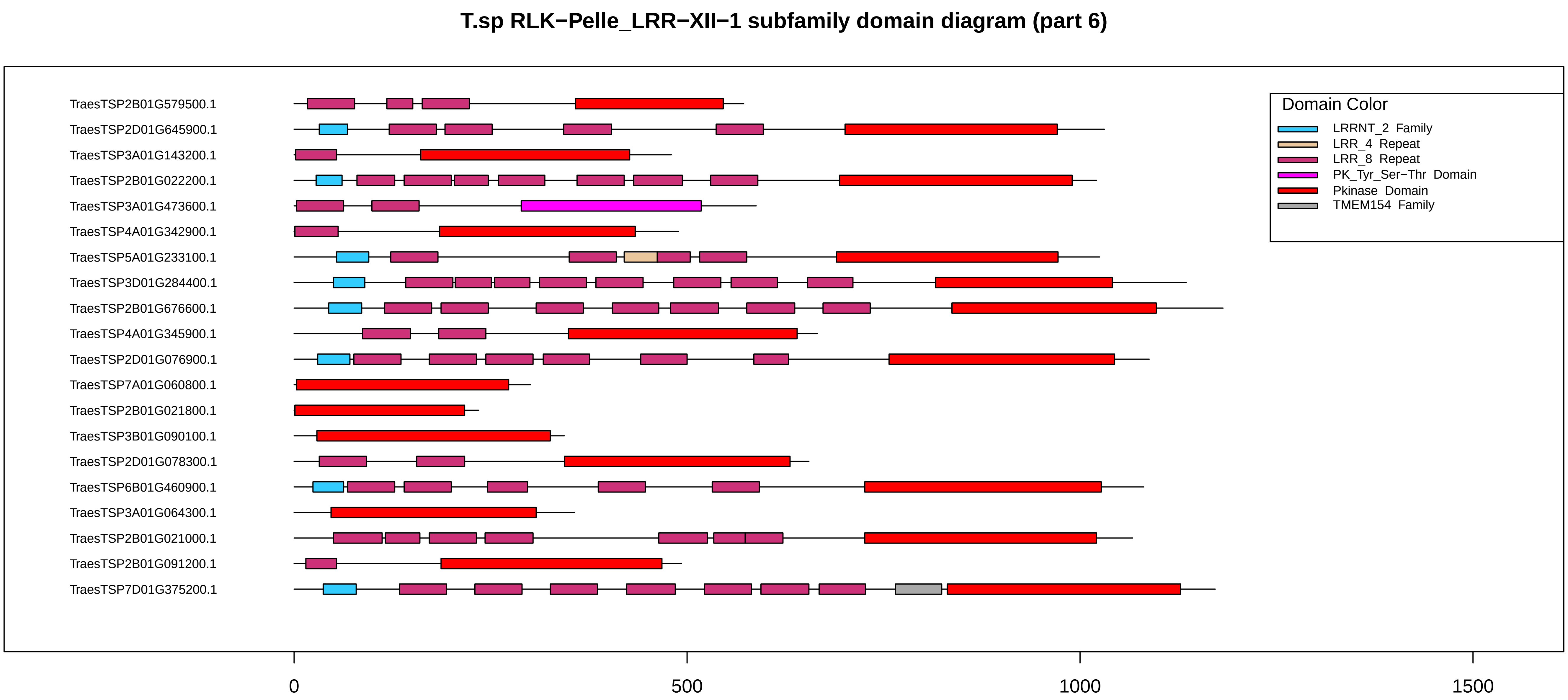
T.sp RLK−Pelle_LRR−XII−1 subfamily domain diagram (part 6)
TraesTSP2B01G579500.1
TraesTSP2D01G645900.1
TraesTSP3A01G143200.1
TraesTSP2B01G022200.1
TraesTSP3A01G473600.1
TraesTSP4A01G342900.1
TraesTSP5A01G233100.1
TraesTSP3D01G284400.1
TraesTSP2B01G676600.1
TraesTSP4A01G345900.1
TraesTSP2D01G076900.1
TraesTSP7A01G060800.1
TraesTSP2B01G021800.1
TraesTSP3B01G090100.1
TraesTSP2D01G078300.1
TraesTSP6B01G460900.1
TraesTSP3A01G064300.1
TraesTSP2B01G021000.1
TraesTSP2B01G091200.1
TraesTSP7D01G375200.1
Domain Color
LRRNT_2 Family
LRR_4 Repeat
LRR_8 Repeat
PK_Tyr_Ser−Thr Domain
Pkinase Domain
TMEM154 Family
0
500
1000
1500

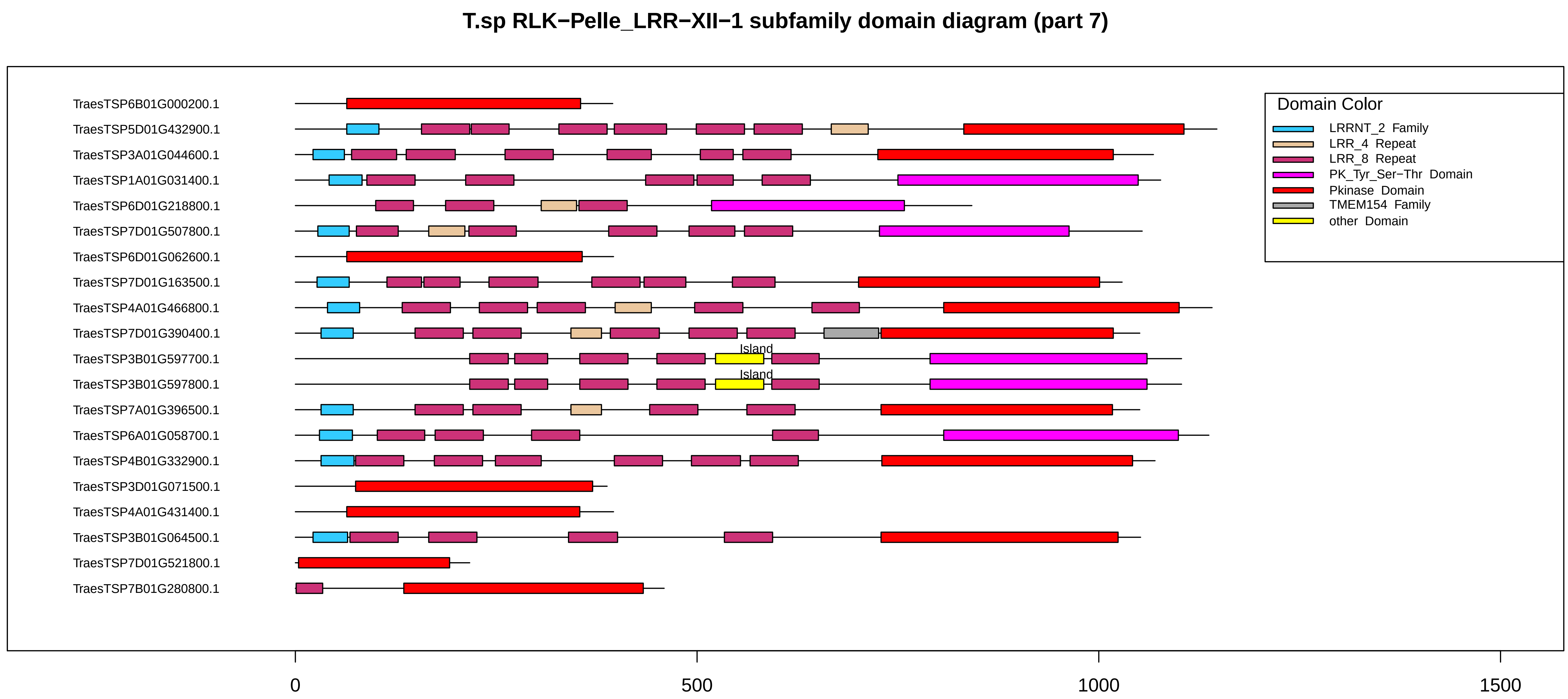
T.sp RLK−Pelle_LRR−XII−1 subfamily domain diagram (part 7)
TraesTSP6B01G000200.1
TraesTSP5D01G432900.1
TraesTSP3A01G044600.1
TraesTSP1A01G031400.1
TraesTSP6D01G218800.1
TraesTSP7D01G507800.1
TraesTSP6D01G062600.1
TraesTSP7D01G163500.1
TraesTSP4A01G466800.1
TraesTSP7D01G390400.1
TraesTSP3B01G597700.1
TraesTSP3B01G597800.1
TraesTSP7A01G396500.1
TraesTSP6A01G058700.1
TraesTSP4B01G332900.1
TraesTSP3D01G071500.1
TraesTSP4A01G431400.1
TraesTSP3B01G064500.1
TraesTSP7D01G521800.1
TraesTSP7B01G280800.1
Island
Island
Domain Color
LRRNT_2 Family
LRR_4 Repeat
LRR_8 Repeat
PK_Tyr_Ser−Thr Domain
Pkinase Domain
TMEM154 Family
other Domain
0
500
1000
1500

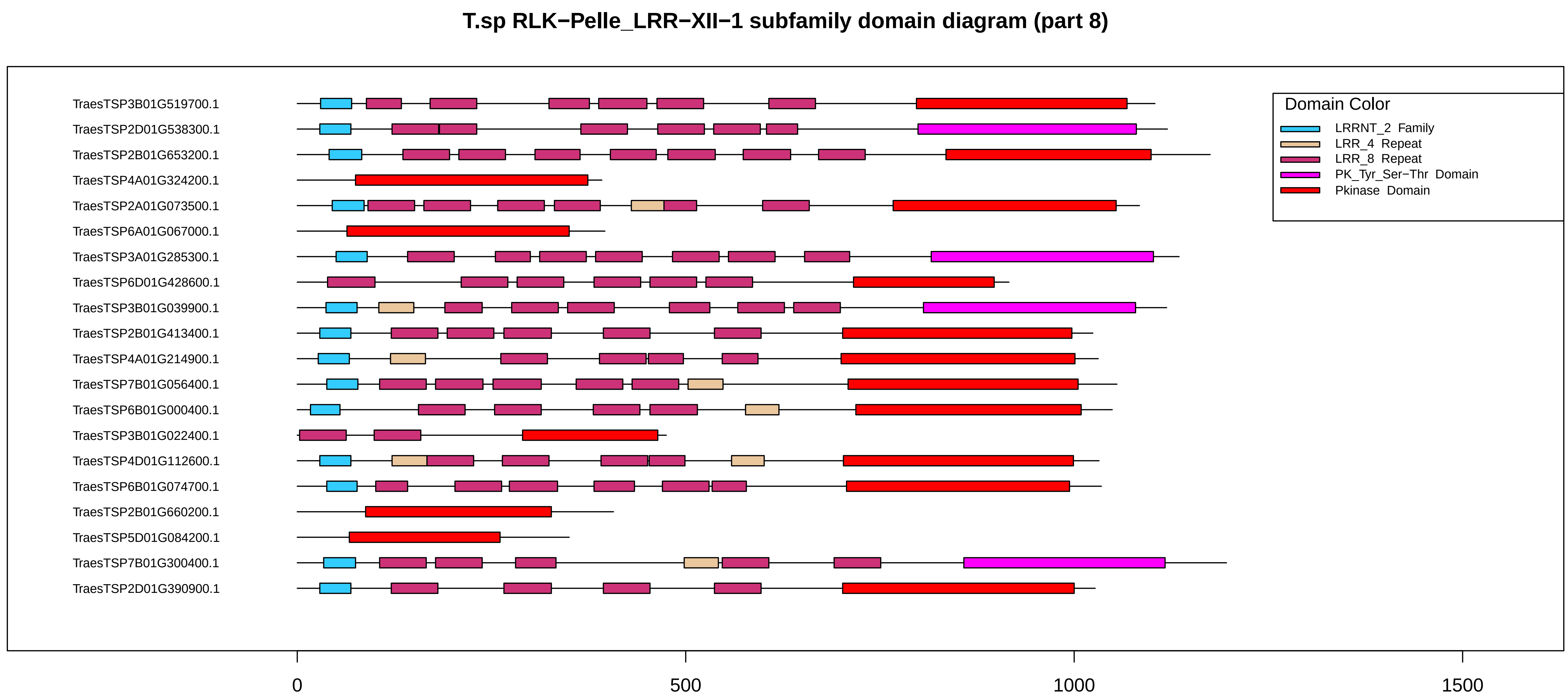
T.sp RLK−Pelle_LRR−XII−1 subfamily domain diagram (part 8)
TraesTSP3B01G519700.1
TraesTSP2D01G538300.1
TraesTSP2B01G653200.1
TraesTSP4A01G324200.1
TraesTSP2A01G073500.1
TraesTSP6A01G067000.1
TraesTSP3A01G285300.1
TraesTSP6D01G428600.1
TraesTSP3B01G039900.1
TraesTSP2B01G413400.1
TraesTSP4A01G214900.1
TraesTSP7B01G056400.1
TraesTSP6B01G000400.1
TraesTSP3B01G022400.1
TraesTSP4D01G112600.1
TraesTSP6B01G074700.1
TraesTSP2B01G660200.1
TraesTSP5D01G084200.1
TraesTSP7B01G300400.1
TraesTSP2D01G390900.1
Domain Color
LRRNT_2 Family
LRR_4 Repeat
LRR_8 Repeat
PK_Tyr_Ser−Thr Domain
Pkinase Domain
0
500
1000
1500

T.sp RLK−Pelle_LRR−XII−1 subfamily domain diagram (part 9)

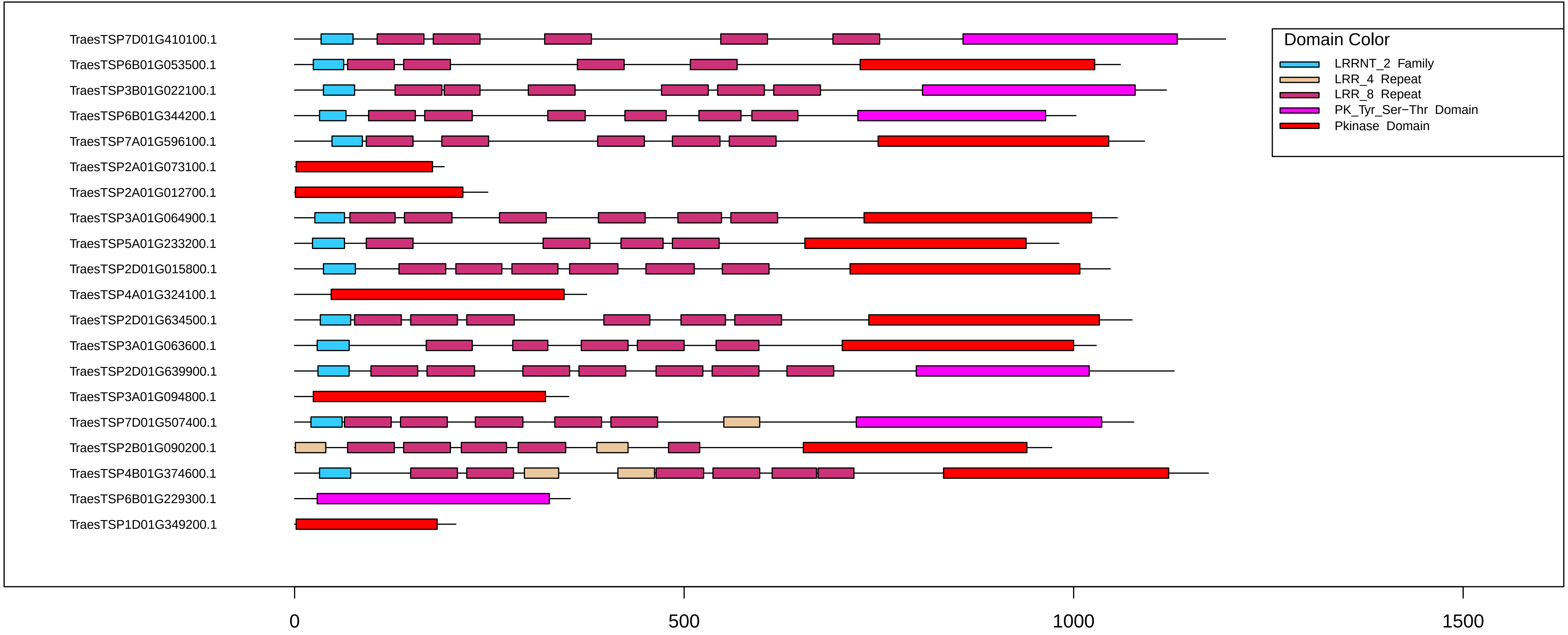

T.sp RLK−Pelle_LRR−XII−1 subfamily domain diagram (part 10)

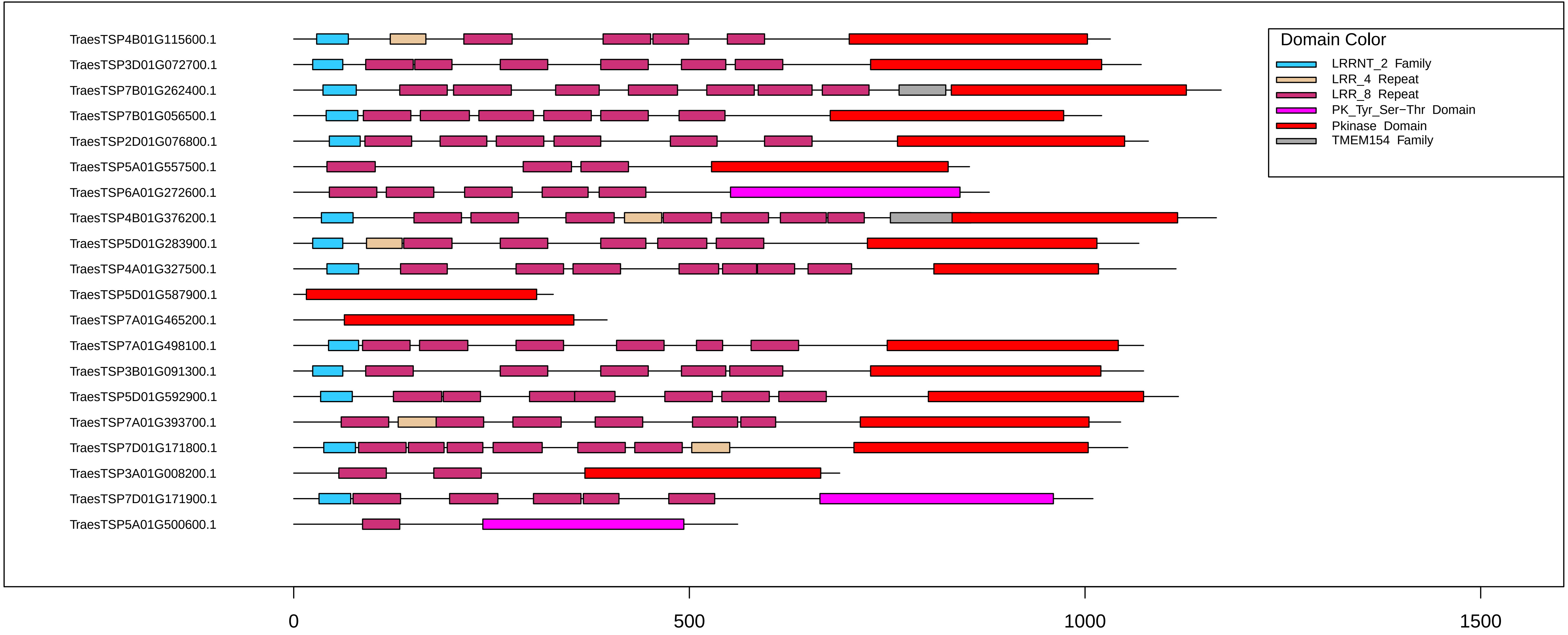

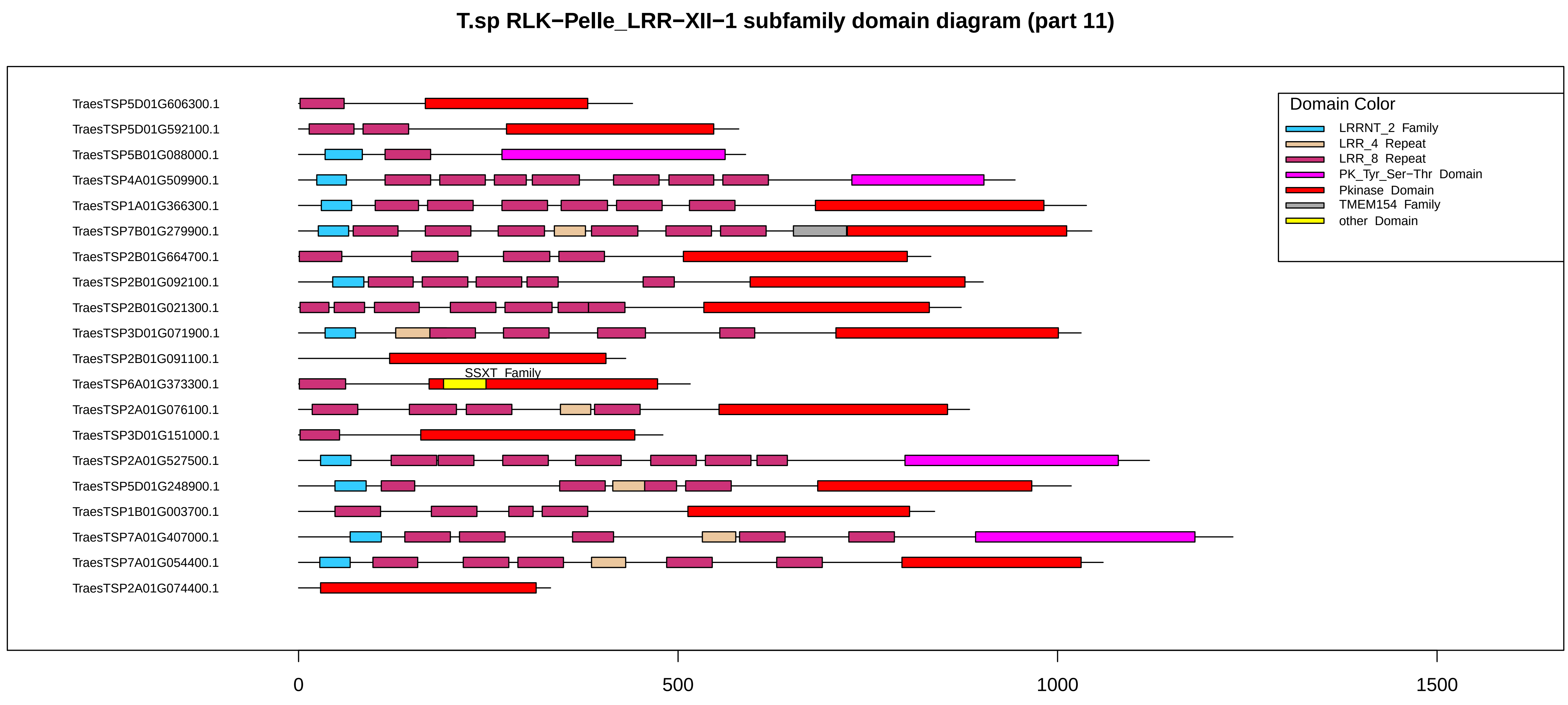
T.sp RLK−Pelle_LRR−XII−1 subfamily domain diagram (part 11)
TraesTSP5D01G606300.1
TraesTSP5D01G592100.1
TraesTSP5B01G088000.1
TraesTSP4A01G509900.1
TraesTSP1A01G366300.1
TraesTSP7B01G279900.1
TraesTSP2B01G664700.1
TraesTSP2B01G092100.1
TraesTSP2B01G021300.1
TraesTSP3D01G071900.1
TraesTSP2B01G091100.1
TraesTSP6A01G373300.1
SSXT_Family
TraesTSP2A01G076100.1
TraesTSP3D01G151000.1
TraesTSP2A01G527500.1
TraesTSP5D01G248900.1
TraesTSP1B01G003700.1
TraesTSP7A01G407000.1
TraesTSP7A01G054400.1
TraesTSP2A01G074400.1
Domain Color
LRRNT_2 Family
LRR_4 Repeat
LRR_8 Repeat
PK_Tyr_Ser−Thr Domain
Pkinase Domain
TMEM154 Family
other Domain
0
500
1000
1500

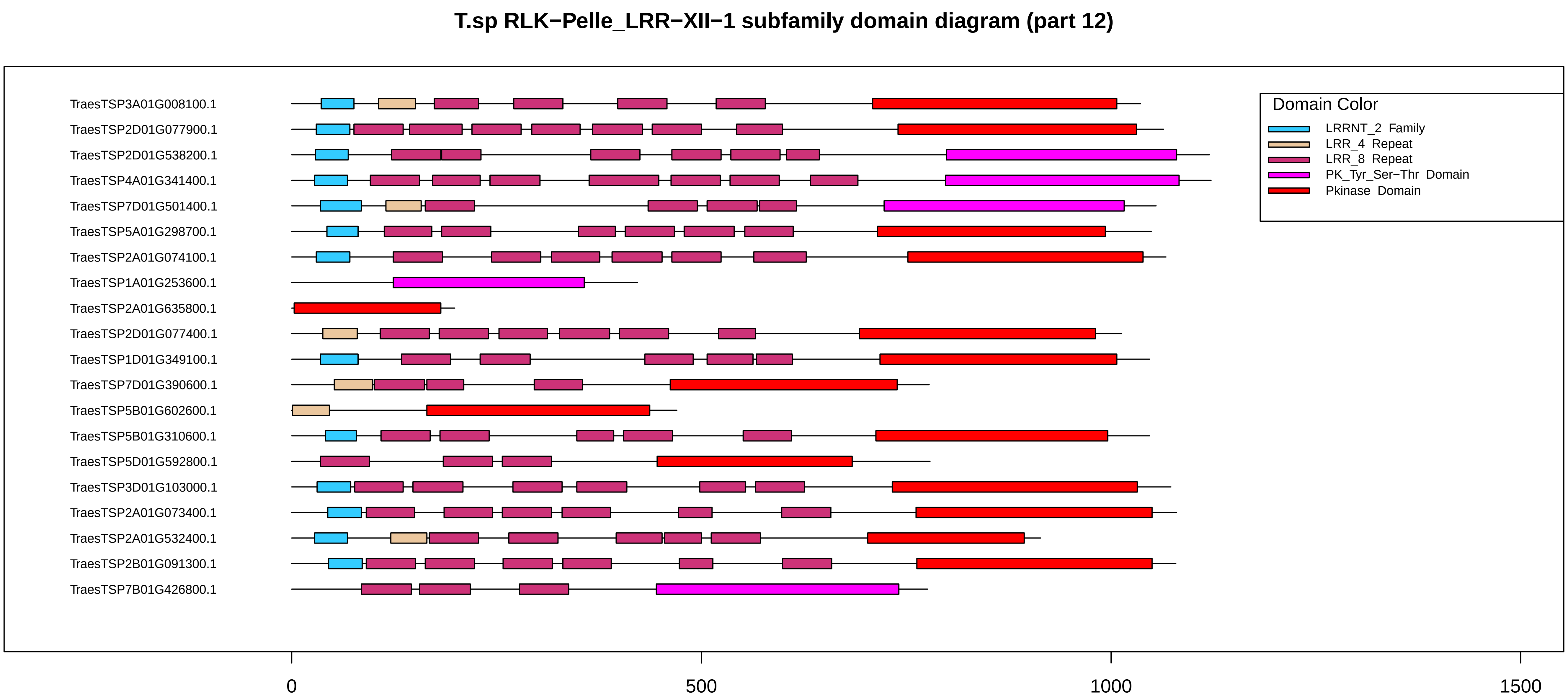
T.sp RLK−Pelle_LRR−XII−1 subfamily domain diagram (part 12)
TraesTSP3A01G008100.1
TraesTSP2D01G077900.1
TraesTSP2D01G538200.1
TraesTSP4A01G341400.1
TraesTSP7D01G501400.1
TraesTSP5A01G298700.1
TraesTSP2A01G074100.1
TraesTSP1A01G253600.1
TraesTSP2A01G635800.1
TraesTSP2D01G077400.1
TraesTSP1D01G349100.1
TraesTSP7D01G390600.1
TraesTSP5B01G602600.1
TraesTSP5B01G310600.1
TraesTSP5D01G592800.1
TraesTSP3D01G103000.1
TraesTSP2A01G073400.1
TraesTSP2A01G532400.1
TraesTSP2B01G091300.1
TraesTSP7B01G426800.1
Domain Color
LRRNT_2 Family
LRR_4 Repeat
LRR_8 Repeat
PK_Tyr_Ser−Thr Domain
Pkinase Domain
0
500
1000
1500

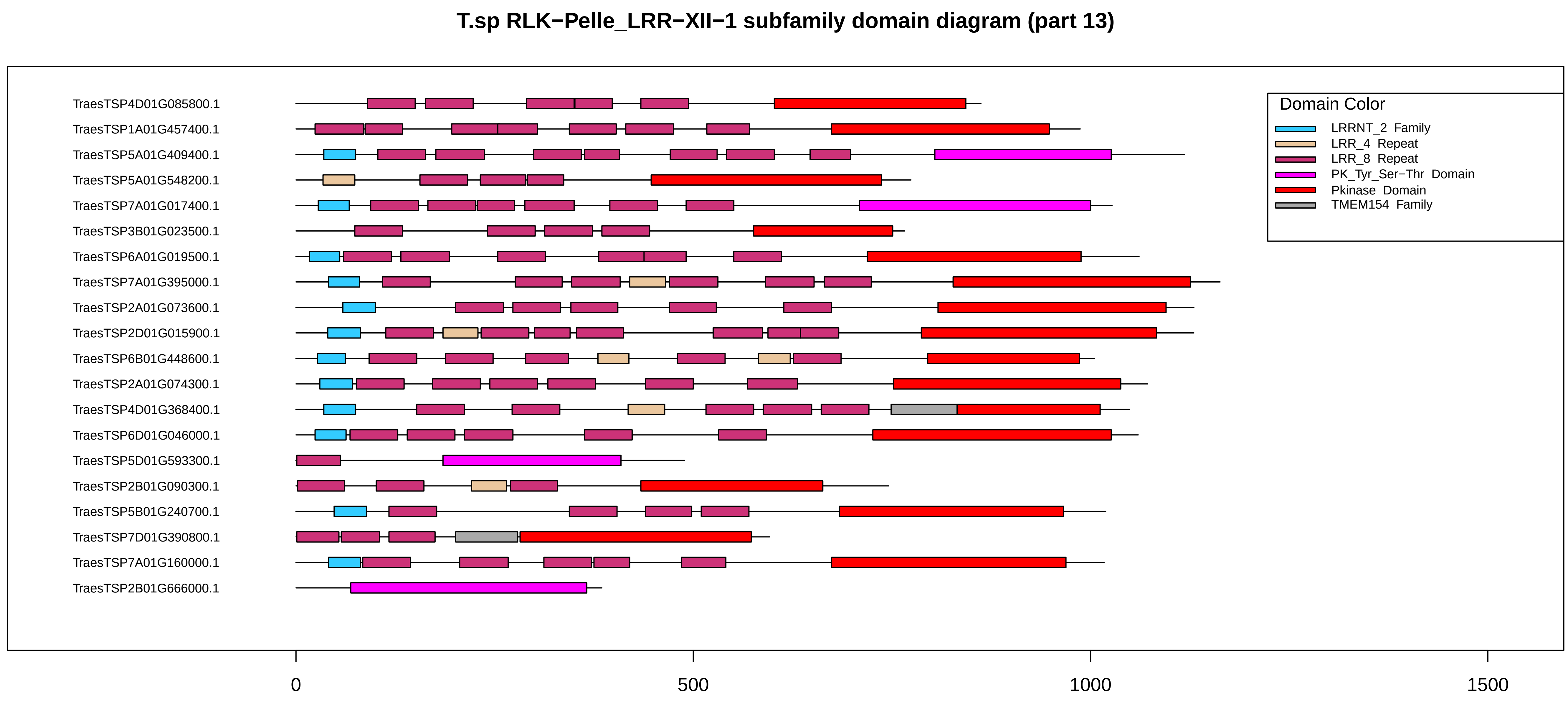
T.sp RLK−Pelle_LRR−XII−1 subfamily domain diagram (part 13)
TraesTSP4D01G085800.1
TraesTSP1A01G457400.1
TraesTSP5A01G409400.1
TraesTSP5A01G548200.1
TraesTSP7A01G017400.1
TraesTSP3B01G023500.1
TraesTSP6A01G019500.1
TraesTSP7A01G395000.1
TraesTSP2A01G073600.1
TraesTSP2D01G015900.1
TraesTSP6B01G448600.1
TraesTSP2A01G074300.1
TraesTSP4D01G368400.1
TraesTSP6D01G046000.1
TraesTSP5D01G593300.1
TraesTSP2B01G090300.1
TraesTSP5B01G240700.1
TraesTSP7D01G390800.1
TraesTSP7A01G160000.1
TraesTSP2B01G666000.1
Domain Color
LRRNT_2 Family
LRR_4 Repeat
LRR_8 Repeat
PK_Tyr_Ser−Thr Domain
Pkinase Domain
TMEM154 Family
0
500
1000
1500

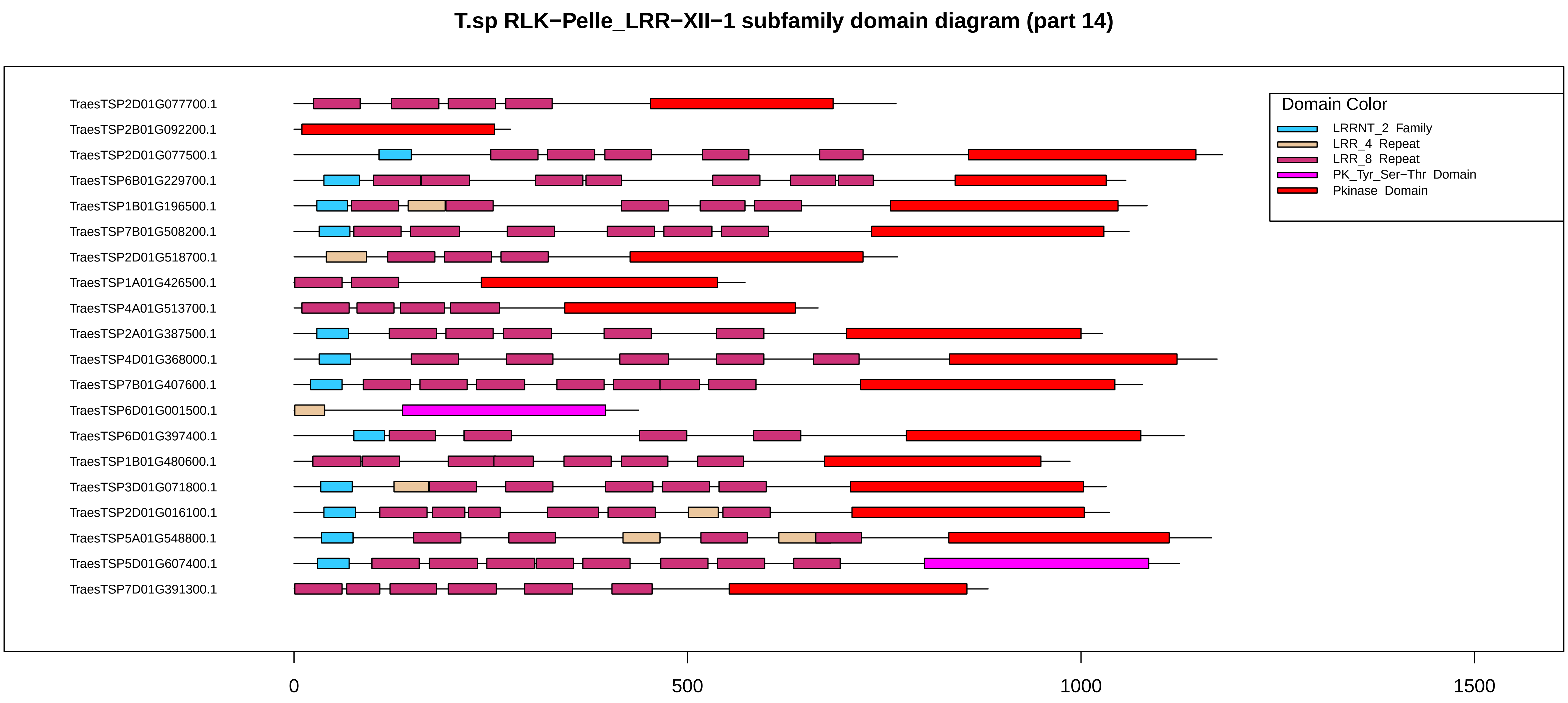
T.sp RLK−Pelle_LRR−XII−1 subfamily domain diagram (part 14)
TraesTSP2D01G077700.1
TraesTSP2B01G092200.1
TraesTSP2D01G077500.1
TraesTSP6B01G229700.1
TraesTSP1B01G196500.1
TraesTSP7B01G508200.1
TraesTSP2D01G518700.1
TraesTSP1A01G426500.1
TraesTSP4A01G513700.1
TraesTSP2A01G387500.1
TraesTSP4D01G368000.1
TraesTSP7B01G407600.1
TraesTSP6D01G001500.1
TraesTSP6D01G397400.1
TraesTSP1B01G480600.1
TraesTSP3D01G071800.1
TraesTSP2D01G016100.1
TraesTSP5A01G548800.1
TraesTSP5D01G607400.1
TraesTSP7D01G391300.1
Domain Color
LRRNT_2 Family
LRR_4 Repeat
LRR_8 Repeat
PK_Tyr_Ser−Thr Domain
Pkinase Domain
0
500
1000
1500

T.sp RLK−Pelle_LRR−XII−1 subfamily domain diagram (part 15)

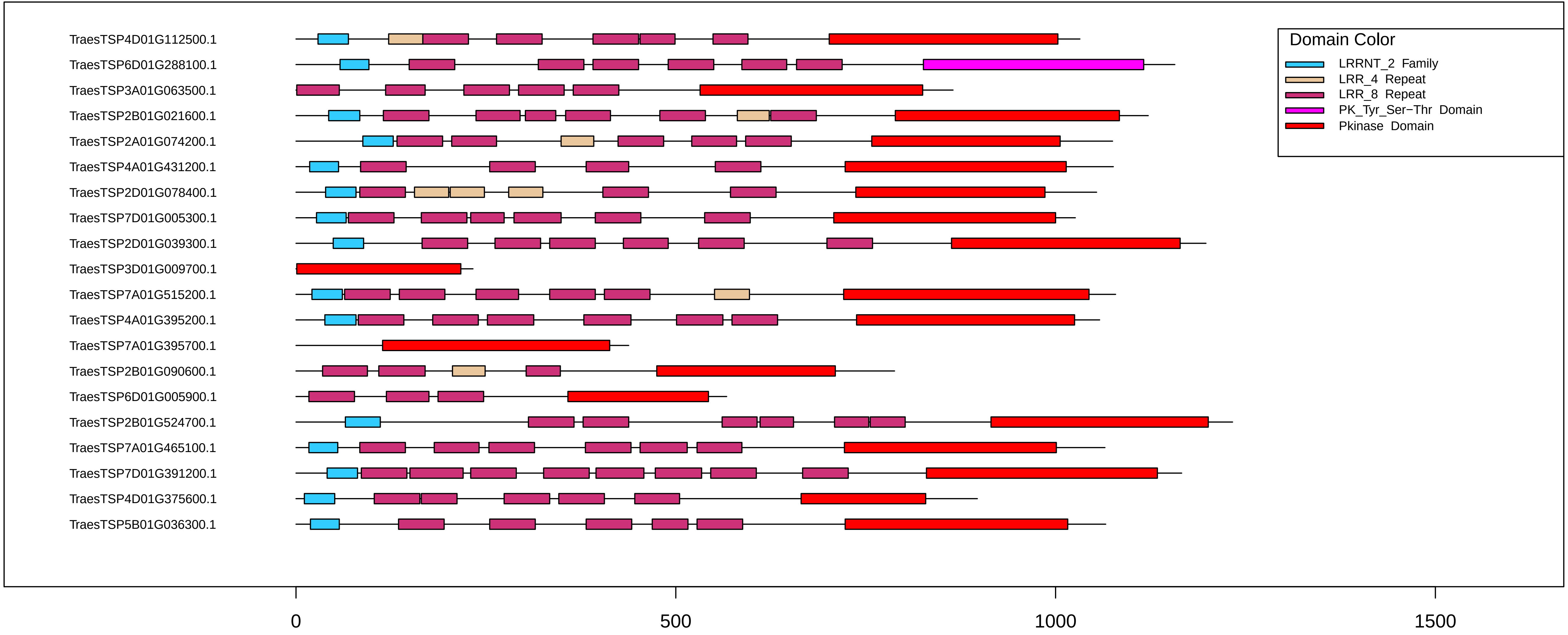

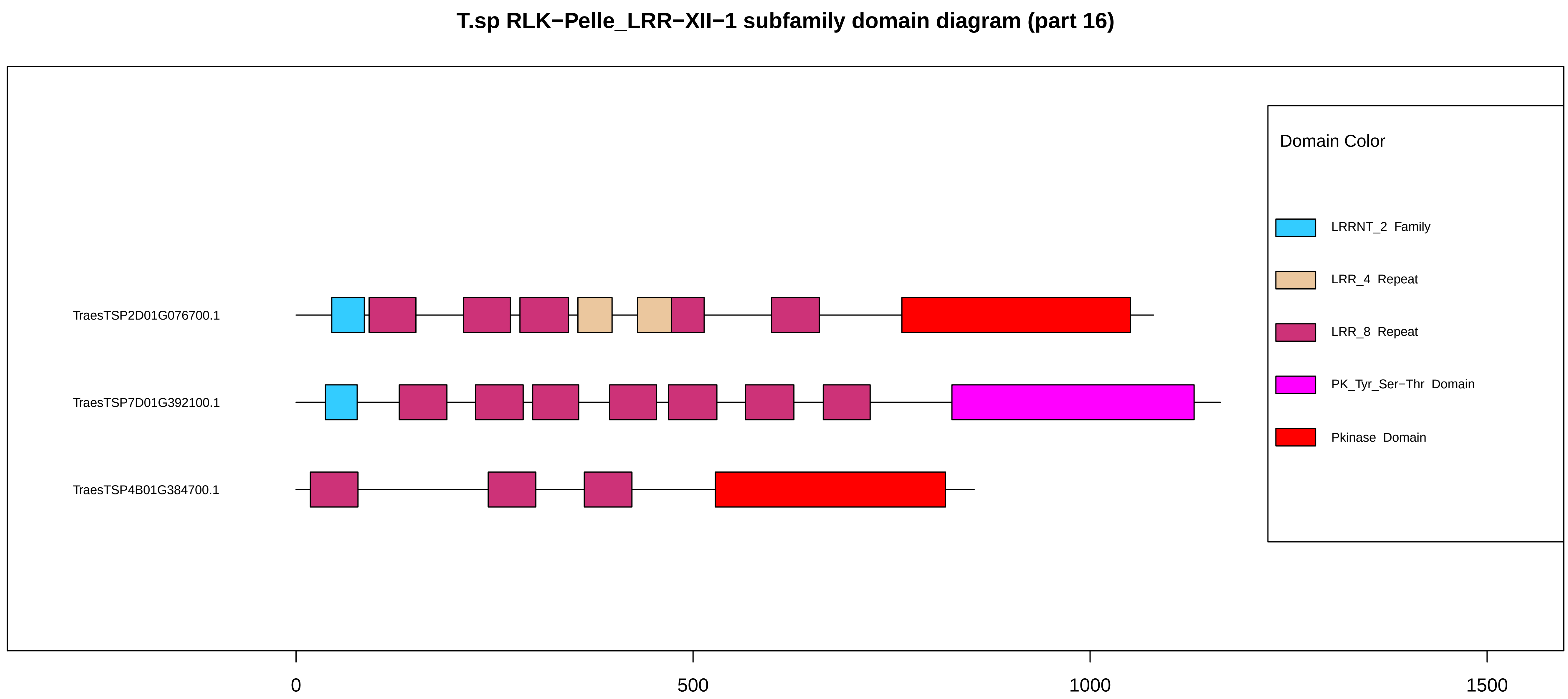
T.sp RLK−Pelle_LRR−XII−1 subfamily domain diagram (part 16)
TraesTSP2D01G076700.1
TraesTSP7D01G392100.1
TraesTSP4B01G384700.1
0
500
1000
1500
Domain Color
LRRNT_2 Family
LRR_4 Repeat
LRR_8 Repeat
PK_Tyr_Ser−Thr Domain
Pkinase Domain

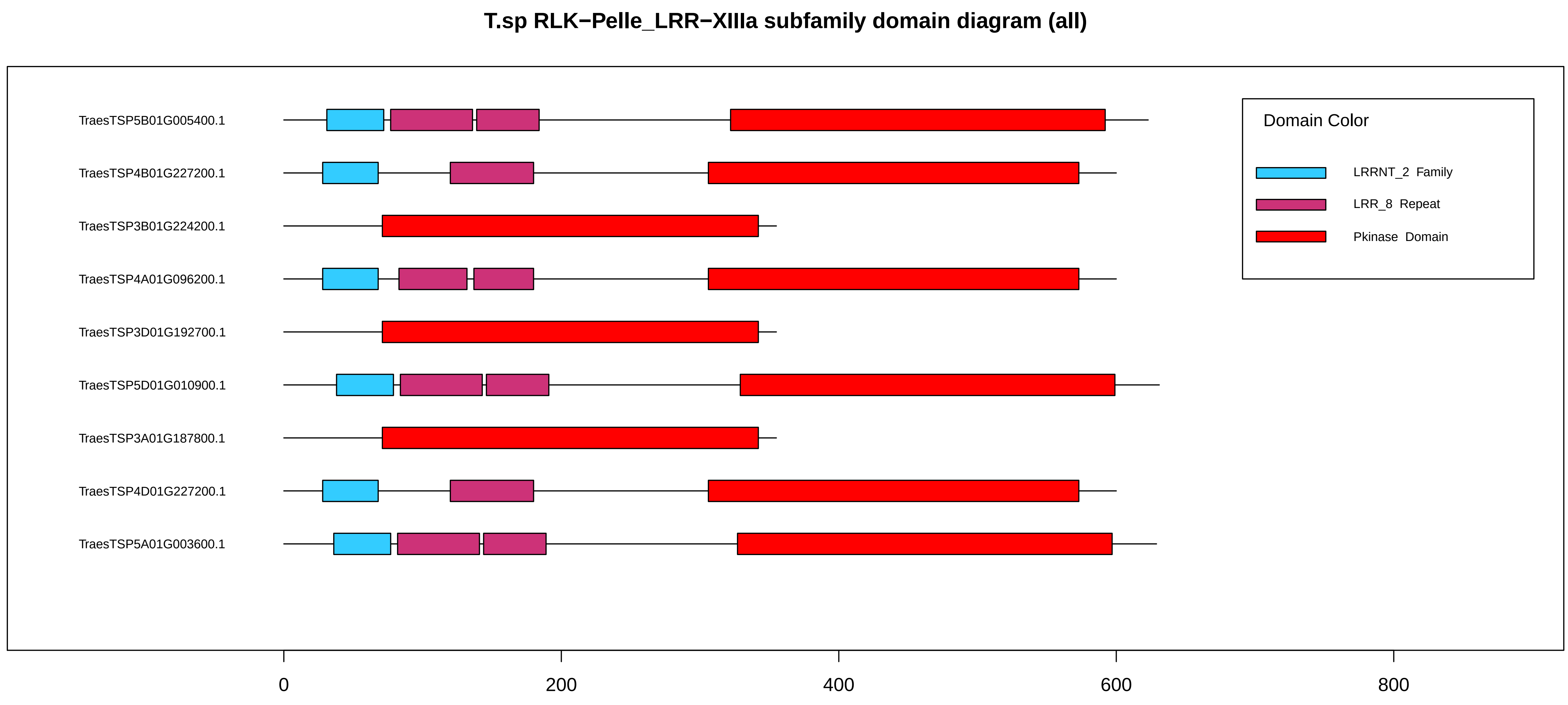
T.sp RLK−Pelle_LRR−XIIIa subfamily domain diagram (all)
TraesTSP5B01G005400.1
TraesTSP4B01G227200.1
TraesTSP3B01G224200.1
TraesTSP4A01G096200.1
TraesTSP3D01G192700.1
TraesTSP5D01G010900.1
TraesTSP3A01G187800.1
TraesTSP4D01G227200.1
TraesTSP5A01G003600.1
Domain Color
LRRNT_2 Family
LRR_8 Repeat
Pkinase Domain
0
200
400
600
800

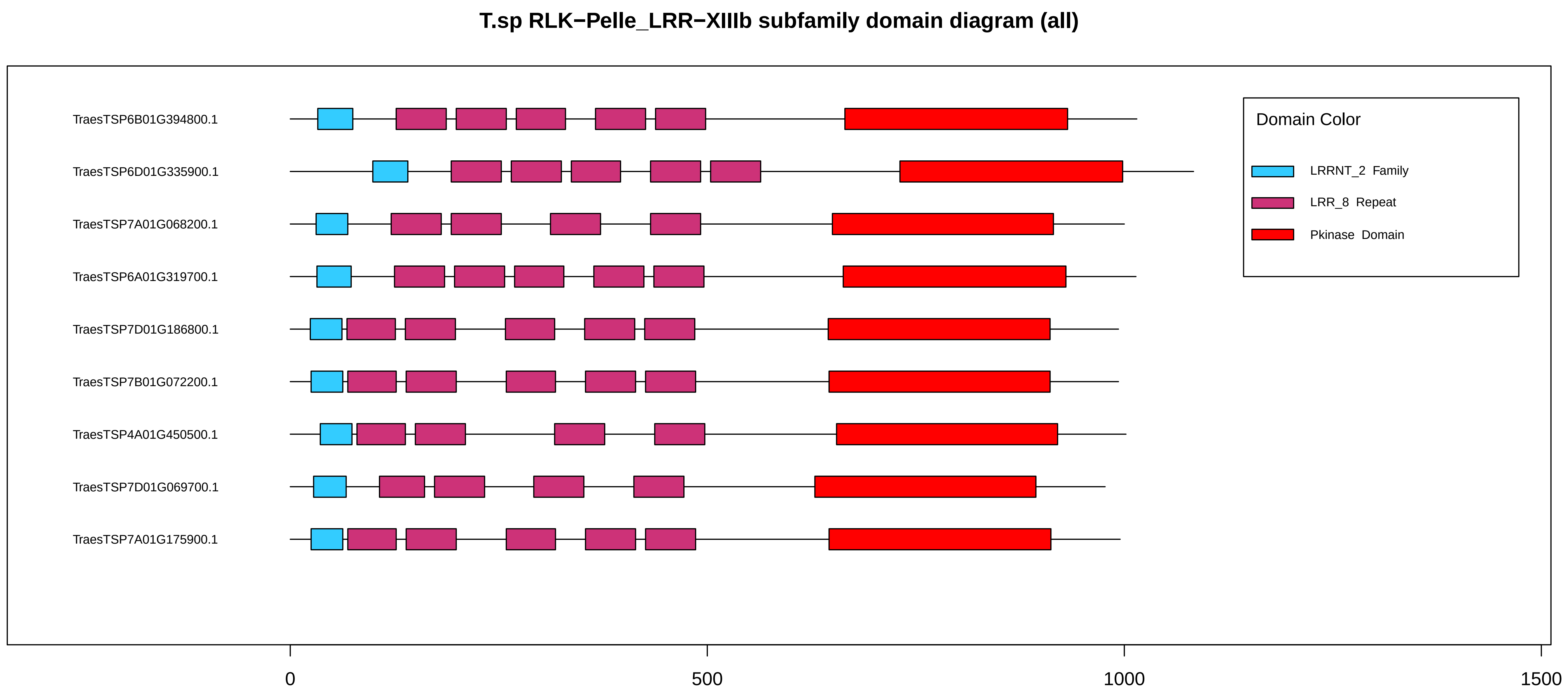

T.sp RLK−Pelle_LRR−XIIIb subfamily domain diagram (all)
TraesTSP6B01G394800.1
TraesTSP6D01G335900.1
TraesTSP7A01G068200.1
TraesTSP6A01G319700.1
TraesTSP7D01G186800.1
TraesTSP7B01G072200.1
TraesTSP4A01G450500.1
TraesTSP7D01G069700.1
TraesTSP7A01G175900.1
Domain Color
LRRNT_2 Family
LRR_8 Repeat
Pkinase Domain
0
500
1000
1500

T.sp RLK−Pelle_LRR−XIV subfamily domain diagram (all)

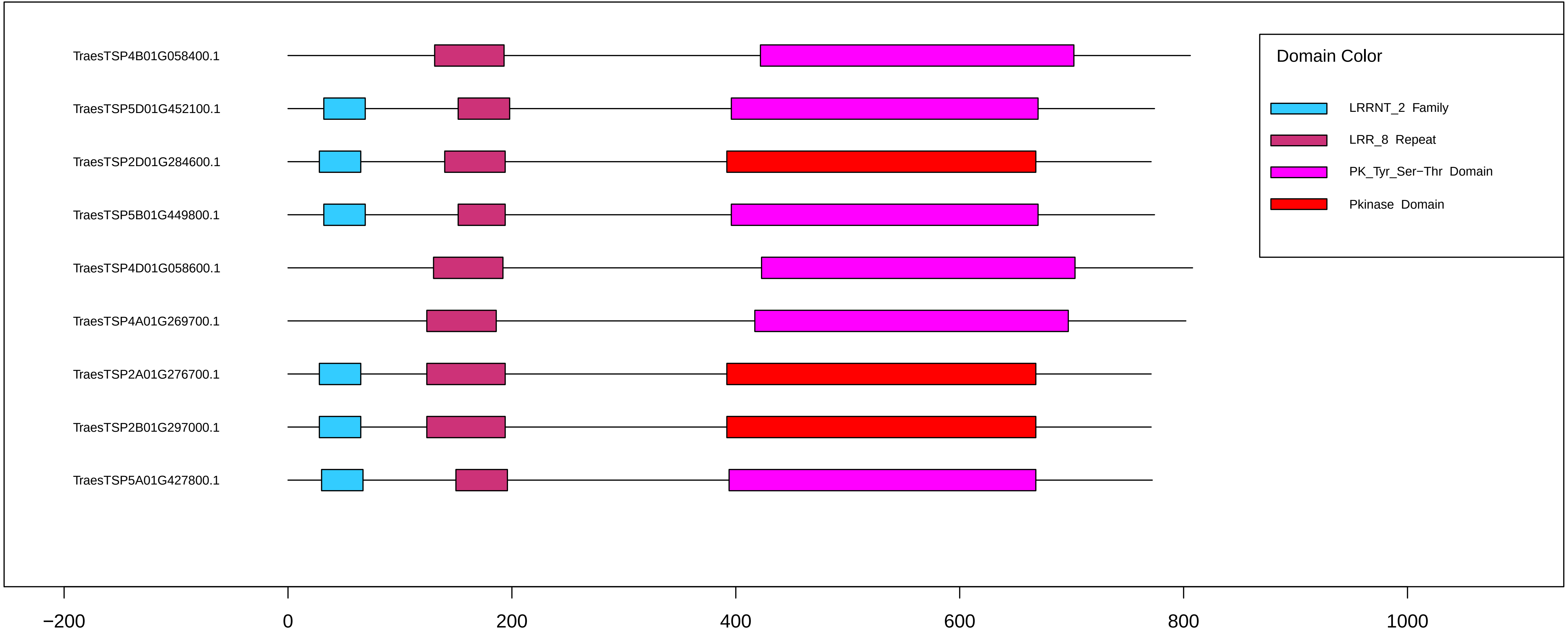

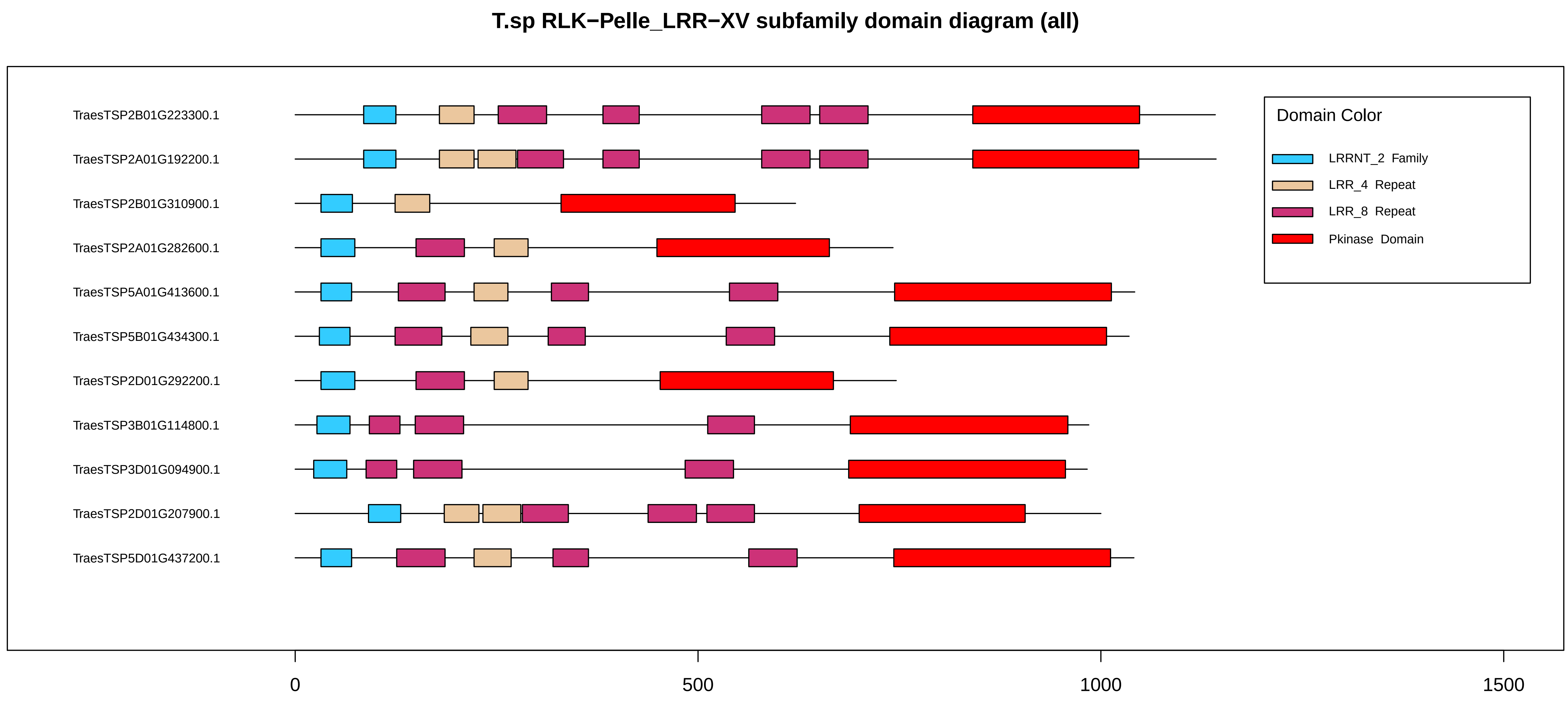
T.sp RLK−Pelle_LRR−XV subfamily domain diagram (all)
TraesTSP2B01G223300.1
TraesTSP2A01G192200.1
TraesTSP2B01G310900.1
TraesTSP2A01G282600.1
TraesTSP5A01G413600.1
TraesTSP5B01G434300.1
TraesTSP2D01G292200.1
TraesTSP3B01G114800.1
TraesTSP3D01G094900.1
TraesTSP2D01G207900.1
TraesTSP5D01G437200.1
Domain Color
LRRNT_2 Family
LRR_4 Repeat
LRR_8 Repeat
Pkinase Domain
0
500
1000
1500

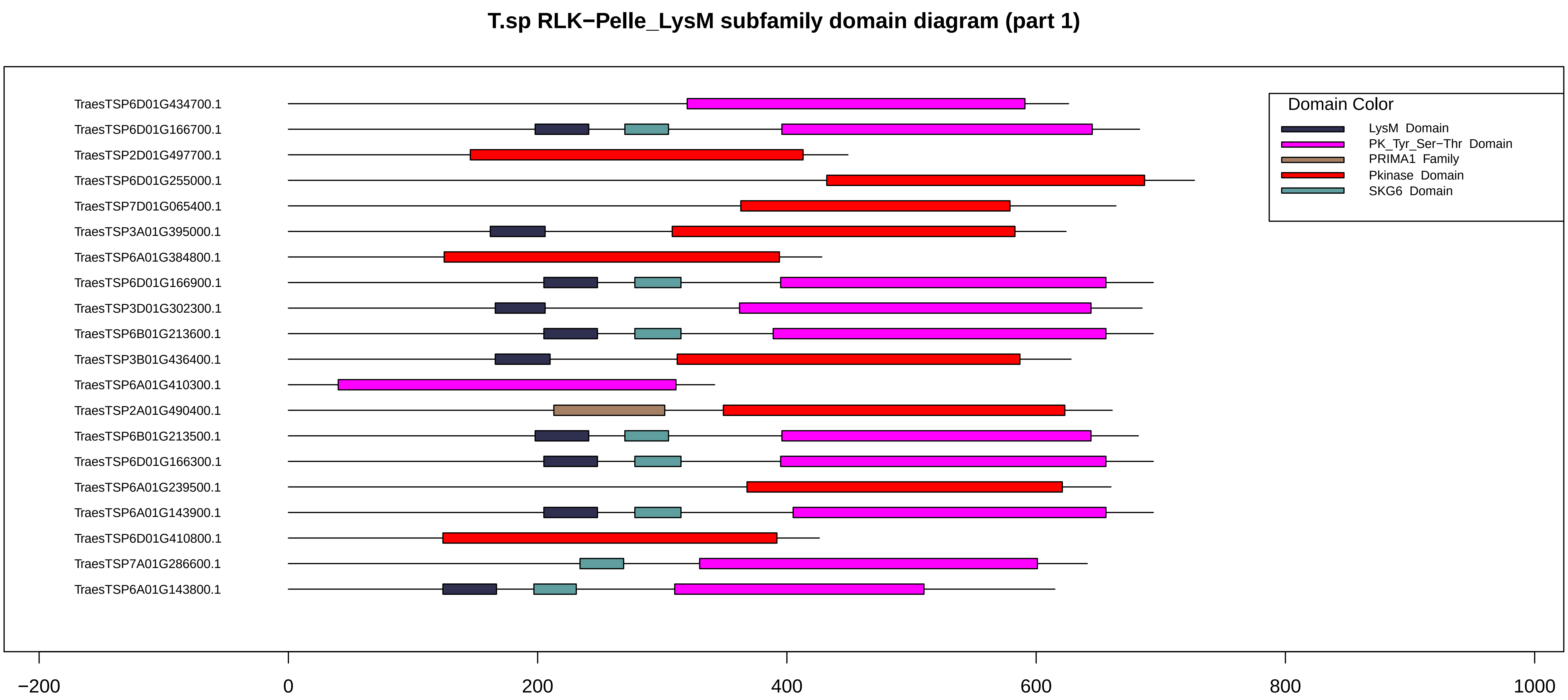
T.sp RLK−Pelle_LysM subfamily domain diagram (part 1)
TraesTSP6D01G434700.1
TraesTSP6D01G166700.1
TraesTSP2D01G497700.1
TraesTSP6D01G255000.1
TraesTSP7D01G065400.1
TraesTSP3A01G395000.1
TraesTSP6A01G384800.1
TraesTSP6D01G166900.1
TraesTSP3D01G302300.1
TraesTSP6B01G213600.1
TraesTSP3B01G436400.1
TraesTSP6A01G410300.1
TraesTSP2A01G490400.1
TraesTSP6B01G213500.1
TraesTSP6D01G166300.1
TraesTSP6A01G239500.1
TraesTSP6A01G143900.1
TraesTSP6D01G410800.1
TraesTSP7A01G286600.1
TraesTSP6A01G143800.1
Domain Color
LysM Domain
PK_Tyr_Ser−Thr Domain
PRIMA1 Family
Pkinase Domain
SKG6 Domain
−200
0
200
400
600
800
1000

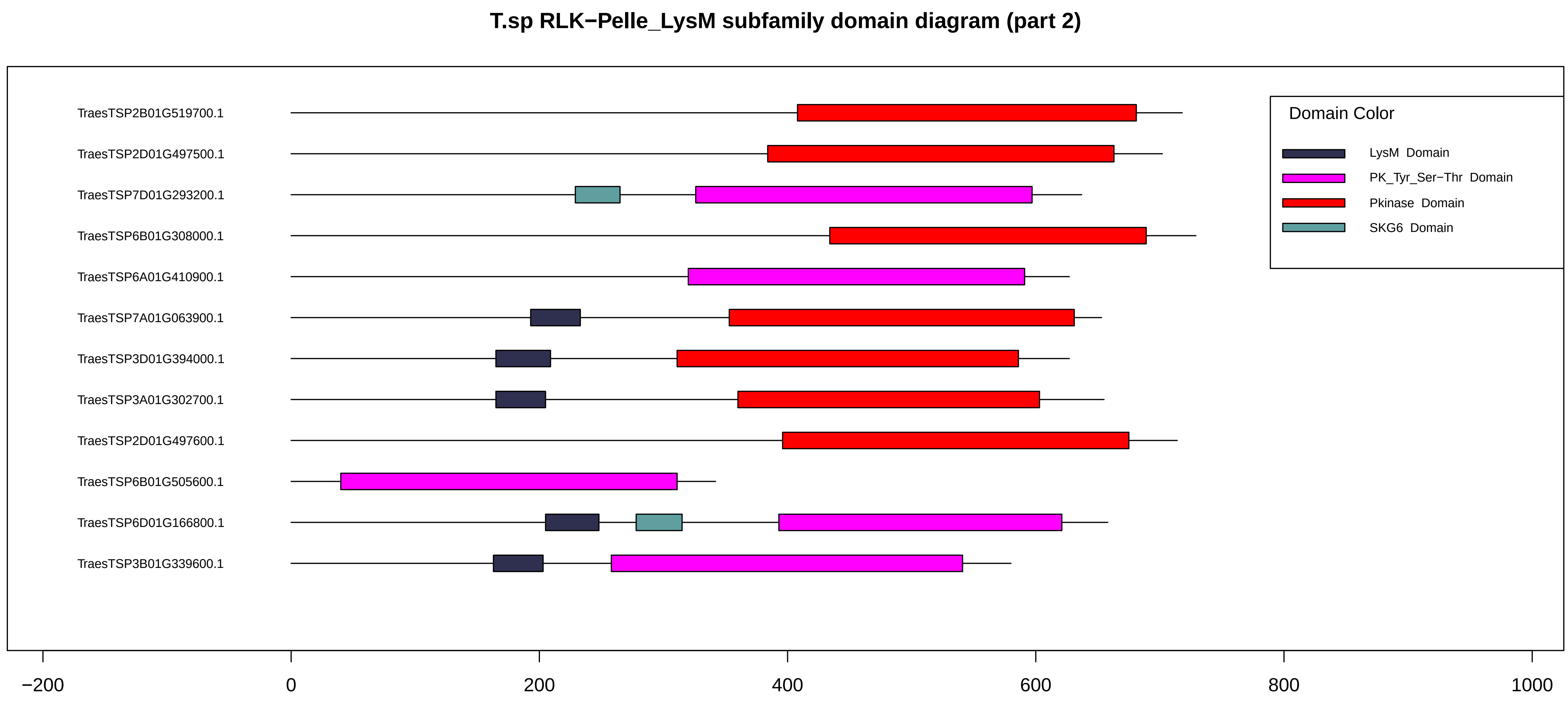
T.sp RLK−Pelle_LysM subfamily domain diagram (part 2)
TraesTSP2B01G519700.1
TraesTSP2D01G497500.1
TraesTSP7D01G293200.1
TraesTSP6B01G308000.1
TraesTSP6A01G410900.1
TraesTSP7A01G063900.1
TraesTSP3D01G394000.1
TraesTSP3A01G302700.1
TraesTSP2D01G497600.1
TraesTSP6B01G505600.1
TraesTSP6D01G166800.1
TraesTSP3B01G339600.1
Domain Color
LysM Domain
PK_Tyr_Ser−Thr Domain
Pkinase Domain
SKG6 Domain
−200
0
200
400
600
800
1000

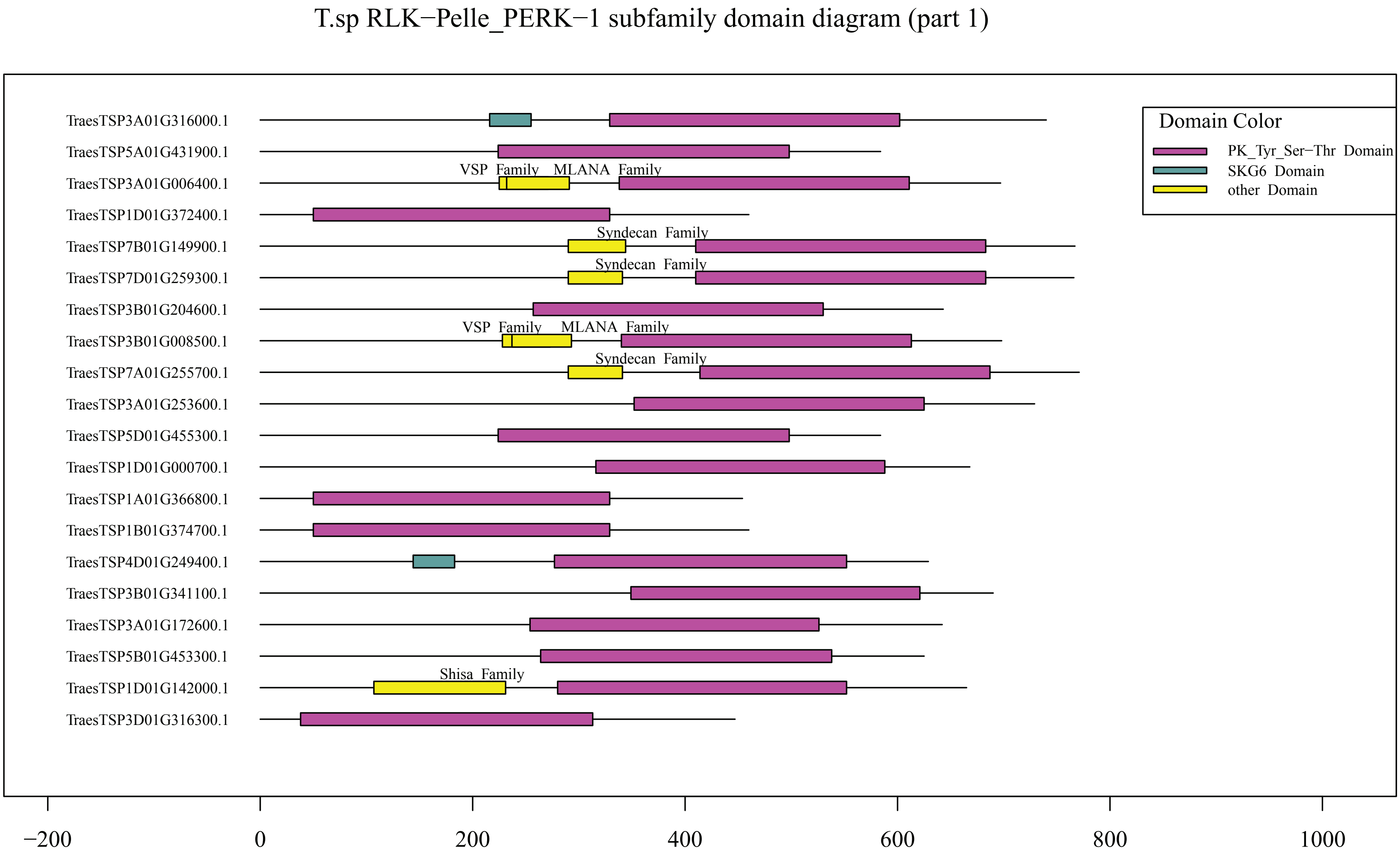
T.sp RLK−Pelle_PERK−1 subfamily domain diagram (part 1)
TraesTSP3A01G316000.1
TraesTSP5A01G431900.1
TraesTSP3A01G006400.1
VSP Family
MLANA Family
TraesTSP1D01G372400.1
TraesTSP7B01G149900.1
Syndecan Family
TraesTSP7D01G259300.1
Syndecan Family
TraesTSP3B01G204600.1
TraesTSP3B01G008500.1
VSP Family
MLANA Family
TraesTSP7A01G255700.1
Syndecan Family
TraesTSP3A01G253600.1
TraesTSP5D01G455300.1
TraesTSP1D01G000700.1
TraesTSP1A01G366800.1
TraesTSP1B01G374700.1
TraesTSP4D01G249400.1
TraesTSP3B01G341100.1
TraesTSP3A01G172600.1
TraesTSP5B01G453300.1
TraesTSP1D01G142000.1
Shisa Family
TraesTSP3D01G316300.1
Domain Color
PK_Tyr_Ser−Thr Domain
SKG6 Domain
other Domain
−200
0
200
400
600
800
1000

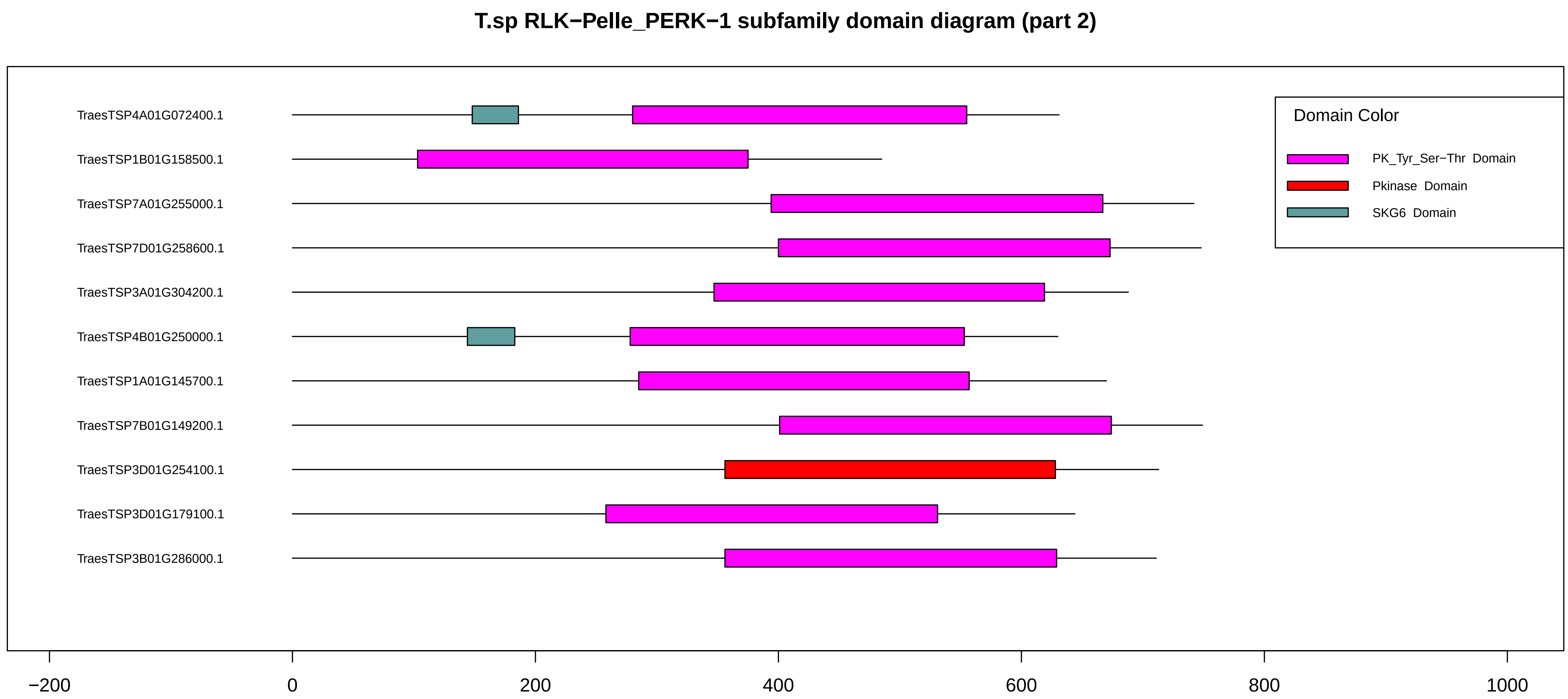
T.sp RLK−Pelle_PERK−1 subfamily domain diagram (part 2)
TraesTSP4A01G072400.1
TraesTSP1B01G158500.1
TraesTSP7A01G255000.1
TraesTSP7D01G258600.1
TraesTSP3A01G304200.1
TraesTSP4B01G250000.1
TraesTSP1A01G145700.1
TraesTSP7B01G149200.1
TraesTSP3D01G254100.1
TraesTSP3D01G179100.1
TraesTSP3B01G286000.1
Domain Color
PK_Tyr_Ser−Thr Domain
Pkinase Domain
SKG6 Domain
−200
0
200
400
600
800
1000

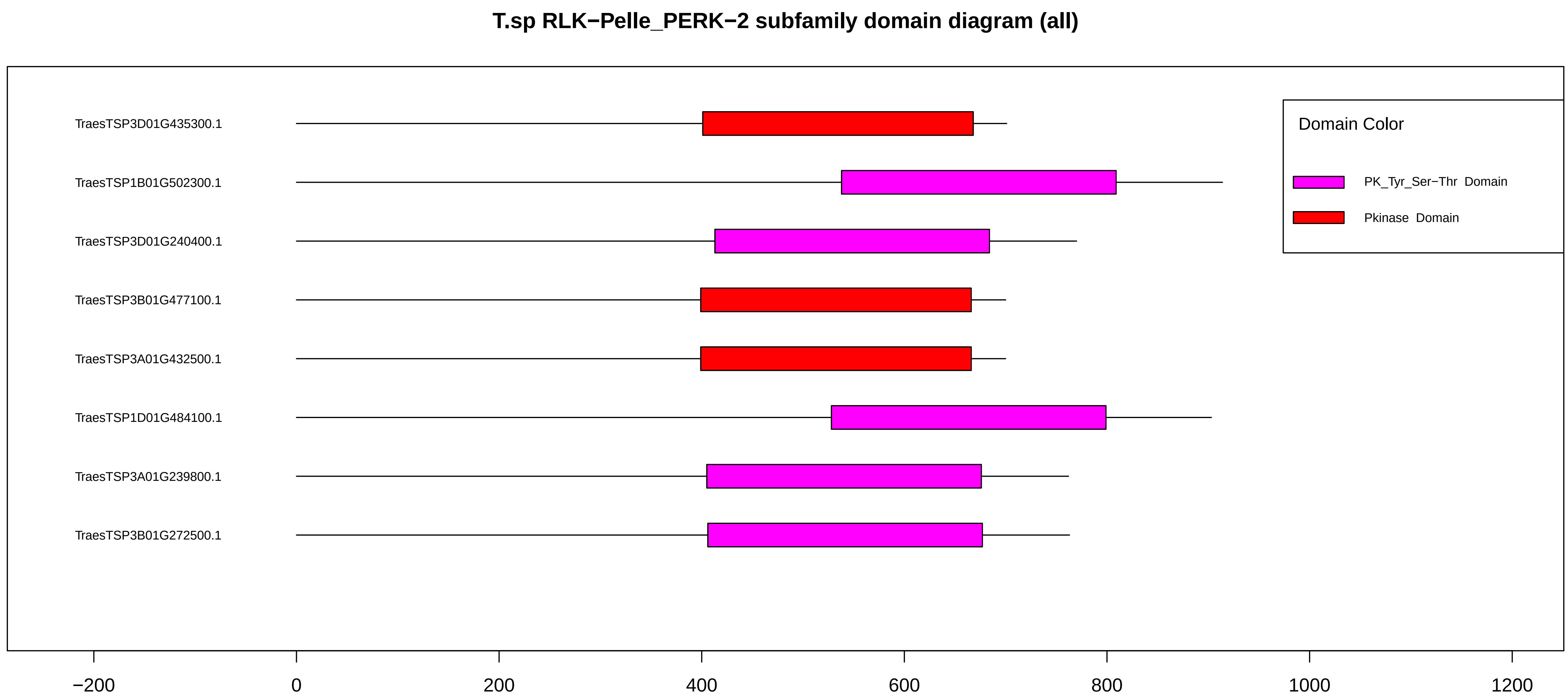
T.sp RLK−Pelle_PERK−2 subfamily domain diagram (all)
TraesTSP3D01G435300.1
TraesTSP1B01G502300.1
TraesTSP3D01G240400.1
TraesTSP3B01G477100.1
TraesTSP3A01G432500.1
TraesTSP1D01G484100.1
TraesTSP3A01G239800.1
TraesTSP3B01G272500.1
Domain Color
PK_Tyr_Ser−Thr Domain
Pkinase Domain
−200
0
200
400
600
800
1000
1200

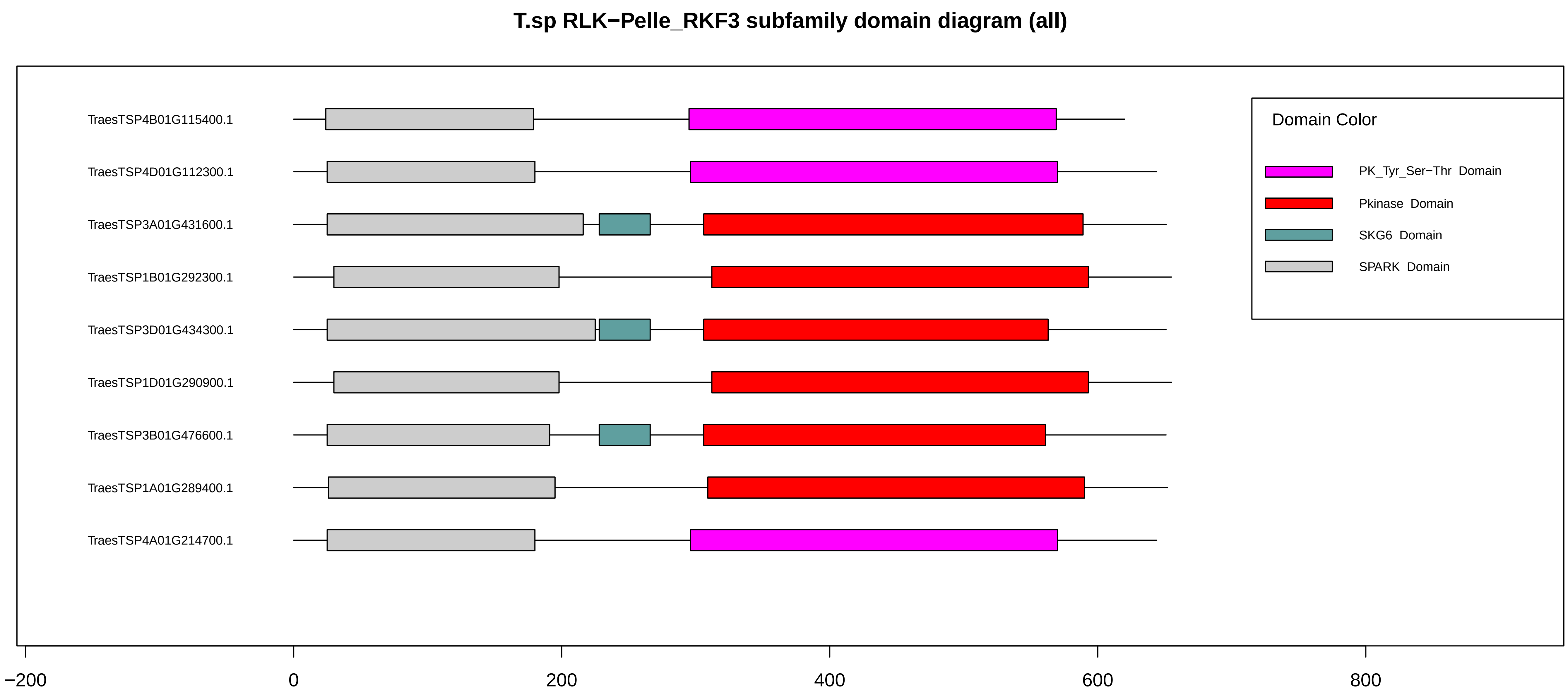
T.sp RLK−Pelle_RKF3 subfamily domain diagram (all)
TraesTSP4B01G115400.1
TraesTSP4D01G112300.1
TraesTSP3A01G431600.1
TraesTSP1B01G292300.1
TraesTSP3D01G434300.1
TraesTSP1D01G290900.1
TraesTSP3B01G476600.1
TraesTSP1A01G289400.1
TraesTSP4A01G214700.1
Domain Color
PK_Tyr_Ser−Thr Domain
Pkinase Domain
SKG6 Domain
SPARK Domain
−200
0
200
400
600
800

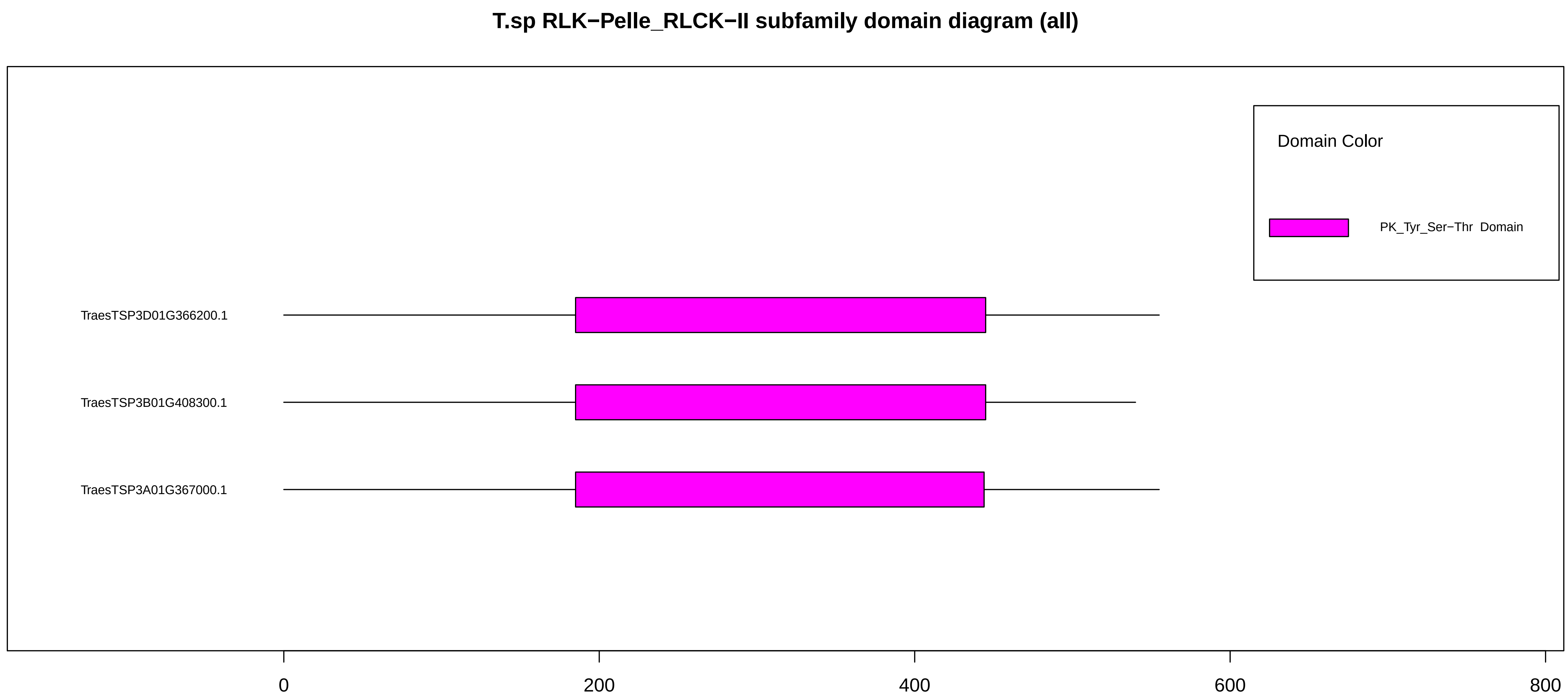

T.sp RLK−Pelle_RLCK−II subfamily domain diagram (all)
Domain Color
PK_Tyr_Ser−Thr Domain
TraesTSP3D01G366200.1
TraesTSP3B01G408300.1
TraesTSP3A01G367000.1
0
200
400
600
800

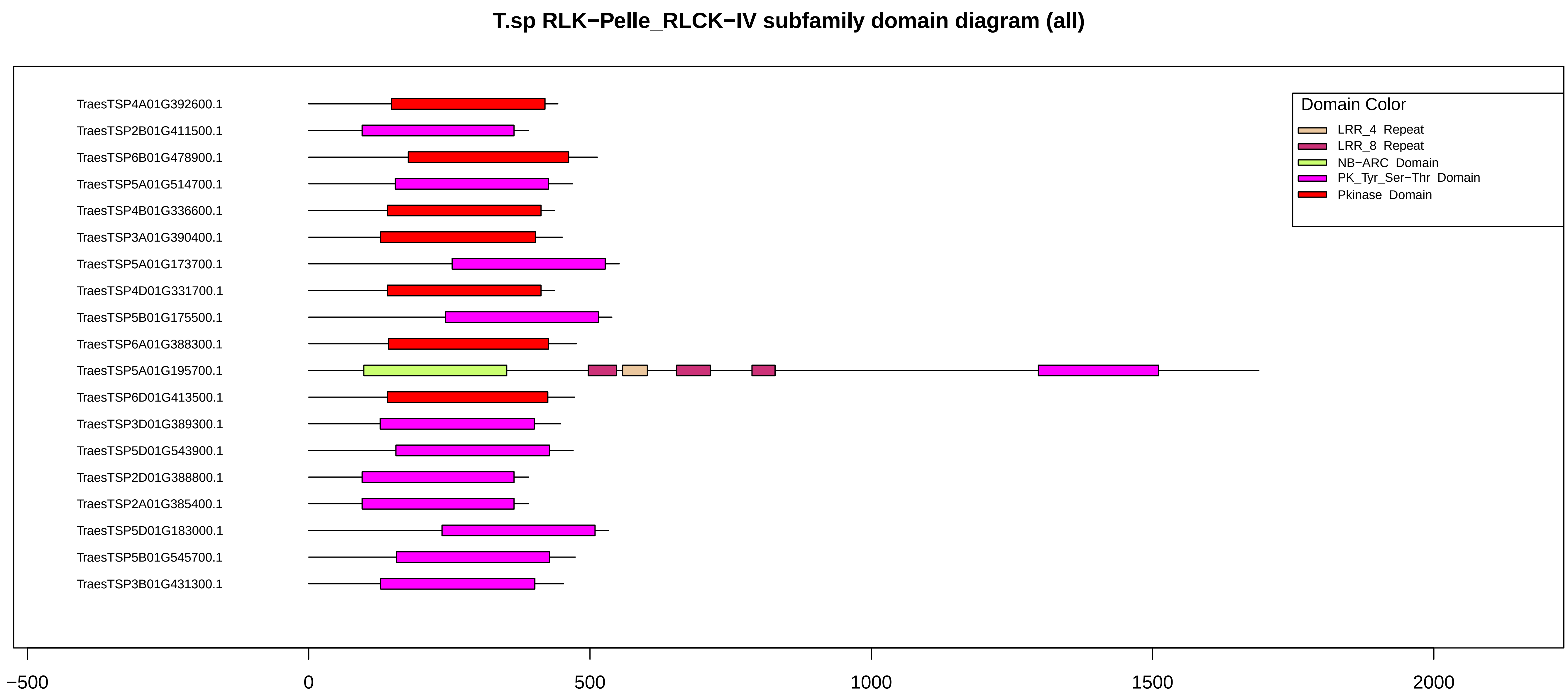
T.sp RLK−Pelle_RLCK−IV subfamily domain diagram (all)
TraesTSP4A01G392600.1
TraesTSP2B01G411500.1
TraesTSP6B01G478900.1
TraesTSP5A01G514700.1
TraesTSP4B01G336600.1
TraesTSP3A01G390400.1
TraesTSP5A01G173700.1
TraesTSP4D01G331700.1
TraesTSP5B01G175500.1
TraesTSP6A01G388300.1
TraesTSP5A01G195700.1
TraesTSP6D01G413500.1
TraesTSP3D01G389300.1
TraesTSP5D01G543900.1
TraesTSP2D01G388800.1
TraesTSP2A01G385400.1
TraesTSP5D01G183000.1
TraesTSP5B01G545700.1
TraesTSP3B01G431300.1
Domain Color
LRR_4 Repeat
LRR_8 Repeat
NB−ARC Domain
PK_Tyr_Ser−Thr Domain
Pkinase Domain
−500
0
500
1000
1500
2000

T.sp RLK−Pelle_RLCK−IXa subfamily domain diagram (all)

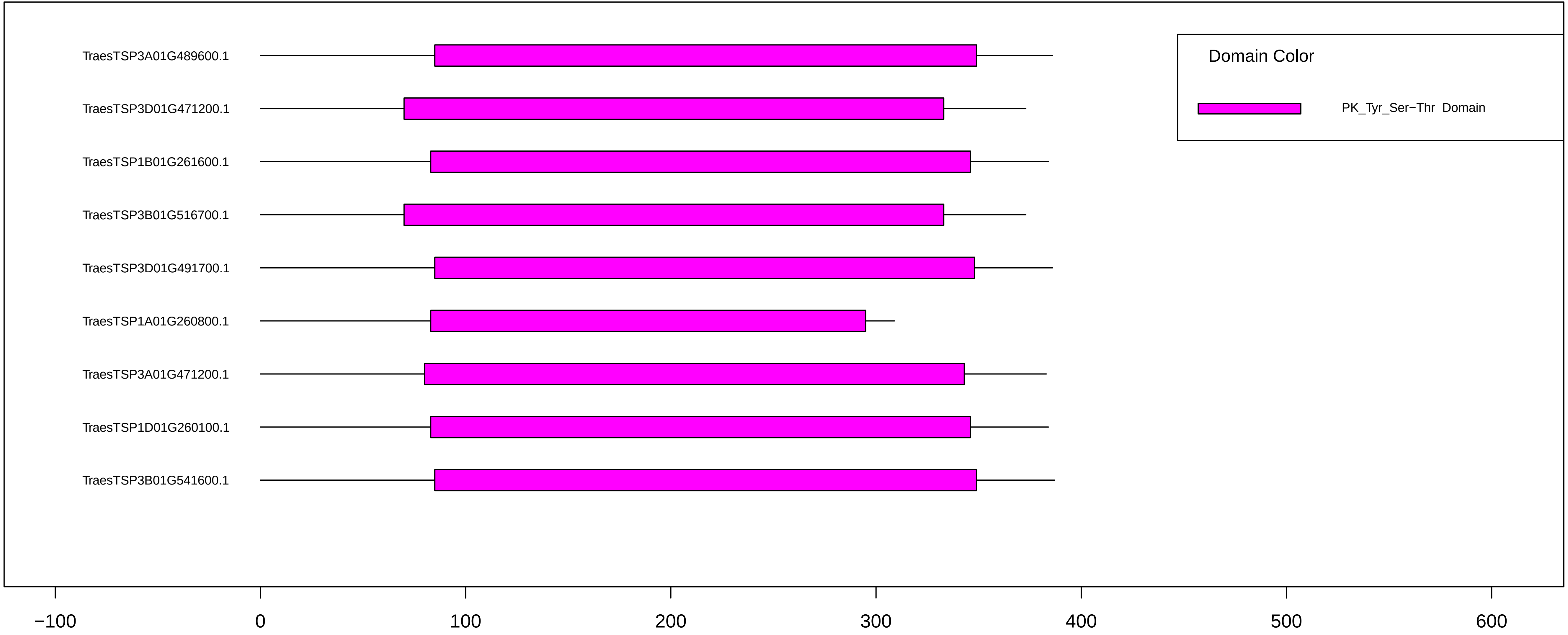

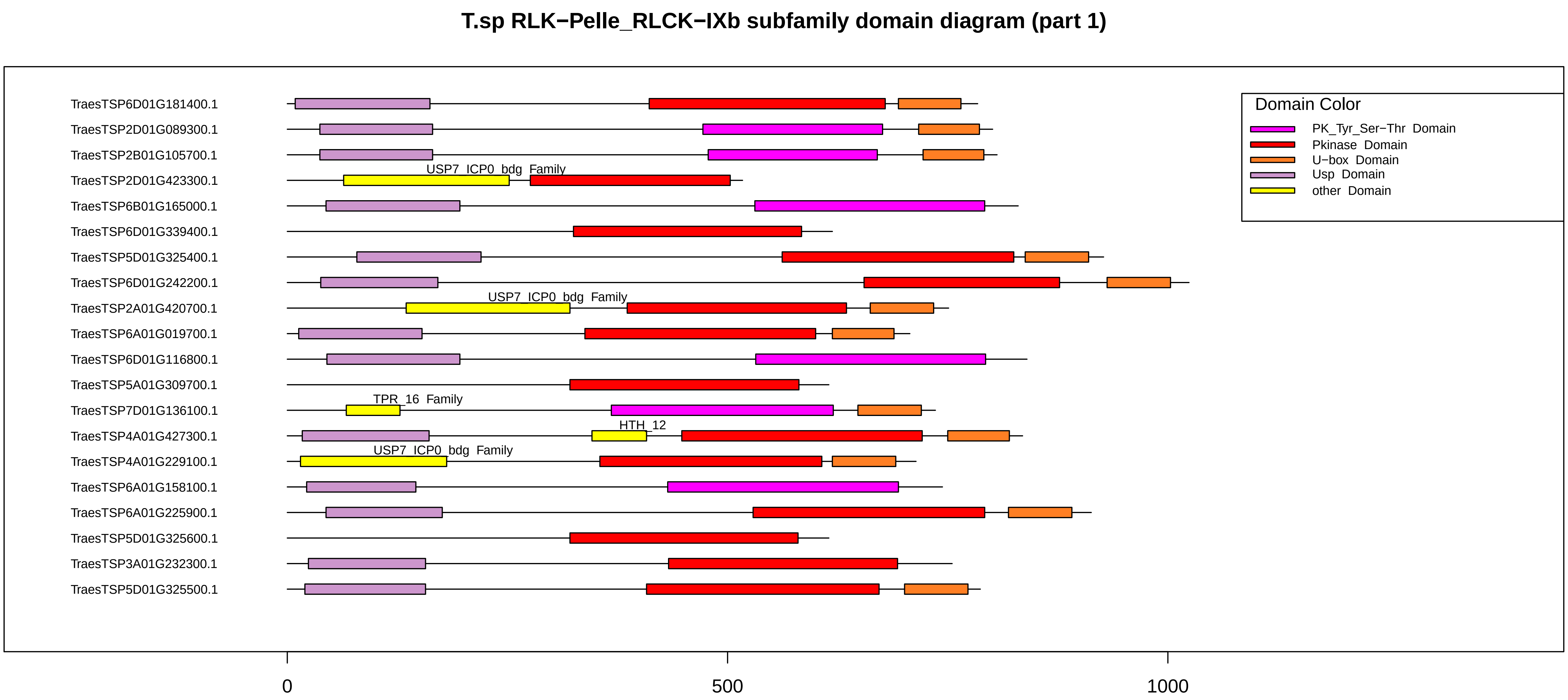
T.sp RLK−Pelle_RLCK−IXb subfamily domain diagram (part 1)
TraesTSP6D01G181400.1
TraesTSP2D01G089300.1
TraesTSP2B01G105700.1
TraesTSP2D01G423300.1
USP7_ICP0_bdg Family
TraesTSP6B01G165000.1
TraesTSP6D01G339400.1
TraesTSP5D01G325400.1
TraesTSP6D01G242200.1
TraesTSP2A01G420700.1
USP7_ICP0_bdg Family
TraesTSP6A01G019700.1
TraesTSP6D01G116800.1
TraesTSP5A01G309700.1
TraesTSP7D01G136100.1
TPR_16 Family
TraesTSP4A01G427300.1
HTH_12
TraesTSP4A01G229100.1
USP7_ICP0_bdg Family
TraesTSP6A01G158100.1
TraesTSP6A01G225900.1
TraesTSP5D01G325600.1
TraesTSP3A01G232300.1
TraesTSP5D01G325500.1
Domain Color
PK_Tyr_Ser−Thr Domain
Pkinase Domain
U−box Domain
Usp Domain
other Domain
0
500
1000

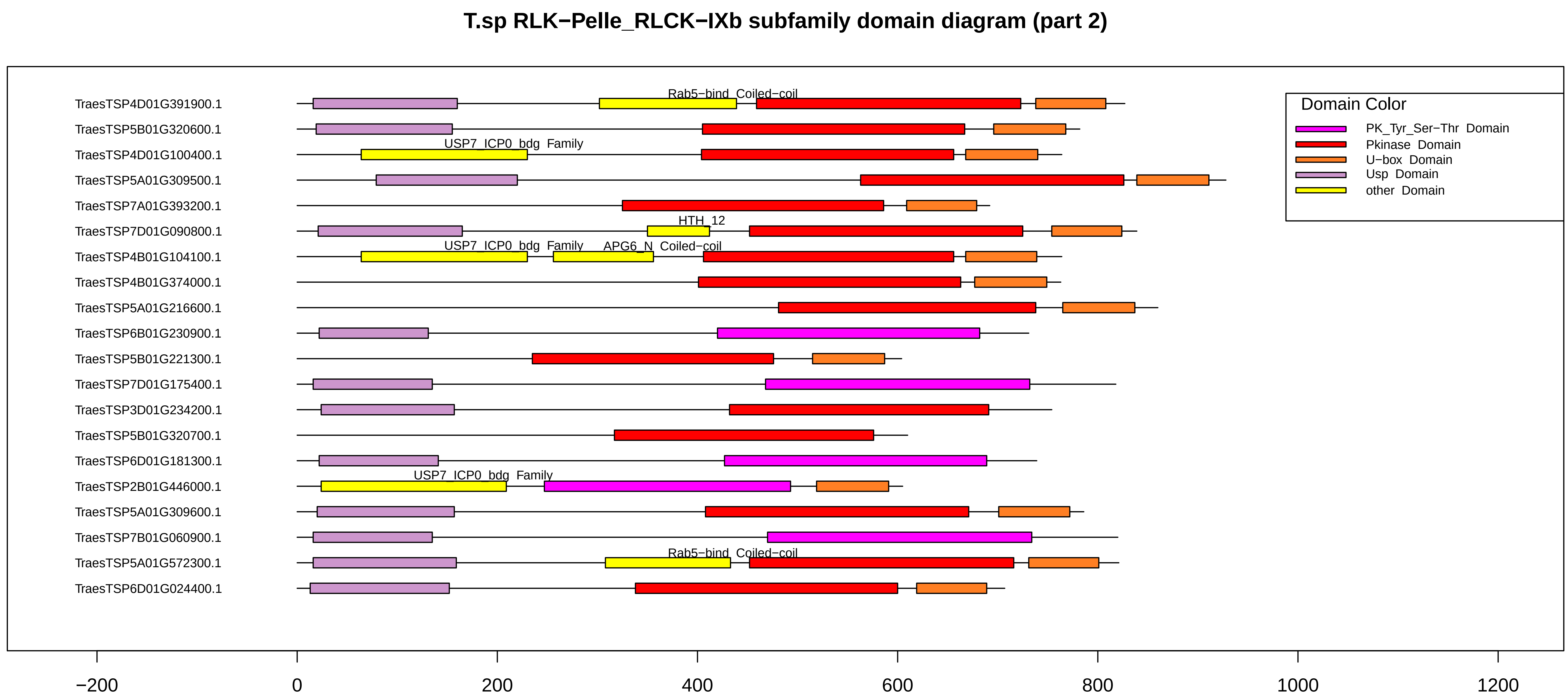
T.sp RLK−Pelle_RLCK−IXb subfamily domain diagram (part 2)
TraesTSP4D01G391900.1
Rab5−bind Coiled−coil
TraesTSP5B01G320600.1
TraesTSP4D01G100400.1
USP7_ICP0_bdg Family
TraesTSP5A01G309500.1
TraesTSP7A01G393200.1
TraesTSP7D01G090800.1
HTH_12
TraesTSP4B01G104100.1
USP7_ICP0_bdg Family
APG6_N Coiled−coil
TraesTSP4B01G374000.1
TraesTSP5A01G216600.1
TraesTSP6B01G230900.1
TraesTSP5B01G221300.1
TraesTSP7D01G175400.1
TraesTSP3D01G234200.1
TraesTSP5B01G320700.1
TraesTSP6D01G181300.1
TraesTSP2B01G446000.1
USP7_ICP0_bdg Family
TraesTSP5A01G309600.1
TraesTSP7B01G060900.1
TraesTSP5A01G572300.1
Rab5−bind Coiled−coil
TraesTSP6D01G024400.1
Domain Color
PK_Tyr_Ser−Thr Domain
Pkinase Domain
U−box Domain
Usp Domain
other Domain
−200
0
200
400
600
800
1000
1200

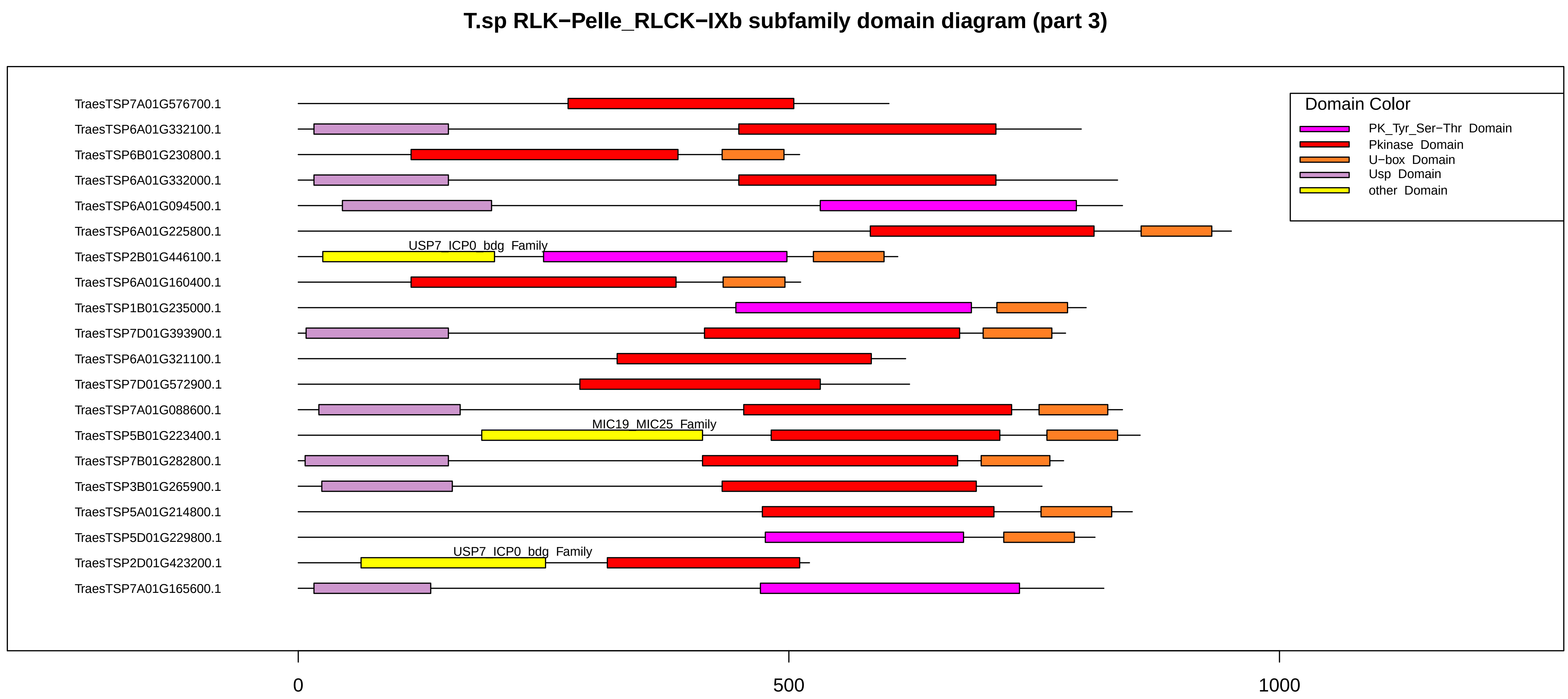

T.sp RLK−Pelle_RLCK−IXb subfamily domain diagram (part 3)
TraesTSP7A01G576700.1
TraesTSP6A01G332100.1
TraesTSP6B01G230800.1
TraesTSP6A01G332000.1
TraesTSP6A01G094500.1
TraesTSP6A01G225800.1
TraesTSP2B01G446100.1
USP7_ICP0_bdg Family
TraesTSP6A01G160400.1
TraesTSP1B01G235000.1
TraesTSP7D01G393900.1
TraesTSP6A01G321100.1
TraesTSP7D01G572900.1
TraesTSP7A01G088600.1
TraesTSP5B01G223400.1
MIC19_MIC25 Family
TraesTSP7B01G282800.1
TraesTSP3B01G265900.1
TraesTSP5A01G214800.1
TraesTSP5D01G229800.1
TraesTSP2D01G423200.1
USP7_ICP0_bdg Family
TraesTSP7A01G165600.1
Domain Color
PK_Tyr_Ser−Thr Domain
Pkinase Domain
U−box Domain
Usp Domain
other Domain
0
500
1000

T.sp RLK−Pelle_RLCK−IXb subfamily domain diagram (part 4)

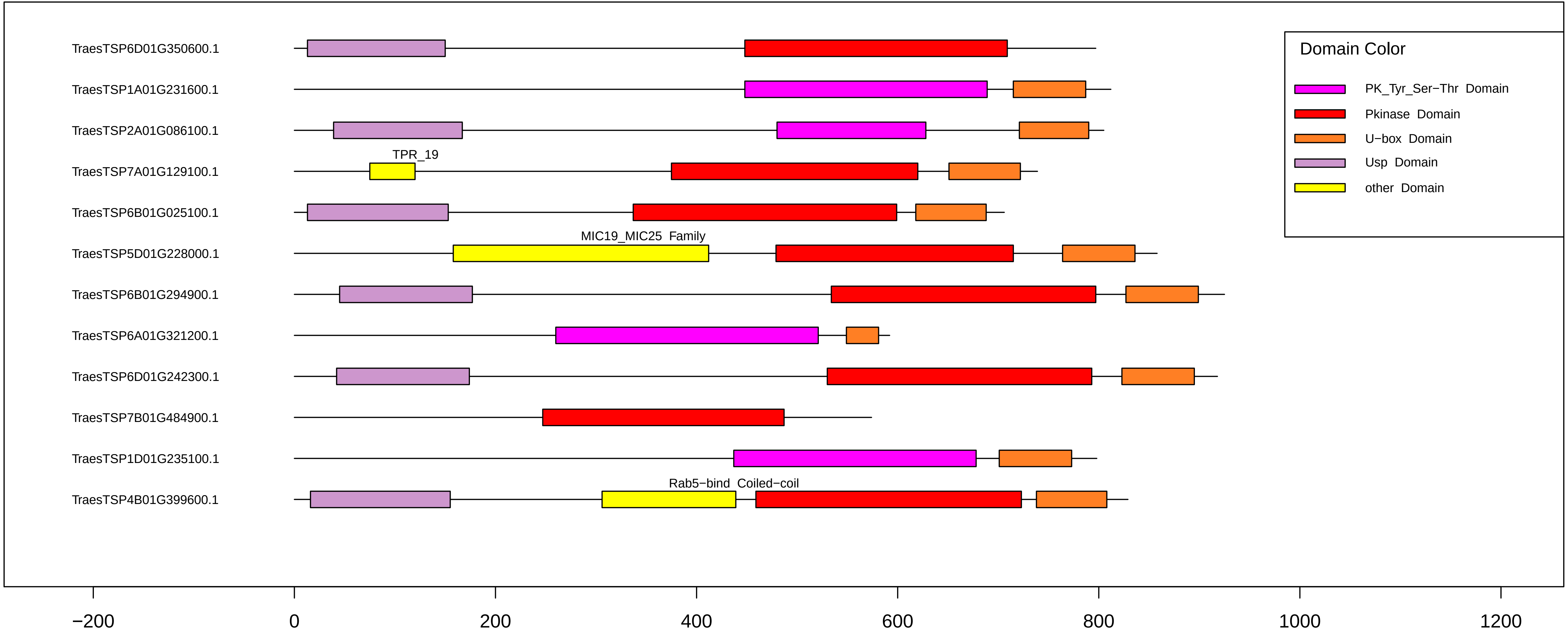

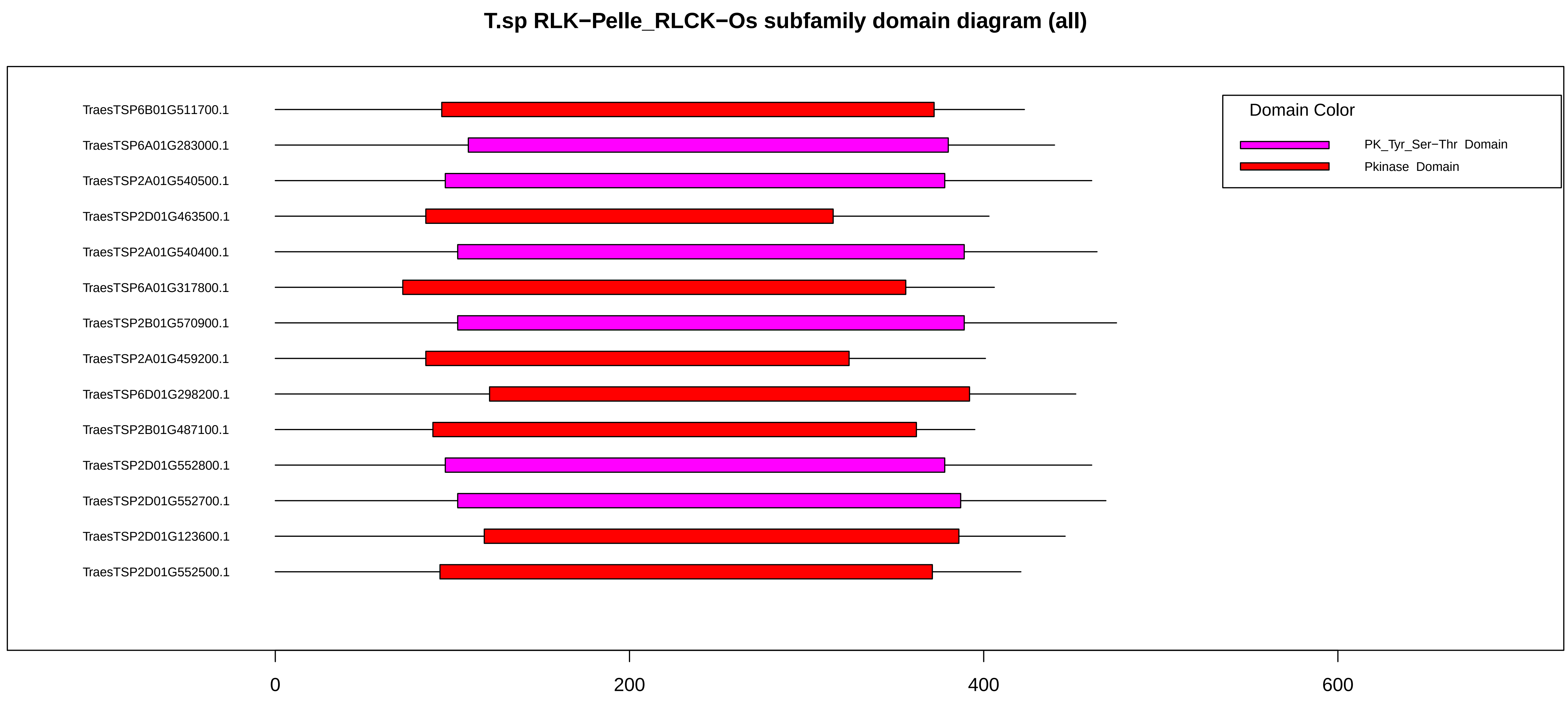

T.sp RLK−Pelle_RLCK−Os subfamily domain diagram (all)
TraesTSP6B01G511700.1
TraesTSP6A01G283000.1
TraesTSP2A01G540500.1
TraesTSP2D01G463500.1
TraesTSP2A01G540400.1
TraesTSP6A01G317800.1
TraesTSP2B01G570900.1
TraesTSP2A01G459200.1
TraesTSP6D01G298200.1
TraesTSP2B01G487100.1
TraesTSP2D01G552800.1
TraesTSP2D01G552700.1
TraesTSP2D01G123600.1
TraesTSP2D01G552500.1
Domain Color
PK_Tyr_Ser−Thr Domain
Pkinase Domain
0
200
400
600

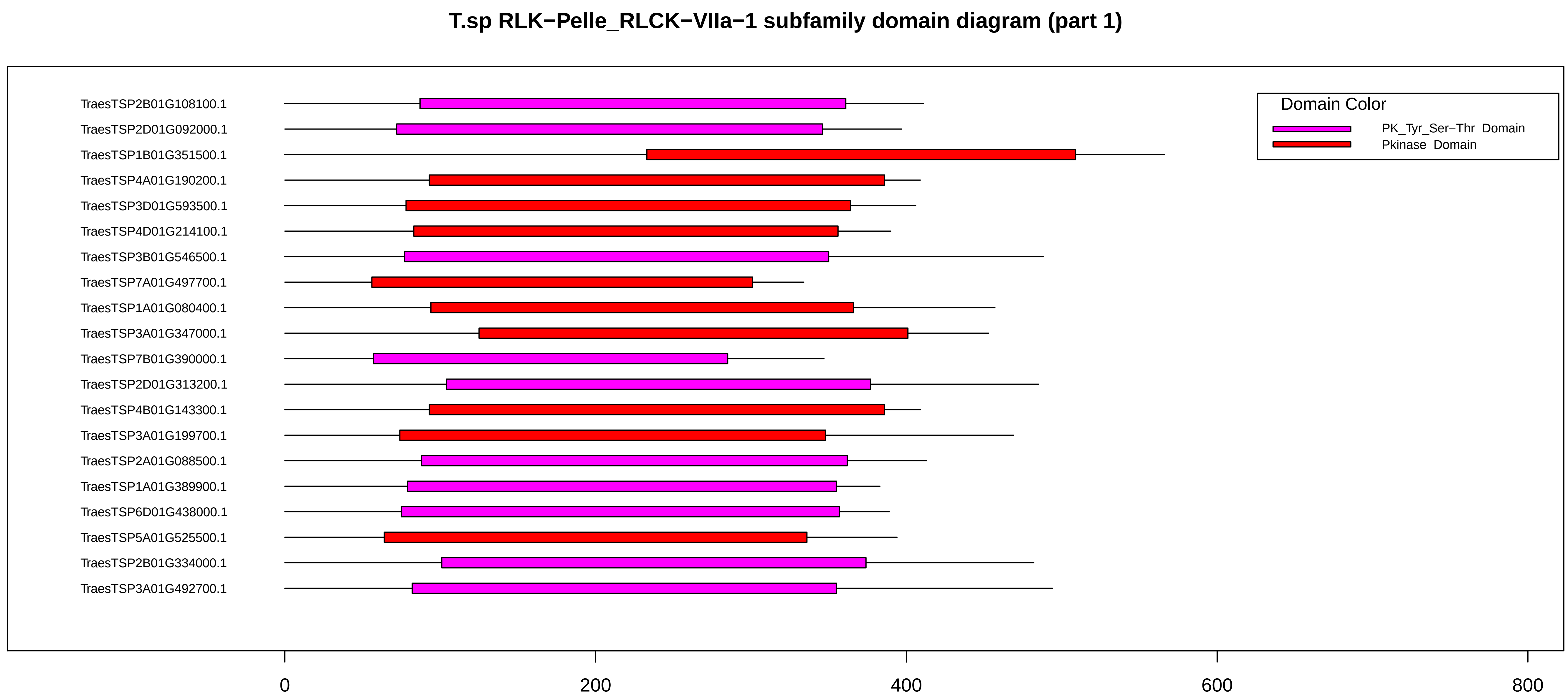
T.sp RLK−Pelle_RLCK−VIIa−1 subfamily domain diagram (part 1)
TraesTSP2B01G108100.1
TraesTSP2D01G092000.1
TraesTSP1B01G351500.1
TraesTSP4A01G190200.1
TraesTSP3D01G593500.1
TraesTSP4D01G214100.1
TraesTSP3B01G546500.1
TraesTSP7A01G497700.1
TraesTSP1A01G080400.1
TraesTSP3A01G347000.1
TraesTSP7B01G390000.1
TraesTSP2D01G313200.1
TraesTSP4B01G143300.1
TraesTSP3A01G199700.1
TraesTSP2A01G088500.1
TraesTSP1A01G389900.1
TraesTSP6D01G438000.1
TraesTSP5A01G525500.1
TraesTSP2B01G334000.1
TraesTSP3A01G492700.1
Domain Color
PK_Tyr_Ser−Thr Domain
Pkinase Domain
0
200
400
600
800

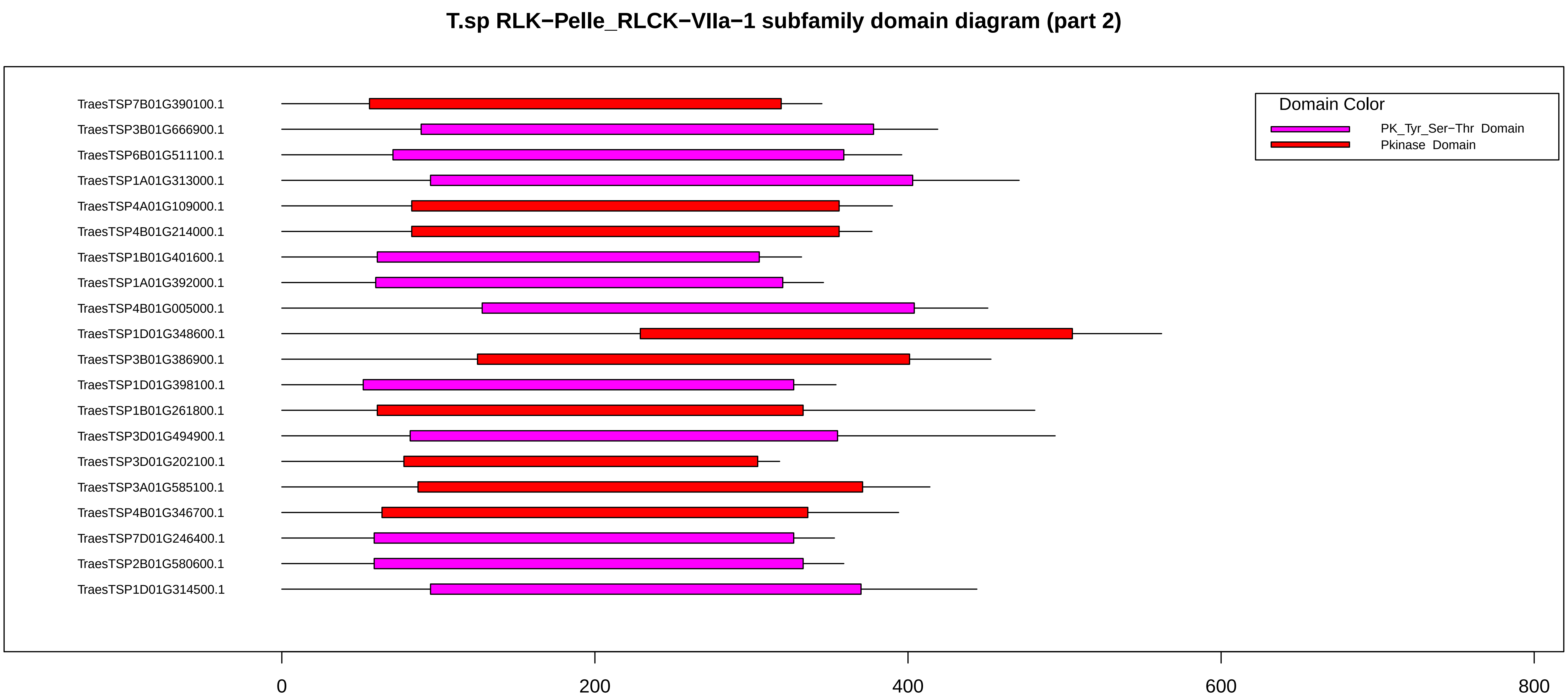
T.sp RLK−Pelle_RLCK−VIIa−1 subfamily domain diagram (part 2)
TraesTSP7B01G390100.1
TraesTSP3B01G666900.1
TraesTSP6B01G511100.1
TraesTSP1A01G313000.1
TraesTSP4A01G109000.1
TraesTSP4B01G214000.1
TraesTSP1B01G401600.1
TraesTSP1A01G392000.1
TraesTSP4B01G005000.1
TraesTSP1D01G348600.1
TraesTSP3B01G386900.1
TraesTSP1D01G398100.1
TraesTSP1B01G261800.1
TraesTSP3D01G494900.1
TraesTSP3D01G202100.1
TraesTSP3A01G585100.1
TraesTSP4B01G346700.1
TraesTSP7D01G246400.1
TraesTSP2B01G580600.1
TraesTSP1D01G314500.1
Domain Color
PK_Tyr_Ser−Thr Domain
Pkinase Domain
0
200
400
600
800

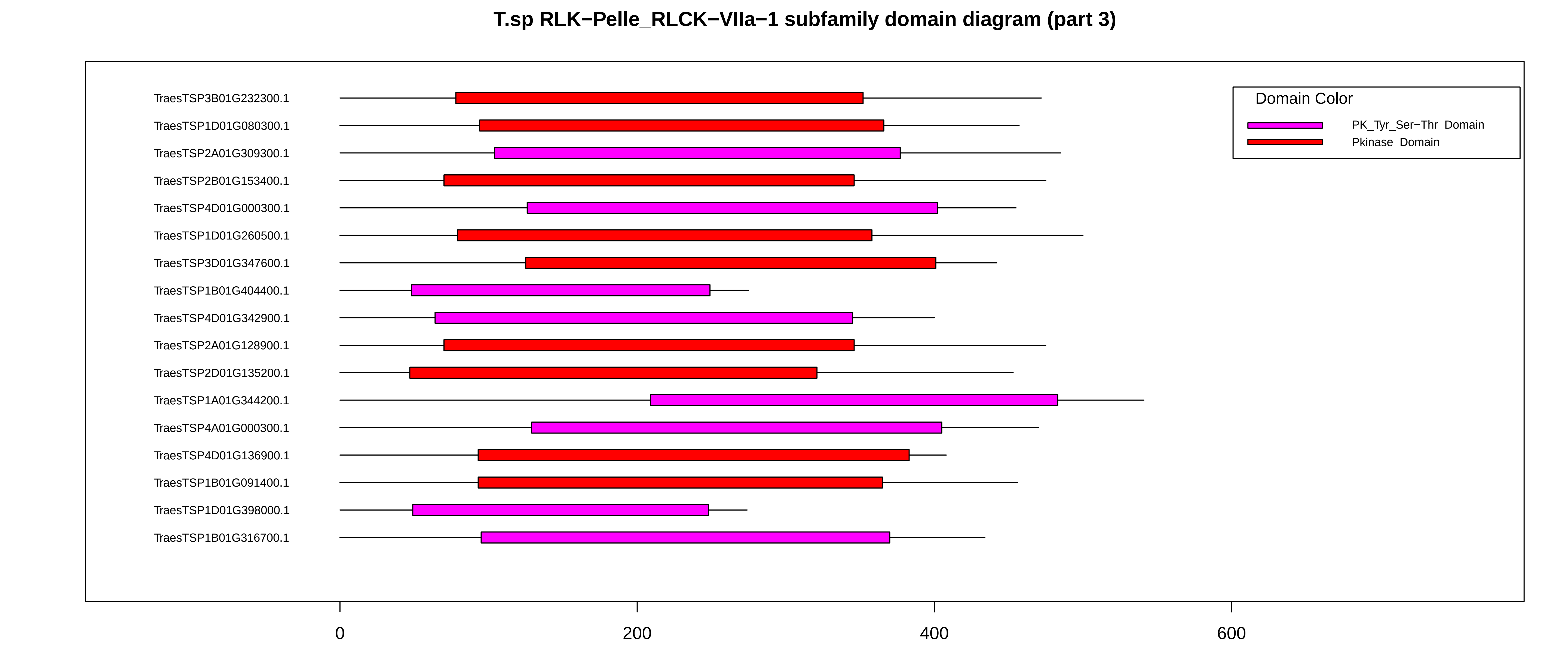
T.sp RLK−Pelle_RLCK−VIIa−1 subfamily domain diagram (part 3)
TraesTSP3B01G232300.1
TraesTSP1D01G080300.1
TraesTSP2A01G309300.1
TraesTSP2B01G153400.1
TraesTSP4D01G000300.1
TraesTSP1D01G260500.1
TraesTSP3D01G347600.1
TraesTSP1B01G404400.1
TraesTSP4D01G342900.1
TraesTSP2A01G128900.1
TraesTSP2D01G135200.1
TraesTSP1A01G344200.1
TraesTSP4A01G000300.1
TraesTSP4D01G136900.1
TraesTSP1B01G091400.1
TraesTSP1D01G398000.1
TraesTSP1B01G316700.1
Domain Color
PK_Tyr_Ser−Thr Domain
Pkinase Domain
0
200
400
600

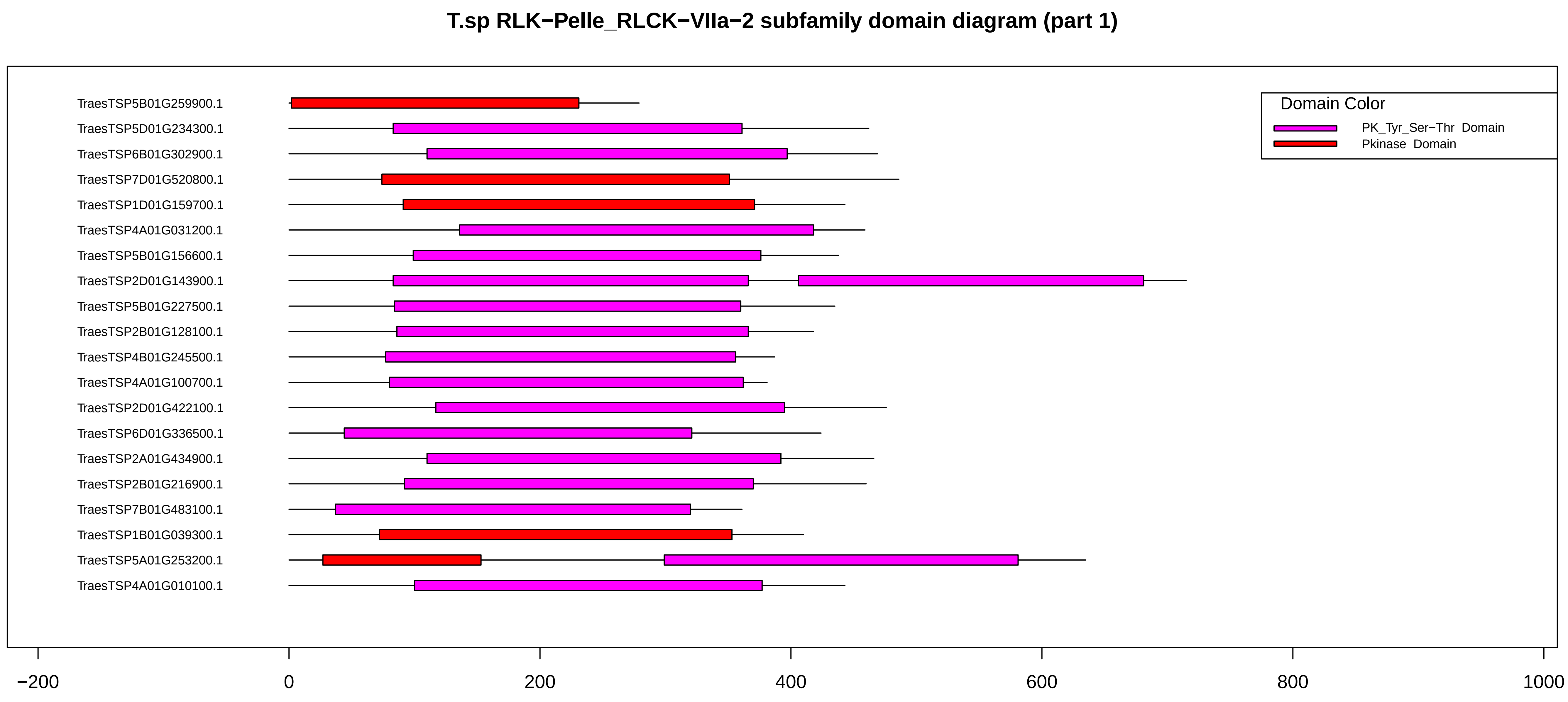
T.sp RLK−Pelle_RLCK−VIIa−2 subfamily domain diagram (part 1)
TraesTSP5B01G259900.1
TraesTSP5D01G234300.1
TraesTSP6B01G302900.1
TraesTSP7D01G520800.1
TraesTSP1D01G159700.1
TraesTSP4A01G031200.1
TraesTSP5B01G156600.1
TraesTSP2D01G143900.1
TraesTSP5B01G227500.1
TraesTSP2B01G128100.1
TraesTSP4B01G245500.1
TraesTSP4A01G100700.1
TraesTSP2D01G422100.1
TraesTSP6D01G336500.1
TraesTSP2A01G434900.1
TraesTSP2B01G216900.1
TraesTSP7B01G483100.1
TraesTSP1B01G039300.1
TraesTSP5A01G253200.1
TraesTSP4A01G010100.1
Domain Color
PK_Tyr_Ser−Thr Domain
Pkinase Domain
−200
0
200
400
600
800
1000

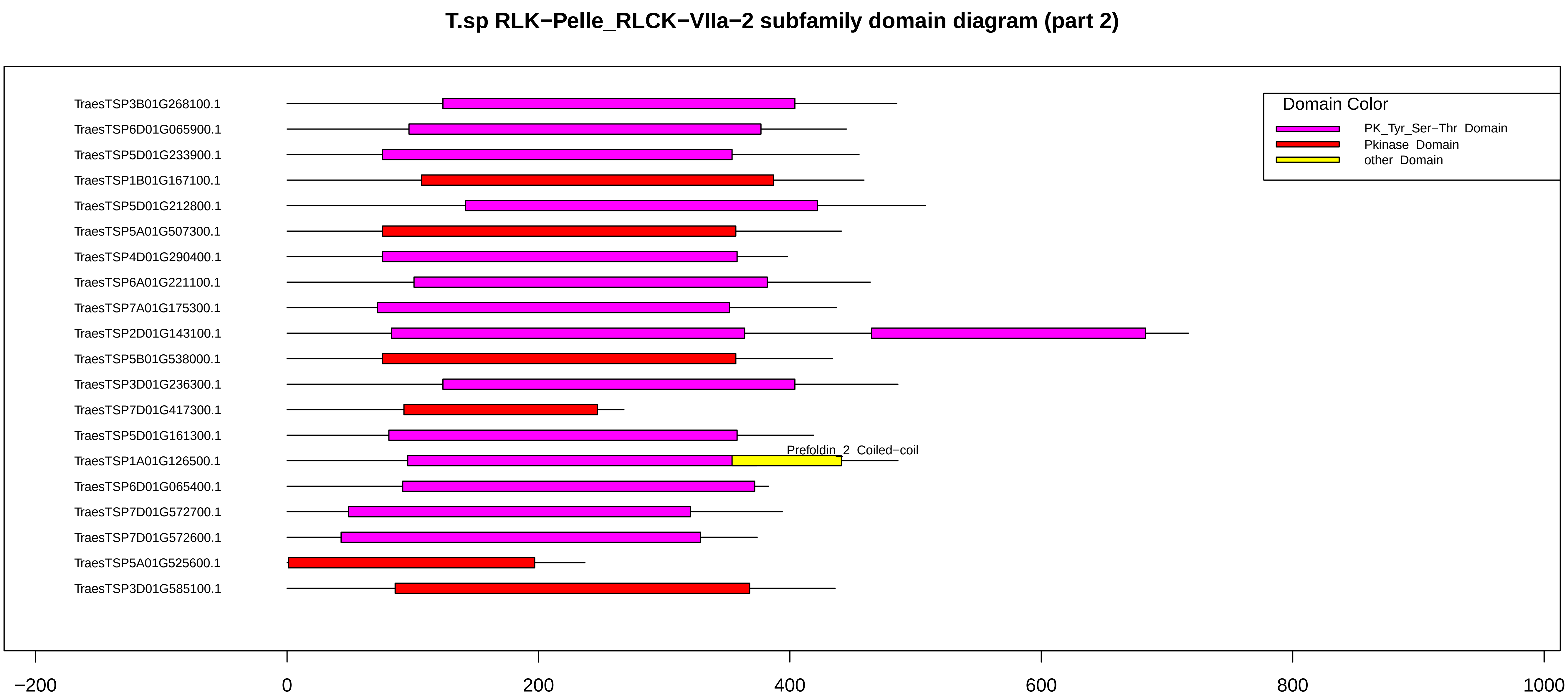
T.sp RLK−Pelle_RLCK−VIIa−2 subfamily domain diagram (part 2)
TraesTSP3B01G268100.1
TraesTSP6D01G065900.1
TraesTSP5D01G233900.1
TraesTSP1B01G167100.1
TraesTSP5D01G212800.1
TraesTSP5A01G507300.1
TraesTSP4D01G290400.1
TraesTSP6A01G221100.1
TraesTSP7A01G175300.1
TraesTSP2D01G143100.1
TraesTSP5B01G538000.1
TraesTSP3D01G236300.1
TraesTSP7D01G417300.1
TraesTSP5D01G161300.1
TraesTSP1A01G126500.1
TraesTSP6D01G065400.1
TraesTSP7D01G572700.1
TraesTSP7D01G572600.1
TraesTSP5A01G525600.1
TraesTSP3D01G585100.1
Prefoldin_2 Coiled−coil
Domain Color
PK_Tyr_Ser−Thr Domain
Pkinase Domain
other Domain
−200
0
200
400
600
800
1000

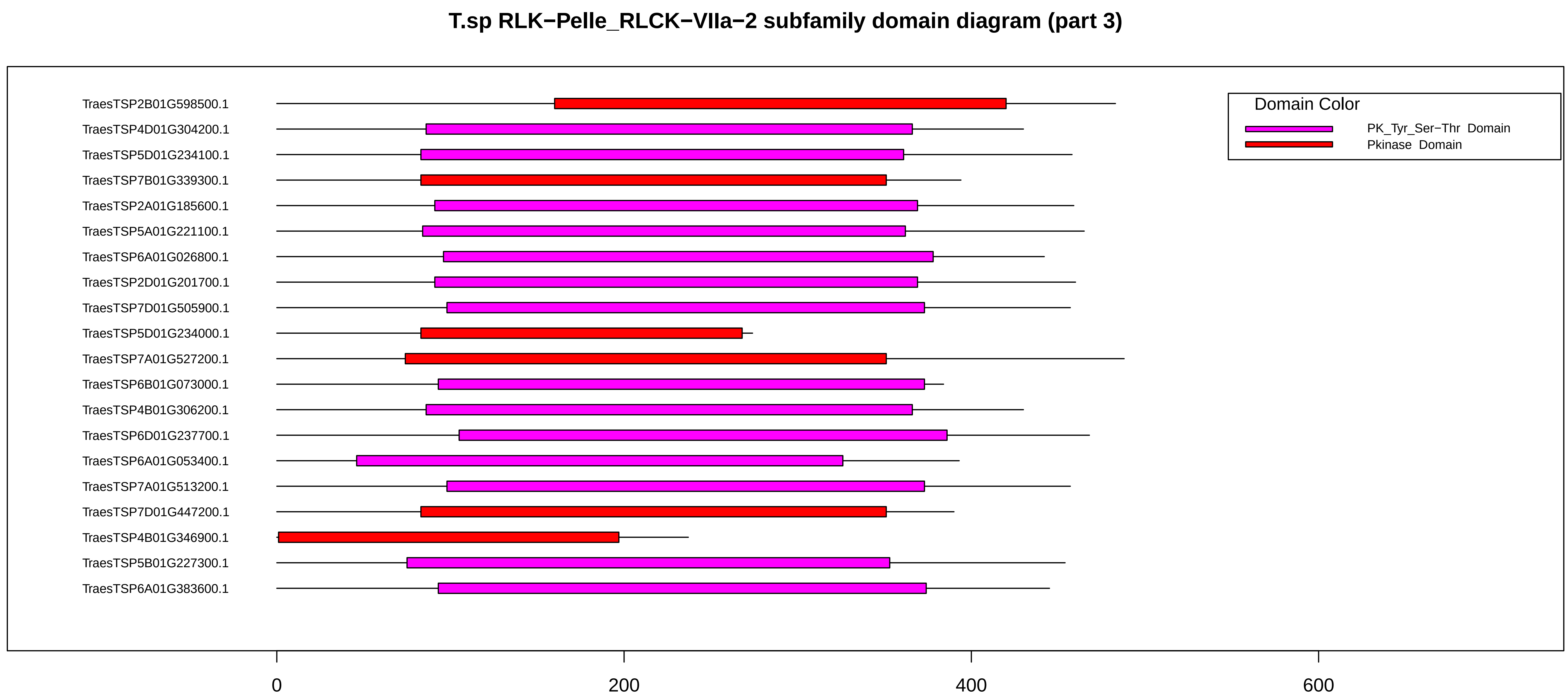
T.sp RLK−Pelle_RLCK−VIIa−2 subfamily domain diagram (part 3)
TraesTSP2B01G598500.1
TraesTSP4D01G304200.1
TraesTSP5D01G234100.1
TraesTSP7B01G339300.1
TraesTSP2A01G185600.1
TraesTSP5A01G221100.1
TraesTSP6A01G026800.1
TraesTSP2D01G201700.1
TraesTSP7D01G505900.1
TraesTSP5D01G234000.1
TraesTSP7A01G527200.1
TraesTSP6B01G073000.1
TraesTSP4B01G306200.1
TraesTSP6D01G237700.1
TraesTSP6A01G053400.1
TraesTSP7A01G513200.1
TraesTSP7D01G447200.1
TraesTSP4B01G346900.1
TraesTSP5B01G227300.1
TraesTSP6A01G383600.1
Domain Color
PK_Tyr_Ser−Thr Domain
Pkinase Domain
0
200
400
600

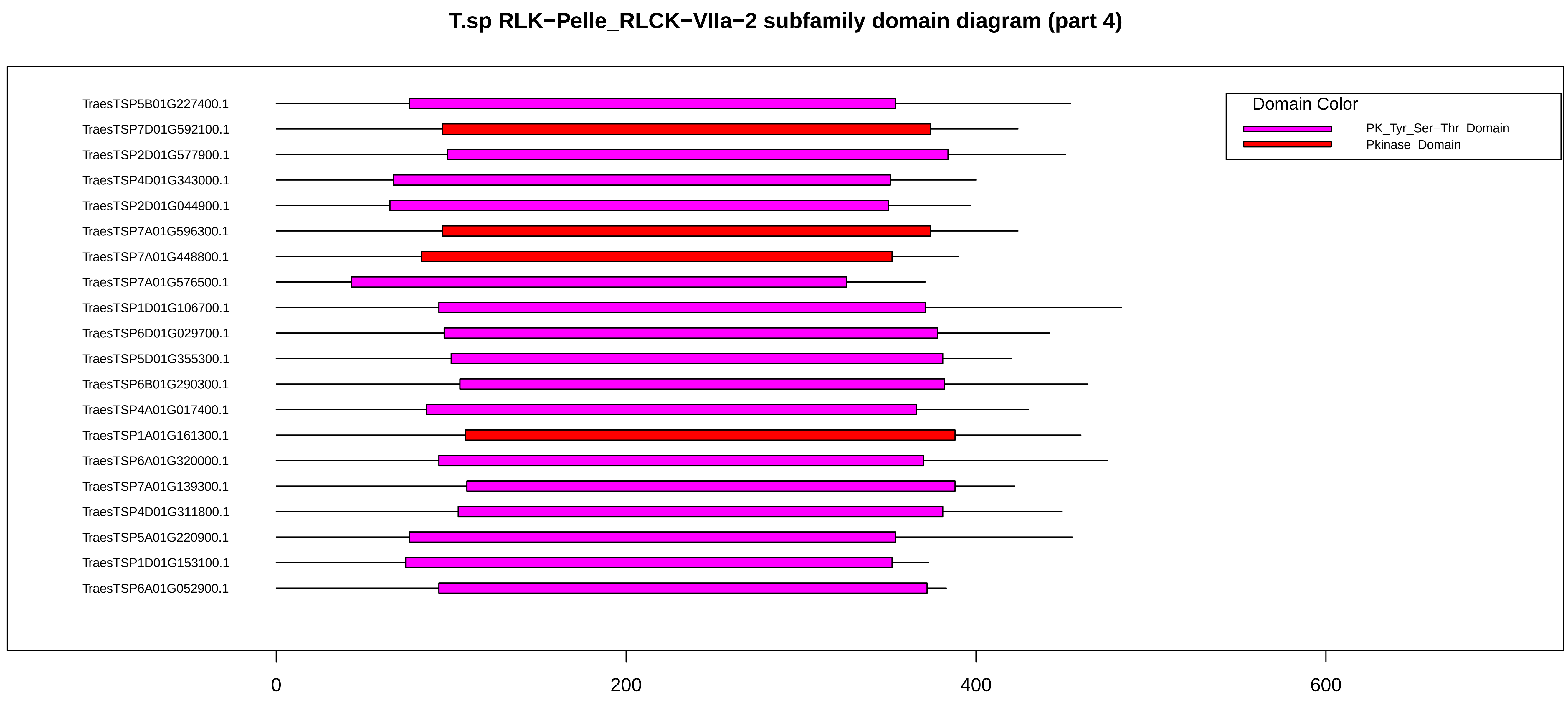

T.sp RLK−Pelle_RLCK−VIIa−2 subfamily domain diagram (part 4)
TraesTSP5B01G227400.1
TraesTSP7D01G592100.1
TraesTSP2D01G577900.1
TraesTSP4D01G343000.1
TraesTSP2D01G044900.1
TraesTSP7A01G596300.1
TraesTSP7A01G448800.1
TraesTSP7A01G576500.1
TraesTSP1D01G106700.1
TraesTSP6D01G029700.1
TraesTSP5D01G355300.1
TraesTSP6B01G290300.1
TraesTSP4A01G017400.1
TraesTSP1A01G161300.1
TraesTSP6A01G320000.1
TraesTSP7A01G139300.1
TraesTSP4D01G311800.1
TraesTSP5A01G220900.1
TraesTSP1D01G153100.1
TraesTSP6A01G052900.1
Domain Color
PK_Tyr_Ser−Thr Domain
Pkinase Domain
0
200
400
600

T.sp RLK−Pelle_RLCK−VIIa−2 subfamily domain diagram (part 5)

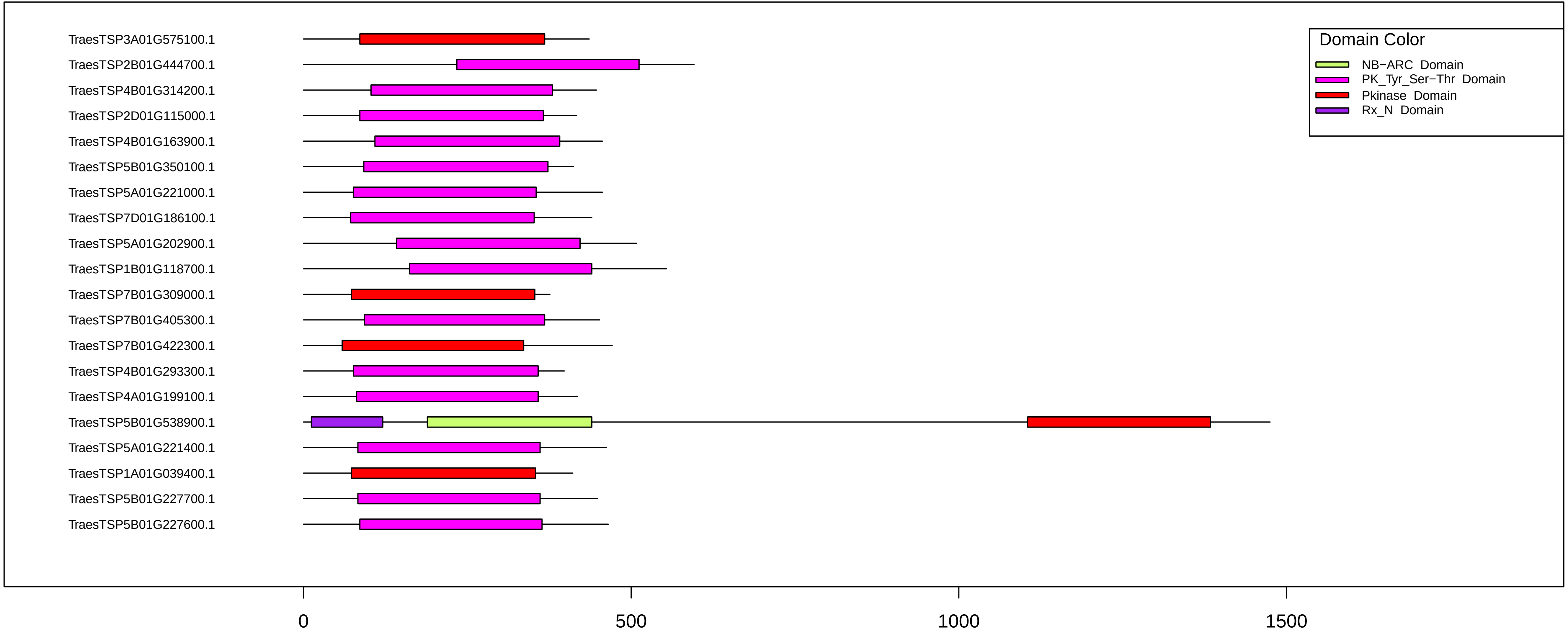

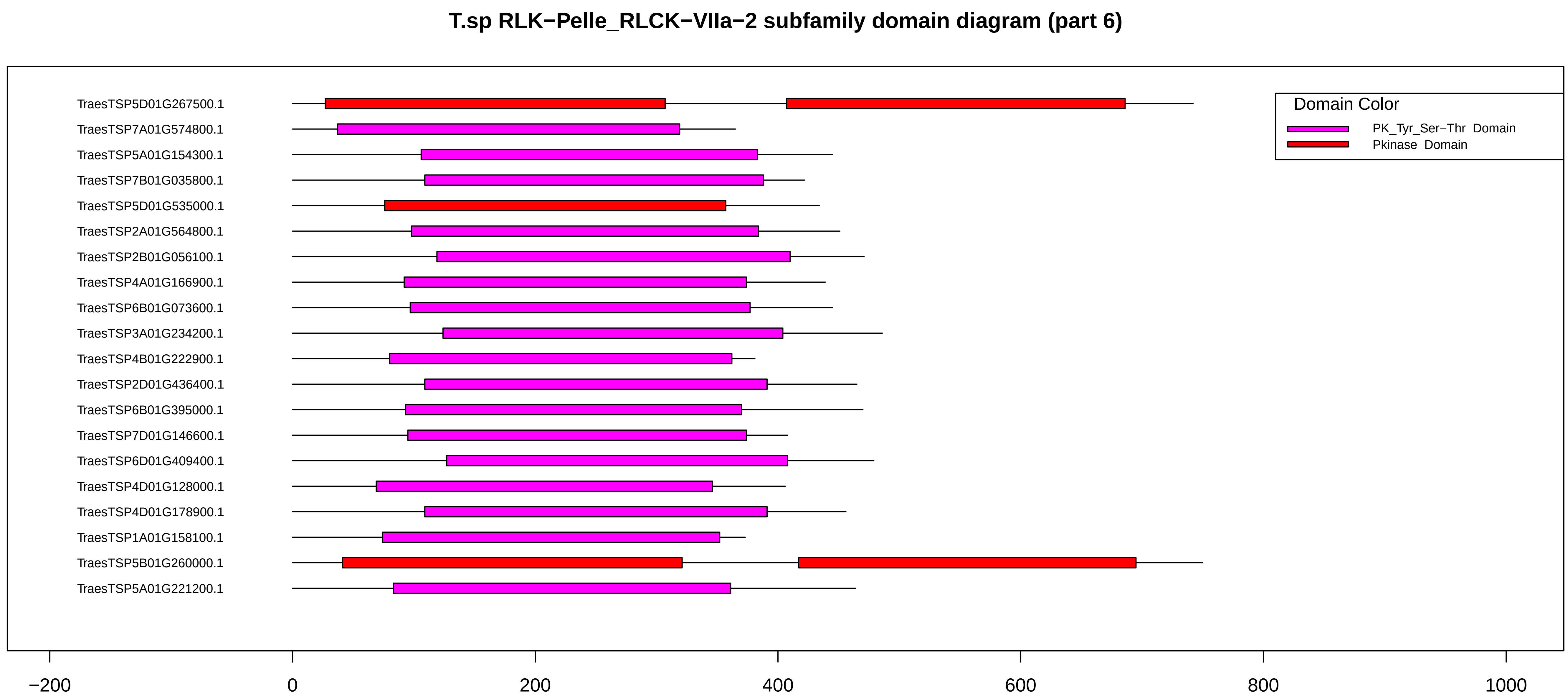
T.sp RLK−Pelle_RLCK−VIIa−2 subfamily domain diagram (part 6)
TraesTSP5D01G267500.1
TraesTSP7A01G574800.1
TraesTSP5A01G154300.1
TraesTSP7B01G035800.1
TraesTSP5D01G535000.1
TraesTSP2A01G564800.1
TraesTSP2B01G056100.1
TraesTSP4A01G166900.1
TraesTSP6B01G073600.1
TraesTSP3A01G234200.1
TraesTSP4B01G222900.1
TraesTSP2D01G436400.1
TraesTSP6B01G395000.1
TraesTSP7D01G146600.1
TraesTSP6D01G409400.1
TraesTSP4D01G128000.1
TraesTSP4D01G178900.1
TraesTSP1A01G158100.1
TraesTSP5B01G260000.1
TraesTSP5A01G221200.1
Domain Color
PK_Tyr_Ser−Thr Domain
Pkinase Domain
−200
0
200
400
600
800
1000

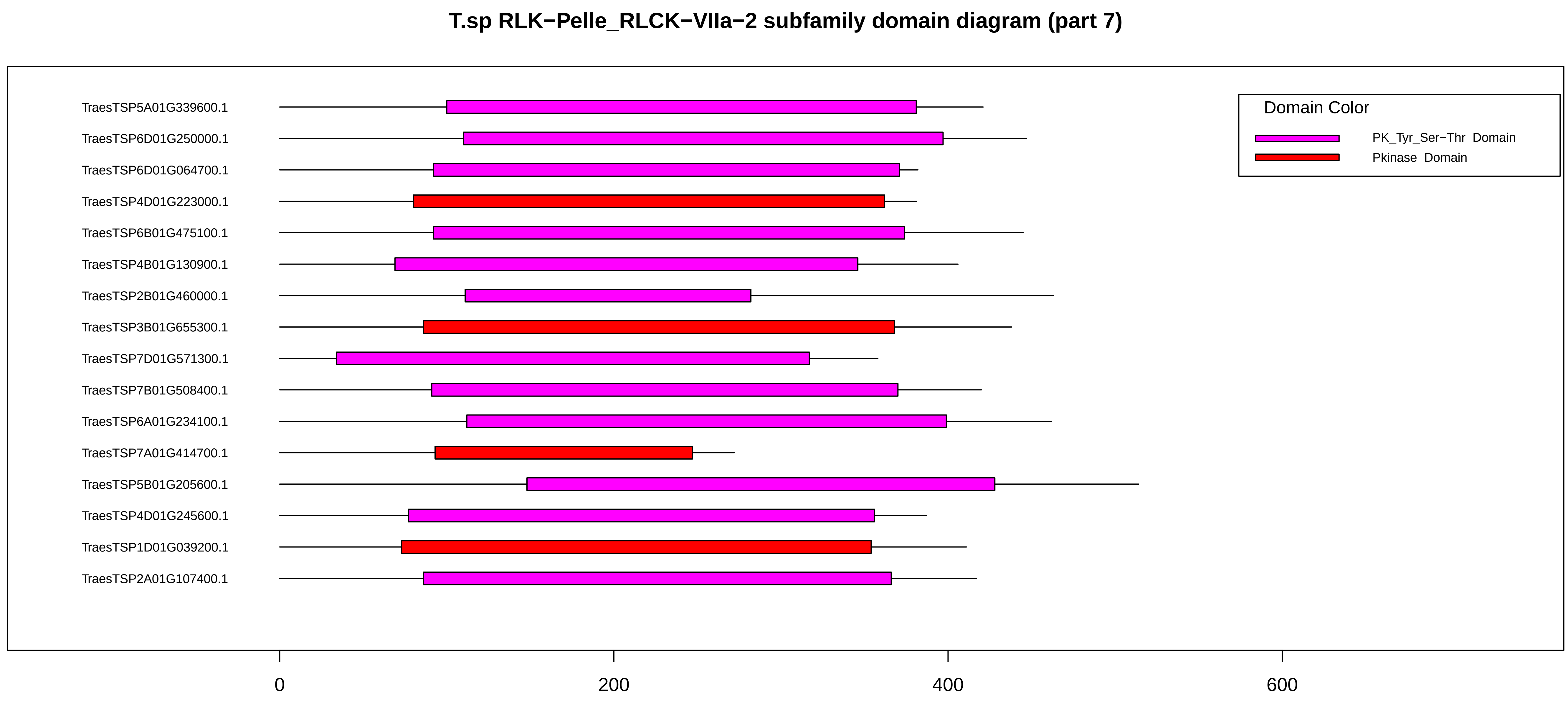

T.sp RLK−Pelle_RLCK−VIIa−2 subfamily domain diagram (part 7)
TraesTSP5A01G339600.1
TraesTSP6D01G250000.1
TraesTSP6D01G064700.1
TraesTSP4D01G223000.1
TraesTSP6B01G475100.1
TraesTSP4B01G130900.1
TraesTSP2B01G460000.1
TraesTSP3B01G655300.1
TraesTSP7D01G571300.1
TraesTSP7B01G508400.1
TraesTSP6A01G234100.1
TraesTSP7A01G414700.1
TraesTSP5B01G205600.1
TraesTSP4D01G245600.1
TraesTSP1D01G039200.1
TraesTSP2A01G107400.1
Domain Color
PK_Tyr_Ser−Thr Domain
Pkinase Domain
0
200
400
600

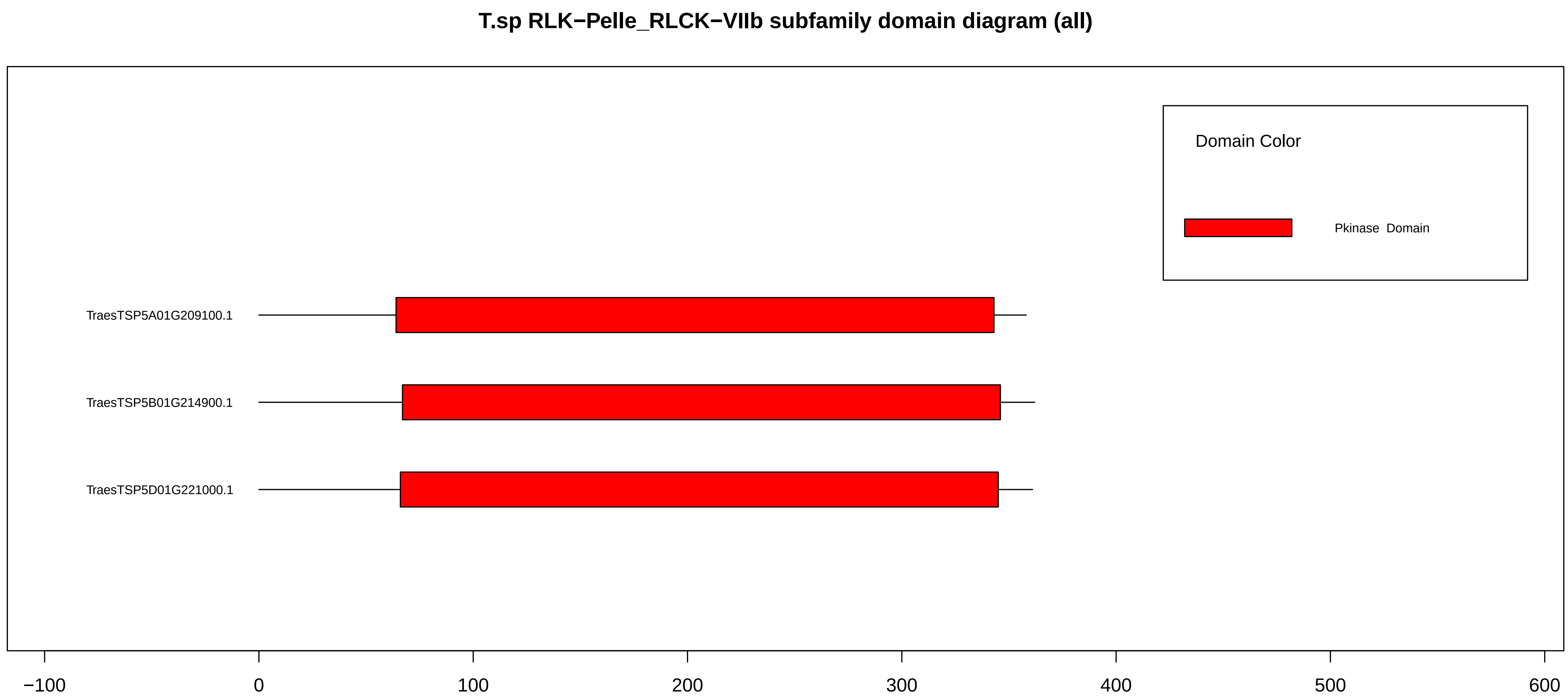
T.sp RLK−Pelle_RLCK−VIIb subfamily domain diagram (all)
Domain Color
Pkinase Domain
TraesTSP5A01G209100.1
TraesTSP5B01G214900.1
TraesTSP5D01G221000.1
−100
0
100
200
300
400
500
600

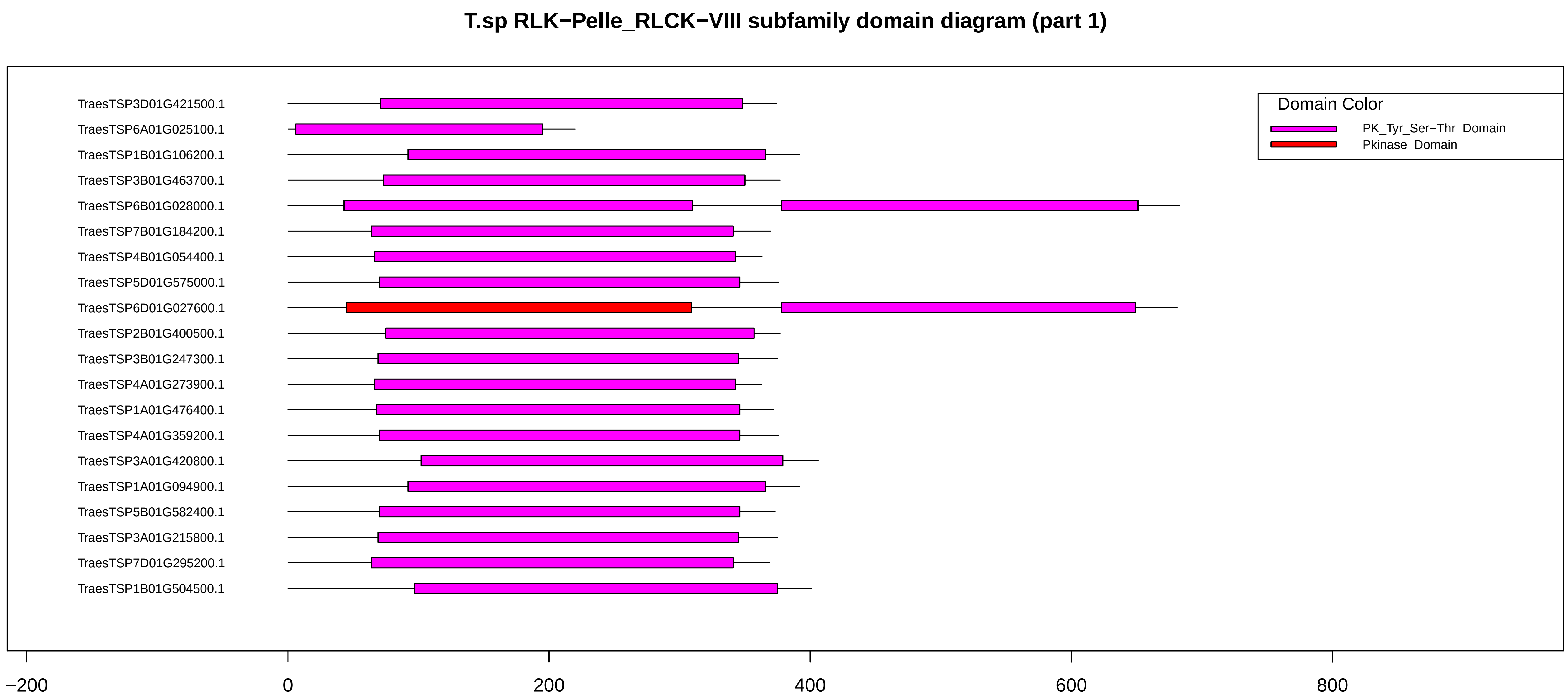

T.sp RLK−Pelle_RLCK−VIII subfamily domain diagram (part 1)
TraesTSP3D01G421500.1
TraesTSP6A01G025100.1
TraesTSP1B01G106200.1
TraesTSP3B01G463700.1
TraesTSP6B01G028000.1
TraesTSP7B01G184200.1
TraesTSP4B01G054400.1
TraesTSP5D01G575000.1
TraesTSP6D01G027600.1
TraesTSP2B01G400500.1
TraesTSP3B01G247300.1
TraesTSP4A01G273900.1
TraesTSP1A01G476400.1
TraesTSP4A01G359200.1
TraesTSP3A01G420800.1
TraesTSP1A01G094900.1
TraesTSP5B01G582400.1
TraesTSP3A01G215800.1
TraesTSP7D01G295200.1
TraesTSP1B01G504500.1
Domain Color
PK_Tyr_Ser−Thr Domain
Pkinase Domain
−200
0
200
400
600
800

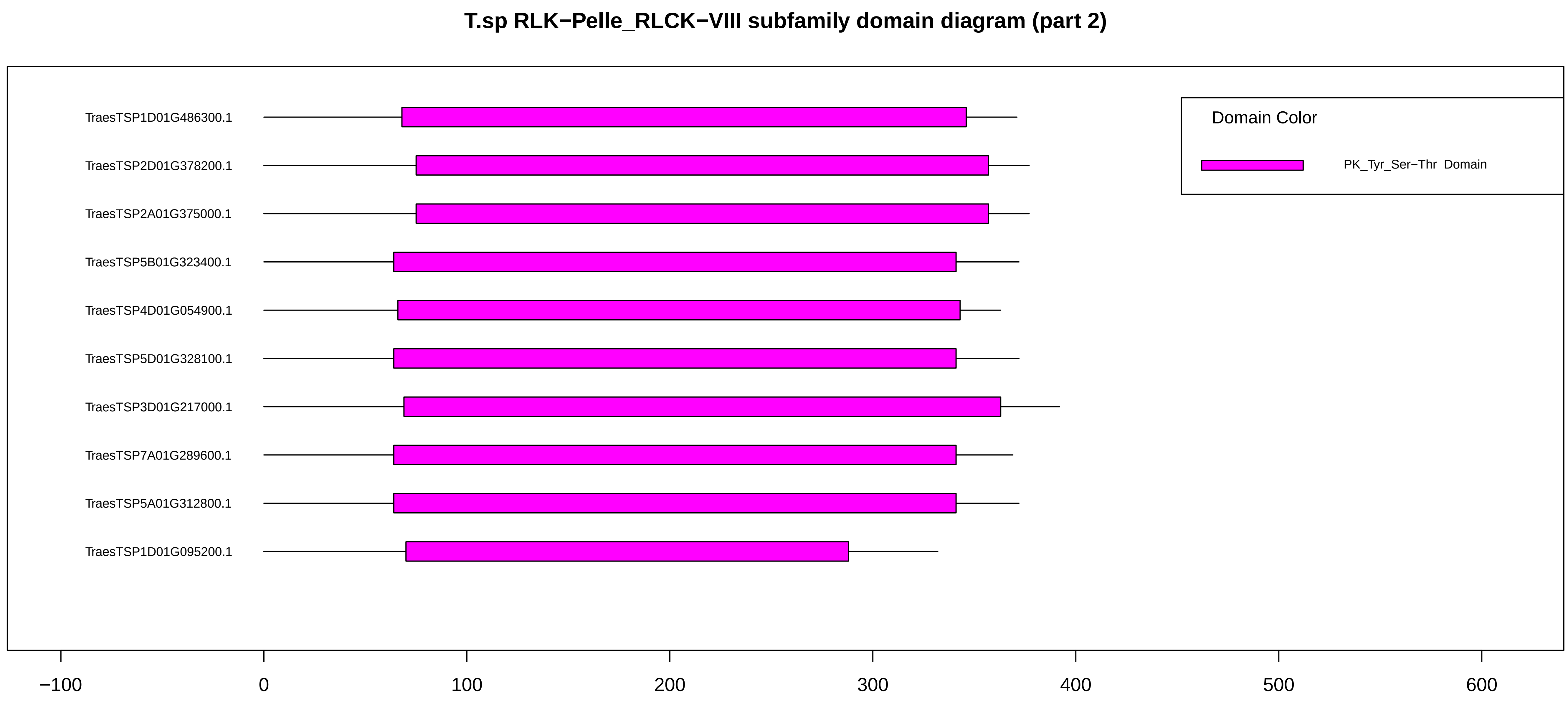
T.sp RLK−Pelle_RLCK−VIII subfamily domain diagram (part 2)
TraesTSP1D01G486300.1
TraesTSP2D01G378200.1
TraesTSP2A01G375000.1
TraesTSP5B01G323400.1
TraesTSP4D01G054900.1
TraesTSP5D01G328100.1
TraesTSP3D01G217000.1
TraesTSP7A01G289600.1
TraesTSP5A01G312800.1
TraesTSP1D01G095200.1
Domain Color
PK_Tyr_Ser−Thr Domain
−100
0
100
200
300
400
500
600

T.sp RLK−Pelle_RLCK−VI subfamily domain diagram (part 1)

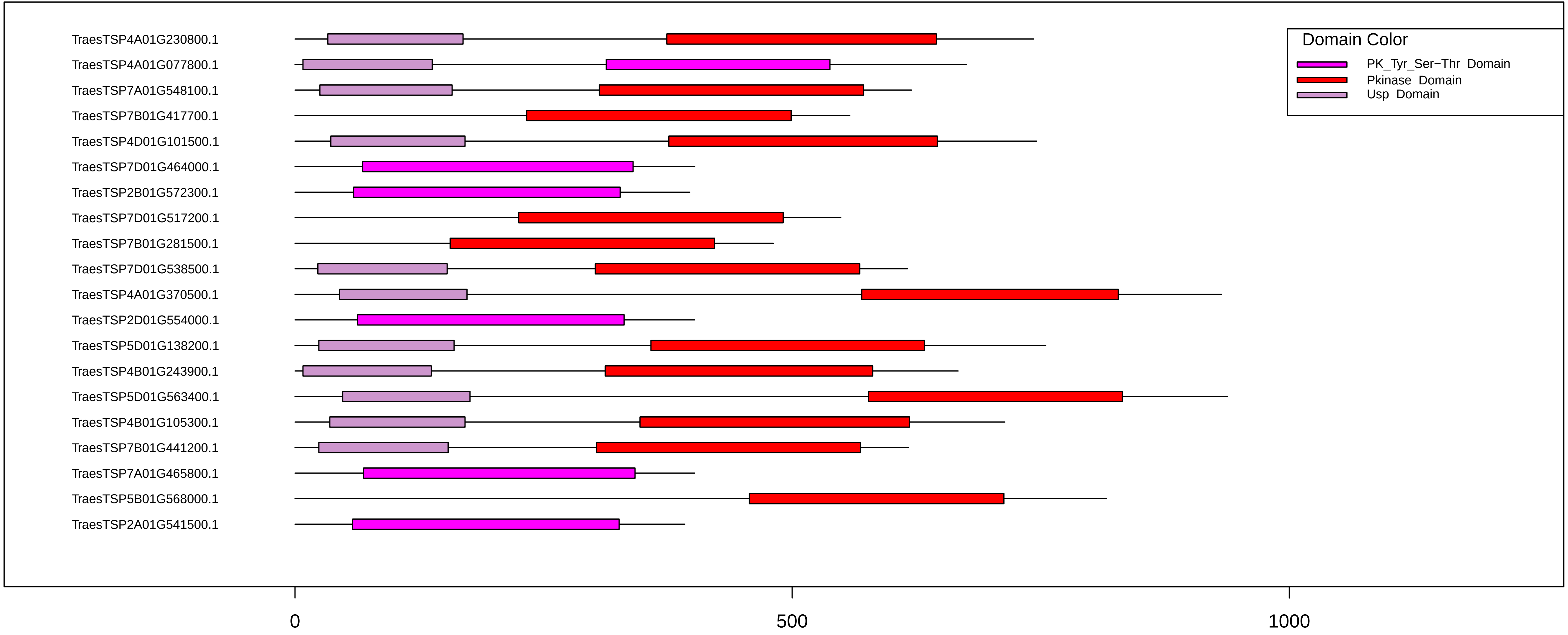

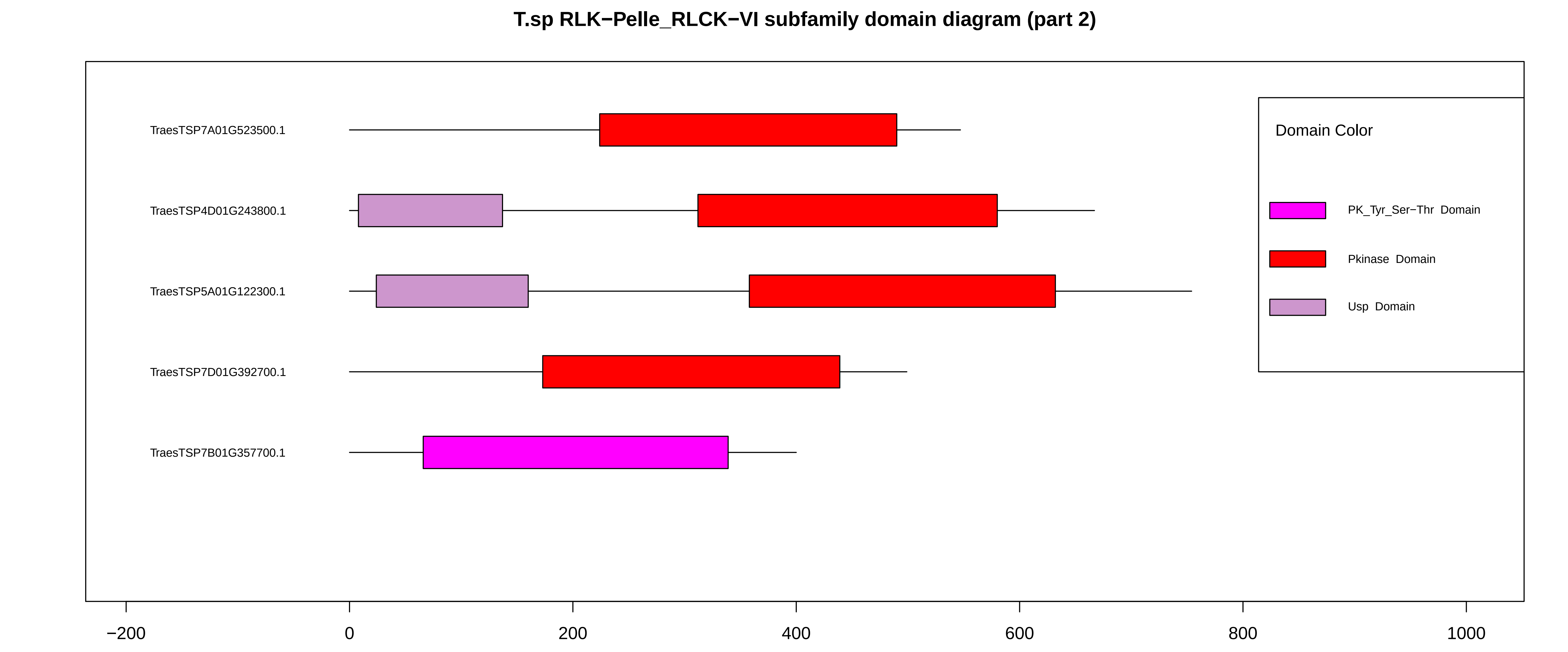
T.sp RLK−Pelle_RLCK−VI subfamily domain diagram (part 2)
TraesTSP7A01G523500.1
TraesTSP4D01G243800.1
TraesTSP5A01G122300.1
TraesTSP7D01G392700.1
TraesTSP7B01G357700.1
Domain Color
PK_Tyr_Ser−Thr Domain
Pkinase Domain
Usp Domain
−200
0
200
400
600
800
1000

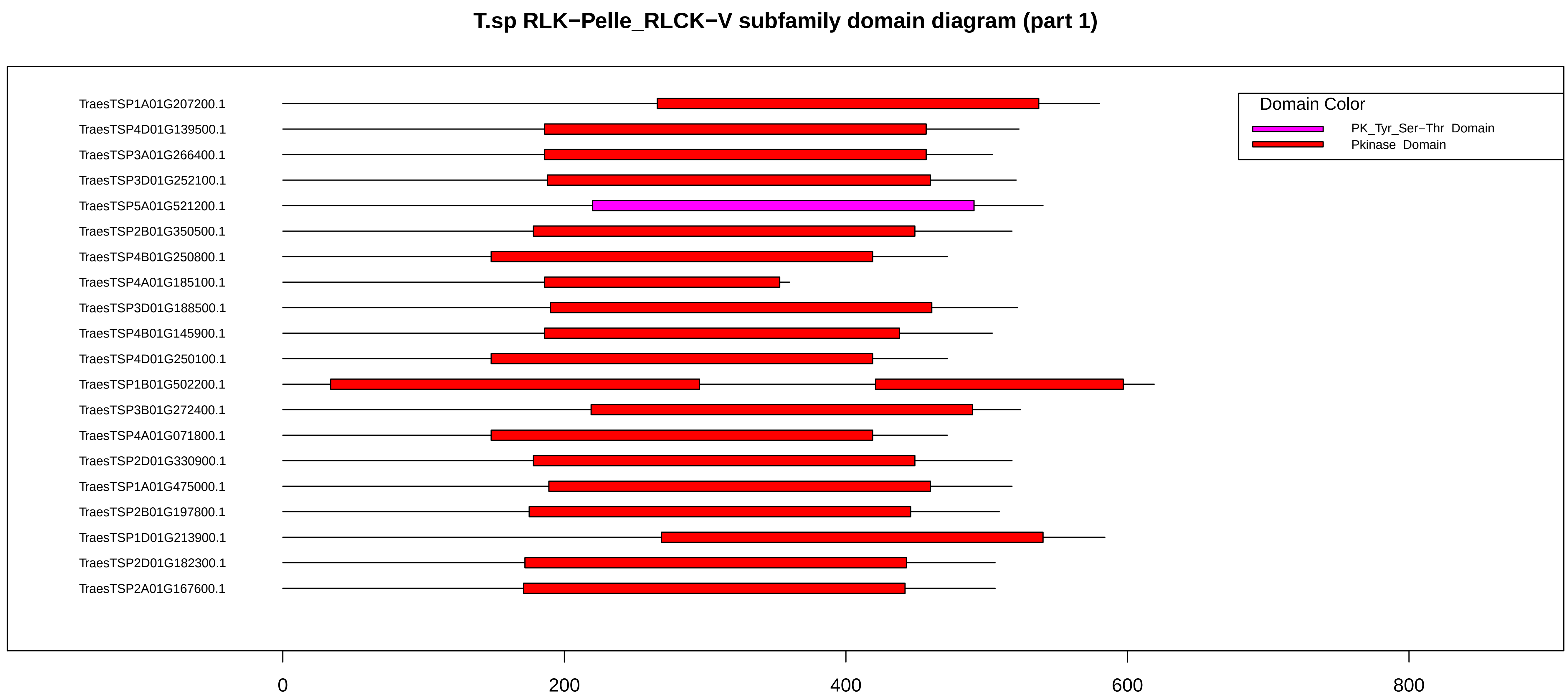
T.sp RLK−Pelle_RLCK−V subfamily domain diagram (part 1)
TraesTSP1A01G207200.1
TraesTSP4D01G139500.1
TraesTSP3A01G266400.1
TraesTSP3D01G252100.1
TraesTSP5A01G521200.1
TraesTSP2B01G350500.1
TraesTSP4B01G250800.1
TraesTSP4A01G185100.1
TraesTSP3D01G188500.1
TraesTSP4B01G145900.1
TraesTSP4D01G250100.1
TraesTSP1B01G502200.1
TraesTSP3B01G272400.1
TraesTSP4A01G071800.1
TraesTSP2D01G330900.1
TraesTSP1A01G475000.1
TraesTSP2B01G197800.1
TraesTSP1D01G213900.1
TraesTSP2D01G182300.1
TraesTSP2A01G167600.1
Domain Color
PK_Tyr_Ser−Thr Domain
Pkinase Domain
0
200
400
600
800

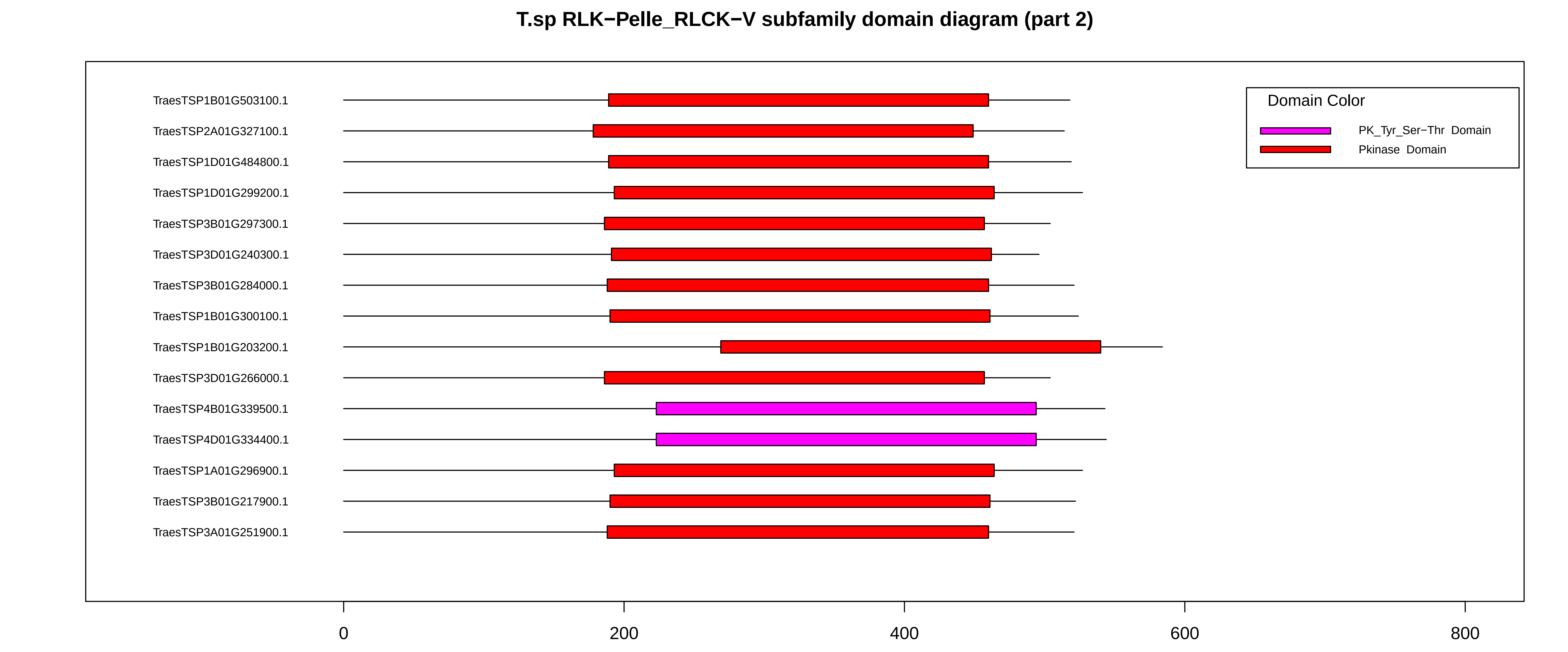
T.sp RLK−Pelle_RLCK−V subfamily domain diagram (part 2)
TraesTSP1B01G503100.1
TraesTSP2A01G327100.1
TraesTSP1D01G484800.1
TraesTSP1D01G299200.1
TraesTSP3B01G297300.1
TraesTSP3D01G240300.1
TraesTSP3B01G284000.1
TraesTSP1B01G300100.1
TraesTSP1B01G203200.1
TraesTSP3D01G266000.1
TraesTSP4B01G339500.1
TraesTSP4D01G334400.1
TraesTSP1A01G296900.1
TraesTSP3B01G217900.1
TraesTSP3A01G251900.1
Domain Color
PK_Tyr_Ser−Thr Domain
Pkinase Domain
0
200
400
600
800

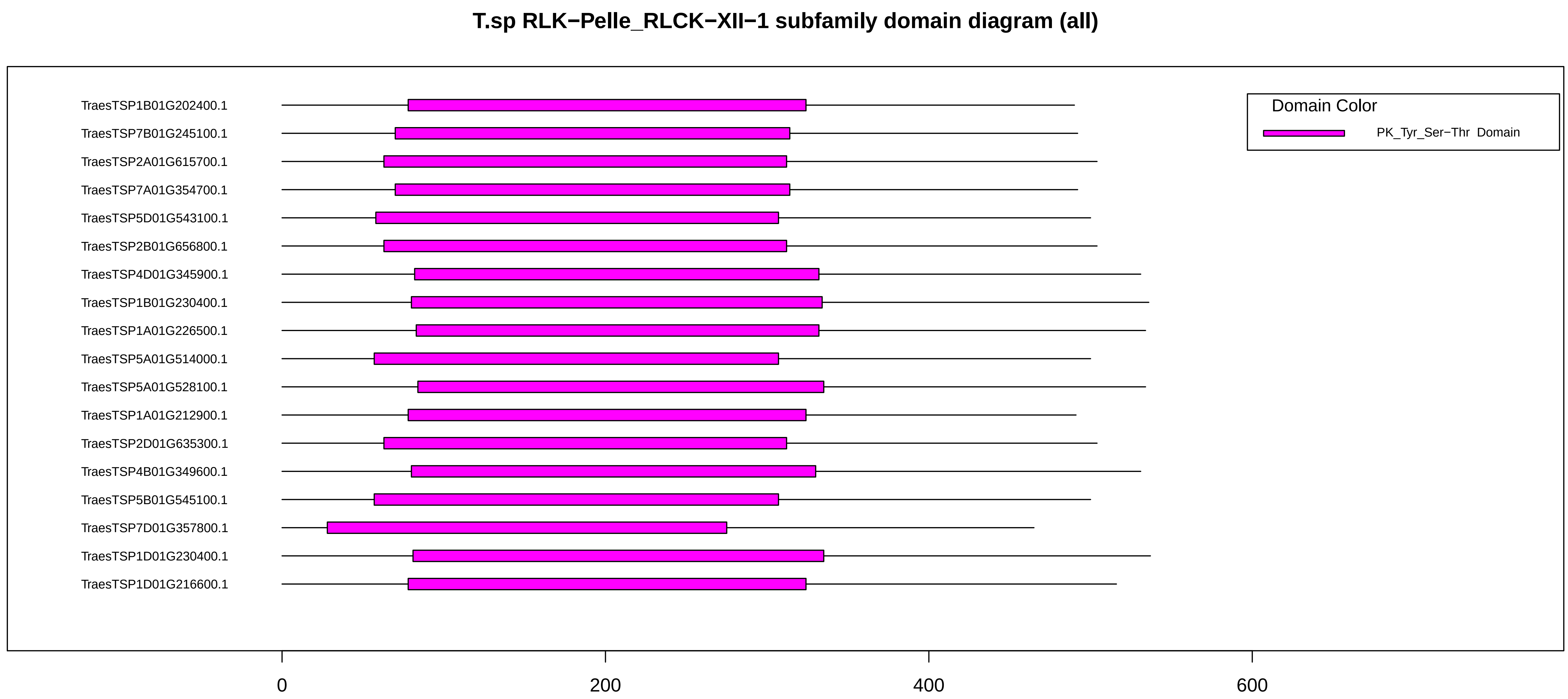
T.sp RLK−Pelle_RLCK−XII−1 subfamily domain diagram (all)
TraesTSP1B01G202400.1
TraesTSP7B01G245100.1
TraesTSP2A01G615700.1
TraesTSP7A01G354700.1
TraesTSP5D01G543100.1
TraesTSP2B01G656800.1
TraesTSP4D01G345900.1
TraesTSP1B01G230400.1
TraesTSP1A01G226500.1
TraesTSP5A01G514000.1
TraesTSP5A01G528100.1
TraesTSP1A01G212900.1
TraesTSP2D01G635300.1
TraesTSP4B01G349600.1
TraesTSP5B01G545100.1
TraesTSP7D01G357800.1
TraesTSP1D01G230400.1
TraesTSP1D01G216600.1
Domain Color
PK_Tyr_Ser−Thr Domain
0
200
400
600

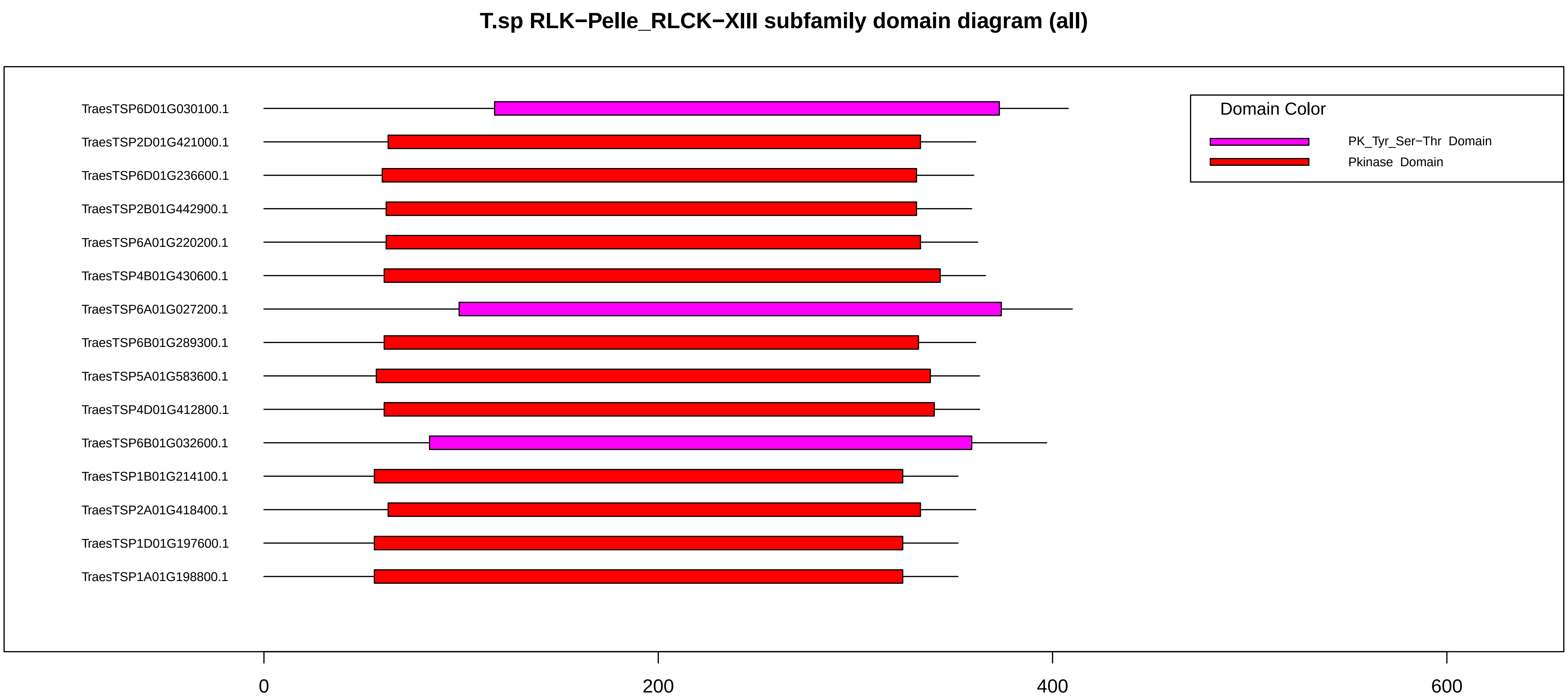
T.sp RLK−Pelle_RLCK−XIII subfamily domain diagram (all)
TraesTSP6D01G030100.1
TraesTSP2D01G421000.1
TraesTSP6D01G236600.1
TraesTSP2B01G442900.1
TraesTSP6A01G220200.1
TraesTSP4B01G430600.1
TraesTSP6A01G027200.1
TraesTSP6B01G289300.1
TraesTSP5A01G583600.1
TraesTSP4D01G412800.1
TraesTSP6B01G032600.1
TraesTSP1B01G214100.1
TraesTSP2A01G418400.1
TraesTSP1D01G197600.1
TraesTSP1A01G198800.1
Domain Color
PK_Tyr_Ser−Thr Domain
Pkinase Domain
0
200
400
600

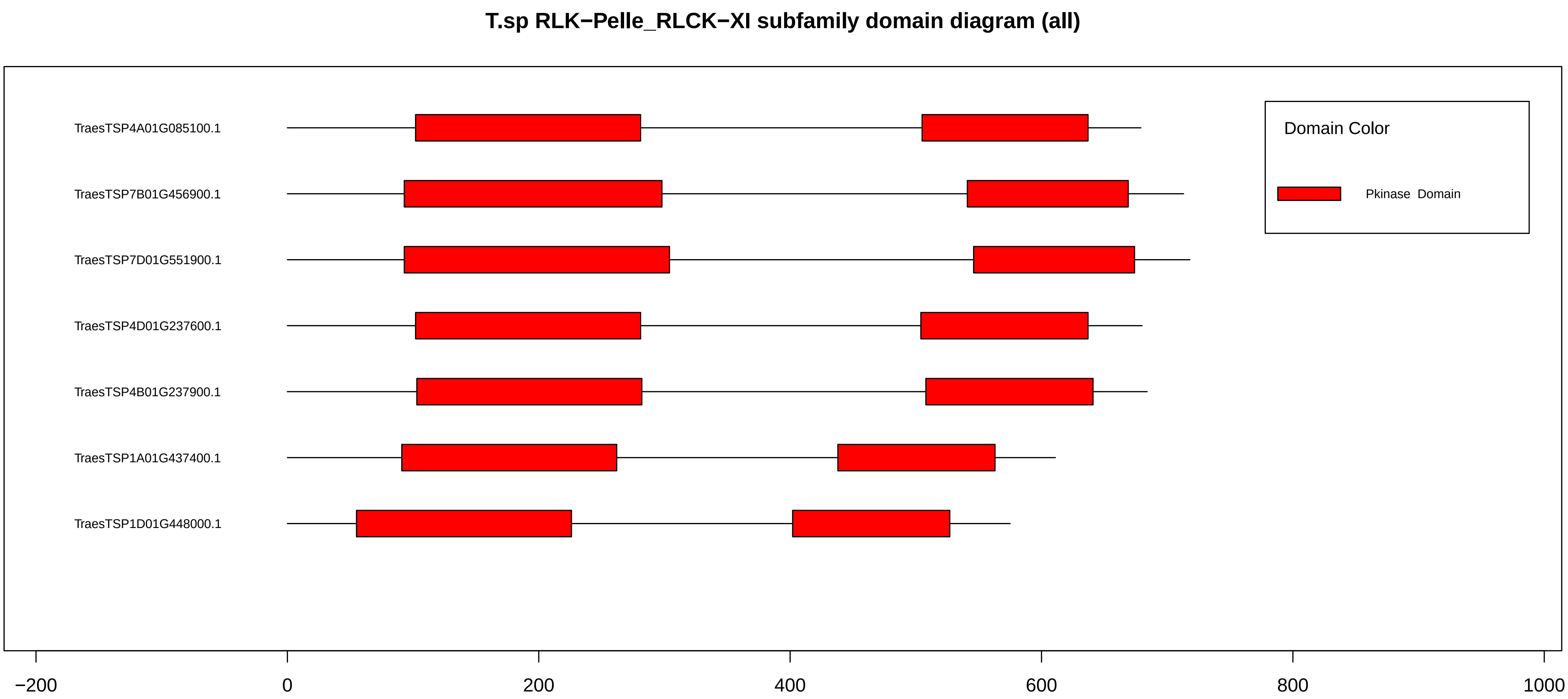
T.sp RLK−Pelle_RLCK−XI subfamily domain diagram (all)
TraesTSP4A01G085100.1
TraesTSP7B01G456900.1
TraesTSP7D01G551900.1
TraesTSP4D01G237600.1
TraesTSP4B01G237900.1
TraesTSP1A01G437400.1
TraesTSP1D01G448000.1
Domain Color
Pkinase Domain
−200
0
200
400
600
800
1000

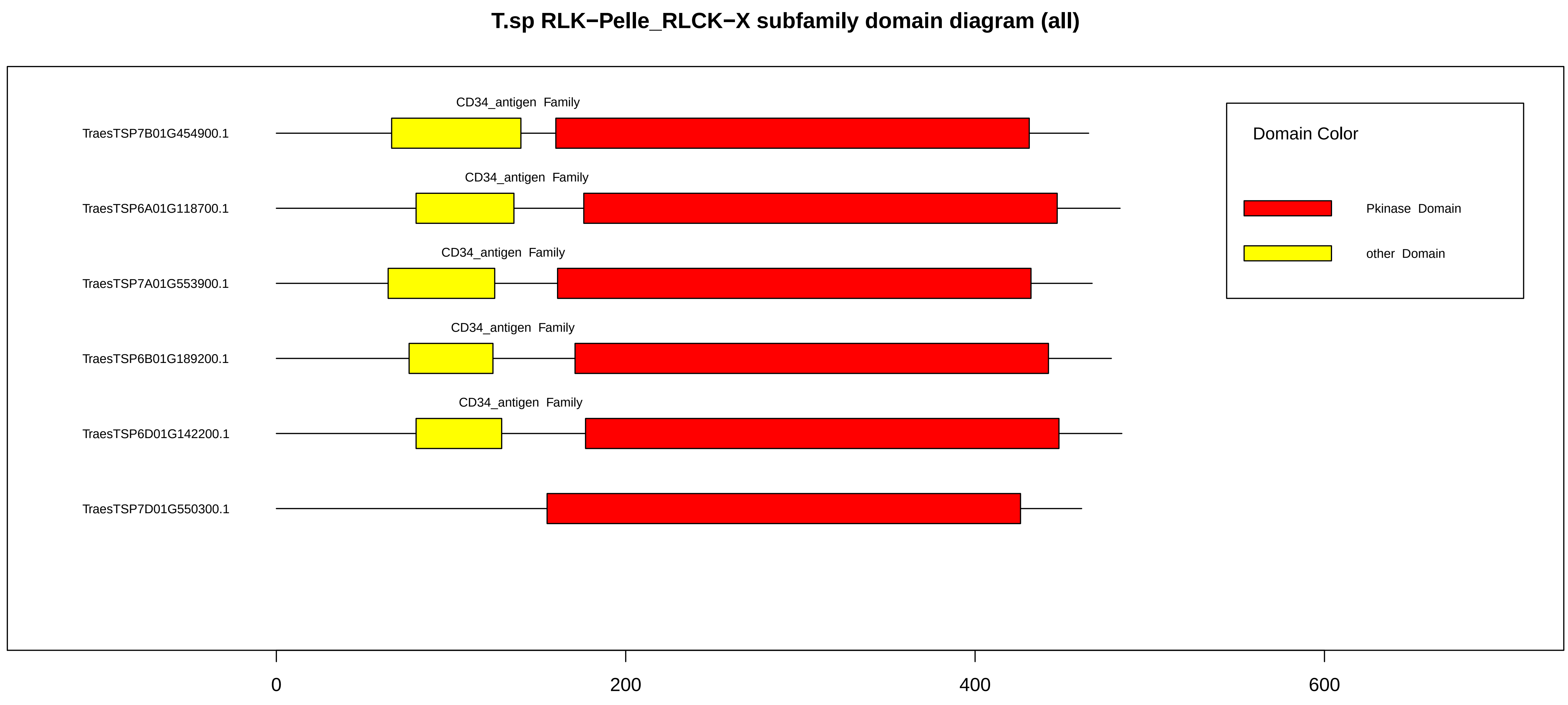
T.sp RLK−Pelle_RLCK−X subfamily domain diagram (all)
TraesTSP7B01G454900.1
CD34_antigen Family
TraesTSP6A01G118700.1
CD34_antigen Family
TraesTSP7A01G553900.1
CD34_antigen Family
TraesTSP6B01G189200.1
CD34_antigen Family
TraesTSP6D01G142200.1
CD34_antigen Family
TraesTSP7D01G550300.1
Domain Color
Pkinase Domain
other Domain
0
200
400
600

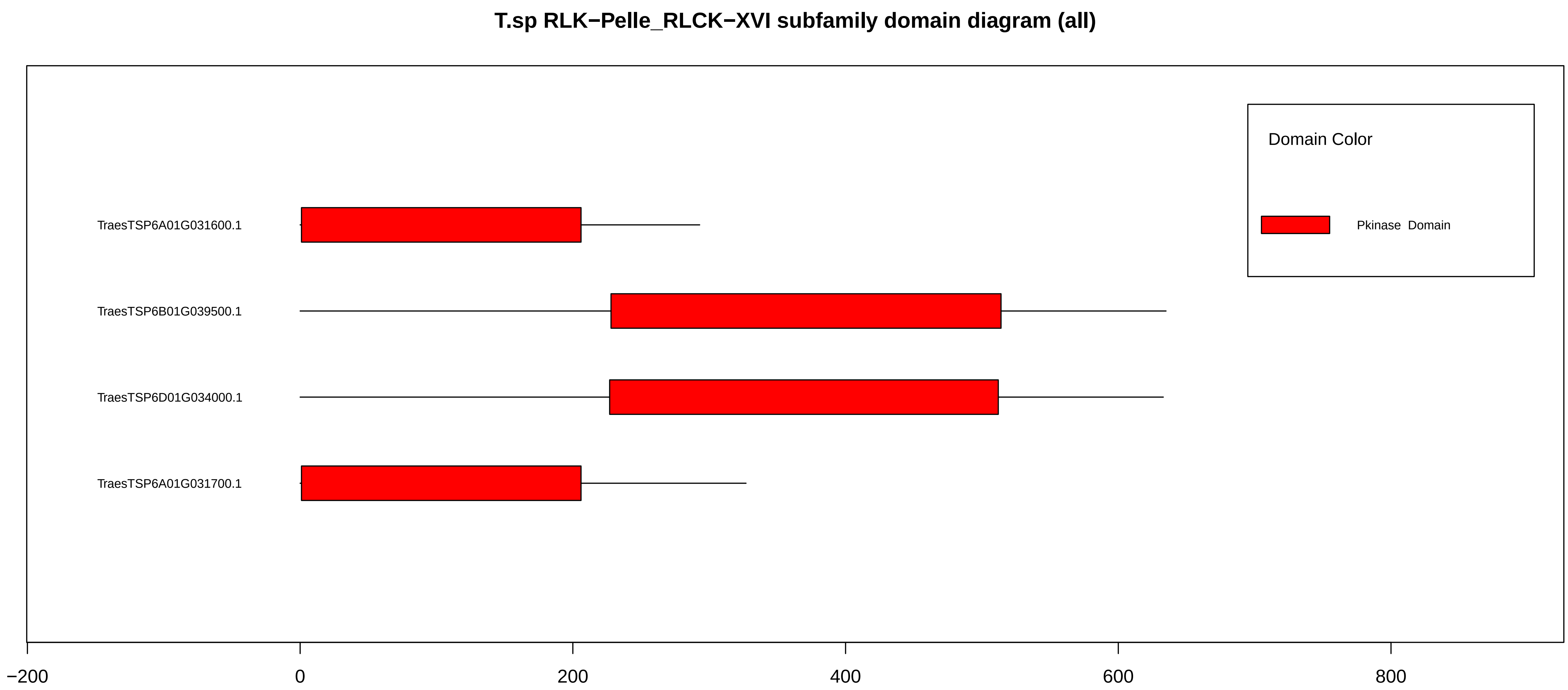
T.sp RLK−Pelle_RLCK−XVI subfamily domain diagram (all)
TraesTSP6A01G031600.1
TraesTSP6B01G039500.1
TraesTSP6D01G034000.1
TraesTSP6A01G031700.1
Domain Color
Pkinase Domain
−200
0
200
400
600
800

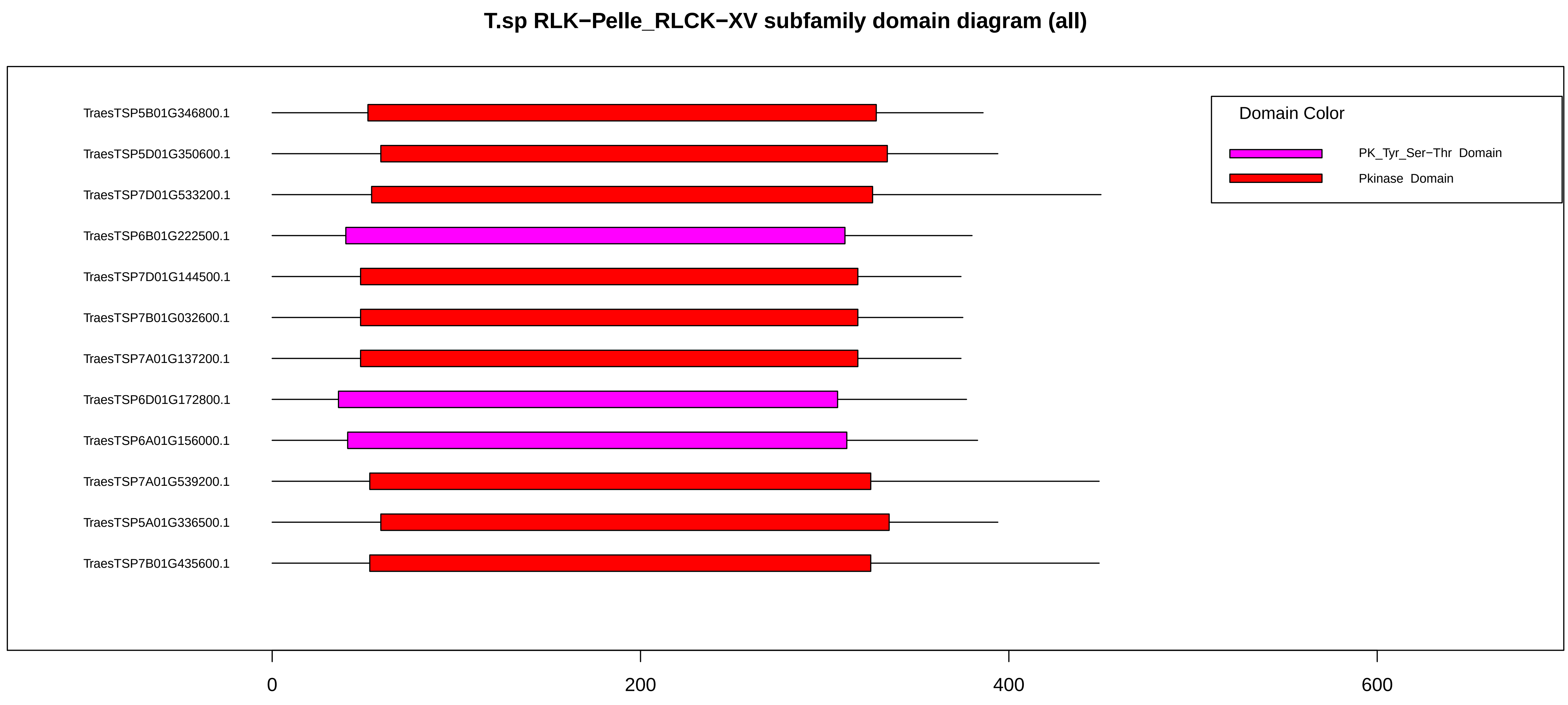

T.sp RLK−Pelle_RLCK−XV subfamily domain diagram (all)
TraesTSP5B01G346800.1
TraesTSP5D01G350600.1
TraesTSP7D01G533200.1
TraesTSP6B01G222500.1
TraesTSP7D01G144500.1
TraesTSP7B01G032600.1
TraesTSP7A01G137200.1
TraesTSP6D01G172800.1
TraesTSP6A01G156000.1
TraesTSP7A01G539200.1
TraesTSP5A01G336500.1
TraesTSP7B01G435600.1
Domain Color
PK_Tyr_Ser−Thr Domain
Pkinase Domain
0
200
400
600

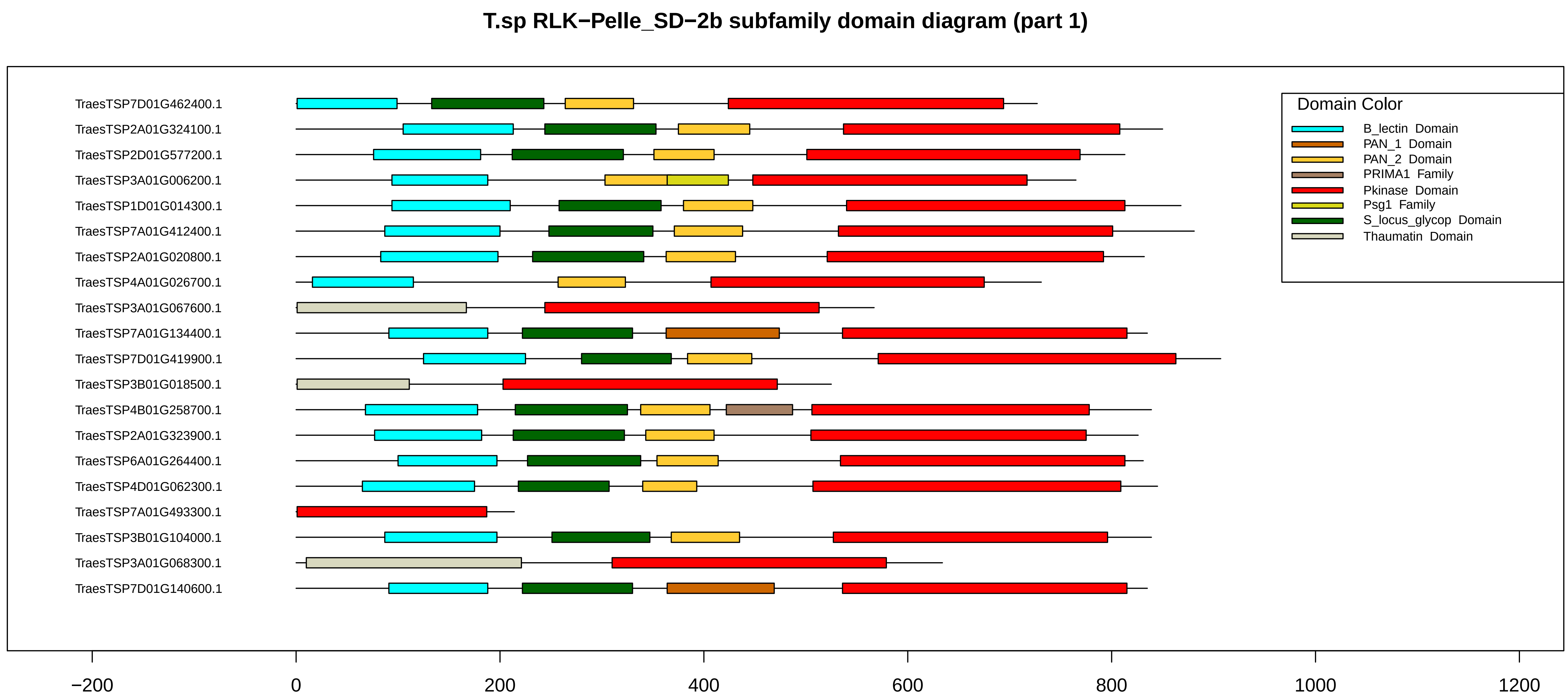
T.sp RLK−Pelle_SD−2b subfamily domain diagram (part 1)
TraesTSP7D01G462400.1
TraesTSP2A01G324100.1
TraesTSP2D01G577200.1
TraesTSP3A01G006200.1
TraesTSP1D01G014300.1
TraesTSP7A01G412400.1
TraesTSP2A01G020800.1
TraesTSP4A01G026700.1
TraesTSP3A01G067600.1
TraesTSP7A01G134400.1
TraesTSP7D01G419900.1
TraesTSP3B01G018500.1
TraesTSP4B01G258700.1
TraesTSP2A01G323900.1
TraesTSP6A01G264400.1
TraesTSP4D01G062300.1
TraesTSP7A01G493300.1
TraesTSP3B01G104000.1
TraesTSP3A01G068300.1
TraesTSP7D01G140600.1
Domain Color
B_lectin Domain
PAN_1 Domain
PAN_2 Domain
PRIMA1 Family
Pkinase Domain
Psg1 Family
S_locus_glycop Domain
Thaumatin Domain
−200
0
200
400
600
800
1000
1200

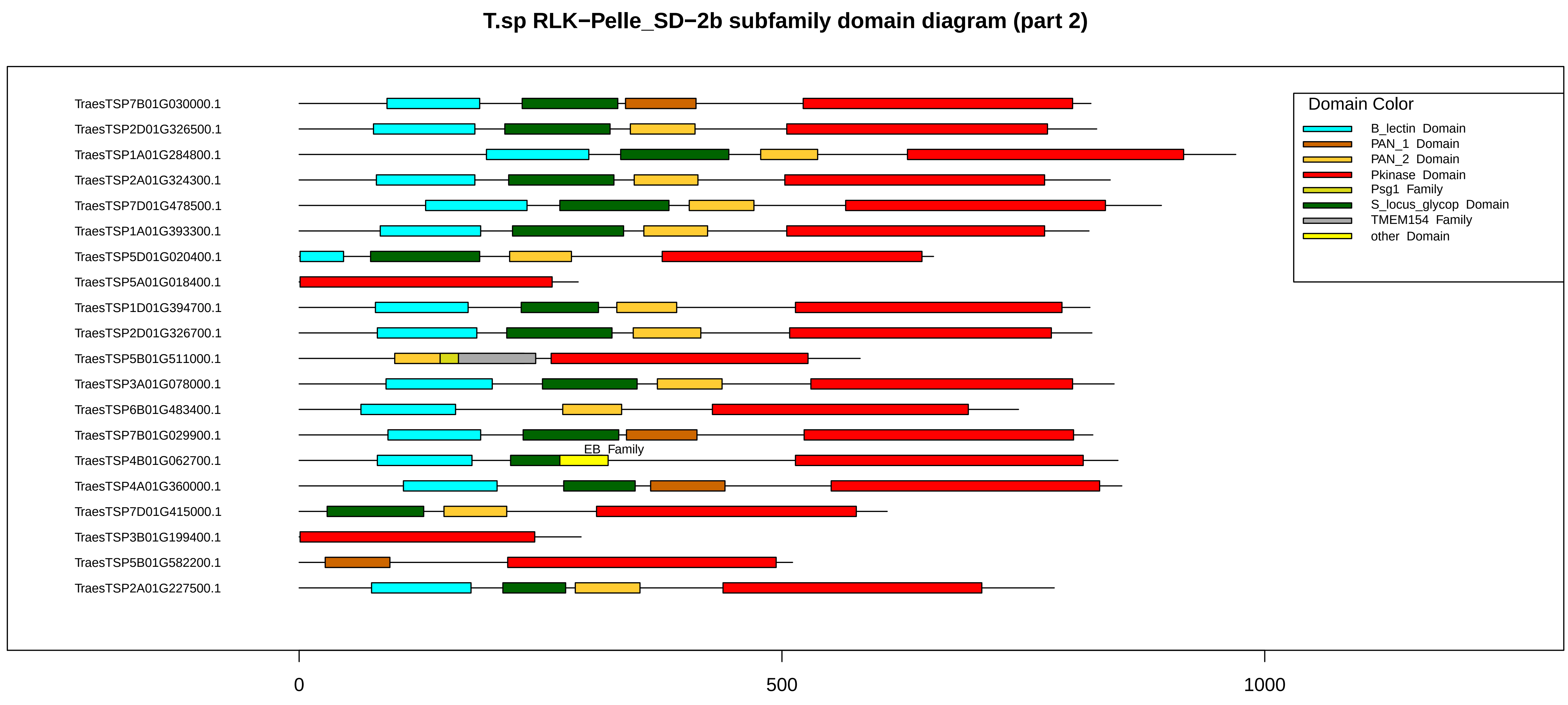
T.sp RLK−Pelle_SD−2b subfamily domain diagram (part 2)
TraesTSP7B01G030000.1
TraesTSP2D01G326500.1
TraesTSP1A01G284800.1
TraesTSP2A01G324300.1
TraesTSP7D01G478500.1
TraesTSP1A01G393300.1
TraesTSP5D01G020400.1
TraesTSP5A01G018400.1
TraesTSP1D01G394700.1
TraesTSP2D01G326700.1
TraesTSP5B01G511000.1
TraesTSP3A01G078000.1
TraesTSP6B01G483400.1
TraesTSP7B01G029900.1
EB_Family
TraesTSP4B01G062700.1
TraesTSP4A01G360000.1
TraesTSP7D01G415000.1
TraesTSP3B01G199400.1
TraesTSP5B01G582200.1
TraesTSP2A01G227500.1
Domain Color
B_lectin Domain
PAN_1 Domain
PAN_2 Domain
Pkinase Domain
Psg1 Family
S_locus_glycop Domain
TMEM154 Family
other Domain
0
500
1000

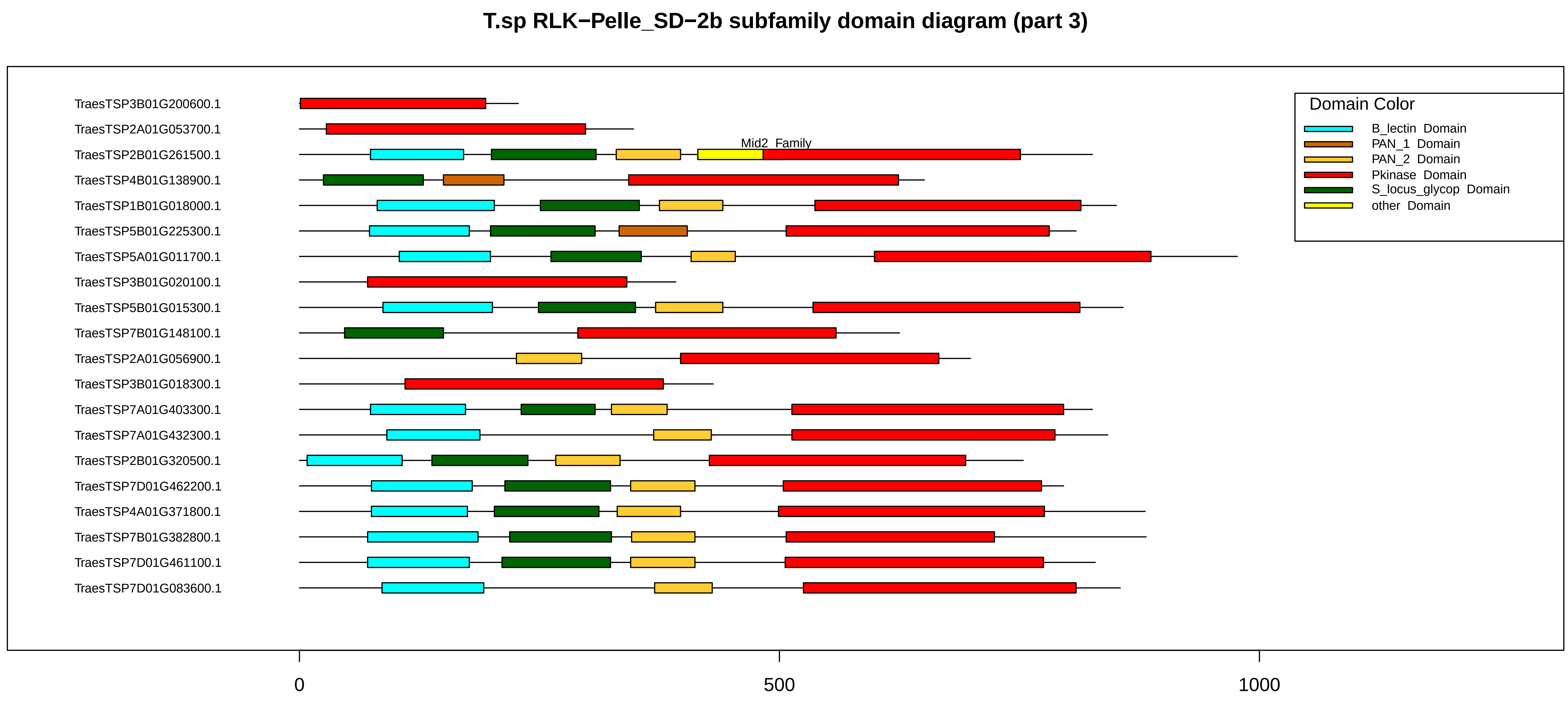
T.sp RLK−Pelle_SD−2b subfamily domain diagram (part 3)
TraesTSP3B01G200600.1
TraesTSP2A01G053700.1
TraesTSP2B01G261500.1
TraesTSP4B01G138900.1
TraesTSP1B01G018000.1
TraesTSP5B01G225300.1
TraesTSP5A01G011700.1
TraesTSP3B01G020100.1
TraesTSP5B01G015300.1
TraesTSP7B01G148100.1
TraesTSP2A01G056900.1
TraesTSP3B01G018300.1
TraesTSP7A01G403300.1
TraesTSP7A01G432300.1
TraesTSP2B01G320500.1
TraesTSP7D01G462200.1
TraesTSP4A01G371800.1
TraesTSP7B01G382800.1
TraesTSP7D01G461100.1
TraesTSP7D01G083600.1
Mid2_Family
Domain Color
B_lectin Domain
PAN_1 Domain
PAN_2 Domain
Pkinase Domain
S_locus_glycop Domain
other Domain
0
500
1000

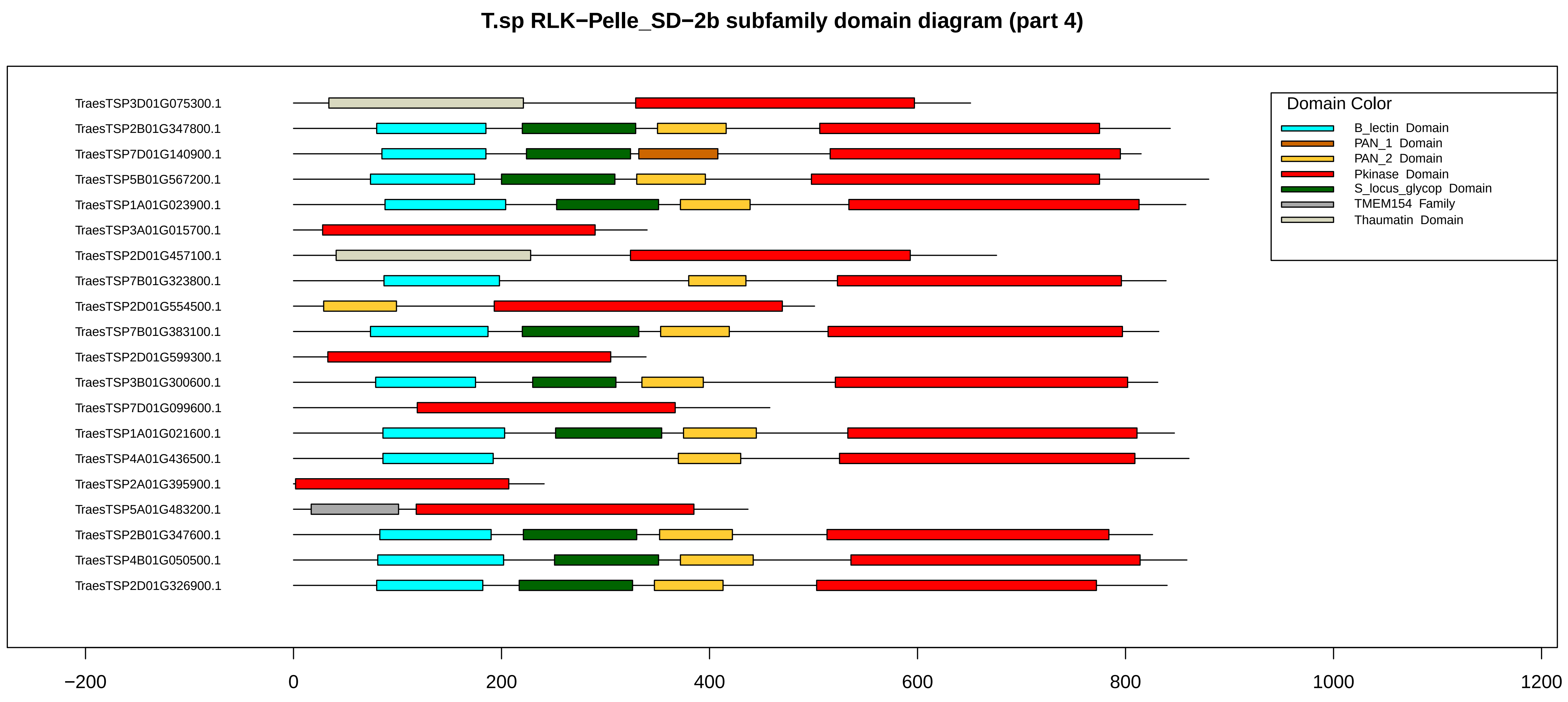
T.sp RLK−Pelle_SD−2b subfamily domain diagram (part 4)
TraesTSP3D01G075300.1
TraesTSP2B01G347800.1
TraesTSP7D01G140900.1
TraesTSP5B01G567200.1
TraesTSP1A01G023900.1
TraesTSP3A01G015700.1
TraesTSP2D01G457100.1
TraesTSP7B01G323800.1
TraesTSP2D01G554500.1
TraesTSP7B01G383100.1
TraesTSP2D01G599300.1
TraesTSP3B01G300600.1
TraesTSP7D01G099600.1
TraesTSP1A01G021600.1
TraesTSP4A01G436500.1
TraesTSP2A01G395900.1
TraesTSP5A01G483200.1
TraesTSP2B01G347600.1
TraesTSP4B01G050500.1
TraesTSP2D01G326900.1
Domain Color
B_lectin Domain
PAN_1 Domain
PAN_2 Domain
Pkinase Domain
S_locus_glycop Domain
TMEM154 Family
Thaumatin Domain
−200
0
200
400
600
800
1000
1200

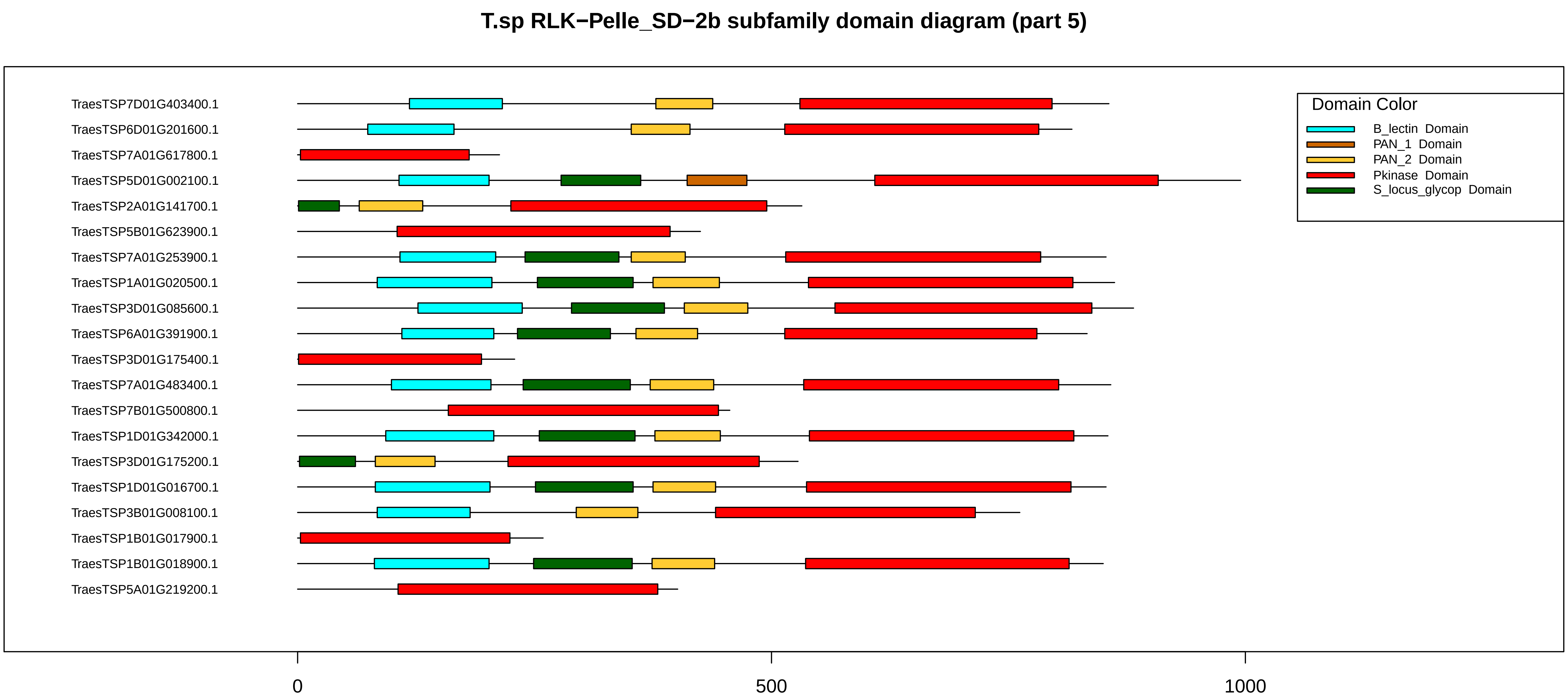
T.sp RLK−Pelle_SD−2b subfamily domain diagram (part 5)
TraesTSP7D01G403400.1
TraesTSP6D01G201600.1
TraesTSP7A01G617800.1
TraesTSP5D01G002100.1
TraesTSP2A01G141700.1
TraesTSP5B01G623900.1
TraesTSP7A01G253900.1
TraesTSP1A01G020500.1
TraesTSP3D01G085600.1
TraesTSP6A01G391900.1
TraesTSP3D01G175400.1
TraesTSP7A01G483400.1
TraesTSP7B01G500800.1
TraesTSP1D01G342000.1
TraesTSP3D01G175200.1
TraesTSP1D01G016700.1
TraesTSP3B01G008100.1
TraesTSP1B01G017900.1
TraesTSP1B01G018900.1
TraesTSP5A01G219200.1
Domain Color
B_lectin Domain
PAN_1 Domain
PAN_2 Domain
Pkinase Domain
S_locus_glycop Domain
0
500
1000

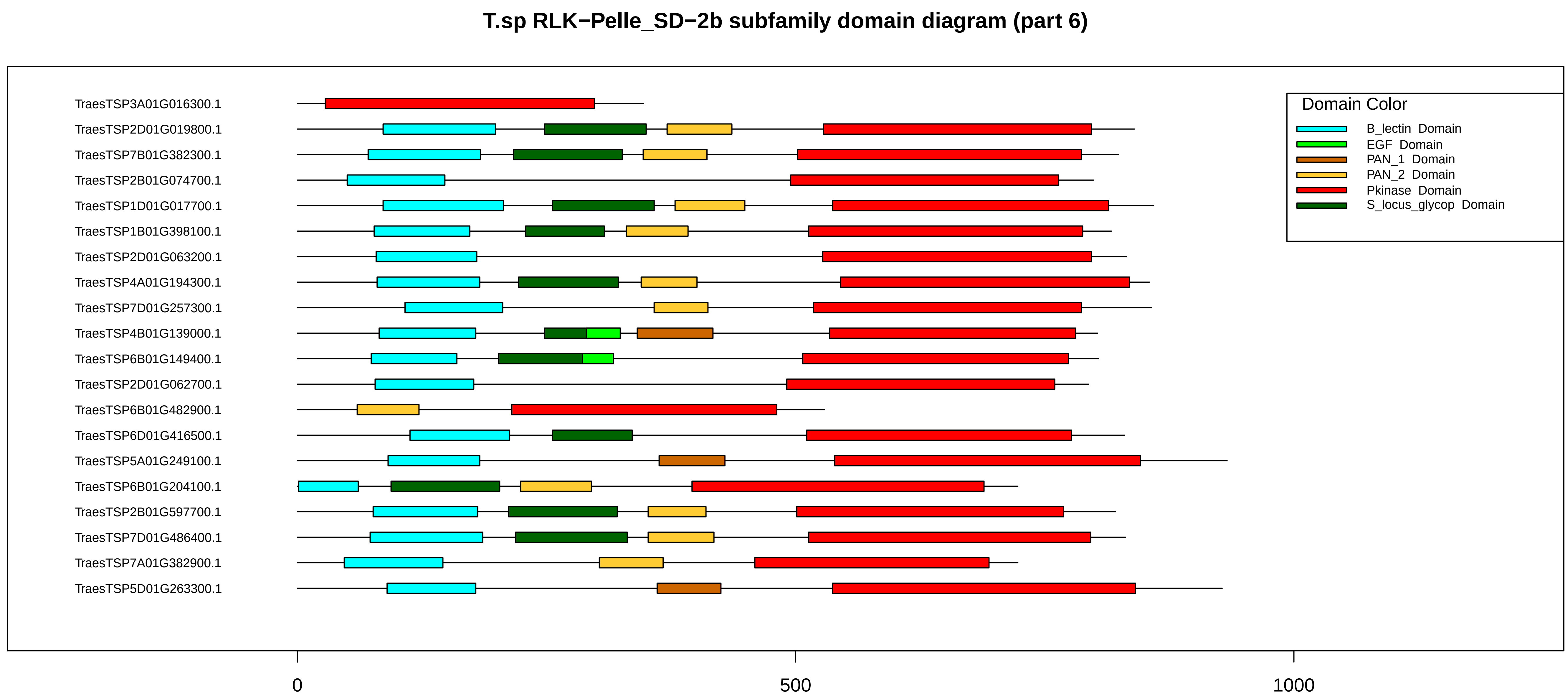
T.sp RLK−Pelle_SD−2b subfamily domain diagram (part 6)
TraesTSP3A01G016300.1
TraesTSP2D01G019800.1
TraesTSP7B01G382300.1
TraesTSP2B01G074700.1
TraesTSP1D01G017700.1
TraesTSP1B01G398100.1
TraesTSP2D01G063200.1
TraesTSP4A01G194300.1
TraesTSP7D01G257300.1
TraesTSP4B01G139000.1
TraesTSP6B01G149400.1
TraesTSP2D01G062700.1
TraesTSP6B01G482900.1
TraesTSP6D01G416500.1
TraesTSP5A01G249100.1
TraesTSP6B01G204100.1
TraesTSP2B01G597700.1
TraesTSP7D01G486400.1
TraesTSP7A01G382900.1
TraesTSP5D01G263300.1
Domain Color
B_lectin Domain
EGF Domain
PAN_1 Domain
PAN_2 Domain
Pkinase Domain
S_locus_glycop Domain
0
500
1000

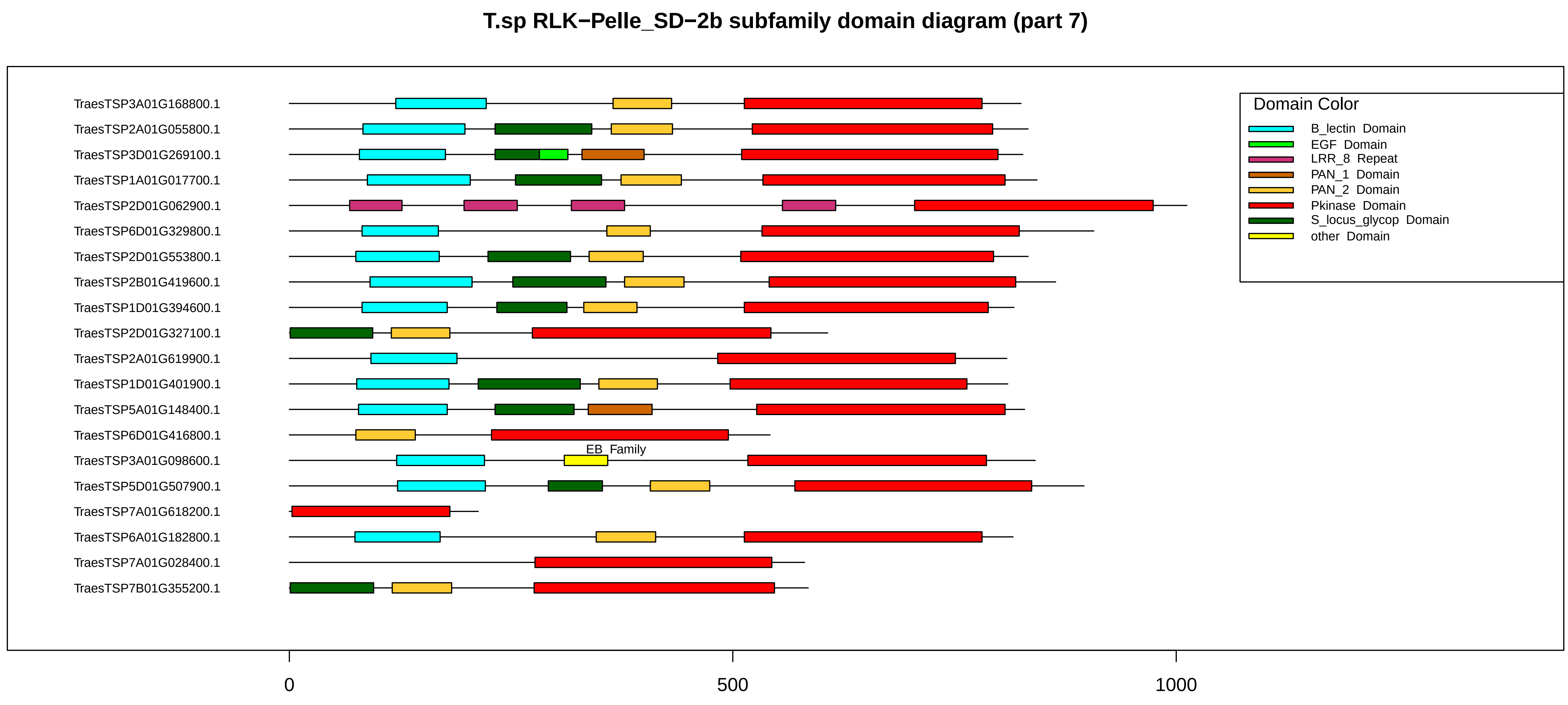

T.sp RLK−Pelle_SD−2b subfamily domain diagram (part 7)
TraesTSP3A01G168800.1
TraesTSP2A01G055800.1
TraesTSP3D01G269100.1
TraesTSP1A01G017700.1
TraesTSP2D01G062900.1
TraesTSP6D01G329800.1
TraesTSP2D01G553800.1
TraesTSP2B01G419600.1
TraesTSP1D01G394600.1
TraesTSP2D01G327100.1
TraesTSP2A01G619900.1
TraesTSP1D01G401900.1
TraesTSP5A01G148400.1
TraesTSP6D01G416800.1
TraesTSP3A01G098600.1
EB_Family
TraesTSP5D01G507900.1
TraesTSP7A01G618200.1
TraesTSP6A01G182800.1
TraesTSP7A01G028400.1
TraesTSP7B01G355200.1
Domain Color
B_lectin Domain
EGF Domain
LRR_8 Repeat
PAN_1 Domain
PAN_2 Domain
Pkinase Domain
S_locus_glycop Domain
other Domain
0
500
1000

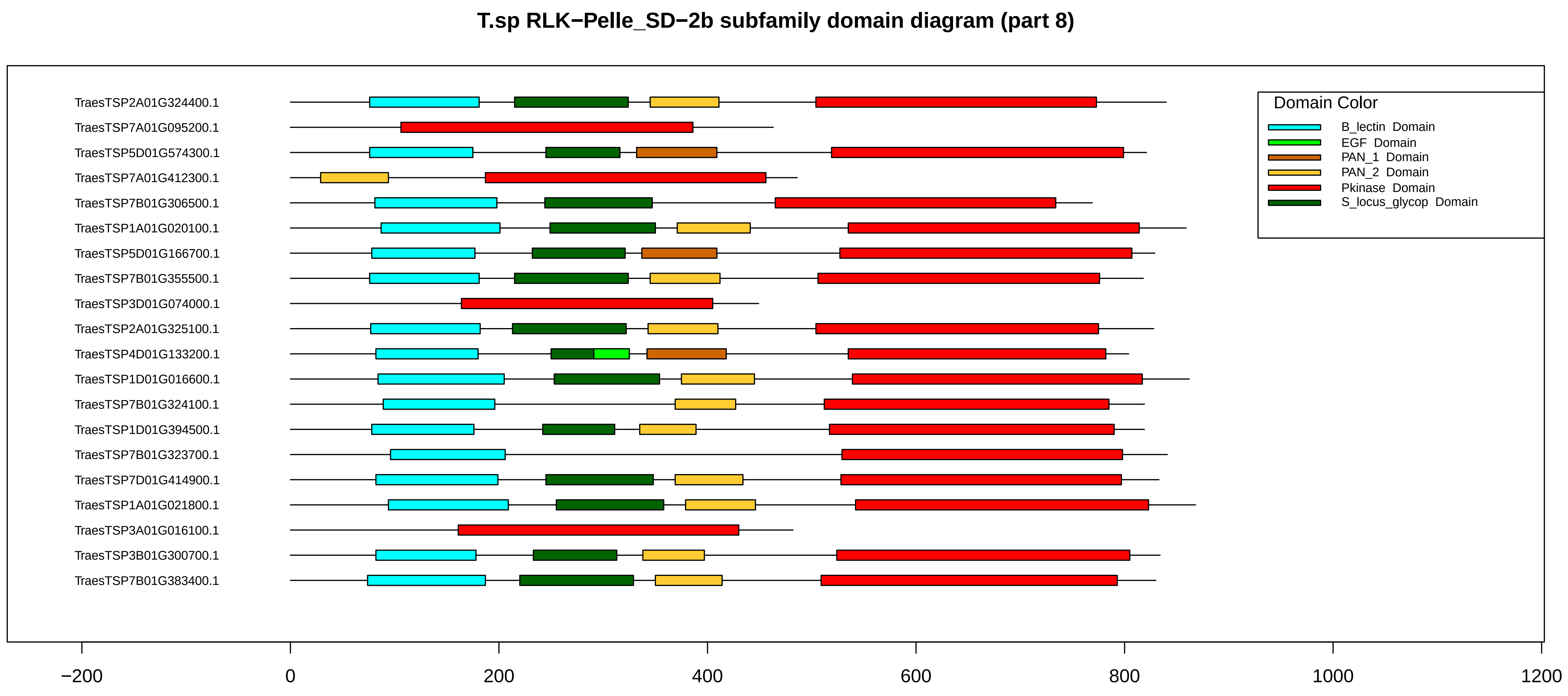

T.sp RLK−Pelle_SD−2b subfamily domain diagram (part 8)
TraesTSP2A01G324400.1
TraesTSP7A01G095200.1
TraesTSP5D01G574300.1
TraesTSP7A01G412300.1
TraesTSP7B01G306500.1
TraesTSP1A01G020100.1
TraesTSP5D01G166700.1
TraesTSP7B01G355500.1
TraesTSP3D01G074000.1
TraesTSP2A01G325100.1
TraesTSP4D01G133200.1
TraesTSP1D01G016600.1
TraesTSP7B01G324100.1
TraesTSP1D01G394500.1
TraesTSP7B01G323700.1
TraesTSP7D01G414900.1
TraesTSP1A01G021800.1
TraesTSP3A01G016100.1
TraesTSP3B01G300700.1
TraesTSP7B01G383400.1
Domain Color
B_lectin Domain
EGF Domain
PAN_1 Domain
PAN_2 Domain
Pkinase Domain
S_locus_glycop Domain
−200
0
200
400
600
800
1000
1200

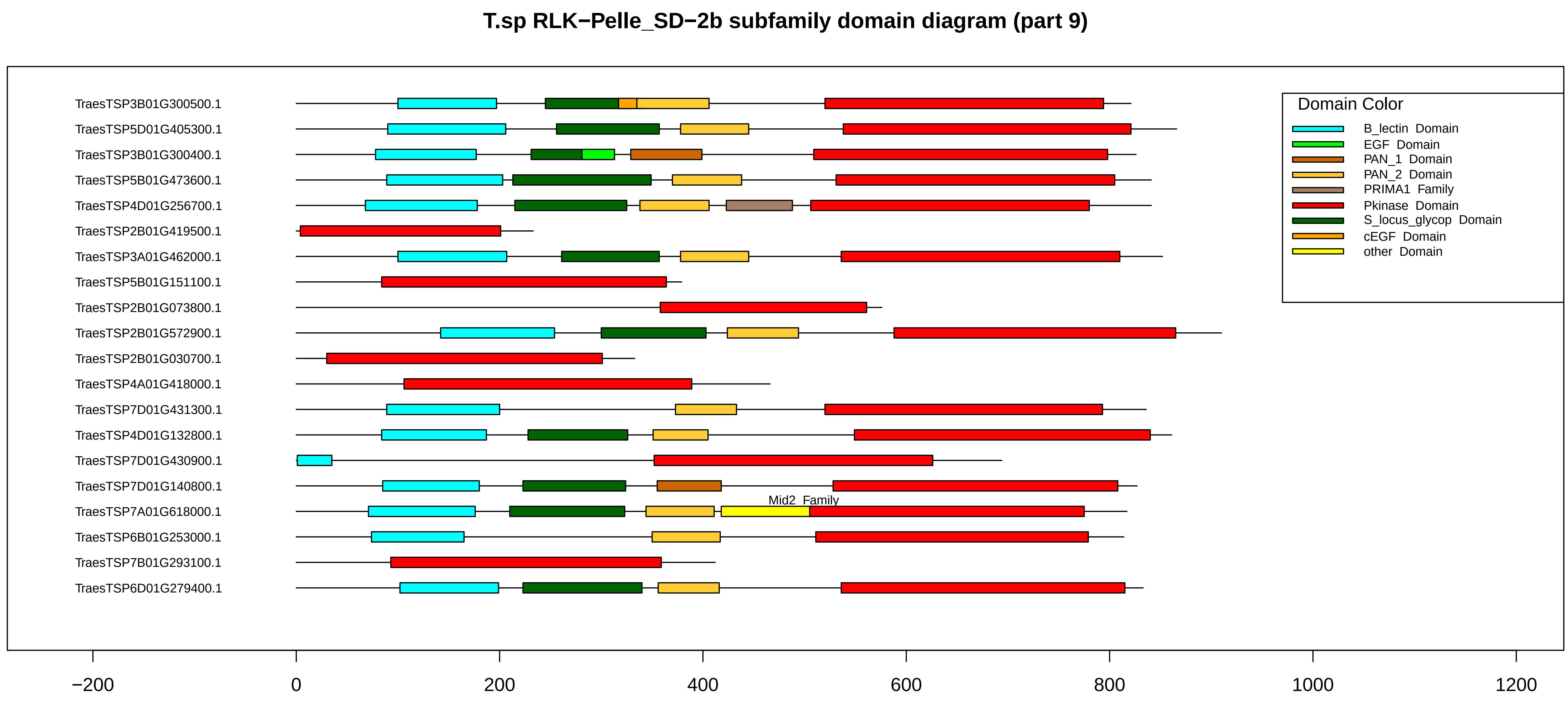
T.sp RLK−Pelle_SD−2b subfamily domain diagram (part 9)
TraesTSP3B01G300500.1
TraesTSP5D01G405300.1
TraesTSP3B01G300400.1
TraesTSP5B01G473600.1
TraesTSP4D01G256700.1
TraesTSP2B01G419500.1
TraesTSP3A01G462000.1
TraesTSP5B01G151100.1
TraesTSP2B01G073800.1
TraesTSP2B01G572900.1
TraesTSP2B01G030700.1
TraesTSP4A01G418000.1
TraesTSP7D01G431300.1
TraesTSP4D01G132800.1
TraesTSP7D01G430900.1
TraesTSP7D01G140800.1
TraesTSP7A01G618000.1
TraesTSP6B01G253000.1
TraesTSP7B01G293100.1
TraesTSP6D01G279400.1
Mid2 Family
Domain Color
B_lectin Domain
EGF Domain
PAN_1 Domain
PAN_2 Domain
PRIMA1 Family
Pkinase Domain
S_locus_glycop Domain
cEGF Domain
other Domain
−200
0
200
400
600
800
1000
1200

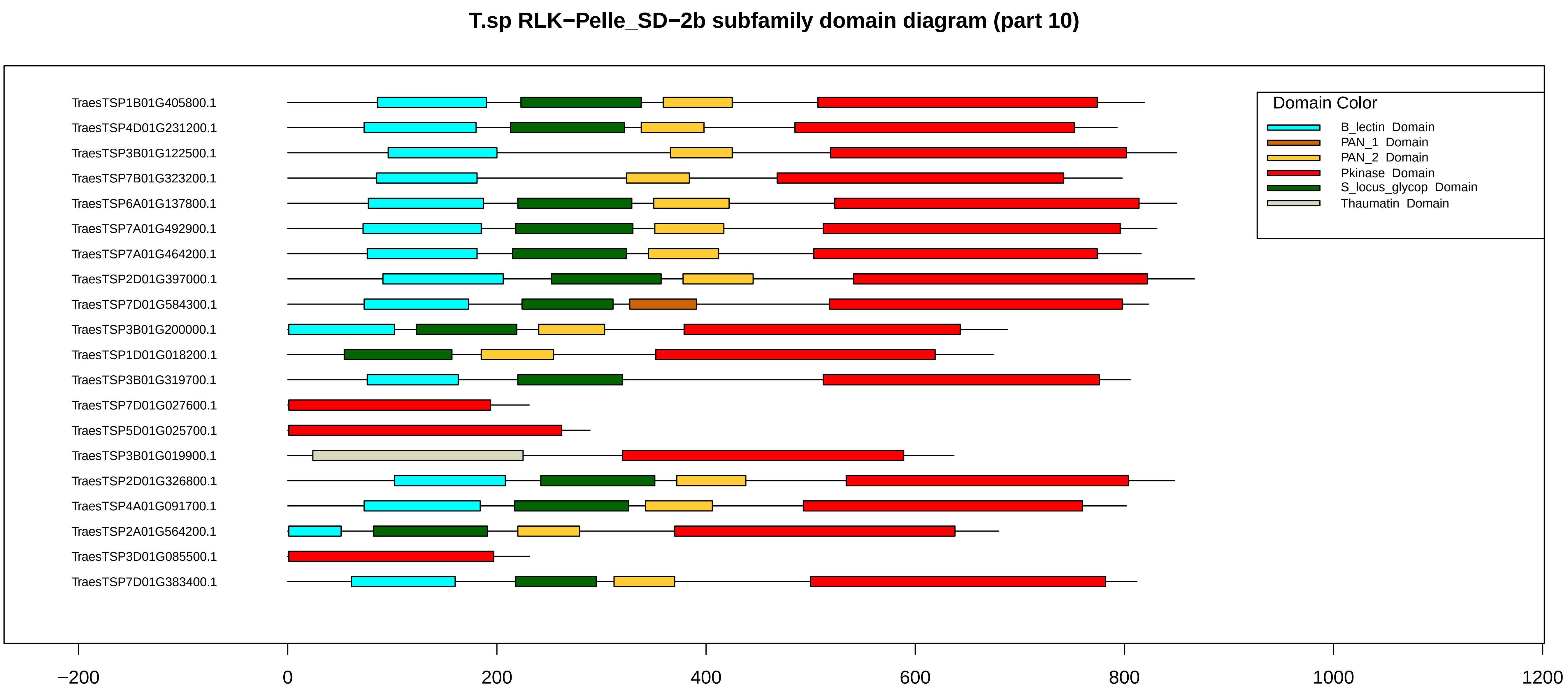

T.sp RLK−Pelle_SD−2b subfamily domain diagram (part 10)
TraesTSP1B01G405800.1
TraesTSP4D01G231200.1
TraesTSP3B01G122500.1
TraesTSP7B01G323200.1
TraesTSP6A01G137800.1
TraesTSP7A01G492900.1
TraesTSP7A01G464200.1
TraesTSP2D01G397000.1
TraesTSP7D01G584300.1
TraesTSP3B01G200000.1
TraesTSP1D01G018200.1
TraesTSP3B01G319700.1
TraesTSP7D01G027600.1
TraesTSP5D01G025700.1
TraesTSP3B01G019900.1
TraesTSP2D01G326800.1
TraesTSP4A01G091700.1
TraesTSP2A01G564200.1
TraesTSP3D01G085500.1
TraesTSP7D01G383400.1
Domain Color
B_lectin Domain
PAN_1 Domain
PAN_2 Domain
Pkinase Domain
S_locus_glycop Domain
Thaumatin Domain
−200
0
200
400
600
800
1000
1200

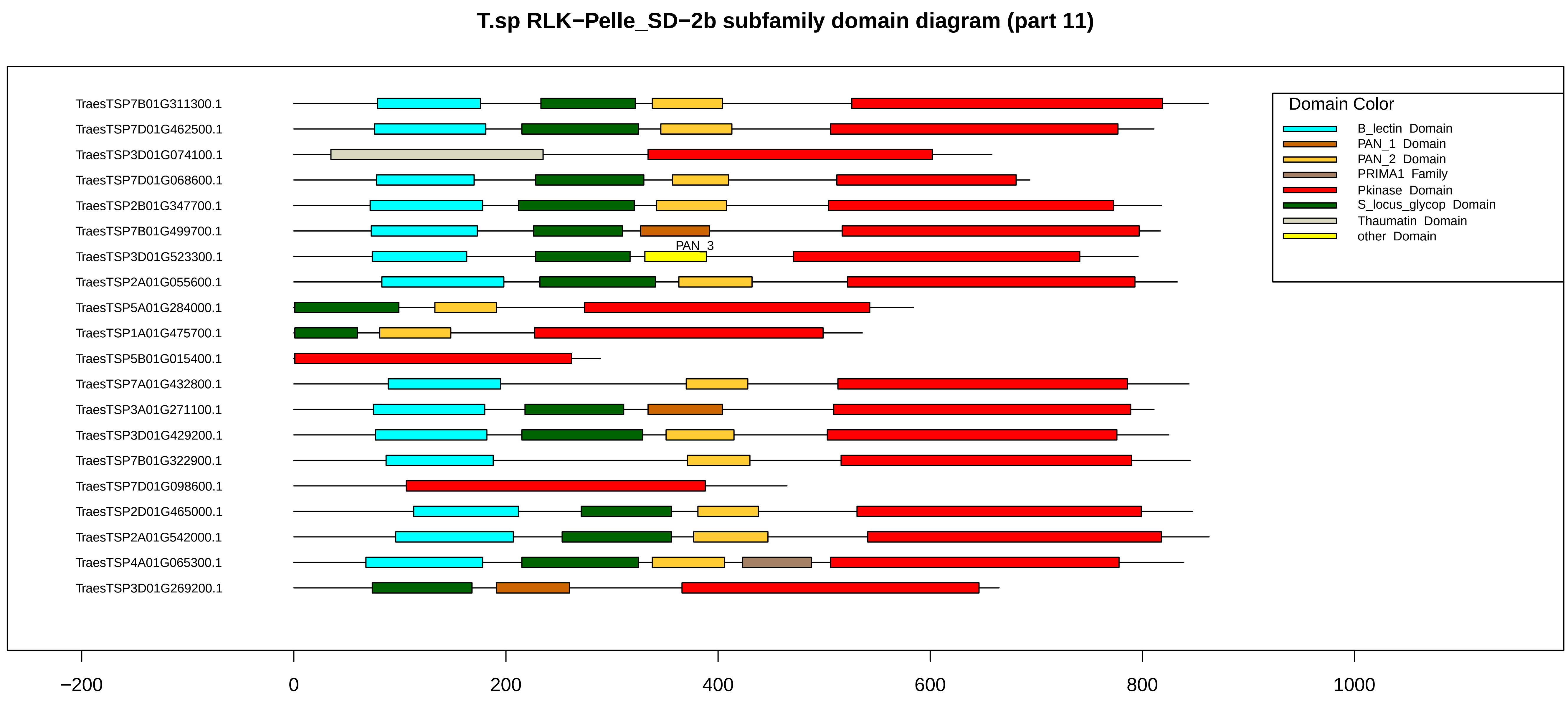
T.sp RLK−Pelle_SD−2b subfamily domain diagram (part 11)
TraesTSP7B01G311300.1
TraesTSP7D01G462500.1
TraesTSP3D01G074100.1
TraesTSP7D01G068600.1
TraesTSP2B01G347700.1
TraesTSP7B01G499700.1
TraesTSP3D01G523300.1
TraesTSP2A01G055600.1
TraesTSP5A01G284000.1
TraesTSP1A01G475700.1
TraesTSP5B01G015400.1
TraesTSP7A01G432800.1
TraesTSP3A01G271100.1
TraesTSP3D01G429200.1
TraesTSP7B01G322900.1
TraesTSP7D01G098600.1
TraesTSP2D01G465000.1
TraesTSP2A01G542000.1
TraesTSP4A01G065300.1
TraesTSP3D01G269200.1
PAN_3
Domain Color
B_lectin Domain
PAN_1 Domain
PAN_2 Domain
PRIMA1 Family
Pkinase Domain
S_locus_glycop Domain
Thaumatin Domain
other Domain
−200
0
200
400
600
800
1000

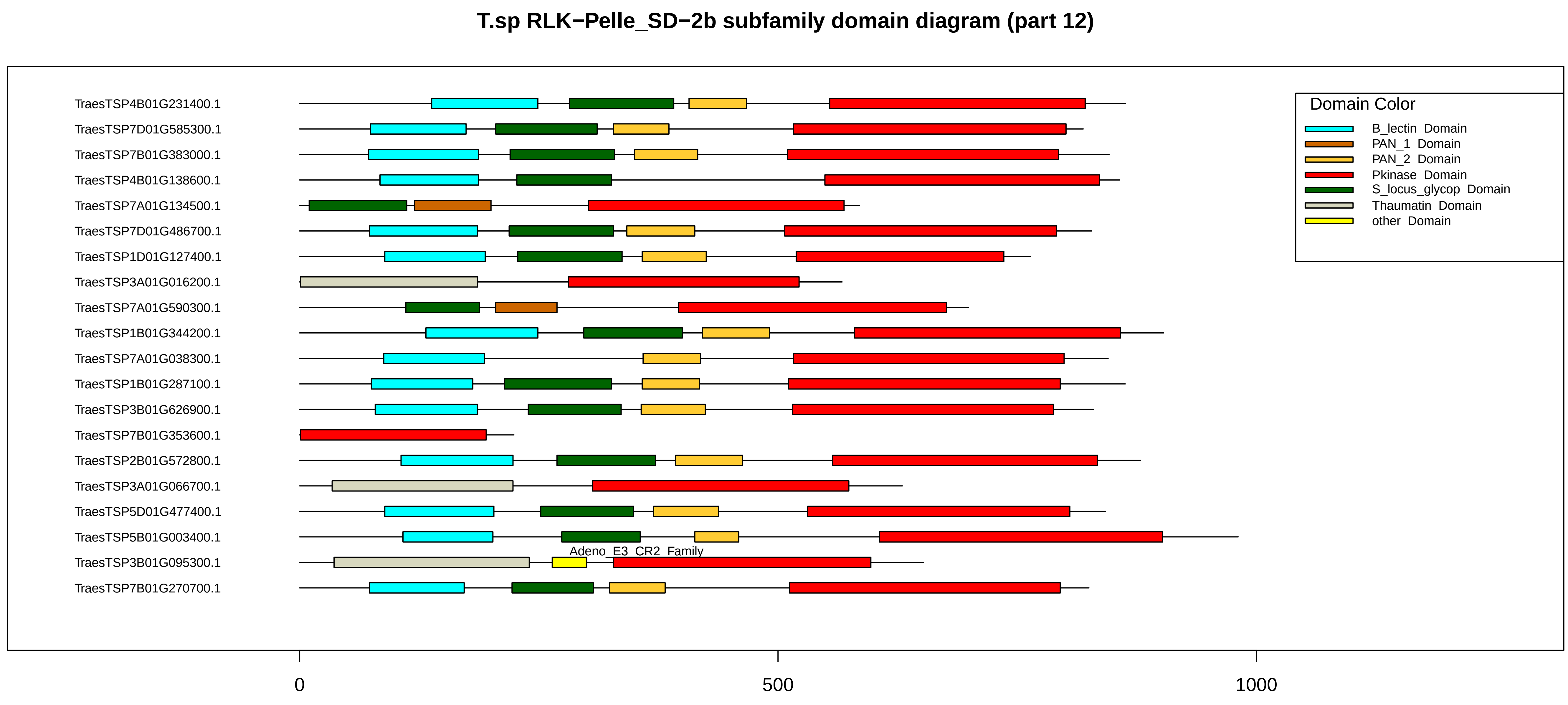

T.sp RLK−Pelle_SD−2b subfamily domain diagram (part 12)
TraesTSP4B01G231400.1
TraesTSP7D01G585300.1
TraesTSP7B01G383000.1
TraesTSP4B01G138600.1
TraesTSP7A01G134500.1
TraesTSP7D01G486700.1
TraesTSP1D01G127400.1
TraesTSP3A01G016200.1
TraesTSP7A01G590300.1
TraesTSP1B01G344200.1
TraesTSP7A01G038300.1
TraesTSP1B01G287100.1
TraesTSP3B01G626900.1
TraesTSP7B01G353600.1
TraesTSP2B01G572800.1
TraesTSP3A01G066700.1
TraesTSP5D01G477400.1
TraesTSP5B01G003400.1
TraesTSP3B01G095300.1
TraesTSP7B01G270700.1
Adeno_E3_CR2_Family
Domain Color
B_lectin Domain
PAN_1 Domain
PAN_2 Domain
Pkinase Domain
S_locus_glycop Domain
Thaumatin Domain
other Domain
0
500
1000

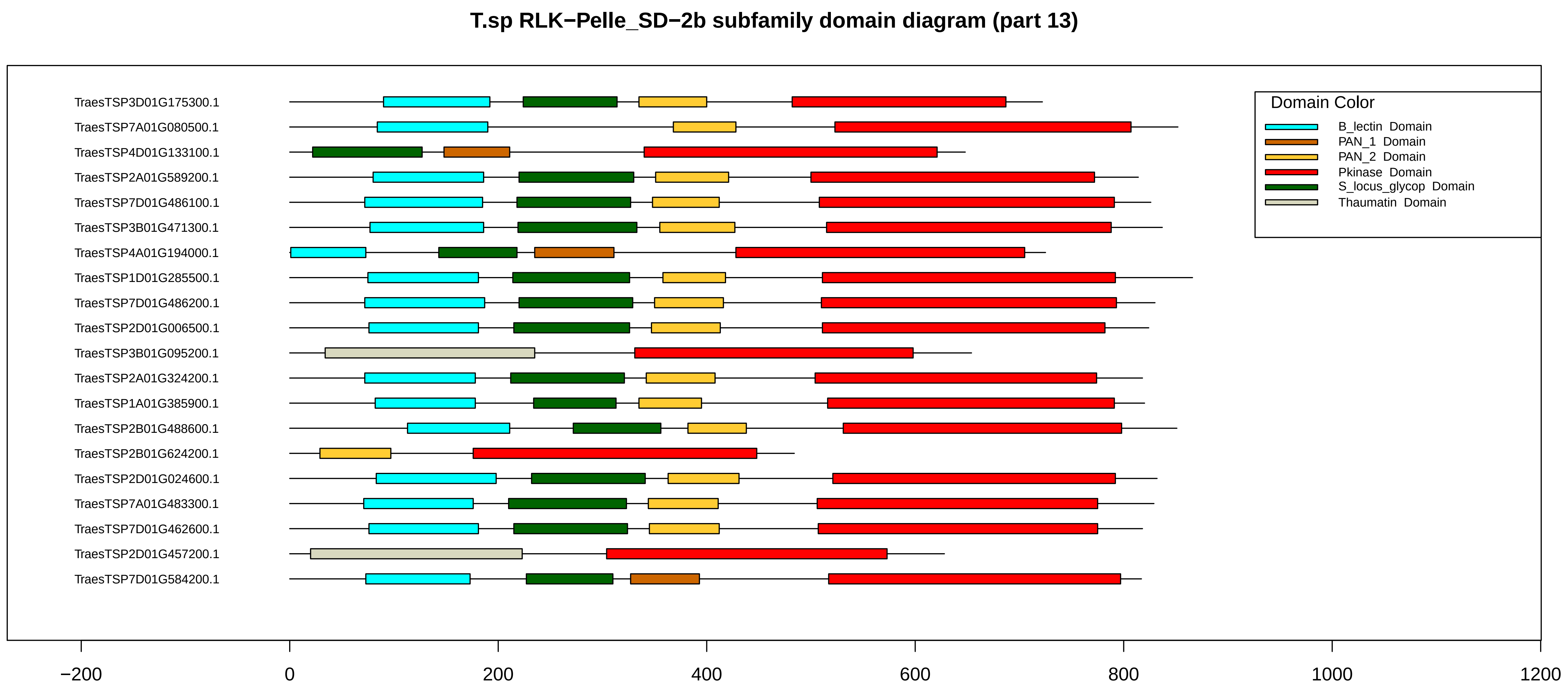
T.sp RLK−Pelle_SD−2b subfamily domain diagram (part 13)
TraesTSP3D01G175300.1
TraesTSP7A01G080500.1
TraesTSP4D01G133100.1
TraesTSP2A01G589200.1
TraesTSP7D01G486100.1
TraesTSP3B01G471300.1
TraesTSP4A01G194000.1
TraesTSP1D01G285500.1
TraesTSP7D01G486200.1
TraesTSP2D01G006500.1
TraesTSP3B01G095200.1
TraesTSP2A01G324200.1
TraesTSP1A01G385900.1
TraesTSP2B01G488600.1
TraesTSP2B01G624200.1
TraesTSP2D01G024600.1
TraesTSP7A01G483300.1
TraesTSP7D01G462600.1
TraesTSP2D01G457200.1
TraesTSP7D01G584200.1
Domain Color
B_lectin Domain
PAN_1 Domain
PAN_2 Domain
Pkinase Domain
S_locus_glycop Domain
Thaumatin Domain
−200
0
200
400
600
800
1000
1200

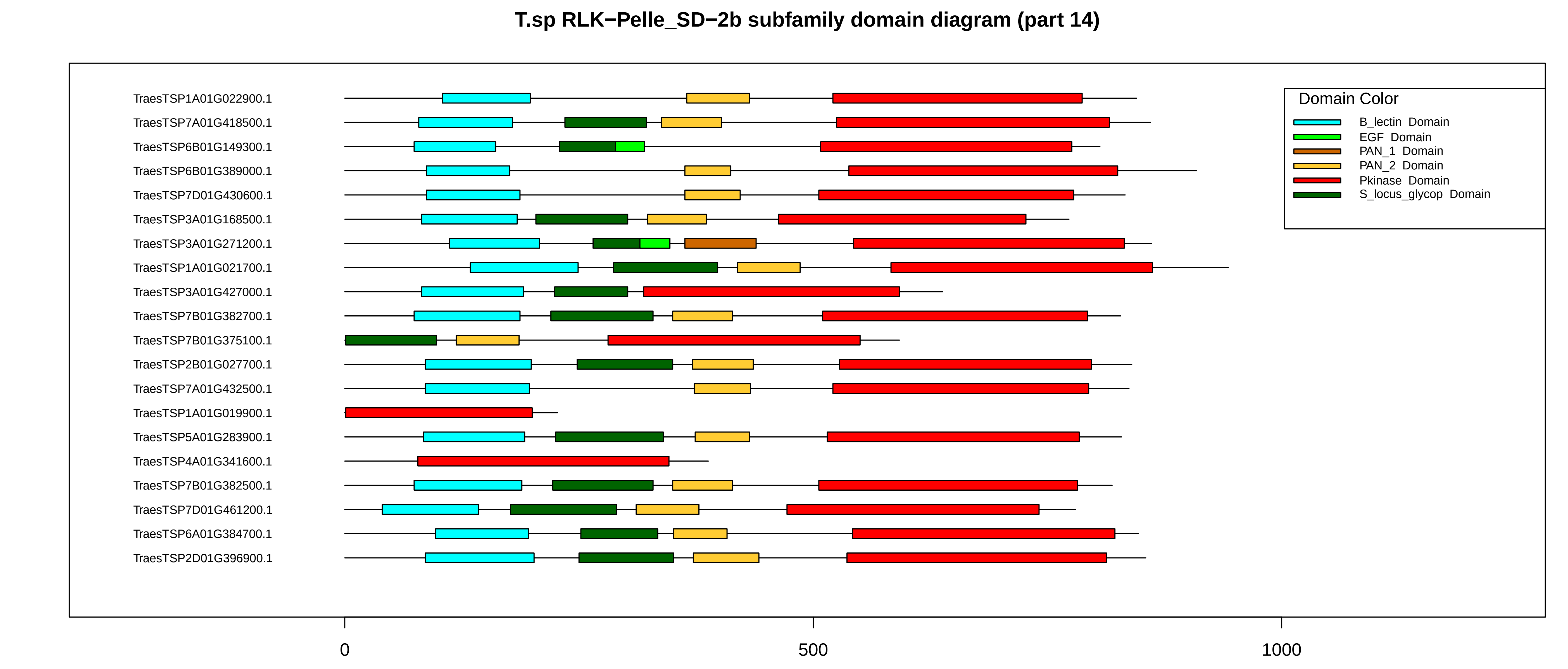
T.sp RLK−Pelle_SD−2b subfamily domain diagram (part 14)
TraesTSP1A01G022900.1
TraesTSP7A01G418500.1
TraesTSP6B01G149300.1
TraesTSP6B01G389000.1
TraesTSP7D01G430600.1
TraesTSP3A01G168500.1
TraesTSP3A01G271200.1
TraesTSP1A01G021700.1
TraesTSP3A01G427000.1
TraesTSP7B01G382700.1
TraesTSP7B01G375100.1
TraesTSP2B01G027700.1
TraesTSP7A01G432500.1
TraesTSP1A01G019900.1
TraesTSP5A01G283900.1
TraesTSP4A01G341600.1
TraesTSP7B01G382500.1
TraesTSP7D01G461200.1
TraesTSP6A01G384700.1
TraesTSP2D01G396900.1
Domain Color
B_lectin Domain
EGF Domain
PAN_1 Domain
PAN_2 Domain
Pkinase Domain
S_locus_glycop Domain
0
500
1000

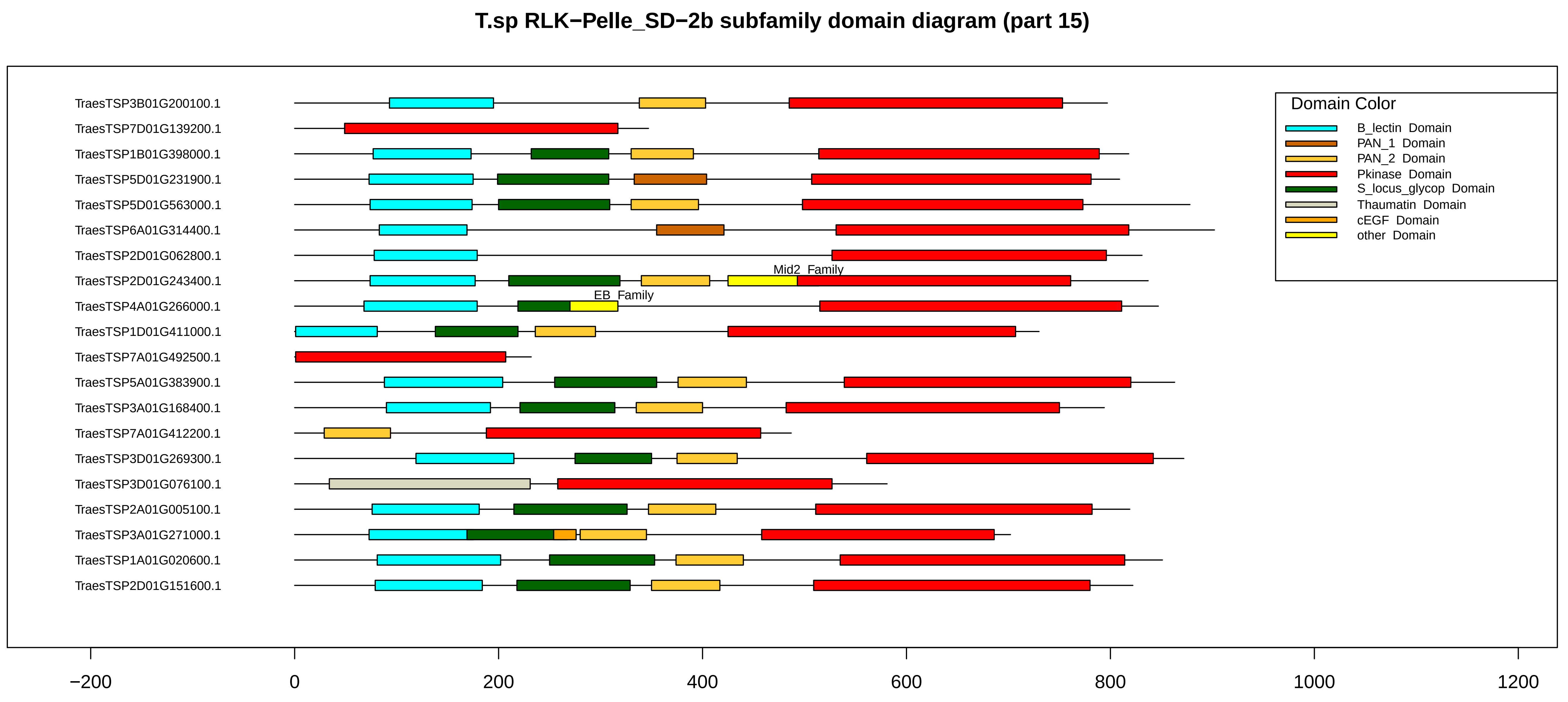
T.sp RLK−Pelle_SD−2b subfamily domain diagram (part 15)
TraesTSP3B01G200100.1
TraesTSP7D01G139200.1
TraesTSP1B01G398000.1
TraesTSP5D01G231900.1
TraesTSP5D01G563000.1
TraesTSP6A01G314400.1
TraesTSP2D01G062800.1
TraesTSP2D01G243400.1
TraesTSP4A01G266000.1
TraesTSP1D01G411000.1
TraesTSP7A01G492500.1
TraesTSP5A01G383900.1
TraesTSP3A01G168400.1
TraesTSP7A01G412200.1
TraesTSP3D01G269300.1
TraesTSP3D01G076100.1
TraesTSP2A01G005100.1
TraesTSP3A01G271000.1
TraesTSP1A01G020600.1
TraesTSP2D01G151600.1
Mid2 Family
EB Family
Domain Color
B_lectin Domain
PAN_1 Domain
PAN_2 Domain
Pkinase Domain
S_locus_glycop Domain
Thaumatin Domain
cEGF Domain
other Domain
−200
0
200
400
600
800
1000
1200

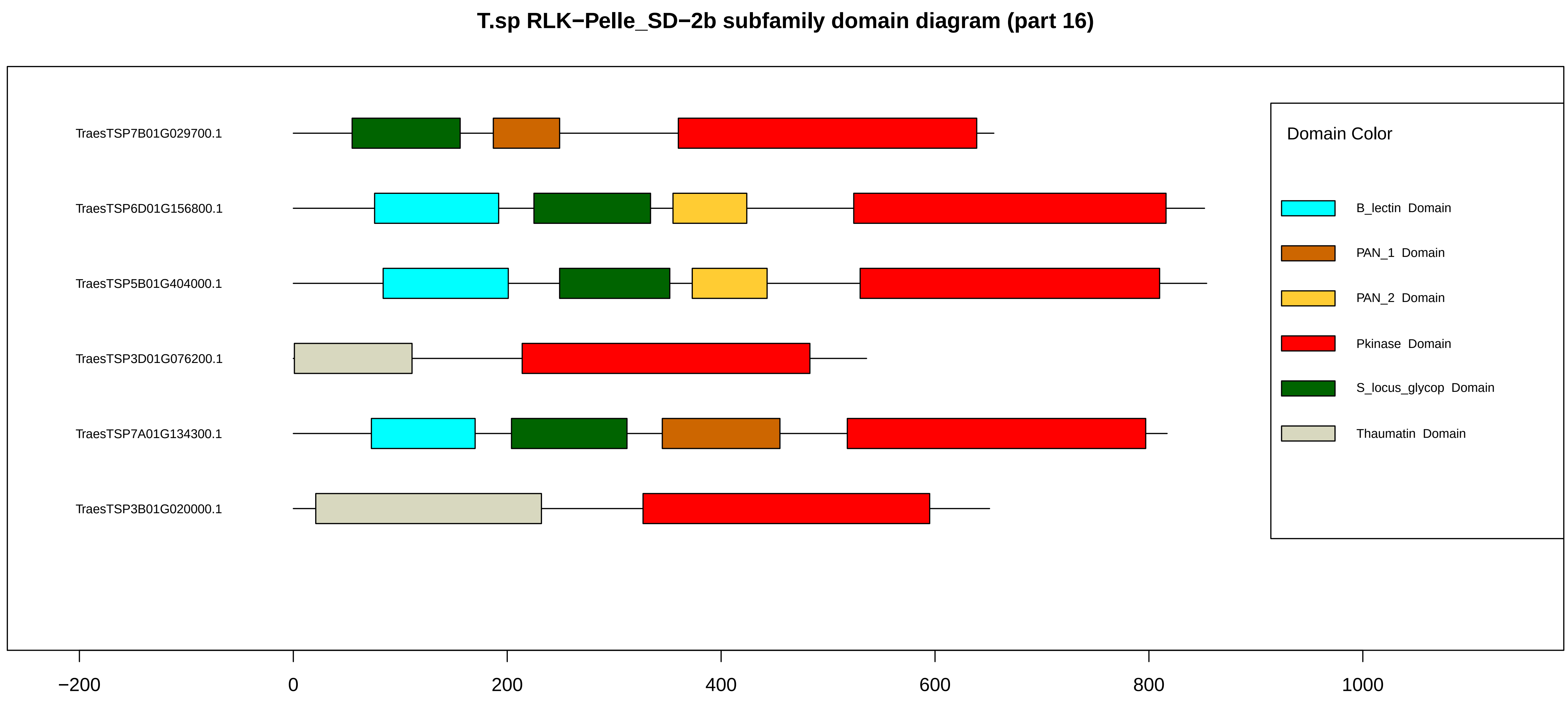
T.sp RLK−Pelle_SD−2b subfamily domain diagram (part 16)
TraesTSP7B01G029700.1
TraesTSP6D01G156800.1
TraesTSP5B01G404000.1
TraesTSP3D01G076200.1
TraesTSP7A01G134300.1
TraesTSP3B01G020000.1
Domain Color
B_lectin Domain
PAN_1 Domain
PAN_2 Domain
Pkinase Domain
S_locus_glycop Domain
Thaumatin Domain
−200
0
200
400
600
800
1000

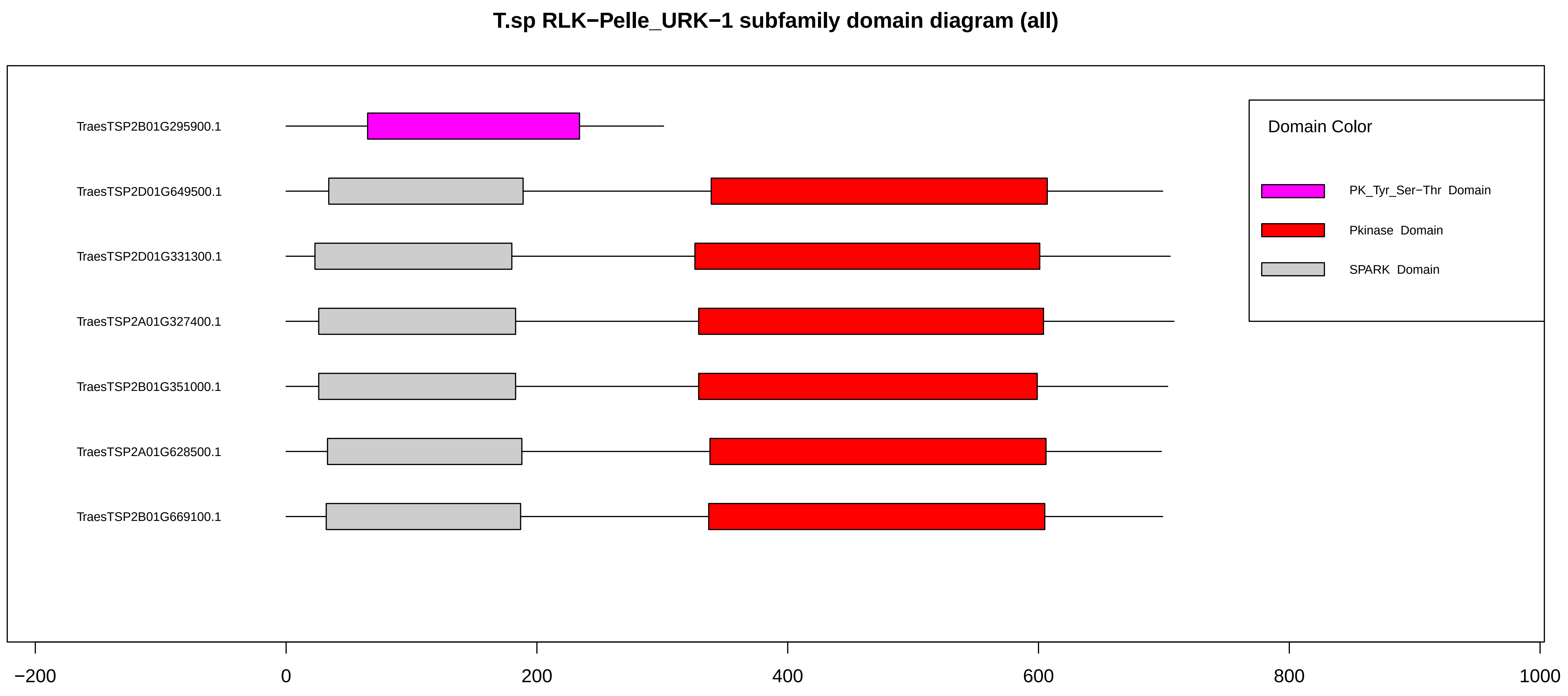

T.sp RLK−Pelle_URK−1 subfamily domain diagram (all)
TraesTSP2B01G295900.1
TraesTSP2D01G649500.1
TraesTSP2D01G331300.1
TraesTSP2A01G327400.1
TraesTSP2B01G351000.1
TraesTSP2A01G628500.1
TraesTSP2B01G669100.1
Domain Color
PK_Tyr_Ser−Thr Domain
Pkinase Domain
SPARK Domain
−200
0
200
400
600
800
1000

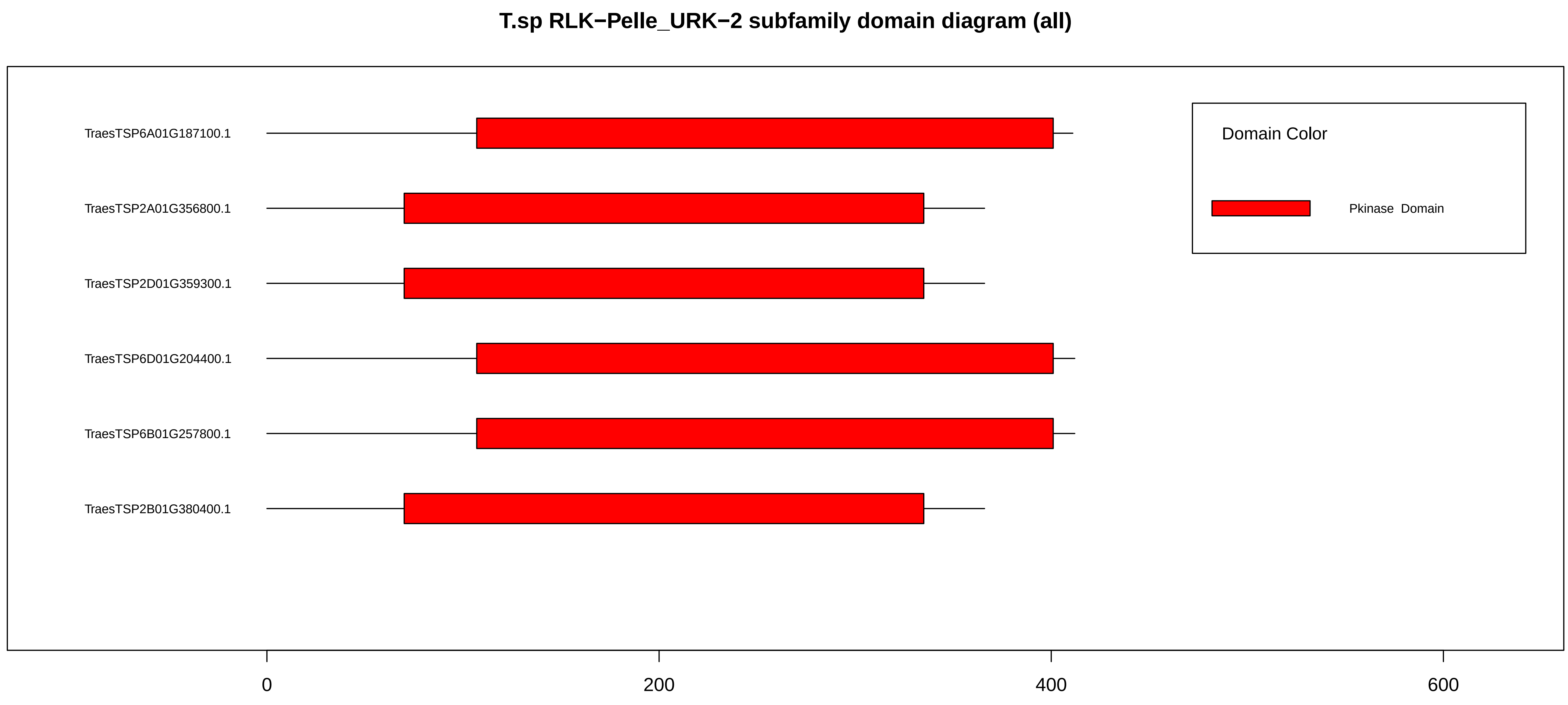
T.sp RLK−Pelle_URK−2 subfamily domain diagram (all)
TraesTSP6A01G187100.1
TraesTSP2A01G356800.1
TraesTSP2D01G359300.1
TraesTSP6D01G204400.1
TraesTSP6B01G257800.1
TraesTSP2B01G380400.1
Domain Color
Pkinase Domain
0
200
400
600

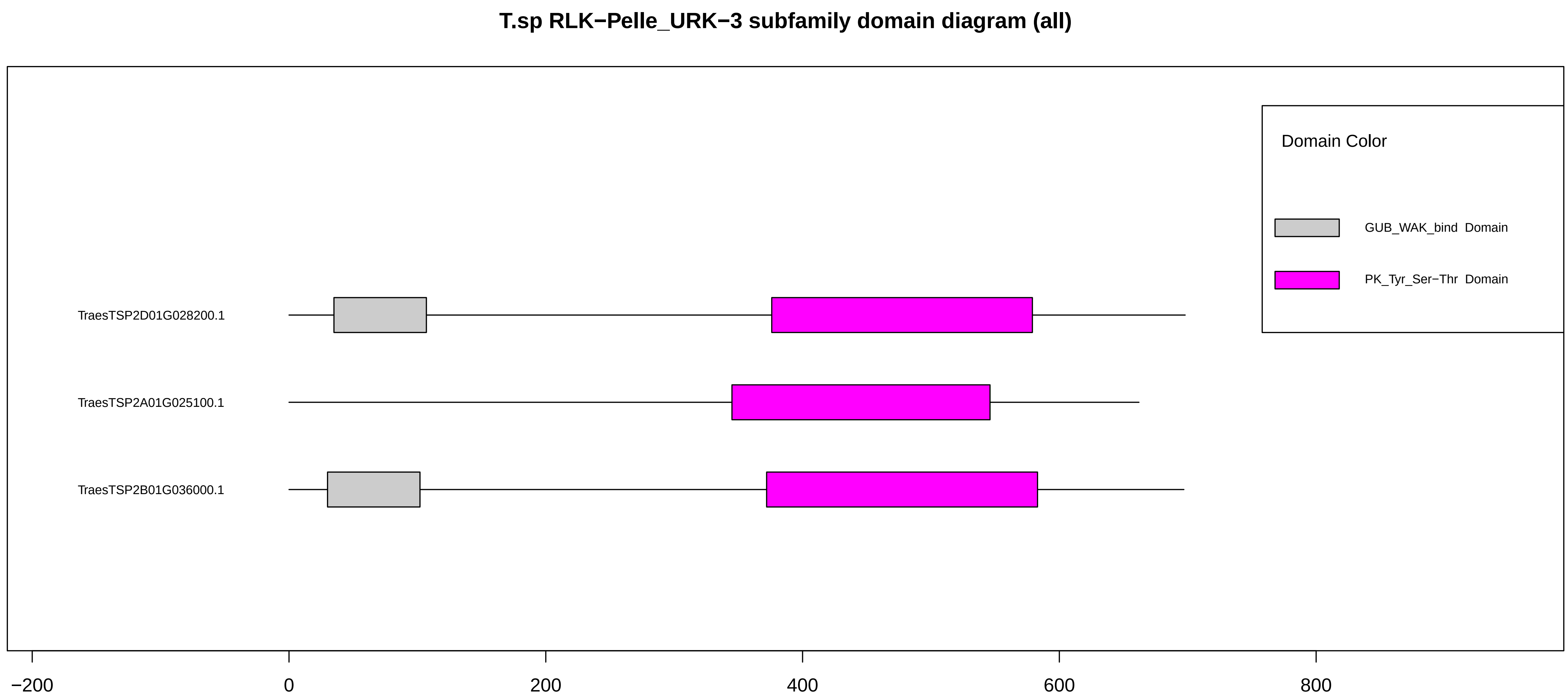

T.sp RLK−Pelle_URK−3 subfamily domain diagram (all)
Domain Color
GUB_WAK_bind Domain
PK_Tyr_Ser−Thr Domain
TraesTSP2D01G028200.1
TraesTSP2A01G025100.1
TraesTSP2B01G036000.1
−200
0
200
400
600
800

T.sp RLK−Pelle_WAK_LRK10L−1 subfamily domain diagram (part 1)

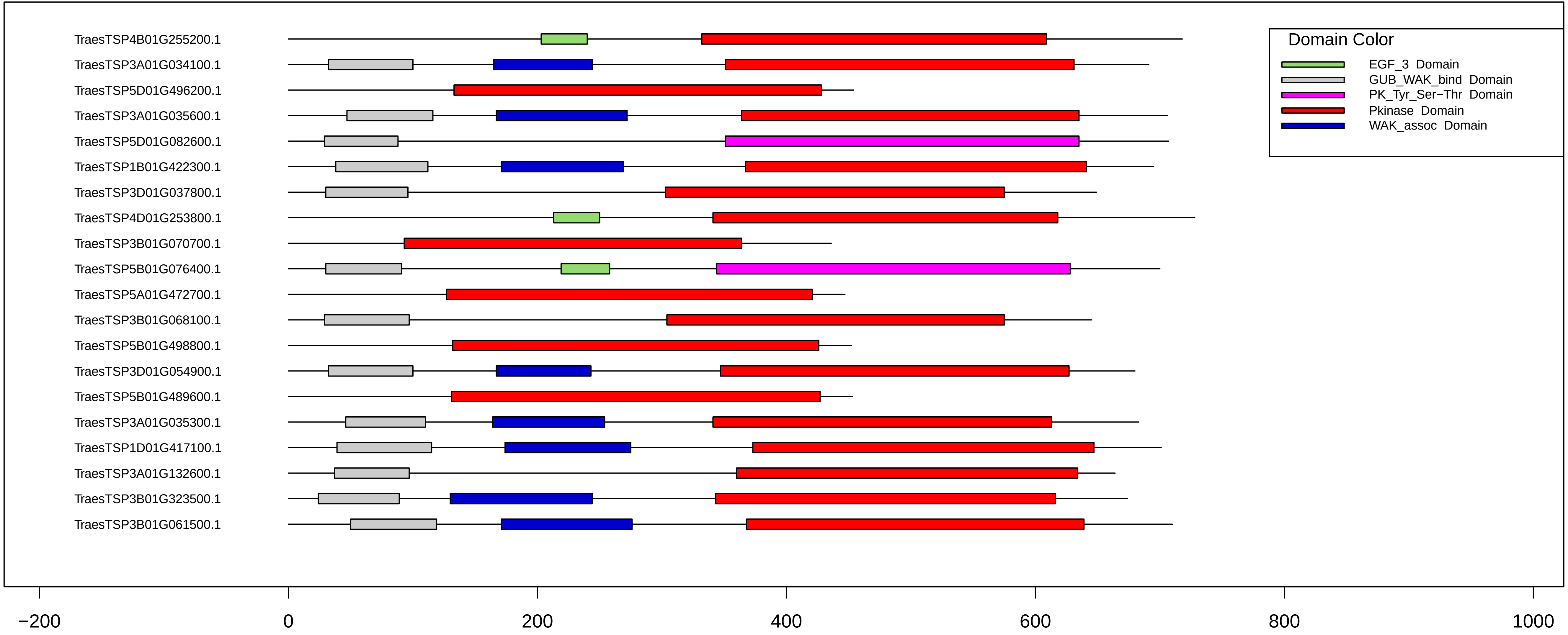

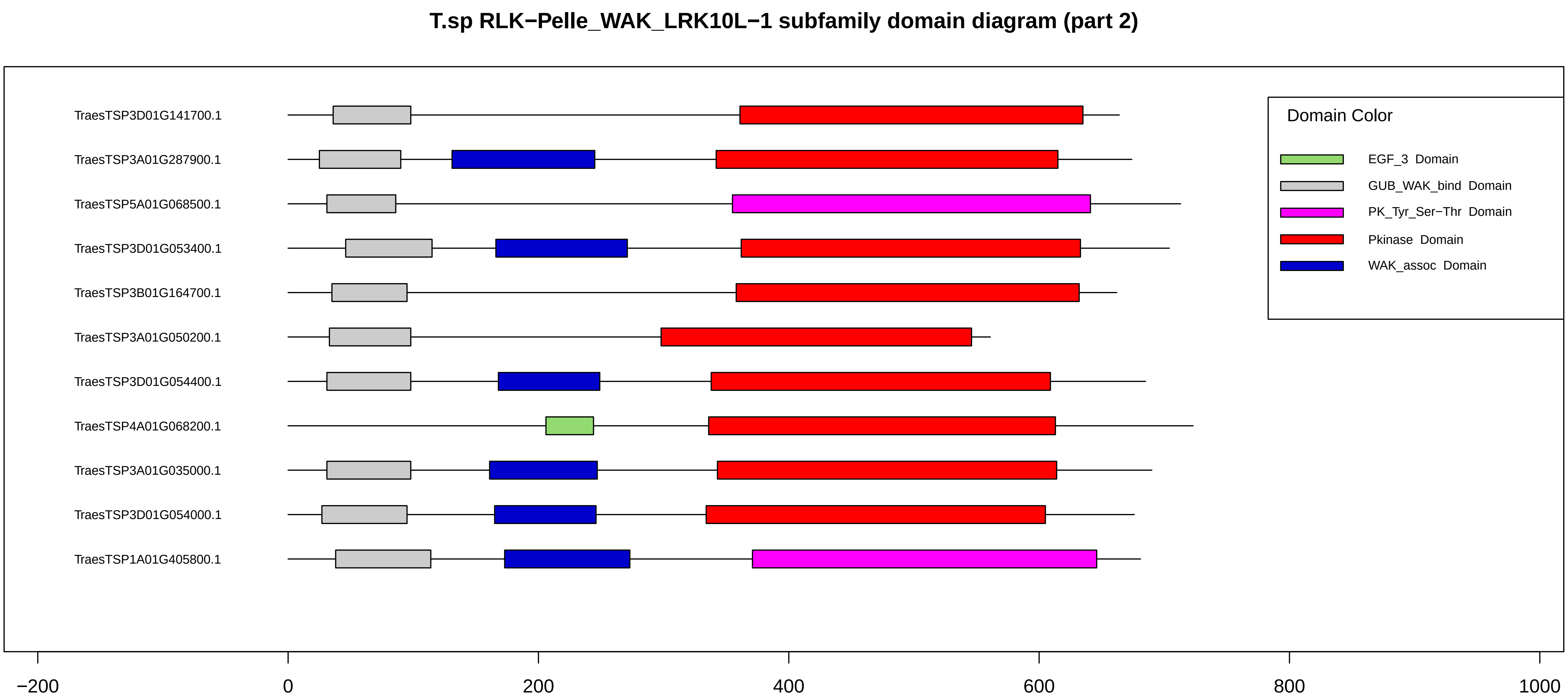
T.sp RLK−Pelle_WAK_LRK10L−1 subfamily domain diagram (part 2)
TraesTSP3D01G141700.1
TraesTSP3A01G287900.1
TraesTSP5A01G068500.1
TraesTSP3D01G053400.1
TraesTSP3B01G164700.1
TraesTSP3A01G050200.1
TraesTSP3D01G054400.1
TraesTSP4A01G068200.1
TraesTSP3A01G035000.1
TraesTSP3D01G054000.1
TraesTSP1A01G405800.1
Domain Color
EGF_3 Domain
GUB_WAK_bind Domain
PK_Tyr_Ser−Thr Domain
Pkinase Domain
WAK_assoc Domain
−200
0
200
400
600
800
1000

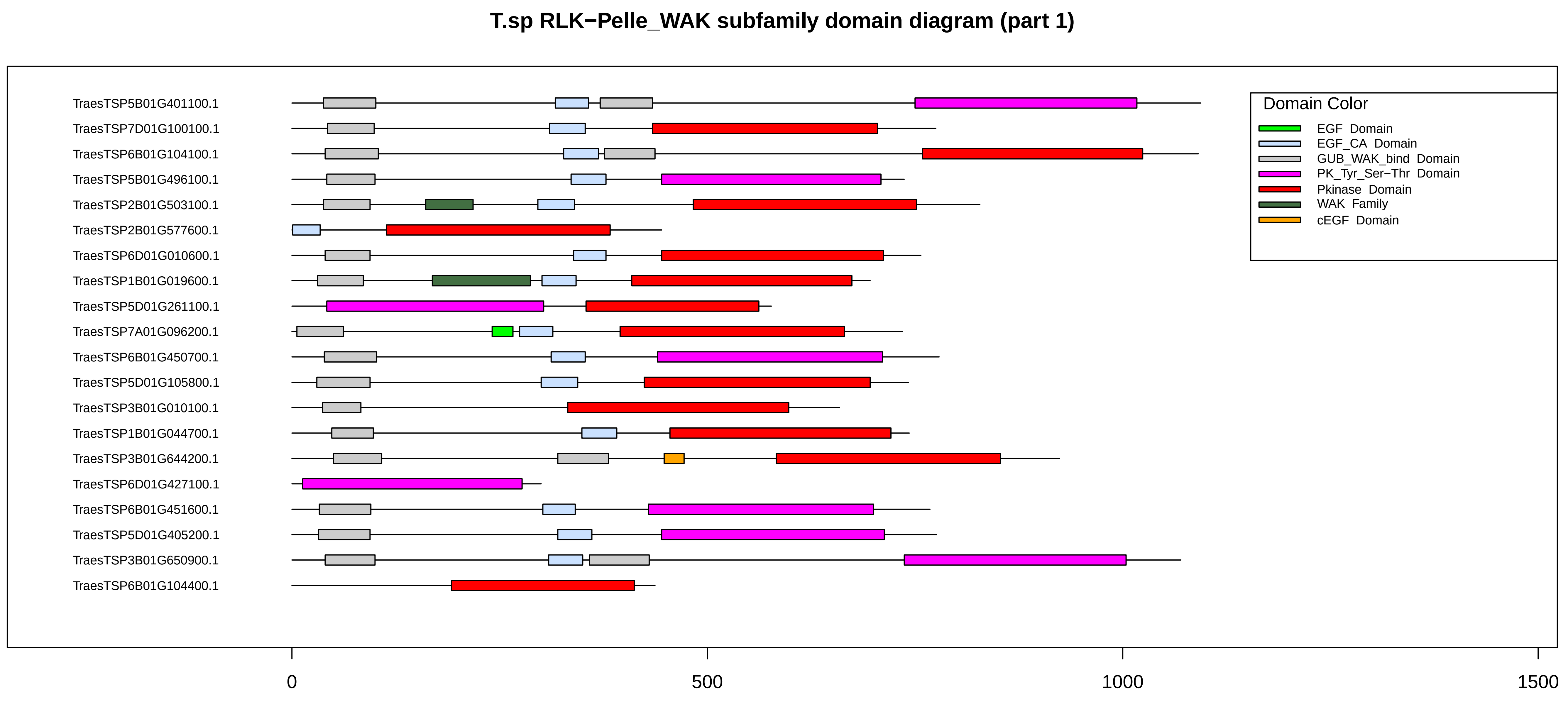
T.sp RLK−Pelle_WAK subfamily domain diagram (part 1)
TraesTSP5B01G401100.1
TraesTSP7D01G100100.1
TraesTSP6B01G104100.1
TraesTSP5B01G496100.1
TraesTSP2B01G503100.1
TraesTSP2B01G577600.1
TraesTSP6D01G010600.1
TraesTSP1B01G019600.1
TraesTSP5D01G261100.1
TraesTSP7A01G096200.1
TraesTSP6B01G450700.1
TraesTSP5D01G105800.1
TraesTSP3B01G010100.1
TraesTSP1B01G044700.1
TraesTSP3B01G644200.1
TraesTSP6D01G427100.1
TraesTSP6B01G451600.1
TraesTSP5D01G405200.1
TraesTSP3B01G650900.1
TraesTSP6B01G104400.1
Domain Color
EGF Domain
EGF_CA Domain
GUB_WAK_bind Domain
PK_Tyr_Ser−Thr Domain
Pkinase Domain
WAK Family
cEGF Domain
0
500
1000
1500

T.sp RLK−Pelle_WAK subfamily domain diagram (part 2)

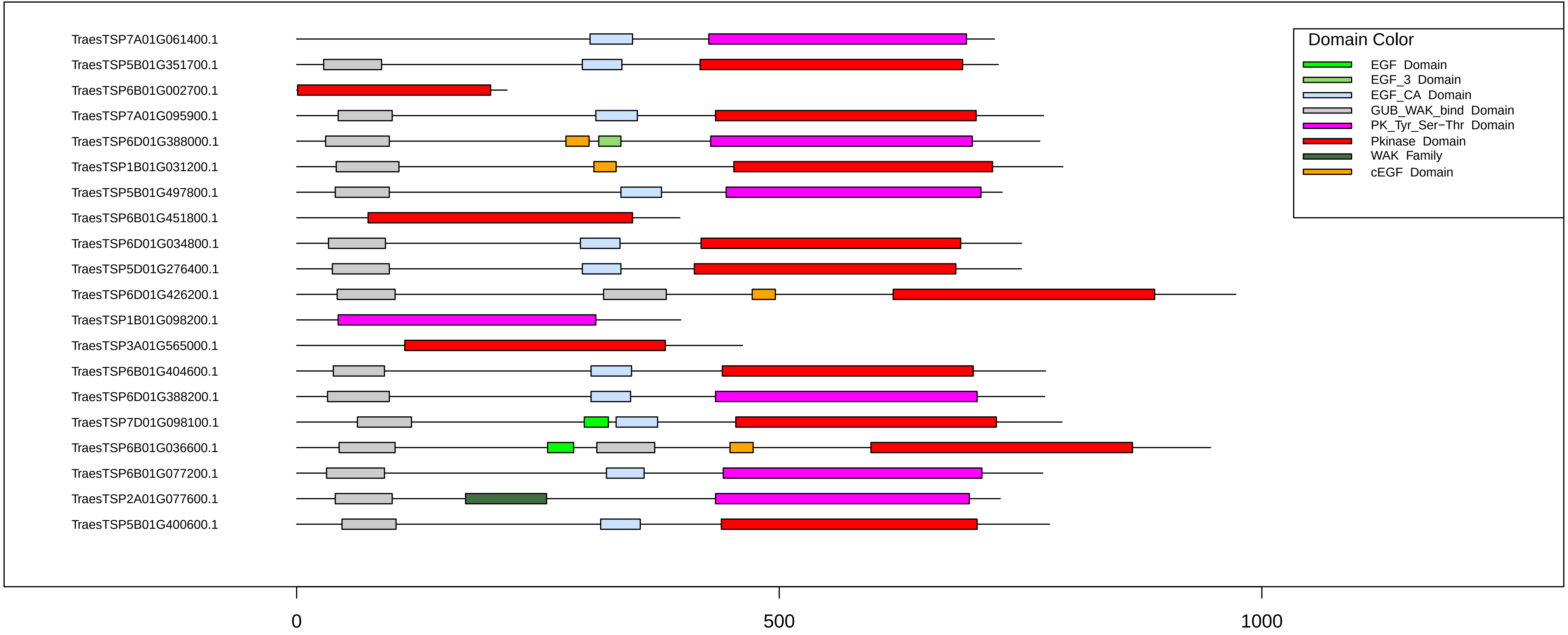

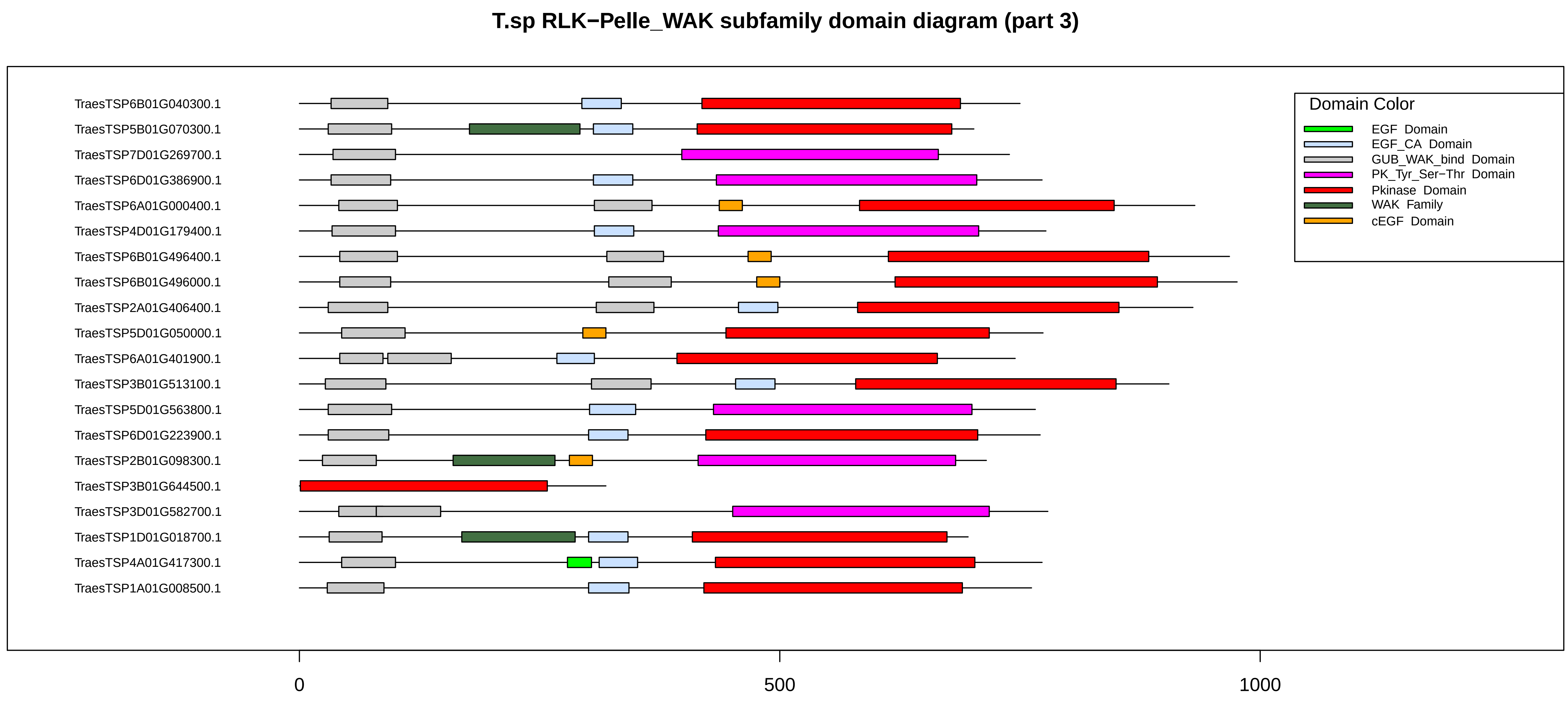
T.sp RLK−Pelle_WAK subfamily domain diagram (part 3)
TraesTSP6B01G040300.1
TraesTSP5B01G070300.1
TraesTSP7D01G269700.1
TraesTSP6D01G386900.1
TraesTSP6A01G000400.1
TraesTSP4D01G179400.1
TraesTSP6B01G496400.1
TraesTSP6B01G496000.1
TraesTSP2A01G406400.1
TraesTSP5D01G050000.1
TraesTSP6A01G401900.1
TraesTSP3B01G513100.1
TraesTSP5D01G563800.1
TraesTSP6D01G223900.1
TraesTSP2B01G098300.1
TraesTSP3B01G644500.1
TraesTSP3D01G582700.1
TraesTSP1D01G018700.1
TraesTSP4A01G417300.1
TraesTSP1A01G008500.1
Domain Color
EGF Domain
EGF_CA Domain
GUB_WAK_bind Domain
PK_Tyr_Ser−Thr Domain
Pkinase Domain
WAK Family
cEGF Domain
0
500
1000

T.sp RLK−Pelle_WAK subfamily domain diagram (part 4)

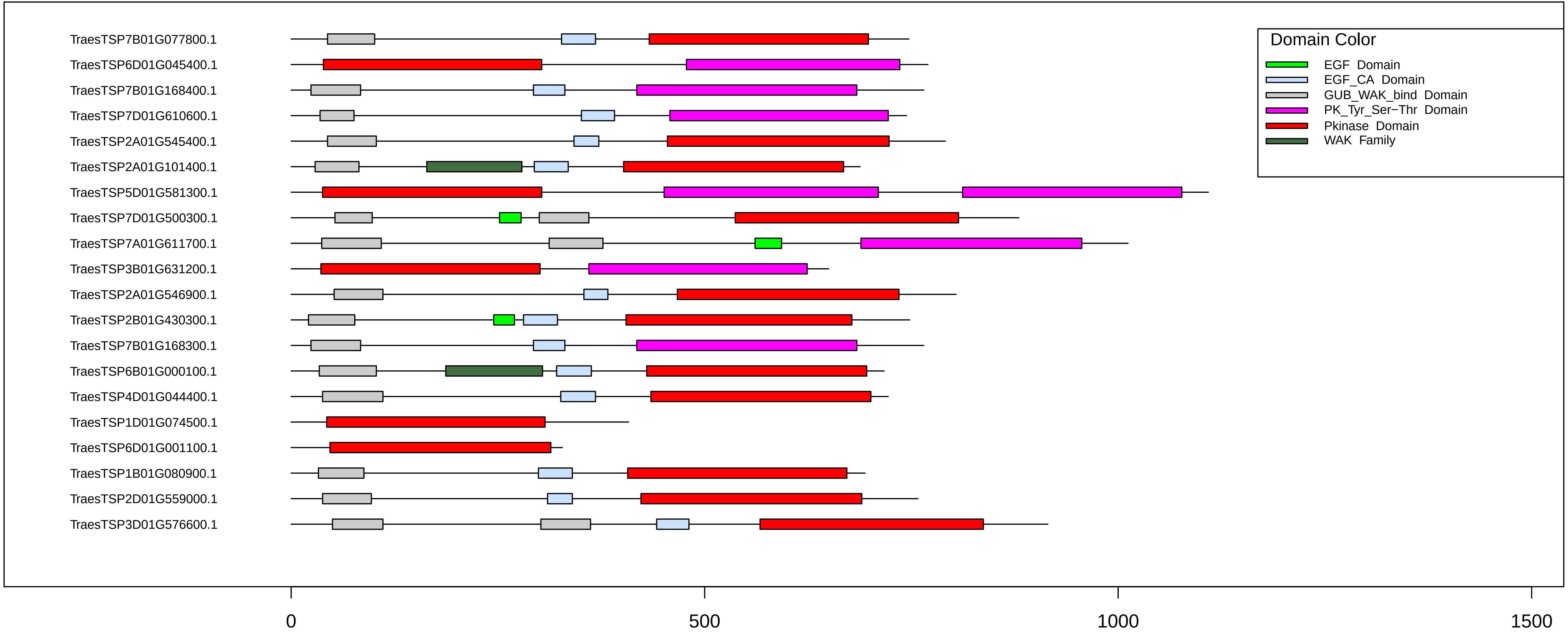

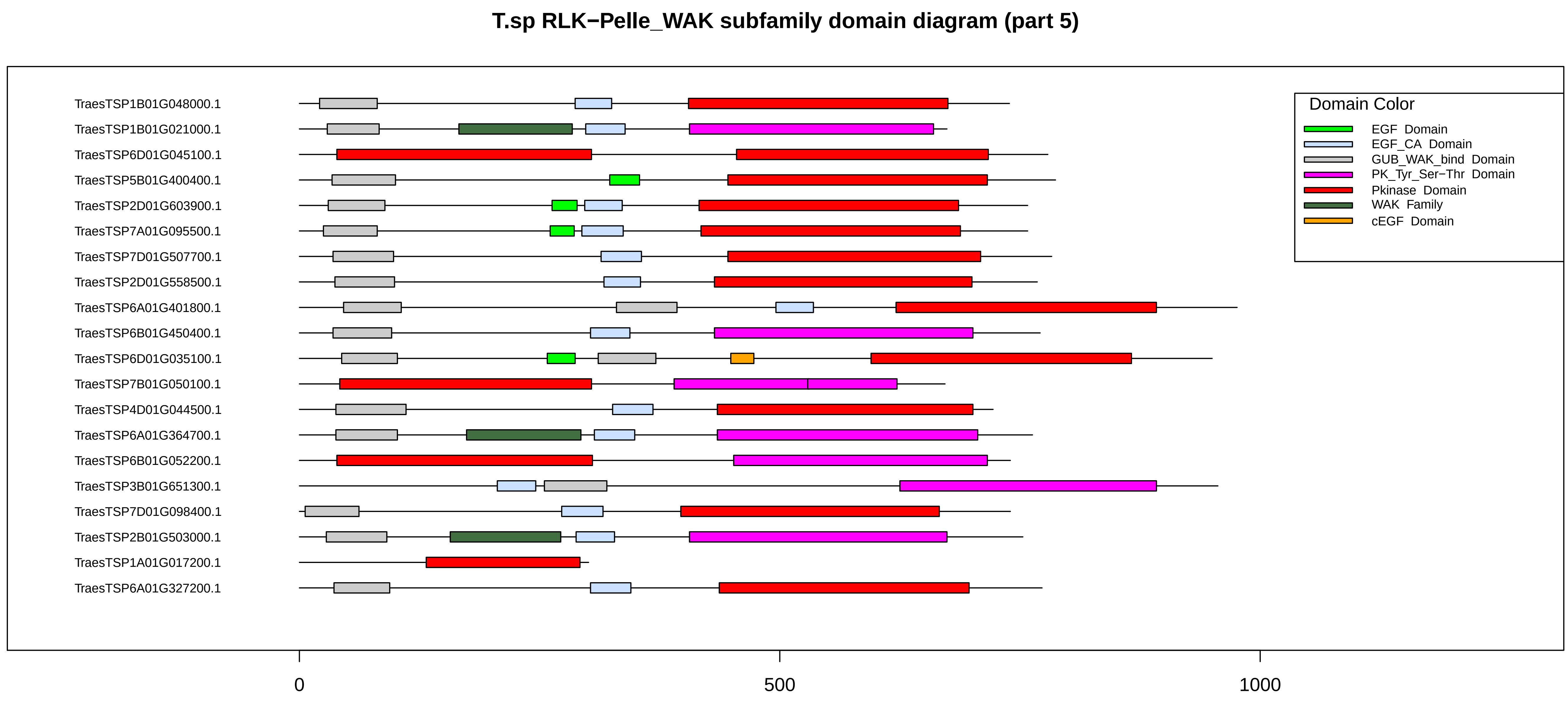
T.sp RLK−Pelle_WAK subfamily domain diagram (part 5)
TraesTSP1B01G048000.1
TraesTSP1B01G021000.1
TraesTSP6D01G045100.1
TraesTSP5B01G400400.1
TraesTSP2D01G603900.1
TraesTSP7A01G095500.1
TraesTSP7D01G507700.1
TraesTSP2D01G558500.1
TraesTSP6A01G401800.1
TraesTSP6B01G450400.1
TraesTSP6D01G035100.1
TraesTSP7B01G050100.1
TraesTSP4D01G044500.1
TraesTSP6A01G364700.1
TraesTSP6B01G052200.1
TraesTSP3B01G651300.1
TraesTSP7D01G098400.1
TraesTSP2B01G503000.1
TraesTSP1A01G017200.1
TraesTSP6A01G327200.1
Domain Color
EGF Domain
EGF_CA Domain
GUB_WAK_bind Domain
PK_Tyr_Ser−Thr Domain
Pkinase Domain
WAK Family
cEGF Domain
0
500
1000

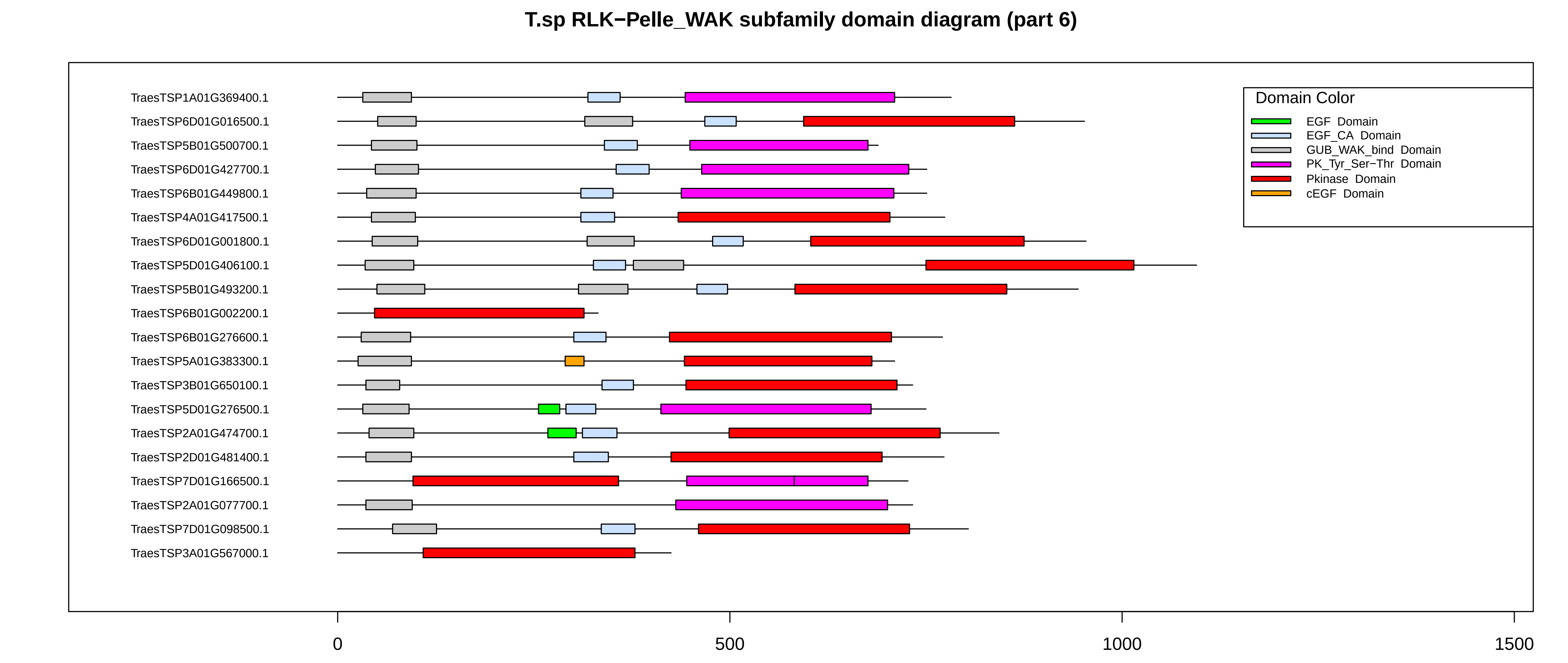

T.sp RLK−Pelle_WAK subfamily domain diagram (part 6)
TraesTSP1A01G369400.1
TraesTSP6D01G016500.1
TraesTSP5B01G500700.1
TraesTSP6D01G427700.1
TraesTSP6B01G449800.1
TraesTSP4A01G417500.1
TraesTSP6D01G001800.1
TraesTSP5D01G406100.1
TraesTSP5B01G493200.1
TraesTSP6B01G002200.1
TraesTSP6B01G276600.1
TraesTSP5A01G383300.1
TraesTSP3B01G650100.1
TraesTSP5D01G276500.1
TraesTSP2A01G474700.1
TraesTSP2D01G481400.1
TraesTSP7D01G166500.1
TraesTSP2A01G077700.1
TraesTSP7D01G098500.1
TraesTSP3A01G567000.1
Domain Color
EGF Domain
EGF_CA Domain
GUB_WAK_bind Domain
PK_Tyr_Ser−Thr Domain
Pkinase Domain
cEGF Domain
0
500
1000
1500

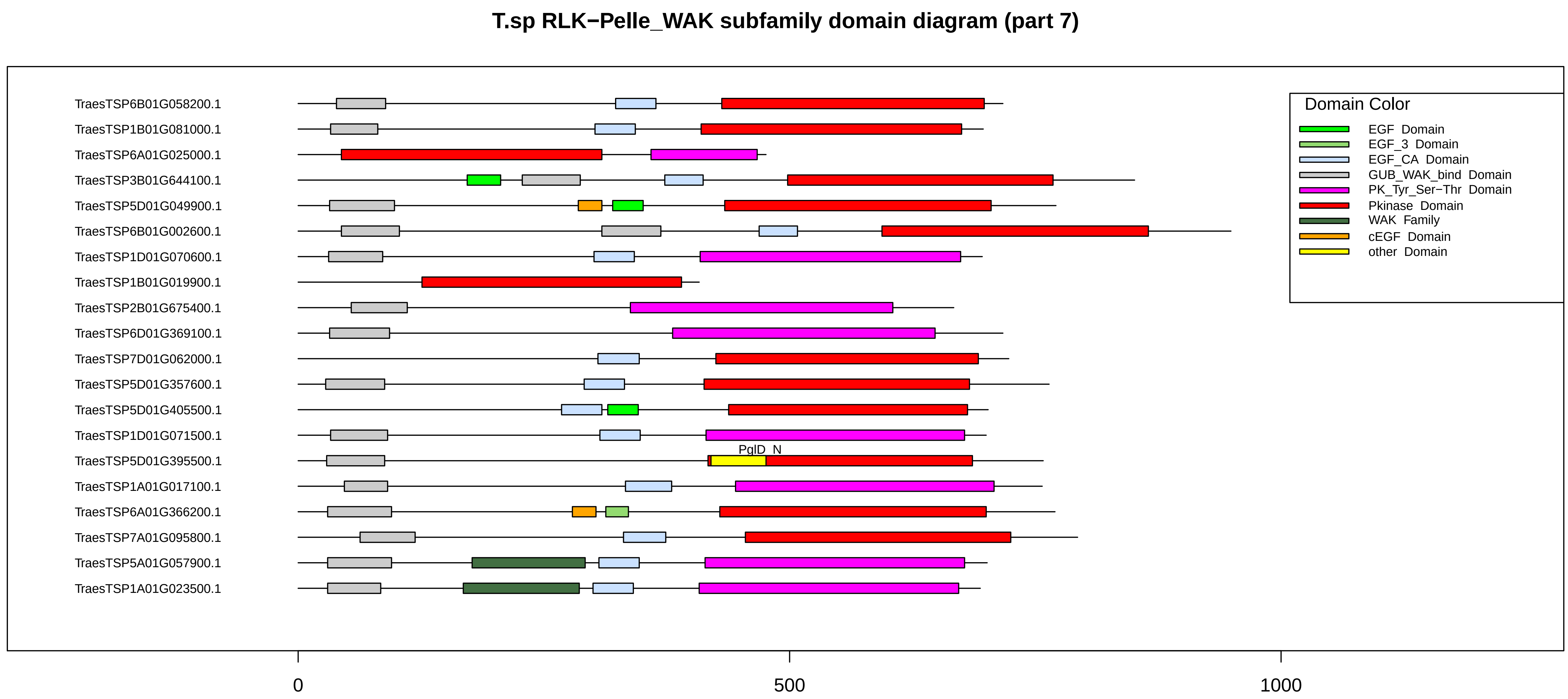
T.sp RLK−Pelle_WAK subfamily domain diagram (part 7)
TraesTSP6B01G058200.1
TraesTSP1B01G081000.1
TraesTSP6A01G025000.1
TraesTSP3B01G644100.1
TraesTSP5D01G049900.1
TraesTSP6B01G002600.1
TraesTSP1D01G070600.1
TraesTSP1B01G019900.1
TraesTSP2B01G675400.1
TraesTSP6D01G369100.1
TraesTSP7D01G062000.1
TraesTSP5D01G357600.1
TraesTSP5D01G405500.1
TraesTSP1D01G071500.1
PglD_N
TraesTSP5D01G395500.1
TraesTSP1A01G017100.1
TraesTSP6A01G366200.1
TraesTSP7A01G095800.1
TraesTSP5A01G057900.1
TraesTSP1A01G023500.1
Domain Color
EGF Domain
EGF_3 Domain
EGF_CA Domain
GUB_WAK_bind Domain
PK_Tyr_Ser−Thr Domain
Pkinase Domain
WAK Family
cEGF Domain
other Domain
0
500
1000

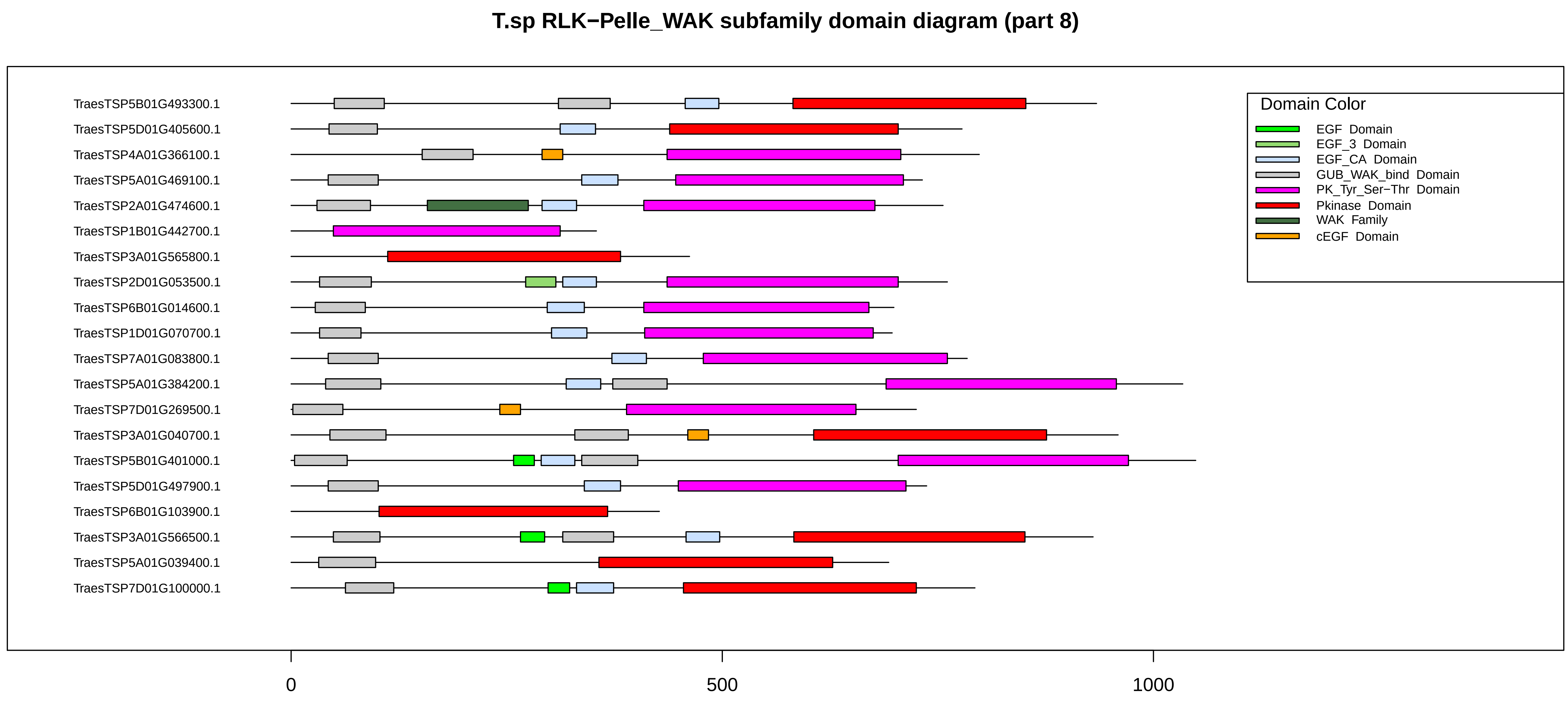

T.sp RLK−Pelle_WAK subfamily domain diagram (part 8)
TraesTSP5B01G493300.1
TraesTSP5D01G405600.1
TraesTSP4A01G366100.1
TraesTSP5A01G469100.1
TraesTSP2A01G474600.1
TraesTSP1B01G442700.1
TraesTSP3A01G565800.1
TraesTSP2D01G053500.1
TraesTSP6B01G014600.1
TraesTSP1D01G070700.1
TraesTSP7A01G083800.1
TraesTSP5A01G384200.1
TraesTSP7D01G269500.1
TraesTSP3A01G040700.1
TraesTSP5B01G401000.1
TraesTSP5D01G497900.1
TraesTSP6B01G103900.1
TraesTSP3A01G566500.1
TraesTSP5A01G039400.1
TraesTSP7D01G100000.1
Domain Color
EGF Domain
EGF_3 Domain
EGF_CA Domain
GUB_WAK_bind Domain
PK_Tyr_Ser−Thr Domain
Pkinase Domain
WAK Family
cEGF Domain
0
500
1000

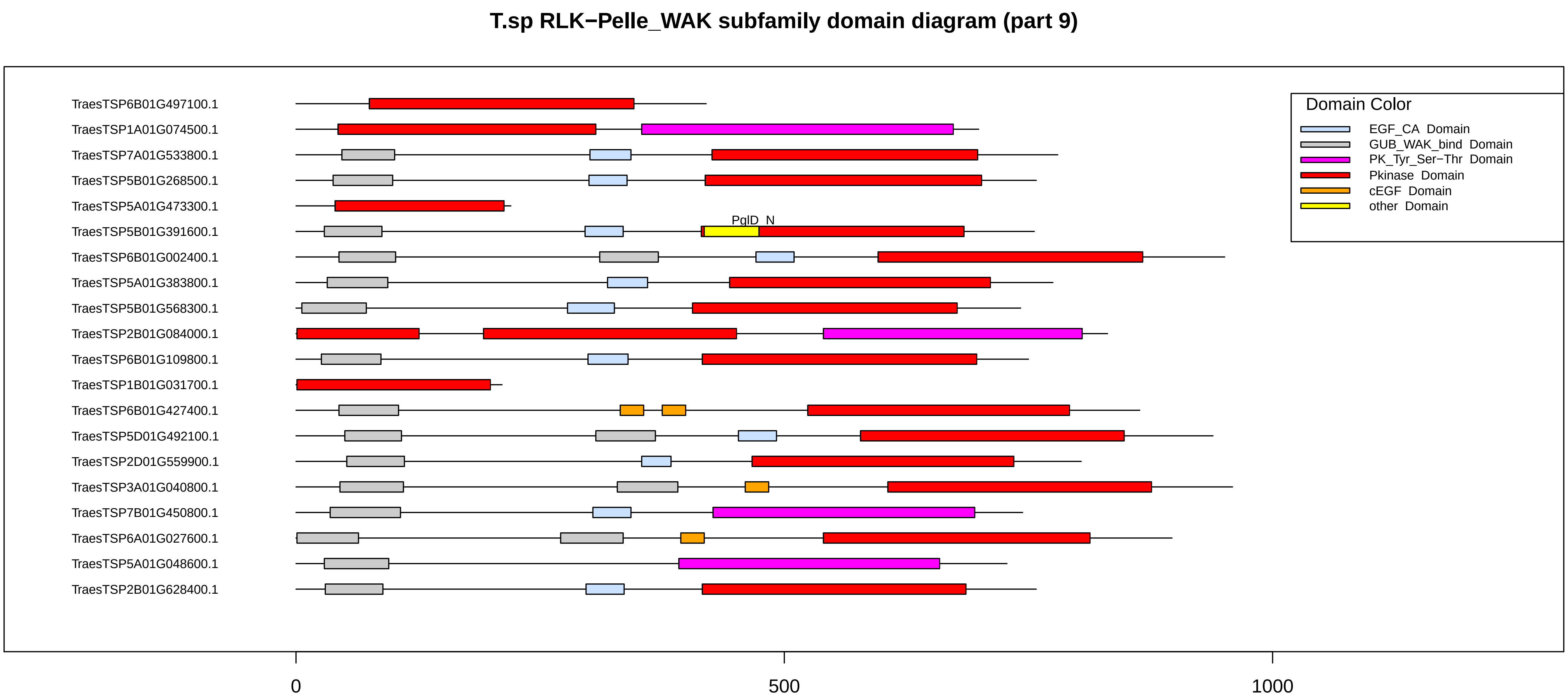
T.sp RLK−Pelle_WAK subfamily domain diagram (part 9)
TraesTSP6B01G497100.1
TraesTSP1A01G074500.1
TraesTSP7A01G533800.1
TraesTSP5B01G268500.1
TraesTSP5A01G473300.1
TraesTSP5B01G391600.1
TraesTSP6B01G002400.1
TraesTSP5A01G383800.1
TraesTSP5B01G568300.1
TraesTSP2B01G084000.1
TraesTSP6B01G109800.1
TraesTSP1B01G031700.1
TraesTSP6B01G427400.1
TraesTSP5D01G492100.1
TraesTSP2D01G559900.1
TraesTSP3A01G040800.1
TraesTSP7B01G450800.1
TraesTSP6A01G027600.1
TraesTSP5A01G048600.1
TraesTSP2B01G628400.1
PglD_N
Domain Color
EGF_CA Domain
GUB_WAK_bind Domain
PK_Tyr_Ser−Thr Domain
Pkinase Domain
cEGF Domain
other Domain
0
500
1000

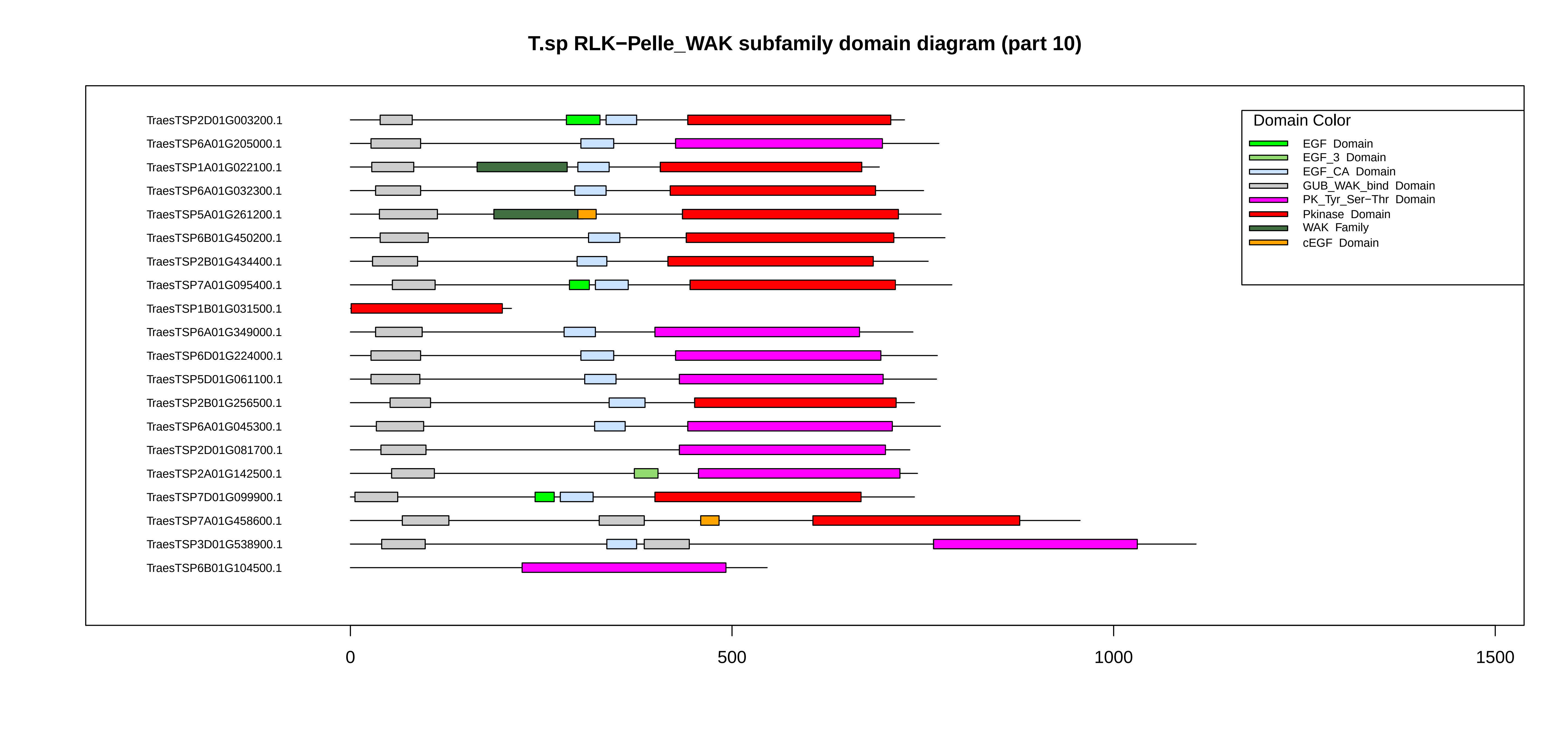
T.sp RLK−Pelle_WAK subfamily domain diagram (part 10)
TraesTSP2D01G003200.1
TraesTSP6A01G205000.1
TraesTSP1A01G022100.1
TraesTSP6A01G032300.1
TraesTSP5A01G261200.1
TraesTSP6B01G450200.1
TraesTSP2B01G434400.1
TraesTSP7A01G095400.1
TraesTSP1B01G031500.1
TraesTSP6A01G349000.1
TraesTSP6D01G224000.1
TraesTSP5D01G061100.1
TraesTSP2B01G256500.1
TraesTSP6A01G045300.1
TraesTSP2D01G081700.1
TraesTSP2A01G142500.1
TraesTSP7D01G099900.1
TraesTSP7A01G458600.1
TraesTSP3D01G538900.1
TraesTSP6B01G104500.1
Domain Color
EGF Domain
EGF_3 Domain
EGF_CA Domain
GUB_WAK_bind Domain
PK_Tyr_Ser−Thr Domain
Pkinase Domain
WAK Family
cEGF Domain
0
500
1000
1500

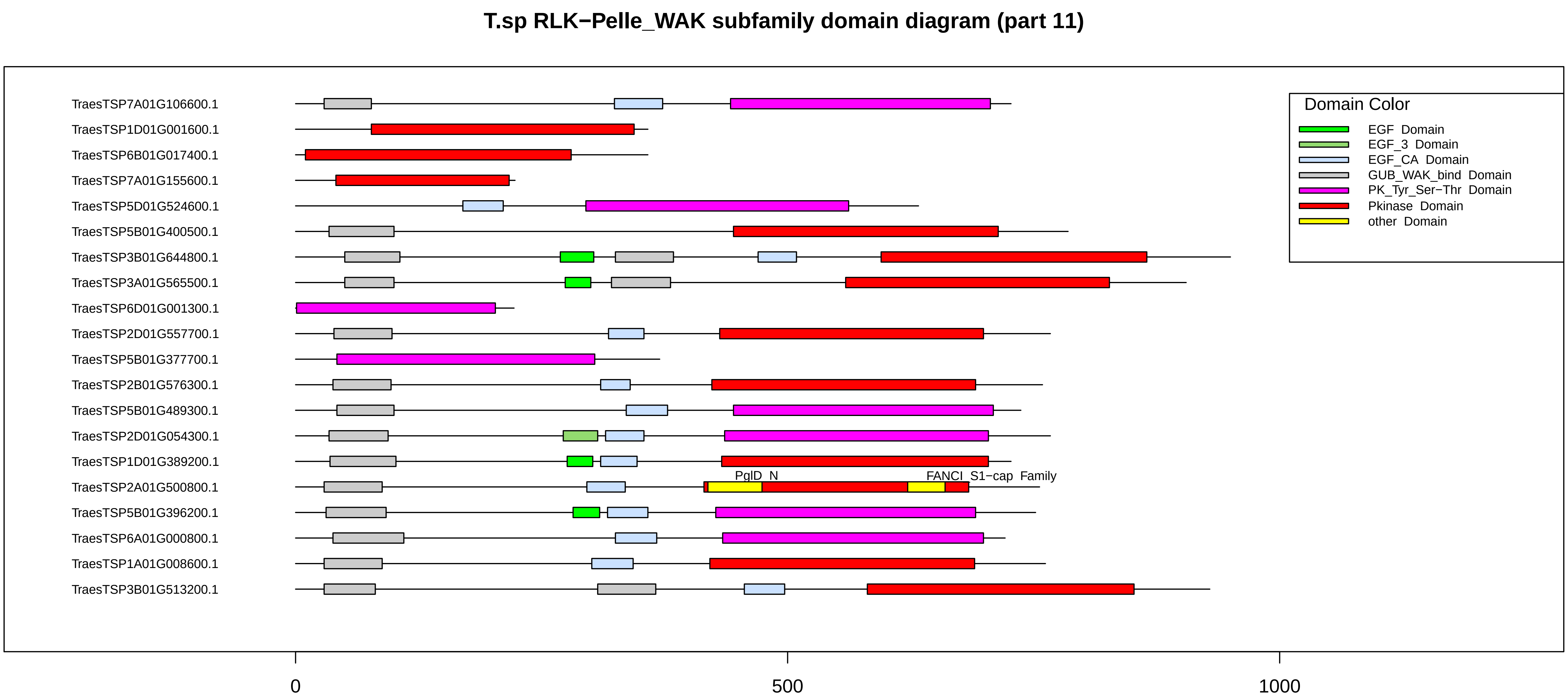
T.sp RLK−Pelle_WAK subfamily domain diagram (part 11)
TraesTSP7A01G106600.1
TraesTSP1D01G001600.1
TraesTSP6B01G017400.1
TraesTSP7A01G155600.1
TraesTSP5D01G524600.1
TraesTSP5B01G400500.1
TraesTSP3B01G644800.1
TraesTSP3A01G565500.1
TraesTSP6D01G001300.1
TraesTSP2D01G557700.1
TraesTSP5B01G377700.1
TraesTSP2B01G576300.1
TraesTSP5B01G489300.1
TraesTSP2D01G054300.1
TraesTSP1D01G389200.1
TraesTSP2A01G500800.1
TraesTSP5B01G396200.1
TraesTSP6A01G000800.1
TraesTSP1A01G008600.1
TraesTSP3B01G513200.1
PglD_N
FANCI_S1−cap Family
Domain Color
EGF Domain
EGF_3 Domain
EGF_CA Domain
GUB_WAK_bind Domain
PK_Tyr_Ser−Thr Domain
Pkinase Domain
other Domain
0
500
1000

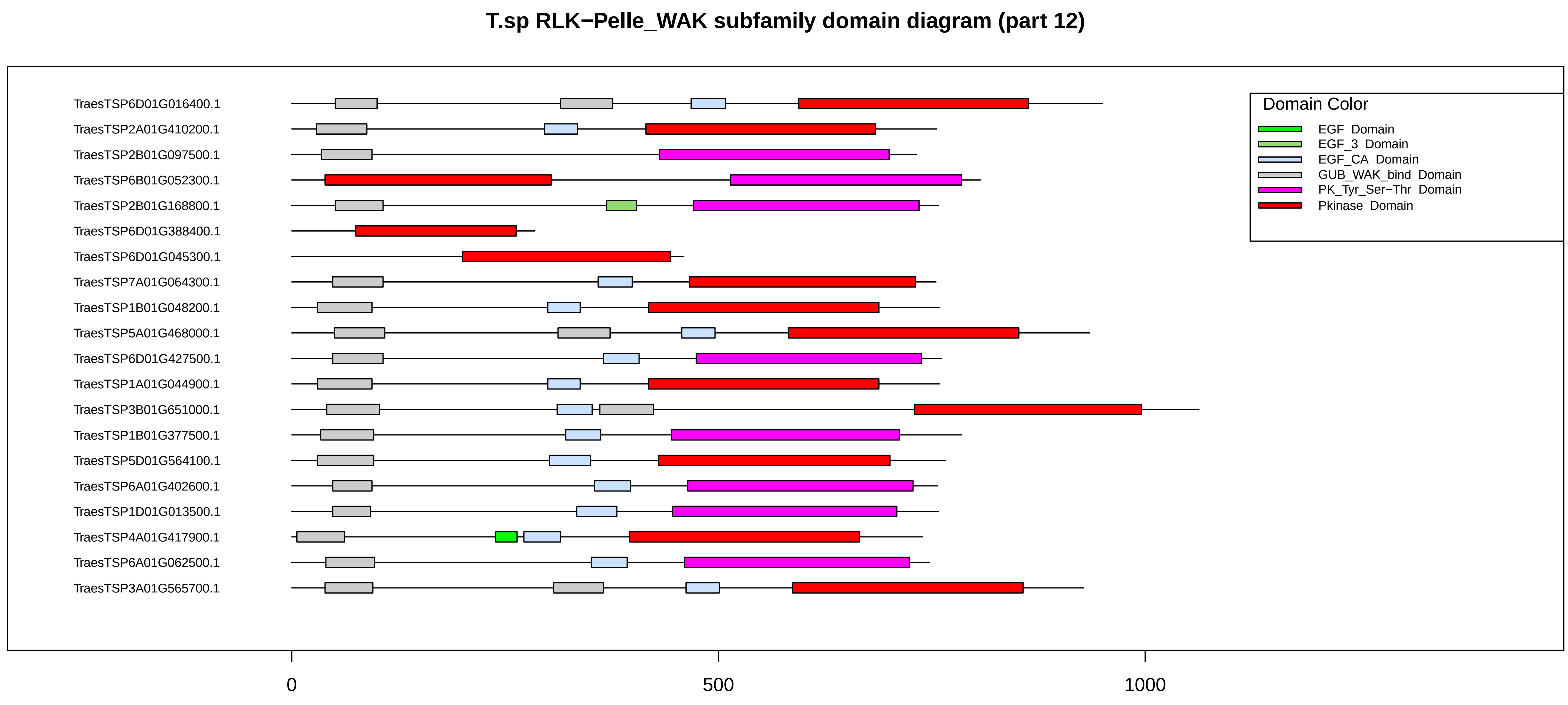
T.sp RLK−Pelle_WAK subfamily domain diagram (part 12)
TraesTSP6D01G016400.1
TraesTSP2A01G410200.1
TraesTSP2B01G097500.1
TraesTSP6B01G052300.1
TraesTSP2B01G168800.1
TraesTSP6D01G388400.1
TraesTSP6D01G045300.1
TraesTSP7A01G064300.1
TraesTSP1B01G048200.1
TraesTSP5A01G468000.1
TraesTSP6D01G427500.1
TraesTSP1A01G044900.1
TraesTSP3B01G651000.1
TraesTSP1B01G377500.1
TraesTSP5D01G564100.1
TraesTSP6A01G402600.1
TraesTSP1D01G013500.1
TraesTSP4A01G417900.1
TraesTSP6A01G062500.1
TraesTSP3A01G565700.1
Domain Color
EGF Domain
EGF_3 Domain
EGF_CA Domain
GUB_WAK_bind Domain
PK_Tyr_Ser−Thr Domain
Pkinase Domain
0
500
1000

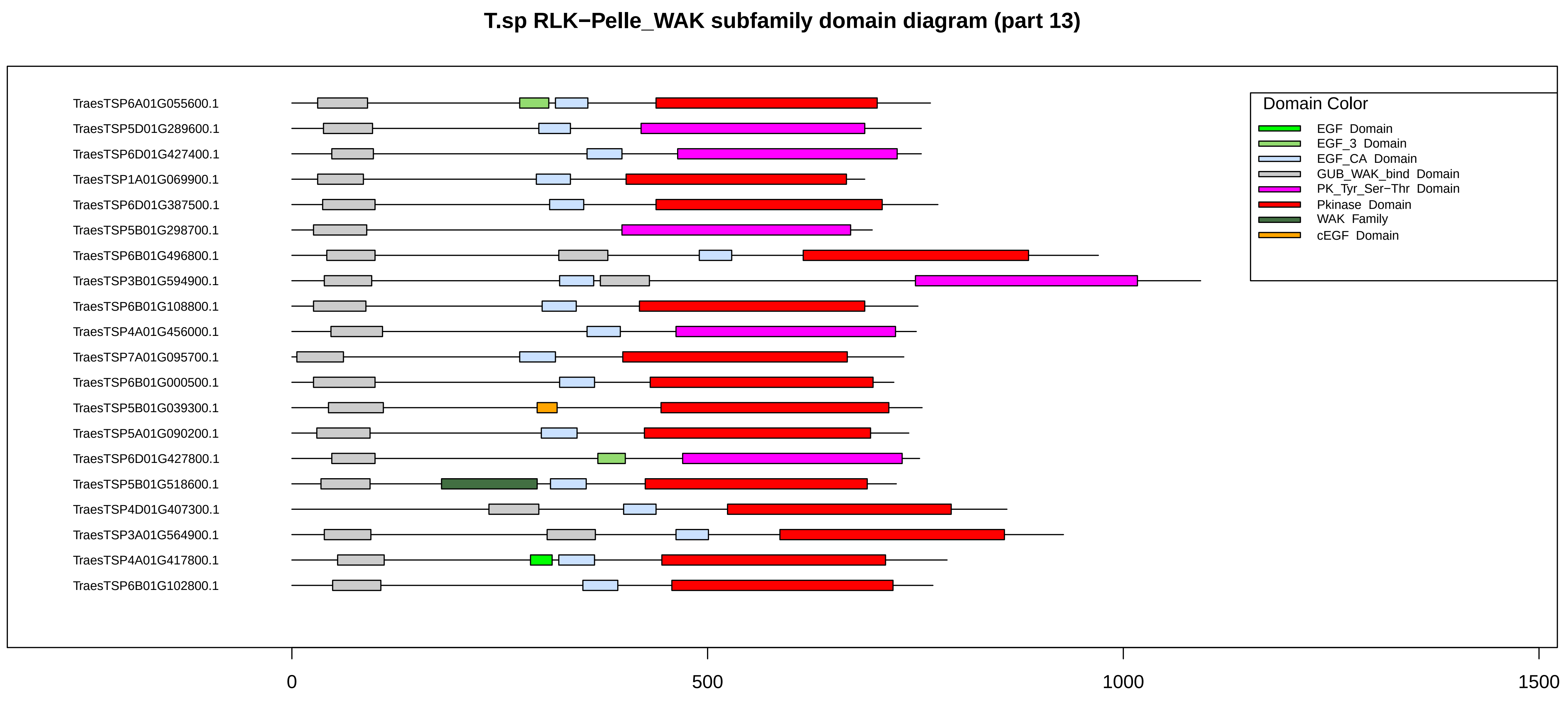

T.sp RLK−Pelle_WAK subfamily domain diagram (part 13)
TraesTSP6A01G055600.1
TraesTSP5D01G289600.1
TraesTSP6D01G427400.1
TraesTSP1A01G069900.1
TraesTSP6D01G387500.1
TraesTSP5B01G298700.1
TraesTSP6B01G496800.1
TraesTSP3B01G594900.1
TraesTSP6B01G108800.1
TraesTSP4A01G456000.1
TraesTSP7A01G095700.1
TraesTSP6B01G000500.1
TraesTSP5B01G039300.1
TraesTSP5A01G090200.1
TraesTSP6D01G427800.1
TraesTSP5B01G518600.1
TraesTSP4D01G407300.1
TraesTSP3A01G564900.1
TraesTSP4A01G417800.1
TraesTSP6B01G102800.1
Domain Color
EGF Domain
EGF_3 Domain
EGF_CA Domain
GUB_WAK_bind Domain
PK_Tyr_Ser−Thr Domain
Pkinase Domain
WAK Family
cEGF Domain
0
500
1000
1500

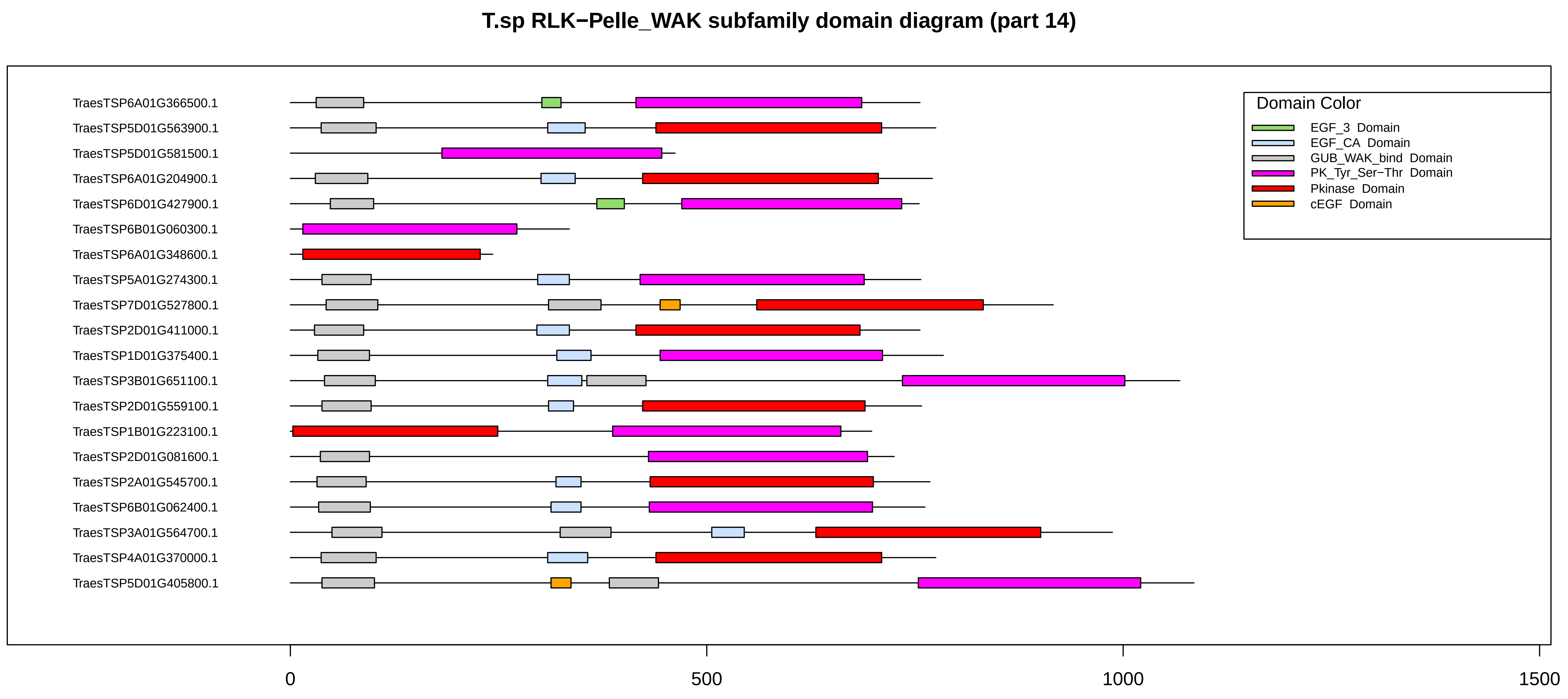

T.sp RLK−Pelle_WAK subfamily domain diagram (part 14)
TraesTSP6A01G366500.1
TraesTSP5D01G563900.1
TraesTSP5D01G581500.1
TraesTSP6A01G204900.1
TraesTSP6D01G427900.1
TraesTSP6B01G060300.1
TraesTSP6A01G348600.1
TraesTSP5A01G274300.1
TraesTSP7D01G527800.1
TraesTSP2D01G411000.1
TraesTSP1D01G375400.1
TraesTSP3B01G651100.1
TraesTSP2D01G559100.1
TraesTSP1B01G223100.1
TraesTSP2D01G081600.1
TraesTSP2A01G545700.1
TraesTSP6B01G062400.1
TraesTSP3A01G564700.1
TraesTSP4A01G370000.1
TraesTSP5D01G405800.1
Domain Color
EGF_3 Domain
EGF_CA Domain
GUB_WAK_bind Domain
PK_Tyr_Ser−Thr Domain
Pkinase Domain
cEGF Domain
0
500
1000
1500

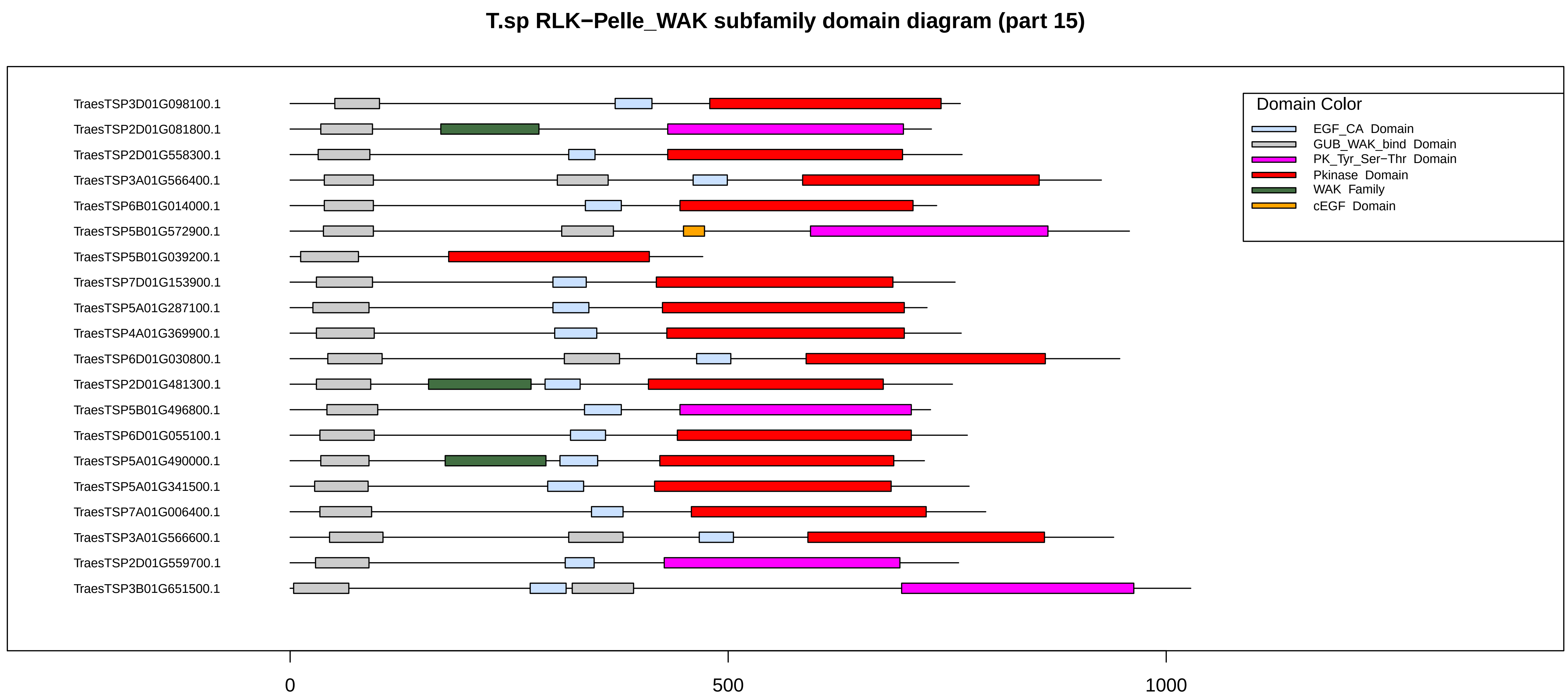

T.sp RLK−Pelle_WAK subfamily domain diagram (part 15)
TraesTSP3D01G098100.1
TraesTSP2D01G081800.1
TraesTSP2D01G558300.1
TraesTSP3A01G566400.1
TraesTSP6B01G014000.1
TraesTSP5B01G572900.1
TraesTSP5B01G039200.1
TraesTSP7D01G153900.1
TraesTSP5A01G287100.1
TraesTSP4A01G369900.1
TraesTSP6D01G030800.1
TraesTSP2D01G481300.1
TraesTSP5B01G496800.1
TraesTSP6D01G055100.1
TraesTSP5A01G490000.1
TraesTSP5A01G341500.1
TraesTSP7A01G006400.1
TraesTSP3A01G566600.1
TraesTSP2D01G559700.1
TraesTSP3B01G651500.1
Domain Color
EGF_CA Domain
GUB_WAK_bind Domain
PK_Tyr_Ser−Thr Domain
Pkinase Domain
WAK Family
cEGF Domain
0
500
1000

T.sp RLK−Pelle_WAK subfamily domain diagram (part 16)

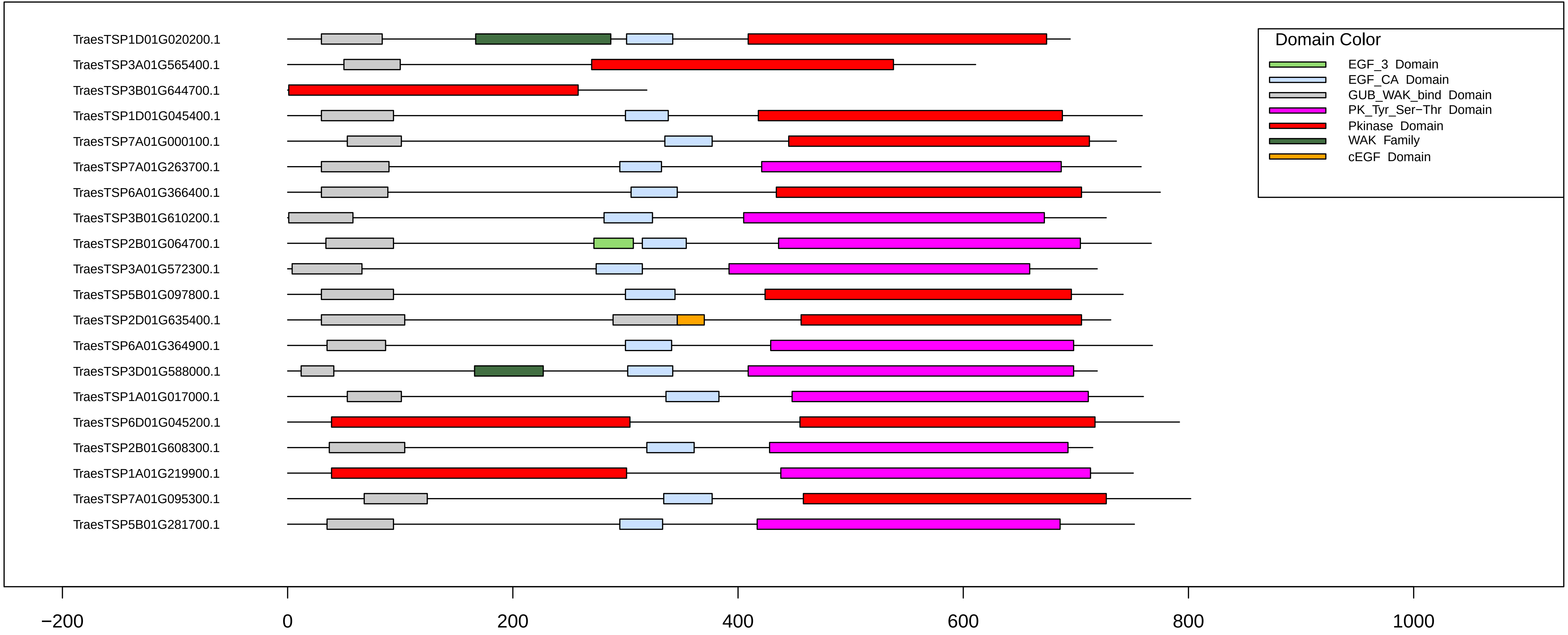

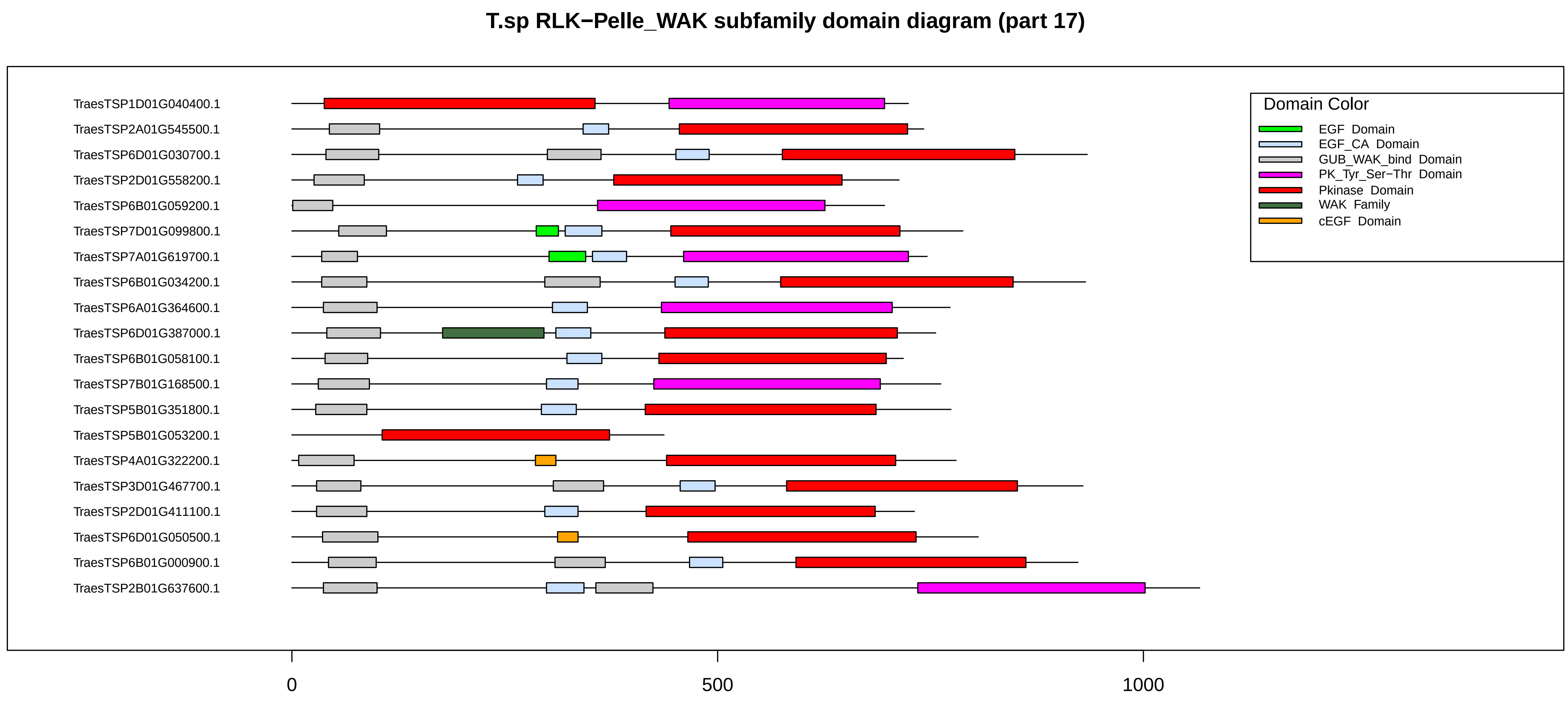
T.sp RLK−Pelle_WAK subfamily domain diagram (part 17)
TraesTSP1D01G040400.1
TraesTSP2A01G545500.1
TraesTSP6D01G030700.1
TraesTSP2D01G558200.1
TraesTSP6B01G059200.1
TraesTSP7D01G099800.1
TraesTSP7A01G619700.1
TraesTSP6B01G034200.1
TraesTSP6A01G364600.1
TraesTSP6D01G387000.1
TraesTSP6B01G058100.1
TraesTSP7B01G168500.1
TraesTSP5B01G351800.1
TraesTSP5B01G053200.1
TraesTSP4A01G322200.1
TraesTSP3D01G467700.1
TraesTSP2D01G411100.1
TraesTSP6D01G050500.1
TraesTSP6B01G000900.1
TraesTSP2B01G637600.1
Domain Color
EGF Domain
EGF_CA Domain
GUB_WAK_bind Domain
PK_Tyr_Ser−Thr Domain
Pkinase Domain
WAK Family
cEGF Domain
0
500
1000

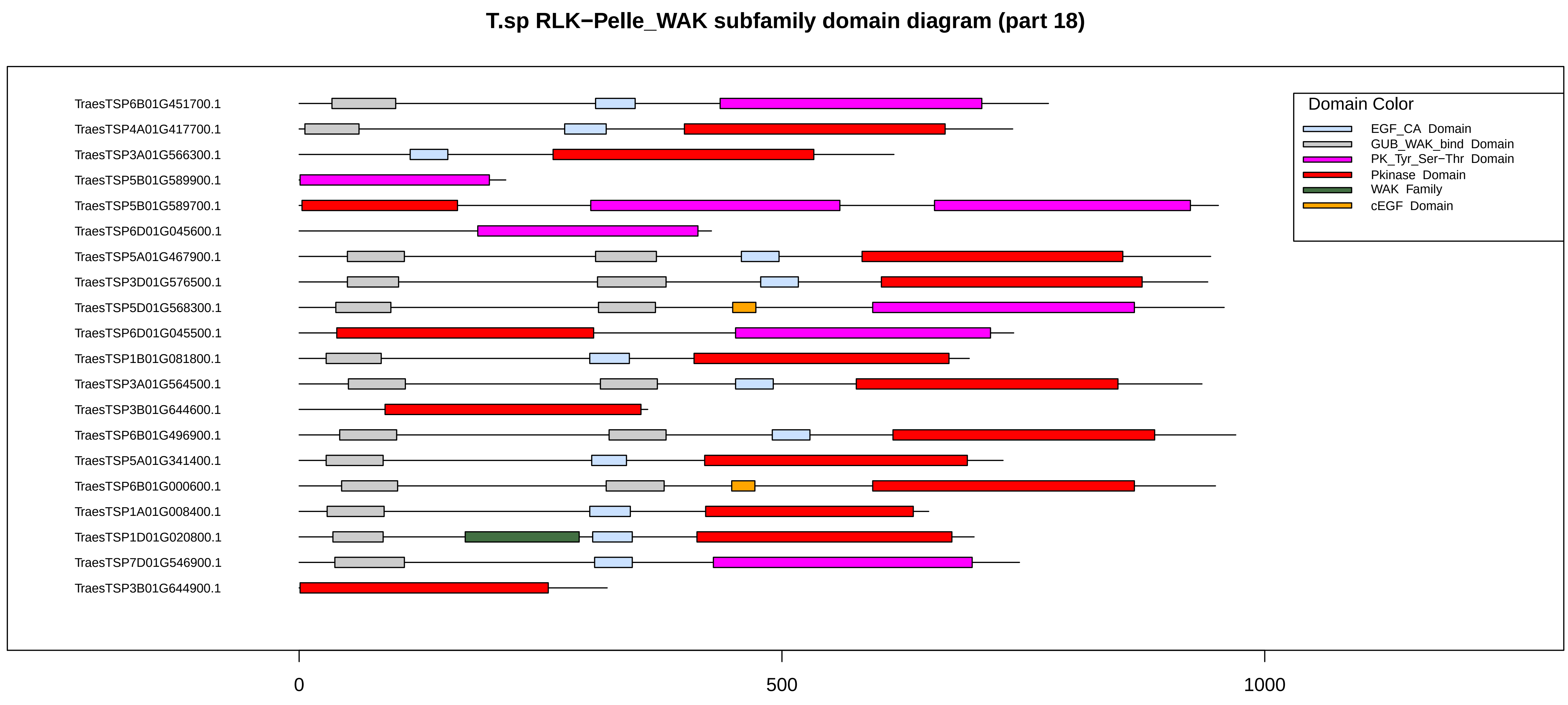

T.sp RLK−Pelle_WAK subfamily domain diagram (part 18)
TraesTSP6B01G451700.1
TraesTSP4A01G417700.1
TraesTSP3A01G566300.1
TraesTSP5B01G589900.1
TraesTSP5B01G589700.1
TraesTSP6D01G045600.1
TraesTSP5A01G467900.1
TraesTSP3D01G576500.1
TraesTSP5D01G568300.1
TraesTSP6D01G045500.1
TraesTSP1B01G081800.1
TraesTSP3A01G564500.1
TraesTSP3B01G644600.1
TraesTSP6B01G496900.1
TraesTSP5A01G341400.1
TraesTSP6B01G000600.1
TraesTSP1A01G008400.1
TraesTSP1D01G020800.1
TraesTSP7D01G546900.1
TraesTSP3B01G644900.1
Domain Color
EGF_CA Domain
GUB_WAK_bind Domain
PK_Tyr_Ser−Thr Domain
Pkinase Domain
WAK Family
cEGF Domain
0
500
1000

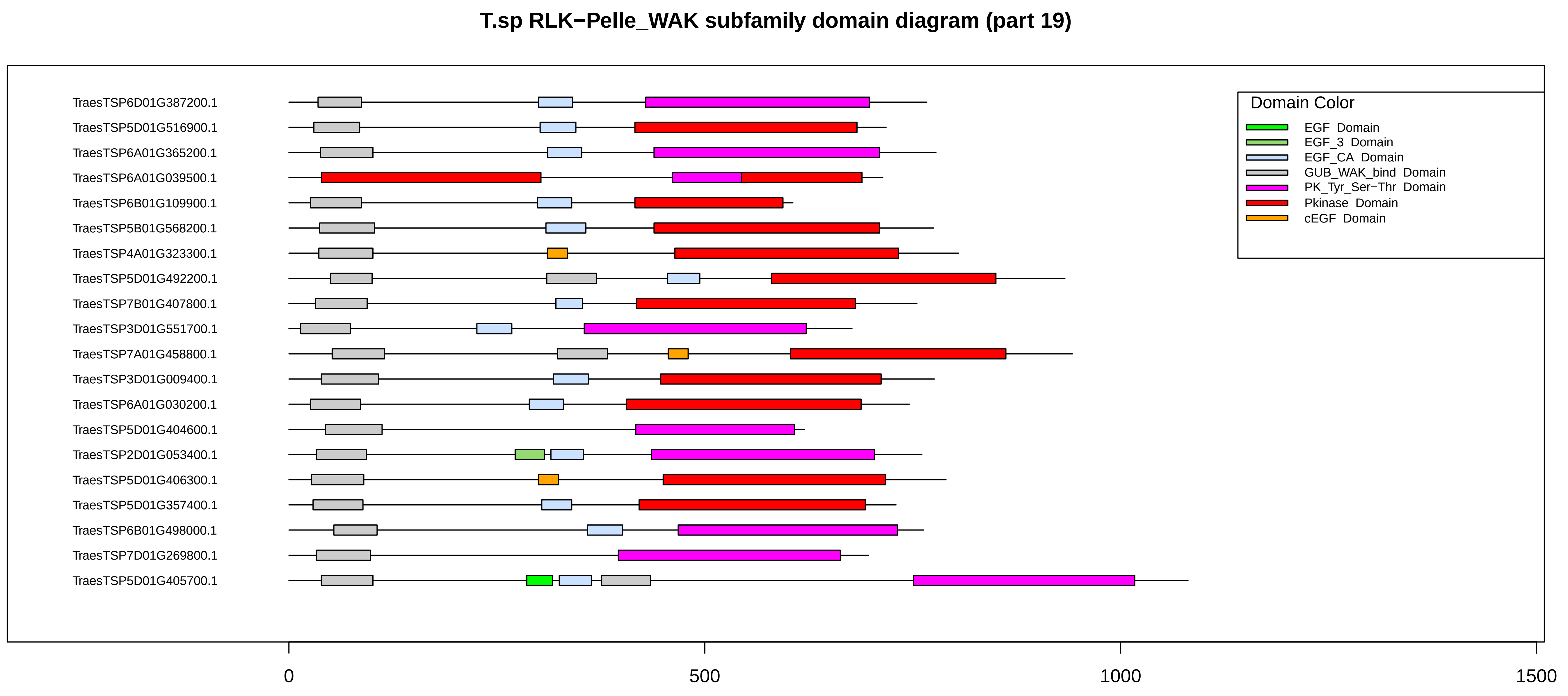

T.sp RLK−Pelle_WAK subfamily domain diagram (part 19)
TraesTSP6D01G387200.1
TraesTSP5D01G516900.1
TraesTSP6A01G365200.1
TraesTSP6A01G039500.1
TraesTSP6B01G109900.1
TraesTSP5B01G568200.1
TraesTSP4A01G323300.1
TraesTSP5D01G492200.1
TraesTSP7B01G407800.1
TraesTSP3D01G551700.1
TraesTSP7A01G458800.1
TraesTSP3D01G009400.1
TraesTSP6A01G030200.1
TraesTSP5D01G404600.1
TraesTSP2D01G053400.1
TraesTSP5D01G406300.1
TraesTSP5D01G357400.1
TraesTSP6B01G498000.1
TraesTSP7D01G269800.1
TraesTSP5D01G405700.1
Domain Color
EGF Domain
EGF_3 Domain
EGF_CA Domain
GUB_WAK_bind Domain
PK_Tyr_Ser−Thr Domain
Pkinase Domain
cEGF Domain
0
500
1000
1500

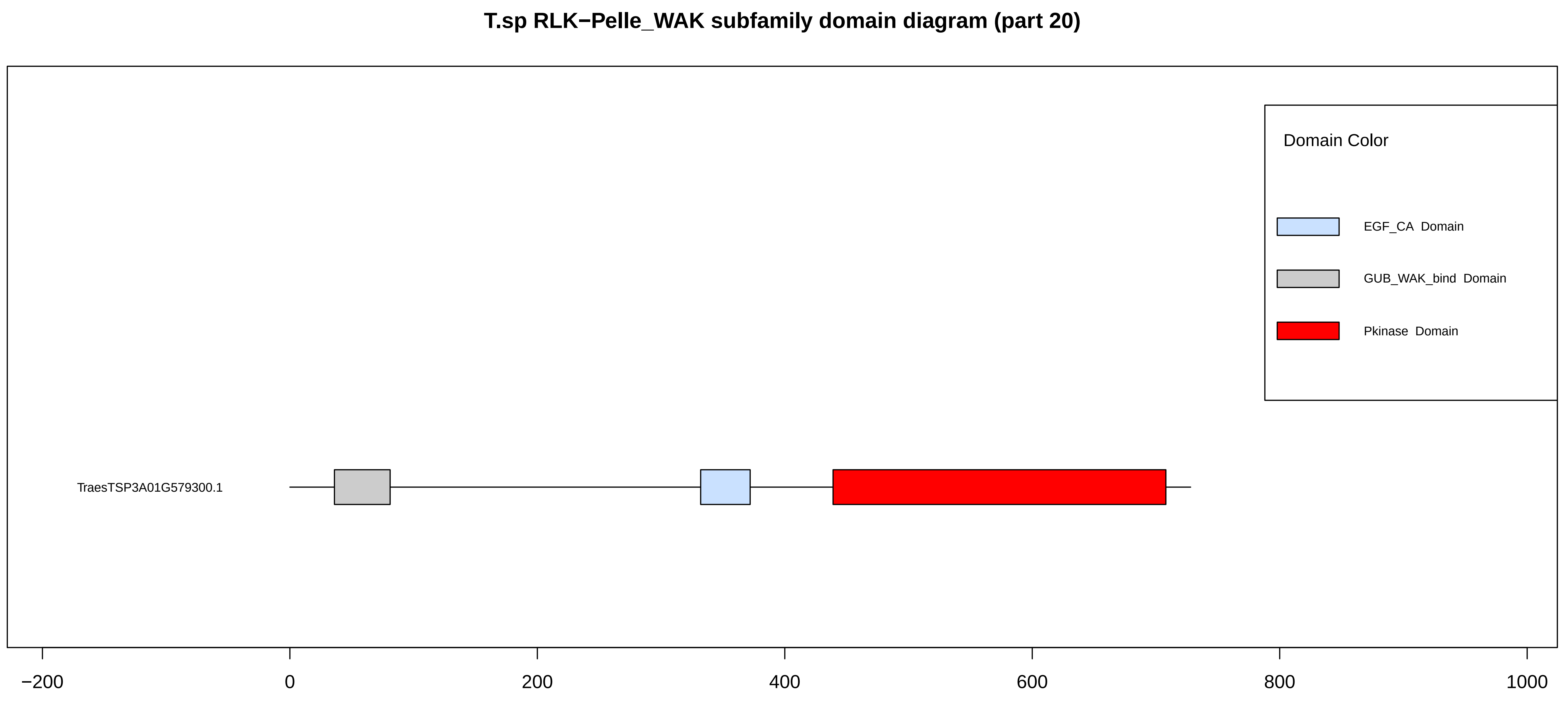

T.sp RLK−Pelle_WAK subfamily domain diagram (part 20)
Domain Color
EGF_CA Domain
GUB_WAK_bind Domain
Pkinase Domain
TraesTSP3A01G579300.1
−200
0
200
400
600
800
1000

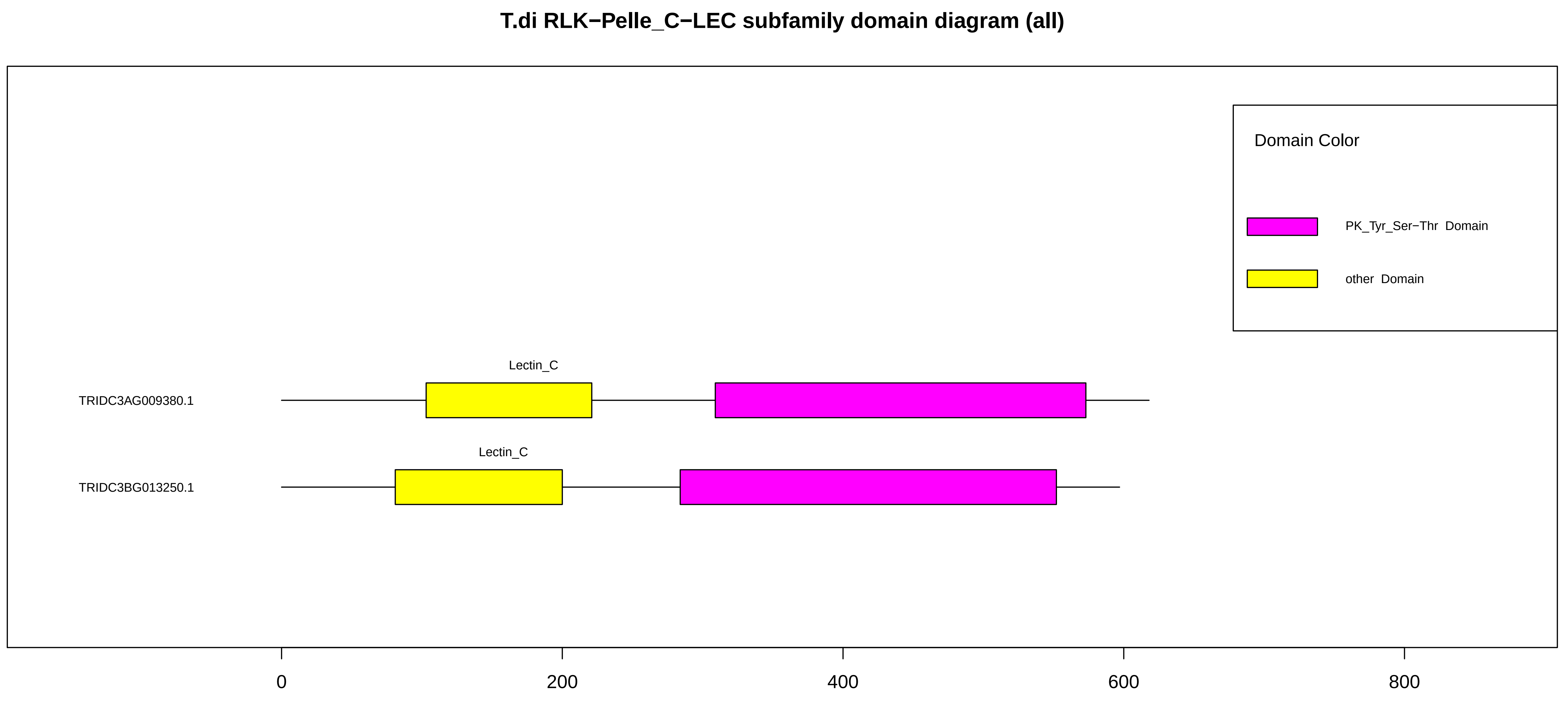

T.di RLK−Pelle_C−LEC subfamily domain diagram (all)
Domain Color
PK_Tyr_Ser−Thr Domain
other Domain
Lectin_C
TRIDC3AG009380.1
Lectin_C
TRIDC3BG013250.1
0
200
400
600
800

T.di RLK−Pelle_CR4L subfamily domain diagram (part 1)

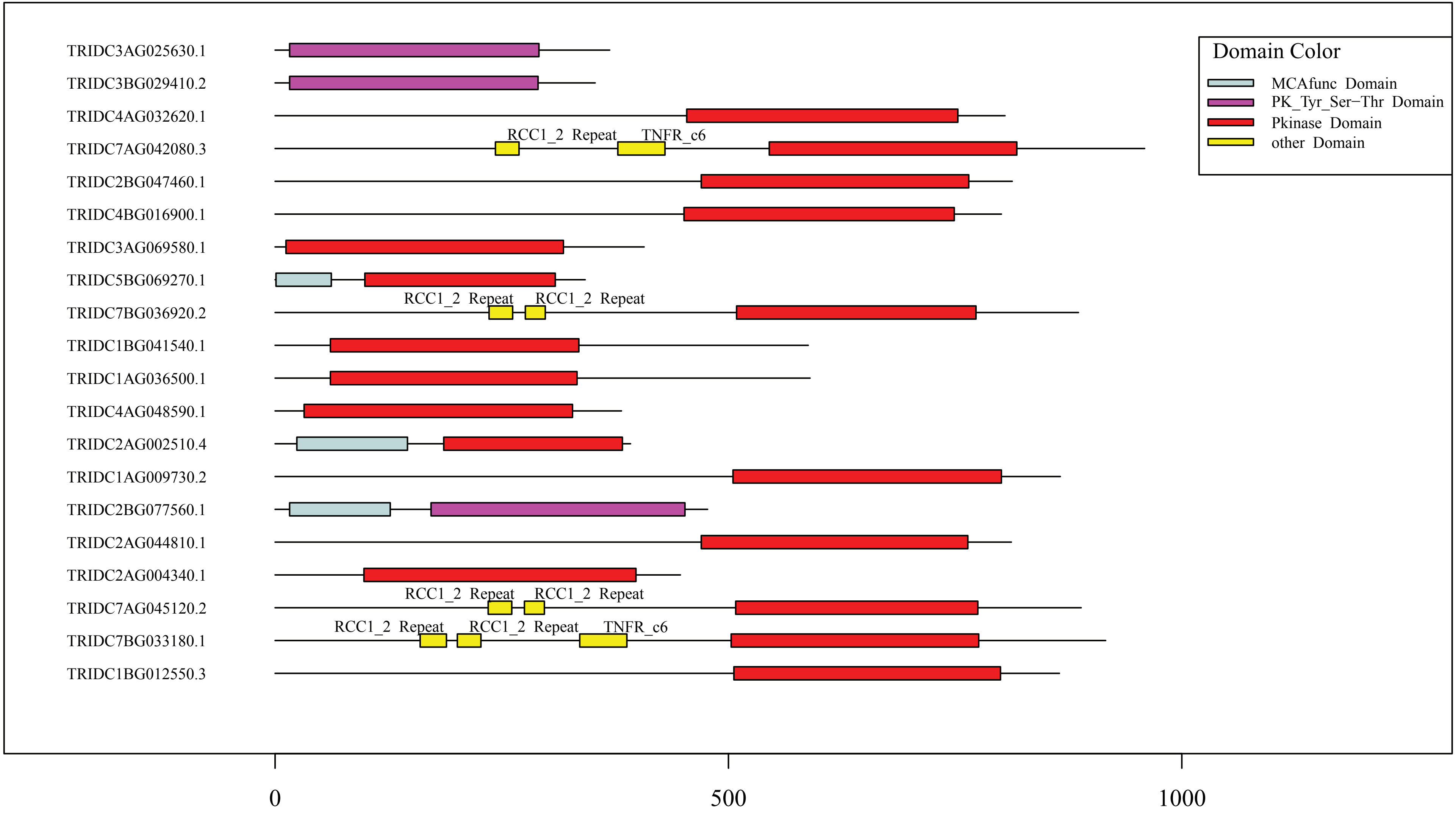

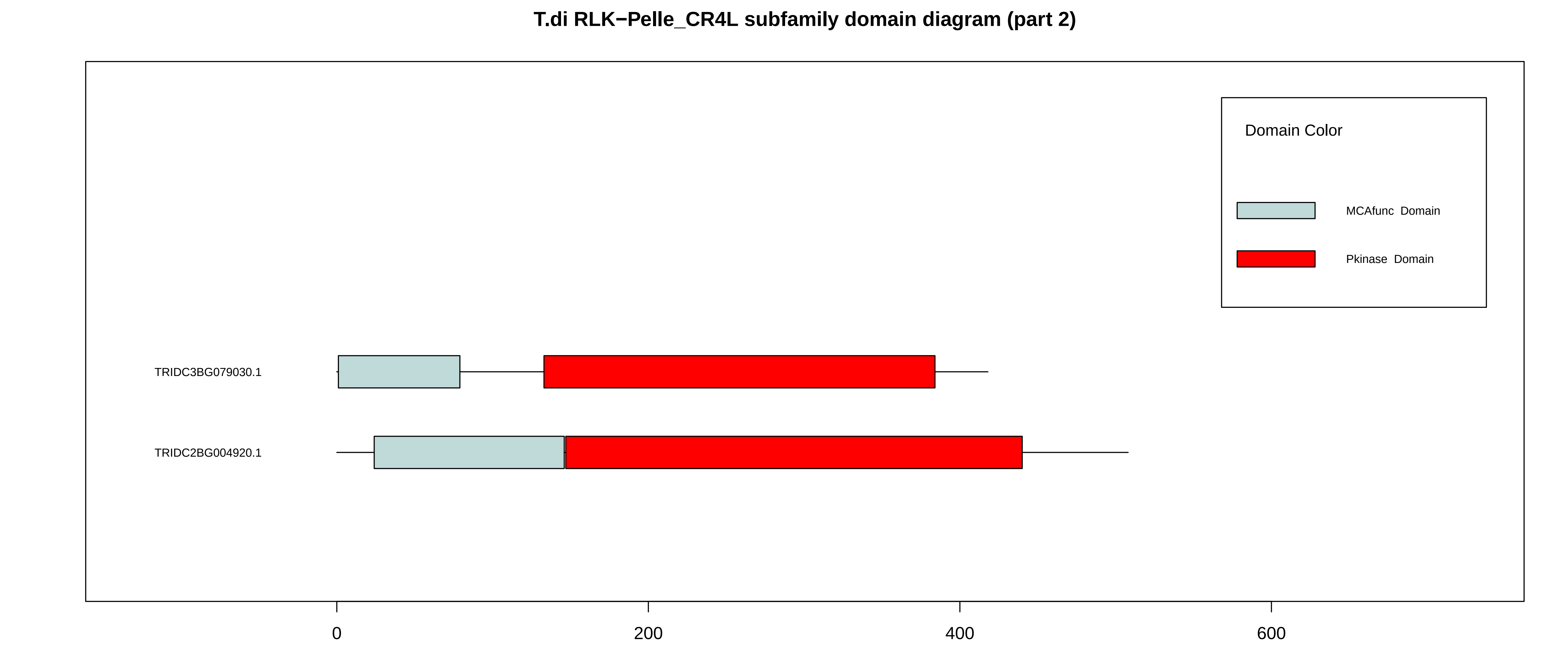

T.di RLK−Pelle_CR4L subfamily domain diagram (part 2)
Domain Color
MCAfunc Domain
Pkinase Domain
TRIDC3BG079030.1
TRIDC2BG004920.1
0
200
400
600

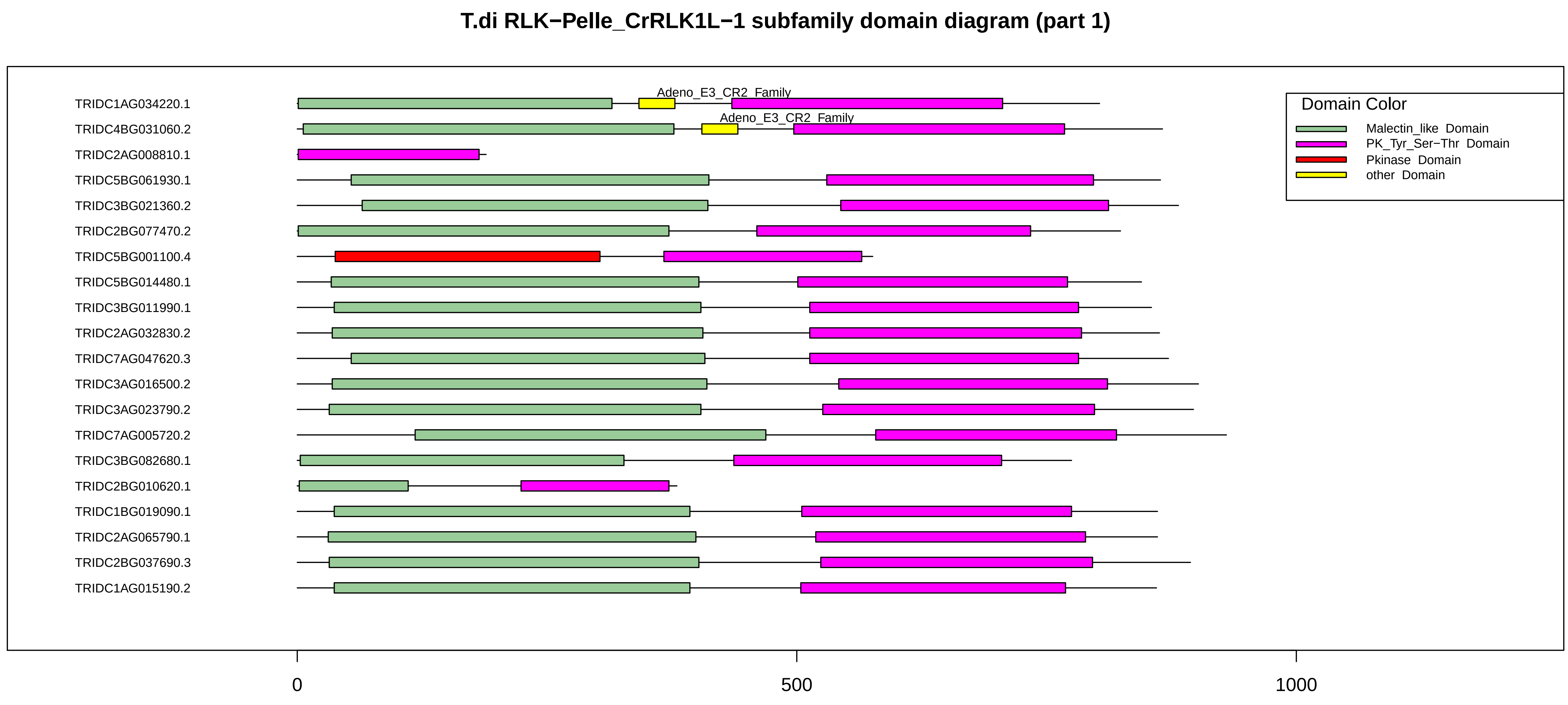
T.di RLK−Pelle_CrRLK1L−1 subfamily domain diagram (part 1)
TRIDC1AG034220.1
TRIDC4BG031060.2
TRIDC2AG008810.1
TRIDC5BG061930.1
TRIDC3BG021360.2
TRIDC2BG077470.2
TRIDC5BG001100.4
TRIDC5BG014480.1
TRIDC3BG011990.1
TRIDC2AG032830.2
TRIDC7AG047620.3
TRIDC3AG016500.2
TRIDC3AG023790.2
TRIDC7AG005720.2
TRIDC3BG082680.1
TRIDC2BG010620.1
TRIDC1BG019090.1
TRIDC2AG065790.1
TRIDC2BG037690.3
TRIDC1AG015190.2
Adeno_E3_CR2_Family
Adeno_E3_CR2_Family
Domain Color
Malectin_like Domain
PK_Tyr_Ser−Thr Domain
Pkinase Domain
other Domain
0
500
1000

T.di RLK−Pelle_CrRLK1L−1 subfamily domain diagram (part 2)

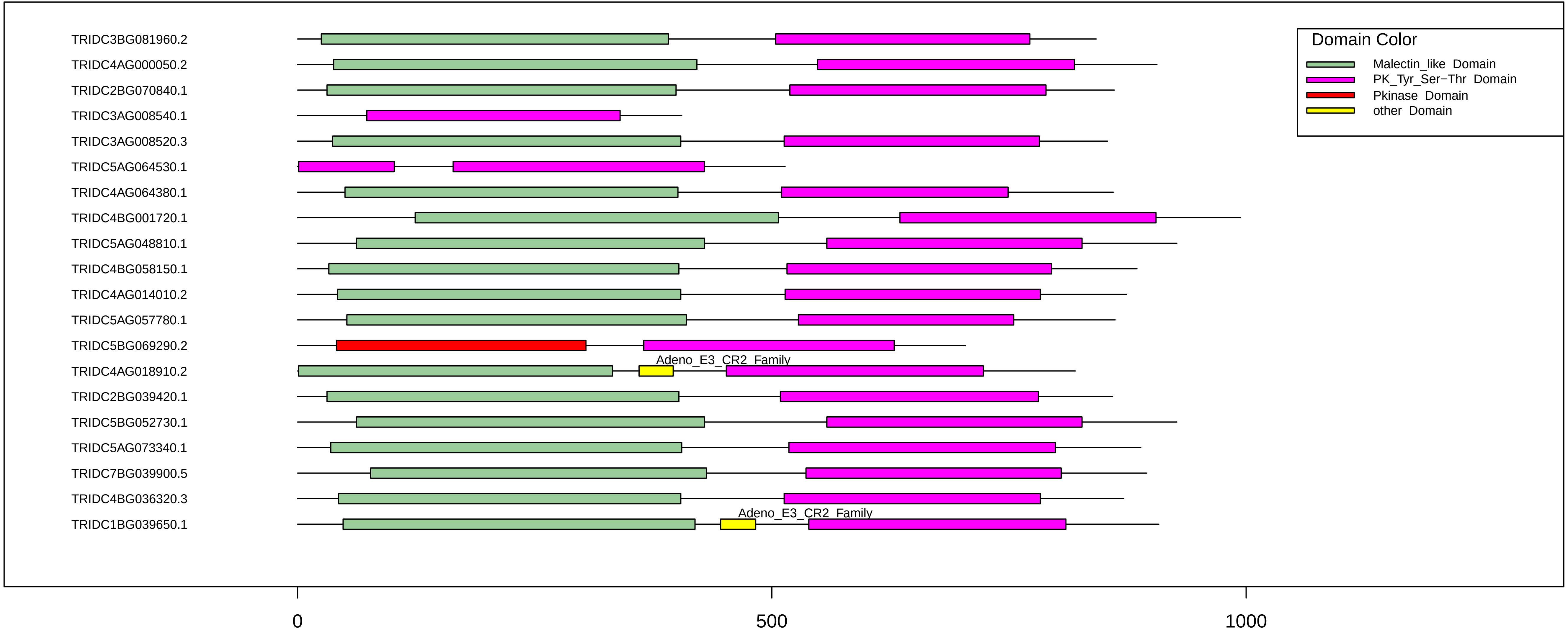

T.di RLK−Pelle_DLSV subfamily domain diagram (part 1)

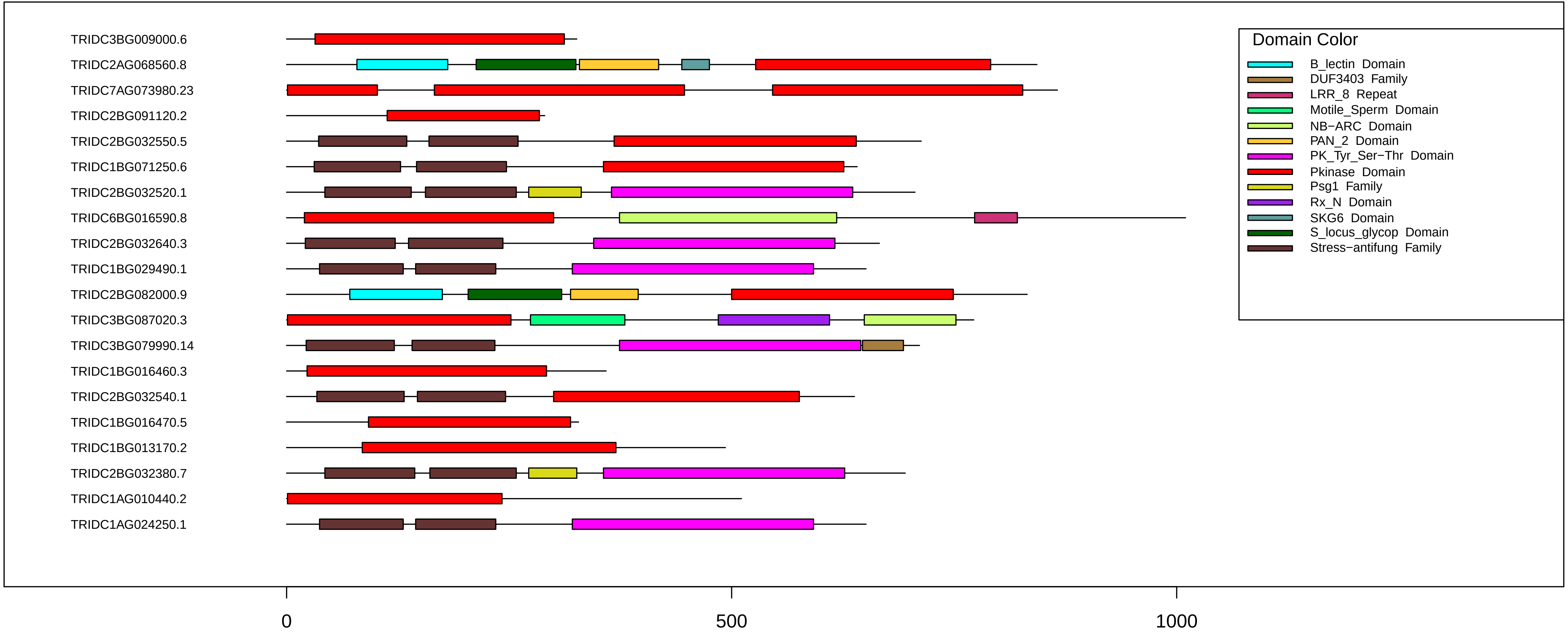

T.di RLK−Pelle_DLSV subfamily domain diagram (part 2)

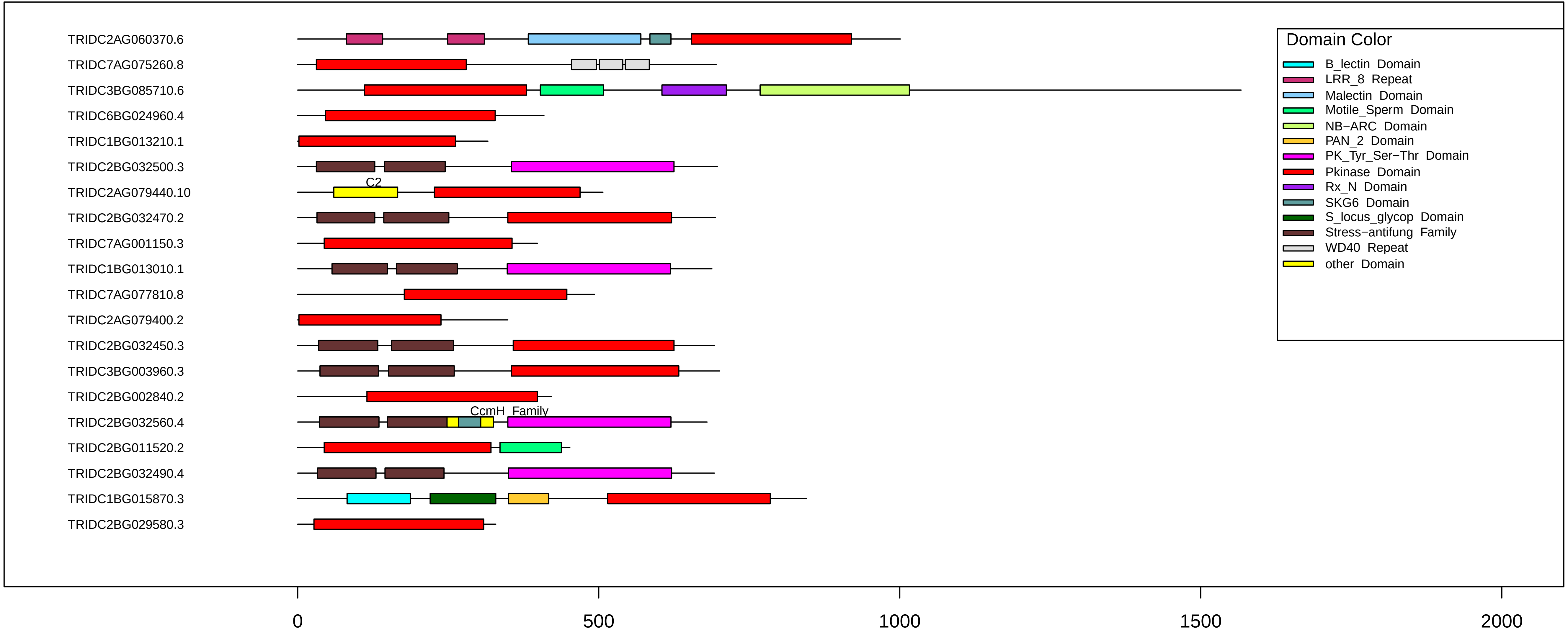

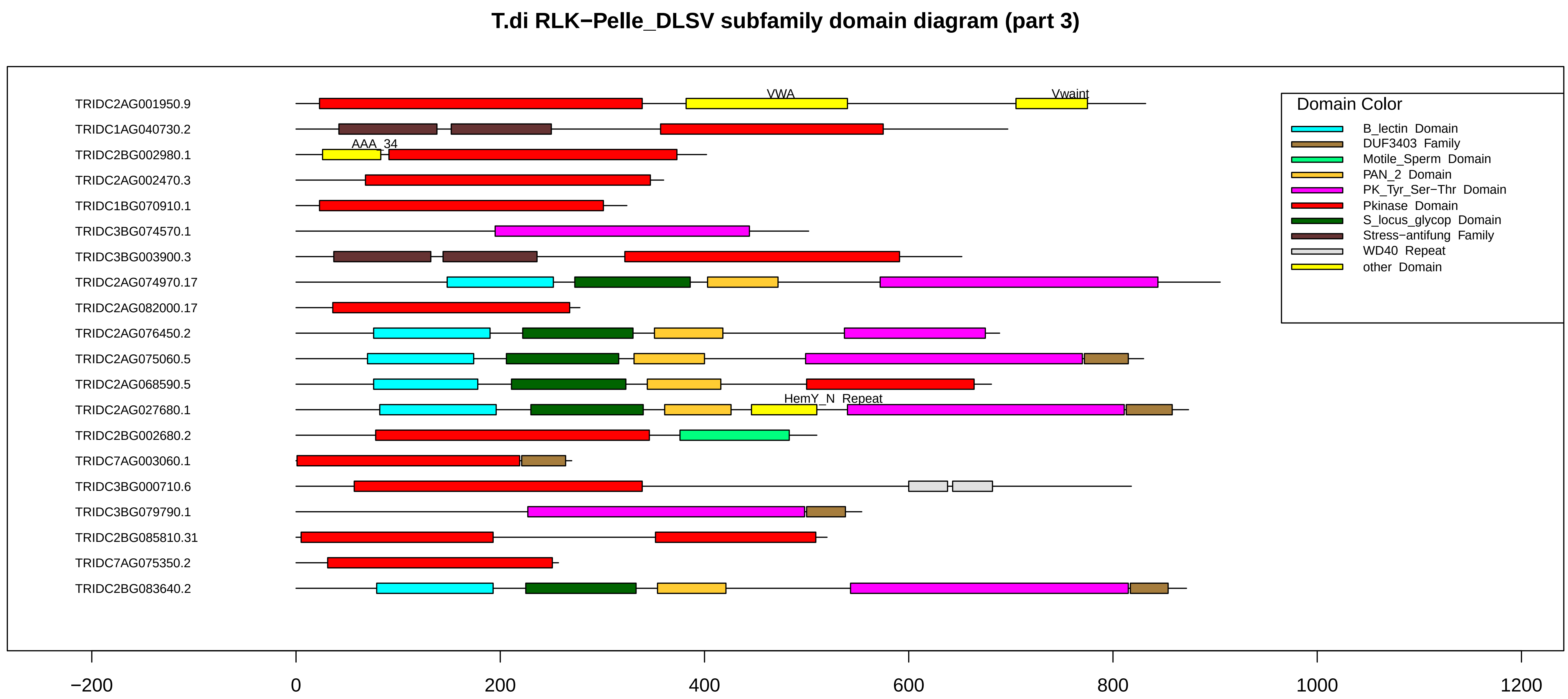

T.di RLK−Pelle_DLSV subfamily domain diagram (part 3)
TRIDC2AG001950.9
TRIDC1AG040730.2
TRIDC2BG002980.1
TRIDC2AG002470.3
TRIDC1BG070910.1
TRIDC3BG074570.1
TRIDC3BG003900.3
TRIDC2AG074970.17
TRIDC2AG082000.17
TRIDC2AG076450.2
TRIDC2AG075060.5
TRIDC2AG068590.5
TRIDC2AG027680.1
TRIDC2BG002680.2
TRIDC7AG003060.1
TRIDC3BG000710.6
TRIDC3BG079790.1
TRIDC2BG085810.31
TRIDC7AG075350.2
TRIDC2BG083640.2
VWA
Vwaint
AAA_34
HemY_N Repeat
Domain Color
B_lectin Domain
DUF3403 Family
Motile_Sperm Domain
PAN_2 Domain
PK_Tyr_Ser−Thr Domain
Pkinase Domain
S_locus_glycop Domain
Stress−antifung Family
WD40 Repeat
other Domain
−200
0
200
400
600
800
1000
1200

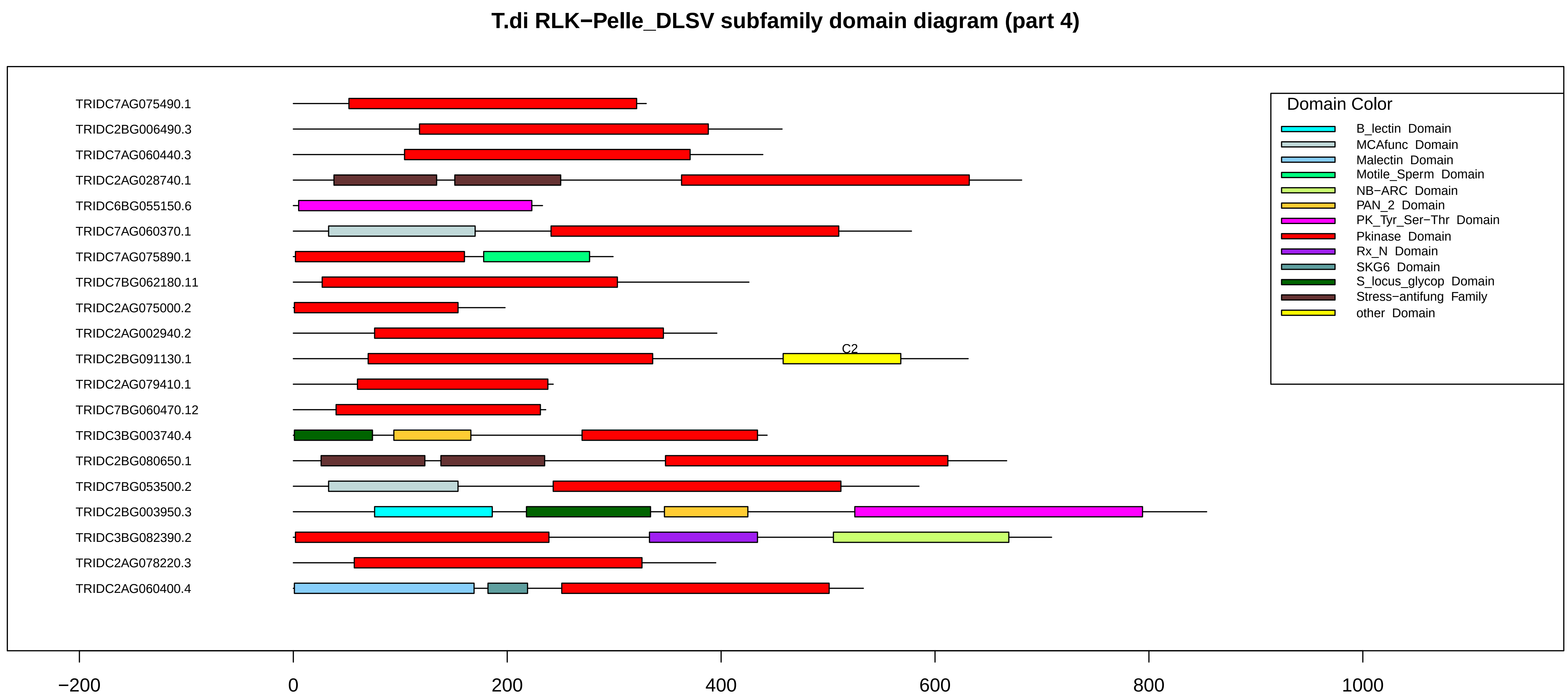

T.di RLK−Pelle_DLSV subfamily domain diagram (part 4)
TRIDC7AG075490.1
TRIDC2BG006490.3
TRIDC7AG060440.3
TRIDC2AG028740.1
TRIDC6BG055150.6
TRIDC7AG060370.1
TRIDC7AG075890.1
TRIDC7BG062180.11
TRIDC2AG075000.2
TRIDC2AG002940.2
TRIDC2BG091130.1
TRIDC2AG079410.1
TRIDC7BG060470.12
TRIDC3BG003740.4
TRIDC2BG080650.1
TRIDC7BG053500.2
TRIDC2BG003950.3
TRIDC3BG082390.2
TRIDC2AG078220.3
TRIDC2AG060400.4
C2
Domain Color
B_lectin Domain
MCAfunc Domain
Malectin Domain
Motile_Sperm Domain
NB−ARC Domain
PAN_2 Domain
PK_Tyr_Ser−Thr Domain
Pkinase Domain
Rx_N Domain
SKG6 Domain
S_locus_glycop Domain
Stress−antifung Family
other Domain
−200
0
200
400
600
800
1000

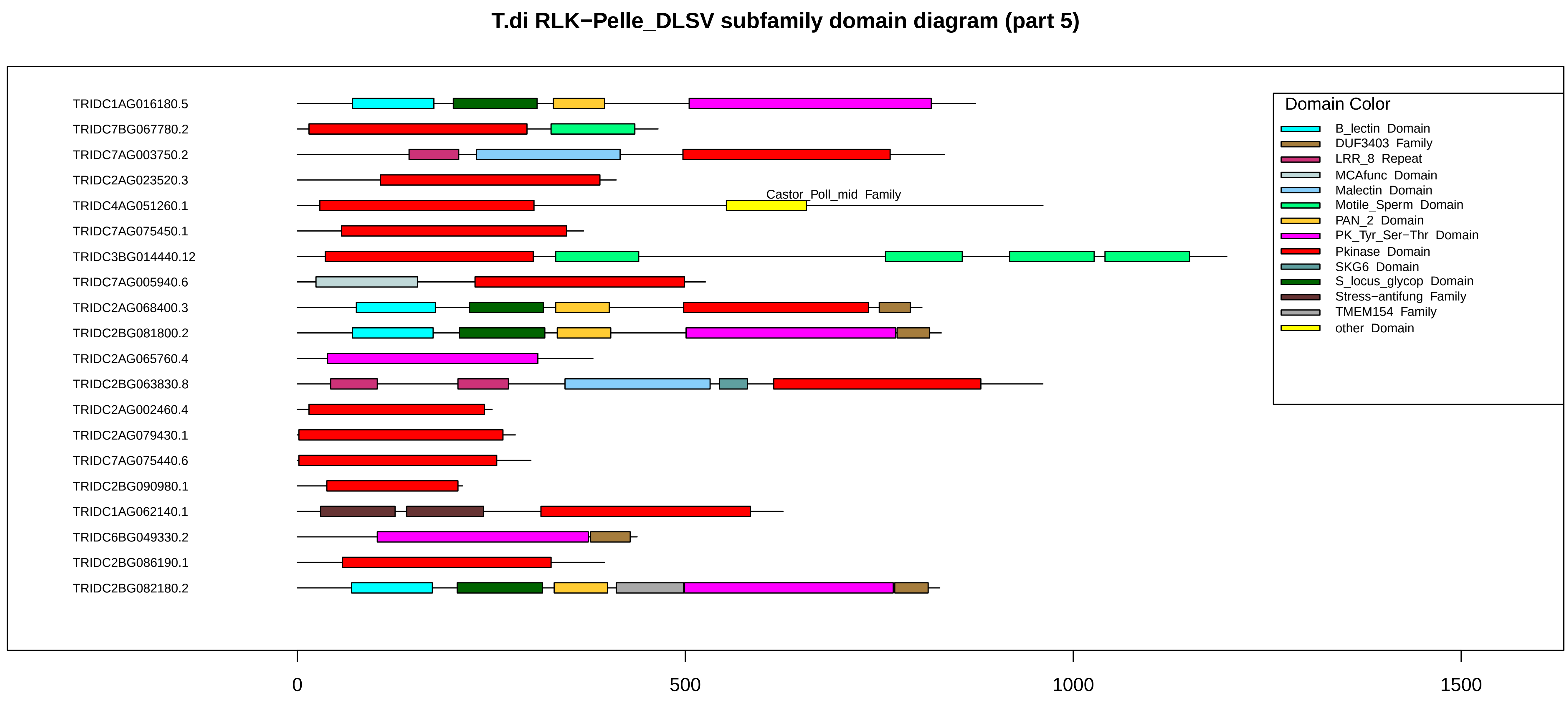
T.di RLK−Pelle_DLSV subfamily domain diagram (part 5)
TRIDC1AG016180.5
TRIDC7BG067780.2
TRIDC7AG003750.2
TRIDC2AG023520.3
TRIDC4AG051260.1
TRIDC7AG075450.1
TRIDC3BG014440.12
TRIDC7AG005940.6
TRIDC2AG068400.3
TRIDC2BG081800.2
TRIDC2AG065760.4
TRIDC2BG063830.8
TRIDC2AG002460.4
TRIDC2AG079430.1
TRIDC7AG075440.6
TRIDC2BG090980.1
TRIDC1AG062140.1
TRIDC6BG049330.2
TRIDC2BG086190.1
TRIDC2BG082180.2
Castor_Poll_mid Family
Domain Color
B_lectin Domain
DUF3403 Family
LRR_8 Repeat
MCAfunc Domain
Malectin Domain
Motile_Sperm Domain
PAN_2 Domain
PK_Tyr_Ser−Thr Domain
Pkinase Domain
SKG6 Domain
S_locus_glycop Domain
Stress−antifung Family
TMEM154 Family
other Domain
0
500
1000
1500

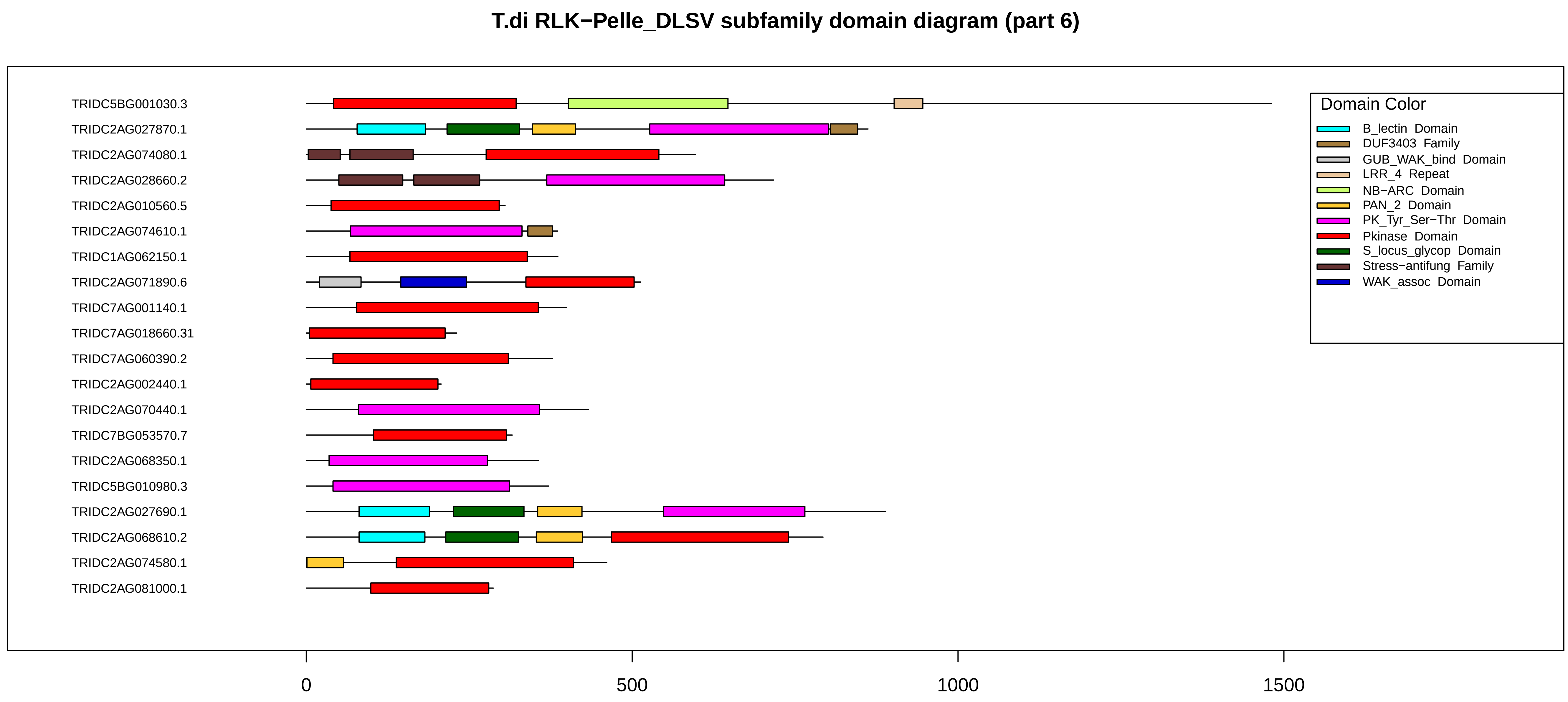
T.di RLK−Pelle_DLSV subfamily domain diagram (part 6)
TRIDC5BG001030.3
TRIDC2AG027870.1
TRIDC2AG074080.1
TRIDC2AG028660.2
TRIDC2AG010560.5
TRIDC2AG074610.1
TRIDC1AG062150.1
TRIDC2AG071890.6
TRIDC7AG001140.1
TRIDC7AG018660.31
TRIDC7AG060390.2
TRIDC2AG002440.1
TRIDC2AG070440.1
TRIDC7BG053570.7
TRIDC2AG068350.1
TRIDC5BG010980.3
TRIDC2AG027690.1
TRIDC2AG068610.2
TRIDC2AG074580.1
TRIDC2AG081000.1
Domain Color
B_lectin Domain
DUF3403 Family
GUB_WAK_bind Domain
LRR_4 Repeat
NB−ARC Domain
PAN_2 Domain
PK_Tyr_Ser−Thr Domain
Pkinase Domain
S_locus_glycop Domain
Stress−antifung Family
WAK_assoc Domain
0
500
1000
1500

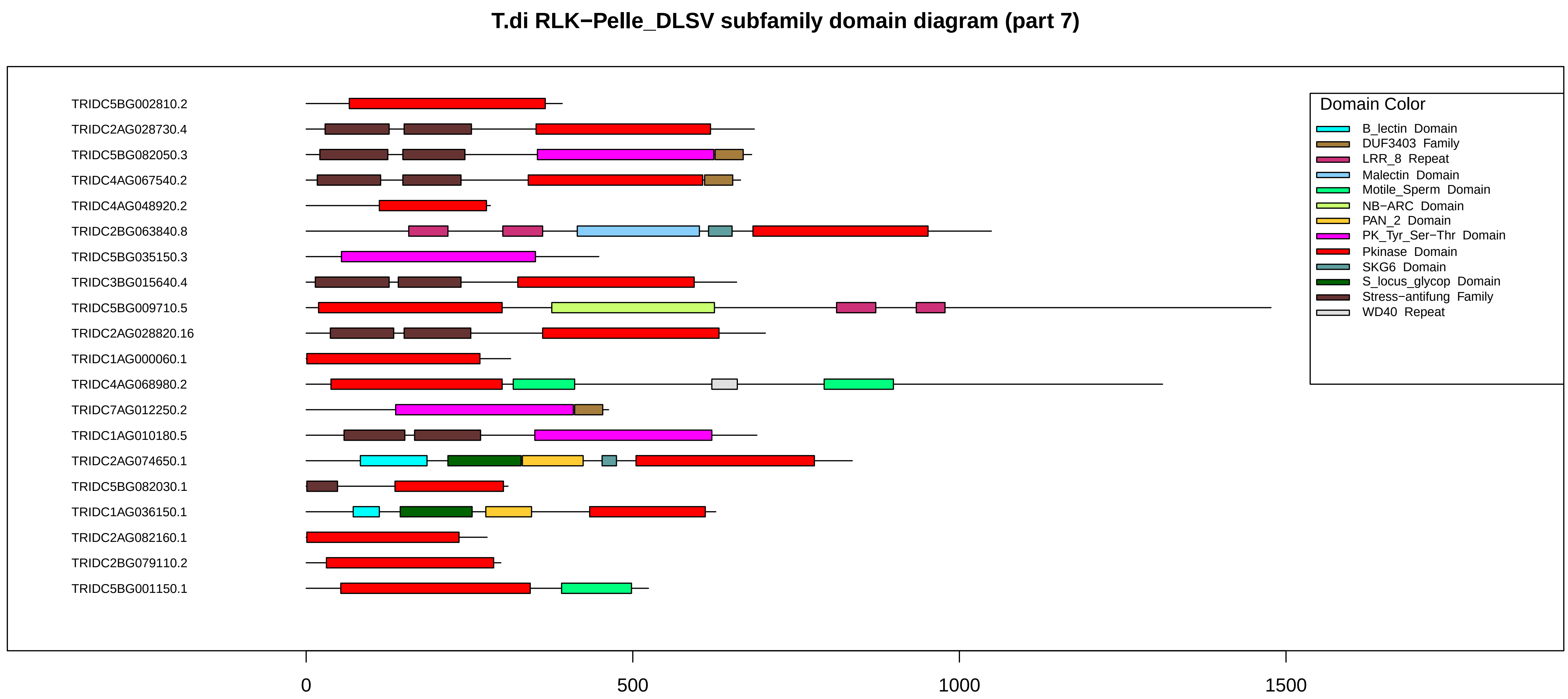

T.di RLK−Pelle_DLSV subfamily domain diagram (part 7)
TRIDC5BG002810.2
TRIDC2AG028730.4
TRIDC5BG082050.3
TRIDC4AG067540.2
TRIDC4AG048920.2
TRIDC2BG063840.8
TRIDC5BG035150.3
TRIDC3BG015640.4
TRIDC5BG009710.5
TRIDC2AG028820.16
TRIDC1AG000060.1
TRIDC4AG068980.2
TRIDC7AG012250.2
TRIDC1AG010180.5
TRIDC2AG074650.1
TRIDC5BG082030.1
TRIDC1AG036150.1
TRIDC2AG082160.1
TRIDC2BG079110.2
TRIDC5BG001150.1
Domain Color
B_lectin Domain
DUF3403 Family
LRR_8 Repeat
Malectin Domain
Motile_Sperm Domain
NB−ARC Domain
PAN_2 Domain
PK_Tyr_Ser−Thr Domain
Pkinase Domain
SKG6 Domain
S_locus_glycop Domain
Stress−antifung Family
WD40 Repeat
0
500
1000
1500

T.di RLK−Pelle_DLSV subfamily domain diagram (part 8)

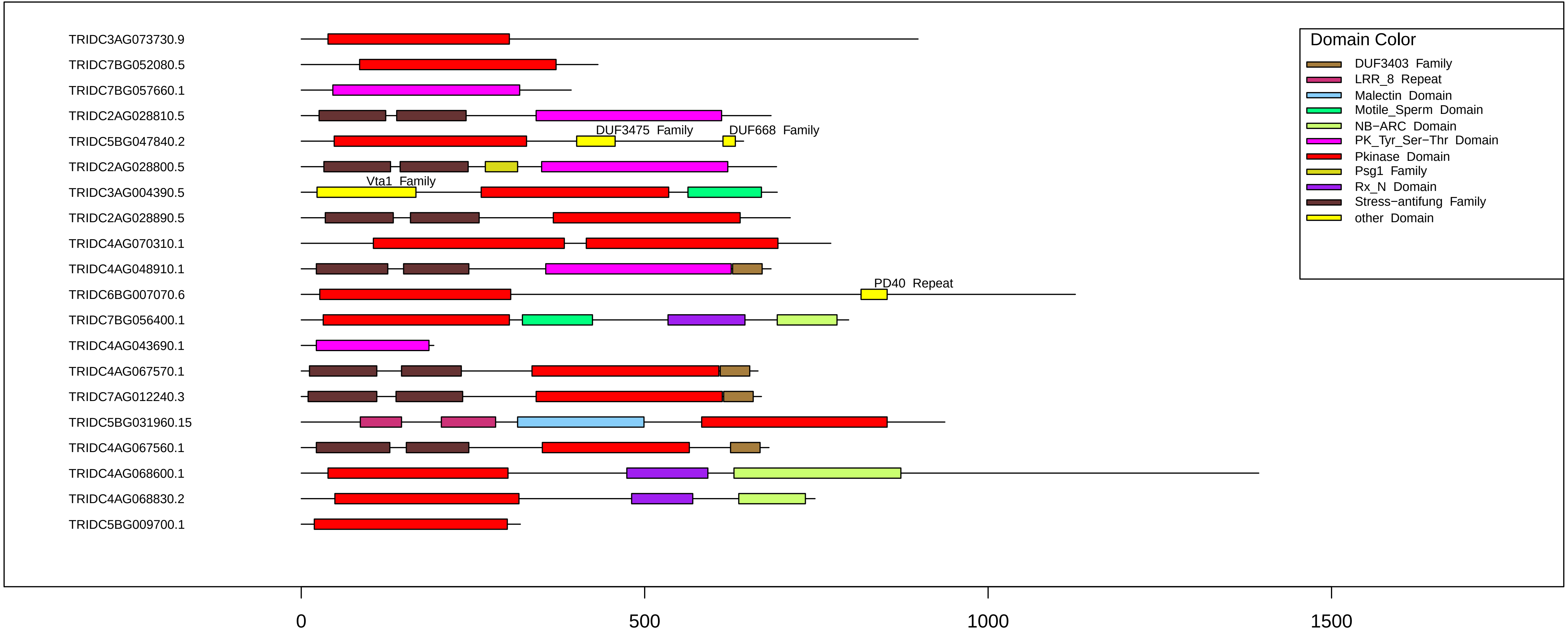

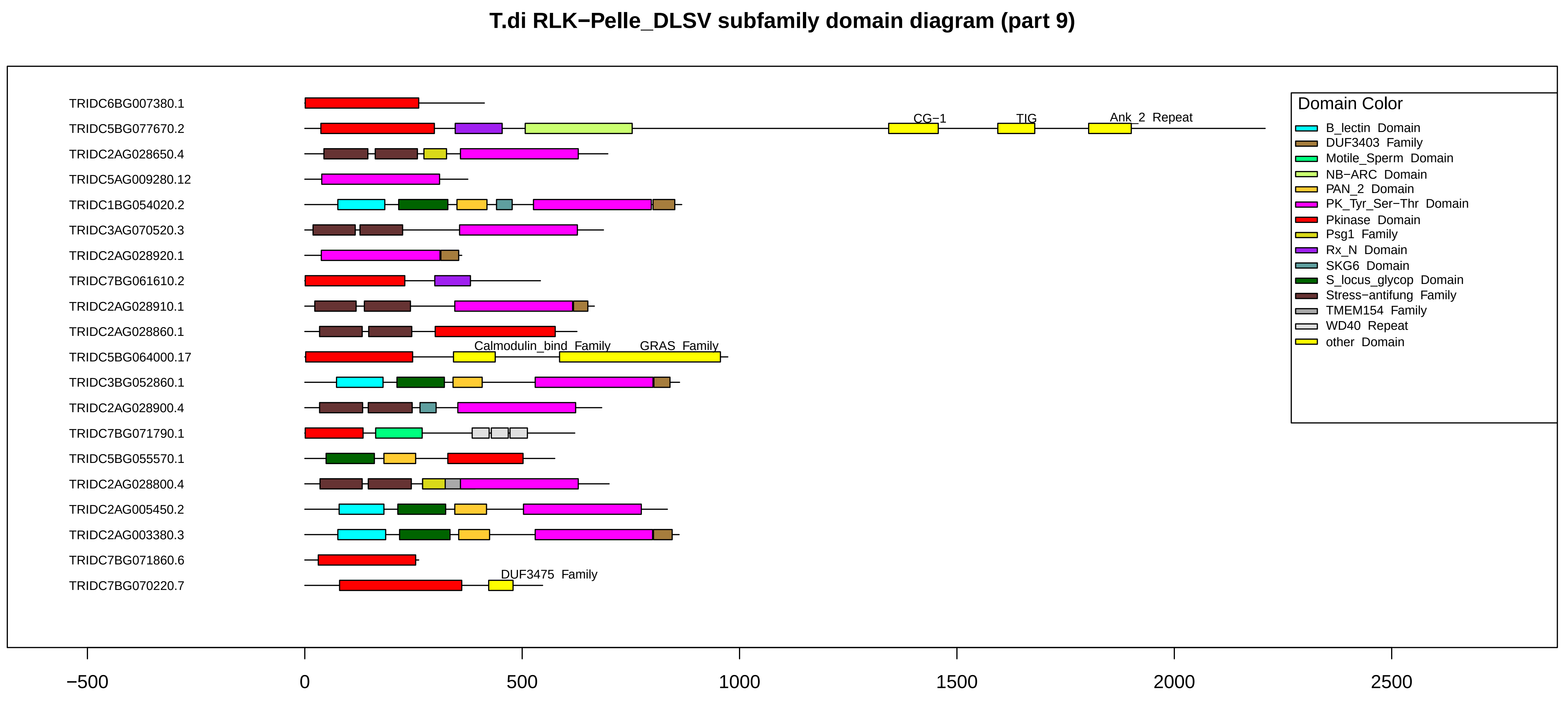

T.di RLK−Pelle_DLSV subfamily domain diagram (part 9)
TRIDC6BG007380.1
TRIDC5BG077670.2
TRIDC2AG028650.4
TRIDC5AG009280.12
TRIDC1BG054020.2
TRIDC3AG070520.3
TRIDC2AG028920.1
TRIDC7BG061610.2
TRIDC2AG028910.1
TRIDC2AG028860.1
TRIDC5BG064000.17
TRIDC3BG052860.1
TRIDC2AG028900.4
TRIDC7BG071790.1
TRIDC5BG055570.1
TRIDC2AG028800.4
TRIDC2AG005450.2
TRIDC2AG003380.3
TRIDC7BG071860.6
TRIDC7BG070220.7
CG−1
TIG
Ank_2 Repeat
Calmodulin_bind Family
GRAS Family
DUF3475 Family
Domain Color
B_lectin Domain
DUF3403 Family
Motile_Sperm Domain
NB−ARC Domain
PAN_2 Domain
PK_Tyr_Ser−Thr Domain
Pkinase Domain
Psg1 Family
Rx_N Domain
SKG6 Domain
S_locus_glycop Domain
Stress−antifung Family
TMEM154 Family
WD40 Repeat
other Domain
−500
0
500
1000
1500
2000
2500

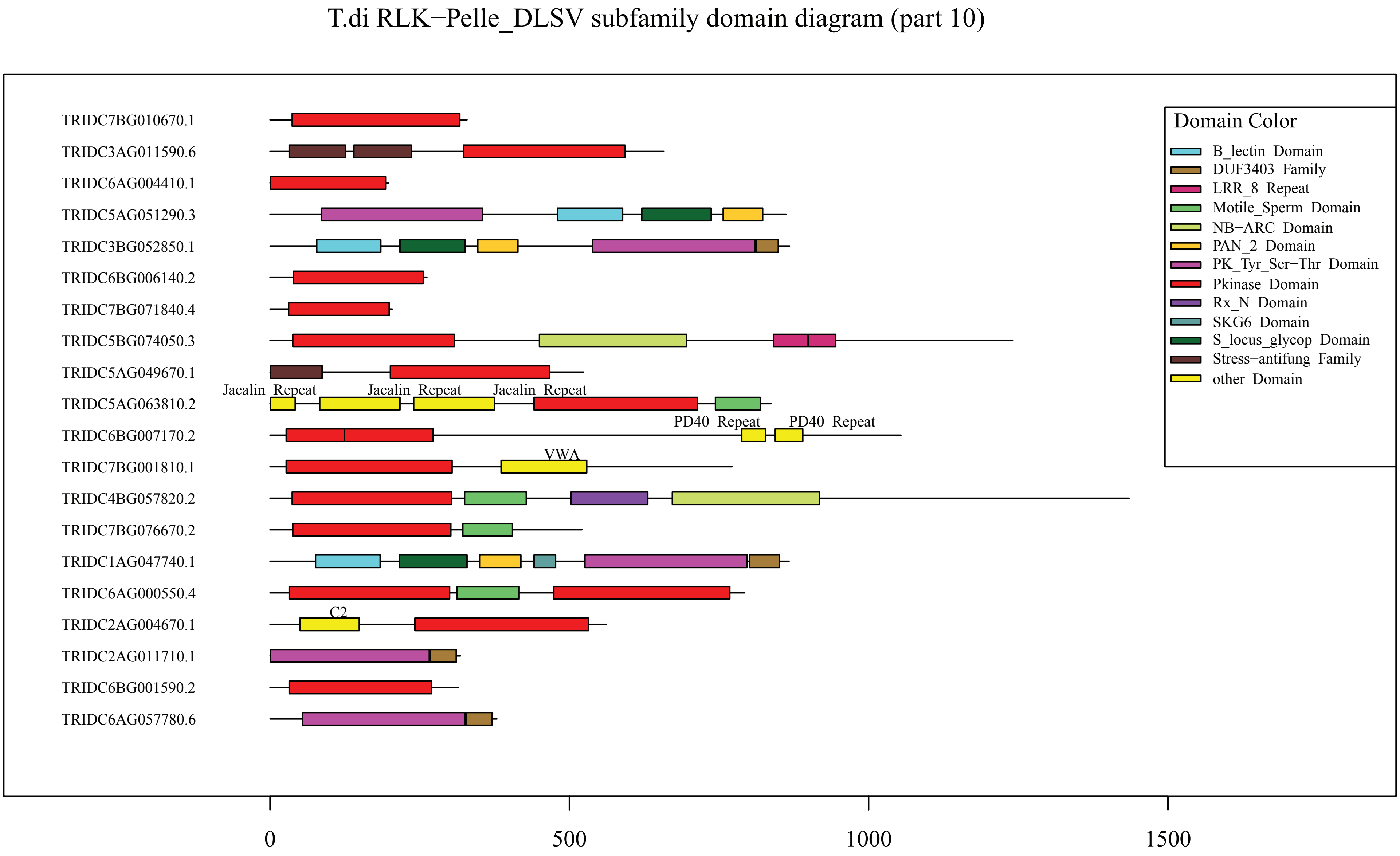
T.di RLK−Pelle_DLSV subfamily domain diagram (part 10)
TRIDC7BG010670.1
TRIDC3AG011590.6
TRIDC6AG004410.1
TRIDC5AG051290.3
TRIDC3BG052850.1
TRIDC6BG006140.2
TRIDC7BG071840.4
TRIDC5BG074050.3
TRIDC5AG049670.1
TRIDC5AG063810.2
TRIDC6BG007170.2
TRIDC7BG001810.1
TRIDC4BG057820.2
TRIDC7BG076670.2
TRIDC1AG047740.1
TRIDC6AG000550.4
TRIDC2AG004670.1
TRIDC2AG011710.1
TRIDC6BG001590.2
TRIDC6AG057780.6
Jacalin Repeat
Jacalin Repeat
Jacalin Repeat
PD40 Repeat
PD40 Repeat
VWA
C2
Domain Color
B_lectin Domain
DUF3403 Family
LRR_8 Repeat
Motile_Sperm Domain
NB−ARC Domain
PAN_2 Domain
PK_Tyr_Ser−Thr Domain
Pkinase Domain
Rx_N Domain
SKG6 Domain
S_locus_glycop Domain
Stress−antifung Family
other Domain
0
500
1000
1500

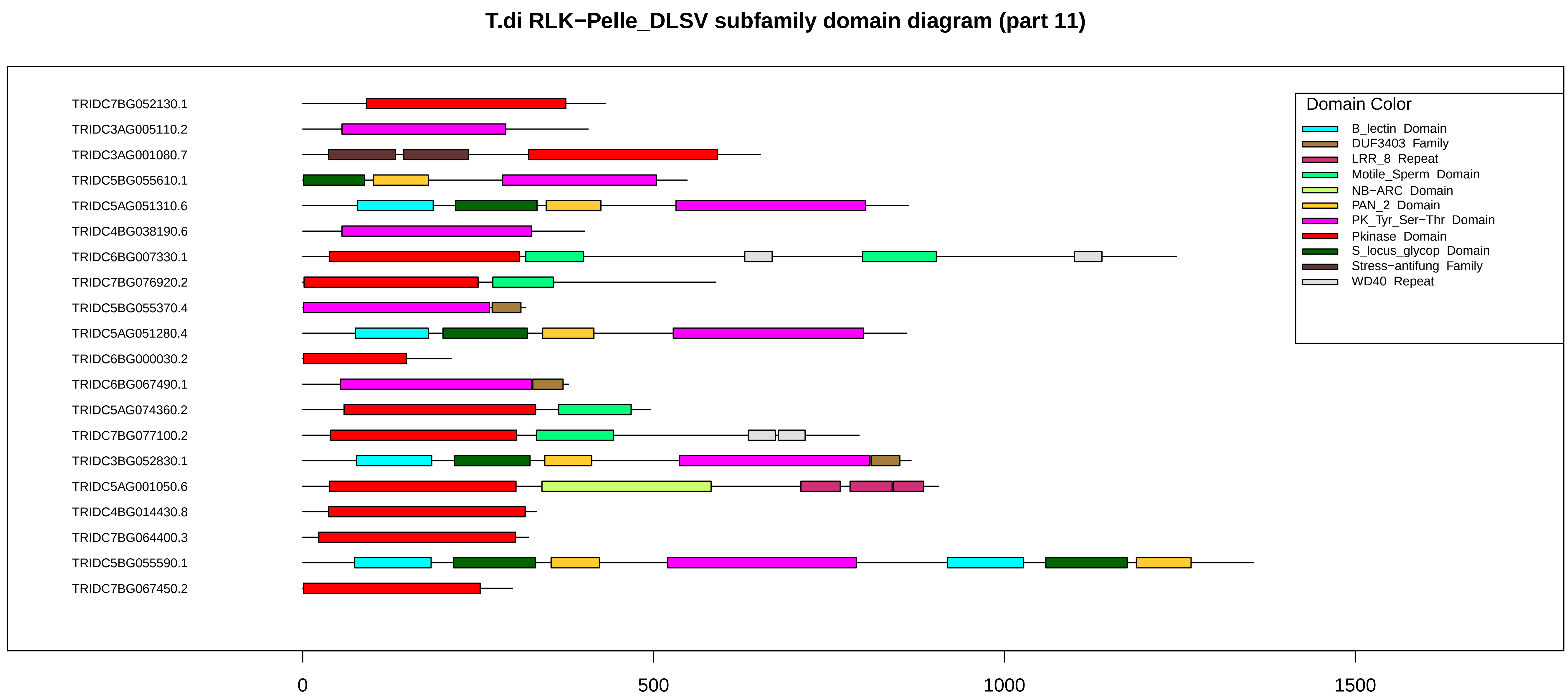
T.di RLK−Pelle_DLSV subfamily domain diagram (part 11)
TRIDC7BG052130.1
TRIDC3AG005110.2
TRIDC3AG001080.7
TRIDC5BG055610.1
TRIDC5AG051310.6
TRIDC4BG038190.6
TRIDC6BG007330.1
TRIDC7BG076920.2
TRIDC5BG055370.4
TRIDC5AG051280.4
TRIDC6BG000030.2
TRIDC6BG067490.1
TRIDC5AG074360.2
TRIDC7BG077100.2
TRIDC3BG052830.1
TRIDC5AG001050.6
TRIDC4BG014430.8
TRIDC7BG064400.3
TRIDC5BG055590.1
TRIDC7BG067450.2
Domain Color
B_lectin Domain
DUF3403 Family
LRR_8 Repeat
Motile_Sperm Domain
NB−ARC Domain
PAN_2 Domain
PK_Tyr_Ser−Thr Domain
Pkinase Domain
S_locus_glycop Domain
Stress−antifung Family
WD40 Repeat
0
500
1000
1500

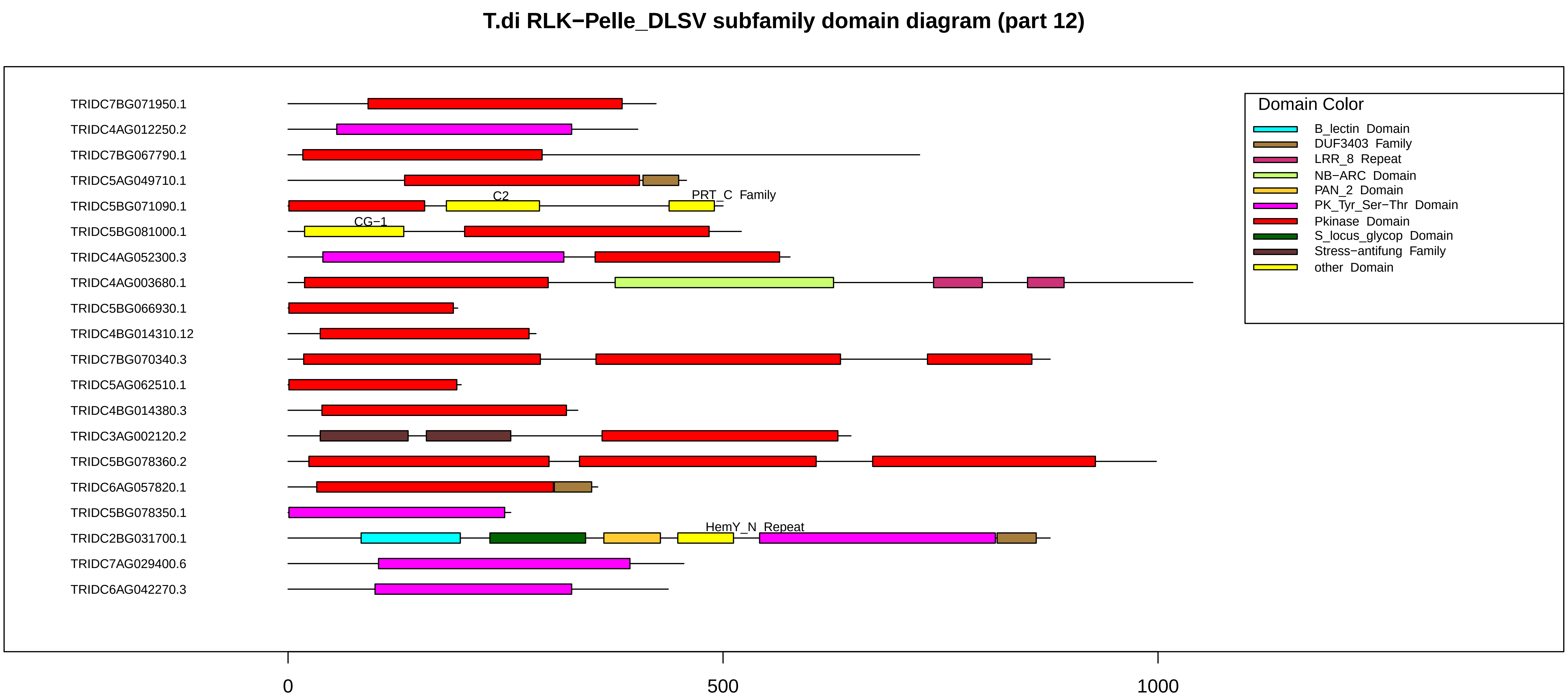
T.di RLK−Pelle_DLSV subfamily domain diagram (part 12)
TRIDC7BG071950.1
TRIDC4AG012250.2
TRIDC7BG067790.1
TRIDC5AG049710.1
TRIDC5BG071090.1
TRIDC5BG081000.1
TRIDC4AG052300.3
TRIDC4AG003680.1
TRIDC5BG066930.1
TRIDC4BG014310.12
TRIDC7BG070340.3
TRIDC5AG062510.1
TRIDC4BG014380.3
TRIDC3AG002120.2
TRIDC5BG078360.2
TRIDC6AG057820.1
TRIDC5BG078350.1
TRIDC2BG031700.1
TRIDC7AG029400.6
TRIDC6AG042270.3
C2
PRT_C Family
CG−1
HemY_N Repeat
Domain Color
B_lectin Domain
DUF3403 Family
LRR_8 Repeat
NB−ARC Domain
PAN_2 Domain
PK_Tyr_Ser−Thr Domain
Pkinase Domain
S_locus_glycop Domain
Stress−antifung Family
other Domain
0
500
1000

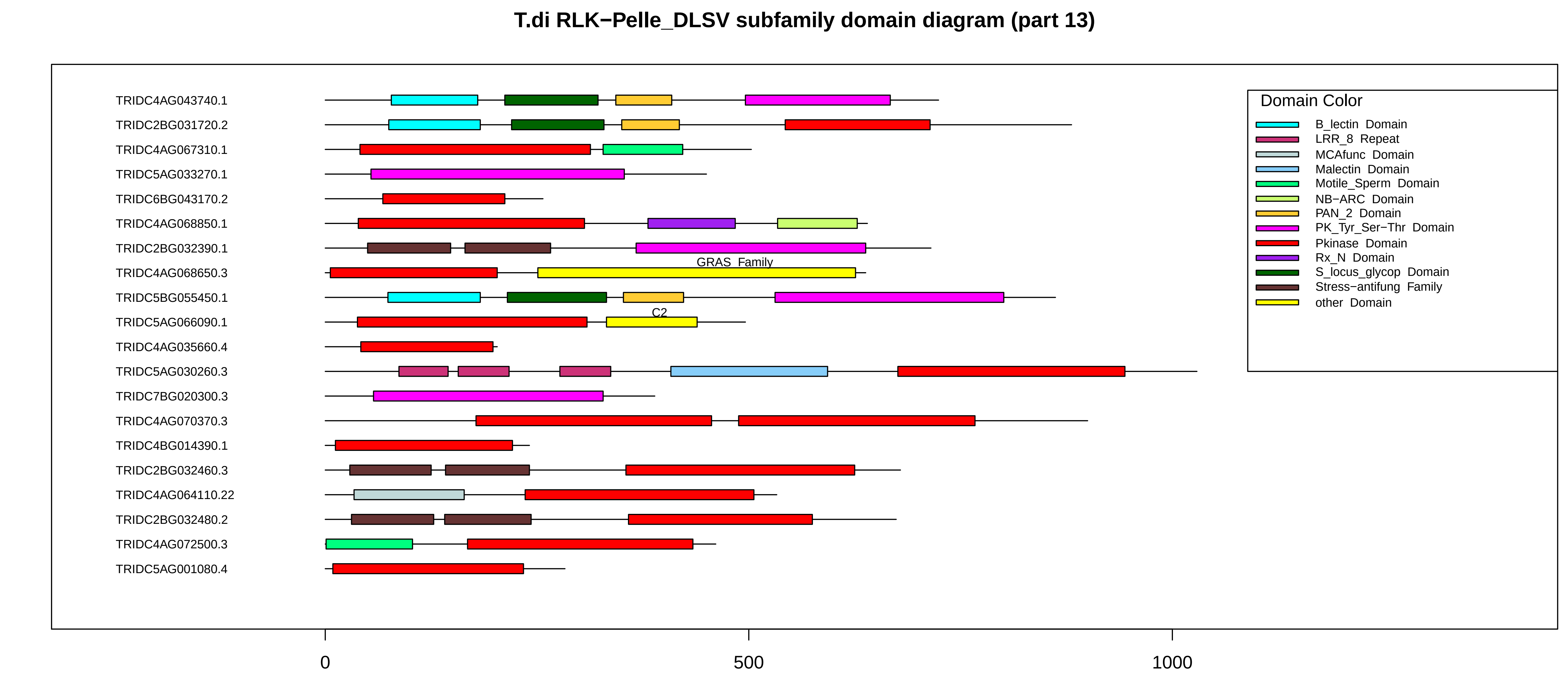
T.di RLK−Pelle_DLSV subfamily domain diagram (part 13)
TRIDC4AG043740.1
TRIDC2BG031720.2
TRIDC4AG067310.1
TRIDC5AG033270.1
TRIDC6BG043170.2
TRIDC4AG068850.1
TRIDC2BG032390.1
TRIDC4AG068650.3
GRAS_Family
TRIDC5BG055450.1
TRIDC5AG066090.1
C2
TRIDC4AG035660.4
TRIDC5AG030260.3
TRIDC7BG020300.3
TRIDC4AG070370.3
TRIDC4BG014390.1
TRIDC2BG032460.3
TRIDC4AG064110.22
TRIDC2BG032480.2
TRIDC4AG072500.3
TRIDC5AG001080.4
Domain Color
B_lectin Domain
LRR_8 Repeat
MCAfunc Domain
Malectin Domain
Motile_Sperm Domain
NB−ARC Domain
PAN_2 Domain
PK_Tyr_Ser−Thr Domain
Pkinase Domain
Rx_N Domain
S_locus_glycop Domain
Stress−antifung Family
other Domain
0
500
1000

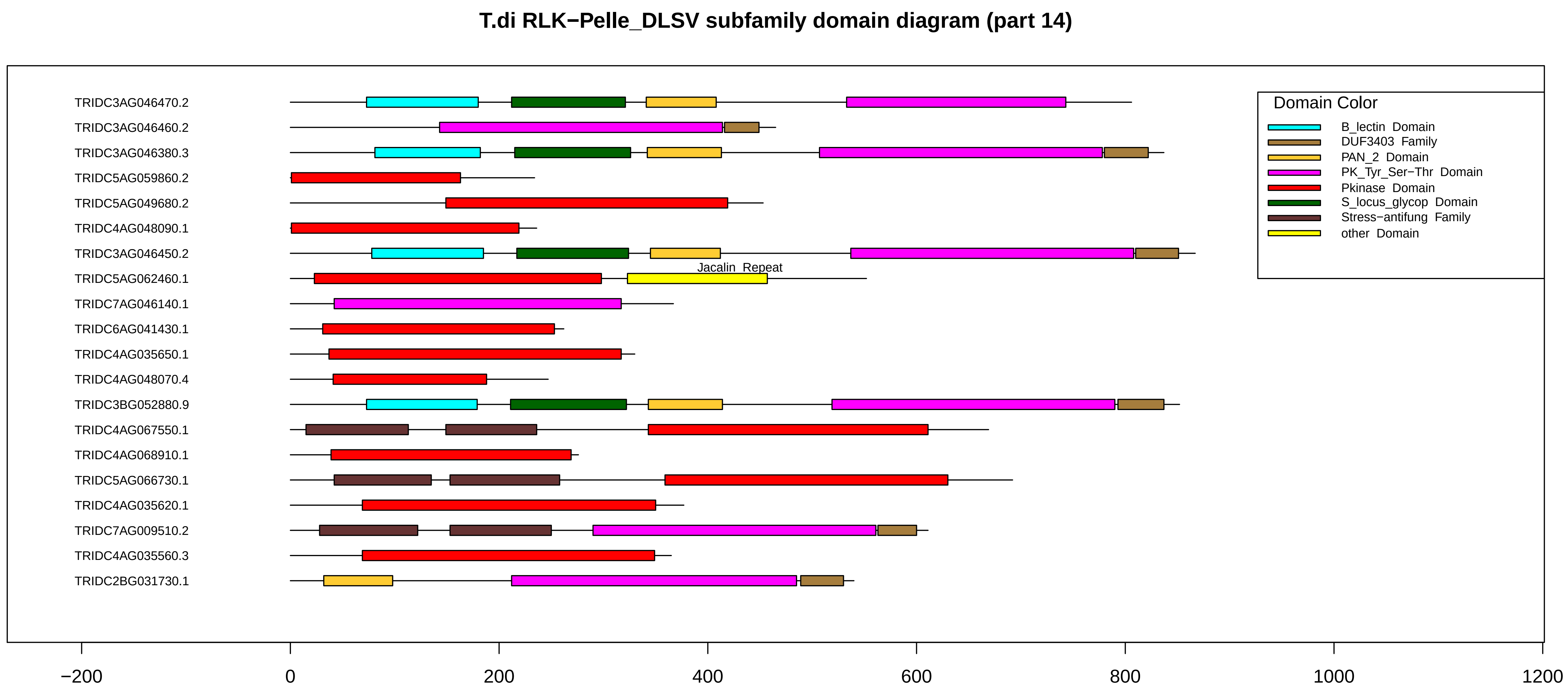
T.di RLK−Pelle_DLSV subfamily domain diagram (part 14)
TRIDC3AG046470.2
TRIDC3AG046460.2
TRIDC3AG046380.3
TRIDC5AG059860.2
TRIDC5AG049680.2
TRIDC4AG048090.1
TRIDC3AG046450.2
TRIDC5AG062460.1
TRIDC7AG046140.1
TRIDC6AG041430.1
TRIDC4AG035650.1
TRIDC4AG048070.4
TRIDC3BG052880.9
TRIDC4AG067550.1
TRIDC4AG068910.1
TRIDC5AG066730.1
TRIDC4AG035620.1
TRIDC7AG009510.2
TRIDC4AG035560.3
TRIDC2BG031730.1
Jacalin Repeat
Domain Color
B_lectin Domain
DUF3403 Family
PAN_2 Domain
PK_Tyr_Ser−Thr Domain
Pkinase Domain
S_locus_glycop Domain
Stress−antifung Family
other Domain
−200
0
200
400
600
800
1000
1200

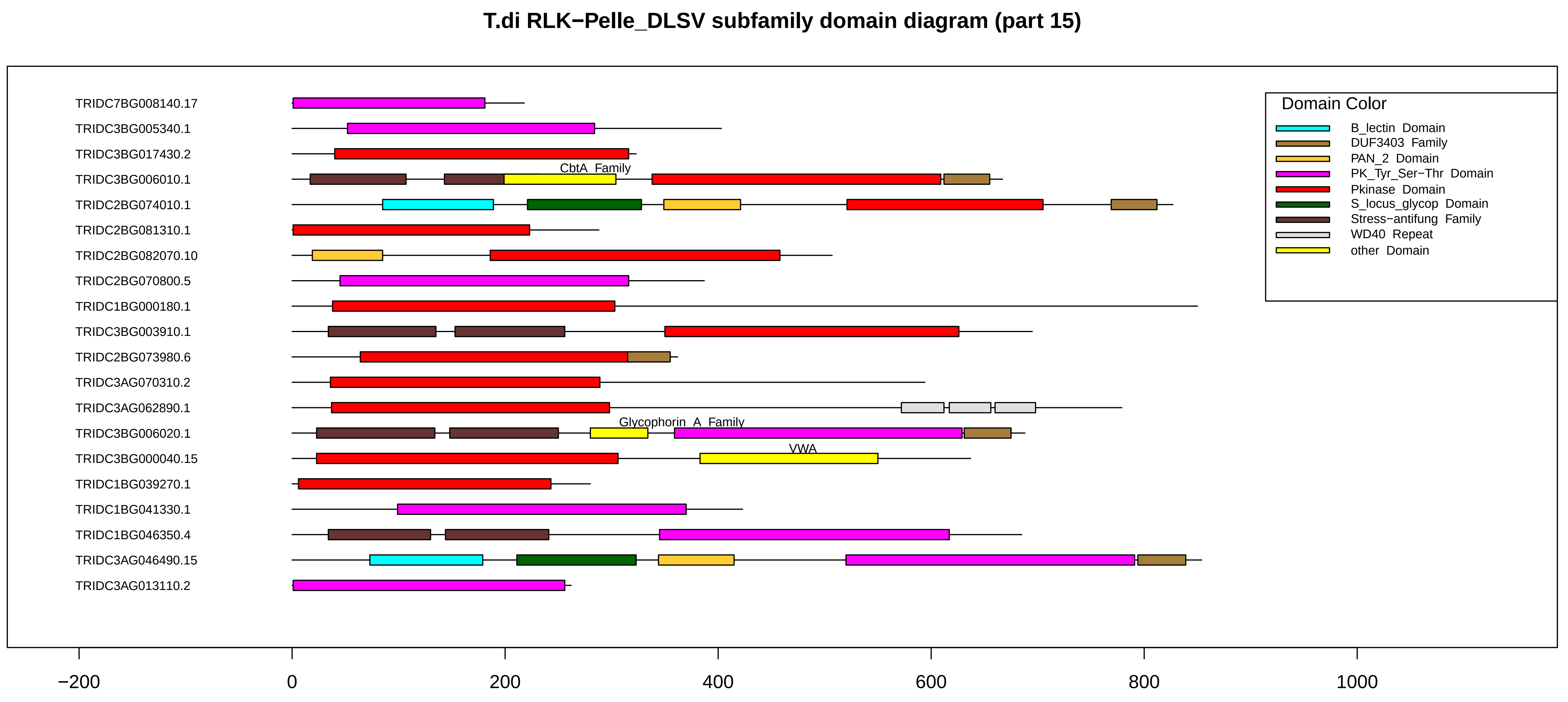

T.di RLK−Pelle_DLSV subfamily domain diagram (part 15)
TRIDC7BG008140.17
TRIDC3BG005340.1
TRIDC3BG017430.2
TRIDC3BG006010.1
TRIDC2BG074010.1
TRIDC2BG081310.1
TRIDC2BG082070.10
TRIDC2BG070800.5
TRIDC1BG000180.1
TRIDC3BG003910.1
TRIDC2BG073980.6
TRIDC3AG070310.2
TRIDC3AG062890.1
TRIDC3BG006020.1
TRIDC3BG000040.15
TRIDC1BG039270.1
TRIDC1BG041330.1
TRIDC1BG046350.4
TRIDC3AG046490.15
TRIDC3AG013110.2
CbtA Family
Glycophorin A Family
VWA
Domain Color
B_lectin Domain
DUF3403 Family
PAN_2 Domain
PK_Tyr_Ser−Thr Domain
Pkinase Domain
S_locus_glycop Domain
Stress−antifung Family
WD40 Repeat
other Domain
−200
0
200
400
600
800
1000

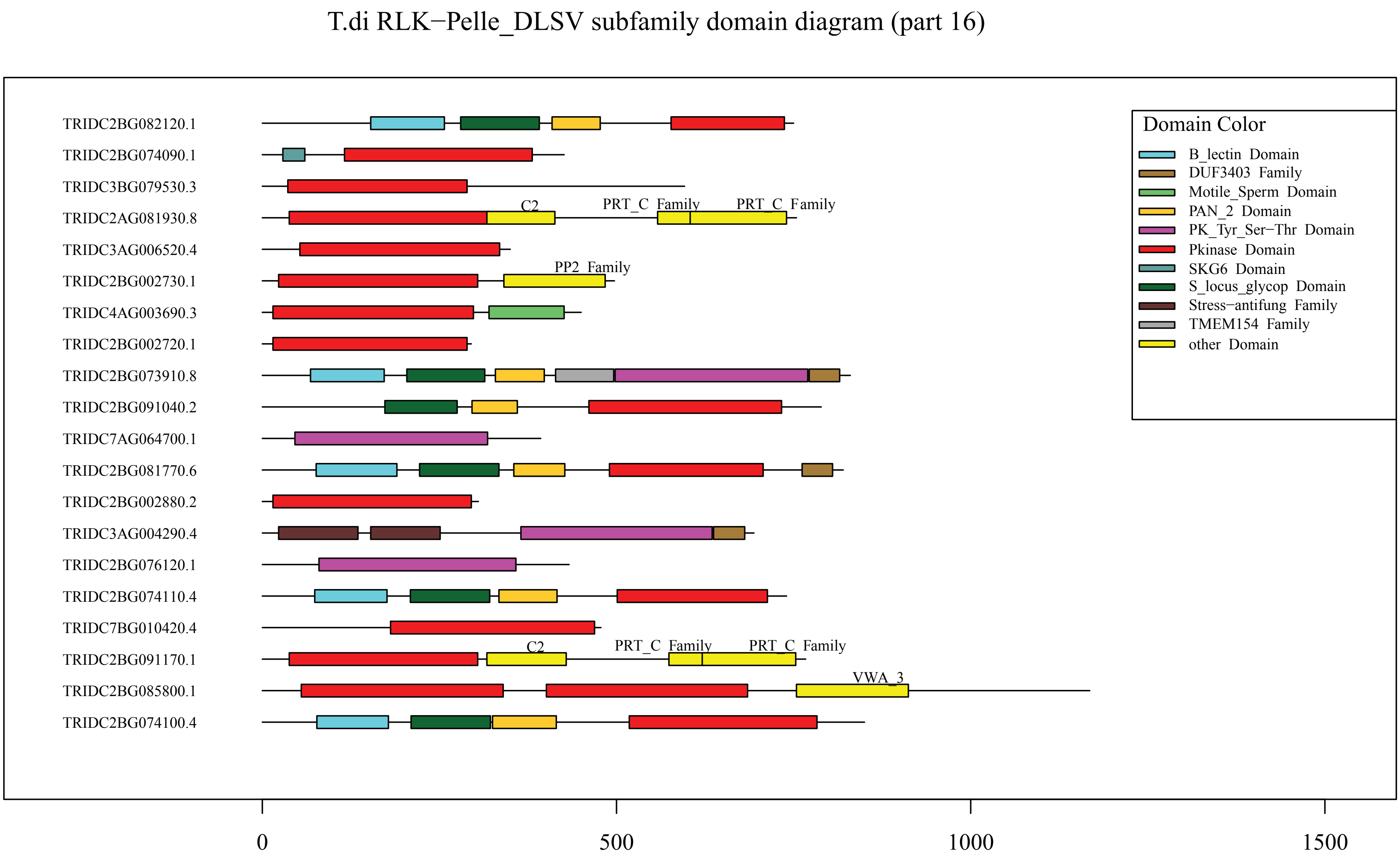

T.di RLK−Pelle_DLSV subfamily domain diagram (part 16)
TRIDC2BG082120.1
TRIDC2BG074090.1
TRIDC3BG079530.3
TRIDC2AG081930.8
TRIDC3AG006520.4
TRIDC2BG002730.1
TRIDC4AG003690.3
TRIDC2BG002720.1
TRIDC2BG073910.8
TRIDC2BG091040.2
TRIDC7AG064700.1
TRIDC2BG081770.6
TRIDC2BG002880.2
TRIDC3AG004290.4
TRIDC2BG076120.1
TRIDC2BG074110.4
TRIDC7BG010420.4
TRIDC2BG091170.1
TRIDC2BG085800.1
TRIDC2BG074100.4
C2
PRT_C Family
PRT_C Family
PP2 Family
C2
PRT_C Family
PRT_C Family
VWA_3
Domain Color
B_lectin Domain
DUF3403 Family
Motile_Sperm Domain
PAN_2 Domain
PK_Tyr_Ser−Thr Domain
Pkinase Domain
SKG6 Domain
S_locus_glycop Domain
Stress−antifung Family
TMEM154 Family
other Domain
0
500
1000
1500

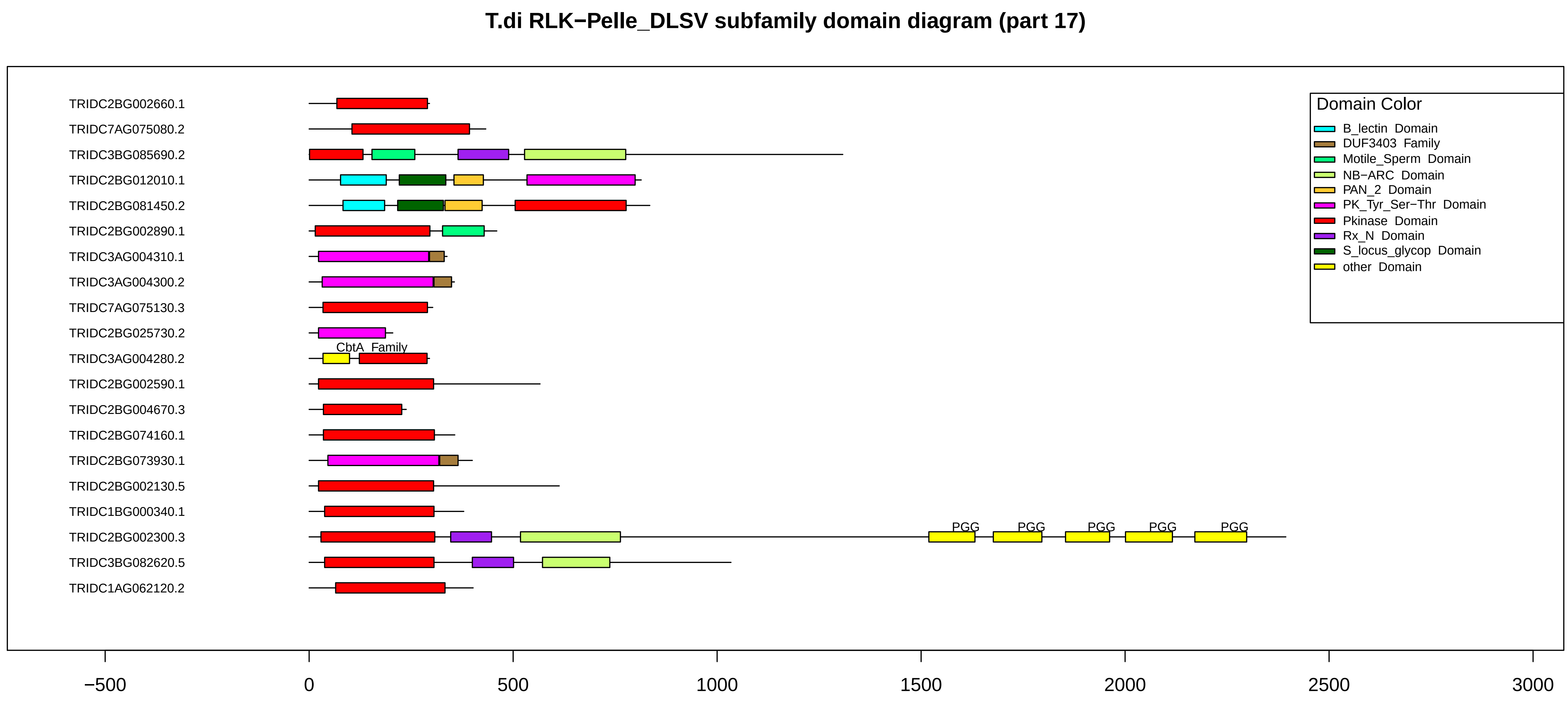
T.di RLK−Pelle_DLSV subfamily domain diagram (part 17)
TRIDC2BG002660.1
TRIDC7AG075080.2
TRIDC3BG085690.2
TRIDC2BG012010.1
TRIDC2BG081450.2
TRIDC2BG002890.1
TRIDC3AG004310.1
TRIDC3AG004300.2
TRIDC7AG075130.3
TRIDC2BG025730.2
TRIDC3AG004280.2
CbtA_Family
TRIDC2BG002590.1
TRIDC2BG004670.3
TRIDC2BG074160.1
TRIDC2BG073930.1
TRIDC2BG002130.5
TRIDC1BG000340.1
TRIDC2BG002300.3
PGG
PGG
PGG
PGG
PGG
TRIDC3BG082620.5
TRIDC1AG062120.2
Domain Color
B_lectin Domain
DUF3403 Family
Motile_Sperm Domain
NB−ARC Domain
PAN_2 Domain
PK_Tyr_Ser−Thr Domain
Pkinase Domain
Rx_N Domain
S_locus_glycop Domain
other Domain
−500
0
500
1000
1500
2000
2500
3000

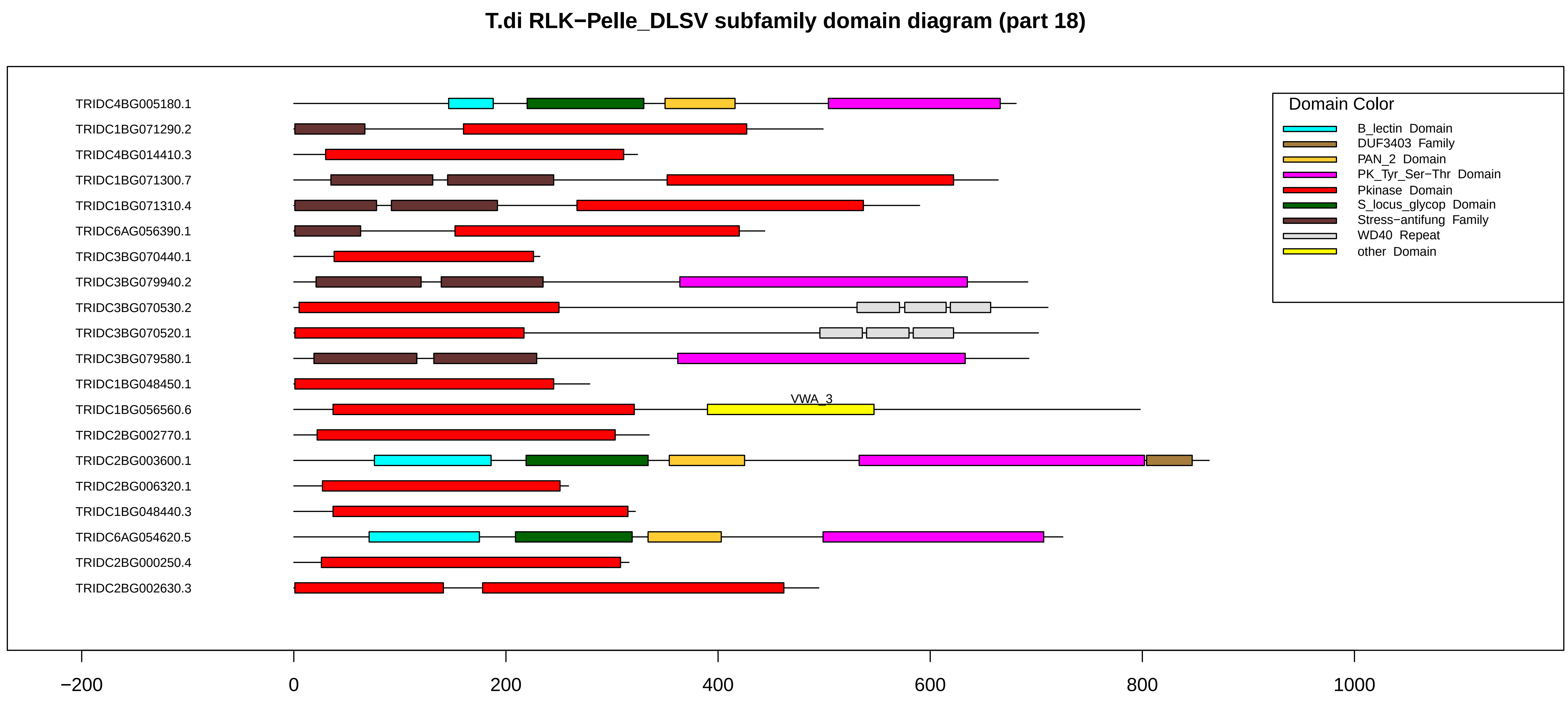

T.di RLK−Pelle_DLSV subfamily domain diagram (part 18)
TRIDC4BG005180.1
TRIDC1BG071290.2
TRIDC4BG014410.3
TRIDC1BG071300.7
TRIDC1BG071310.4
TRIDC6AG056390.1
TRIDC3BG070440.1
TRIDC3BG079940.2
TRIDC3BG070530.2
TRIDC3BG070520.1
TRIDC3BG079580.1
TRIDC1BG048450.1
TRIDC1BG056560.6
TRIDC2BG002770.1
TRIDC2BG003600.1
TRIDC2BG006320.1
TRIDC1BG048440.3
TRIDC6AG054620.5
TRIDC2BG000250.4
TRIDC2BG002630.3
VWA_3
Domain Color
B_lectin Domain
DUF3403 Family
PAN_2 Domain
PK_Tyr_Ser−Thr Domain
Pkinase Domain
S_locus_glycop Domain
Stress−antifung Family
WD40 Repeat
other Domain
−200
0
200
400
600
800
1000

T.di RLK−Pelle_DLSV subfamily domain diagram (part 19)

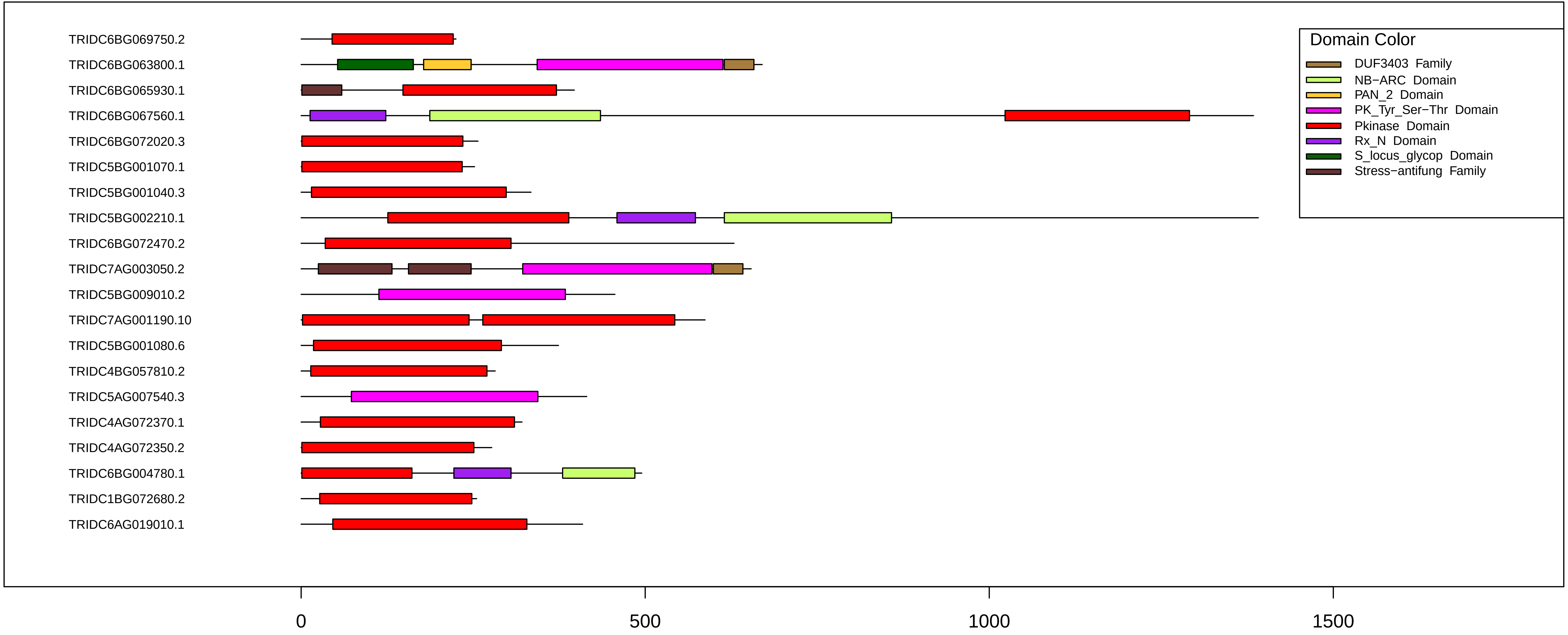

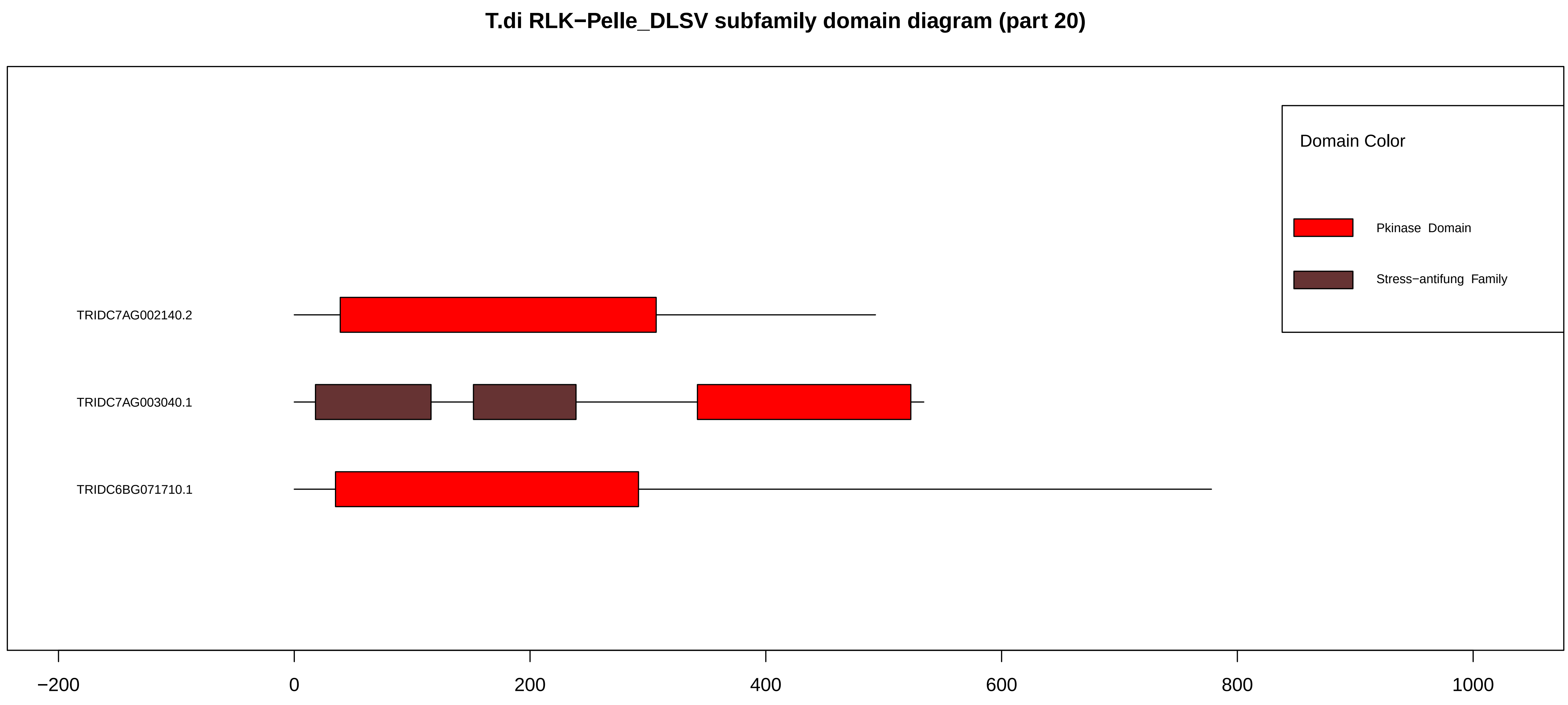
T.di RLK−Pelle_DLSV subfamily domain diagram (part 20)
Domain Color
Pkinase Domain
Stress−antifung Family
TRIDC7AG002140.2
TRIDC7AG003040.1
TRIDC6BG071710.1
−200
0
200
400
600
800
1000

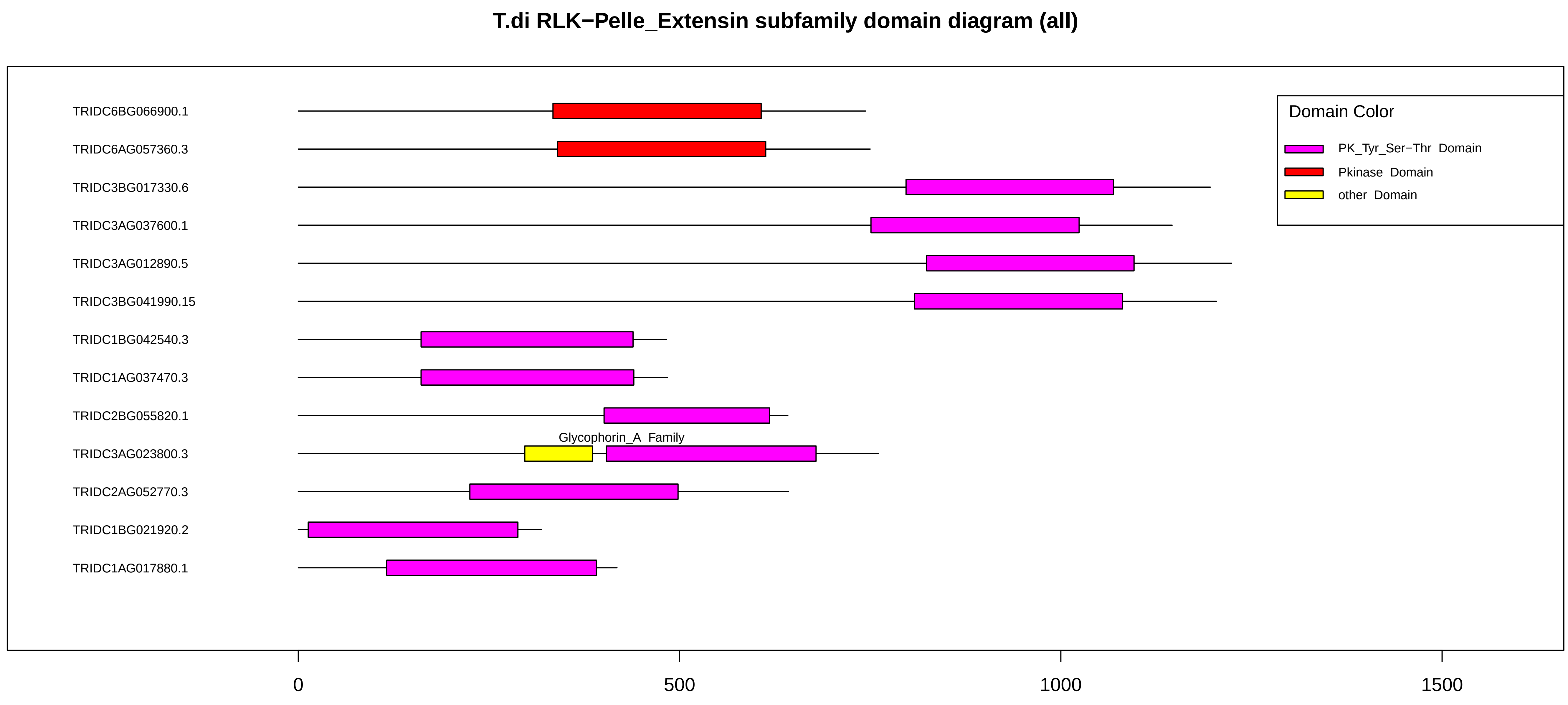
T.di RLK−Pelle_Extensin subfamily domain diagram (all)
TRIDC6BG066900.1
TRIDC6AG057360.3
TRIDC3BG017330.6
TRIDC3AG037600.1
TRIDC3AG012890.5
TRIDC3BG041990.15
TRIDC1BG042540.3
TRIDC1AG037470.3
TRIDC2BG055820.1
Glycophorin_A Family
TRIDC3AG023800.3
TRIDC2AG052770.3
TRIDC1BG021920.2
TRIDC1AG017880.1
Domain Color
PK_Tyr_Ser−Thr Domain
Pkinase Domain
other Domain
0
500
1000
1500

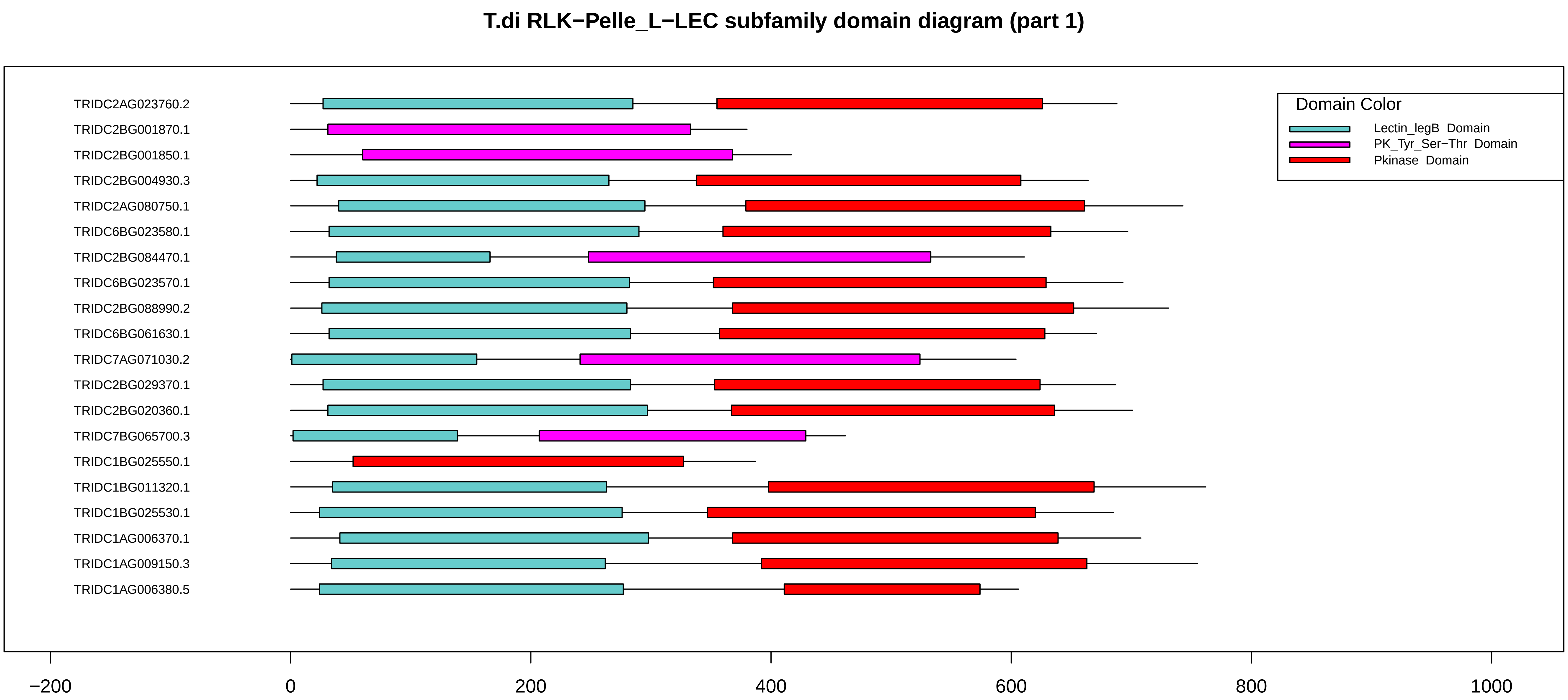

T.di RLK−Pelle_L−LEC subfamily domain diagram (part 1)
TRIDC2AG023760.2
TRIDC2BG001870.1
TRIDC2BG001850.1
TRIDC2BG004930.3
TRIDC2AG080750.1
TRIDC6BG023580.1
TRIDC2BG084470.1
TRIDC6BG023570.1
TRIDC2BG088990.2
TRIDC6BG061630.1
TRIDC7AG071030.2
TRIDC2BG029370.1
TRIDC2BG020360.1
TRIDC7BG065700.3
TRIDC1BG025550.1
TRIDC1BG011320.1
TRIDC1BG025530.1
TRIDC1AG006370.1
TRIDC1AG009150.3
TRIDC1AG006380.5
Domain Color
Lectin_legB Domain
PK_Tyr_Ser−Thr Domain
Pkinase Domain
−200
0
200
400
600
800
1000

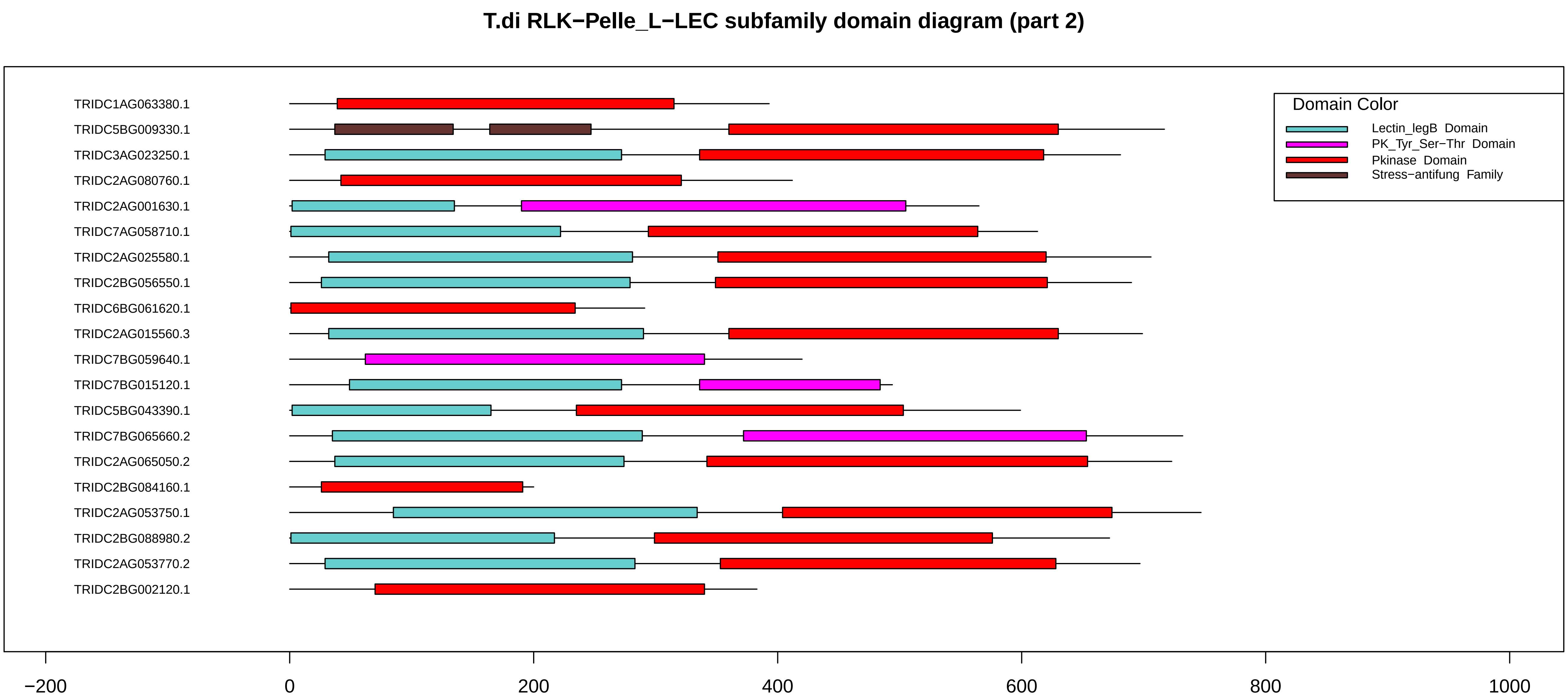
T.di RLK−Pelle_L−LEC subfamily domain diagram (part 2)
TRIDC1AG063380.1
TRIDC5BG009330.1
TRIDC3AG023250.1
TRIDC2AG080760.1
TRIDC2AG001630.1
TRIDC7AG058710.1
TRIDC2AG025580.1
TRIDC2BG056550.1
TRIDC6BG061620.1
TRIDC2AG015560.3
TRIDC7BG059640.1
TRIDC7BG015120.1
TRIDC5BG043390.1
TRIDC7BG065660.2
TRIDC2AG065050.2
TRIDC2BG084160.1
TRIDC2AG053750.1
TRIDC2BG088980.2
TRIDC2AG053770.2
TRIDC2BG002120.1
Domain Color
Lectin_legB Domain
PK_Tyr_Ser−Thr Domain
Pkinase Domain
Stress−antifung Family
−200
0
200
400
600
800
1000

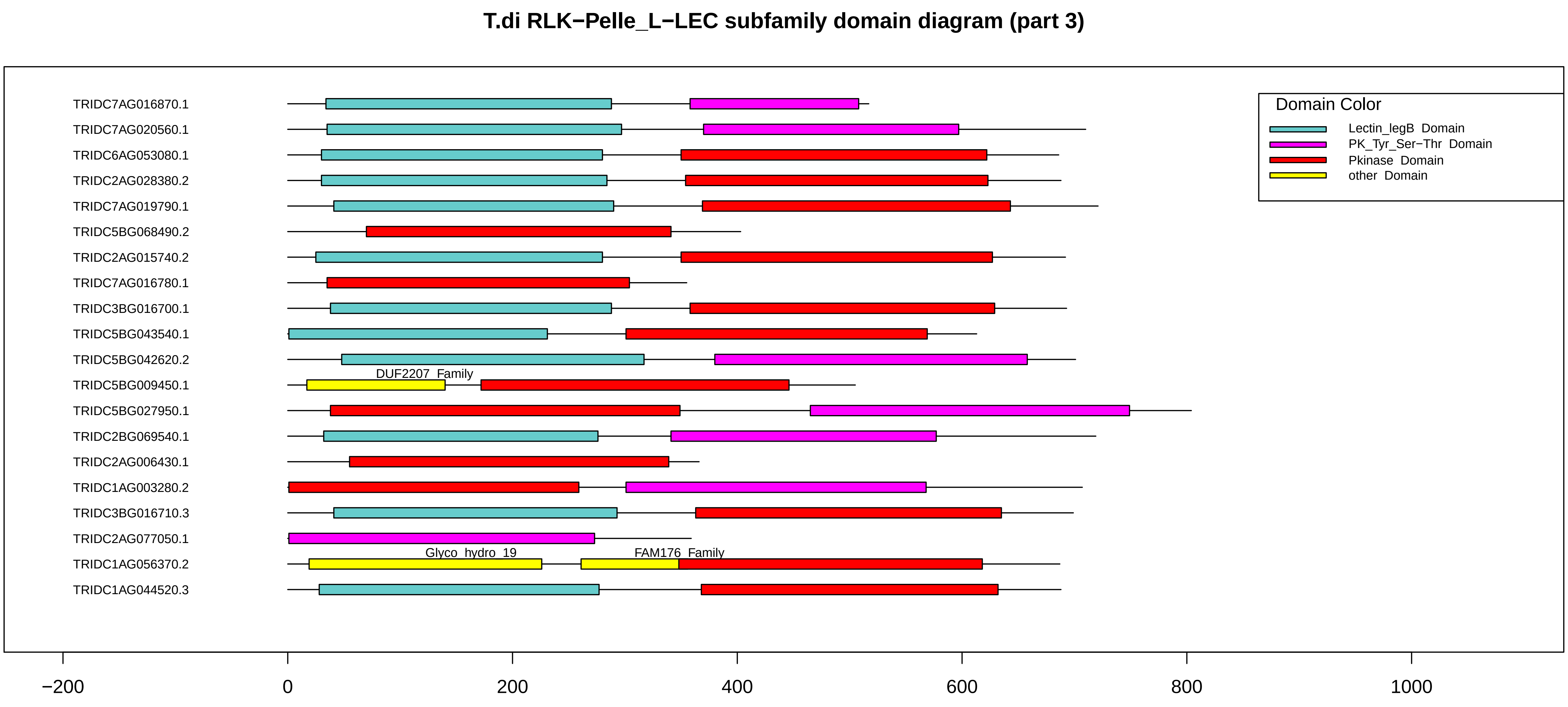
T.di RLK−Pelle_L−LEC subfamily domain diagram (part 3)
TRIDC7AG016870.1
TRIDC7AG020560.1
TRIDC6AG053080.1
TRIDC2AG028380.2
TRIDC7AG019790.1
TRIDC5BG068490.2
TRIDC2AG015740.2
TRIDC7AG016780.1
TRIDC3BG016700.1
TRIDC5BG043540.1
TRIDC5BG042620.2
TRIDC5BG009450.1
TRIDC5BG027950.1
TRIDC2BG069540.1
TRIDC2AG006430.1
TRIDC1AG003280.2
TRIDC3BG016710.3
TRIDC2AG077050.1
TRIDC1AG056370.2
TRIDC1AG044520.3
DUF2207_Family
Glyco_hydro_19
FAM176_Family
Domain Color
Lectin_legB Domain
PK_Tyr_Ser−Thr Domain
Pkinase Domain
other Domain
−200
0
200
400
600
800
1000

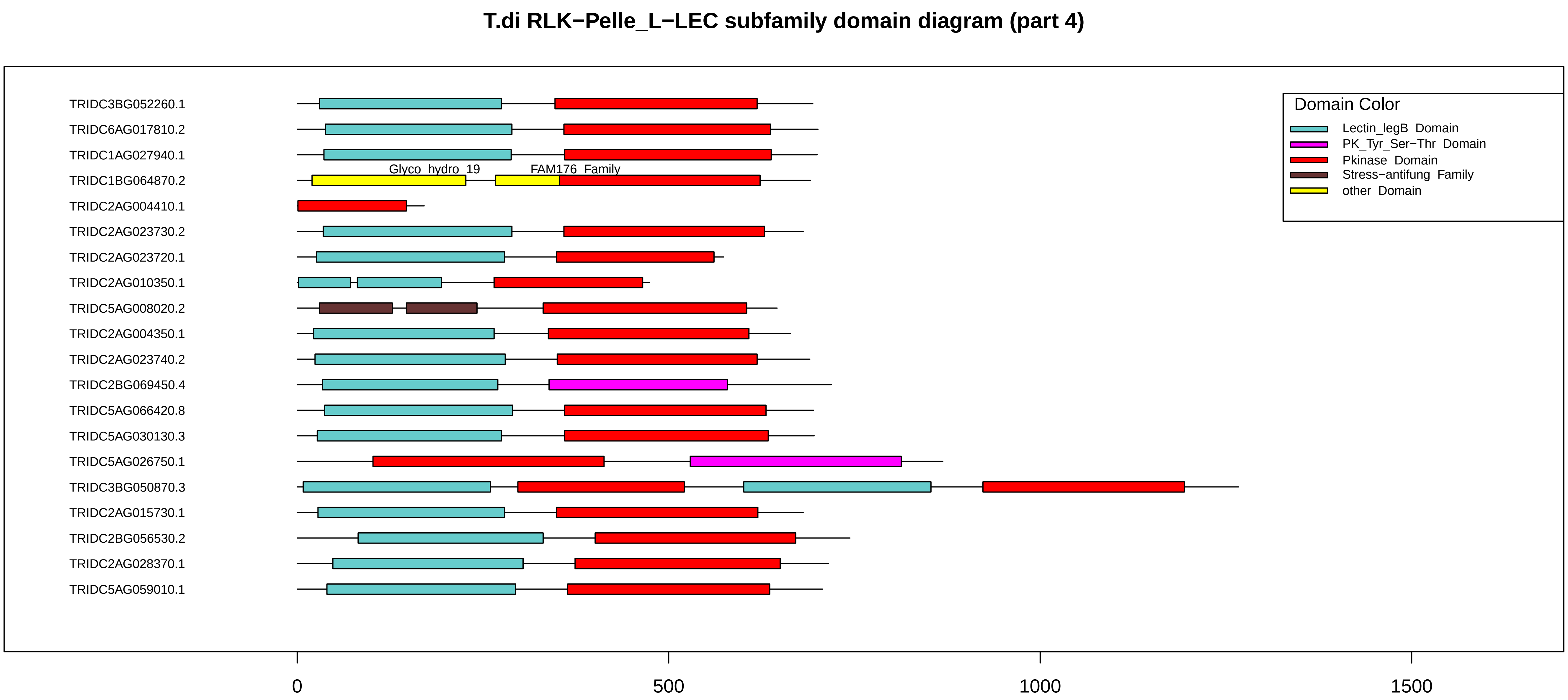
T.di RLK−Pelle_L−LEC subfamily domain diagram (part 4)
TRIDC3BG052260.1
TRIDC6AG017810.2
TRIDC1AG027940.1
TRIDC1BG064870.2
TRIDC2AG004410.1
TRIDC2AG023730.2
TRIDC2AG023720.1
TRIDC2AG010350.1
TRIDC5AG008020.2
TRIDC2AG004350.1
TRIDC2AG023740.2
TRIDC2BG069450.4
TRIDC5AG066420.8
TRIDC5AG030130.3
TRIDC5AG026750.1
TRIDC3BG050870.3
TRIDC2AG015730.1
TRIDC2BG056530.2
TRIDC2AG028370.1
TRIDC5AG059010.1
Glyco_hydro_19
FAM176_Family
Domain Color
Lectin_legB Domain
PK_Tyr_Ser−Thr Domain
Pkinase Domain
Stress−antifung Family
other Domain
0
500
1000
1500

T.di RLK−Pelle_L−LEC subfamily domain diagram (part 5)

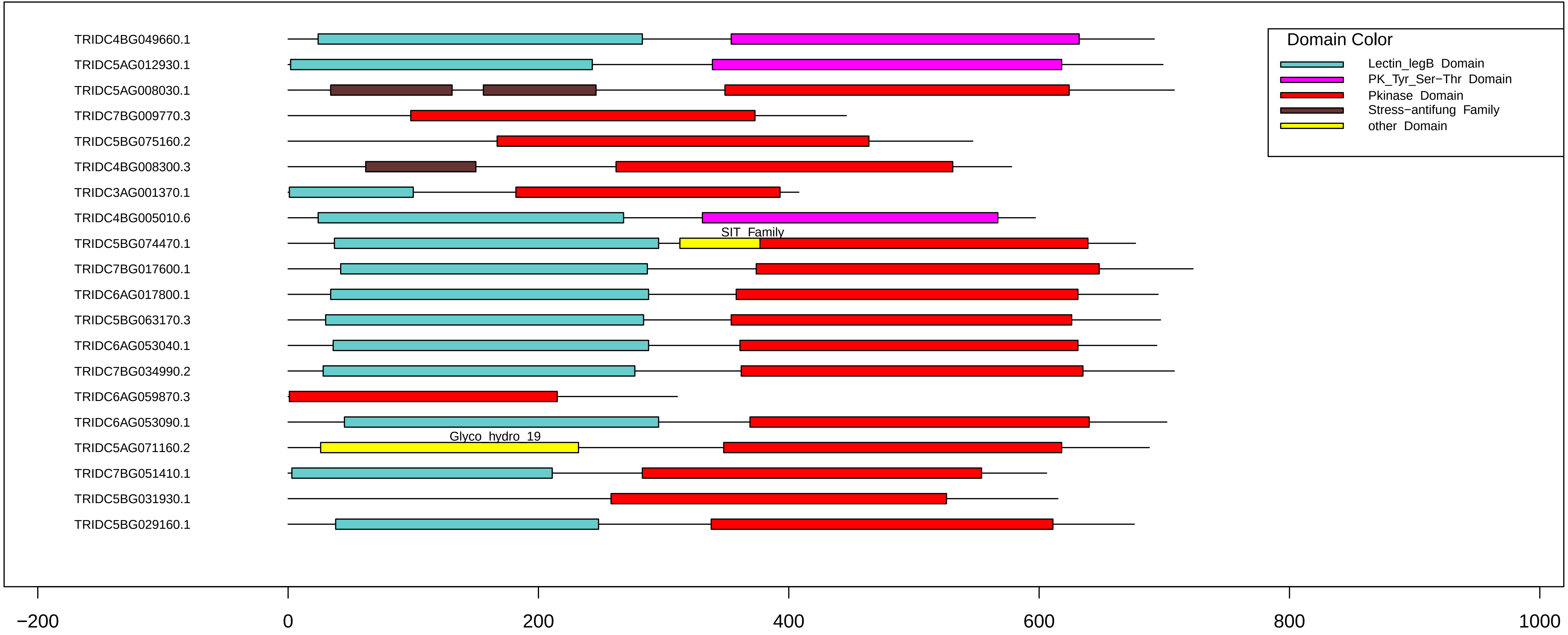

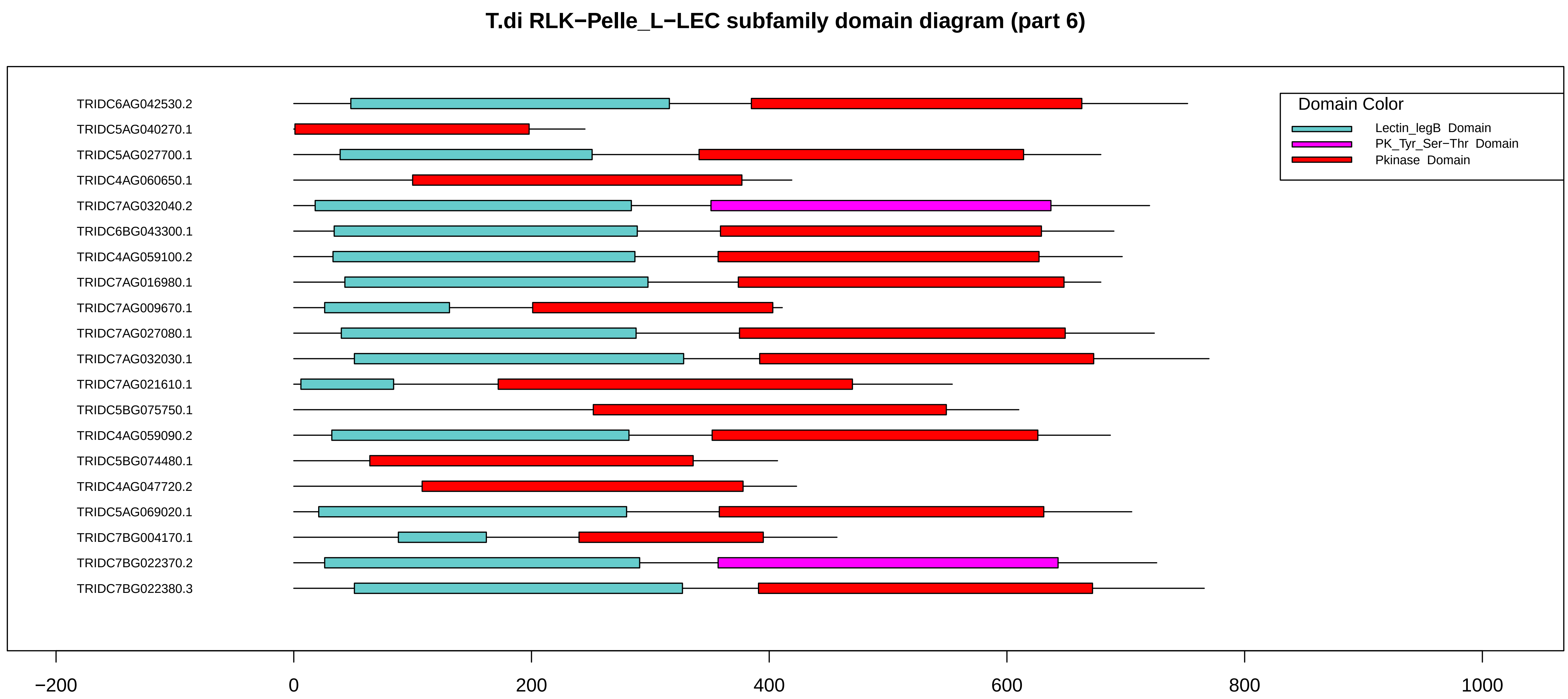

T.di RLK−Pelle_L−LEC subfamily domain diagram (part 6)
TRIDC6AG042530.2
TRIDC5AG040270.1
TRIDC5AG027700.1
TRIDC4AG060650.1
TRIDC7AG032040.2
TRIDC6BG043300.1
TRIDC4AG059100.2
TRIDC7AG016980.1
TRIDC7AG009670.1
TRIDC7AG027080.1
TRIDC7AG032030.1
TRIDC7AG021610.1
TRIDC5BG075750.1
TRIDC4AG059090.2
TRIDC5BG074480.1
TRIDC4AG047720.2
TRIDC5AG069020.1
TRIDC7BG004170.1
TRIDC7BG022370.2
TRIDC7BG022380.3
Domain Color
Lectin_legB Domain
PK_Tyr_Ser−Thr Domain
Pkinase Domain
−200
0
200
400
600
800
1000

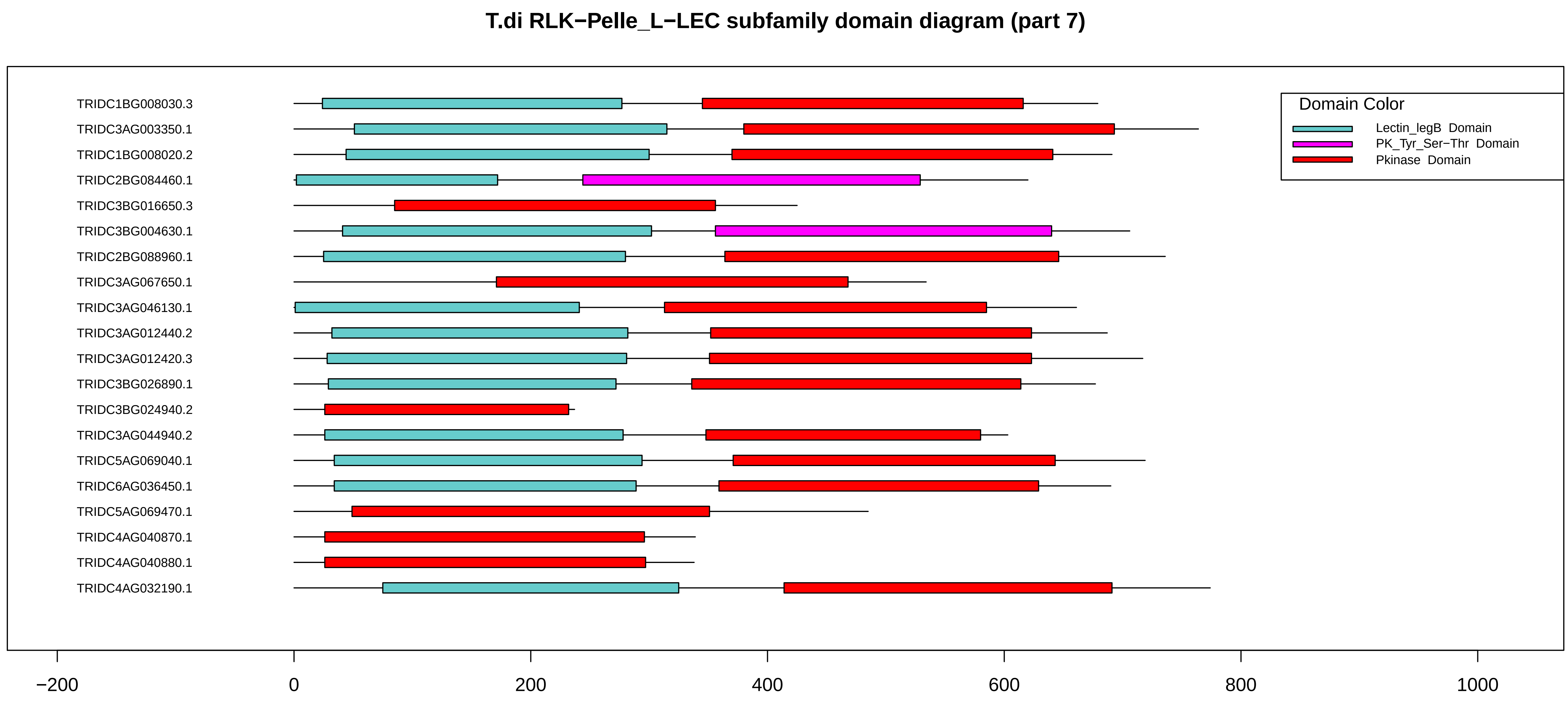

T.di RLK−Pelle_L−LEC subfamily domain diagram (part 7)
TRIDC1BG008030.3
TRIDC3AG003350.1
TRIDC1BG008020.2
TRIDC2BG084460.1
TRIDC3BG016650.3
TRIDC3BG004630.1
TRIDC2BG088960.1
TRIDC3AG067650.1
TRIDC3AG046130.1
TRIDC3AG012440.2
TRIDC3AG012420.3
TRIDC3BG026890.1
TRIDC3BG024940.2
TRIDC3AG044940.2
TRIDC5AG069040.1
TRIDC6AG036450.1
TRIDC5AG069470.1
TRIDC4AG040870.1
TRIDC4AG040880.1
TRIDC4AG032190.1
Domain Color
Lectin_legB Domain
PK_Tyr_Ser−Thr Domain
Pkinase Domain
−200
0
200
400
600
800
1000

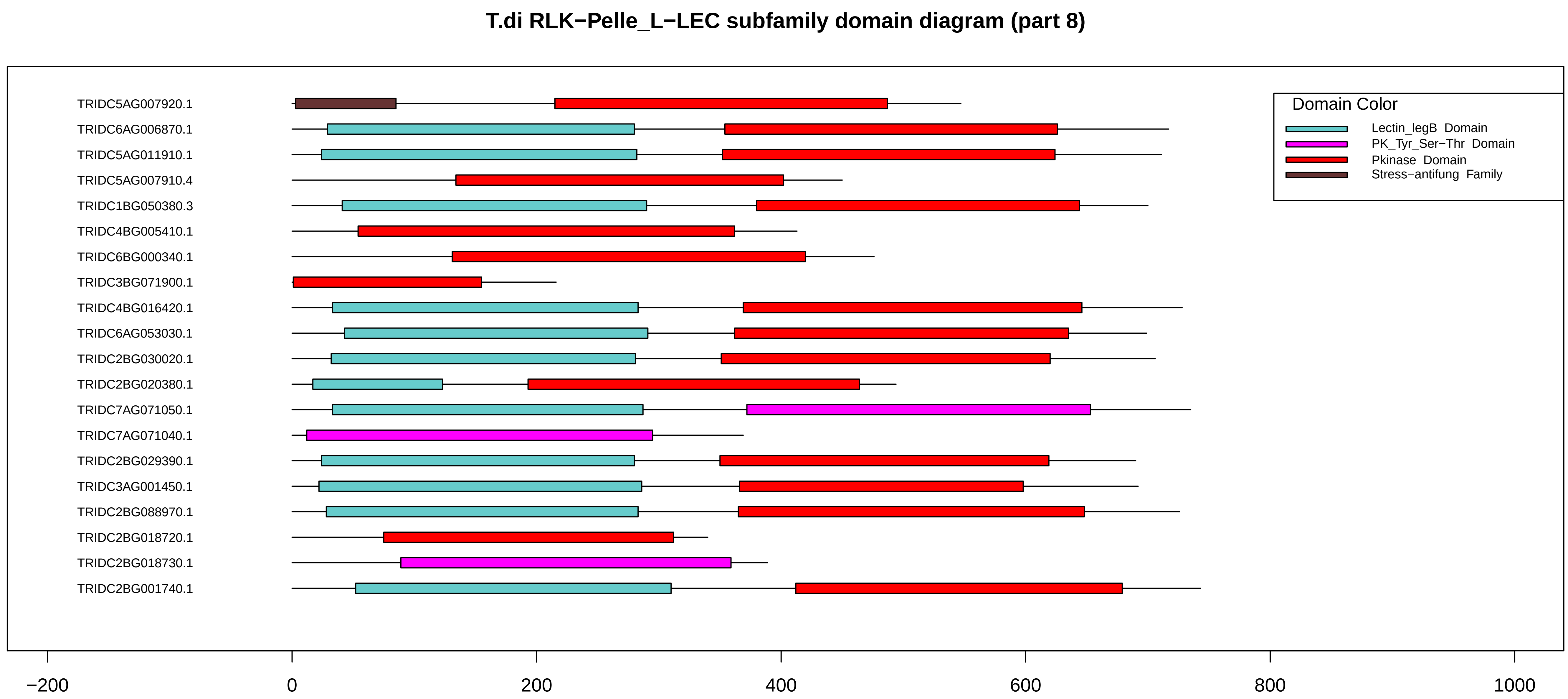
T.di RLK−Pelle_L−LEC subfamily domain diagram (part 8)
TRIDC5AG007920.1
TRIDC6AG006870.1
TRIDC5AG011910.1
TRIDC5AG007910.4
TRIDC1BG050380.3
TRIDC4BG005410.1
TRIDC6BG000340.1
TRIDC3BG071900.1
TRIDC4BG016420.1
TRIDC6AG053030.1
TRIDC2BG030020.1
TRIDC2BG020380.1
TRIDC7AG071050.1
TRIDC7AG071040.1
TRIDC2BG029390.1
TRIDC3AG001450.1
TRIDC2BG088970.1
TRIDC2BG018720.1
TRIDC2BG018730.1
TRIDC2BG001740.1
Domain Color
Lectin_legB Domain
PK_Tyr_Ser−Thr Domain
Pkinase Domain
Stress−antifung Family
−200
0
200
400
600
800
1000

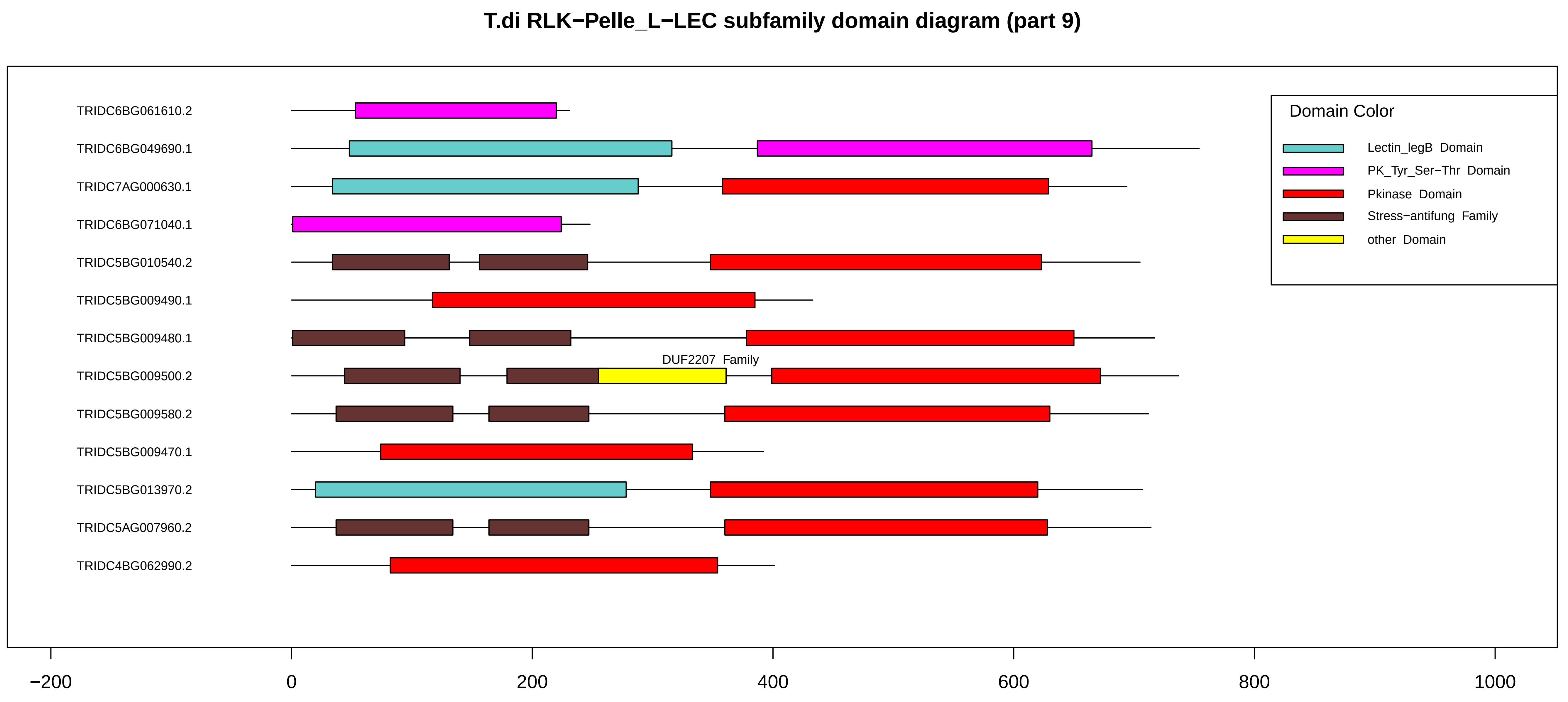
T.di RLK−Pelle_L−LEC subfamily domain diagram (part 9)
TRIDC6BG061610.2
TRIDC6BG049690.1
TRIDC7AG000630.1
TRIDC6BG071040.1
TRIDC5BG010540.2
TRIDC5BG009490.1
TRIDC5BG009480.1
TRIDC5BG009500.2
TRIDC5BG009580.2
TRIDC5BG009470.1
TRIDC5BG013970.2
TRIDC5AG007960.2
TRIDC4BG062990.2
DUF2207 Family
Domain Color
Lectin_legB Domain
PK_Tyr_Ser−Thr Domain
Pkinase Domain
Stress−antifung Family
other Domain
−200
0
200
400
600
800
1000

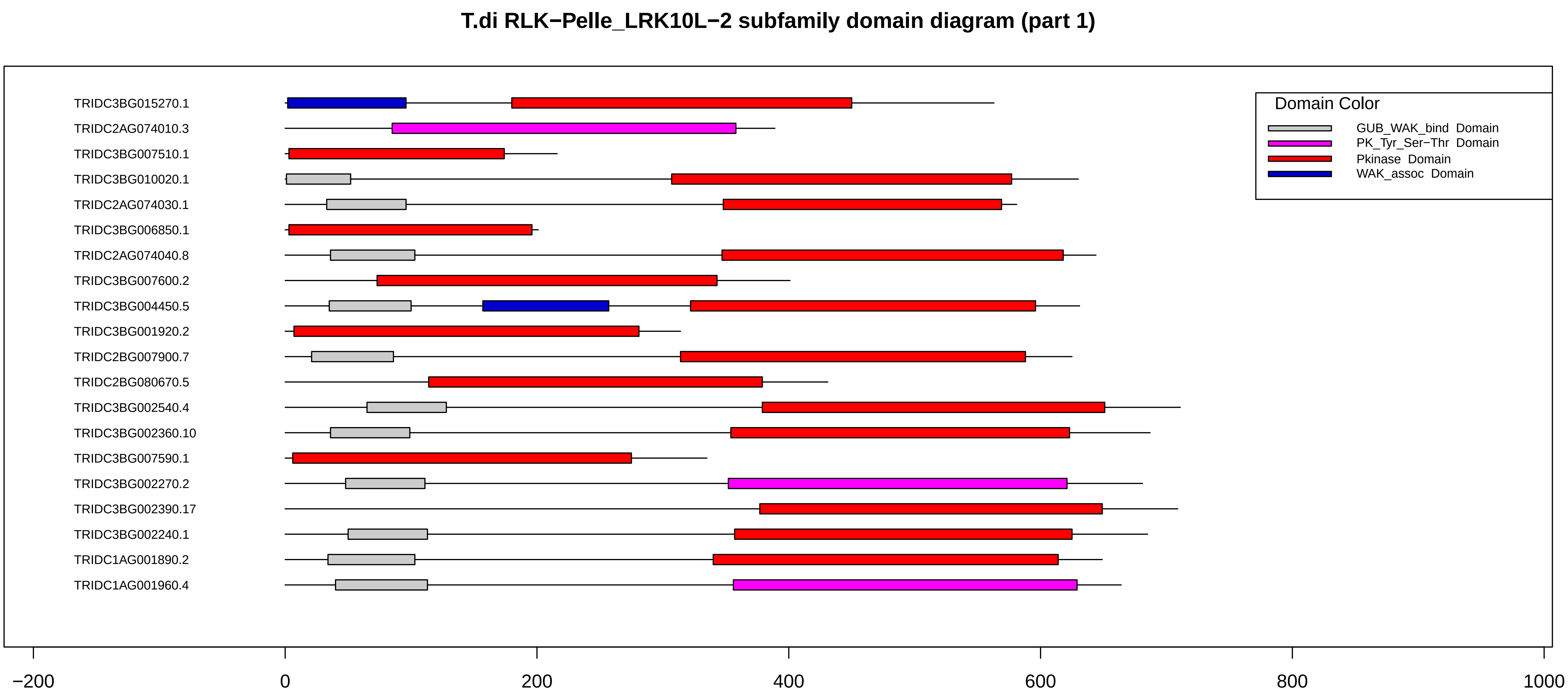
T.di RLK−Pelle_LRK10L−2 subfamily domain diagram (part 1)
TRIDC3BG015270.1
TRIDC2AG074010.3
TRIDC3BG007510.1
TRIDC3BG010020.1
TRIDC2AG074030.1
TRIDC3BG006850.1
TRIDC2AG074040.8
TRIDC3BG007600.2
TRIDC3BG004450.5
TRIDC3BG001920.2
TRIDC2BG007900.7
TRIDC2BG080670.5
TRIDC3BG002540.4
TRIDC3BG002360.10
TRIDC3BG007590.1
TRIDC3BG002270.2
TRIDC3BG002390.17
TRIDC3BG002240.1
TRIDC1AG001890.2
TRIDC1AG001960.4
Domain Color
GUB_WAK_bind Domain
PK_Tyr_Ser−Thr Domain
Pkinase Domain
WAK_assoc Domain
−200
0
200
400
600
800
1000

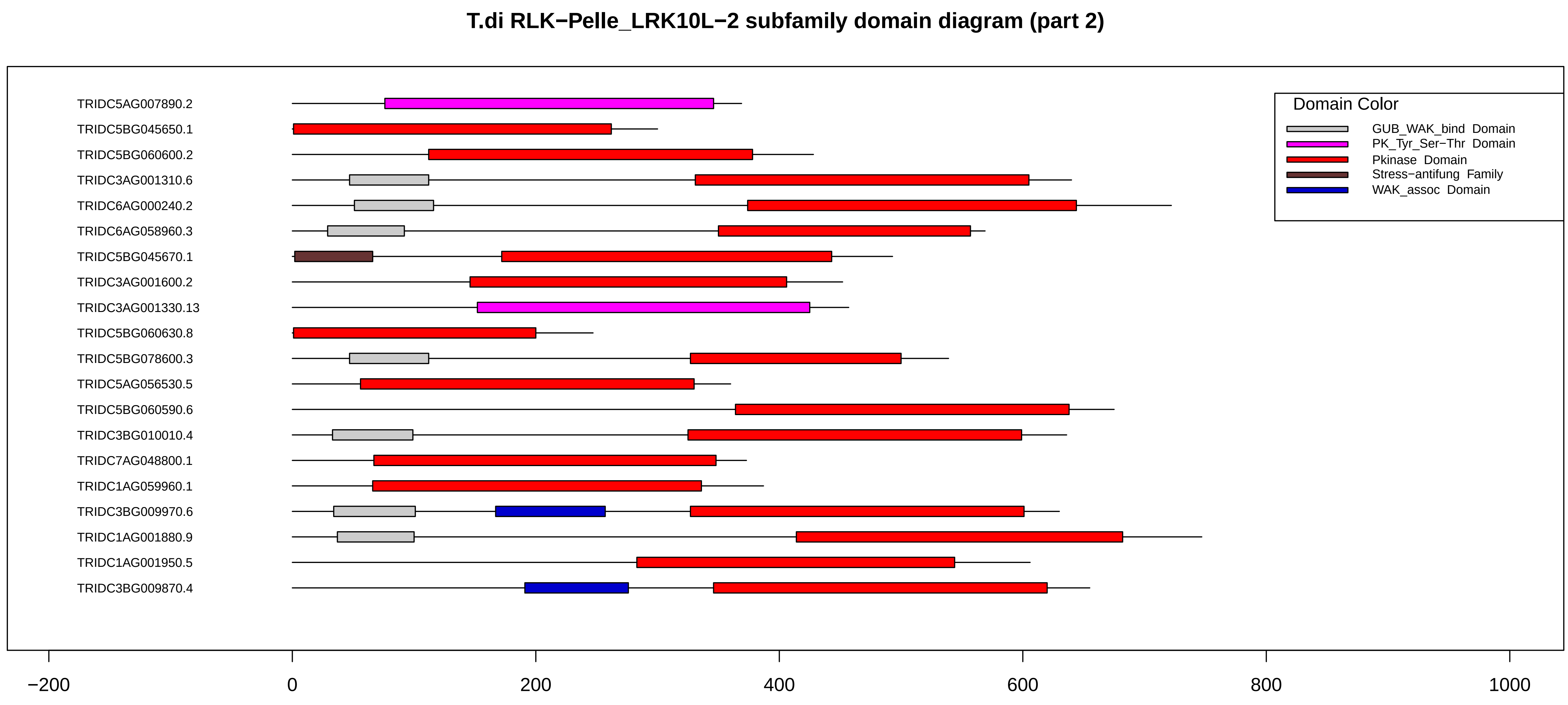
T.di RLK−Pelle_LRK10L−2 subfamily domain diagram (part 2)
TRIDC5AG007890.2
TRIDC5BG045650.1
TRIDC5BG060600.2
TRIDC3AG001310.6
TRIDC6AG000240.2
TRIDC6AG058960.3
TRIDC5BG045670.1
TRIDC3AG001600.2
TRIDC3AG001330.13
TRIDC5BG060630.8
TRIDC5BG078600.3
TRIDC5AG056530.5
TRIDC5BG060590.6
TRIDC3BG010010.4
TRIDC7AG048800.1
TRIDC1AG059960.1
TRIDC3BG009970.6
TRIDC1AG001880.9
TRIDC1AG001950.5
TRIDC3BG009870.4
Domain Color
GUB_WAK_bind Domain
PK_Tyr_Ser−Thr Domain
Pkinase Domain
Stress−antifung Family
WAK_assoc Domain
−200
0
200
400
600
800
1000

T.di RLK−Pelle_LRK10L−2 subfamily domain diagram (part 3)

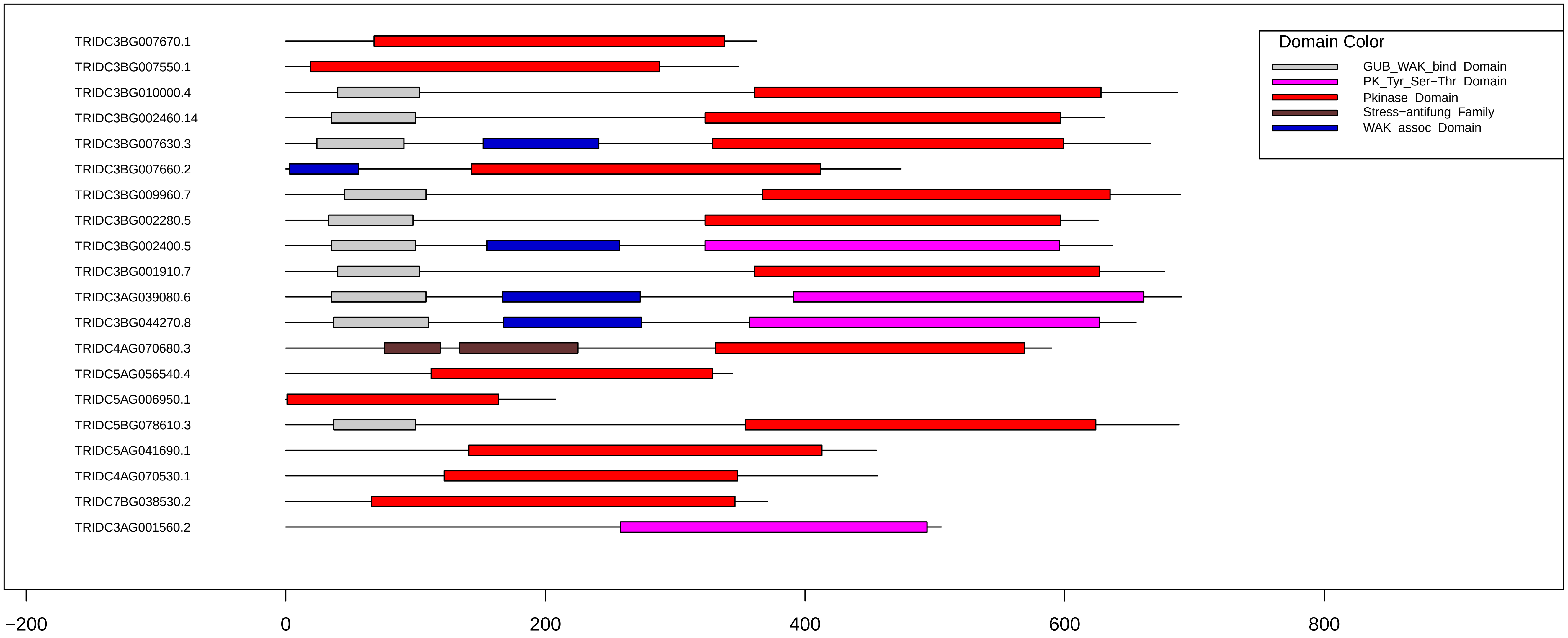

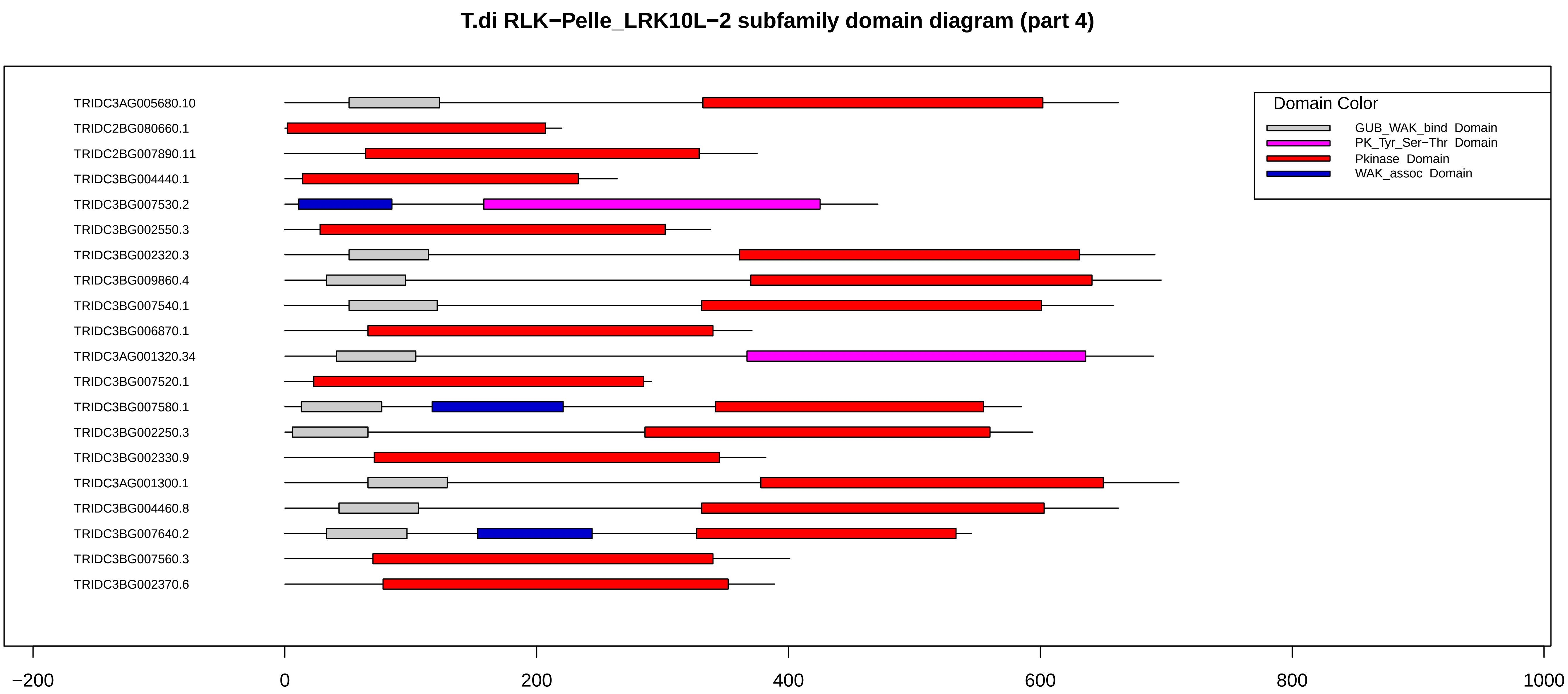
T.di RLK−Pelle_LRK10L−2 subfamily domain diagram (part 4)
TRIDC3AG005680.10
TRIDC2BG080660.1
TRIDC2BG007890.11
TRIDC3BG004440.1
TRIDC3BG007530.2
TRIDC3BG002550.3
TRIDC3BG002320.3
TRIDC3BG009860.4
TRIDC3BG007540.1
TRIDC3BG006870.1
TRIDC3AG001320.34
TRIDC3BG007520.1
TRIDC3BG007580.1
TRIDC3BG002250.3
TRIDC3BG002330.9
TRIDC3AG001300.1
TRIDC3BG004460.8
TRIDC3BG007640.2
TRIDC3BG007560.3
TRIDC3BG002370.6
Domain Color
GUB_WAK_bind Domain
PK_Tyr_Ser−Thr Domain
Pkinase Domain
WAK_assoc Domain
−200
0
200
400
600
800
1000

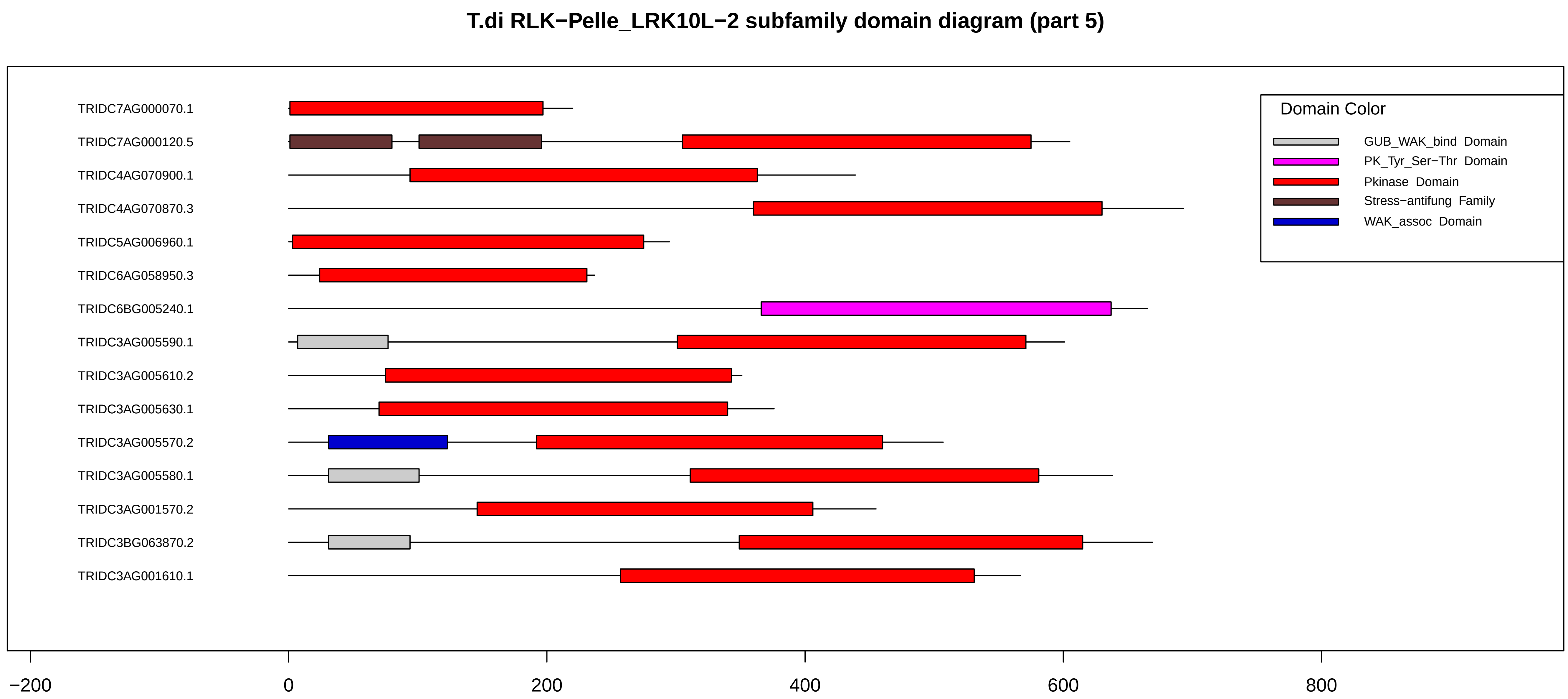
T.di RLK−Pelle_LRK10L−2 subfamily domain diagram (part 5)
TRIDC7AG000070.1
TRIDC7AG000120.5
TRIDC4AG070900.1
TRIDC4AG070870.3
TRIDC5AG006960.1
TRIDC6AG058950.3
TRIDC6BG005240.1
TRIDC3AG005590.1
TRIDC3AG005610.2
TRIDC3AG005630.1
TRIDC3AG005570.2
TRIDC3AG005580.1
TRIDC3AG001570.2
TRIDC3BG063870.2
TRIDC3AG001610.1
Domain Color
GUB_WAK_bind Domain
PK_Tyr_Ser−Thr Domain
Pkinase Domain
Stress−antifung Family
WAK_assoc Domain
−200
0
200
400
600
800

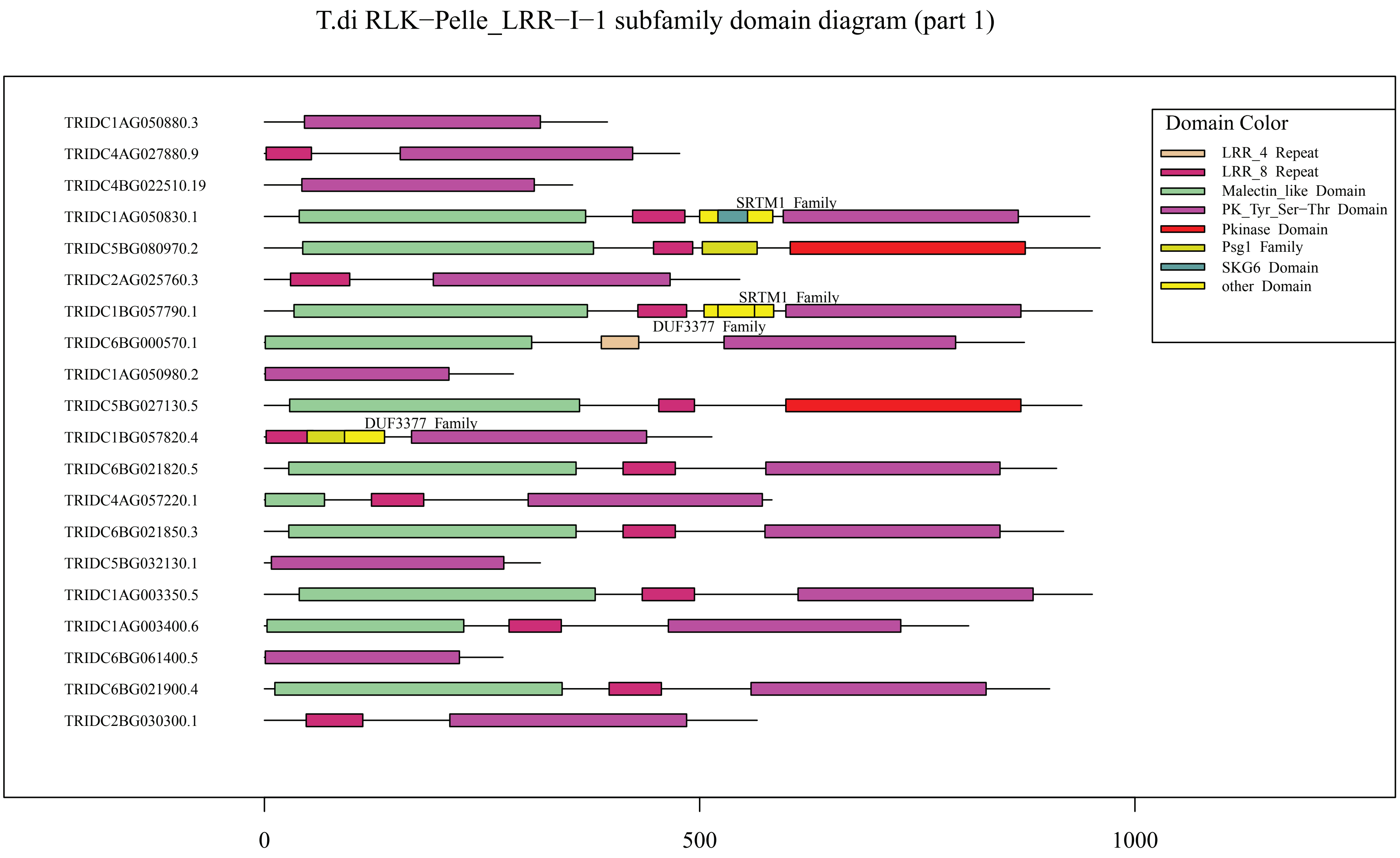
T.di RLK−Pelle_LRR−I−1 subfamily domain diagram (part 1)
TRIDC1AG050880.3
TRIDC4AG027880.9
TRIDC4BG022510.19
TRIDC1AG050830.1
TRIDC5BG080970.2
TRIDC2AG025760.3
TRIDC1BG057790.1
TRIDC6BG000570.1
TRIDC1AG050980.2
TRIDC5BG027130.5
TRIDC1BG057820.4
TRIDC6BG021820.5
TRIDC4AG057220.1
TRIDC6BG021850.3
TRIDC5BG032130.1
TRIDC1AG003350.5
TRIDC1AG003400.6
TRIDC6BG061400.5
TRIDC6BG021900.4
TRIDC2BG030300.1
SRTM1_Family
SRTM1_Family
DUF3377_Family
DUF3377_Family
Domain Color
LRR_4 Repeat
LRR_8 Repeat
Malectin_like Domain
PK_Tyr_Ser−Thr Domain
Pkinase Domain
Psg1 Family
SKG6 Domain
other Domain
0
500
1000

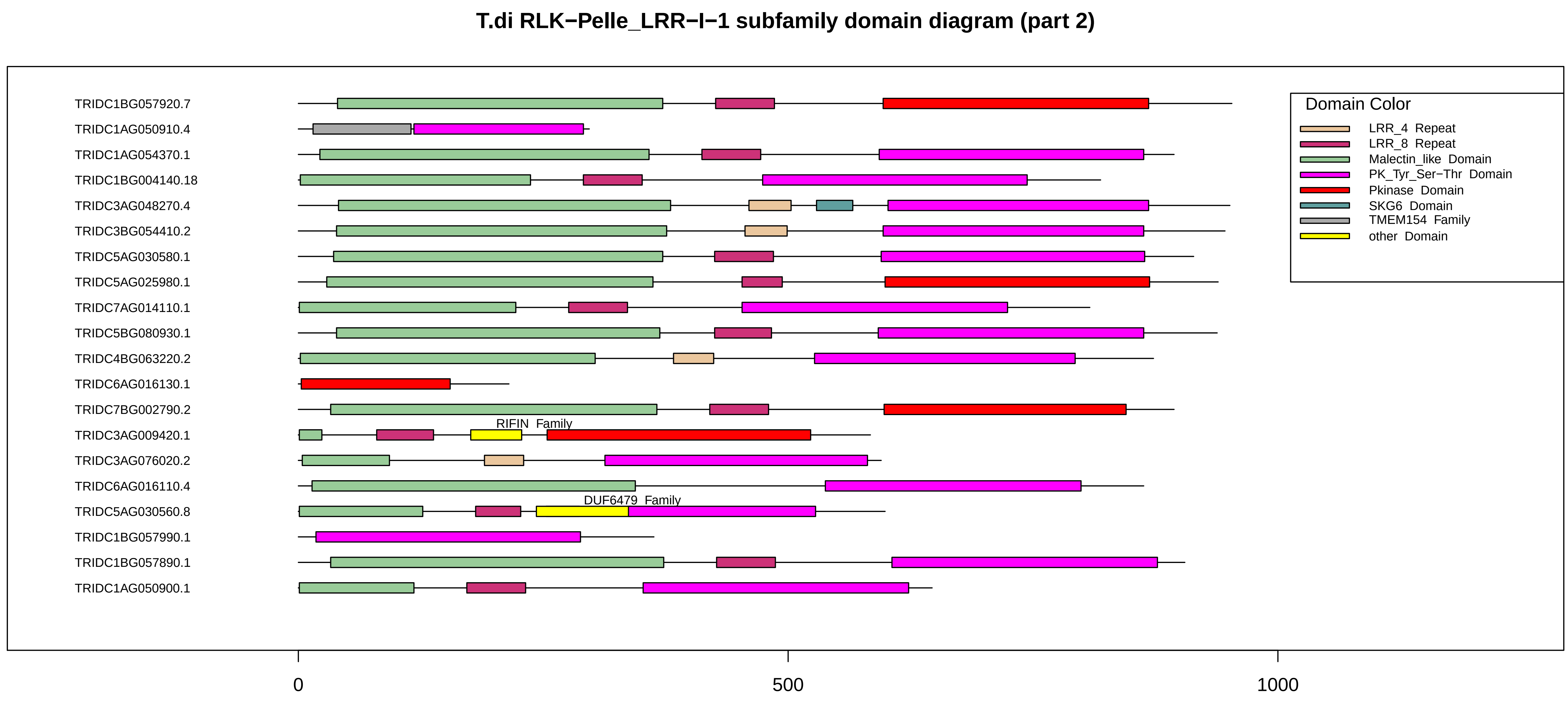

T.di RLK−Pelle_LRR−I−1 subfamily domain diagram (part 2)
TRIDC1BG057920.7
TRIDC1AG050910.4
TRIDC1AG054370.1
TRIDC1BG004140.18
TRIDC3AG048270.4
TRIDC3BG054410.2
TRIDC5AG030580.1
TRIDC5AG025980.1
TRIDC7AG014110.1
TRIDC5BG080930.1
TRIDC4BG063220.2
TRIDC6AG016130.1
TRIDC7BG002790.2
TRIDC3AG009420.1
RIFIN Family
TRIDC3AG076020.2
TRIDC6AG016110.4
TRIDC5AG030560.8
DUF6479 Family
TRIDC1BG057990.1
TRIDC1BG057890.1
TRIDC1AG050900.1
Domain Color
LRR_4 Repeat
LRR_8 Repeat
Malectin_like Domain
PK_Tyr_Ser−Thr Domain
Pkinase Domain
SKG6 Domain
TMEM154 Family
other Domain
0
500
1000

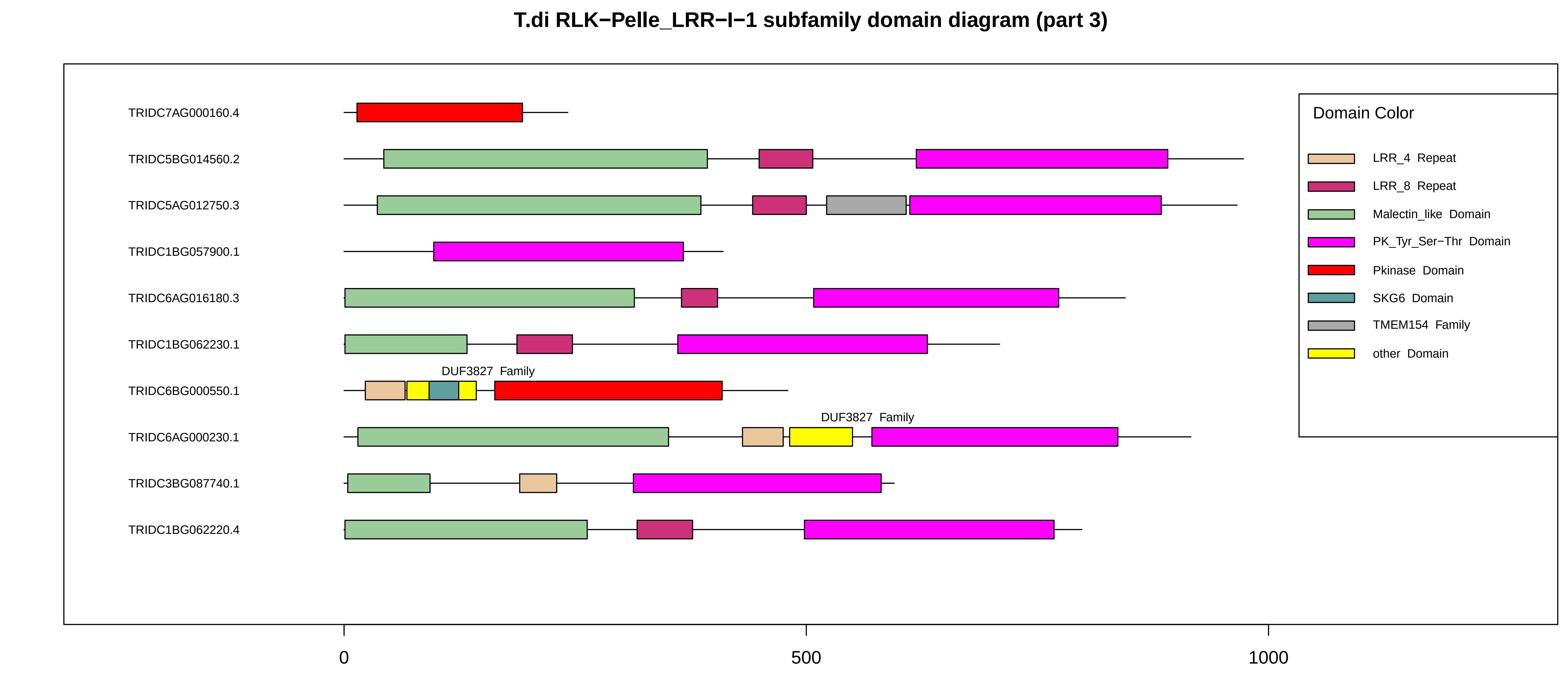

T.di RLK−Pelle_LRR−I−1 subfamily domain diagram (part 3)
TRIDC7AG000160.4
TRIDC5BG014560.2
TRIDC5AG012750.3
TRIDC1BG057900.1
TRIDC6AG016180.3
TRIDC1BG062230.1
TRIDC6BG000550.1
TRIDC6AG000230.1
TRIDC3BG087740.1
TRIDC1BG062220.4
DUF3827 Family
DUF3827 Family
Domain Color
LRR_4 Repeat
LRR_8 Repeat
Malectin_like Domain
PK_Tyr_Ser−Thr Domain
Pkinase Domain
SKG6 Domain
TMEM154 Family
other Domain
0
500
1000

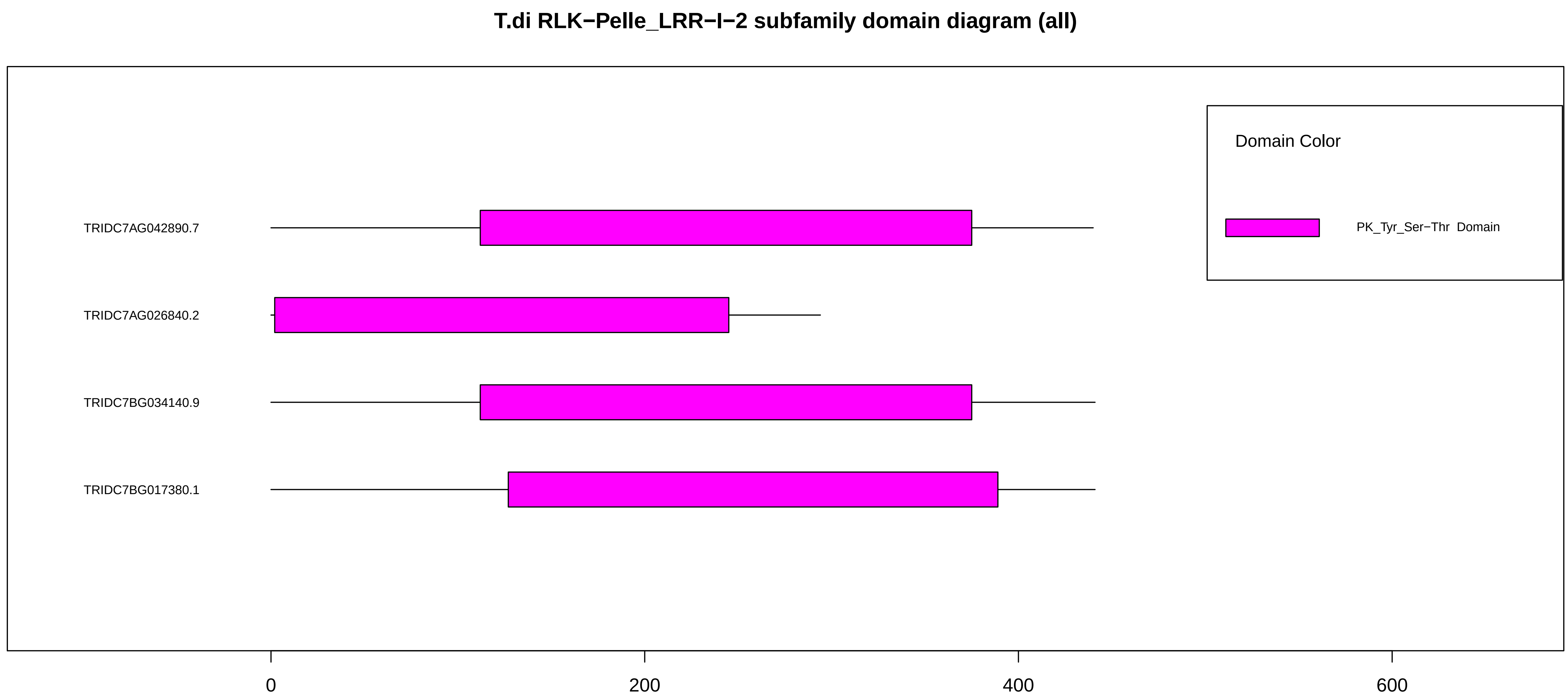
T.di RLK−Pelle_LRR−I−2 subfamily domain diagram (all)
Domain Color
PK_Tyr_Ser−Thr Domain
TRIDC7AG042890.7
TRIDC7AG026840.2
TRIDC7BG034140.9
TRIDC7BG017380.1
0
200
400
600

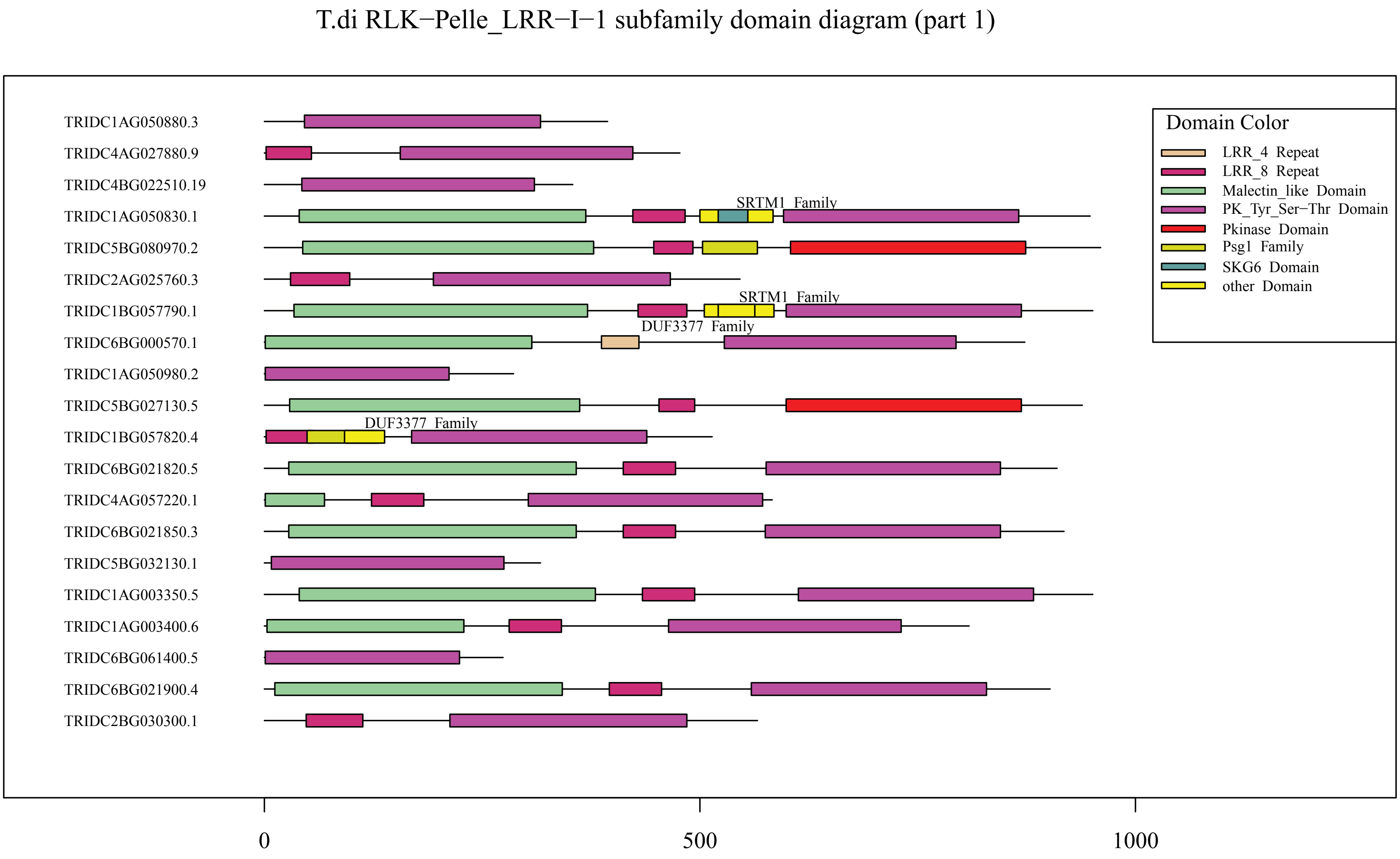
T.di RLK−Pelle_LRR−I−1 subfamily domain diagram (part 1)
TRIDC1AG050880.3
TRIDC4AG027880.9
TRIDC4BG022510.19
TRIDC1AG050830.1
TRIDC5BG080970.2
TRIDC2AG025760.3
TRIDC1BG057790.1
TRIDC6BG000570.1
TRIDC1AG050980.2
TRIDC5BG027130.5
TRIDC1BG057820.4
TRIDC6BG021820.5
TRIDC4AG057220.1
TRIDC6BG021850.3
TRIDC5BG032130.1
TRIDC1AG003350.5
TRIDC1AG003400.6
TRIDC6BG061400.5
TRIDC6BG021900.4
TRIDC2BG030300.1
SRTM1 Family
SRTM1 Family
DUF3377 Family
DUF3377 Family
Domain Color
LRR_4 Repeat
LRR_8 Repeat
Malectin_like Domain
PK_Tyr_Ser−Thr Domain
Pkinase Domain
Psg1 Family
SKG6 Domain
other Domain
0
500
1000

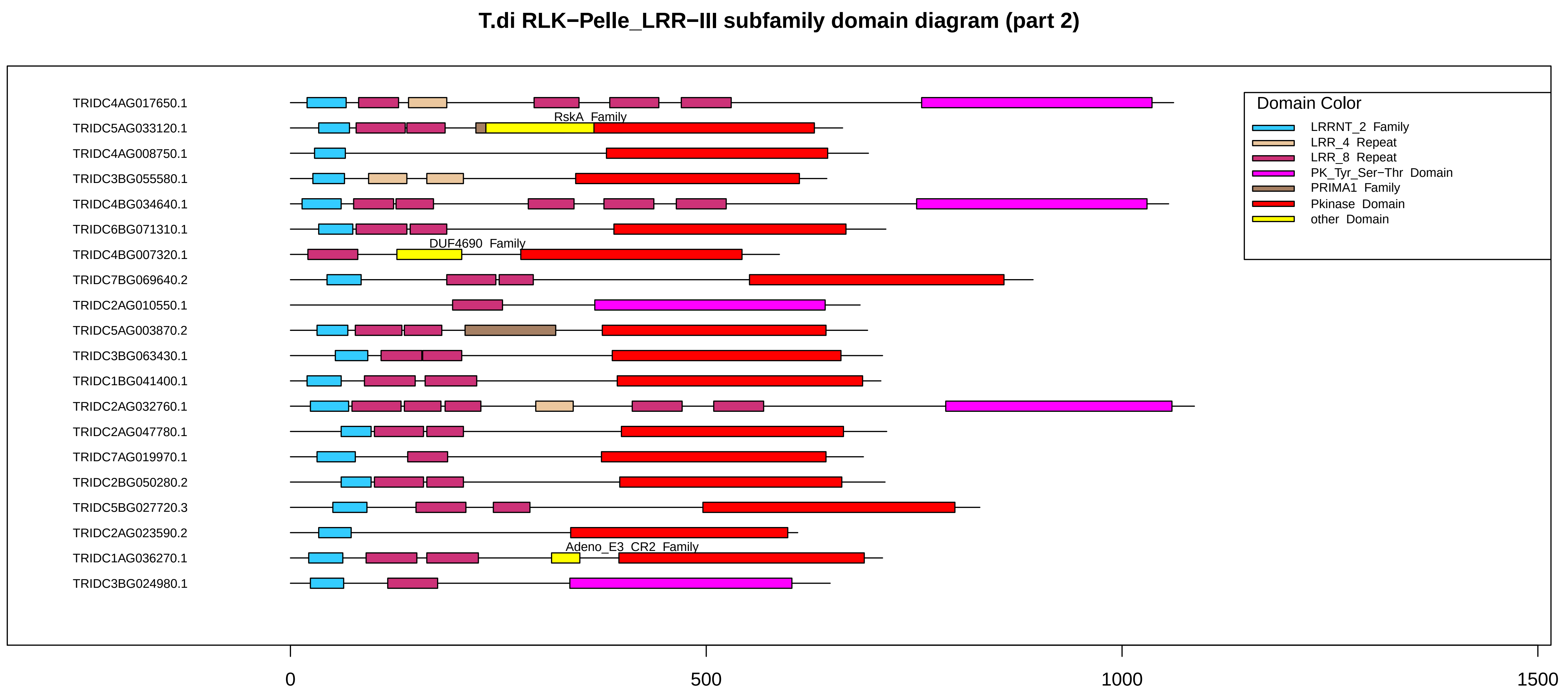
T.di RLK−Pelle_LRR−III subfamily domain diagram (part 2)
TRIDC4AG017650.1
TRIDC5AG033120.1
TRIDC4AG008750.1
TRIDC3BG055580.1
TRIDC4BG034640.1
TRIDC6BG071310.1
TRIDC4BG007320.1
TRIDC7BG069640.2
TRIDC2AG010550.1
TRIDC5AG003870.2
TRIDC3BG063430.1
TRIDC1BG041400.1
TRIDC2AG032760.1
TRIDC2AG047780.1
TRIDC7AG019970.1
TRIDC2BG050280.2
TRIDC5BG027720.3
TRIDC2AG023590.2
TRIDC1AG036270.1
TRIDC3BG024980.1
RskA Family
DUF4690 Family
Adeno_E3_CR2 Family
Domain Color
LRRNT_2 Family
LRR_4 Repeat
LRR_8 Repeat
PK_Tyr_Ser−Thr Domain
PRIMA1 Family
Pkinase Domain
other Domain
0
500
1000
1500

T.di RLK−Pelle_LRR−III subfamily domain diagram (part 3)

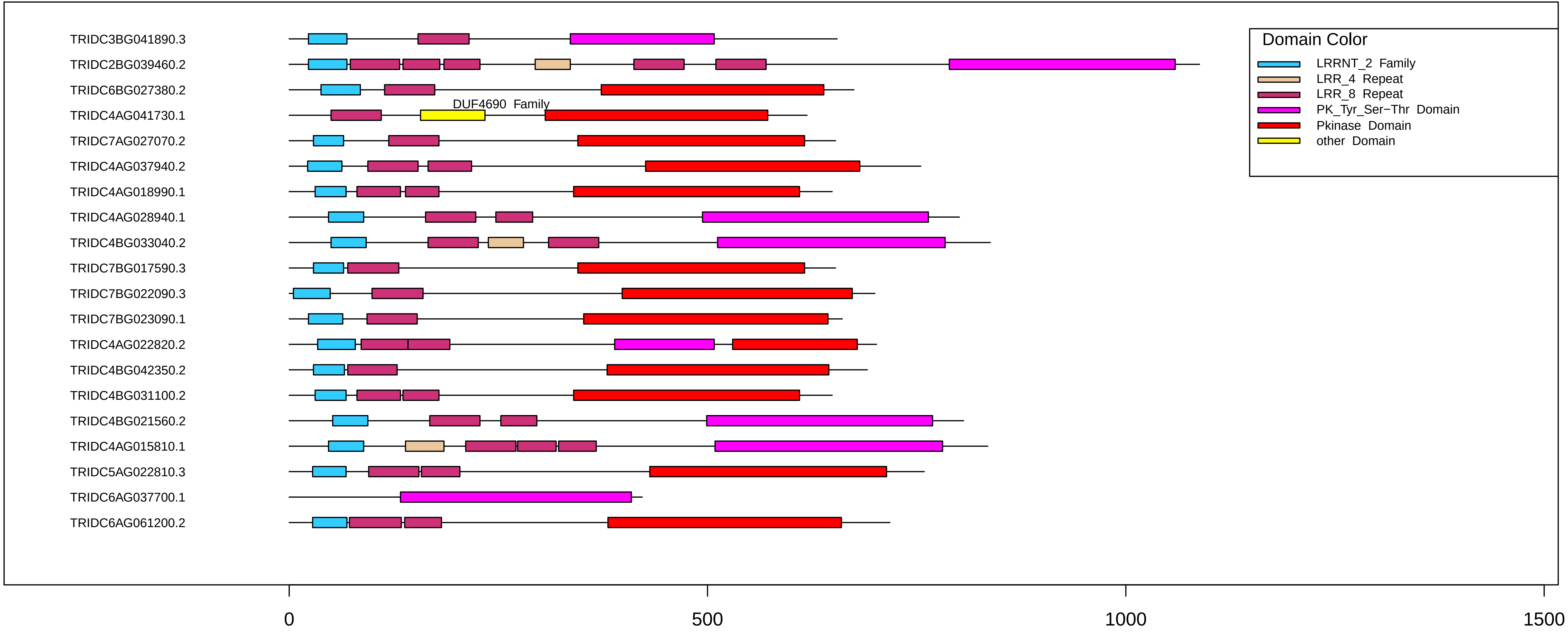

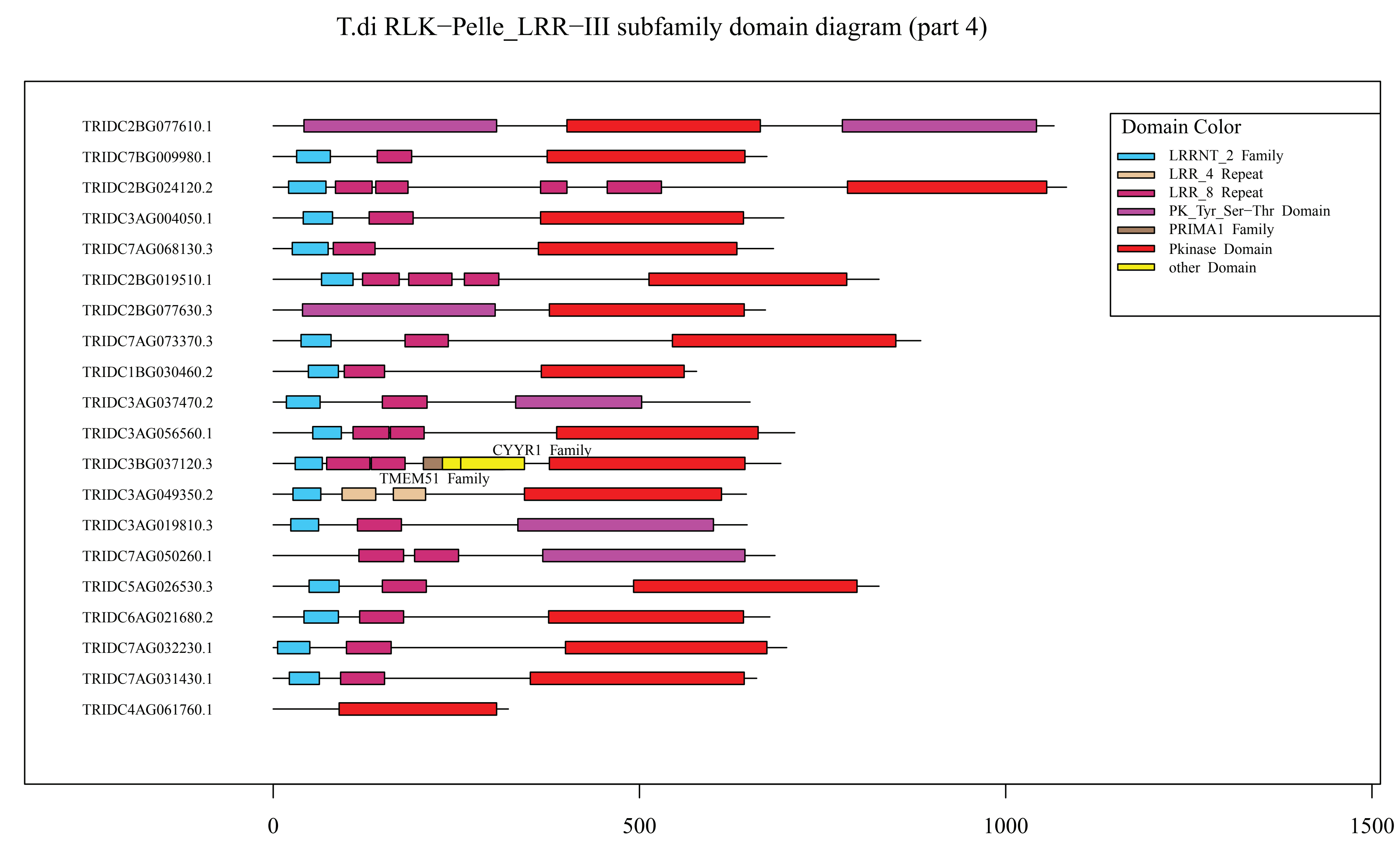
T.di RLK−Pelle_LRR−III subfamily domain diagram (part 4)
TRIDC2BG077610.1
TRIDC7BG009980.1
TRIDC2BG024120.2
TRIDC3AG004050.1
TRIDC7AG068130.3
TRIDC2BG019510.1
TRIDC2BG077630.3
TRIDC7AG073370.3
TRIDC1BG030460.2
TRIDC3AG037470.2
TRIDC3AG056560.1
TRIDC3BG037120.3
TRIDC3AG049350.2
TRIDC3AG019810.3
TRIDC7AG050260.1
TRIDC5AG026530.3
TRIDC6AG021680.2
TRIDC7AG032230.1
TRIDC7AG031430.1
TRIDC4AG061760.1
CYYR1 Family
TMEM51 Family
Domain Color
LRRNT_2 Family
LRR_4 Repeat
LRR_8 Repeat
PK_Tyr_Ser−Thr Domain
PRIMA1 Family
Pkinase Domain
other Domain
0
500
1000
1500

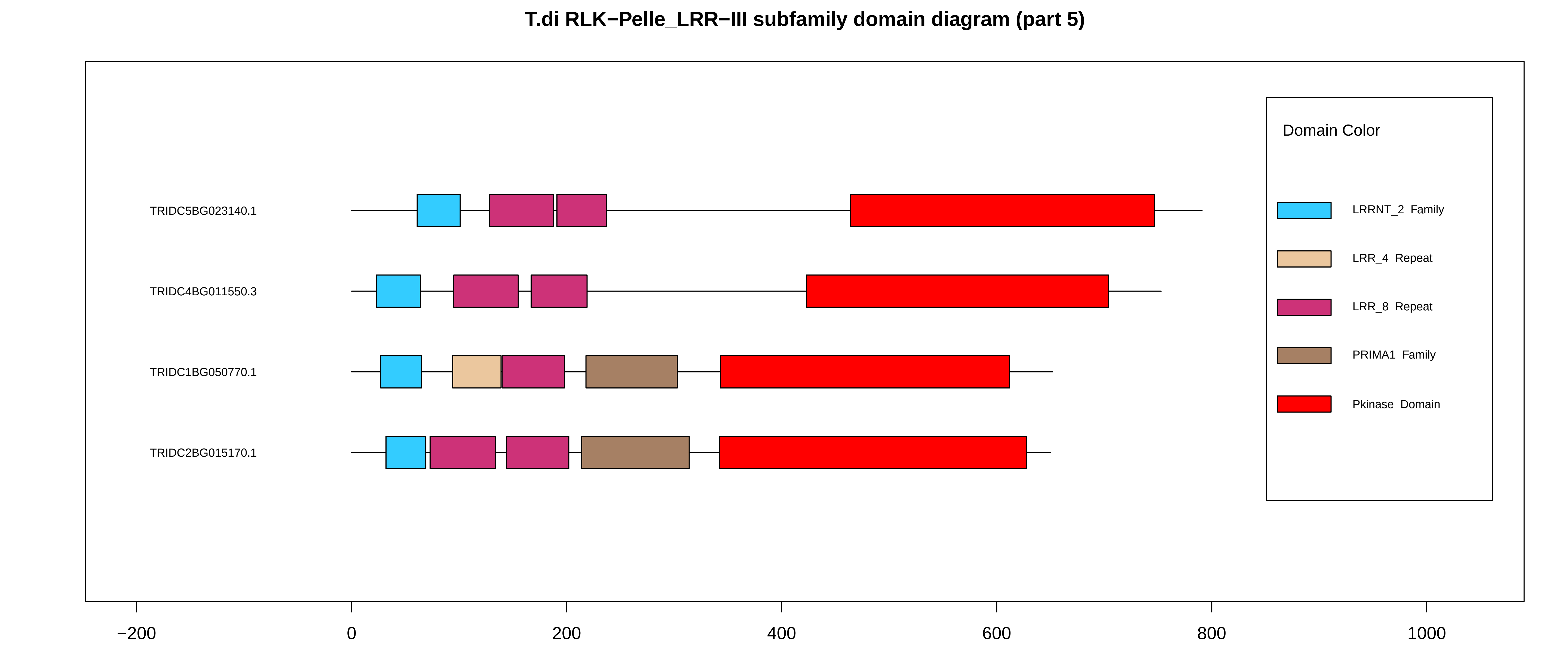
T.di RLK−Pelle_LRR−III subfamily domain diagram (part 5)
TRIDC5BG023140.1
TRIDC4BG011550.3
TRIDC1BG050770.1
TRIDC2BG015170.1
Domain Color
LRRNT_2 Family
LRR_4 Repeat
LRR_8 Repeat
PRIMA1 Family
Pkinase Domain
−200
0
200
400
600
800
1000

T.di RLK−Pelle_LRR−II subfamily domain diagram (part 1)

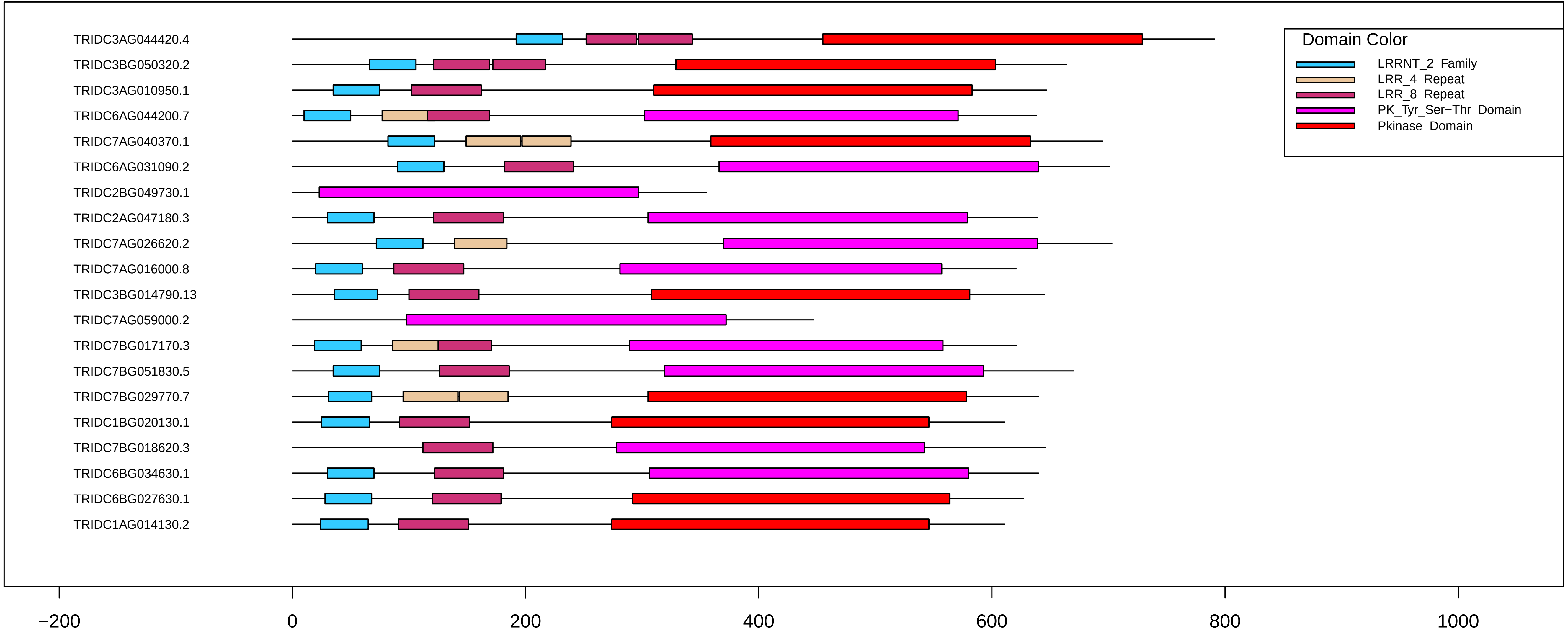

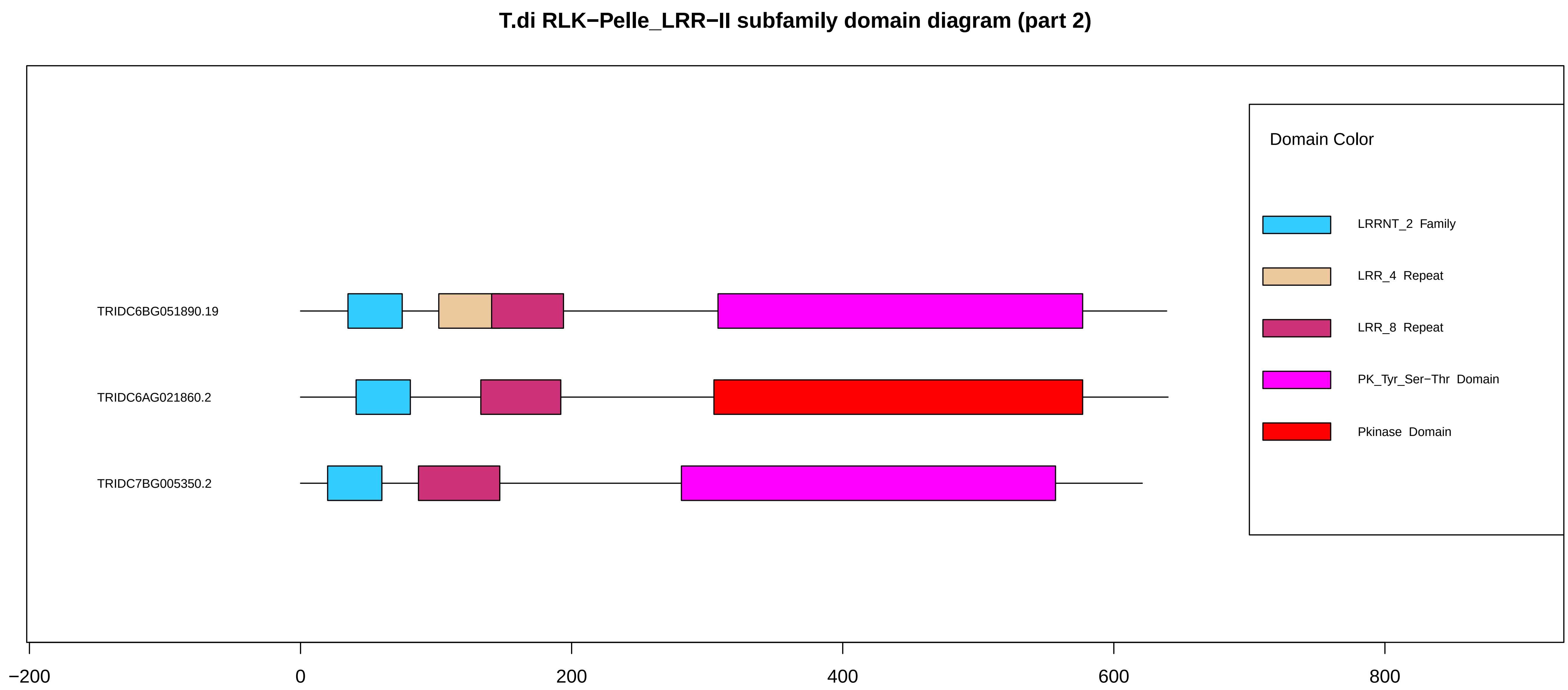
T.di RLK−Pelle_LRR−II subfamily domain diagram (part 2)
TRIDC6BG051890.19
TRIDC6AG021860.2
TRIDC7BG005350.2
Domain Color
LRRNT_2 Family
LRR_4 Repeat
LRR_8 Repeat
PK_Tyr_Ser−Thr Domain
Pkinase Domain
−200
0
200
400
600
800

T.di RLK−Pelle_LRR−IV subfamily domain diagram (all)

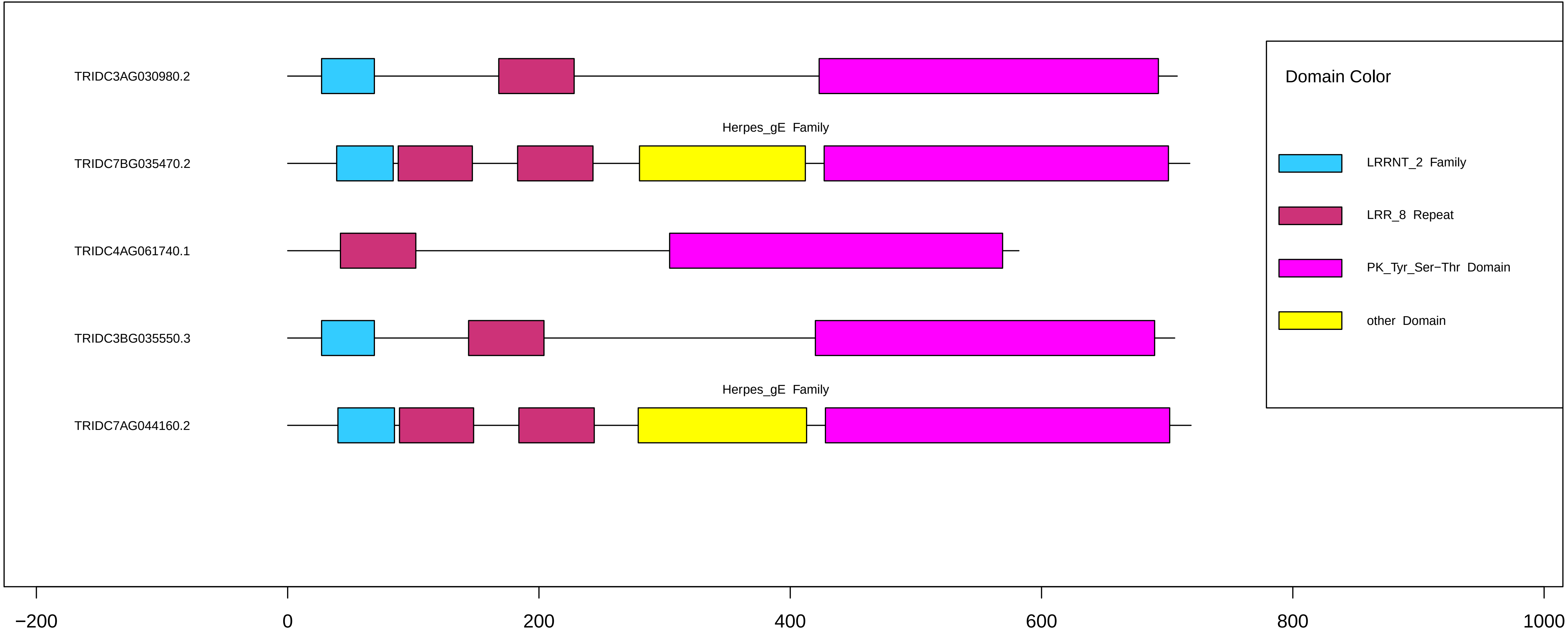

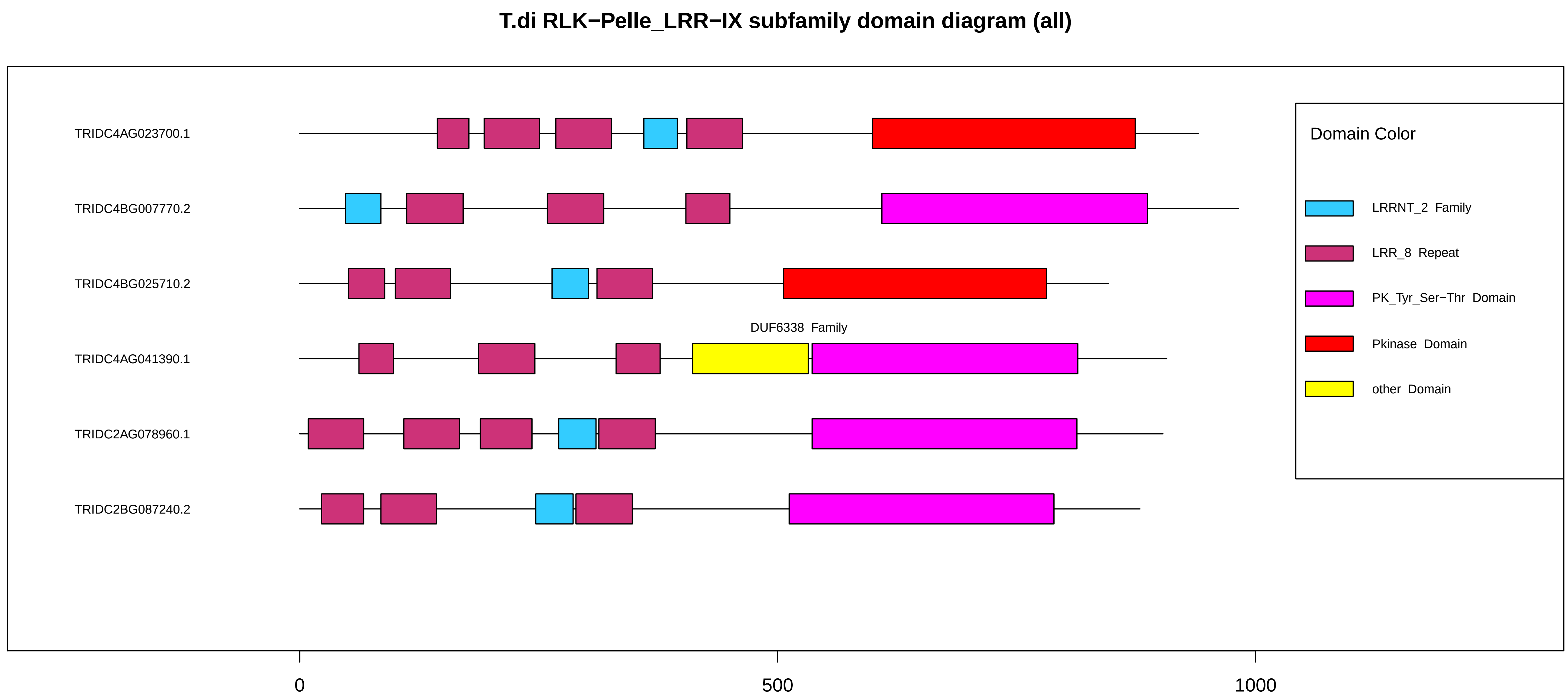
T.di RLK−Pelle_LRR−IX subfamily domain diagram (all)
TRIDC4AG023700.1
TRIDC4BG007770.2
TRIDC4BG025710.2
TRIDC4AG041390.1
TRIDC2AG078960.1
TRIDC2BG087240.2
DUF6338 Family
Domain Color
LRRNT_2 Family
LRR_8 Repeat
PK_Tyr_Ser−Thr Domain
Pkinase Domain
other Domain
0
500
1000

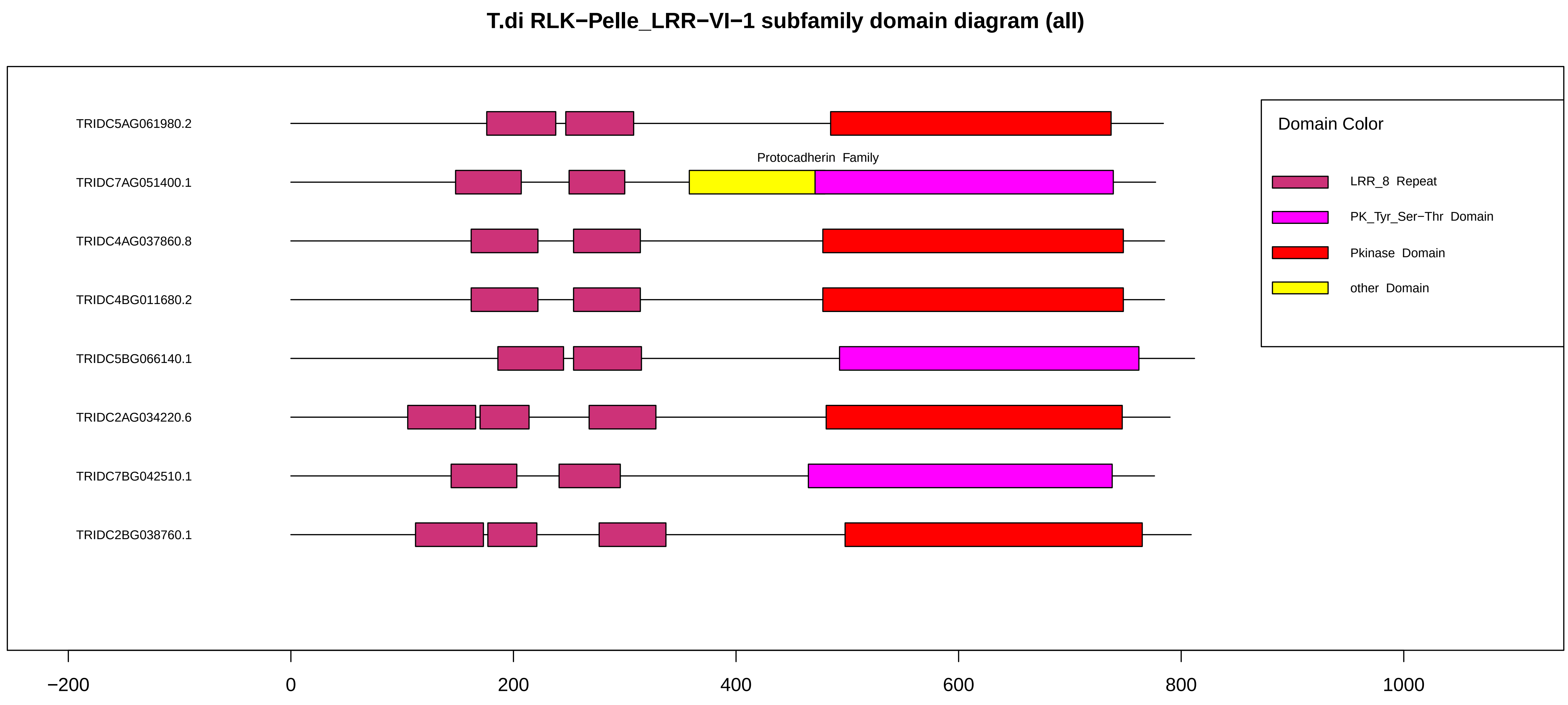
T.di RLK−Pelle_LRR−VI−1 subfamily domain diagram (all)
TRIDC5AG061980.2
TRIDC7AG051400.1
Protocadherin Family
TRIDC4AG037860.8
TRIDC4BG011680.2
TRIDC5BG066140.1
TRIDC2AG034220.6
TRIDC7BG042510.1
TRIDC2BG038760.1
Domain Color
LRR_8 Repeat
PK_Tyr_Ser−Thr Domain
Pkinase Domain
other Domain
−200
0
200
400
600
800
1000

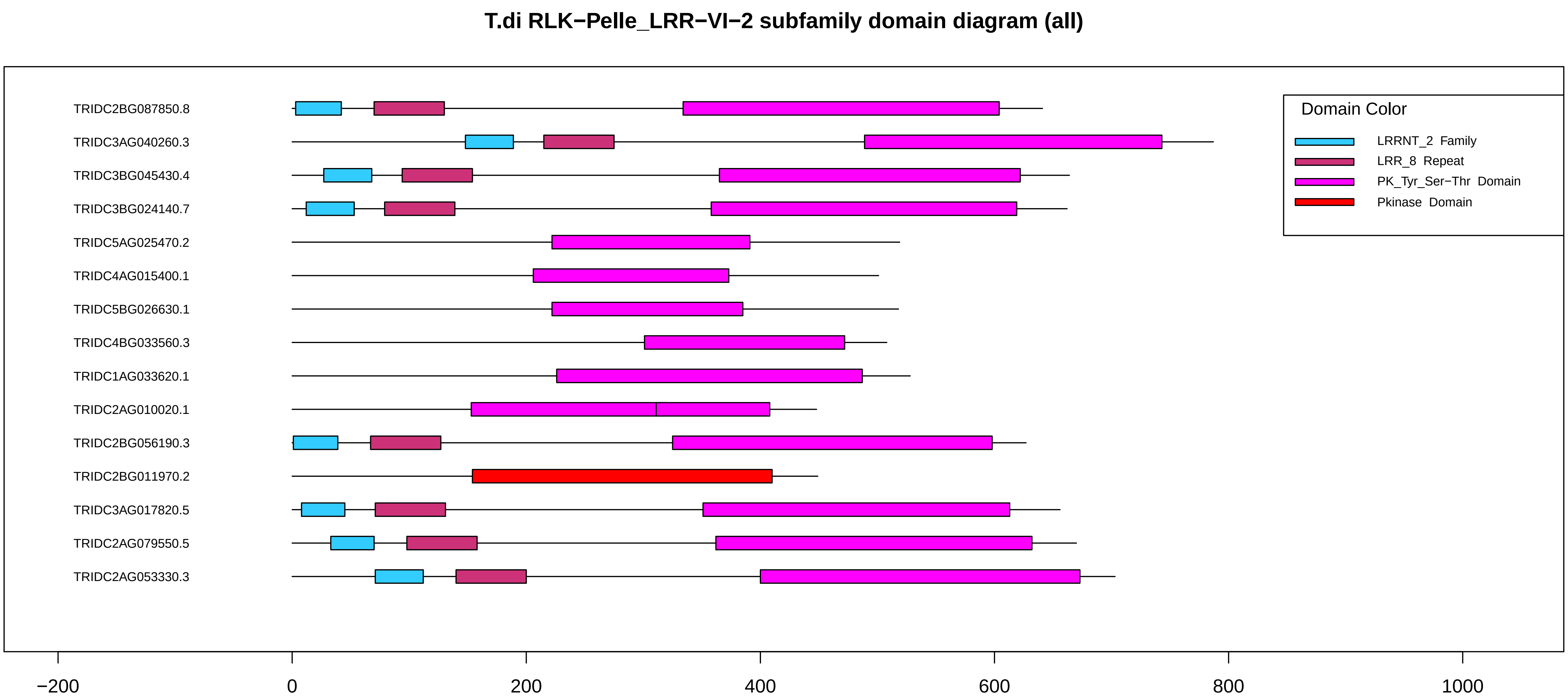
T.di RLK−Pelle_LRR−VI−2 subfamily domain diagram (all)
TRIDC2BG087850.8
TRIDC3AG040260.3
TRIDC3BG045430.4
TRIDC3BG024140.7
TRIDC5AG025470.2
TRIDC4AG015400.1
TRIDC5BG026630.1
TRIDC4BG033560.3
TRIDC1AG033620.1
TRIDC2AG010020.1
TRIDC2BG056190.3
TRIDC2BG011970.2
TRIDC3AG017820.5
TRIDC2AG079550.5
TRIDC2AG053330.3
Domain Color
LRRNT_2 Family
LRR_8 Repeat
PK_Tyr_Ser−Thr Domain
Pkinase Domain
−200
0
200
400
600
800
1000

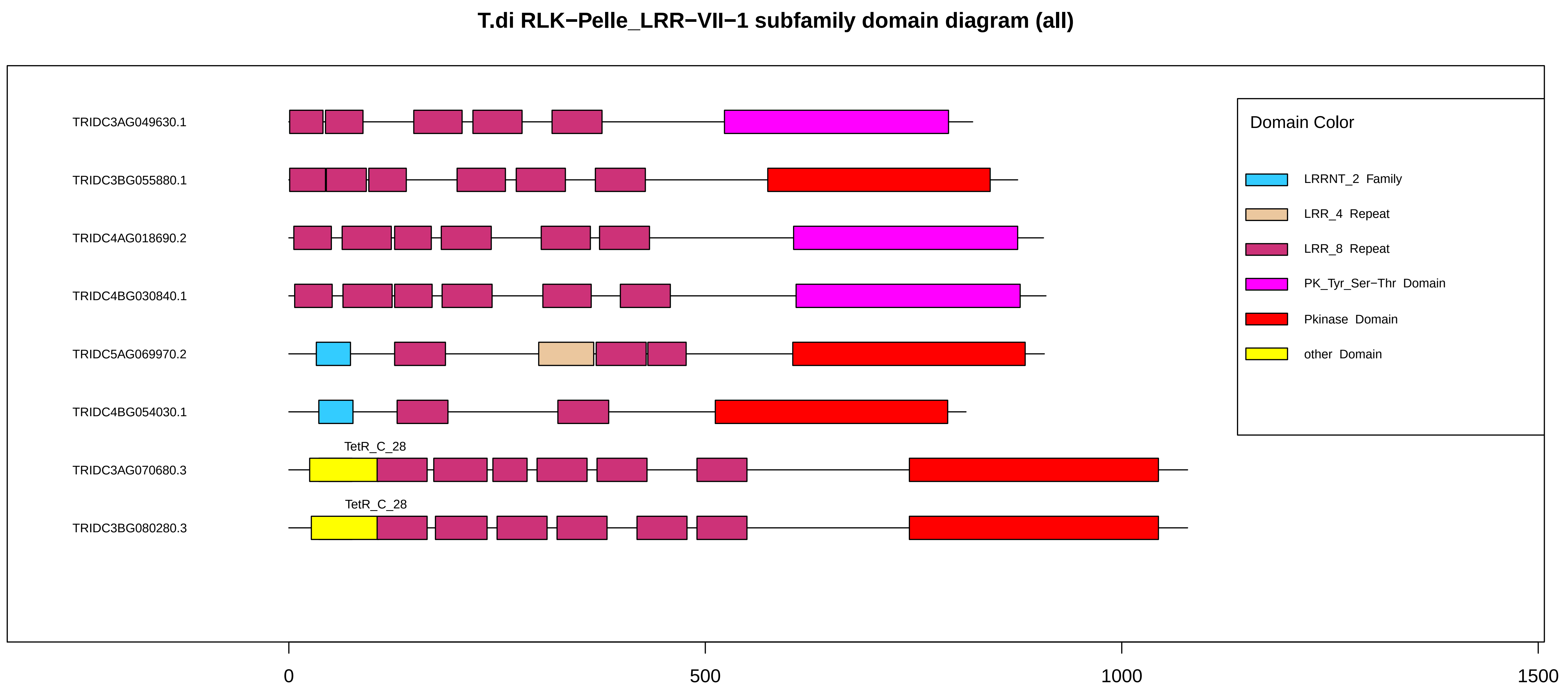
T.di RLK−Pelle_LRR−VII−1 subfamily domain diagram (all)
TRIDC3AG049630.1
TRIDC3BG055880.1
TRIDC4AG018690.2
TRIDC4BG030840.1
TRIDC5AG069970.2
TRIDC4BG054030.1
TetR_C_28
TRIDC3AG070680.3
TetR_C_28
TRIDC3BG080280.3
Domain Color
LRRNT_2 Family
LRR_4 Repeat
LRR_8 Repeat
PK_Tyr_Ser−Thr Domain
Pkinase Domain
other Domain
0
500
1000
1500

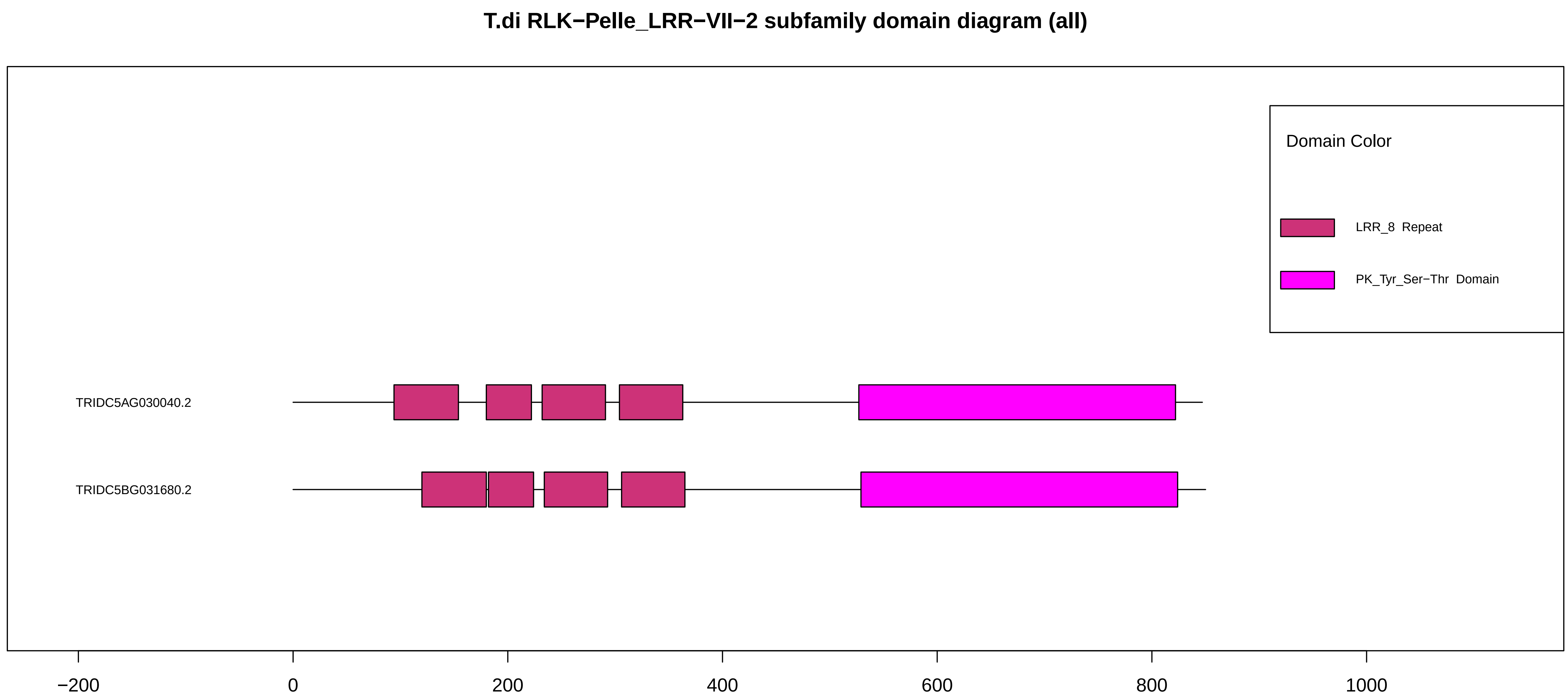
T.di RLK−Pelle_LRR−VII−2 subfamily domain diagram (all)
Domain Color
LRR_8 Repeat
PK_Tyr_Ser−Thr Domain
TRIDC5AG030040.2
TRIDC5BG031680.2
−200
0
200
400
600
800
1000

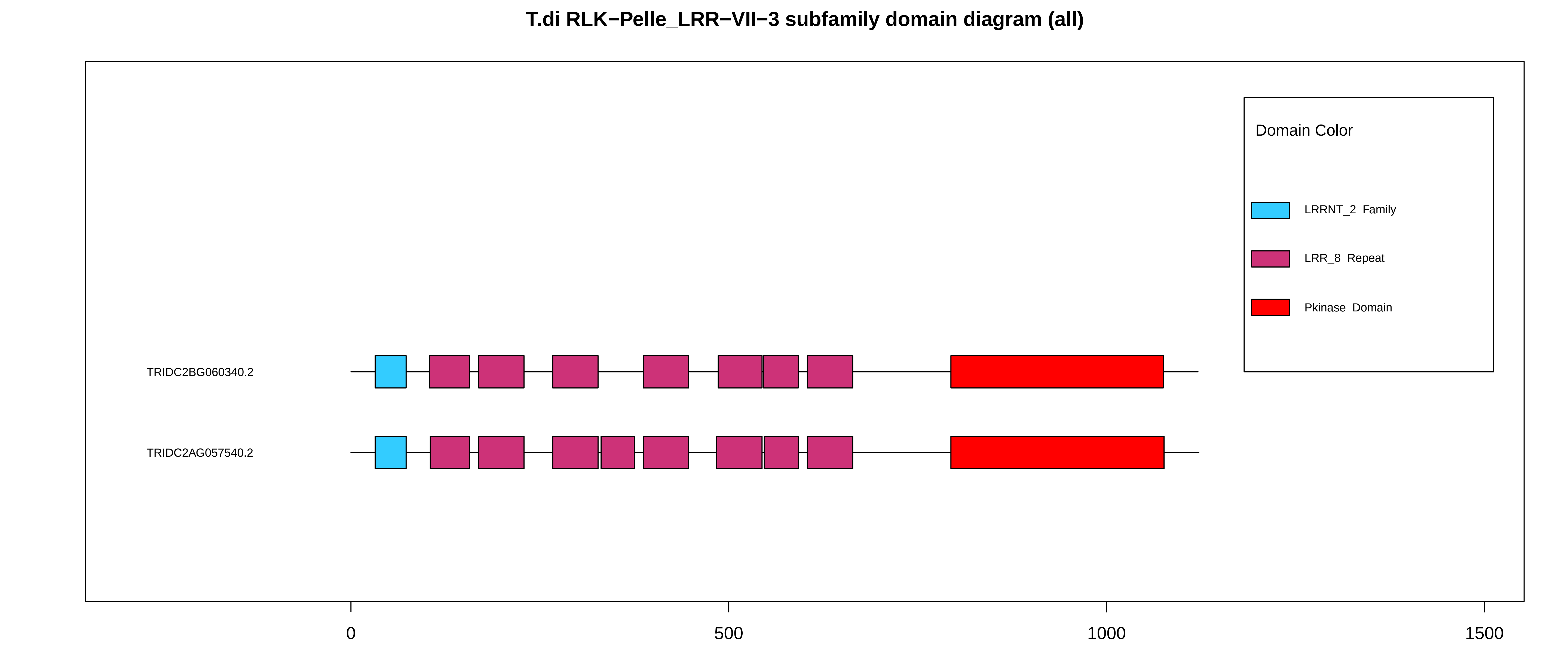

T.di RLK−Pelle_LRR−VII−3 subfamily domain diagram (all)
Domain Color
LRRNT_2 Family
LRR_8 Repeat
Pkinase Domain
TRIDC2BG060340.2
TRIDC2AG057540.2
0
500
1000
1500

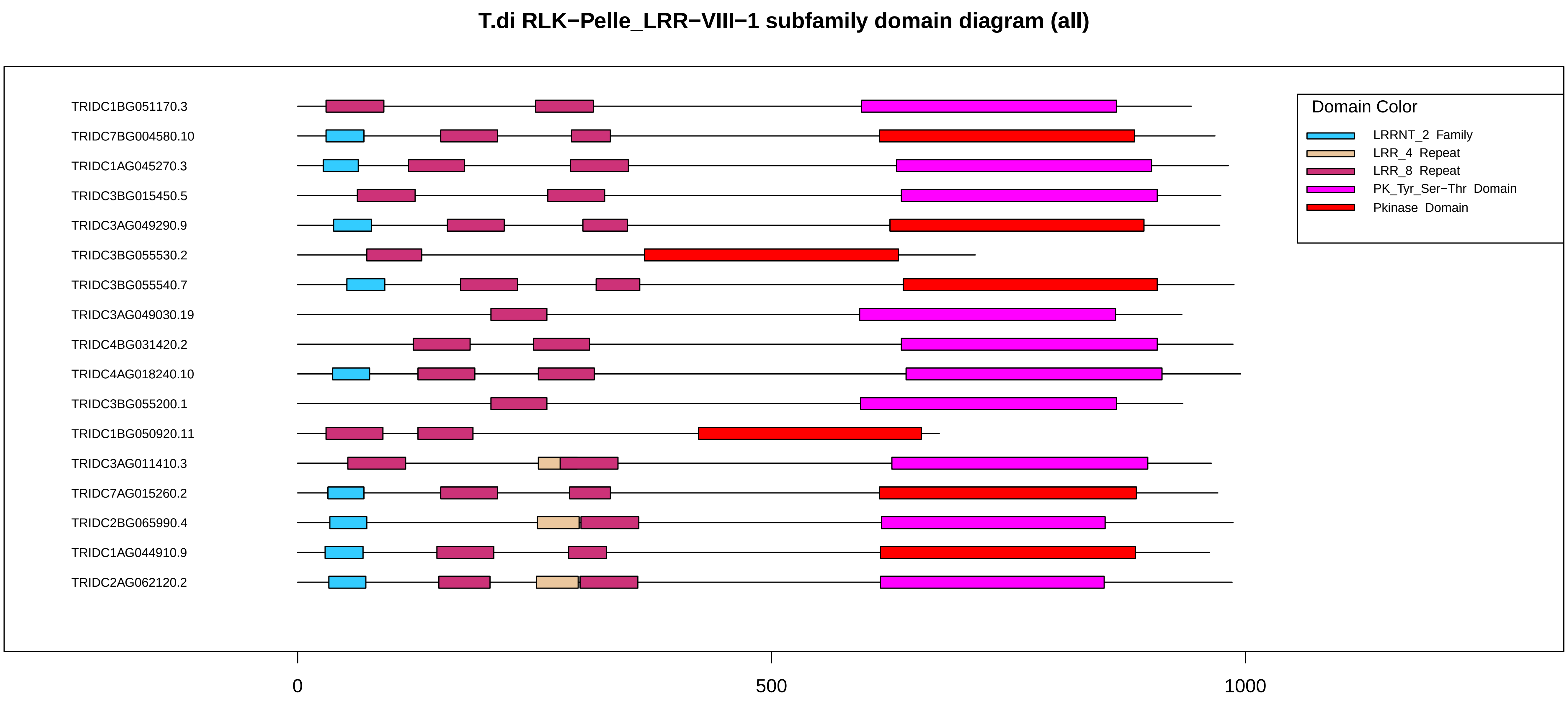
T.di RLK−Pelle_LRR−VIII−1 subfamily domain diagram (all)
TRIDC1BG051170.3
TRIDC7BG004580.10
TRIDC1AG045270.3
TRIDC3BG015450.5
TRIDC3AG049290.9
TRIDC3BG055530.2
TRIDC3BG055540.7
TRIDC3AG049030.19
TRIDC4BG031420.2
TRIDC4AG018240.10
TRIDC3BG055200.1
TRIDC1BG050920.11
TRIDC3AG011410.3
TRIDC7AG015260.2
TRIDC2BG065990.4
TRIDC1AG044910.9
TRIDC2AG062120.2
Domain Color
LRRNT_2 Family
LRR_4 Repeat
LRR_8 Repeat
PK_Tyr_Ser−Thr Domain
Pkinase Domain
0
500
1000

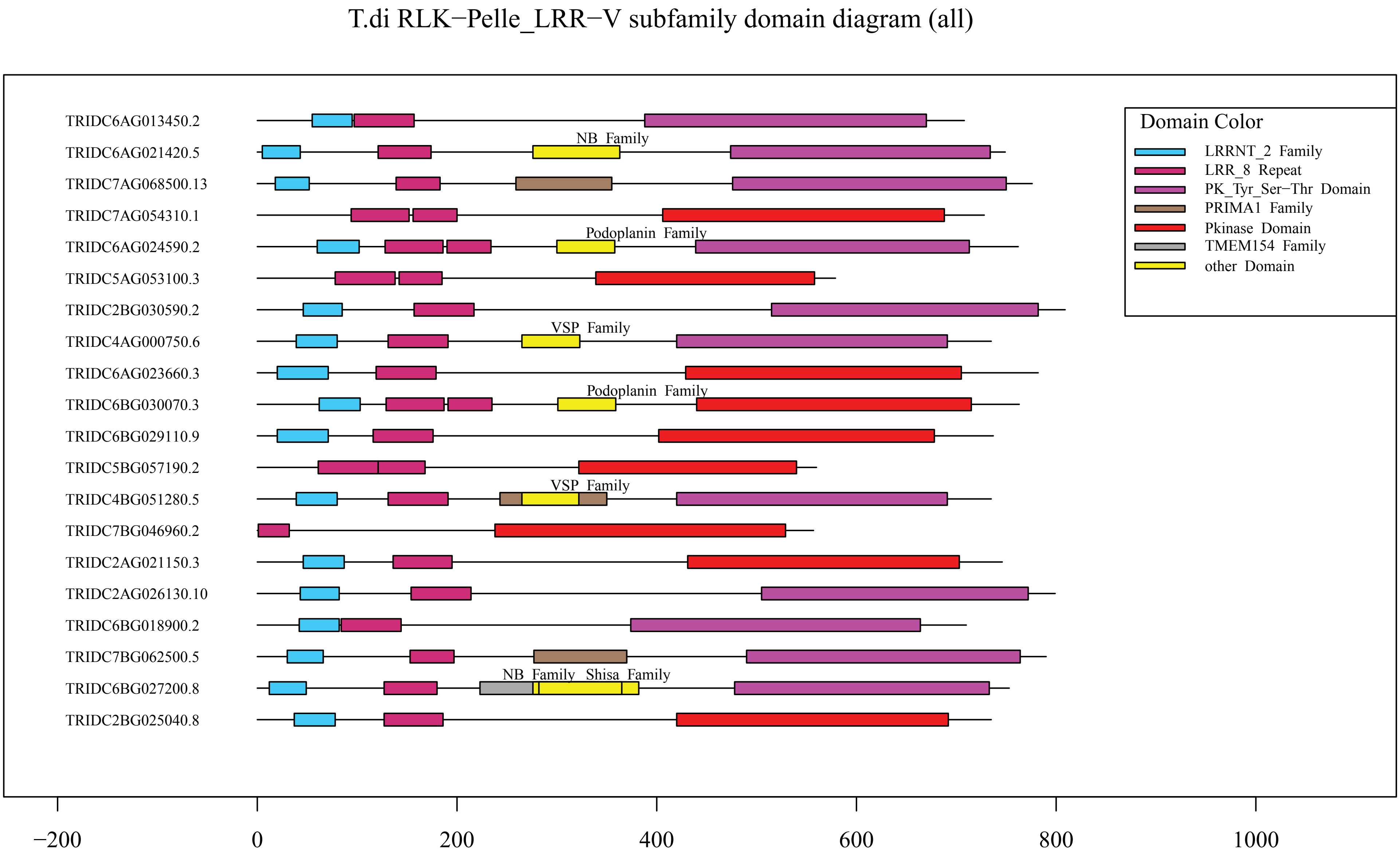
T.di RLK−Pelle_LRR−V subfamily domain diagram (all)
TRIDC6AG013450.2
TRIDC6AG021420.5
TRIDC7AG068500.13
TRIDC7AG054310.1
TRIDC6AG024590.2
TRIDC5AG053100.3
TRIDC2BG030590.2
TRIDC4AG000750.6
TRIDC6AG023660.3
TRIDC6BG030070.3
TRIDC6BG029110.9
TRIDC5BG057190.2
TRIDC4BG051280.5
TRIDC7BG046960.2
TRIDC2AG021150.3
TRIDC2AG026130.10
TRIDC6BG018900.2
TRIDC7BG062500.5
TRIDC6BG027200.8
TRIDC2BG025040.8
NB Family
Podoplanin Family
VSP Family
Podoplanin Family
VSP Family
NB Family Shisa Family
Domain Color
LRRNT_2 Family
LRR_8 Repeat
PK_Tyr_Ser−Thr Domain
PRIMA1 Family
Pkinase Domain
TMEM154 Family
other Domain
−200
0
200
400
600
800
1000

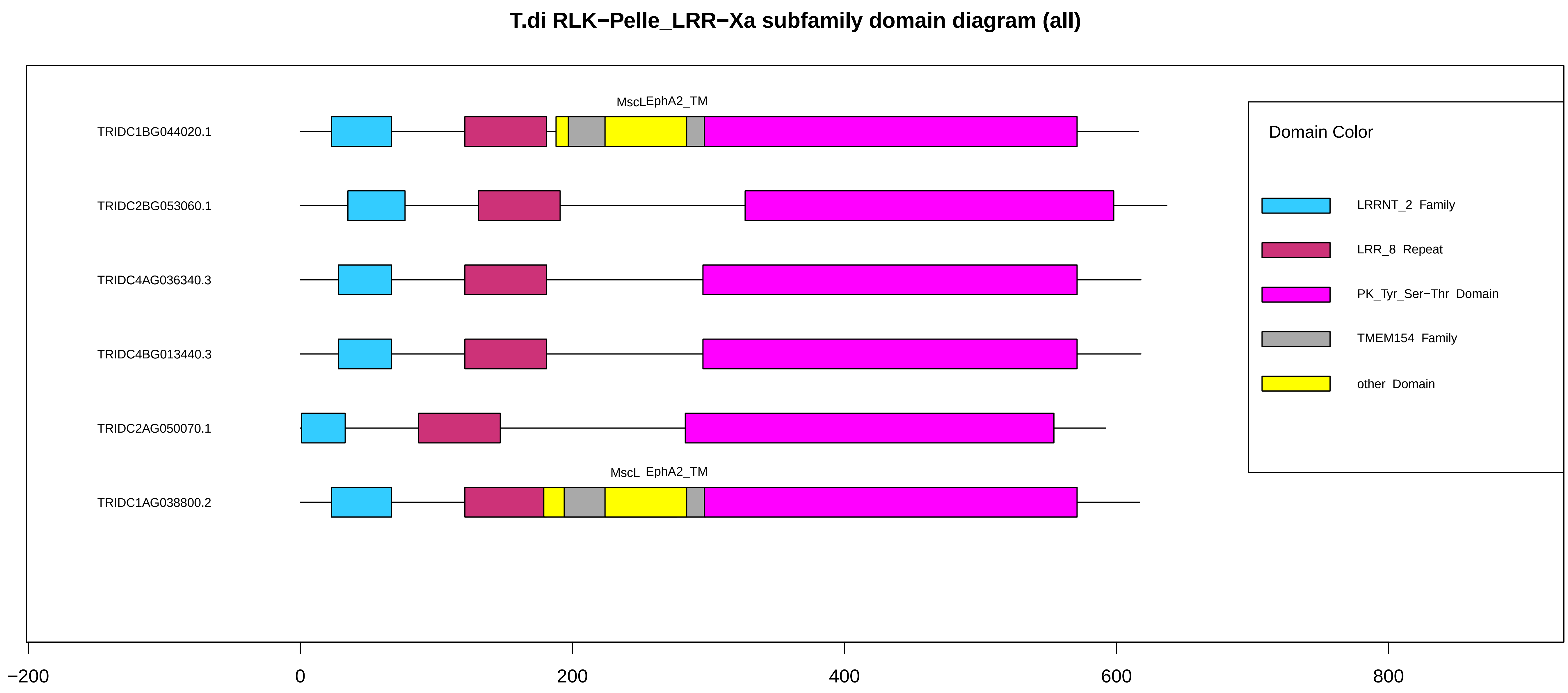
T.di RLK−Pelle_LRR−Xa subfamily domain diagram (all)
MscL
EphA2_TM
TRIDC1BG044020.1
TRIDC2BG053060.1
TRIDC4AG036340.3
TRIDC4BG013440.3
TRIDC2AG050070.1
MscL
EphA2_TM
TRIDC1AG038800.2
Domain Color
LRRNT_2 Family
LRR_8 Repeat
PK_Tyr_Ser−Thr Domain
TMEM154 Family
other Domain
−200
0
200
400
600
800

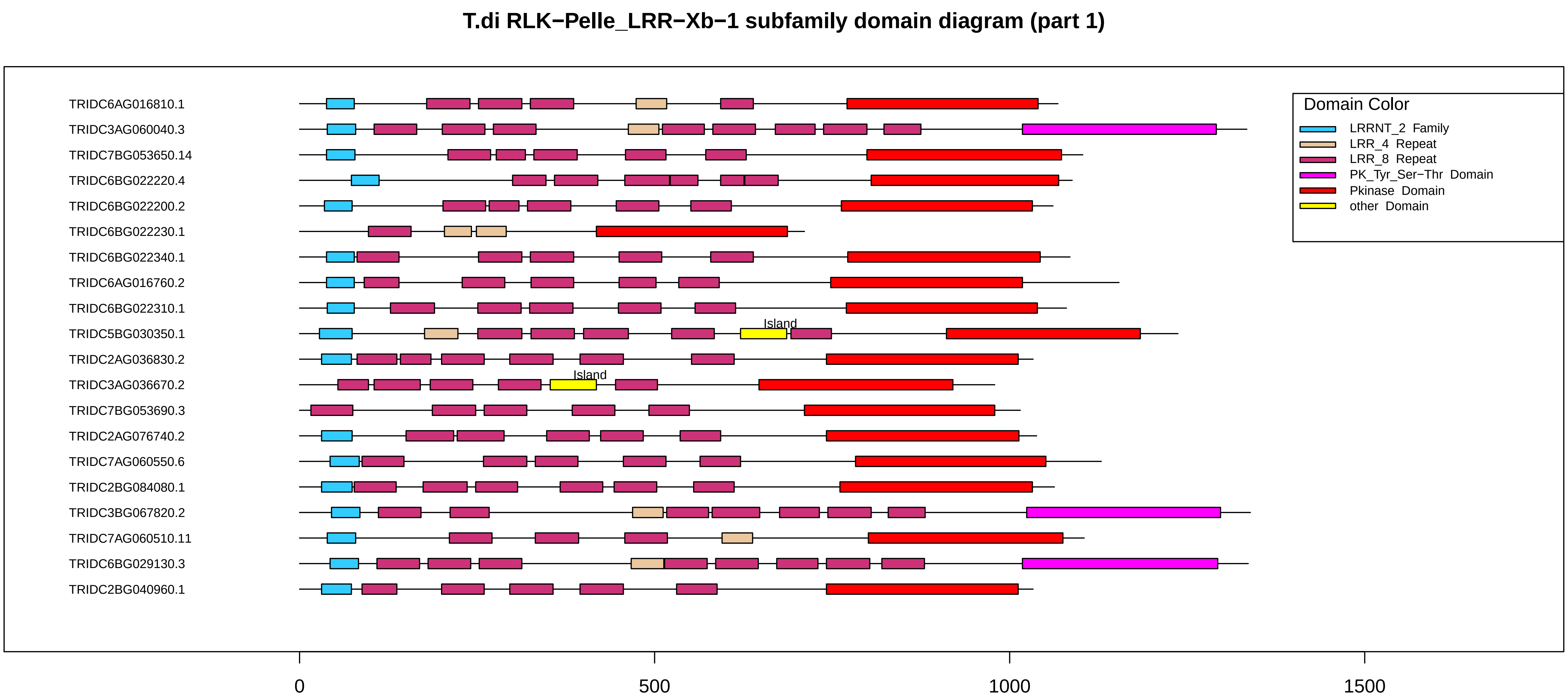
T.di RLK−Pelle_LRR−Xb−1 subfamily domain diagram (part 1)
TRIDC6AG016810.1
TRIDC3AG060040.3
TRIDC7BG053650.14
TRIDC6BG022220.4
TRIDC6BG022200.2
TRIDC6BG022230.1
TRIDC6BG022340.1
TRIDC6AG016760.2
TRIDC6BG022310.1
TRIDC5BG030350.1
TRIDC2AG036830.2
TRIDC3AG036670.2
TRIDC7BG053690.3
TRIDC2AG076740.2
TRIDC7AG060550.6
TRIDC2BG084080.1
TRIDC3BG067820.2
TRIDC7AG060510.11
TRIDC6BG029130.3
TRIDC2BG040960.1
Island
Island
Domain Color
LRRNT_2 Family
LRR_4 Repeat
LRR_8 Repeat
PK_Tyr_Ser−Thr Domain
Pkinase Domain
other Domain
0
500
1000
1500

T.di RLK−Pelle_LRR−Xb−1 subfamily domain diagram (part 2)

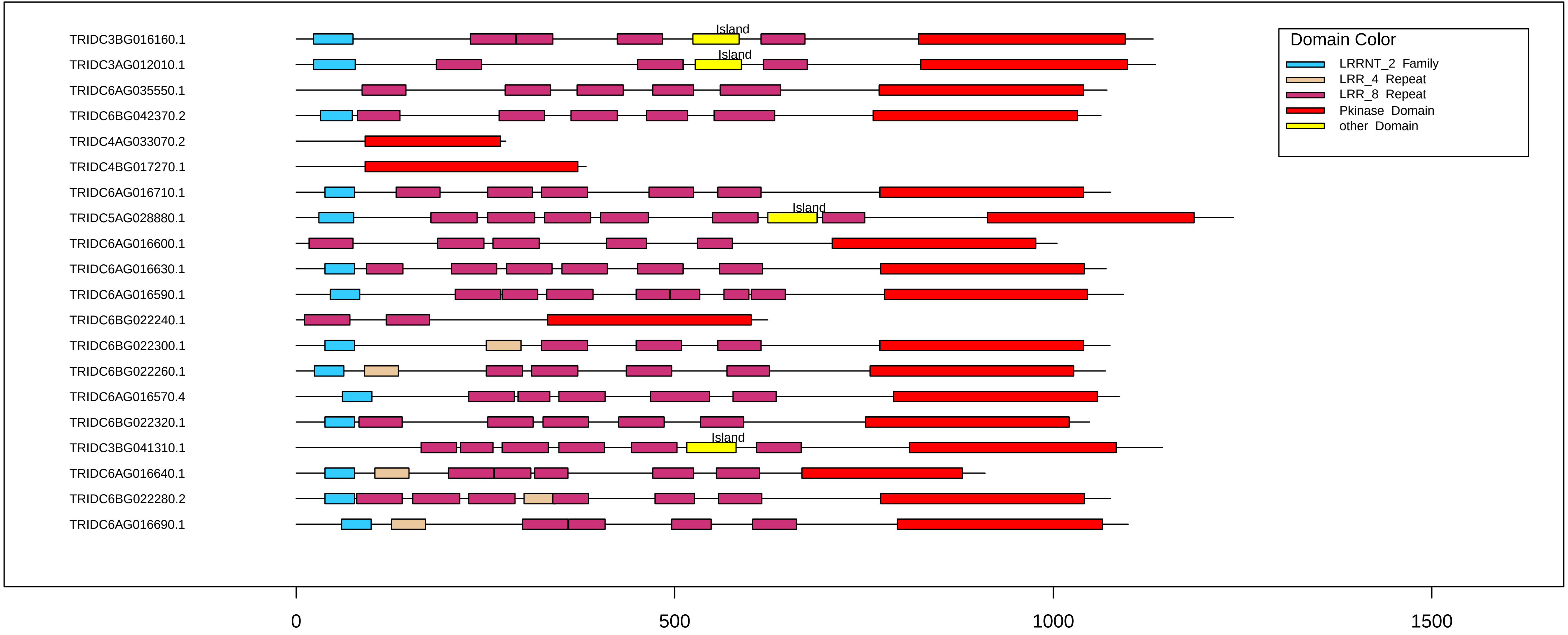

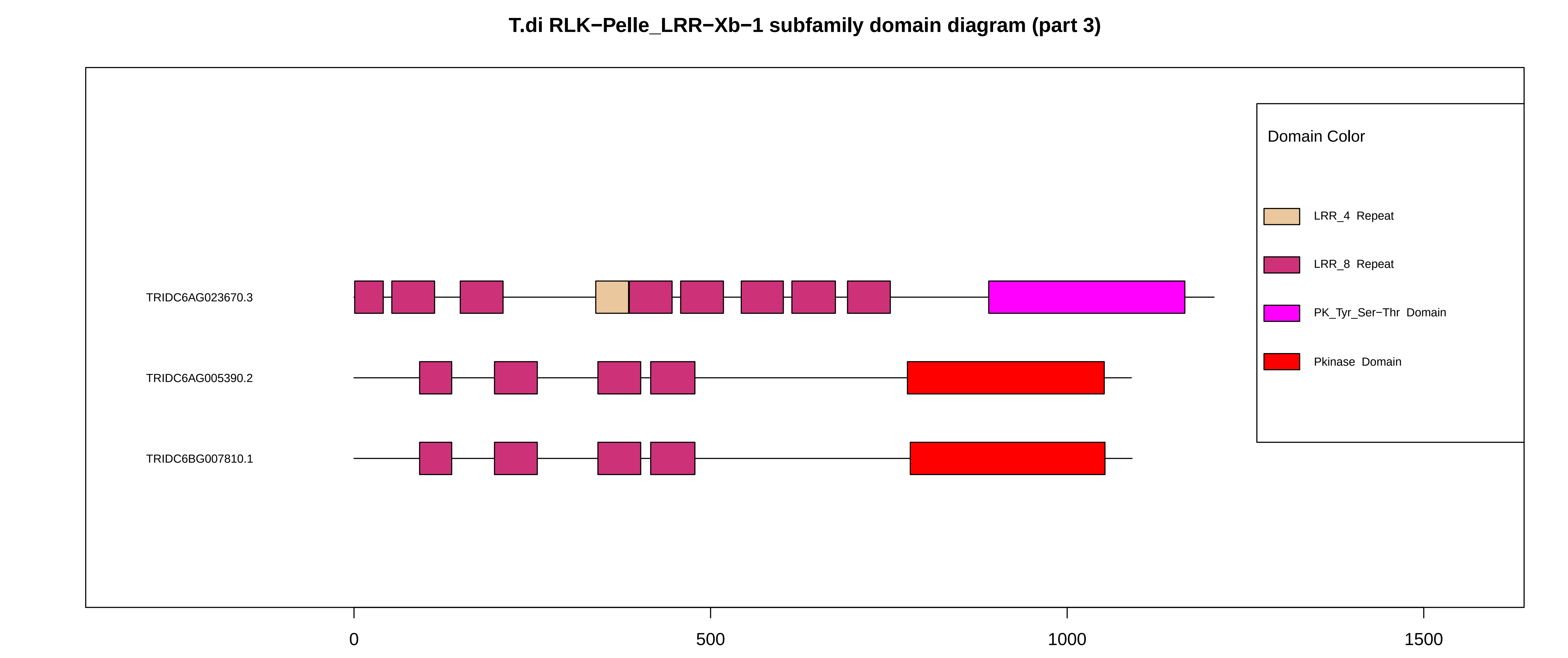
T.di RLK−Pelle_LRR−Xb−1 subfamily domain diagram (part 3)
TRIDC6AG023670.3
TRIDC6AG005390.2
TRIDC6BG007810.1
Domain Color
LRR_4 Repeat
LRR_8 Repeat
PK_Tyr_Ser−Thr Domain
Pkinase Domain
0
500
1000
1500

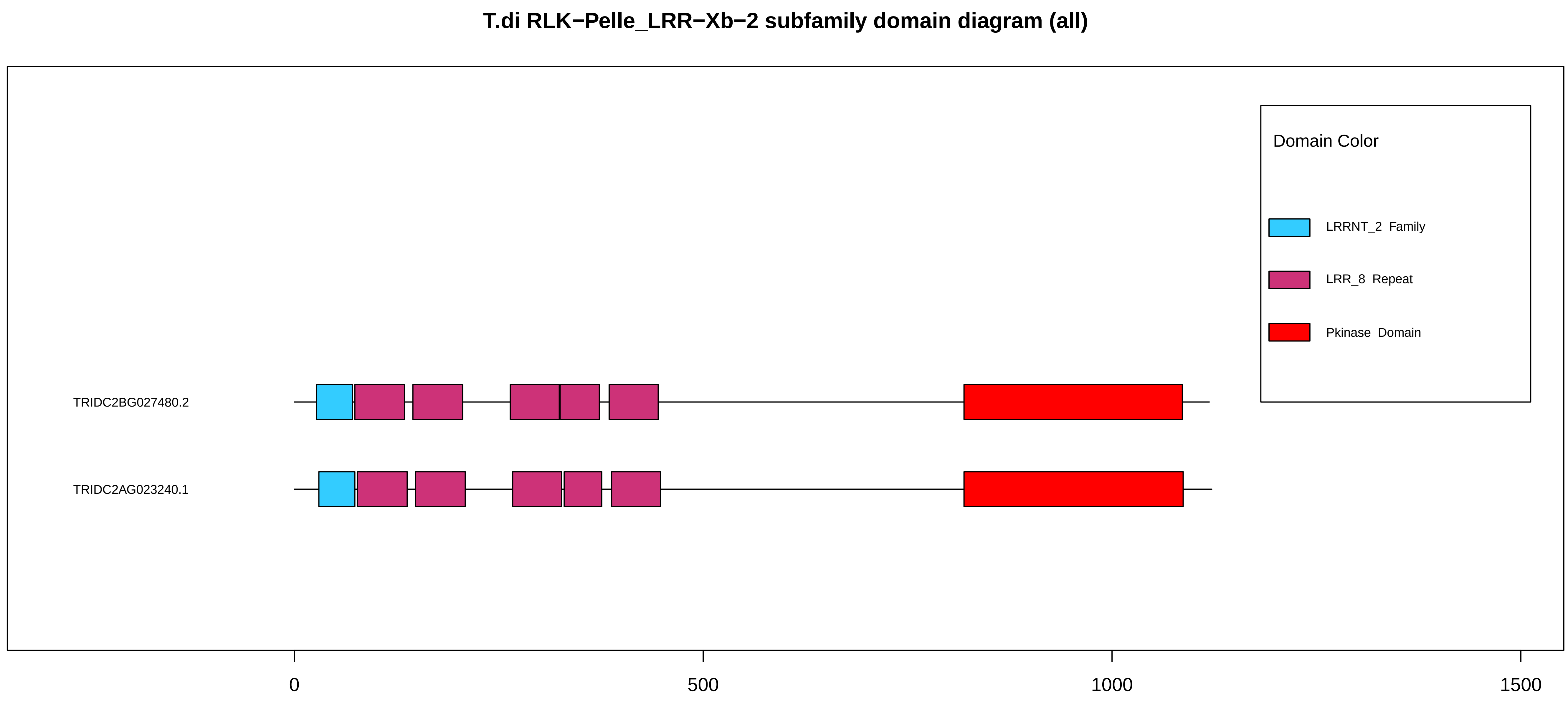
T.di RLK−Pelle_LRR−Xb−2 subfamily domain diagram (all)
Domain Color
LRRNT_2 Family
LRR_8 Repeat
Pkinase Domain
TRIDC2BG027480.2
TRIDC2AG023240.1
0
500
1000
1500

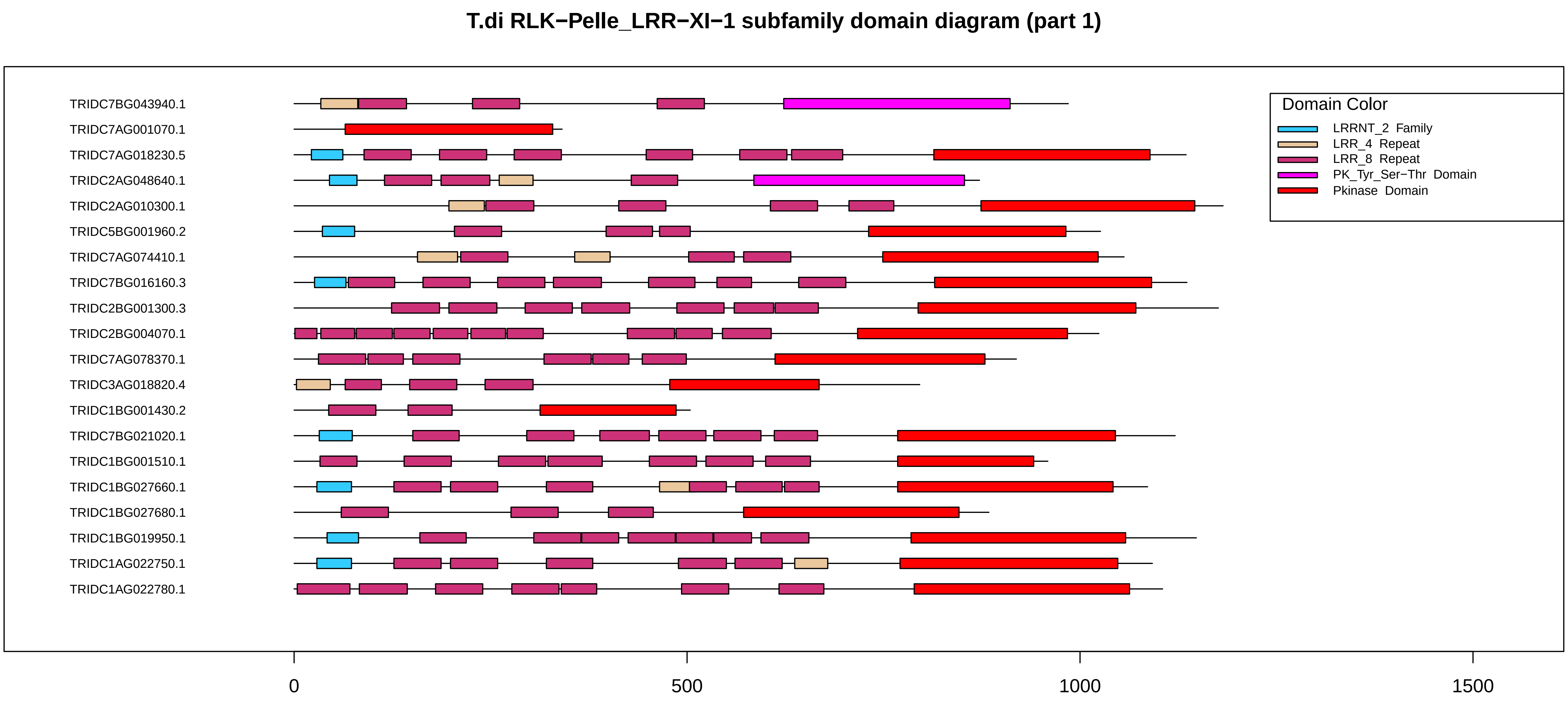

T.di RLK−Pelle_LRR−XI−1 subfamily domain diagram (part 1)
TRIDC7BG043940.1
TRIDC7AG001070.1
TRIDC7AG018230.5
TRIDC2AG048640.1
TRIDC2AG010300.1
TRIDC5BG001960.2
TRIDC7AG074410.1
TRIDC7BG016160.3
TRIDC2BG001300.3
TRIDC2BG004070.1
TRIDC7AG078370.1
TRIDC3AG018820.4
TRIDC1BG001430.2
TRIDC7BG021020.1
TRIDC1BG001510.1
TRIDC1BG027660.1
TRIDC1BG027680.1
TRIDC1BG019950.1
TRIDC1AG022750.1
TRIDC1AG022780.1
Domain Color
LRRNT_2 Family
LRR_4 Repeat
LRR_8 Repeat
PK_Tyr_Ser−Thr Domain
Pkinase Domain
0
500
1000
1500

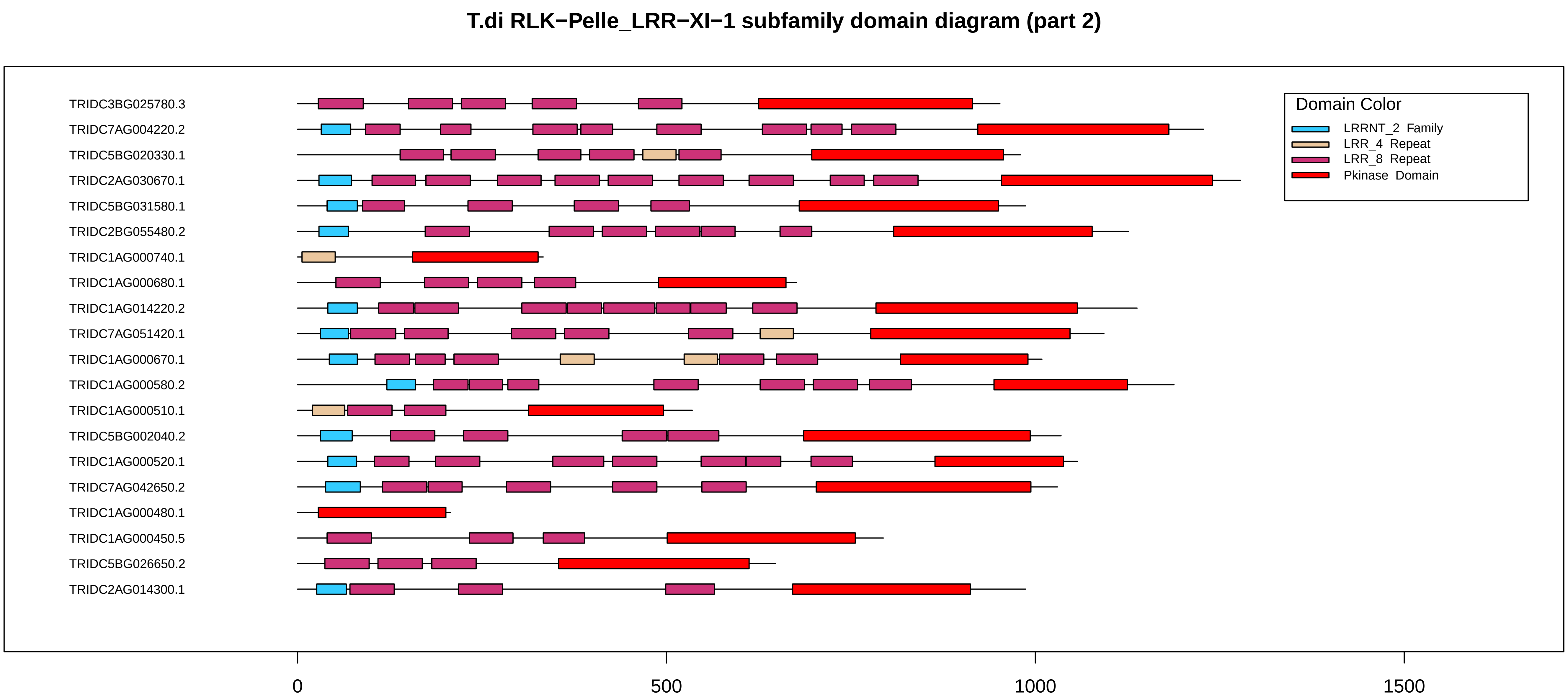
T.di RLK−Pelle_LRR−XI−1 subfamily domain diagram (part 2)
TRIDC3BG025780.3
TRIDC7AG004220.2
TRIDC5BG020330.1
TRIDC2AG030670.1
TRIDC5BG031580.1
TRIDC2BG055480.2
TRIDC1AG000740.1
TRIDC1AG000680.1
TRIDC1AG014220.2
TRIDC7AG051420.1
TRIDC1AG000670.1
TRIDC1AG000580.2
TRIDC1AG000510.1
TRIDC5BG002040.2
TRIDC1AG000520.1
TRIDC7AG042650.2
TRIDC1AG000480.1
TRIDC1AG000450.5
TRIDC5BG026650.2
TRIDC2AG014300.1
Domain Color
LRRNT_2 Family
LRR_4 Repeat
LRR_8 Repeat
Pkinase Domain
0
500
1000
1500

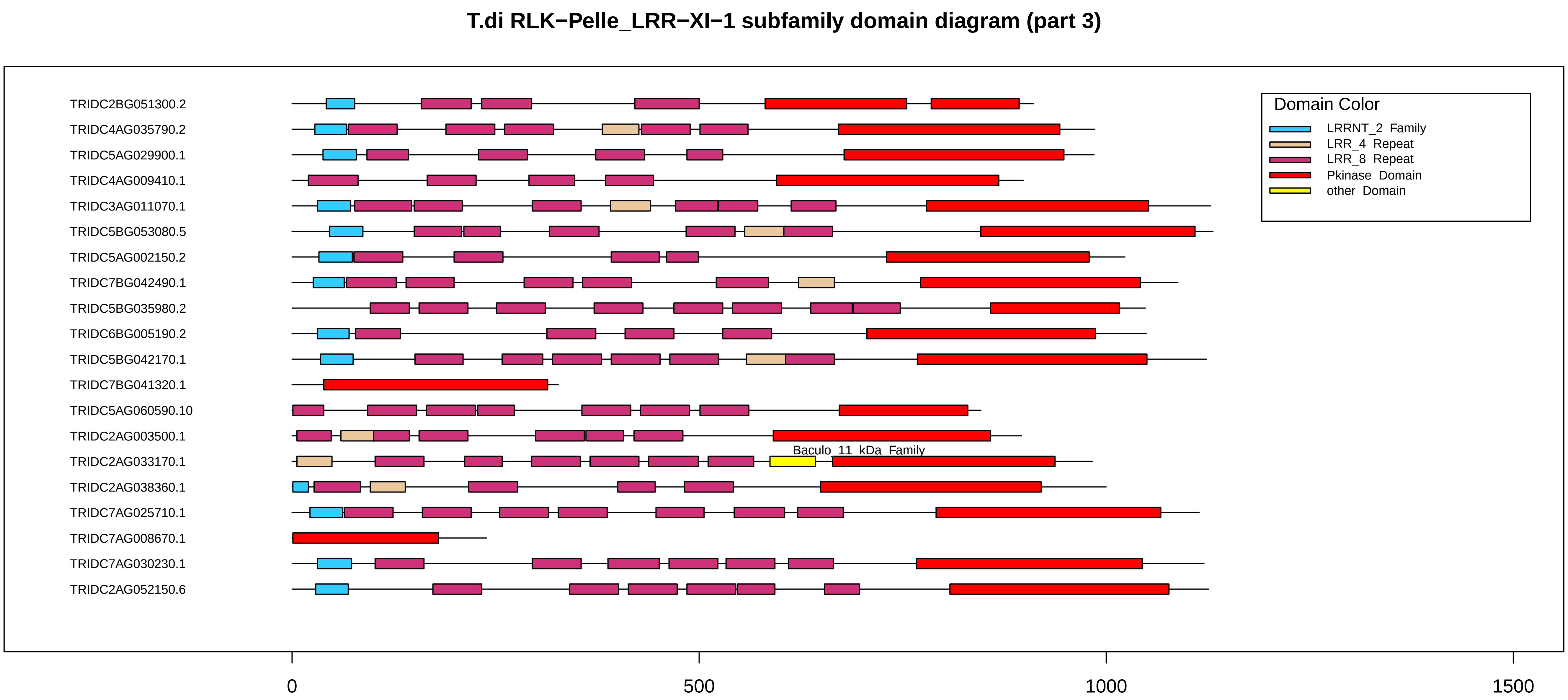
T.di RLK−Pelle_LRR−XI−1 subfamily domain diagram (part 3)
TRIDC2BG051300.2
TRIDC4AG035790.2
TRIDC5AG029900.1
TRIDC4AG009410.1
TRIDC3AG011070.1
TRIDC5BG053080.5
TRIDC5AG002150.2
TRIDC7BG042490.1
TRIDC5BG035980.2
TRIDC6BG005190.2
TRIDC5BG042170.1
TRIDC7BG041320.1
TRIDC5AG060590.10
TRIDC2AG003500.1
TRIDC2AG033170.1
TRIDC2AG038360.1
TRIDC7AG025710.1
TRIDC7AG008670.1
TRIDC7AG030230.1
TRIDC2AG052150.6
Baculo_11_kDa_Family
Domain Color
LRRNT_2 Family
LRR_4 Repeat
LRR_8 Repeat
Pkinase Domain
other Domain
0
500
1000
1500

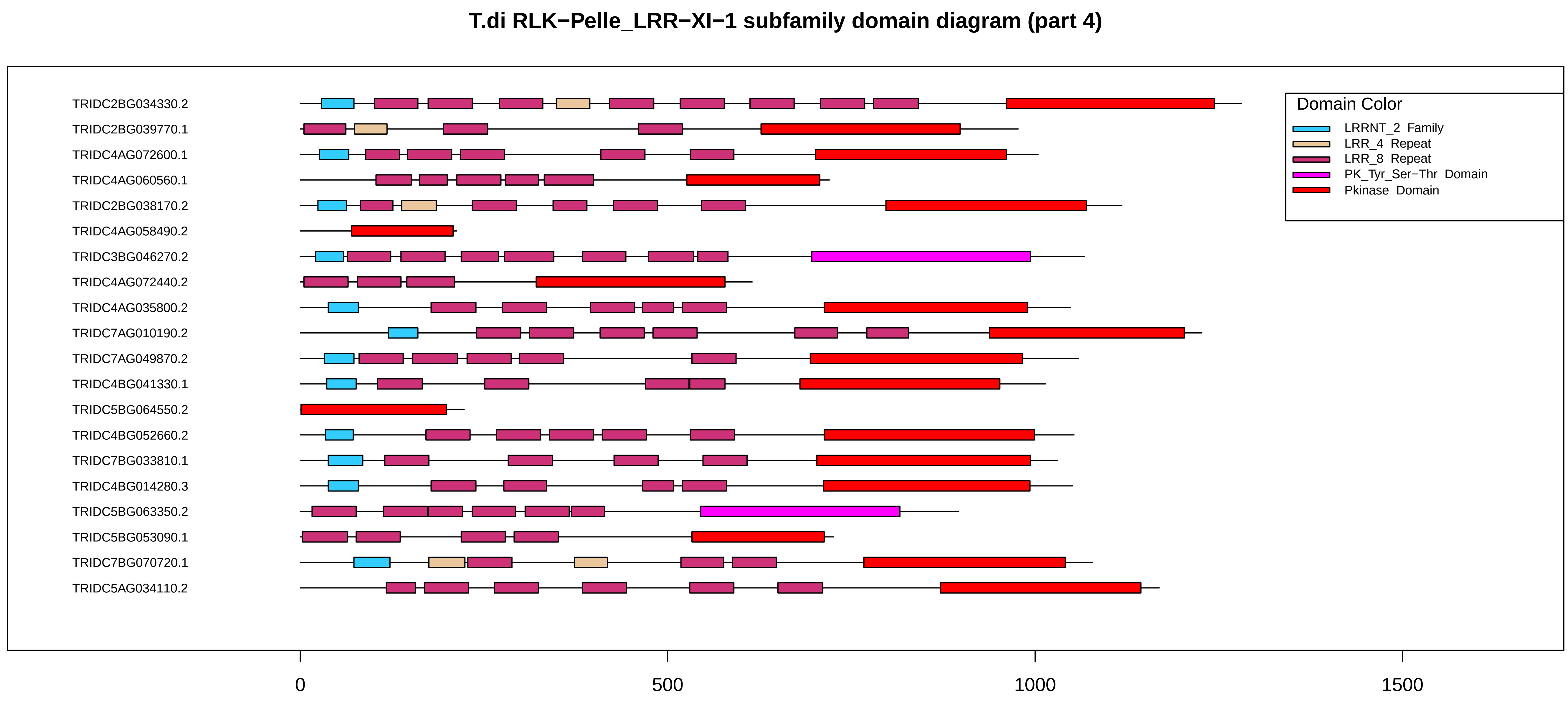
T.di RLK−Pelle_LRR−XI−1 subfamily domain diagram (part 4)
TRIDC2BG034330.2
TRIDC2BG039770.1
TRIDC4AG072600.1
TRIDC4AG060560.1
TRIDC2BG038170.2
TRIDC4AG058490.2
TRIDC3BG046270.2
TRIDC4AG072440.2
TRIDC4AG035800.2
TRIDC7AG010190.2
TRIDC7AG049870.2
TRIDC4BG041330.1
TRIDC5BG064550.2
TRIDC4BG052660.2
TRIDC7BG033810.1
TRIDC4BG014280.3
TRIDC5BG063350.2
TRIDC5BG053090.1
TRIDC7BG070720.1
TRIDC5AG034110.2
Domain Color
LRRNT_2 Family
LRR_4 Repeat
LRR_8 Repeat
PK_Tyr_Ser−Thr Domain
Pkinase Domain
0
500
1000
1500

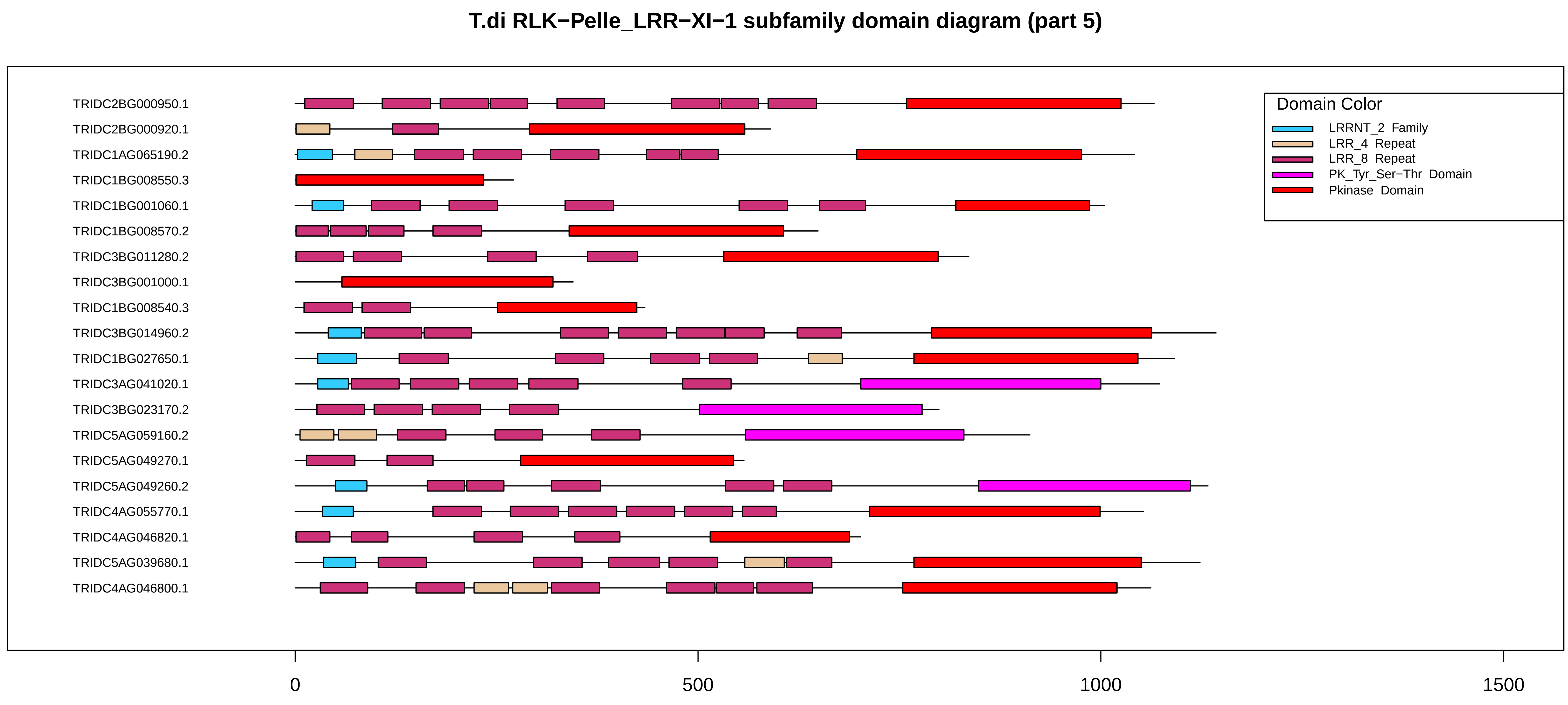
T.di RLK−Pelle_LRR−XI−1 subfamily domain diagram (part 5)
TRIDC2BG000950.1
TRIDC2BG000920.1
TRIDC1AG065190.2
TRIDC1BG008550.3
TRIDC1BG001060.1
TRIDC1BG008570.2
TRIDC3BG011280.2
TRIDC3BG001000.1
TRIDC1BG008540.3
TRIDC3BG014960.2
TRIDC1BG027650.1
TRIDC3AG041020.1
TRIDC3BG023170.2
TRIDC5AG059160.2
TRIDC5AG049270.1
TRIDC5AG049260.2
TRIDC4AG055770.1
TRIDC4AG046820.1
TRIDC5AG039680.1
TRIDC4AG046800.1
Domain Color
LRRNT_2 Family
LRR_4 Repeat
LRR_8 Repeat
PK_Tyr_Ser−Thr Domain
Pkinase Domain
0
500
1000
1500

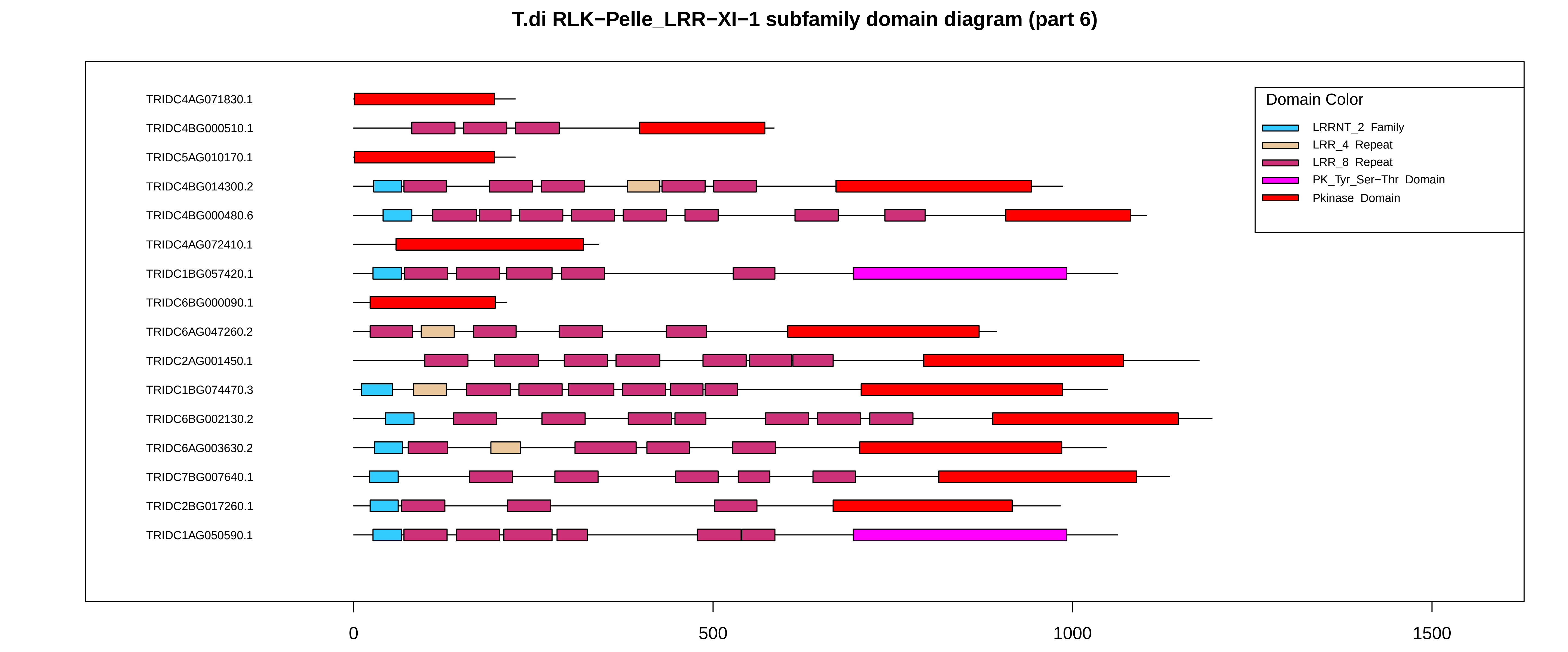
T.di RLK−Pelle_LRR−XI−1 subfamily domain diagram (part 6)
TRIDC4AG071830.1
TRIDC4BG000510.1
TRIDC5AG010170.1
TRIDC4BG014300.2
TRIDC4BG000480.6
TRIDC4AG072410.1
TRIDC1BG057420.1
TRIDC6BG000090.1
TRIDC6AG047260.2
TRIDC2AG001450.1
TRIDC1BG074470.3
TRIDC6BG002130.2
TRIDC6AG003630.2
TRIDC7BG007640.1
TRIDC2BG017260.1
TRIDC1AG050590.1
Domain Color
LRRNT_2 Family
LRR_4 Repeat
LRR_8 Repeat
PK_Tyr_Ser−Thr Domain
Pkinase Domain
0
500
1000
1500

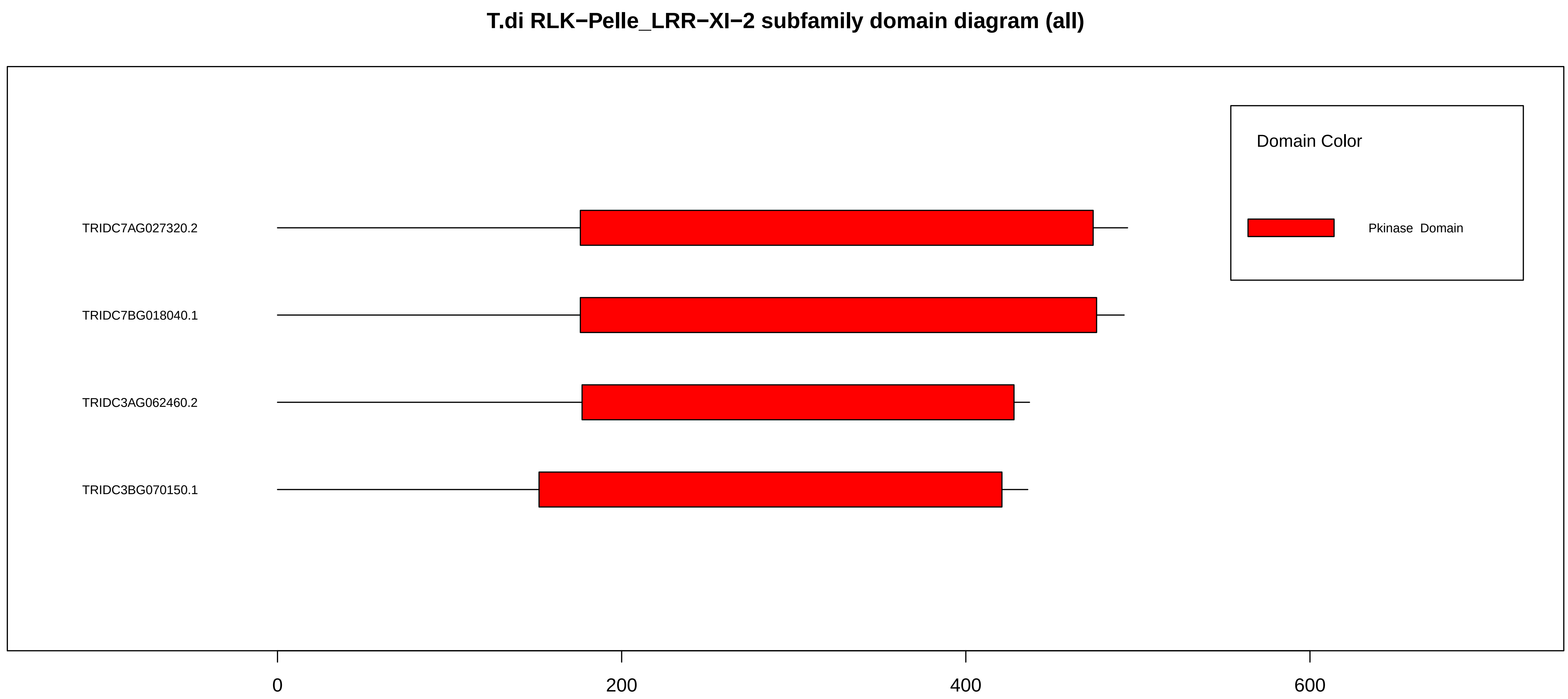

T.di RLK−Pelle_LRR−XI−2 subfamily domain diagram (all)
Domain Color
Pkinase Domain
TRIDC7AG027320.2
TRIDC7BG018040.1
TRIDC3AG062460.2
TRIDC3BG070150.1
0
200
400
600

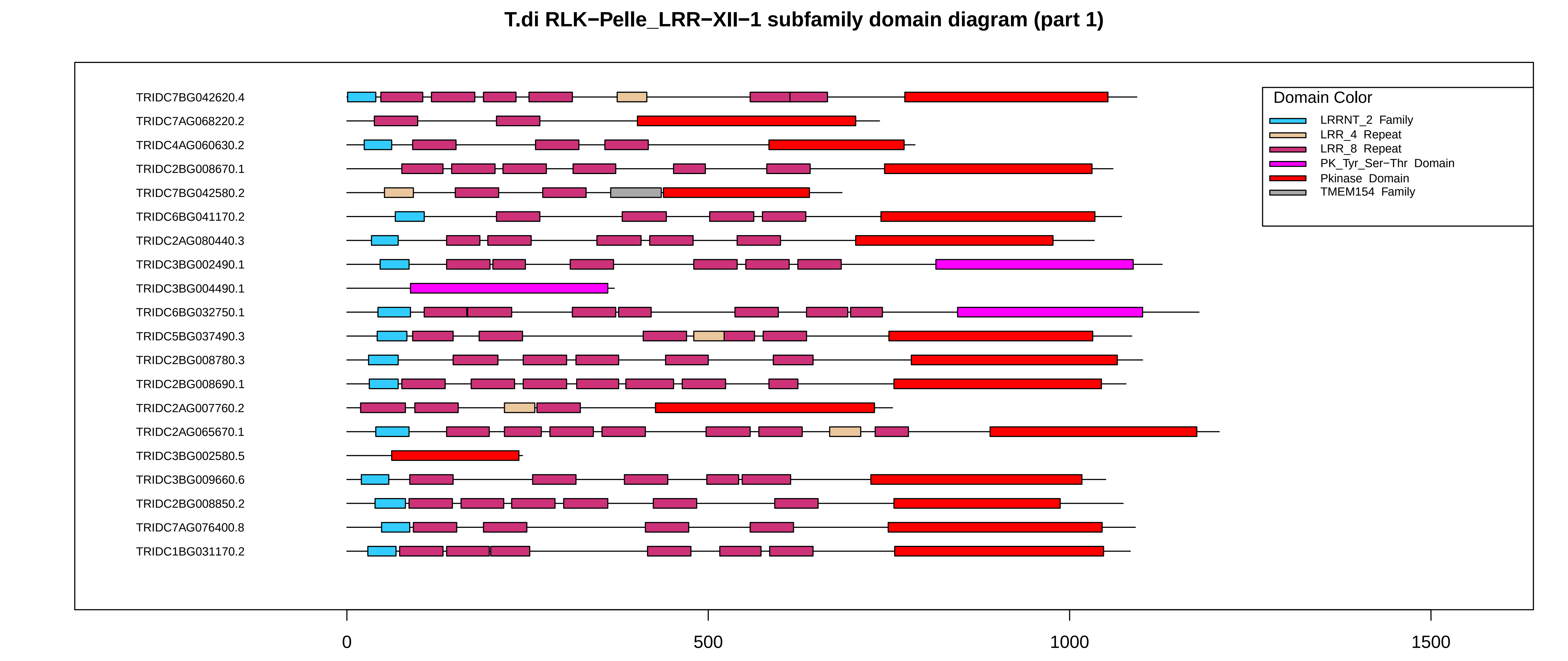
T.di RLK−Pelle_LRR−XII−1 subfamily domain diagram (part 1)
TRIDC7BG042620.4
TRIDC7AG068220.2
TRIDC4AG060630.2
TRIDC2BG008670.1
TRIDC7BG042580.2
TRIDC6BG041170.2
TRIDC2AG080440.3
TRIDC3BG002490.1
TRIDC3BG004490.1
TRIDC6BG032750.1
TRIDC5BG037490.3
TRIDC2BG008780.3
TRIDC2BG008690.1
TRIDC2AG007760.2
TRIDC2AG065670.1
TRIDC3BG002580.5
TRIDC3BG009660.6
TRIDC2BG008850.2
TRIDC7AG076400.8
TRIDC1BG031170.2
Domain Color
LRRNT_2 Family
LRR_4 Repeat
LRR_8 Repeat
PK_Tyr_Ser−Thr Domain
Pkinase Domain
TMEM154 Family
0
500
1000
1500

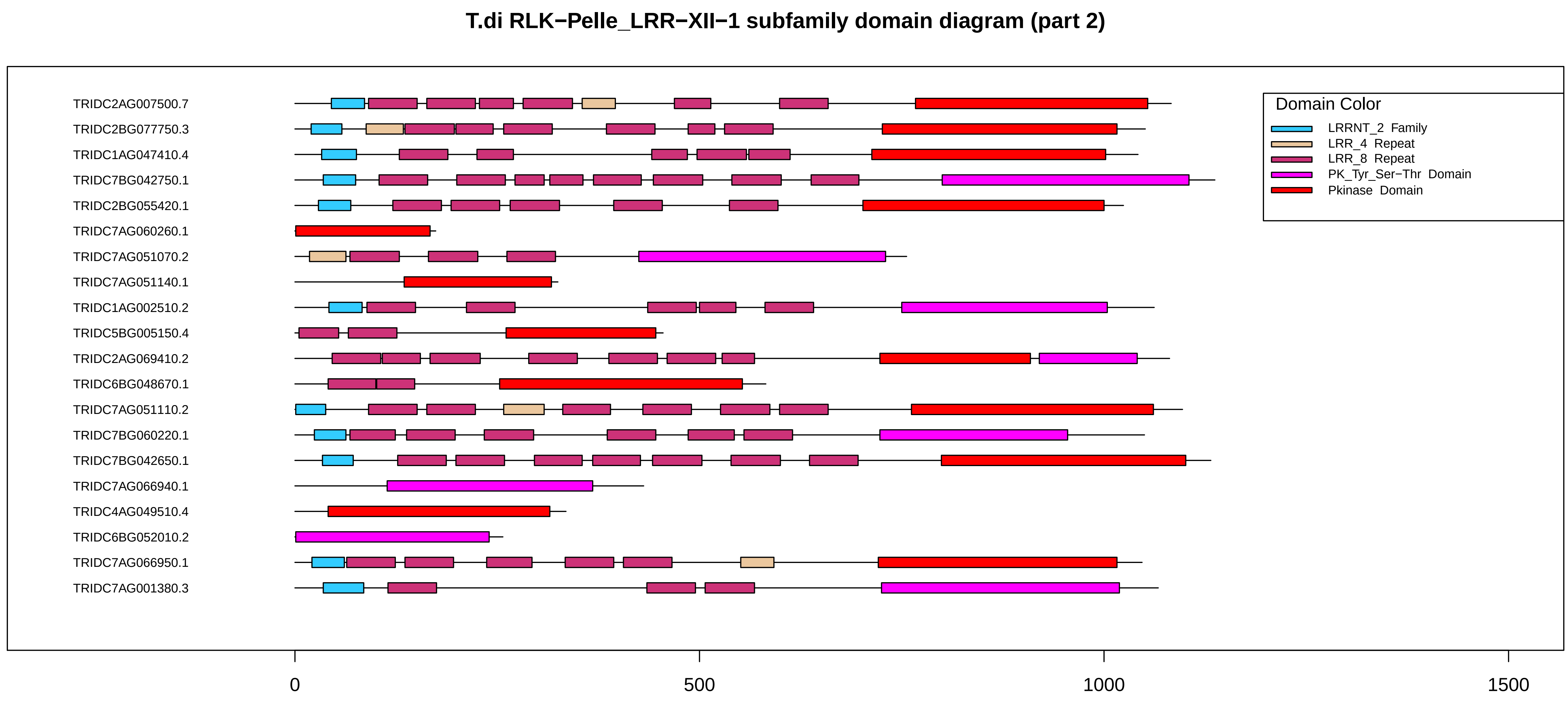
T.di RLK−Pelle_LRR−XII−1 subfamily domain diagram (part 2)
TRIDC2AG007500.7
TRIDC2BG077750.3
TRIDC1AG047410.4
TRIDC7BG042750.1
TRIDC2BG055420.1
TRIDC7AG060260.1
TRIDC7AG051070.2
TRIDC7AG051140.1
TRIDC1AG002510.2
TRIDC5BG005150.4
TRIDC2AG069410.2
TRIDC6BG048670.1
TRIDC7AG051110.2
TRIDC7BG060220.1
TRIDC7BG042650.1
TRIDC7AG066940.1
TRIDC4AG049510.4
TRIDC6BG052010.2
TRIDC7AG066950.1
TRIDC7AG001380.3
Domain Color
LRRNT_2 Family
LRR_4 Repeat
LRR_8 Repeat
PK_Tyr_Ser−Thr Domain
Pkinase Domain
0
500
1000
1500

T.di RLK−Pelle_LRR−XII−1 subfamily domain diagram (part 3)

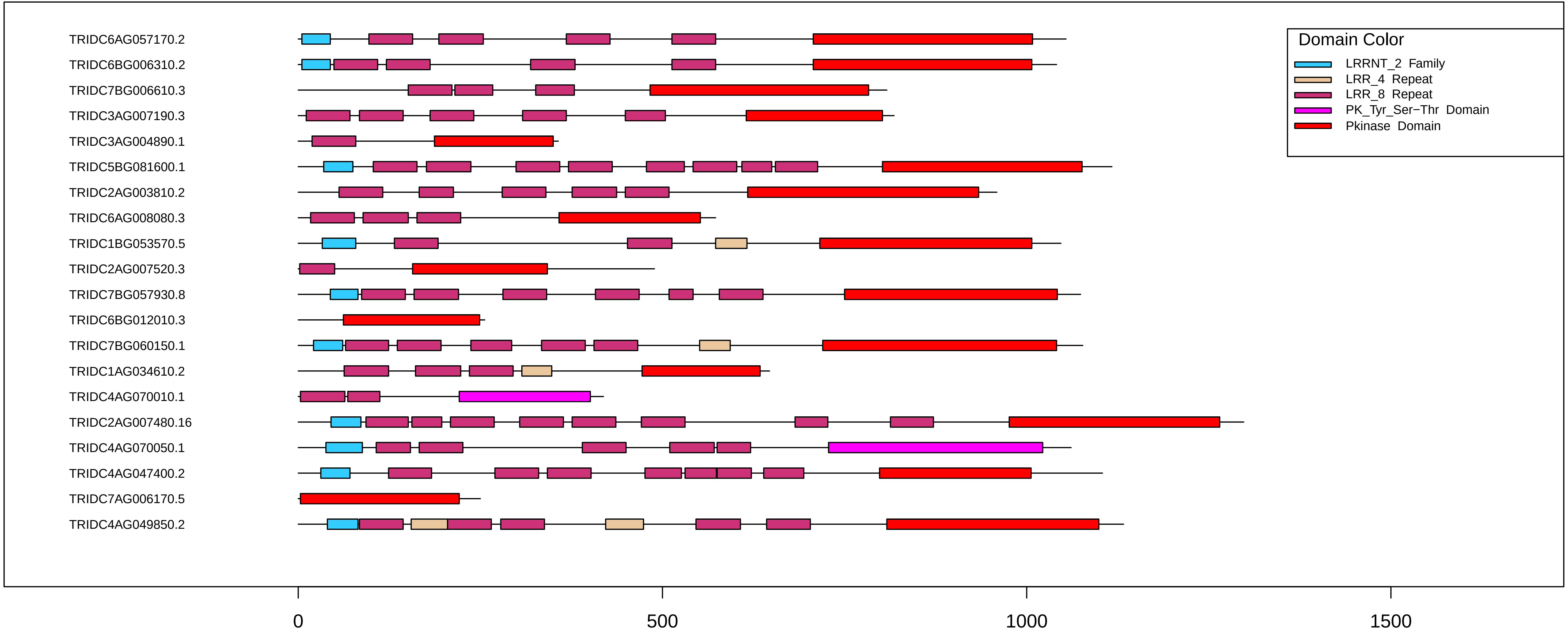

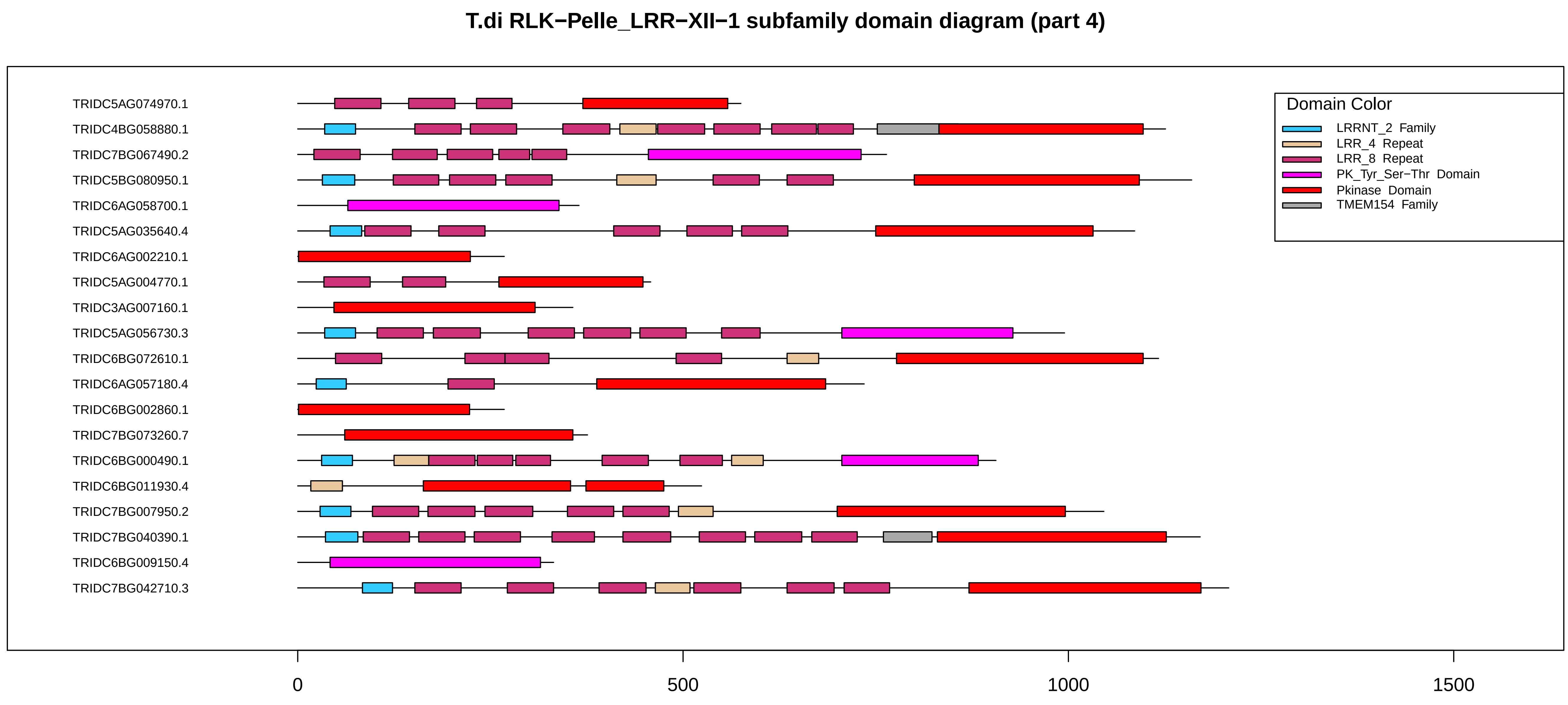
T.di RLK−Pelle_LRR−XII−1 subfamily domain diagram (part 4)
TRIDC5AG074970.1
TRIDC4BG058880.1
TRIDC7BG067490.2
TRIDC5BG080950.1
TRIDC6AG058700.1
TRIDC5AG035640.4
TRIDC6AG002210.1
TRIDC5AG004770.1
TRIDC3AG007160.1
TRIDC5AG056730.3
TRIDC6BG072610.1
TRIDC6AG057180.4
TRIDC6BG002860.1
TRIDC7BG073260.7
TRIDC6BG000490.1
TRIDC6BG011930.4
TRIDC7BG007950.2
TRIDC7BG040390.1
TRIDC6BG009150.4
TRIDC7BG042710.3
Domain Color
LRRNT_2 Family
LRR_4 Repeat
LRR_8 Repeat
PK_Tyr_Ser−Thr Domain
Pkinase Domain
TMEM154 Family
0
500
1000
1500

T.di RLK−Pelle_LRR−XII−1 subfamily domain diagram (part 5)

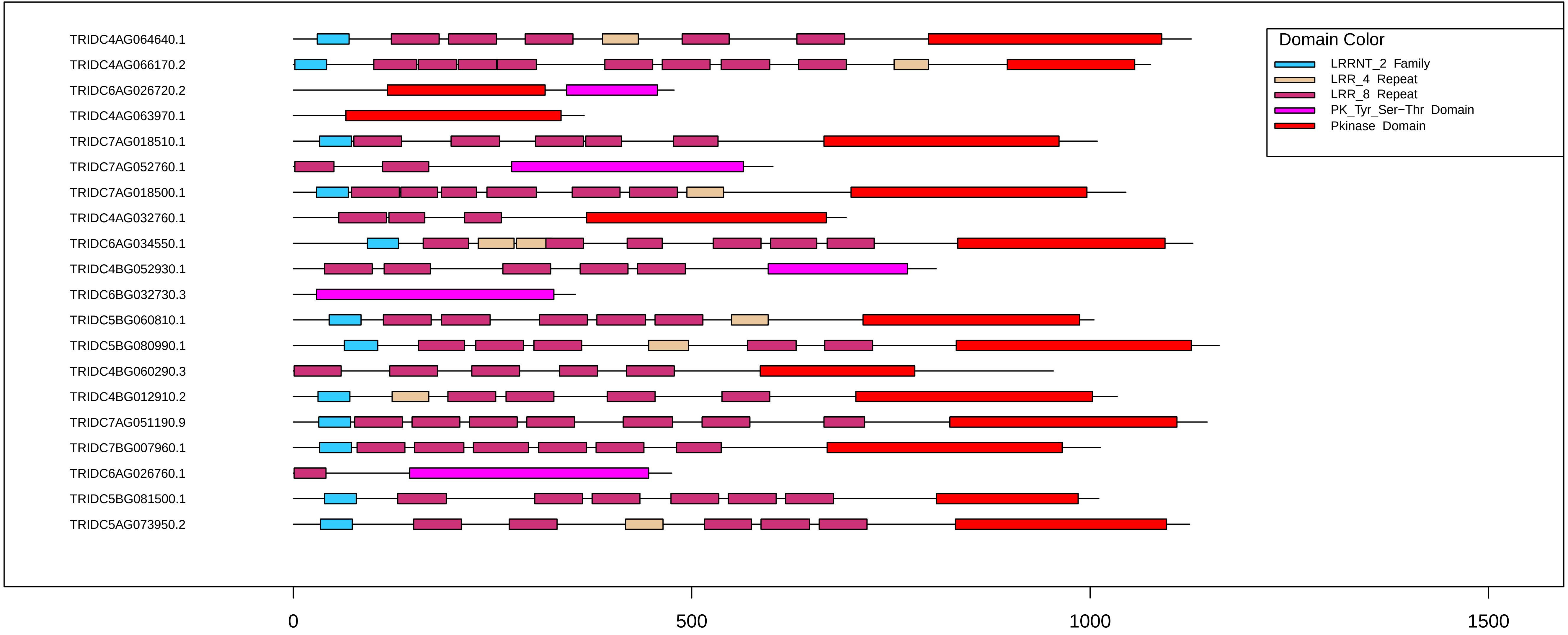

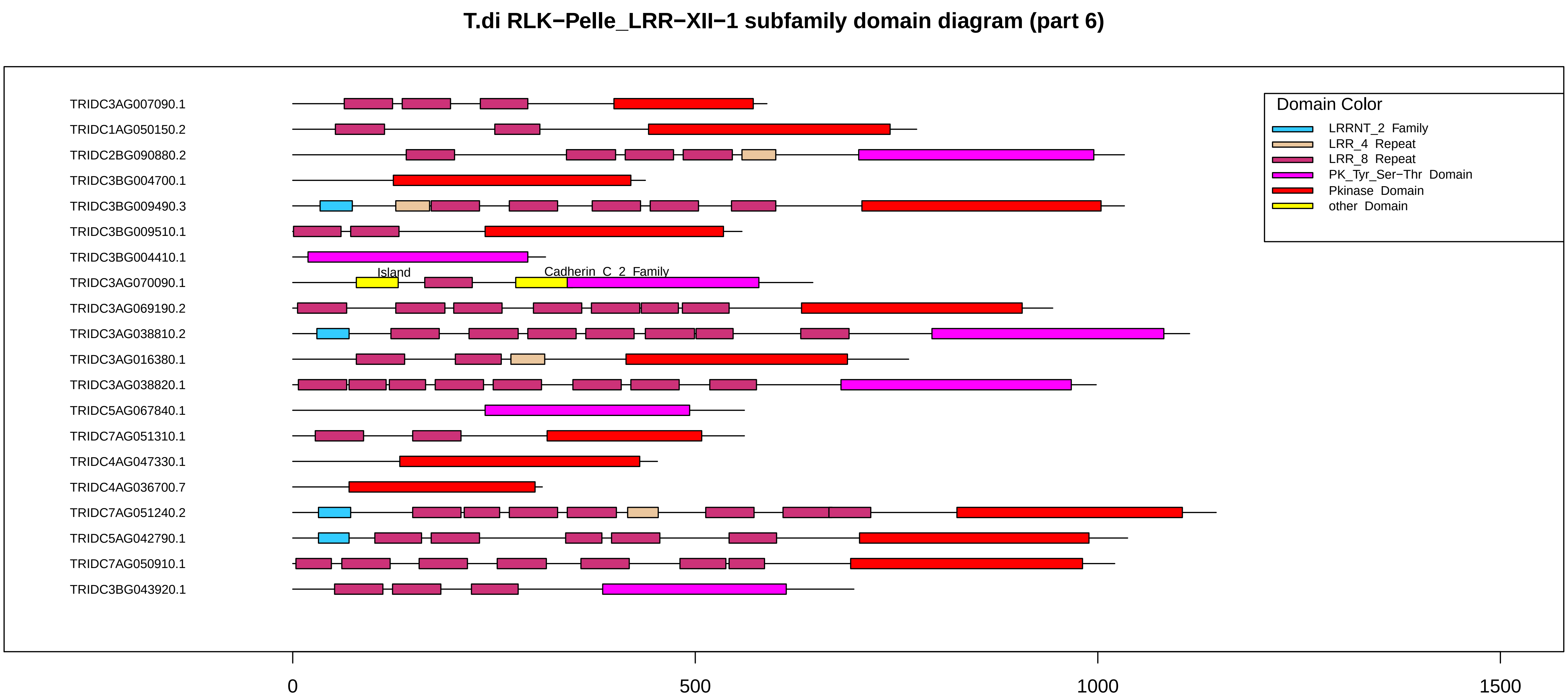
T.di RLK−Pelle_LRR−XII−1 subfamily domain diagram (part 6)
TRIDC3AG007090.1
TRIDC1AG050150.2
TRIDC2BG090880.2
TRIDC3BG004700.1
TRIDC3BG009490.3
TRIDC3BG009510.1
TRIDC3BG004410.1
TRIDC3AG070090.1
TRIDC3AG069190.2
TRIDC3AG038810.2
TRIDC3AG016380.1
TRIDC3AG038820.1
TRIDC5AG067840.1
TRIDC7AG051310.1
TRIDC4AG047330.1
TRIDC4AG036700.7
TRIDC7AG051240.2
TRIDC5AG042790.1
TRIDC7AG050910.1
TRIDC3BG043920.1
Island
Cadherin_C_2_Family
Domain Color
LRRNT_2 Family
LRR_4 Repeat
LRR_8 Repeat
PK_Tyr_Ser−Thr Domain
Pkinase Domain
other Domain
0
500
1000
1500

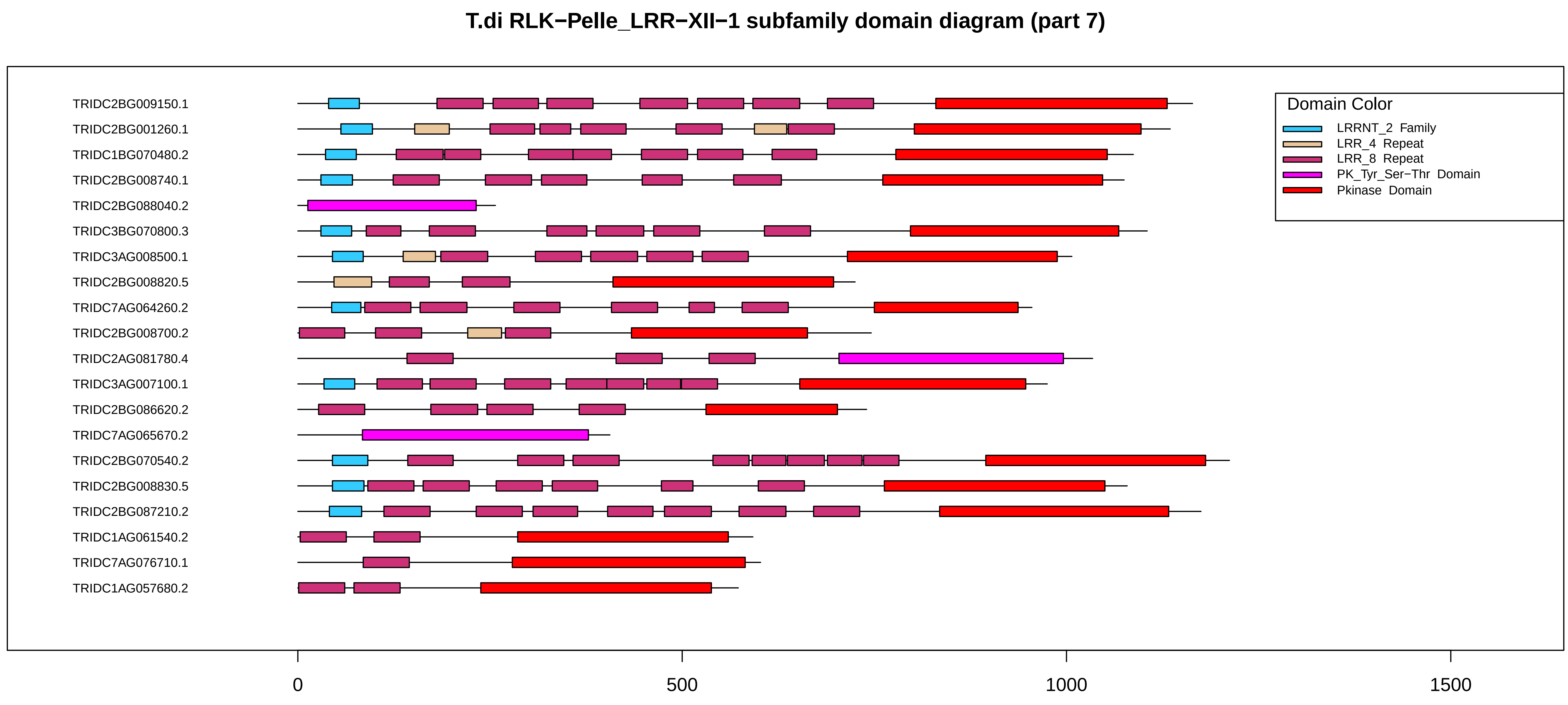
T.di RLK−Pelle_LRR−XII−1 subfamily domain diagram (part 7)
TRIDC2BG009150.1
TRIDC2BG001260.1
TRIDC1BG070480.2
TRIDC2BG008740.1
TRIDC2BG088040.2
TRIDC3BG070800.3
TRIDC3AG008500.1
TRIDC2BG008820.5
TRIDC7AG064260.2
TRIDC2BG008700.2
TRIDC2AG081780.4
TRIDC3AG007100.1
TRIDC2BG086620.2
TRIDC7AG065670.2
TRIDC2BG070540.2
TRIDC2BG008830.5
TRIDC2BG087210.2
TRIDC1AG061540.2
TRIDC7AG076710.1
TRIDC1AG057680.2
Domain Color
LRRNT_2 Family
LRR_4 Repeat
LRR_8 Repeat
PK_Tyr_Ser−Thr Domain
Pkinase Domain
0
500
1000
1500

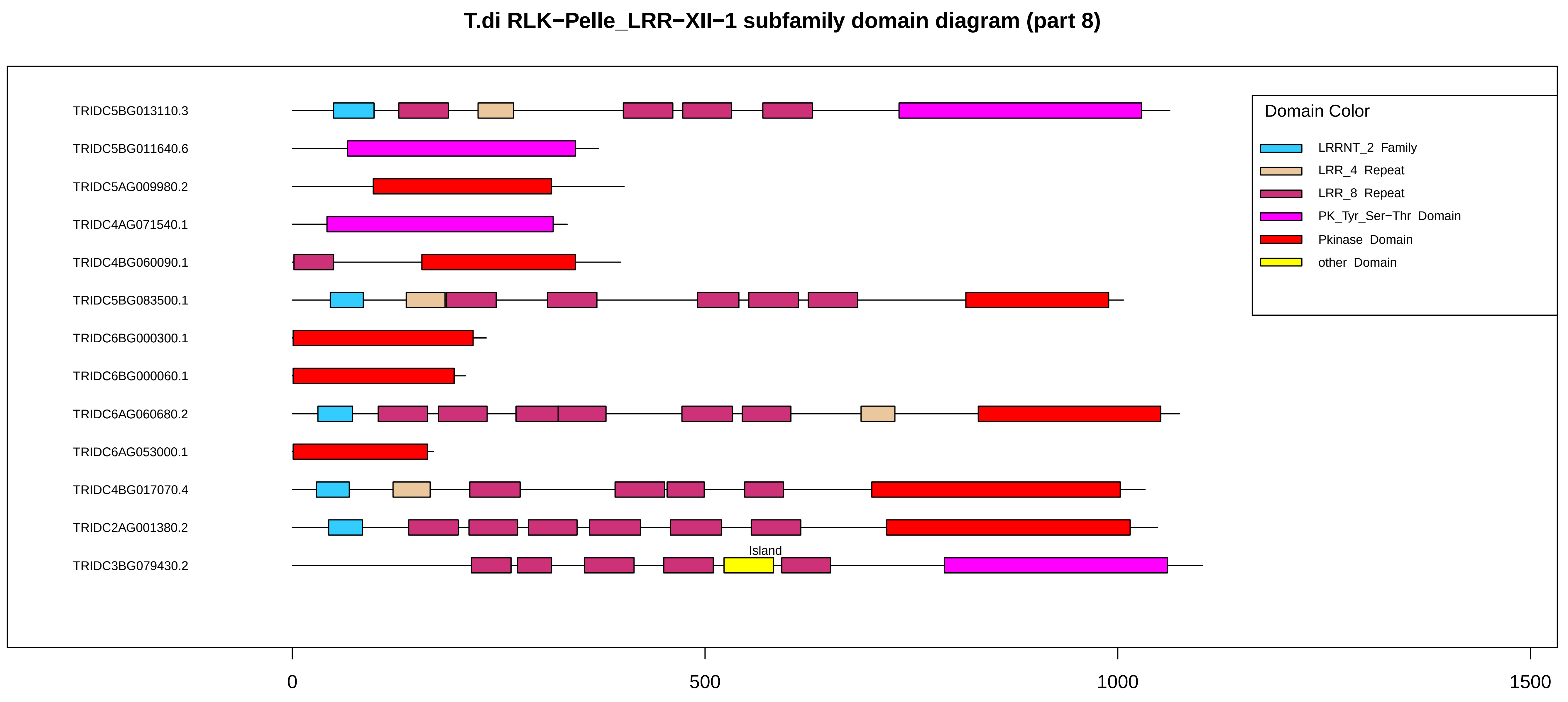

T.di RLK−Pelle_LRR−XII−1 subfamily domain diagram (part 8)
TRIDC5BG013110.3
TRIDC5BG011640.6
TRIDC5AG009980.2
TRIDC4AG071540.1
TRIDC4BG060090.1
TRIDC5BG083500.1
TRIDC6BG000300.1
TRIDC6BG000060.1
TRIDC6AG060680.2
TRIDC6AG053000.1
TRIDC4BG017070.4
TRIDC2AG001380.2
TRIDC3BG079430.2
Island
Domain Color
LRRNT_2 Family
LRR_4 Repeat
LRR_8 Repeat
PK_Tyr_Ser−Thr Domain
Pkinase Domain
other Domain
0
500
1000
1500

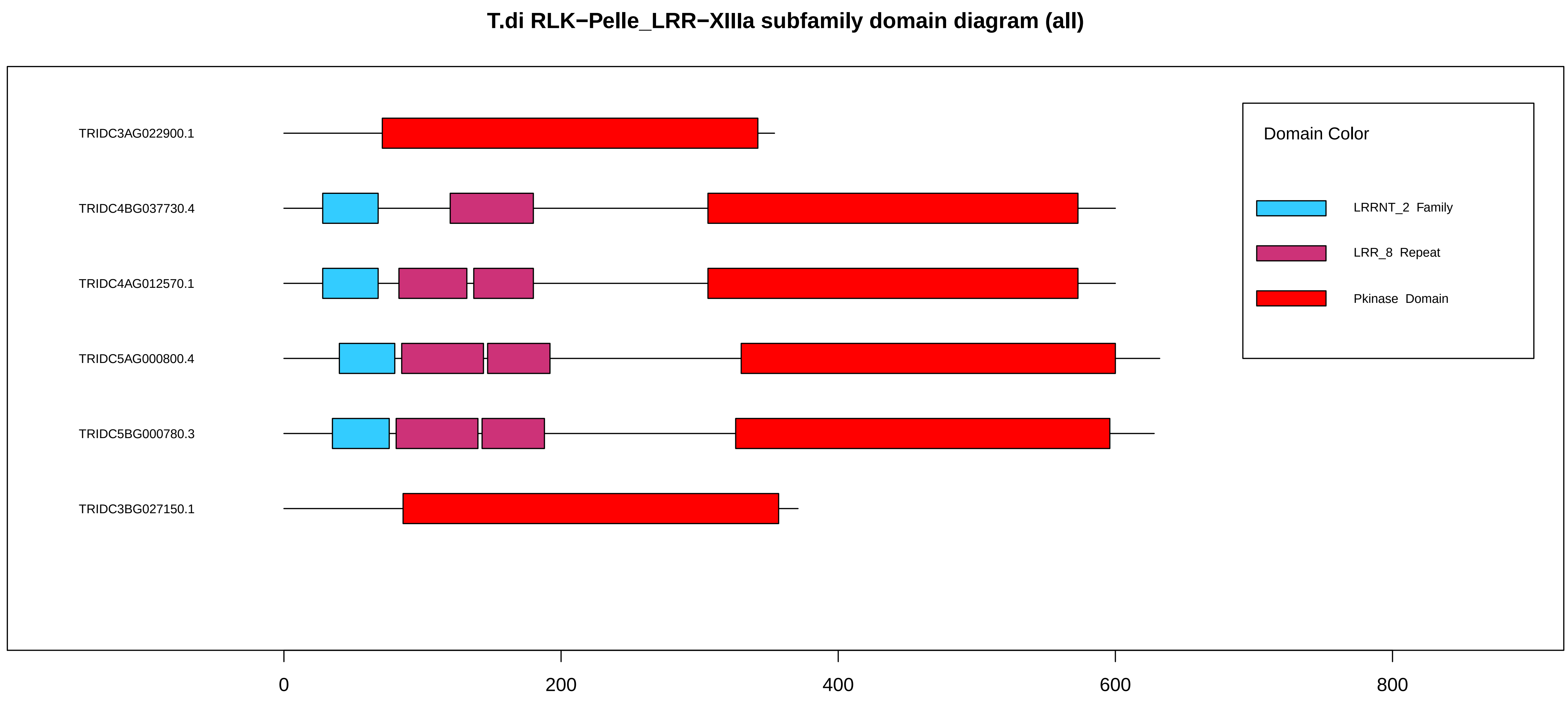
T.di RLK−Pelle_LRR−XIIIa subfamily domain diagram (all)
TRIDC3AG022900.1
TRIDC4BG037730.4
TRIDC4AG012570.1
TRIDC5AG000800.4
TRIDC5BG000780.3
TRIDC3BG027150.1
Domain Color
LRRNT_2 Family
LRR_8 Repeat
Pkinase Domain
0
200
400
600
800

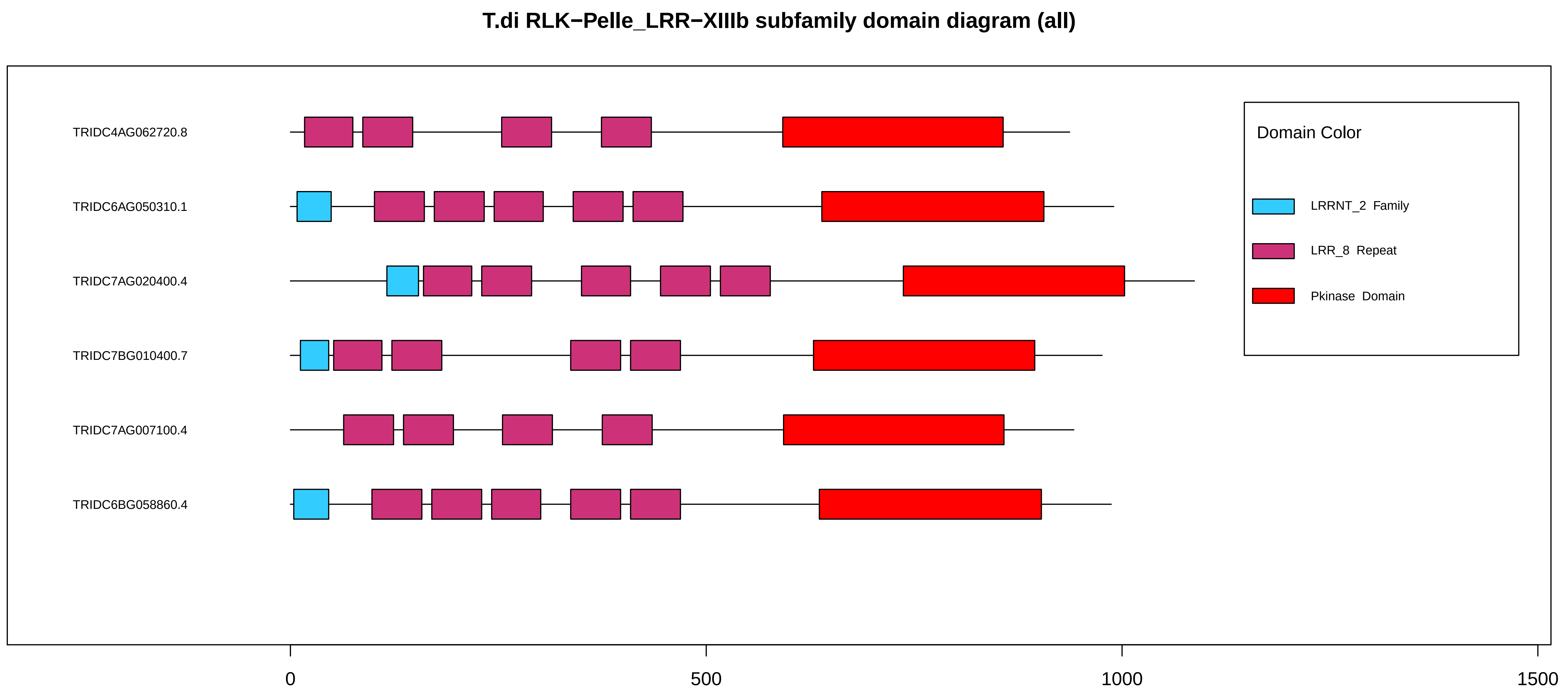
T.di RLK−Pelle_LRR−XIIIb subfamily domain diagram (all)
TRIDC4AG062720.8
TRIDC6AG050310.1
TRIDC7AG020400.4
TRIDC7BG010400.7
TRIDC7AG007100.4
TRIDC6BG058860.4
Domain Color
LRRNT_2 Family
LRR_8 Repeat
Pkinase Domain
0
500
1000
1500

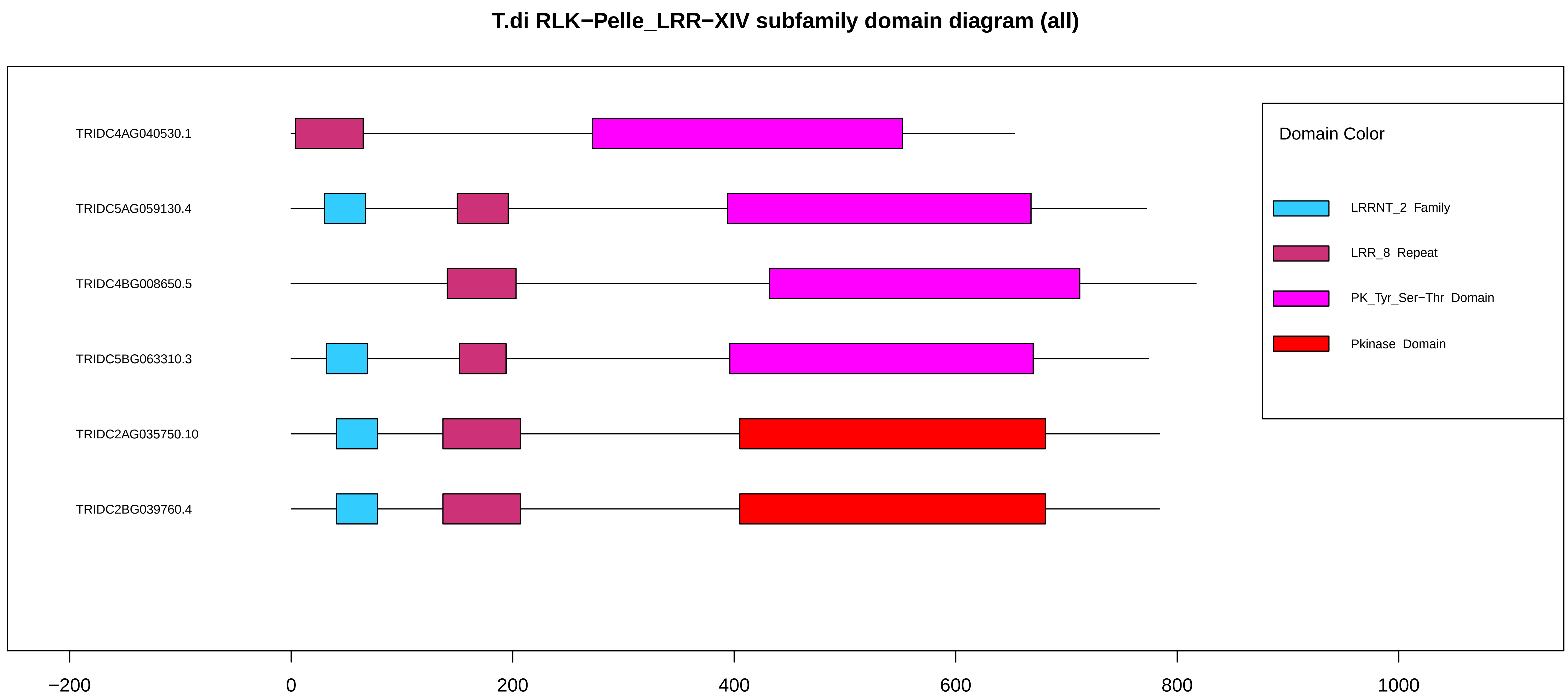
T.di RLK−Pelle_LRR−XIV subfamily domain diagram (all)
TRIDC4AG040530.1
TRIDC5AG059130.4
TRIDC4BG008650.5
TRIDC5BG063310.3
TRIDC2AG035750.10
TRIDC2BG039760.4
Domain Color
LRRNT_2 Family
LRR_8 Repeat
PK_Tyr_Ser−Thr Domain
Pkinase Domain
−200
0
200
400
600
800
1000

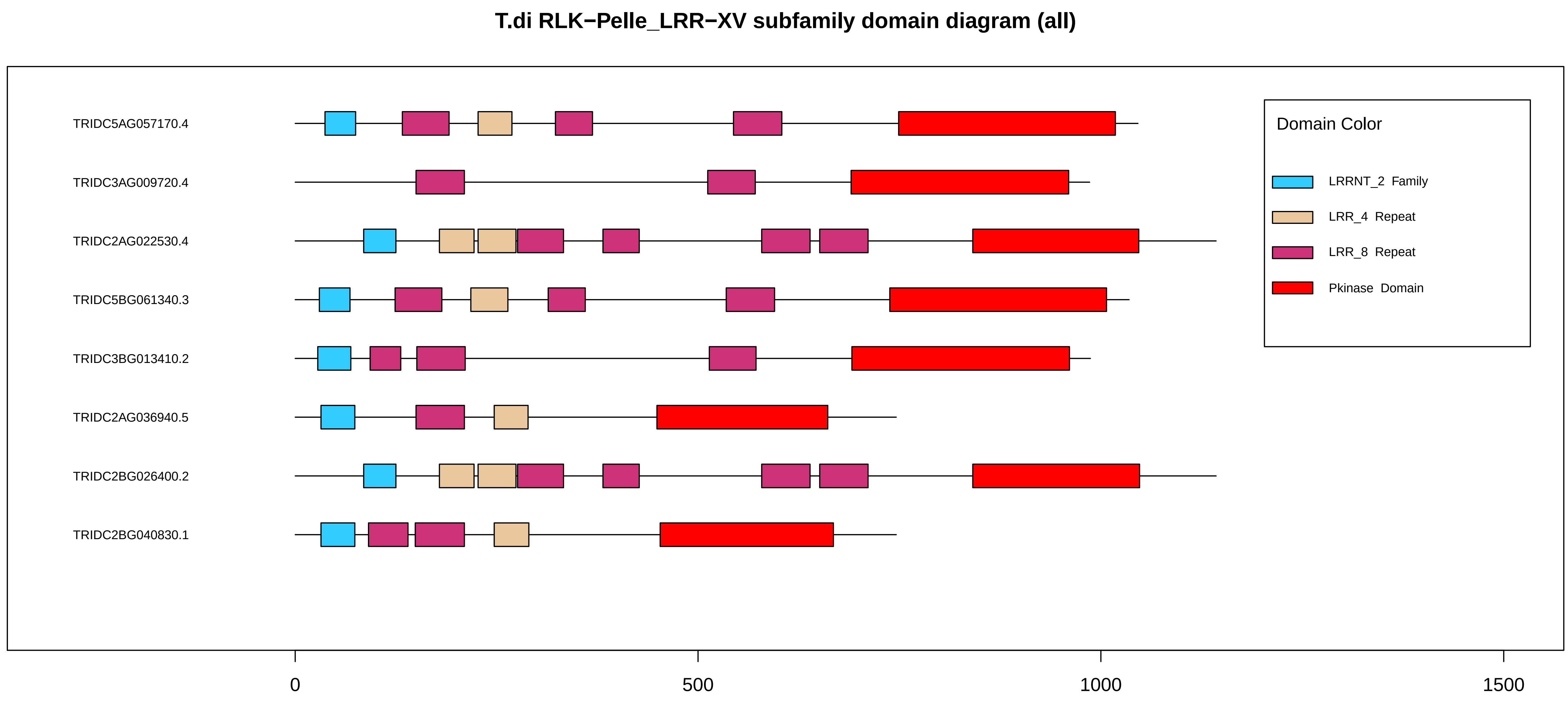

T.di RLK−Pelle_LRR−XV subfamily domain diagram (all)
TRIDC5AG057170.4
TRIDC3AG009720.4
TRIDC2AG022530.4
TRIDC5BG061340.3
TRIDC3BG013410.2
TRIDC2AG036940.5
TRIDC2BG026400.2
TRIDC2BG040830.1
Domain Color
LRRNT_2 Family
LRR_4 Repeat
LRR_8 Repeat
Pkinase Domain
0
500
1000
1500

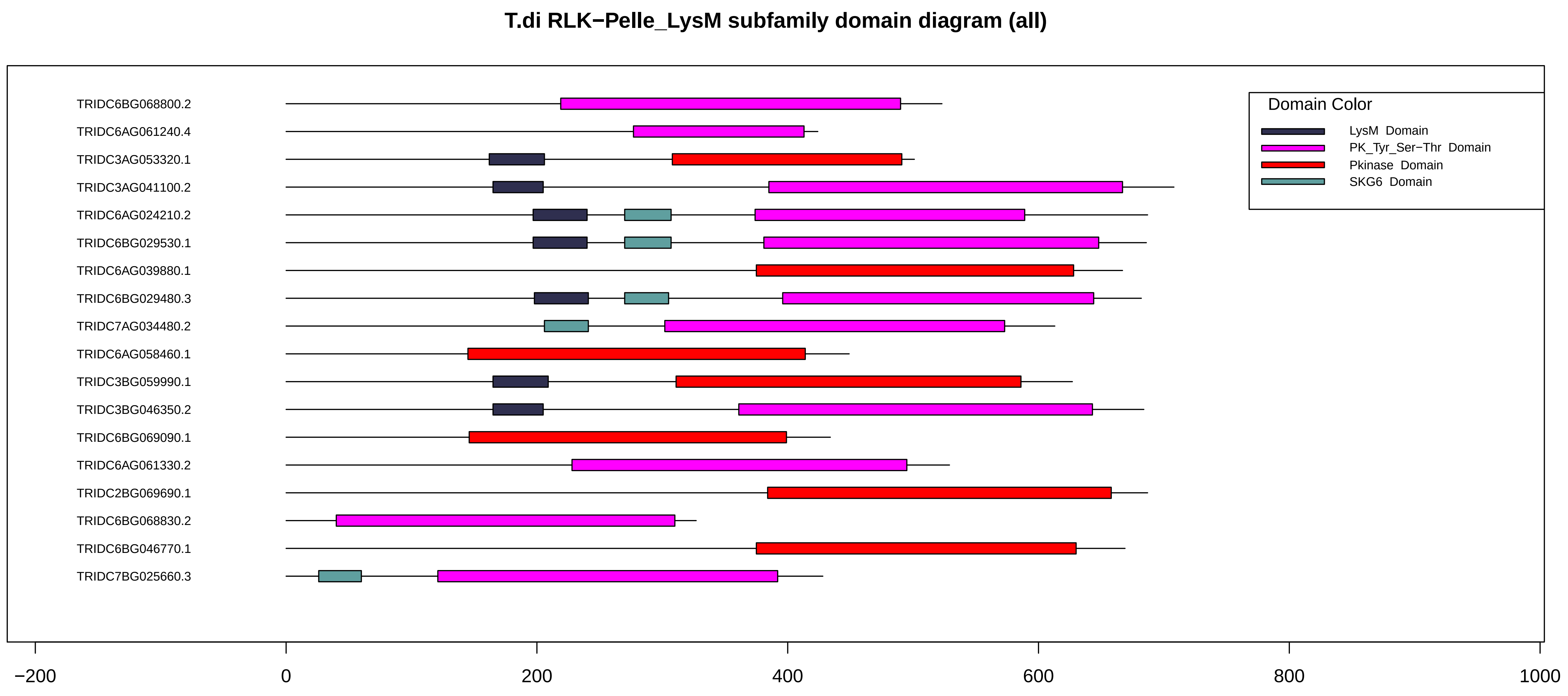
T.di RLK−Pelle_LysM subfamily domain diagram (all)
TRIDC6BG068800.2
TRIDC6AG061240.4
TRIDC3AG053320.1
TRIDC3AG041100.2
TRIDC6AG024210.2
TRIDC6BG029530.1
TRIDC6AG039880.1
TRIDC6BG029480.3
TRIDC7AG034480.2
TRIDC6AG058460.1
TRIDC3BG059990.1
TRIDC3BG046350.2
TRIDC6BG069090.1
TRIDC6AG061330.2
TRIDC2BG069690.1
TRIDC6BG068830.2
TRIDC6BG046770.1
TRIDC7BG025660.3
Domain Color
LysM Domain
PK_Tyr_Ser−Thr Domain
Pkinase Domain
SKG6 Domain
−200
0
200
400
600
800
1000

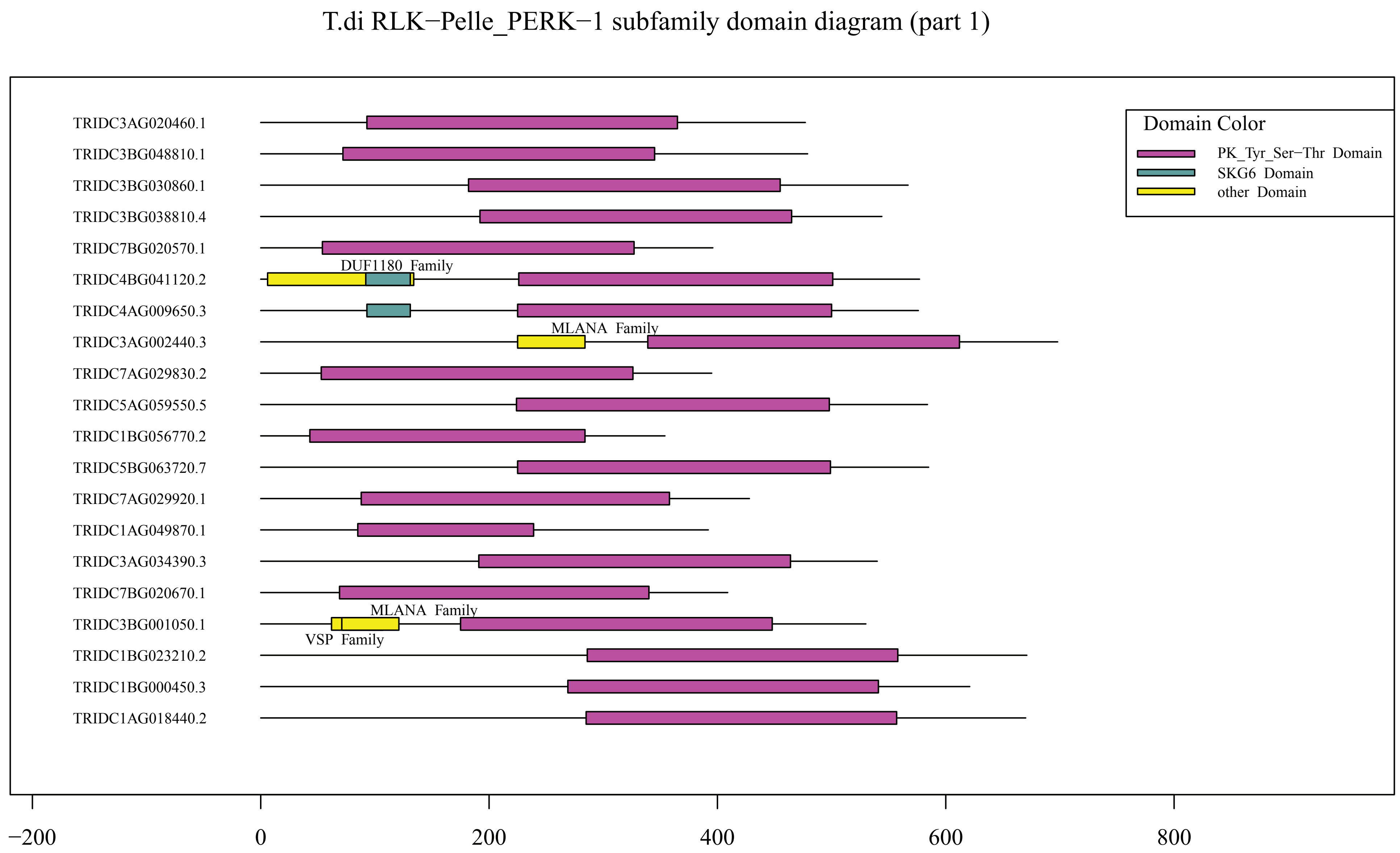
T.di RLK−Pelle_PERK−1 subfamily domain diagram (part 1)
Domain Color
PK_Tyr_Ser−Thr Domain
SKG6 Domain
other Domain
TRIDC3AG020460.1
TRIDC3BG048810.1
TRIDC3BG030860.1
TRIDC3BG038810.4
TRIDC7BG020570.1
TRIDC4BG041120.2
DUF1180 Family
TRIDC4AG009650.3
TRIDC3AG002440.3
MLANA Family
TRIDC7AG029830.2
TRIDC5AG059550.5
TRIDC1BG056770.2
TRIDC5BG063720.7
TRIDC7AG029920.1
TRIDC1AG049870.1
TRIDC3AG034390.3
TRIDC7BG020670.1
TRIDC3BG001050.1
MLANA Family
VSP Family
TRIDC1BG023210.2
TRIDC1BG000450.3
TRIDC1AG018440.2
−200
0
200
400
600
800

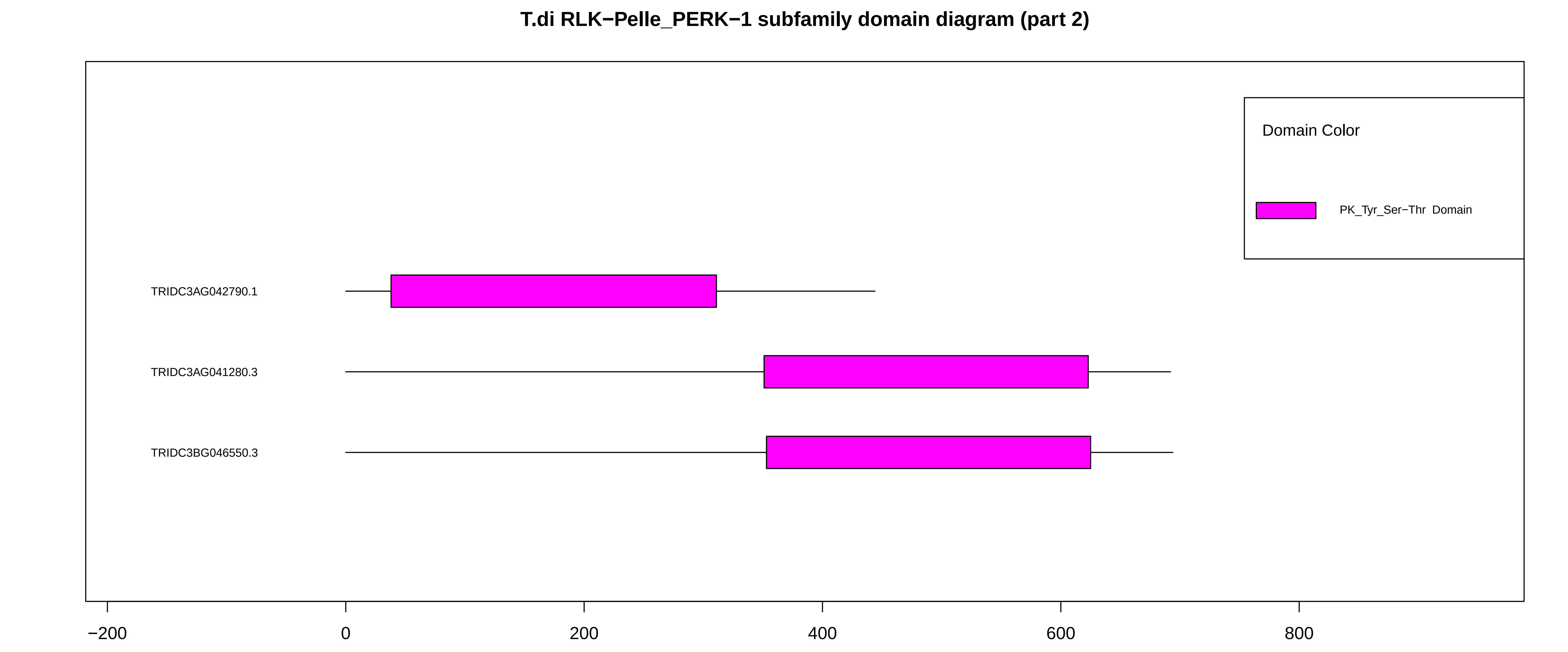

T.di RLK−Pelle_PERK−1 subfamily domain diagram (part 2)
Domain Color
PK_Tyr_Ser−Thr Domain
TRIDC3AG042790.1
TRIDC3AG041280.3
TRIDC3BG046550.3
−200
0
200
400
600
800

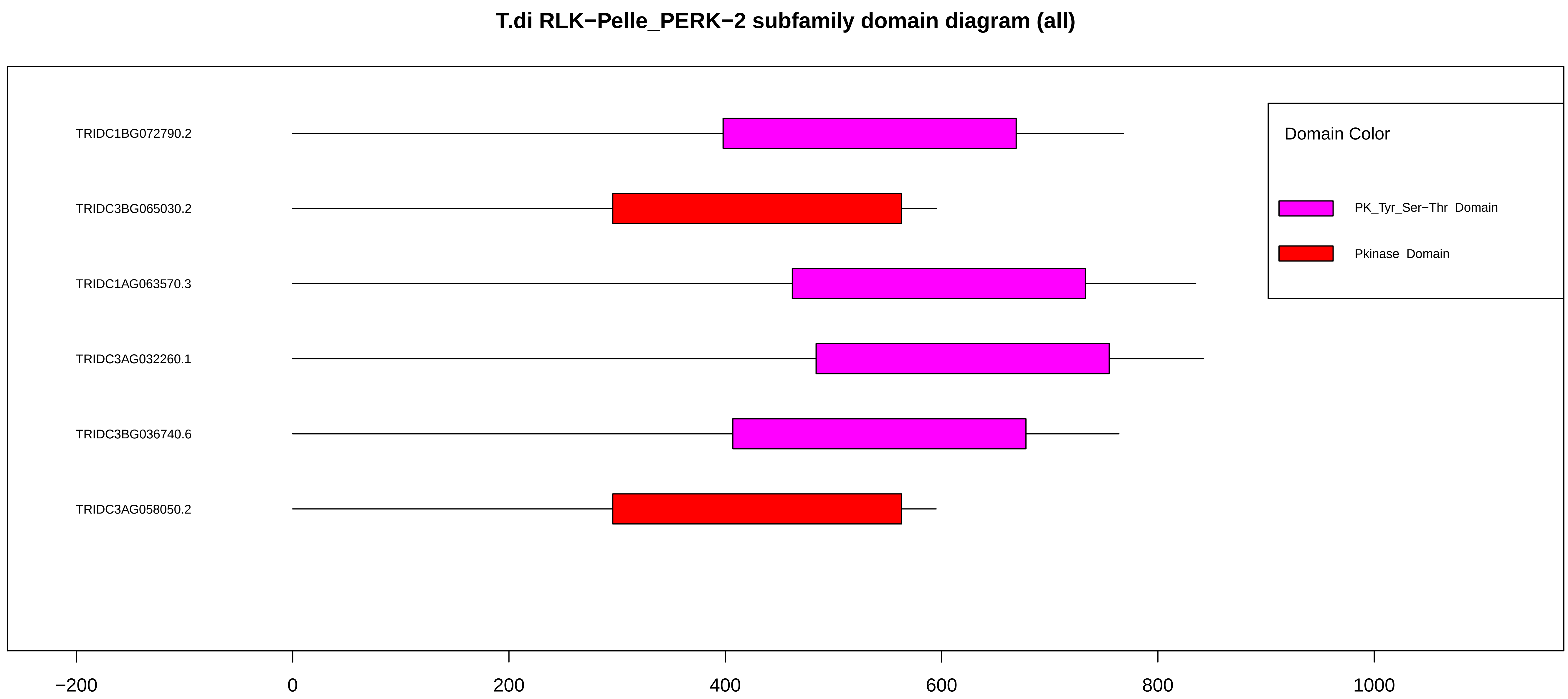

T.di RLK−Pelle_PERK−2 subfamily domain diagram (all)
TRIDC1BG072790.2
TRIDC3BG065030.2
TRIDC1AG063570.3
TRIDC3AG032260.1
TRIDC3BG036740.6
TRIDC3AG058050.2
Domain Color
PK_Tyr_Ser−Thr Domain
Pkinase Domain
−200
0
200
400
600
800
1000

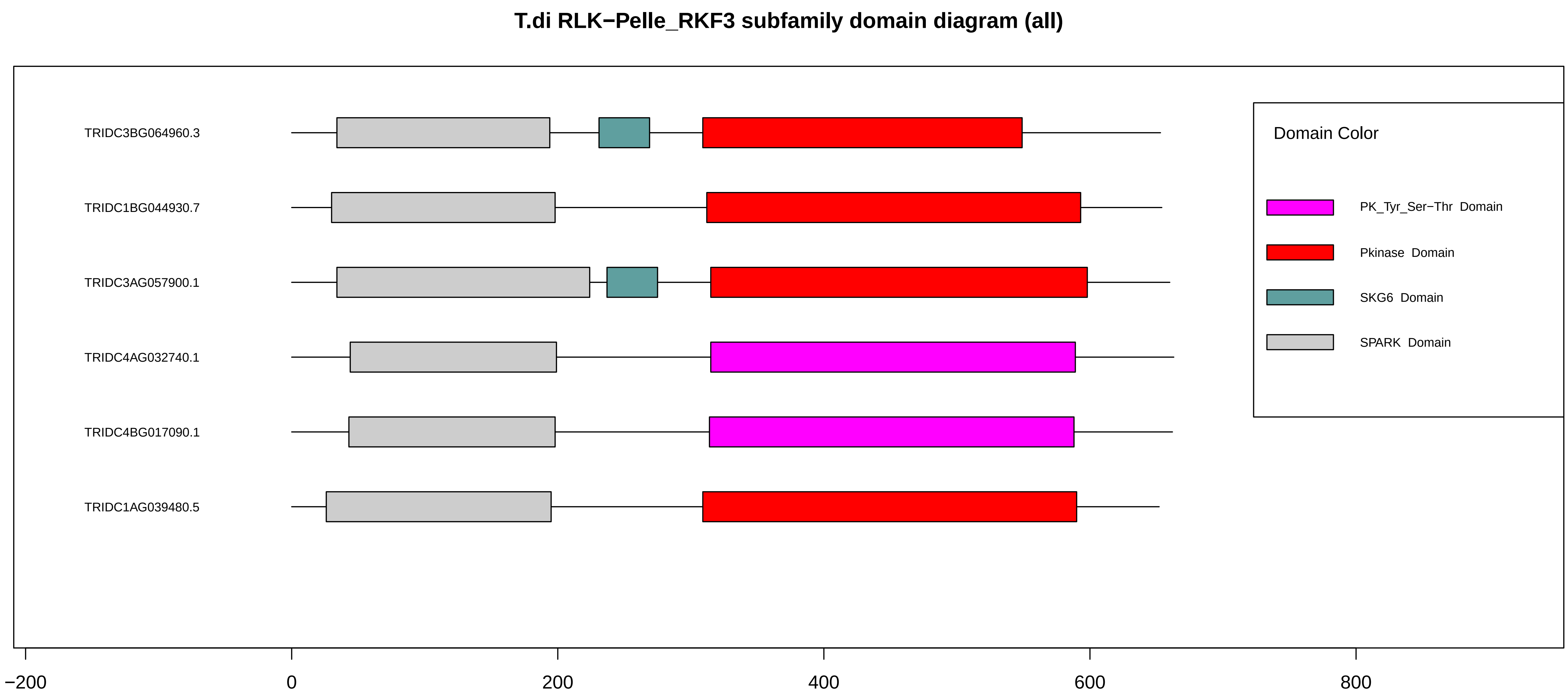

T.di RLK−Pelle_RKF3 subfamily domain diagram (all)
TRIDC3BG064960.3
TRIDC1BG044930.7
TRIDC3AG057900.1
TRIDC4AG032740.1
TRIDC4BG017090.1
TRIDC1AG039480.5
Domain Color
PK_Tyr_Ser−Thr Domain
Pkinase Domain
SKG6 Domain
SPARK Domain
−200
0
200
400
600
800

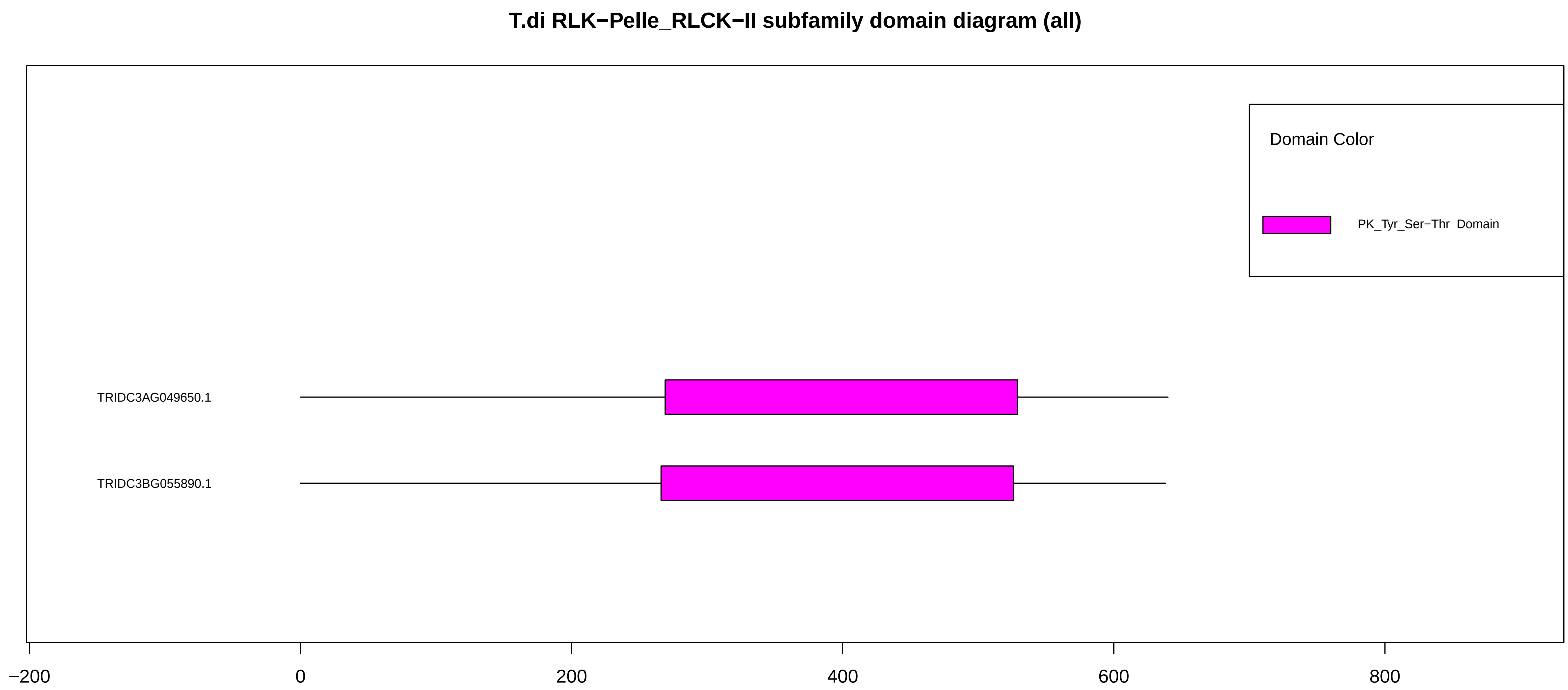
T.di RLK−Pelle_RLCK−II subfamily domain diagram (all)
Domain Color
PK_Tyr_Ser−Thr Domain
TRIDC3AG049650.1
TRIDC3BG055890.1
−200
0
200
400
600
800

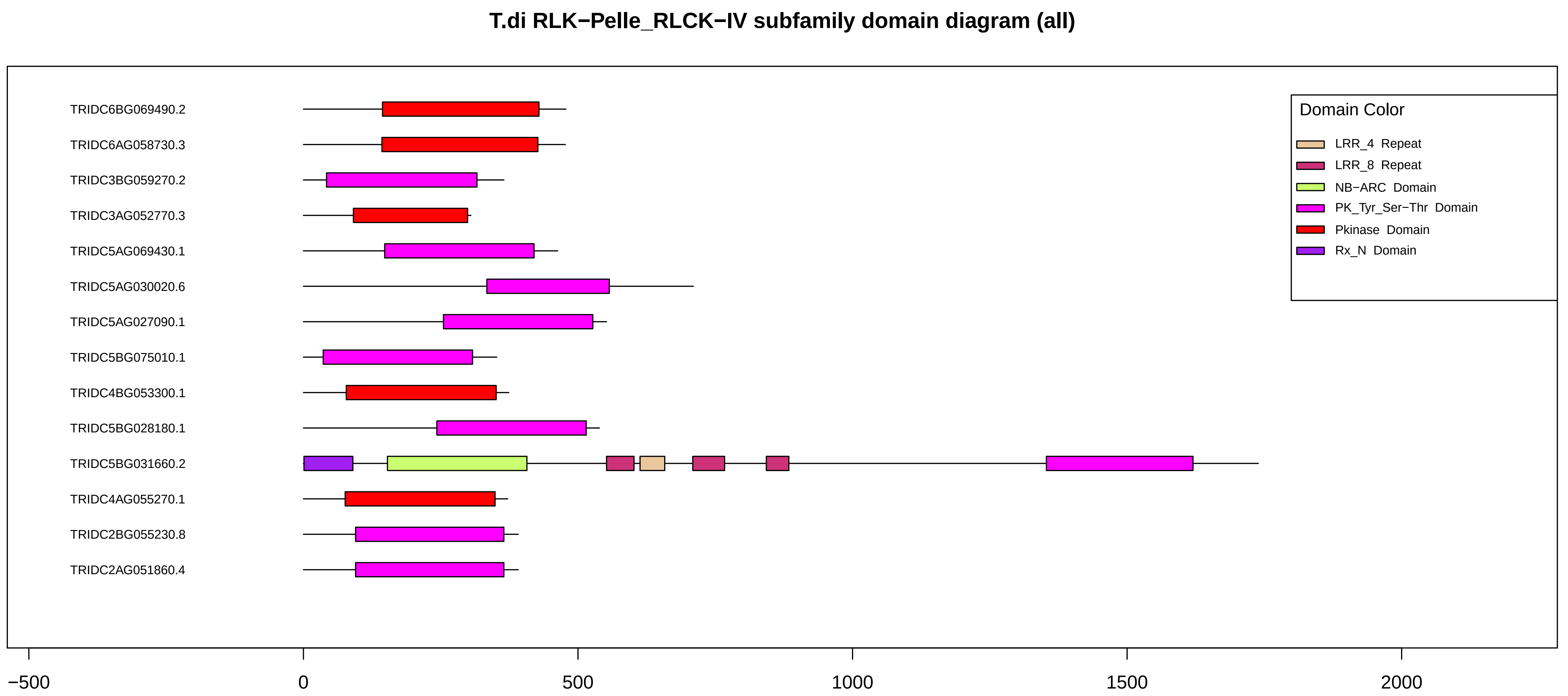

T.di RLK−Pelle_RLCK−IV subfamily domain diagram (all)
TRIDC6BG069490.2
TRIDC6AG058730.3
TRIDC3BG059270.2
TRIDC3AG052770.3
TRIDC5AG069430.1
TRIDC5AG030020.6
TRIDC5AG027090.1
TRIDC5BG075010.1
TRIDC4BG053300.1
TRIDC5BG028180.1
TRIDC5BG031660.2
TRIDC4AG055270.1
TRIDC2BG055230.8
TRIDC2AG051860.4
Domain Color
LRR_4 Repeat
LRR_8 Repeat
NB−ARC Domain
PK_Tyr_Ser−Thr Domain
Pkinase Domain
Rx_N Domain
−500
0
500
1000
1500
2000

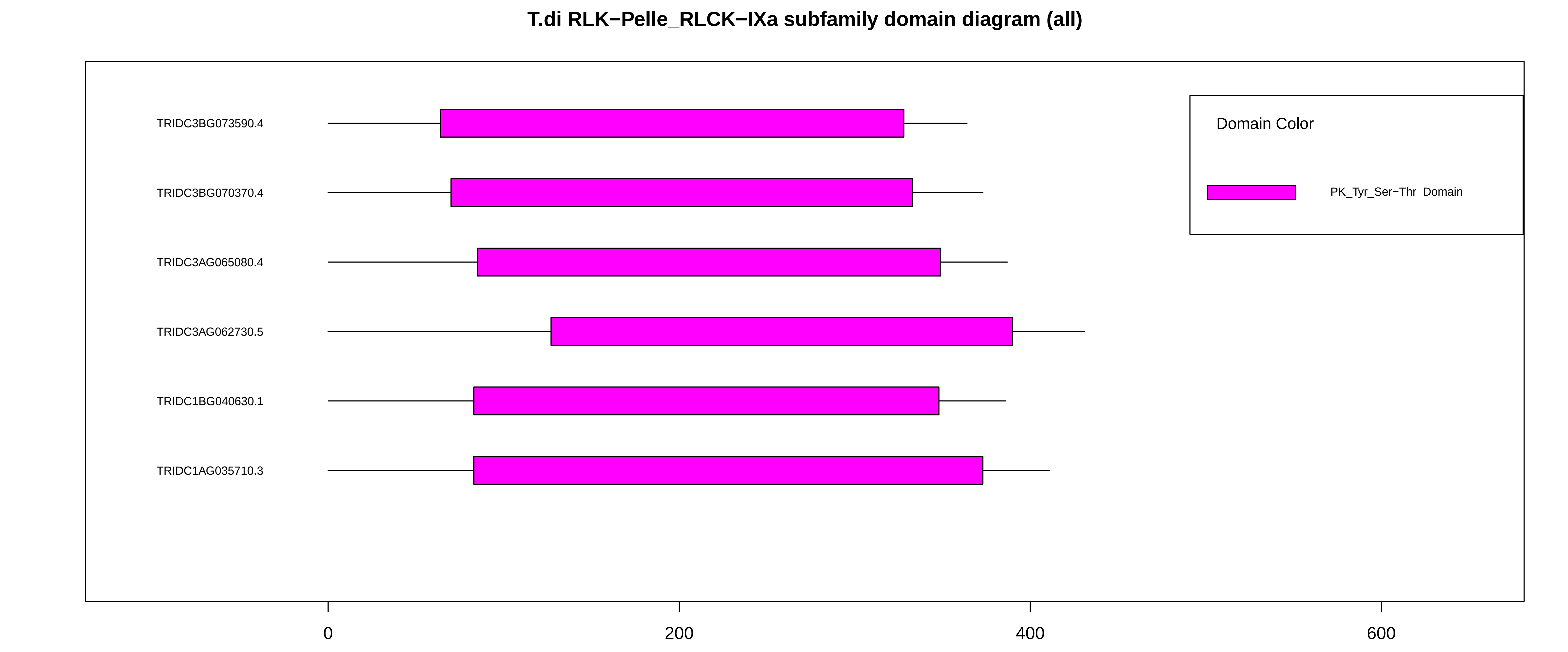

T.di RLK−Pelle_RLCK−IXa subfamily domain diagram (all)
TRIDC3BG073590.4
TRIDC3BG070370.4
TRIDC3AG065080.4
TRIDC3AG062730.5
TRIDC1BG040630.1
TRIDC1AG035710.3
Domain Color
PK_Tyr_Ser−Thr Domain
0
200
400
600

T.di RLK−Pelle_RLCK−IXb subfamily domain diagram (part 1)

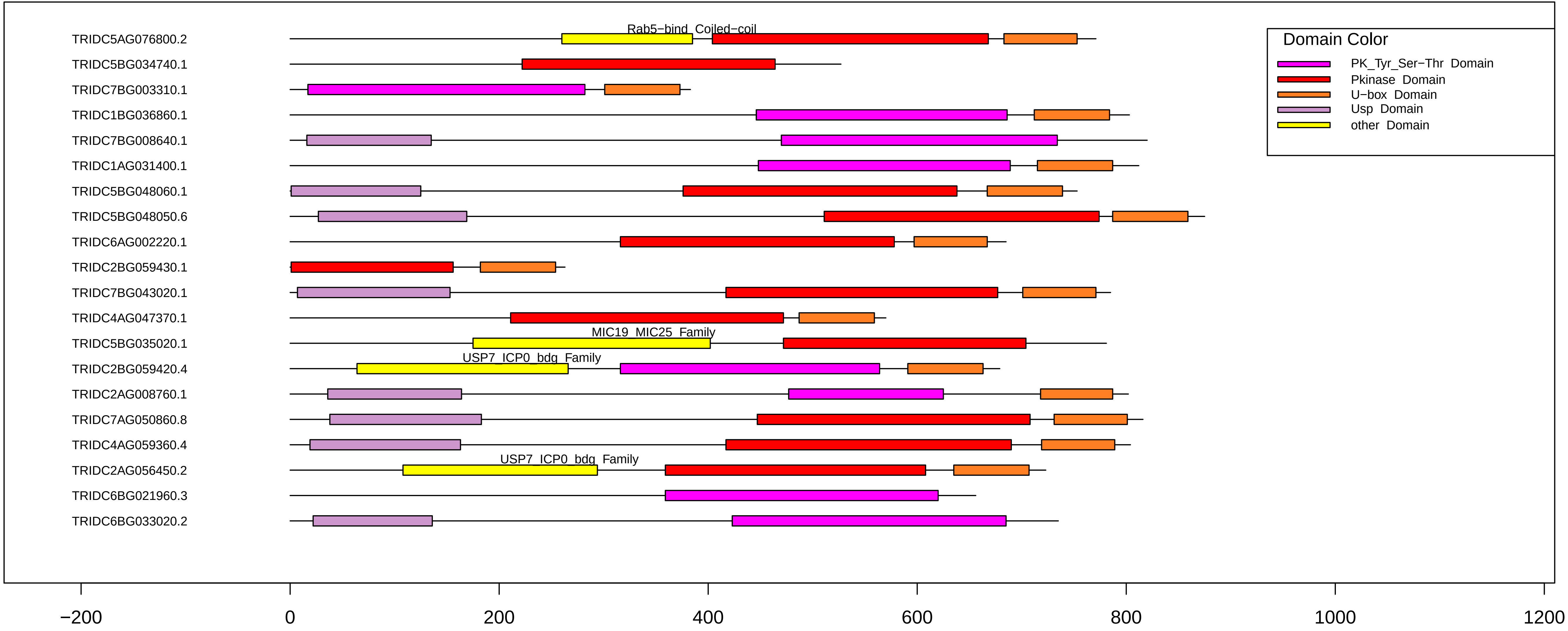

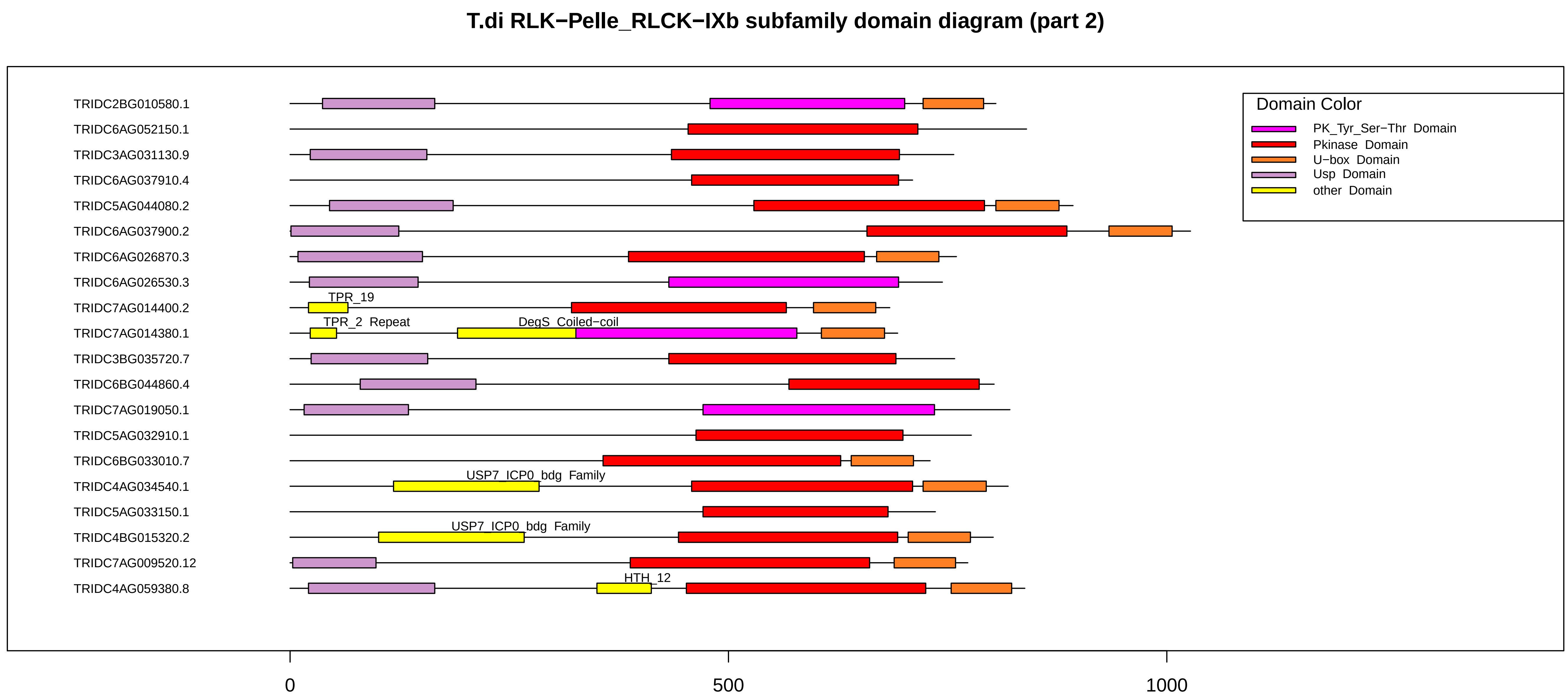
T.di RLK−Pelle_RLCK−IXb subfamily domain diagram (part 2)
TRIDC2BG010580.1
TRIDC6AG052150.1
TRIDC3AG031130.9
TRIDC6AG037910.4
TRIDC5AG044080.2
TRIDC6AG037900.2
TRIDC6AG026870.3
TRIDC6AG026530.3
TRIDC7AG014400.2
TRIDC7AG014380.1
TRIDC3BG035720.7
TRIDC6BG044860.4
TRIDC7AG019050.1
TRIDC5AG032910.1
TRIDC6BG033010.7
TRIDC4AG034540.1
TRIDC5AG033150.1
TRIDC4BG015320.2
TRIDC7AG009520.12
TRIDC4AG059380.8
TPR_19
TPR_2 Repeat
DegS Coiled−coil
USP7_ICP0_bdg Family
USP7_ICP0_bdg Family
HTH_12
Domain Color
PK_Tyr_Ser−Thr Domain
Pkinase Domain
U−box Domain
Usp Domain
other Domain
0
500
1000

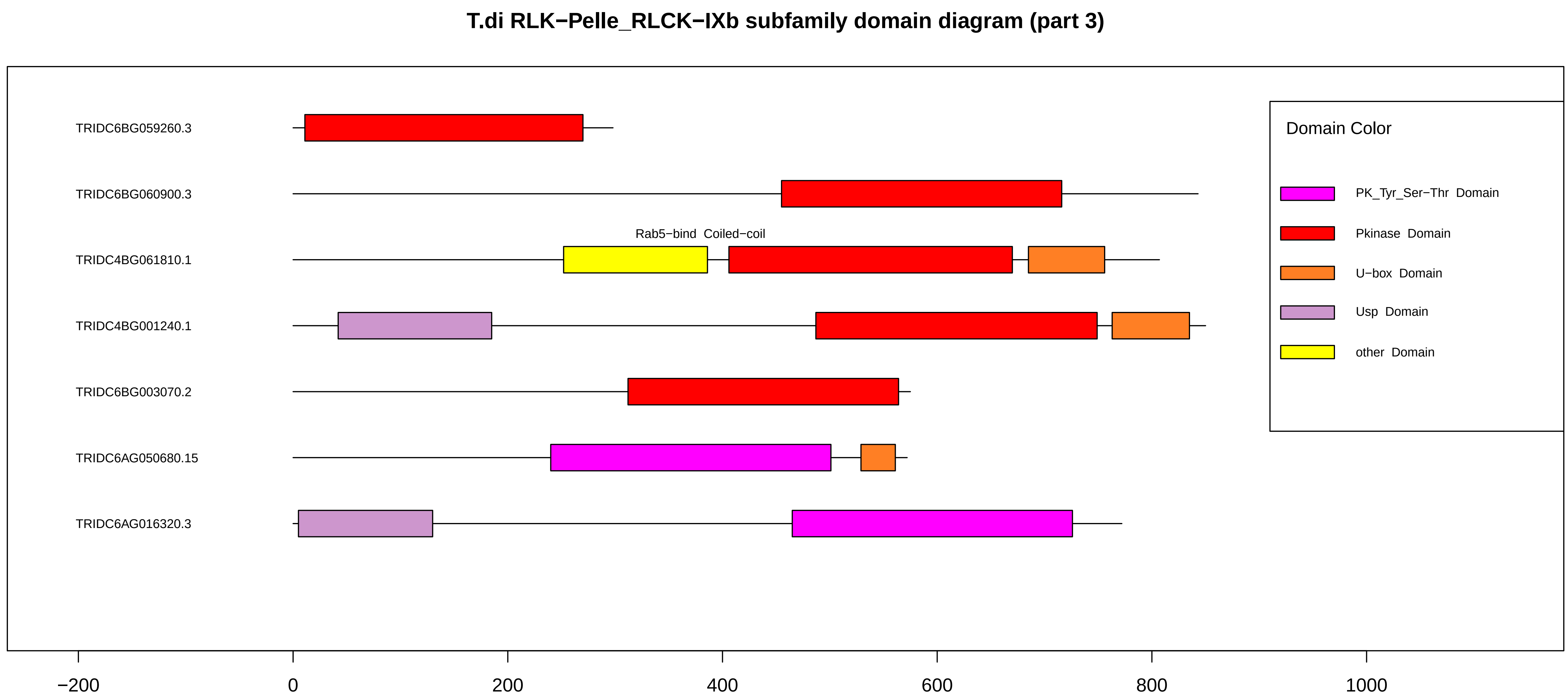
T.di RLK−Pelle_RLCK−IXb subfamily domain diagram (part 3)
TRIDC6BG059260.3
TRIDC6BG060900.3
Rab5−bind Coiled−coil
TRIDC4BG061810.1
TRIDC4BG001240.1
TRIDC6BG003070.2
TRIDC6AG050680.15
TRIDC6AG016320.3
Domain Color
PK_Tyr_Ser−Thr Domain
Pkinase Domain
U−box Domain
Usp Domain
other Domain
−200
0
200
400
600
800
1000

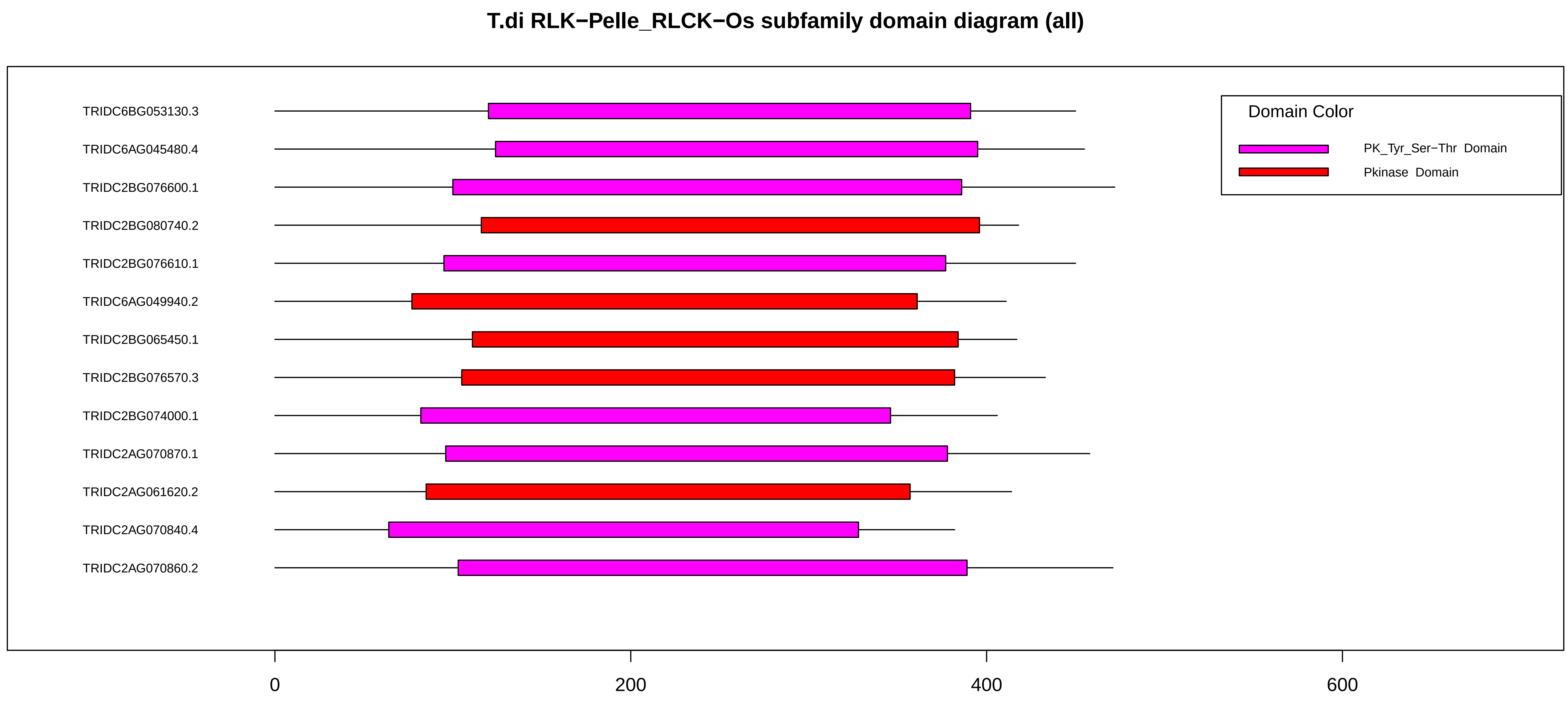

T.di RLK−Pelle_RLCK−Os subfamily domain diagram (all)
TRIDC6BG053130.3
TRIDC6AG045480.4
TRIDC2BG076600.1
TRIDC2BG080740.2
TRIDC2BG076610.1
TRIDC6AG049940.2
TRIDC2BG065450.1
TRIDC2BG076570.3
TRIDC2BG074000.1
TRIDC2AG070870.1
TRIDC2AG061620.2
TRIDC2AG070840.4
TRIDC2AG070860.2
Domain Color
PK_Tyr_Ser−Thr Domain
Pkinase Domain
0
200
400
600

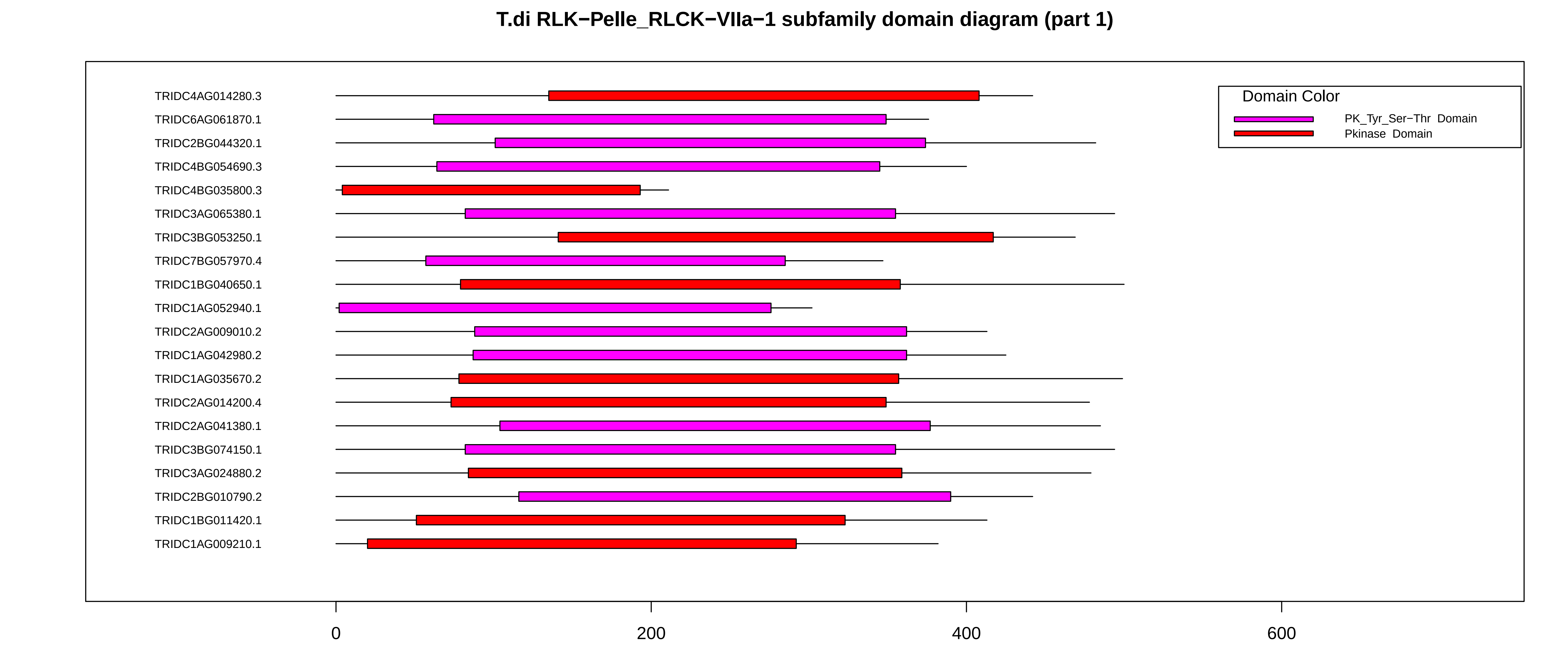

T.di RLK−Pelle_RLCK−VIIa−1 subfamily domain diagram (part 1)
TRIDC4AG014280.3
TRIDC6AG061870.1
TRIDC2BG044320.1
TRIDC4BG054690.3
TRIDC4BG035800.3
TRIDC3AG065380.1
TRIDC3BG053250.1
TRIDC7BG057970.4
TRIDC1BG040650.1
TRIDC1AG052940.1
TRIDC2AG009010.2
TRIDC1AG042980.2
TRIDC1AG035670.2
TRIDC2AG014200.4
TRIDC2AG041380.1
TRIDC3BG074150.1
TRIDC3AG024880.2
TRIDC2BG010790.2
TRIDC1BG011420.1
TRIDC1AG009210.1
Domain Color
PK_Tyr_Ser−Thr Domain
Pkinase Domain
0
200
400
600

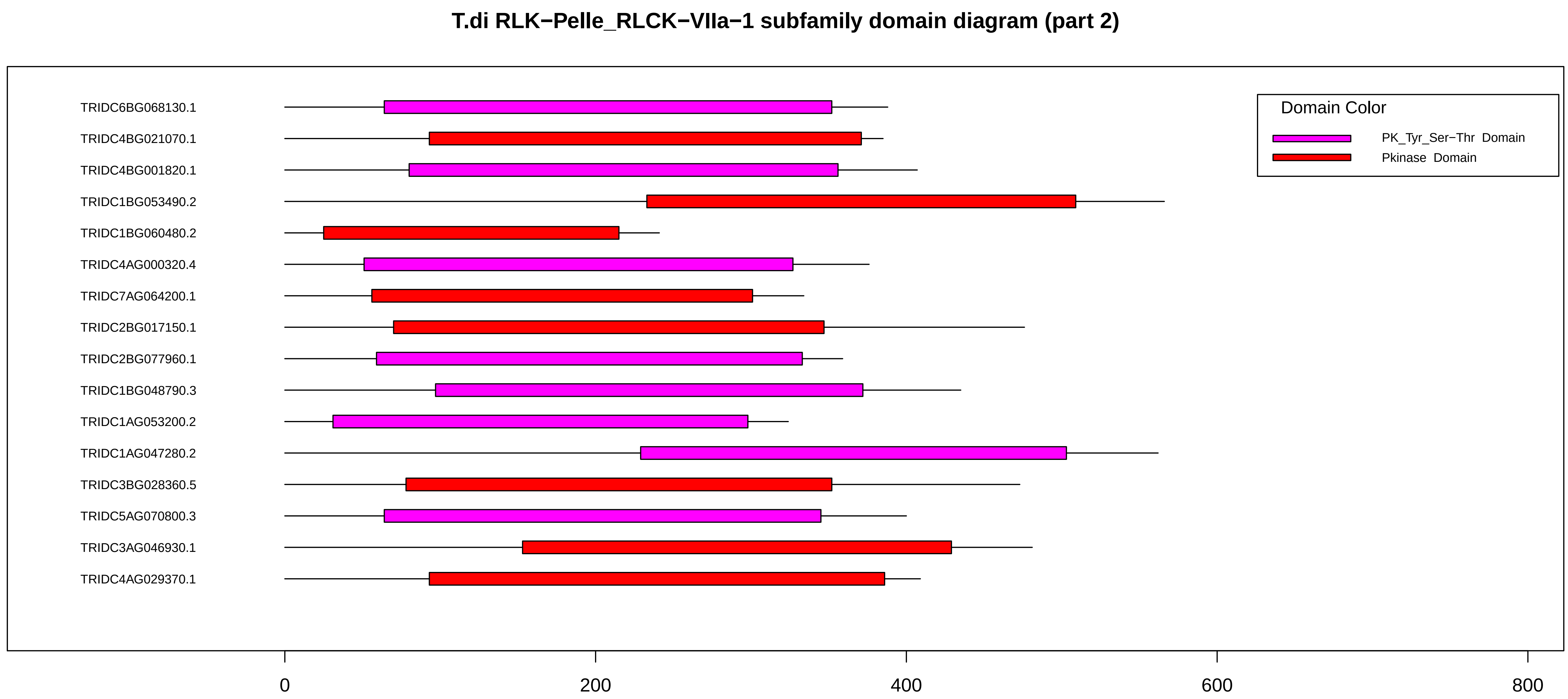

T.di RLK−Pelle_RLCK−VIIa−1 subfamily domain diagram (part 2)
TRIDC6BG068130.1
TRIDC4BG021070.1
TRIDC4BG001820.1
TRIDC1BG053490.2
TRIDC1BG060480.2
TRIDC4AG000320.4
TRIDC7AG064200.1
TRIDC2BG017150.1
TRIDC2BG077960.1
TRIDC1BG048790.3
TRIDC1AG053200.2
TRIDC1AG047280.2
TRIDC3BG028360.5
TRIDC5AG070800.3
TRIDC3AG046930.1
TRIDC4AG029370.1
Domain Color
PK_Tyr_Ser−Thr Domain
Pkinase Domain
0
200
400
600
800

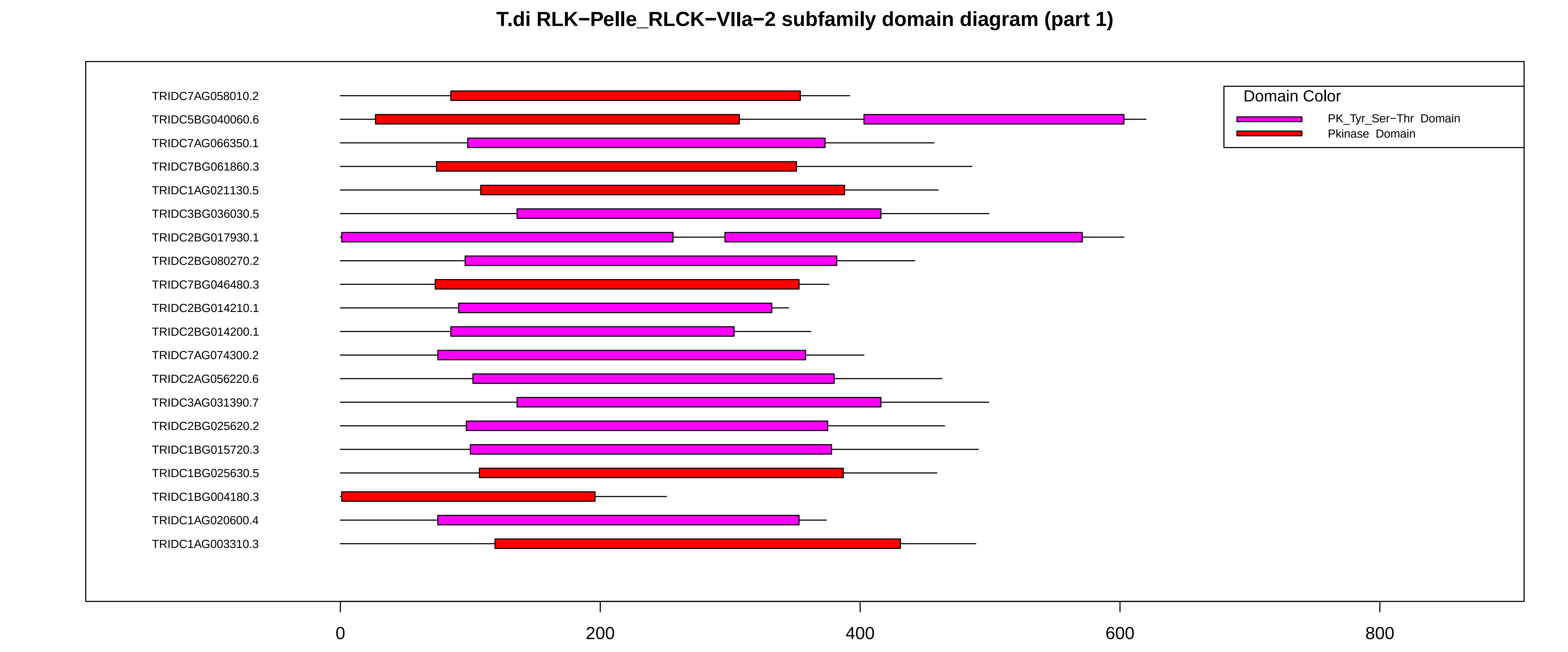
T.di RLK−Pelle_RLCK−VIIa−2 subfamily domain diagram (part 1)
TRIDC7AG058010.2
TRIDC5BG040060.6
TRIDC7AG066350.1
TRIDC7BG061860.3
TRIDC1AG021130.5
TRIDC3BG036030.5
TRIDC2BG017930.1
TRIDC2BG080270.2
TRIDC7BG046480.3
TRIDC2BG014210.1
TRIDC2BG014200.1
TRIDC7AG074300.2
TRIDC2AG056220.6
TRIDC3AG031390.7
TRIDC2BG025620.2
TRIDC1BG015720.3
TRIDC1BG025630.5
TRIDC1BG004180.3
TRIDC1AG020600.4
TRIDC1AG003310.3
Domain Color
PK_Tyr_Ser−Thr Domain
Pkinase Domain
0
200
400
600
800

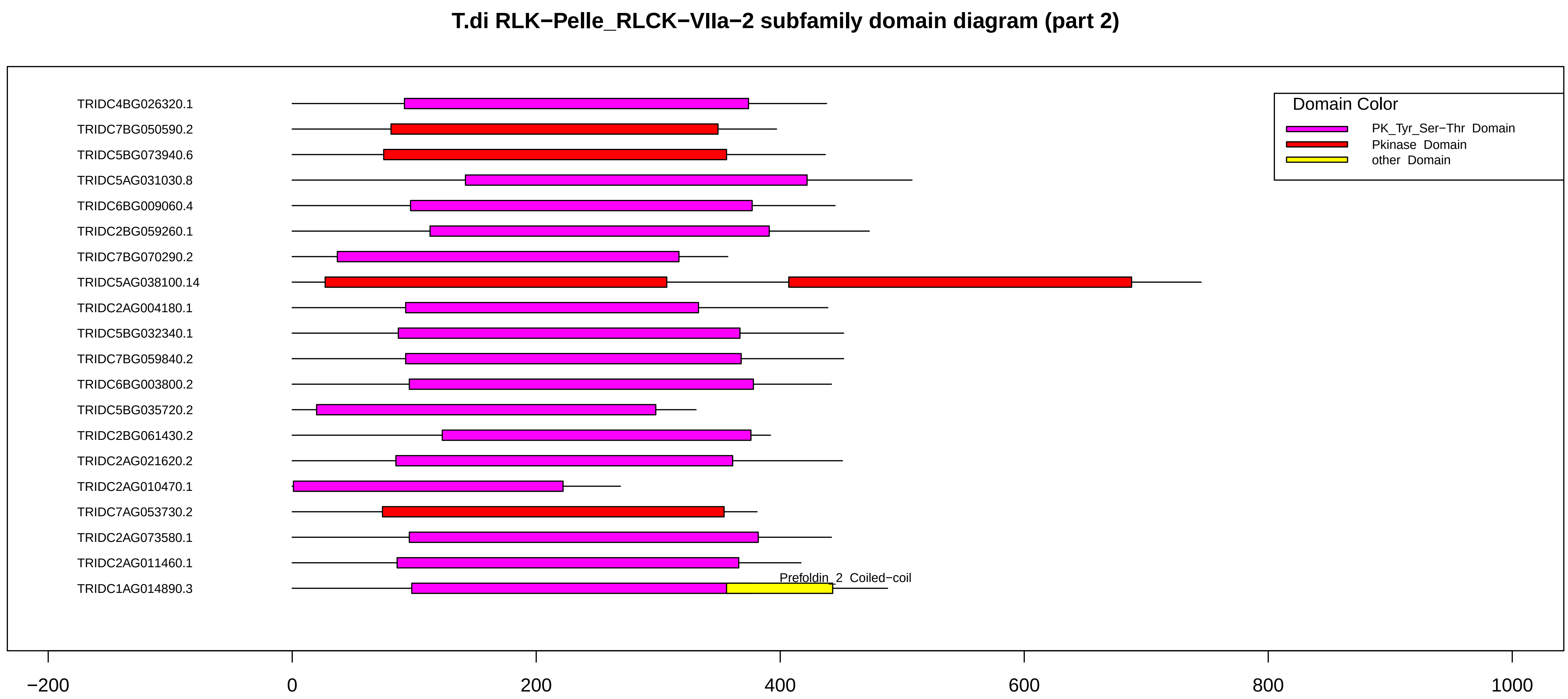
T.di RLK−Pelle_RLCK−VIIa−2 subfamily domain diagram (part 2)
TRIDC4BG026320.1
TRIDC7BG050590.2
TRIDC5BG073940.6
TRIDC5AG031030.8
TRIDC6BG009060.4
TRIDC2BG059260.1
TRIDC7BG070290.2
TRIDC5AG038100.14
TRIDC2AG004180.1
TRIDC5BG032340.1
TRIDC7BG059840.2
TRIDC6BG003800.2
TRIDC5BG035720.2
TRIDC2BG061430.2
TRIDC2AG021620.2
TRIDC2AG010470.1
TRIDC7AG053730.2
TRIDC2AG073580.1
TRIDC2AG011460.1
TRIDC1AG014890.3
Prefoldin_2 Coiled−coil
Domain Color
PK_Tyr_Ser−Thr Domain
Pkinase Domain
other Domain
−200
0
200
400
600
800
1000

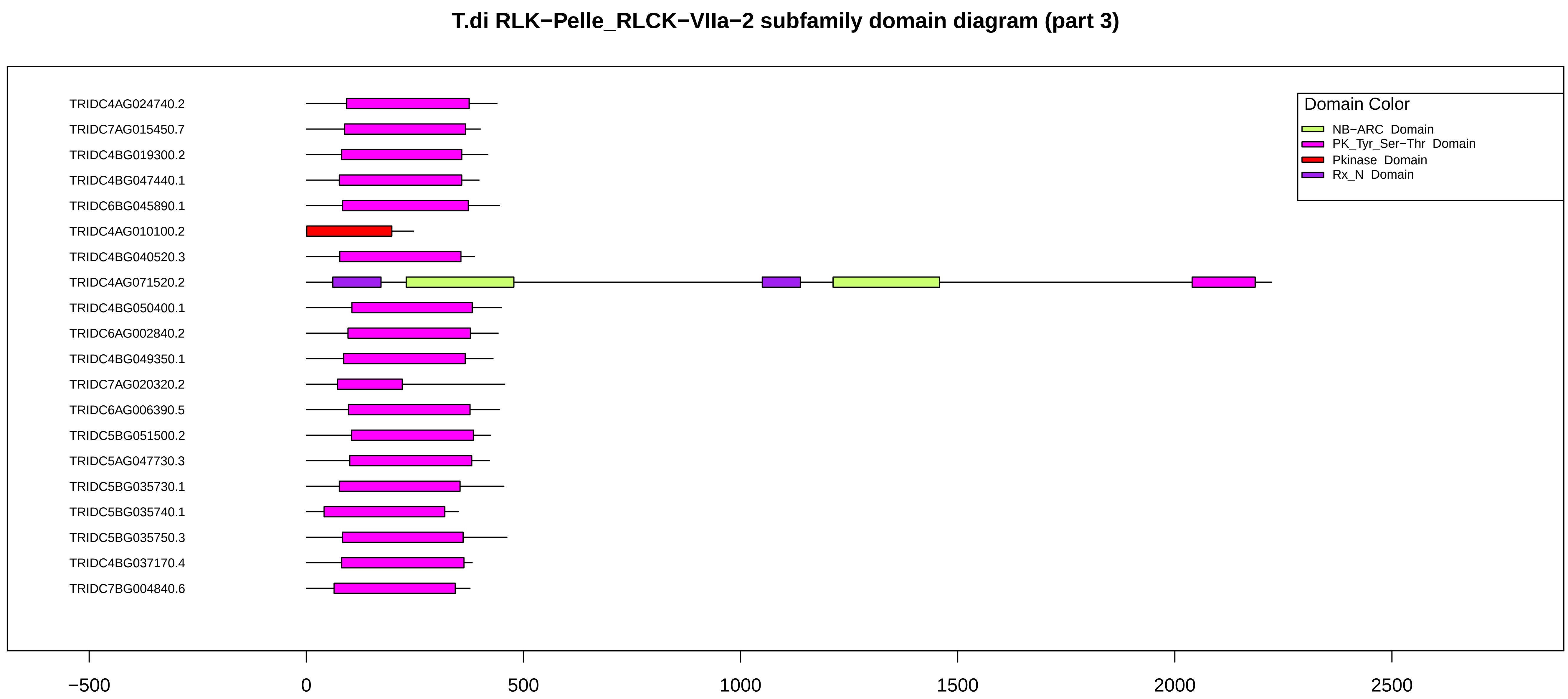
T.di RLK−Pelle_RLCK−VIIa−2 subfamily domain diagram (part 3)
TRIDC4AG024740.2
TRIDC7AG015450.7
TRIDC4BG019300.2
TRIDC4BG047440.1
TRIDC6BG045890.1
TRIDC4AG010100.2
TRIDC4BG040520.3
TRIDC4AG071520.2
TRIDC4BG050400.1
TRIDC6AG002840.2
TRIDC4BG049350.1
TRIDC7AG020320.2
TRIDC6AG006390.5
TRIDC5BG051500.2
TRIDC5AG047730.3
TRIDC5BG035730.1
TRIDC5BG035740.1
TRIDC5BG035750.3
TRIDC4BG037170.4
TRIDC7BG004840.6
Domain Color
NB−ARC Domain
PK_Tyr_Ser−Thr Domain
Pkinase Domain
Rx_N Domain
−500
0
500
1000
1500
2000
2500

T.di RLK−Pelle_RLCK−VIIa−2 subfamily domain diagram (part 4)

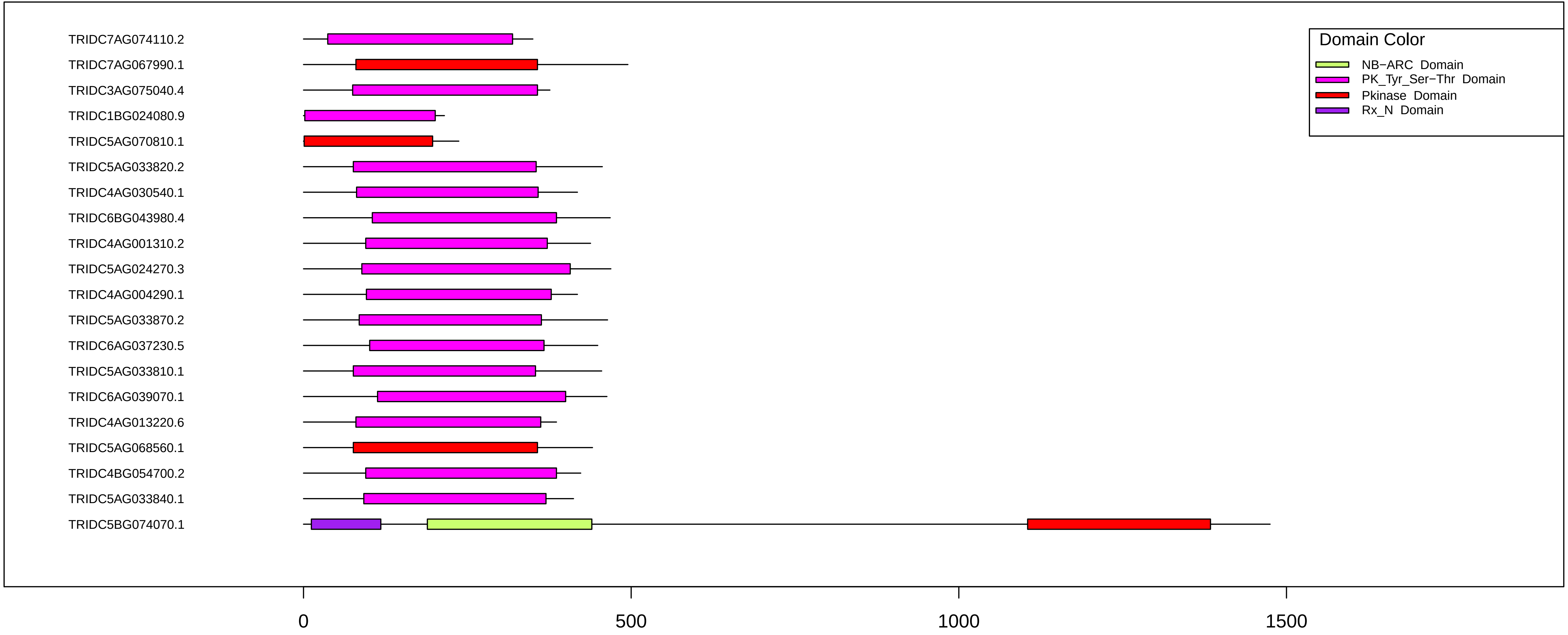

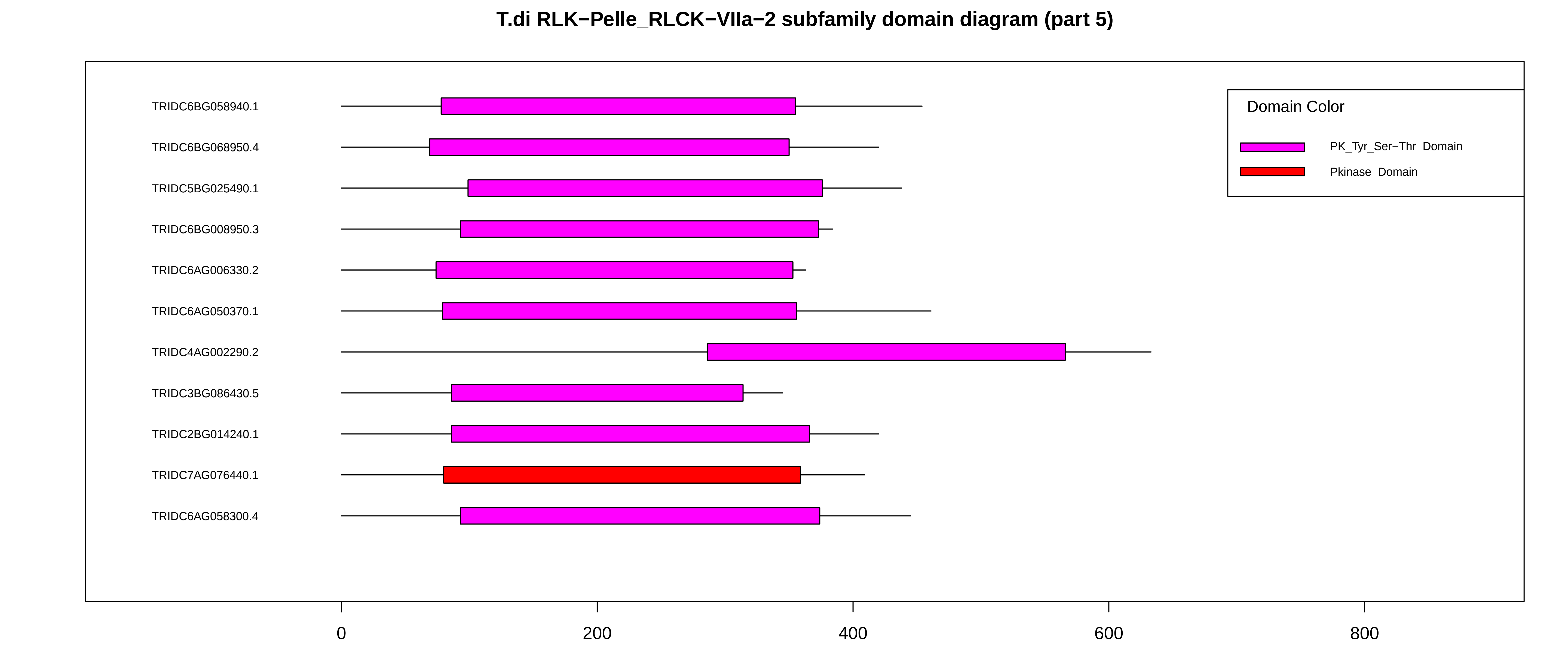
T.di RLK−Pelle_RLCK−VIIa−2 subfamily domain diagram (part 5)
TRIDC6BG058940.1
TRIDC6BG068950.4
TRIDC5BG025490.1
TRIDC6BG008950.3
TRIDC6AG006330.2
TRIDC6AG050370.1
TRIDC4AG002290.2
TRIDC3BG086430.5
TRIDC2BG014240.1
TRIDC7AG076440.1
TRIDC6AG058300.4
Domain Color
PK_Tyr_Ser−Thr Domain
Pkinase Domain
0
200
400
600
800

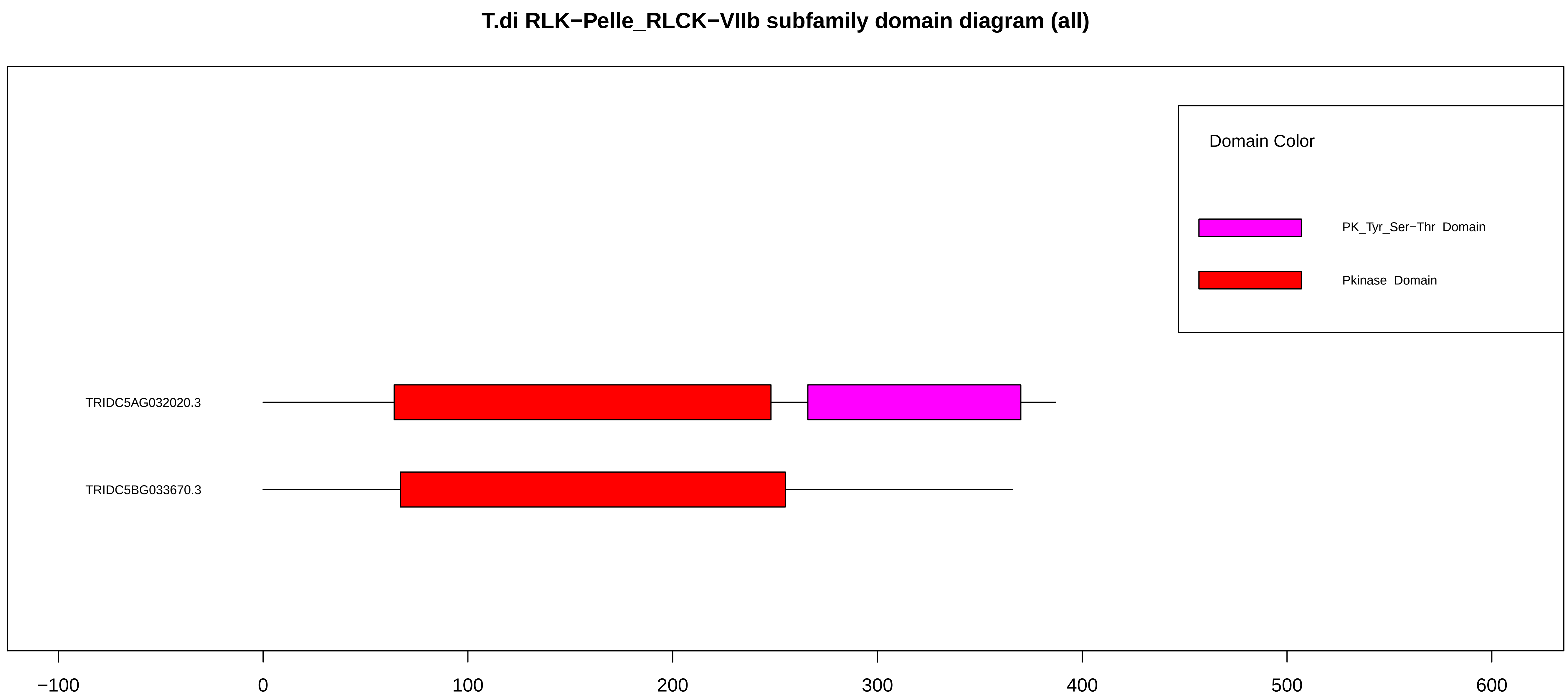
T.di RLK−Pelle_RLCK−VIIb subfamily domain diagram (all)
Domain Color
PK_Tyr_Ser−Thr Domain
Pkinase Domain
TRIDC5AG032020.3
TRIDC5BG033670.3
−100
0
100
200
300
400
500
600

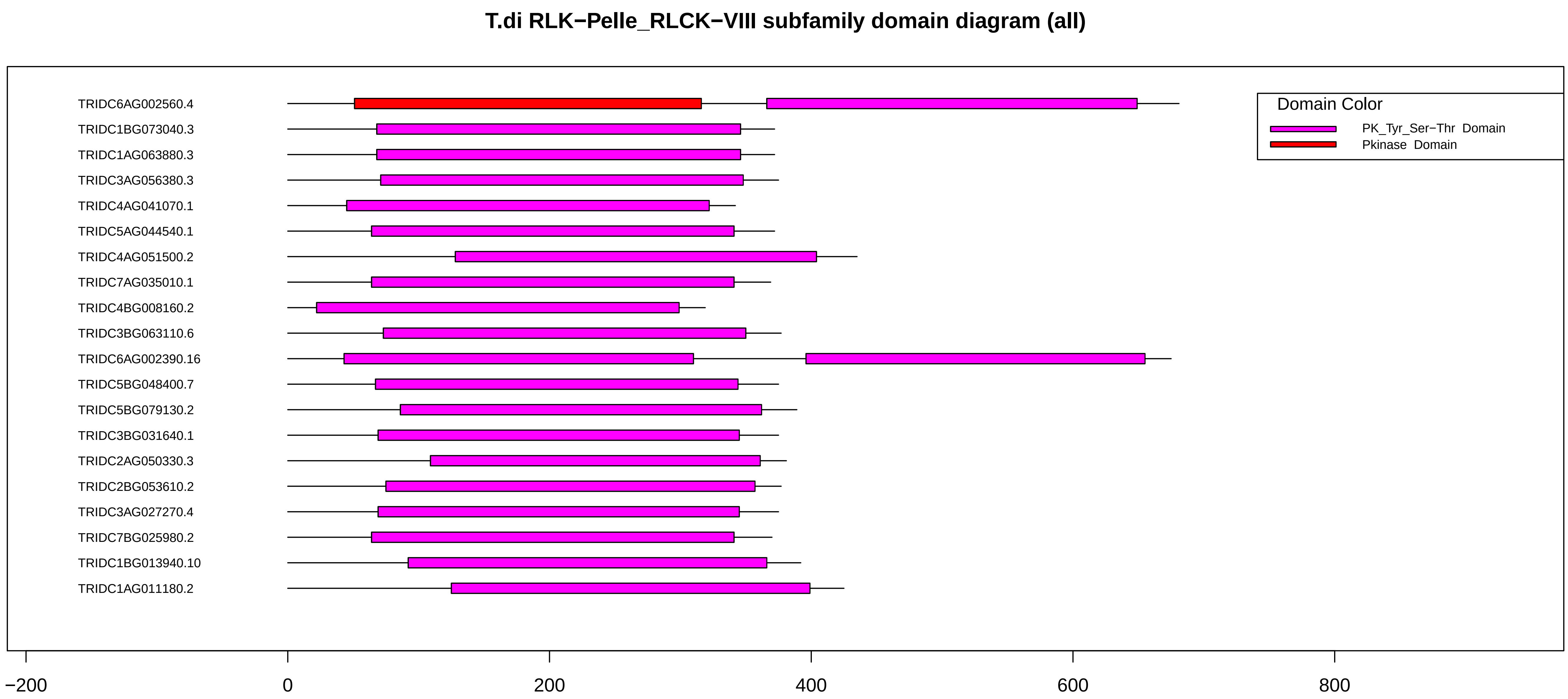
T.di RLK−Pelle_RLCK−VIII subfamily domain diagram (all)
TRIDC6AG002560.4
TRIDC1BG073040.3
TRIDC1AG063880.3
TRIDC3AG056380.3
TRIDC4AG041070.1
TRIDC5AG044540.1
TRIDC4AG051500.2
TRIDC7AG035010.1
TRIDC4BG008160.2
TRIDC3BG063110.6
TRIDC6AG002390.16
TRIDC5BG048400.7
TRIDC5BG079130.2
TRIDC3BG031640.1
TRIDC2AG050330.3
TRIDC2BG053610.2
TRIDC3AG027270.4
TRIDC7BG025980.2
TRIDC1BG013940.10
TRIDC1AG011180.2
Domain Color
PK_Tyr_Ser−Thr Domain
Pkinase Domain
−200
0
200
400
600
800

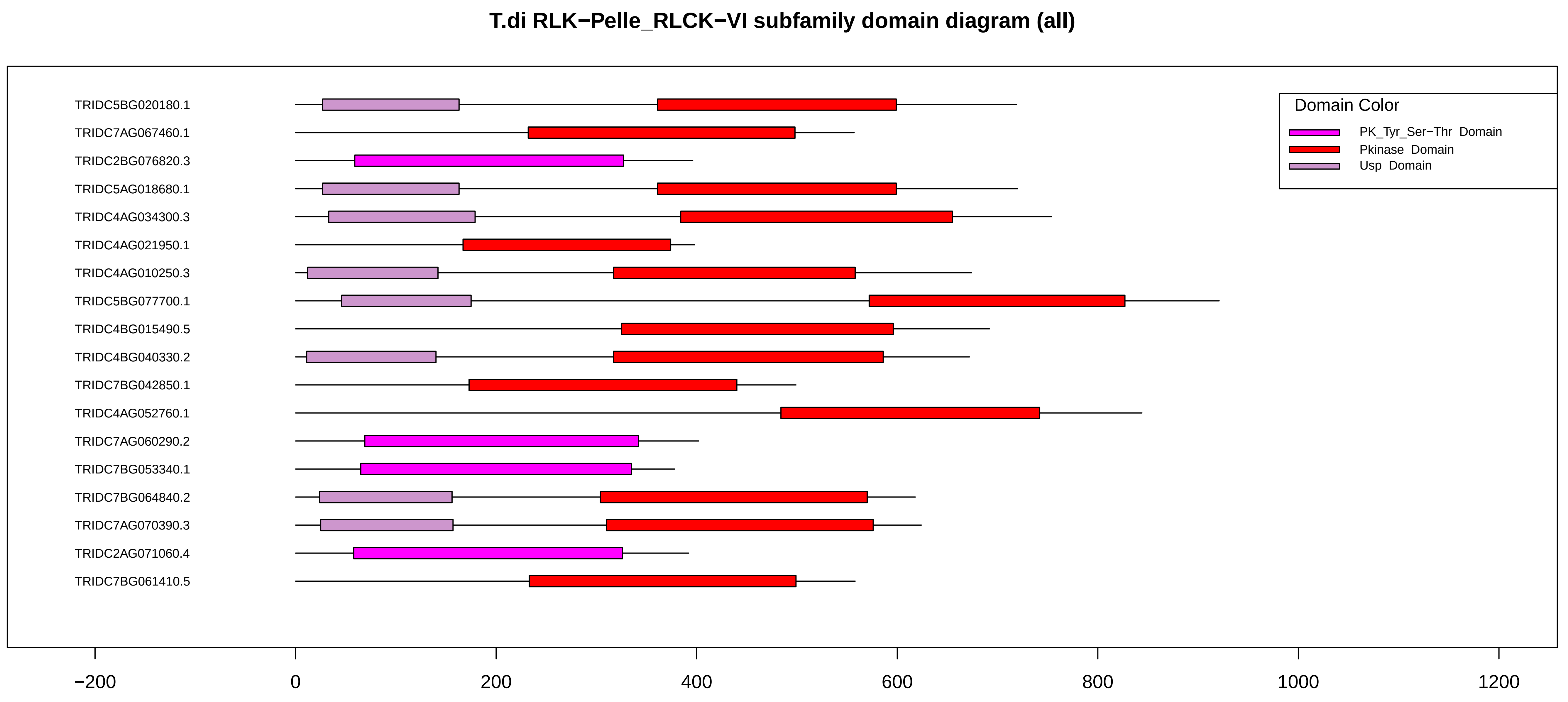

T.di RLK−Pelle_RLCK−VI subfamily domain diagram (all)
TRIDC5BG020180.1
TRIDC7AG067460.1
TRIDC2BG076820.3
TRIDC5AG018680.1
TRIDC4AG034300.3
TRIDC4AG021950.1
TRIDC4AG010250.3
TRIDC5BG077700.1
TRIDC4BG015490.5
TRIDC4BG040330.2
TRIDC7BG042850.1
TRIDC4AG052760.1
TRIDC7AG060290.2
TRIDC7BG053340.1
TRIDC7BG064840.2
TRIDC7AG070390.3
TRIDC2AG071060.4
TRIDC7BG061410.5
Domain Color
PK_Tyr_Ser−Thr Domain
Pkinase Domain
Usp Domain
−200
0
200
400
600
800
1000
1200

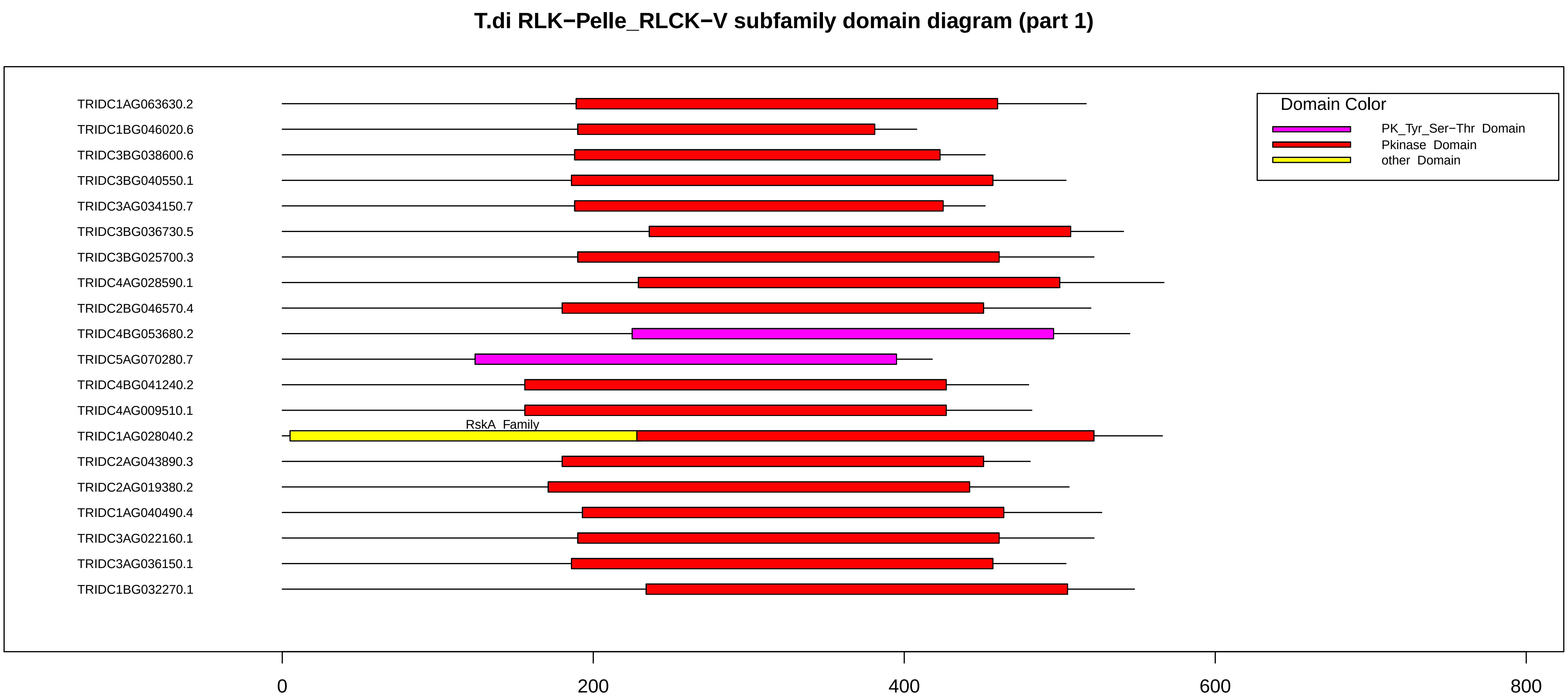
T.di RLK−Pelle_RLCK−V subfamily domain diagram (part 1)
TRIDC1AG063630.2
TRIDC1BG046020.6
TRIDC3BG038600.6
TRIDC3BG040550.1
TRIDC3AG034150.7
TRIDC3BG036730.5
TRIDC3BG025700.3
TRIDC4AG028590.1
TRIDC2BG046570.4
TRIDC4BG053680.2
TRIDC5AG070280.7
TRIDC4BG041240.2
TRIDC4AG009510.1
TRIDC1AG028040.2
RskA_Family
TRIDC2AG043890.3
TRIDC2AG019380.2
TRIDC1AG040490.4
TRIDC3AG022160.1
TRIDC3AG036150.1
TRIDC1BG032270.1
Domain Color
PK_Tyr_Ser−Thr Domain
Pkinase Domain
other Domain
0
200
400
600
800

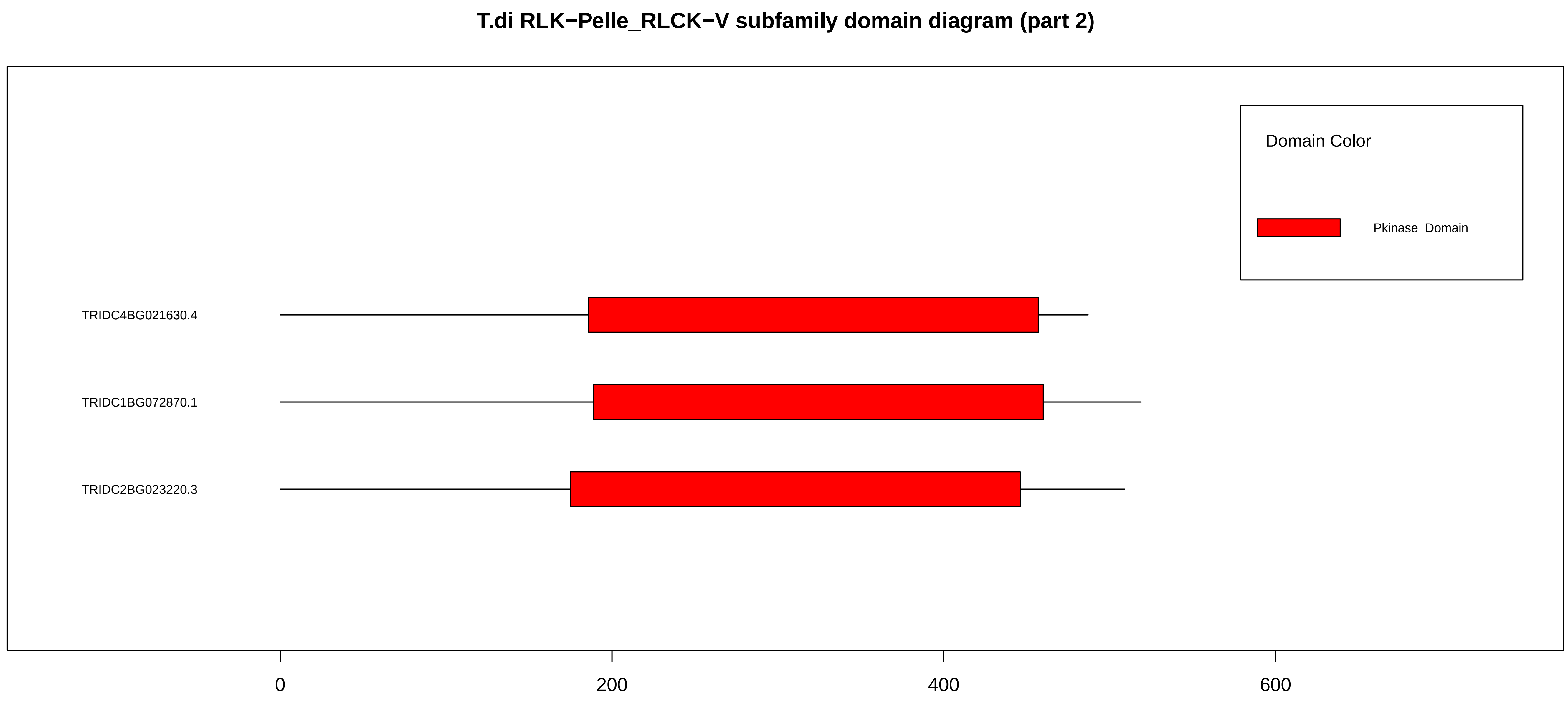

T.di RLK−Pelle_RLCK−V subfamily domain diagram (part 2)
Domain Color
Pkinase Domain
TRIDC4BG021630.4
TRIDC1BG072870.1
TRIDC2BG023220.3
0
200
400
600

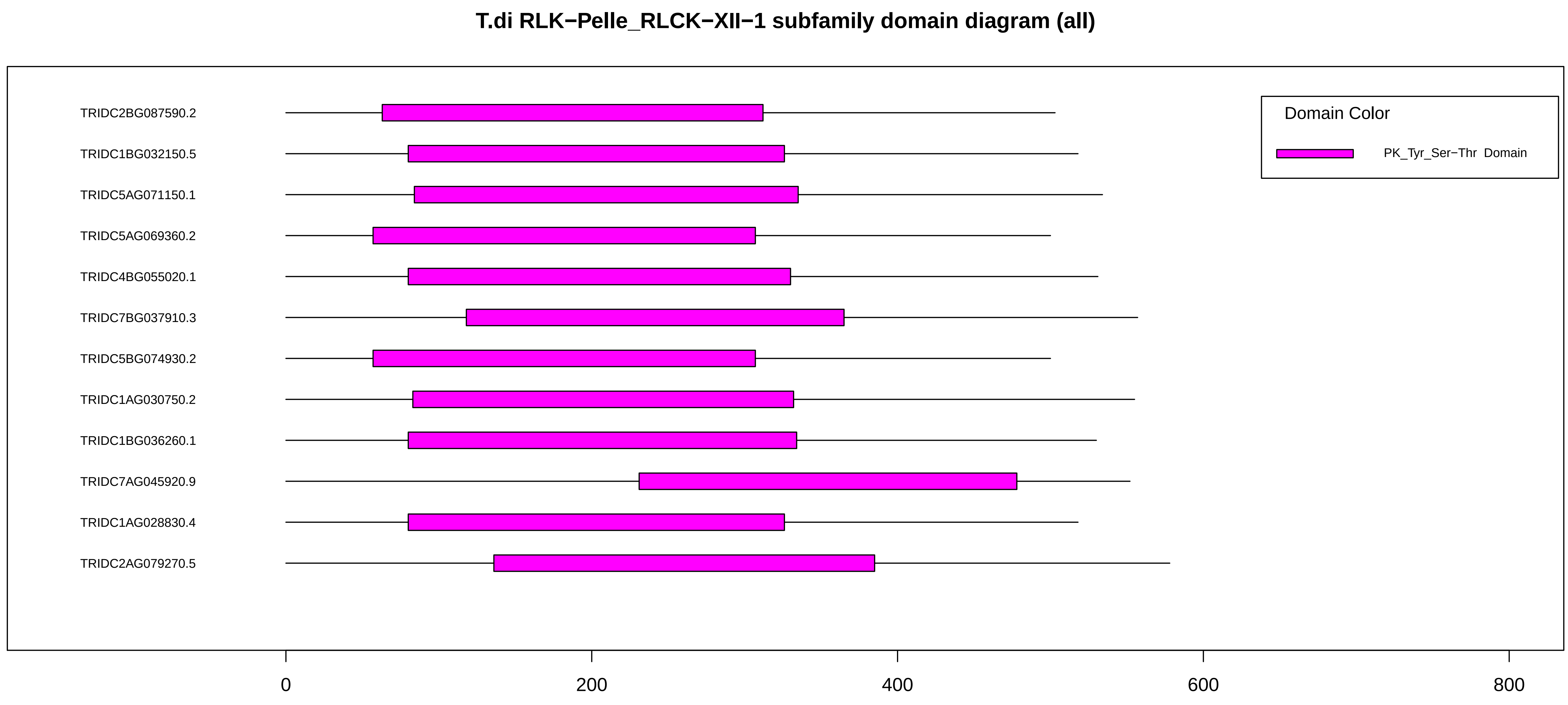
T.di RLK−Pelle_RLCK−XII−1 subfamily domain diagram (all)
TRIDC2BG087590.2
TRIDC1BG032150.5
TRIDC5AG071150.1
TRIDC5AG069360.2
TRIDC4BG055020.1
TRIDC7BG037910.3
TRIDC5BG074930.2
TRIDC1AG030750.2
TRIDC1BG036260.1
TRIDC7AG045920.9
TRIDC1AG028830.4
TRIDC2AG079270.5
Domain Color
PK_Tyr_Ser−Thr Domain
0
200
400
600
800

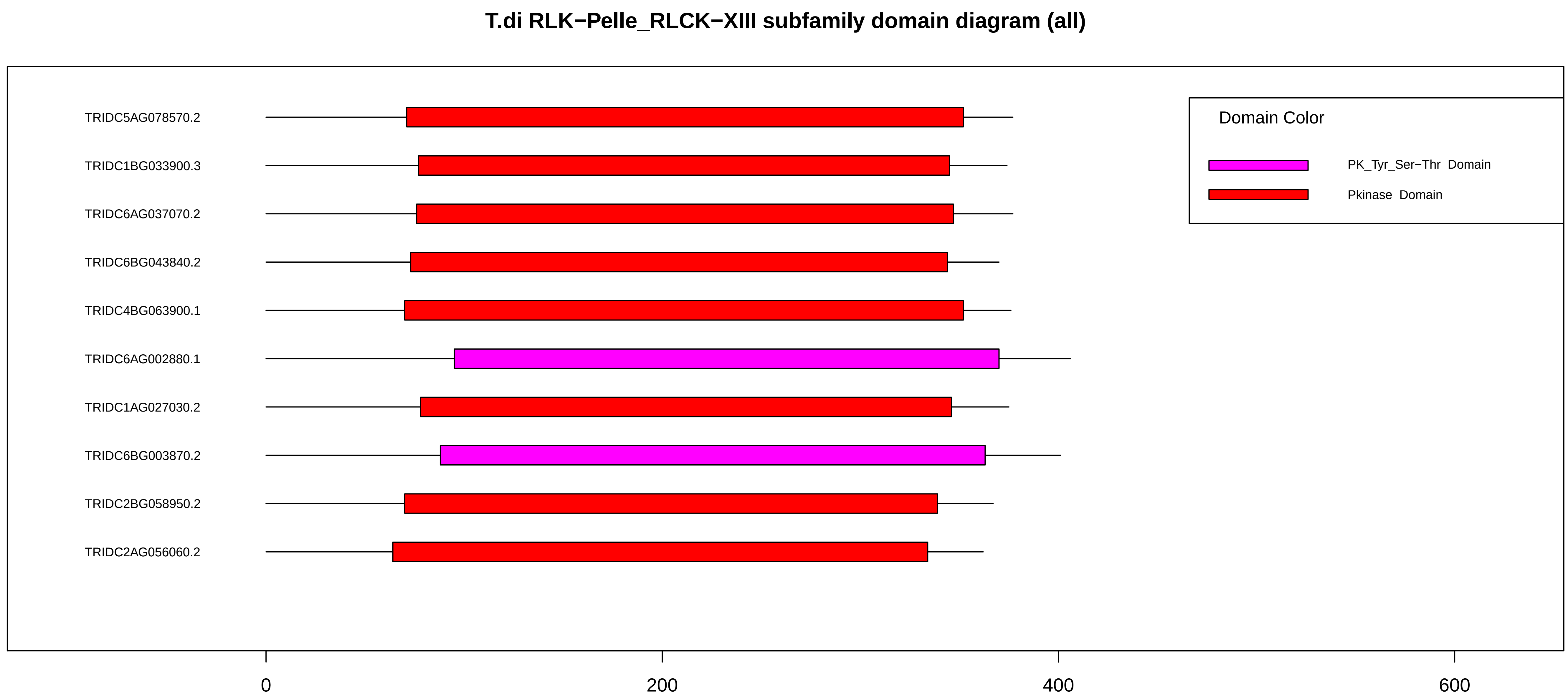
T.di RLK−Pelle_RLCK−XIII subfamily domain diagram (all)
TRIDC5AG078570.2
TRIDC1BG033900.3
TRIDC6AG037070.2
TRIDC6BG043840.2
TRIDC4BG063900.1
TRIDC6AG002880.1
TRIDC1AG027030.2
TRIDC6BG003870.2
TRIDC2BG058950.2
TRIDC2AG056060.2
Domain Color
PK_Tyr_Ser−Thr Domain
Pkinase Domain
0
200
400
600

T.di RLK−Pelle_RLCK−XI subfamily domain diagram (all)

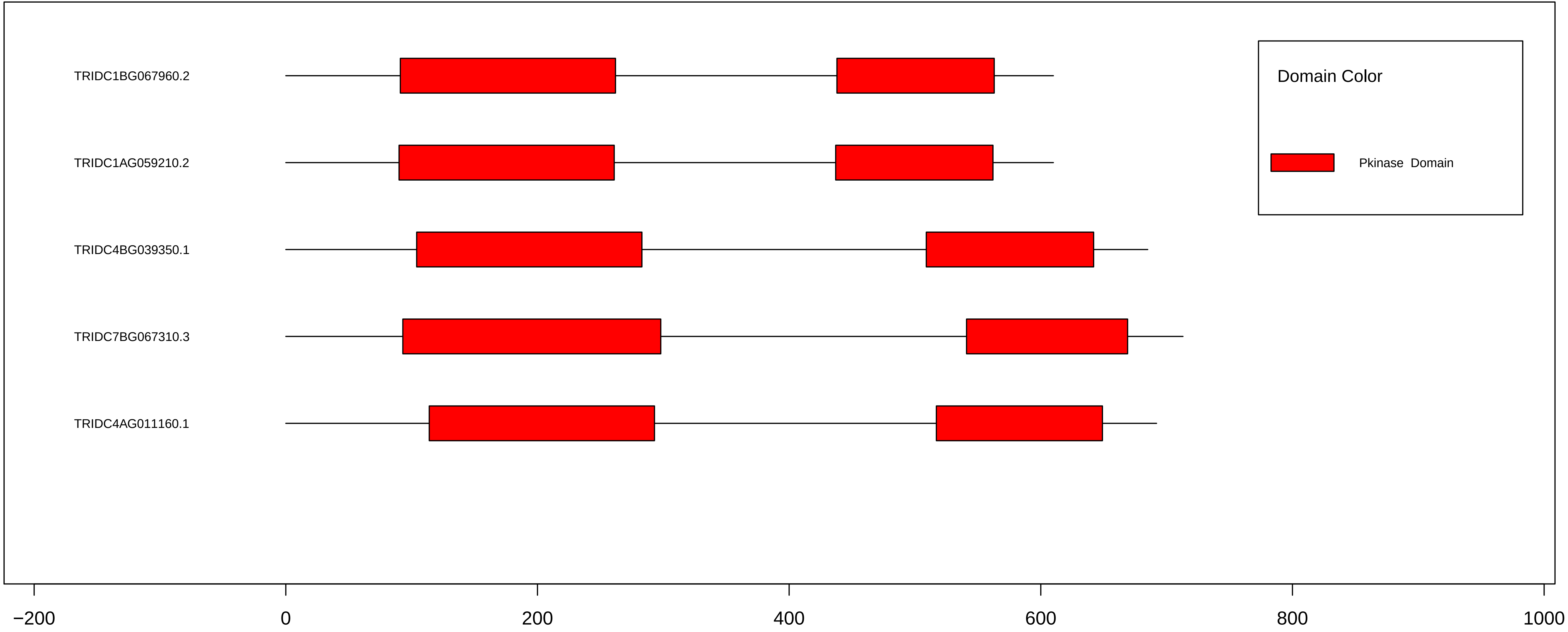

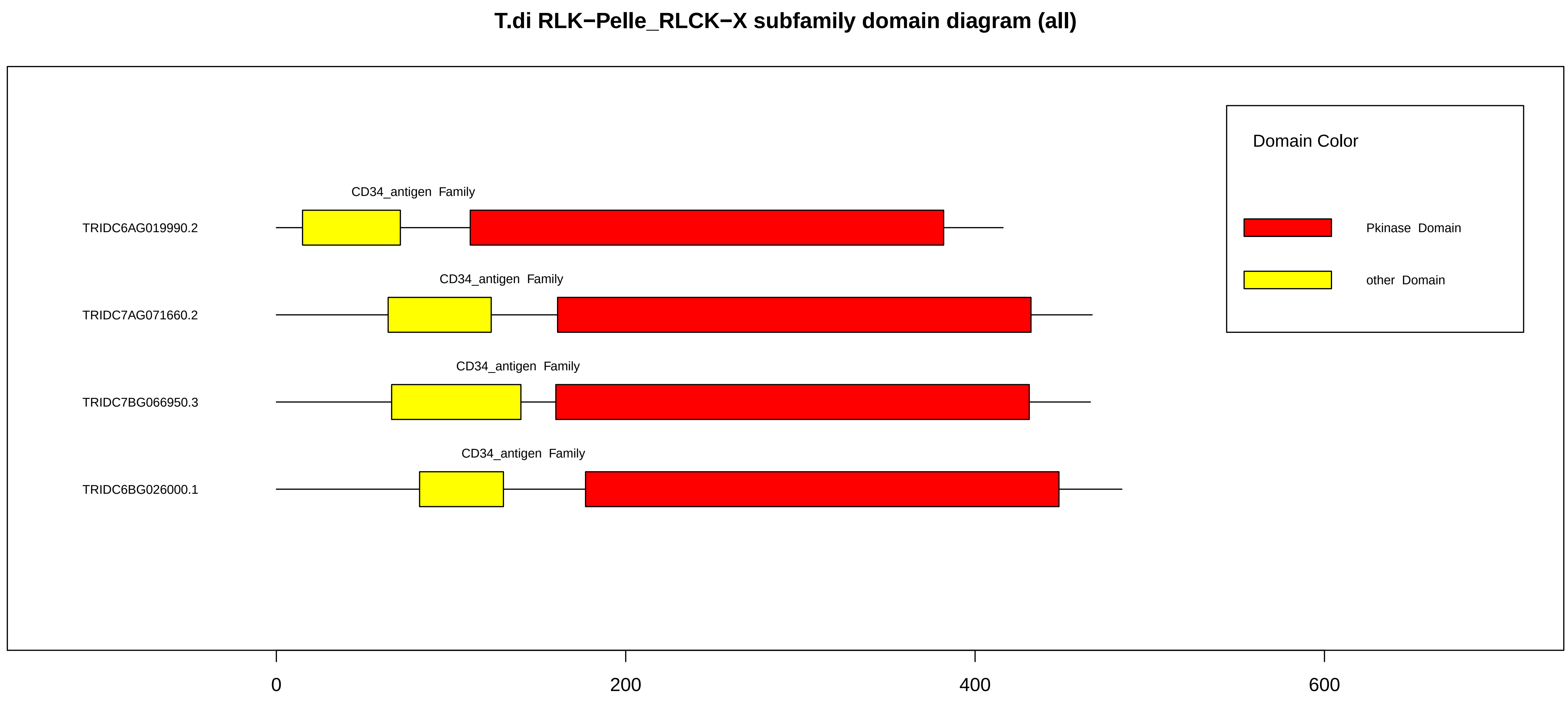

T.di RLK−Pelle_RLCK−X subfamily domain diagram (all)
Domain Color
Pkinase Domain
other Domain
CD34_antigen Family
TRIDC6AG019990.2
CD34_antigen Family
TRIDC7AG071660.2
CD34_antigen Family
TRIDC7BG066950.3
CD34_antigen Family
TRIDC6BG026000.1
0
200
400
600

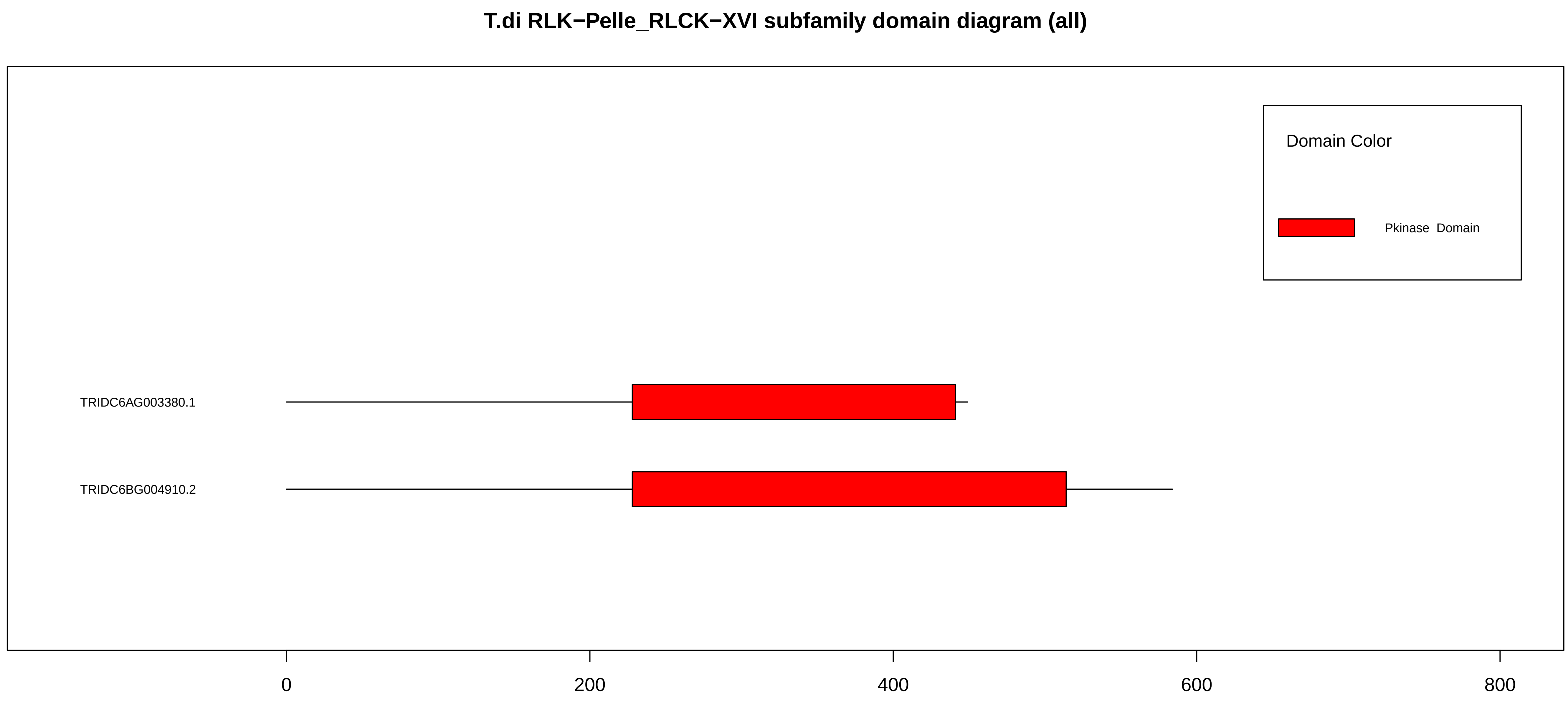

T.di RLK−Pelle_RLCK−XVI subfamily domain diagram (all)
Domain Color
Pkinase Domain
TRIDC6AG003380.1
TRIDC6BG004910.2
0
200
400
600
800

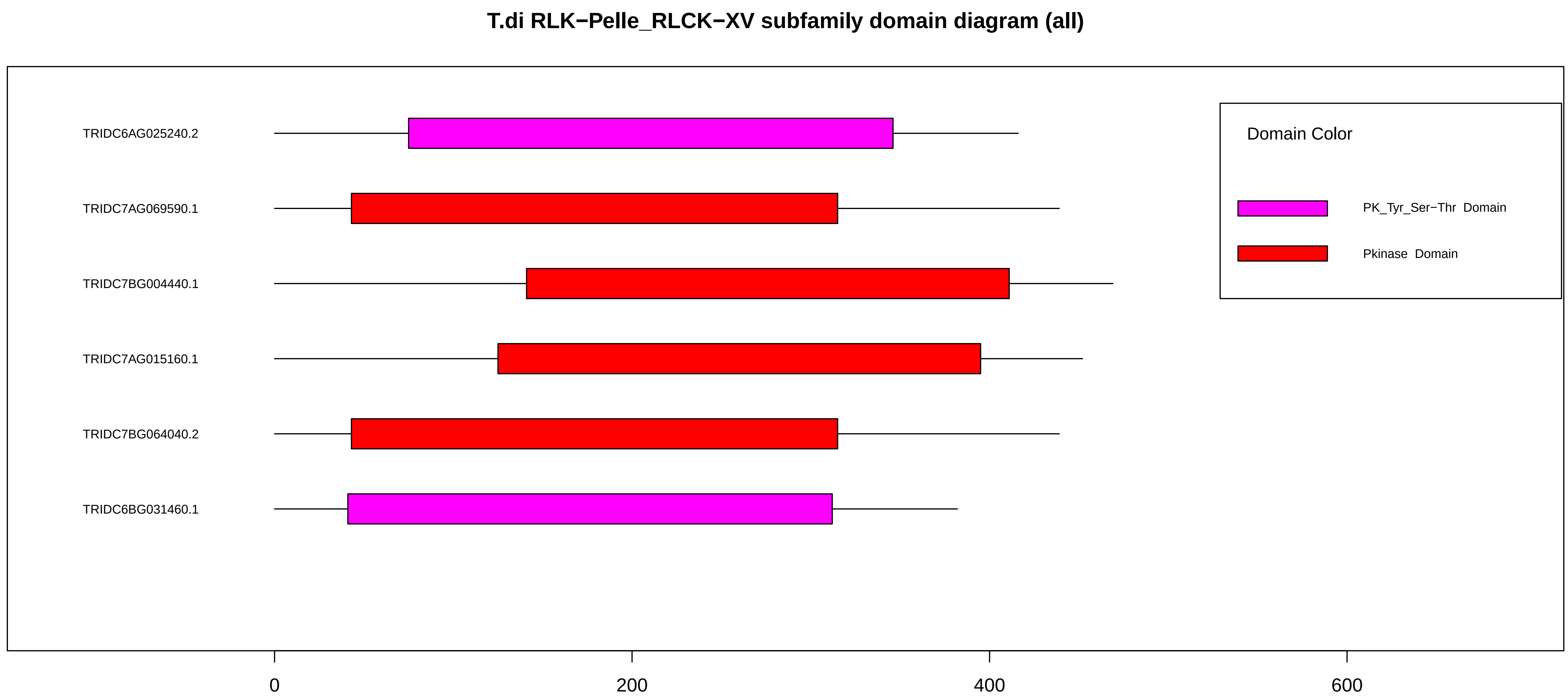
T.di RLK−Pelle_RLCK−XV subfamily domain diagram (all)
TRIDC6AG025240.2
TRIDC7AG069590.1
TRIDC7BG004440.1
TRIDC7AG015160.1
TRIDC7BG064040.2
TRIDC6BG031460.1
Domain Color
PK_Tyr_Ser−Thr Domain
Pkinase Domain
0
200
400
600

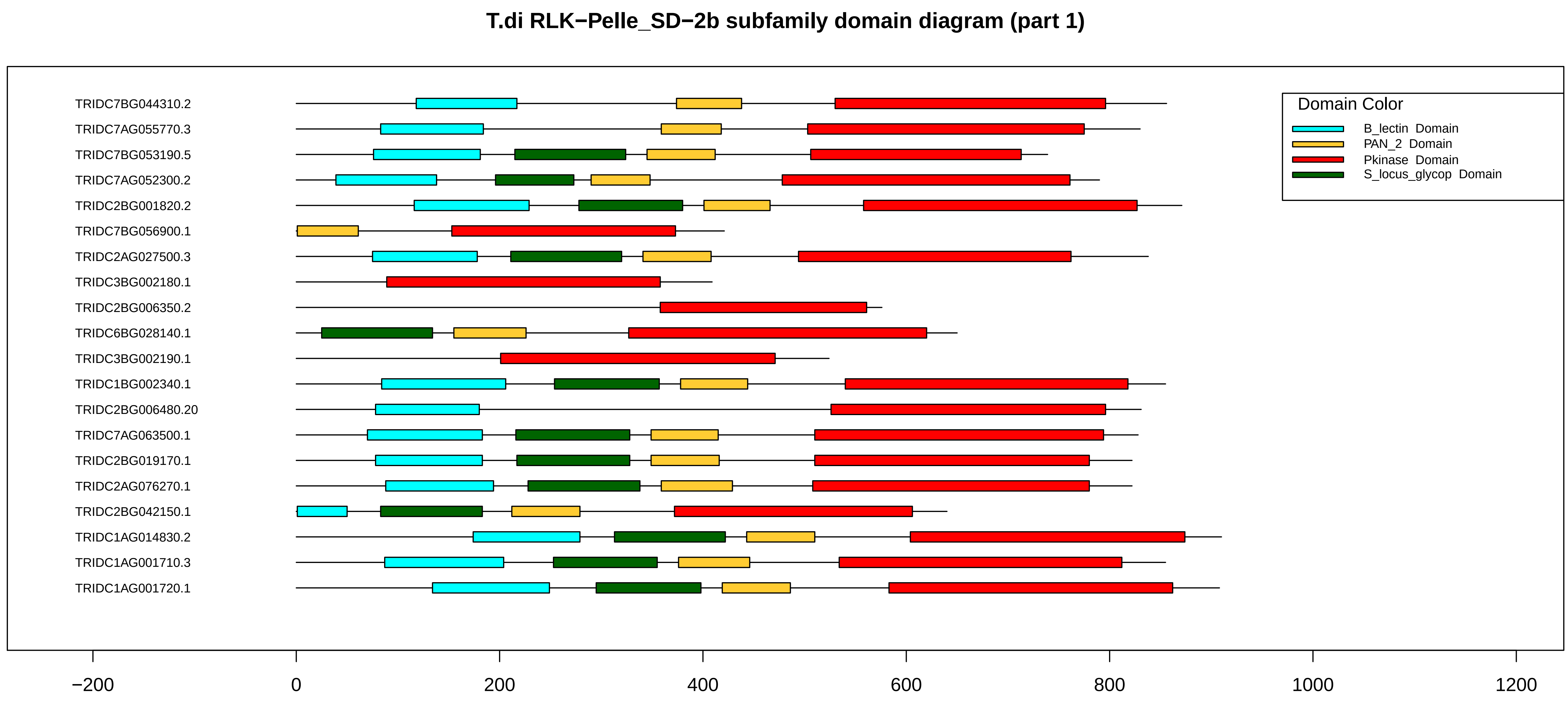
T.di RLK−Pelle_SD−2b subfamily domain diagram (part 1)
TRIDC7BG044310.2
TRIDC7AG055770.3
TRIDC7BG053190.5
TRIDC7AG052300.2
TRIDC2BG001820.2
TRIDC7BG056900.1
TRIDC2AG027500.3
TRIDC3BG002180.1
TRIDC2BG006350.2
TRIDC6BG028140.1
TRIDC3BG002190.1
TRIDC1BG002340.1
TRIDC2BG006480.20
TRIDC7AG063500.1
TRIDC2BG019170.1
TRIDC2AG076270.1
TRIDC2BG042150.1
TRIDC1AG014830.2
TRIDC1AG001710.3
TRIDC1AG001720.1
Domain Color
B_lectin Domain
PAN_2 Domain
Pkinase Domain
S_locus_glycop Domain
−200
0
200
400
600
800
1000
1200

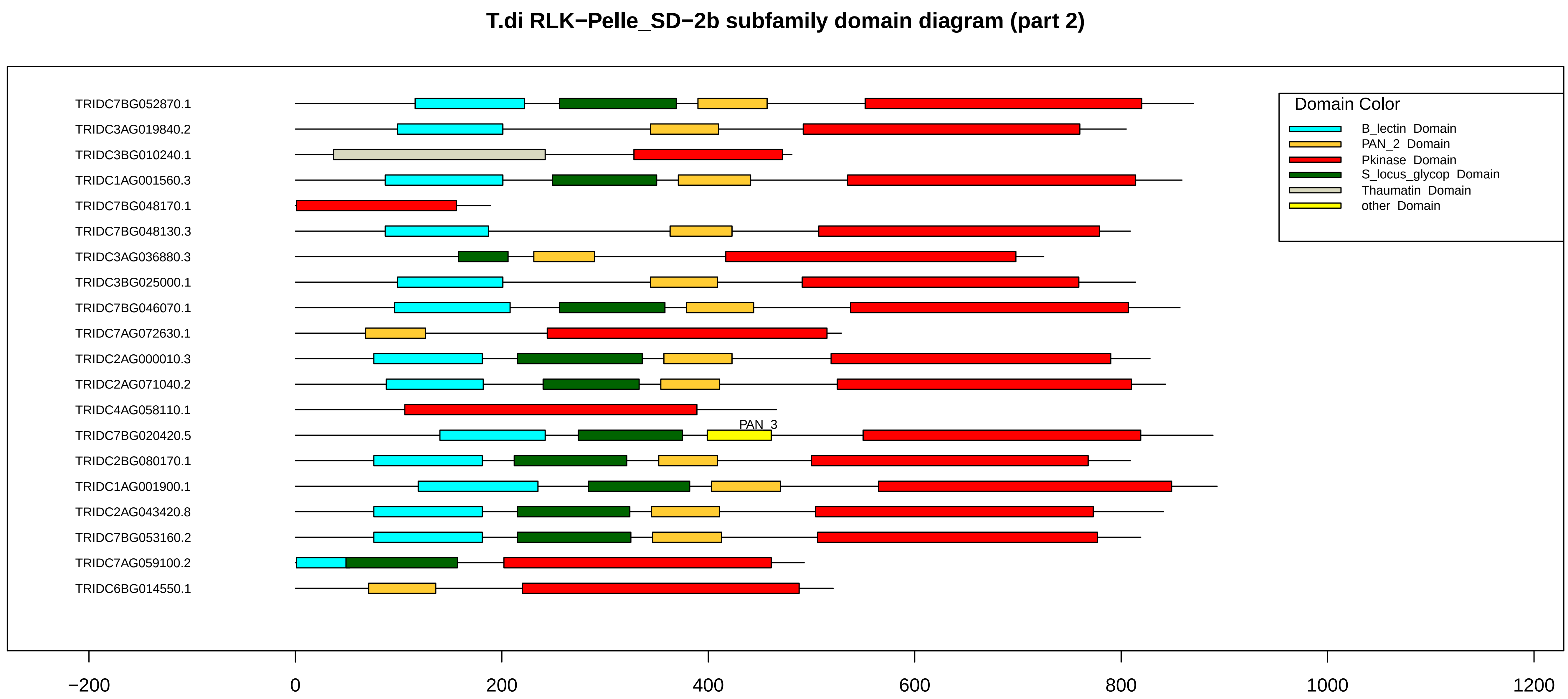
T.di RLK−Pelle_SD−2b subfamily domain diagram (part 2)
TRIDC7BG052870.1
TRIDC3AG019840.2
TRIDC3BG010240.1
TRIDC1AG001560.3
TRIDC7BG048170.1
TRIDC7BG048130.3
TRIDC3AG036880.3
TRIDC3BG025000.1
TRIDC7BG046070.1
TRIDC7AG072630.1
TRIDC2AG000010.3
TRIDC2AG071040.2
TRIDC4AG058110.1
TRIDC7BG020420.5
TRIDC2BG080170.1
TRIDC1AG001900.1
TRIDC2AG043420.8
TRIDC7BG053160.2
TRIDC7AG059100.2
TRIDC6BG014550.1
PAN_3
Domain Color
B_lectin Domain
PAN_2 Domain
Pkinase Domain
S_locus_glycop Domain
Thaumatin Domain
other Domain
−200
0
200
400
600
800
1000
1200

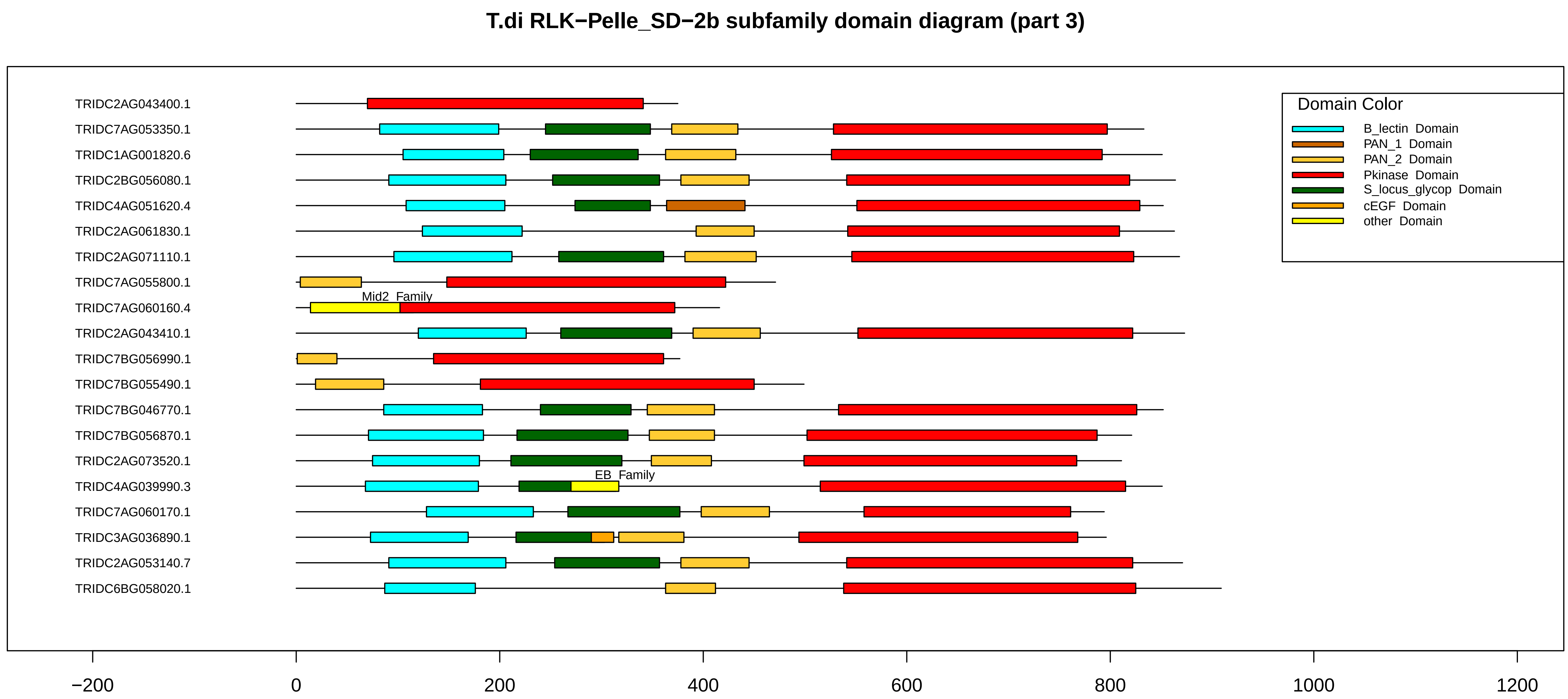
T.di RLK−Pelle_SD−2b subfamily domain diagram (part 3)
TRIDC2AG043400.1
TRIDC7AG053350.1
TRIDC1AG001820.6
TRIDC2BG056080.1
TRIDC4AG051620.4
TRIDC2AG061830.1
TRIDC2AG071110.1
TRIDC7AG055800.1
TRIDC7AG060160.4
TRIDC2AG043410.1
TRIDC7BG056990.1
TRIDC7BG055490.1
TRIDC7BG046770.1
TRIDC7BG056870.1
TRIDC2AG073520.1
TRIDC4AG039990.3
TRIDC7AG060170.1
TRIDC3AG036890.1
TRIDC2AG053140.7
TRIDC6BG058020.1
Mid2_Family
EB_Family
Domain Color
B_lectin Domain
PAN_1 Domain
PAN_2 Domain
Pkinase Domain
S_locus_glycop Domain
cEGF Domain
other Domain
−200
0
200
400
600
800
1000
1200

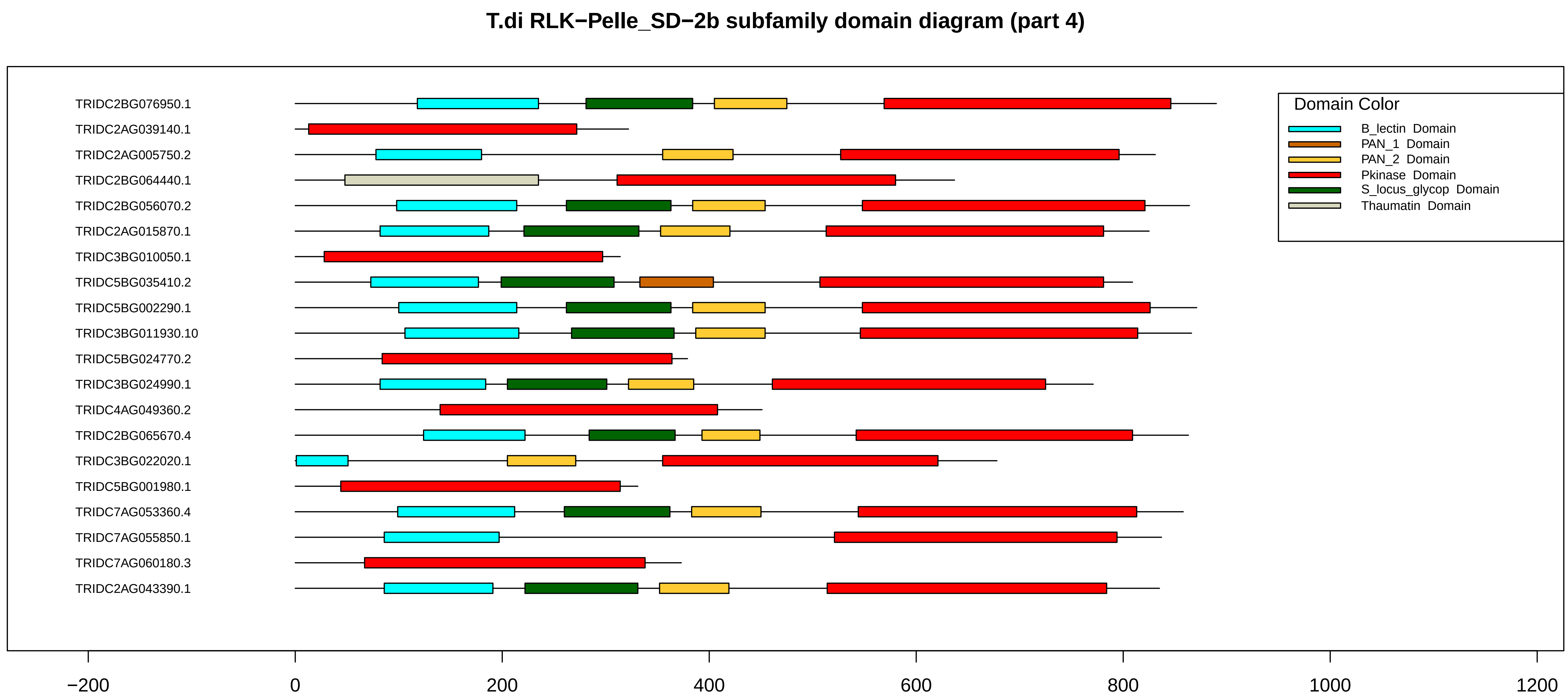
T.di RLK−Pelle_SD−2b subfamily domain diagram (part 4)
TRIDC2BG076950.1
TRIDC2AG039140.1
TRIDC2AG005750.2
TRIDC2BG064440.1
TRIDC2BG056070.2
TRIDC2AG015870.1
TRIDC3BG010050.1
TRIDC5BG035410.2
TRIDC5BG002290.1
TRIDC3BG011930.10
TRIDC5BG024770.2
TRIDC3BG024990.1
TRIDC4AG049360.2
TRIDC2BG065670.4
TRIDC3BG022020.1
TRIDC5BG001980.1
TRIDC7AG053360.4
TRIDC7AG055850.1
TRIDC7AG060180.3
TRIDC2AG043390.1
Domain Color
B_lectin Domain
PAN_1 Domain
PAN_2 Domain
Pkinase Domain
S_locus_glycop Domain
Thaumatin Domain
−200
0
200
400
600
800
1000
1200

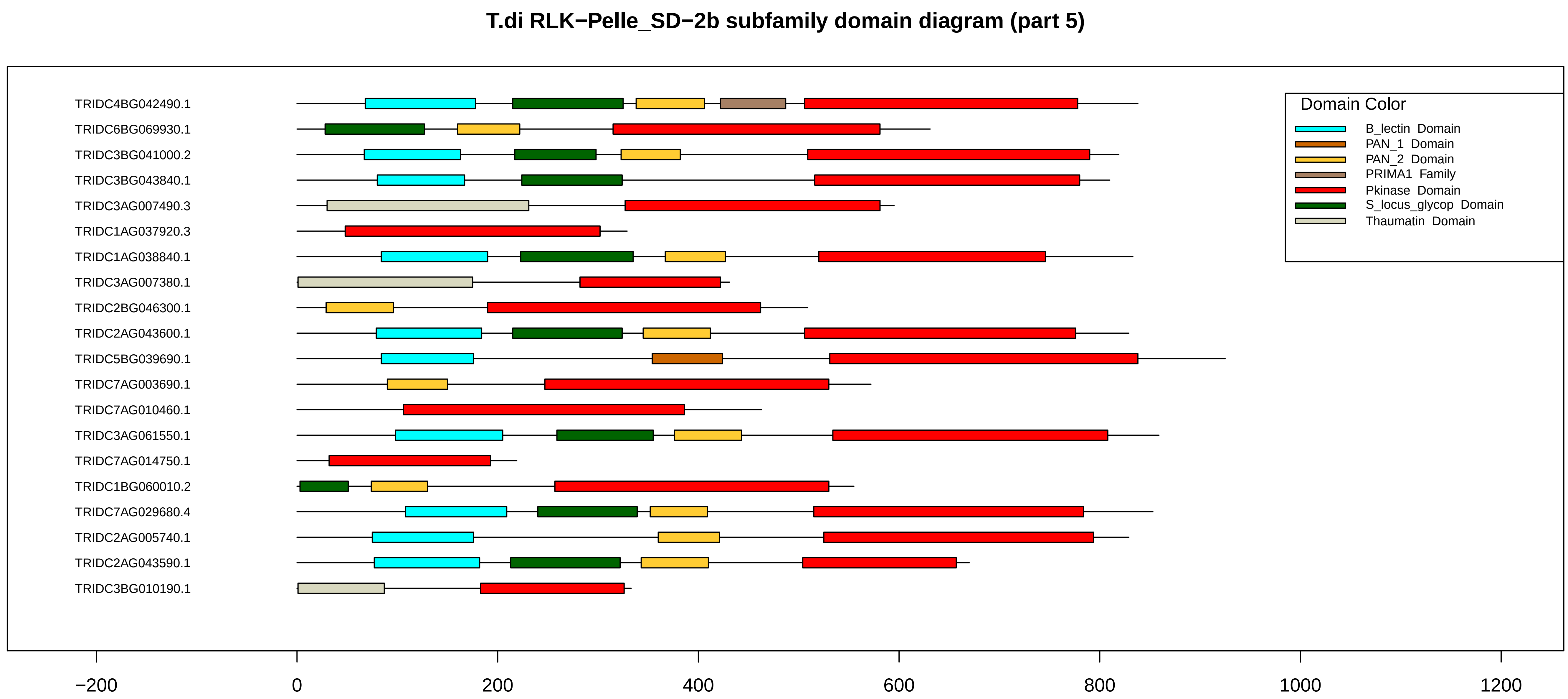

T.di RLK−Pelle_SD−2b subfamily domain diagram (part 5)
TRIDC4BG042490.1
TRIDC6BG069930.1
TRIDC3BG041000.2
TRIDC3BG043840.1
TRIDC3AG007490.3
TRIDC1AG037920.3
TRIDC1AG038840.1
TRIDC3AG007380.1
TRIDC2BG046300.1
TRIDC2AG043600.1
TRIDC5BG039690.1
TRIDC7AG003690.1
TRIDC7AG010460.1
TRIDC3AG061550.1
TRIDC7AG014750.1
TRIDC1BG060010.2
TRIDC7AG029680.4
TRIDC2AG005740.1
TRIDC2AG043590.1
TRIDC3BG010190.1
Domain Color
B_lectin Domain
PAN_1 Domain
PAN_2 Domain
PRIMA1 Family
Pkinase Domain
S_locus_glycop Domain
Thaumatin Domain
−200
0
200
400
600
800
1000
1200

T.di RLK−Pelle_SD−2b subfamily domain diagram (part 6)

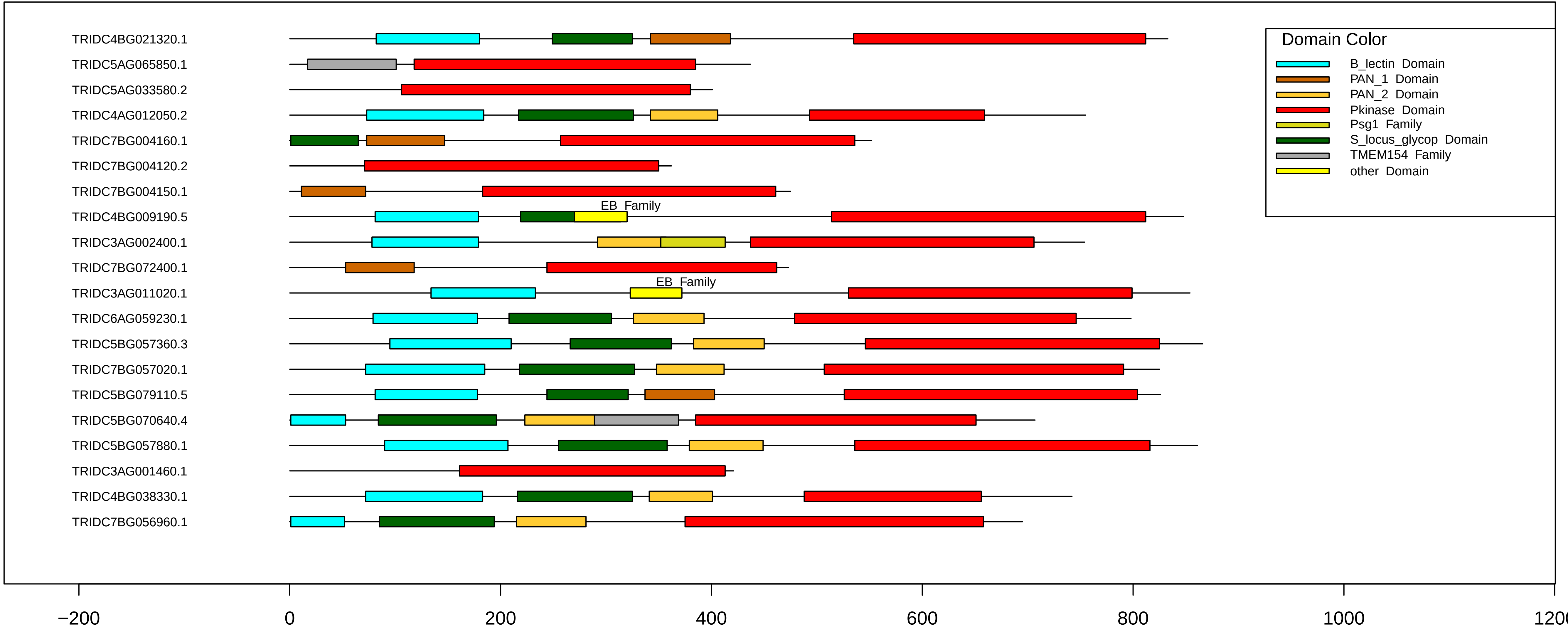

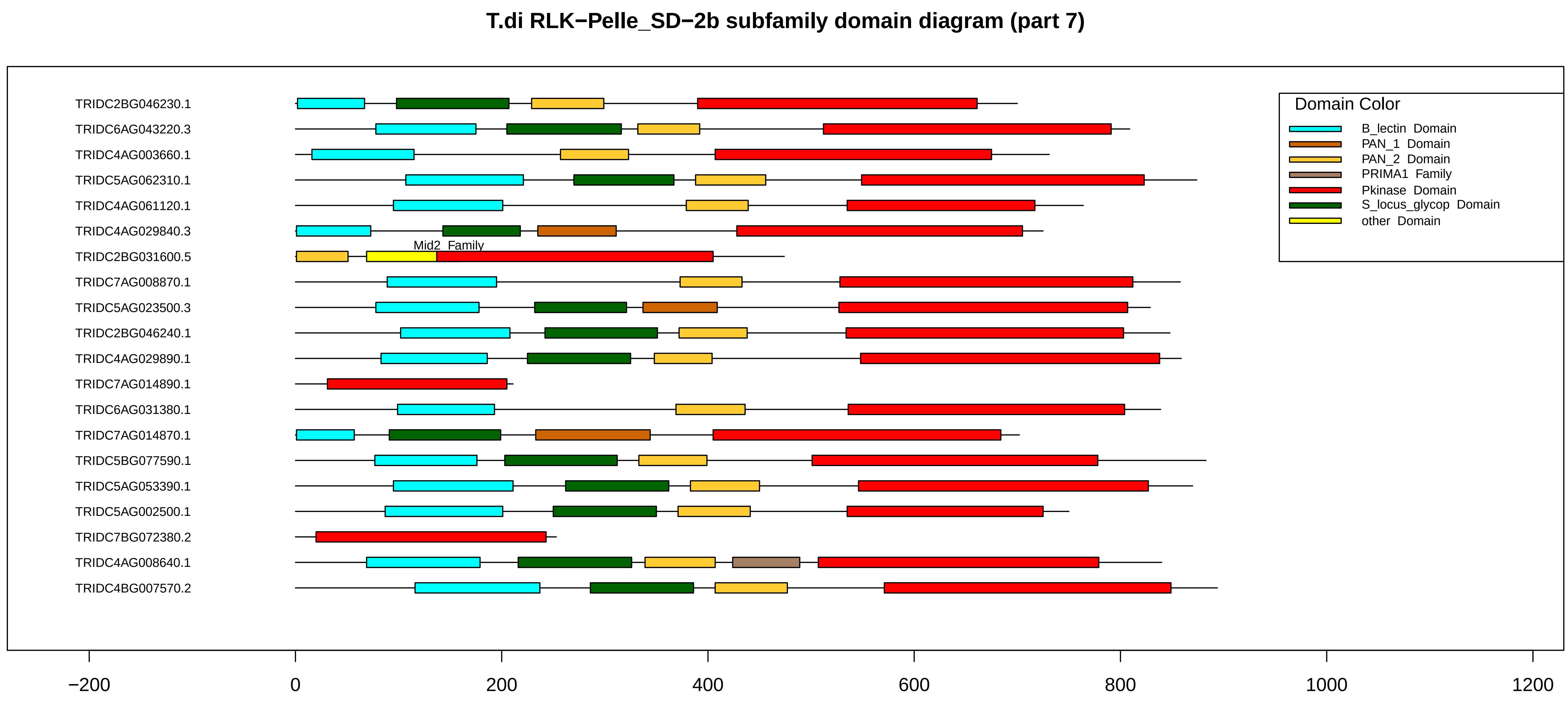
T.di RLK−Pelle_SD−2b subfamily domain diagram (part 7)
TRIDC2BG046230.1
TRIDC6AG043220.3
TRIDC4AG003660.1
TRIDC5AG062310.1
TRIDC4AG061120.1
TRIDC4AG029840.3
TRIDC2BG031600.5
Mid2_Family
TRIDC7AG008870.1
TRIDC5AG023500.3
TRIDC2BG046240.1
TRIDC4AG029890.1
TRIDC7AG014890.1
TRIDC6AG031380.1
TRIDC7AG014870.1
TRIDC5BG077590.1
TRIDC5AG053390.1
TRIDC5AG002500.1
TRIDC7BG072380.2
TRIDC4AG008640.1
TRIDC4BG007570.2
Domain Color
B_lectin Domain
PAN_1 Domain
PAN_2 Domain
PRIMA1 Family
Pkinase Domain
S_locus_glycop Domain
other Domain
−200
0
200
400
600
800
1000
1200

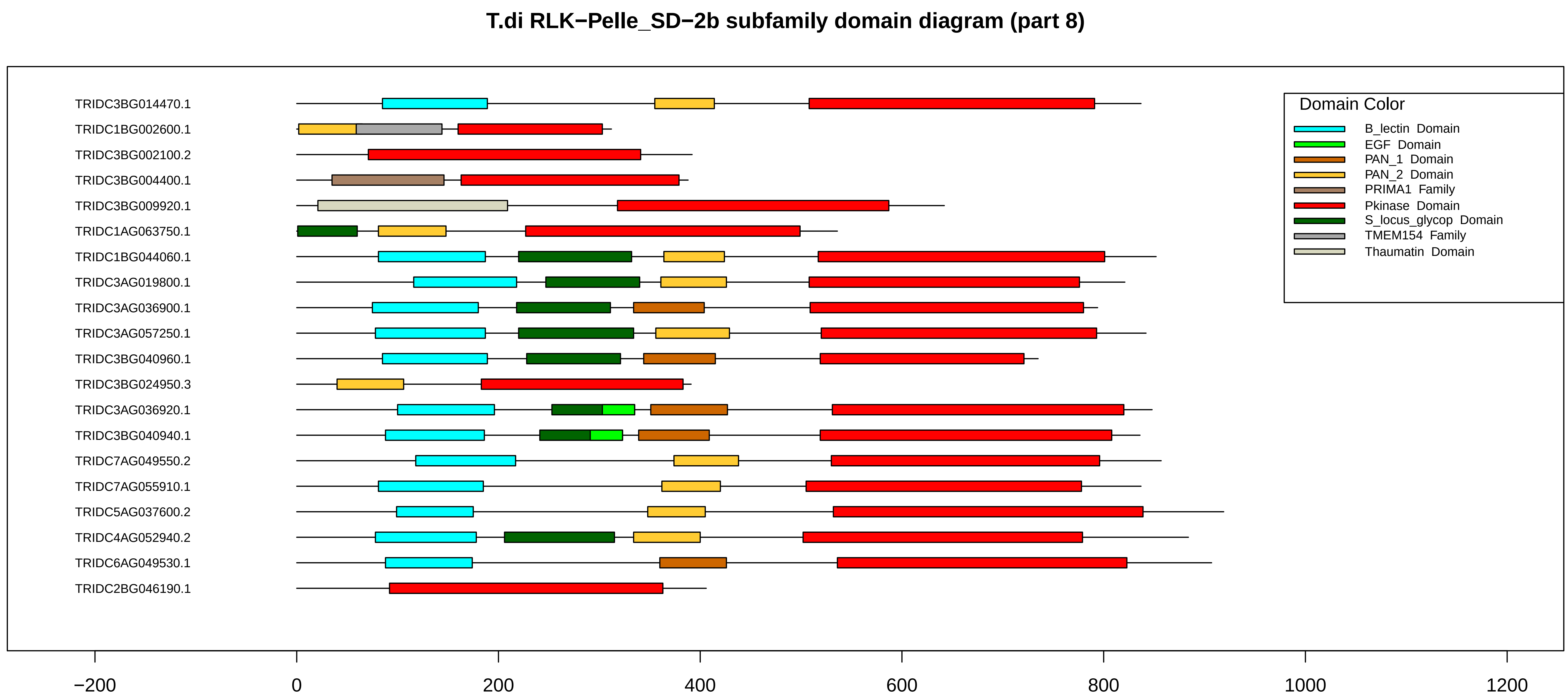

T.di RLK−Pelle_SD−2b subfamily domain diagram (part 8)
TRIDC3BG014470.1
TRIDC1BG002600.1
TRIDC3BG002100.2
TRIDC3BG004400.1
TRIDC3BG009920.1
TRIDC1AG063750.1
TRIDC1BG044060.1
TRIDC3AG019800.1
TRIDC3AG036900.1
TRIDC3AG057250.1
TRIDC3BG040960.1
TRIDC3BG024950.3
TRIDC3AG036920.1
TRIDC3BG040940.1
TRIDC7AG049550.2
TRIDC7AG055910.1
TRIDC5AG037600.2
TRIDC4AG052940.2
TRIDC6AG049530.1
TRIDC2BG046190.1
Domain Color
B_lectin Domain
EGF Domain
PAN_1 Domain
PAN_2 Domain
PRIMA1 Family
Pkinase Domain
S_locus_glycop Domain
TMEM154 Family
Thaumatin Domain
−200
0
200
400
600
800
1000
1200

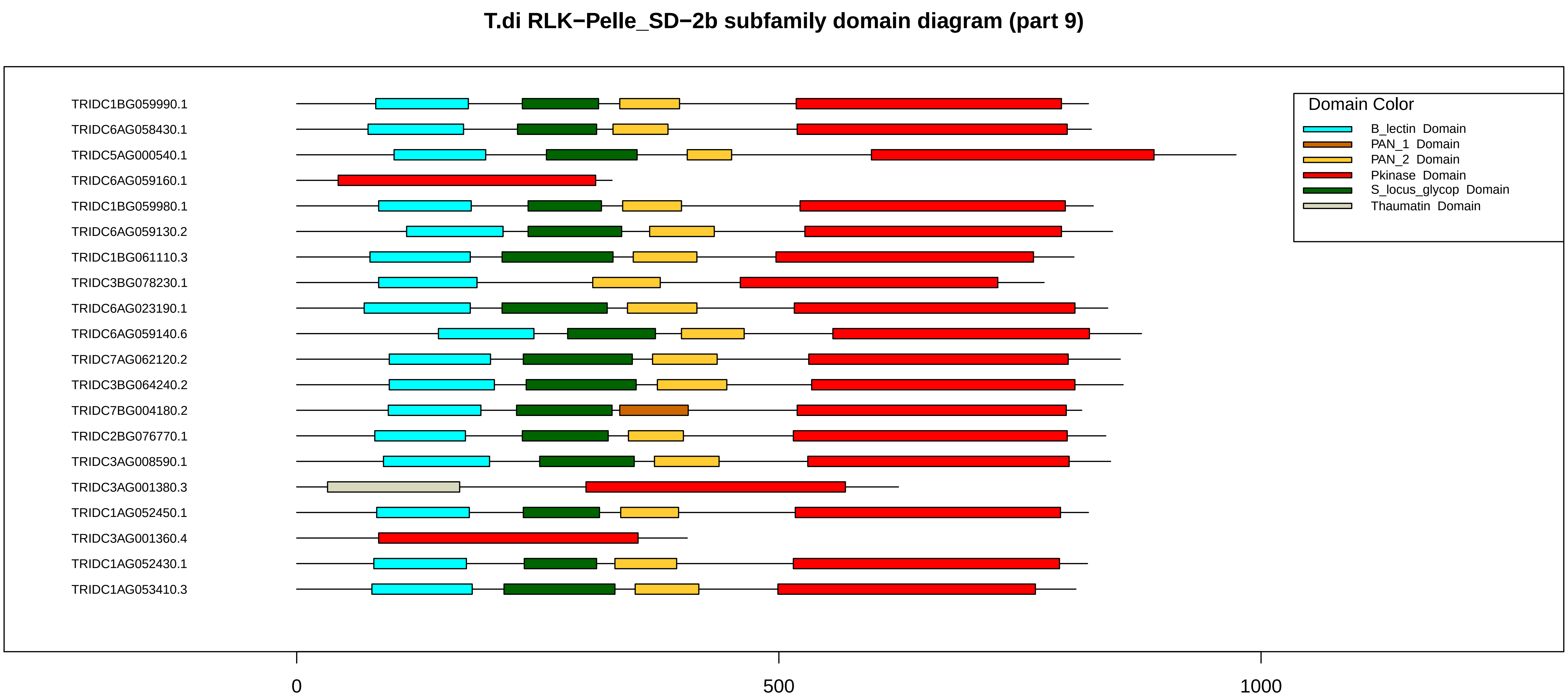
T.di RLK−Pelle_SD−2b subfamily domain diagram (part 9)
TRIDC1BG059990.1
TRIDC6AG058430.1
TRIDC5AG000540.1
TRIDC6AG059160.1
TRIDC1BG059980.1
TRIDC6AG059130.2
TRIDC1BG061110.3
TRIDC3BG078230.1
TRIDC6AG023190.1
TRIDC6AG059140.6
TRIDC7AG062120.2
TRIDC3BG064240.2
TRIDC7BG004180.2
TRIDC2BG076770.1
TRIDC3AG008590.1
TRIDC3AG001380.3
TRIDC1AG052450.1
TRIDC3AG001360.4
TRIDC1AG052430.1
TRIDC1AG053410.3
Domain Color
B_lectin Domain
PAN_1 Domain
PAN_2 Domain
Pkinase Domain
S_locus_glycop Domain
Thaumatin Domain
0
500
1000

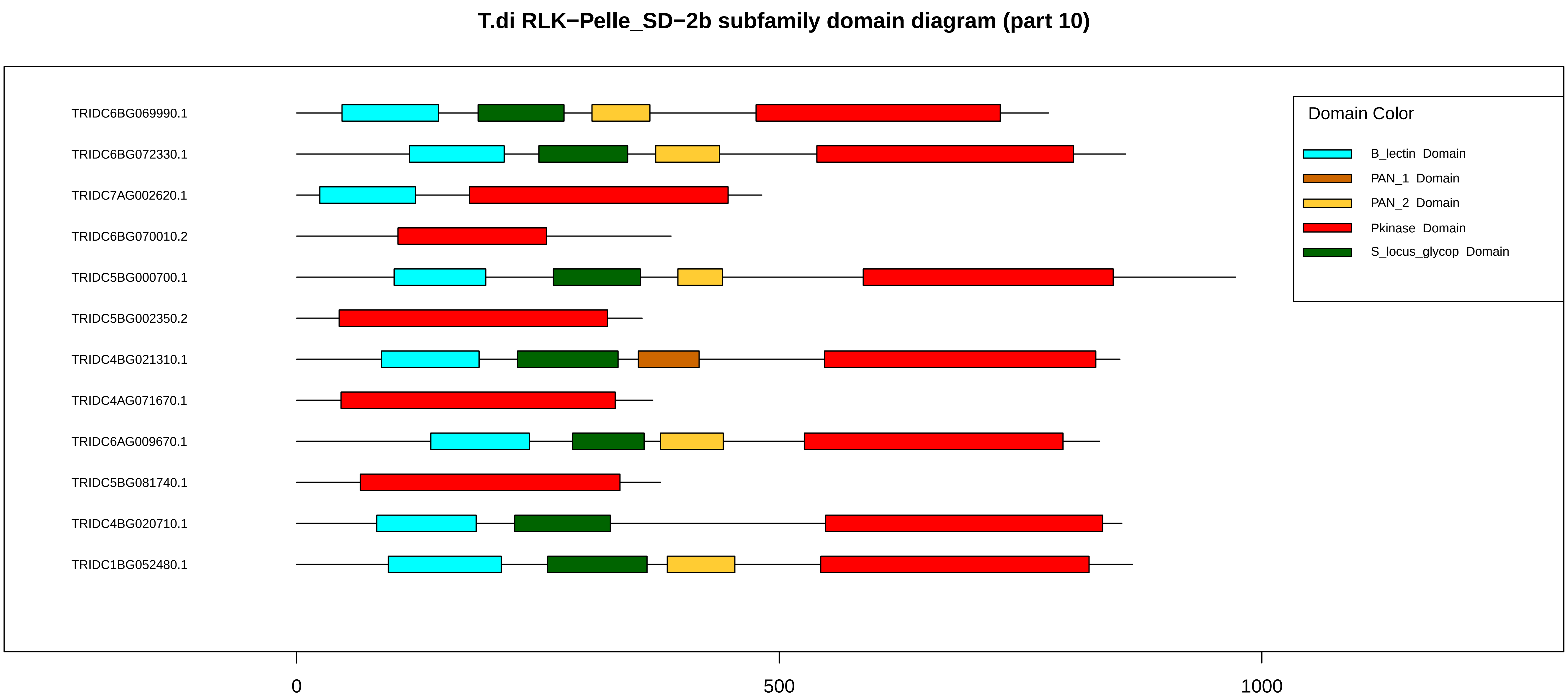

T.di RLK−Pelle_SD−2b subfamily domain diagram (part 10)
TRIDC6BG069990.1
TRIDC6BG072330.1
TRIDC7AG002620.1
TRIDC6BG070010.2
TRIDC5BG000700.1
TRIDC5BG002350.2
TRIDC4BG021310.1
TRIDC4AG071670.1
TRIDC6AG009670.1
TRIDC5BG081740.1
TRIDC4BG020710.1
TRIDC1BG052480.1
Domain Color
B_lectin Domain
PAN_1 Domain
PAN_2 Domain
Pkinase Domain
S_locus_glycop Domain
0
500
1000

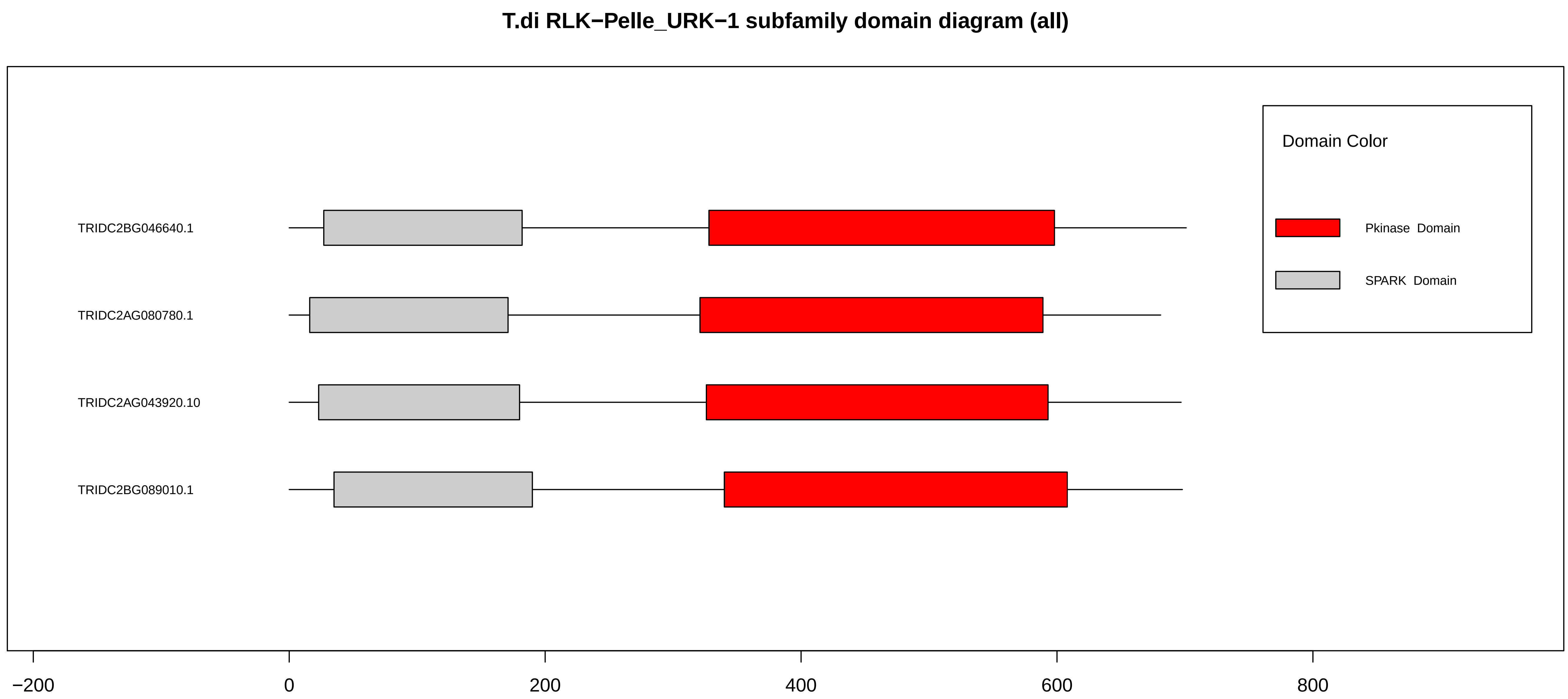
T.di RLK−Pelle_URK−1 subfamily domain diagram (all)
TRIDC2BG046640.1
TRIDC2AG080780.1
TRIDC2AG043920.10
TRIDC2BG089010.1
Domain Color
Pkinase Domain
SPARK Domain
−200
0
200
400
600
800

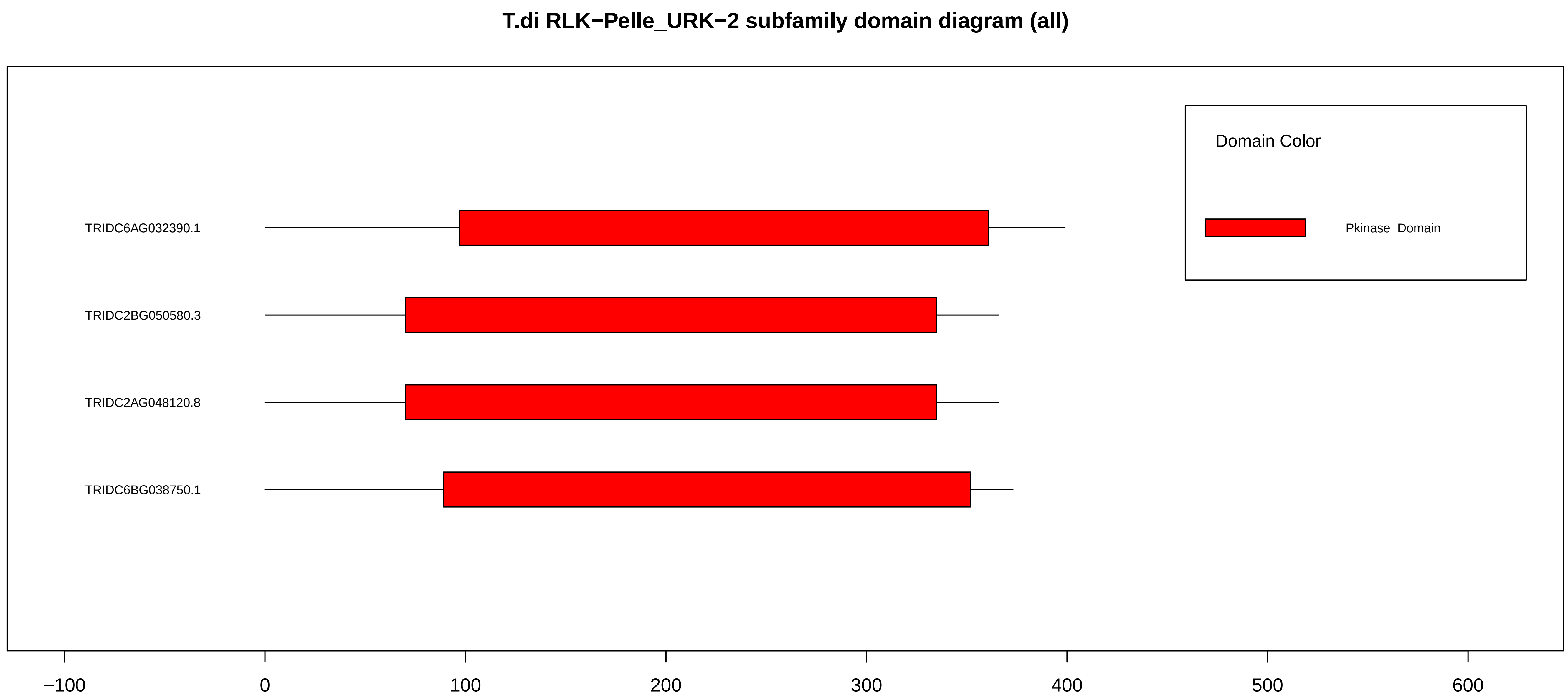
T.di RLK−Pelle_URK−2 subfamily domain diagram (all)
TRIDC6AG032390.1
TRIDC2BG050580.3
TRIDC2AG048120.8
TRIDC6BG038750.1
Domain Color
Pkinase Domain
−100
0
100
200
300
400
500
600

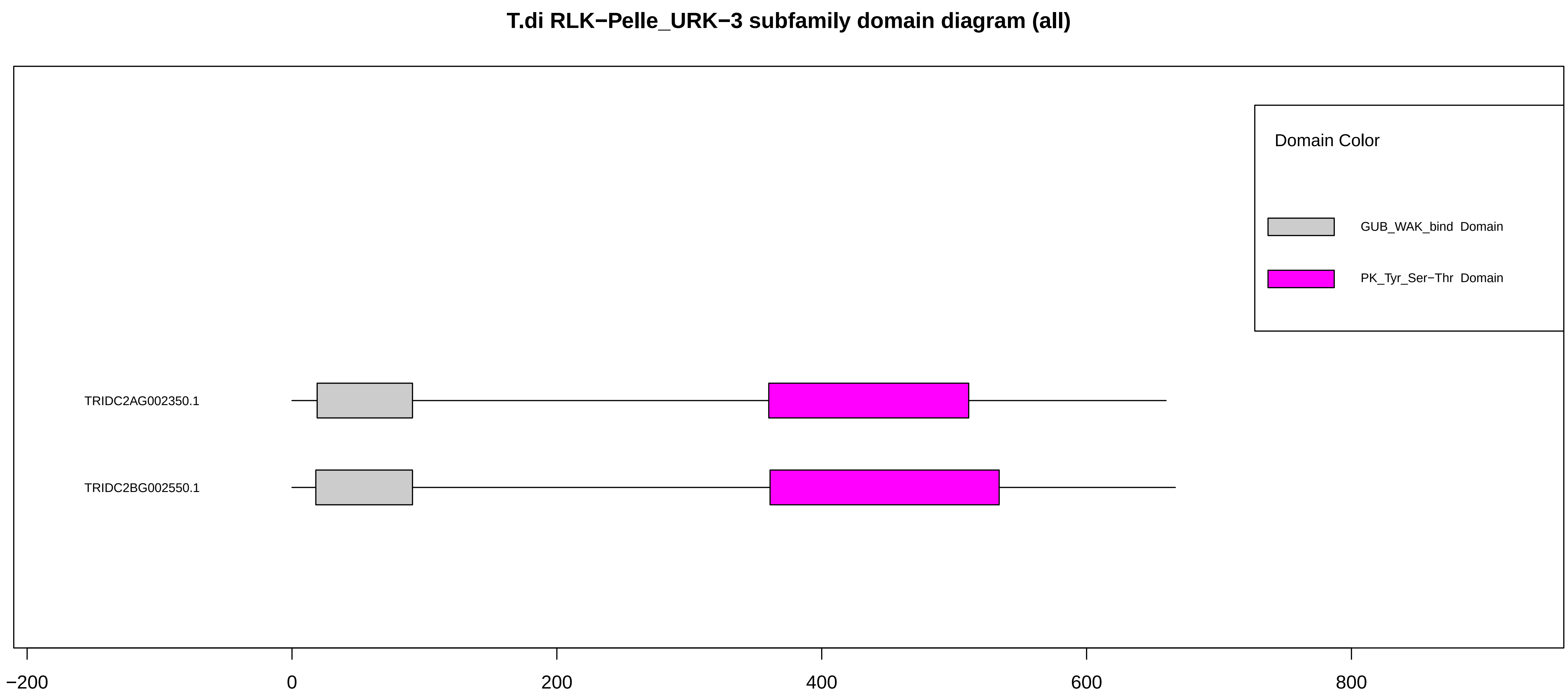

T.di RLK−Pelle_URK−3 subfamily domain diagram (all)
Domain Color
GUB_WAK_bind Domain
PK_Tyr_Ser−Thr Domain
TRIDC2AG002350.1
TRIDC2BG002550.1
−200
0
200
400
600
800

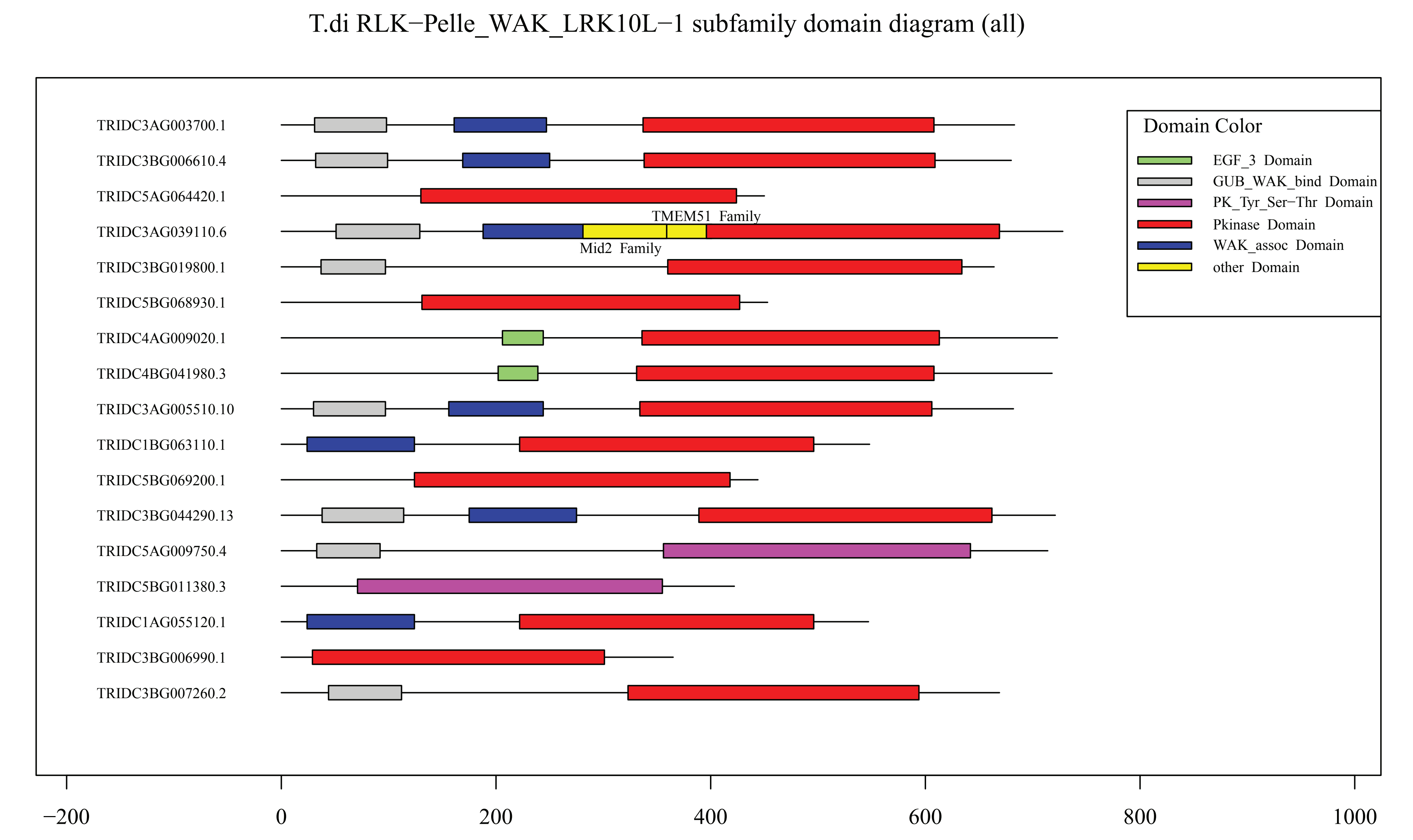
T.di RLK−Pelle_WAK_LRK10L−1 subfamily domain diagram (all)
TRIDC3AG003700.1
TRIDC3BG006610.4
TRIDC5AG064420.1
TRIDC3AG039110.6
TMEM51 Family
Mid2 Family
TRIDC3BG019800.1
TRIDC5BG068930.1
TRIDC4AG009020.1
TRIDC4BG041980.3
TRIDC3AG005510.10
TRIDC1BG063110.1
TRIDC5BG069200.1
TRIDC3BG044290.13
TRIDC5AG009750.4
TRIDC5BG011380.3
TRIDC1AG055120.1
TRIDC3BG006990.1
TRIDC3BG007260.2
Domain Color
EGF_3 Domain
GUB_WAK_bind Domain
PK_Tyr_Ser−Thr Domain
Pkinase Domain
WAK_assoc Domain
other Domain
−200
0
200
400
600
800
1000

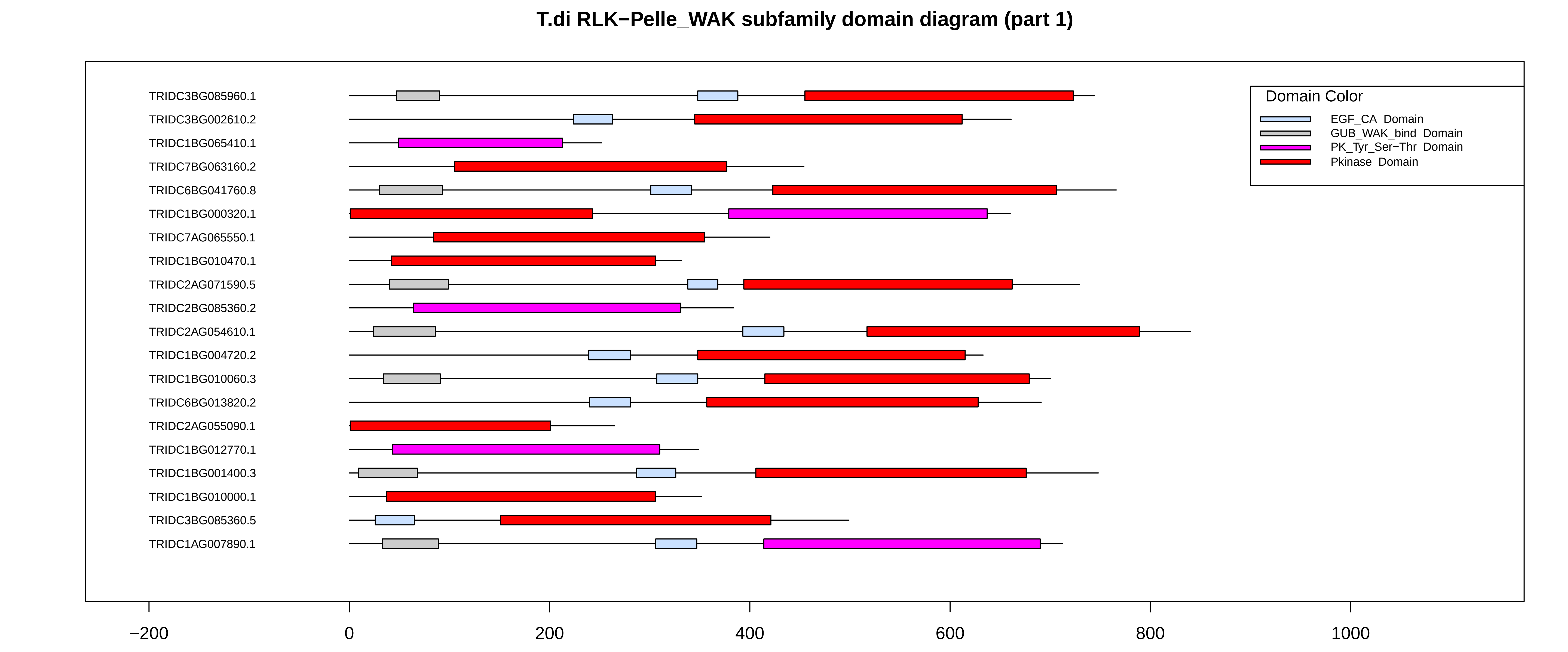
T.di RLK−Pelle_WAK subfamily domain diagram (part 1)
TRIDC3BG085960.1
TRIDC3BG002610.2
TRIDC1BG065410.1
TRIDC7BG063160.2
TRIDC6BG041760.8
TRIDC1BG000320.1
TRIDC7AG065550.1
TRIDC1BG010470.1
TRIDC2AG071590.5
TRIDC2BG085360.2
TRIDC2AG054610.1
TRIDC1BG004720.2
TRIDC1BG010060.3
TRIDC6BG013820.2
TRIDC2AG055090.1
TRIDC1BG012770.1
TRIDC1BG001400.3
TRIDC1BG010000.1
TRIDC3BG085360.5
TRIDC1AG007890.1
Domain Color
EGF_CA Domain
GUB_WAK_bind Domain
PK_Tyr_Ser−Thr Domain
Pkinase Domain
−200
0
200
400
600
800
1000

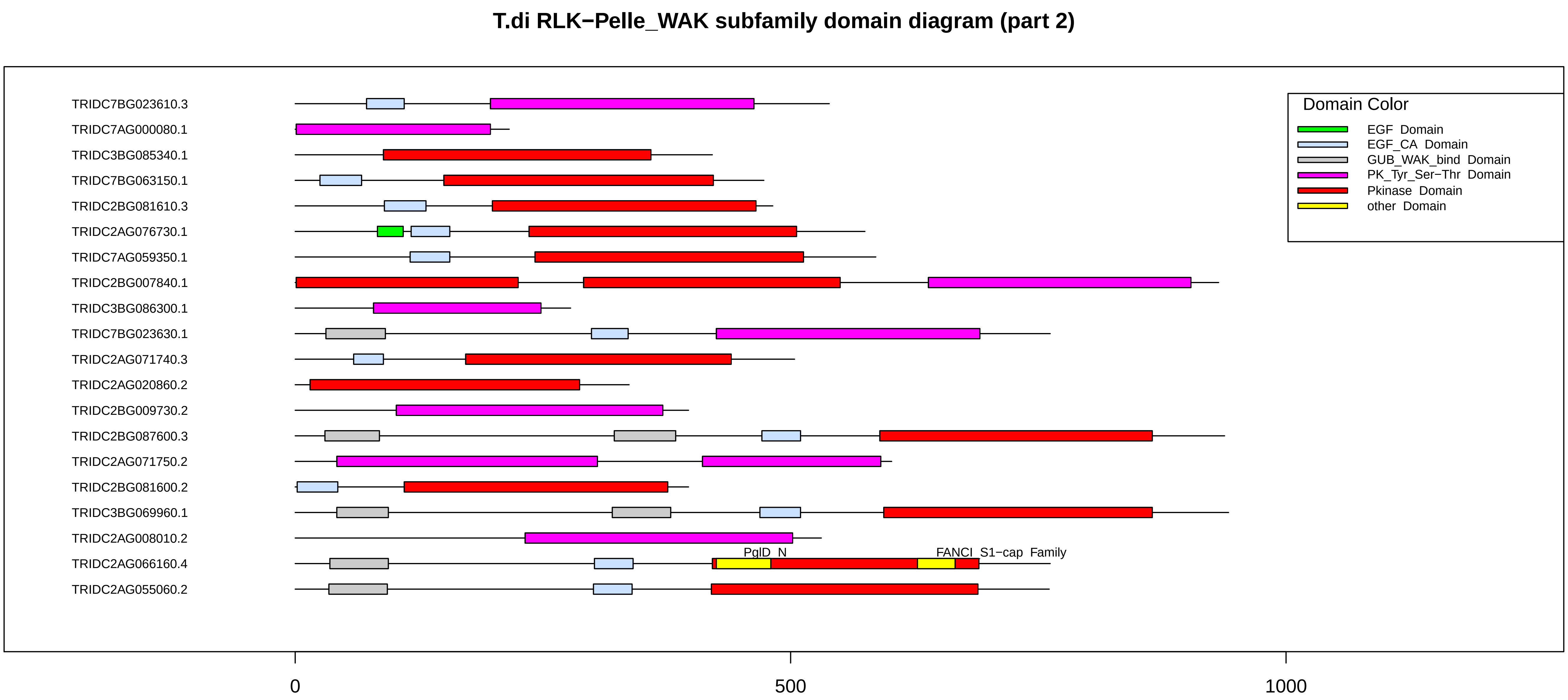
T.di RLK−Pelle_WAK subfamily domain diagram (part 2)
TRIDC7BG023610.3
TRIDC7AG000080.1
TRIDC3BG085340.1
TRIDC7BG063150.1
TRIDC2BG081610.3
TRIDC2AG076730.1
TRIDC7AG059350.1
TRIDC2BG007840.1
TRIDC3BG086300.1
TRIDC7BG023630.1
TRIDC2AG071740.3
TRIDC2AG020860.2
TRIDC2BG009730.2
TRIDC2BG087600.3
TRIDC2AG071750.2
TRIDC2BG081600.2
TRIDC3BG069960.1
TRIDC2AG008010.2
TRIDC2AG066160.4
TRIDC2AG055060.2
PglD_N
FANCI_S1−cap_Family
Domain Color
EGF_Domain
EGF_CA_Domain
GUB_WAK_bind_Domain
PK_Tyr_Ser−Thr_Domain
Pkinase_Domain
other_Domain
0
500
1000

T.di RLK−Pelle_WAK subfamily domain diagram (part 3)

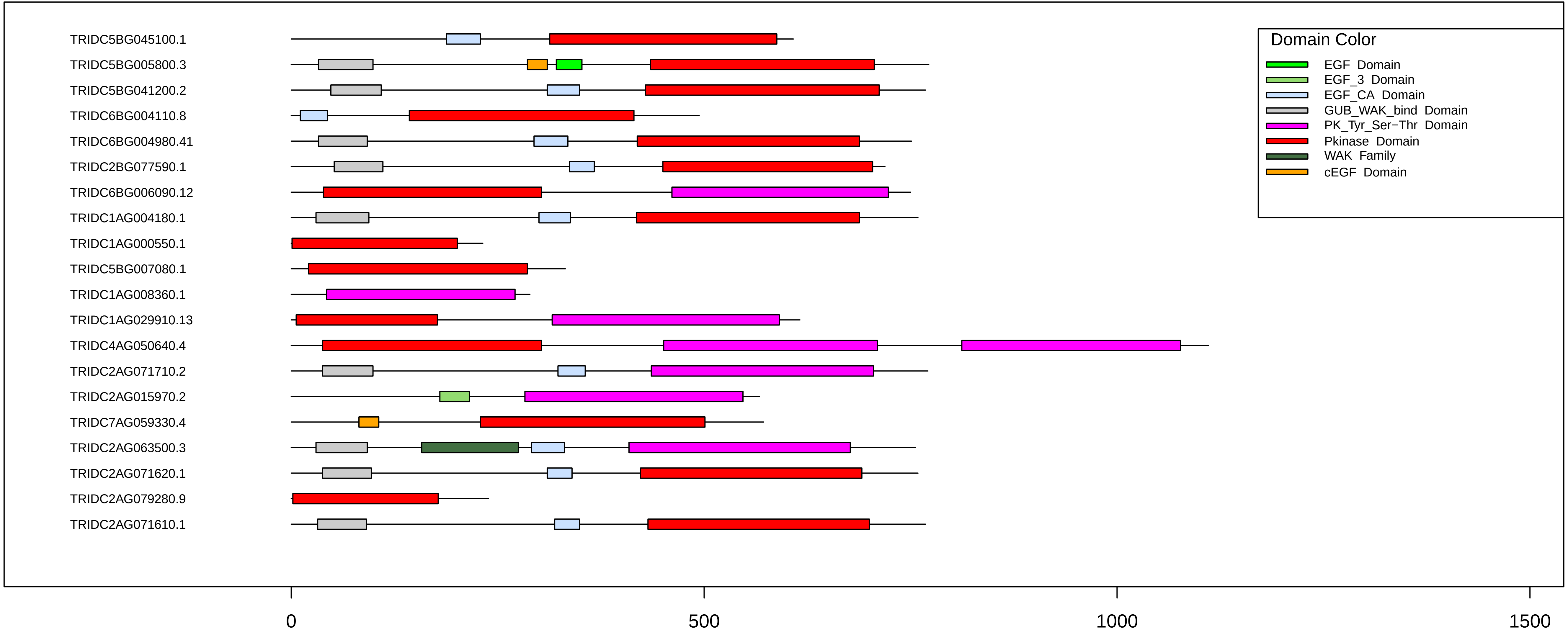

T.di RLK−Pelle_WAK subfamily domain diagram (part 4)

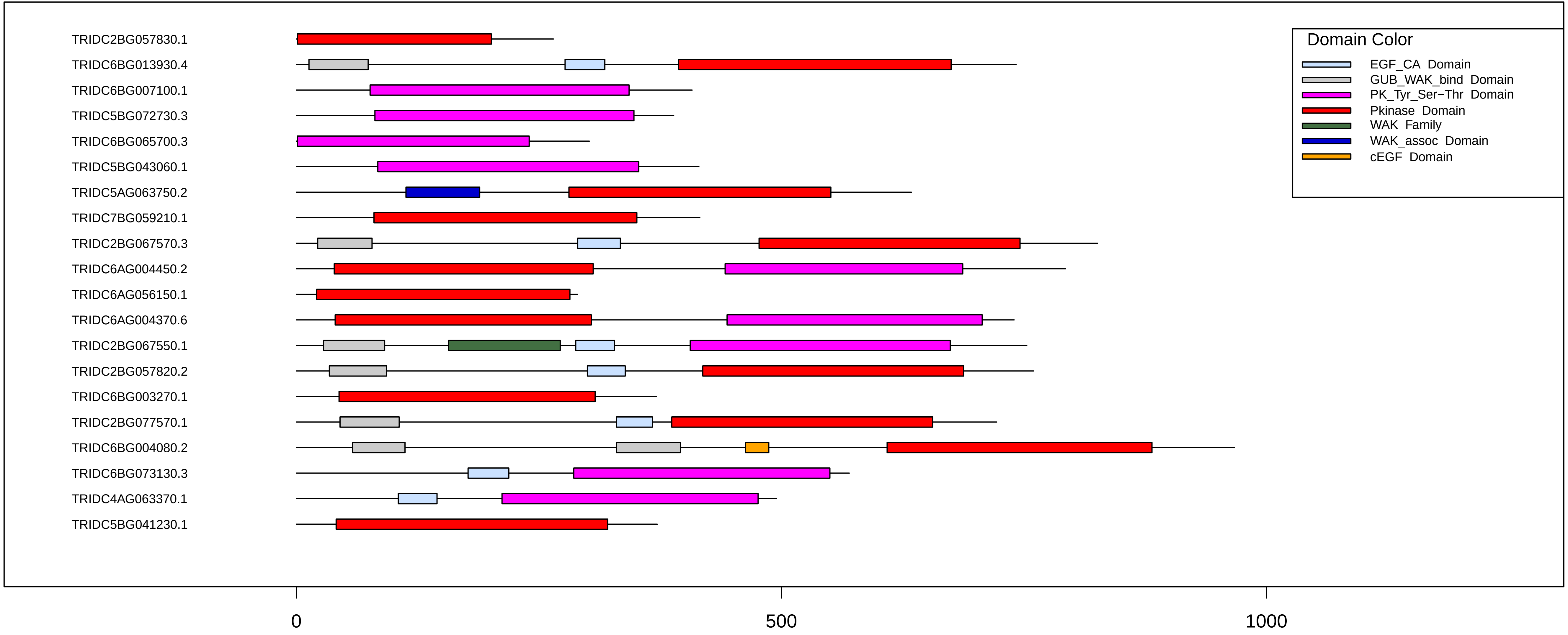

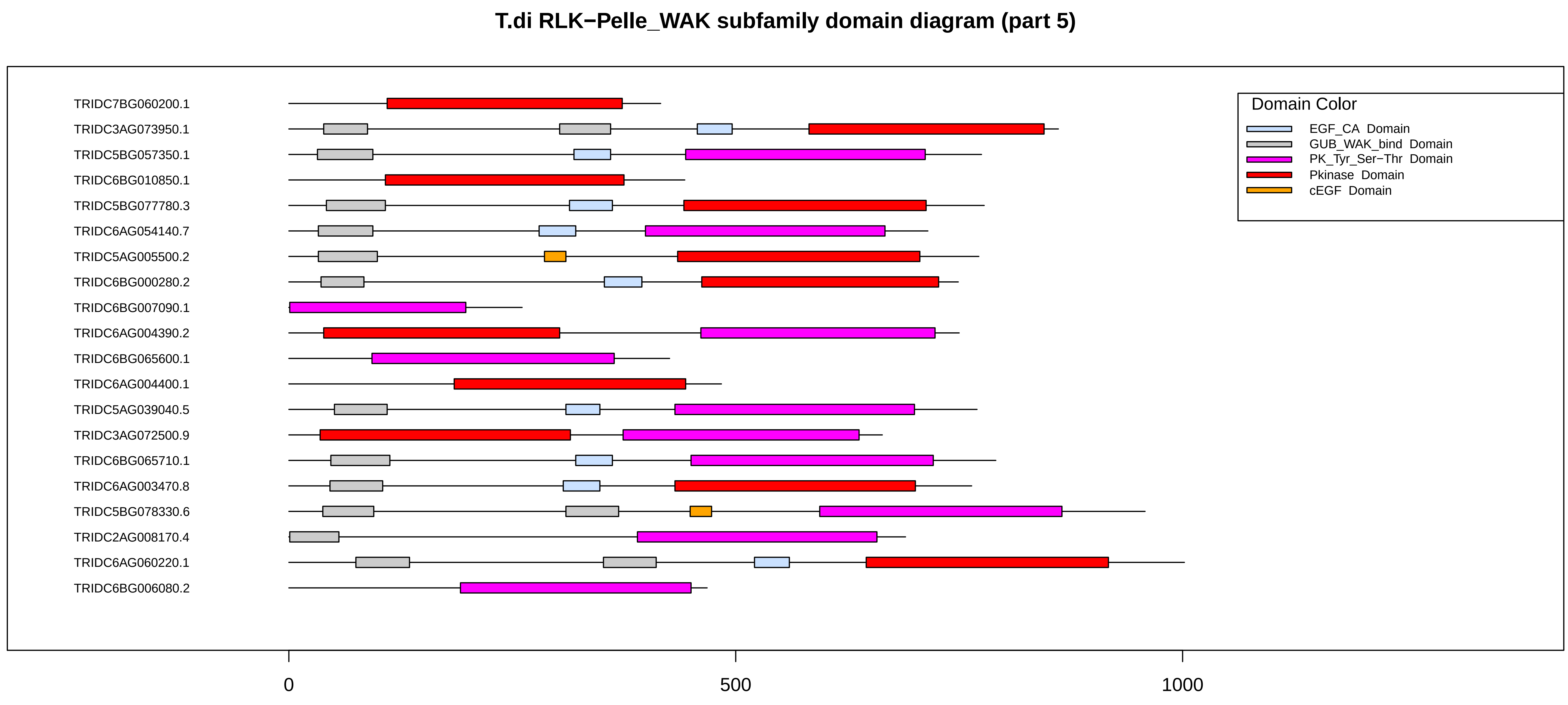

T.di RLK−Pelle_WAK subfamily domain diagram (part 5)
TRIDC7BG060200.1
TRIDC3AG073950.1
TRIDC5BG057350.1
TRIDC6BG010850.1
TRIDC5BG077780.3
TRIDC6AG054140.7
TRIDC5AG005500.2
TRIDC6BG000280.2
TRIDC6BG007090.1
TRIDC6AG004390.2
TRIDC6BG065600.1
TRIDC6AG004400.1
TRIDC5AG039040.5
TRIDC3AG072500.9
TRIDC6BG065710.1
TRIDC6AG003470.8
TRIDC5BG078330.6
TRIDC2AG008170.4
TRIDC6AG060220.1
TRIDC6BG006080.2
Domain Color
EGF_CA Domain
GUB_WAK_bind Domain
PK_Tyr_Ser−Thr Domain
Pkinase Domain
cEGF Domain
0
500
1000

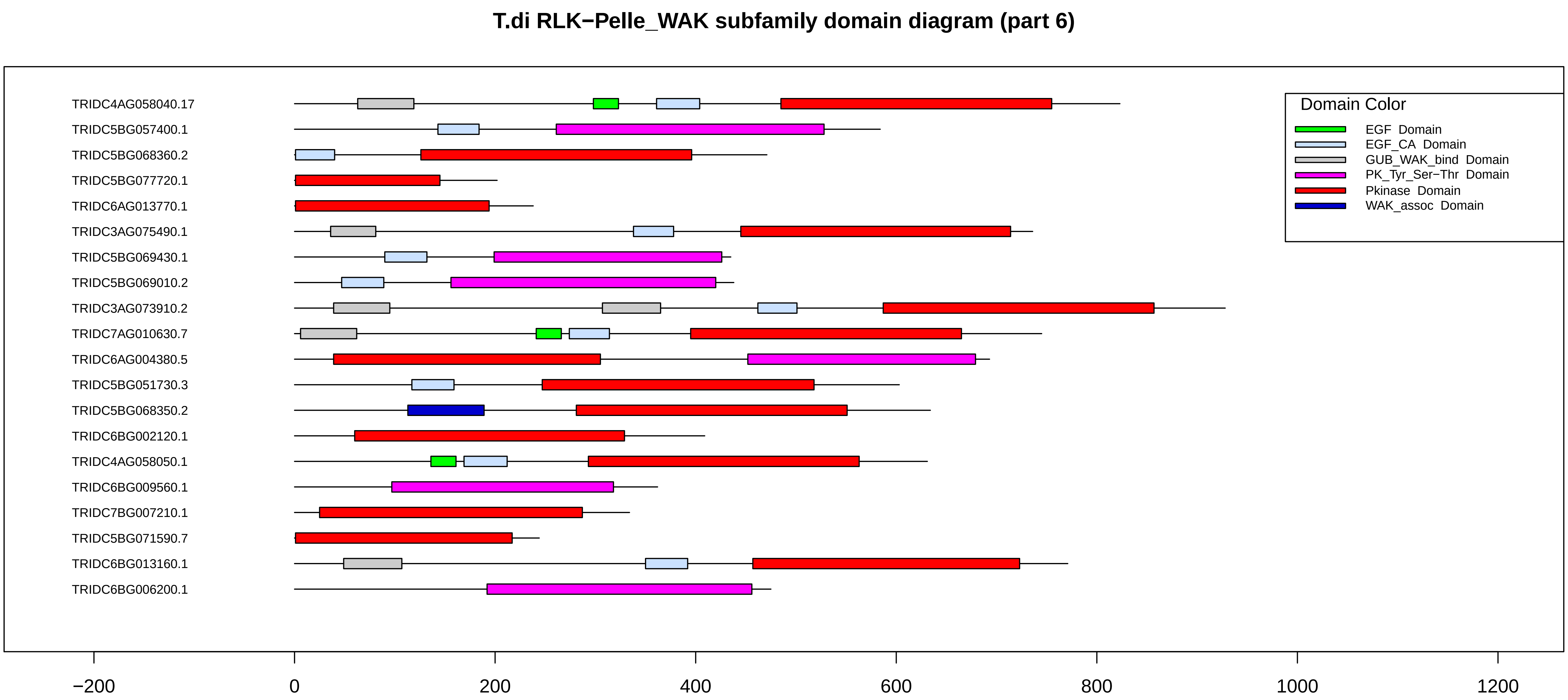
T.di RLK−Pelle_WAK subfamily domain diagram (part 6)
TRIDC4AG058040.17
TRIDC5BG057400.1
TRIDC5BG068360.2
TRIDC5BG077720.1
TRIDC6AG013770.1
TRIDC3AG075490.1
TRIDC5BG069430.1
TRIDC5BG069010.2
TRIDC3AG073910.2
TRIDC7AG010630.7
TRIDC6AG004380.5
TRIDC5BG051730.3
TRIDC5BG068350.2
TRIDC6BG002120.1
TRIDC4AG058050.1
TRIDC6BG009560.1
TRIDC7BG007210.1
TRIDC5BG071590.7
TRIDC6BG013160.1
TRIDC6BG006200.1
Domain Color
EGF Domain
EGF_CA Domain
GUB_WAK_bind Domain
PK_Tyr_Ser−Thr Domain
Pkinase Domain
WAK_assoc Domain
−200
0
200
400
600
800
1000
1200

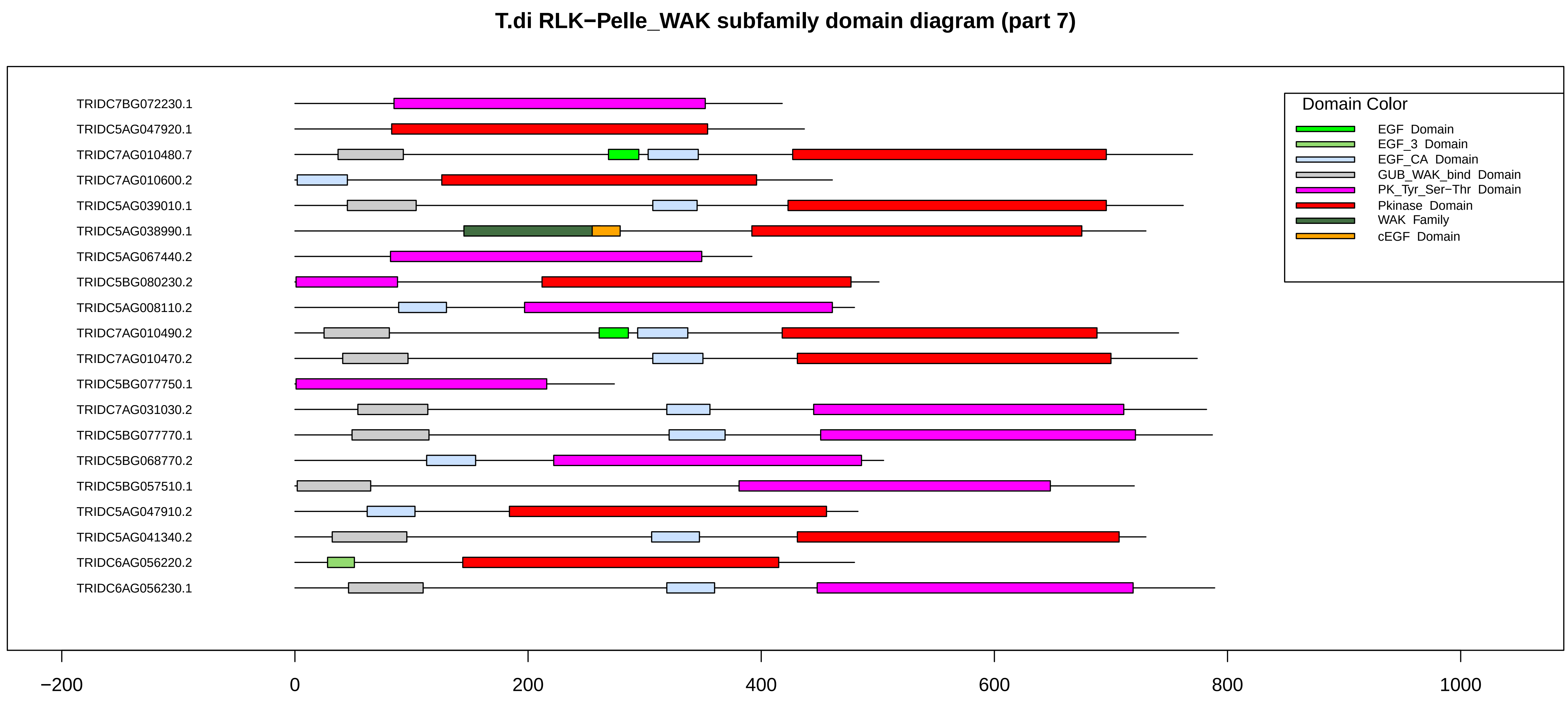
T.di RLK−Pelle_WAK subfamily domain diagram (part 7)
TRIDC7BG072230.1
TRIDC5AG047920.1
TRIDC7AG010480.7
TRIDC7AG010600.2
TRIDC5AG039010.1
TRIDC5AG038990.1
TRIDC5AG067440.2
TRIDC5BG080230.2
TRIDC5AG008110.2
TRIDC7AG010490.2
TRIDC7AG010470.2
TRIDC5BG077750.1
TRIDC7AG031030.2
TRIDC5BG077770.1
TRIDC5BG068770.2
TRIDC5BG057510.1
TRIDC5AG047910.2
TRIDC5AG041340.2
TRIDC6AG056220.2
TRIDC6AG056230.1
Domain Color
EGF Domain
EGF_3 Domain
EGF_CA Domain
GUB_WAK_bind Domain
PK_Tyr_Ser−Thr Domain
Pkinase Domain
WAK Family
cEGF Domain
−200
0
200
400
600
800
1000

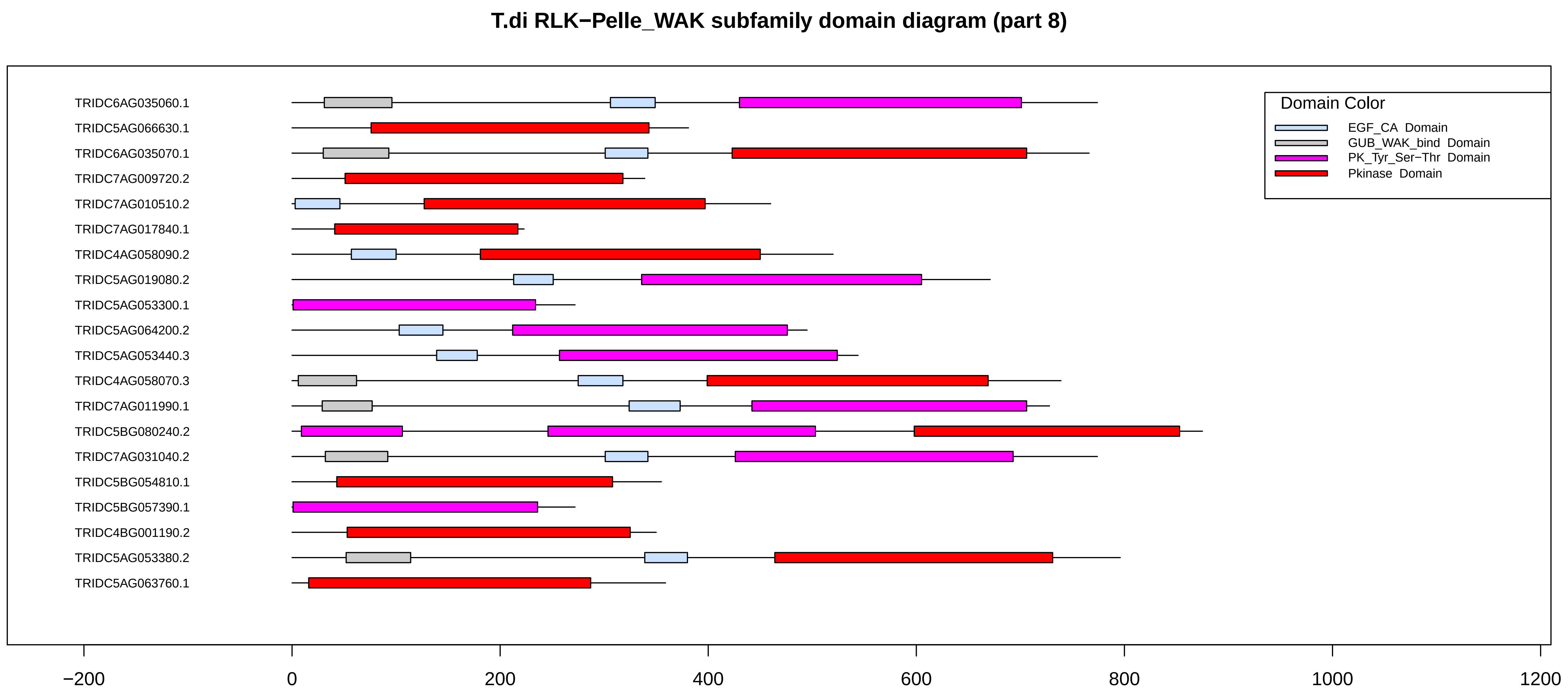

T.di RLK−Pelle_WAK subfamily domain diagram (part 8)
TRIDC6AG035060.1
TRIDC5AG066630.1
TRIDC6AG035070.1
TRIDC7AG009720.2
TRIDC7AG010510.2
TRIDC7AG017840.1
TRIDC4AG058090.2
TRIDC5AG019080.2
TRIDC5AG053300.1
TRIDC5AG064200.2
TRIDC5AG053440.3
TRIDC4AG058070.3
TRIDC7AG011990.1
TRIDC5BG080240.2
TRIDC7AG031040.2
TRIDC5BG054810.1
TRIDC5BG057390.1
TRIDC4BG001190.2
TRIDC5AG053380.2
TRIDC5AG063760.1
Domain Color
EGF_CA Domain
GUB_WAK_bind Domain
PK_Tyr_Ser−Thr Domain
Pkinase Domain
−200
0
200
400
600
800
1000
1200

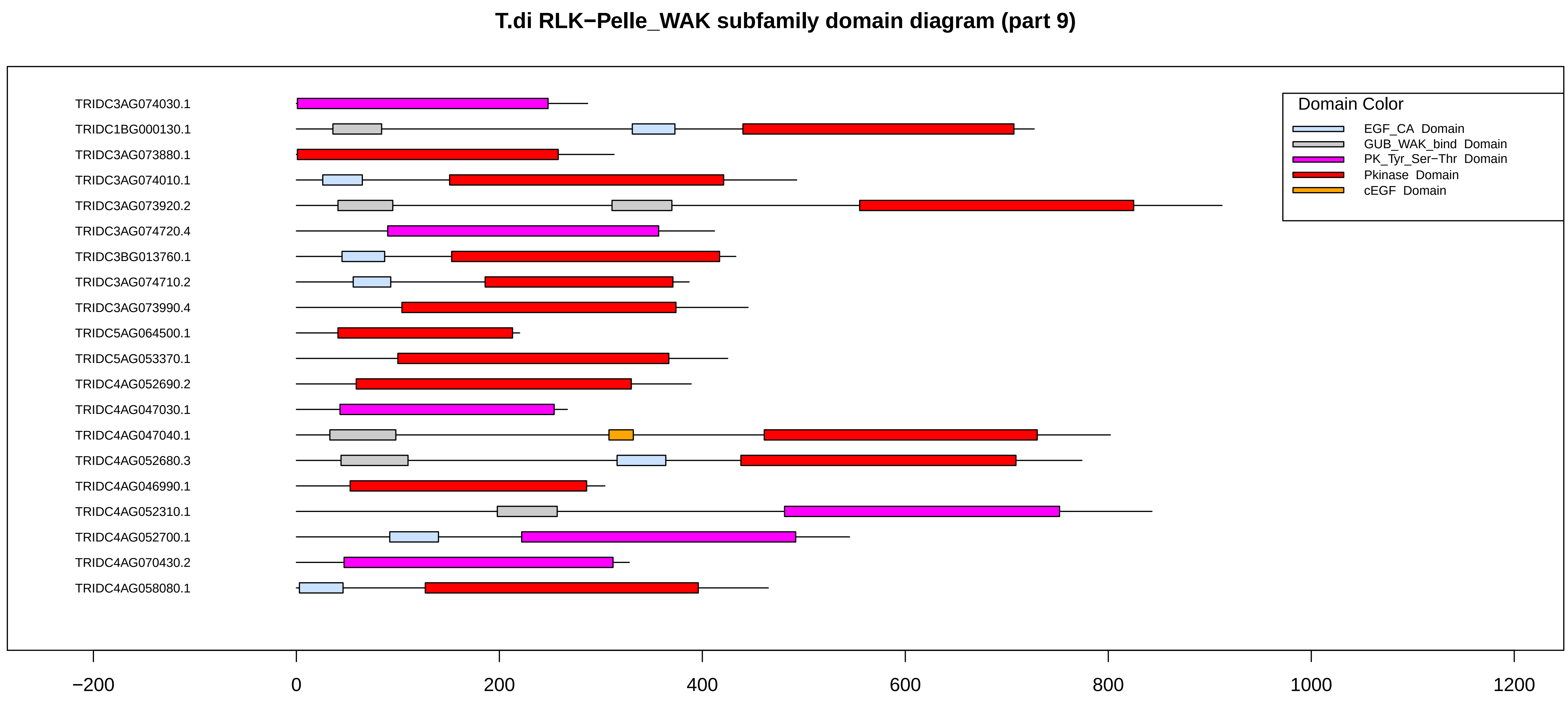
T.di RLK−Pelle_WAK subfamily domain diagram (part 9)
TRIDC3AG074030.1
TRIDC1BG000130.1
TRIDC3AG073880.1
TRIDC3AG074010.1
TRIDC3AG073920.2
TRIDC3AG074720.4
TRIDC3BG013760.1
TRIDC3AG074710.2
TRIDC3AG073990.4
TRIDC5AG064500.1
TRIDC5AG053370.1
TRIDC4AG052690.2
TRIDC4AG047030.1
TRIDC4AG047040.1
TRIDC4AG052680.3
TRIDC4AG046990.1
TRIDC4AG052310.1
TRIDC4AG052700.1
TRIDC4AG070430.2
TRIDC4AG058080.1
Domain Color
EGF_CA Domain
GUB_WAK_bind Domain
PK_Tyr_Ser−Thr Domain
Pkinase Domain
cEGF Domain
−200
0
200
400
600
800
1000
1200

T.di RLK−Pelle_WAK subfamily domain diagram (part 10)

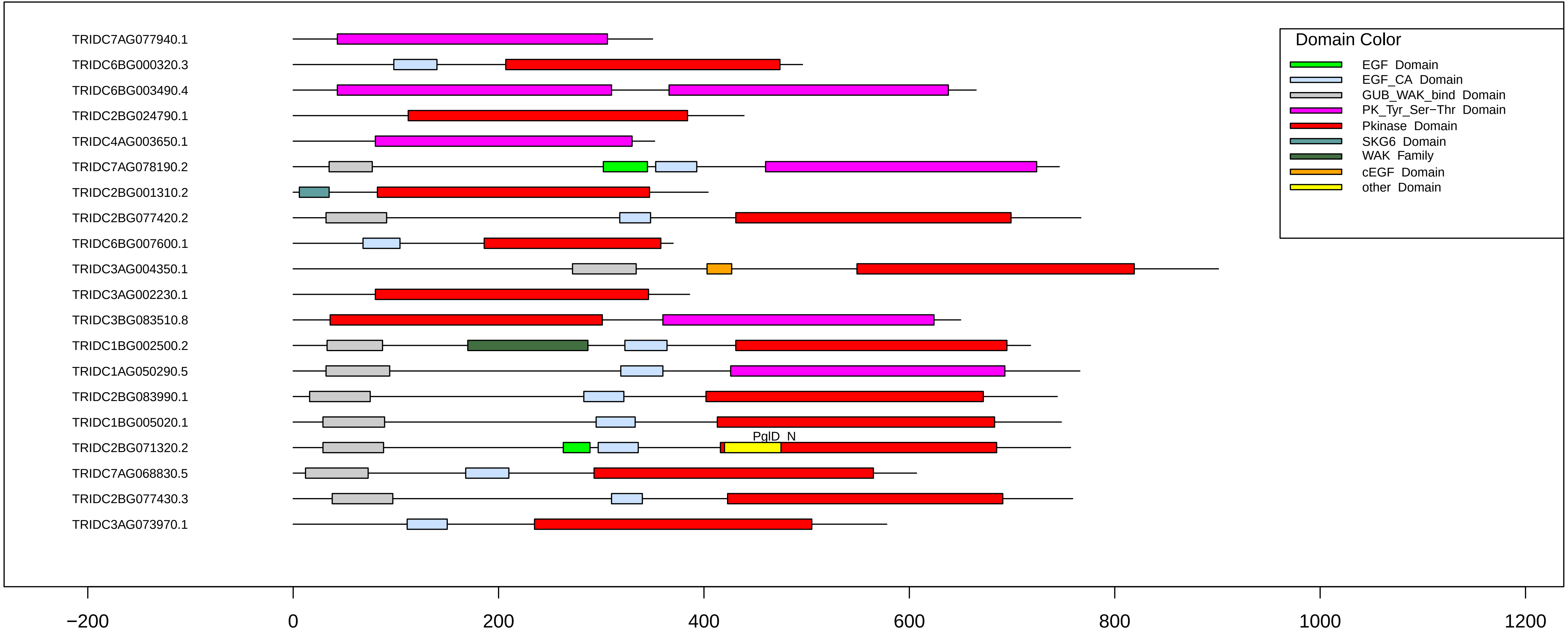

T.di RLK−Pelle_WAK subfamily domain diagram (part 11)

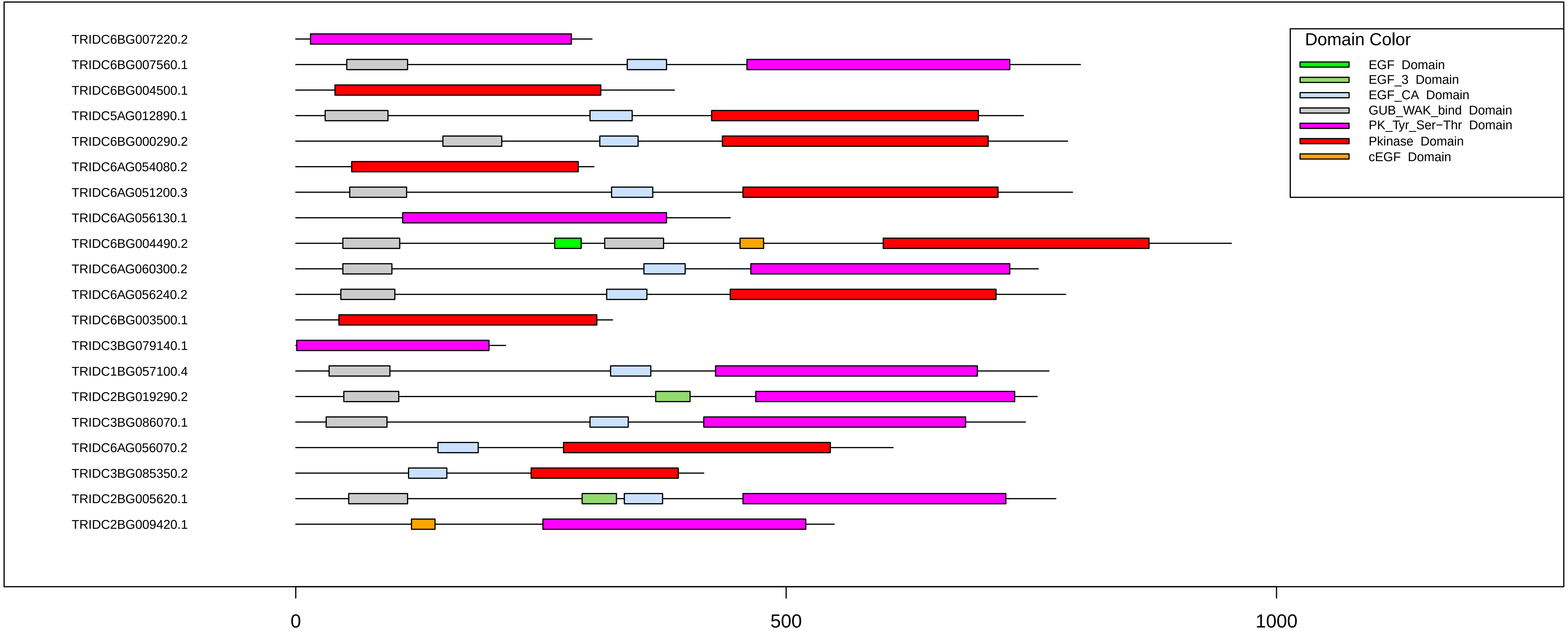

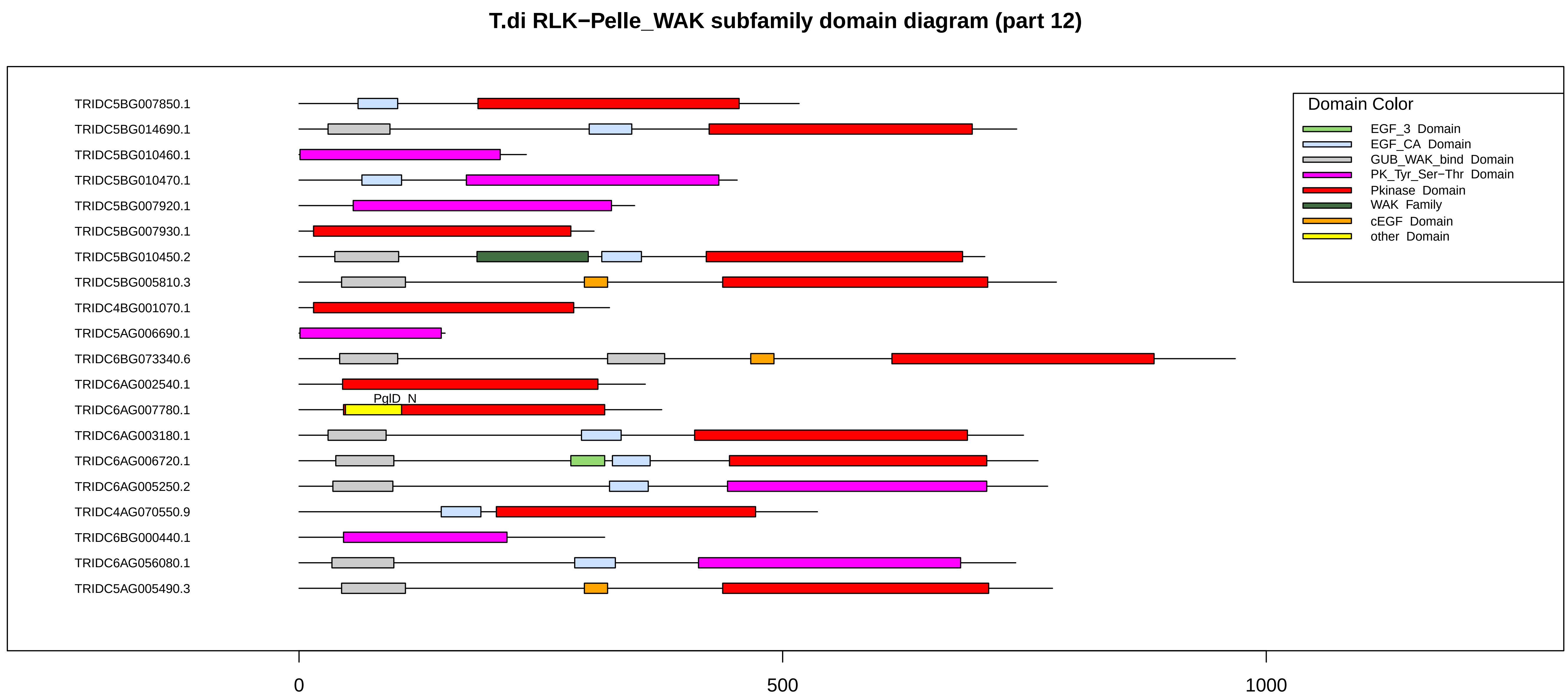

T.di RLK−Pelle_WAK subfamily domain diagram (part 12)
TRIDC5BG007850.1
TRIDC5BG014690.1
TRIDC5BG010460.1
TRIDC5BG010470.1
TRIDC5BG007920.1
TRIDC5BG007930.1
TRIDC5BG010450.2
TRIDC5BG005810.3
TRIDC4BG001070.1
TRIDC5AG006690.1
TRIDC6BG073340.6
TRIDC6AG002540.1
TRIDC6AG007780.1
PglD_N
TRIDC6AG003180.1
TRIDC6AG006720.1
TRIDC6AG005250.2
TRIDC4AG070550.9
TRIDC6BG000440.1
TRIDC6AG056080.1
TRIDC5AG005490.3
Domain Color
EGF_3 Domain
EGF_CA Domain
GUB_WAK_bind Domain
PK_Tyr_Ser−Thr Domain
Pkinase Domain
WAK Family
cEGF Domain
other Domain
0
500
1000

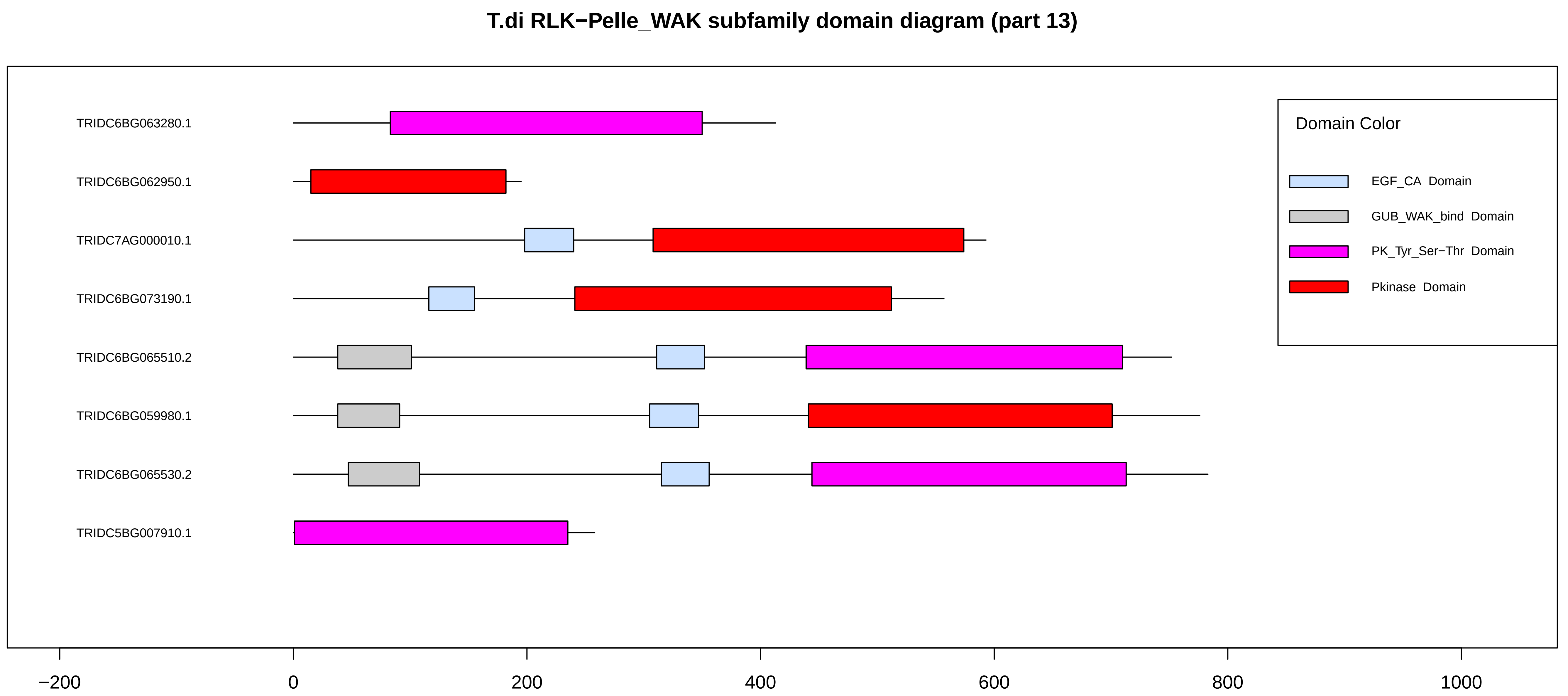

T.di RLK−Pelle_WAK subfamily domain diagram (part 13)
TRIDC6BG063280.1
TRIDC6BG062950.1
TRIDC7AG000010.1
TRIDC6BG073190.1
TRIDC6BG065510.2
TRIDC6BG059980.1
TRIDC6BG065530.2
TRIDC5BG007910.1
Domain Color
EGF_CA Domain
GUB_WAK_bind Domain
PK_Tyr_Ser−Thr Domain
Pkinase Domain
−200
0
200
400
600
800
1000

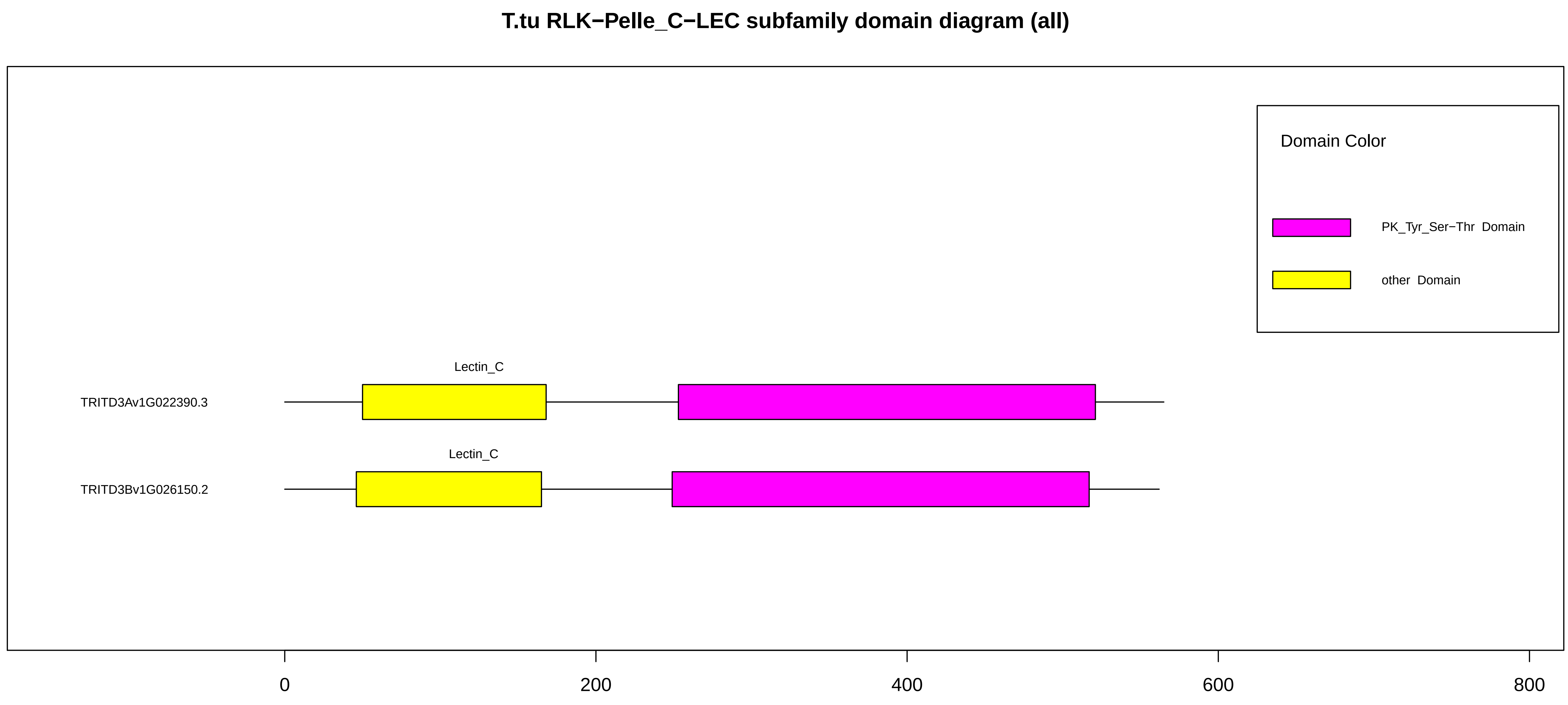

T.tu RLK−Pelle_C−LEC subfamily domain diagram (all)
Domain Color
PK_Tyr_Ser−Thr Domain
other Domain
Lectin_C
TRITD3Av1G022390.3
Lectin_C
TRITD3Bv1G026150.2
0
200
400
600
800

T.tu RLK−Pelle_CR4L subfamily domain diagram (part 1)

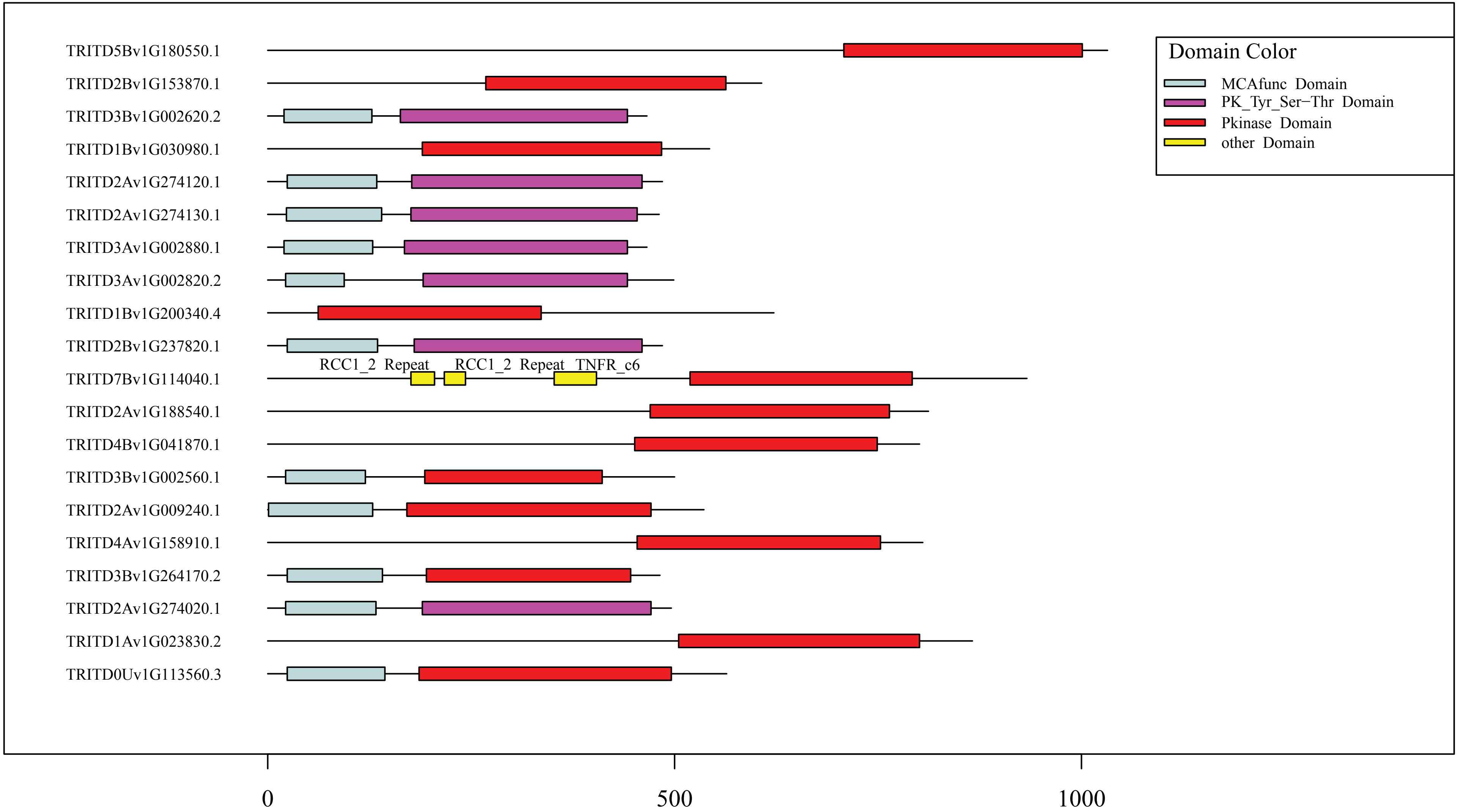

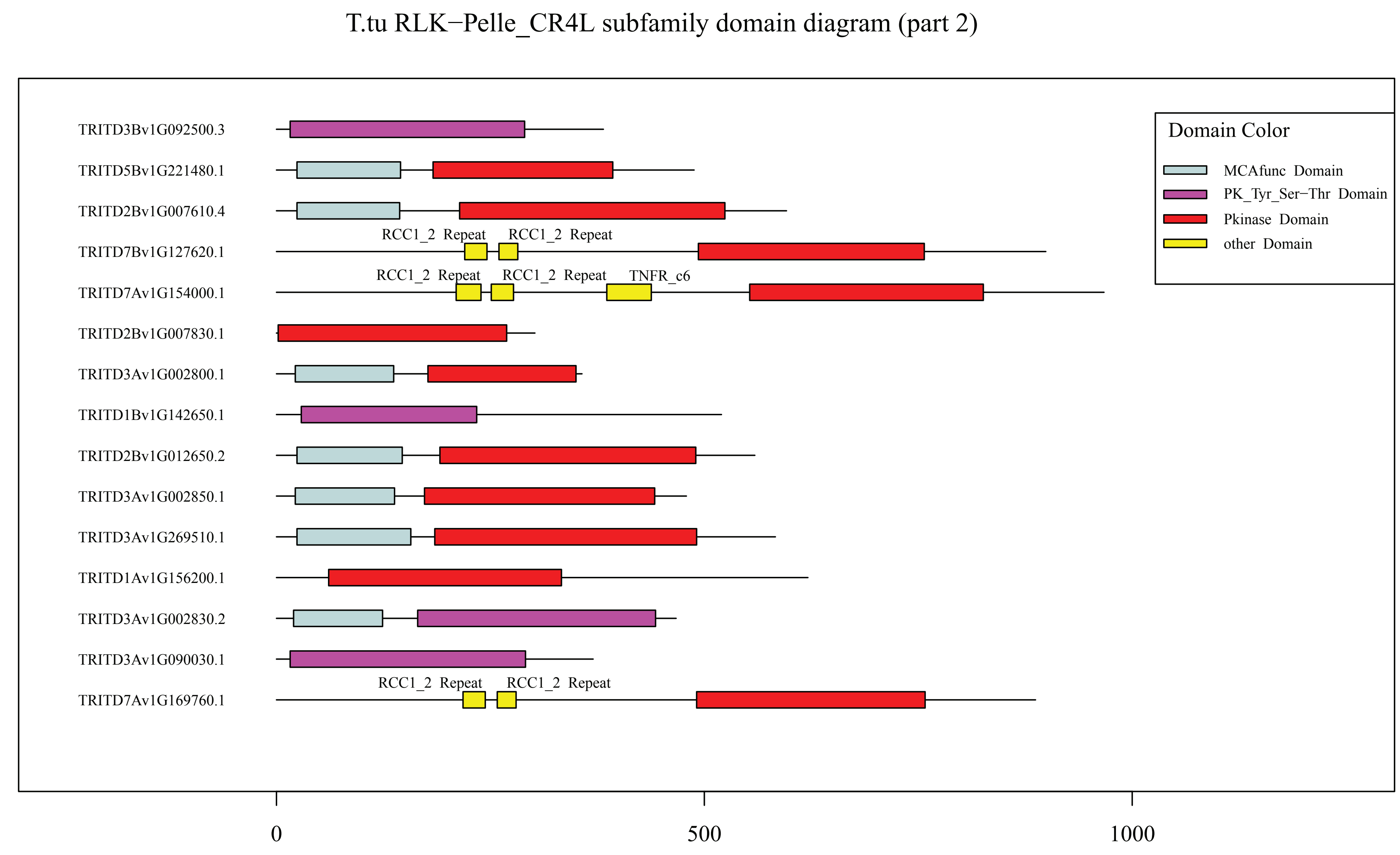
T.tu RLK−Pelle_CR4L subfamily domain diagram (part 2)
TRITD3Bv1G092500.3
TRITD5Bv1G221480.1
TRITD2Bv1G007610.4
RCC1_2 Repeat
RCC1_2 Repeat
TRITD7Bv1G127620.1
RCC1_2 Repeat
RCC1_2 Repeat
TNFR_c6
TRITD7Av1G154000.1
TRITD2Bv1G007830.1
TRITD3Av1G002800.1
TRITD1Bv1G142650.1
TRITD2Bv1G012650.2
TRITD3Av1G002850.1
TRITD3Av1G269510.1
TRITD1Av1G156200.1
TRITD3Av1G002830.2
TRITD3Av1G090030.1
RCC1_2 Repeat
RCC1_2 Repeat
TRITD7Av1G169760.1
Domain Color
MCAfunc Domain
PK_Tyr_Ser−Thr Domain
Pkinase Domain
other Domain
0
500
1000

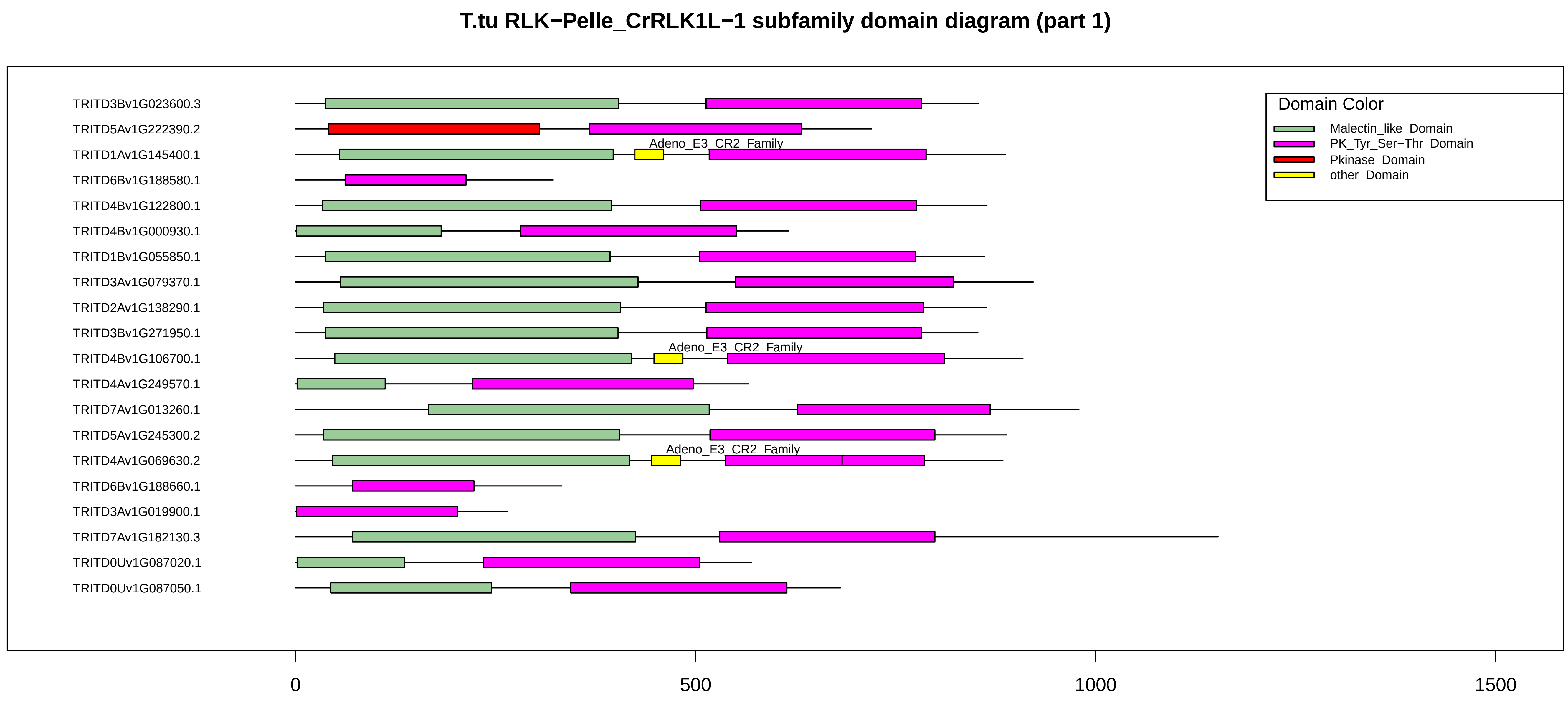
T.tu RLK−Pelle_CrRLK1L−1 subfamily domain diagram (part 1)
TRITD3Bv1G023600.3
TRITD5Av1G222390.2
TRITD1Av1G145400.1
TRITD6Bv1G188580.1
TRITD4Bv1G122800.1
TRITD4Bv1G000930.1
TRITD1Bv1G055850.1
TRITD3Av1G079370.1
TRITD2Av1G138290.1
TRITD3Bv1G271950.1
TRITD4Bv1G106700.1
TRITD4Av1G249570.1
TRITD7Av1G013260.1
TRITD5Av1G245300.2
TRITD4Av1G069630.2
TRITD6Bv1G188660.1
TRITD3Av1G019900.1
TRITD7Av1G182130.3
TRITD0Uv1G087020.1
TRITD0Uv1G087050.1
Adeno_E3_CR2_Family
Adeno_E3_CR2_Family
Adeno_E3_CR2_Family
Domain Color
Malectin_like Domain
PK_Tyr_Ser−Thr Domain
Pkinase Domain
other Domain
0
500
1000
1500

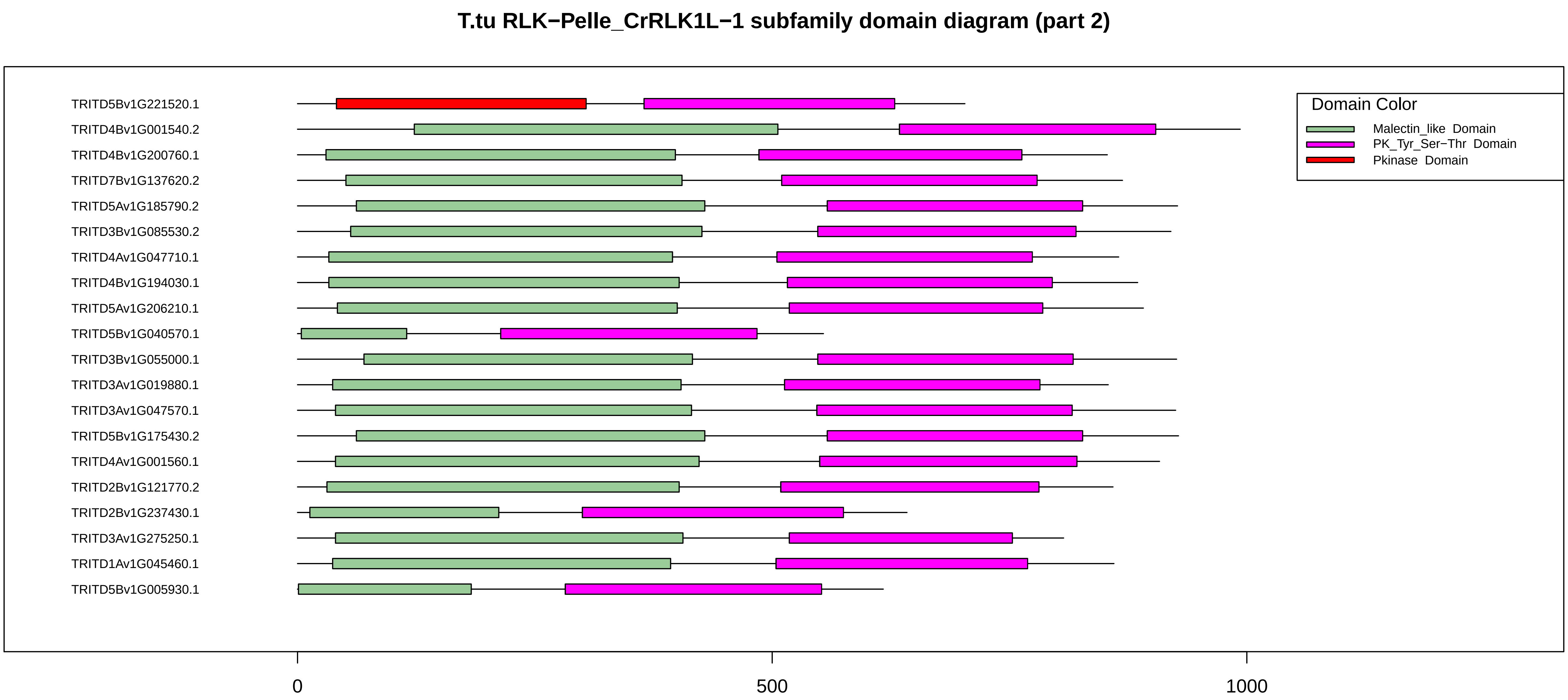

T.tu RLK−Pelle_CrRLK1L−1 subfamily domain diagram (part 2)
TRITD5Bv1G221520.1
TRITD4Bv1G001540.2
TRITD4Bv1G200760.1
TRITD7Bv1G137620.2
TRITD5Av1G185790.2
TRITD3Bv1G085530.2
TRITD4Av1G047710.1
TRITD4Bv1G194030.1
TRITD5Av1G206210.1
TRITD5Bv1G040570.1
TRITD3Bv1G055000.1
TRITD3Av1G019880.1
TRITD3Av1G047570.1
TRITD5Bv1G175430.2
TRITD4Av1G001560.1
TRITD2Bv1G121770.2
TRITD2Bv1G237430.1
TRITD3Av1G275250.1
TRITD1Av1G045460.1
TRITD5Bv1G005930.1
Domain Color
Malectin_like Domain
PK_Tyr_Ser−Thr Domain
Pkinase Domain
0
500
1000

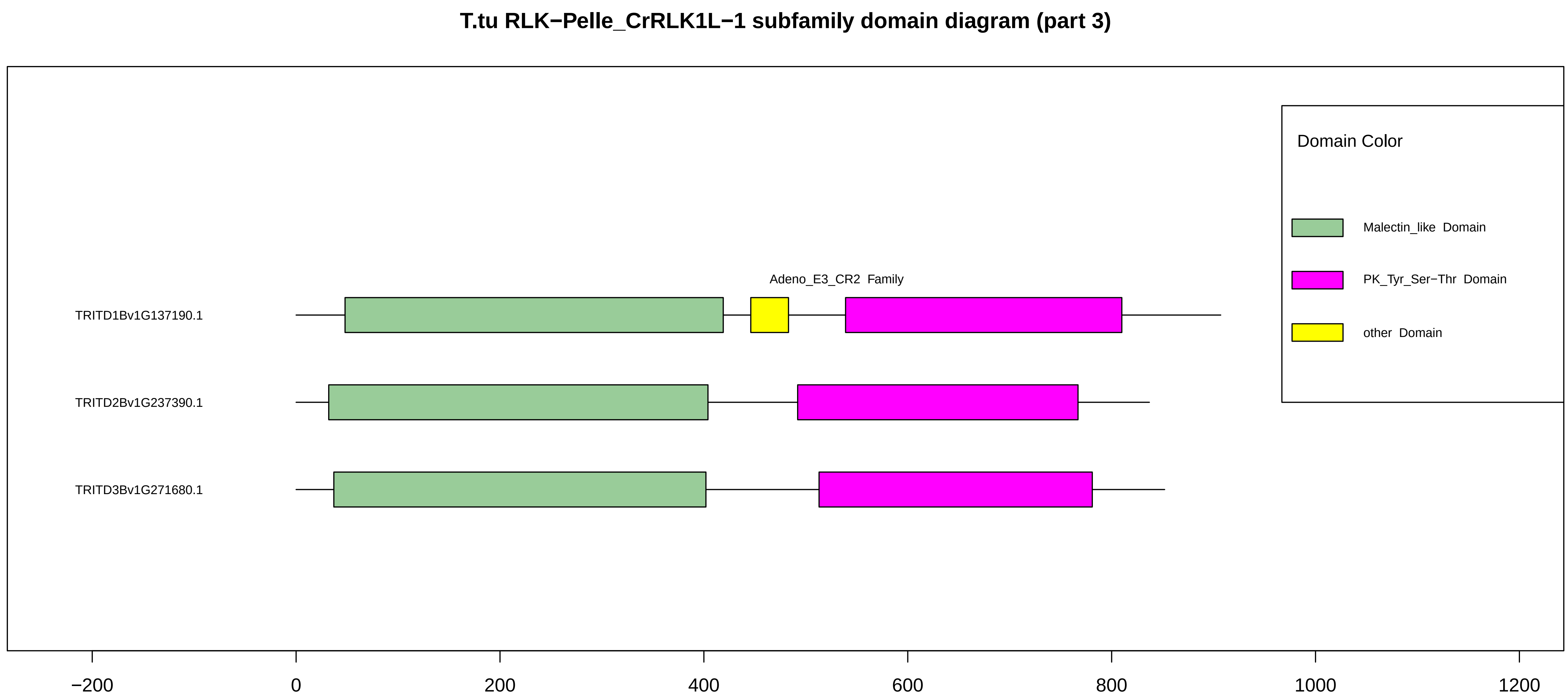
T.tu RLK−Pelle_CrRLK1L−1 subfamily domain diagram (part 3)
Domain Color
Malectin_like Domain
PK_Tyr_Ser−Thr Domain
other Domain
Adeno_E3_CR2 Family
TRITD1Bv1G137190.1
TRITD2Bv1G237390.1
TRITD3Bv1G271680.1
−200
0
200
400
600
800
1000
1200

T.tu RLK−Pelle_DLSV subfamily domain diagram (part 1)

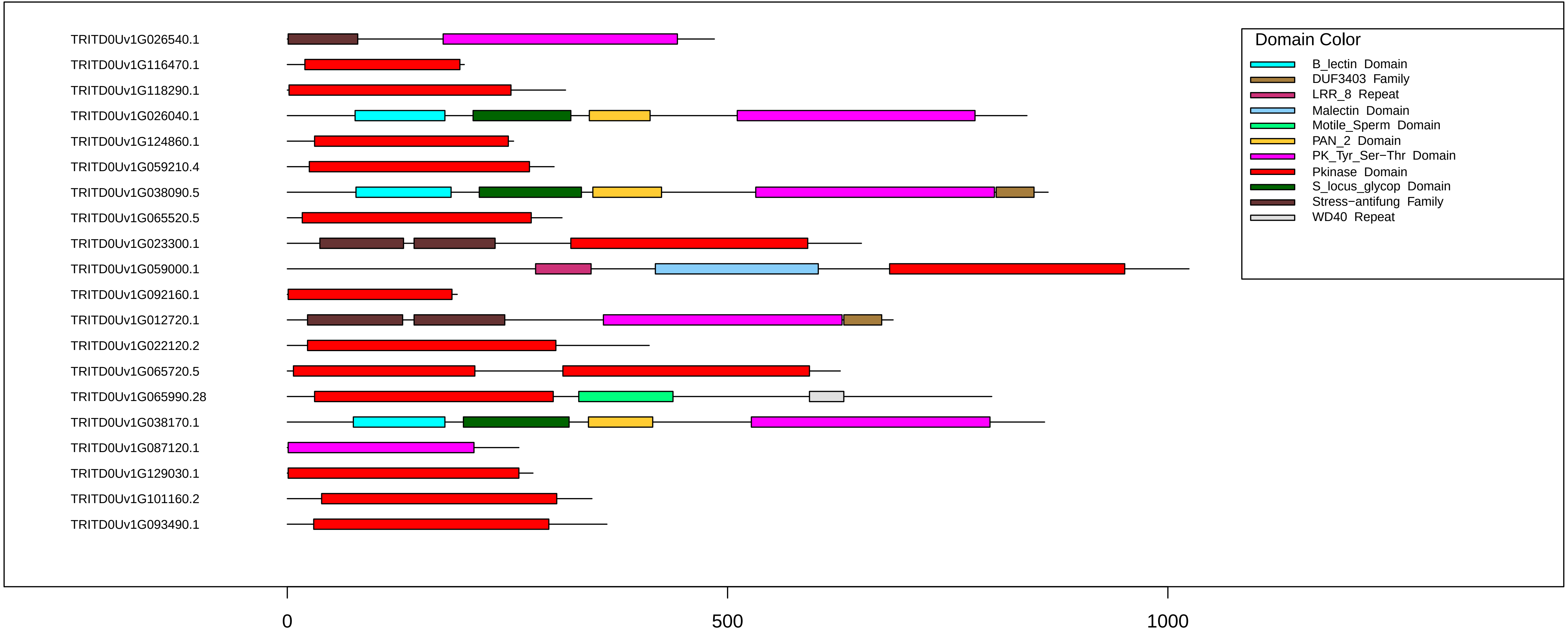

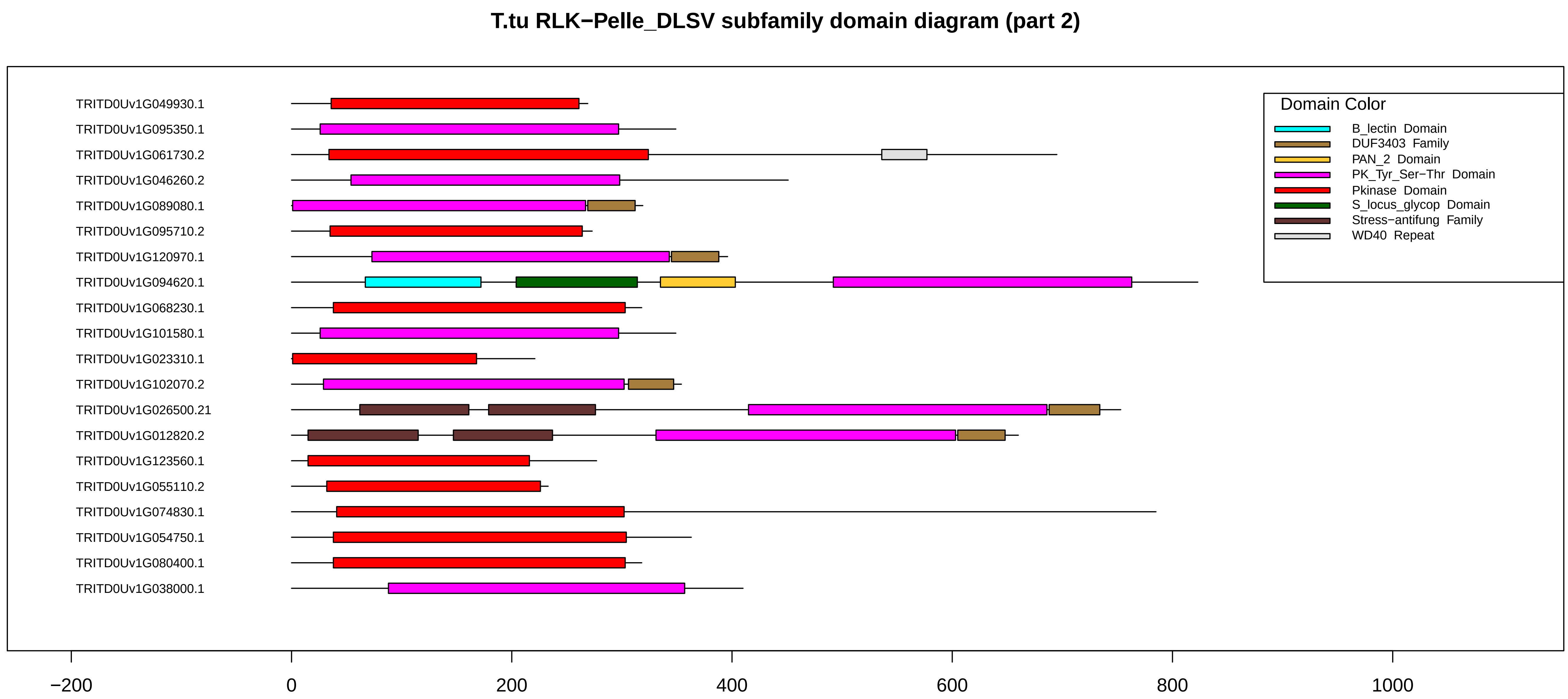
T.tu RLK−Pelle_DLSV subfamily domain diagram (part 2)
TRITD0Uv1G049930.1
TRITD0Uv1G095350.1
TRITD0Uv1G061730.2
TRITD0Uv1G046260.2
TRITD0Uv1G089080.1
TRITD0Uv1G095710.2
TRITD0Uv1G120970.1
TRITD0Uv1G094620.1
TRITD0Uv1G068230.1
TRITD0Uv1G101580.1
TRITD0Uv1G023310.1
TRITD0Uv1G102070.2
TRITD0Uv1G026500.21
TRITD0Uv1G012820.2
TRITD0Uv1G123560.1
TRITD0Uv1G055110.2
TRITD0Uv1G074830.1
TRITD0Uv1G054750.1
TRITD0Uv1G080400.1
TRITD0Uv1G038000.1
Domain Color
B_lectin Domain
DUF3403 Family
PAN_2 Domain
PK_Tyr_Ser−Thr Domain
Pkinase Domain
S_locus_glycop Domain
Stress−antifung Family
WD40 Repeat
−200
0
200
400
600
800
1000

T.tu RLK−Pelle_DLSV subfamily domain diagram (part 3)

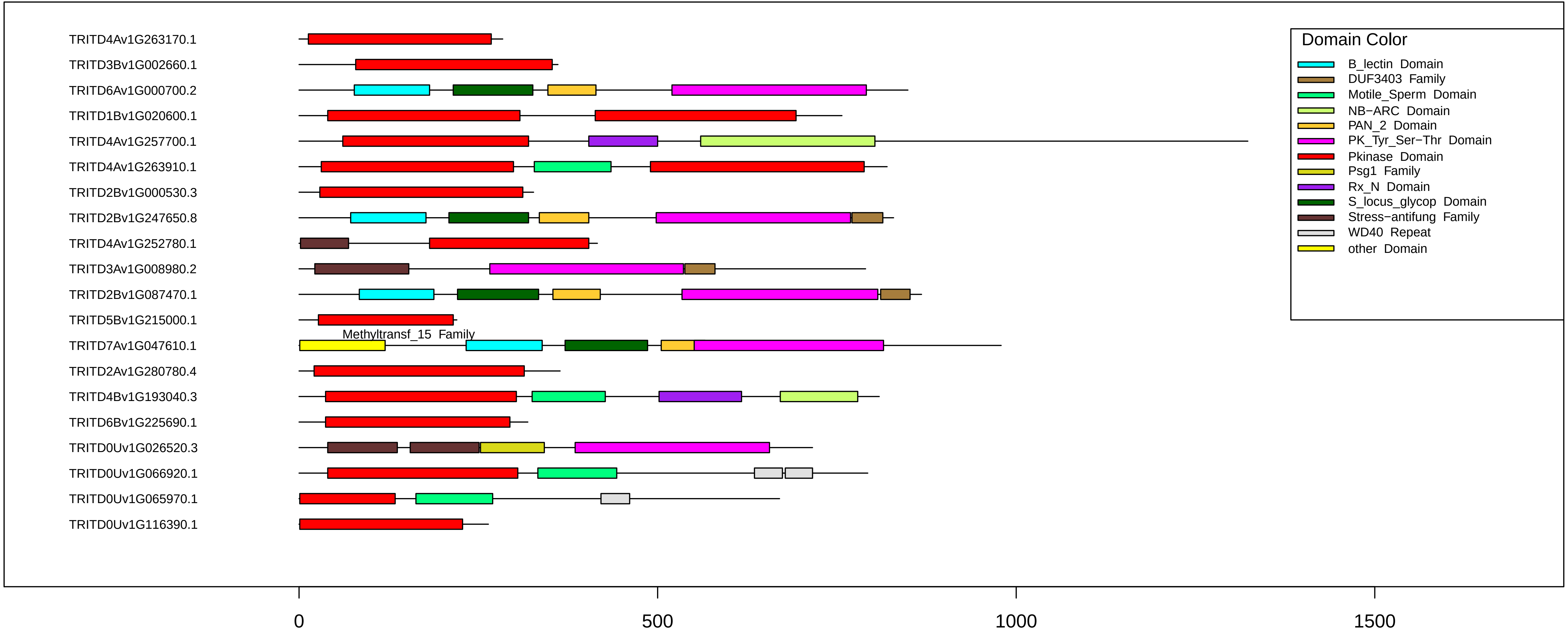

T.tu RLK−Pelle_DLSV subfamily domain diagram (part 4)

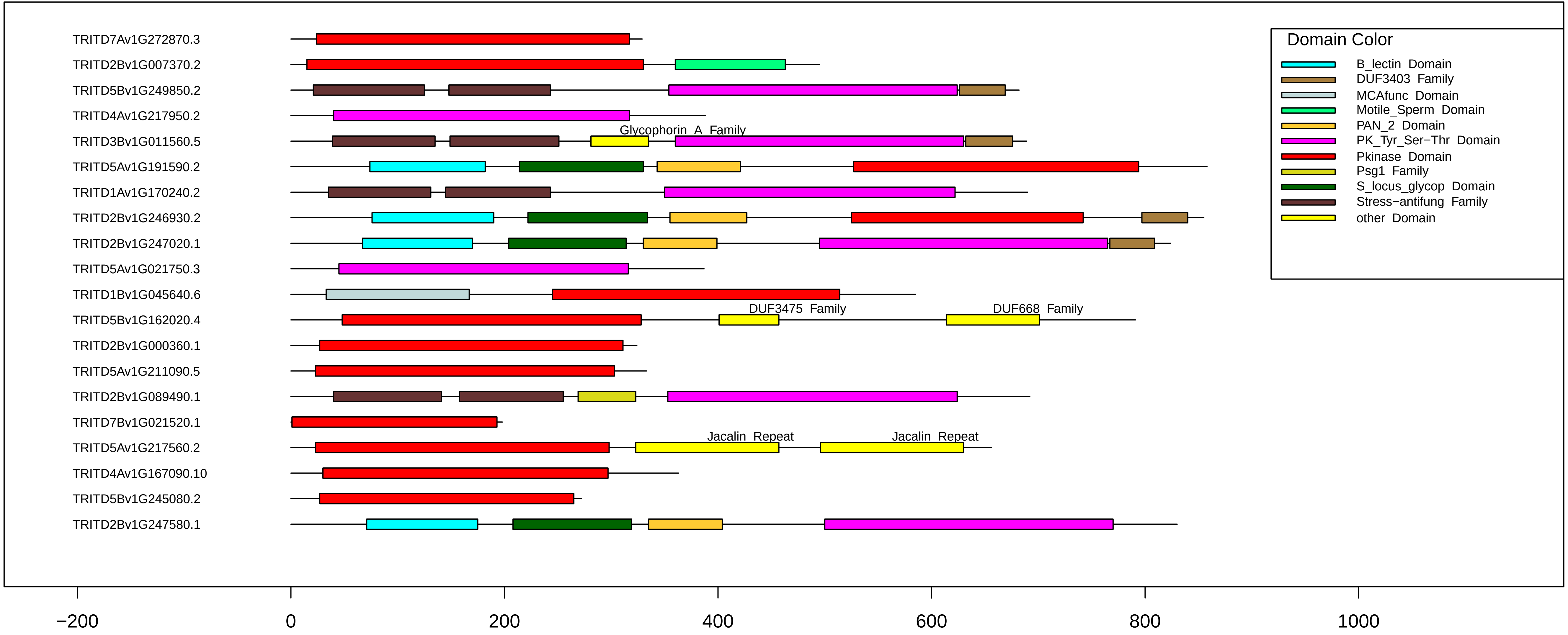

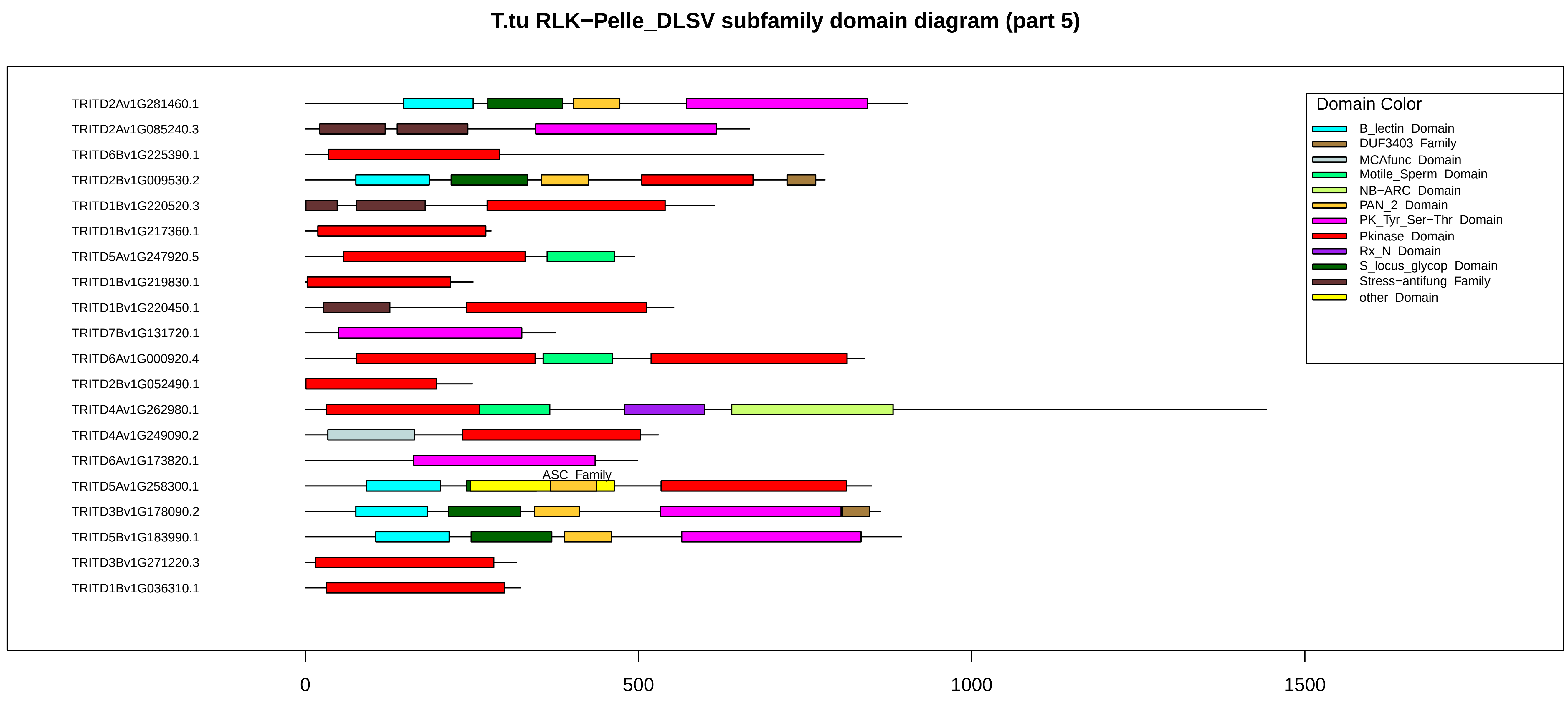
T.tu RLK−Pelle_DLSV subfamily domain diagram (part 5)
TRITD2Av1G281460.1
TRITD2Av1G085240.3
TRITD6Bv1G225390.1
TRITD2Bv1G009530.2
TRITD1Bv1G220520.3
TRITD1Bv1G217360.1
TRITD5Av1G247920.5
TRITD1Bv1G219830.1
TRITD1Bv1G220450.1
TRITD7Bv1G131720.1
TRITD6Av1G000920.4
TRITD2Bv1G052490.1
TRITD4Av1G262980.1
TRITD4Av1G249090.2
TRITD6Av1G173820.1
TRITD5Av1G258300.1
TRITD3Bv1G178090.2
TRITD5Bv1G183990.1
TRITD3Bv1G271220.3
TRITD1Bv1G036310.1
ASC_Family
Domain Color
B_lectin Domain
DUF3403 Family
MCAfunc Domain
Motile_Sperm Domain
NB−ARC Domain
PAN_2 Domain
PK_Tyr_Ser−Thr Domain
Pkinase Domain
Rx_N Domain
S_locus_glycop Domain
Stress−antifung Family
other Domain
0
500
1000
1500

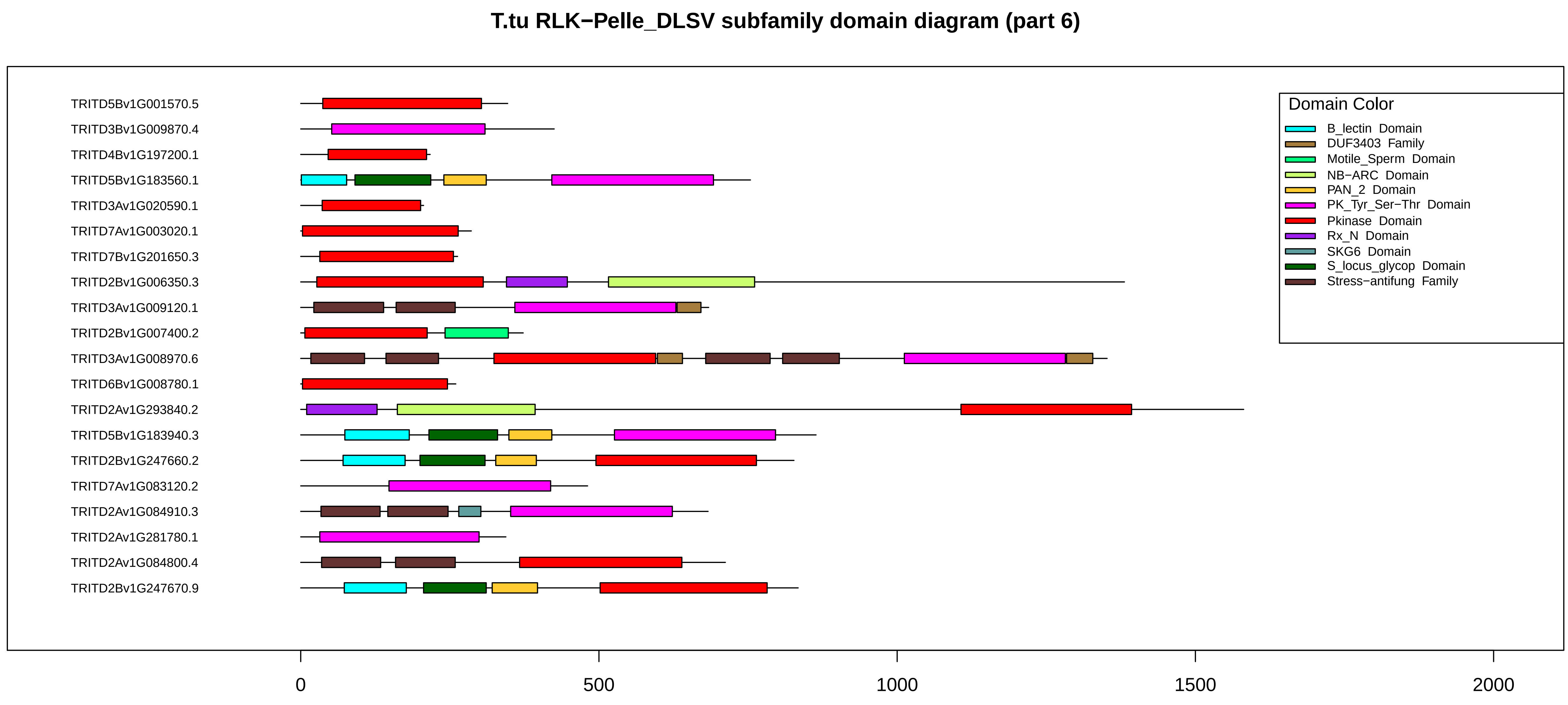
T.tu RLK−Pelle_DLSV subfamily domain diagram (part 6)
TRITD5Bv1G001570.5
TRITD3Bv1G009870.4
TRITD4Bv1G197200.1
TRITD5Bv1G183560.1
TRITD3Av1G020590.1
TRITD7Av1G003020.1
TRITD7Bv1G201650.3
TRITD2Bv1G006350.3
TRITD3Av1G009120.1
TRITD2Bv1G007400.2
TRITD3Av1G008970.6
TRITD6Bv1G008780.1
TRITD2Av1G293840.2
TRITD5Bv1G183940.3
TRITD2Bv1G247660.2
TRITD7Av1G083120.2
TRITD2Av1G084910.3
TRITD2Av1G281780.1
TRITD2Av1G084800.4
TRITD2Bv1G247670.9
Domain Color
B_lectin Domain
DUF3403 Family
Motile_Sperm Domain
NB−ARC Domain
PAN_2 Domain
PK_Tyr_Ser−Thr Domain
Pkinase Domain
Rx_N Domain
SKG6 Domain
S_locus_glycop Domain
Stress−antifung Family
0
500
1000
1500
2000

T.tu RLK−Pelle_DLSV subfamily domain diagram (part 7)

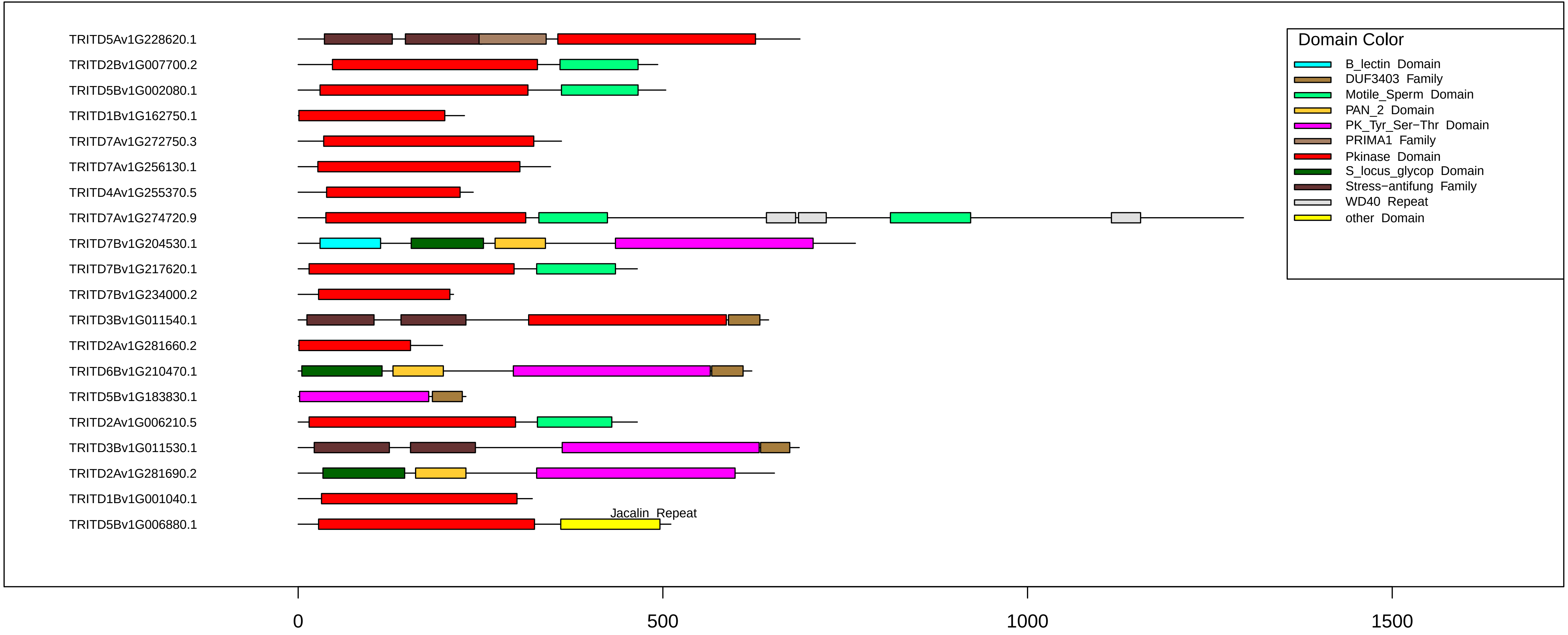

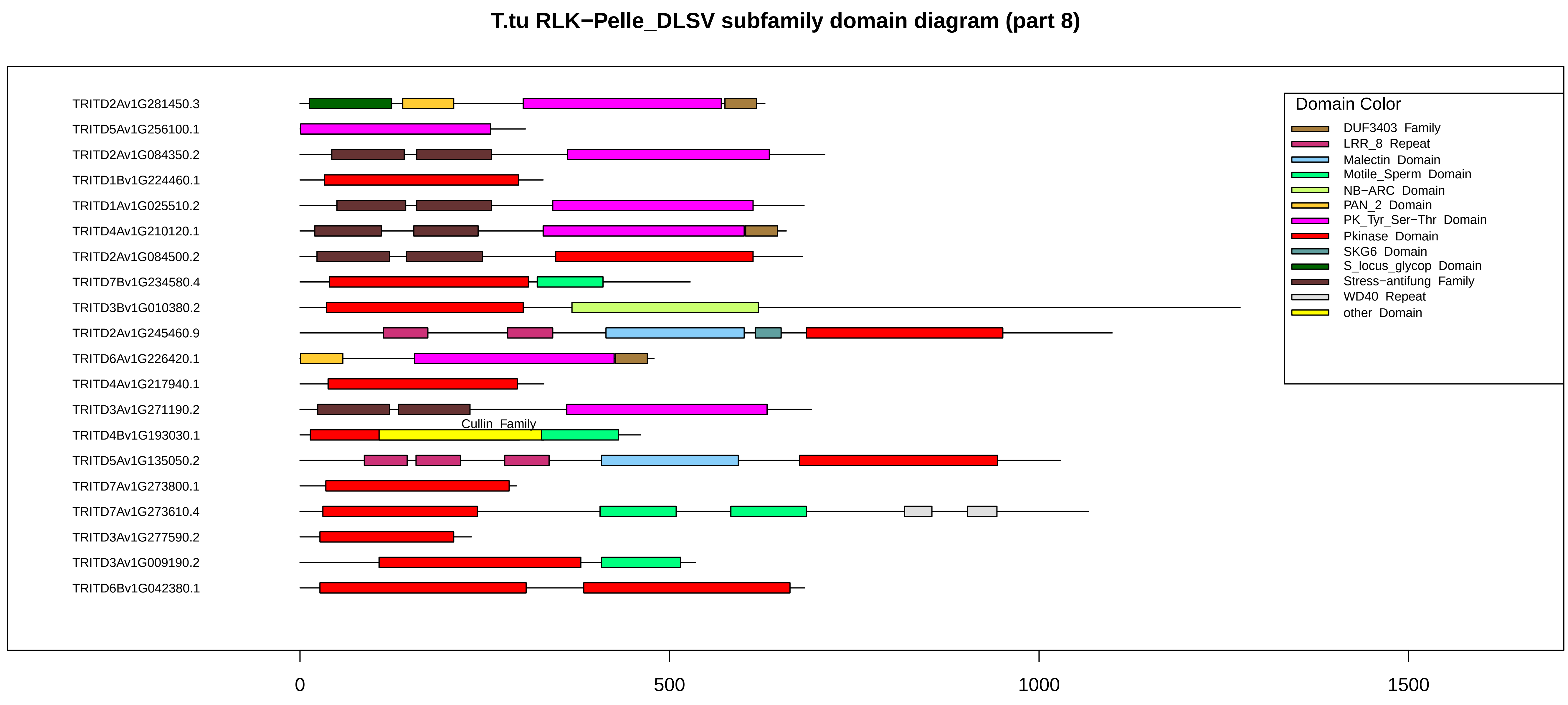
T.tu RLK−Pelle_DLSV subfamily domain diagram (part 8)
TRITD2Av1G281450.3
TRITD5Av1G256100.1
TRITD2Av1G084350.2
TRITD1Bv1G224460.1
TRITD1Av1G025510.2
TRITD4Av1G210120.1
TRITD2Av1G084500.2
TRITD7Bv1G234580.4
TRITD3Bv1G010380.2
TRITD2Av1G245460.9
TRITD6Av1G226420.1
TRITD4Av1G217940.1
TRITD3Av1G271190.2
TRITD4Bv1G193030.1
TRITD5Av1G135050.2
TRITD7Av1G273800.1
TRITD7Av1G273610.4
TRITD3Av1G277590.2
TRITD3Av1G009190.2
TRITD6Bv1G042380.1
Cullin Family
Domain Color
DUF3403 Family
LRR_8 Repeat
Malectin Domain
Motile_Sperm Domain
NB−ARC Domain
PAN_2 Domain
PK_Tyr_Ser−Thr Domain
Pkinase Domain
SKG6 Domain
S_locus_glycop Domain
Stress−antifung Family
WD40 Repeat
other Domain
0
500
1000
1500

T.tu RLK−Pelle_DLSV subfamily domain diagram (part 9)

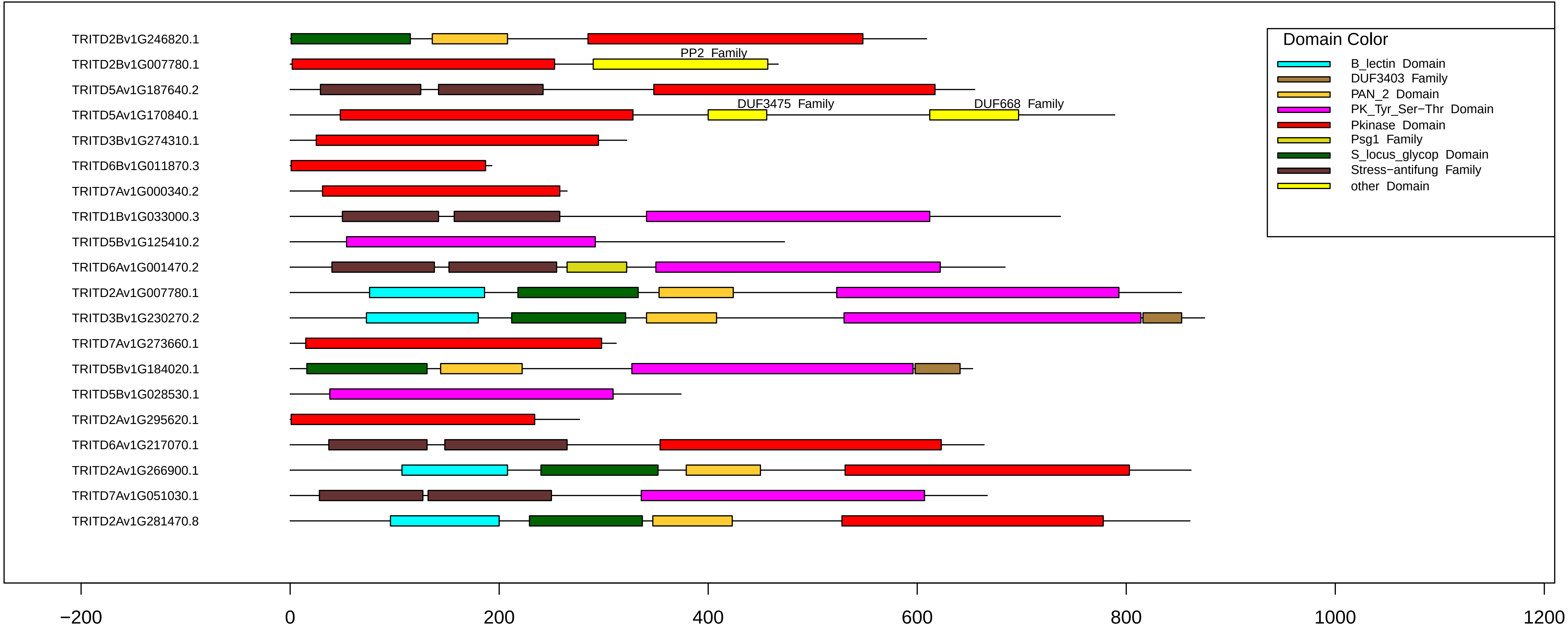

T.tu RLK−Pelle_DLSV subfamily domain diagram (part 10)

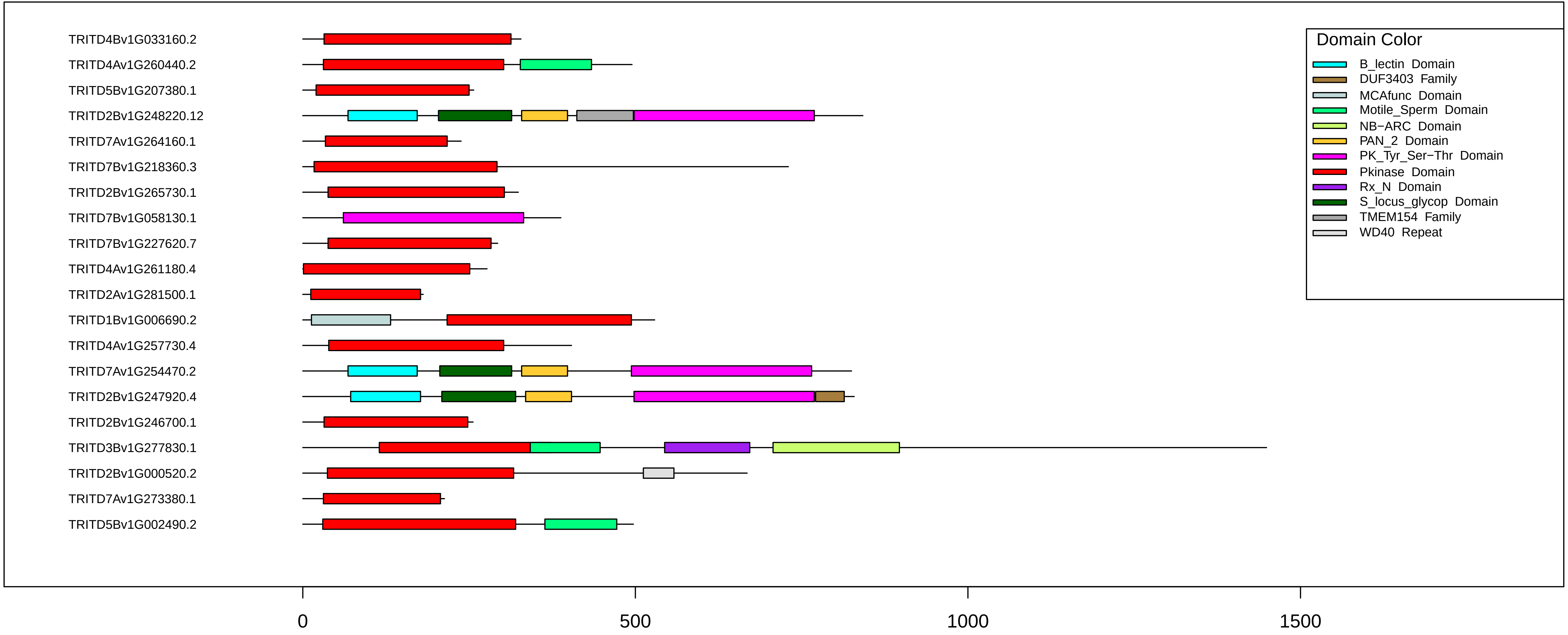

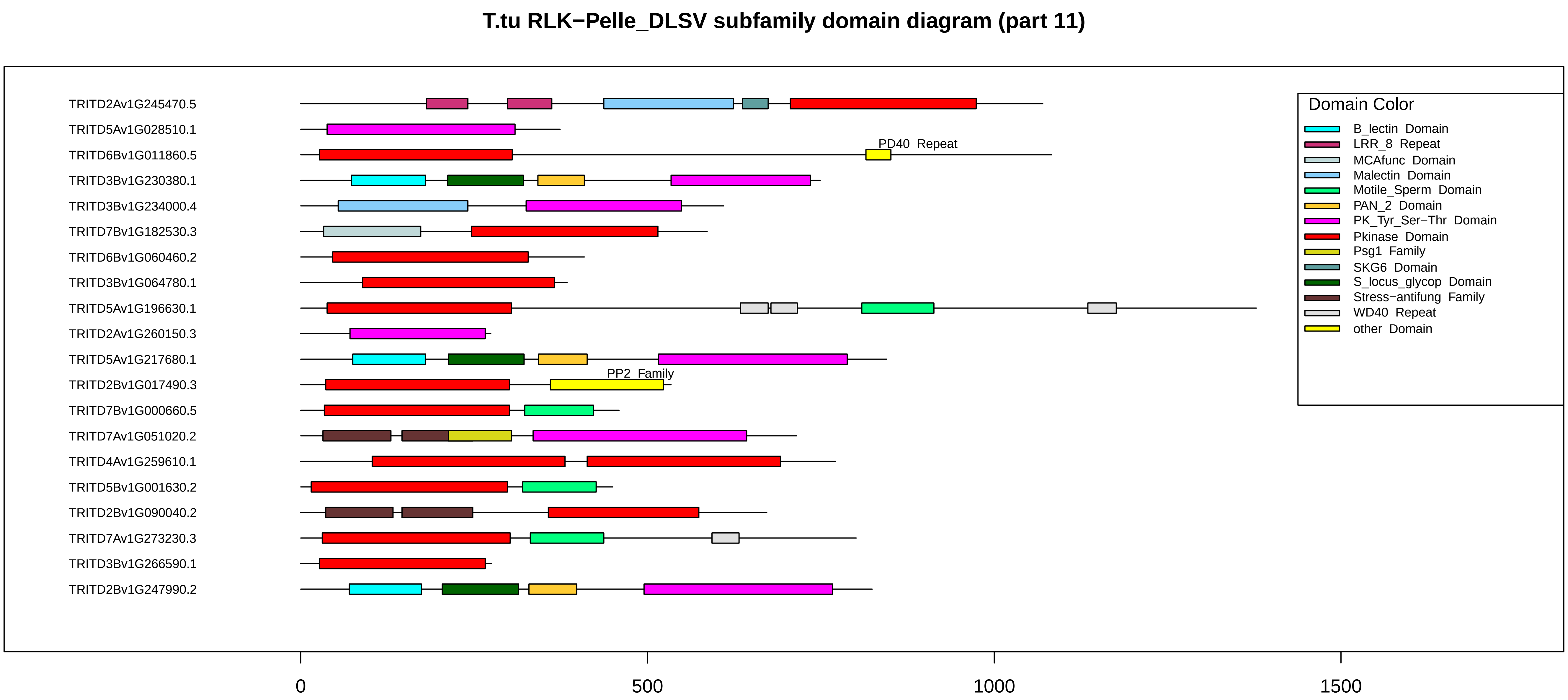

T.tu RLK−Pelle_DLSV subfamily domain diagram (part 11)
TRITD2Av1G245470.5
TRITD5Av1G028510.1
TRITD6Bv1G011860.5
TRITD3Bv1G230380.1
TRITD3Bv1G234000.4
TRITD7Bv1G182530.3
TRITD6Bv1G060460.2
TRITD3Bv1G064780.1
TRITD5Av1G196630.1
TRITD2Av1G260150.3
TRITD5Av1G217680.1
TRITD2Bv1G017490.3
TRITD7Bv1G000660.5
TRITD7Av1G051020.2
TRITD4Av1G259610.1
TRITD5Bv1G001630.2
TRITD2Bv1G090040.2
TRITD7Av1G273230.3
TRITD3Bv1G266590.1
TRITD2Bv1G247990.2
PD40 Repeat
PP2 Family
Domain Color
B_lectin Domain
LRR_8 Repeat
MCAfunc Domain
Malectin Domain
Motile_Sperm Domain
PAN_2 Domain
PK_Tyr_Ser−Thr Domain
Pkinase Domain
Psg1 Family
SKG6 Domain
S_locus_glycop Domain
Stress−antifung Family
WD40 Repeat
other Domain
0
500
1000
1500

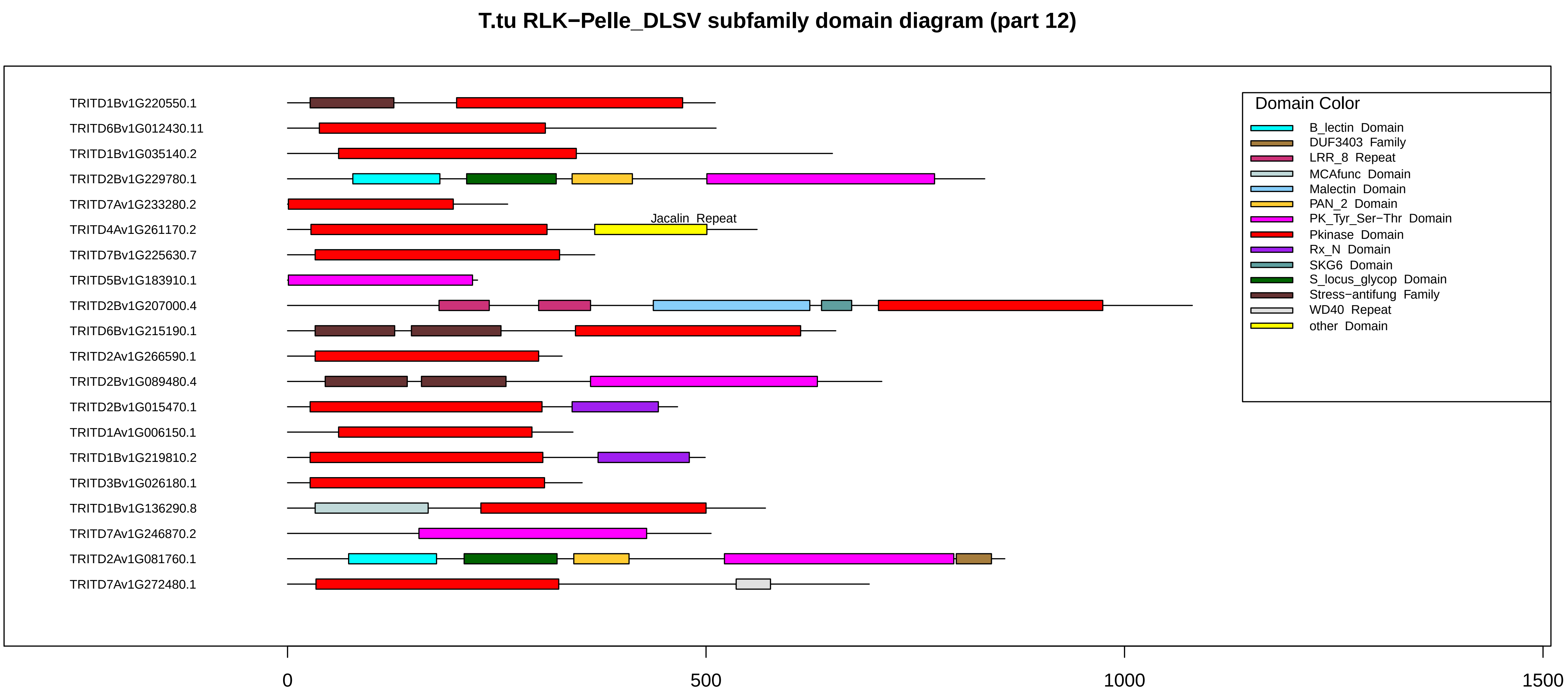
T.tu RLK−Pelle_DLSV subfamily domain diagram (part 12)
TRITD1Bv1G220550.1
TRITD6Bv1G012430.11
TRITD1Bv1G035140.2
TRITD2Bv1G229780.1
TRITD7Av1G233280.2
TRITD4Av1G261170.2
Jacalin_Repeat
TRITD7Bv1G225630.7
TRITD5Bv1G183910.1
TRITD2Bv1G207000.4
TRITD6Bv1G215190.1
TRITD2Av1G266590.1
TRITD2Bv1G089480.4
TRITD2Bv1G015470.1
TRITD1Av1G006150.1
TRITD1Bv1G219810.2
TRITD3Bv1G026180.1
TRITD1Bv1G136290.8
TRITD7Av1G246870.2
TRITD2Av1G081760.1
TRITD7Av1G272480.1
Domain Color
B_lectin Domain
DUF3403 Family
LRR_8 Repeat
MCAfunc Domain
Malectin Domain
PAN_2 Domain
PK_Tyr_Ser−Thr Domain
Pkinase Domain
Rx_N Domain
SKG6 Domain
S_locus_glycop Domain
Stress−antifung Family
WD40 Repeat
other Domain
0
500
1000
1500

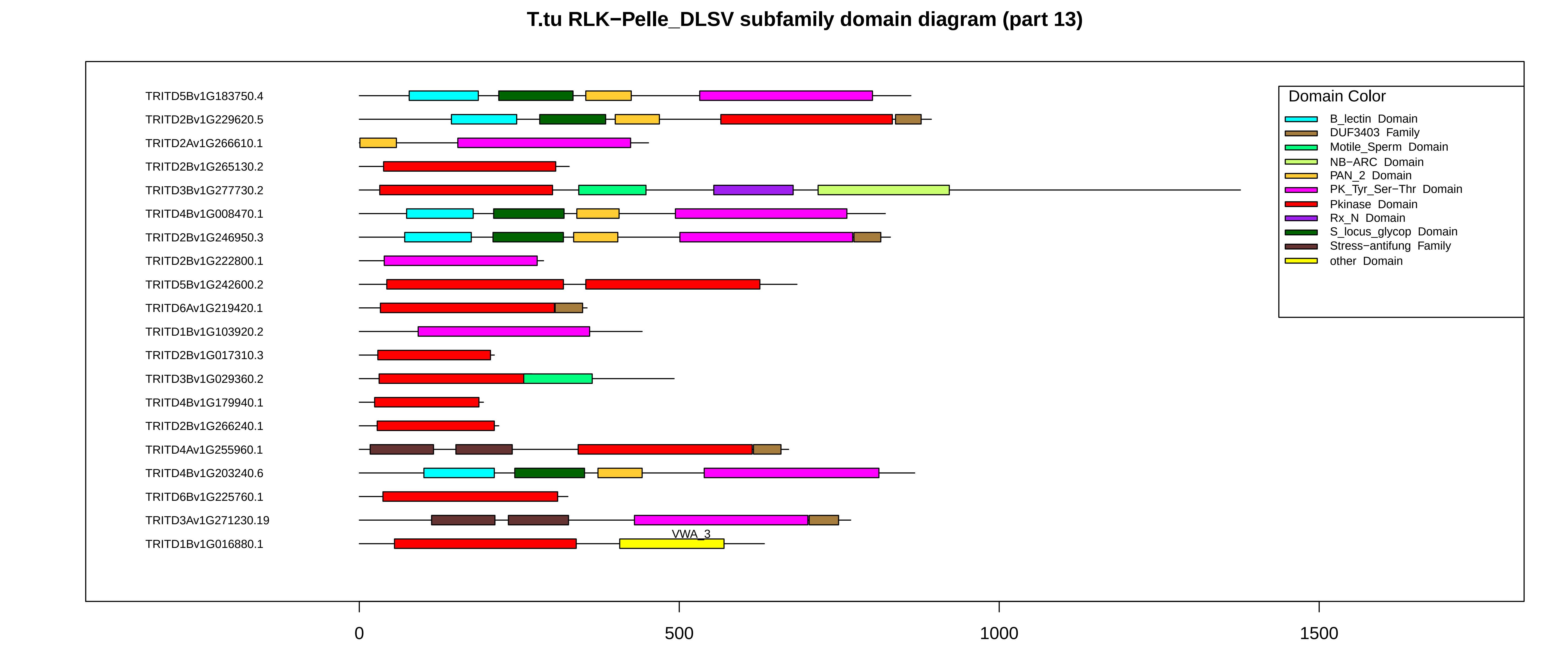
T.tu RLK−Pelle_DLSV subfamily domain diagram (part 13)
TRITD5Bv1G183750.4
TRITD2Bv1G229620.5
TRITD2Av1G266610.1
TRITD2Bv1G265130.2
TRITD3Bv1G277730.2
TRITD4Bv1G008470.1
TRITD2Bv1G246950.3
TRITD2Bv1G222800.1
TRITD5Bv1G242600.2
TRITD6Av1G219420.1
TRITD1Bv1G103920.2
TRITD2Bv1G017310.3
TRITD3Bv1G029360.2
TRITD4Bv1G179940.1
TRITD2Bv1G266240.1
TRITD4Av1G255960.1
TRITD4Bv1G203240.6
TRITD6Bv1G225760.1
TRITD3Av1G271230.19
TRITD1Bv1G016880.1
VWA_3
Domain Color
B_lectin Domain
DUF3403 Family
Motile_Sperm Domain
NB−ARC Domain
PAN_2 Domain
PK_Tyr_Ser−Thr Domain
Pkinase Domain
Rx_N Domain
S_locus_glycop Domain
Stress−antifung Family
other Domain
0
500
1000
1500

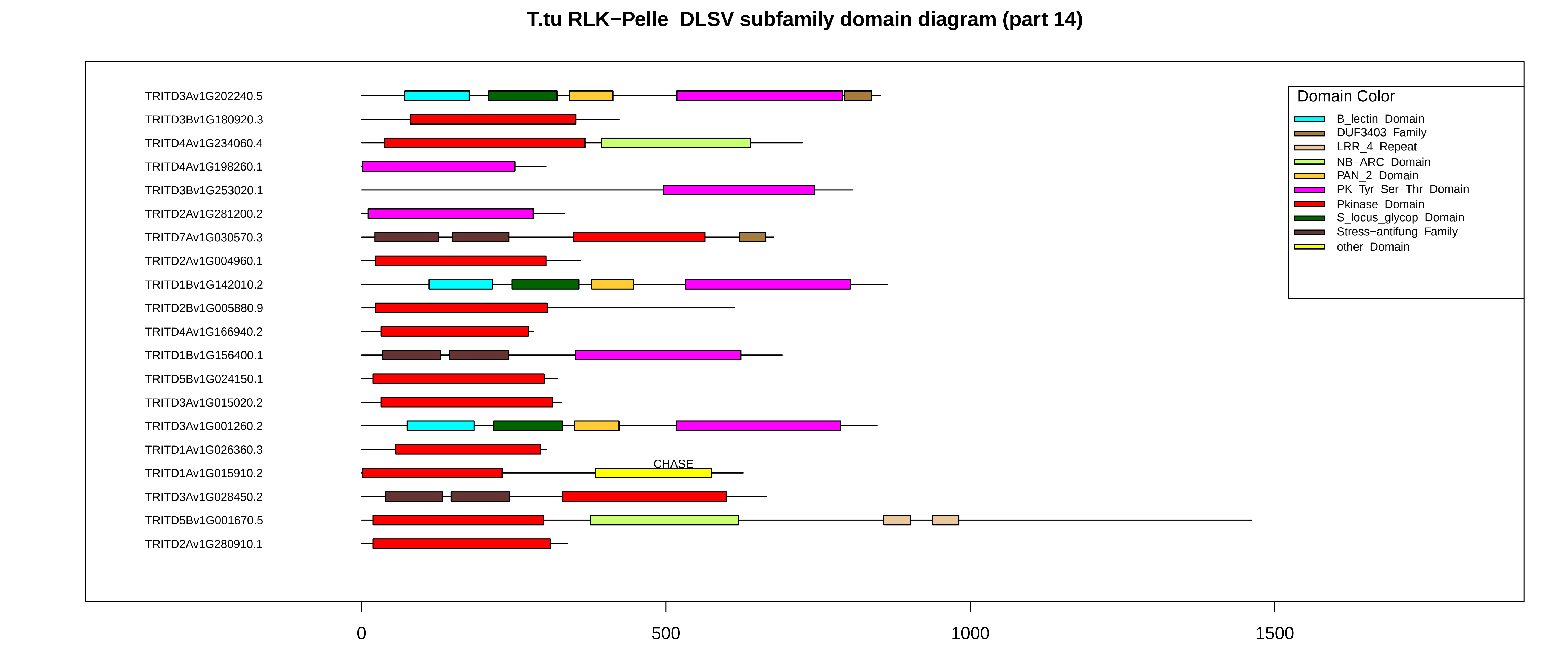
T.tu RLK−Pelle_DLSV subfamily domain diagram (part 14)
TRITD3Av1G202240.5
TRITD3Bv1G180920.3
TRITD4Av1G234060.4
TRITD4Av1G198260.1
TRITD3Bv1G253020.1
TRITD2Av1G281200.2
TRITD7Av1G030570.3
TRITD2Av1G004960.1
TRITD1Bv1G142010.2
TRITD2Bv1G005880.9
TRITD4Av1G166940.2
TRITD1Bv1G156400.1
TRITD5Bv1G024150.1
TRITD3Av1G015020.2
TRITD3Av1G001260.2
TRITD1Av1G026360.3
TRITD1Av1G015910.2
TRITD3Av1G028450.2
TRITD5Bv1G001670.5
TRITD2Av1G280910.1
CHASE
Domain Color
B_lectin Domain
DUF3403 Family
LRR_4 Repeat
NB−ARC Domain
PAN_2 Domain
PK_Tyr_Ser−Thr Domain
Pkinase Domain
S_locus_glycop Domain
Stress−antifung Family
other Domain
0
500
1000
1500

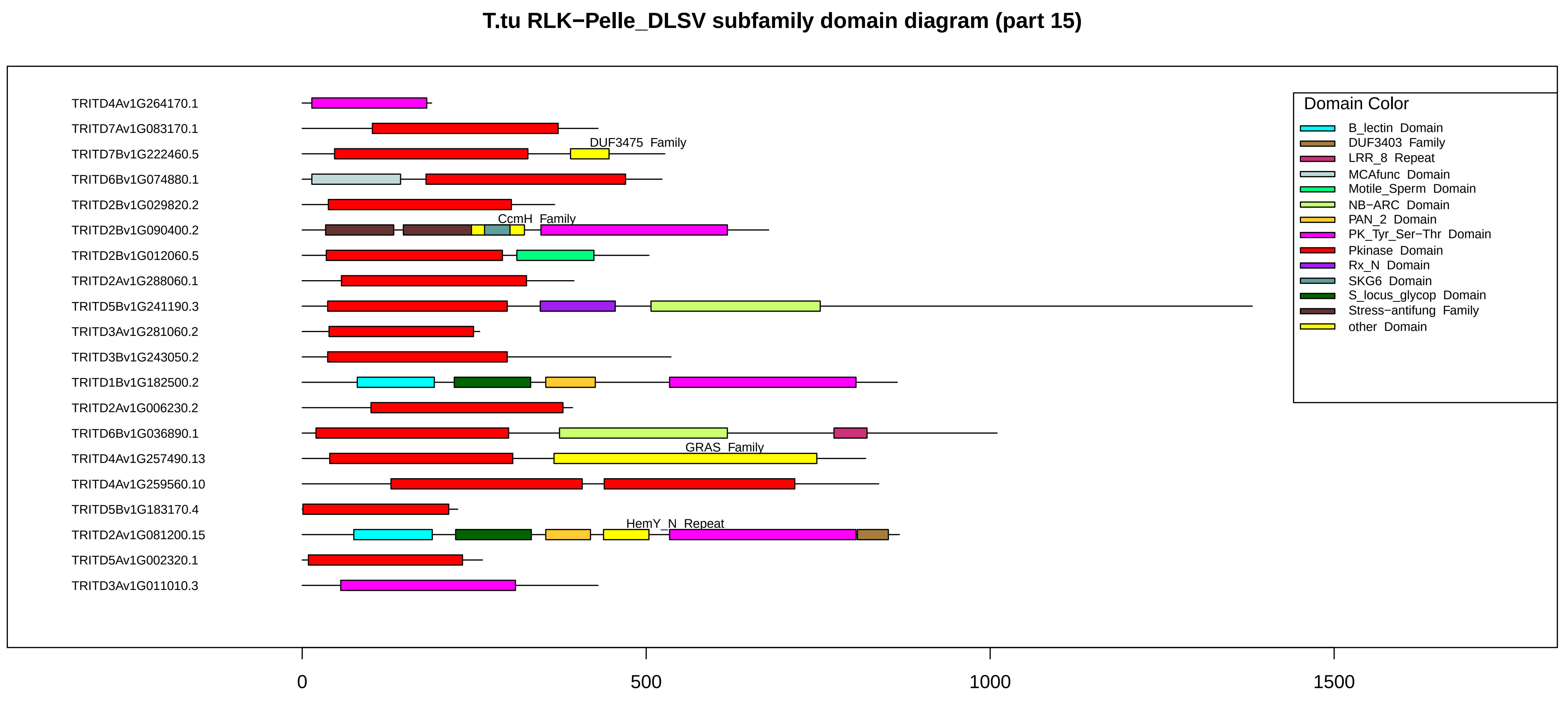

T.tu RLK−Pelle_DLSV subfamily domain diagram (part 15)
TRITD4Av1G264170.1
TRITD7Av1G083170.1
TRITD7Bv1G222460.5
DUF3475 Family
TRITD6Bv1G074880.1
TRITD2Bv1G029820.2
TRITD2Bv1G090400.2
CcmH Family
TRITD2Bv1G012060.5
TRITD2Av1G288060.1
TRITD5Bv1G241190.3
TRITD3Av1G281060.2
TRITD3Bv1G243050.2
TRITD1Bv1G182500.2
TRITD2Av1G006230.2
TRITD6Bv1G036890.1
TRITD4Av1G257490.13
GRAS Family
TRITD4Av1G259560.10
TRITD5Bv1G183170.4
TRITD2Av1G081200.15
HemY_N Repeat
TRITD5Av1G002320.1
TRITD3Av1G011010.3
0
500
1000
1500
Domain Color
B_lectin Domain
DUF3403 Family
LRR_8 Repeat
MCAfunc Domain
Motile_Sperm Domain
NB−ARC Domain
PAN_2 Domain
PK_Tyr_Ser−Thr Domain
Pkinase Domain
Rx_N Domain
SKG6 Domain
S_locus_glycop Domain
Stress−antifung Family
other Domain

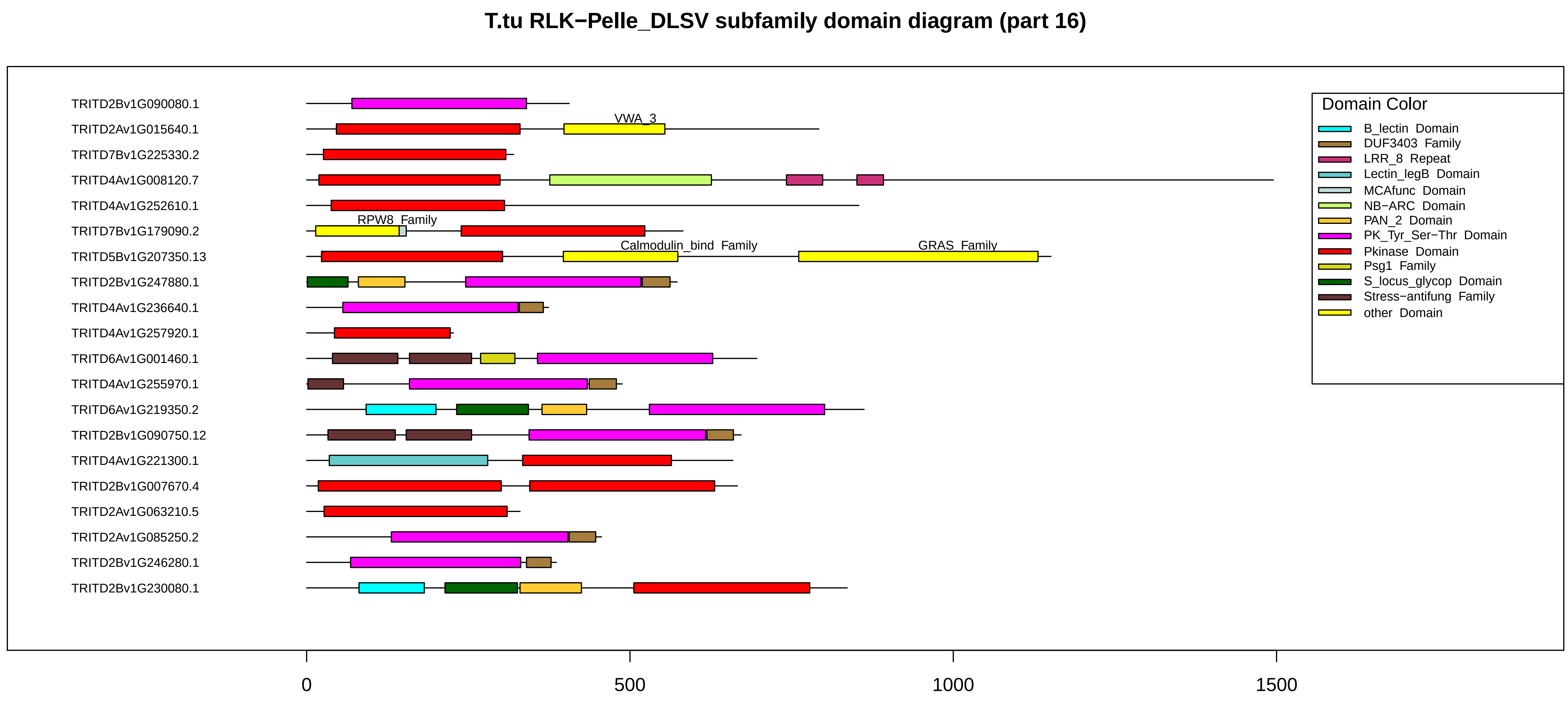
T.tu RLK−Pelle_DLSV subfamily domain diagram (part 16)
TRITD2Bv1G090080.1
TRITD2Av1G015640.1
VWA_3
TRITD7Bv1G225330.2
TRITD4Av1G008120.7
TRITD4Av1G252610.1
TRITD7Bv1G179090.2
RPW8_Family
TRITD5Bv1G207350.13
Calmodulin_bind_Family
GRAS_Family
TRITD2Bv1G247880.1
TRITD4Av1G236640.1
TRITD4Av1G257920.1
TRITD6Av1G001460.1
TRITD4Av1G255970.1
TRITD6Av1G219350.2
TRITD2Bv1G090750.12
TRITD4Av1G221300.1
TRITD2Bv1G007670.4
TRITD2Av1G063210.5
TRITD2Av1G085250.2
TRITD2Bv1G246280.1
TRITD2Bv1G230080.1
Domain Color
B_lectin Domain
DUF3403 Family
LRR_8 Repeat
Lectin_legB Domain
MCAfunc Domain
NB−ARC Domain
PAN_2 Domain
PK_Tyr_Ser−Thr Domain
Pkinase Domain
Psg1 Family
S_locus_glycop Domain
Stress−antifung Family
other Domain
0
500
1000
1500

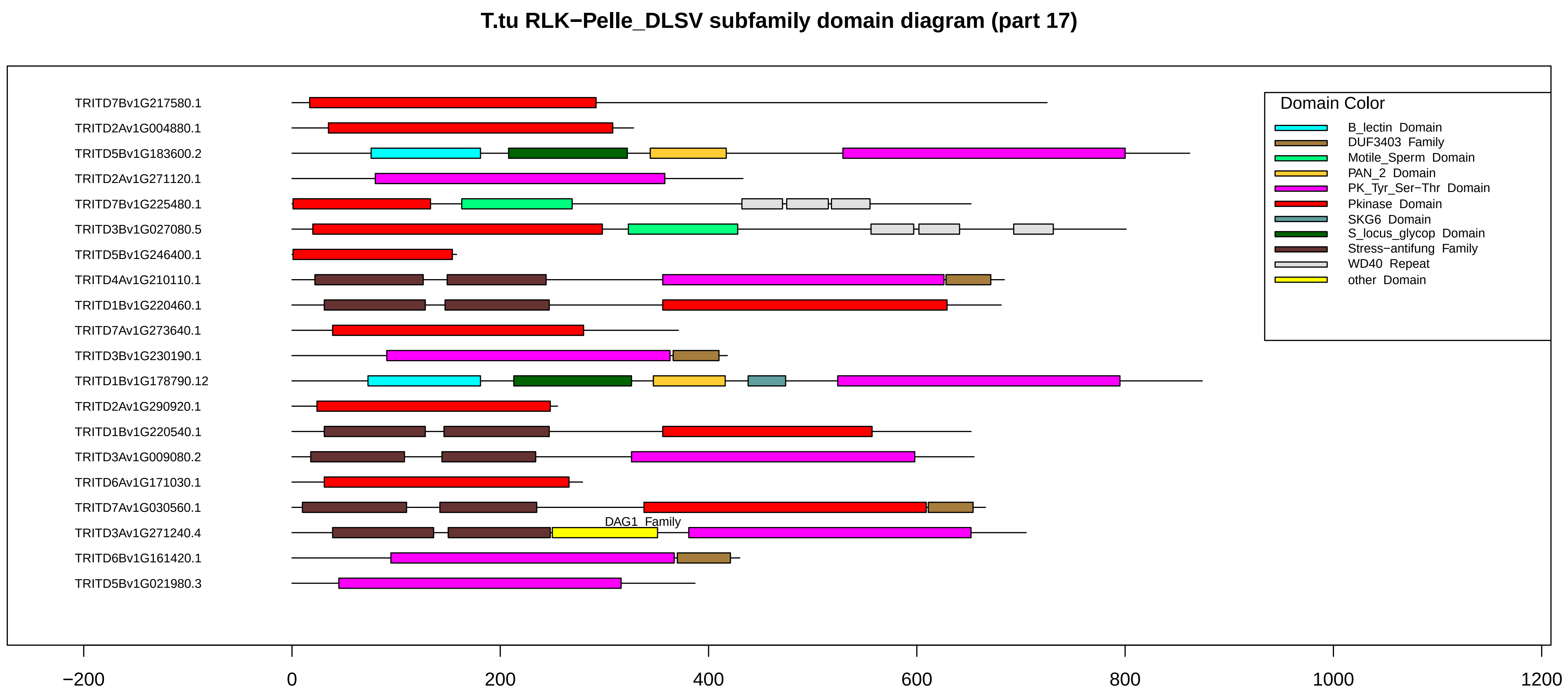
T.tu RLK−Pelle_DLSV subfamily domain diagram (part 17)
TRITD7Bv1G217580.1
TRITD2Av1G004880.1
TRITD5Bv1G183600.2
TRITD2Av1G271120.1
TRITD7Bv1G225480.1
TRITD3Bv1G027080.5
TRITD5Bv1G246400.1
TRITD4Av1G210110.1
TRITD1Bv1G220460.1
TRITD7Av1G273640.1
TRITD3Bv1G230190.1
TRITD1Bv1G178790.12
TRITD2Av1G290920.1
TRITD1Bv1G220540.1
TRITD3Av1G009080.2
TRITD6Av1G171030.1
TRITD7Av1G030560.1
TRITD3Av1G271240.4
TRITD6Bv1G161420.1
TRITD5Bv1G021980.3
DAG1 Family
Domain Color
B_lectin Domain
DUF3403 Family
Motile_Sperm Domain
PAN_2 Domain
PK_Tyr_Ser−Thr Domain
Pkinase Domain
SKG6 Domain
S_locus_glycop Domain
Stress−antifung Family
WD40 Repeat
other Domain
−200
0
200
400
600
800
1000
1200

T.tu RLK−Pelle_DLSV subfamily domain diagram (part 18)

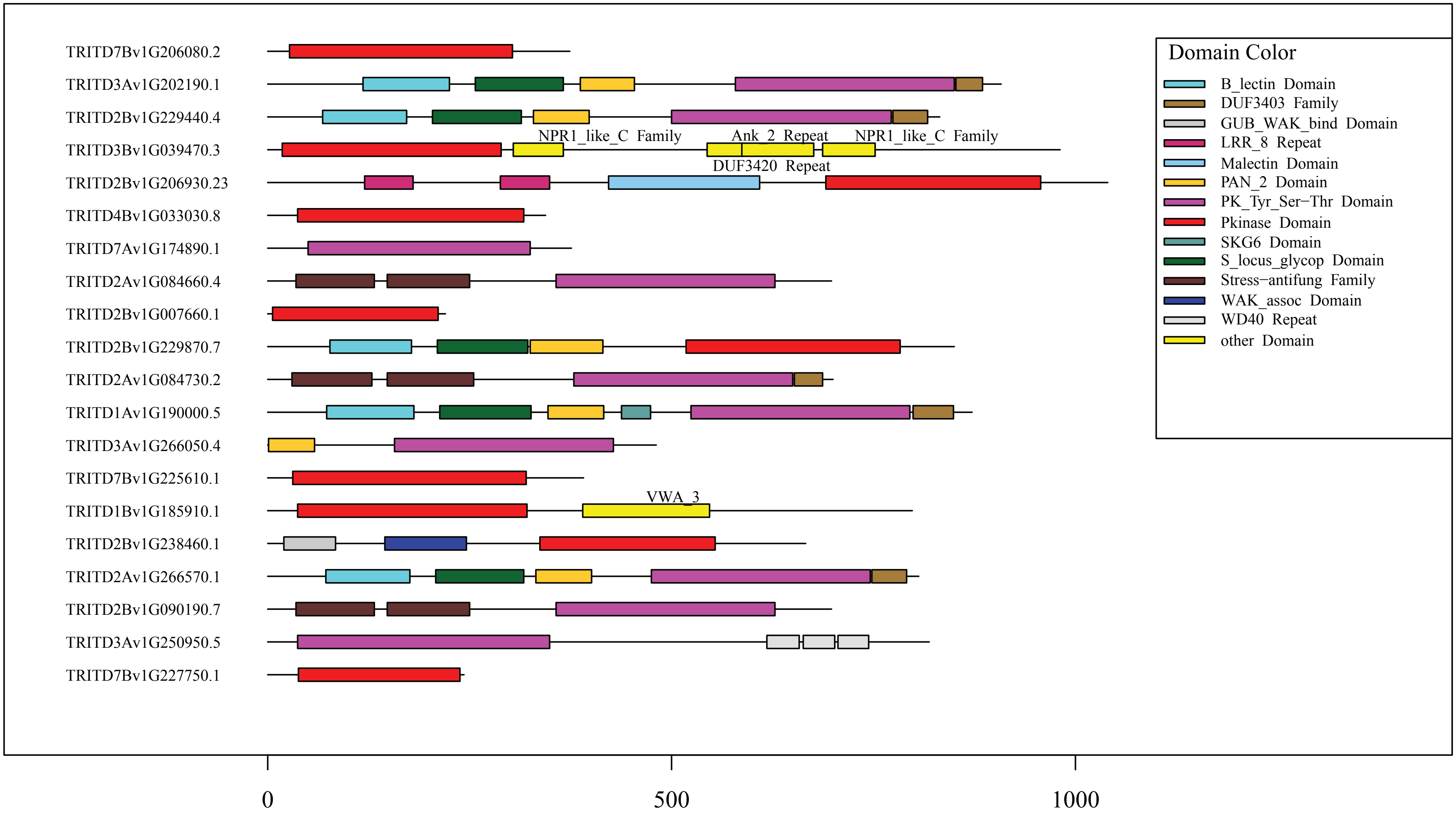

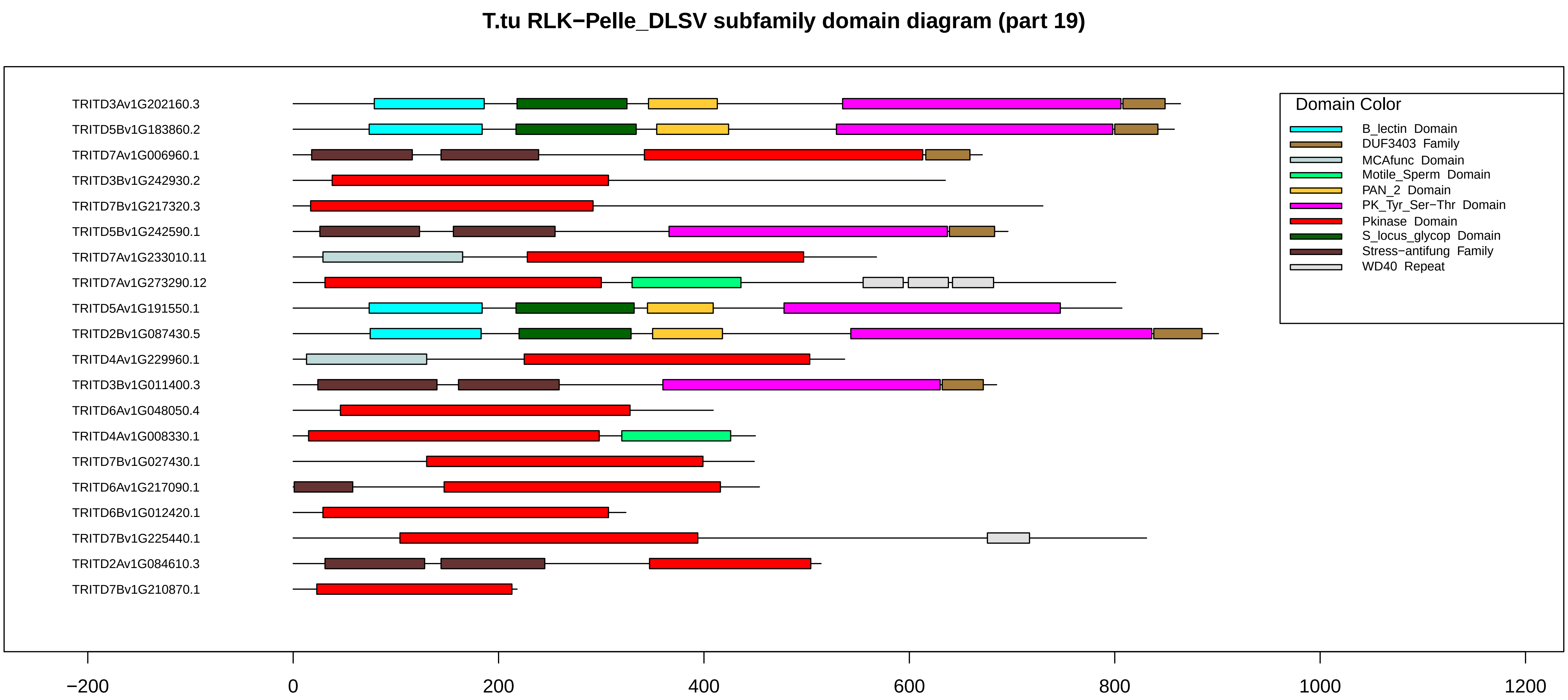
T.tu RLK−Pelle_DLSV subfamily domain diagram (part 19)
TRITD3Av1G202160.3
TRITD5Bv1G183860.2
TRITD7Av1G006960.1
TRITD3Bv1G242930.2
TRITD7Bv1G217320.3
TRITD5Bv1G242590.1
TRITD7Av1G233010.11
TRITD7Av1G273290.12
TRITD5Av1G191550.1
TRITD2Bv1G087430.5
TRITD4Av1G229960.1
TRITD3Bv1G011400.3
TRITD6Av1G048050.4
TRITD4Av1G008330.1
TRITD7Bv1G027430.1
TRITD6Av1G217090.1
TRITD6Bv1G012420.1
TRITD7Bv1G225440.1
TRITD2Av1G084610.3
TRITD7Bv1G210870.1
Domain Color
B_lectin Domain
DUF3403 Family
MCAfunc Domain
Motile_Sperm Domain
PAN_2 Domain
PK_Tyr_Ser−Thr Domain
Pkinase Domain
S_locus_glycop Domain
Stress−antifung Family
WD40 Repeat
−200
0
200
400
600
800
1000
1200

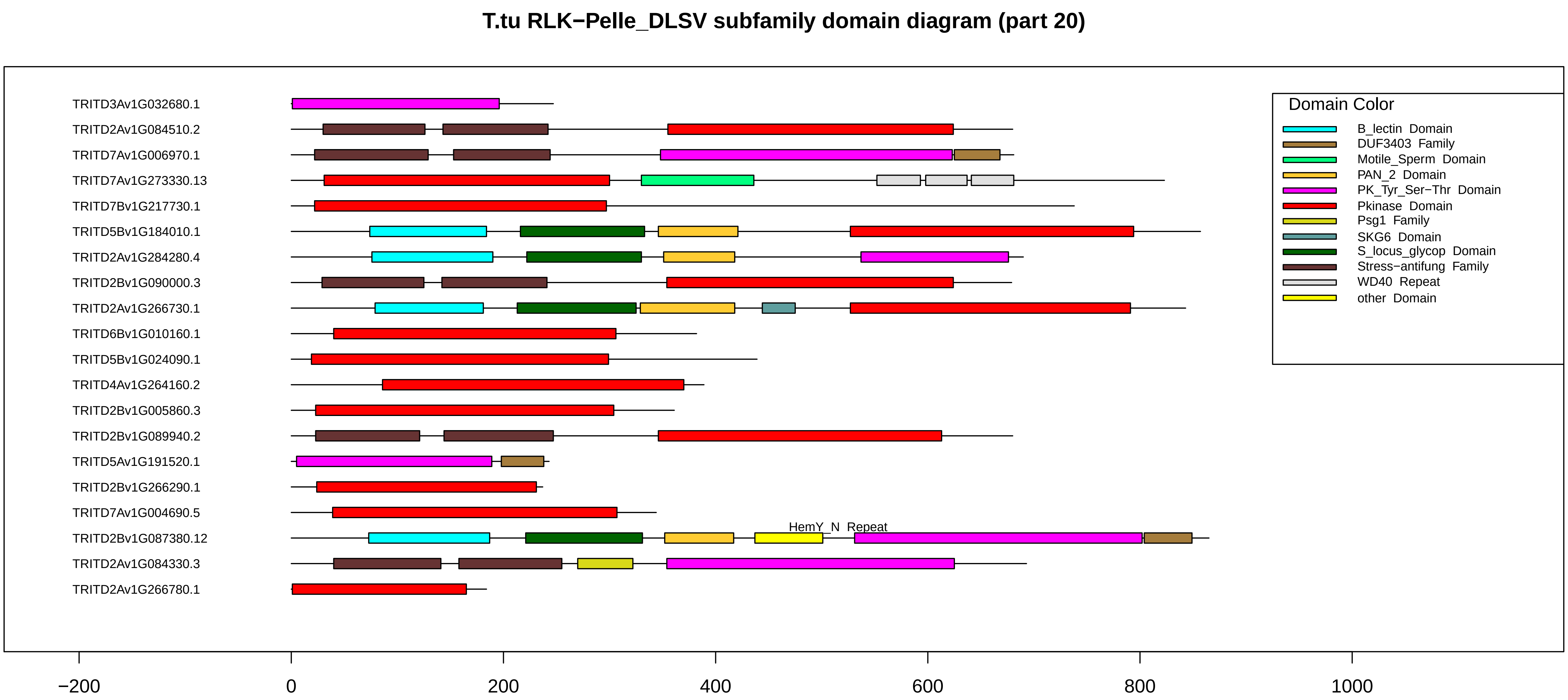

T.tu RLK−Pelle_DLSV subfamily domain diagram (part 20)
TRITD3Av1G032680.1
TRITD2Av1G084510.2
TRITD7Av1G006970.1
TRITD7Av1G273330.13
TRITD7Bv1G217730.1
TRITD5Bv1G184010.1
TRITD2Av1G284280.4
TRITD2Bv1G090000.3
TRITD2Av1G266730.1
TRITD6Bv1G010160.1
TRITD5Bv1G024090.1
TRITD4Av1G264160.2
TRITD2Bv1G005860.3
TRITD2Bv1G089940.2
TRITD5Av1G191520.1
TRITD2Bv1G266290.1
TRITD7Av1G004690.5
HemY_N Repeat
TRITD2Bv1G087380.12
TRITD2Av1G084330.3
TRITD2Av1G266780.1
Domain Color
B_lectin Domain
DUF3403 Family
Motile_Sperm Domain
PAN_2 Domain
PK_Tyr_Ser−Thr Domain
Pkinase Domain
Psg1 Family
SKG6 Domain
S_locus_glycop Domain
Stress−antifung Family
WD40 Repeat
other Domain
−200
0
200
400
600
800
1000

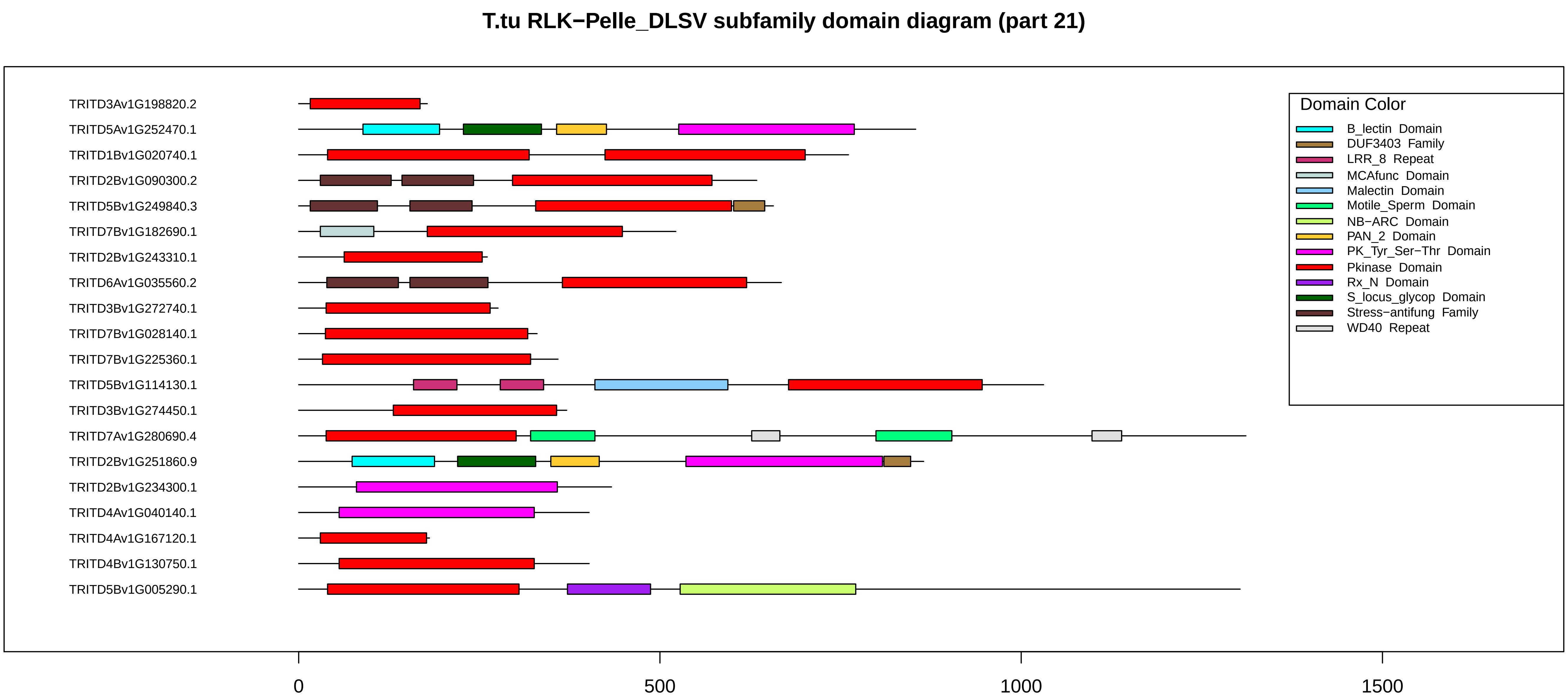
T.tu RLK−Pelle_DLSV subfamily domain diagram (part 21)
TRITD3Av1G198820.2
TRITD5Av1G252470.1
TRITD1Bv1G020740.1
TRITD2Bv1G090300.2
TRITD5Bv1G249840.3
TRITD7Bv1G182690.1
TRITD2Bv1G243310.1
TRITD6Av1G035560.2
TRITD3Bv1G272740.1
TRITD7Bv1G028140.1
TRITD7Bv1G225360.1
TRITD5Bv1G114130.1
TRITD3Bv1G274450.1
TRITD7Av1G280690.4
TRITD2Bv1G251860.9
TRITD2Bv1G234300.1
TRITD4Av1G040140.1
TRITD4Av1G167120.1
TRITD4Bv1G130750.1
TRITD5Bv1G005290.1
Domain Color
B_lectin Domain
DUF3403 Family
LRR_8 Repeat
MCAfunc Domain
Malectin Domain
Motile_Sperm Domain
NB−ARC Domain
PAN_2 Domain
PK_Tyr_Ser−Thr Domain
Pkinase Domain
Rx_N Domain
S_locus_glycop Domain
Stress−antifung Family
WD40 Repeat
0
500
1000
1500

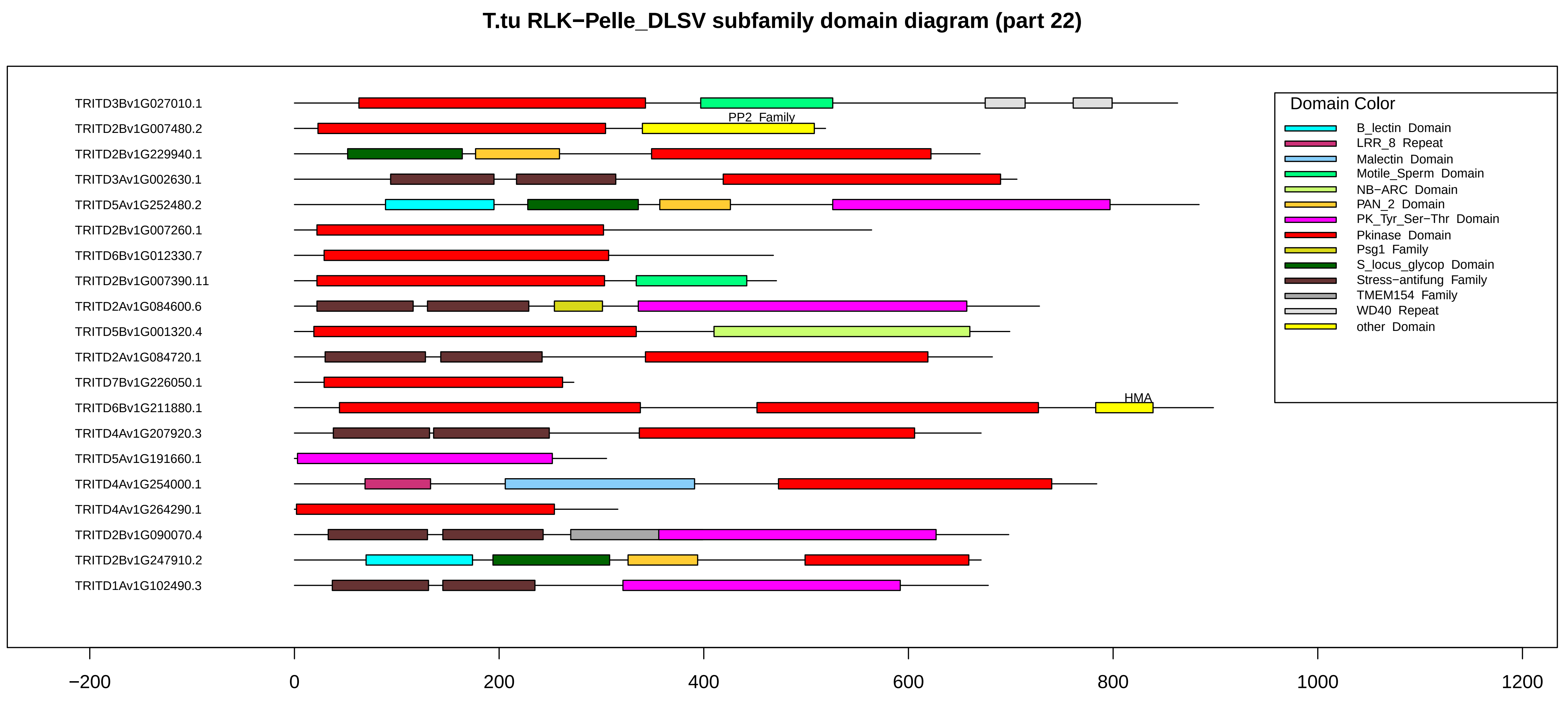

T.tu RLK−Pelle_DLSV subfamily domain diagram (part 22)
TRITD3Bv1G027010.1
TRITD2Bv1G007480.2
TRITD2Bv1G229940.1
TRITD3Av1G002630.1
TRITD5Av1G252480.2
TRITD2Bv1G007260.1
TRITD6Bv1G012330.7
TRITD2Bv1G007390.11
TRITD2Av1G084600.6
TRITD5Bv1G001320.4
TRITD2Av1G084720.1
TRITD7Bv1G226050.1
TRITD6Bv1G211880.1
TRITD4Av1G207920.3
TRITD5Av1G191660.1
TRITD4Av1G254000.1
TRITD4Av1G264290.1
TRITD2Bv1G090070.4
TRITD2Bv1G247910.2
TRITD1Av1G102490.3
PP2_Family
HMA
Domain Color
B_lectin Domain
LRR_8 Repeat
Malectin Domain
Motile_Sperm Domain
NB−ARC Domain
PAN_2 Domain
PK_Tyr_Ser−Thr Domain
Pkinase Domain
Psg1 Family
S_locus_glycop Domain
Stress−antifung Family
TMEM154 Family
WD40 Repeat
other Domain
−200
0
200
400
600
800
1000
1200

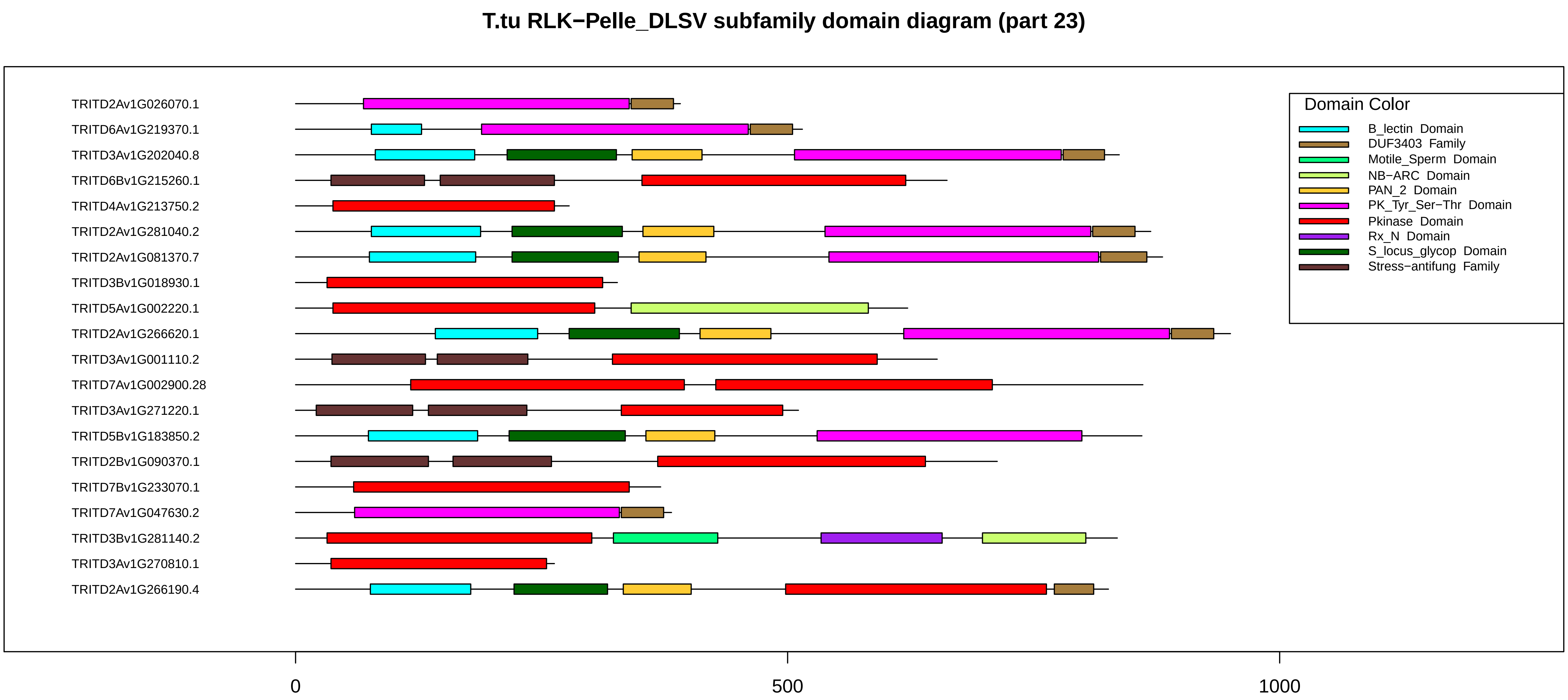
T.tu RLK−Pelle_DLSV subfamily domain diagram (part 23)
TRITD2Av1G026070.1
TRITD6Av1G219370.1
TRITD3Av1G202040.8
TRITD6Bv1G215260.1
TRITD4Av1G213750.2
TRITD2Av1G281040.2
TRITD2Av1G081370.7
TRITD3Bv1G018930.1
TRITD5Av1G002220.1
TRITD2Av1G266620.1
TRITD3Av1G001110.2
TRITD7Av1G002900.28
TRITD3Av1G271220.1
TRITD5Bv1G183850.2
TRITD2Bv1G090370.1
TRITD7Bv1G233070.1
TRITD7Av1G047630.2
TRITD3Bv1G281140.2
TRITD3Av1G270810.1
TRITD2Av1G266190.4
0
500
1000
Domain Color
B_lectin Domain
DUF3403 Family
Motile_Sperm Domain
NB−ARC Domain
PAN_2 Domain
PK_Tyr_Ser−Thr Domain
Pkinase Domain
Rx_N Domain
S_locus_glycop Domain
Stress−antifung Family

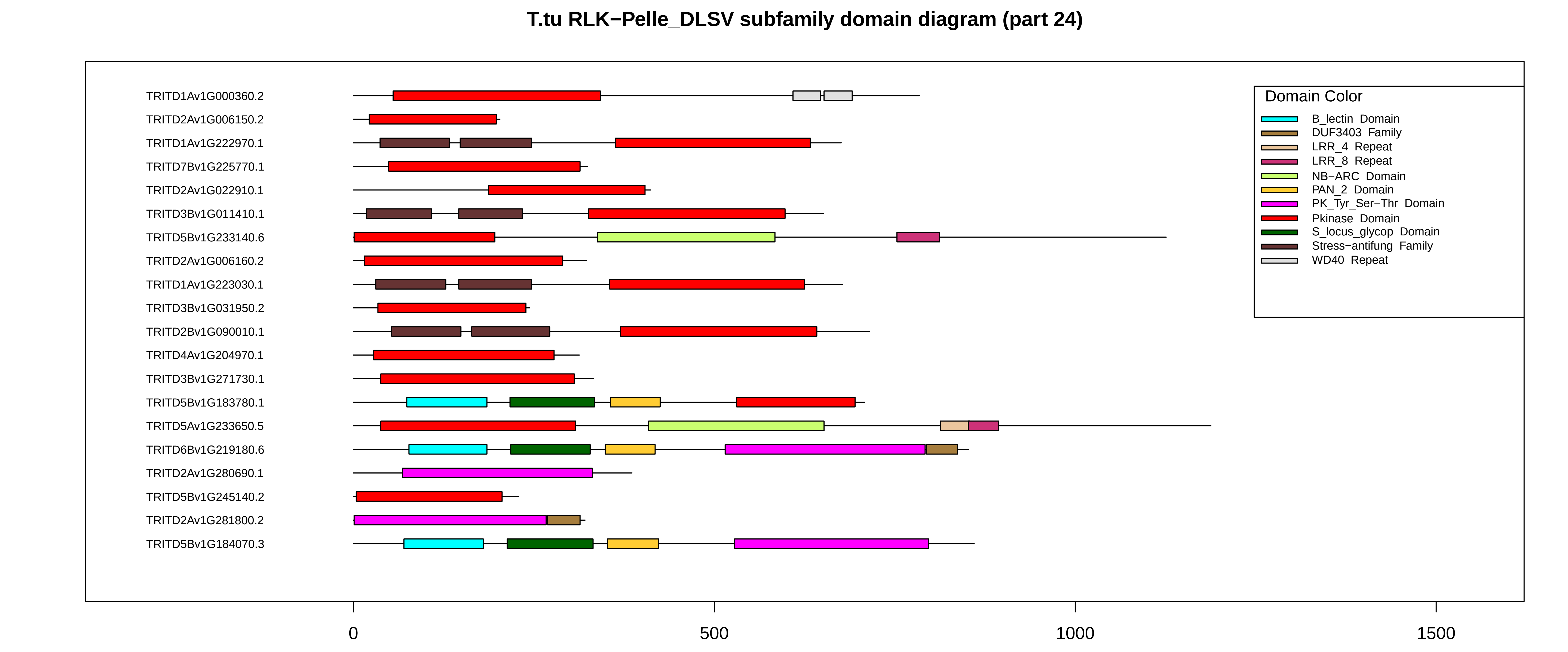
T.tu RLK−Pelle_DLSV subfamily domain diagram (part 24)
TRITD1Av1G000360.2
TRITD2Av1G006150.2
TRITD1Av1G222970.1
TRITD7Bv1G225770.1
TRITD2Av1G022910.1
TRITD3Bv1G011410.1
TRITD5Bv1G233140.6
TRITD2Av1G006160.2
TRITD1Av1G223030.1
TRITD3Bv1G031950.2
TRITD2Bv1G090010.1
TRITD4Av1G204970.1
TRITD3Bv1G271730.1
TRITD5Bv1G183780.1
TRITD5Av1G233650.5
TRITD6Bv1G219180.6
TRITD2Av1G280690.1
TRITD5Bv1G245140.2
TRITD2Av1G281800.2
TRITD5Bv1G184070.3
Domain Color
B_lectin Domain
DUF3403 Family
LRR_4 Repeat
LRR_8 Repeat
NB−ARC Domain
PAN_2 Domain
PK_Tyr_Ser−Thr Domain
Pkinase Domain
S_locus_glycop Domain
Stress−antifung Family
WD40 Repeat
0
500
1000
1500

T.tu RLK−Pelle_DLSV subfamily domain diagram (part 25)

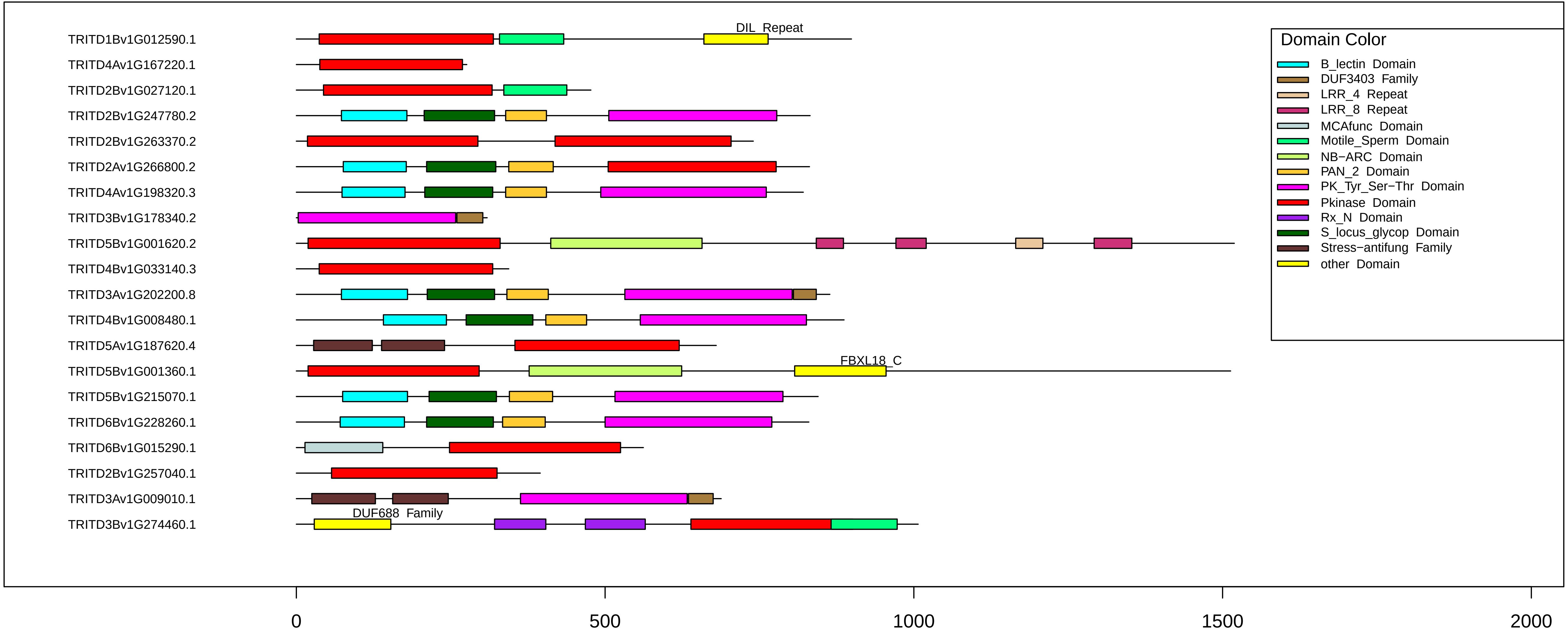

T.tu RLK−Pelle_DLSV subfamily domain diagram (part 26)

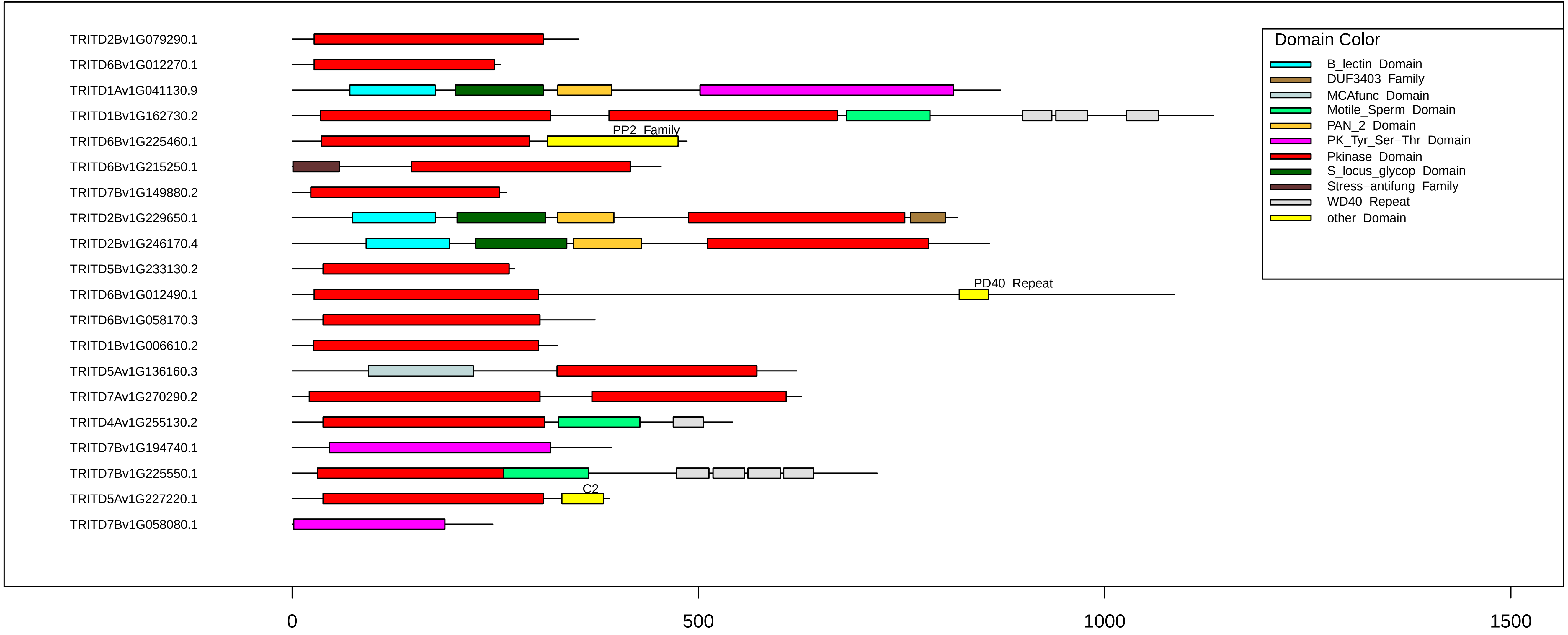

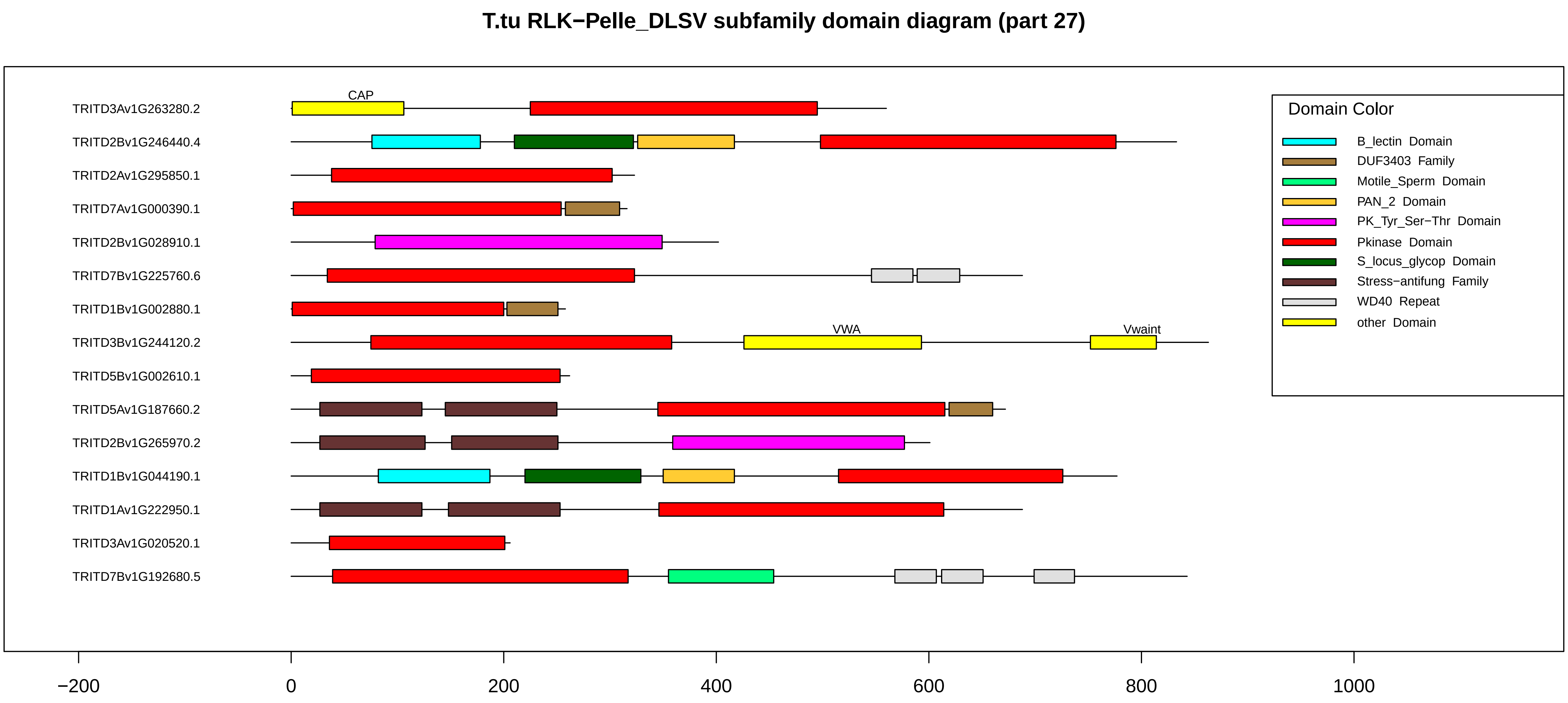

T.tu RLK−Pelle_DLSV subfamily domain diagram (part 27)
TRITD3Av1G263280.2
CAP
TRITD2Bv1G246440.4
TRITD2Av1G295850.1
TRITD7Av1G000390.1
TRITD2Bv1G028910.1
TRITD7Bv1G225760.6
TRITD1Bv1G002880.1
TRITD3Bv1G244120.2
VWA
Vwaint
TRITD5Bv1G002610.1
TRITD5Av1G187660.2
TRITD2Bv1G265970.2
TRITD1Bv1G044190.1
TRITD1Av1G222950.1
TRITD3Av1G020520.1
TRITD7Bv1G192680.5
Domain Color
B_lectin Domain
DUF3403 Family
Motile_Sperm Domain
PAN_2 Domain
PK_Tyr_Ser−Thr Domain
Pkinase Domain
S_locus_glycop Domain
Stress−antifung Family
WD40 Repeat
other Domain
−200
0
200
400
600
800
1000

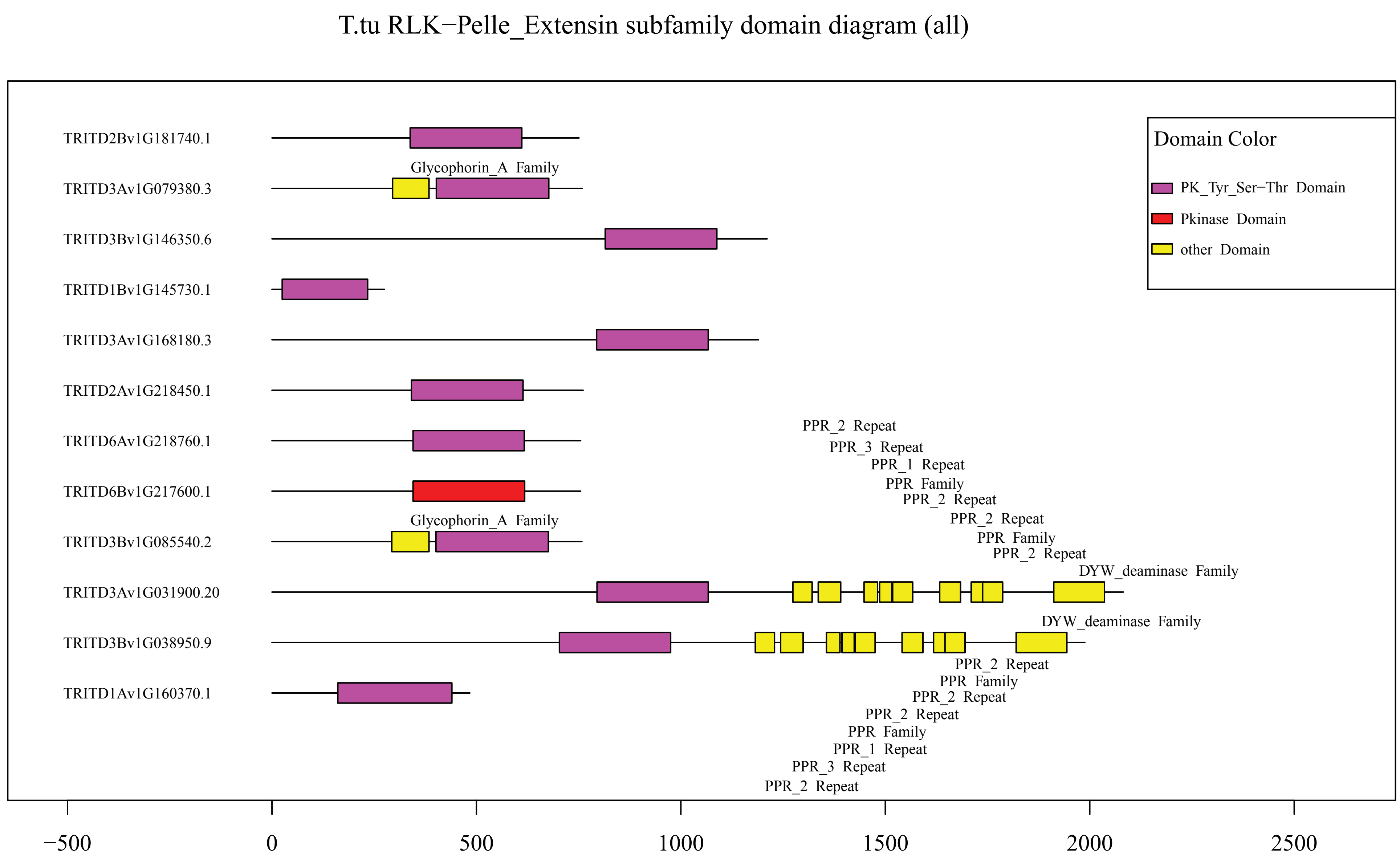
T.tu RLK−Pelle_Extensin subfamily domain diagram (all)
TRITD2Bv1G181740.1
TRITD3Av1G079380.3
TRITD3Bv1G146350.6
TRITD1Bv1G145730.1
TRITD3Av1G168180.3
TRITD2Av1G218450.1
TRITD6Av1G218760.1
TRITD6Bv1G217600.1
TRITD3Bv1G085540.2
TRITD3Av1G031900.20
TRITD3Bv1G038950.9
TRITD1Av1G160370.1
Glycophorin_A Family
Glycophorin_A Family
PPR_2 Repeat
PPR_3 Repeat
PPR_1 Repeat
PPR Family
PPR_2 Repeat
PPR_2 Repeat
PPR Family
PPR_2 Repeat
DYW_deaminase Family
DYW_deaminase Family
PPR_2 Repeat
PPR Family
PPR_2 Repeat
PPR_2 Repeat
PPR Family
PPR_1 Repeat
PPR_3 Repeat
PPR_2 Repeat
Domain Color
PK_Tyr_Ser−Thr Domain
Pkinase Domain
other Domain
−500
0
500
1000
1500
2000
2500

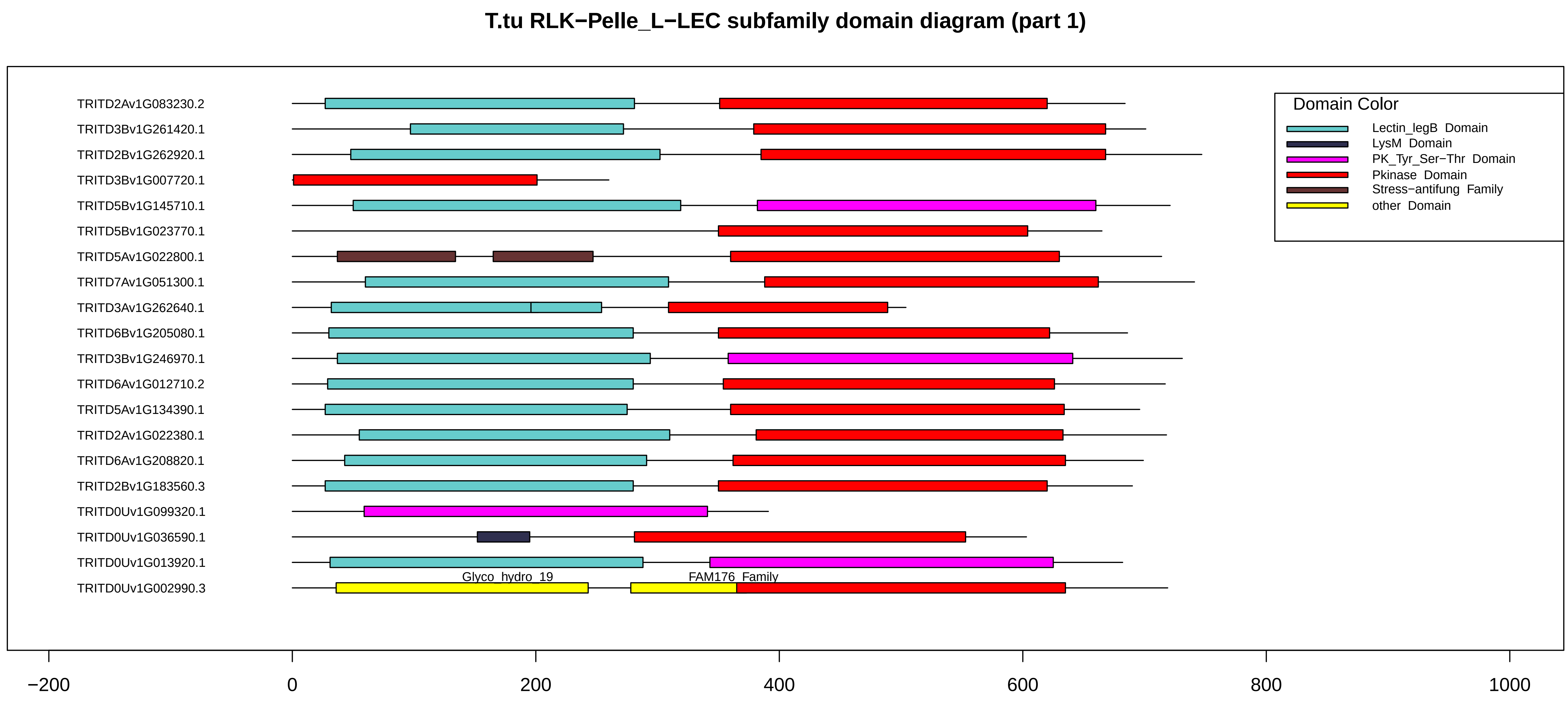

T.tu RLK−Pelle_L−LEC subfamily domain diagram (part 1)
TRITD2Av1G083230.2
TRITD3Bv1G261420.1
TRITD2Bv1G262920.1
TRITD3Bv1G007720.1
TRITD5Bv1G145710.1
TRITD5Bv1G023770.1
TRITD5Av1G022800.1
TRITD7Av1G051300.1
TRITD3Av1G262640.1
TRITD6Bv1G205080.1
TRITD3Bv1G246970.1
TRITD6Av1G012710.2
TRITD5Av1G134390.1
TRITD2Av1G022380.1
TRITD6Av1G208820.1
TRITD2Bv1G183560.3
TRITD0Uv1G099320.1
TRITD0Uv1G036590.1
TRITD0Uv1G013920.1
TRITD0Uv1G002990.3
Glyco_hydro_19
FAM176_Family
Domain Color
Lectin_legB Domain
LysM Domain
PK_Tyr_Ser−Thr Domain
Pkinase Domain
Stress−antifung Family
other Domain
−200
0
200
400
600
800
1000

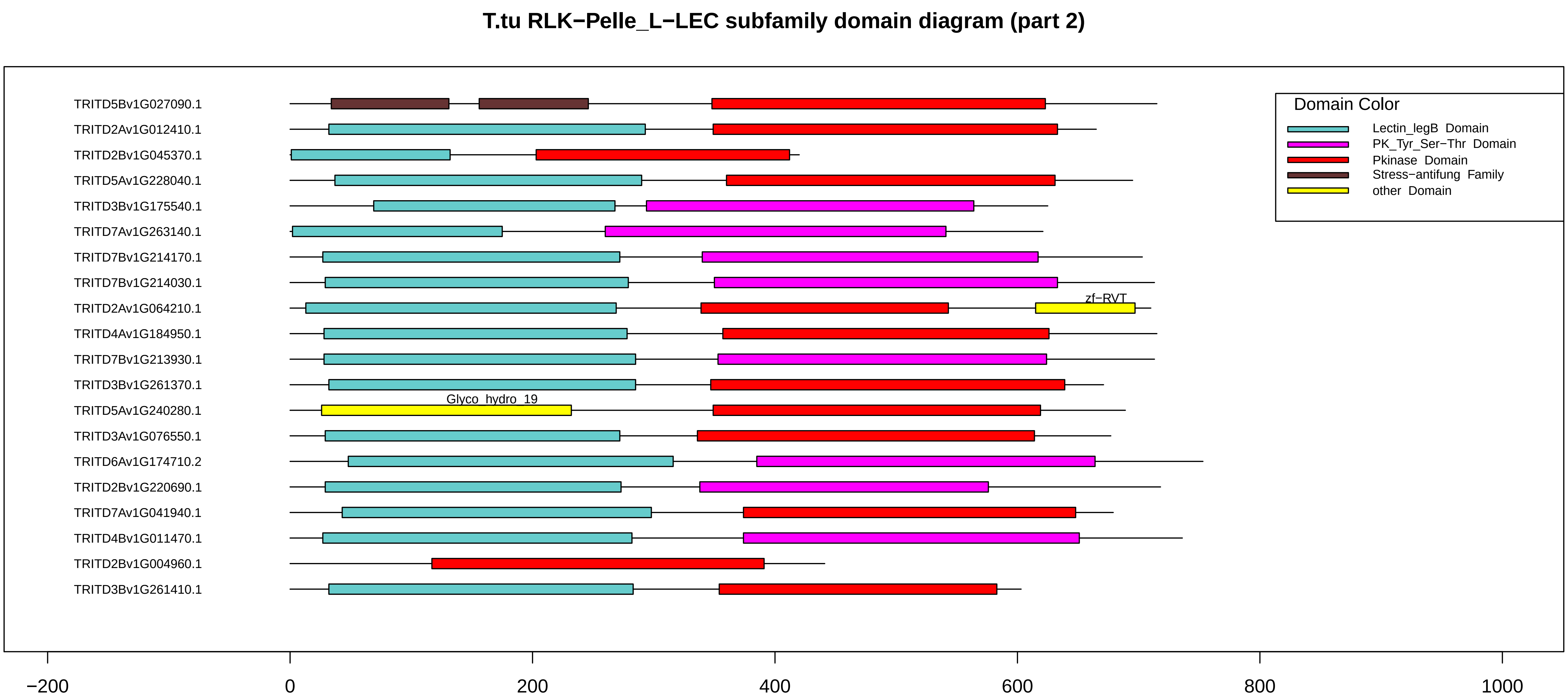
T.tu RLK−Pelle_L−LEC subfamily domain diagram (part 2)
TRITD5Bv1G027090.1
TRITD2Av1G012410.1
TRITD2Bv1G045370.1
TRITD5Av1G228040.1
TRITD3Bv1G175540.1
TRITD7Av1G263140.1
TRITD7Bv1G214170.1
TRITD7Bv1G214030.1
TRITD2Av1G064210.1
TRITD4Av1G184950.1
TRITD7Bv1G213930.1
TRITD3Bv1G261370.1
TRITD5Av1G240280.1
TRITD3Av1G076550.1
TRITD6Av1G174710.2
TRITD2Bv1G220690.1
TRITD7Av1G041940.1
TRITD4Bv1G011470.1
TRITD2Bv1G004960.1
TRITD3Bv1G261410.1
zf−RVT
Glyco_hydro_19
Domain Color
Lectin_legB Domain
PK_Tyr_Ser−Thr Domain
Pkinase Domain
Stress−antifung Family
other Domain
−200
0
200
400
600
800
1000

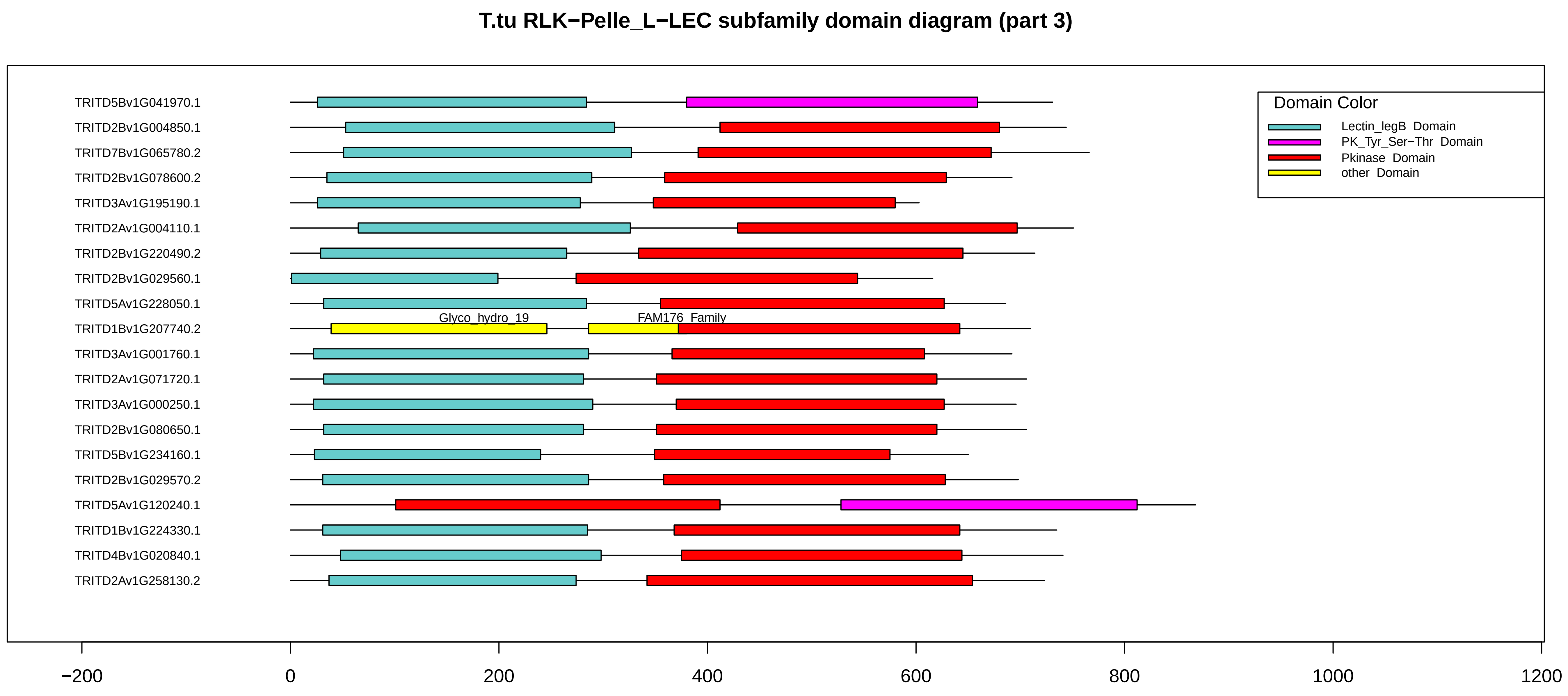
T.tu RLK−Pelle_L−LEC subfamily domain diagram (part 3)
TRITD5Bv1G041970.1
TRITD2Bv1G004850.1
TRITD7Bv1G065780.2
TRITD2Bv1G078600.2
TRITD3Av1G195190.1
TRITD2Av1G004110.1
TRITD2Bv1G220490.2
TRITD2Bv1G029560.1
TRITD5Av1G228050.1
TRITD1Bv1G207740.2
Glyco_hydro_19
FAM176_Family
TRITD3Av1G001760.1
TRITD2Av1G071720.1
TRITD3Av1G000250.1
TRITD2Bv1G080650.1
TRITD5Bv1G234160.1
TRITD2Bv1G029570.2
TRITD5Av1G120240.1
TRITD1Bv1G224330.1
TRITD4Bv1G020840.1
TRITD2Av1G258130.2
Domain Color
Lectin_legB Domain
PK_Tyr_Ser−Thr Domain
Pkinase Domain
other Domain
−200
0
200
400
600
800
1000
1200

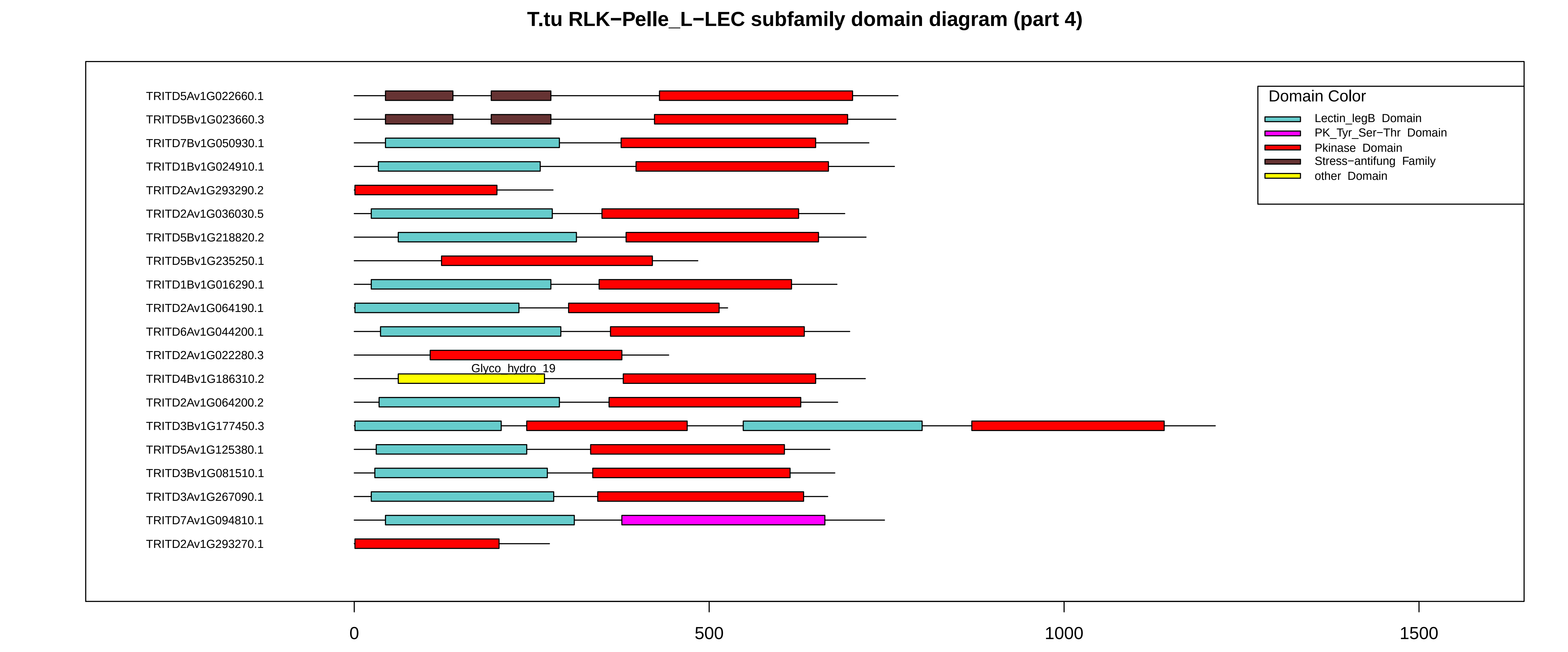
T.tu RLK−Pelle_L−LEC subfamily domain diagram (part 4)
TRITD5Av1G022660.1
TRITD5Bv1G023660.3
TRITD7Bv1G050930.1
TRITD1Bv1G024910.1
TRITD2Av1G293290.2
TRITD2Av1G036030.5
TRITD5Bv1G218820.2
TRITD5Bv1G235250.1
TRITD1Bv1G016290.1
TRITD2Av1G064190.1
TRITD6Av1G044200.1
TRITD2Av1G022280.3
TRITD4Bv1G186310.2
Glyco_hydro_19
TRITD2Av1G064200.2
TRITD3Bv1G177450.3
TRITD5Av1G125380.1
TRITD3Bv1G081510.1
TRITD3Av1G267090.1
TRITD7Av1G094810.1
TRITD2Av1G293270.1
Domain Color
Lectin_legB Domain
PK_Tyr_Ser−Thr Domain
Pkinase Domain
Stress−antifung Family
other Domain
0
500
1000
1500

T.tu RLK−Pelle_L−LEC subfamily domain diagram (part 5)

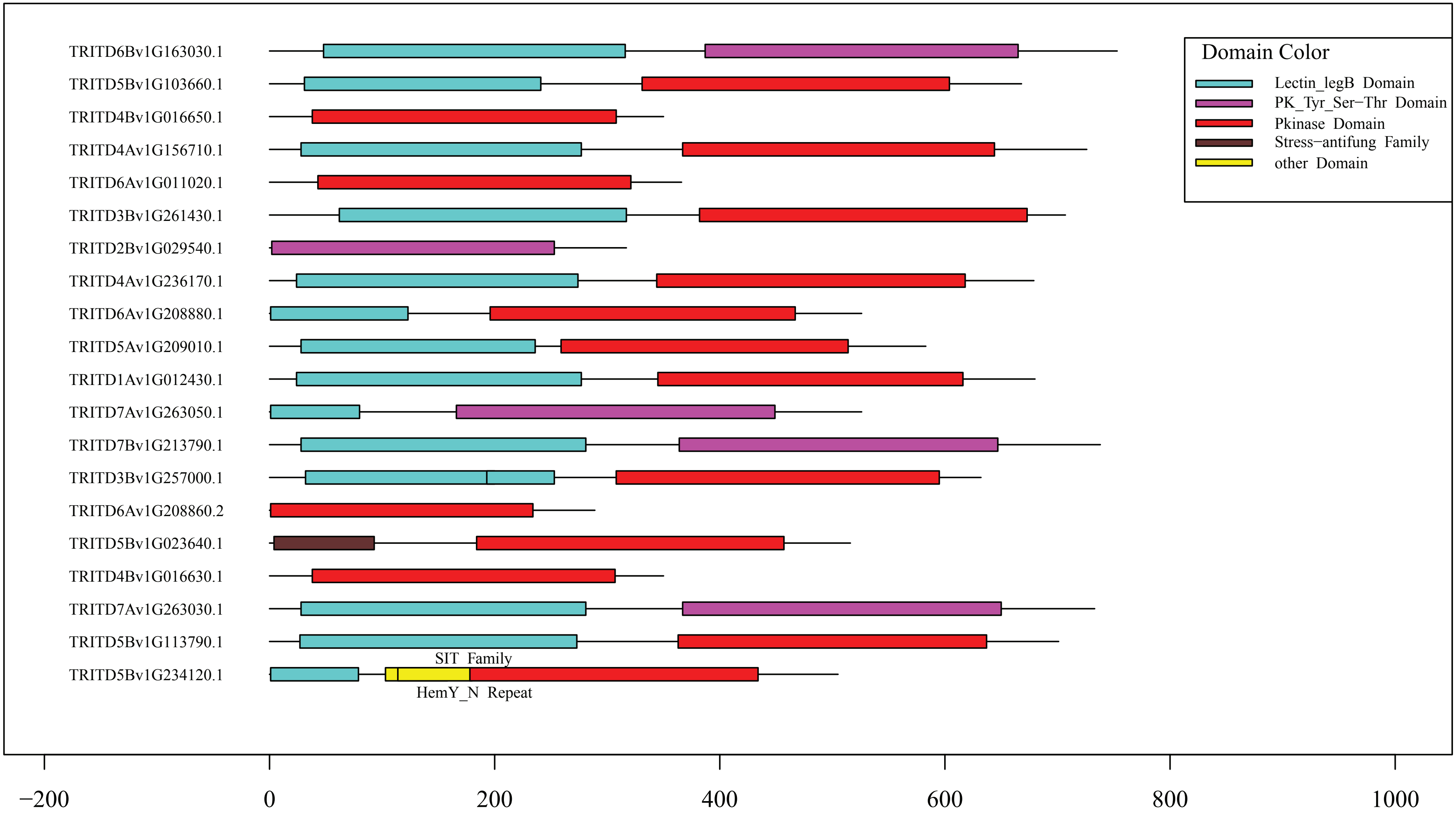

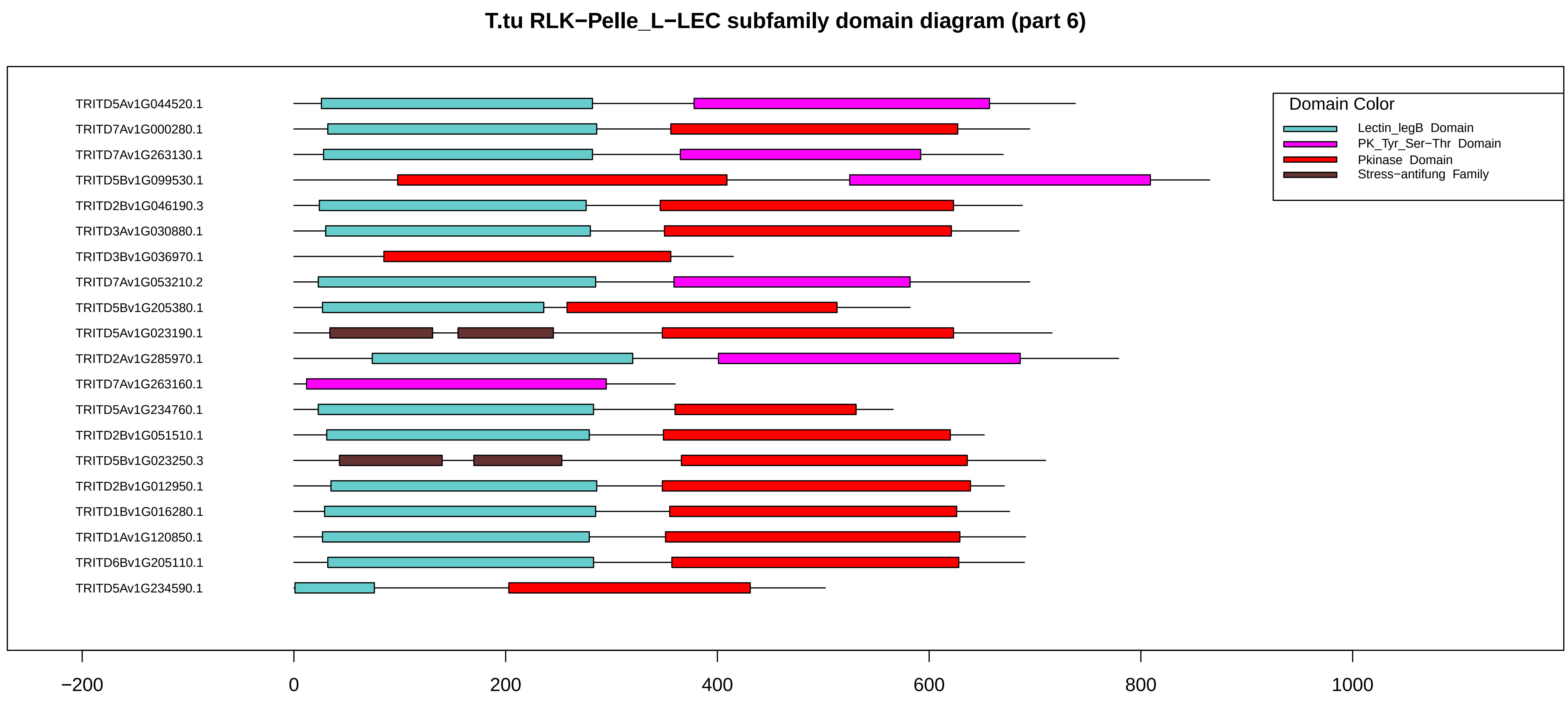
T.tu RLK−Pelle_L−LEC subfamily domain diagram (part 6)
TRITD5Av1G044520.1
TRITD7Av1G000280.1
TRITD7Av1G263130.1
TRITD5Bv1G099530.1
TRITD2Bv1G046190.3
TRITD3Av1G030880.1
TRITD3Bv1G036970.1
TRITD7Av1G053210.2
TRITD5Bv1G205380.1
TRITD5Av1G023190.1
TRITD2Av1G285970.1
TRITD7Av1G263160.1
TRITD5Av1G234760.1
TRITD2Bv1G051510.1
TRITD5Bv1G023250.3
TRITD2Bv1G012950.1
TRITD1Bv1G016280.1
TRITD1Av1G120850.1
TRITD6Bv1G205110.1
TRITD5Av1G234590.1
Domain Color
Lectin_legB Domain
PK_Tyr_Ser−Thr Domain
Pkinase Domain
Stress−antifung Family
−200
0
200
400
600
800
1000

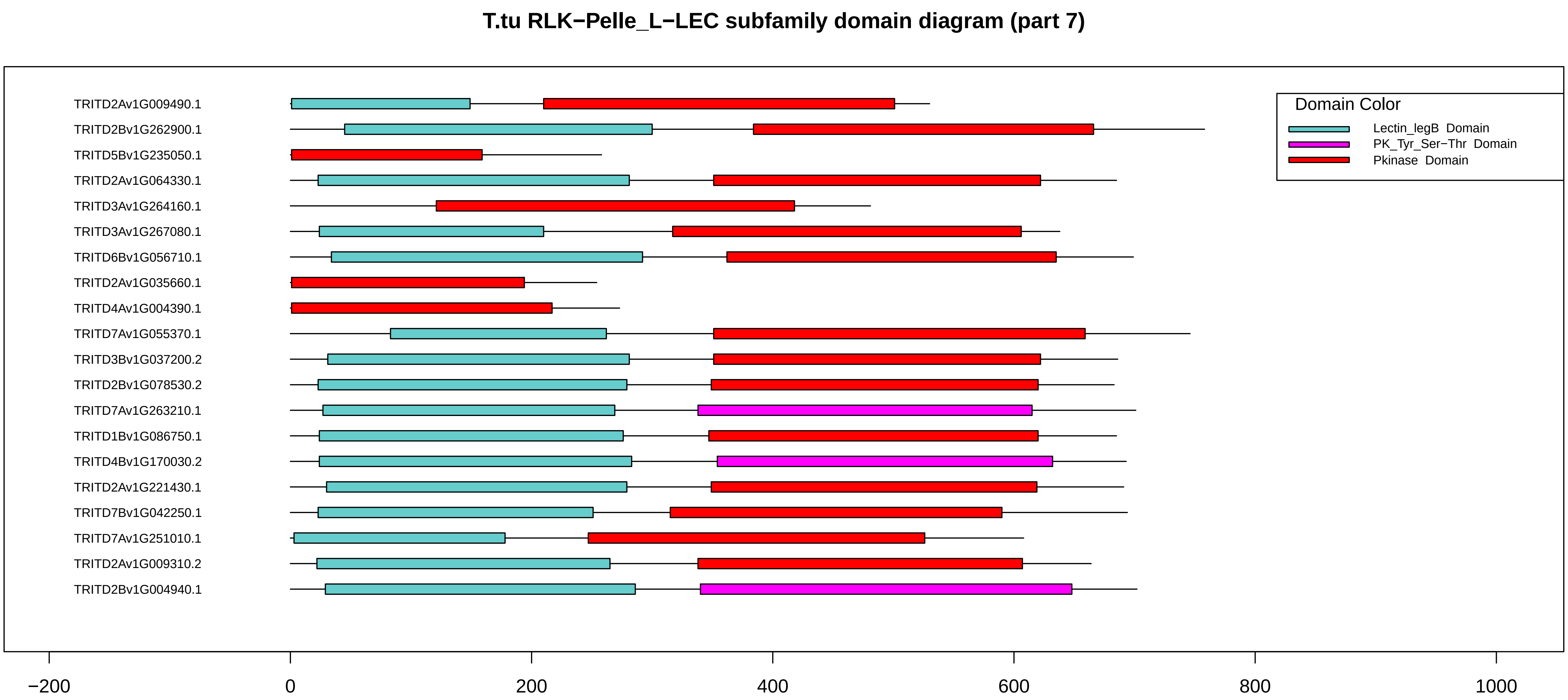

T.tu RLK−Pelle_L−LEC subfamily domain diagram (part 7)
TRITD2Av1G009490.1
TRITD2Bv1G262900.1
TRITD5Bv1G235050.1
TRITD2Av1G064330.1
TRITD3Av1G264160.1
TRITD3Av1G267080.1
TRITD6Bv1G056710.1
TRITD2Av1G035660.1
TRITD4Av1G004390.1
TRITD7Av1G055370.1
TRITD3Bv1G037200.2
TRITD2Bv1G078530.2
TRITD7Av1G263210.1
TRITD1Bv1G086750.1
TRITD4Bv1G170030.2
TRITD2Av1G221430.1
TRITD7Bv1G042250.1
TRITD7Av1G251010.1
TRITD2Av1G009310.2
TRITD2Bv1G004940.1
Domain Color
Lectin_legB Domain
PK_Tyr_Ser−Thr Domain
Pkinase Domain
−200
0
200
400
600
800
1000

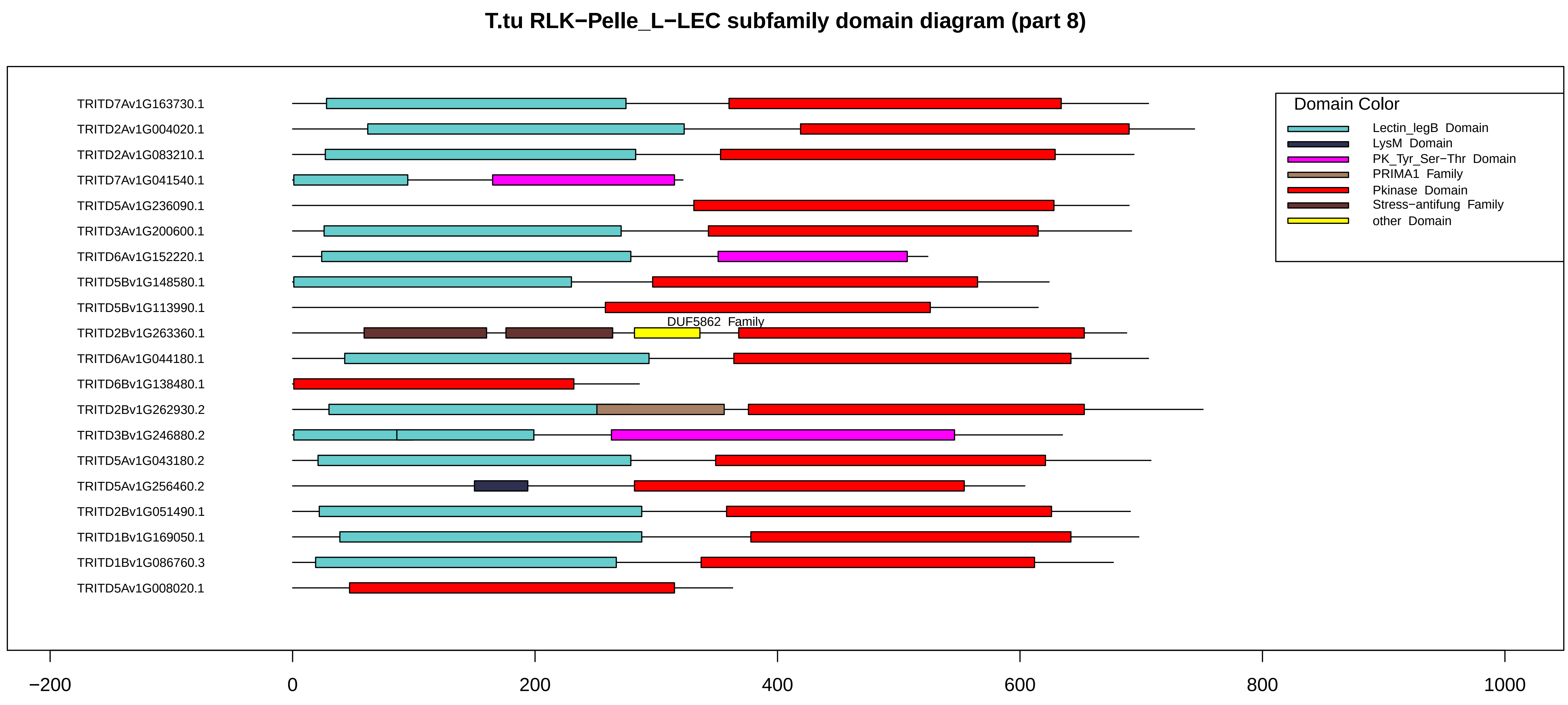
T.tu RLK−Pelle_L−LEC subfamily domain diagram (part 8)
TRITD7Av1G163730.1
TRITD2Av1G004020.1
TRITD2Av1G083210.1
TRITD7Av1G041540.1
TRITD5Av1G236090.1
TRITD3Av1G200600.1
TRITD6Av1G152220.1
TRITD5Bv1G148580.1
TRITD5Bv1G113990.1
TRITD2Bv1G263360.1
TRITD6Av1G044180.1
TRITD6Bv1G138480.1
TRITD2Bv1G262930.2
TRITD3Bv1G246880.2
TRITD5Av1G043180.2
TRITD5Av1G256460.2
TRITD2Bv1G051490.1
TRITD1Bv1G169050.1
TRITD1Bv1G086760.3
TRITD5Av1G008020.1
DUF5862 Family
Domain Color
Lectin_legB Domain
LysM Domain
PK_Tyr_Ser−Thr Domain
PRIMA1 Family
Pkinase Domain
Stress−antifung Family
other Domain
−200
0
200
400
600
800
1000

T.tu RLK−Pelle_L−LEC subfamily domain diagram (part 9)

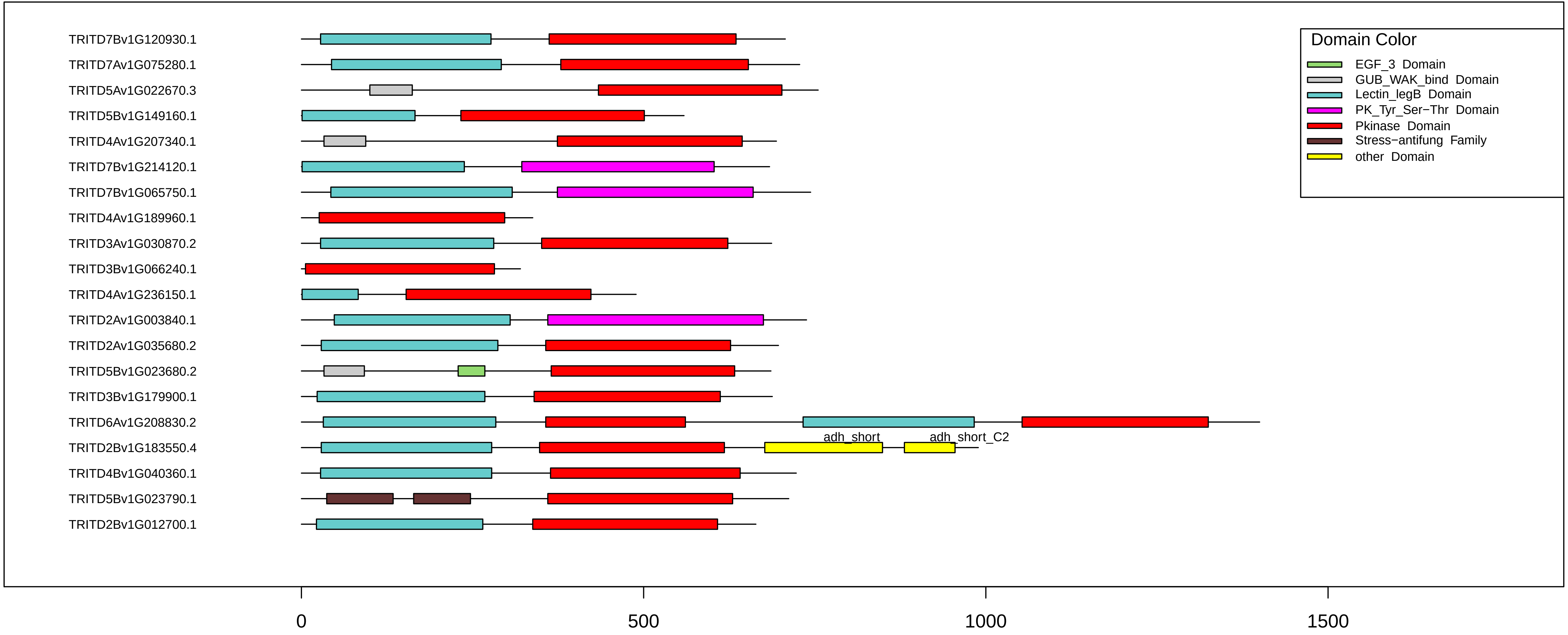

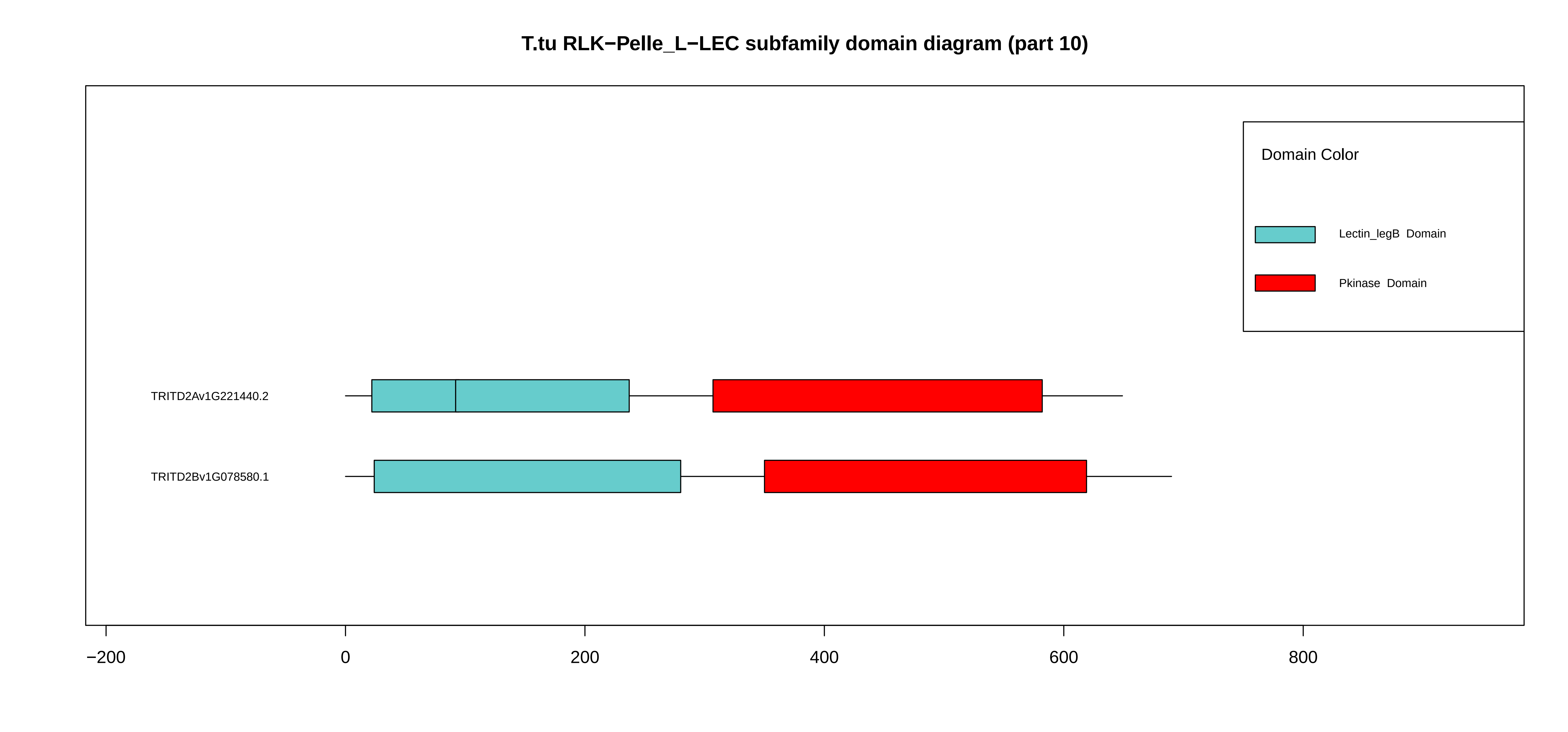
T.tu RLK−Pelle_L−LEC subfamily domain diagram (part 10)
Domain Color
Lectin_legB Domain
Pkinase Domain
TRITD2Av1G221440.2
TRITD2Bv1G078580.1
−200
0
200
400
600
800

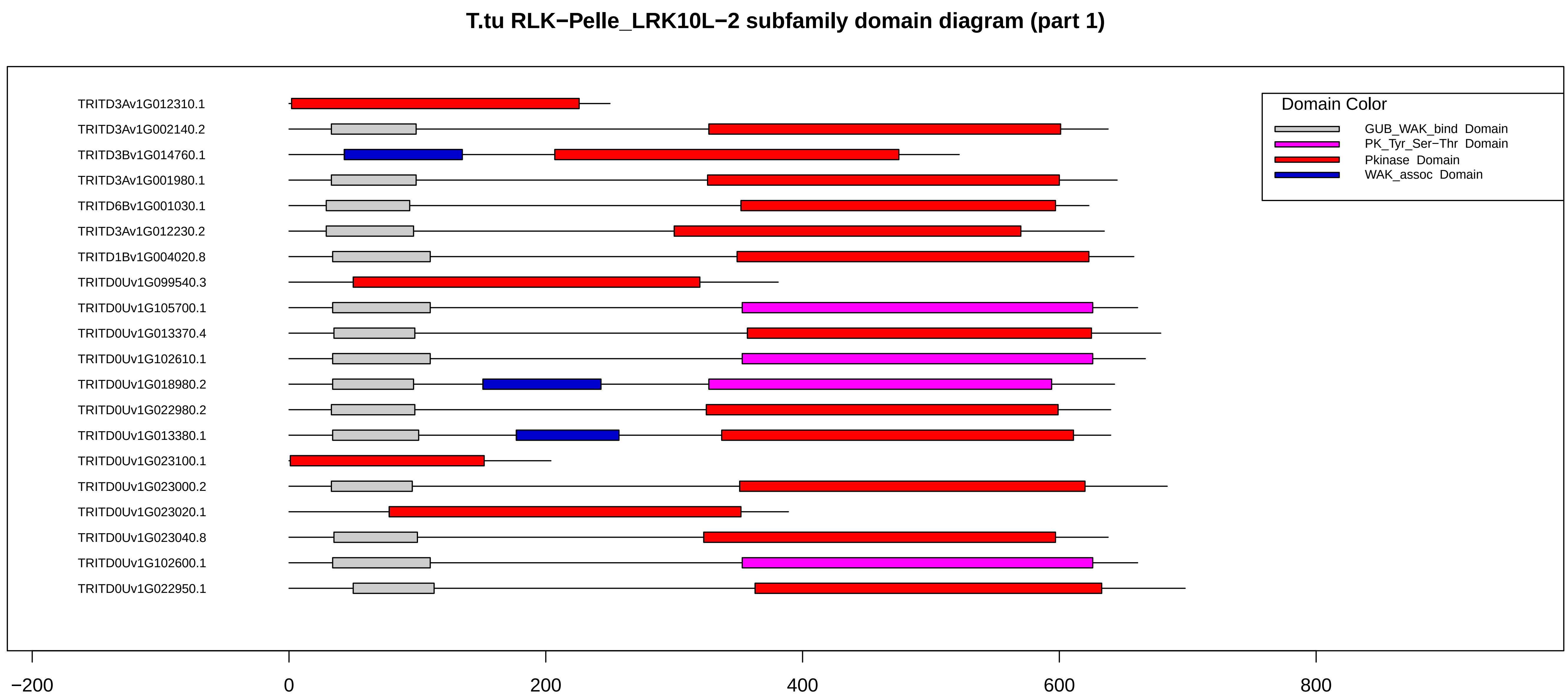
T.tu RLK−Pelle_LRK10L−2 subfamily domain diagram (part 1)
TRITD3Av1G012310.1
TRITD3Av1G002140.2
TRITD3Bv1G014760.1
TRITD3Av1G001980.1
TRITD6Bv1G001030.1
TRITD3Av1G012230.2
TRITD1Bv1G004020.8
TRITD0Uv1G099540.3
TRITD0Uv1G105700.1
TRITD0Uv1G013370.4
TRITD0Uv1G102610.1
TRITD0Uv1G018980.2
TRITD0Uv1G022980.2
TRITD0Uv1G013380.1
TRITD0Uv1G023100.1
TRITD0Uv1G023000.2
TRITD0Uv1G023020.1
TRITD0Uv1G023040.8
TRITD0Uv1G102600.1
TRITD0Uv1G022950.1
Domain Color
GUB_WAK_bind Domain
PK_Tyr_Ser−Thr Domain
Pkinase Domain
WAK_assoc Domain
−200
0
200
400
600
800

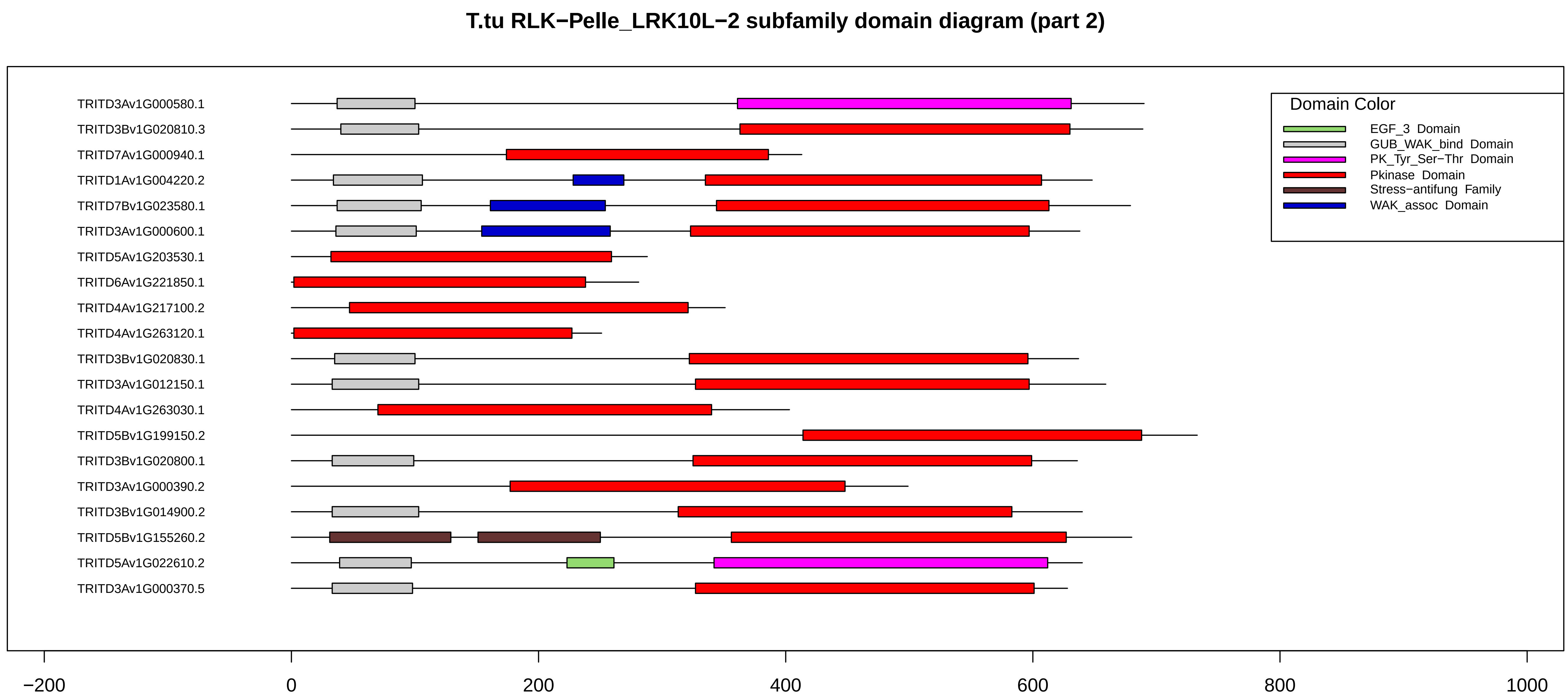
T.tu RLK−Pelle_LRK10L−2 subfamily domain diagram (part 2)
TRITD3Av1G000580.1
TRITD3Bv1G020810.3
TRITD7Av1G000940.1
TRITD1Av1G004220.2
TRITD7Bv1G023580.1
TRITD3Av1G000600.1
TRITD5Av1G203530.1
TRITD6Av1G221850.1
TRITD4Av1G217100.2
TRITD4Av1G263120.1
TRITD3Bv1G020830.1
TRITD3Av1G012150.1
TRITD4Av1G263030.1
TRITD5Bv1G199150.2
TRITD3Bv1G020800.1
TRITD3Av1G000390.2
TRITD3Bv1G014900.2
TRITD5Bv1G155260.2
TRITD5Av1G022610.2
TRITD3Av1G000370.5
Domain Color
EGF_3 Domain
GUB_WAK_bind Domain
PK_Tyr_Ser−Thr Domain
Pkinase Domain
Stress−antifung Family
WAK_assoc Domain
−200
0
200
400
600
800
1000

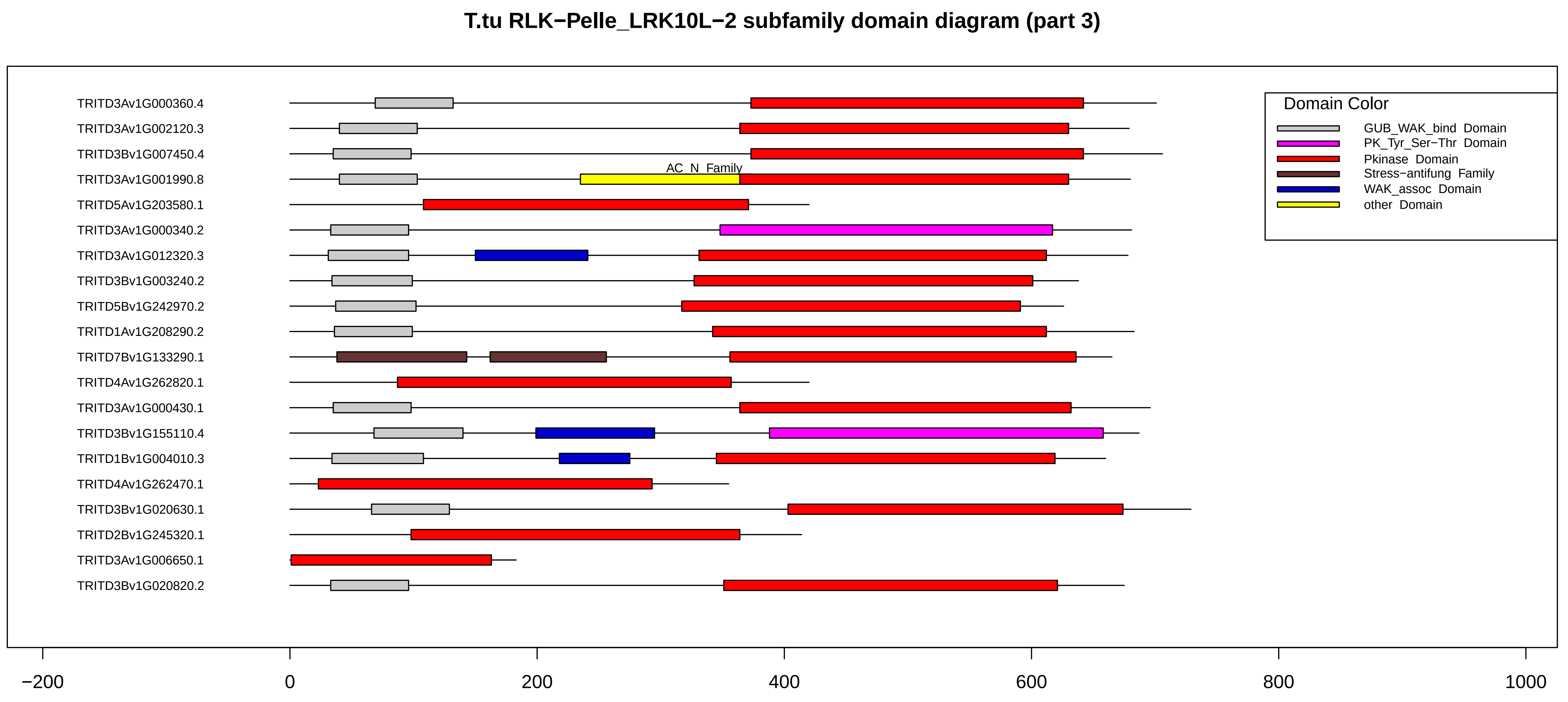
T.tu RLK−Pelle_LRK10L−2 subfamily domain diagram (part 3)
TRITD3Av1G000360.4
TRITD3Av1G002120.3
TRITD3Bv1G007450.4
TRITD3Av1G001990.8
TRITD5Av1G203580.1
TRITD3Av1G000340.2
TRITD3Av1G012320.3
TRITD3Bv1G003240.2
TRITD5Bv1G242970.2
TRITD1Av1G208290.2
TRITD7Bv1G133290.1
TRITD4Av1G262820.1
TRITD3Av1G000430.1
TRITD3Bv1G155110.4
TRITD1Bv1G004010.3
TRITD4Av1G262470.1
TRITD3Bv1G020630.1
TRITD2Bv1G245320.1
TRITD3Av1G006650.1
TRITD3Bv1G020820.2
AC_N_Family
Domain Color
GUB_WAK_bind Domain
PK_Tyr_Ser−Thr Domain
Pkinase Domain
Stress−antifung Family
WAK_assoc Domain
other Domain
−200
0
200
400
600
800
1000

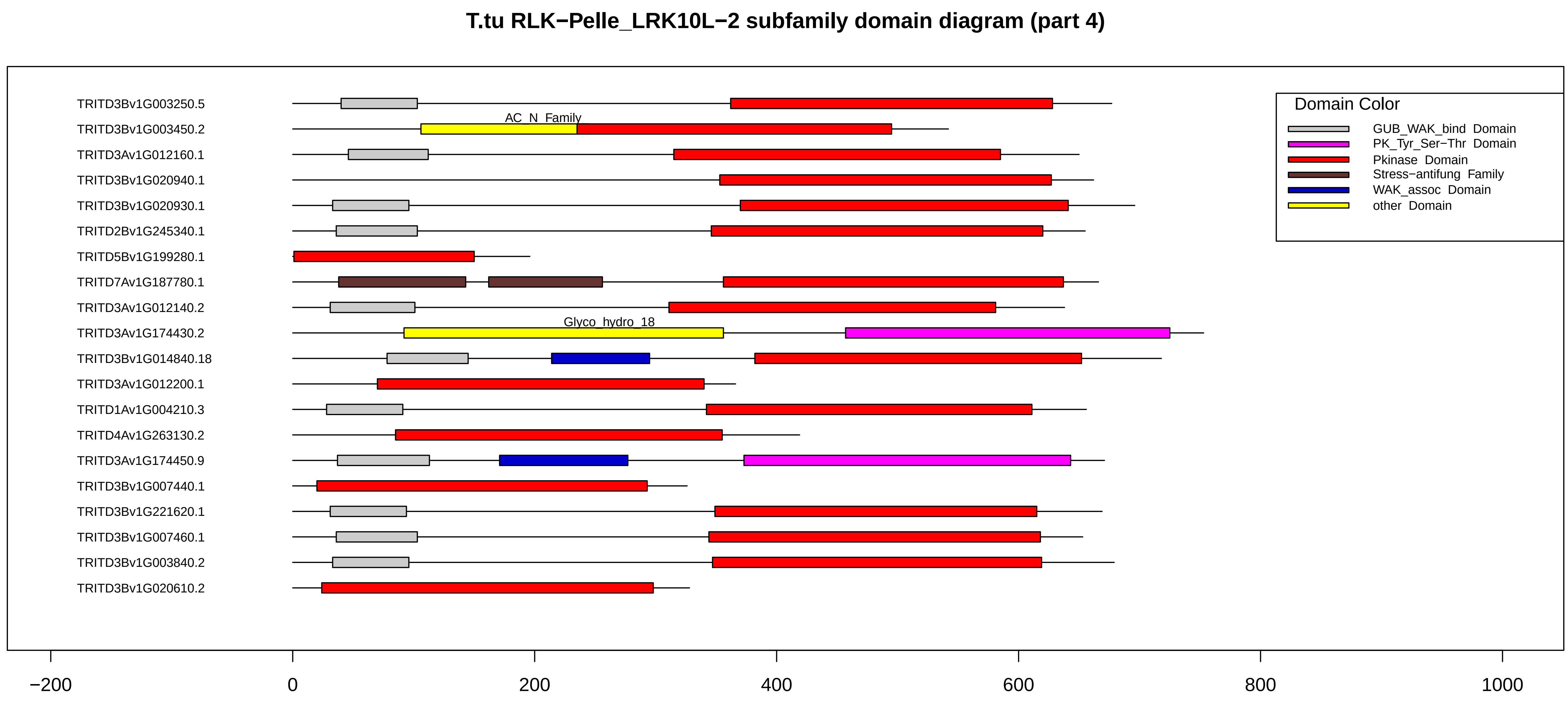

T.tu RLK−Pelle_LRK10L−2 subfamily domain diagram (part 4)
TRITD3Bv1G003250.5
TRITD3Bv1G003450.2
TRITD3Av1G012160.1
TRITD3Bv1G020940.1
TRITD3Bv1G020930.1
TRITD2Bv1G245340.1
TRITD5Bv1G199280.1
TRITD7Av1G187780.1
TRITD3Av1G012140.2
TRITD3Av1G174430.2
TRITD3Bv1G014840.18
TRITD3Av1G012200.1
TRITD1Av1G004210.3
TRITD4Av1G263130.2
TRITD3Av1G174450.9
TRITD3Bv1G007440.1
TRITD3Bv1G221620.1
TRITD3Bv1G007460.1
TRITD3Bv1G003840.2
TRITD3Bv1G020610.2
AC_N_Family
Glyco_hydro_18
Domain Color
GUB_WAK_bind Domain
PK_Tyr_Ser−Thr Domain
Pkinase Domain
Stress−antifung Family
WAK_assoc Domain
other Domain
−200
0
200
400
600
800
1000

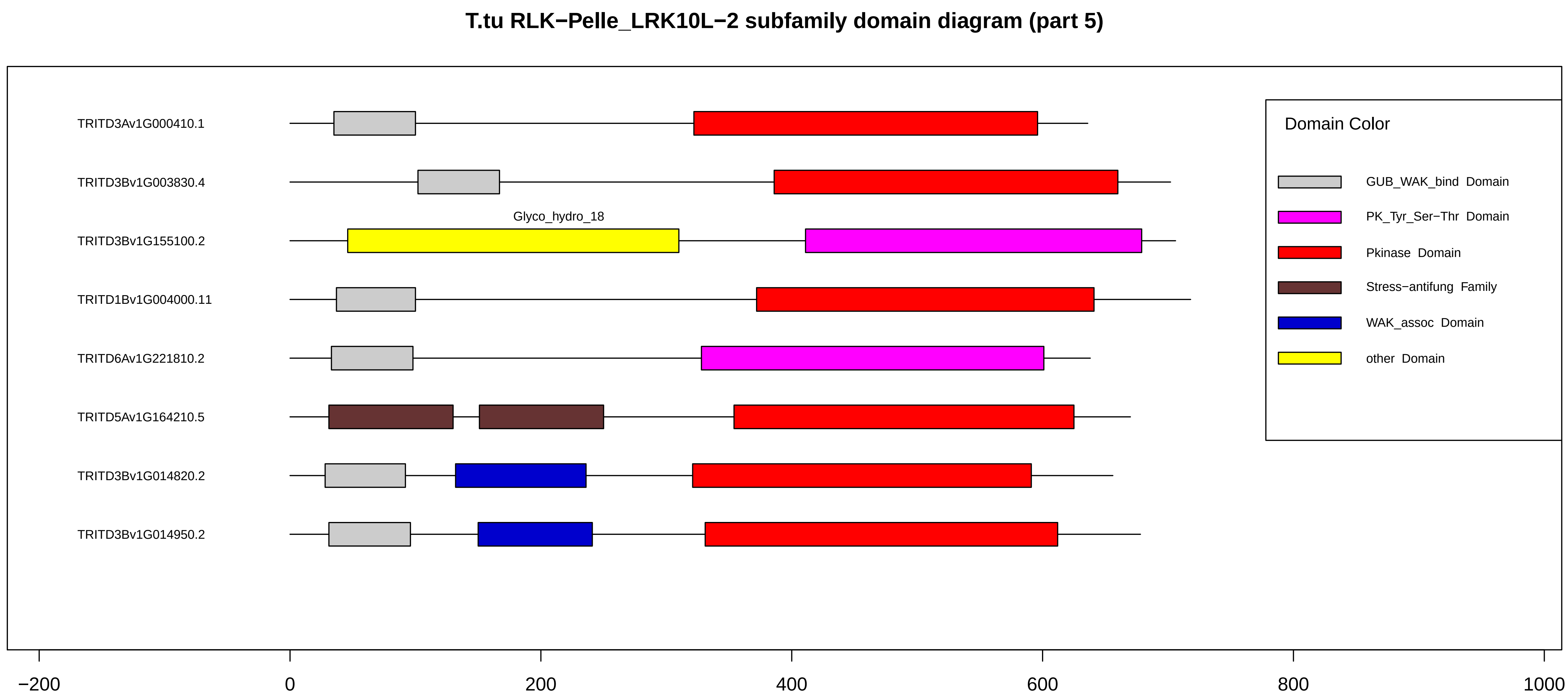
T.tu RLK−Pelle_LRK10L−2 subfamily domain diagram (part 5)
TRITD3Av1G000410.1
TRITD3Bv1G003830.4
Glyco_hydro_18
TRITD3Bv1G155100.2
TRITD1Bv1G004000.11
TRITD6Av1G221810.2
TRITD5Av1G164210.5
TRITD3Bv1G014820.2
TRITD3Bv1G014950.2
Domain Color
GUB_WAK_bind Domain
PK_Tyr_Ser−Thr Domain
Pkinase Domain
Stress−antifung Family
WAK_assoc Domain
other Domain
−200
0
200
400
600
800
1000

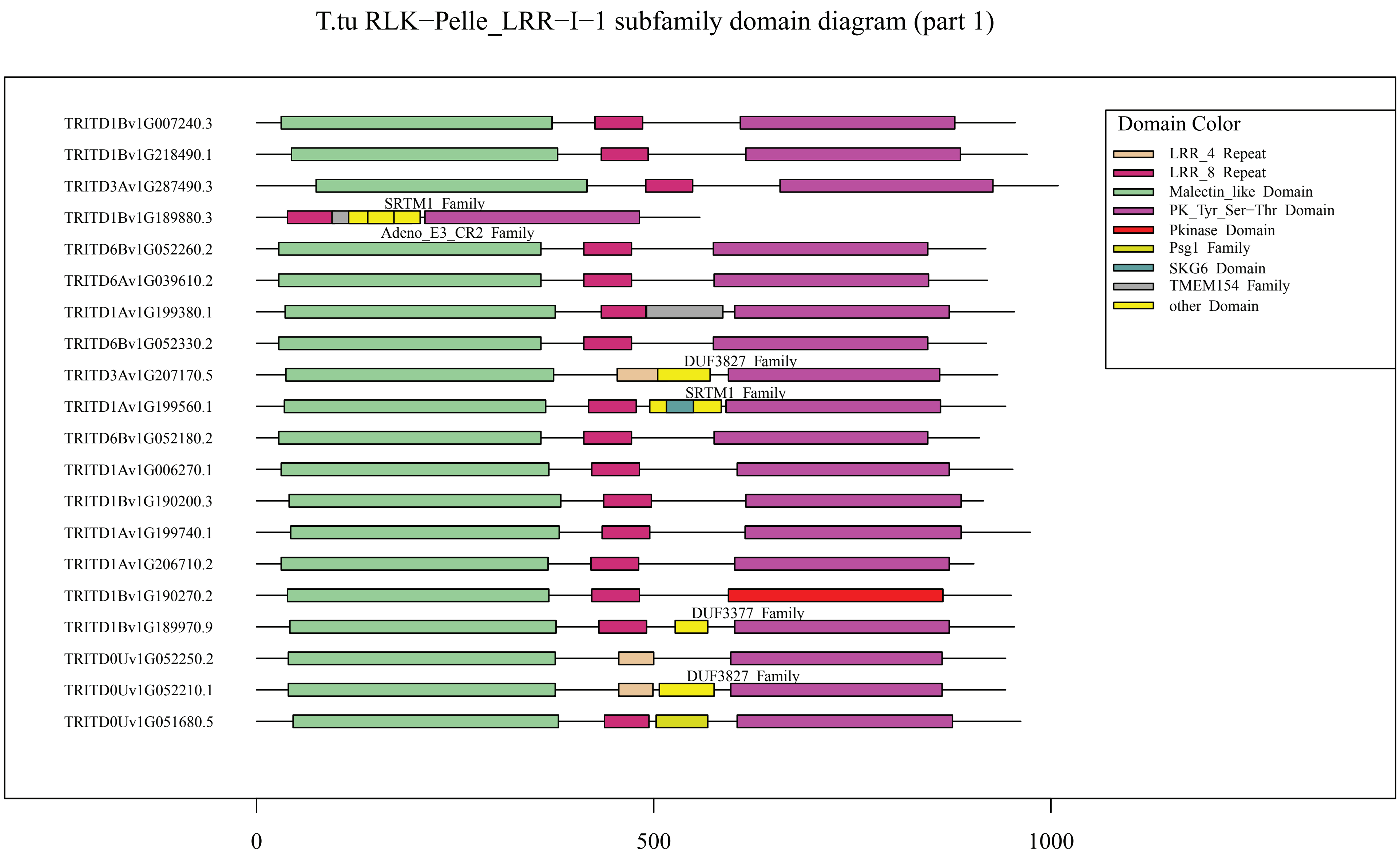

T.tu RLK−Pelle_LRR−I−1 subfamily domain diagram (part 1)
TRITD1Bv1G007240.3
TRITD1Bv1G218490.1
TRITD3Av1G287490.3
TRITD1Bv1G189880.3
TRITD6Bv1G052260.2
TRITD6Av1G039610.2
TRITD1Av1G199380.1
TRITD6Bv1G052330.2
TRITD3Av1G207170.5
TRITD1Av1G199560.1
TRITD6Bv1G052180.2
TRITD1Av1G006270.1
TRITD1Bv1G190200.3
TRITD1Av1G199740.1
TRITD1Av1G206710.2
TRITD1Bv1G190270.2
TRITD1Bv1G189970.9
TRITD0Uv1G052250.2
TRITD0Uv1G052210.1
TRITD0Uv1G051680.5
SRTM1_Family
Adeno_E3_CR2_Family
DUF3827_Family
SRTM1_Family
DUF3377_Family
DUF3827_Family
Domain Color
LRR_4_Repeat
LRR_8_Repeat
Malectin_like_Domain
PK_Tyr_Ser−Thr_Domain
Pkinase_Domain
Psg1_Family
SKG6_Domain
TMEM154_Family
other_Domain
0
500
1000

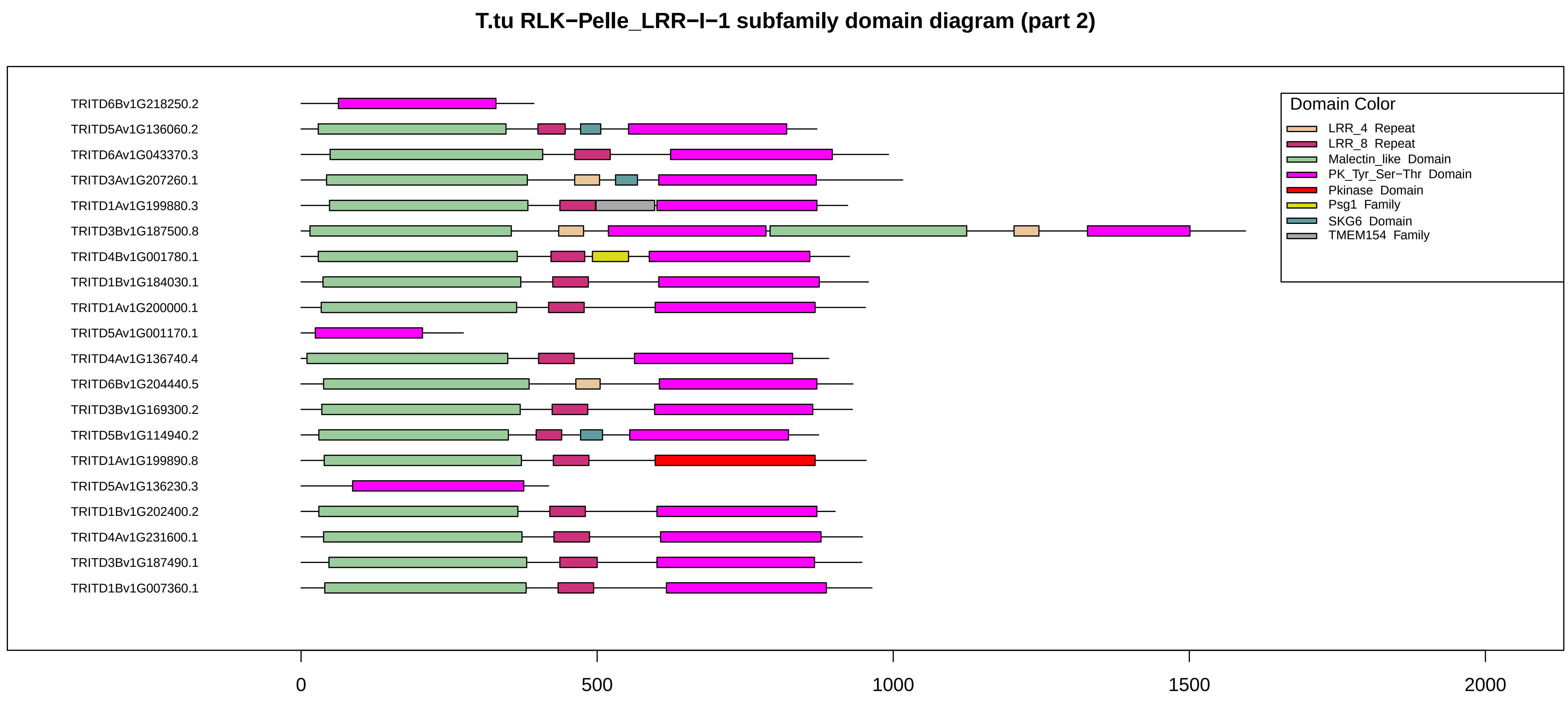
T.tu RLK−Pelle_LRR−I−1 subfamily domain diagram (part 2)
TRITD6Bv1G218250.2
TRITD5Av1G136060.2
TRITD6Av1G043370.3
TRITD3Av1G207260.1
TRITD1Av1G199880.3
TRITD3Bv1G187500.8
TRITD4Bv1G001780.1
TRITD1Bv1G184030.1
TRITD1Av1G200000.1
TRITD5Av1G001170.1
TRITD4Av1G136740.4
TRITD6Bv1G204440.5
TRITD3Bv1G169300.2
TRITD5Bv1G114940.2
TRITD1Av1G199890.8
TRITD5Av1G136230.3
TRITD1Bv1G202400.2
TRITD4Av1G231600.1
TRITD3Bv1G187490.1
TRITD1Bv1G007360.1
Domain Color
LRR_4 Repeat
LRR_8 Repeat
Malectin_like Domain
PK_Tyr_Ser−Thr Domain
Pkinase Domain
Psg1 Family
SKG6 Domain
TMEM154 Family
0
500
1000
1500
2000

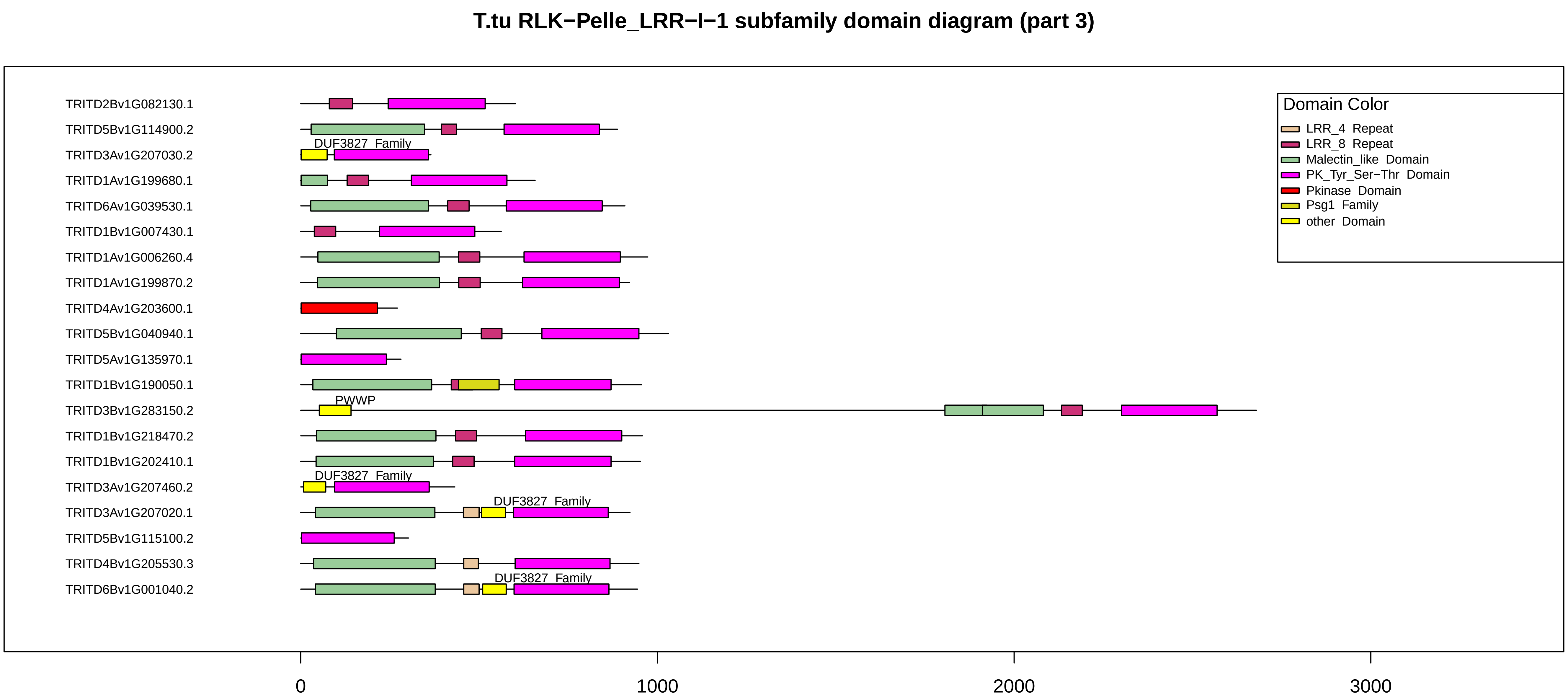
T.tu RLK−Pelle_LRR−I−1 subfamily domain diagram (part 3)
TRITD2Bv1G082130.1
TRITD5Bv1G114900.2
DUF3827_Family
TRITD3Av1G207030.2
TRITD1Av1G199680.1
TRITD6Av1G039530.1
TRITD1Bv1G007430.1
TRITD1Av1G006260.4
TRITD1Av1G199870.2
TRITD4Av1G203600.1
TRITD5Bv1G040940.1
TRITD5Av1G135970.1
TRITD1Bv1G190050.1
PWWP
TRITD3Bv1G283150.2
TRITD1Bv1G218470.2
TRITD1Bv1G202410.1
DUF3827_Family
TRITD3Av1G207460.2
DUF3827_Family
TRITD3Av1G207020.1
TRITD5Bv1G115100.2
TRITD4Bv1G205530.3
DUF3827_Family
TRITD6Bv1G001040.2
Domain Color
LRR_4 Repeat
LRR_8 Repeat
Malectin_like Domain
PK_Tyr_Ser−Thr Domain
Pkinase Domain
Psg1 Family
other Domain
0
1000
2000
3000

T.tu RLK−Pelle_LRR−I−1 subfamily domain diagram (part 4)

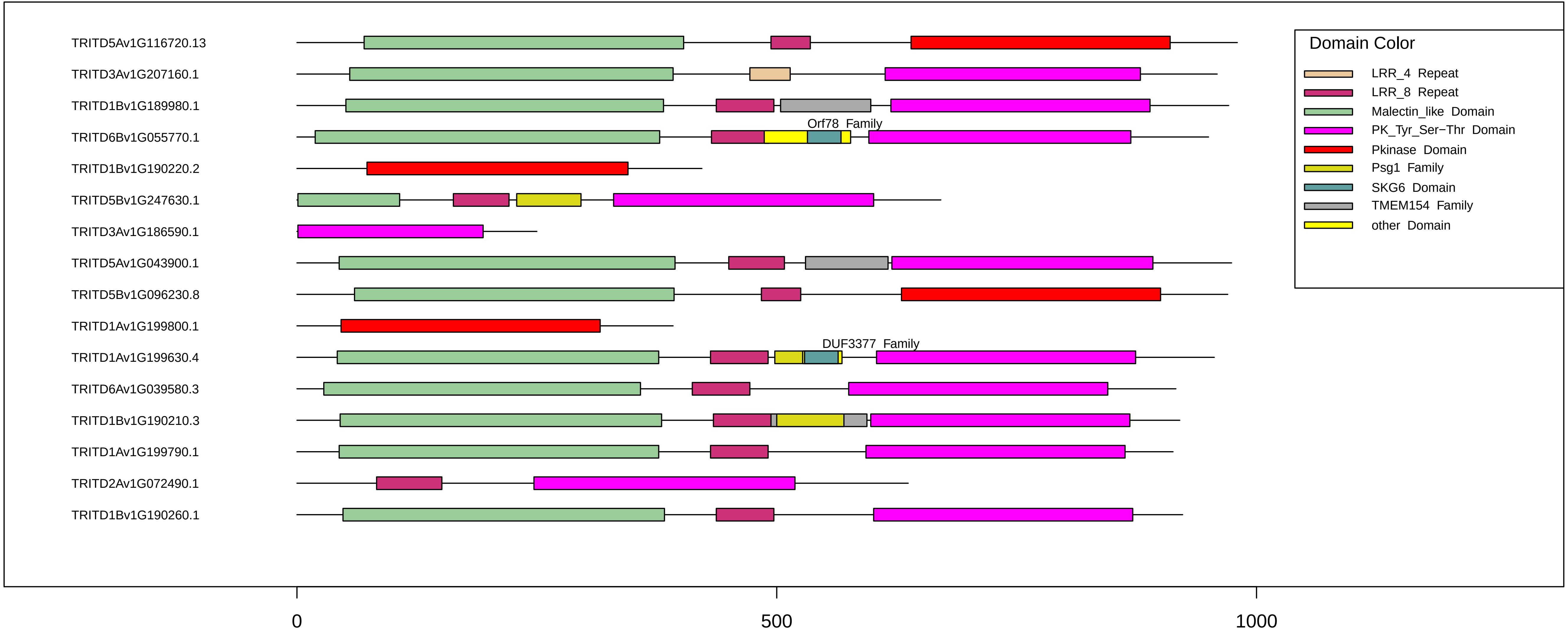

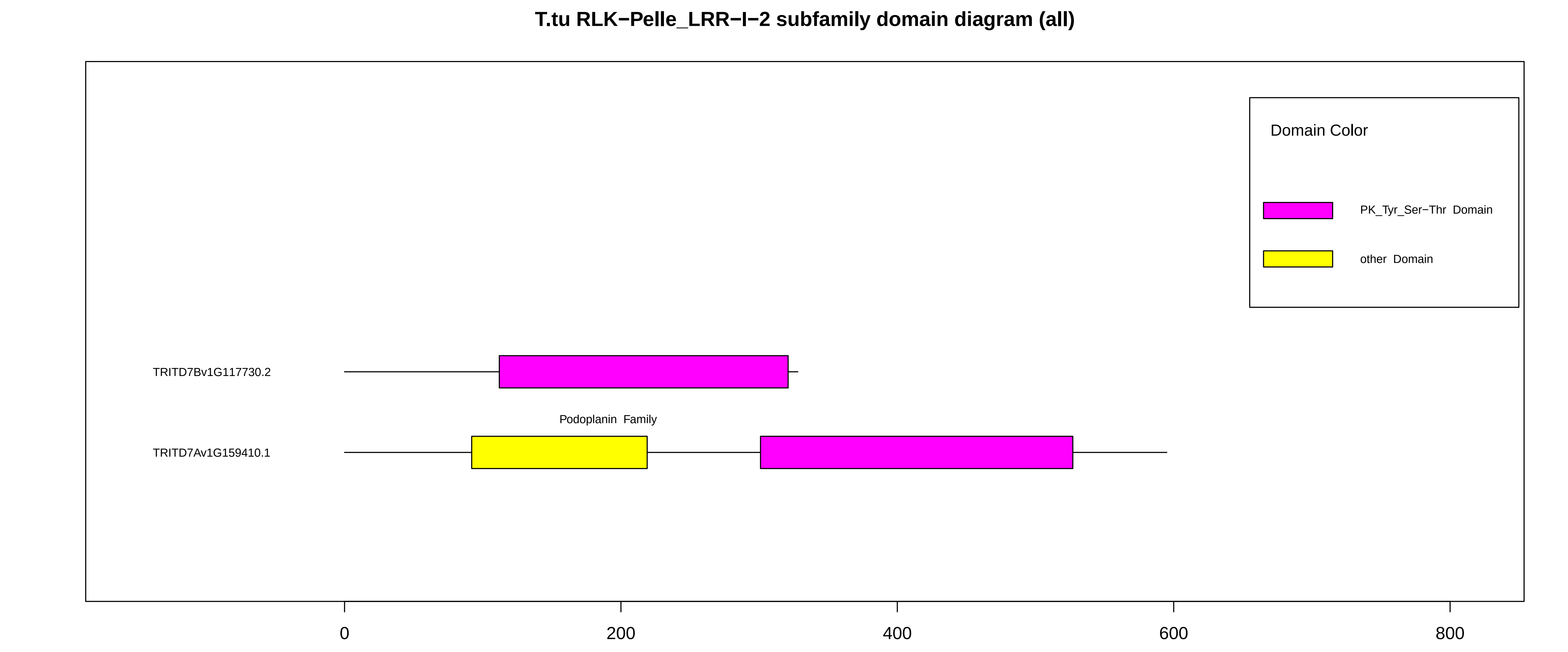

T.tu RLK−Pelle_LRR−I−2 subfamily domain diagram (all)
Domain Color
PK_Tyr_Ser−Thr Domain
other Domain
TRITD7Bv1G117730.2
Podoplanin Family
TRITD7Av1G159410.1
0
200
400
600
800

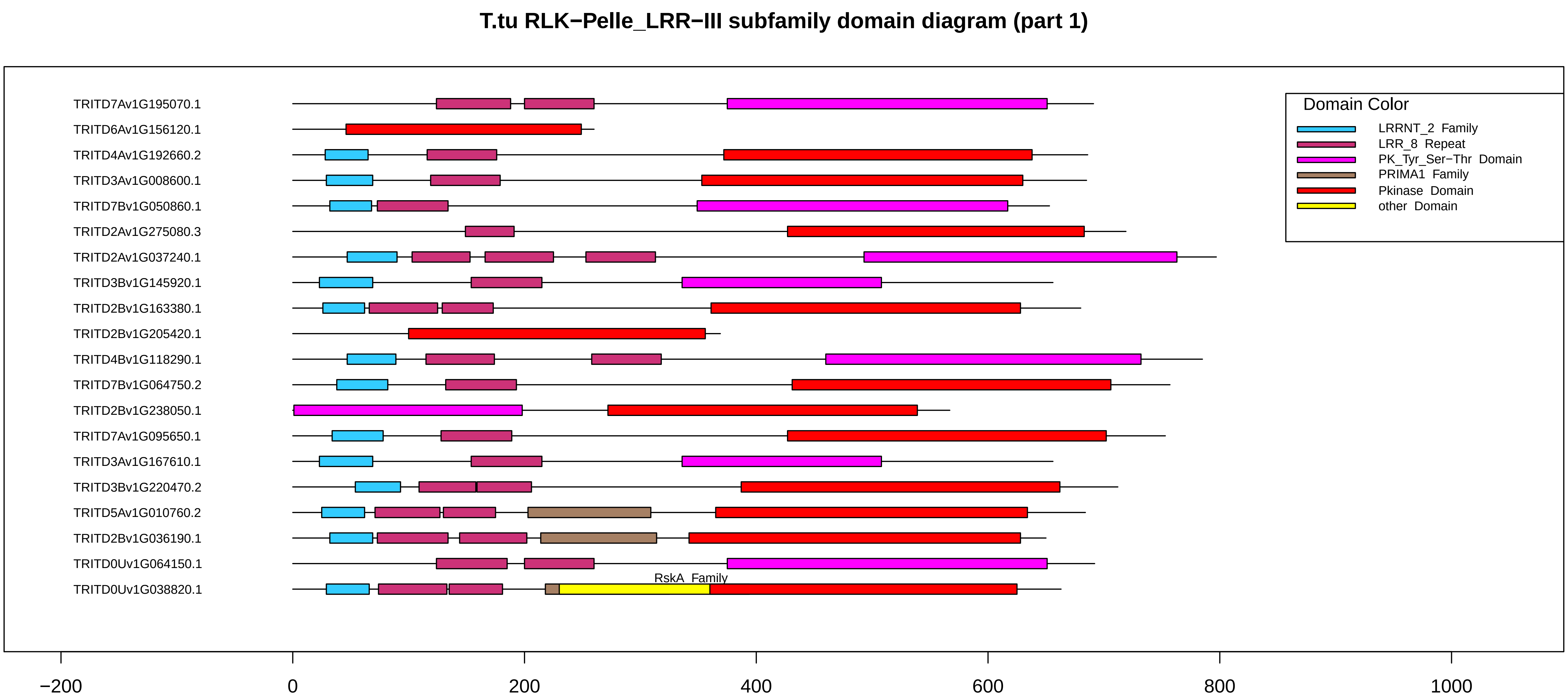
T.tu RLK−Pelle_LRR−III subfamily domain diagram (part 1)
TRITD7Av1G195070.1
TRITD6Av1G156120.1
TRITD4Av1G192660.2
TRITD3Av1G008600.1
TRITD7Bv1G050860.1
TRITD2Av1G275080.3
TRITD2Av1G037240.1
TRITD3Bv1G145920.1
TRITD2Bv1G163380.1
TRITD2Bv1G205420.1
TRITD4Bv1G118290.1
TRITD7Bv1G064750.2
TRITD2Bv1G238050.1
TRITD7Av1G095650.1
TRITD3Av1G167610.1
TRITD3Bv1G220470.2
TRITD5Av1G010760.2
TRITD2Bv1G036190.1
TRITD0Uv1G064150.1
TRITD0Uv1G038820.1
RskA Family
Domain Color
LRRNT_2 Family
LRR_8 Repeat
PK_Tyr_Ser−Thr Domain
PRIMA1 Family
Pkinase Domain
other Domain
−200
0
200
400
600
800
1000

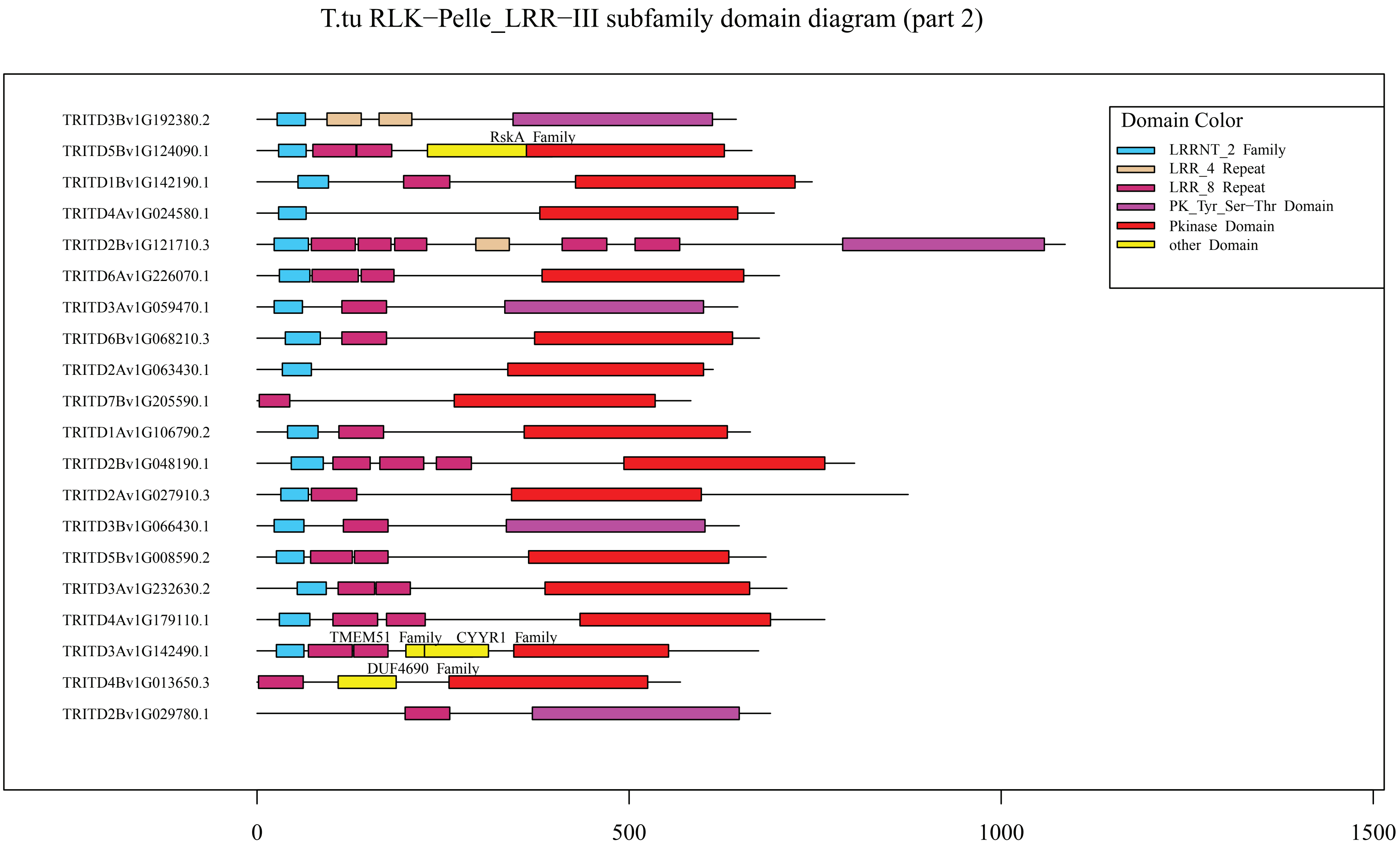
T.tu RLK−Pelle_LRR−III subfamily domain diagram (part 2)
TRITD3Bv1G192380.2
TRITD5Bv1G124090.1
RskA Family
TRITD1Bv1G142190.1
TRITD4Av1G024580.1
TRITD2Bv1G121710.3
TRITD6Av1G226070.1
TRITD3Av1G059470.1
TRITD6Bv1G068210.3
TRITD2Av1G063430.1
TRITD7Bv1G205590.1
TRITD1Av1G106790.2
TRITD2Bv1G048190.1
TRITD2Av1G027910.3
TRITD3Bv1G066430.1
TRITD5Bv1G008590.2
TRITD3Av1G232630.2
TRITD4Av1G179110.1
TMEM51 Family
CYYR1 Family
TRITD3Av1G142490.1
DUF4690 Family
TRITD4Bv1G013650.3
TRITD2Bv1G029780.1
Domain Color
LRRNT_2 Family
LRR_4 Repeat
LRR_8 Repeat
PK_Tyr_Ser−Thr Domain
Pkinase Domain
other Domain
0
500
1000
1500

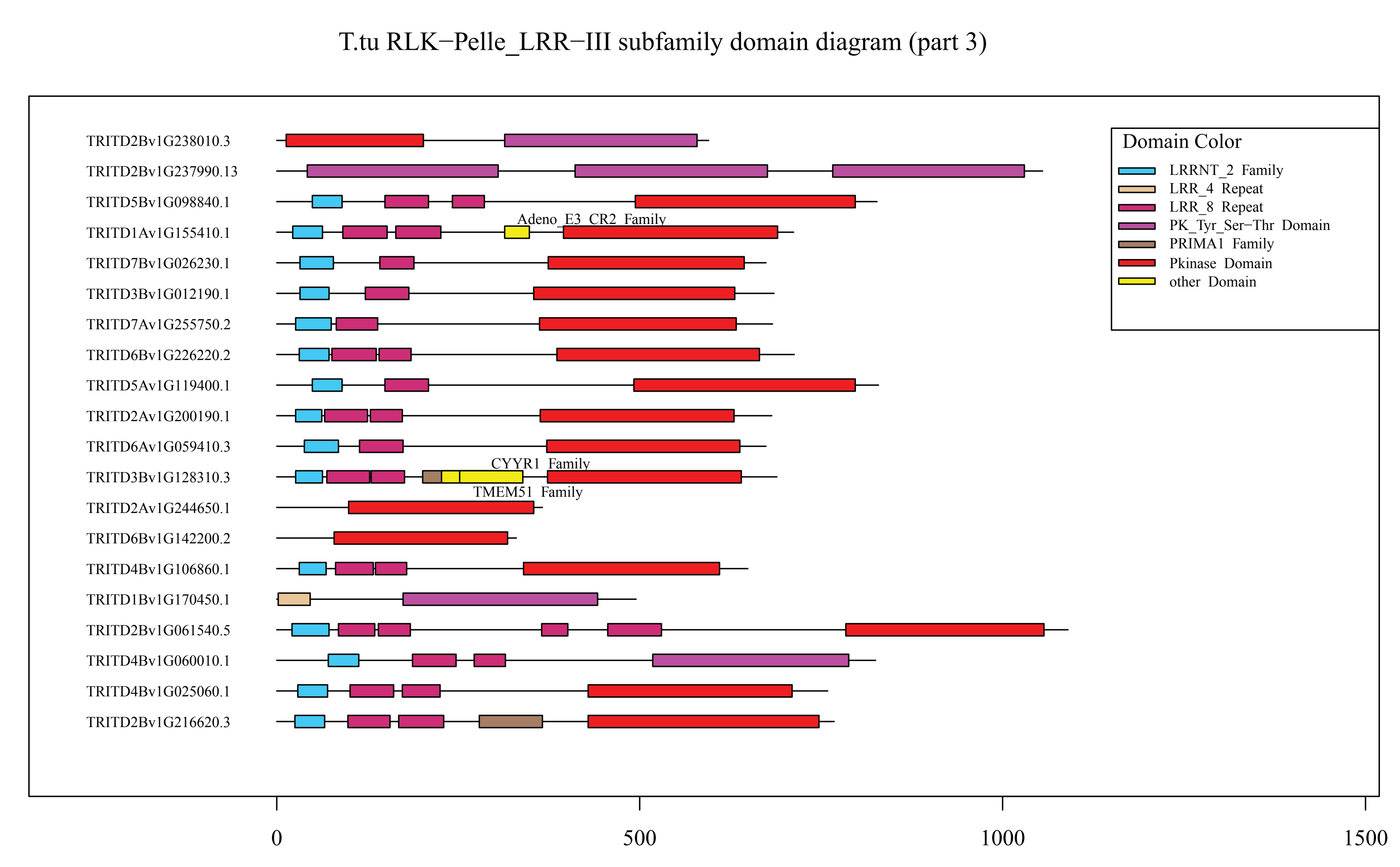
T.tu RLK−Pelle_LRR−III subfamily domain diagram (part 3)
TRITD2Bv1G238010.3
TRITD2Bv1G237990.13
TRITD5Bv1G098840.1
TRITD1Av1G155410.1
TRITD7Bv1G026230.1
TRITD3Bv1G012190.1
TRITD7Av1G255750.2
TRITD6Bv1G226220.2
TRITD5Av1G119400.1
TRITD2Av1G200190.1
TRITD6Av1G059410.3
TRITD3Bv1G128310.3
TRITD2Av1G244650.1
TRITD6Bv1G142200.2
TRITD4Bv1G106860.1
TRITD1Bv1G170450.1
TRITD2Bv1G061540.5
TRITD4Bv1G060010.1
TRITD4Bv1G025060.1
TRITD2Bv1G216620.3
Adeno_E3_CR2 Family
CYYR1 Family
TMEM51 Family
Domain Color
LRRNT_2 Family
LRR_4 Repeat
LRR_8 Repeat
PK_Tyr_Ser−Thr Domain
PRIMA1 Family
Pkinase Domain
other Domain
0
500
1000
1500

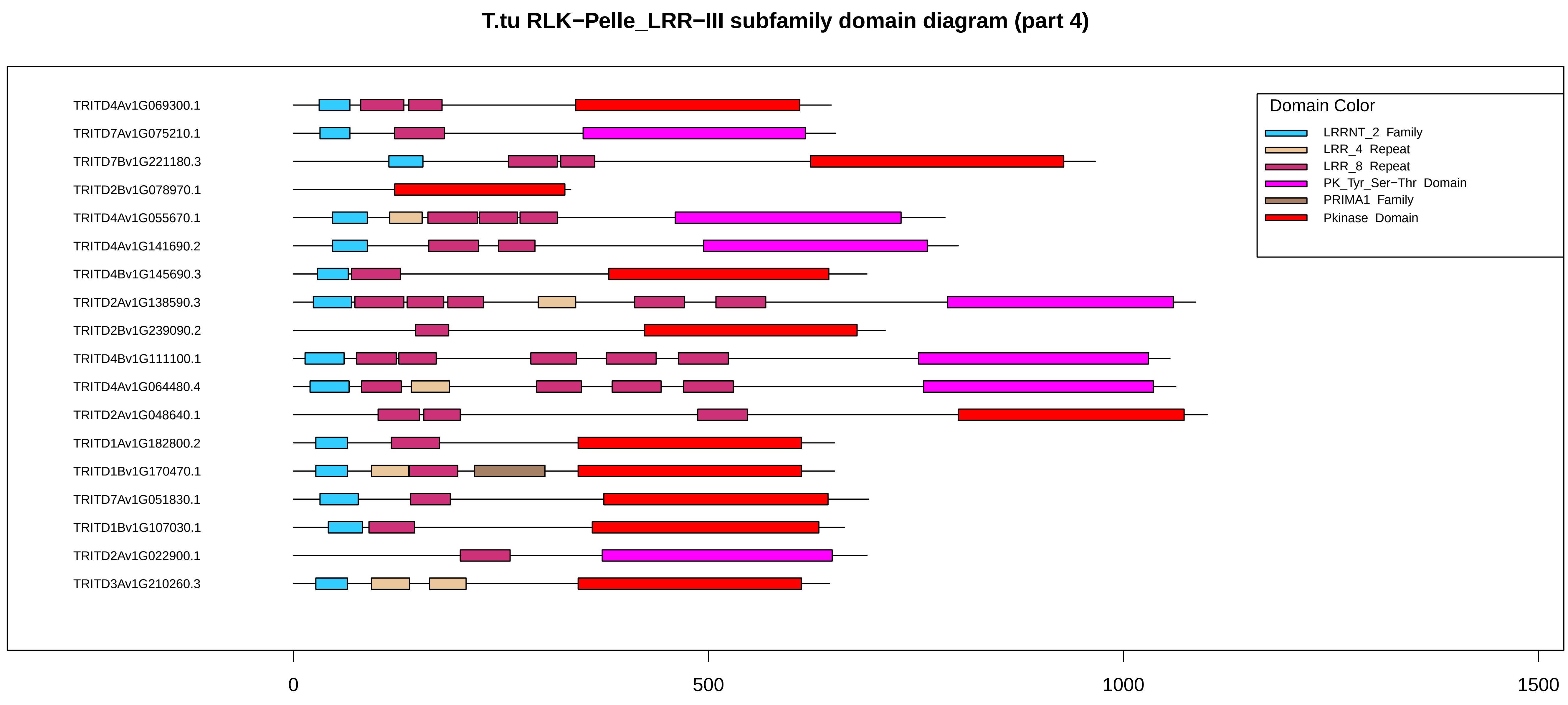
T.tu RLK−Pelle_LRR−III subfamily domain diagram (part 4)
TRITD4Av1G069300.1
TRITD7Av1G075210.1
TRITD7Bv1G221180.3
TRITD2Bv1G078970.1
TRITD4Av1G055670.1
TRITD4Av1G141690.2
TRITD4Bv1G145690.3
TRITD2Av1G138590.3
TRITD2Bv1G239090.2
TRITD4Bv1G111100.1
TRITD4Av1G064480.4
TRITD2Av1G048640.1
TRITD1Av1G182800.2
TRITD1Bv1G170470.1
TRITD7Av1G051830.1
TRITD1Bv1G107030.1
TRITD2Av1G022900.1
TRITD3Av1G210260.3
Domain Color
LRRNT_2 Family
LRR_4 Repeat
LRR_8 Repeat
PK_Tyr_Ser−Thr Domain
PRIMA1 Family
Pkinase Domain
0
500
1000
1500

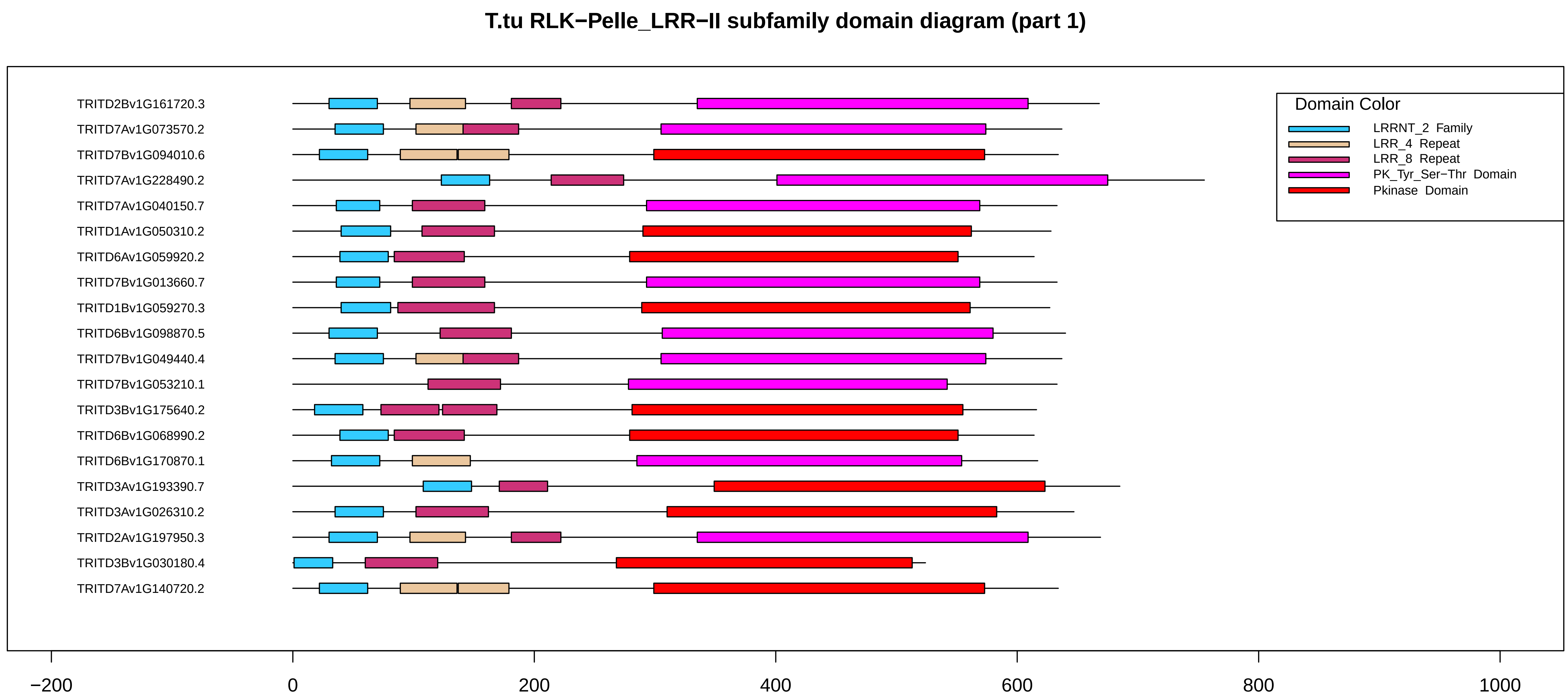
T.tu RLK−Pelle_LRR−II subfamily domain diagram (part 1)
TRITD2Bv1G161720.3
TRITD7Av1G073570.2
TRITD7Bv1G094010.6
TRITD7Av1G228490.2
TRITD7Av1G040150.7
TRITD1Av1G050310.2
TRITD6Av1G059920.2
TRITD7Bv1G013660.7
TRITD1Bv1G059270.3
TRITD6Bv1G098870.5
TRITD7Bv1G049440.4
TRITD7Bv1G053210.1
TRITD3Bv1G175640.2
TRITD6Bv1G068990.2
TRITD6Bv1G170870.1
TRITD3Av1G193390.7
TRITD3Av1G026310.2
TRITD2Av1G197950.3
TRITD3Bv1G030180.4
TRITD7Av1G140720.2
Domain Color
LRRNT_2 Family
LRR_4 Repeat
LRR_8 Repeat
PK_Tyr_Ser−Thr Domain
Pkinase Domain
−200
0
200
400
600
800
1000

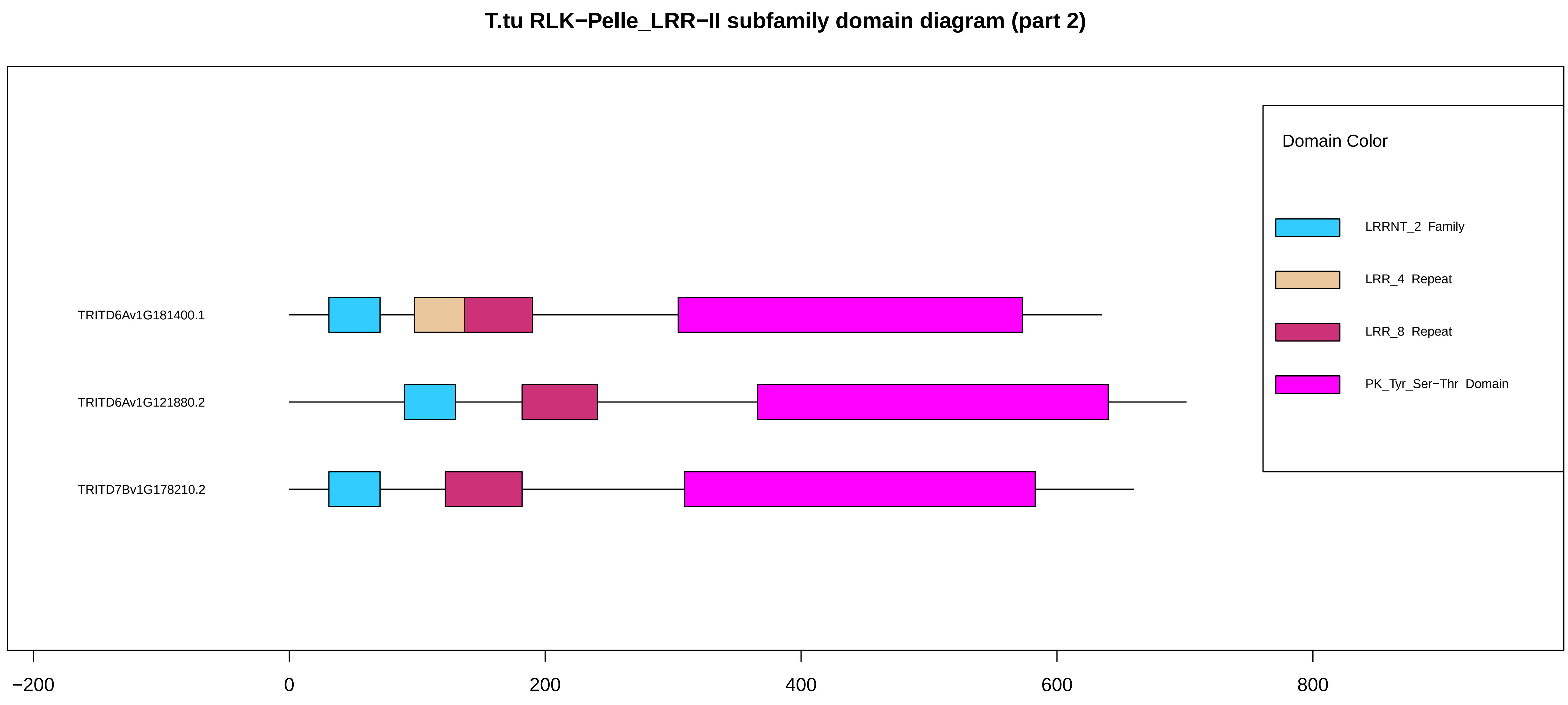
T.tu RLK−Pelle_LRR−II subfamily domain diagram (part 2)
TRITD6Av1G181400.1
TRITD6Av1G121880.2
TRITD7Bv1G178210.2
Domain Color
LRRNT_2 Family
LRR_4 Repeat
LRR_8 Repeat
PK_Tyr_Ser−Thr Domain
−200
0
200
400
600
800

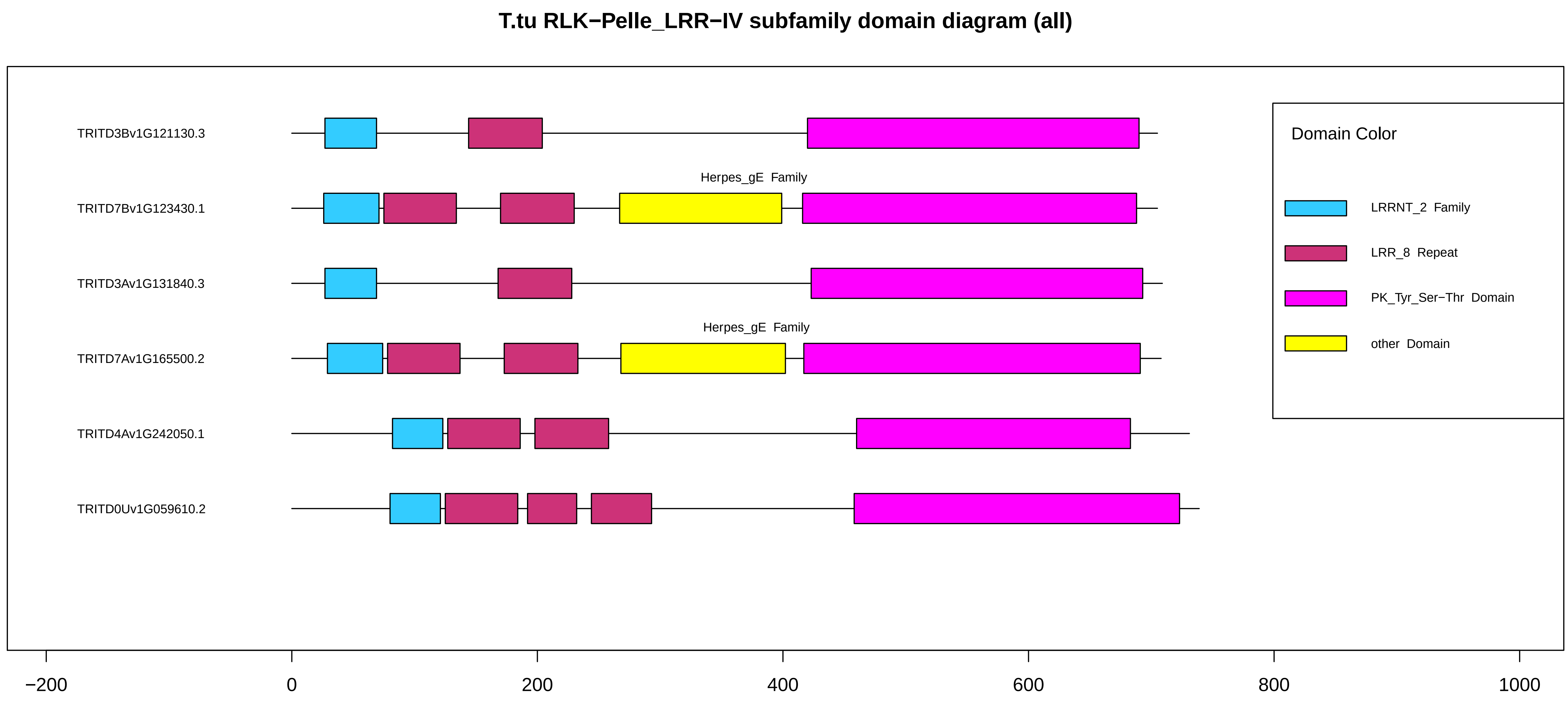
T.tu RLK−Pelle_LRR−IV subfamily domain diagram (all)
TRITD3Bv1G121130.3
TRITD7Bv1G123430.1
Herpes_gE Family
TRITD3Av1G131840.3
TRITD7Av1G165500.2
Herpes_gE Family
TRITD4Av1G242050.1
TRITD0Uv1G059610.2
Domain Color
LRRNT_2 Family
LRR_8 Repeat
PK_Tyr_Ser−Thr Domain
other Domain
−200
0
200
400
600
800
1000

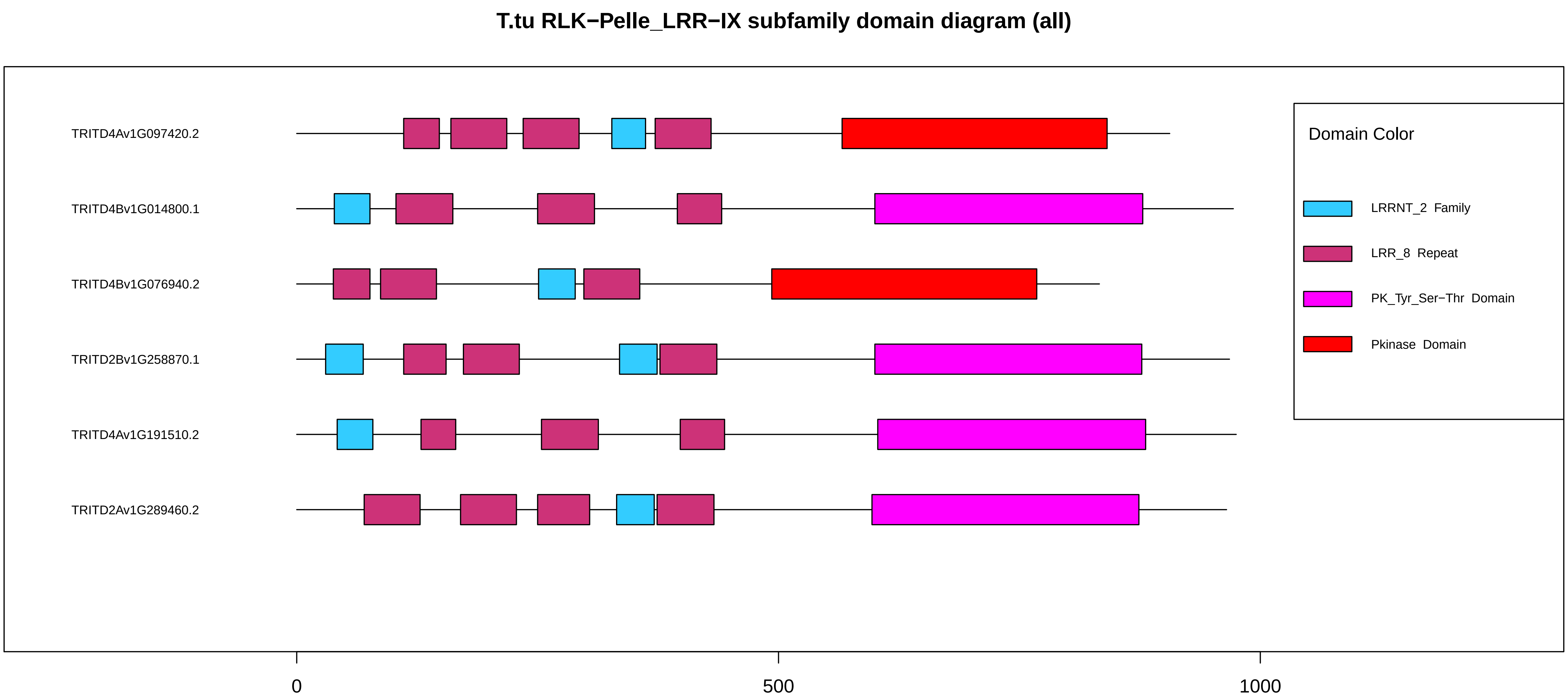
T.tu RLK−Pelle_LRR−IX subfamily domain diagram (all)
TRITD4Av1G097420.2
TRITD4Bv1G014800.1
TRITD4Bv1G076940.2
TRITD2Bv1G258870.1
TRITD4Av1G191510.2
TRITD2Av1G289460.2
Domain Color
LRRNT_2 Family
LRR_8 Repeat
PK_Tyr_Ser−Thr Domain
Pkinase Domain
0
500
1000

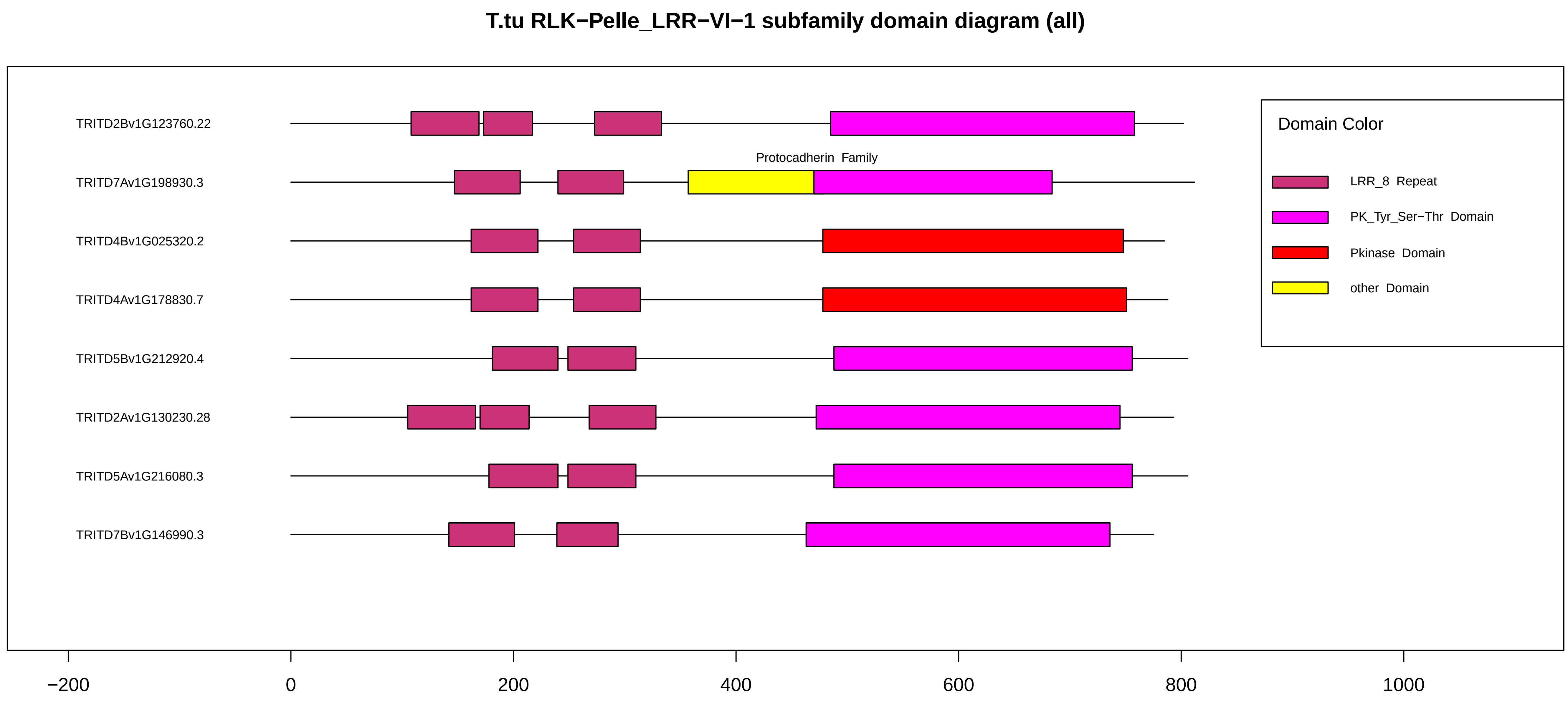

T.tu RLK−Pelle_LRR−VI−1 subfamily domain diagram (all)
TRITD2Bv1G123760.22
TRITD7Av1G198930.3
TRITD4Bv1G025320.2
TRITD4Av1G178830.7
TRITD5Bv1G212920.4
TRITD2Av1G130230.28
TRITD5Av1G216080.3
TRITD7Bv1G146990.3
Protocadherin Family
Domain Color
LRR_8 Repeat
PK_Tyr_Ser−Thr Domain
Pkinase Domain
other Domain
−200
0
200
400
600
800
1000

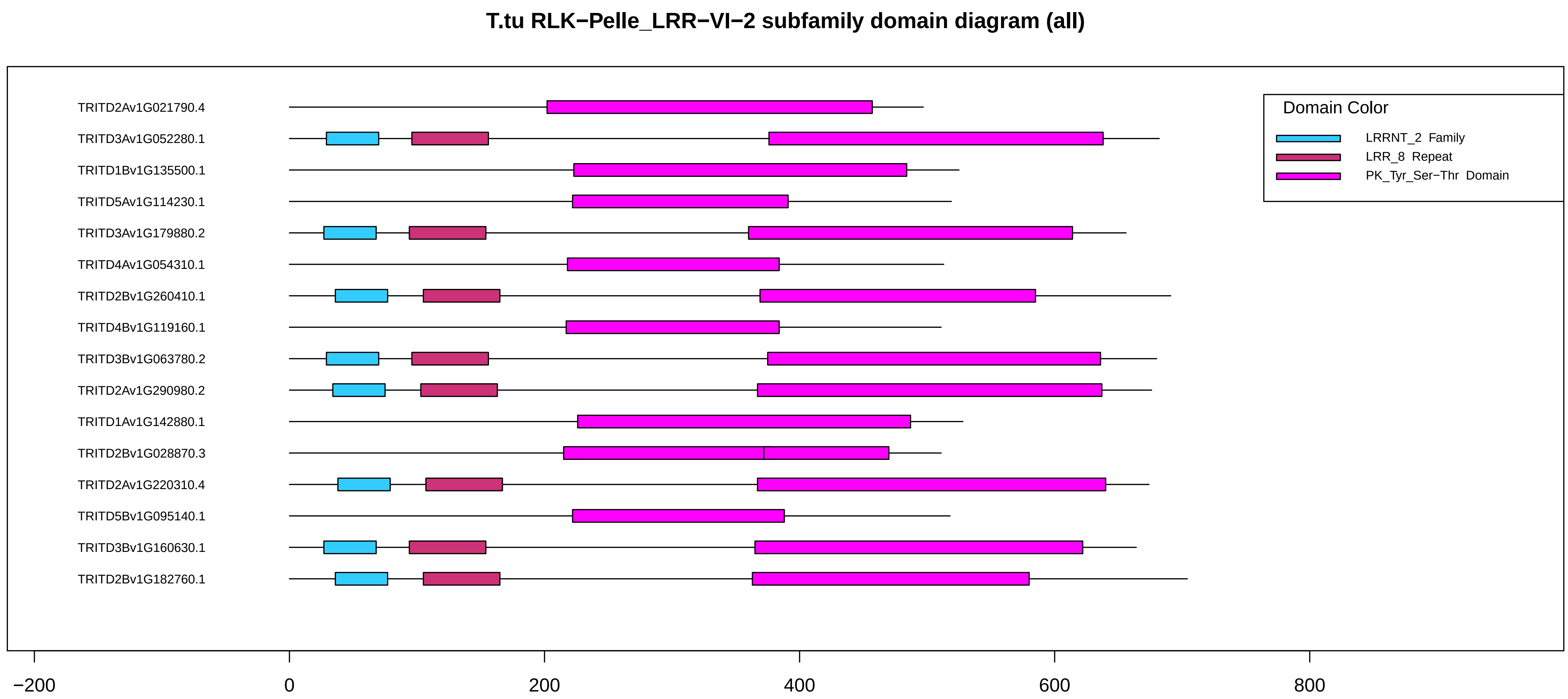

T.tu RLK−Pelle_LRR−VI−2 subfamily domain diagram (all)
TRITD2Av1G021790.4
TRITD3Av1G052280.1
TRITD1Bv1G135500.1
TRITD5Av1G114230.1
TRITD3Av1G179880.2
TRITD4Av1G054310.1
TRITD2Bv1G260410.1
TRITD4Bv1G119160.1
TRITD3Bv1G063780.2
TRITD2Av1G290980.2
TRITD1Av1G142880.1
TRITD2Bv1G028870.3
TRITD2Av1G220310.4
TRITD5Bv1G095140.1
TRITD3Bv1G160630.1
TRITD2Bv1G182760.1
Domain Color
LRRNT_2 Family
LRR_8 Repeat
PK_Tyr_Ser−Thr Domain
−200
0
200
400
600
800

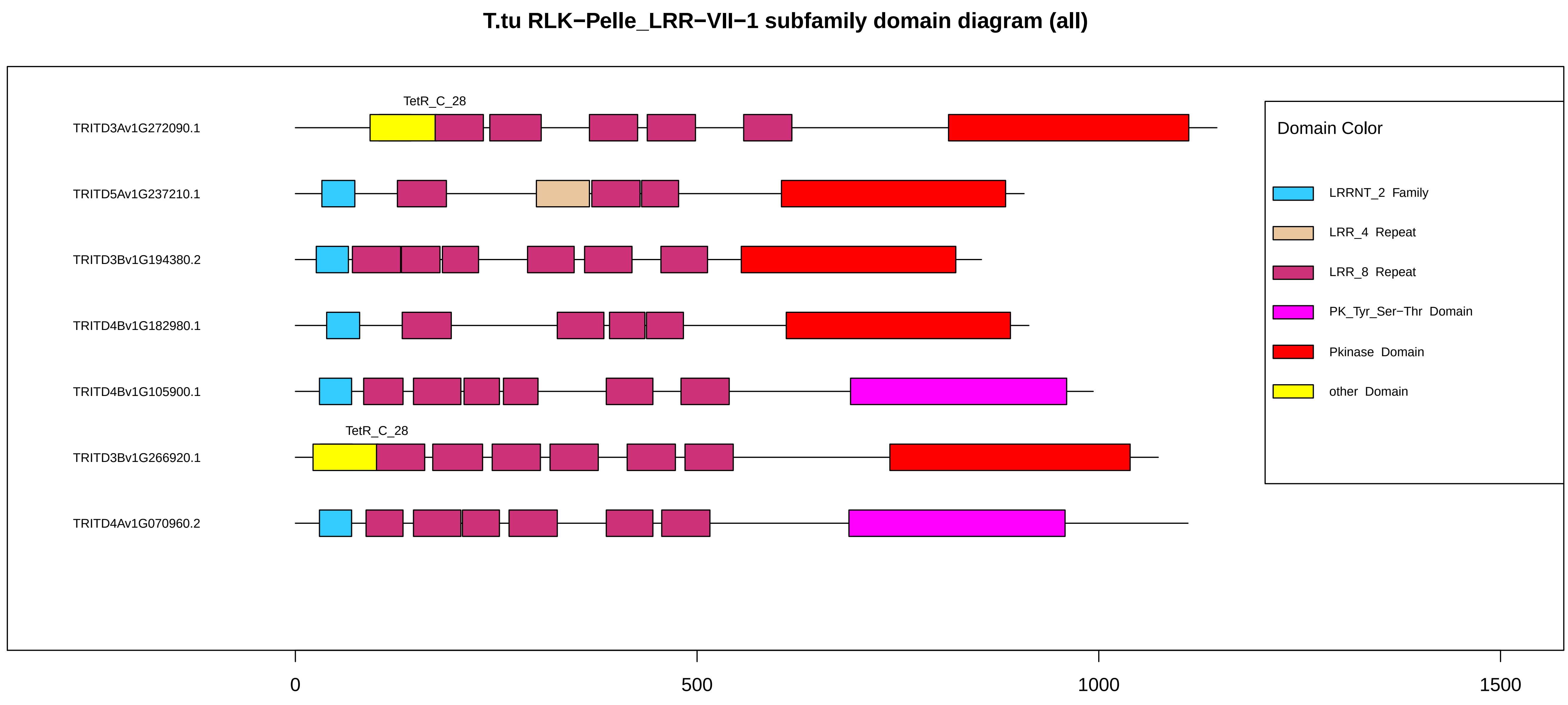

T.tu RLK−Pelle_LRR−VII−1 subfamily domain diagram (all)
TetR_C_28
TRITD3Av1G272090.1
TRITD5Av1G237210.1
TRITD3Bv1G194380.2
TRITD4Bv1G182980.1
TRITD4Bv1G105900.1
TetR_C_28
TRITD3Bv1G266920.1
TRITD4Av1G070960.2
Domain Color
LRRNT_2 Family
LRR_4 Repeat
LRR_8 Repeat
PK_Tyr_Ser−Thr Domain
Pkinase Domain
other Domain
0
500
1000
1500

T.tu RLK−Pelle_LRR−VII−2 subfamily domain diagram (all)

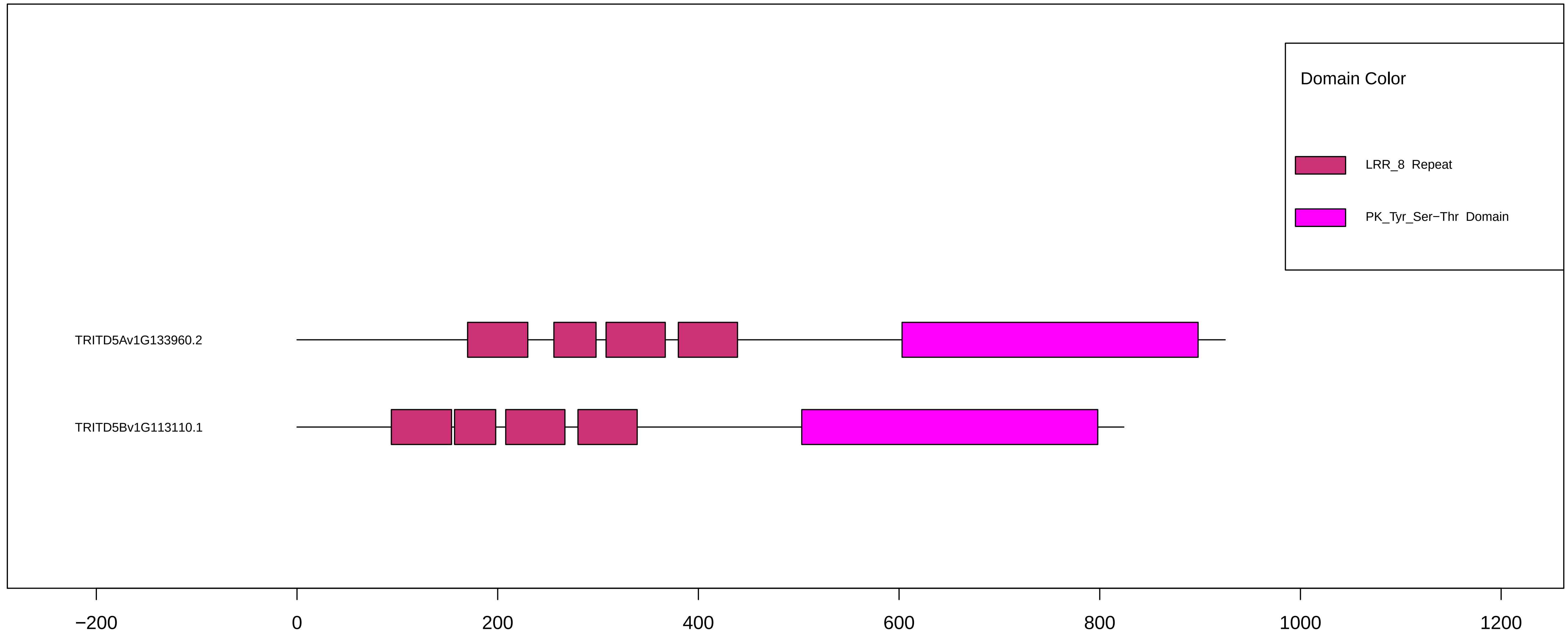

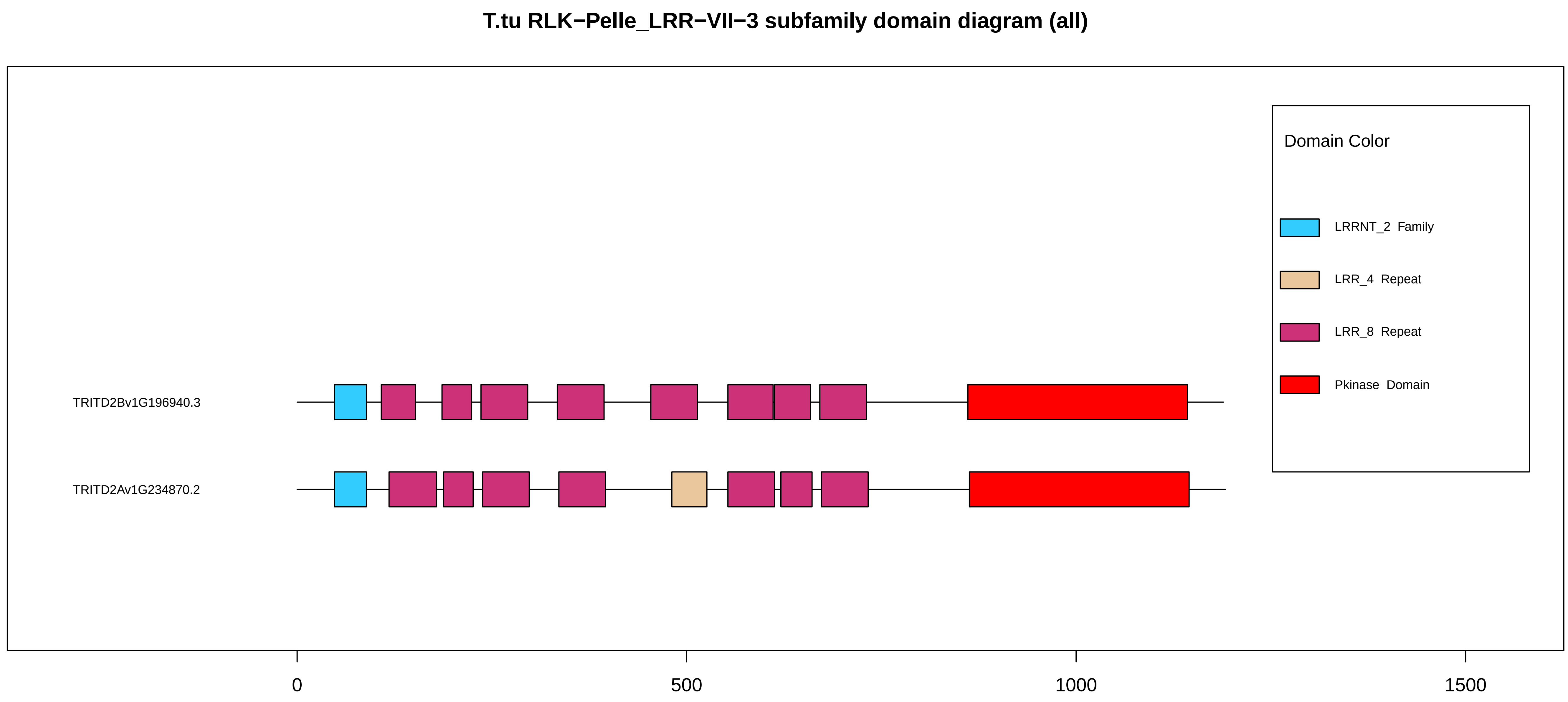
T.tu RLK−Pelle_LRR−VII−3 subfamily domain diagram (all)
TRITD2Bv1G196940.3
TRITD2Av1G234870.2
Domain Color
LRRNT_2 Family
LRR_4 Repeat
LRR_8 Repeat
Pkinase Domain
0
500
1000
1500

T.tu RLK−Pelle_LRR−VIII−1 subfamily domain diagram (part 1)

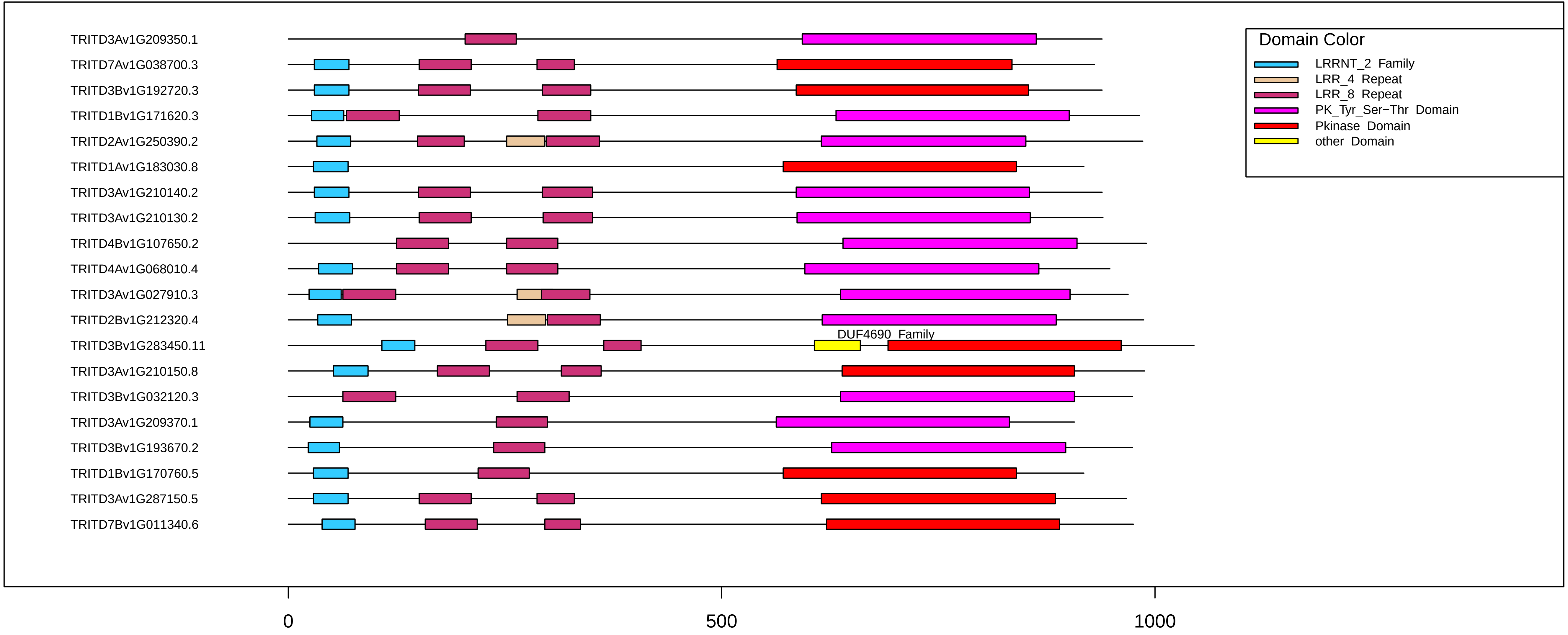

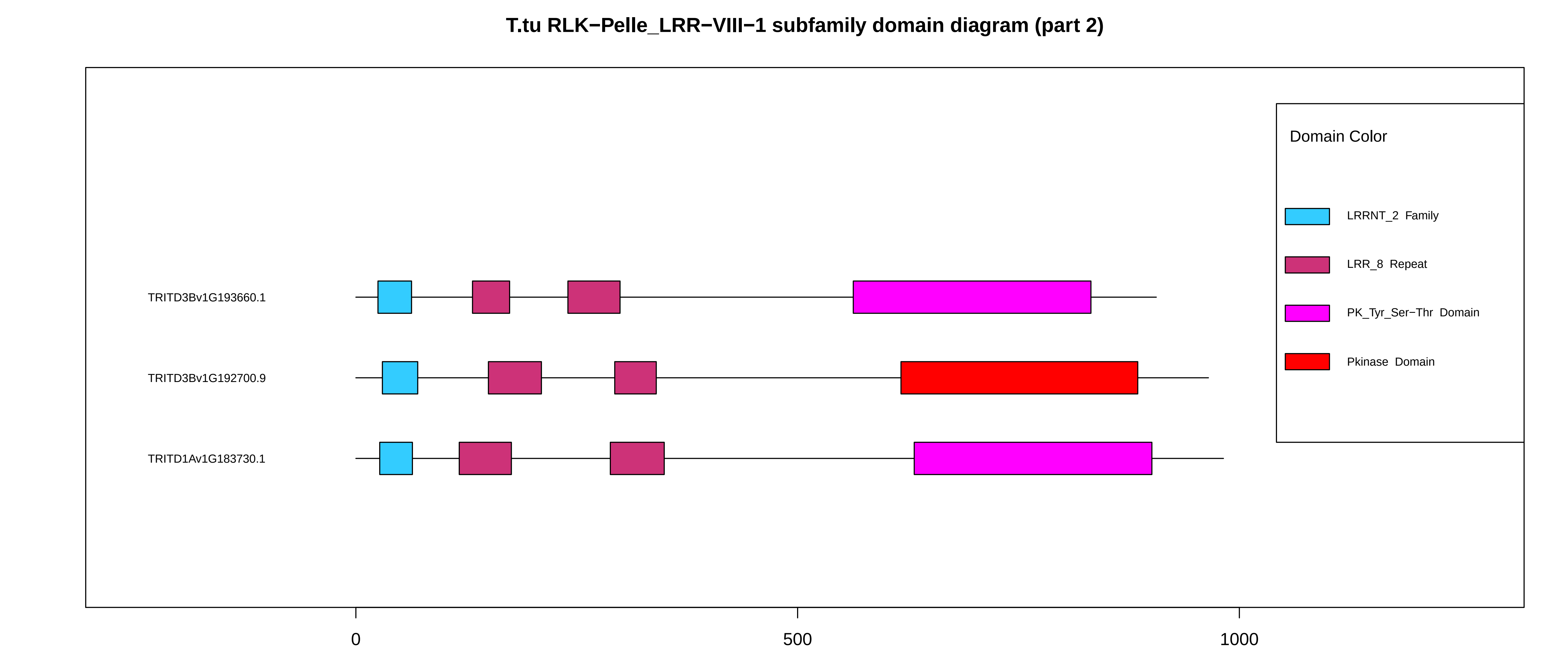
T.tu RLK−Pelle_LRR−VIII−1 subfamily domain diagram (part 2)
TRITD3Bv1G193660.1
TRITD3Bv1G192700.9
TRITD1Av1G183730.1
Domain Color
LRRNT_2 Family
LRR_8 Repeat
PK_Tyr_Ser−Thr Domain
Pkinase Domain
0
500
1000

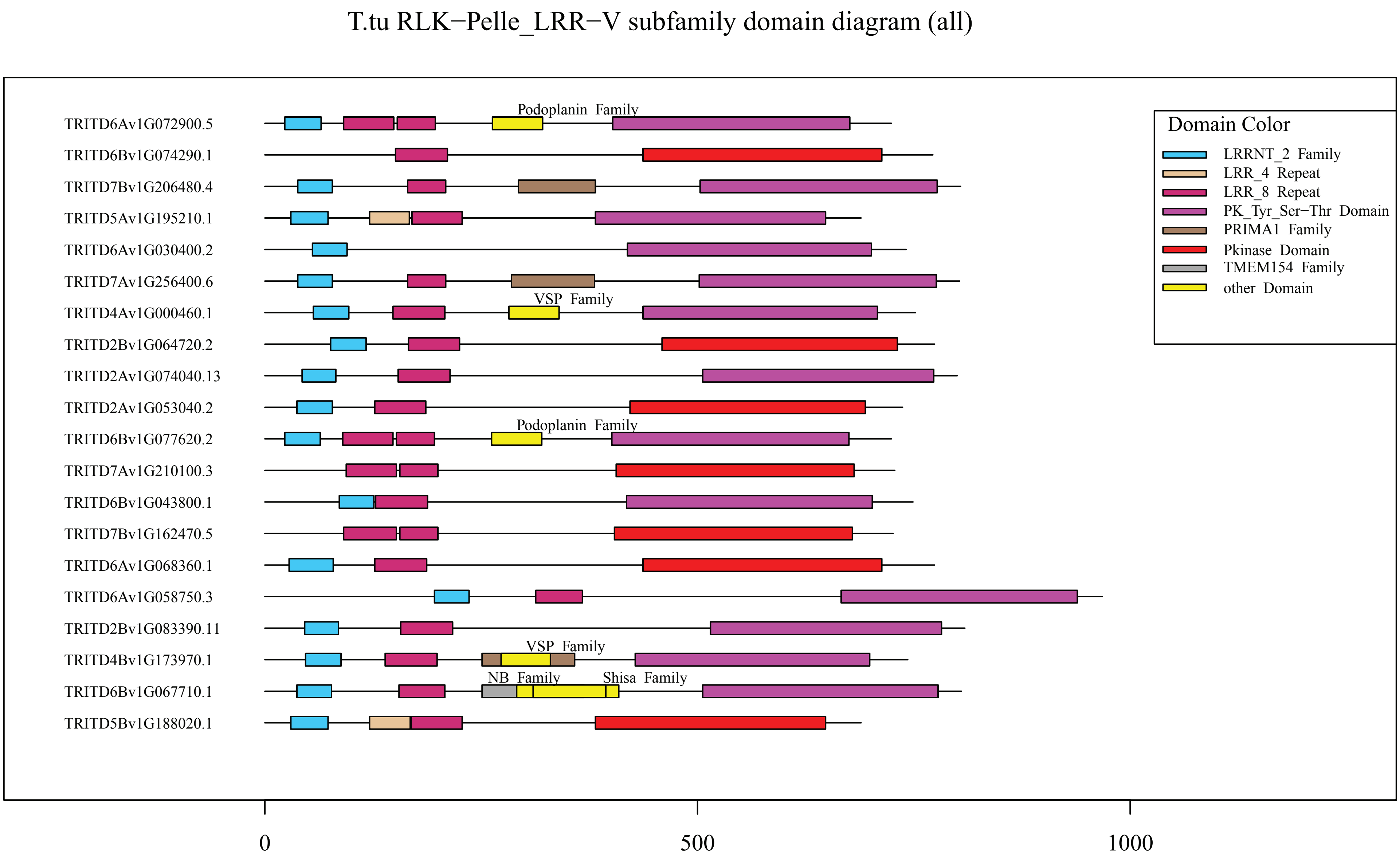

T.tu RLK−Pelle_LRR−V subfamily domain diagram (all)
TRITD6Av1G072900.5
TRITD6Bv1G074290.1
TRITD7Bv1G206480.4
TRITD5Av1G195210.1
TRITD6Av1G030400.2
TRITD7Av1G256400.6
TRITD4Av1G000460.1
TRITD2Bv1G064720.2
TRITD2Av1G074040.13
TRITD2Av1G053040.2
TRITD6Bv1G077620.2
TRITD7Av1G210100.3
TRITD6Bv1G043800.1
TRITD7Bv1G162470.5
TRITD6Av1G068360.1
TRITD6Av1G058750.3
TRITD2Bv1G083390.11
TRITD4Bv1G173970.1
TRITD6Bv1G067710.1
TRITD5Bv1G188020.1
Podoplanin Family
VSP Family
Podoplanin Family
VSP Family
NB Family
Shisa Family
Domain Color
LRRNT_2 Family
LRR_4 Repeat
LRR_8 Repeat
PK_Tyr_Ser−Thr Domain
PRIMA1 Family
Pkinase Domain
TMEM154 Family
other Domain
0
500
1000

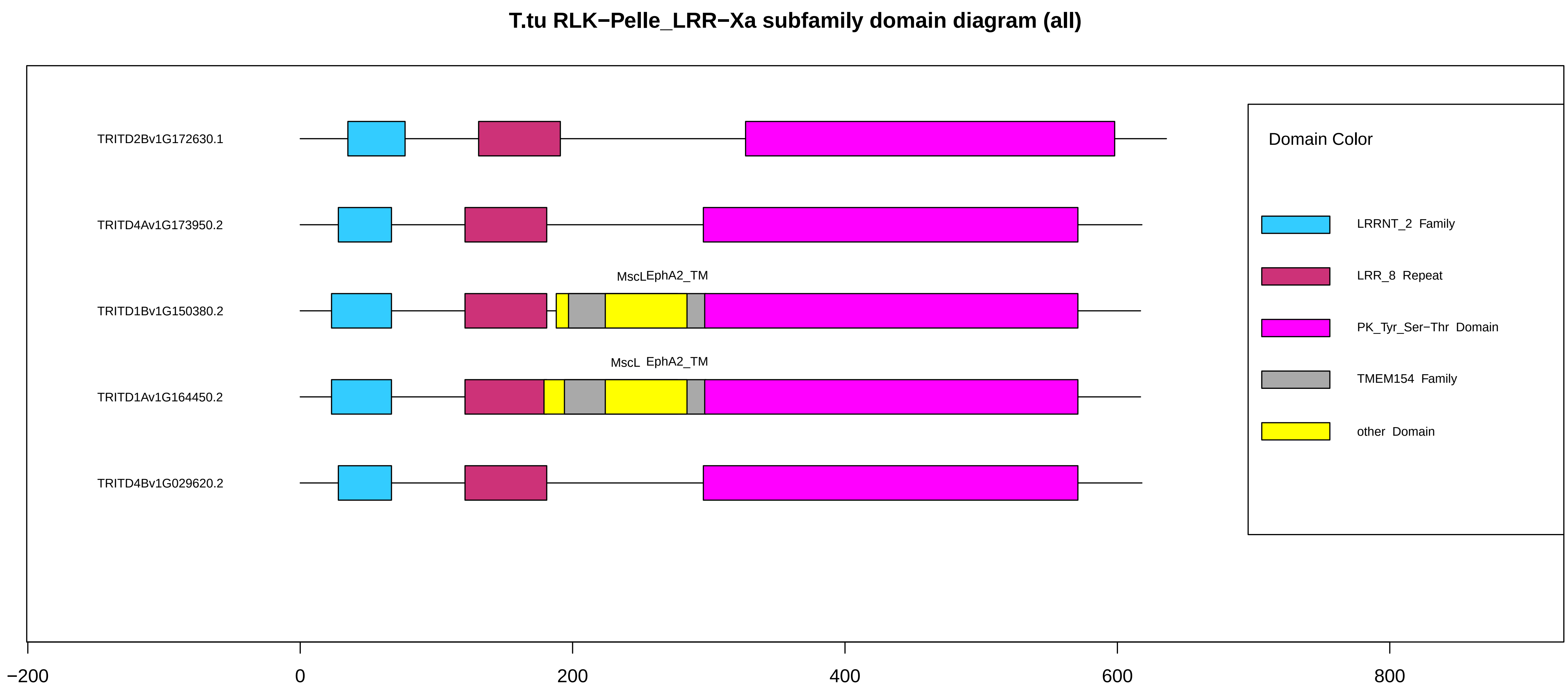

T.tu RLK−Pelle_LRR−Xa subfamily domain diagram (all)
TRITD2Bv1G172630.1
TRITD4Av1G173950.2
MscLEphA2_TM
TRITD1Bv1G150380.2
MscL EphA2_TM
TRITD1Av1G164450.2
TRITD4Bv1G029620.2
Domain Color
LRRNT_2 Family
LRR_8 Repeat
PK_Tyr_Ser−Thr Domain
TMEM154 Family
other Domain
−200
0
200
400
600
800

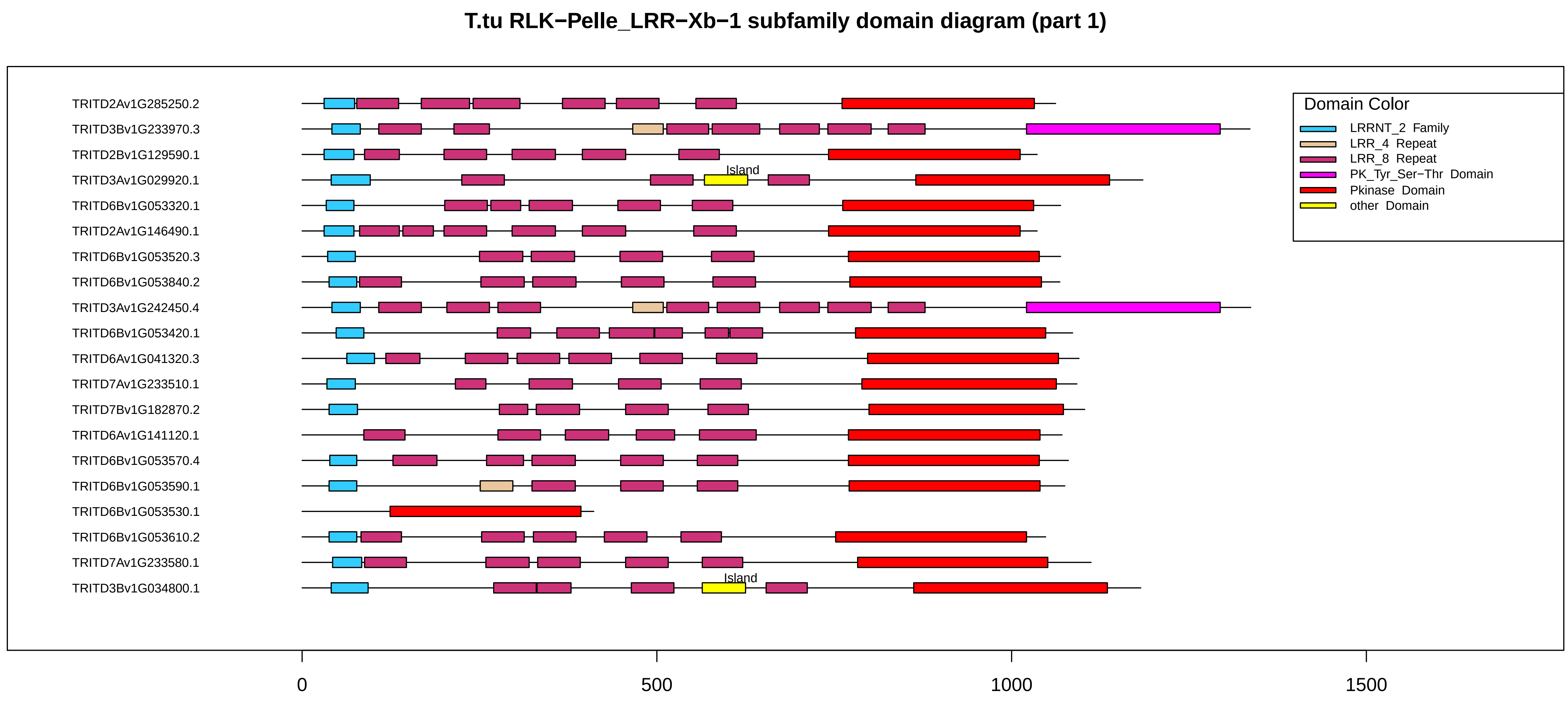

T.tu RLK−Pelle_LRR−Xb−1 subfamily domain diagram (part 1)
TRITD2Av1G285250.2
TRITD3Bv1G233970.3
TRITD2Bv1G129590.1
TRITD3Av1G029920.1
Island
TRITD6Bv1G053320.1
TRITD2Av1G146490.1
TRITD6Bv1G053520.3
TRITD6Bv1G053840.2
TRITD3Av1G242450.4
TRITD6Bv1G053420.1
TRITD6Av1G041320.3
TRITD7Av1G233510.1
TRITD7Bv1G182870.2
TRITD6Av1G141120.1
TRITD6Bv1G053570.4
TRITD6Bv1G053590.1
TRITD6Bv1G053530.1
TRITD6Bv1G053610.2
TRITD7Av1G233580.1
TRITD3Bv1G034800.1
Island
Domain Color
LRRNT_2 Family
LRR_4 Repeat
LRR_8 Repeat
PK_Tyr_Ser−Thr Domain
Pkinase Domain
other Domain
0
500
1000
1500

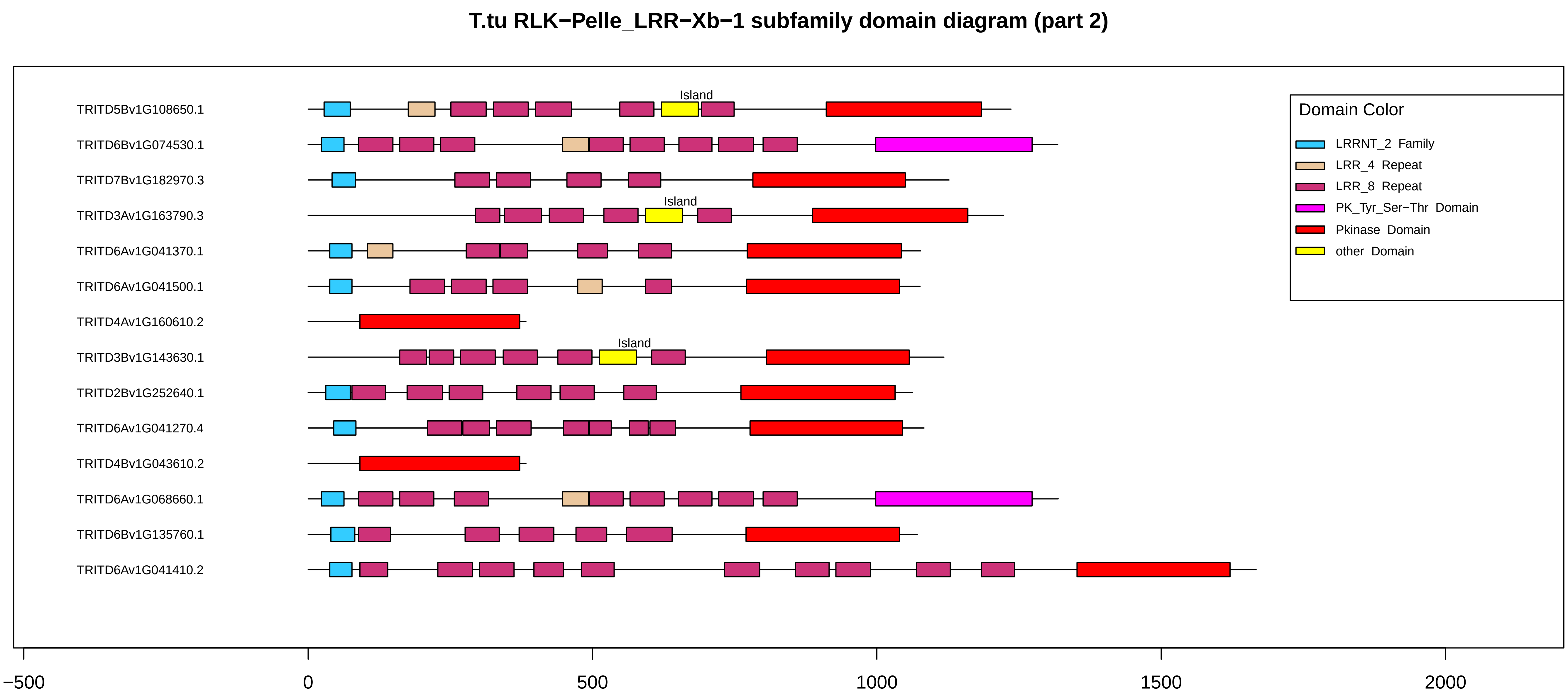
T.tu RLK−Pelle_LRR−Xb−1 subfamily domain diagram (part 2)
TRITD5Bv1G108650.1
TRITD6Bv1G074530.1
TRITD7Bv1G182970.3
TRITD3Av1G163790.3
TRITD6Av1G041370.1
TRITD6Av1G041500.1
TRITD4Av1G160610.2
TRITD3Bv1G143630.1
TRITD2Bv1G252640.1
TRITD6Av1G041270.4
TRITD4Bv1G043610.2
TRITD6Av1G068660.1
TRITD6Bv1G135760.1
TRITD6Av1G041410.2
Island
Island
Island
Domain Color
LRRNT_2 Family
LRR_4 Repeat
LRR_8 Repeat
PK_Tyr_Ser−Thr Domain
Pkinase Domain
other Domain
−500
0
500
1000
1500
2000

T.tu RLK−Pelle_LRR−Xb−2 subfamily domain diagram (all)

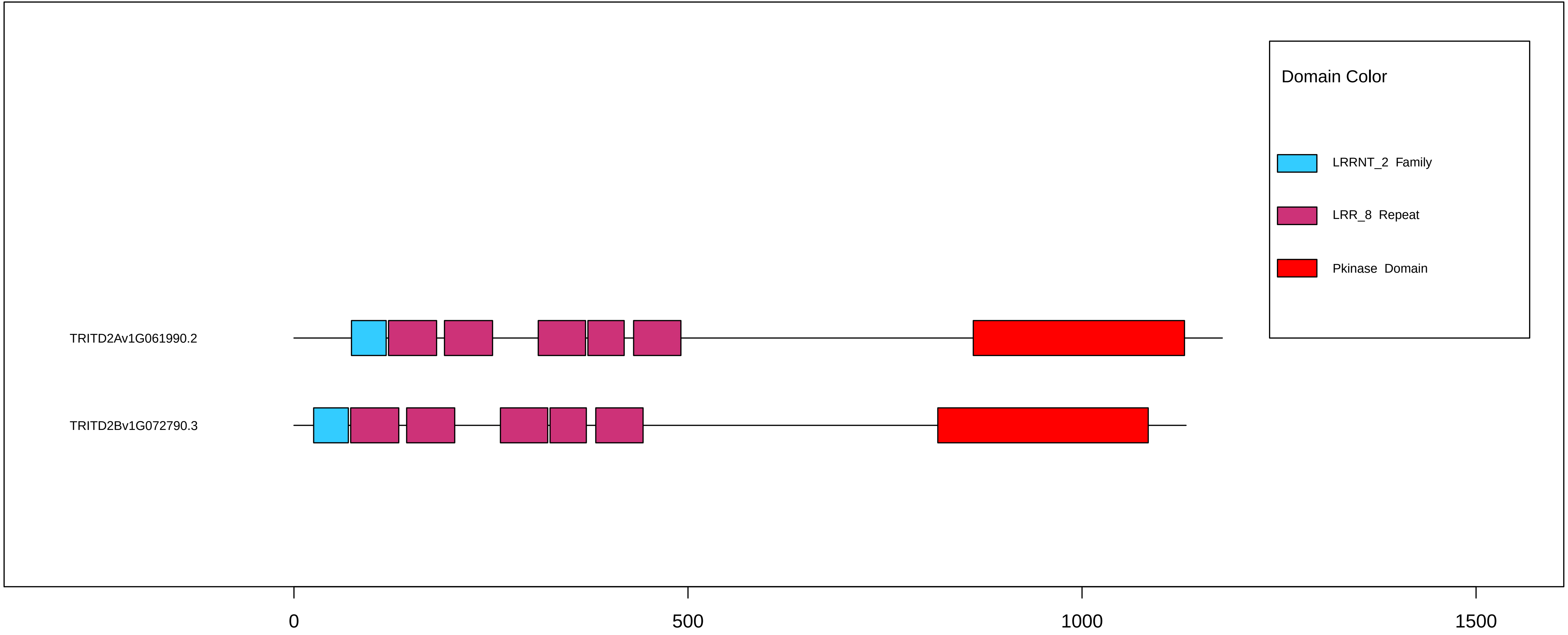

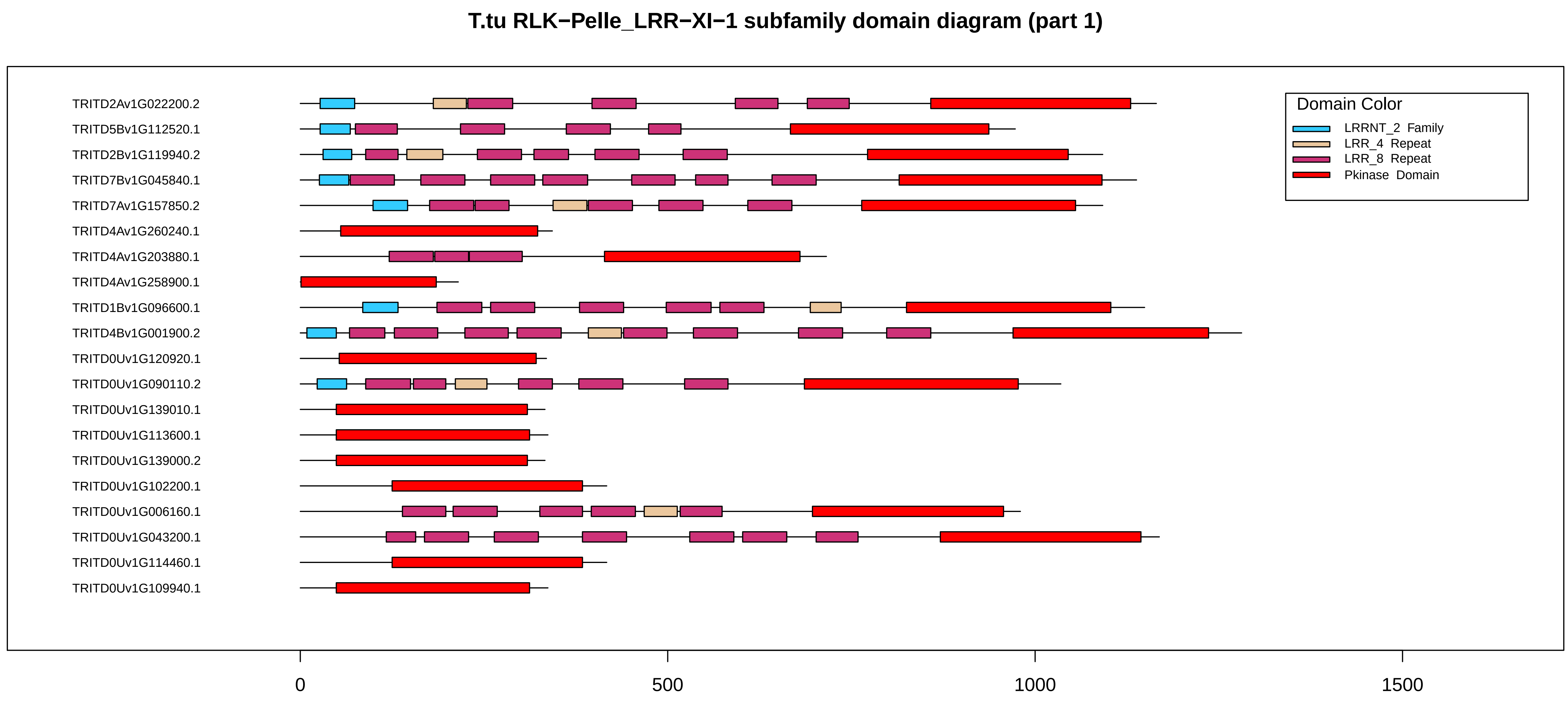
T.tu RLK−Pelle_LRR−XI−1 subfamily domain diagram (part 1)
TRITD2Av1G022200.2
TRITD5Bv1G112520.1
TRITD2Bv1G119940.2
TRITD7Bv1G045840.1
TRITD7Av1G157850.2
TRITD4Av1G260240.1
TRITD4Av1G203880.1
TRITD4Av1G258900.1
TRITD1Bv1G096600.1
TRITD4Bv1G001900.2
TRITD0Uv1G120920.1
TRITD0Uv1G090110.2
TRITD0Uv1G139010.1
TRITD0Uv1G113600.1
TRITD0Uv1G139000.2
TRITD0Uv1G102200.1
TRITD0Uv1G006160.1
TRITD0Uv1G043200.1
TRITD0Uv1G114460.1
TRITD0Uv1G109940.1
Domain Color
LRRNT_2 Family
LRR_4 Repeat
LRR_8 Repeat
Pkinase Domain
0
500
1000
1500

T.tu RLK−Pelle_LRR−XI−1 subfamily domain diagram (part 2)

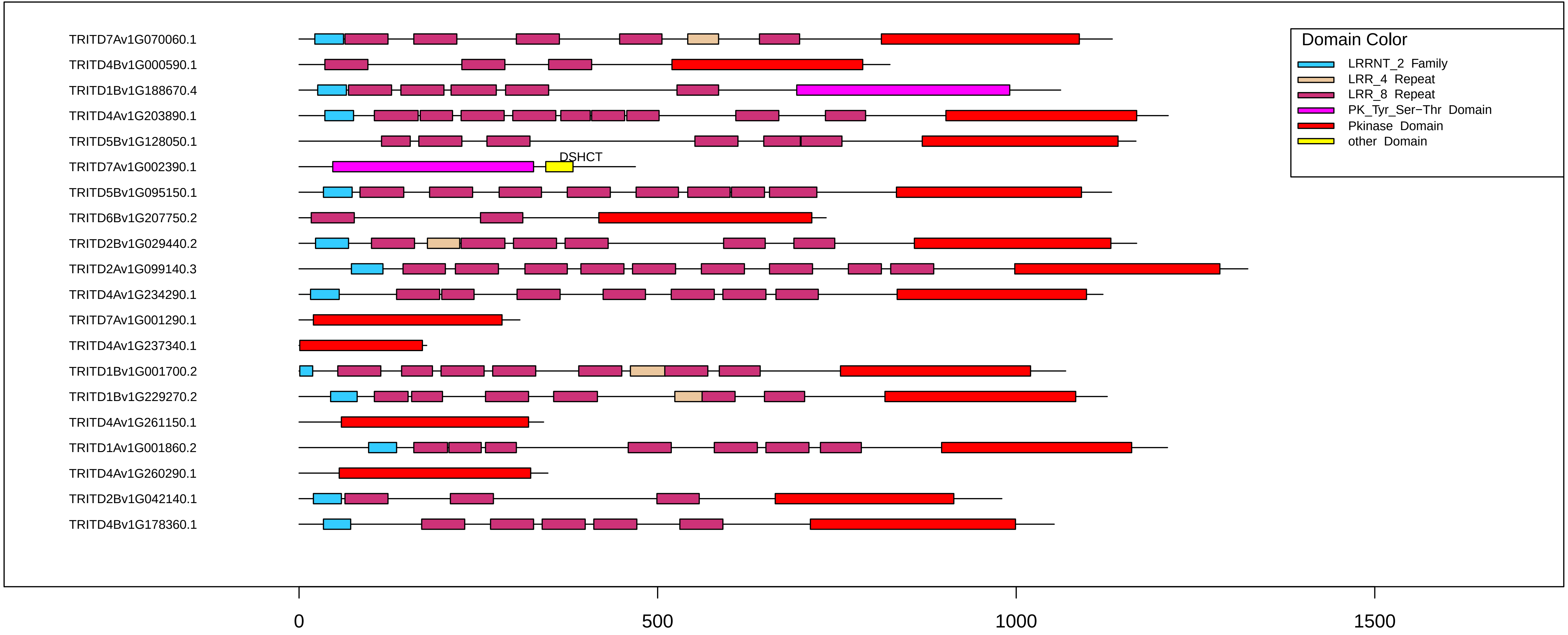

T.tu RLK−Pelle_LRR−XI−1 subfamily domain diagram (part 3)

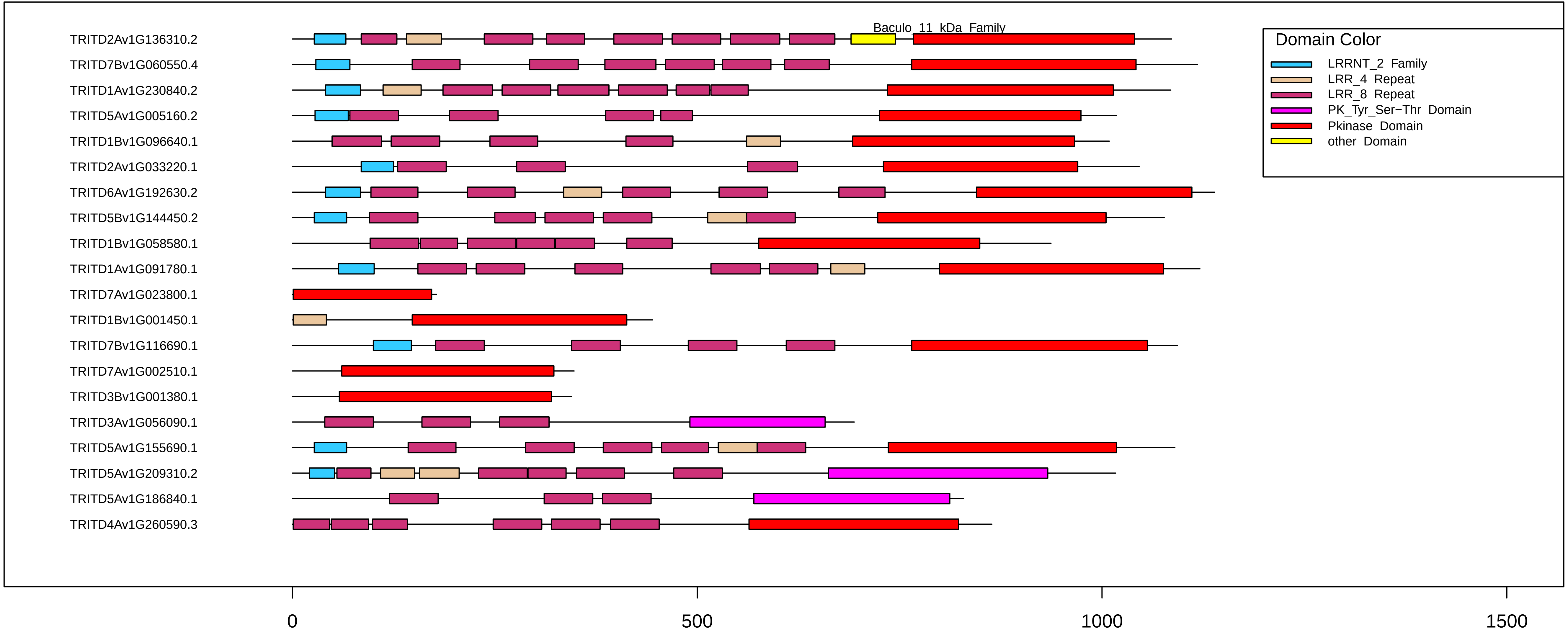

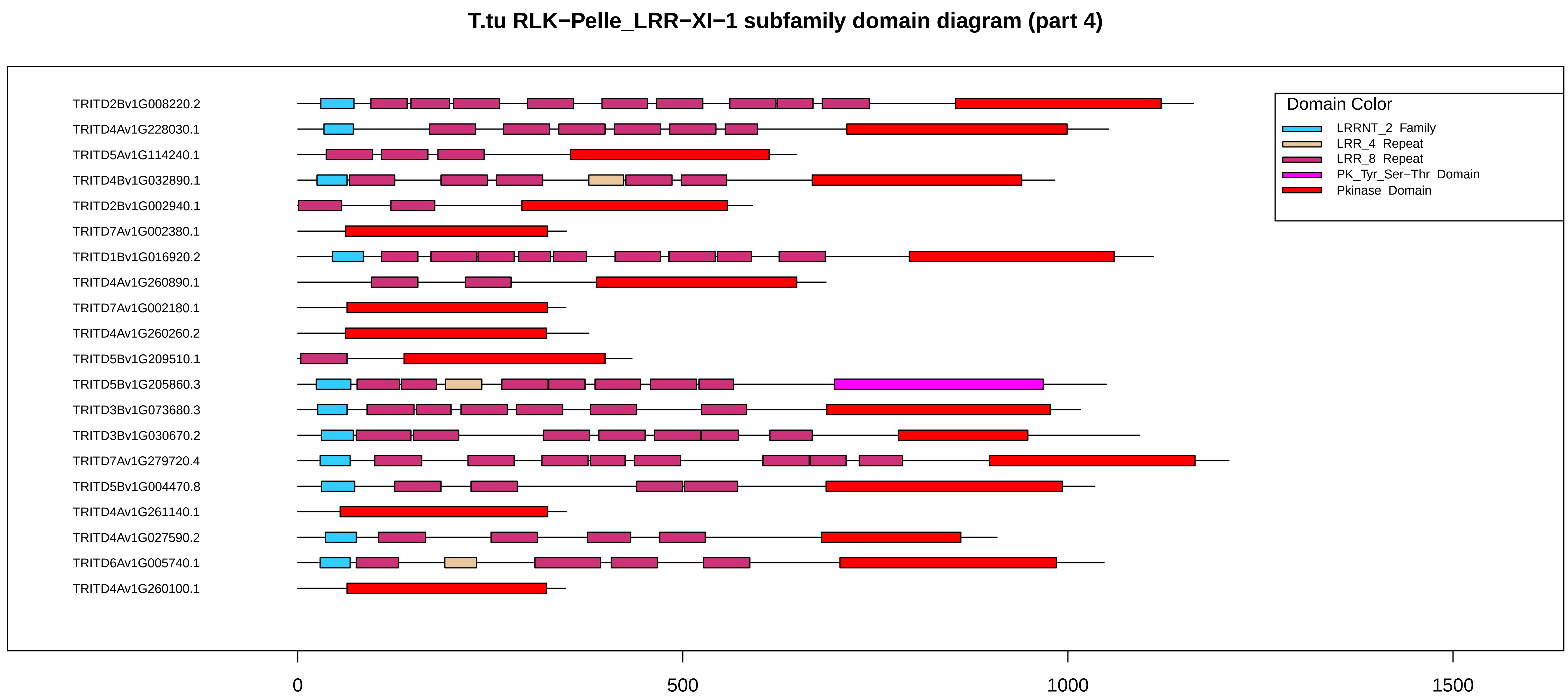
T.tu RLK−Pelle_LRR−XI−1 subfamily domain diagram (part 4)
TRITD2Bv1G008220.2
TRITD4Av1G228030.1
TRITD5Av1G114240.1
TRITD4Bv1G032890.1
TRITD2Bv1G002940.1
TRITD7Av1G002380.1
TRITD1Bv1G016920.2
TRITD4Av1G260890.1
TRITD7Av1G002180.1
TRITD4Av1G260260.2
TRITD5Bv1G209510.1
TRITD5Bv1G205860.3
TRITD3Bv1G073680.3
TRITD3Bv1G030670.2
TRITD7Av1G279720.4
TRITD5Bv1G004470.8
TRITD4Av1G261140.1
TRITD4Av1G027590.2
TRITD6Av1G005740.1
TRITD4Av1G260100.1
Domain Color
LRRNT_2 Family
LRR_4 Repeat
LRR_8 Repeat
PK_Tyr_Ser−Thr Domain
Pkinase Domain
0
500
1000
1500

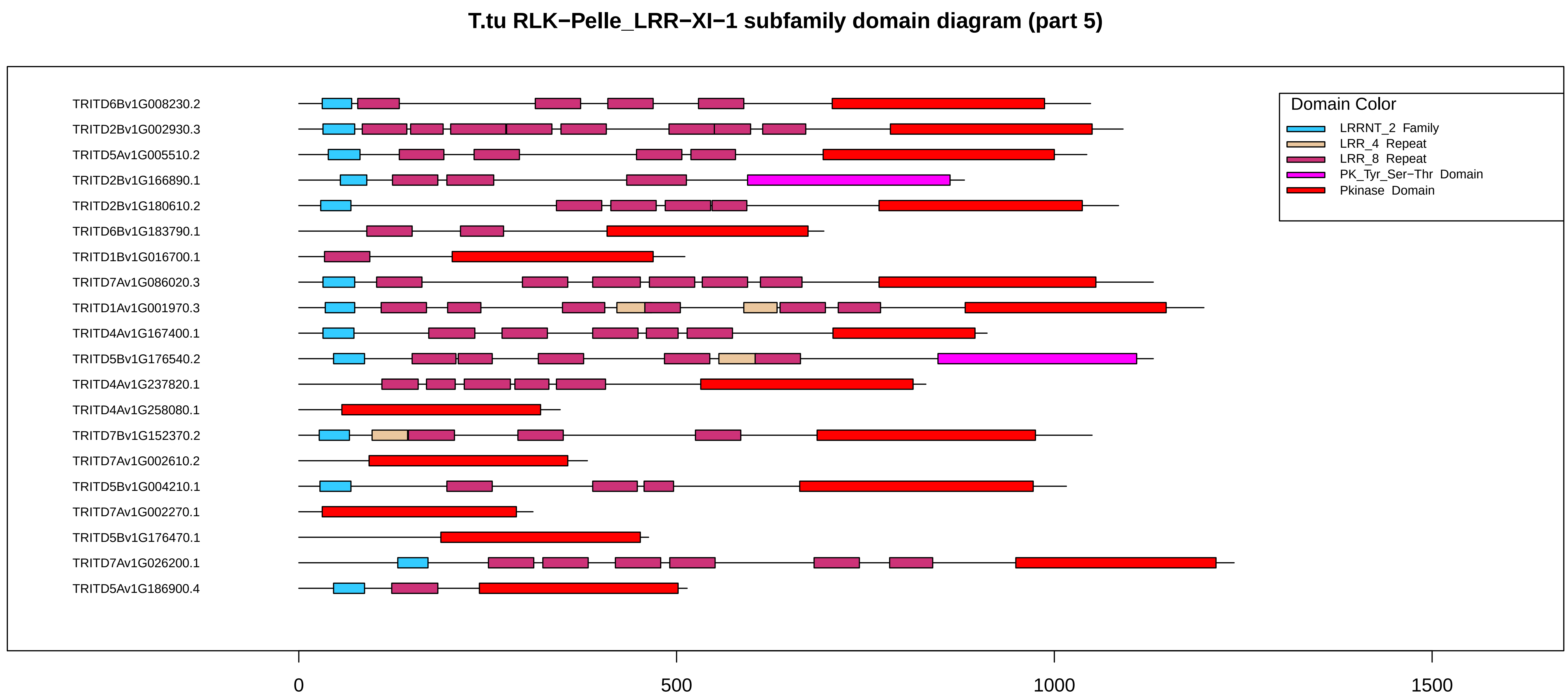
T.tu RLK−Pelle_LRR−XI−1 subfamily domain diagram (part 5)
TRITD6Bv1G008230.2
TRITD2Bv1G002930.3
TRITD5Av1G005510.2
TRITD2Bv1G166890.1
TRITD2Bv1G180610.2
TRITD6Bv1G183790.1
TRITD1Bv1G016700.1
TRITD7Av1G086020.3
TRITD1Av1G001970.3
TRITD4Av1G167400.1
TRITD5Bv1G176540.2
TRITD4Av1G237820.1
TRITD4Av1G258080.1
TRITD7Bv1G152370.2
TRITD7Av1G002610.2
TRITD5Bv1G004210.1
TRITD7Av1G002270.1
TRITD5Bv1G176470.1
TRITD7Av1G026200.1
TRITD5Av1G186900.4
Domain Color
LRRNT_2 Family
LRR_4 Repeat
LRR_8 Repeat
PK_Tyr_Ser−Thr Domain
Pkinase Domain
0
500
1000
1500

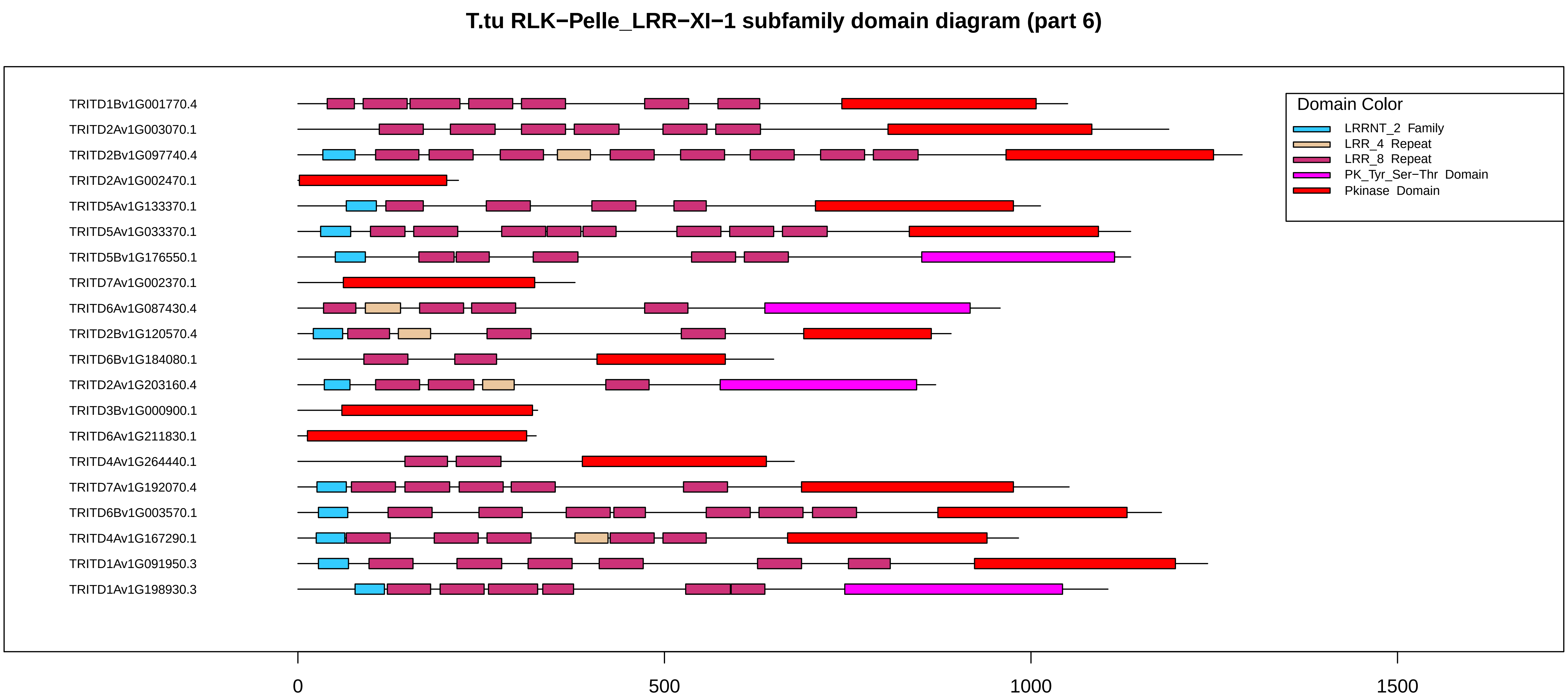
T.tu RLK−Pelle_LRR−XI−1 subfamily domain diagram (part 6)
TRITD1Bv1G001770.4
TRITD2Av1G003070.1
TRITD2Bv1G097740.4
TRITD2Av1G002470.1
TRITD5Av1G133370.1
TRITD5Av1G033370.1
TRITD5Bv1G176550.1
TRITD7Av1G002370.1
TRITD6Av1G087430.4
TRITD2Bv1G120570.4
TRITD6Bv1G184080.1
TRITD2Av1G203160.4
TRITD3Bv1G000900.1
TRITD6Av1G211830.1
TRITD4Av1G264440.1
TRITD7Av1G192070.4
TRITD6Bv1G003570.1
TRITD4Av1G167290.1
TRITD1Av1G091950.3
TRITD1Av1G198930.3
Domain Color
LRRNT_2 Family
LRR_4 Repeat
LRR_8 Repeat
PK_Tyr_Ser−Thr Domain
Pkinase Domain
0
500
1000
1500

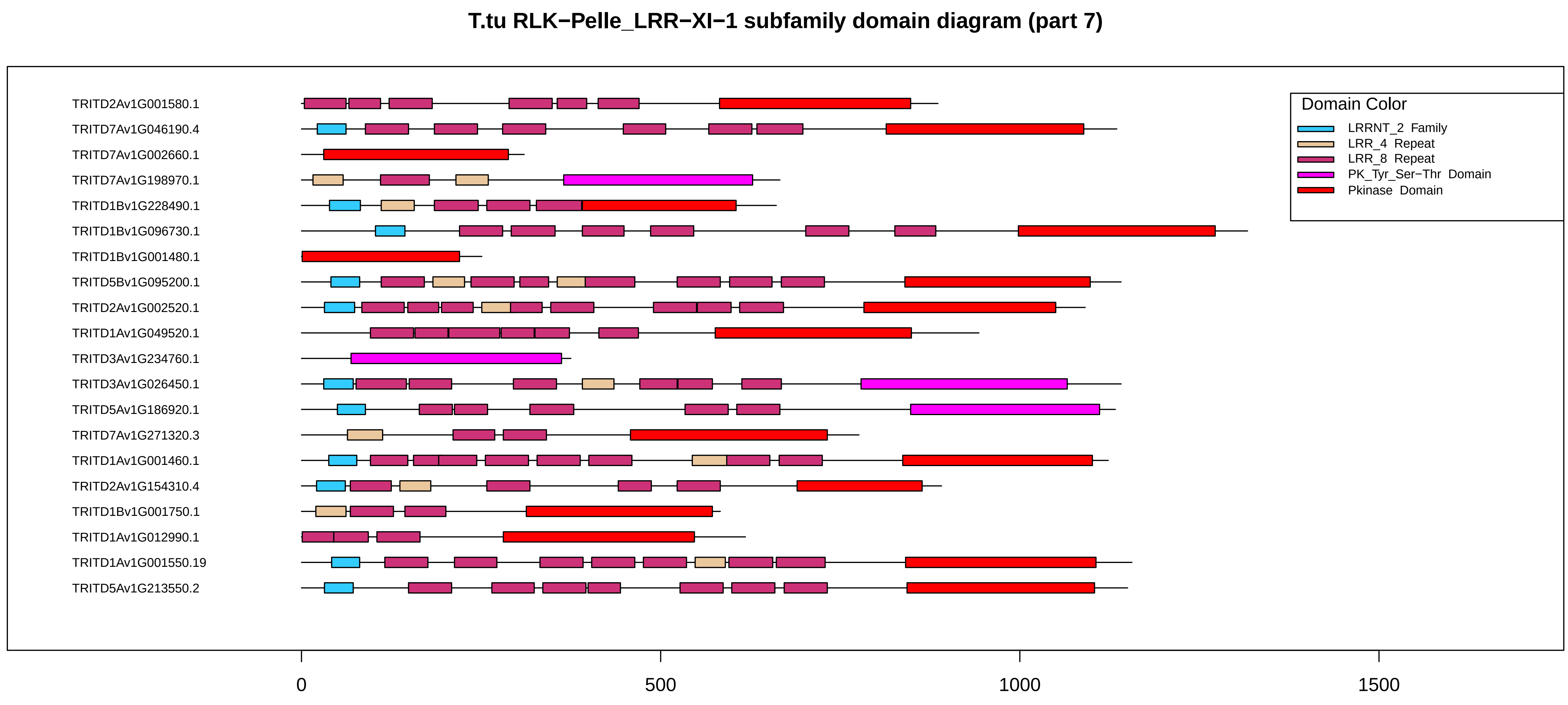
T.tu RLK−Pelle_LRR−XI−1 subfamily domain diagram (part 7)
TRITD2Av1G001580.1
TRITD7Av1G046190.4
TRITD7Av1G002660.1
TRITD7Av1G198970.1
TRITD1Bv1G228490.1
TRITD1Bv1G096730.1
TRITD1Bv1G001480.1
TRITD5Bv1G095200.1
TRITD2Av1G002520.1
TRITD1Av1G049520.1
TRITD3Av1G234760.1
TRITD3Av1G026450.1
TRITD5Av1G186920.1
TRITD7Av1G271320.3
TRITD1Av1G001460.1
TRITD2Av1G154310.4
TRITD1Bv1G001750.1
TRITD1Av1G012990.1
TRITD1Av1G001550.19
TRITD5Av1G213550.2
Domain Color
LRRNT_2 Family
LRR_4 Repeat
LRR_8 Repeat
PK_Tyr_Ser−Thr Domain
Pkinase Domain
0
500
1000
1500

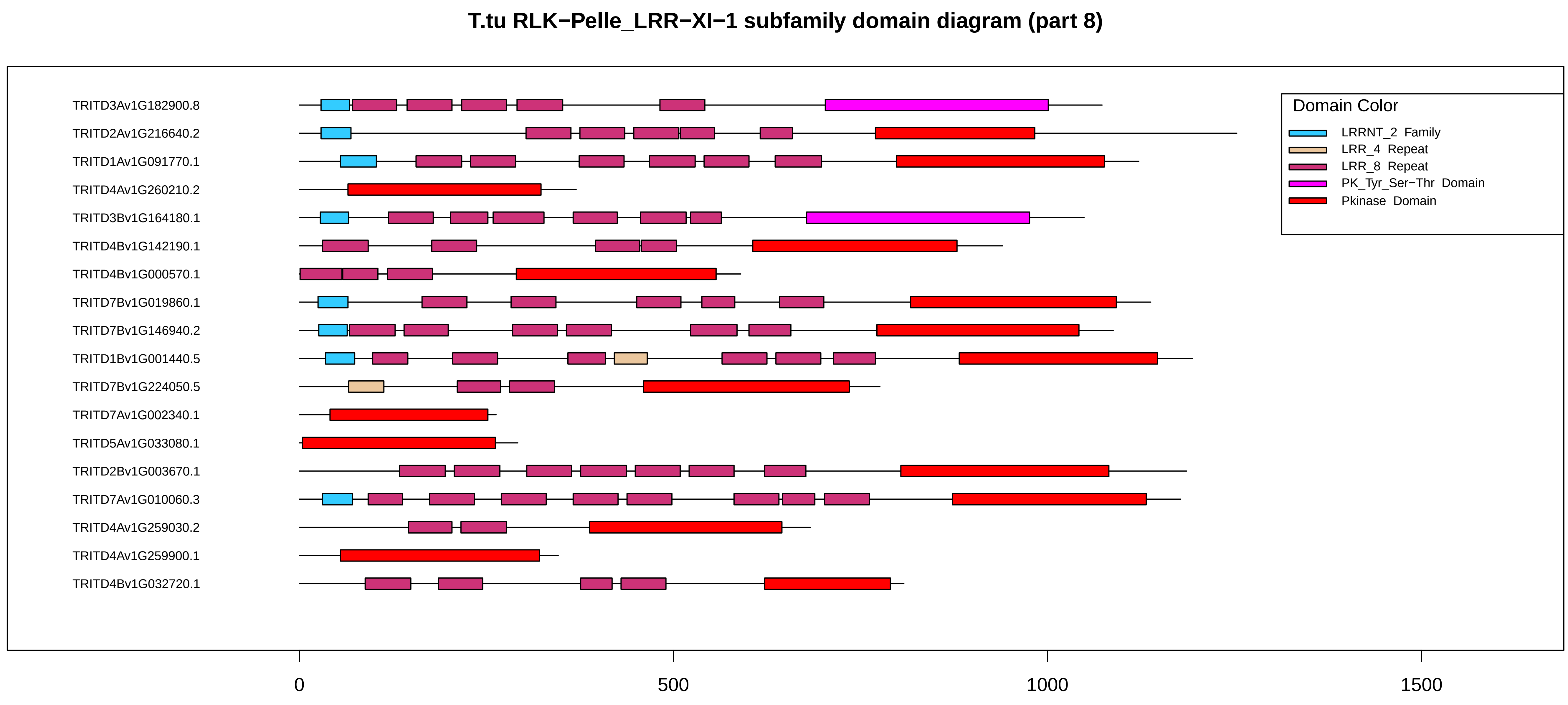

T.tu RLK−Pelle_LRR−XI−1 subfamily domain diagram (part 8)
TRITD3Av1G182900.8
TRITD2Av1G216640.2
TRITD1Av1G091770.1
TRITD4Av1G260210.2
TRITD3Bv1G164180.1
TRITD4Bv1G142190.1
TRITD4Bv1G000570.1
TRITD7Bv1G019860.1
TRITD7Bv1G146940.2
TRITD1Bv1G001440.5
TRITD7Bv1G224050.5
TRITD7Av1G002340.1
TRITD5Av1G033080.1
TRITD2Bv1G003670.1
TRITD7Av1G010060.3
TRITD4Av1G259030.2
TRITD4Av1G259900.1
TRITD4Bv1G032720.1
Domain Color
LRRNT_2 Family
LRR_4 Repeat
LRR_8 Repeat
PK_Tyr_Ser−Thr Domain
Pkinase Domain
0
500
1000
1500

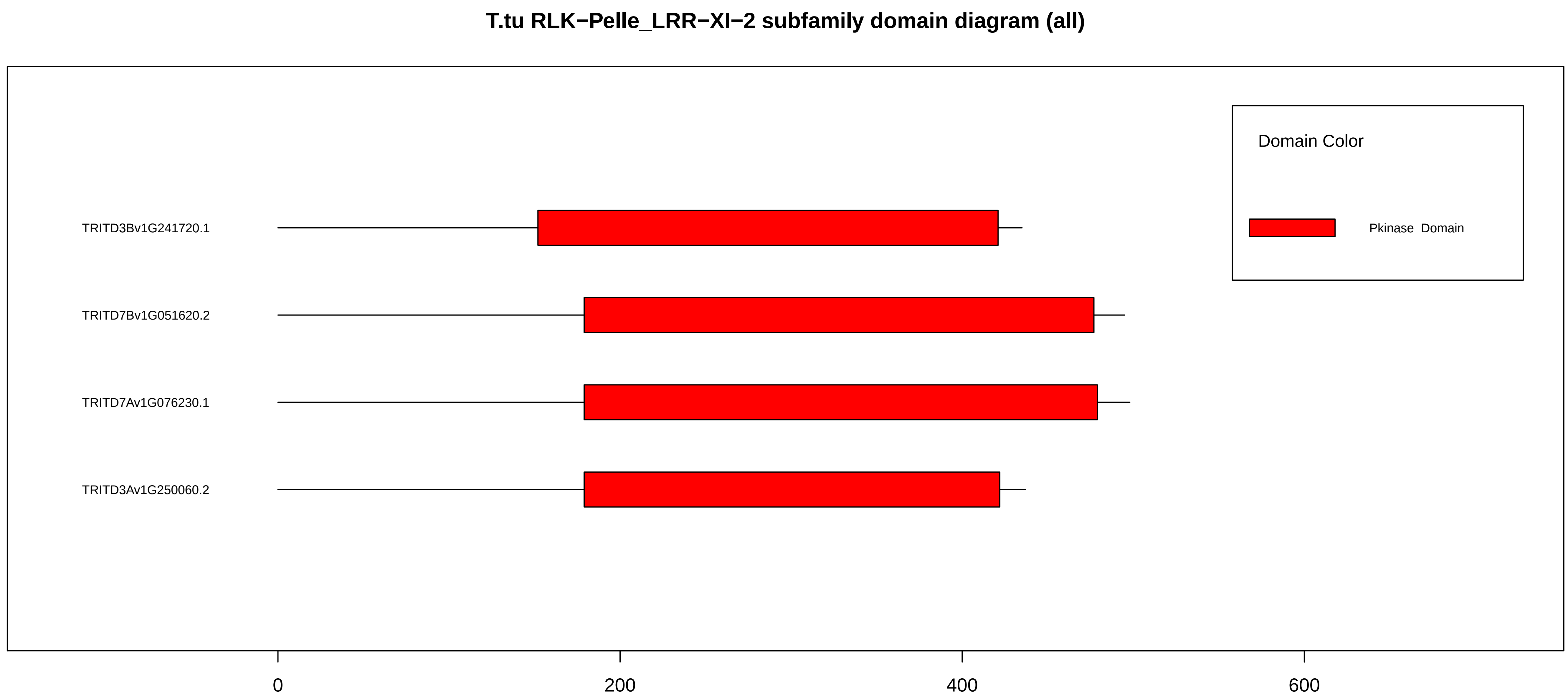

T.tu RLK−Pelle_LRR−XI−2 subfamily domain diagram (all)
TRITD3Bv1G241720.1
TRITD7Bv1G051620.2
TRITD7Av1G076230.1
TRITD3Av1G250060.2
Domain Color
Pkinase Domain
0
200
400
600

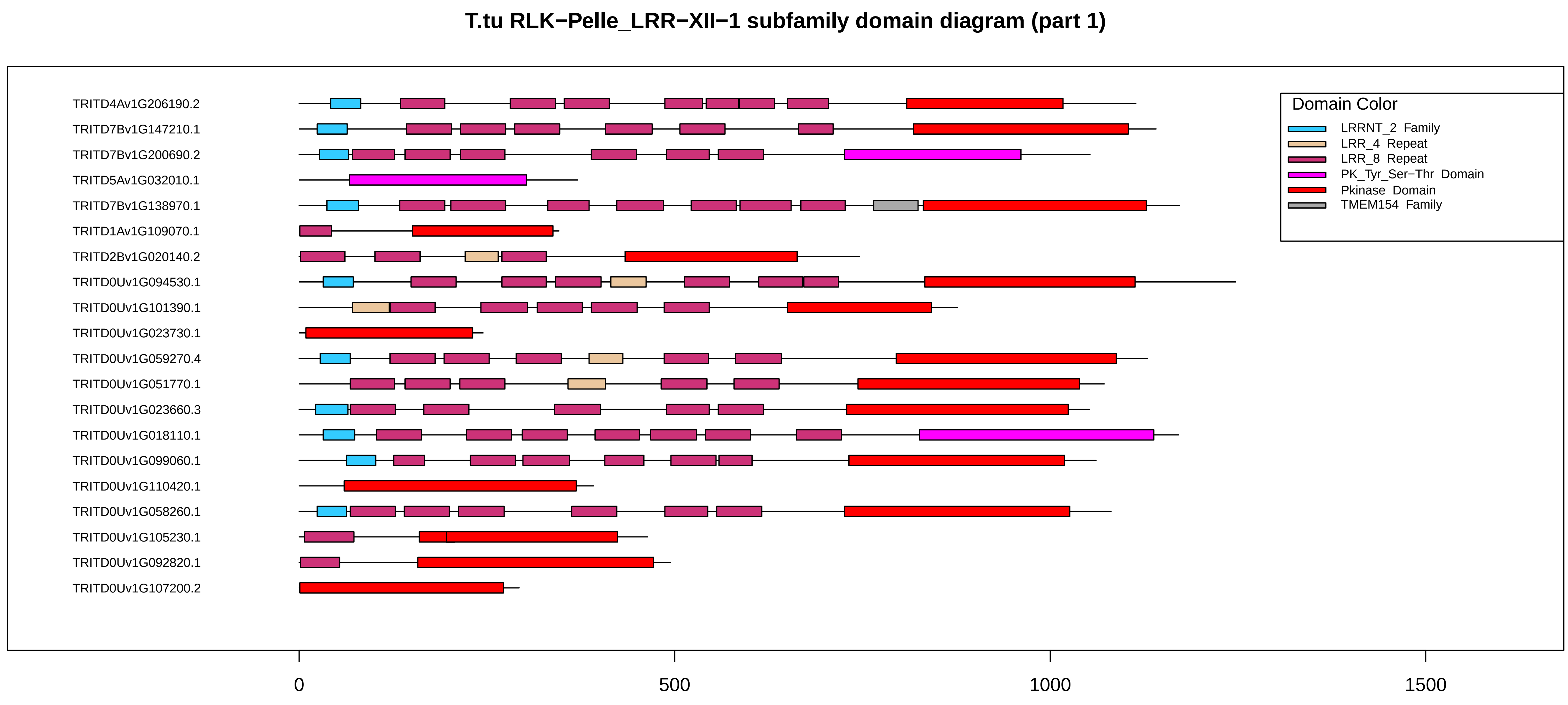
T.tu RLK−Pelle_LRR−XII−1 subfamily domain diagram (part 1)
TRITD4Av1G206190.2
TRITD7Bv1G147210.1
TRITD7Bv1G200690.2
TRITD5Av1G032010.1
TRITD7Bv1G138970.1
TRITD1Av1G109070.1
TRITD2Bv1G020140.2
TRITD0Uv1G094530.1
TRITD0Uv1G101390.1
TRITD0Uv1G023730.1
TRITD0Uv1G059270.4
TRITD0Uv1G051770.1
TRITD0Uv1G023660.3
TRITD0Uv1G018110.1
TRITD0Uv1G099060.1
TRITD0Uv1G110420.1
TRITD0Uv1G058260.1
TRITD0Uv1G105230.1
TRITD0Uv1G092820.1
TRITD0Uv1G107200.2
Domain Color
LRRNT_2 Family
LRR_4 Repeat
LRR_8 Repeat
PK_Tyr_Ser−Thr Domain
Pkinase Domain
TMEM154 Family
0
500
1000
1500

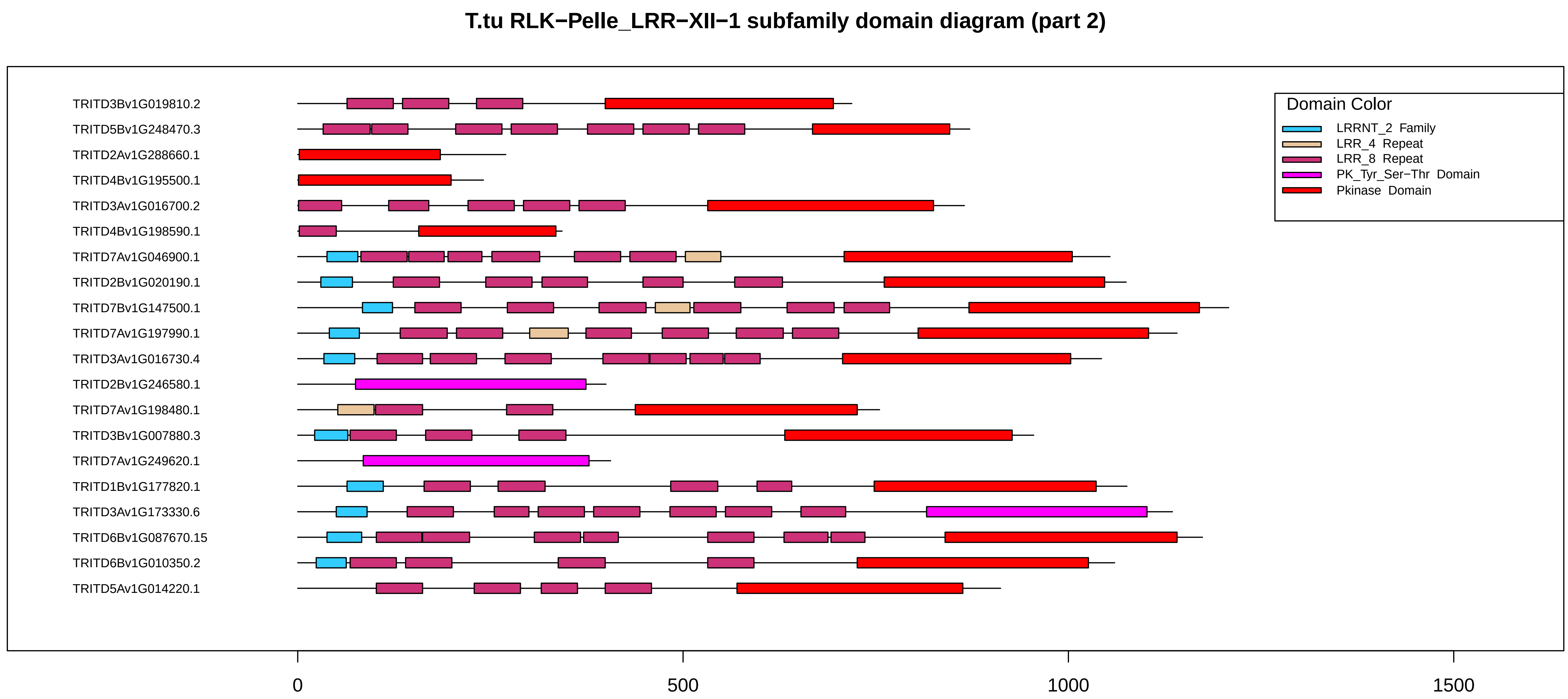
T.tu RLK−Pelle_LRR−XII−1 subfamily domain diagram (part 2)
TRITD3Bv1G019810.2
TRITD5Bv1G248470.3
TRITD2Av1G288660.1
TRITD4Bv1G195500.1
TRITD3Av1G016700.2
TRITD4Bv1G198590.1
TRITD7Av1G046900.1
TRITD2Bv1G020190.1
TRITD7Bv1G147500.1
TRITD7Av1G197990.1
TRITD3Av1G016730.4
TRITD2Bv1G246580.1
TRITD7Av1G198480.1
TRITD3Bv1G007880.3
TRITD7Av1G249620.1
TRITD1Bv1G177820.1
TRITD3Av1G173330.6
TRITD6Bv1G087670.15
TRITD6Bv1G010350.2
TRITD5Av1G014220.1
Domain Color
LRRNT_2 Family
LRR_4 Repeat
LRR_8 Repeat
PK_Tyr_Ser−Thr Domain
Pkinase Domain
0
500
1000
1500

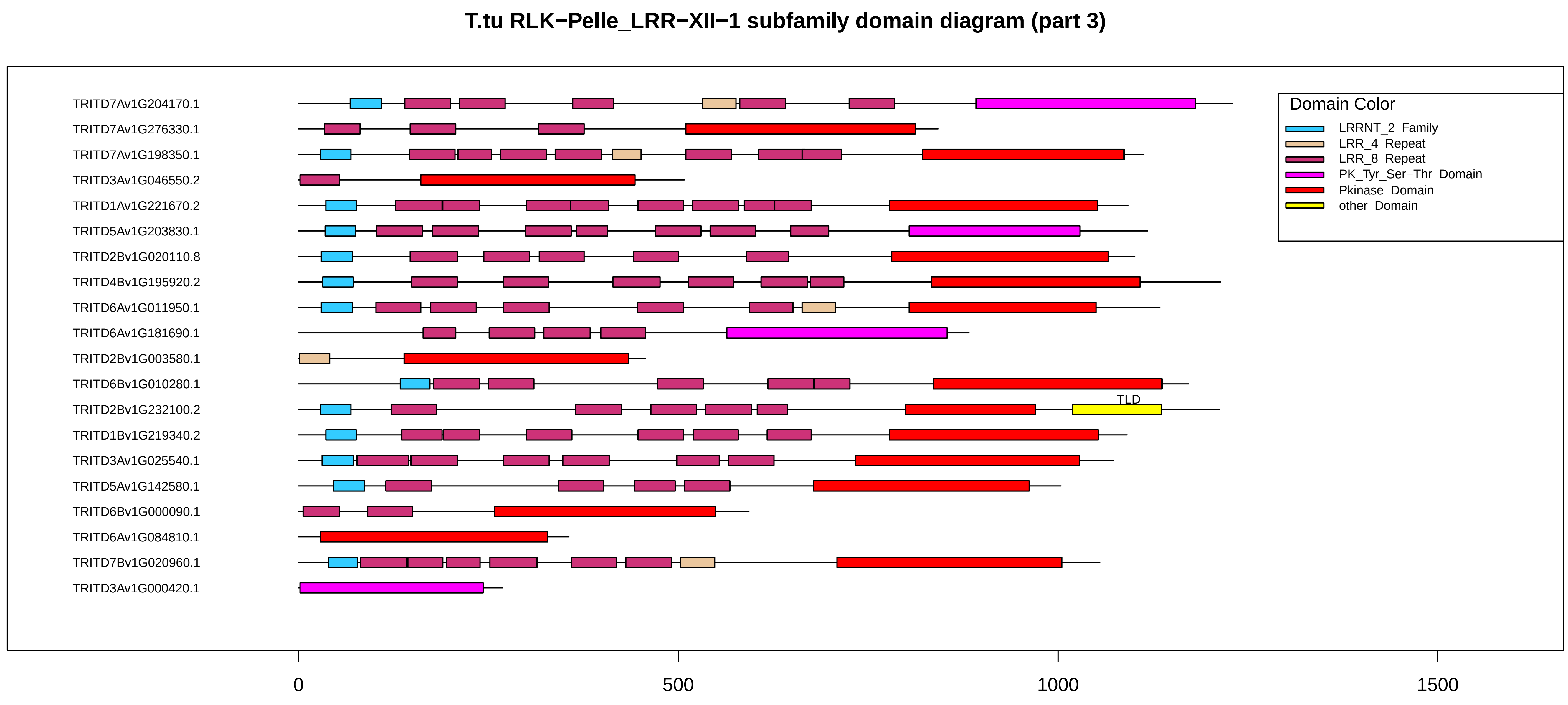
T.tu RLK−Pelle_LRR−XII−1 subfamily domain diagram (part 3)
TRITD7Av1G204170.1
TRITD7Av1G276330.1
TRITD7Av1G198350.1
TRITD3Av1G046550.2
TRITD1Av1G221670.2
TRITD5Av1G203830.1
TRITD2Bv1G020110.8
TRITD4Bv1G195920.2
TRITD6Av1G011950.1
TRITD6Av1G181690.1
TRITD2Bv1G003580.1
TRITD6Bv1G010280.1
TRITD2Bv1G232100.2
TRITD1Bv1G219340.2
TRITD3Av1G025540.1
TRITD5Av1G142580.1
TRITD6Bv1G000090.1
TRITD6Av1G084810.1
TRITD7Bv1G020960.1
TRITD3Av1G000420.1
TLD
Domain Color
LRRNT_2 Family
LRR_4 Repeat
LRR_8 Repeat
PK_Tyr_Ser−Thr Domain
Pkinase Domain
other Domain
0
500
1000
1500

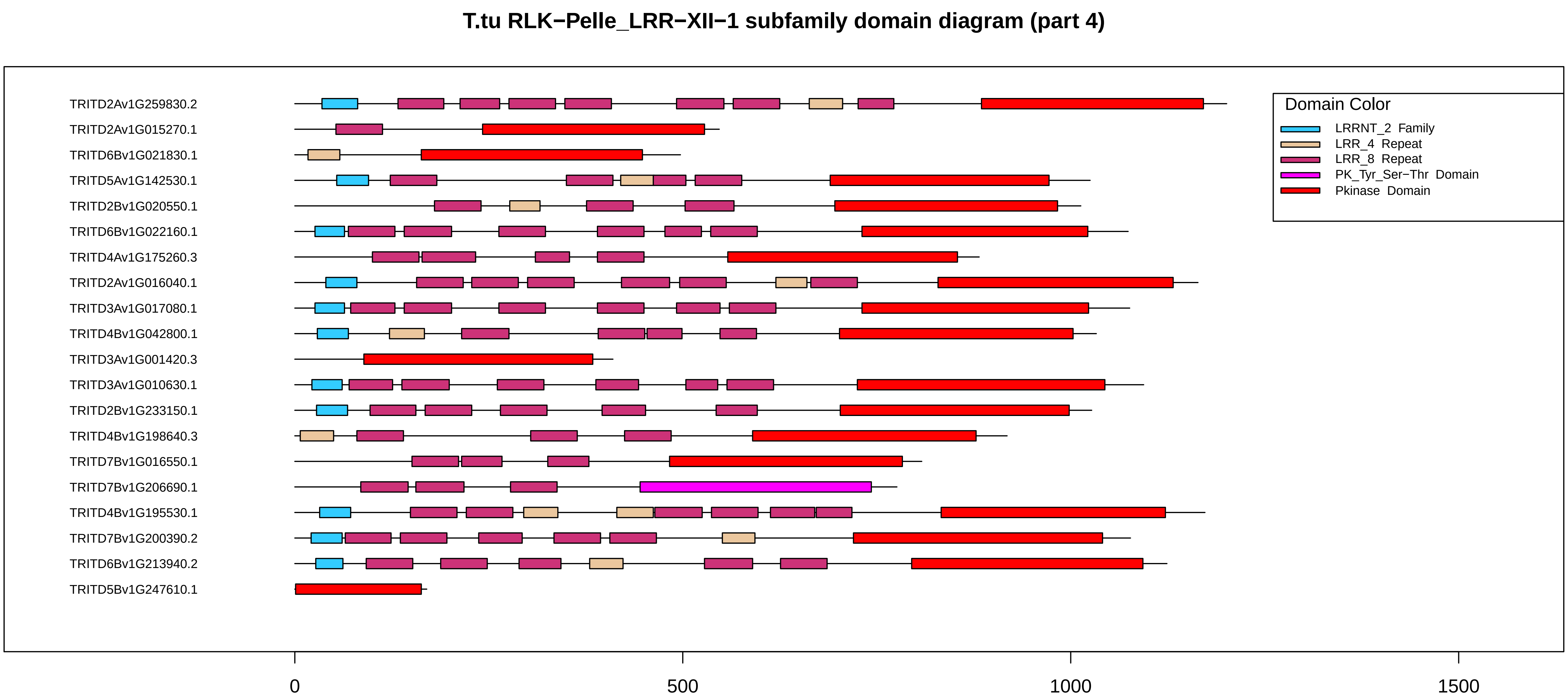
T.tu RLK−Pelle_LRR−XII−1 subfamily domain diagram (part 4)
TRITD2Av1G259830.2
TRITD2Av1G015270.1
TRITD6Bv1G021830.1
TRITD5Av1G142530.1
TRITD2Bv1G020550.1
TRITD6Bv1G022160.1
TRITD4Av1G175260.3
TRITD2Av1G016040.1
TRITD3Av1G017080.1
TRITD4Bv1G042800.1
TRITD3Av1G001420.3
TRITD3Av1G010630.1
TRITD2Bv1G233150.1
TRITD4Bv1G198640.3
TRITD7Bv1G016550.1
TRITD7Bv1G206690.1
TRITD4Bv1G195530.1
TRITD7Bv1G200390.2
TRITD6Bv1G213940.2
TRITD5Bv1G247610.1
Domain Color
LRRNT_2 Family
LRR_4 Repeat
LRR_8 Repeat
PK_Tyr_Ser−Thr Domain
Pkinase Domain
0
500
1000
1500

T.tu RLK−Pelle_LRR−XII−1 subfamily domain diagram (part 5)

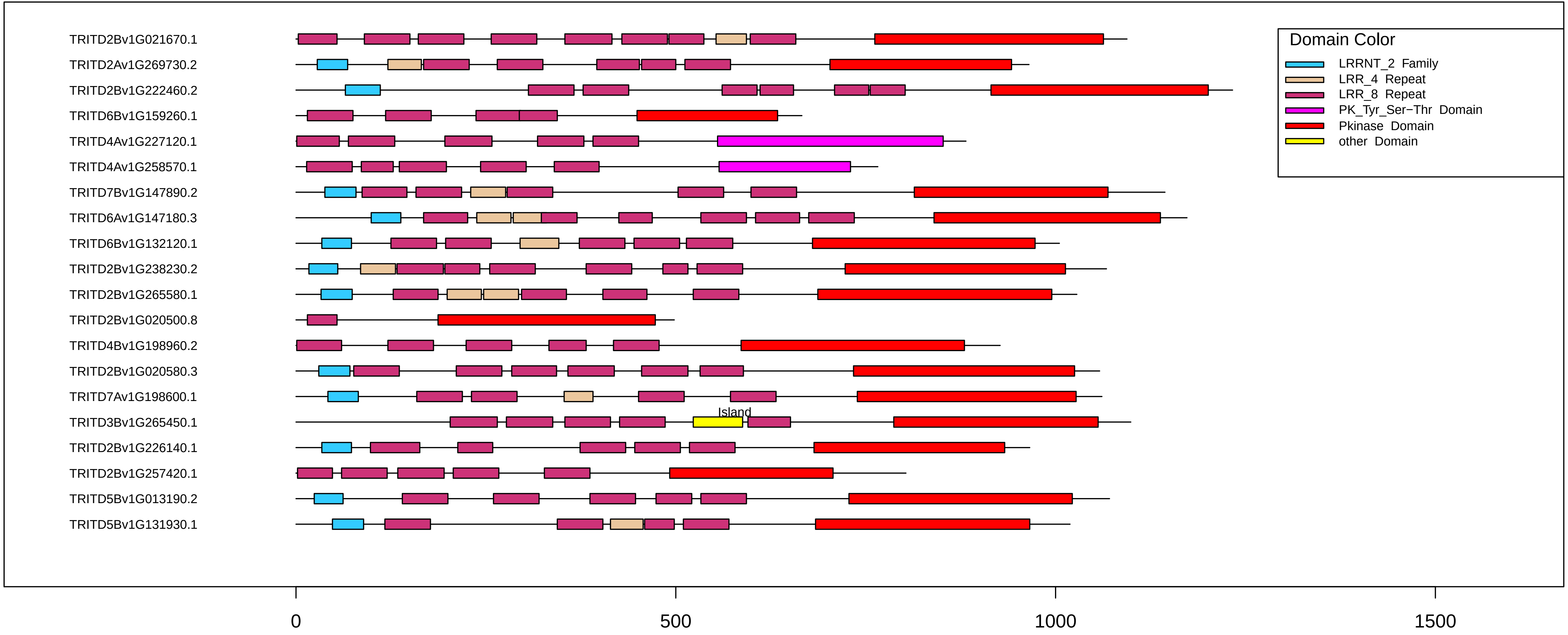

T.tu RLK−Pelle_LRR−XII−1 subfamily domain diagram (part 6)

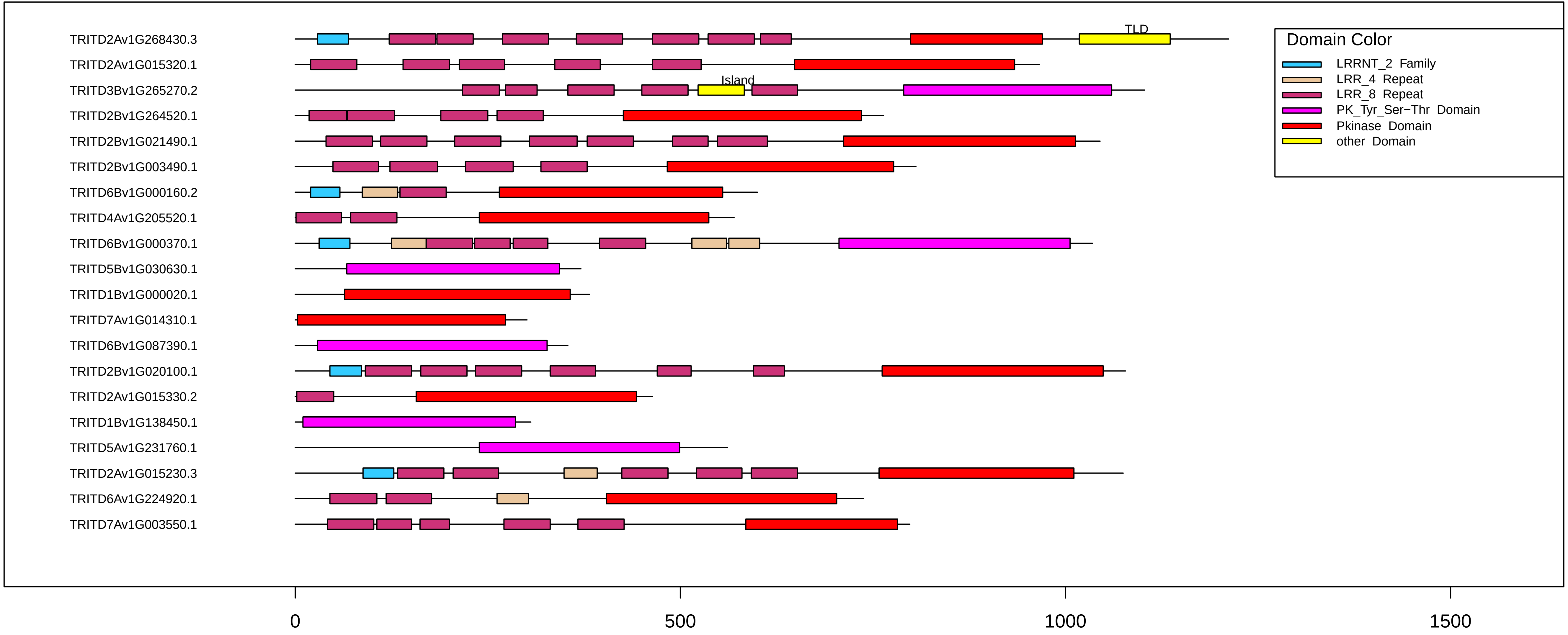

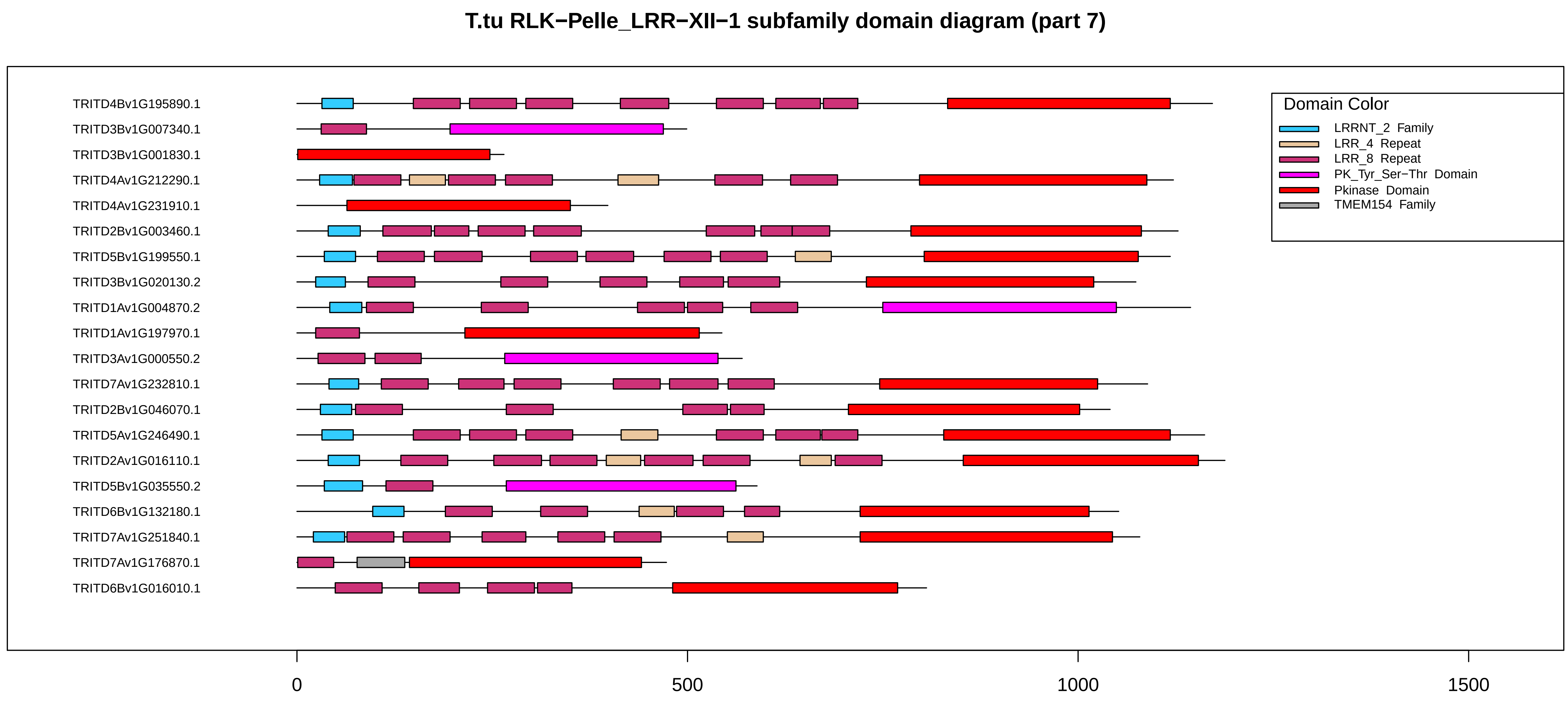
T.tu RLK−Pelle_LRR−XII−1 subfamily domain diagram (part 7)
TRITD4Bv1G195890.1
TRITD3Bv1G007340.1
TRITD3Bv1G001830.1
TRITD4Av1G212290.1
TRITD4Av1G231910.1
TRITD2Bv1G003460.1
TRITD5Bv1G199550.1
TRITD3Bv1G020130.2
TRITD1Av1G004870.2
TRITD1Av1G197970.1
TRITD3Av1G000550.2
TRITD7Av1G232810.1
TRITD2Bv1G046070.1
TRITD5Av1G246490.1
TRITD2Av1G016110.1
TRITD5Bv1G035550.2
TRITD6Bv1G132180.1
TRITD7Av1G251840.1
TRITD7Av1G176870.1
TRITD6Bv1G016010.1
Domain Color
LRRNT_2 Family
LRR_4 Repeat
LRR_8 Repeat
PK_Tyr_Ser−Thr Domain
Pkinase Domain
TMEM154 Family
0
500
1000
1500

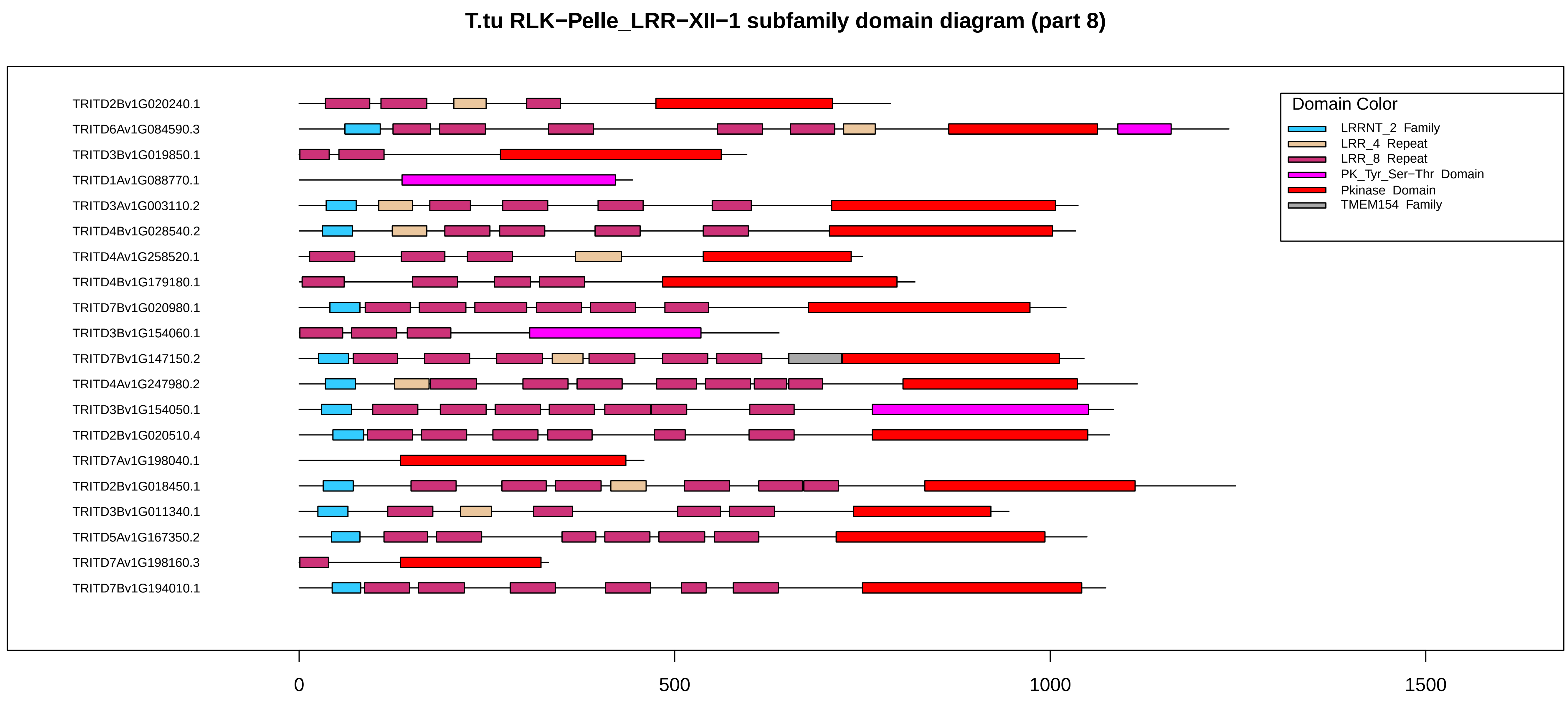
T.tu RLK−Pelle_LRR−XII−1 subfamily domain diagram (part 8)
TRITD2Bv1G020240.1
TRITD6Av1G084590.3
TRITD3Bv1G019850.1
TRITD1Av1G088770.1
TRITD3Av1G003110.2
TRITD4Bv1G028540.2
TRITD4Av1G258520.1
TRITD4Bv1G179180.1
TRITD7Bv1G020980.1
TRITD3Bv1G154060.1
TRITD7Bv1G147150.2
TRITD4Av1G247980.2
TRITD3Bv1G154050.1
TRITD2Bv1G020510.4
TRITD7Av1G198040.1
TRITD2Bv1G018450.1
TRITD3Bv1G011340.1
TRITD5Av1G167350.2
TRITD7Av1G198160.3
TRITD7Bv1G194010.1
Domain Color
LRRNT_2 Family
LRR_4 Repeat
LRR_8 Repeat
PK_Tyr_Ser−Thr Domain
Pkinase Domain
TMEM154 Family
0
500
1000
1500

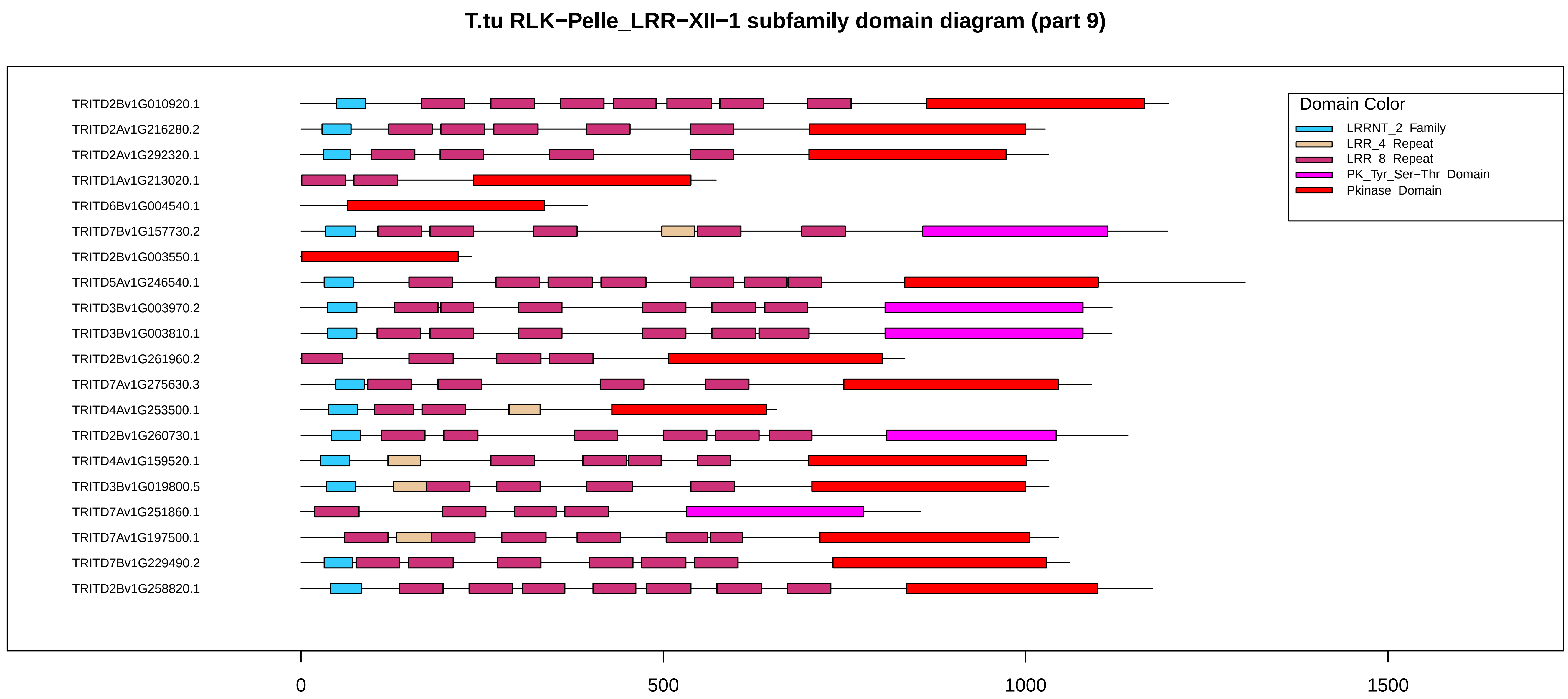
T.tu RLK−Pelle_LRR−XII−1 subfamily domain diagram (part 9)
TRITD2Bv1G010920.1
TRITD2Av1G216280.2
TRITD2Av1G292320.1
TRITD1Av1G213020.1
TRITD6Bv1G004540.1
TRITD7Bv1G157730.2
TRITD2Bv1G003550.1
TRITD5Av1G246540.1
TRITD3Bv1G003970.2
TRITD3Bv1G003810.1
TRITD2Bv1G261960.2
TRITD7Av1G275630.3
TRITD4Av1G253500.1
TRITD2Bv1G260730.1
TRITD4Av1G159520.1
TRITD3Bv1G019800.5
TRITD7Av1G251860.1
TRITD7Av1G197500.1
TRITD7Bv1G229490.2
TRITD2Bv1G258820.1
Domain Color
LRRNT_2 Family
LRR_4 Repeat
LRR_8 Repeat
PK_Tyr_Ser−Thr Domain
Pkinase Domain
0
500
1000
1500

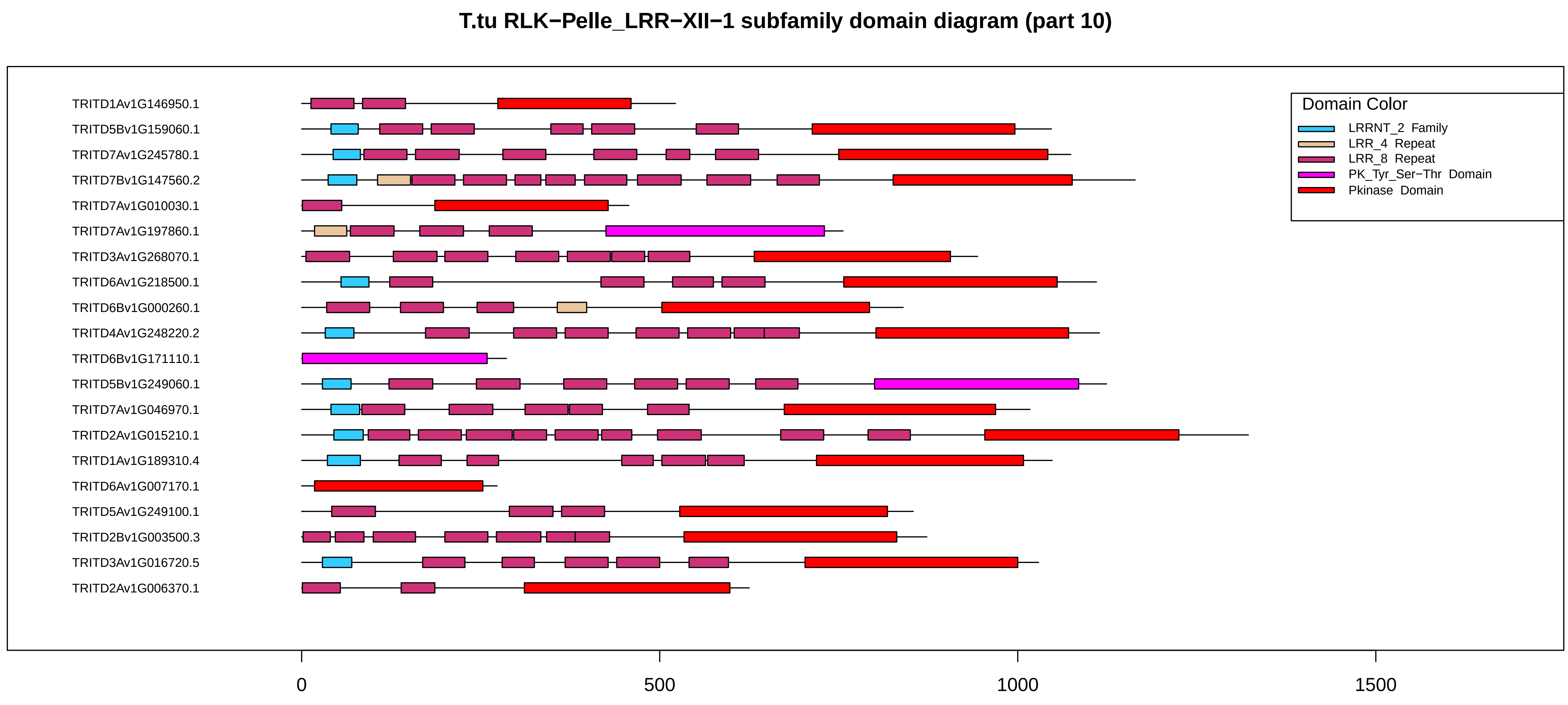
T.tu RLK−Pelle_LRR−XII−1 subfamily domain diagram (part 10)
TRITD1Av1G146950.1
TRITD5Bv1G159060.1
TRITD7Av1G245780.1
TRITD7Bv1G147560.2
TRITD7Av1G010030.1
TRITD7Av1G197860.1
TRITD3Av1G268070.1
TRITD6Av1G218500.1
TRITD6Bv1G000260.1
TRITD4Av1G248220.2
TRITD6Bv1G171110.1
TRITD5Bv1G249060.1
TRITD7Av1G046970.1
TRITD2Av1G015210.1
TRITD1Av1G189310.4
TRITD6Av1G007170.1
TRITD5Av1G249100.1
TRITD2Bv1G003500.3
TRITD3Av1G016720.5
TRITD2Av1G006370.1
Domain Color
LRRNT_2 Family
LRR_4 Repeat
LRR_8 Repeat
PK_Tyr_Ser−Thr Domain
Pkinase Domain
0
500
1000
1500

T.tu RLK−Pelle_LRR−XII−1 subfamily domain diagram (part 11)

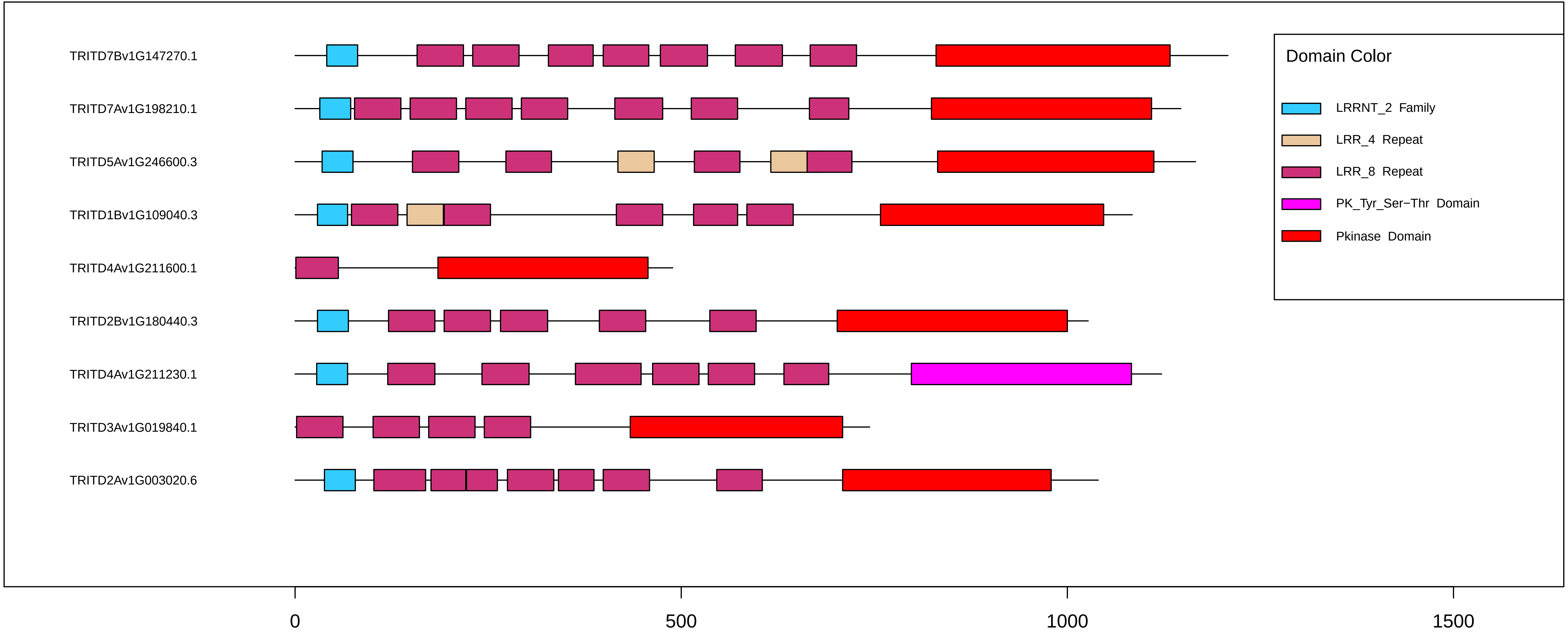

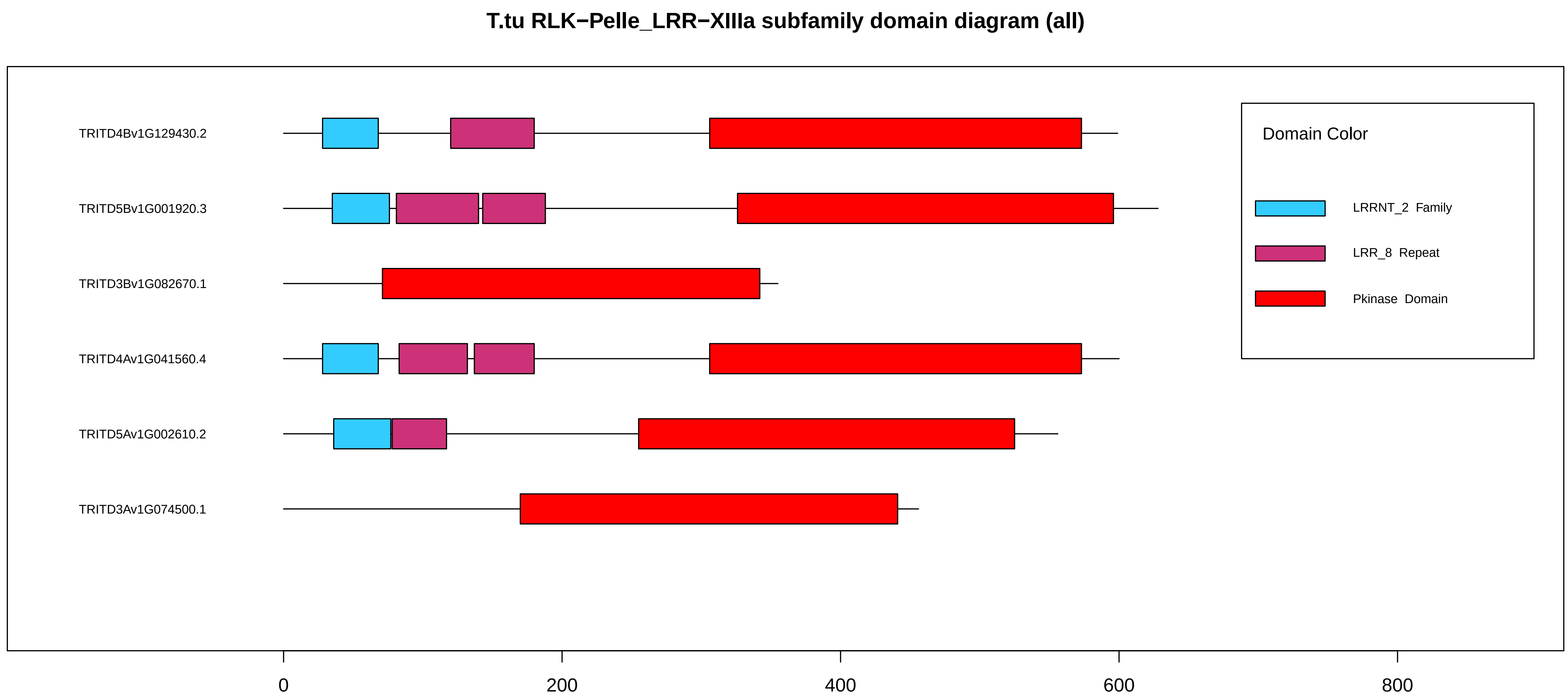
T.tu RLK−Pelle_LRR−XIIIa subfamily domain diagram (all)
TRITD4Bv1G129430.2
TRITD5Bv1G001920.3
TRITD3Bv1G082670.1
TRITD4Av1G041560.4
TRITD5Av1G002610.2
TRITD3Av1G074500.1
Domain Color
LRRNT_2 Family
LRR_8 Repeat
Pkinase Domain
0
200
400
600
800

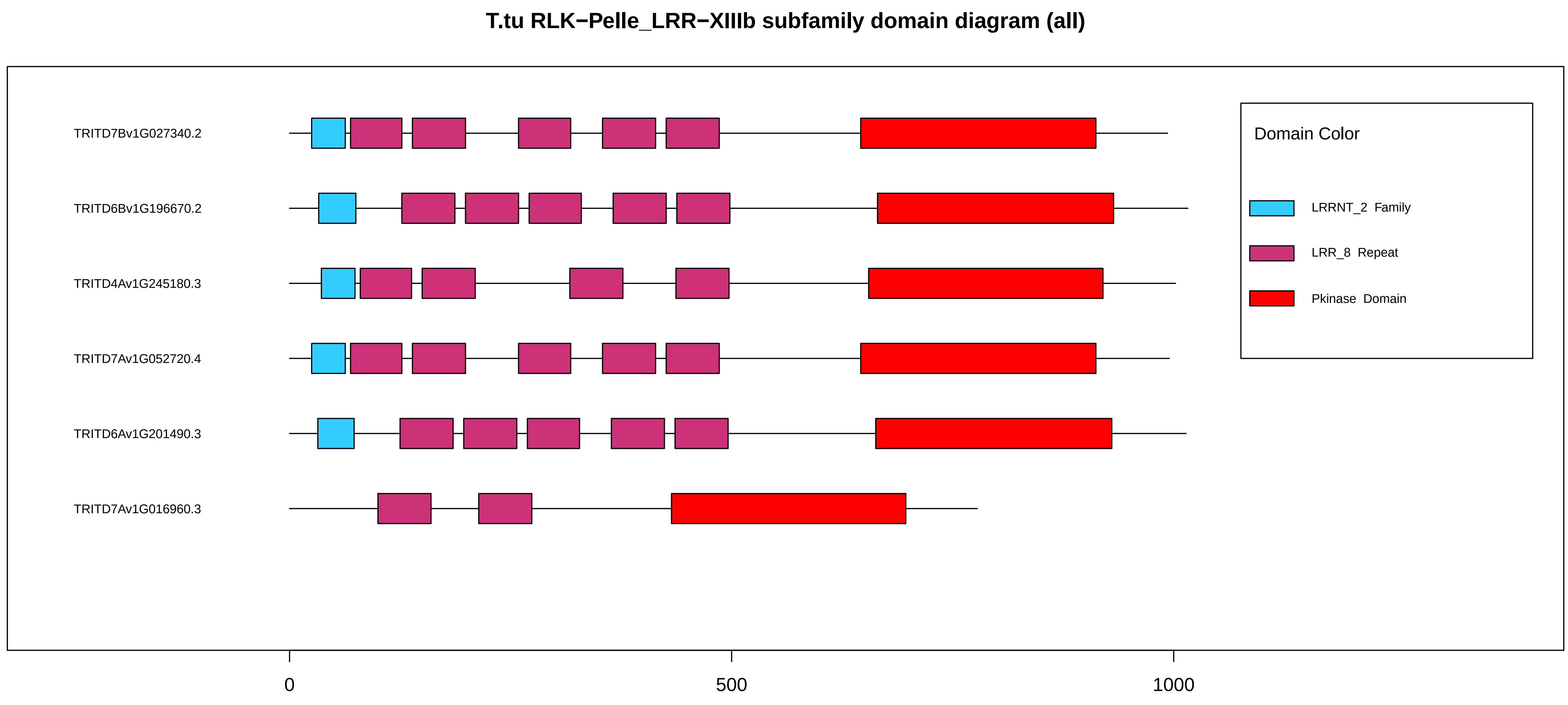
T.tu RLK−Pelle_LRR−XIIIb subfamily domain diagram (all)
TRITD7Bv1G027340.2
TRITD6Bv1G196670.2
TRITD4Av1G245180.3
TRITD7Av1G052720.4
TRITD6Av1G201490.3
TRITD7Av1G016960.3
Domain Color
LRRNT_2 Family
LRR_8 Repeat
Pkinase Domain
0
500
1000

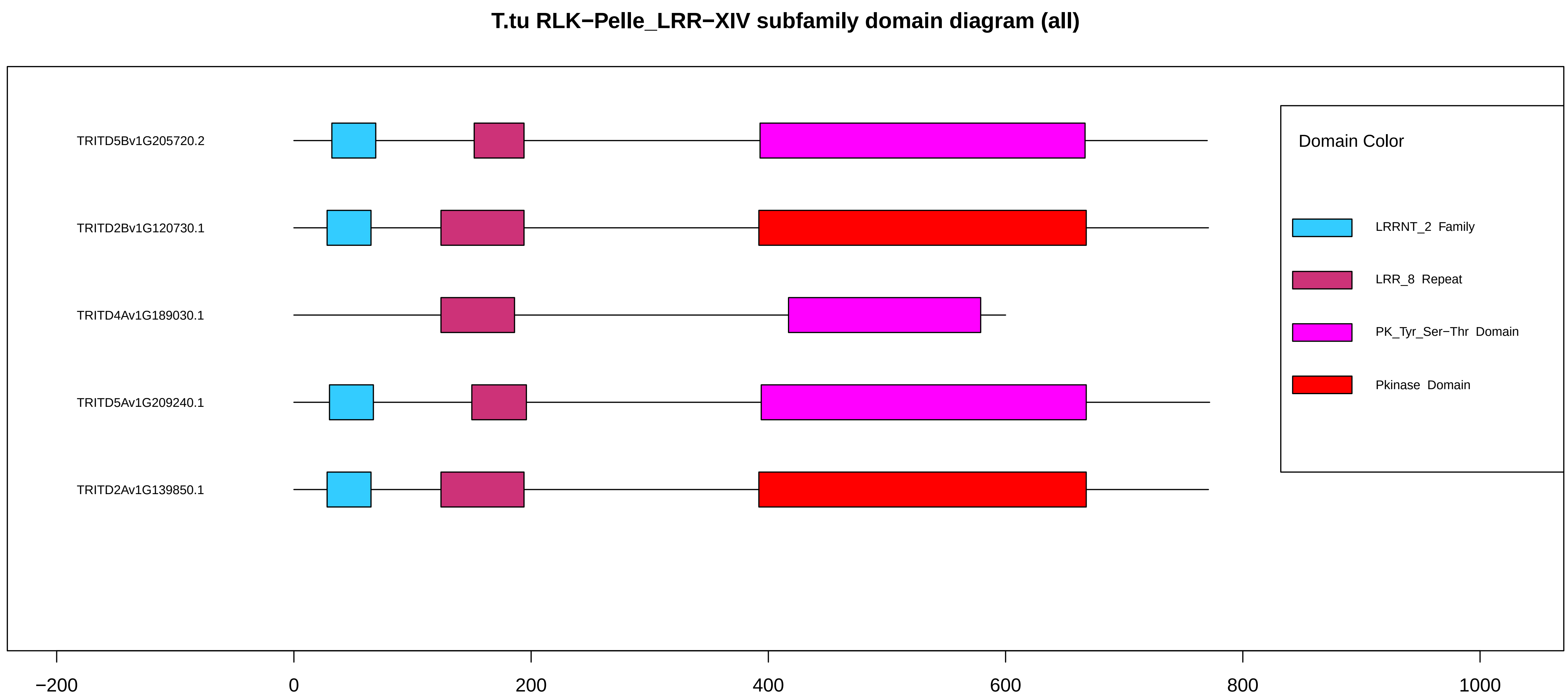
T.tu RLK−Pelle_LRR−XIV subfamily domain diagram (all)
TRITD5Bv1G205720.2
TRITD2Bv1G120730.1
TRITD4Av1G189030.1
TRITD5Av1G209240.1
TRITD2Av1G139850.1
Domain Color
LRRNT_2 Family
LRR_8 Repeat
PK_Tyr_Ser−Thr Domain
Pkinase Domain
−200
0
200
400
600
800
1000

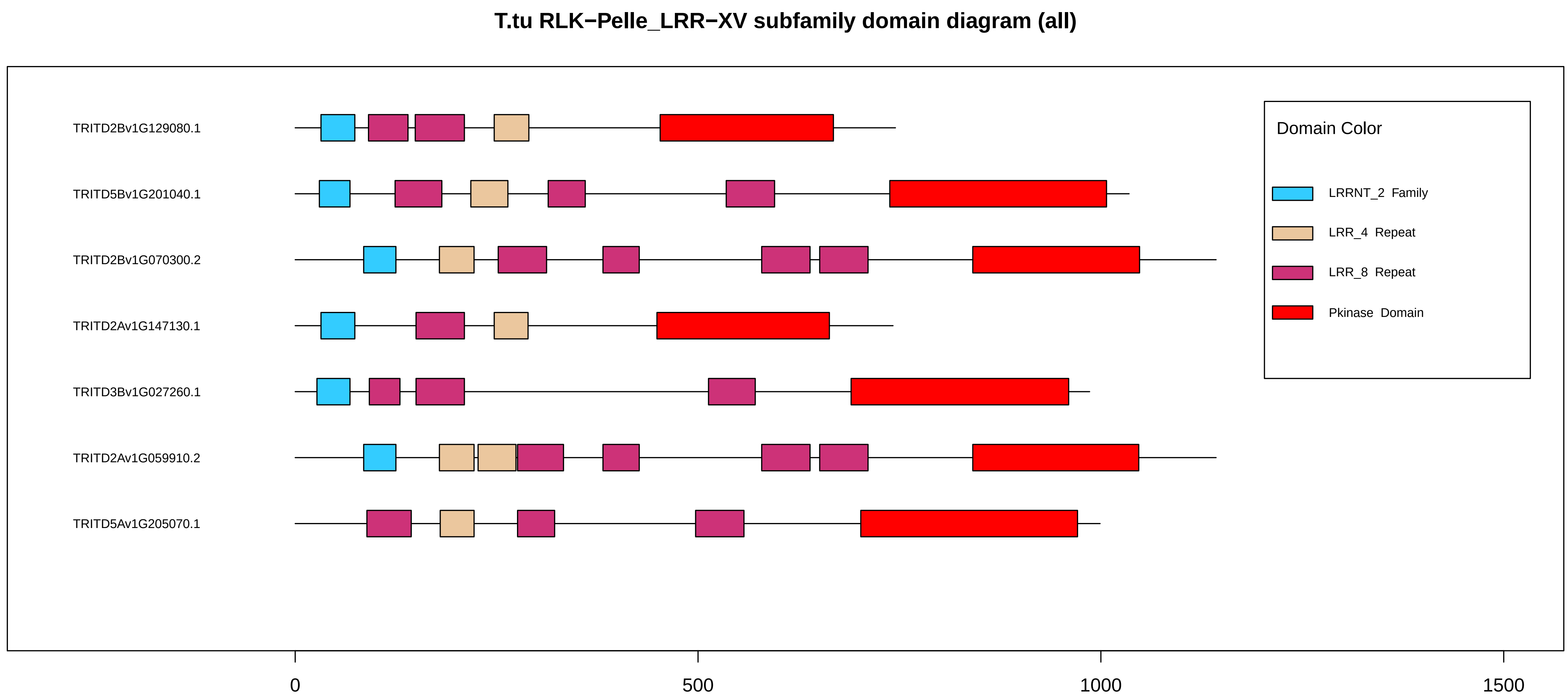
T.tu RLK−Pelle_LRR−XV subfamily domain diagram (all)
TRITD2Bv1G129080.1
TRITD5Bv1G201040.1
TRITD2Bv1G070300.2
TRITD2Av1G147130.1
TRITD3Bv1G027260.1
TRITD2Av1G059910.2
TRITD5Av1G205070.1
Domain Color
LRRNT_2 Family
LRR_4 Repeat
LRR_8 Repeat
Pkinase Domain
0
500
1000
1500

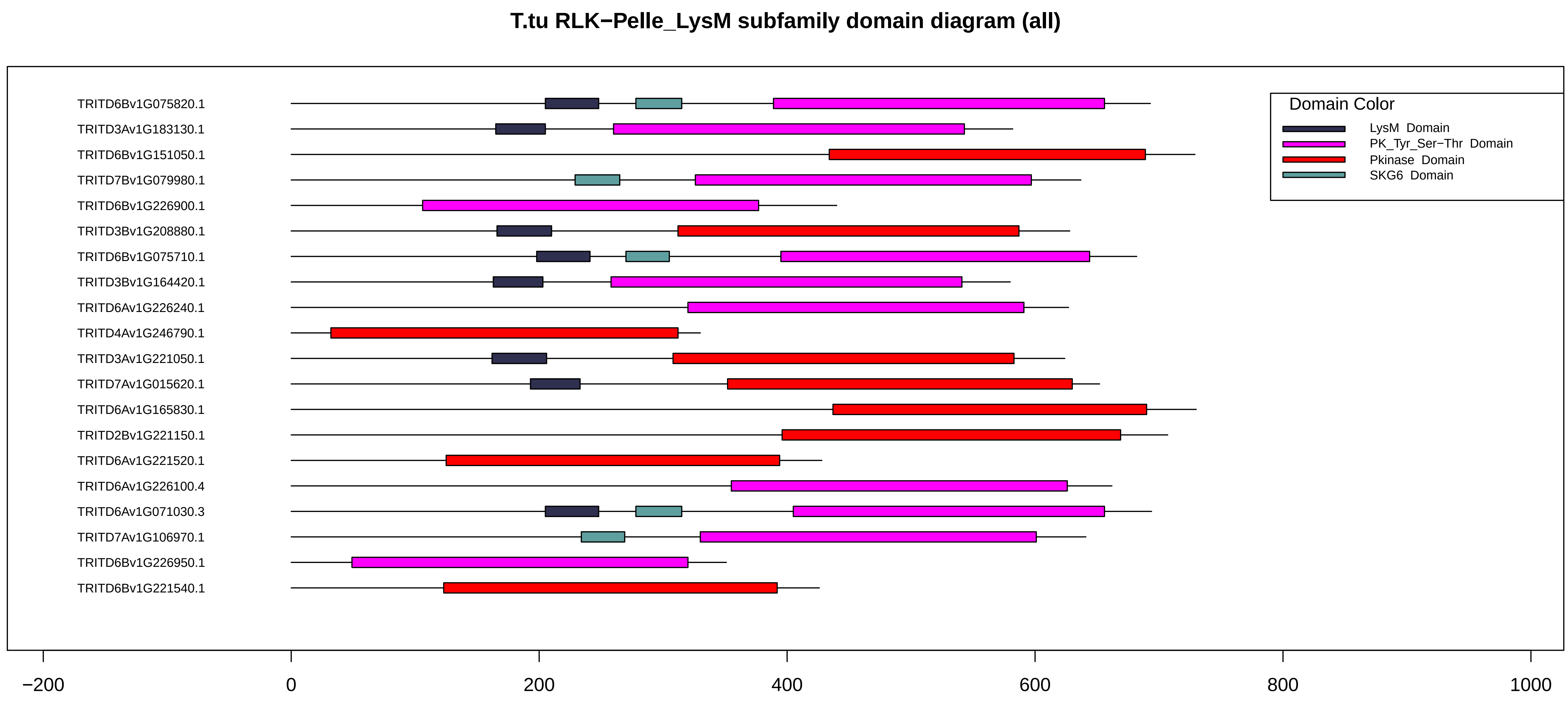
T.tu RLK−Pelle_LysM subfamily domain diagram (all)
TRITD6Bv1G075820.1
TRITD3Av1G183130.1
TRITD6Bv1G151050.1
TRITD7Bv1G079980.1
TRITD6Bv1G226900.1
TRITD3Bv1G208880.1
TRITD6Bv1G075710.1
TRITD3Bv1G164420.1
TRITD6Av1G226240.1
TRITD4Av1G246790.1
TRITD3Av1G221050.1
TRITD7Av1G015620.1
TRITD6Av1G165830.1
TRITD2Bv1G221150.1
TRITD6Av1G221520.1
TRITD6Av1G226100.4
TRITD6Av1G071030.3
TRITD7Av1G106970.1
TRITD6Bv1G226950.1
TRITD6Bv1G221540.1
Domain Color
LysM Domain
PK_Tyr_Ser−Thr Domain
Pkinase Domain
SKG6 Domain
−200
0
200
400
600
800
1000

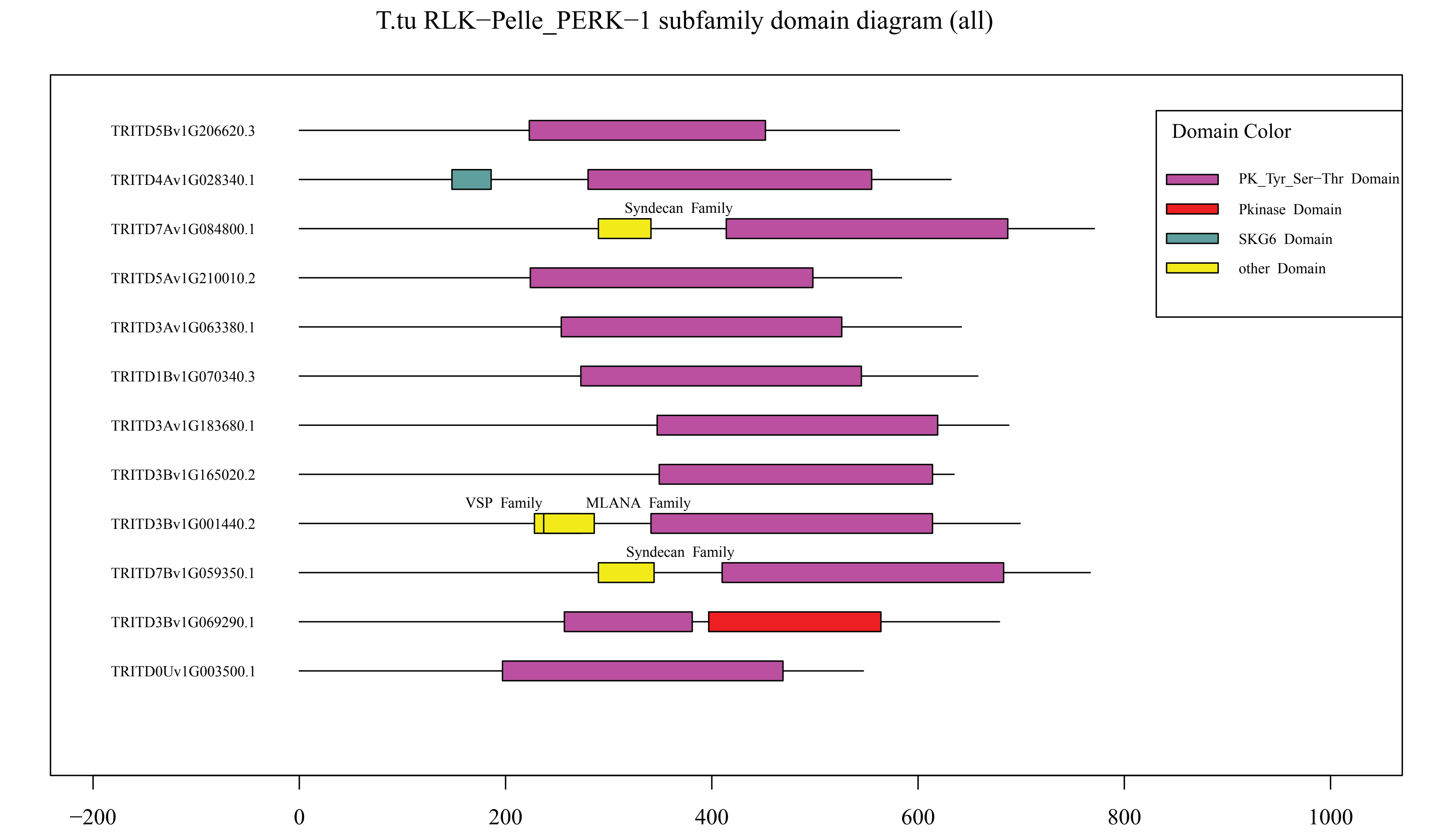

T.tu RLK−Pelle_PERK−1 subfamily domain diagram (all)
TRITD5Bv1G206620.3
TRITD4Av1G028340.1
TRITD7Av1G084800.1
Syndecan Family
TRITD5Av1G210010.2
TRITD3Av1G063380.1
TRITD1Bv1G070340.3
TRITD3Av1G183680.1
TRITD3Bv1G165020.2
TRITD3Bv1G001440.2
VSP Family
MLANA Family
TRITD7Bv1G059350.1
Syndecan Family
TRITD3Bv1G069290.1
TRITD0Uv1G003500.1
Domain Color
PK_Tyr_Ser−Thr Domain
Pkinase Domain
SKG6 Domain
other Domain
−200
0
200
400
600
800
1000

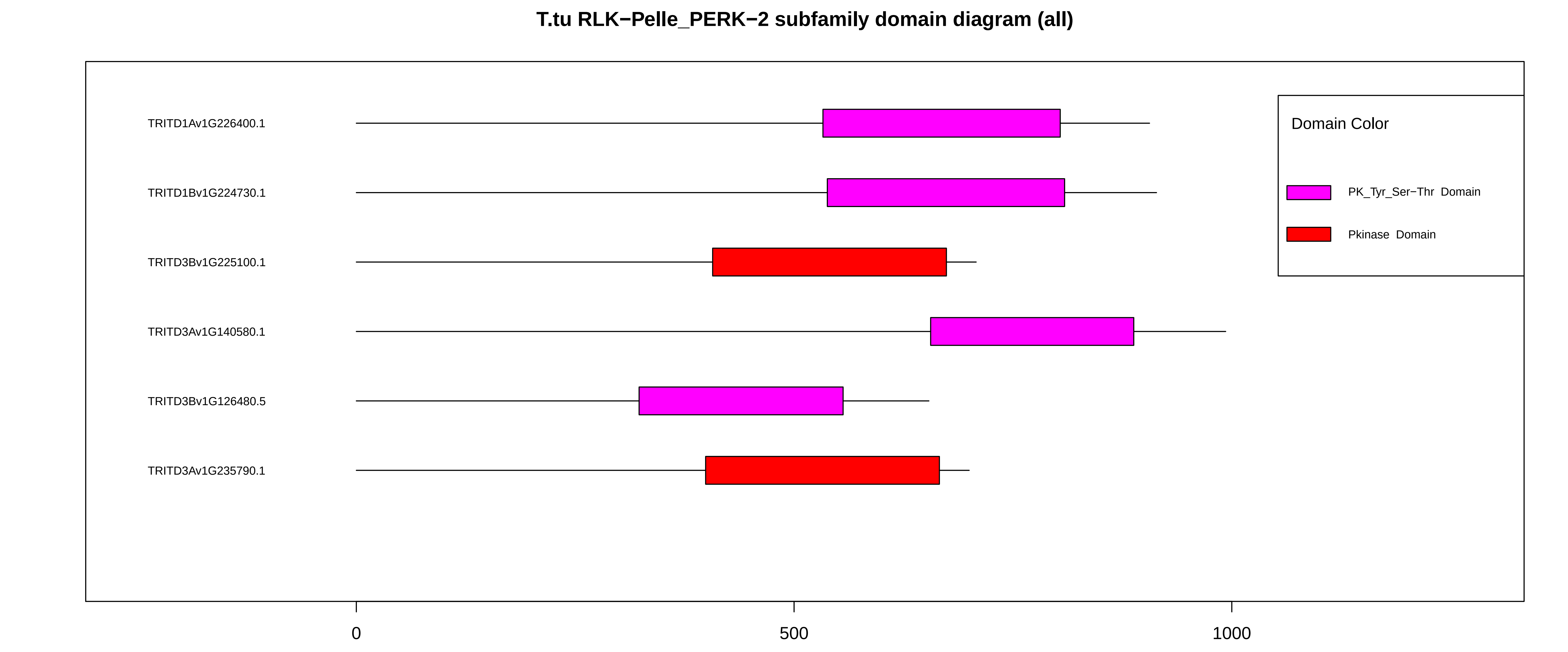

T.tu RLK−Pelle_PERK−2 subfamily domain diagram (all)
TRITD1Av1G226400.1
TRITD1Bv1G224730.1
TRITD3Bv1G225100.1
TRITD3Av1G140580.1
TRITD3Bv1G126480.5
TRITD3Av1G235790.1
Domain Color
PK_Tyr_Ser−Thr Domain
Pkinase Domain
0
500
1000

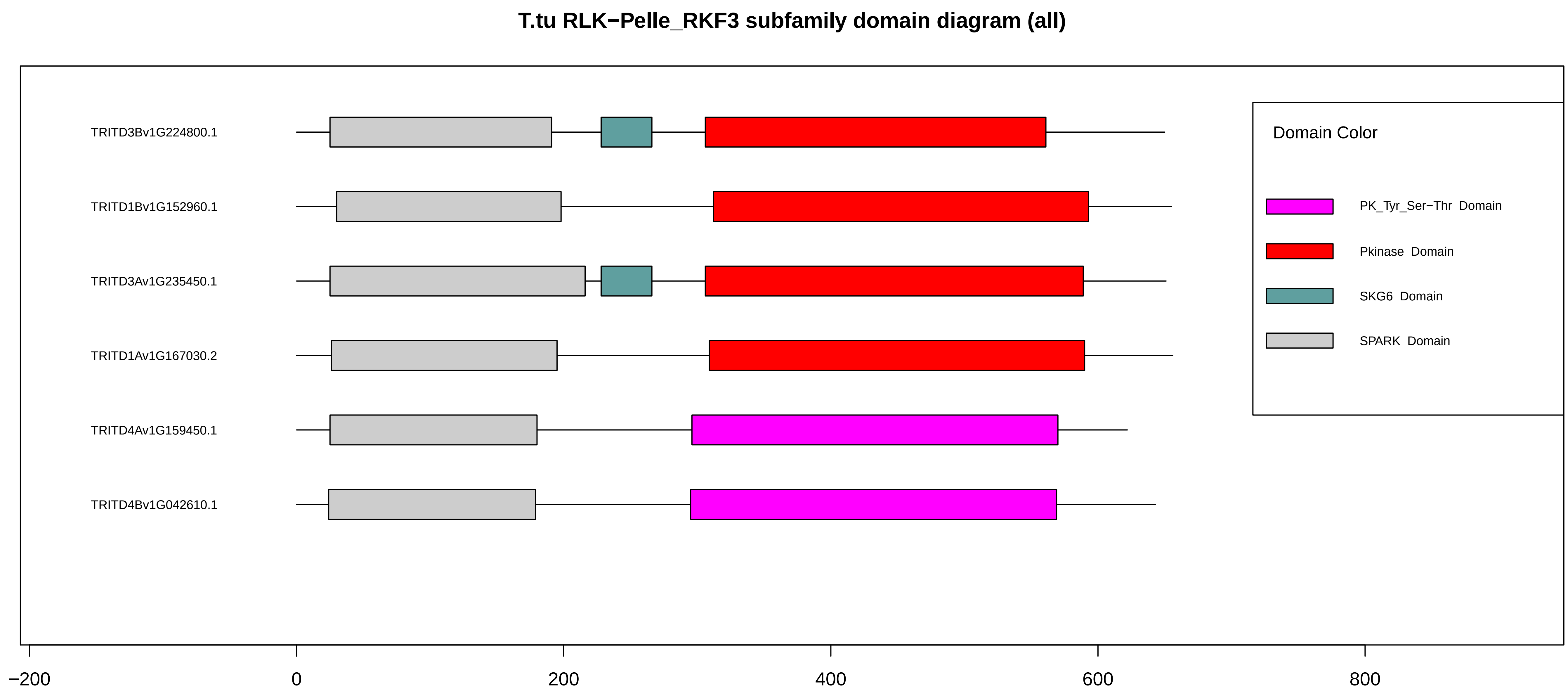

T.tu RLK−Pelle_RKF3 subfamily domain diagram (all)
TRITD3Bv1G224800.1
TRITD1Bv1G152960.1
TRITD3Av1G235450.1
TRITD1Av1G167030.2
TRITD4Av1G159450.1
TRITD4Bv1G042610.1
Domain Color
PK_Tyr_Ser−Thr Domain
Pkinase Domain
SKG6 Domain
SPARK Domain
−200
0
200
400
600
800

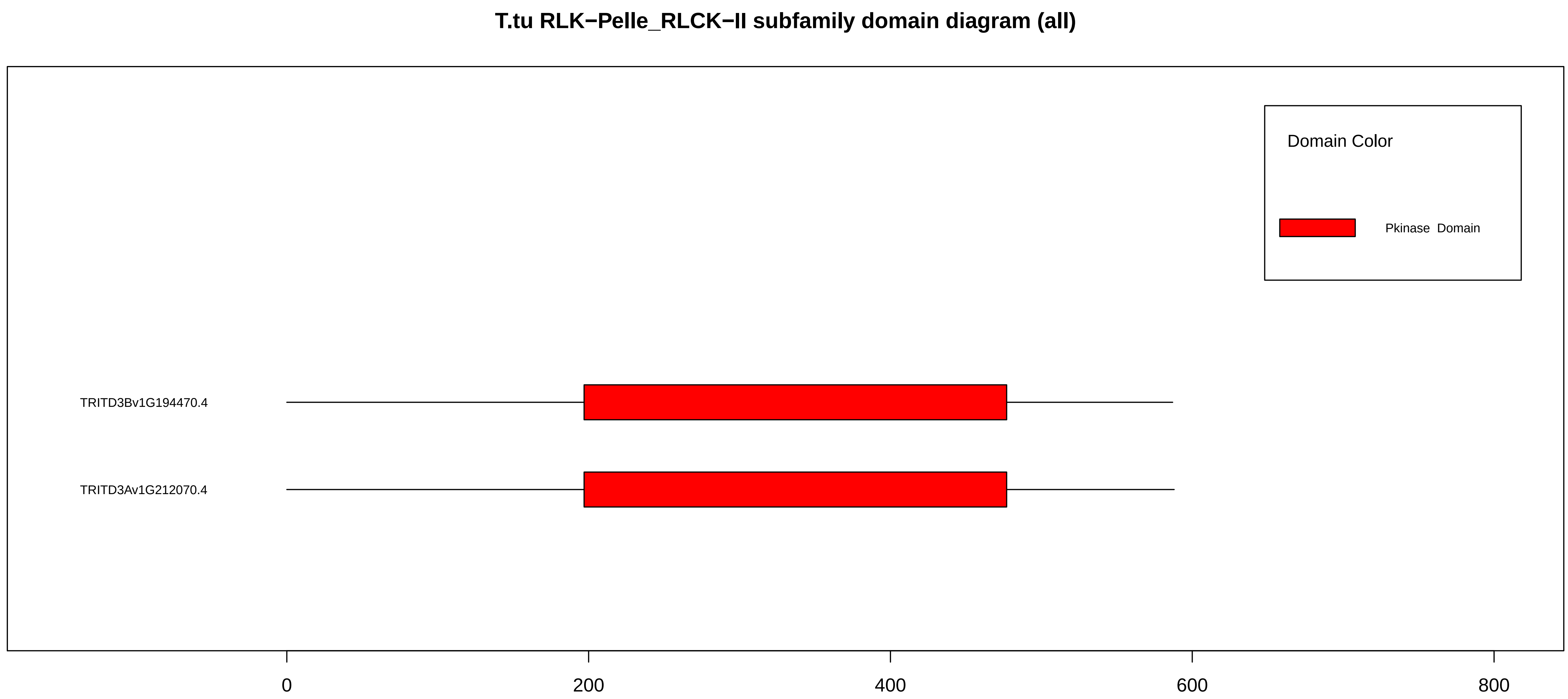
T.tu RLK−Pelle_RLCK−II subfamily domain diagram (all)
Domain Color
Pkinase Domain
TRITD3Bv1G194470.4
TRITD3Av1G212070.4
0
200
400
600
800

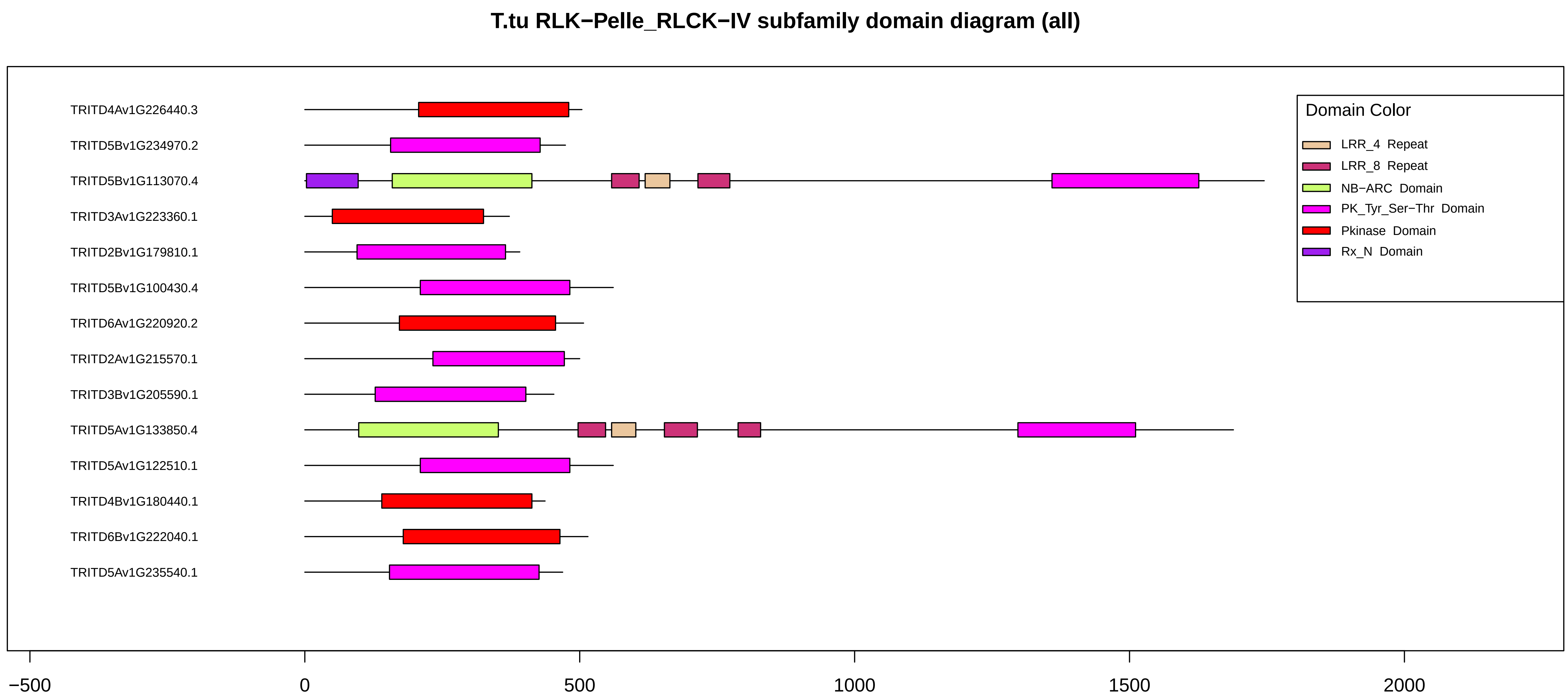

T.tu RLK−Pelle_RLCK−IV subfamily domain diagram (all)
TRITD4Av1G226440.3
TRITD5Bv1G234970.2
TRITD5Bv1G113070.4
TRITD3Av1G223360.1
TRITD2Bv1G179810.1
TRITD5Bv1G100430.4
TRITD6Av1G220920.2
TRITD2Av1G215570.1
TRITD3Bv1G205590.1
TRITD5Av1G133850.4
TRITD5Av1G122510.1
TRITD4Bv1G180440.1
TRITD6Bv1G222040.1
TRITD5Av1G235540.1
Domain Color
LRR_4 Repeat
LRR_8 Repeat
NB−ARC Domain
PK_Tyr_Ser−Thr Domain
Pkinase Domain
Rx_N Domain
−500
0
500
1000
1500
2000

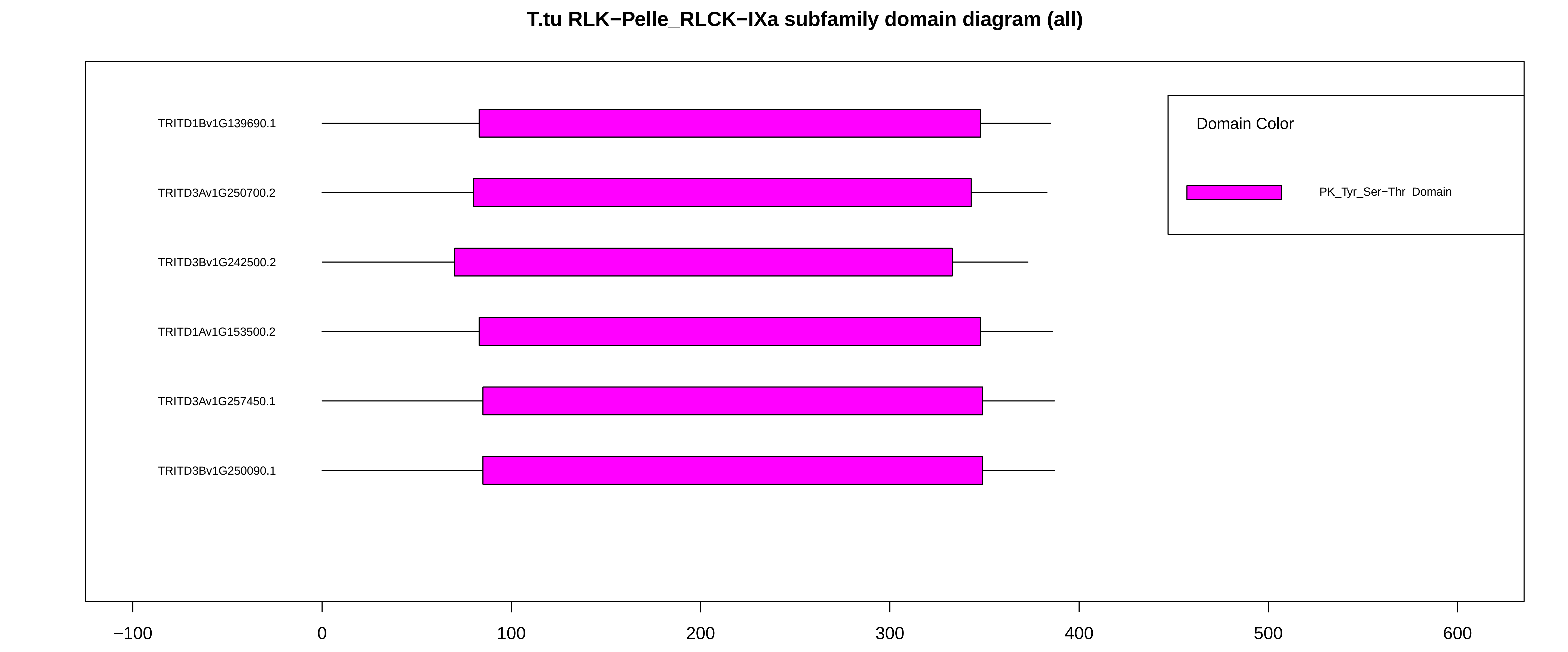

T.tu RLK−Pelle_RLCK−IXa subfamily domain diagram (all)
TRITD1Bv1G139690.1
TRITD3Av1G250700.2
TRITD3Bv1G242500.2
TRITD1Av1G153500.2
TRITD3Av1G257450.1
TRITD3Bv1G250090.1
Domain Color
PK_Tyr_Ser−Thr Domain
−100
0
100
200
300
400
500
600

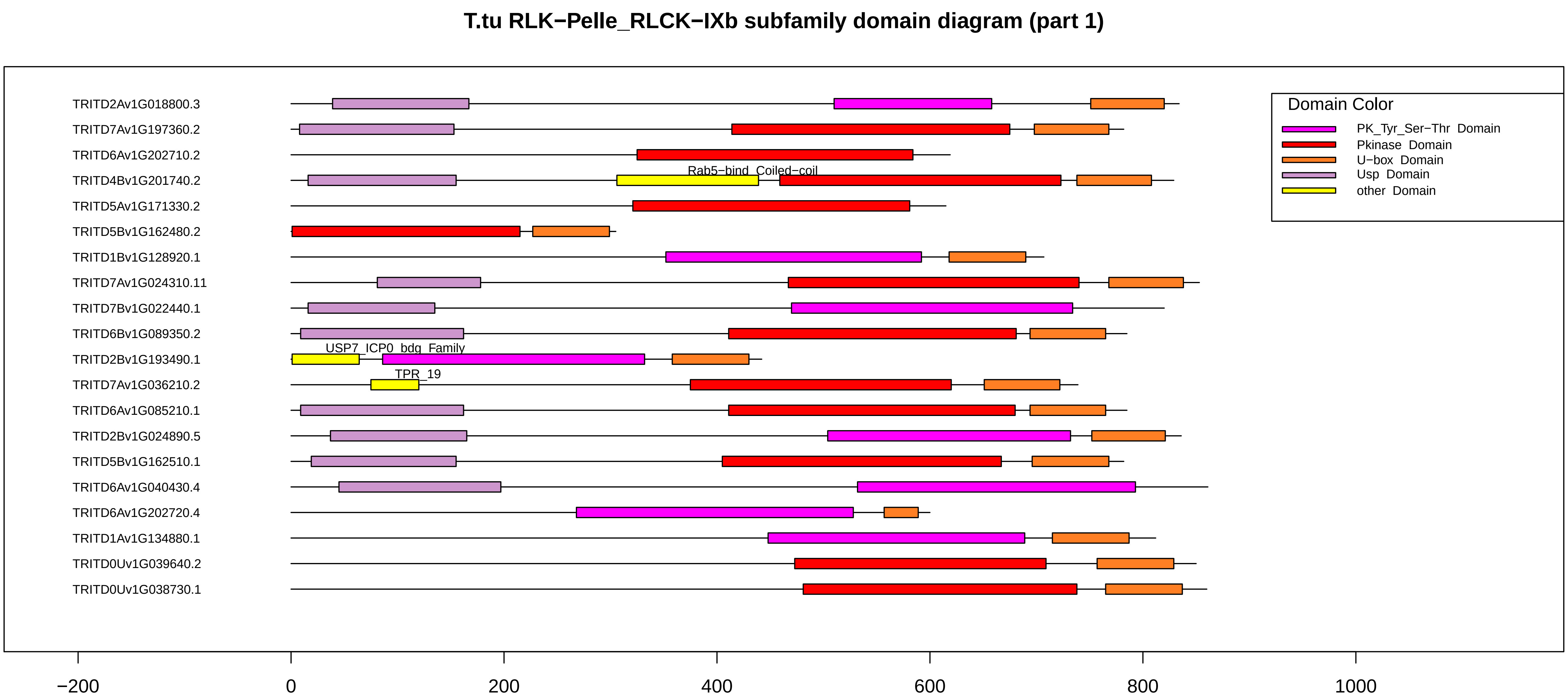
T.tu RLK−Pelle_RLCK−IXb subfamily domain diagram (part 1)
TRITD2Av1G018800.3
TRITD7Av1G197360.2
TRITD6Av1G202710.2
TRITD4Bv1G201740.2
TRITD5Av1G171330.2
TRITD5Bv1G162480.2
TRITD1Bv1G128920.1
TRITD7Av1G024310.11
TRITD7Bv1G022440.1
TRITD6Bv1G089350.2
TRITD2Bv1G193490.1
TRITD7Av1G036210.2
TRITD6Av1G085210.1
TRITD2Bv1G024890.5
TRITD5Bv1G162510.1
TRITD6Av1G040430.4
TRITD6Av1G202720.4
TRITD1Av1G134880.1
TRITD0Uv1G039640.2
TRITD0Uv1G038730.1
Rab5−bind_Coiled−coil
USP7_ICP0_bdg_Family
TPR_19
Domain Color
PK_Tyr_Ser−Thr Domain
Pkinase Domain
U−box Domain
Usp Domain
other Domain
−200
0
200
400
600
800
1000

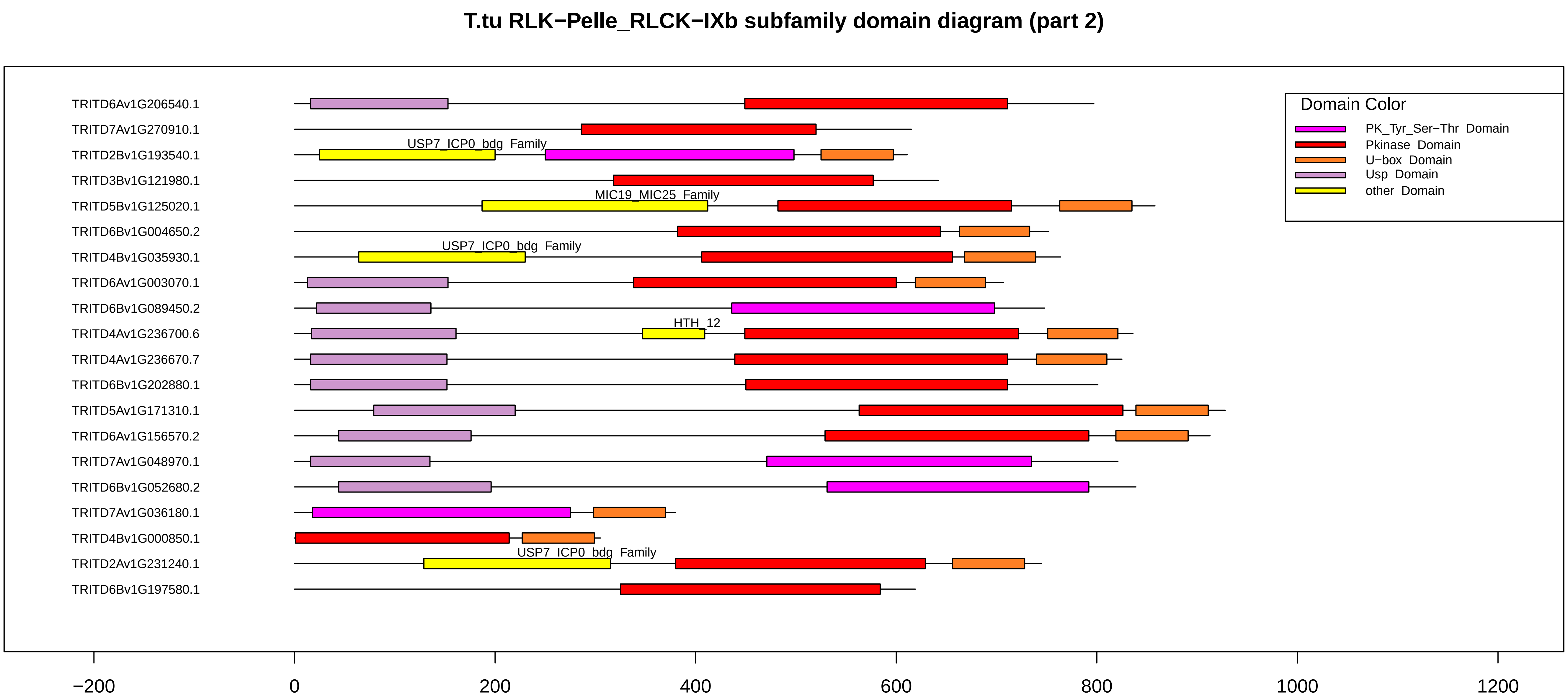
T.tu RLK−Pelle_RLCK−IXb subfamily domain diagram (part 2)
TRITD6Av1G206540.1
TRITD7Av1G270910.1
TRITD2Bv1G193540.1
TRITD3Bv1G121980.1
TRITD5Bv1G125020.1
TRITD6Bv1G004650.2
TRITD4Bv1G035930.1
TRITD6Av1G003070.1
TRITD6Bv1G089450.2
TRITD4Av1G236700.6
TRITD4Av1G236670.7
TRITD6Bv1G202880.1
TRITD5Av1G171310.1
TRITD6Av1G156570.2
TRITD7Av1G048970.1
TRITD6Bv1G052680.2
TRITD7Av1G036180.1
TRITD4Bv1G000850.1
TRITD2Av1G231240.1
TRITD6Bv1G197580.1
USP7_ICP0_bdg Family
MIC19_MIC25 Family
USP7_ICP0_bdg Family
HTH_12
USP7_ICP0_bdg Family
Domain Color
PK_Tyr_Ser−Thr Domain
Pkinase Domain
U−box Domain
Usp Domain
other Domain
−200
0
200
400
600
800
1000
1200

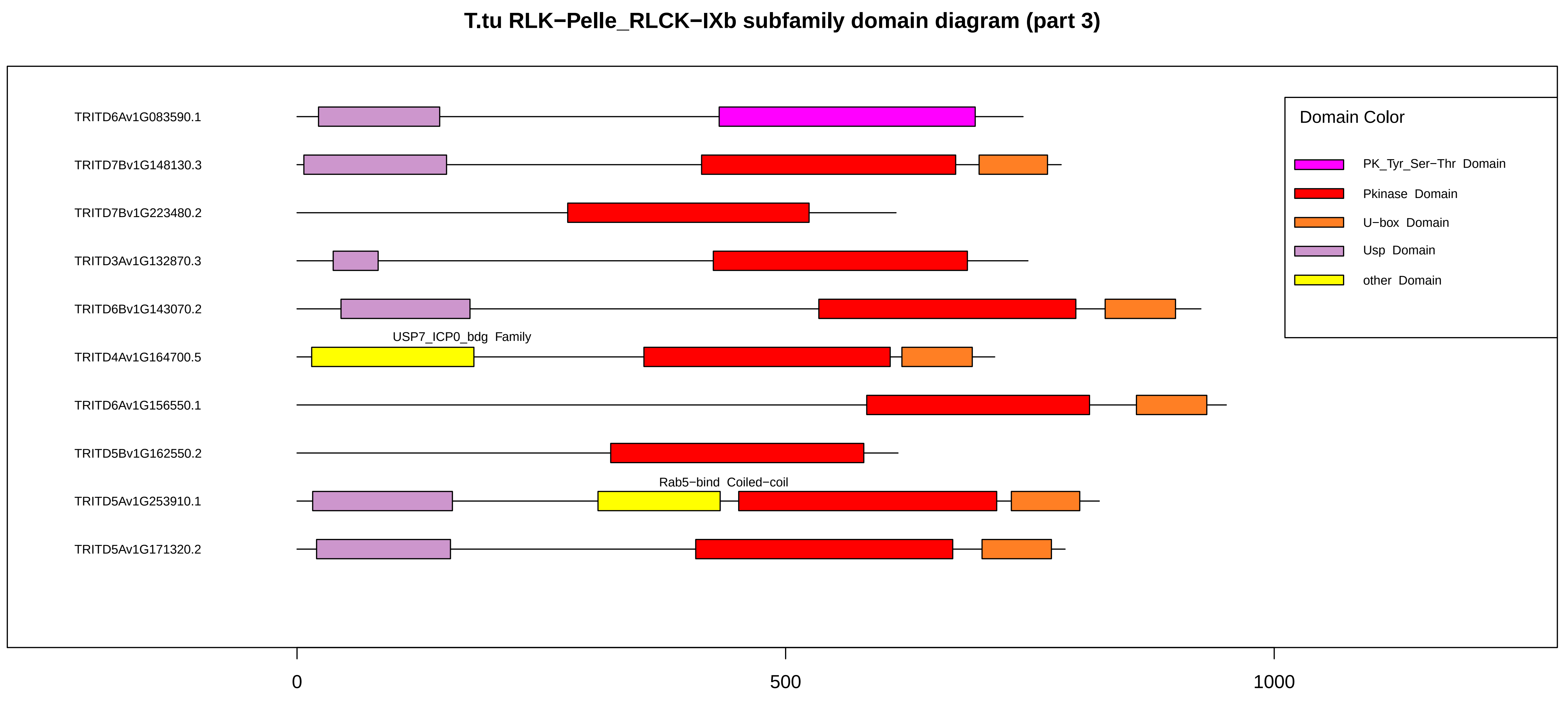
T.tu RLK−Pelle_RLCK−IXb subfamily domain diagram (part 3)
TRITD6Av1G083590.1
TRITD7Bv1G148130.3
TRITD7Bv1G223480.2
TRITD3Av1G132870.3
TRITD6Bv1G143070.2
USP7_ICP0_bdg Family
TRITD4Av1G164700.5
TRITD6Av1G156550.1
TRITD5Bv1G162550.2
Rab5−bind Coiled−coil
TRITD5Av1G253910.1
TRITD5Av1G171320.2
Domain Color
PK_Tyr_Ser−Thr Domain
Pkinase Domain
U−box Domain
Usp Domain
other Domain
0
500
1000

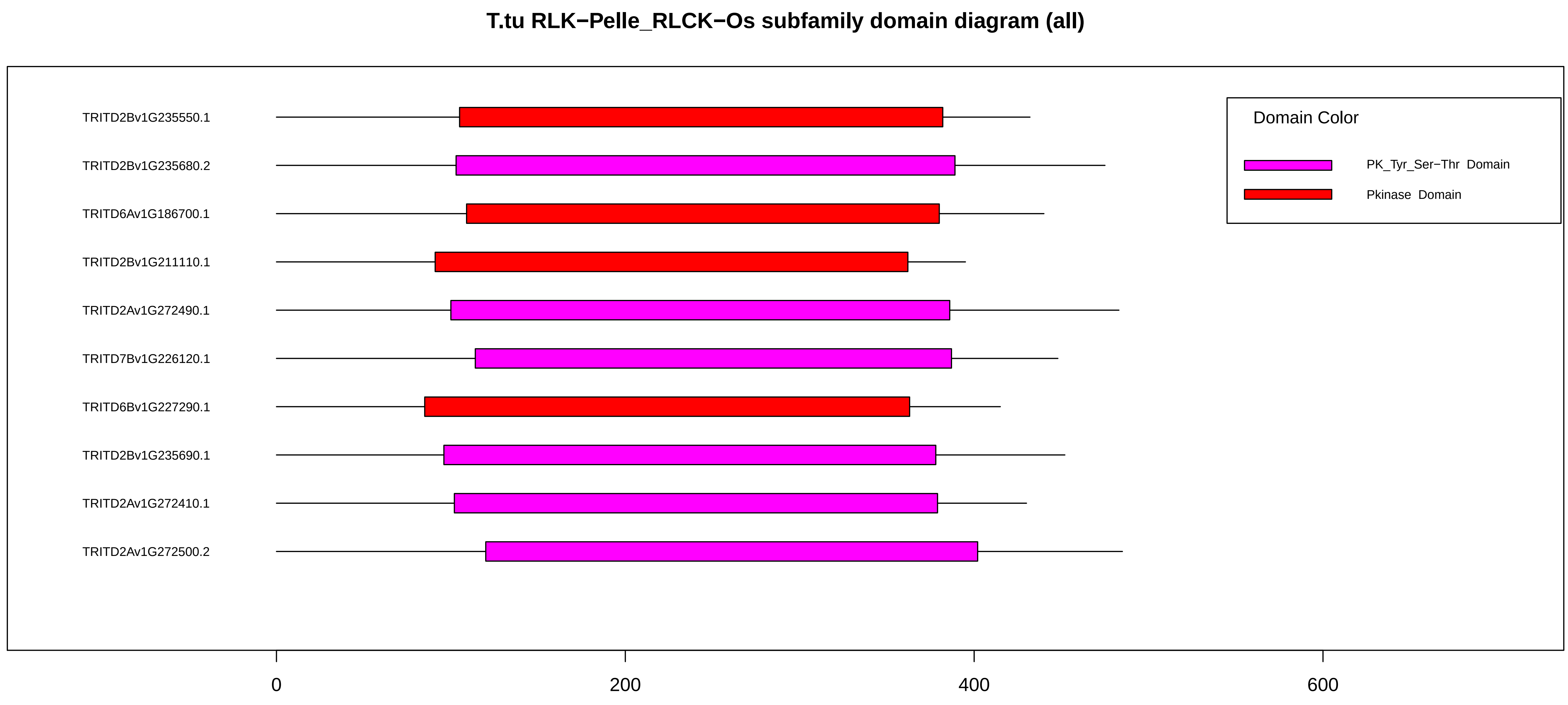
T.tu RLK−Pelle_RLCK−Os subfamily domain diagram (all)
TRITD2Bv1G235550.1
TRITD2Bv1G235680.2
TRITD6Av1G186700.1
TRITD2Bv1G211110.1
TRITD2Av1G272490.1
TRITD7Bv1G226120.1
TRITD6Bv1G227290.1
TRITD2Bv1G235690.1
TRITD2Av1G272410.1
TRITD2Av1G272500.2
Domain Color
PK_Tyr_Ser−Thr Domain
Pkinase Domain
0
200
400
600

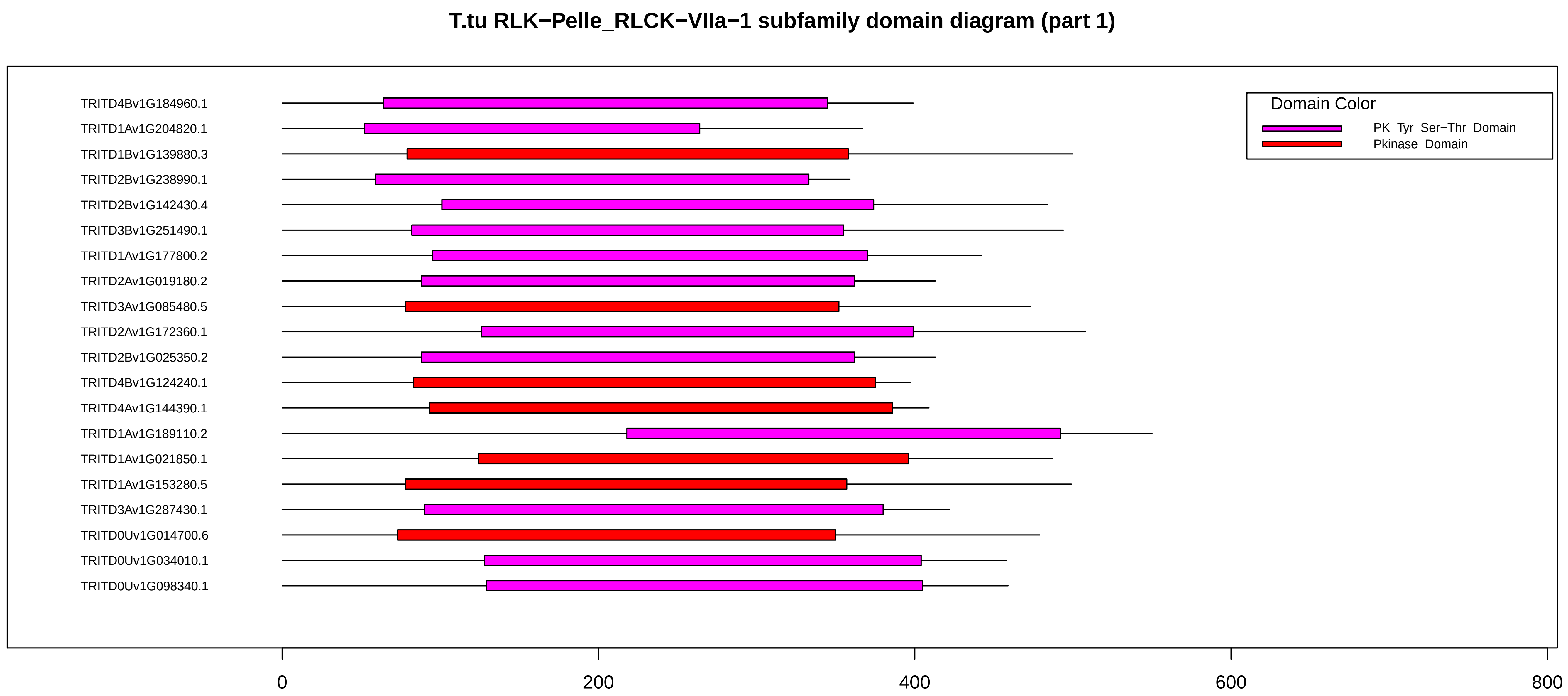
T.tu RLK−Pelle_RLCK−VIIa−1 subfamily domain diagram (part 1)
TRITD4Bv1G184960.1
TRITD1Av1G204820.1
TRITD1Bv1G139880.3
TRITD2Bv1G238990.1
TRITD2Bv1G142430.4
TRITD3Bv1G251490.1
TRITD1Av1G177800.2
TRITD2Av1G019180.2
TRITD3Av1G085480.5
TRITD2Av1G172360.1
TRITD2Bv1G025350.2
TRITD4Bv1G124240.1
TRITD4Av1G144390.1
TRITD1Av1G189110.2
TRITD1Av1G021850.1
TRITD1Av1G153280.5
TRITD3Av1G287430.1
TRITD0Uv1G014700.6
TRITD0Uv1G034010.1
TRITD0Uv1G098340.1
Domain Color
PK_Tyr_Ser−Thr Domain
Pkinase Domain
0
200
400
600
800

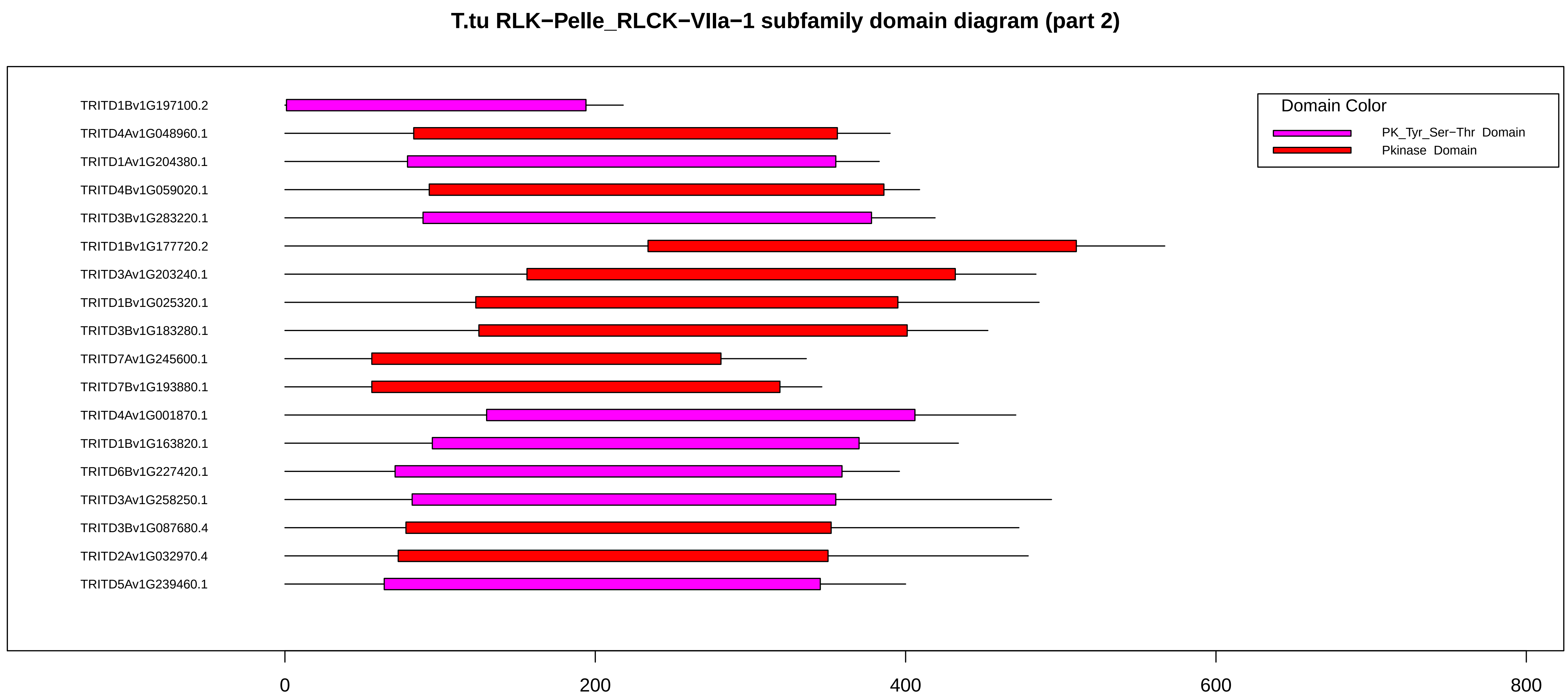

T.tu RLK−Pelle_RLCK−VIIa−1 subfamily domain diagram (part 2)
TRITD1Bv1G197100.2
TRITD4Av1G048960.1
TRITD1Av1G204380.1
TRITD4Bv1G059020.1
TRITD3Bv1G283220.1
TRITD1Bv1G177720.2
TRITD3Av1G203240.1
TRITD1Bv1G025320.1
TRITD3Bv1G183280.1
TRITD7Av1G245600.1
TRITD7Bv1G193880.1
TRITD4Av1G001870.1
TRITD1Bv1G163820.1
TRITD6Bv1G227420.1
TRITD3Av1G258250.1
TRITD3Bv1G087680.4
TRITD2Av1G032970.4
TRITD5Av1G239460.1
Domain Color
PK_Tyr_Ser−Thr Domain
Pkinase Domain
0
200
400
600
800

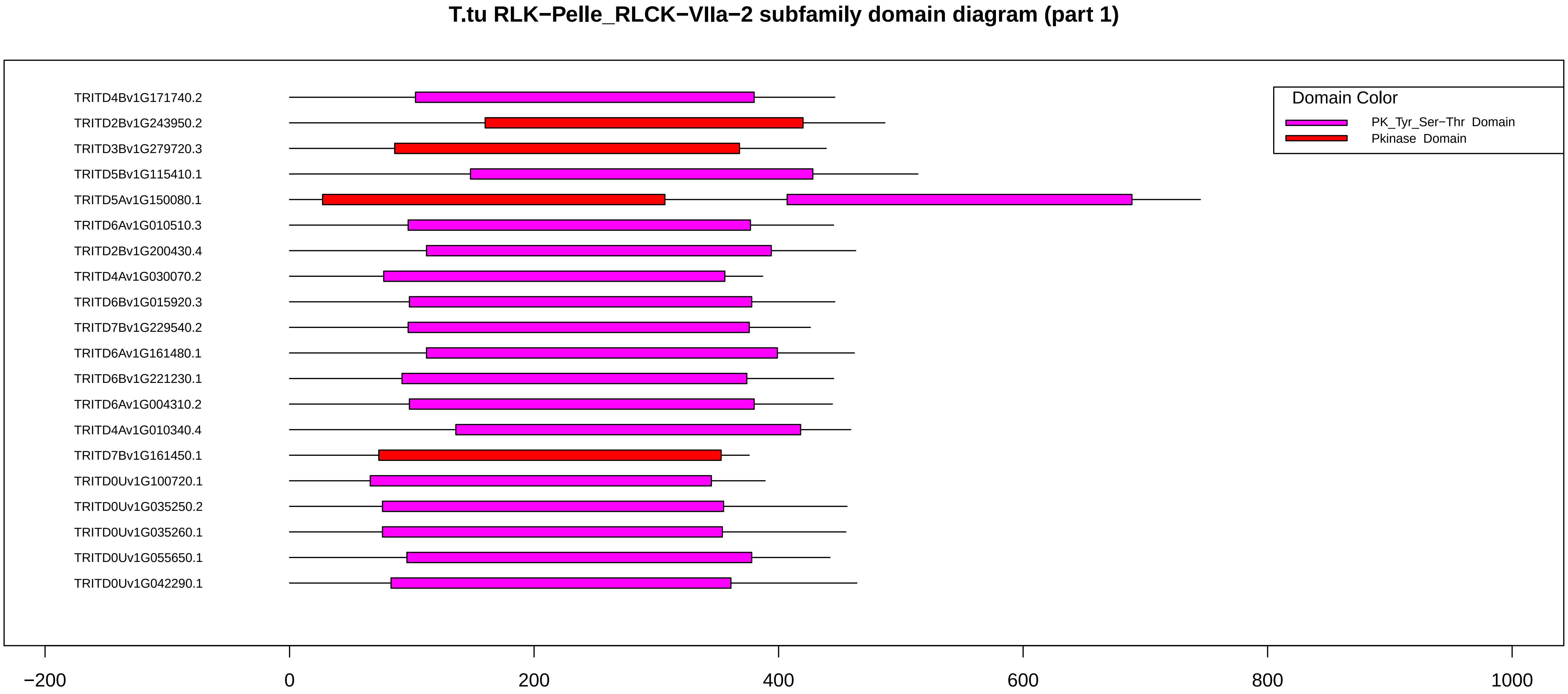
T.tu RLK−Pelle_RLCK−VIIa−2 subfamily domain diagram (part 1)
TRITD4Bv1G171740.2
TRITD2Bv1G243950.2
TRITD3Bv1G279720.3
TRITD5Bv1G115410.1
TRITD5Av1G150080.1
TRITD6Av1G010510.3
TRITD2Bv1G200430.4
TRITD4Av1G030070.2
TRITD6Bv1G015920.3
TRITD7Bv1G229540.2
TRITD6Av1G161480.1
TRITD6Bv1G221230.1
TRITD6Av1G004310.2
TRITD4Av1G010340.4
TRITD7Bv1G161450.1
TRITD0Uv1G100720.1
TRITD0Uv1G035250.2
TRITD0Uv1G035260.1
TRITD0Uv1G055650.1
TRITD0Uv1G042290.1
Domain Color
PK_Tyr_Ser−Thr Domain
Pkinase Domain
−200
0
200
400
600
800
1000

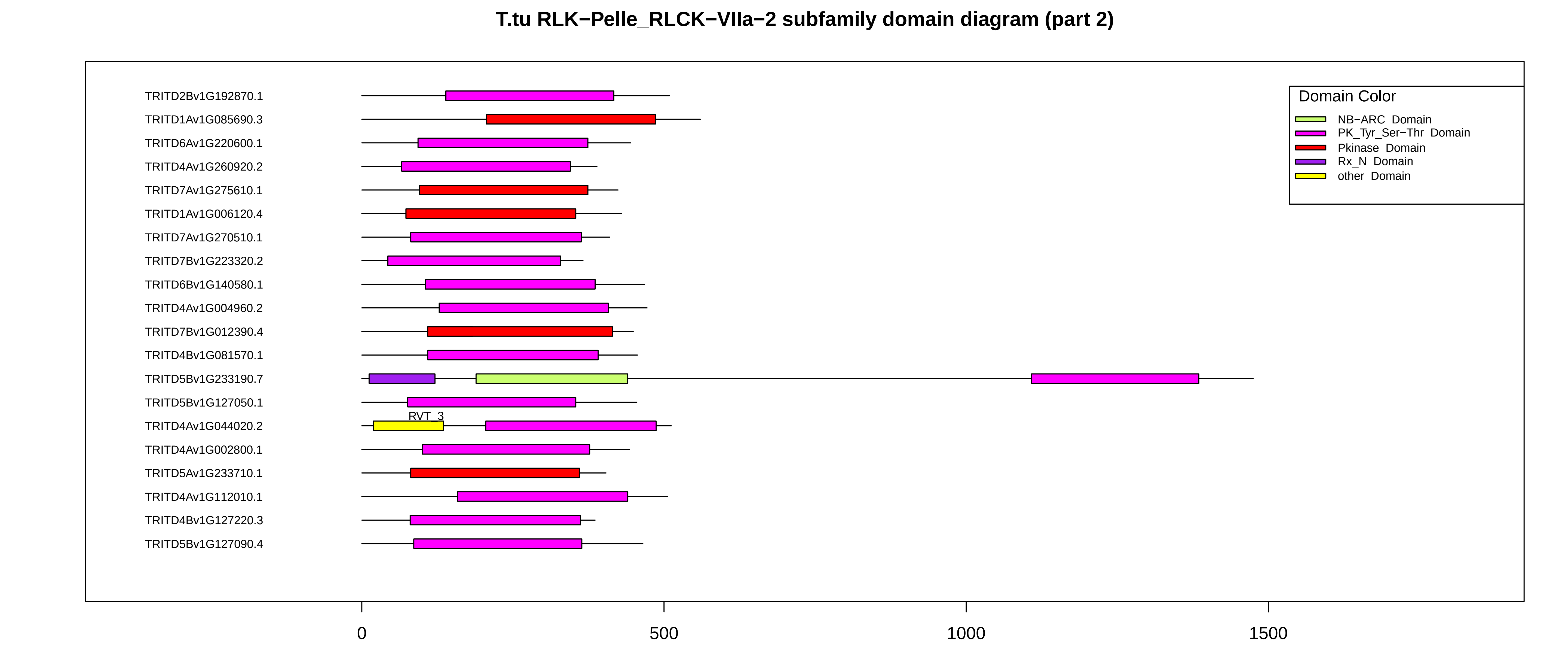
T.tu RLK−Pelle_RLCK−VIIa−2 subfamily domain diagram (part 2)
TRITD2Bv1G192870.1
TRITD1Av1G085690.3
TRITD6Av1G220600.1
TRITD4Av1G260920.2
TRITD7Av1G275610.1
TRITD1Av1G006120.4
TRITD7Av1G270510.1
TRITD7Bv1G223320.2
TRITD6Bv1G140580.1
TRITD4Av1G004960.2
TRITD7Bv1G012390.4
TRITD4Bv1G081570.1
TRITD5Bv1G233190.7
TRITD5Bv1G127050.1
RVT_3
TRITD4Av1G044020.2
TRITD4Av1G002800.1
TRITD5Av1G233710.1
TRITD4Av1G112010.1
TRITD4Bv1G127220.3
TRITD5Bv1G127090.4
Domain Color
NB−ARC Domain
PK_Tyr_Ser−Thr Domain
Pkinase Domain
Rx_N Domain
other Domain
0
500
1000
1500

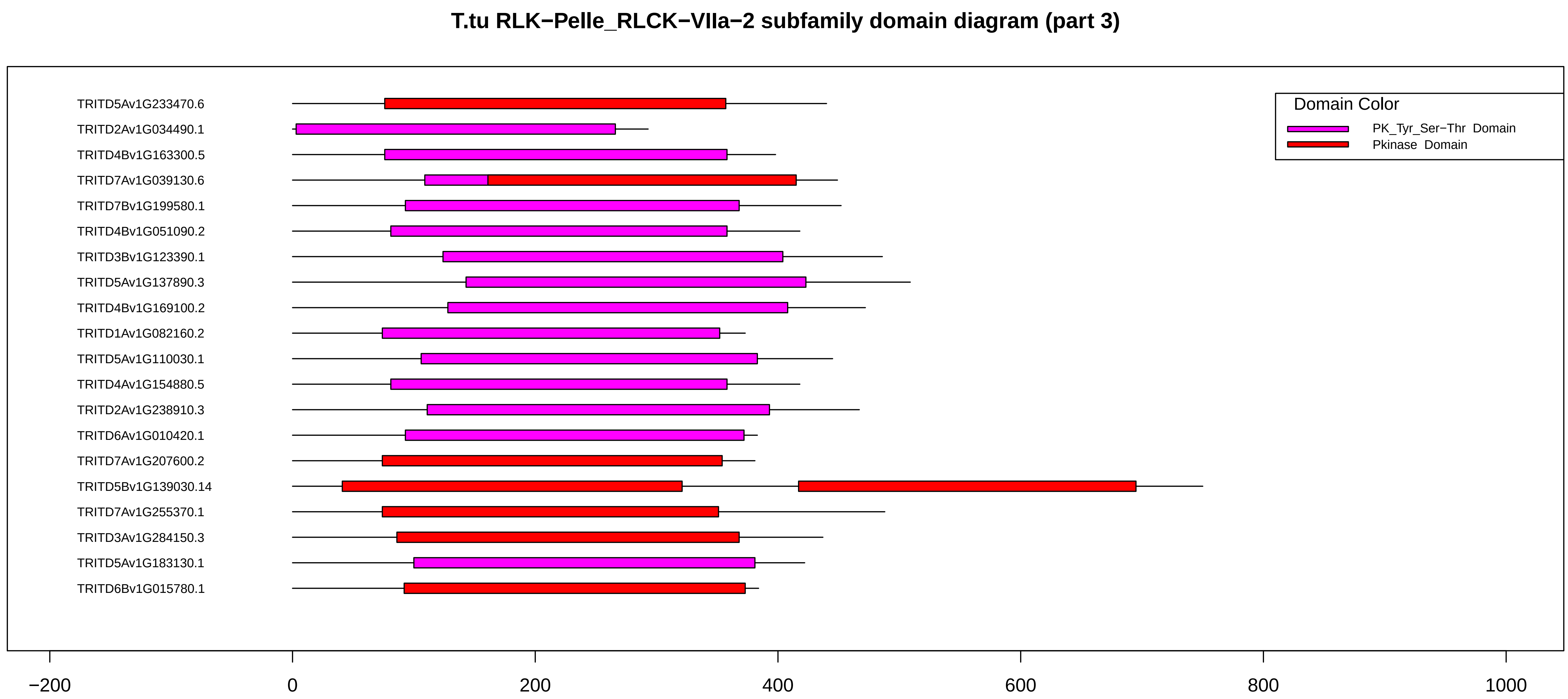

T.tu RLK−Pelle_RLCK−VIIa−2 subfamily domain diagram (part 3)
TRITD5Av1G233470.6
TRITD2Av1G034490.1
TRITD4Bv1G163300.5
TRITD7Av1G039130.6
TRITD7Bv1G199580.1
TRITD4Bv1G051090.2
TRITD3Bv1G123390.1
TRITD5Av1G137890.3
TRITD4Bv1G169100.2
TRITD1Av1G082160.2
TRITD5Av1G110030.1
TRITD4Av1G154880.5
TRITD2Av1G238910.3
TRITD6Av1G010420.1
TRITD7Av1G207600.2
TRITD5Bv1G139030.14
TRITD7Av1G255370.1
TRITD3Av1G284150.3
TRITD5Av1G183130.1
TRITD6Bv1G015780.1
Domain Color
PK_Tyr_Ser−Thr Domain
Pkinase Domain
−200
0
200
400
600
800
1000

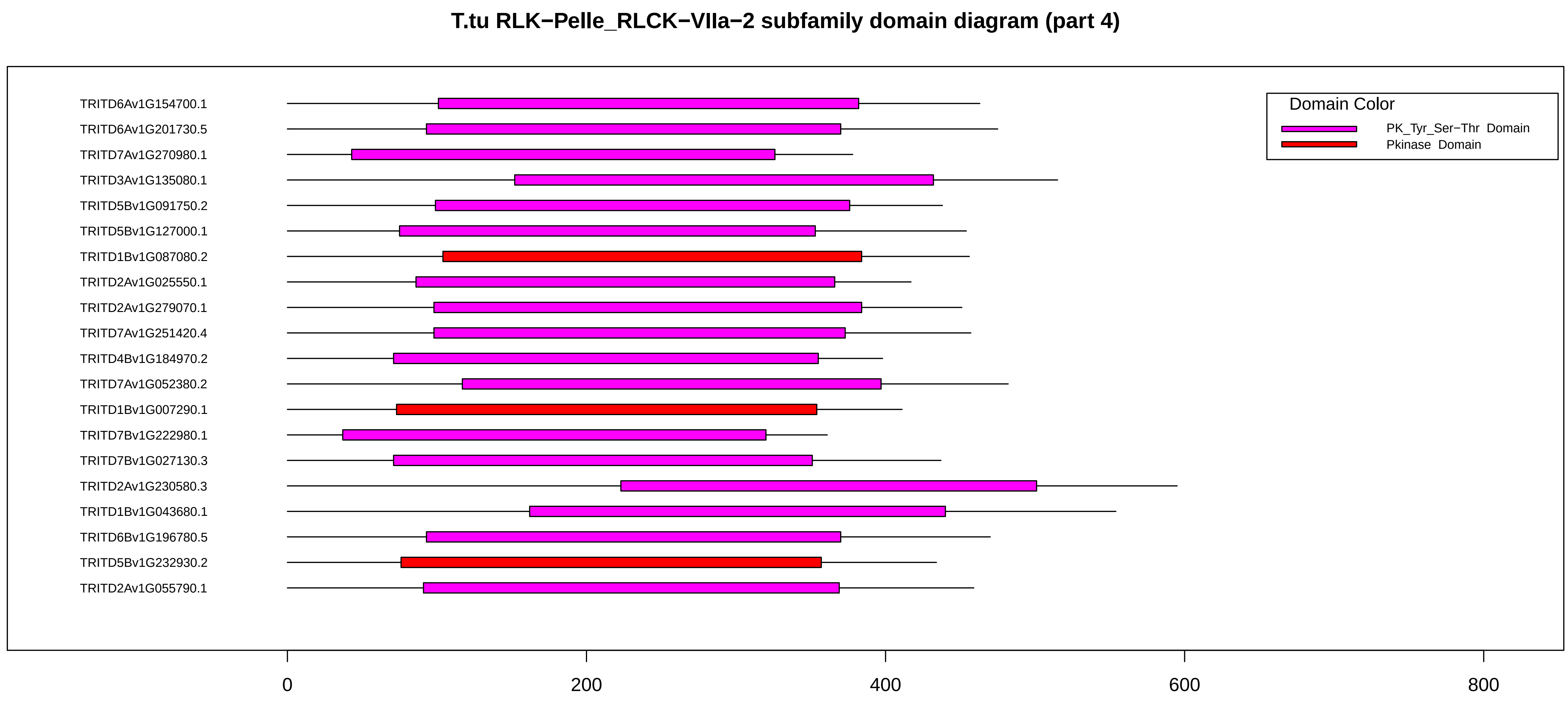
T.tu RLK−Pelle_RLCK−VIIa−2 subfamily domain diagram (part 4)
TRITD6Av1G154700.1
TRITD6Av1G201730.5
TRITD7Av1G270980.1
TRITD3Av1G135080.1
TRITD5Bv1G091750.2
TRITD5Bv1G127000.1
TRITD1Bv1G087080.2
TRITD2Av1G025550.1
TRITD2Av1G279070.1
TRITD7Av1G251420.4
TRITD4Bv1G184970.2
TRITD7Av1G052380.2
TRITD1Bv1G007290.1
TRITD7Bv1G222980.1
TRITD7Bv1G027130.3
TRITD2Av1G230580.3
TRITD1Bv1G043680.1
TRITD6Bv1G196780.5
TRITD5Bv1G232930.2
TRITD2Av1G055790.1
Domain Color
PK_Tyr_Ser−Thr Domain
Pkinase Domain
0
200
400
600
800

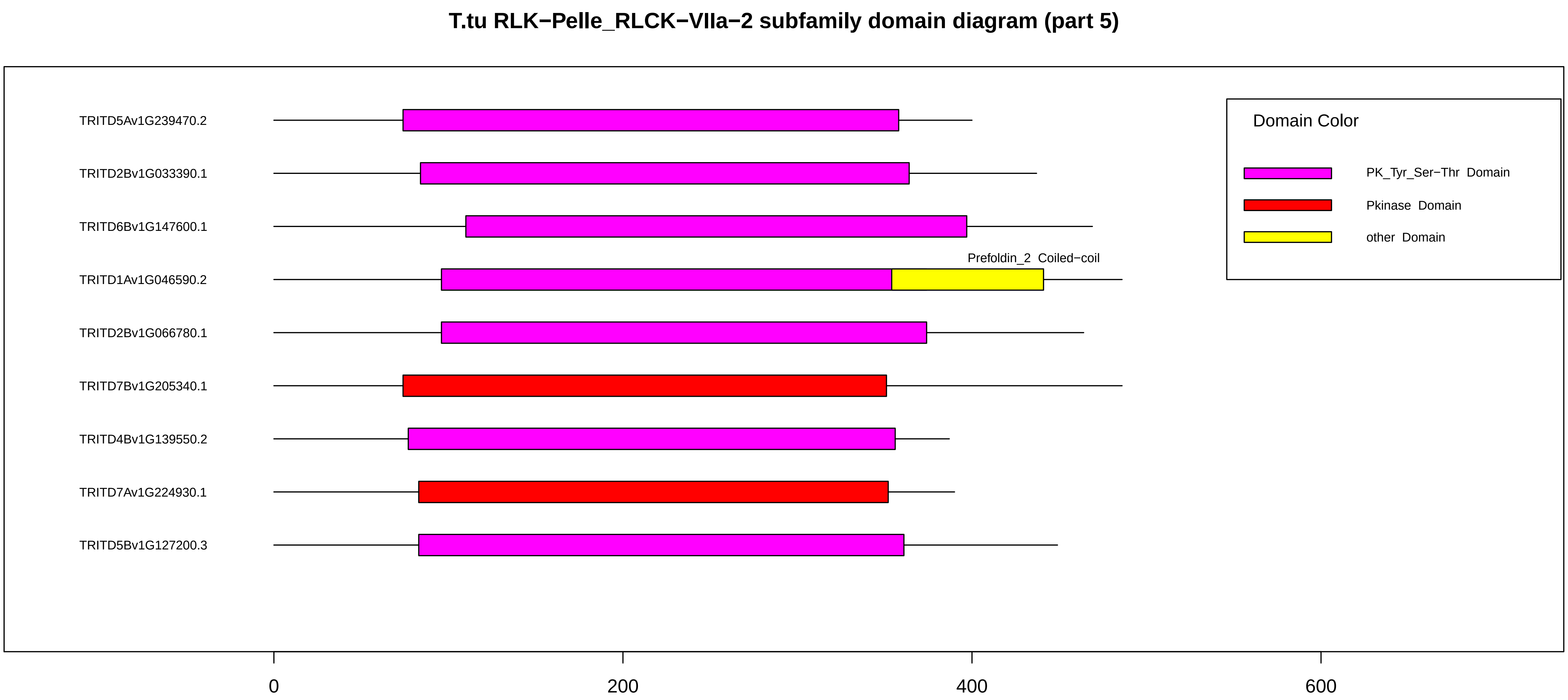

T.tu RLK−Pelle_RLCK−VIIa−2 subfamily domain diagram (part 5)
TRITD5Av1G239470.2
TRITD2Bv1G033390.1
TRITD6Bv1G147600.1
TRITD1Av1G046590.2
TRITD2Bv1G066780.1
TRITD7Bv1G205340.1
TRITD4Bv1G139550.2
TRITD7Av1G224930.1
TRITD5Bv1G127200.3
Prefoldin_2 Coiled−coil
Domain Color
PK_Tyr_Ser−Thr Domain
Pkinase Domain
other Domain
0
200
400
600

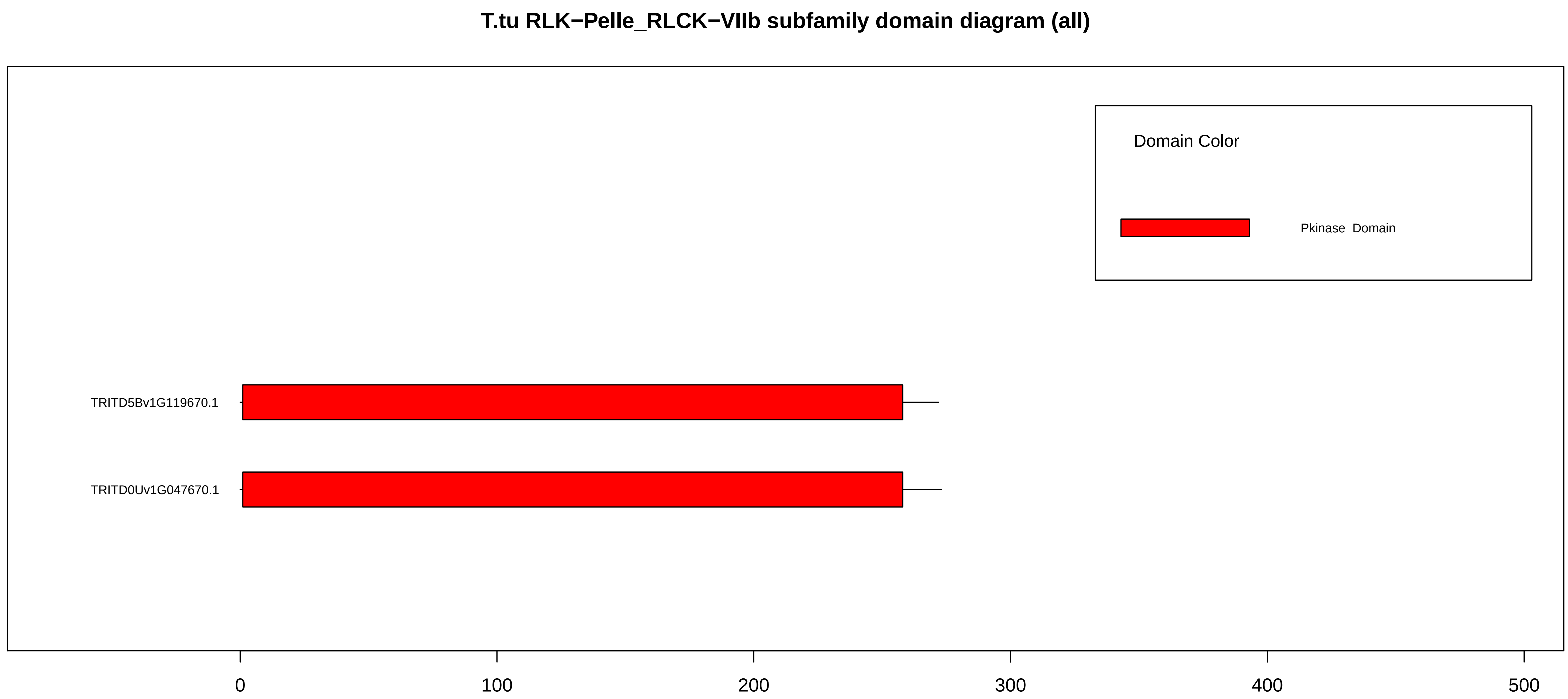

T.tu RLK−Pelle_RLCK−VIIb subfamily domain diagram (all)
Domain Color
Pkinase Domain
TRITD5Bv1G119670.1
TRITD0Uv1G047670.1
0
100
200
300
400
500

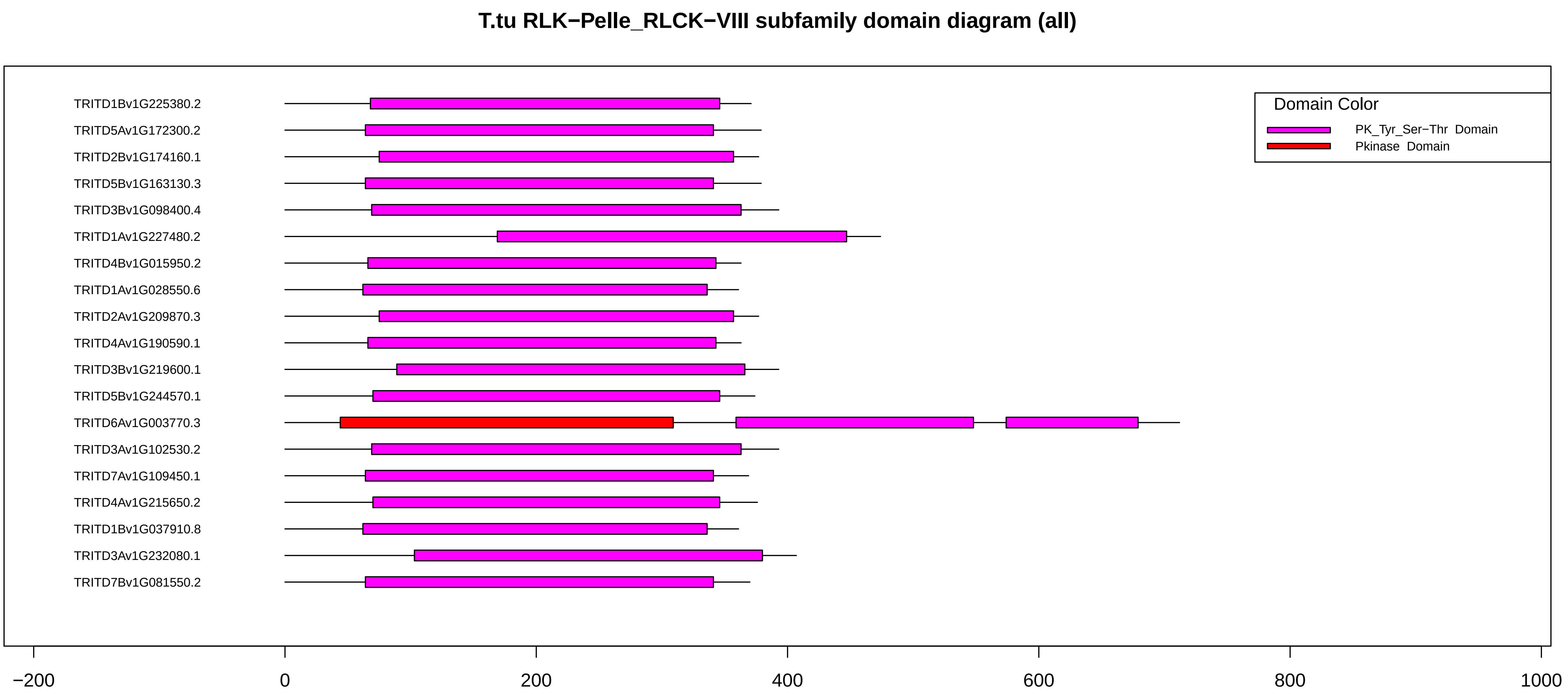
T.tu RLK−Pelle_RLCK−VIII subfamily domain diagram (all)
Domain Color
PK_Tyr_Ser−Thr Domain
Pkinase Domain
TRITD1Bv1G225380.2
TRITD5Av1G172300.2
TRITD2Bv1G174160.1
TRITD5Bv1G163130.3
TRITD3Bv1G098400.4
TRITD1Av1G227480.2
TRITD4Bv1G015950.2
TRITD1Av1G028550.6
TRITD2Av1G209870.3
TRITD4Av1G190590.1
TRITD3Bv1G219600.1
TRITD5Bv1G244570.1
TRITD6Av1G003770.3
TRITD3Av1G102530.2
TRITD7Av1G109450.1
TRITD4Av1G215650.2
TRITD1Bv1G037910.8
TRITD3Av1G232080.1
TRITD7Bv1G081550.2
−200
0
200
400
600
800
1000

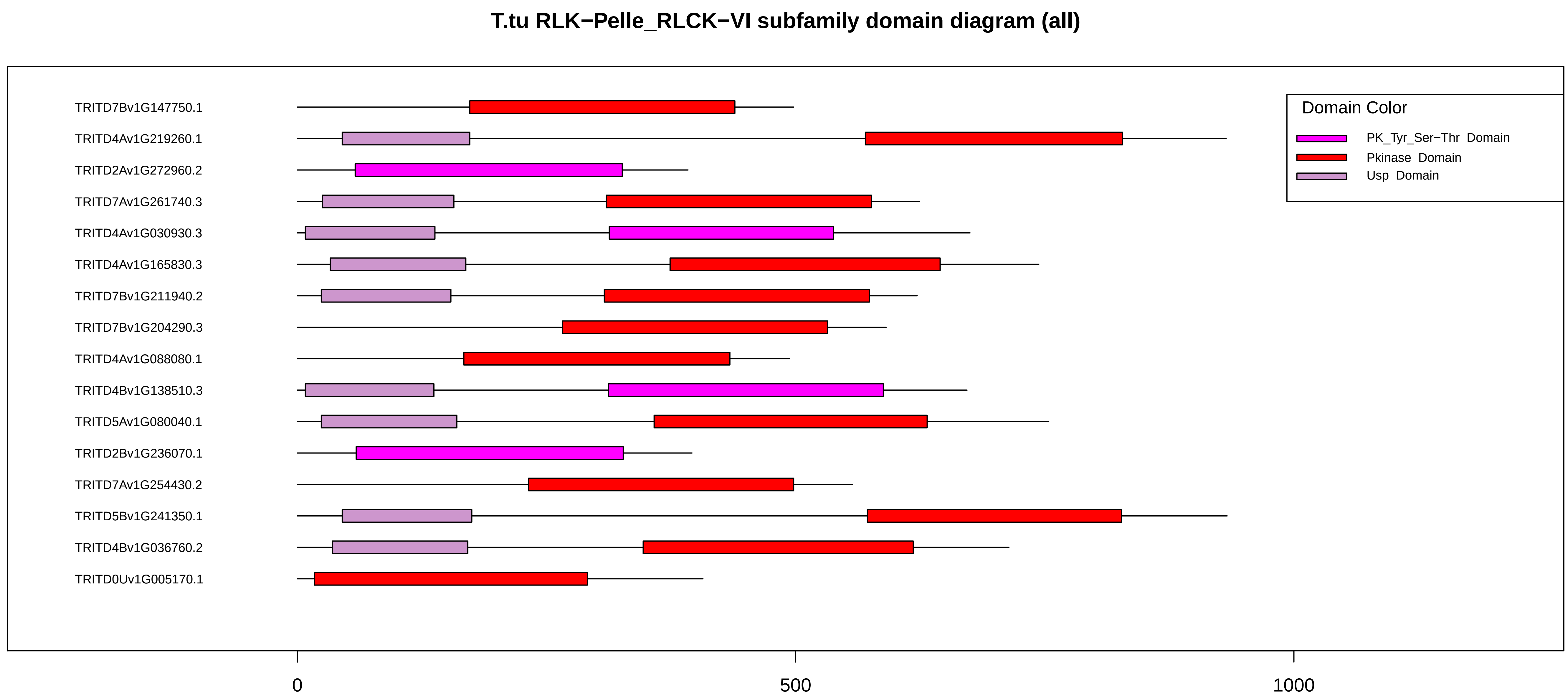
T.tu RLK−Pelle_RLCK−VI subfamily domain diagram (all)
TRITD7Bv1G147750.1
TRITD4Av1G219260.1
TRITD2Av1G272960.2
TRITD7Av1G261740.3
TRITD4Av1G030930.3
TRITD4Av1G165830.3
TRITD7Bv1G211940.2
TRITD7Bv1G204290.3
TRITD4Av1G088080.1
TRITD4Bv1G138510.3
TRITD5Av1G080040.1
TRITD2Bv1G236070.1
TRITD7Av1G254430.2
TRITD5Bv1G241350.1
TRITD4Bv1G036760.2
TRITD0Uv1G005170.1
Domain Color
PK_Tyr_Ser−Thr Domain
Pkinase Domain
Usp Domain
0
500
1000

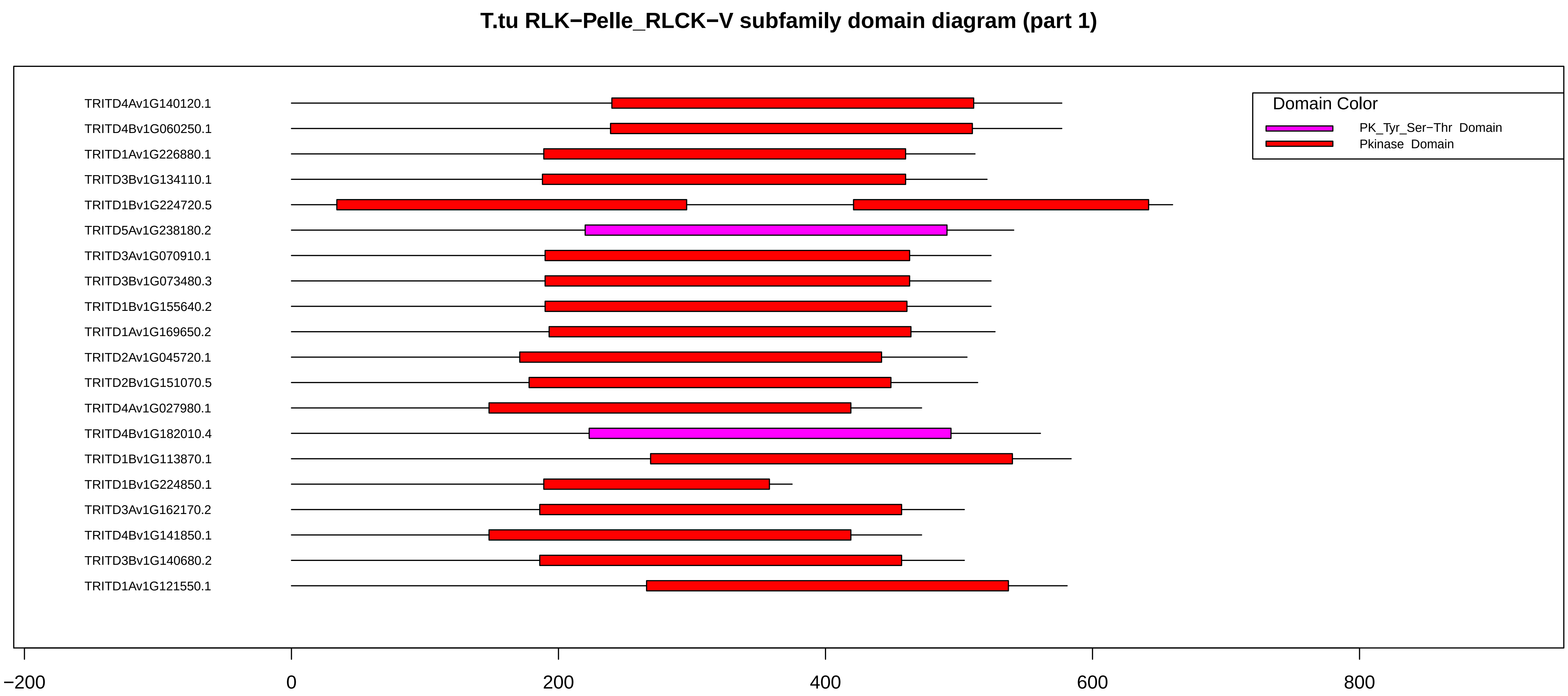
T.tu RLK−Pelle_RLCK−V subfamily domain diagram (part 1)
TRITD4Av1G140120.1
TRITD4Bv1G060250.1
TRITD1Av1G226880.1
TRITD3Bv1G134110.1
TRITD1Bv1G224720.5
TRITD5Av1G238180.2
TRITD3Av1G070910.1
TRITD3Bv1G073480.3
TRITD1Bv1G155640.2
TRITD1Av1G169650.2
TRITD2Av1G045720.1
TRITD2Bv1G151070.5
TRITD4Av1G027980.1
TRITD4Bv1G182010.4
TRITD1Bv1G113870.1
TRITD1Bv1G224850.1
TRITD3Av1G162170.2
TRITD4Bv1G141850.1
TRITD3Bv1G140680.2
TRITD1Av1G121550.1
Domain Color
PK_Tyr_Ser−Thr Domain
Pkinase Domain
−200
0
200
400
600
800

T.tu RLK−Pelle_RLCK−V subfamily domain diagram (part 2)

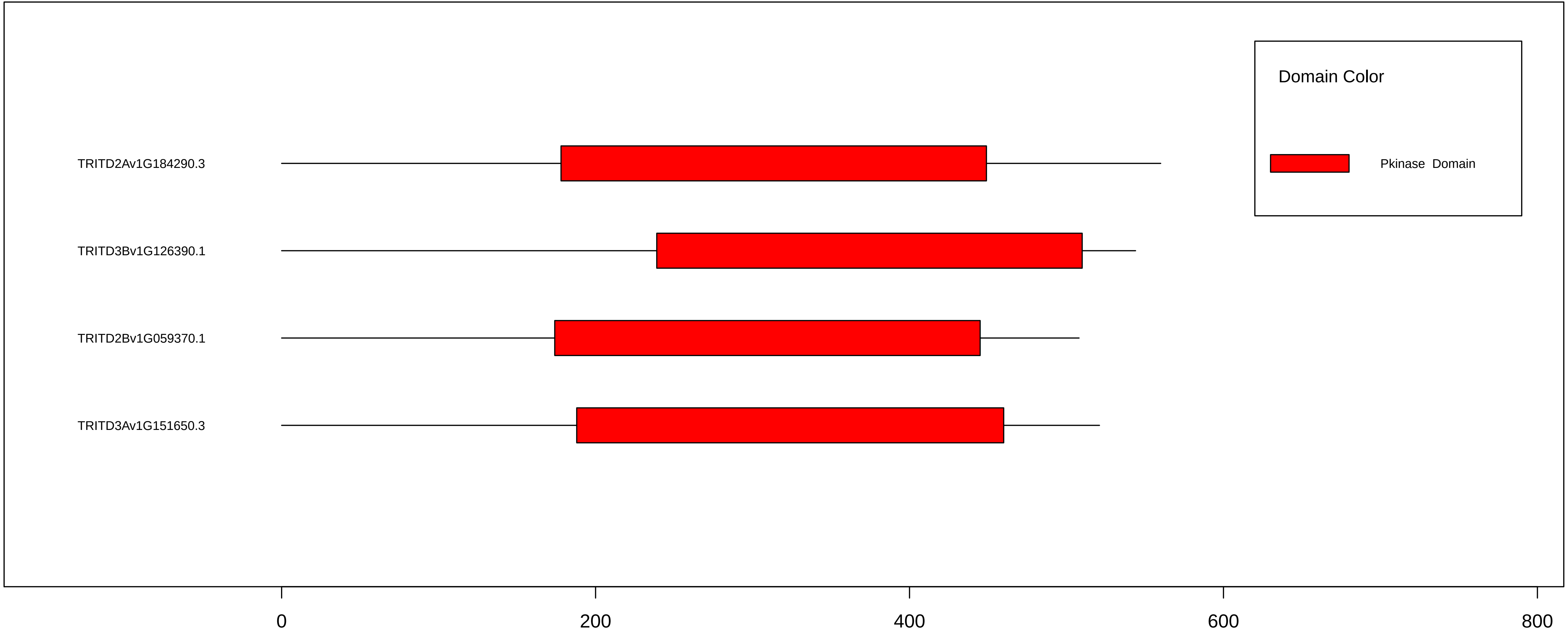

T.tu RLK−Pelle_RLCK−XII−1 subfamily domain diagram (all)

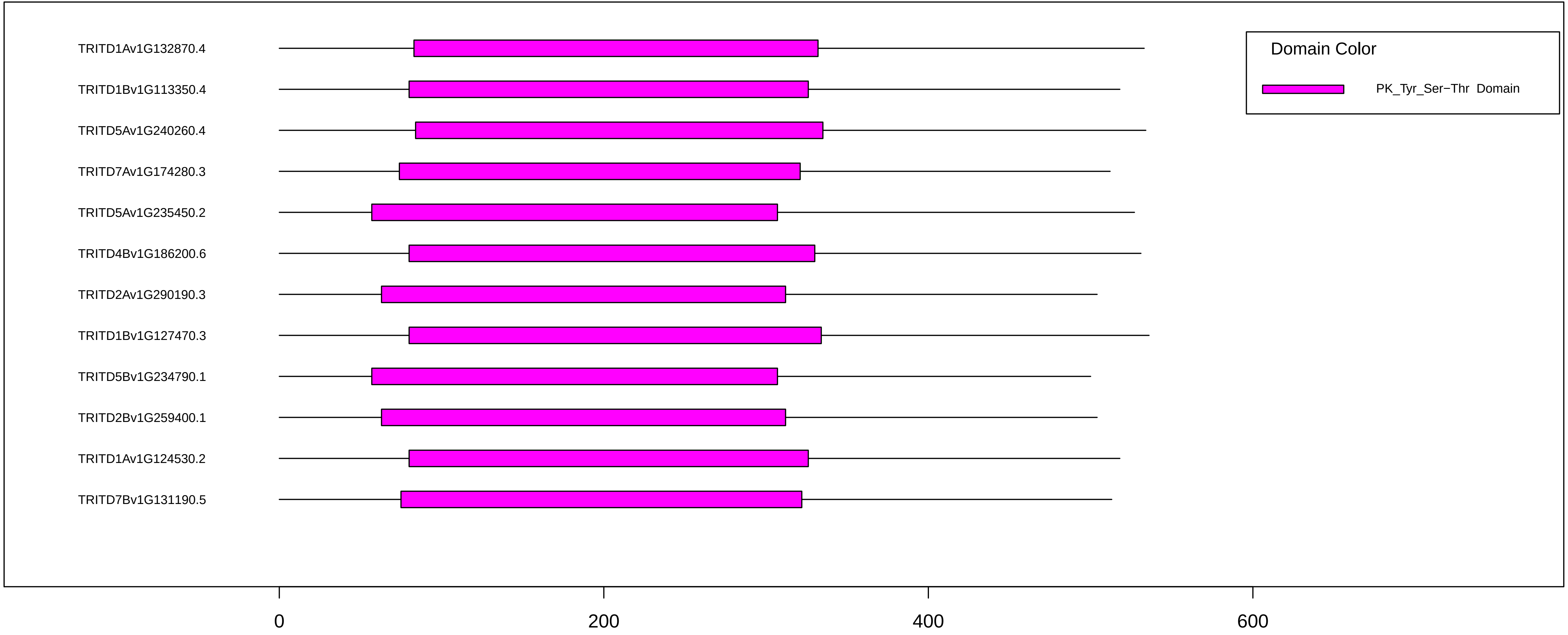

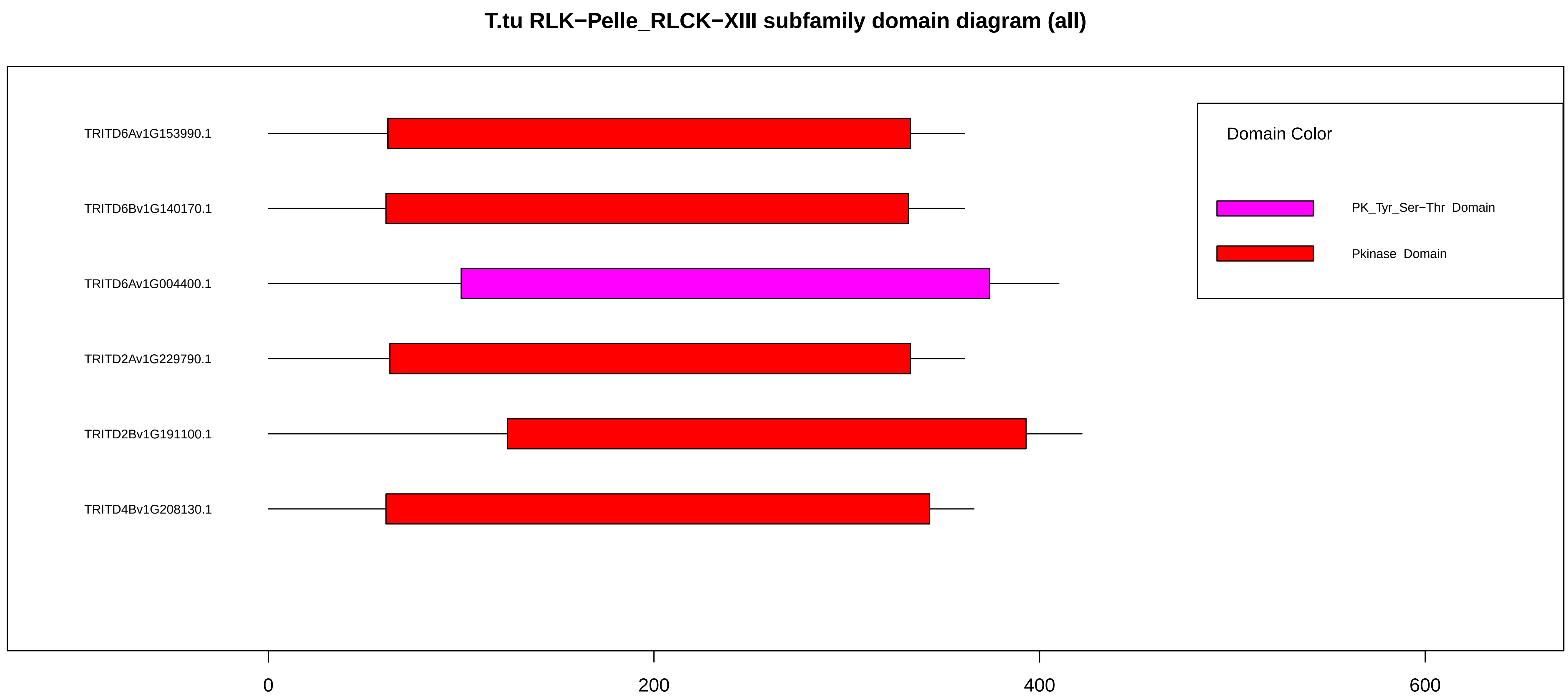
T.tu RLK−Pelle_RLCK−XIII subfamily domain diagram (all)
TRITD6Av1G153990.1
TRITD6Bv1G140170.1
TRITD6Av1G004400.1
TRITD2Av1G229790.1
TRITD2Bv1G191100.1
TRITD4Bv1G208130.1
Domain Color
PK_Tyr_Ser−Thr Domain
Pkinase Domain
0
200
400
600

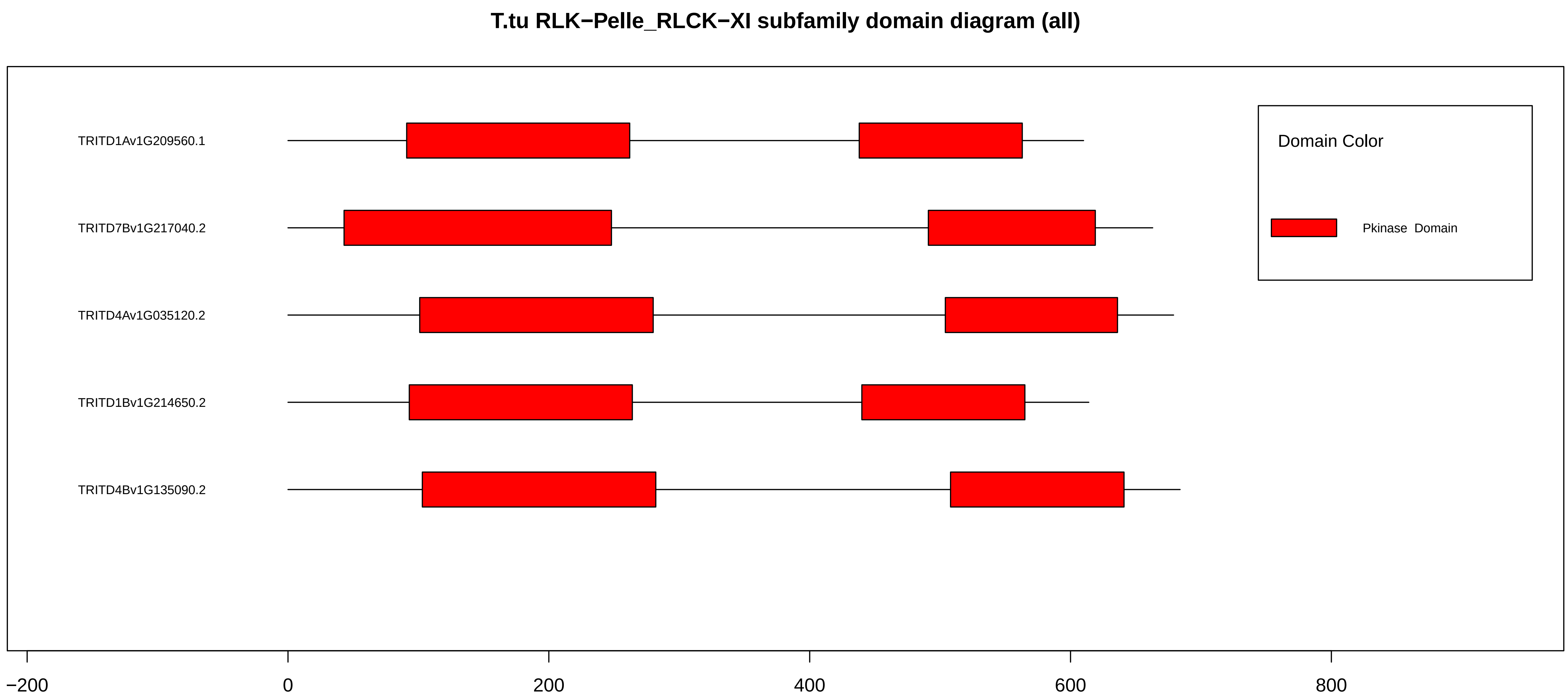

T.tu RLK−Pelle_RLCK−XI subfamily domain diagram (all)
TRITD1Av1G209560.1
TRITD7Bv1G217040.2
TRITD4Av1G035120.2
TRITD1Bv1G214650.2
TRITD4Bv1G135090.2
Domain Color
Pkinase Domain
−200
0
200
400
600
800

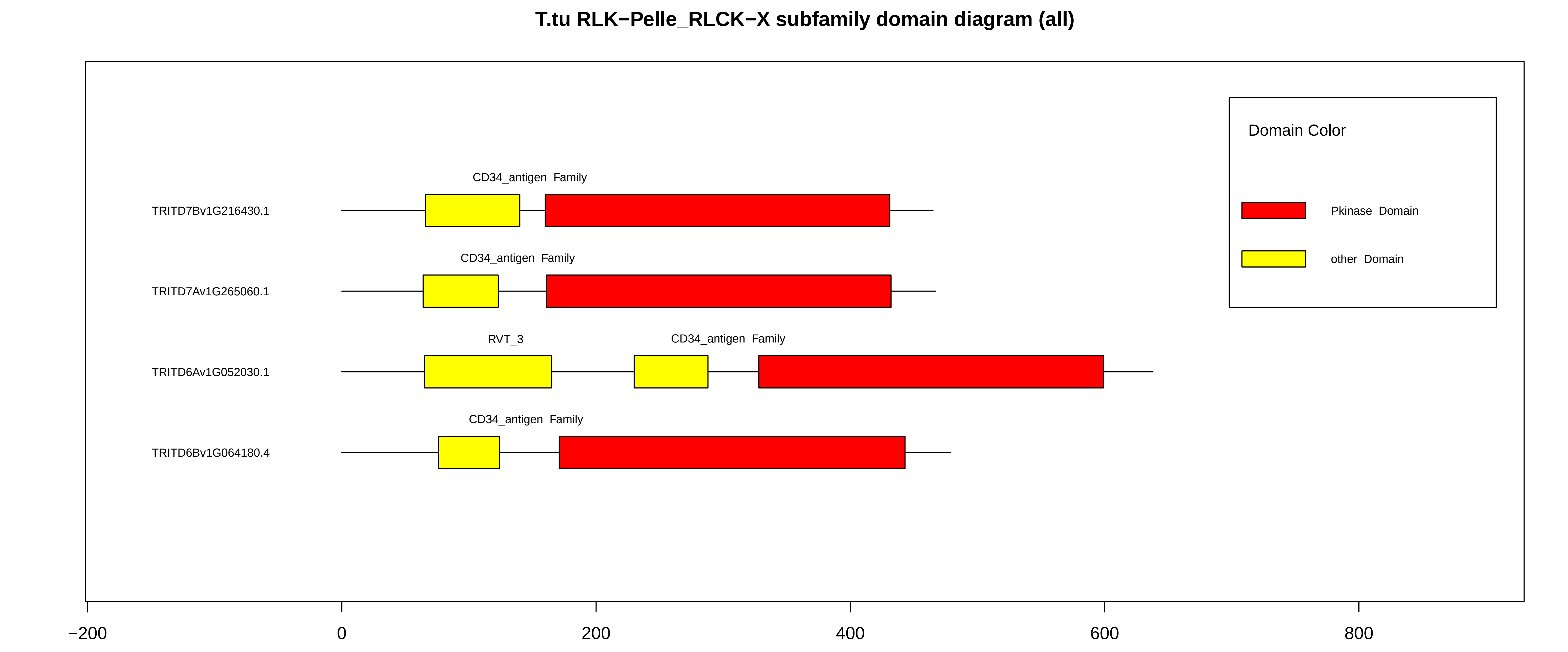
T.tu RLK−Pelle_RLCK−X subfamily domain diagram (all)
TRITD7Bv1G216430.1
CD34_antigen Family
TRITD7Av1G265060.1
CD34_antigen Family
TRITD6Av1G052030.1
RVT_3
CD34_antigen Family
TRITD6Bv1G064180.4
CD34_antigen Family
Domain Color
Pkinase Domain
other Domain
−200
0
200
400
600
800

T.tu RLK−Pelle_RLCK−XVI subfamily domain diagram (all)

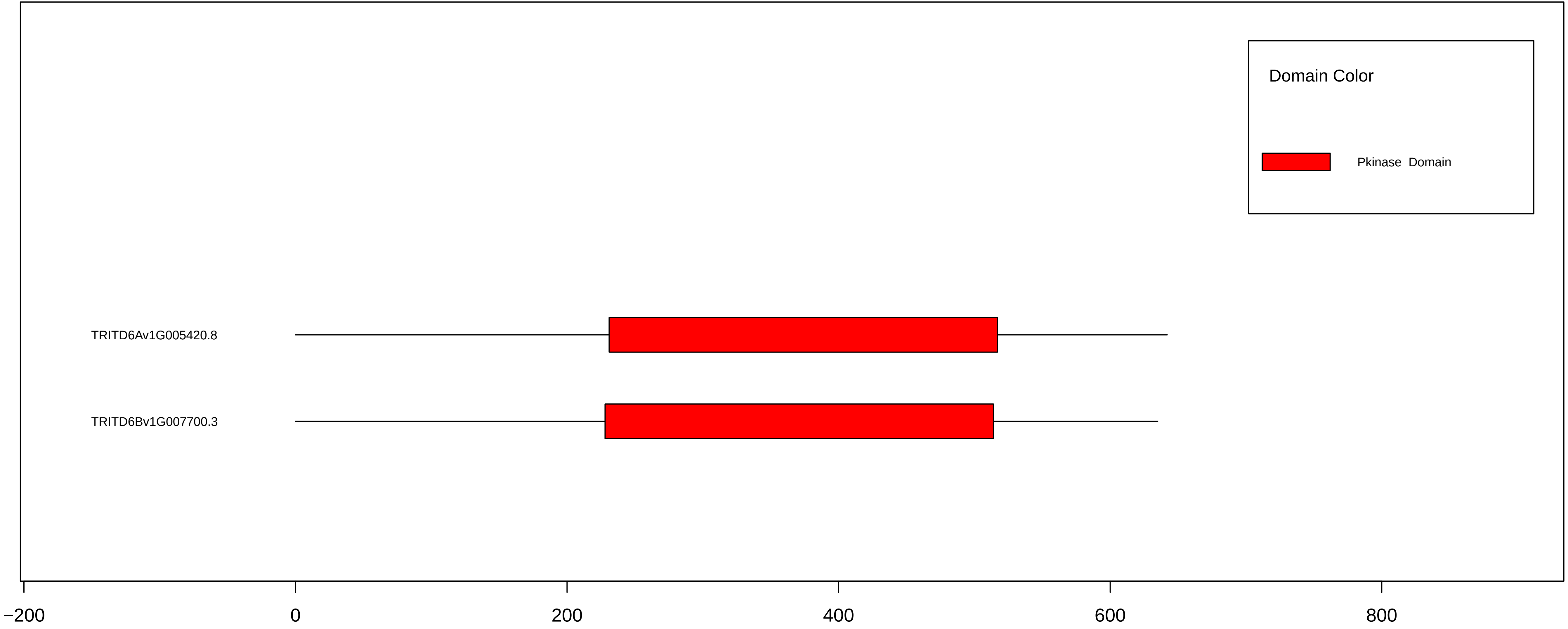

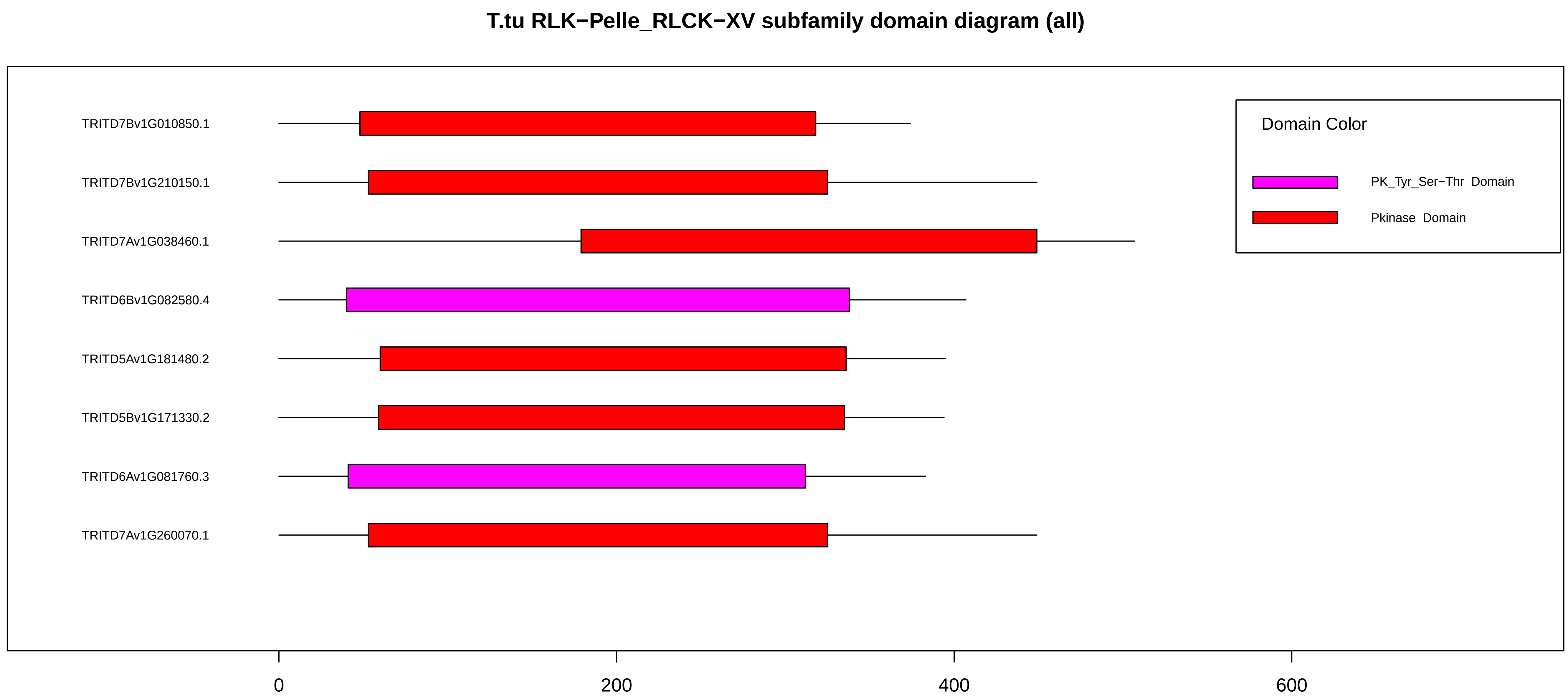
T.tu RLK−Pelle_RLCK−XV subfamily domain diagram (all)
TRITD7Bv1G010850.1
TRITD7Bv1G210150.1
TRITD7Av1G038460.1
TRITD6Bv1G082580.4
TRITD5Av1G181480.2
TRITD5Bv1G171330.2
TRITD6Av1G081760.3
TRITD7Av1G260070.1
Domain Color
PK_Tyr_Ser−Thr Domain
Pkinase Domain
0
200
400
600

T.tu RLK−Pelle_SD−2b subfamily domain diagram (part 1)

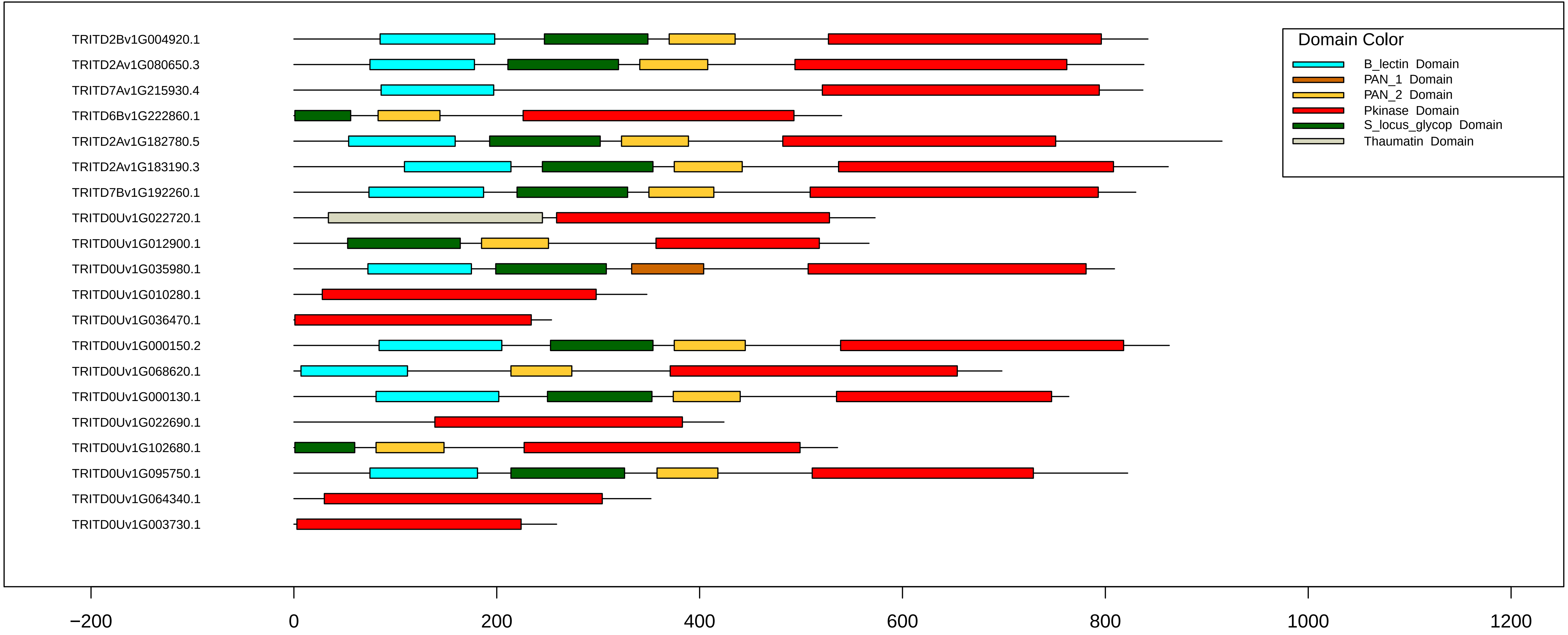

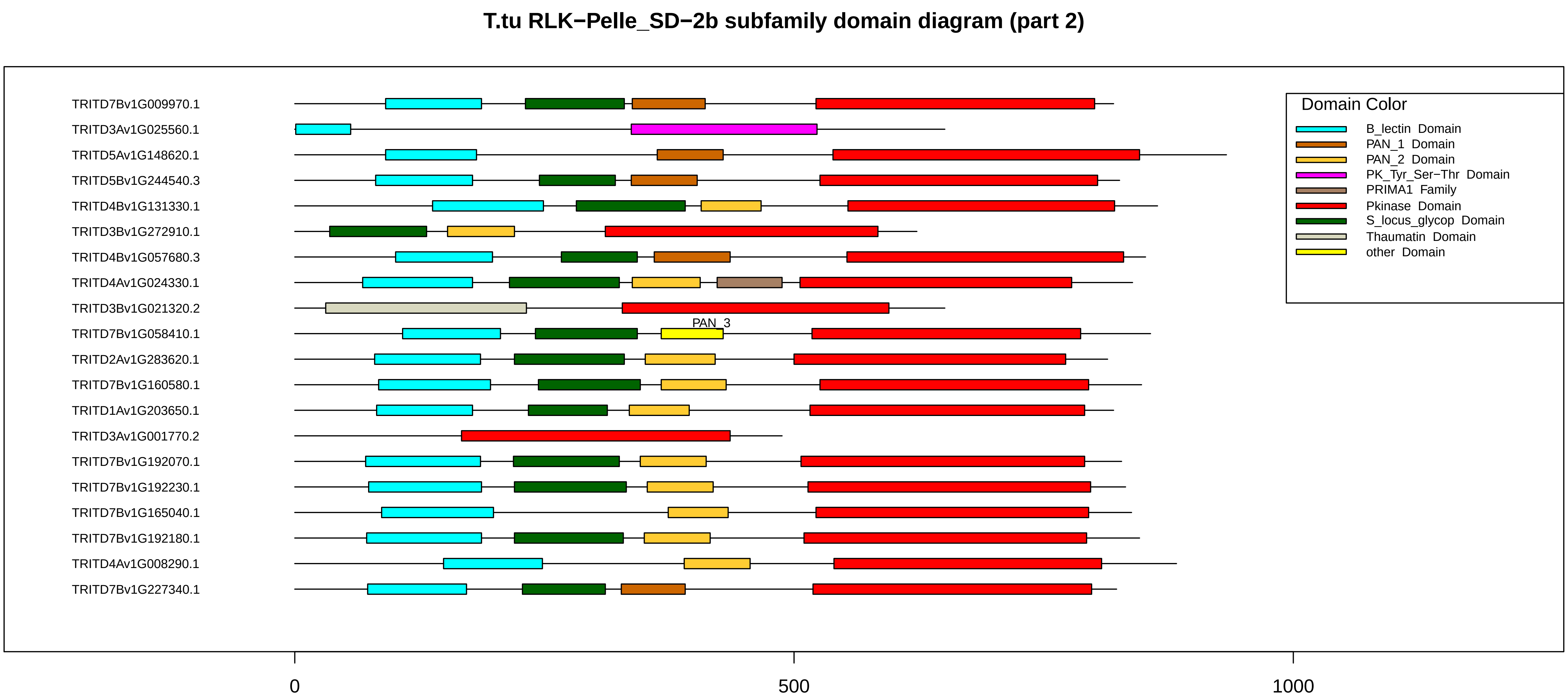

T.tu RLK−Pelle_SD−2b subfamily domain diagram (part 2)
TRITD7Bv1G009970.1
TRITD3Av1G025560.1
TRITD5Av1G148620.1
TRITD5Bv1G244540.3
TRITD4Bv1G131330.1
TRITD3Bv1G272910.1
TRITD4Bv1G057680.3
TRITD4Av1G024330.1
TRITD3Bv1G021320.2
TRITD7Bv1G058410.1
TRITD2Av1G283620.1
TRITD7Bv1G160580.1
TRITD1Av1G203650.1
TRITD3Av1G001770.2
TRITD7Bv1G192070.1
TRITD7Bv1G192230.1
TRITD7Bv1G165040.1
TRITD7Bv1G192180.1
TRITD4Av1G008290.1
TRITD7Bv1G227340.1
PAN_3
Domain Color
B_lectin Domain
PAN_1 Domain
PAN_2 Domain
PK_Tyr_Ser−Thr Domain
PRIMA1 Family
Pkinase Domain
S_locus_glycop Domain
Thaumatin Domain
other Domain
0
500
1000

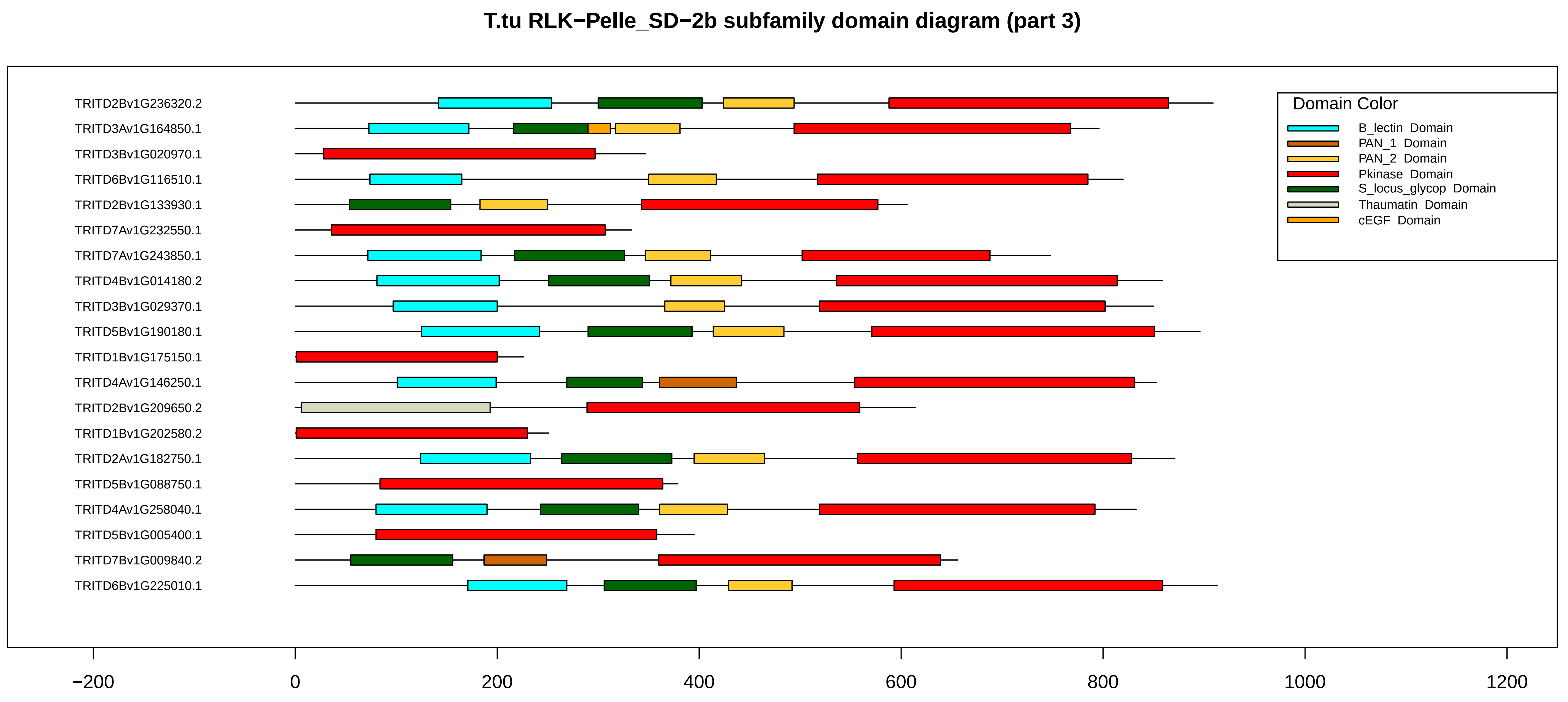
T.tu RLK−Pelle_SD−2b subfamily domain diagram (part 3)
TRITD2Bv1G236320.2
TRITD3Av1G164850.1
TRITD3Bv1G020970.1
TRITD6Bv1G116510.1
TRITD2Bv1G133930.1
TRITD7Av1G232550.1
TRITD7Av1G243850.1
TRITD4Bv1G014180.2
TRITD3Bv1G029370.1
TRITD5Bv1G190180.1
TRITD1Bv1G175150.1
TRITD4Av1G146250.1
TRITD2Bv1G209650.2
TRITD1Bv1G202580.2
TRITD2Av1G182750.1
TRITD5Bv1G088750.1
TRITD4Av1G258040.1
TRITD5Bv1G005400.1
TRITD7Bv1G009840.2
TRITD6Bv1G225010.1
Domain Color
B_lectin Domain
PAN_1 Domain
PAN_2 Domain
Pkinase Domain
S_locus_glycop Domain
Thaumatin Domain
cEGF Domain
−200
0
200
400
600
800
1000
1200

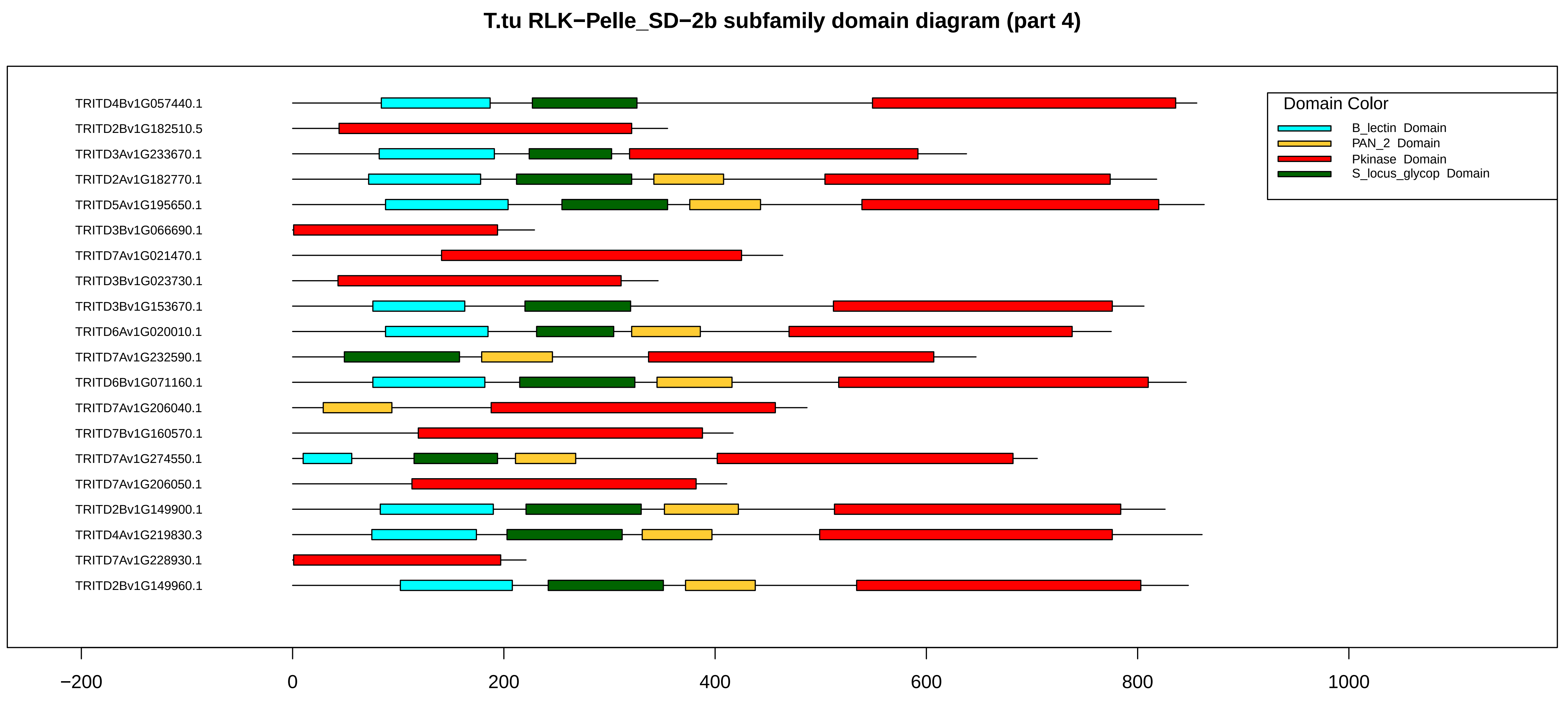
T.tu RLK−Pelle_SD−2b subfamily domain diagram (part 4)
TRITD4Bv1G057440.1
TRITD2Bv1G182510.5
TRITD3Av1G233670.1
TRITD2Av1G182770.1
TRITD5Av1G195650.1
TRITD3Bv1G066690.1
TRITD7Av1G021470.1
TRITD3Bv1G023730.1
TRITD3Bv1G153670.1
TRITD6Av1G020010.1
TRITD7Av1G232590.1
TRITD6Bv1G071160.1
TRITD7Av1G206040.1
TRITD7Bv1G160570.1
TRITD7Av1G274550.1
TRITD7Av1G206050.1
TRITD2Bv1G149900.1
TRITD4Av1G219830.3
TRITD7Av1G228930.1
TRITD2Bv1G149960.1
Domain Color
B_lectin Domain
PAN_2 Domain
Pkinase Domain
S_locus_glycop Domain
−200
0
200
400
600
800
1000

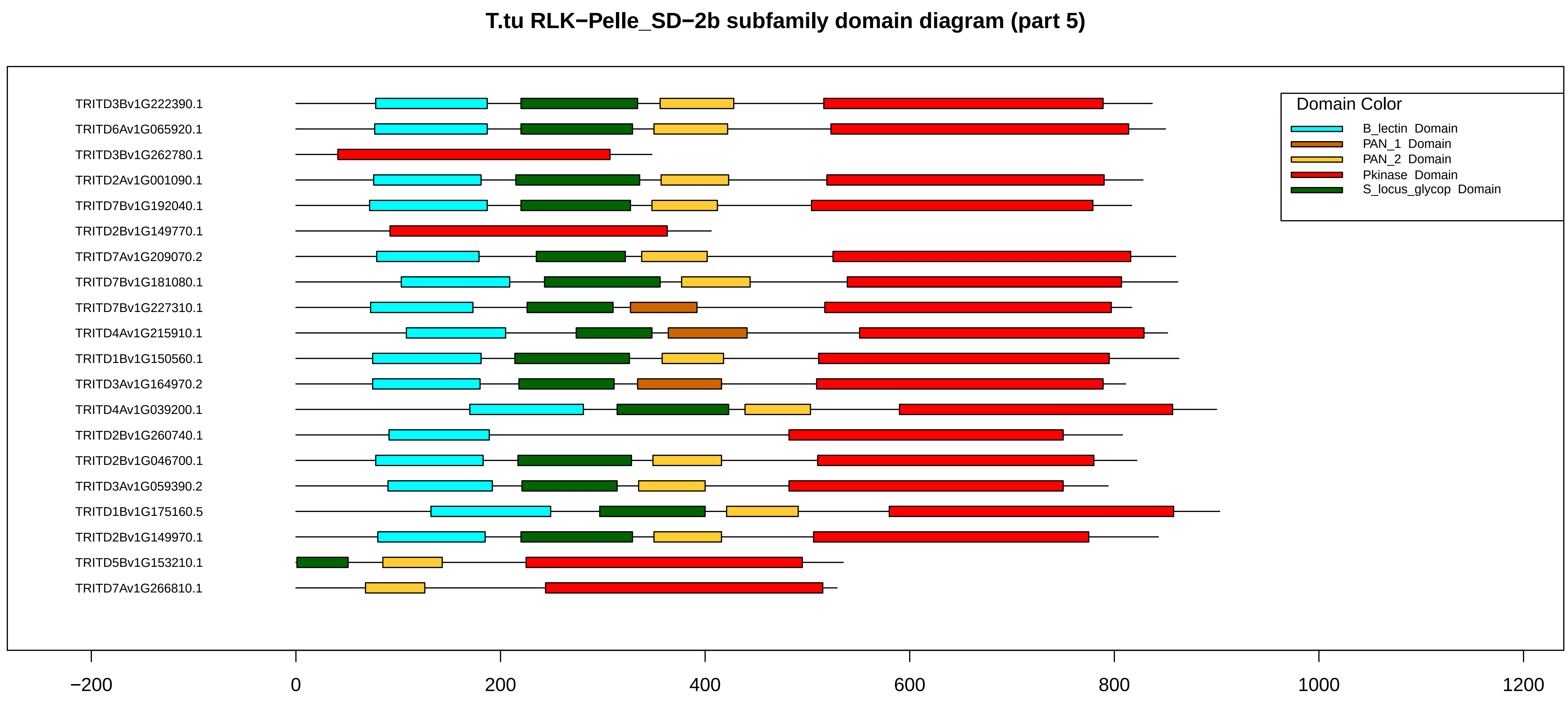

T.tu RLK−Pelle_SD−2b subfamily domain diagram (part 5)
TRITD3Bv1G222390.1
TRITD6Av1G065920.1
TRITD3Bv1G262780.1
TRITD2Av1G001090.1
TRITD7Bv1G192040.1
TRITD2Bv1G149770.1
TRITD7Av1G209070.2
TRITD7Bv1G181080.1
TRITD7Bv1G227310.1
TRITD4Av1G215910.1
TRITD1Bv1G150560.1
TRITD3Av1G164970.2
TRITD4Av1G039200.1
TRITD2Bv1G260740.1
TRITD2Bv1G046700.1
TRITD3Av1G059390.2
TRITD1Bv1G175160.5
TRITD2Bv1G149970.1
TRITD5Bv1G153210.1
TRITD7Av1G266810.1
Domain Color
B_lectin Domain
PAN_1 Domain
PAN_2 Domain
Pkinase Domain
S_locus_glycop Domain
−200
0
200
400
600
800
1000
1200

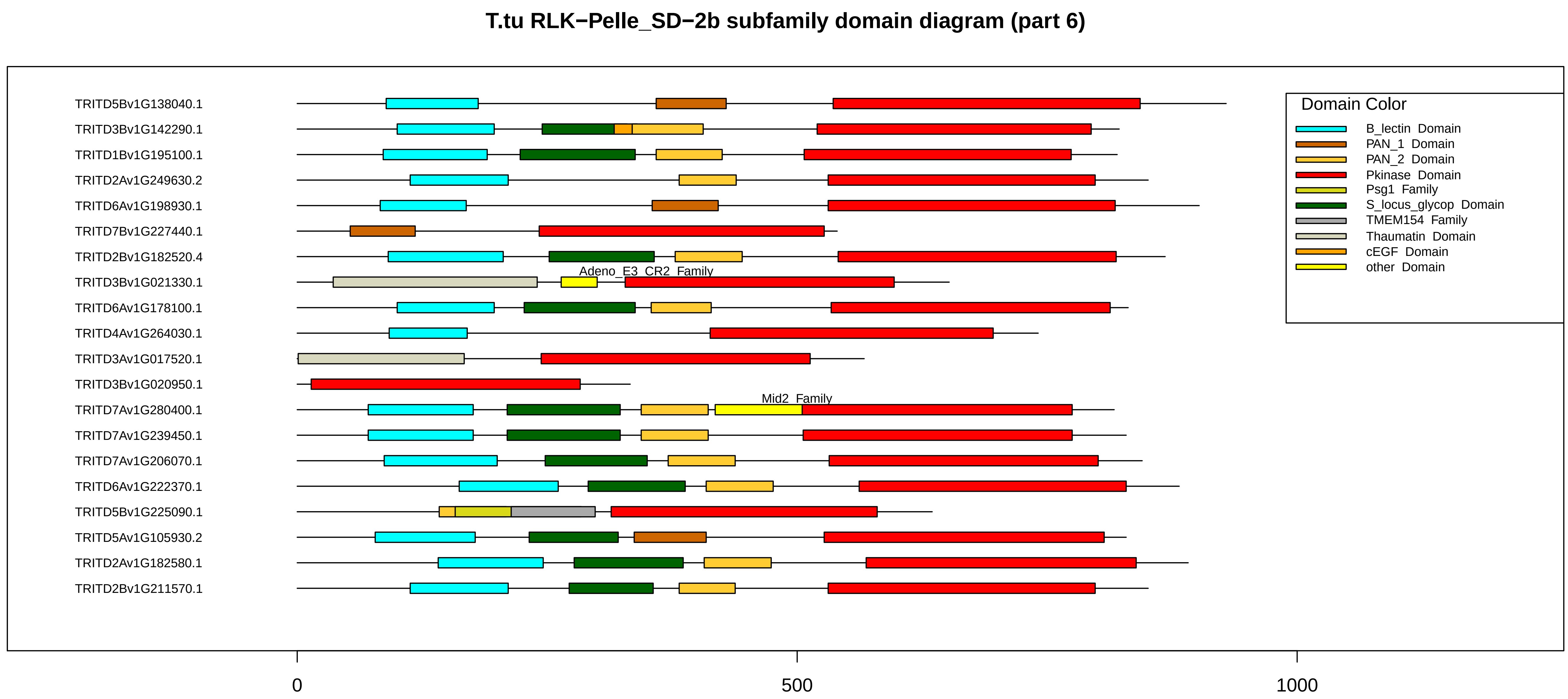

T.tu RLK−Pelle_SD−2b subfamily domain diagram (part 6)
TRITD5Bv1G138040.1
TRITD3Bv1G142290.1
TRITD1Bv1G195100.1
TRITD2Av1G249630.2
TRITD6Av1G198930.1
TRITD7Bv1G227440.1
TRITD2Bv1G182520.4
TRITD3Bv1G021330.1
Adeno_E3_CR2_Family
TRITD6Av1G178100.1
TRITD4Av1G264030.1
TRITD3Av1G017520.1
TRITD3Bv1G020950.1
TRITD7Av1G280400.1
Mid2_Family
TRITD7Av1G239450.1
TRITD7Av1G206070.1
TRITD6Av1G222370.1
TRITD5Bv1G225090.1
TRITD5Av1G105930.2
TRITD2Av1G182580.1
TRITD2Bv1G211570.1
Domain Color
B_lectin Domain
PAN_1 Domain
PAN_2 Domain
Pkinase Domain
Psg1 Family
S_locus_glycop Domain
TMEM154 Family
Thaumatin Domain
cEGF Domain
other Domain
0
500
1000

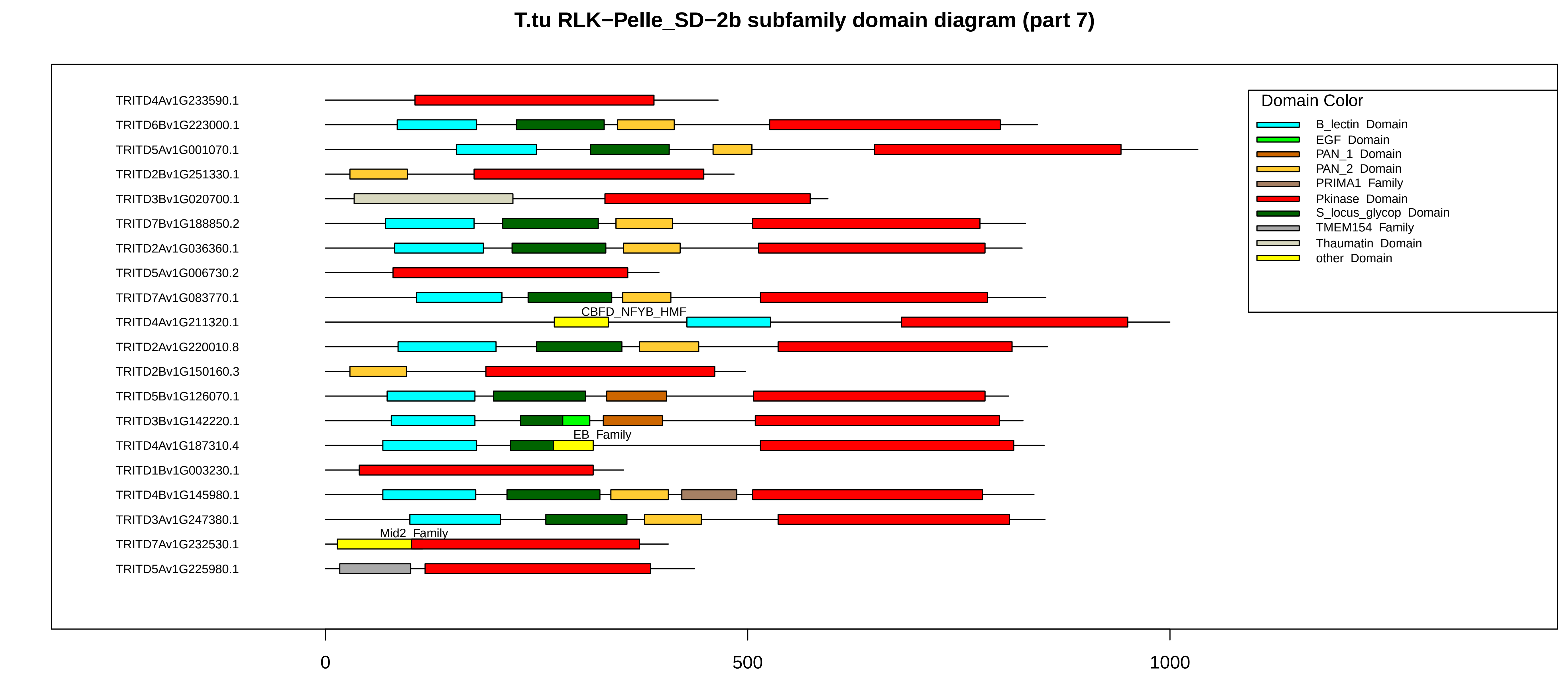
T.tu RLK−Pelle_SD−2b subfamily domain diagram (part 7)
TRITD4Av1G233590.1
TRITD6Bv1G223000.1
TRITD5Av1G001070.1
TRITD2Bv1G251330.1
TRITD3Bv1G020700.1
TRITD7Bv1G188850.2
TRITD2Av1G036360.1
TRITD5Av1G006730.2
TRITD7Av1G083770.1
CBFD_NFYB_HMF
TRITD4Av1G211320.1
TRITD2Av1G220010.8
TRITD2Bv1G150160.3
TRITD5Bv1G126070.1
TRITD3Bv1G142220.1
EB_Family
TRITD4Av1G187310.4
TRITD1Bv1G003230.1
TRITD4Bv1G145980.1
TRITD3Av1G247380.1
Mid2_Family
TRITD7Av1G232530.1
TRITD5Av1G225980.1
0
500
1000
Domain Color
B_lectin Domain
EGF Domain
PAN_1 Domain
PAN_2 Domain
PRIMA1 Family
Pkinase Domain
S_locus_glycop Domain
TMEM154 Family
Thaumatin Domain
other Domain

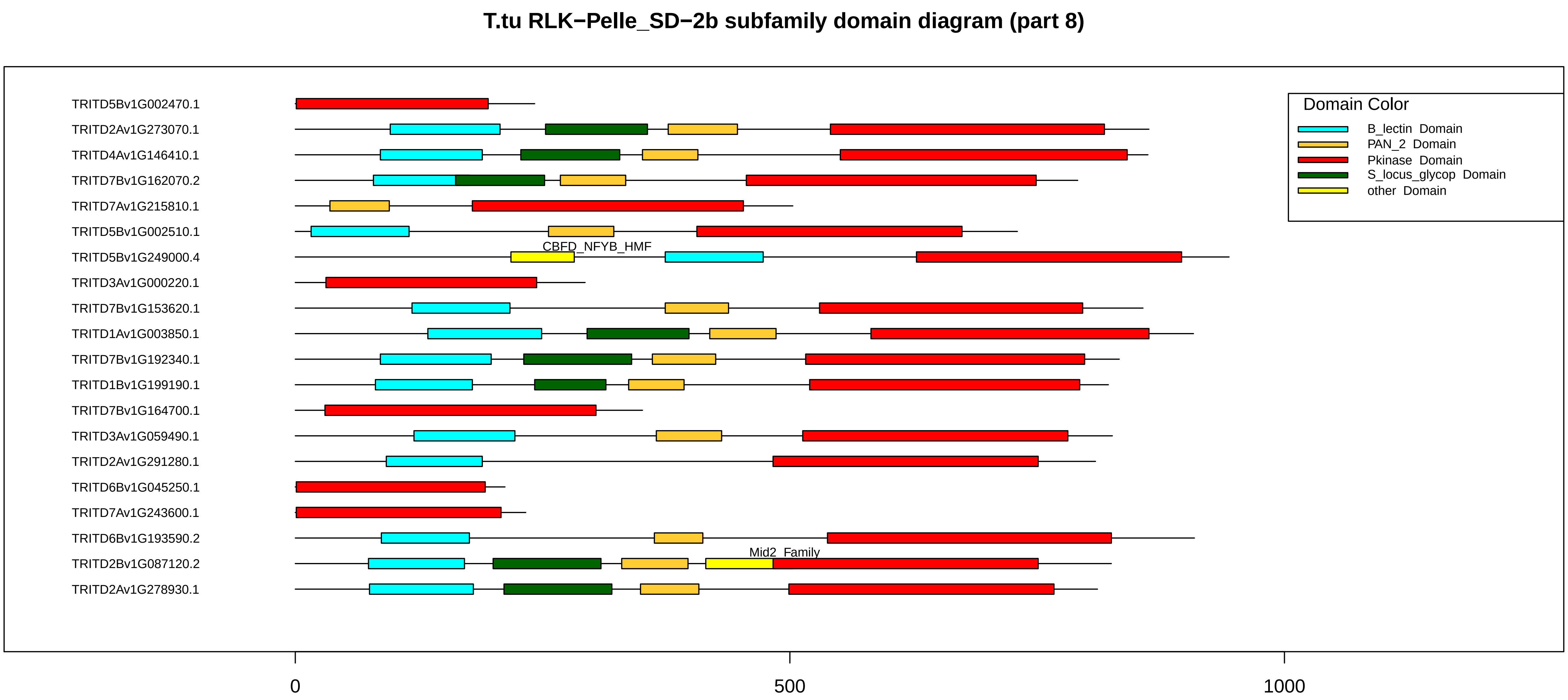
T.tu RLK−Pelle_SD−2b subfamily domain diagram (part 8)
TRITD5Bv1G002470.1
TRITD2Av1G273070.1
TRITD4Av1G146410.1
TRITD7Bv1G162070.2
TRITD7Av1G215810.1
TRITD5Bv1G002510.1
TRITD5Bv1G249000.4
TRITD3Av1G000220.1
TRITD7Bv1G153620.1
TRITD1Av1G003850.1
TRITD7Bv1G192340.1
TRITD1Bv1G199190.1
TRITD7Bv1G164700.1
TRITD3Av1G059490.1
TRITD2Av1G291280.1
TRITD6Bv1G045250.1
TRITD7Av1G243600.1
TRITD6Bv1G193590.2
TRITD2Bv1G087120.2
TRITD2Av1G278930.1
CBFD_NFYB_HMF
Mid2_Family
Domain Color
B_lectin Domain
PAN_2 Domain
Pkinase Domain
S_locus_glycop Domain
other Domain
0
500
1000

T.tu RLK−Pelle_SD−2b subfamily domain diagram (part 9)

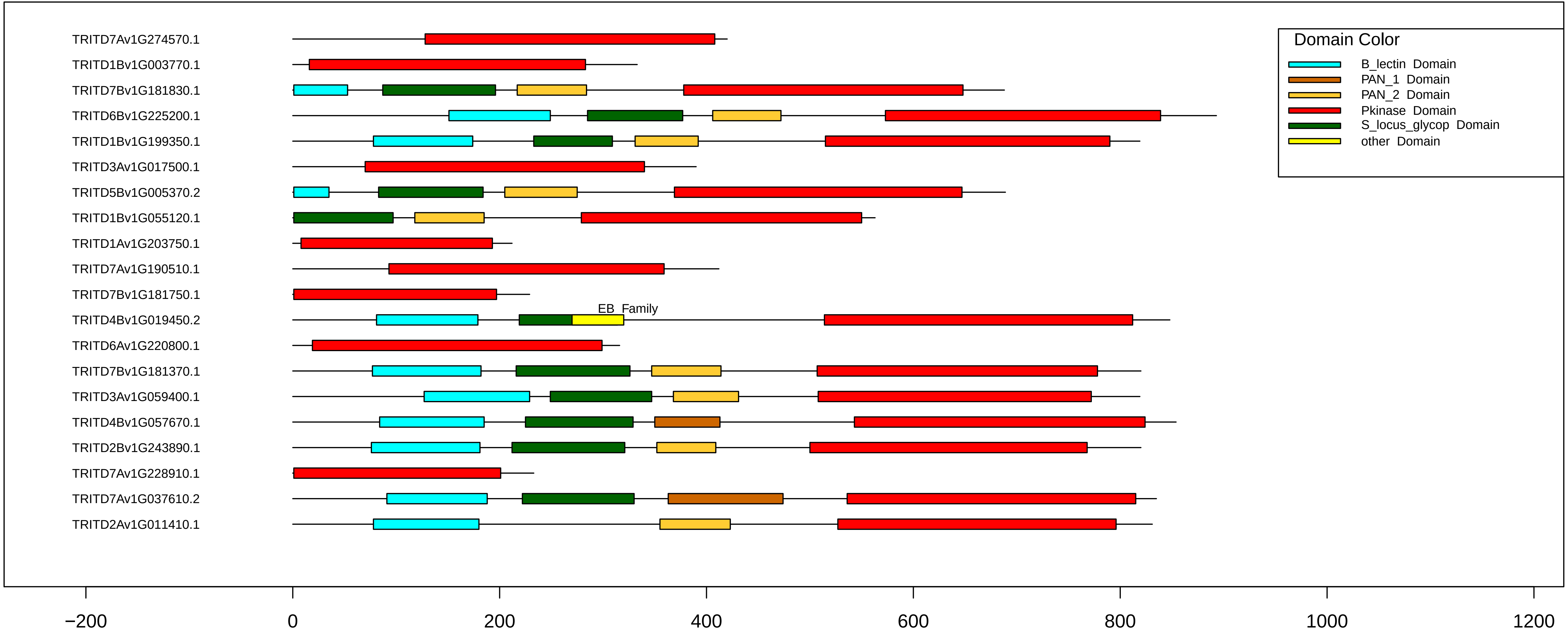

T.tu RLK−Pelle_SD−2b subfamily domain diagram (part 10)

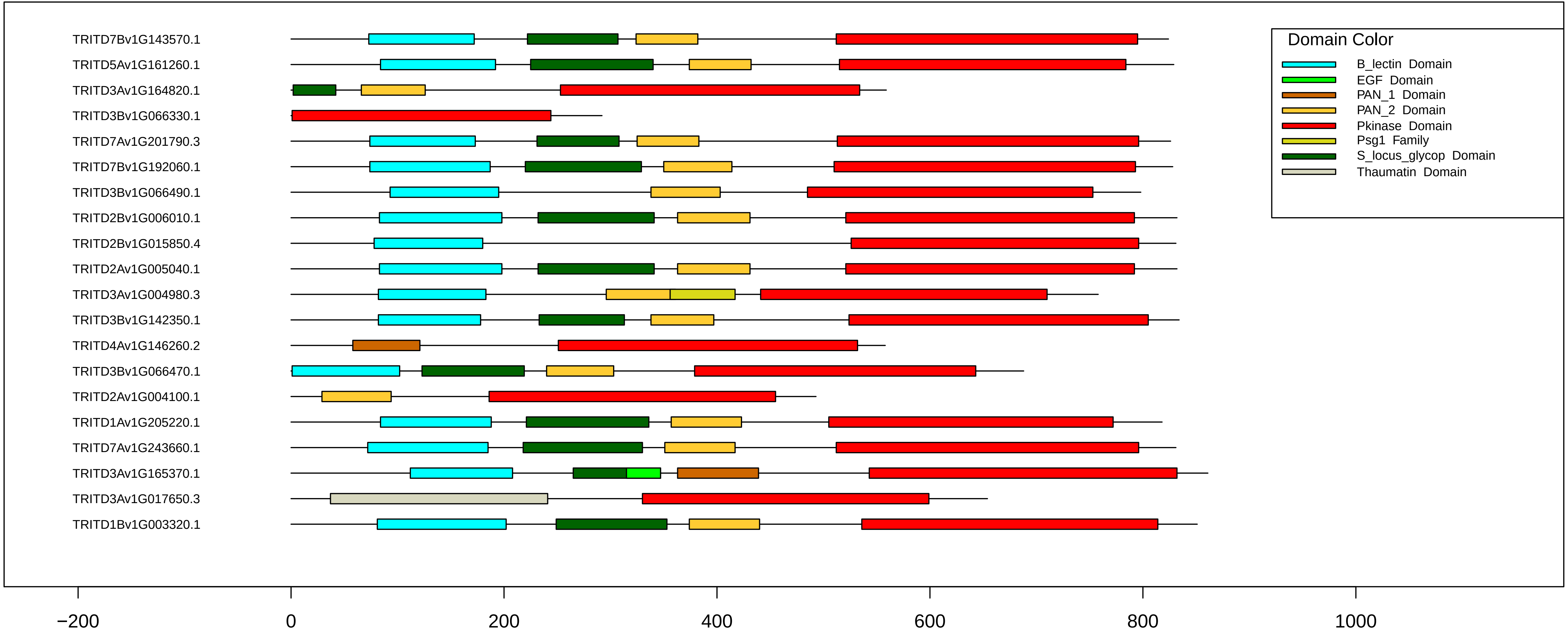

T.tu RLK−Pelle_SD−2b subfamily domain diagram (part 11)

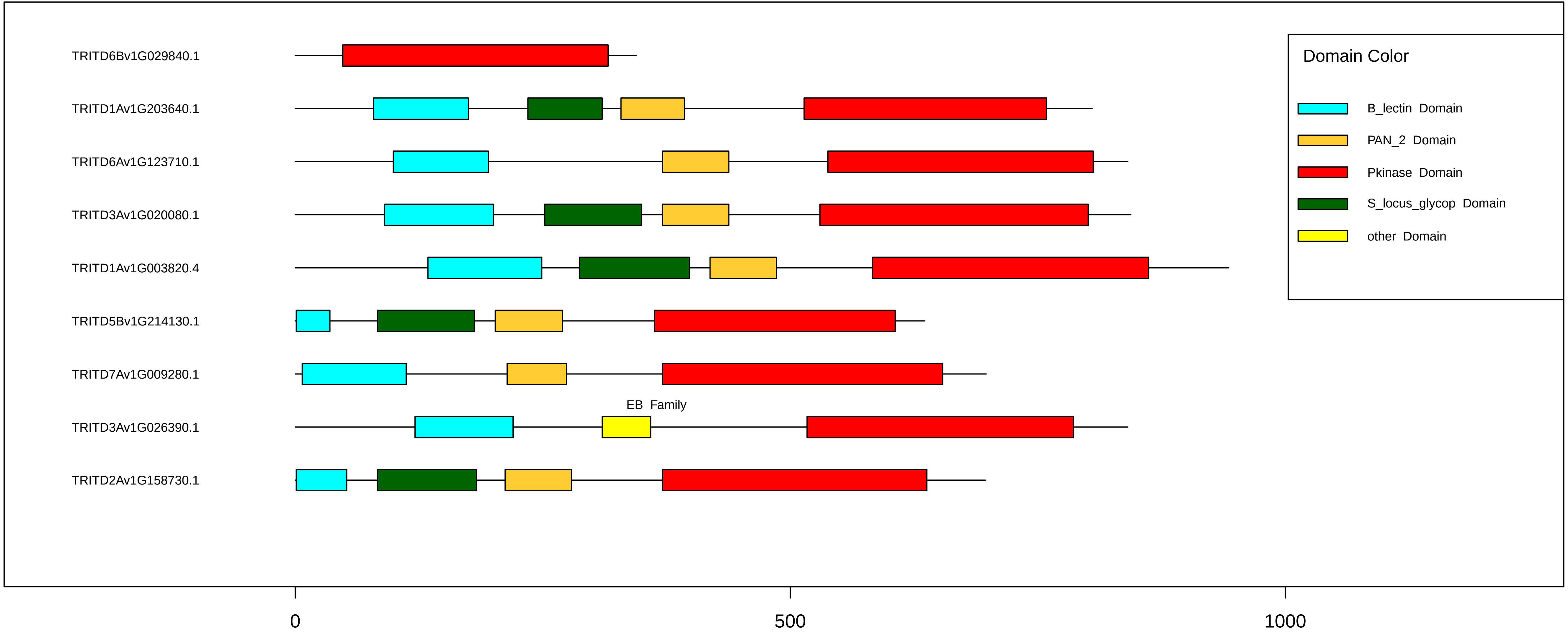

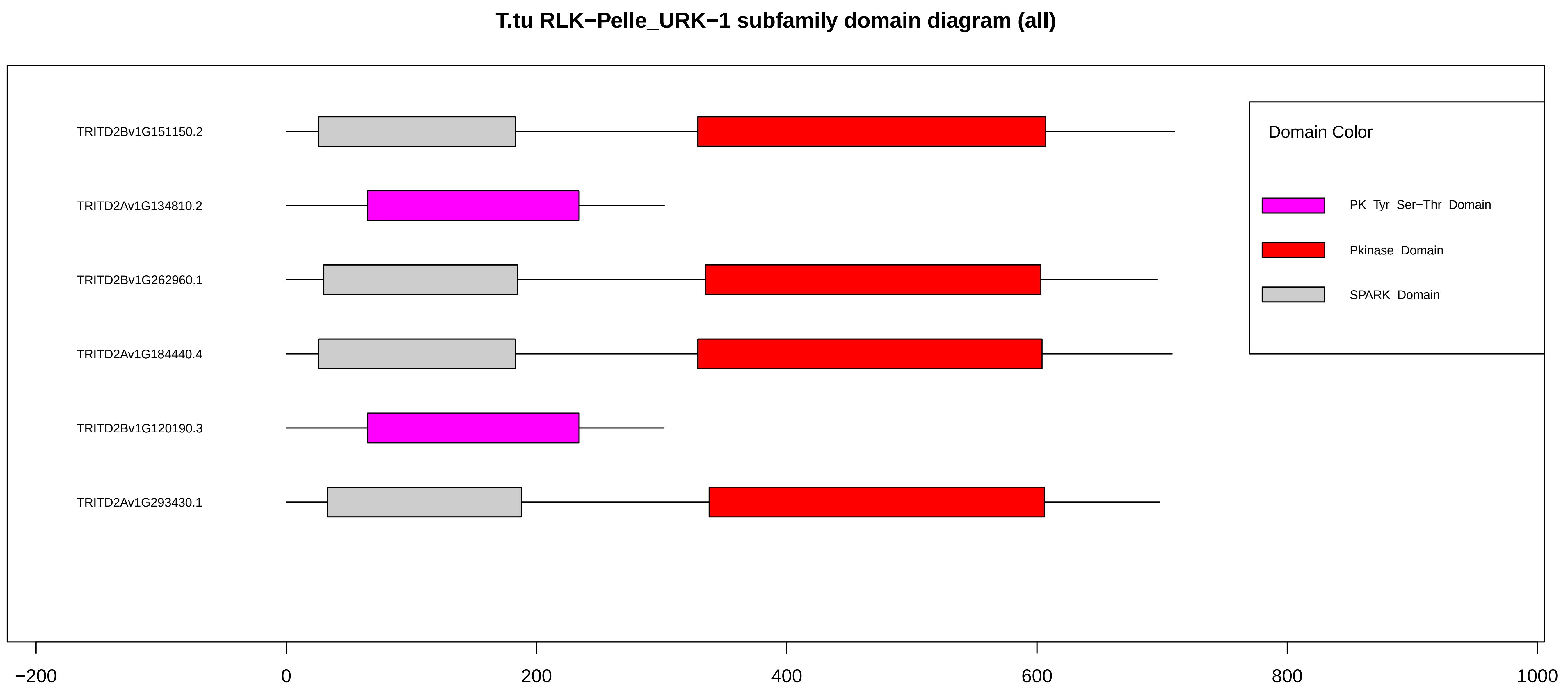

T.tu RLK−Pelle_URK−1 subfamily domain diagram (all)
TRITD2Bv1G151150.2
TRITD2Av1G134810.2
TRITD2Bv1G262960.1
TRITD2Av1G184440.4
TRITD2Bv1G120190.3
TRITD2Av1G293430.1
Domain Color
PK_Tyr_Ser−Thr Domain
Pkinase Domain
SPARK Domain
−200
0
200
400
600
800
1000

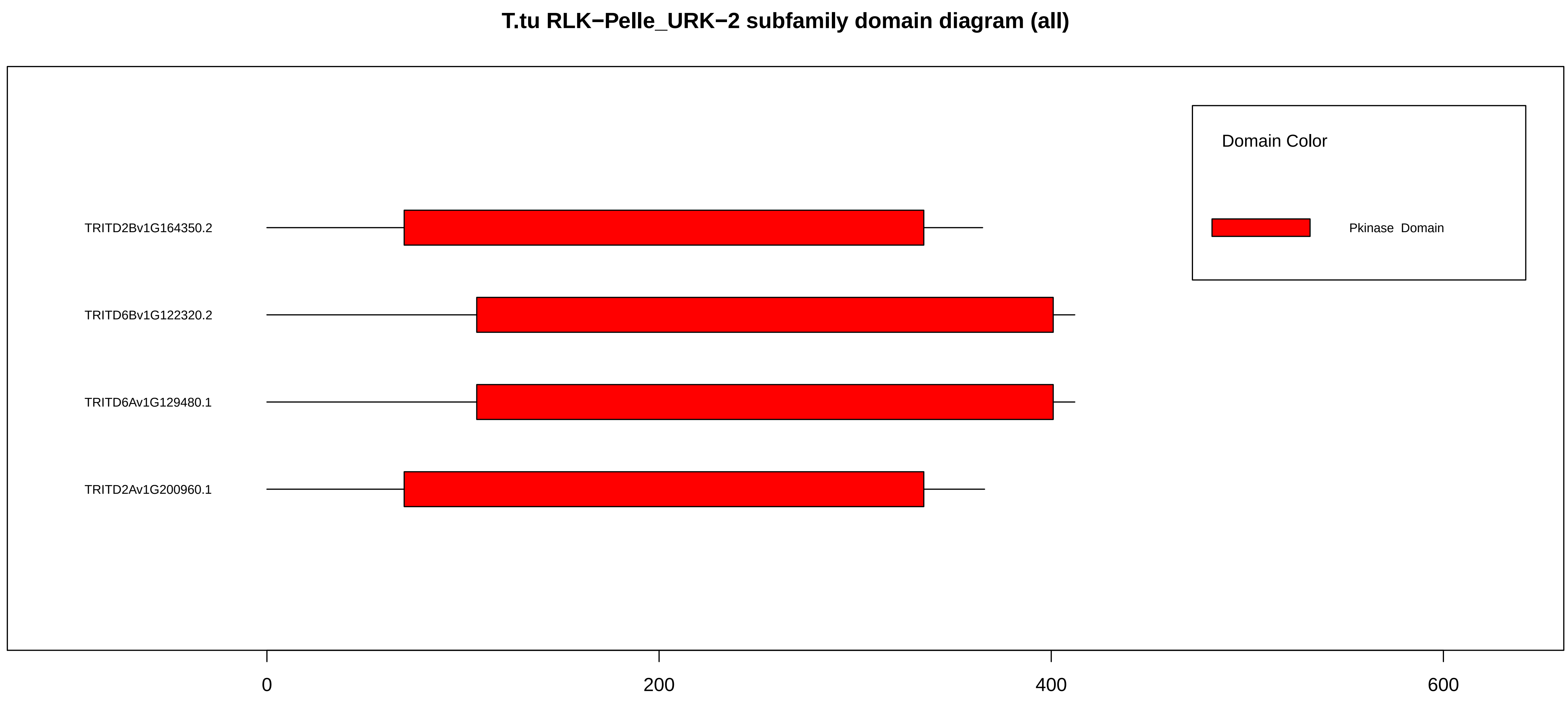
T.tu RLK−Pelle_URK−2 subfamily domain diagram (all)
Domain Color
Pkinase Domain
TRITD2Bv1G164350.2
TRITD6Bv1G122320.2
TRITD6Av1G129480.1
TRITD2Av1G200960.1
0
200
400
600

T.tu RLK−Pelle_URK−3 subfamily domain diagram (all)

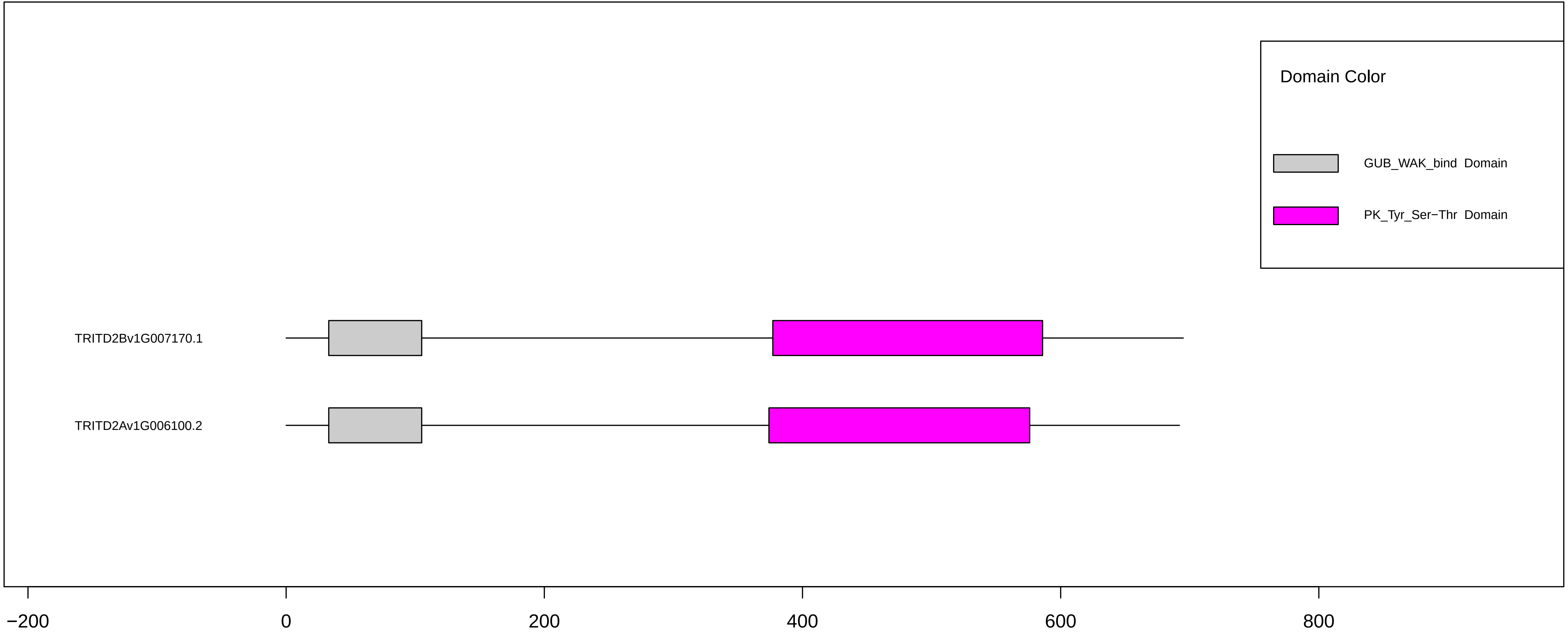

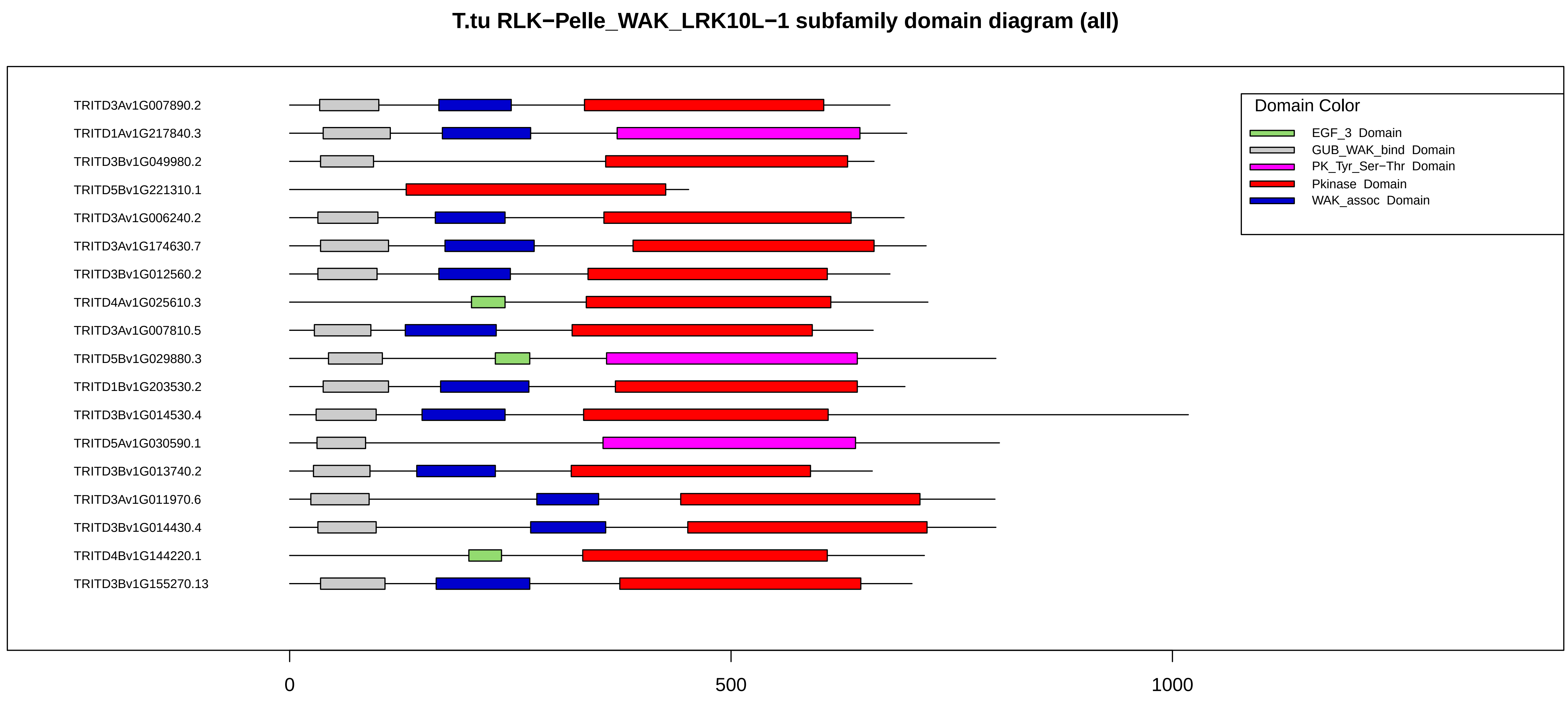
T.tu RLK−Pelle_WAK_LRK10L−1 subfamily domain diagram (all)
TRITD3Av1G007890.2
TRITD1Av1G217840.3
TRITD3Bv1G049980.2
TRITD5Bv1G221310.1
TRITD3Av1G006240.2
TRITD3Av1G174630.7
TRITD3Bv1G012560.2
TRITD4Av1G025610.3
TRITD3Av1G007810.5
TRITD5Bv1G029880.3
TRITD1Bv1G203530.2
TRITD3Bv1G014530.4
TRITD5Av1G030590.1
TRITD3Bv1G013740.2
TRITD3Av1G011970.6
TRITD3Bv1G014430.4
TRITD4Bv1G144220.1
TRITD3Bv1G155270.13
Domain Color
EGF_3 Domain
GUB_WAK_bind Domain
PK_Tyr_Ser−Thr Domain
Pkinase Domain
WAK_assoc Domain
0
500
1000

T.tu RLK−Pelle_WAK subfamily domain diagram (part 1)

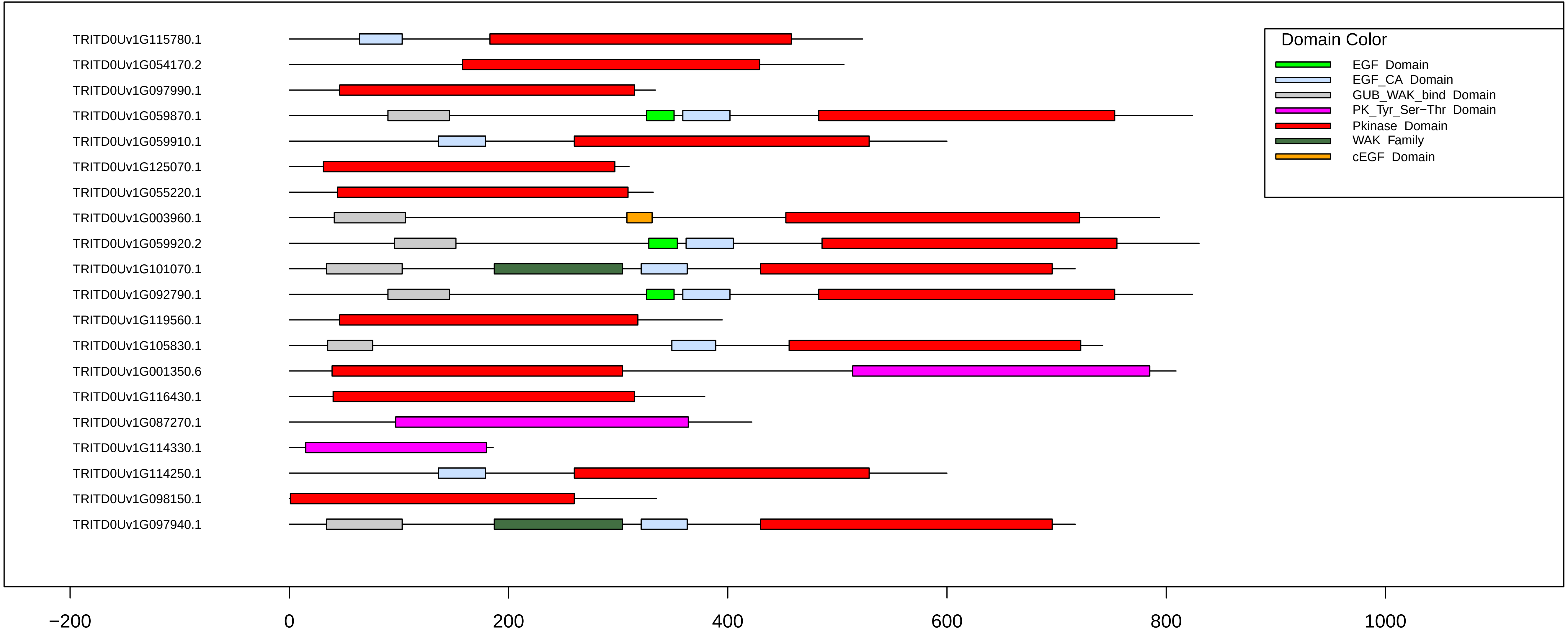

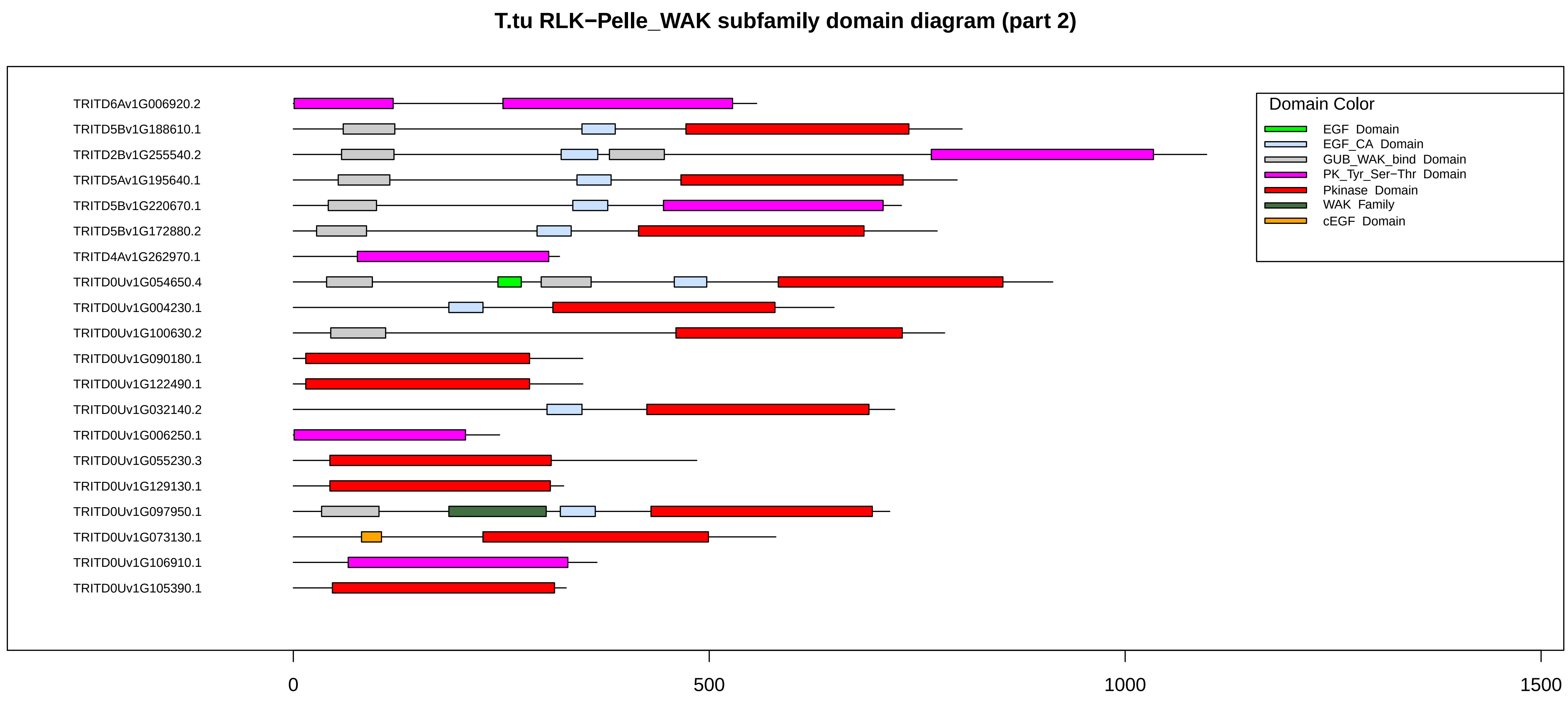

T.tu RLK−Pelle_WAK subfamily domain diagram (part 2)
TRITD6Av1G006920.2
TRITD5Bv1G188610.1
TRITD2Bv1G255540.2
TRITD5Av1G195640.1
TRITD5Bv1G220670.1
TRITD5Bv1G172880.2
TRITD4Av1G262970.1
TRITD0Uv1G054650.4
TRITD0Uv1G004230.1
TRITD0Uv1G100630.2
TRITD0Uv1G090180.1
TRITD0Uv1G122490.1
TRITD0Uv1G032140.2
TRITD0Uv1G006250.1
TRITD0Uv1G055230.3
TRITD0Uv1G129130.1
TRITD0Uv1G097950.1
TRITD0Uv1G073130.1
TRITD0Uv1G106910.1
TRITD0Uv1G105390.1
Domain Color
EGF Domain
EGF_CA Domain
GUB_WAK_bind Domain
PK_Tyr_Ser−Thr Domain
Pkinase Domain
WAK Family
cEGF Domain
0
500
1000
1500

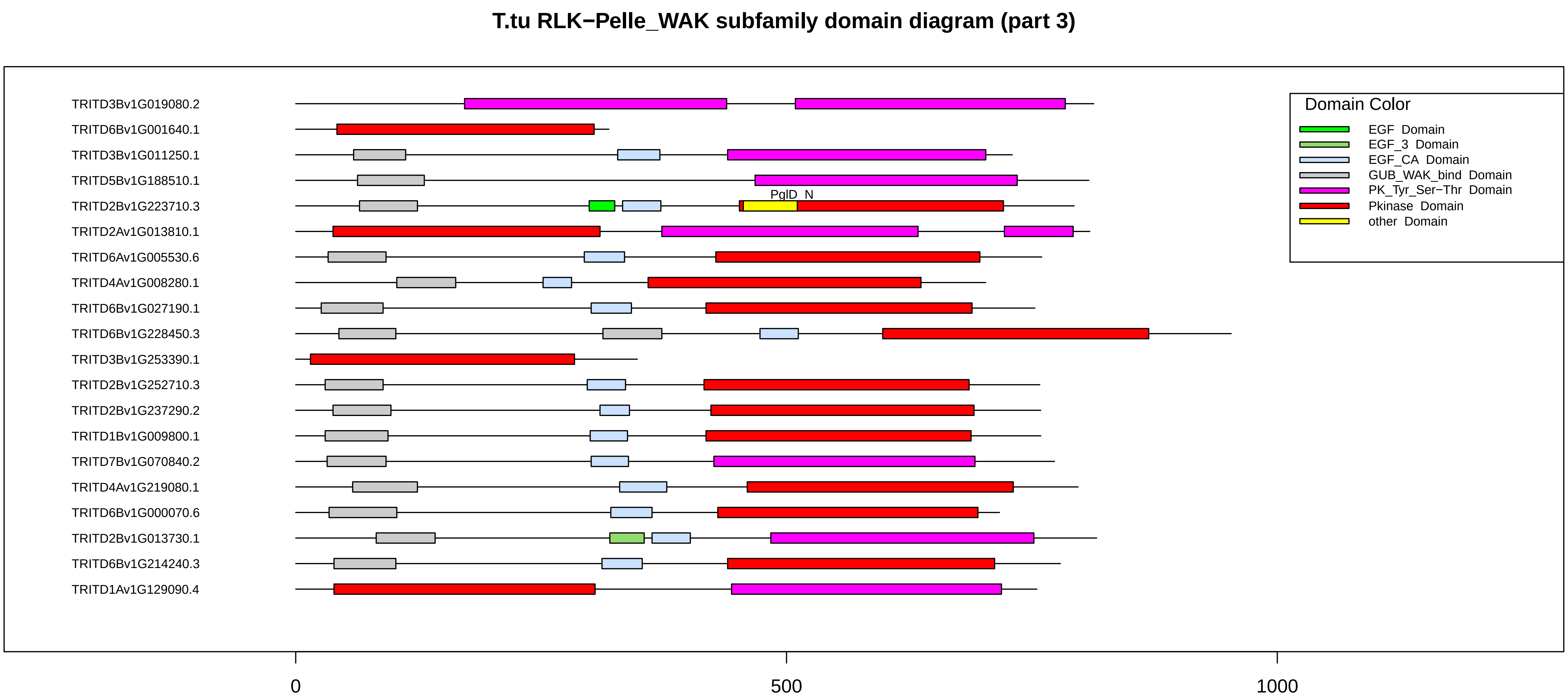
T.tu RLK−Pelle_WAK subfamily domain diagram (part 3)
TRITD3Bv1G019080.2
TRITD6Bv1G001640.1
TRITD3Bv1G011250.1
TRITD5Bv1G188510.1
TRITD2Bv1G223710.3
TRITD2Av1G013810.1
TRITD6Av1G005530.6
TRITD4Av1G008280.1
TRITD6Bv1G027190.1
TRITD6Bv1G228450.3
TRITD3Bv1G253390.1
TRITD2Bv1G252710.3
TRITD2Bv1G237290.2
TRITD1Bv1G009800.1
TRITD7Bv1G070840.2
TRITD4Av1G219080.1
TRITD6Bv1G000070.6
TRITD2Bv1G013730.1
TRITD6Bv1G214240.3
TRITD1Av1G129090.4
PglD_N
Domain Color
EGF Domain
EGF_3 Domain
EGF_CA Domain
GUB_WAK_bind Domain
PK_Tyr_Ser−Thr Domain
Pkinase Domain
other Domain
0
500
1000

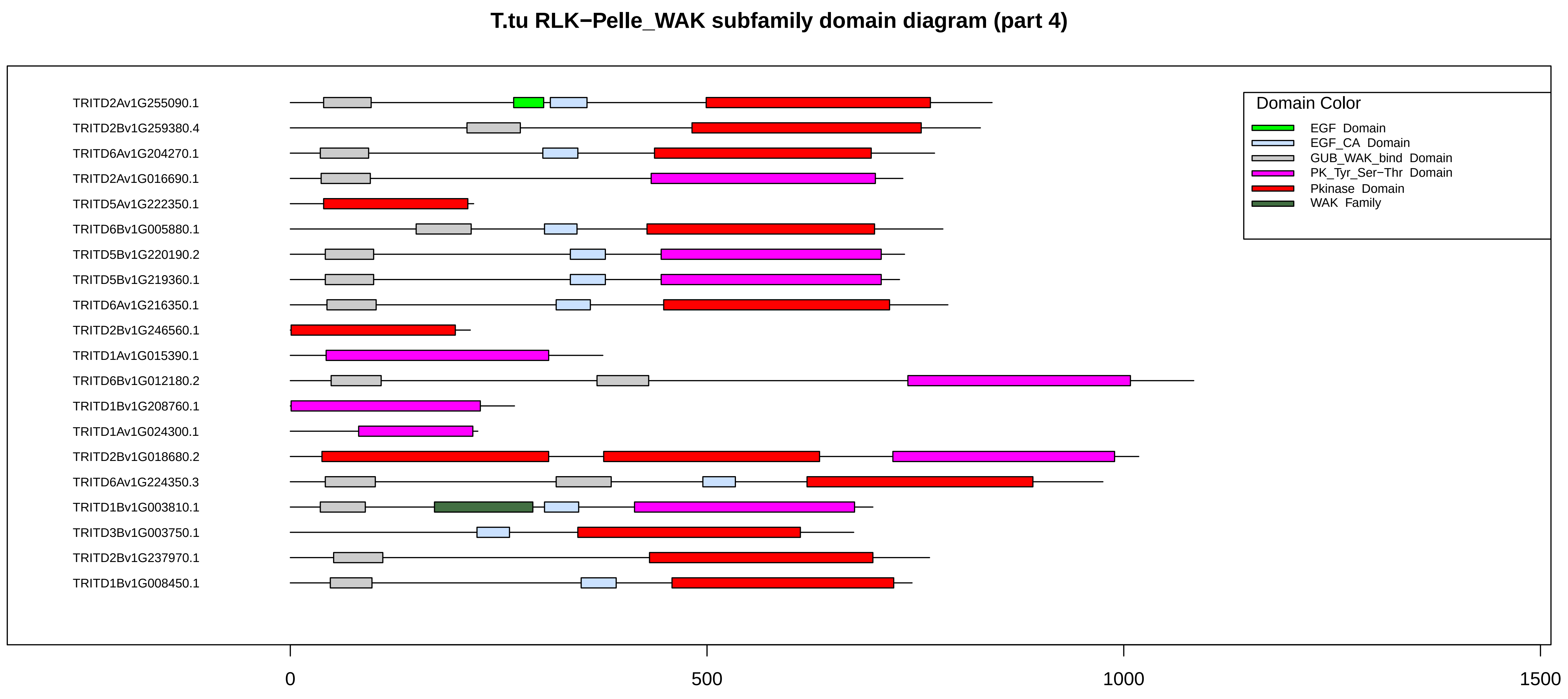
T.tu RLK−Pelle_WAK subfamily domain diagram (part 4)
TRITD2Av1G255090.1
TRITD2Bv1G259380.4
TRITD6Av1G204270.1
TRITD2Av1G016690.1
TRITD5Av1G222350.1
TRITD6Bv1G005880.1
TRITD5Bv1G220190.2
TRITD5Bv1G219360.1
TRITD6Av1G216350.1
TRITD2Bv1G246560.1
TRITD1Av1G015390.1
TRITD6Bv1G012180.2
TRITD1Bv1G208760.1
TRITD1Av1G024300.1
TRITD2Bv1G018680.2
TRITD6Av1G224350.3
TRITD1Bv1G003810.1
TRITD3Bv1G003750.1
TRITD2Bv1G237970.1
TRITD1Bv1G008450.1
Domain Color
EGF Domain
EGF_CA Domain
GUB_WAK_bind Domain
PK_Tyr_Ser−Thr Domain
Pkinase Domain
WAK Family
0
500
1000
1500

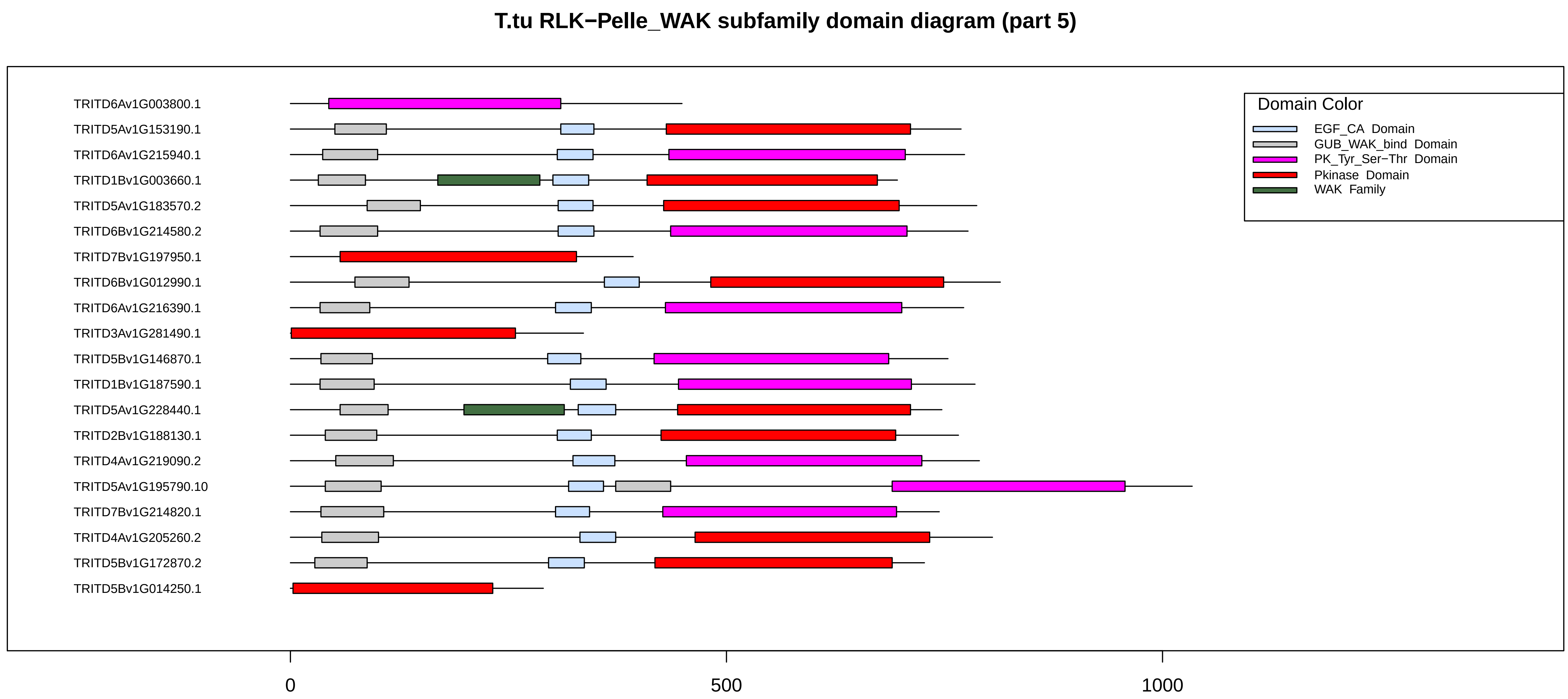
T.tu RLK−Pelle_WAK subfamily domain diagram (part 5)
TRITD6Av1G003800.1
TRITD5Av1G153190.1
TRITD6Av1G215940.1
TRITD1Bv1G003660.1
TRITD5Av1G183570.2
TRITD6Bv1G214580.2
TRITD7Bv1G197950.1
TRITD6Bv1G012990.1
TRITD6Av1G216390.1
TRITD3Av1G281490.1
TRITD5Bv1G146870.1
TRITD1Bv1G187590.1
TRITD5Av1G228440.1
TRITD2Bv1G188130.1
TRITD4Av1G219090.2
TRITD5Av1G195790.10
TRITD7Bv1G214820.1
TRITD4Av1G205260.2
TRITD5Bv1G172870.2
TRITD5Bv1G014250.1
Domain Color
EGF_CA Domain
GUB_WAK_bind Domain
PK_Tyr_Ser−Thr Domain
Pkinase Domain
WAK Family
0
500
1000

T.tu RLK−Pelle_WAK subfamily domain diagram (part 6)

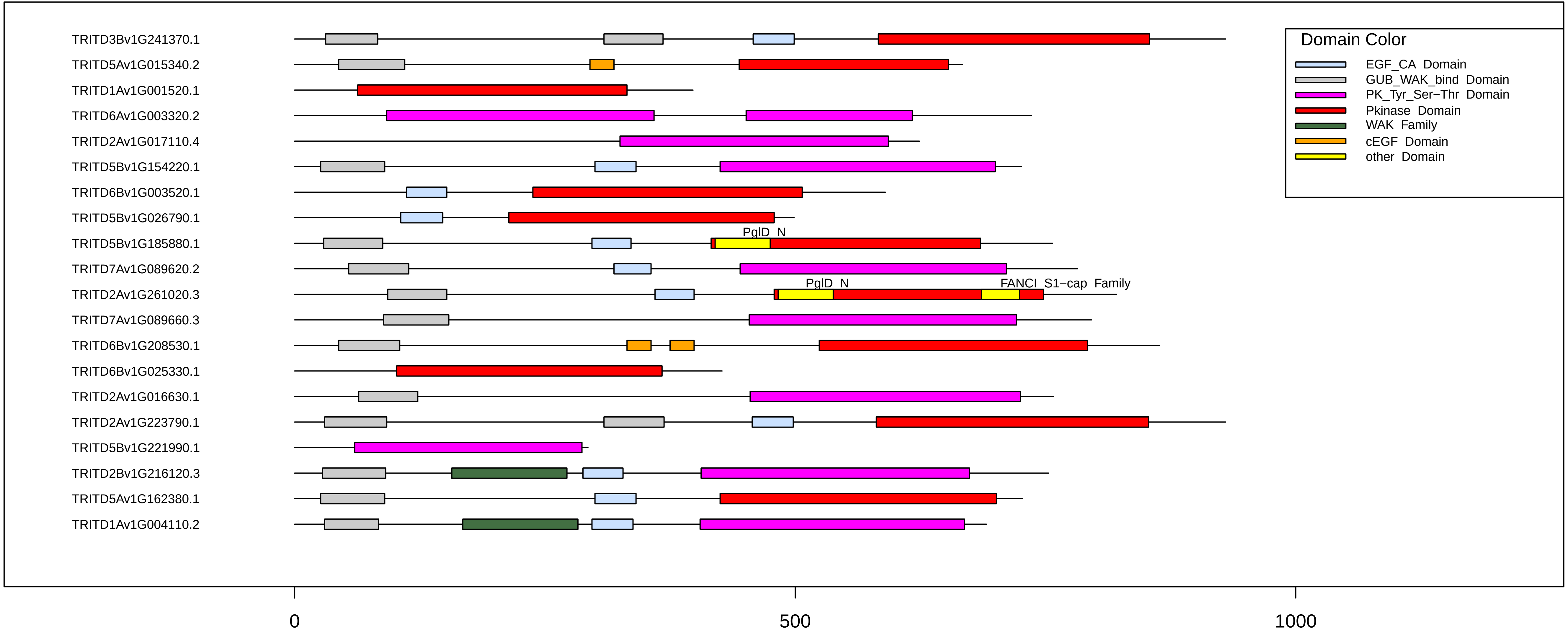

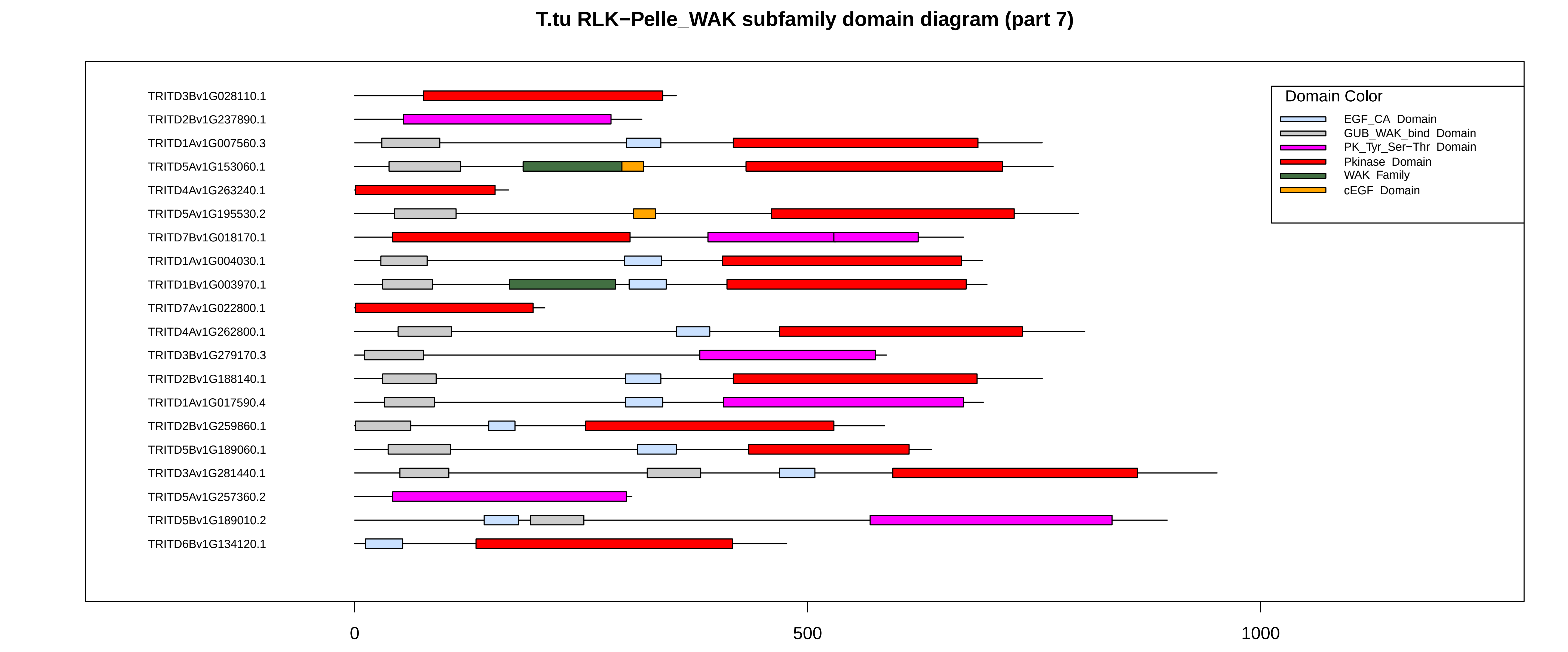
T.tu RLK−Pelle_WAK subfamily domain diagram (part 7)
TRITD3Bv1G028110.1
TRITD2Bv1G237890.1
TRITD1Av1G007560.3
TRITD5Av1G153060.1
TRITD4Av1G263240.1
TRITD5Av1G195530.2
TRITD7Bv1G018170.1
TRITD1Av1G004030.1
TRITD1Bv1G003970.1
TRITD7Av1G022800.1
TRITD4Av1G262800.1
TRITD3Bv1G279170.3
TRITD2Bv1G188140.1
TRITD1Av1G017590.4
TRITD2Bv1G259860.1
TRITD5Bv1G189060.1
TRITD3Av1G281440.1
TRITD5Av1G257360.2
TRITD5Bv1G189010.2
TRITD6Bv1G134120.1
Domain Color
EGF_CA Domain
GUB_WAK_bind Domain
PK_Tyr_Ser−Thr Domain
Pkinase Domain
WAK Family
cEGF Domain
0
500
1000

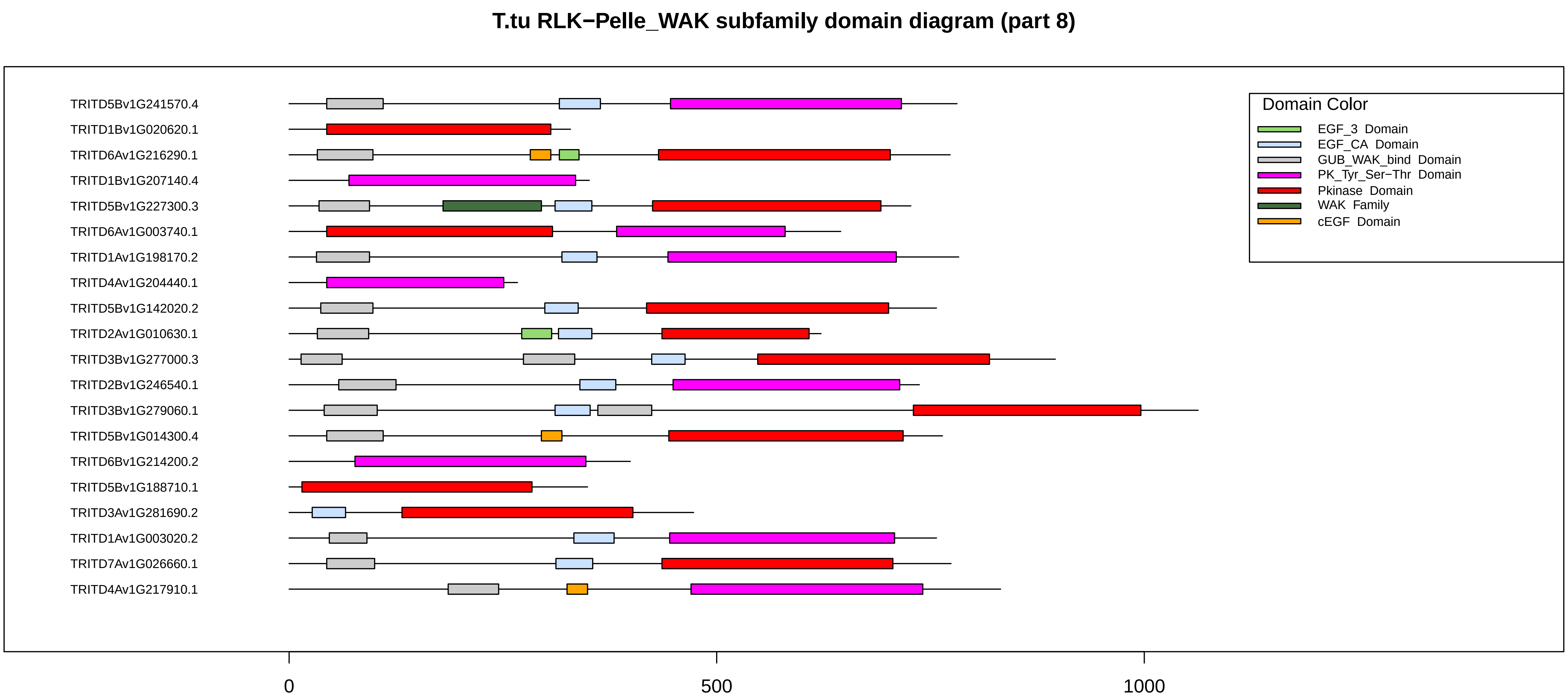
T.tu RLK−Pelle_WAK subfamily domain diagram (part 8)
TRITD5Bv1G241570.4
TRITD1Bv1G020620.1
TRITD6Av1G216290.1
TRITD1Bv1G207140.4
TRITD5Bv1G227300.3
TRITD6Av1G003740.1
TRITD1Av1G198170.2
TRITD4Av1G204440.1
TRITD5Bv1G142020.2
TRITD2Av1G010630.1
TRITD3Bv1G277000.3
TRITD2Bv1G246540.1
TRITD3Bv1G279060.1
TRITD5Bv1G014300.4
TRITD6Bv1G214200.2
TRITD5Bv1G188710.1
TRITD3Av1G281690.2
TRITD1Av1G003020.2
TRITD7Av1G026660.1
TRITD4Av1G217910.1
Domain Color
EGF_3 Domain
EGF_CA Domain
GUB_WAK_bind Domain
PK_Tyr_Ser−Thr Domain
Pkinase Domain
WAK Family
cEGF Domain
0
500
1000

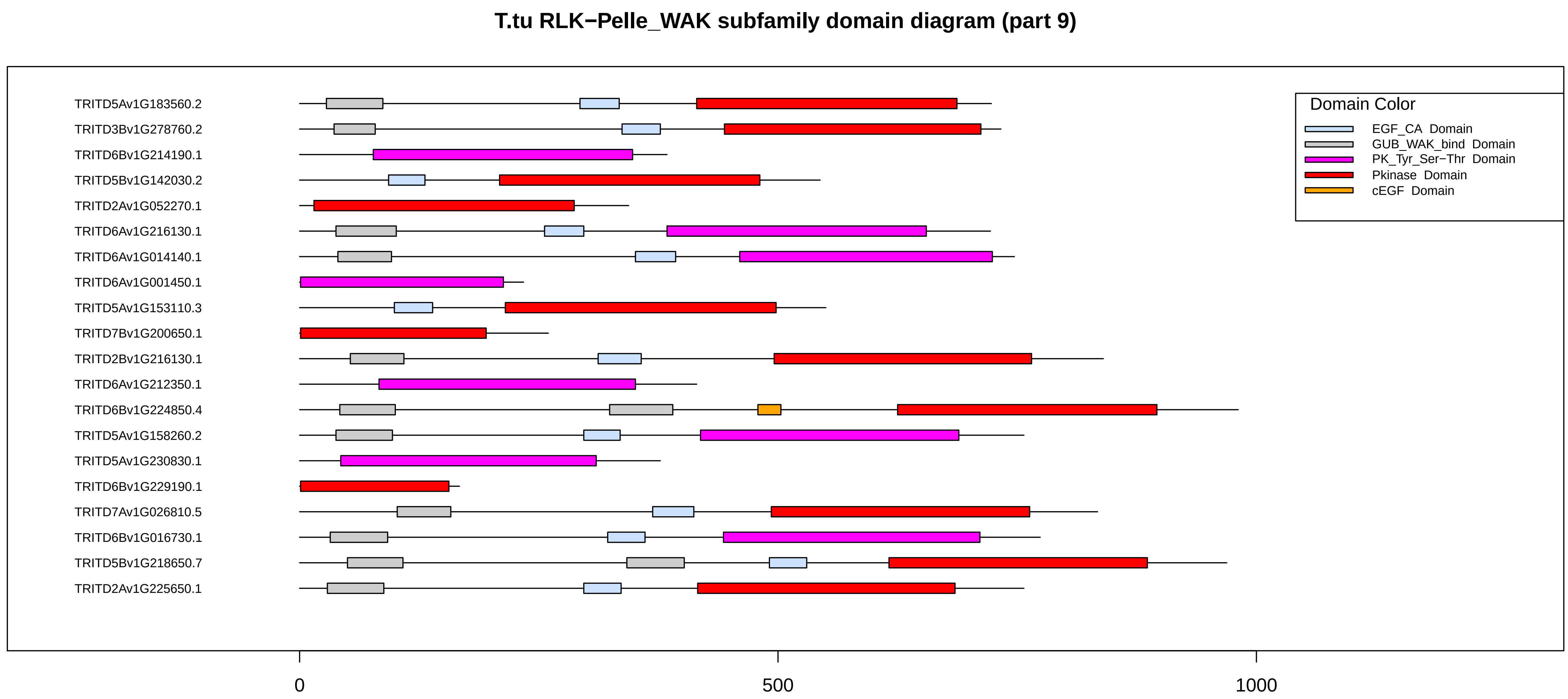
T.tu RLK−Pelle_WAK subfamily domain diagram (part 9)
TRITD5Av1G183560.2
TRITD3Bv1G278760.2
TRITD6Bv1G214190.1
TRITD5Bv1G142030.2
TRITD2Av1G052270.1
TRITD6Av1G216130.1
TRITD6Av1G014140.1
TRITD6Av1G001450.1
TRITD5Av1G153110.3
TRITD7Bv1G200650.1
TRITD2Bv1G216130.1
TRITD6Av1G212350.1
TRITD6Bv1G224850.4
TRITD5Av1G158260.2
TRITD5Av1G230830.1
TRITD6Bv1G229190.1
TRITD7Av1G026810.5
TRITD6Bv1G016730.1
TRITD5Bv1G218650.7
TRITD2Av1G225650.1
Domain Color
EGF_CA Domain
GUB_WAK_bind Domain
PK_Tyr_Ser−Thr Domain
Pkinase Domain
cEGF Domain
0
500
1000

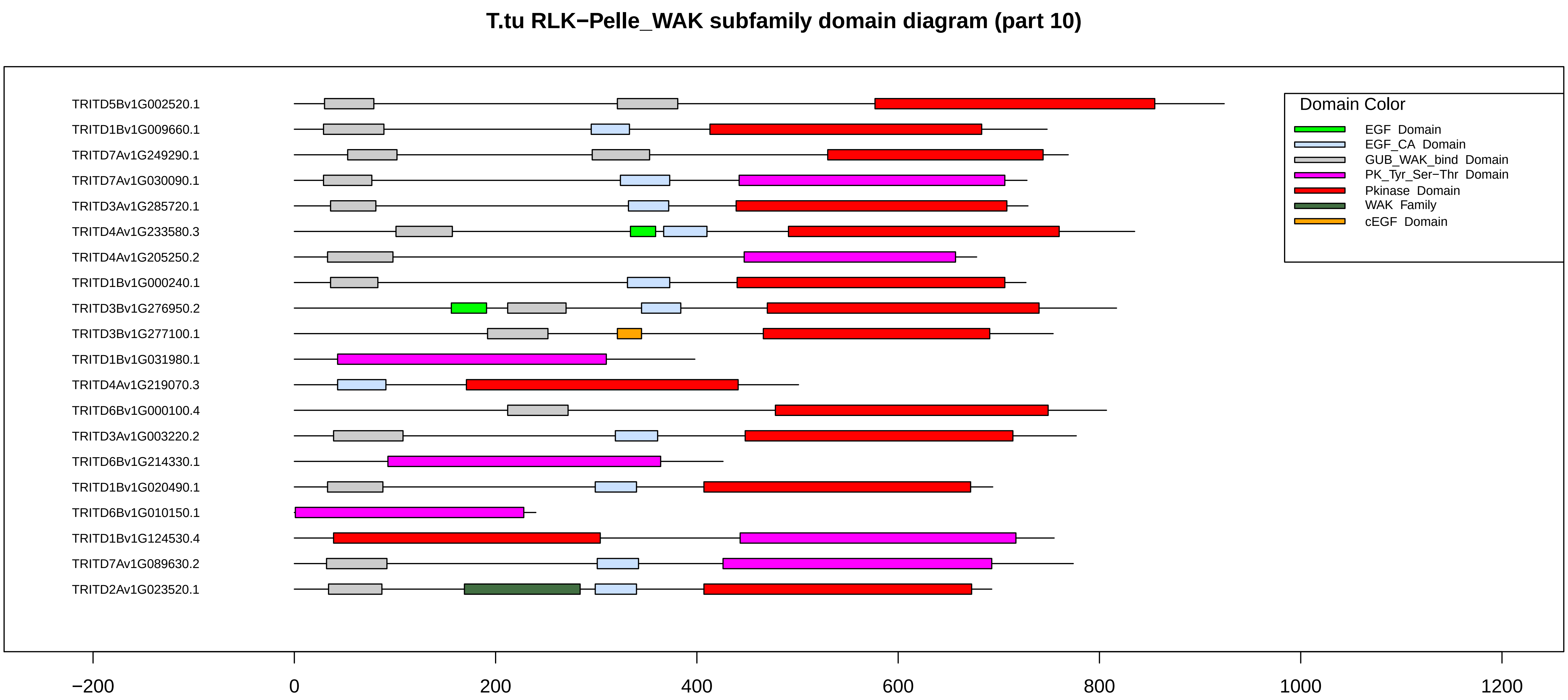

T.tu RLK−Pelle_WAK subfamily domain diagram (part 10)
TRITD5Bv1G002520.1
TRITD1Bv1G009660.1
TRITD7Av1G249290.1
TRITD7Av1G030090.1
TRITD3Av1G285720.1
TRITD4Av1G233580.3
TRITD4Av1G205250.2
TRITD1Bv1G000240.1
TRITD3Bv1G276950.2
TRITD3Bv1G277100.1
TRITD1Bv1G031980.1
TRITD4Av1G219070.3
TRITD6Bv1G000100.4
TRITD3Av1G003220.2
TRITD6Bv1G214330.1
TRITD1Bv1G020490.1
TRITD6Bv1G010150.1
TRITD1Bv1G124530.4
TRITD7Av1G089630.2
TRITD2Av1G023520.1
Domain Color
EGF Domain
EGF_CA Domain
GUB_WAK_bind Domain
PK_Tyr_Ser−Thr Domain
Pkinase Domain
WAK Family
cEGF Domain
−200
0
200
400
600
800
1000
1200

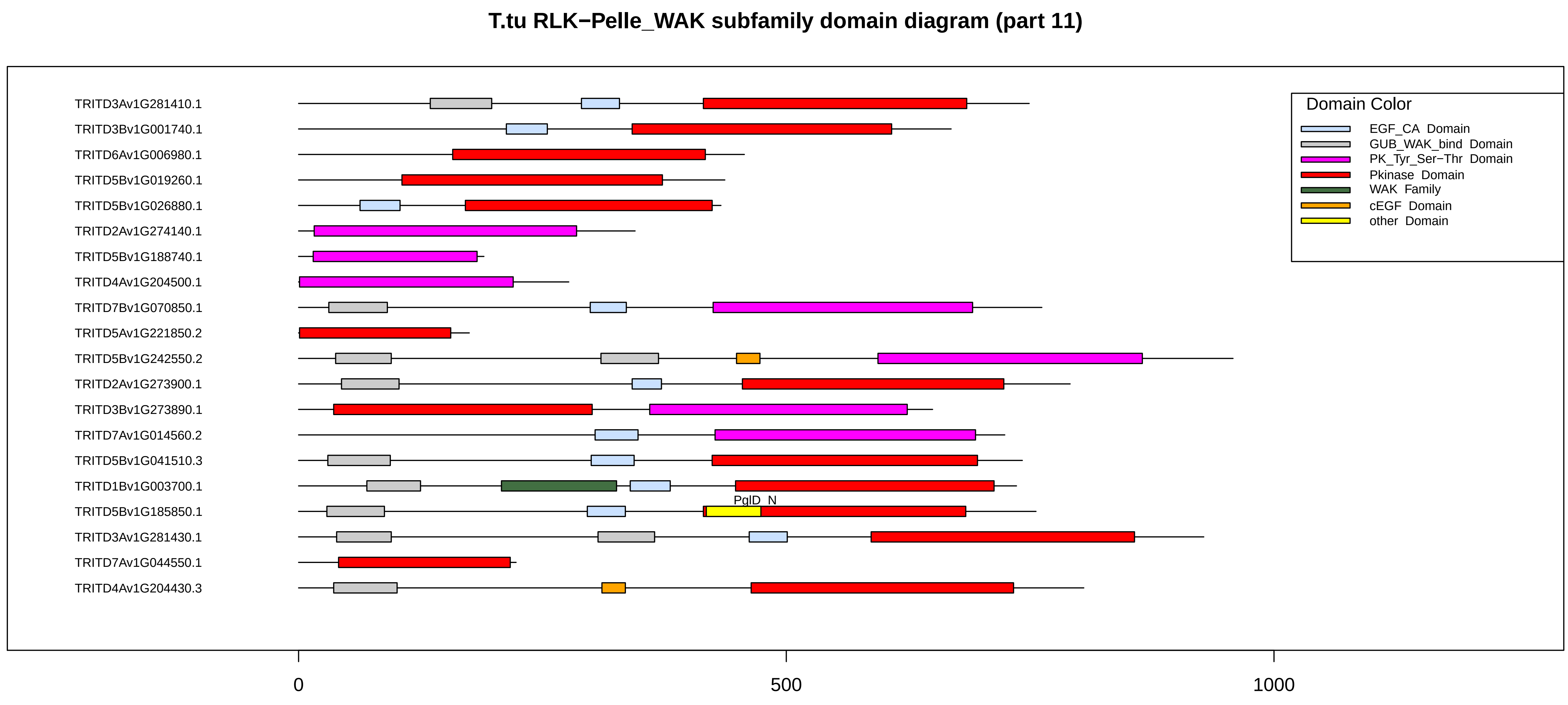
T.tu RLK−Pelle_WAK subfamily domain diagram (part 11)
TRITD3Av1G281410.1
TRITD3Bv1G001740.1
TRITD6Av1G006980.1
TRITD5Bv1G019260.1
TRITD5Bv1G026880.1
TRITD2Av1G274140.1
TRITD5Bv1G188740.1
TRITD4Av1G204500.1
TRITD7Bv1G070850.1
TRITD5Av1G221850.2
TRITD5Bv1G242550.2
TRITD2Av1G273900.1
TRITD3Bv1G273890.1
TRITD7Av1G014560.2
TRITD5Bv1G041510.3
TRITD1Bv1G003700.1
TRITD5Bv1G185850.1
TRITD3Av1G281430.1
TRITD7Av1G044550.1
TRITD4Av1G204430.3
PglD_N
Domain Color
EGF_CA Domain
GUB_WAK_bind Domain
PK_Tyr_Ser−Thr Domain
Pkinase Domain
WAK Family
cEGF Domain
other Domain
0
500
1000

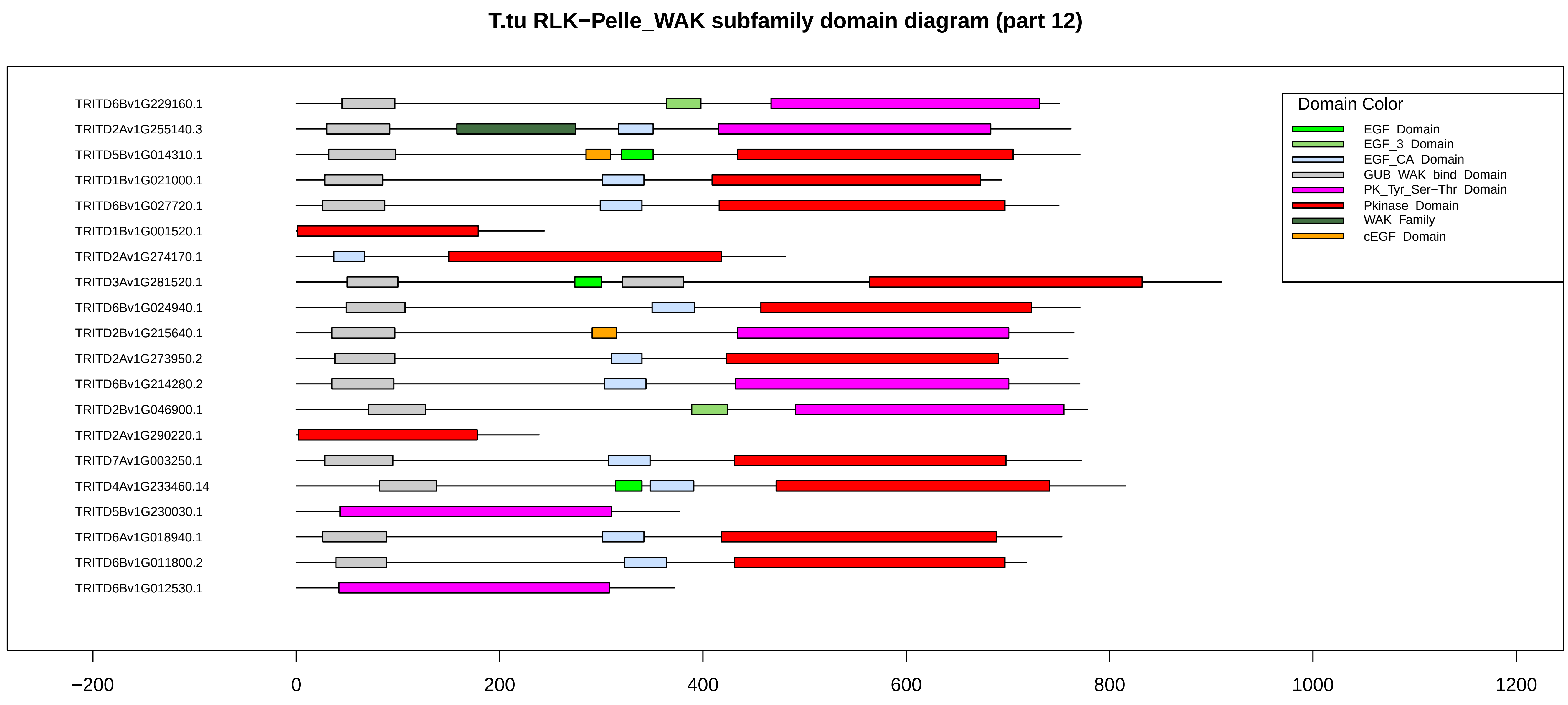
T.tu RLK−Pelle_WAK subfamily domain diagram (part 12)
TRITD6Bv1G229160.1
TRITD2Av1G255140.3
TRITD5Bv1G014310.1
TRITD1Bv1G021000.1
TRITD6Bv1G027720.1
TRITD1Bv1G001520.1
TRITD2Av1G274170.1
TRITD3Av1G281520.1
TRITD6Bv1G024940.1
TRITD2Bv1G215640.1
TRITD2Av1G273950.2
TRITD6Bv1G214280.2
TRITD2Bv1G046900.1
TRITD2Av1G290220.1
TRITD7Av1G003250.1
TRITD4Av1G233460.14
TRITD5Bv1G230030.1
TRITD6Av1G018940.1
TRITD6Bv1G011800.2
TRITD6Bv1G012530.1
Domain Color
EGF Domain
EGF_3 Domain
EGF_CA Domain
GUB_WAK_bind Domain
PK_Tyr_Ser−Thr Domain
Pkinase Domain
WAK Family
cEGF Domain
−200
0
200
400
600
800
1000
1200

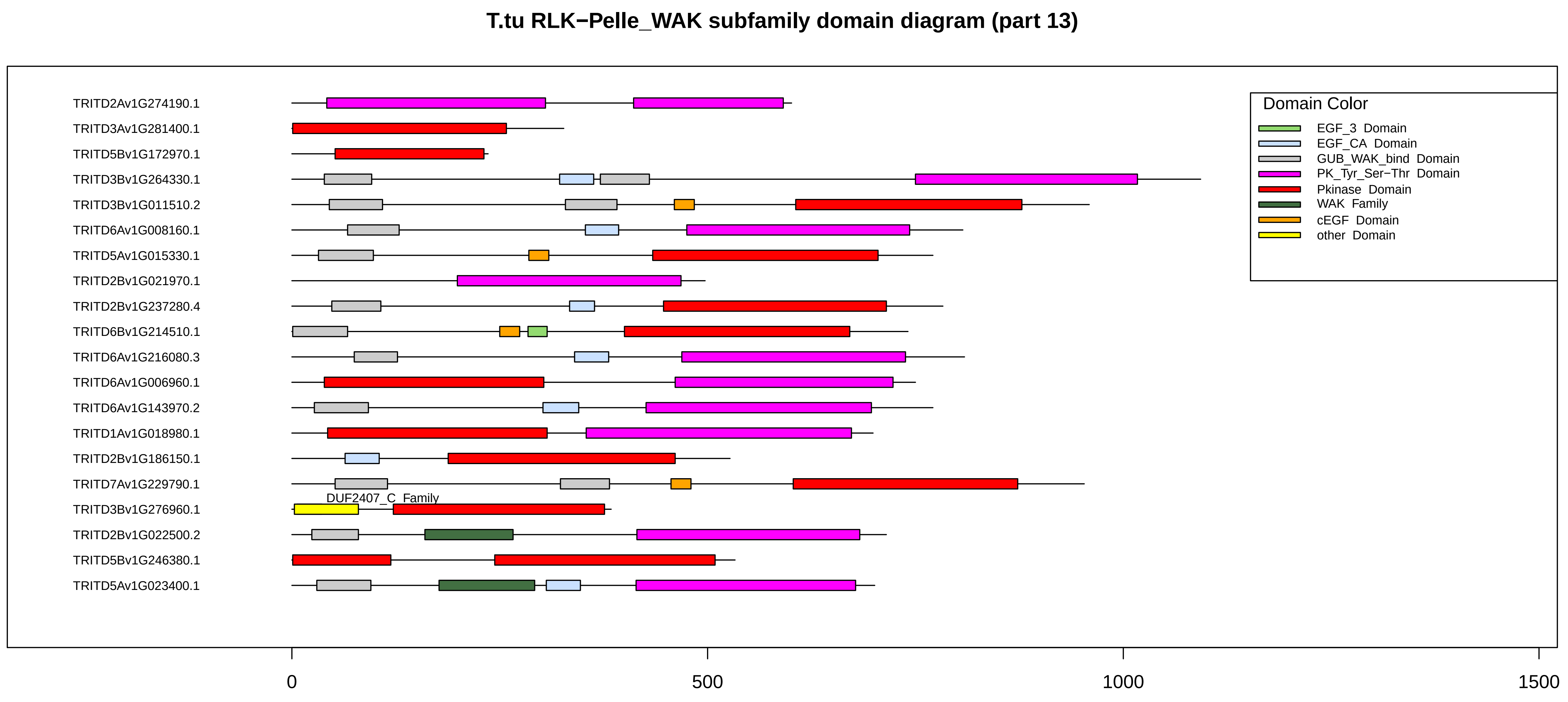
T.tu RLK−Pelle_WAK subfamily domain diagram (part 13)
TRITD2Av1G274190.1
TRITD3Av1G281400.1
TRITD5Bv1G172970.1
TRITD3Bv1G264330.1
TRITD3Bv1G011510.2
TRITD6Av1G008160.1
TRITD5Av1G015330.1
TRITD2Bv1G021970.1
TRITD2Bv1G237280.4
TRITD6Bv1G214510.1
TRITD6Av1G216080.3
TRITD6Av1G006960.1
TRITD6Av1G143970.2
TRITD1Av1G018980.1
TRITD2Bv1G186150.1
TRITD7Av1G229790.1
DUF2407_C_Family
TRITD3Bv1G276960.1
TRITD2Bv1G022500.2
TRITD5Bv1G246380.1
TRITD5Av1G023400.1
Domain Color
EGF_3 Domain
EGF_CA Domain
GUB_WAK_bind Domain
PK_Tyr_Ser−Thr Domain
Pkinase Domain
WAK Family
cEGF Domain
other Domain
0
500
1000
1500

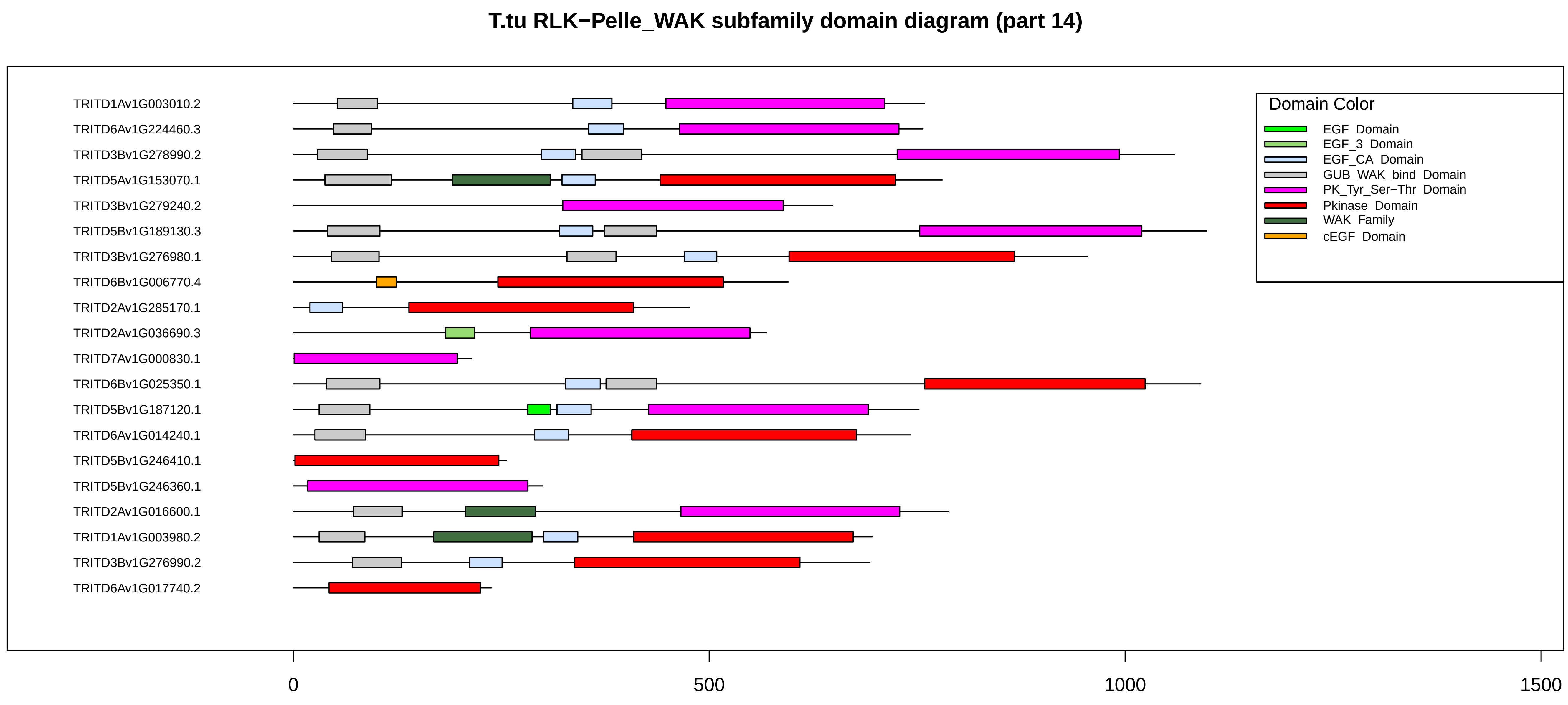
T.tu RLK−Pelle_WAK subfamily domain diagram (part 14)
TRITD1Av1G003010.2
TRITD6Av1G224460.3
TRITD3Bv1G278990.2
TRITD5Av1G153070.1
TRITD3Bv1G279240.2
TRITD5Bv1G189130.3
TRITD3Bv1G276980.1
TRITD6Bv1G006770.4
TRITD2Av1G285170.1
TRITD2Av1G036690.3
TRITD7Av1G000830.1
TRITD6Bv1G025350.1
TRITD5Bv1G187120.1
TRITD6Av1G014240.1
TRITD5Bv1G246410.1
TRITD5Bv1G246360.1
TRITD2Av1G016600.1
TRITD1Av1G003980.2
TRITD3Bv1G276990.2
TRITD6Av1G017740.2
Domain Color
EGF Domain
EGF_3 Domain
EGF_CA Domain
GUB_WAK_bind Domain
PK_Tyr_Ser−Thr Domain
Pkinase Domain
WAK Family
cEGF Domain
0
500
1000
1500

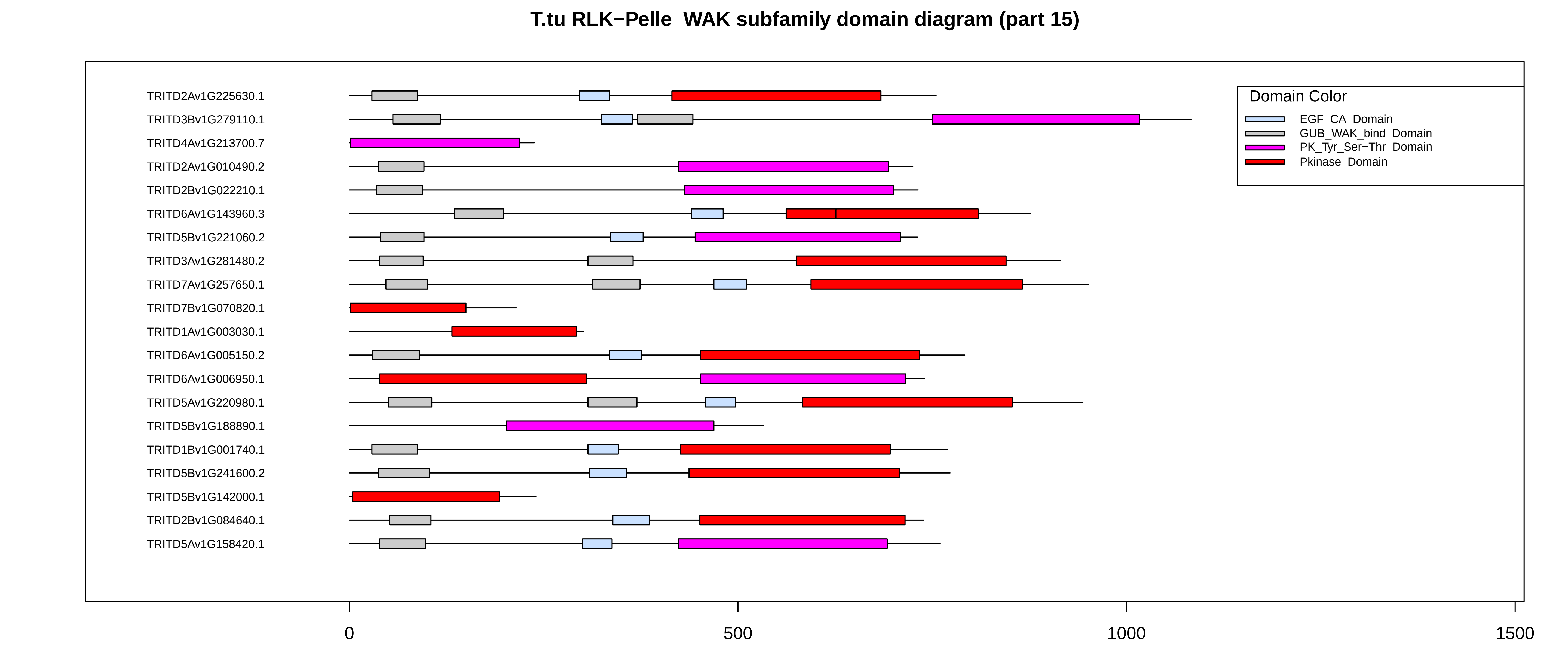
T.tu RLK−Pelle_WAK subfamily domain diagram (part 15)
TRITD2Av1G225630.1
TRITD3Bv1G279110.1
TRITD4Av1G213700.7
TRITD2Av1G010490.2
TRITD2Bv1G022210.1
TRITD6Av1G143960.3
TRITD5Bv1G221060.2
TRITD3Av1G281480.2
TRITD7Av1G257650.1
TRITD7Bv1G070820.1
TRITD1Av1G003030.1
TRITD6Av1G005150.2
TRITD6Av1G006950.1
TRITD5Av1G220980.1
TRITD5Bv1G188890.1
TRITD1Bv1G001740.1
TRITD5Bv1G241600.2
TRITD5Bv1G142000.1
TRITD2Bv1G084640.1
TRITD5Av1G158420.1
Domain Color
EGF_CA Domain
GUB_WAK_bind Domain
PK_Tyr_Ser−Thr Domain
Pkinase Domain
0
500
1000
1500

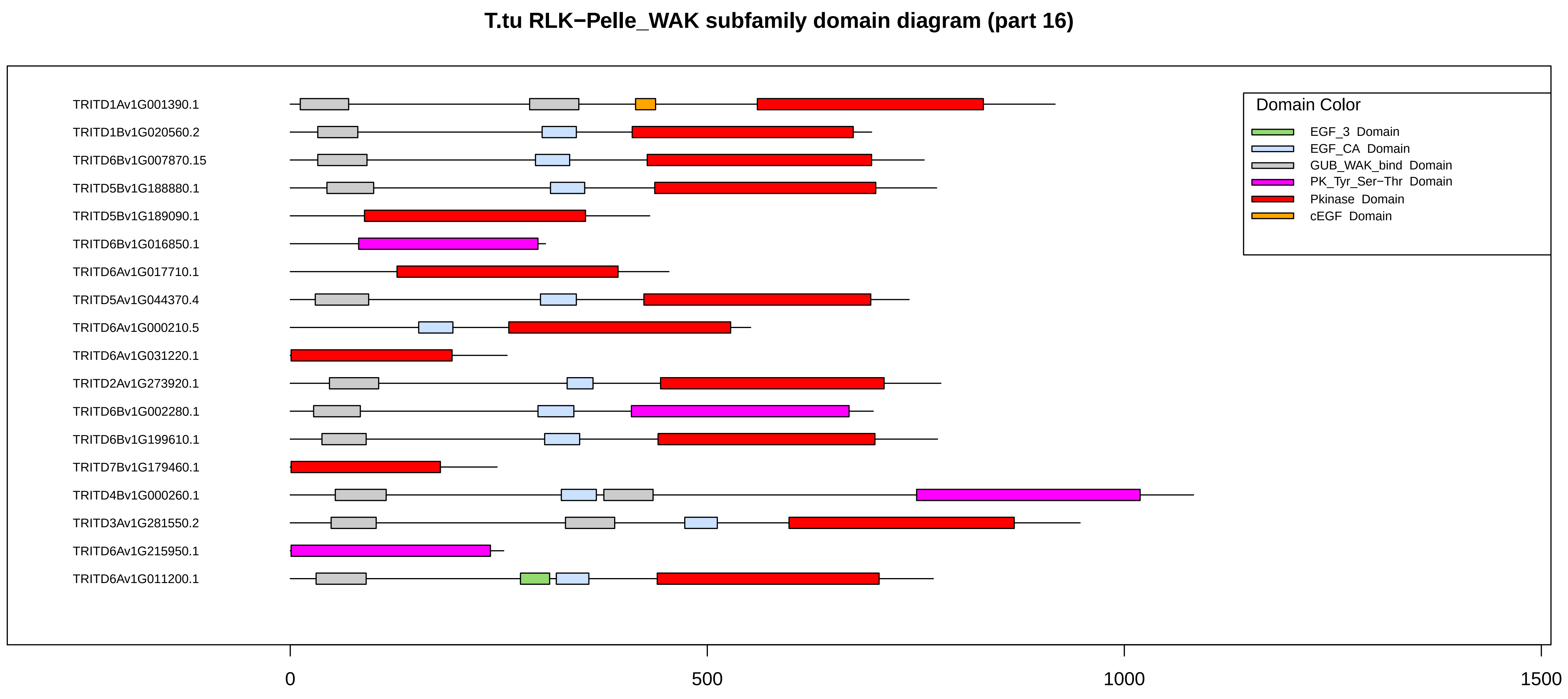
T.tu RLK−Pelle_WAK subfamily domain diagram (part 16)
TRITD1Av1G001390.1
TRITD1Bv1G020560.2
TRITD6Bv1G007870.15
TRITD5Bv1G188880.1
TRITD5Bv1G189090.1
TRITD6Bv1G016850.1
TRITD6Av1G017710.1
TRITD5Av1G044370.4
TRITD6Av1G000210.5
TRITD6Av1G031220.1
TRITD2Av1G273920.1
TRITD6Bv1G002280.1
TRITD6Bv1G199610.1
TRITD7Bv1G179460.1
TRITD4Bv1G000260.1
TRITD3Av1G281550.2
TRITD6Av1G215950.1
TRITD6Av1G011200.1
Domain Color
EGF_3 Domain
EGF_CA Domain
GUB_WAK_bind Domain
PK_Tyr_Ser−Thr Domain
Pkinase Domain
cEGF Domain
0
500
1000
1500

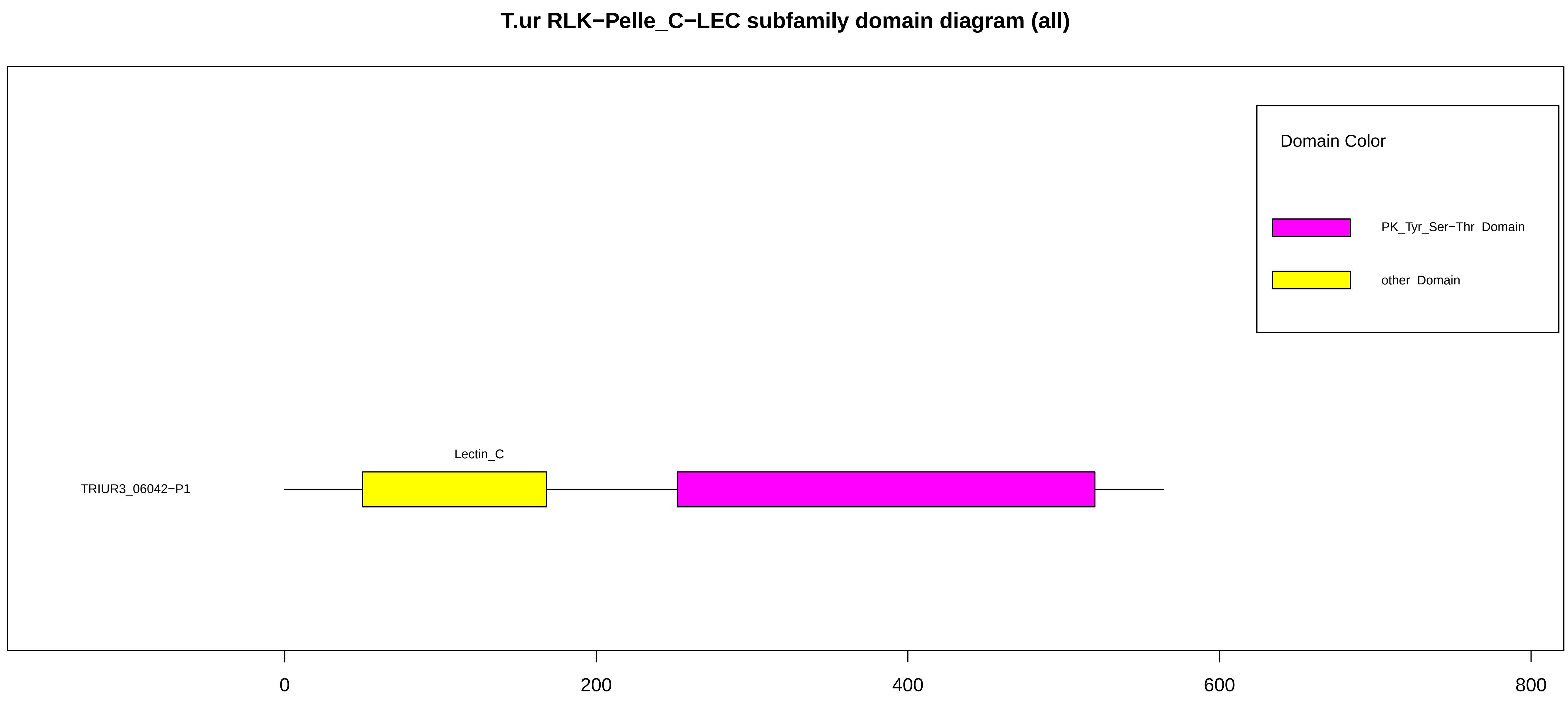
T.ur RLK−Pelle_C−LEC subfamily domain diagram (all)
Domain Color
PK_Tyr_Ser−Thr Domain
other Domain
Lectin_C
TRIUR3_06042−P1
0
200
400
600
800

T.ur RLK−Pelle_CR4L subfamily domain diagram (all)

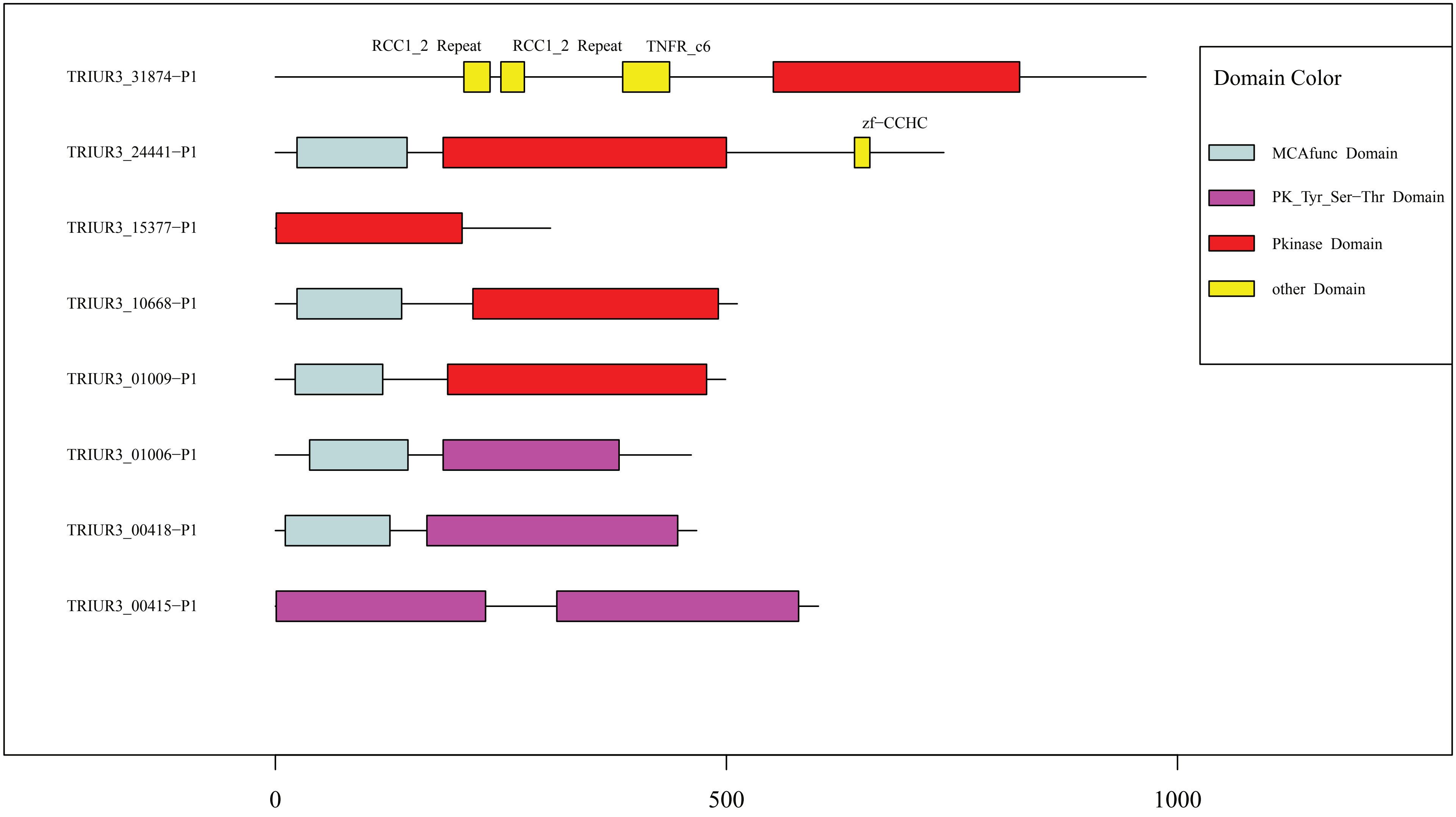

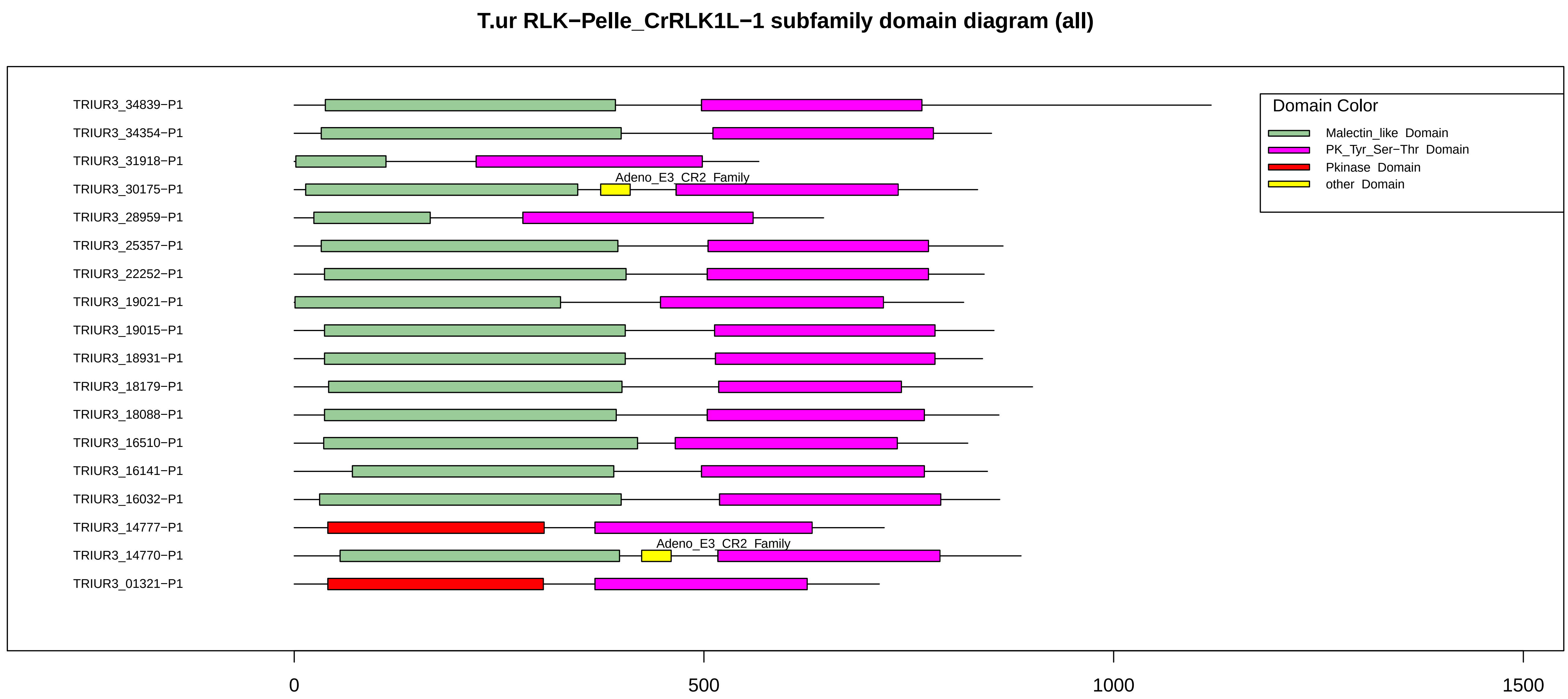

T.ur RLK−Pelle_CrRLK1L−1 subfamily domain diagram (all)
TRIUR3_34839−P1
TRIUR3_34354−P1
TRIUR3_31918−P1
TRIUR3_30175−P1
TRIUR3_28959−P1
TRIUR3_25357−P1
TRIUR3_22252−P1
TRIUR3_19021−P1
TRIUR3_19015−P1
TRIUR3_18931−P1
TRIUR3_18179−P1
TRIUR3_18088−P1
TRIUR3_16510−P1
TRIUR3_16141−P1
TRIUR3_16032−P1
TRIUR3_14777−P1
TRIUR3_14770−P1
TRIUR3_01321−P1
Adeno_E3_CR2_Family
Adeno_E3_CR2_Family
Domain Color
Malectin_like Domain
PK_Tyr_Ser−Thr Domain
Pkinase Domain
other Domain
0
500
1000
1500

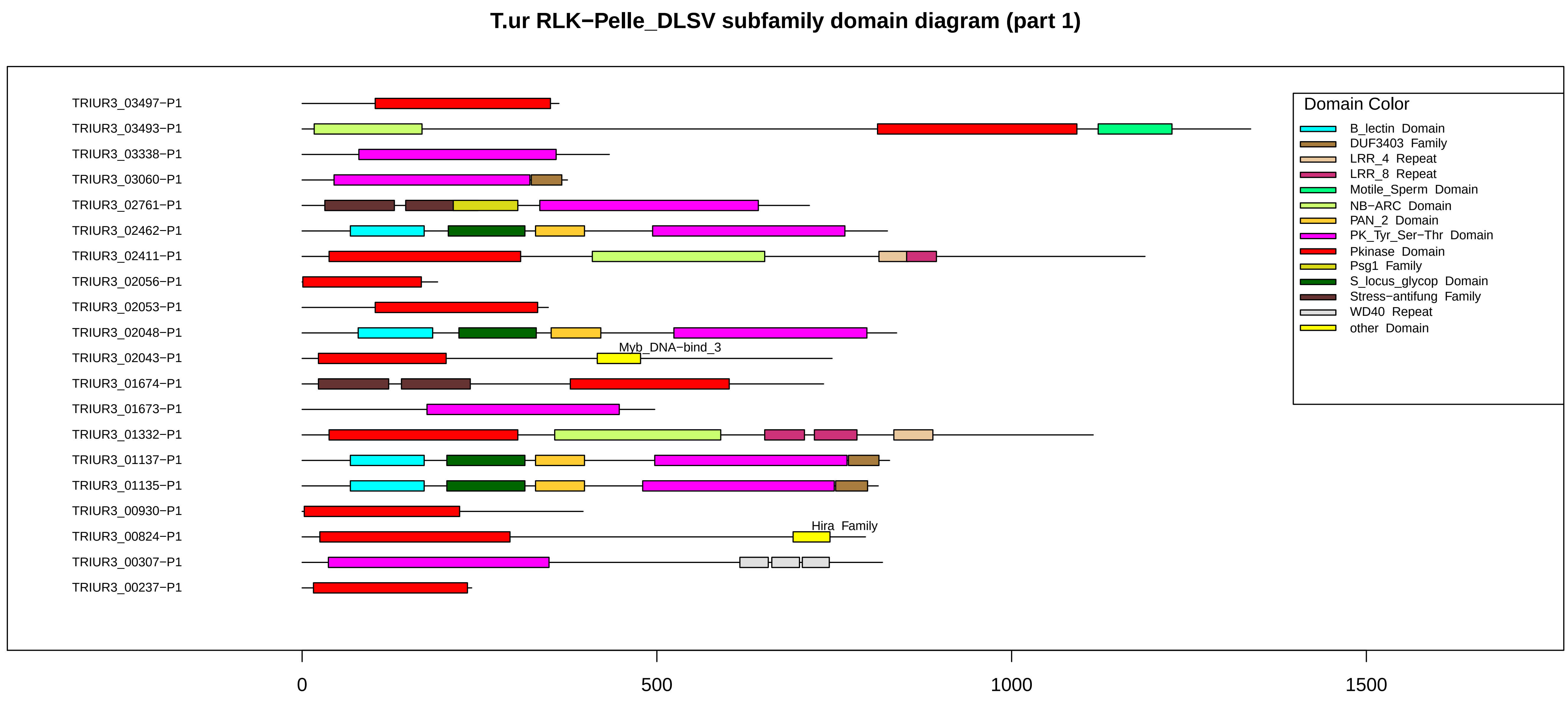

T.ur RLK−Pelle_DLSV subfamily domain diagram (part 1)
TRIUR3_03497−P1
TRIUR3_03493−P1
TRIUR3_03338−P1
TRIUR3_03060−P1
TRIUR3_02761−P1
TRIUR3_02462−P1
TRIUR3_02411−P1
TRIUR3_02056−P1
TRIUR3_02053−P1
TRIUR3_02048−P1
TRIUR3_02043−P1
TRIUR3_01674−P1
TRIUR3_01673−P1
TRIUR3_01332−P1
TRIUR3_01137−P1
TRIUR3_01135−P1
TRIUR3_00930−P1
TRIUR3_00824−P1
TRIUR3_00307−P1
TRIUR3_00237−P1
Myb_DNA−bind_3
Hira Family
Domain Color
B_lectin Domain
DUF3403 Family
LRR_4 Repeat
LRR_8 Repeat
Motile_Sperm Domain
NB−ARC Domain
PAN_2 Domain
PK_Tyr_Ser−Thr Domain
Pkinase Domain
Psg1 Family
S_locus_glycop Domain
Stress−antifung Family
WD40 Repeat
other Domain
0
500
1000
1500

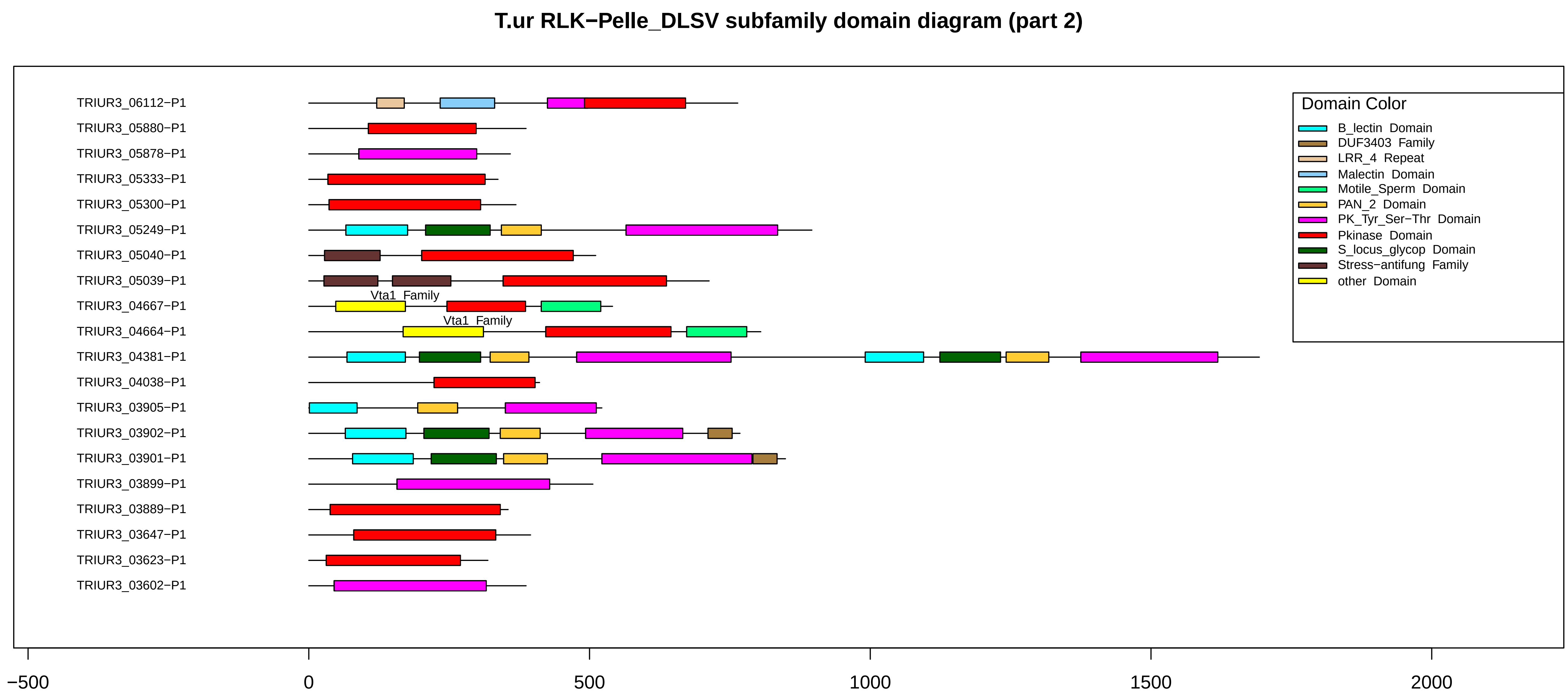
T.ur RLK−Pelle_DLSV subfamily domain diagram (part 2)
TRIUR3_06112−P1
TRIUR3_05880−P1
TRIUR3_05878−P1
TRIUR3_05333−P1
TRIUR3_05300−P1
TRIUR3_05249−P1
TRIUR3_05040−P1
TRIUR3_05039−P1
TRIUR3_04667−P1
TRIUR3_04664−P1
TRIUR3_04381−P1
TRIUR3_04038−P1
TRIUR3_03905−P1
TRIUR3_03902−P1
TRIUR3_03901−P1
TRIUR3_03899−P1
TRIUR3_03889−P1
TRIUR3_03647−P1
TRIUR3_03623−P1
TRIUR3_03602−P1
Vta1 Family
Vta1 Family
Domain Color
B_lectin Domain
DUF3403 Family
LRR_4 Repeat
Malectin Domain
Motile_Sperm Domain
PAN_2 Domain
PK_Tyr_Ser−Thr Domain
Pkinase Domain
S_locus_glycop Domain
Stress−antifung Family
other Domain
−500
0
500
1000
1500
2000

T.ur RLK−Pelle_DLSV subfamily domain diagram (part 3)

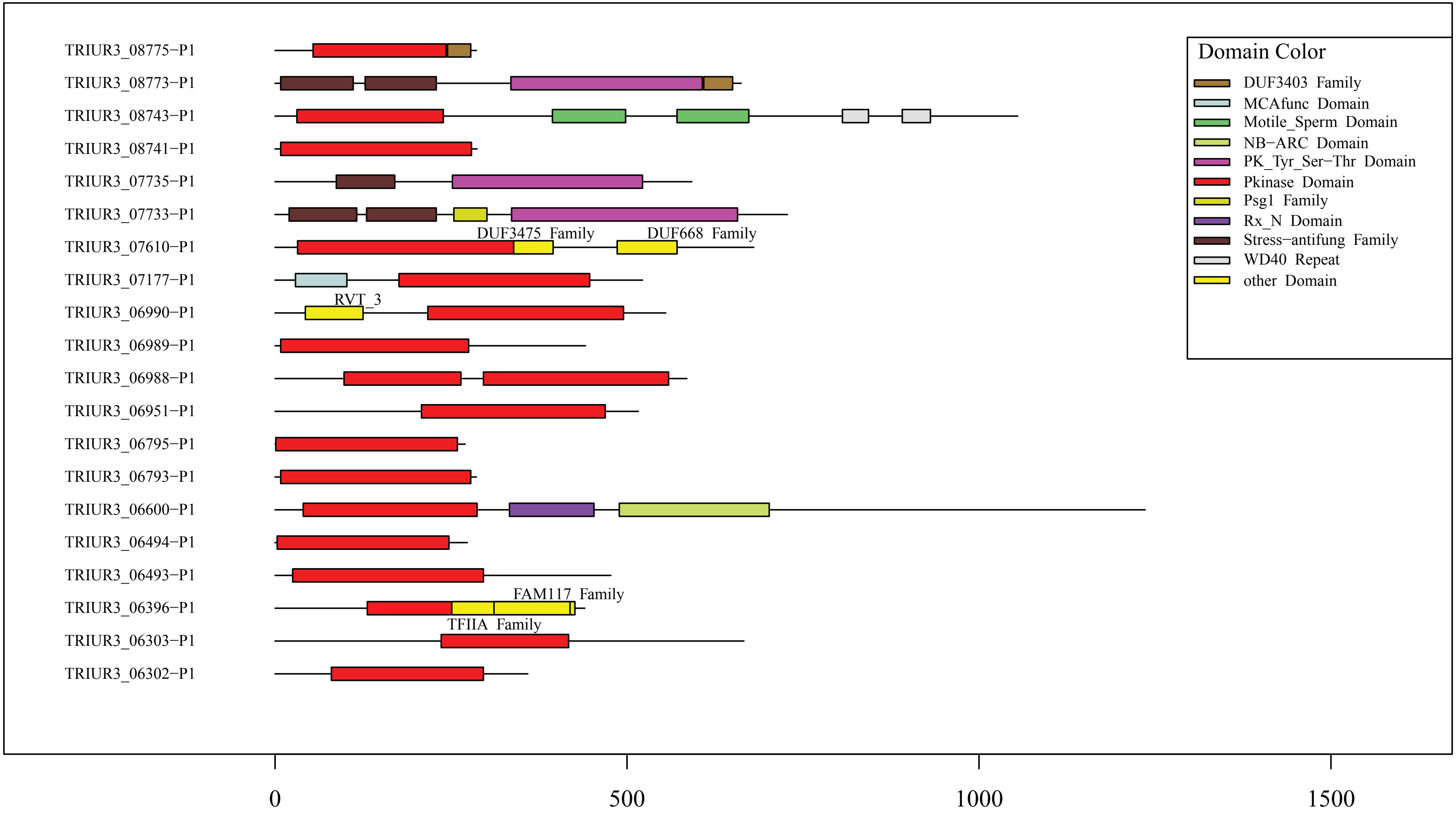

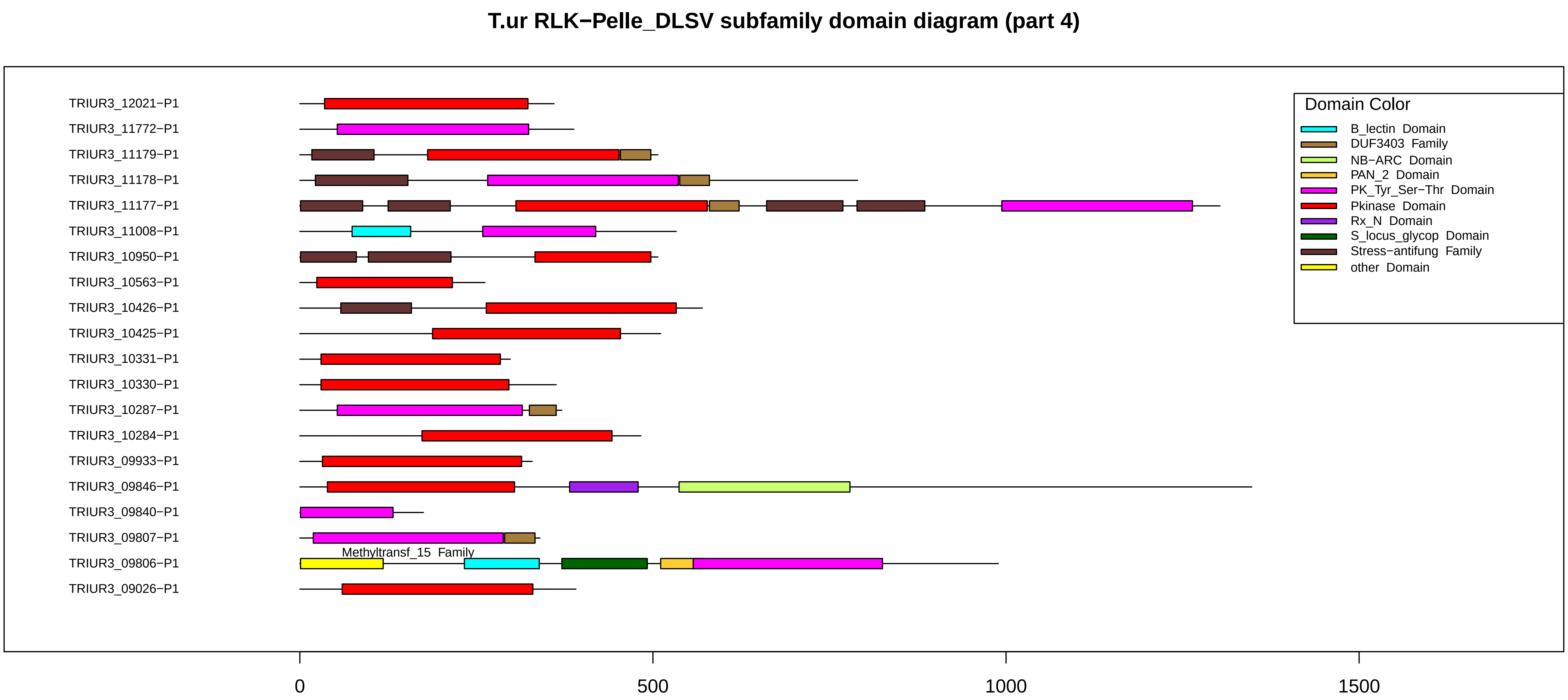

T.ur RLK−Pelle_DLSV subfamily domain diagram (part 4)
TRIUR3_12021−P1
TRIUR3_11772−P1
TRIUR3_11179−P1
TRIUR3_11178−P1
TRIUR3_11177−P1
TRIUR3_11008−P1
TRIUR3_10950−P1
TRIUR3_10563−P1
TRIUR3_10426−P1
TRIUR3_10425−P1
TRIUR3_10331−P1
TRIUR3_10330−P1
TRIUR3_10287−P1
TRIUR3_10284−P1
TRIUR3_09933−P1
TRIUR3_09846−P1
TRIUR3_09840−P1
TRIUR3_09807−P1
TRIUR3_09806−P1
Methyltransf_15 Family
TRIUR3_09026−P1
Domain Color
B_lectin Domain
DUF3403 Family
NB−ARC Domain
PAN_2 Domain
PK_Tyr_Ser−Thr Domain
Pkinase Domain
Rx_N Domain
S_locus_glycop Domain
Stress−antifung Family
other Domain
0
500
1000
1500

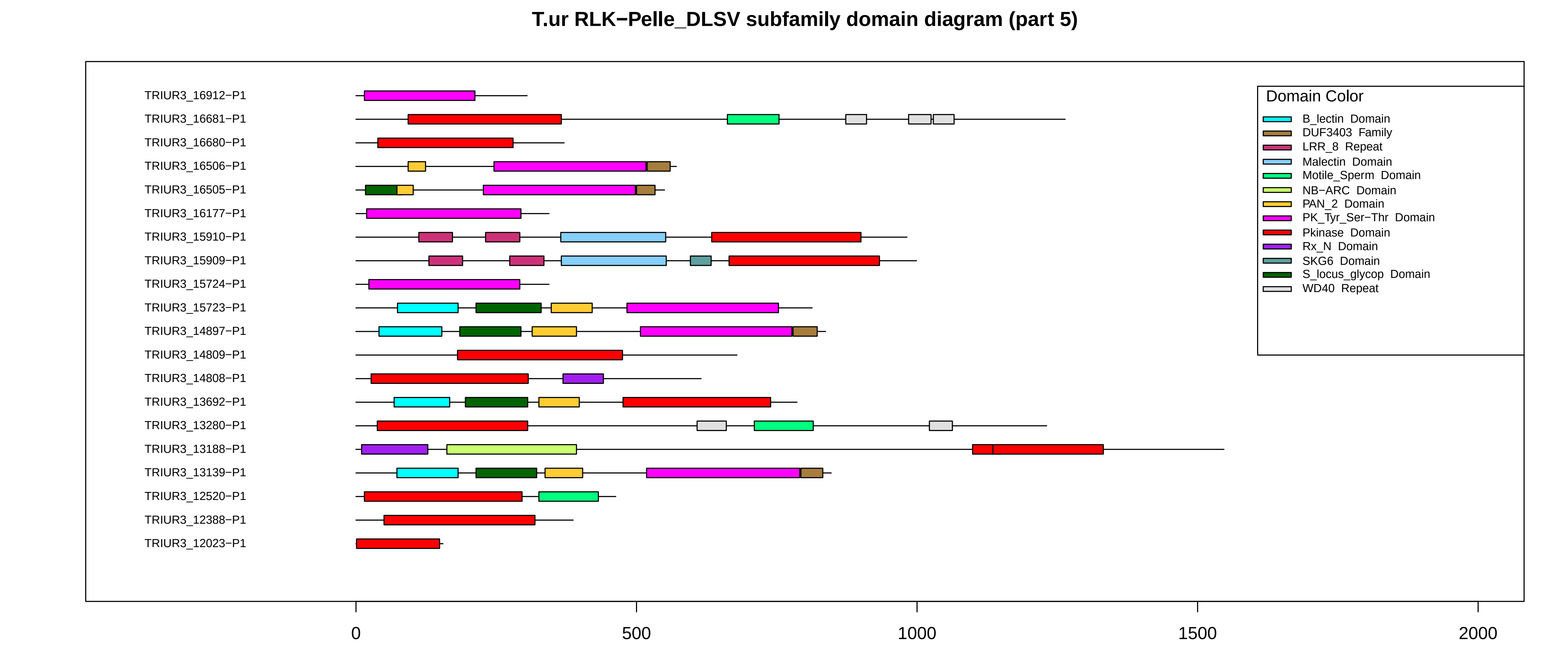

T.ur RLK−Pelle_DLSV subfamily domain diagram (part 5)
TRIUR3_16912−P1
TRIUR3_16681−P1
TRIUR3_16680−P1
TRIUR3_16506−P1
TRIUR3_16505−P1
TRIUR3_16177−P1
TRIUR3_15910−P1
TRIUR3_15909−P1
TRIUR3_15724−P1
TRIUR3_15723−P1
TRIUR3_14897−P1
TRIUR3_14809−P1
TRIUR3_14808−P1
TRIUR3_13692−P1
TRIUR3_13280−P1
TRIUR3_13188−P1
TRIUR3_13139−P1
TRIUR3_12520−P1
TRIUR3_12388−P1
TRIUR3_12023−P1
Domain Color
B_lectin Domain
DUF3403 Family
LRR_8 Repeat
Malectin Domain
Motile_Sperm Domain
NB−ARC Domain
PAN_2 Domain
PK_Tyr_Ser−Thr Domain
Pkinase Domain
Rx_N Domain
SKG6 Domain
S_locus_glycop Domain
WD40 Repeat
0
500
1000
1500
2000

T.ur RLK−Pelle_DLSV subfamily domain diagram (part 6)

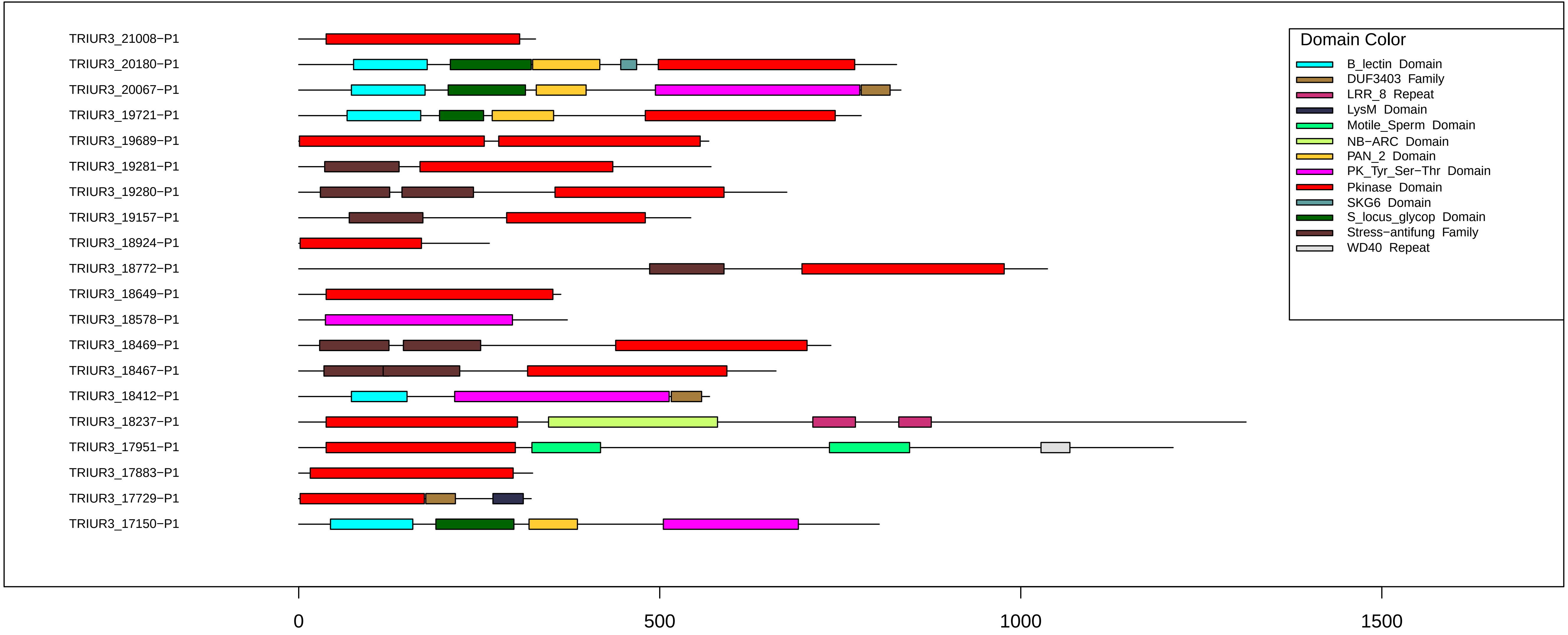

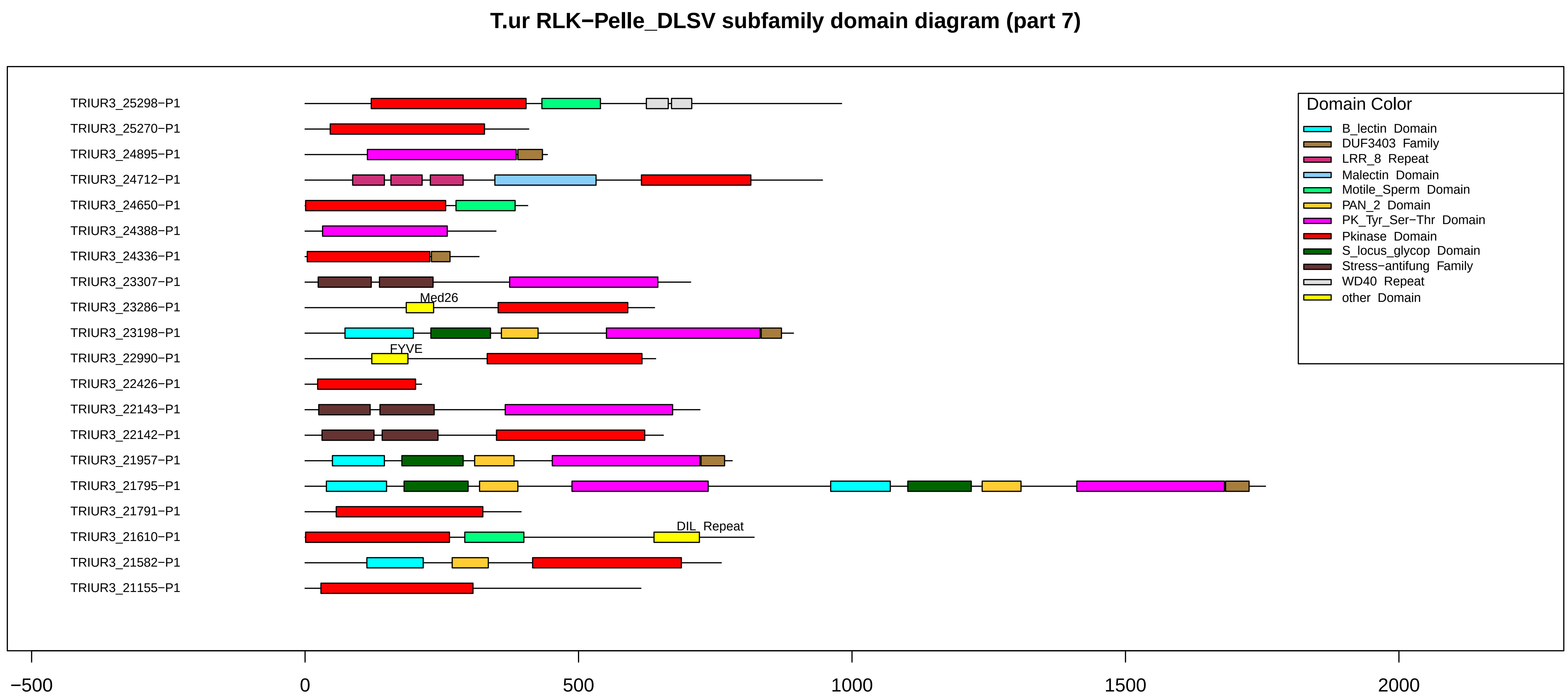
T.ur RLK−Pelle_DLSV subfamily domain diagram (part 7)
TRIUR3_25298−P1
TRIUR3_25270−P1
TRIUR3_24895−P1
TRIUR3_24712−P1
TRIUR3_24650−P1
TRIUR3_24388−P1
TRIUR3_24336−P1
TRIUR3_23307−P1
TRIUR3_23286−P1
Med26
TRIUR3_23198−P1
TRIUR3_22990−P1
FYVE
TRIUR3_22426−P1
TRIUR3_22143−P1
TRIUR3_22142−P1
TRIUR3_21957−P1
TRIUR3_21795−P1
TRIUR3_21791−P1
TRIUR3_21610−P1
DIL Repeat
TRIUR3_21582−P1
TRIUR3_21155−P1
Domain Color
B_lectin Domain
DUF3403 Family
LRR_8 Repeat
Malectin Domain
Motile_Sperm Domain
PAN_2 Domain
PK_Tyr_Ser−Thr Domain
Pkinase Domain
S_locus_glycop Domain
Stress−antifung Family
WD40 Repeat
other Domain
−500
0
500
1000
1500
2000

T.ur RLK−Pelle_DLSV subfamily domain diagram (part 8)

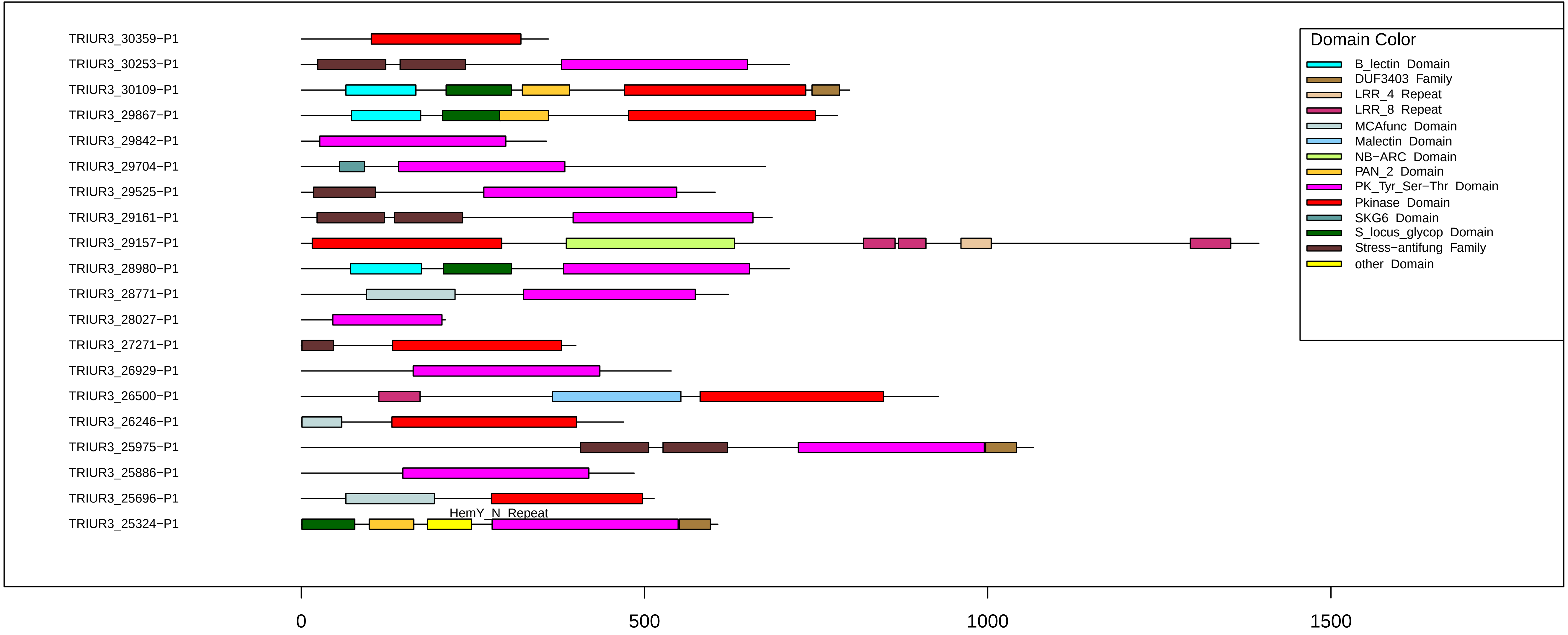

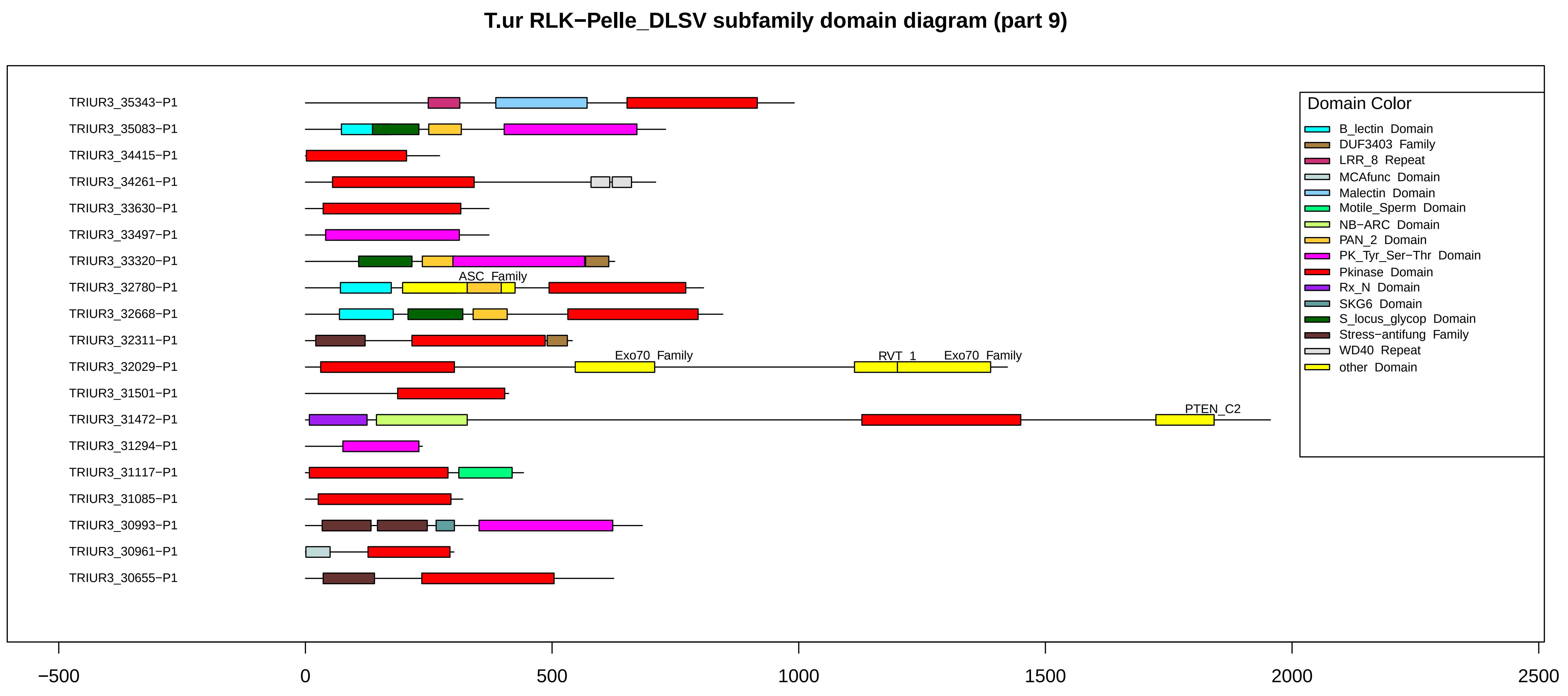

T.ur RLK−Pelle_DLSV subfamily domain diagram (part 9)
TRIUR3_35343−P1
TRIUR3_35083−P1
TRIUR3_34415−P1
TRIUR3_34261−P1
TRIUR3_33630−P1
TRIUR3_33497−P1
TRIUR3_33320−P1
TRIUR3_32780−P1
TRIUR3_32668−P1
TRIUR3_32311−P1
TRIUR3_32029−P1
TRIUR3_31501−P1
TRIUR3_31472−P1
TRIUR3_31294−P1
TRIUR3_31117−P1
TRIUR3_31085−P1
TRIUR3_30993−P1
TRIUR3_30961−P1
TRIUR3_30655−P1
ASC_Family
Exo70_Family
RVT_1
Exo70_Family
PTEN_C2
Domain Color
B_lectin Domain
DUF3403 Family
LRR_8 Repeat
MCAfunc Domain
Malectin Domain
Motile_Sperm Domain
NB−ARC Domain
PAN_2 Domain
PK_Tyr_Ser−Thr Domain
Pkinase Domain
Rx_N Domain
SKG6 Domain
S_locus_glycop Domain
Stress−antifung Family
WD40 Repeat
other Domain
−500
0
500
1000
1500
2000
2500

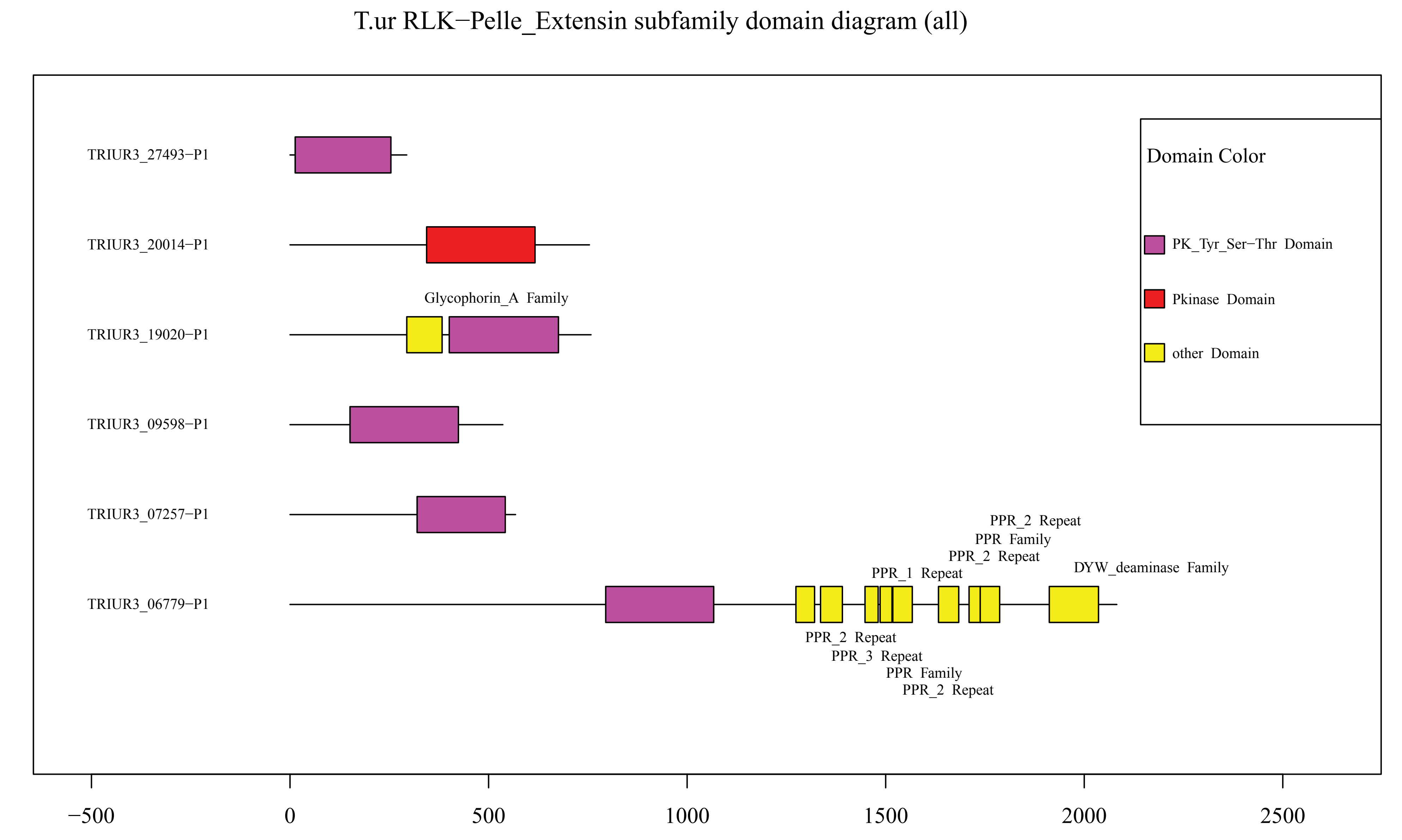

T.ur RLK−Pelle_Extensin subfamily domain diagram (all)
TRIUR3_27493−P1
TRIUR3_20014−P1
TRIUR3_19020−P1
TRIUR3_09598−P1
TRIUR3_07257−P1
TRIUR3_06779−P1
Glycophorin_A Family
PPR_2 Repeat
PPR Family
PPR_2 Repeat
PPR_1 Repeat
DYW_deaminase Family
PPR_2 Repeat
PPR_3 Repeat
PPR Family
PPR_2 Repeat
Domain Color
PK_Tyr_Ser−Thr Domain
Pkinase Domain
other Domain
−500
0
500
1000
1500
2000
2500

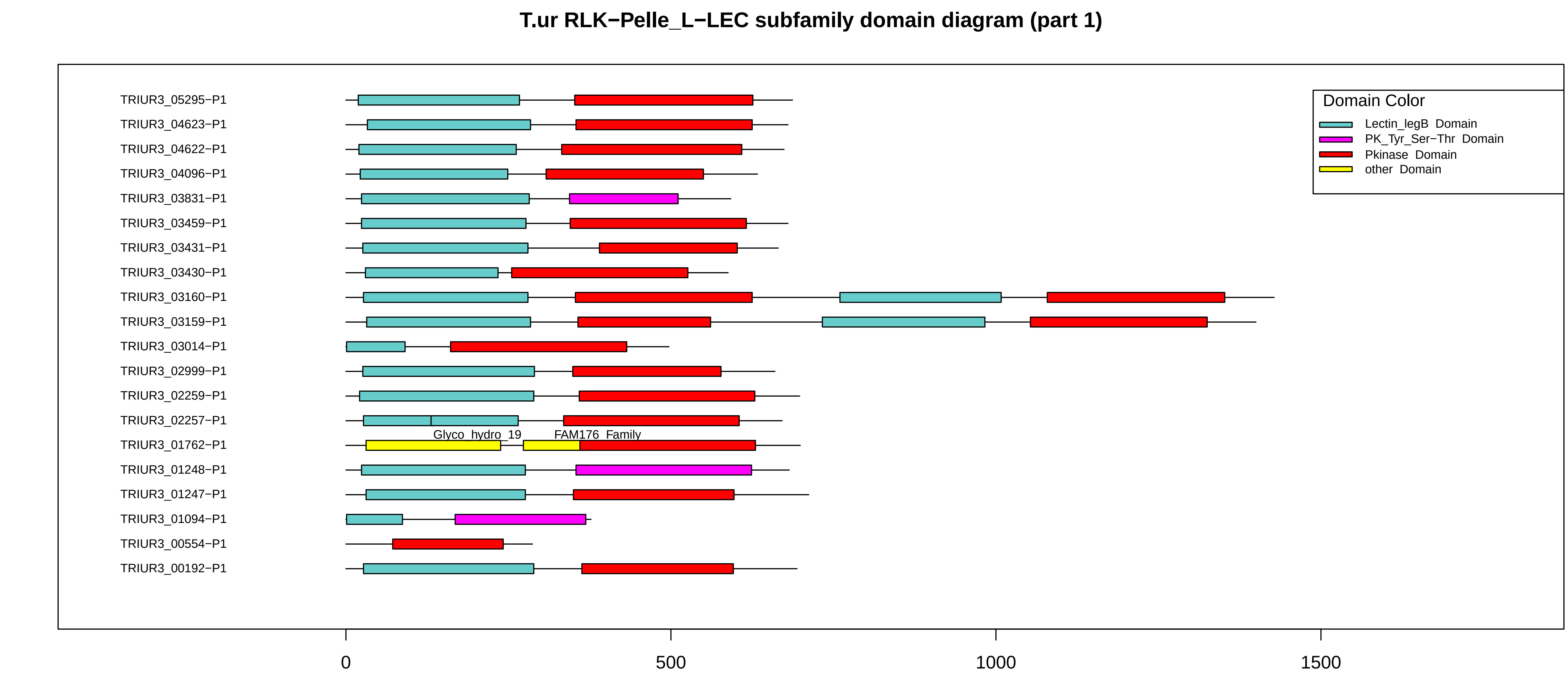
T.ur RLK−Pelle_L−LEC subfamily domain diagram (part 1)
TRIUR3_05295−P1
TRIUR3_04623−P1
TRIUR3_04622−P1
TRIUR3_04096−P1
TRIUR3_03831−P1
TRIUR3_03459−P1
TRIUR3_03431−P1
TRIUR3_03430−P1
TRIUR3_03160−P1
TRIUR3_03159−P1
TRIUR3_03014−P1
TRIUR3_02999−P1
TRIUR3_02259−P1
TRIUR3_02257−P1
TRIUR3_01762−P1
TRIUR3_01248−P1
TRIUR3_01247−P1
TRIUR3_01094−P1
TRIUR3_00554−P1
TRIUR3_00192−P1
Glyco_hydro_19
FAM176_Family
Domain Color
Lectin_legB Domain
PK_Tyr_Ser−Thr Domain
Pkinase Domain
other Domain
0
500
1000
1500

T.ur RLK−Pelle_L−LEC subfamily domain diagram (part 2)

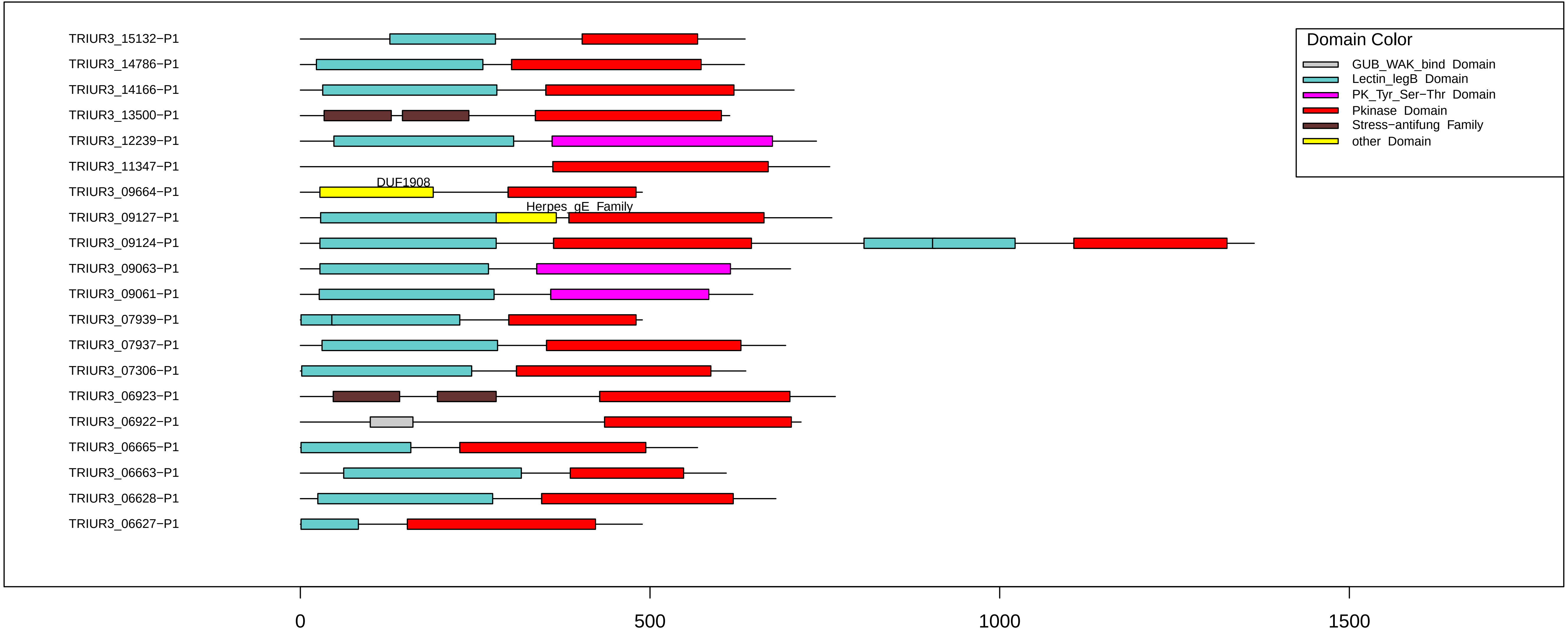

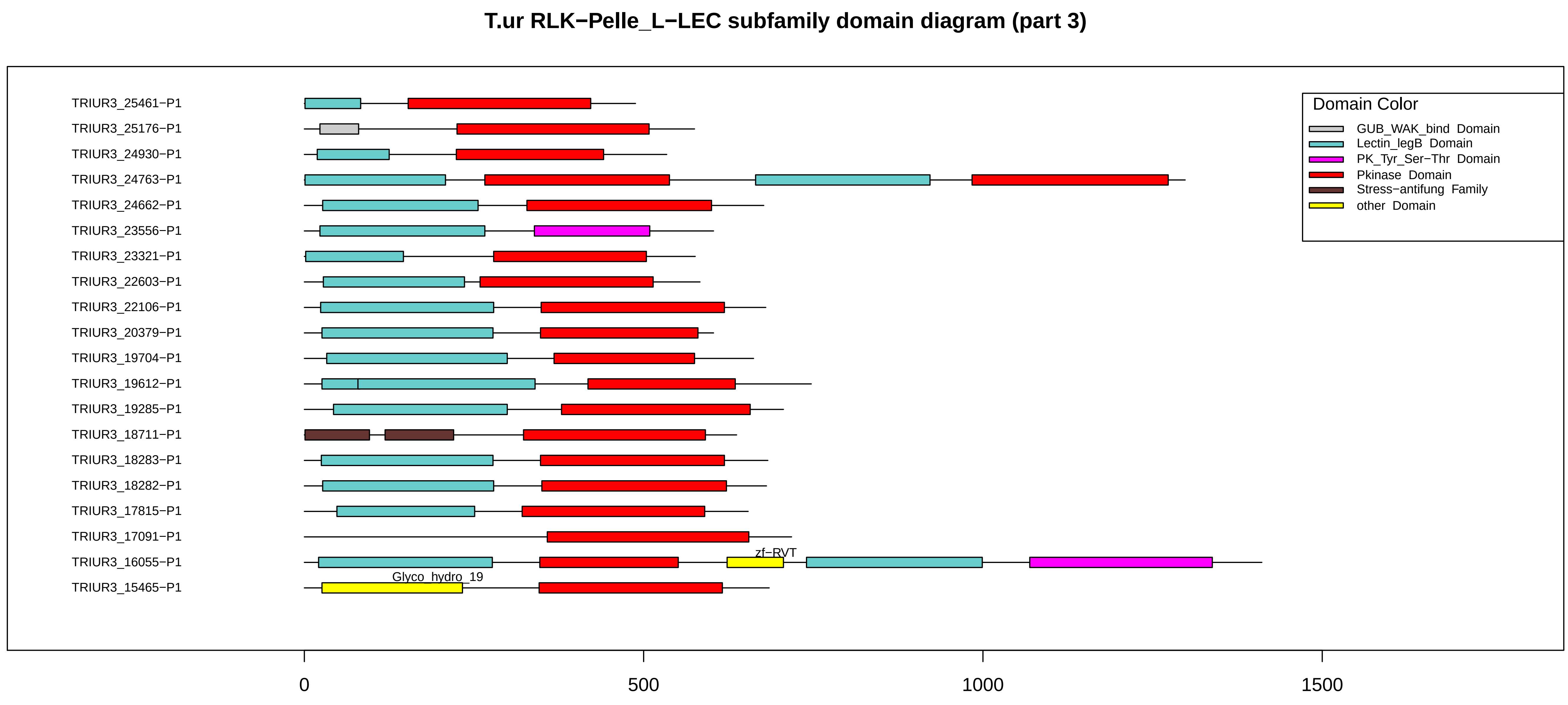
T.ur RLK−Pelle_L−LEC subfamily domain diagram (part 3)
TRIUR3_25461−P1
TRIUR3_25176−P1
TRIUR3_24930−P1
TRIUR3_24763−P1
TRIUR3_24662−P1
TRIUR3_23556−P1
TRIUR3_23321−P1
TRIUR3_22603−P1
TRIUR3_22106−P1
TRIUR3_20379−P1
TRIUR3_19704−P1
TRIUR3_19612−P1
TRIUR3_19285−P1
TRIUR3_18711−P1
TRIUR3_18283−P1
TRIUR3_18282−P1
TRIUR3_17815−P1
TRIUR3_17091−P1
TRIUR3_16055−P1
TRIUR3_15465−P1
zf−RVT
Glyco_hydro_19
Domain Color
GUB_WAK_bind Domain
Lectin_legB Domain
PK_Tyr_Ser−Thr Domain
Pkinase Domain
Stress−antifung Family
other Domain
0
500
1000
1500

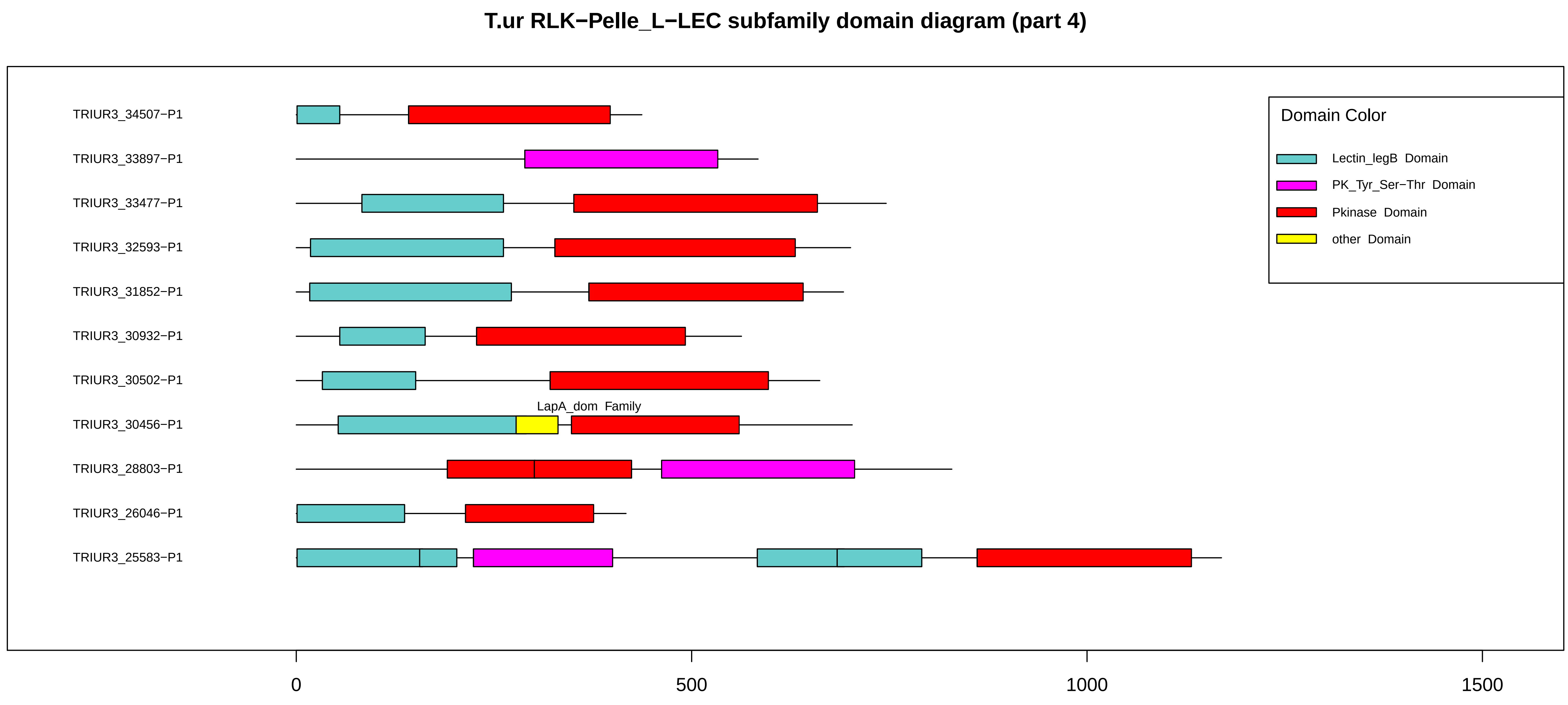
T.ur RLK−Pelle_L−LEC subfamily domain diagram (part 4)
TRIUR3_34507−P1
TRIUR3_33897−P1
TRIUR3_33477−P1
TRIUR3_32593−P1
TRIUR3_31852−P1
TRIUR3_30932−P1
TRIUR3_30502−P1
LapA_dom Family
TRIUR3_30456−P1
TRIUR3_28803−P1
TRIUR3_26046−P1
TRIUR3_25583−P1
Domain Color
Lectin_legB Domain
PK_Tyr_Ser−Thr Domain
Pkinase Domain
other Domain
0
500
1000
1500

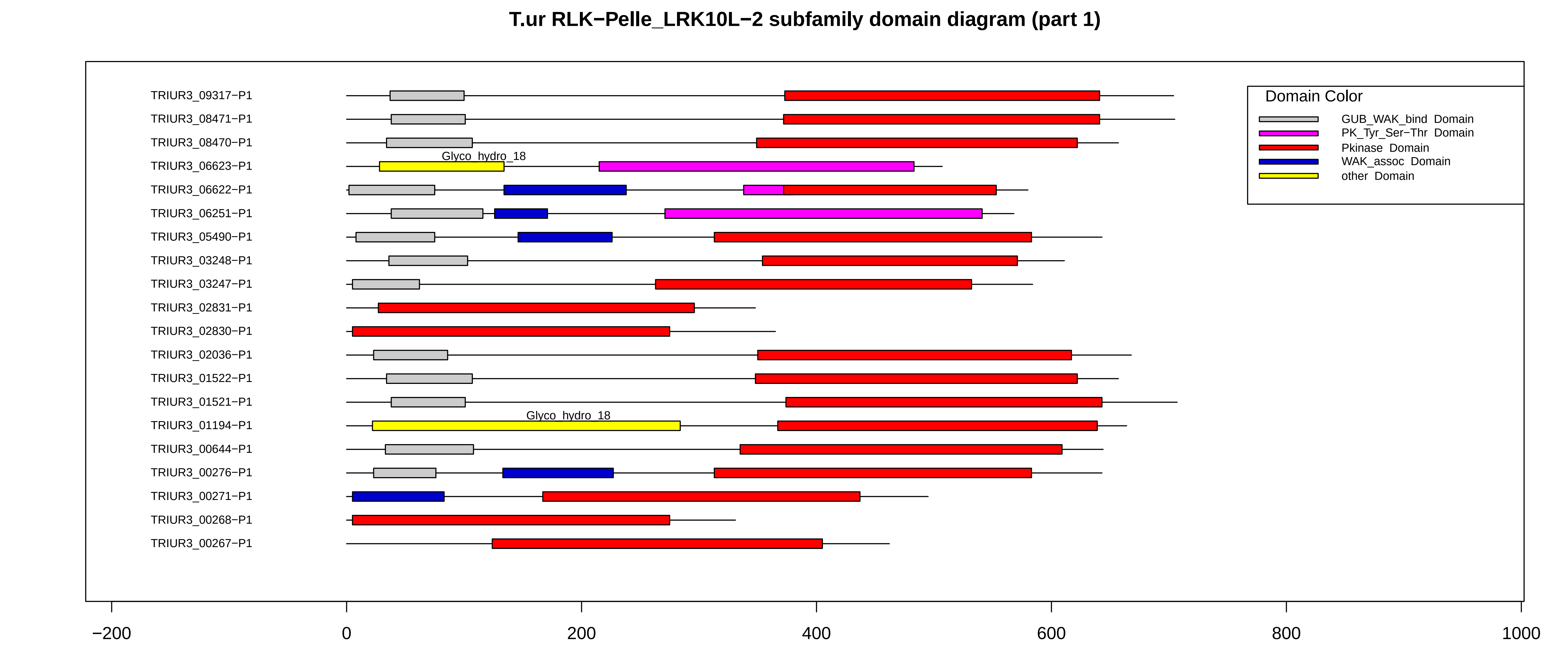

T.ur RLK−Pelle_LRK10L−2 subfamily domain diagram (part 1)
TRIUR3_09317−P1
TRIUR3_08471−P1
TRIUR3_08470−P1
TRIUR3_06623−P1
Glyco_hydro_18
TRIUR3_06622−P1
TRIUR3_06251−P1
TRIUR3_05490−P1
TRIUR3_03248−P1
TRIUR3_03247−P1
TRIUR3_02831−P1
TRIUR3_02830−P1
TRIUR3_02036−P1
TRIUR3_01522−P1
TRIUR3_01521−P1
TRIUR3_01194−P1
Glyco_hydro_18
TRIUR3_00644−P1
TRIUR3_00276−P1
TRIUR3_00271−P1
TRIUR3_00268−P1
TRIUR3_00267−P1
Domain Color
GUB_WAK_bind Domain
PK_Tyr_Ser−Thr Domain
Pkinase Domain
WAK_assoc Domain
other Domain
−200
0
200
400
600
800
1000

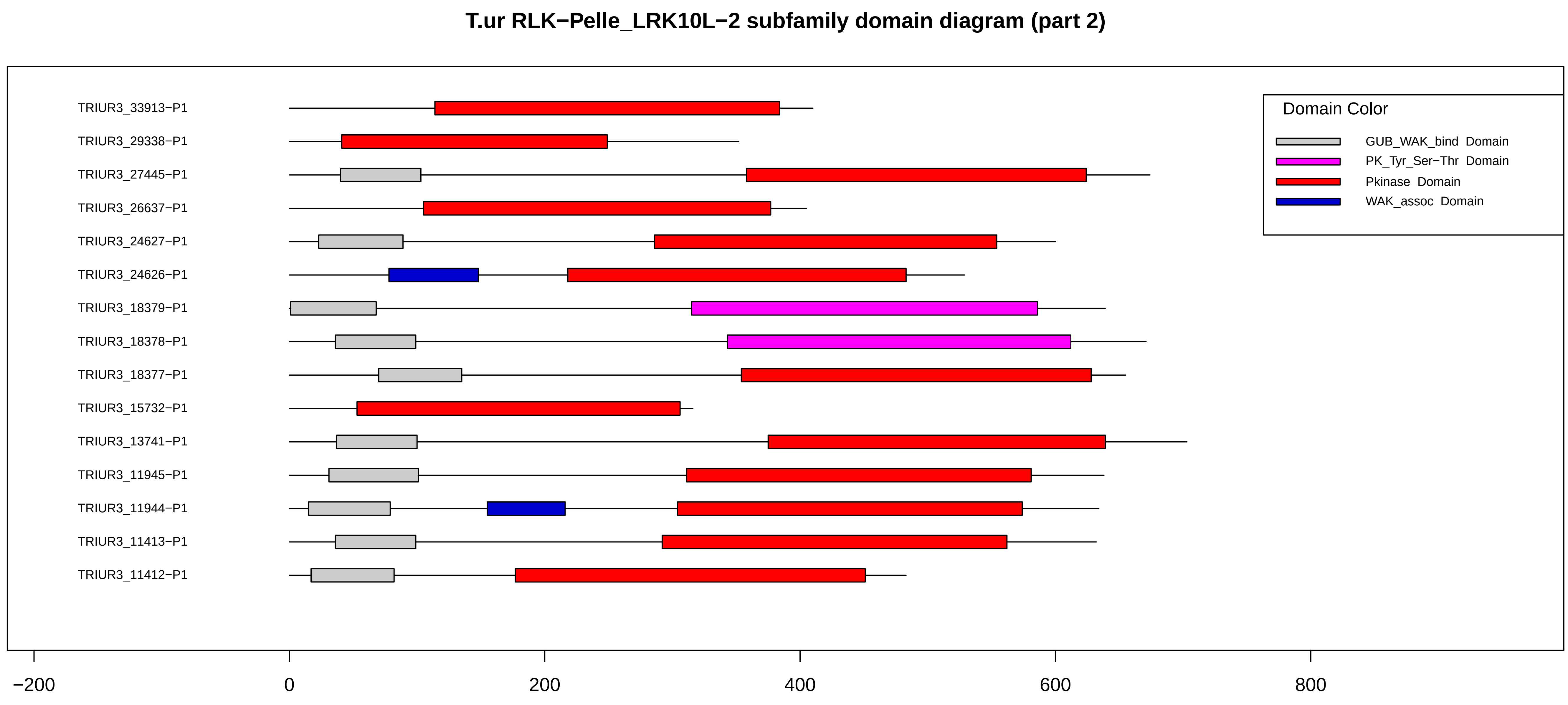

T.ur RLK−Pelle_LRK10L−2 subfamily domain diagram (part 2)
TRIUR3_33913−P1
TRIUR3_29338−P1
TRIUR3_27445−P1
TRIUR3_26637−P1
TRIUR3_24627−P1
TRIUR3_24626−P1
TRIUR3_18379−P1
TRIUR3_18378−P1
TRIUR3_18377−P1
TRIUR3_15732−P1
TRIUR3_13741−P1
TRIUR3_11945−P1
TRIUR3_11944−P1
TRIUR3_11413−P1
TRIUR3_11412−P1
Domain Color
GUB_WAK_bind Domain
PK_Tyr_Ser−Thr Domain
Pkinase Domain
WAK_assoc Domain
−200
0
200
400
600
800

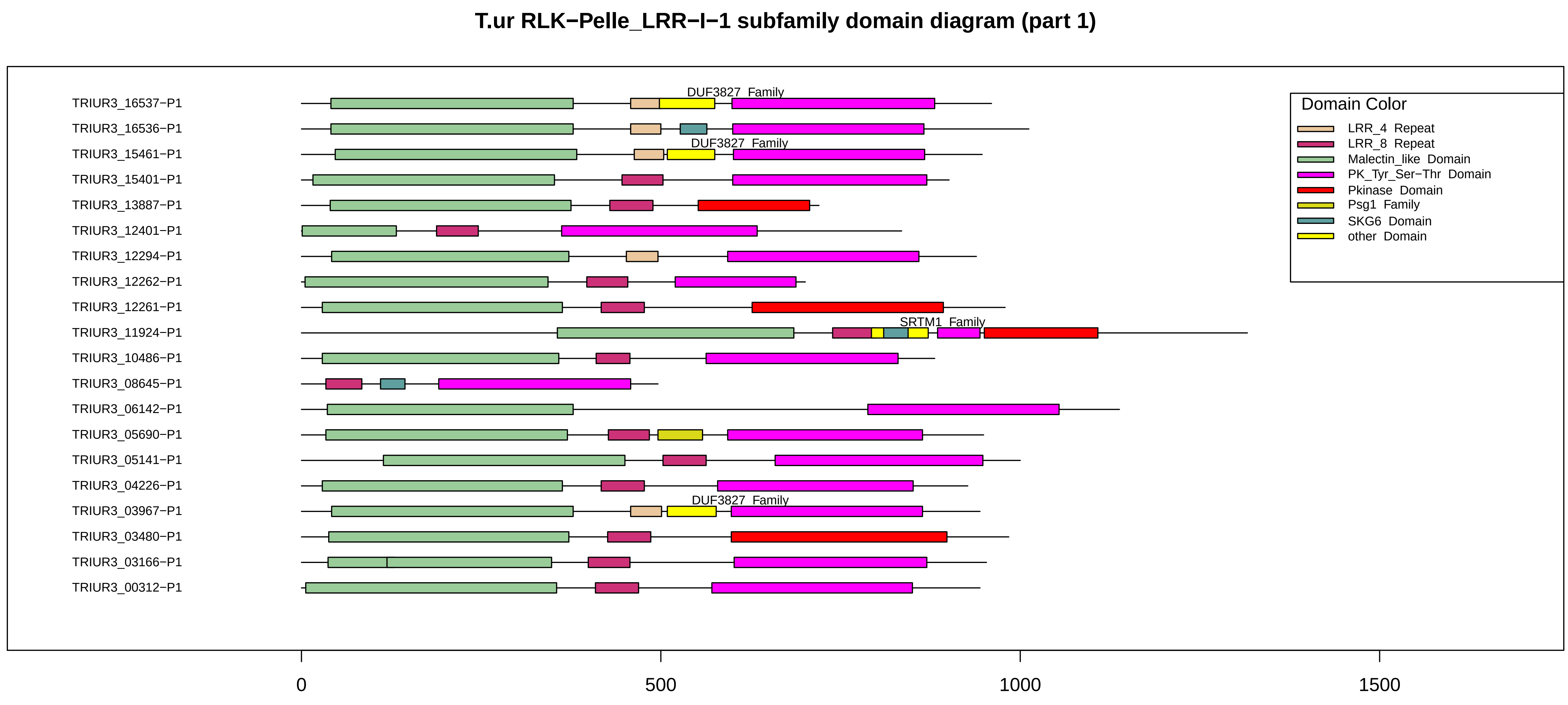
T.ur RLK−Pelle_LRR−I−1 subfamily domain diagram (part 1)
TRIUR3_16537−P1
TRIUR3_16536−P1
TRIUR3_15461−P1
TRIUR3_15401−P1
TRIUR3_13887−P1
TRIUR3_12401−P1
TRIUR3_12294−P1
TRIUR3_12262−P1
TRIUR3_12261−P1
TRIUR3_11924−P1
TRIUR3_10486−P1
TRIUR3_08645−P1
TRIUR3_06142−P1
TRIUR3_05690−P1
TRIUR3_05141−P1
TRIUR3_04226−P1
TRIUR3_03967−P1
TRIUR3_03480−P1
TRIUR3_03166−P1
TRIUR3_00312−P1
DUF3827_Family
DUF3827_Family
SRTM1_Family
DUF3827_Family
Domain Color
LRR_4_Repeat
LRR_8_Repeat
Malectin_like_Domain
PK_Tyr_Ser−Thr_Domain
Pkinase_Domain
Psg1_Family
SKG6_Domain
other_Domain
0
500
1000
1500

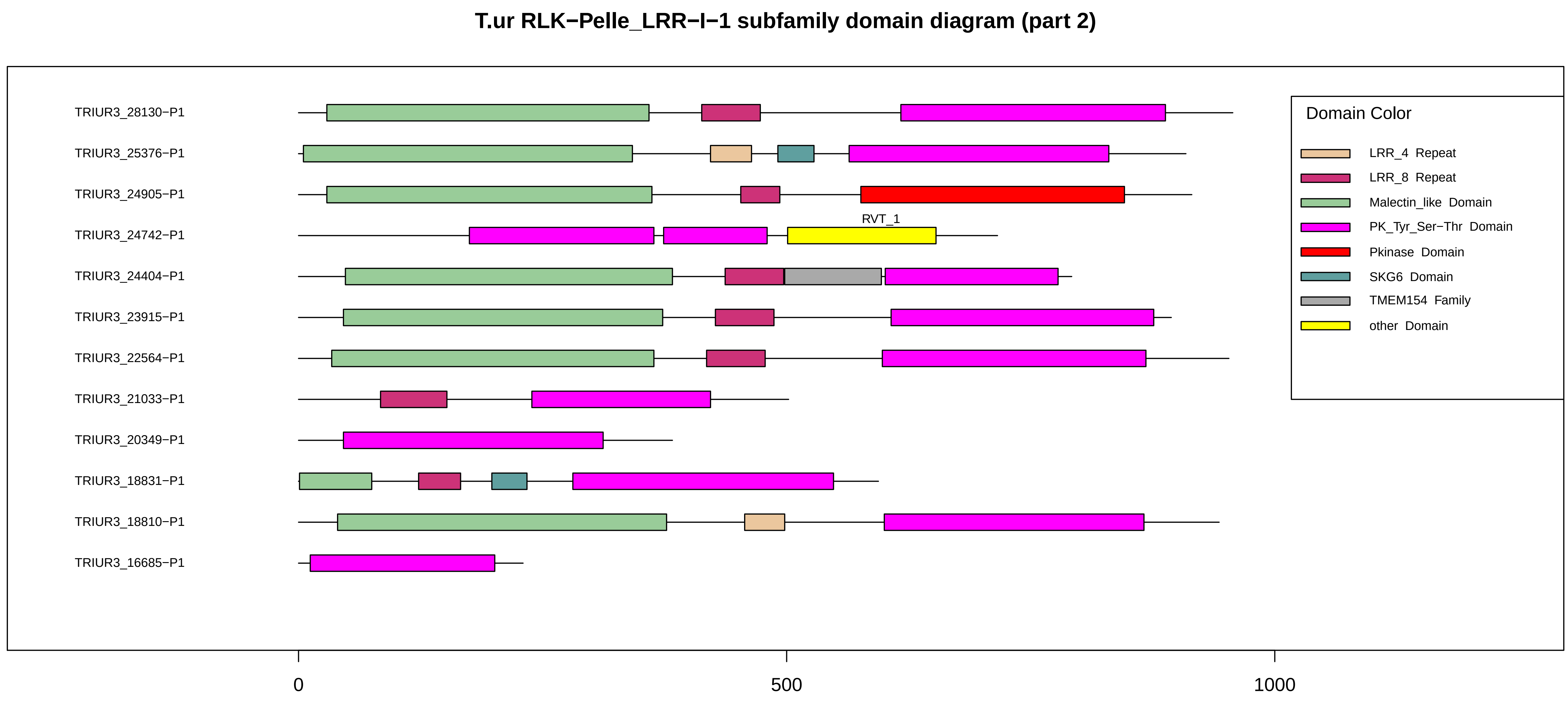

T.ur RLK−Pelle_LRR−I−1 subfamily domain diagram (part 2)
TRIUR3_28130−P1
TRIUR3_25376−P1
TRIUR3_24905−P1
TRIUR3_24742−P1
TRIUR3_24404−P1
TRIUR3_23915−P1
TRIUR3_22564−P1
TRIUR3_21033−P1
TRIUR3_20349−P1
TRIUR3_18831−P1
TRIUR3_18810−P1
TRIUR3_16685−P1
RVT_1
Domain Color
LRR_4 Repeat
LRR_8 Repeat
Malectin_like Domain
PK_Tyr_Ser−Thr Domain
Pkinase Domain
SKG6 Domain
TMEM154 Family
other Domain
0
500
1000

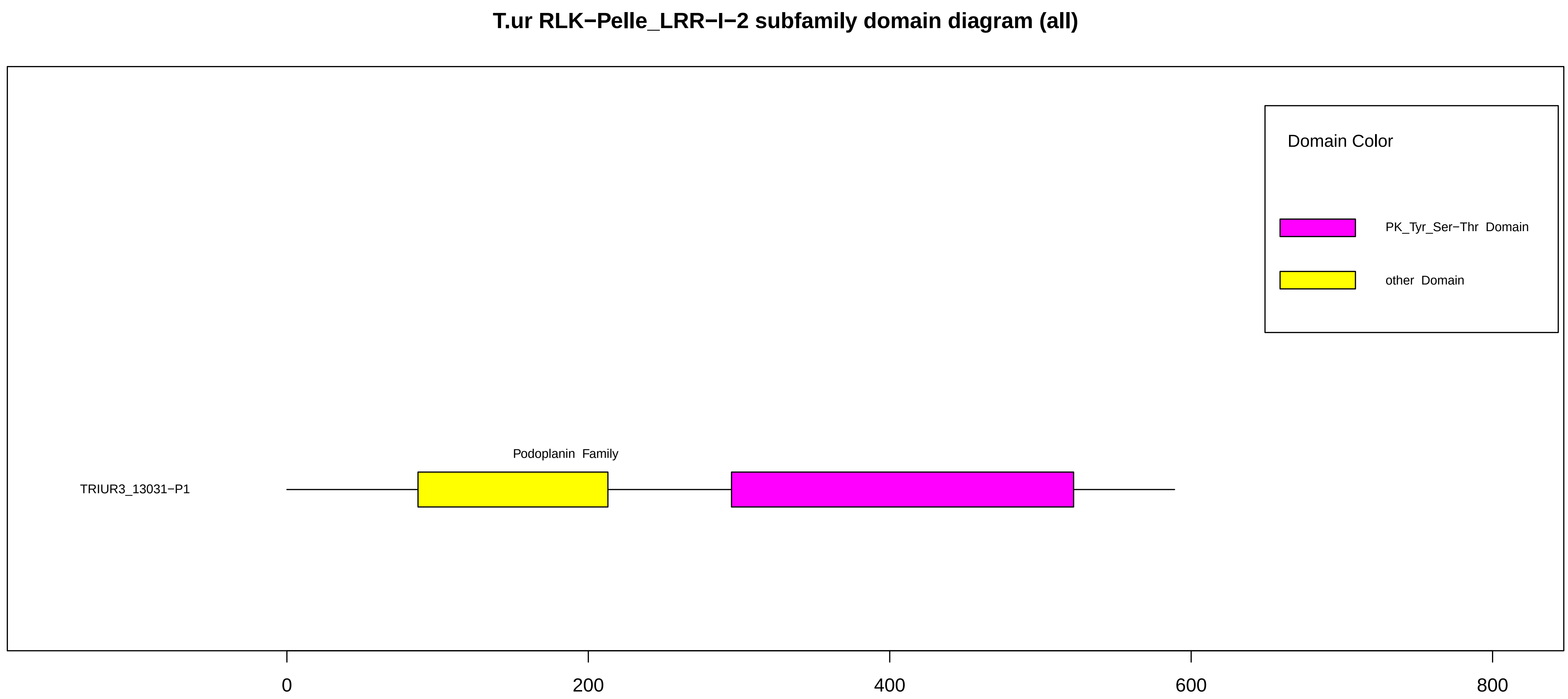

T.ur RLK−Pelle_LRR−I−2 subfamily domain diagram (all)
Domain Color
PK_Tyr_Ser−Thr Domain
other Domain
Podoplanin Family
TRIUR3_13031−P1
0
200
400
600
800

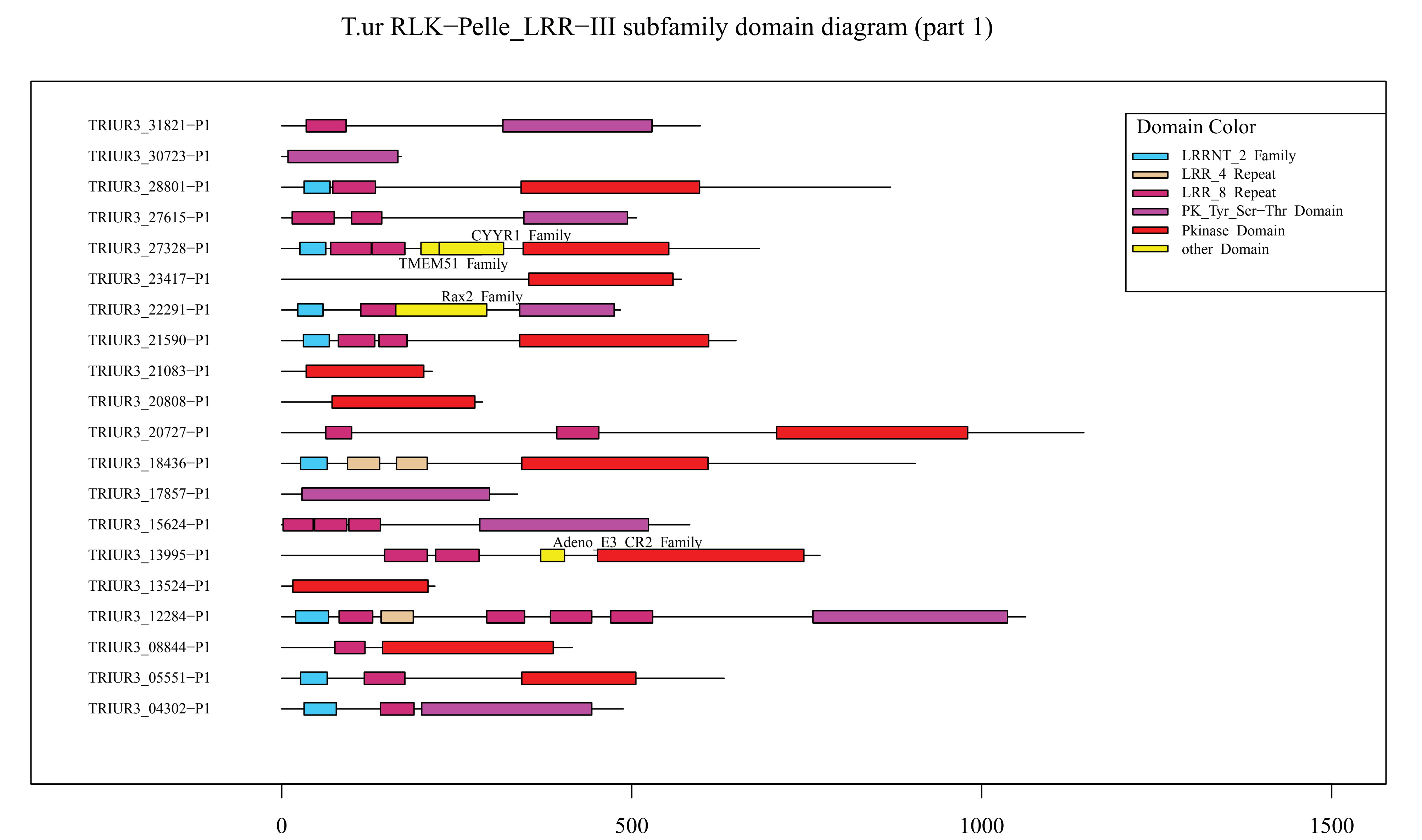

T.ur RLK−Pelle_LRR−III subfamily domain diagram (part 1)
TRIUR3_31821−P1
TRIUR3_30723−P1
TRIUR3_28801−P1
TRIUR3_27615−P1
TRIUR3_27328−P1
TRIUR3_23417−P1
TRIUR3_22291−P1
TRIUR3_21590−P1
TRIUR3_21083−P1
TRIUR3_20808−P1
TRIUR3_20727−P1
TRIUR3_18436−P1
TRIUR3_17857−P1
TRIUR3_15624−P1
TRIUR3_13995−P1
TRIUR3_13524−P1
TRIUR3_12284−P1
TRIUR3_08844−P1
TRIUR3_05551−P1
TRIUR3_04302−P1
CYYR1 Family
TMEM51 Family
Rax2 Family
Adeno_E3_CR2 Family
Domain Color
LRRNT_2 Family
LRR_4 Repeat
LRR_8 Repeat
PK_Tyr_Ser−Thr Domain
Pkinase Domain
other Domain
0
500
1000
1500

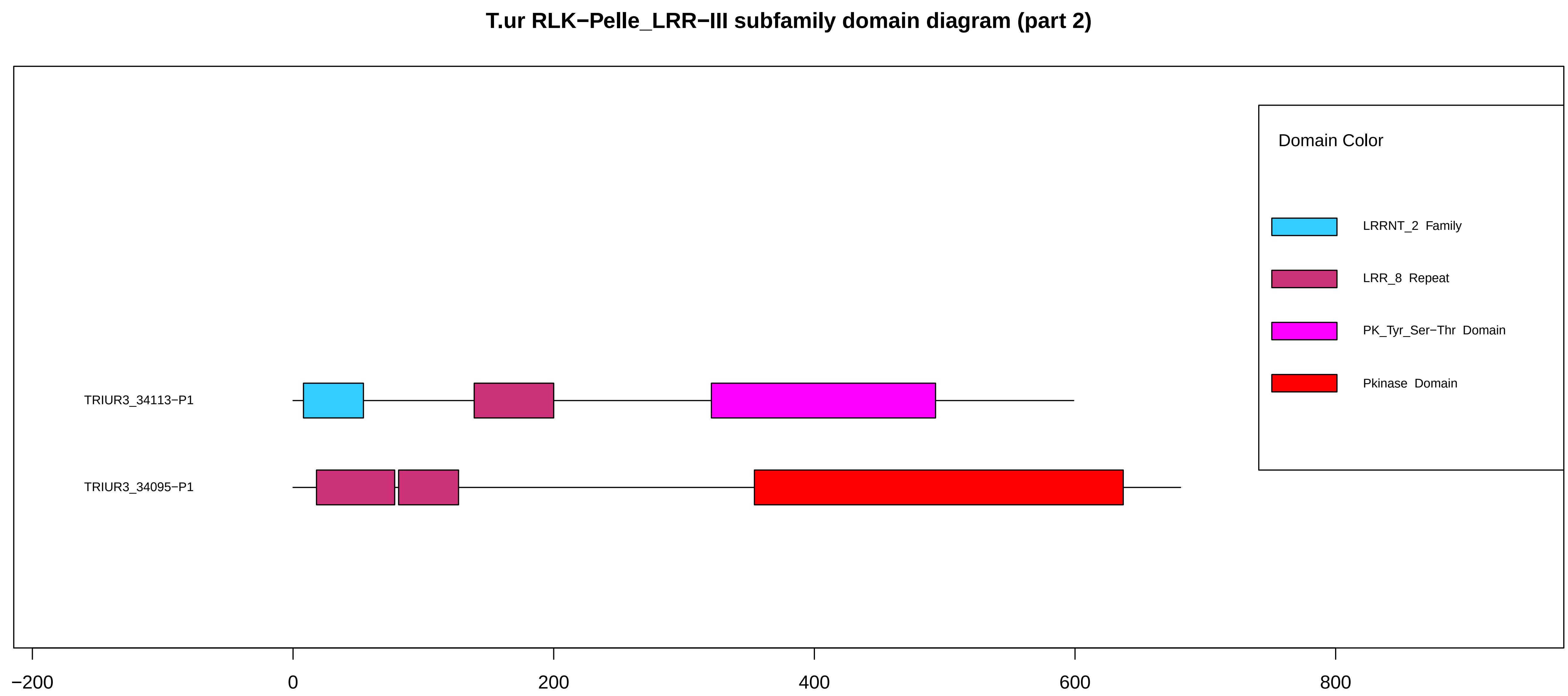
T.ur RLK−Pelle_LRR−III subfamily domain diagram (part 2)
Domain Color
LRRNT_2 Family
LRR_8 Repeat
PK_Tyr_Ser−Thr Domain
Pkinase Domain
TRIUR3_34113−P1
TRIUR3_34095−P1
−200
0
200
400
600
800

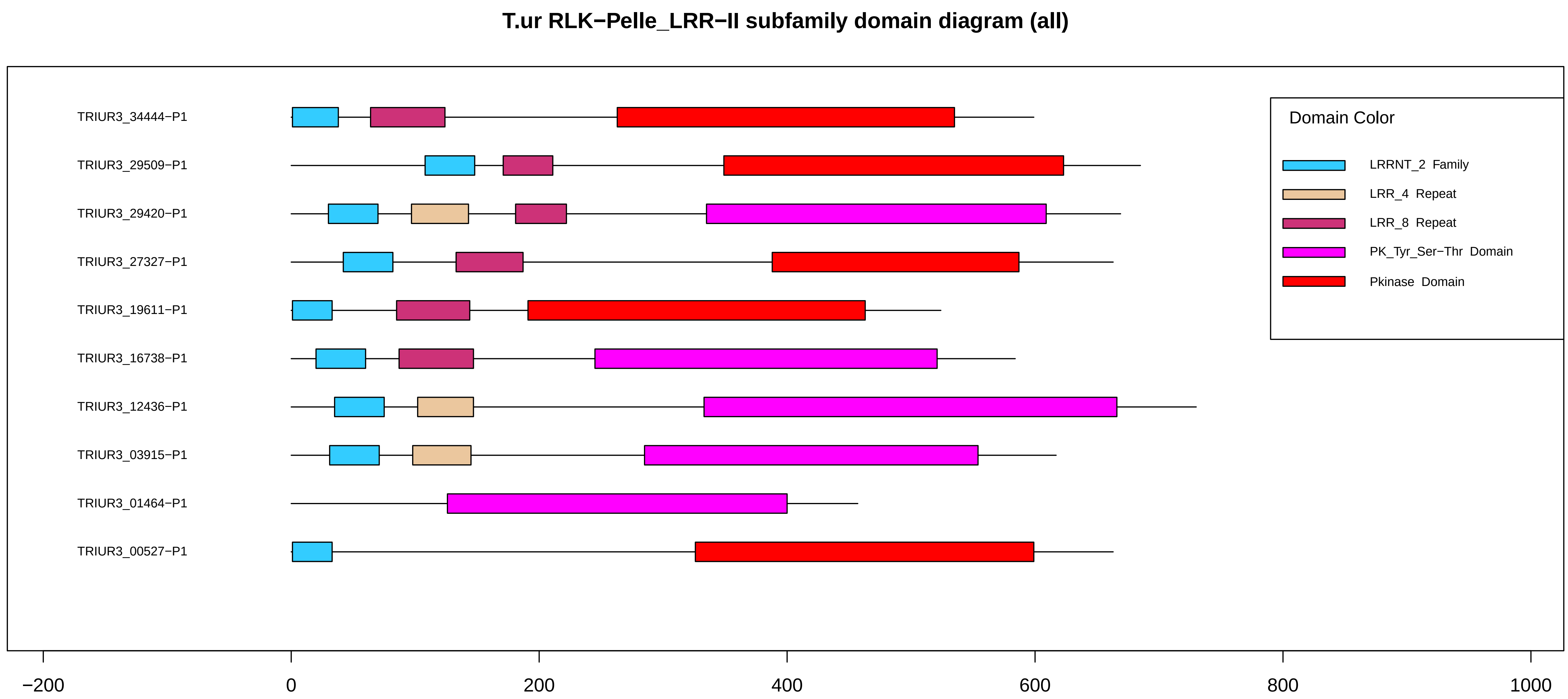
T.ur RLK−Pelle_LRR−II subfamily domain diagram (all)
TRIUR3_34444−P1
TRIUR3_29509−P1
TRIUR3_29420−P1
TRIUR3_27327−P1
TRIUR3_19611−P1
TRIUR3_16738−P1
TRIUR3_12436−P1
TRIUR3_03915−P1
TRIUR3_01464−P1
TRIUR3_00527−P1
Domain Color
LRRNT_2 Family
LRR_4 Repeat
LRR_8 Repeat
PK_Tyr_Ser−Thr Domain
Pkinase Domain
−200
0
200
400
600
800
1000

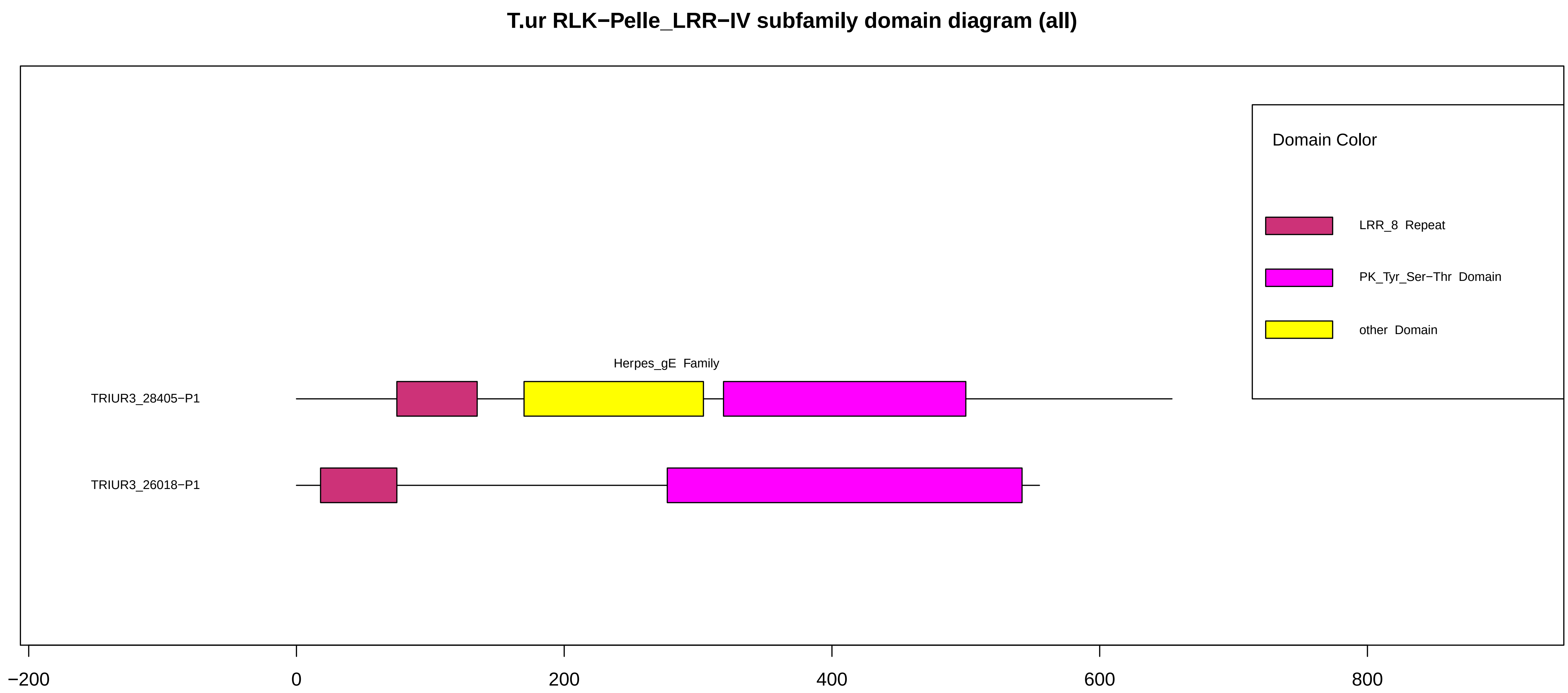

T.ur RLK−Pelle_LRR−IV subfamily domain diagram (all)
Domain Color
LRR_8 Repeat
PK_Tyr_Ser−Thr Domain
other Domain
Herpes_gE Family
TRIUR3_28405−P1
TRIUR3_26018−P1
−200
0
200
400
600
800

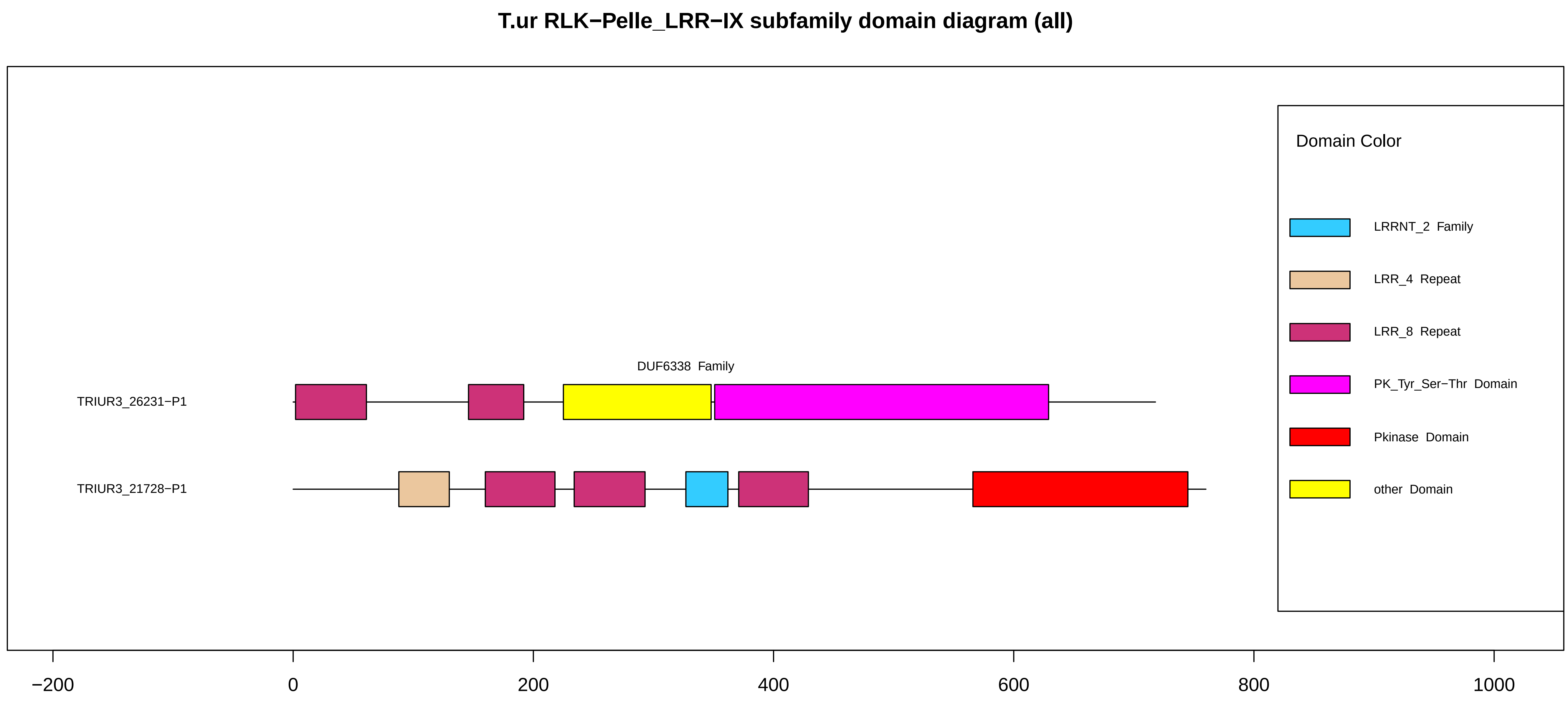
T.ur RLK−Pelle_LRR−IX subfamily domain diagram (all)
DUF6338 Family
TRIUR3_26231−P1
TRIUR3_21728−P1
Domain Color
LRRNT_2 Family
LRR_4 Repeat
LRR_8 Repeat
PK_Tyr_Ser−Thr Domain
Pkinase Domain
other Domain
−200
0
200
400
600
800
1000

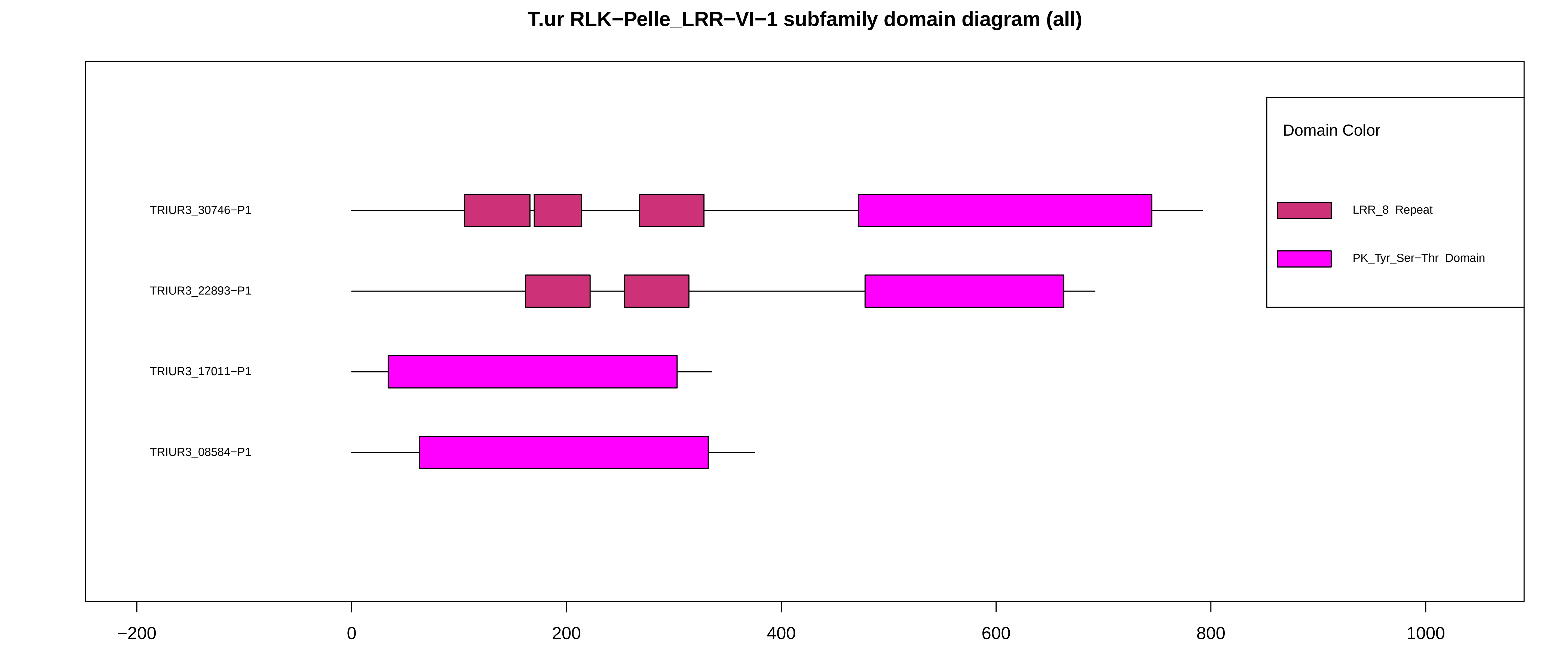
T.ur RLK−Pelle_LRR−VI−1 subfamily domain diagram (all)
TRIUR3_30746−P1
TRIUR3_22893−P1
TRIUR3_17011−P1
TRIUR3_08584−P1
Domain Color
LRR_8 Repeat
PK_Tyr_Ser−Thr Domain
−200
0
200
400
600
800
1000

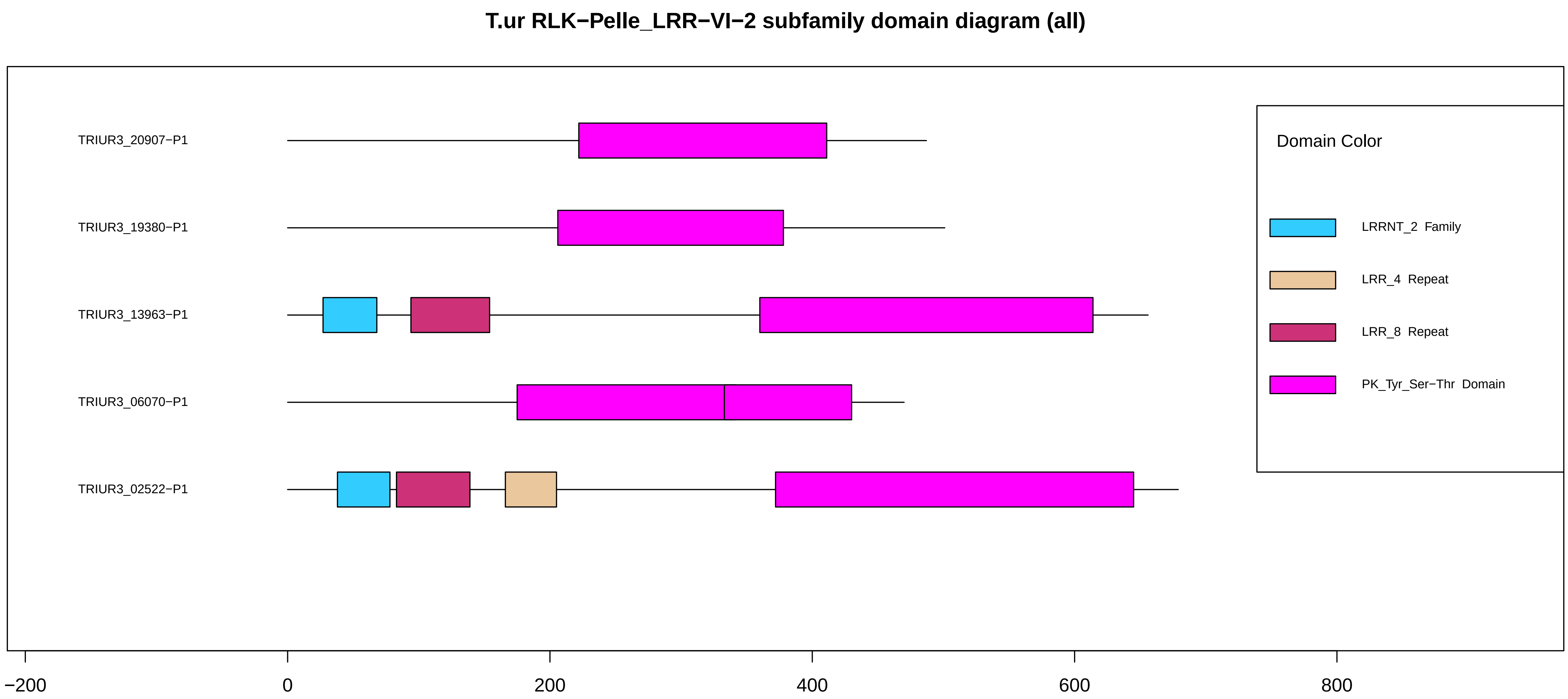
T.ur RLK−Pelle_LRR−VI−2 subfamily domain diagram (all)
TRIUR3_20907−P1
TRIUR3_19380−P1
TRIUR3_13963−P1
TRIUR3_06070−P1
TRIUR3_02522−P1
Domain Color
LRRNT_2 Family
LRR_4 Repeat
LRR_8 Repeat
PK_Tyr_Ser−Thr Domain
−200
0
200
400
600
800

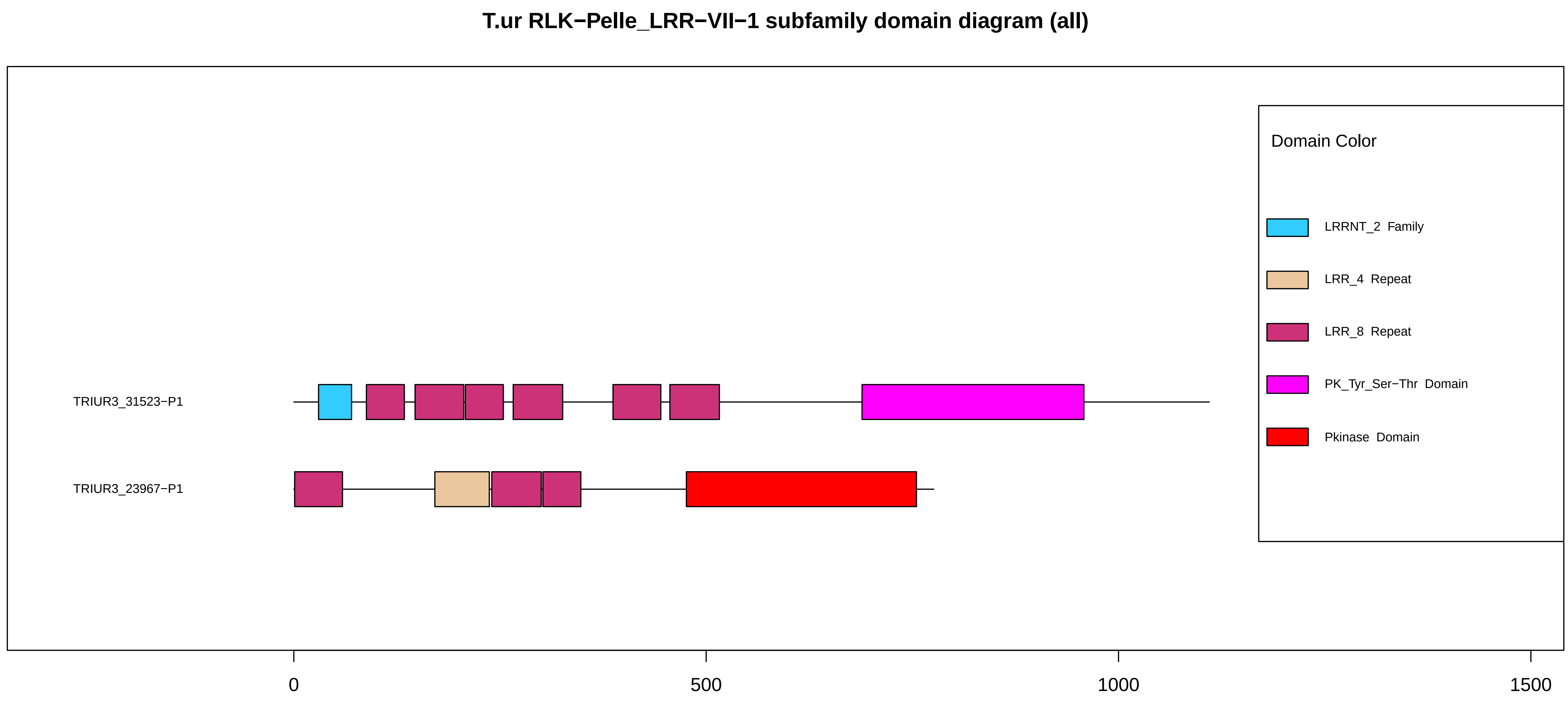
T.ur RLK−Pelle_LRR−VII−1 subfamily domain diagram (all)
TRIUR3_31523−P1
TRIUR3_23967−P1
Domain Color
LRRNT_2 Family
LRR_4 Repeat
LRR_8 Repeat
PK_Tyr_Ser−Thr Domain
Pkinase Domain
0
500
1000
1500

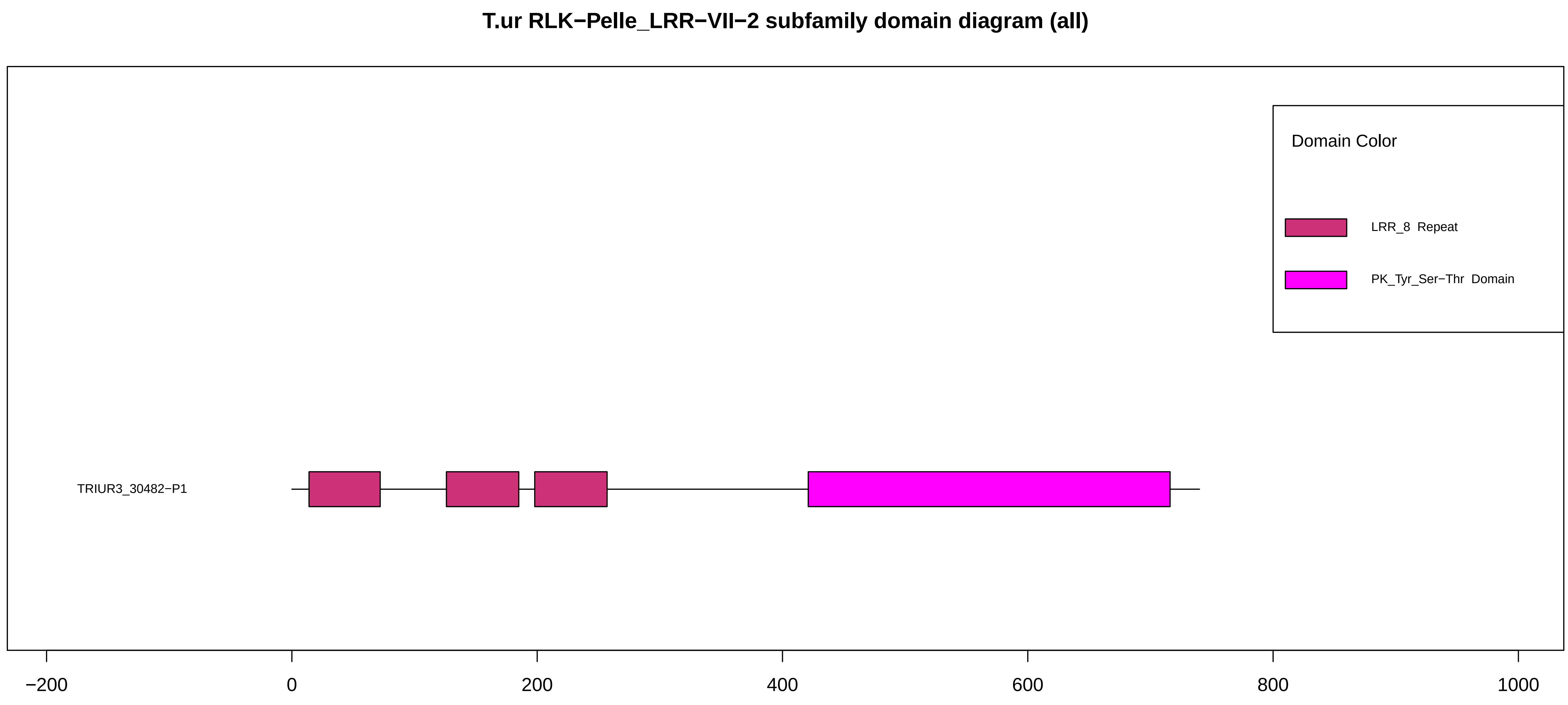

T.ur RLK−Pelle_LRR−VII−2 subfamily domain diagram (all)
Domain Color
LRR_8 Repeat
PK_Tyr_Ser−Thr Domain
TRIUR3_30482−P1
−200
0
200
400
600
800
1000

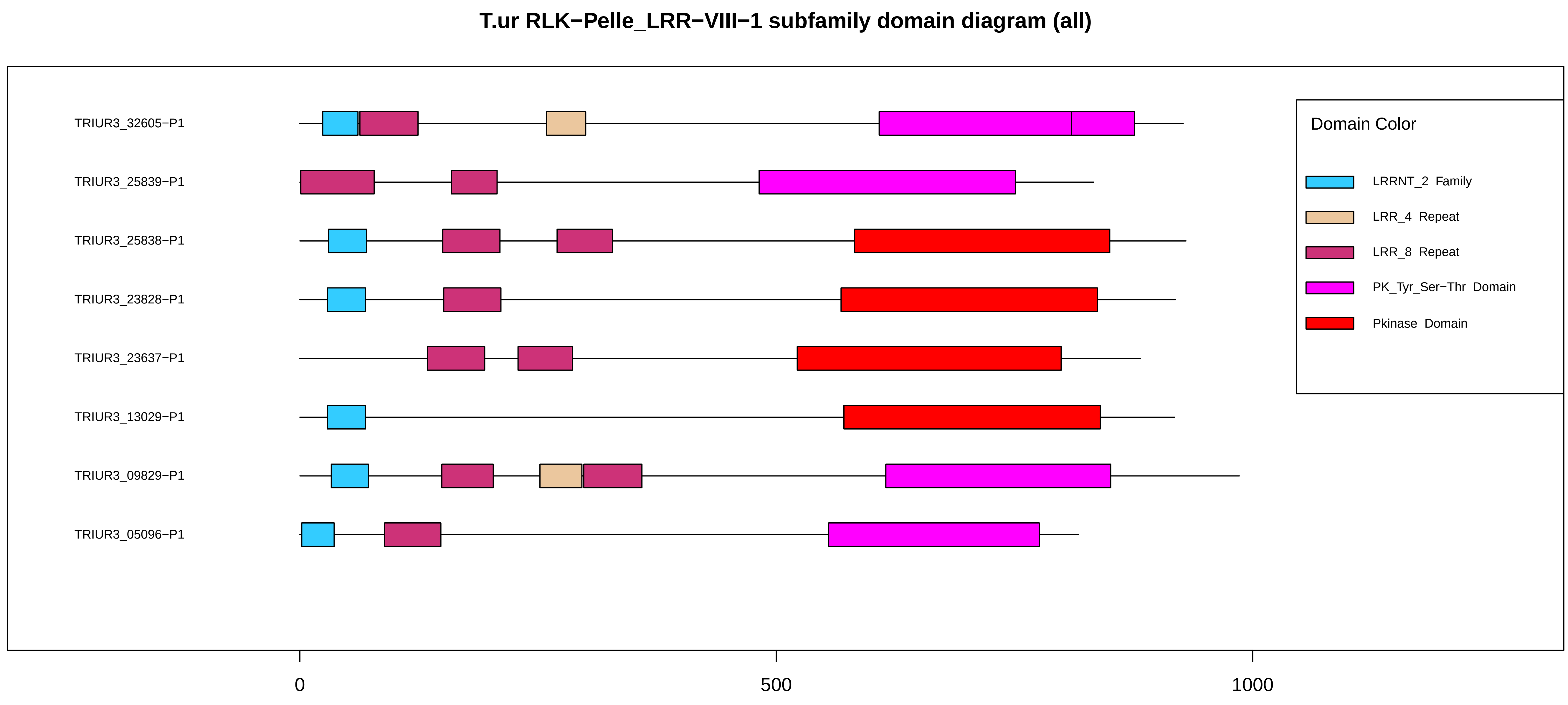

T.ur RLK−Pelle_LRR−VIII−1 subfamily domain diagram (all)
TRIUR3_32605−P1
TRIUR3_25839−P1
TRIUR3_25838−P1
TRIUR3_23828−P1
TRIUR3_23637−P1
TRIUR3_13029−P1
TRIUR3_09829−P1
TRIUR3_05096−P1
Domain Color
LRRNT_2 Family
LRR_4 Repeat
LRR_8 Repeat
PK_Tyr_Ser−Thr Domain
Pkinase Domain
0
500
1000

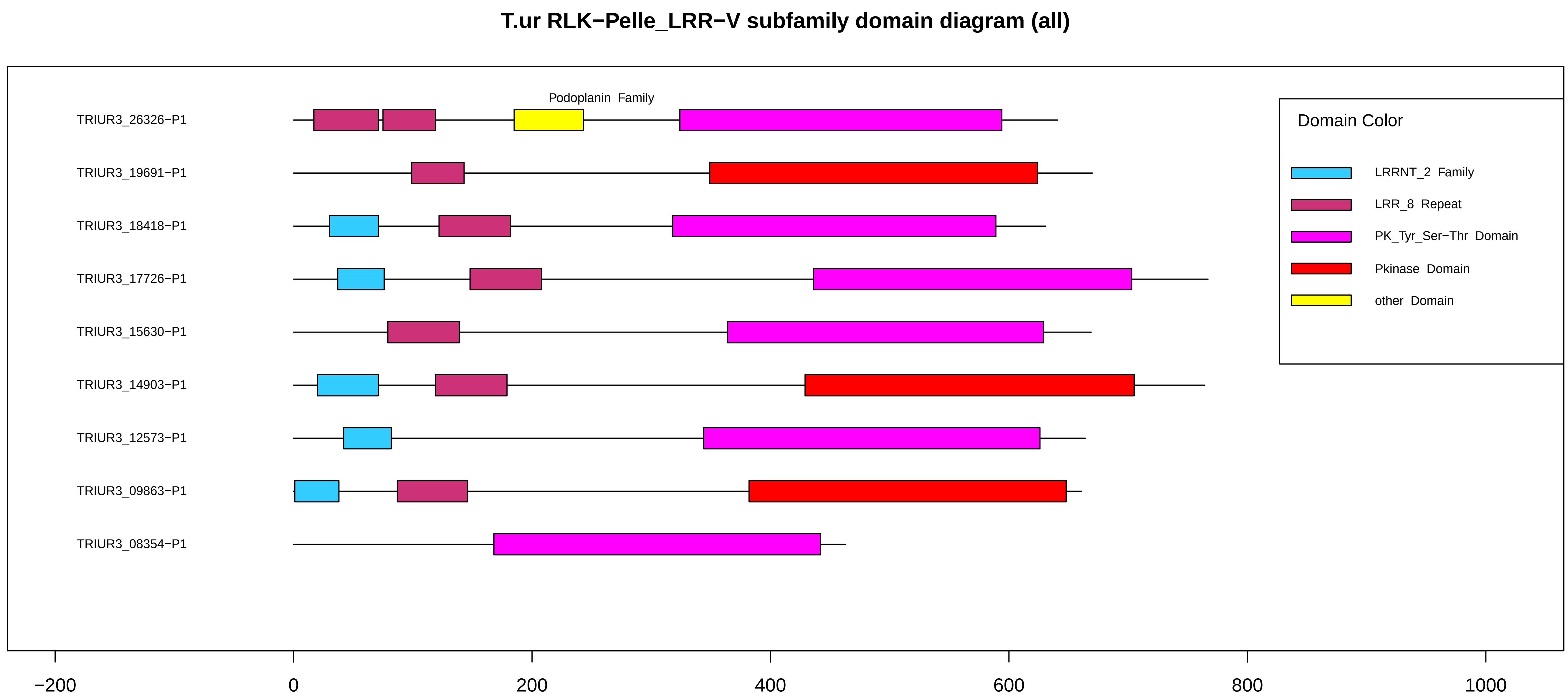
T.ur RLK−Pelle_LRR−V subfamily domain diagram (all)
Podoplanin Family
TRIUR3_26326−P1
TRIUR3_19691−P1
TRIUR3_18418−P1
TRIUR3_17726−P1
TRIUR3_15630−P1
TRIUR3_14903−P1
TRIUR3_12573−P1
TRIUR3_09863−P1
TRIUR3_08354−P1
Domain Color
LRRNT_2 Family
LRR_8 Repeat
PK_Tyr_Ser−Thr Domain
Pkinase Domain
other Domain
−200
0
200
400
600
800
1000

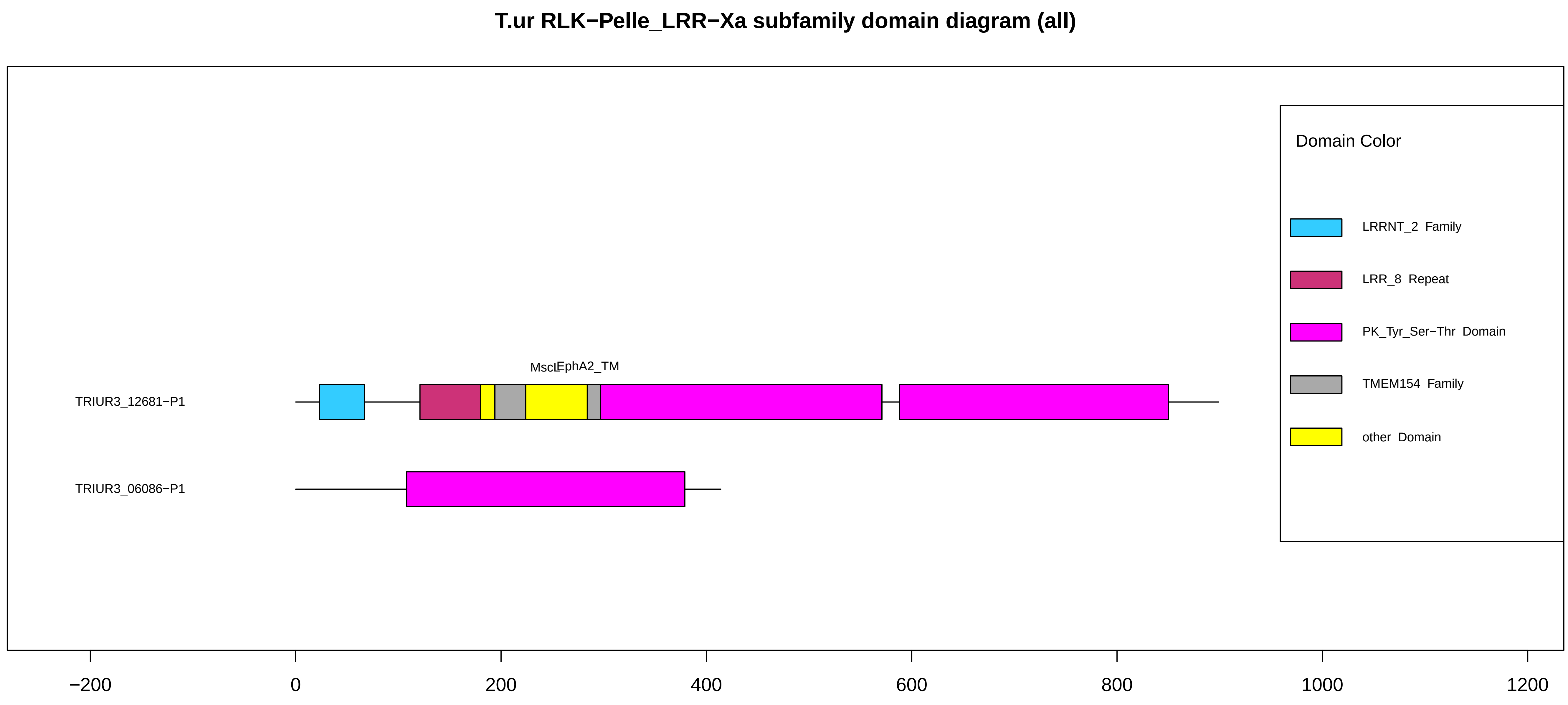

T.ur RLK−Pelle_LRR−Xa subfamily domain diagram (all)
TRIUR3_12681−P1
TRIUR3_06086−P1
MscL
EphA2_TM
Domain Color
LRRNT_2 Family
LRR_8 Repeat
PK_Tyr_Ser−Thr Domain
TMEM154 Family
other Domain
−200
0
200
400
600
800
1000
1200

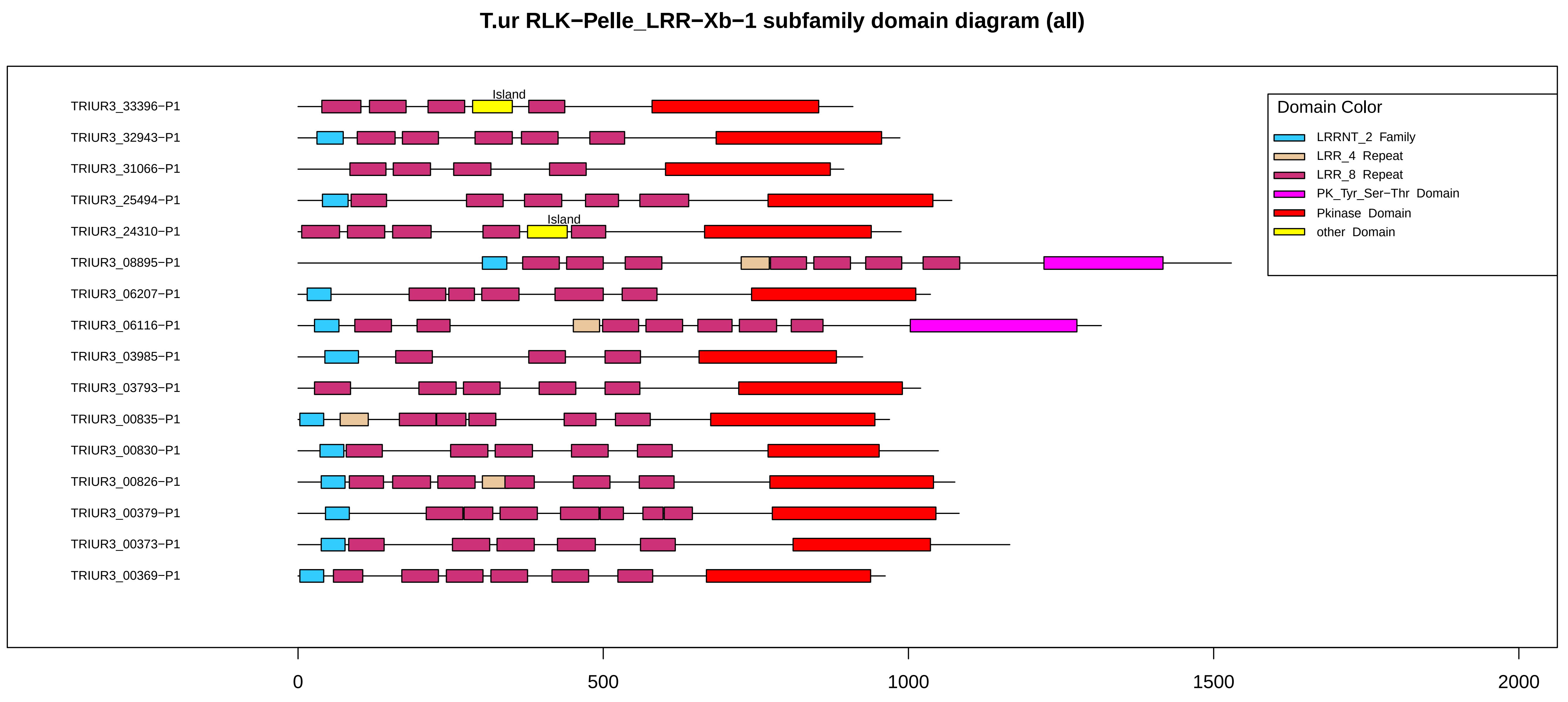

T.ur RLK−Pelle_LRR−Xb−1 subfamily domain diagram (all)
TRIUR3_33396−P1
TRIUR3_32943−P1
TRIUR3_31066−P1
TRIUR3_25494−P1
TRIUR3_24310−P1
TRIUR3_08895−P1
TRIUR3_06207−P1
TRIUR3_06116−P1
TRIUR3_03985−P1
TRIUR3_03793−P1
TRIUR3_00835−P1
TRIUR3_00830−P1
TRIUR3_00826−P1
TRIUR3_00379−P1
TRIUR3_00373−P1
TRIUR3_00369−P1
Island
Island
Domain Color
LRRNT_2 Family
LRR_4 Repeat
LRR_8 Repeat
PK_Tyr_Ser−Thr Domain
Pkinase Domain
other Domain
0
500
1000
1500
2000

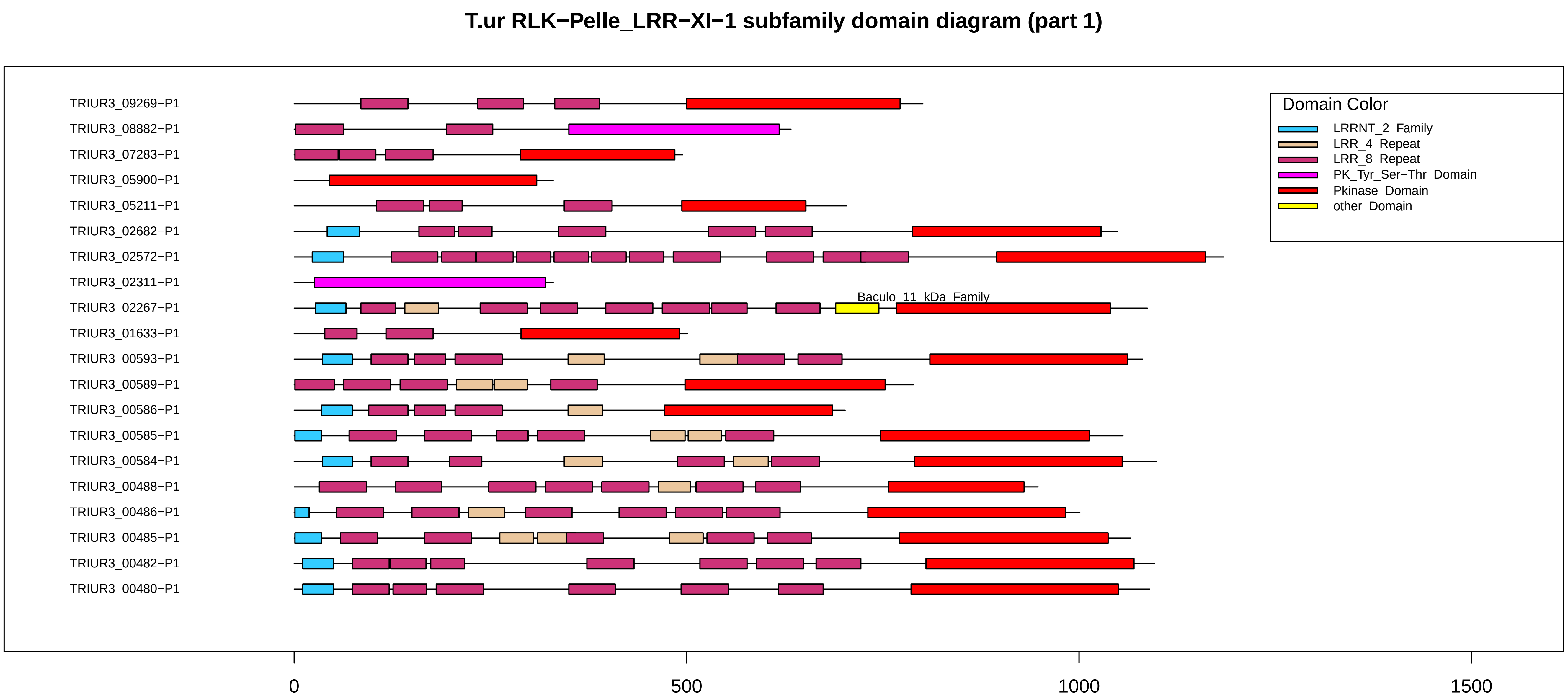
T.ur RLK−Pelle_LRR−XI−1 subfamily domain diagram (part 1)
TRIUR3_09269−P1
TRIUR3_08882−P1
TRIUR3_07283−P1
TRIUR3_05900−P1
TRIUR3_05211−P1
TRIUR3_02682−P1
TRIUR3_02572−P1
TRIUR3_02311−P1
TRIUR3_02267−P1
TRIUR3_01633−P1
TRIUR3_00593−P1
TRIUR3_00589−P1
TRIUR3_00586−P1
TRIUR3_00585−P1
TRIUR3_00584−P1
TRIUR3_00488−P1
TRIUR3_00486−P1
TRIUR3_00485−P1
TRIUR3_00482−P1
TRIUR3_00480−P1
Baculo_11_kDa_Family
Domain Color
LRRNT_2 Family
LRR_4 Repeat
LRR_8 Repeat
PK_Tyr_Ser−Thr Domain
Pkinase Domain
other Domain
0
500
1000
1500

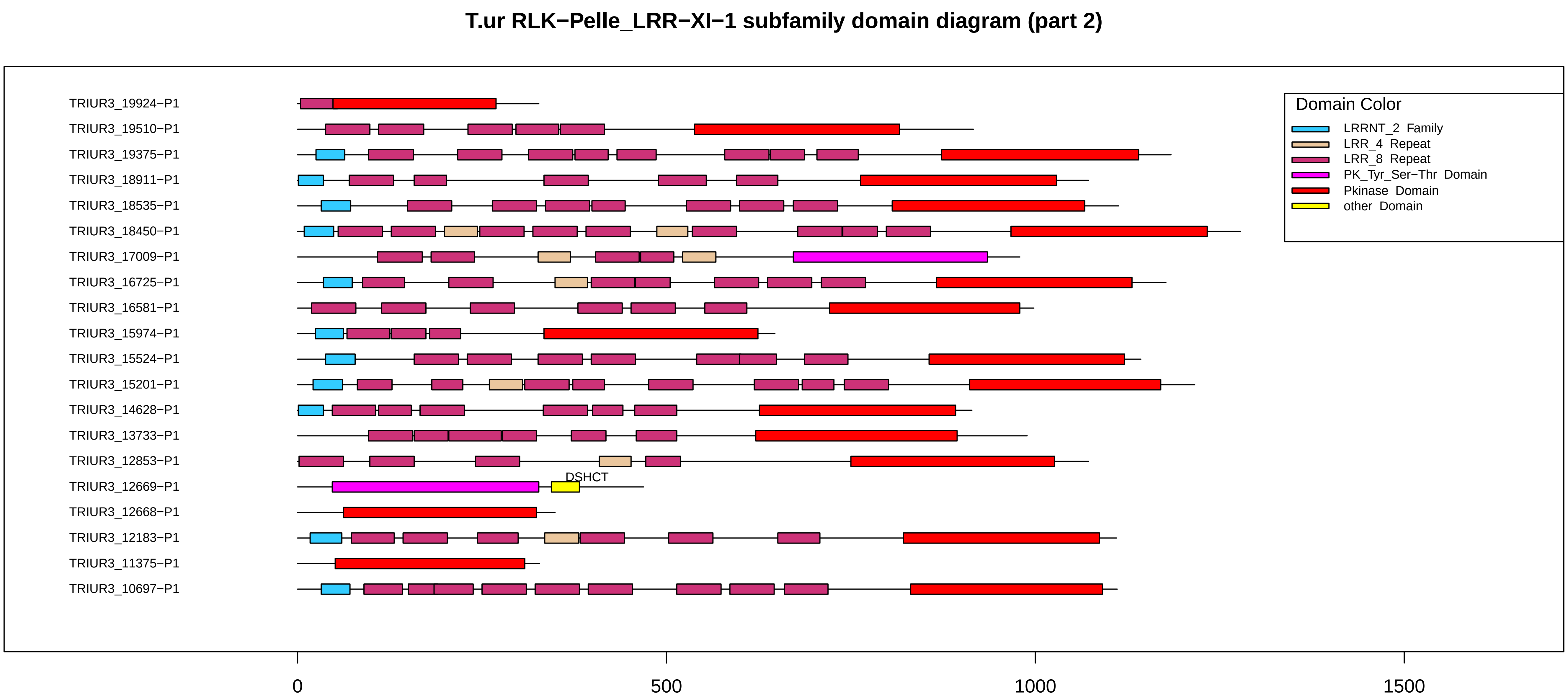
T.ur RLK−Pelle_LRR−XI−1 subfamily domain diagram (part 2)
TRIUR3_19924−P1
TRIUR3_19510−P1
TRIUR3_19375−P1
TRIUR3_18911−P1
TRIUR3_18535−P1
TRIUR3_18450−P1
TRIUR3_17009−P1
TRIUR3_16725−P1
TRIUR3_16581−P1
TRIUR3_15974−P1
TRIUR3_15524−P1
TRIUR3_15201−P1
TRIUR3_14628−P1
TRIUR3_13733−P1
TRIUR3_12853−P1
TRIUR3_12669−P1
DSHCT
TRIUR3_12668−P1
TRIUR3_12183−P1
TRIUR3_11375−P1
TRIUR3_10697−P1
Domain Color
LRRNT_2 Family
LRR_4 Repeat
LRR_8 Repeat
PK_Tyr_Ser−Thr Domain
Pkinase Domain
other Domain
0
500
1000
1500

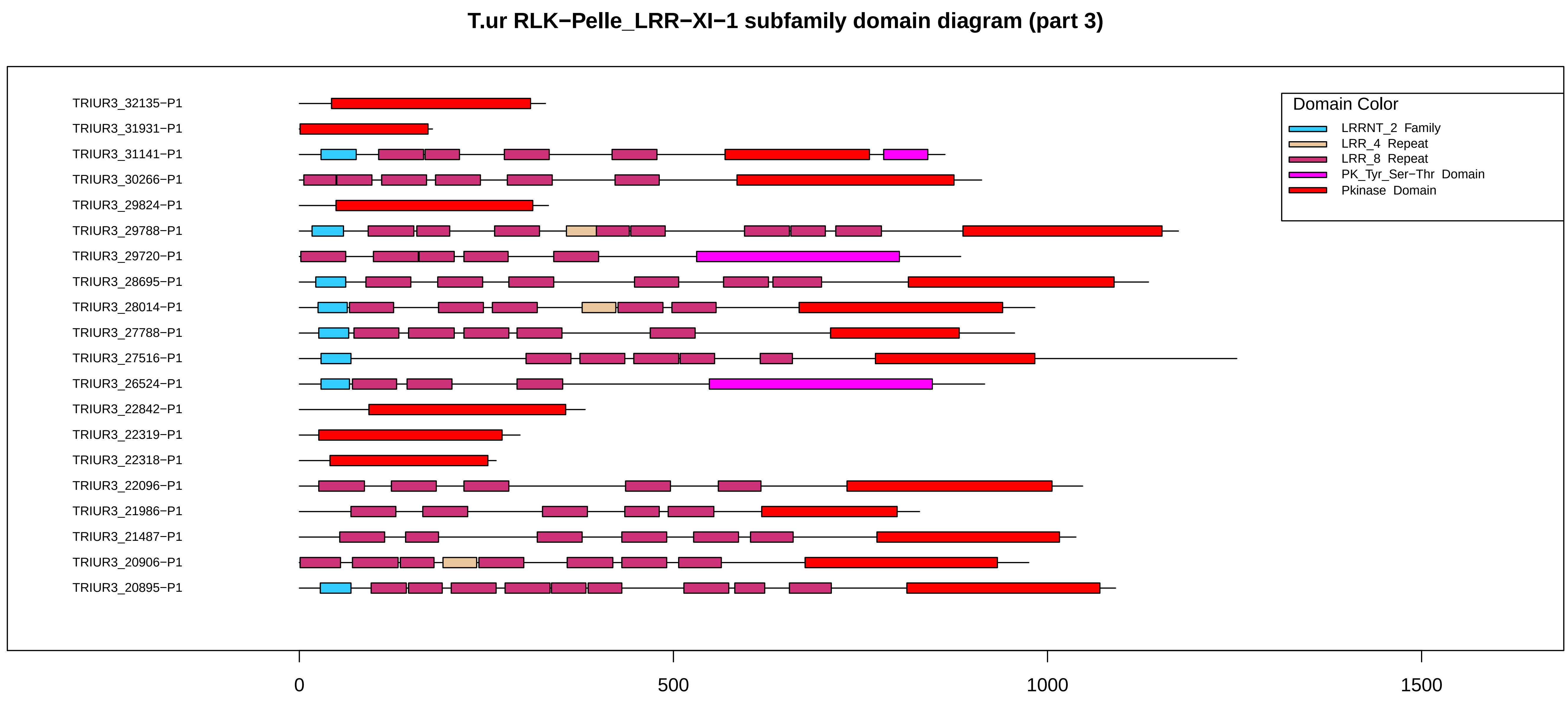

T.ur RLK−Pelle_LRR−XI−1 subfamily domain diagram (part 3)
TRIUR3_32135−P1
TRIUR3_31931−P1
TRIUR3_31141−P1
TRIUR3_30266−P1
TRIUR3_29824−P1
TRIUR3_29788−P1
TRIUR3_29720−P1
TRIUR3_28695−P1
TRIUR3_28014−P1
TRIUR3_27788−P1
TRIUR3_27516−P1
TRIUR3_26524−P1
TRIUR3_22842−P1
TRIUR3_22319−P1
TRIUR3_22318−P1
TRIUR3_22096−P1
TRIUR3_21986−P1
TRIUR3_21487−P1
TRIUR3_20906−P1
TRIUR3_20895−P1
Domain Color
LRRNT_2 Family
LRR_4 Repeat
LRR_8 Repeat
PK_Tyr_Ser−Thr Domain
Pkinase Domain
0
500
1000
1500

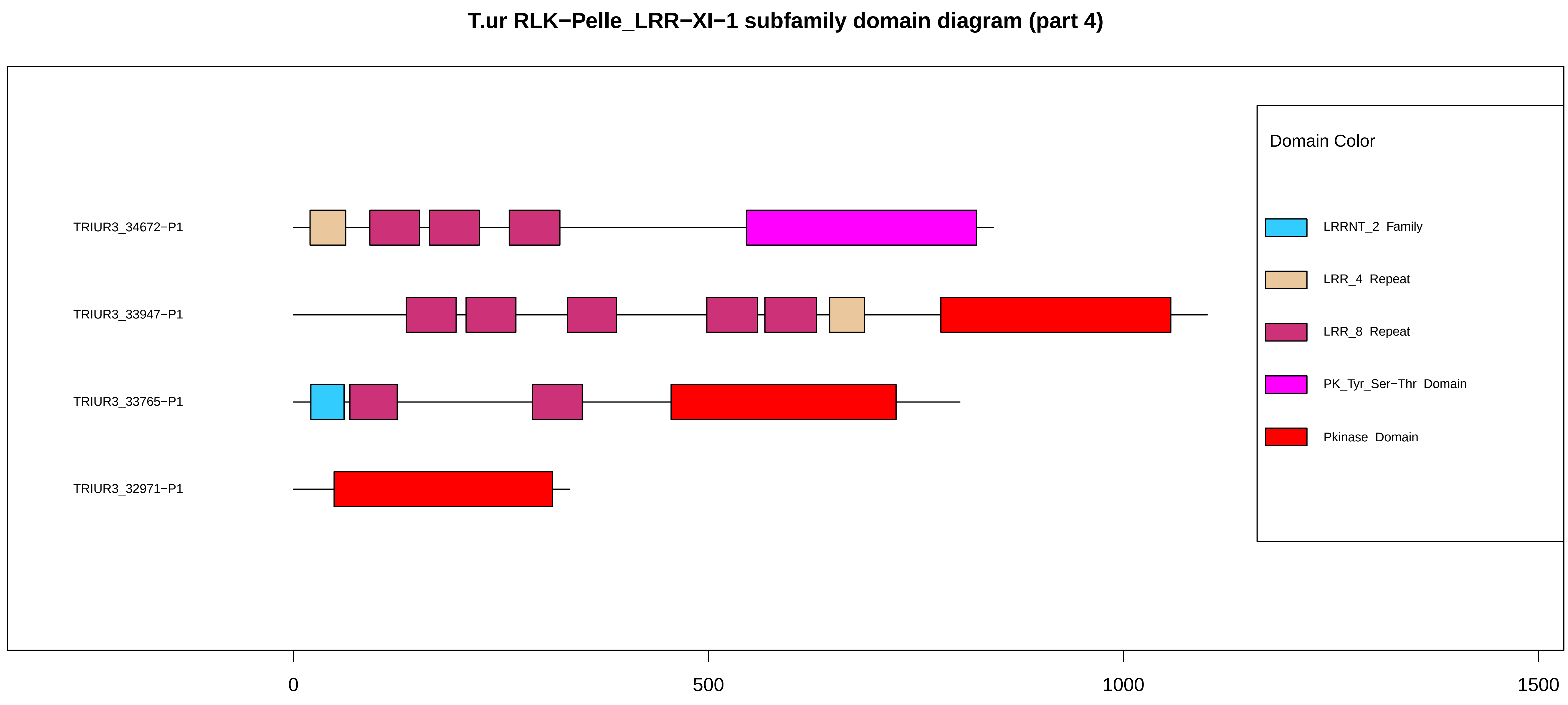

T.ur RLK−Pelle_LRR−XI−1 subfamily domain diagram (part 4)
TRIUR3_34672−P1
TRIUR3_33947−P1
TRIUR3_33765−P1
TRIUR3_32971−P1
Domain Color
LRRNT_2 Family
LRR_4 Repeat
LRR_8 Repeat
PK_Tyr_Ser−Thr Domain
Pkinase Domain
0
500
1000
1500

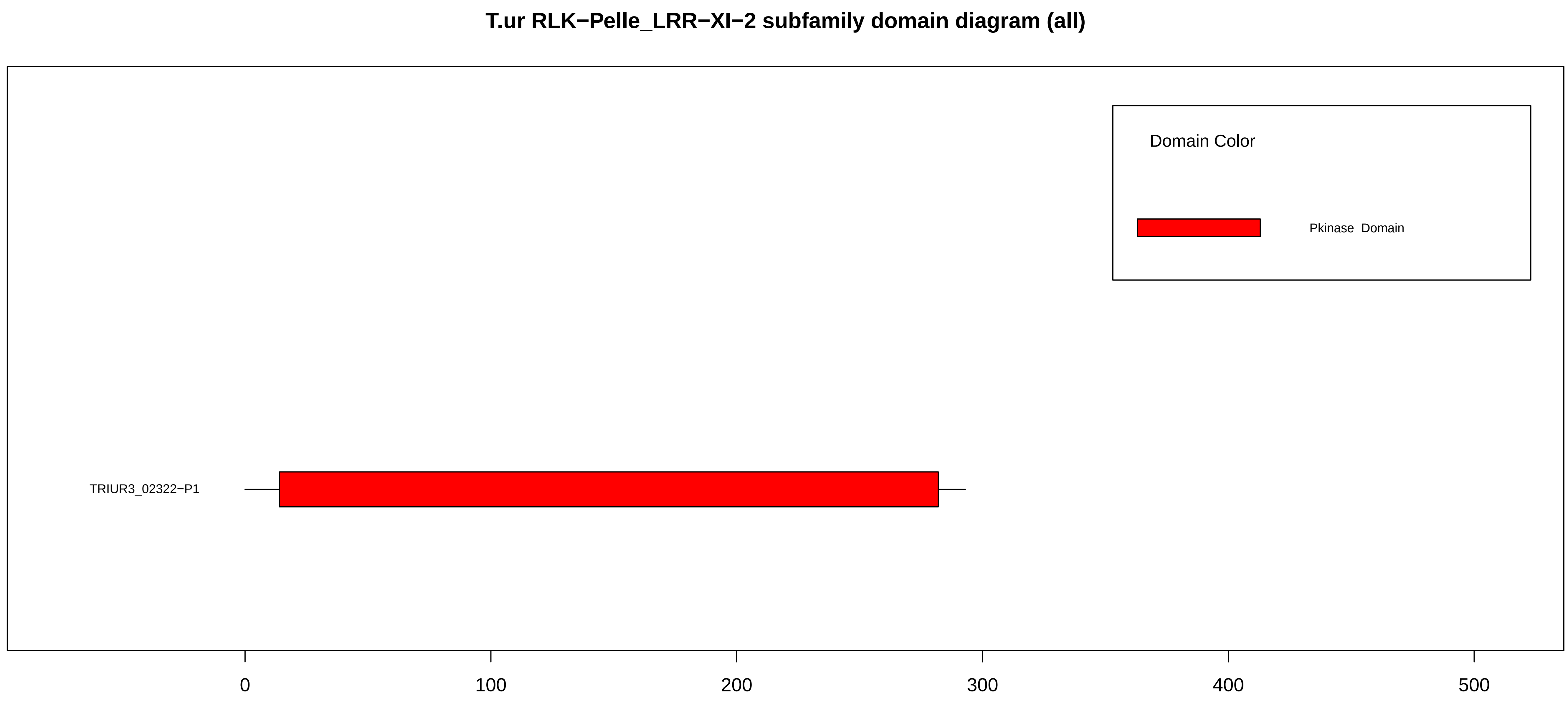
T.ur RLK−Pelle_LRR−XI−2 subfamily domain diagram (all)
Domain Color
Pkinase Domain
TRIUR3_02322−P1
0
100
200
300
400
500

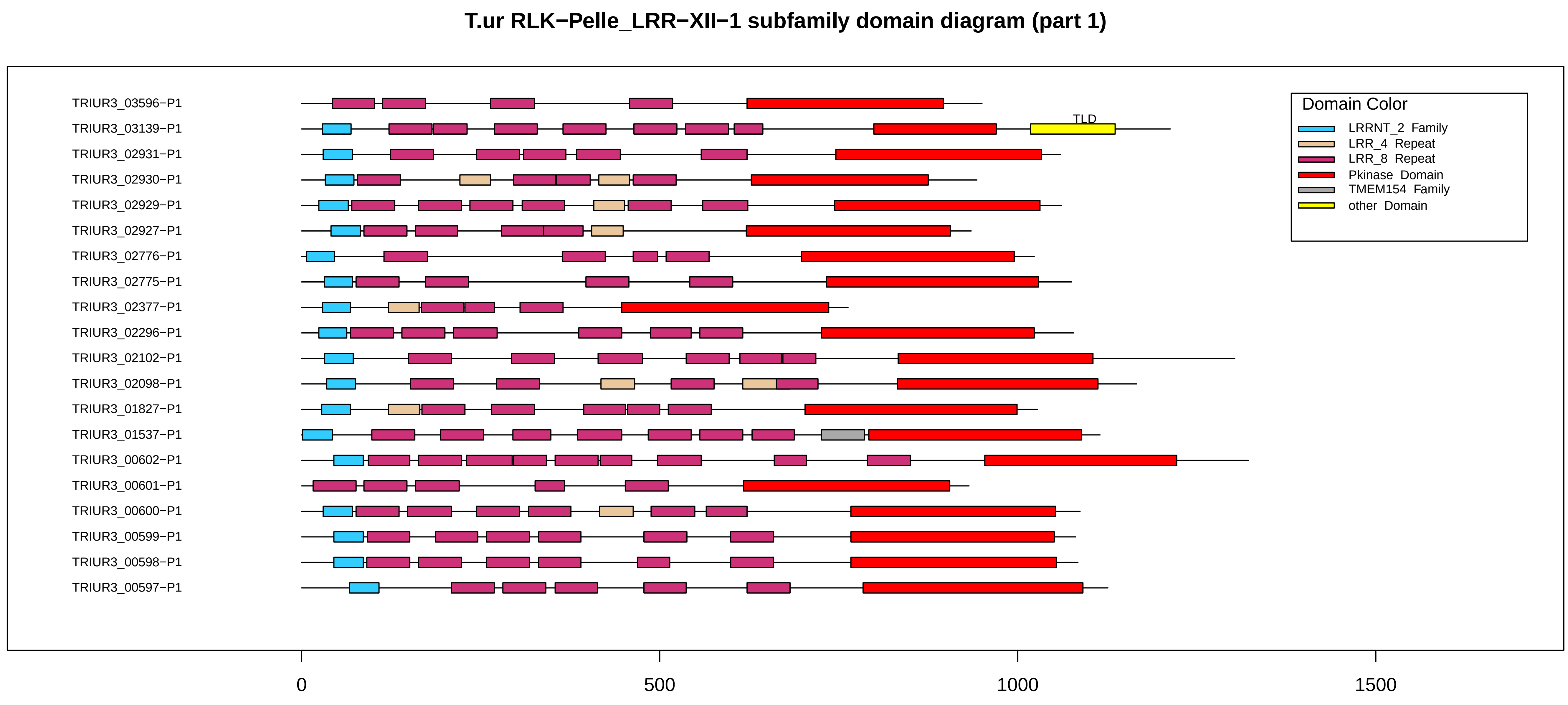
T.ur RLK−Pelle_LRR−XII−1 subfamily domain diagram (part 1)
TRIUR3_03596−P1
TRIUR3_03139−P1
TRIUR3_02931−P1
TRIUR3_02930−P1
TRIUR3_02929−P1
TRIUR3_02927−P1
TRIUR3_02776−P1
TRIUR3_02775−P1
TRIUR3_02377−P1
TRIUR3_02296−P1
TRIUR3_02102−P1
TRIUR3_02098−P1
TRIUR3_01827−P1
TRIUR3_01537−P1
TRIUR3_00602−P1
TRIUR3_00601−P1
TRIUR3_00600−P1
TRIUR3_00599−P1
TRIUR3_00598−P1
TRIUR3_00597−P1
TLD
Domain Color
LRRNT_2 Family
LRR_4 Repeat
LRR_8 Repeat
Pkinase Domain
TMEM154 Family
other Domain
0
500
1000
1500

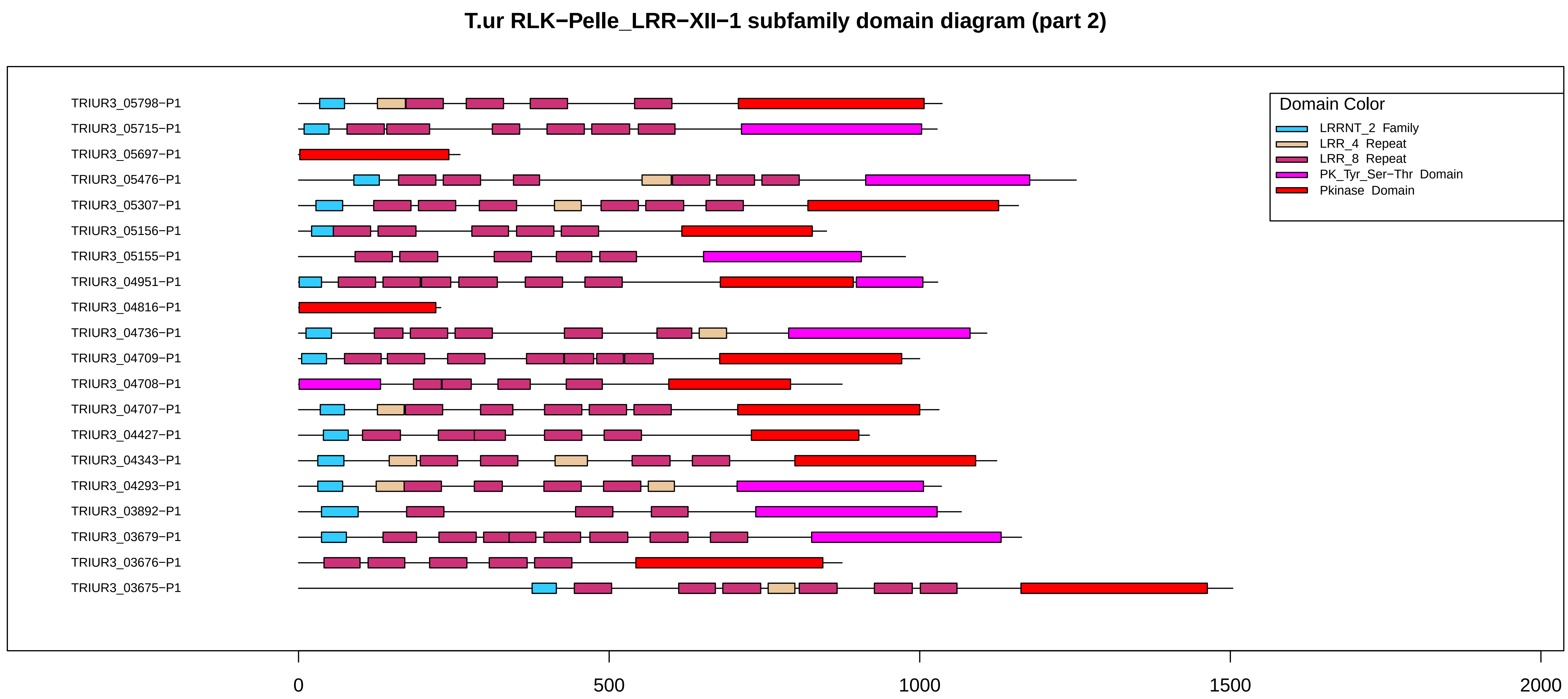
T.ur RLK−Pelle_LRR−XII−1 subfamily domain diagram (part 2)
TRIUR3_05798−P1
TRIUR3_05715−P1
TRIUR3_05697−P1
TRIUR3_05476−P1
TRIUR3_05307−P1
TRIUR3_05156−P1
TRIUR3_05155−P1
TRIUR3_04951−P1
TRIUR3_04816−P1
TRIUR3_04736−P1
TRIUR3_04709−P1
TRIUR3_04708−P1
TRIUR3_04707−P1
TRIUR3_04427−P1
TRIUR3_04343−P1
TRIUR3_04293−P1
TRIUR3_03892−P1
TRIUR3_03679−P1
TRIUR3_03676−P1
TRIUR3_03675−P1
Domain Color
LRRNT_2 Family
LRR_4 Repeat
LRR_8 Repeat
PK_Tyr_Ser−Thr Domain
Pkinase Domain
0
500
1000
1500
2000

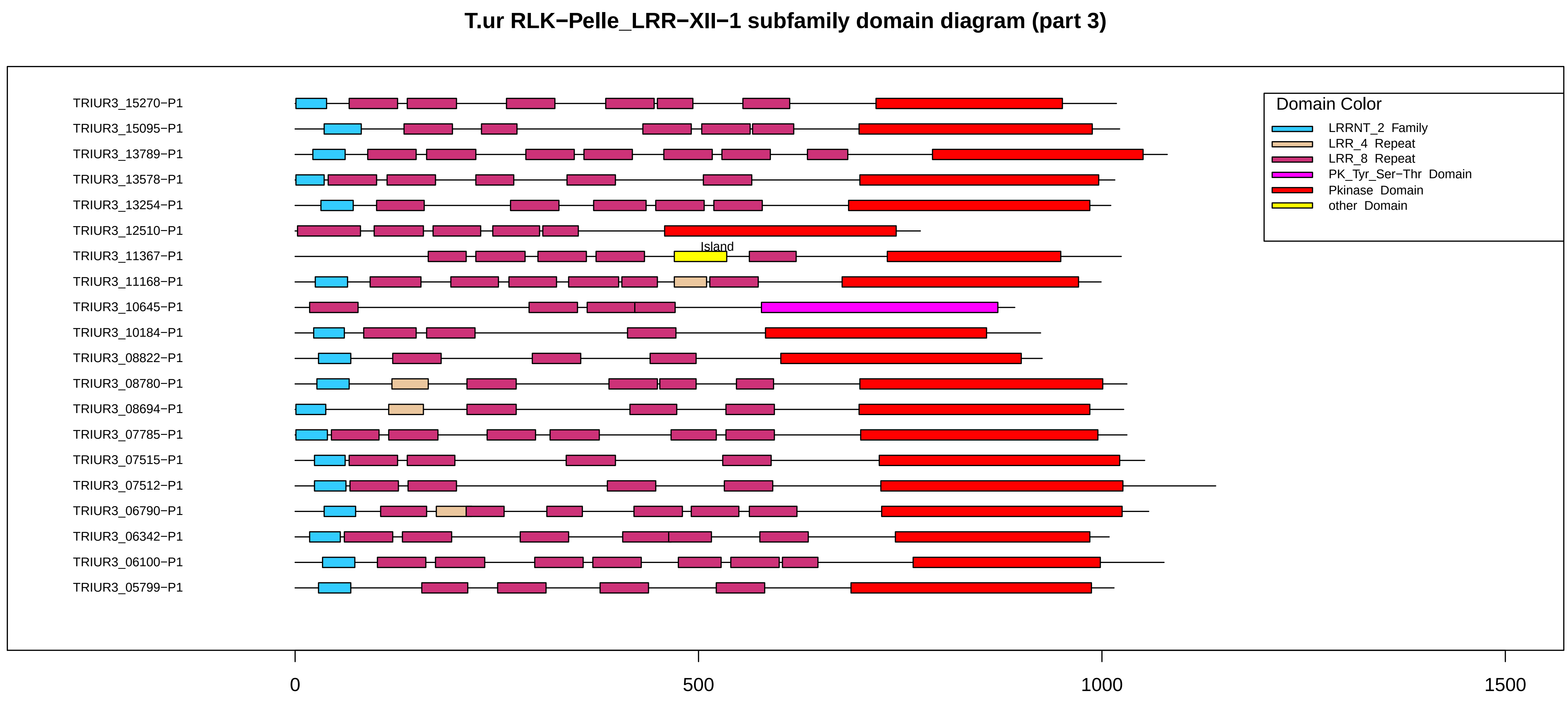
T.ur RLK−Pelle_LRR−XII−1 subfamily domain diagram (part 3)
TRIUR3_15270−P1
TRIUR3_15095−P1
TRIUR3_13789−P1
TRIUR3_13578−P1
TRIUR3_13254−P1
TRIUR3_12510−P1
TRIUR3_11367−P1
TRIUR3_11168−P1
TRIUR3_10645−P1
TRIUR3_10184−P1
TRIUR3_08822−P1
TRIUR3_08780−P1
TRIUR3_08694−P1
TRIUR3_07785−P1
TRIUR3_07515−P1
TRIUR3_07512−P1
TRIUR3_06790−P1
TRIUR3_06342−P1
TRIUR3_06100−P1
TRIUR3_05799−P1
Island
Domain Color
LRRNT_2 Family
LRR_4 Repeat
LRR_8 Repeat
PK_Tyr_Ser−Thr Domain
Pkinase Domain
other Domain
0
500
1000
1500

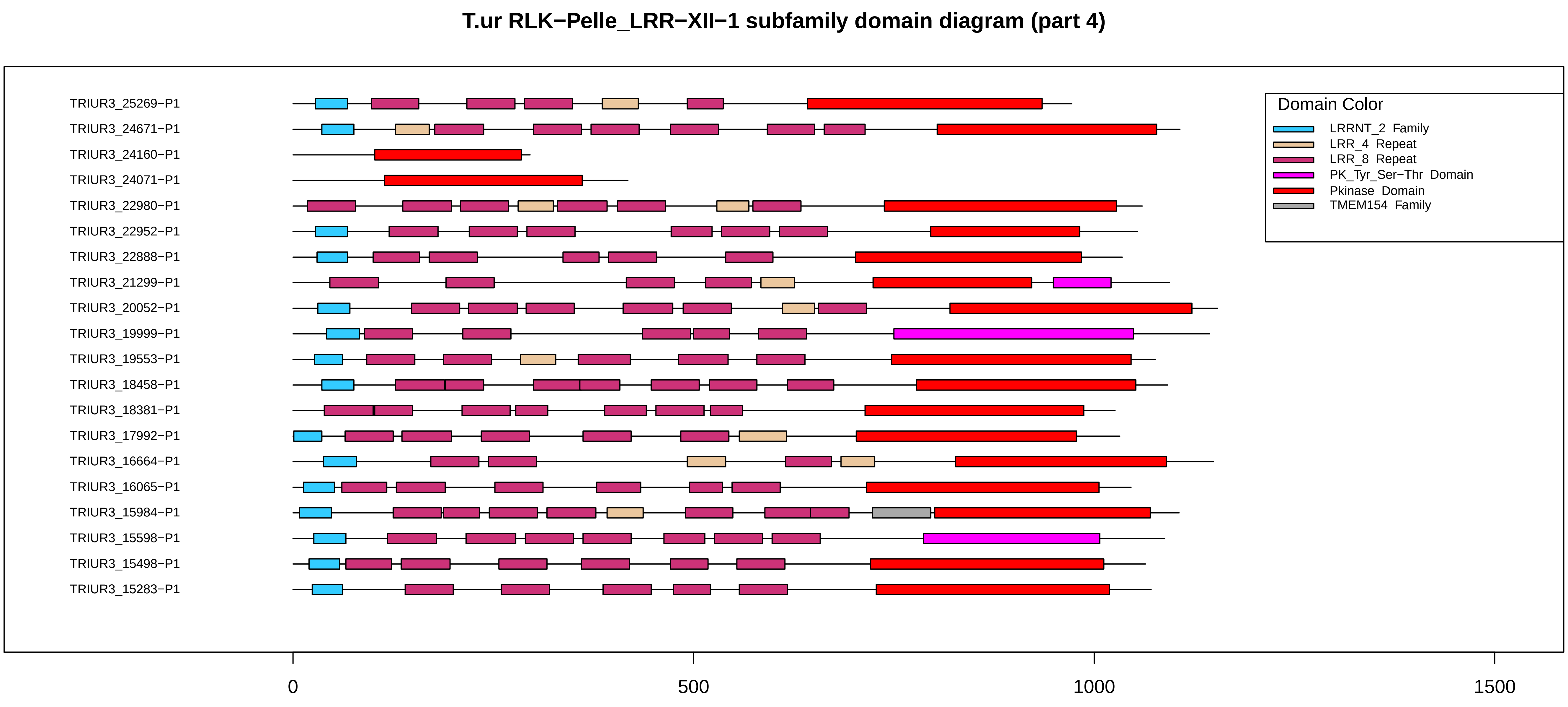
T.ur RLK−Pelle_LRR−XII−1 subfamily domain diagram (part 4)
TRIUR3_25269−P1
TRIUR3_24671−P1
TRIUR3_24160−P1
TRIUR3_24071−P1
TRIUR3_22980−P1
TRIUR3_22952−P1
TRIUR3_22888−P1
TRIUR3_21299−P1
TRIUR3_20052−P1
TRIUR3_19999−P1
TRIUR3_19553−P1
TRIUR3_18458−P1
TRIUR3_18381−P1
TRIUR3_17992−P1
TRIUR3_16664−P1
TRIUR3_16065−P1
TRIUR3_15984−P1
TRIUR3_15598−P1
TRIUR3_15498−P1
TRIUR3_15283−P1
Domain Color
LRRNT_2 Family
LRR_4 Repeat
LRR_8 Repeat
PK_Tyr_Ser−Thr Domain
Pkinase Domain
TMEM154 Family
0
500
1000
1500

T.ur RLK−Pelle_LRR−XII−1 subfamily domain diagram (part 5)

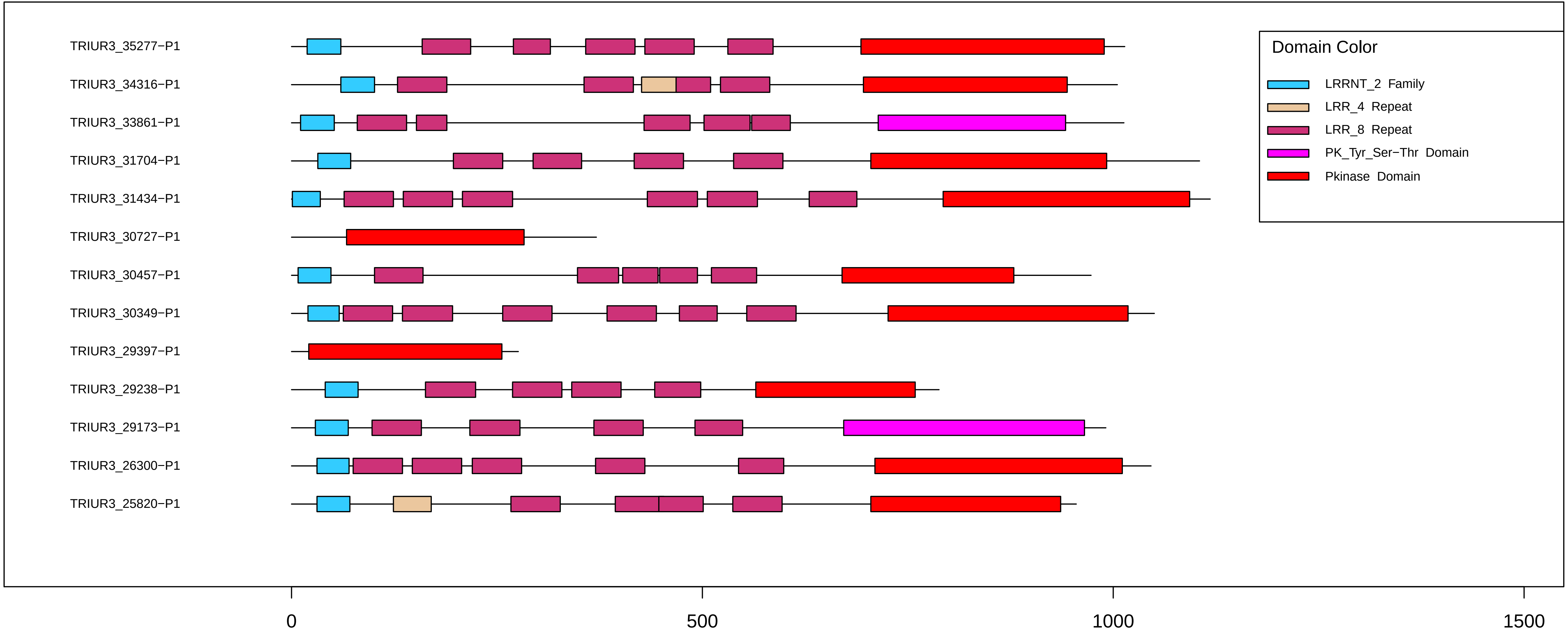

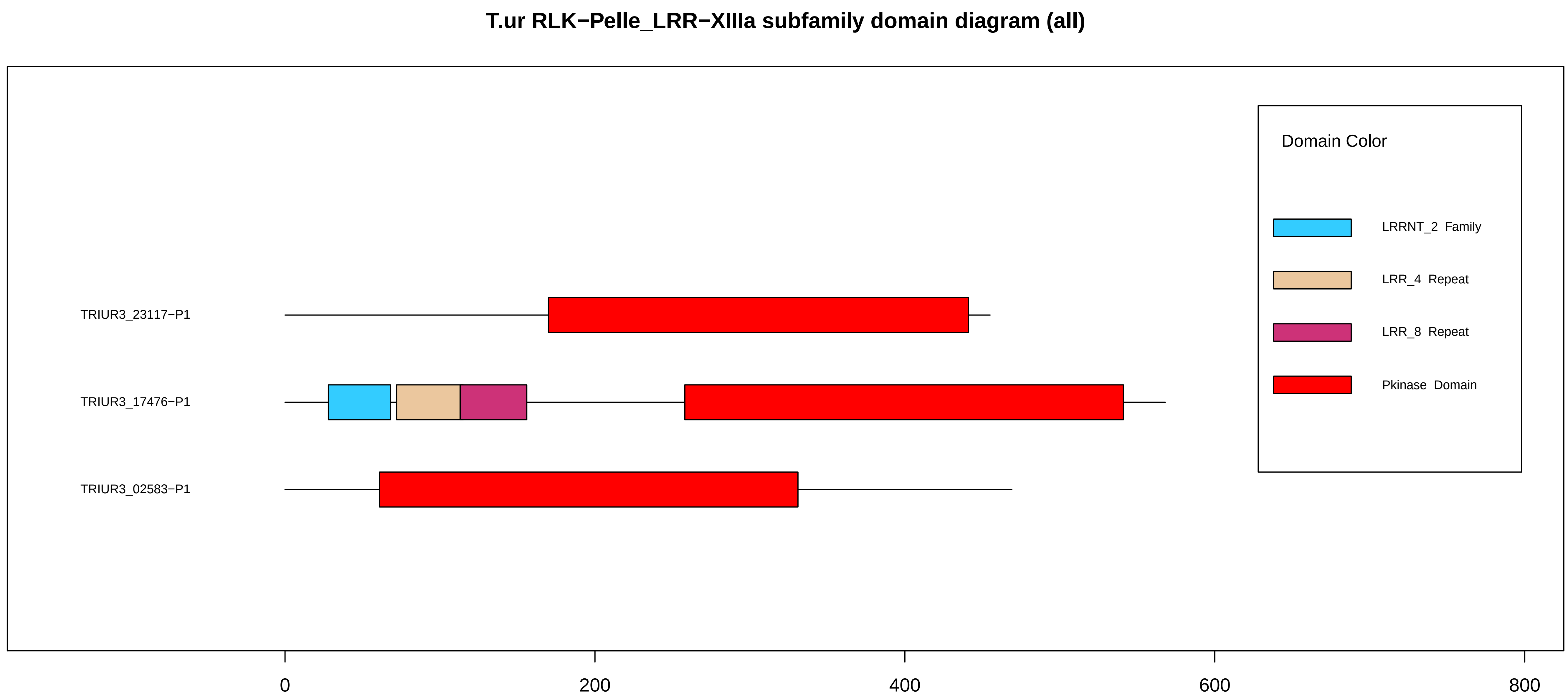
T.ur RLK−Pelle_LRR−XIIIa subfamily domain diagram (all)
TRIUR3_23117−P1
TRIUR3_17476−P1
TRIUR3_02583−P1
Domain Color
LRRNT_2 Family
LRR_4 Repeat
LRR_8 Repeat
Pkinase Domain
0
200
400
600
800

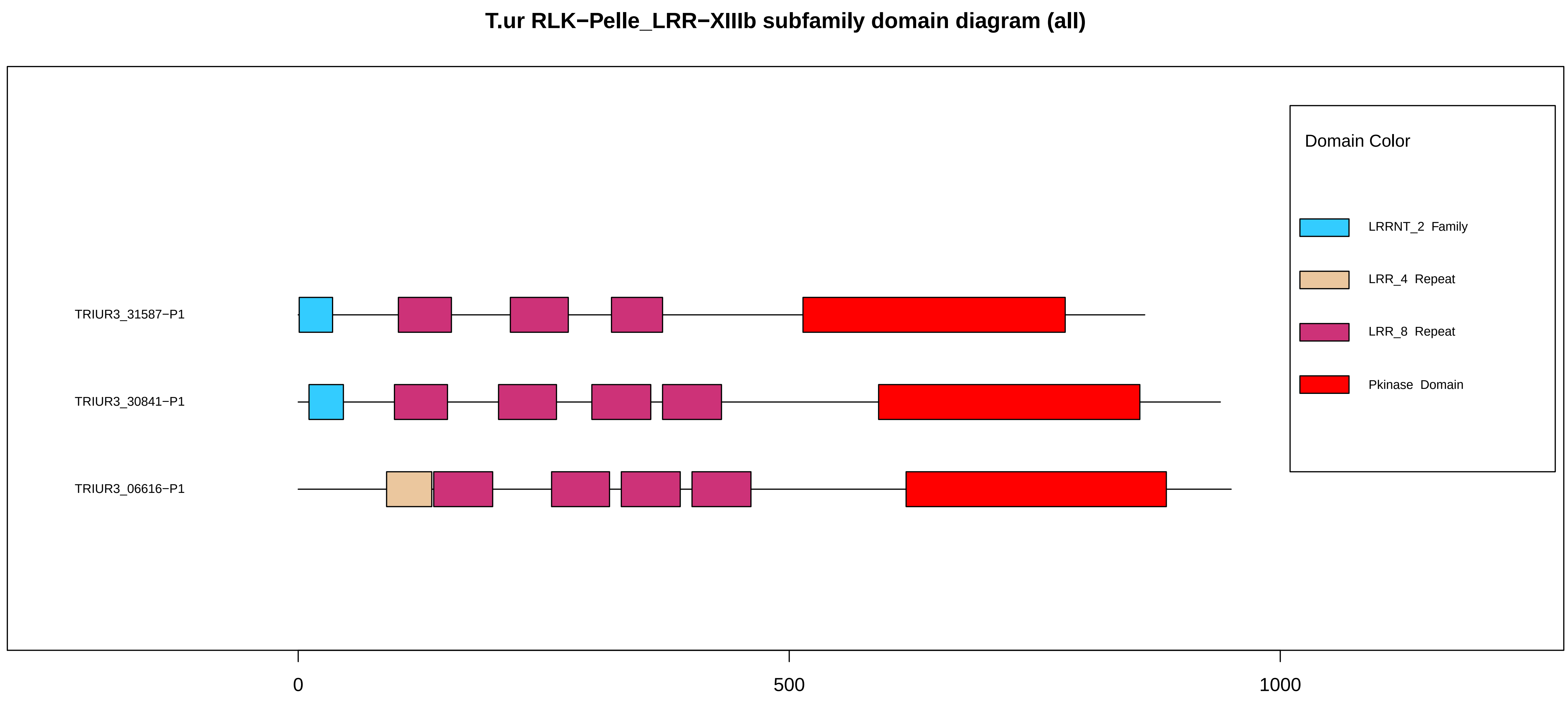

T.ur RLK−Pelle_LRR−XIIIb subfamily domain diagram (all)
TRIUR3_31587−P1
TRIUR3_30841−P1
TRIUR3_06616−P1
0
500
1000
Domain Color
LRRNT_2 Family
LRR_4 Repeat
LRR_8 Repeat
Pkinase Domain

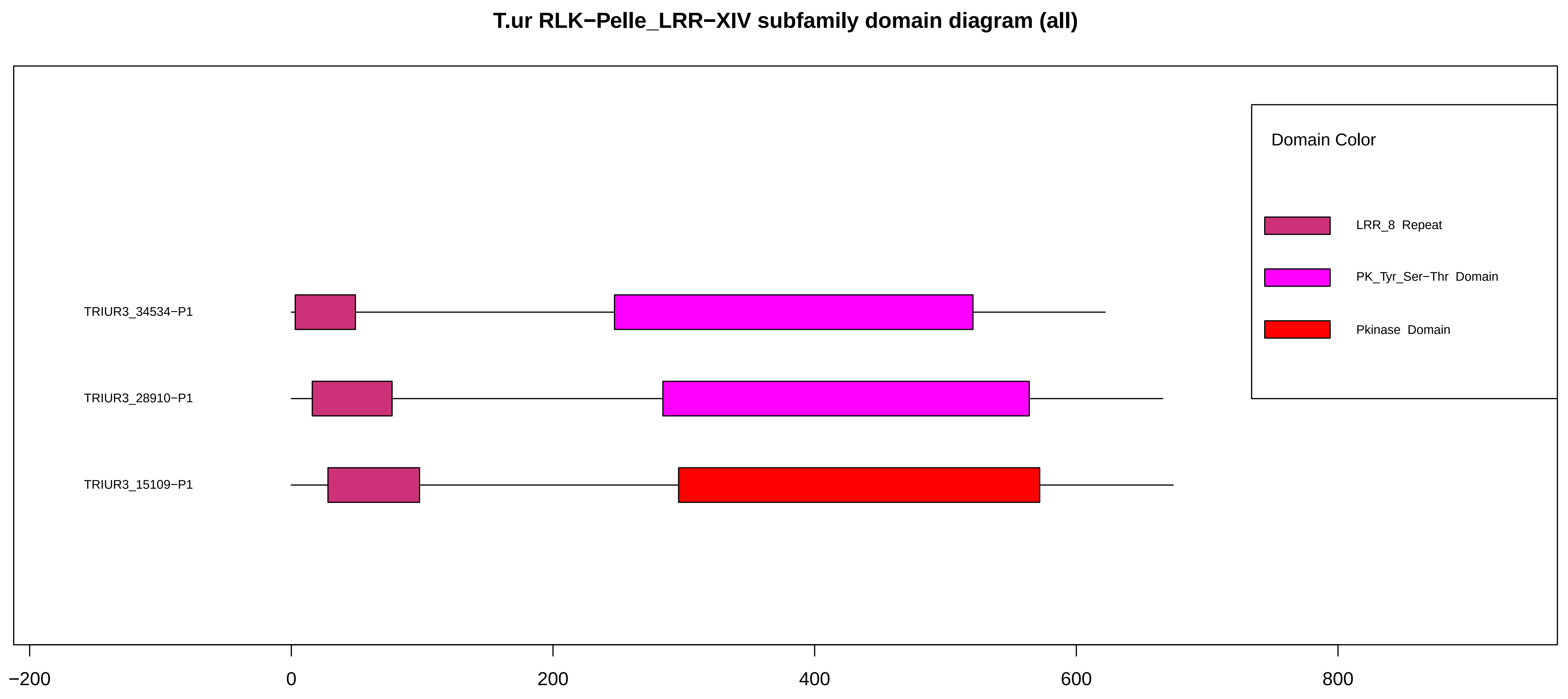
T.ur RLK−Pelle_LRR−XIV subfamily domain diagram (all)
Domain Color
LRR_8 Repeat
PK_Tyr_Ser−Thr Domain
Pkinase Domain
TRIUR3_34534−P1
TRIUR3_28910−P1
TRIUR3_15109−P1
−200
0
200
400
600
800

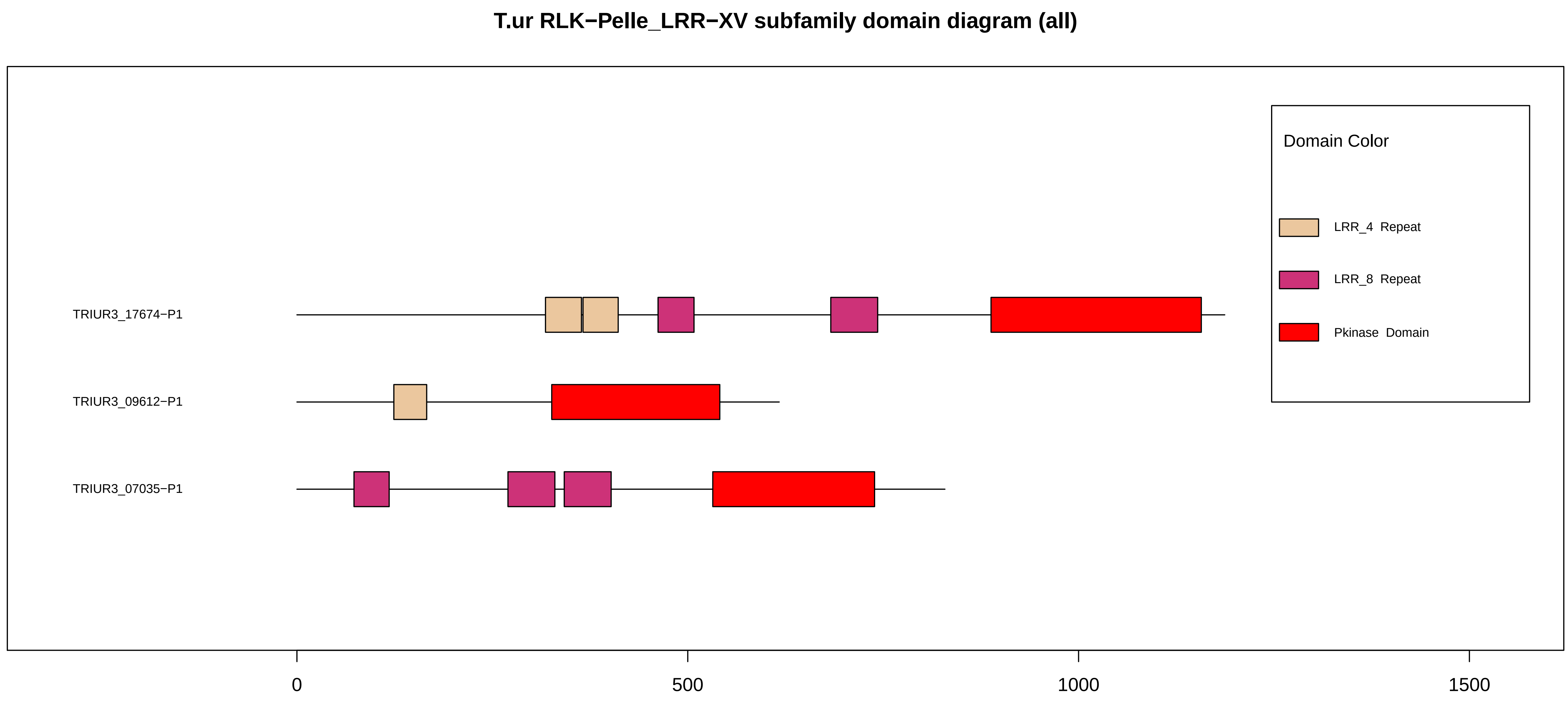

T.ur RLK−Pelle_LRR−XV subfamily domain diagram (all)
Domain Color
LRR_4 Repeat
LRR_8 Repeat
Pkinase Domain
TRIUR3_17674−P1
TRIUR3_09612−P1
TRIUR3_07035−P1
0
500
1000
1500

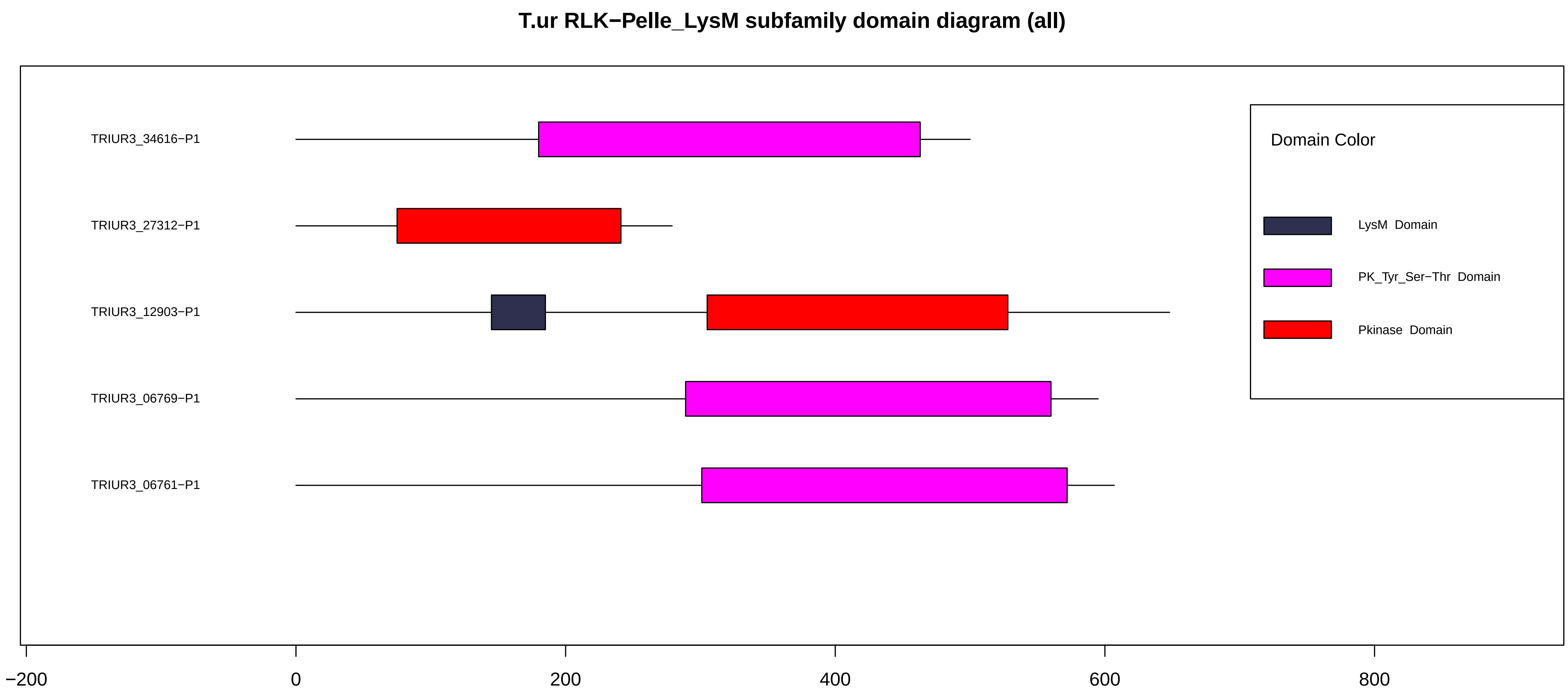

T.ur RLK−Pelle_LysM subfamily domain diagram (all)
TRIUR3_34616−P1
TRIUR3_27312−P1
TRIUR3_12903−P1
TRIUR3_06769−P1
TRIUR3_06761−P1
Domain Color
LysM Domain
PK_Tyr_Ser−Thr Domain
Pkinase Domain
−200
0
200
400
600
800

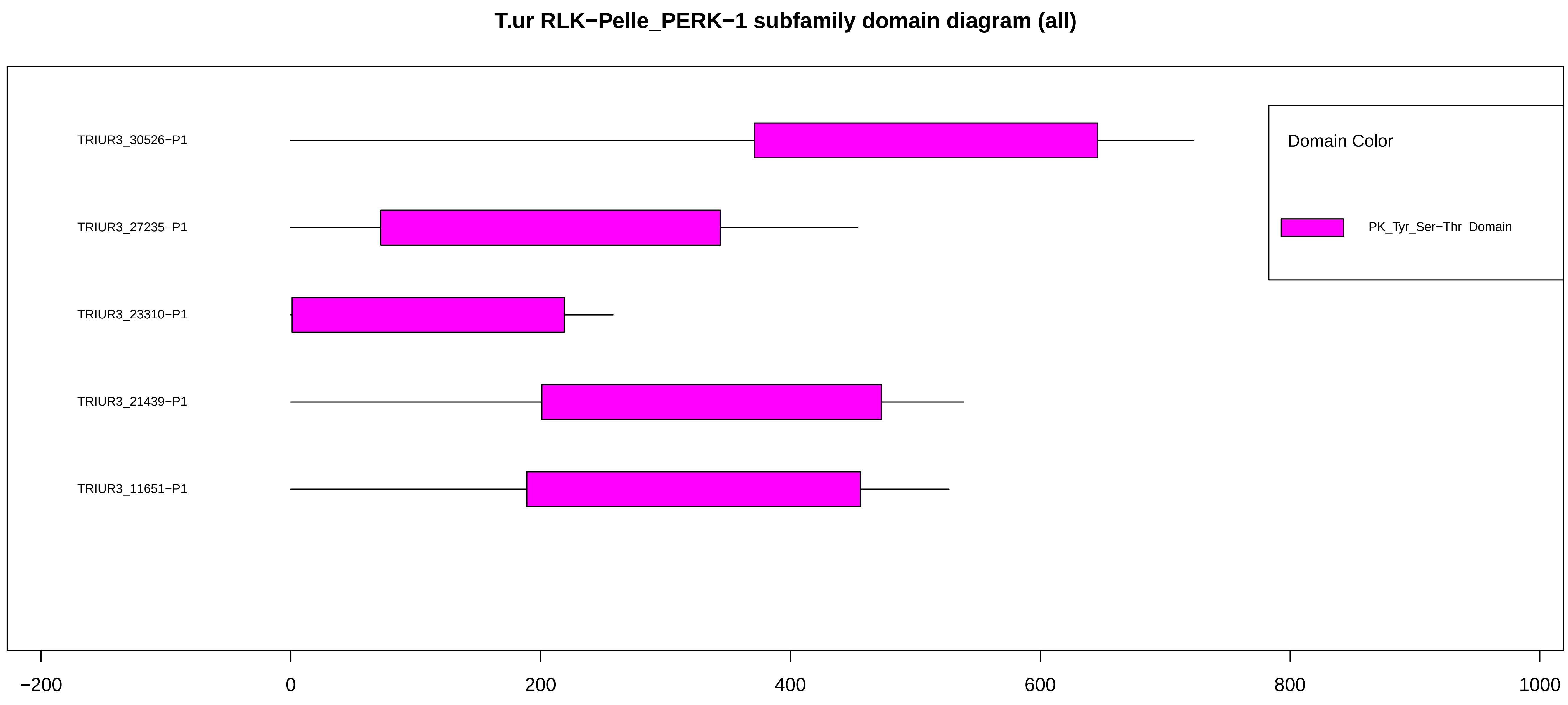

T.ur RLK−Pelle_PERK−1 subfamily domain diagram (all)
TRIUR3_30526−P1
TRIUR3_27235−P1
TRIUR3_23310−P1
TRIUR3_21439−P1
TRIUR3_11651−P1
Domain Color
PK_Tyr_Ser−Thr Domain
−200
0
200
400
600
800
1000

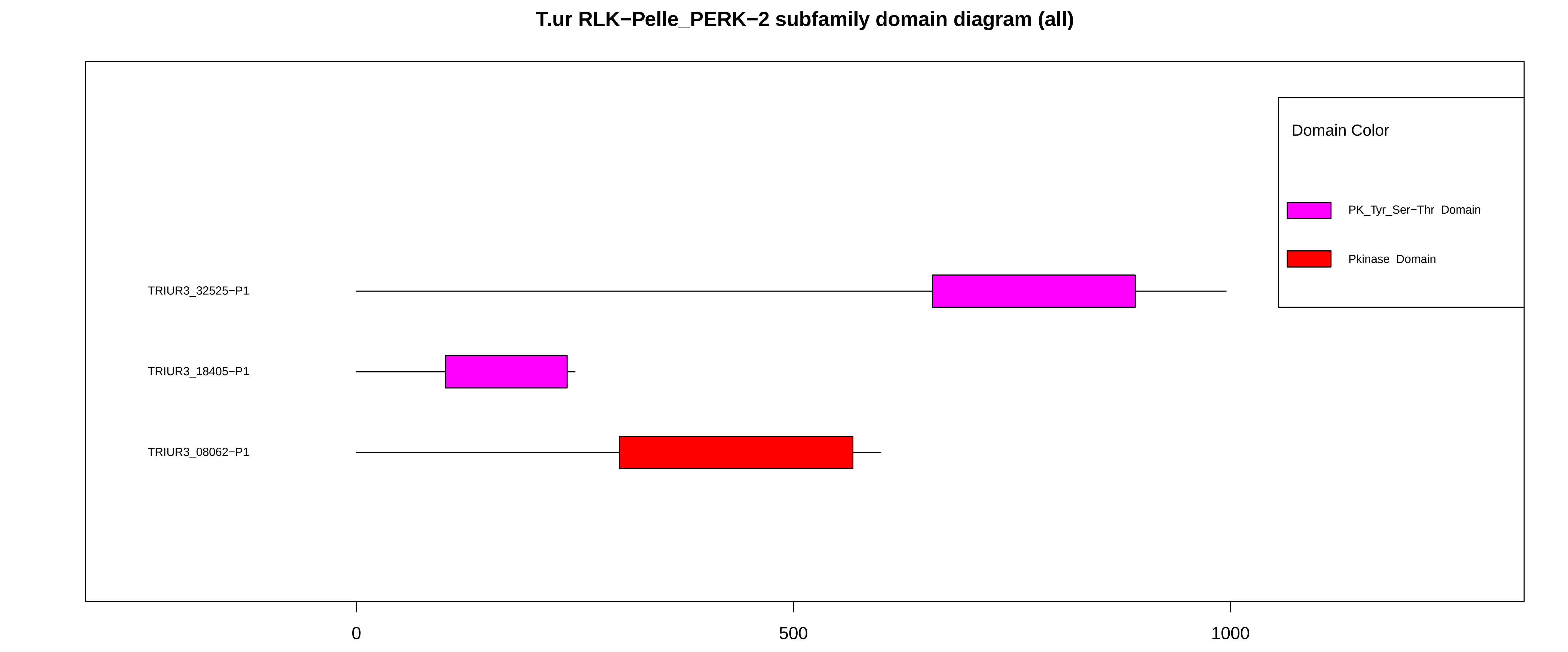
T.ur RLK−Pelle_PERK−2 subfamily domain diagram (all)
Domain Color
PK_Tyr_Ser−Thr Domain
Pkinase Domain
TRIUR3_32525−P1
TRIUR3_18405−P1
TRIUR3_08062−P1
0
500
1000

T.ur RLK−Pelle_RKF3 subfamily domain diagram (all)

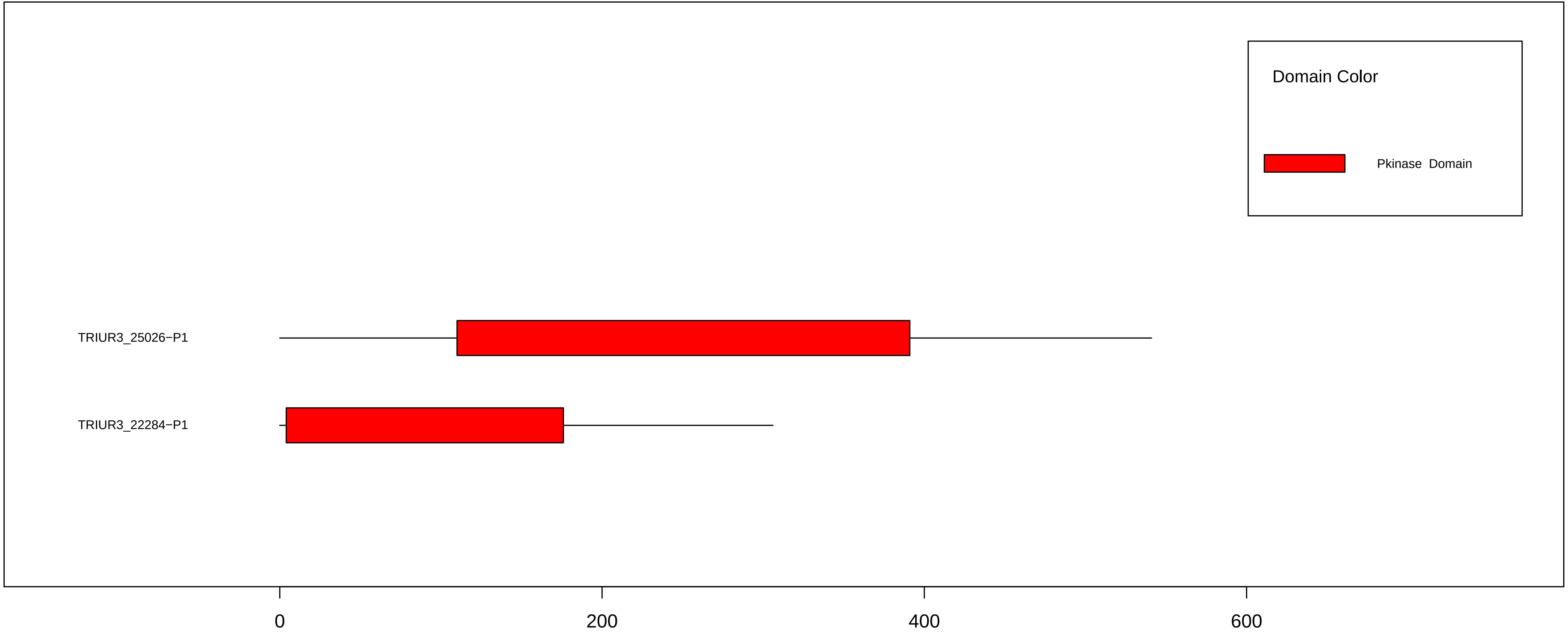

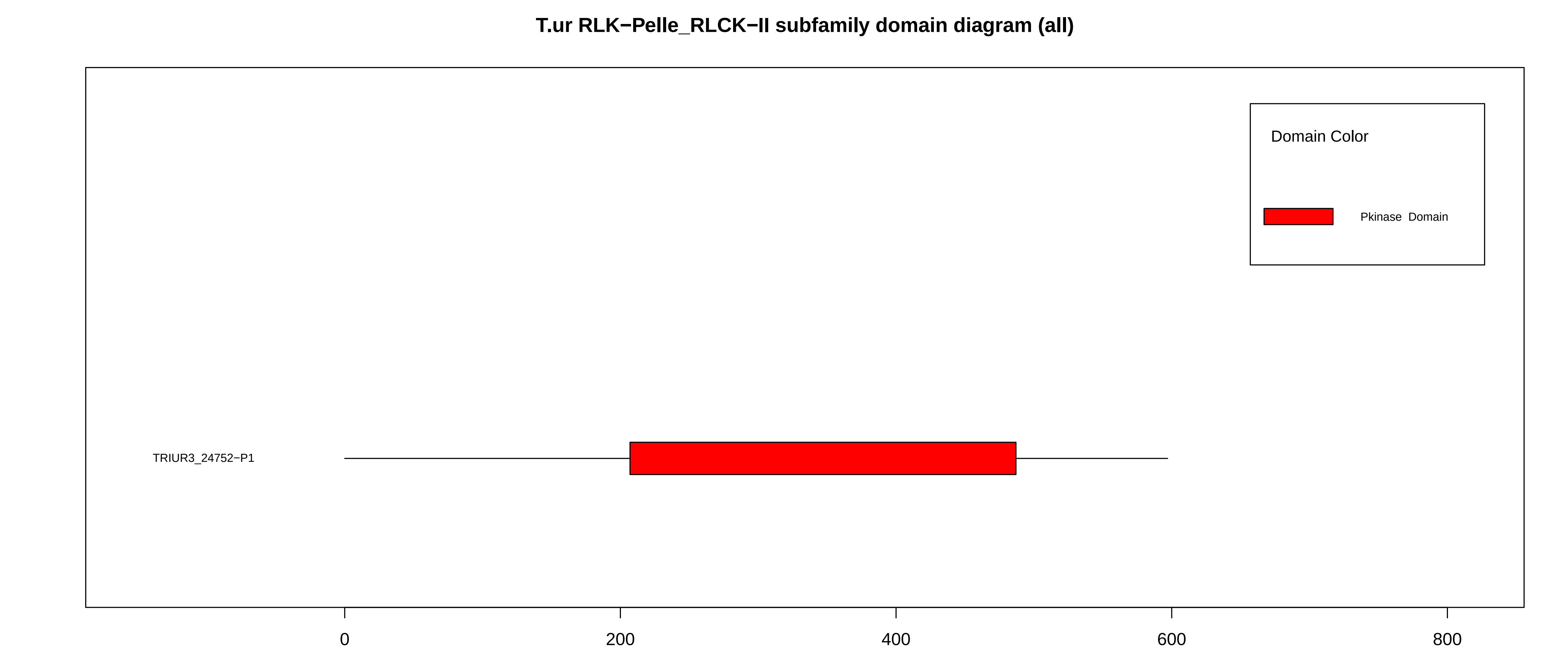
T.ur RLK−Pelle_RLCK−II subfamily domain diagram (all)
Domain Color
Pkinase Domain
TRIUR3_24752−P1
0
200
400
600
800

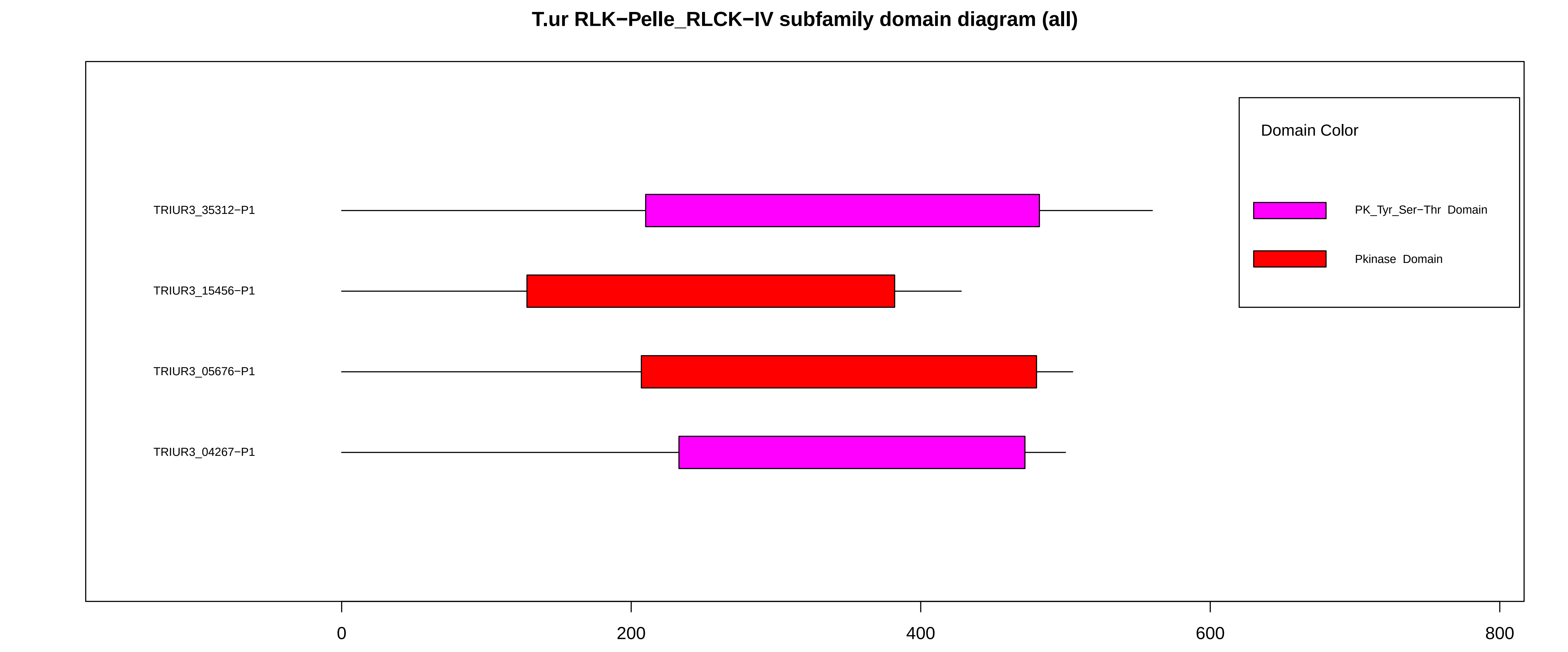
T.ur RLK−Pelle_RLCK−IV subfamily domain diagram (all)
TRIUR3_35312−P1
TRIUR3_15456−P1
TRIUR3_05676−P1
TRIUR3_04267−P1
Domain Color
PK_Tyr_Ser−Thr Domain
Pkinase Domain
0
200
400
600
800

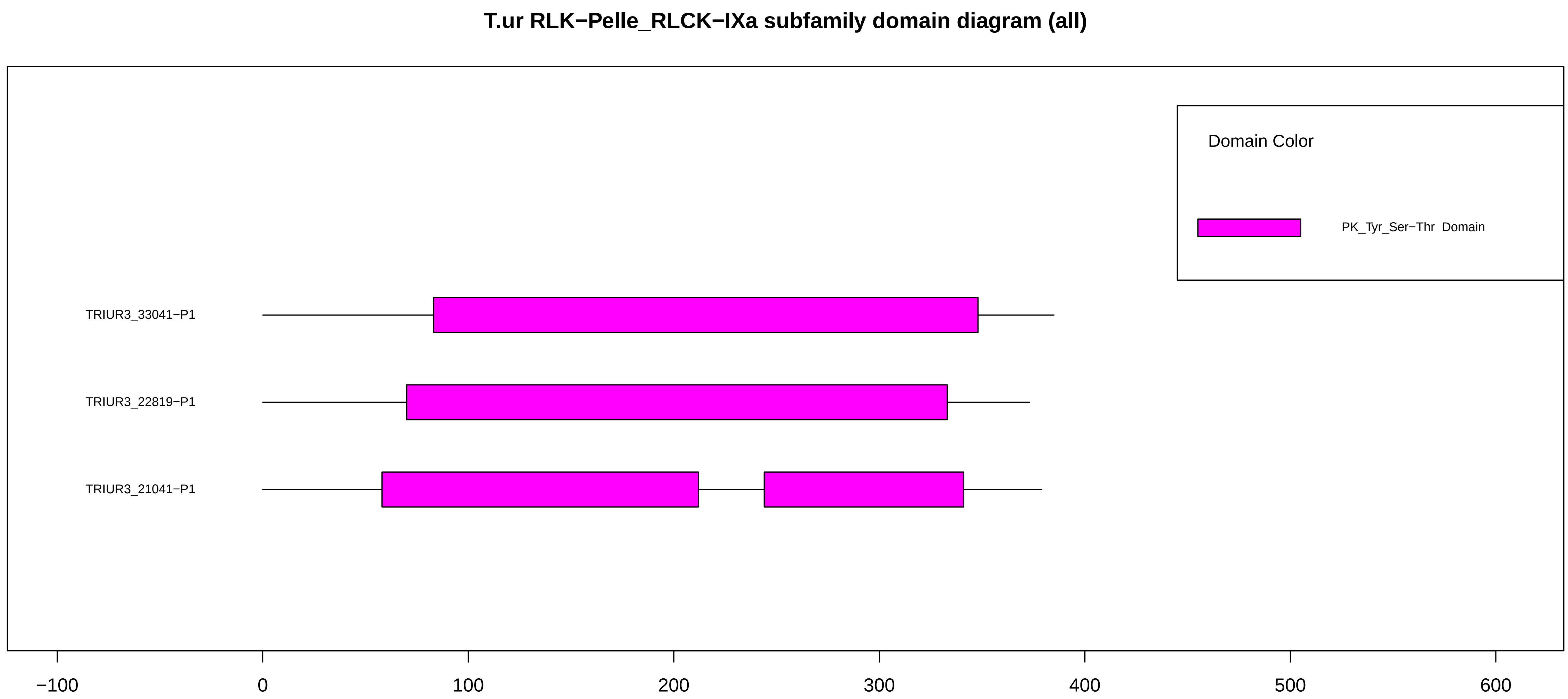
T.ur RLK−Pelle_RLCK−IXa subfamily domain diagram (all)
Domain Color
PK_Tyr_Ser−Thr Domain
TRIUR3_33041−P1
TRIUR3_22819−P1
TRIUR3_21041−P1
−100
0
100
200
300
400
500
600

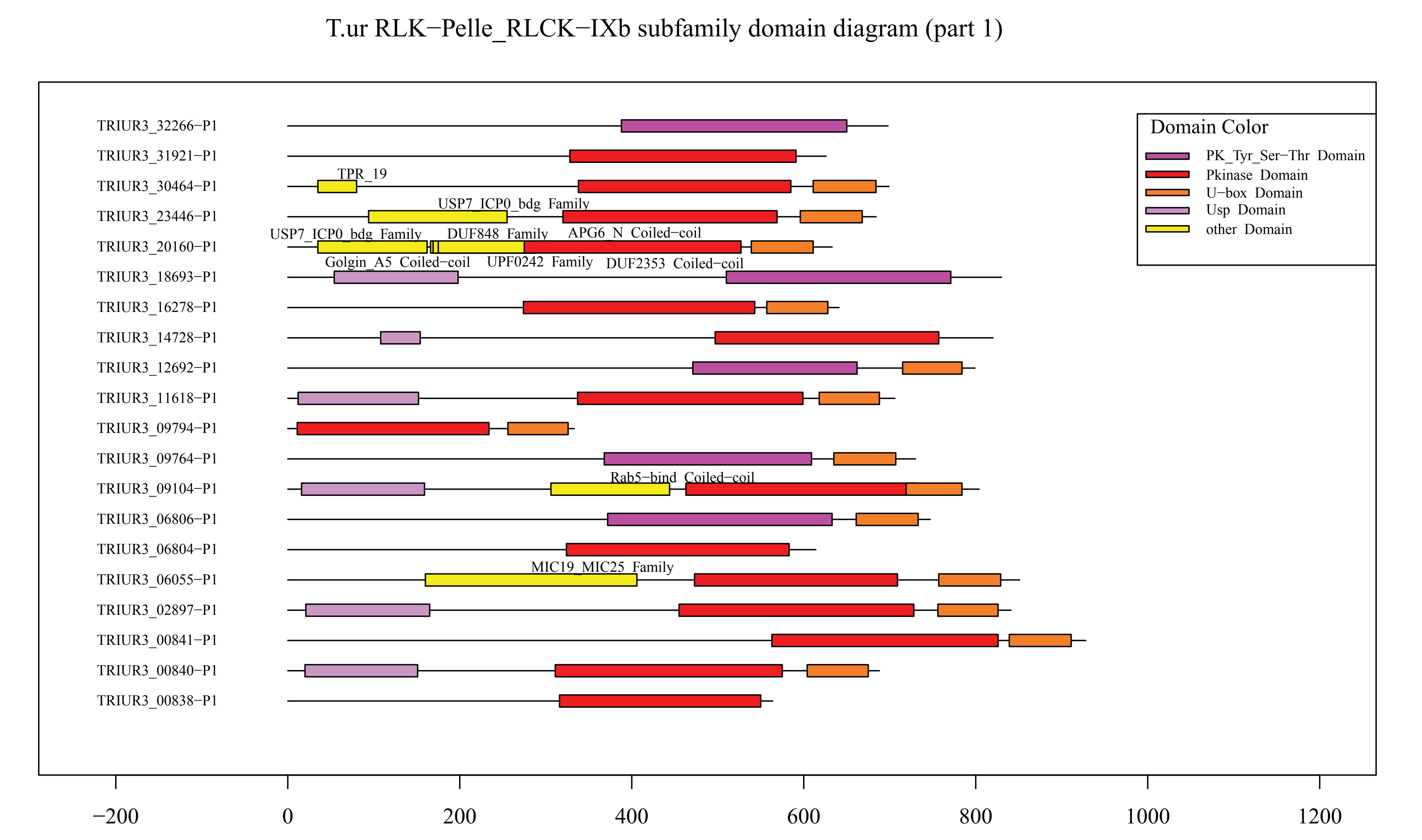

T.ur RLK−Pelle_RLCK−IXb subfamily domain diagram (part 1)
TRIUR3_32266−P1
TRIUR3_31921−P1
TRIUR3_30464−P1
TRIUR3_23446−P1
TRIUR3_20160−P1
TRIUR3_18693−P1
TRIUR3_16278−P1
TRIUR3_14728−P1
TRIUR3_12692−P1
TRIUR3_11618−P1
TRIUR3_09794−P1
TRIUR3_09764−P1
TRIUR3_09104−P1
TRIUR3_06806−P1
TRIUR3_06804−P1
TRIUR3_06055−P1
TRIUR3_02897−P1
TRIUR3_00841−P1
TRIUR3_00840−P1
TRIUR3_00838−P1
TPR_19
USP7_ICP0_bdg Family
USP7_ICP0_bdg Family
DUF848 Family
APG6_N Coiled−coil
Golgin_A5 Coiled−coil
UPF0242 Family
DUF2353 Coiled−coil
Rab5−bind Coiled−coil
MIC19_MIC25 Family
Domain Color
PK_Tyr_Ser−Thr Domain
Pkinase Domain
U−box Domain
Usp Domain
other Domain
−200
0
200
400
600
800
1000
1200

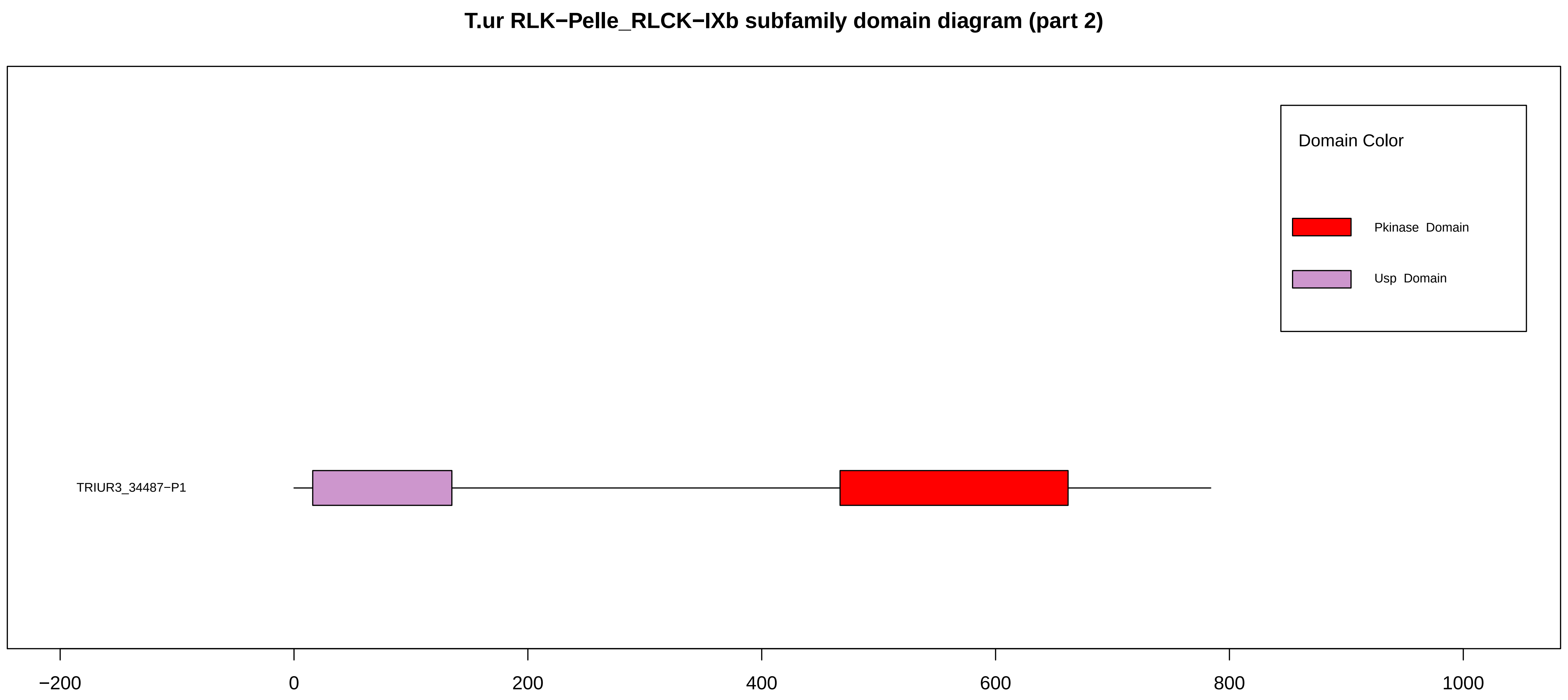
T.ur RLK−Pelle_RLCK−IXb subfamily domain diagram (part 2)
Domain Color
Pkinase Domain
Usp Domain
TRIUR3_34487−P1
−200
0
200
400
600
800
1000

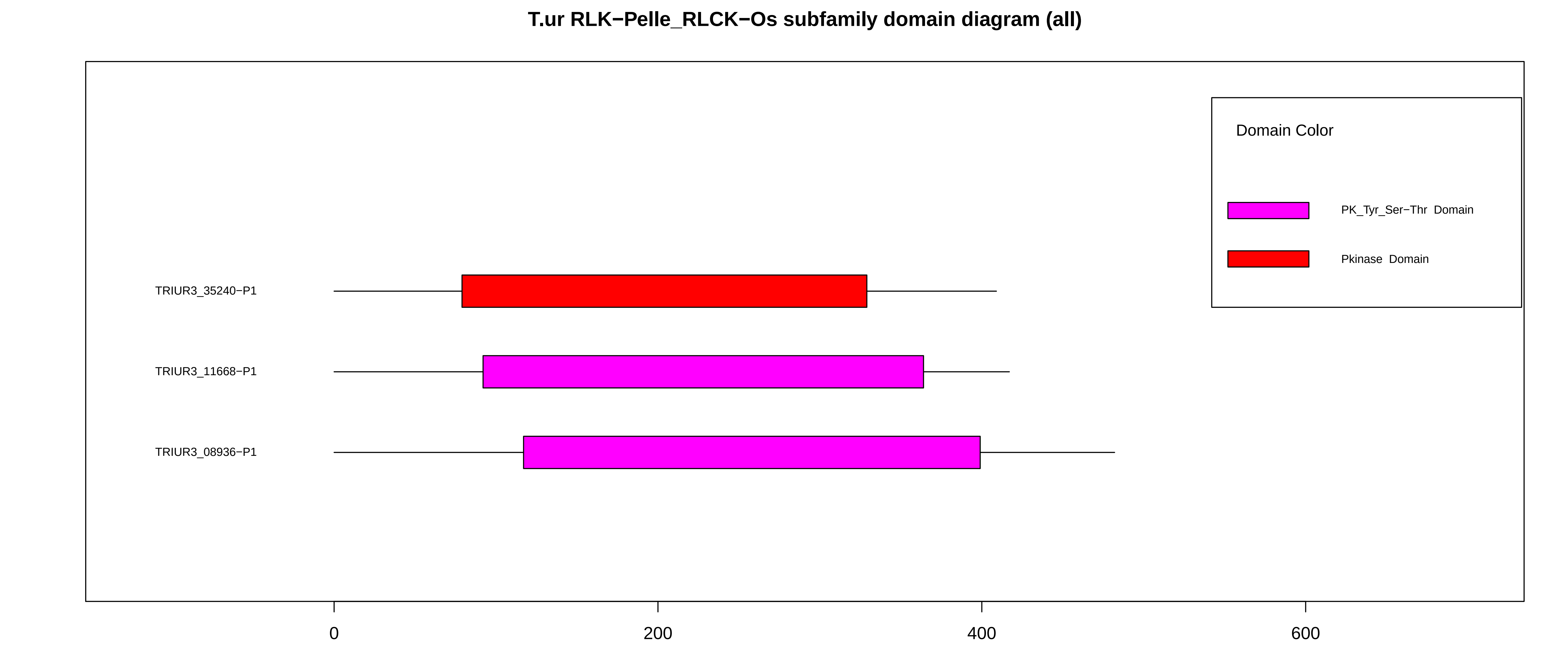

T.ur RLK−Pelle_RLCK−Os subfamily domain diagram (all)
Domain Color
PK_Tyr_Ser−Thr Domain
Pkinase Domain
TRIUR3_35240−P1
TRIUR3_11668−P1
TRIUR3_08936−P1
0
200
400
600

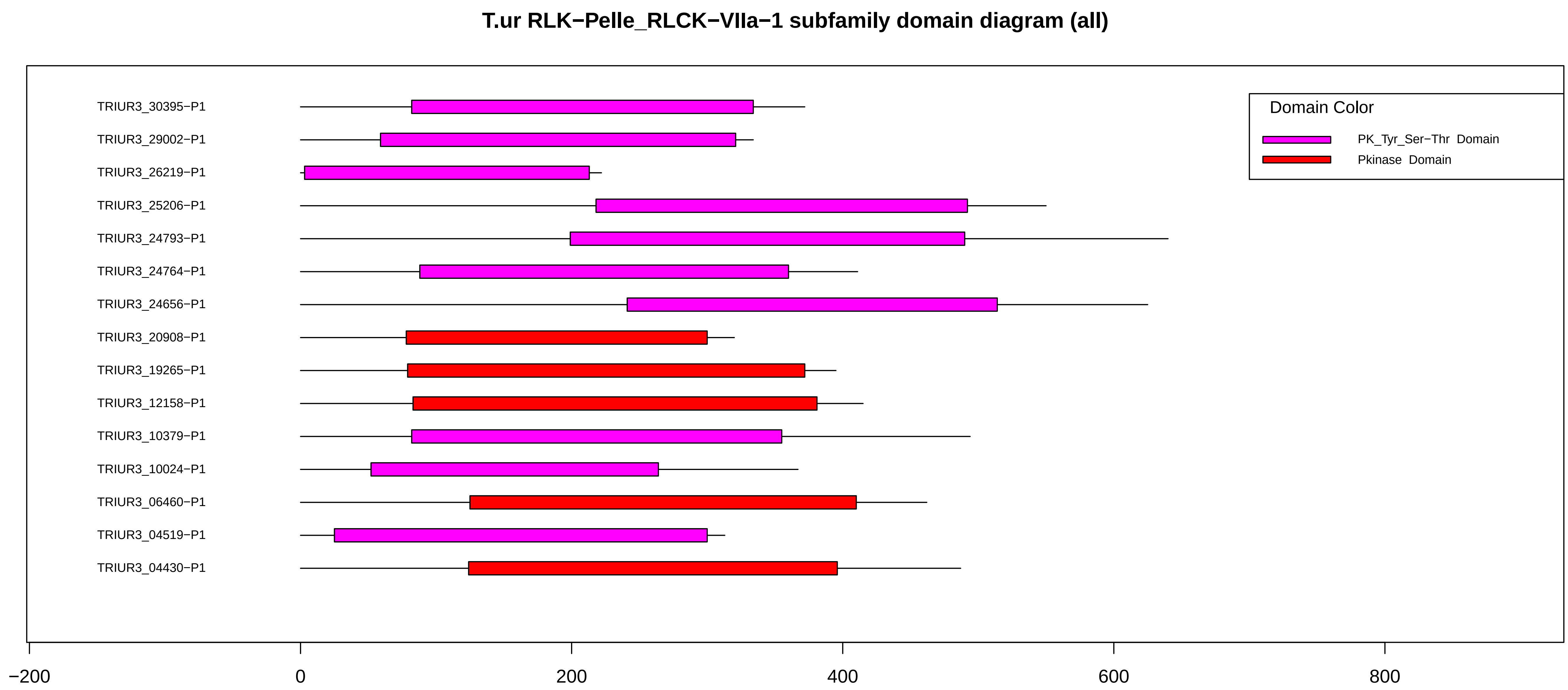

T.ur RLK−Pelle_RLCK−VIIa−1 subfamily domain diagram (all)
TRIUR3_30395−P1
TRIUR3_29002−P1
TRIUR3_26219−P1
TRIUR3_25206−P1
TRIUR3_24793−P1
TRIUR3_24764−P1
TRIUR3_24656−P1
TRIUR3_20908−P1
TRIUR3_19265−P1
TRIUR3_12158−P1
TRIUR3_10379−P1
TRIUR3_10024−P1
TRIUR3_06460−P1
TRIUR3_04519−P1
TRIUR3_04430−P1
Domain Color
PK_Tyr_Ser−Thr Domain
Pkinase Domain
−200
0
200
400
600
800

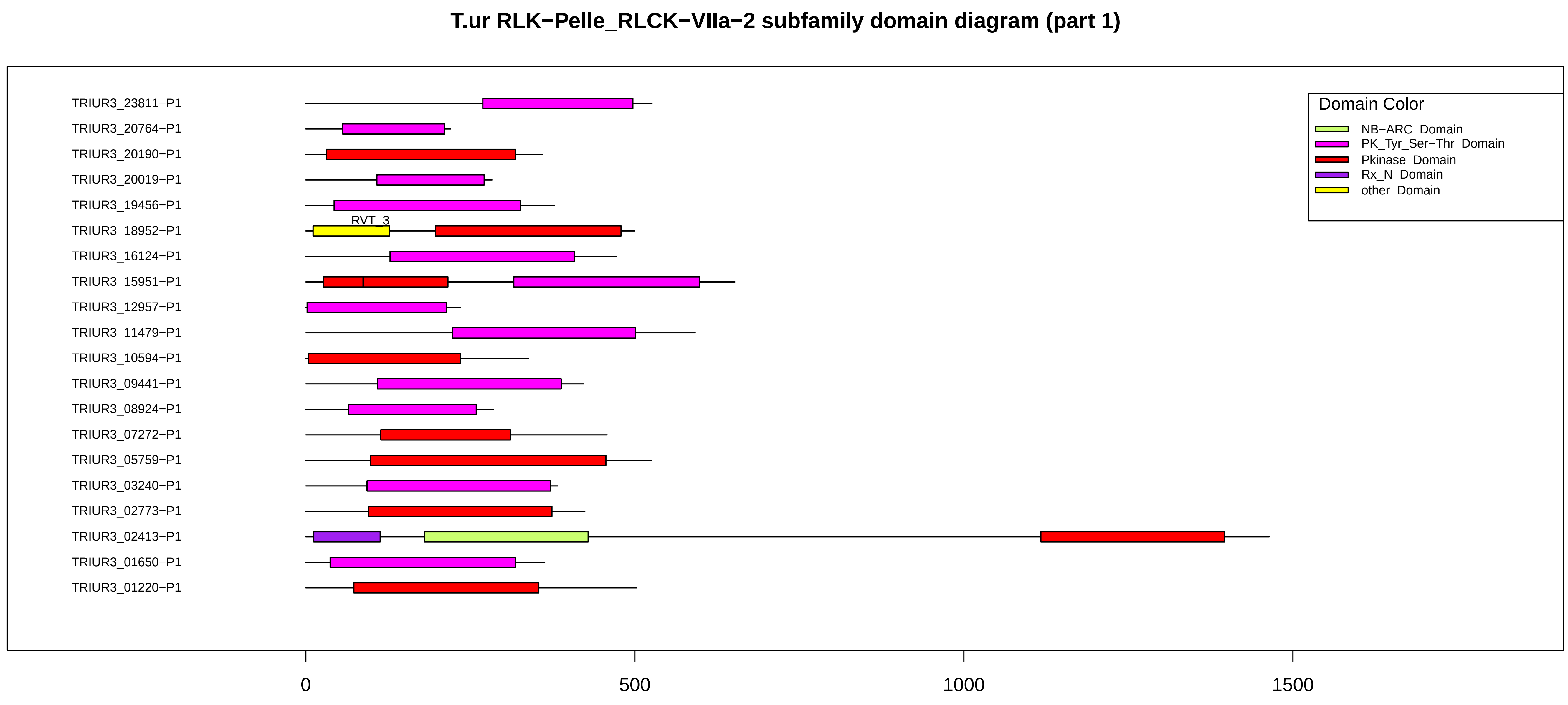

T.ur RLK−Pelle_RLCK−VIIa−2 subfamily domain diagram (part 1)
TRIUR3_23811−P1
TRIUR3_20764−P1
TRIUR3_20190−P1
TRIUR3_20019−P1
TRIUR3_19456−P1
TRIUR3_18952−P1
RVT_3
TRIUR3_16124−P1
TRIUR3_15951−P1
TRIUR3_12957−P1
TRIUR3_11479−P1
TRIUR3_10594−P1
TRIUR3_09441−P1
TRIUR3_08924−P1
TRIUR3_07272−P1
TRIUR3_05759−P1
TRIUR3_03240−P1
TRIUR3_02773−P1
TRIUR3_02413−P1
TRIUR3_01650−P1
TRIUR3_01220−P1
Domain Color
NB−ARC Domain
PK_Tyr_Ser−Thr Domain
Pkinase Domain
Rx_N Domain
other Domain
0
500
1000
1500

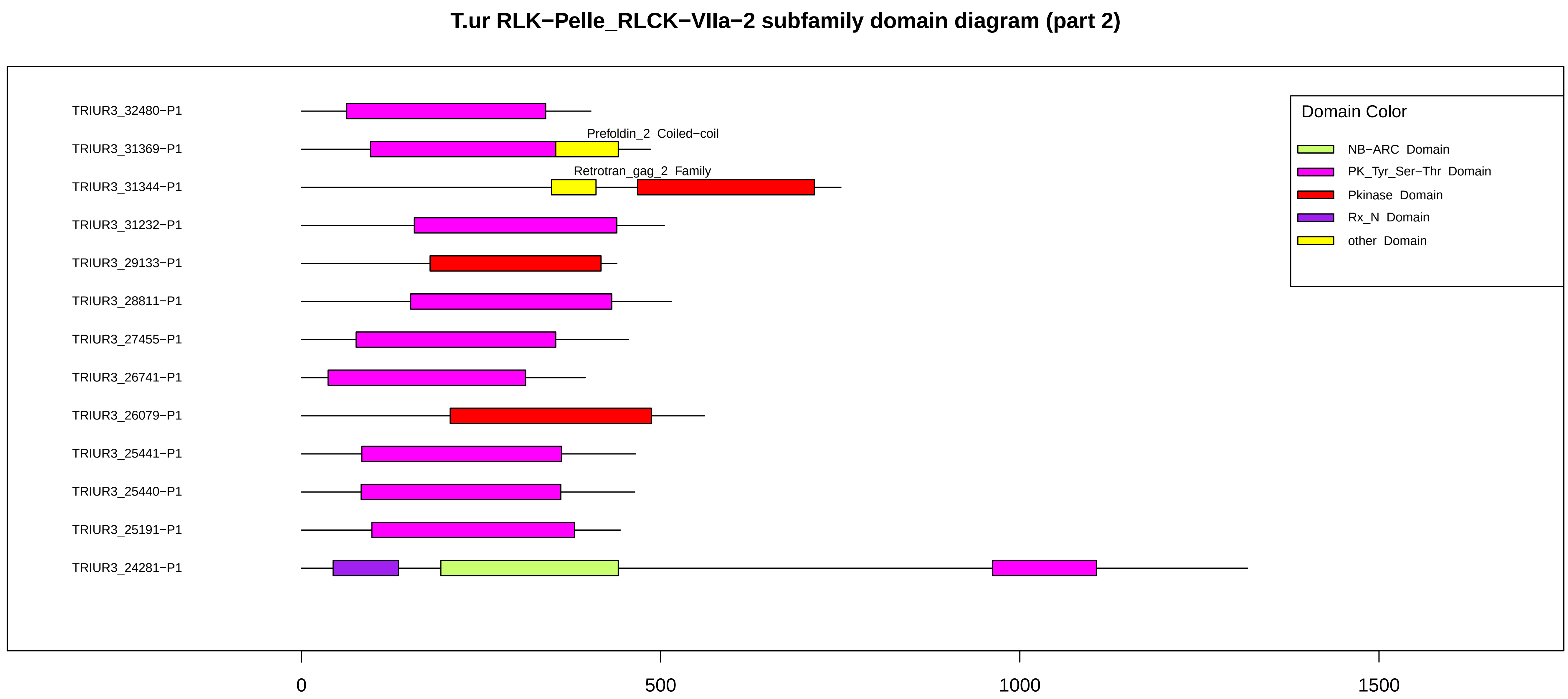

T.ur RLK−Pelle_RLCK−VIIa−2 subfamily domain diagram (part 2)
TRIUR3_32480−P1
TRIUR3_31369−P1
TRIUR3_31344−P1
TRIUR3_31232−P1
TRIUR3_29133−P1
TRIUR3_28811−P1
TRIUR3_27455−P1
TRIUR3_26741−P1
TRIUR3_26079−P1
TRIUR3_25441−P1
TRIUR3_25440−P1
TRIUR3_25191−P1
TRIUR3_24281−P1
Prefoldin_2 Coiled−coil
Retrotran_gag_2 Family
Domain Color
NB−ARC Domain
PK_Tyr_Ser−Thr Domain
Pkinase Domain
Rx_N Domain
other Domain
0
500
1000
1500

T.ur RLK−Pelle_RLCK−VIIb subfamily domain diagram (all)

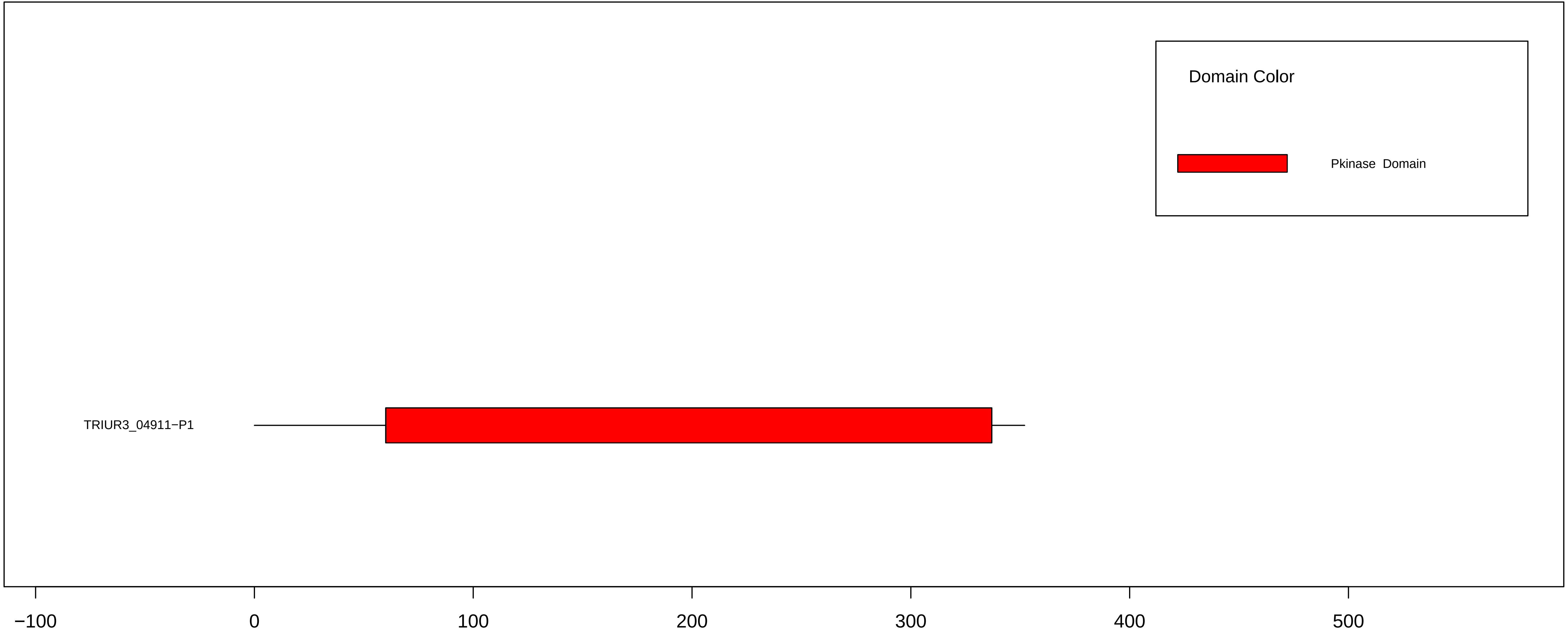

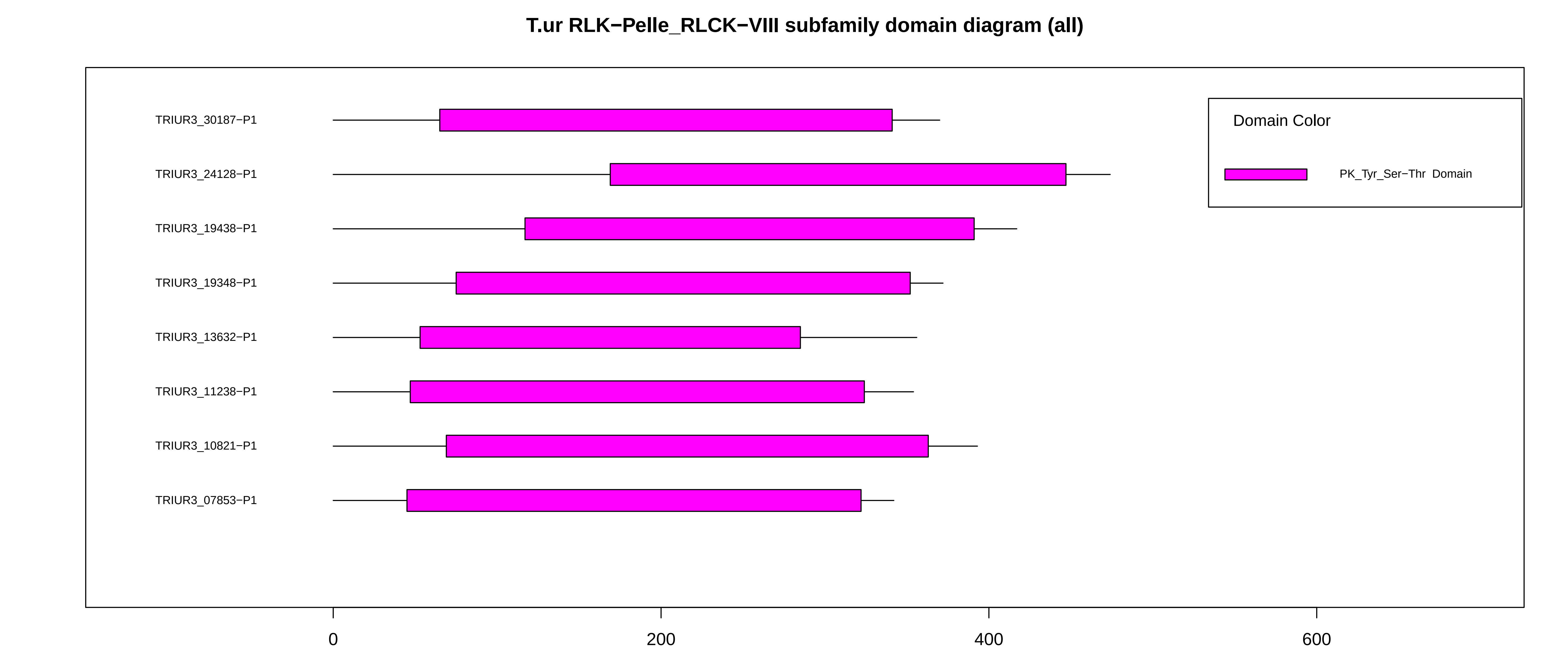
T.ur RLK−Pelle_RLCK−VIII subfamily domain diagram (all)
TRIUR3_30187−P1
TRIUR3_24128−P1
TRIUR3_19438−P1
TRIUR3_19348−P1
TRIUR3_13632−P1
TRIUR3_11238−P1
TRIUR3_10821−P1
TRIUR3_07853−P1
Domain Color
PK_Tyr_Ser−Thr Domain
0
200
400
600

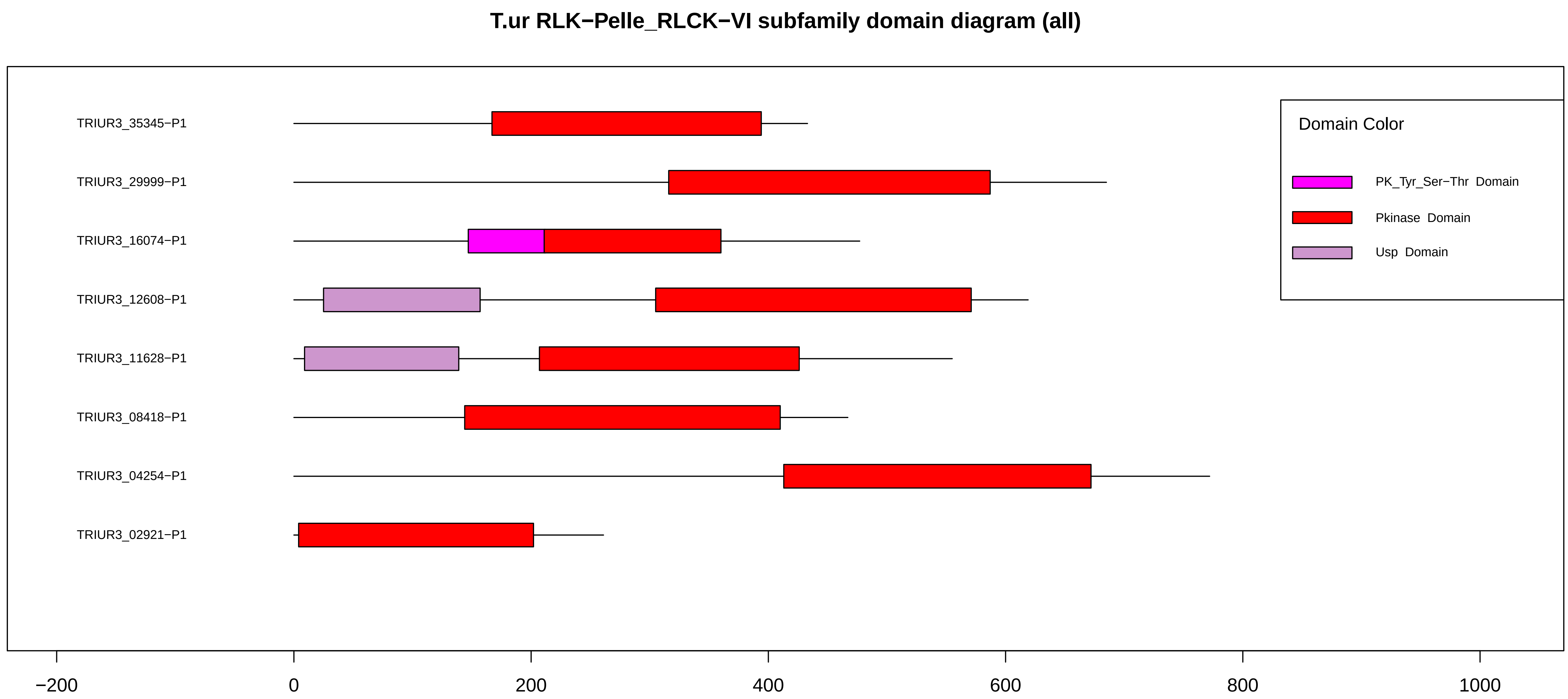
T.ur RLK−Pelle_RLCK−VI subfamily domain diagram (all)
TRIUR3_35345−P1
TRIUR3_29999−P1
TRIUR3_16074−P1
TRIUR3_12608−P1
TRIUR3_11628−P1
TRIUR3_08418−P1
TRIUR3_04254−P1
TRIUR3_02921−P1
Domain Color
PK_Tyr_Ser−Thr Domain
Pkinase Domain
Usp Domain
−200
0
200
400
600
800
1000

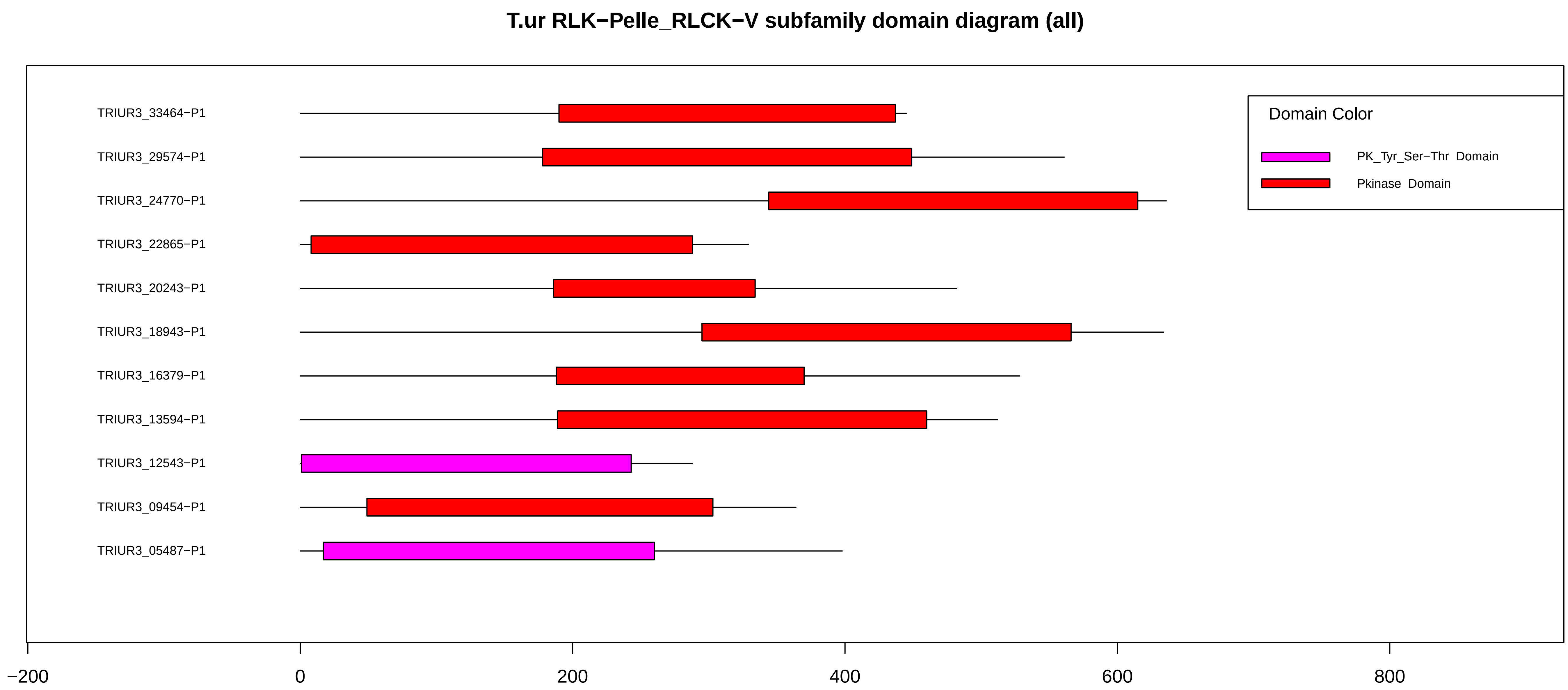

T.ur RLK−Pelle_RLCK−V subfamily domain diagram (all)
TRIUR3_33464−P1
TRIUR3_29574−P1
TRIUR3_24770−P1
TRIUR3_22865−P1
TRIUR3_20243−P1
TRIUR3_18943−P1
TRIUR3_16379−P1
TRIUR3_13594−P1
TRIUR3_12543−P1
TRIUR3_09454−P1
TRIUR3_05487−P1
Domain Color
PK_Tyr_Ser−Thr Domain
Pkinase Domain
−200
0
200
400
600
800

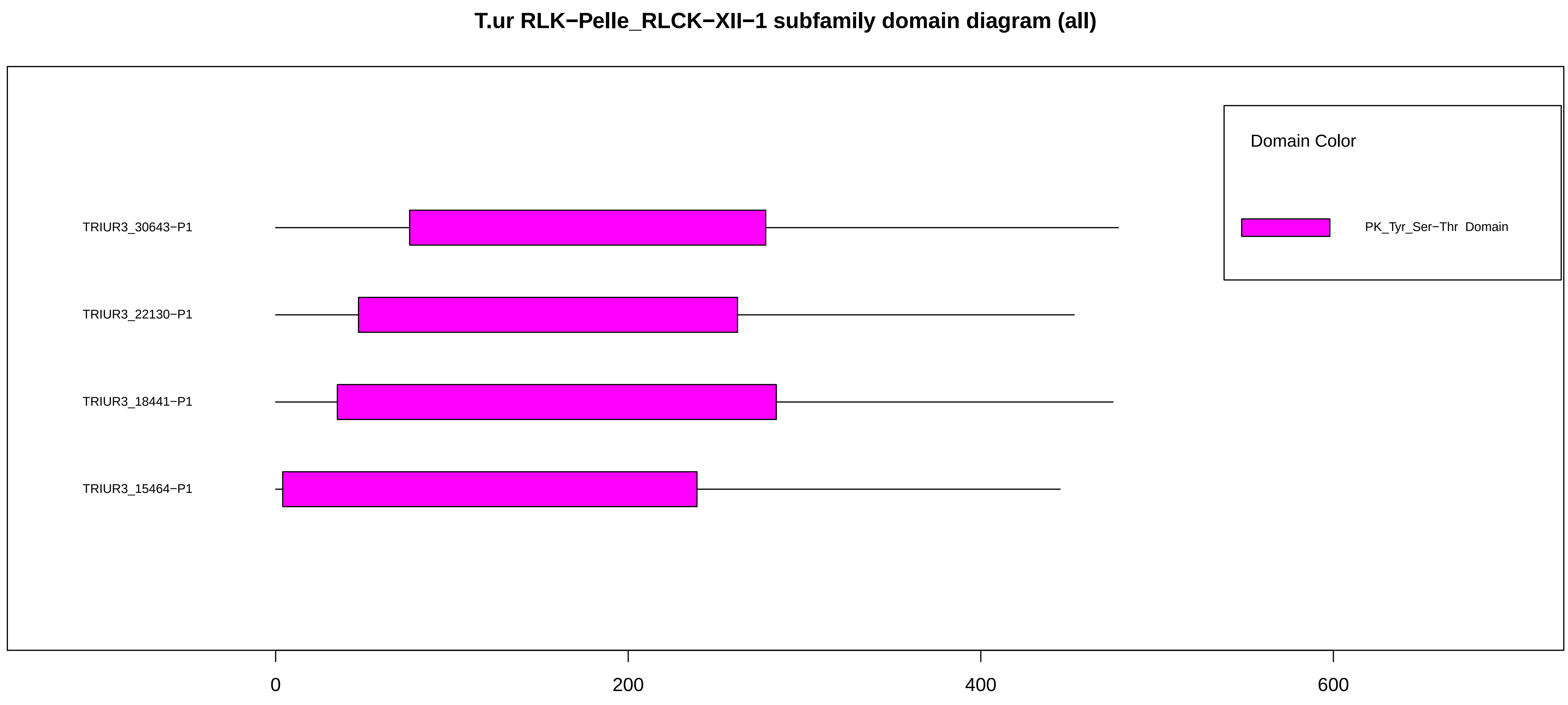
T.ur RLK−Pelle_RLCK−XII−1 subfamily domain diagram (all)
TRIUR3_30643−P1
TRIUR3_22130−P1
TRIUR3_18441−P1
TRIUR3_15464−P1
Domain Color
PK_Tyr_Ser−Thr Domain
0
200
400
600

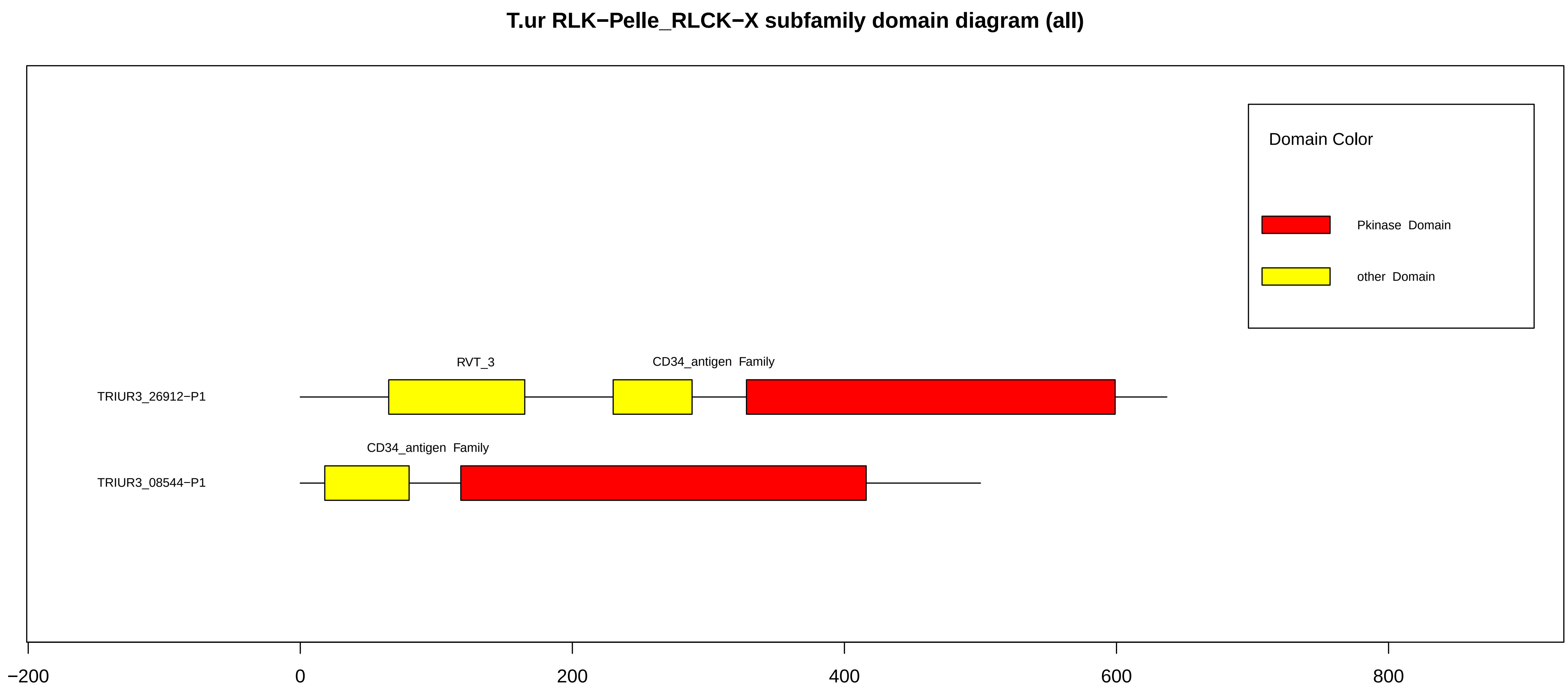
T.ur RLK−Pelle_RLCK−X subfamily domain diagram (all)
Domain Color
Pkinase Domain
other Domain
RVT_3
CD34_antigen Family
TRIUR3_26912−P1
CD34_antigen Family
TRIUR3_08544−P1
−200
0
200
400
600
800

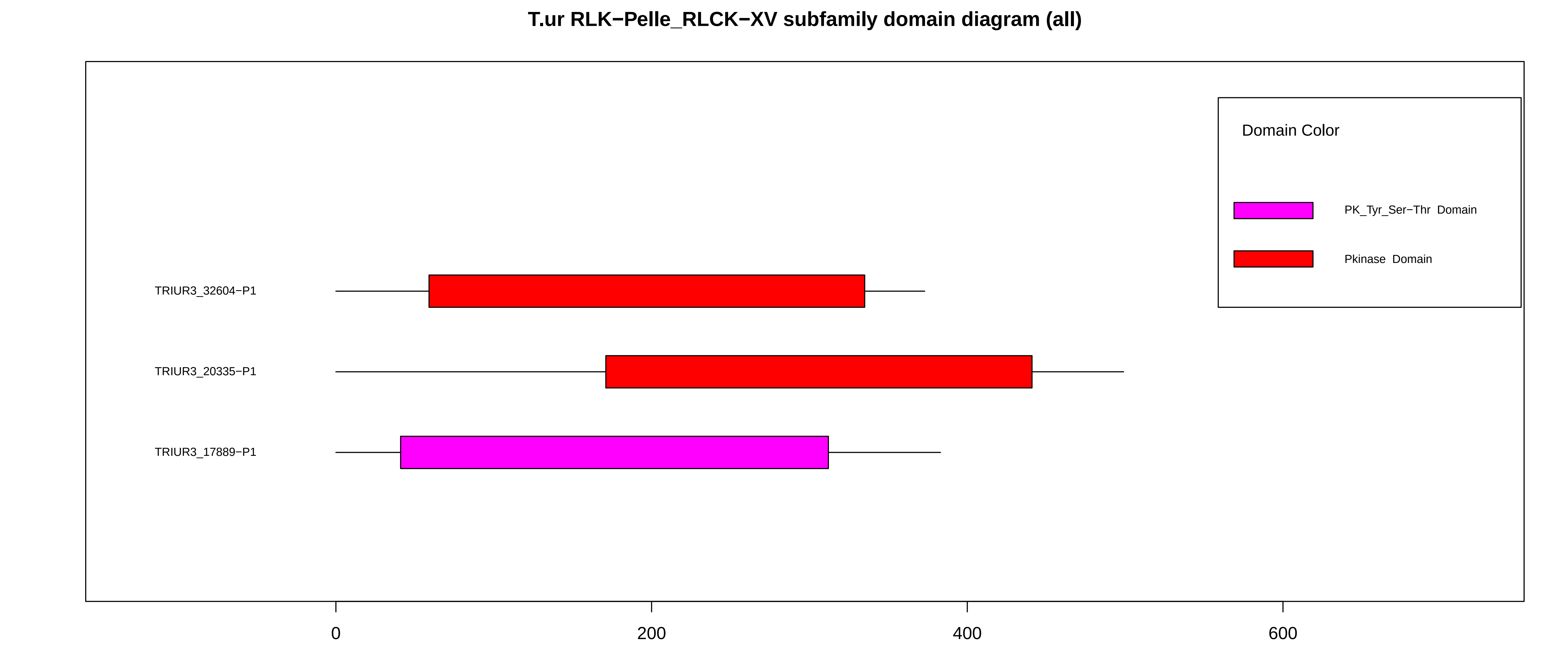

T.ur RLK−Pelle_RLCK−XV subfamily domain diagram (all)
Domain Color
PK_Tyr_Ser−Thr Domain
Pkinase Domain
TRIUR3_32604−P1
TRIUR3_20335−P1
TRIUR3_17889−P1
0
200
400
600

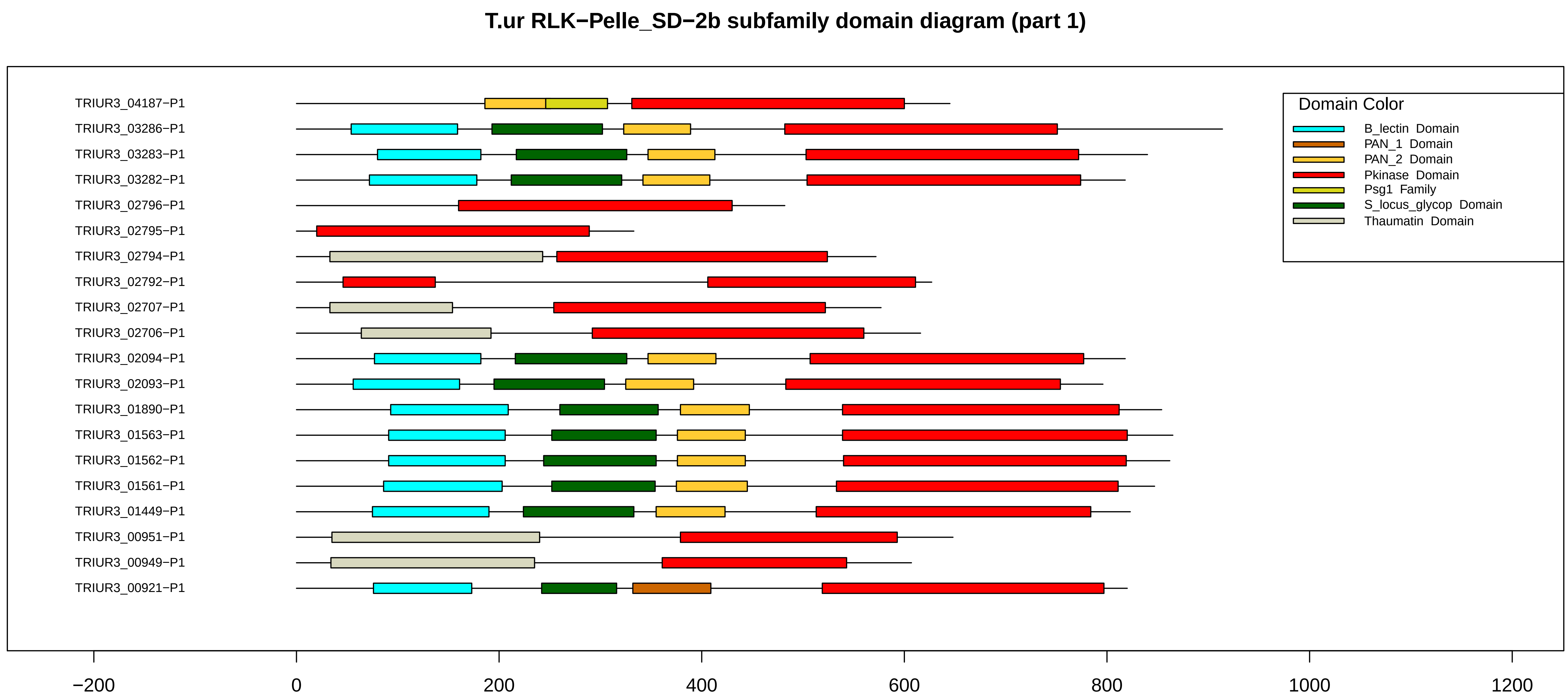

T.ur RLK−Pelle_SD−2b subfamily domain diagram (part 1)
TRIUR3_04187−P1
TRIUR3_03286−P1
TRIUR3_03283−P1
TRIUR3_03282−P1
TRIUR3_02796−P1
TRIUR3_02795−P1
TRIUR3_02794−P1
TRIUR3_02792−P1
TRIUR3_02707−P1
TRIUR3_02706−P1
TRIUR3_02094−P1
TRIUR3_02093−P1
TRIUR3_01890−P1
TRIUR3_01563−P1
TRIUR3_01562−P1
TRIUR3_01561−P1
TRIUR3_01449−P1
TRIUR3_00951−P1
TRIUR3_00949−P1
TRIUR3_00921−P1
Domain Color
B_lectin Domain
PAN_1 Domain
PAN_2 Domain
Pkinase Domain
Psg1 Family
S_locus_glycop Domain
Thaumatin Domain
−200
0
200
400
600
800
1000
1200

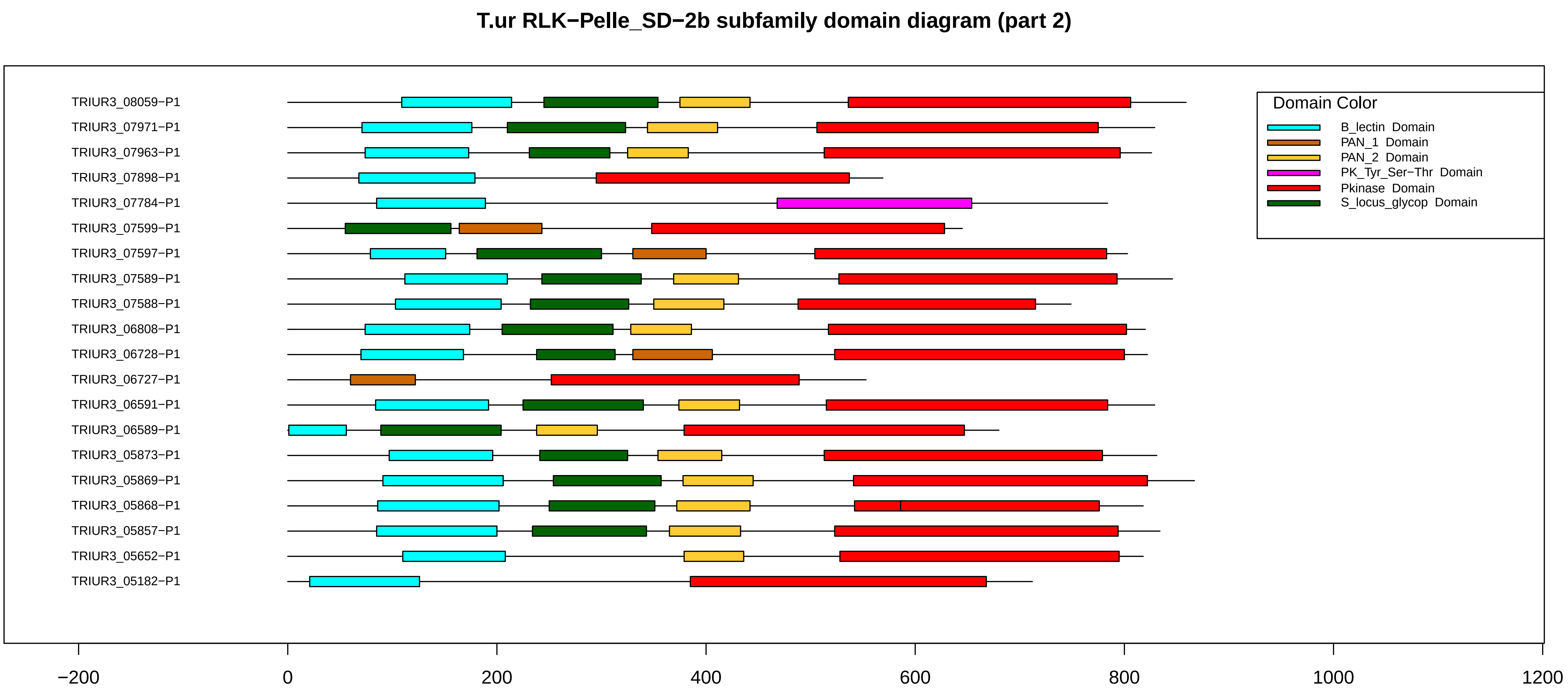
T.ur RLK−Pelle_SD−2b subfamily domain diagram (part 2)
TRIUR3_08059−P1
TRIUR3_07971−P1
TRIUR3_07963−P1
TRIUR3_07898−P1
TRIUR3_07784−P1
TRIUR3_07599−P1
TRIUR3_07597−P1
TRIUR3_07589−P1
TRIUR3_07588−P1
TRIUR3_06808−P1
TRIUR3_06728−P1
TRIUR3_06727−P1
TRIUR3_06591−P1
TRIUR3_06589−P1
TRIUR3_05873−P1
TRIUR3_05869−P1
TRIUR3_05868−P1
TRIUR3_05857−P1
TRIUR3_05652−P1
TRIUR3_05182−P1
Domain Color
B_lectin Domain
PAN_1 Domain
PAN_2 Domain
PK_Tyr_Ser−Thr Domain
Pkinase Domain
S_locus_glycop Domain
−200
0
200
400
600
800
1000
1200

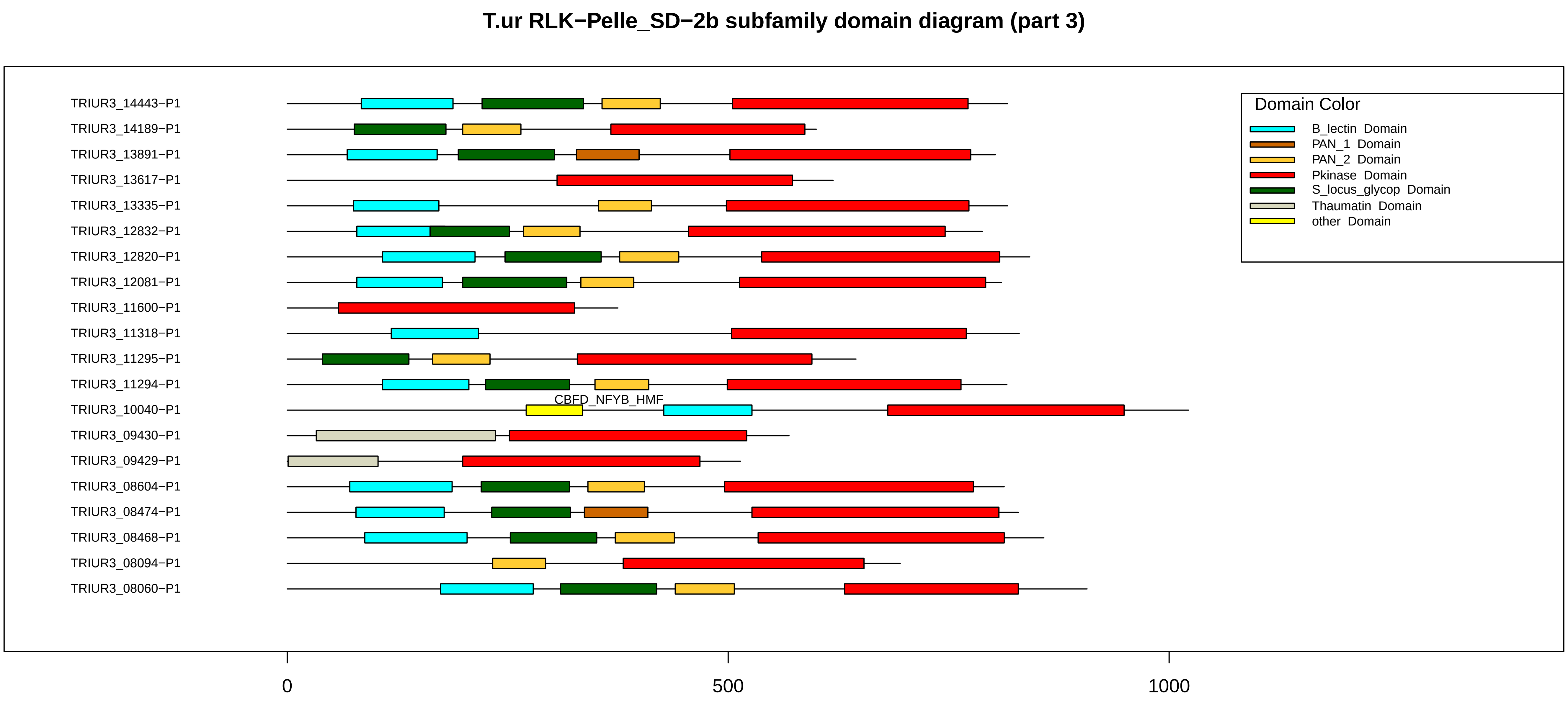

T.ur RLK−Pelle_SD−2b subfamily domain diagram (part 3)
TRIUR3_14443−P1
TRIUR3_14189−P1
TRIUR3_13891−P1
TRIUR3_13617−P1
TRIUR3_13335−P1
TRIUR3_12832−P1
TRIUR3_12820−P1
TRIUR3_12081−P1
TRIUR3_11600−P1
TRIUR3_11318−P1
TRIUR3_11295−P1
TRIUR3_11294−P1
TRIUR3_10040−P1
CBFD_NFYB_HMF
TRIUR3_09430−P1
TRIUR3_09429−P1
TRIUR3_08604−P1
TRIUR3_08474−P1
TRIUR3_08468−P1
TRIUR3_08094−P1
TRIUR3_08060−P1
Domain Color
B_lectin Domain
PAN_1 Domain
PAN_2 Domain
Pkinase Domain
S_locus_glycop Domain
Thaumatin Domain
other Domain
0
500
1000

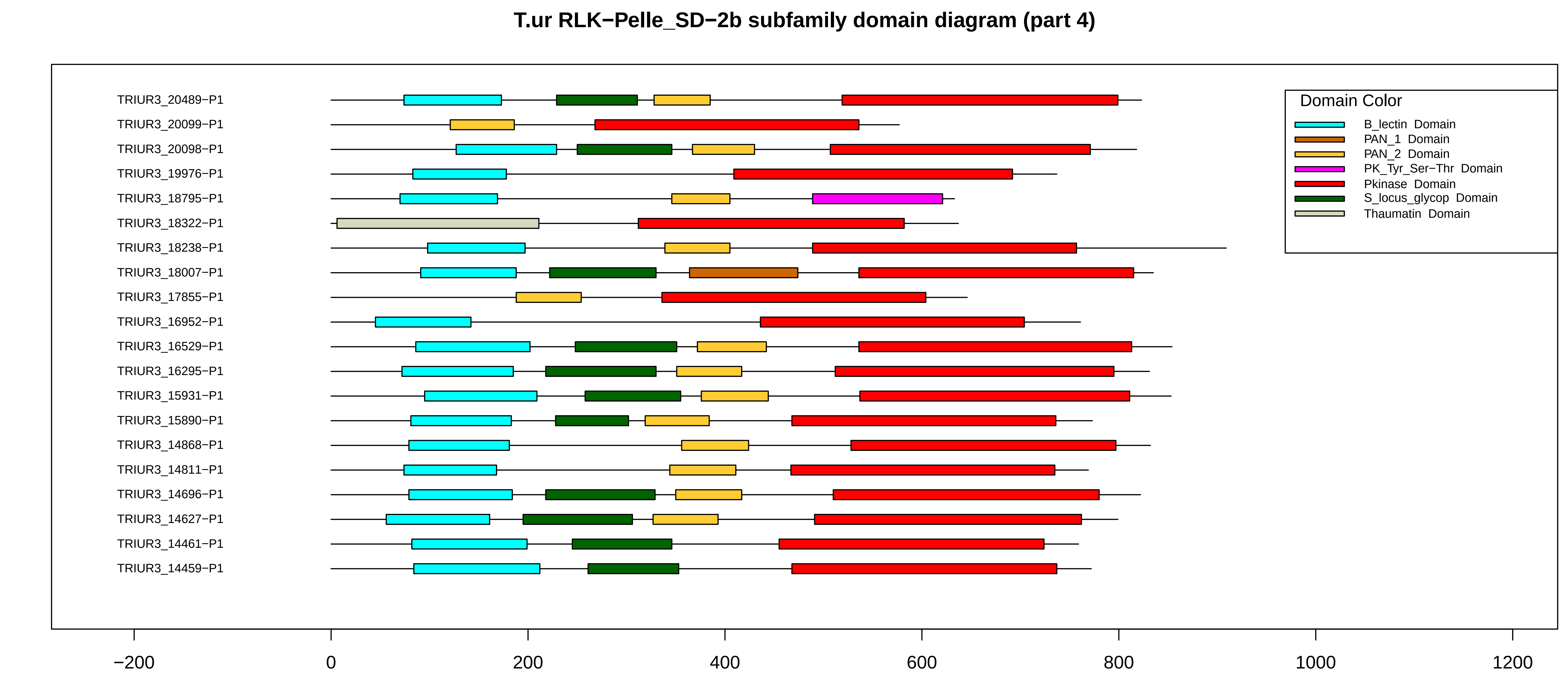
T.ur RLK−Pelle_SD−2b subfamily domain diagram (part 4)
TRIUR3_20489−P1
TRIUR3_20099−P1
TRIUR3_20098−P1
TRIUR3_19976−P1
TRIUR3_18795−P1
TRIUR3_18322−P1
TRIUR3_18238−P1
TRIUR3_18007−P1
TRIUR3_17855−P1
TRIUR3_16952−P1
TRIUR3_16529−P1
TRIUR3_16295−P1
TRIUR3_15931−P1
TRIUR3_15890−P1
TRIUR3_14868−P1
TRIUR3_14811−P1
TRIUR3_14696−P1
TRIUR3_14627−P1
TRIUR3_14461−P1
TRIUR3_14459−P1
Domain Color
B_lectin Domain
PAN_1 Domain
PAN_2 Domain
PK_Tyr_Ser−Thr Domain
Pkinase Domain
S_locus_glycop Domain
Thaumatin Domain
−200
0
200
400
600
800
1000
1200

T.ur RLK−Pelle_SD−2b subfamily domain diagram (part 5)

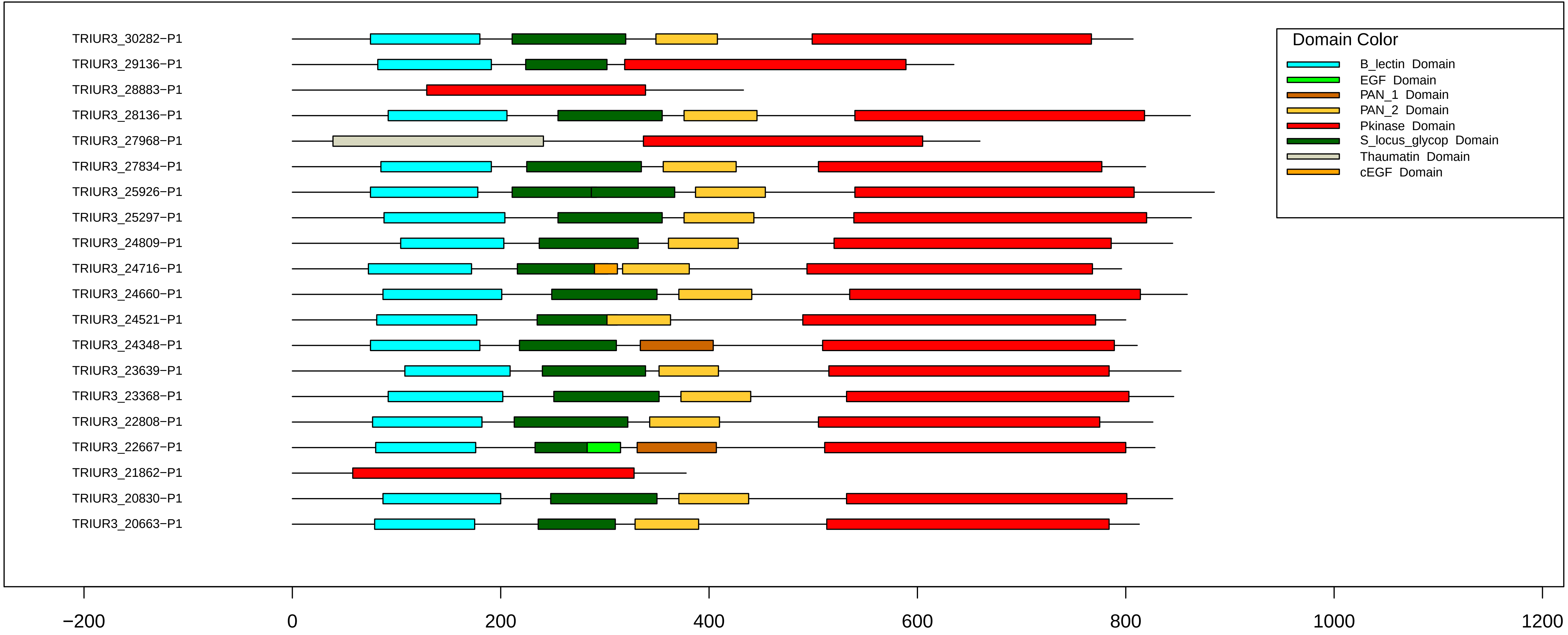

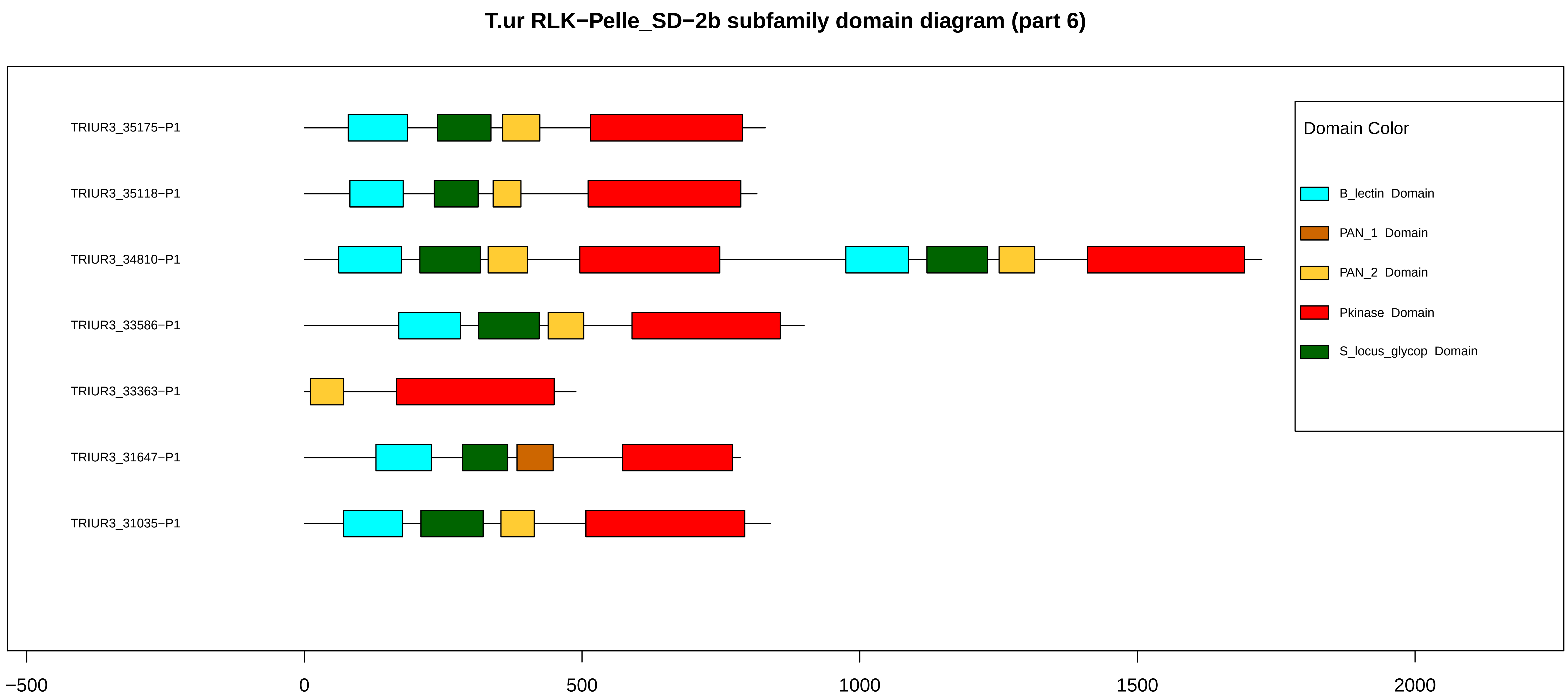

T.ur RLK−Pelle_SD−2b subfamily domain diagram (part 6)
TRIUR3_35175−P1
TRIUR3_35118−P1
TRIUR3_34810−P1
TRIUR3_33586−P1
TRIUR3_33363−P1
TRIUR3_31647−P1
TRIUR3_31035−P1
Domain Color
B_lectin Domain
PAN_1 Domain
PAN_2 Domain
Pkinase Domain
S_locus_glycop Domain
−500
0
500
1000
1500
2000

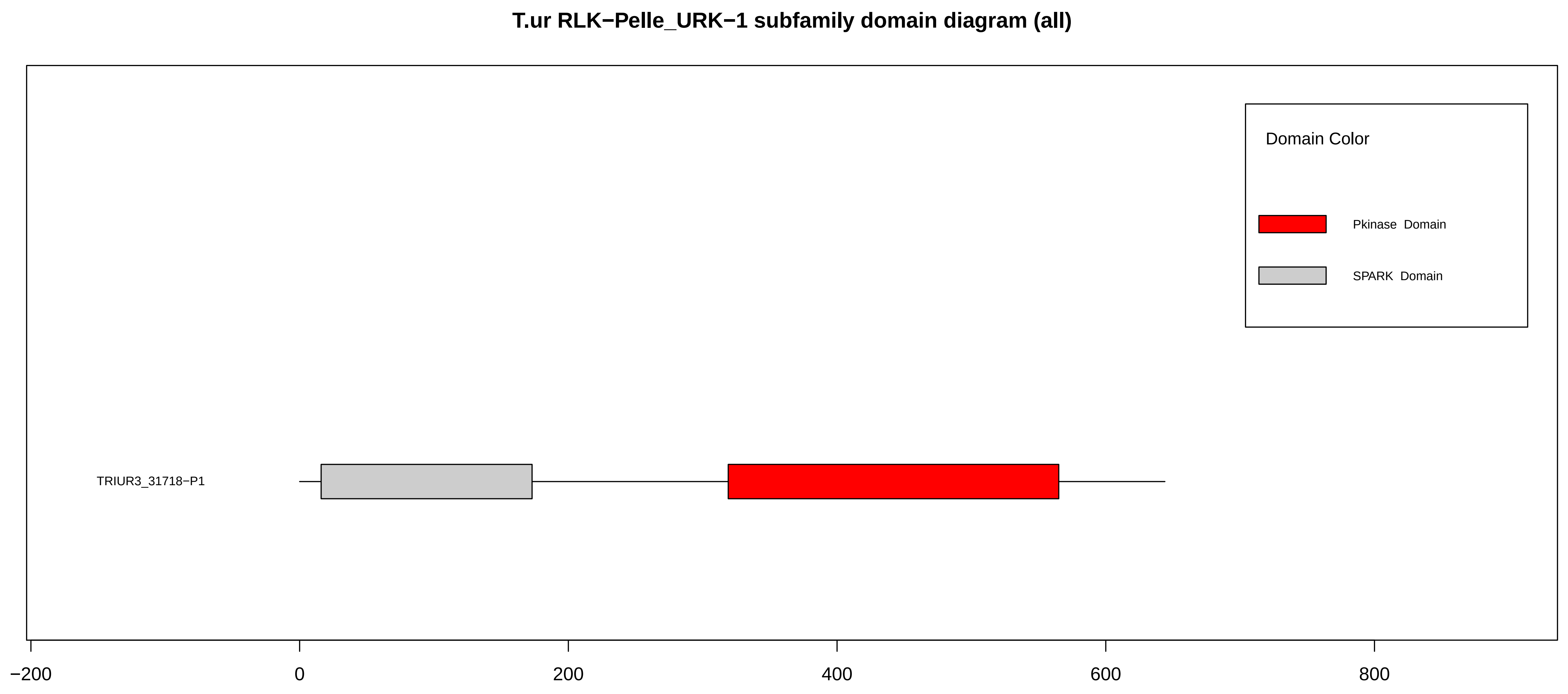

T.ur RLK−Pelle_URK−1 subfamily domain diagram (all)
Domain Color
Pkinase Domain
SPARK Domain
TRIUR3_31718−P1
−200
0
200
400
600
800

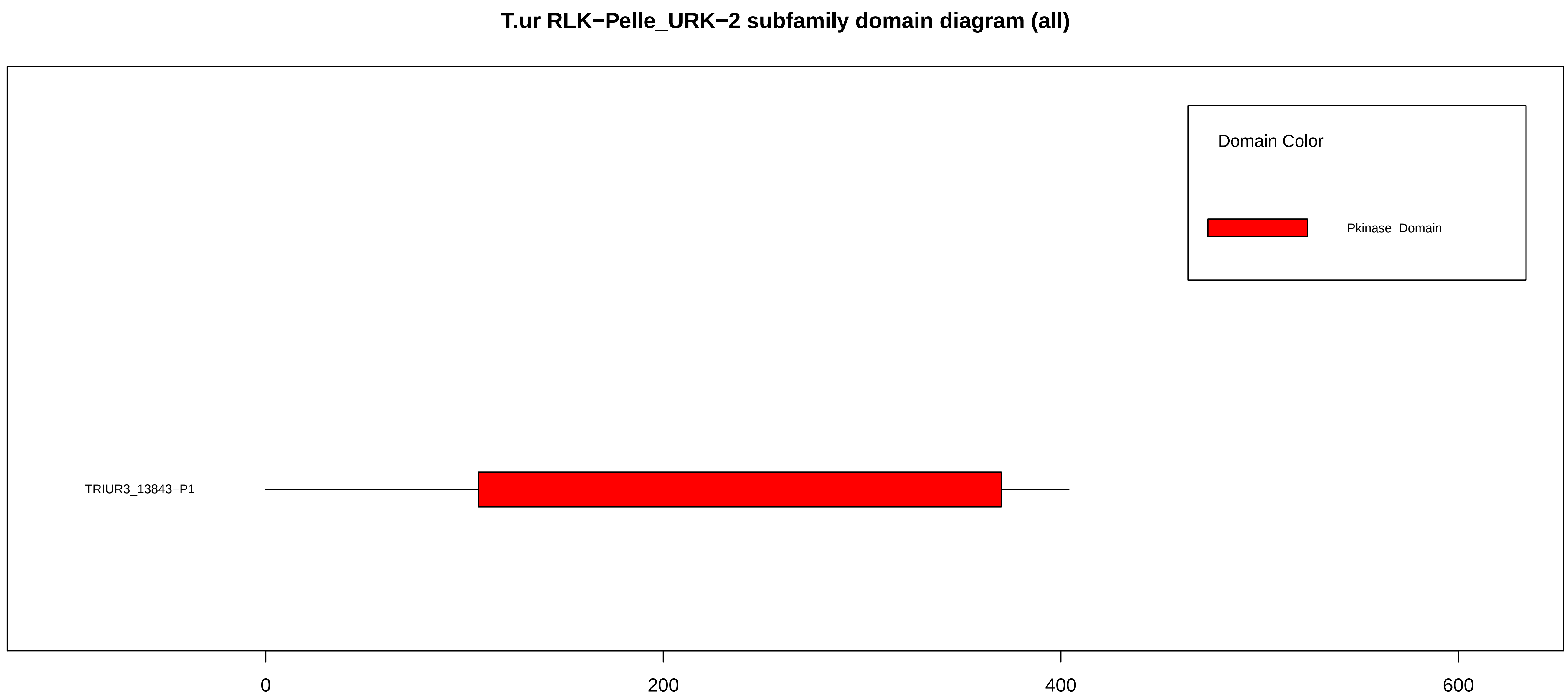
T.ur RLK−Pelle_URK−2 subfamily domain diagram (all)
Domain Color
Pkinase Domain
TRIUR3_13843−P1
0
200
400
600

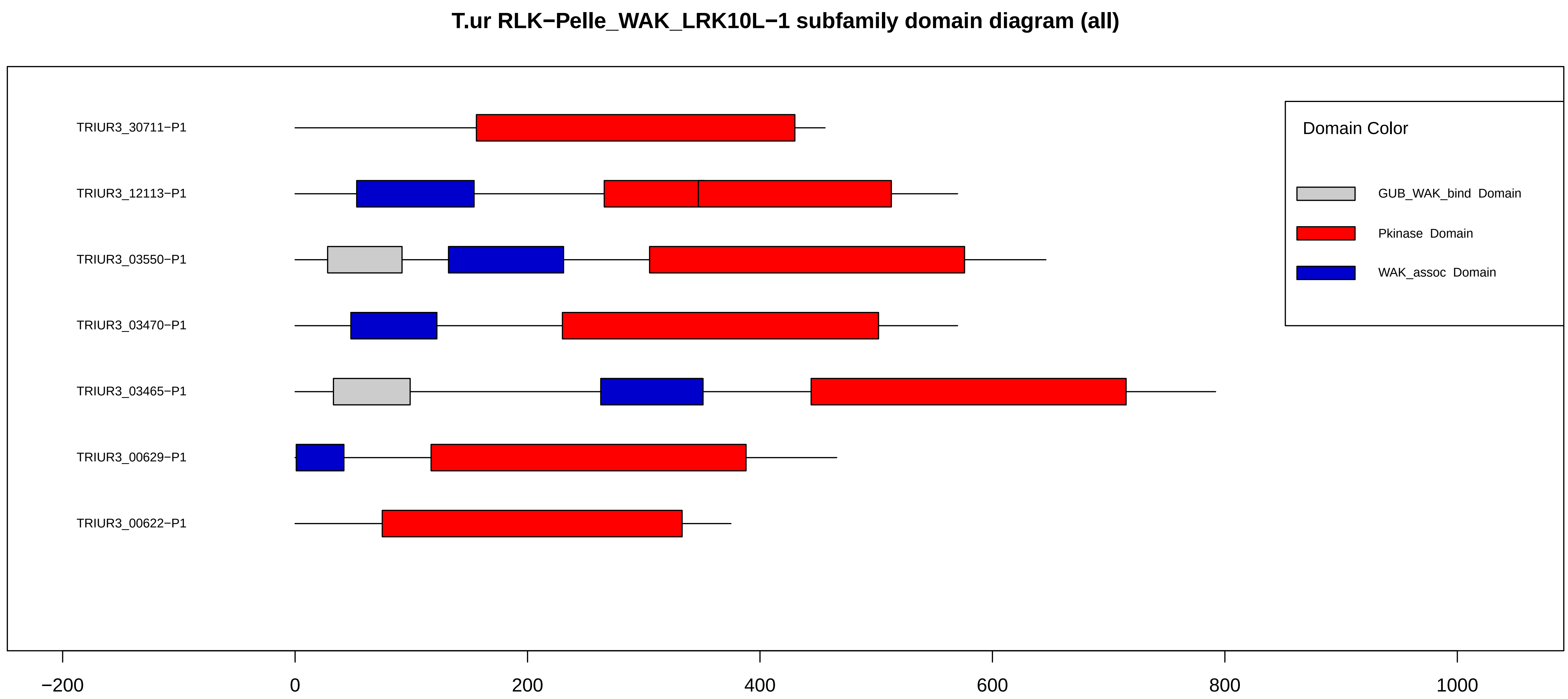

T.ur RLK−Pelle_WAK_LRK10L−1 subfamily domain diagram (all)
TRIUR3_30711−P1
TRIUR3_12113−P1
TRIUR3_03550−P1
TRIUR3_03470−P1
TRIUR3_03465−P1
TRIUR3_00629−P1
TRIUR3_00622−P1
Domain Color
GUB_WAK_bind Domain
Pkinase Domain
WAK_assoc Domain
−200
0
200
400
600
800
1000

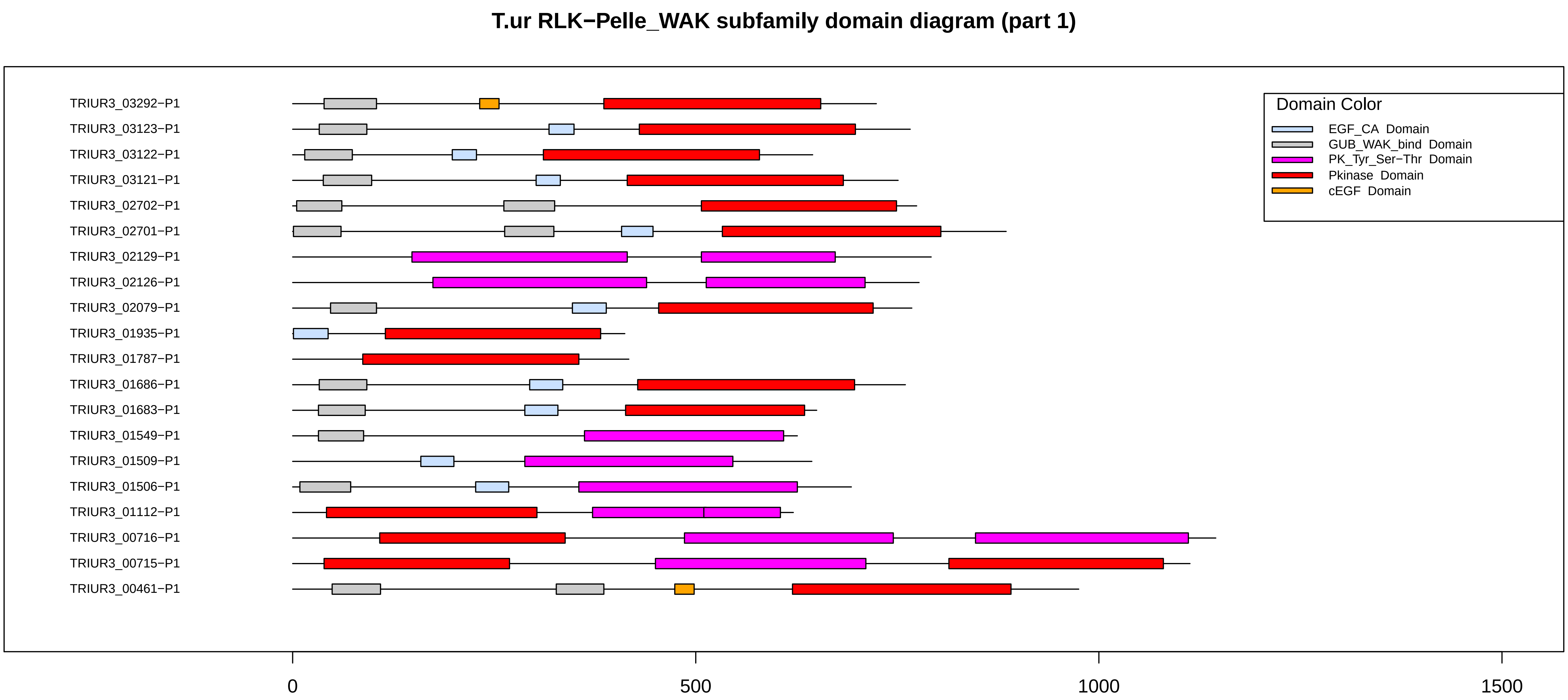
T.ur RLK−Pelle_WAK subfamily domain diagram (part 1)
TRIUR3_03292−P1
TRIUR3_03123−P1
TRIUR3_03122−P1
TRIUR3_03121−P1
TRIUR3_02702−P1
TRIUR3_02701−P1
TRIUR3_02129−P1
TRIUR3_02126−P1
TRIUR3_02079−P1
TRIUR3_01935−P1
TRIUR3_01787−P1
TRIUR3_01686−P1
TRIUR3_01683−P1
TRIUR3_01549−P1
TRIUR3_01509−P1
TRIUR3_01506−P1
TRIUR3_01112−P1
TRIUR3_00716−P1
TRIUR3_00715−P1
TRIUR3_00461−P1
Domain Color
EGF_CA Domain
GUB_WAK_bind Domain
PK_Tyr_Ser−Thr Domain
Pkinase Domain
cEGF Domain
0
500
1000
1500

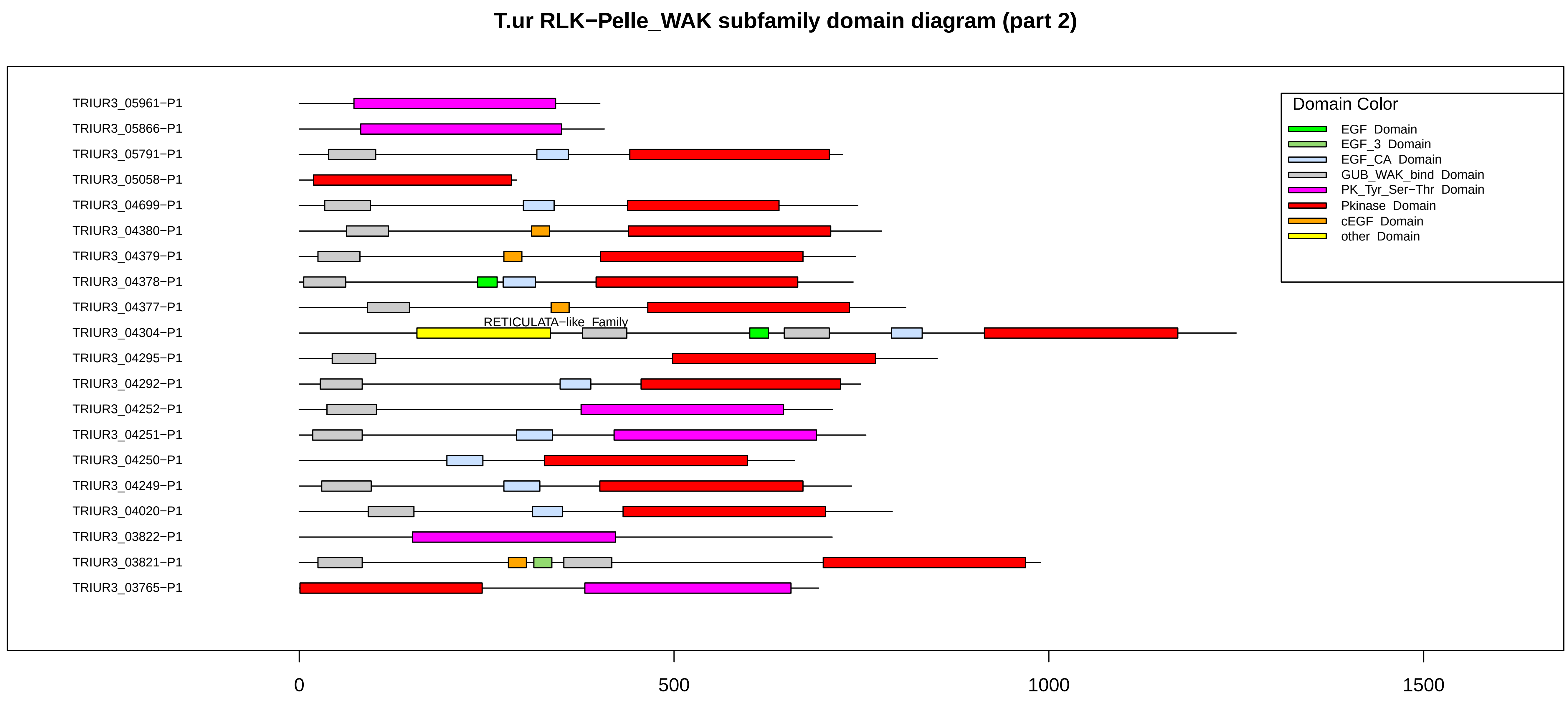

T.ur RLK−Pelle_WAK subfamily domain diagram (part 2)
TRIUR3_05961−P1
TRIUR3_05866−P1
TRIUR3_05791−P1
TRIUR3_05058−P1
TRIUR3_04699−P1
TRIUR3_04380−P1
TRIUR3_04379−P1
TRIUR3_04378−P1
TRIUR3_04377−P1
TRIUR3_04304−P1
RETICULATA−like_Family
TRIUR3_04295−P1
TRIUR3_04292−P1
TRIUR3_04252−P1
TRIUR3_04251−P1
TRIUR3_04250−P1
TRIUR3_04249−P1
TRIUR3_04020−P1
TRIUR3_03822−P1
TRIUR3_03821−P1
TRIUR3_03765−P1
Domain Color
EGF Domain
EGF_3 Domain
EGF_CA Domain
GUB_WAK_bind Domain
PK_Tyr_Ser−Thr Domain
Pkinase Domain
cEGF Domain
other Domain
0
500
1000
1500

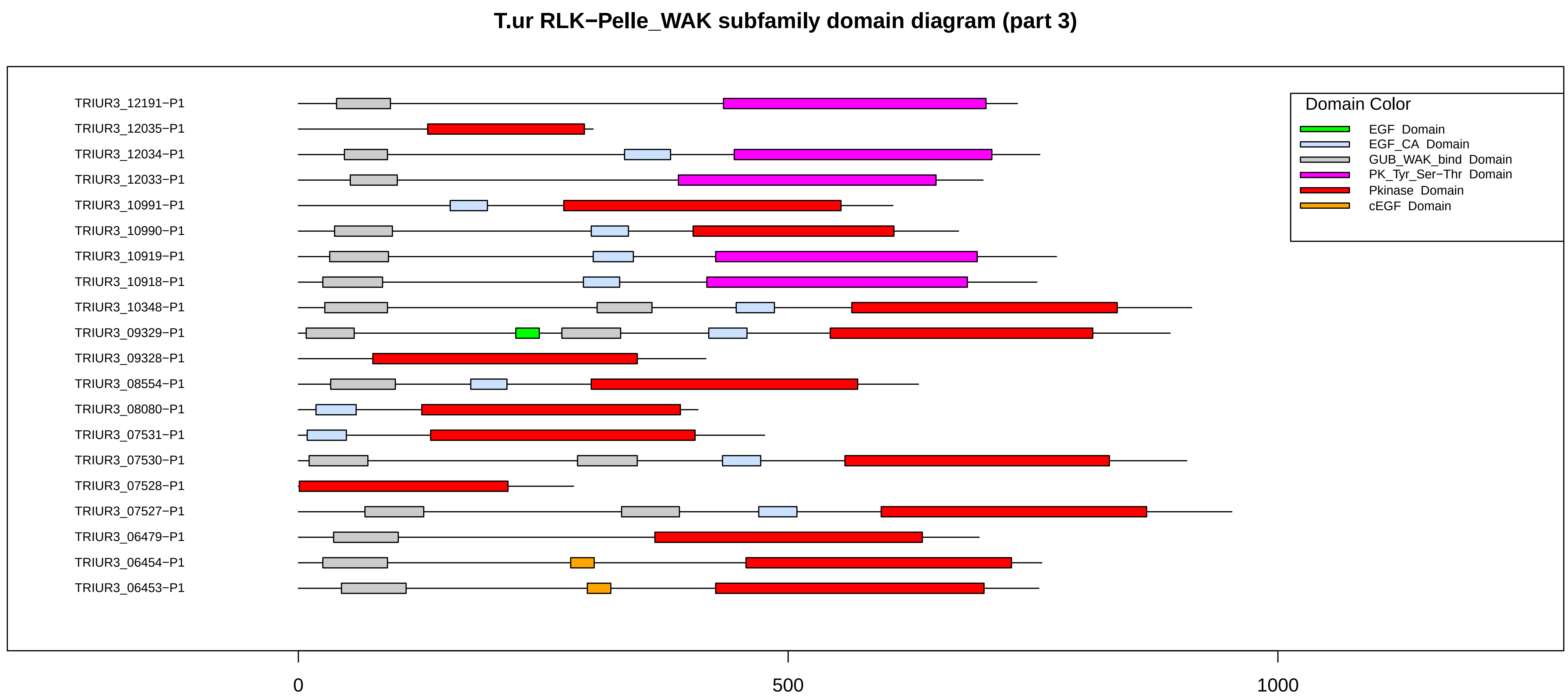
T.ur RLK−Pelle_WAK subfamily domain diagram (part 3)
TRIUR3_12191−P1
TRIUR3_12035−P1
TRIUR3_12034−P1
TRIUR3_12033−P1
TRIUR3_10991−P1
TRIUR3_10990−P1
TRIUR3_10919−P1
TRIUR3_10918−P1
TRIUR3_10348−P1
TRIUR3_09329−P1
TRIUR3_09328−P1
TRIUR3_08554−P1
TRIUR3_08080−P1
TRIUR3_07531−P1
TRIUR3_07530−P1
TRIUR3_07528−P1
TRIUR3_07527−P1
TRIUR3_06479−P1
TRIUR3_06454−P1
TRIUR3_06453−P1
Domain Color
EGF Domain
EGF_CA Domain
GUB_WAK_bind Domain
PK_Tyr_Ser−Thr Domain
Pkinase Domain
cEGF Domain
0
500
1000

T.ur RLK−Pelle_WAK subfamily domain diagram (part 4)

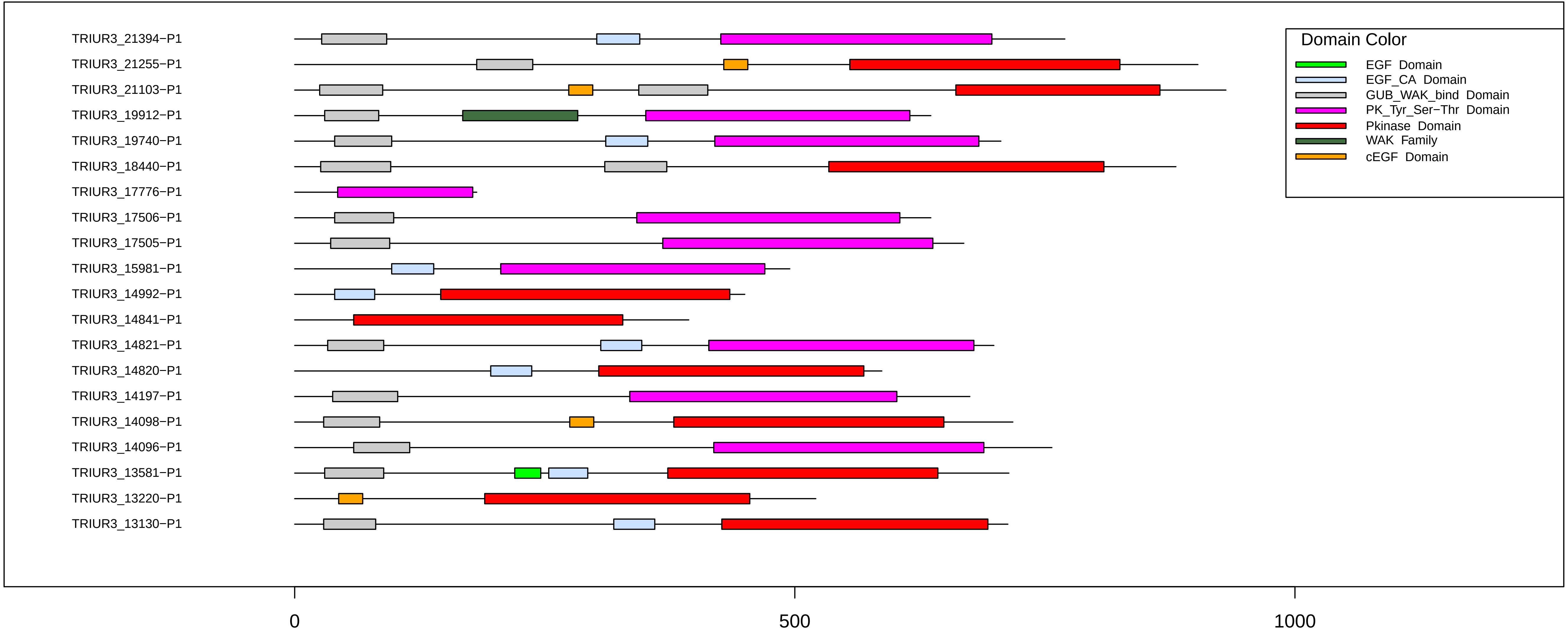

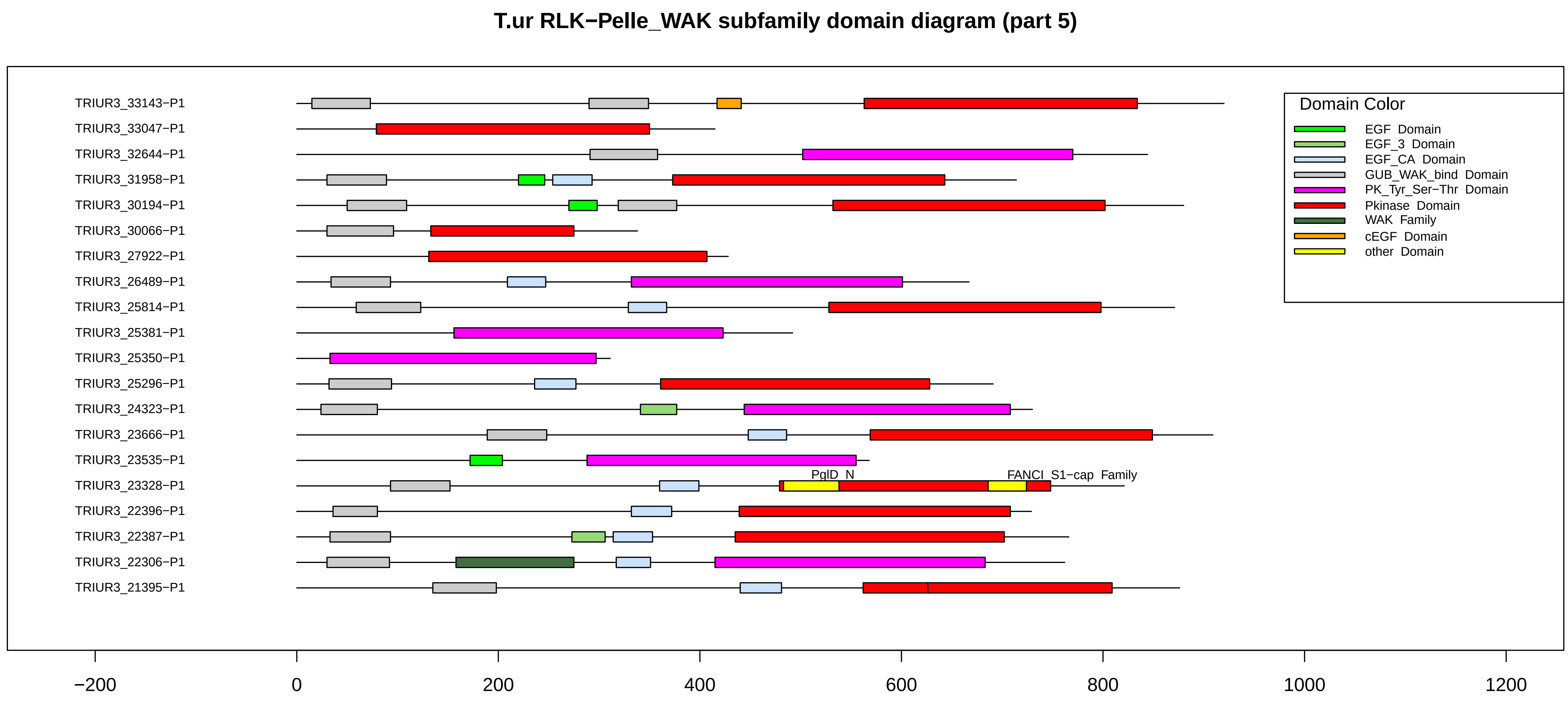

T.ur RLK−Pelle_WAK subfamily domain diagram (part 5)
TRIUR3_33143−P1
TRIUR3_33047−P1
TRIUR3_32644−P1
TRIUR3_31958−P1
TRIUR3_30194−P1
TRIUR3_30066−P1
TRIUR3_27922−P1
TRIUR3_26489−P1
TRIUR3_25814−P1
TRIUR3_25381−P1
TRIUR3_25350−P1
TRIUR3_25296−P1
TRIUR3_24323−P1
TRIUR3_23666−P1
TRIUR3_23535−P1
TRIUR3_23328−P1
TRIUR3_22396−P1
TRIUR3_22387−P1
TRIUR3_22306−P1
TRIUR3_21395−P1
PglD_N
FANCI_S1−cap Family
Domain Color
EGF Domain
EGF_3 Domain
EGF_CA Domain
GUB_WAK_bind Domain
PK_Tyr_Ser−Thr Domain
Pkinase Domain
WAK Family
cEGF Domain
other Domain
−200
0
200
400
600
800
1000
1200

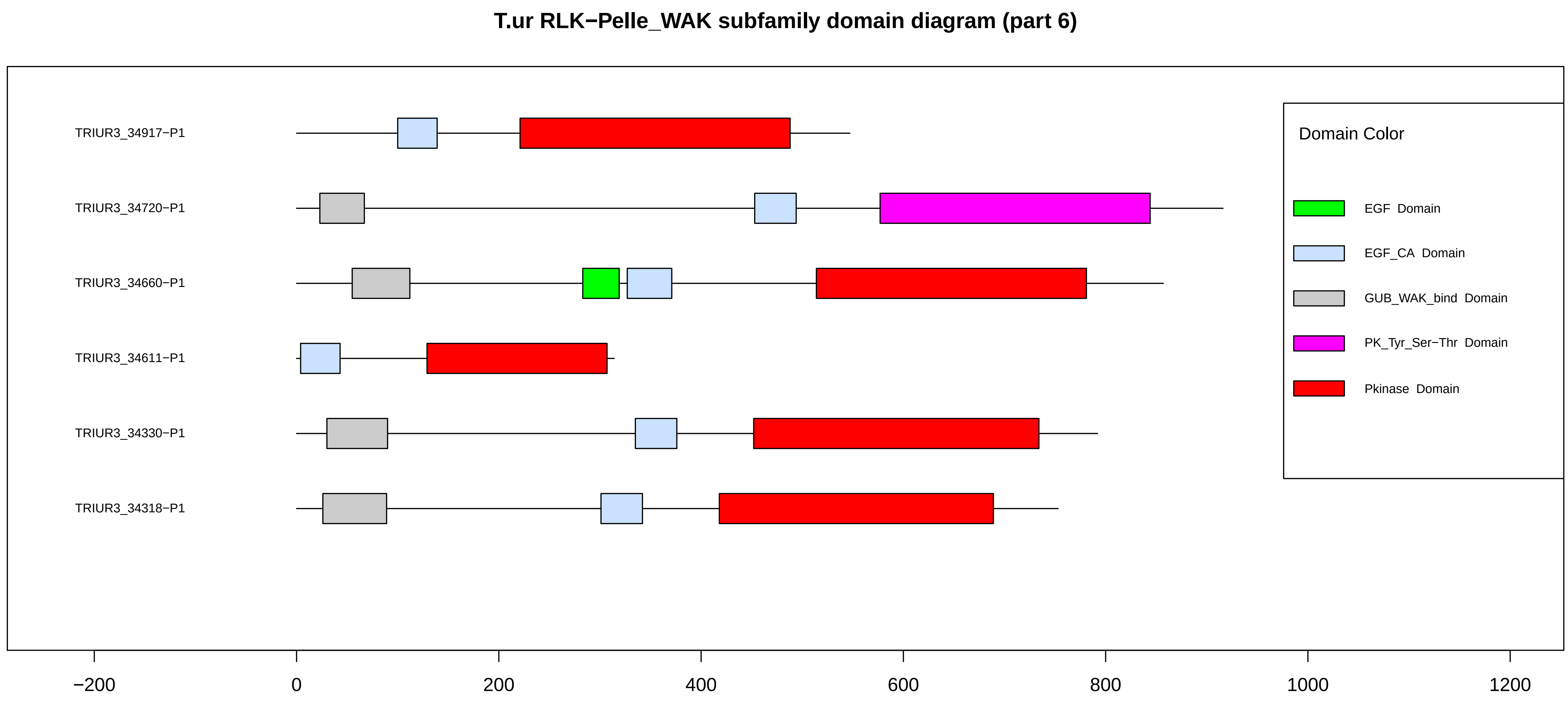
T.ur RLK−Pelle_WAK subfamily domain diagram (part 6)
TRIUR3_34917−P1
TRIUR3_34720−P1
TRIUR3_34660−P1
TRIUR3_34611−P1
TRIUR3_34330−P1
TRIUR3_34318−P1
Domain Color
EGF Domain
EGF_CA Domain
GUB_WAK_bind Domain
PK_Tyr_Ser−Thr Domain
Pkinase Domain
−200
0
200
400
600
800
1000
1200

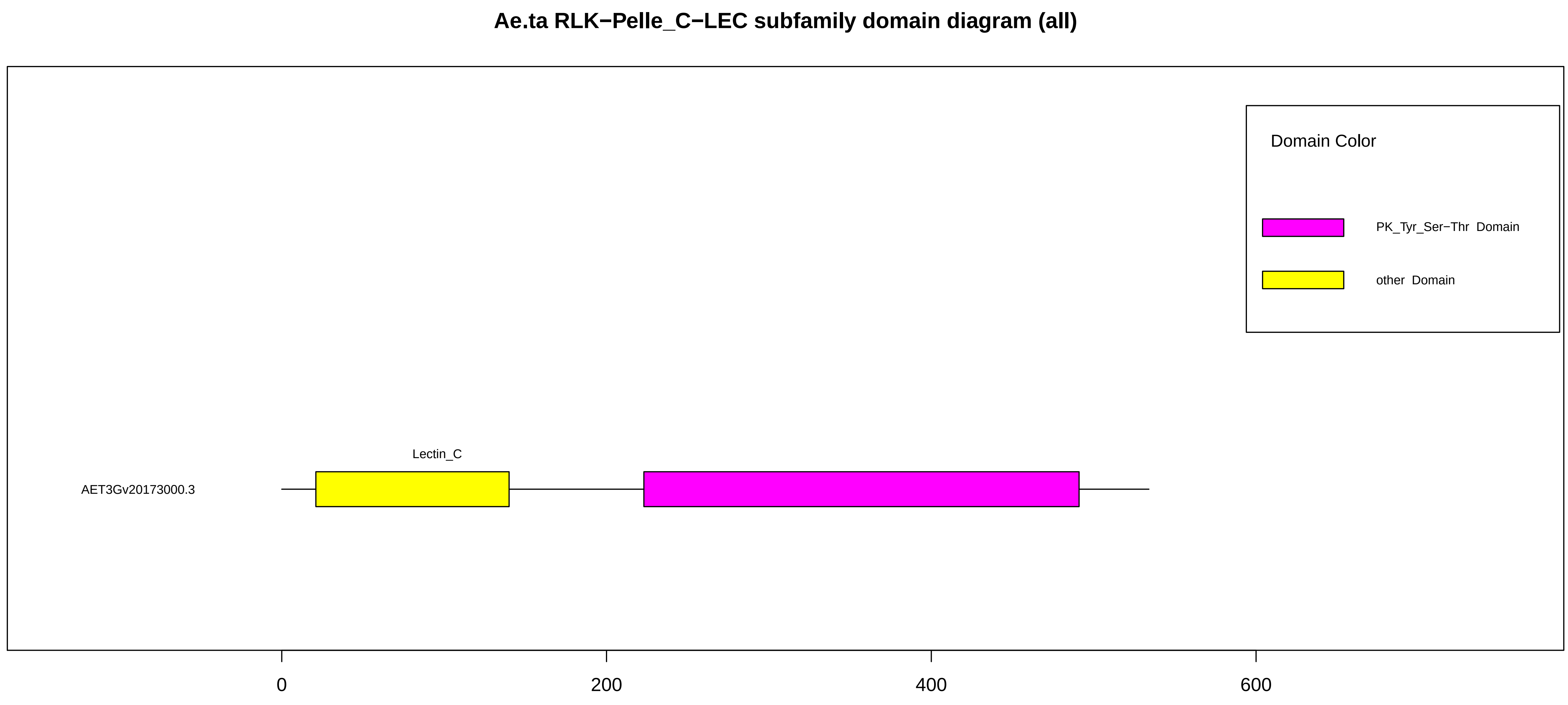
Ae.ta RLK−Pelle_C−LEC subfamily domain diagram (all)
Domain Color
PK_Tyr_Ser−Thr Domain
other Domain
Lectin_C
AET3Gv20173000.3
0
200
400
600

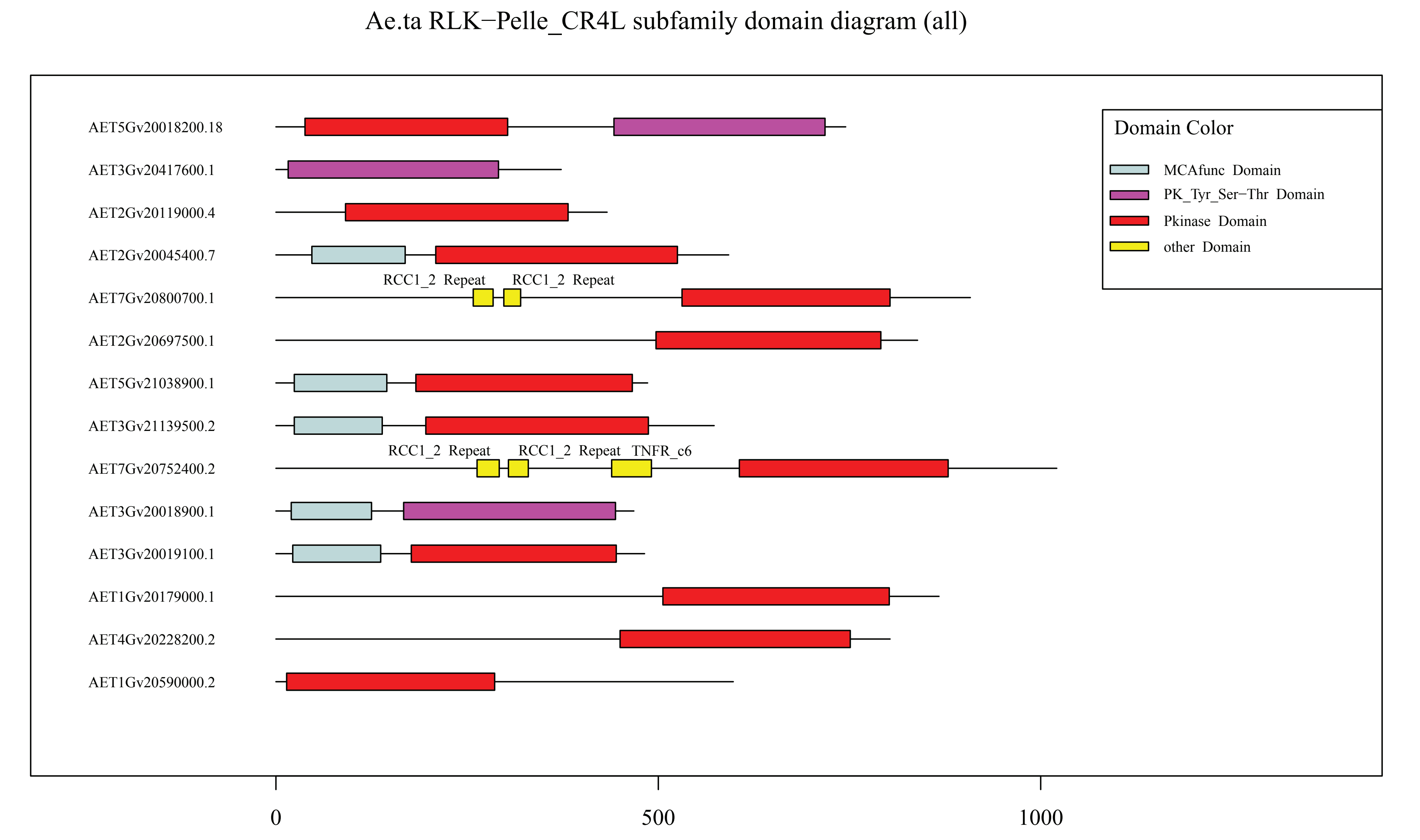
Ae.ta RLK−Pelle_CR4L subfamily domain diagram (all)
AET5Gv20018200.18
AET3Gv20417600.1
AET2Gv20119000.4
AET2Gv20045400.7
AET7Gv20800700.1
AET2Gv20697500.1
AET5Gv21038900.1
AET3Gv21139500.2
AET7Gv20752400.2
AET3Gv20018900.1
AET3Gv20019100.1
AET1Gv20179000.1
AET4Gv20228200.2
AET1Gv20590000.2
RCC1_2 Repeat
RCC1_2 Repeat
RCC1_2 Repeat
RCC1_2 Repeat
TNFR_c6
Domain Color
MCAfunc Domain
PK_Tyr_Ser−Thr Domain
Pkinase Domain
other Domain
0
500
1000

Ae.ta RLK−Pelle_CrRLK1L−1 subfamily domain diagram (part 1)

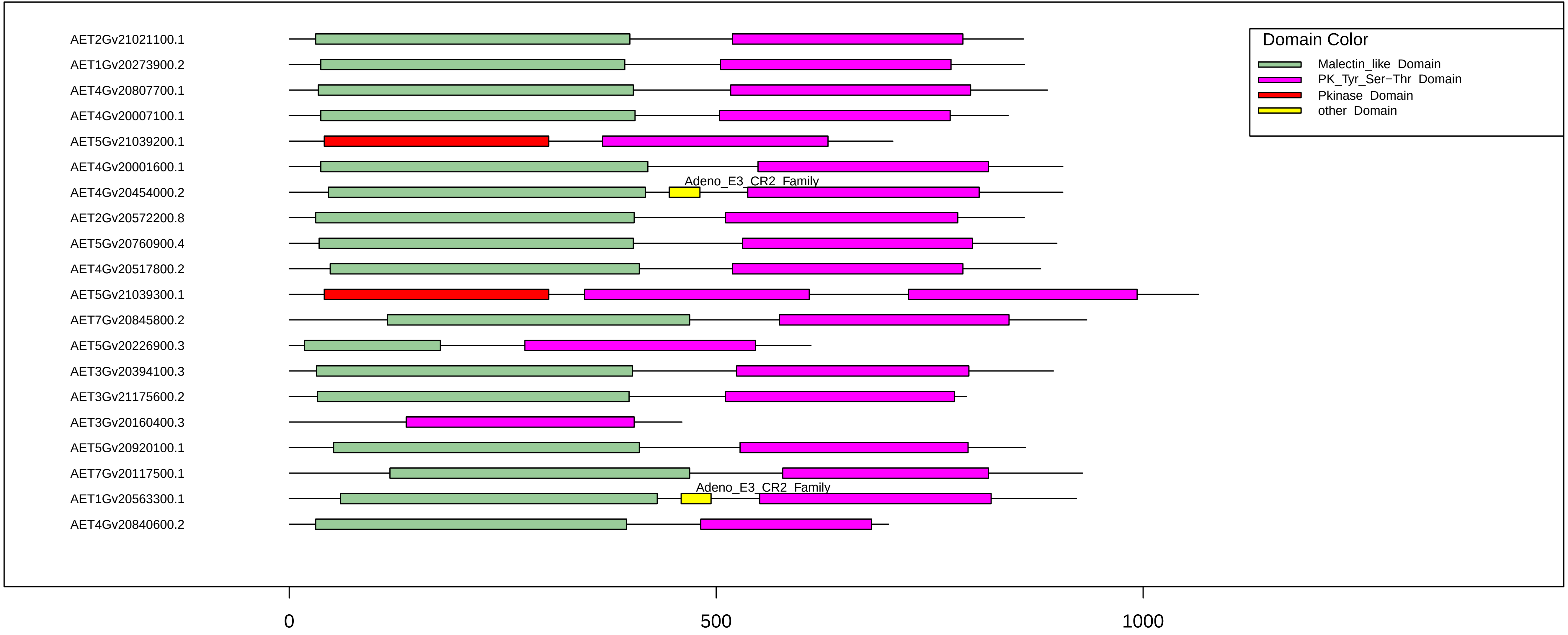

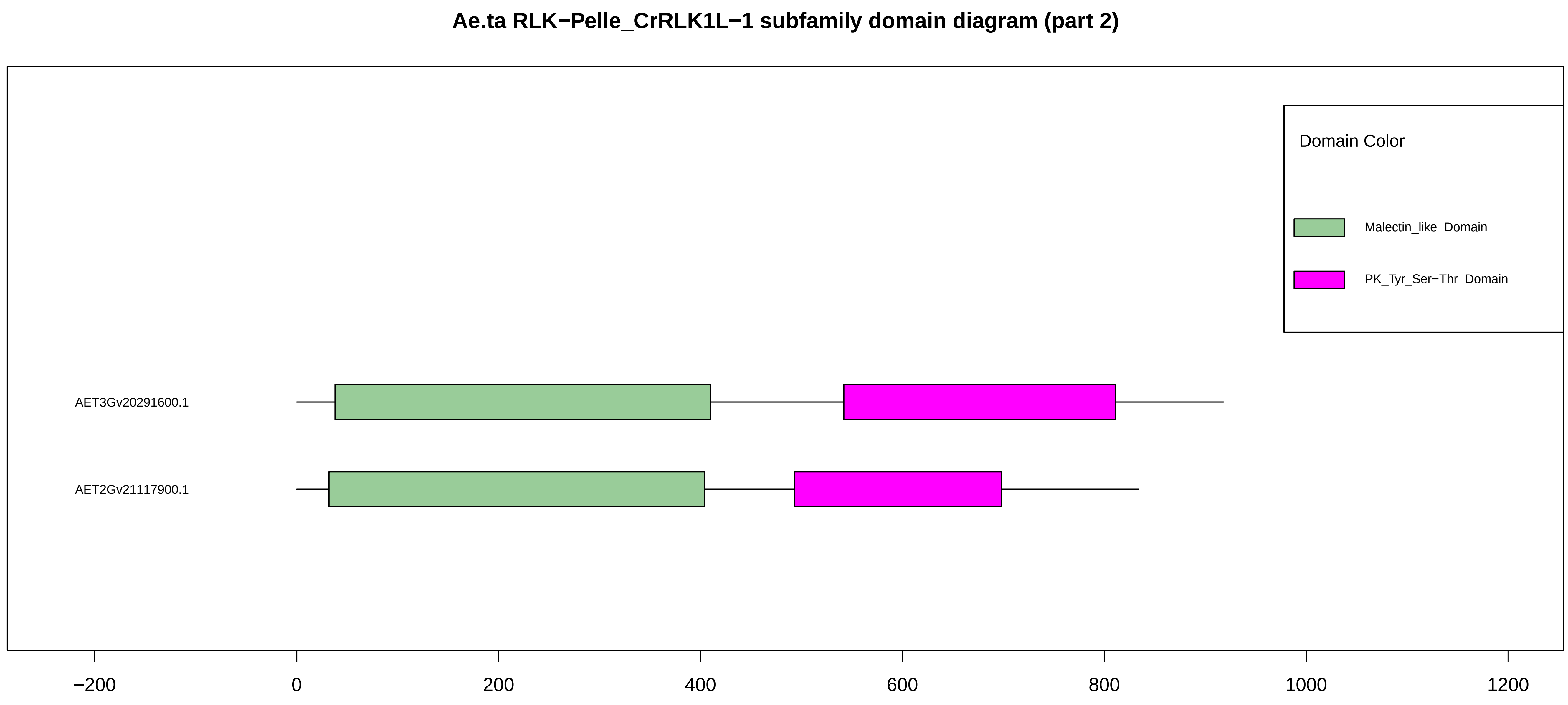
Ae.ta RLK−Pelle_CrRLK1L−1 subfamily domain diagram (part 2)
Domain Color
Malectin_like Domain
PK_Tyr_Ser−Thr Domain
AET3Gv20291600.1
AET2Gv21117900.1
−200
0
200
400
600
800
1000
1200

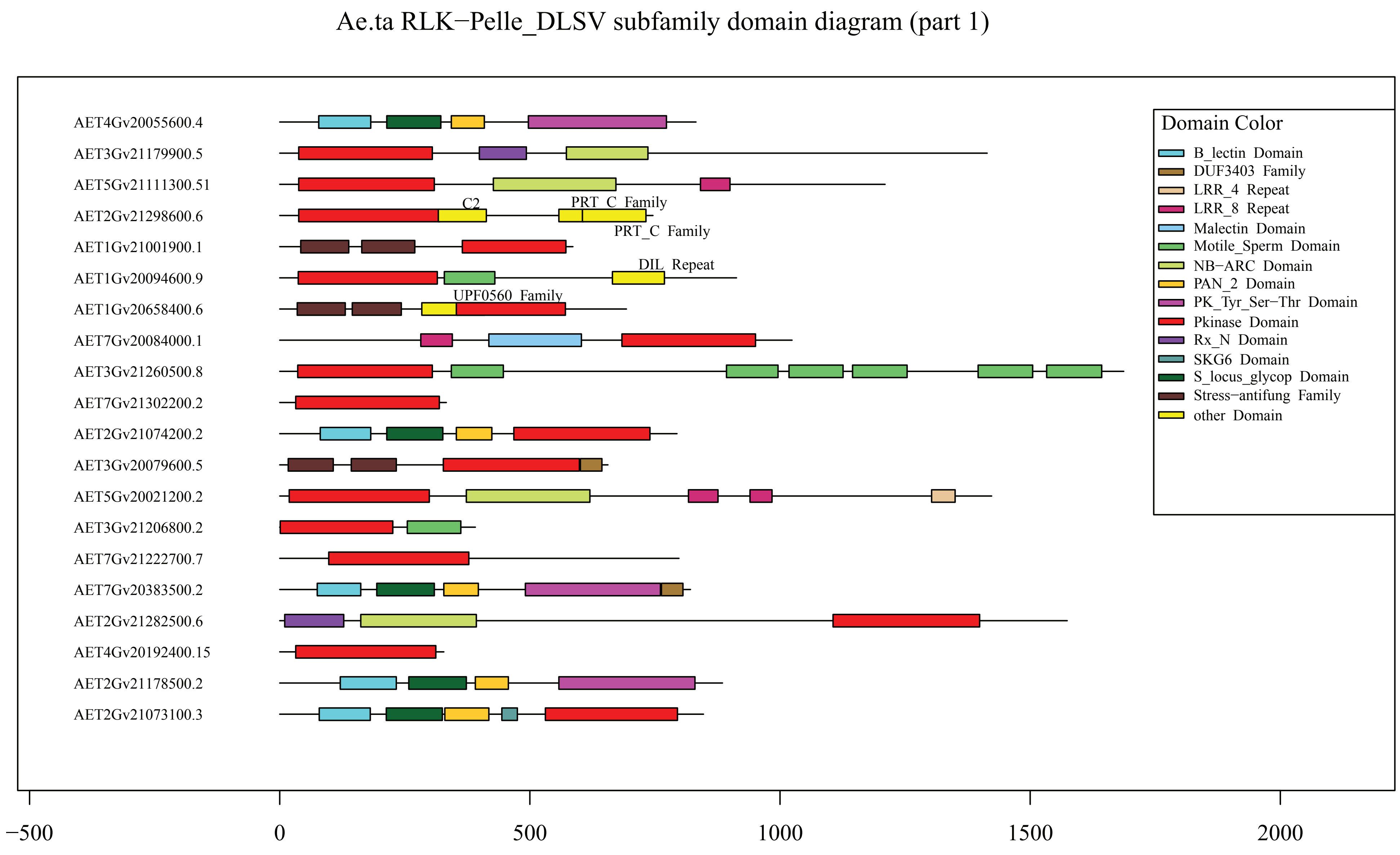

Ae.ta RLK−Pelle_DLSV subfamily domain diagram (part 1)
AET4Gv20055600.4
AET3Gv21179900.5
AET5Gv21111300.51
AET2Gv21298600.6
AET1Gv21001900.1
AET1Gv20094600.9
AET1Gv20658400.6
AET7Gv20084000.1
AET3Gv21260500.8
AET7Gv21302200.2
AET2Gv21074200.2
AET3Gv20079600.5
AET5Gv20021200.2
AET3Gv21206800.2
AET7Gv21222700.7
AET7Gv20383500.2
AET2Gv21282500.6
AET4Gv20192400.15
AET2Gv21178500.2
AET2Gv21073100.3
C2
PRT_C Family
PRT_C Family
DIL Repeat
UPF0560 Family
Domain Color
B_lectin Domain
DUF3403 Family
LRR_4 Repeat
LRR_8 Repeat
Malectin Domain
Motile_Sperm Domain
NB−ARC Domain
PAN_2 Domain
PK_Tyr_Ser−Thr Domain
Pkinase Domain
Rx_N Domain
SKG6 Domain
S_locus_glycop Domain
Stress−antifung Family
other Domain
−500
0
500
1000
1500
2000

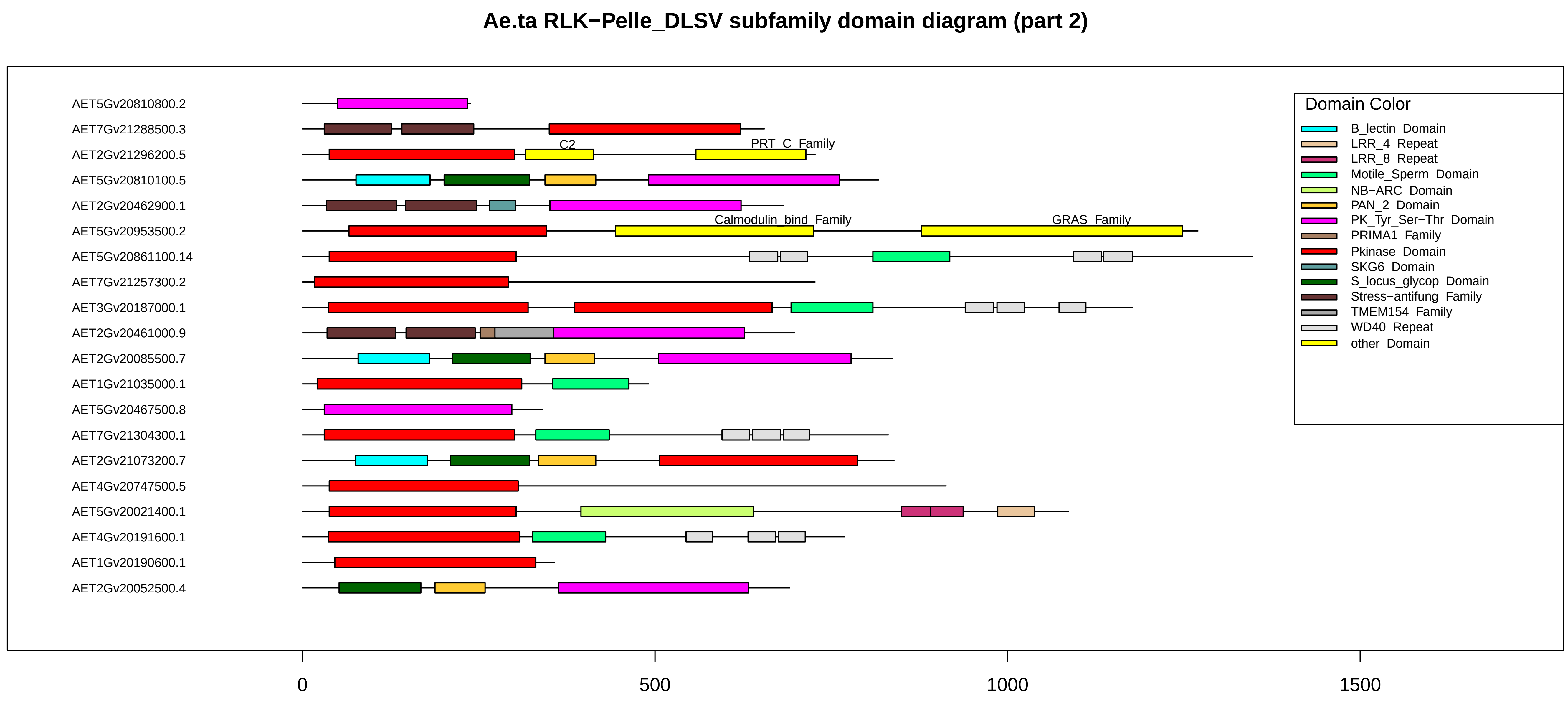
Ae.ta RLK−Pelle_DLSV subfamily domain diagram (part 2)
AET5Gv20810800.2
AET7Gv21288500.3
AET2Gv21296200.5
AET5Gv20810100.5
AET2Gv20462900.1
AET5Gv20953500.2
AET5Gv20861100.14
AET7Gv21257300.2
AET3Gv20187000.1
AET2Gv20461000.9
AET2Gv20085500.7
AET1Gv21035000.1
AET5Gv20467500.8
AET7Gv21304300.1
AET2Gv21073200.7
AET4Gv20747500.5
AET5Gv20021400.1
AET4Gv20191600.1
AET1Gv20190600.1
AET2Gv20052500.4
C2
PRT_C_Family
Calmodulin_bind_Family
GRAS_Family
Domain Color
B_lectin Domain
LRR_4 Repeat
LRR_8 Repeat
Motile_Sperm Domain
NB−ARC Domain
PAN_2 Domain
PK_Tyr_Ser−Thr Domain
PRIMA1 Family
Pkinase Domain
SKG6 Domain
S_locus_glycop Domain
Stress−antifung Family
TMEM154 Family
WD40 Repeat
other Domain
0
500
1000
1500

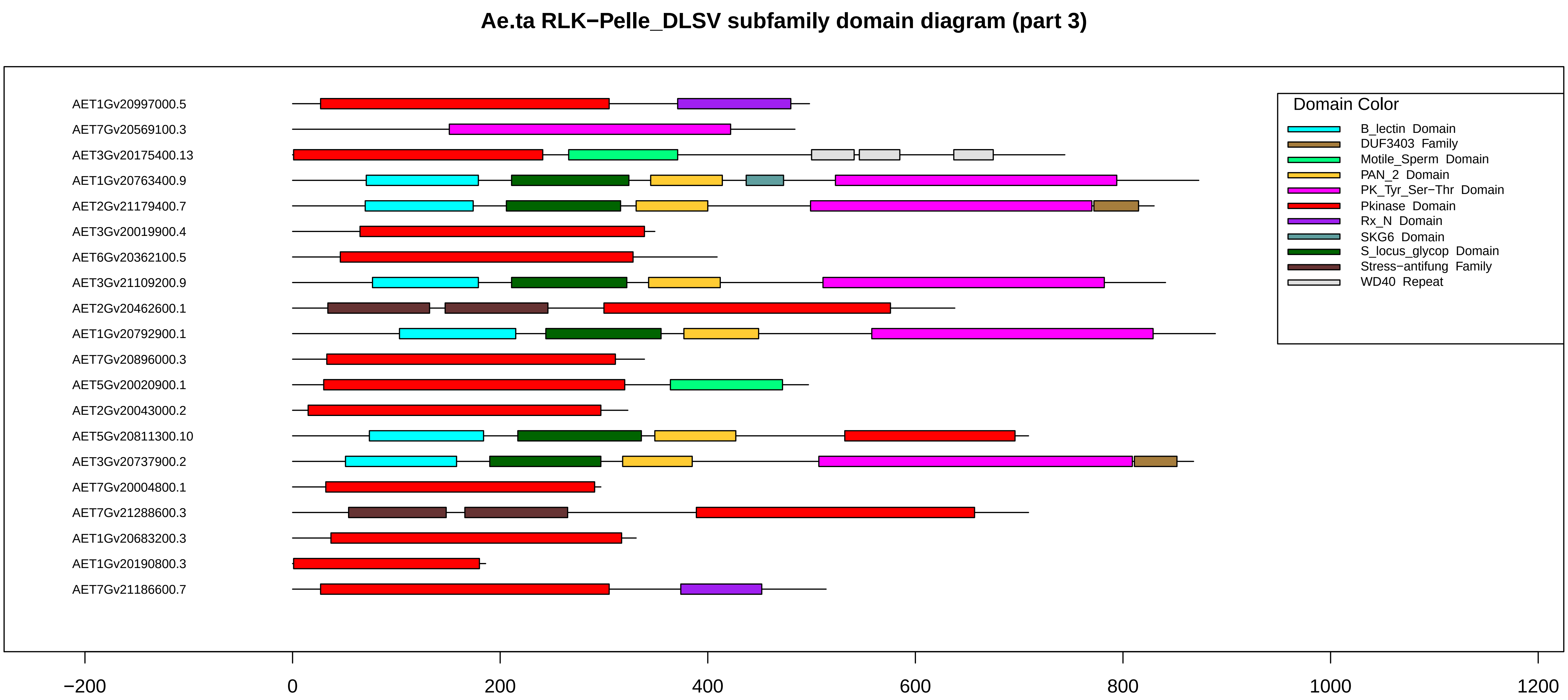

Ae.ta RLK−Pelle_DLSV subfamily domain diagram (part 3)
AET1Gv20997000.5
AET7Gv20569100.3
AET3Gv20175400.13
AET1Gv20763400.9
AET2Gv21179400.7
AET3Gv20019900.4
AET6Gv20362100.5
AET3Gv21109200.9
AET2Gv20462600.1
AET1Gv20792900.1
AET7Gv20896000.3
AET5Gv20020900.1
AET2Gv20043000.2
AET5Gv20811300.10
AET3Gv20737900.2
AET7Gv20004800.1
AET7Gv21288600.3
AET1Gv20683200.3
AET1Gv20190800.3
AET7Gv21186600.7
Domain Color
B_lectin Domain
DUF3403 Family
Motile_Sperm Domain
PAN_2 Domain
PK_Tyr_Ser−Thr Domain
Pkinase Domain
Rx_N Domain
SKG6 Domain
S_locus_glycop Domain
Stress−antifung Family
WD40 Repeat
−200
0
200
400
600
800
1000
1200

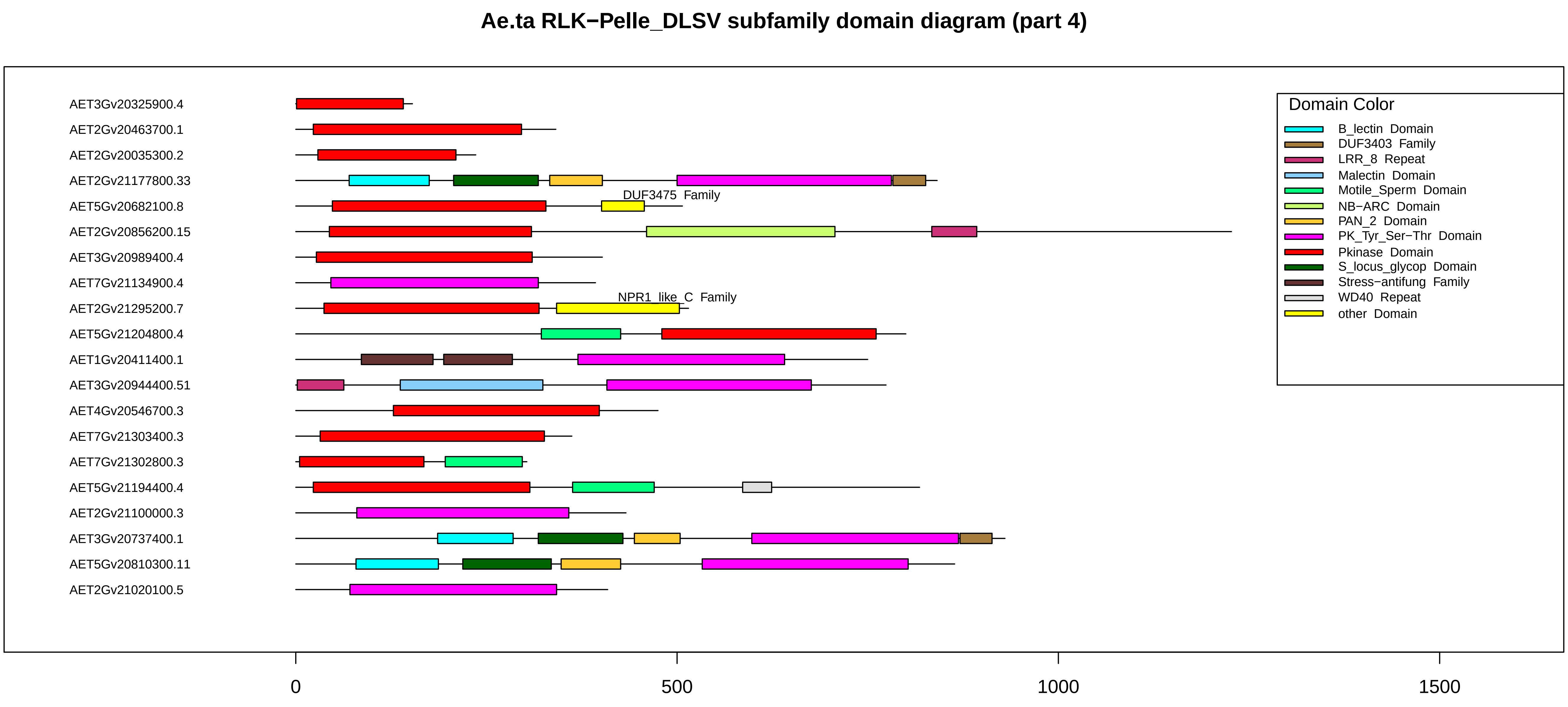

Ae.ta RLK−Pelle_DLSV subfamily domain diagram (part 4)
AET3Gv20325900.4
AET2Gv20463700.1
AET2Gv20035300.2
AET2Gv21177800.33
AET5Gv20682100.8
AET2Gv20856200.15
AET3Gv20989400.4
AET7Gv21134900.4
AET2Gv21295200.7
AET5Gv21204800.4
AET1Gv20411400.1
AET3Gv20944400.51
AET4Gv20546700.3
AET7Gv21303400.3
AET7Gv21302800.3
AET5Gv21194400.4
AET2Gv21100000.3
AET3Gv20737400.1
AET5Gv20810300.11
AET2Gv21020100.5
DUF3475 Family
NPR1_like_C Family
Domain Color
B_lectin Domain
DUF3403 Family
LRR_8 Repeat
Malectin Domain
Motile_Sperm Domain
NB−ARC Domain
PAN_2 Domain
PK_Tyr_Ser−Thr Domain
Pkinase Domain
S_locus_glycop Domain
Stress−antifung Family
WD40 Repeat
other Domain
0
500
1000
1500

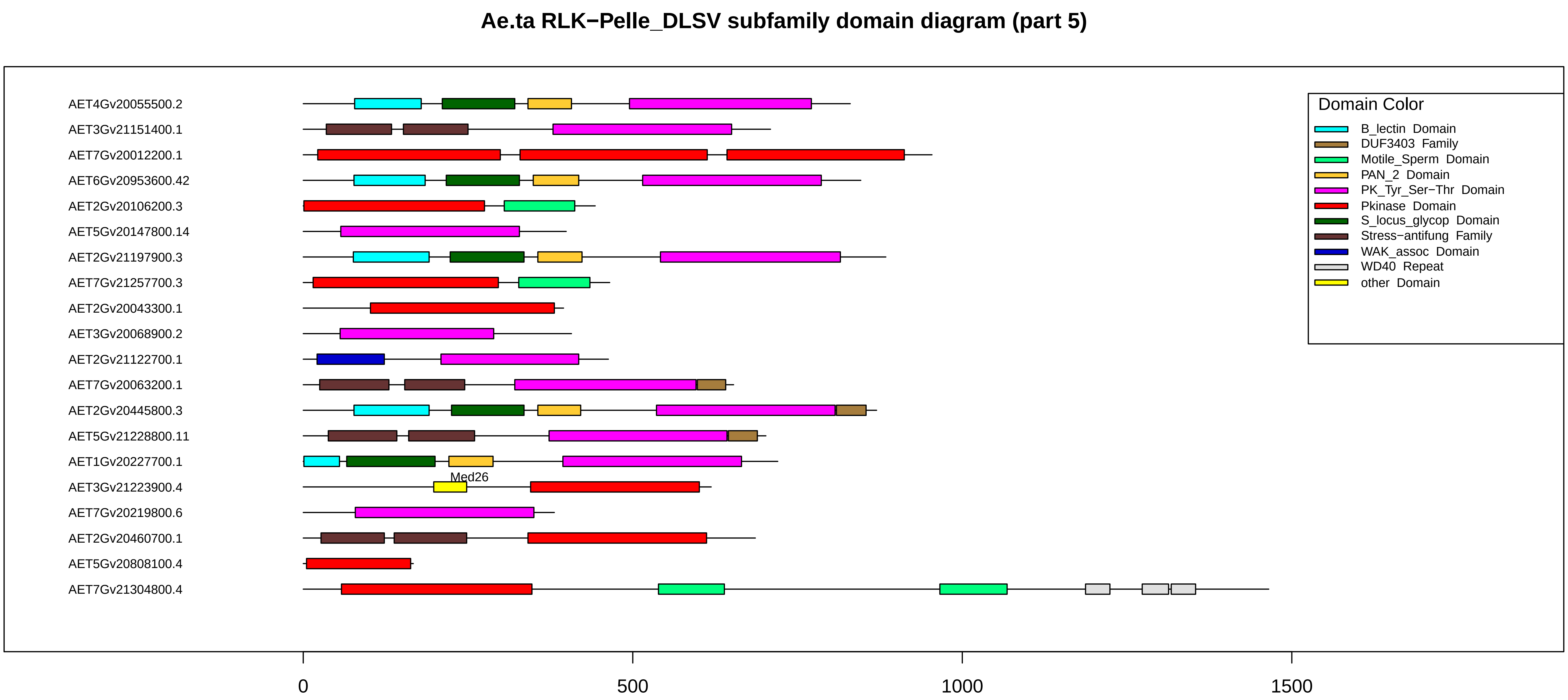

Ae.ta RLK−Pelle_DLSV subfamily domain diagram (part 5)
AET4Gv20055500.2
AET3Gv21151400.1
AET7Gv20012200.1
AET6Gv20953600.42
AET2Gv20106200.3
AET5Gv20147800.14
AET2Gv21197900.3
AET7Gv21257700.3
AET2Gv20043300.1
AET3Gv20068900.2
AET2Gv21122700.1
AET7Gv20063200.1
AET2Gv20445800.3
AET5Gv21228800.11
AET1Gv20227700.1
AET3Gv21223900.4
Med26
AET7Gv20219800.6
AET2Gv20460700.1
AET5Gv20808100.4
AET7Gv21304800.4
Domain Color
B_lectin Domain
DUF3403 Family
Motile_Sperm Domain
PAN_2 Domain
PK_Tyr_Ser−Thr Domain
Pkinase Domain
S_locus_glycop Domain
Stress−antifung Family
WAK_assoc Domain
WD40 Repeat
other Domain
0
500
1000
1500

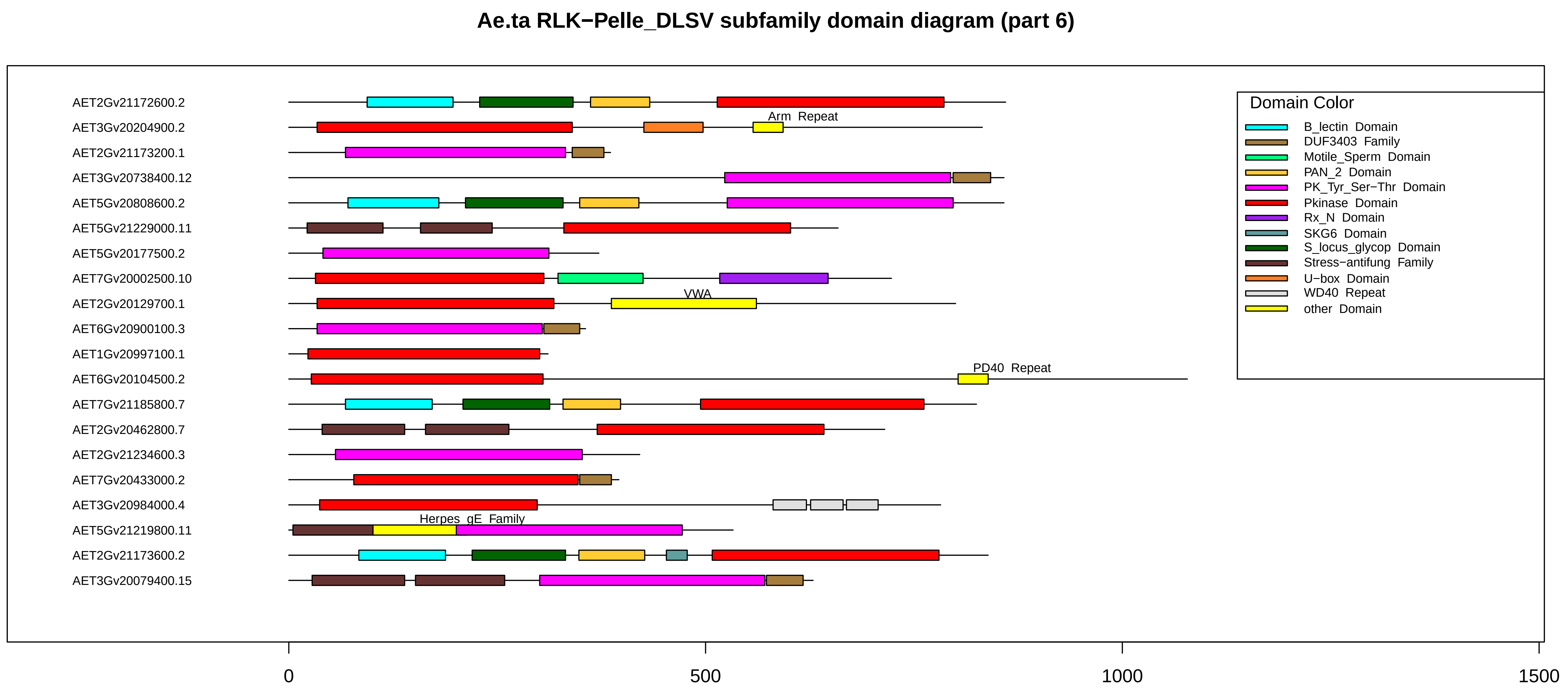
Ae.ta RLK−Pelle_DLSV subfamily domain diagram (part 6)
AET2Gv21172600.2
AET3Gv20204900.2
AET2Gv21173200.1
AET3Gv20738400.12
AET5Gv20808600.2
AET5Gv21229000.11
AET5Gv20177500.2
AET7Gv20002500.10
AET2Gv20129700.1
AET6Gv20900100.3
AET1Gv20997100.1
AET6Gv20104500.2
AET7Gv21185800.7
AET2Gv20462800.7
AET2Gv21234600.3
AET7Gv20433000.2
AET3Gv20984000.4
AET5Gv21219800.11
AET2Gv21173600.2
AET3Gv20079400.15
Arm Repeat
VWA
PD40 Repeat
Herpes gE Family
Domain Color
B_lectin Domain
DUF3403 Family
Motile_Sperm Domain
PAN_2 Domain
PK_Tyr_Ser−Thr Domain
Pkinase Domain
Rx_N Domain
SKG6 Domain
S_locus_glycop Domain
Stress−antifung Family
U−box Domain
WD40 Repeat
other Domain
0
500
1000
1500

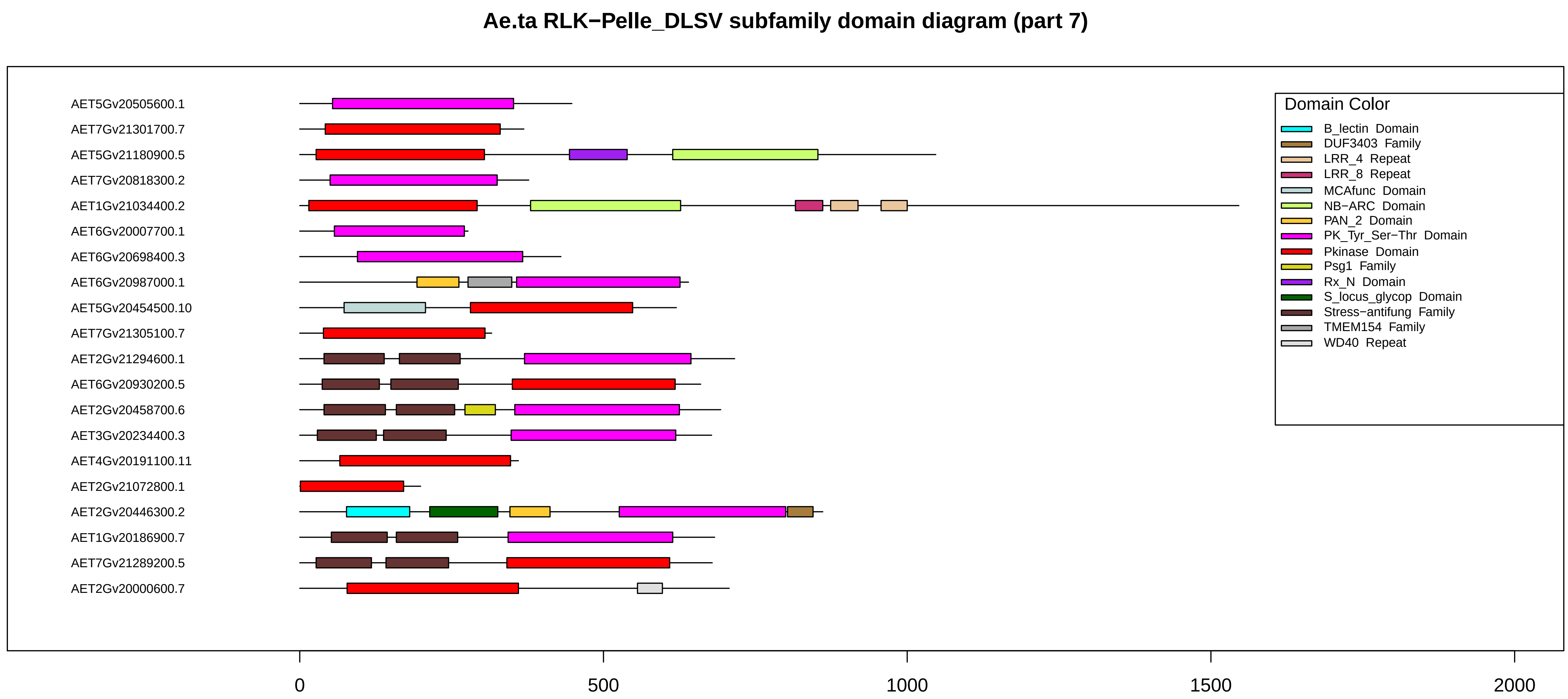
Ae.ta RLK−Pelle_DLSV subfamily domain diagram (part 7)
AET5Gv20505600.1
AET7Gv21301700.7
AET5Gv21180900.5
AET7Gv20818300.2
AET1Gv21034400.2
AET6Gv20007700.1
AET6Gv20698400.3
AET6Gv20987000.1
AET5Gv20454500.10
AET7Gv21305100.7
AET2Gv21294600.1
AET6Gv20930200.5
AET2Gv20458700.6
AET3Gv20234400.3
AET4Gv20191100.11
AET2Gv21072800.1
AET2Gv20446300.2
AET1Gv20186900.7
AET7Gv21289200.5
AET2Gv20000600.7
Domain Color
B_lectin Domain
DUF3403 Family
LRR_4 Repeat
LRR_8 Repeat
MCAfunc Domain
NB−ARC Domain
PAN_2 Domain
PK_Tyr_Ser−Thr Domain
Pkinase Domain
Psg1 Family
Rx_N Domain
S_locus_glycop Domain
Stress−antifung Family
TMEM154 Family
WD40 Repeat
0
500
1000
1500
2000

Ae.ta RLK−Pelle_DLSV subfamily domain diagram (part 8)

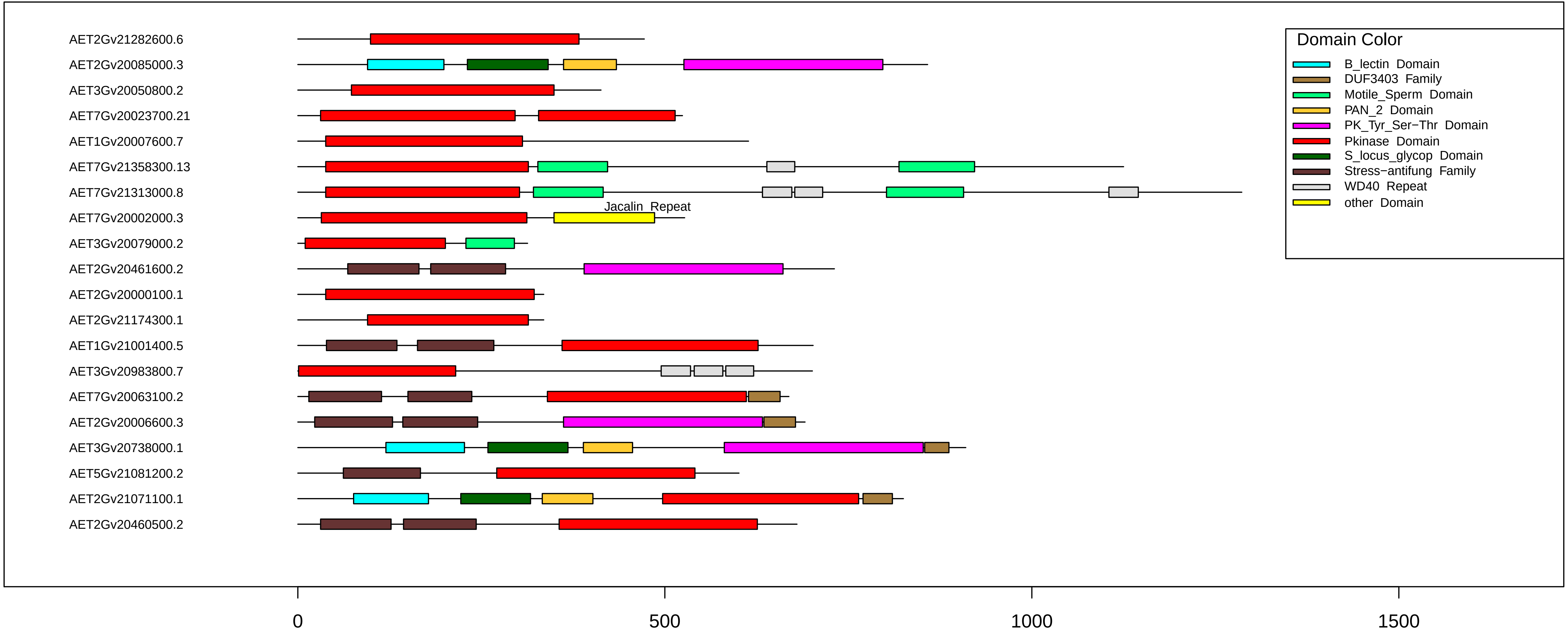

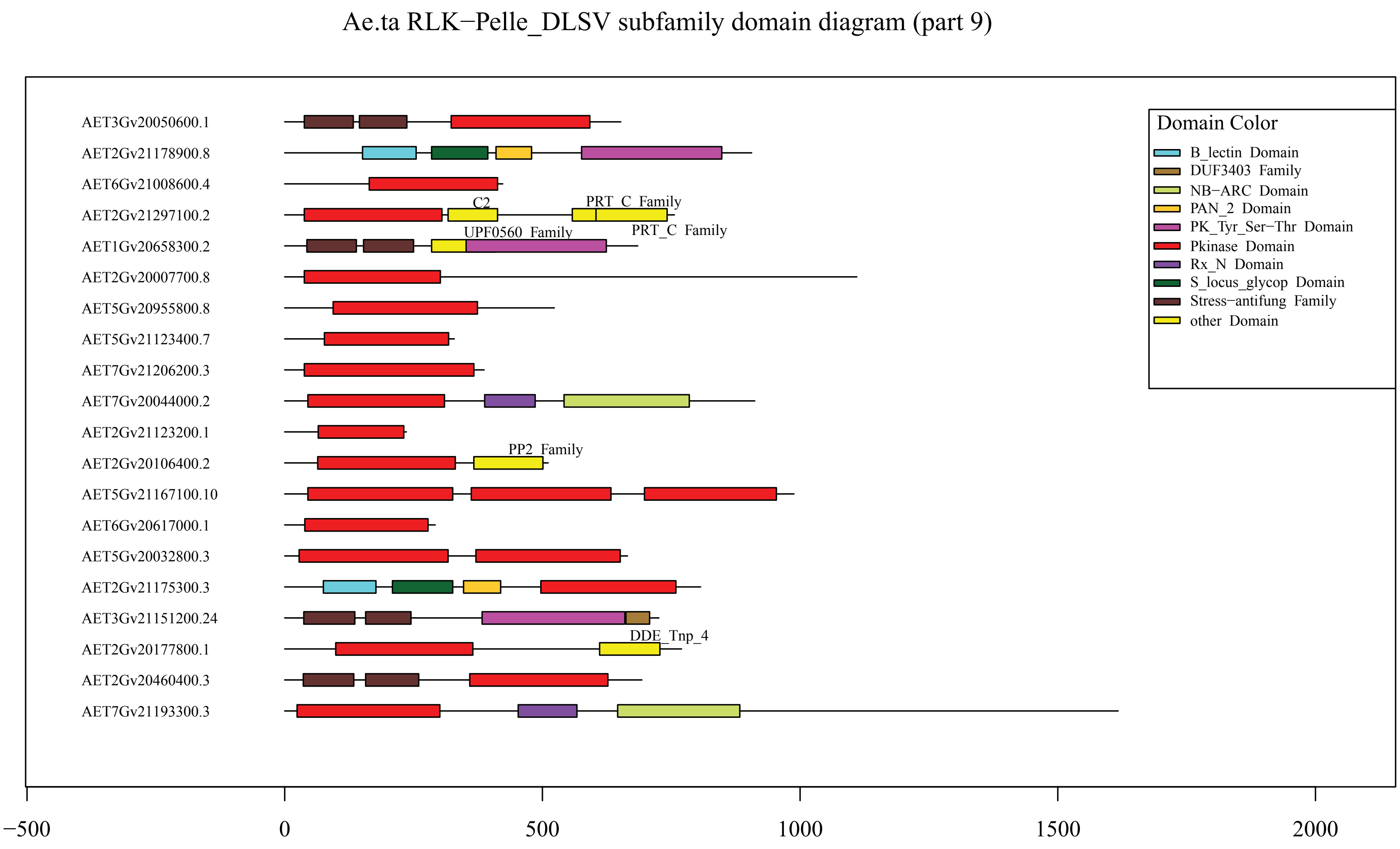

Ae.ta RLK−Pelle_DLSV subfamily domain diagram (part 9)
AET3Gv20050600.1
AET2Gv21178900.8
AET6Gv21008600.4
AET2Gv21297100.2
AET1Gv20658300.2
AET2Gv20007700.8
AET5Gv20955800.8
AET5Gv21123400.7
AET7Gv21206200.3
AET7Gv20044000.2
AET2Gv21123200.1
AET2Gv20106400.2
AET5Gv21167100.10
AET6Gv20617000.1
AET5Gv20032800.3
AET2Gv21175300.3
AET3Gv21151200.24
AET2Gv20177800.1
AET2Gv20460400.3
AET7Gv21193300.3
C2
PRT_C_Family
UPF0560_Family
PRT_C_Family
PP2_Family
DDE_Tnp_4
Domain Color
B_lectin Domain
DUF3403 Family
NB−ARC Domain
PAN_2 Domain
PK_Tyr_Ser−Thr Domain
Pkinase Domain
Rx_N Domain
S_locus_glycop Domain
Stress−antifung Family
other Domain
−500
0
500
1000
1500
2000

Ae.ta RLK−Pelle_DLSV subfamily domain diagram (part 10)

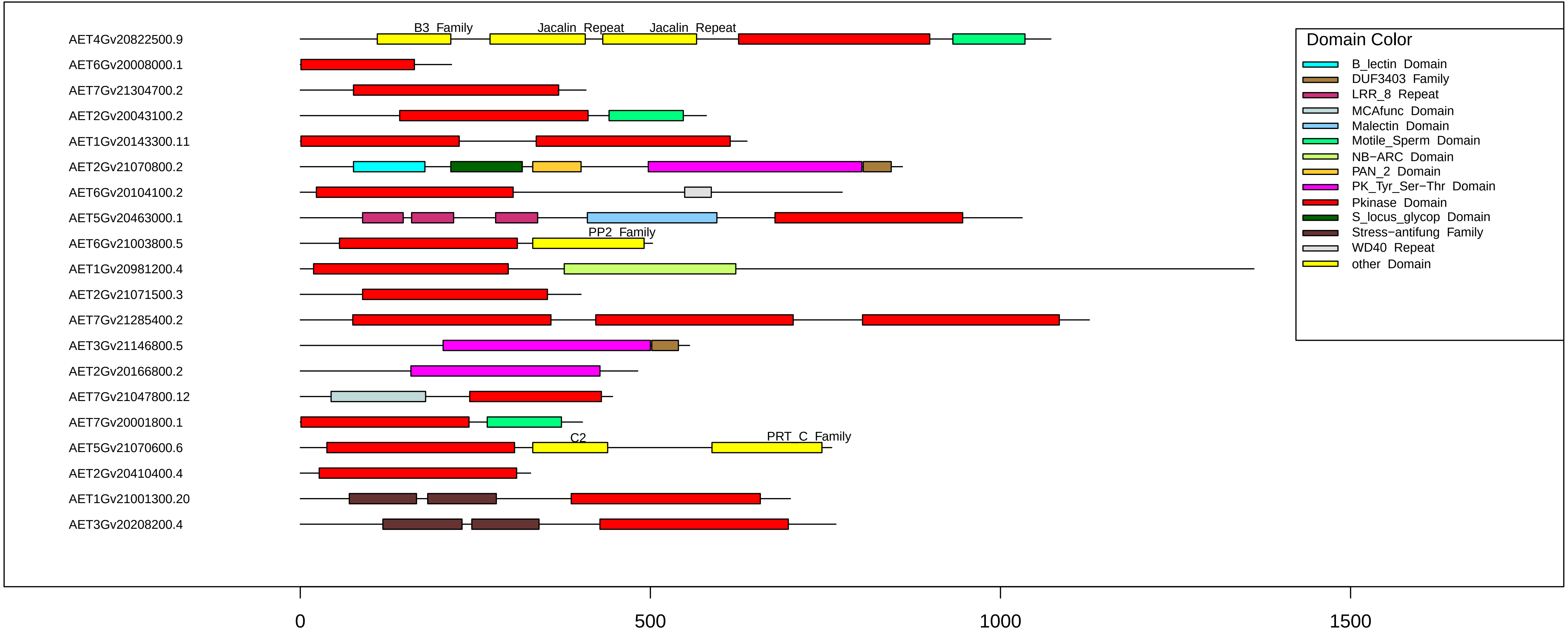

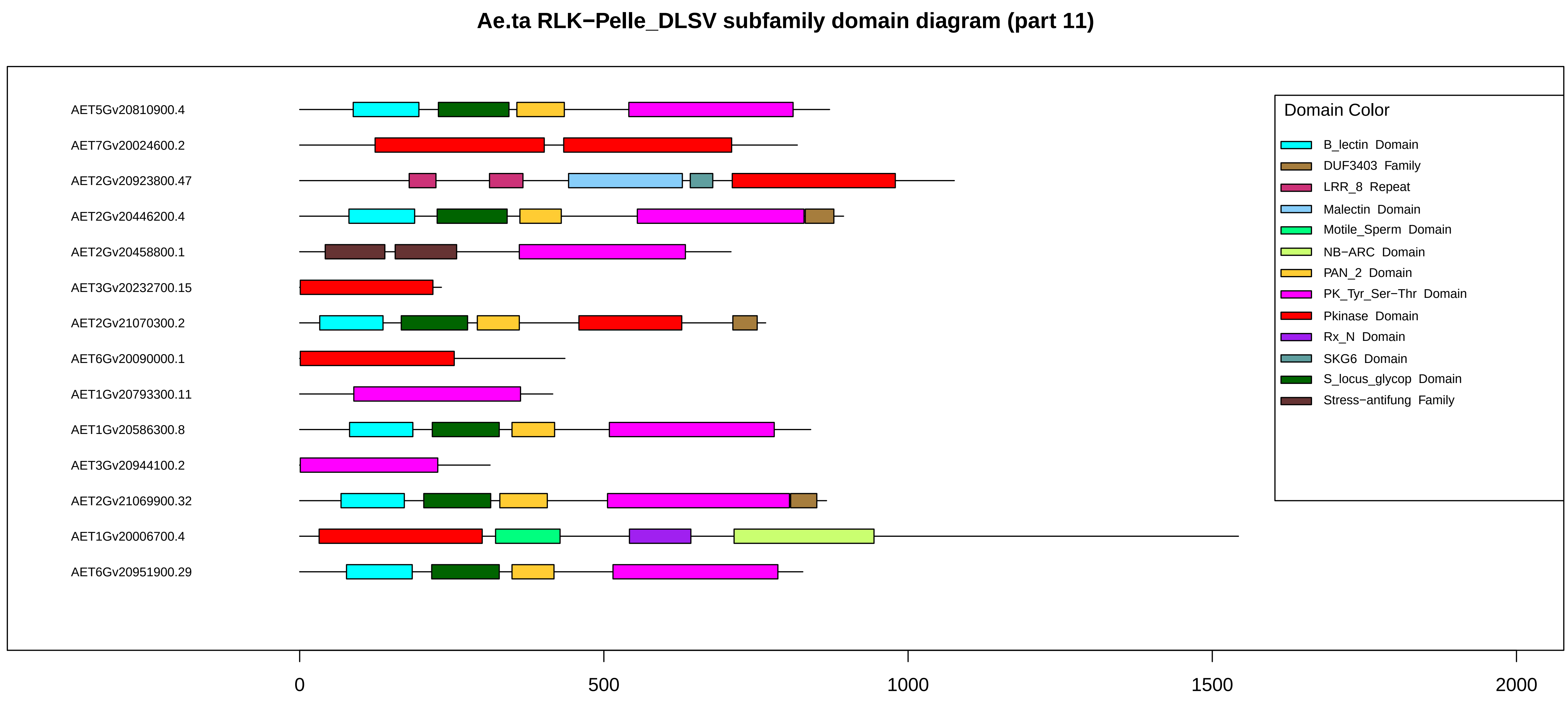
Ae.ta RLK−Pelle_DLSV subfamily domain diagram (part 11)
AET5Gv20810900.4
AET7Gv20024600.2
AET2Gv20923800.47
AET2Gv20446200.4
AET2Gv20458800.1
AET3Gv20232700.15
AET2Gv21070300.2
AET6Gv20090000.1
AET1Gv20793300.11
AET1Gv20586300.8
AET3Gv20944100.2
AET2Gv21069900.32
AET1Gv20006700.4
AET6Gv20951900.29
Domain Color
B_lectin Domain
DUF3403 Family
LRR_8 Repeat
Malectin Domain
Motile_Sperm Domain
NB−ARC Domain
PAN_2 Domain
PK_Tyr_Ser−Thr Domain
Pkinase Domain
Rx_N Domain
SKG6 Domain
S_locus_glycop Domain
Stress−antifung Family
0
500
1000
1500
2000

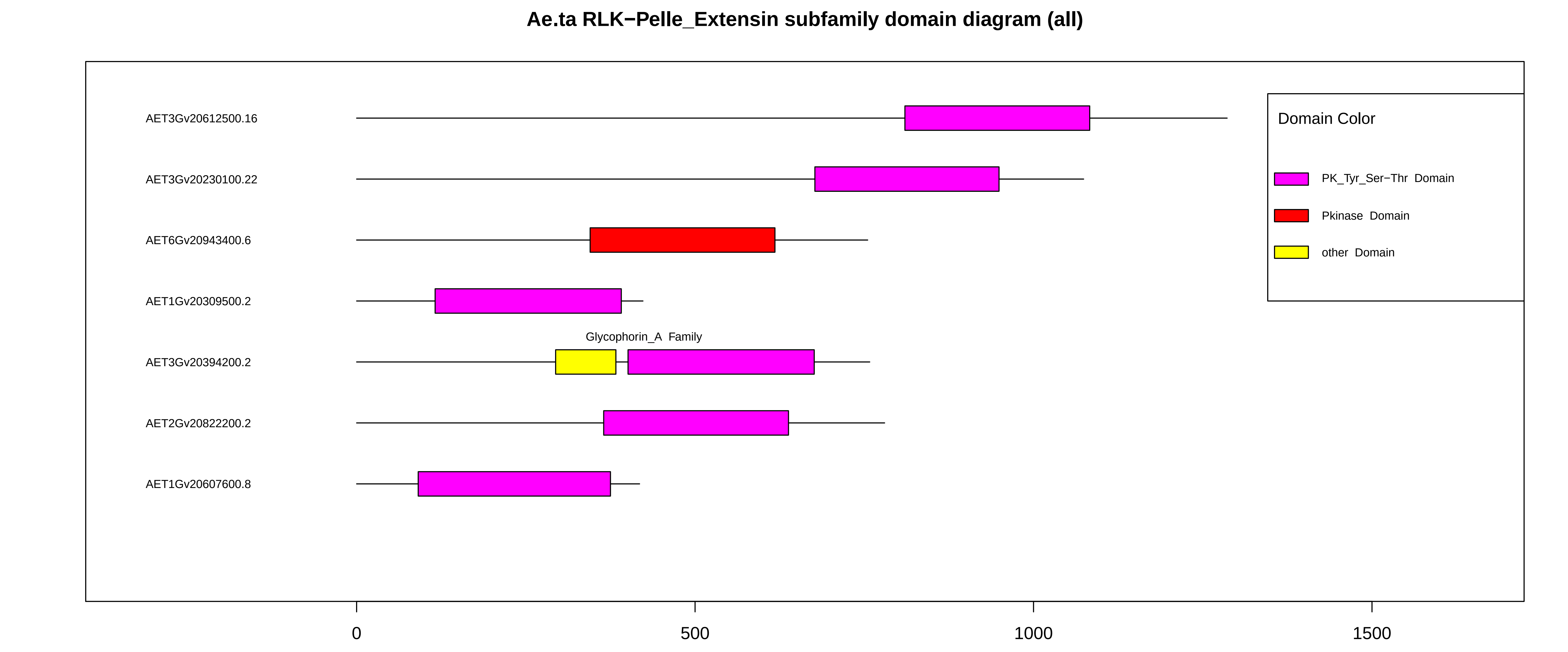

Ae.ta RLK−Pelle_Extensin subfamily domain diagram (all)
AET3Gv20612500.16
AET3Gv20230100.22
AET6Gv20943400.6
AET1Gv20309500.2
Glycophorin_A Family
AET3Gv20394200.2
AET2Gv20822200.2
AET1Gv20607600.8
Domain Color
PK_Tyr_Ser−Thr Domain
Pkinase Domain
other Domain
0
500
1000
1500

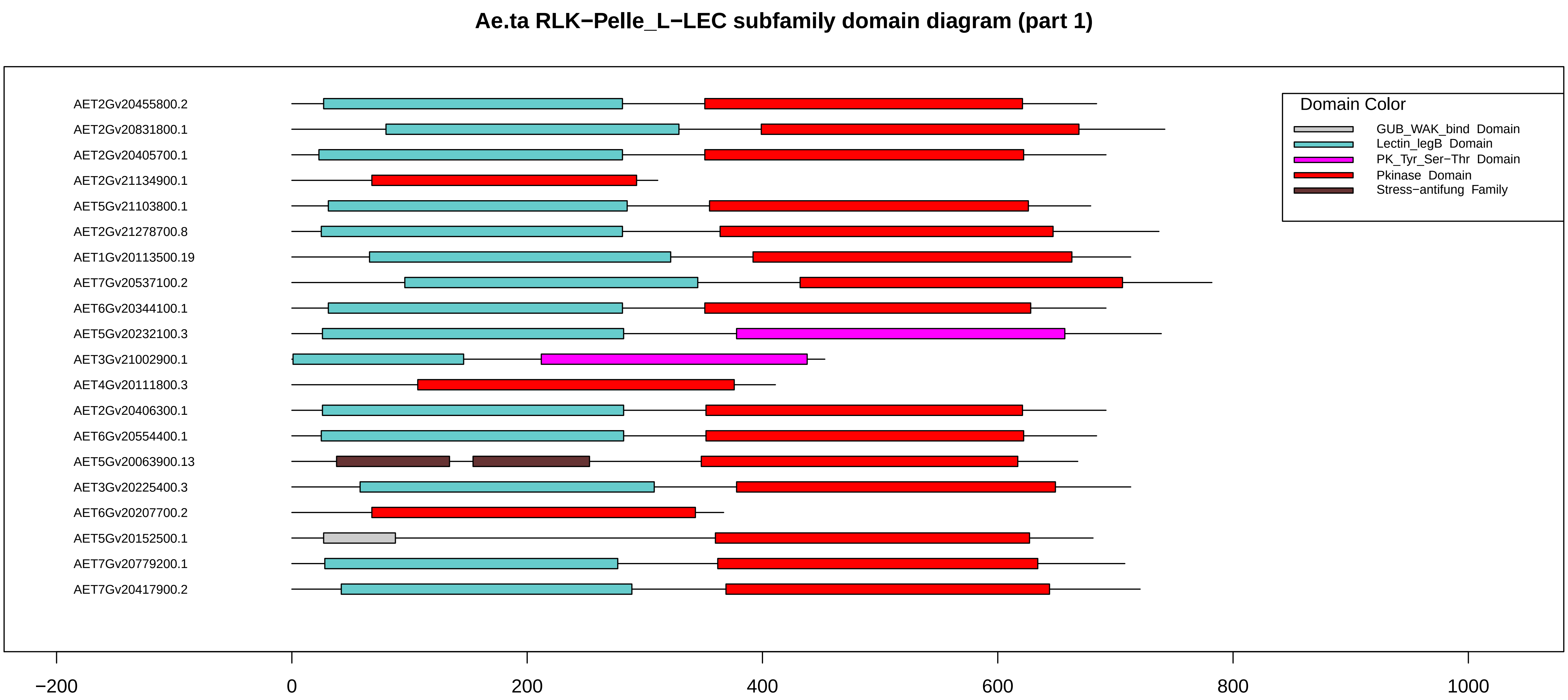

Ae.ta RLK−Pelle_L−LEC subfamily domain diagram (part 1)
AET2Gv20455800.2
AET2Gv20831800.1
AET2Gv20405700.1
AET2Gv21134900.1
AET5Gv21103800.1
AET2Gv21278700.8
AET1Gv20113500.19
AET7Gv20537100.2
AET6Gv20344100.1
AET5Gv20232100.3
AET3Gv21002900.1
AET4Gv20111800.3
AET2Gv20406300.1
AET6Gv20554400.1
AET5Gv20063900.13
AET3Gv20225400.3
AET6Gv20207700.2
AET5Gv20152500.1
AET7Gv20779200.1
AET7Gv20417900.2
Domain Color
GUB_WAK_bind Domain
Lectin_legB Domain
PK_Tyr_Ser−Thr Domain
Pkinase Domain
Stress−antifung Family
−200
0
200
400
600
800
1000

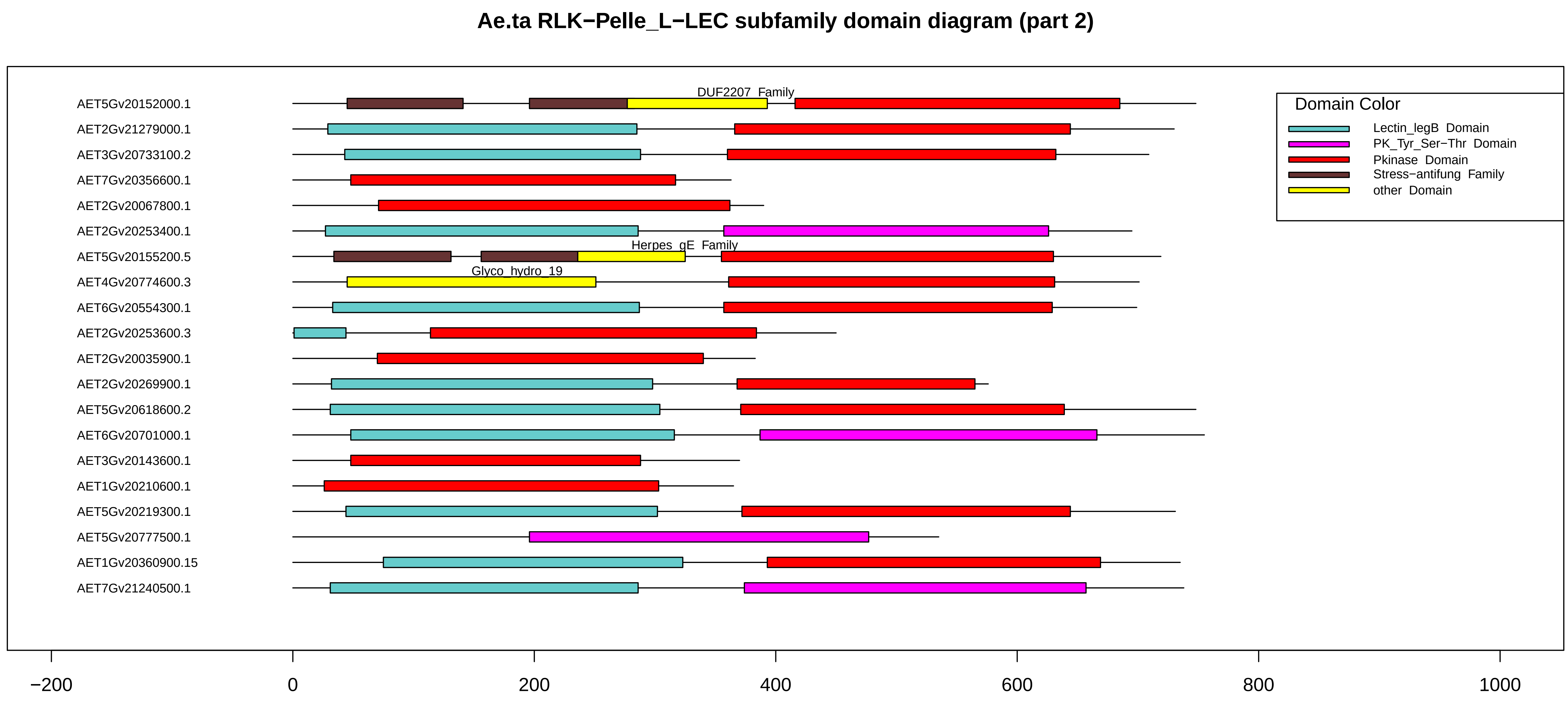

Ae.ta RLK−Pelle_L−LEC subfamily domain diagram (part 2)
AET5Gv20152000.1
AET2Gv21279000.1
AET3Gv20733100.2
AET7Gv20356600.1
AET2Gv20067800.1
AET2Gv20253400.1
AET5Gv20155200.5
AET4Gv20774600.3
AET6Gv20554300.1
AET2Gv20253600.3
AET2Gv20035900.1
AET2Gv20269900.1
AET5Gv20618600.2
AET6Gv20701000.1
AET3Gv20143600.1
AET1Gv20210600.1
AET5Gv20219300.1
AET5Gv20777500.1
AET1Gv20360900.15
AET7Gv21240500.1
DUF2207_Family
Herpes_gE_Family
Glyco_hydro_19
Domain Color
Lectin_legB Domain
PK_Tyr_Ser−Thr Domain
Pkinase Domain
Stress−antifung Family
other Domain
−200
0
200
400
600
800
1000

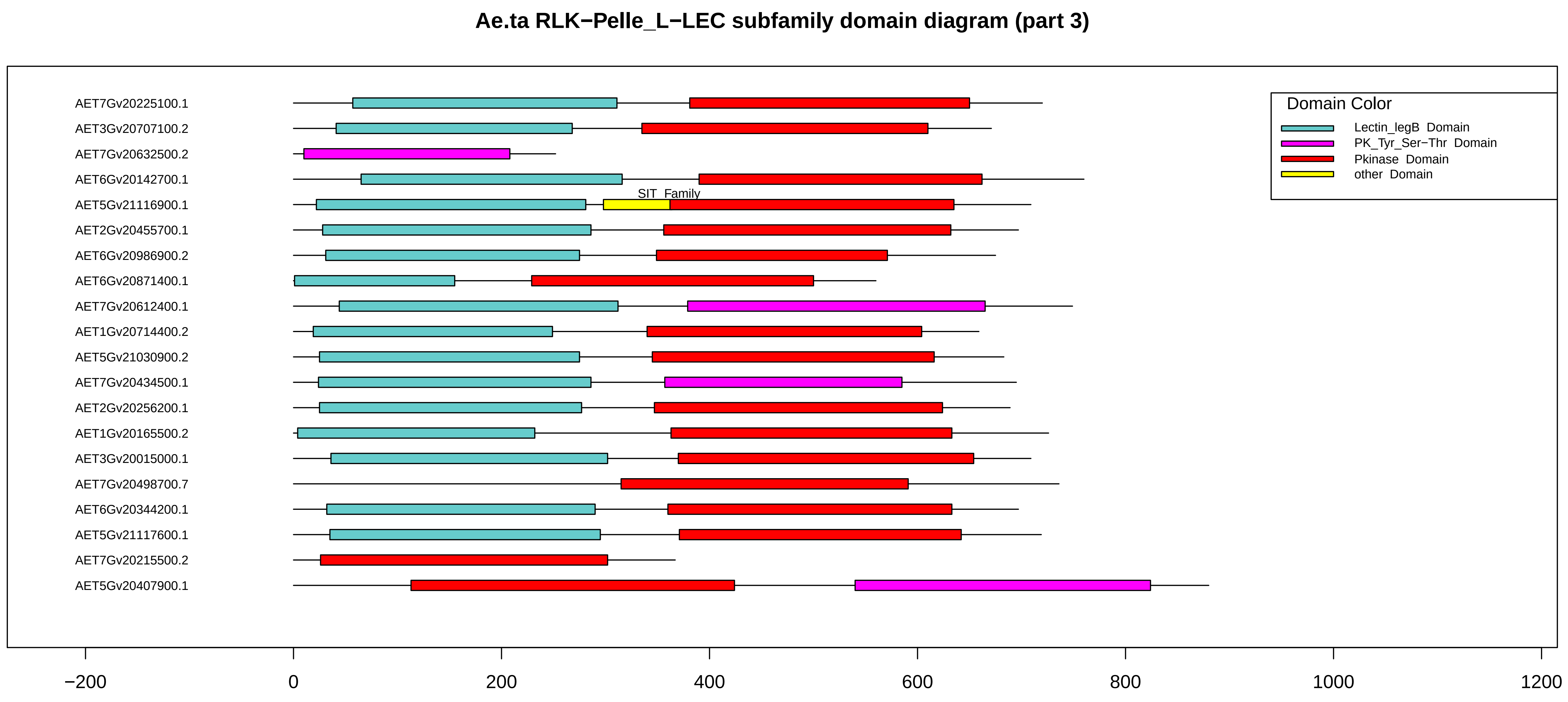

Ae.ta RLK−Pelle_L−LEC subfamily domain diagram (part 3)
AET7Gv20225100.1
AET3Gv20707100.2
AET7Gv20632500.2
AET6Gv20142700.1
AET5Gv21116900.1
AET2Gv20455700.1
AET6Gv20986900.2
AET6Gv20871400.1
AET7Gv20612400.1
AET1Gv20714400.2
AET5Gv21030900.2
AET7Gv20434500.1
AET2Gv20256200.1
AET1Gv20165500.2
AET3Gv20015000.1
AET7Gv20498700.7
AET6Gv20344200.1
AET5Gv21117600.1
AET7Gv20215500.2
AET5Gv20407900.1
SIT_Family
Domain Color
Lectin_legB Domain
PK_Tyr_Ser−Thr Domain
Pkinase Domain
other Domain
−200
0
200
400
600
800
1000
1200

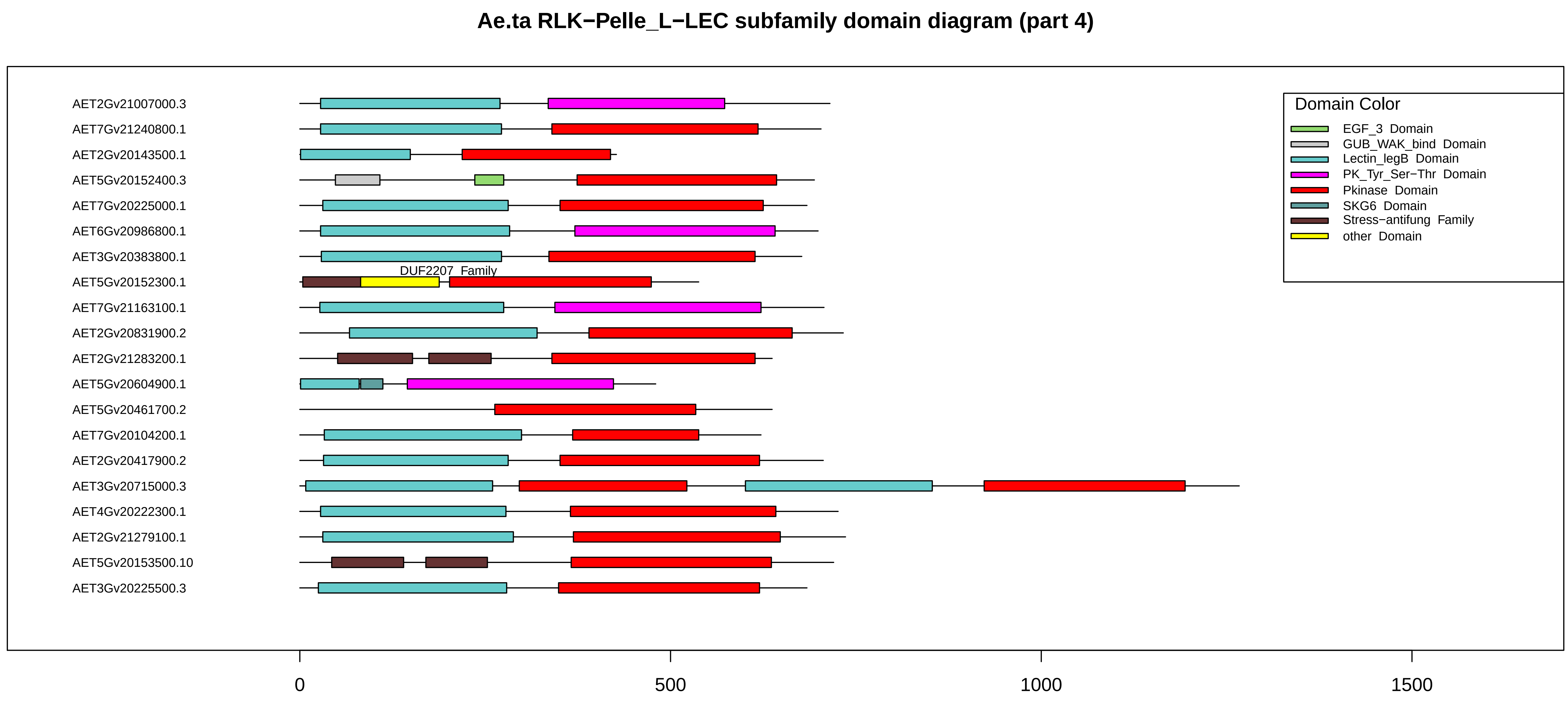
Ae.ta RLK−Pelle_L−LEC subfamily domain diagram (part 4)
AET2Gv21007000.3
AET7Gv21240800.1
AET2Gv20143500.1
AET5Gv20152400.3
AET7Gv20225000.1
AET6Gv20986800.1
AET3Gv20383800.1
AET5Gv20152300.1
DUF2207_Family
AET7Gv21163100.1
AET2Gv20831900.2
AET2Gv21283200.1
AET5Gv20604900.1
AET5Gv20461700.2
AET7Gv20104200.1
AET2Gv20417900.2
AET3Gv20715000.3
AET4Gv20222300.1
AET2Gv21279100.1
AET5Gv20153500.10
AET3Gv20225500.3
Domain Color
EGF_3 Domain
GUB_WAK_bind Domain
Lectin_legB Domain
PK_Tyr_Ser−Thr Domain
Pkinase Domain
SKG6 Domain
Stress−antifung Family
other Domain
0
500
1000
1500

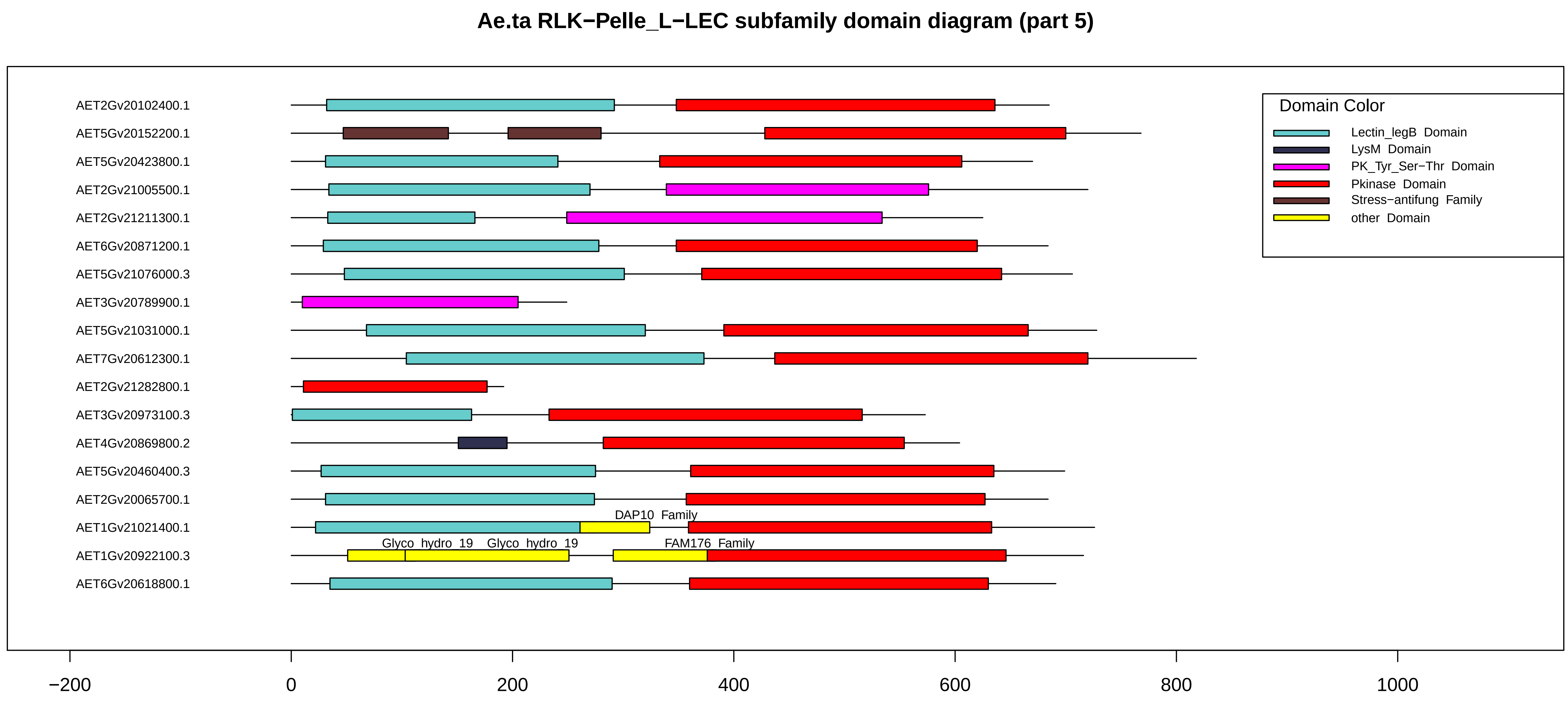
Ae.ta RLK−Pelle_L−LEC subfamily domain diagram (part 5)
AET2Gv20102400.1
AET5Gv20152200.1
AET5Gv20423800.1
AET2Gv21005500.1
AET2Gv21211300.1
AET6Gv20871200.1
AET5Gv21076000.3
AET3Gv20789900.1
AET5Gv21031000.1
AET7Gv20612300.1
AET2Gv21282800.1
AET3Gv20973100.3
AET4Gv20869800.2
AET5Gv20460400.3
AET2Gv20065700.1
AET1Gv21021400.1
AET1Gv20922100.3
AET6Gv20618800.1
DAP10 Family
Glyco hydro 19
Glyco hydro 19
FAM176 Family
Domain Color
Lectin_legB Domain
LysM Domain
PK_Tyr_Ser−Thr Domain
Pkinase Domain
Stress−antifung Family
other Domain
−200
0
200
400
600
800
1000

Ae.ta RLK−Pelle_LRK10L−2 subfamily domain diagram (part 1)

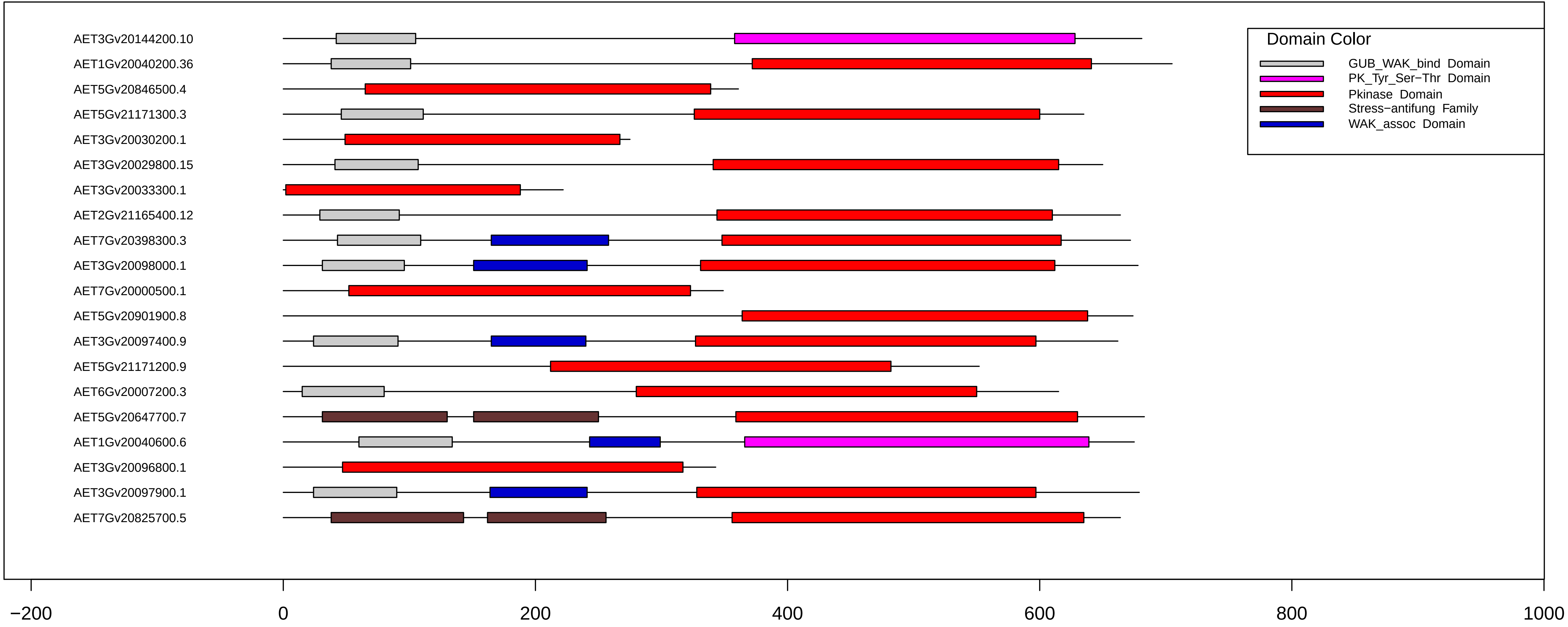

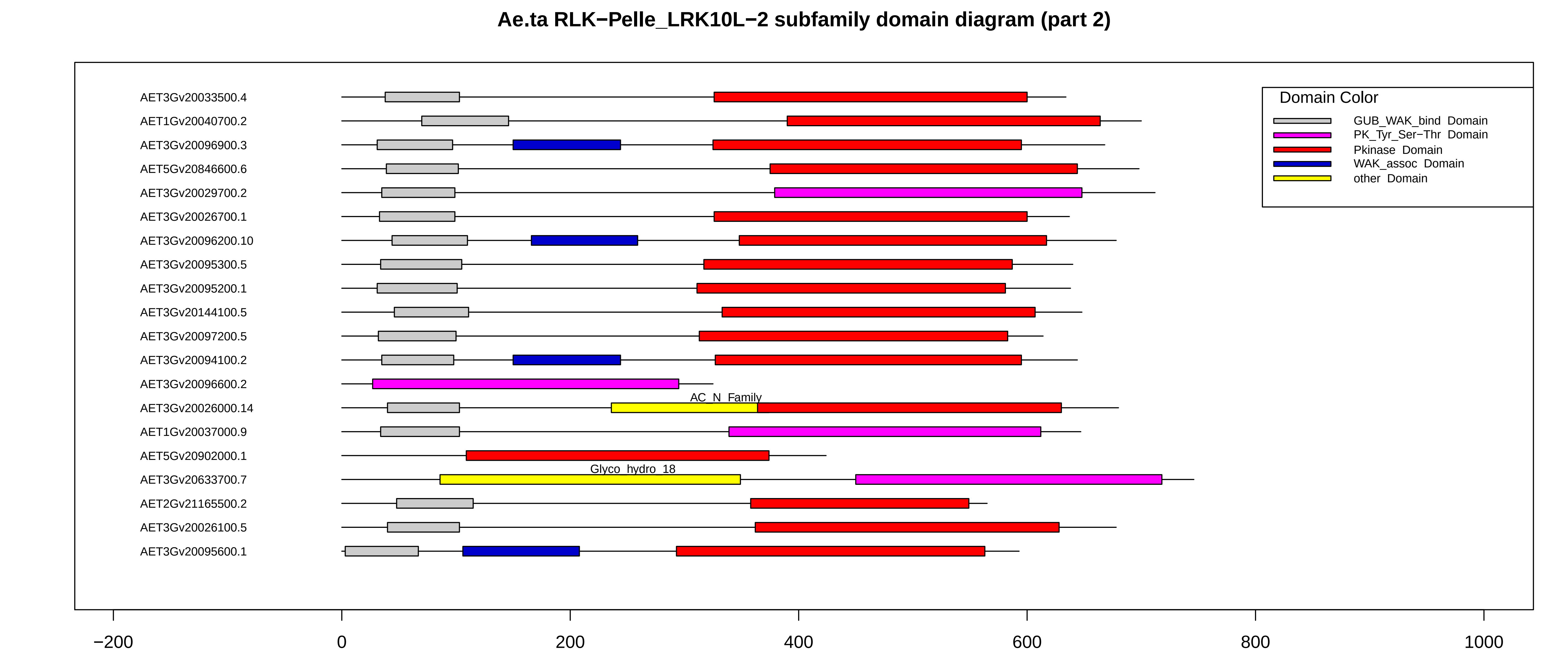

Ae.ta RLK−Pelle_LRK10L−2 subfamily domain diagram (part 2)
AET3Gv20033500.4
AET1Gv20040700.2
AET3Gv20096900.3
AET5Gv20846600.6
AET3Gv20029700.2
AET3Gv20026700.1
AET3Gv20096200.10
AET3Gv20095300.5
AET3Gv20095200.1
AET3Gv20144100.5
AET3Gv20097200.5
AET3Gv20094100.2
AET3Gv20096600.2
AC_N_Family
AET3Gv20026000.14
AET1Gv20037000.9
AET5Gv20902000.1
Glyco_hydro_18
AET3Gv20633700.7
AET2Gv21165500.2
AET3Gv20026100.5
AET3Gv20095600.1
Domain Color
GUB_WAK_bind Domain
PK_Tyr_Ser−Thr Domain
Pkinase Domain
WAK_assoc Domain
other Domain
−200
0
200
400
600
800
1000

Ae.ta RLK−Pelle_LRR−I−1 subfamily domain diagram (part 1)

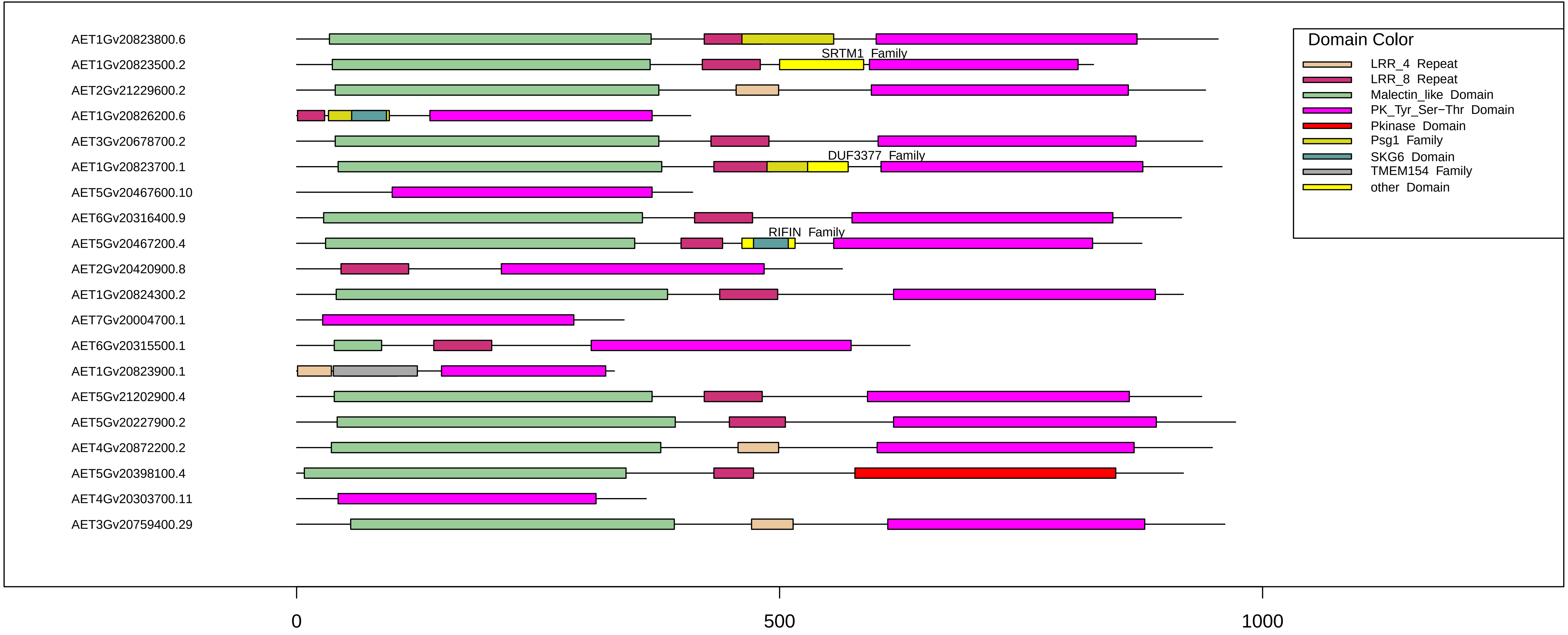

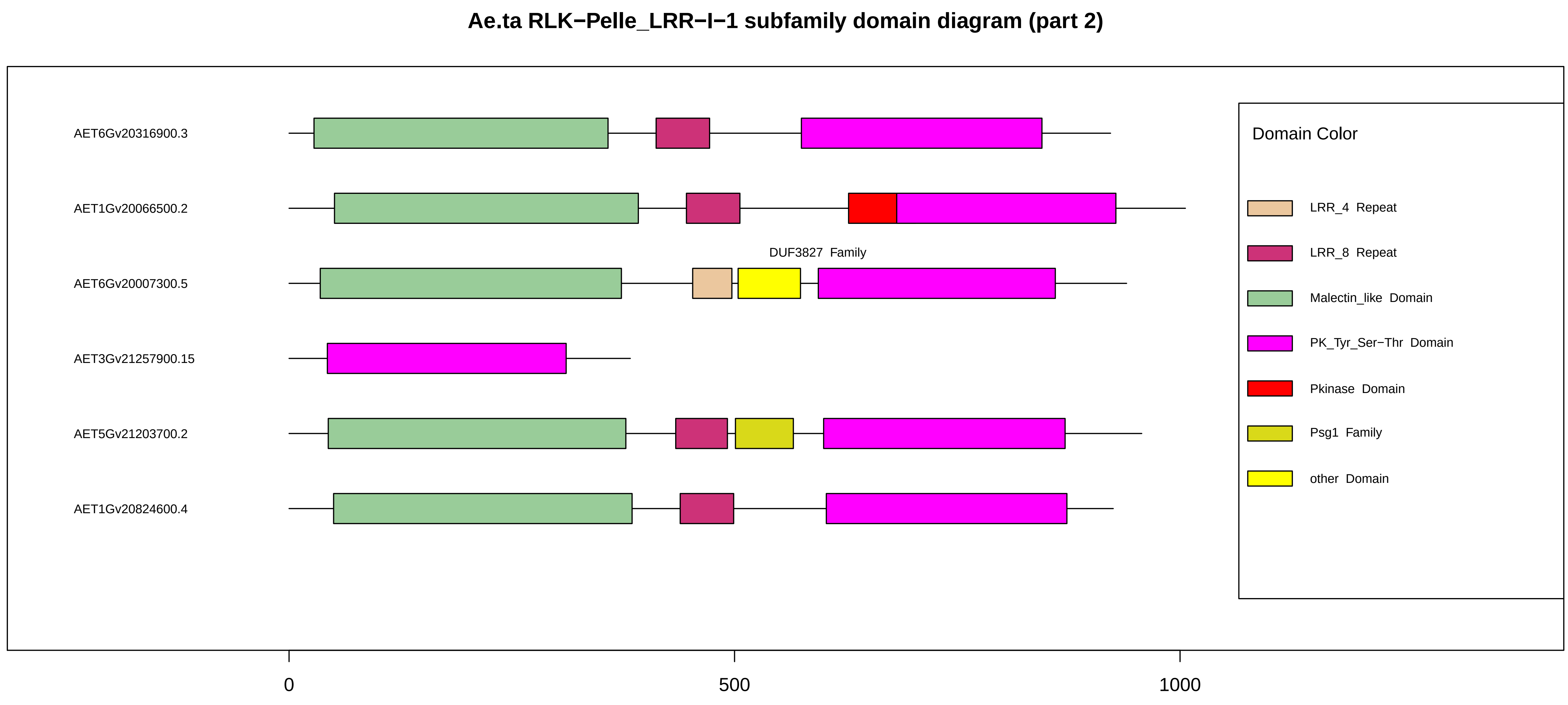
Ae.ta RLK−Pelle_LRR−I−1 subfamily domain diagram (part 2)
AET6Gv20316900.3
AET1Gv20066500.2
DUF3827 Family
AET6Gv20007300.5
AET3Gv21257900.15
AET5Gv21203700.2
AET1Gv20824600.4
Domain Color
LRR_4 Repeat
LRR_8 Repeat
Malectin_like Domain
PK_Tyr_Ser−Thr Domain
Pkinase Domain
Psg1 Family
other Domain
0
500
1000

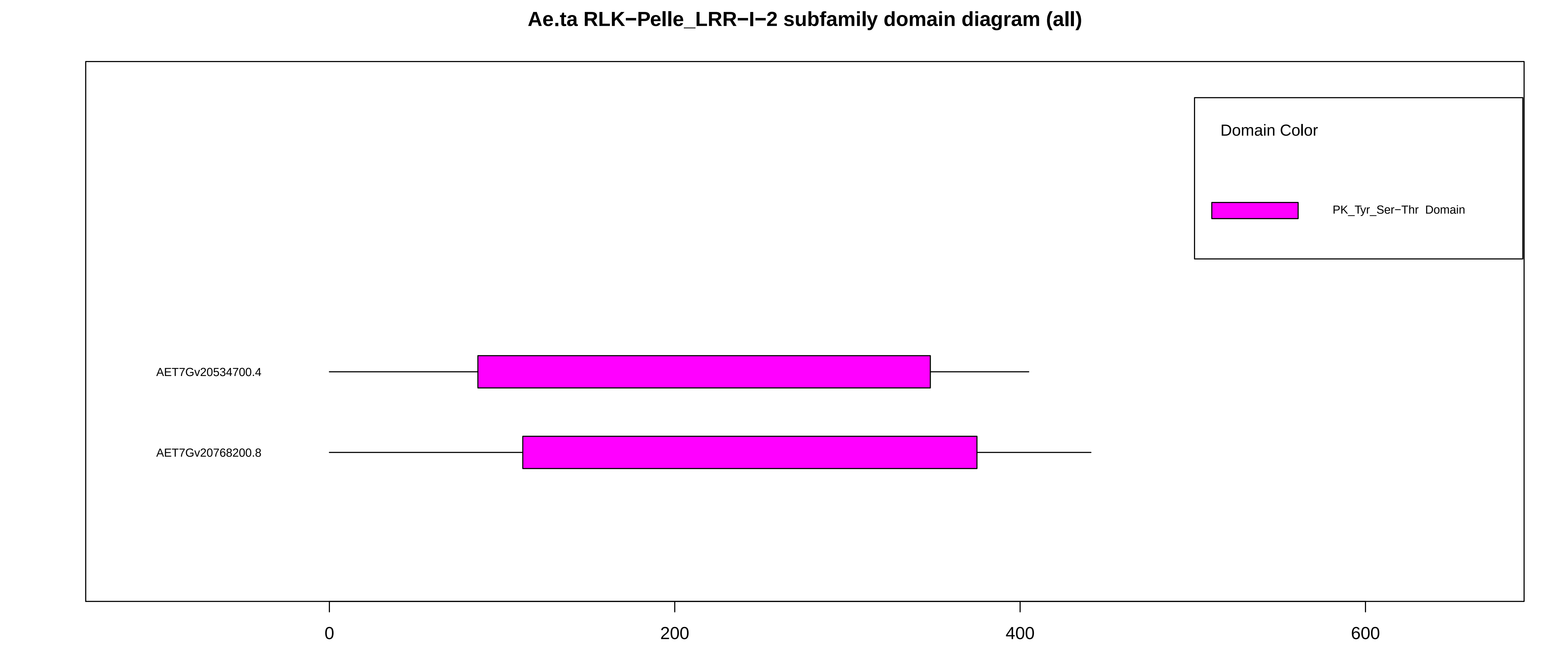

Ae.ta RLK−Pelle_LRR−I−2 subfamily domain diagram (all)
Domain Color
PK_Tyr_Ser−Thr Domain
AET7Gv20534700.4
AET7Gv20768200.8
0
200
400
600

Ae.ta RLK−Pelle_LRR−III subfamily domain diagram (part 1)

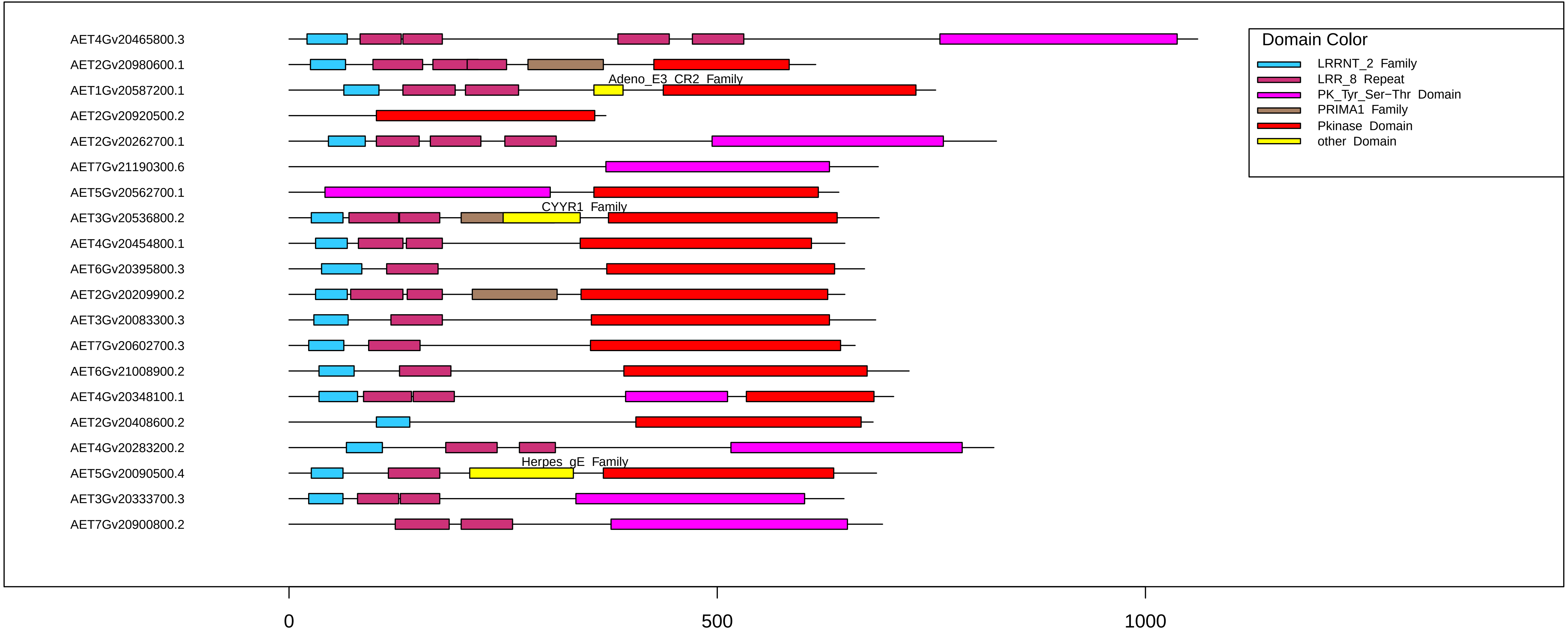

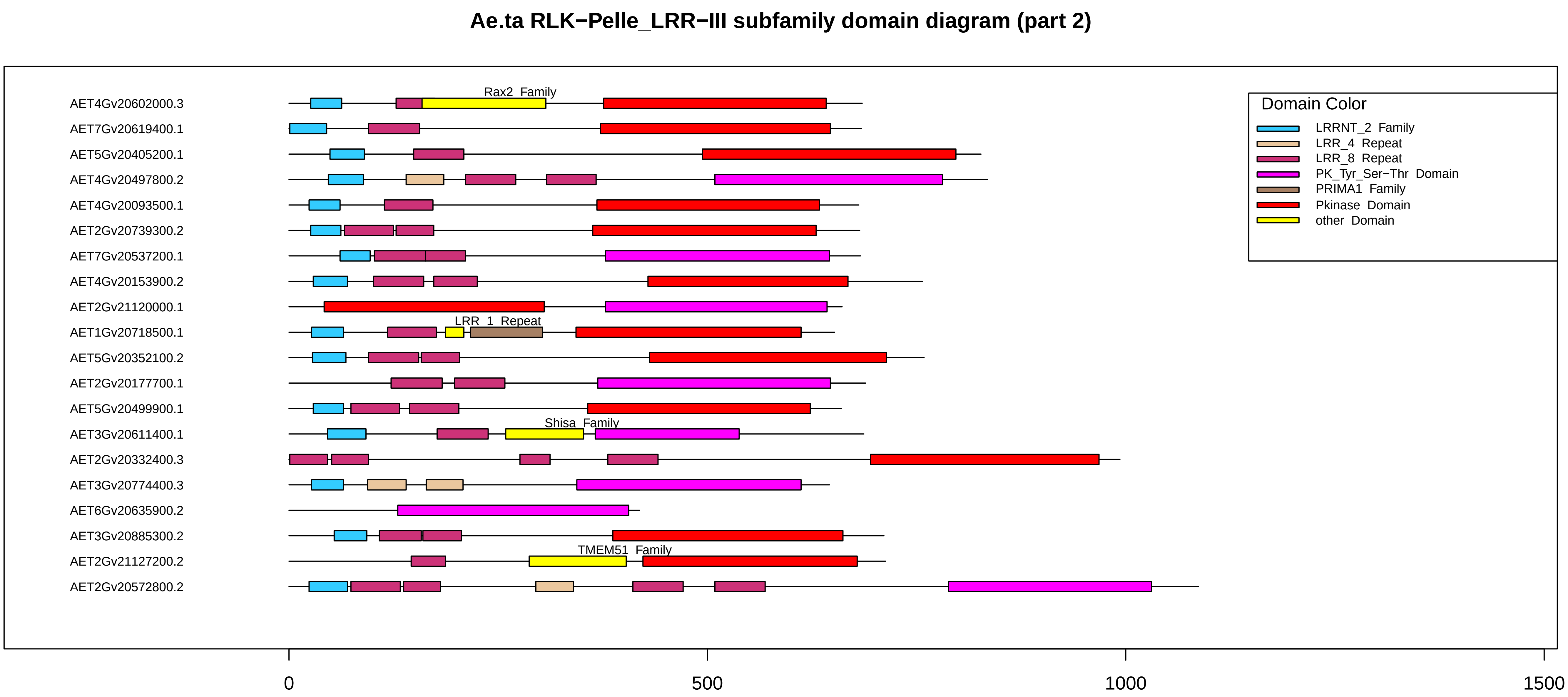
Ae.ta RLK−Pelle_LRR−III subfamily domain diagram (part 2)
AET4Gv20602000.3
AET7Gv20619400.1
AET5Gv20405200.1
AET4Gv20497800.2
AET4Gv20093500.1
AET2Gv20739300.2
AET7Gv20537200.1
AET4Gv20153900.2
AET2Gv21120000.1
AET1Gv20718500.1
AET5Gv20352100.2
AET2Gv20177700.1
AET5Gv20499900.1
AET3Gv20611400.1
AET2Gv20332400.3
AET3Gv20774400.3
AET6Gv20635900.2
AET3Gv20885300.2
AET2Gv21127200.2
AET2Gv20572800.2
Rax2 Family
LRR_1 Repeat
Shisa Family
TMEM51 Family
Domain Color
LRRNT_2 Family
LRR_4 Repeat
LRR_8 Repeat
PK_Tyr_Ser−Thr Domain
PRIMA1 Family
Pkinase Domain
other Domain
0
500
1000
1500

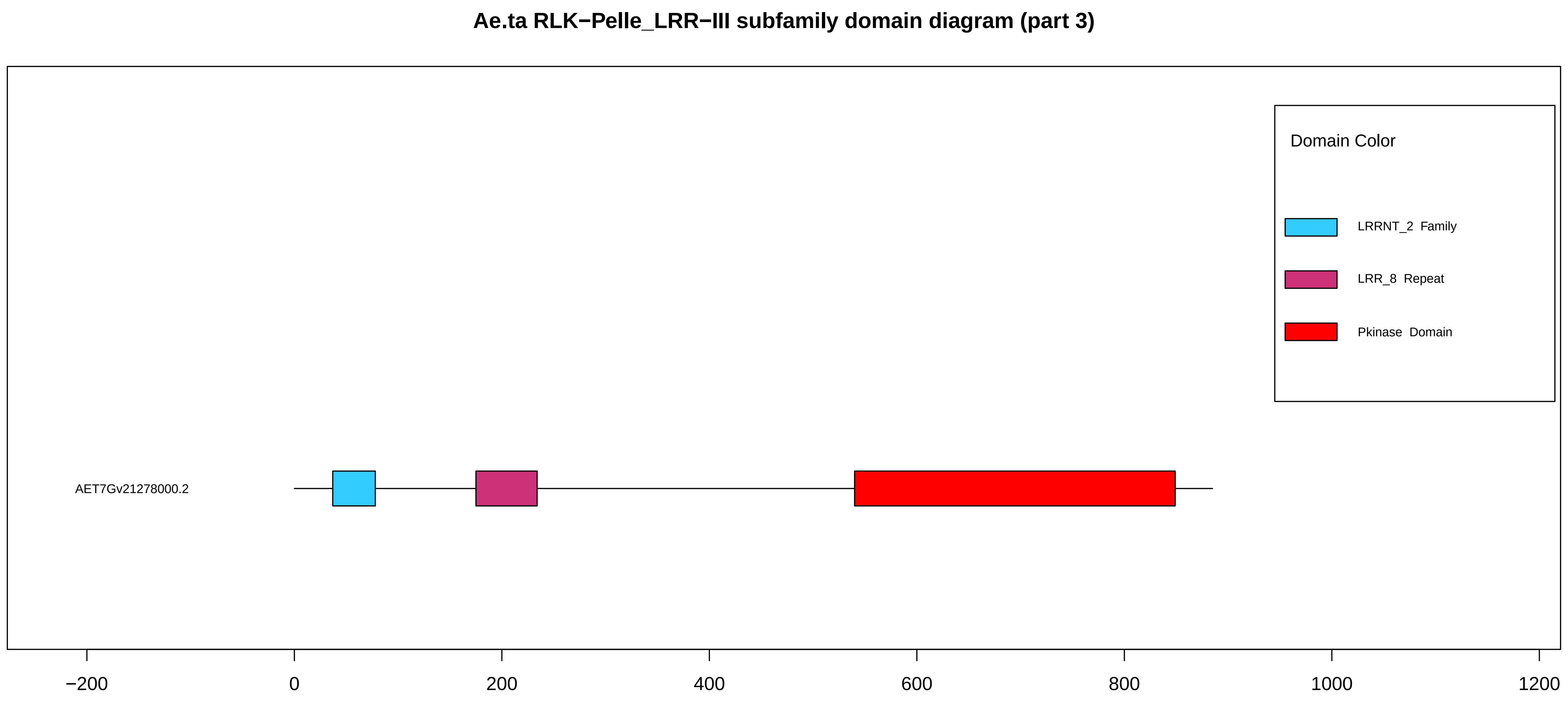

Ae.ta RLK−Pelle_LRR−III subfamily domain diagram (part 3)
Domain Color
LRRNT_2 Family
LRR_8 Repeat
Pkinase Domain
AET7Gv21278000.2
−200
0
200
400
600
800
1000
1200

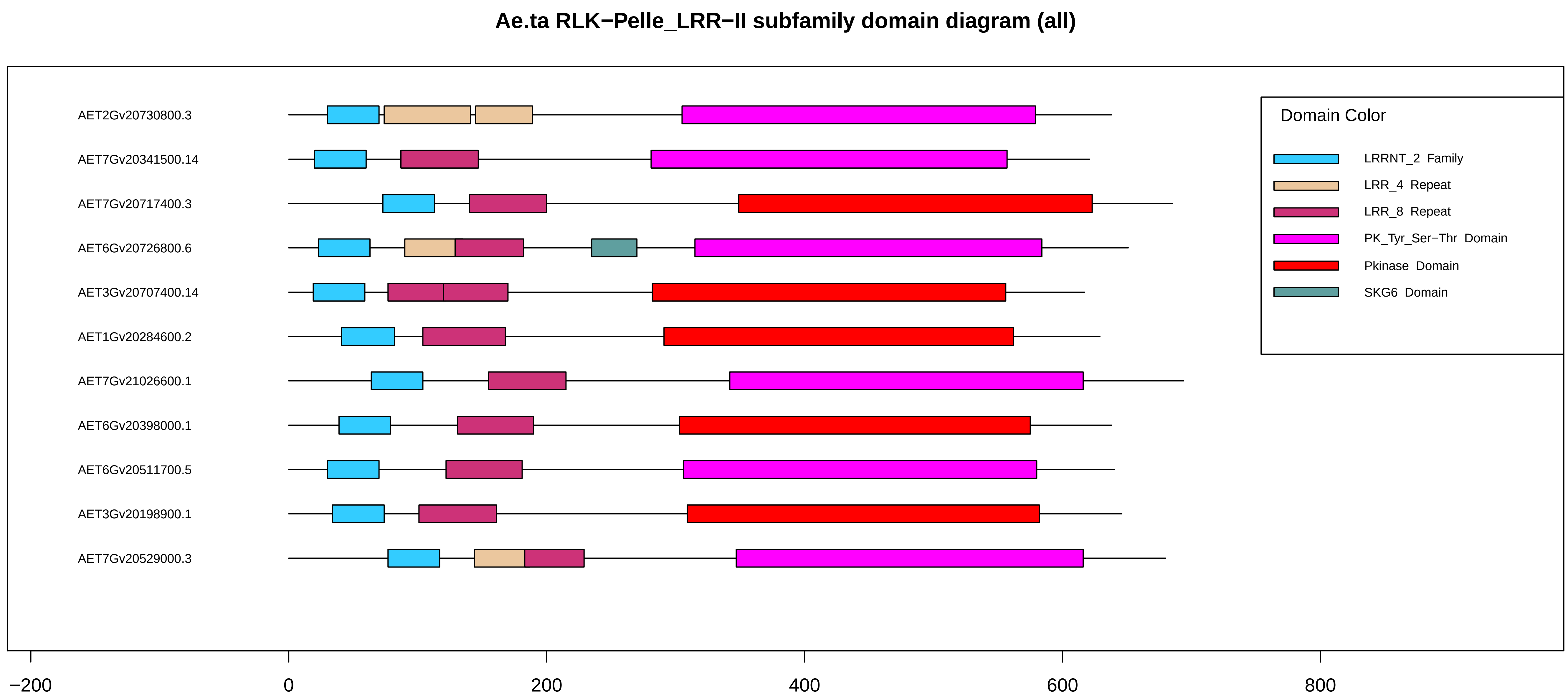
Ae.ta RLK−Pelle_LRR−II subfamily domain diagram (all)
AET2Gv20730800.3
AET7Gv20341500.14
AET7Gv20717400.3
AET6Gv20726800.6
AET3Gv20707400.14
AET1Gv20284600.2
AET7Gv21026600.1
AET6Gv20398000.1
AET6Gv20511700.5
AET3Gv20198900.1
AET7Gv20529000.3
Domain Color
LRRNT_2 Family
LRR_4 Repeat
LRR_8 Repeat
PK_Tyr_Ser−Thr Domain
Pkinase Domain
SKG6 Domain
−200
0
200
400
600
800

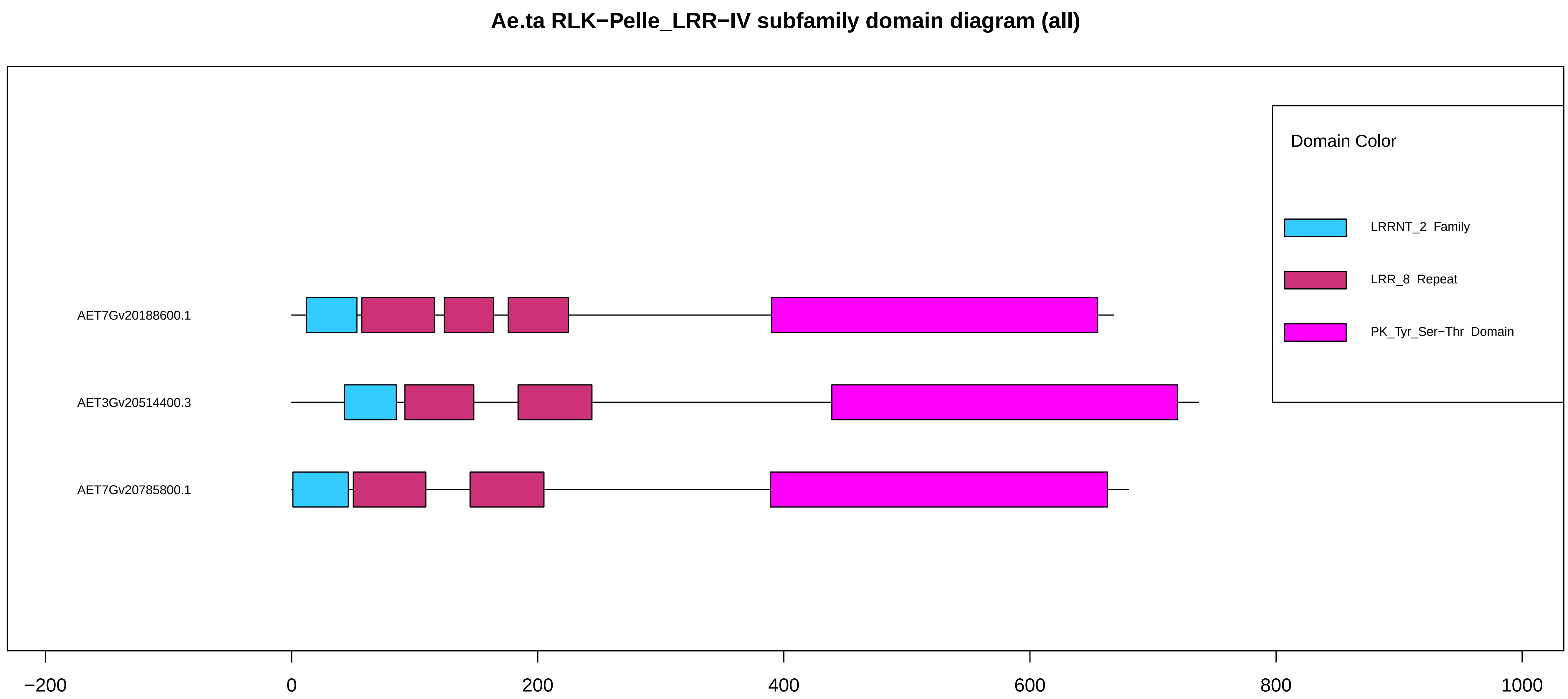

Ae.ta RLK−Pelle_LRR−IV subfamily domain diagram (all)
Domain Color
LRRNT_2 Family
LRR_8 Repeat
PK_Tyr_Ser−Thr Domain
AET7Gv20188600.1
AET3Gv20514400.3
AET7Gv20785800.1
−200
0
200
400
600
800
1000

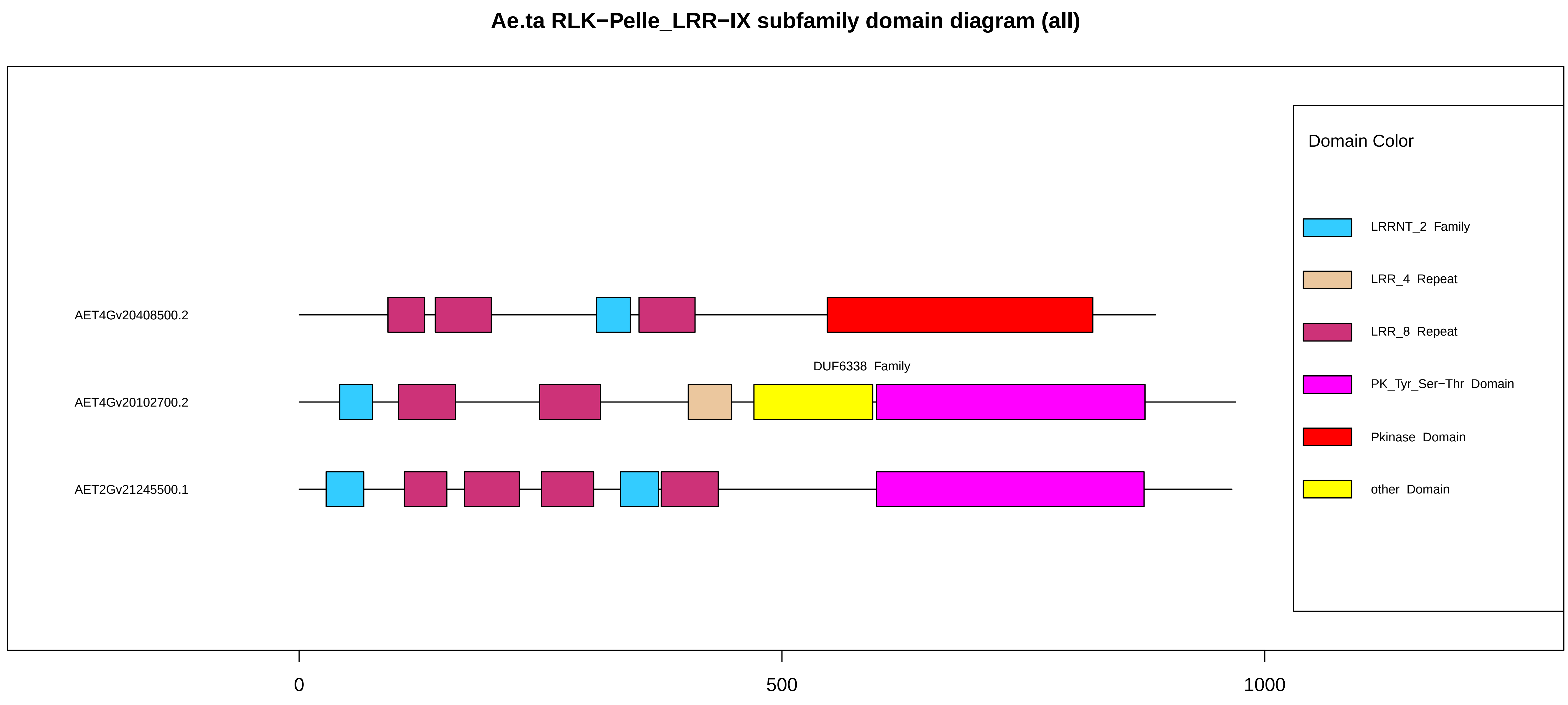
Ae.ta RLK−Pelle_LRR−IX subfamily domain diagram (all)
AET4Gv20408500.2
AET4Gv20102700.2
AET2Gv21245500.1
DUF6338 Family
Domain Color
LRRNT_2 Family
LRR_4 Repeat
LRR_8 Repeat
PK_Tyr_Ser−Thr Domain
Pkinase Domain
other Domain
0
500
1000

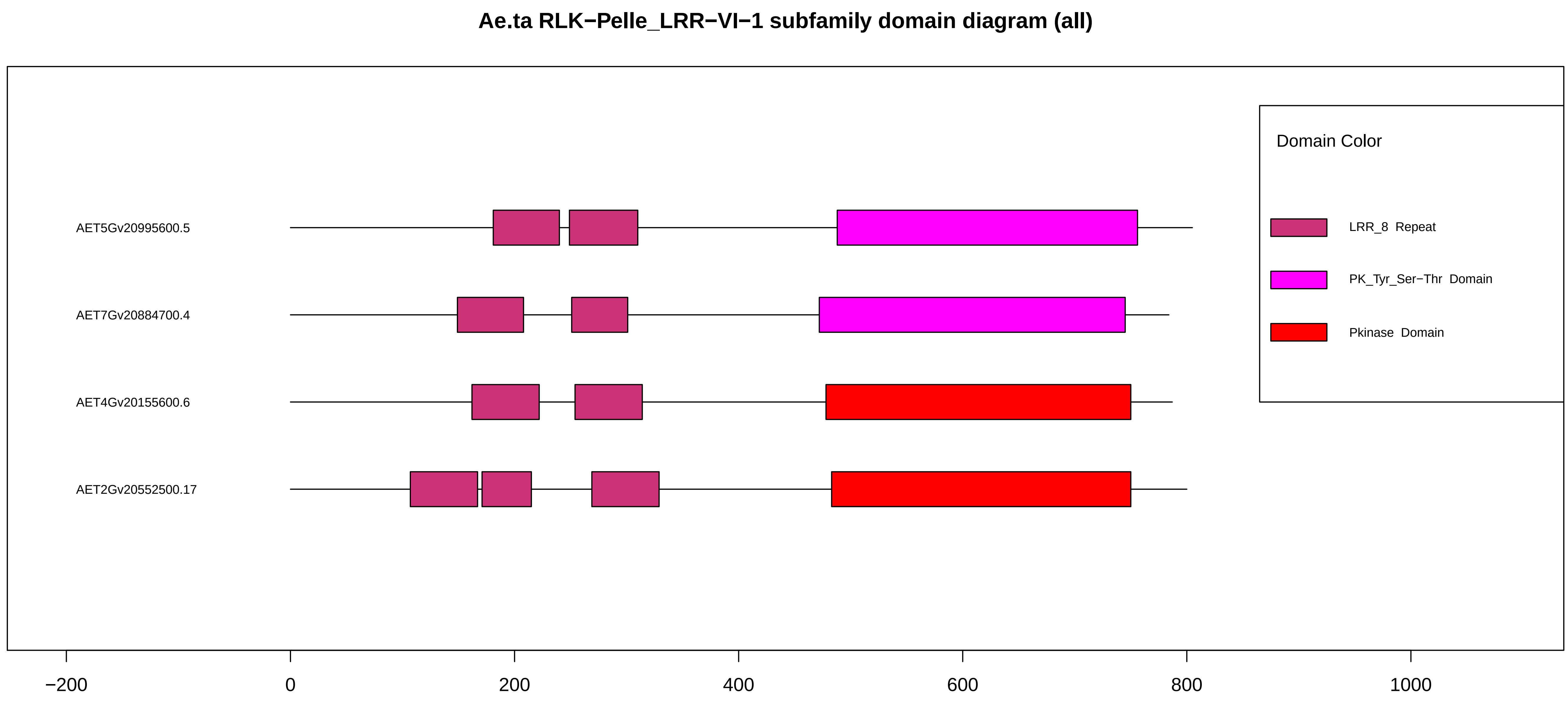

Ae.ta RLK−Pelle_LRR−VI−1 subfamily domain diagram (all)
AET5Gv20995600.5
AET7Gv20884700.4
AET4Gv20155600.6
AET2Gv20552500.17
Domain Color
LRR_8 Repeat
PK_Tyr_Ser−Thr Domain
Pkinase Domain
−200
0
200
400
600
800
1000

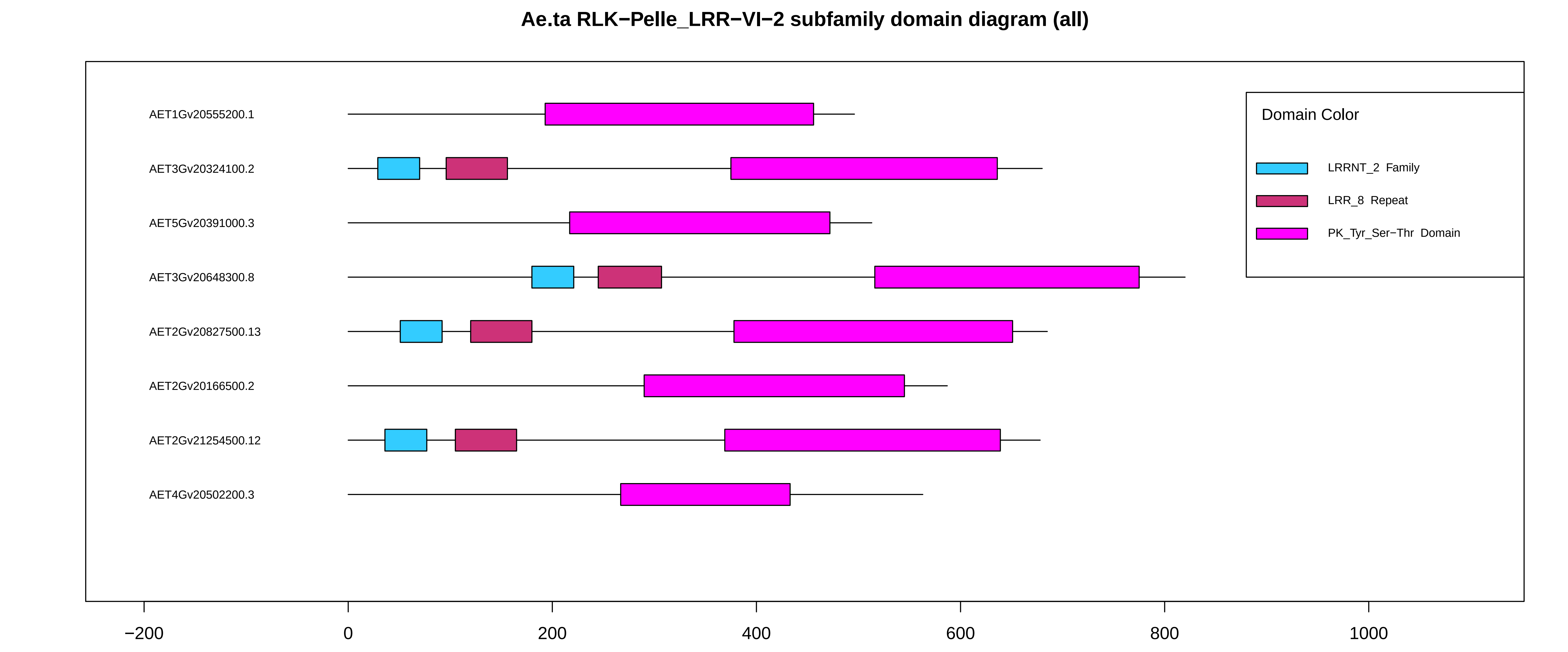

Ae.ta RLK−Pelle_LRR−VI−2 subfamily domain diagram (all)
AET1Gv20555200.1
AET3Gv20324100.2
AET5Gv20391000.3
AET3Gv20648300.8
AET2Gv20827500.13
AET2Gv20166500.2
AET2Gv21254500.12
AET4Gv20502200.3
Domain Color
LRRNT_2 Family
LRR_8 Repeat
PK_Tyr_Ser−Thr Domain
−200
0
200
400
600
800
1000

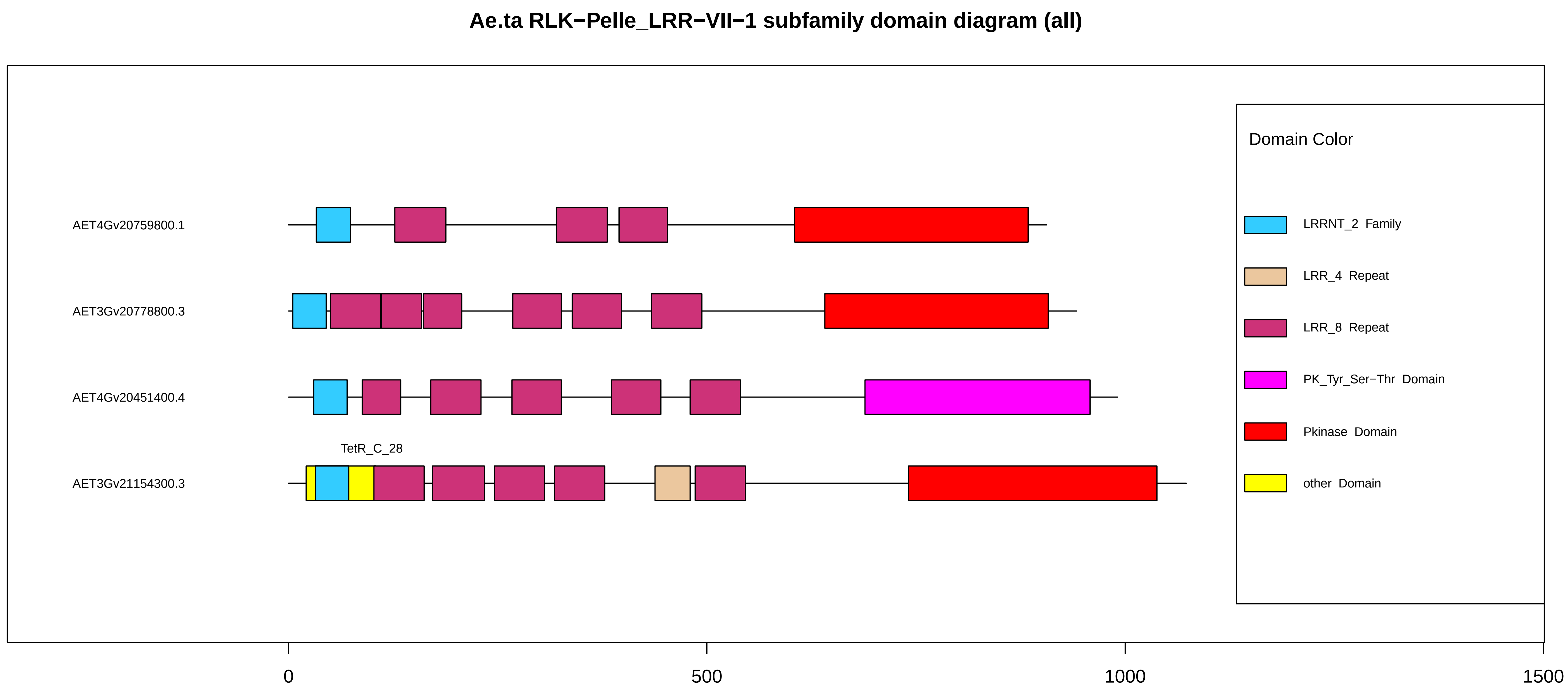
Ae.ta RLK−Pelle_LRR−VII−1 subfamily domain diagram (all)
AET4Gv20759800.1
AET3Gv20778800.3
AET4Gv20451400.4
AET3Gv21154300.3
TetR_C_28
Domain Color
LRRNT_2 Family
LRR_4 Repeat
LRR_8 Repeat
PK_Tyr_Ser−Thr Domain
Pkinase Domain
other Domain
0
500
1000
1500

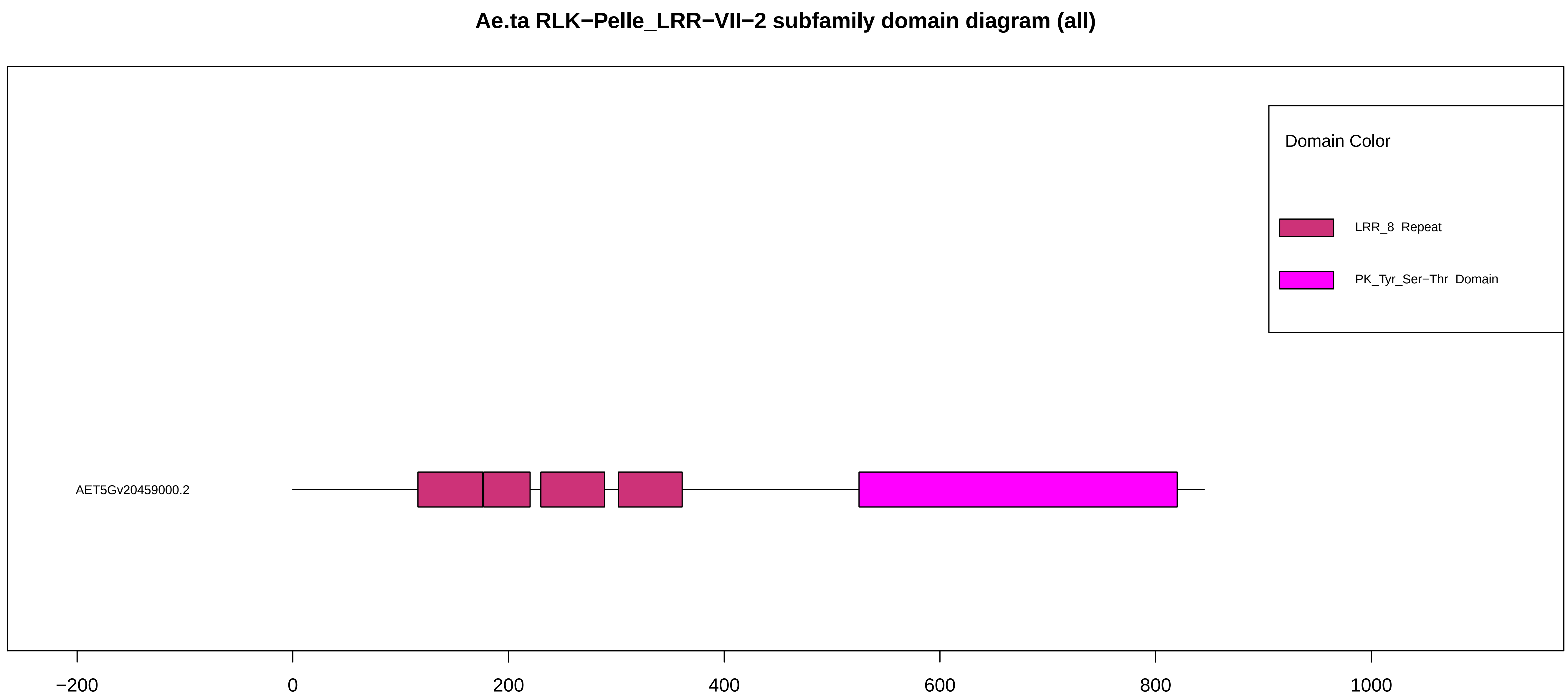

Ae.ta RLK−Pelle_LRR−VII−2 subfamily domain diagram (all)
Domain Color
LRR_8 Repeat
PK_Tyr_Ser−Thr Domain
AET5Gv20459000.2
−200
0
200
400
600
800
1000

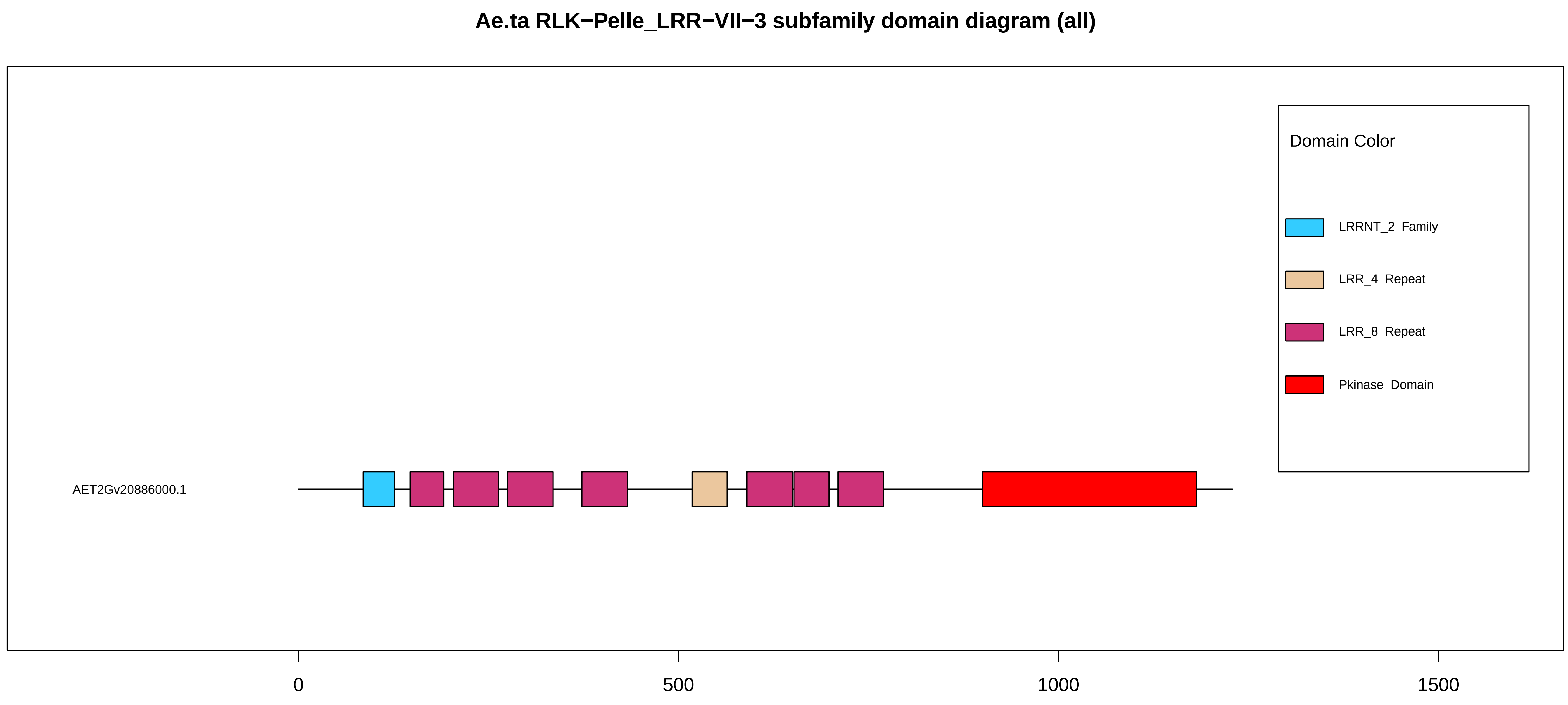

Ae.ta RLK−Pelle_LRR−VII−3 subfamily domain diagram (all)
Domain Color
LRRNT_2 Family
LRR_4 Repeat
LRR_8 Repeat
Pkinase Domain
AET2Gv20886000.1
0
500
1000
1500

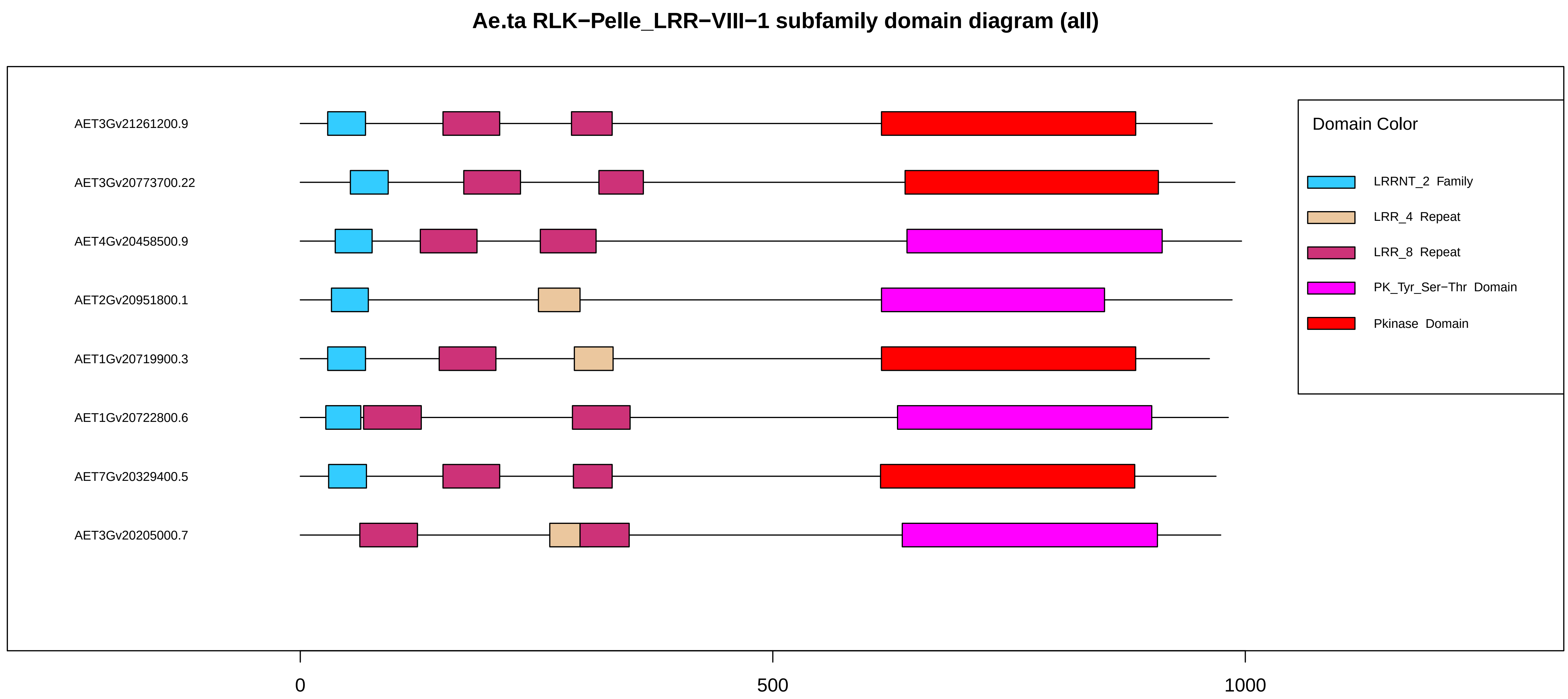

Ae.ta RLK−Pelle_LRR−VIII−1 subfamily domain diagram (all)
AET3Gv21261200.9
AET3Gv20773700.22
AET4Gv20458500.9
AET2Gv20951800.1
AET1Gv20719900.3
AET1Gv20722800.6
AET7Gv20329400.5
AET3Gv20205000.7
Domain Color
LRRNT_2 Family
LRR_4 Repeat
LRR_8 Repeat
PK_Tyr_Ser−Thr Domain
Pkinase Domain
0
500
1000

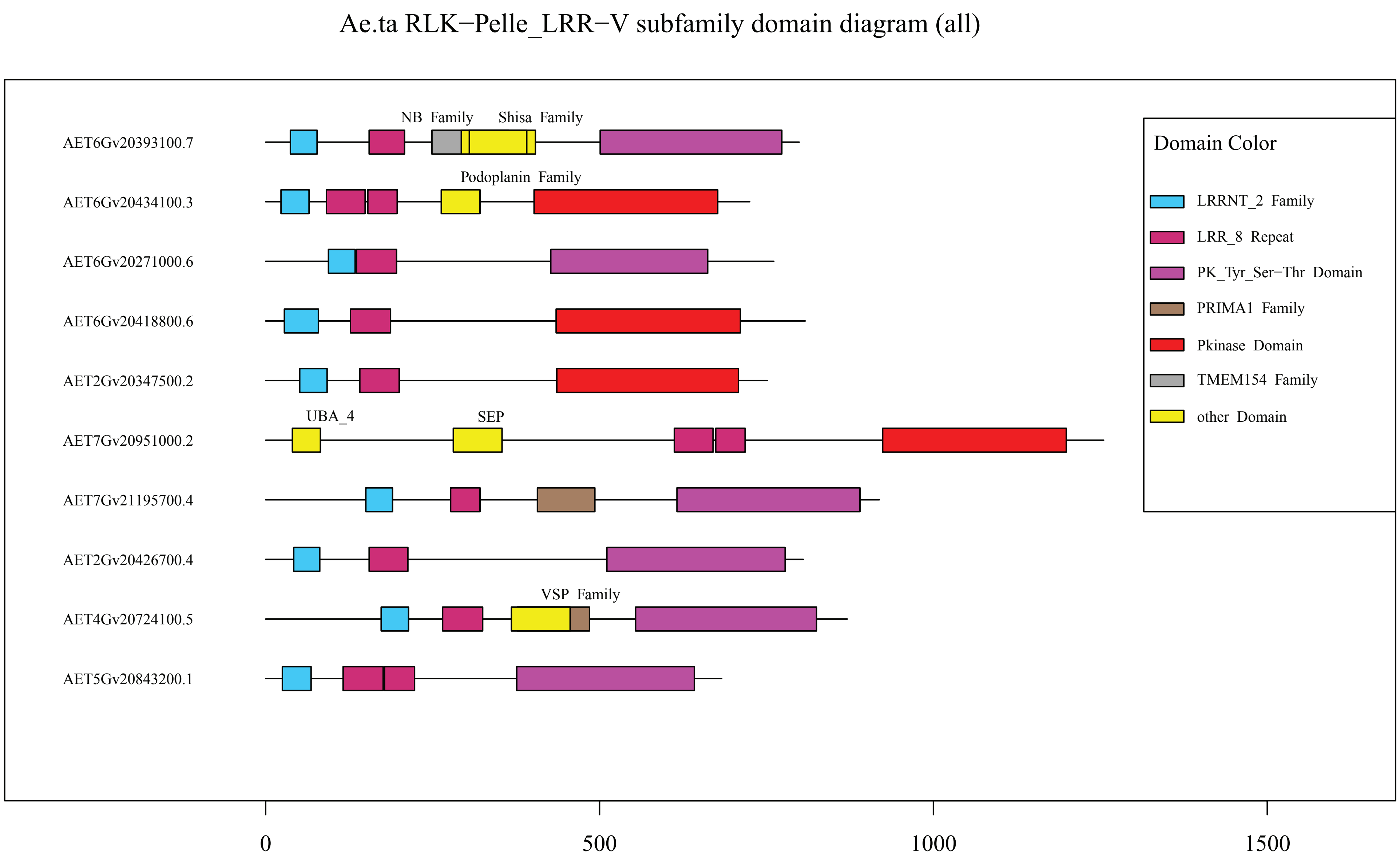
Ae.ta RLK−Pelle_LRR−V subfamily domain diagram (all)
AET6Gv20393100.7
NB Family
Shisa Family
AET6Gv20434100.3
Podoplanin Family
AET6Gv20271000.6
AET6Gv20418800.6
AET2Gv20347500.2
AET7Gv20951000.2
UBA_4
SEP
AET7Gv21195700.4
AET2Gv20426700.4
AET4Gv20724100.5
VSP Family
AET5Gv20843200.1
Domain Color
LRRNT_2 Family
LRR_8 Repeat
PK_Tyr_Ser−Thr Domain
PRIMA1 Family
Pkinase Domain
TMEM154 Family
other Domain
0
500
1000
1500

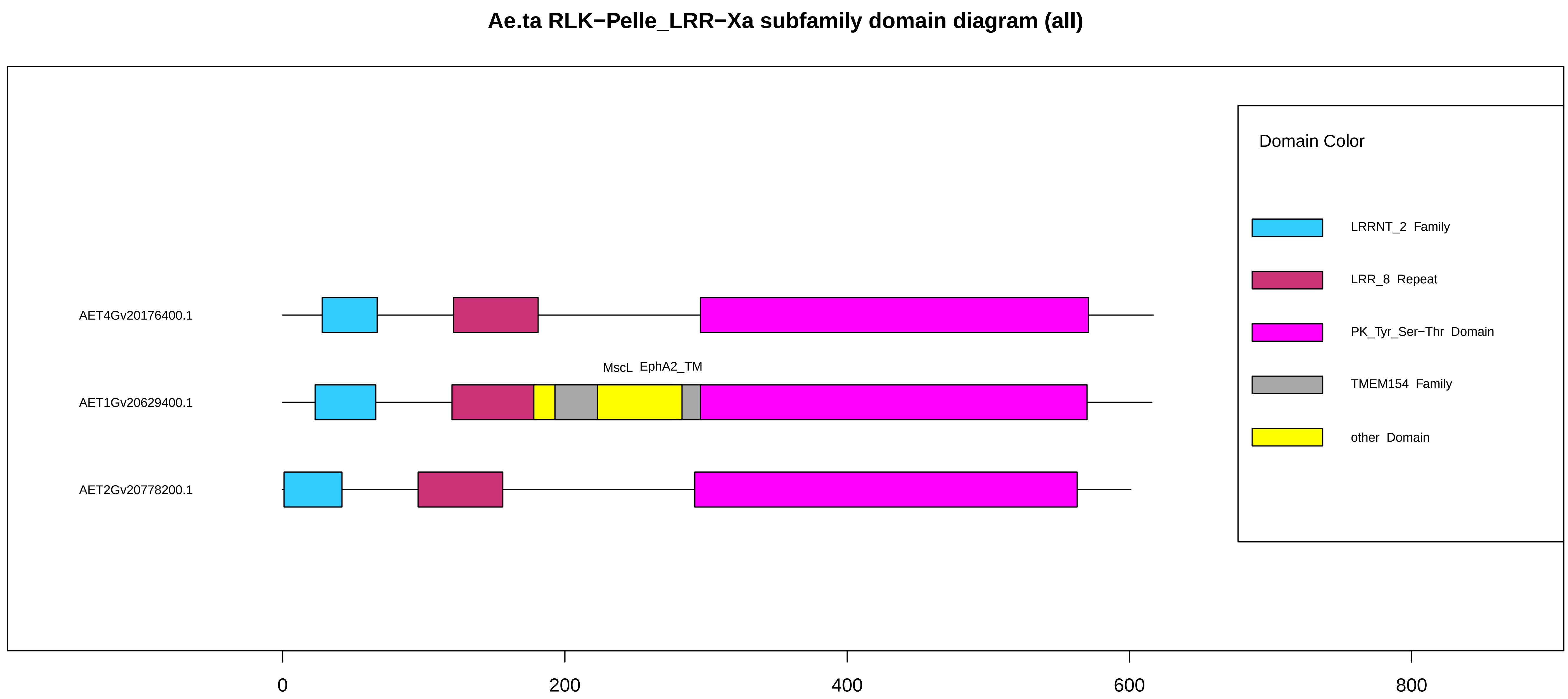

Ae.ta RLK−Pelle_LRR−Xa subfamily domain diagram (all)
AET4Gv20176400.1
AET1Gv20629400.1
AET2Gv20778200.1
MscL
EphA2_TM
0
200
400
600
800
Domain Color
LRRNT_2 Family
LRR_8 Repeat
PK_Tyr_Ser−Thr Domain
TMEM154 Family
other Domain

Ae.ta RLK−Pelle_LRR−Xb−1 subfamily domain diagram (part 1)

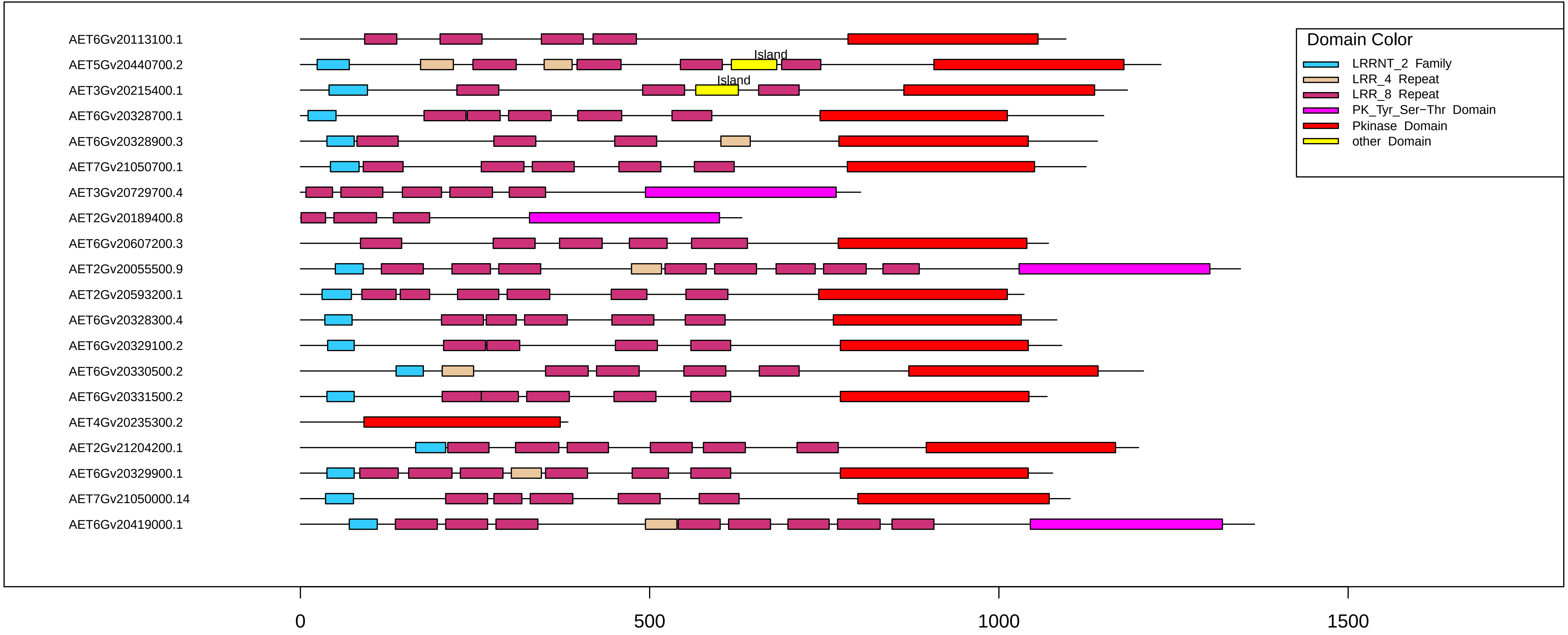

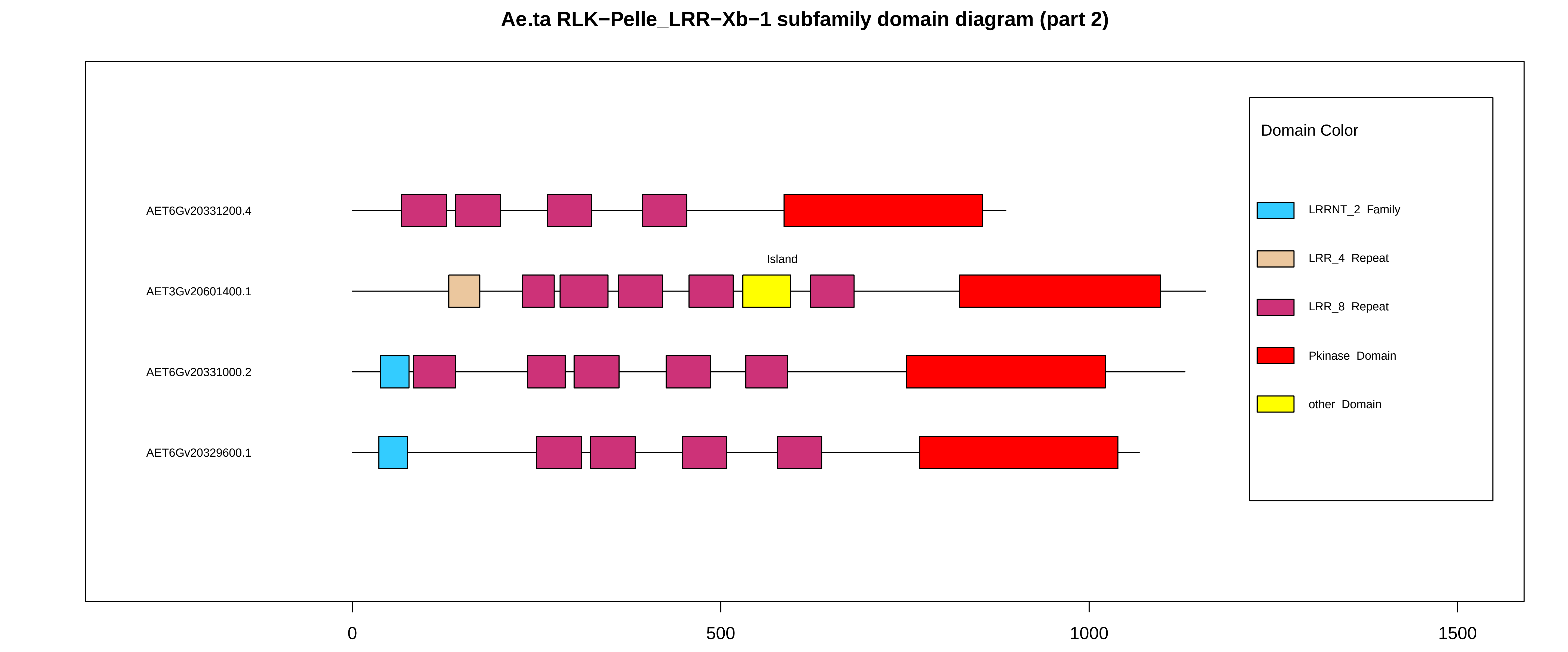
Ae.ta RLK−Pelle_LRR−Xb−1 subfamily domain diagram (part 2)
AET6Gv20331200.4
AET3Gv20601400.1
AET6Gv20331000.2
AET6Gv20329600.1
Island
Domain Color
LRRNT_2 Family
LRR_4 Repeat
LRR_8 Repeat
Pkinase Domain
other Domain
0
500
1000
1500

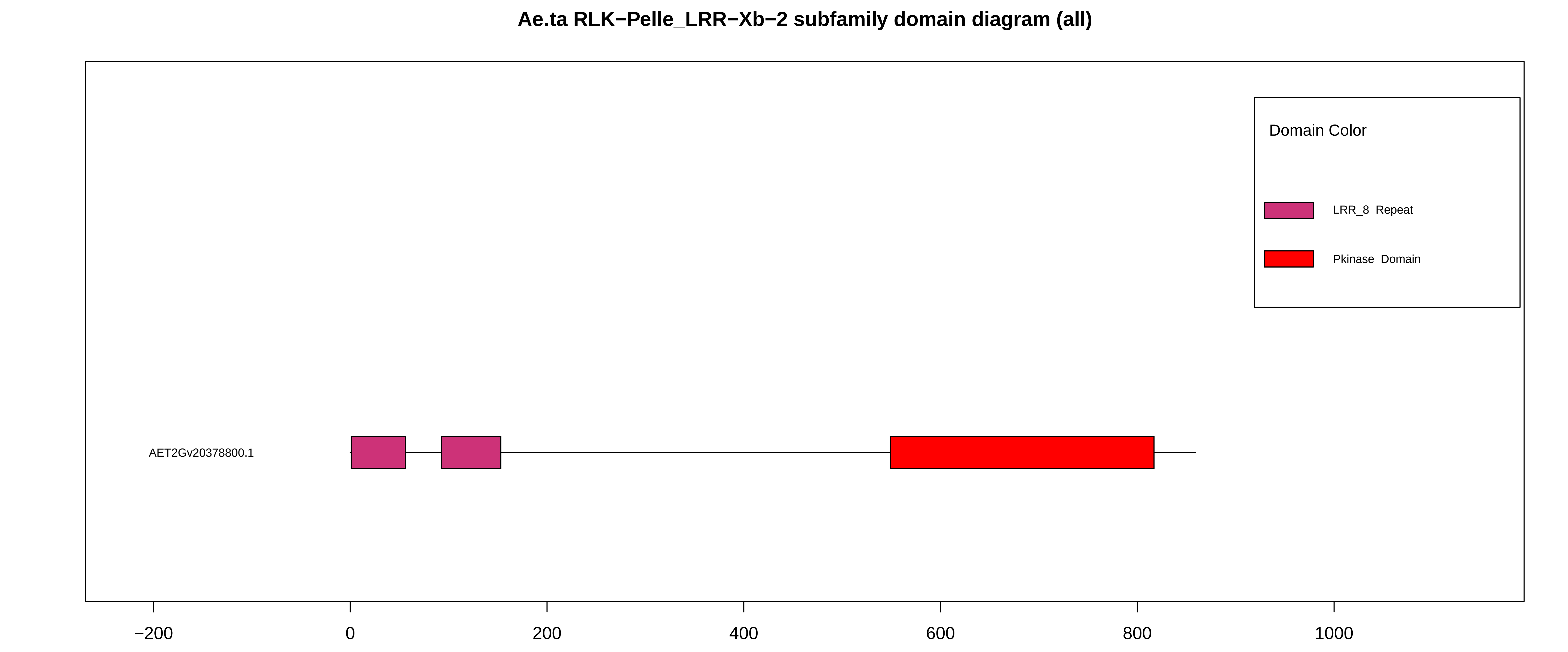
Ae.ta RLK−Pelle_LRR−Xb−2 subfamily domain diagram (all)
Domain Color
LRR_8 Repeat
Pkinase Domain
AET2Gv20378800.1
−200
0
200
400
600
800
1000

Ae.ta RLK−Pelle_LRR−XI−1 subfamily domain diagram (part 1)

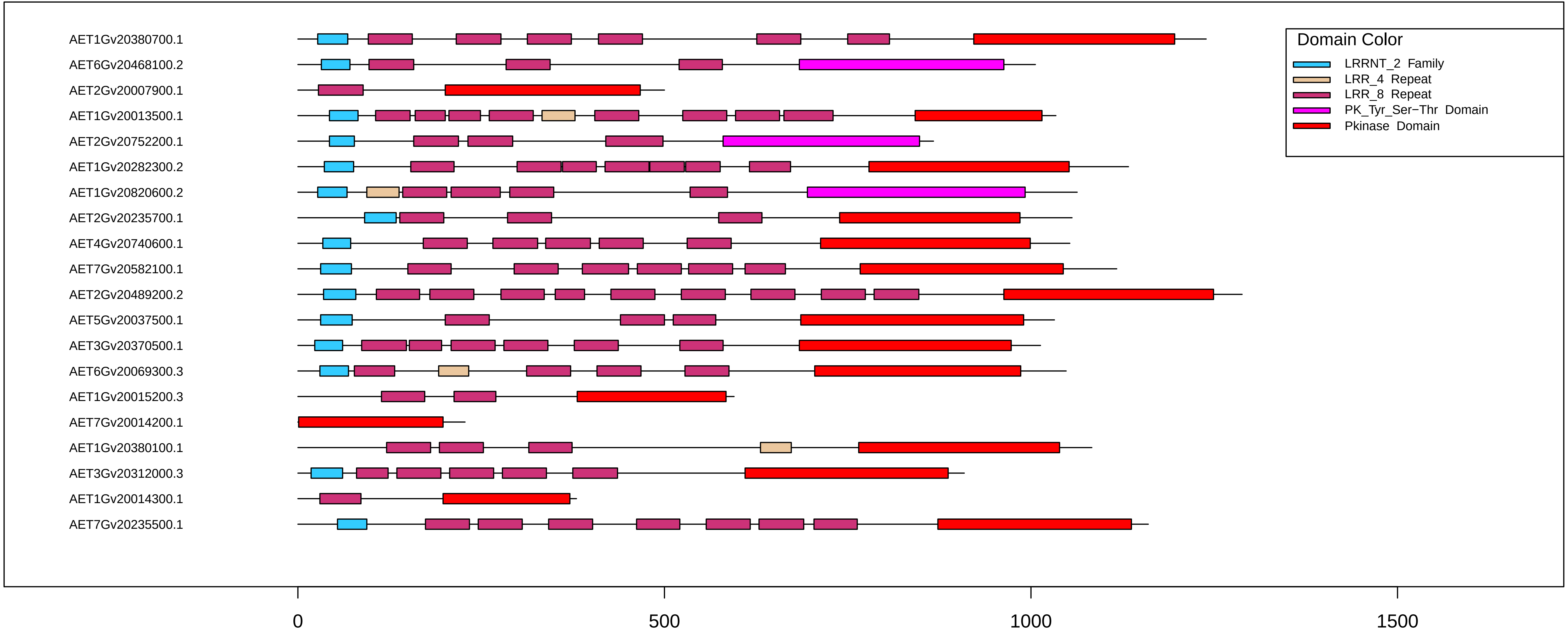

Ae.ta RLK−Pelle_LRR−XI−1 subfamily domain diagram (part 2)

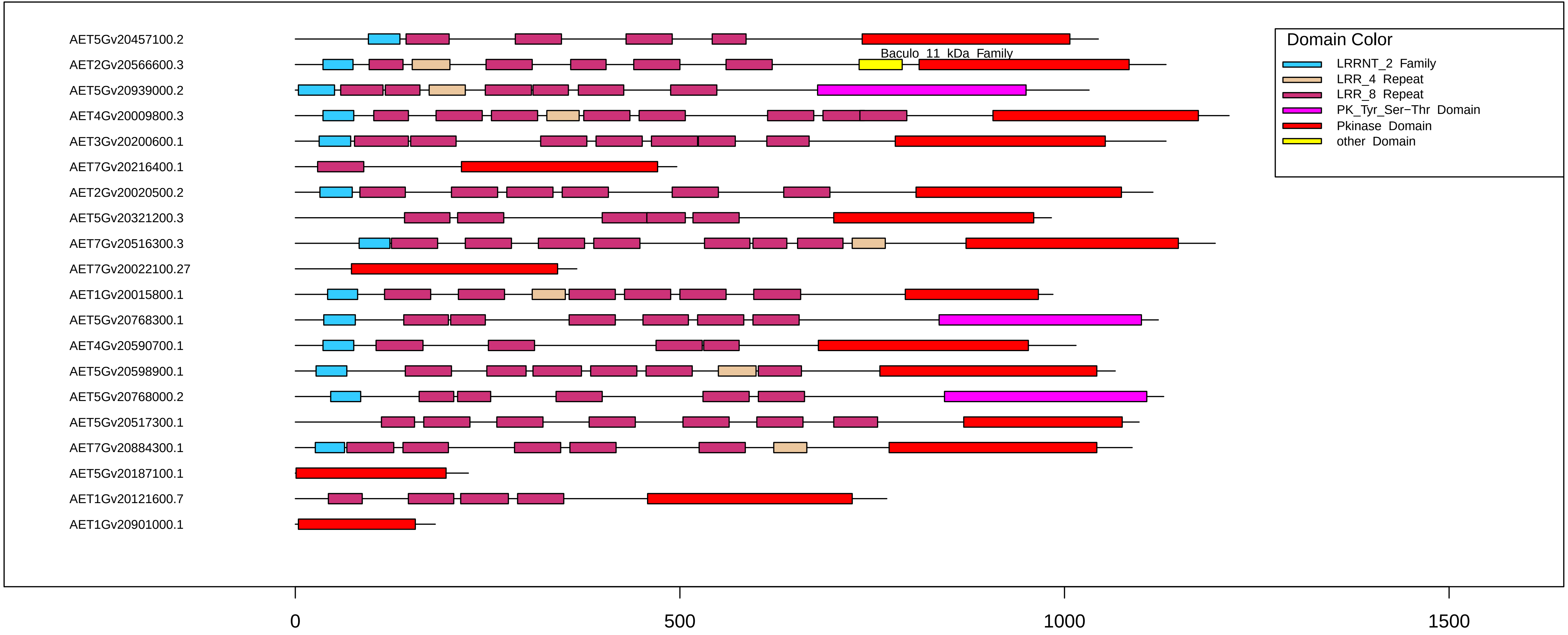

Ae.ta RLK−Pelle_LRR−XI−1 subfamily domain diagram (part 3)

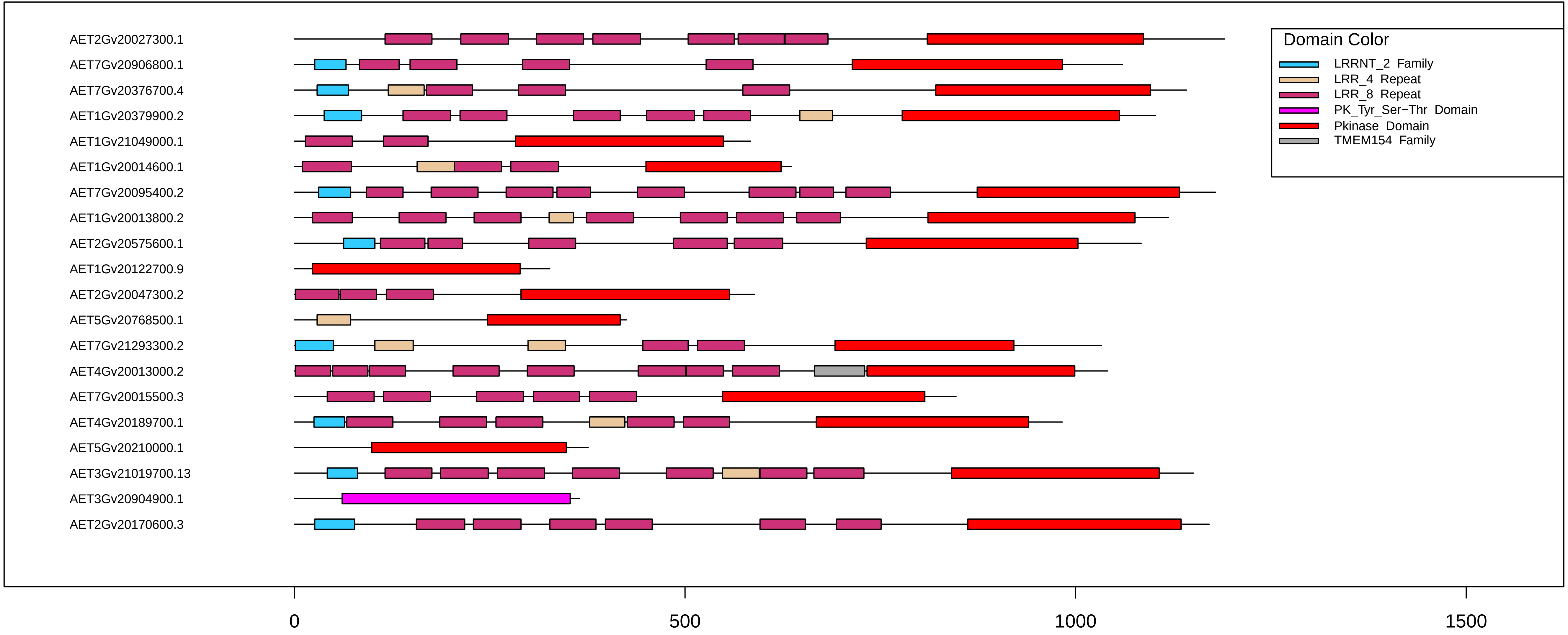

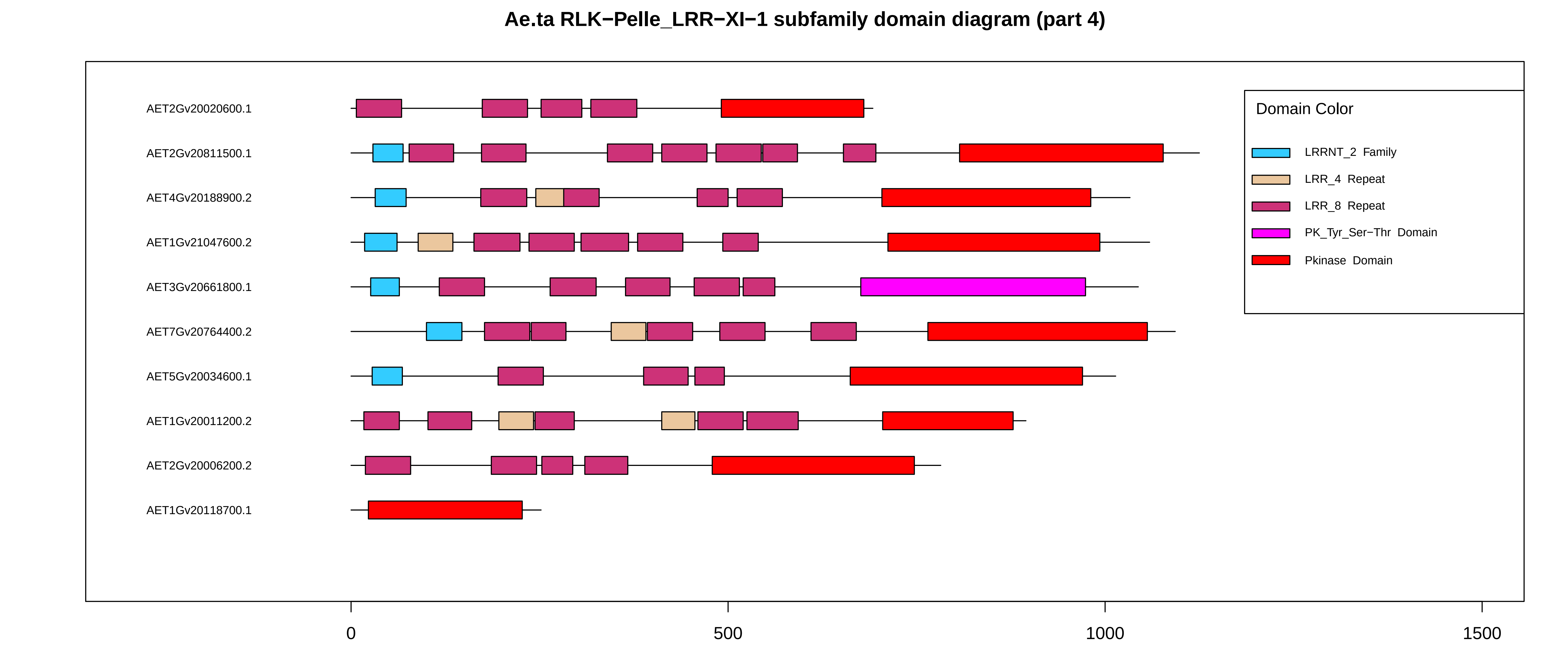
Ae.ta RLK−Pelle_LRR−XI−1 subfamily domain diagram (part 4)
AET2Gv20020600.1
AET2Gv20811500.1
AET4Gv20188900.2
AET1Gv21047600.2
AET3Gv20661800.1
AET7Gv20764400.2
AET5Gv20034600.1
AET1Gv20011200.2
AET2Gv20006200.2
AET1Gv20118700.1
Domain Color
LRRNT_2 Family
LRR_4 Repeat
LRR_8 Repeat
PK_Tyr_Ser−Thr Domain
Pkinase Domain
0
500
1000
1500

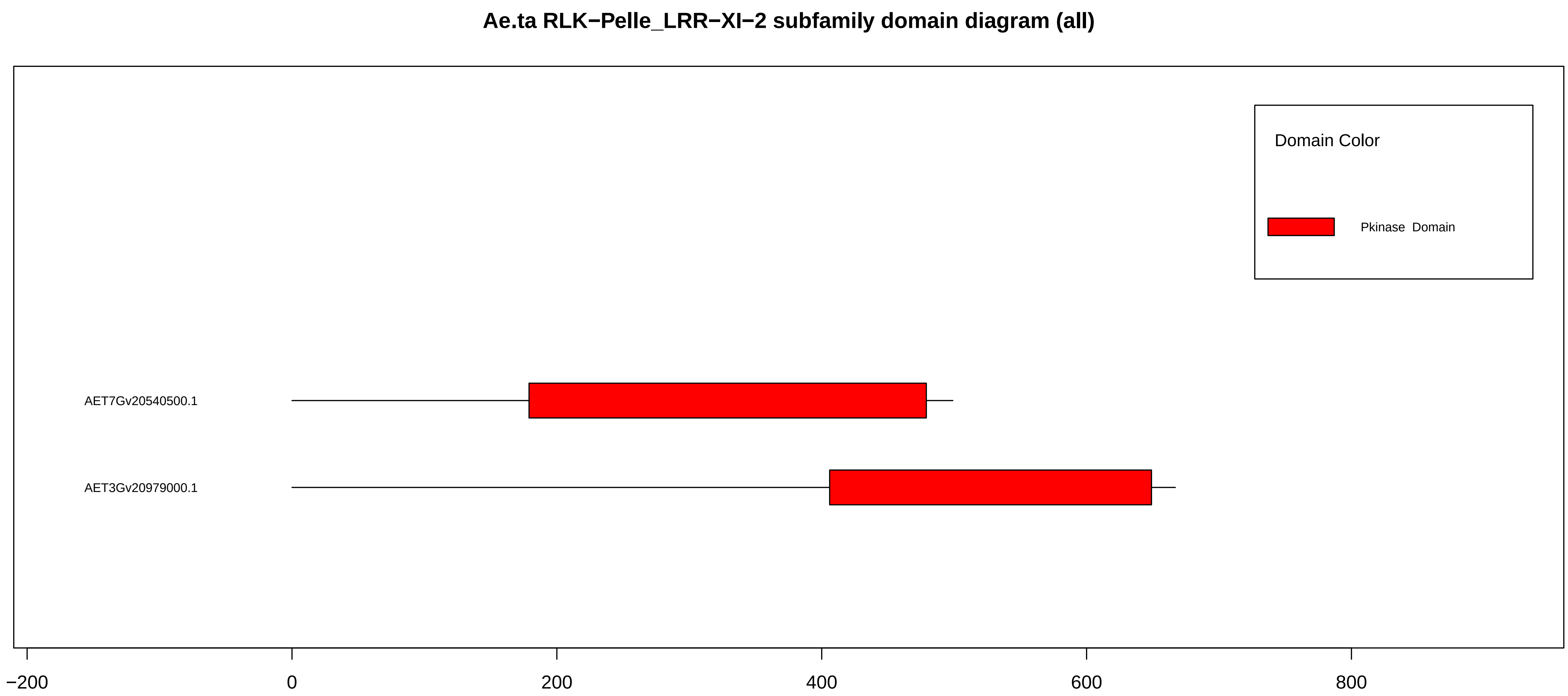
Ae.ta RLK−Pelle_LRR−XI−2 subfamily domain diagram (all)
Domain Color
Pkinase Domain
AET7Gv20540500.1
AET3Gv20979000.1
−200
0
200
400
600
800

Ae.ta RLK−Pelle_LRR−XII−1 subfamily domain diagram (part 1)

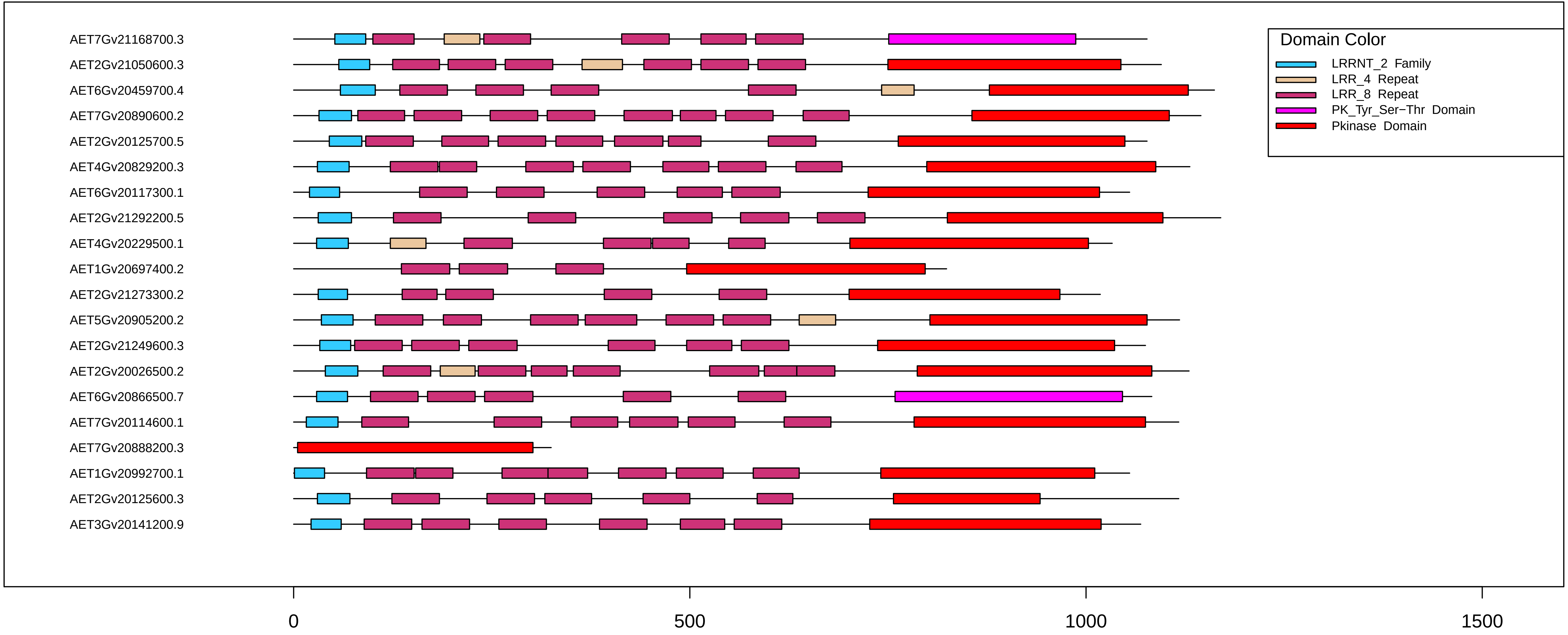

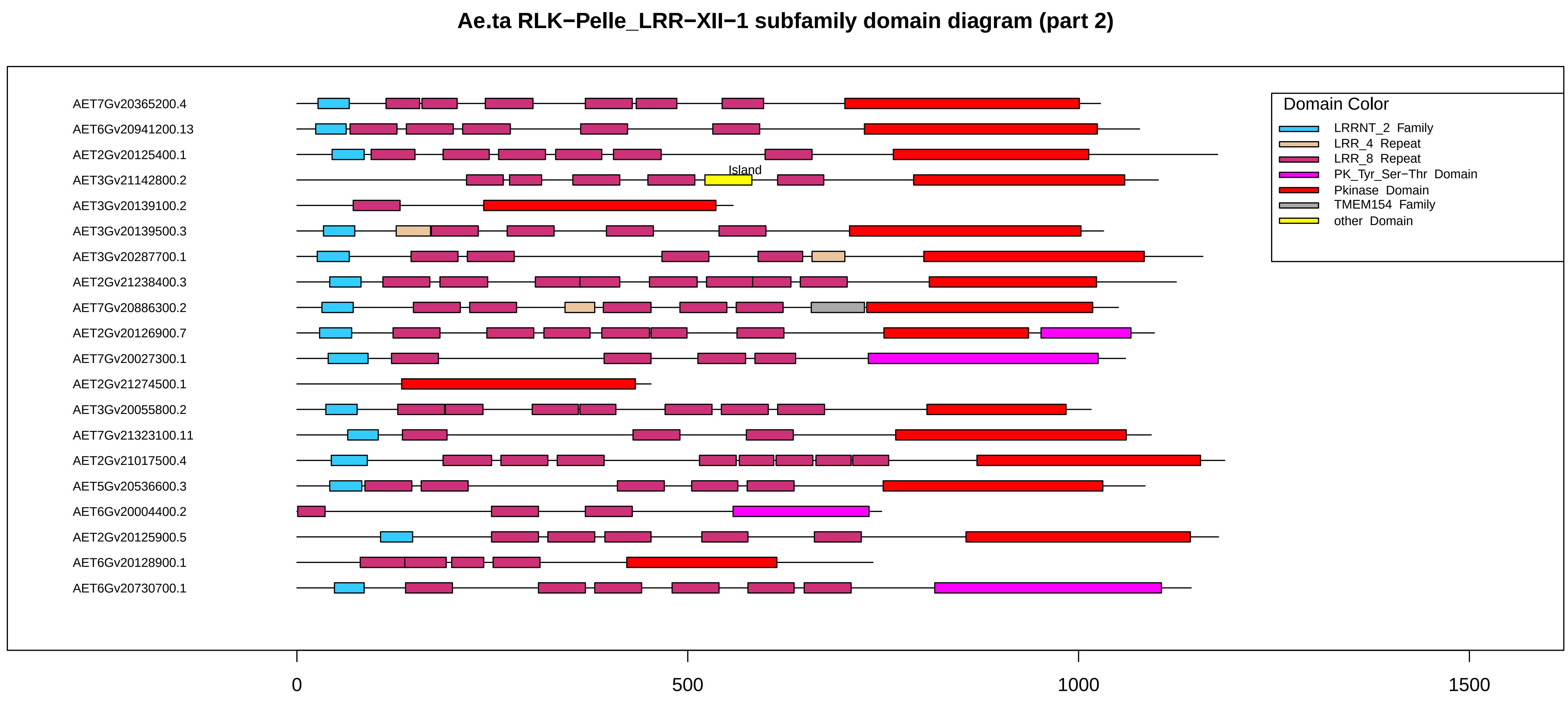
Ae.ta RLK−Pelle_LRR−XII−1 subfamily domain diagram (part 2)
AET7Gv20365200.4
AET6Gv20941200.13
AET2Gv20125400.1
AET3Gv21142800.2
AET3Gv20139100.2
AET3Gv20139500.3
AET3Gv20287700.1
AET2Gv21238400.3
AET7Gv20886300.2
AET2Gv20126900.7
AET7Gv20027300.1
AET2Gv21274500.1
AET3Gv20055800.2
AET7Gv21323100.11
AET2Gv21017500.4
AET5Gv20536600.3
AET6Gv20004400.2
AET2Gv20125900.5
AET6Gv20128900.1
AET6Gv20730700.1
Island
Domain Color
LRRNT_2 Family
LRR_4 Repeat
LRR_8 Repeat
PK_Tyr_Ser−Thr Domain
Pkinase Domain
TMEM154 Family
other Domain
0
500
1000
1500

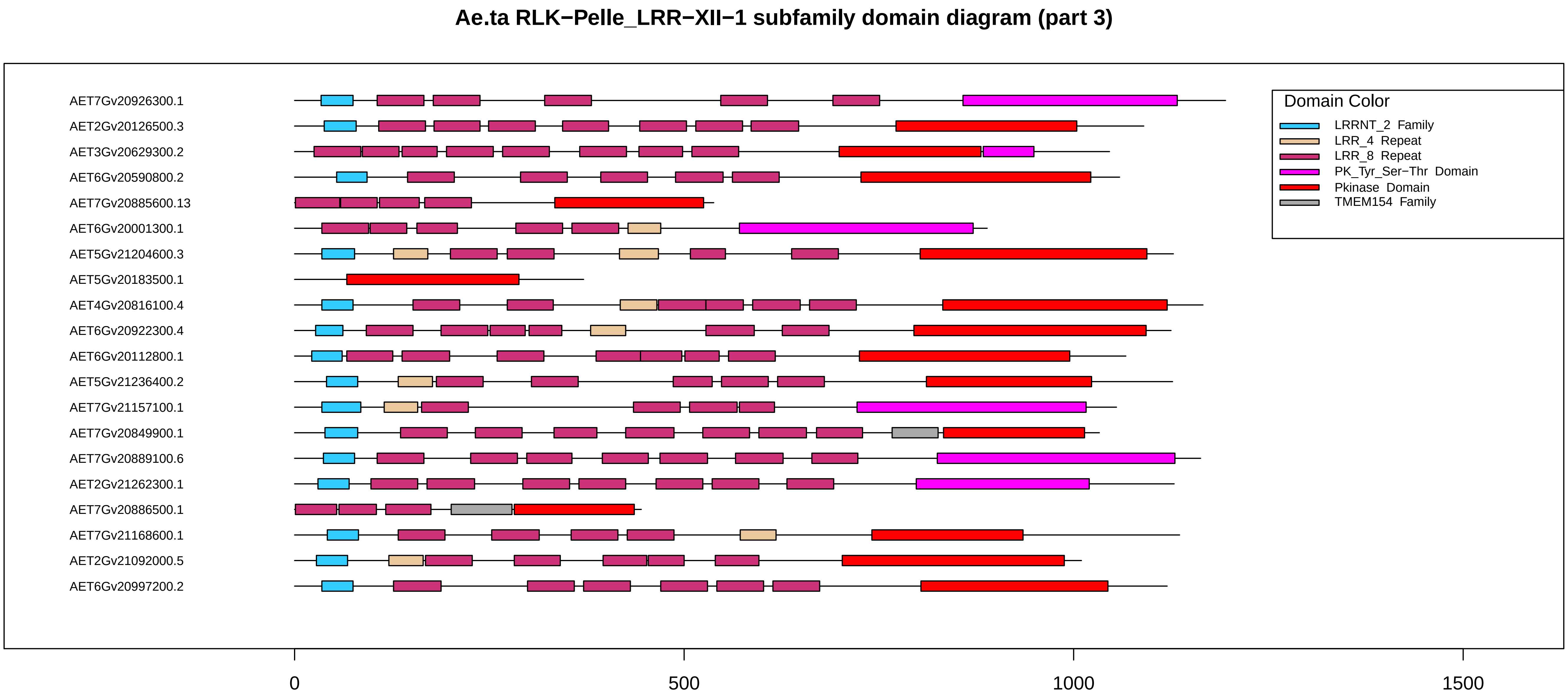
Ae.ta RLK−Pelle_LRR−XII−1 subfamily domain diagram (part 3)
AET7Gv20926300.1
AET2Gv20126500.3
AET3Gv20629300.2
AET6Gv20590800.2
AET7Gv20885600.13
AET6Gv20001300.1
AET5Gv21204600.3
AET5Gv20183500.1
AET4Gv20816100.4
AET6Gv20922300.4
AET6Gv20112800.1
AET5Gv21236400.2
AET7Gv21157100.1
AET7Gv20849900.1
AET7Gv20889100.6
AET2Gv21262300.1
AET7Gv20886500.1
AET7Gv21168600.1
AET2Gv21092000.5
AET6Gv20997200.2
Domain Color
LRRNT_2 Family
LRR_4 Repeat
LRR_8 Repeat
PK_Tyr_Ser−Thr Domain
Pkinase Domain
TMEM154 Family
0
500
1000
1500

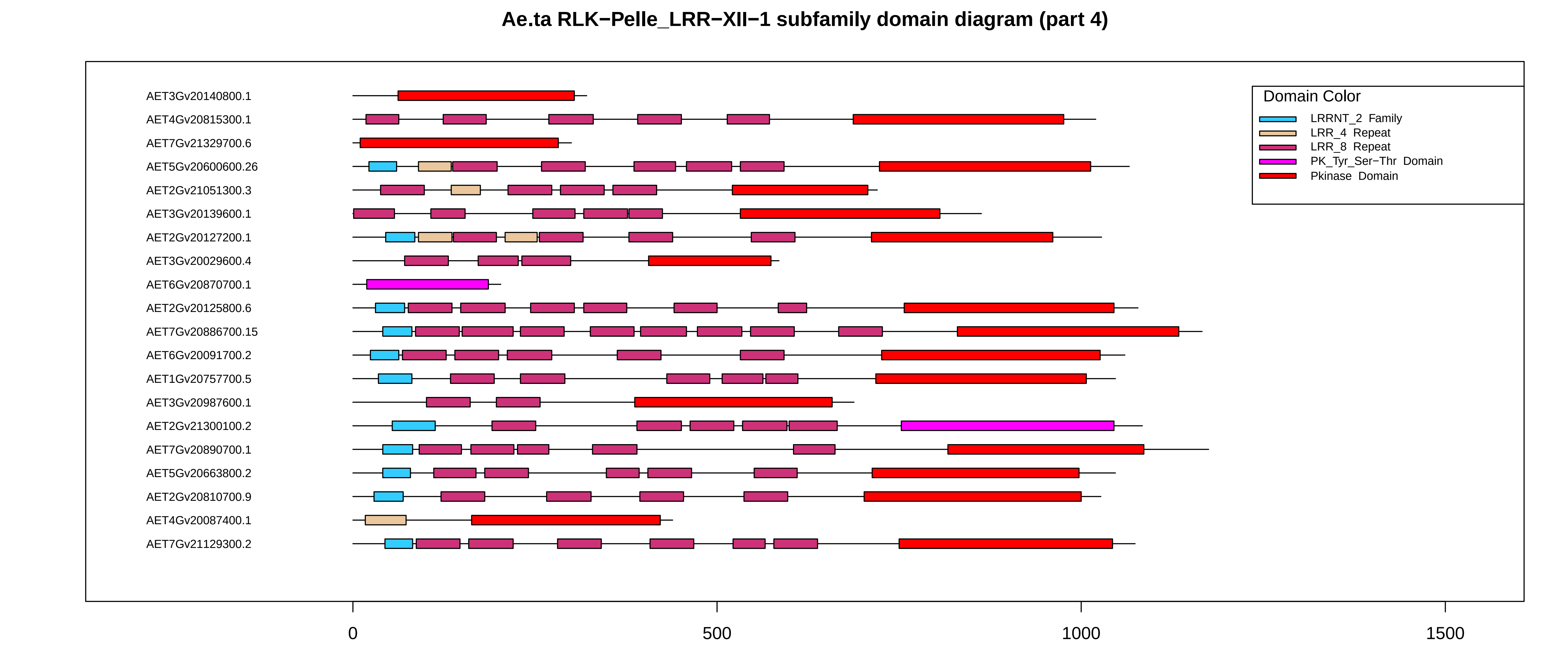
Ae.ta RLK−Pelle_LRR−XII−1 subfamily domain diagram (part 4)
AET3Gv20140800.1
AET4Gv20815300.1
AET7Gv21329700.6
AET5Gv20600600.26
AET2Gv21051300.3
AET3Gv20139600.1
AET2Gv20127200.1
AET3Gv20029600.4
AET6Gv20870700.1
AET2Gv20125800.6
AET7Gv20886700.15
AET6Gv20091700.2
AET1Gv20757700.5
AET3Gv20987600.1
AET2Gv21300100.2
AET7Gv20890700.1
AET5Gv20663800.2
AET2Gv20810700.9
AET4Gv20087400.1
AET7Gv21129300.2
Domain Color
LRRNT_2 Family
LRR_4 Repeat
LRR_8 Repeat
PK_Tyr_Ser−Thr Domain
Pkinase Domain
0
500
1000
1500

Ae.ta RLK−Pelle_LRR−XII−1 subfamily domain diagram (part 5)

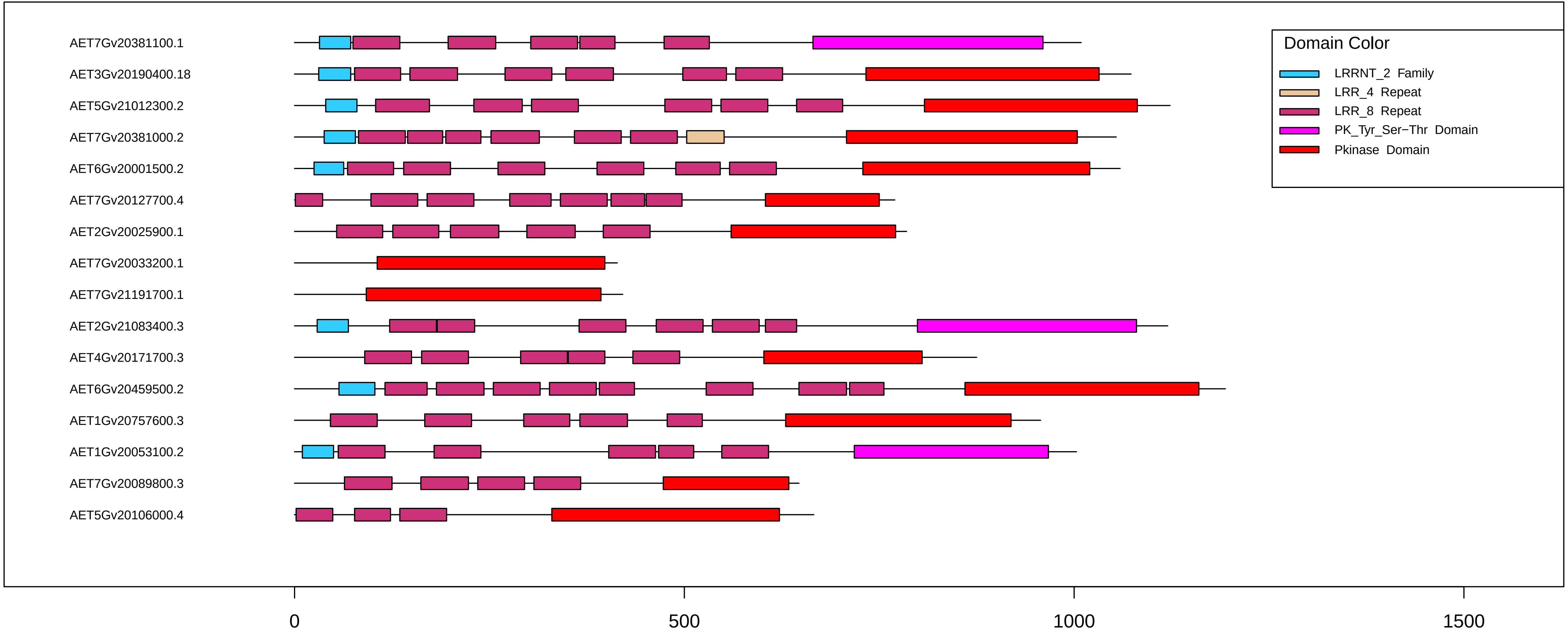

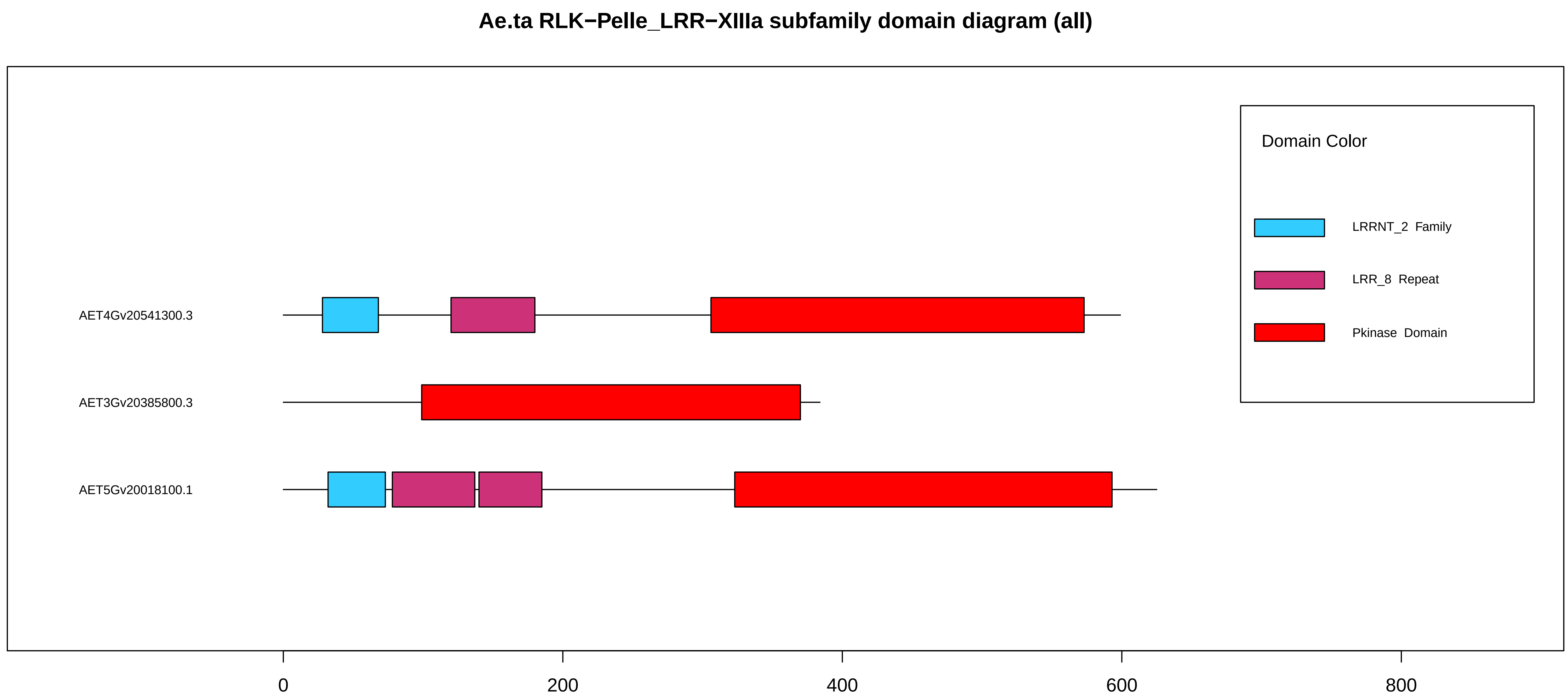
Ae.ta RLK−Pelle_LRR−XIIIa subfamily domain diagram (all)
Domain Color
LRRNT_2 Family
LRR_8 Repeat
Pkinase Domain
AET4Gv20541300.3
AET3Gv20385800.3
AET5Gv20018100.1
0
200
400
600
800

Ae.ta RLK−Pelle_LRR−XIIIb subfamily domain diagram (all)

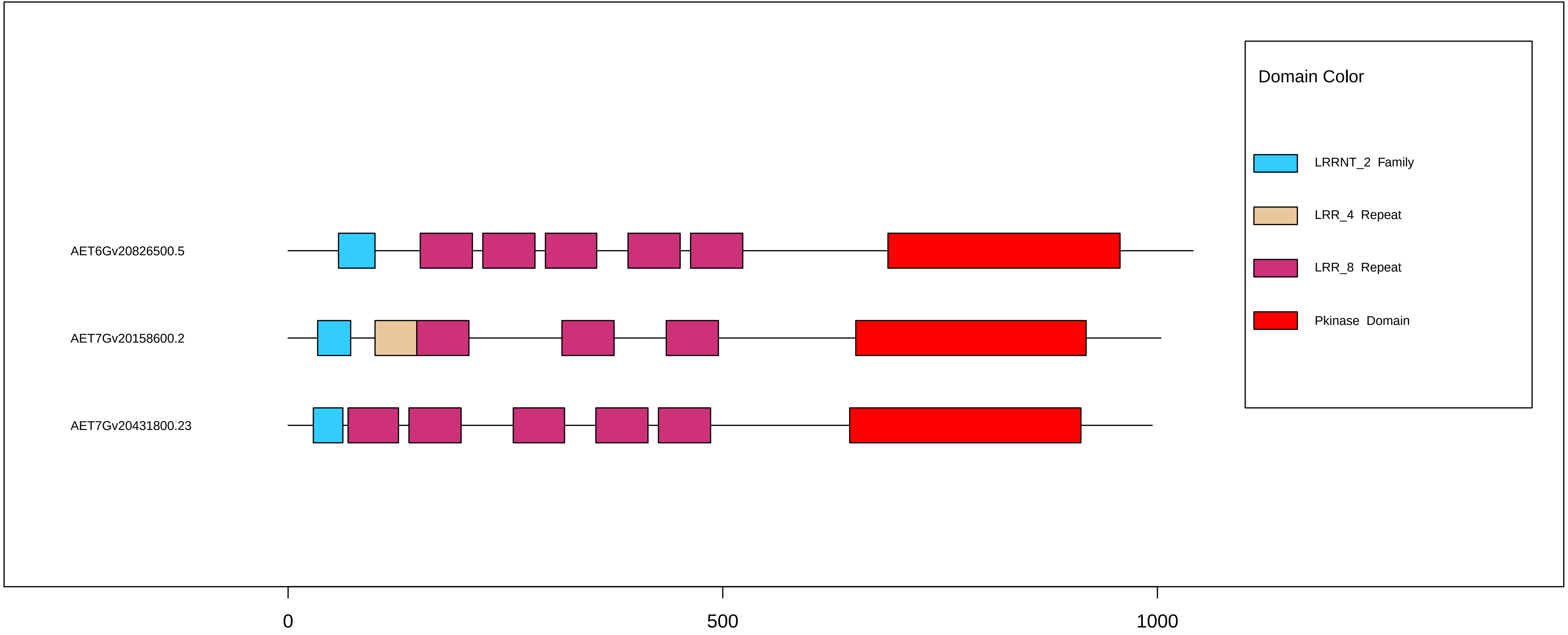

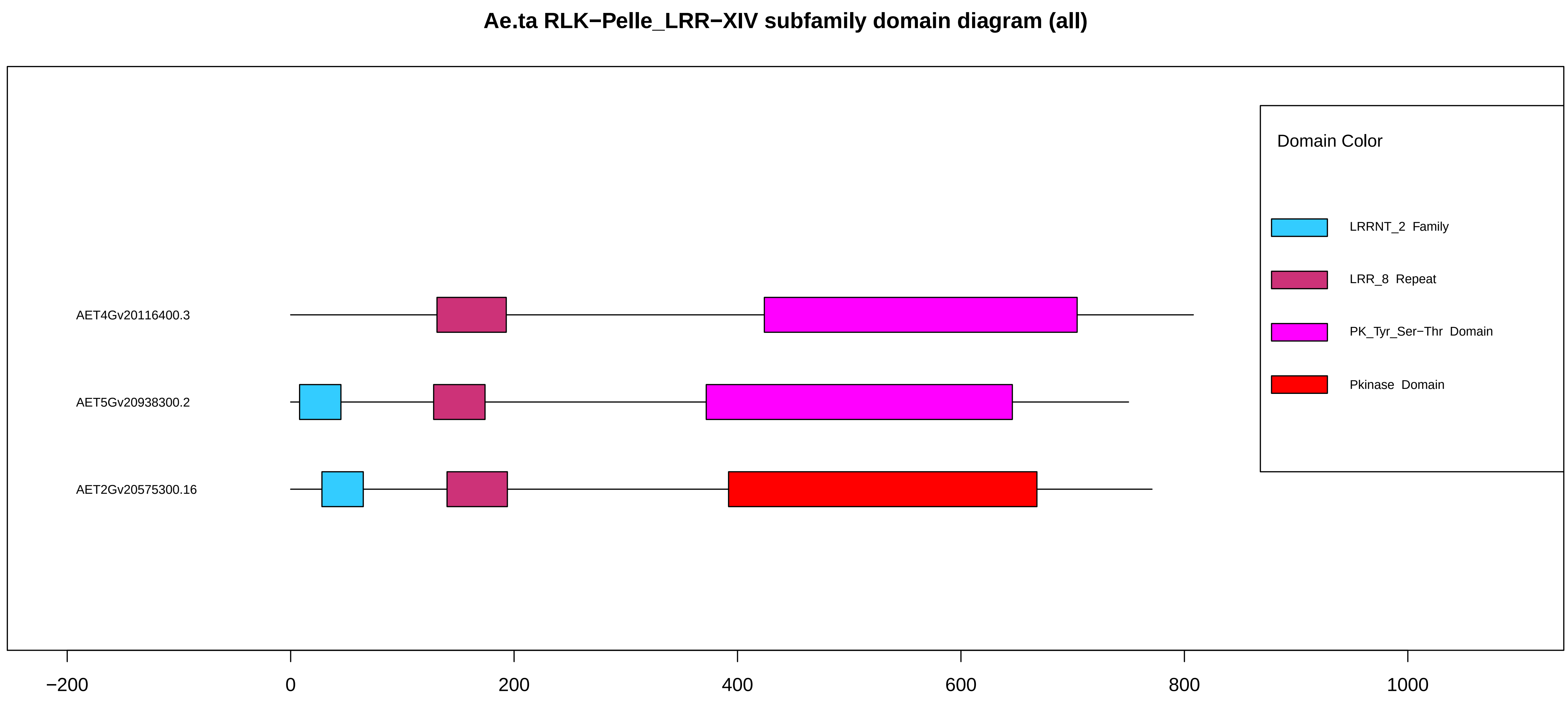
Ae.ta RLK−Pelle_LRR−XIV subfamily domain diagram (all)
Domain Color
LRRNT_2 Family
LRR_8 Repeat
PK_Tyr_Ser−Thr Domain
Pkinase Domain
AET4Gv20116400.3
AET5Gv20938300.2
AET2Gv20575300.16
−200
0
200
400
600
800
1000

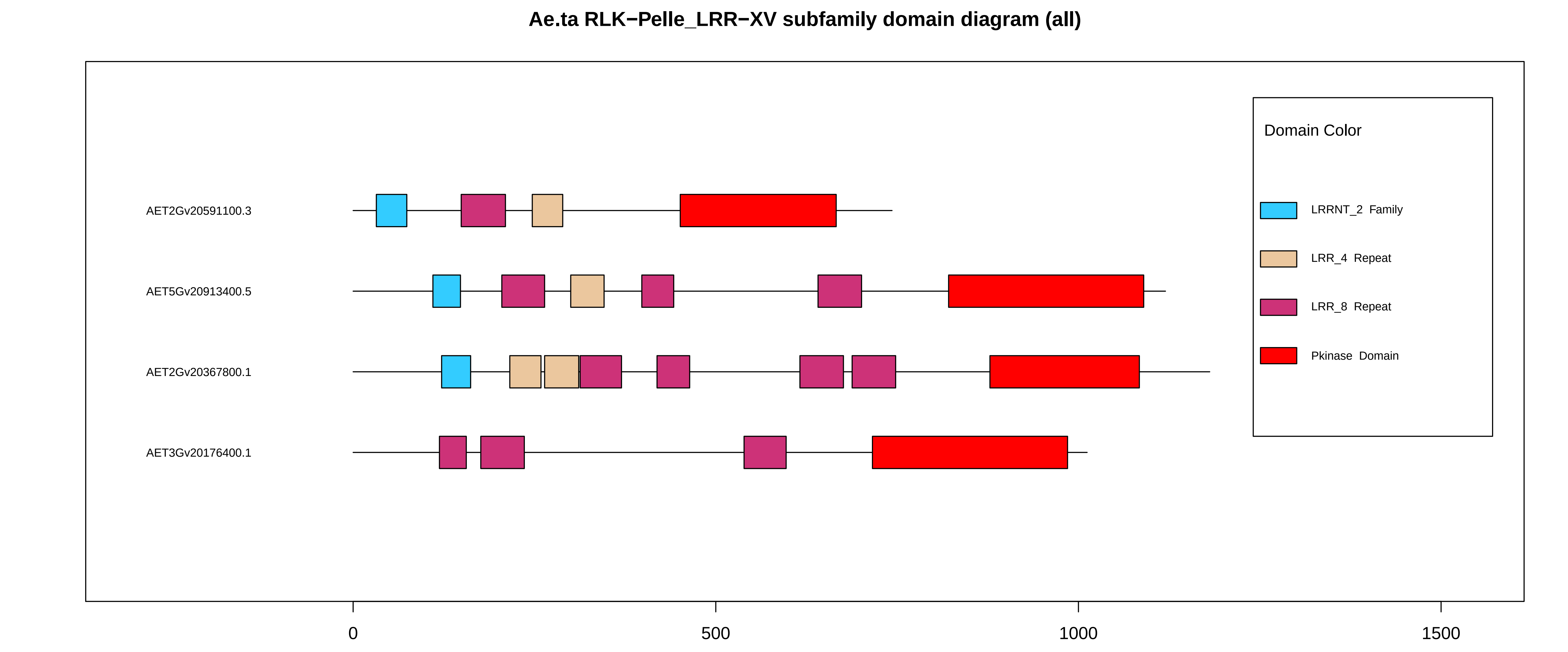
Ae.ta RLK−Pelle_LRR−XV subfamily domain diagram (all)
AET2Gv20591100.3
AET5Gv20913400.5
AET2Gv20367800.1
AET3Gv20176400.1
Domain Color
LRRNT_2 Family
LRR_4 Repeat
LRR_8 Repeat
Pkinase Domain
0
500
1000
1500

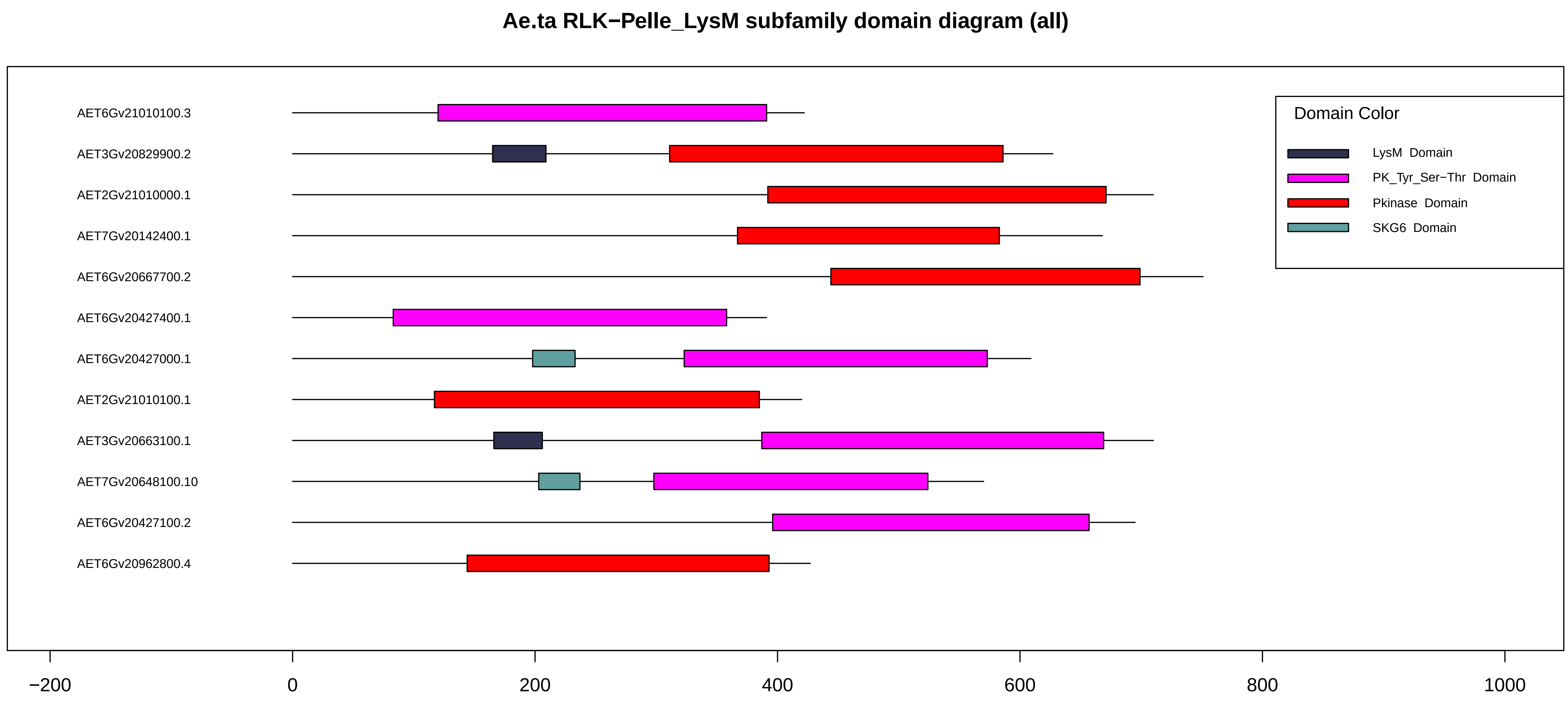
Ae.ta RLK−Pelle_LysM subfamily domain diagram (all)
AET6Gv21010100.3
AET3Gv20829900.2
AET2Gv21010000.1
AET7Gv20142400.1
AET6Gv20667700.2
AET6Gv20427400.1
AET6Gv20427000.1
AET2Gv21010100.1
AET3Gv20663100.1
AET7Gv20648100.10
AET6Gv20427100.2
AET6Gv20962800.4
Domain Color
LysM Domain
PK_Tyr_Ser−Thr Domain
Pkinase Domain
SKG6 Domain
−200
0
200
400
600
800
1000

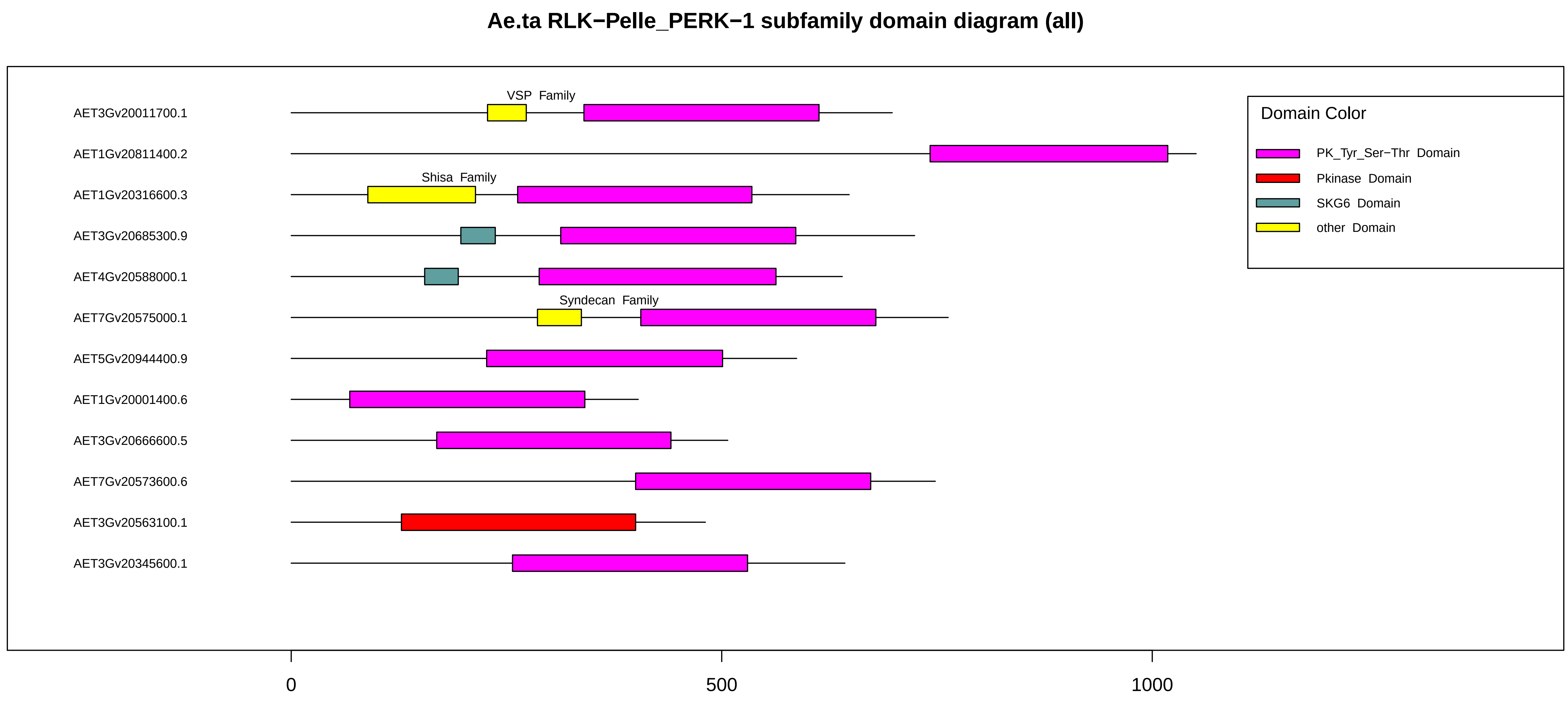

Ae.ta RLK−Pelle_PERK−1 subfamily domain diagram (all)
AET3Gv20011700.1
AET1Gv20811400.2
AET1Gv20316600.3
AET3Gv20685300.9
AET4Gv20588000.1
AET7Gv20575000.1
AET5Gv20944400.9
AET1Gv20001400.6
AET3Gv20666600.5
AET7Gv20573600.6
AET3Gv20563100.1
AET3Gv20345600.1
VSP Family
Shisa Family
Syndecan Family
Domain Color
PK_Tyr_Ser−Thr Domain
Pkinase Domain
SKG6 Domain
other Domain
0
500
1000

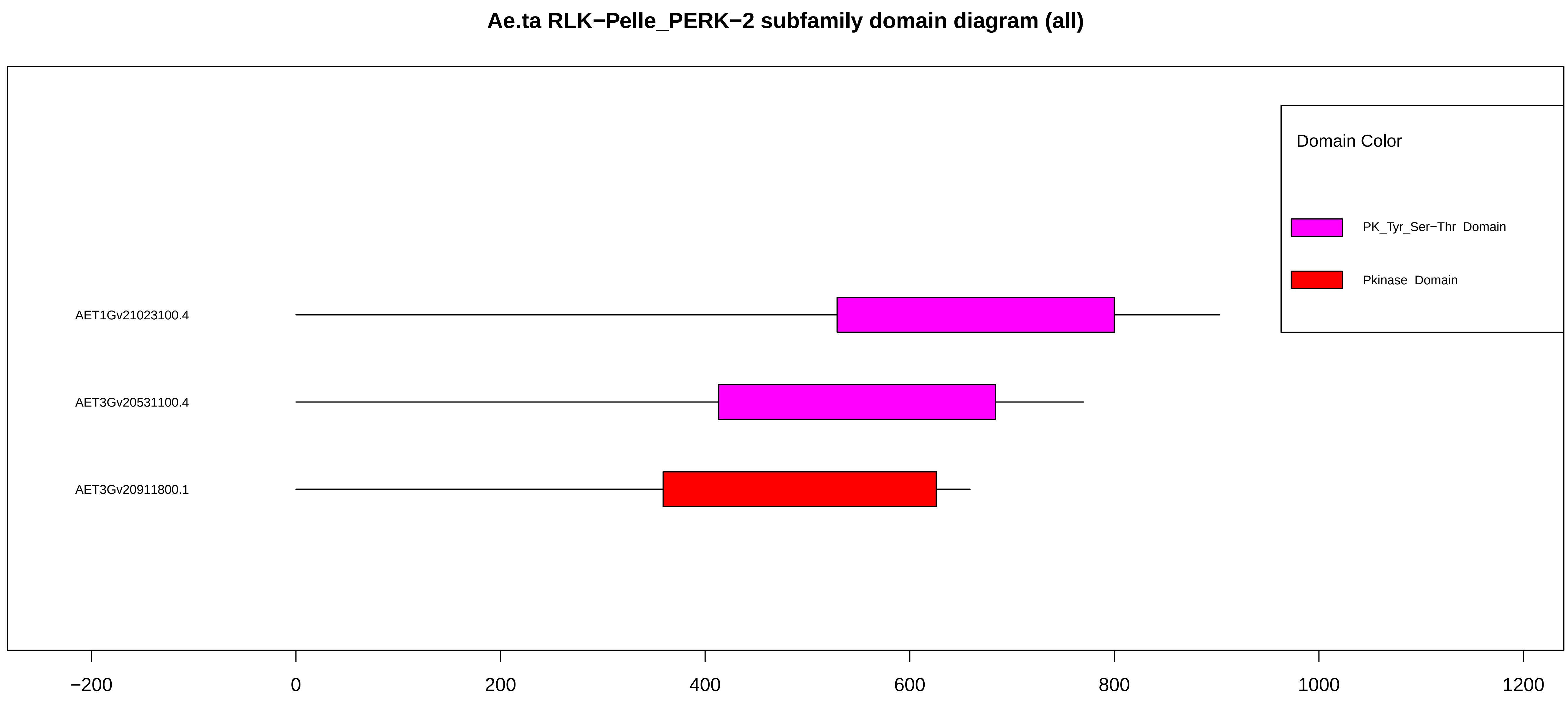

Ae.ta RLK−Pelle_PERK−2 subfamily domain diagram (all)
Domain Color
PK_Tyr_Ser−Thr Domain
Pkinase Domain
AET1Gv21023100.4
AET3Gv20531100.4
AET3Gv20911800.1
−200
0
200
400
600
800
1000
1200

Ae.ta RLK−Pelle_RKF3 subfamily domain diagram (all)

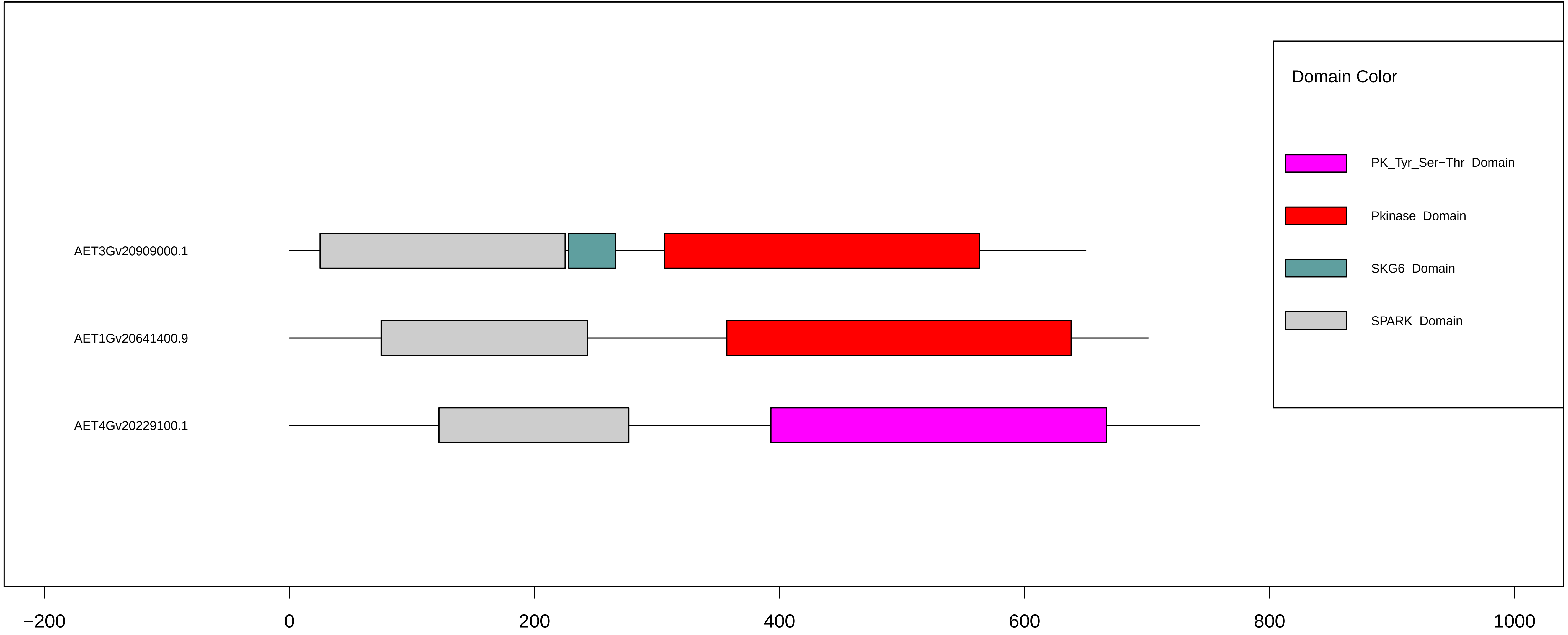

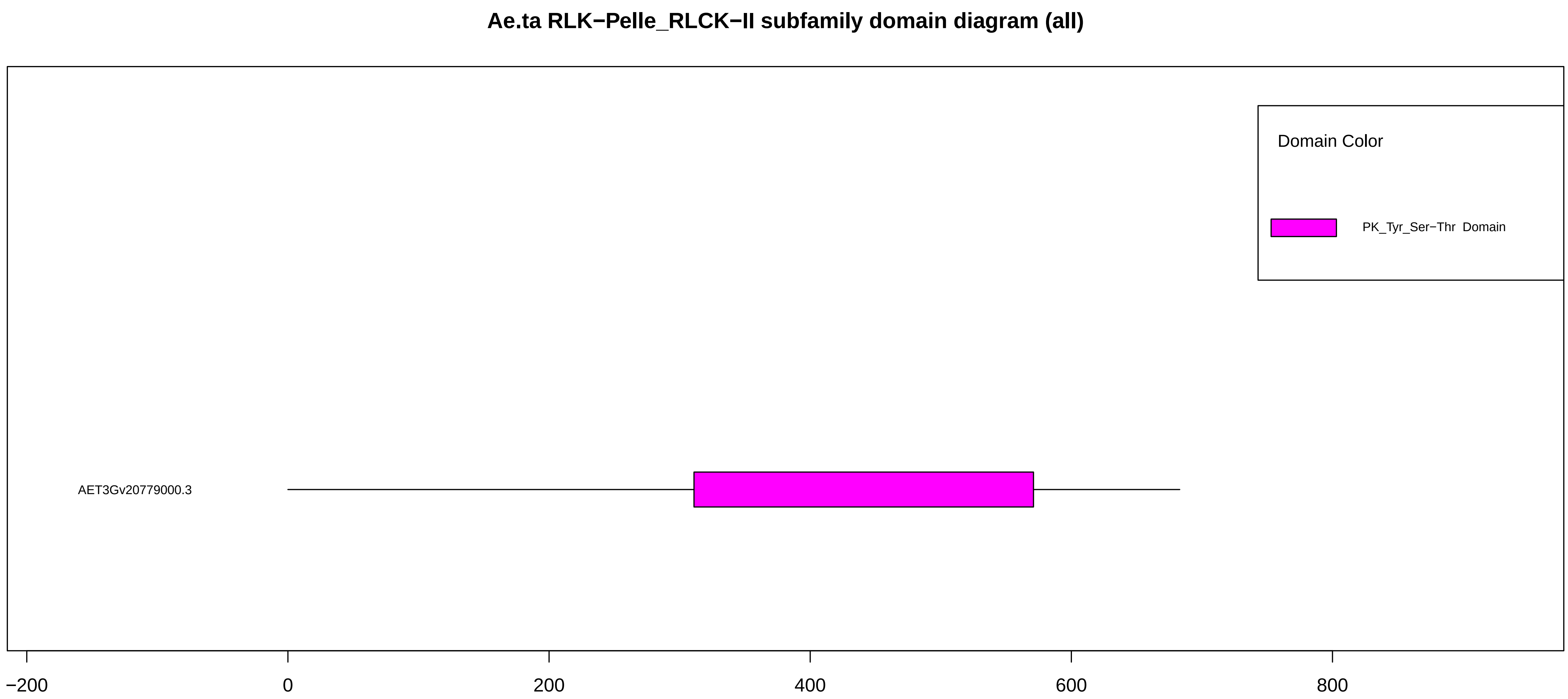
Ae.ta RLK−Pelle_RLCK−II subfamily domain diagram (all)
Domain Color
PK_Tyr_Ser−Thr Domain
AET3Gv20779000.3
−200
0
200
400
600
800

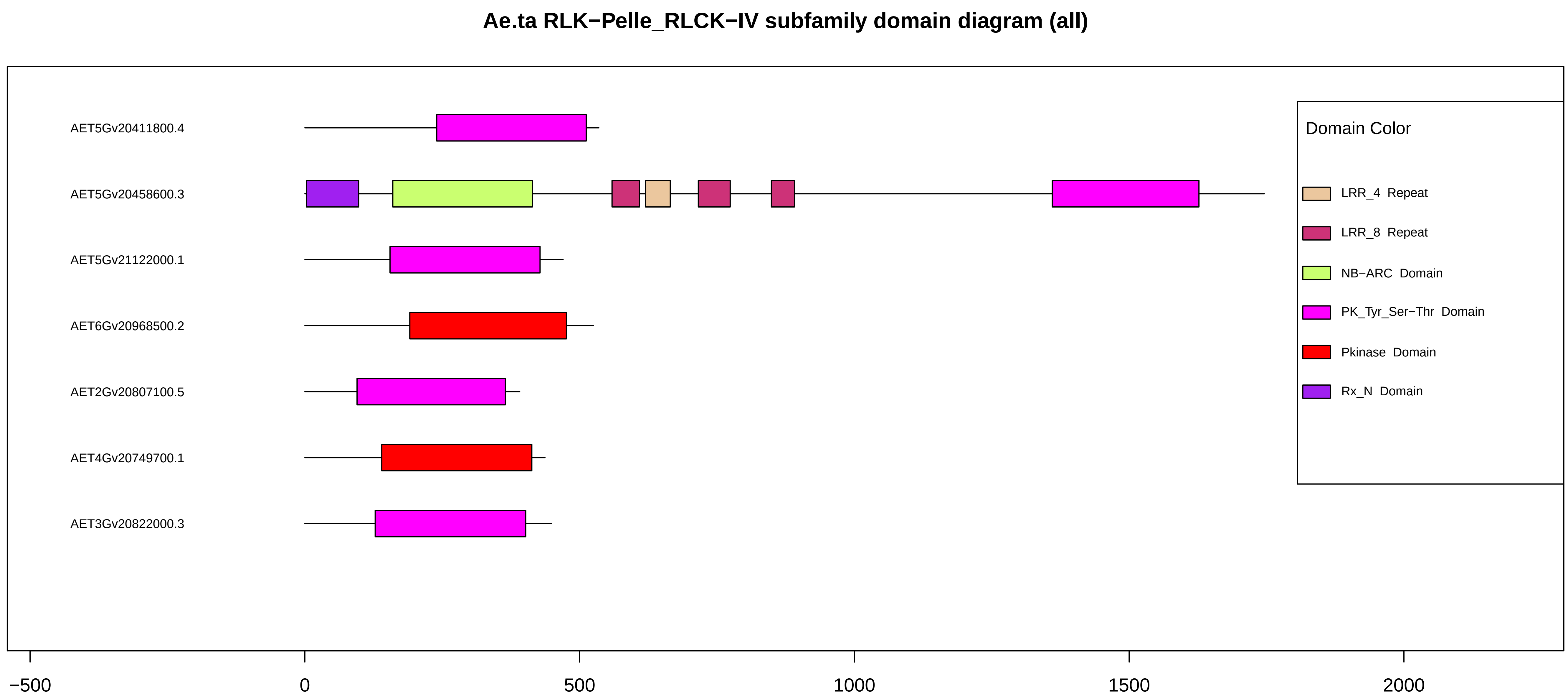

Ae.ta RLK−Pelle_RLCK−IV subfamily domain diagram (all)
AET5Gv20411800.4
AET5Gv20458600.3
AET5Gv21122000.1
AET6Gv20968500.2
AET2Gv20807100.5
AET4Gv20749700.1
AET3Gv20822000.3
Domain Color
LRR_4 Repeat
LRR_8 Repeat
NB−ARC Domain
PK_Tyr_Ser−Thr Domain
Pkinase Domain
Rx_N Domain
−500
0
500
1000
1500
2000

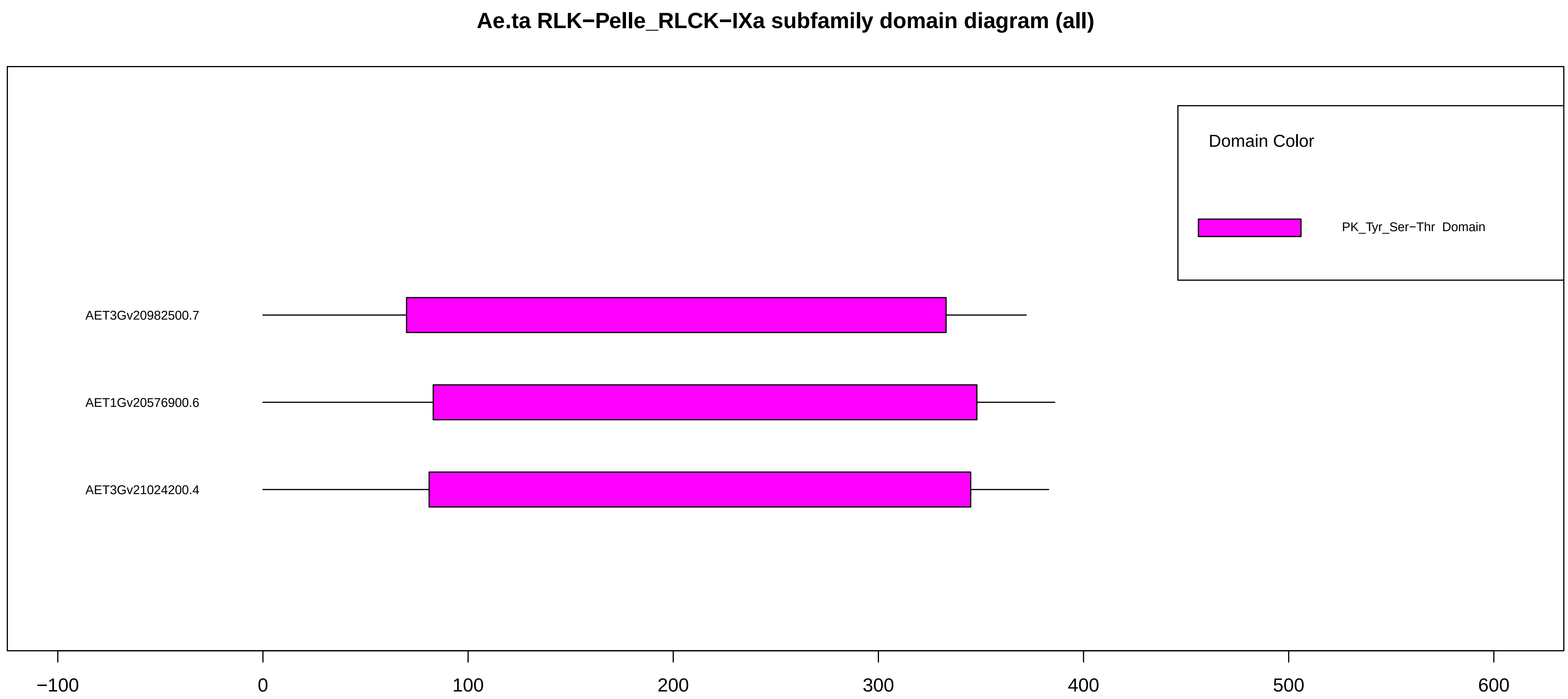

Ae.ta RLK−Pelle_RLCK−IXa subfamily domain diagram (all)
Domain Color
PK_Tyr_Ser−Thr Domain
AET3Gv20982500.7
AET1Gv20576900.6
AET3Gv21024200.4
−100
0
100
200
300
400
500
600

Ae.ta RLK−Pelle_RLCK−IXb subfamily domain diagram (part 1)

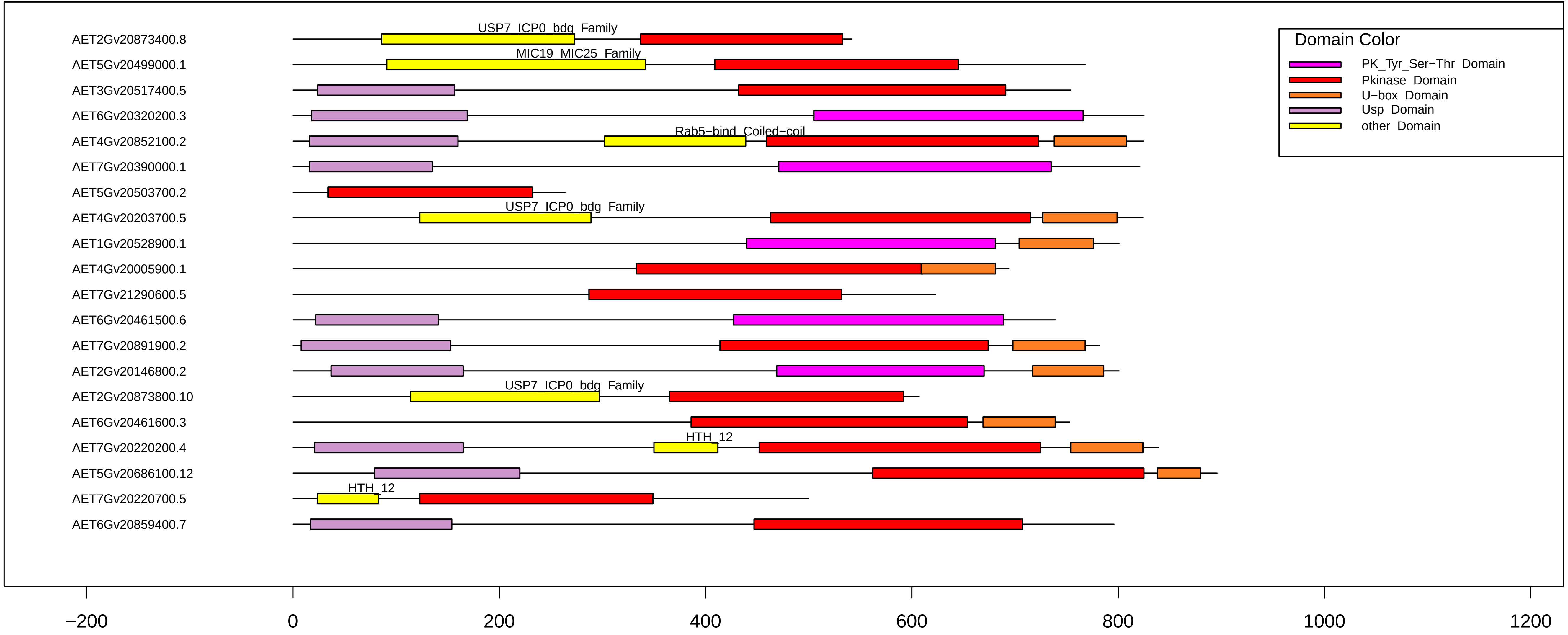

Ae.ta RLK−Pelle_RLCK−IXb subfamily domain diagram (part 2)

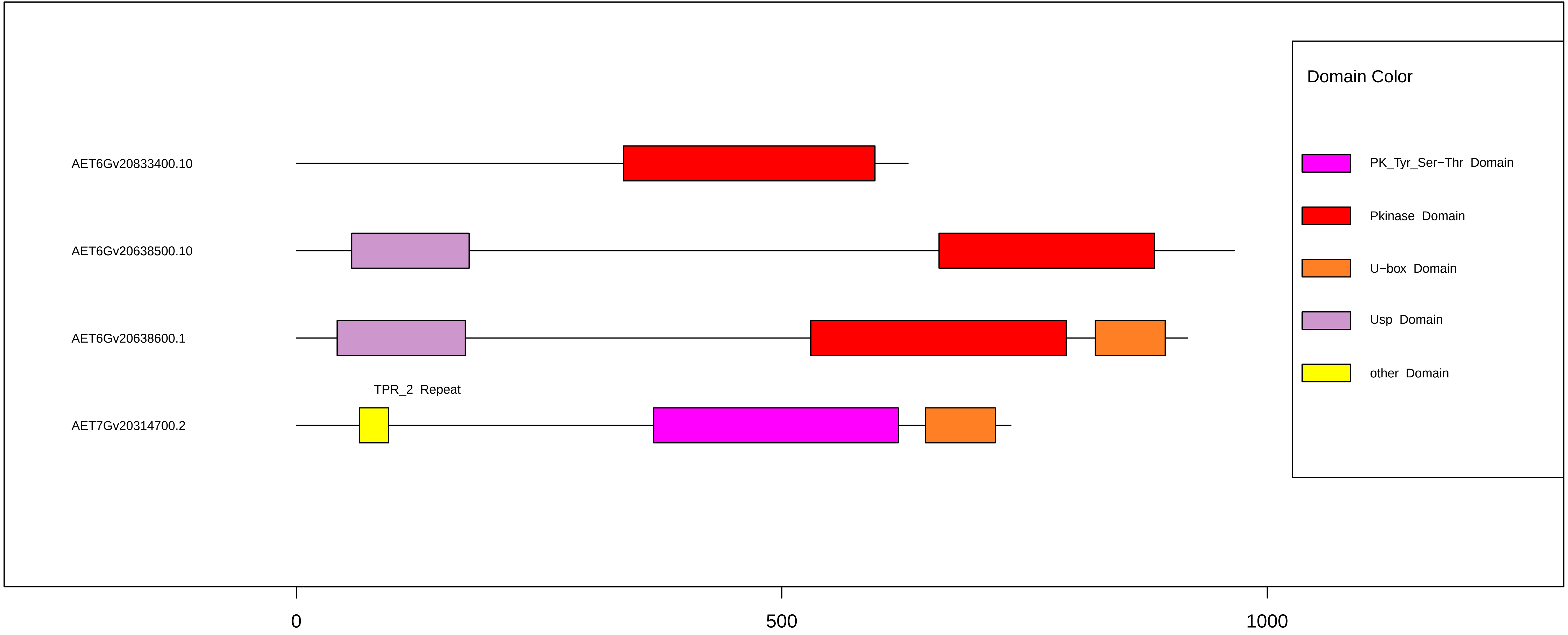

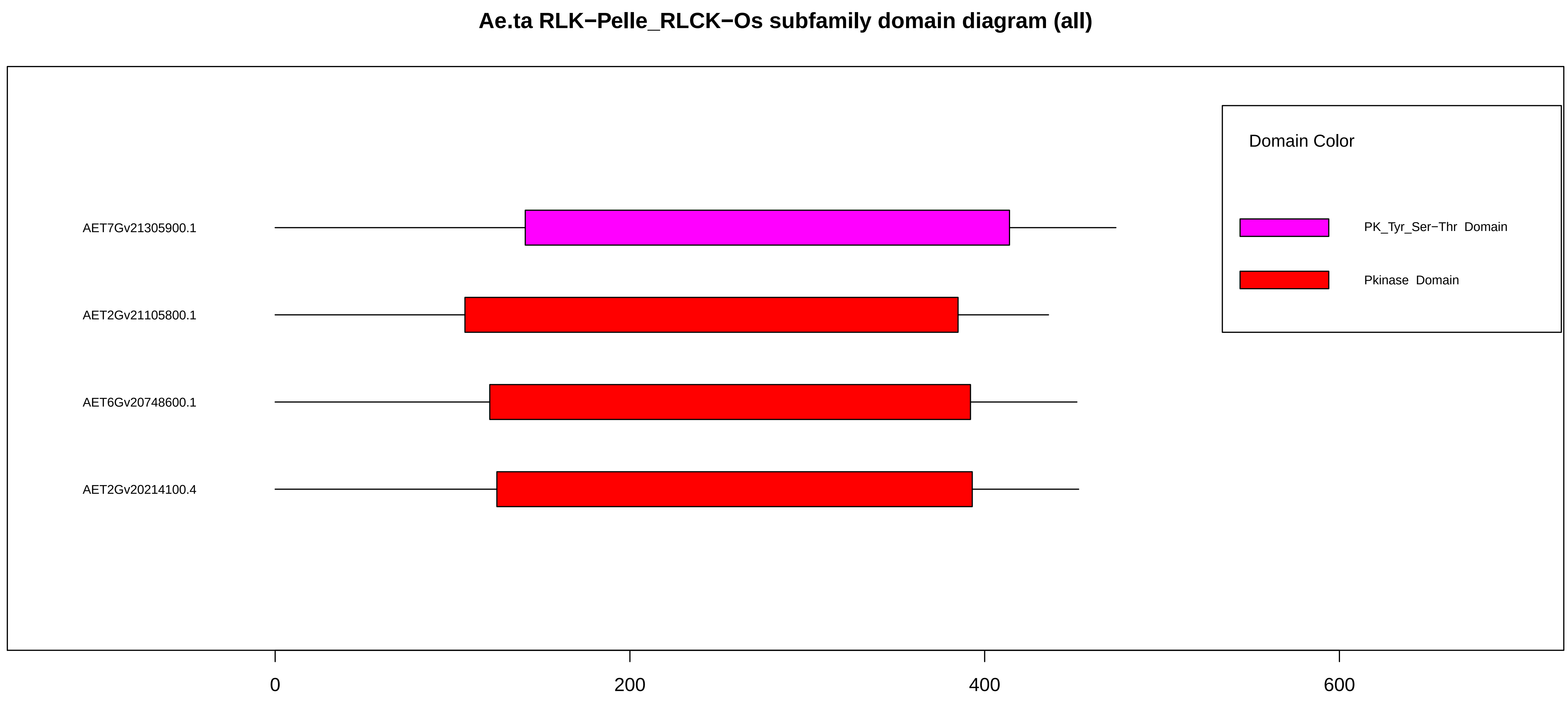
Ae.ta RLK−Pelle_RLCK−Os subfamily domain diagram (all)
AET7Gv21305900.1
AET2Gv21105800.1
AET6Gv20748600.1
AET2Gv20214100.4
Domain Color
PK_Tyr_Ser−Thr Domain
Pkinase Domain
0
200
400
600

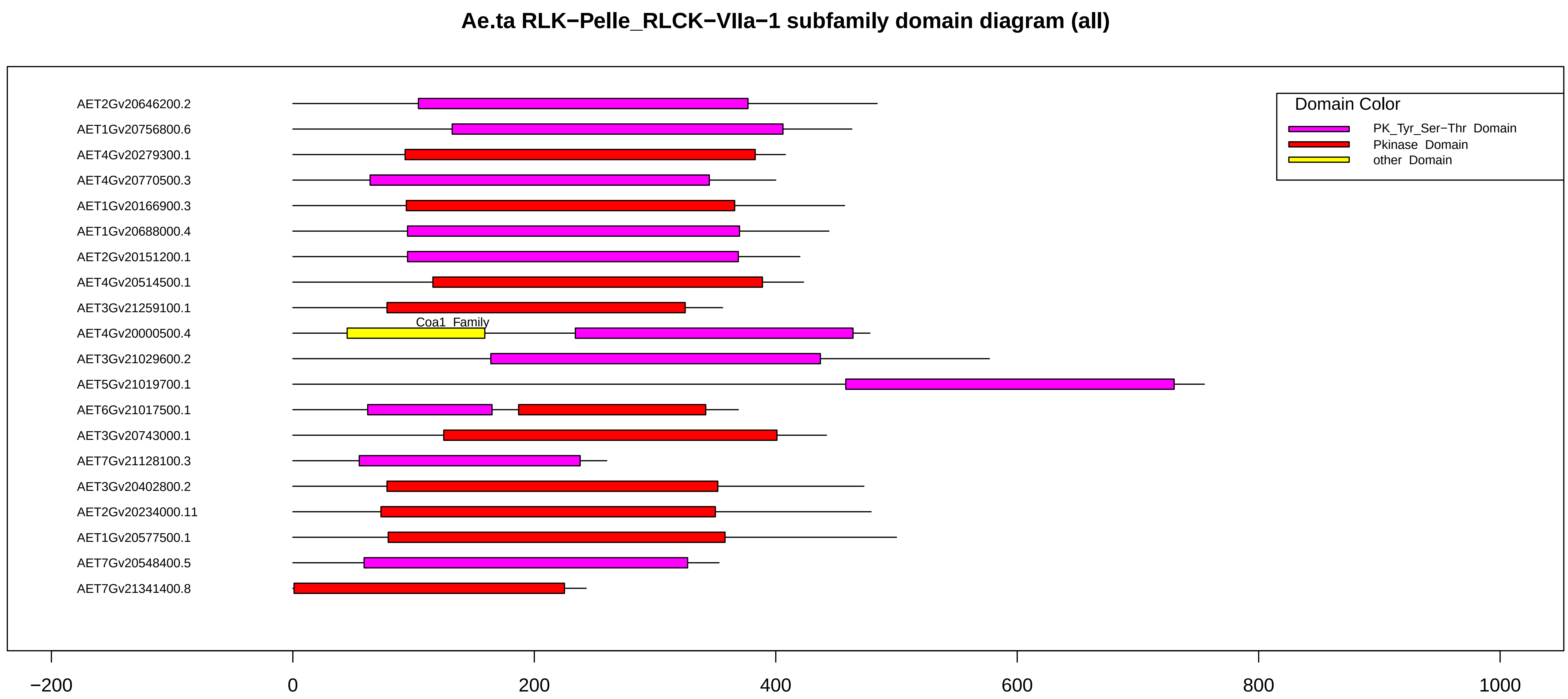

Ae.ta RLK−Pelle_RLCK−VIIa−1 subfamily domain diagram (all)
AET2Gv20646200.2
AET1Gv20756800.6
AET4Gv20279300.1
AET4Gv20770500.3
AET1Gv20166900.3
AET1Gv20688000.4
AET2Gv20151200.1
AET4Gv20514500.1
AET3Gv21259100.1
AET4Gv20000500.4
Coa1_Family
AET3Gv21029600.2
AET5Gv21019700.1
AET6Gv21017500.1
AET3Gv20743000.1
AET7Gv21128100.3
AET3Gv20402800.2
AET2Gv20234000.11
AET1Gv20577500.1
AET7Gv20548400.5
AET7Gv21341400.8
Domain Color
PK_Tyr_Ser−Thr Domain
Pkinase Domain
other Domain
−200
0
200
400
600
800
1000

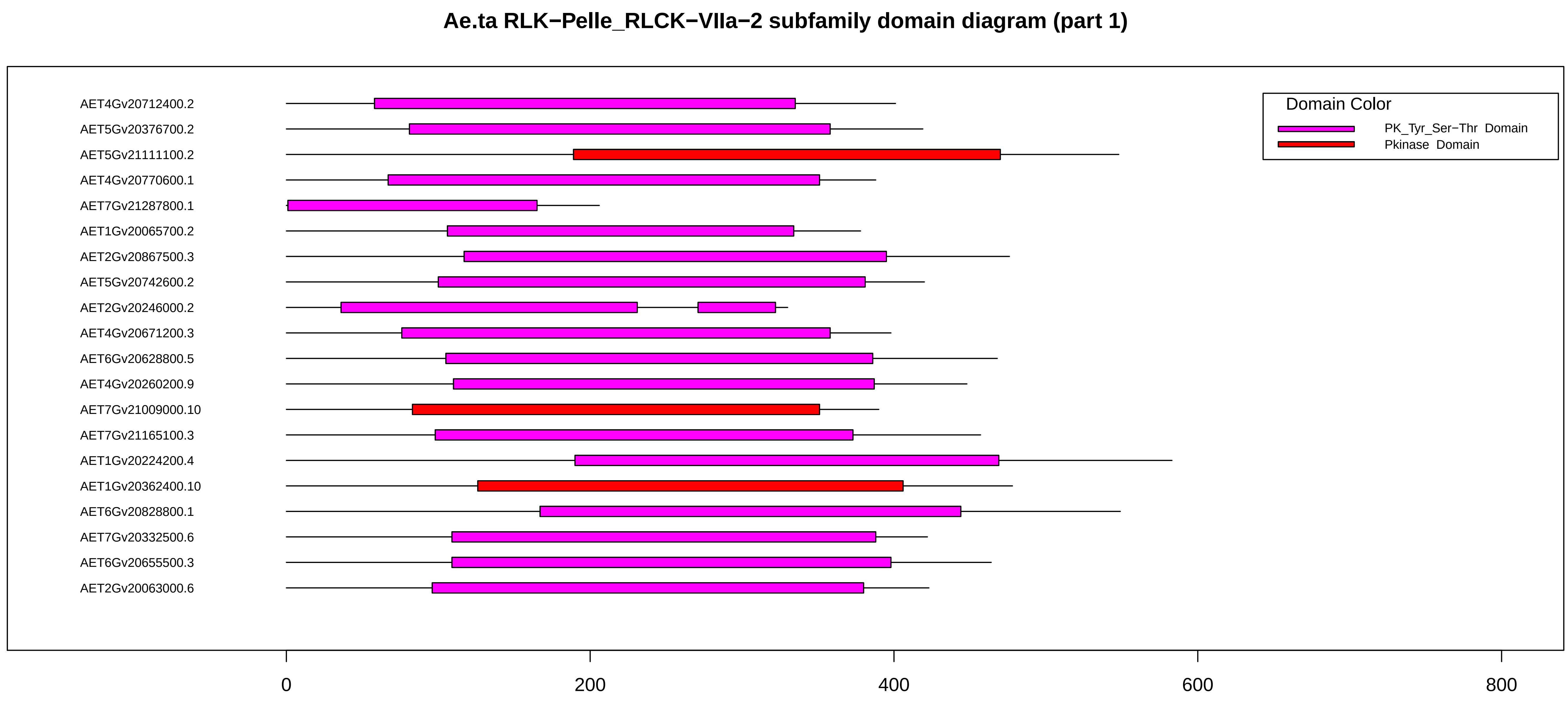
Ae.ta RLK−Pelle_RLCK−VIIa−2 subfamily domain diagram (part 1)
Domain Color
PK_Tyr_Ser−Thr Domain
Pkinase Domain
AET4Gv20712400.2
AET5Gv20376700.2
AET5Gv21111100.2
AET4Gv20770600.1
AET7Gv21287800.1
AET1Gv20065700.2
AET2Gv20867500.3
AET5Gv20742600.2
AET2Gv20246000.2
AET4Gv20671200.3
AET6Gv20628800.5
AET4Gv20260200.9
AET7Gv21009000.10
AET7Gv21165100.3
AET1Gv20224200.4
AET1Gv20362400.10
AET6Gv20828800.1
AET7Gv20332500.6
AET6Gv20655500.3
AET2Gv20063000.6
0
200
400
600
800

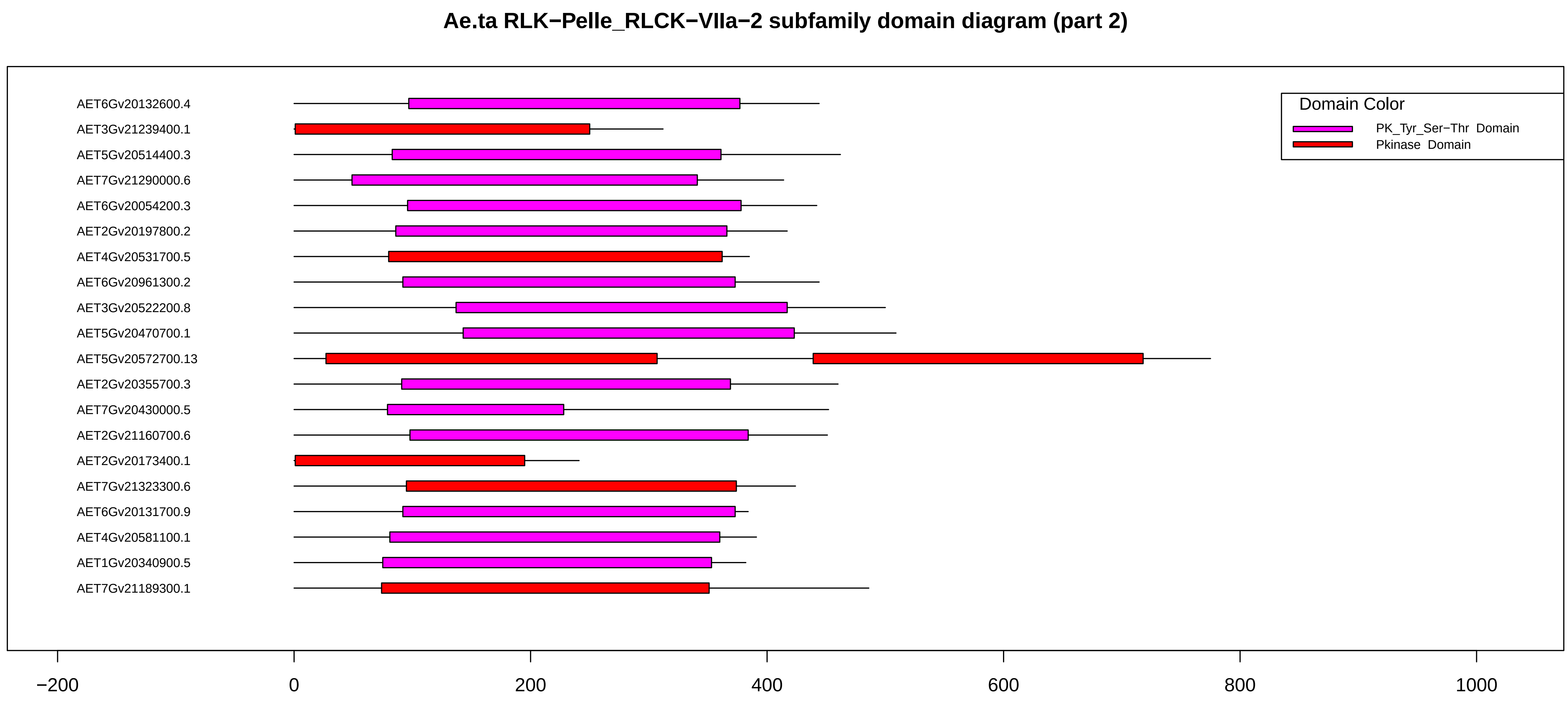

Ae.ta RLK−Pelle_RLCK−VIIa−2 subfamily domain diagram (part 2)
AET6Gv20132600.4
AET3Gv21239400.1
AET5Gv20514400.3
AET7Gv21290000.6
AET6Gv20054200.3
AET2Gv20197800.2
AET4Gv20531700.5
AET6Gv20961300.2
AET3Gv20522200.8
AET5Gv20470700.1
AET5Gv20572700.13
AET2Gv20355700.3
AET7Gv20430000.5
AET2Gv21160700.6
AET2Gv20173400.1
AET7Gv21323300.6
AET6Gv20131700.9
AET4Gv20581100.1
AET1Gv20340900.5
AET7Gv21189300.1
Domain Color
PK_Tyr_Ser−Thr Domain
Pkinase Domain
−200
0
200
400
600
800
1000

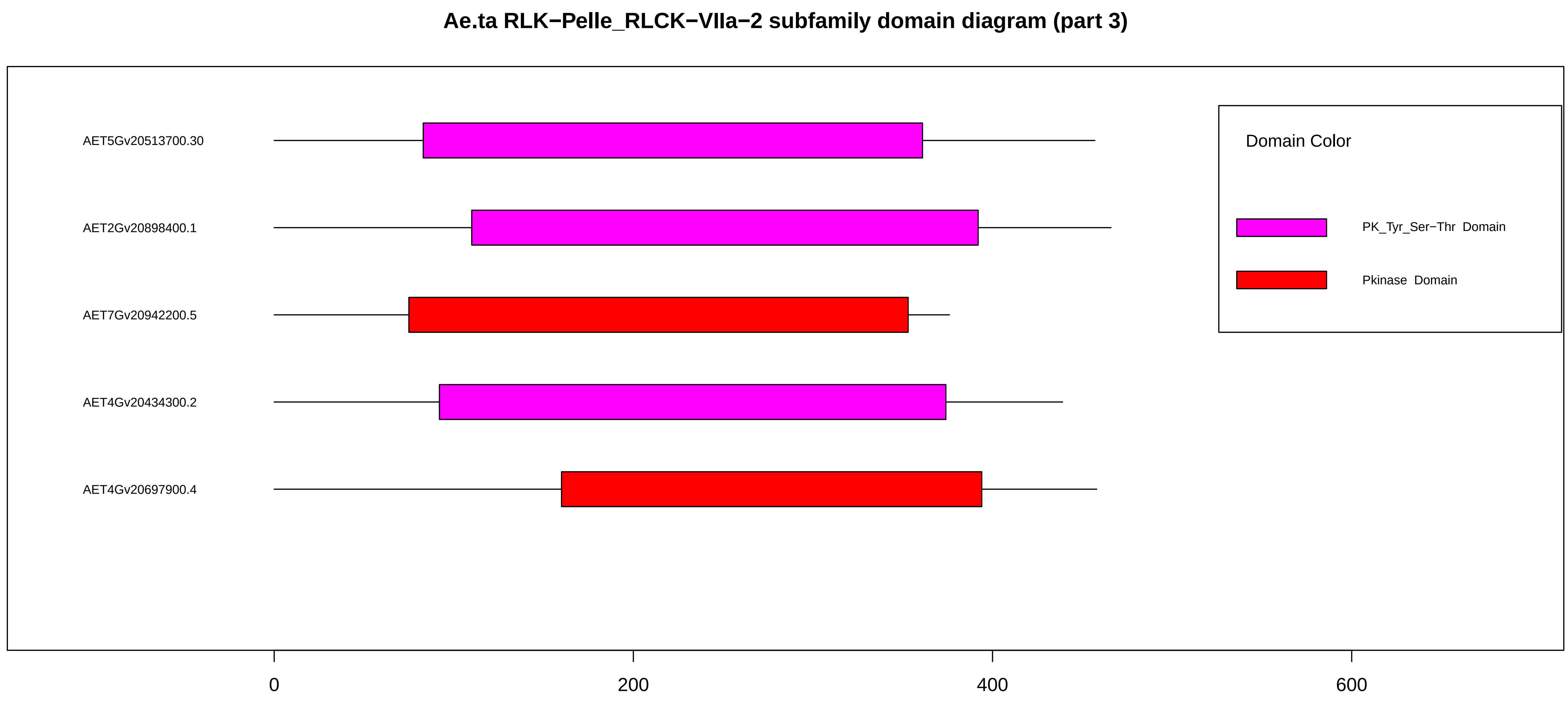
Ae.ta RLK−Pelle_RLCK−VIIa−2 subfamily domain diagram (part 3)
AET5Gv20513700.30
AET2Gv20898400.1
AET7Gv20942200.5
AET4Gv20434300.2
AET4Gv20697900.4
Domain Color
PK_Tyr_Ser−Thr Domain
Pkinase Domain
0
200
400
600

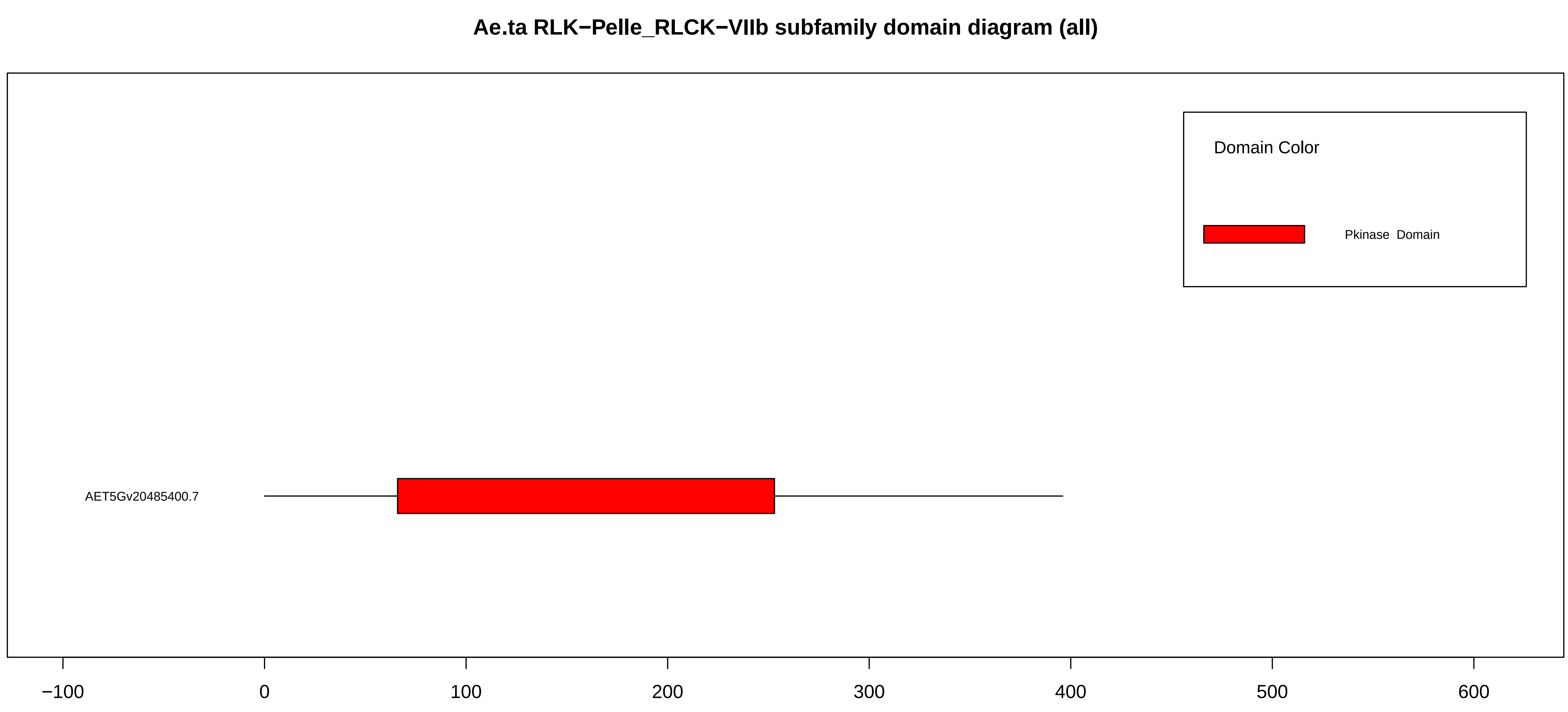
Ae.ta RLK−Pelle_RLCK−VIIb subfamily domain diagram (all)
Domain Color
Pkinase Domain
AET5Gv20485400.7
−100
0
100
200
300
400
500
600

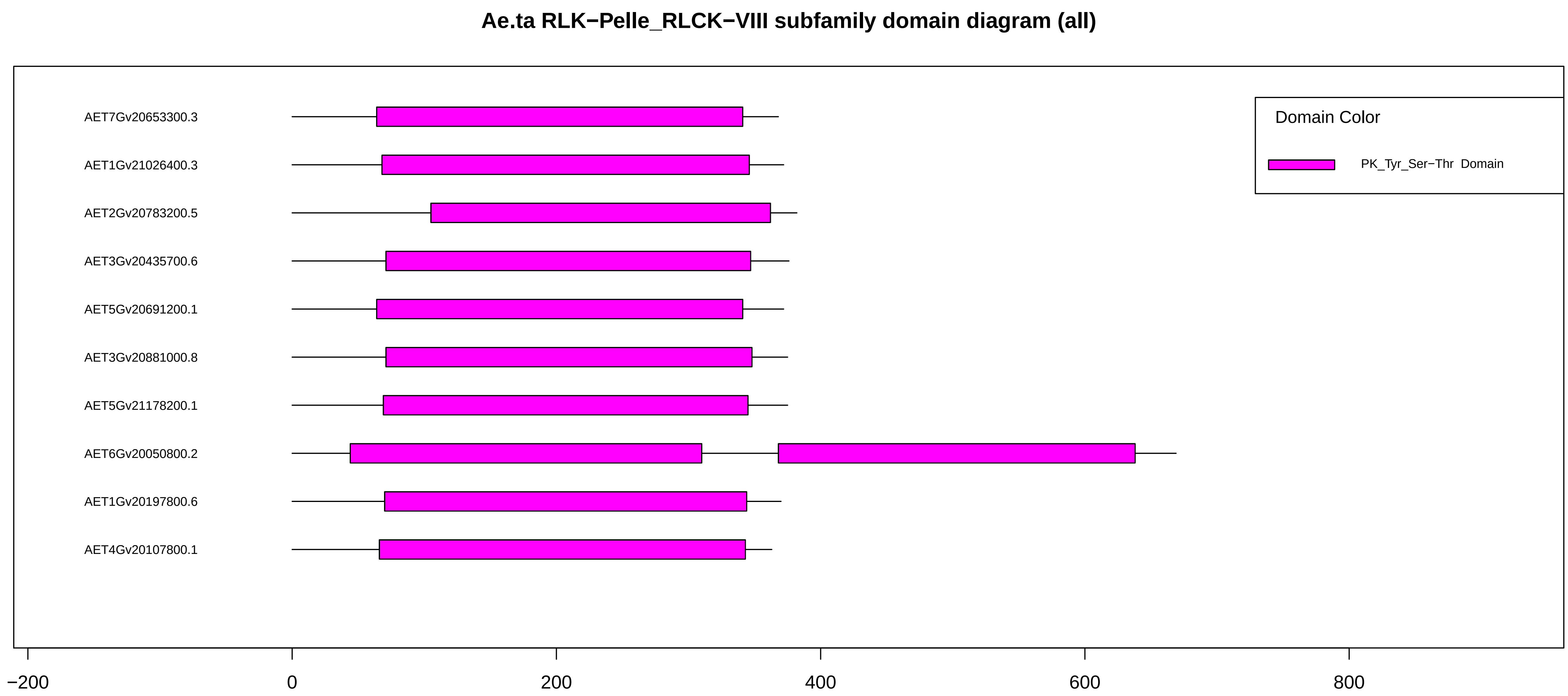

Ae.ta RLK−Pelle_RLCK−VIII subfamily domain diagram (all)
AET7Gv20653300.3
AET1Gv21026400.3
AET2Gv20783200.5
AET3Gv20435700.6
AET5Gv20691200.1
AET3Gv20881000.8
AET5Gv21178200.1
AET6Gv20050800.2
AET1Gv20197800.6
AET4Gv20107800.1
Domain Color
PK_Tyr_Ser−Thr Domain
−200
0
200
400
600
800

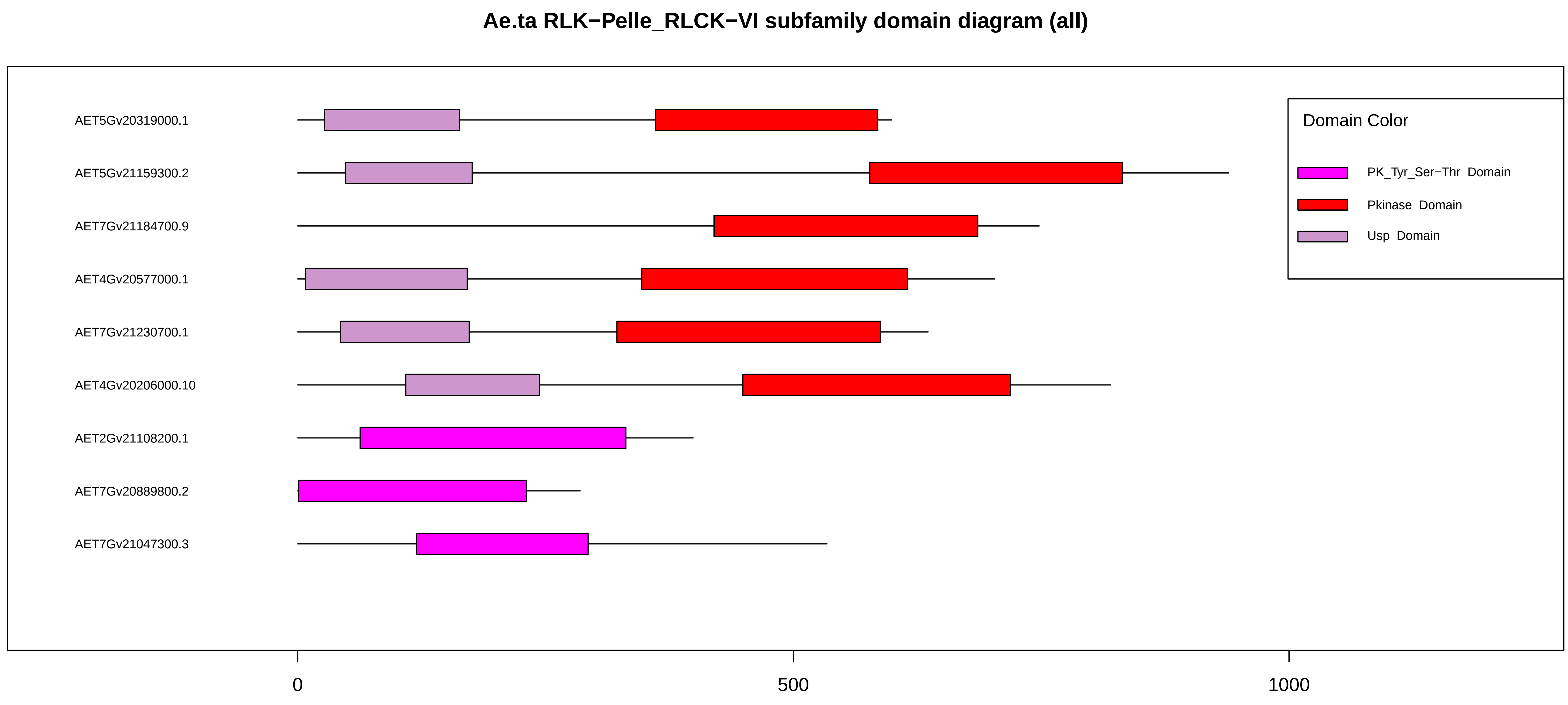
Ae.ta RLK−Pelle_RLCK−VI subfamily domain diagram (all)
AET5Gv20319000.1
AET5Gv21159300.2
AET7Gv21184700.9
AET4Gv20577000.1
AET7Gv21230700.1
AET4Gv20206000.10
AET2Gv21108200.1
AET7Gv20889800.2
AET7Gv21047300.3
Domain Color
PK_Tyr_Ser−Thr Domain
Pkinase Domain
Usp Domain
0
500
1000

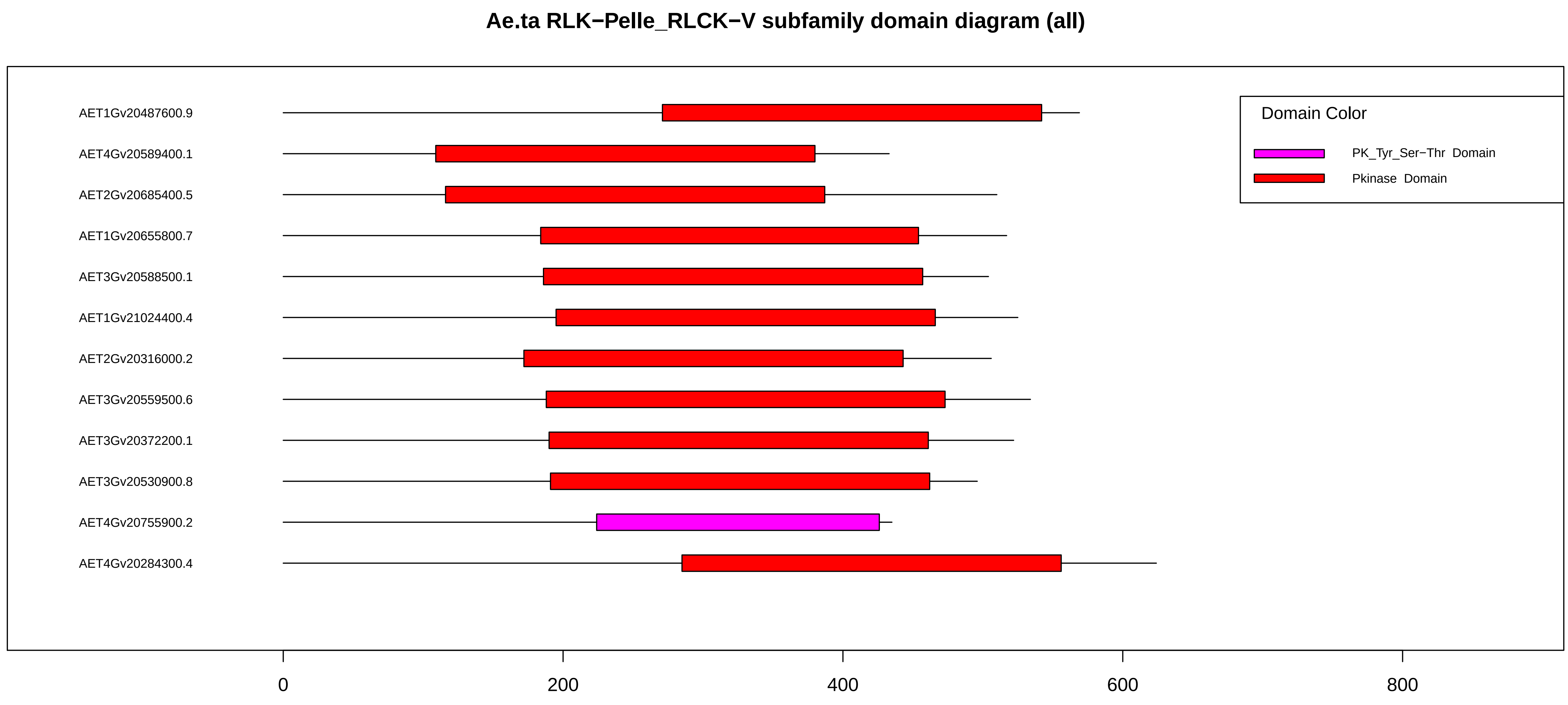
Ae.ta RLK−Pelle_RLCK−V subfamily domain diagram (all)
AET1Gv20487600.9
AET4Gv20589400.1
AET2Gv20685400.5
AET1Gv20655800.7
AET3Gv20588500.1
AET1Gv21024400.4
AET2Gv20316000.2
AET3Gv20559500.6
AET3Gv20372200.1
AET3Gv20530900.8
AET4Gv20755900.2
AET4Gv20284300.4
Domain Color
PK_Tyr_Ser−Thr Domain
Pkinase Domain
0
200
400
600
800

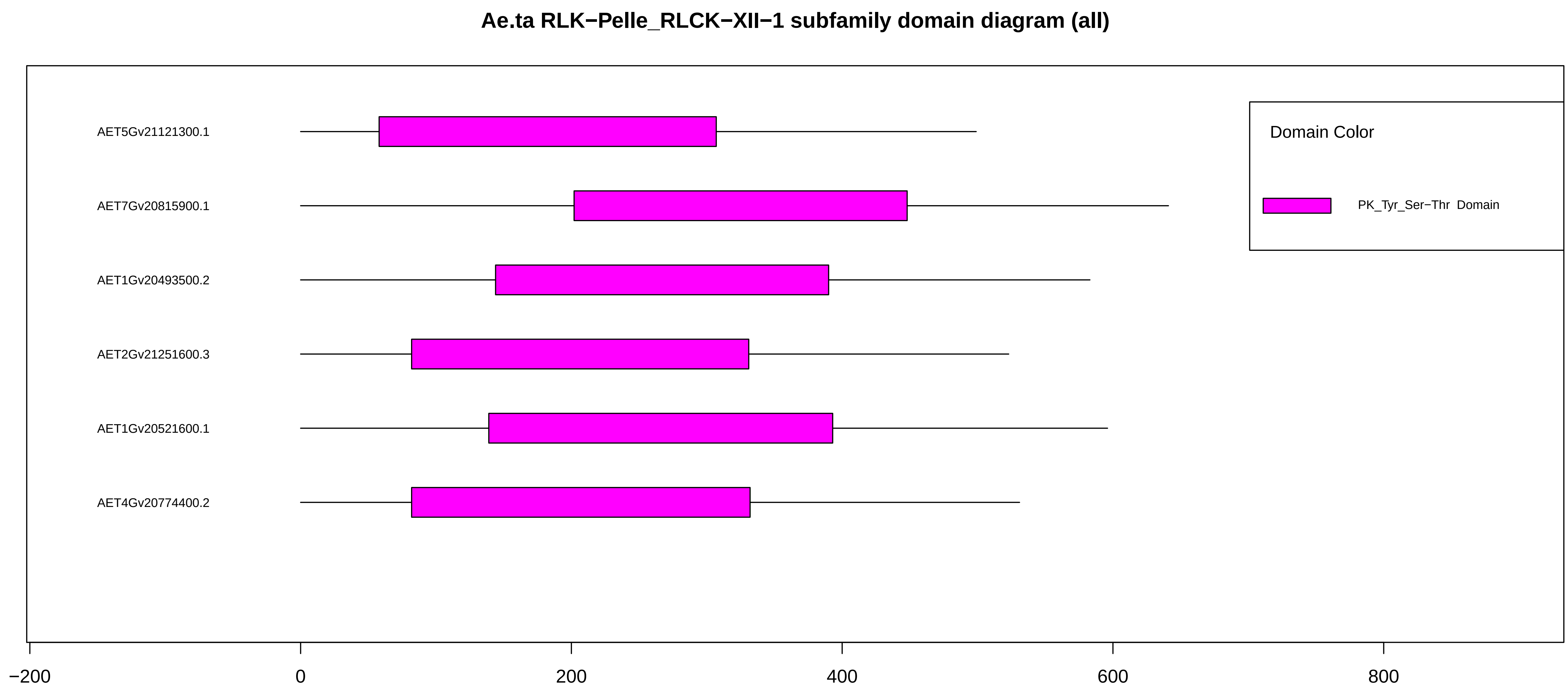
Ae.ta RLK−Pelle_RLCK−XII−1 subfamily domain diagram (all)
AET5Gv21121300.1
AET7Gv20815900.1
AET1Gv20493500.2
AET2Gv21251600.3
AET1Gv20521600.1
AET4Gv20774400.2
Domain Color
PK_Tyr_Ser−Thr Domain
−200
0
200
400
600
800

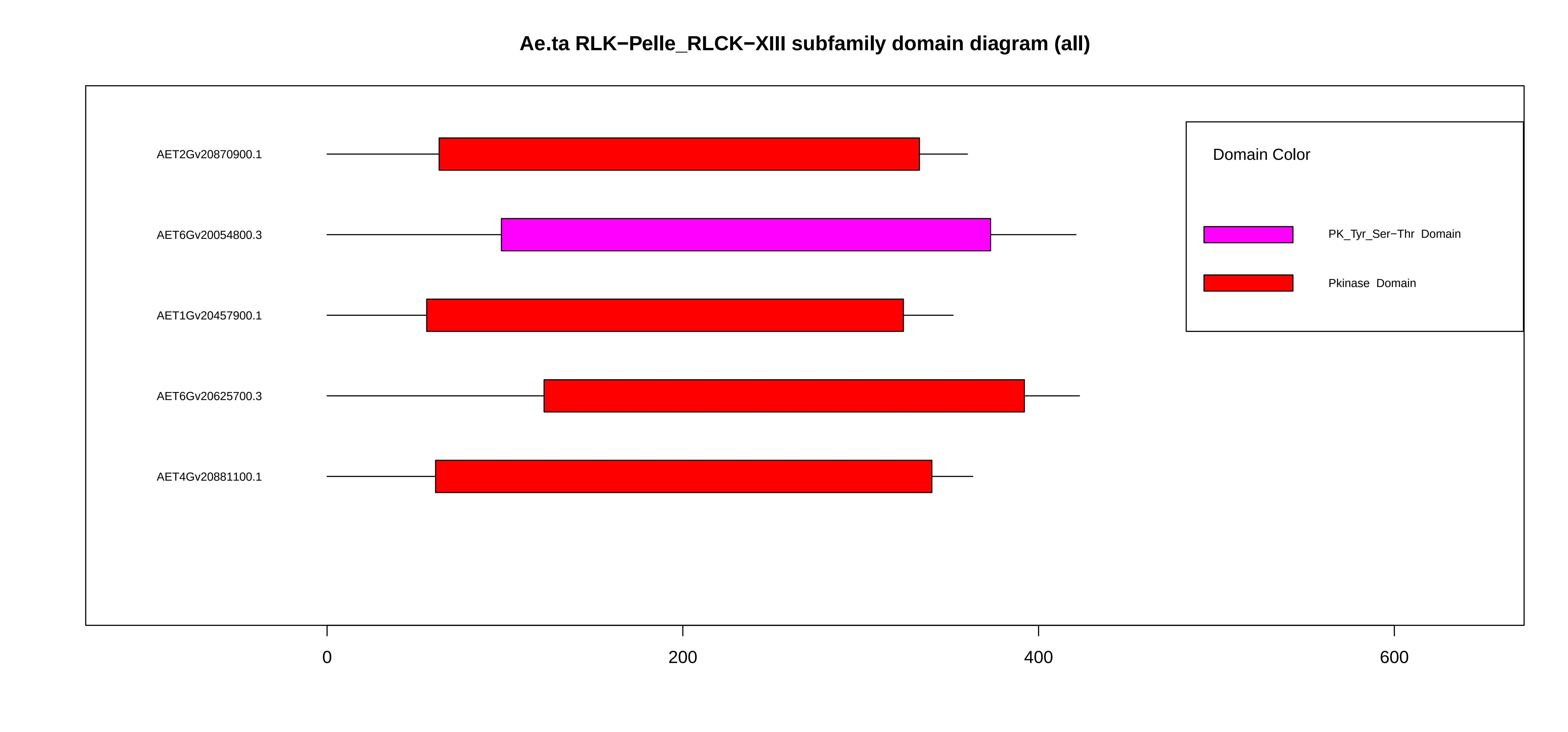
Ae.ta RLK−Pelle_RLCK−XIII subfamily domain diagram (all)
AET2Gv20870900.1
AET6Gv20054800.3
AET1Gv20457900.1
AET6Gv20625700.3
AET4Gv20881100.1
Domain Color
PK_Tyr_Ser−Thr Domain
Pkinase Domain
0
200
400
600

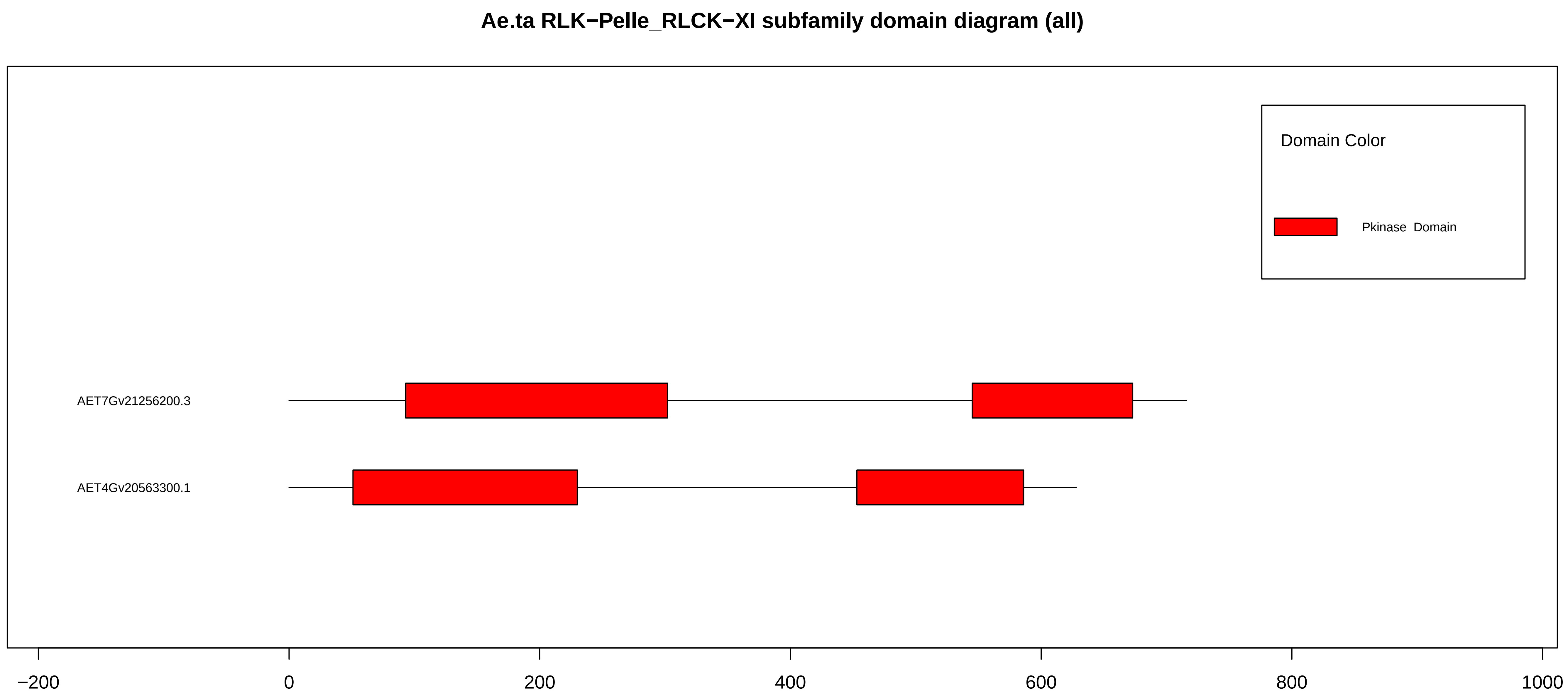
Ae.ta RLK−Pelle_RLCK−XI subfamily domain diagram (all)
Domain Color
Pkinase Domain
AET7Gv21256200.3
AET4Gv20563300.1
−200
0
200
400
600
800
1000

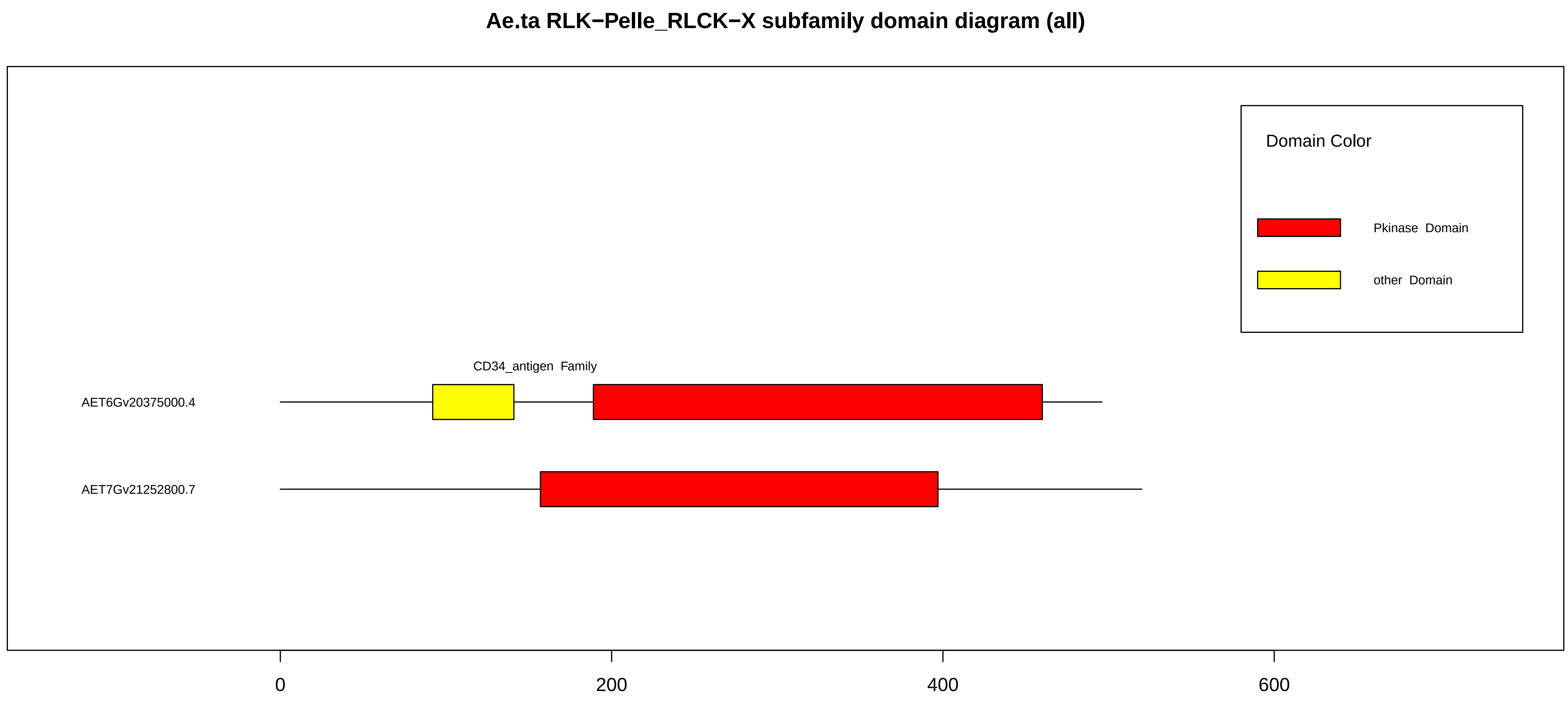
Ae.ta RLK−Pelle_RLCK−X subfamily domain diagram (all)
Domain Color
Pkinase Domain
other Domain
CD34_antigen Family
AET6Gv20375000.4
AET7Gv21252800.7
0
200
400
600

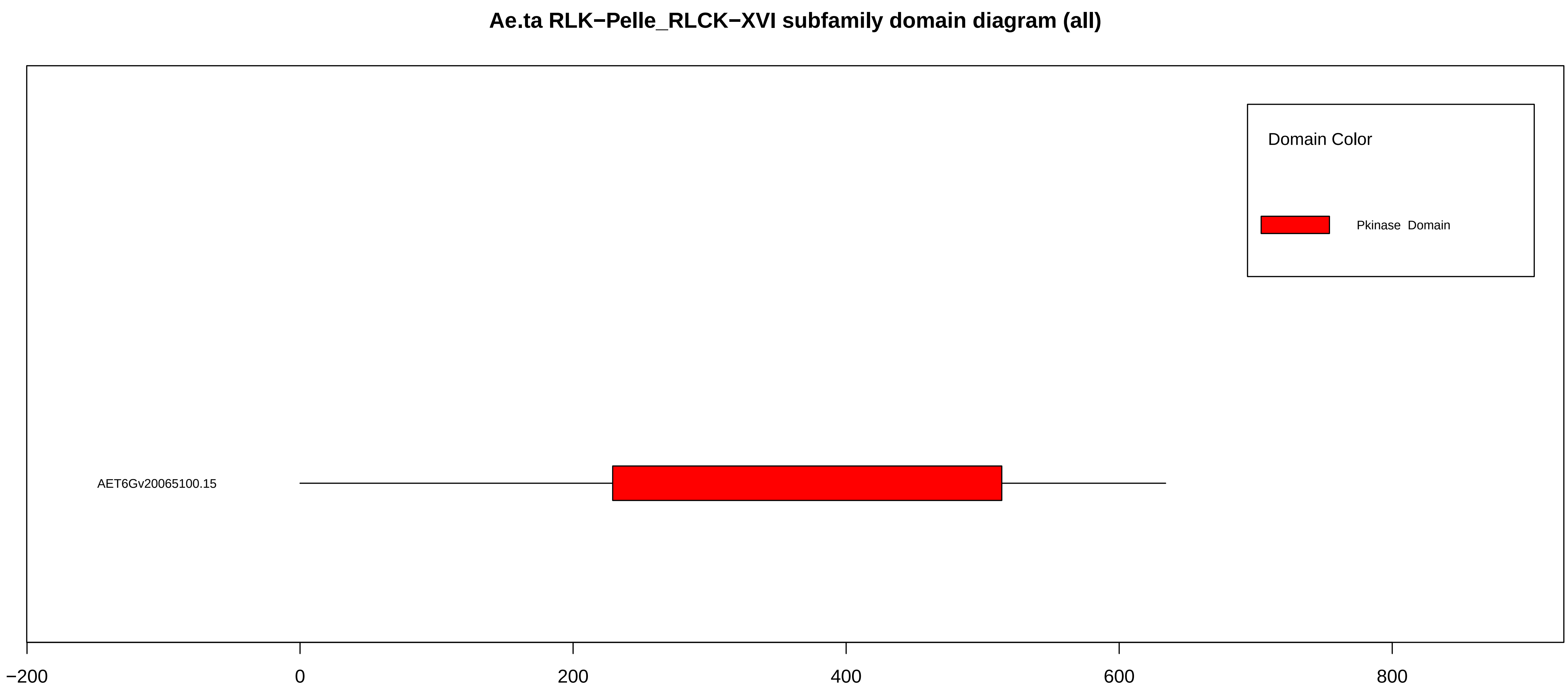

Ae.ta RLK−Pelle_RLCK−XVI subfamily domain diagram (all)
Domain Color
Pkinase Domain
AET6Gv20065100.15
−200
0
200
400
600
800

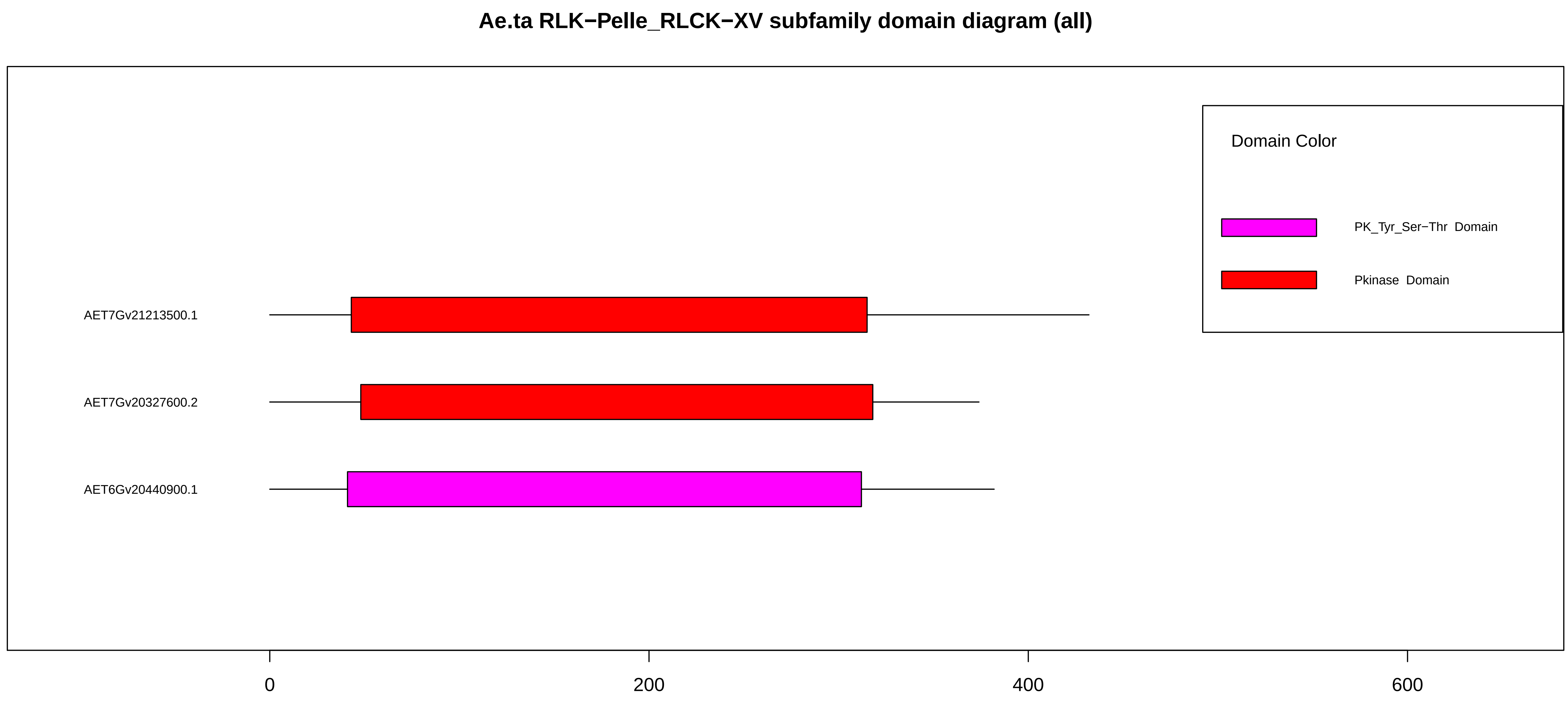
Ae.ta RLK−Pelle_RLCK−XV subfamily domain diagram (all)
Domain Color
PK_Tyr_Ser−Thr Domain
Pkinase Domain
AET7Gv21213500.1
AET7Gv20327600.2
AET6Gv20440900.1
0
200
400
600

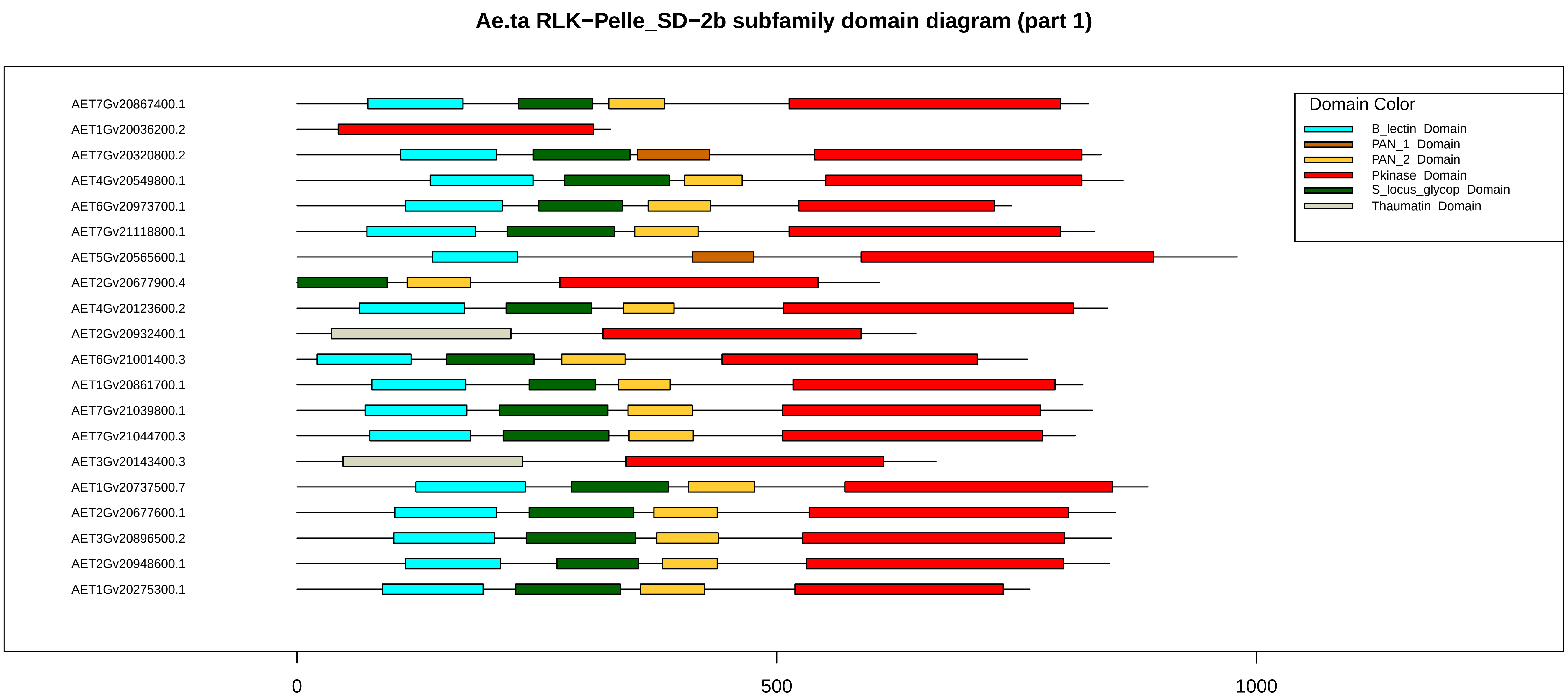
Ae.ta RLK−Pelle_SD−2b subfamily domain diagram (part 1)
AET7Gv20867400.1
AET1Gv20036200.2
AET7Gv20320800.2
AET4Gv20549800.1
AET6Gv20973700.1
AET7Gv21118800.1
AET5Gv20565600.1
AET2Gv20677900.4
AET4Gv20123600.2
AET2Gv20932400.1
AET6Gv21001400.3
AET1Gv20861700.1
AET7Gv21039800.1
AET7Gv21044700.3
AET3Gv20143400.3
AET1Gv20737500.7
AET2Gv20677600.1
AET3Gv20896500.2
AET2Gv20948600.1
AET1Gv20275300.1
Domain Color
B_lectin Domain
PAN_1 Domain
PAN_2 Domain
Pkinase Domain
S_locus_glycop Domain
Thaumatin Domain
0
500
1000

Ae.ta RLK−Pelle_SD−2b subfamily domain diagram (part 2)

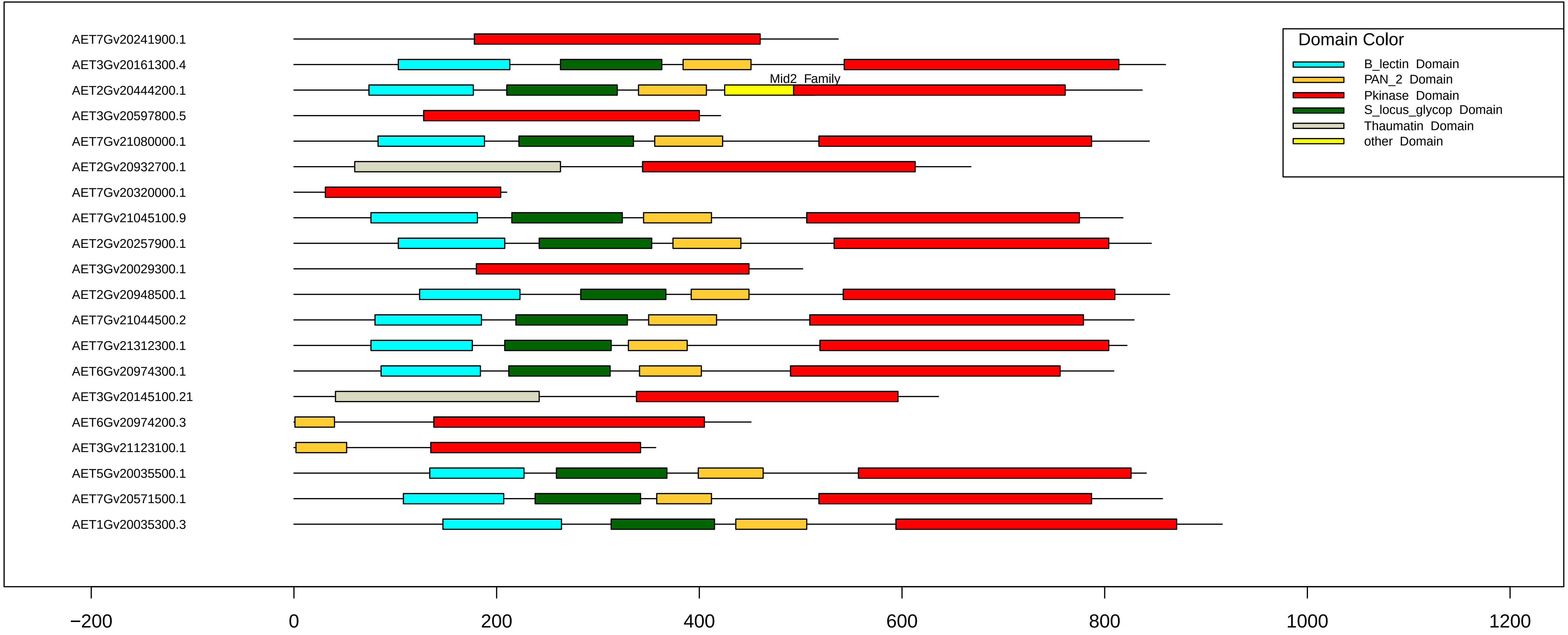

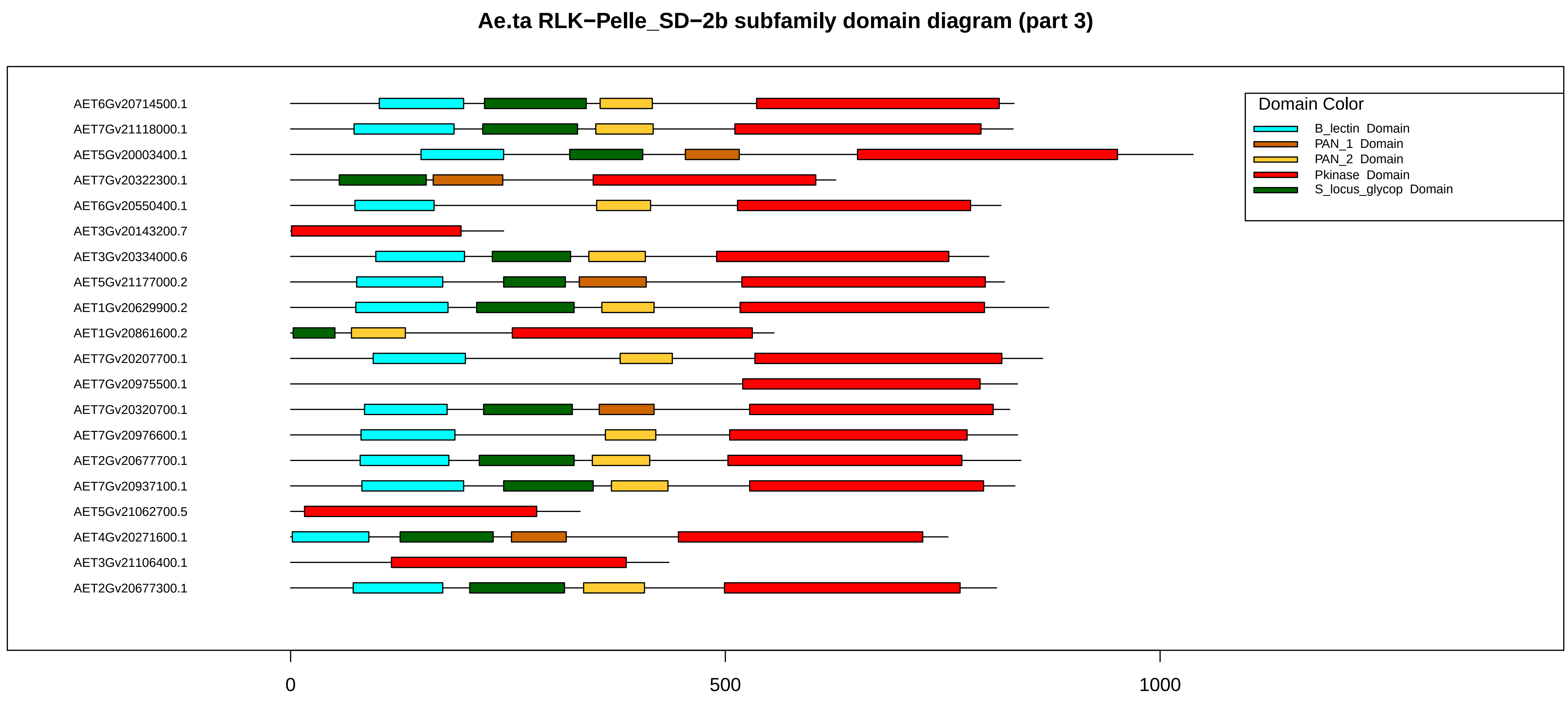
Ae.ta RLK−Pelle_SD−2b subfamily domain diagram (part 3)
AET6Gv20714500.1
AET7Gv21118000.1
AET5Gv20003400.1
AET7Gv20322300.1
AET6Gv20550400.1
AET3Gv20143200.7
AET3Gv20334000.6
AET5Gv21177000.2
AET1Gv20629900.2
AET1Gv20861600.2
AET7Gv20207700.1
AET7Gv20975500.1
AET7Gv20320700.1
AET7Gv20976600.1
AET2Gv20677700.1
AET7Gv20937100.1
AET5Gv21062700.5
AET4Gv20271600.1
AET3Gv21106400.1
AET2Gv20677300.1
Domain Color
B_lectin Domain
PAN_1 Domain
PAN_2 Domain
Pkinase Domain
S_locus_glycop Domain
0
500
1000

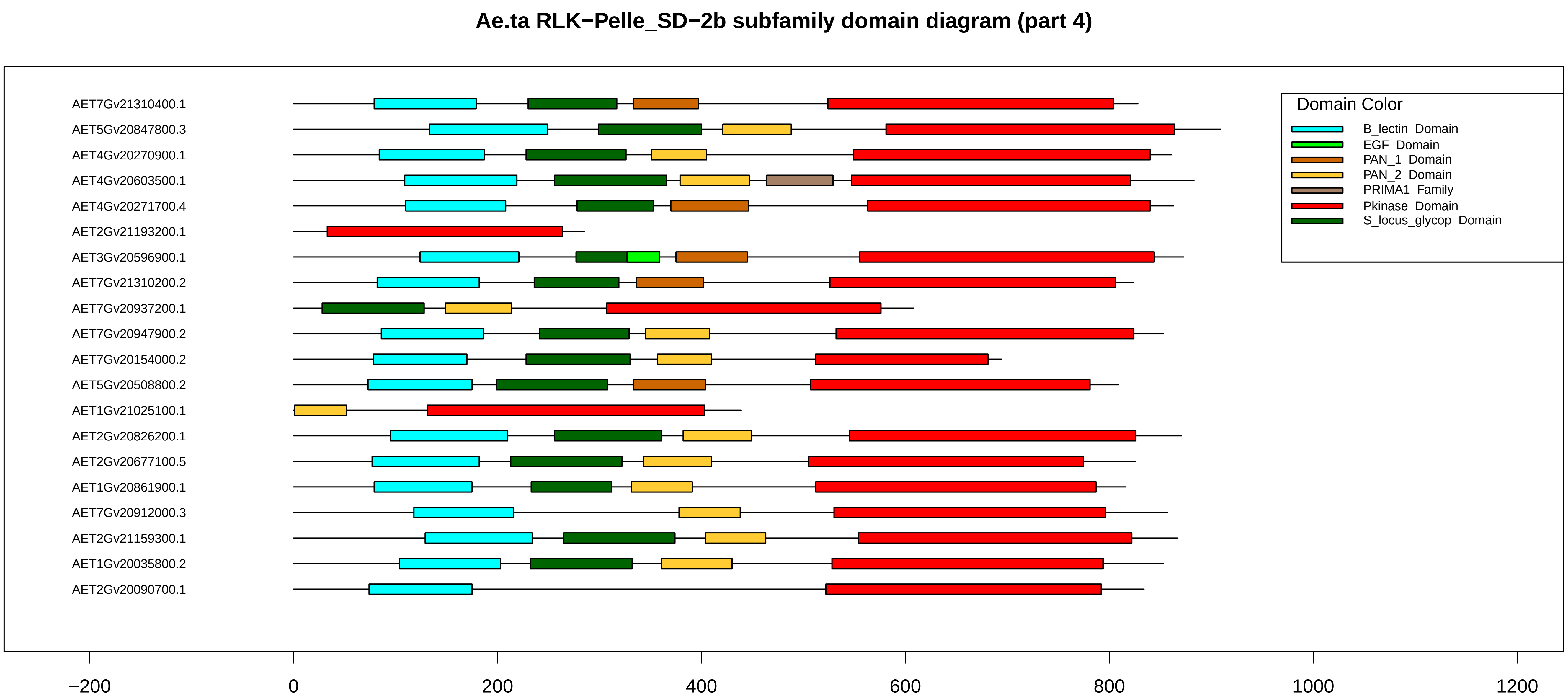
Ae.ta RLK−Pelle_SD−2b subfamily domain diagram (part 4)
AET7Gv21310400.1
AET5Gv20847800.3
AET4Gv20270900.1
AET4Gv20603500.1
AET4Gv20271700.4
AET2Gv21193200.1
AET3Gv20596900.1
AET7Gv21310200.2
AET7Gv20937200.1
AET7Gv20947900.2
AET7Gv20154000.2
AET5Gv20508800.2
AET1Gv21025100.1
AET2Gv20826200.1
AET2Gv20677100.5
AET1Gv20861900.1
AET7Gv20912000.3
AET2Gv21159300.1
AET1Gv20035800.2
AET2Gv20090700.1
Domain Color
B_lectin Domain
EGF Domain
PAN_1 Domain
PAN_2 Domain
PRIMA1 Family
Pkinase Domain
S_locus_glycop Domain
−200
0
200
400
600
800
1000
1200

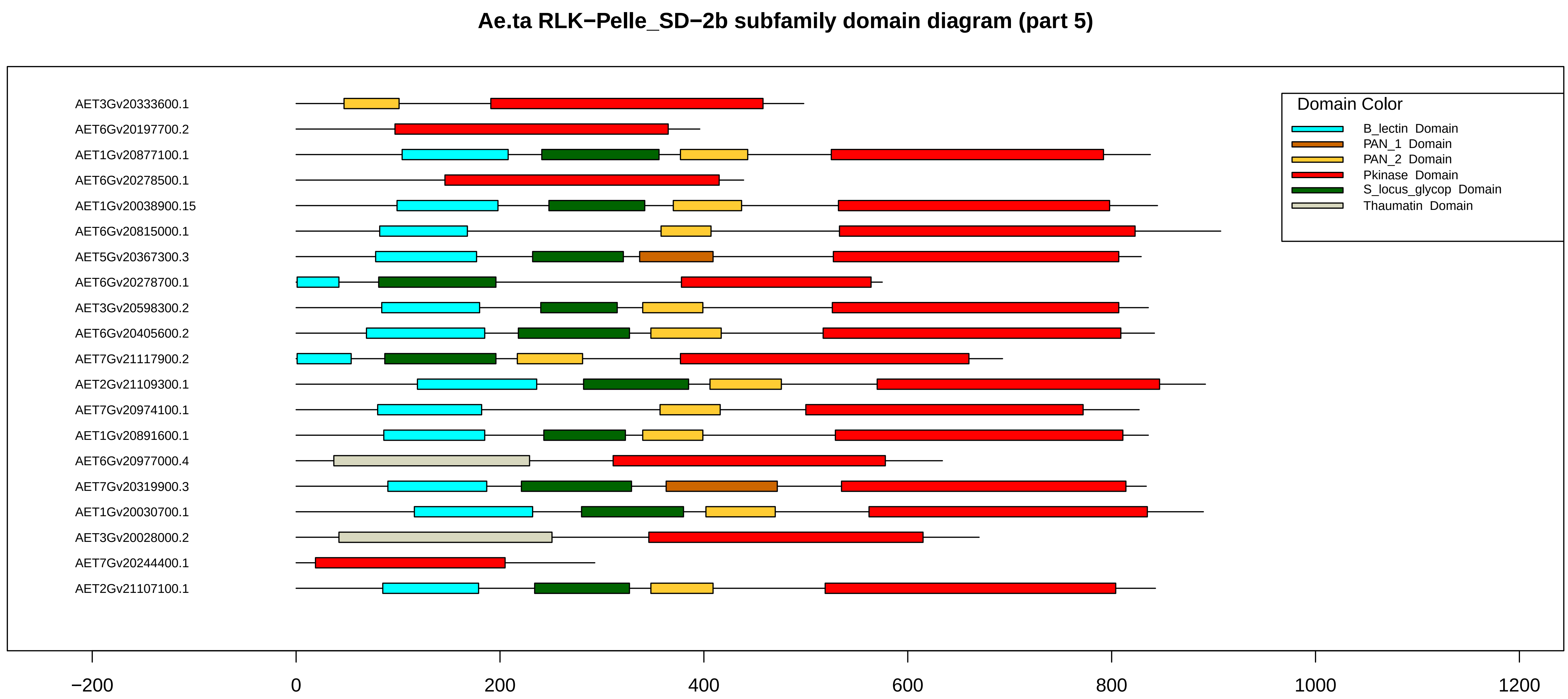

Ae.ta RLK−Pelle_SD−2b subfamily domain diagram (part 5)
AET3Gv20333600.1
AET6Gv20197700.2
AET1Gv20877100.1
AET6Gv20278500.1
AET1Gv20038900.15
AET6Gv20815000.1
AET5Gv20367300.3
AET6Gv20278700.1
AET3Gv20598300.2
AET6Gv20405600.2
AET7Gv21117900.2
AET2Gv21109300.1
AET7Gv20974100.1
AET1Gv20891600.1
AET6Gv20977000.4
AET7Gv20319900.3
AET1Gv20030700.1
AET3Gv20028000.2
AET7Gv20244400.1
AET2Gv21107100.1
Domain Color
B_lectin Domain
PAN_1 Domain
PAN_2 Domain
Pkinase Domain
S_locus_glycop Domain
Thaumatin Domain
−200
0
200
400
600
800
1000
1200

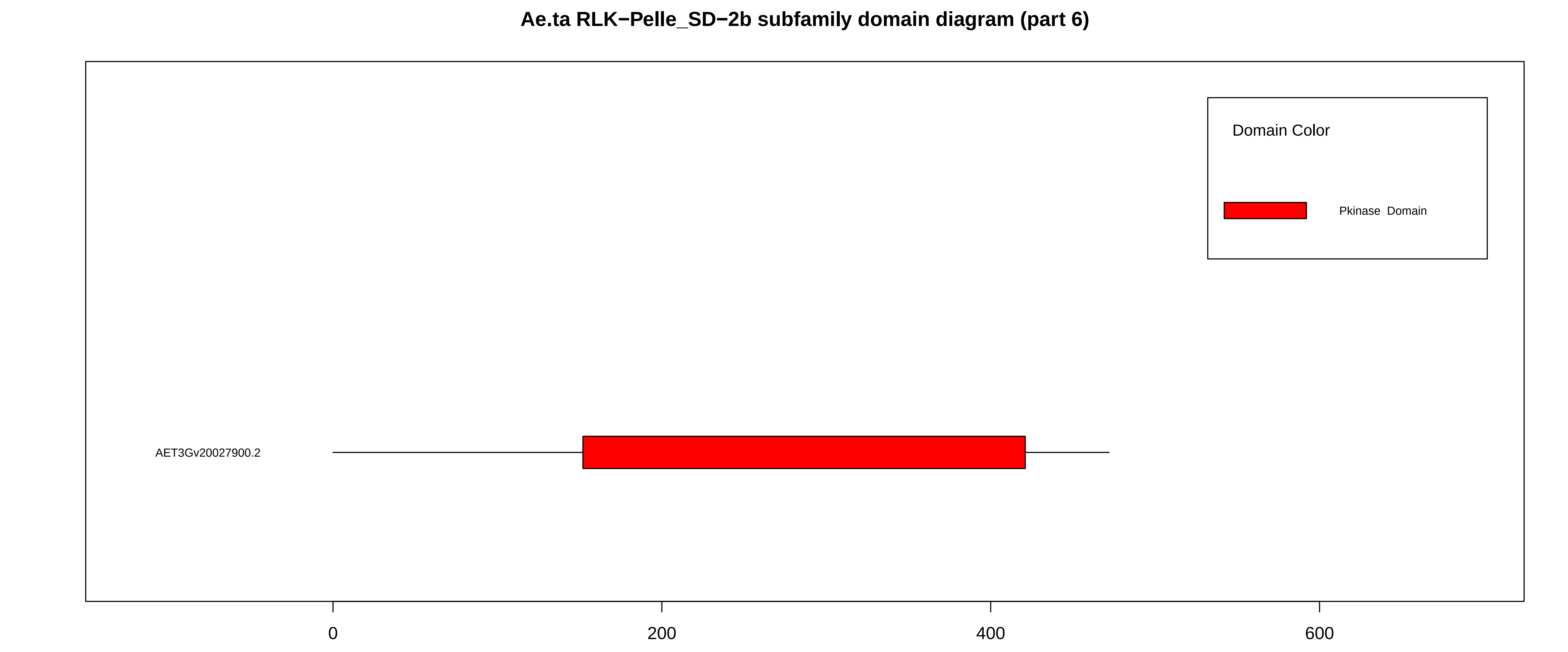

Ae.ta RLK−Pelle_SD−2b subfamily domain diagram (part 6)
Domain Color
Pkinase Domain
AET3Gv20027900.2
0
200
400
600

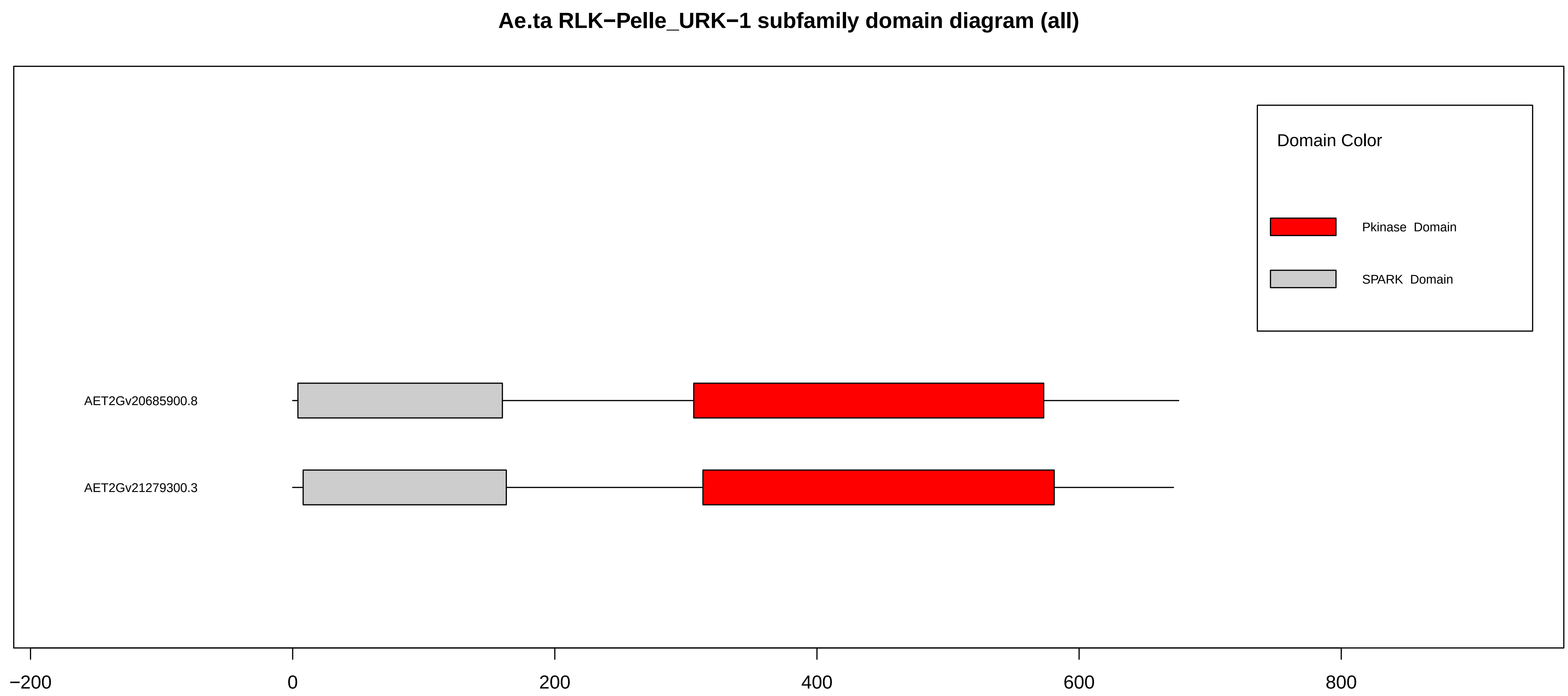
Ae.ta RLK−Pelle_URK−1 subfamily domain diagram (all)
Domain Color
Pkinase Domain
SPARK Domain
AET2Gv20685900.8
AET2Gv21279300.3
−200
0
200
400
600
800

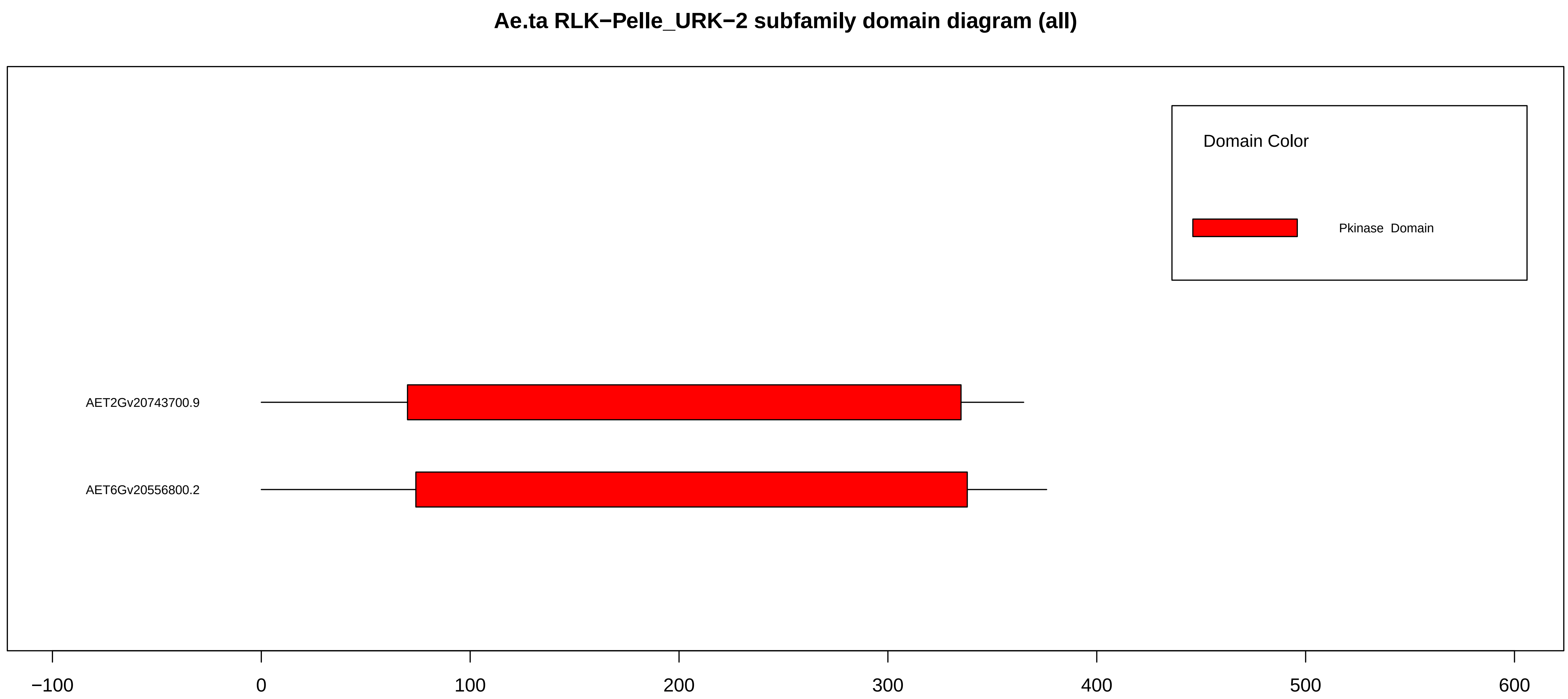

Ae.ta RLK−Pelle_URK−2 subfamily domain diagram (all)
Domain Color
Pkinase Domain
AET2Gv20743700.9
AET6Gv20556800.2
−100
0
100
200
300
400
500
600

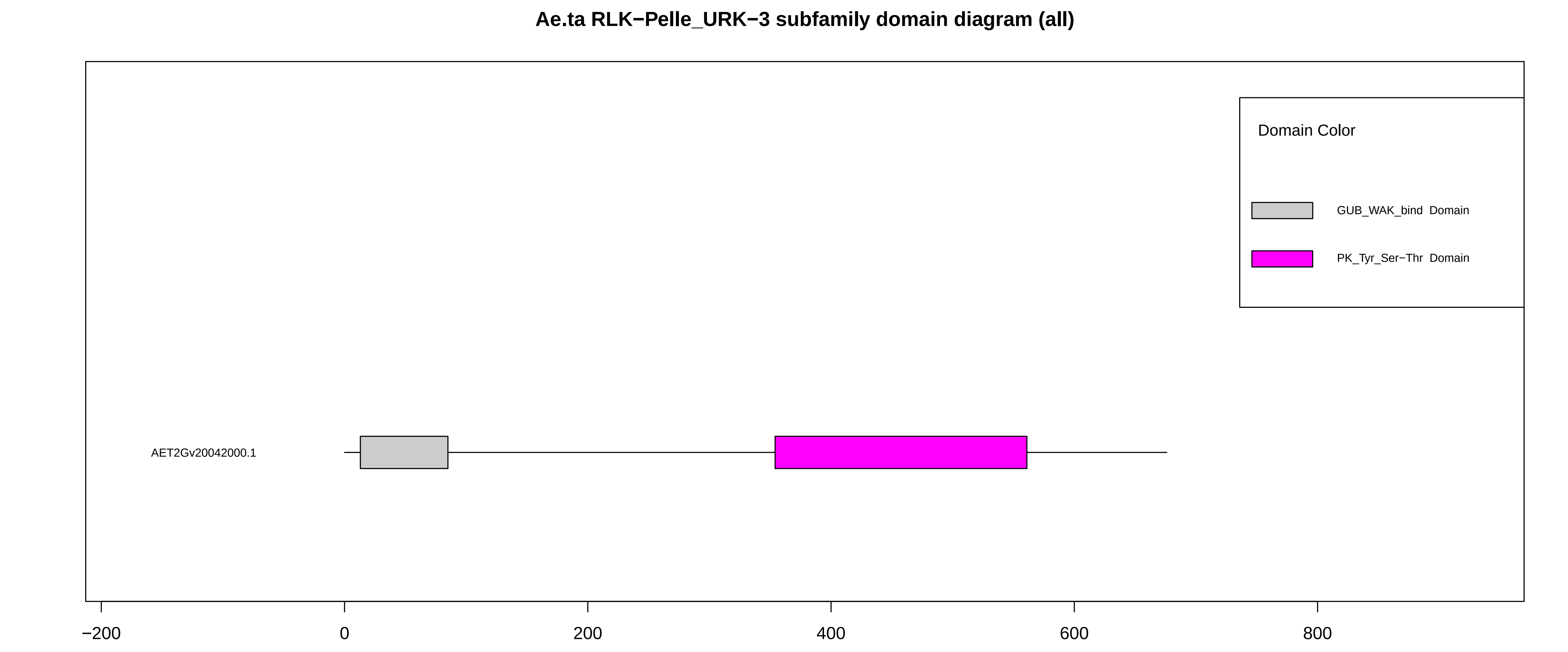

Ae.ta RLK−Pelle_URK−3 subfamily domain diagram (all)
Domain Color
GUB_WAK_bind Domain
PK_Tyr_Ser−Thr Domain
AET2Gv20042000.1
−200
0
200
400
600
800

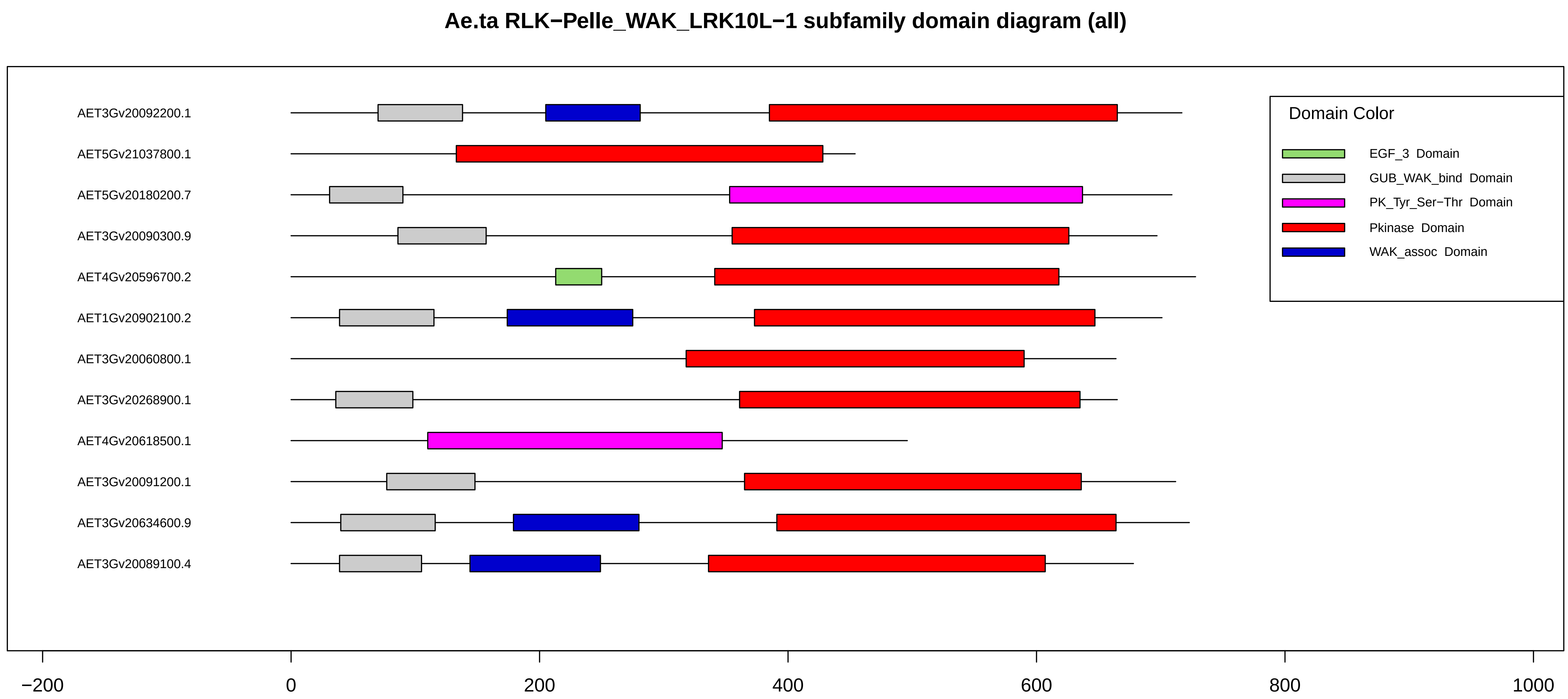

Ae.ta RLK−Pelle_WAK_LRK10L−1 subfamily domain diagram (all)
AET3Gv20092200.1
AET5Gv21037800.1
AET5Gv20180200.7
AET3Gv20090300.9
AET4Gv20596700.2
AET1Gv20902100.2
AET3Gv20060800.1
AET3Gv20268900.1
AET4Gv20618500.1
AET3Gv20091200.1
AET3Gv20634600.9
AET3Gv20089100.4
Domain Color
EGF_3 Domain
GUB_WAK_bind Domain
PK_Tyr_Ser−Thr Domain
Pkinase Domain
WAK_assoc Domain
−200
0
200
400
600
800
1000

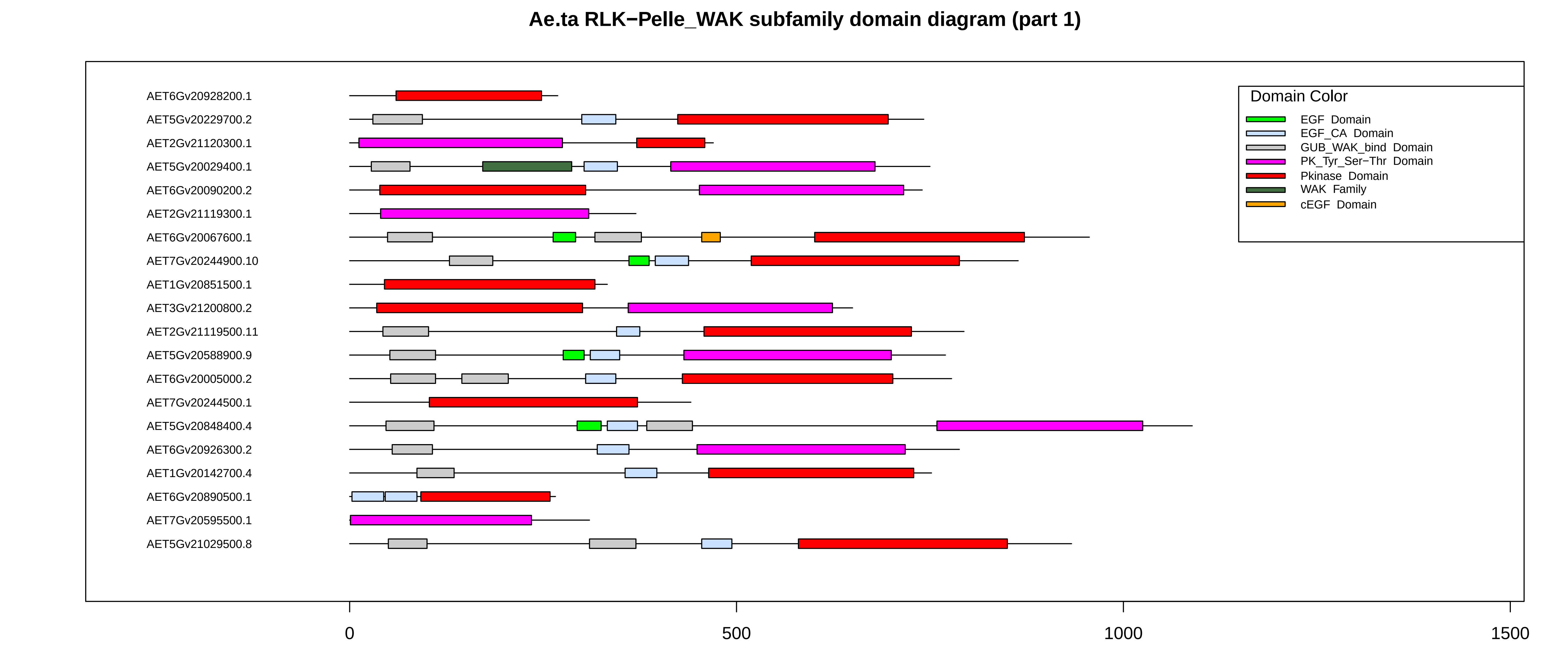

Ae.ta RLK−Pelle_WAK subfamily domain diagram (part 1)
AET6Gv20928200.1
AET5Gv20229700.2
AET2Gv21120300.1
AET5Gv20029400.1
AET6Gv20090200.2
AET2Gv21119300.1
AET6Gv20067600.1
AET7Gv20244900.10
AET1Gv20851500.1
AET3Gv21200800.2
AET2Gv21119500.11
AET5Gv20588900.9
AET6Gv20005000.2
AET7Gv20244500.1
AET5Gv20848400.4
AET6Gv20926300.2
AET1Gv20142700.4
AET6Gv20890500.1
AET7Gv20595500.1
AET5Gv21029500.8
Domain Color
EGF Domain
EGF_CA Domain
GUB_WAK_bind Domain
PK_Tyr_Ser−Thr Domain
Pkinase Domain
WAK Family
cEGF Domain
0
500
1000
1500

Ae.ta RLK−Pelle_WAK subfamily domain diagram (part 2)

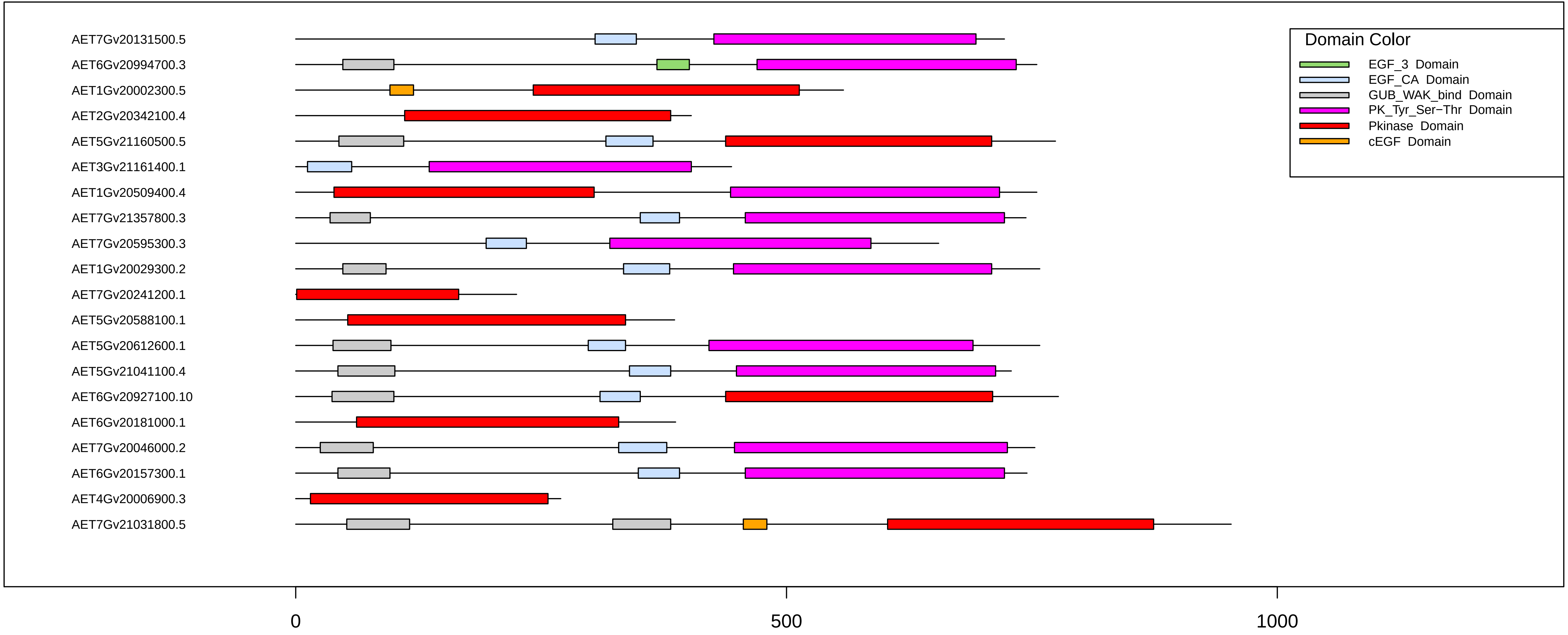

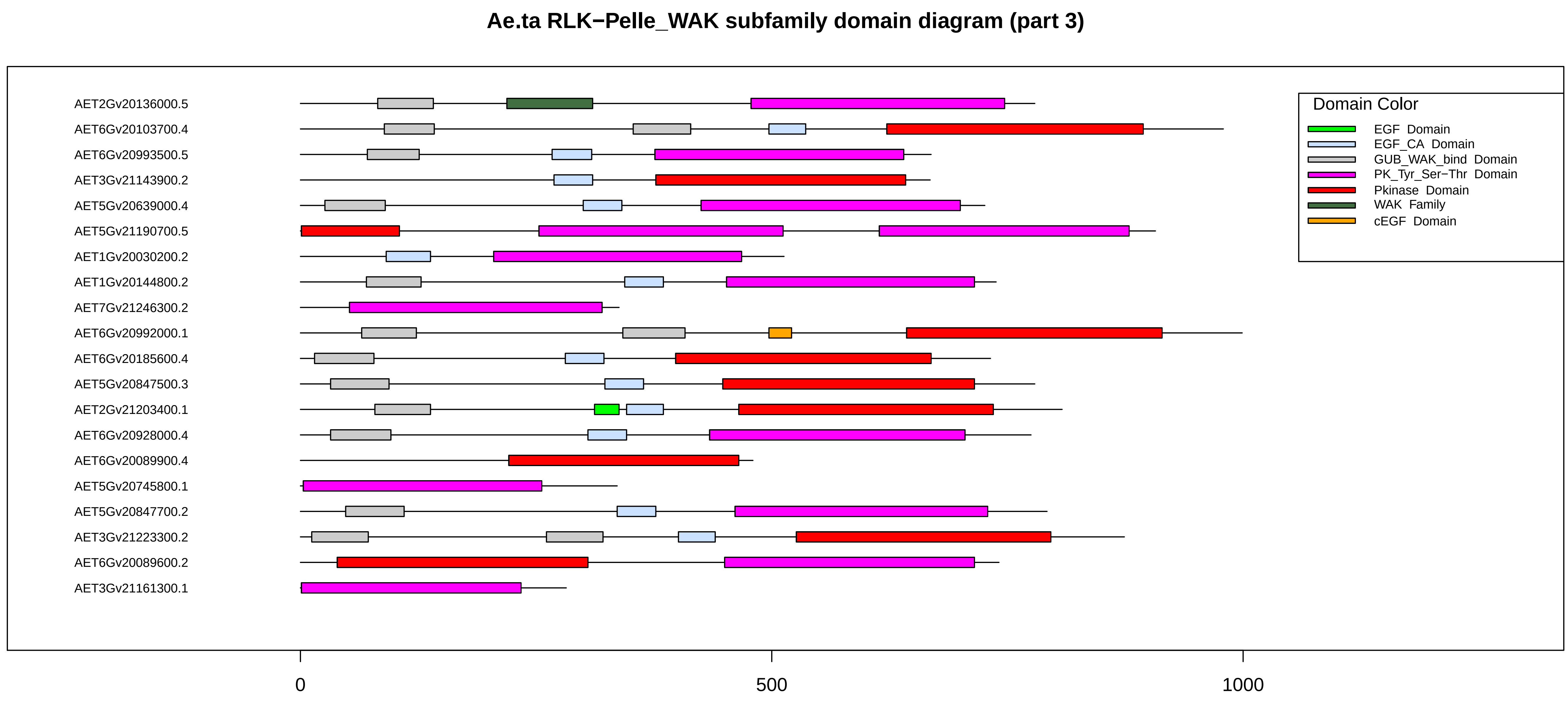

Ae.ta RLK−Pelle_WAK subfamily domain diagram (part 3)
AET2Gv20136000.5
AET6Gv20103700.4
AET6Gv20993500.5
AET3Gv21143900.2
AET5Gv20639000.4
AET5Gv21190700.5
AET1Gv20030200.2
AET1Gv20144800.2
AET7Gv21246300.2
AET6Gv20992000.1
AET6Gv20185600.4
AET5Gv20847500.3
AET2Gv21203400.1
AET6Gv20928000.4
AET6Gv20089900.4
AET5Gv20745800.1
AET5Gv20847700.2
AET3Gv21223300.2
AET6Gv20089600.2
AET3Gv21161300.1
Domain Color
EGF Domain
EGF_CA Domain
GUB_WAK_bind Domain
PK_Tyr_Ser−Thr Domain
Pkinase Domain
WAK Family
cEGF Domain
0
500
1000

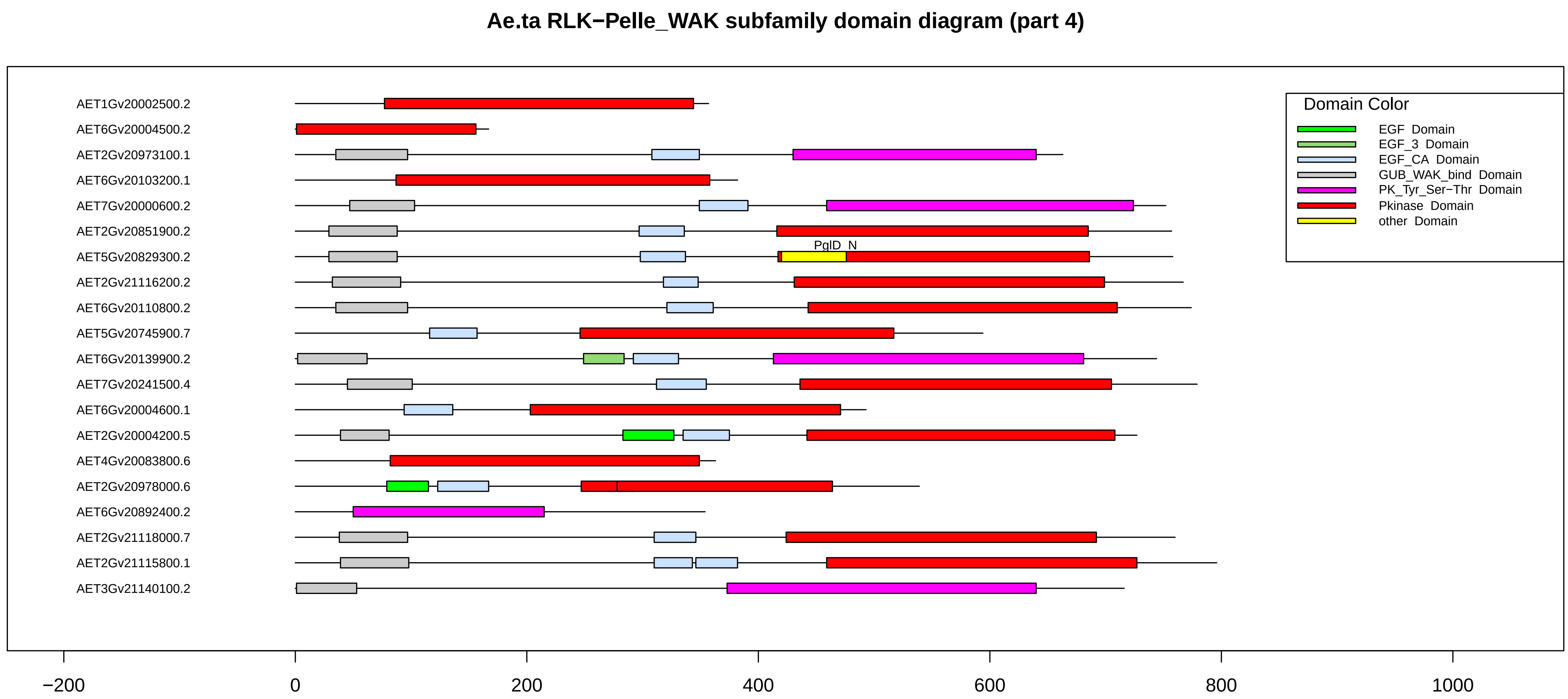
Ae.ta RLK−Pelle_WAK subfamily domain diagram (part 4)
AET1Gv20002500.2
AET6Gv20004500.2
AET2Gv20973100.1
AET6Gv20103200.1
AET7Gv20000600.2
AET2Gv20851900.2
AET5Gv20829300.2
AET2Gv21116200.2
AET6Gv20110800.2
AET5Gv20745900.7
AET6Gv20139900.2
AET7Gv20241500.4
AET6Gv20004600.1
AET2Gv20004200.5
AET4Gv20083800.6
AET2Gv20978000.6
AET6Gv20892400.2
AET2Gv21118000.7
AET2Gv21115800.1
AET3Gv21140100.2
PglD_N
Domain Color
EGF Domain
EGF_3 Domain
EGF_CA Domain
GUB_WAK_bind Domain
PK_Tyr_Ser−Thr Domain
Pkinase Domain
other Domain
−200
0
200
400
600
800
1000

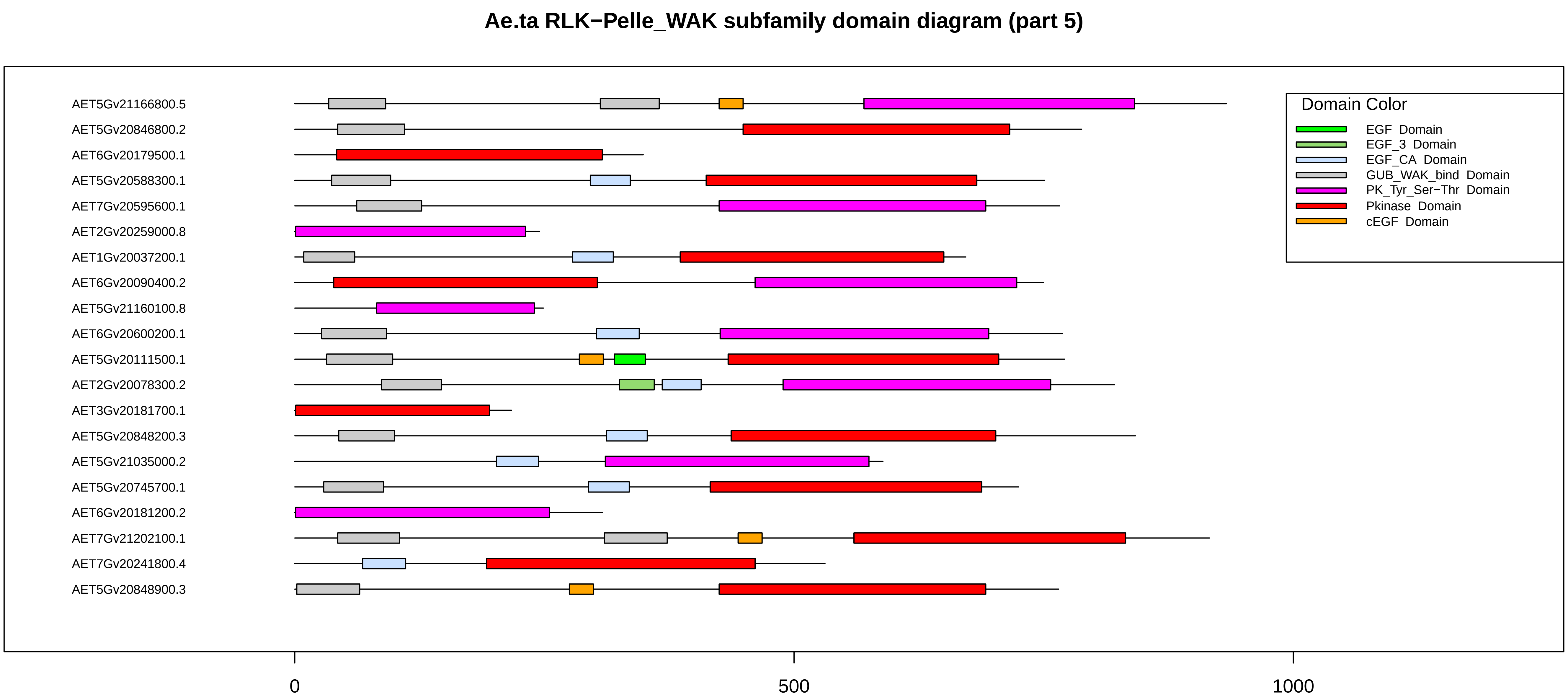
Ae.ta RLK−Pelle_WAK subfamily domain diagram (part 5)
AET5Gv21166800.5
AET5Gv20846800.2
AET6Gv20179500.1
AET5Gv20588300.1
AET7Gv20595600.1
AET2Gv20259000.8
AET1Gv20037200.1
AET6Gv20090400.2
AET5Gv21160100.8
AET6Gv20600200.1
AET5Gv20111500.1
AET2Gv20078300.2
AET3Gv20181700.1
AET5Gv20848200.3
AET5Gv21035000.2
AET5Gv20745700.1
AET6Gv20181200.2
AET7Gv21202100.1
AET7Gv20241800.4
AET5Gv20848900.3
Domain Color
EGF Domain
EGF_3 Domain
EGF_CA Domain
GUB_WAK_bind Domain
PK_Tyr_Ser−Thr Domain
Pkinase Domain
cEGF Domain
0
500
1000

Ae.ta RLK−Pelle_WAK subfamily domain diagram (part 6)

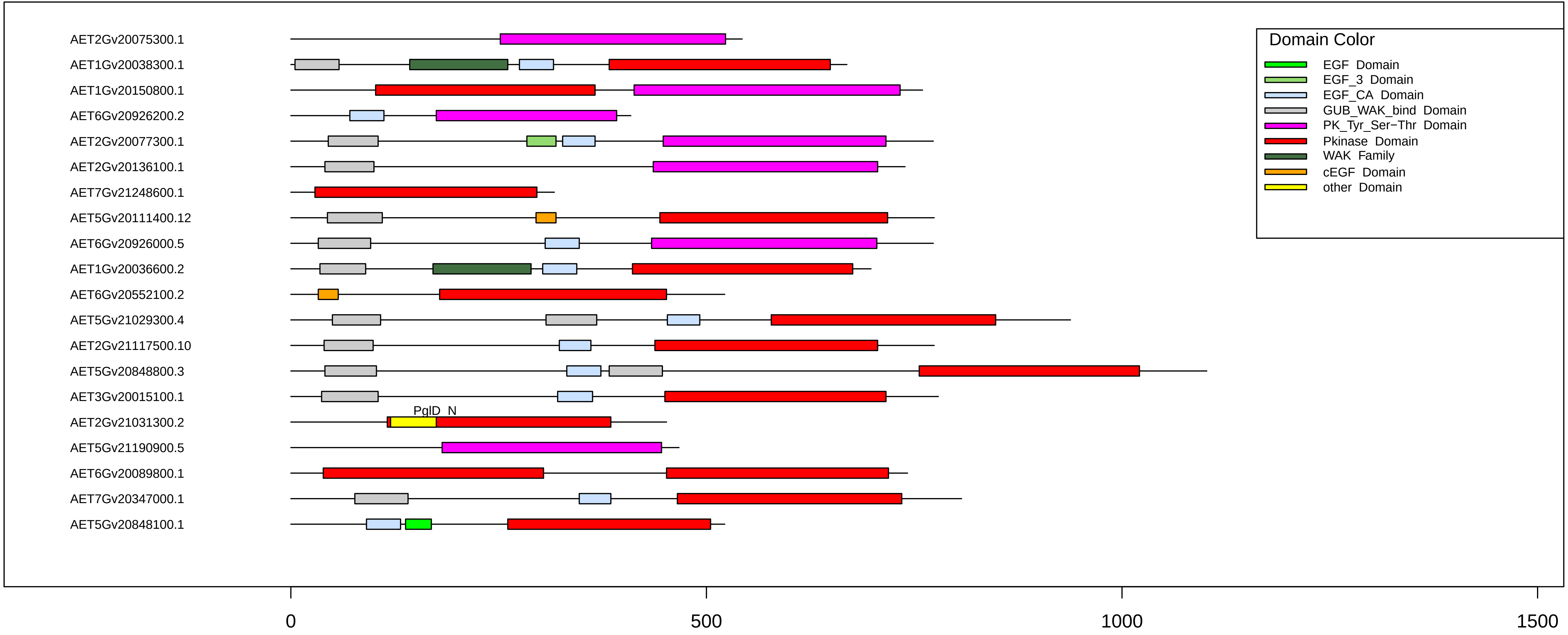

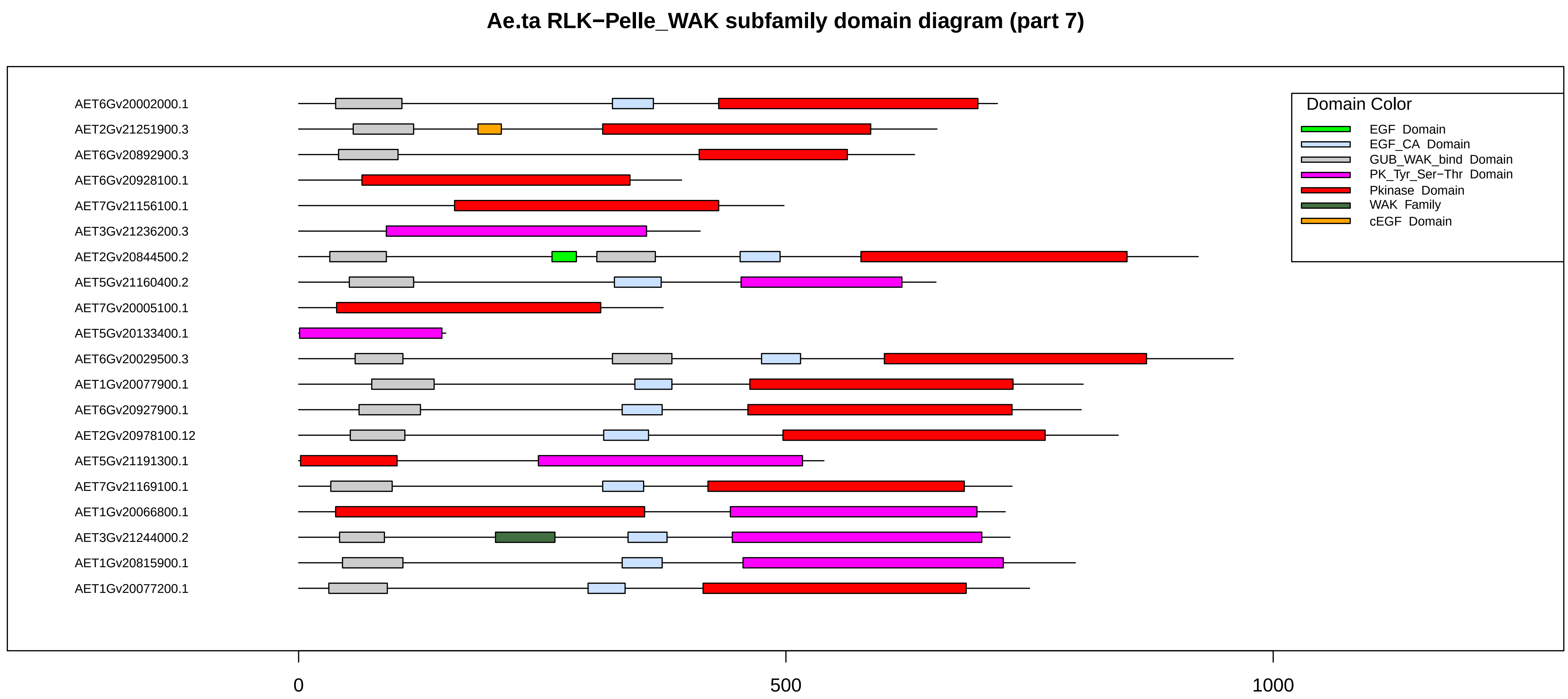

Ae.ta RLK−Pelle_WAK subfamily domain diagram (part 7)
AET6Gv20002000.1
AET2Gv21251900.3
AET6Gv20892900.3
AET6Gv20928100.1
AET7Gv21156100.1
AET3Gv21236200.3
AET2Gv20844500.2
AET5Gv21160400.2
AET7Gv20005100.1
AET5Gv20133400.1
AET6Gv20029500.3
AET1Gv20077900.1
AET6Gv20927900.1
AET2Gv20978100.12
AET5Gv21191300.1
AET7Gv21169100.1
AET1Gv20066800.1
AET3Gv21244000.2
AET1Gv20815900.1
AET1Gv20077200.1
Domain Color
EGF Domain
EGF_CA Domain
GUB_WAK_bind Domain
PK_Tyr_Ser−Thr Domain
Pkinase Domain
WAK Family
cEGF Domain
0
500
1000

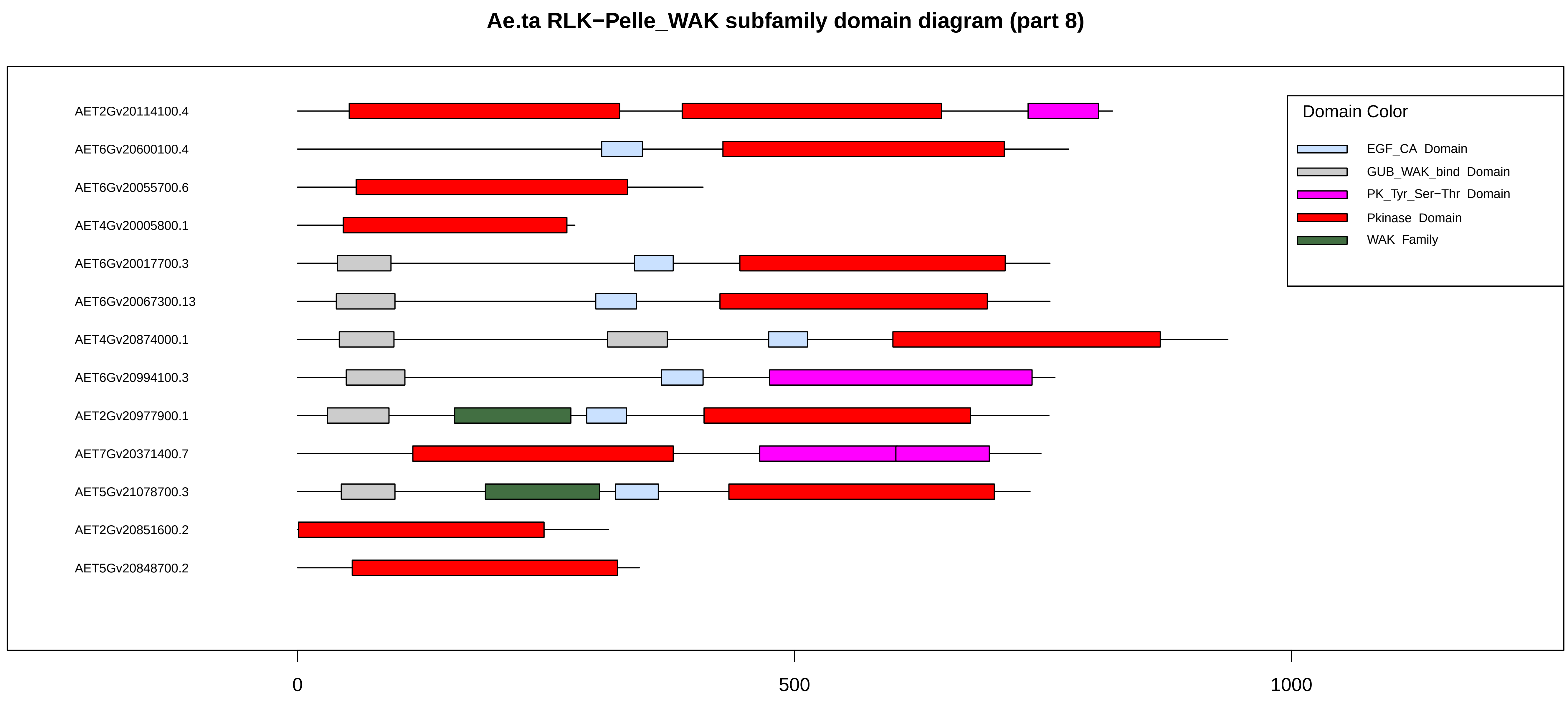
Ae.ta RLK−Pelle_WAK subfamily domain diagram (part 8)
AET2Gv20114100.4
AET6Gv20600100.4
AET6Gv20055700.6
AET4Gv20005800.1
AET6Gv20017700.3
AET6Gv20067300.13
AET4Gv20874000.1
AET6Gv20994100.3
AET2Gv20977900.1
AET7Gv20371400.7
AET5Gv21078700.3
AET2Gv20851600.2
AET5Gv20848700.2
Domain Color
EGF_CA Domain
GUB_WAK_bind Domain
PK_Tyr_Ser−Thr Domain
Pkinase Domain
WAK Family
0
500
1000

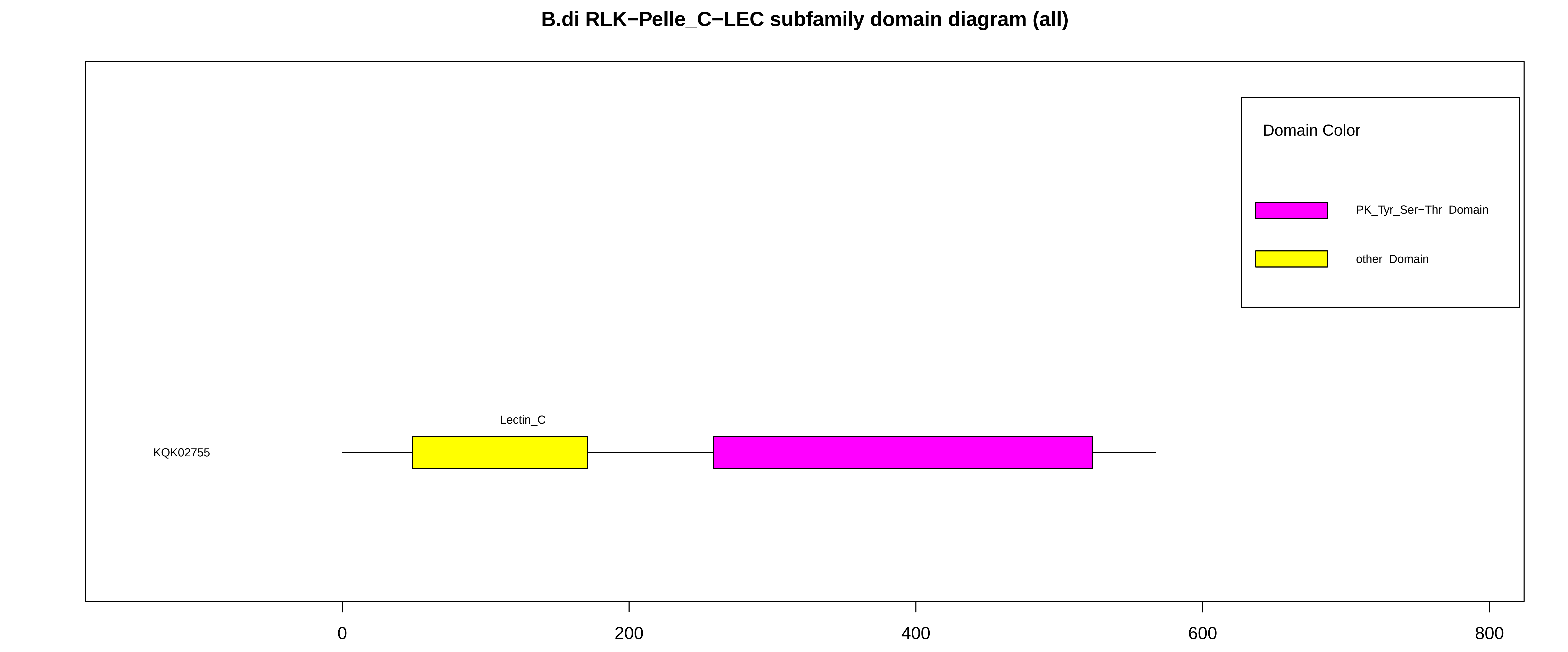
B.di RLK−Pelle_C−LEC subfamily domain diagram (all)
Domain Color
PK_Tyr_Ser−Thr Domain
other Domain
Lectin_C
KQK02755
0
200
400
600
800

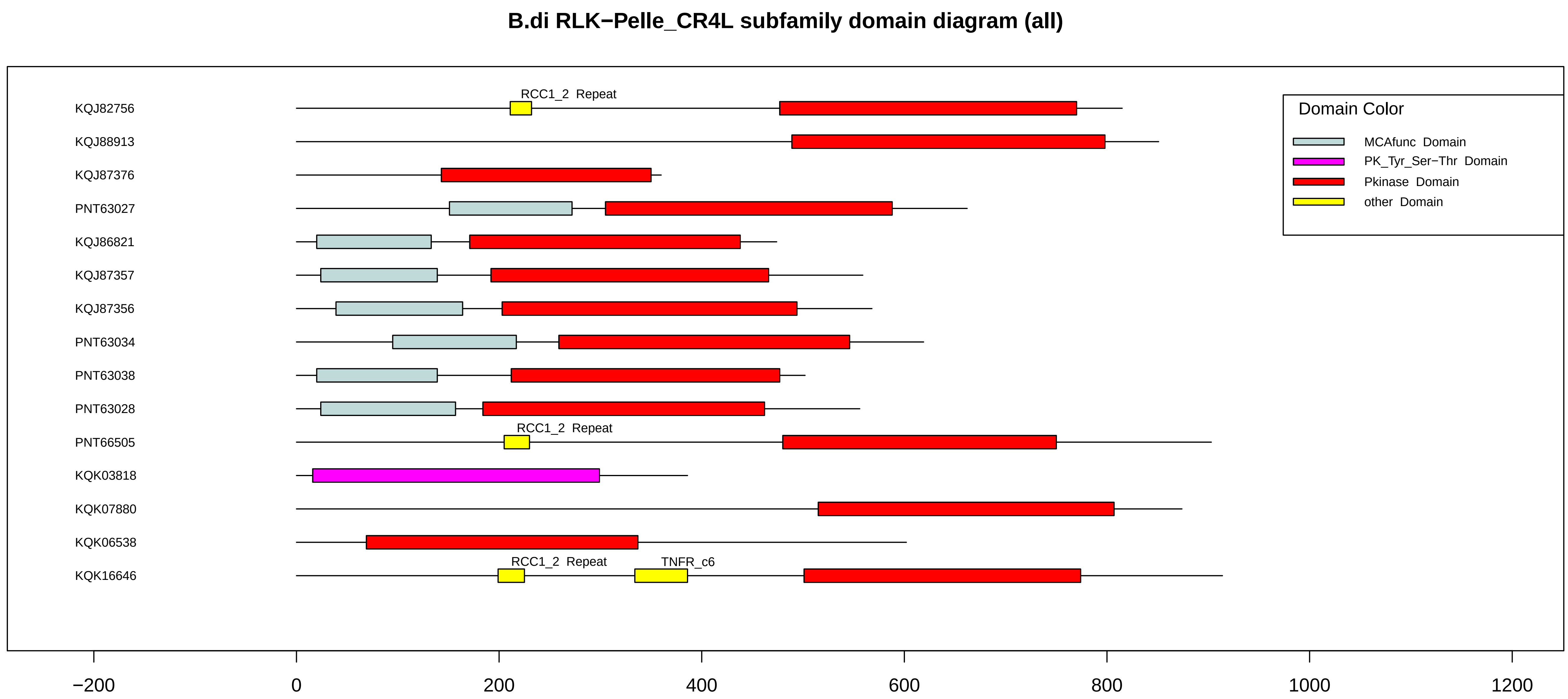
B.di RLK−Pelle_CR4L subfamily domain diagram (all)
KQJ82756
KQJ88913
KQJ87376
PNT63027
KQJ86821
KQJ87357
KQJ87356
PNT63034
PNT63038
PNT63028
PNT66505
KQK03818
KQK07880
KQK06538
KQK16646
RCC1_2 Repeat
RCC1_2 Repeat
RCC1_2 Repeat
TNFR_c6
Domain Color
MCAfunc Domain
PK_Tyr_Ser−Thr Domain
Pkinase Domain
other Domain
−200
0
200
400
600
800
1000
1200

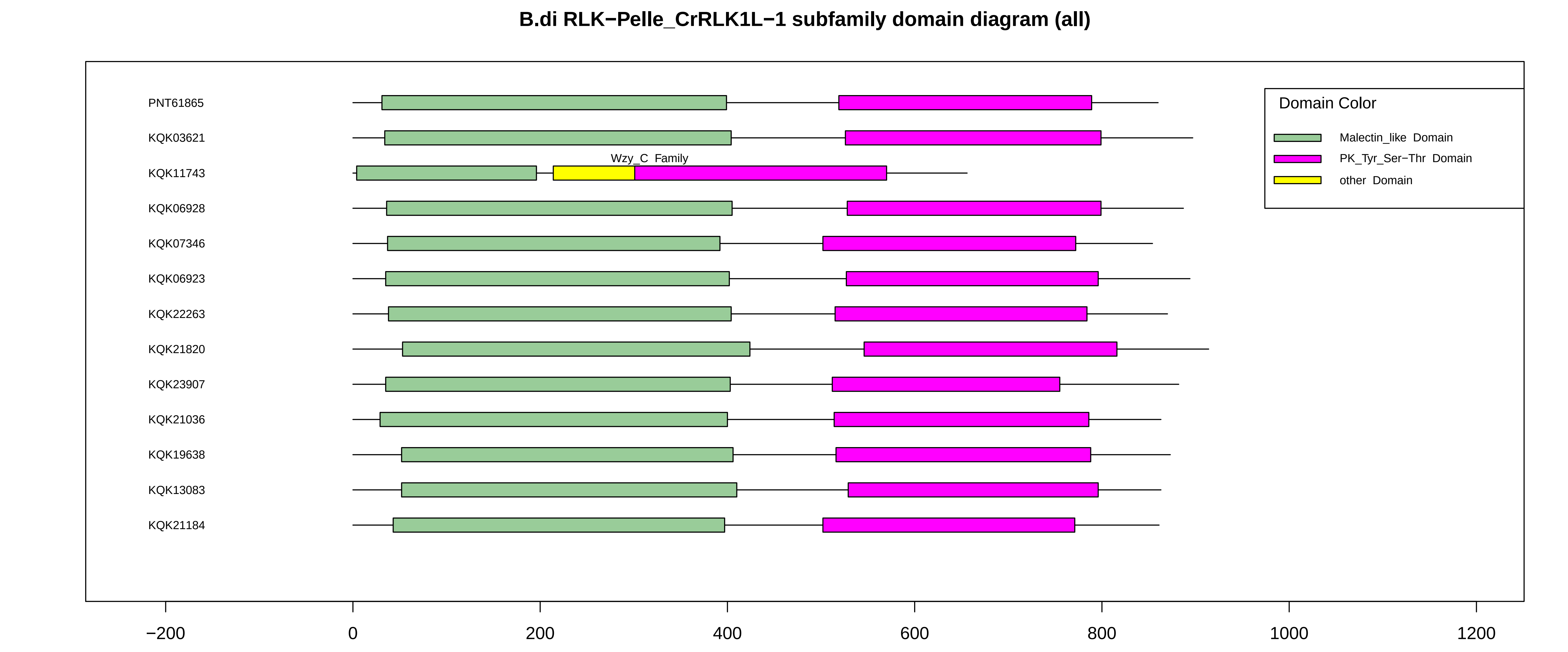

B.di RLK−Pelle_CrRLK1L−1 subfamily domain diagram (all)
PNT61865
KQK03621
KQK11743
KQK06928
KQK07346
KQK06923
KQK22263
KQK21820
KQK23907
KQK21036
KQK19638
KQK13083
KQK21184
Wzy_C Family
Domain Color
Malectin_like Domain
PK_Tyr_Ser−Thr Domain
other Domain
−200
0
200
400
600
800
1000
1200

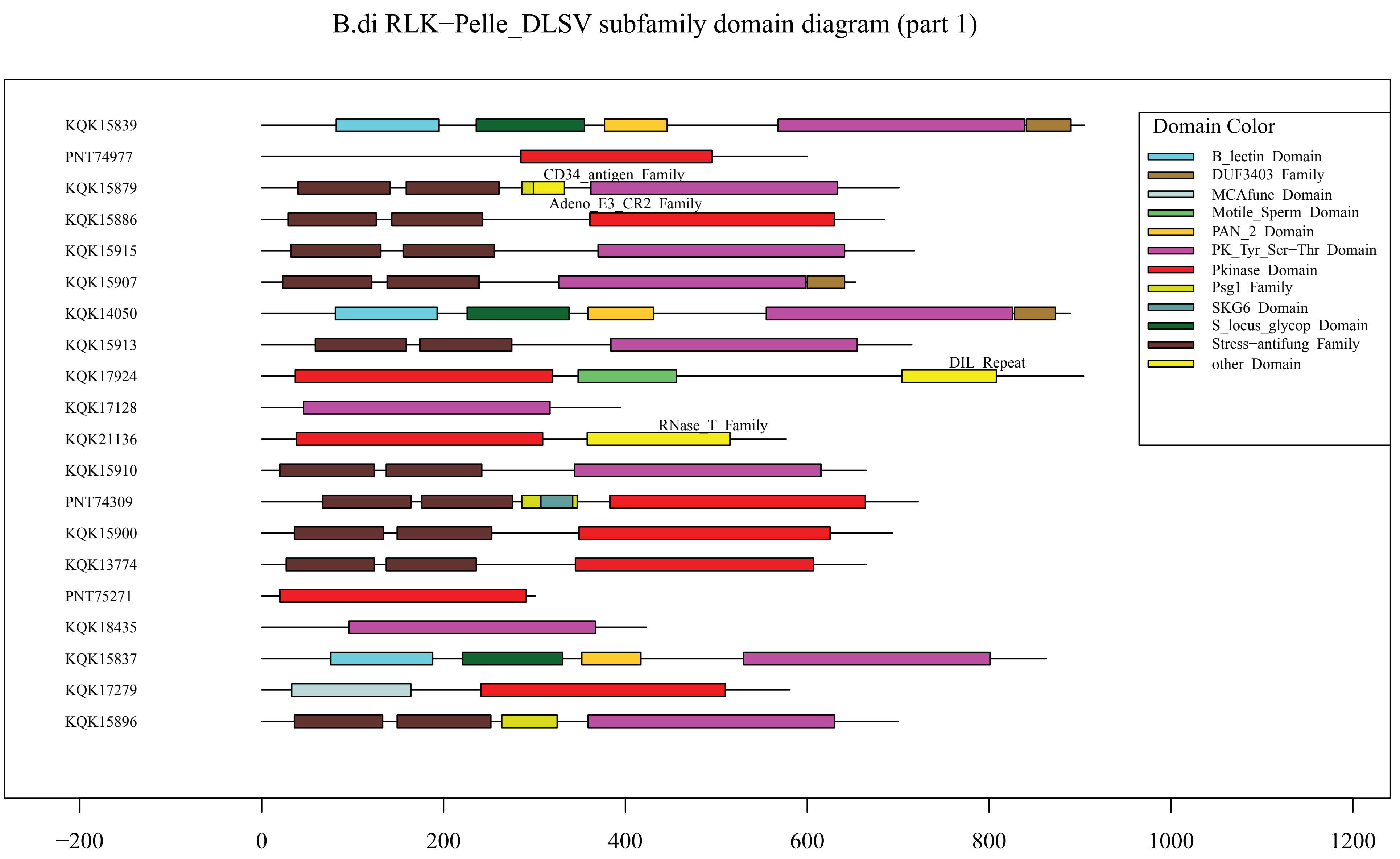
B.di RLK−Pelle_DLSV subfamily domain diagram (part 1)
KQK15839
PNT74977
KQK15879
KQK15886
KQK15915
KQK15907
KQK14050
KQK15913
KQK17924
KQK17128
KQK21136
KQK15910
PNT74309
KQK15900
KQK13774
PNT75271
KQK18435
KQK15837
KQK17279
KQK15896
CD34_antigen_Family
Adeno_E3_CR2_Family
DIL_Repeat
RNase_T_Family
Domain Color
B_lectin Domain
DUF3403 Family
MCAfunc Domain
Motile_Sperm Domain
PAN_2 Domain
PK_Tyr_Ser−Thr Domain
Pkinase Domain
Psg1 Family
SKG6 Domain
S_locus_glycop Domain
Stress−antifung Family
other Domain
−200
0
200
400
600
800
1000
1200

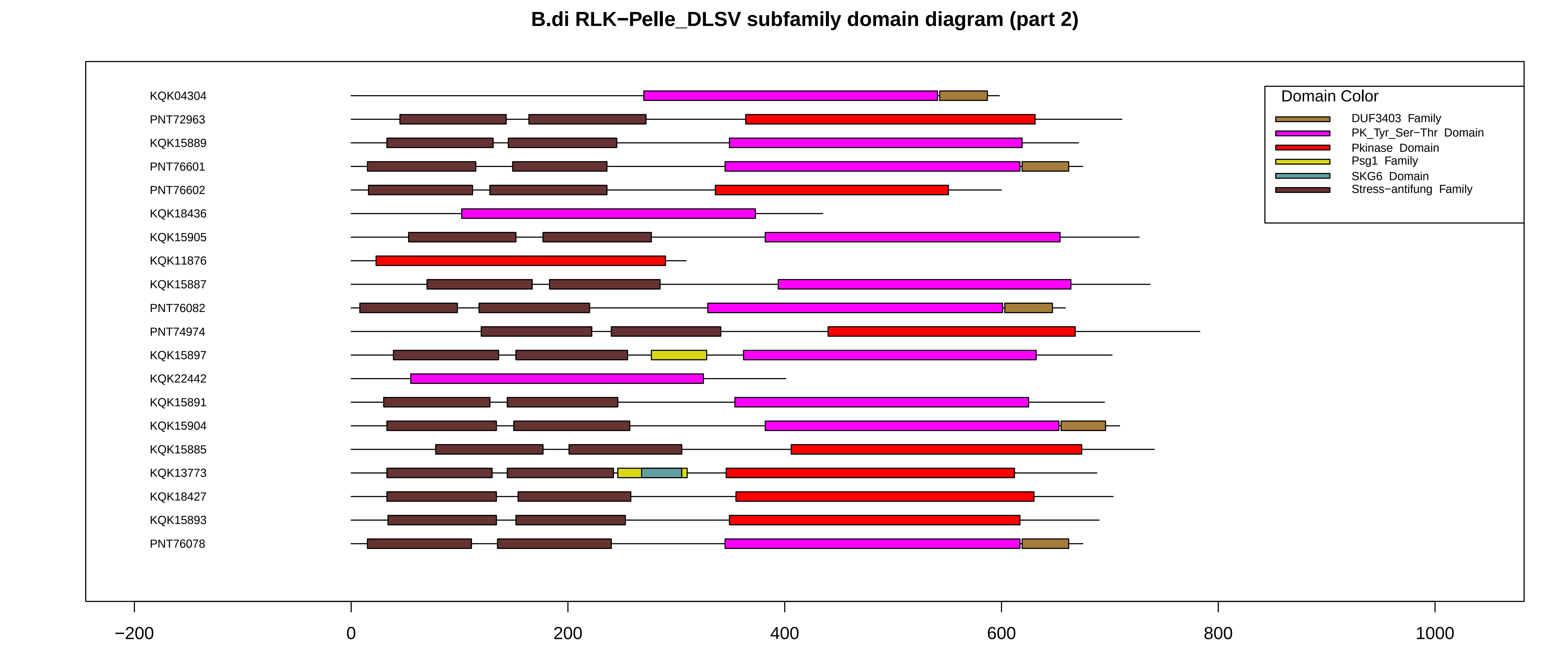

B.di RLK−Pelle_DLSV subfamily domain diagram (part 2)
KQK04304
PNT72963
KQK15889
PNT76601
PNT76602
KQK18436
KQK15905
KQK11876
KQK15887
PNT76082
PNT74974
KQK15897
KQK22442
KQK15891
KQK15904
KQK15885
KQK13773
KQK18427
KQK15893
PNT76078
Domain Color
DUF3403 Family
PK_Tyr_Ser−Thr Domain
Pkinase Domain
Psg1 Family
SKG6 Domain
Stress−antifung Family
−200
0
200
400
600
800
1000

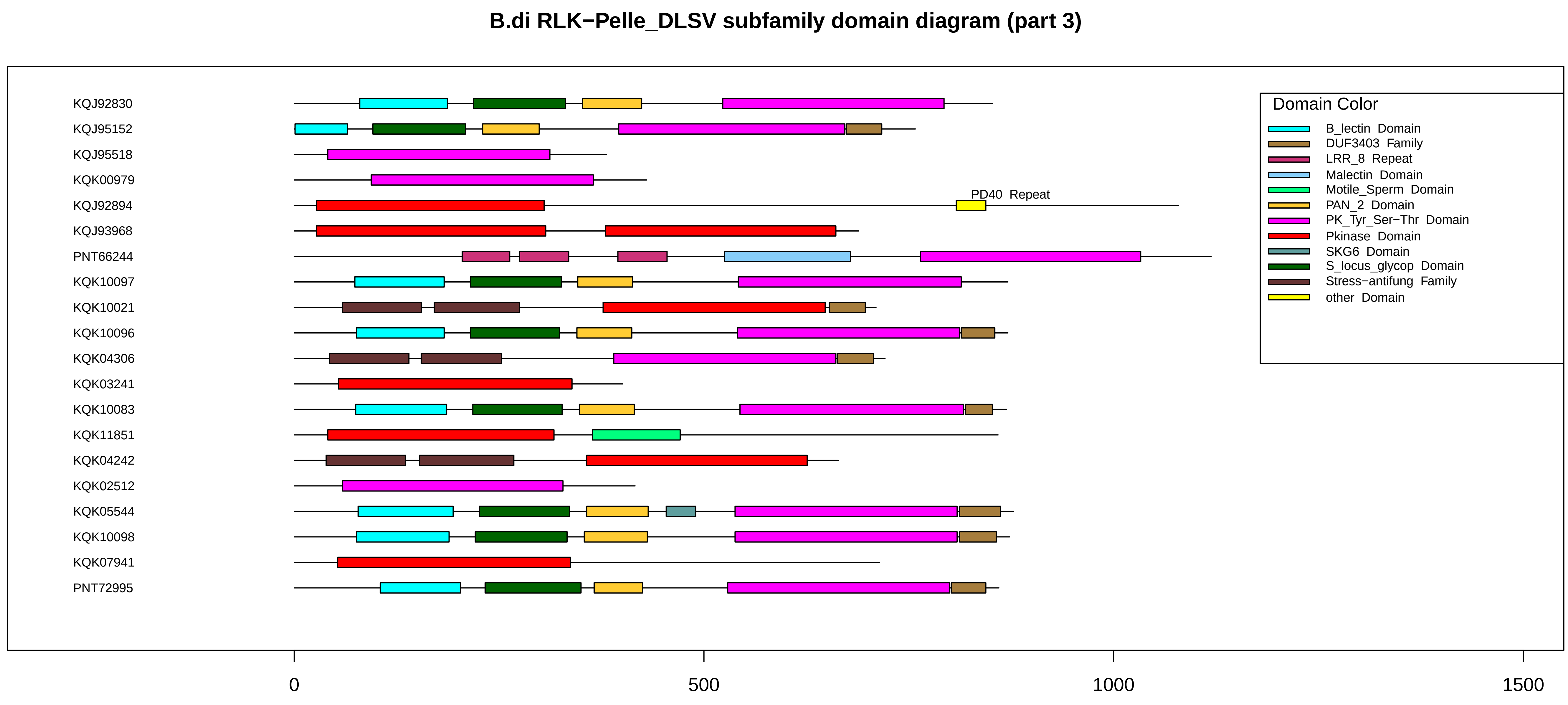

B.di RLK−Pelle_DLSV subfamily domain diagram (part 3)
KQJ92830
KQJ95152
KQJ95518
KQK00979
KQJ92894
KQJ93968
PNT66244
KQK10097
KQK10021
KQK10096
KQK04306
KQK03241
KQK10083
KQK11851
KQK04242
KQK02512
KQK05544
KQK10098
KQK07941
PNT72995
PD40 Repeat
Domain Color
B_lectin Domain
DUF3403 Family
LRR_8 Repeat
Malectin Domain
Motile_Sperm Domain
PAN_2 Domain
PK_Tyr_Ser−Thr Domain
Pkinase Domain
SKG6 Domain
S_locus_glycop Domain
Stress−antifung Family
other Domain
0
500
1000
1500

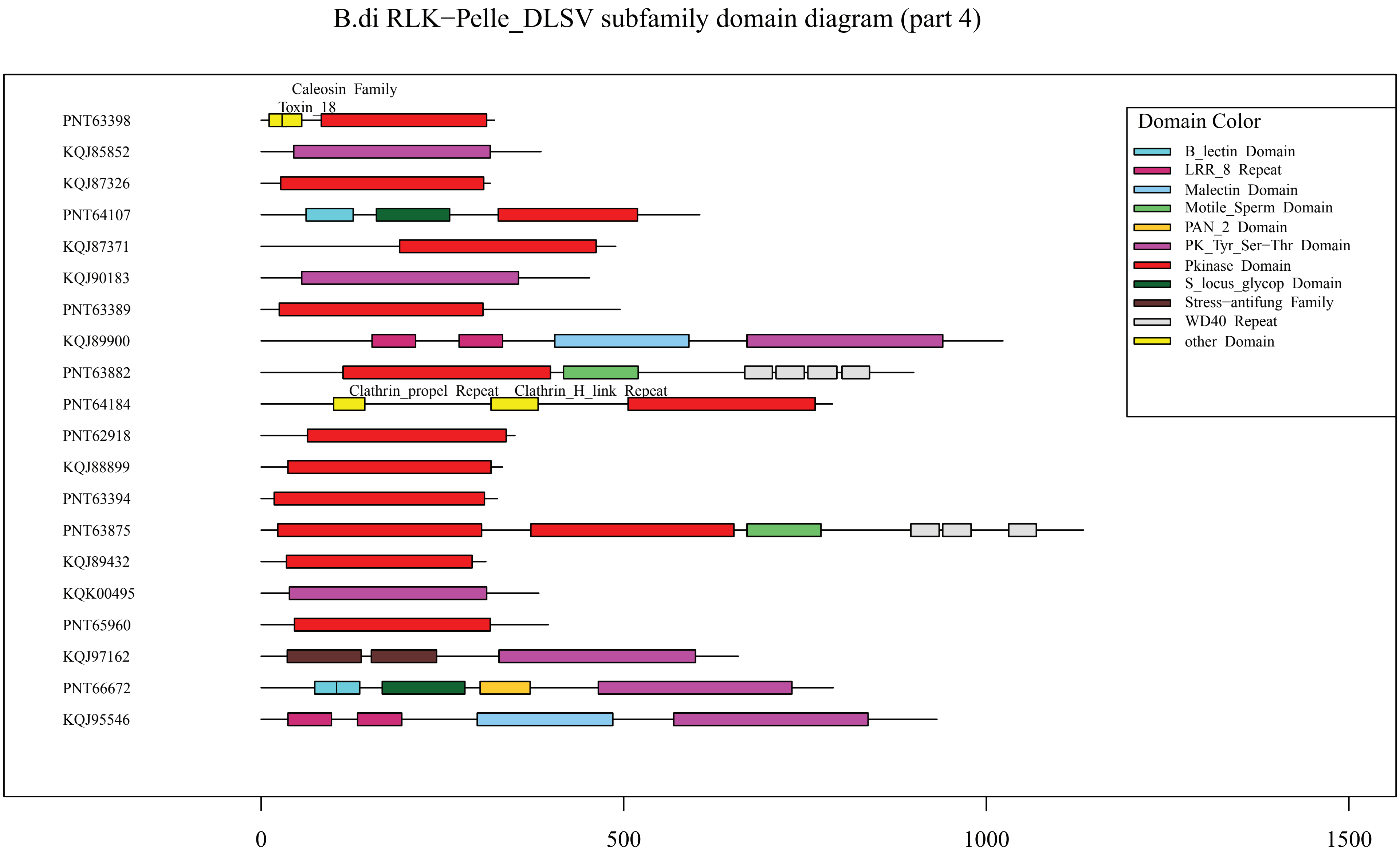
B.di RLK−Pelle_DLSV subfamily domain diagram (part 4)
Caleosin Family
Toxin_18
PNT63398
KQJ85852
KQJ87326
PNT64107
KQJ87371
KQJ90183
PNT63389
KQJ89900
PNT63882
Clathrin_propel Repeat
Clathrin_H_link Repeat
PNT64184
PNT62918
KQJ88899
PNT63394
PNT63875
KQJ89432
KQK00495
PNT65960
KQJ97162
PNT66672
KQJ95546
Domain Color
B_lectin Domain
LRR_8 Repeat
Malectin Domain
Motile_Sperm Domain
PAN_2 Domain
PK_Tyr_Ser−Thr Domain
Pkinase Domain
S_locus_glycop Domain
Stress−antifung Family
WD40 Repeat
other Domain
0
500
1000
1500

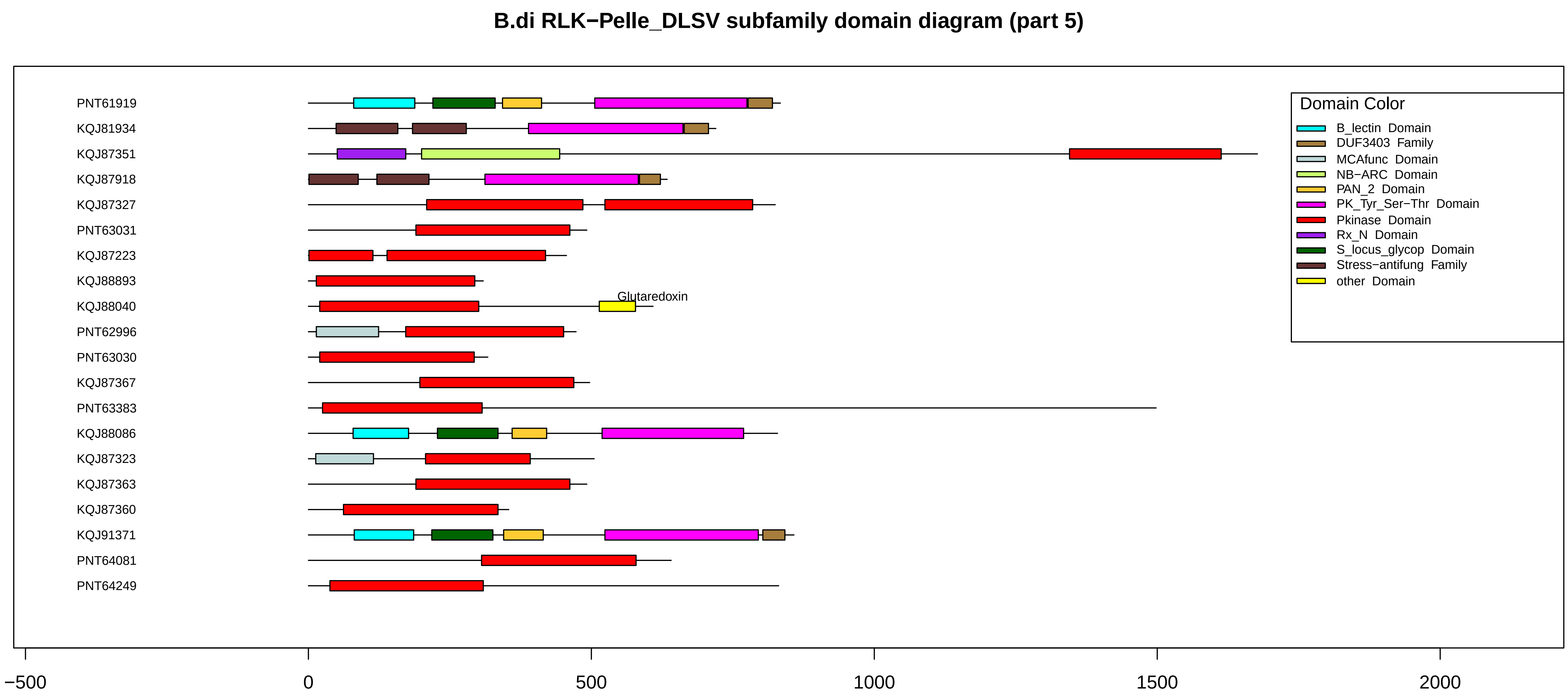
B.di RLK−Pelle_DLSV subfamily domain diagram (part 5)
PNT61919
KQJ81934
KQJ87351
KQJ87918
KQJ87327
PNT63031
KQJ87223
KQJ88893
KQJ88040
PNT62996
PNT63030
KQJ87367
PNT63383
KQJ88086
KQJ87323
KQJ87363
KQJ87360
KQJ91371
PNT64081
PNT64249
Glutaredoxin
Domain Color
B_lectin Domain
DUF3403 Family
MCAfunc Domain
NB−ARC Domain
PAN_2 Domain
PK_Tyr_Ser−Thr Domain
Pkinase Domain
Rx_N Domain
S_locus_glycop Domain
Stress−antifung Family
other Domain
−500
0
500
1000
1500
2000

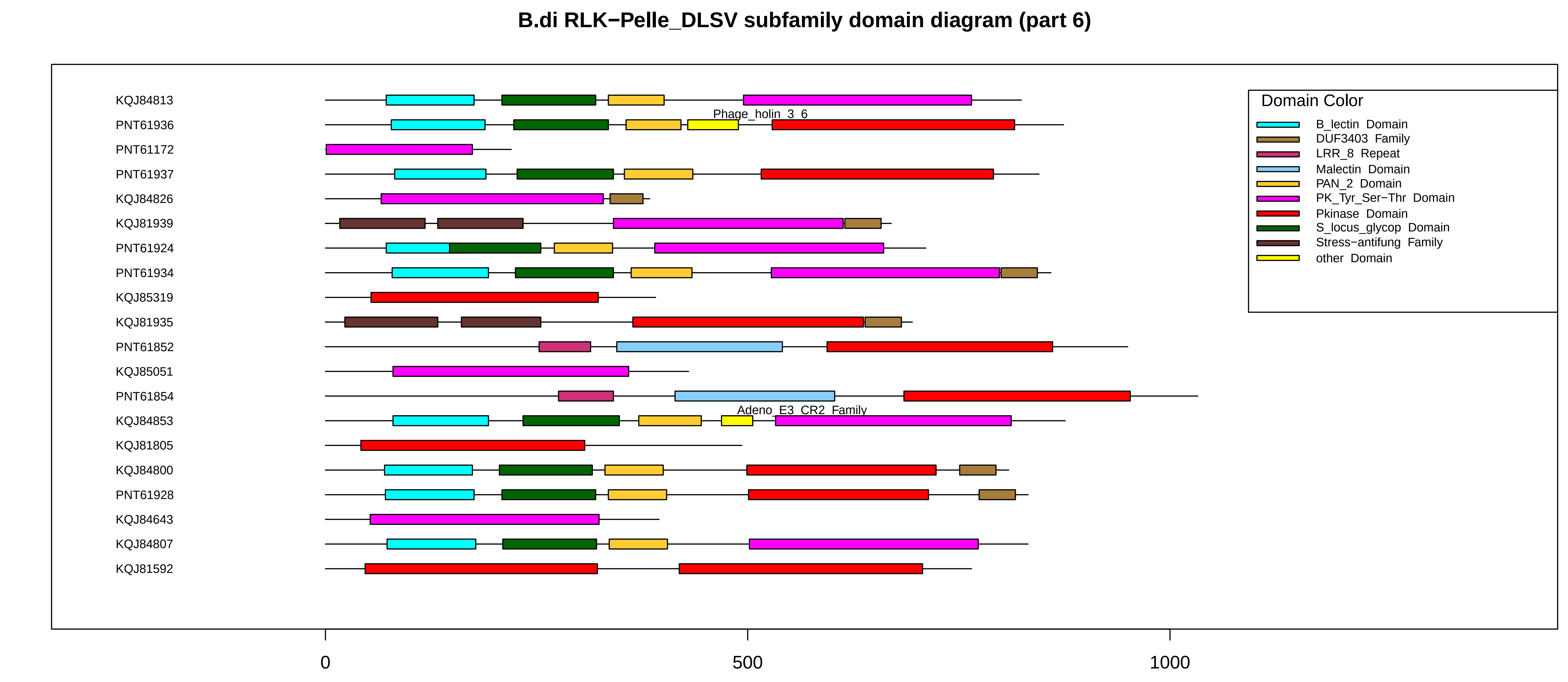
B.di RLK−Pelle_DLSV subfamily domain diagram (part 6)
KQJ84813
PNT61936
PNT61172
PNT61937
KQJ84826
KQJ81939
PNT61924
PNT61934
KQJ85319
KQJ81935
PNT61852
KQJ85051
PNT61854
KQJ84853
KQJ81805
KQJ84800
PNT61928
KQJ84643
KQJ84807
KQJ81592
Phage_holin_3_6
Adeno_E3_CR2_Family
Domain Color
B_lectin Domain
DUF3403 Family
LRR_8 Repeat
Malectin Domain
PAN_2 Domain
PK_Tyr_Ser−Thr Domain
Pkinase Domain
S_locus_glycop Domain
Stress−antifung Family
other Domain
0
500
1000

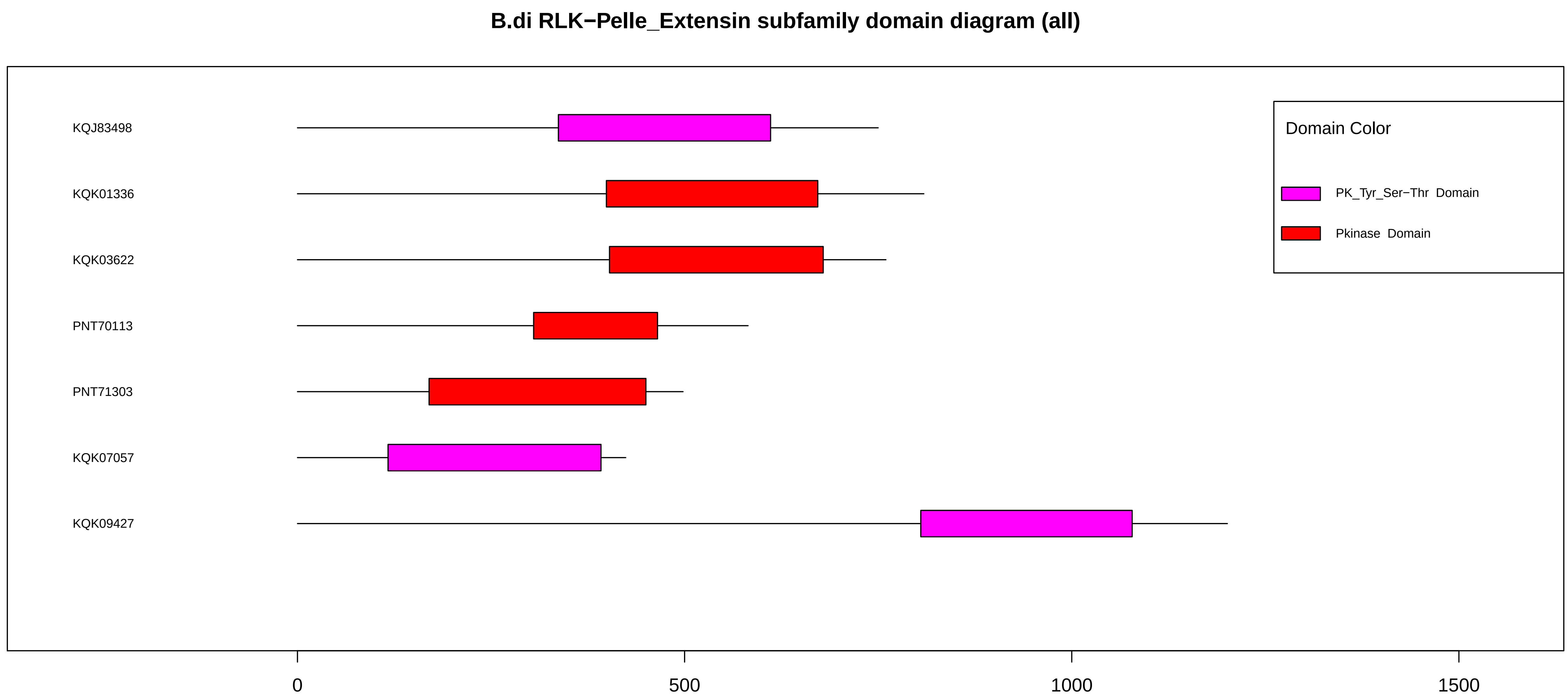
B.di RLK−Pelle_Extensin subfamily domain diagram (all)
KQJ83498
KQK01336
KQK03622
PNT70113
PNT71303
KQK07057
KQK09427
Domain Color
PK_Tyr_Ser−Thr Domain
Pkinase Domain
0
500
1000
1500

B.di RLK−Pelle_L−LEC subfamily domain diagram (part 1)

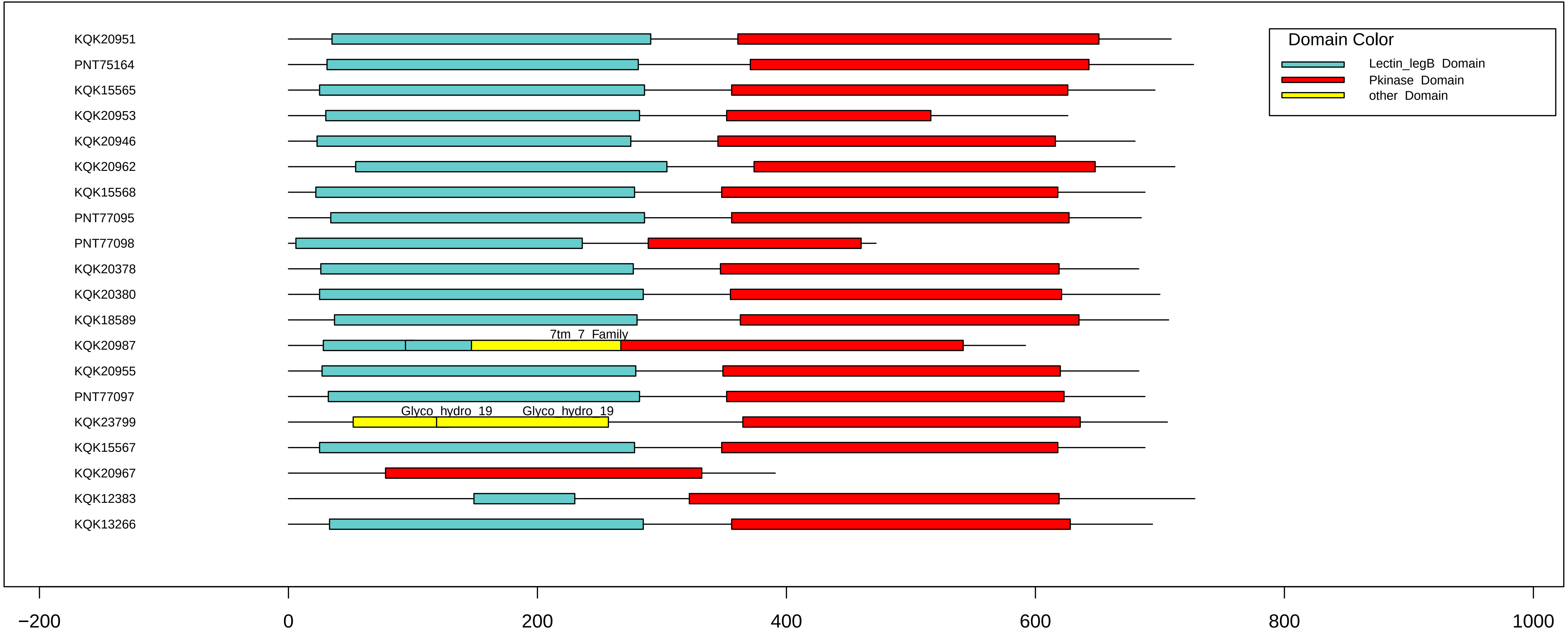

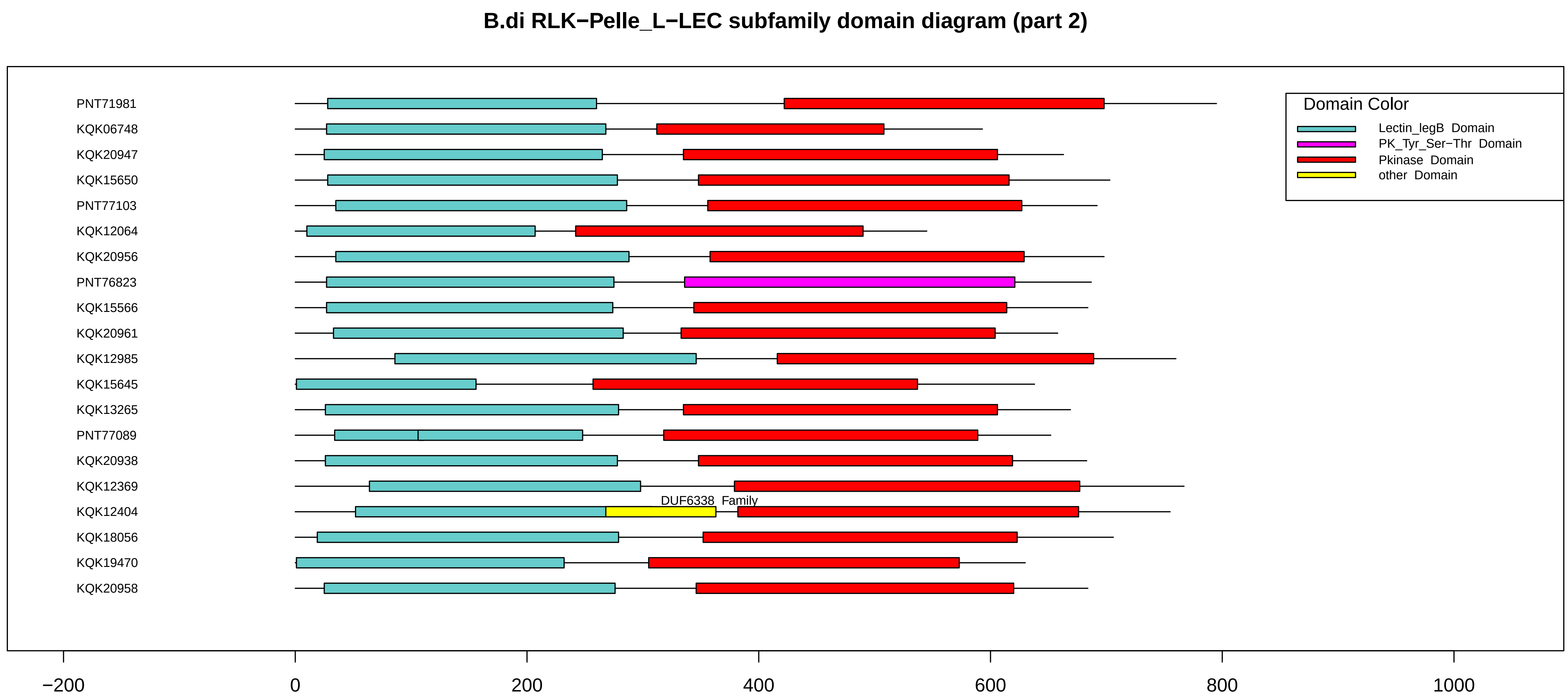

B.di RLK−Pelle_L−LEC subfamily domain diagram (part 2)
PNT71981
KQK06748
KQK20947
KQK15650
PNT77103
KQK12064
KQK20956
PNT76823
KQK15566
KQK20961
KQK12985
KQK15645
KQK13265
PNT77089
KQK20938
KQK12369
KQK12404
KQK18056
KQK19470
KQK20958
DUF6338 Family
Domain Color
Lectin_legB Domain
PK_Tyr_Ser−Thr Domain
Pkinase Domain
other Domain
−200
0
200
400
600
800
1000

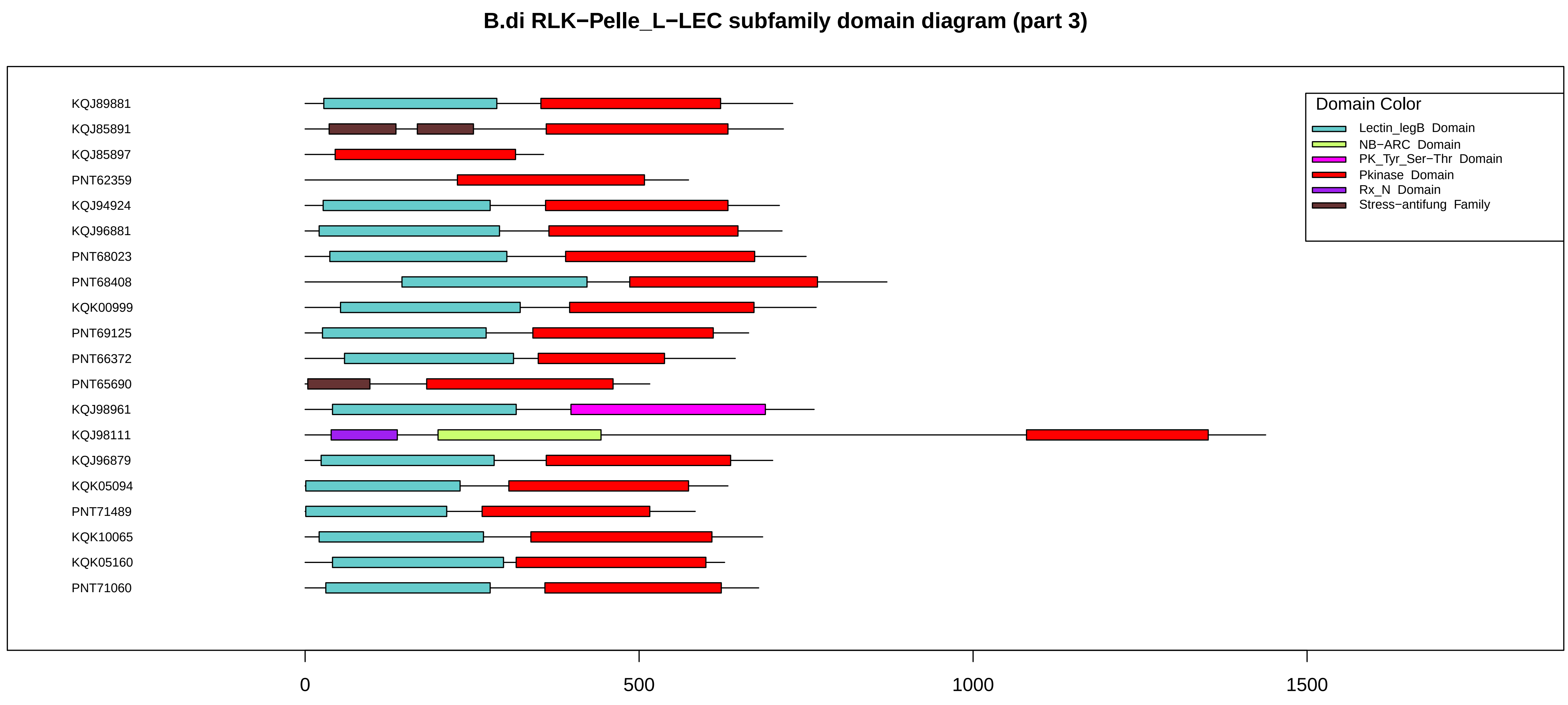
B.di RLK−Pelle_L−LEC subfamily domain diagram (part 3)
KQJ89881
KQJ85891
KQJ85897
PNT62359
KQJ94924
KQJ96881
PNT68023
PNT68408
KQK00999
PNT69125
PNT66372
PNT65690
KQJ98961
KQJ98111
KQJ96879
KQK05094
PNT71489
KQK10065
KQK05160
PNT71060
Domain Color
Lectin_legB Domain
NB−ARC Domain
PK_Tyr_Ser−Thr Domain
Pkinase Domain
Rx_N Domain
Stress−antifung Family
0
500
1000
1500

B.di RLK−Pelle_L−LEC subfamily domain diagram (part 4)

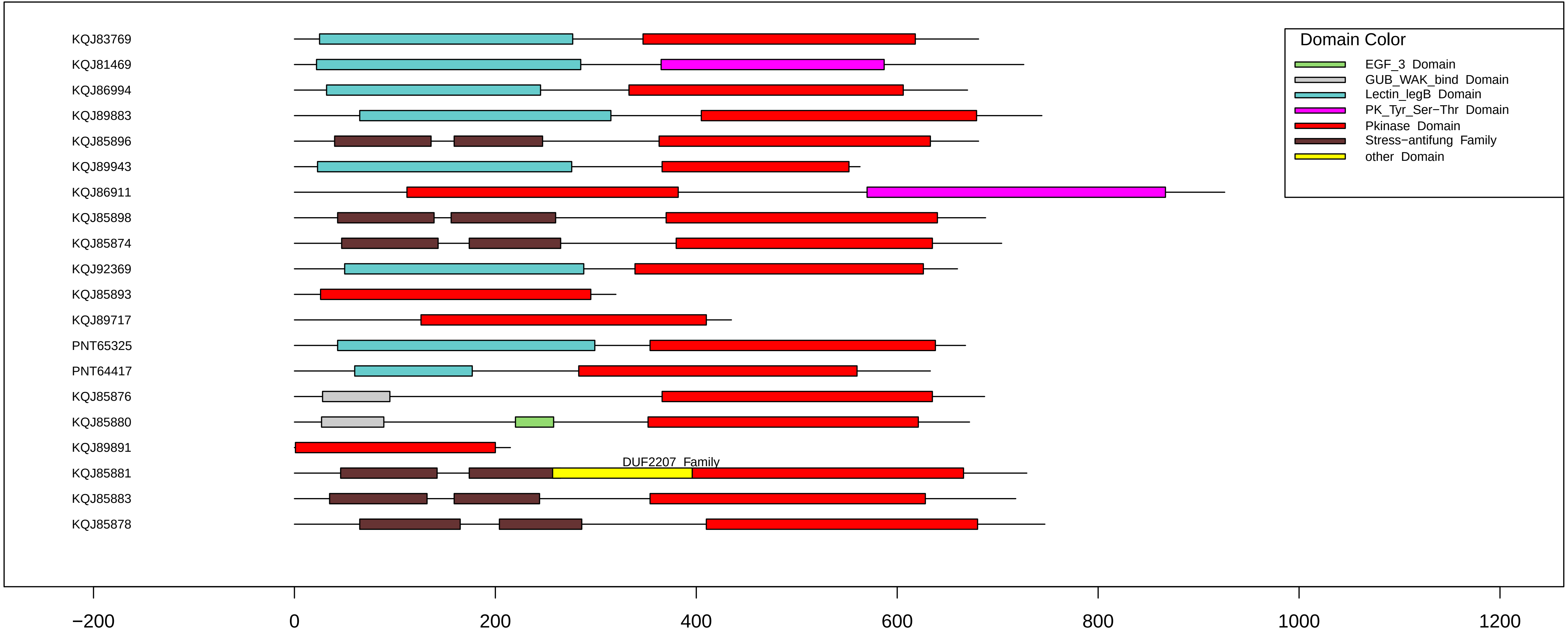

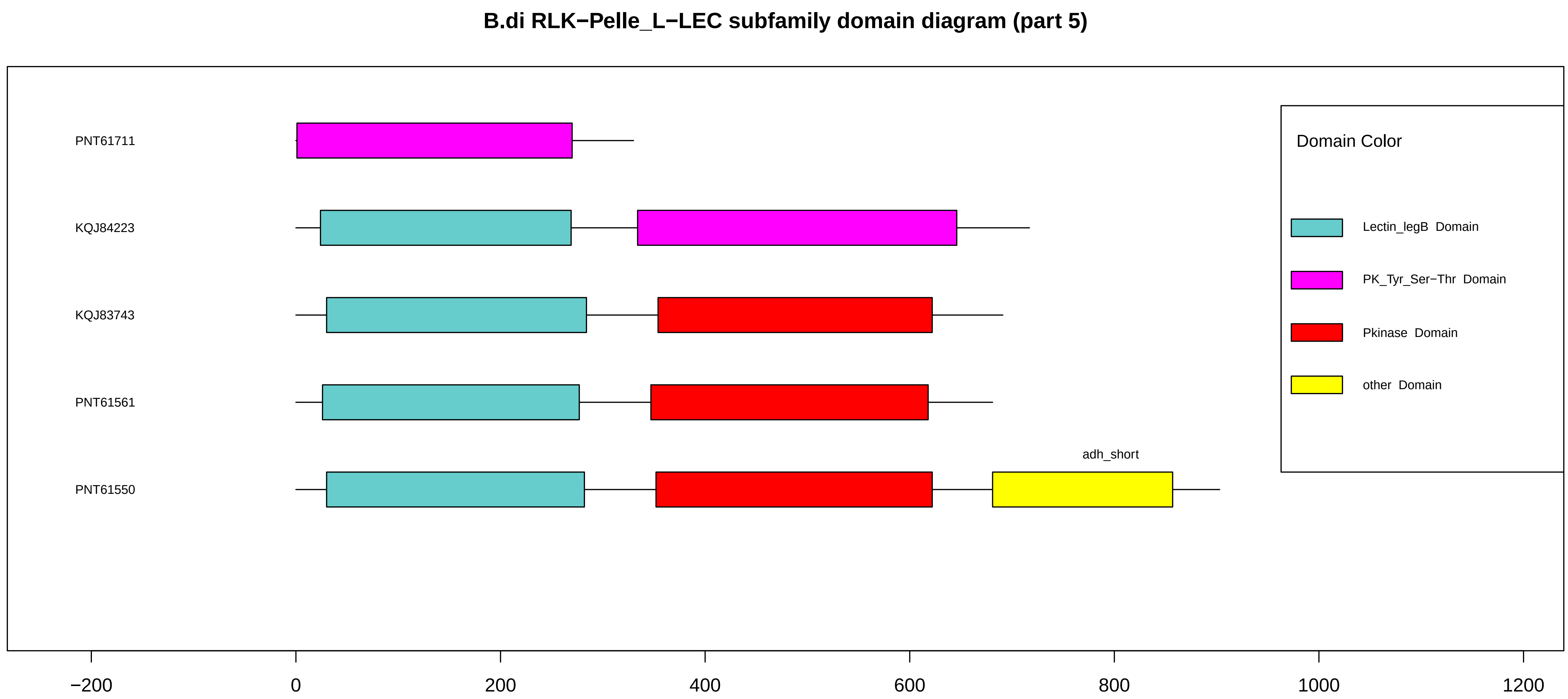
B.di RLK−Pelle_L−LEC subfamily domain diagram (part 5)
PNT61711
KQJ84223
KQJ83743
PNT61561
PNT61550
adh_short
Domain Color
Lectin_legB Domain
PK_Tyr_Ser−Thr Domain
Pkinase Domain
other Domain
−200
0
200
400
600
800
1000
1200

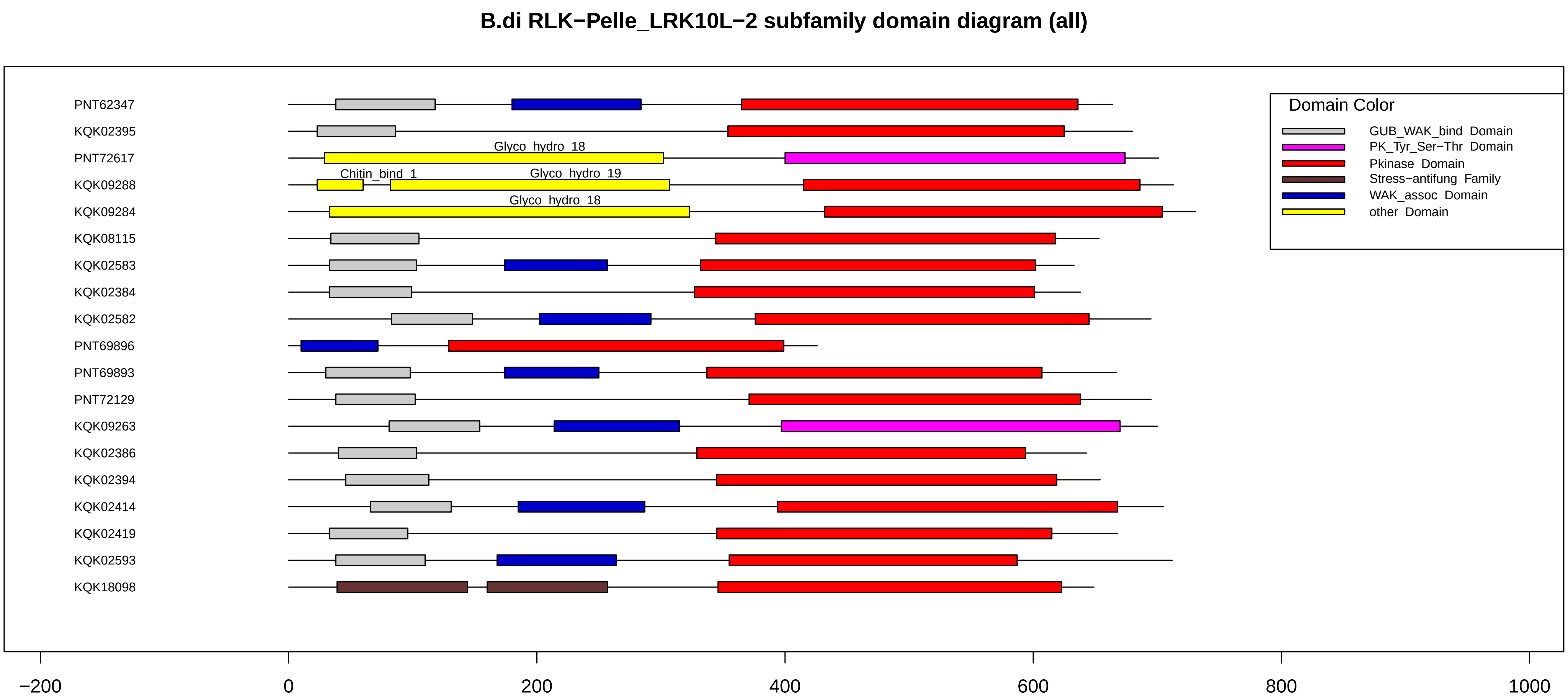

B.di RLK−Pelle_LRK10L−2 subfamily domain diagram (all)
PNT62347
KQK02395
PNT72617
KQK09288
KQK09284
KQK08115
KQK02583
KQK02384
KQK02582
PNT69896
PNT69893
PNT72129
KQK09263
KQK02386
KQK02394
KQK02414
KQK02419
KQK02593
KQK18098
Glyco_hydro_18
Chitin_bind_1
Glyco_hydro_19
Glyco_hydro_18
Domain Color
GUB_WAK_bind Domain
PK_Tyr_Ser−Thr Domain
Pkinase Domain
Stress−antifung Family
WAK_assoc Domain
other Domain
−200
0
200
400
600
800
1000

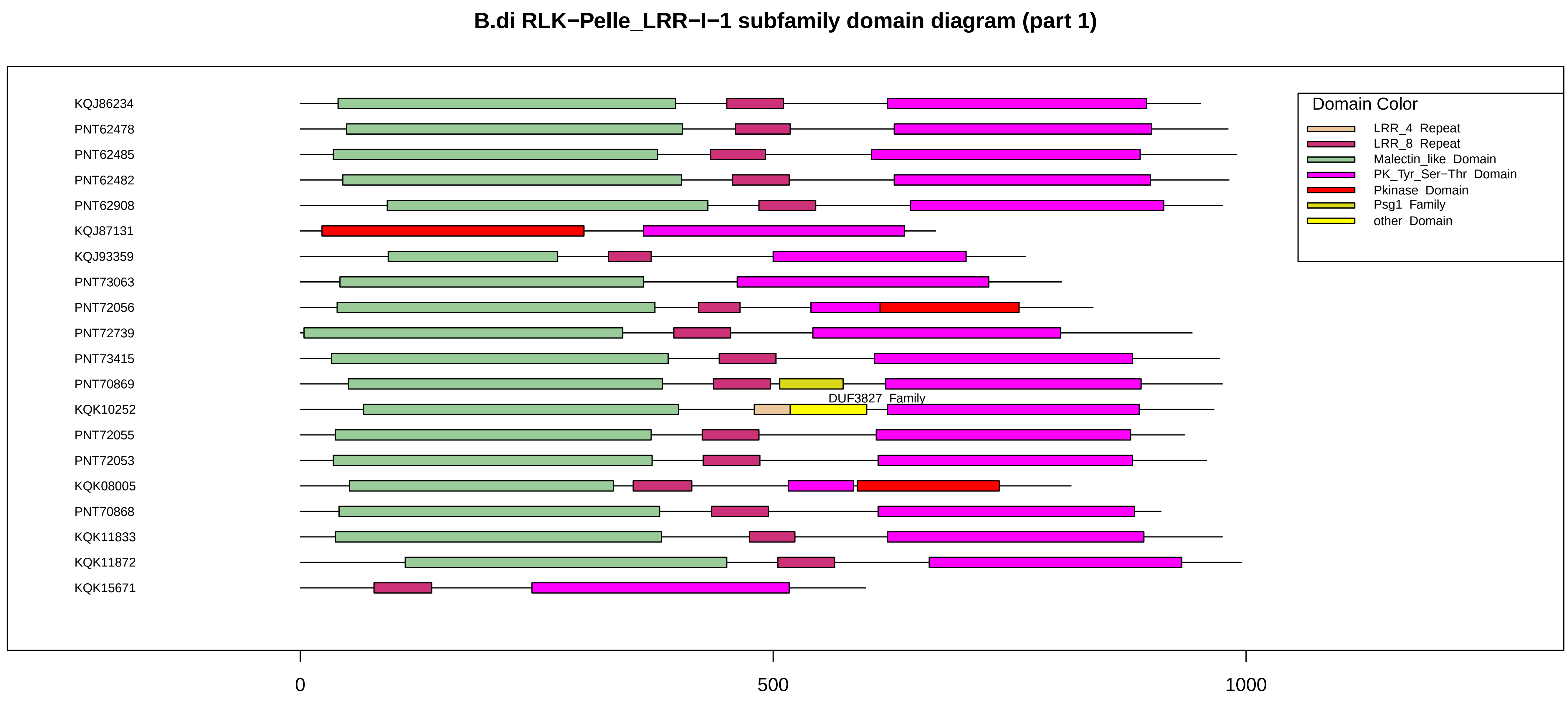
B.di RLK−Pelle_LRR−I−1 subfamily domain diagram (part 1)
KQJ86234
PNT62478
PNT62485
PNT62482
PNT62908
KQJ87131
KQJ93359
PNT73063
PNT72056
PNT72739
PNT73415
PNT70869
KQK10252
PNT72055
PNT72053
KQK08005
PNT70868
KQK11833
KQK11872
KQK15671
DUF3827 Family
Domain Color
LRR_4 Repeat
LRR_8 Repeat
Malectin_like Domain
PK_Tyr_Ser−Thr Domain
Pkinase Domain
Psg1 Family
other Domain
0
500
1000

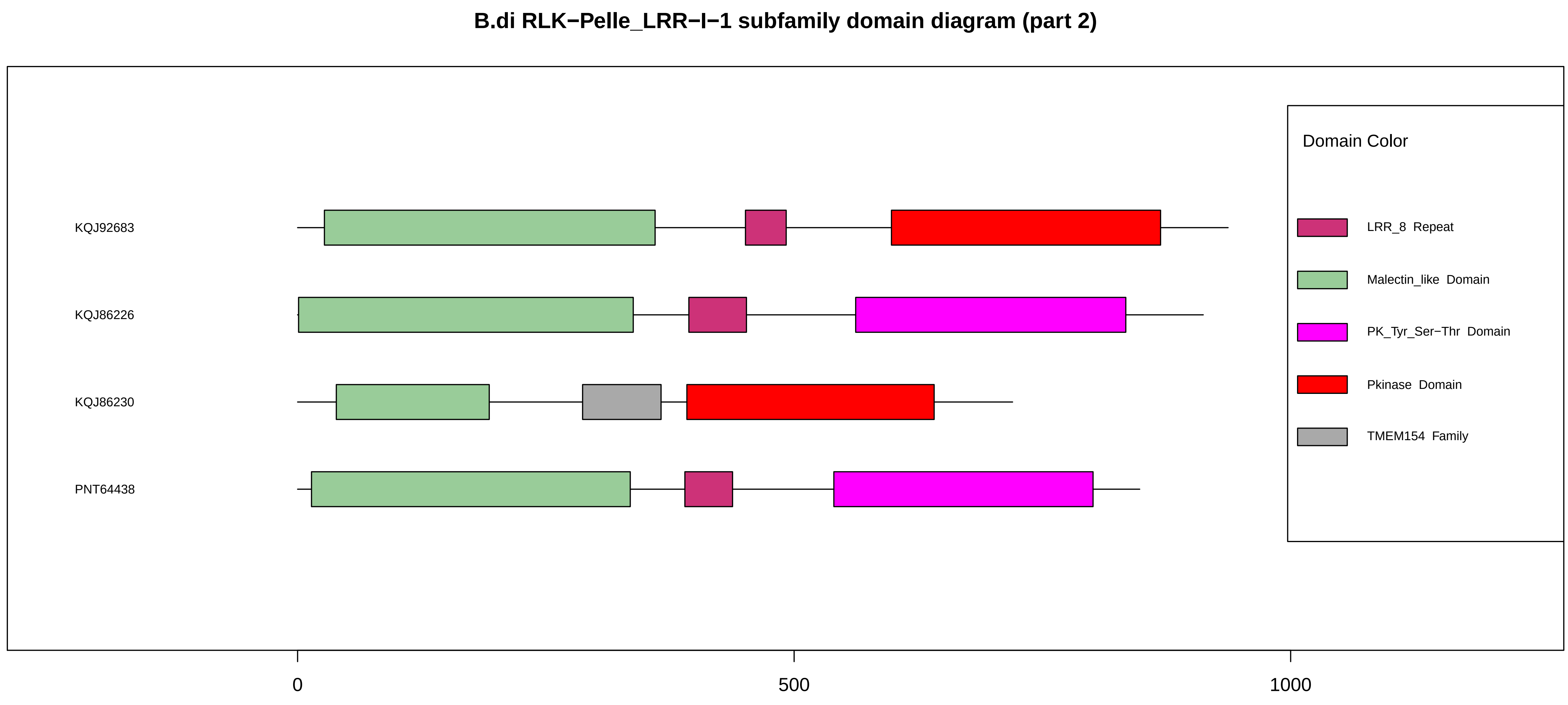
B.di RLK−Pelle_LRR−I−1 subfamily domain diagram (part 2)
KQJ92683
KQJ86226
KQJ86230
PNT64438
Domain Color
LRR_8 Repeat
Malectin_like Domain
PK_Tyr_Ser−Thr Domain
Pkinase Domain
TMEM154 Family
0
500
1000

B.di RLK−Pelle_LRR−I−2 subfamily domain diagram (all)

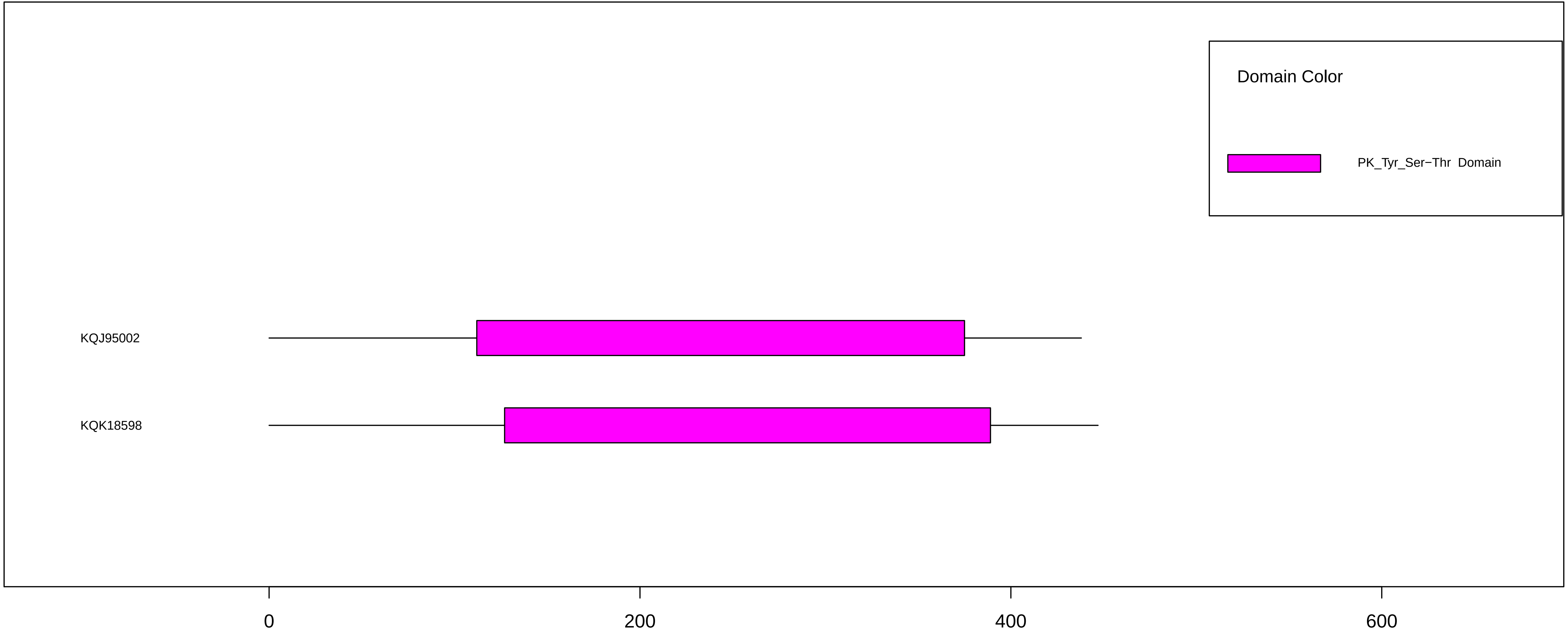

B.di RLK−Pelle_LRR−III subfamily domain diagram (part 1)

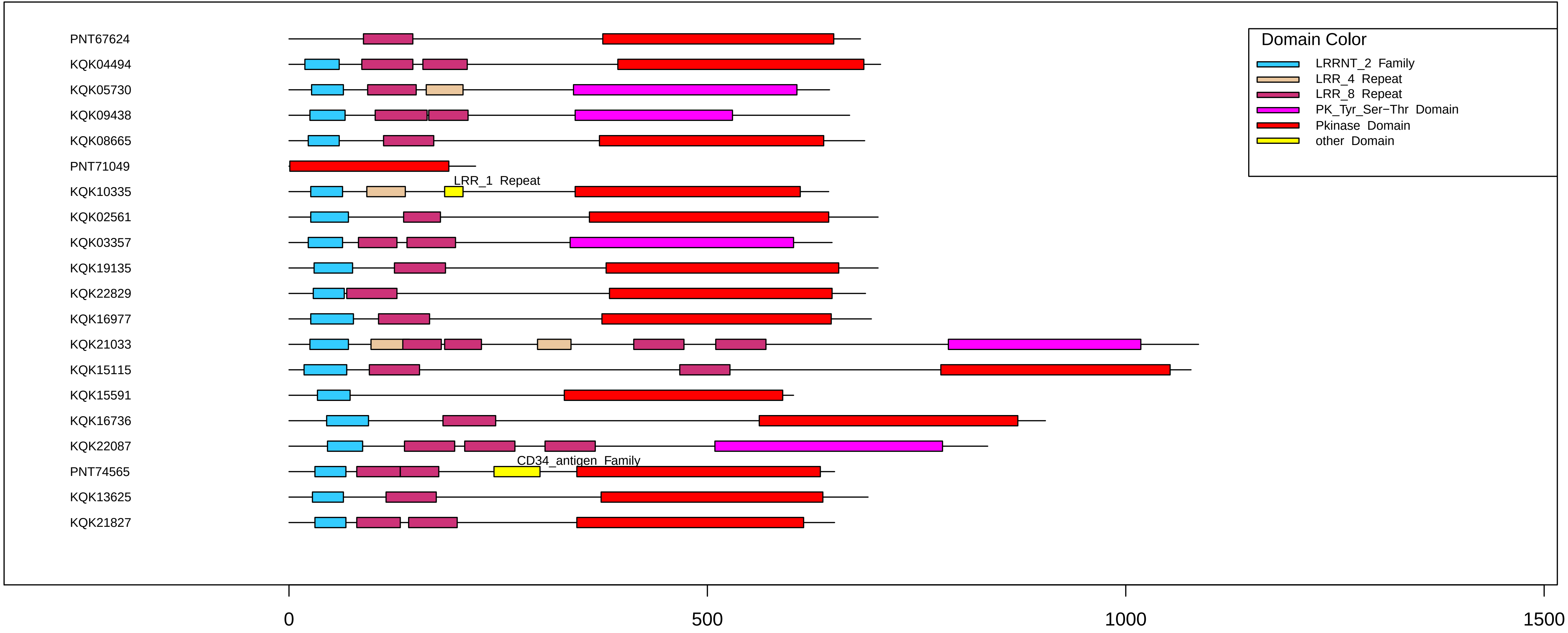

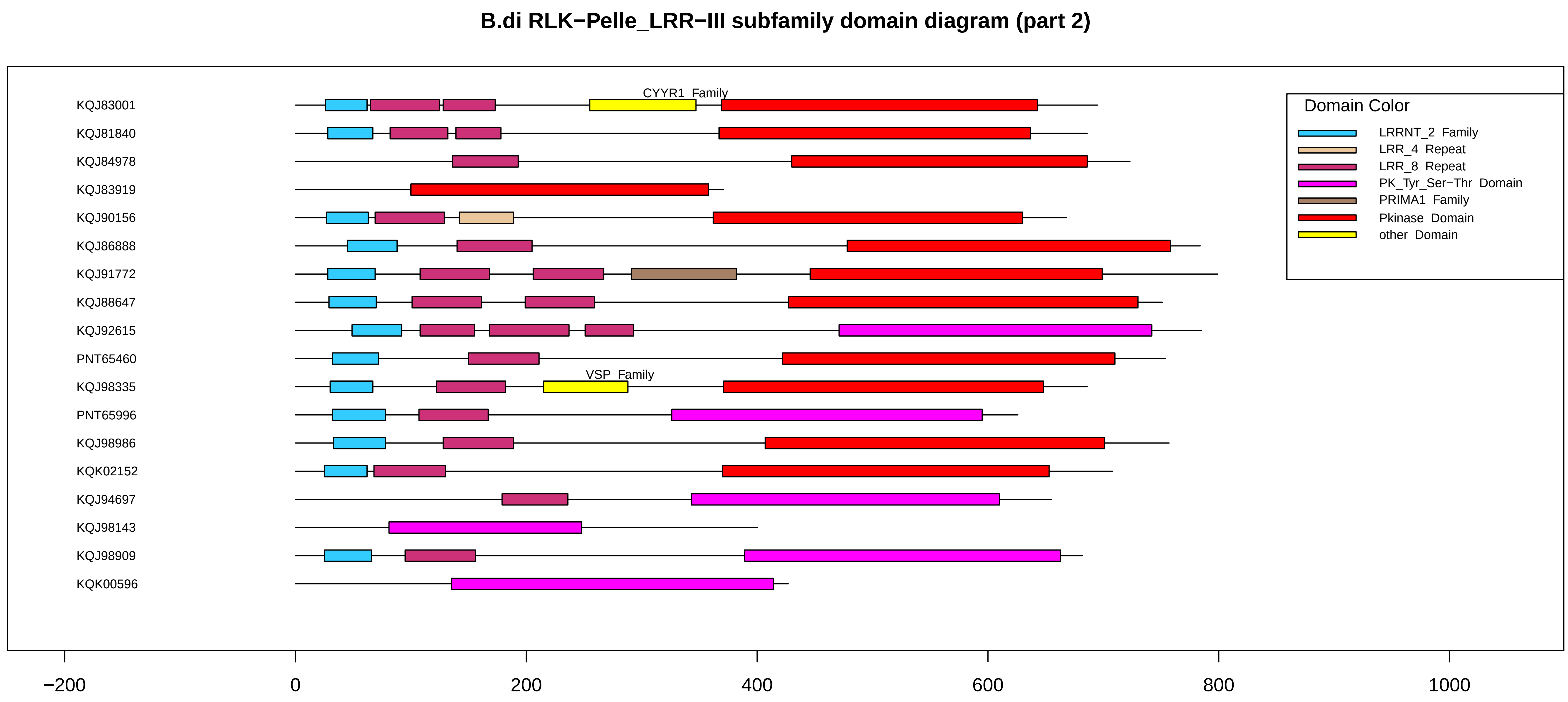
B.di RLK−Pelle_LRR−III subfamily domain diagram (part 2)
KQJ83001
KQJ81840
KQJ84978
KQJ83919
KQJ90156
KQJ86888
KQJ91772
KQJ88647
KQJ92615
PNT65460
KQJ98335
PNT65996
KQJ98986
KQK02152
KQJ94697
KQJ98143
KQJ98909
KQK00596
CYYR1 Family
VSP Family
Domain Color
LRRNT_2 Family
LRR_4 Repeat
LRR_8 Repeat
PK_Tyr_Ser−Thr Domain
PRIMA1 Family
Pkinase Domain
other Domain
−200
0
200
400
600
800
1000

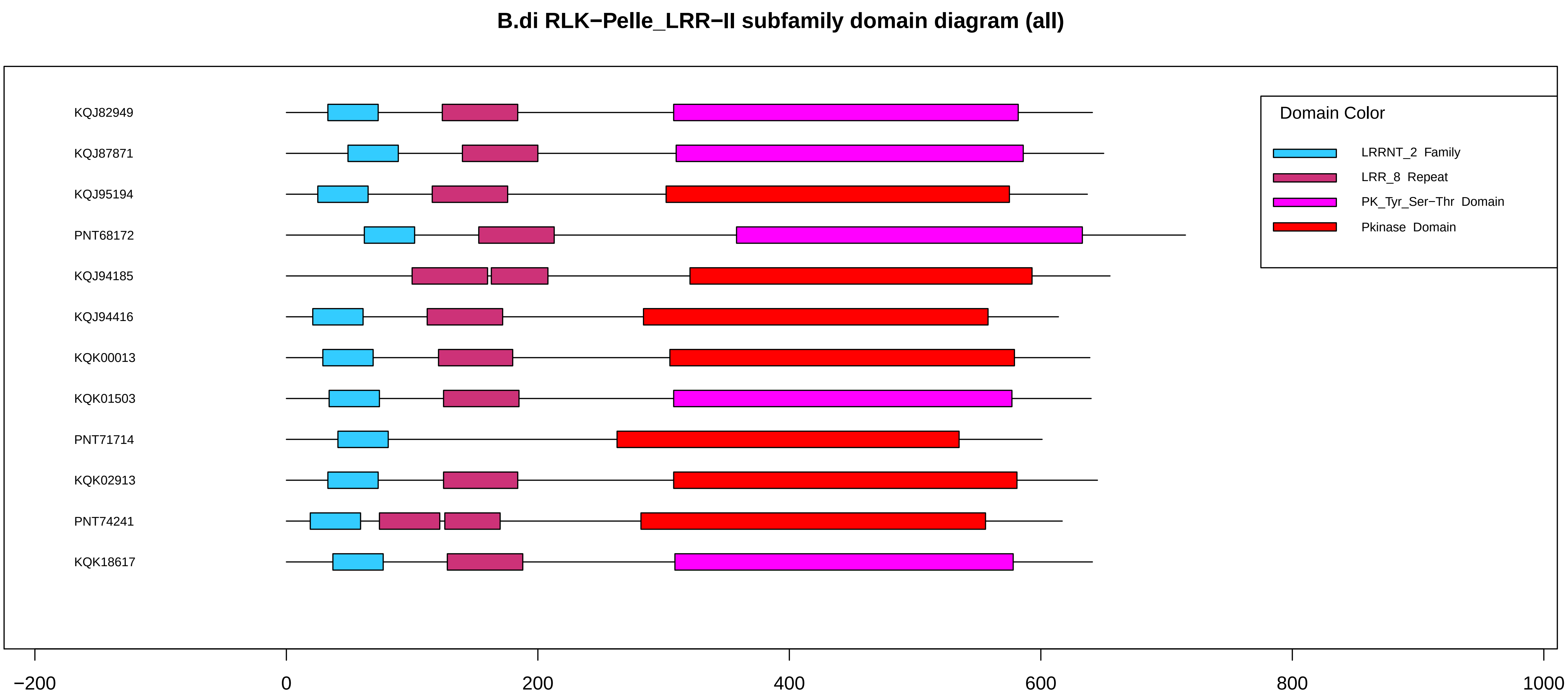
B.di RLK−Pelle_LRR−II subfamily domain diagram (all)
KQJ82949
KQJ87871
KQJ95194
PNT68172
KQJ94185
KQJ94416
KQK00013
KQK01503
PNT71714
KQK02913
PNT74241
KQK18617
Domain Color
LRRNT_2 Family
LRR_8 Repeat
PK_Tyr_Ser−Thr Domain
Pkinase Domain
−200
0
200
400
600
800
1000

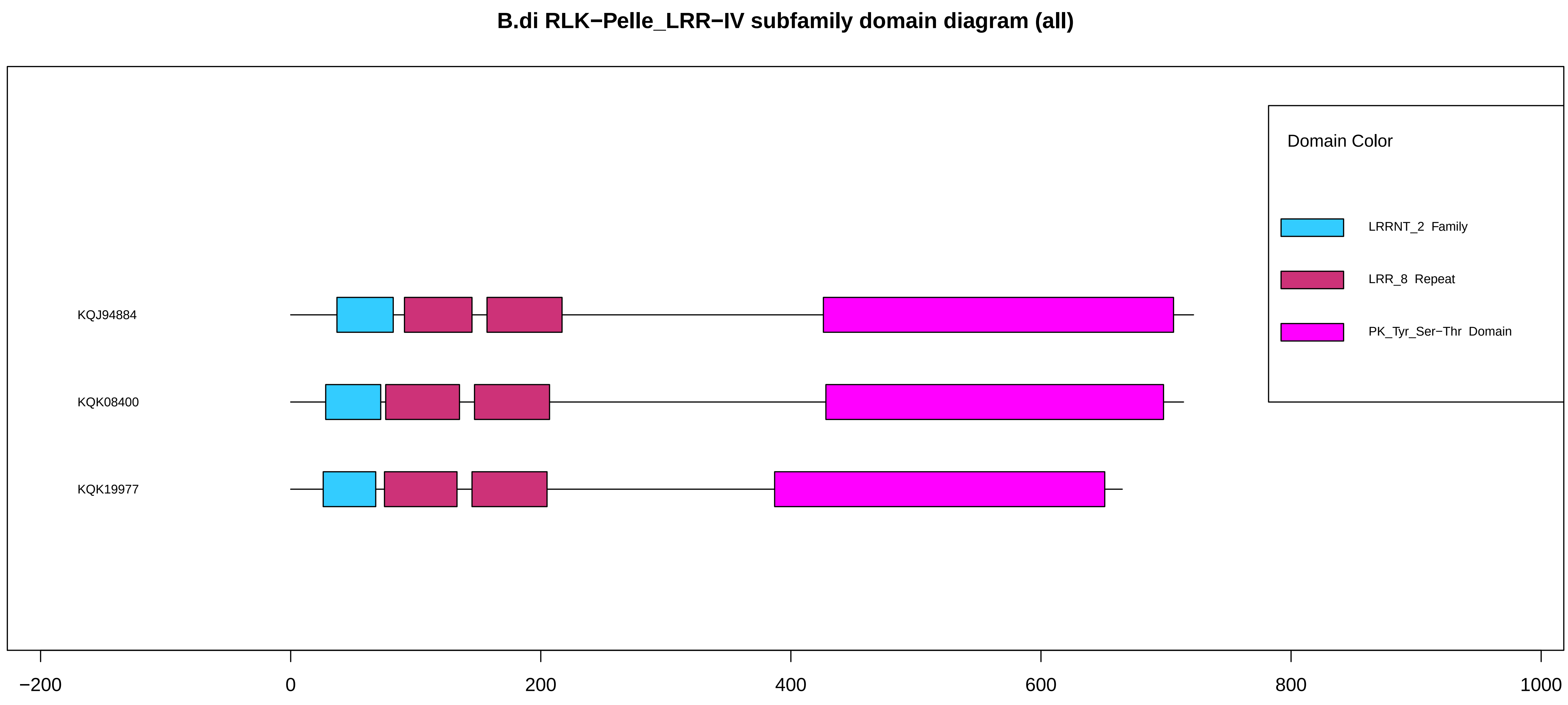
B.di RLK−Pelle_LRR−IV subfamily domain diagram (all)
Domain Color
LRRNT_2 Family
LRR_8 Repeat
PK_Tyr_Ser−Thr Domain
KQJ94884
KQK08400
KQK19977
−200
0
200
400
600
800
1000

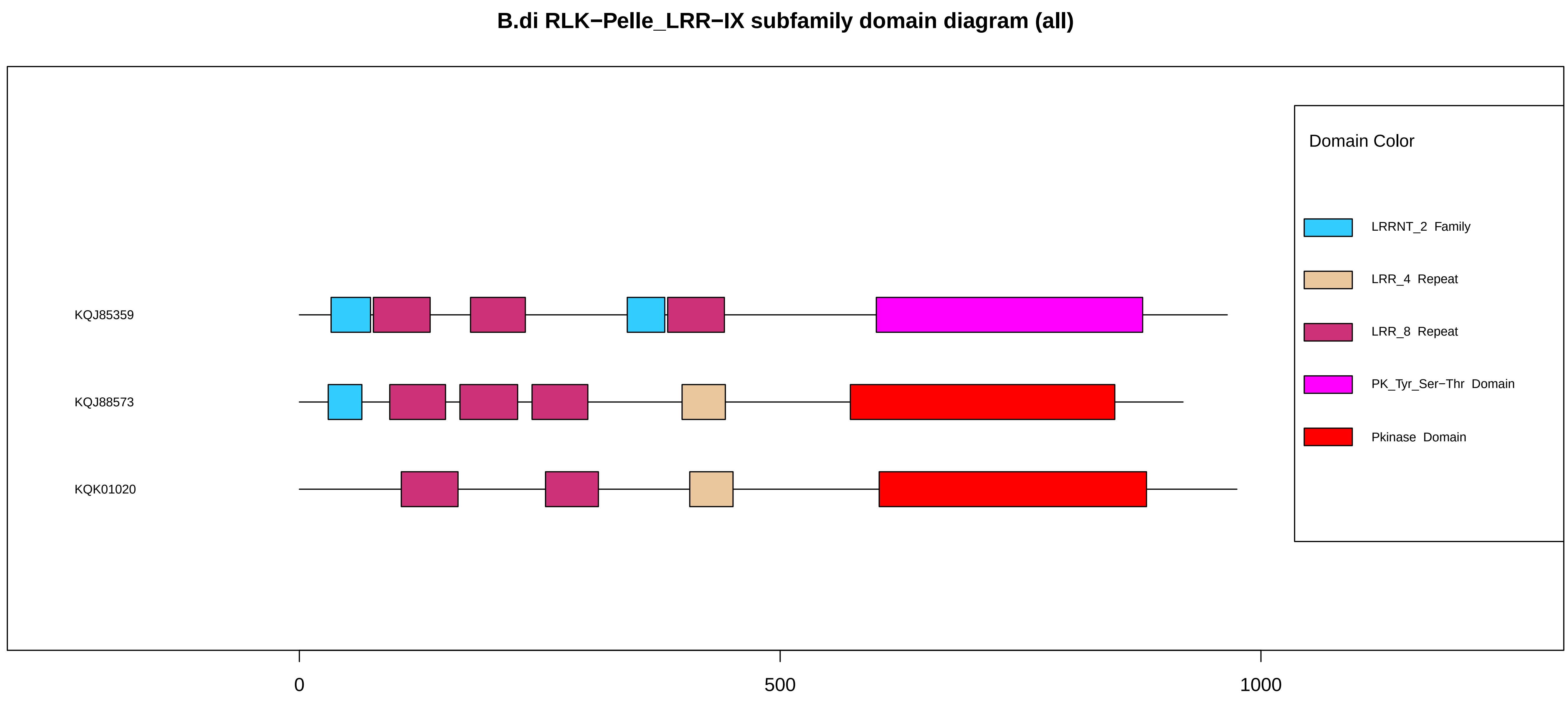
B.di RLK−Pelle_LRR−IX subfamily domain diagram (all)
KQJ85359
KQJ88573
KQK01020
Domain Color
LRRNT_2 Family
LRR_4 Repeat
LRR_8 Repeat
PK_Tyr_Ser−Thr Domain
Pkinase Domain
0
500
1000

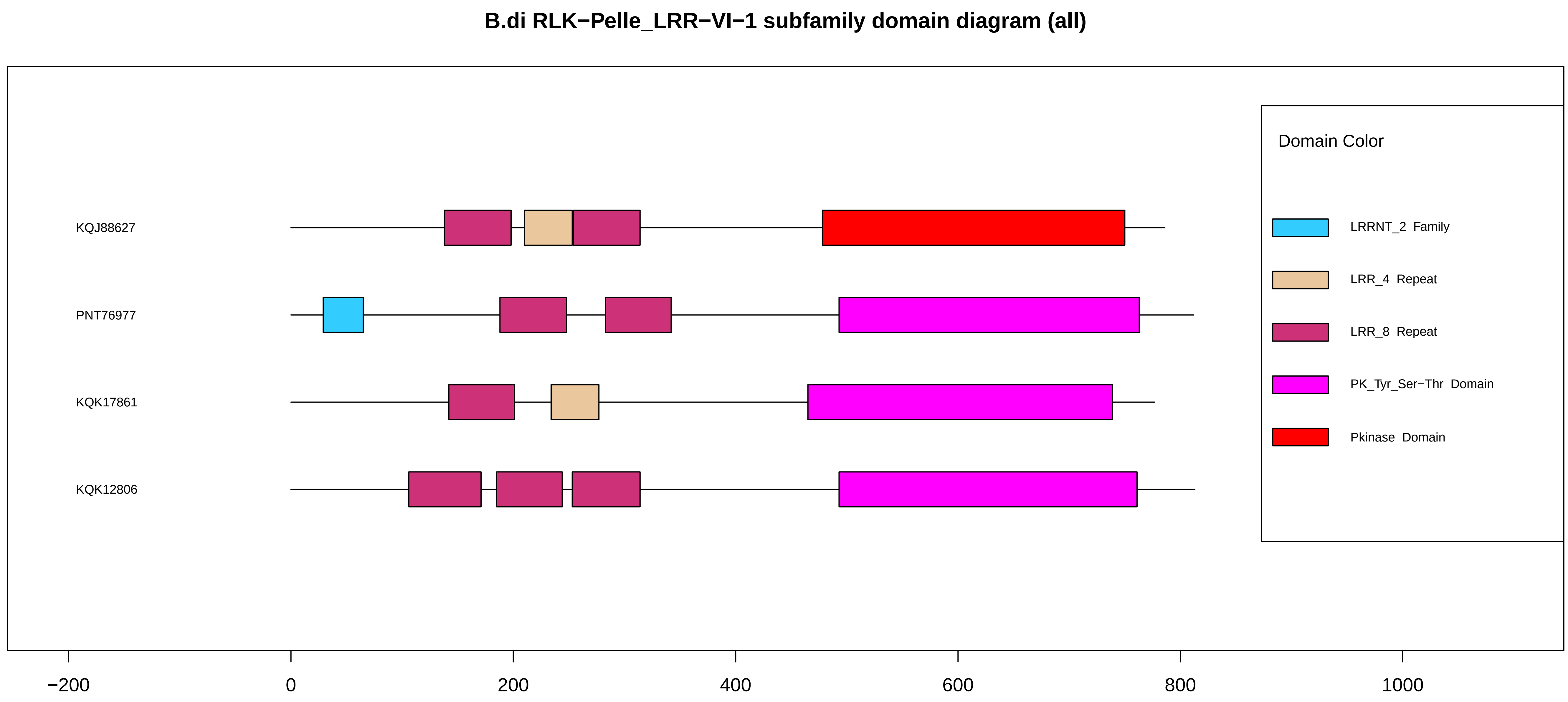
B.di RLK−Pelle_LRR−VI−1 subfamily domain diagram (all)
KQJ88627
PNT76977
KQK17861
KQK12806
Domain Color
LRRNT_2 Family
LRR_4 Repeat
LRR_8 Repeat
PK_Tyr_Ser−Thr Domain
Pkinase Domain
−200
0
200
400
600
800
1000

B.di RLK−Pelle_LRR−VI−2 subfamily domain diagram (all)

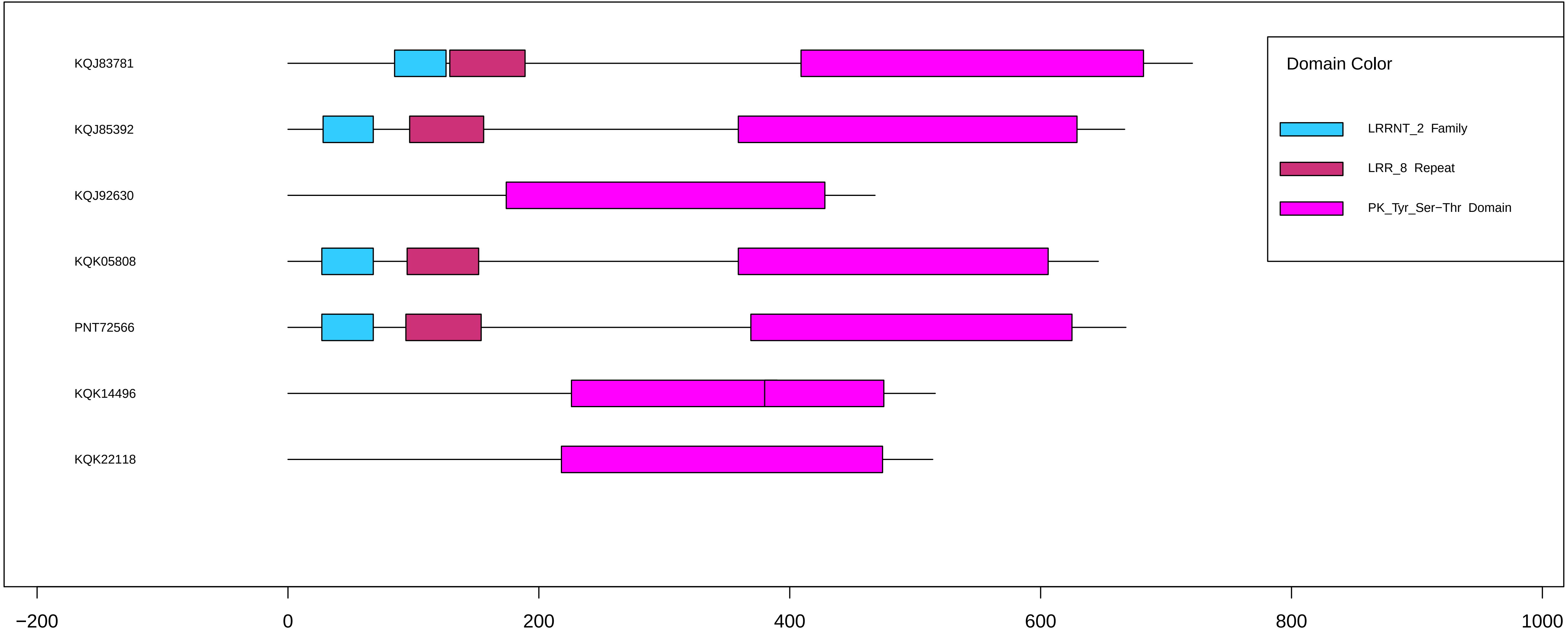

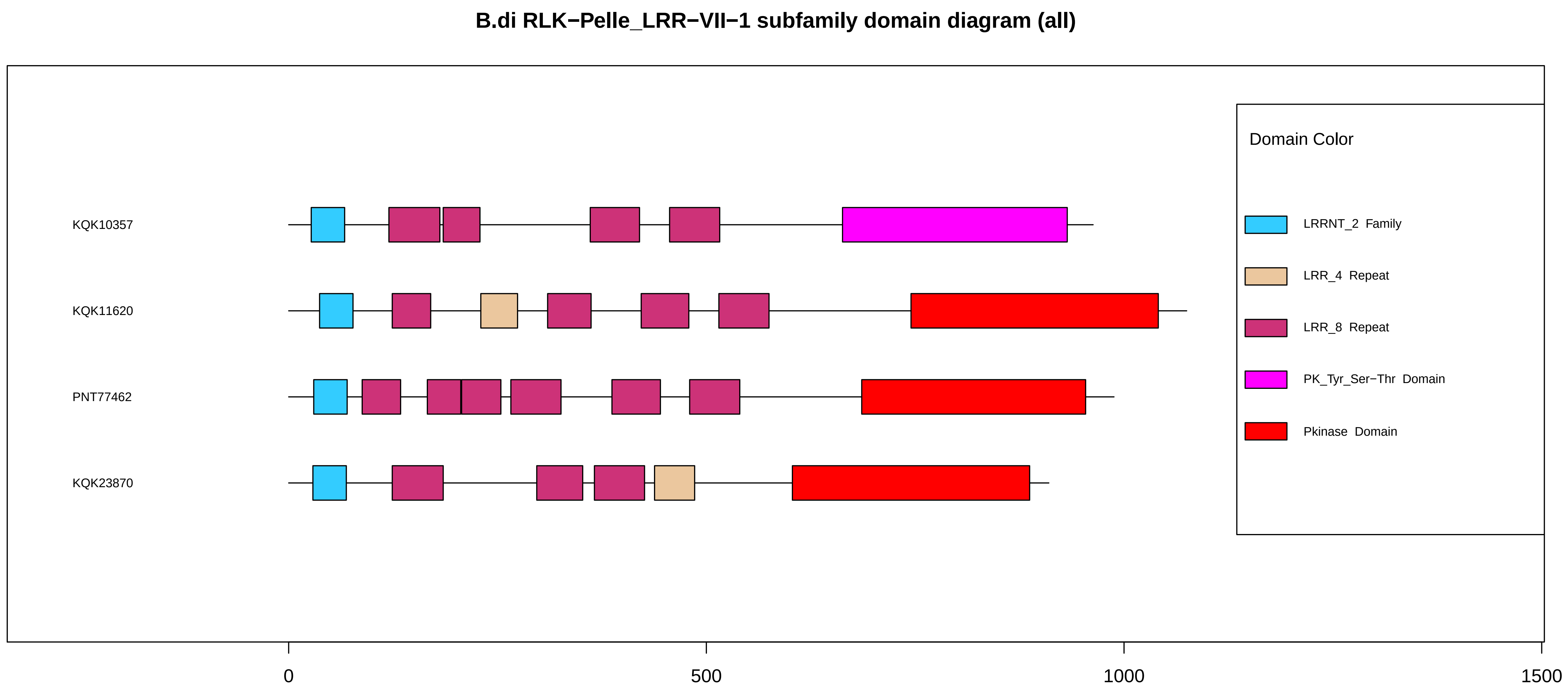

B.di RLK−Pelle_LRR−VII−1 subfamily domain diagram (all)
KQK10357
KQK11620
PNT77462
KQK23870
Domain Color
LRRNT_2 Family
LRR_4 Repeat
LRR_8 Repeat
PK_Tyr_Ser−Thr Domain
Pkinase Domain
0
500
1000
1500

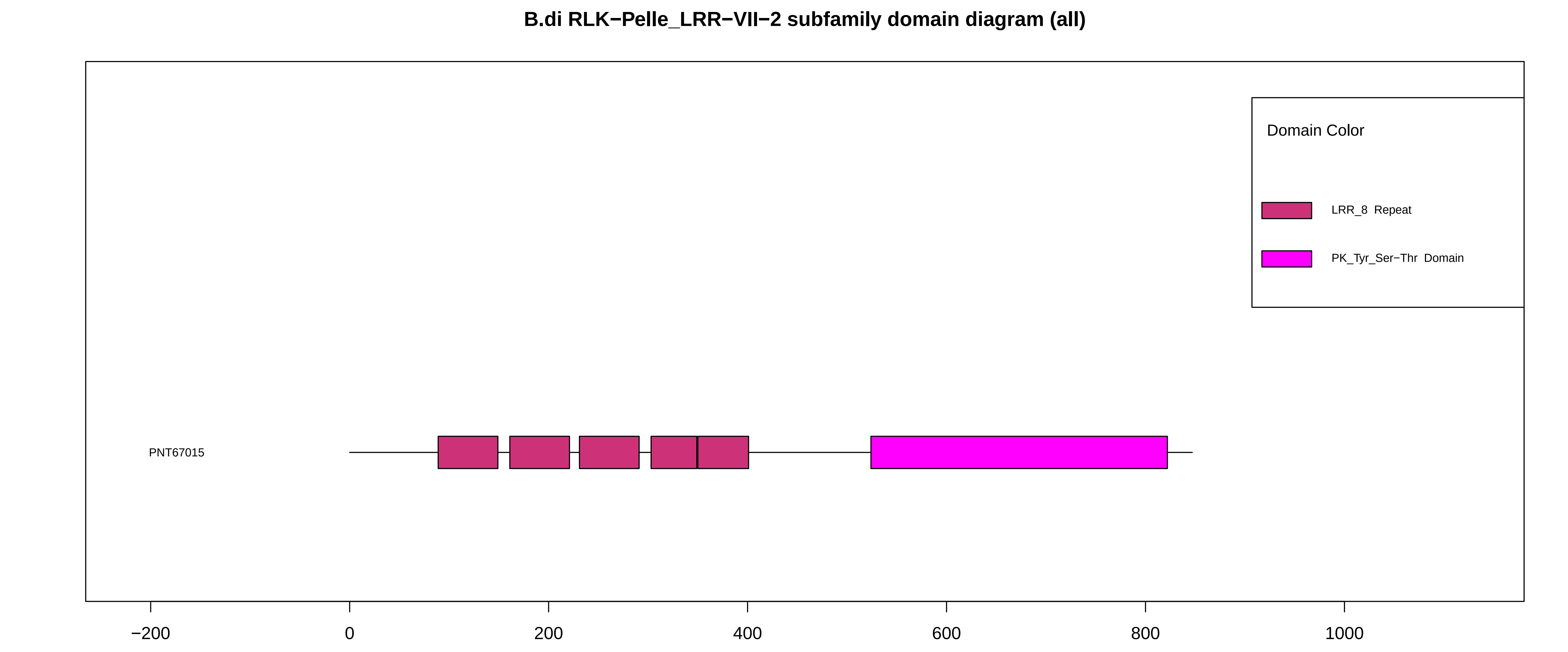
B.di RLK−Pelle_LRR−VII−2 subfamily domain diagram (all)
Domain Color
LRR_8 Repeat
PK_Tyr_Ser−Thr Domain
PNT67015
−200
0
200
400
600
800
1000

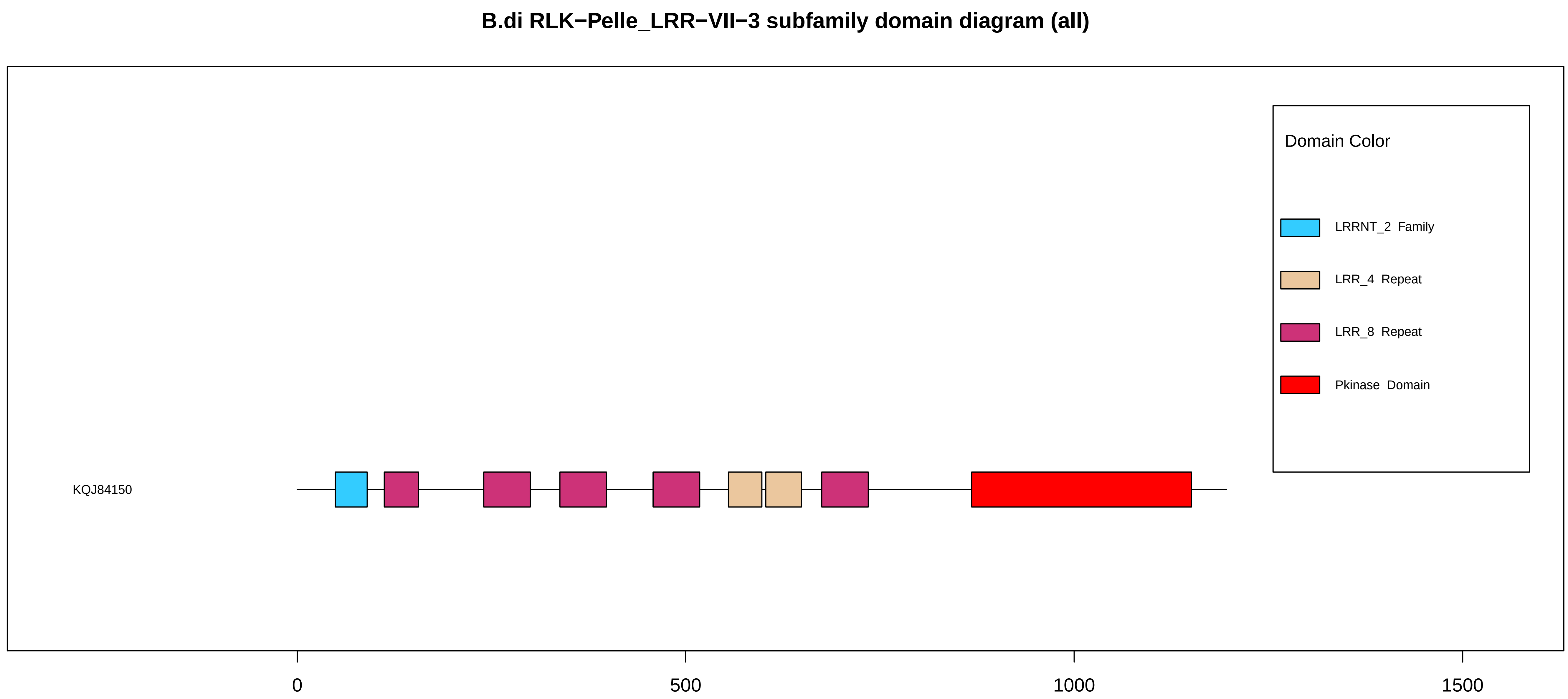

B.di RLK−Pelle_LRR−VII−3 subfamily domain diagram (all)
Domain Color
LRRNT_2 Family
LRR_4 Repeat
LRR_8 Repeat
Pkinase Domain
KQJ84150
0
500
1000
1500

B.di RLK−Pelle_LRR−VIII−1 subfamily domain diagram (all)

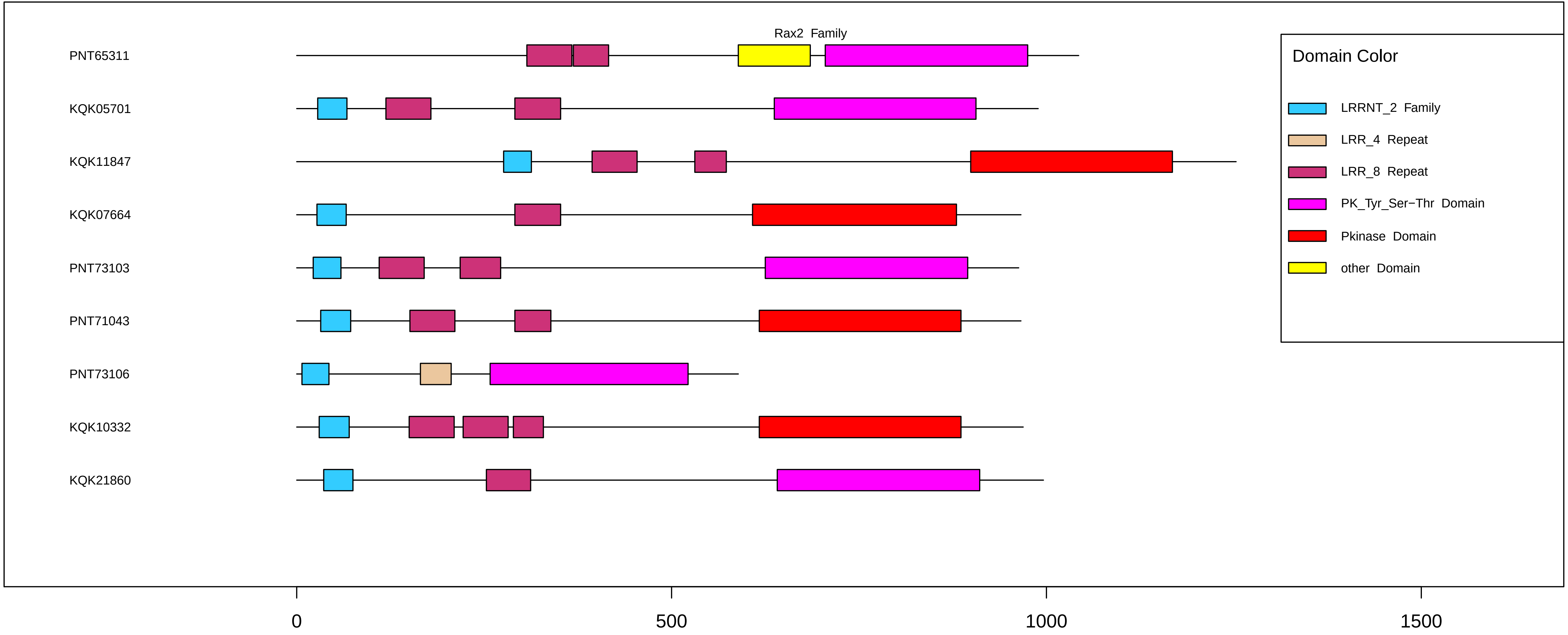

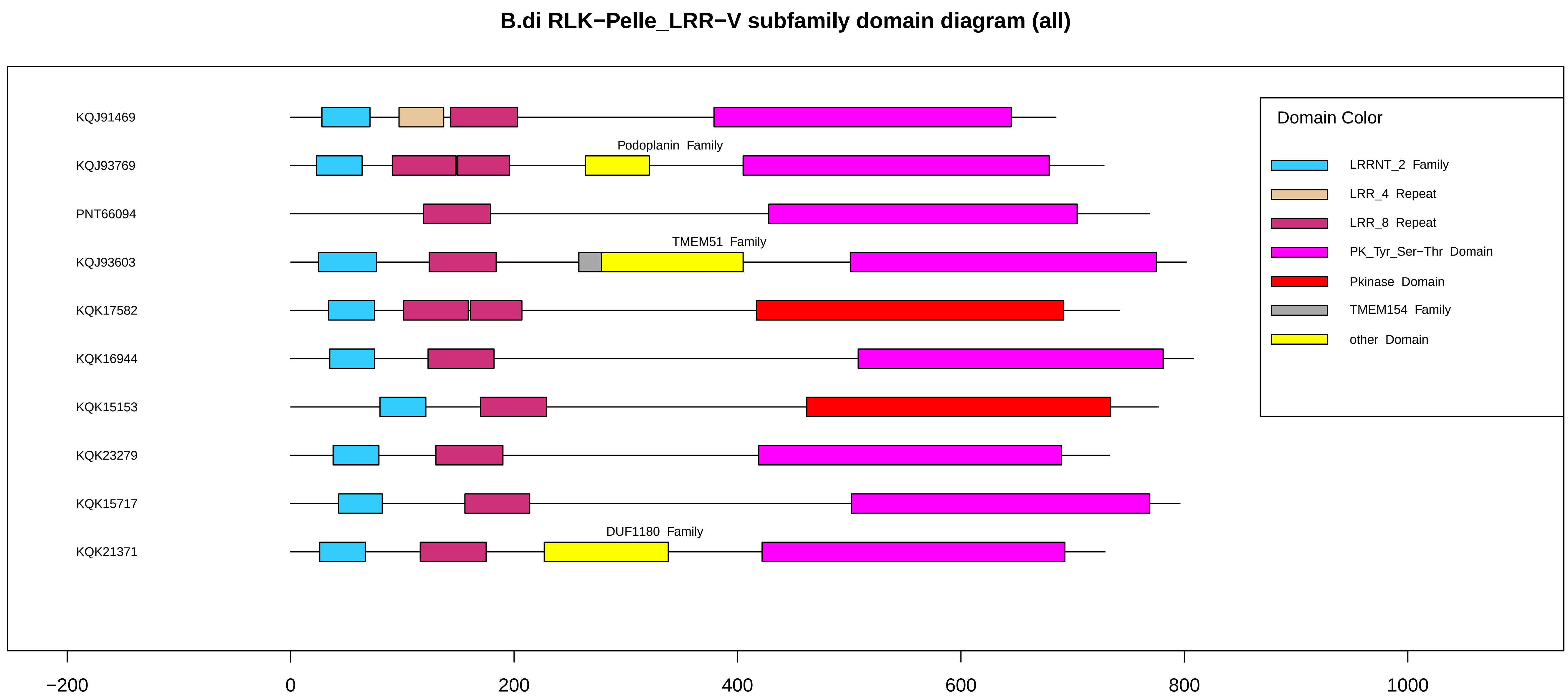

B.di RLK−Pelle_LRR−V subfamily domain diagram (all)
KQJ91469
KQJ93769
PNT66094
KQJ93603
KQK17582
KQK16944
KQK15153
KQK23279
KQK15717
KQK21371
Podoplanin Family
TMEM51 Family
DUF1180 Family
Domain Color
LRRNT_2 Family
LRR_4 Repeat
LRR_8 Repeat
PK_Tyr_Ser−Thr Domain
Pkinase Domain
TMEM154 Family
other Domain
−200
0
200
400
600
800
1000

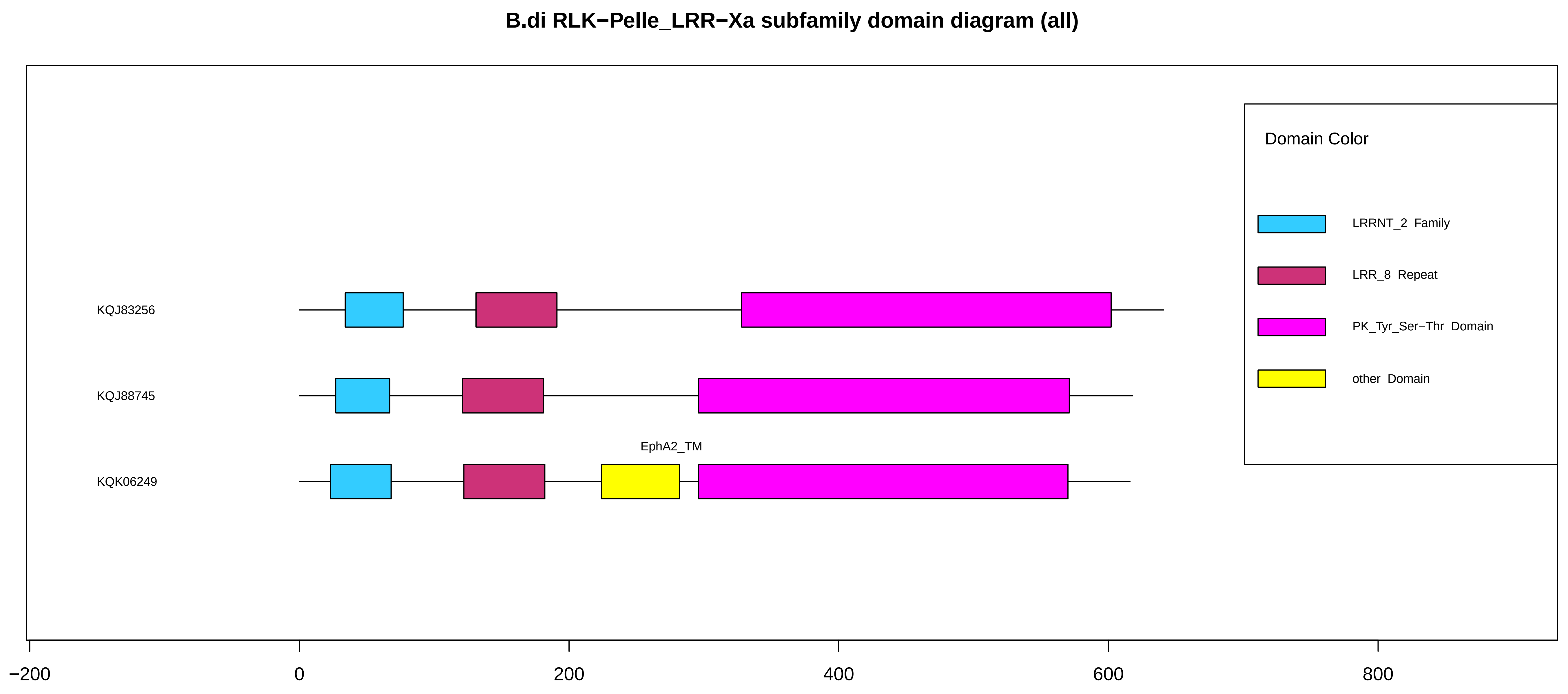
B.di RLK−Pelle_LRR−Xa subfamily domain diagram (all)
KQJ83256
KQJ88745
KQK06249
EphA2_TM
Domain Color
LRRNT_2 Family
LRR_8 Repeat
PK_Tyr_Ser−Thr Domain
other Domain
−200
0
200
400
600
800

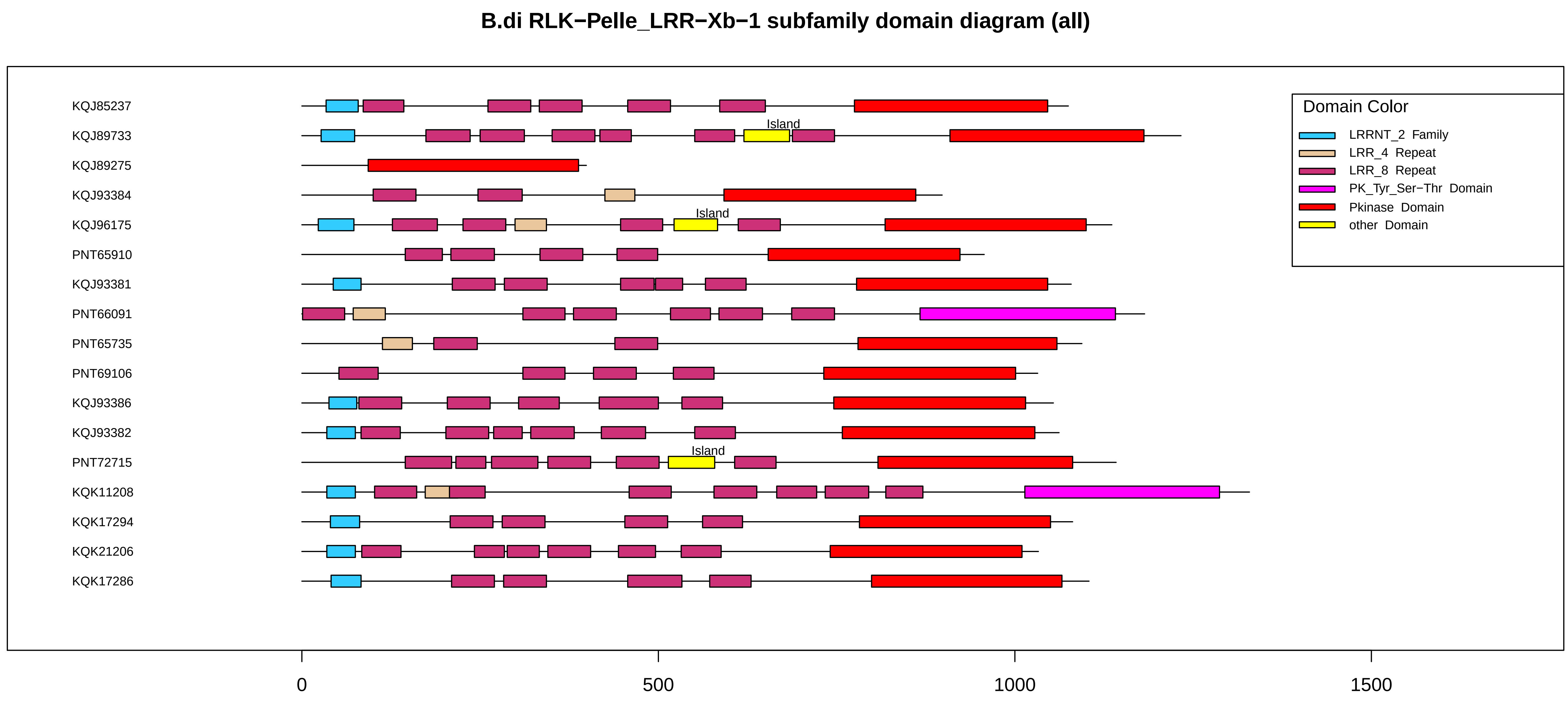
B.di RLK−Pelle_LRR−Xb−1 subfamily domain diagram (all)
KQJ85237
KQJ89733
KQJ89275
KQJ93384
KQJ96175
PNT65910
KQJ93381
PNT66091
PNT65735
PNT69106
KQJ93386
KQJ93382
PNT72715
KQK11208
KQK17294
KQK21206
KQK17286
Island
Island
Island
Domain Color
LRRNT_2 Family
LRR_4 Repeat
LRR_8 Repeat
PK_Tyr_Ser−Thr Domain
Pkinase Domain
other Domain
0
500
1000
1500

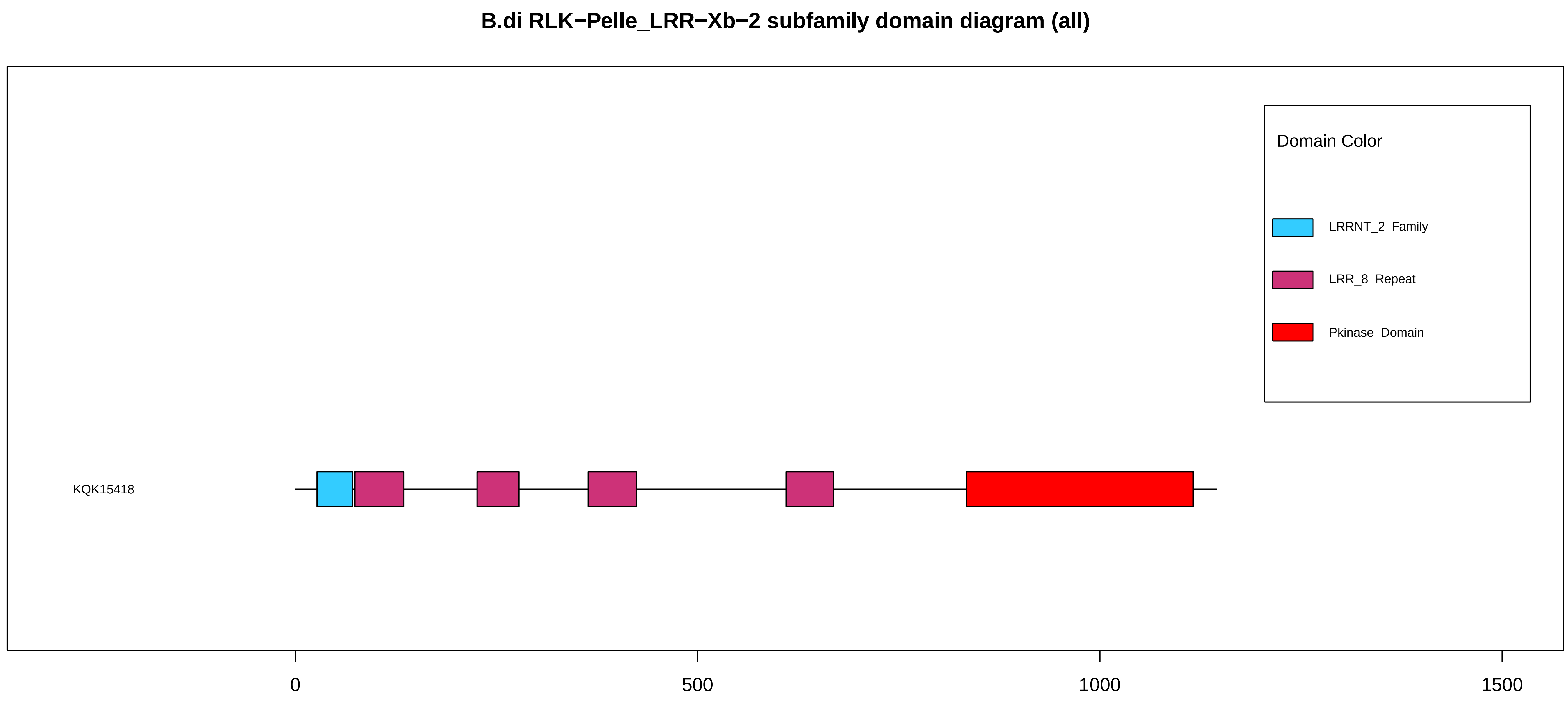
B.di RLK−Pelle_LRR−Xb−2 subfamily domain diagram (all)
Domain Color
LRRNT_2 Family
LRR_8 Repeat
Pkinase Domain
KQK15418
0
500
1000
1500

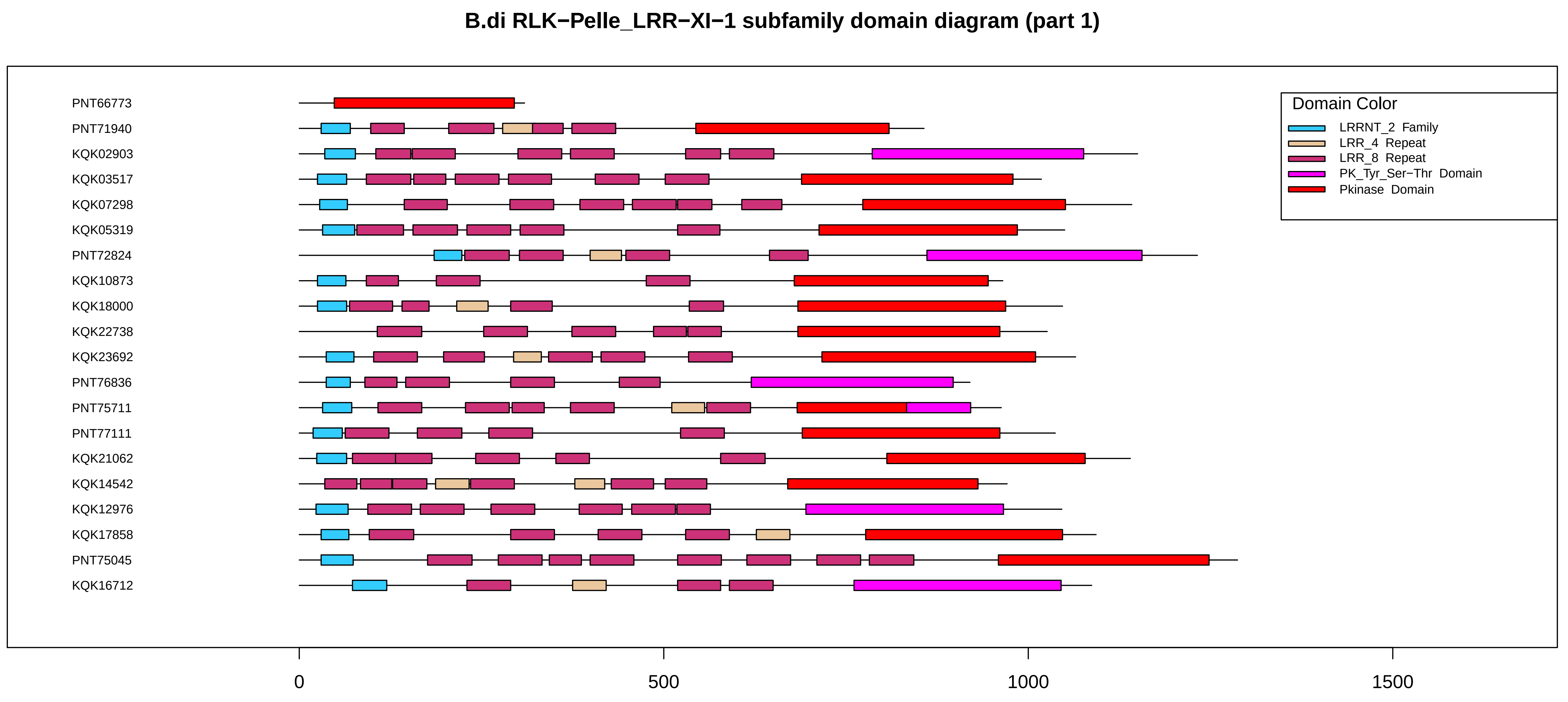
B.di RLK−Pelle_LRR−XI−1 subfamily domain diagram (part 1)
PNT66773
PNT71940
KQK02903
KQK03517
KQK07298
KQK05319
PNT72824
KQK10873
KQK18000
KQK22738
KQK23692
PNT76836
PNT75711
PNT77111
KQK21062
KQK14542
KQK12976
KQK17858
PNT75045
KQK16712
Domain Color
LRRNT_2 Family
LRR_4 Repeat
LRR_8 Repeat
PK_Tyr_Ser−Thr Domain
Pkinase Domain
0
500
1000
1500

B.di RLK−Pelle_LRR−XI−1 subfamily domain diagram (part 2)

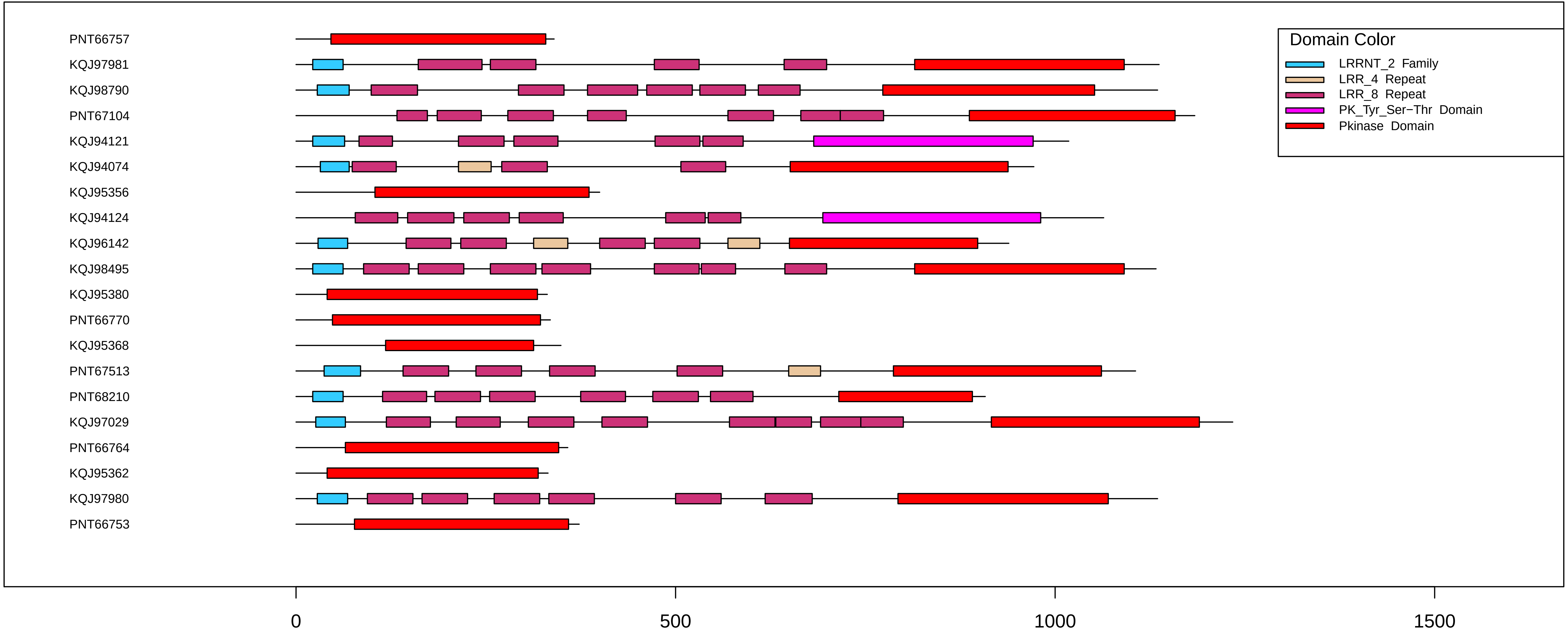

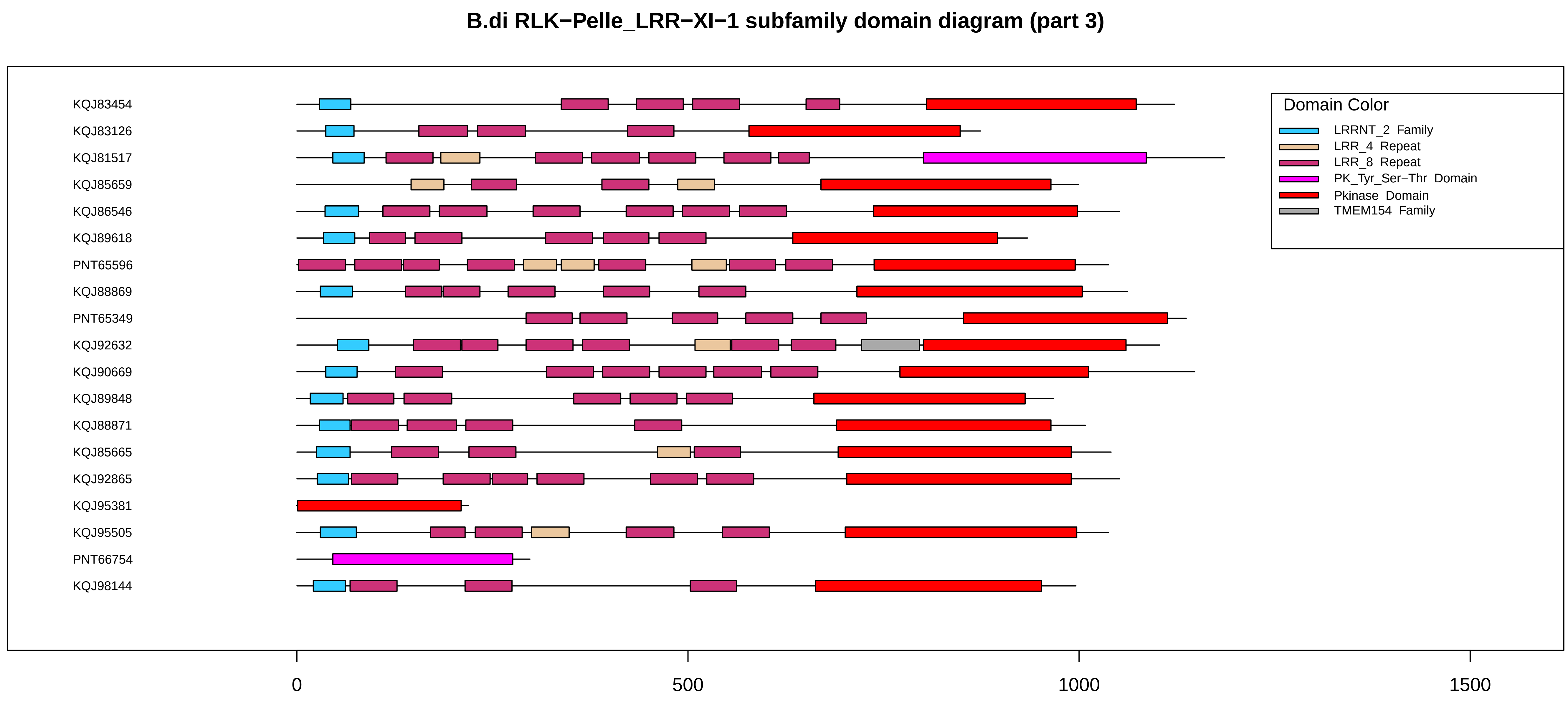
B.di RLK−Pelle_LRR−XI−1 subfamily domain diagram (part 3)
KQJ83454
KQJ83126
KQJ81517
KQJ85659
KQJ86546
KQJ89618
PNT65596
KQJ88869
PNT65349
KQJ92632
KQJ90669
KQJ89848
KQJ88871
KQJ85665
KQJ92865
KQJ95381
KQJ95505
PNT66754
KQJ98144
Domain Color
LRRNT_2 Family
LRR_4 Repeat
LRR_8 Repeat
PK_Tyr_Ser−Thr Domain
Pkinase Domain
TMEM154 Family
0
500
1000
1500

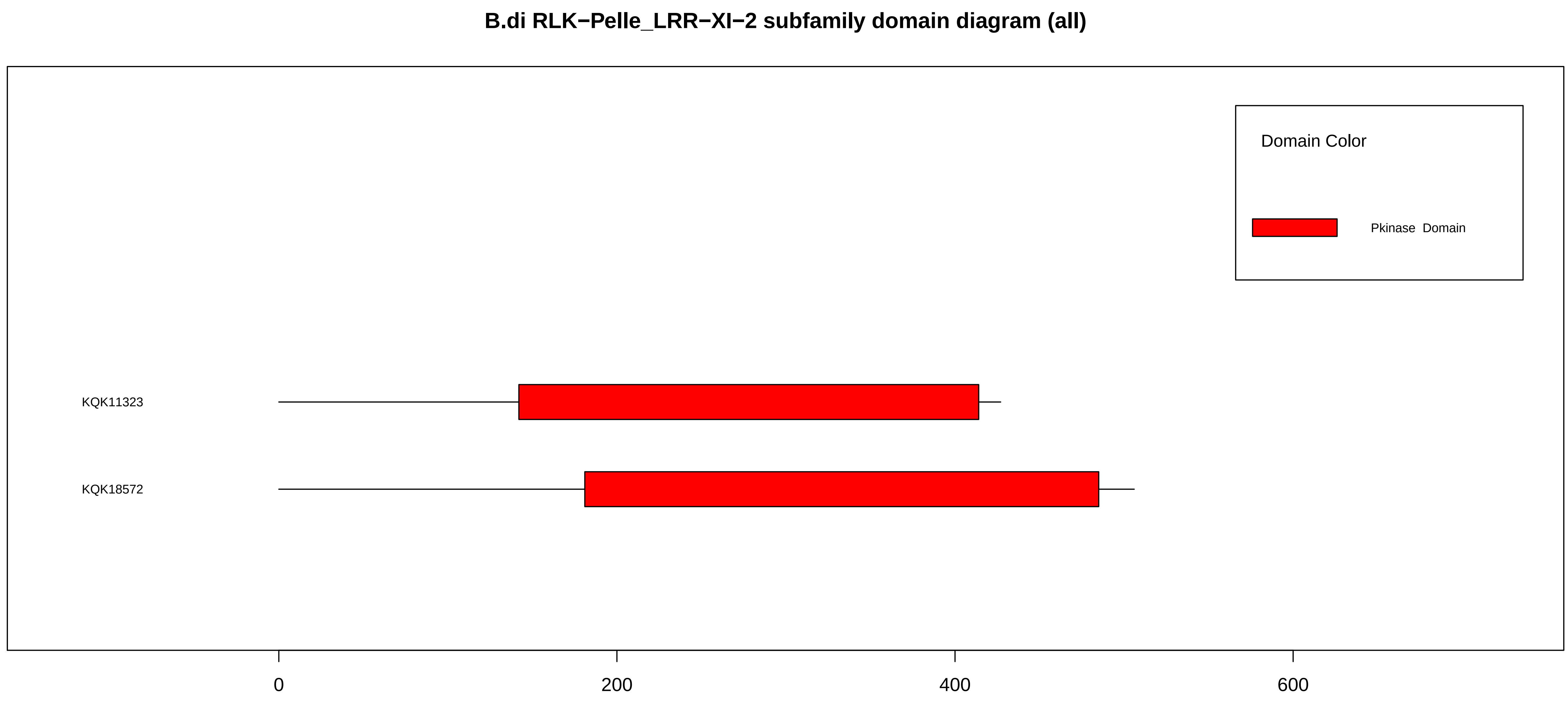

B.di RLK−Pelle_LRR−XI−2 subfamily domain diagram (all)
Domain Color
Pkinase Domain
KQK11323
KQK18572
0
200
400
600

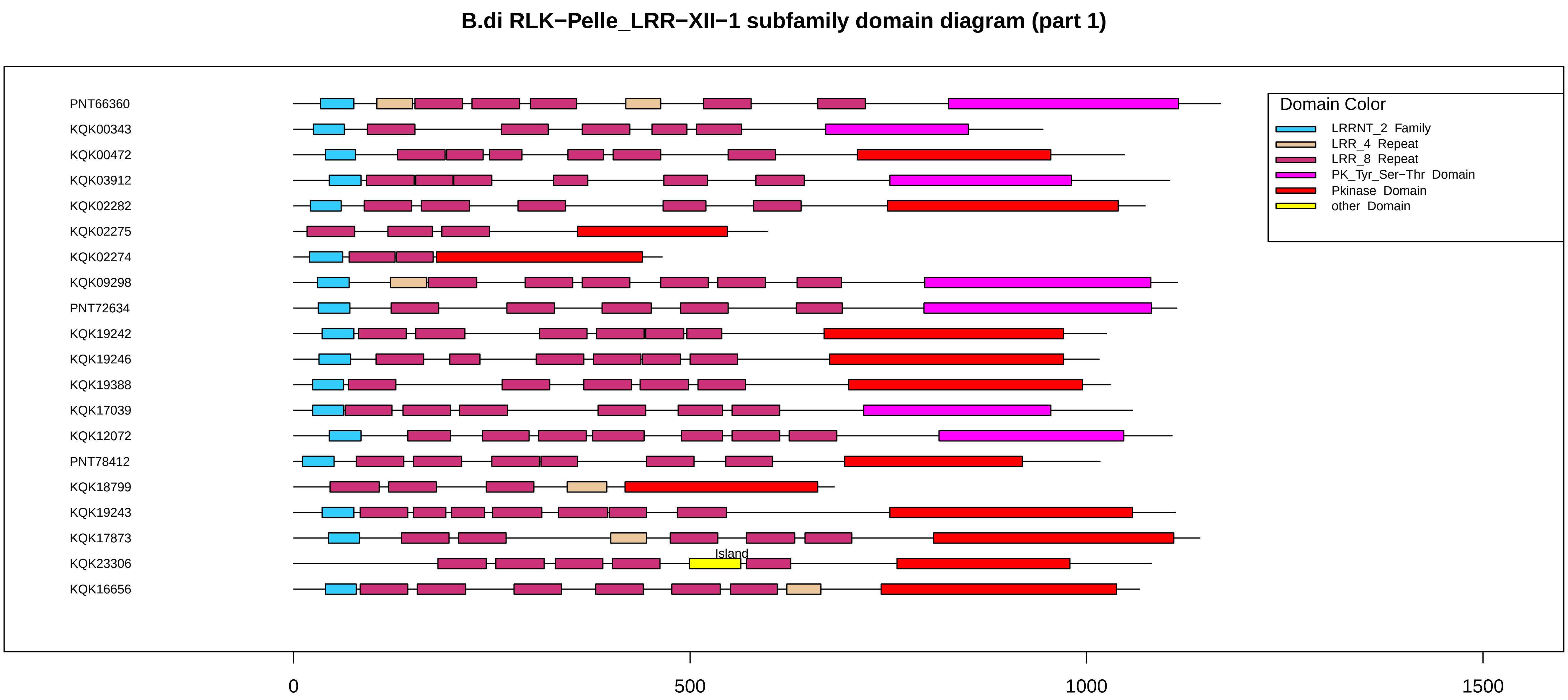
B.di RLK−Pelle_LRR−XII−1 subfamily domain diagram (part 1)
PNT66360
KQK00343
KQK00472
KQK03912
KQK02282
KQK02275
KQK02274
KQK09298
PNT72634
KQK19242
KQK19246
KQK19388
KQK17039
KQK12072
PNT78412
KQK18799
KQK19243
KQK17873
KQK23306
KQK16656
Island
Domain Color
LRRNT_2 Family
LRR_4 Repeat
LRR_8 Repeat
PK_Tyr_Ser−Thr Domain
Pkinase Domain
other Domain
0
500
1000
1500

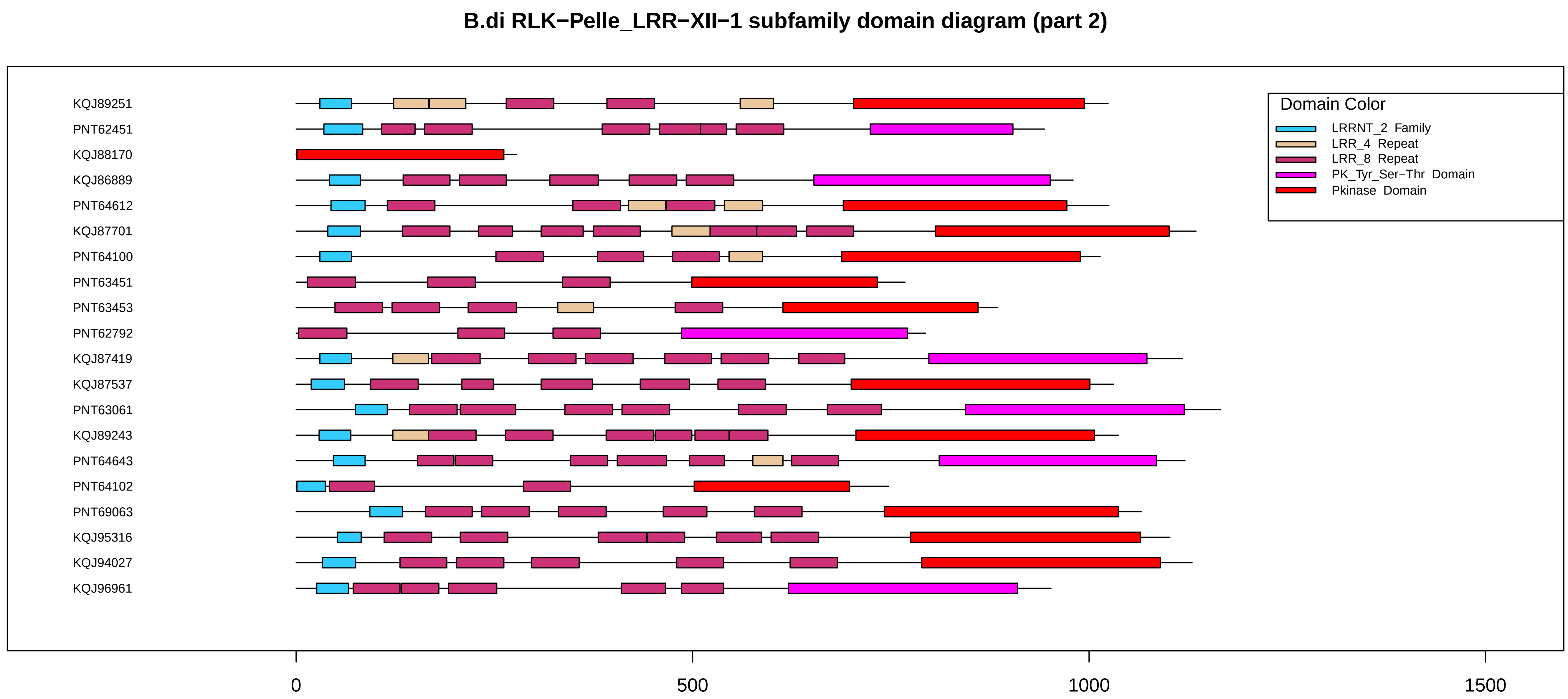

B.di RLK−Pelle_LRR−XII−1 subfamily domain diagram (part 2)
KQJ89251
PNT62451
KQJ88170
KQJ86889
PNT64612
KQJ87701
PNT64100
PNT63451
PNT63453
PNT62792
KQJ87419
KQJ87537
PNT63061
KQJ89243
PNT64643
PNT64102
PNT69063
KQJ95316
KQJ94027
KQJ96961
Domain Color
LRRNT_2 Family
LRR_4 Repeat
LRR_8 Repeat
PK_Tyr_Ser−Thr Domain
Pkinase Domain
0
500
1000
1500

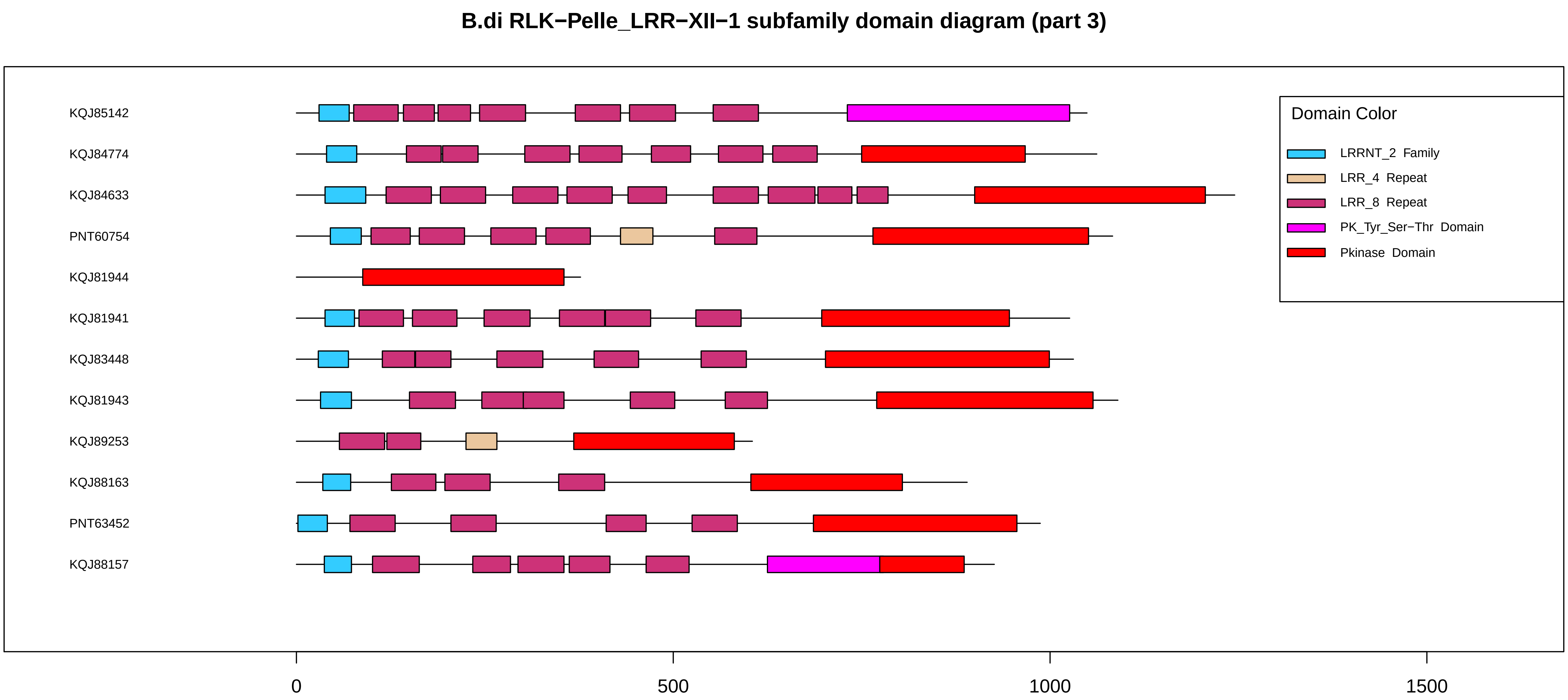
B.di RLK−Pelle_LRR−XII−1 subfamily domain diagram (part 3)
KQJ85142
KQJ84774
KQJ84633
PNT60754
KQJ81944
KQJ81941
KQJ83448
KQJ81943
KQJ89253
KQJ88163
PNT63452
KQJ88157
Domain Color
LRRNT_2 Family
LRR_4 Repeat
LRR_8 Repeat
PK_Tyr_Ser−Thr Domain
Pkinase Domain
0
500
1000
1500

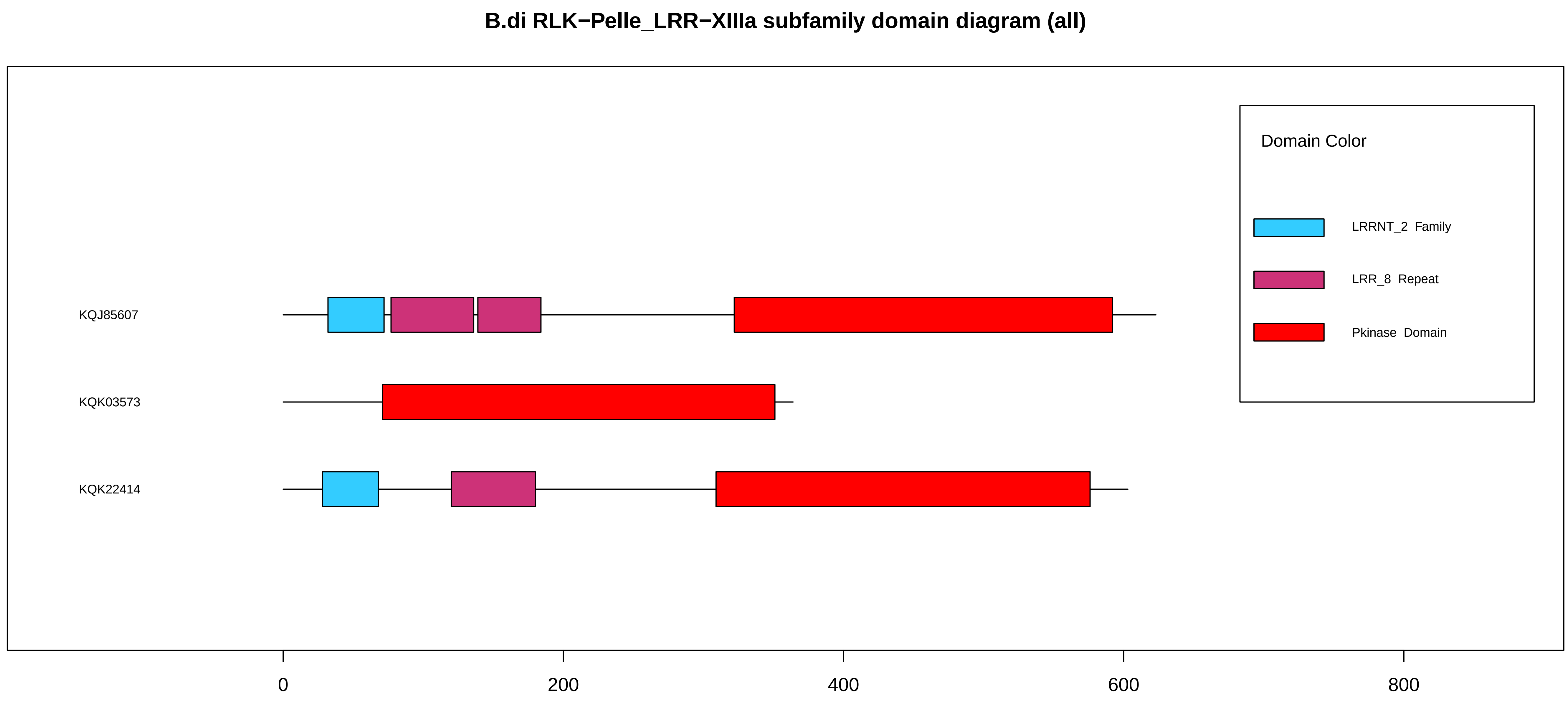
B.di RLK−Pelle_LRR−XIIIa subfamily domain diagram (all)
Domain Color
LRRNT_2 Family
LRR_8 Repeat
Pkinase Domain
KQJ85607
KQK03573
KQK22414
0
200
400
600
800

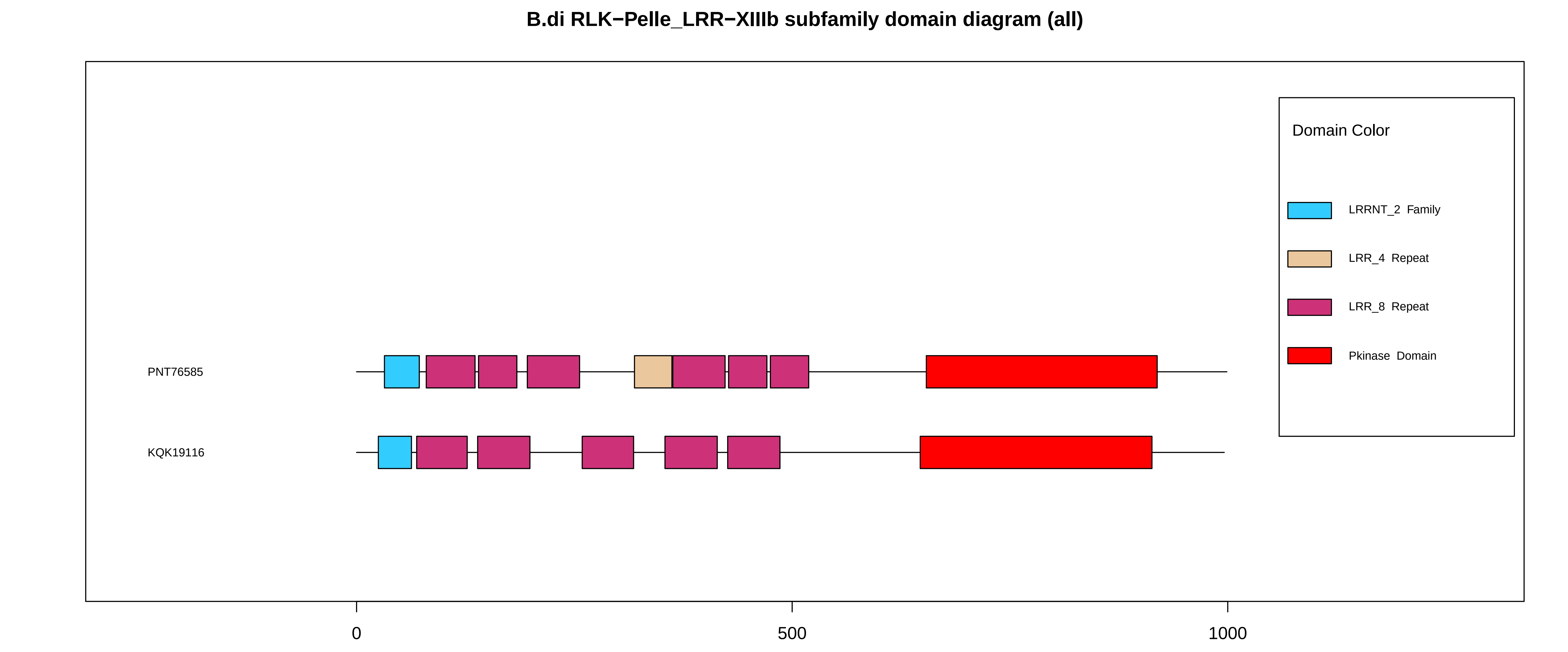
B.di RLK−Pelle_LRR−XIIIb subfamily domain diagram (all)
PNT76585
KQK19116
Domain Color
LRRNT_2 Family
LRR_4 Repeat
LRR_8 Repeat
Pkinase Domain
0
500
1000

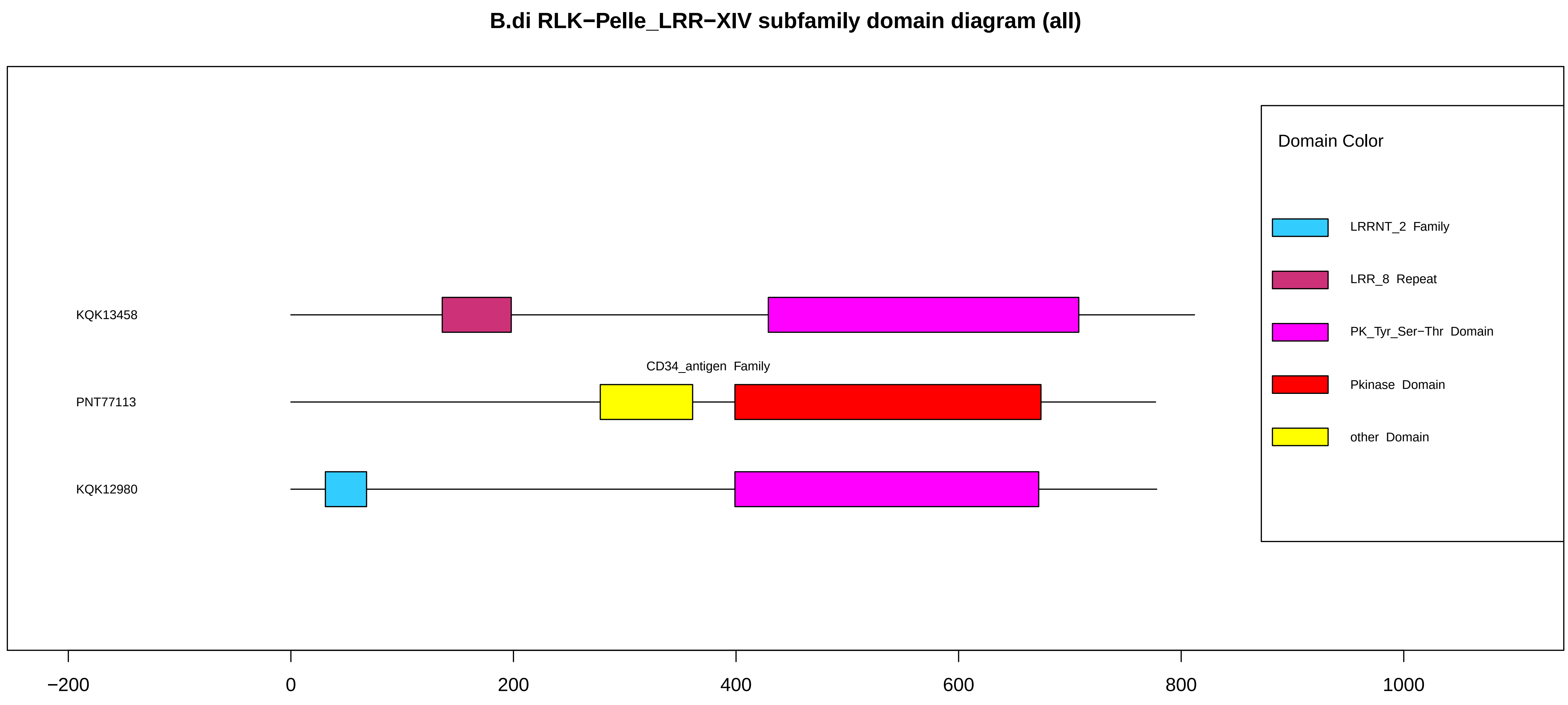
B.di RLK−Pelle_LRR−XIV subfamily domain diagram (all)
KQK13458
CD34_antigen Family
PNT77113
KQK12980
Domain Color
LRRNT_2 Family
LRR_8 Repeat
PK_Tyr_Ser−Thr Domain
Pkinase Domain
other Domain
−200
0
200
400
600
800
1000

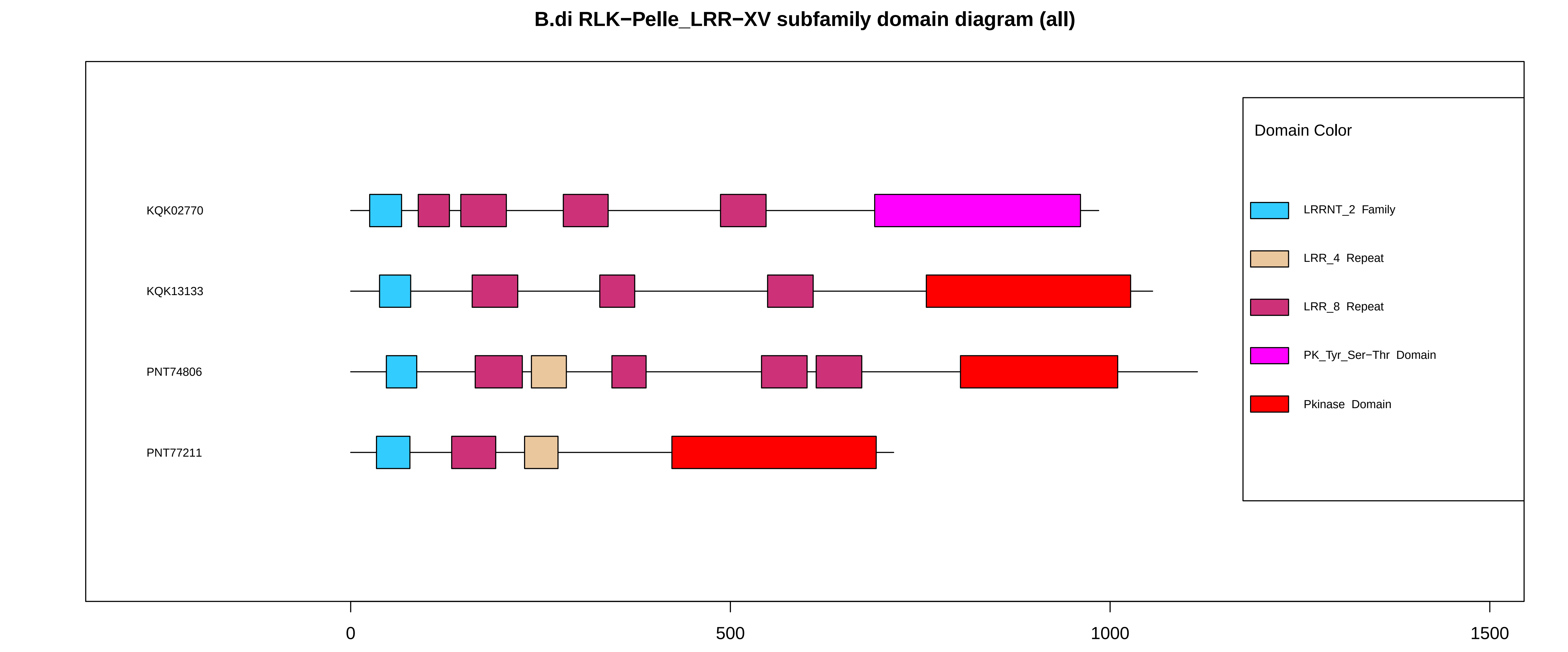
B.di RLK−Pelle_LRR−XV subfamily domain diagram (all)
KQK02770
KQK13133
PNT74806
PNT77211
Domain Color
LRRNT_2 Family
LRR_4 Repeat
LRR_8 Repeat
PK_Tyr_Ser−Thr Domain
Pkinase Domain
0
500
1000
1500

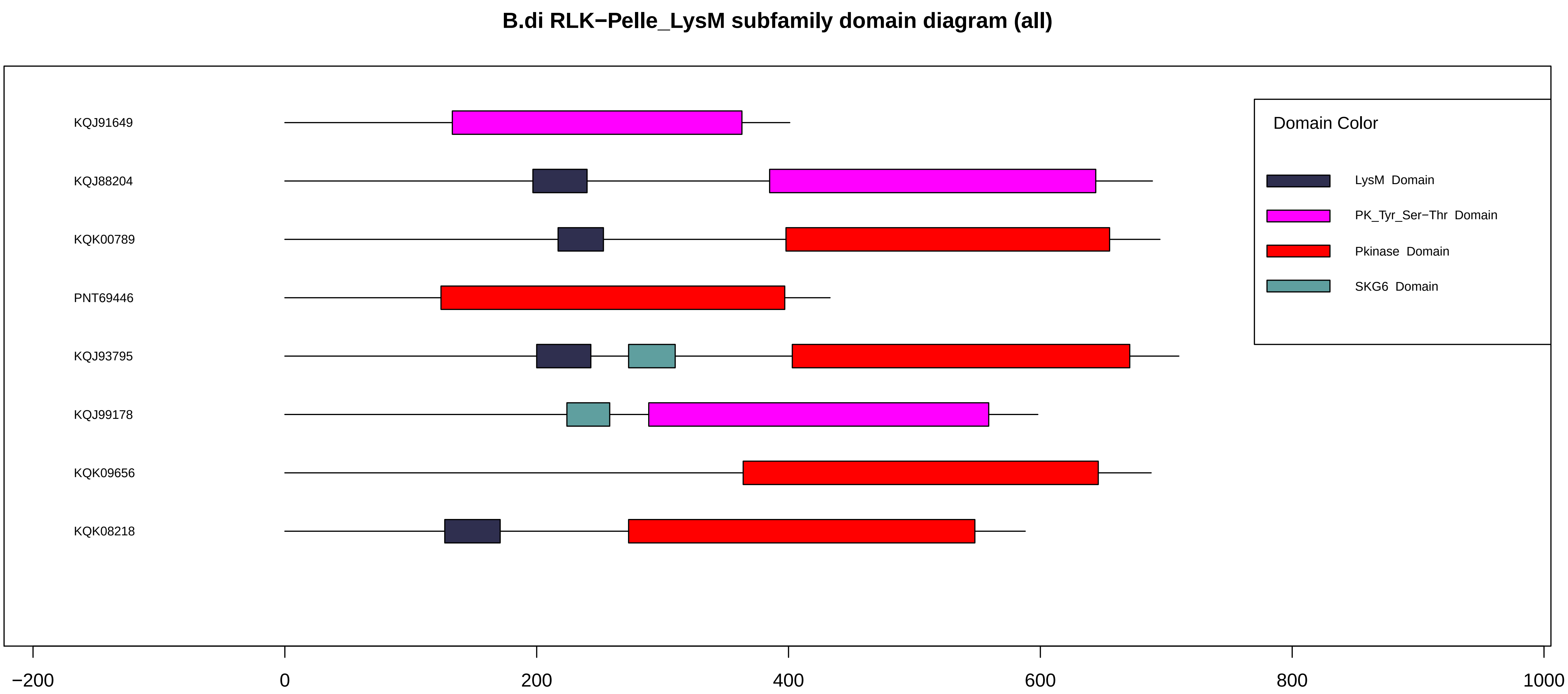
B.di RLK−Pelle_LysM subfamily domain diagram (all)
KQJ91649
KQJ88204
KQK00789
PNT69446
KQJ93795
KQJ99178
KQK09656
KQK08218
Domain Color
LysM Domain
PK_Tyr_Ser−Thr Domain
Pkinase Domain
SKG6 Domain
−200
0
200
400
600
800
1000

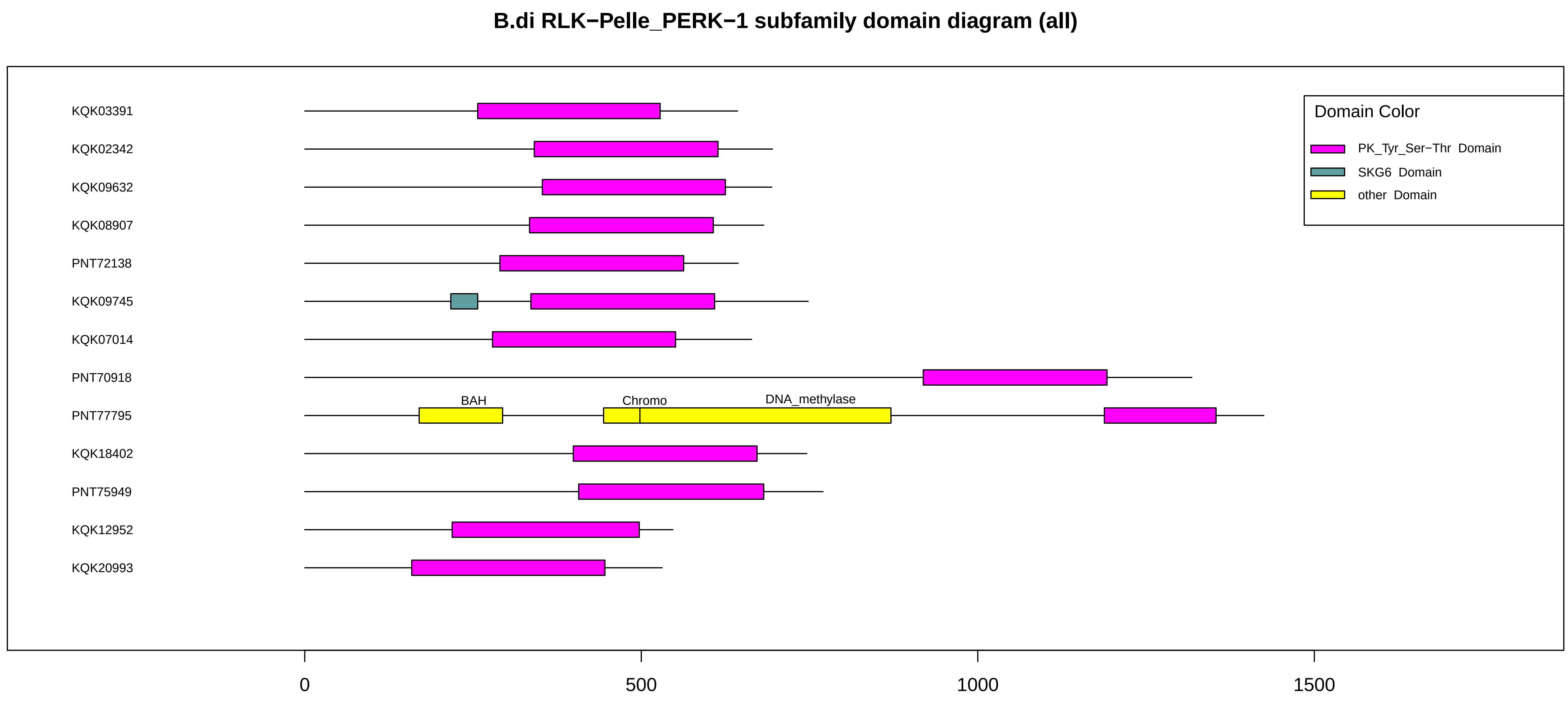

B.di RLK−Pelle_PERK−1 subfamily domain diagram (all)
KQK03391
KQK02342
KQK09632
KQK08907
PNT72138
KQK09745
KQK07014
PNT70918
PNT77795
KQK18402
PNT75949
KQK12952
KQK20993
BAH
Chromo
DNA_methylase
Domain Color
PK_Tyr_Ser−Thr Domain
SKG6 Domain
other Domain
0
500
1000
1500

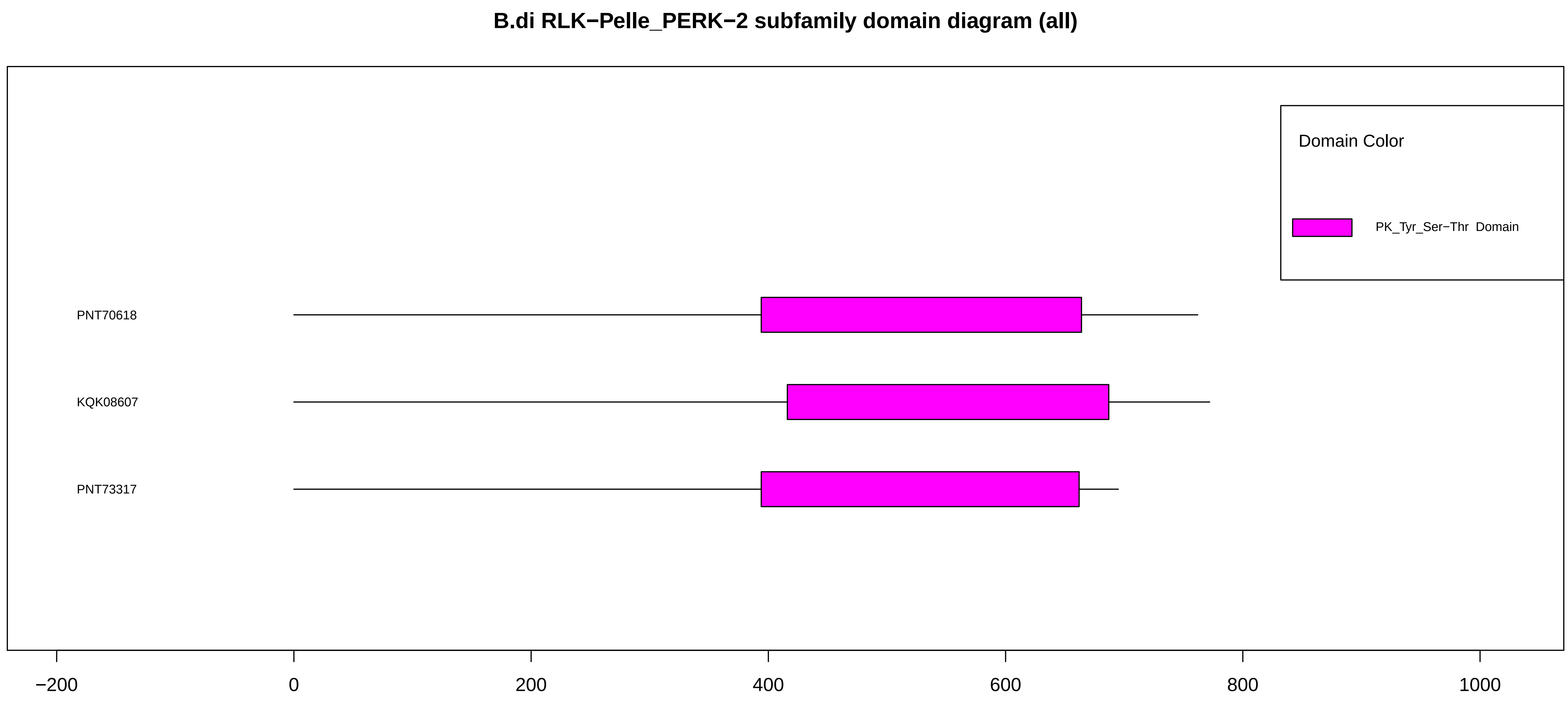

B.di RLK−Pelle_PERK−2 subfamily domain diagram (all)
Domain Color
PK_Tyr_Ser−Thr Domain
PNT70618
KQK08607
PNT73317
−200
0
200
400
600
800
1000

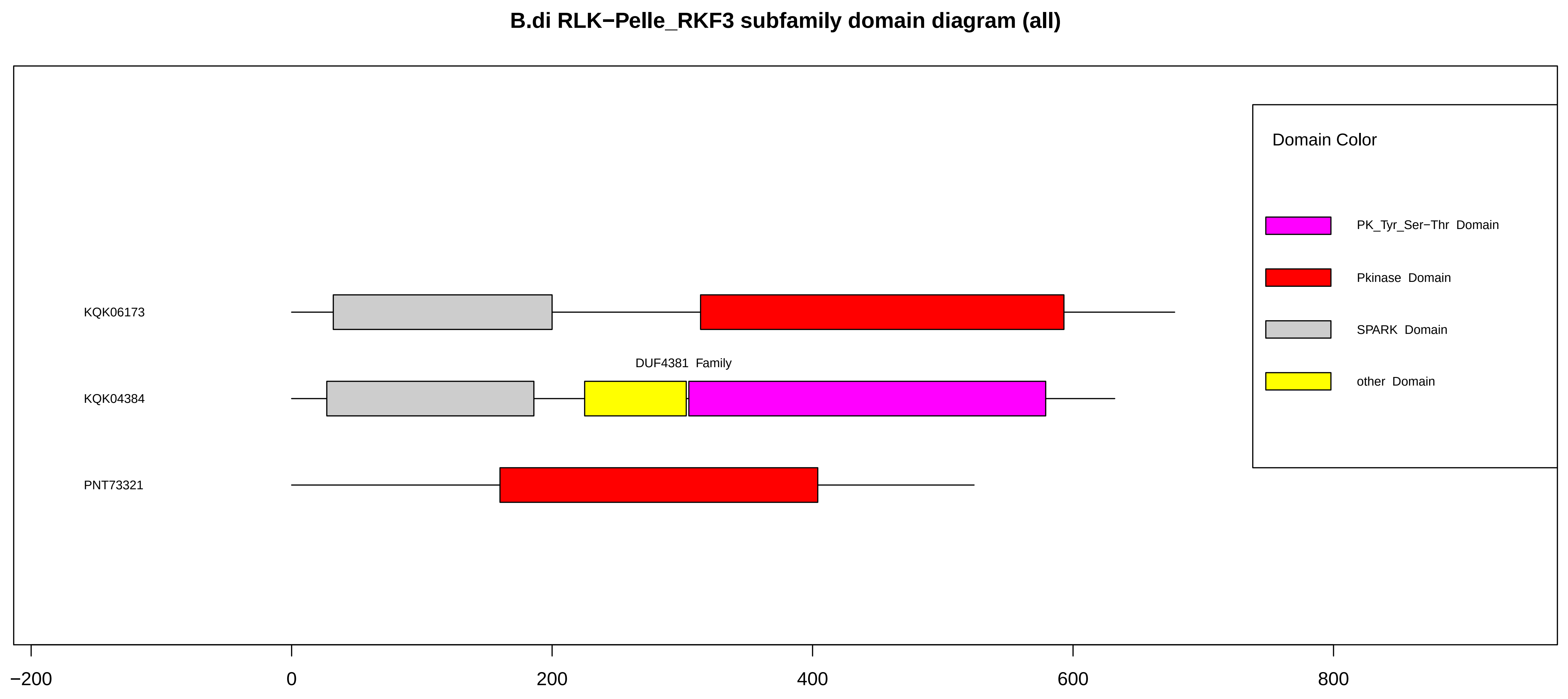

B.di RLK−Pelle_RKF3 subfamily domain diagram (all)
KQK06173
KQK04384
PNT73321
DUF4381 Family
Domain Color
PK_Tyr_Ser−Thr Domain
Pkinase Domain
SPARK Domain
other Domain
−200
0
200
400
600
800

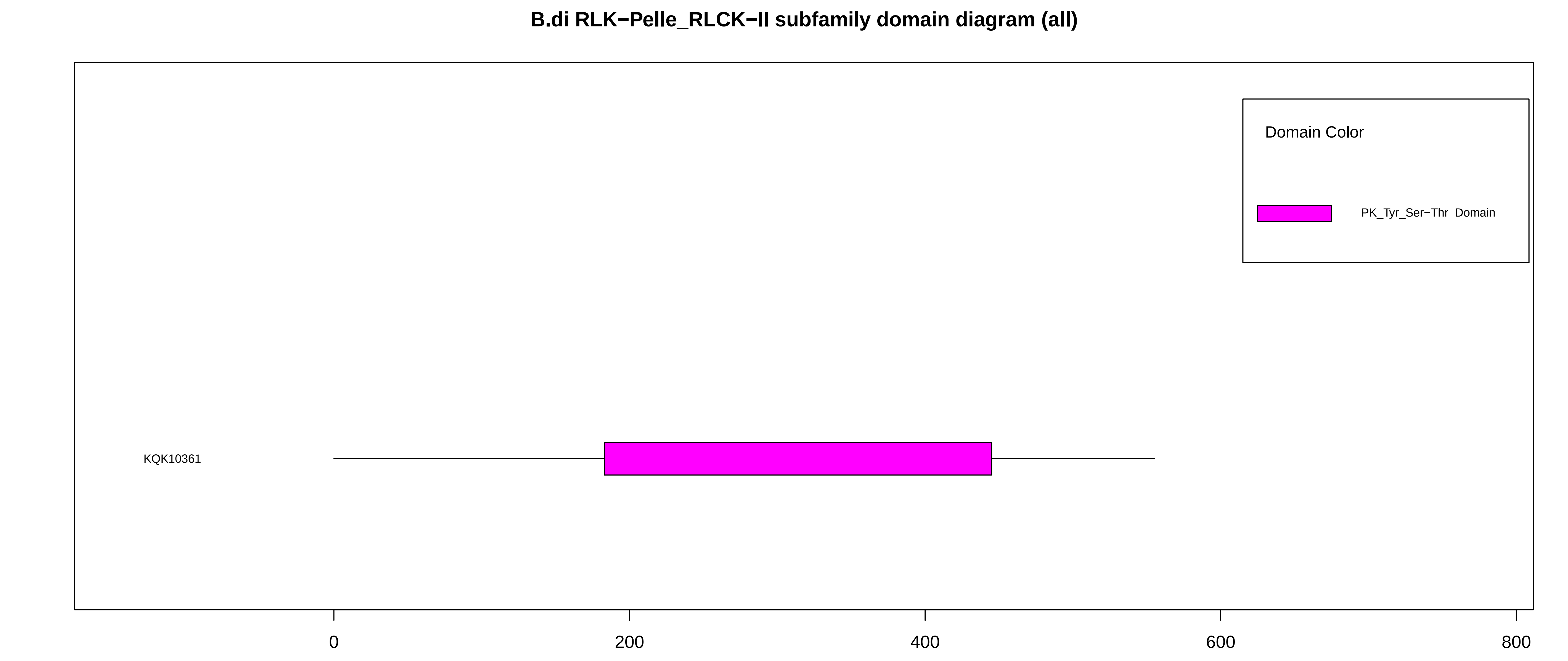
B.di RLK−Pelle_RLCK−II subfamily domain diagram (all)
Domain Color
PK_Tyr_Ser−Thr Domain
KQK10361
0
200
400
600
800

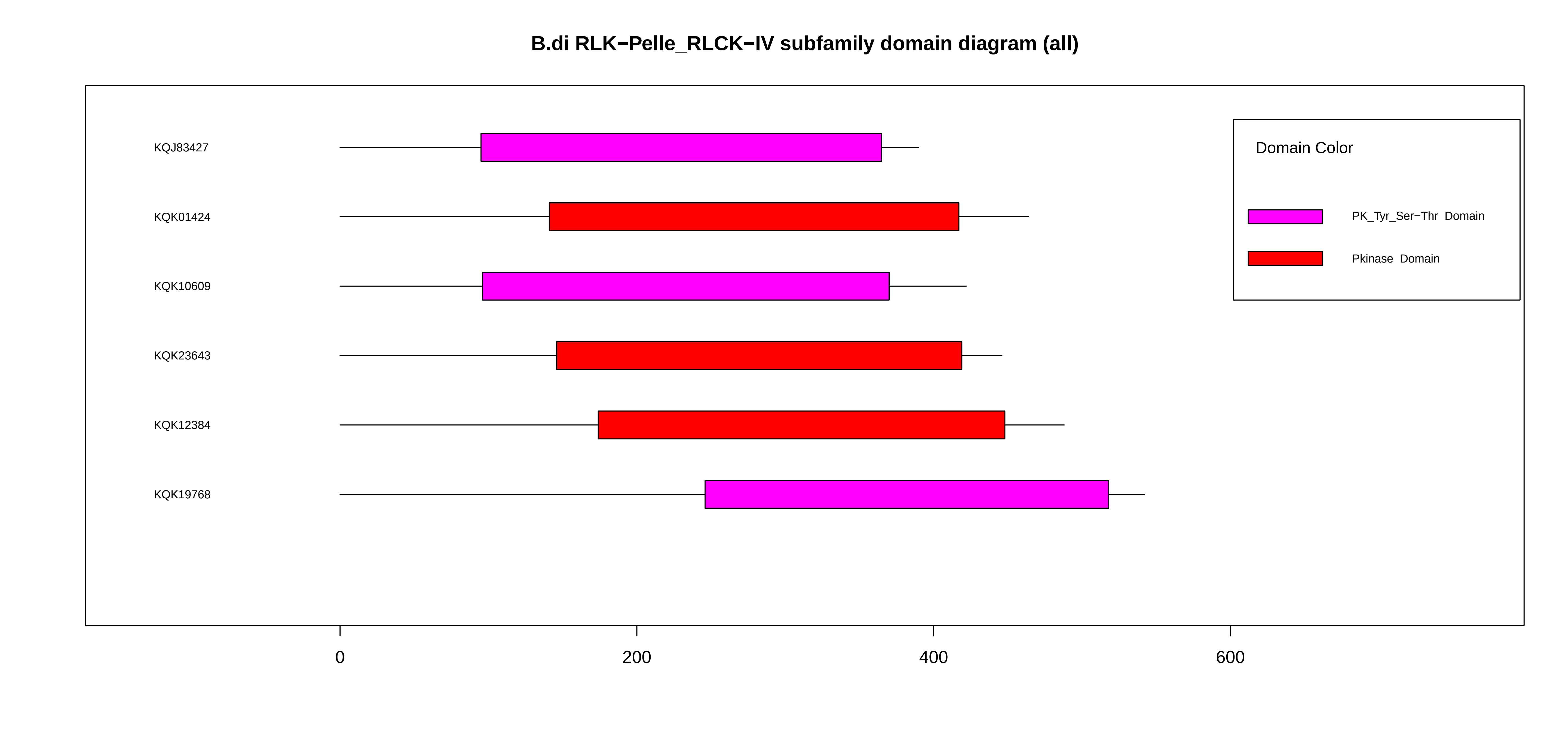
B.di RLK−Pelle_RLCK−IV subfamily domain diagram (all)
KQJ83427
KQK01424
KQK10609
KQK23643
KQK12384
KQK19768
Domain Color
PK_Tyr_Ser−Thr Domain
Pkinase Domain
0
200
400
600

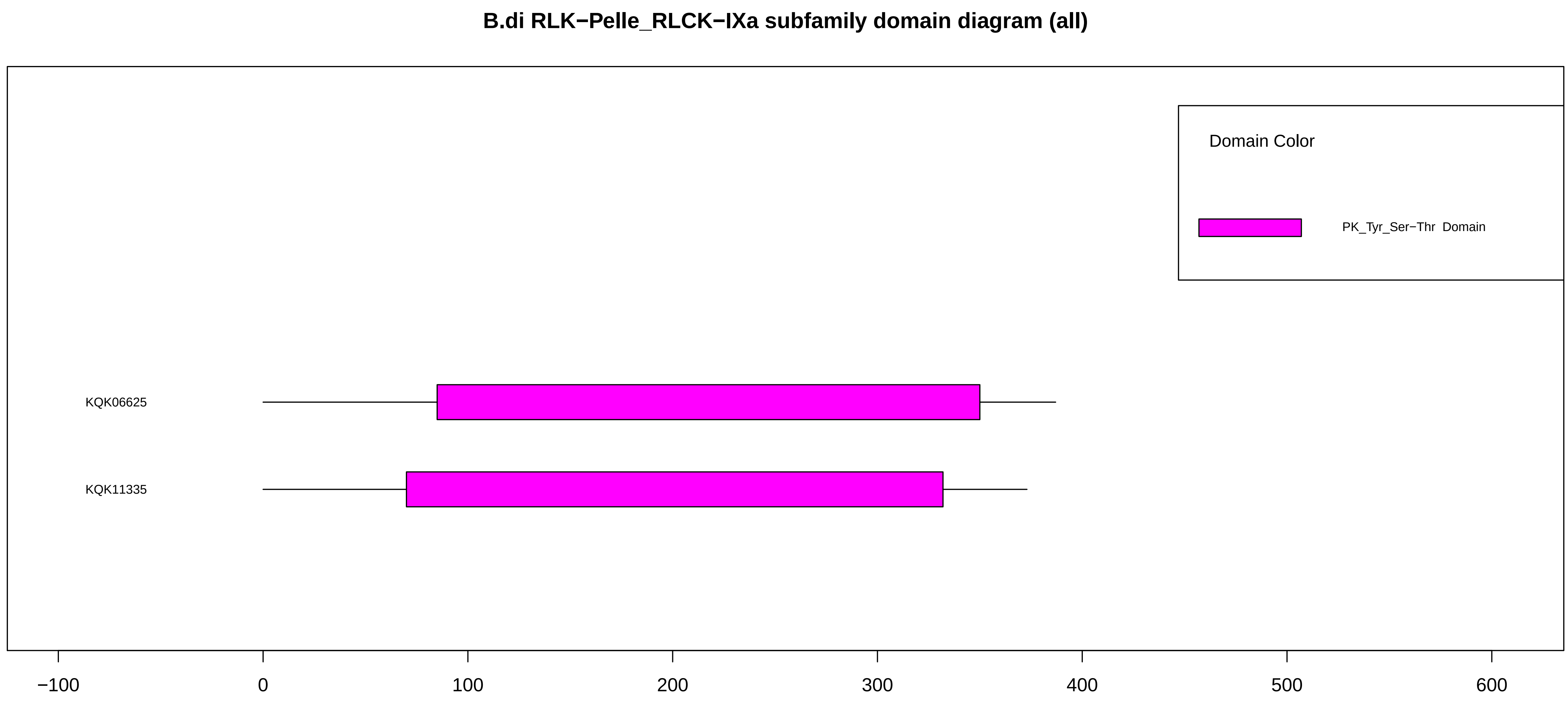

B.di RLK−Pelle_RLCK−IXa subfamily domain diagram (all)
Domain Color
PK_Tyr_Ser−Thr Domain
KQK06625
KQK11335
−100
0
100
200
300
400
500
600

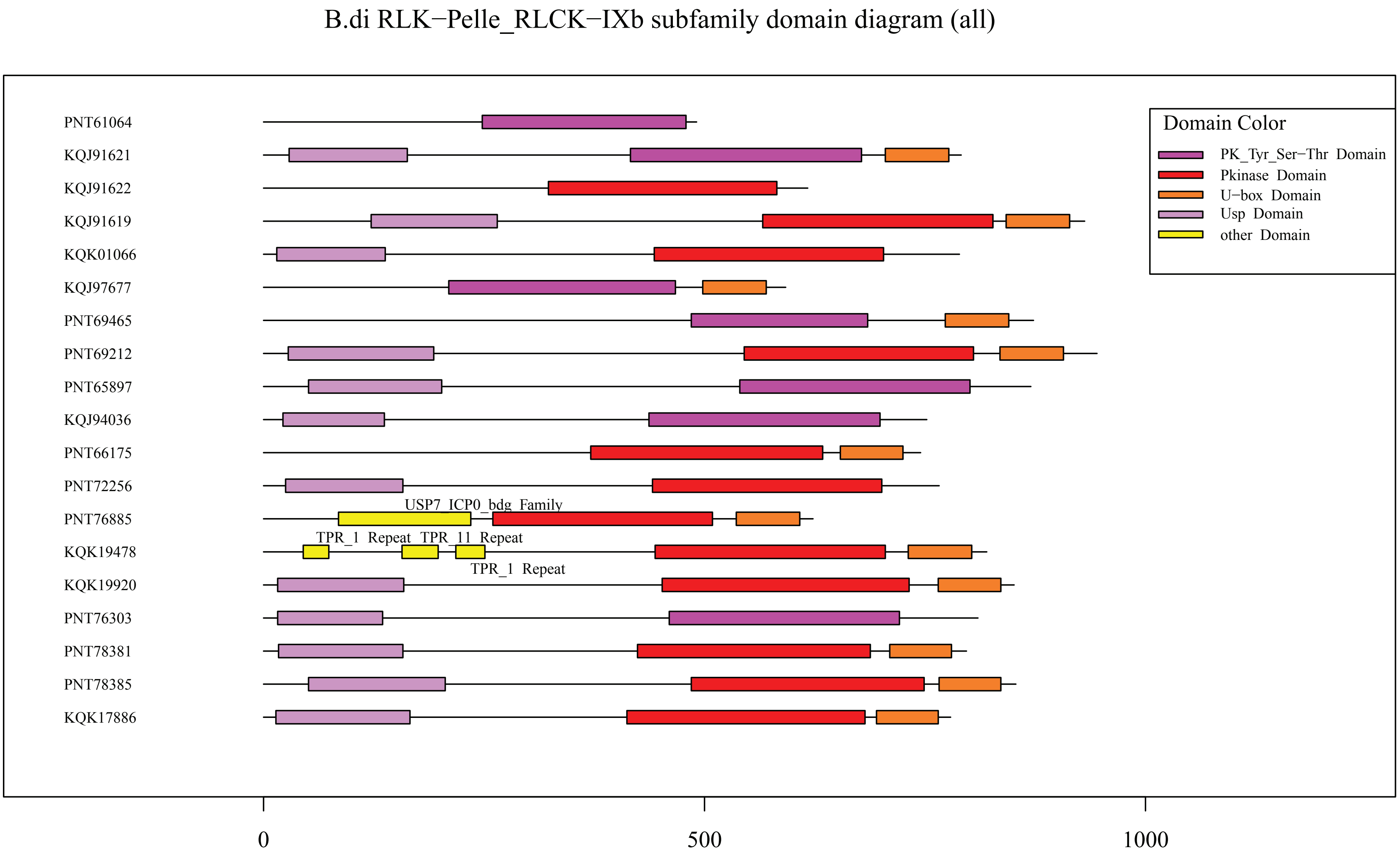

B.di RLK−Pelle_RLCK−IXb subfamily domain diagram (all)
PNT61064
KQJ91621
KQJ91622
KQJ91619
KQK01066
KQJ97677
PNT69465
PNT69212
PNT65897
KQJ94036
PNT66175
PNT72256
PNT76885
KQK19478
KQK19920
PNT76303
PNT78381
PNT78385
KQK17886
USP7_ICP0_bdg_Family
TPR_1_Repeat
TPR_11_Repeat
TPR_1_Repeat
Domain Color
PK_Tyr_Ser−Thr Domain
Pkinase Domain
U−box Domain
Usp Domain
other Domain
0
500
1000

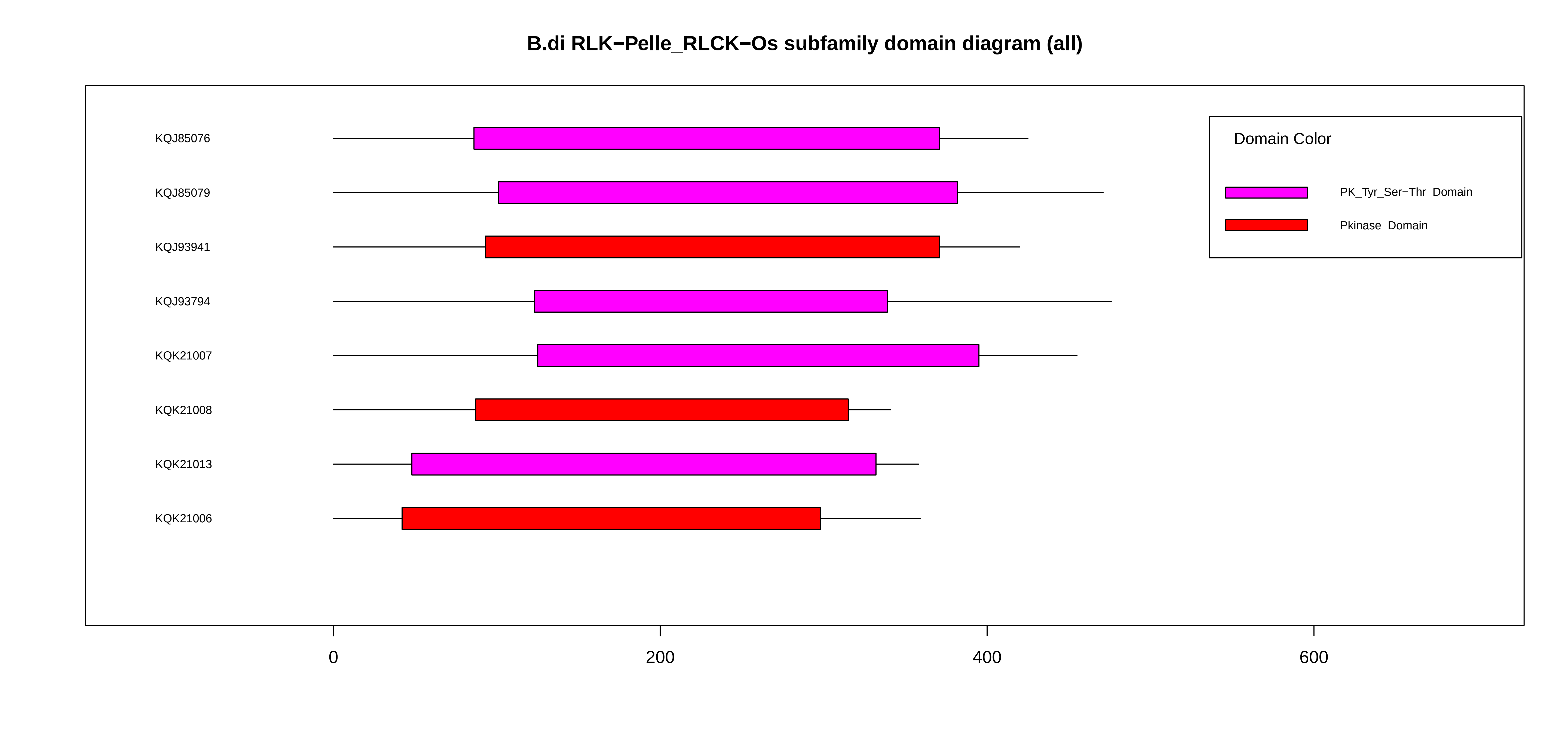

B.di RLK−Pelle_RLCK−Os subfamily domain diagram (all)
KQJ85076
KQJ85079
KQJ93941
KQJ93794
KQK21007
KQK21008
KQK21013
KQK21006
Domain Color
PK_Tyr_Ser−Thr Domain
Pkinase Domain
0
200
400
600

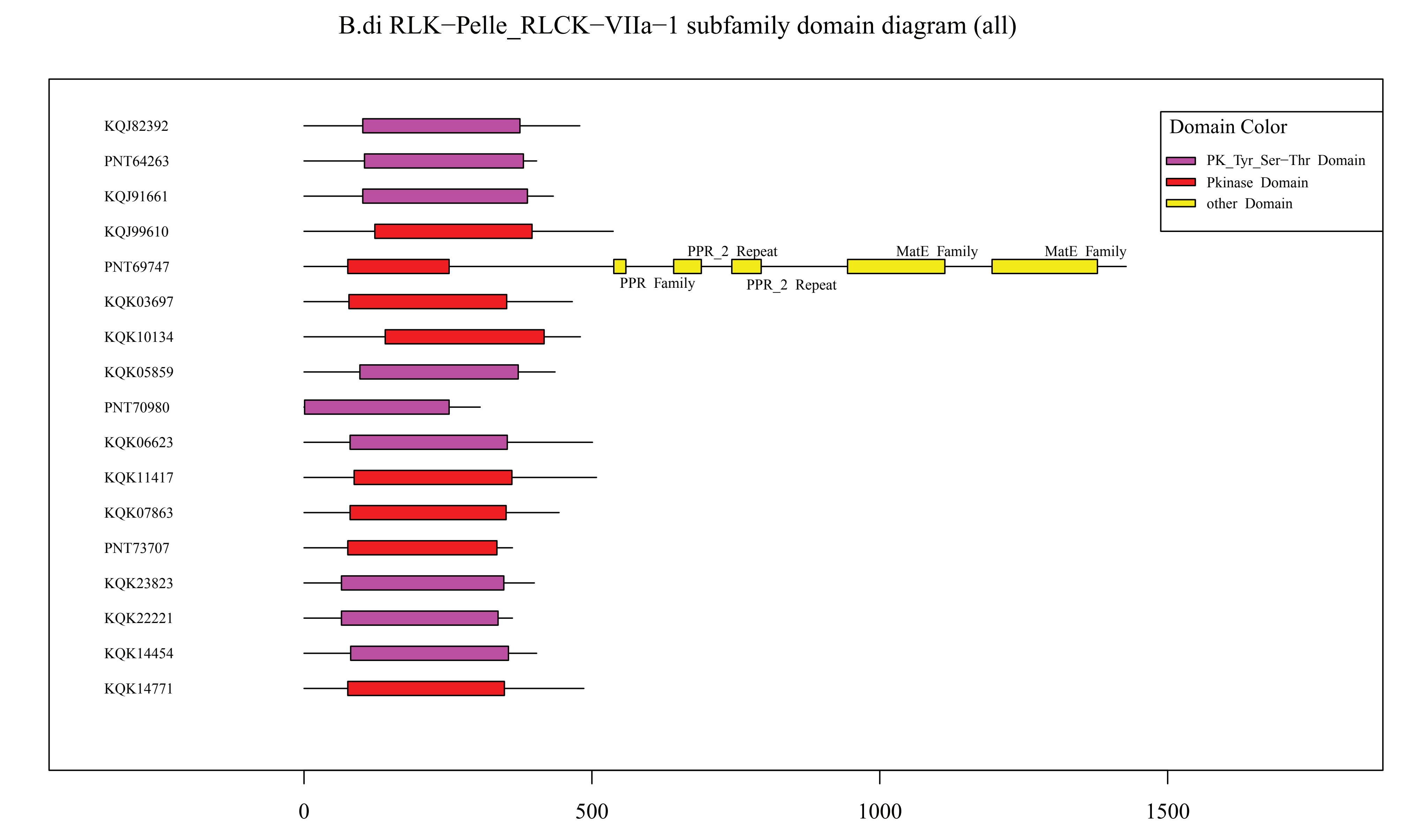
B.di RLK−Pelle_RLCK−VIIa−1 subfamily domain diagram (all)
KQJ82392
PNT64263
KQJ91661
KQJ99610
PNT69747
KQK03697
KQK10134
KQK05859
PNT70980
KQK06623
KQK11417
KQK07863
PNT73707
KQK23823
KQK22221
KQK14454
KQK14771
PPR Family
PPR_2 Repeat
PPR_2 Repeat
MatE Family
MatE Family
Domain Color
PK_Tyr_Ser−Thr Domain
Pkinase Domain
other Domain
0
500
1000
1500

B.di RLK−Pelle_RLCK−VIIa−2 subfamily domain diagram (part 1)

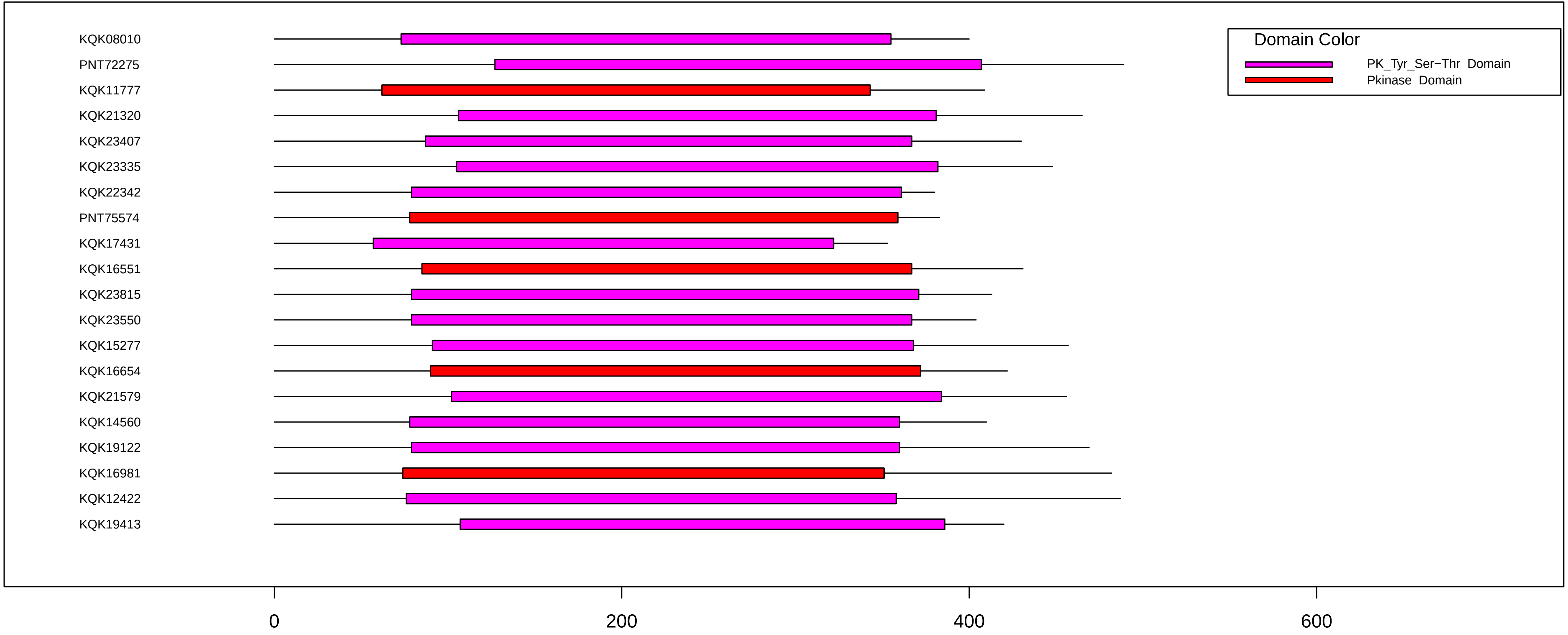

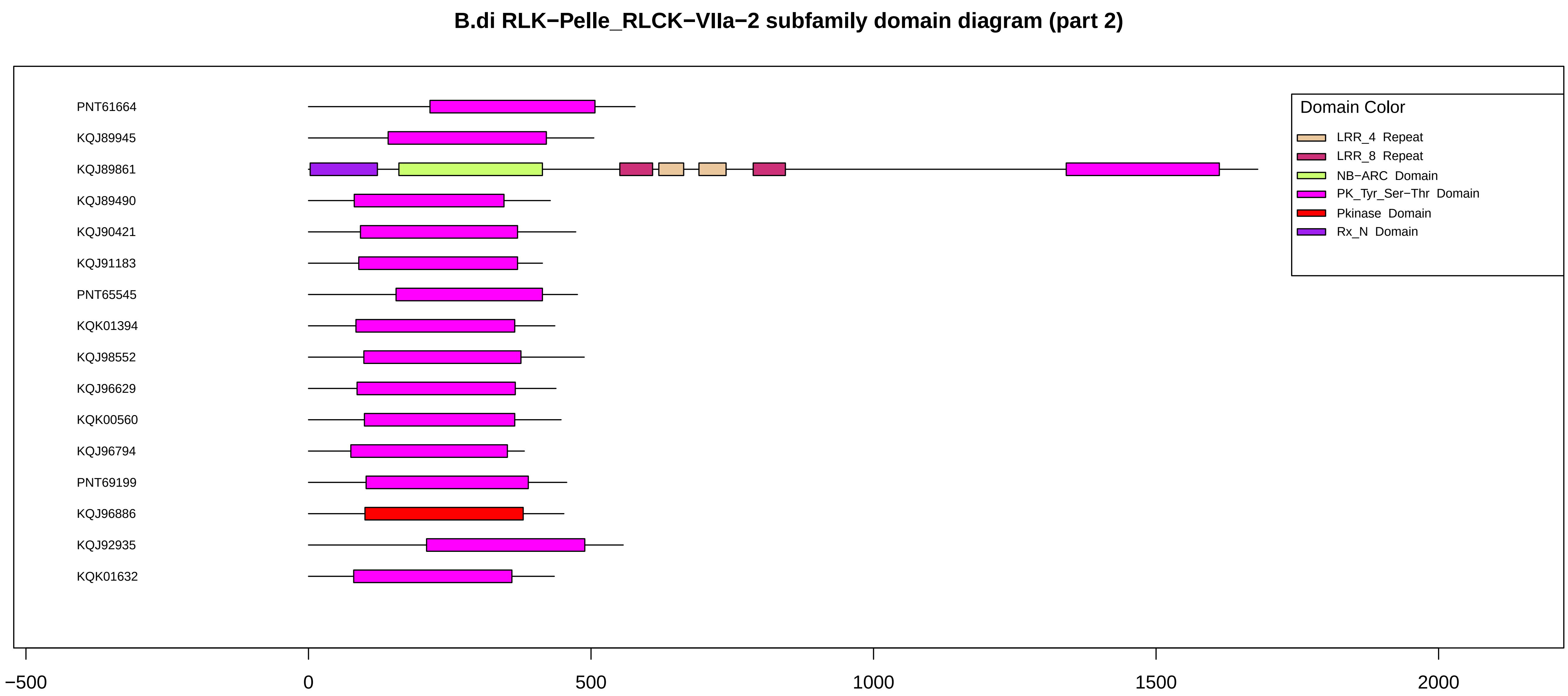
B.di RLK−Pelle_RLCK−VIIa−2 subfamily domain diagram (part 2)
PNT61664
KQJ89945
KQJ89861
KQJ89490
KQJ90421
KQJ91183
PNT65545
KQK01394
KQJ98552
KQJ96629
KQK00560
KQJ96794
PNT69199
KQJ96886
KQJ92935
KQK01632
Domain Color
LRR_4 Repeat
LRR_8 Repeat
NB−ARC Domain
PK_Tyr_Ser−Thr Domain
Pkinase Domain
Rx_N Domain
−500
0
500
1000
1500
2000

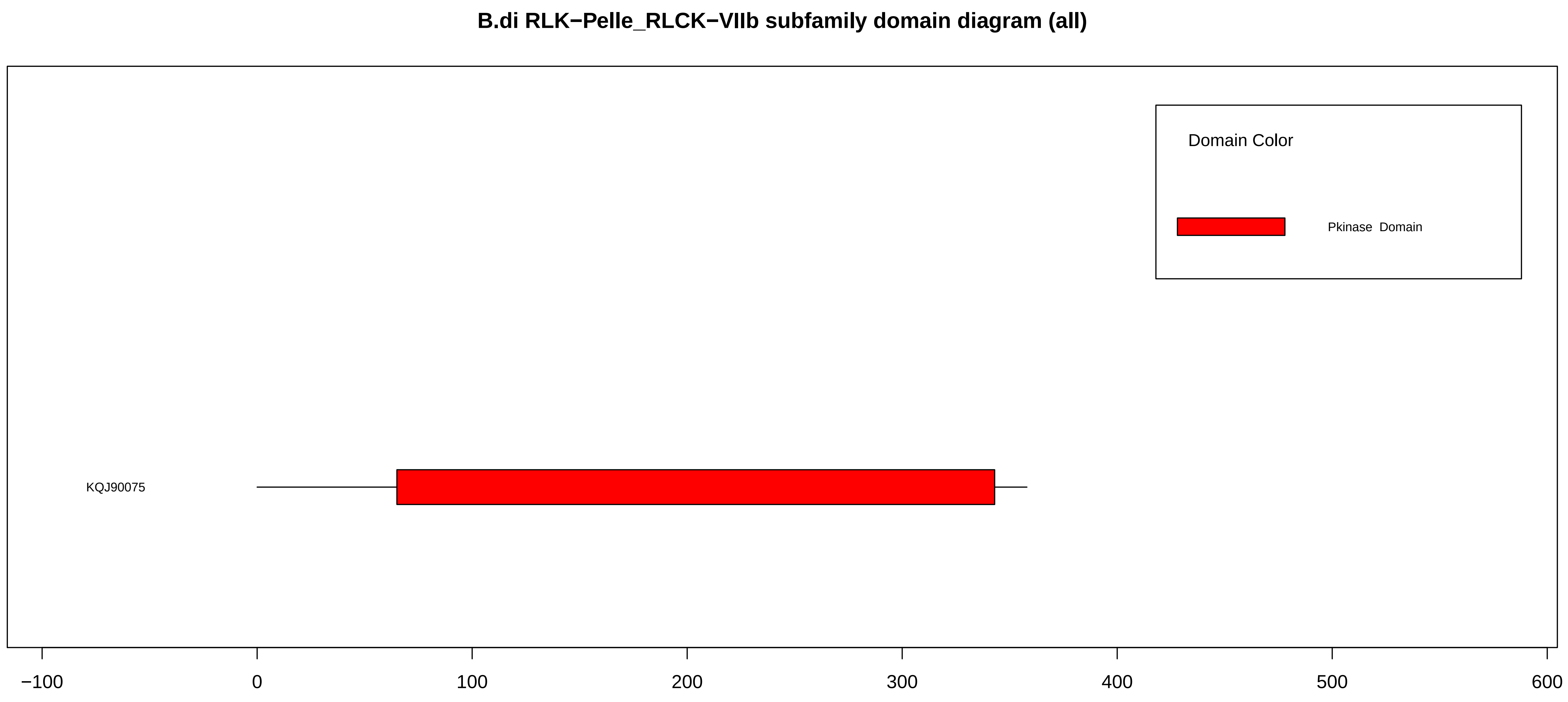
B.di RLK−Pelle_RLCK−VIIb subfamily domain diagram (all)
Domain Color
Pkinase Domain
KQJ90075
−100
0
100
200
300
400
500
600

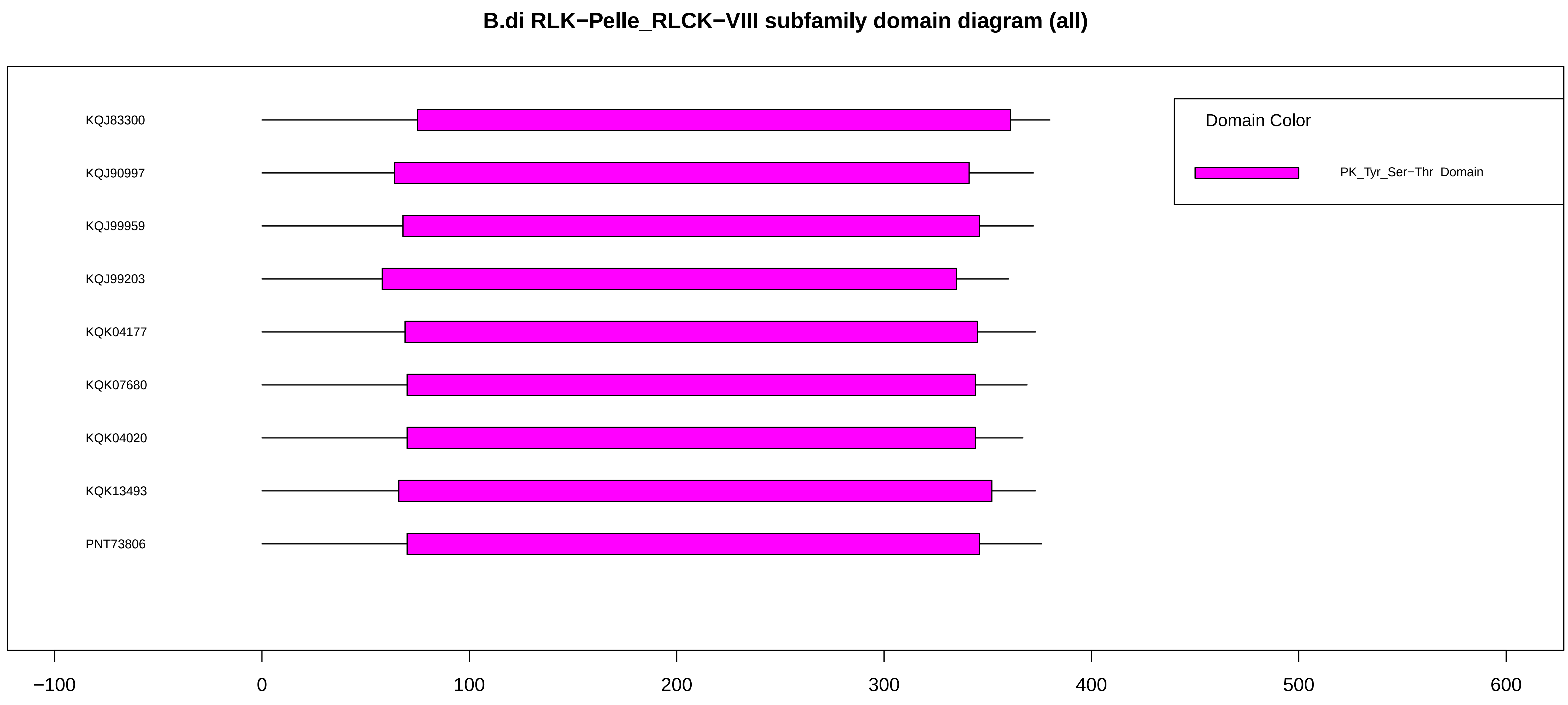
B.di RLK−Pelle_RLCK−VIII subfamily domain diagram (all)
KQJ83300
KQJ90997
KQJ99959
KQJ99203
KQK04177
KQK07680
KQK04020
KQK13493
PNT73806
Domain Color
PK_Tyr_Ser−Thr Domain
−100
0
100
200
300
400
500
600

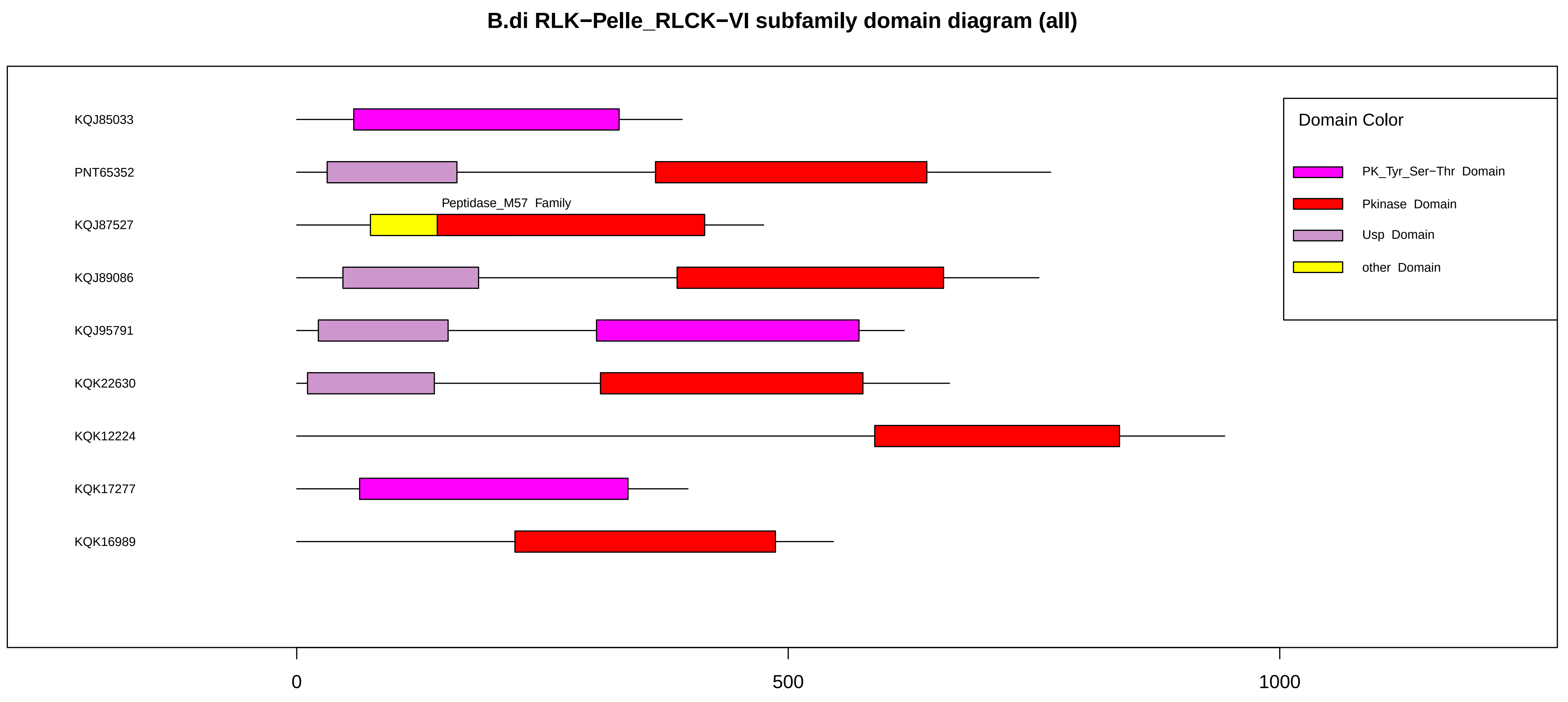
B.di RLK−Pelle_RLCK−VI subfamily domain diagram (all)
KQJ85033
PNT65352
KQJ87527
KQJ89086
KQJ95791
KQK22630
KQK12224
KQK17277
KQK16989
Peptidase_M57 Family
Domain Color
PK_Tyr_Ser−Thr Domain
Pkinase Domain
Usp Domain
other Domain
0
500
1000

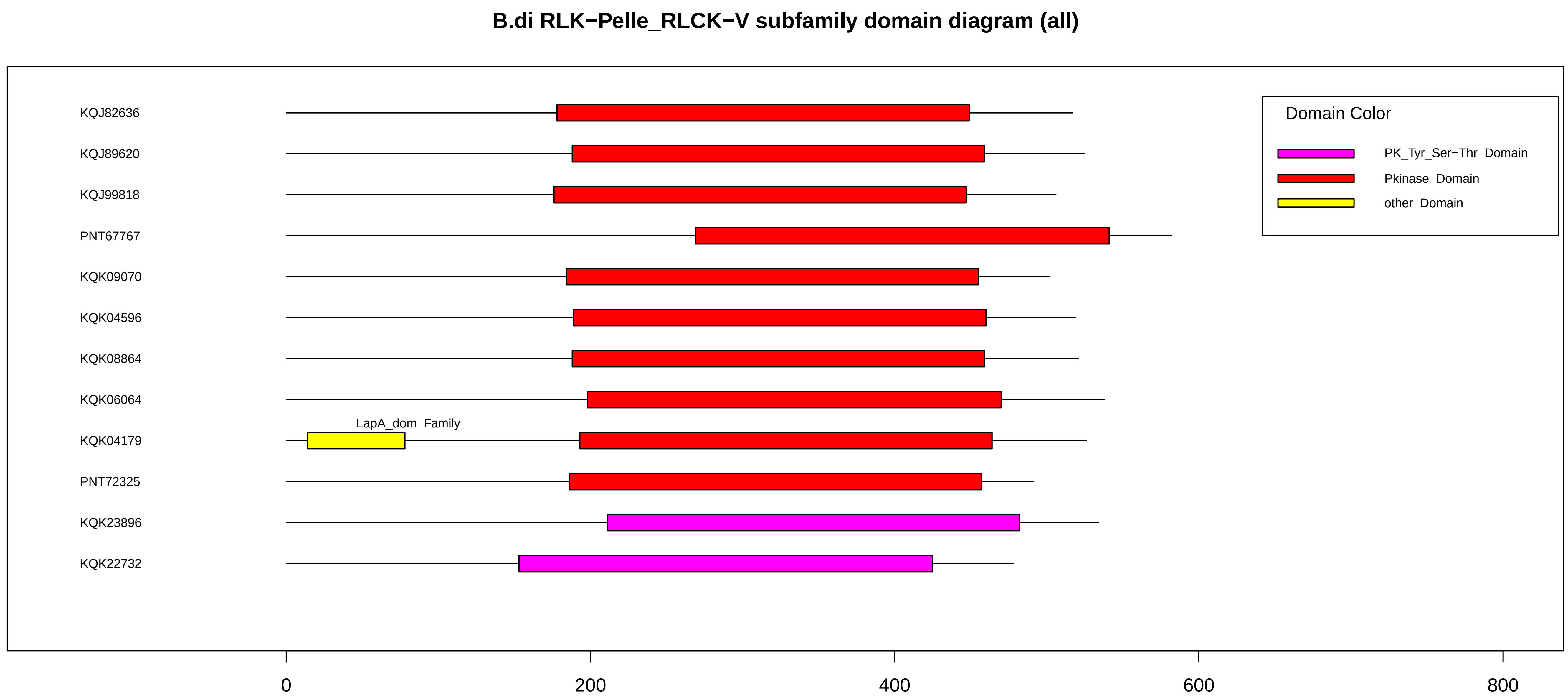
B.di RLK−Pelle_RLCK−V subfamily domain diagram (all)
KQJ82636
KQJ89620
KQJ99818
PNT67767
KQK09070
KQK04596
KQK08864
KQK06064
KQK04179
PNT72325
KQK23896
KQK22732
LapA_dom Family
Domain Color
PK_Tyr_Ser−Thr Domain
Pkinase Domain
other Domain
0
200
400
600
800

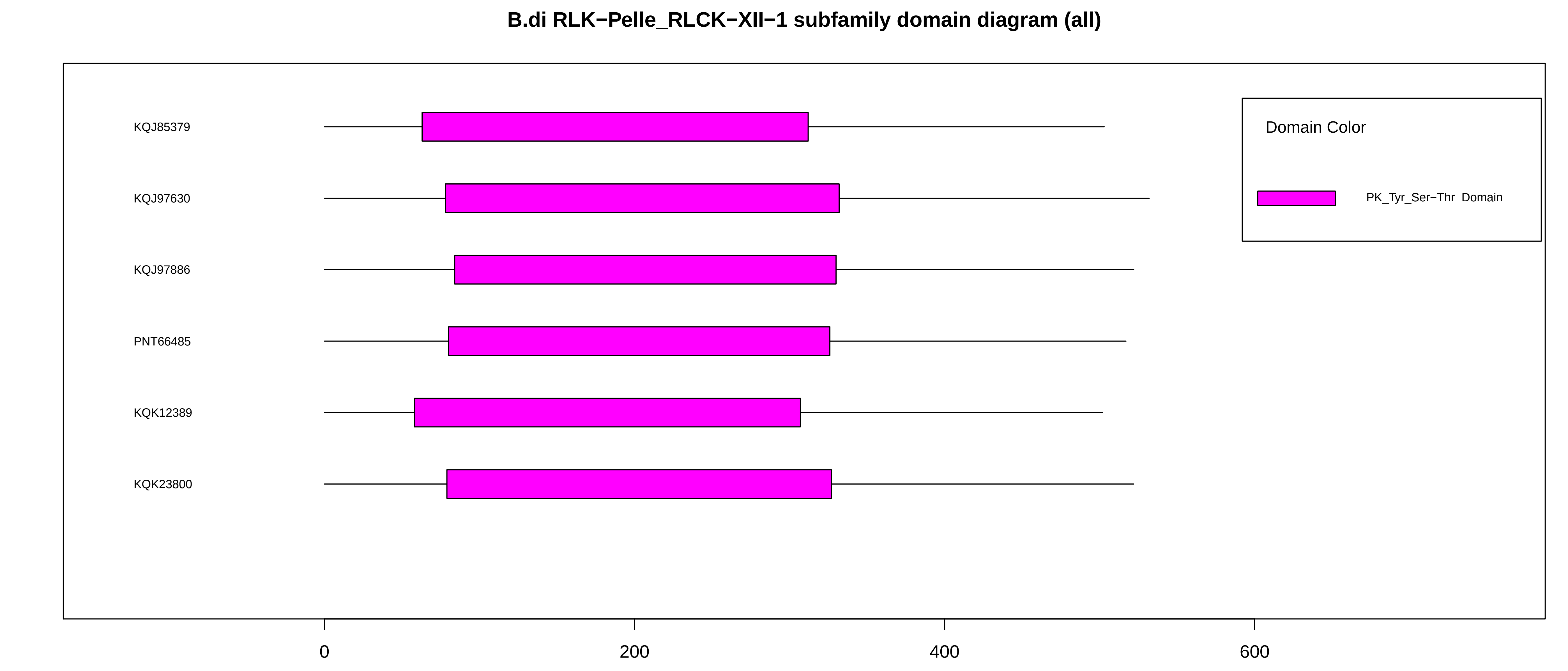
B.di RLK−Pelle_RLCK−XII−1 subfamily domain diagram (all)
KQJ85379
KQJ97630
KQJ97886
PNT66485
KQK12389
KQK23800
Domain Color
PK_Tyr_Ser−Thr Domain
0
200
400
600

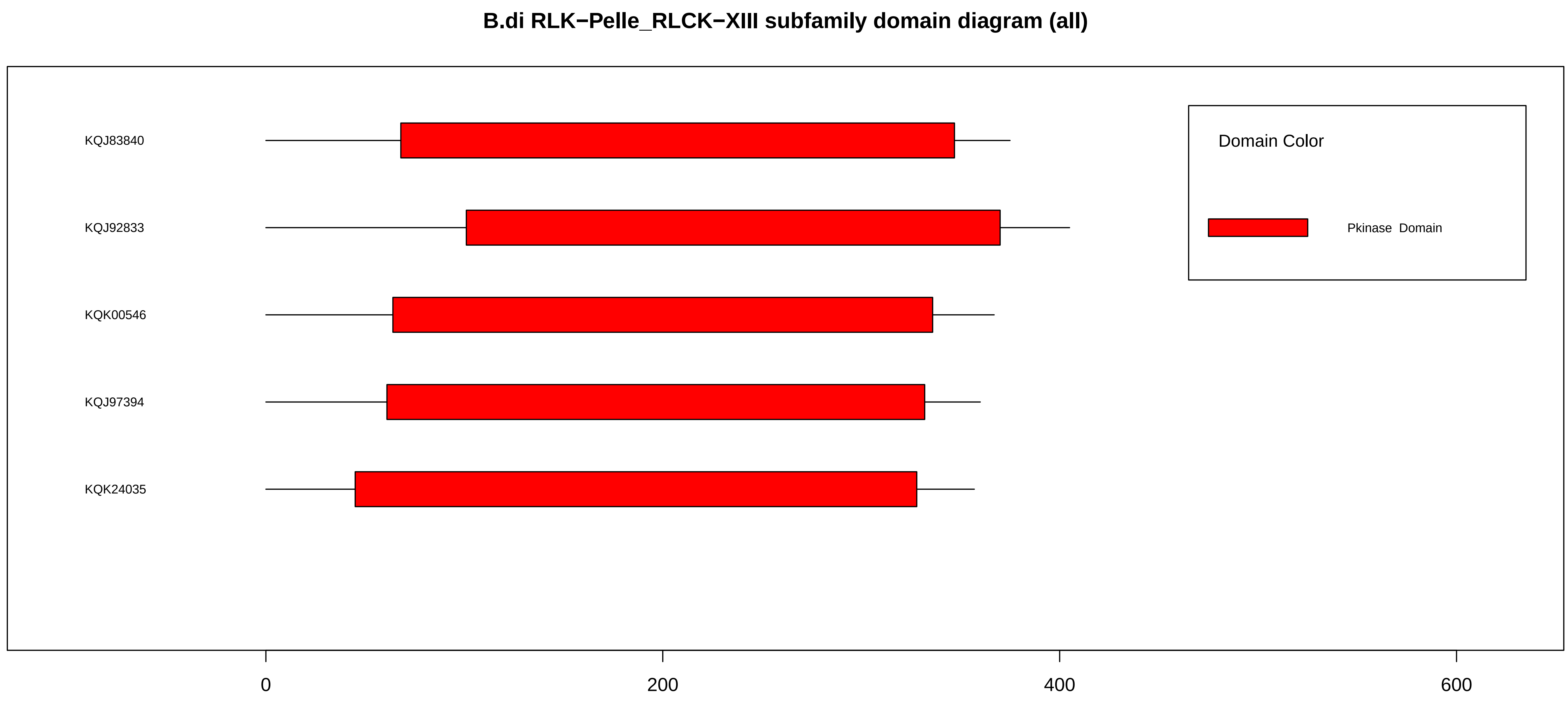
B.di RLK−Pelle_RLCK−XIII subfamily domain diagram (all)
KQJ83840
KQJ92833
KQK00546
KQJ97394
KQK24035
Domain Color
Pkinase Domain
0
200
400
600

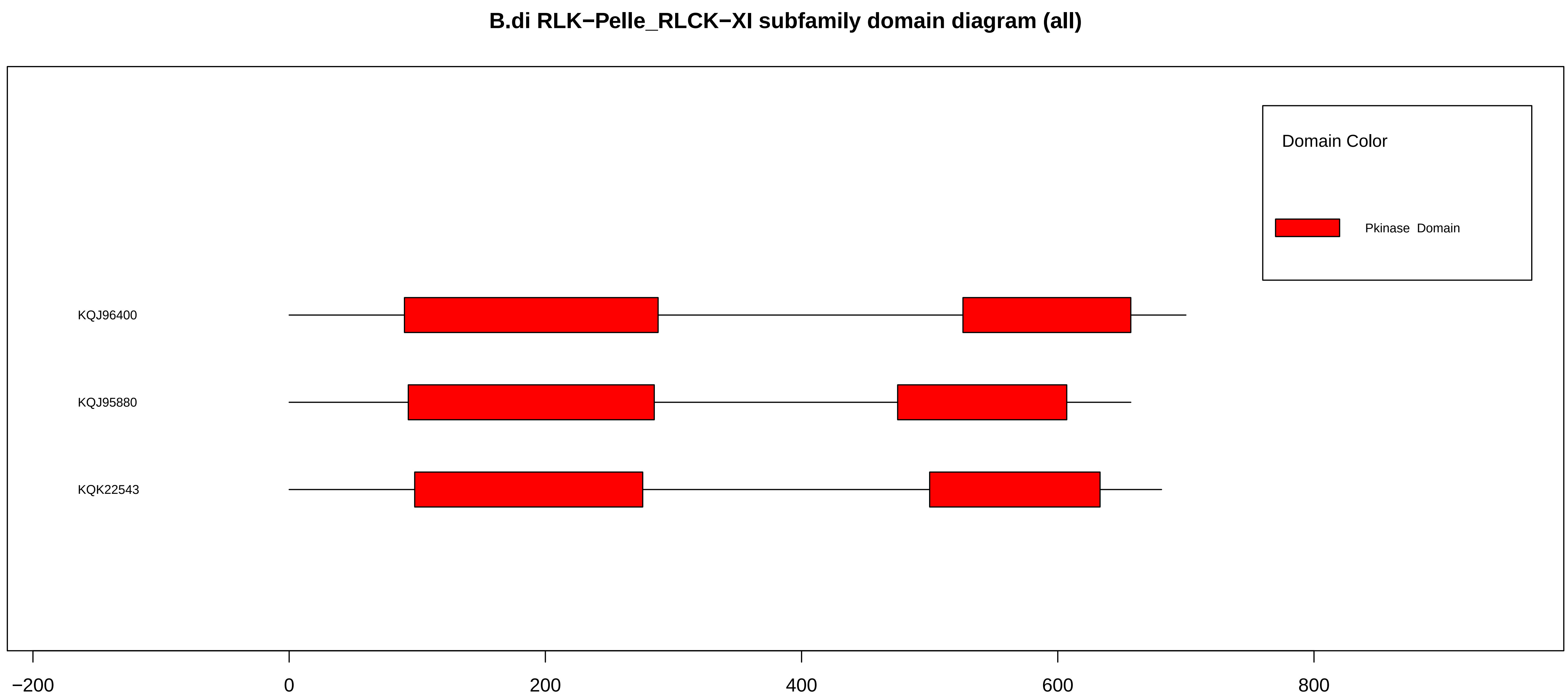
B.di RLK−Pelle_RLCK−XI subfamily domain diagram (all)
Domain Color
Pkinase Domain
KQJ96400
KQJ95880
KQK22543
−200
0
200
400
600
800

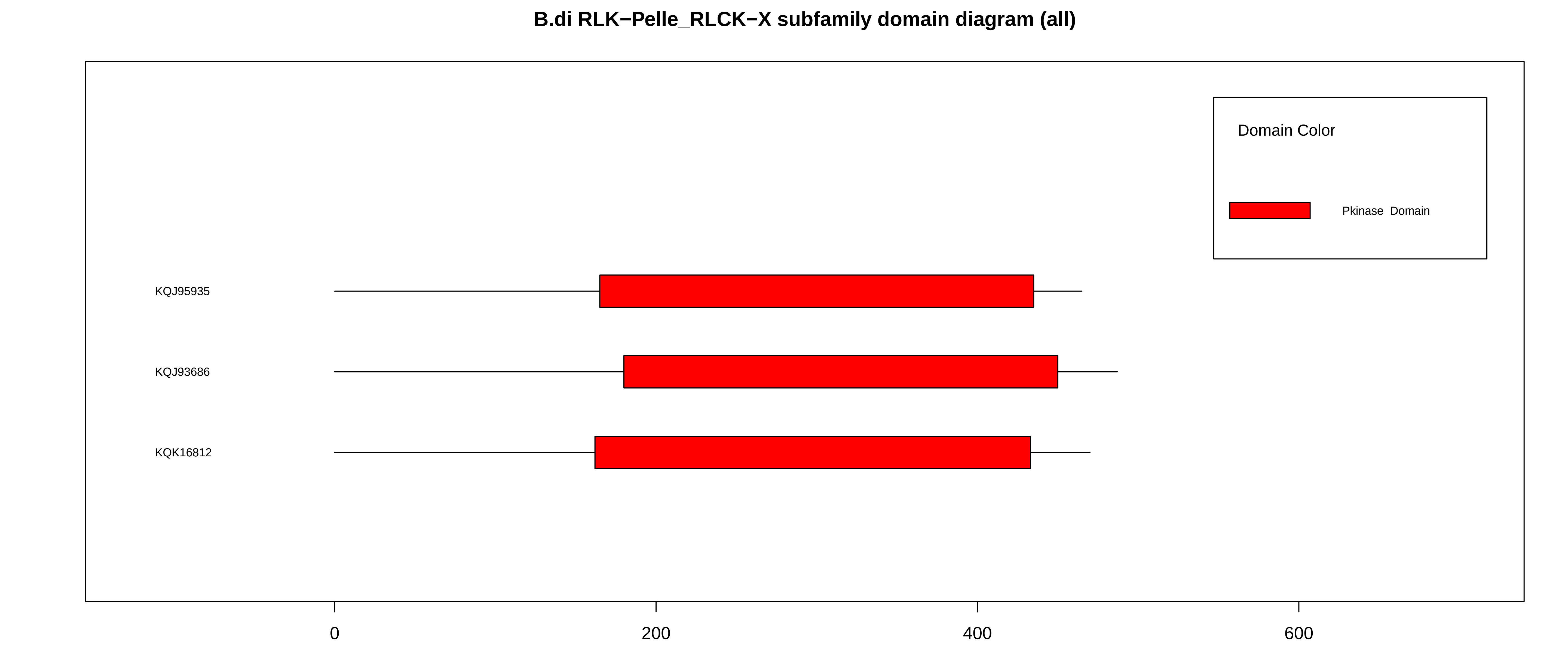
B.di RLK−Pelle_RLCK−X subfamily domain diagram (all)
Domain Color
Pkinase Domain
KQJ95935
KQJ93686
KQK16812
0
200
400
600

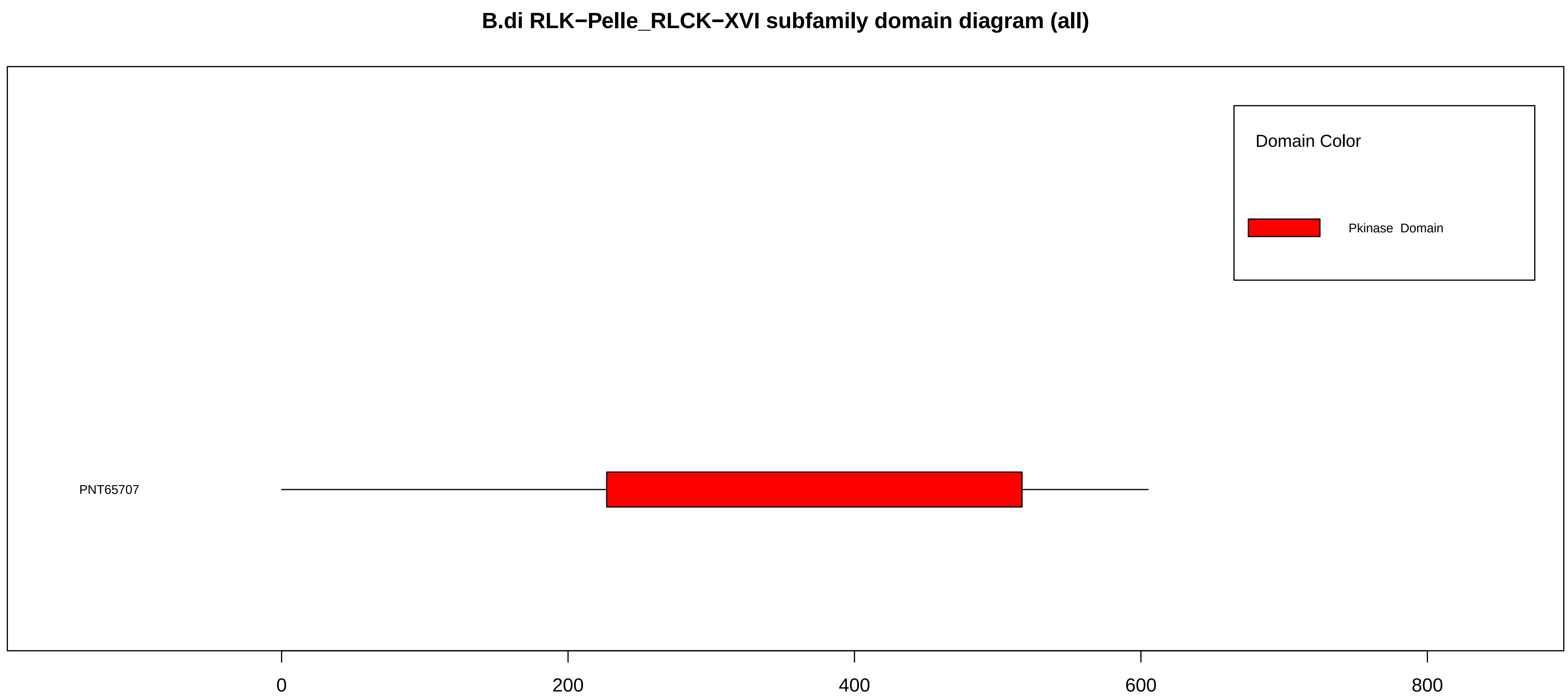
B.di RLK−Pelle_RLCK−XVI subfamily domain diagram (all)
Domain Color
Pkinase Domain
PNT65707
0
200
400
600
800

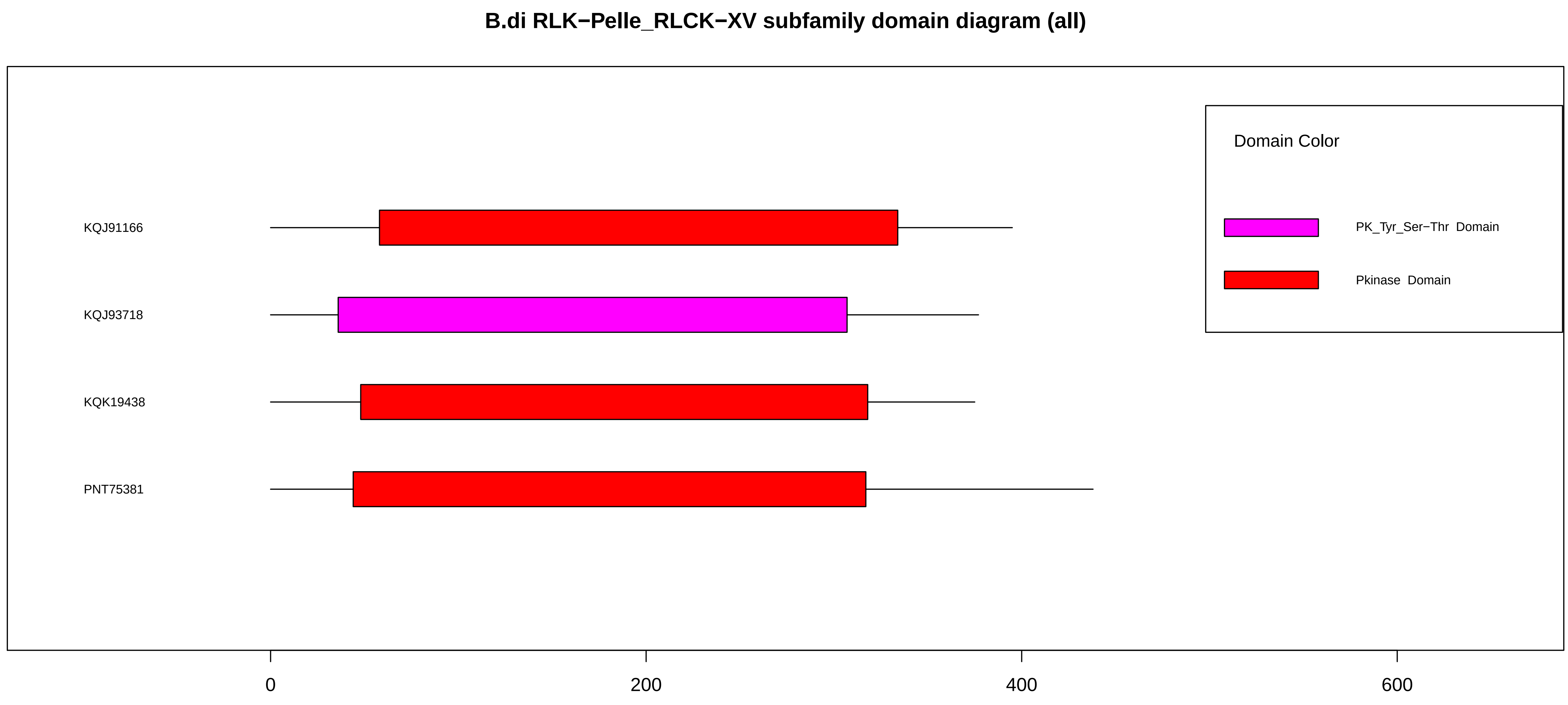
B.di RLK−Pelle_RLCK−XV subfamily domain diagram (all)
KQJ91166
KQJ93718
KQK19438
PNT75381
Domain Color
PK_Tyr_Ser−Thr Domain
Pkinase Domain
0
200
400
600

B.di RLK−Pelle_SD−2b subfamily domain diagram (part 1)

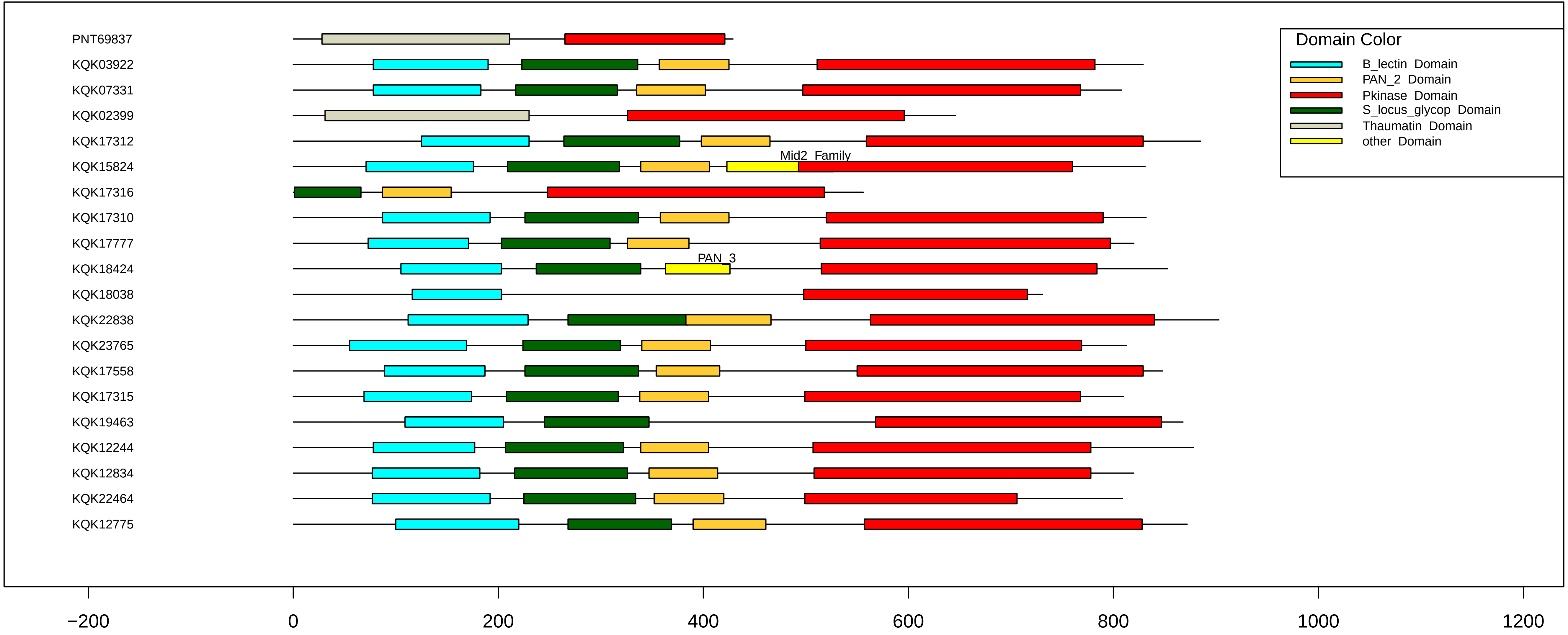

B.di RLK−Pelle_SD−2b subfamily domain diagram (part 2)

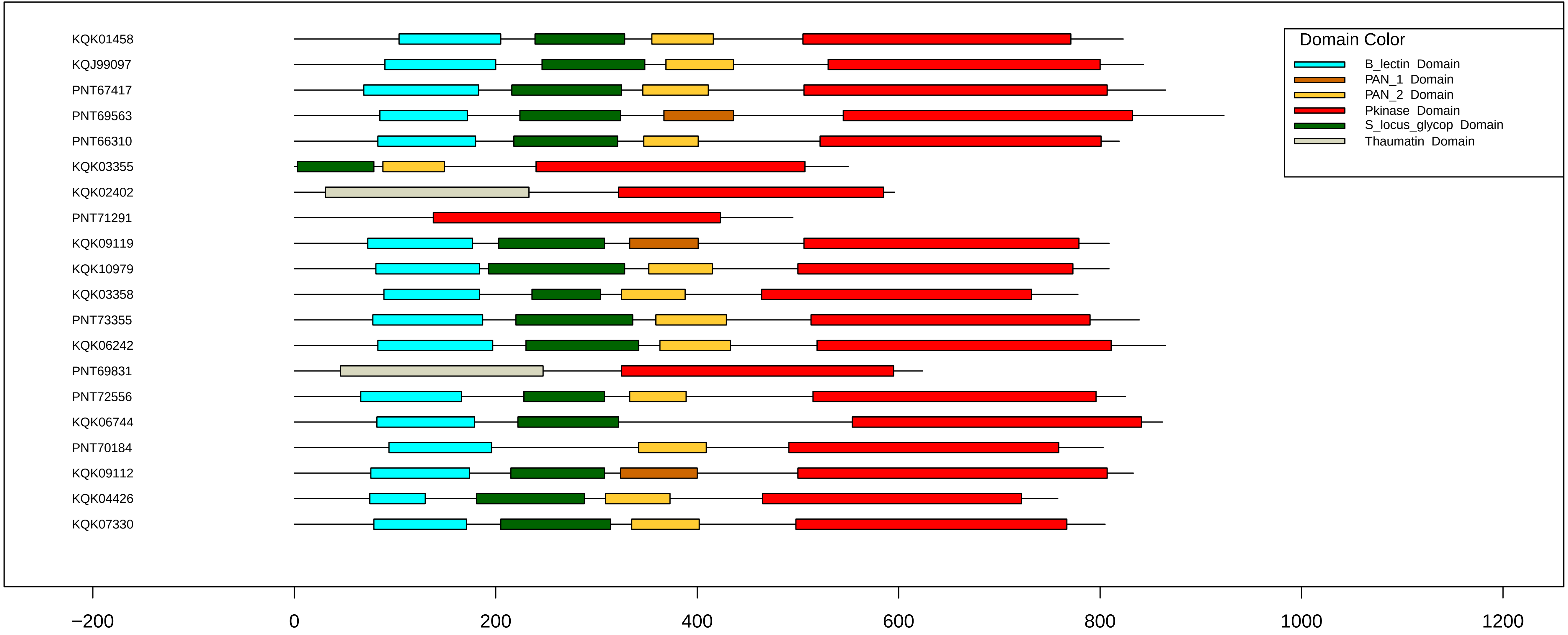

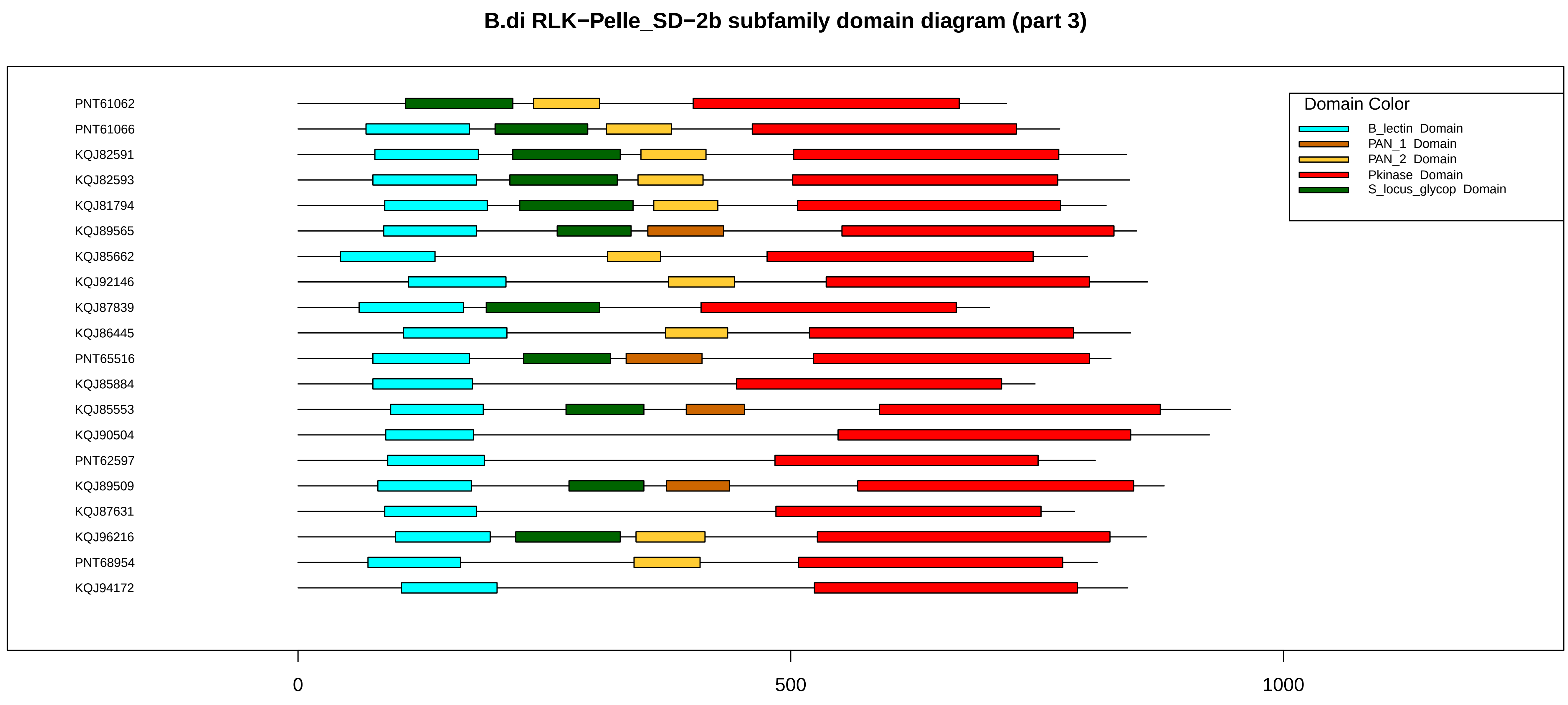
B.di RLK−Pelle_SD−2b subfamily domain diagram (part 3)
PNT61062
PNT61066
KQJ82591
KQJ82593
KQJ81794
KQJ89565
KQJ85662
KQJ92146
KQJ87839
KQJ86445
PNT65516
KQJ85884
KQJ85553
KQJ90504
PNT62597
KQJ89509
KQJ87631
KQJ96216
PNT68954
KQJ94172
Domain Color
B_lectin Domain
PAN_1 Domain
PAN_2 Domain
Pkinase Domain
S_locus_glycop Domain
0
500
1000

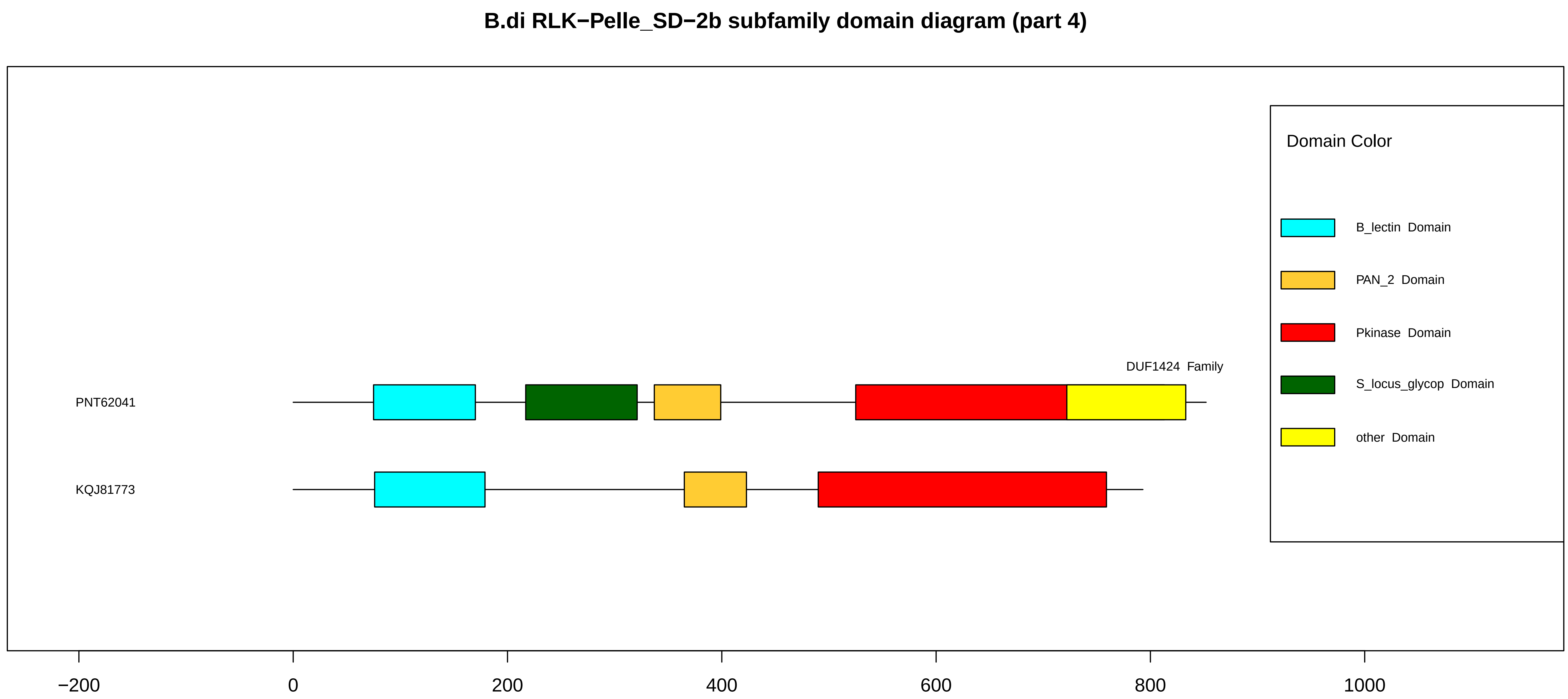
B.di RLK−Pelle_SD−2b subfamily domain diagram (part 4)
Domain Color
B_lectin Domain
PAN_2 Domain
Pkinase Domain
S_locus_glycop Domain
other Domain
DUF1424 Family
PNT62041
KQJ81773
−200
0
200
400
600
800
1000

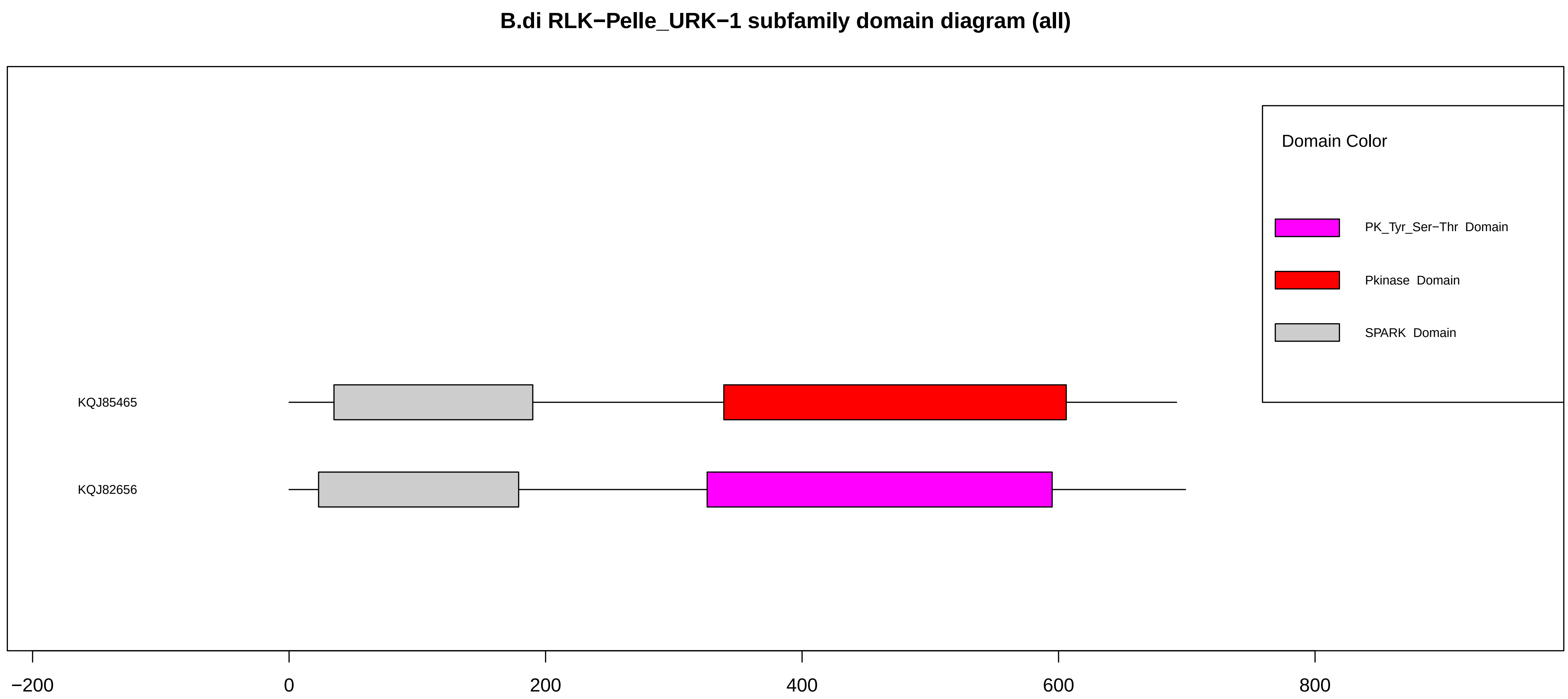

B.di RLK−Pelle_URK−1 subfamily domain diagram (all)
Domain Color
PK_Tyr_Ser−Thr Domain
Pkinase Domain
SPARK Domain
KQJ85465
KQJ82656
−200
0
200
400
600
800

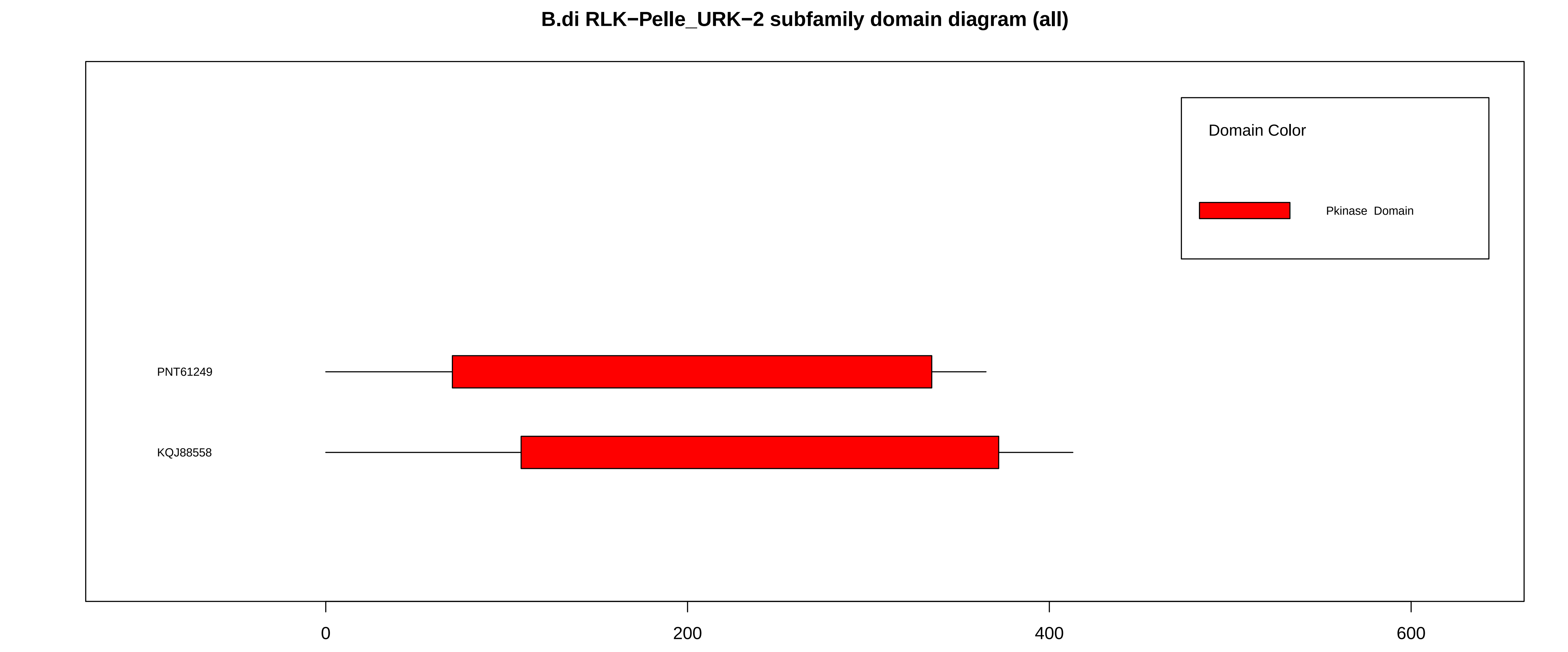
B.di RLK−Pelle_URK−2 subfamily domain diagram (all)
Domain Color
Pkinase Domain
PNT61249
KQJ88558
0
200
400
600

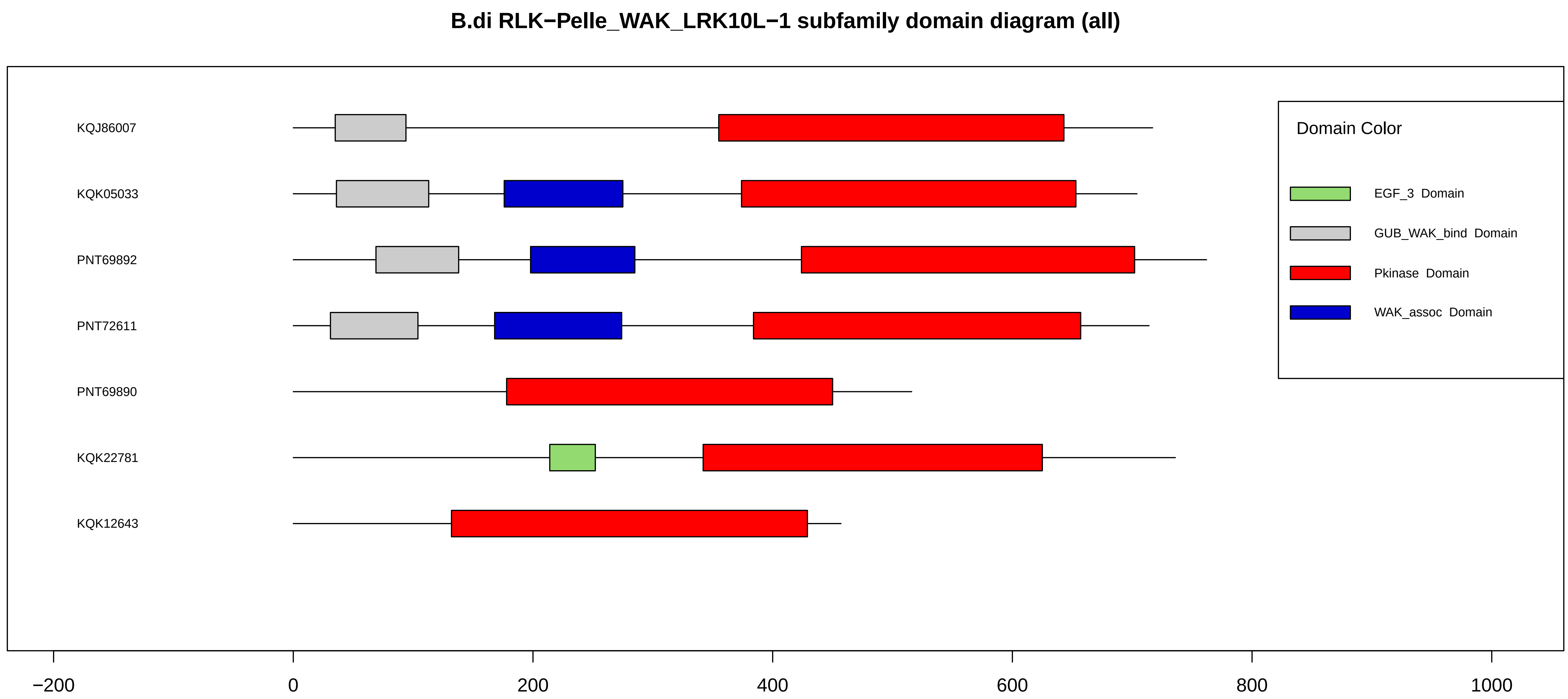
B.di RLK−Pelle_WAK_LRK10L−1 subfamily domain diagram (all)
KQJ86007
KQK05033
PNT69892
PNT72611
PNT69890
KQK22781
KQK12643
Domain Color
EGF_3 Domain
GUB_WAK_bind Domain
Pkinase Domain
WAK_assoc Domain
−200
0
200
400
600
800
1000

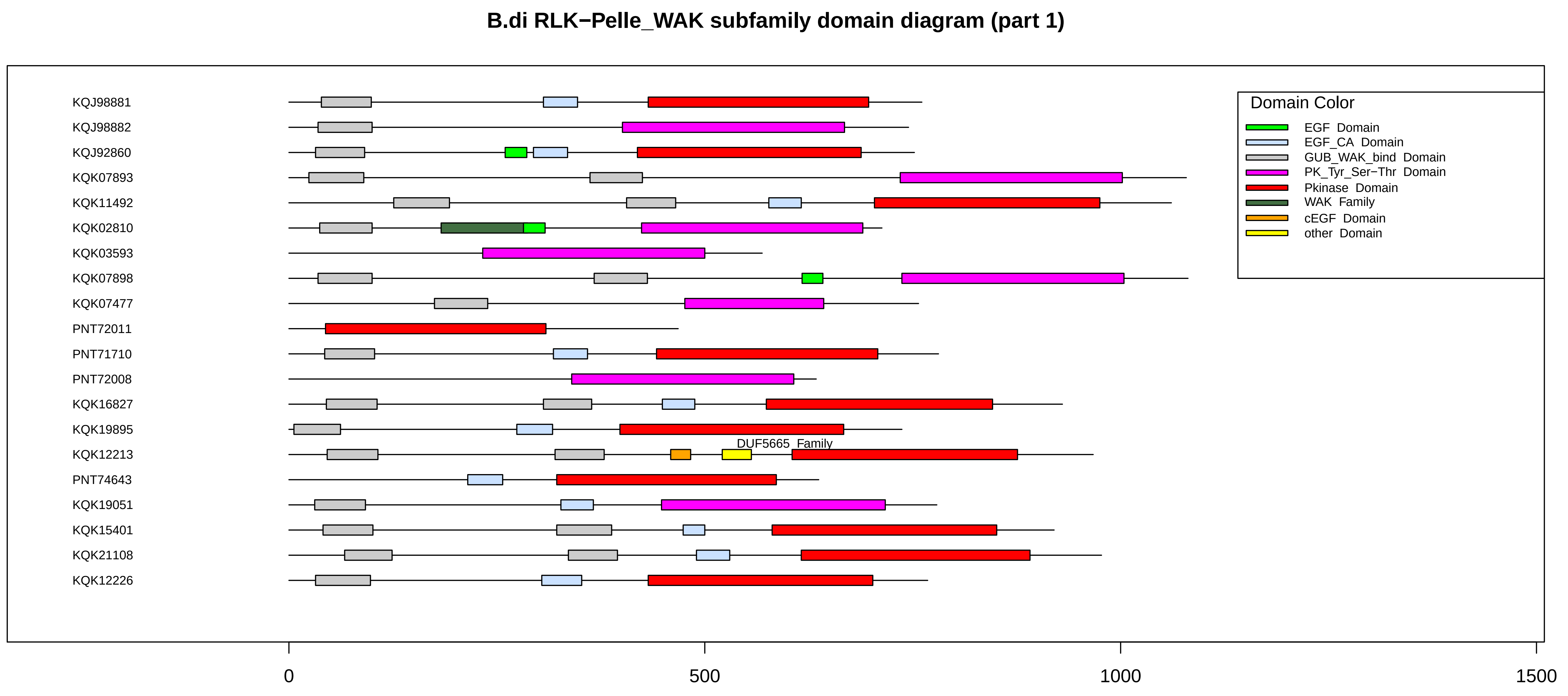
B.di RLK−Pelle_WAK subfamily domain diagram (part 1)
KQJ98881
KQJ98882
KQJ92860
KQK07893
KQK11492
KQK02810
KQK03593
KQK07898
KQK07477
PNT72011
PNT71710
PNT72008
KQK16827
KQK19895
KQK12213
PNT74643
KQK19051
KQK15401
KQK21108
KQK12226
DUF5665 Family
Domain Color
EGF Domain
EGF_CA Domain
GUB_WAK_bind Domain
PK_Tyr_Ser−Thr Domain
Pkinase Domain
WAK Family
cEGF Domain
other Domain
0
500
1000
1500

B.di RLK−Pelle_WAK subfamily domain diagram (part 2)

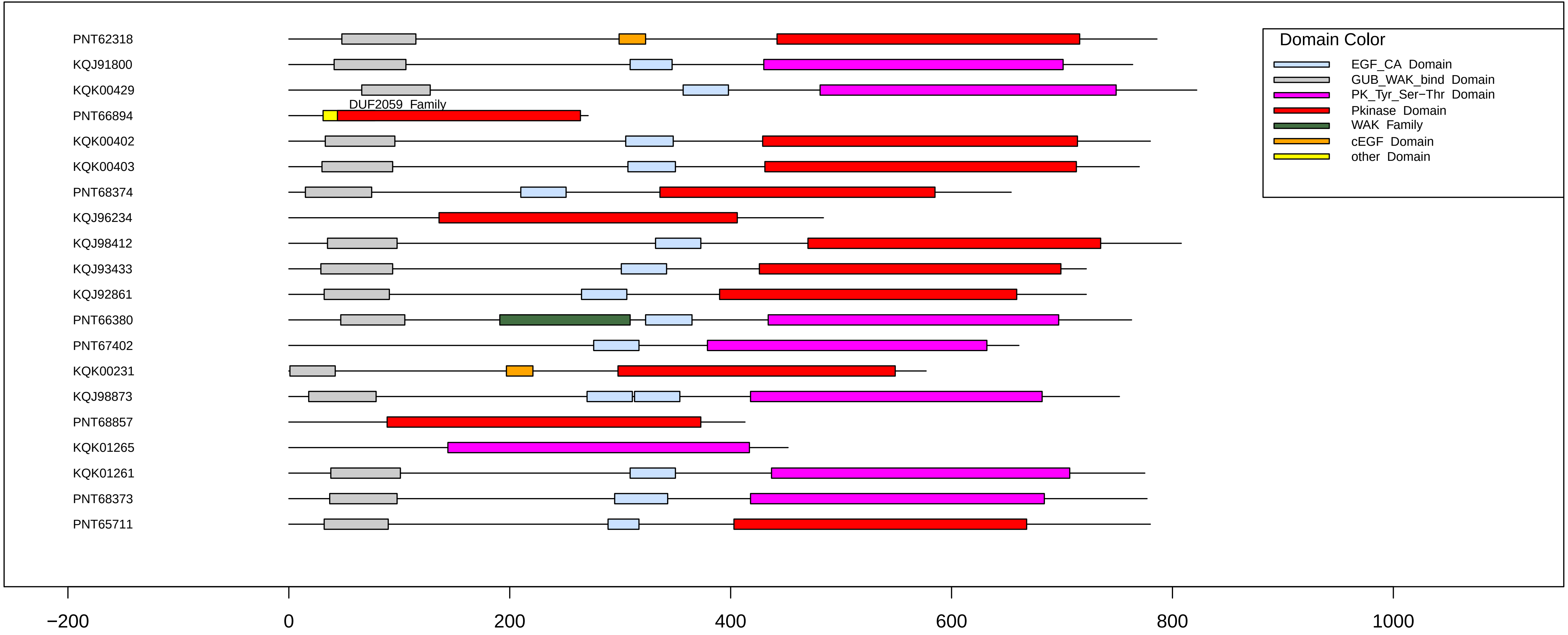

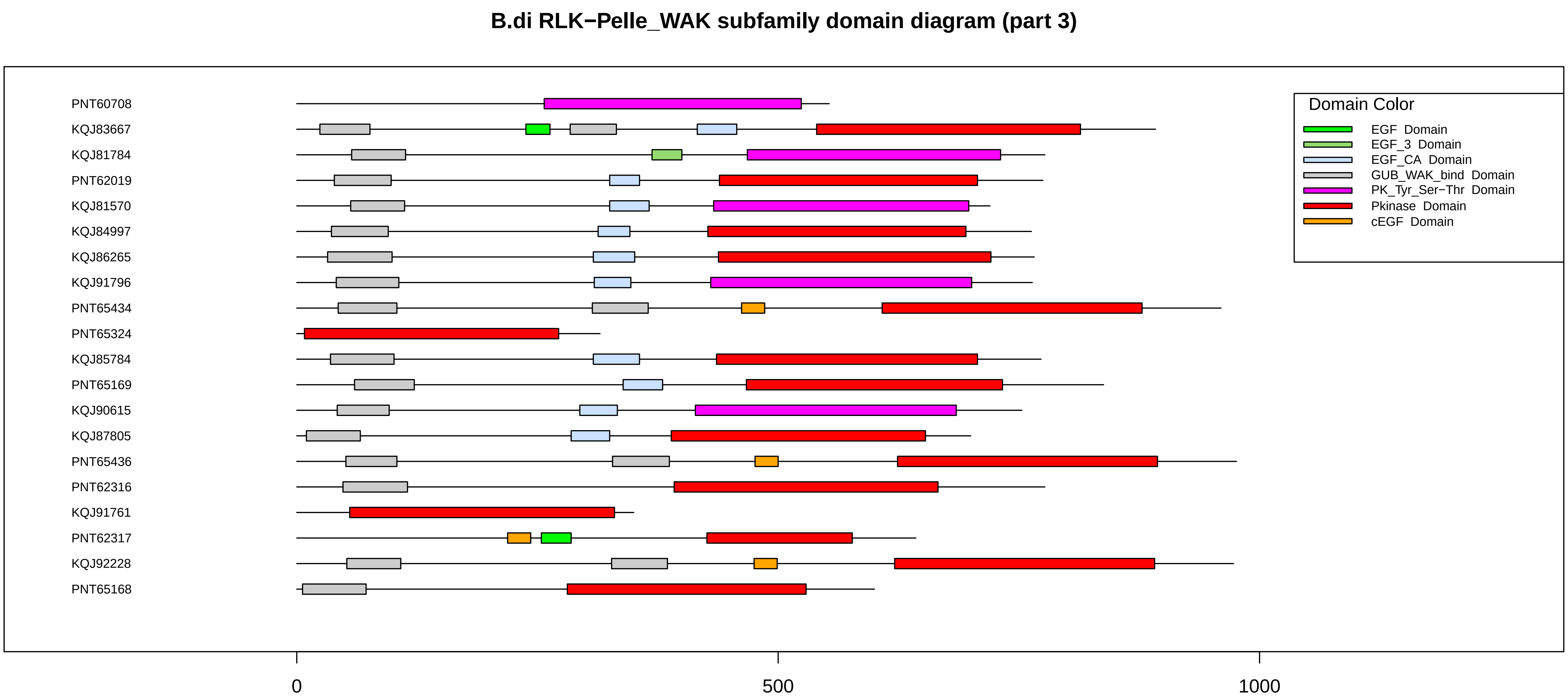
B.di RLK−Pelle_WAK subfamily domain diagram (part 3)
PNT60708
KQJ83667
KQJ81784
PNT62019
KQJ81570
KQJ84997
KQJ86265
KQJ91796
PNT65434
PNT65324
KQJ85784
PNT65169
KQJ90615
KQJ87805
PNT65436
PNT62316
KQJ91761
PNT62317
KQJ92228
PNT65168
Domain Color
EGF Domain
EGF_3 Domain
EGF_CA Domain
GUB_WAK_bind Domain
PK_Tyr_Ser−Thr Domain
Pkinase Domain
cEGF Domain
0
500
1000

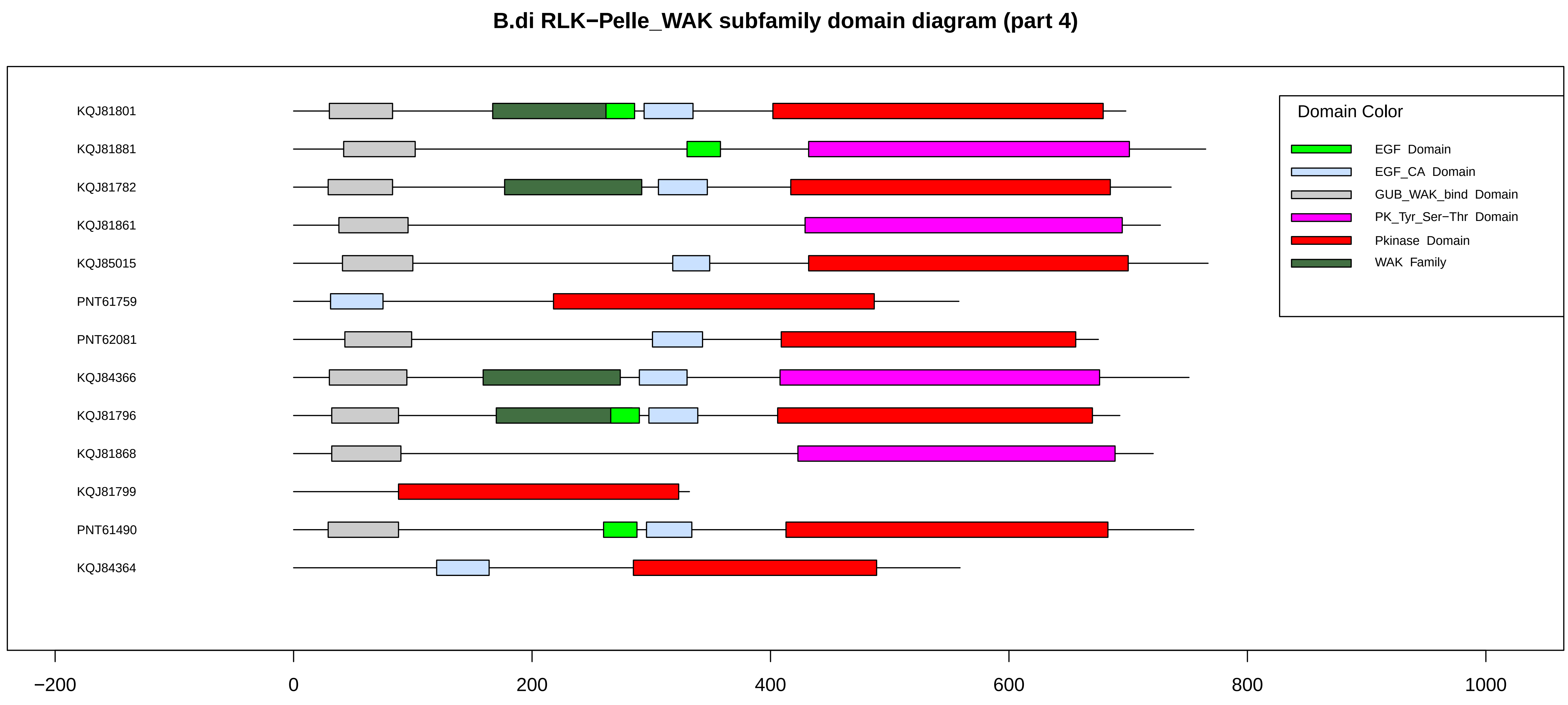
B.di RLK−Pelle_WAK subfamily domain diagram (part 4)
KQJ81801
KQJ81881
KQJ81782
KQJ81861
KQJ85015
PNT61759
PNT62081
KQJ84366
KQJ81796
KQJ81868
KQJ81799
PNT61490
KQJ84364
Domain Color
EGF Domain
EGF_CA Domain
GUB_WAK_bind Domain
PK_Tyr_Ser−Thr Domain
Pkinase Domain
WAK Family
−200
0
200
400
600
800
1000

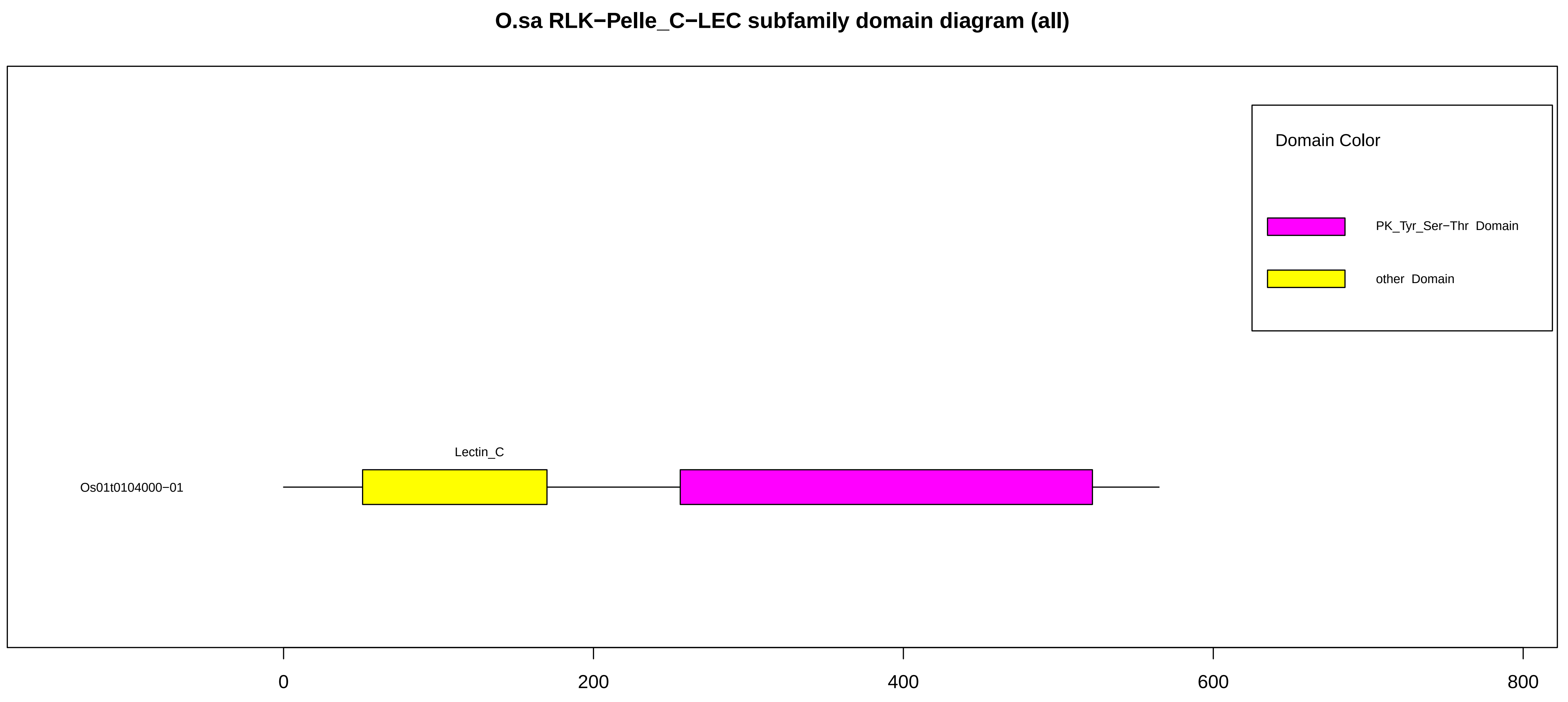

O.sa RLK−Pelle_C−LEC subfamily domain diagram (all)
Domain Color
PK_Tyr_Ser−Thr Domain
other Domain
Lectin_C
Os01t0104000−01
0
200
400
600
800

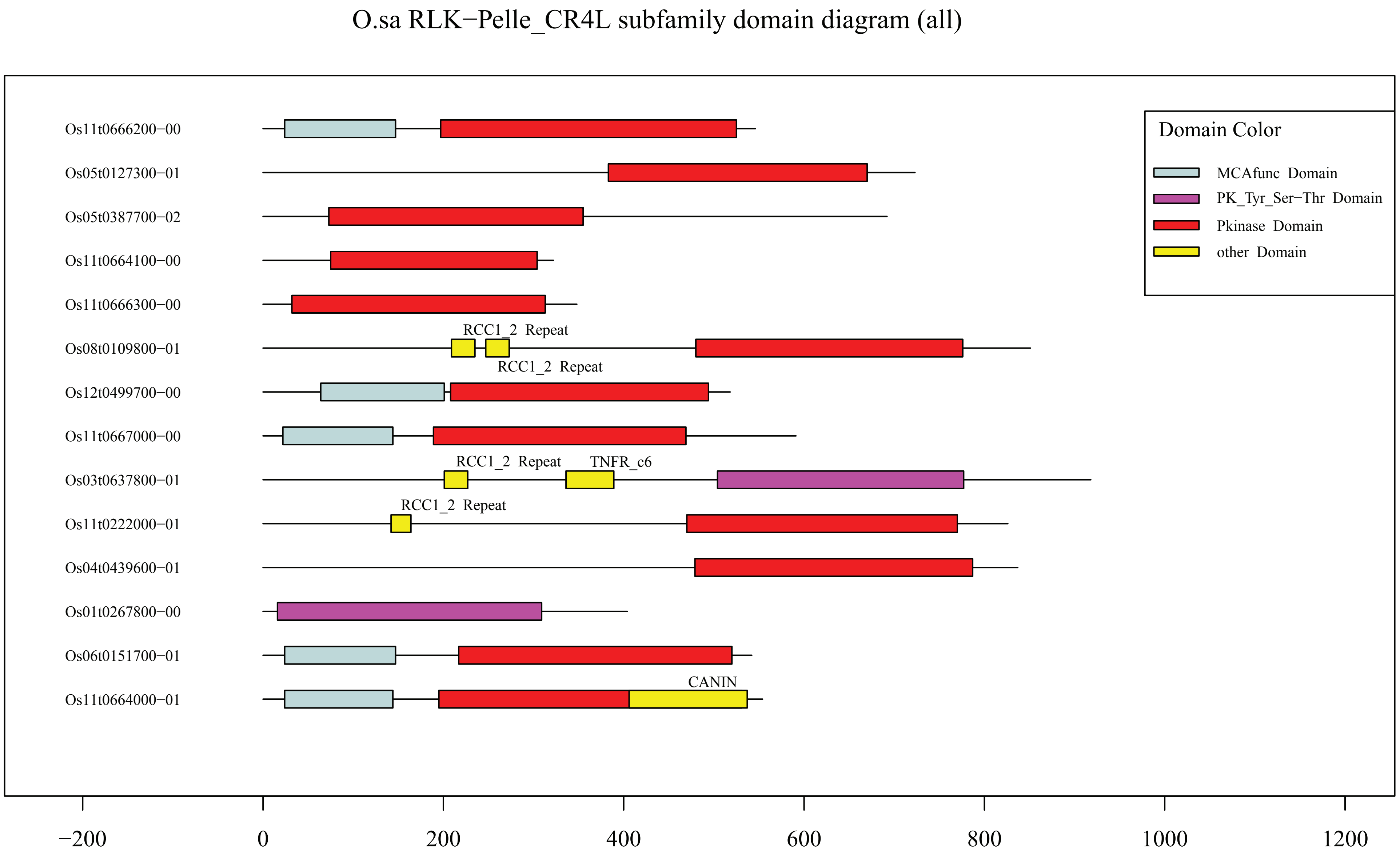

O.sa RLK−Pelle_CR4L subfamily domain diagram (all)
Os11t0666200−00
Os05t0127300−01
Os05t0387700−02
Os11t0664100−00
Os11t0666300−00
Os08t0109800−01
Os12t0499700−00
Os11t0667000−00
Os03t0637800−01
Os11t0222000−01
Os04t0439600−01
Os01t0267800−00
Os06t0151700−01
Os11t0664000−01
RCC1_2 Repeat
RCC1_2 Repeat
RCC1_2 Repeat
TNFR_c6
RCC1_2 Repeat
CANIN
Domain Color
MCAfunc Domain
PK_Tyr_Ser−Thr Domain
Pkinase Domain
other Domain
−200
0
200
400
600
800
1000
1200

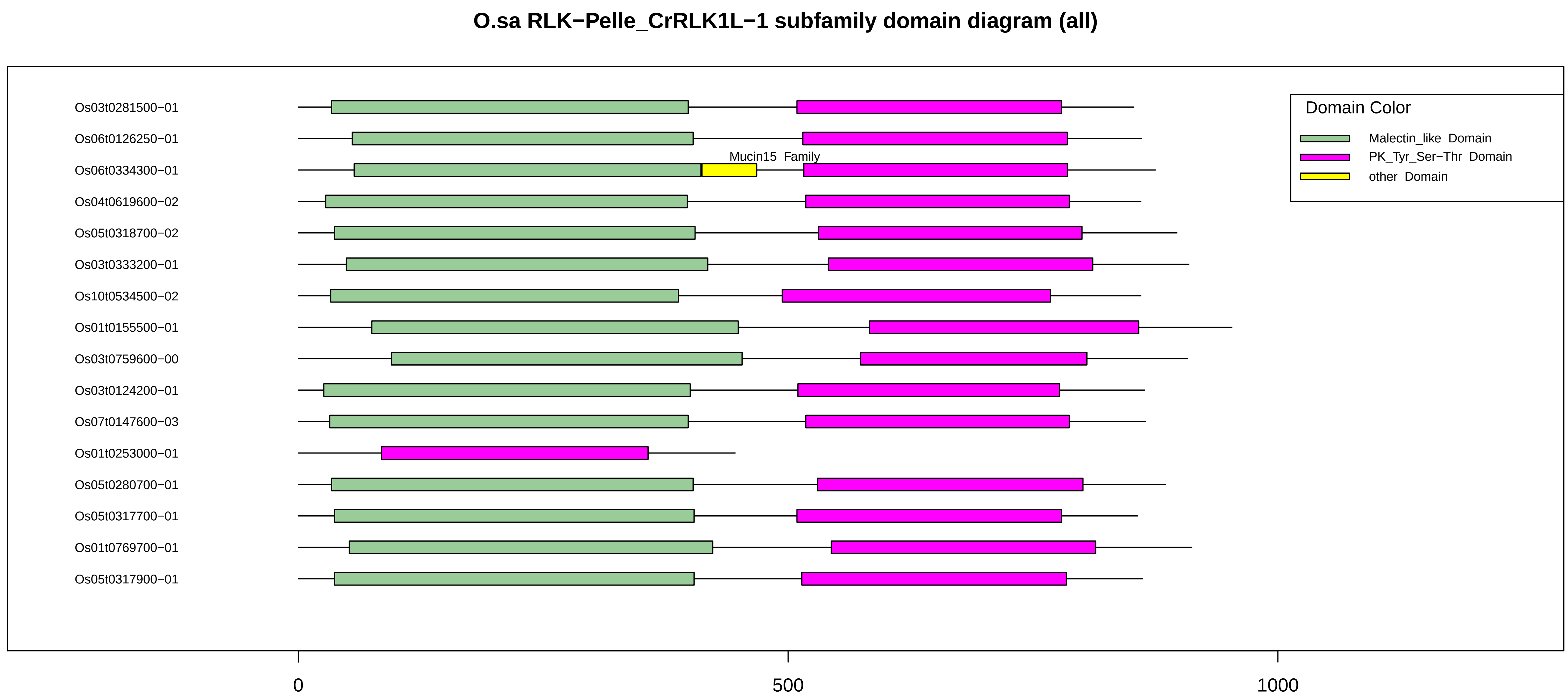

O.sa RLK−Pelle_CrRLK1L−1 subfamily domain diagram (all)
Os03t0281500−01
Os06t0126250−01
Os06t0334300−01
Os04t0619600−02
Os05t0318700−02
Os03t0333200−01
Os10t0534500−02
Os01t0155500−01
Os03t0759600−00
Os03t0124200−01
Os07t0147600−03
Os01t0253000−01
Os05t0280700−01
Os05t0317700−01
Os01t0769700−01
Os05t0317900−01
Mucin15 Family
Domain Color
Malectin_like Domain
PK_Tyr_Ser−Thr Domain
other Domain
0
500
1000

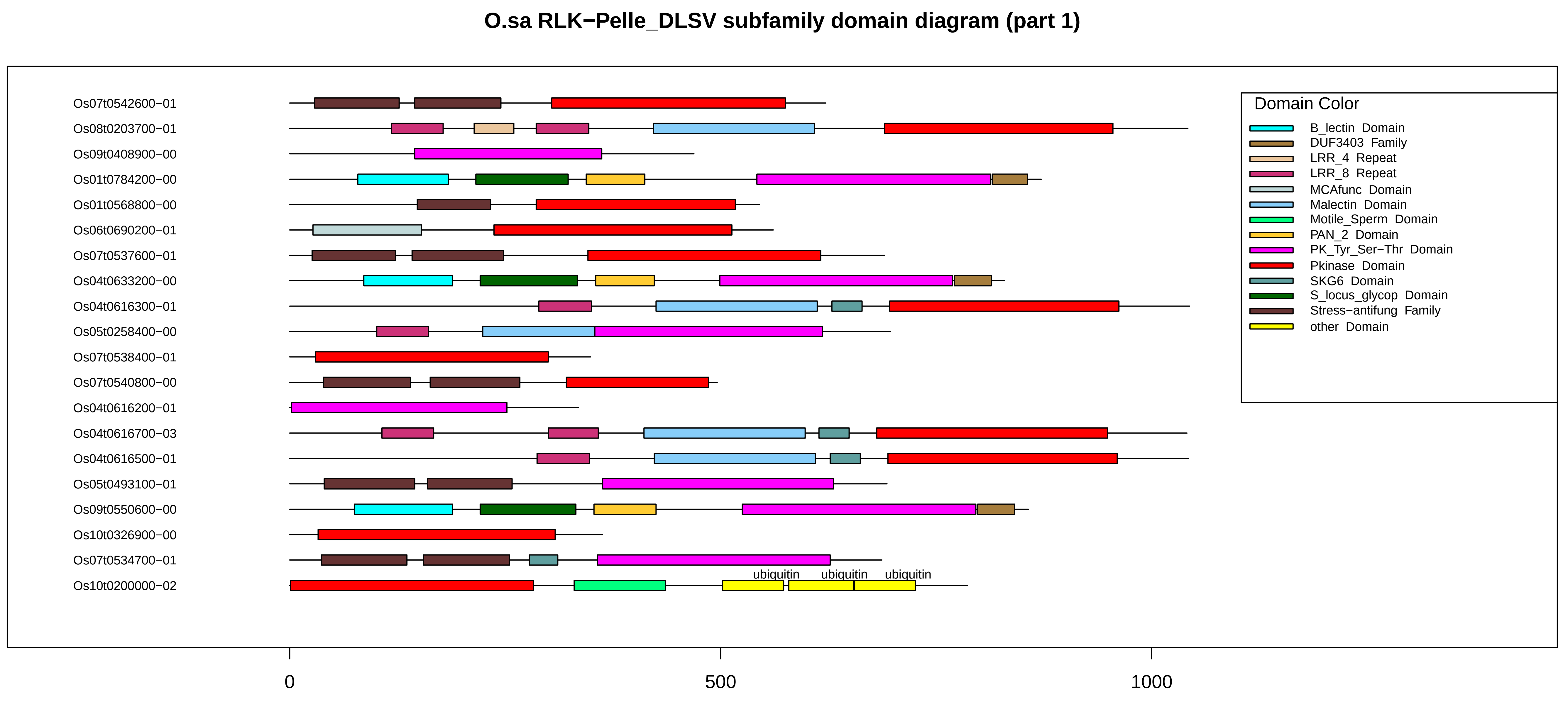
O.sa RLK−Pelle_DLSV subfamily domain diagram (part 1)
Os07t0542600−01
Os08t0203700−01
Os09t0408900−00
Os01t0784200−00
Os01t0568800−00
Os06t0690200−01
Os07t0537600−01
Os04t0633200−00
Os04t0616300−01
Os05t0258400−00
Os07t0538400−01
Os07t0540800−00
Os04t0616200−01
Os04t0616700−03
Os04t0616500−01
Os05t0493100−01
Os09t0550600−00
Os10t0326900−00
Os07t0534700−01
Os10t0200000−02
ubiquitin
ubiquitin
ubiquitin
Domain Color
B_lectin Domain
DUF3403 Family
LRR_4 Repeat
LRR_8 Repeat
MCAfunc Domain
Malectin Domain
Motile_Sperm Domain
PAN_2 Domain
PK_Tyr_Ser−Thr Domain
Pkinase Domain
SKG6 Domain
S_locus_glycop Domain
Stress−antifung Family
other Domain
0
500
1000

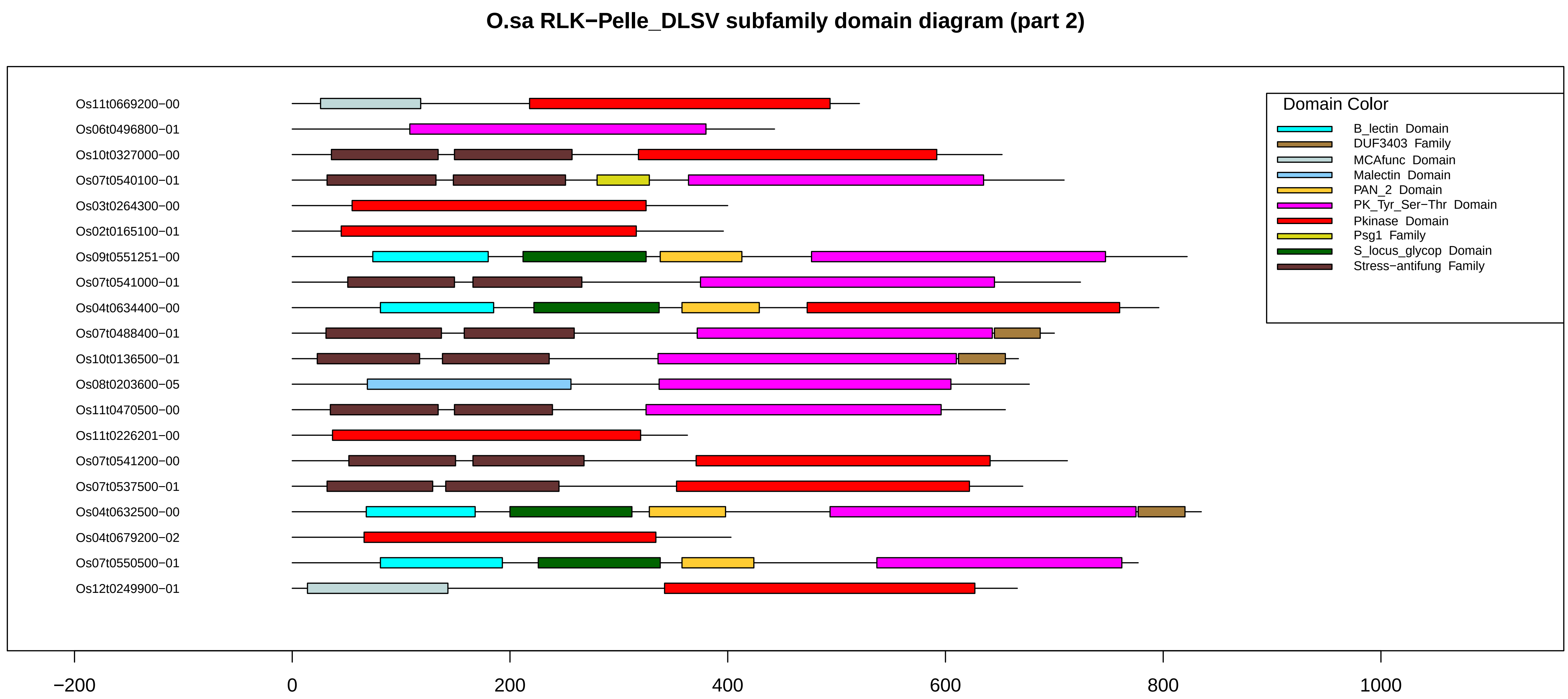
O.sa RLK−Pelle_DLSV subfamily domain diagram (part 2)
Os11t0669200−00
Os06t0496800−01
Os10t0327000−00
Os07t0540100−01
Os03t0264300−00
Os02t0165100−01
Os09t0551251−00
Os07t0541000−01
Os04t0634400−00
Os07t0488400−01
Os10t0136500−01
Os08t0203600−05
Os11t0470500−00
Os11t0226201−00
Os07t0541200−00
Os07t0537500−01
Os04t0632500−00
Os04t0679200−02
Os07t0550500−01
Os12t0249900−01
Domain Color
B_lectin Domain
DUF3403 Family
MCAfunc Domain
Malectin Domain
PAN_2 Domain
PK_Tyr_Ser−Thr Domain
Pkinase Domain
Psg1 Family
S_locus_glycop Domain
Stress−antifung Family
−200
0
200
400
600
800
1000

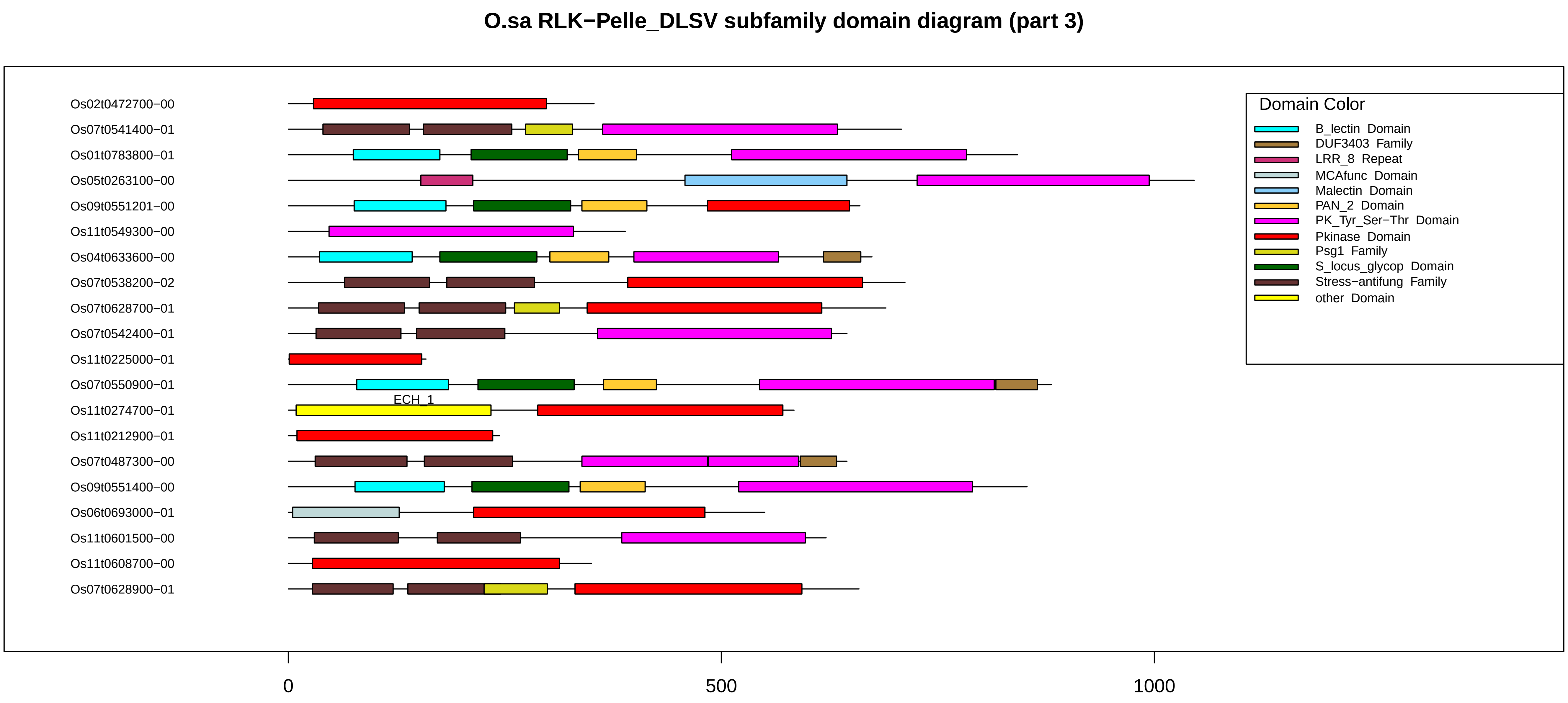
O.sa RLK−Pelle_DLSV subfamily domain diagram (part 3)
Os02t0472700−00
Os07t0541400−01
Os01t0783800−01
Os05t0263100−00
Os09t0551201−00
Os11t0549300−00
Os04t0633600−00
Os07t0538200−02
Os07t0628700−01
Os07t0542400−01
Os11t0225000−01
Os07t0550900−01
ECH_1
Os11t0274700−01
Os11t0212900−01
Os07t0487300−00
Os09t0551400−00
Os06t0693000−01
Os11t0601500−00
Os11t0608700−00
Os07t0628900−01
Domain Color
B_lectin Domain
DUF3403 Family
LRR_8 Repeat
MCAfunc Domain
Malectin Domain
PAN_2 Domain
PK_Tyr_Ser−Thr Domain
Pkinase Domain
Psg1 Family
S_locus_glycop Domain
Stress−antifung Family
other Domain
0
500
1000

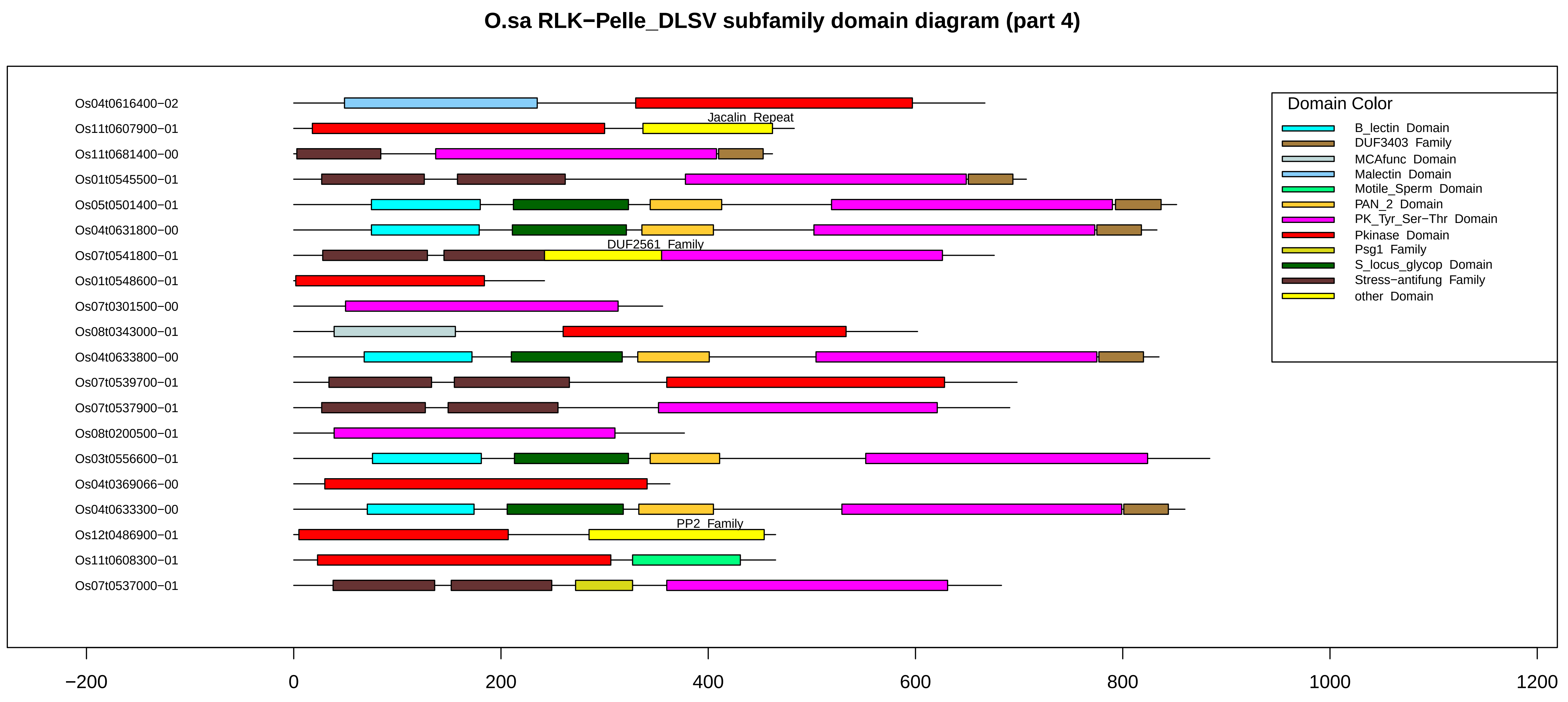
O.sa RLK−Pelle_DLSV subfamily domain diagram (part 4)
Os04t0616400−02
Os11t0607900−01
Jacalin_Repeat
Os11t0681400−00
Os01t0545500−01
Os05t0501400−01
Os04t0631800−00
Os07t0541800−01
DUF2561_Family
Os01t0548600−01
Os07t0301500−00
Os08t0343000−01
Os04t0633800−00
Os07t0539700−01
Os07t0537900−01
Os08t0200500−01
Os03t0556600−01
Os04t0369066−00
Os04t0633300−00
Os12t0486900−01
PP2_Family
Os11t0608300−01
Os07t0537000−01
Domain Color
B_lectin_Domain
DUF3403_Family
MCAfunc_Domain
Malectin_Domain
Motile_Sperm_Domain
PAN_2_Domain
PK_Tyr_Ser−Thr_Domain
Pkinase_Domain
Psg1_Family
S_locus_glycop_Domain
Stress−antifung_Family
other_Domain
−200
0
200
400
600
800
1000
1200

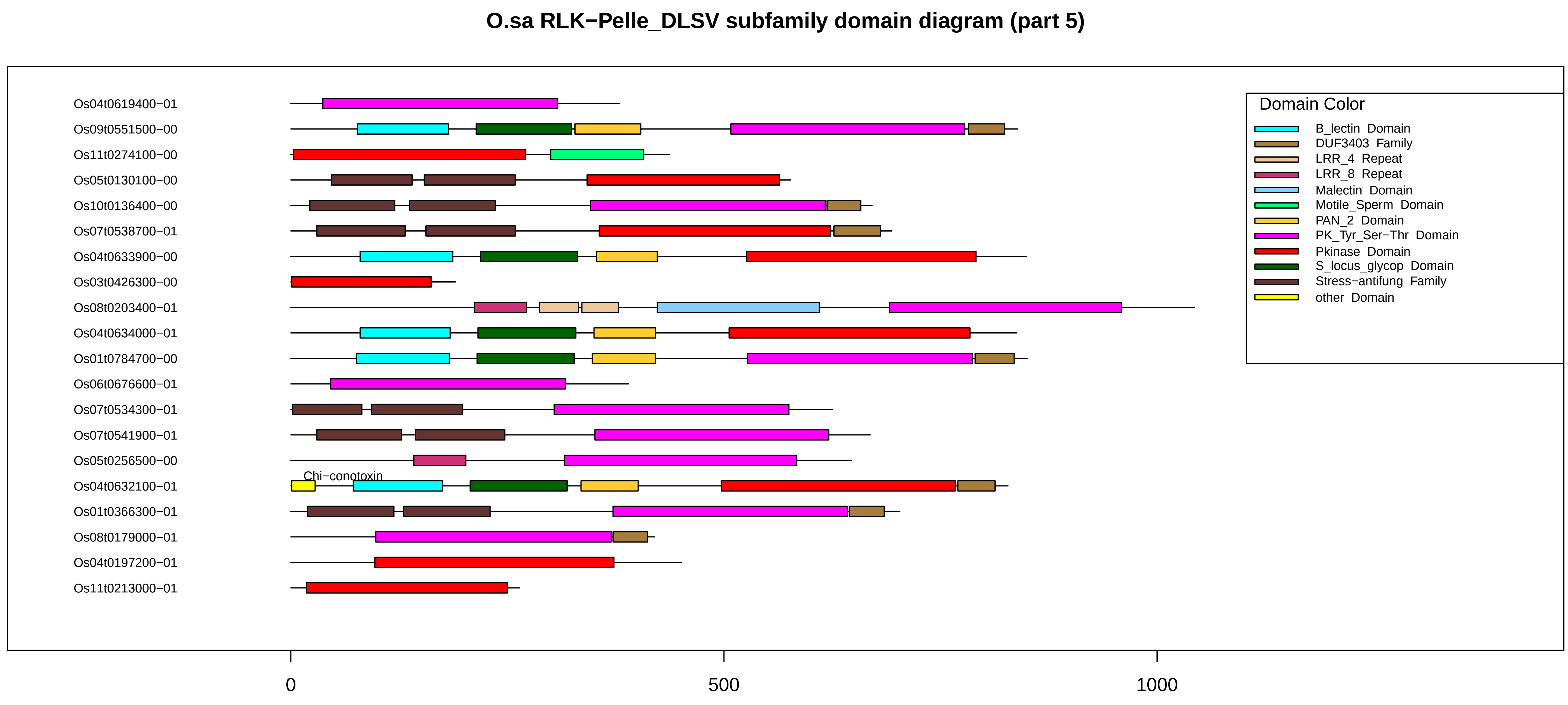
O.sa RLK−Pelle_DLSV subfamily domain diagram (part 5)
Os04t0619400−01
Os09t0551500−00
Os11t0274100−00
Os05t0130100−00
Os10t0136400−00
Os07t0538700−01
Os04t0633900−00
Os03t0426300−00
Os08t0203400−01
Os04t0634000−01
Os01t0784700−00
Os06t0676600−01
Os07t0534300−01
Os07t0541900−01
Os05t0256500−00
Chi−conotoxin
Os04t0632100−01
Os01t0366300−01
Os08t0179000−01
Os04t0197200−01
Os11t0213000−01
0
500
1000
Domain Color
B_lectin Domain
DUF3403 Family
LRR_4 Repeat
LRR_8 Repeat
Malectin Domain
Motile_Sperm Domain
PAN_2 Domain
PK_Tyr_Ser−Thr Domain
Pkinase Domain
S_locus_glycop Domain
Stress−antifung Family
other Domain

O.sa RLK−Pelle_DLSV subfamily domain diagram (part 6)

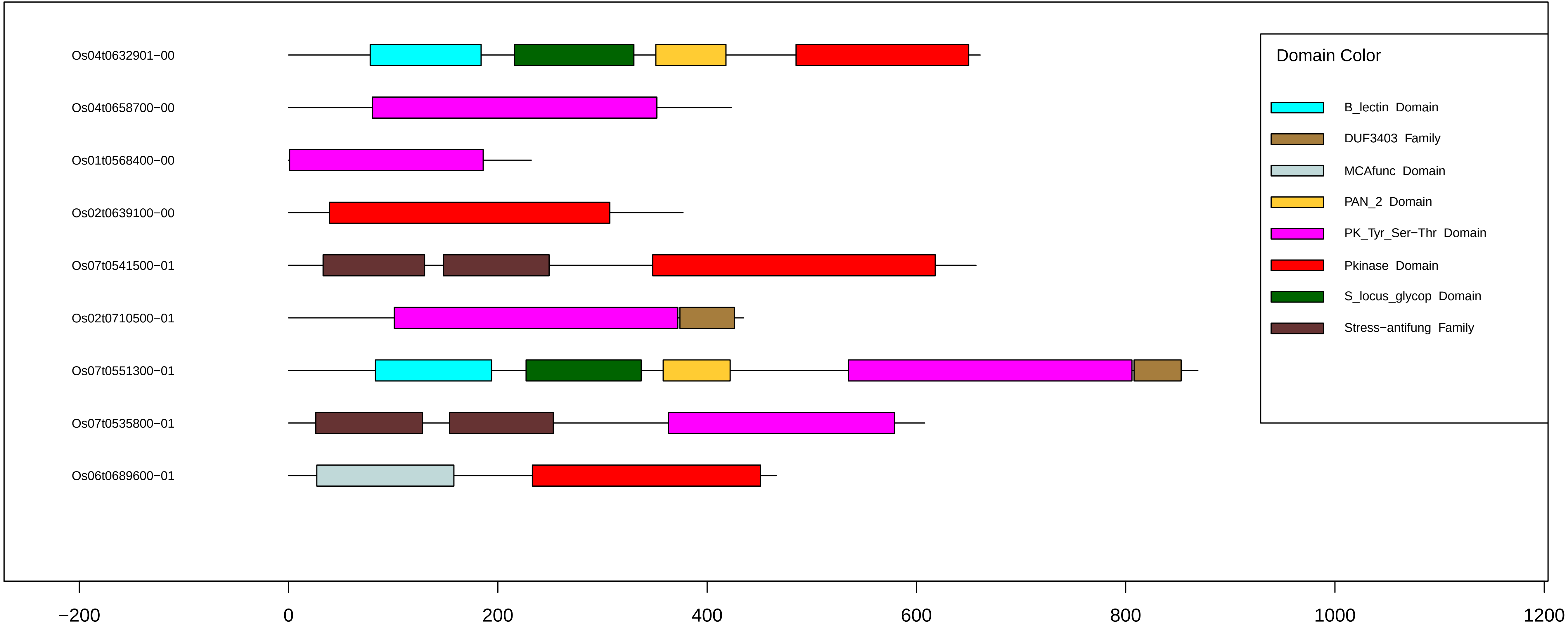

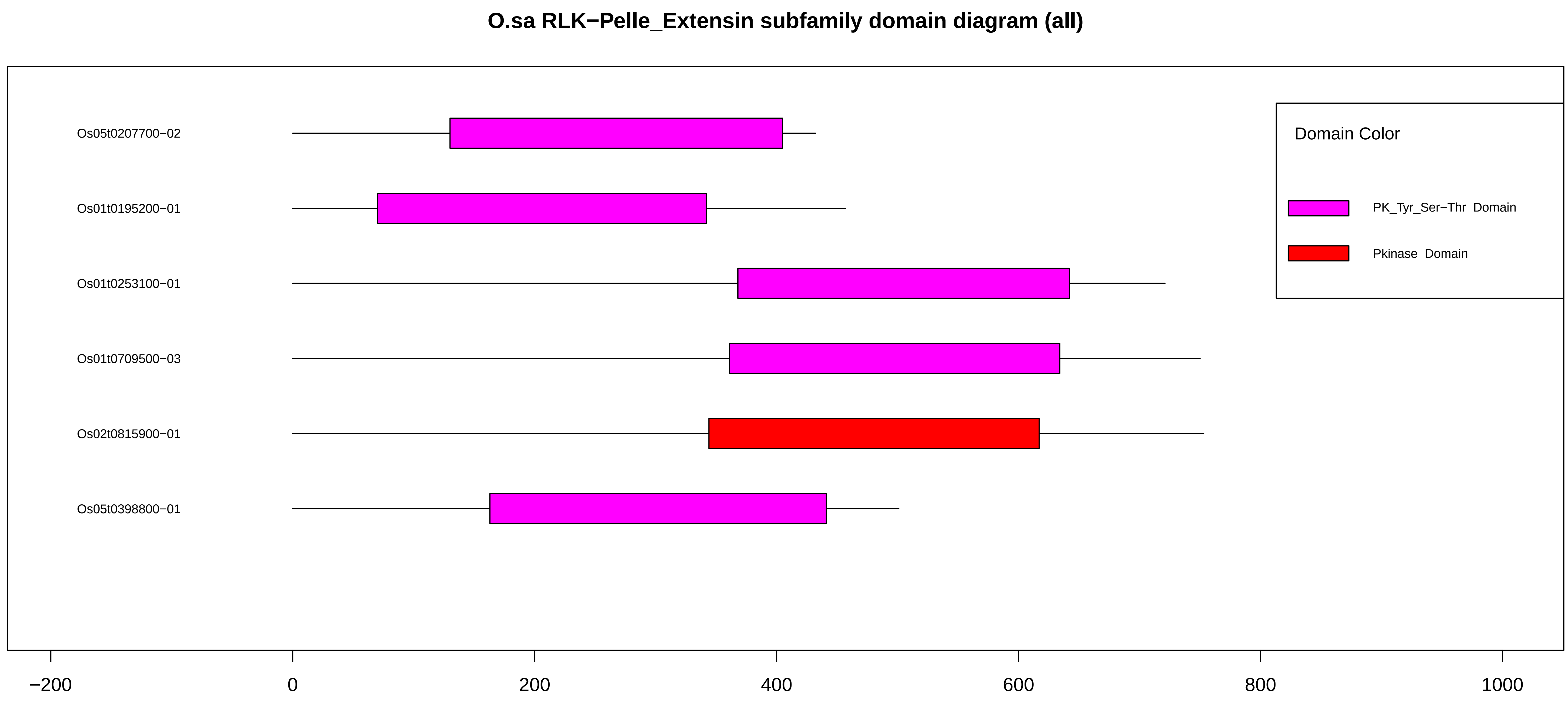
O.sa RLK−Pelle_Extensin subfamily domain diagram (all)
Os05t0207700−02
Os01t0195200−01
Os01t0253100−01
Os01t0709500−03
Os02t0815900−01
Os05t0398800−01
Domain Color
PK_Tyr_Ser−Thr Domain
Pkinase Domain
−200
0
200
400
600
800
1000

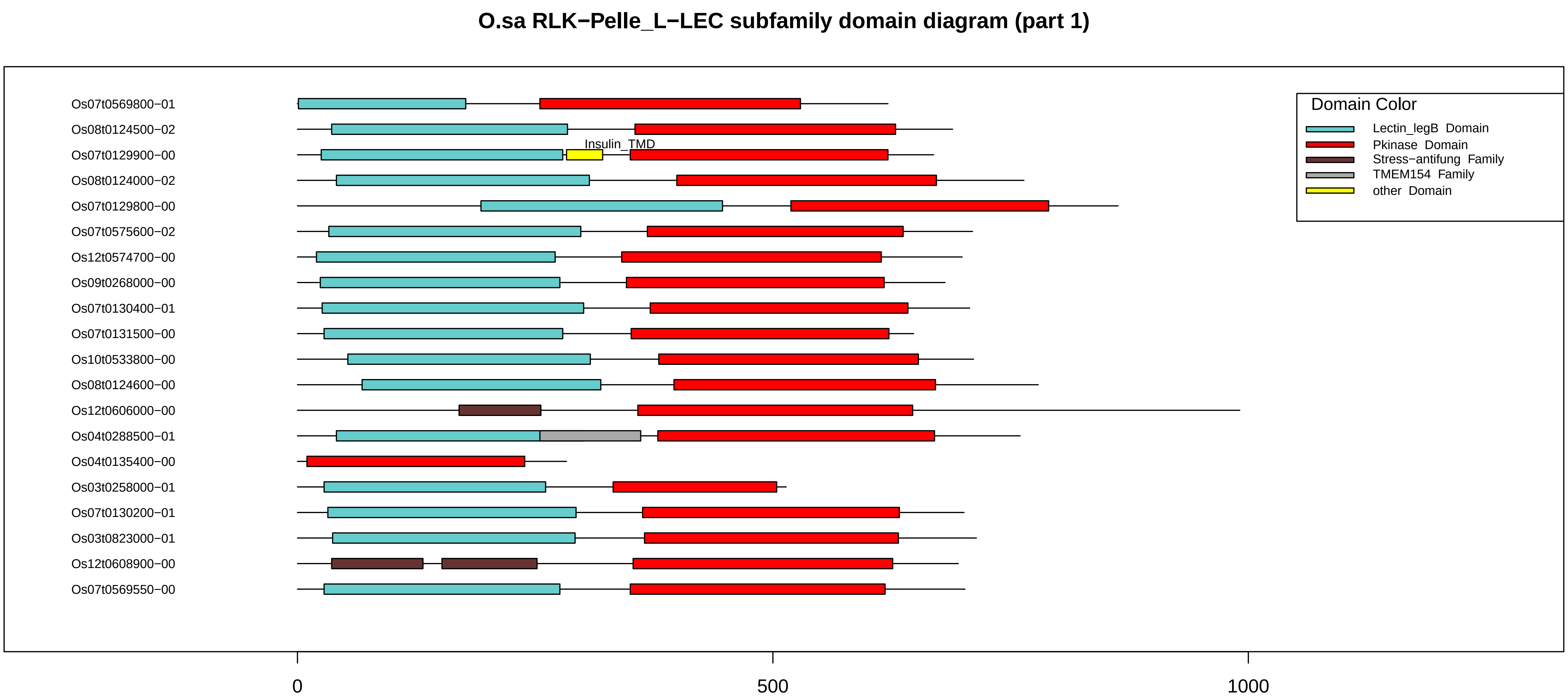
O.sa RLK−Pelle_L−LEC subfamily domain diagram (part 1)
Os07t0569800−01
Os08t0124500−02
Os07t0129900−00
Insulin_TMD
Os08t0124000−02
Os07t0129800−00
Os07t0575600−02
Os12t0574700−00
Os09t0268000−00
Os07t0130400−01
Os07t0131500−00
Os10t0533800−00
Os08t0124600−00
Os12t0606000−00
Os04t0288500−01
Os04t0135400−00
Os03t0258000−01
Os07t0130200−01
Os03t0823000−01
Os12t0608900−00
Os07t0569550−00
Domain Color
Lectin_legB Domain
Pkinase Domain
Stress−antifung Family
TMEM154 Family
other Domain
0
500
1000

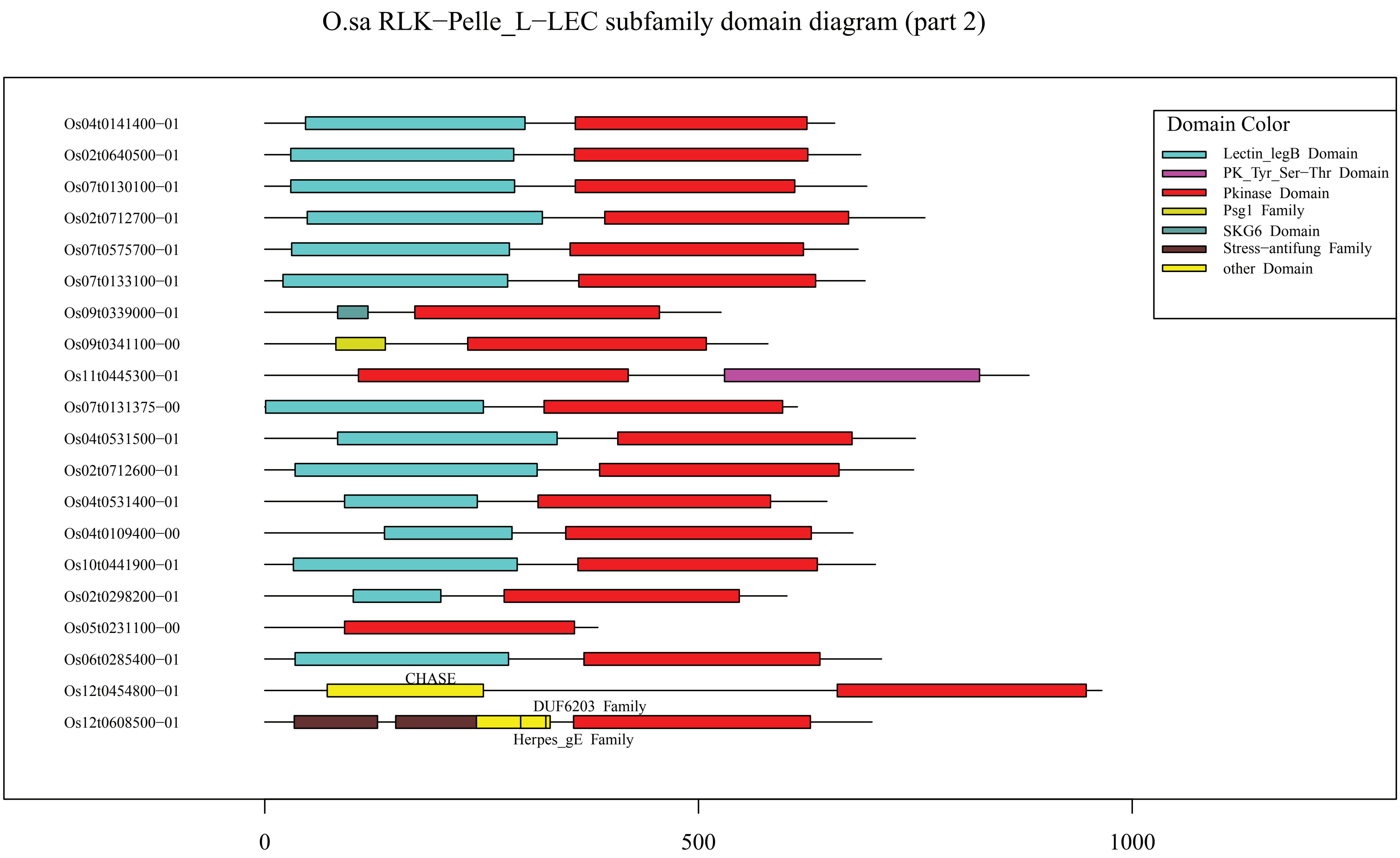
O.sa RLK−Pelle_L−LEC subfamily domain diagram (part 2)
Os04t0141400−01
Os02t0640500−01
Os07t0130100−01
Os02t0712700−01
Os07t0575700−01
Os07t0133100−01
Os09t0339000−01
Os09t0341100−00
Os11t0445300−01
Os07t0131375−00
Os04t0531500−01
Os02t0712600−01
Os04t0531400−01
Os04t0109400−00
Os10t0441900−01
Os02t0298200−01
Os05t0231100−00
Os06t0285400−01
Os12t0454800−01
Os12t0608500−01
CHASE
DUF6203 Family
Herpes_gE Family
Domain Color
Lectin_legB Domain
PK_Tyr_Ser−Thr Domain
Pkinase Domain
Psg1 Family
SKG6 Domain
Stress−antifung Family
other Domain
0
500
1000

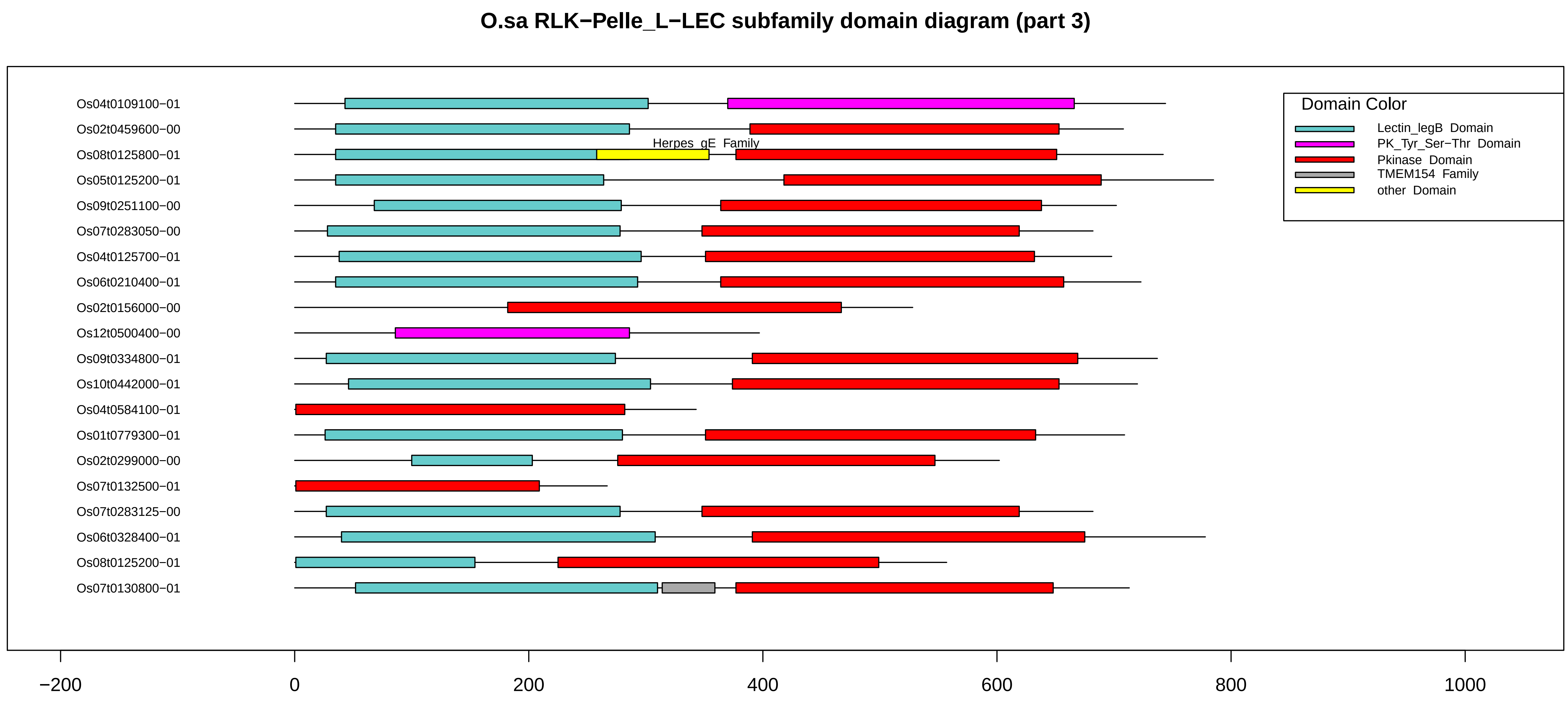
O.sa RLK−Pelle_L−LEC subfamily domain diagram (part 3)
Os04t0109100−01
Os02t0459600−00
Os08t0125800−01
Os05t0125200−01
Os09t0251100−00
Os07t0283050−00
Os04t0125700−01
Os06t0210400−01
Os02t0156000−00
Os12t0500400−00
Os09t0334800−01
Os10t0442000−01
Os04t0584100−01
Os01t0779300−01
Os02t0299000−00
Os07t0132500−01
Os07t0283125−00
Os06t0328400−01
Os08t0125200−01
Os07t0130800−01
Herpes_gE_Family
Domain Color
Lectin_legB Domain
PK_Tyr_Ser−Thr Domain
Pkinase Domain
TMEM154 Family
other Domain
−200
0
200
400
600
800
1000

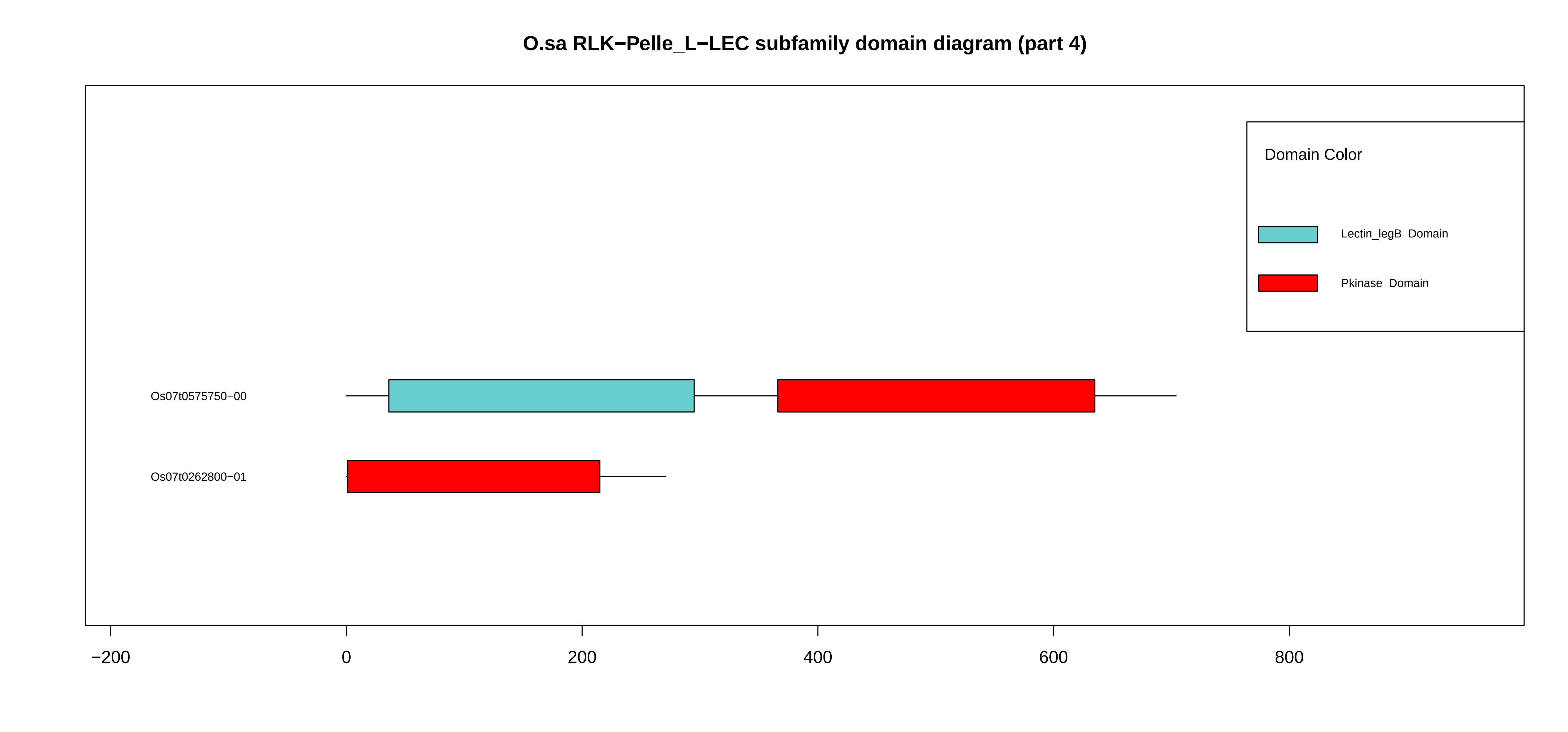
O.sa RLK−Pelle_L−LEC subfamily domain diagram (part 4)
Domain Color
Lectin_legB Domain
Pkinase Domain
Os07t0575750−00
Os07t0262800−01
−200
0
200
400
600
800

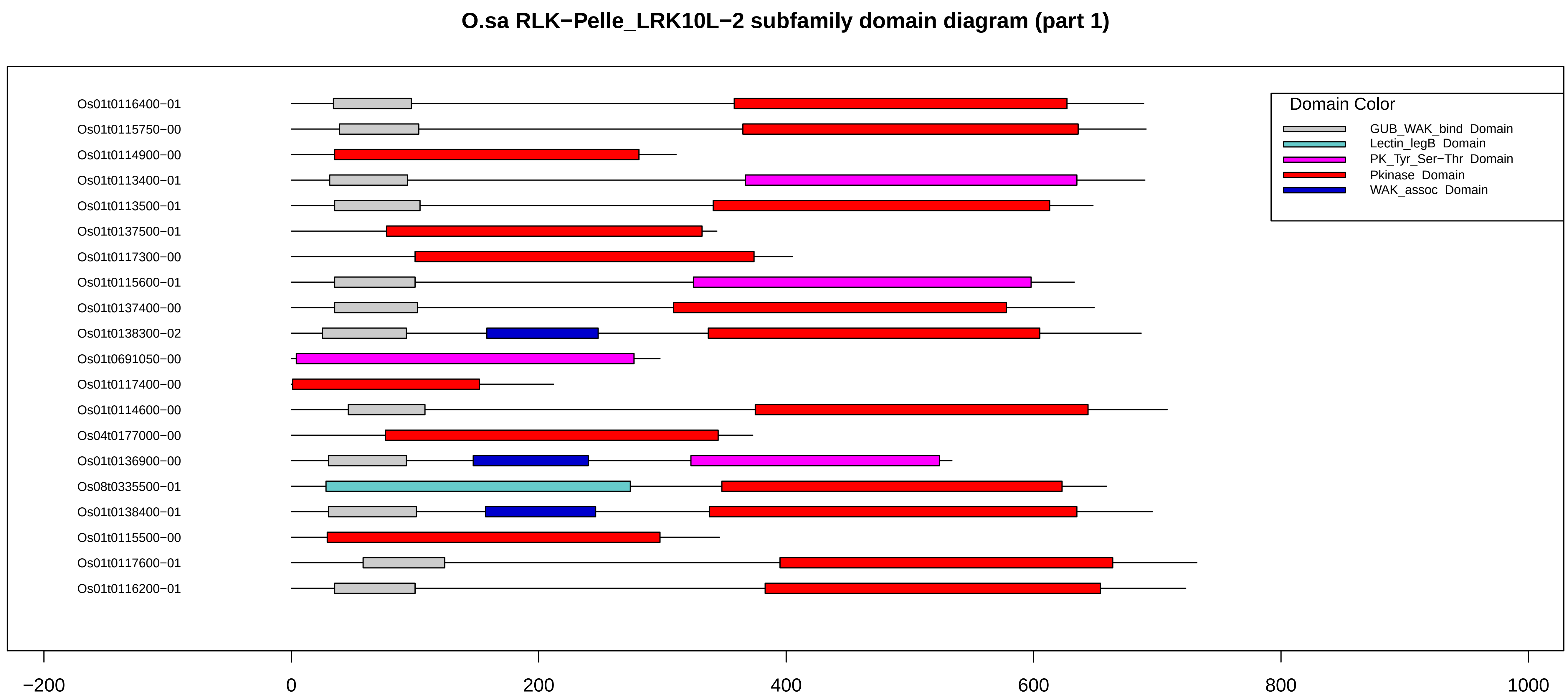

O.sa RLK−Pelle_LRK10L−2 subfamily domain diagram (part 1)
Os01t0116400−01
Os01t0115750−00
Os01t0114900−00
Os01t0113400−01
Os01t0113500−01
Os01t0137500−01
Os01t0117300−00
Os01t0115600−01
Os01t0137400−00
Os01t0138300−02
Os01t0691050−00
Os01t0117400−00
Os01t0114600−00
Os04t0177000−00
Os01t0136900−00
Os08t0335500−01
Os01t0138400−01
Os01t0115500−00
Os01t0117600−01
Os01t0116200−01
Domain Color
GUB_WAK_bind Domain
Lectin_legB Domain
PK_Tyr_Ser−Thr Domain
Pkinase Domain
WAK_assoc Domain
−200
0
200
400
600
800
1000

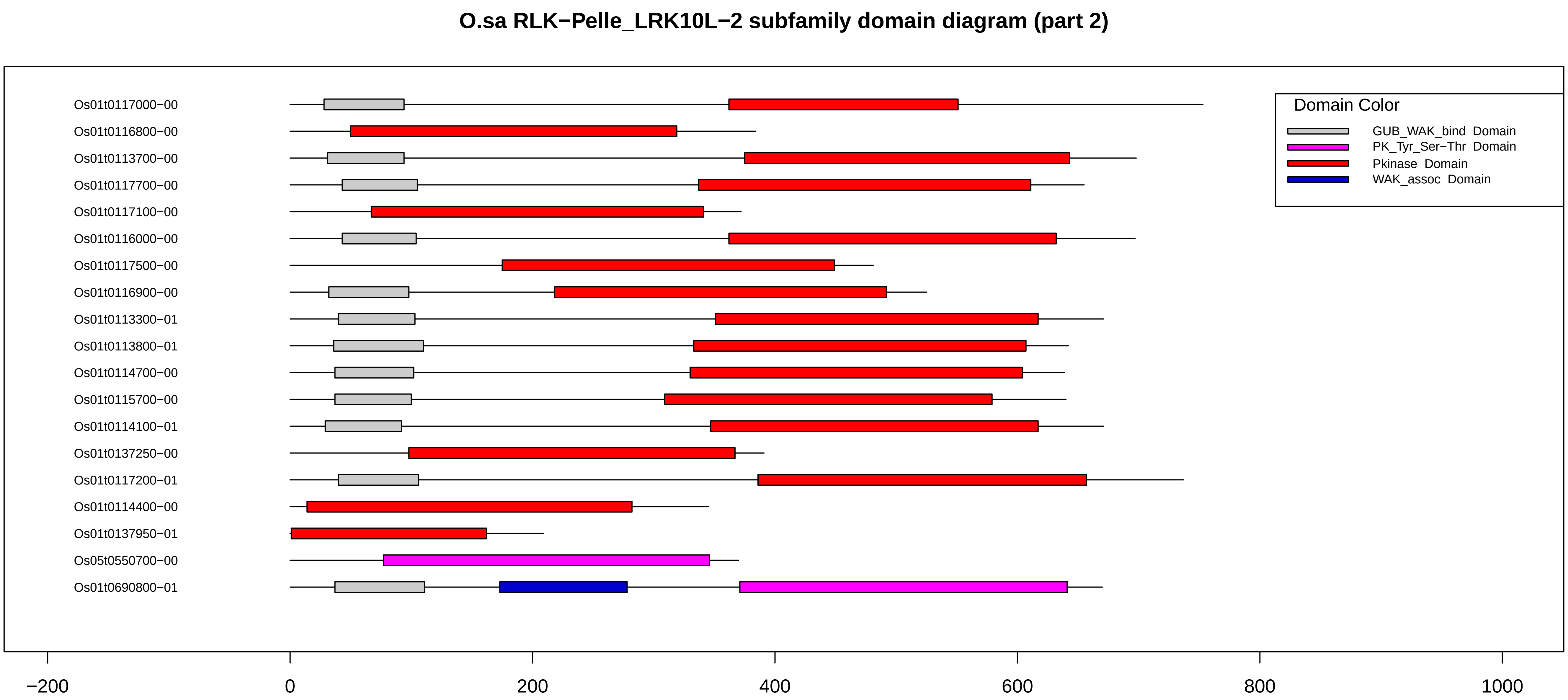
O.sa RLK−Pelle_LRK10L−2 subfamily domain diagram (part 2)
Domain Color
GUB_WAK_bind Domain
PK_Tyr_Ser−Thr Domain
Pkinase Domain
WAK_assoc Domain
Os01t0117000−00
Os01t0116800−00
Os01t0113700−00
Os01t0117700−00
Os01t0117100−00
Os01t0116000−00
Os01t0117500−00
Os01t0116900−00
Os01t0113300−01
Os01t0113800−01
Os01t0114700−00
Os01t0115700−00
Os01t0114100−01
Os01t0137250−00
Os01t0117200−01
Os01t0114400−00
Os01t0137950−01
Os05t0550700−00
Os01t0690800−01
−200
0
200
400
600
800
1000

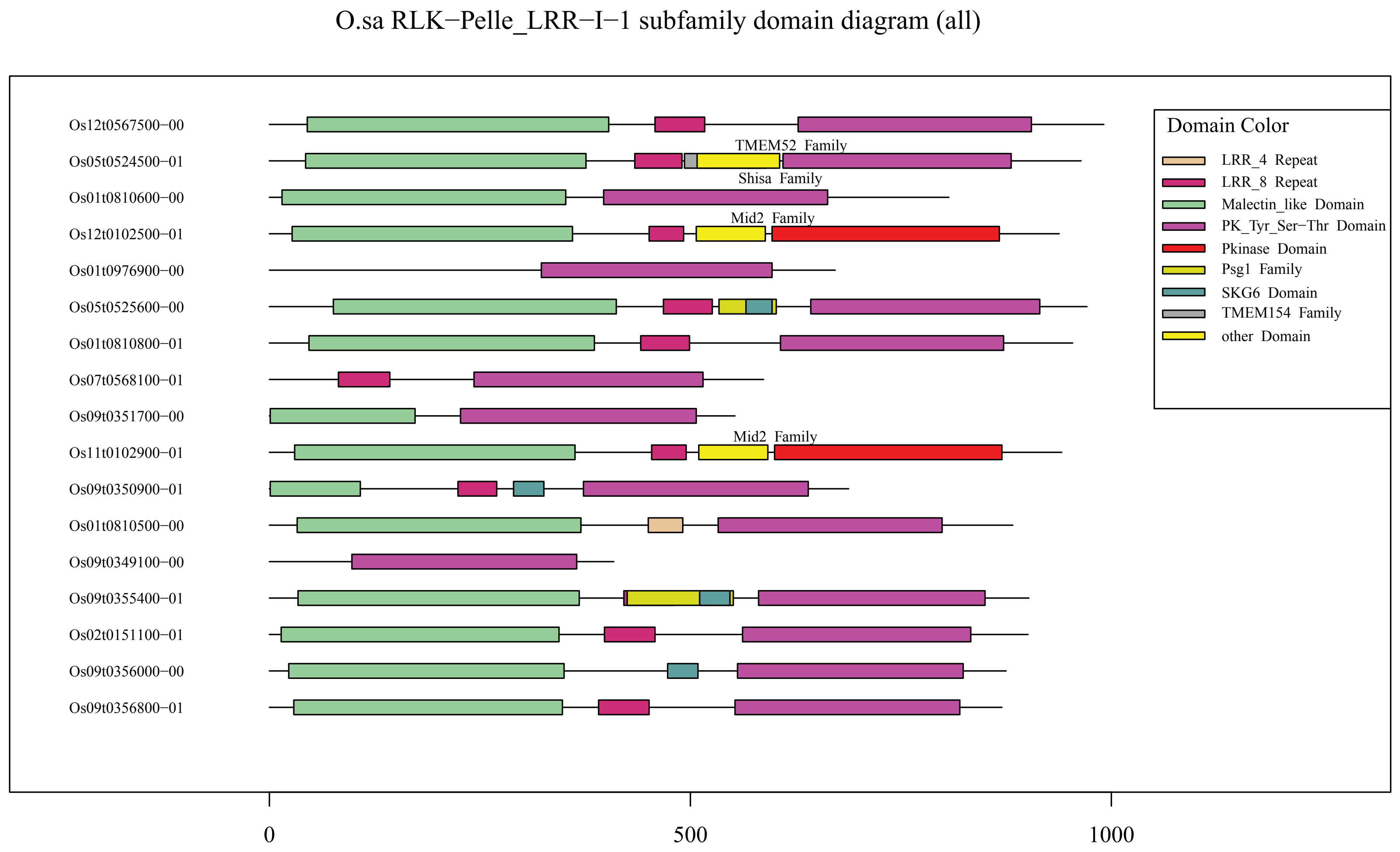
O.sa RLK−Pelle_LRR−I−1 subfamily domain diagram (all)
Os12t0567500−00
Os05t0524500−01
Os01t0810600−00
Os12t0102500−01
Os01t0976900−00
Os05t0525600−00
Os01t0810800−01
Os07t0568100−01
Os09t0351700−00
Os11t0102900−01
Os09t0350900−01
Os01t0810500−00
Os09t0349100−00
Os09t0355400−01
Os02t0151100−01
Os09t0356000−00
Os09t0356800−01
TMEM52 Family
Shisa Family
Mid2 Family
Mid2 Family
Domain Color
LRR_4 Repeat
LRR_8 Repeat
Malectin_like Domain
PK_Tyr_Ser−Thr Domain
Pkinase Domain
Psg1 Family
SKG6 Domain
TMEM154 Family
other Domain
0
500
1000

O.sa RLK−Pelle_LRR−I−2 subfamily domain diagram (all)

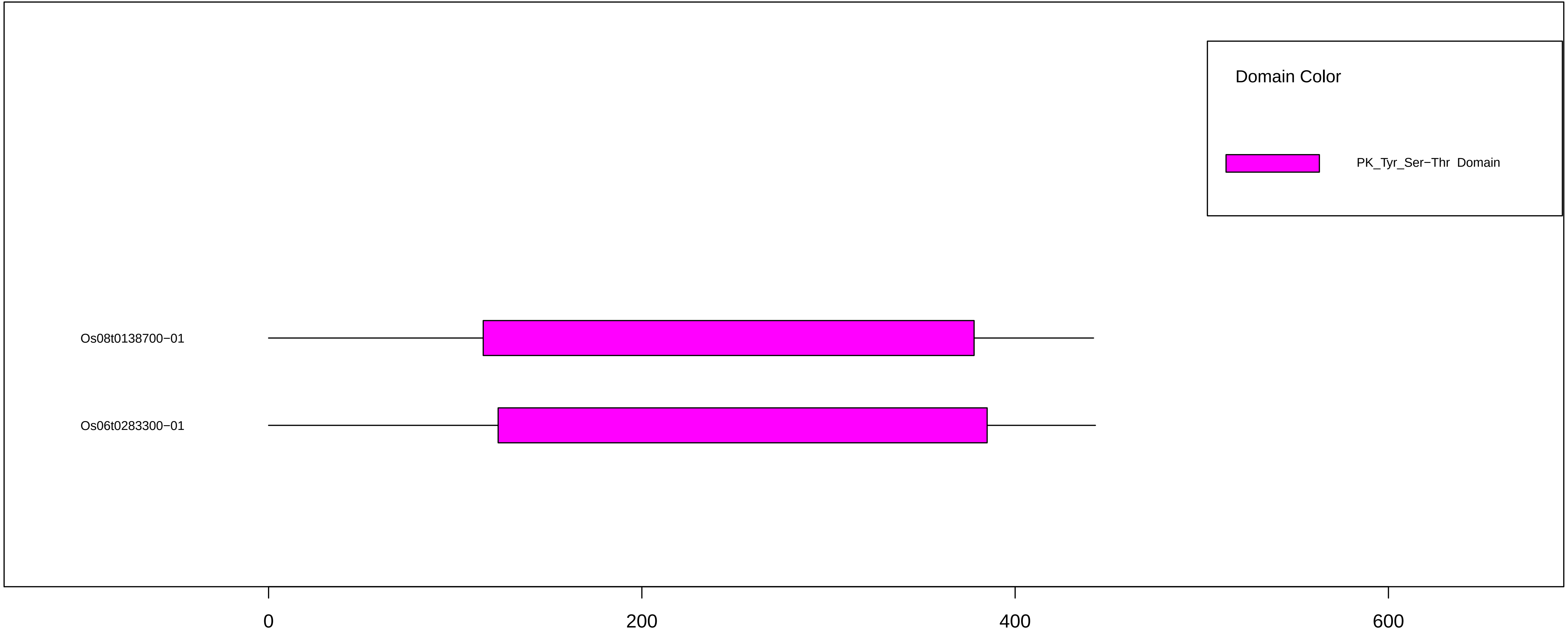

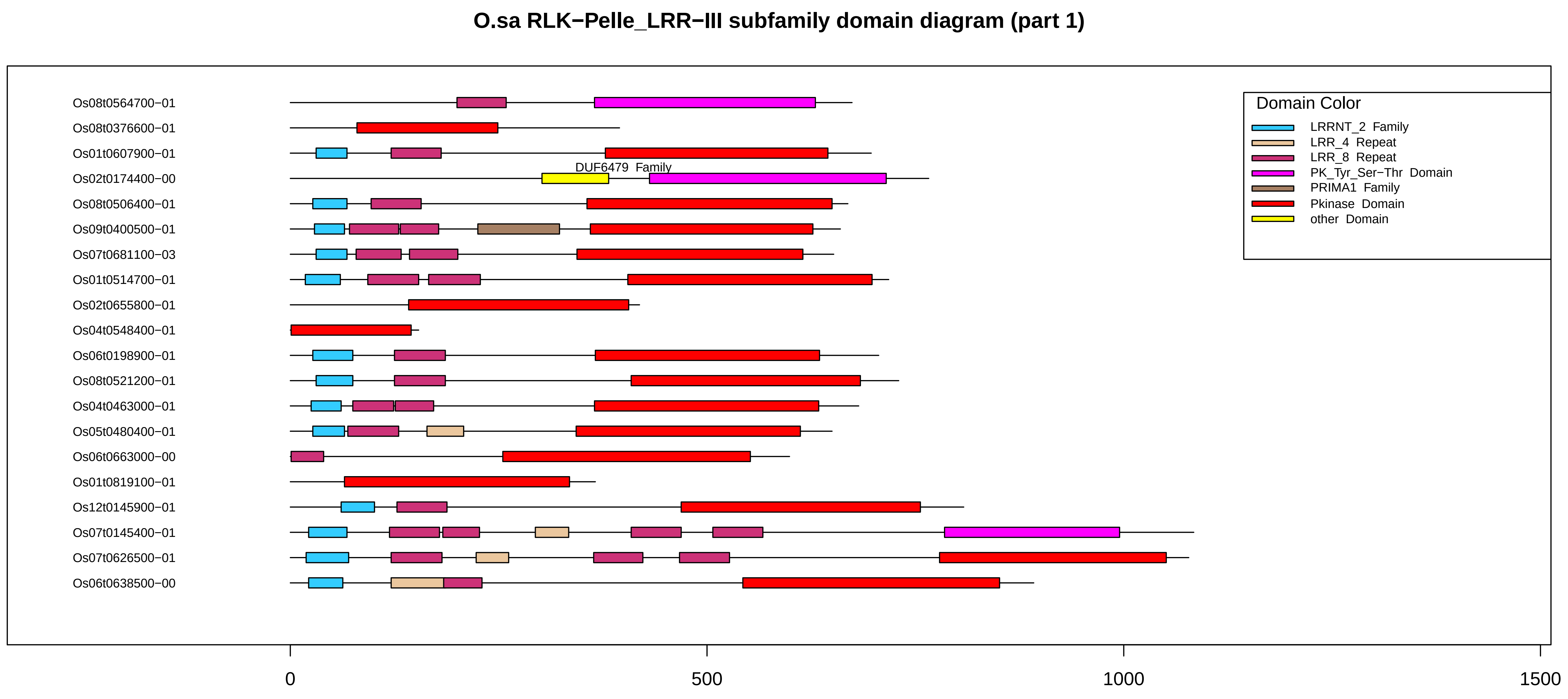
O.sa RLK−Pelle_LRR−III subfamily domain diagram (part 1)
Os08t0564700−01
Os08t0376600−01
Os01t0607900−01
Os02t0174400−00
Os08t0506400−01
Os09t0400500−01
Os07t0681100−03
Os01t0514700−01
Os02t0655800−01
Os04t0548400−01
Os06t0198900−01
Os08t0521200−01
Os04t0463000−01
Os05t0480400−01
Os06t0663000−00
Os01t0819100−01
Os12t0145900−01
Os07t0145400−01
Os07t0626500−01
Os06t0638500−00
DUF6479 Family
Domain Color
LRRNT_2 Family
LRR_4 Repeat
LRR_8 Repeat
PK_Tyr_Ser−Thr Domain
PRIMA1 Family
Pkinase Domain
other Domain
0
500
1000
1500

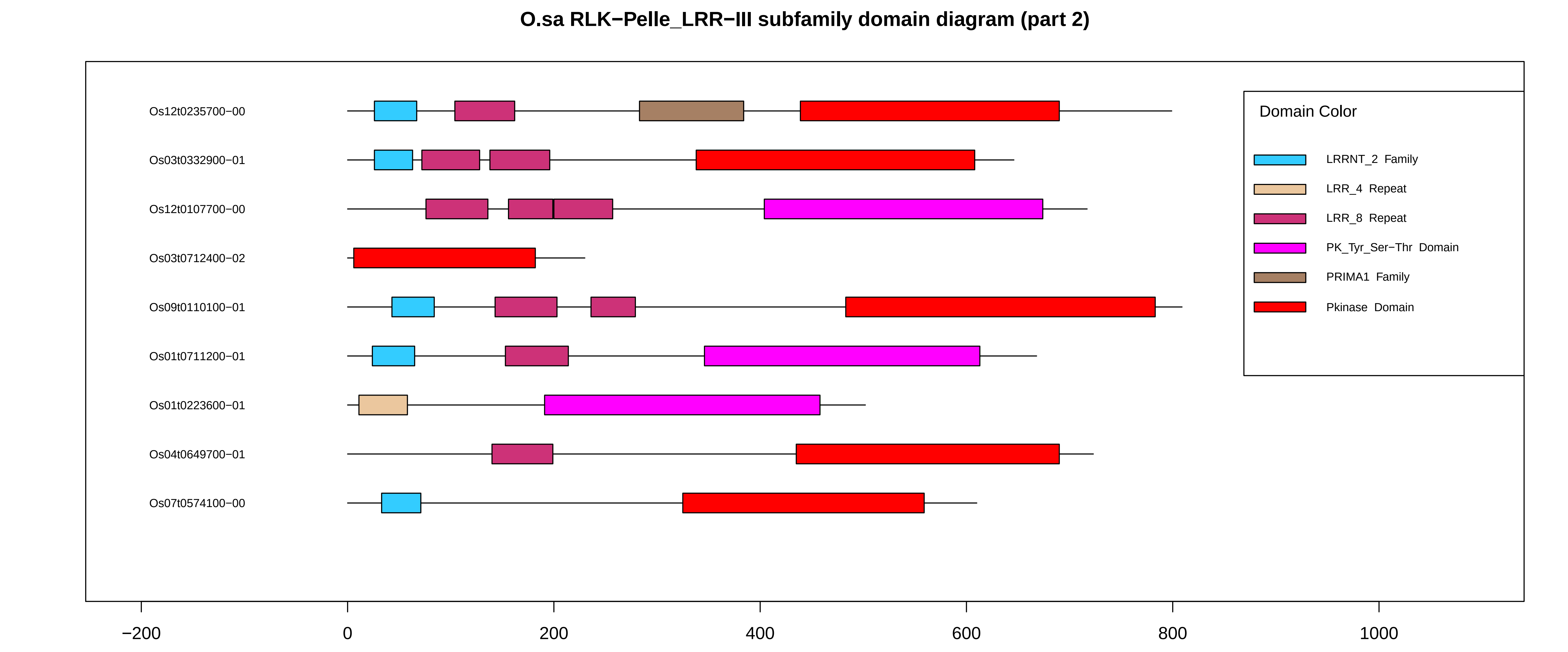
O.sa RLK−Pelle_LRR−III subfamily domain diagram (part 2)
Os12t0235700−00
Os03t0332900−01
Os12t0107700−00
Os03t0712400−02
Os09t0110100−01
Os01t0711200−01
Os01t0223600−01
Os04t0649700−01
Os07t0574100−00
Domain Color
LRRNT_2 Family
LRR_4 Repeat
LRR_8 Repeat
PK_Tyr_Ser−Thr Domain
PRIMA1 Family
Pkinase Domain
−200
0
200
400
600
800
1000

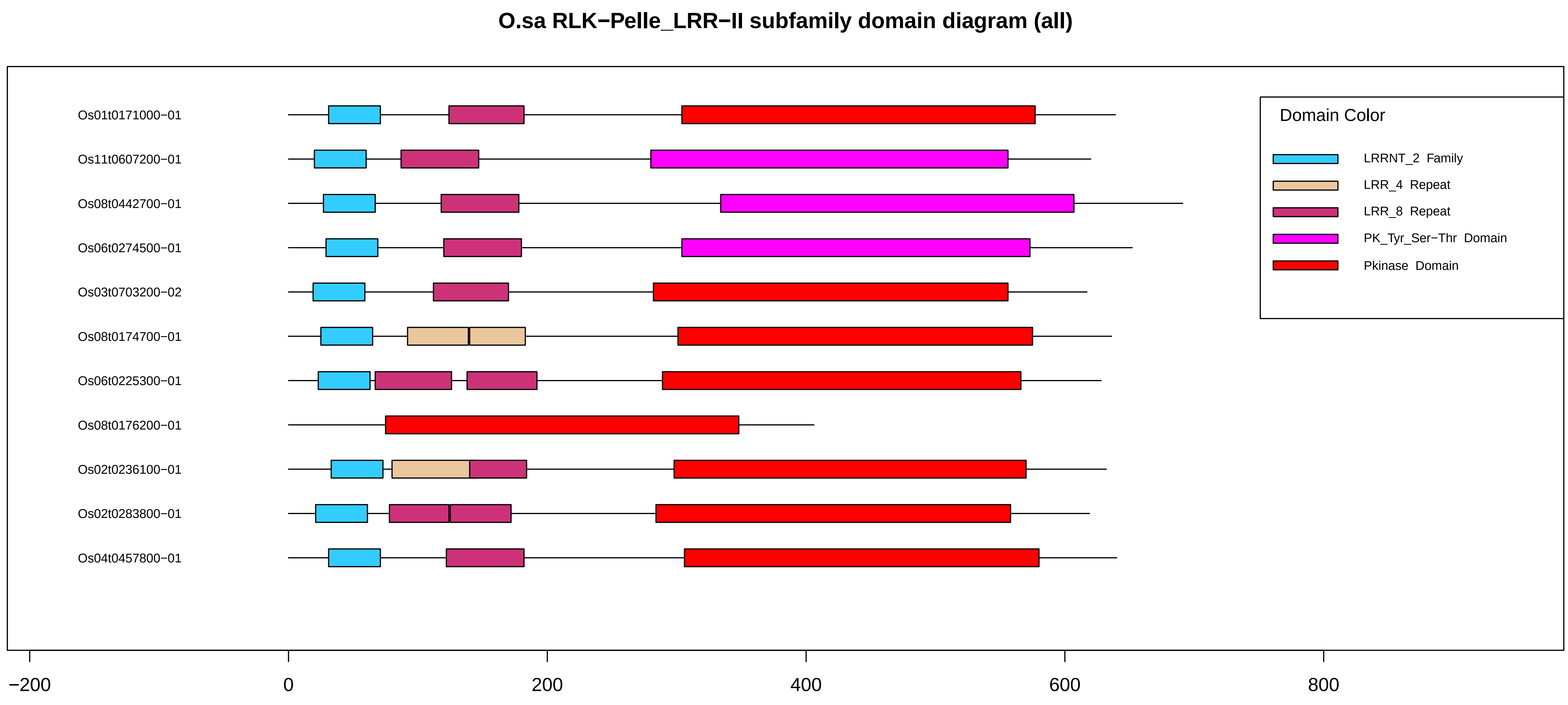

O.sa RLK−Pelle_LRR−II subfamily domain diagram (all)
Os01t0171000−01
Os11t0607200−01
Os08t0442700−01
Os06t0274500−01
Os03t0703200−02
Os08t0174700−01
Os06t0225300−01
Os08t0176200−01
Os02t0236100−01
Os02t0283800−01
Os04t0457800−01
Domain Color
LRRNT_2 Family
LRR_4 Repeat
LRR_8 Repeat
PK_Tyr_Ser−Thr Domain
Pkinase Domain
−200
0
200
400
600
800

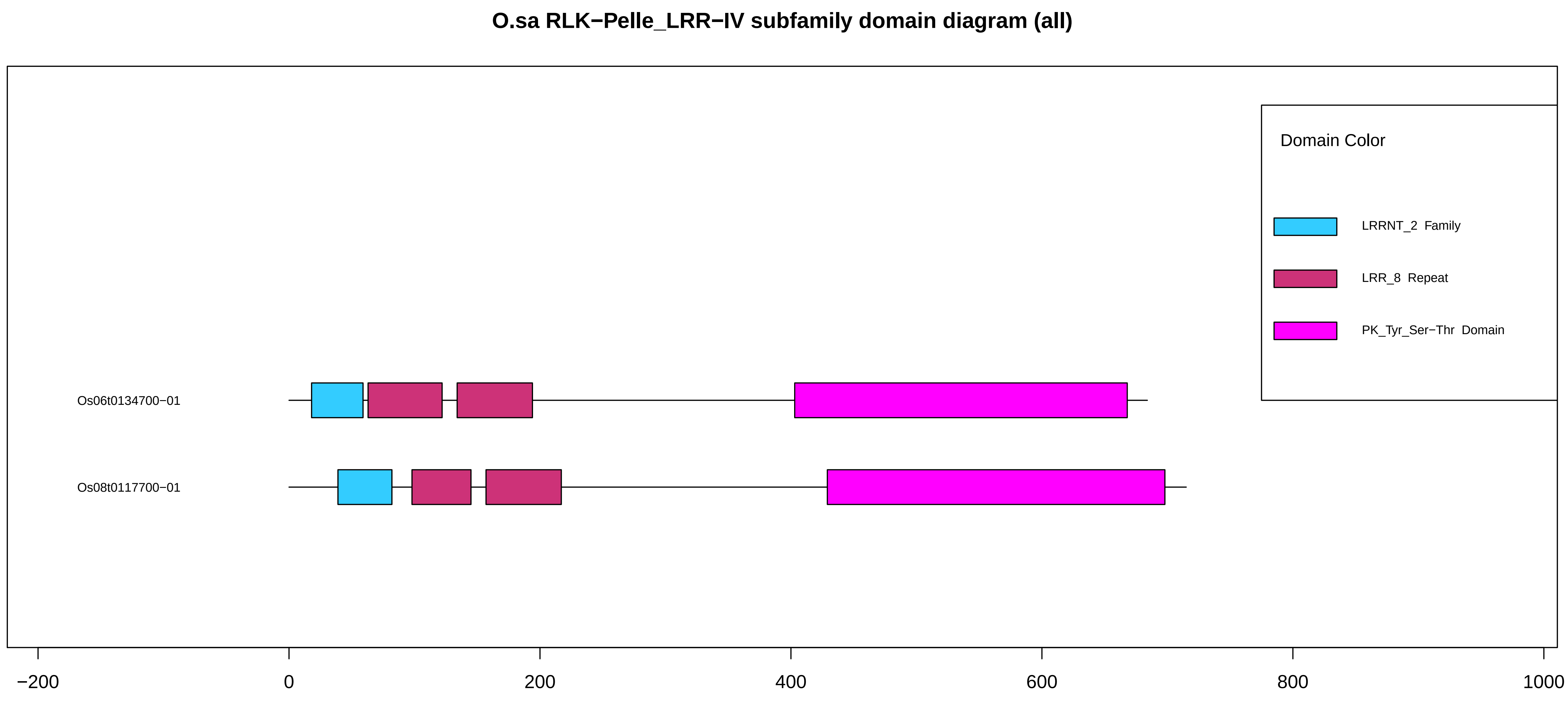
O.sa RLK−Pelle_LRR−IV subfamily domain diagram (all)
Domain Color
LRRNT_2 Family
LRR_8 Repeat
PK_Tyr_Ser−Thr Domain
Os06t0134700−01
Os08t0117700−01
−200
0
200
400
600
800
1000

O.sa RLK−Pelle_LRR−IX subfamily domain diagram (all)

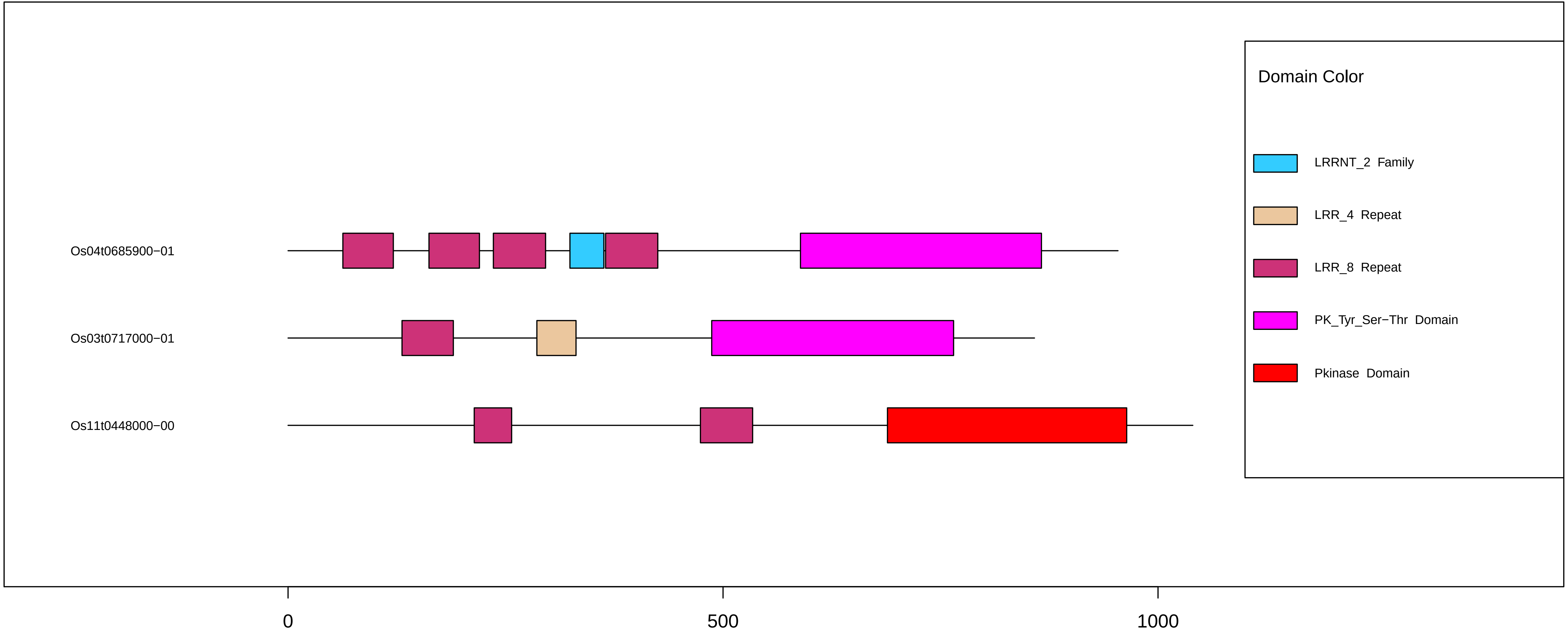

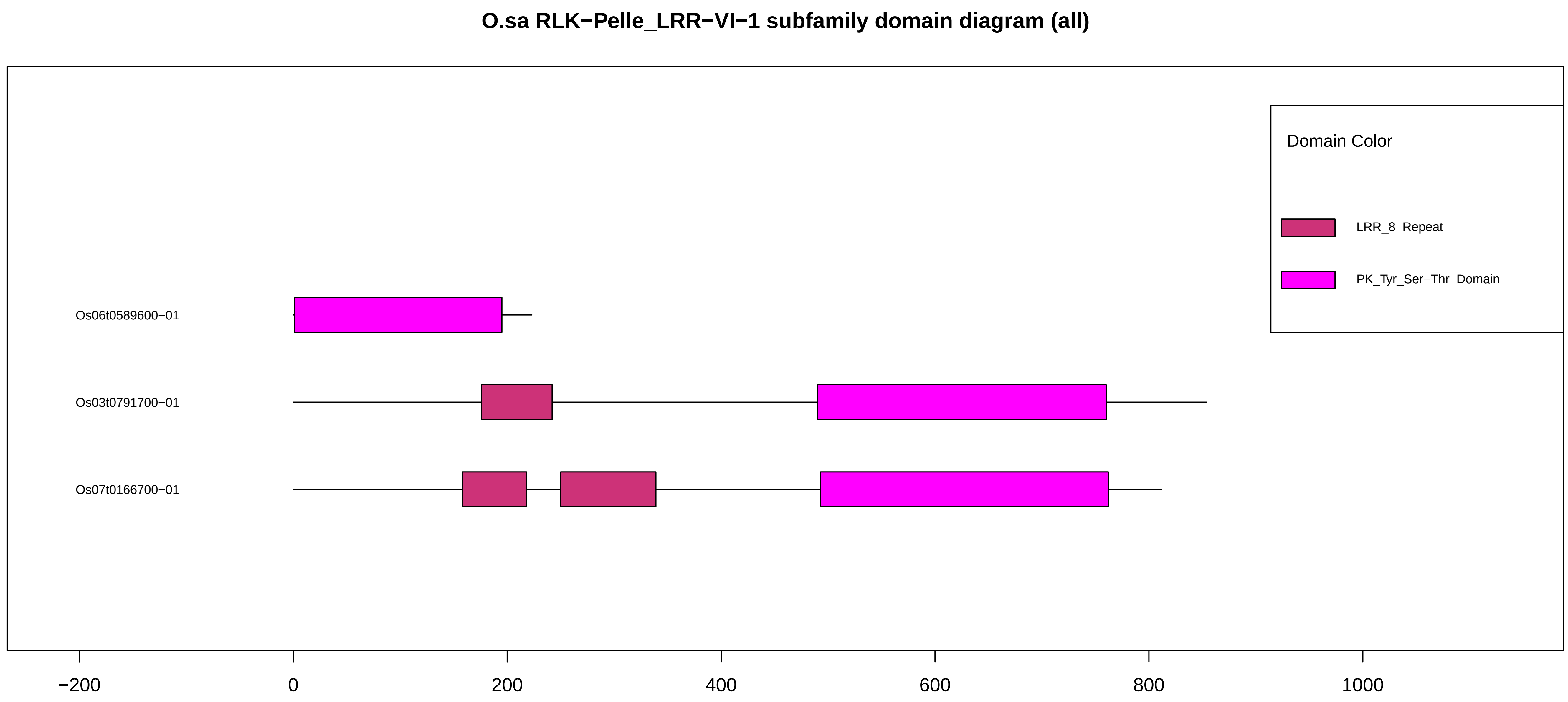
O.sa RLK−Pelle_LRR−VI−1 subfamily domain diagram (all)
Domain Color
LRR_8 Repeat
PK_Tyr_Ser−Thr Domain
Os06t0589600−01
Os03t0791700−01
Os07t0166700−01
−200
0
200
400
600
800
1000

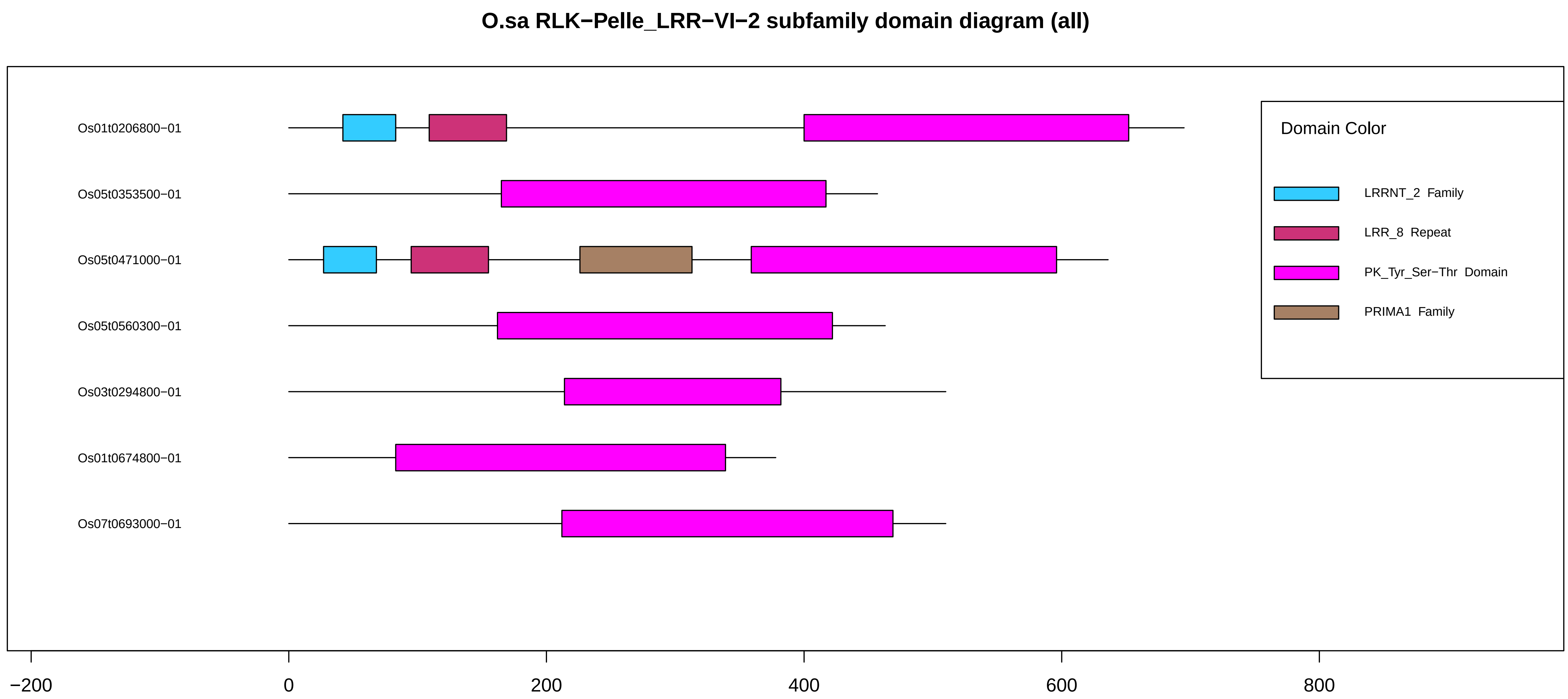

O.sa RLK−Pelle_LRR−VI−2 subfamily domain diagram (all)
Os01t0206800−01
Os05t0353500−01
Os05t0471000−01
Os05t0560300−01
Os03t0294800−01
Os01t0674800−01
Os07t0693000−01
Domain Color
LRRNT_2 Family
LRR_8 Repeat
PK_Tyr_Ser−Thr Domain
PRIMA1 Family
−200
0
200
400
600
800

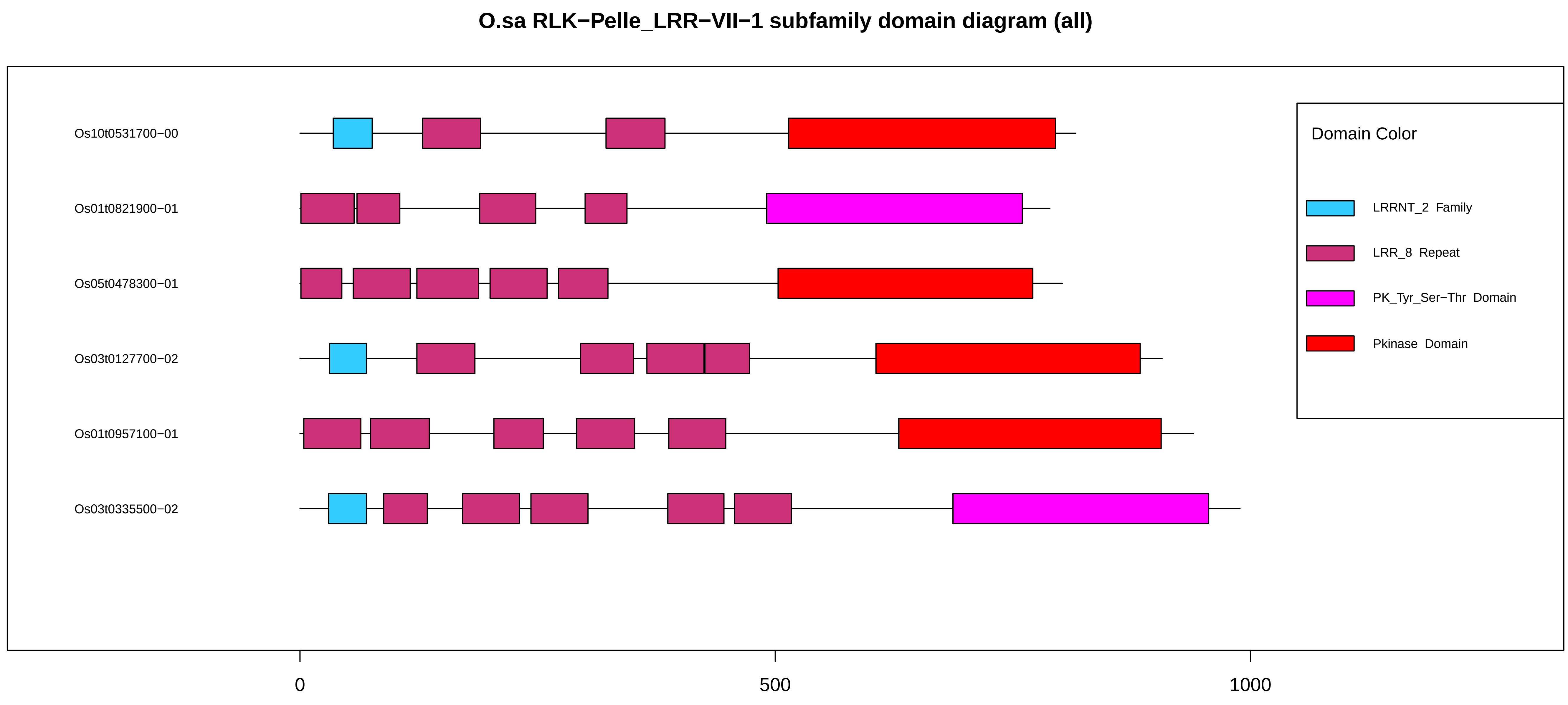
O.sa RLK−Pelle_LRR−VII−1 subfamily domain diagram (all)
Os10t0531700−00
Os01t0821900−01
Os05t0478300−01
Os03t0127700−02
Os01t0957100−01
Os03t0335500−02
Domain Color
LRRNT_2 Family
LRR_8 Repeat
PK_Tyr_Ser−Thr Domain
Pkinase Domain
0
500
1000

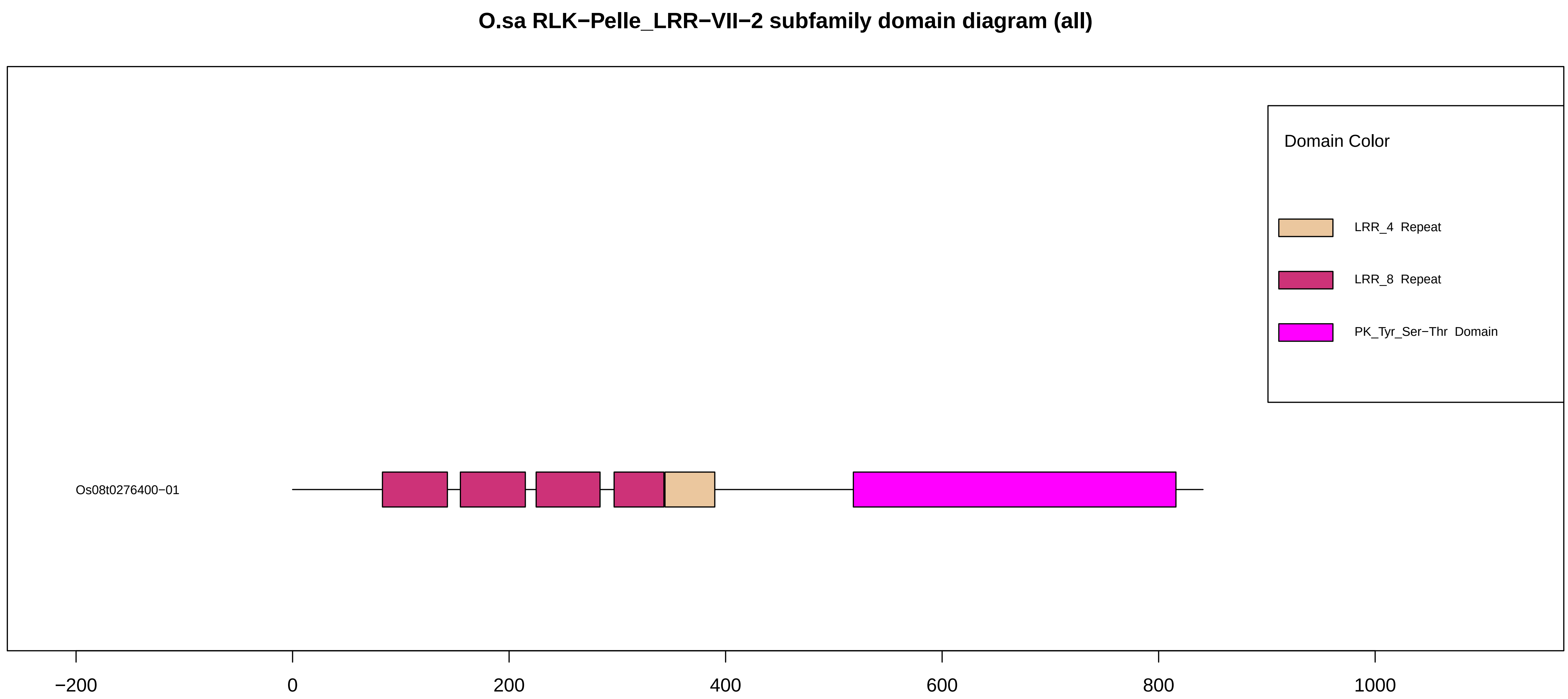
O.sa RLK−Pelle_LRR−VII−2 subfamily domain diagram (all)
Domain Color
LRR_4 Repeat
LRR_8 Repeat
PK_Tyr_Ser−Thr Domain
Os08t0276400−01
−200
0
200
400
600
800
1000

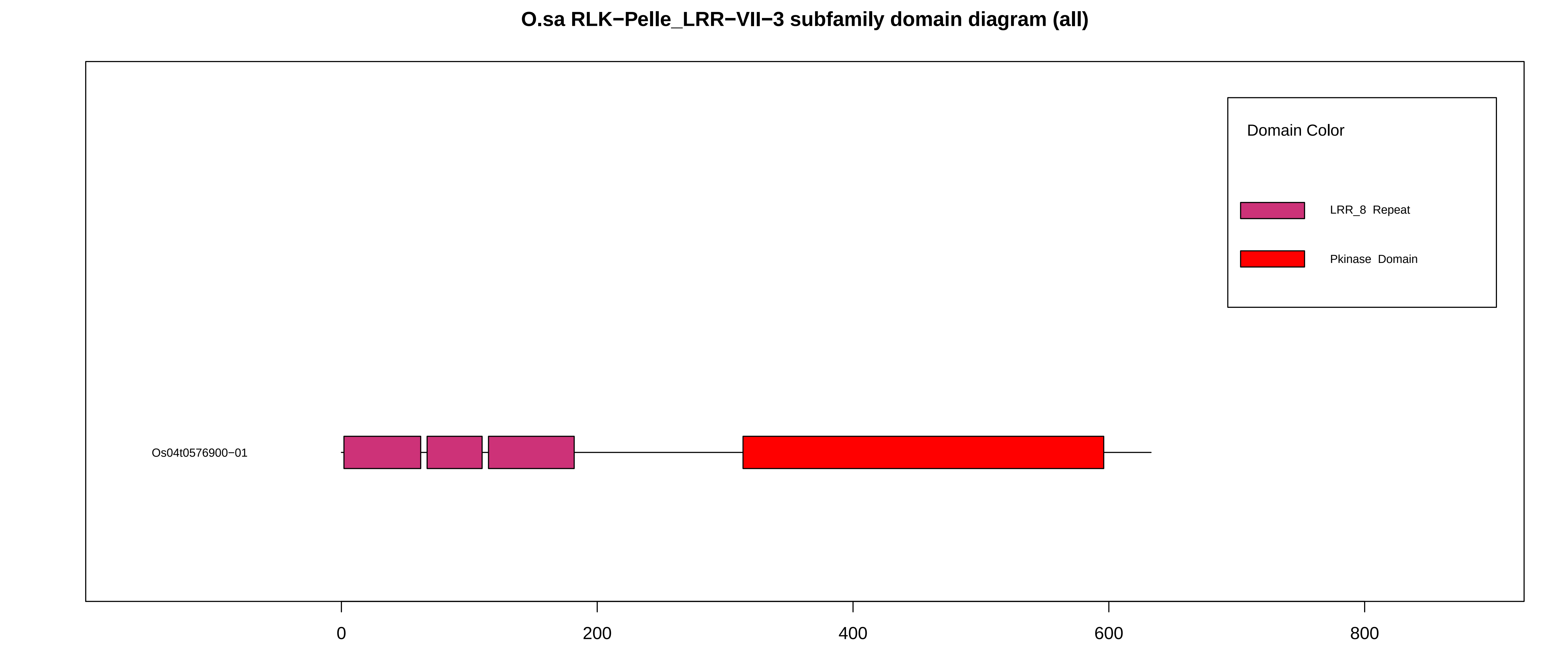

O.sa RLK−Pelle_LRR−VII−3 subfamily domain diagram (all)
Domain Color
LRR_8 Repeat
Pkinase Domain
Os04t0576900−01
0
200
400
600
800

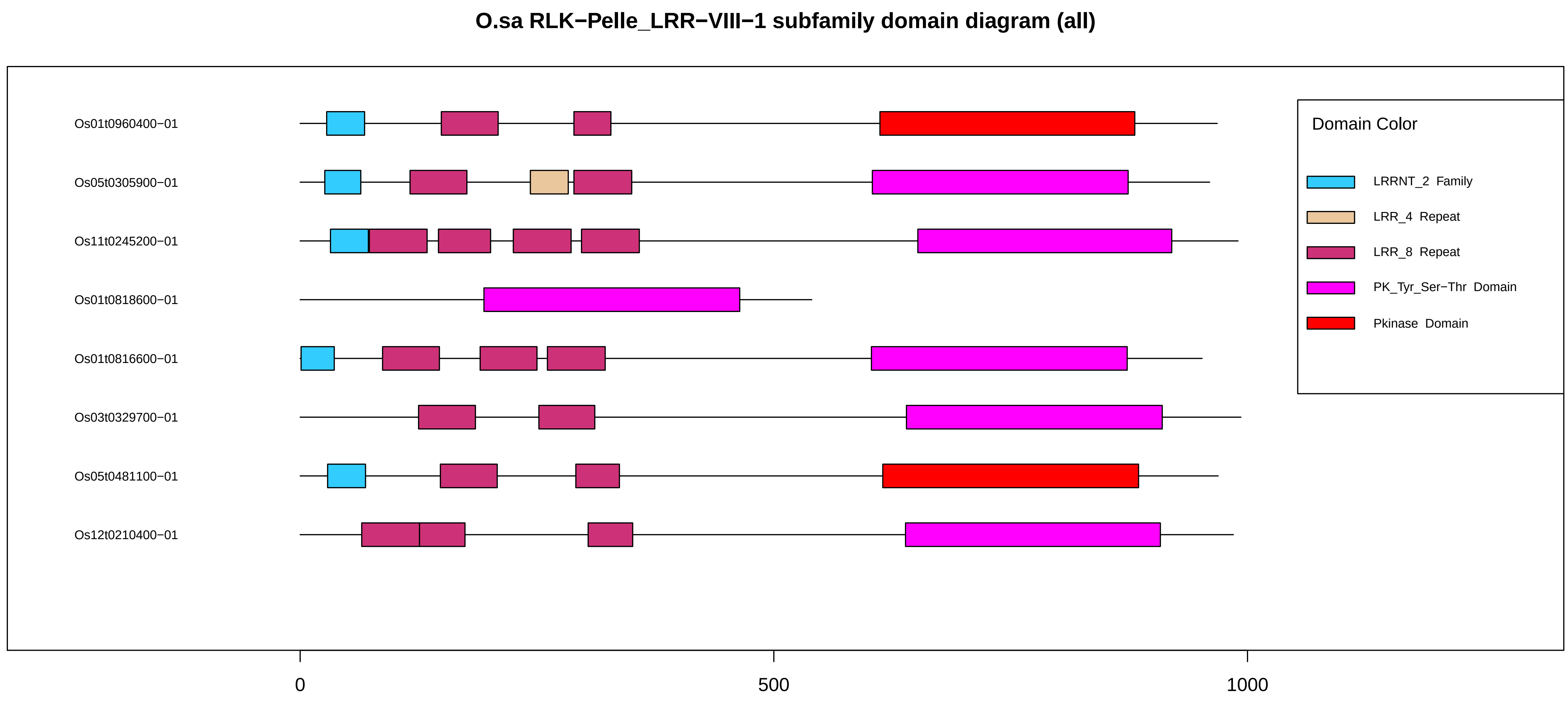
O.sa RLK−Pelle_LRR−VIII−1 subfamily domain diagram (all)
Os01t0960400−01
Os05t0305900−01
Os11t0245200−01
Os01t0818600−01
Os01t0816600−01
Os03t0329700−01
Os05t0481100−01
Os12t0210400−01
Domain Color
LRRNT_2 Family
LRR_4 Repeat
LRR_8 Repeat
PK_Tyr_Ser−Thr Domain
Pkinase Domain
0
500
1000

O.sa RLK−Pelle_LRR−V subfamily domain diagram (all)

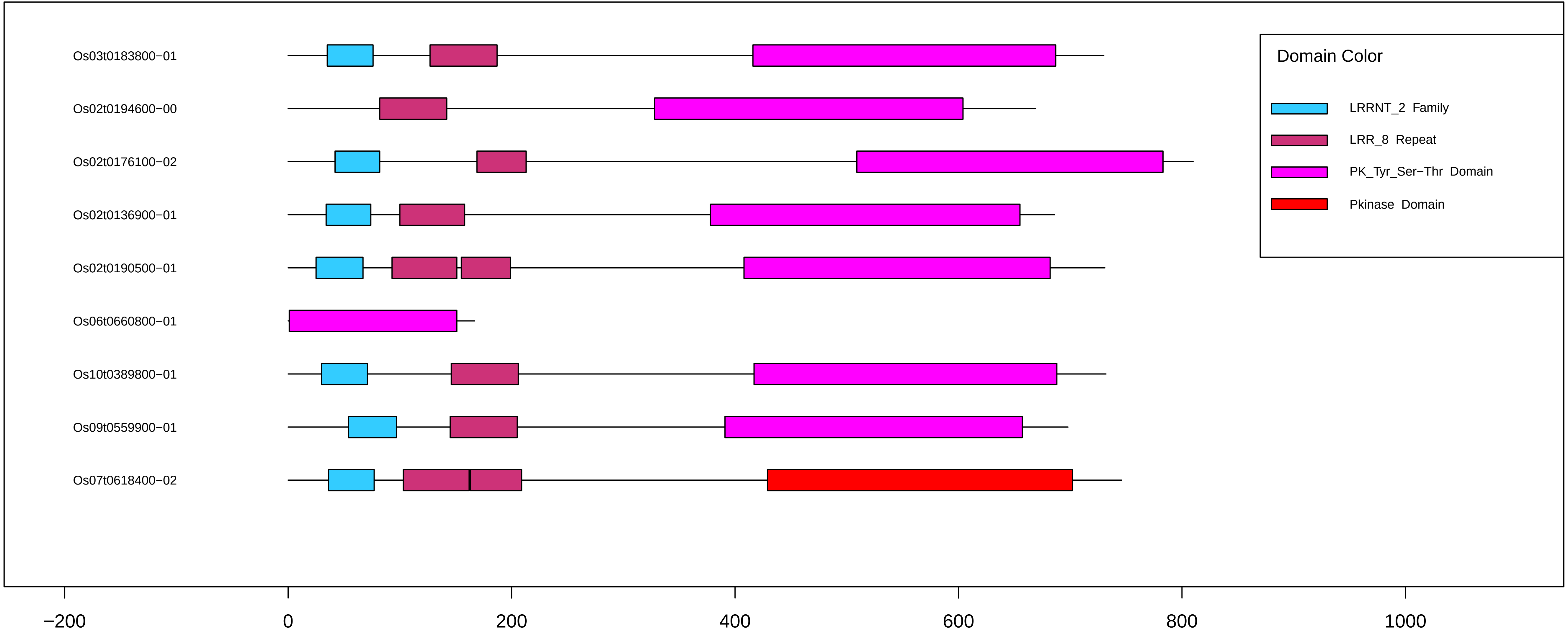

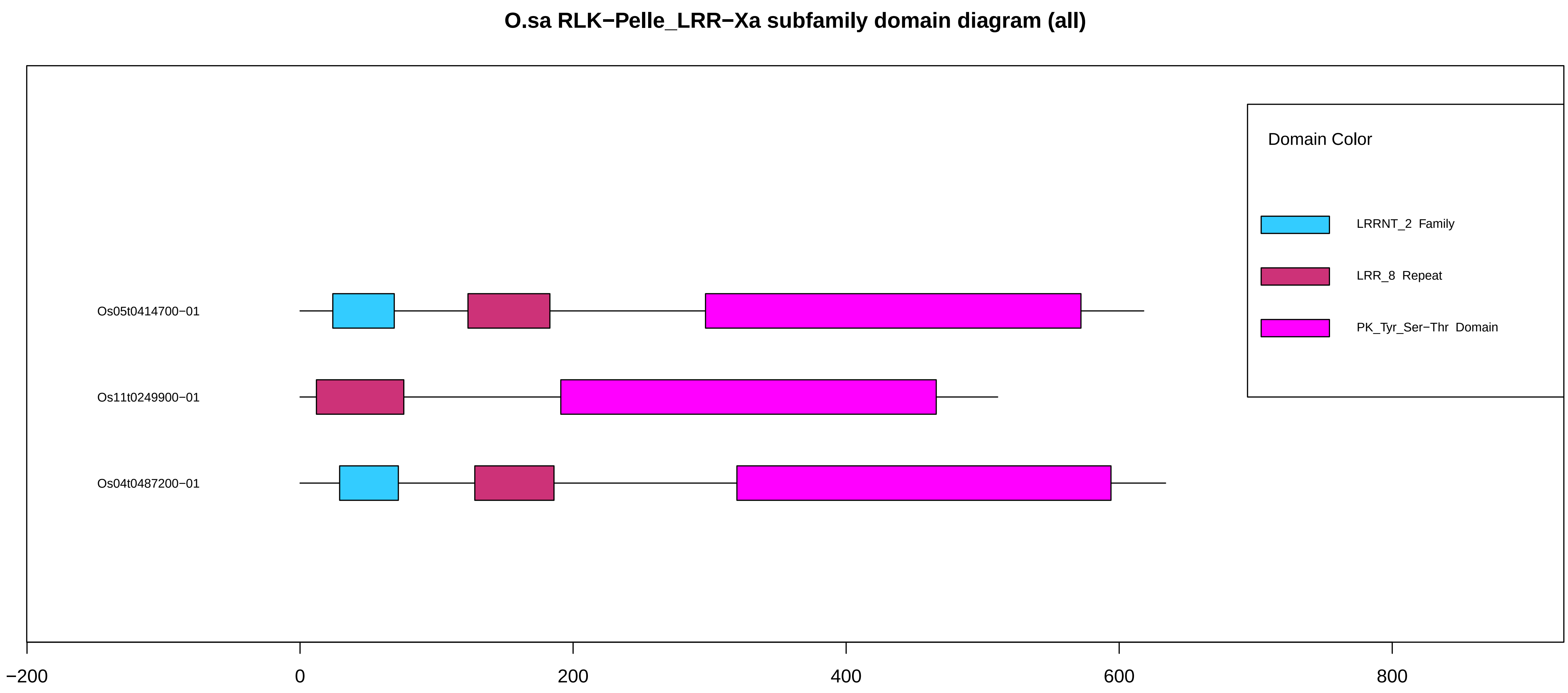
O.sa RLK−Pelle_LRR−Xa subfamily domain diagram (all)
Domain Color
LRRNT_2 Family
LRR_8 Repeat
PK_Tyr_Ser−Thr Domain
Os05t0414700−01
Os11t0249900−01
Os04t0487200−01
−200
0
200
400
600
800

O.sa RLK−Pelle_LRR−Xb−1 subfamily domain diagram (part 1)

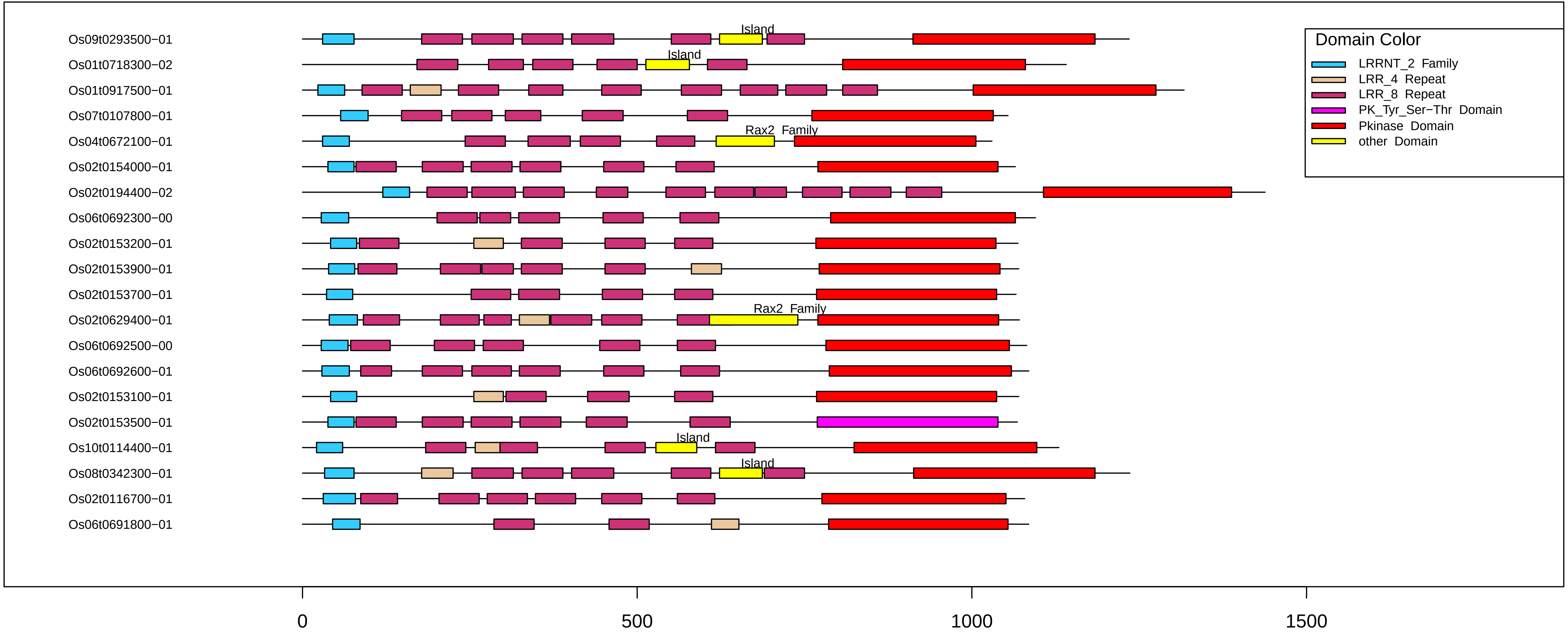

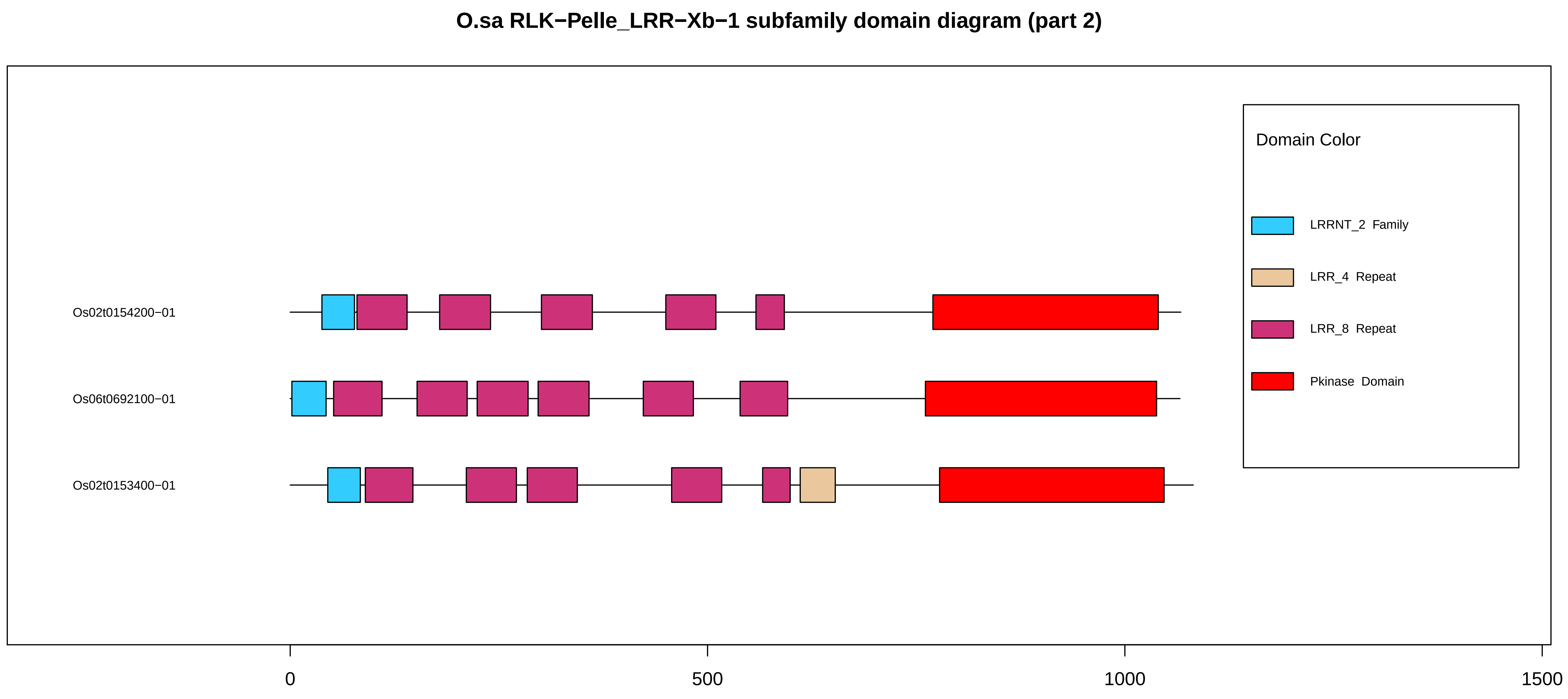

O.sa RLK−Pelle_LRR−Xb−1 subfamily domain diagram (part 2)
Os02t0154200−01
Os06t0692100−01
Os02t0153400−01
Domain Color
LRRNT_2 Family
LRR_4 Repeat
LRR_8 Repeat
Pkinase Domain
0
500
1000
1500

O.sa RLK−Pelle_LRR−Xb−2 subfamily domain diagram (all)

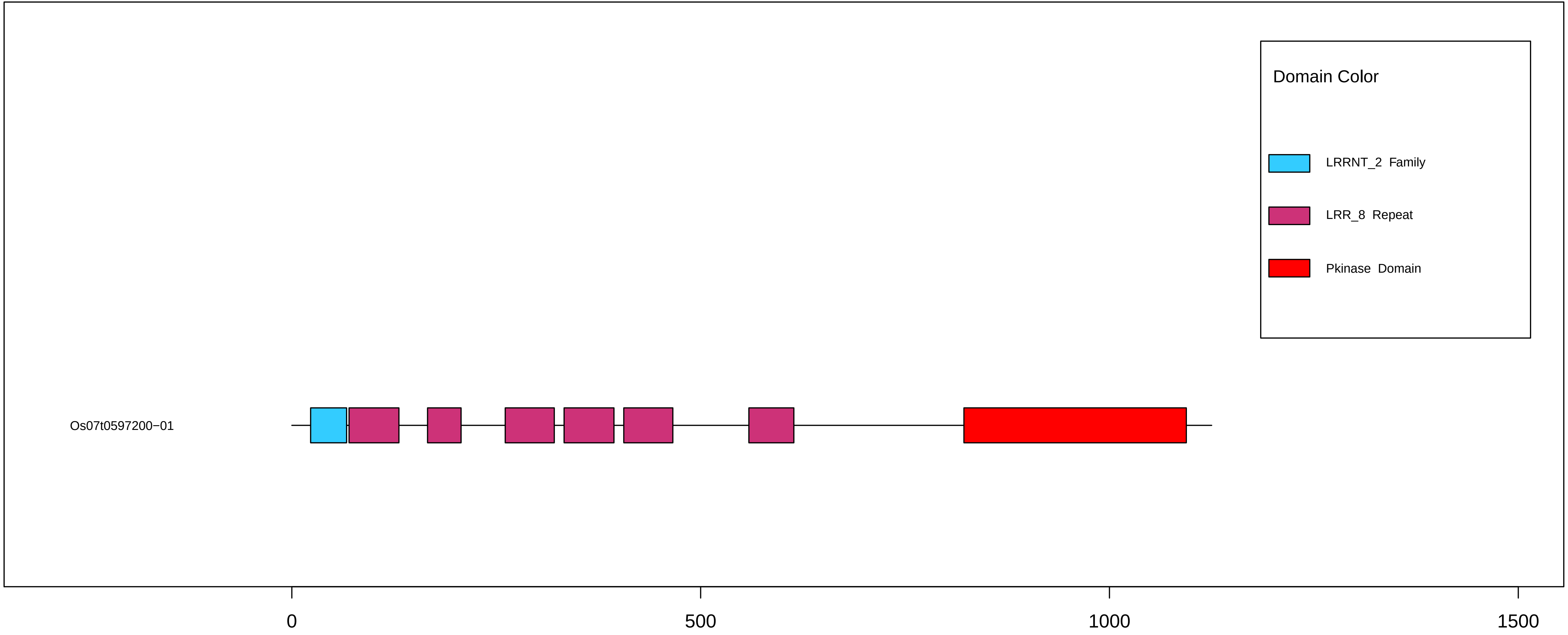

O.sa RLK−Pelle_LRR−XI−1 subfamily domain diagram (part 1)

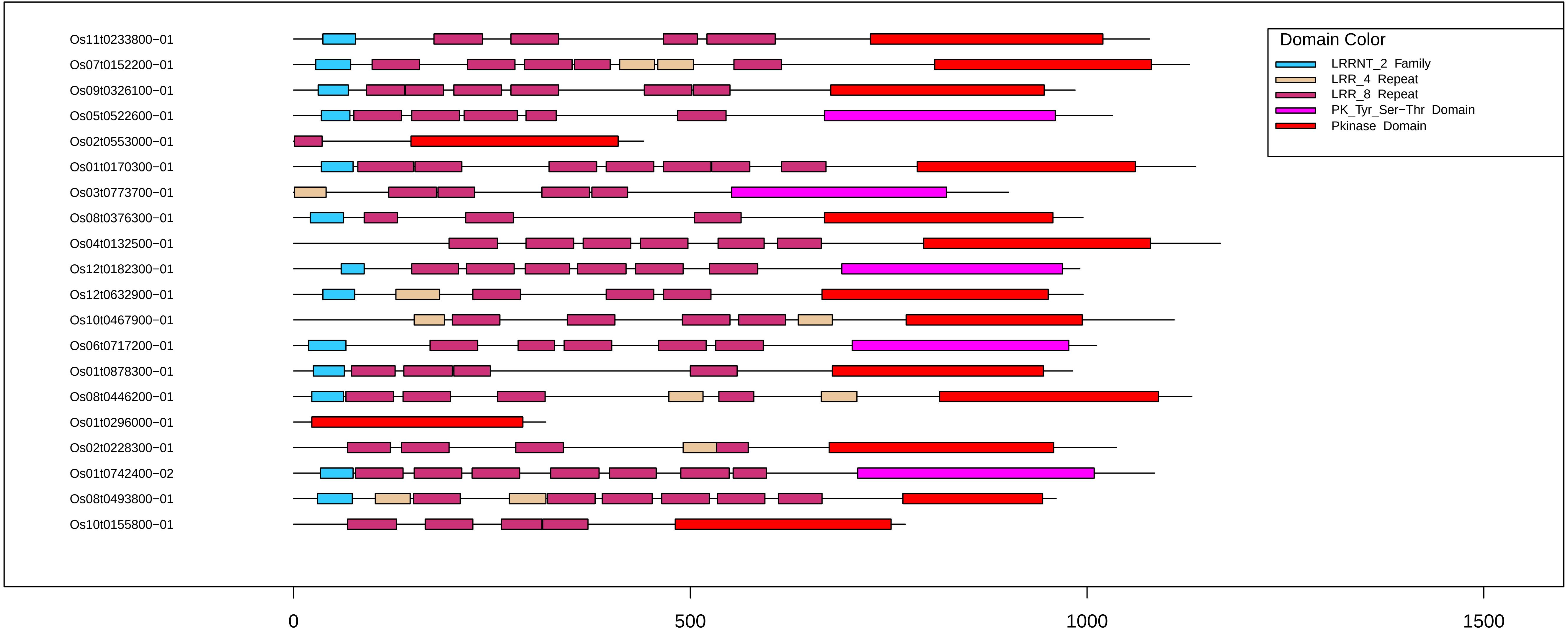

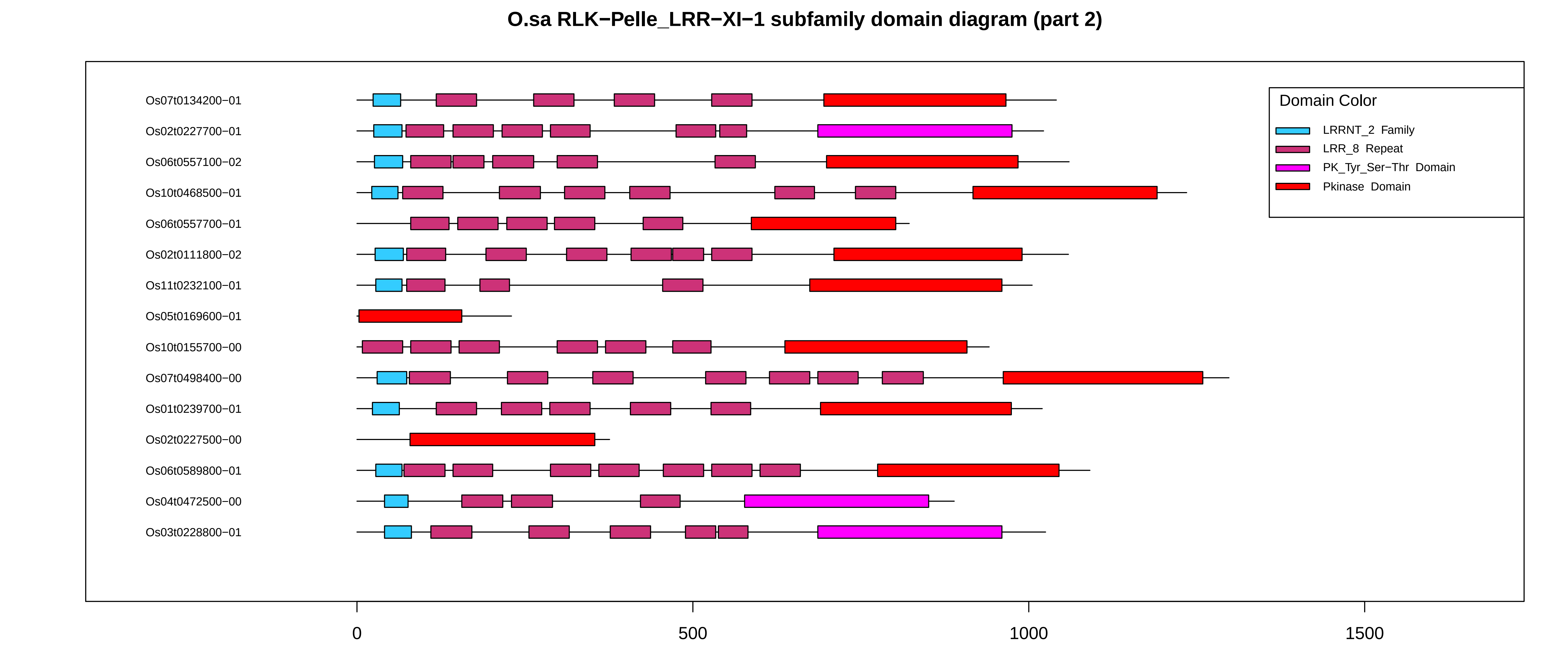
O.sa RLK−Pelle_LRR−XI−1 subfamily domain diagram (part 2)
Os07t0134200−01
Os02t0227700−01
Os06t0557100−02
Os10t0468500−01
Os06t0557700−01
Os02t0111800−02
Os11t0232100−01
Os05t0169600−01
Os10t0155700−00
Os07t0498400−00
Os01t0239700−01
Os02t0227500−00
Os06t0589800−01
Os04t0472500−00
Os03t0228800−01
Domain Color
LRRNT_2 Family
LRR_8 Repeat
PK_Tyr_Ser−Thr Domain
Pkinase Domain
0
500
1000
1500

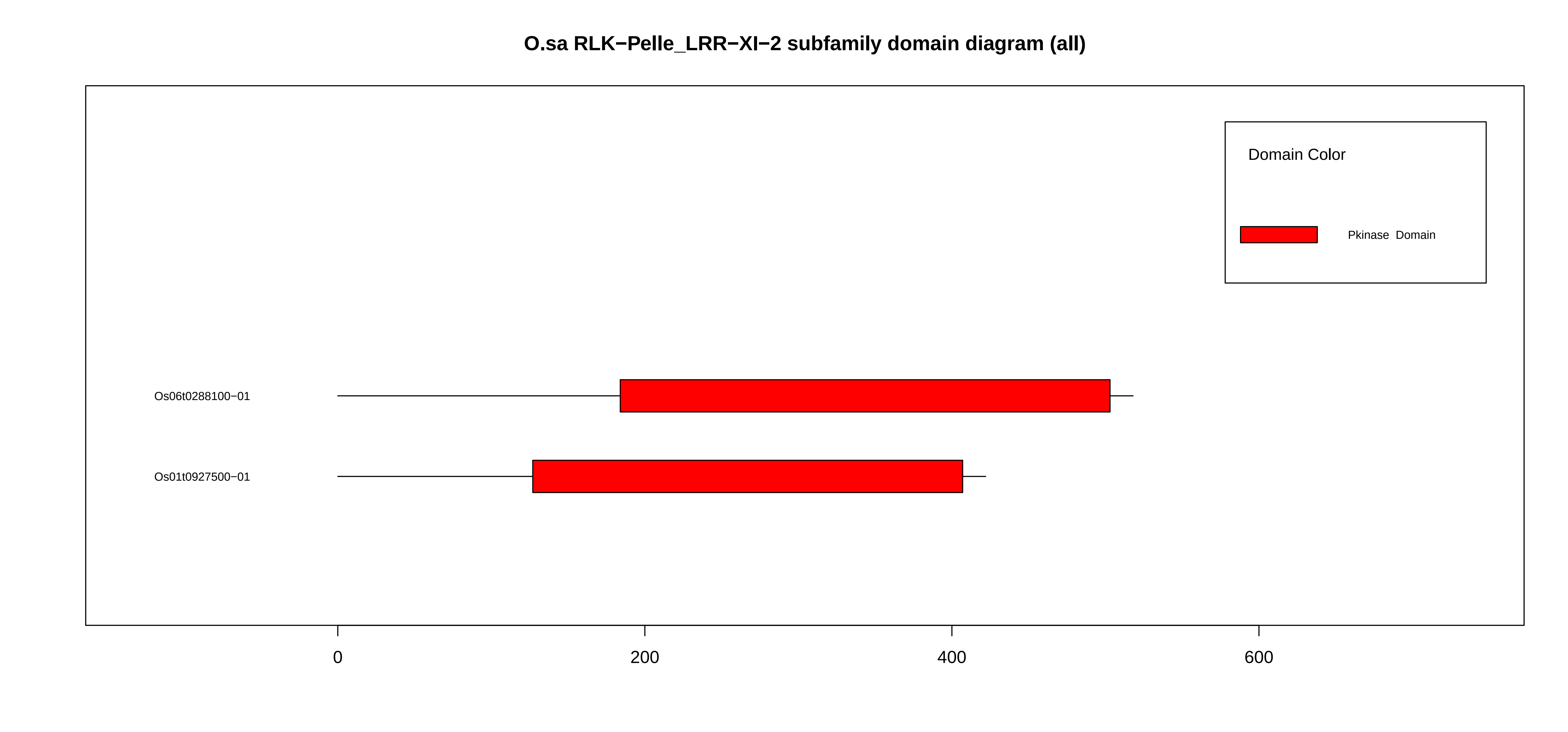
O.sa RLK−Pelle_LRR−XI−2 subfamily domain diagram (all)
Domain Color
Pkinase Domain
Os06t0288100−01
Os01t0927500−01
0
200
400
600

O.sa RLK−Pelle_LRR−XII−1 subfamily domain diagram (part 1)

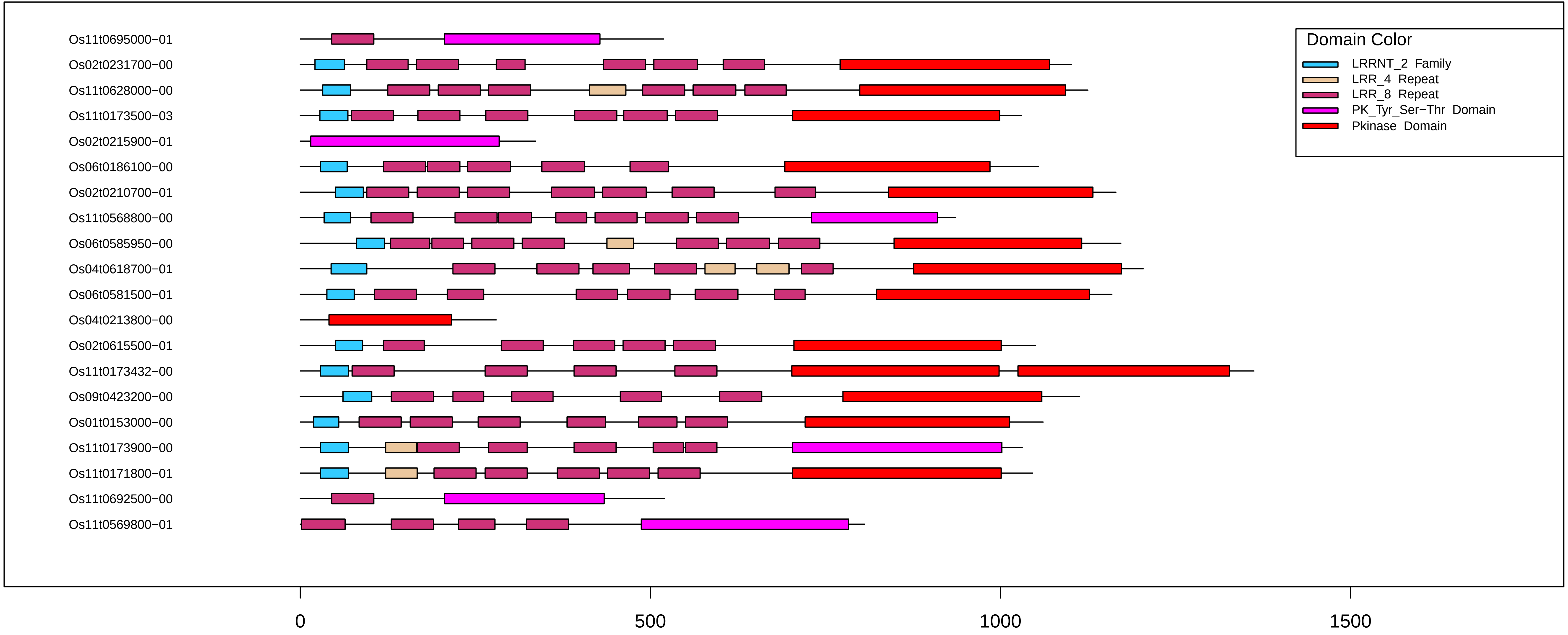

O.sa RLK−Pelle_LRR−XII−1 subfamily domain diagram (part 2)

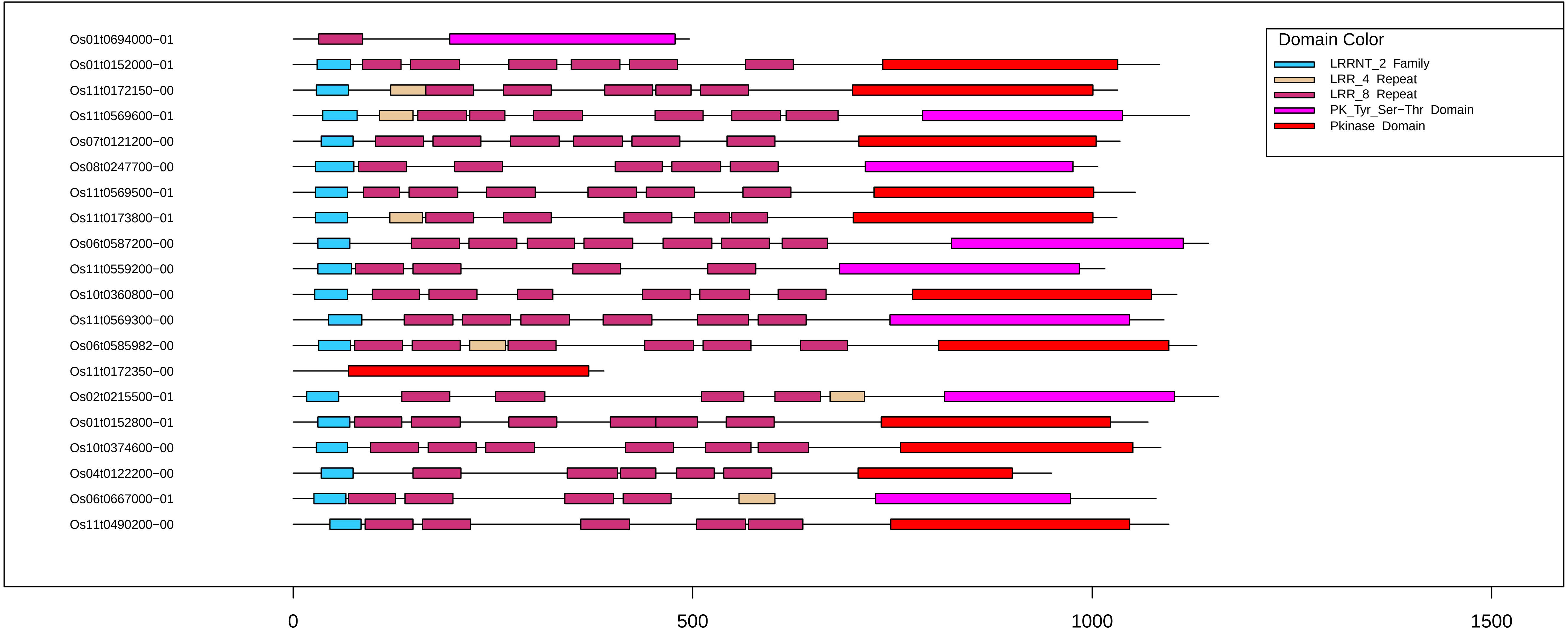

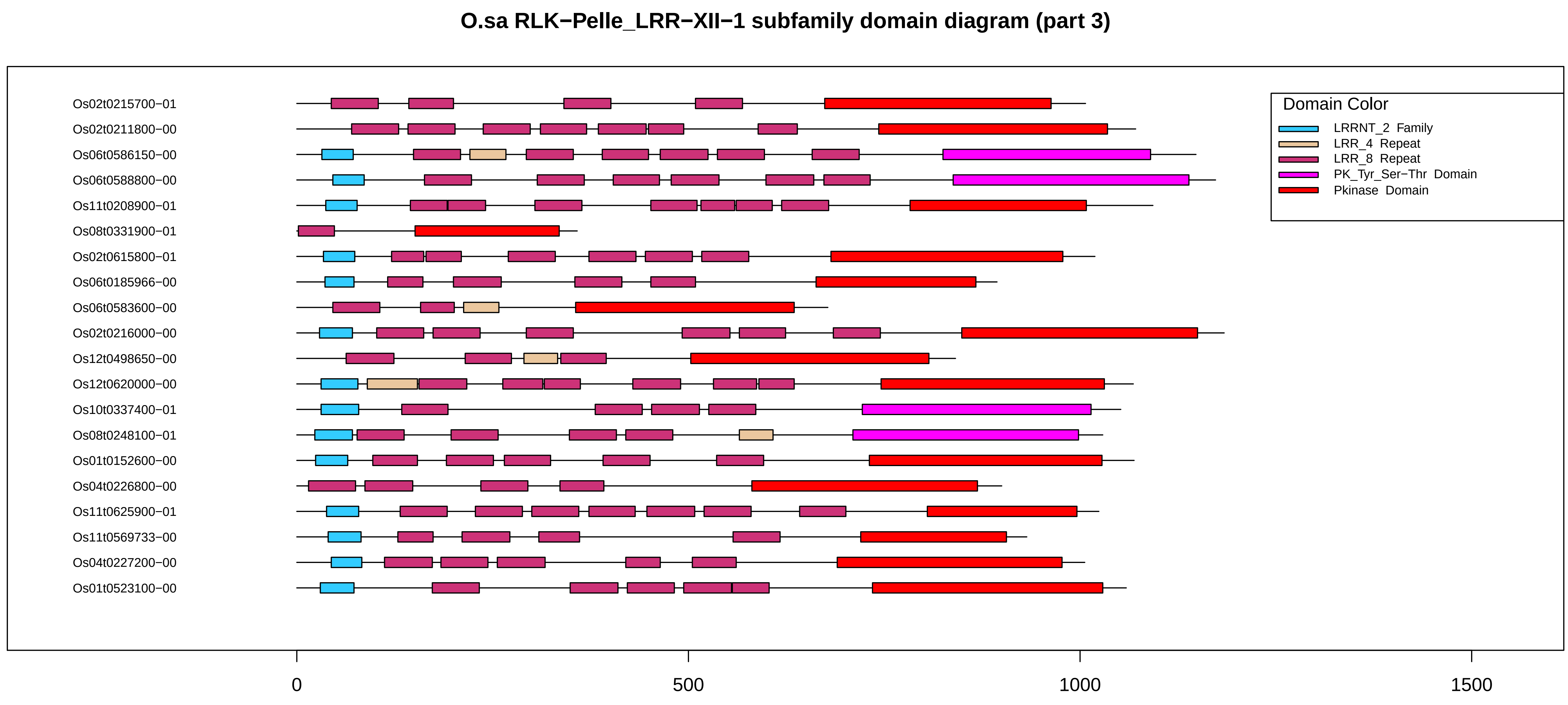
O.sa RLK−Pelle_LRR−XII−1 subfamily domain diagram (part 3)
Os02t0215700−01
Os02t0211800−00
Os06t0586150−00
Os06t0588800−00
Os11t0208900−01
Os08t0331900−01
Os02t0615800−01
Os06t0185966−00
Os06t0583600−00
Os02t0216000−00
Os12t0498650−00
Os12t0620000−00
Os10t0337400−01
Os08t0248100−01
Os01t0152600−00
Os04t0226800−00
Os11t0625900−01
Os11t0569733−00
Os04t0227200−00
Os01t0523100−00
Domain Color
LRRNT_2 Family
LRR_4 Repeat
LRR_8 Repeat
PK_Tyr_Ser−Thr Domain
Pkinase Domain
0
500
1000
1500

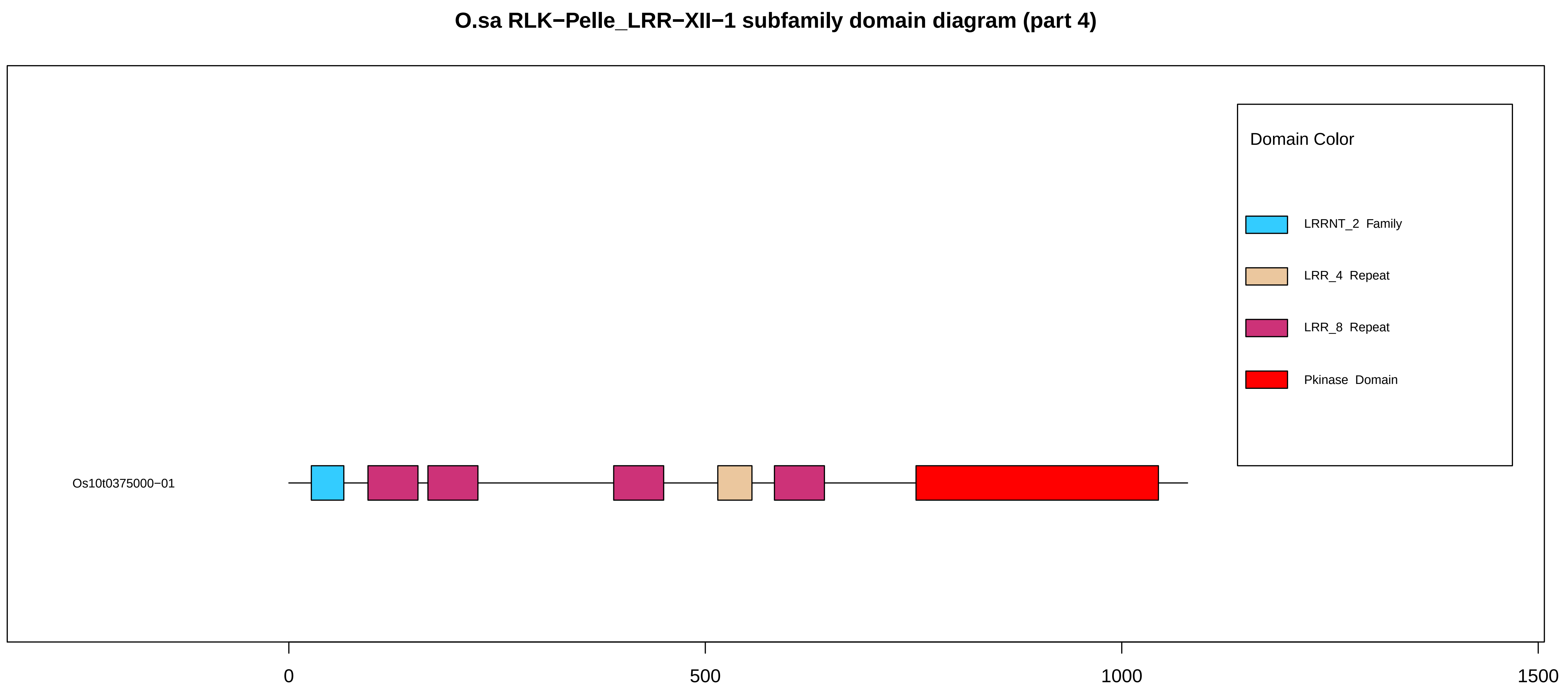
O.sa RLK−Pelle_LRR−XII−1 subfamily domain diagram (part 4)
Domain Color
LRRNT_2 Family
LRR_4 Repeat
LRR_8 Repeat
Pkinase Domain
Os10t0375000−01
0
500
1000
1500

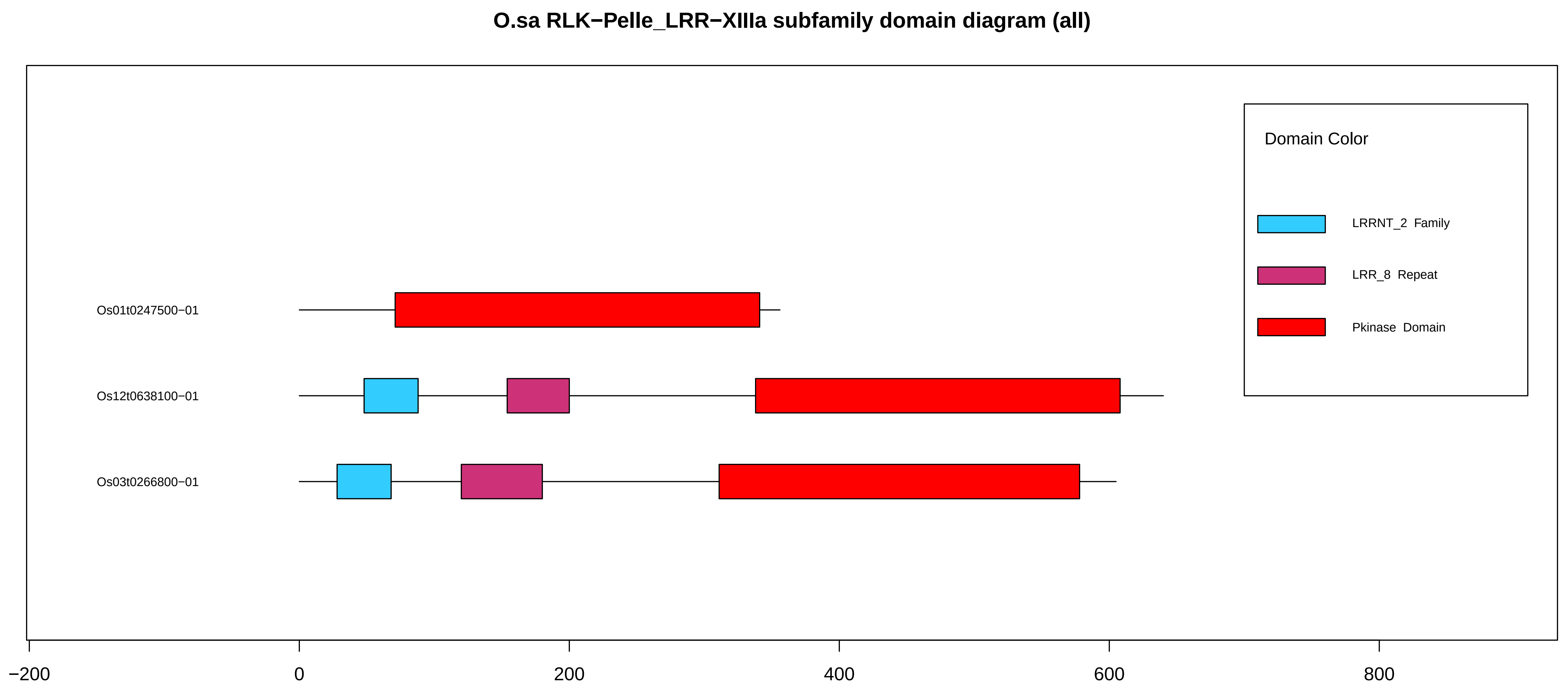

O.sa RLK−Pelle_LRR−XIIIa subfamily domain diagram (all)
Domain Color
LRRNT_2 Family
LRR_8 Repeat
Pkinase Domain
Os01t0247500−01
Os12t0638100−01
Os03t0266800−01
−200
0
200
400
600
800

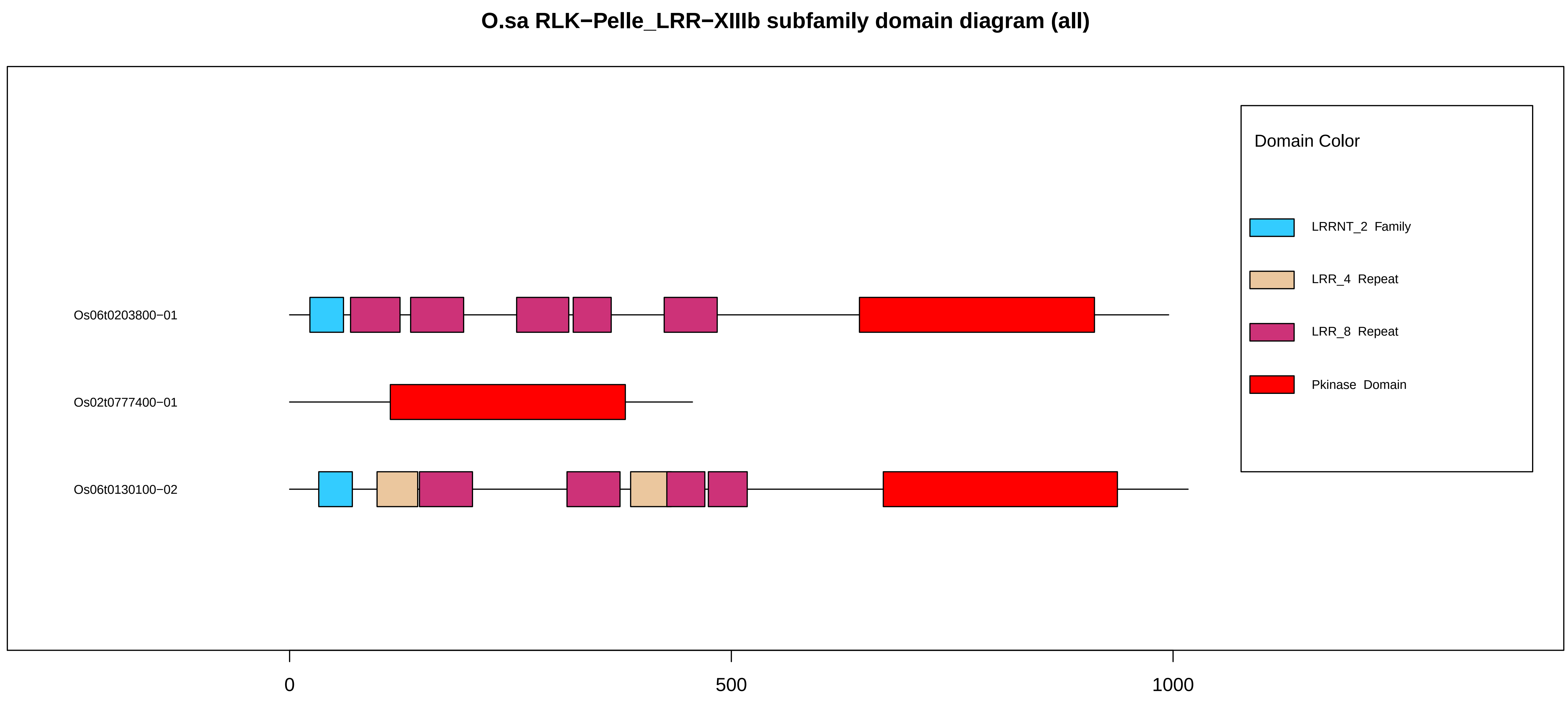
O.sa RLK−Pelle_LRR−XIIIb subfamily domain diagram (all)
Os06t0203800−01
Os02t0777400−01
Os06t0130100−02
Domain Color
LRRNT_2 Family
LRR_4 Repeat
LRR_8 Repeat
Pkinase Domain
0
500
1000

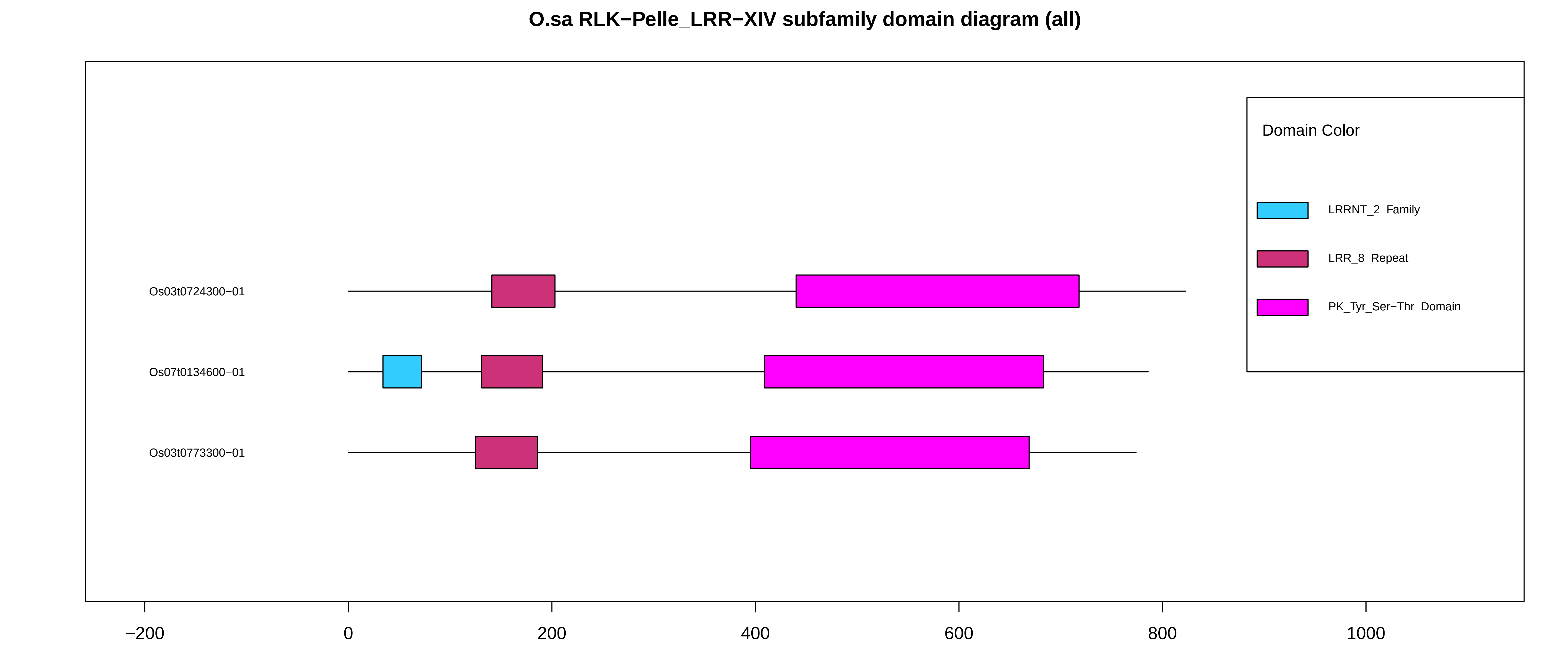
O.sa RLK−Pelle_LRR−XIV subfamily domain diagram (all)
Domain Color
LRRNT_2 Family
LRR_8 Repeat
PK_Tyr_Ser−Thr Domain
Os03t0724300−01
Os07t0134600−01
Os03t0773300−01
−200
0
200
400
600
800
1000

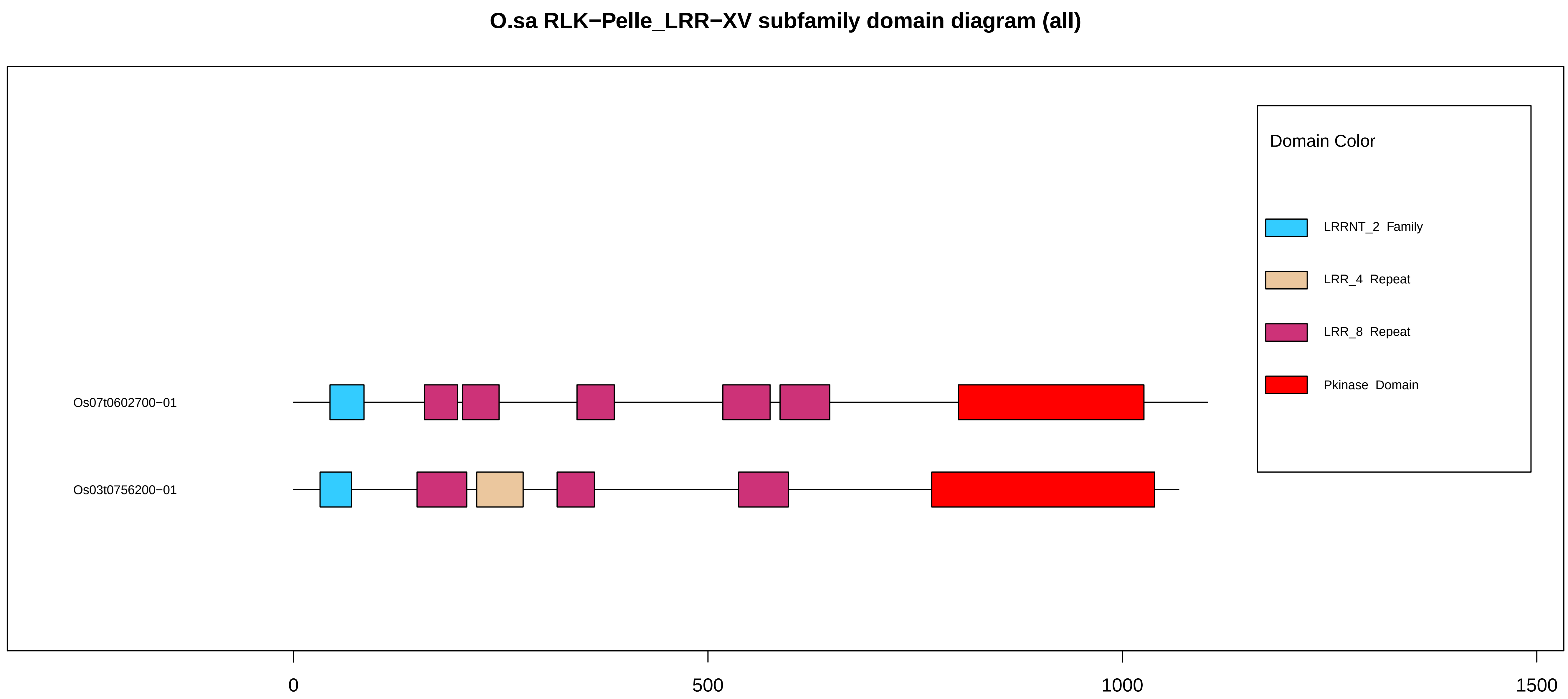

O.sa RLK−Pelle_LRR−XV subfamily domain diagram (all)
Domain Color
LRRNT_2 Family
LRR_4 Repeat
LRR_8 Repeat
Pkinase Domain
Os07t0602700−01
Os03t0756200−01
0
500
1000
1500

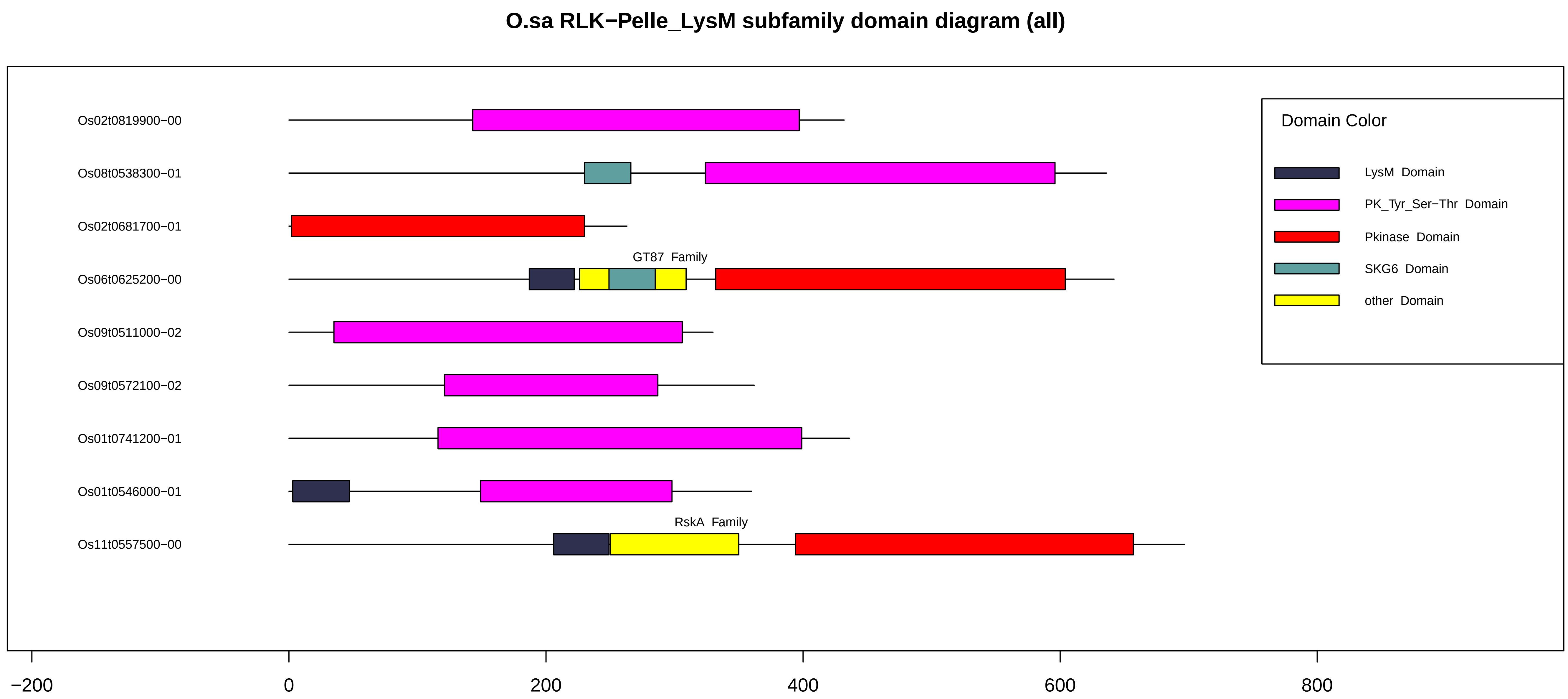
O.sa RLK−Pelle_LysM subfamily domain diagram (all)
Os02t0819900−00
Os08t0538300−01
Os02t0681700−01
Os06t0625200−00
Os09t0511000−02
Os09t0572100−02
Os01t0741200−01
Os01t0546000−01
Os11t0557500−00
GT87 Family
RskA Family
Domain Color
LysM Domain
PK_Tyr_Ser−Thr Domain
Pkinase Domain
SKG6 Domain
other Domain
−200
0
200
400
600
800

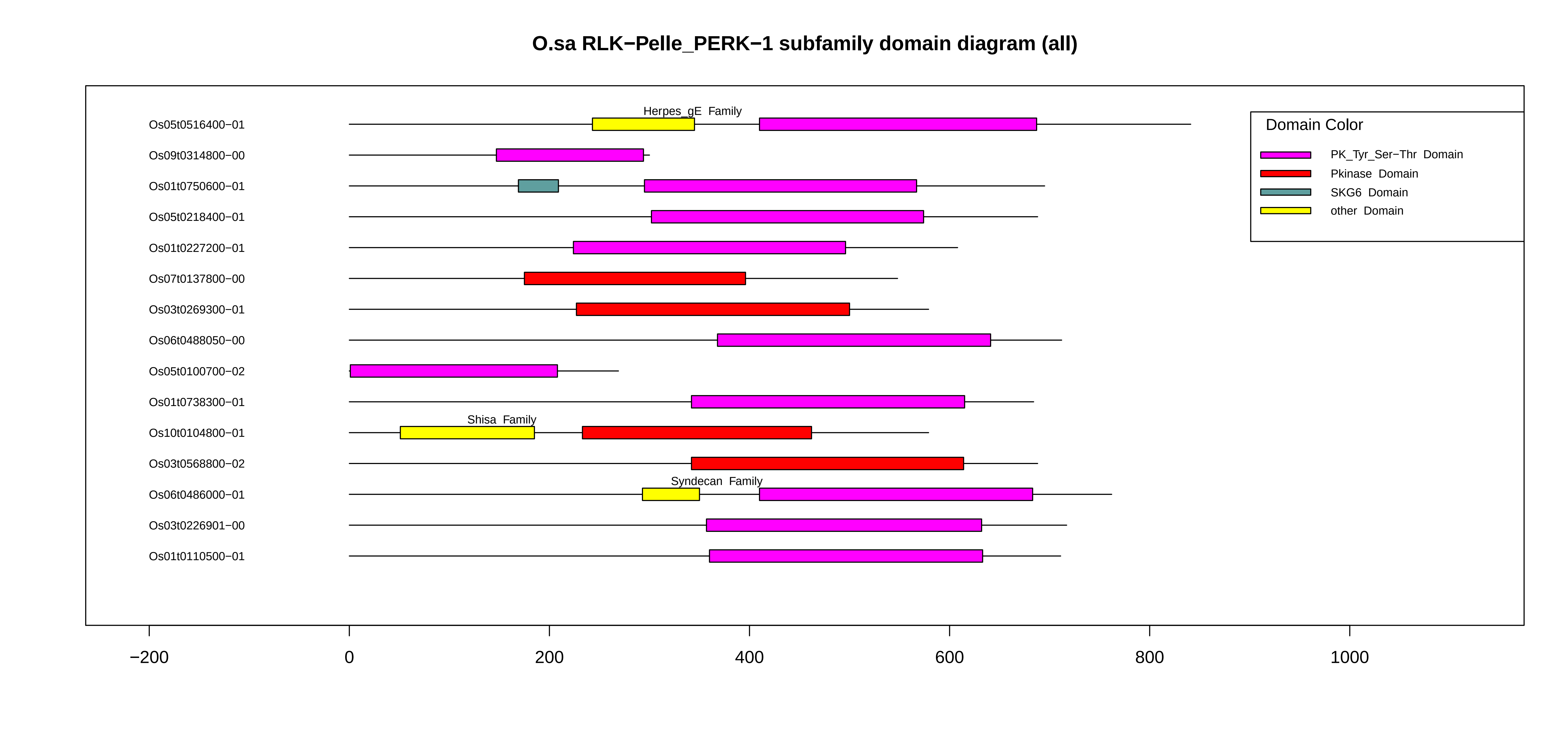
O.sa RLK−Pelle_PERK−1 subfamily domain diagram (all)
Os05t0516400−01
Os09t0314800−00
Os01t0750600−01
Os05t0218400−01
Os01t0227200−01
Os07t0137800−00
Os03t0269300−01
Os06t0488050−00
Os05t0100700−02
Os01t0738300−01
Os10t0104800−01
Os03t0568800−02
Os06t0486000−01
Os03t0226901−00
Os01t0110500−01
Herpes_gE Family
Shisa Family
Syndecan Family
Domain Color
PK_Tyr_Ser−Thr Domain
Pkinase Domain
SKG6 Domain
other Domain
−200
0
200
400
600
800
1000

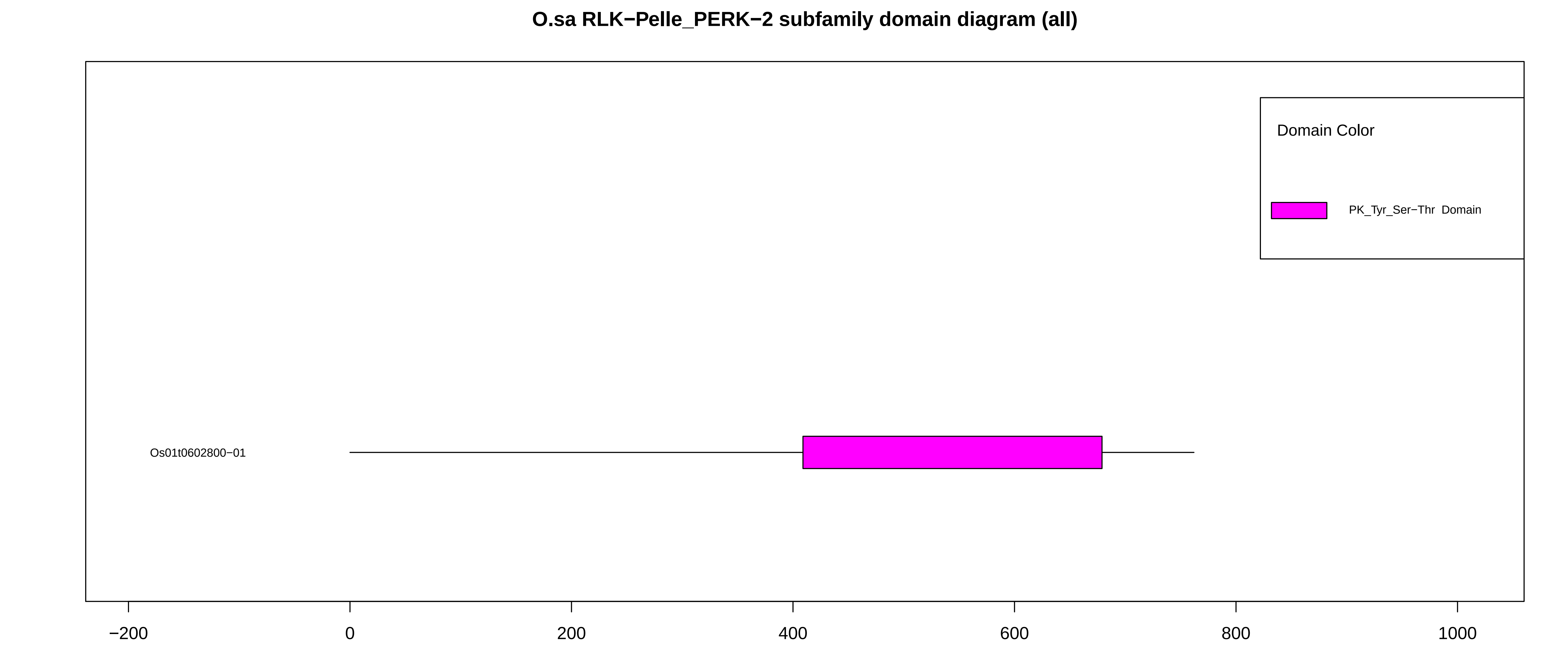
O.sa RLK−Pelle_PERK−2 subfamily domain diagram (all)
Domain Color
PK_Tyr_Ser−Thr Domain
Os01t0602800−01
−200
0
200
400
600
800
1000

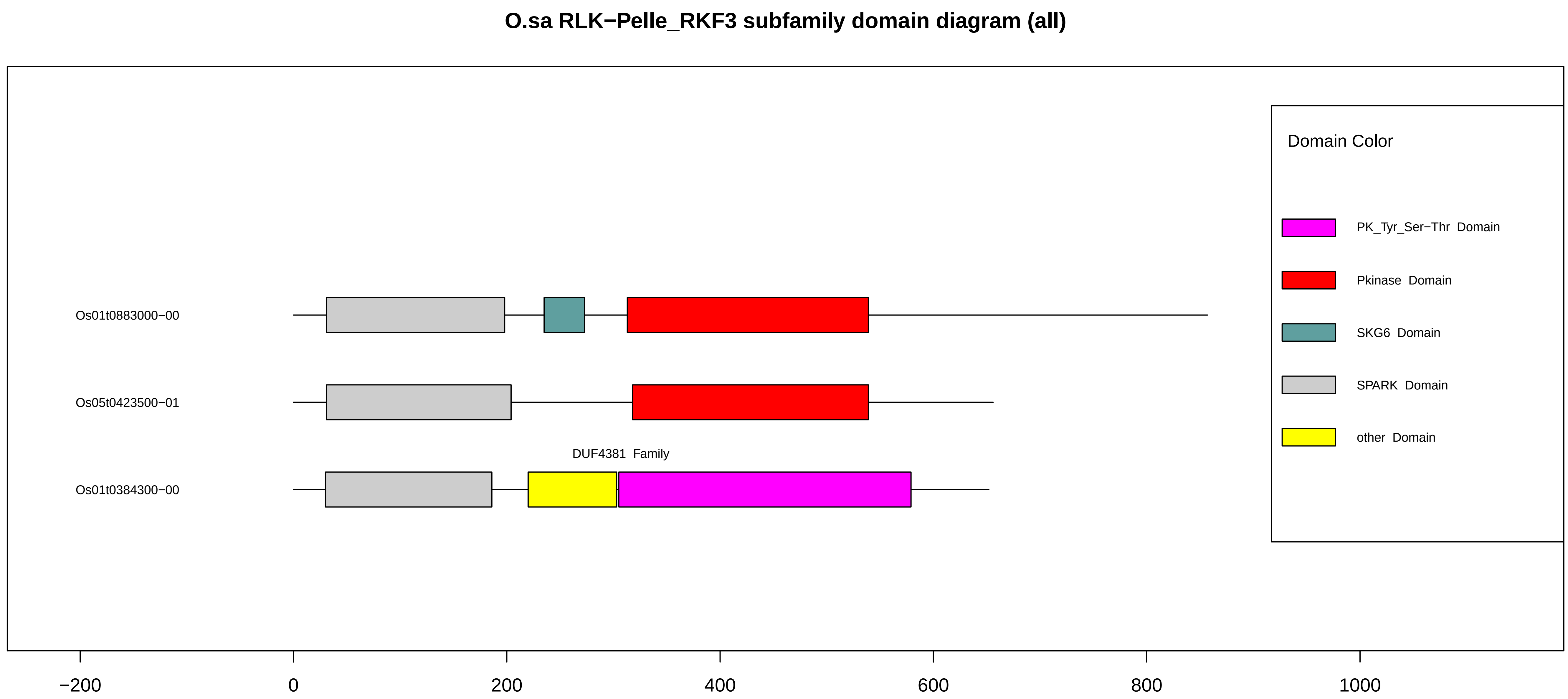
O.sa RLK−Pelle_RKF3 subfamily domain diagram (all)
Os01t0883000−00
Os05t0423500−01
Os01t0384300−00
DUF4381 Family
Domain Color
PK_Tyr_Ser−Thr Domain
Pkinase Domain
SKG6 Domain
SPARK Domain
other Domain
−200
0
200
400
600
800
1000

O.sa RLK−Pelle_RLCK−II subfamily domain diagram (all)

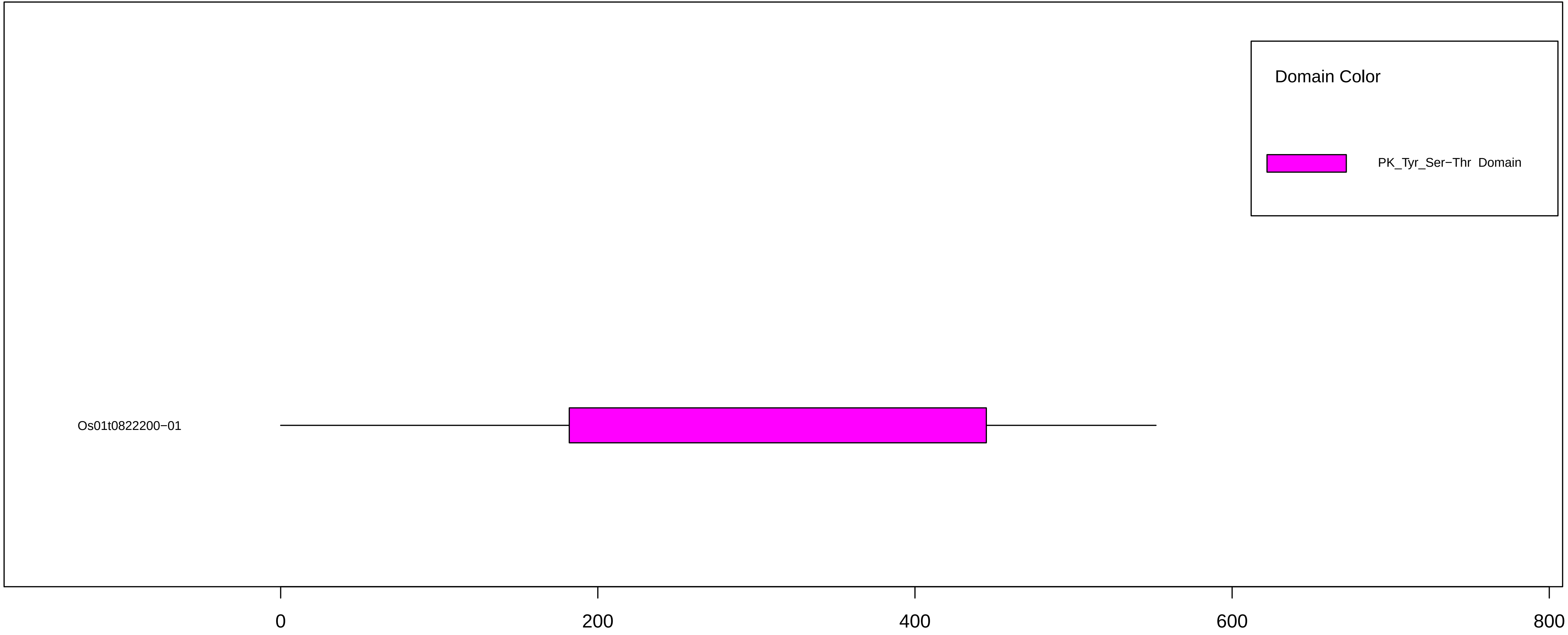

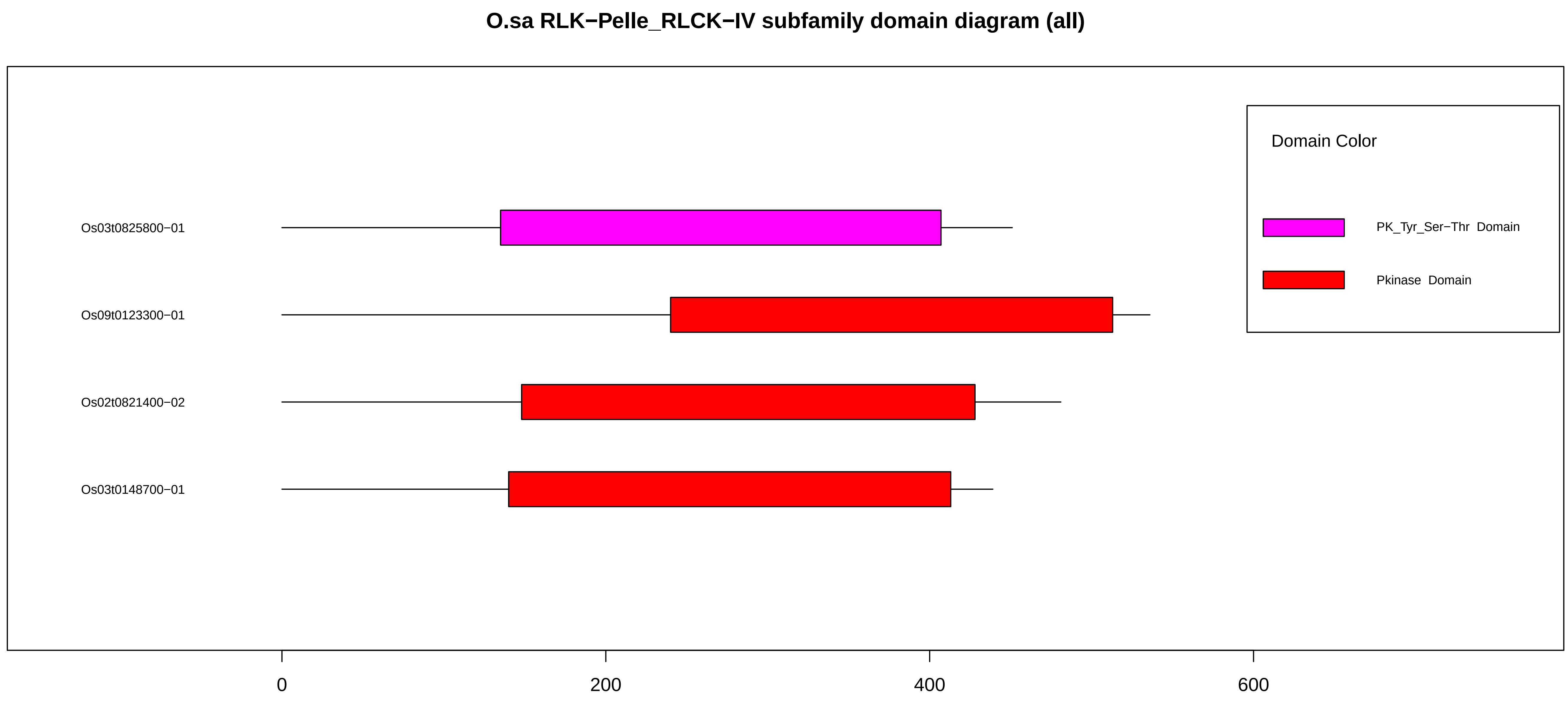
O.sa RLK−Pelle_RLCK−IV subfamily domain diagram (all)
Domain Color
PK_Tyr_Ser−Thr Domain
Pkinase Domain
Os03t0825800−01
Os09t0123300−01
Os02t0821400−02
Os03t0148700−01
0
200
400
600

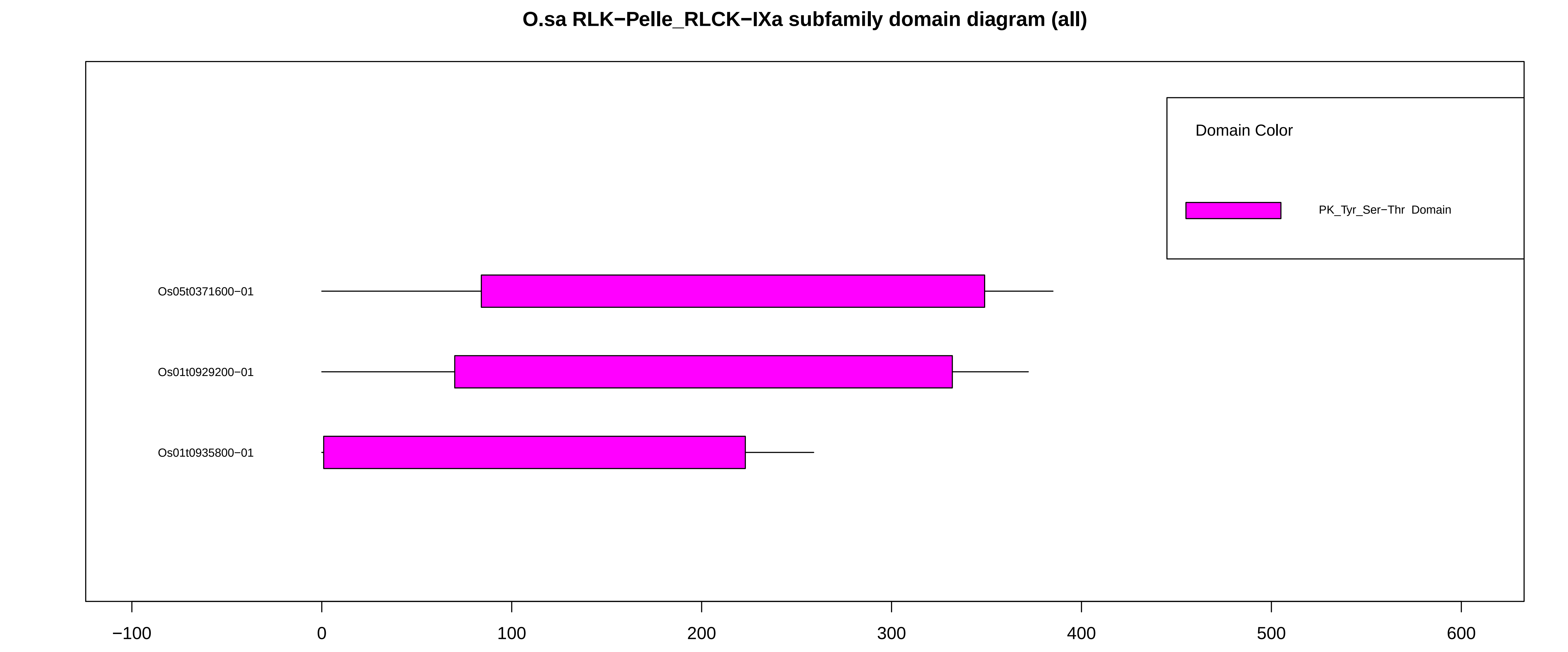
O.sa RLK−Pelle_RLCK−IXa subfamily domain diagram (all)
Domain Color
PK_Tyr_Ser−Thr Domain
Os05t0371600−01
Os01t0929200−01
Os01t0935800−01
−100
0
100
200
300
400
500
600

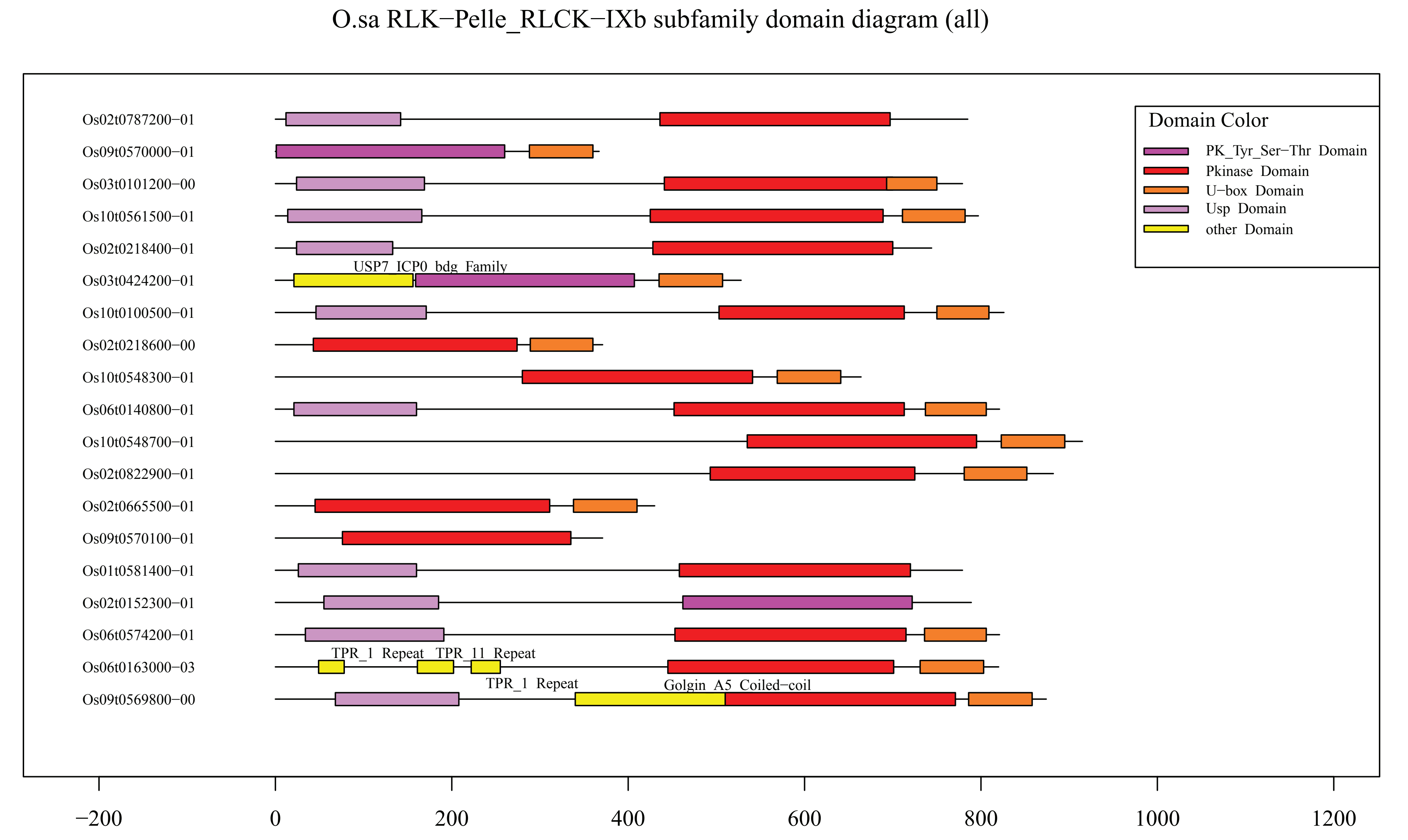
O.sa RLK−Pelle_RLCK−IXb subfamily domain diagram (all)
Domain Color
PK_Tyr_Ser−Thr Domain
Pkinase Domain
U−box Domain
Usp Domain
other Domain
Os02t0787200−01
Os09t0570000−01
Os03t0101200−00
Os10t0561500−01
Os02t0218400−01
Os03t0424200−01
USP7_ICP0_bdg_Family
Os10t0100500−01
Os02t0218600−00
Os10t0548300−01
Os06t0140800−01
Os10t0548700−01
Os02t0822900−01
Os02t0665500−01
Os09t0570100−01
Os01t0581400−01
Os02t0152300−01
Os06t0574200−01
Os06t0163000−03
TPR_1 Repeat
TPR_11 Repeat
TPR_1 Repeat
Os09t0569800−00
Golgin_A5
Coiled−coil
−200
0
200
400
600
800
1000
1200

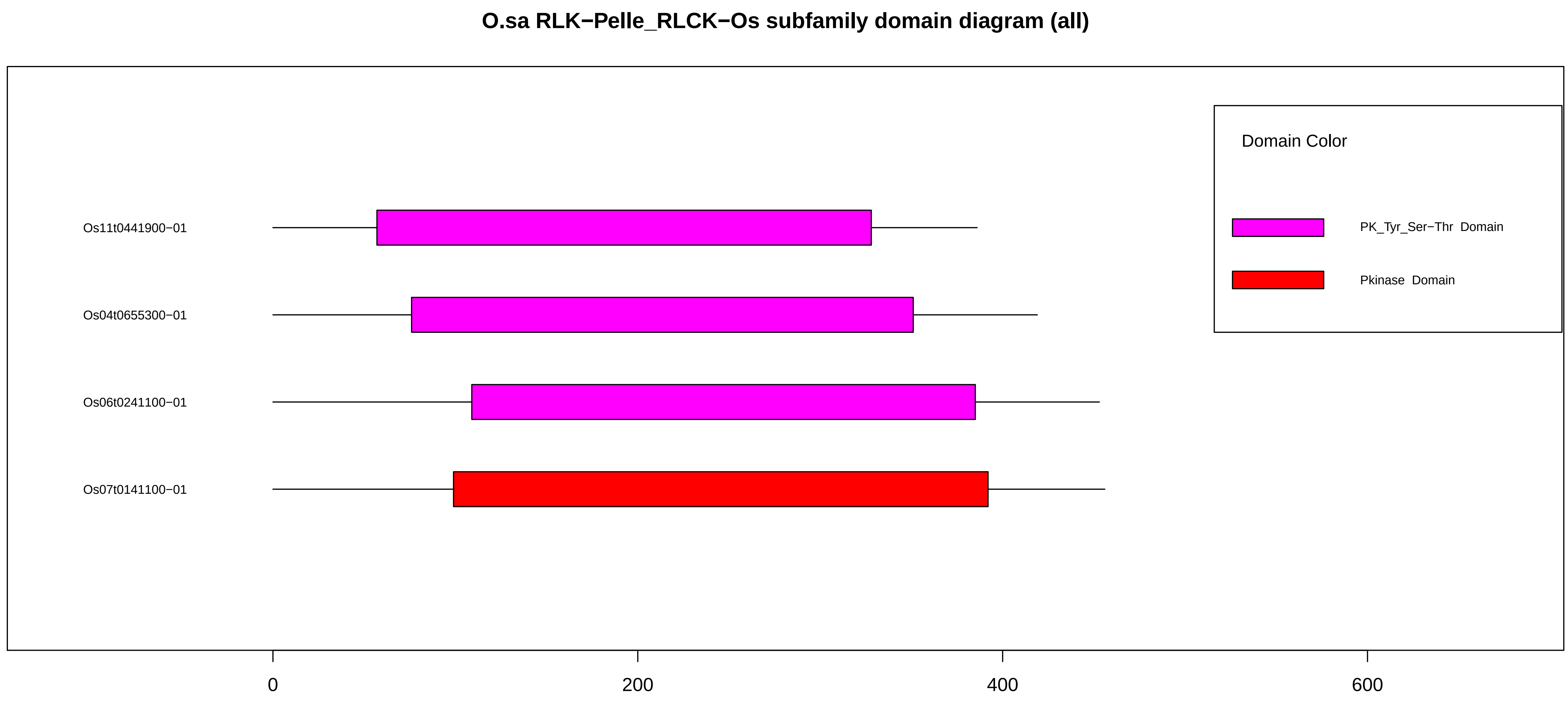
O.sa RLK−Pelle_RLCK−Os subfamily domain diagram (all)
Os11t0441900−01
Os04t0655300−01
Os06t0241100−01
Os07t0141100−01
Domain Color
PK_Tyr_Ser−Thr Domain
Pkinase Domain
0
200
400
600

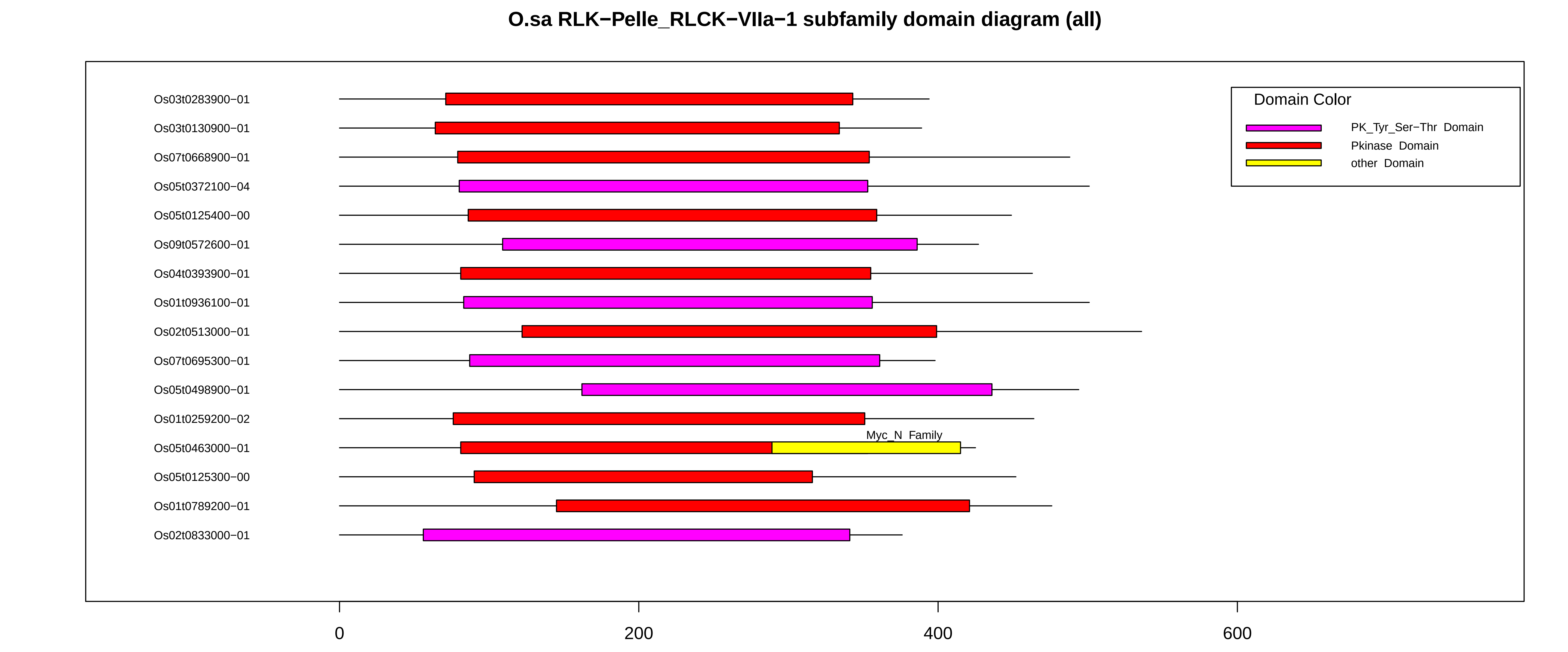
O.sa RLK−Pelle_RLCK−VIIa−1 subfamily domain diagram (all)
Os03t0283900−01
Os03t0130900−01
Os07t0668900−01
Os05t0372100−04
Os05t0125400−00
Os09t0572600−01
Os04t0393900−01
Os01t0936100−01
Os02t0513000−01
Os07t0695300−01
Os05t0498900−01
Os01t0259200−02
Os05t0463000−01
Os05t0125300−00
Os01t0789200−01
Os02t0833000−01
Myc_N Family
Domain Color
PK_Tyr_Ser−Thr Domain
Pkinase Domain
other Domain
0
200
400
600

O.sa RLK−Pelle_RLCK−VIIa−2 subfamily domain diagram (part 1)

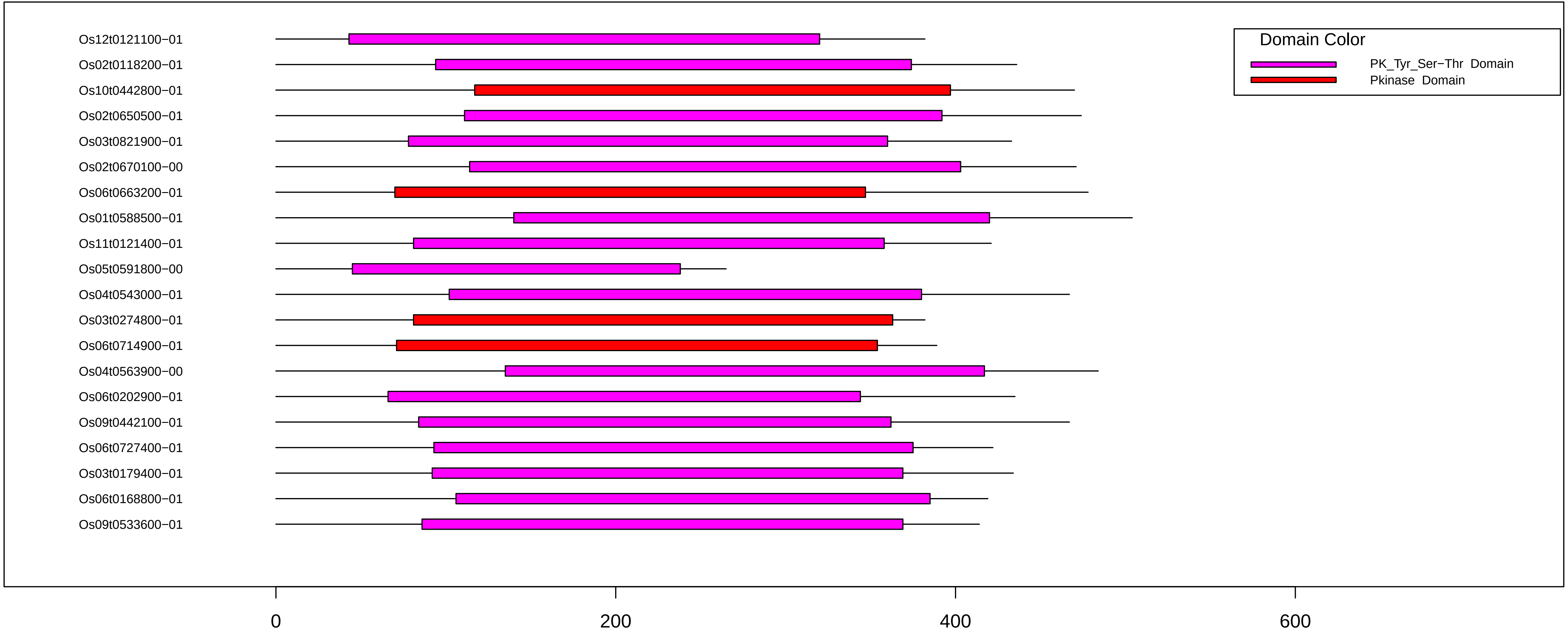

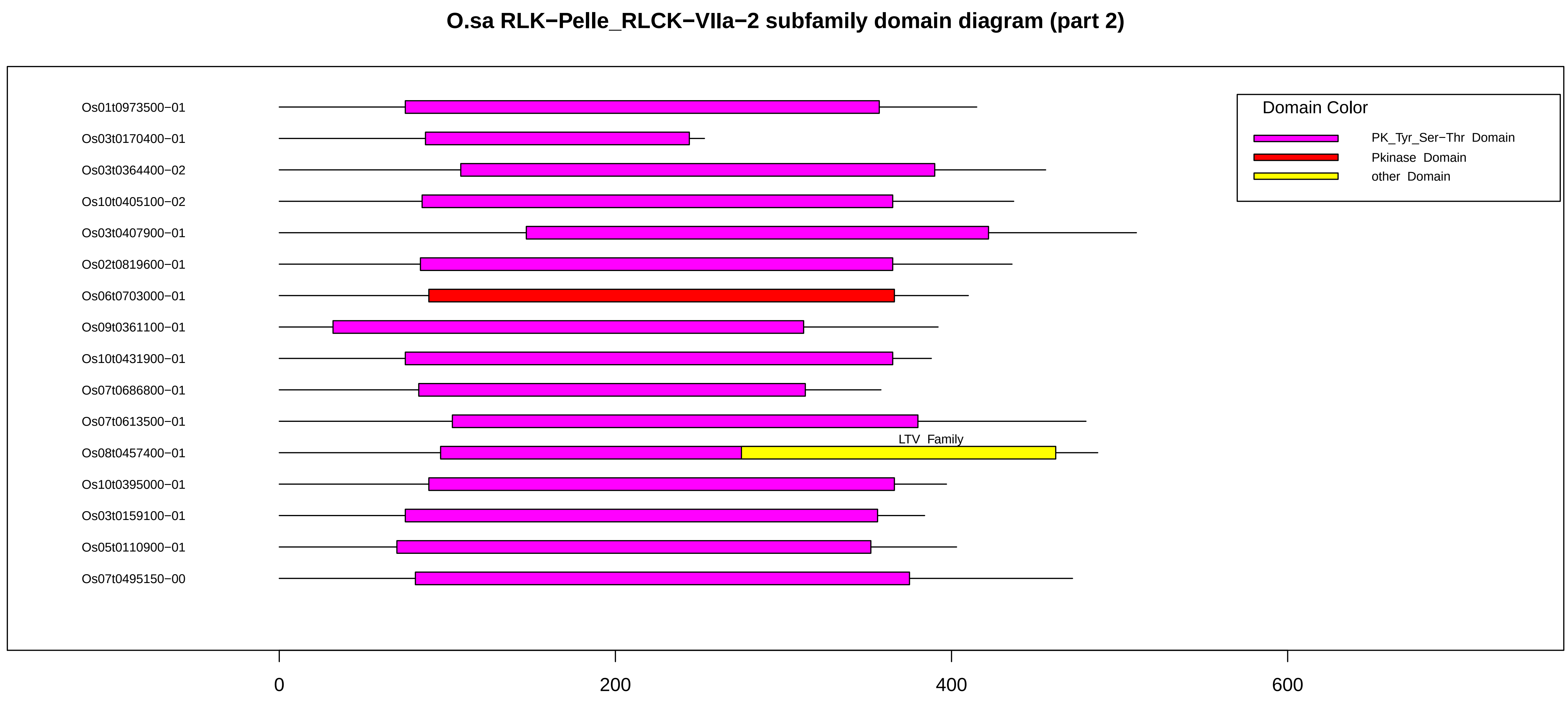
O.sa RLK−Pelle_RLCK−VIIa−2 subfamily domain diagram (part 2)
Os01t0973500−01
Os03t0170400−01
Os03t0364400−02
Os10t0405100−02
Os03t0407900−01
Os02t0819600−01
Os06t0703000−01
Os09t0361100−01
Os10t0431900−01
Os07t0686800−01
Os07t0613500−01
Os08t0457400−01
Os10t0395000−01
Os03t0159100−01
Os05t0110900−01
Os07t0495150−00
LTV Family
Domain Color
PK_Tyr_Ser−Thr Domain
Pkinase Domain
other Domain
0
200
400
600

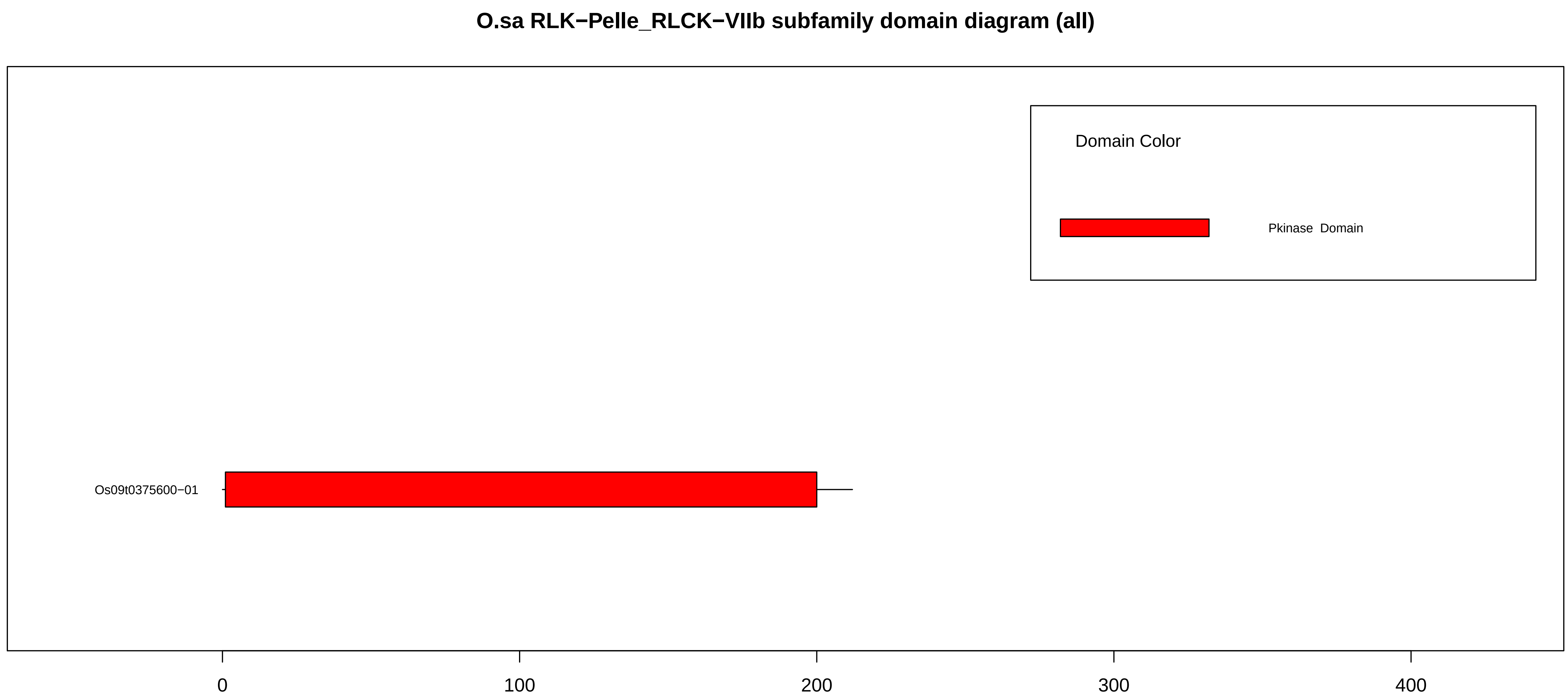

O.sa RLK−Pelle_RLCK−VIIb subfamily domain diagram (all)
Domain Color
Pkinase Domain
Os09t0375600−01
0
100
200
300
400

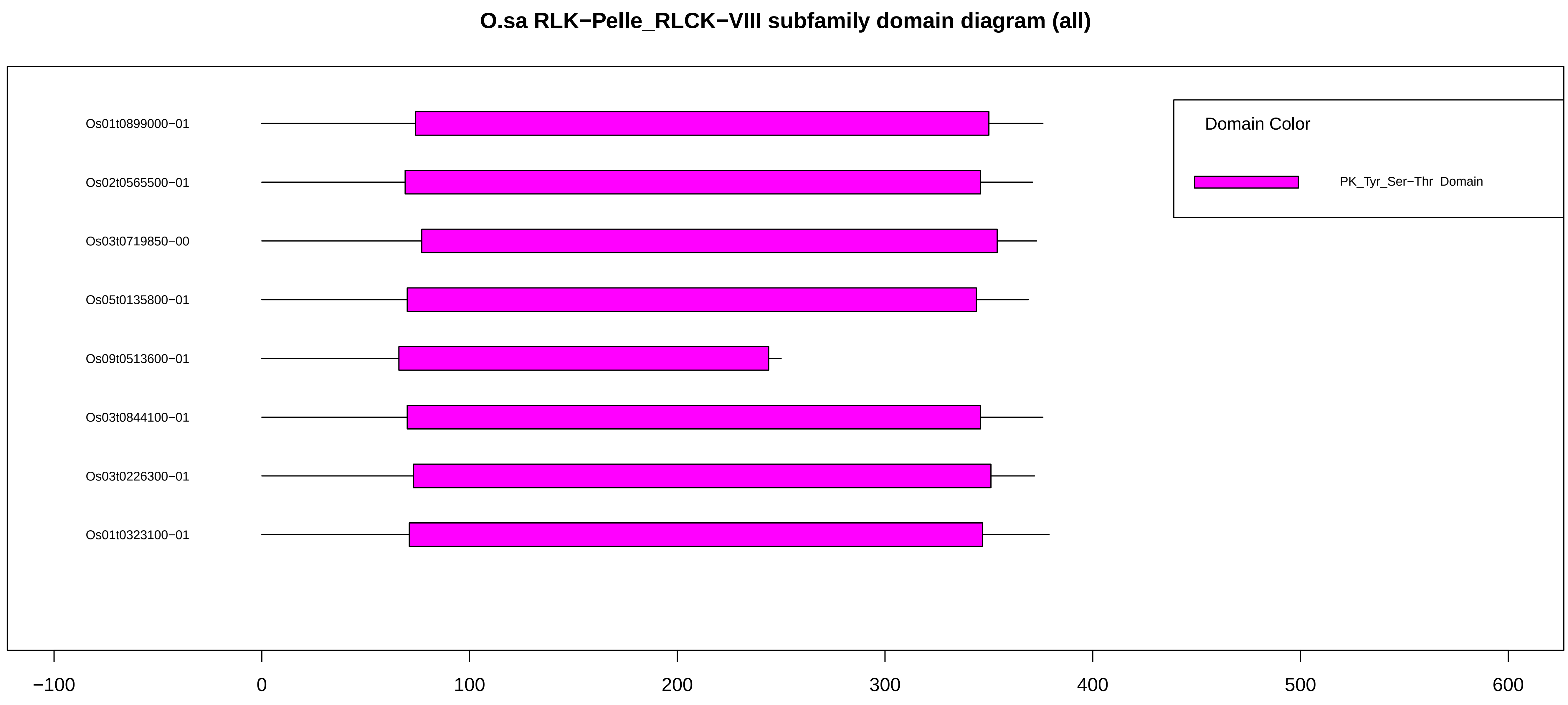

O.sa RLK−Pelle_RLCK−VIII subfamily domain diagram (all)
Os01t0899000−01
Os02t0565500−01
Os03t0719850−00
Os05t0135800−01
Os09t0513600−01
Os03t0844100−01
Os03t0226300−01
Os01t0323100−01
Domain Color
PK_Tyr_Ser−Thr Domain
−100
0
100
200
300
400
500
600

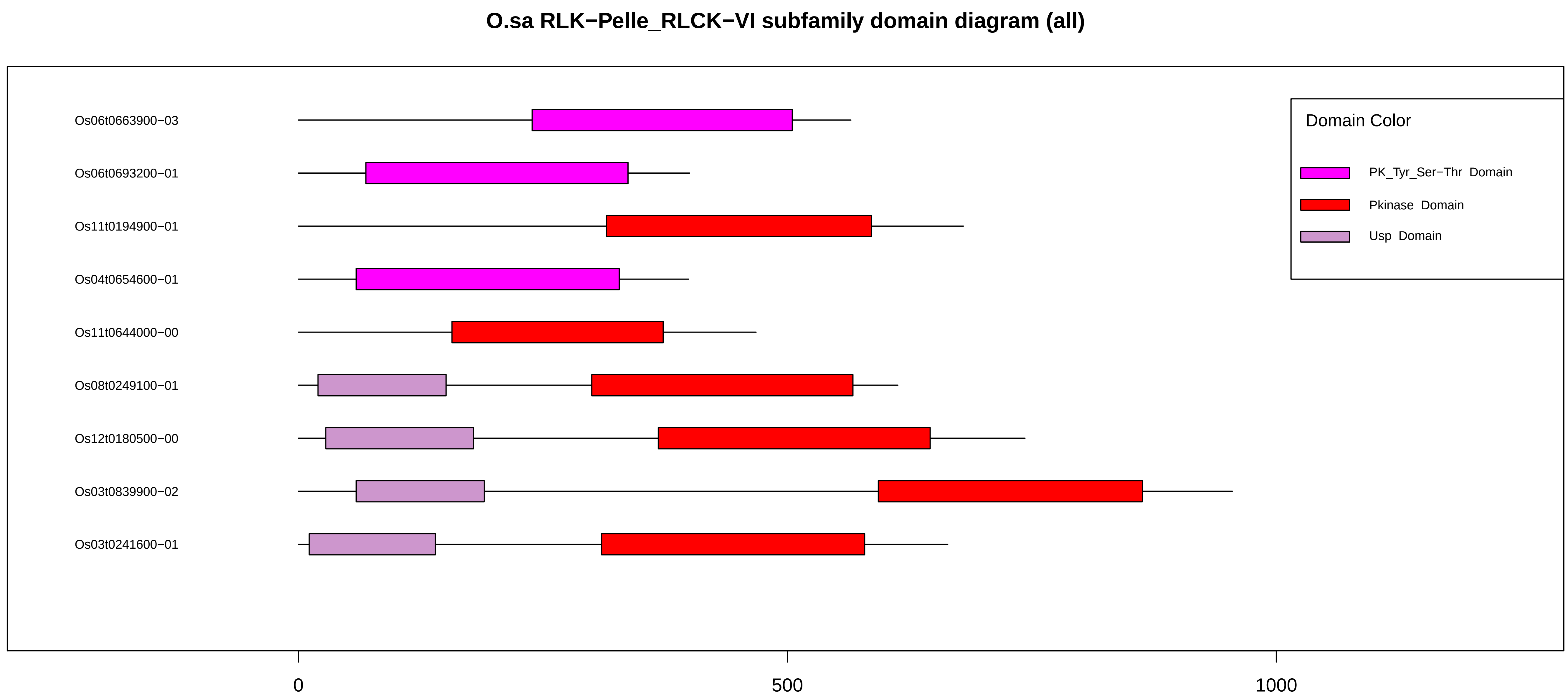
O.sa RLK−Pelle_RLCK−VI subfamily domain diagram (all)
Os06t0663900−03
Os06t0693200−01
Os11t0194900−01
Os04t0654600−01
Os11t0644000−00
Os08t0249100−01
Os12t0180500−00
Os03t0839900−02
Os03t0241600−01
Domain Color
PK_Tyr_Ser−Thr Domain
Pkinase Domain
Usp Domain
0
500
1000

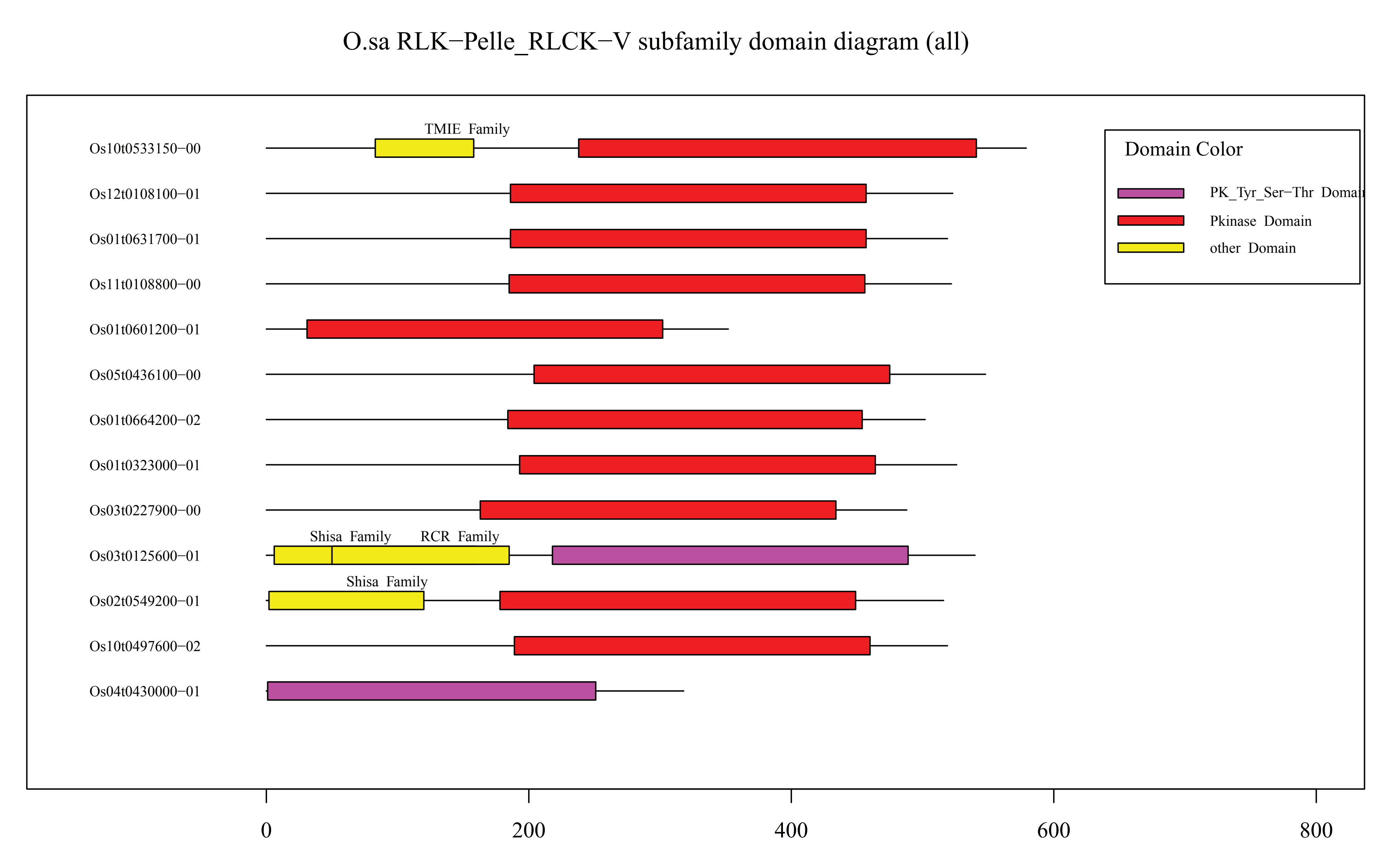

O.sa RLK−Pelle_RLCK−V subfamily domain diagram (all)
TMIE Family
Os10t0533150−00
Os12t0108100−01
Os01t0631700−01
Os11t0108800−00
Os01t0601200−01
Os05t0436100−00
Os01t0664200−02
Os01t0323000−01
Os03t0227900−00
Shisa Family
RCR Family
Os03t0125600−01
Shisa Family
Os02t0549200−01
Os10t0497600−02
Os04t0430000−01
Domain Color
PK_Tyr_Ser−Thr Domain
Pkinase Domain
other Domain
0
200
400
600
800

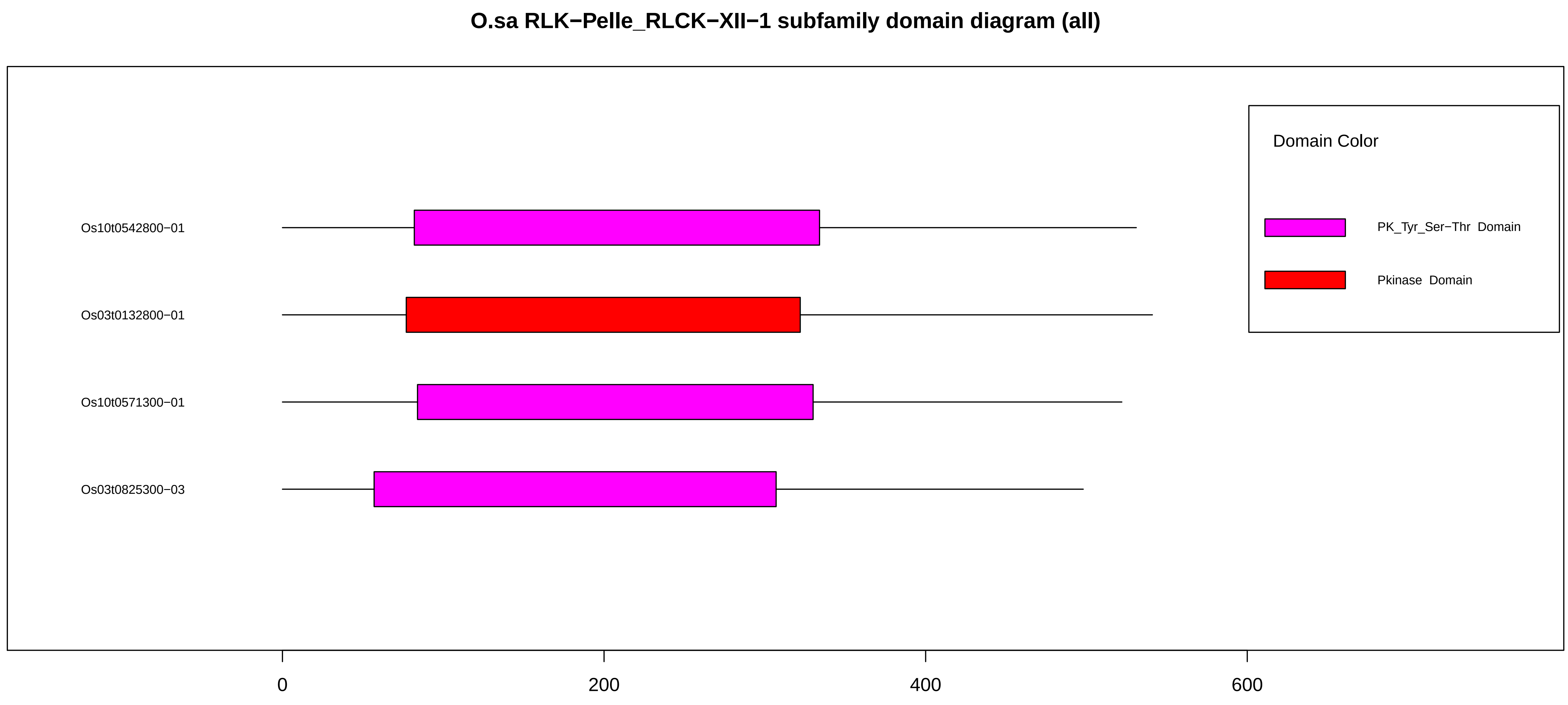
O.sa RLK−Pelle_RLCK−XII−1 subfamily domain diagram (all)
Domain Color
PK_Tyr_Ser−Thr Domain
Pkinase Domain
Os10t0542800−01
Os03t0132800−01
Os10t0571300−01
Os03t0825300−03
0
200
400
600

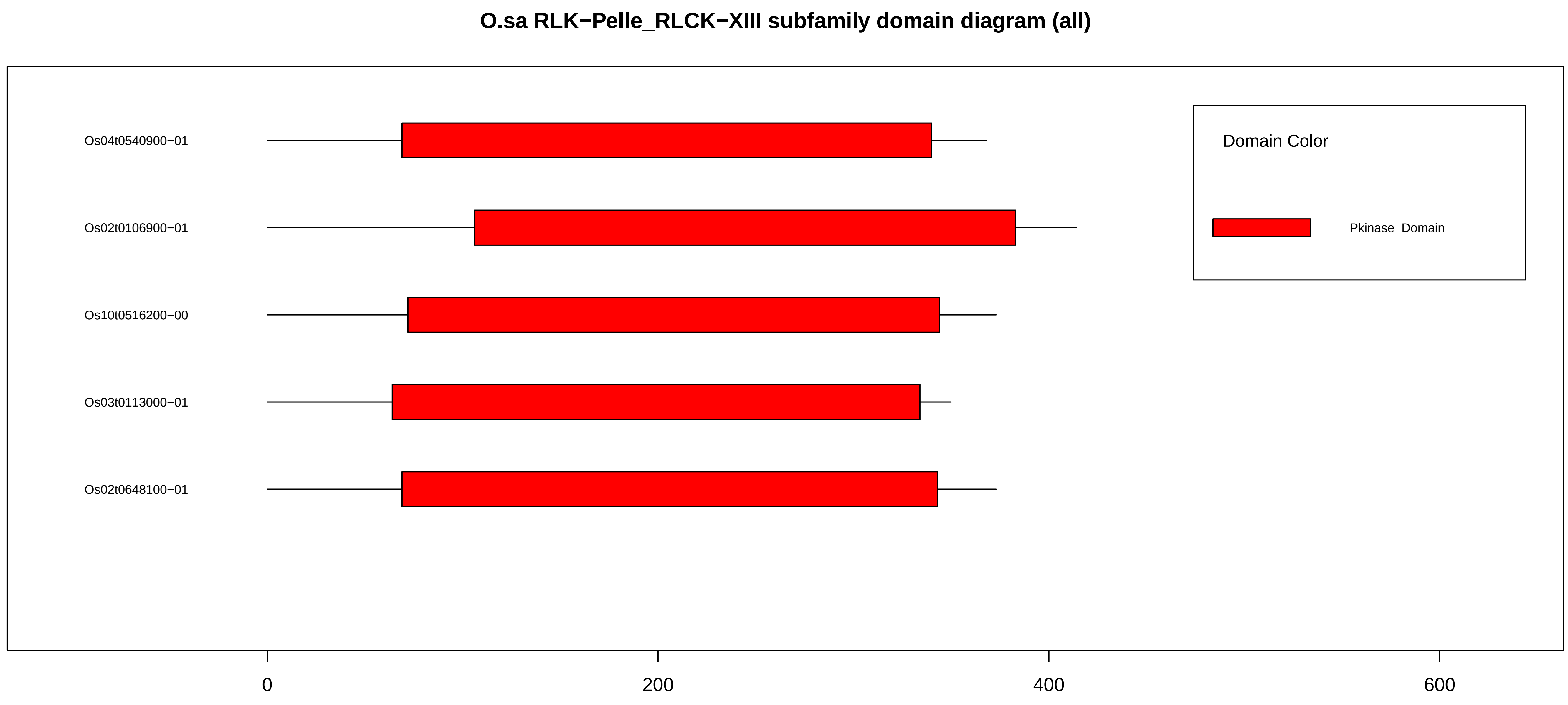

O.sa RLK−Pelle_RLCK−XIII subfamily domain diagram (all)
Os04t0540900−01
Os02t0106900−01
Os10t0516200−00
Os03t0113000−01
Os02t0648100−01
Domain Color
Pkinase Domain
0
200
400
600

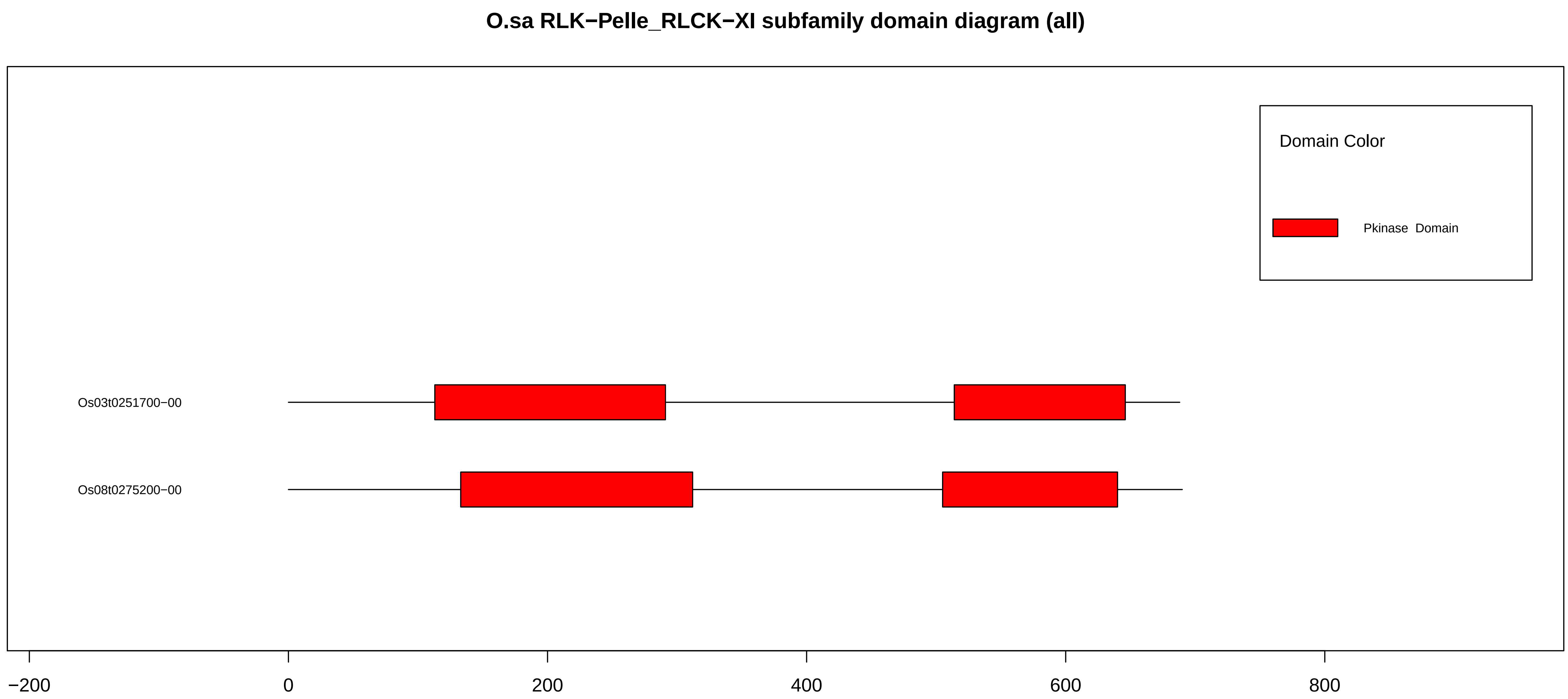

O.sa RLK−Pelle_RLCK−XI subfamily domain diagram (all)
Domain Color
Pkinase Domain
Os03t0251700−00
Os08t0275200−00
−200
0
200
400
600
800

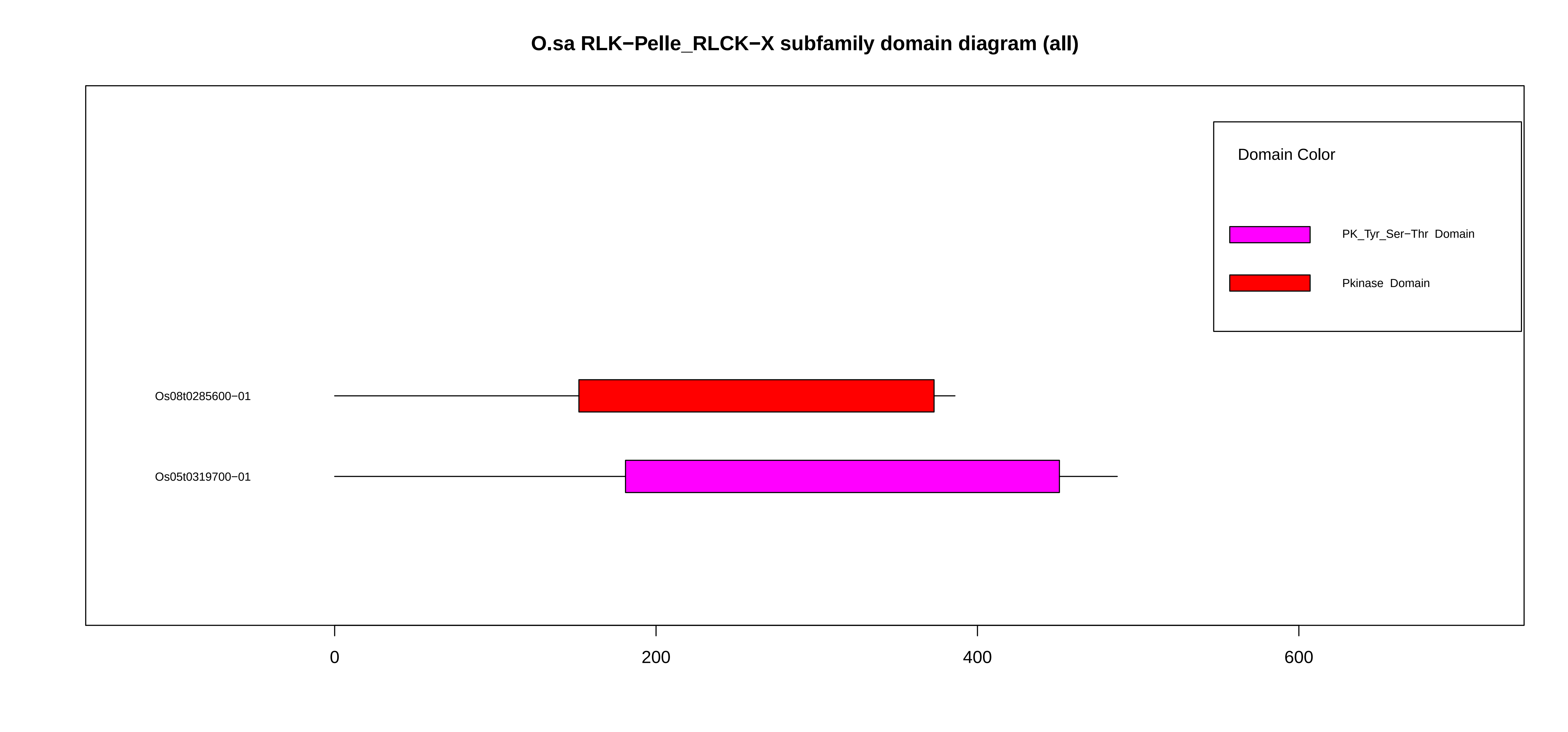

O.sa RLK−Pelle_RLCK−X subfamily domain diagram (all)
Domain Color
PK_Tyr_Ser−Thr Domain
Pkinase Domain
Os08t0285600−01
Os05t0319700−01
0
200
400
600

O.sa RLK−Pelle_RLCK−XVI subfamily domain diagram (all)

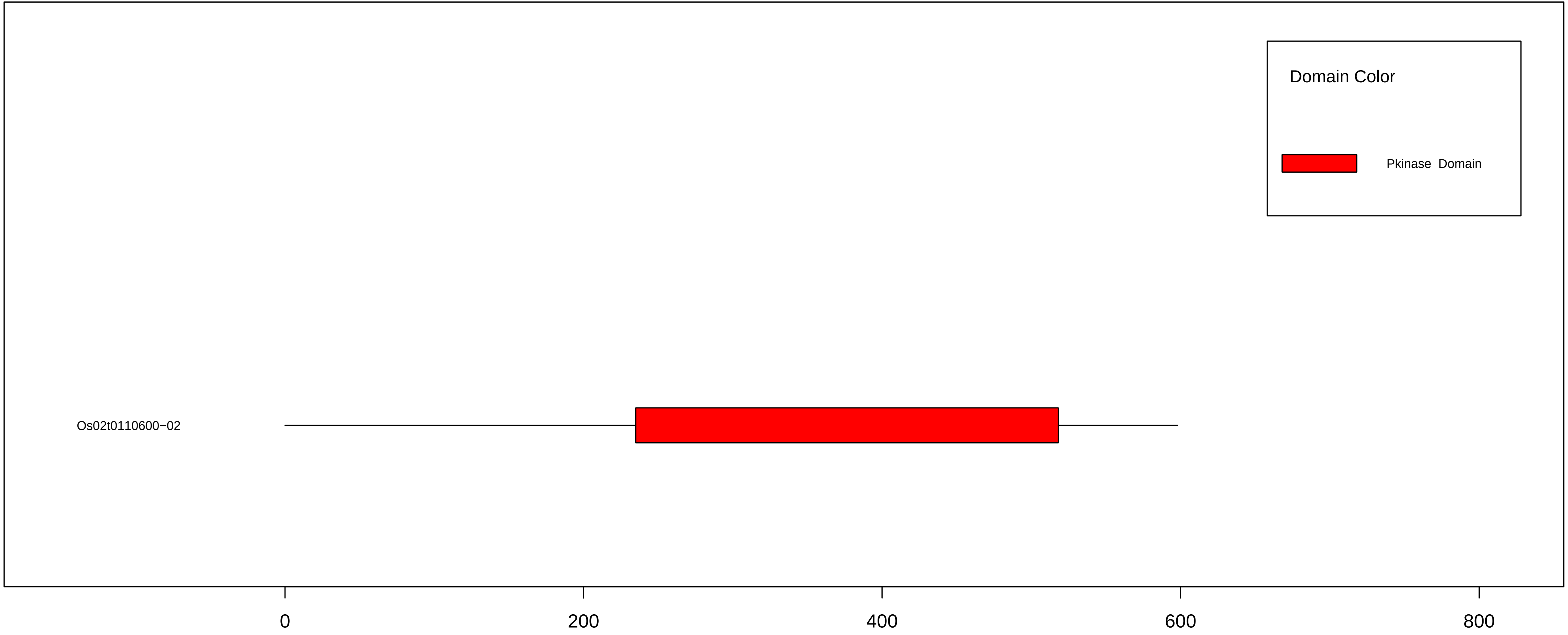

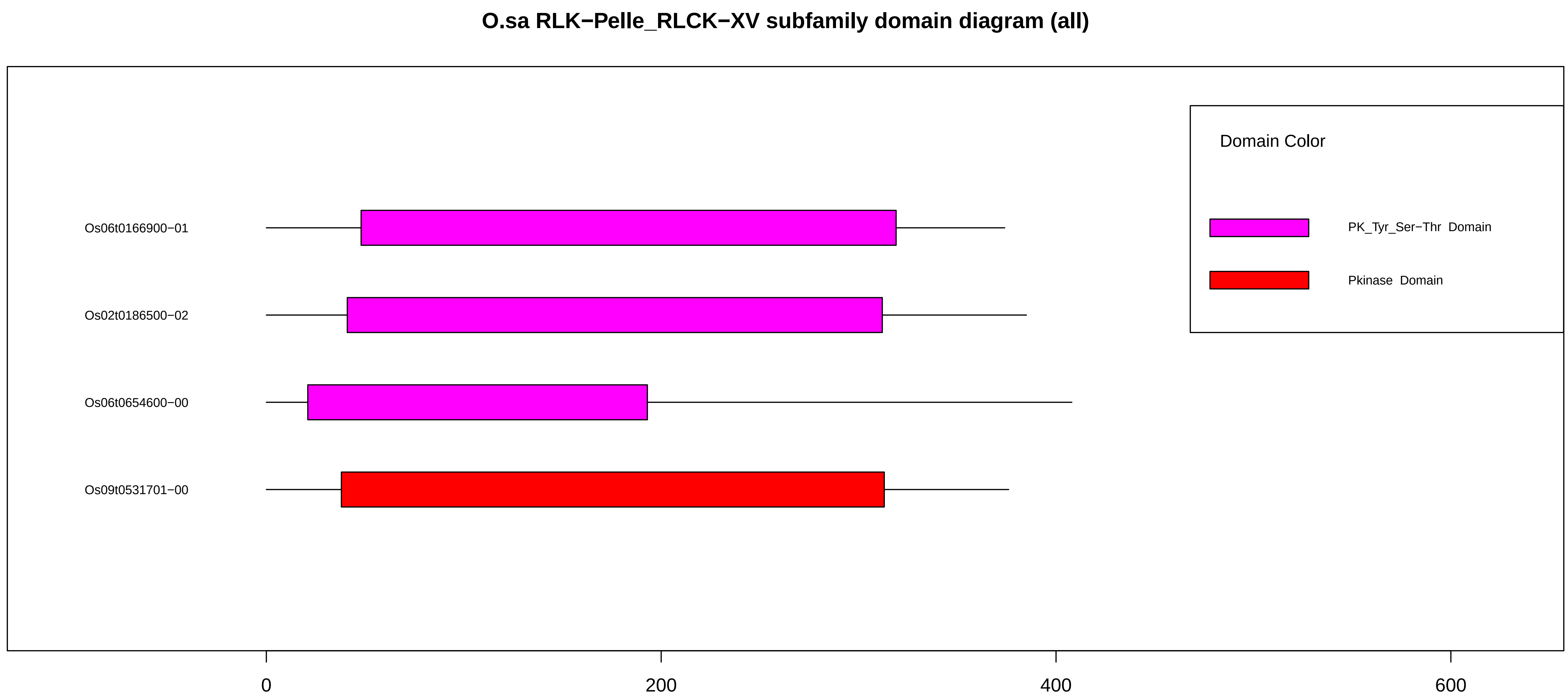
O.sa RLK−Pelle_RLCK−XV subfamily domain diagram (all)
Domain Color
PK_Tyr_Ser−Thr Domain
Pkinase Domain
Os06t0166900−01
Os02t0186500−02
Os06t0654600−00
Os09t0531701−00
0
200
400
600

O.sa RLK−Pelle_SD−2b subfamily domain diagram (part 1)

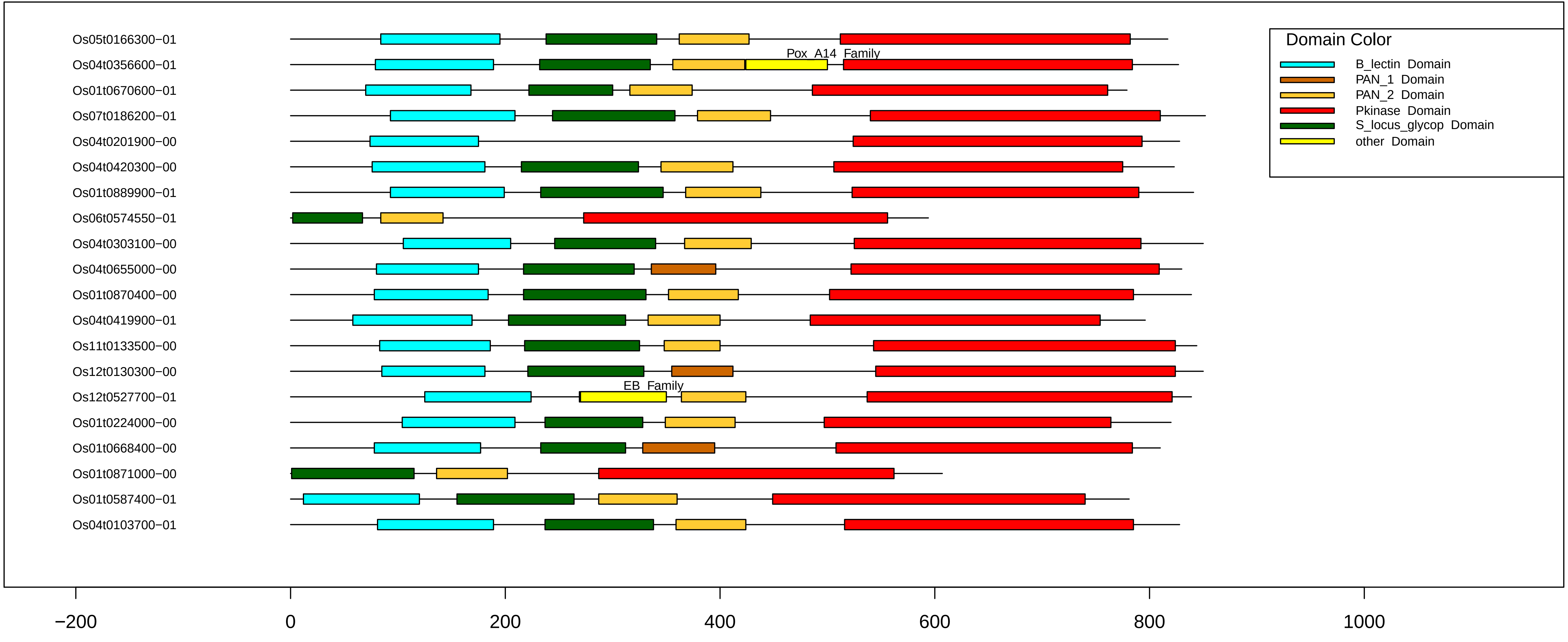

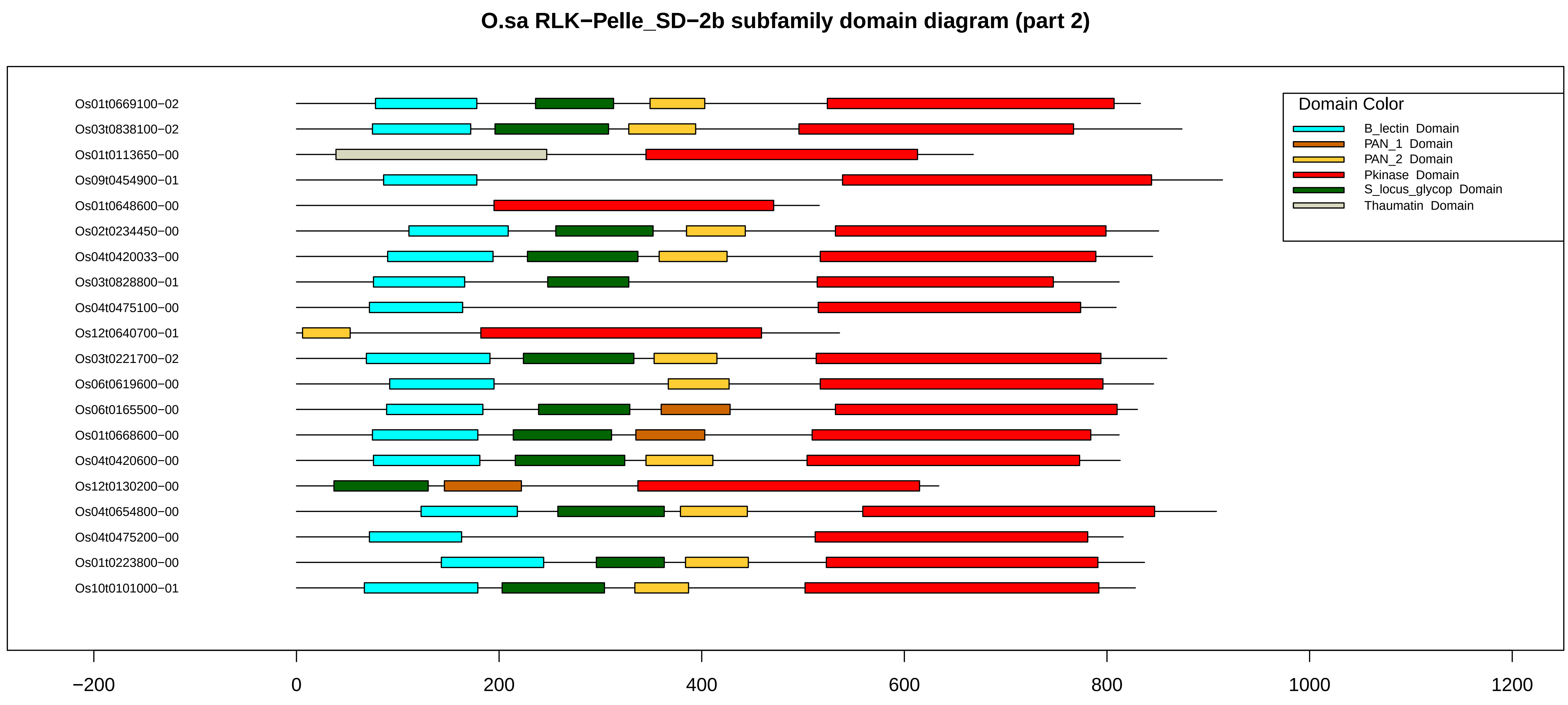
O.sa RLK−Pelle_SD−2b subfamily domain diagram (part 2)
Os01t0669100−02
Os03t0838100−02
Os01t0113650−00
Os09t0454900−01
Os01t0648600−00
Os02t0234450−00
Os04t0420033−00
Os03t0828800−01
Os04t0475100−00
Os12t0640700−01
Os03t0221700−02
Os06t0619600−00
Os06t0165500−00
Os01t0668600−00
Os04t0420600−00
Os12t0130200−00
Os04t0654800−00
Os04t0475200−00
Os01t0223800−00
Os10t0101000−01
Domain Color
B_lectin Domain
PAN_1 Domain
PAN_2 Domain
Pkinase Domain
S_locus_glycop Domain
Thaumatin Domain
−200
0
200
400
600
800
1000
1200

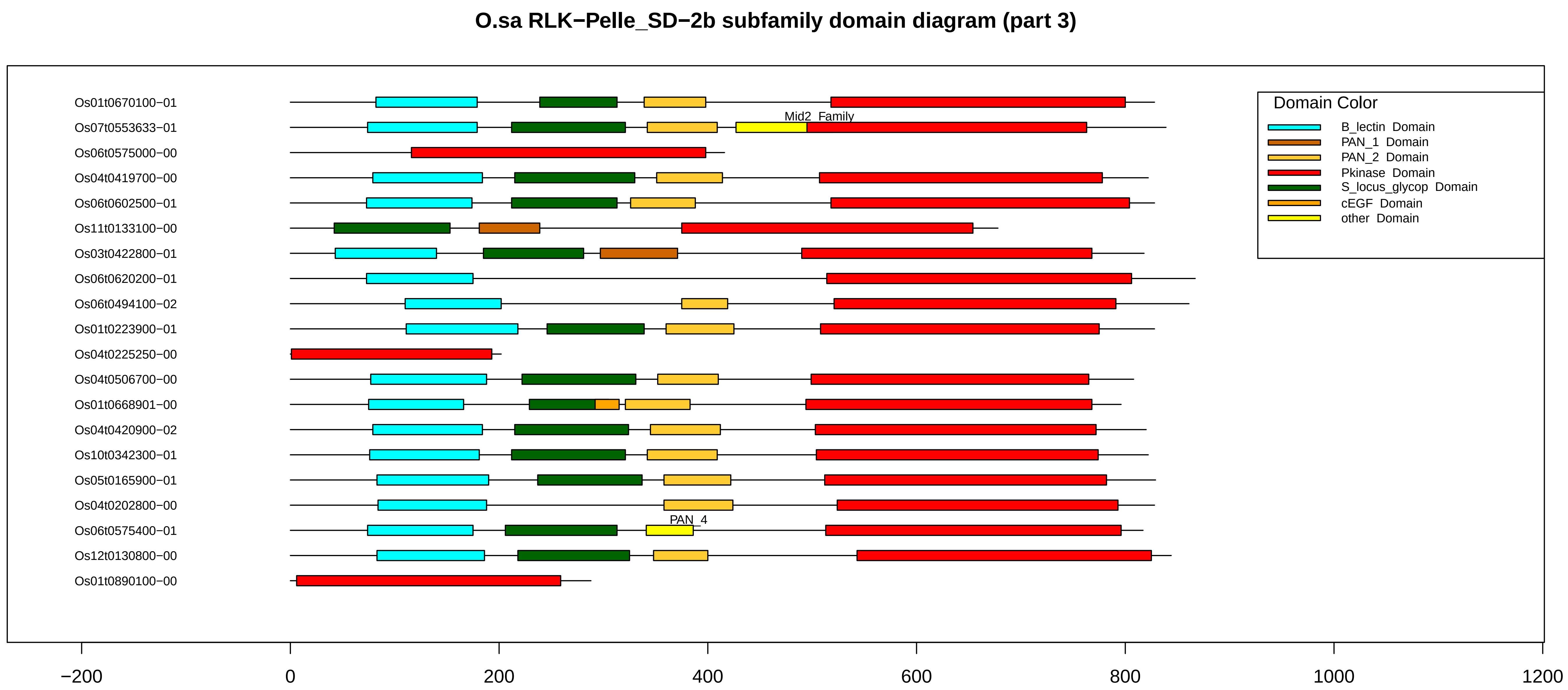
O.sa RLK−Pelle_SD−2b subfamily domain diagram (part 3)
Os01t0670100−01
Os07t0553633−01
Os06t0575000−00
Os04t0419700−00
Os06t0602500−01
Os11t0133100−00
Os03t0422800−01
Os06t0620200−01
Os06t0494100−02
Os01t0223900−01
Os04t0225250−00
Os04t0506700−00
Os01t0668901−00
Os04t0420900−02
Os10t0342300−01
Os05t0165900−01
Os04t0202800−00
Os06t0575400−01
Os12t0130800−00
Os01t0890100−00
Mid2_Family
PAN_4
Domain Color
B_lectin Domain
PAN_1 Domain
PAN_2 Domain
Pkinase Domain
S_locus_glycop Domain
cEGF Domain
other Domain
−200
0
200
400
600
800
1000
1200

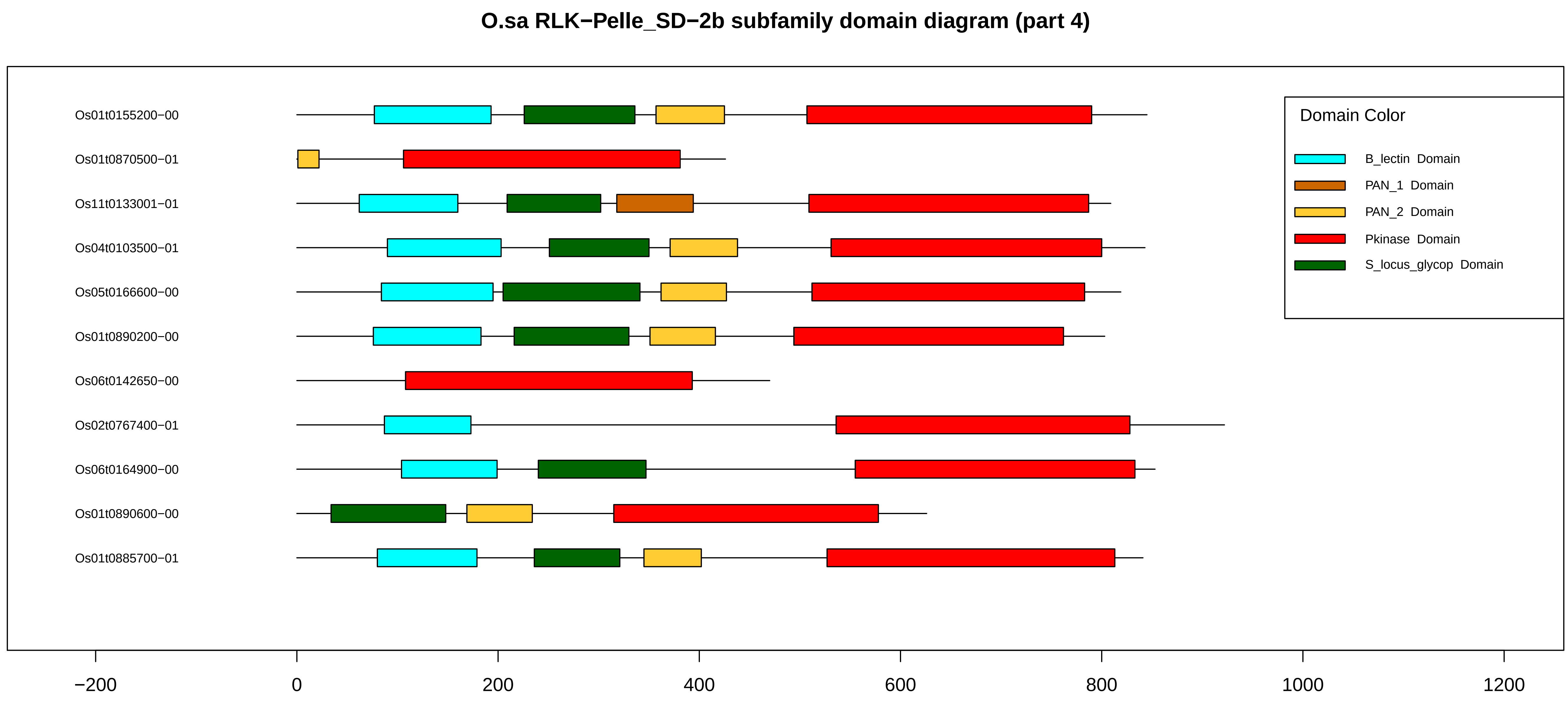

O.sa RLK−Pelle_SD−2b subfamily domain diagram (part 4)
Os01t0155200−00
Os01t0870500−01
Os11t0133001−01
Os04t0103500−01
Os05t0166600−00
Os01t0890200−00
Os06t0142650−00
Os02t0767400−01
Os06t0164900−00
Os01t0890600−00
Os01t0885700−01
Domain Color
B_lectin Domain
PAN_1 Domain
PAN_2 Domain
Pkinase Domain
S_locus_glycop Domain
−200
0
200
400
600
800
1000
1200

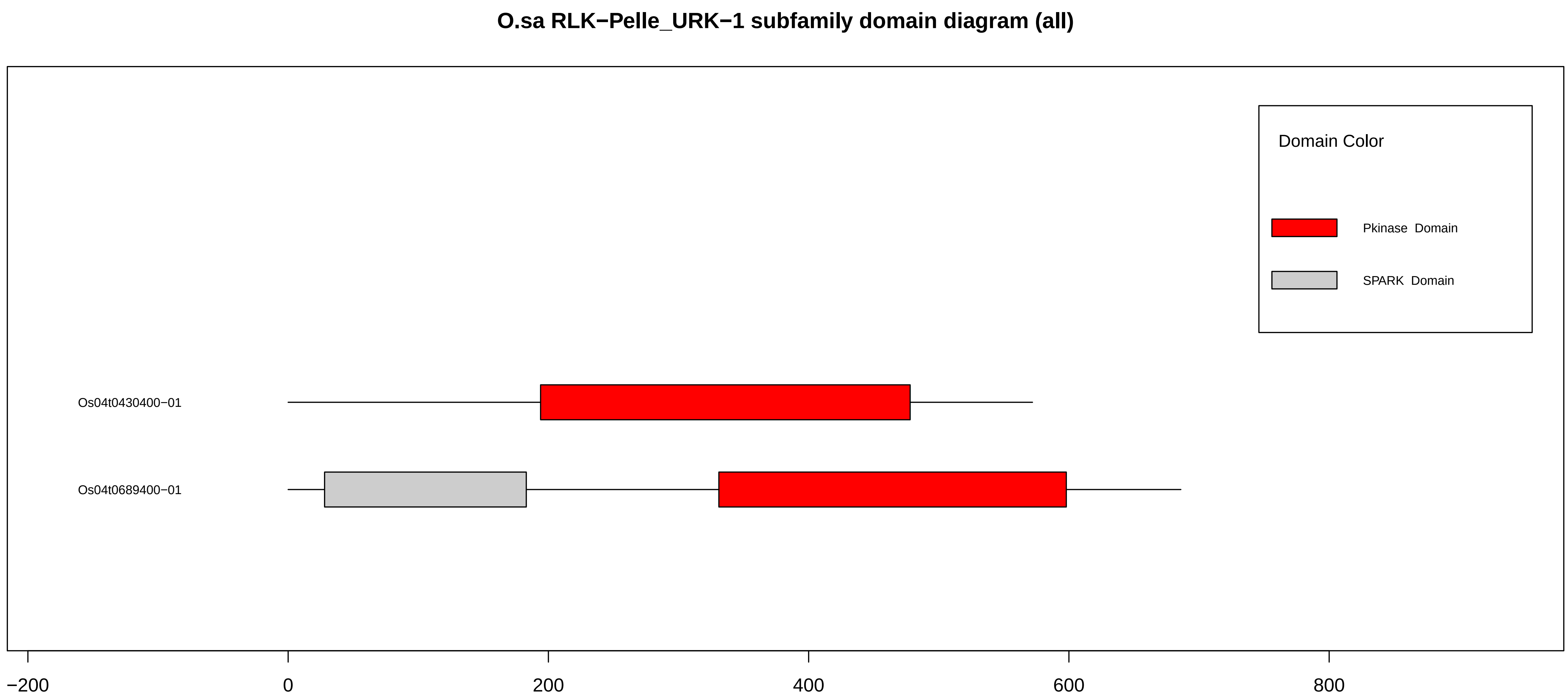

O.sa RLK−Pelle_URK−1 subfamily domain diagram (all)
Domain Color
Pkinase Domain
SPARK Domain
Os04t0430400−01
Os04t0689400−01
−200
0
200
400
600
800

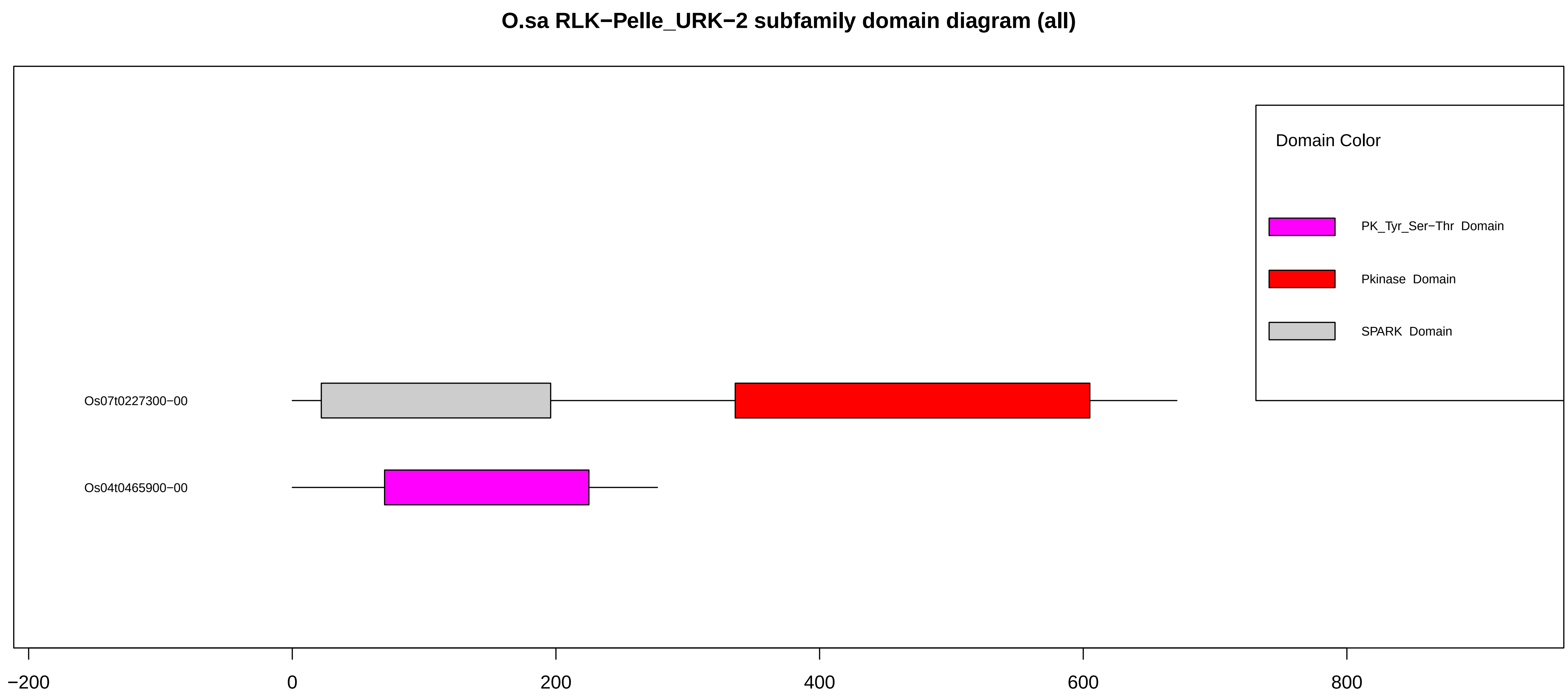

O.sa RLK−Pelle_URK−2 subfamily domain diagram (all)
Domain Color
PK_Tyr_Ser−Thr Domain
Pkinase Domain
SPARK Domain
Os07t0227300−00
Os04t0465900−00
−200
0
200
400
600
800

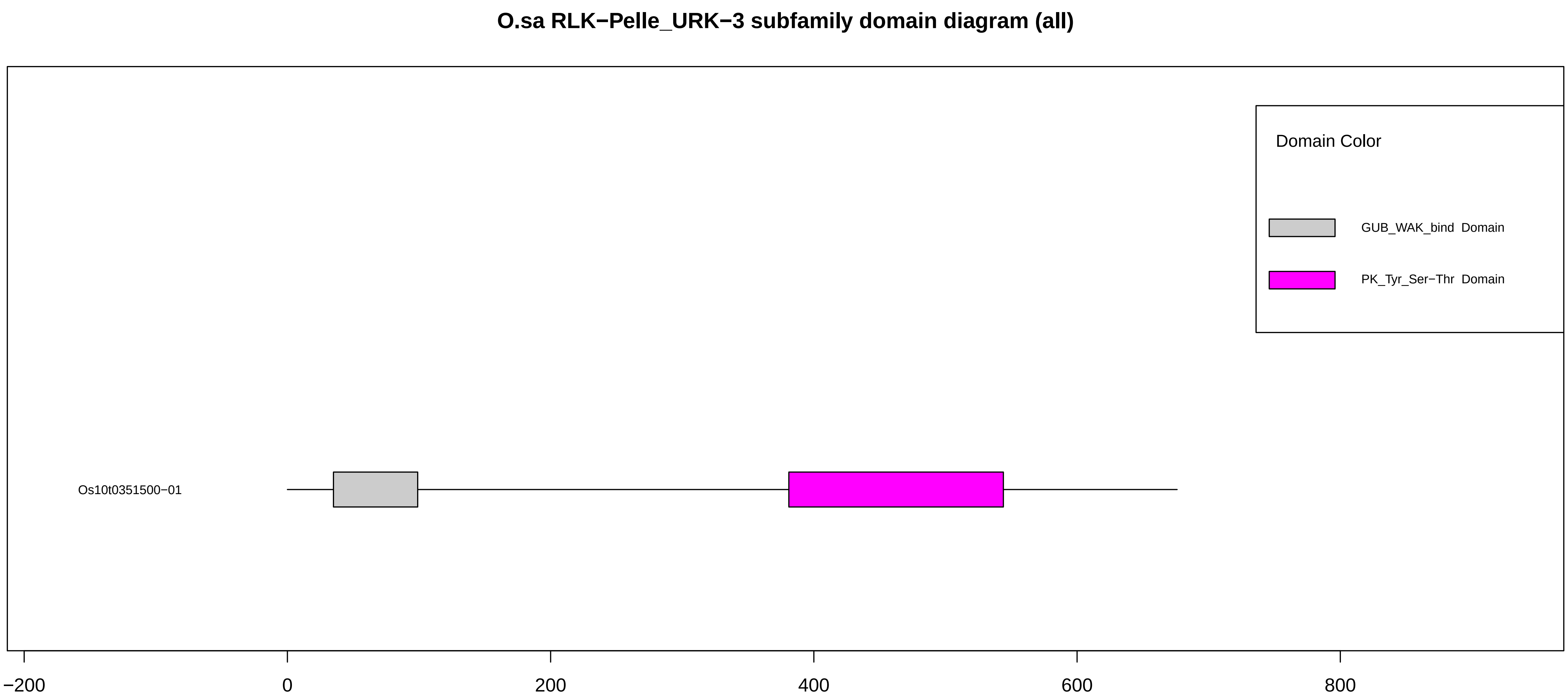
O.sa RLK−Pelle_URK−3 subfamily domain diagram (all)
Domain Color
GUB_WAK_bind Domain
PK_Tyr_Ser−Thr Domain
Os10t0351500−01
−200
0
200
400
600
800

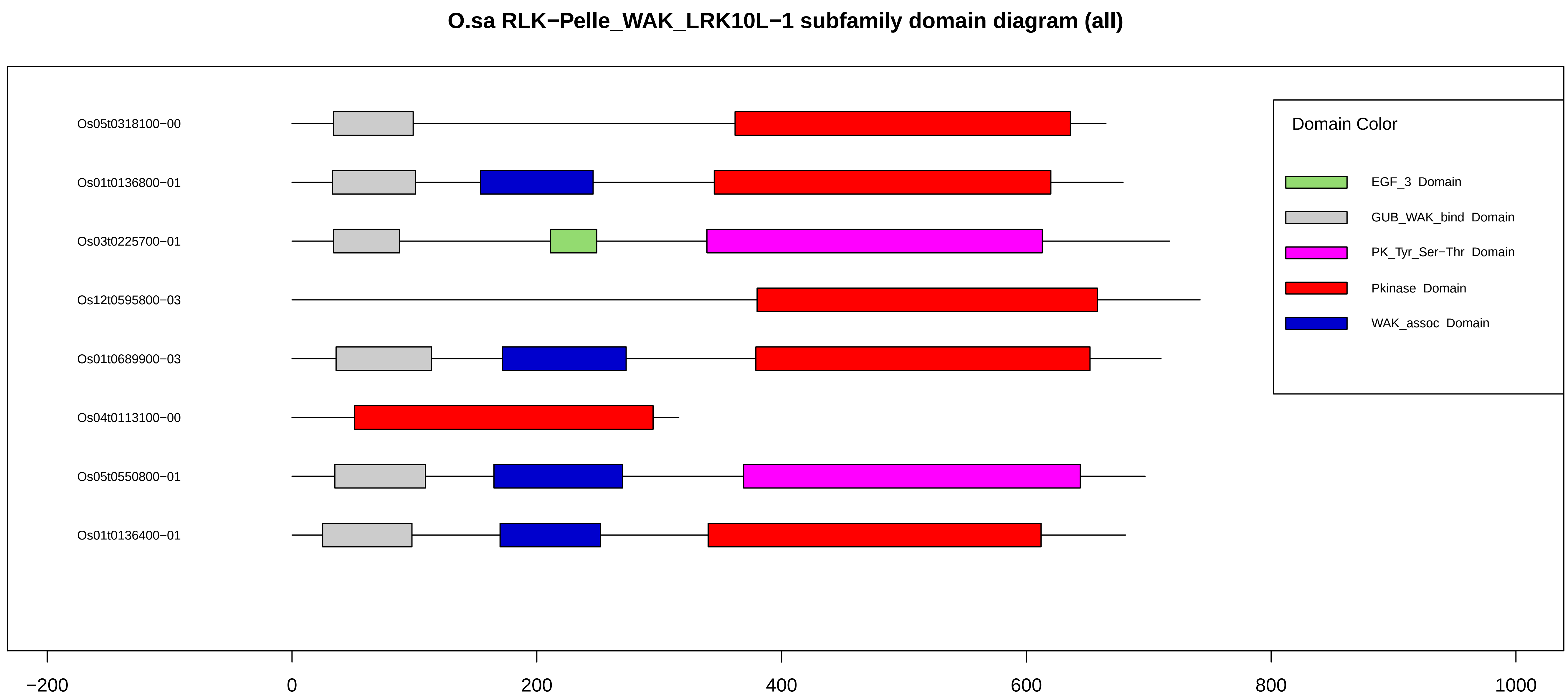
O.sa RLK−Pelle_WAK_LRK10L−1 subfamily domain diagram (all)
Os05t0318100−00
Os01t0136800−01
Os03t0225700−01
Os12t0595800−03
Os01t0689900−03
Os04t0113100−00
Os05t0550800−01
Os01t0136400−01
Domain Color
EGF_3 Domain
GUB_WAK_bind Domain
PK_Tyr_Ser−Thr Domain
Pkinase Domain
WAK_assoc Domain
−200
0
200
400
600
800
1000

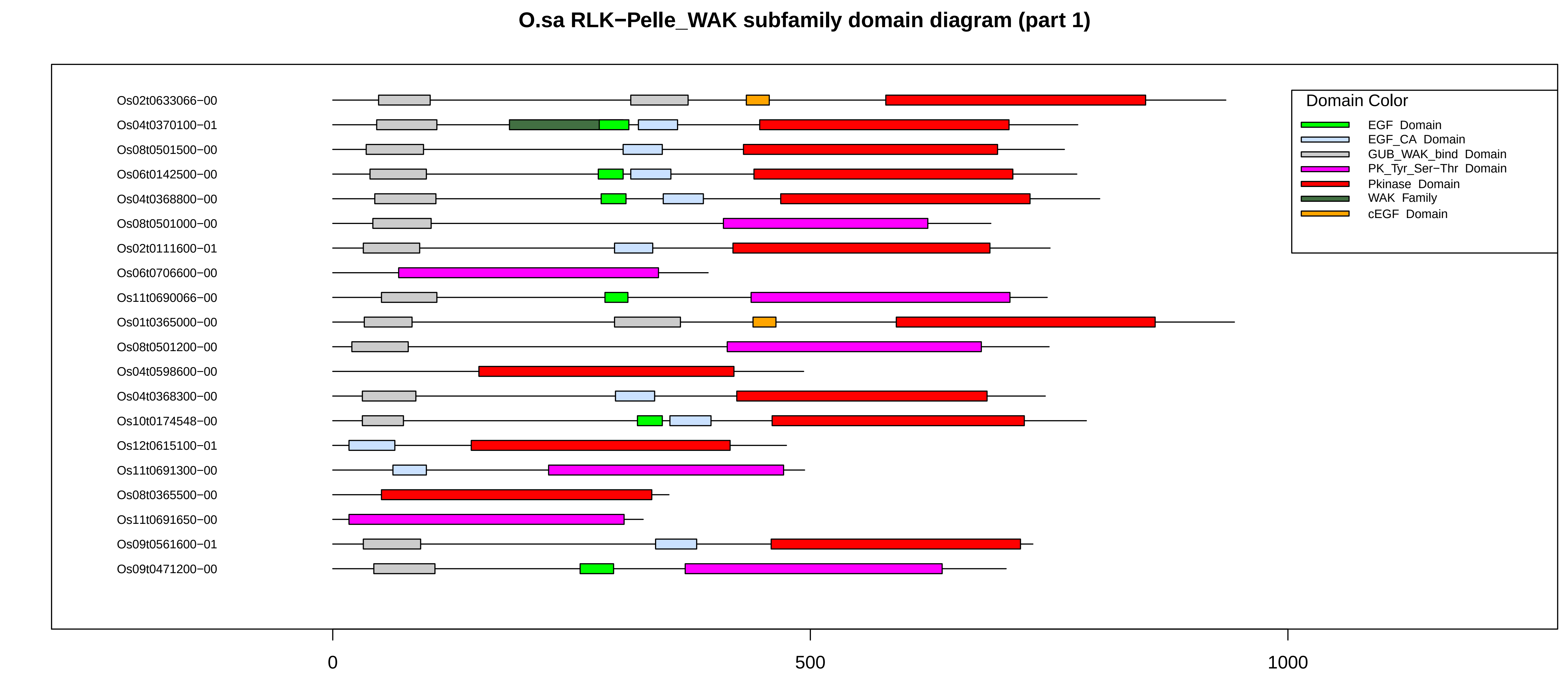
O.sa RLK−Pelle_WAK subfamily domain diagram (part 1)
Os02t0633066−00
Os04t0370100−01
Os08t0501500−00
Os06t0142500−00
Os04t0368800−00
Os08t0501000−00
Os02t0111600−01
Os06t0706600−00
Os11t0690066−00
Os01t0365000−00
Os08t0501200−00
Os04t0598600−00
Os04t0368300−00
Os10t0174548−00
Os12t0615100−01
Os11t0691300−00
Os08t0365500−00
Os11t0691650−00
Os09t0561600−01
Os09t0471200−00
Domain Color
EGF Domain
EGF_CA Domain
GUB_WAK_bind Domain
PK_Tyr_Ser−Thr Domain
Pkinase Domain
WAK Family
cEGF Domain
0
500
1000

O.sa RLK−Pelle_WAK subfamily domain diagram (part 2)

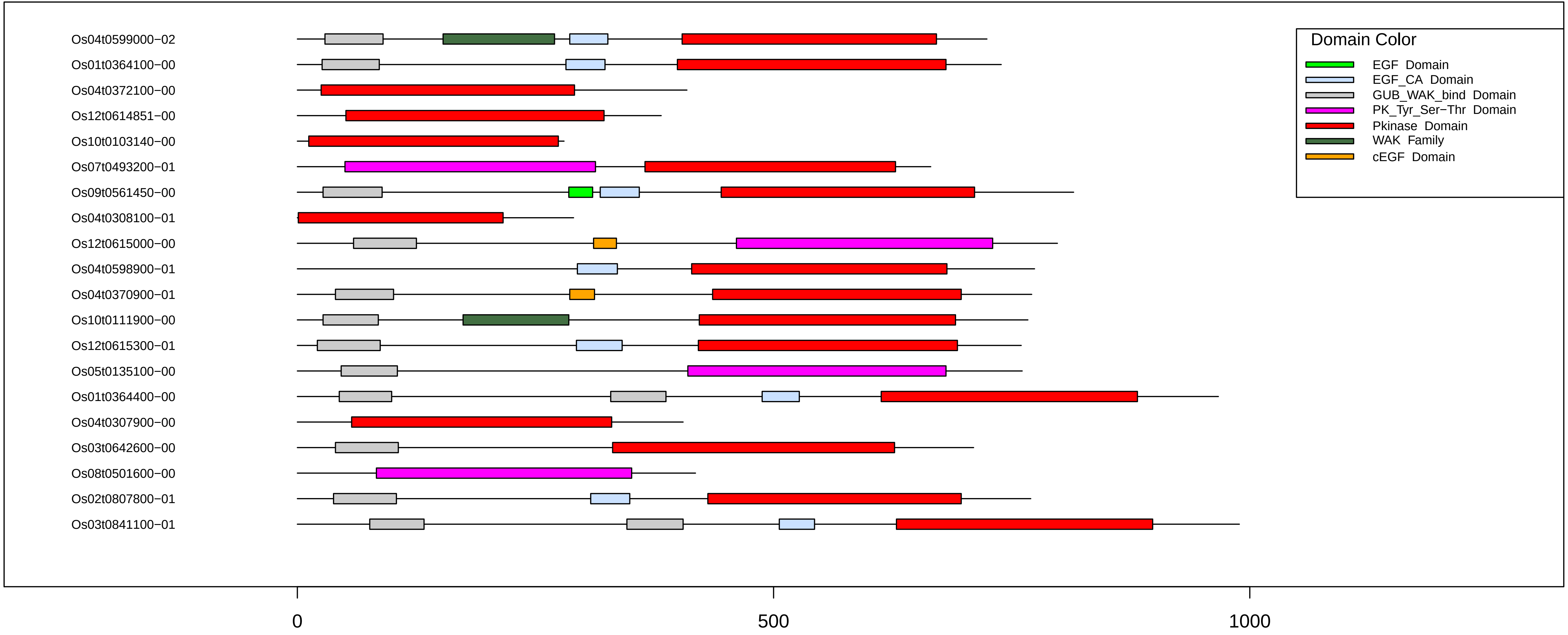

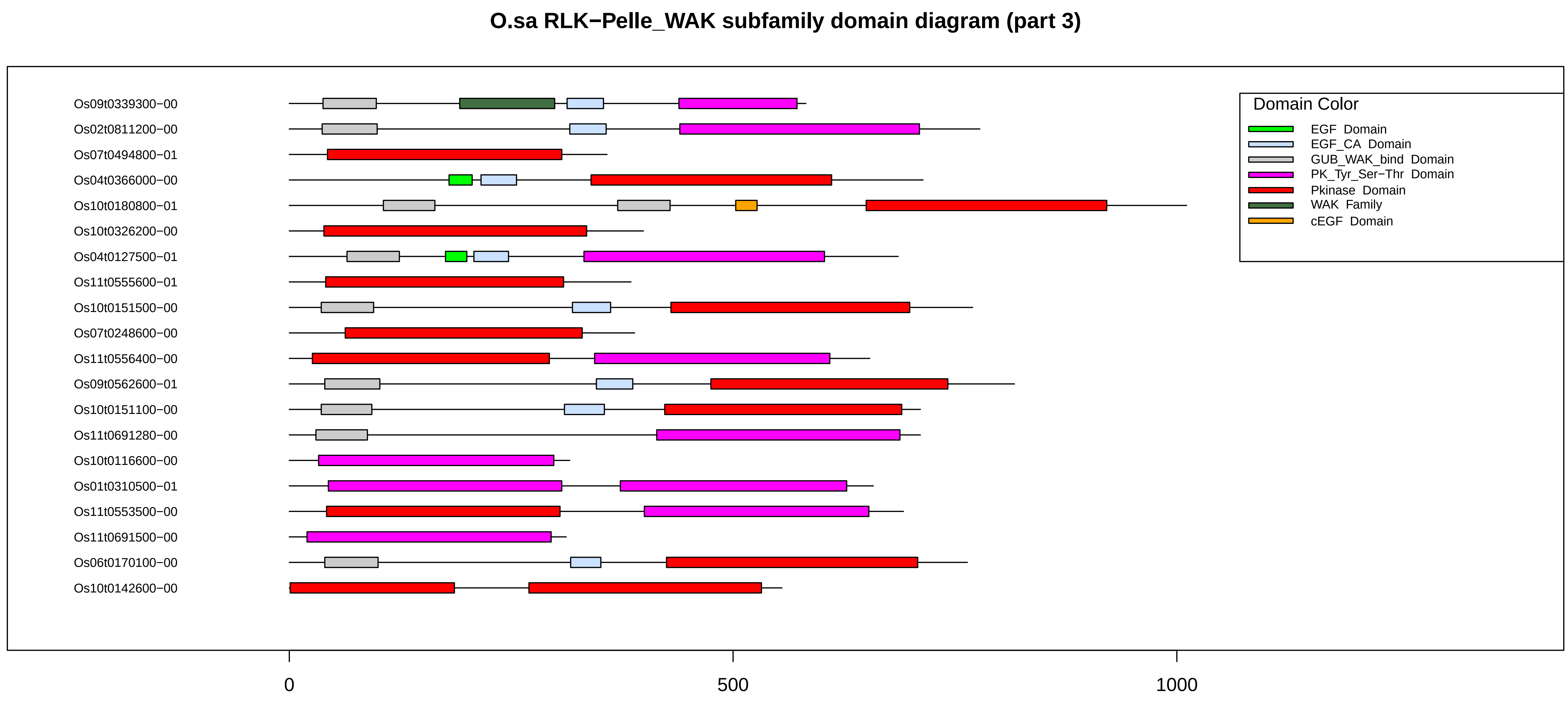

O.sa RLK−Pelle_WAK subfamily domain diagram (part 3)
Os09t0339300−00
Os02t0811200−00
Os07t0494800−01
Os04t0366000−00
Os10t0180800−01
Os10t0326200−00
Os04t0127500−01
Os11t0555600−01
Os10t0151500−00
Os07t0248600−00
Os11t0556400−00
Os09t0562600−01
Os10t0151100−00
Os11t0691280−00
Os10t0116600−00
Os01t0310500−01
Os11t0553500−00
Os11t0691500−00
Os06t0170100−00
Os10t0142600−00
Domain Color
EGF Domain
EGF_CA Domain
GUB_WAK_bind Domain
PK_Tyr_Ser−Thr Domain
Pkinase Domain
WAK Family
cEGF Domain
0
500
1000

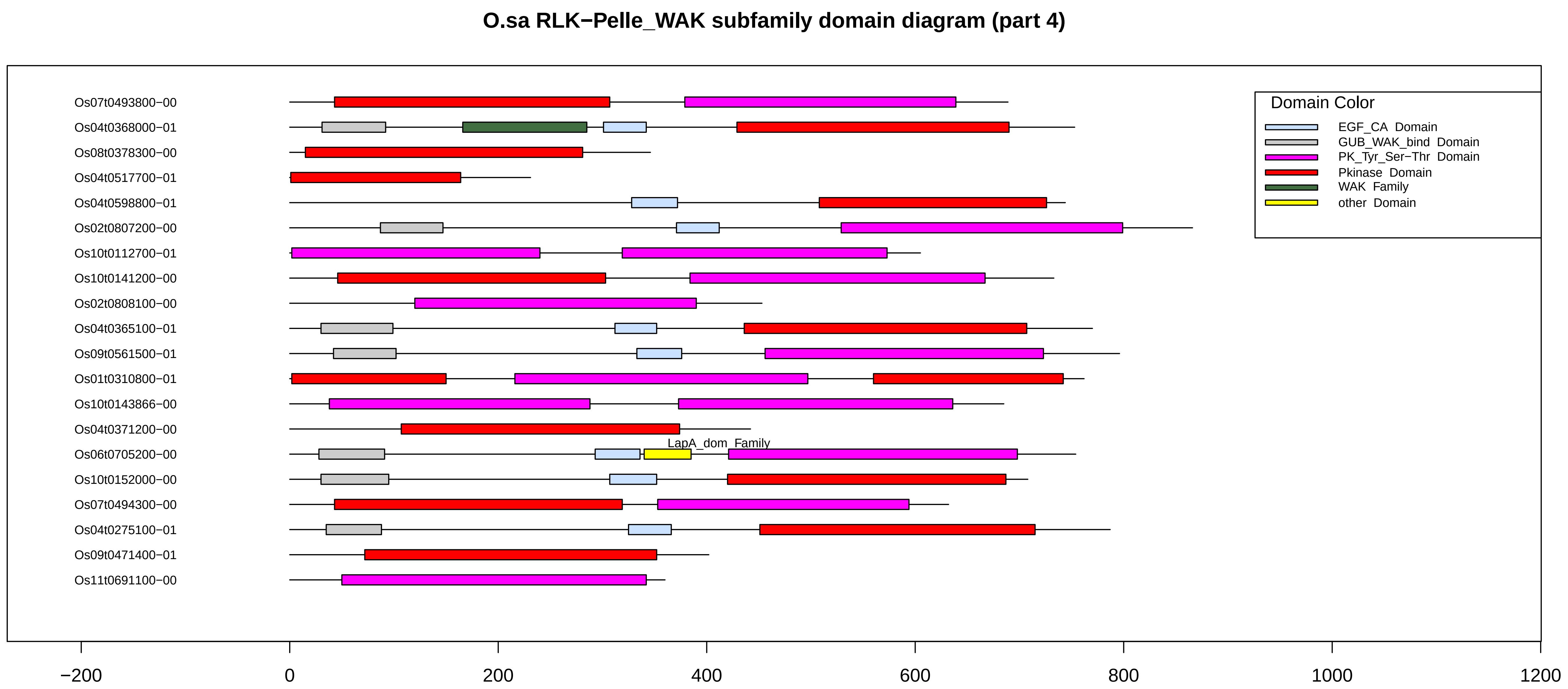
O.sa RLK−Pelle_WAK subfamily domain diagram (part 4)
Os07t0493800−00
Os04t0368000−01
Os08t0378300−00
Os04t0517700−01
Os04t0598800−01
Os02t0807200−00
Os10t0112700−01
Os10t0141200−00
Os02t0808100−00
Os04t0365100−01
Os09t0561500−01
Os01t0310800−01
Os10t0143866−00
Os04t0371200−00
LapA_dom_Family
Os06t0705200−00
Os10t0152000−00
Os07t0494300−00
Os04t0275100−01
Os09t0471400−01
Os11t0691100−00
Domain Color
EGF_CA Domain
GUB_WAK_bind Domain
PK_Tyr_Ser−Thr Domain
Pkinase Domain
WAK Family
other Domain
−200
0
200
400
600
800
1000
1200

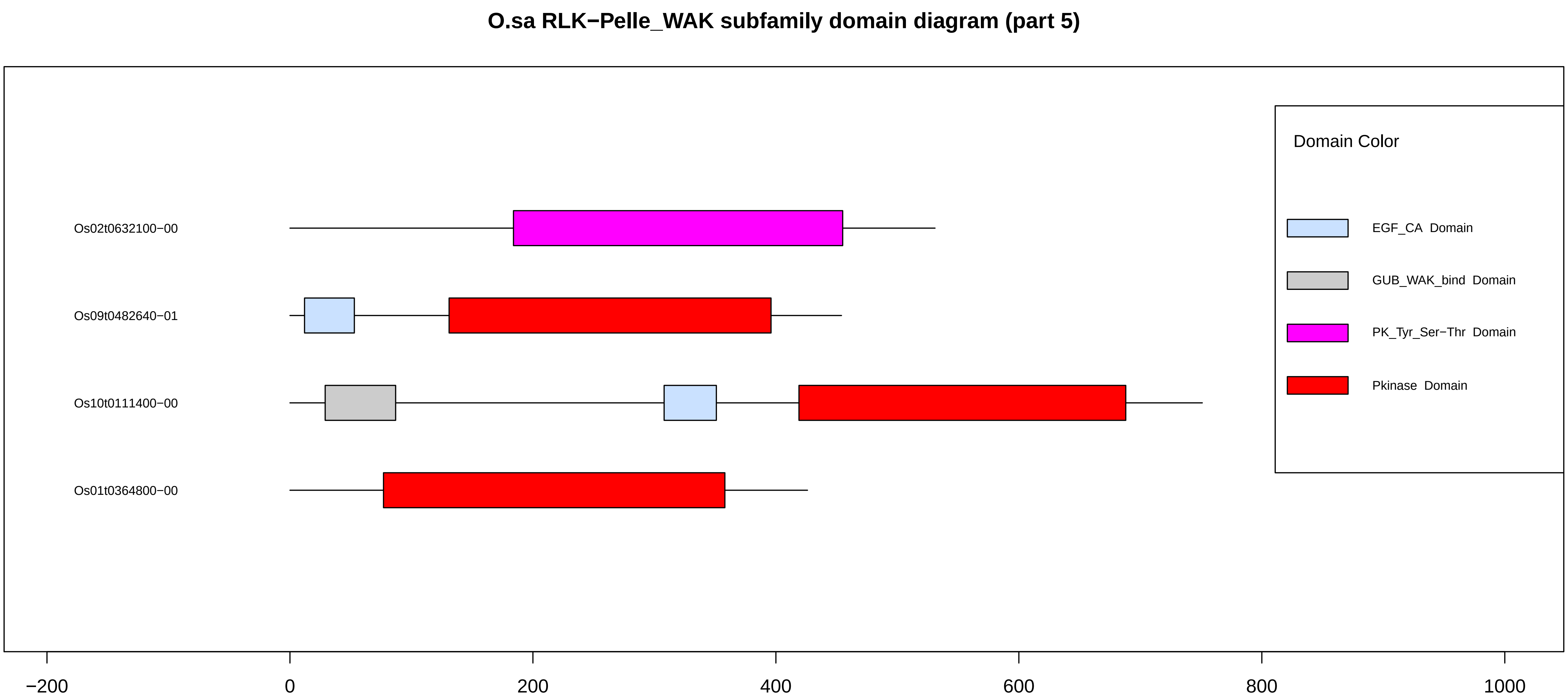

O.sa RLK−Pelle_WAK subfamily domain diagram (part 5)
Os02t0632100−00
Os09t0482640−01
Os10t0111400−00
Os01t0364800−00
Domain Color
EGF_CA Domain
GUB_WAK_bind Domain
PK_Tyr_Ser−Thr Domain
Pkinase Domain
−200
0
200
400
600
800
1000

Z.ma RLK−Pelle_C−LEC subfamily domain diagram (all)

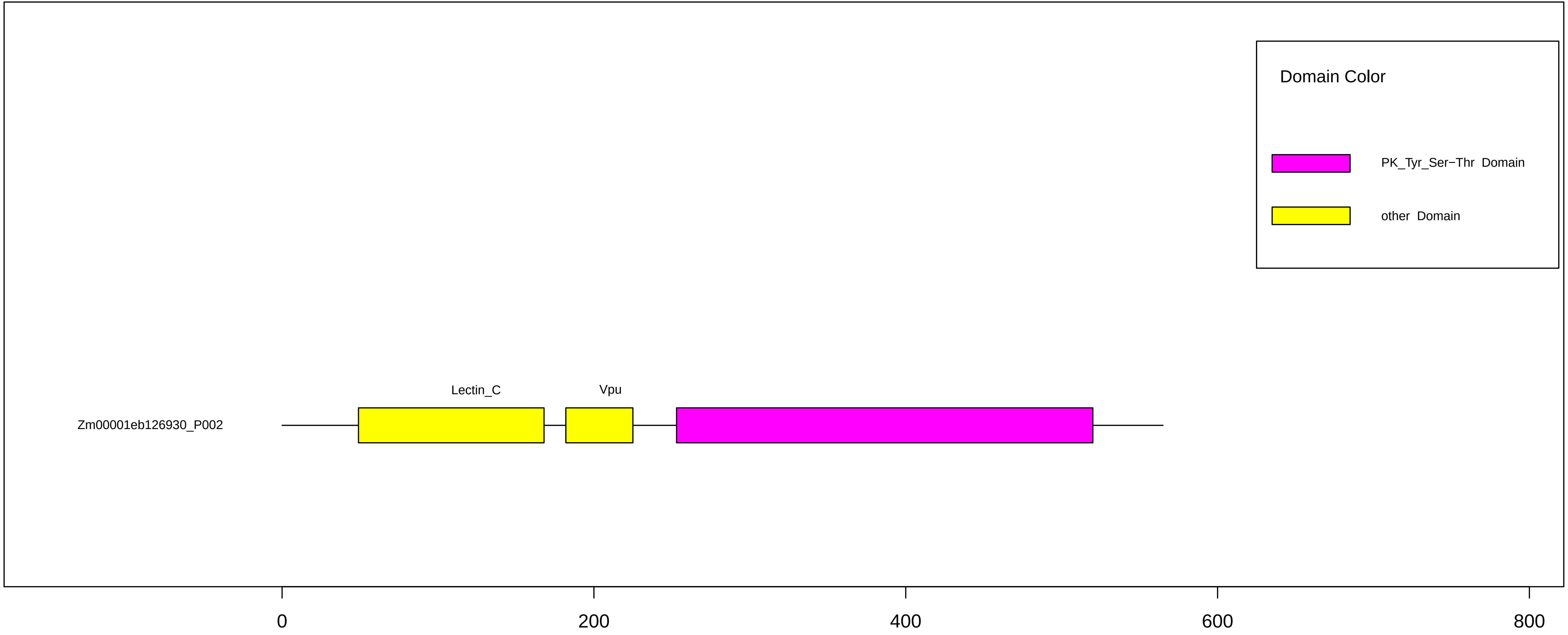

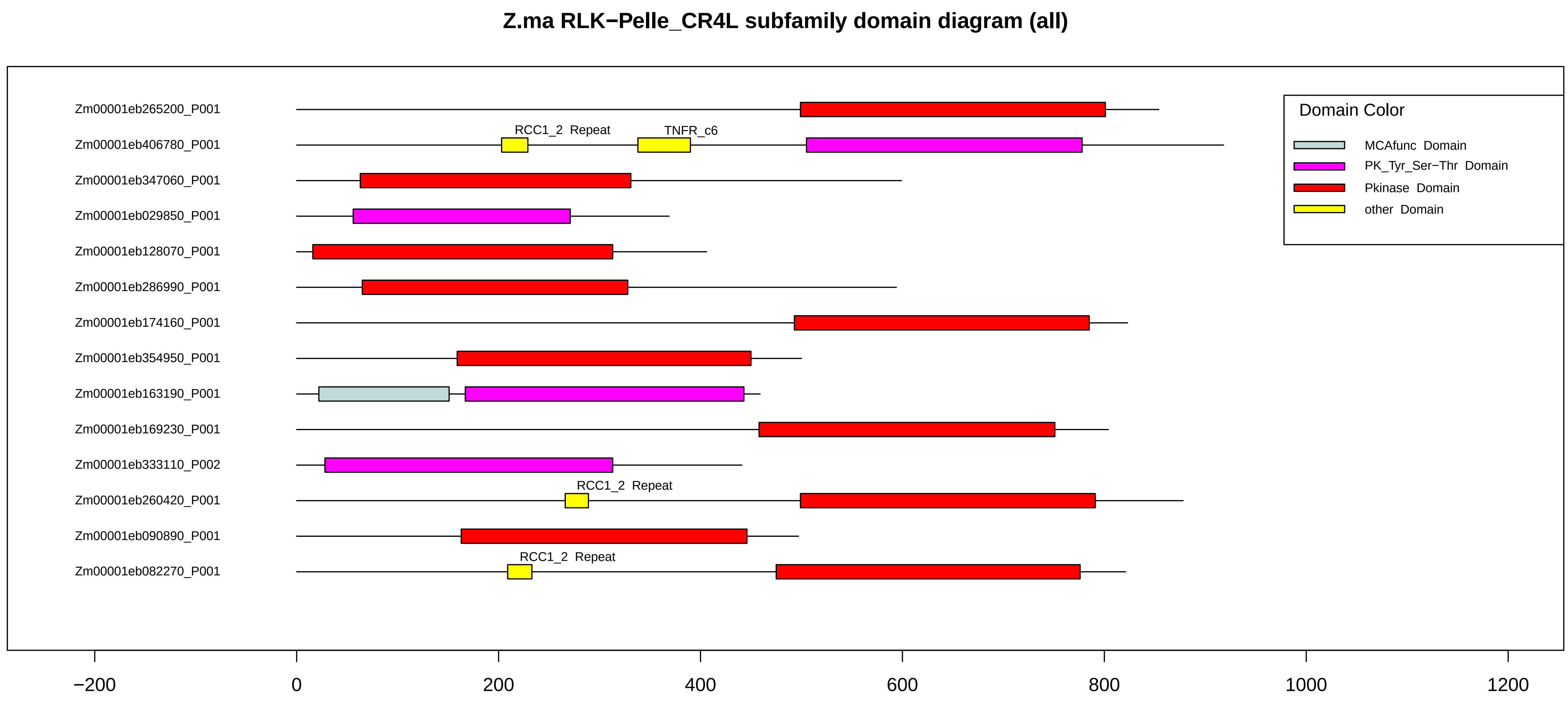

Z.ma RLK−Pelle_CR4L subfamily domain diagram (all)
Zm00001eb265200_P001
Zm00001eb406780_P001
Zm00001eb347060_P001
Zm00001eb029850_P001
Zm00001eb128070_P001
Zm00001eb286990_P001
Zm00001eb174160_P001
Zm00001eb354950_P001
Zm00001eb163190_P001
Zm00001eb169230_P001
Zm00001eb333110_P002
Zm00001eb260420_P001
Zm00001eb090890_P001
Zm00001eb082270_P001
RCC1_2 Repeat
TNFR_c6
RCC1_2 Repeat
RCC1_2 Repeat
Domain Color
MCAfunc Domain
PK_Tyr_Ser−Thr Domain
Pkinase Domain
other Domain
−200
0
200
400
600
800
1000
1200

Z.ma RLK−Pelle_CrRLK1L−1 subfamily domain diagram (all)

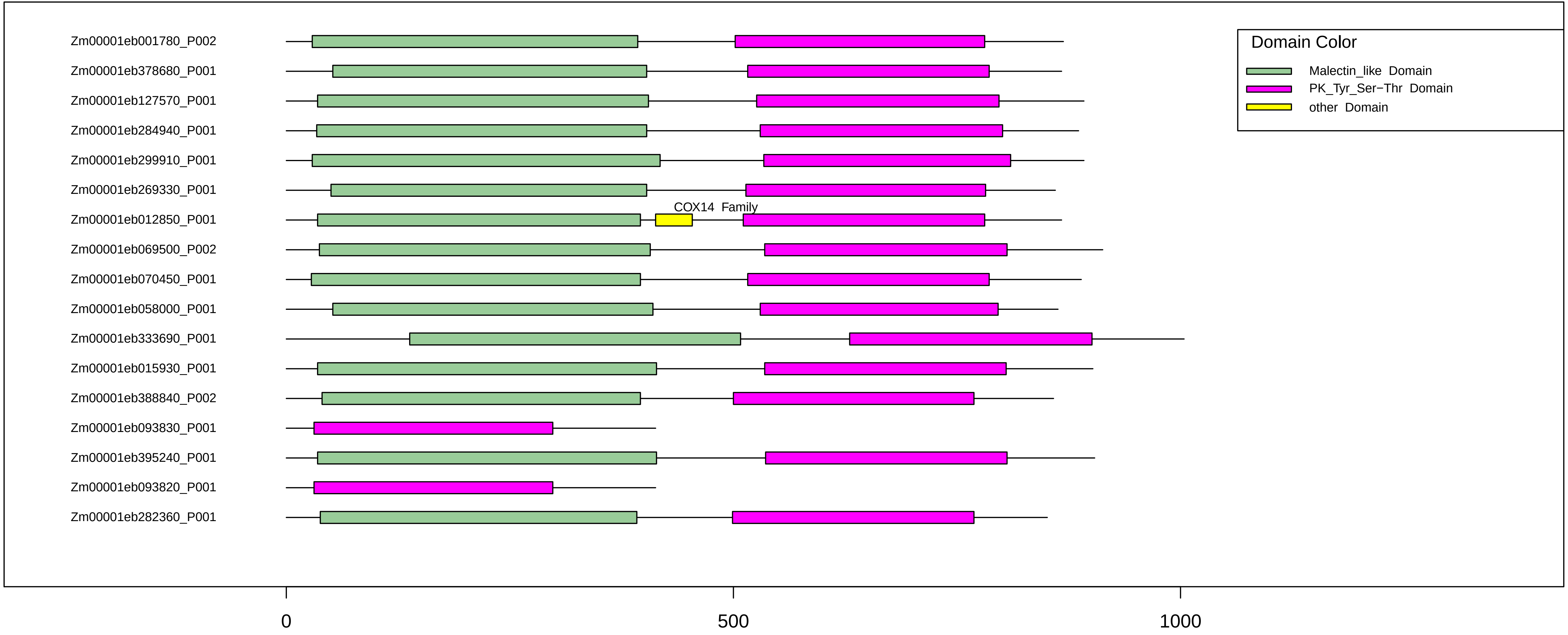

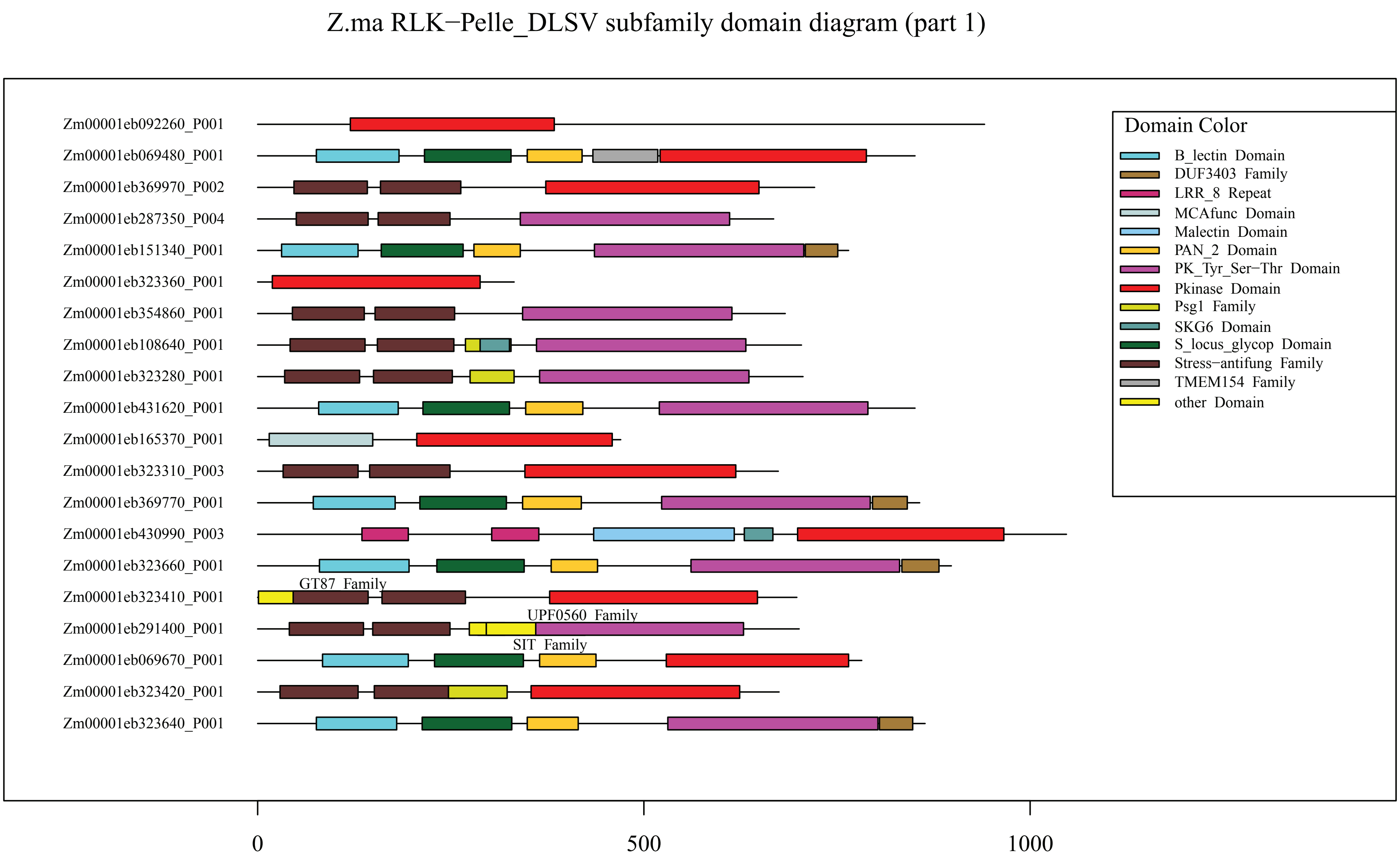

Z.ma RLK−Pelle_DLSV subfamily domain diagram (part 1)
Zm00001eb092260_P001
Zm00001eb069480_P001
Zm00001eb369970_P002
Zm00001eb287350_P004
Zm00001eb151340_P001
Zm00001eb323360_P001
Zm00001eb354860_P001
Zm00001eb108640_P001
Zm00001eb323280_P001
Zm00001eb431620_P001
Zm00001eb165370_P001
Zm00001eb323310_P003
Zm00001eb369770_P001
Zm00001eb430990_P003
Zm00001eb323660_P001
Zm00001eb323410_P001
Zm00001eb291400_P001
Zm00001eb069670_P001
Zm00001eb323420_P001
Zm00001eb323640_P001
GT87 Family
UPF0560 Family
SIT Family
Domain Color
B_lectin Domain
DUF3403 Family
LRR_8 Repeat
MCAfunc Domain
Malectin Domain
PAN_2 Domain
PK_Tyr_Ser−Thr Domain
Pkinase Domain
Psg1 Family
SKG6 Domain
S_locus_glycop Domain
Stress−antifung Family
TMEM154 Family
other Domain
0
500
1000

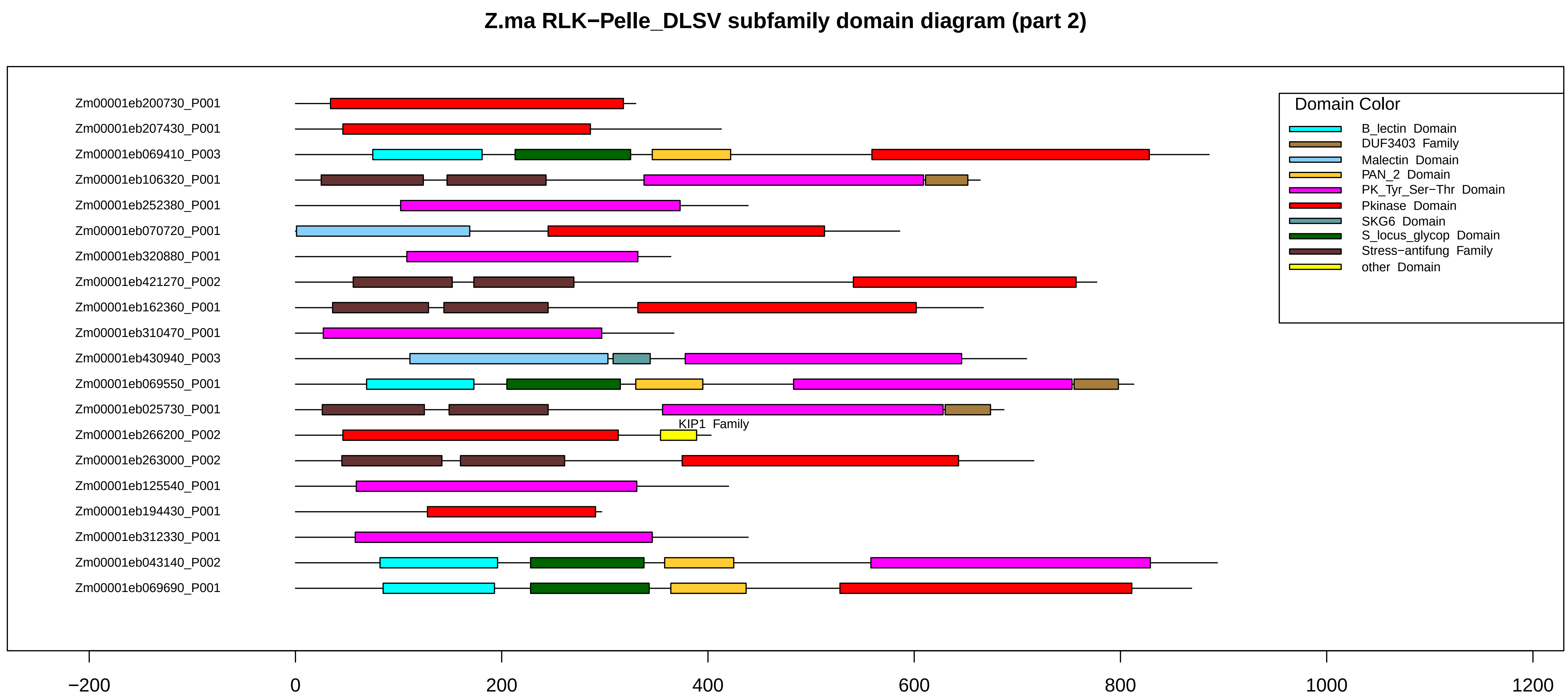

Z.ma RLK−Pelle_DLSV subfamily domain diagram (part 2)
Zm00001eb200730_P001
Zm00001eb207430_P001
Zm00001eb069410_P003
Zm00001eb106320_P001
Zm00001eb252380_P001
Zm00001eb070720_P001
Zm00001eb320880_P001
Zm00001eb421270_P002
Zm00001eb162360_P001
Zm00001eb310470_P001
Zm00001eb430940_P003
Zm00001eb069550_P001
Zm00001eb025730_P001
Zm00001eb266200_P002
Zm00001eb263000_P002
Zm00001eb125540_P001
Zm00001eb194430_P001
Zm00001eb312330_P001
Zm00001eb043140_P002
Zm00001eb069690_P001
KIP1 Family
Domain Color
B_lectin Domain
DUF3403 Family
Malectin Domain
PAN_2 Domain
PK_Tyr_Ser−Thr Domain
Pkinase Domain
SKG6 Domain
S_locus_glycop Domain
Stress−antifung Family
other Domain
−200
0
200
400
600
800
1000
1200

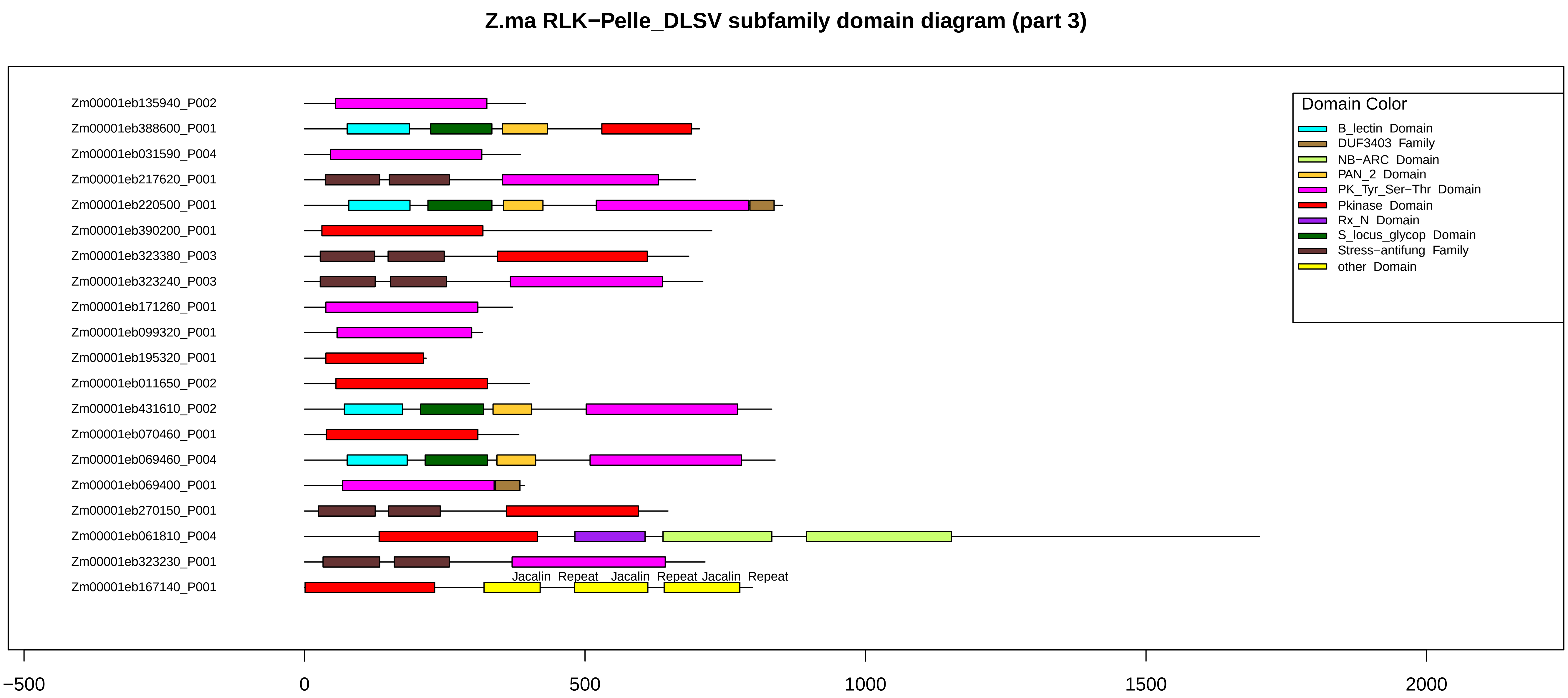

Z.ma RLK−Pelle_DLSV subfamily domain diagram (part 3)
Zm00001eb135940_P002
Zm00001eb388600_P001
Zm00001eb031590_P004
Zm00001eb217620_P001
Zm00001eb220500_P001
Zm00001eb390200_P001
Zm00001eb323380_P003
Zm00001eb323240_P003
Zm00001eb171260_P001
Zm00001eb099320_P001
Zm00001eb195320_P001
Zm00001eb011650_P002
Zm00001eb431610_P002
Zm00001eb070460_P001
Zm00001eb069460_P004
Zm00001eb069400_P001
Zm00001eb270150_P001
Zm00001eb061810_P004
Zm00001eb323230_P001
Zm00001eb167140_P001
Jacalin Repeat
Jacalin Repeat
Jacalin Repeat
Domain Color
B_lectin Domain
DUF3403 Family
NB−ARC Domain
PAN_2 Domain
PK_Tyr_Ser−Thr Domain
Pkinase Domain
Rx_N Domain
S_locus_glycop Domain
Stress−antifung Family
other Domain
−500
0
500
1000
1500
2000

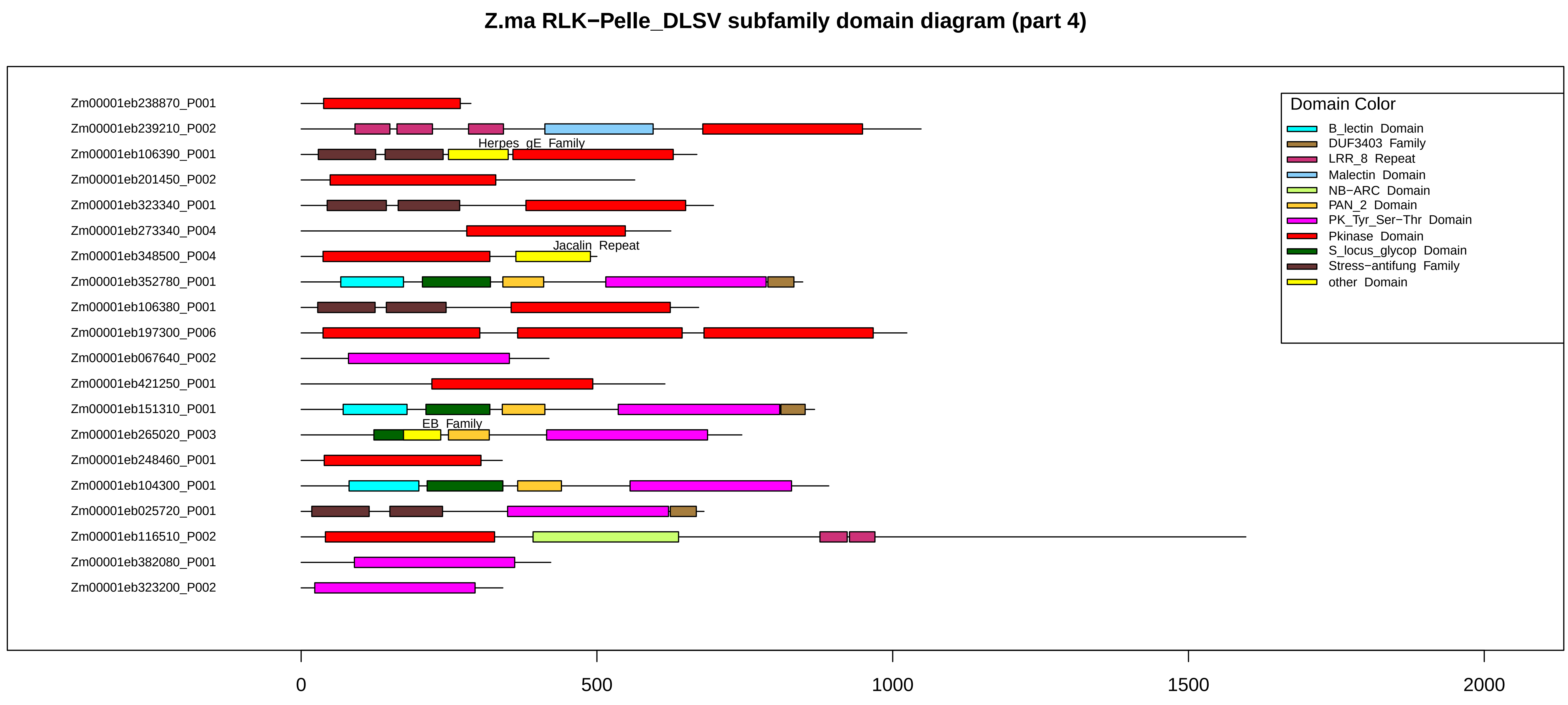
Z.ma RLK−Pelle_DLSV subfamily domain diagram (part 4)
Zm00001eb238870_P001
Zm00001eb239210_P002
Zm00001eb106390_P001
Zm00001eb201450_P002
Zm00001eb323340_P001
Zm00001eb273340_P004
Zm00001eb348500_P004
Zm00001eb352780_P001
Zm00001eb106380_P001
Zm00001eb197300_P006
Zm00001eb067640_P002
Zm00001eb421250_P001
Zm00001eb151310_P001
Zm00001eb265020_P003
Zm00001eb248460_P001
Zm00001eb104300_P001
Zm00001eb025720_P001
Zm00001eb116510_P002
Zm00001eb382080_P001
Zm00001eb323200_P002
Herpes_gE Family
Jacalin Repeat
EB Family
Domain Color
B_lectin Domain
DUF3403 Family
LRR_8 Repeat
Malectin Domain
NB−ARC Domain
PAN_2 Domain
PK_Tyr_Ser−Thr Domain
Pkinase Domain
S_locus_glycop Domain
Stress−antifung Family
other Domain
0
500
1000
1500
2000

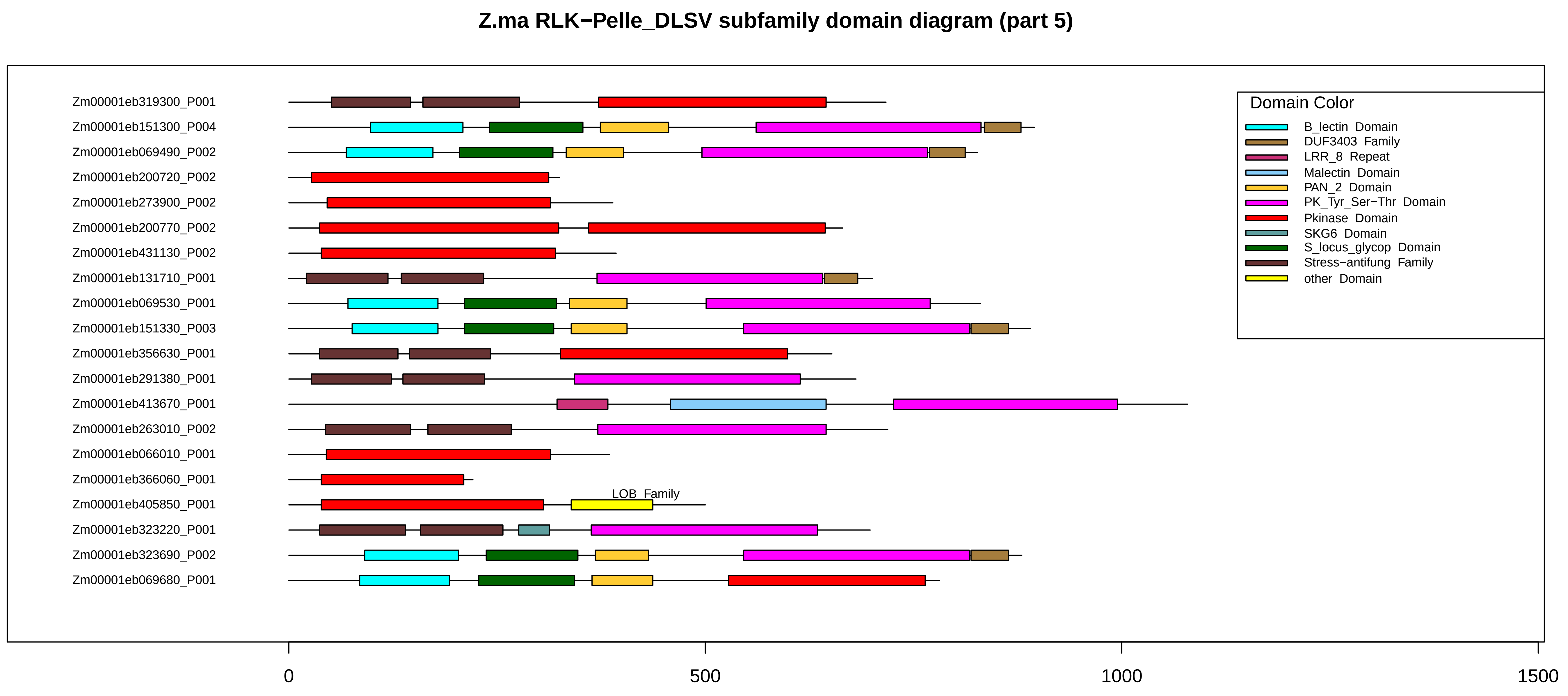
Z.ma RLK−Pelle_DLSV subfamily domain diagram (part 5)
Zm00001eb319300_P001
Zm00001eb151300_P004
Zm00001eb069490_P002
Zm00001eb200720_P002
Zm00001eb273900_P002
Zm00001eb200770_P002
Zm00001eb431130_P002
Zm00001eb131710_P001
Zm00001eb069530_P001
Zm00001eb151330_P003
Zm00001eb356630_P001
Zm00001eb291380_P001
Zm00001eb413670_P001
Zm00001eb263010_P002
Zm00001eb066010_P001
Zm00001eb366060_P001
Zm00001eb405850_P001
Zm00001eb323220_P001
Zm00001eb323690_P002
Zm00001eb069680_P001
LOB Family
Domain Color
B_lectin Domain
DUF3403 Family
LRR_8 Repeat
Malectin Domain
PAN_2 Domain
PK_Tyr_Ser−Thr Domain
Pkinase Domain
SKG6 Domain
S_locus_glycop Domain
Stress−antifung Family
other Domain
0
500
1000
1500

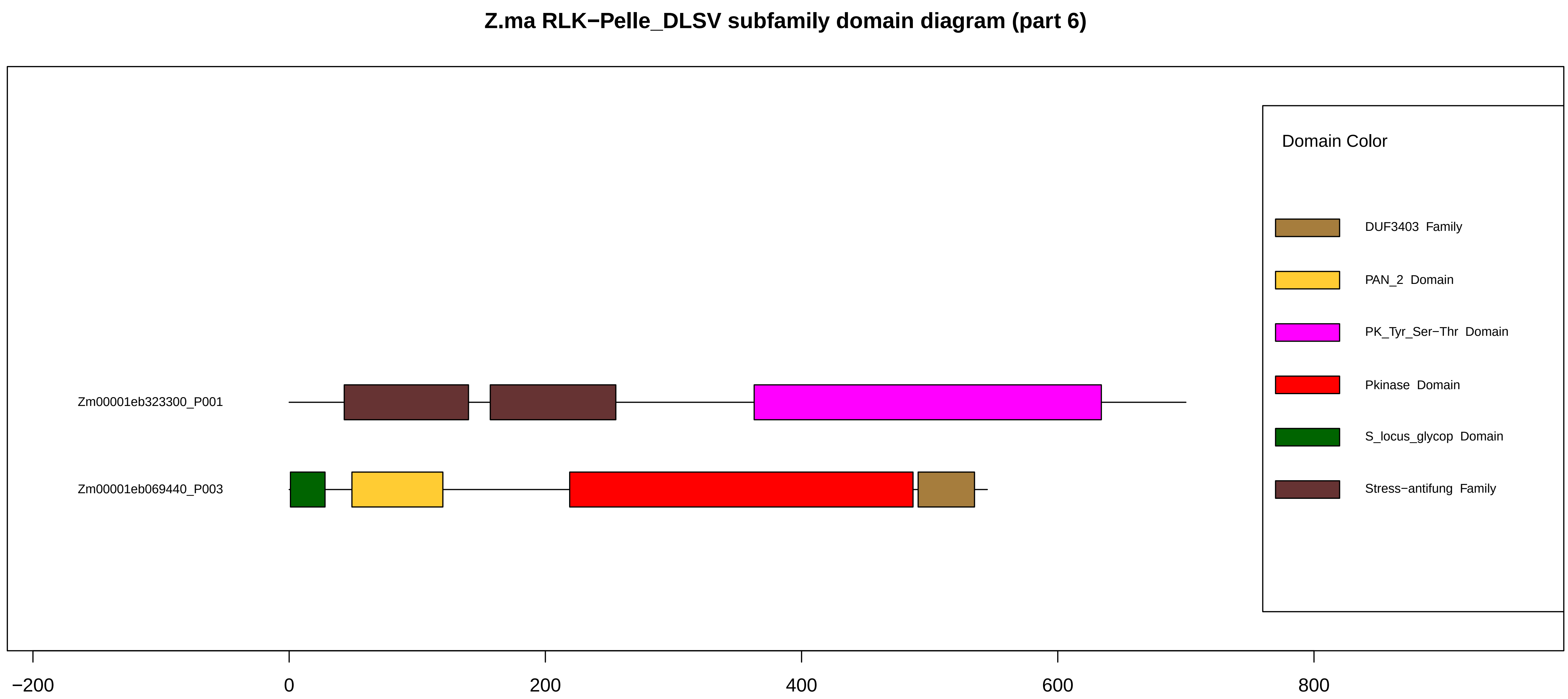

Z.ma RLK−Pelle_DLSV subfamily domain diagram (part 6)
Zm00001eb323300_P001
Zm00001eb069440_P003
Domain Color
DUF3403 Family
PAN_2 Domain
PK_Tyr_Ser−Thr Domain
Pkinase Domain
S_locus_glycop Domain
Stress−antifung Family
−200
0
200
400
600
800

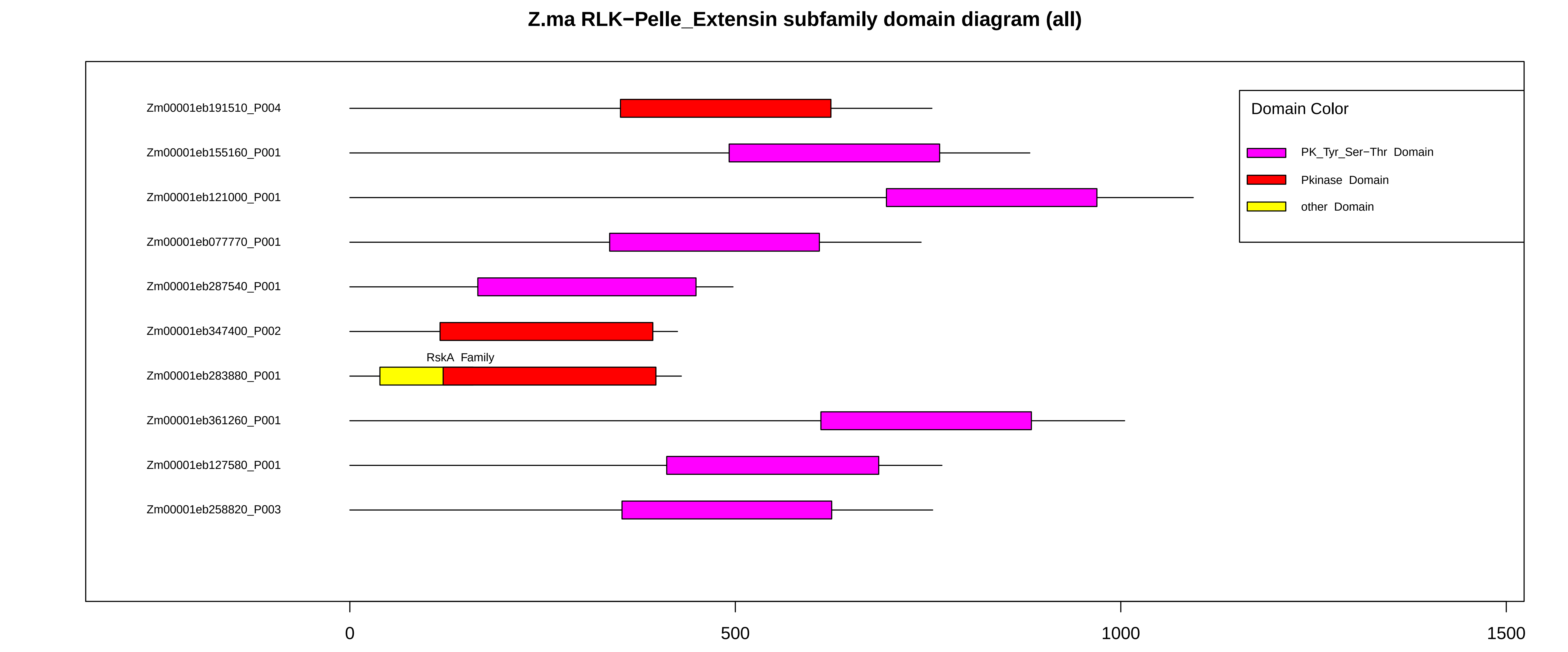
Z.ma RLK−Pelle_Extensin subfamily domain diagram (all)
Zm00001eb191510_P004
Zm00001eb155160_P001
Zm00001eb121000_P001
Zm00001eb077770_P001
Zm00001eb287540_P001
Zm00001eb347400_P002
RskA Family
Zm00001eb283880_P001
Zm00001eb361260_P001
Zm00001eb127580_P001
Zm00001eb258820_P003
Domain Color
PK_Tyr_Ser−Thr Domain
Pkinase Domain
other Domain
0
500
1000
1500

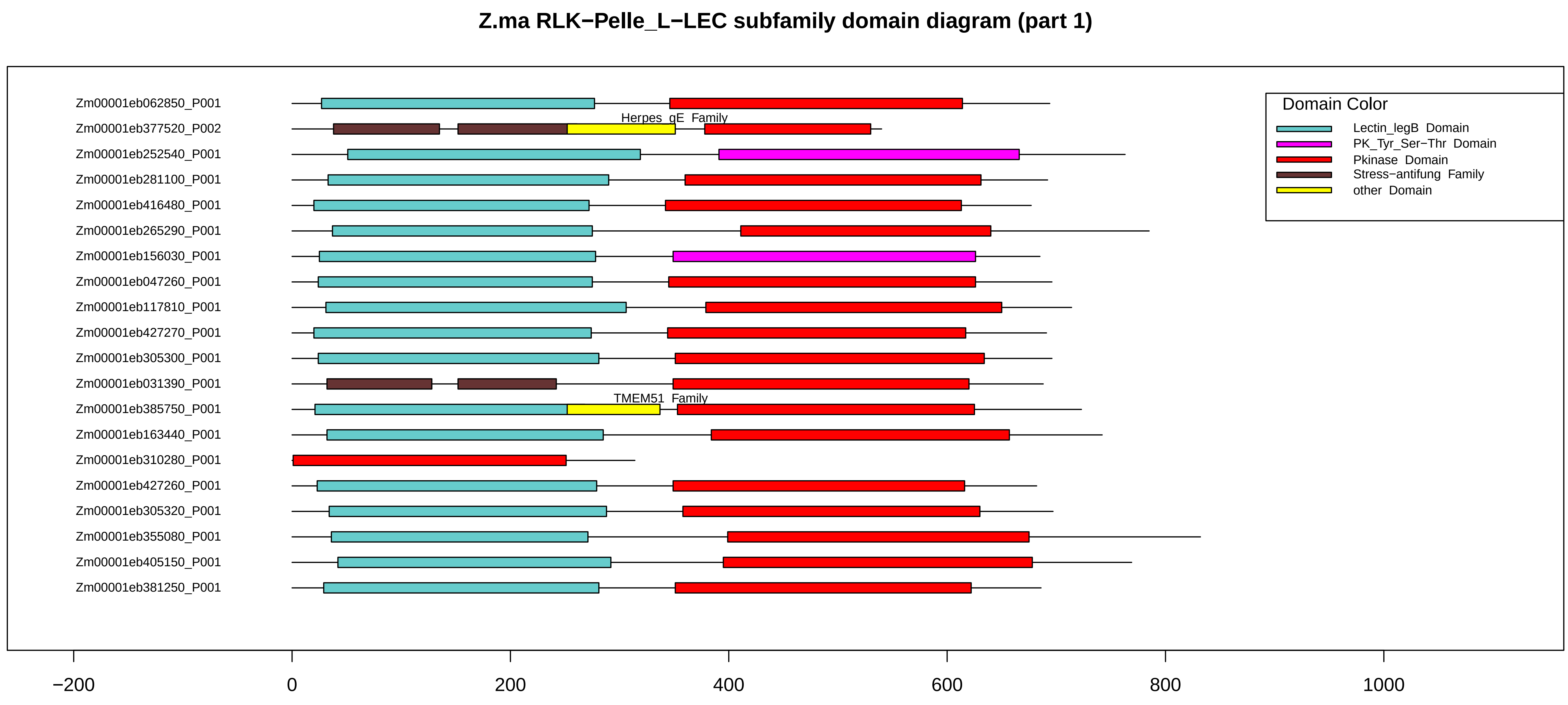
Z.ma RLK−Pelle_L−LEC subfamily domain diagram (part 1)
Zm00001eb062850_P001
Zm00001eb377520_P002
Zm00001eb252540_P001
Zm00001eb281100_P001
Zm00001eb416480_P001
Zm00001eb265290_P001
Zm00001eb156030_P001
Zm00001eb047260_P001
Zm00001eb117810_P001
Zm00001eb427270_P001
Zm00001eb305300_P001
Zm00001eb031390_P001
Zm00001eb385750_P001
Zm00001eb163440_P001
Zm00001eb310280_P001
Zm00001eb427260_P001
Zm00001eb305320_P001
Zm00001eb355080_P001
Zm00001eb405150_P001
Zm00001eb381250_P001
Herpes_gE Family
TMEM51 Family
Domain Color
Lectin_legB Domain
PK_Tyr_Ser−Thr Domain
Pkinase Domain
Stress−antifung Family
other Domain
−200
0
200
400
600
800
1000

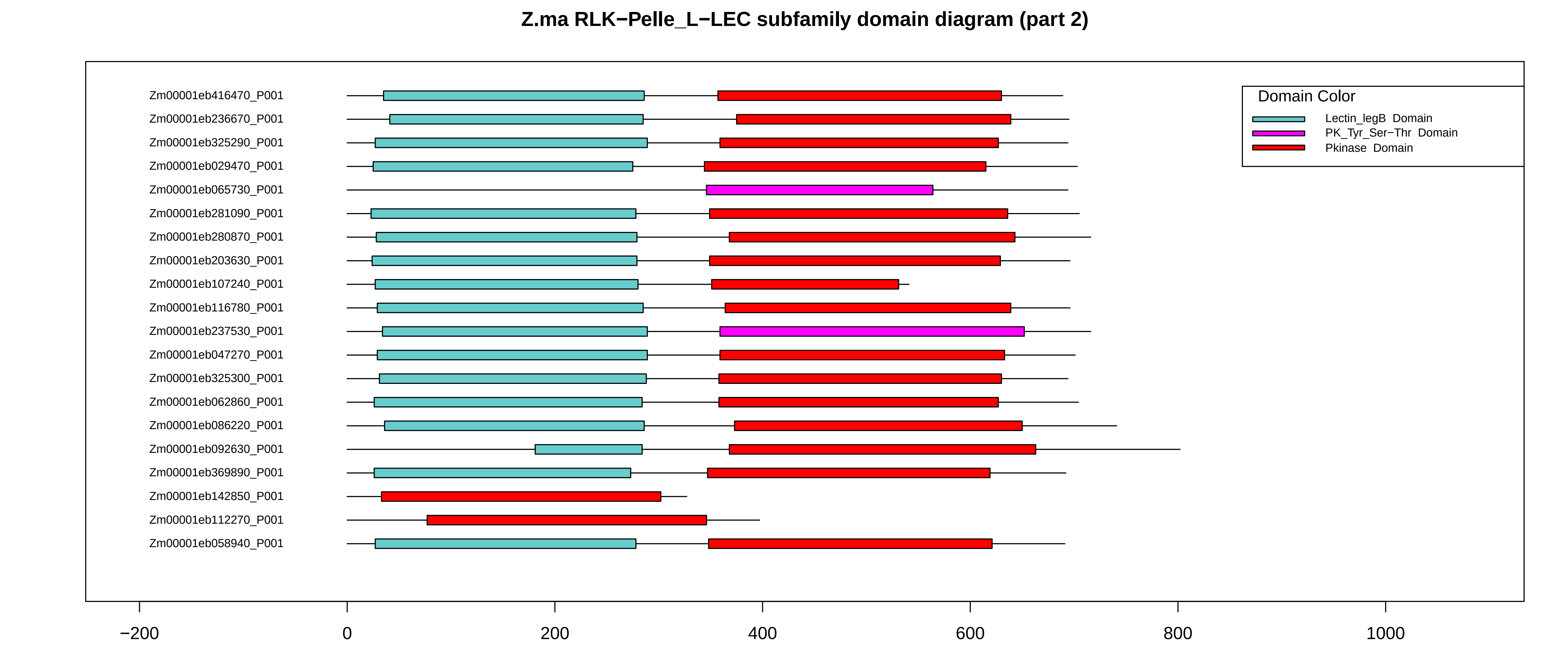
Z.ma RLK−Pelle_L−LEC subfamily domain diagram (part 2)
Zm00001eb416470_P001
Zm00001eb236670_P001
Zm00001eb325290_P001
Zm00001eb029470_P001
Zm00001eb065730_P001
Zm00001eb281090_P001
Zm00001eb280870_P001
Zm00001eb203630_P001
Zm00001eb107240_P001
Zm00001eb116780_P001
Zm00001eb237530_P001
Zm00001eb047270_P001
Zm00001eb325300_P001
Zm00001eb062860_P001
Zm00001eb086220_P001
Zm00001eb092630_P001
Zm00001eb369890_P001
Zm00001eb142850_P001
Zm00001eb112270_P001
Zm00001eb058940_P001
Domain Color
Lectin_legB Domain
PK_Tyr_Ser−Thr Domain
Pkinase Domain
−200
0
200
400
600
800
1000

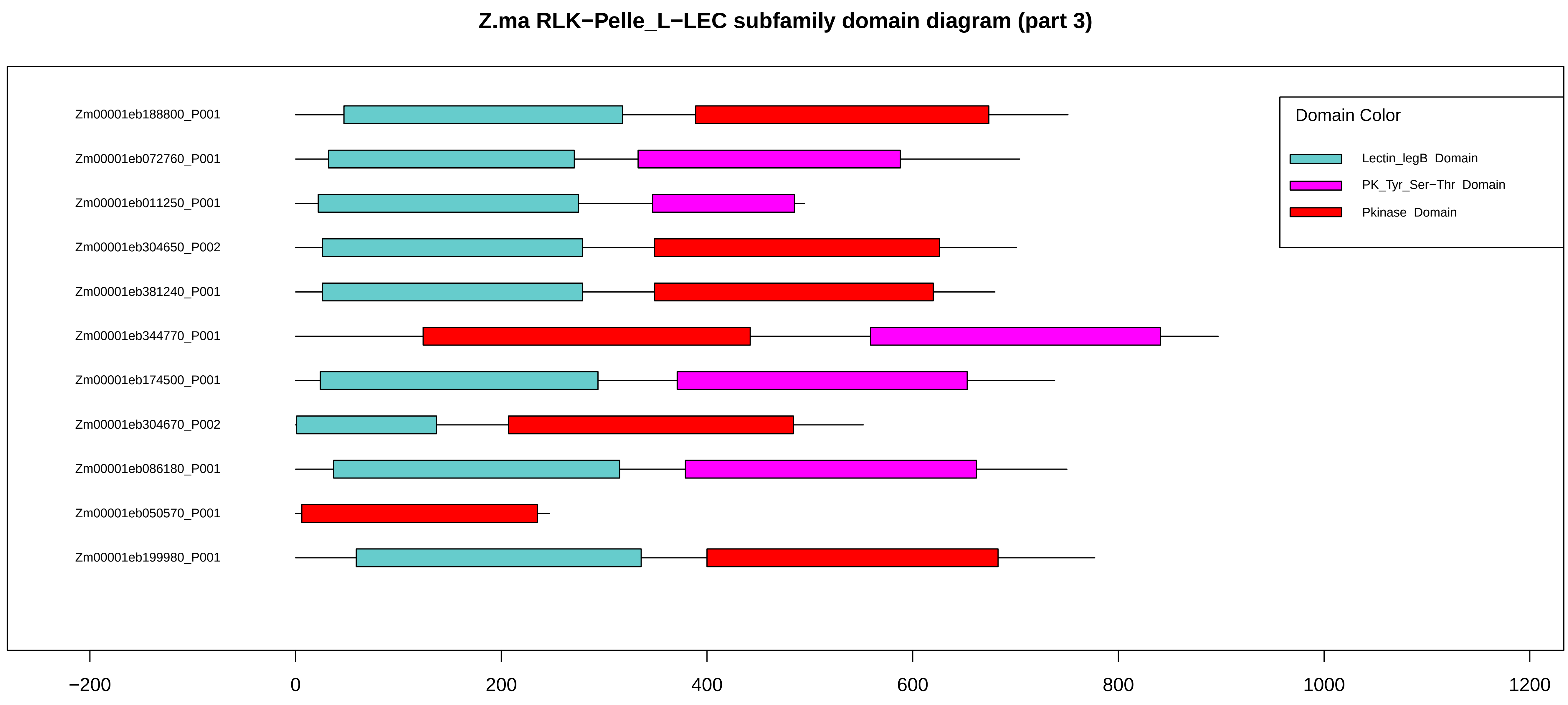

Z.ma RLK−Pelle_L−LEC subfamily domain diagram (part 3)
Zm00001eb188800_P001
Zm00001eb072760_P001
Zm00001eb011250_P001
Zm00001eb304650_P002
Zm00001eb381240_P001
Zm00001eb344770_P001
Zm00001eb174500_P001
Zm00001eb304670_P002
Zm00001eb086180_P001
Zm00001eb050570_P001
Zm00001eb199980_P001
Domain Color
Lectin_legB Domain
PK_Tyr_Ser−Thr Domain
Pkinase Domain
−200
0
200
400
600
800
1000
1200

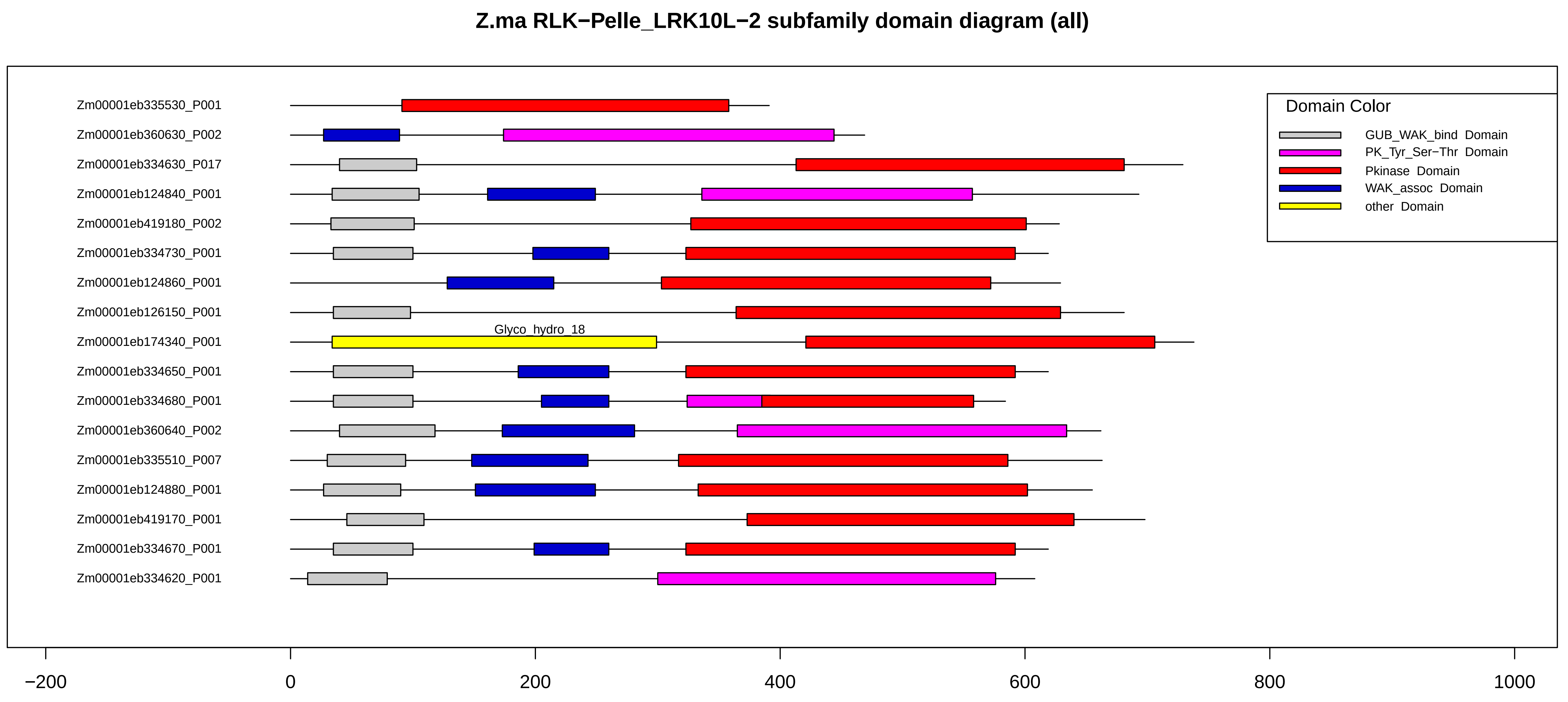

Z.ma RLK−Pelle_LRK10L−2 subfamily domain diagram (all)
Zm00001eb335530_P001
Zm00001eb360630_P002
Zm00001eb334630_P017
Zm00001eb124840_P001
Zm00001eb419180_P002
Zm00001eb334730_P001
Zm00001eb124860_P001
Zm00001eb126150_P001
Zm00001eb174340_P001
Zm00001eb334650_P001
Zm00001eb334680_P001
Zm00001eb360640_P002
Zm00001eb335510_P007
Zm00001eb124880_P001
Zm00001eb419170_P001
Zm00001eb334670_P001
Zm00001eb334620_P001
Glyco_hydro_18
Domain Color
GUB_WAK_bind Domain
PK_Tyr_Ser−Thr Domain
Pkinase Domain
WAK_assoc Domain
other Domain
−200
0
200
400
600
800
1000

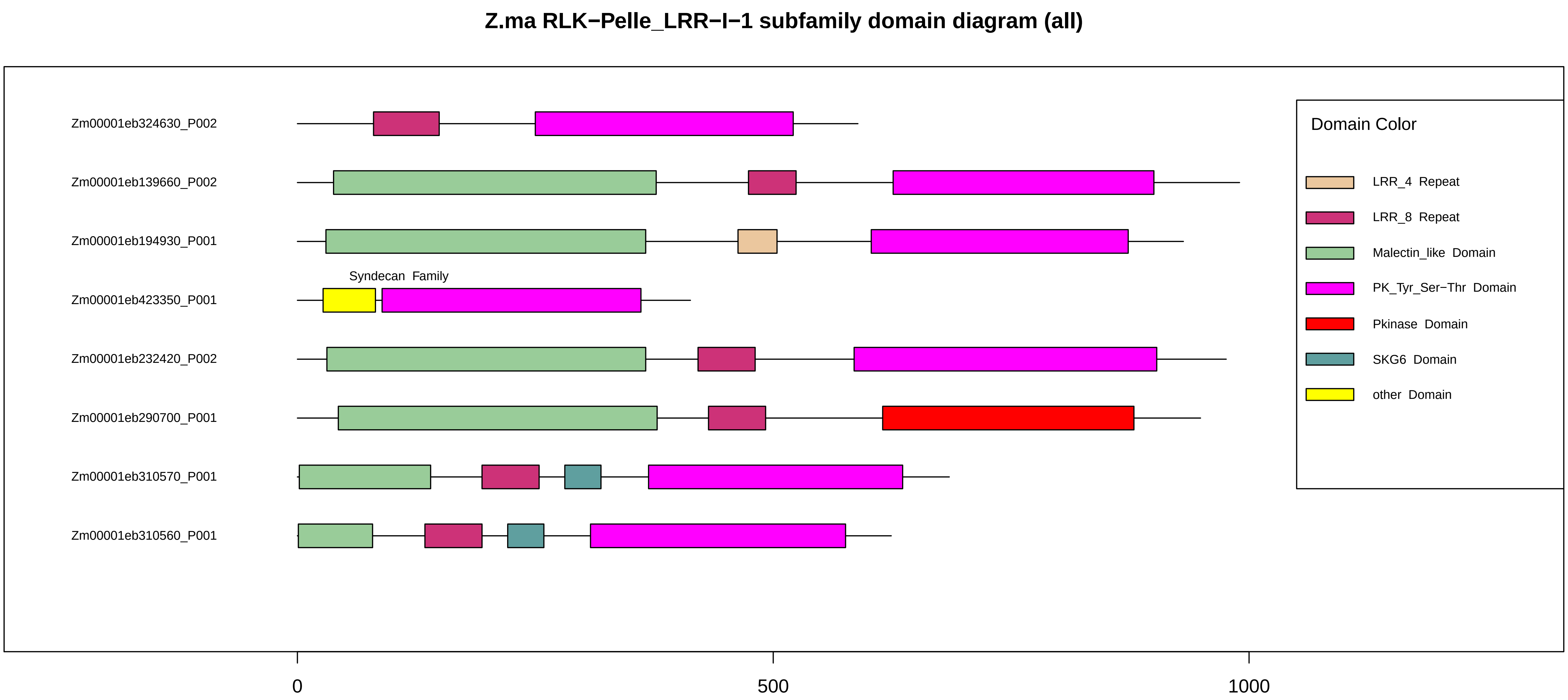
Z.ma RLK−Pelle_LRR−I−1 subfamily domain diagram (all)
Zm00001eb324630_P002
Zm00001eb139660_P002
Zm00001eb194930_P001
Syndecan Family
Zm00001eb423350_P001
Zm00001eb232420_P002
Zm00001eb290700_P001
Zm00001eb310570_P001
Zm00001eb310560_P001
Domain Color
LRR_4 Repeat
LRR_8 Repeat
Malectin_like Domain
PK_Tyr_Ser−Thr Domain
Pkinase Domain
SKG6 Domain
other Domain
0
500
1000

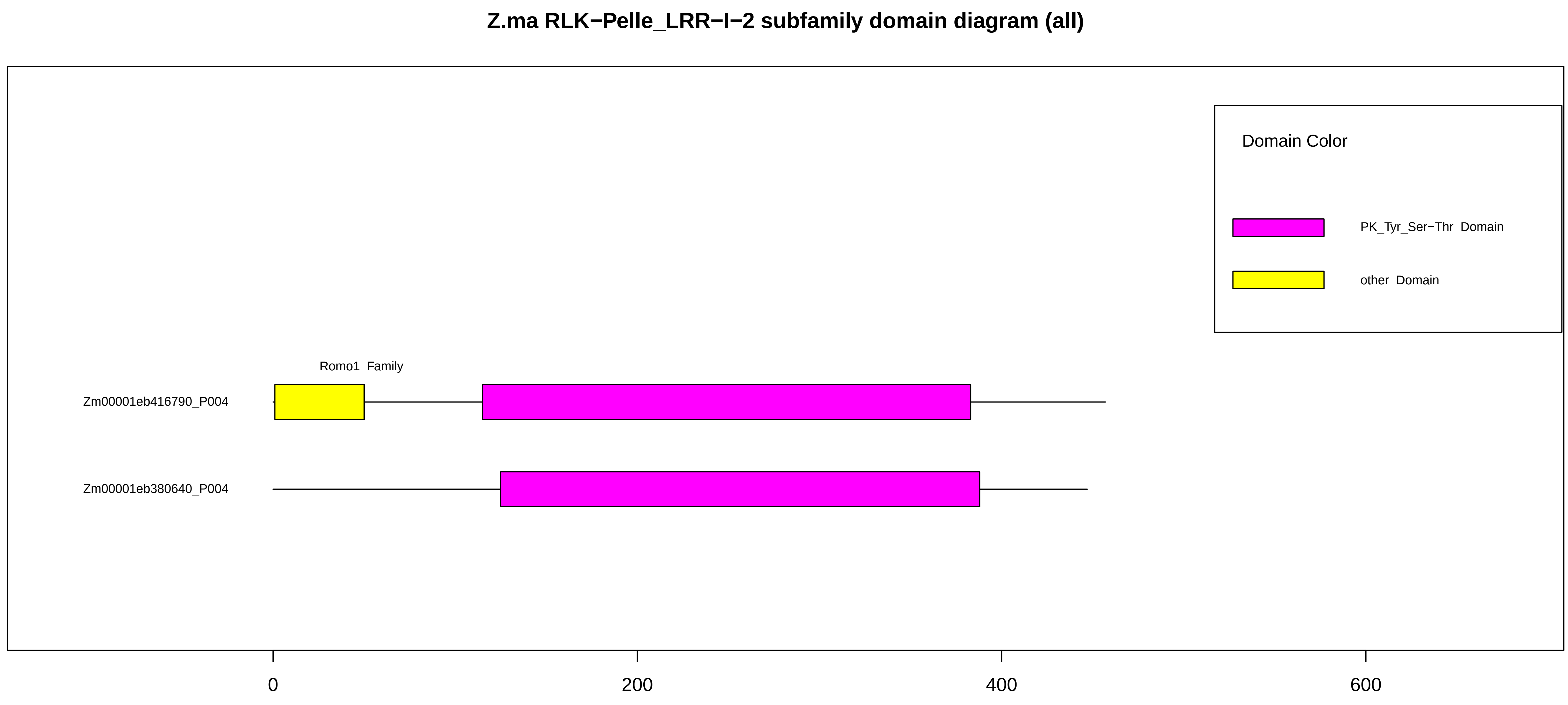
Z.ma RLK−Pelle_LRR−I−2 subfamily domain diagram (all)
Domain Color
PK_Tyr_Ser−Thr Domain
other Domain
Romo1 Family
Zm00001eb416790_P004
Zm00001eb380640_P004
0
200
400
600

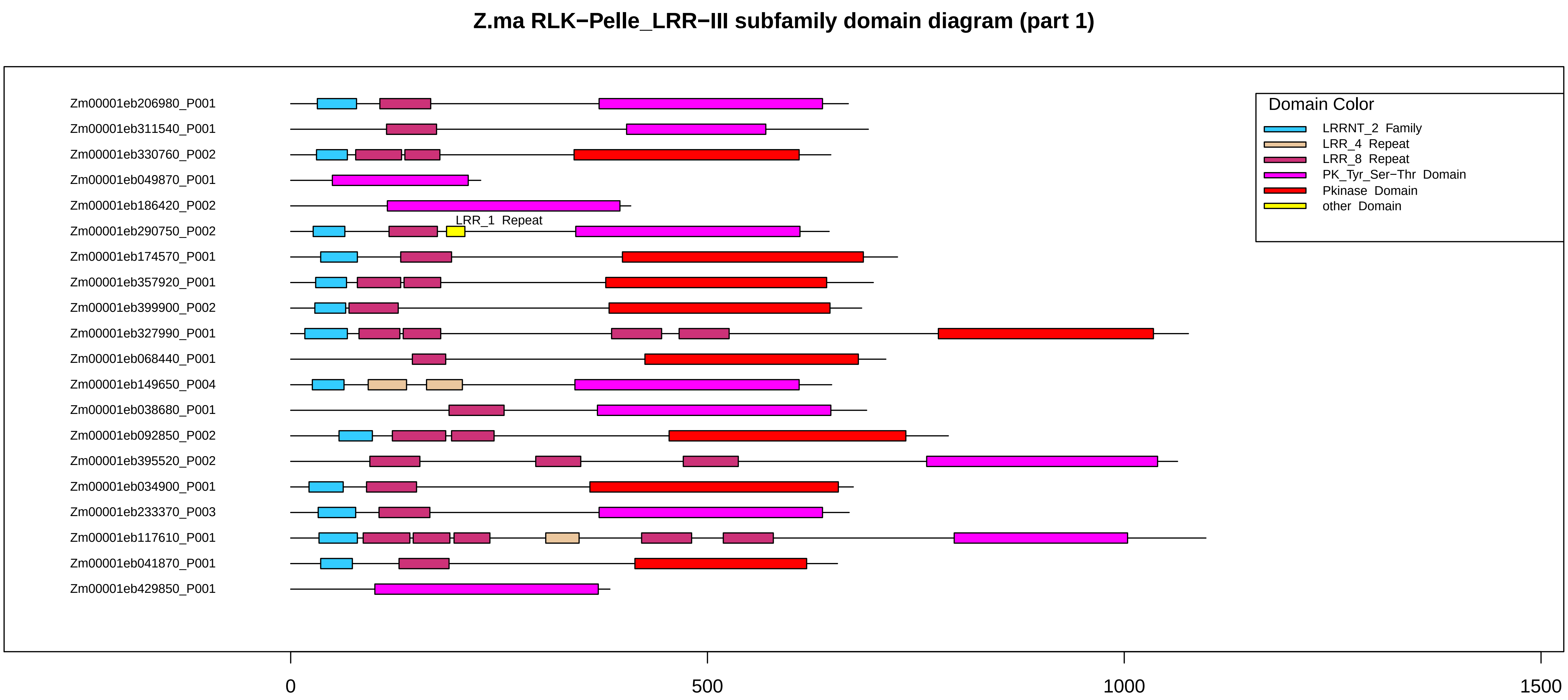
Z.ma RLK−Pelle_LRR−III subfamily domain diagram (part 1)
Zm00001eb206980_P001
Zm00001eb311540_P001
Zm00001eb330760_P002
Zm00001eb049870_P001
Zm00001eb186420_P002
Zm00001eb290750_P002
Zm00001eb174570_P001
Zm00001eb357920_P001
Zm00001eb399900_P002
Zm00001eb327990_P001
Zm00001eb068440_P001
Zm00001eb149650_P004
Zm00001eb038680_P001
Zm00001eb092850_P002
Zm00001eb395520_P002
Zm00001eb034900_P001
Zm00001eb233370_P003
Zm00001eb117610_P001
Zm00001eb041870_P001
Zm00001eb429850_P001
LRR_1 Repeat
Domain Color
LRRNT_2 Family
LRR_4 Repeat
LRR_8 Repeat
PK_Tyr_Ser−Thr Domain
Pkinase Domain
other Domain
0
500
1000
1500

Z.ma RLK−Pelle_LRR−III subfamily domain diagram (part 2)

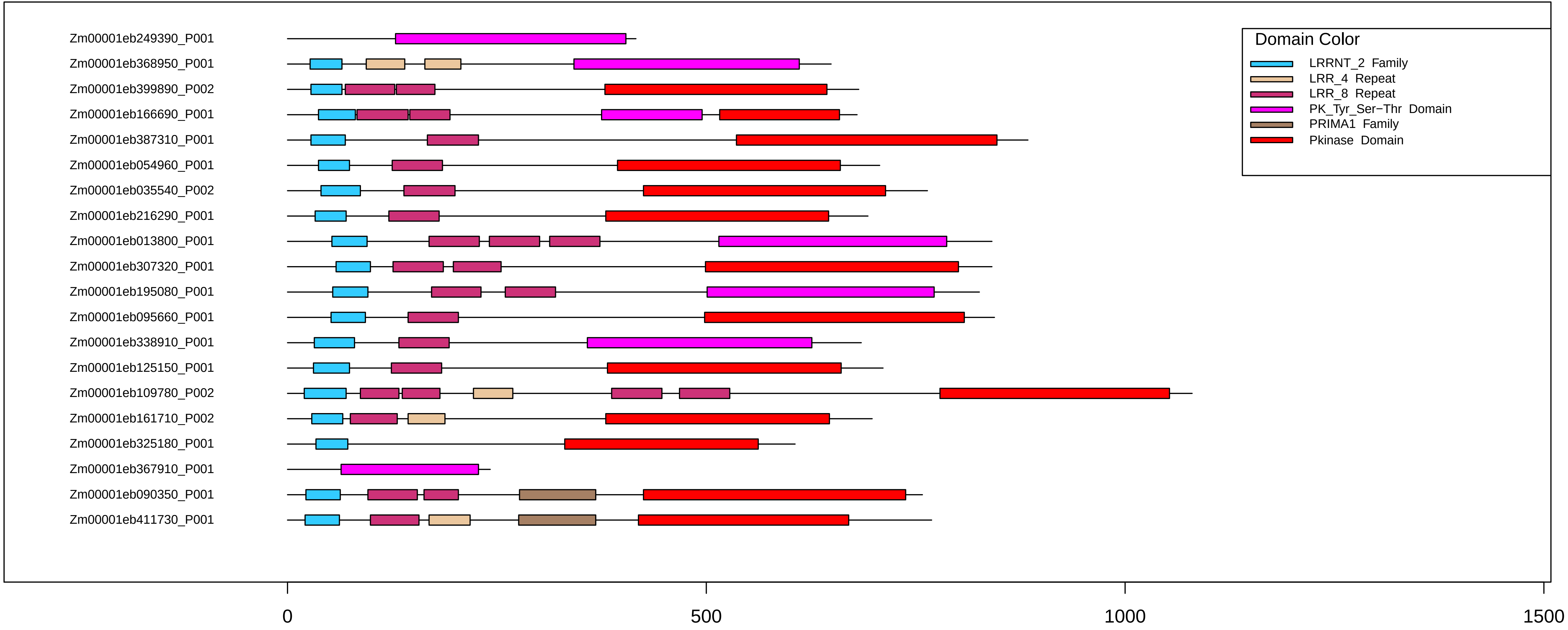

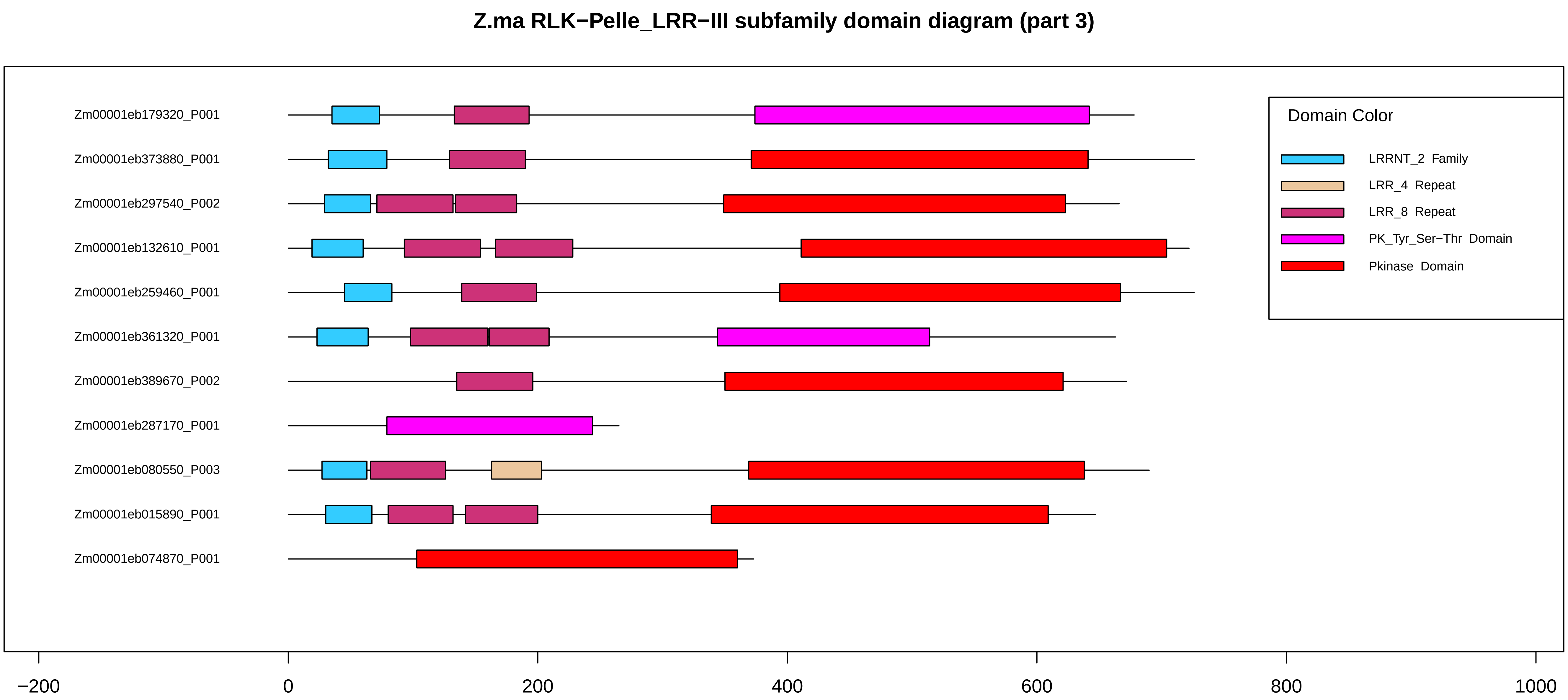
Z.ma RLK−Pelle_LRR−III subfamily domain diagram (part 3)
Zm00001eb179320_P001
Zm00001eb373880_P001
Zm00001eb297540_P002
Zm00001eb132610_P001
Zm00001eb259460_P001
Zm00001eb361320_P001
Zm00001eb389670_P002
Zm00001eb287170_P001
Zm00001eb080550_P003
Zm00001eb015890_P001
Zm00001eb074870_P001
Domain Color
LRRNT_2 Family
LRR_4 Repeat
LRR_8 Repeat
PK_Tyr_Ser−Thr Domain
Pkinase Domain
−200
0
200
400
600
800
1000

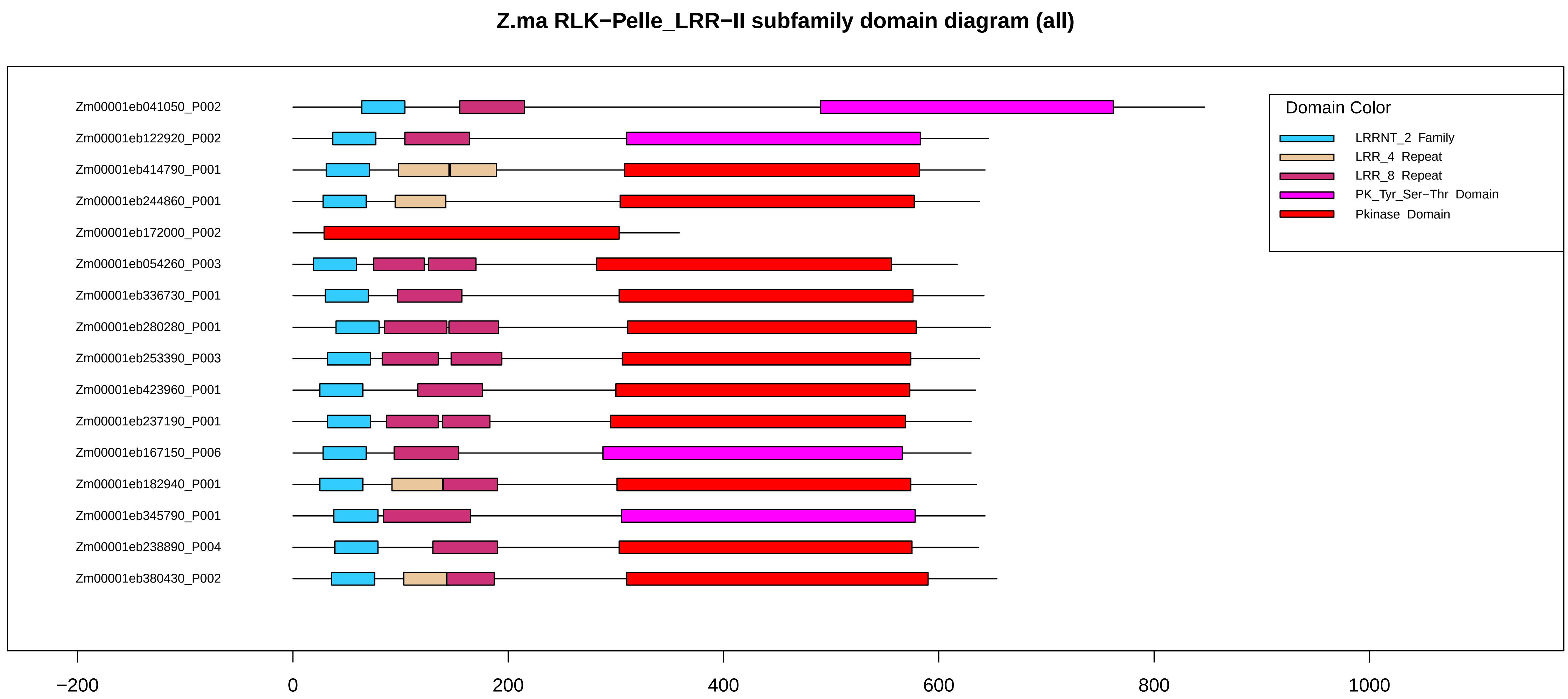
Z.ma RLK−Pelle_LRR−II subfamily domain diagram (all)
Zm00001eb041050_P002
Zm00001eb122920_P002
Zm00001eb414790_P001
Zm00001eb244860_P001
Zm00001eb172000_P002
Zm00001eb054260_P003
Zm00001eb336730_P001
Zm00001eb280280_P001
Zm00001eb253390_P003
Zm00001eb423960_P001
Zm00001eb237190_P001
Zm00001eb167150_P006
Zm00001eb182940_P001
Zm00001eb345790_P001
Zm00001eb238890_P004
Zm00001eb380430_P002
Domain Color
LRRNT_2 Family
LRR_4 Repeat
LRR_8 Repeat
PK_Tyr_Ser−Thr Domain
Pkinase Domain
−200
0
200
400
600
800
1000

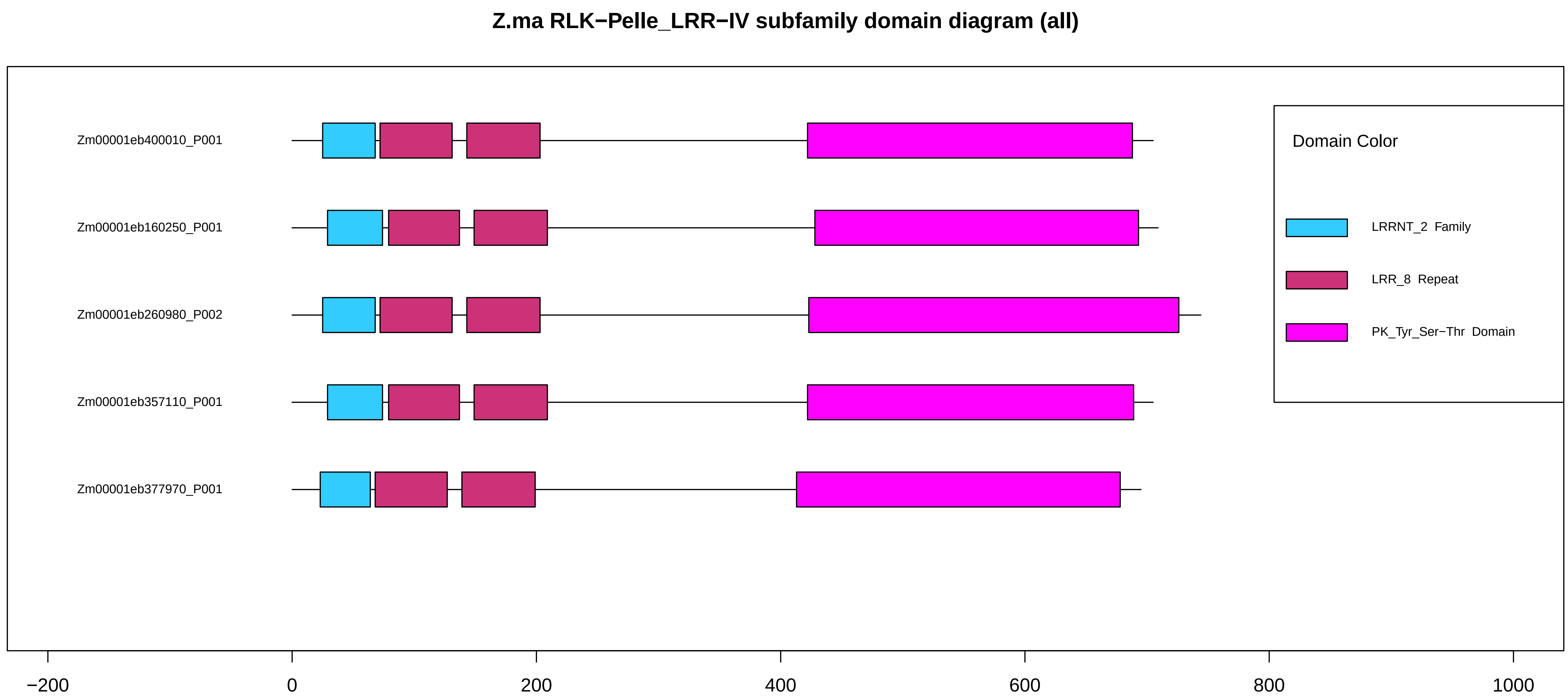
Z.ma RLK−Pelle_LRR−IV subfamily domain diagram (all)
Zm00001eb400010_P001
Zm00001eb160250_P001
Zm00001eb260980_P002
Zm00001eb357110_P001
Zm00001eb377970_P001
Domain Color
LRRNT_2 Family
LRR_8 Repeat
PK_Tyr_Ser−Thr Domain
−200
0
200
400
600
800
1000

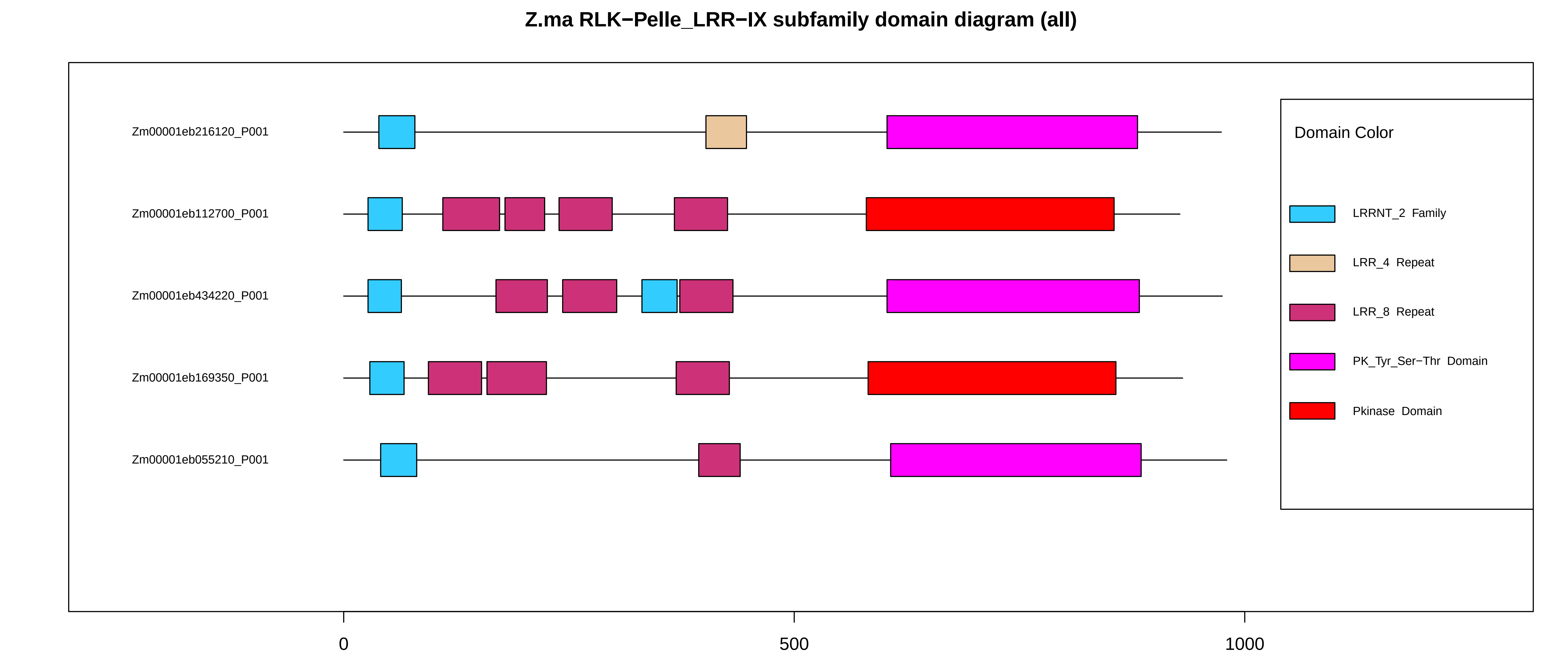
Z.ma RLK−Pelle_LRR−IX subfamily domain diagram (all)
Zm00001eb216120_P001
Zm00001eb112700_P001
Zm00001eb434220_P001
Zm00001eb169350_P001
Zm00001eb055210_P001
Domain Color
LRRNT_2 Family
LRR_4 Repeat
LRR_8 Repeat
PK_Tyr_Ser−Thr Domain
Pkinase Domain
0
500
1000

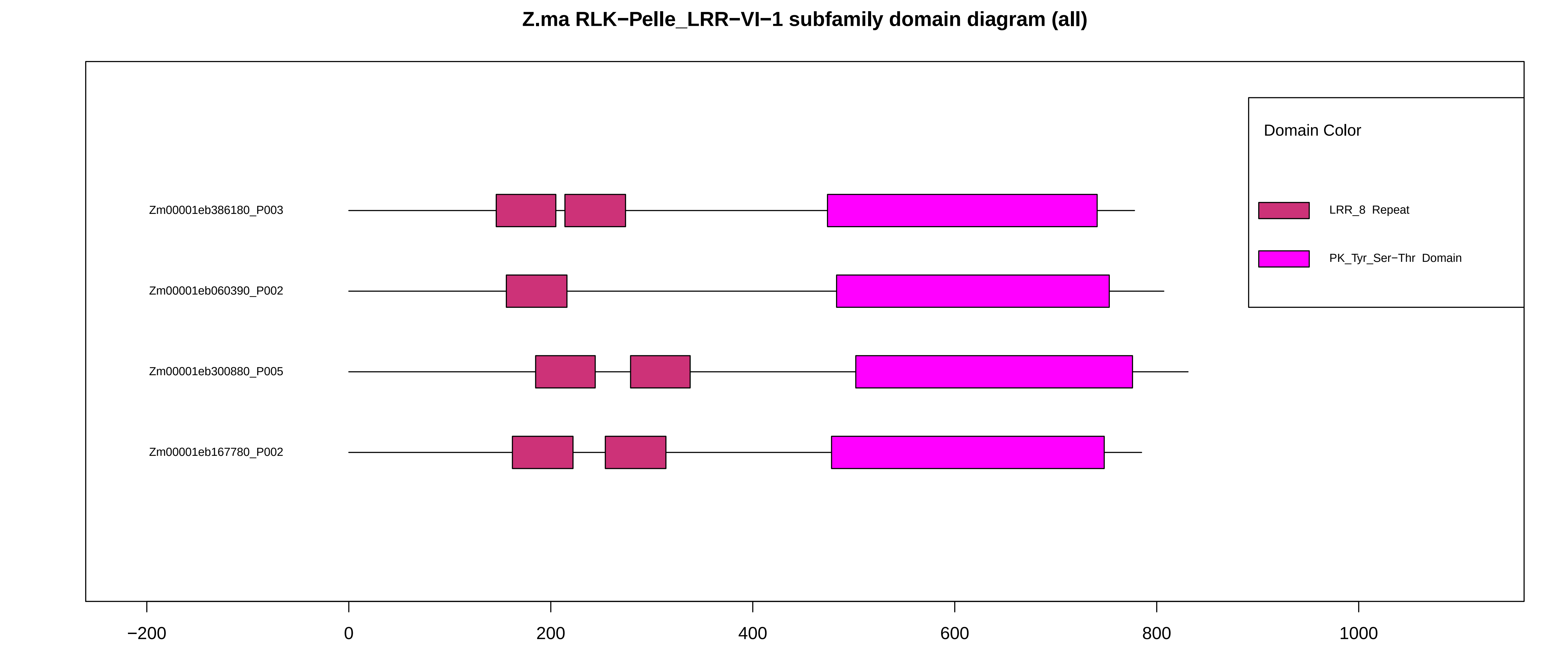
Z.ma RLK−Pelle_LRR−VI−1 subfamily domain diagram (all)
Zm00001eb386180_P003
Zm00001eb060390_P002
Zm00001eb300880_P005
Zm00001eb167780_P002
Domain Color
LRR_8 Repeat
PK_Tyr_Ser−Thr Domain
−200
0
200
400
600
800
1000

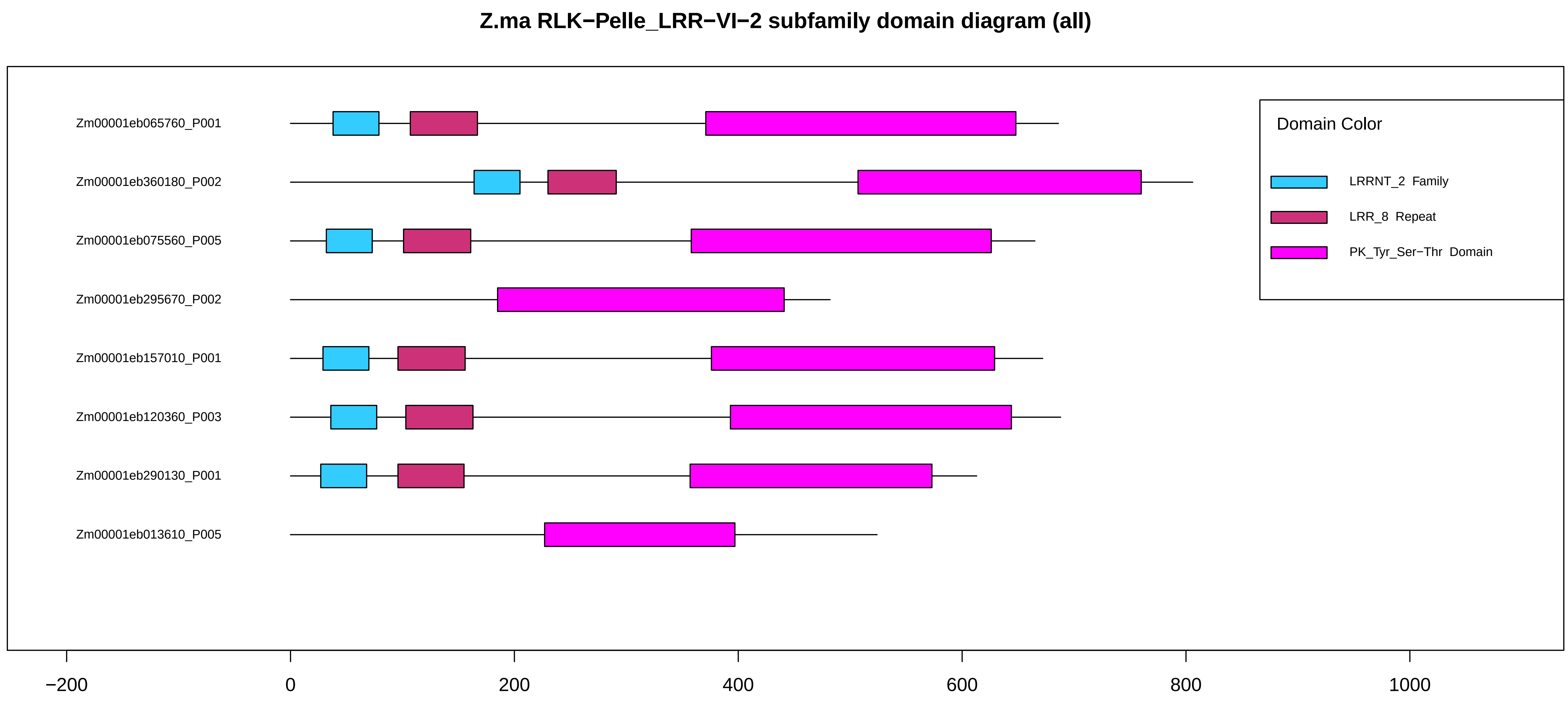

Z.ma RLK−Pelle_LRR−VI−2 subfamily domain diagram (all)
Zm00001eb065760_P001
Zm00001eb360180_P002
Zm00001eb075560_P005
Zm00001eb295670_P002
Zm00001eb157010_P001
Zm00001eb120360_P003
Zm00001eb290130_P001
Zm00001eb013610_P005
Domain Color
LRRNT_2 Family
LRR_8 Repeat
PK_Tyr_Ser−Thr Domain
−200
0
200
400
600
800
1000

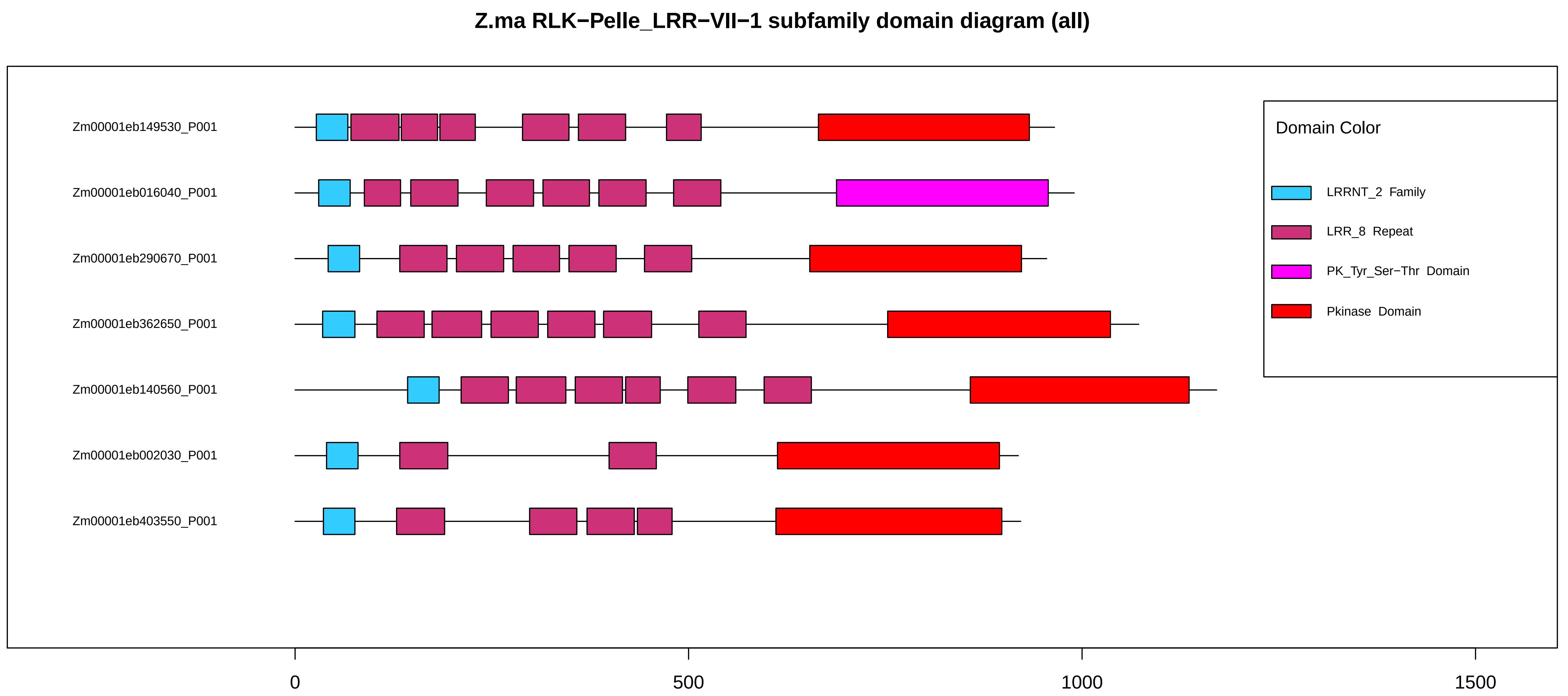

Z.ma RLK−Pelle_LRR−VII−1 subfamily domain diagram (all)
Zm00001eb149530_P001
Zm00001eb016040_P001
Zm00001eb290670_P001
Zm00001eb362650_P001
Zm00001eb140560_P001
Zm00001eb002030_P001
Zm00001eb403550_P001
Domain Color
LRRNT_2 Family
LRR_8 Repeat
PK_Tyr_Ser−Thr Domain
Pkinase Domain
0
500
1000
1500

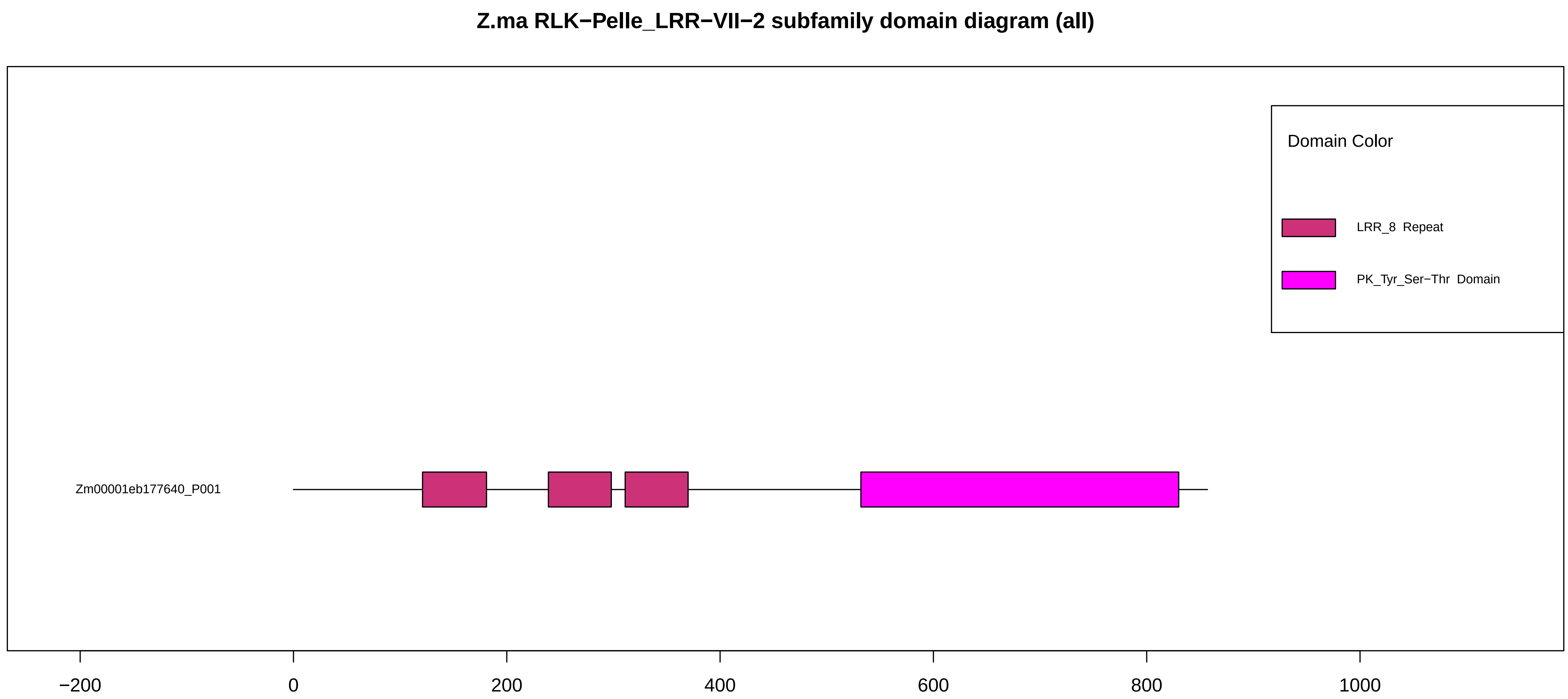

Z.ma RLK−Pelle_LRR−VII−2 subfamily domain diagram (all)
Domain Color
LRR_8 Repeat
PK_Tyr_Ser−Thr Domain
Zm00001eb177640_P001
−200
0
200
400
600
800
1000

Z.ma RLK−Pelle_LRR−VII−3 subfamily domain diagram (all)

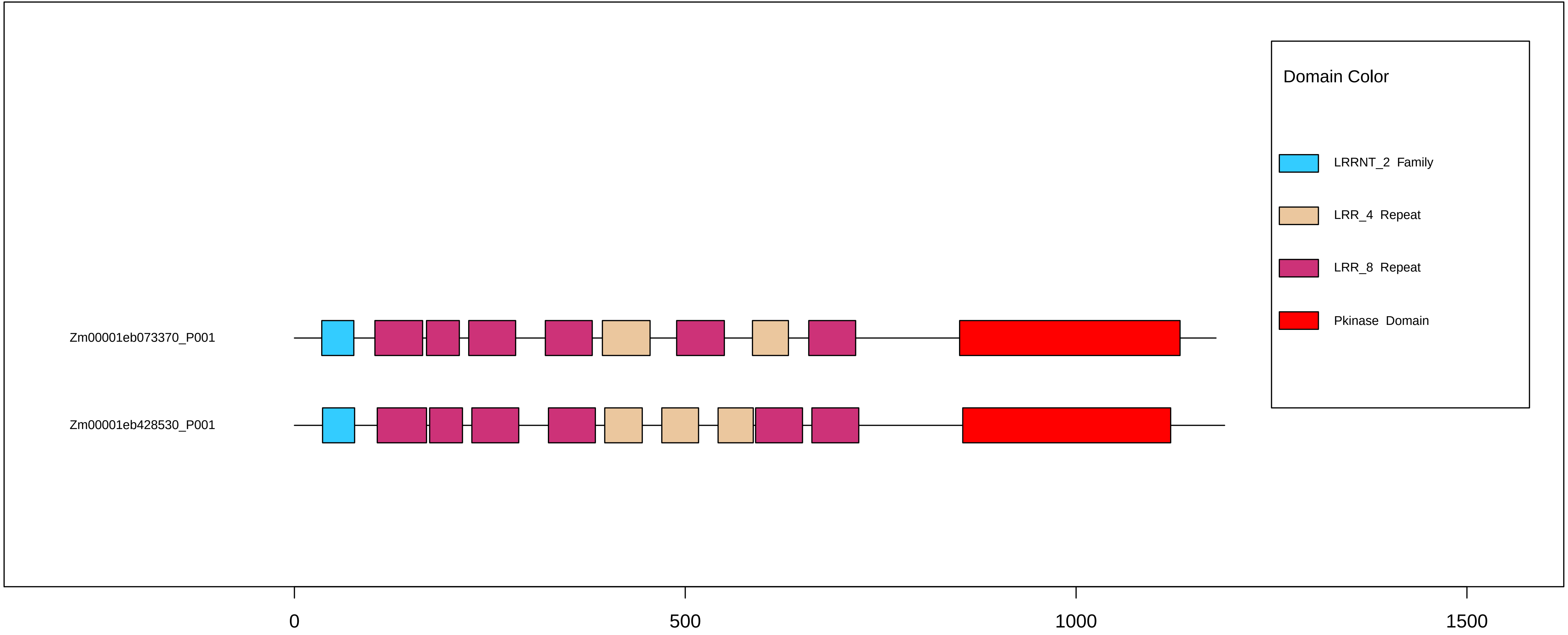

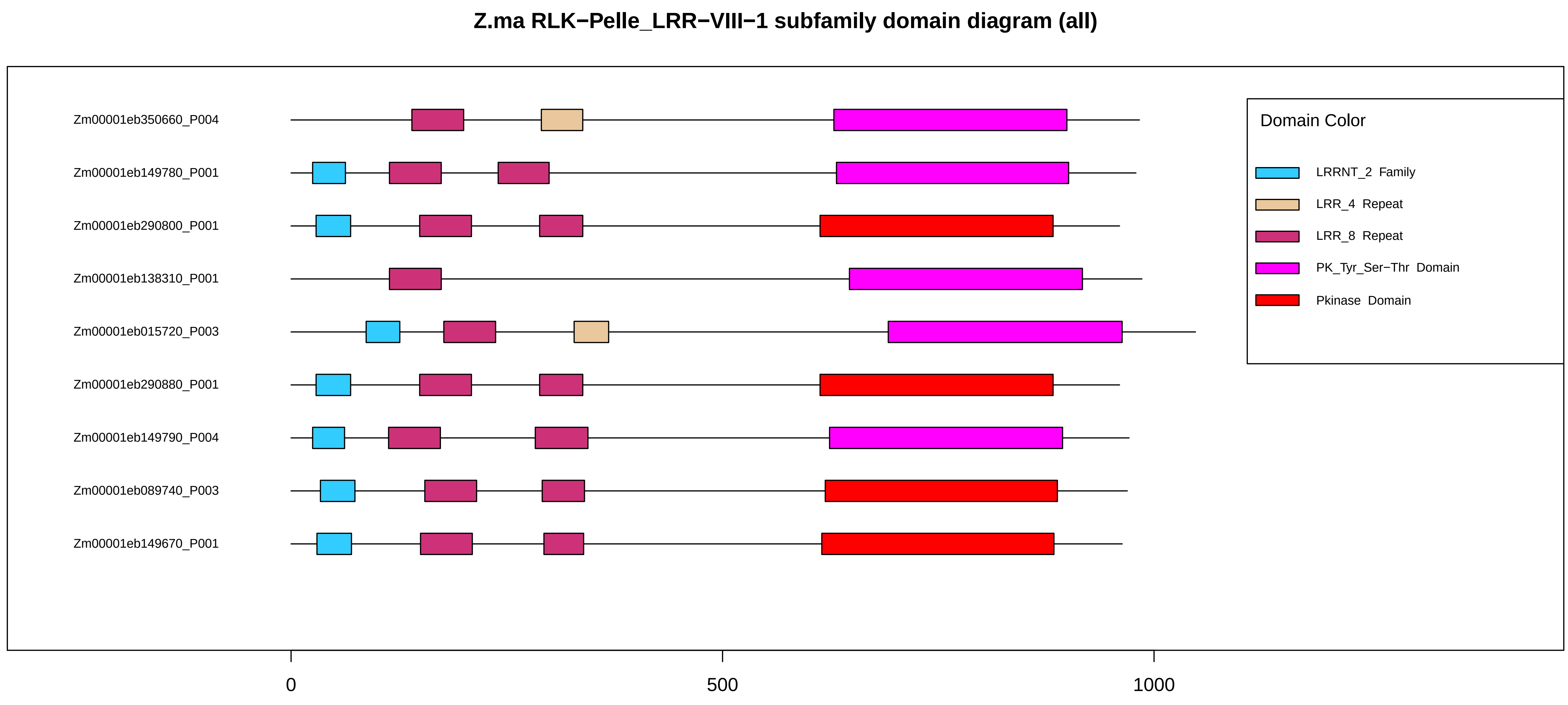

Z.ma RLK−Pelle_LRR−VIII−1 subfamily domain diagram (all)
Zm00001eb350660_P004
Zm00001eb149780_P001
Zm00001eb290800_P001
Zm00001eb138310_P001
Zm00001eb015720_P003
Zm00001eb290880_P001
Zm00001eb149790_P004
Zm00001eb089740_P003
Zm00001eb149670_P001
Domain Color
LRRNT_2 Family
LRR_4 Repeat
LRR_8 Repeat
PK_Tyr_Ser−Thr Domain
Pkinase Domain
0
500
1000

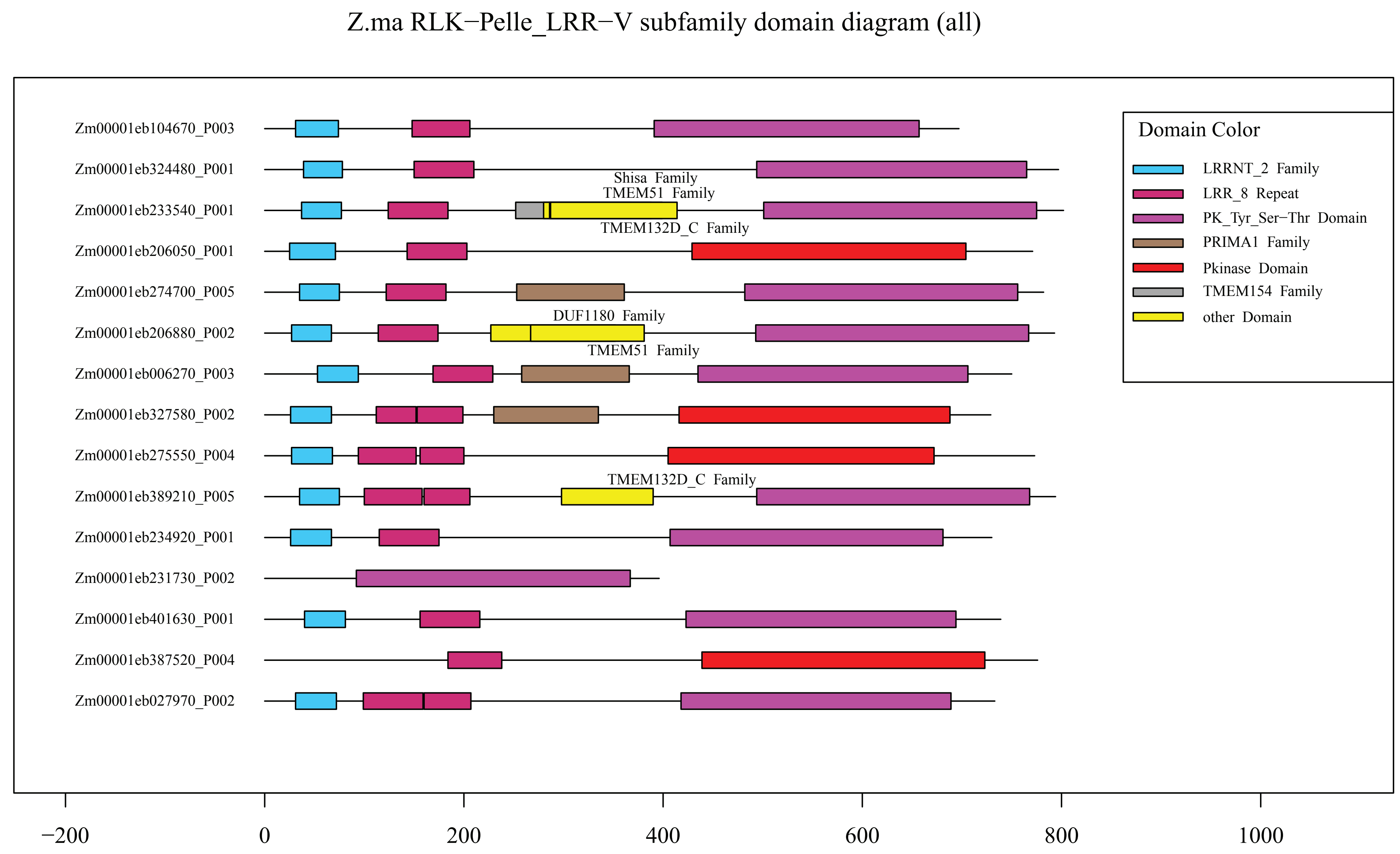

Z.ma RLK−Pelle_LRR−V subfamily domain diagram (all)
Zm00001eb104670_P003
Zm00001eb324480_P001
Zm00001eb233540_P001
Zm00001eb206050_P001
Zm00001eb274700_P005
Zm00001eb206880_P002
Zm00001eb006270_P003
Zm00001eb327580_P002
Zm00001eb275550_P004
Zm00001eb389210_P005
Zm00001eb234920_P001
Zm00001eb231730_P002
Zm00001eb401630_P001
Zm00001eb387520_P004
Zm00001eb027970_P002
Shisa Family
TMEM51 Family
TMEM132D_C Family
DUF1180 Family
TMEM51 Family
TMEM132D_C Family
Domain Color
LRRNT_2 Family
LRR_8 Repeat
PK_Tyr_Ser−Thr Domain
PRIMA1 Family
Pkinase Domain
TMEM154 Family
other Domain
−200
0
200
400
600
800
1000

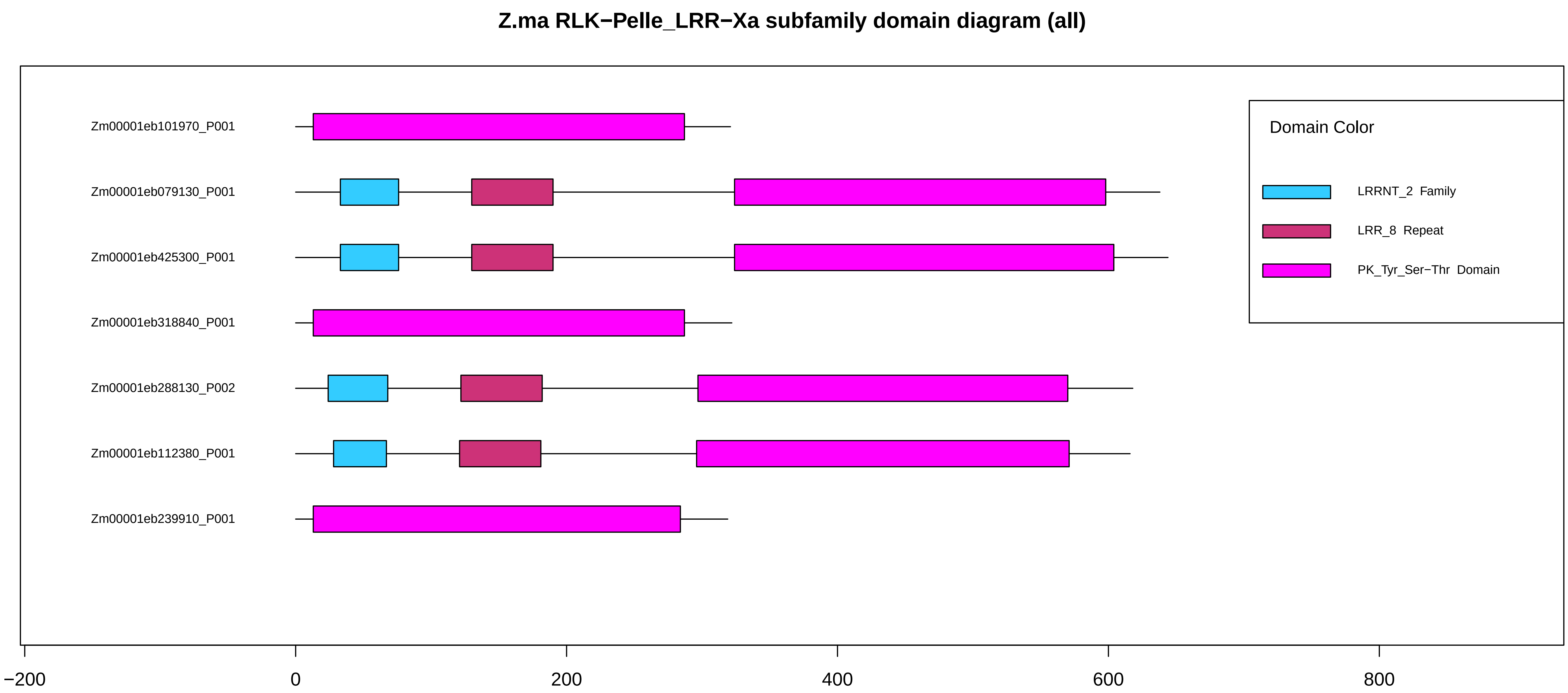
Z.ma RLK−Pelle_LRR−Xa subfamily domain diagram (all)
Zm00001eb101970_P001
Zm00001eb079130_P001
Zm00001eb425300_P001
Zm00001eb318840_P001
Zm00001eb288130_P002
Zm00001eb112380_P001
Zm00001eb239910_P001
Domain Color
LRRNT_2 Family
LRR_8 Repeat
PK_Tyr_Ser−Thr Domain
−200
0
200
400
600
800

Z.ma RLK−Pelle_LRR−Xb−1 subfamily domain diagram (all)

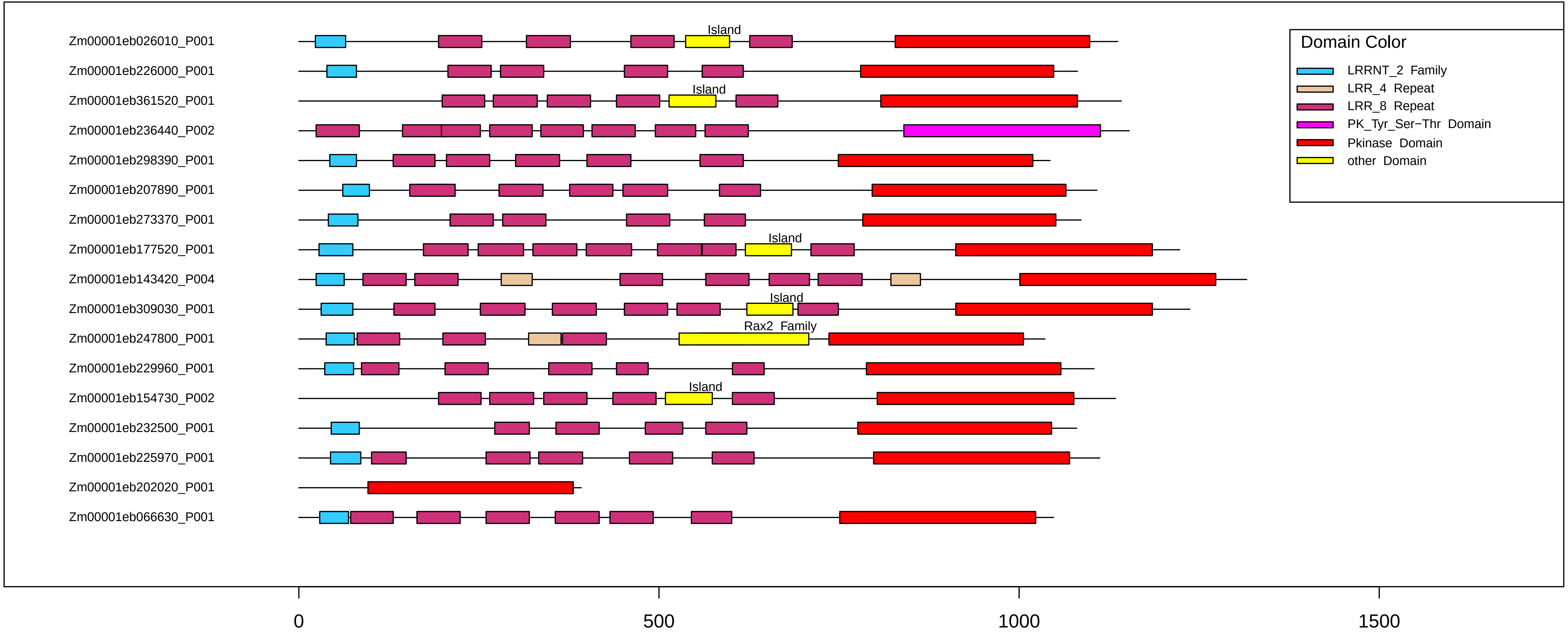

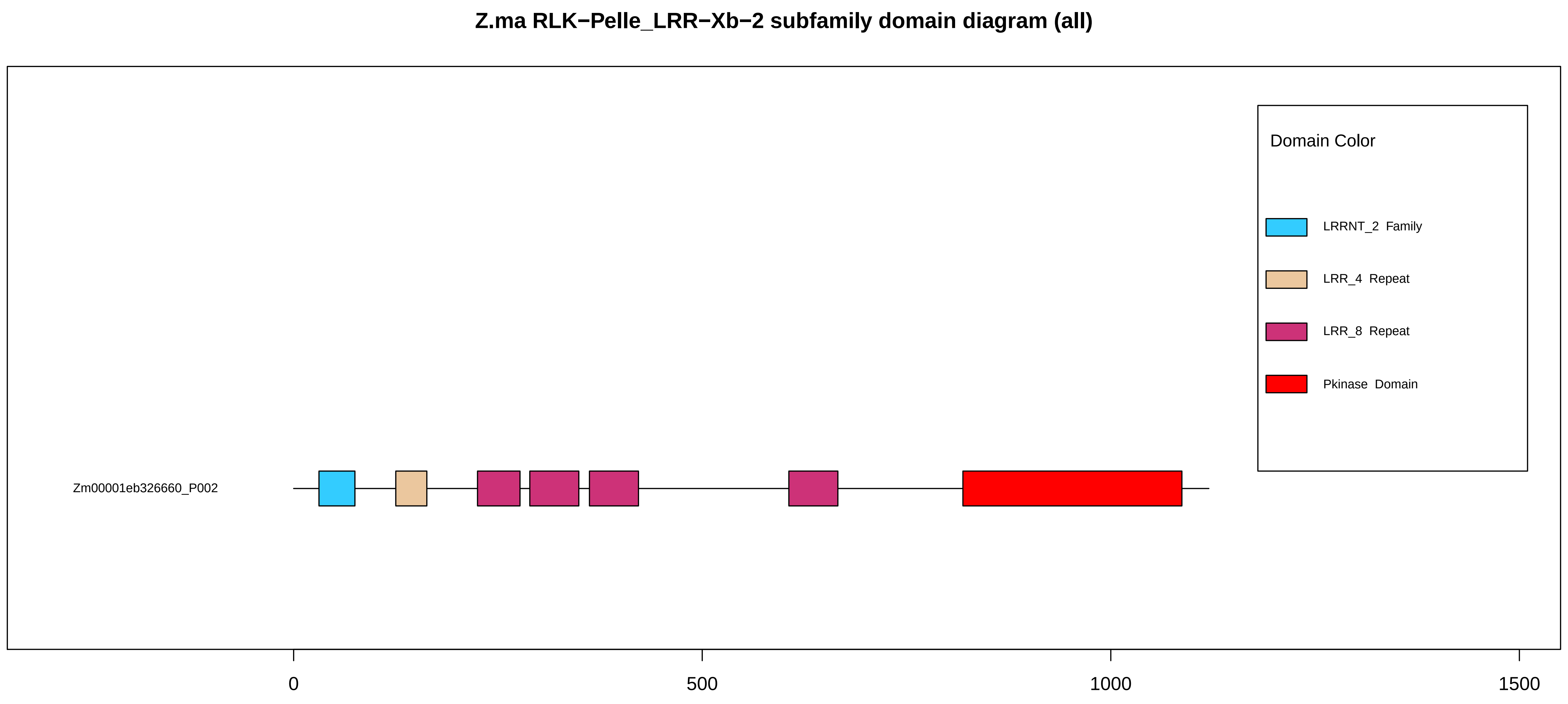
Z.ma RLK−Pelle_LRR−Xb−2 subfamily domain diagram (all)
Domain Color
LRRNT_2 Family
LRR_4 Repeat
LRR_8 Repeat
Pkinase Domain
Zm00001eb326660_P002
0
500
1000
1500

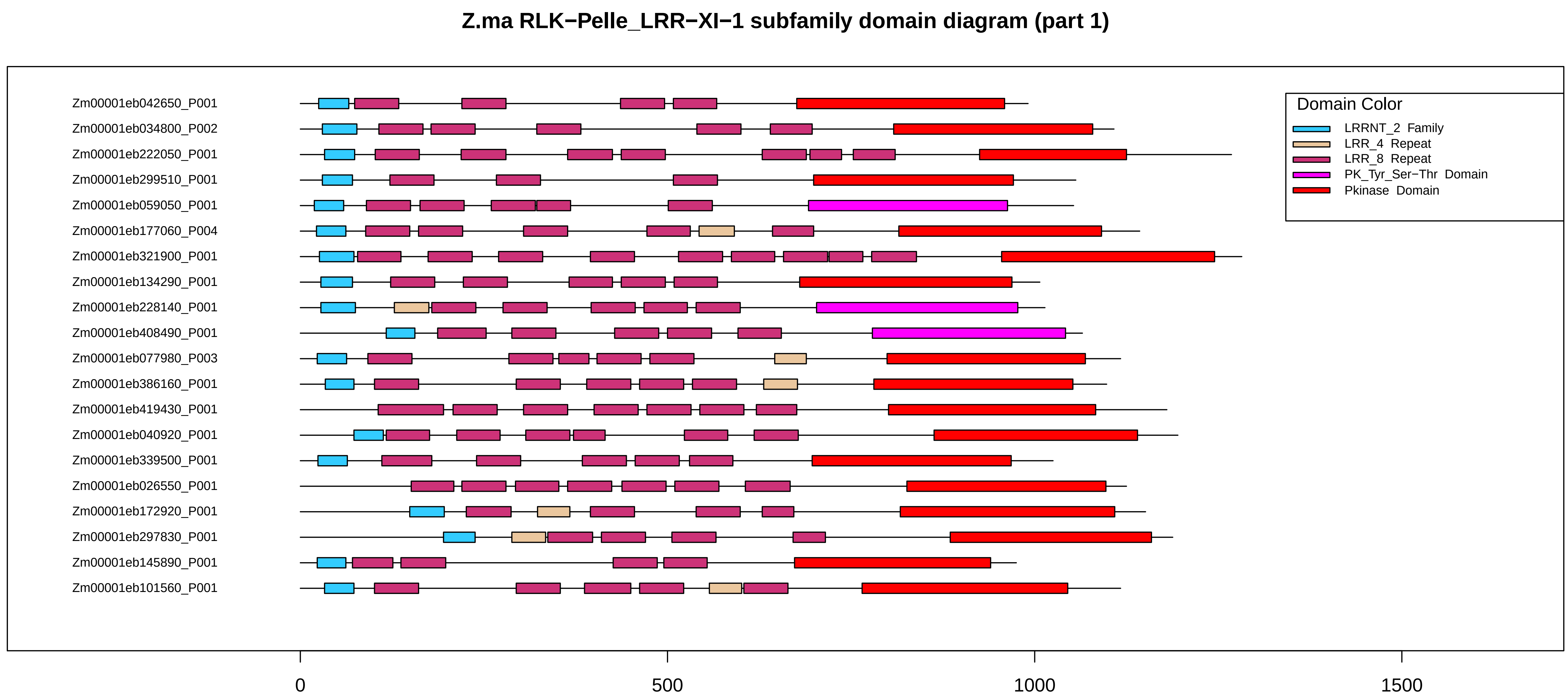
Z.ma RLK−Pelle_LRR−XI−1 subfamily domain diagram (part 1)
Zm00001eb042650_P001
Zm00001eb034800_P002
Zm00001eb222050_P001
Zm00001eb299510_P001
Zm00001eb059050_P001
Zm00001eb177060_P004
Zm00001eb321900_P001
Zm00001eb134290_P001
Zm00001eb228140_P001
Zm00001eb408490_P001
Zm00001eb077980_P003
Zm00001eb386160_P001
Zm00001eb419430_P001
Zm00001eb040920_P001
Zm00001eb339500_P001
Zm00001eb026550_P001
Zm00001eb172920_P001
Zm00001eb297830_P001
Zm00001eb145890_P001
Zm00001eb101560_P001
Domain Color
LRRNT_2 Family
LRR_4 Repeat
LRR_8 Repeat
PK_Tyr_Ser−Thr Domain
Pkinase Domain
0
500
1000
1500

Z.ma RLK−Pelle_LRR−XI−1 subfamily domain diagram (part 2)

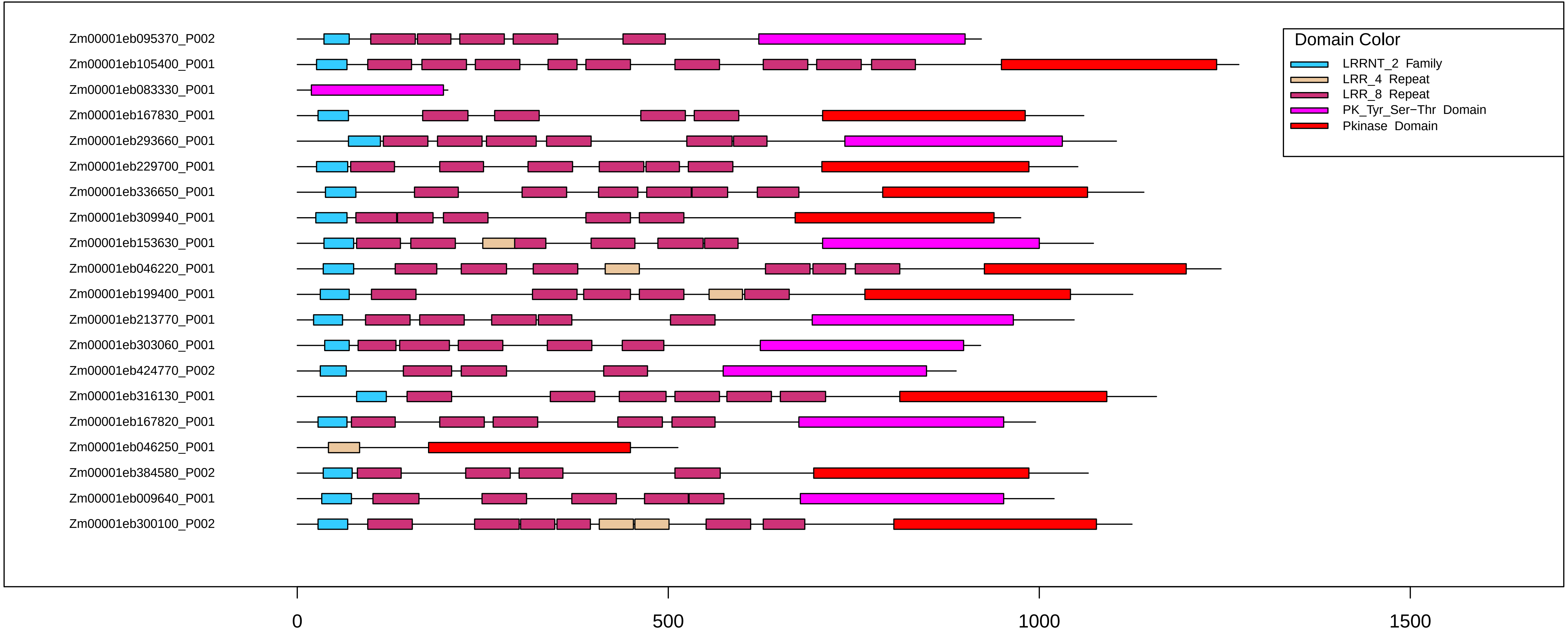

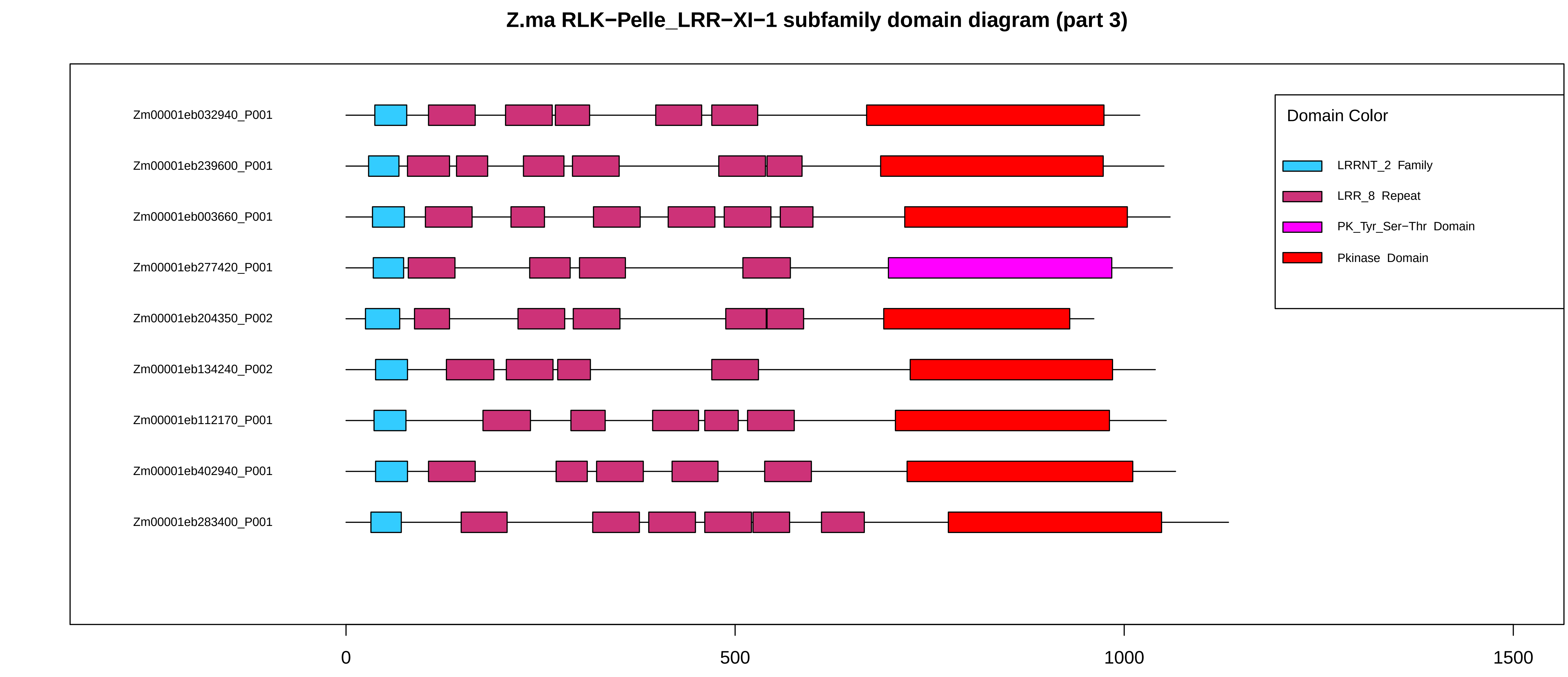

Z.ma RLK−Pelle_LRR−XI−1 subfamily domain diagram (part 3)
Zm00001eb032940_P001
Zm00001eb239600_P001
Zm00001eb003660_P001
Zm00001eb277420_P001
Zm00001eb204350_P002
Zm00001eb134240_P002
Zm00001eb112170_P001
Zm00001eb402940_P001
Zm00001eb283400_P001
Domain Color
LRRNT_2 Family
LRR_8 Repeat
PK_Tyr_Ser−Thr Domain
Pkinase Domain
0
500
1000
1500

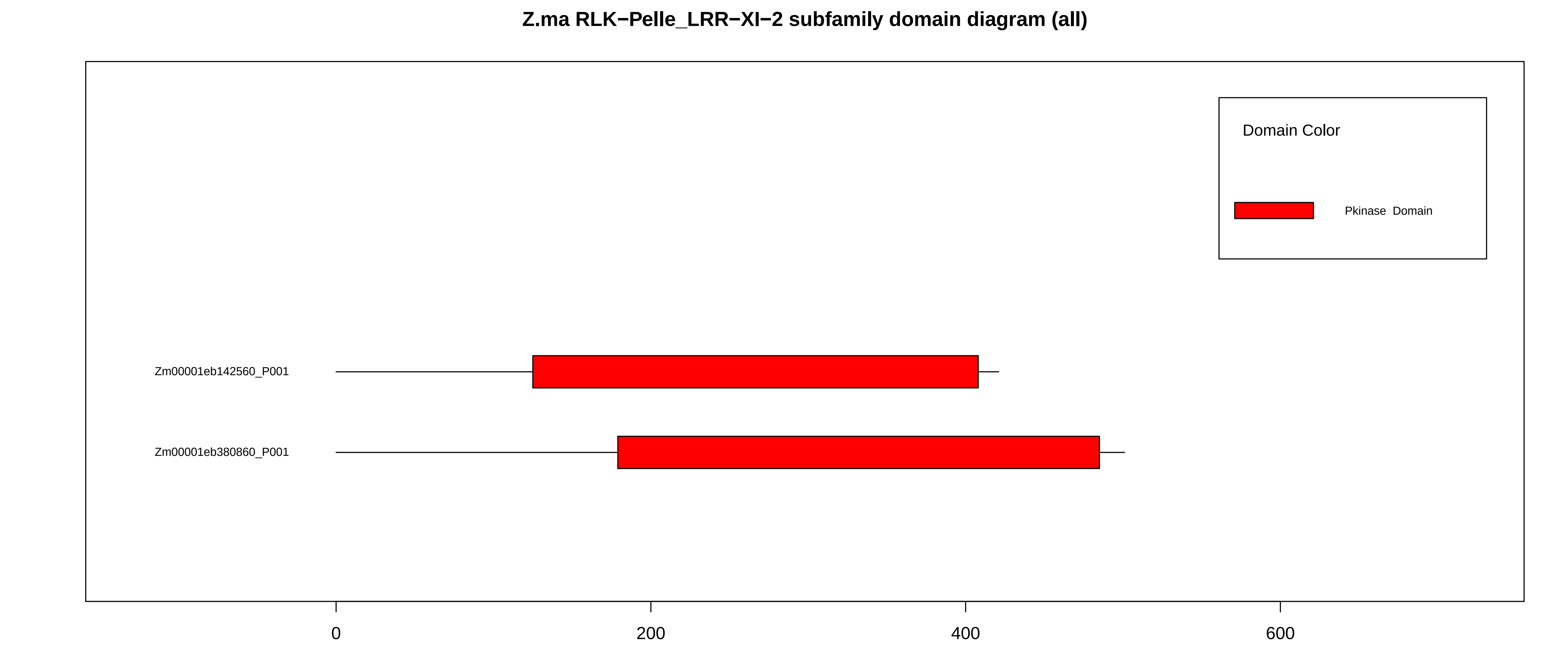
Z.ma RLK−Pelle_LRR−XI−2 subfamily domain diagram (all)
Domain Color
Pkinase Domain
Zm00001eb142560_P001
Zm00001eb380860_P001
0
200
400
600

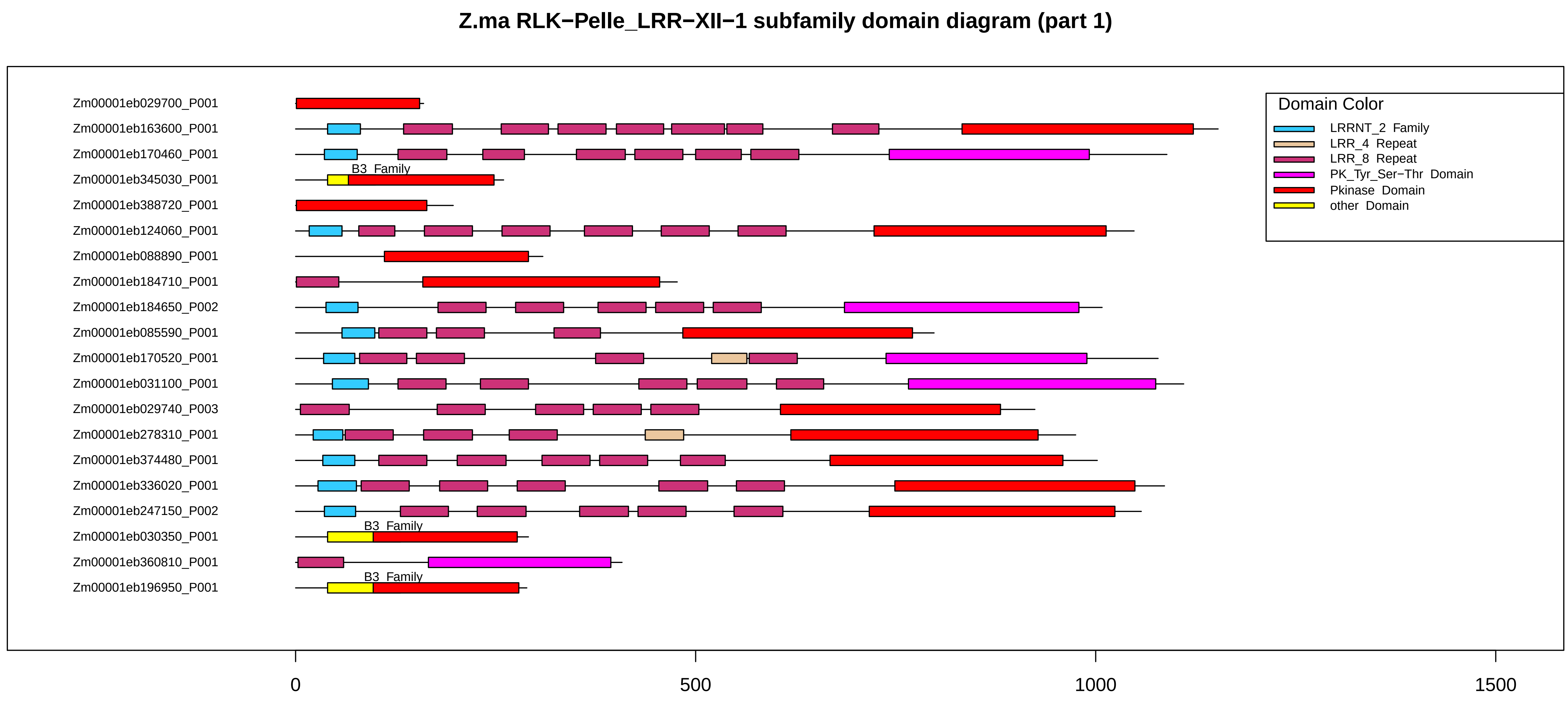

Z.ma RLK−Pelle_LRR−XII−1 subfamily domain diagram (part 1)
Zm00001eb029700_P001
Zm00001eb163600_P001
Zm00001eb170460_P001
Zm00001eb345030_P001
Zm00001eb388720_P001
Zm00001eb124060_P001
Zm00001eb088890_P001
Zm00001eb184710_P001
Zm00001eb184650_P002
Zm00001eb085590_P001
Zm00001eb170520_P001
Zm00001eb031100_P001
Zm00001eb029740_P003
Zm00001eb278310_P001
Zm00001eb374480_P001
Zm00001eb336020_P001
Zm00001eb247150_P002
Zm00001eb030350_P001
Zm00001eb360810_P001
Zm00001eb196950_P001
B3 Family
B3 Family
B3 Family
Domain Color
LRRNT_2 Family
LRR_4 Repeat
LRR_8 Repeat
PK_Tyr_Ser−Thr Domain
Pkinase Domain
other Domain
0
500
1000
1500

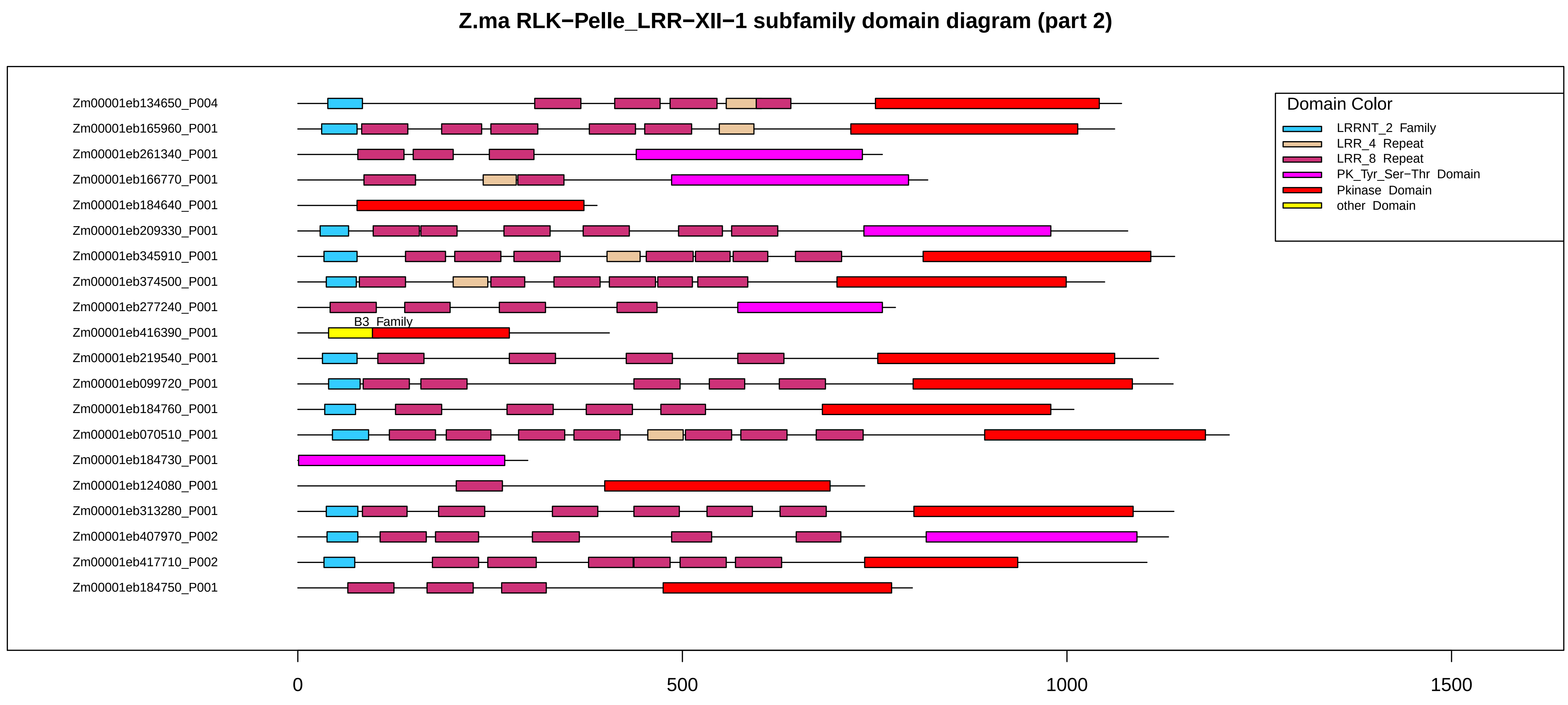
Z.ma RLK−Pelle_LRR−XII−1 subfamily domain diagram (part 2)
Zm00001eb134650_P004
Zm00001eb165960_P001
Zm00001eb261340_P001
Zm00001eb166770_P001
Zm00001eb184640_P001
Zm00001eb209330_P001
Zm00001eb345910_P001
Zm00001eb374500_P001
Zm00001eb277240_P001
Zm00001eb416390_P001
B3_Family
Zm00001eb219540_P001
Zm00001eb099720_P001
Zm00001eb184760_P001
Zm00001eb070510_P001
Zm00001eb184730_P001
Zm00001eb124080_P001
Zm00001eb313280_P001
Zm00001eb407970_P002
Zm00001eb417710_P002
Zm00001eb184750_P001
Domain Color
LRRNT_2 Family
LRR_4 Repeat
LRR_8 Repeat
PK_Tyr_Ser−Thr Domain
Pkinase Domain
other Domain
0
500
1000
1500

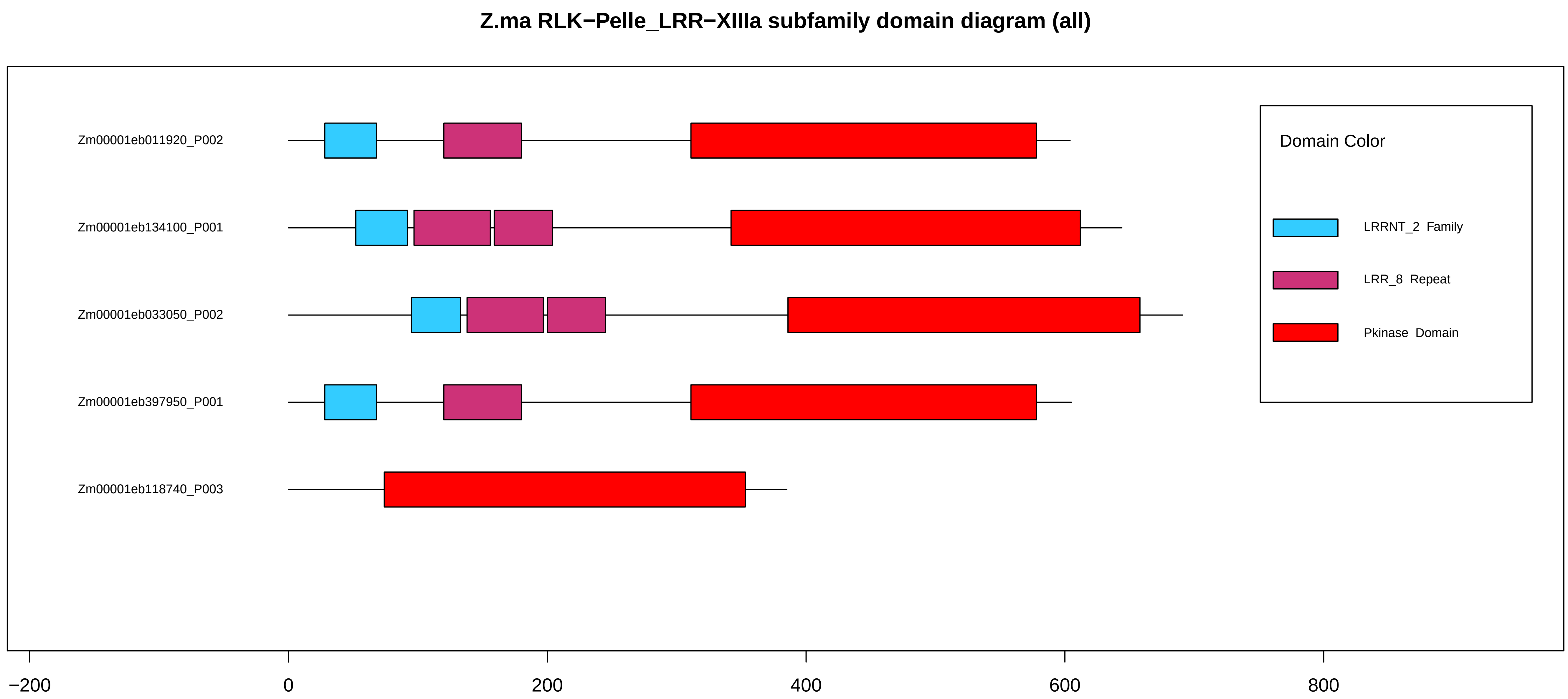
Z.ma RLK−Pelle_LRR−XIIIa subfamily domain diagram (all)
Zm00001eb011920_P002
Zm00001eb134100_P001
Zm00001eb033050_P002
Zm00001eb397950_P001
Zm00001eb118740_P003
Domain Color
LRRNT_2 Family
LRR_8 Repeat
Pkinase Domain
−200
0
200
400
600
800

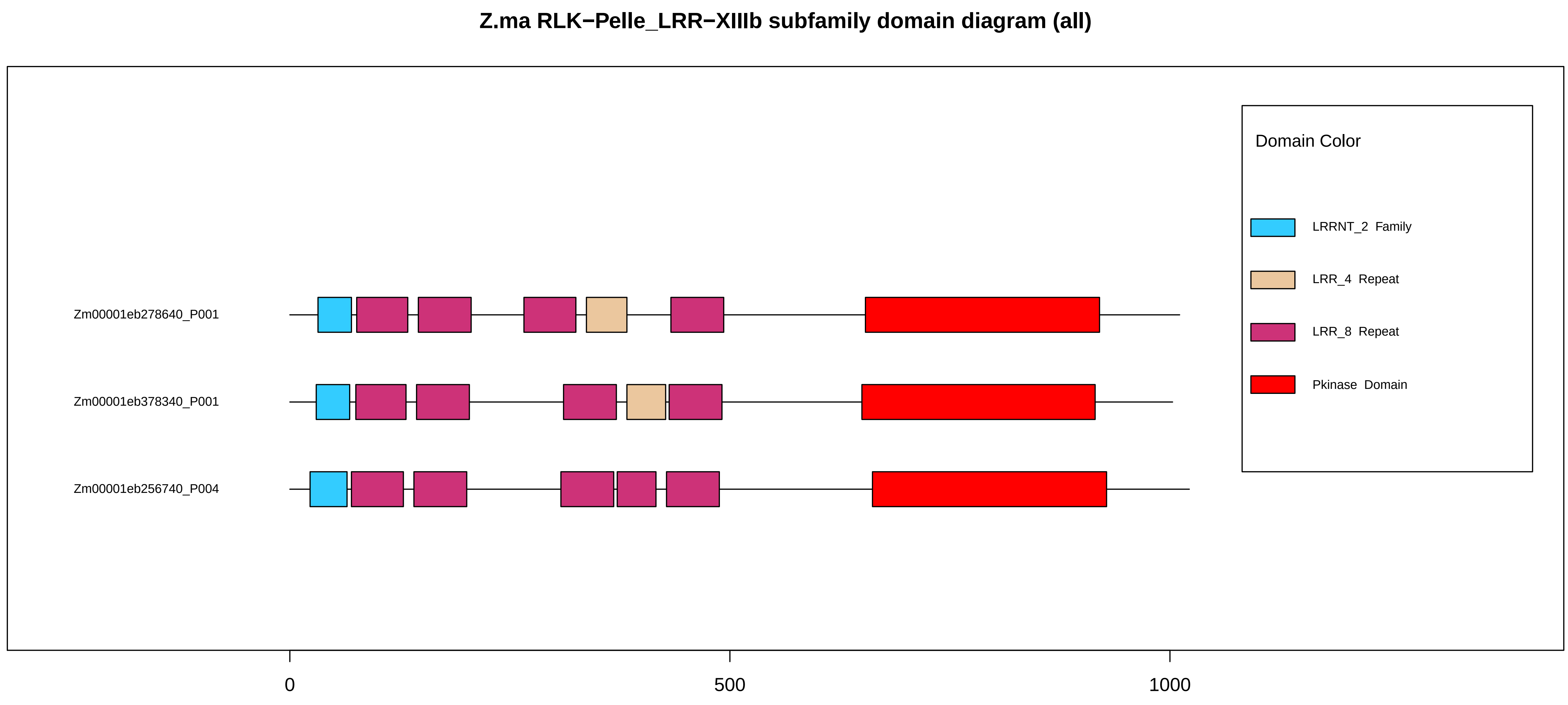
Z.ma RLK−Pelle_LRR−XIIIb subfamily domain diagram (all)
Zm00001eb278640_P001
Zm00001eb378340_P001
Zm00001eb256740_P004
Domain Color
LRRNT_2 Family
LRR_4 Repeat
LRR_8 Repeat
Pkinase Domain
0
500
1000

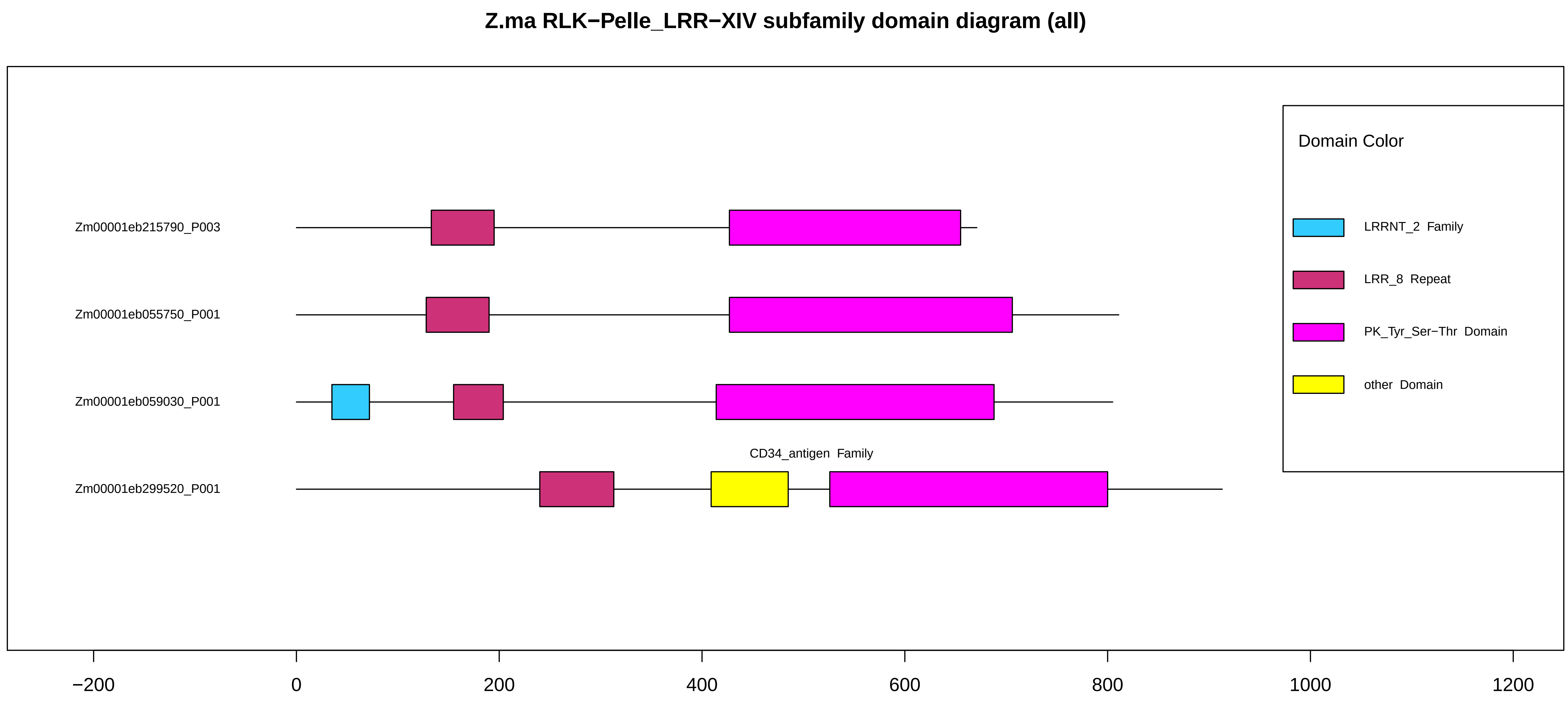

Z.ma RLK−Pelle_LRR−XIV subfamily domain diagram (all)
Zm00001eb215790_P003
Zm00001eb055750_P001
Zm00001eb059030_P001
Zm00001eb299520_P001
CD34_antigen Family
Domain Color
LRRNT_2 Family
LRR_8 Repeat
PK_Tyr_Ser−Thr Domain
other Domain
−200
0
200
400
600
800
1000
1200

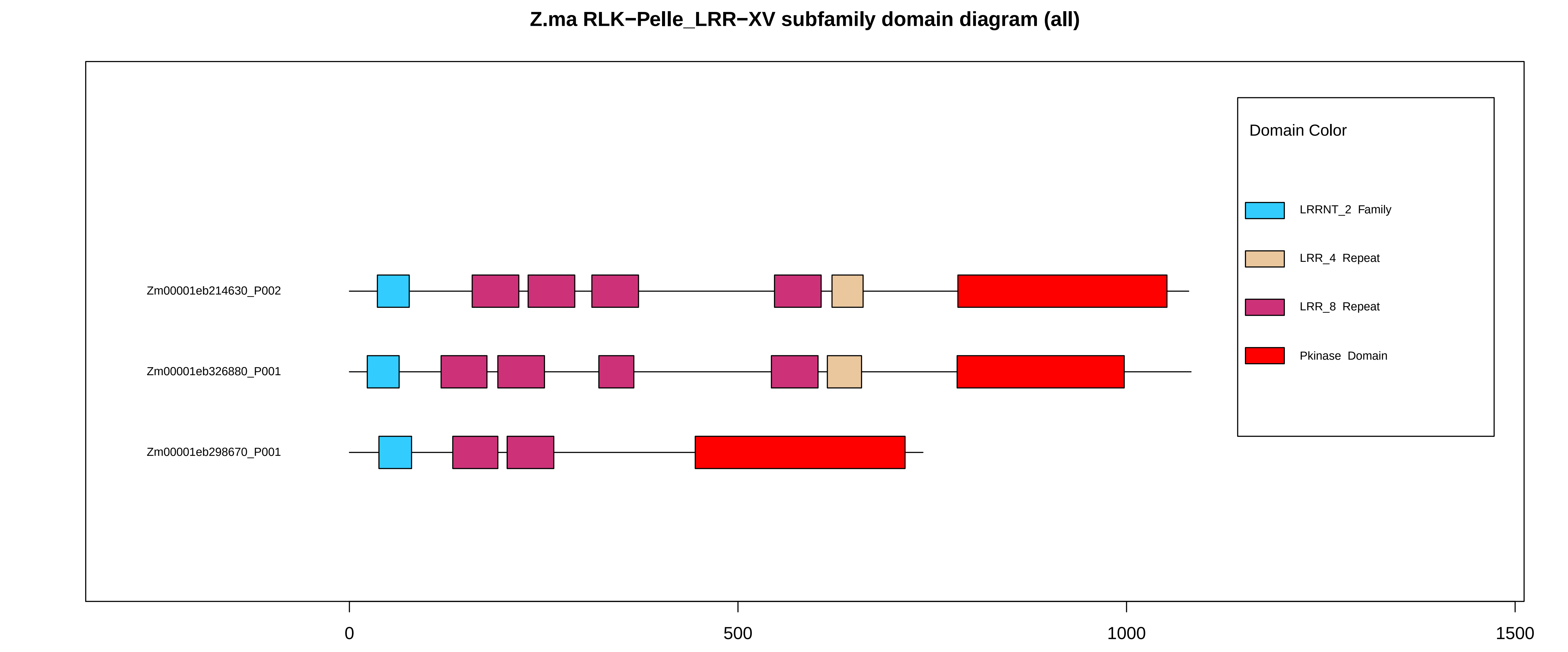

Z.ma RLK−Pelle_LRR−XV subfamily domain diagram (all)
Domain Color
LRRNT_2 Family
LRR_4 Repeat
LRR_8 Repeat
Pkinase Domain
Zm00001eb214630_P002
Zm00001eb326880_P001
Zm00001eb298670_P001
0
500
1000
1500

Z.ma RLK−Pelle_LysM subfamily domain diagram (all)

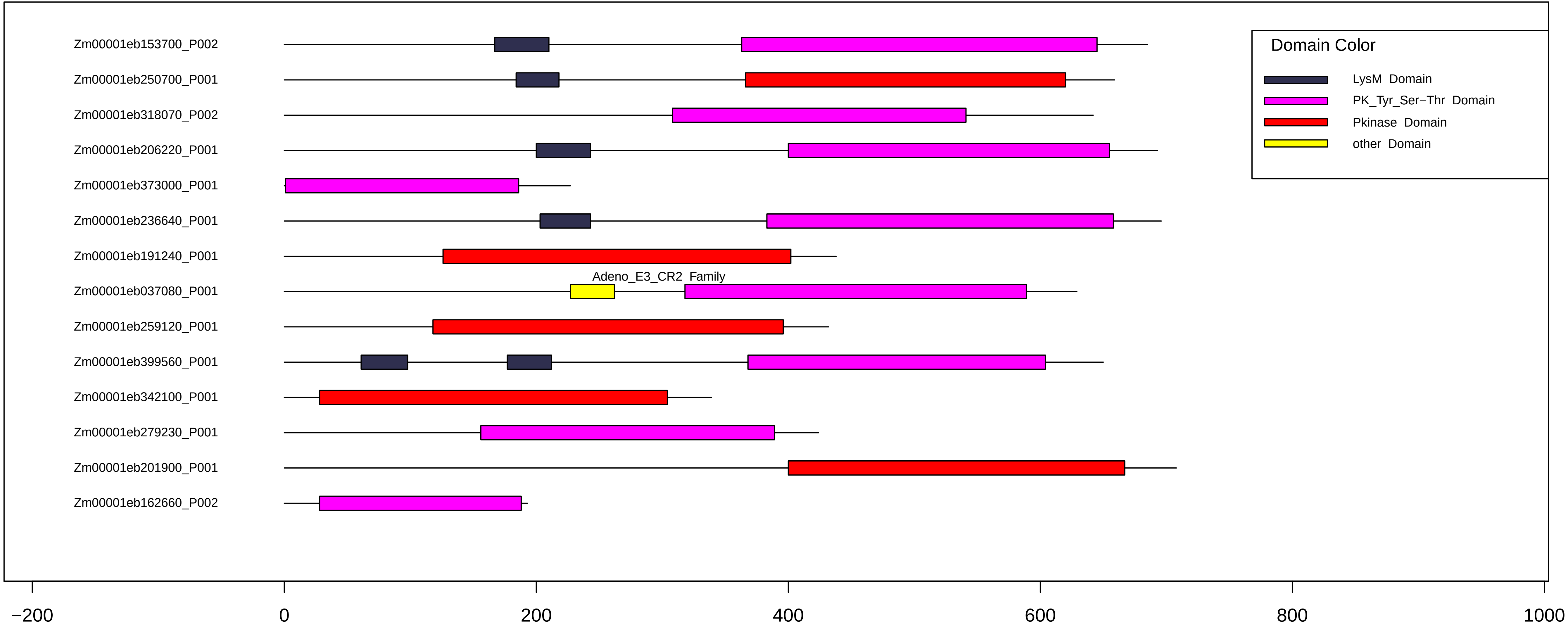

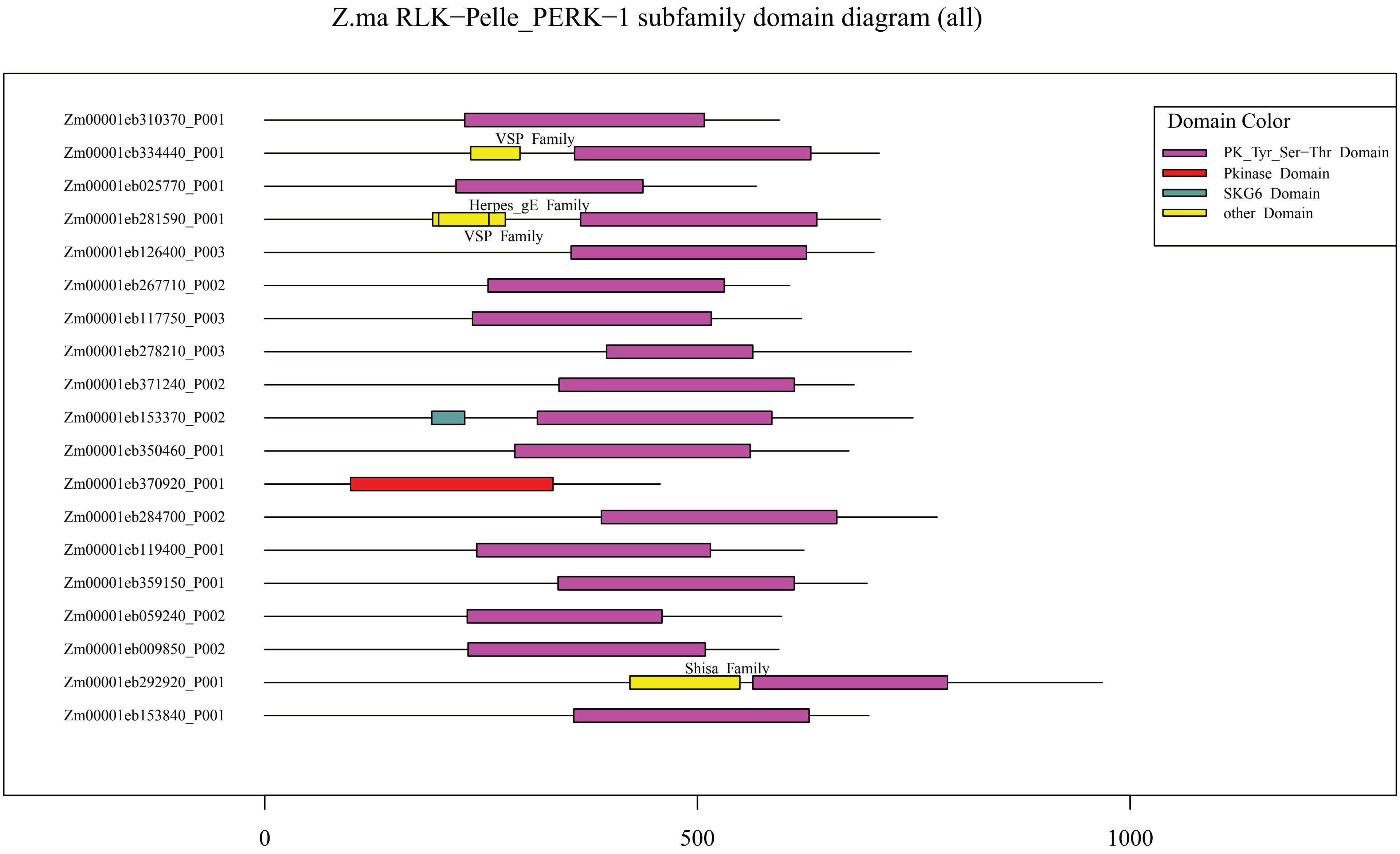

Z.ma RLK−Pelle_PERK−1 subfamily domain diagram (all)
Zm00001eb310370_P001
Zm00001eb334440_P001
VSP Family
Zm00001eb025770_P001
Zm00001eb281590_P001
Herpes_gE Family
VSP Family
Zm00001eb126400_P003
Zm00001eb267710_P002
Zm00001eb117750_P003
Zm00001eb278210_P003
Zm00001eb371240_P002
Zm00001eb153370_P002
Zm00001eb350460_P001
Zm00001eb370920_P001
Zm00001eb284700_P002
Zm00001eb119400_P001
Zm00001eb359150_P001
Zm00001eb059240_P002
Zm00001eb009850_P002
Zm00001eb292920_P001
Shisa Family
Zm00001eb153840_P001
Domain Color
PK_Tyr_Ser−Thr Domain
Pkinase Domain
SKG6 Domain
other Domain
0
500
1000

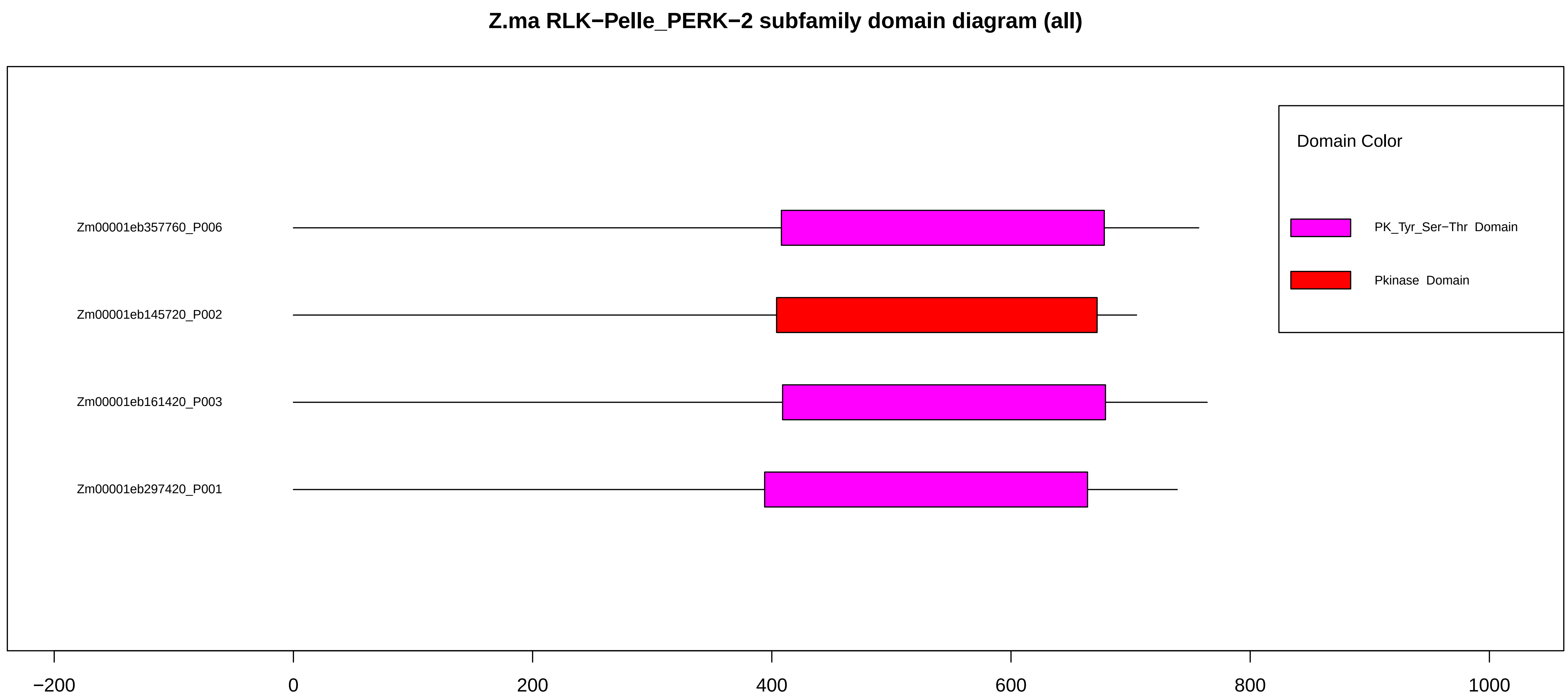

Z.ma RLK−Pelle_PERK−2 subfamily domain diagram (all)
Zm00001eb357760_P006
Zm00001eb145720_P002
Zm00001eb161420_P003
Zm00001eb297420_P001
Domain Color
PK_Tyr_Ser−Thr Domain
Pkinase Domain
−200
0
200
400
600
800
1000

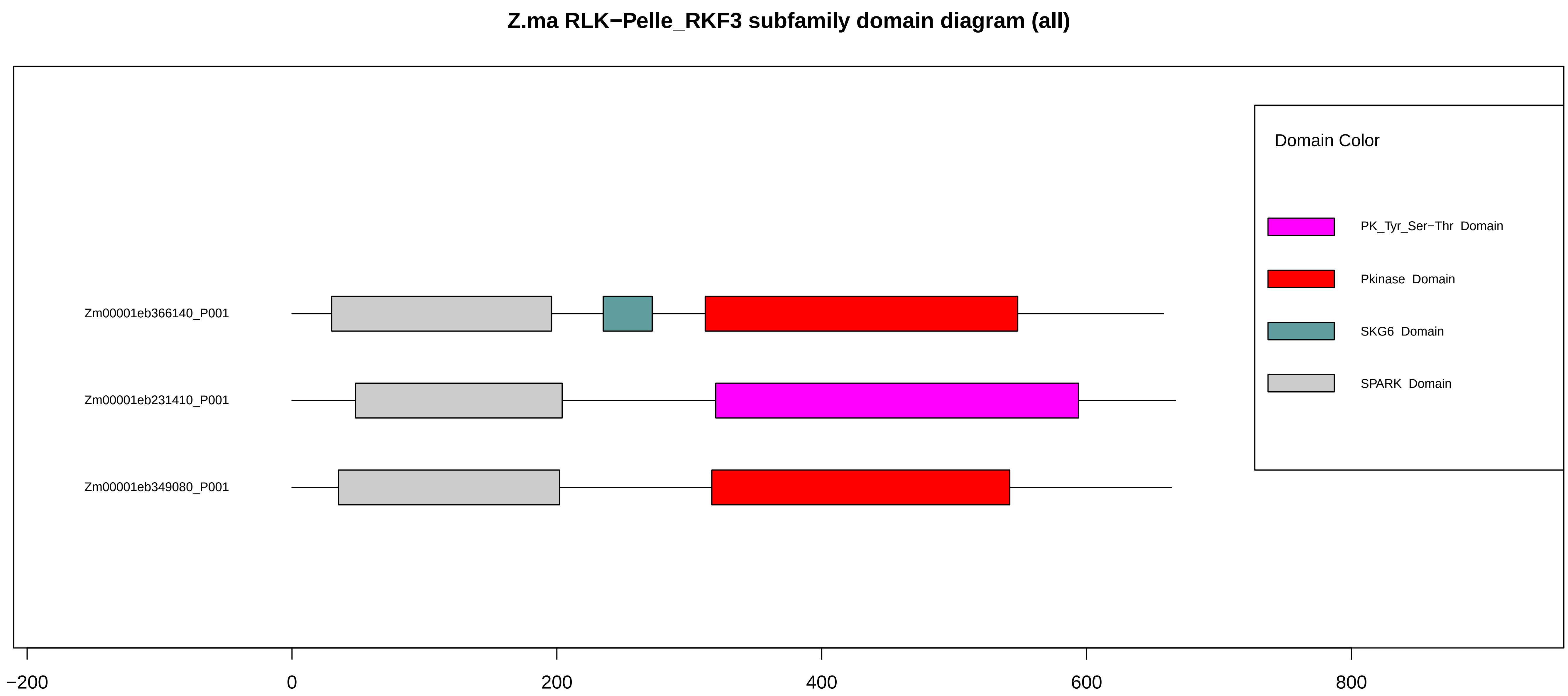

Z.ma RLK−Pelle_RKF3 subfamily domain diagram (all)
Zm00001eb366140_P001
Zm00001eb231410_P001
Zm00001eb349080_P001
Domain Color
PK_Tyr_Ser−Thr Domain
Pkinase Domain
SKG6 Domain
SPARK Domain
−200
0
200
400
600
800

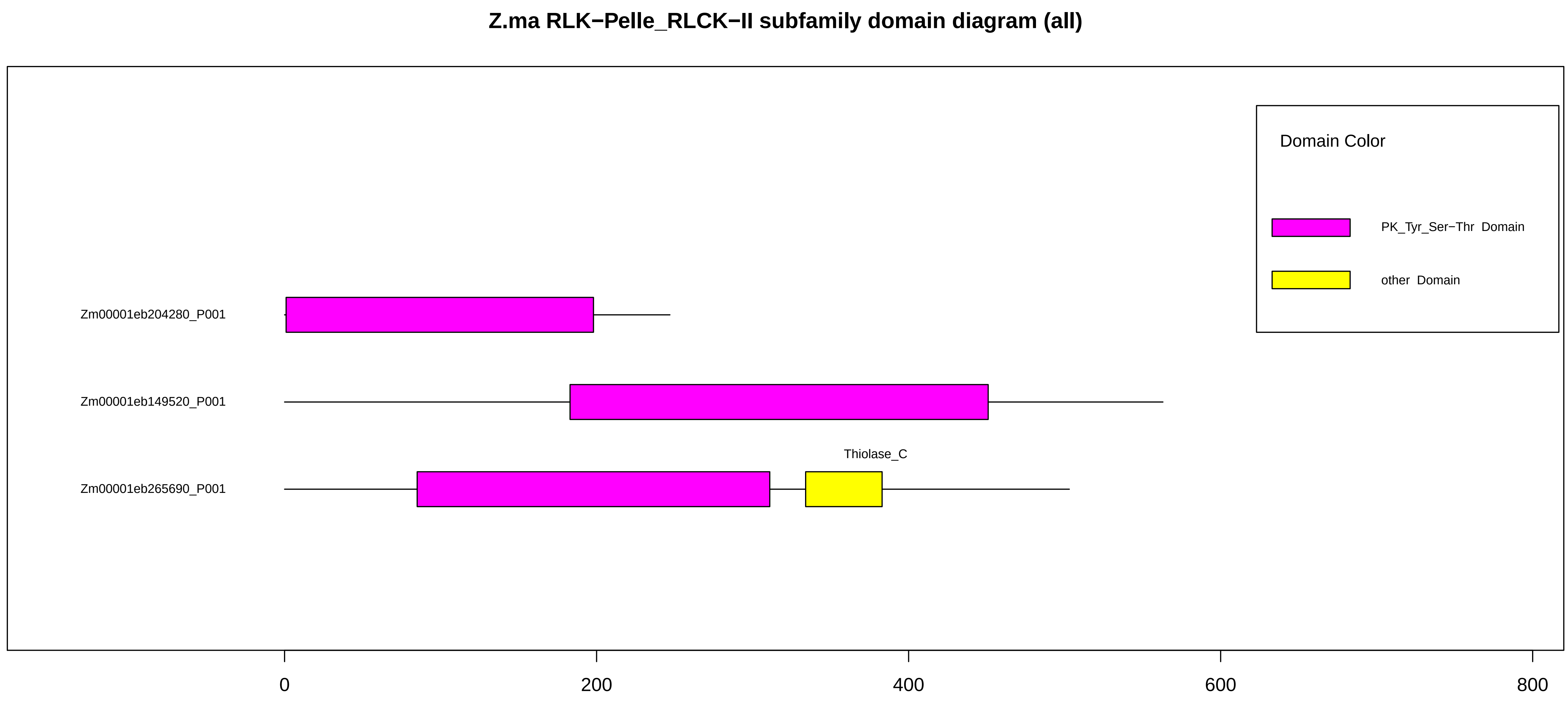
Z.ma RLK−Pelle_RLCK−II subfamily domain diagram (all)
Domain Color
PK_Tyr_Ser−Thr Domain
other Domain
Zm00001eb204280_P001
Zm00001eb149520_P001
Zm00001eb265690_P001
Thiolase_C
0
200
400
600
800

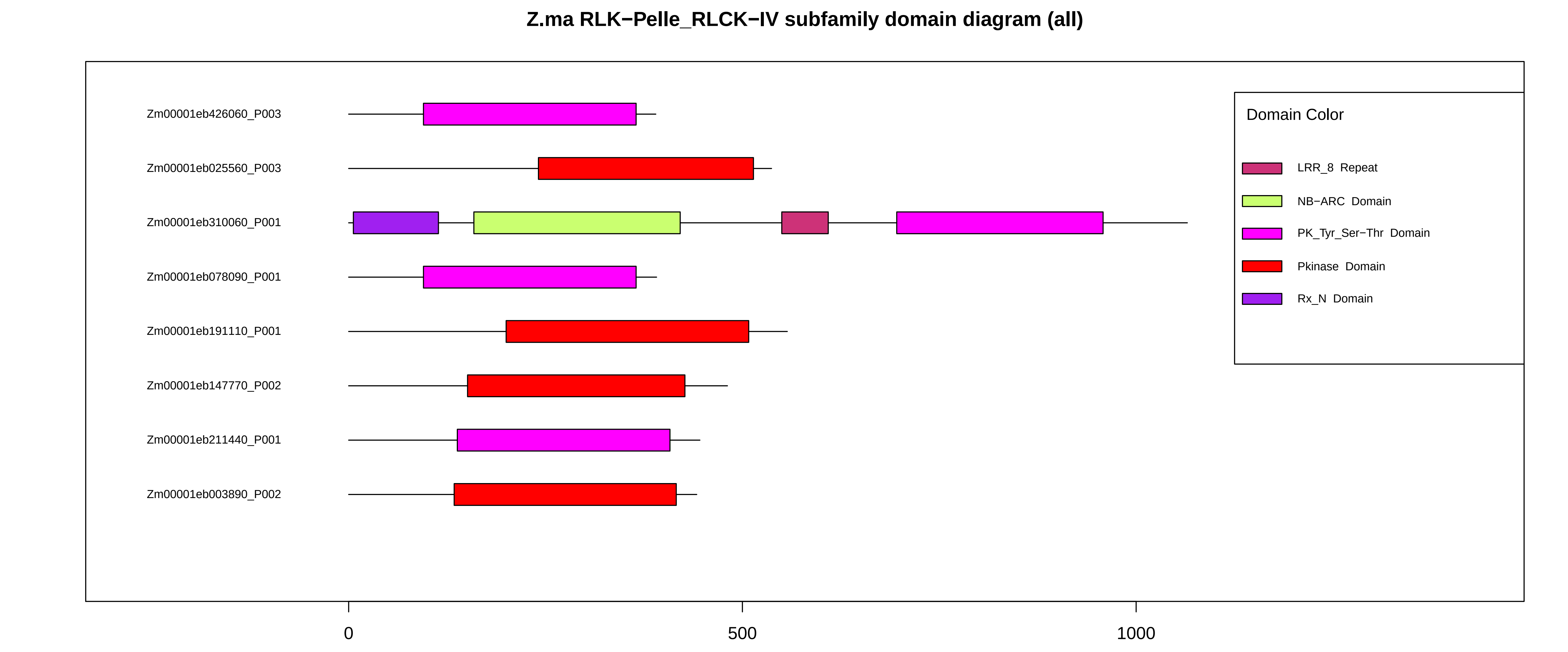

Z.ma RLK−Pelle_RLCK−IV subfamily domain diagram (all)
Zm00001eb426060_P003
Zm00001eb025560_P003
Zm00001eb310060_P001
Zm00001eb078090_P001
Zm00001eb191110_P001
Zm00001eb147770_P002
Zm00001eb211440_P001
Zm00001eb003890_P002
Domain Color
LRR_8 Repeat
NB−ARC Domain
PK_Tyr_Ser−Thr Domain
Pkinase Domain
Rx_N Domain
0
500
1000

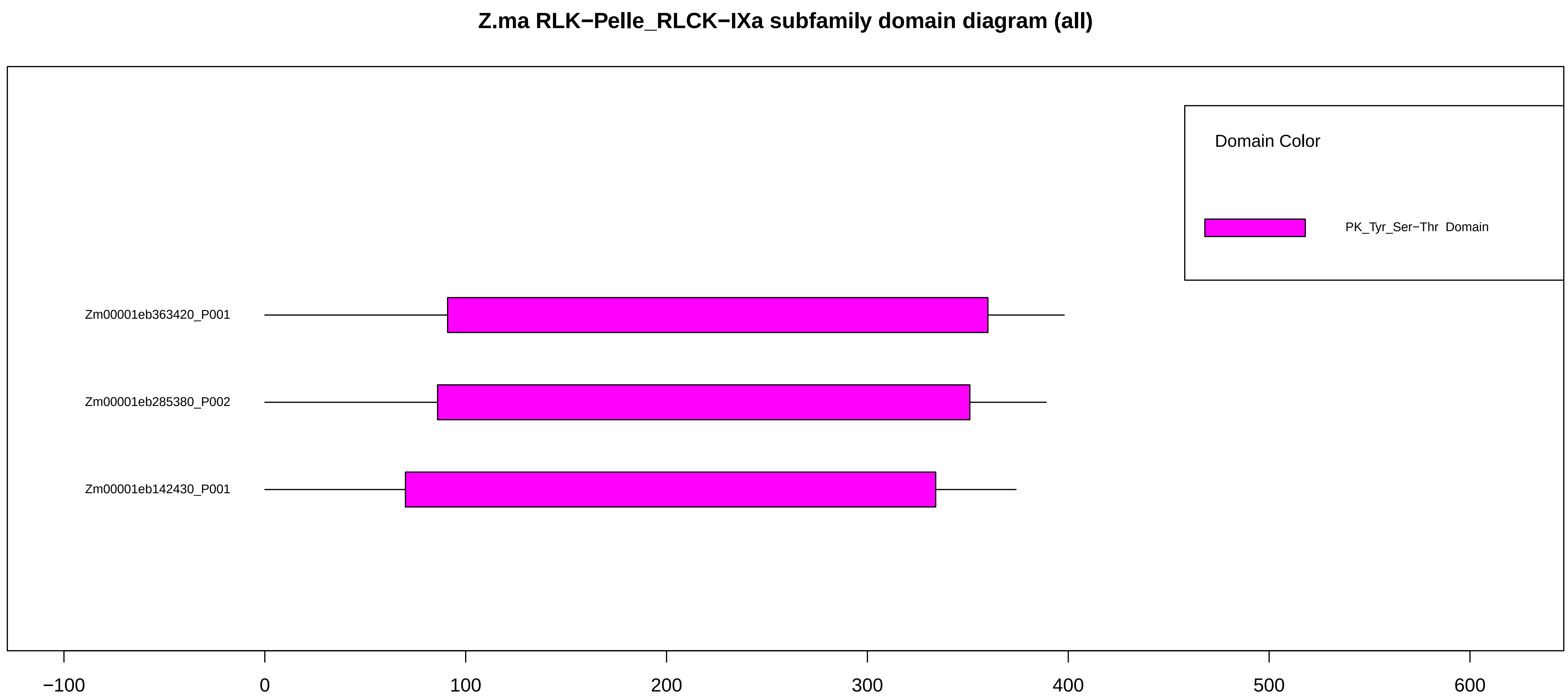
Z.ma RLK−Pelle_RLCK−IXa subfamily domain diagram (all)
Domain Color
PK_Tyr_Ser−Thr Domain
Zm00001eb363420_P001
Zm00001eb285380_P002
Zm00001eb142430_P001
−100
0
100
200
300
400
500
600

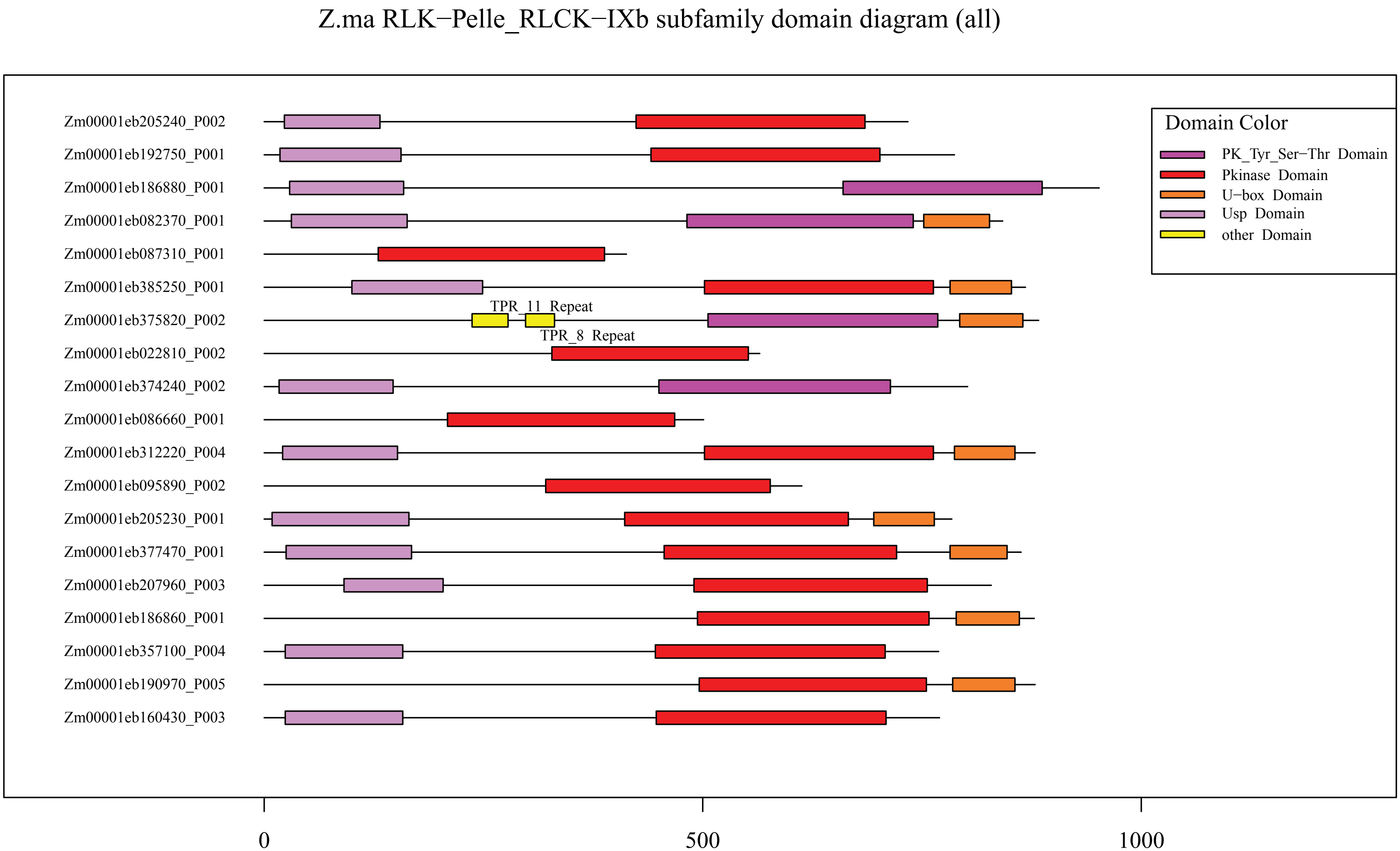

Z.ma RLK−Pelle_RLCK−IXb subfamily domain diagram (all)
Zm00001eb205240_P002
Zm00001eb192750_P001
Zm00001eb186880_P001
Zm00001eb082370_P001
Zm00001eb087310_P001
Zm00001eb385250_P001
Zm00001eb375820_P002
Zm00001eb022810_P002
Zm00001eb374240_P002
Zm00001eb086660_P001
Zm00001eb312220_P004
Zm00001eb095890_P002
Zm00001eb205230_P001
Zm00001eb377470_P001
Zm00001eb207960_P003
Zm00001eb186860_P001
Zm00001eb357100_P004
Zm00001eb190970_P005
Zm00001eb160430_P003
TPR_11 Repeat
TPR_8 Repeat
Domain Color
PK_Tyr_Ser−Thr Domain
Pkinase Domain
U−box Domain
Usp Domain
other Domain
0
500
1000

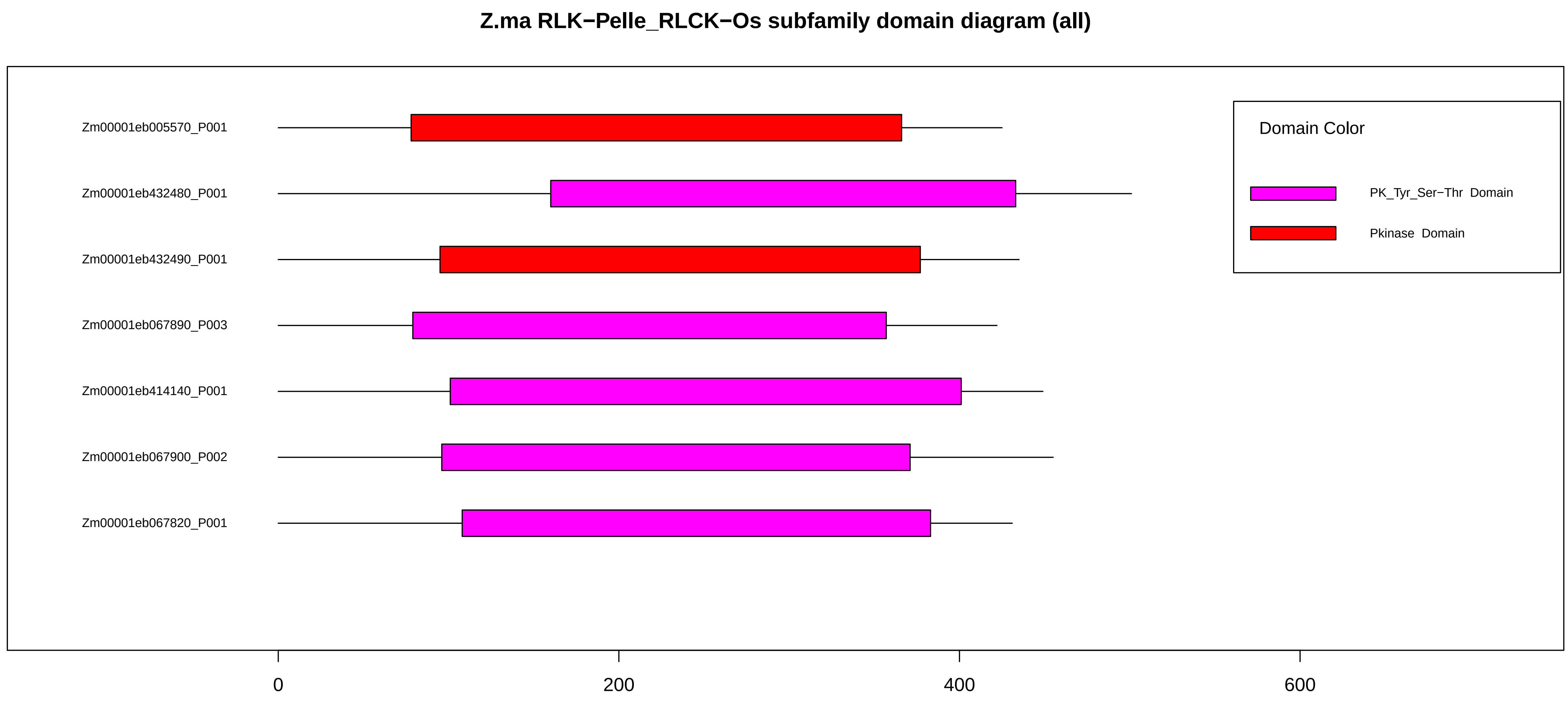
Z.ma RLK−Pelle_RLCK−Os subfamily domain diagram (all)
Zm00001eb005570_P001
Zm00001eb432480_P001
Zm00001eb432490_P001
Zm00001eb067890_P003
Zm00001eb414140_P001
Zm00001eb067900_P002
Zm00001eb067820_P001
Domain Color
PK_Tyr_Ser−Thr Domain
Pkinase Domain
0
200
400
600

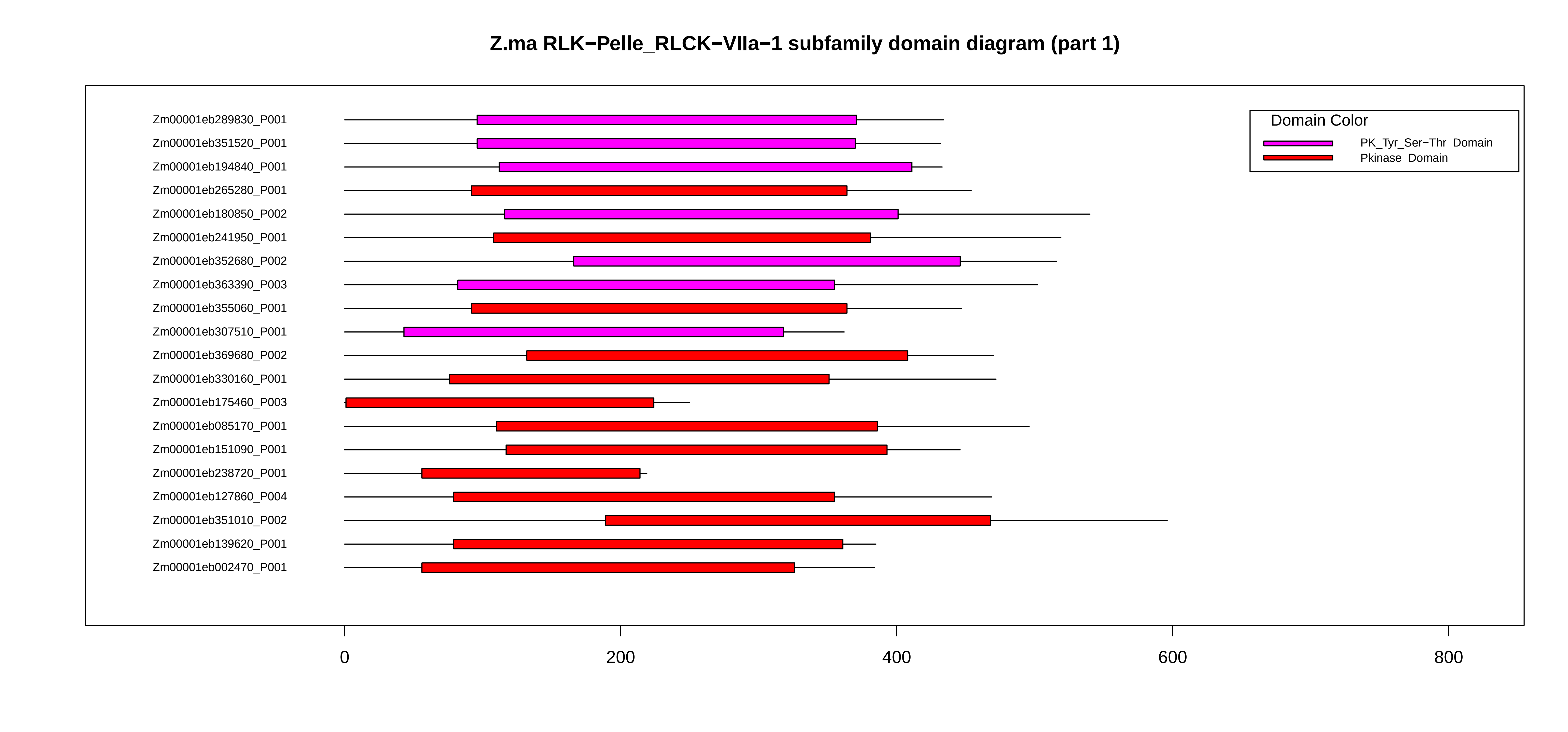

Z.ma RLK−Pelle_RLCK−VIIa−1 subfamily domain diagram (part 1)
Domain Color
PK_Tyr_Ser−Thr Domain
Pkinase Domain
Zm00001eb289830_P001
Zm00001eb351520_P001
Zm00001eb194840_P001
Zm00001eb265280_P001
Zm00001eb180850_P002
Zm00001eb241950_P001
Zm00001eb352680_P002
Zm00001eb363390_P003
Zm00001eb355060_P001
Zm00001eb307510_P001
Zm00001eb369680_P002
Zm00001eb330160_P001
Zm00001eb175460_P003
Zm00001eb085170_P001
Zm00001eb151090_P001
Zm00001eb238720_P001
Zm00001eb127860_P004
Zm00001eb351010_P002
Zm00001eb139620_P001
Zm00001eb002470_P001
0
200
400
600
800

Z.ma RLK−Pelle_RLCK−VIIa−1 subfamily domain diagram (part 2)

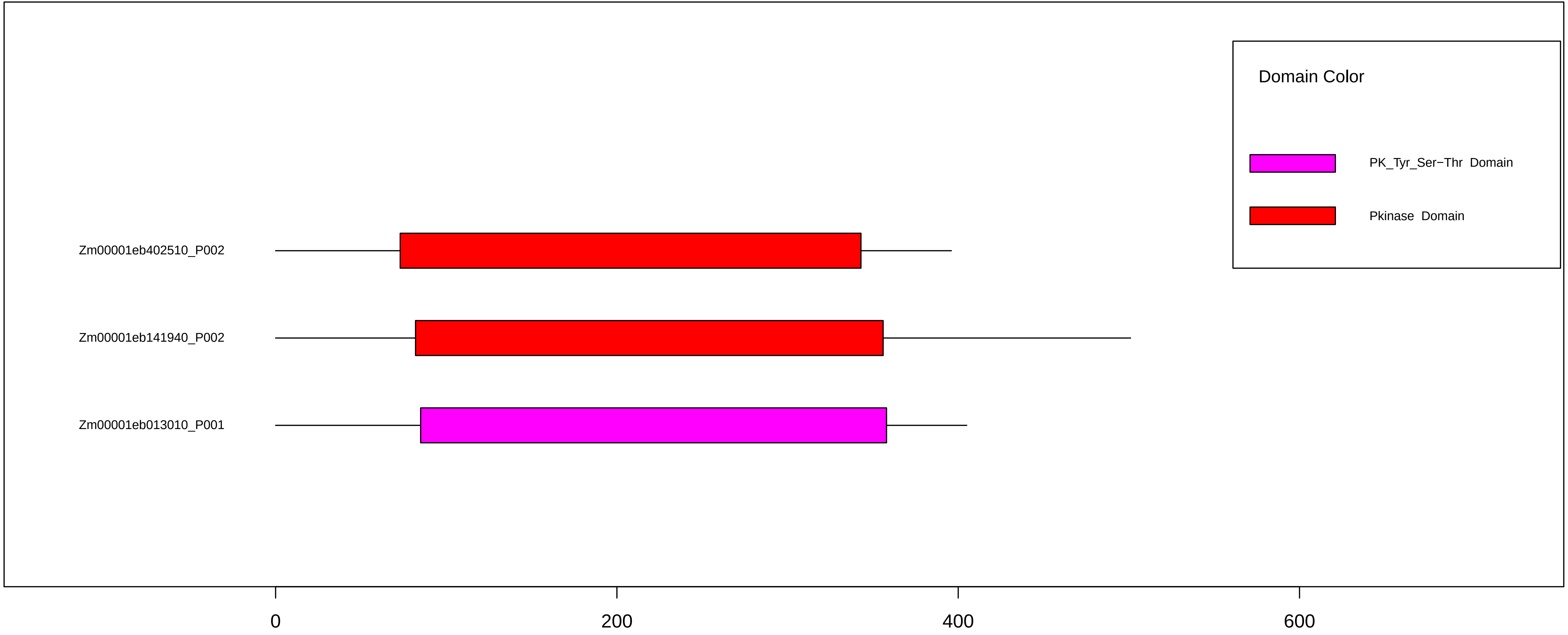

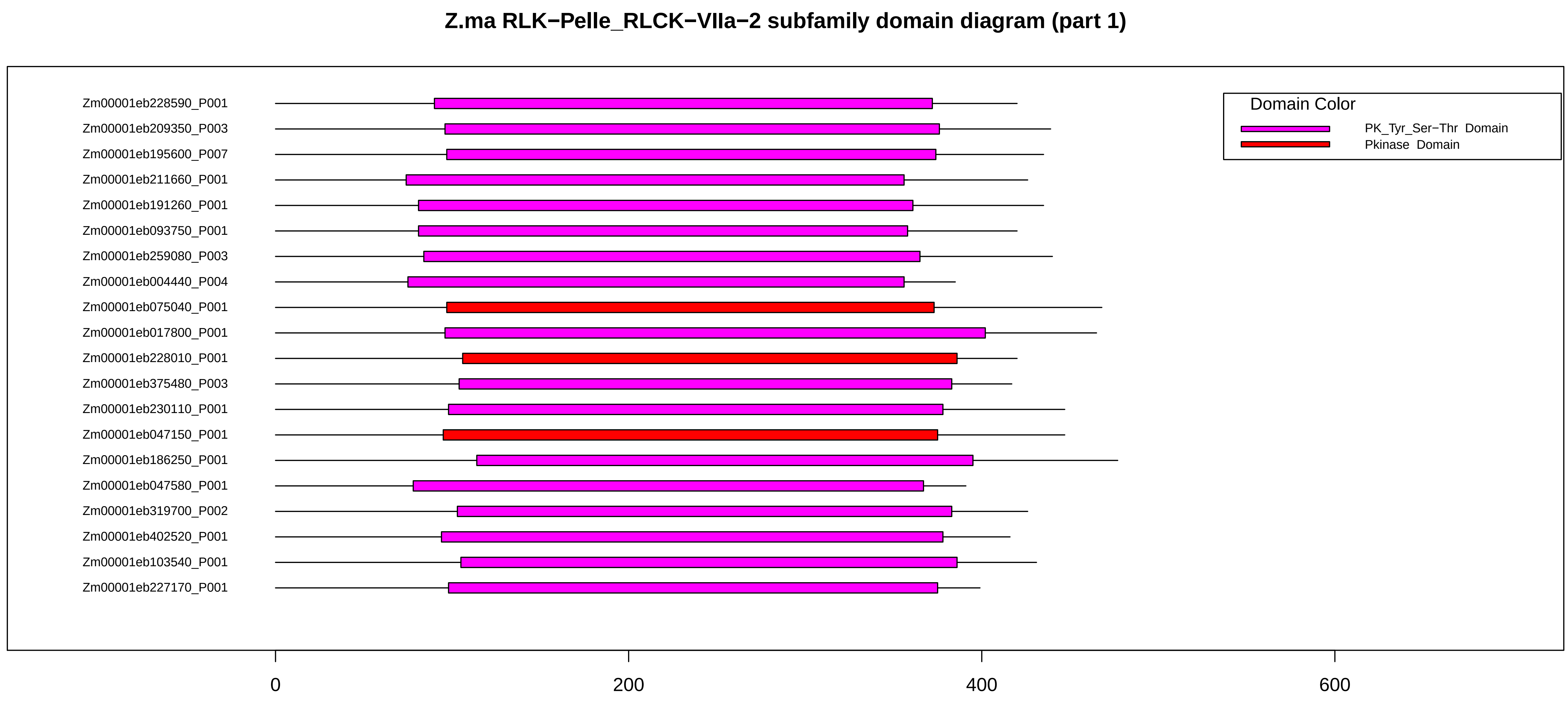
Z.ma RLK−Pelle_RLCK−VIIa−2 subfamily domain diagram (part 1)
Zm00001eb228590_P001
Zm00001eb209350_P003
Zm00001eb195600_P007
Zm00001eb211660_P001
Zm00001eb191260_P001
Zm00001eb093750_P001
Zm00001eb259080_P003
Zm00001eb004440_P004
Zm00001eb075040_P001
Zm00001eb017800_P001
Zm00001eb228010_P001
Zm00001eb375480_P003
Zm00001eb230110_P001
Zm00001eb047150_P001
Zm00001eb186250_P001
Zm00001eb047580_P001
Zm00001eb319700_P002
Zm00001eb402520_P001
Zm00001eb103540_P001
Zm00001eb227170_P001
Domain Color
PK_Tyr_Ser−Thr Domain
Pkinase Domain
0
200
400
600

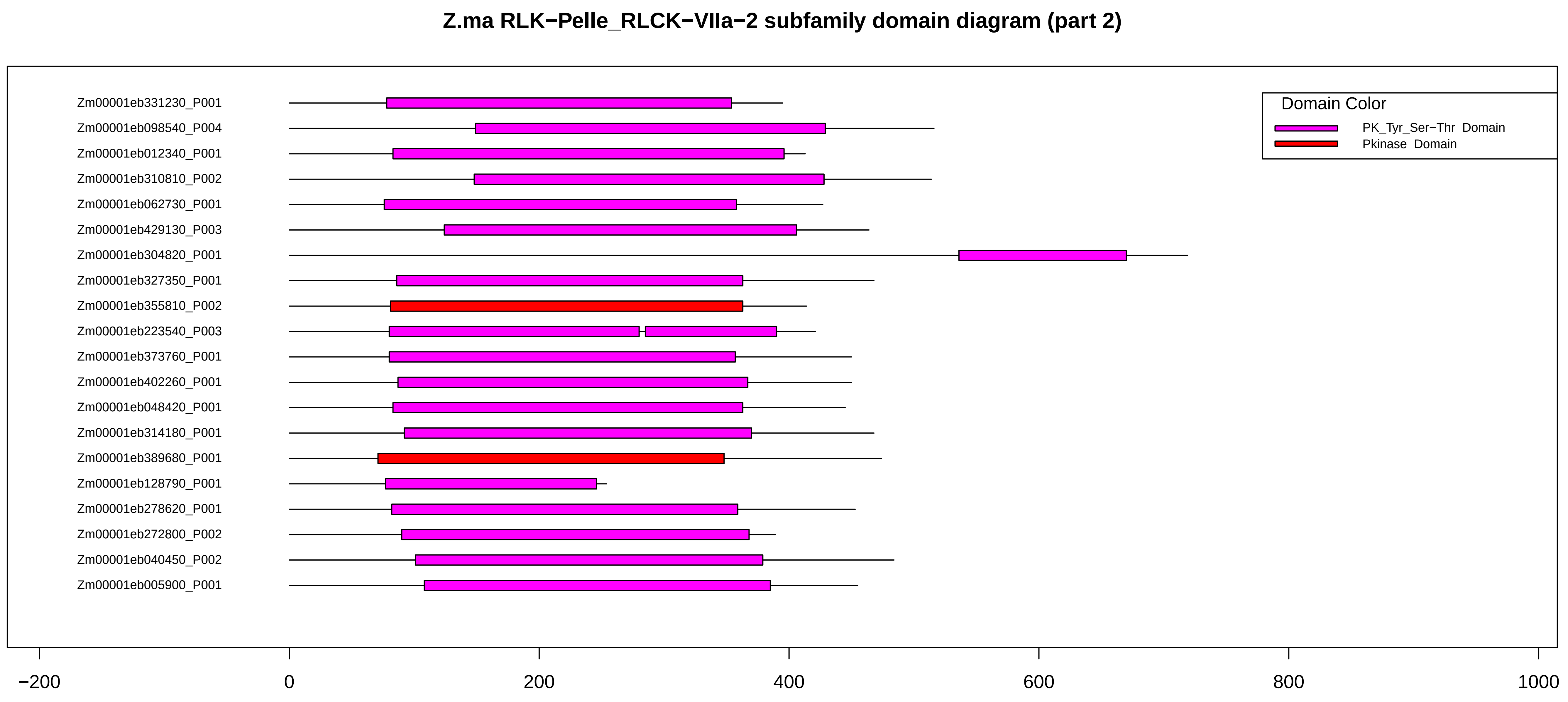
Z.ma RLK−Pelle_RLCK−VIIa−2 subfamily domain diagram (part 2)
Zm00001eb331230_P001
Zm00001eb098540_P004
Zm00001eb012340_P001
Zm00001eb310810_P002
Zm00001eb062730_P001
Zm00001eb429130_P003
Zm00001eb304820_P001
Zm00001eb327350_P001
Zm00001eb355810_P002
Zm00001eb223540_P003
Zm00001eb373760_P001
Zm00001eb402260_P001
Zm00001eb048420_P001
Zm00001eb314180_P001
Zm00001eb389680_P001
Zm00001eb128790_P001
Zm00001eb278620_P001
Zm00001eb272800_P002
Zm00001eb040450_P002
Zm00001eb005900_P001
Domain Color
PK_Tyr_Ser−Thr Domain
Pkinase Domain
−200
0
200
400
600
800
1000

Z.ma RLK−Pelle_RLCK−VIIa−2 subfamily domain diagram (part 3)

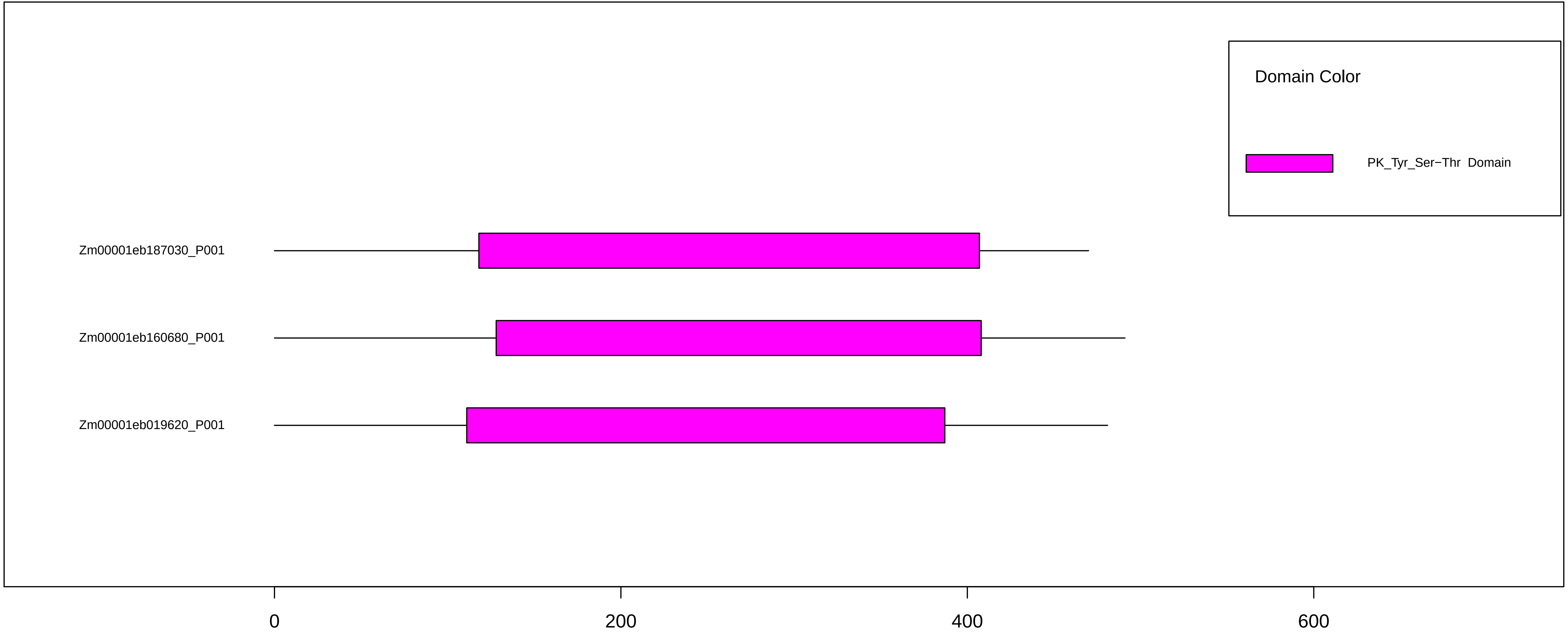

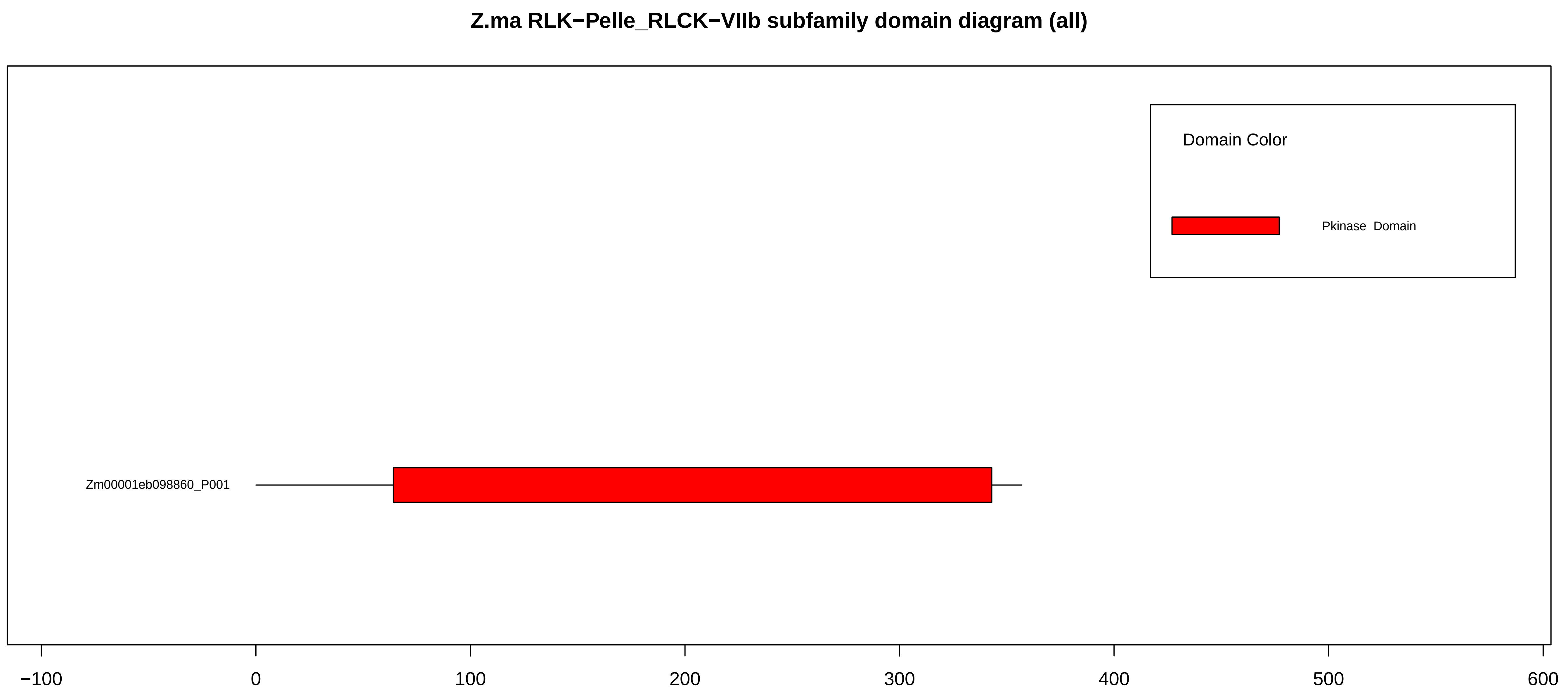

Z.ma RLK−Pelle_RLCK−VIIb subfamily domain diagram (all)
Domain Color
Pkinase Domain
Zm00001eb098860_P001
−100
0
100
200
300
400
500
600

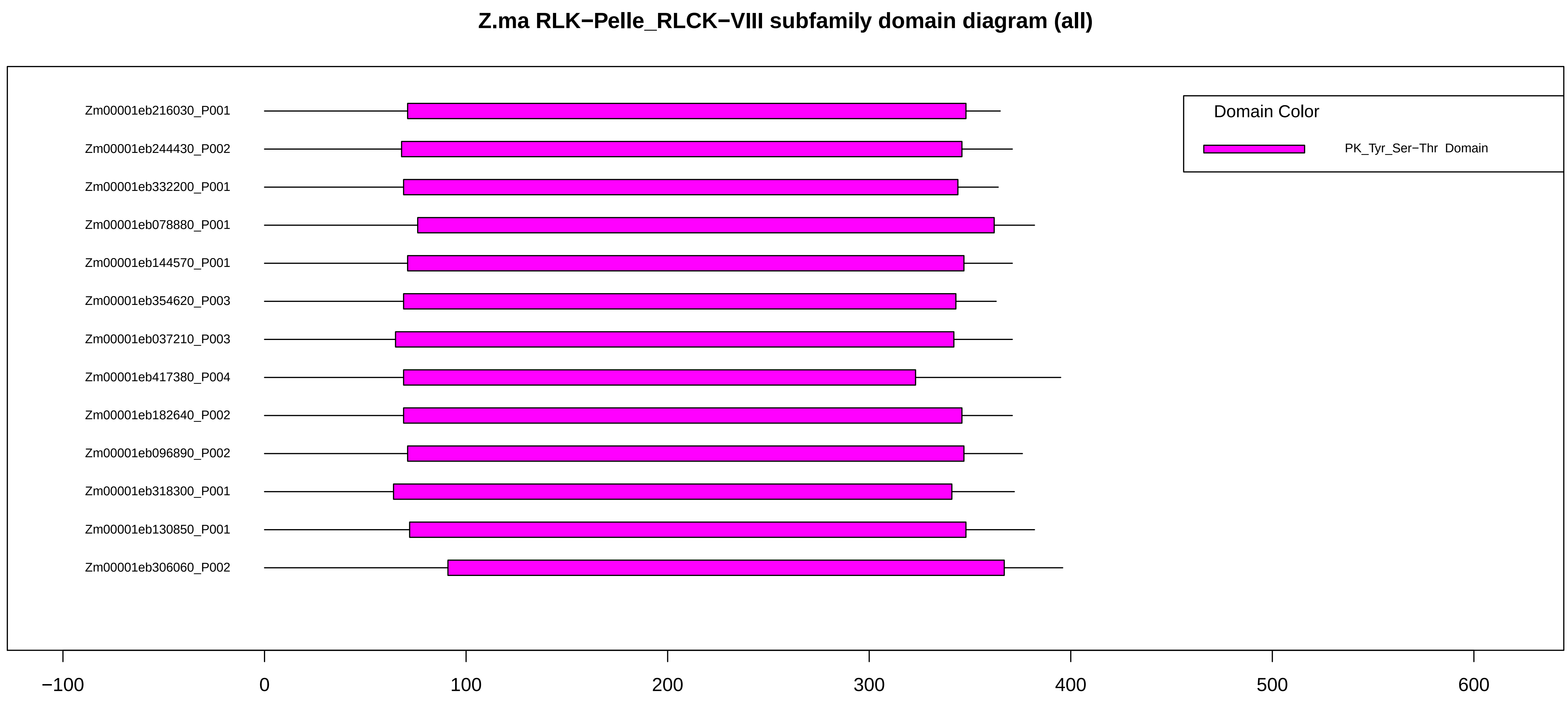

Z.ma RLK−Pelle_RLCK−VIII subfamily domain diagram (all)
Zm00001eb216030_P001
Zm00001eb244430_P002
Zm00001eb332200_P001
Zm00001eb078880_P001
Zm00001eb144570_P001
Zm00001eb354620_P003
Zm00001eb037210_P003
Zm00001eb417380_P004
Zm00001eb182640_P002
Zm00001eb096890_P002
Zm00001eb318300_P001
Zm00001eb130850_P001
Zm00001eb306060_P002
Domain Color
PK_Tyr_Ser−Thr Domain
−100
0
100
200
300
400
500
600

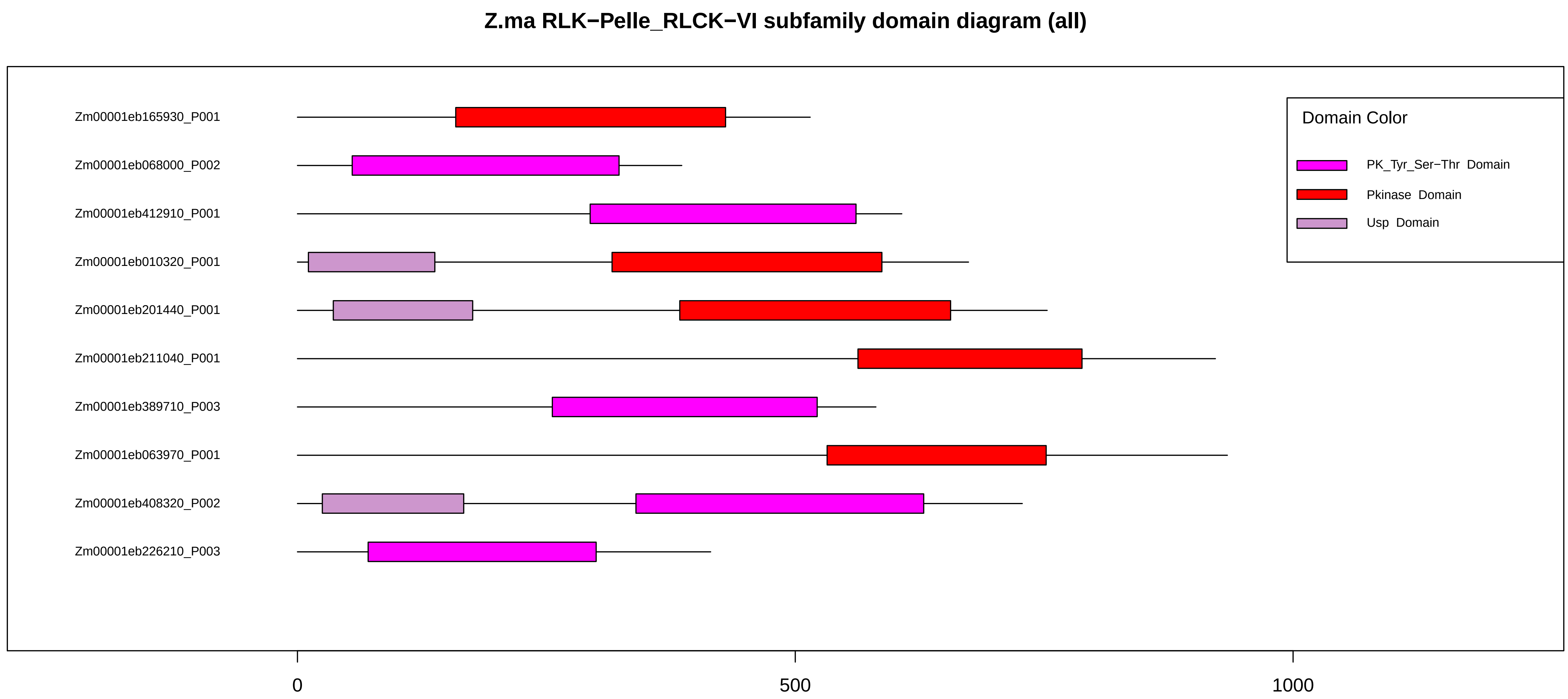
Z.ma RLK−Pelle_RLCK−VI subfamily domain diagram (all)
Zm00001eb165930_P001
Zm00001eb068000_P002
Zm00001eb412910_P001
Zm00001eb010320_P001
Zm00001eb201440_P001
Zm00001eb211040_P001
Zm00001eb389710_P003
Zm00001eb063970_P001
Zm00001eb408320_P002
Zm00001eb226210_P003
Domain Color
PK_Tyr_Ser−Thr Domain
Pkinase Domain
Usp Domain
0
500
1000

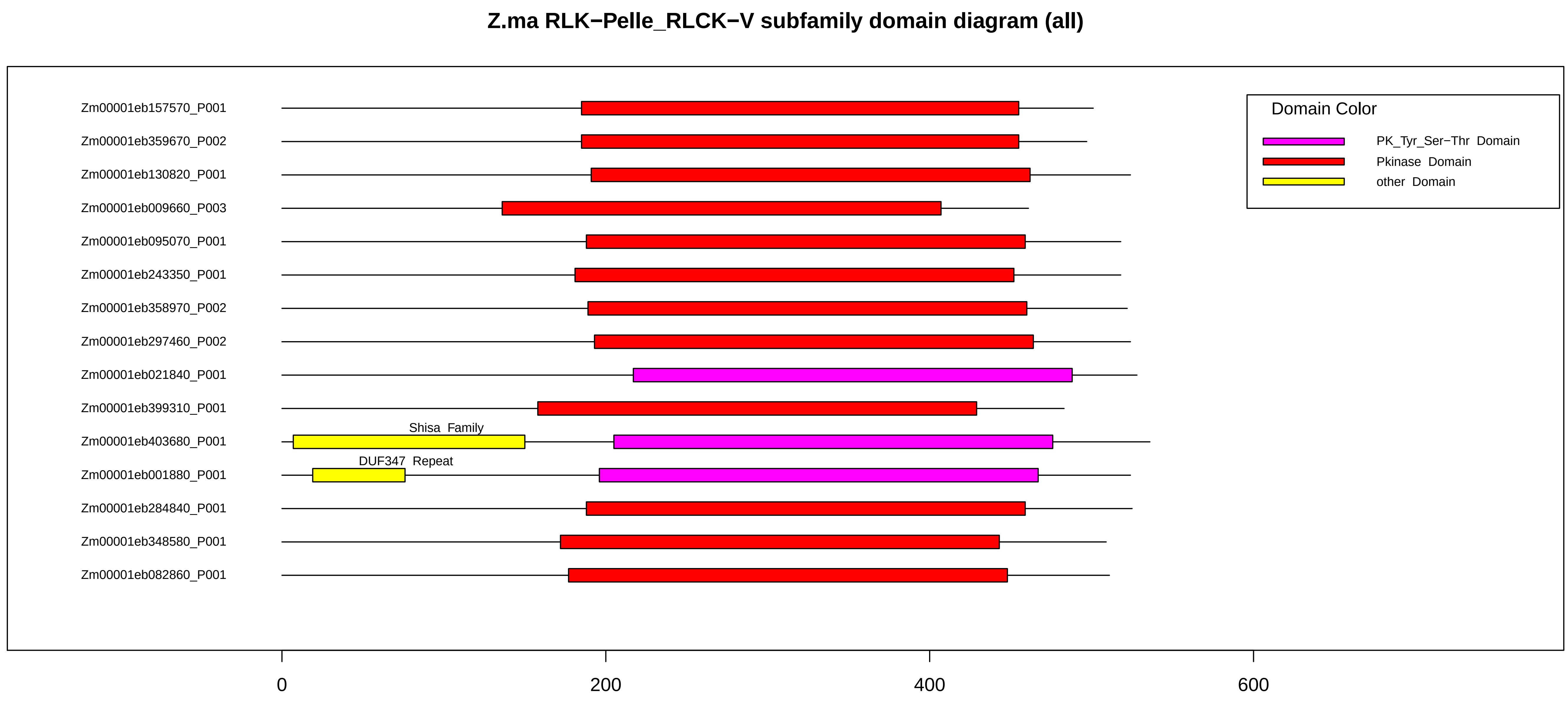
Z.ma RLK−Pelle_RLCK−V subfamily domain diagram (all)
Zm00001eb157570_P001
Zm00001eb359670_P002
Zm00001eb130820_P001
Zm00001eb009660_P003
Zm00001eb095070_P001
Zm00001eb243350_P001
Zm00001eb358970_P002
Zm00001eb297460_P002
Zm00001eb021840_P001
Zm00001eb399310_P001
Zm00001eb403680_P001
Shisa Family
Zm00001eb001880_P001
DUF347 Repeat
Zm00001eb284840_P001
Zm00001eb348580_P001
Zm00001eb082860_P001
Domain Color
PK_Tyr_Ser−Thr Domain
Pkinase Domain
other Domain
0
200
400
600

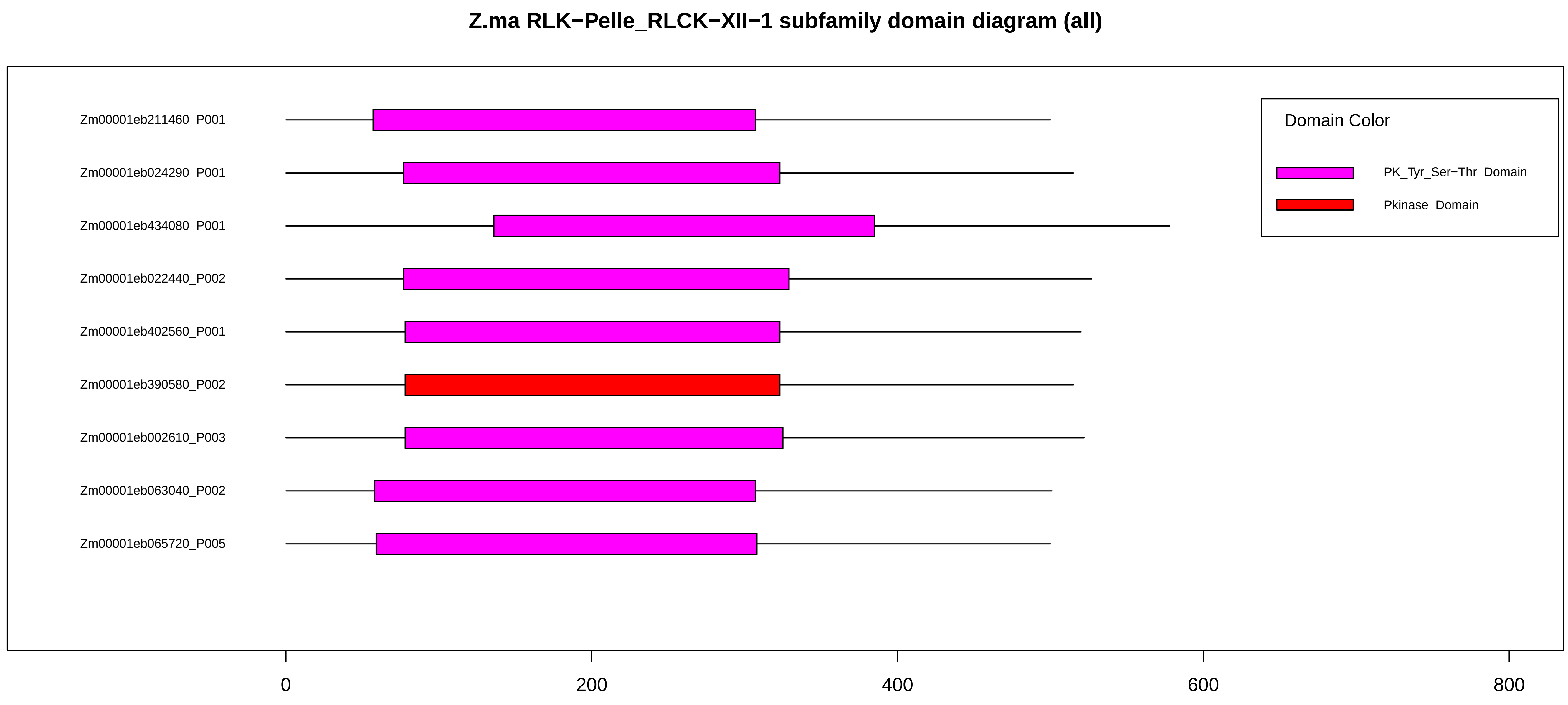
Z.ma RLK−Pelle_RLCK−XII−1 subfamily domain diagram (all)
Zm00001eb211460_P001
Zm00001eb024290_P001
Zm00001eb434080_P001
Zm00001eb022440_P002
Zm00001eb402560_P001
Zm00001eb390580_P002
Zm00001eb002610_P003
Zm00001eb063040_P002
Zm00001eb065720_P005
Domain Color
PK_Tyr_Ser−Thr Domain
Pkinase Domain
0
200
400
600
800

Z.ma RLK−Pelle_RLCK−XIII subfamily domain diagram (all)

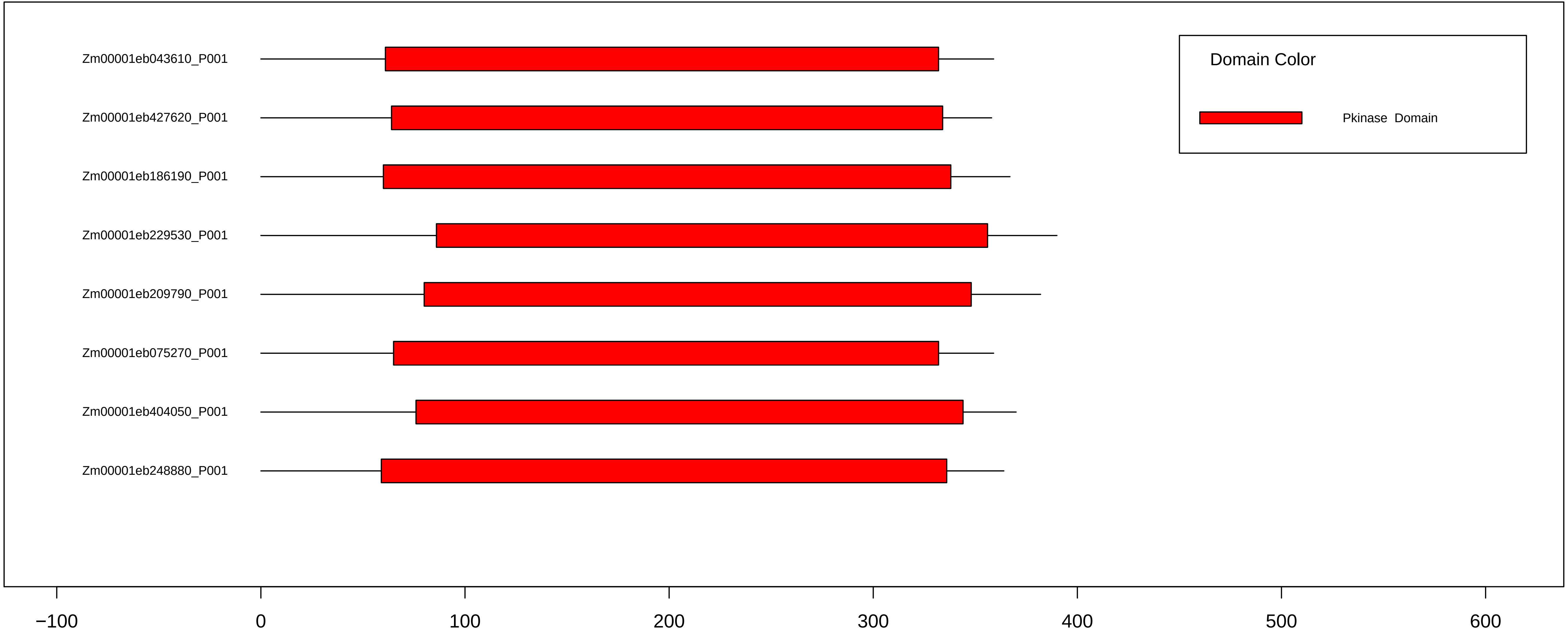

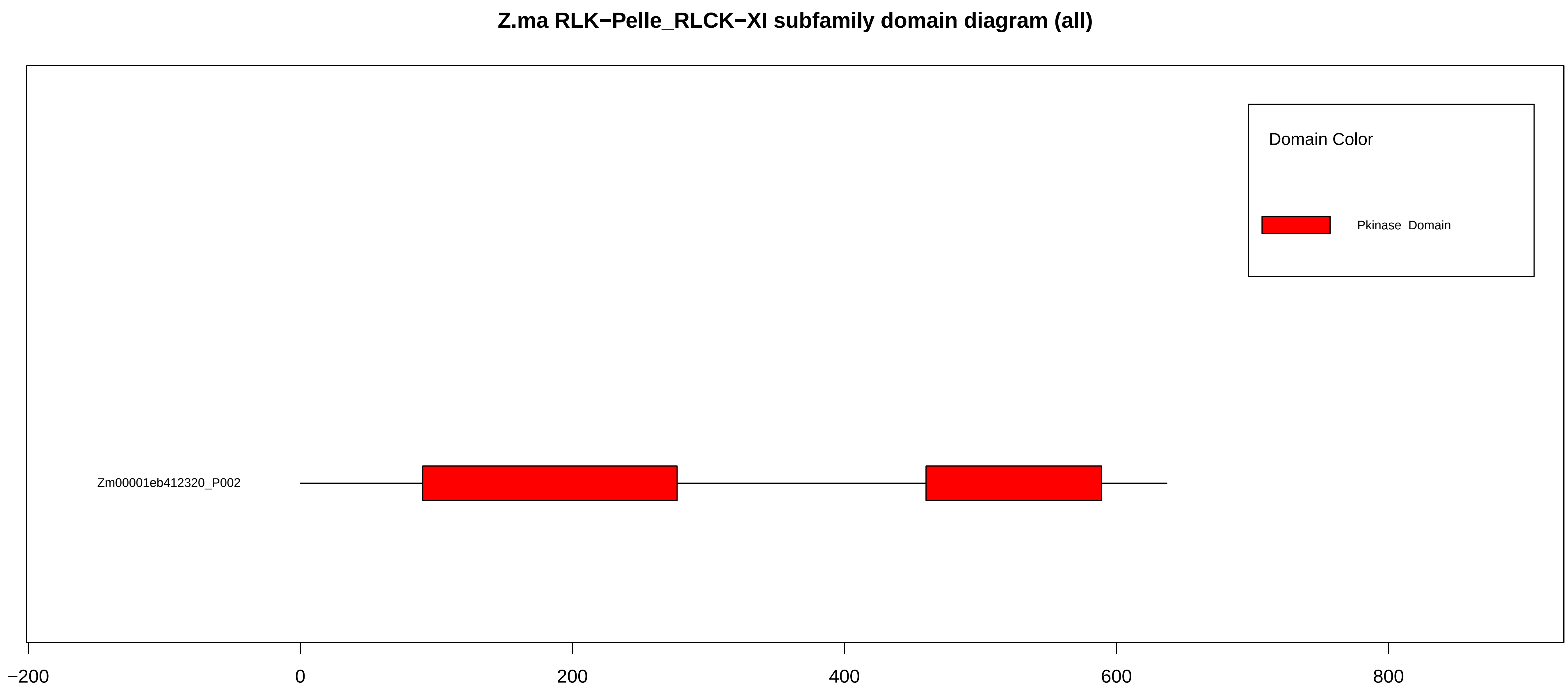

Z.ma RLK−Pelle_RLCK−XI subfamily domain diagram (all)
Domain Color
Pkinase Domain
Zm00001eb412320_P002
−200
0
200
400
600
800

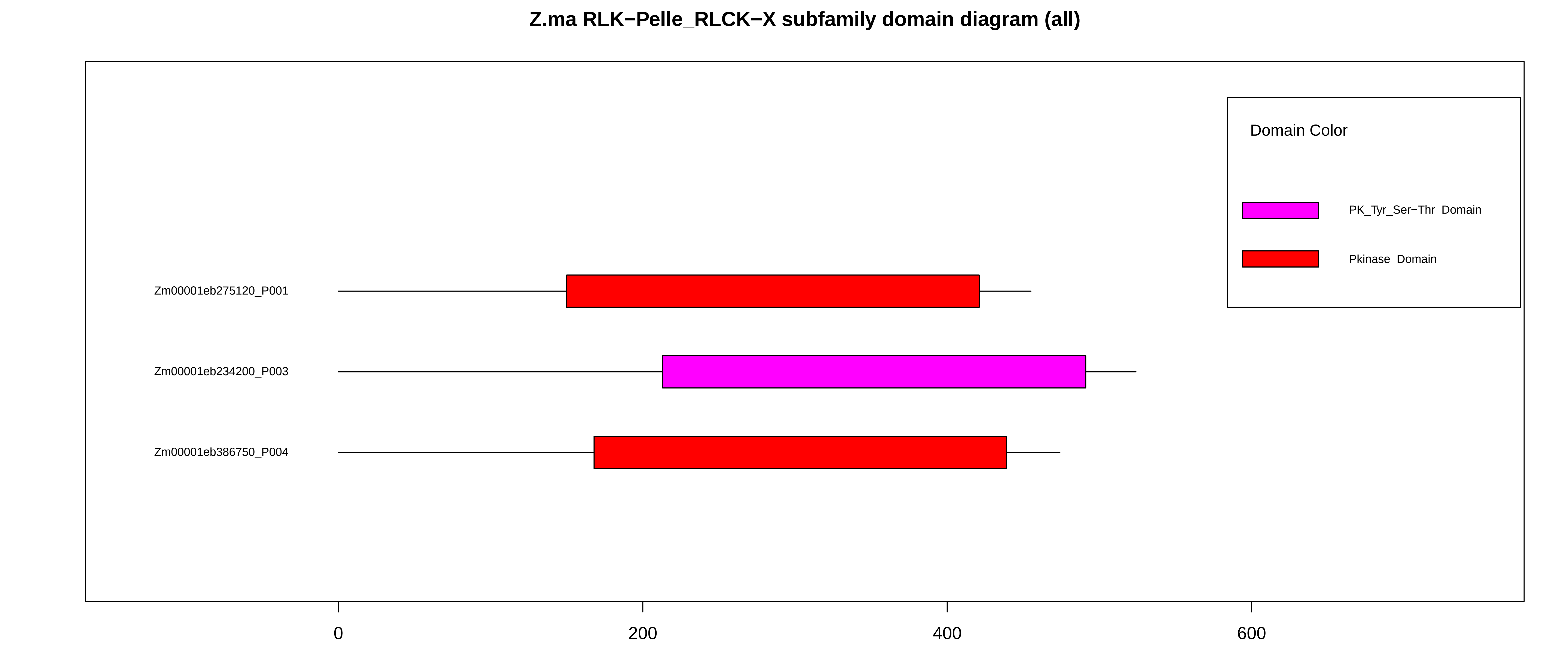
Z.ma RLK−Pelle_RLCK−X subfamily domain diagram (all)
Domain Color
PK_Tyr_Ser−Thr Domain
Pkinase Domain
Zm00001eb275120_P001
Zm00001eb234200_P003
Zm00001eb386750_P004
0
200
400
600

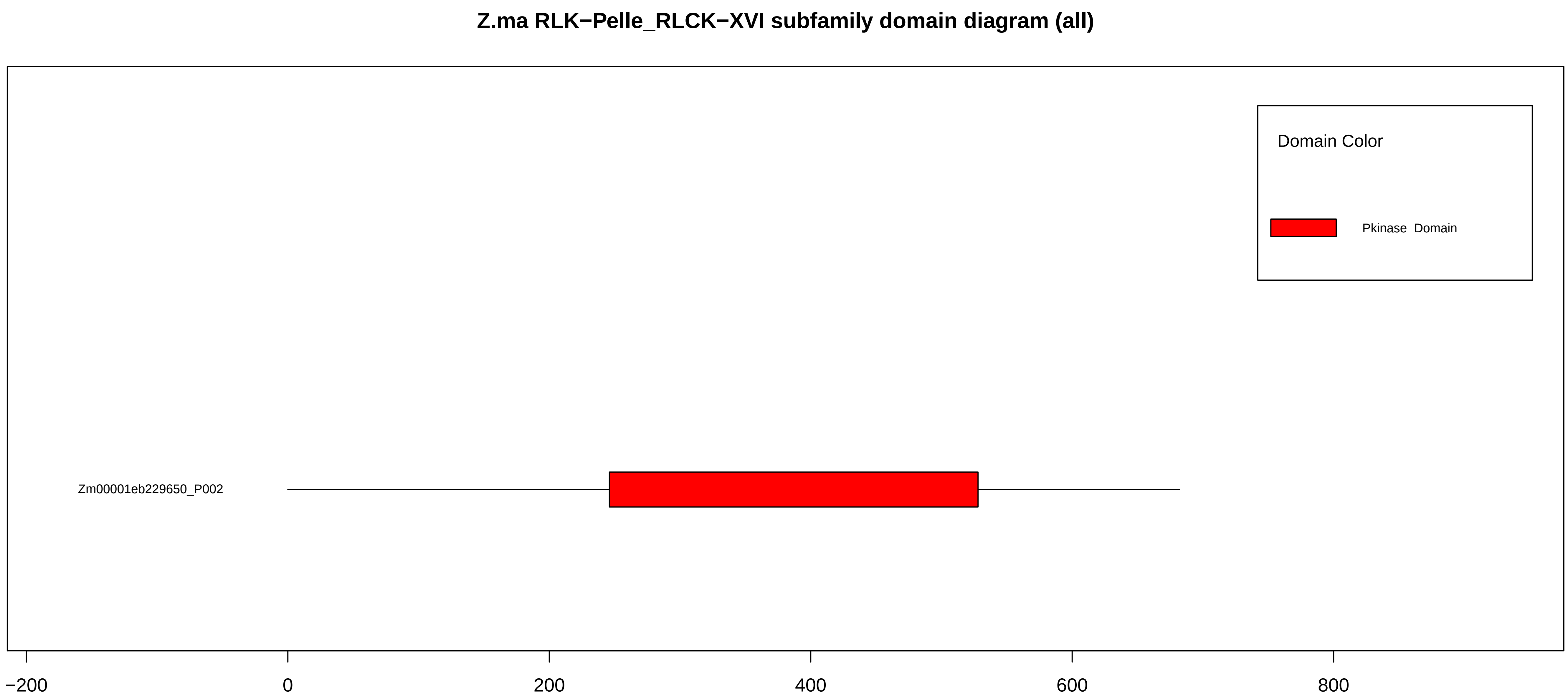
Z.ma RLK−Pelle_RLCK−XVI subfamily domain diagram (all)
Domain Color
Pkinase Domain
Zm00001eb229650_P002
−200
0
200
400
600
800

Z.ma RLK−Pelle_RLCK−XV subfamily domain diagram (all)

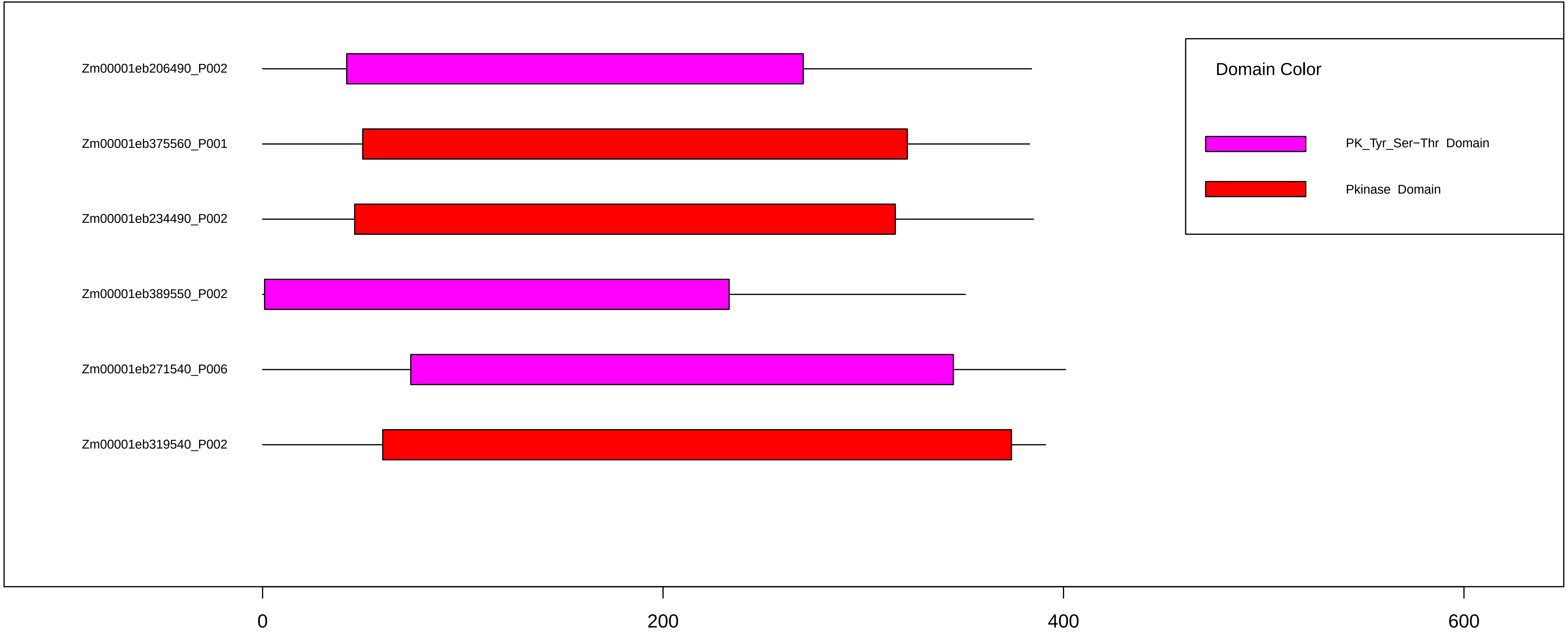

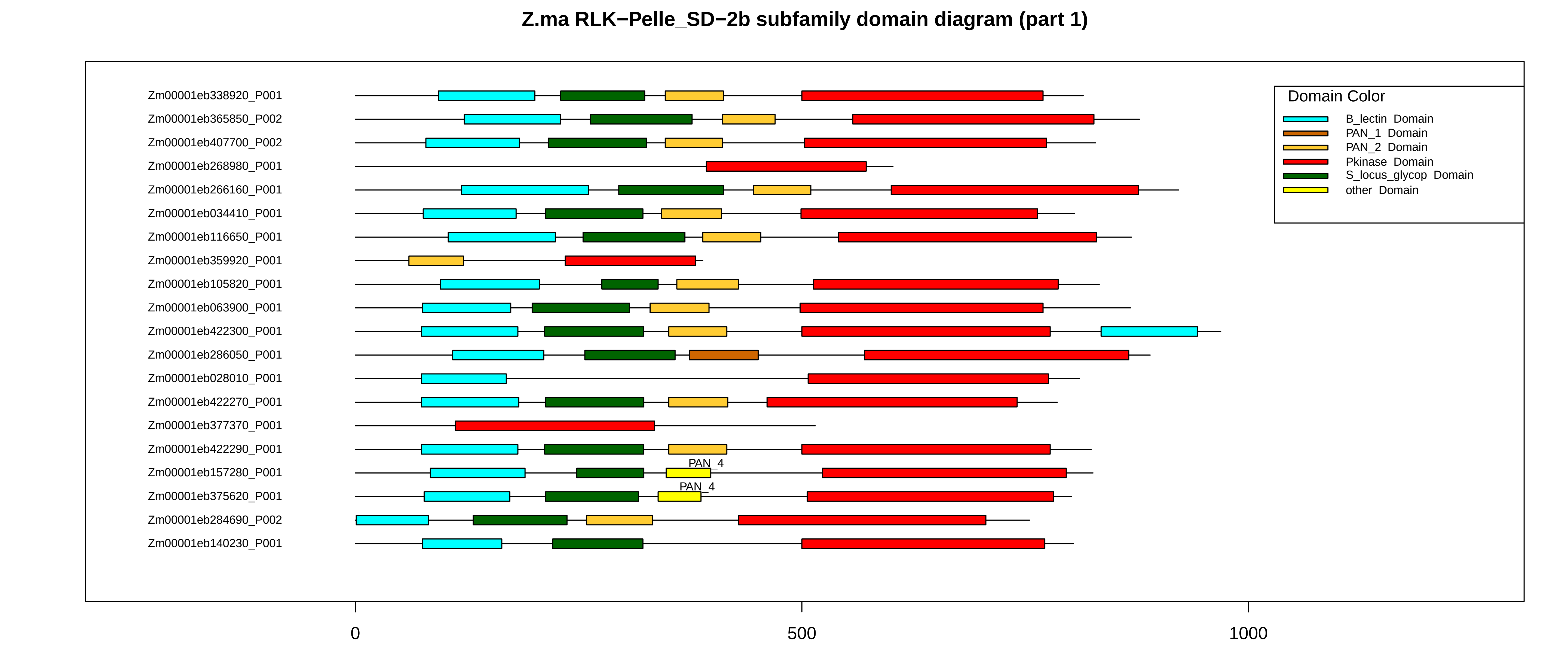
Z.ma RLK−Pelle_SD−2b subfamily domain diagram (part 1)
Zm00001eb338920_P001
Zm00001eb365850_P002
Zm00001eb407700_P002
Zm00001eb268980_P001
Zm00001eb266160_P001
Zm00001eb034410_P001
Zm00001eb116650_P001
Zm00001eb359920_P001
Zm00001eb105820_P001
Zm00001eb063900_P001
Zm00001eb422300_P001
Zm00001eb286050_P001
Zm00001eb028010_P001
Zm00001eb422270_P001
Zm00001eb377370_P001
Zm00001eb422290_P001
Zm00001eb157280_P001
PAN_4
Zm00001eb375620_P001
PAN_4
Zm00001eb284690_P002
Zm00001eb140230_P001
Domain Color
B_lectin Domain
PAN_1 Domain
PAN_2 Domain
Pkinase Domain
S_locus_glycop Domain
other Domain
0
500
1000

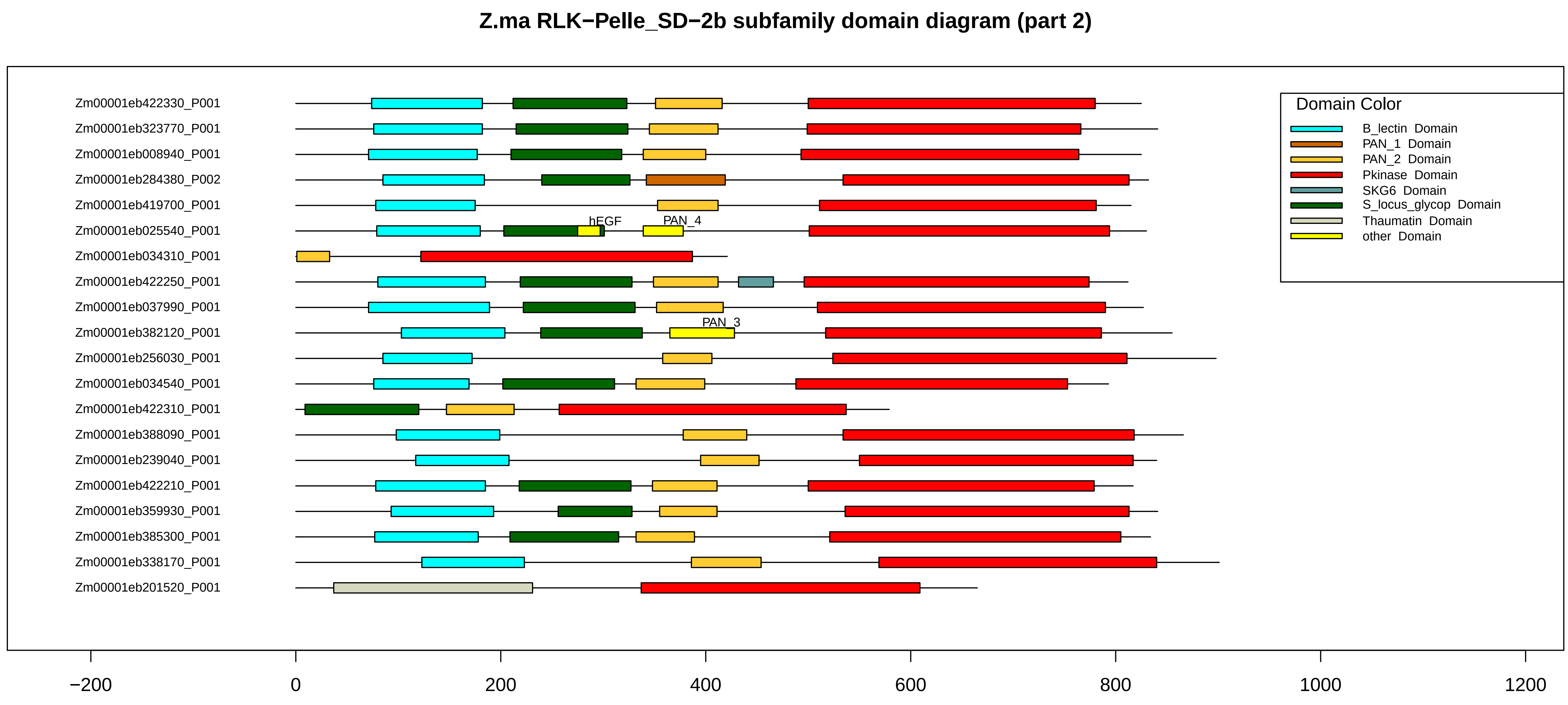

Z.ma RLK−Pelle_SD−2b subfamily domain diagram (part 2)
Zm00001eb422330_P001
Zm00001eb323770_P001
Zm00001eb008940_P001
Zm00001eb284380_P002
Zm00001eb419700_P001
Zm00001eb025540_P001
Zm00001eb034310_P001
Zm00001eb422250_P001
Zm00001eb037990_P001
Zm00001eb382120_P001
Zm00001eb256030_P001
Zm00001eb034540_P001
Zm00001eb422310_P001
Zm00001eb388090_P001
Zm00001eb239040_P001
Zm00001eb422210_P001
Zm00001eb359930_P001
Zm00001eb385300_P001
Zm00001eb338170_P001
Zm00001eb201520_P001
hEGF
PAN_4
PAN_3
Domain Color
B_lectin Domain
PAN_1 Domain
PAN_2 Domain
Pkinase Domain
SKG6 Domain
S_locus_glycop Domain
Thaumatin Domain
other Domain
−200
0
200
400
600
800
1000
1200

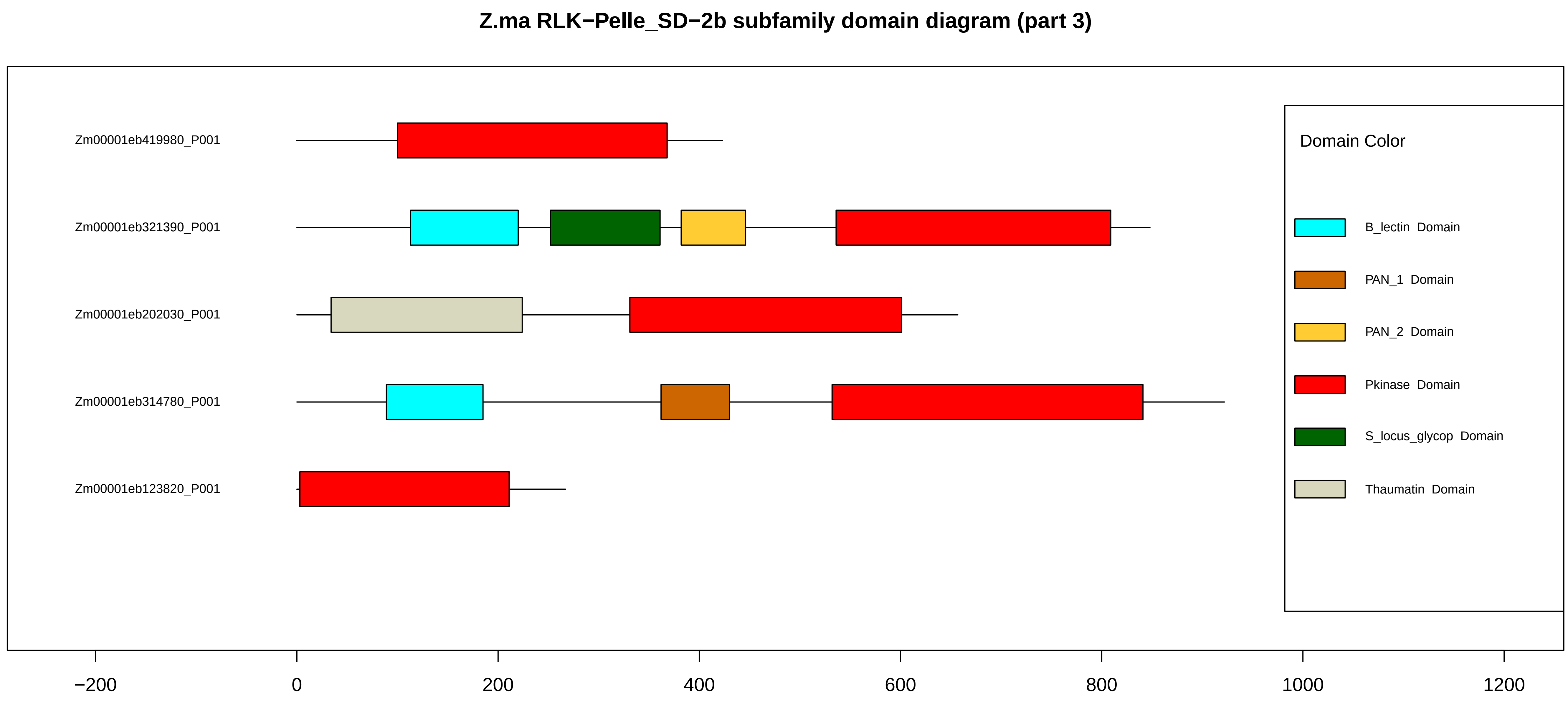
Z.ma RLK−Pelle_SD−2b subfamily domain diagram (part 3)
Zm00001eb419980_P001
Zm00001eb321390_P001
Zm00001eb202030_P001
Zm00001eb314780_P001
Zm00001eb123820_P001
Domain Color
B_lectin Domain
PAN_1 Domain
PAN_2 Domain
Pkinase Domain
S_locus_glycop Domain
Thaumatin Domain
−200
0
200
400
600
800
1000
1200

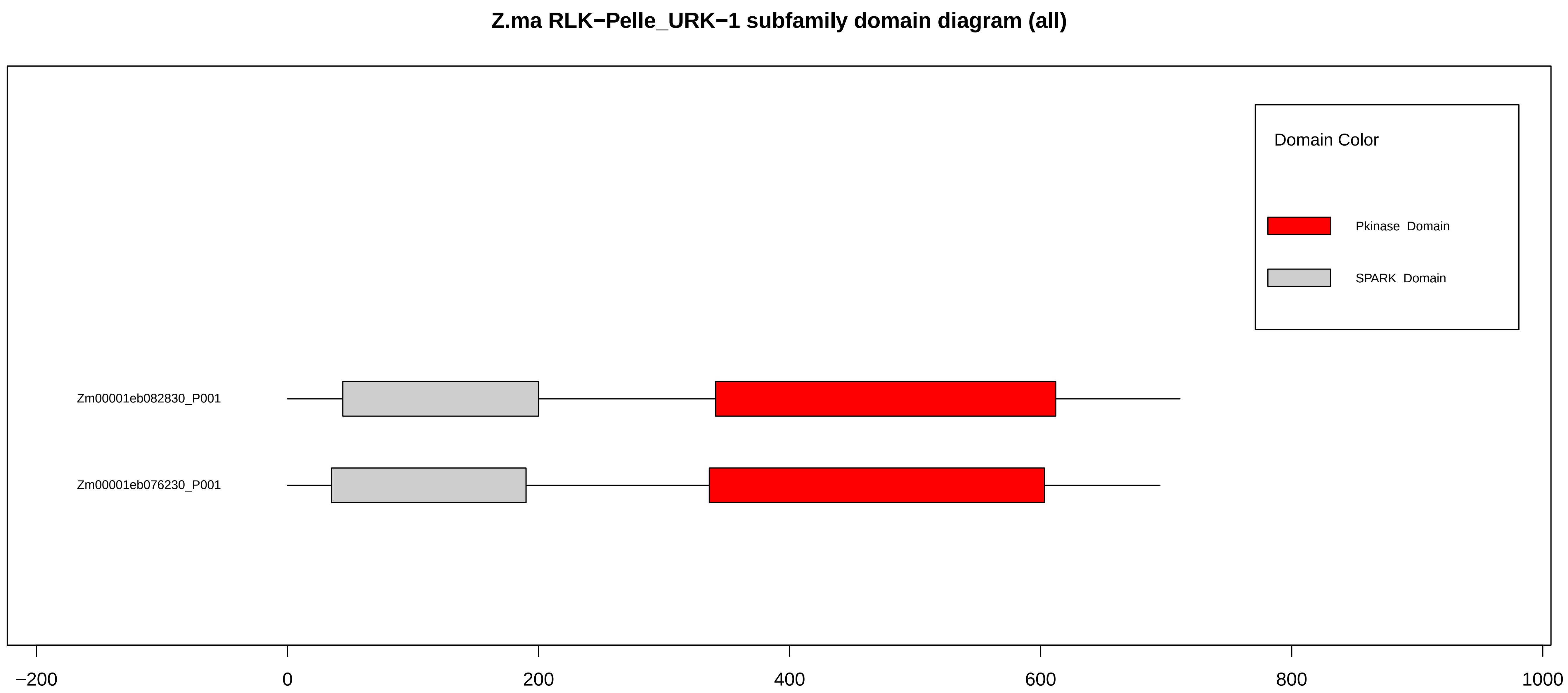
Z.ma RLK−Pelle_URK−1 subfamily domain diagram (all)
Domain Color
Pkinase Domain
SPARK Domain
Zm00001eb082830_P001
Zm00001eb076230_P001
−200
0
200
400
600
800
1000

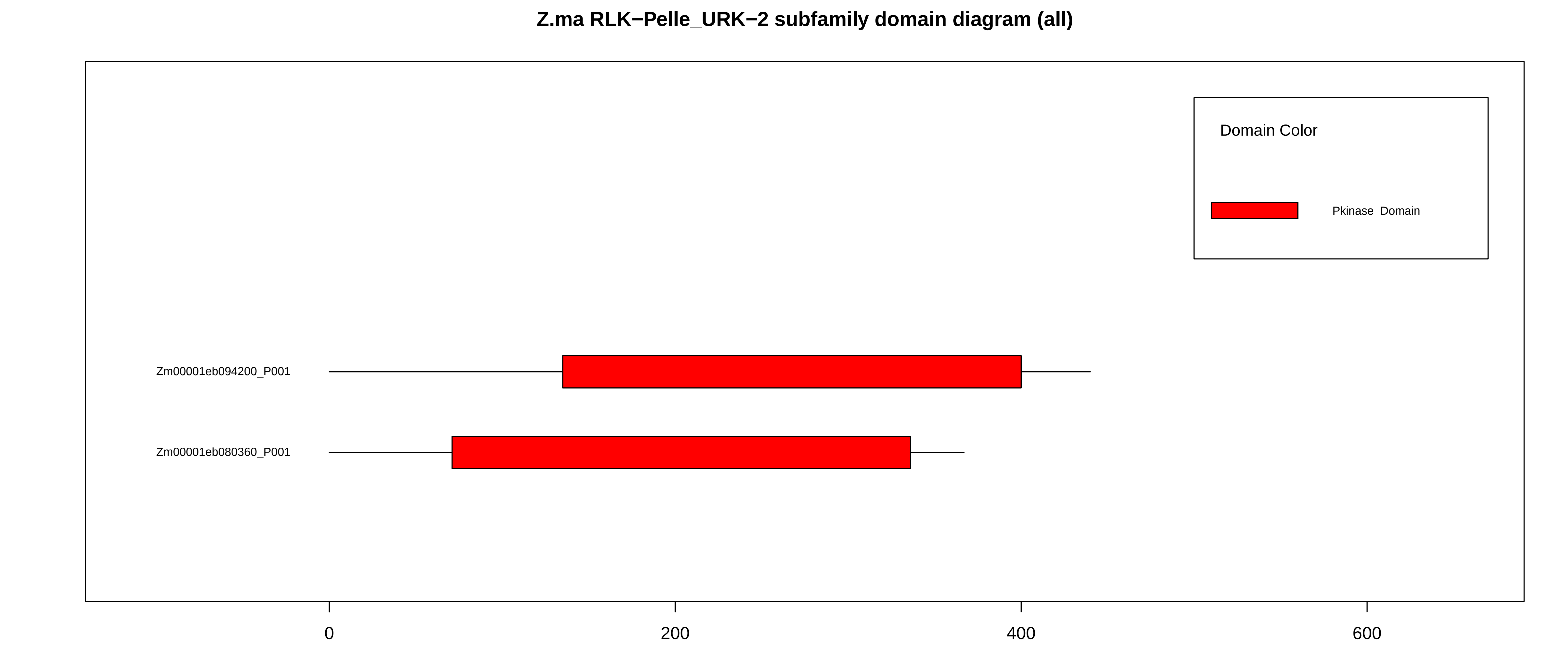
Z.ma RLK−Pelle_URK−2 subfamily domain diagram (all)
Domain Color
Pkinase Domain
Zm00001eb094200_P001
Zm00001eb080360_P001
0
200
400
600

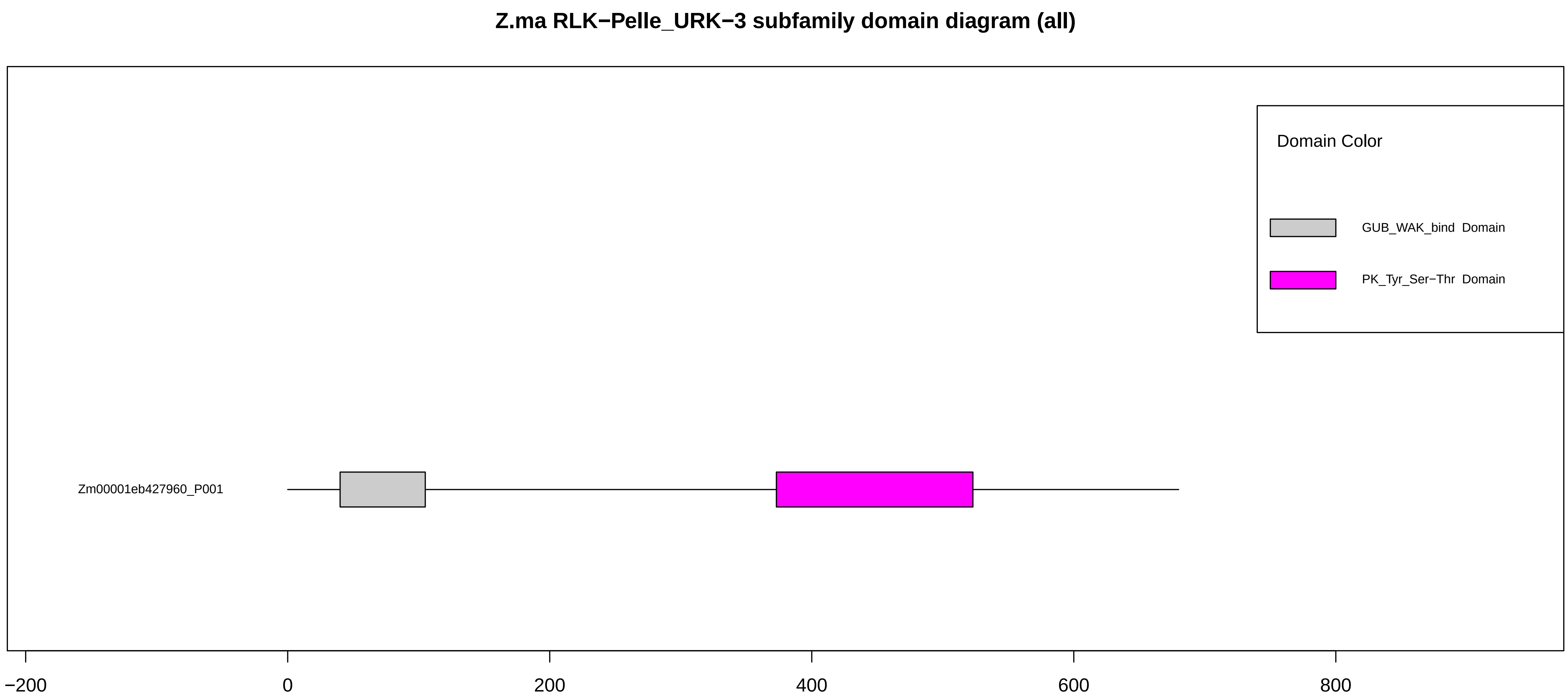

Z.ma RLK−Pelle_URK−3 subfamily domain diagram (all)
Domain Color
GUB_WAK_bind Domain
PK_Tyr_Ser−Thr Domain
Zm00001eb427960_P001
−200
0
200
400
600
800

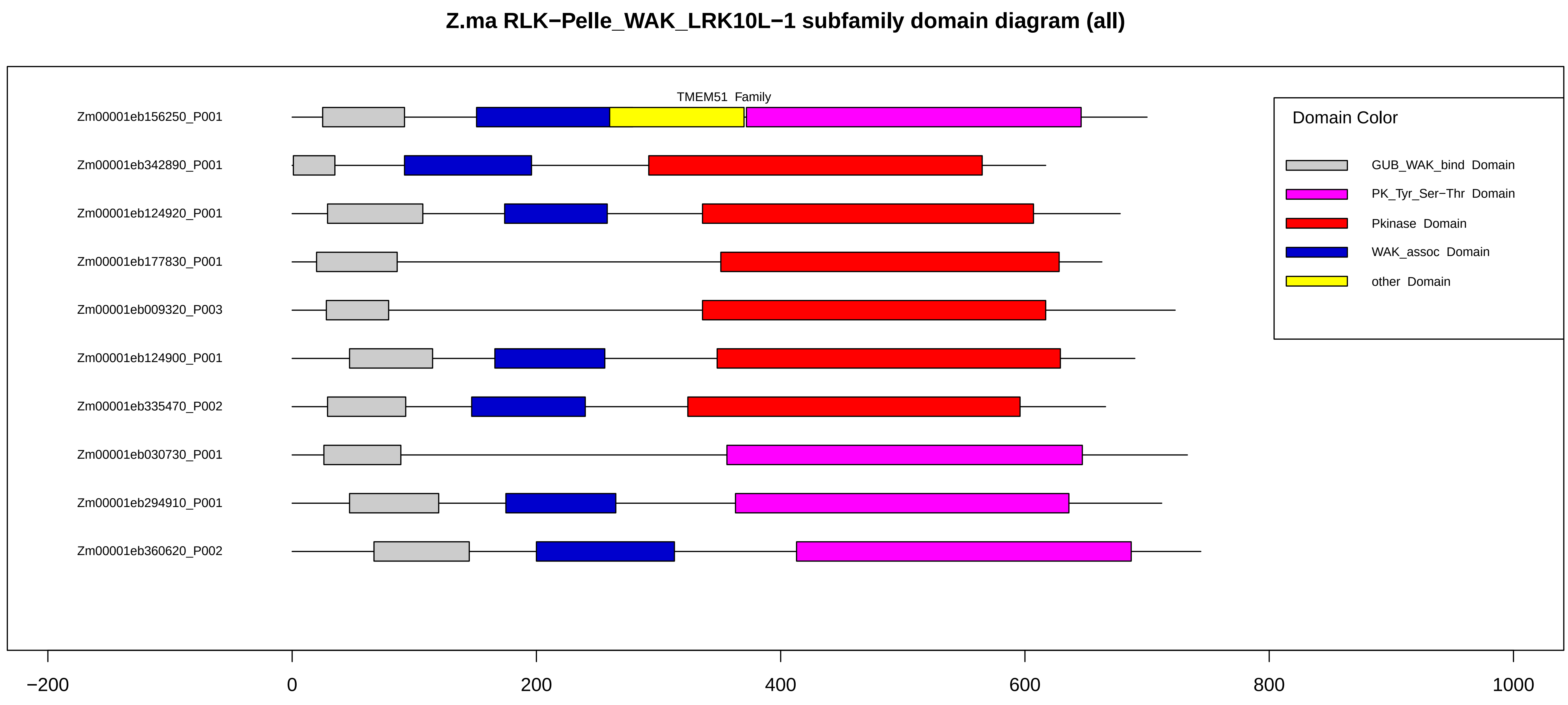
Z.ma RLK−Pelle_WAK_LRK10L−1 subfamily domain diagram (all)
TMEM51 Family
Zm00001eb156250_P001
Zm00001eb342890_P001
Zm00001eb124920_P001
Zm00001eb177830_P001
Zm00001eb009320_P003
Zm00001eb124900_P001
Zm00001eb335470_P002
Zm00001eb030730_P001
Zm00001eb294910_P001
Zm00001eb360620_P002
Domain Color
GUB_WAK_bind Domain
PK_Tyr_Ser−Thr Domain
Pkinase Domain
WAK_assoc Domain
other Domain
−200
0
200
400
600
800
1000

Z.ma RLK−Pelle_WAK subfamily domain diagram (part 1)

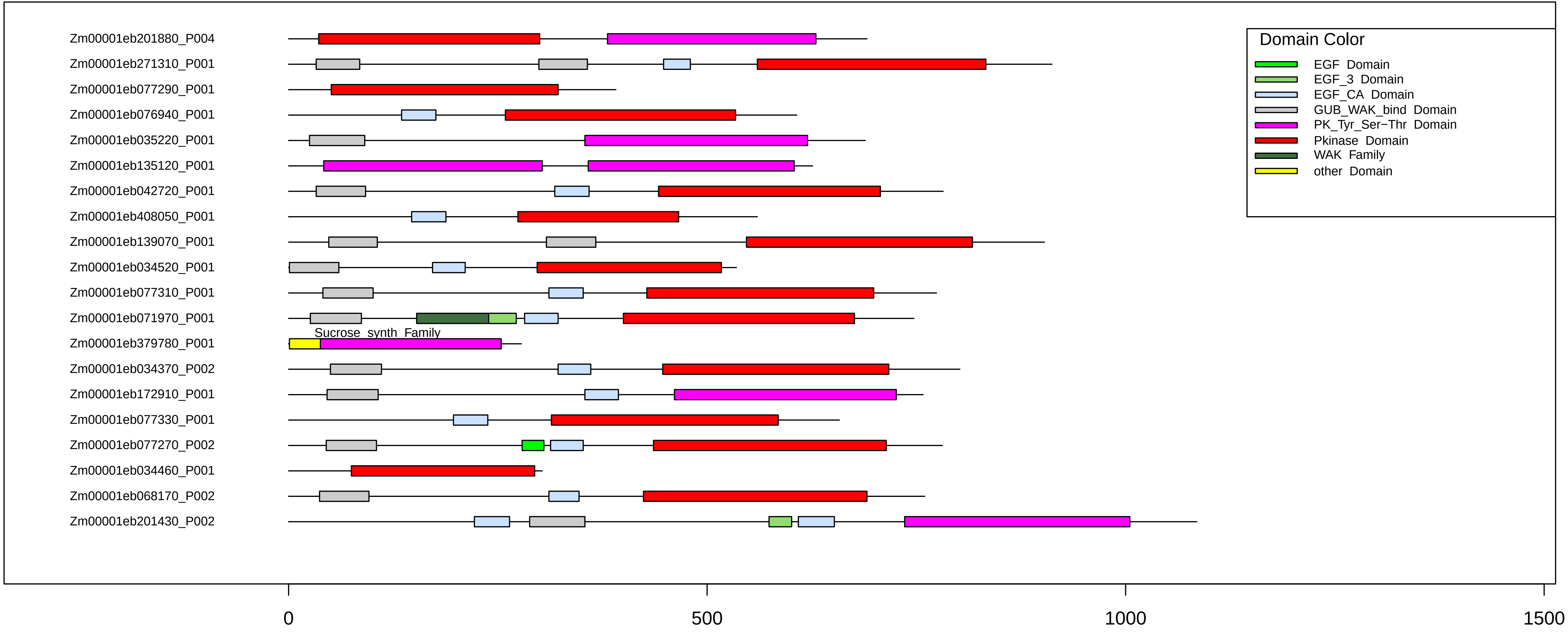

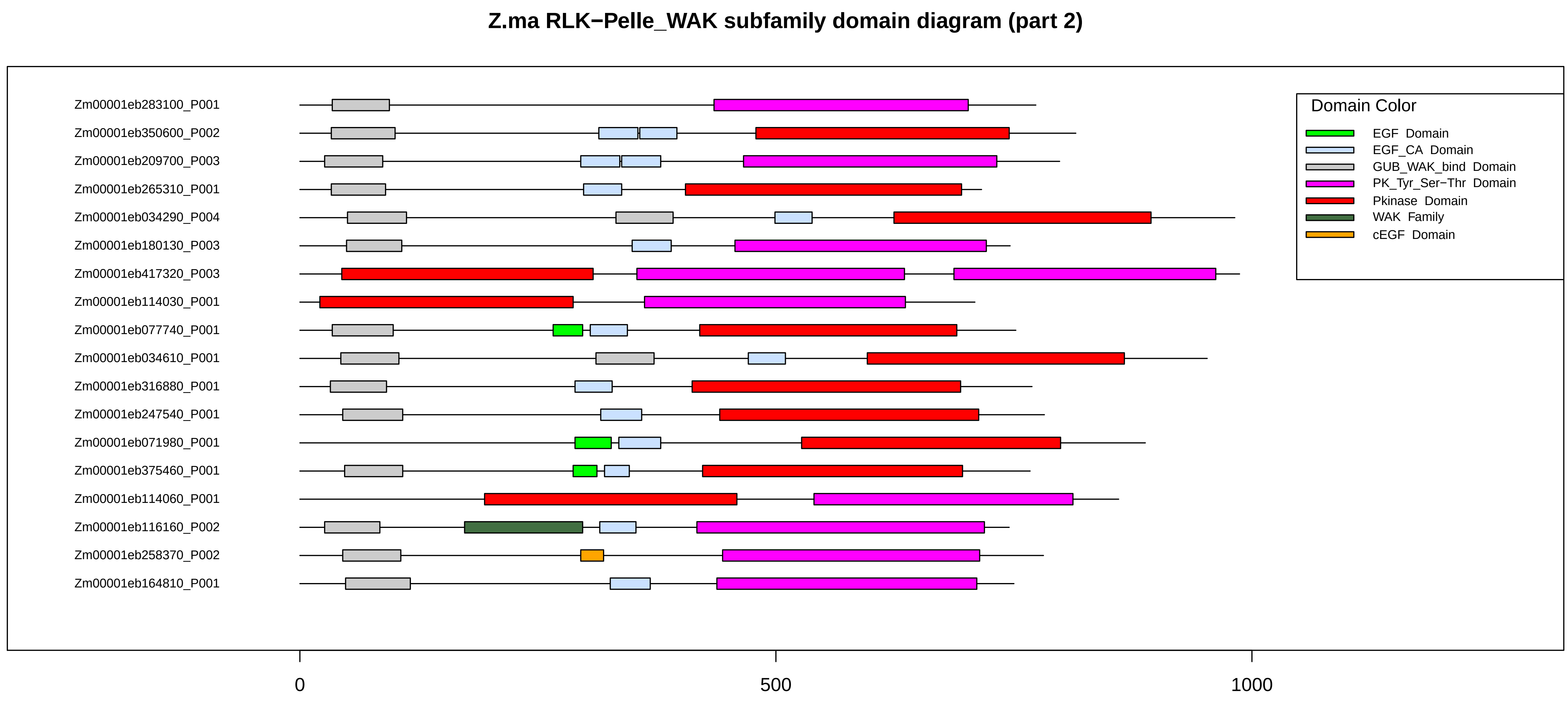
Z.ma RLK−Pelle_WAK subfamily domain diagram (part 2)
Zm00001eb283100_P001
Zm00001eb350600_P002
Zm00001eb209700_P003
Zm00001eb265310_P001
Zm00001eb034290_P004
Zm00001eb180130_P003
Zm00001eb417320_P003
Zm00001eb114030_P001
Zm00001eb077740_P001
Zm00001eb034610_P001
Zm00001eb316880_P001
Zm00001eb247540_P001
Zm00001eb071980_P001
Zm00001eb375460_P001
Zm00001eb114060_P001
Zm00001eb116160_P002
Zm00001eb258370_P002
Zm00001eb164810_P001
Domain Color
EGF Domain
EGF_CA Domain
GUB_WAK_bind Domain
PK_Tyr_Ser−Thr Domain
Pkinase Domain
WAK Family
cEGF Domain
0
500
1000

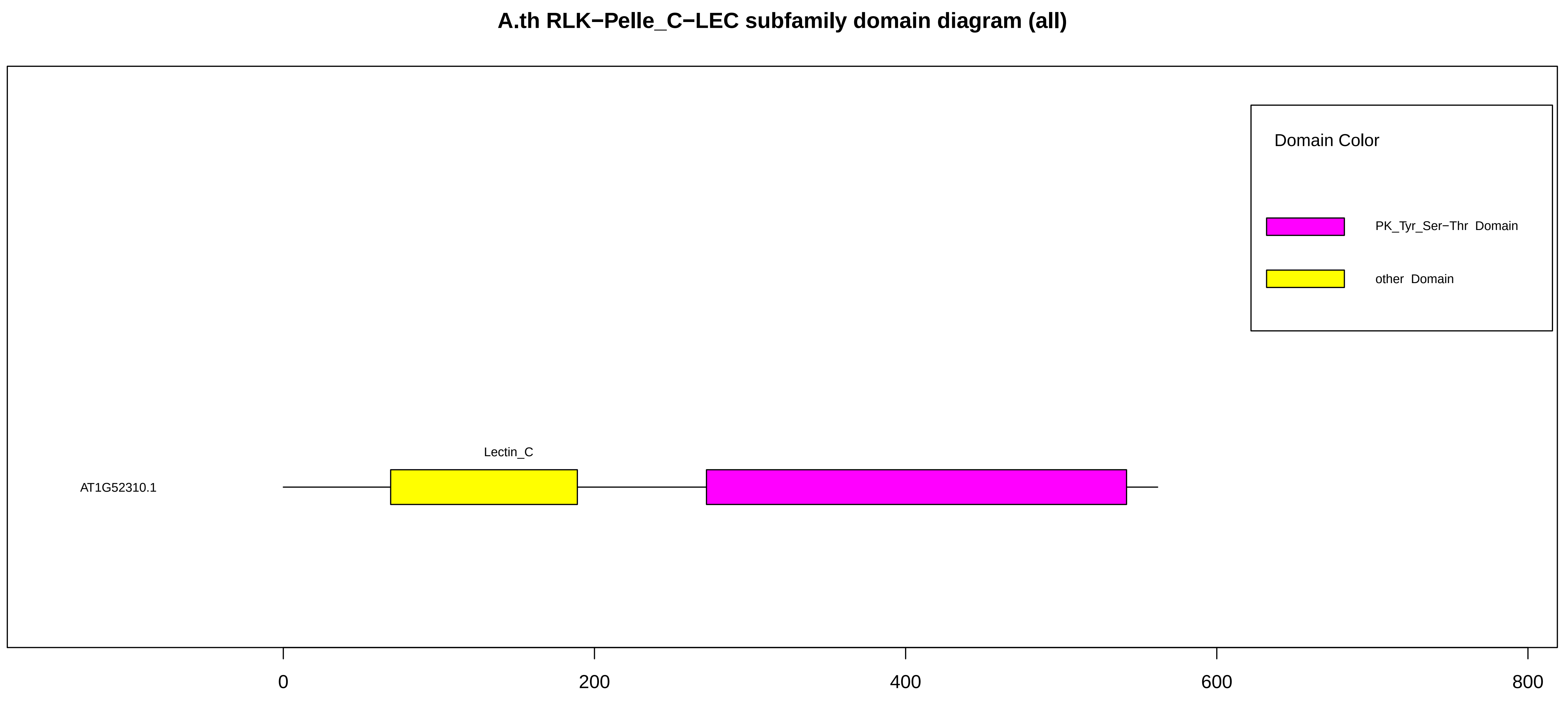
A.th RLK−Pelle_C−LEC subfamily domain diagram (all)
Domain Color
PK_Tyr_Ser−Thr Domain
other Domain
Lectin_C
AT1G52310.1
0
200
400
600
800

A.th RLK−Pelle_CR4L subfamily domain diagram (all)

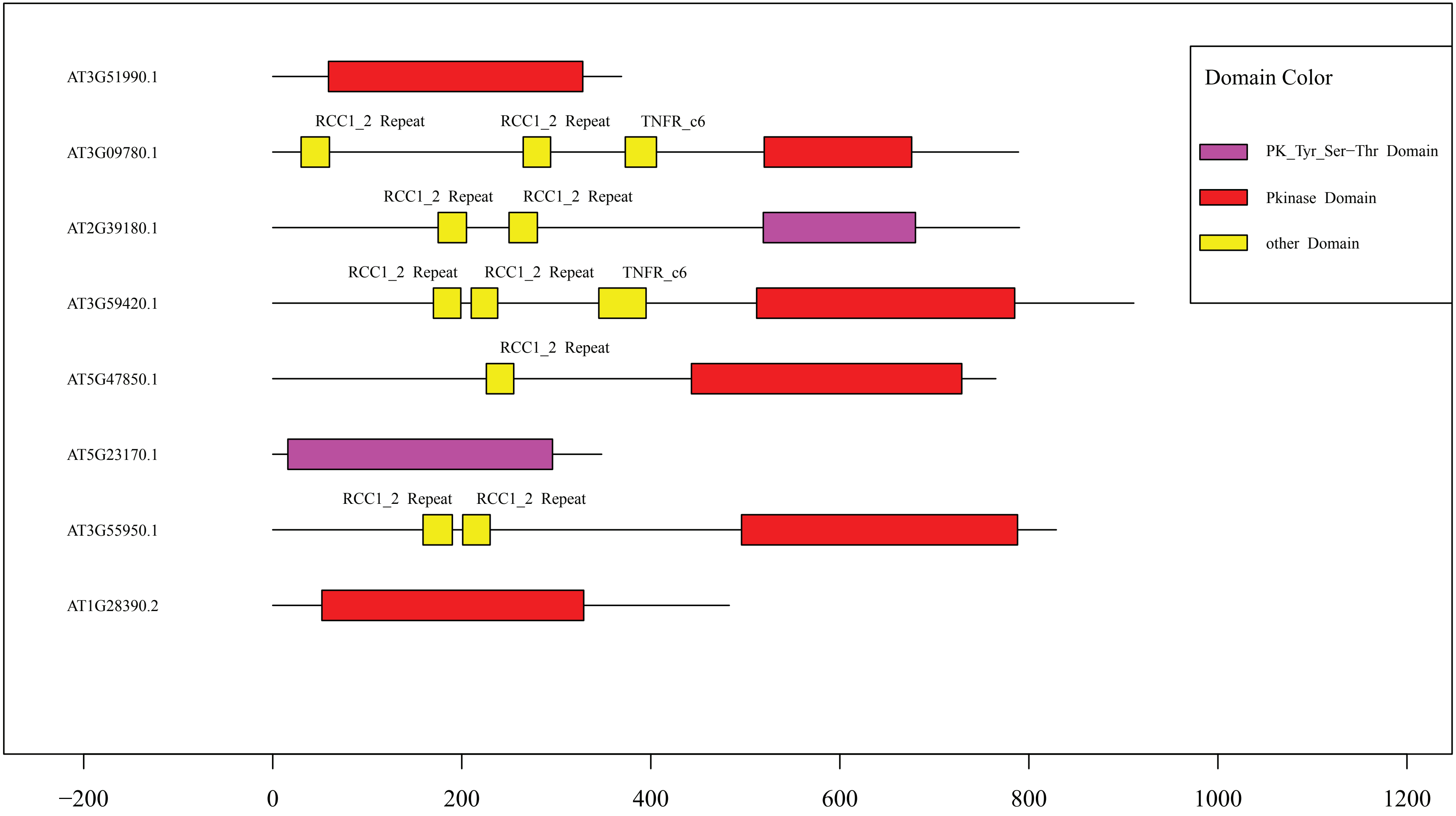

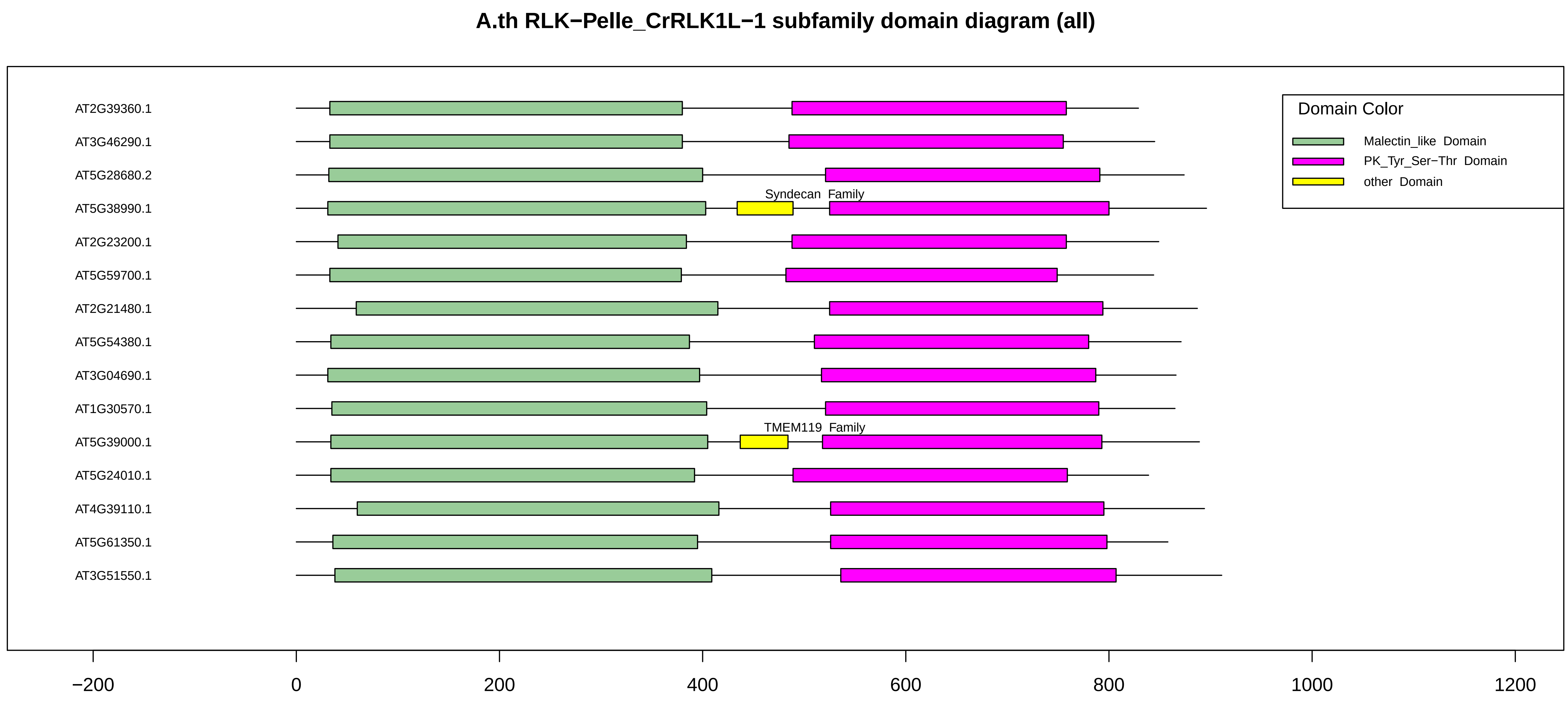

A.th RLK−Pelle_CrRLK1L−1 subfamily domain diagram (all)
AT2G39360.1
AT3G46290.1
AT5G28680.2
AT5G38990.1
AT2G23200.1
AT5G59700.1
AT2G21480.1
AT5G54380.1
AT3G04690.1
AT1G30570.1
AT5G39000.1
AT5G24010.1
AT4G39110.1
AT5G61350.1
AT3G51550.1
Syndecan Family
TMEM119 Family
Domain Color
Malectin_like Domain
PK_Tyr_Ser−Thr Domain
other Domain
−200
0
200
400
600
800
1000
1200

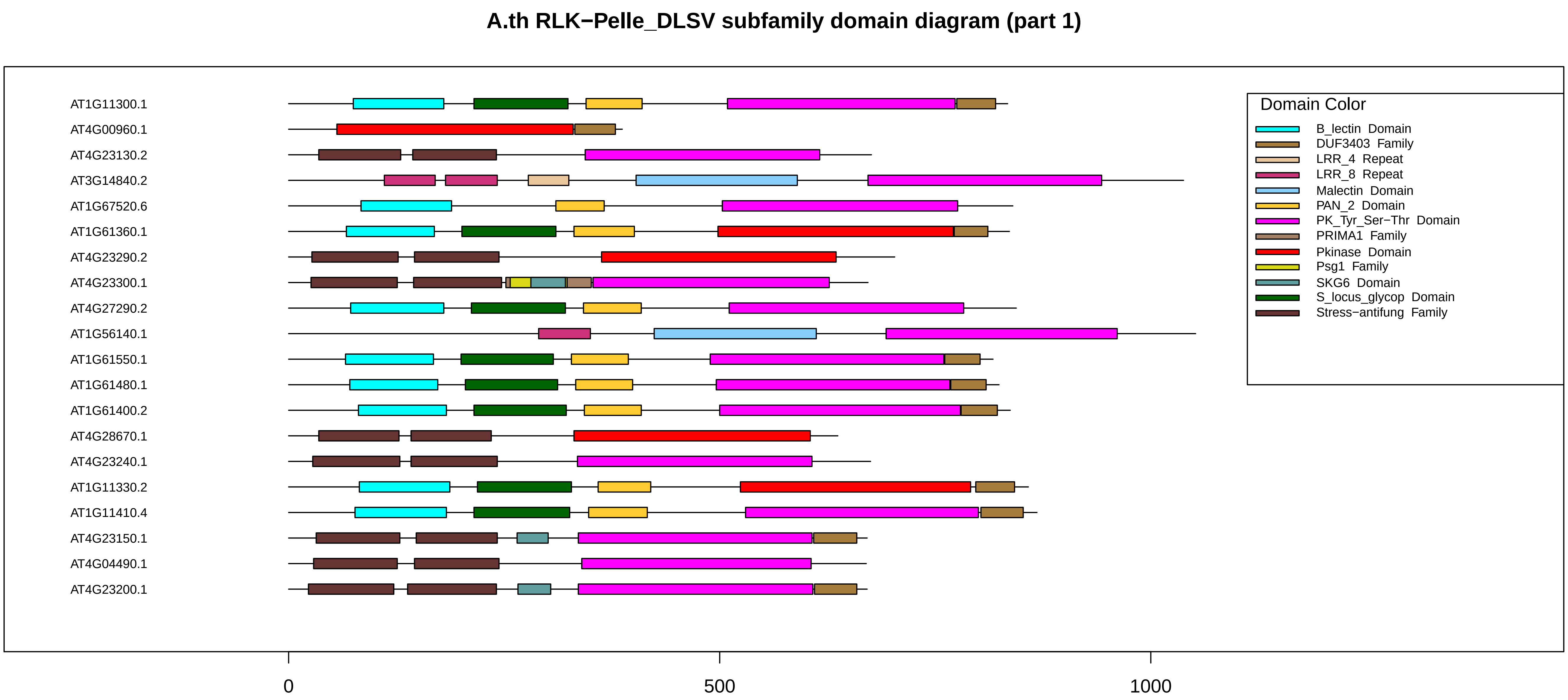
A.th RLK−Pelle_DLSV subfamily domain diagram (part 1)
AT1G11300.1
AT4G00960.1
AT4G23130.2
AT3G14840.2
AT1G67520.6
AT1G61360.1
AT4G23290.2
AT4G23300.1
AT4G27290.2
AT1G56140.1
AT1G61550.1
AT1G61480.1
AT1G61400.2
AT4G28670.1
AT4G23240.1
AT1G11330.2
AT1G11410.4
AT4G23150.1
AT4G04490.1
AT4G23200.1
Domain Color
B_lectin Domain
DUF3403 Family
LRR_4 Repeat
LRR_8 Repeat
Malectin Domain
PAN_2 Domain
PK_Tyr_Ser−Thr Domain
PRIMA1 Family
Pkinase Domain
Psg1 Family
SKG6 Domain
S_locus_glycop Domain
Stress−antifung Family
0
500
1000

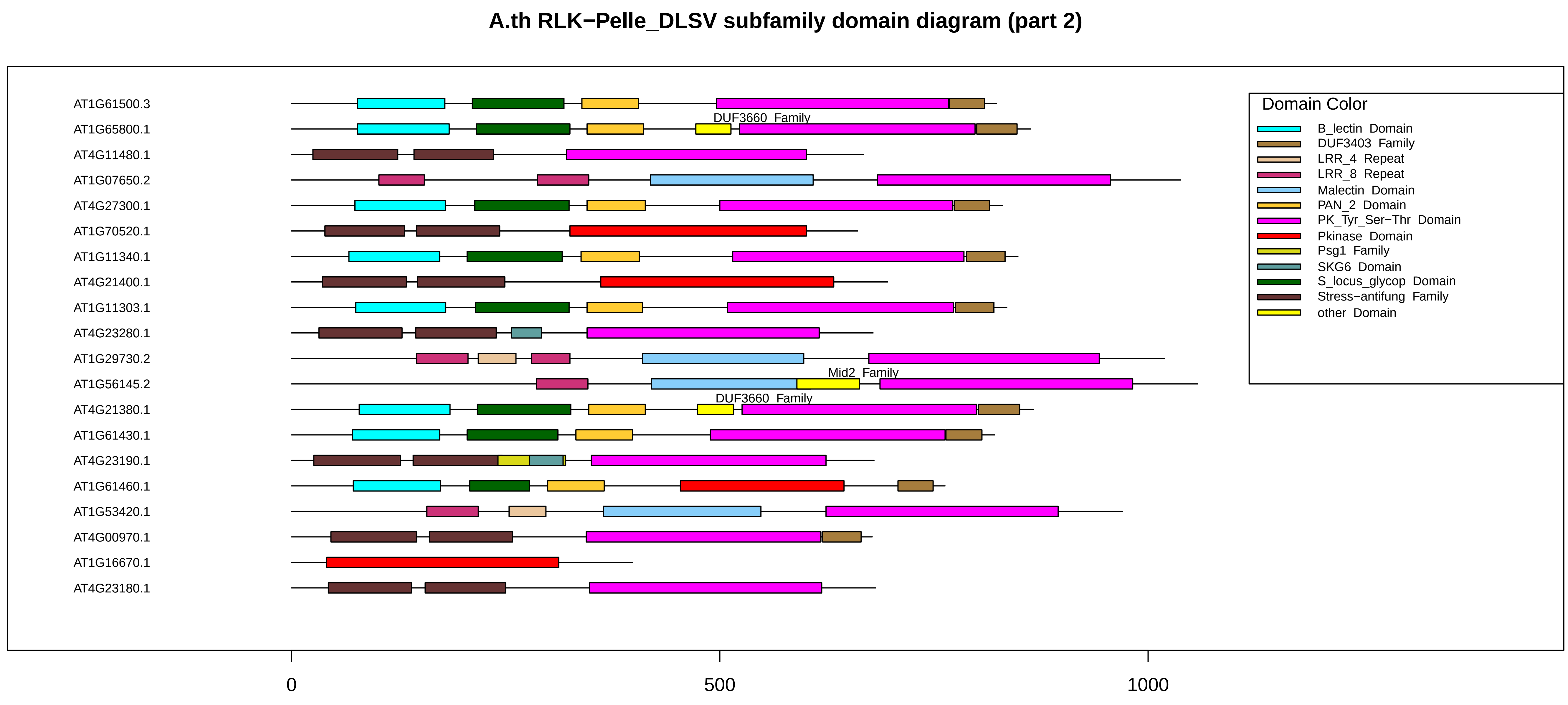
A.th RLK−Pelle_DLSV subfamily domain diagram (part 2)
AT1G61500.3
AT1G65800.1
AT4G11480.1
AT1G07650.2
AT4G27300.1
AT1G70520.1
AT1G11340.1
AT4G21400.1
AT1G11303.1
AT4G23280.1
AT1G29730.2
AT1G56145.2
AT4G21380.1
AT1G61430.1
AT4G23190.1
AT1G61460.1
AT1G53420.1
AT4G00970.1
AT1G16670.1
AT4G23180.1
DUF3660_Family
Mid2_Family
DUF3660_Family
0
500
1000
Domain Color
B_lectin Domain
DUF3403 Family
LRR_4 Repeat
LRR_8 Repeat
Malectin Domain
PAN_2 Domain
PK_Tyr_Ser−Thr Domain
Pkinase Domain
Psg1 Family
SKG6 Domain
S_locus_glycop Domain
Stress−antifung Family
other Domain

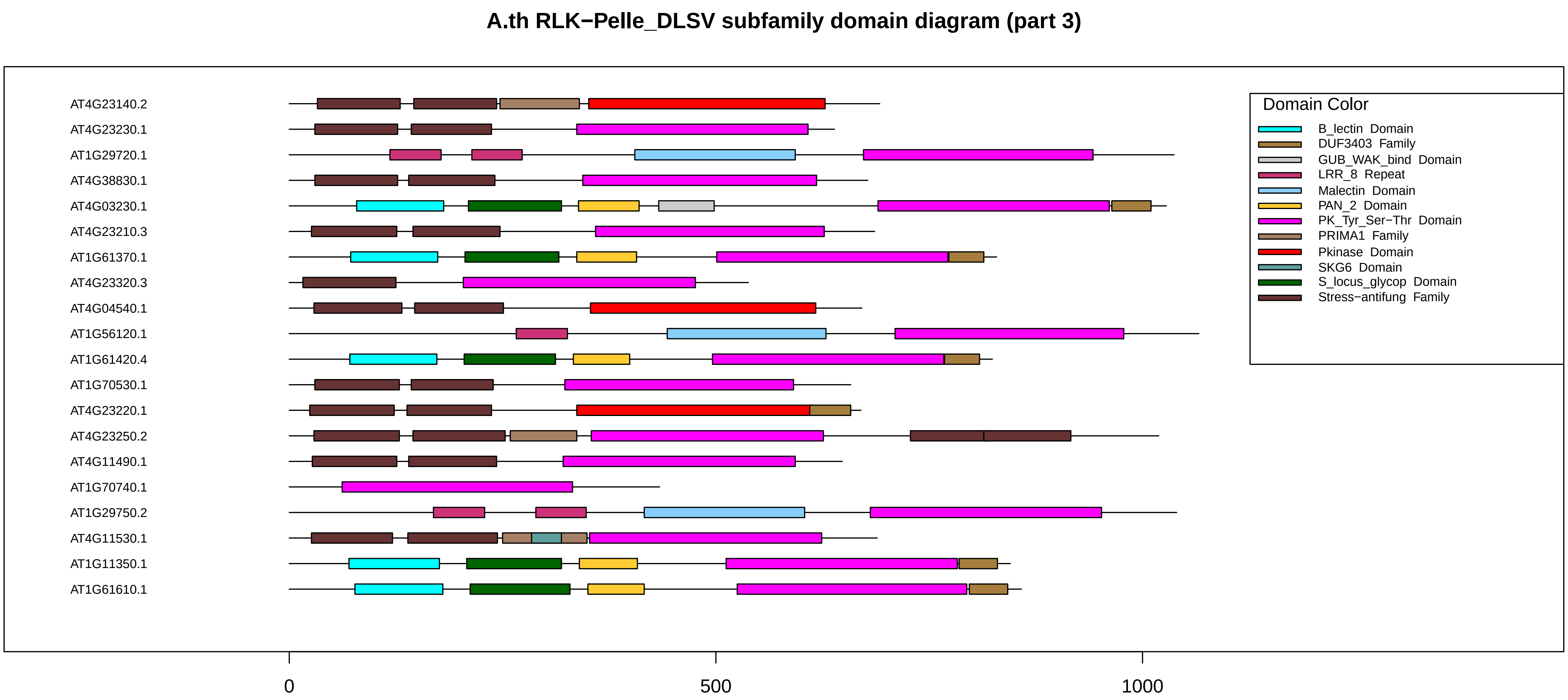

A.th RLK−Pelle_DLSV subfamily domain diagram (part 3)
AT4G23140.2
AT4G23230.1
AT1G29720.1
AT4G38830.1
AT4G03230.1
AT4G23210.3
AT1G61370.1
AT4G23320.3
AT4G04540.1
AT1G56120.1
AT1G61420.4
AT1G70530.1
AT4G23220.1
AT4G23250.2
AT4G11490.1
AT1G70740.1
AT1G29750.2
AT4G11530.1
AT1G11350.1
AT1G61610.1
Domain Color
B_lectin Domain
DUF3403 Family
GUB_WAK_bind Domain
LRR_8 Repeat
Malectin Domain
PAN_2 Domain
PK_Tyr_Ser−Thr Domain
PRIMA1 Family
Pkinase Domain
SKG6 Domain
S_locus_glycop Domain
Stress−antifung Family
0
500
1000

A.th RLK−Pelle_DLSV subfamily domain diagram (part 4)

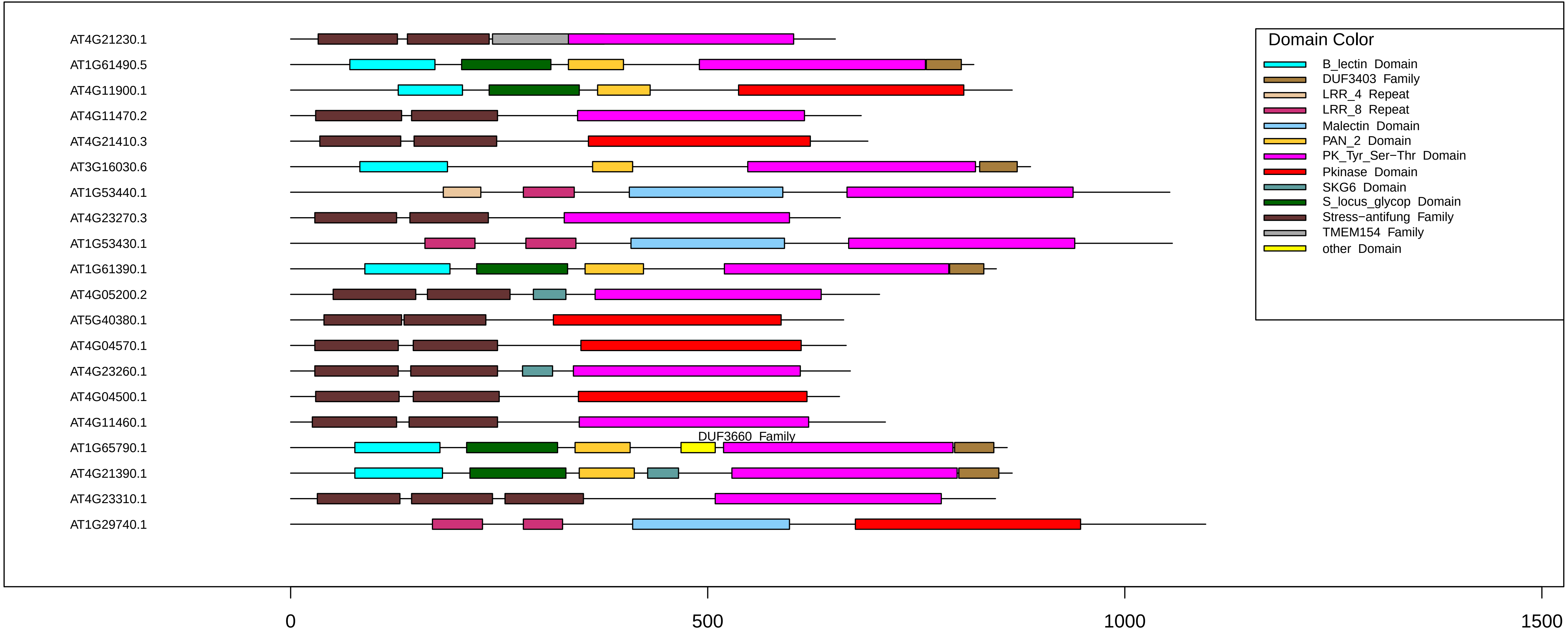

A.th RLK−Pelle_DLSV subfamily domain diagram (part 5)

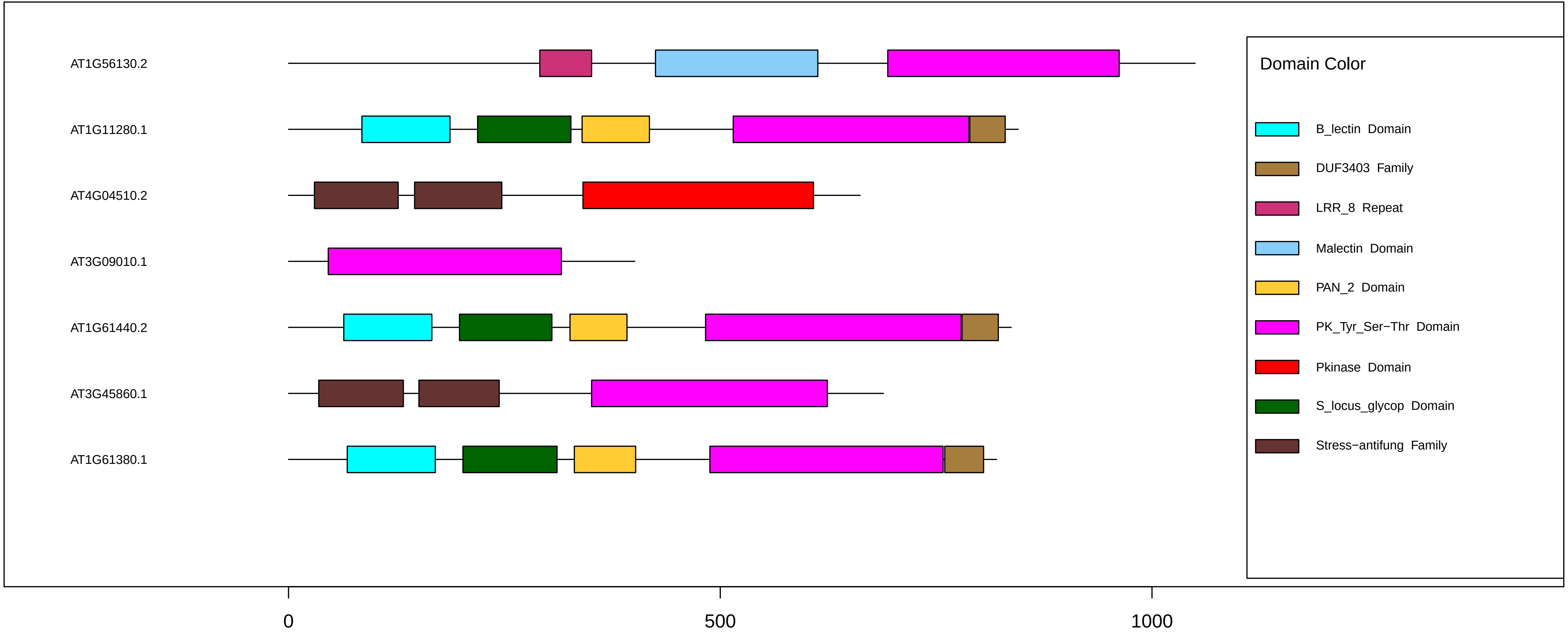

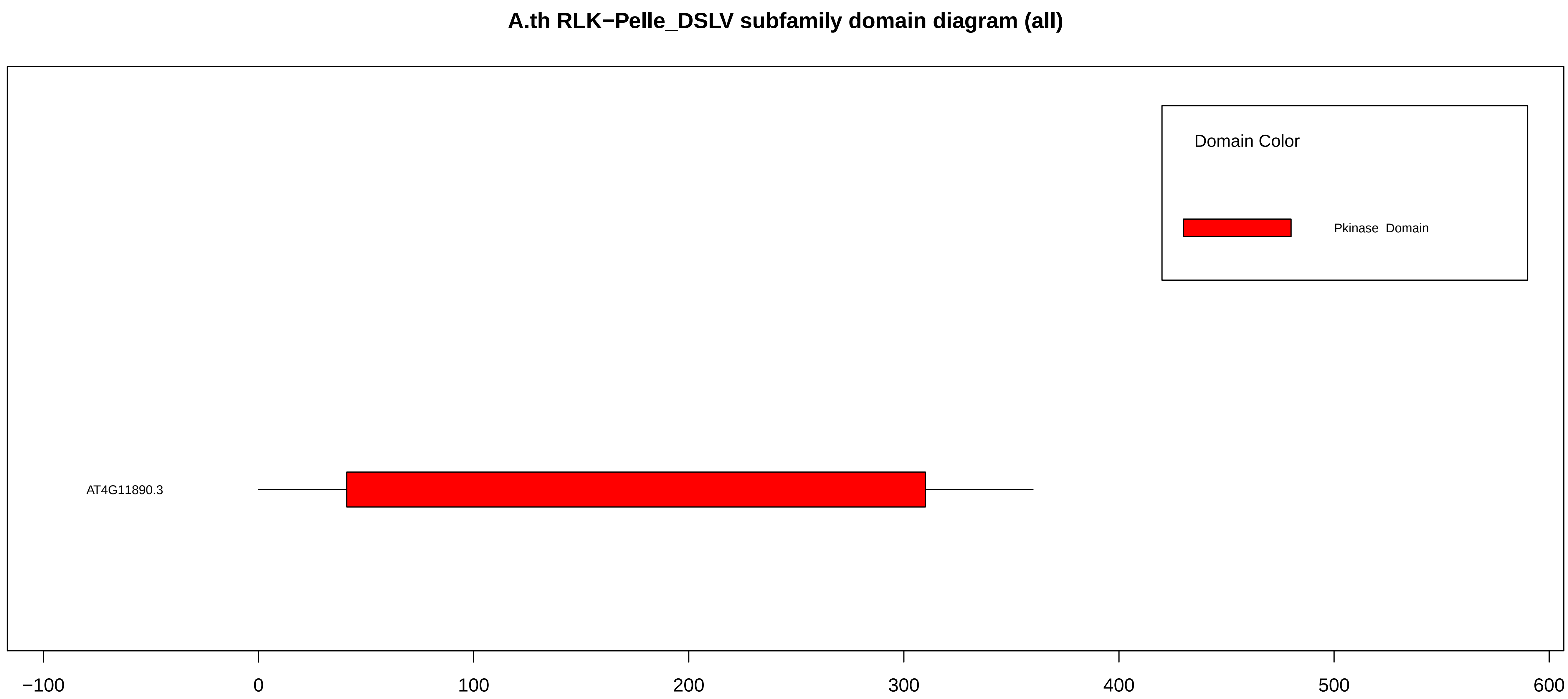

A.th RLK−Pelle_DSLV subfamily domain diagram (all)
Domain Color
Pkinase Domain
AT4G11890.3
−100
0
100
200
300
400
500
600

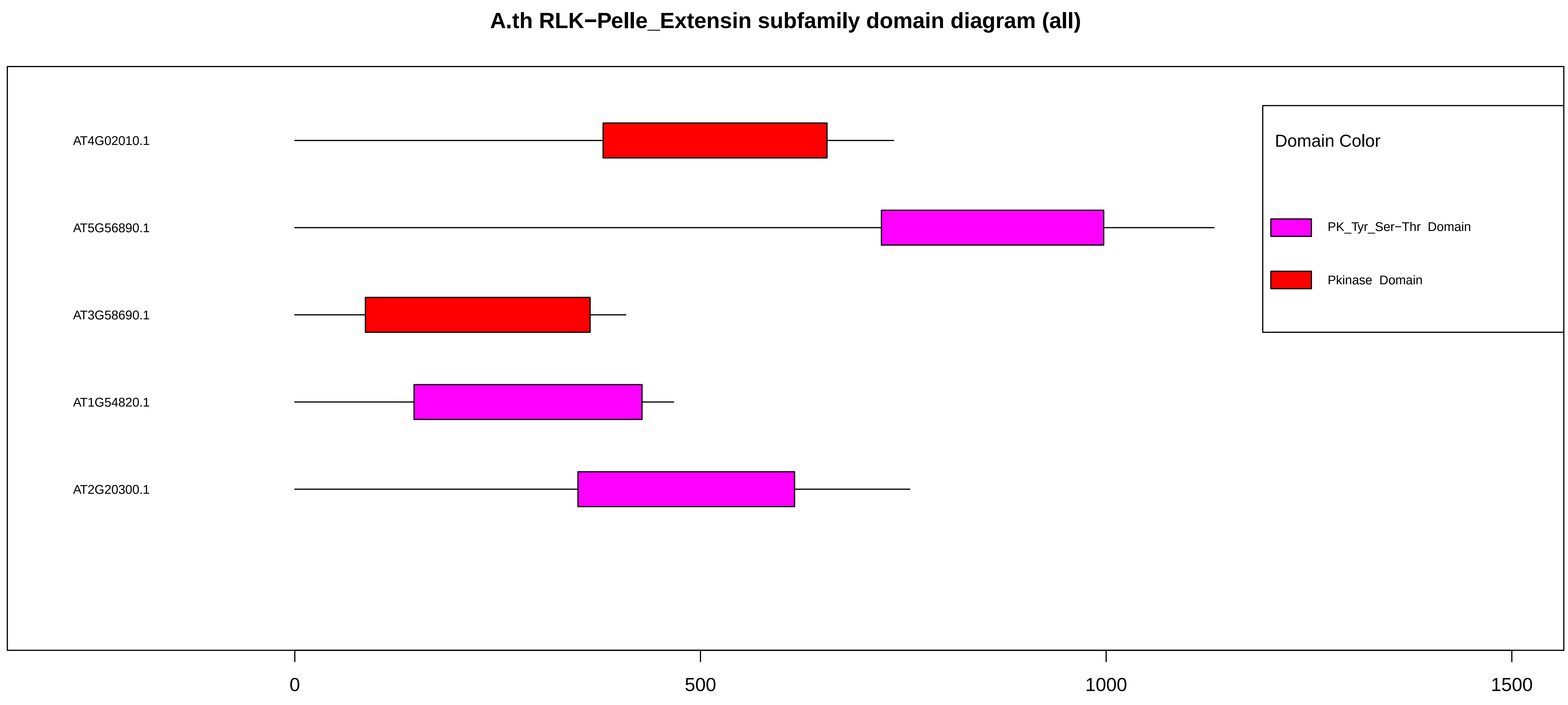

A.th RLK−Pelle_Extensin subfamily domain diagram (all)
AT4G02010.1
AT5G56890.1
AT3G58690.1
AT1G54820.1
AT2G20300.1
Domain Color
PK_Tyr_Ser−Thr Domain
Pkinase Domain
0
500
1000
1500

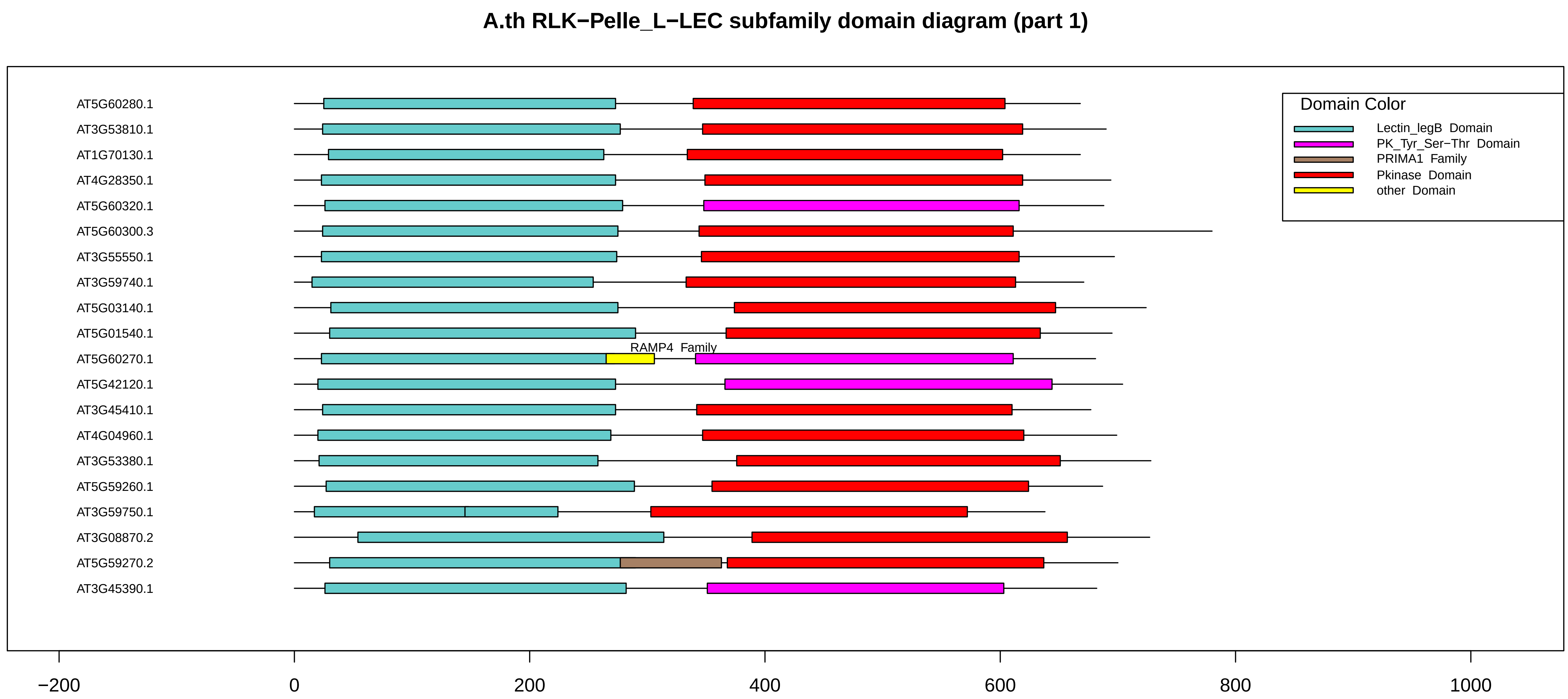

A.th RLK−Pelle_L−LEC subfamily domain diagram (part 1)
AT5G60280.1
AT3G53810.1
AT1G70130.1
AT4G28350.1
AT5G60320.1
AT5G60300.3
AT3G55550.1
AT3G59740.1
AT5G03140.1
AT5G01540.1
AT5G60270.1
AT5G42120.1
AT3G45410.1
AT4G04960.1
AT3G53380.1
AT5G59260.1
AT3G59750.1
AT3G08870.2
AT5G59270.2
AT3G45390.1
RAMP4 Family
Domain Color
Lectin_legB Domain
PK_Tyr_Ser−Thr Domain
PRIMA1 Family
Pkinase Domain
other Domain
−200
0
200
400
600
800
1000

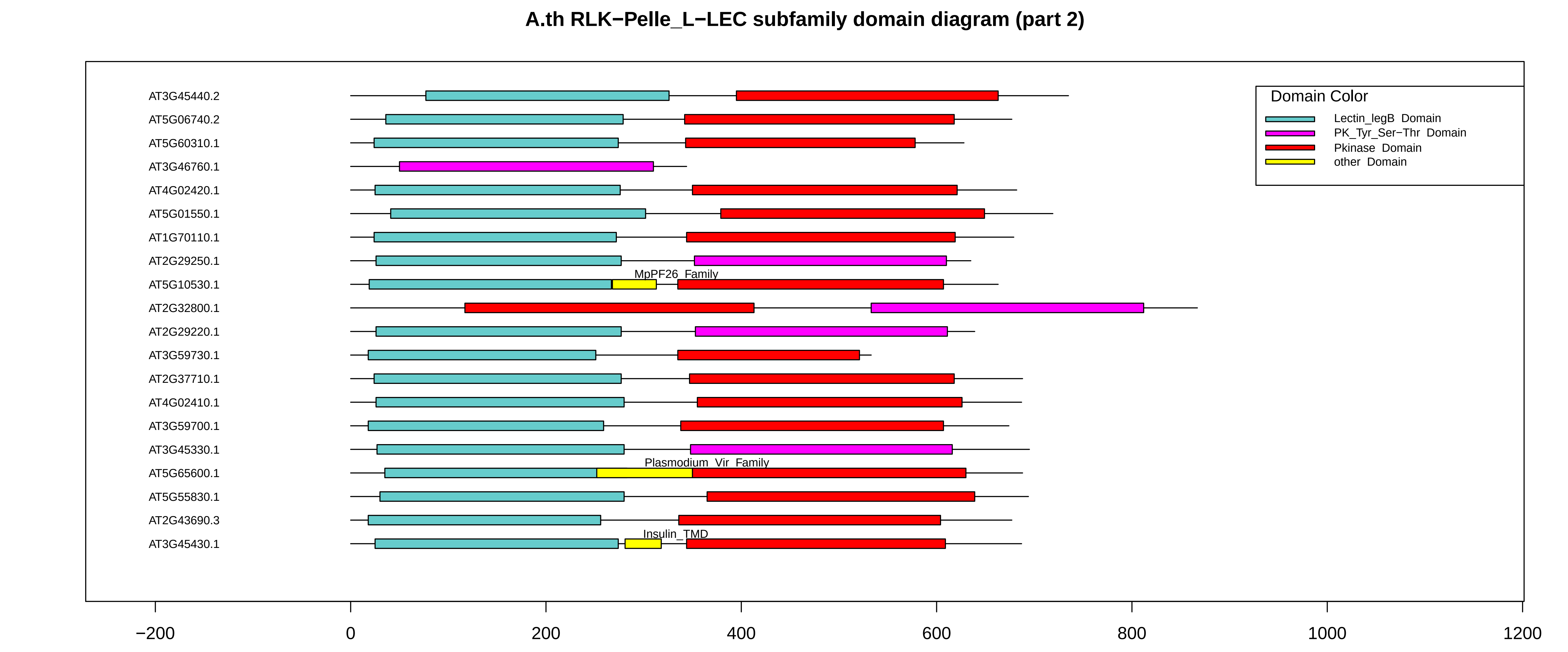
A.th RLK−Pelle_L−LEC subfamily domain diagram (part 2)
AT3G45440.2
AT5G06740.2
AT5G60310.1
AT3G46760.1
AT4G02420.1
AT5G01550.1
AT1G70110.1
AT2G29250.1
AT5G10530.1
MpPF26_Family
AT2G32800.1
AT2G29220.1
AT3G59730.1
AT2G37710.1
AT4G02410.1
AT3G59700.1
AT3G45330.1
AT5G65600.1
Plasmodium_Vir_Family
AT5G55830.1
AT2G43690.3
AT3G45430.1
Insulin_TMD
Domain Color
Lectin_legB Domain
PK_Tyr_Ser−Thr Domain
Pkinase Domain
other Domain
−200
0
200
400
600
800
1000
1200

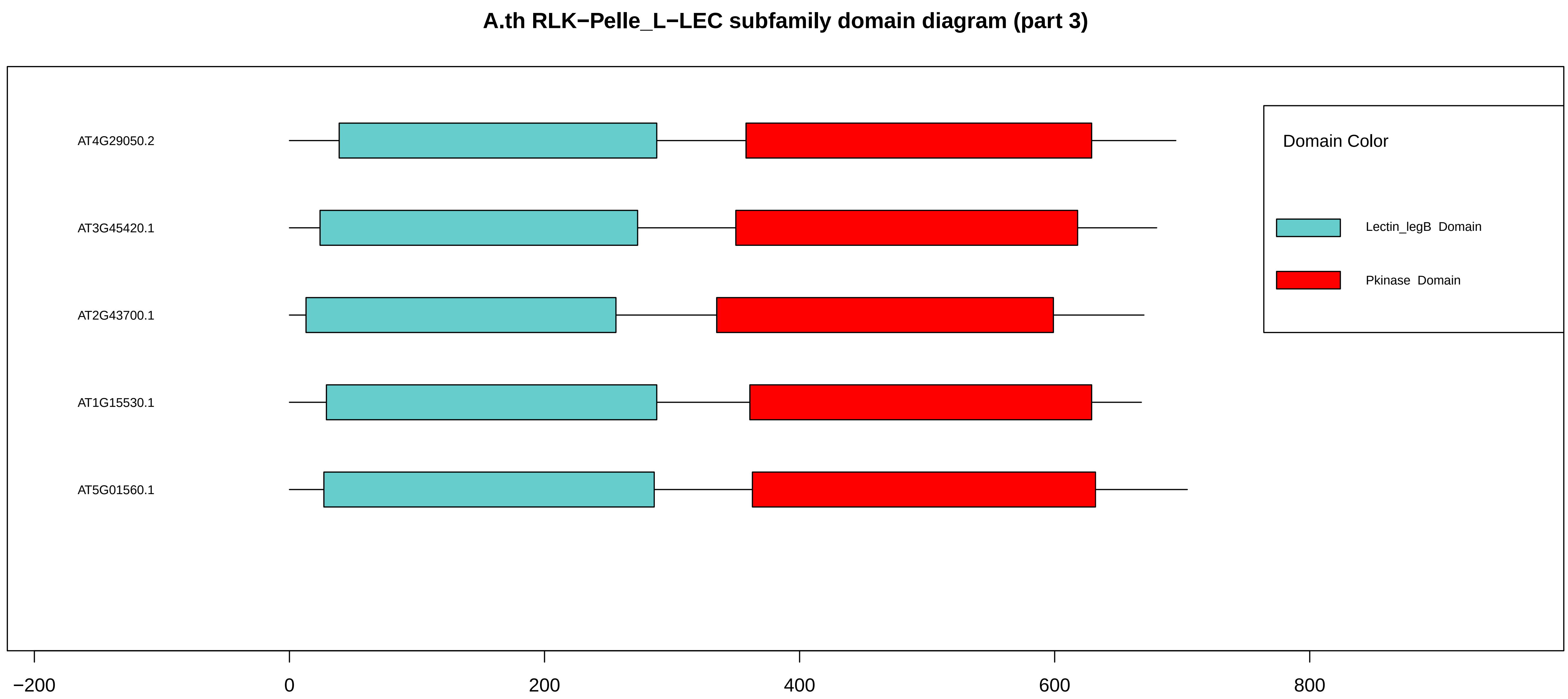

A.th RLK−Pelle_L−LEC subfamily domain diagram (part 3)
AT4G29050.2
AT3G45420.1
AT2G43700.1
AT1G15530.1
AT5G01560.1
Domain Color
Lectin_legB Domain
Pkinase Domain
−200
0
200
400
600
800

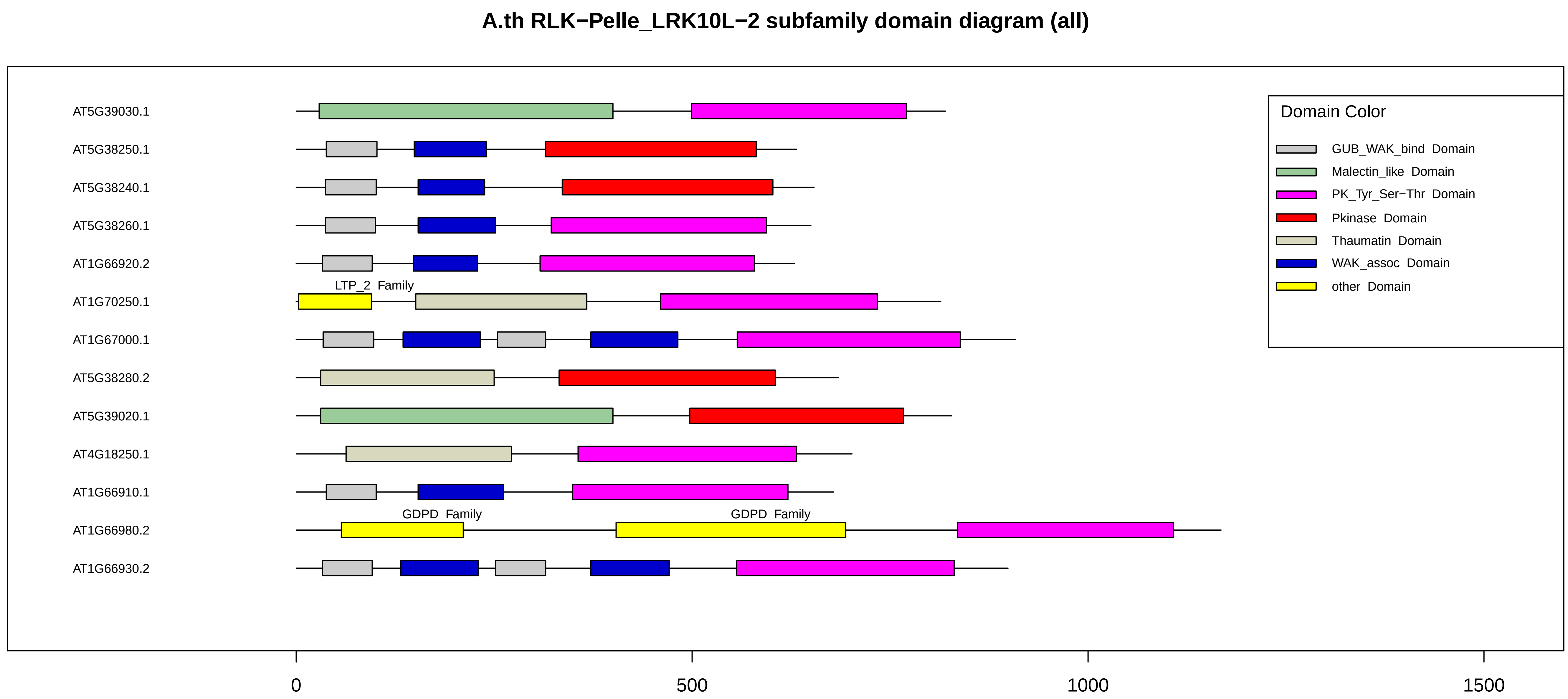
A.th RLK−Pelle_LRK10L−2 subfamily domain diagram (all)
AT5G39030.1
AT5G38250.1
AT5G38240.1
AT5G38260.1
AT1G66920.2
LTP_2 Family
AT1G70250.1
AT1G67000.1
AT5G38280.2
AT5G39020.1
AT4G18250.1
AT1G66910.1
GDPD Family
GDPD Family
AT1G66980.2
AT1G66930.2
Domain Color
GUB_WAK_bind Domain
Malectin_like Domain
PK_Tyr_Ser−Thr Domain
Pkinase Domain
Thaumatin Domain
WAK_assoc Domain
other Domain
0
500
1000
1500

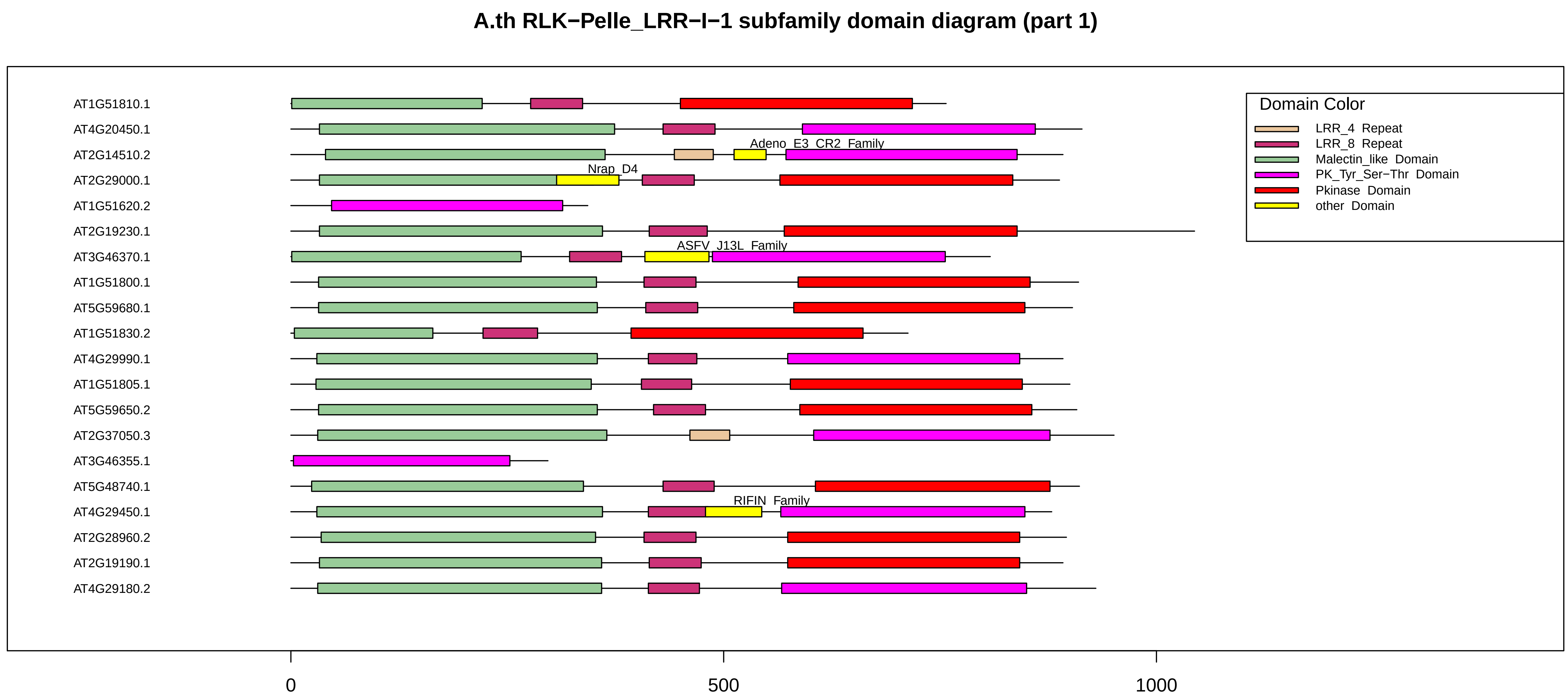
A.th RLK−Pelle_LRR−I−1 subfamily domain diagram (part 1)
AT1G51810.1
AT4G20450.1
AT2G14510.2
AT2G29000.1
AT1G51620.2
AT2G19230.1
AT3G46370.1
AT1G51800.1
AT5G59680.1
AT1G51830.2
AT4G29990.1
AT1G51805.1
AT5G59650.2
AT2G37050.3
AT3G46355.1
AT5G48740.1
AT4G29450.1
AT2G28960.2
AT2G19190.1
AT4G29180.2
Adeno_E3_CR2_Family
Nrap_D4
ASFV_J13L_Family
RIFIN_Family
Domain Color
LRR_4 Repeat
LRR_8 Repeat
Malectin_like Domain
PK_Tyr_Ser−Thr Domain
Pkinase Domain
other Domain
0
500
1000

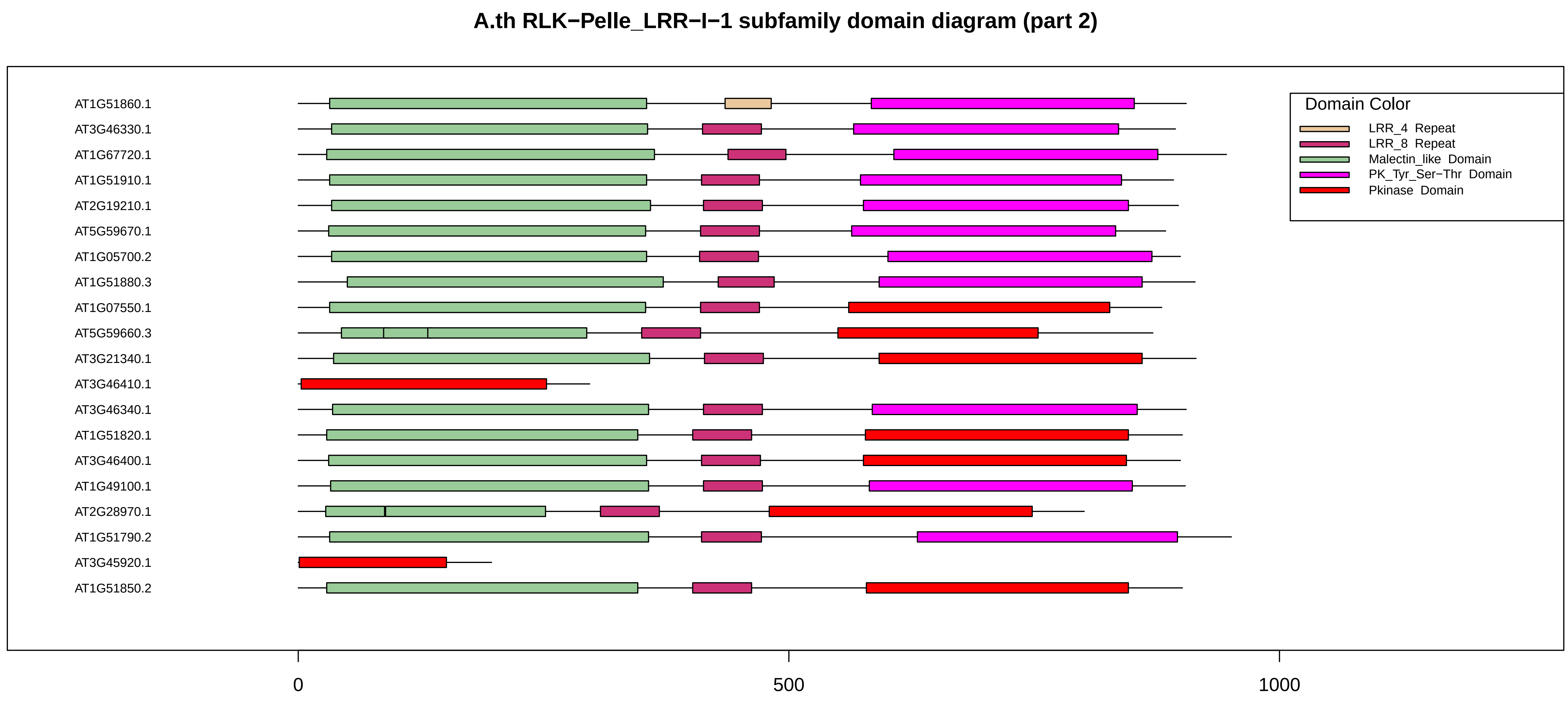
A.th RLK−Pelle_LRR−I−1 subfamily domain diagram (part 2)
AT1G51860.1
AT3G46330.1
AT1G67720.1
AT1G51910.1
AT2G19210.1
AT5G59670.1
AT1G05700.2
AT1G51880.3
AT1G07550.1
AT5G59660.3
AT3G21340.1
AT3G46410.1
AT3G46340.1
AT1G51820.1
AT3G46400.1
AT1G49100.1
AT2G28970.1
AT1G51790.2
AT3G45920.1
AT1G51850.2
Domain Color
LRR_4 Repeat
LRR_8 Repeat
Malectin_like Domain
PK_Tyr_Ser−Thr Domain
Pkinase Domain
0
500
1000

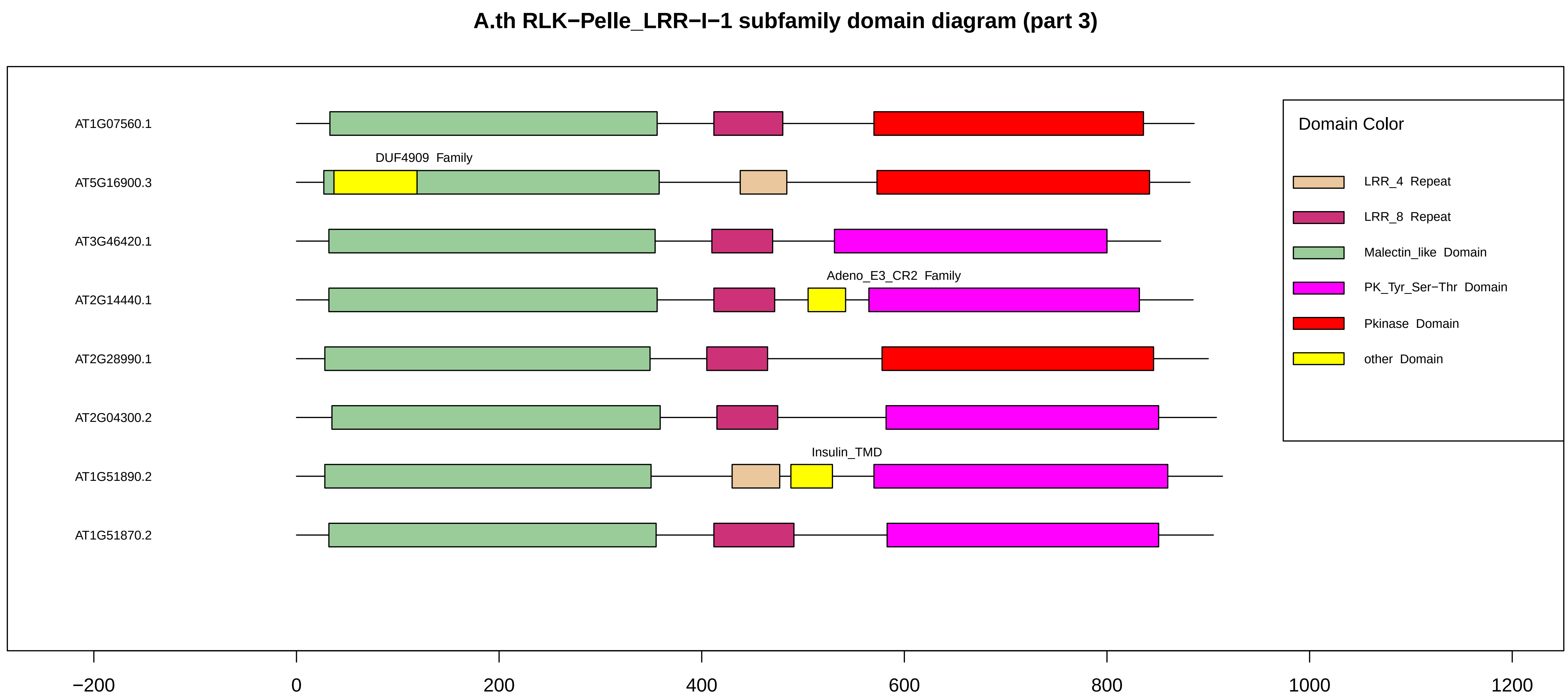
A.th RLK−Pelle_LRR−I−1 subfamily domain diagram (part 3)
AT1G07560.1
AT5G16900.3
AT3G46420.1
AT2G14440.1
AT2G28990.1
AT2G04300.2
AT1G51890.2
AT1G51870.2
DUF4909 Family
Adeno_E3_CR2 Family
Insulin_TMD
Domain Color
LRR_4 Repeat
LRR_8 Repeat
Malectin_like Domain
PK_Tyr_Ser−Thr Domain
Pkinase Domain
other Domain
−200
0
200
400
600
800
1000
1200

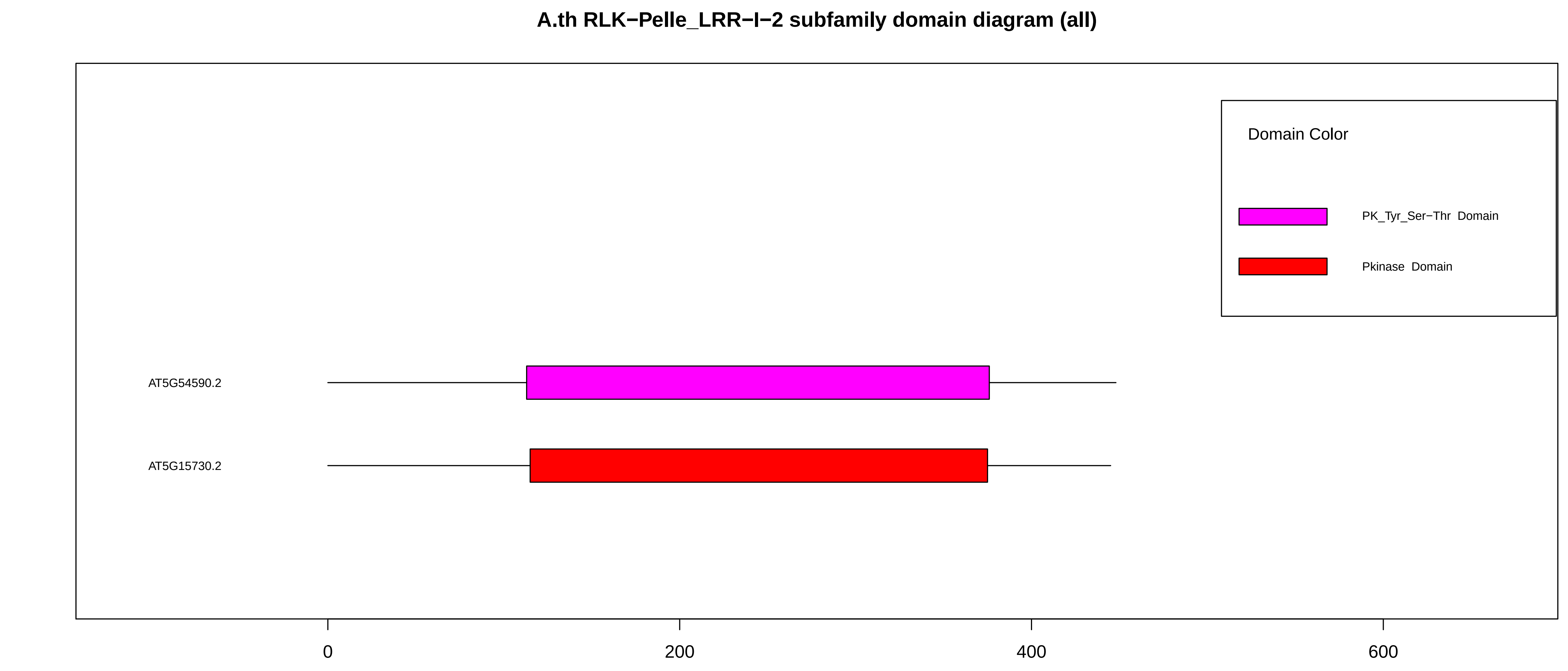

A.th RLK−Pelle_LRR−I−2 subfamily domain diagram (all)
Domain Color
PK_Tyr_Ser−Thr Domain
Pkinase Domain
AT5G54590.2
AT5G15730.2
0
200
400
600

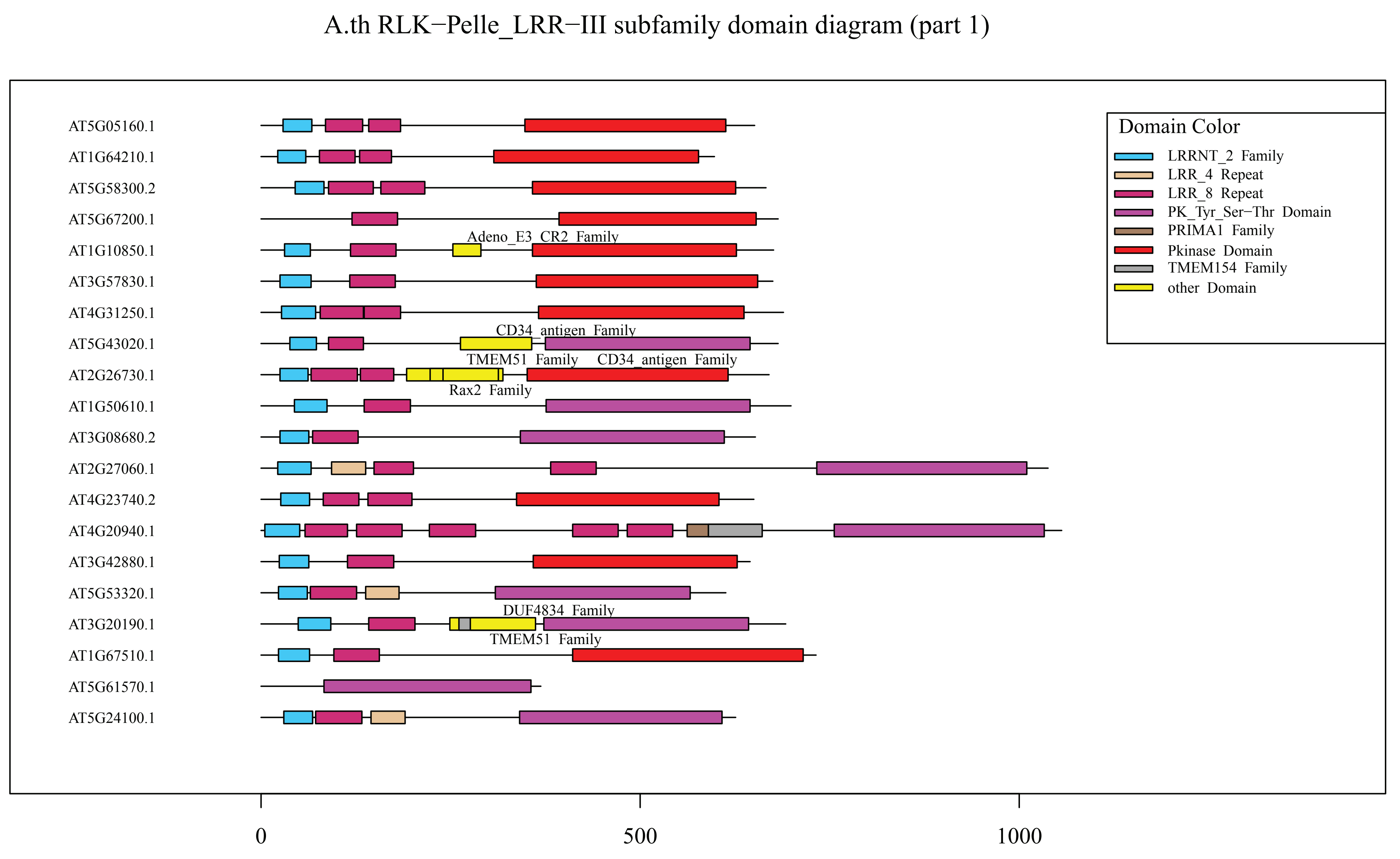
A.th RLK−Pelle_LRR−III subfamily domain diagram (part 1)
AT5G05160.1
AT1G64210.1
AT5G58300.2
AT5G67200.1
AT1G10850.1
AT3G57830.1
AT4G31250.1
AT5G43020.1
AT2G26730.1
AT1G50610.1
AT3G08680.2
AT2G27060.1
AT4G23740.2
AT4G20940.1
AT3G42880.1
AT5G53320.1
AT3G20190.1
AT1G67510.1
AT5G61570.1
AT5G24100.1
Adeno_E3_CR2_Family
CD34_antigen_Family
TMEM51_Family
CD34_antigen_Family
Rax2_Family
DUF4834_Family
TMEM51_Family
Domain Color
LRRNT_2_Family
LRR_4_Repeat
LRR_8_Repeat
PK_Tyr_Ser−Thr_Domain
PRIMA1_Family
Pkinase_Domain
TMEM154_Family
other_Domain
0
500
1000

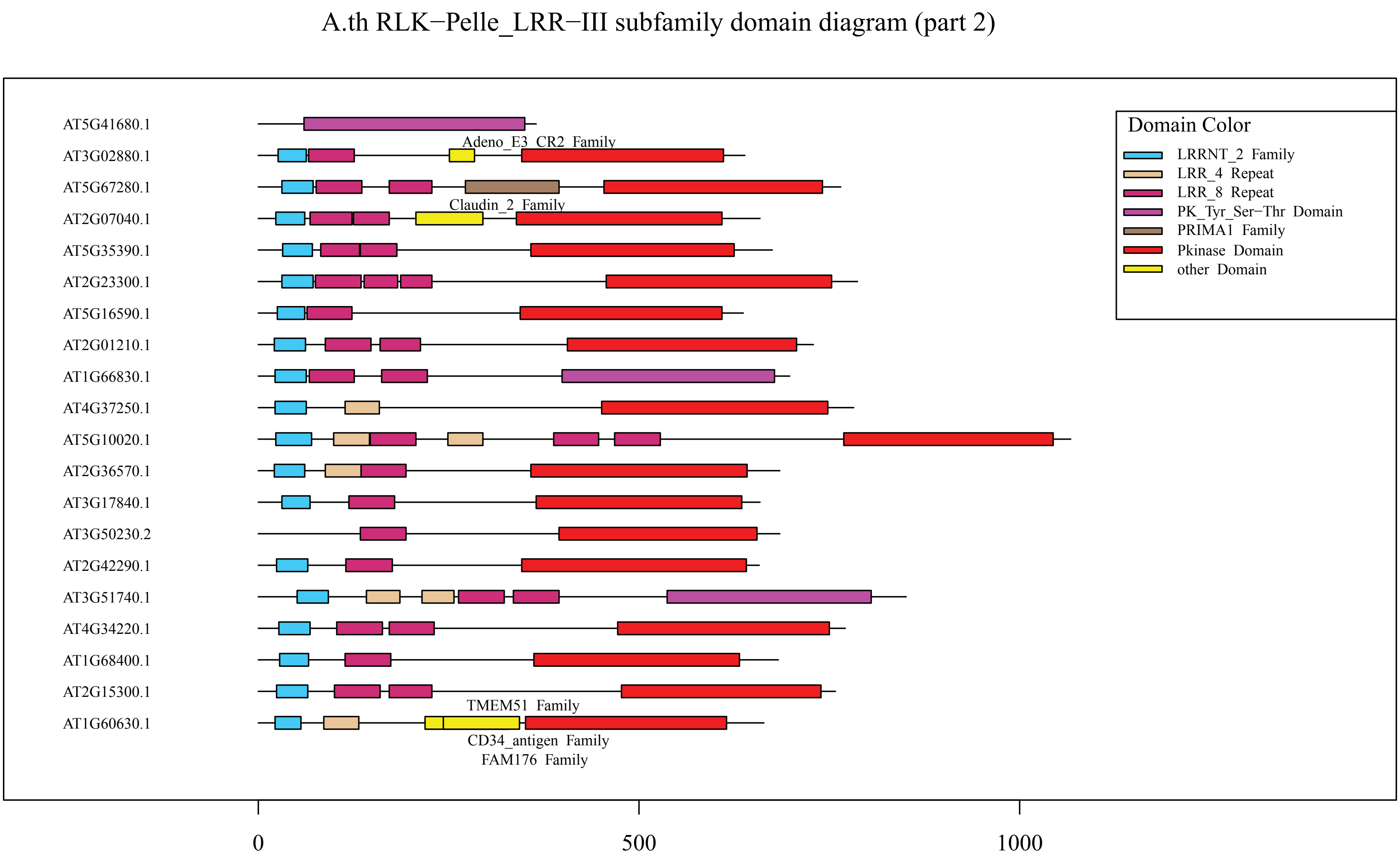

A.th RLK−Pelle_LRR−III subfamily domain diagram (part 2)
AT5G41680.1
AT3G02880.1
AT5G67280.1
AT2G07040.1
AT5G35390.1
AT2G23300.1
AT5G16590.1
AT2G01210.1
AT1G66830.1
AT4G37250.1
AT5G10020.1
AT2G36570.1
AT3G17840.1
AT3G50230.2
AT2G42290.1
AT3G51740.1
AT4G34220.1
AT1G68400.1
AT2G15300.1
AT1G60630.1
Adeno_E3_CR2 Family
Claudin_2 Family
TMEM51 Family
CD34_antigen Family
FAM176 Family
Domain Color
LRRNT_2 Family
LRR_4 Repeat
LRR_8 Repeat
PK_Tyr_Ser−Thr Domain
PRIMA1 Family
Pkinase Domain
other Domain
0
500
1000

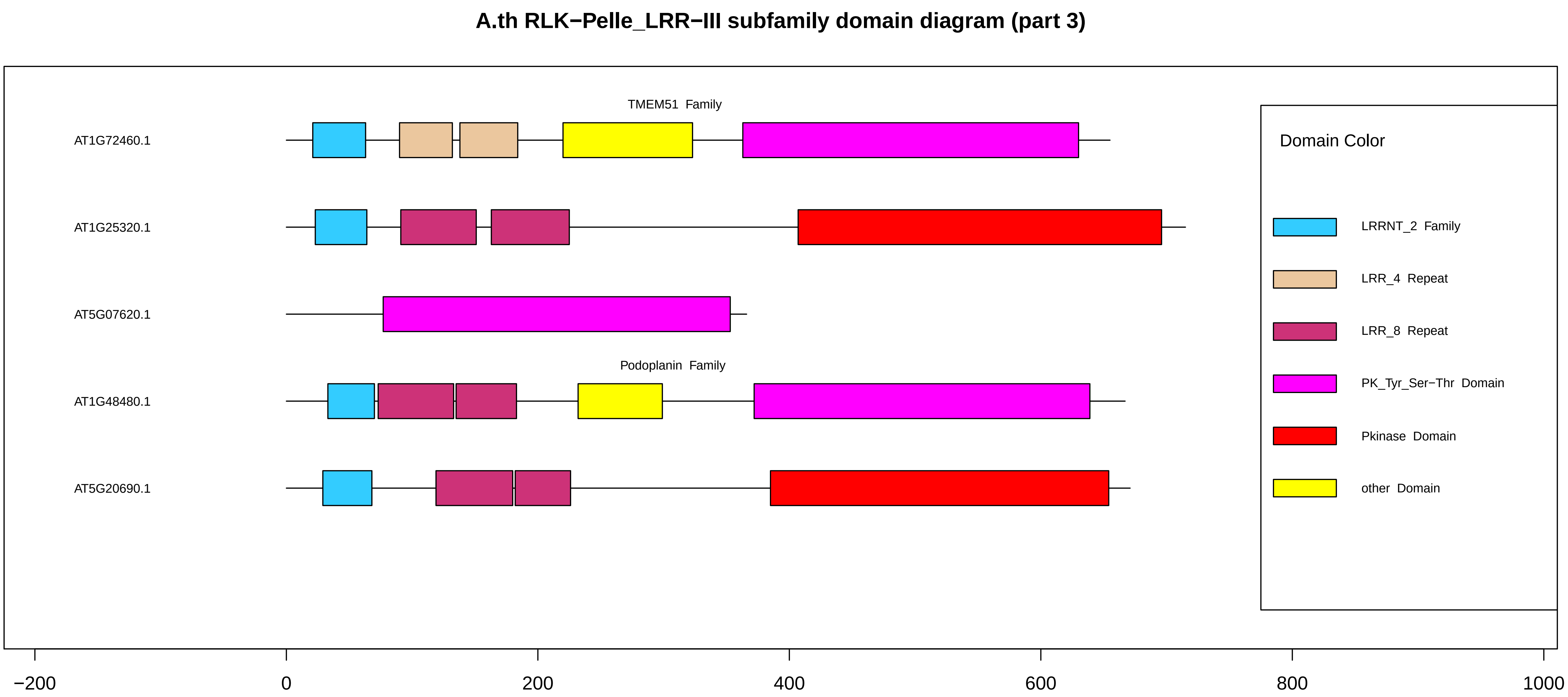

A.th RLK−Pelle_LRR−III subfamily domain diagram (part 3)
TMEM51 Family
AT1G72460.1
AT1G25320.1
AT5G07620.1
Podoplanin Family
AT1G48480.1
AT5G20690.1
Domain Color
LRRNT_2 Family
LRR_4 Repeat
LRR_8 Repeat
PK_Tyr_Ser−Thr Domain
Pkinase Domain
other Domain
−200
0
200
400
600
800
1000

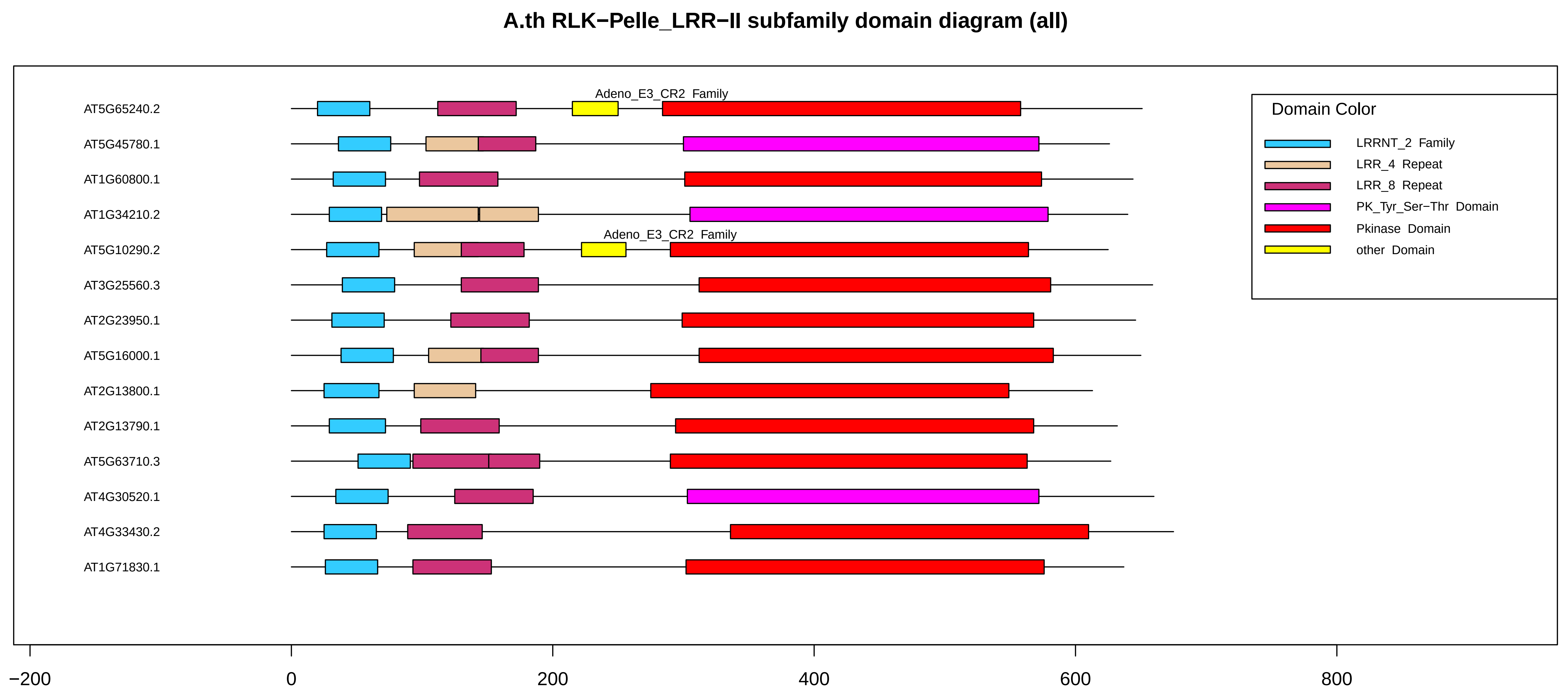
A.th RLK−Pelle_LRR−II subfamily domain diagram (all)
Adeno_E3_CR2 Family
AT5G65240.2
AT5G45780.1
AT1G60800.1
AT1G34210.2
Adeno_E3_CR2 Family
AT5G10290.2
AT3G25560.3
AT2G23950.1
AT5G16000.1
AT2G13800.1
AT2G13790.1
AT5G63710.3
AT4G30520.1
AT4G33430.2
AT1G71830.1
Domain Color
LRRNT_2 Family
LRR_4 Repeat
LRR_8 Repeat
PK_Tyr_Ser−Thr Domain
Pkinase Domain
other Domain
−200
0
200
400
600
800

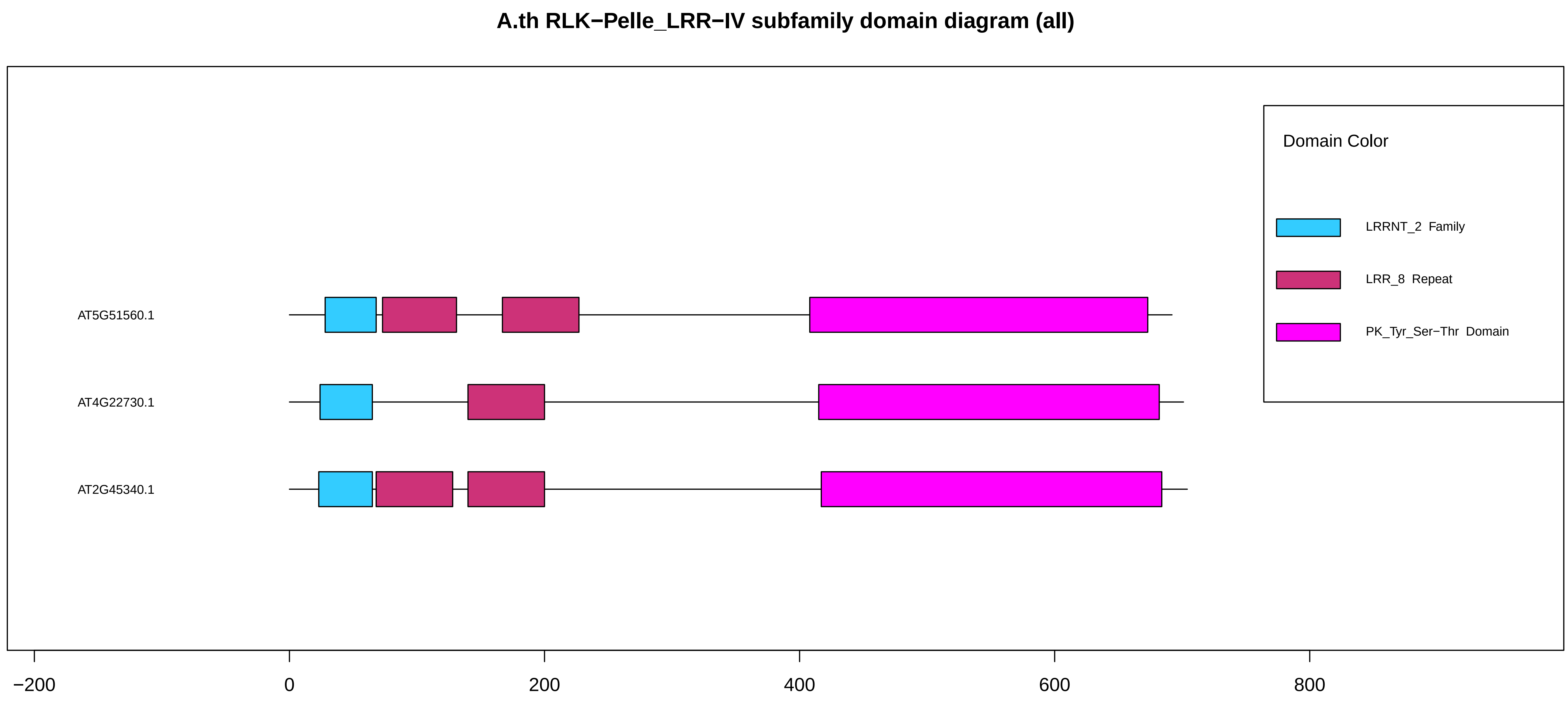
A.th RLK−Pelle_LRR−IV subfamily domain diagram (all)
Domain Color
LRRNT_2 Family
LRR_8 Repeat
PK_Tyr_Ser−Thr Domain
AT5G51560.1
AT4G22730.1
AT2G45340.1
−200
0
200
400
600
800

A.th RLK−Pelle_LRR−IX subfamily domain diagram (all)

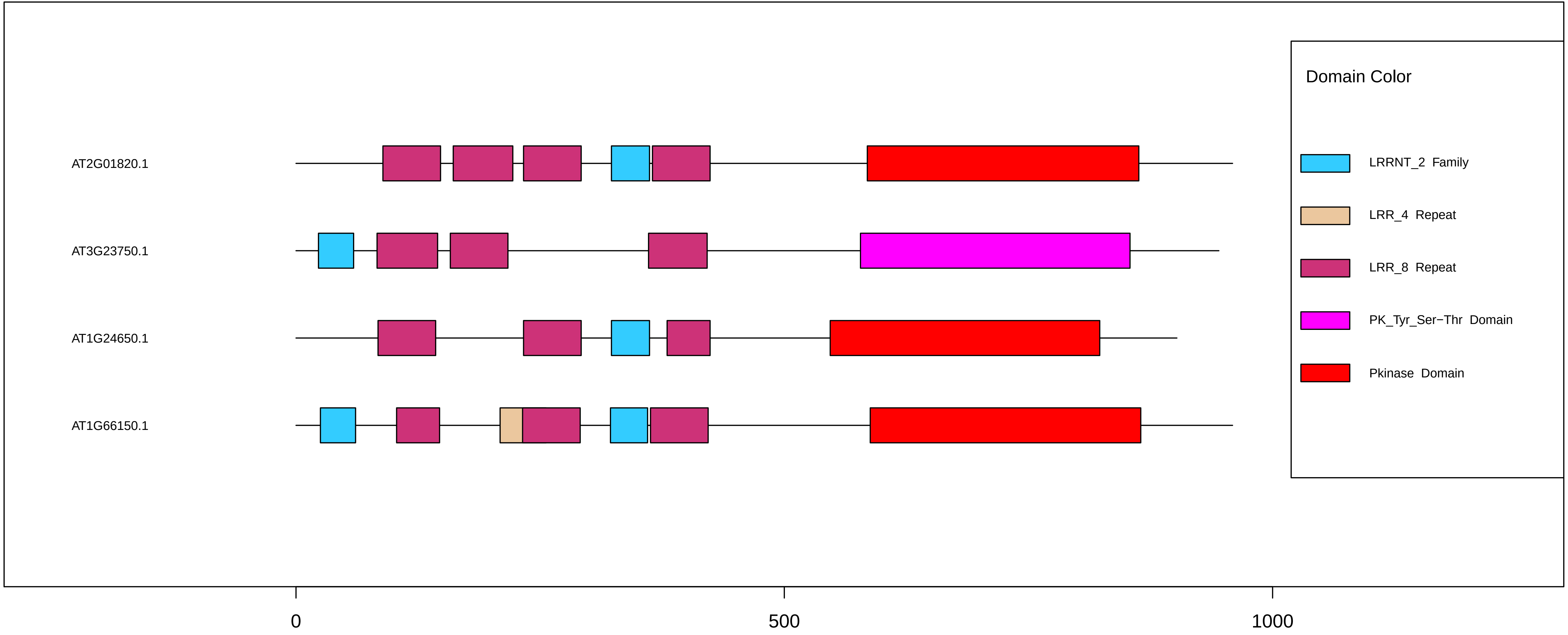

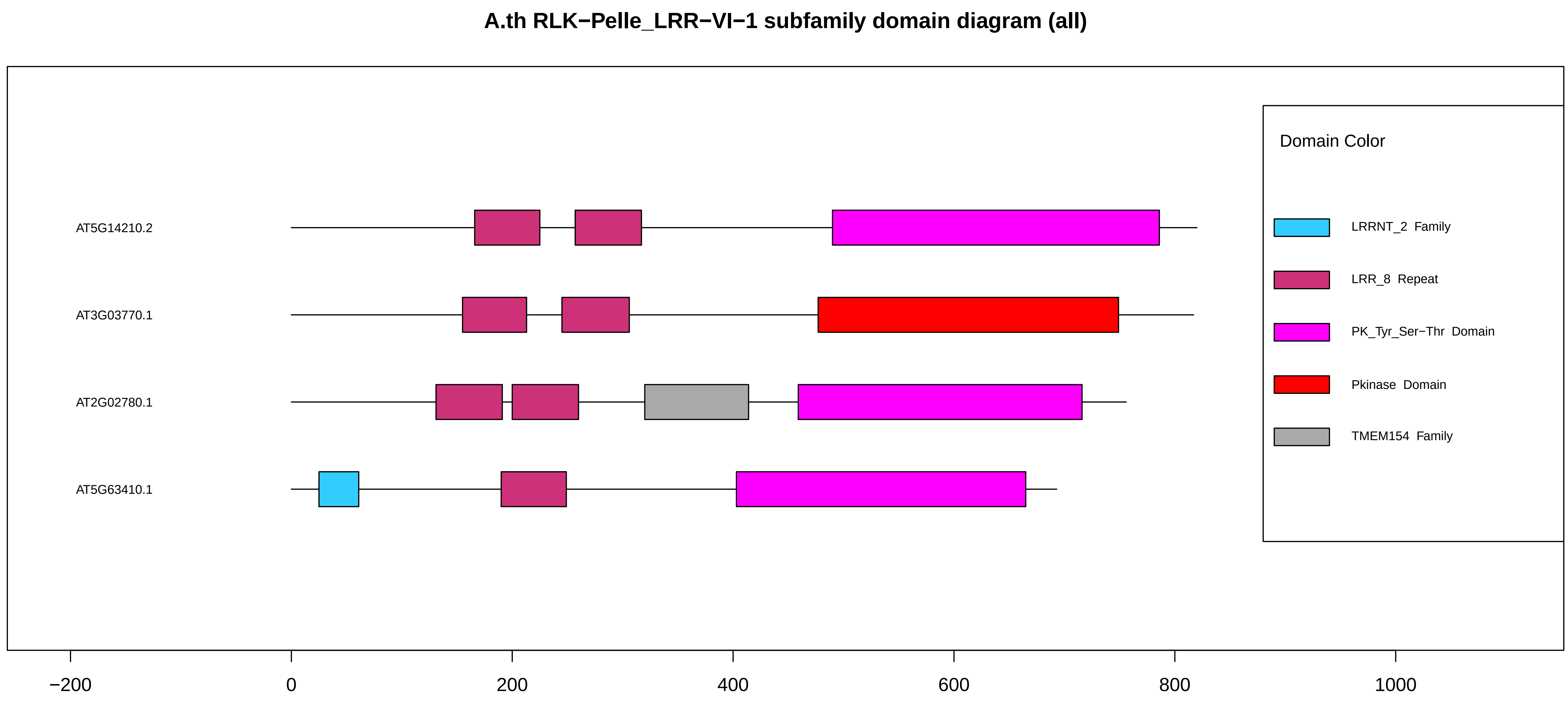
A.th RLK−Pelle_LRR−VI−1 subfamily domain diagram (all)
AT5G14210.2
AT3G03770.1
AT2G02780.1
AT5G63410.1
Domain Color
LRRNT_2 Family
LRR_8 Repeat
PK_Tyr_Ser−Thr Domain
Pkinase Domain
TMEM154 Family
−200
0
200
400
600
800
1000

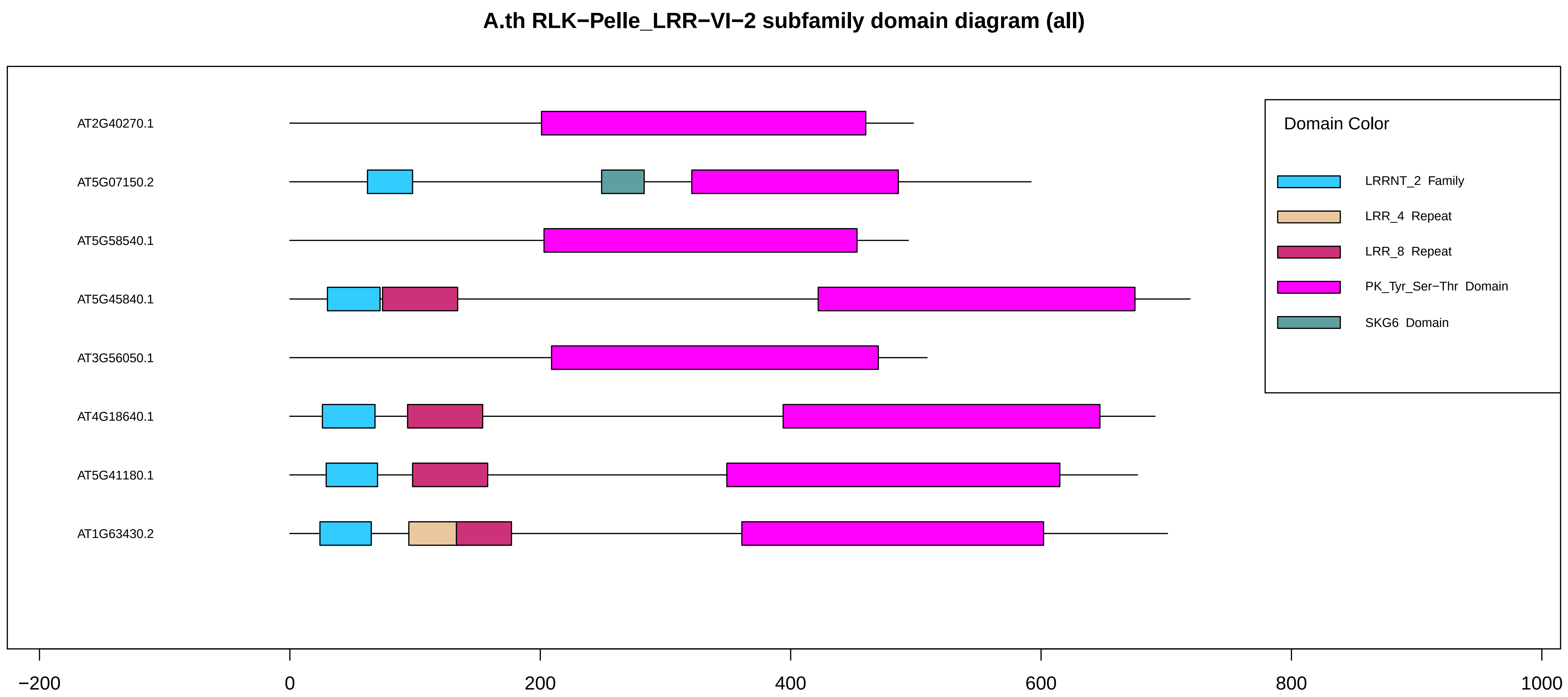
A.th RLK−Pelle_LRR−VI−2 subfamily domain diagram (all)
AT2G40270.1
AT5G07150.2
AT5G58540.1
AT5G45840.1
AT3G56050.1
AT4G18640.1
AT5G41180.1
AT1G63430.2
Domain Color
LRRNT_2 Family
LRR_4 Repeat
LRR_8 Repeat
PK_Tyr_Ser−Thr Domain
SKG6 Domain
−200
0
200
400
600
800
1000

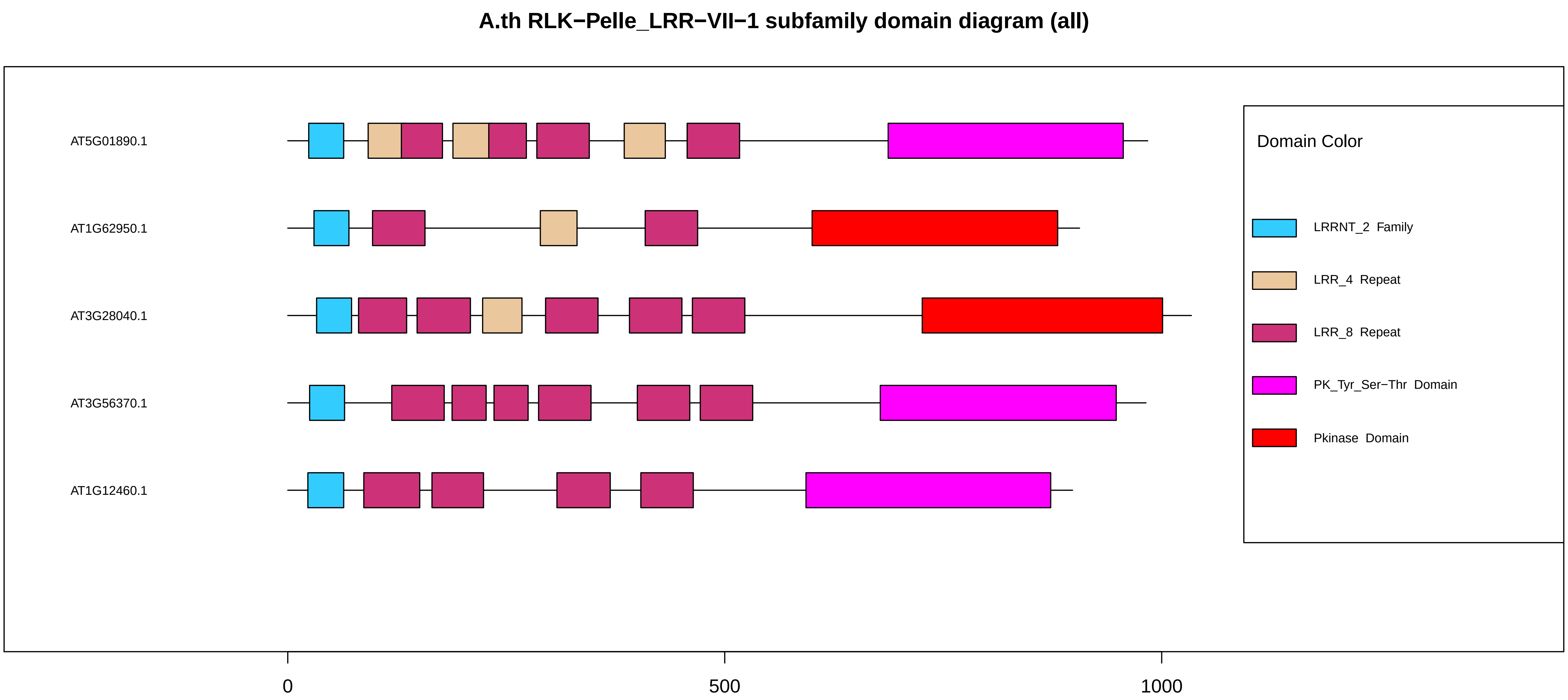
A.th RLK−Pelle_LRR−VII−1 subfamily domain diagram (all)
AT5G01890.1
AT1G62950.1
AT3G28040.1
AT3G56370.1
AT1G12460.1
0
500
1000
Domain Color
LRRNT_2 Family
LRR_4 Repeat
LRR_8 Repeat
PK_Tyr_Ser−Thr Domain
Pkinase Domain

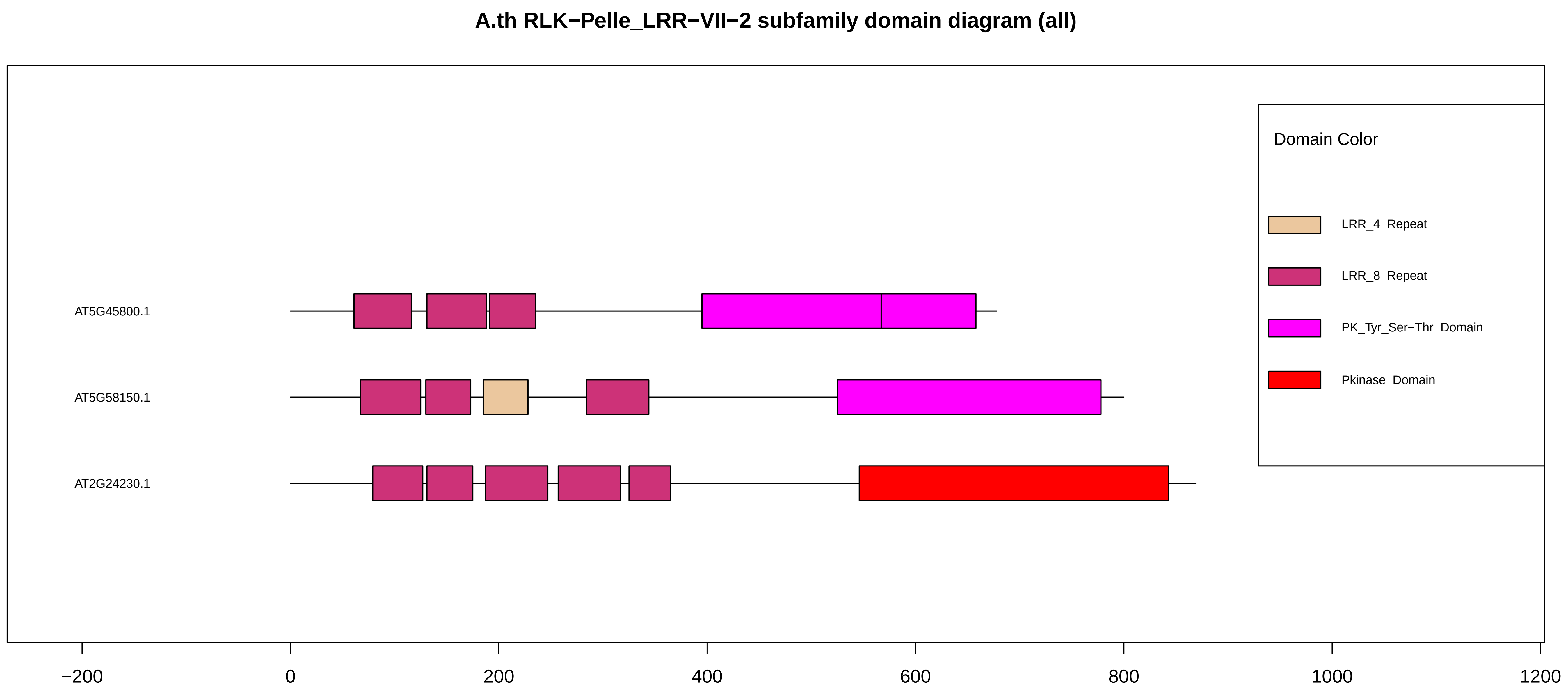
A.th RLK−Pelle_LRR−VII−2 subfamily domain diagram (all)
Domain Color
LRR_4 Repeat
LRR_8 Repeat
PK_Tyr_Ser−Thr Domain
Pkinase Domain
AT5G45800.1
AT5G58150.1
AT2G24230.1
−200
0
200
400
600
800
1000
1200

A.th RLK−Pelle_LRR−VII−3 subfamily domain diagram (all)

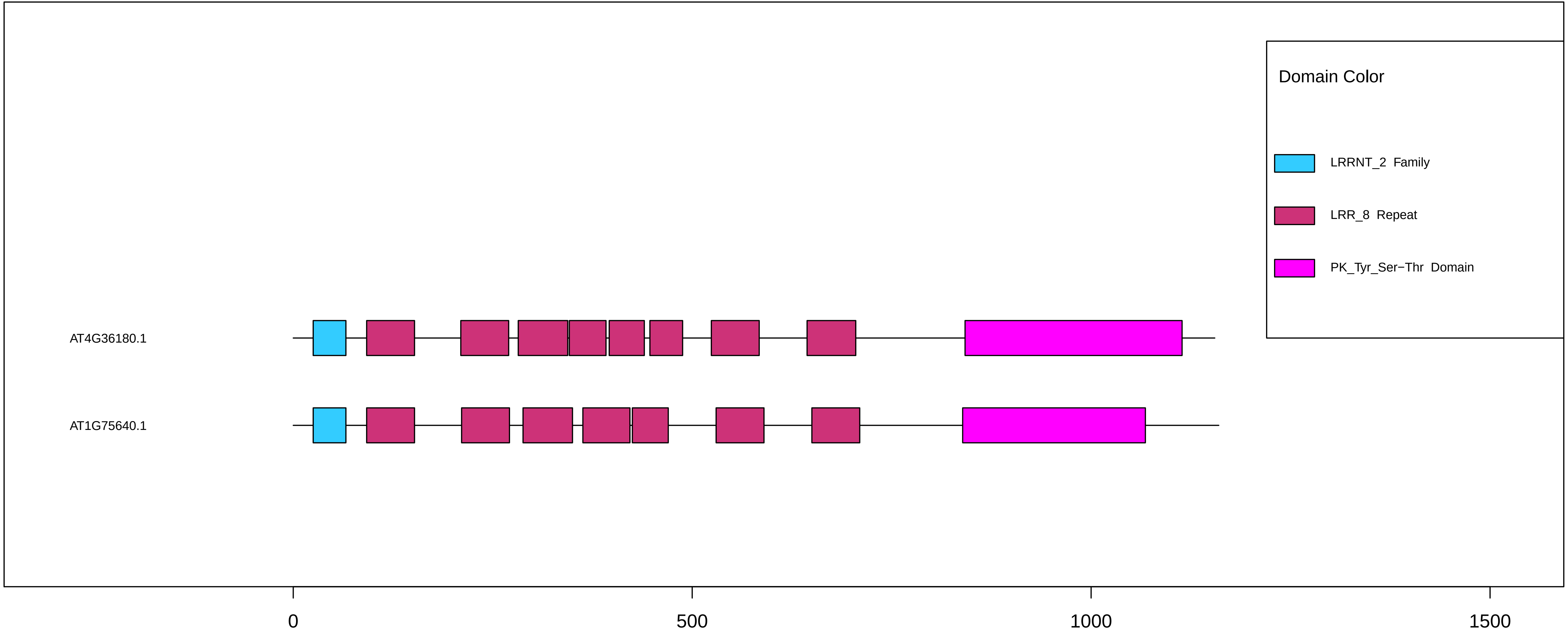

A.th RLK−Pelle_LRR−VIII−1 subfamily domain diagram (all)

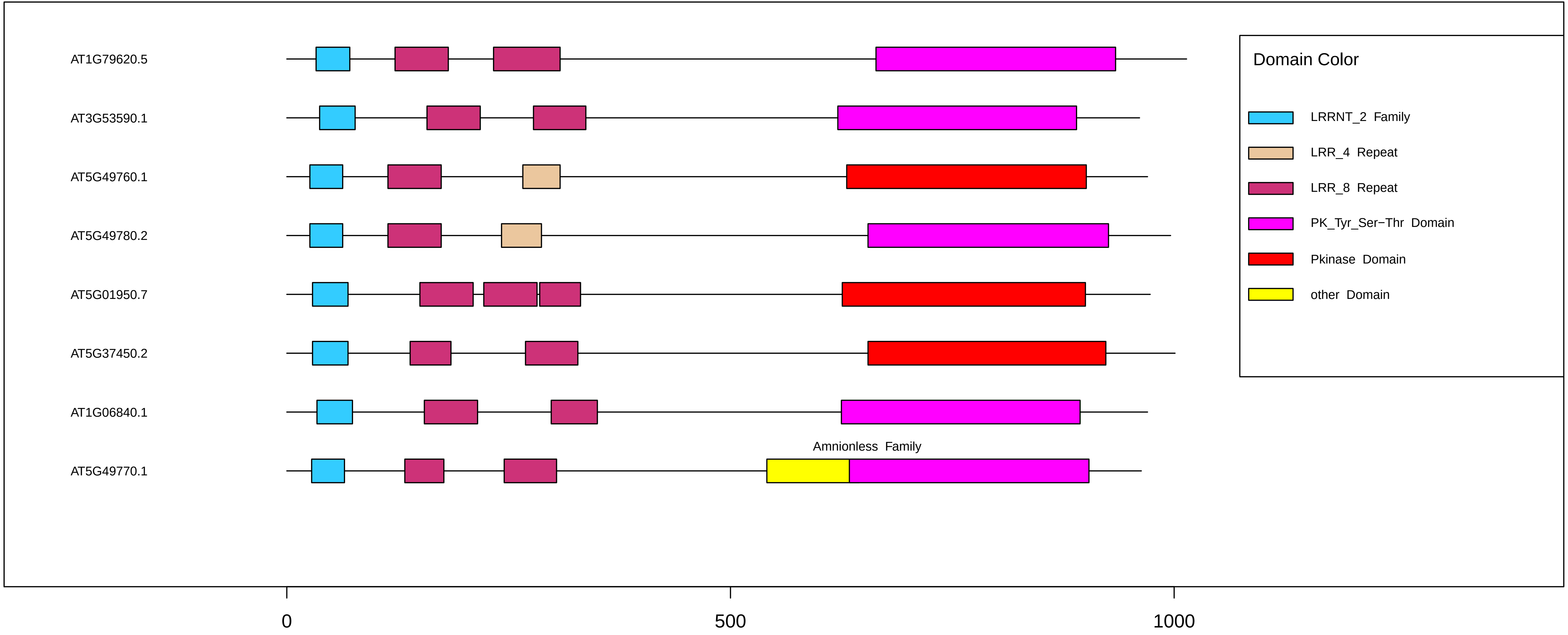

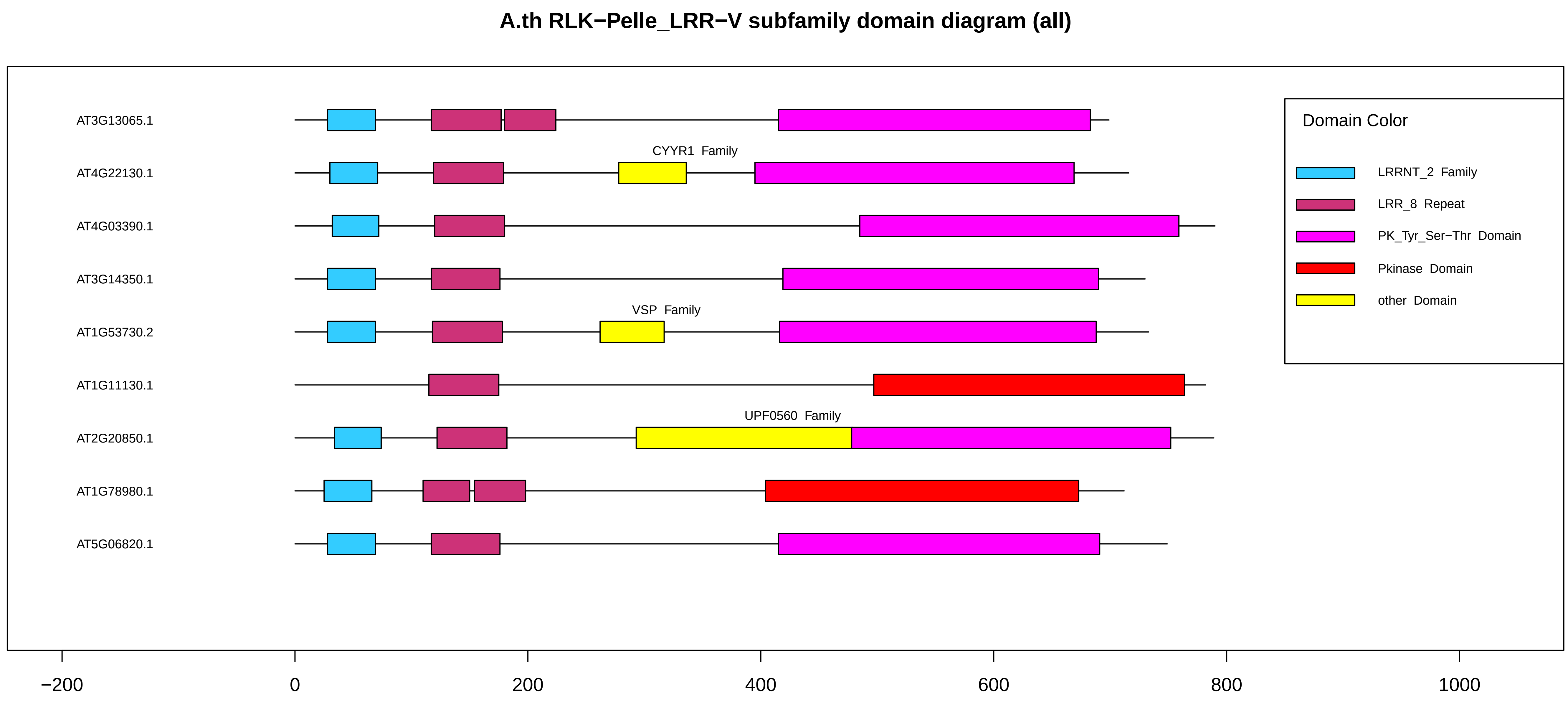

A.th RLK−Pelle_LRR−V subfamily domain diagram (all)
AT3G13065.1
AT4G22130.1
CYYR1 Family
AT4G03390.1
AT3G14350.1
AT1G53730.2
VSP Family
AT1G11130.1
AT2G20850.1
UPF0560 Family
AT1G78980.1
AT5G06820.1
Domain Color
LRRNT_2 Family
LRR_8 Repeat
PK_Tyr_Ser−Thr Domain
Pkinase Domain
other Domain
−200
0
200
400
600
800
1000

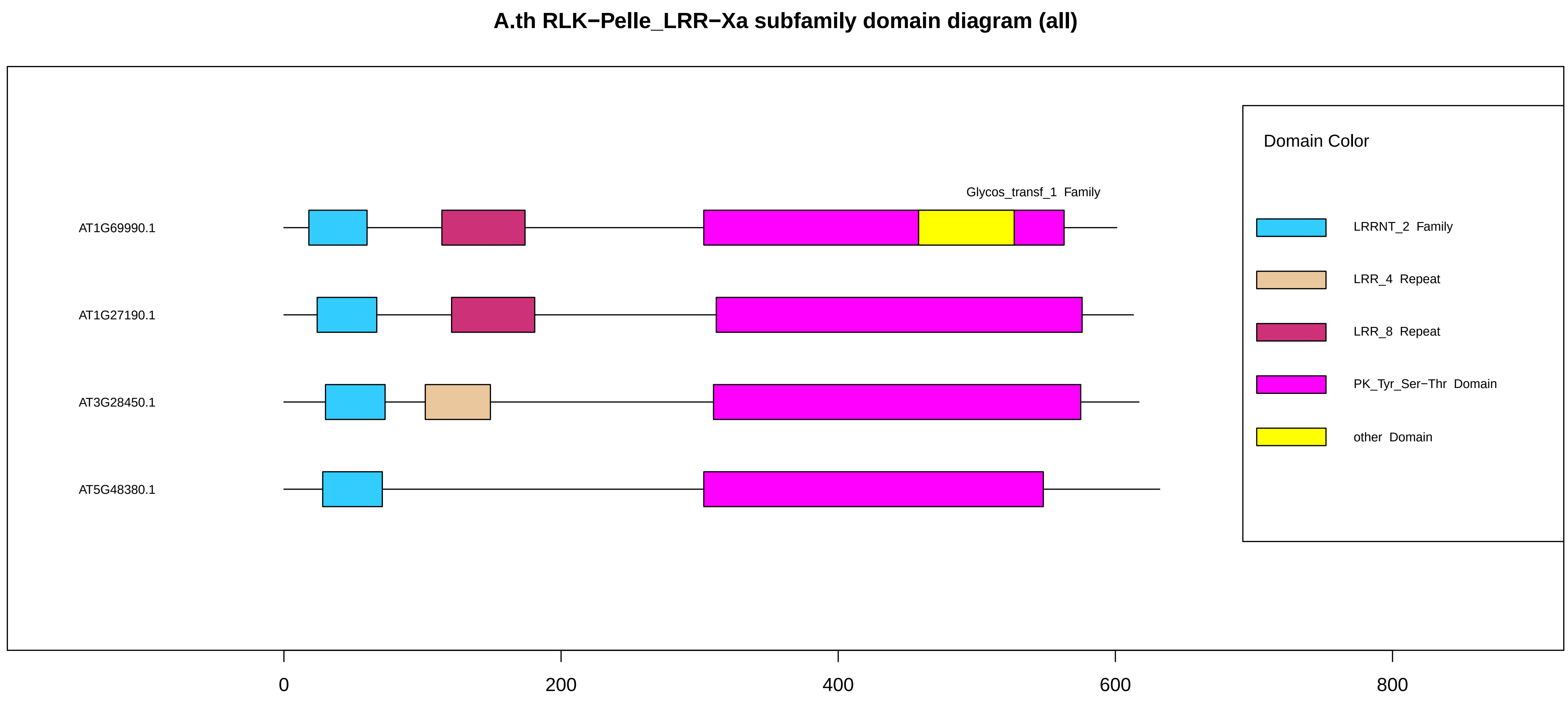
A.th RLK−Pelle_LRR−Xa subfamily domain diagram (all)
Glycos_transf_1 Family
AT1G69990.1
AT1G27190.1
AT3G28450.1
AT5G48380.1
0
200
400
600
800
Domain Color
LRRNT_2 Family
LRR_4 Repeat
LRR_8 Repeat
PK_Tyr_Ser−Thr Domain
other Domain

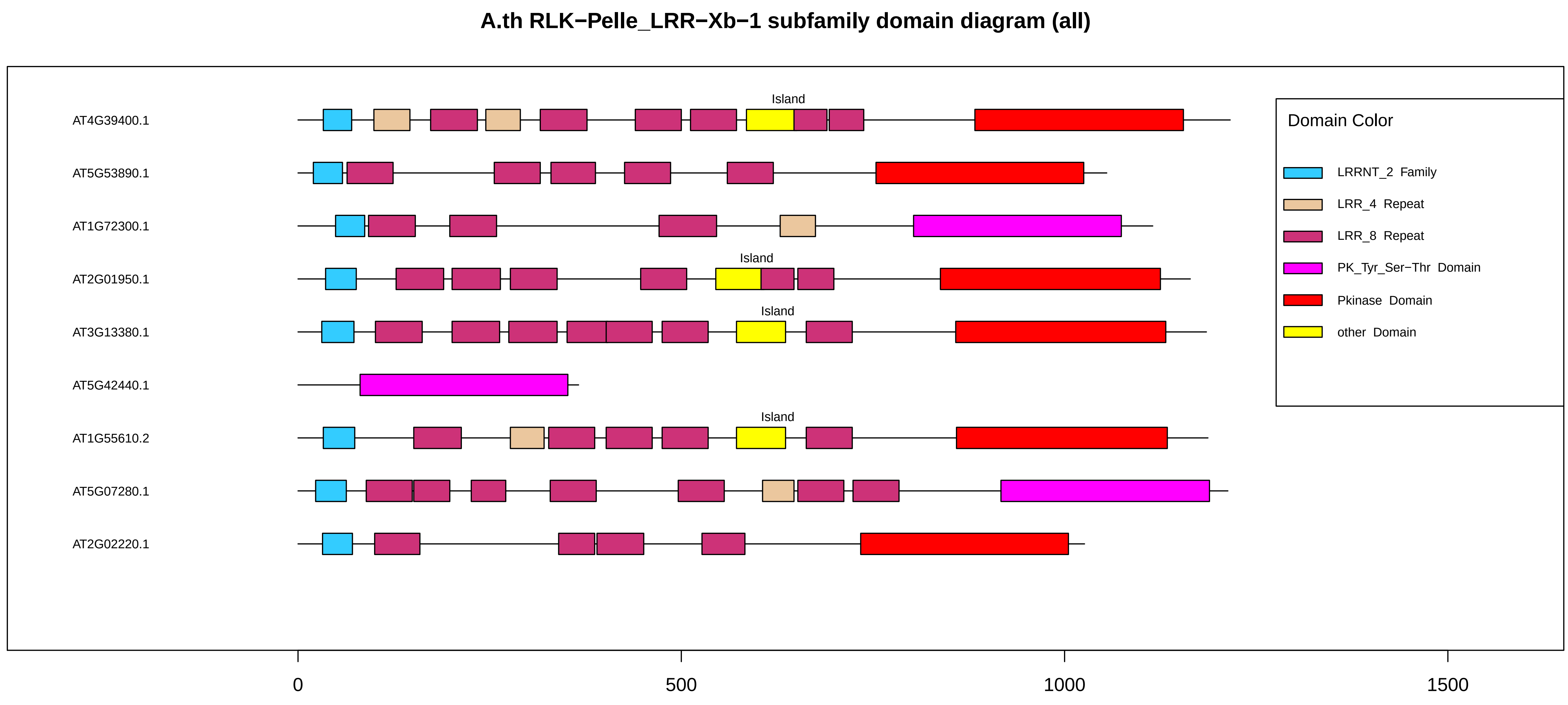
A.th RLK−Pelle_LRR−Xb−1 subfamily domain diagram (all)
AT4G39400.1
AT5G53890.1
AT1G72300.1
AT2G01950.1
AT3G13380.1
AT5G42440.1
AT1G55610.2
AT5G07280.1
AT2G02220.1
Island
Island
Island
Island
Domain Color
LRRNT_2 Family
LRR_4 Repeat
LRR_8 Repeat
PK_Tyr_Ser−Thr Domain
Pkinase Domain
other Domain
0
500
1000
1500

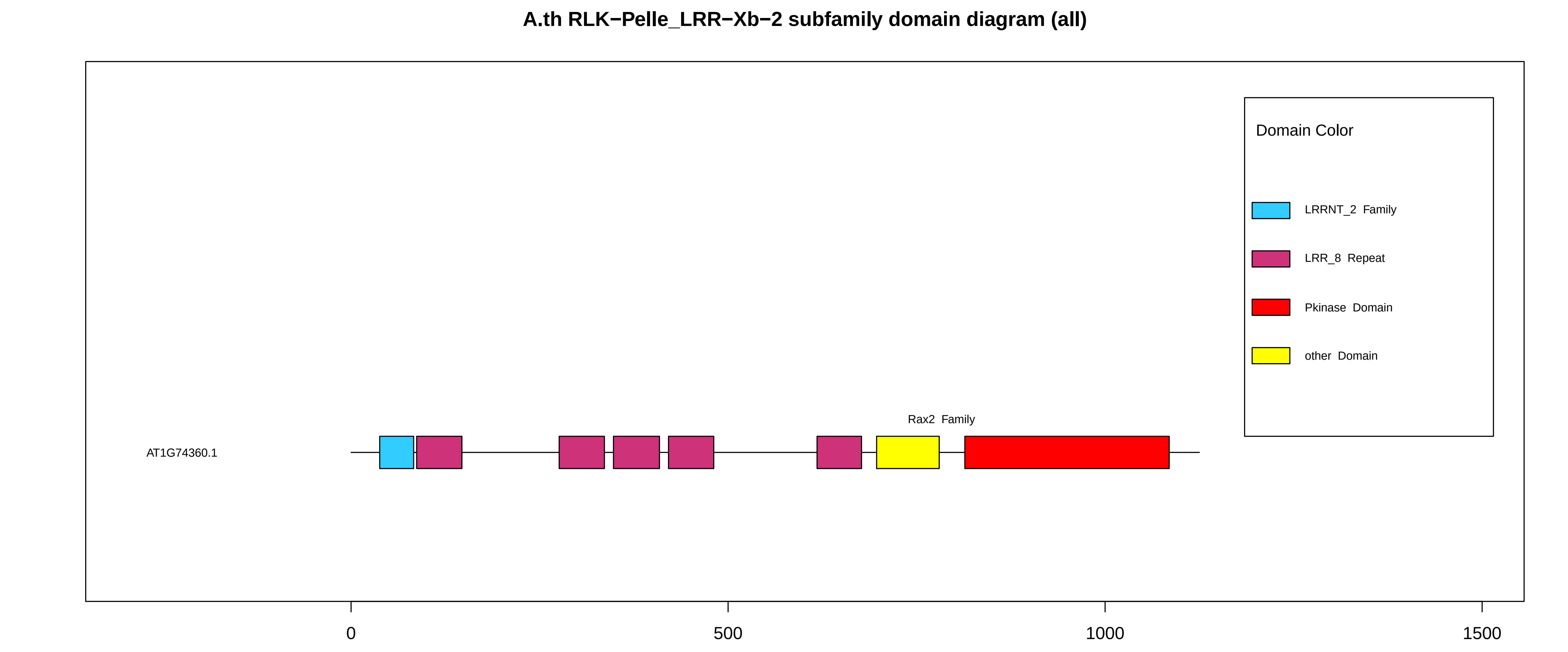
A.th RLK−Pelle_LRR−Xb−2 subfamily domain diagram (all)
Domain Color
LRRNT_2 Family
LRR_8 Repeat
Pkinase Domain
other Domain
Rax2 Family
AT1G74360.1
0
500
1000
1500

A.th RLK−Pelle_LRR−XI−1 subfamily domain diagram (part 1)

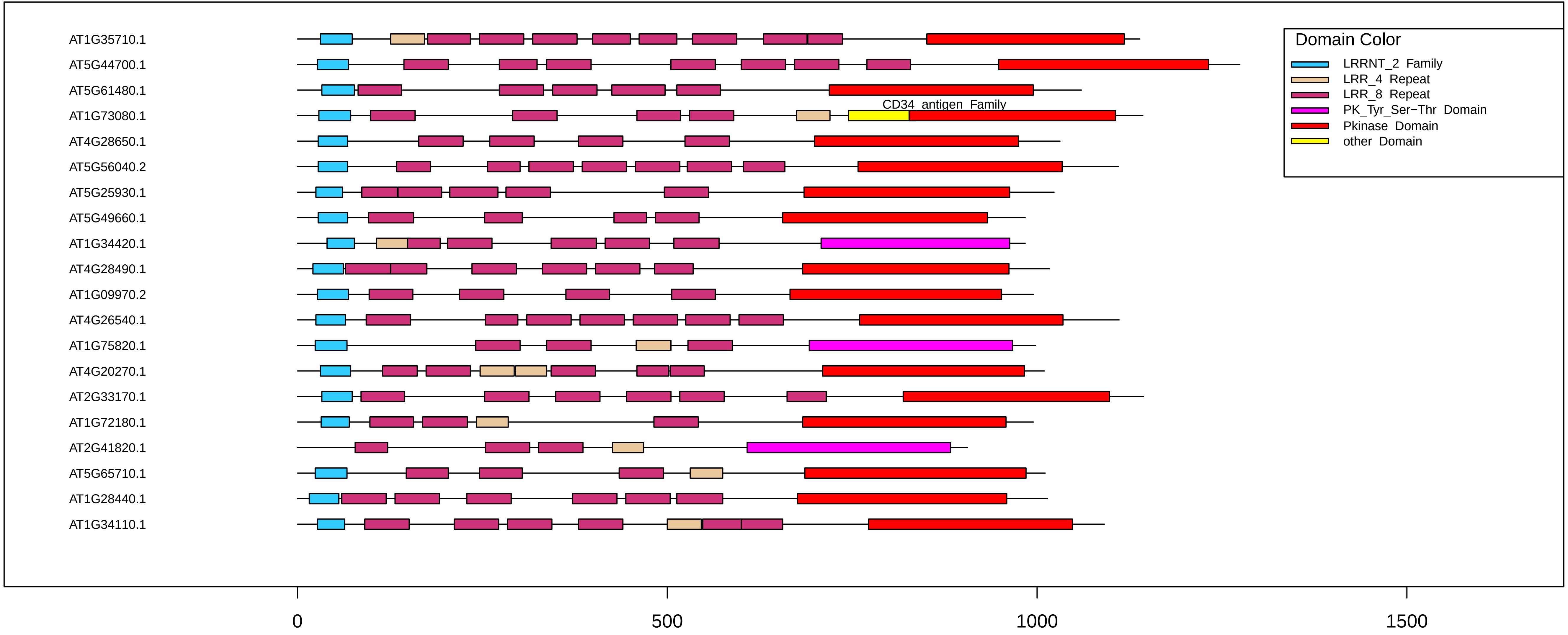

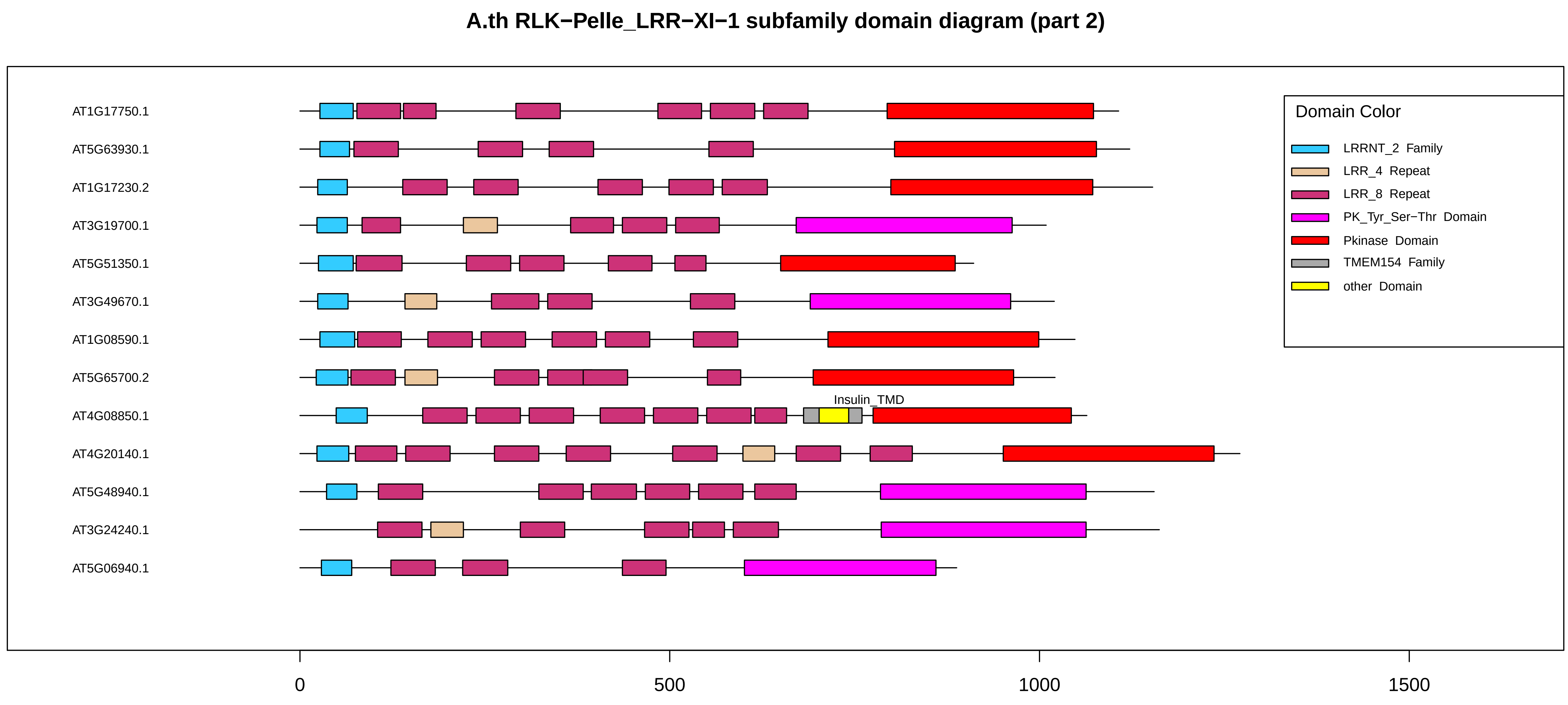
A.th RLK−Pelle_LRR−XI−1 subfamily domain diagram (part 2)
AT1G17750.1
AT5G63930.1
AT1G17230.2
AT3G19700.1
AT5G51350.1
AT3G49670.1
AT1G08590.1
AT5G65700.2
AT4G08850.1
AT4G20140.1
AT5G48940.1
AT3G24240.1
AT5G06940.1
Insulin_TMD
Domain Color
LRRNT_2 Family
LRR_4 Repeat
LRR_8 Repeat
PK_Tyr_Ser−Thr Domain
Pkinase Domain
TMEM154 Family
other Domain
0
500
1000
1500

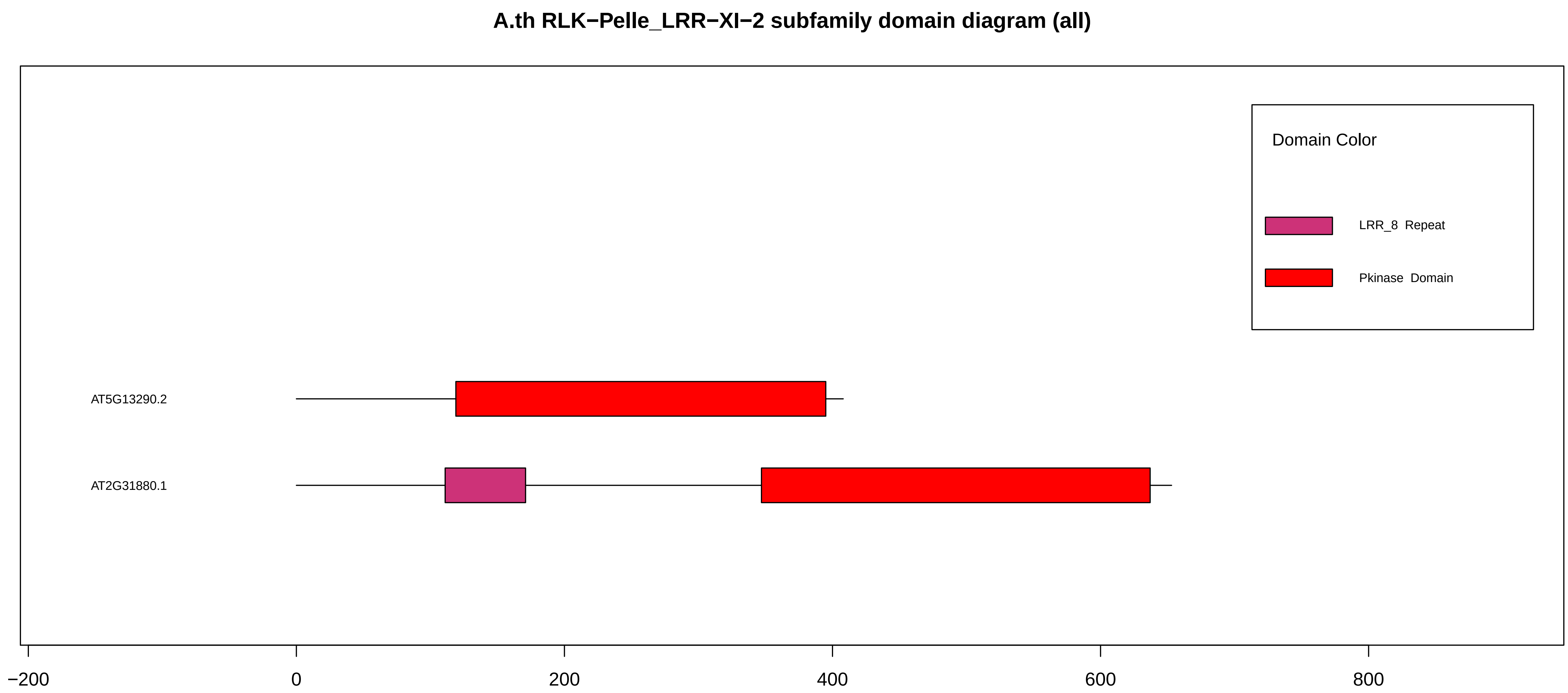
A.th RLK−Pelle_LRR−XI−2 subfamily domain diagram (all)
Domain Color
LRR_8 Repeat
Pkinase Domain
AT5G13290.2
AT2G31880.1
−200
0
200
400
600
800

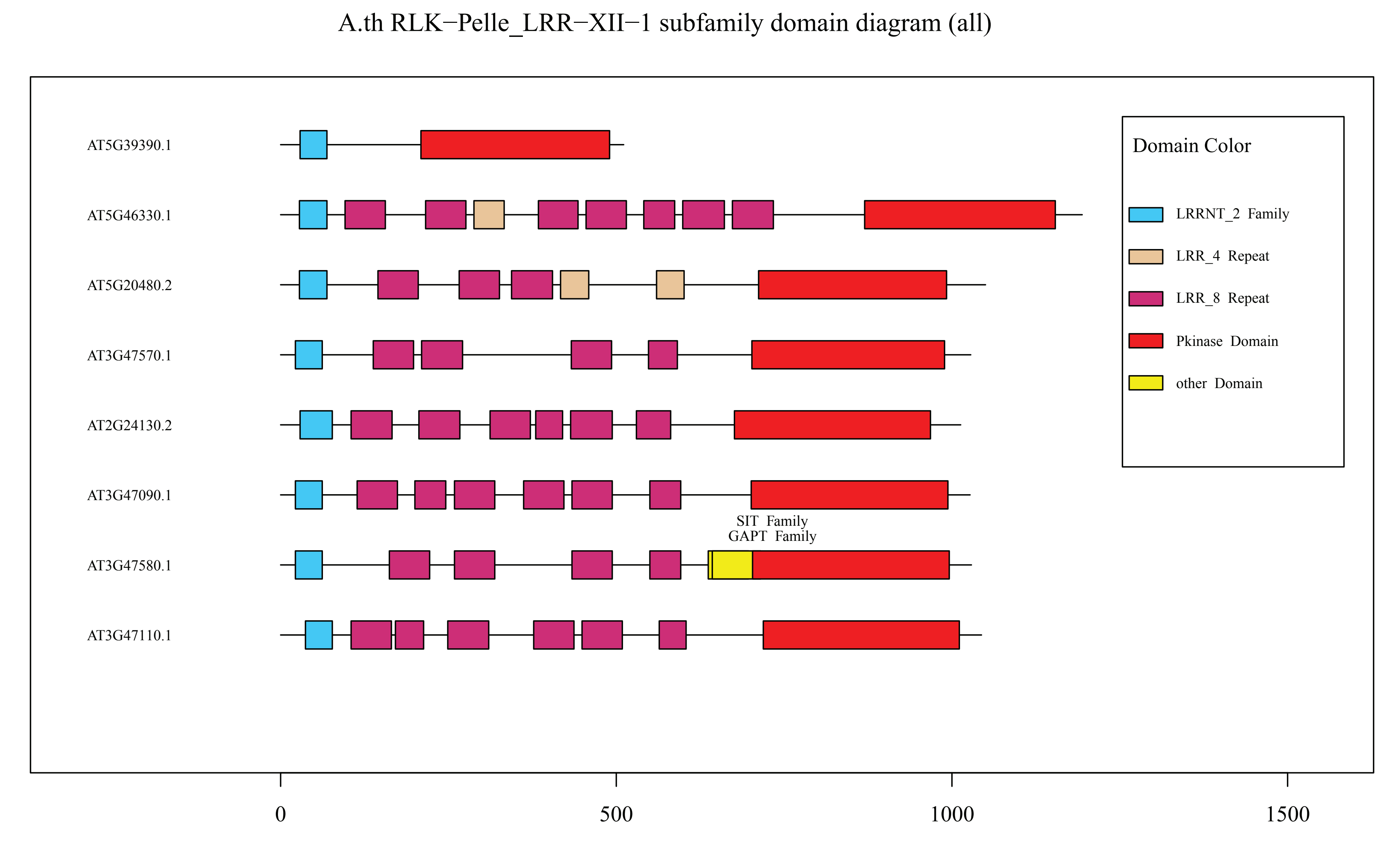
A.th RLK−Pelle_LRR−XII−1 subfamily domain diagram (all)
AT5G39390.1
AT5G46330.1
AT5G20480.2
AT3G47570.1
AT2G24130.2
AT3G47090.1
AT3G47580.1
AT3G47110.1
SIT Family
GAPT Family
Domain Color
LRRNT_2 Family
LRR_4 Repeat
LRR_8 Repeat
Pkinase Domain
other Domain
0
500
1000
1500

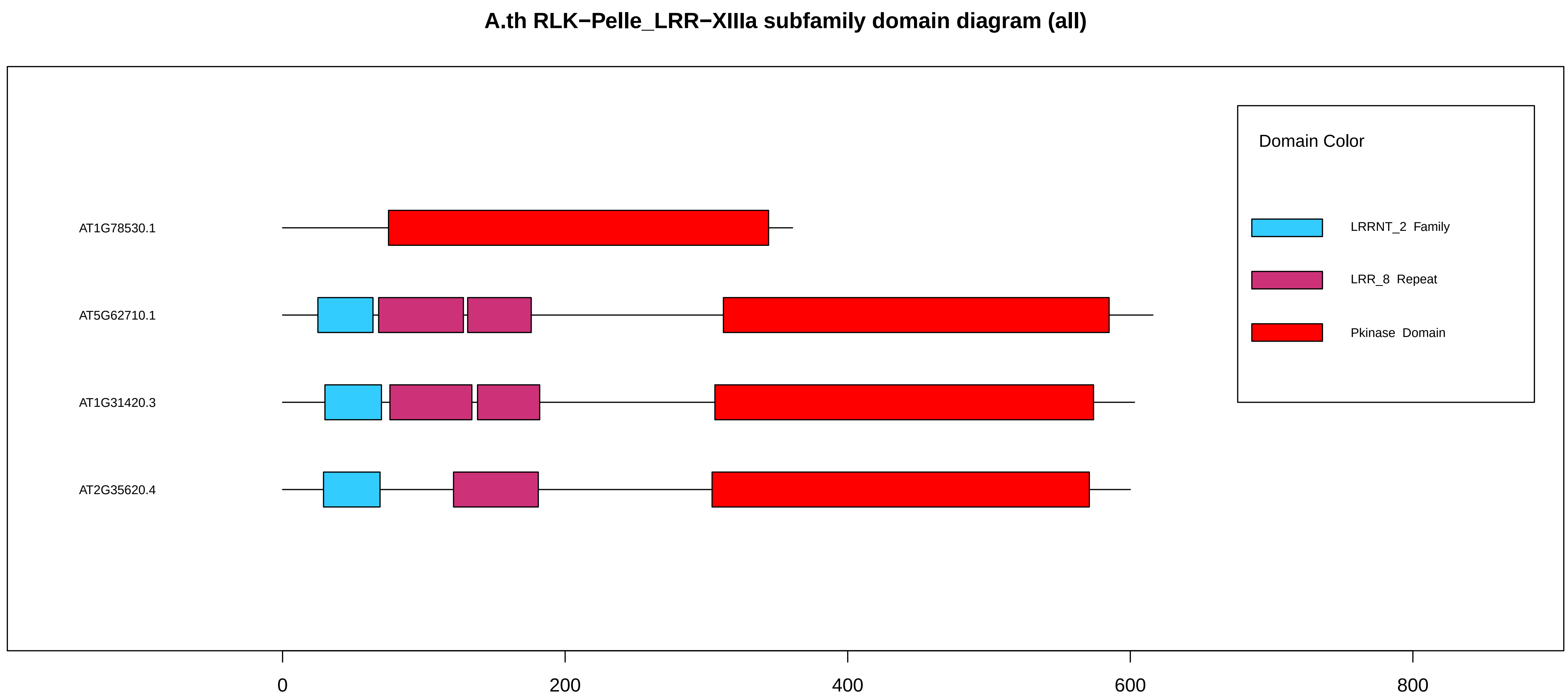
A.th RLK−Pelle_LRR−XIIIa subfamily domain diagram (all)
Domain Color
LRRNT_2 Family
LRR_8 Repeat
Pkinase Domain
AT1G78530.1
AT5G62710.1
AT1G31420.3
AT2G35620.4
0
200
400
600
800

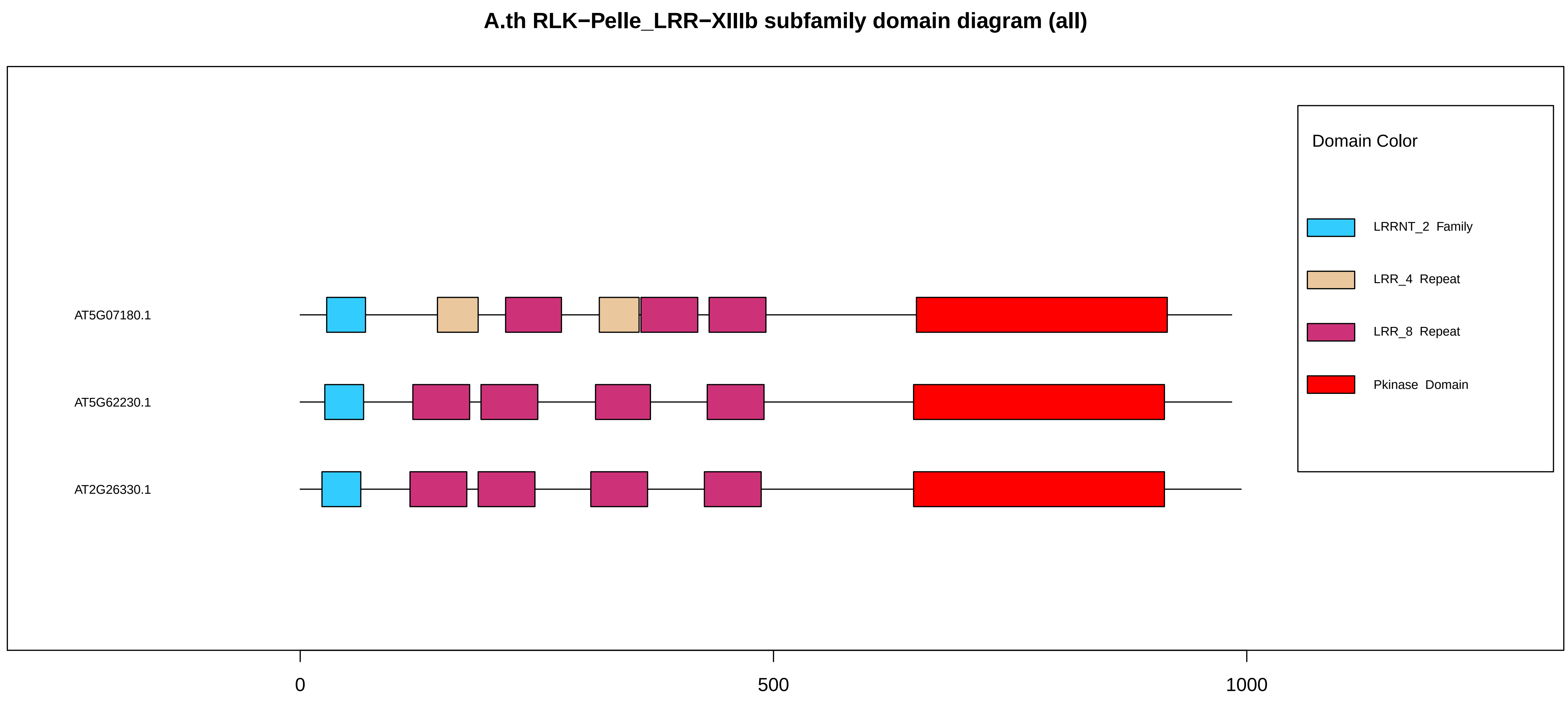
A.th RLK−Pelle_LRR−XIIIb subfamily domain diagram (all)
AT5G07180.1
AT5G62230.1
AT2G26330.1
Domain Color
LRRNT_2 Family
LRR_4 Repeat
LRR_8 Repeat
Pkinase Domain
0
500
1000

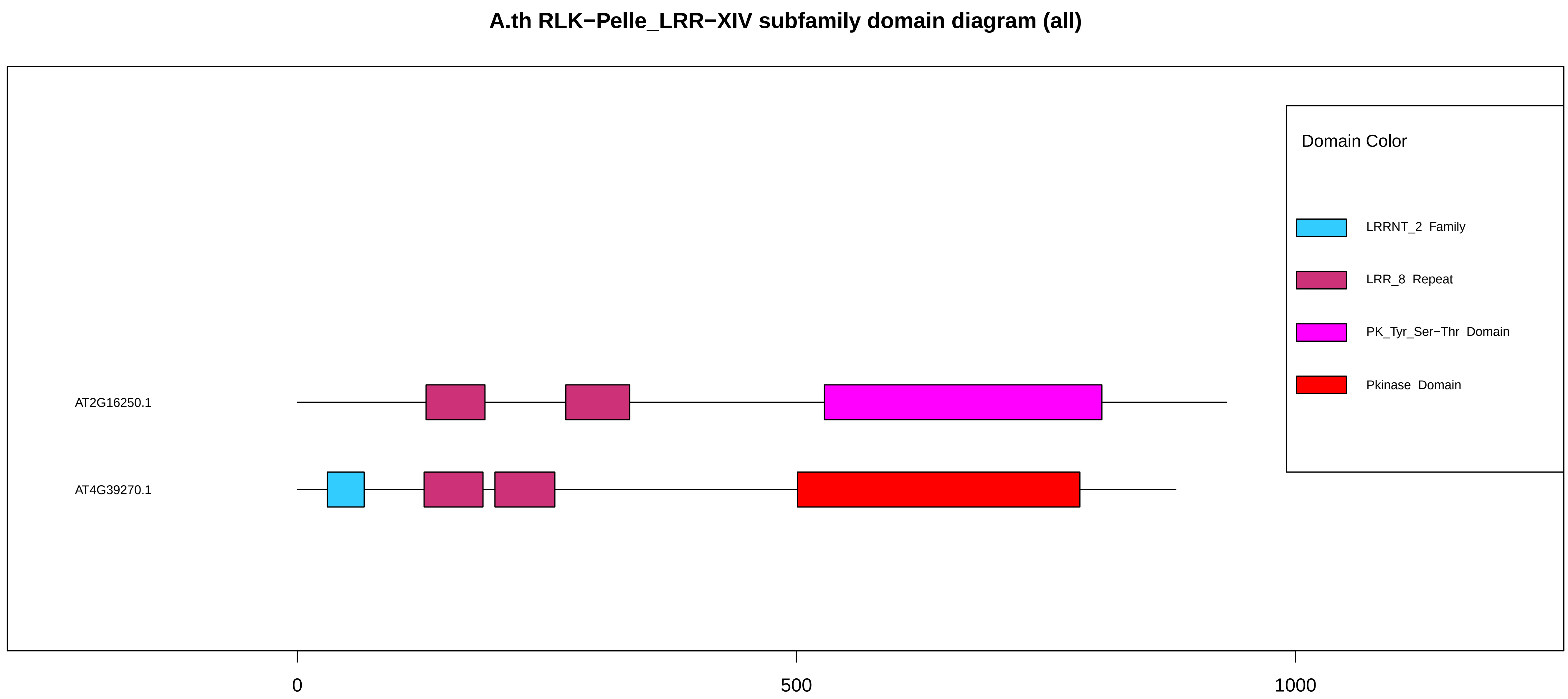
A.th RLK−Pelle_LRR−XIV subfamily domain diagram (all)
Domain Color
LRRNT_2 Family
LRR_8 Repeat
PK_Tyr_Ser−Thr Domain
Pkinase Domain
AT2G16250.1
AT4G39270.1
0
500
1000

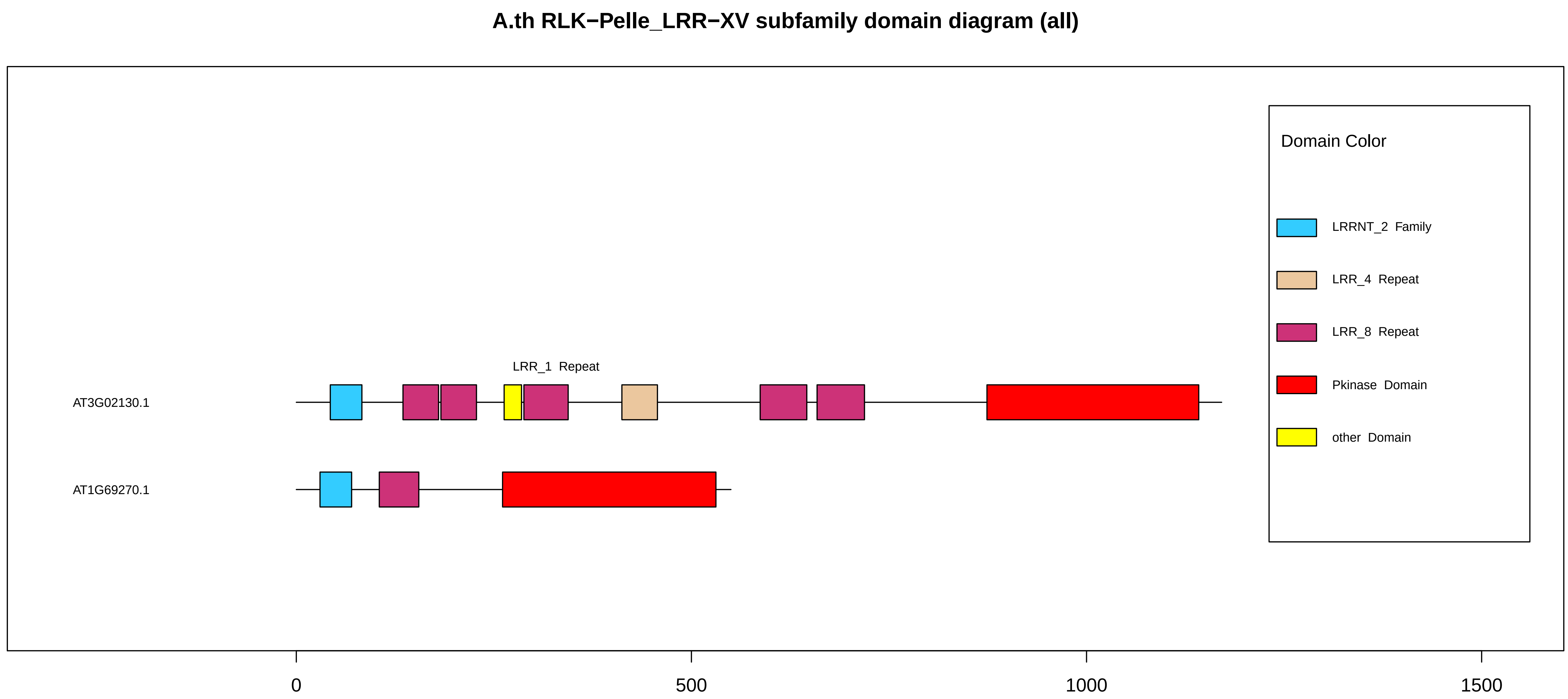
A.th RLK−Pelle_LRR−XV subfamily domain diagram (all)
AT3G02130.1
AT1G69270.1
LRR_1 Repeat
Domain Color
LRRNT_2 Family
LRR_4 Repeat
LRR_8 Repeat
Pkinase Domain
other Domain
0
500
1000
1500

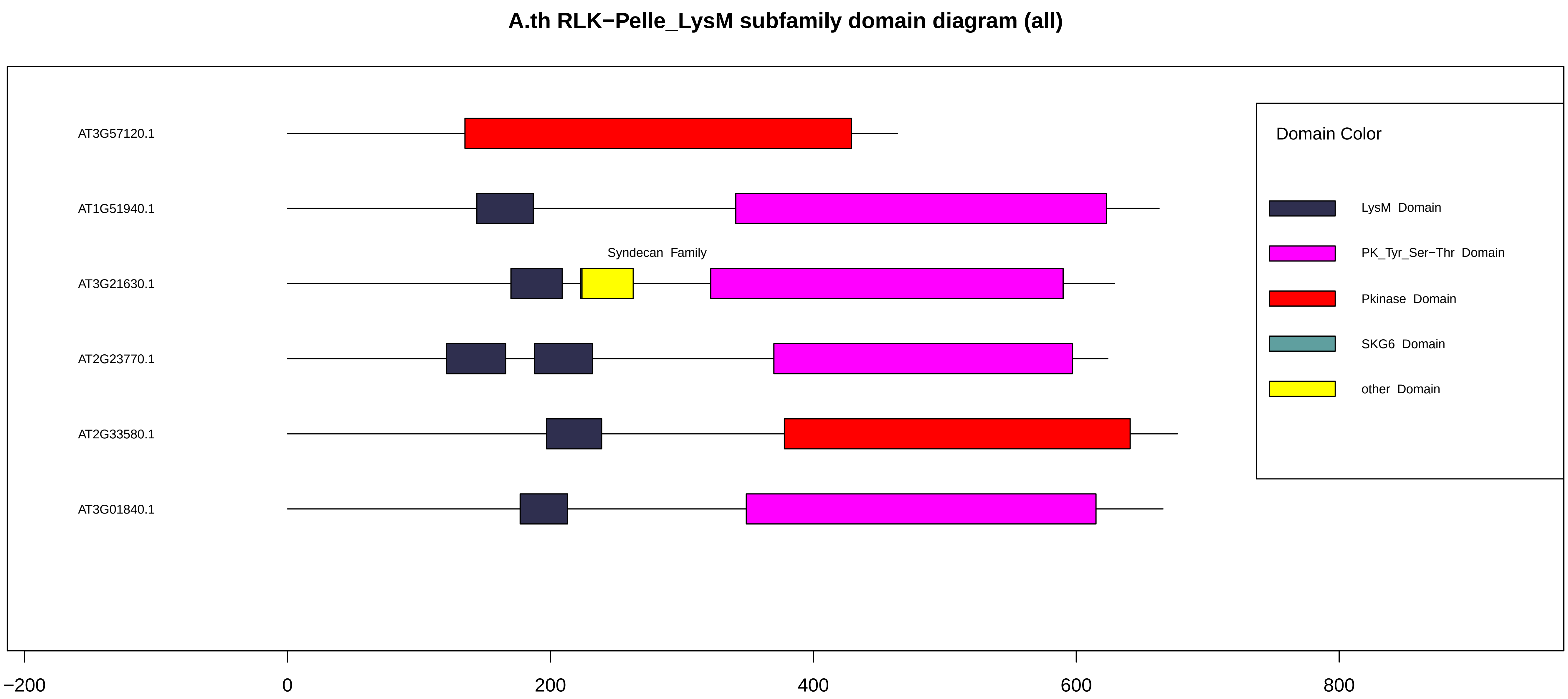

A.th RLK−Pelle_LysM subfamily domain diagram (all)
AT3G57120.1
AT1G51940.1
AT3G21630.1
AT2G23770.1
AT2G33580.1
AT3G01840.1
Syndecan Family
Domain Color
LysM Domain
PK_Tyr_Ser−Thr Domain
Pkinase Domain
SKG6 Domain
other Domain
−200
0
200
400
600
800

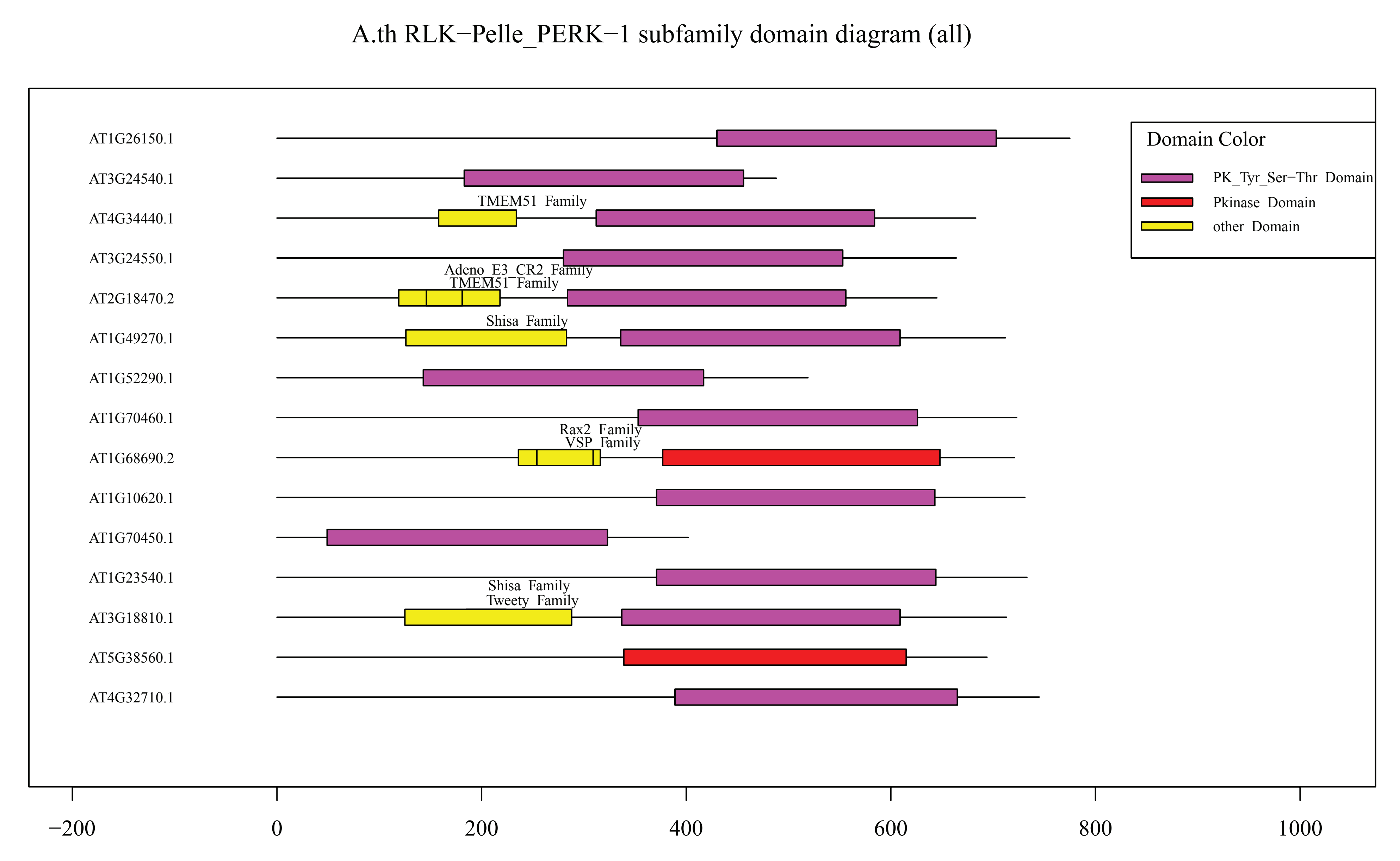

A.th RLK−Pelle_PERK−1 subfamily domain diagram (all)
AT1G26150.1
AT3G24540.1
AT4G34440.1
AT3G24550.1
AT2G18470.2
AT1G49270.1
AT1G52290.1
AT1G70460.1
AT1G68690.2
AT1G10620.1
AT1G70450.1
AT1G23540.1
AT3G18810.1
AT5G38560.1
AT4G32710.1
TMEM51 Family
Adeno_E3_CR2 Family
TMEM51 Family
Shisa Family
Rax2 Family
VSP Family
Shisa Family
Tweety Family
Domain Color
PK_Tyr_Ser−Thr Domain
Pkinase Domain
other Domain
−200
0
200
400
600
800
1000

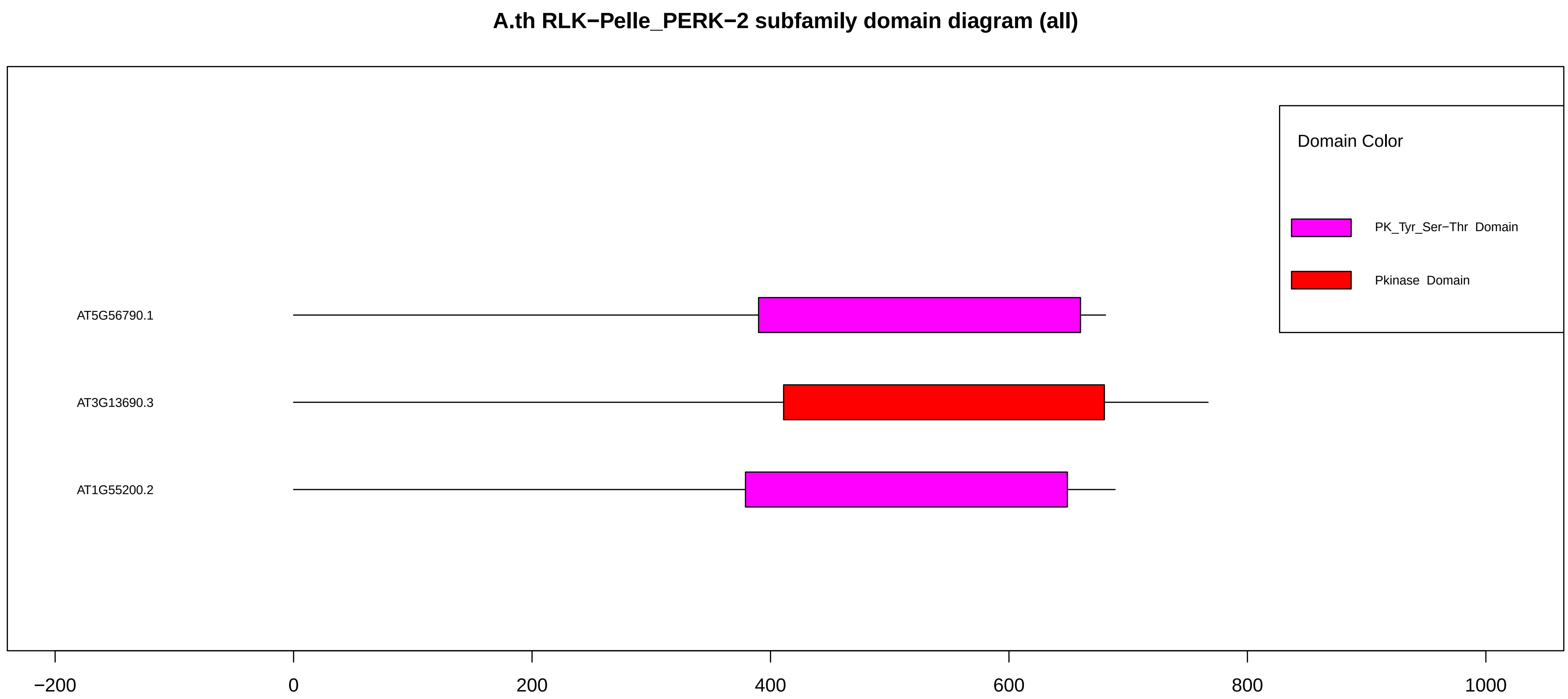
A.th RLK−Pelle_PERK−2 subfamily domain diagram (all)
Domain Color
PK_Tyr_Ser−Thr Domain
Pkinase Domain
AT5G56790.1
AT3G13690.3
AT1G55200.2
−200
0
200
400
600
800
1000

A.th RLK−Pelle_RKF3 subfamily domain diagram (all)

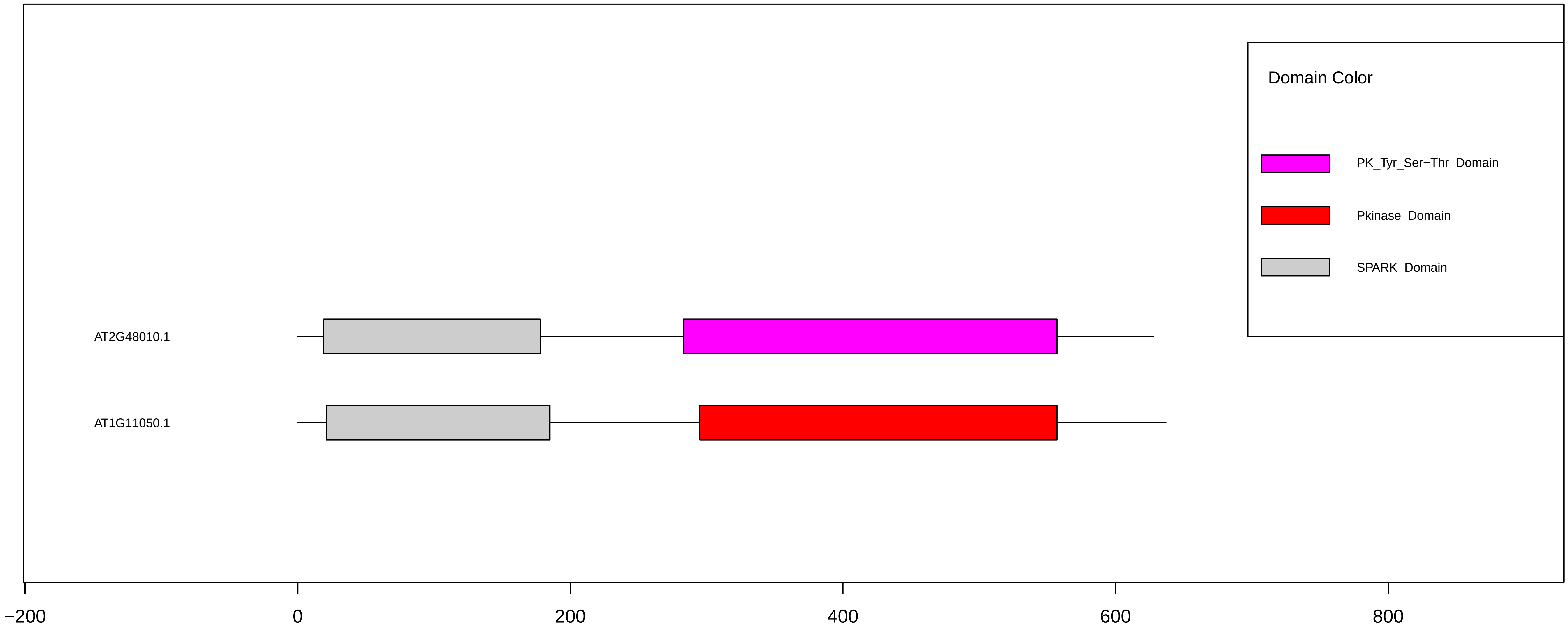

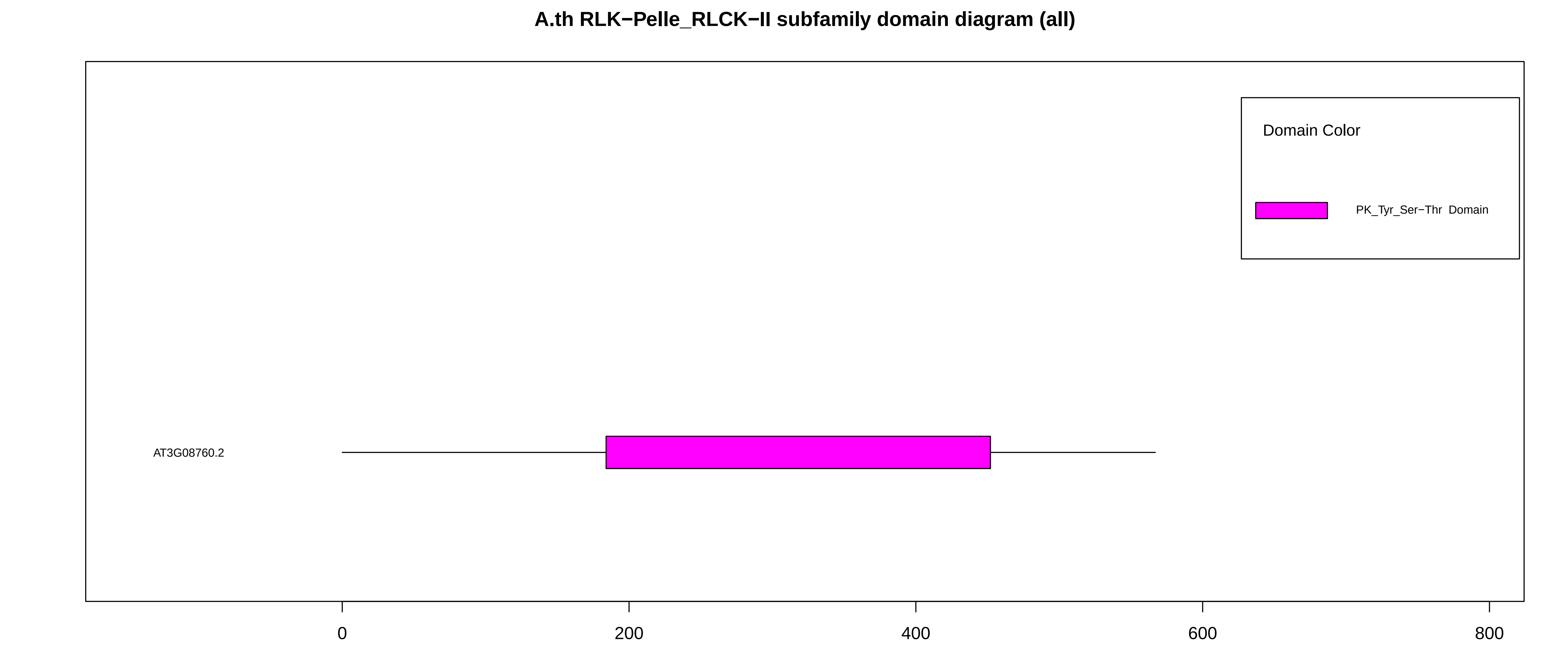

A.th RLK−Pelle_RLCK−II subfamily domain diagram (all)
Domain Color
PK_Tyr_Ser−Thr Domain
AT3G08760.2
0
200
400
600
800

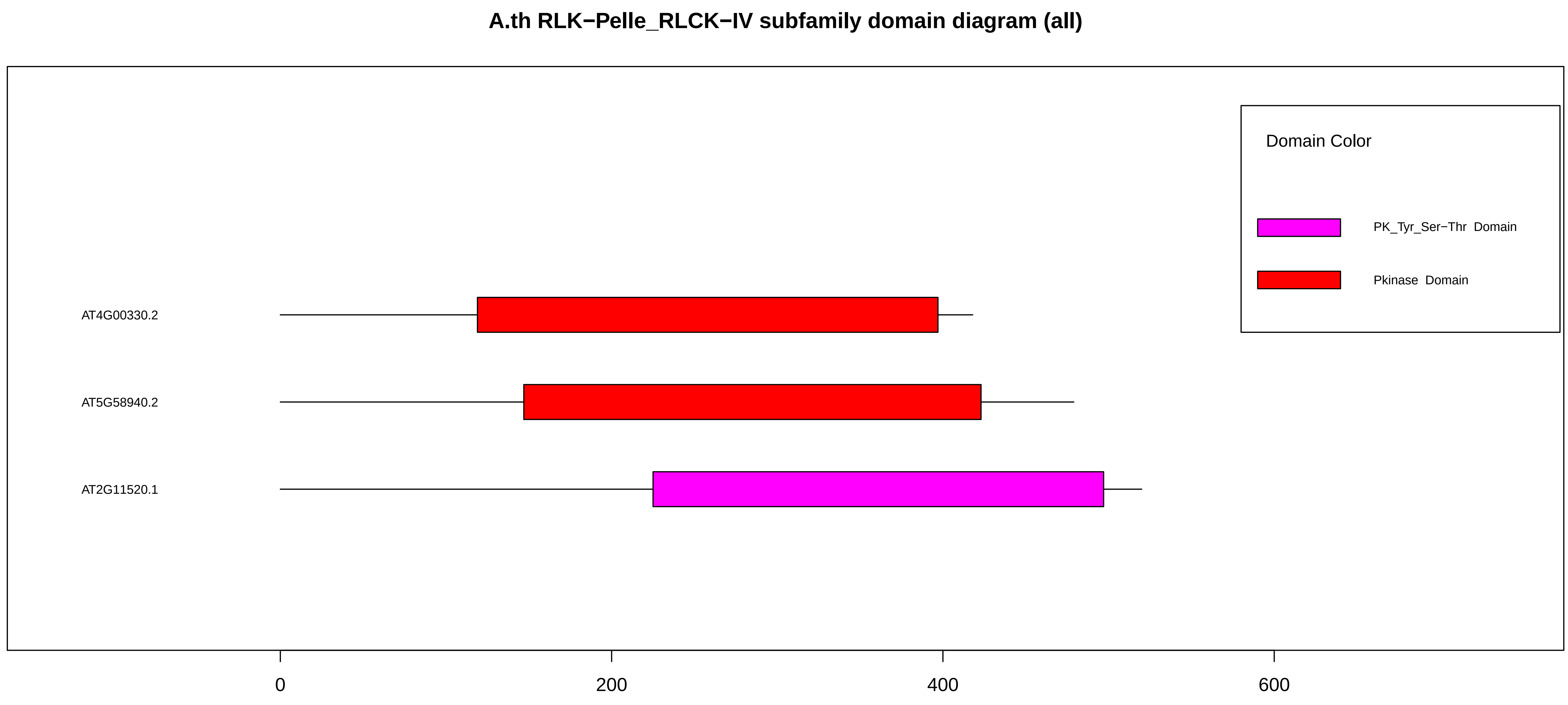
A.th RLK−Pelle_RLCK−IV subfamily domain diagram (all)
Domain Color
PK_Tyr_Ser−Thr Domain
Pkinase Domain
AT4G00330.2
AT5G58940.2
AT2G11520.1
0
200
400
600

A.th RLK−Pelle_RLCK−IXa subfamily domain diagram (all)

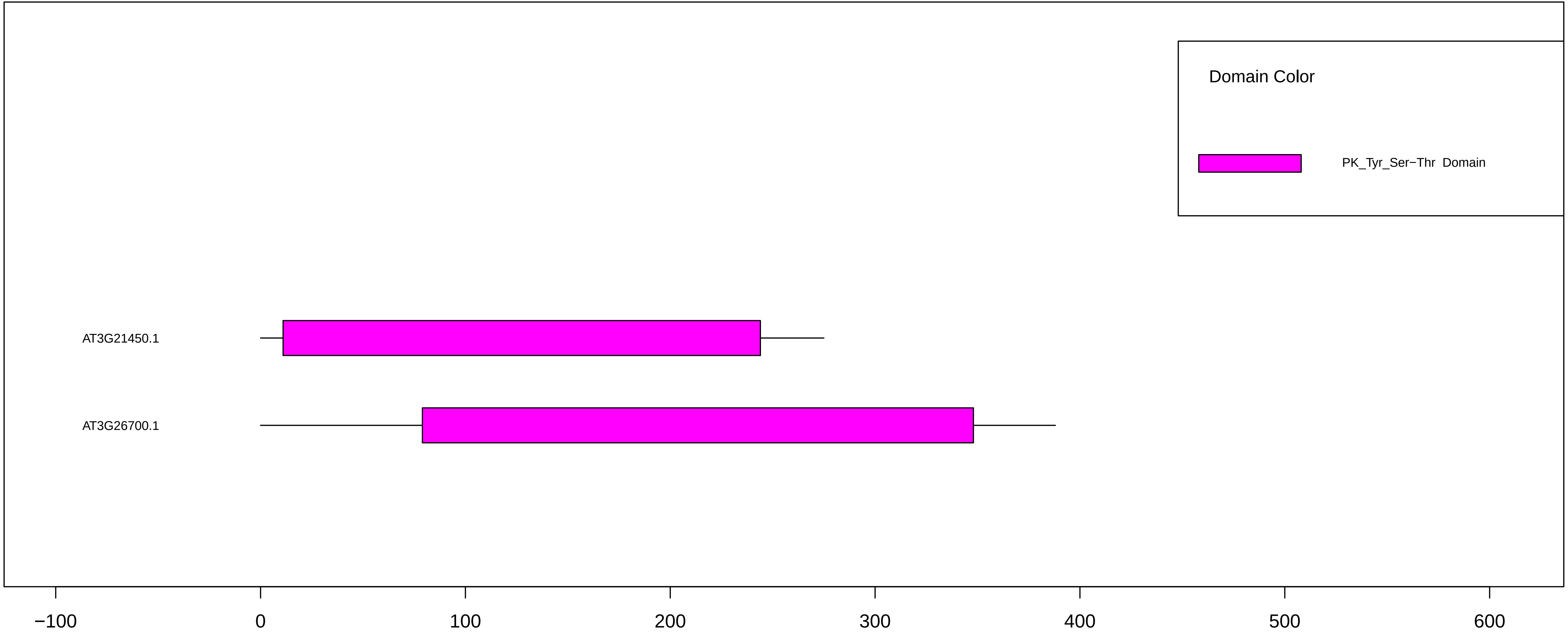

A.th RLK−Pelle_RLCK−IXb subfamily domain diagram (all)

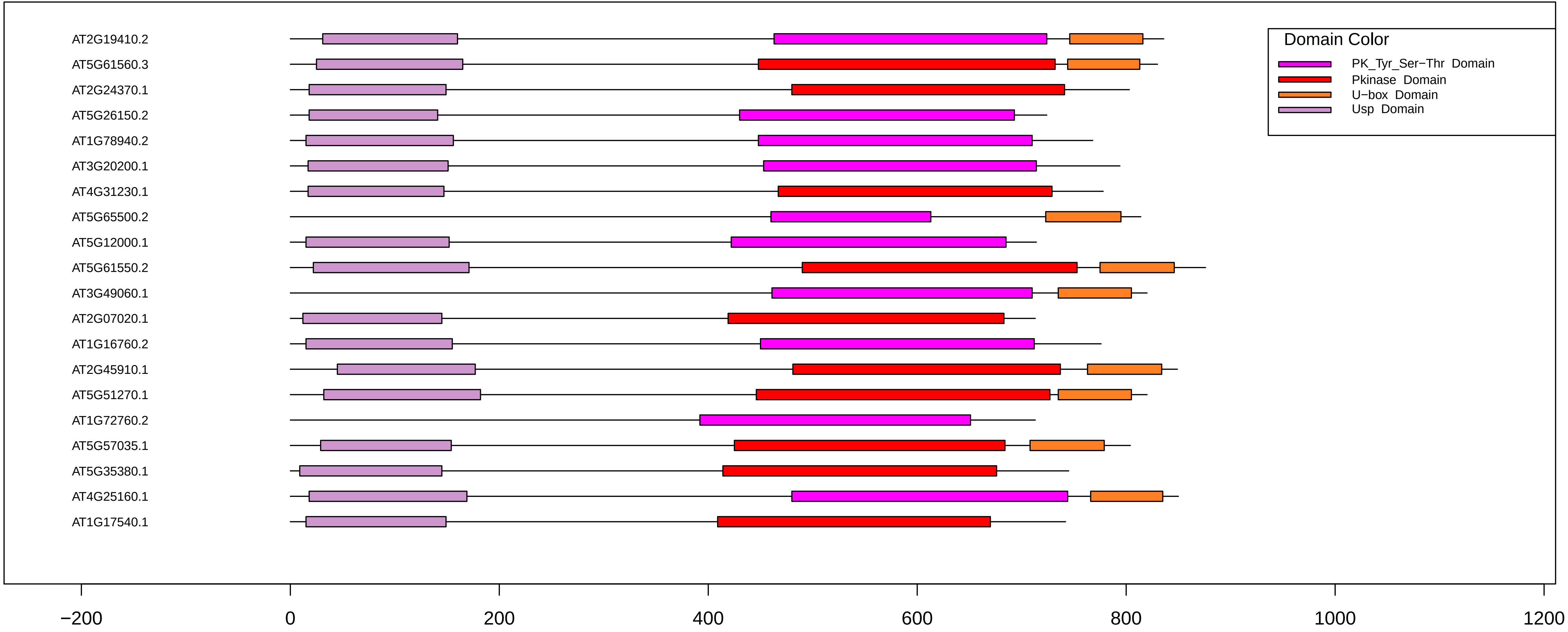

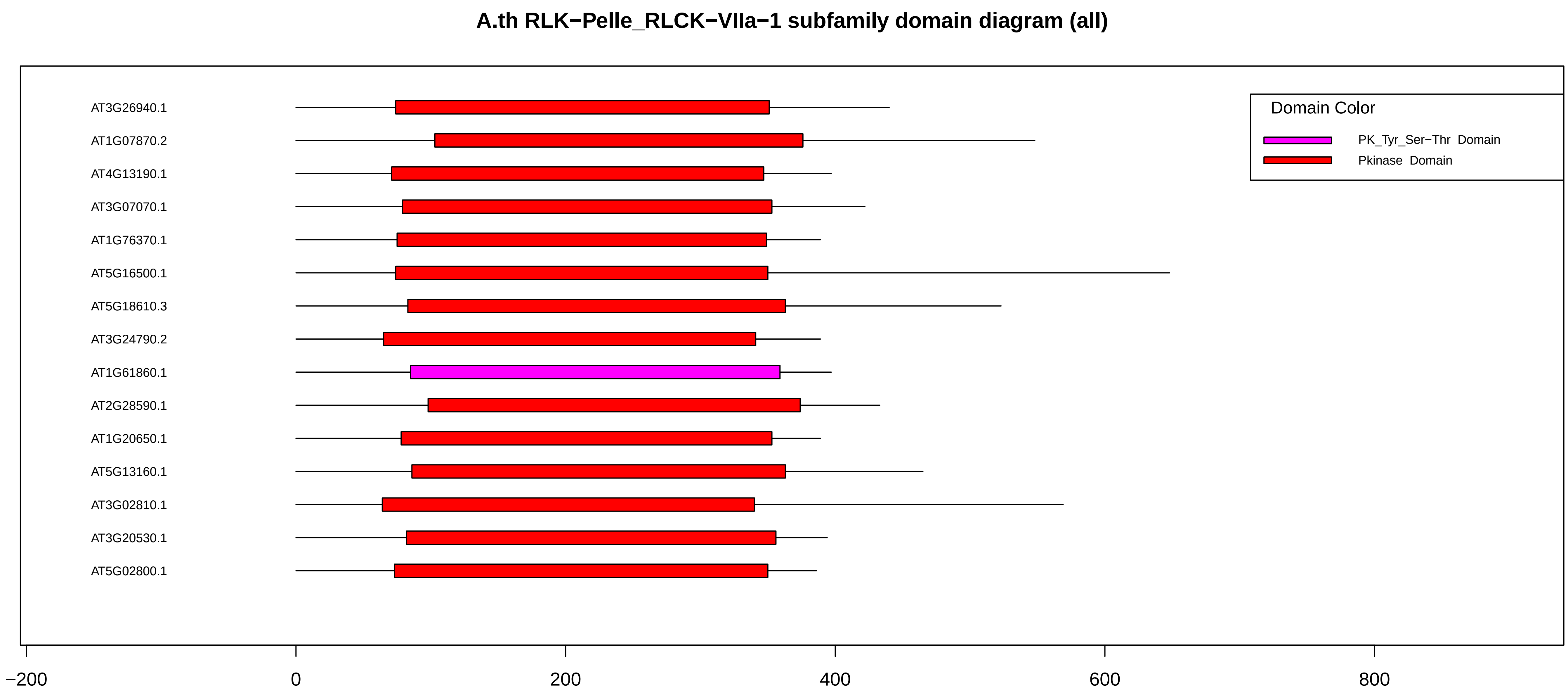
A.th RLK−Pelle_RLCK−VIIa−1 subfamily domain diagram (all)
AT3G26940.1
AT1G07870.2
AT4G13190.1
AT3G07070.1
AT1G76370.1
AT5G16500.1
AT5G18610.3
AT3G24790.2
AT1G61860.1
AT2G28590.1
AT1G20650.1
AT5G13160.1
AT3G02810.1
AT3G20530.1
AT5G02800.1
Domain Color
PK_Tyr_Ser−Thr Domain
Pkinase Domain
−200
0
200
400
600
800

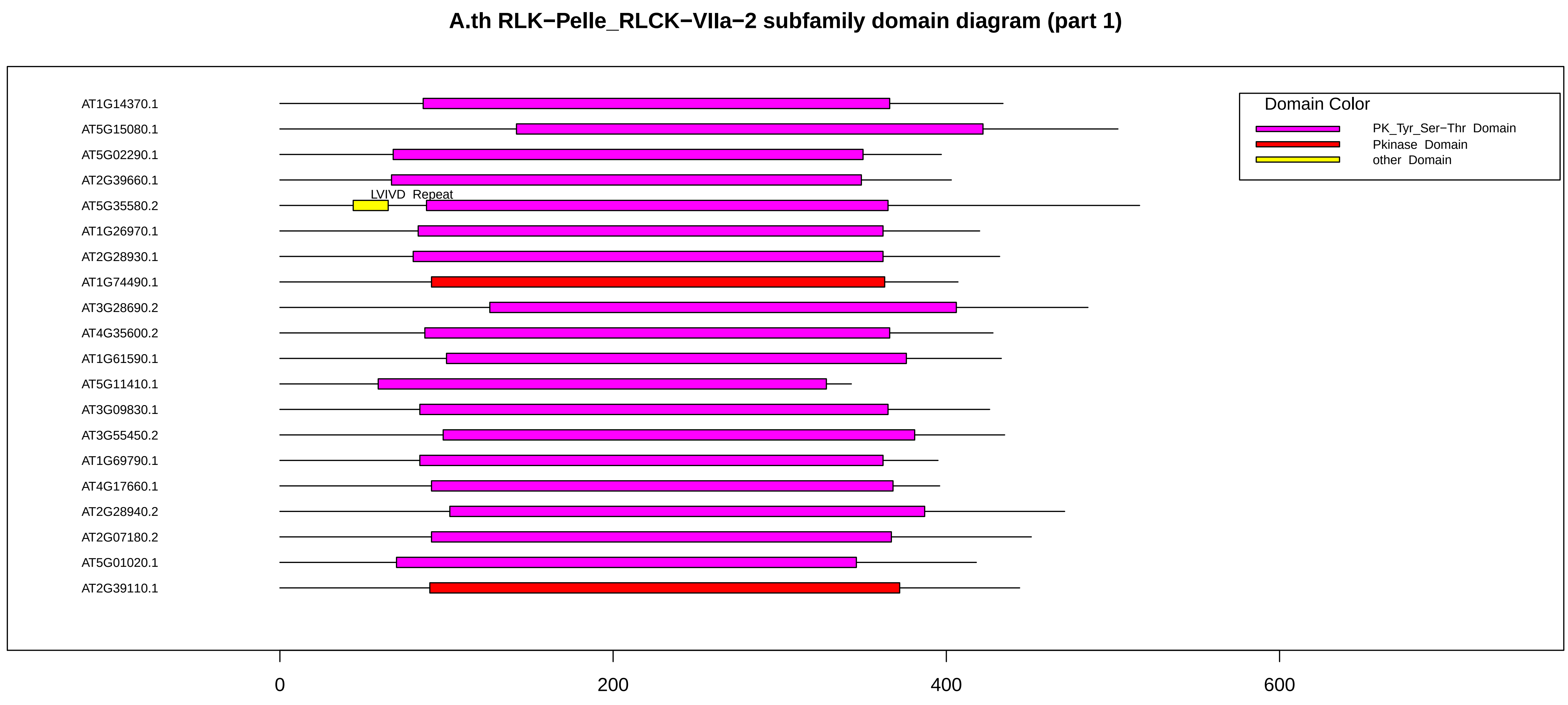
A.th RLK−Pelle_RLCK−VIIa−2 subfamily domain diagram (part 1)
AT1G14370.1
AT5G15080.1
AT5G02290.1
AT2G39660.1
AT5G35580.2
LVIVD Repeat
AT1G26970.1
AT2G28930.1
AT1G74490.1
AT3G28690.2
AT4G35600.2
AT1G61590.1
AT5G11410.1
AT3G09830.1
AT3G55450.2
AT1G69790.1
AT4G17660.1
AT2G28940.2
AT2G07180.2
AT5G01020.1
AT2G39110.1
Domain Color
PK_Tyr_Ser−Thr Domain
Pkinase Domain
other Domain
0
200
400
600

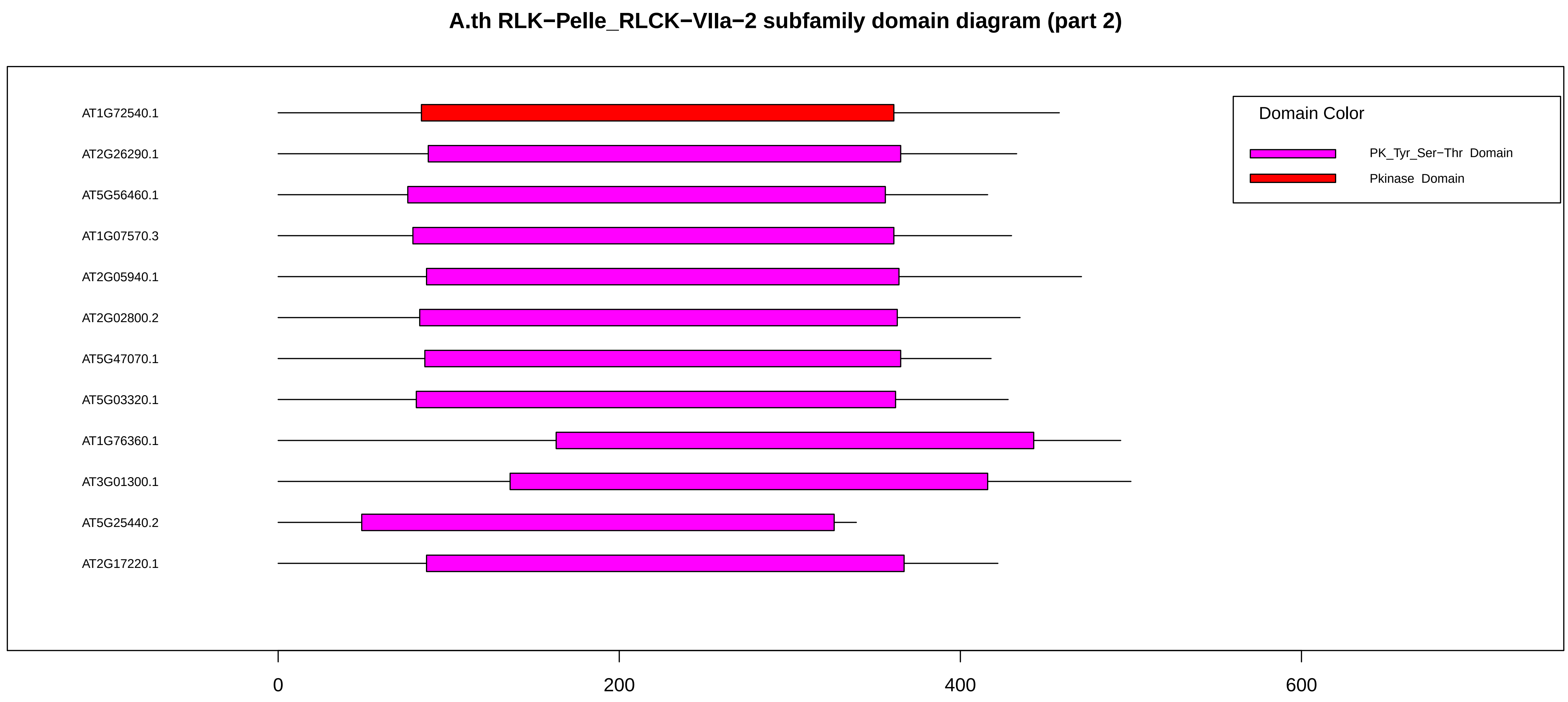
A.th RLK−Pelle_RLCK−VIIa−2 subfamily domain diagram (part 2)
AT1G72540.1
AT2G26290.1
AT5G56460.1
AT1G07570.3
AT2G05940.1
AT2G02800.2
AT5G47070.1
AT5G03320.1
AT1G76360.1
AT3G01300.1
AT5G25440.2
AT2G17220.1
Domain Color
PK_Tyr_Ser−Thr Domain
Pkinase Domain
0
200
400
600

A.th RLK−Pelle_RLCK−VIIb subfamily domain diagram (all)

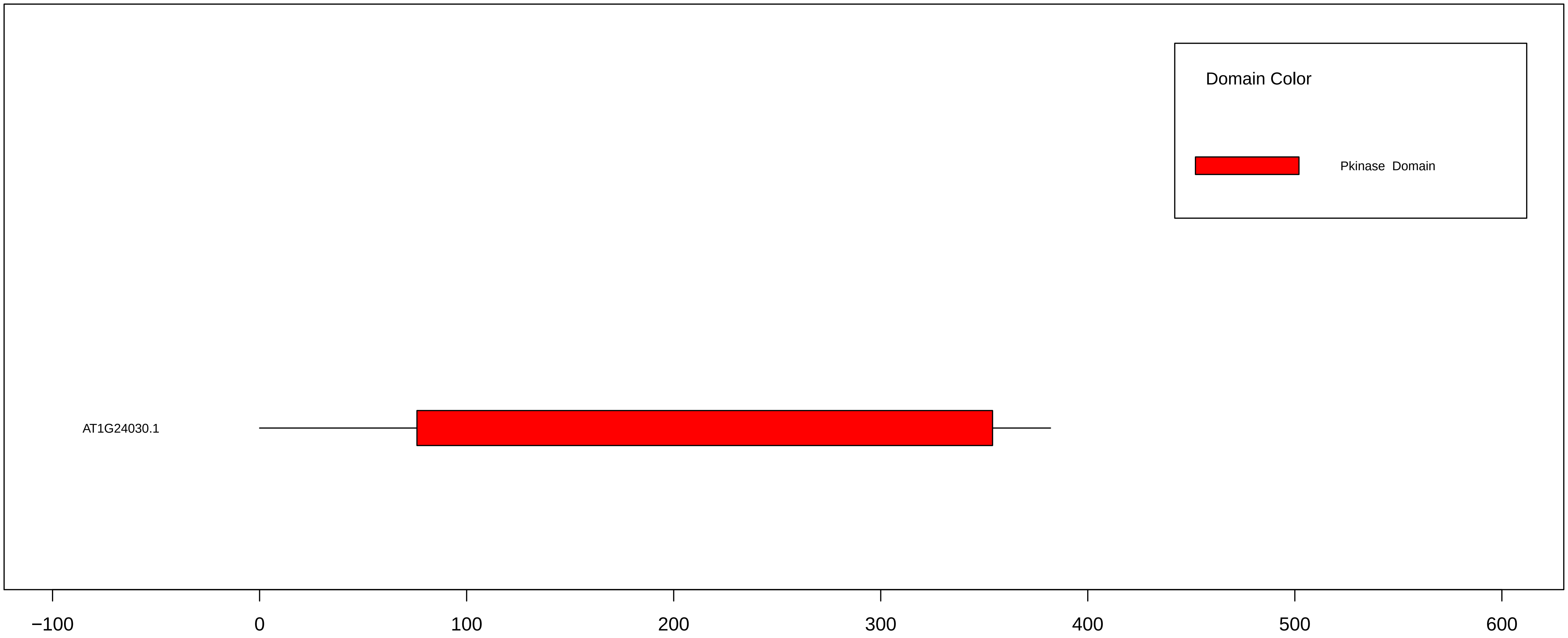

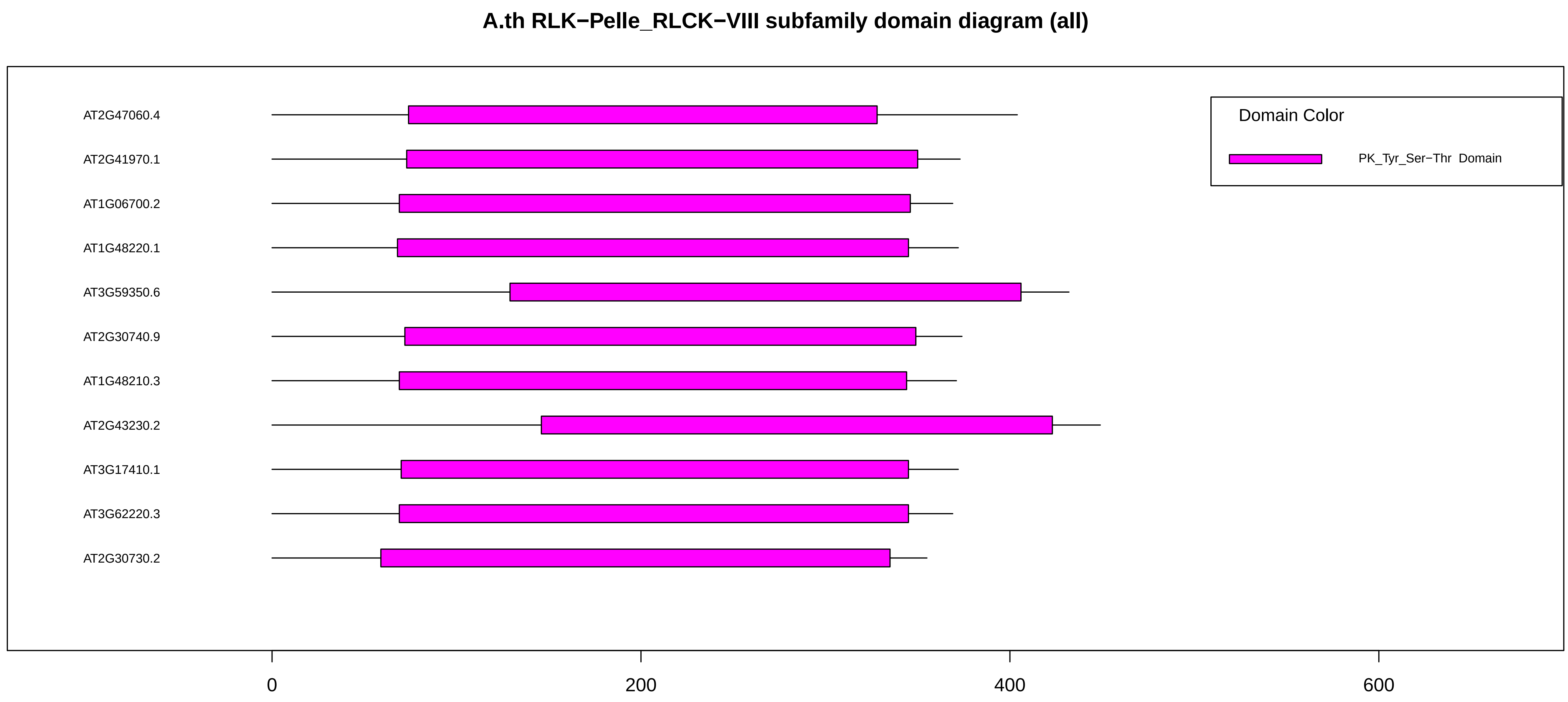
A.th RLK−Pelle_RLCK−VIII subfamily domain diagram (all)
AT2G47060.4
AT2G41970.1
AT1G06700.2
AT1G48220.1
AT3G59350.6
AT2G30740.9
AT1G48210.3
AT2G43230.2
AT3G17410.1
AT3G62220.3
AT2G30730.2
Domain Color
PK_Tyr_Ser−Thr Domain
0
200
400
600

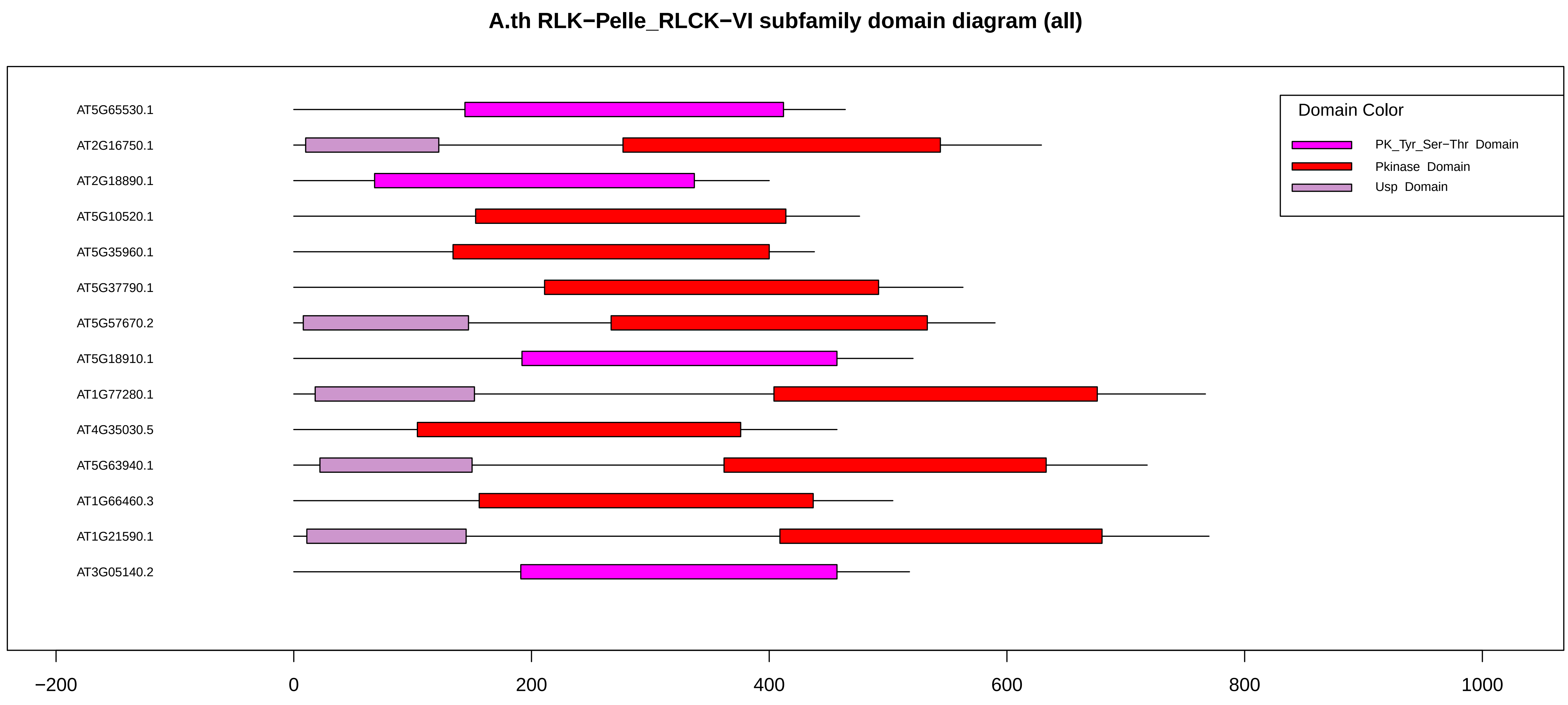
A.th RLK−Pelle_RLCK−VI subfamily domain diagram (all)
AT5G65530.1
AT2G16750.1
AT2G18890.1
AT5G10520.1
AT5G35960.1
AT5G37790.1
AT5G57670.2
AT5G18910.1
AT1G77280.1
AT4G35030.5
AT5G63940.1
AT1G66460.3
AT1G21590.1
AT3G05140.2
Domain Color
PK_Tyr_Ser−Thr Domain
Pkinase Domain
Usp Domain
−200
0
200
400
600
800
1000

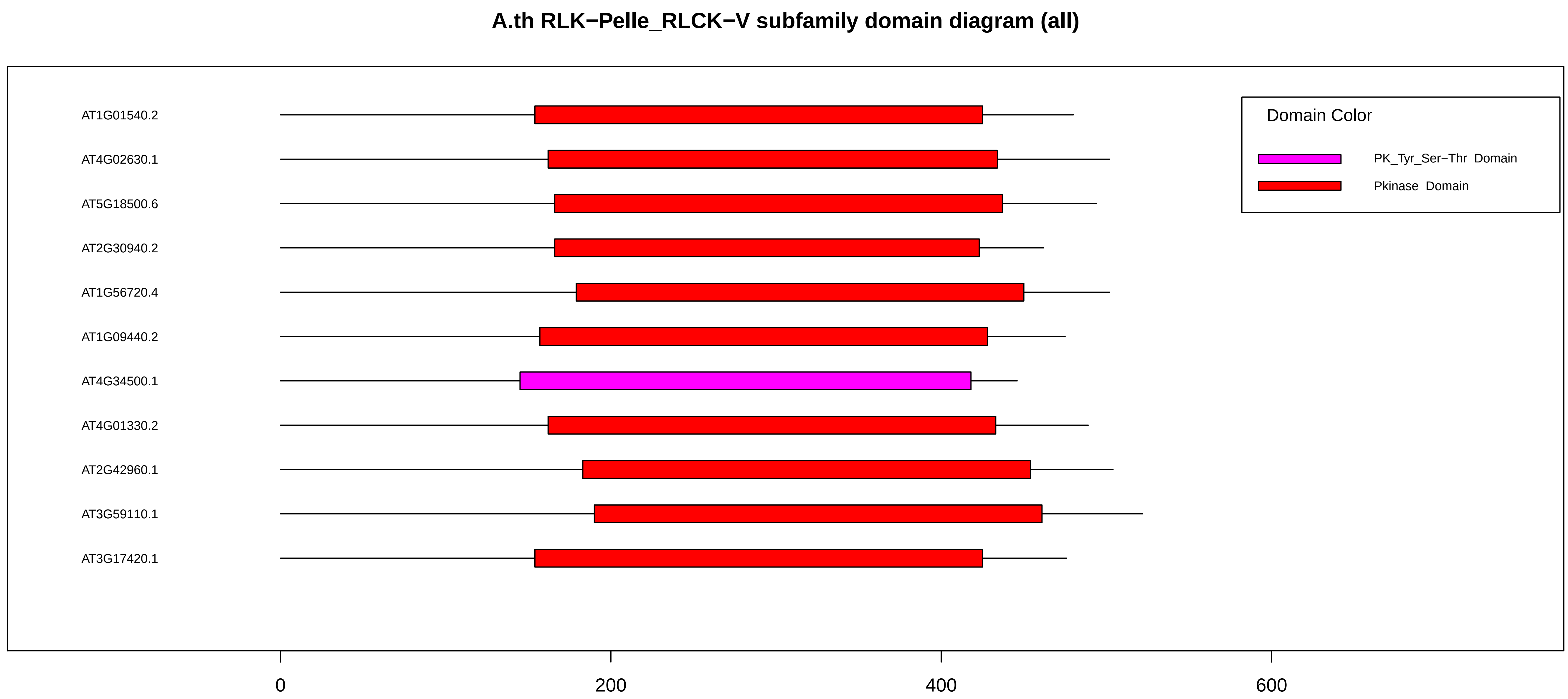
A.th RLK−Pelle_RLCK−V subfamily domain diagram (all)
AT1G01540.2
AT4G02630.1
AT5G18500.6
AT2G30940.2
AT1G56720.4
AT1G09440.2
AT4G34500.1
AT4G01330.2
AT2G42960.1
AT3G59110.1
AT3G17420.1
Domain Color
PK_Tyr_Ser−Thr Domain
Pkinase Domain
0
200
400
600

A.th RLK−Pelle_RLCK−XII−1 subfamily domain diagram (all)

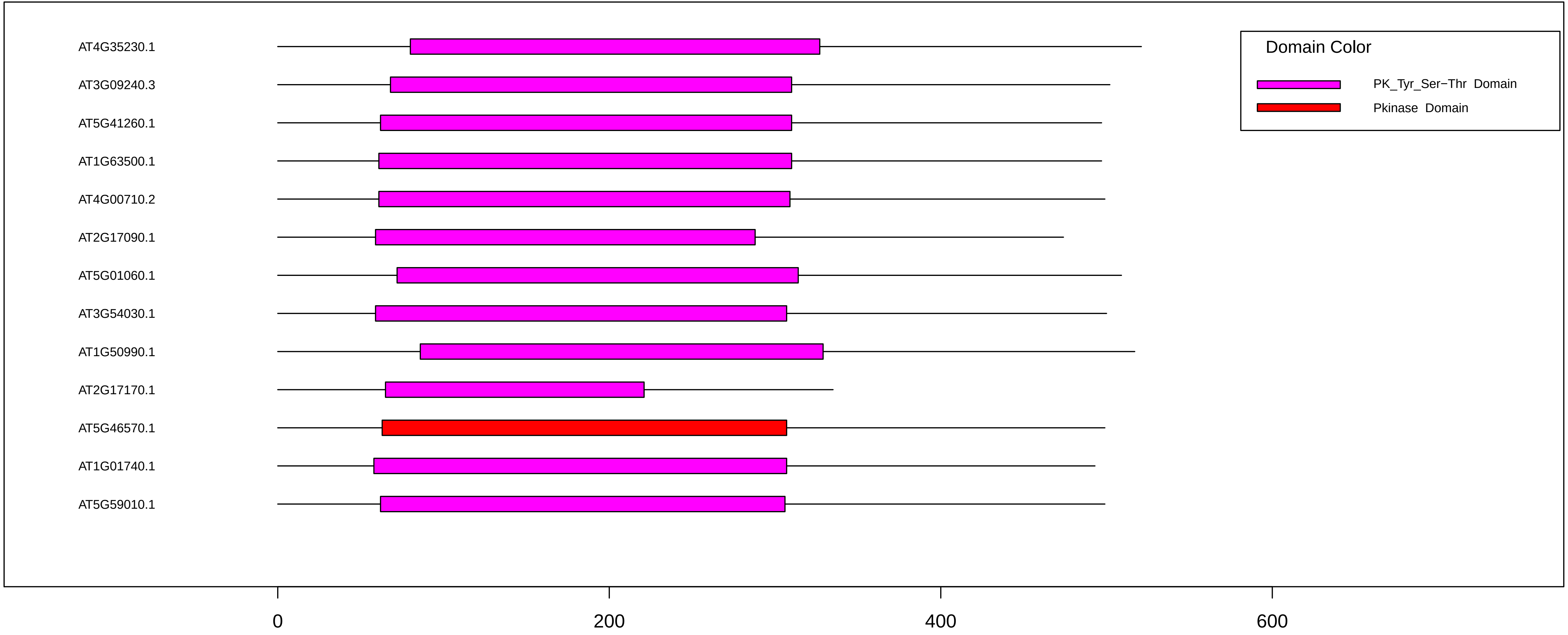

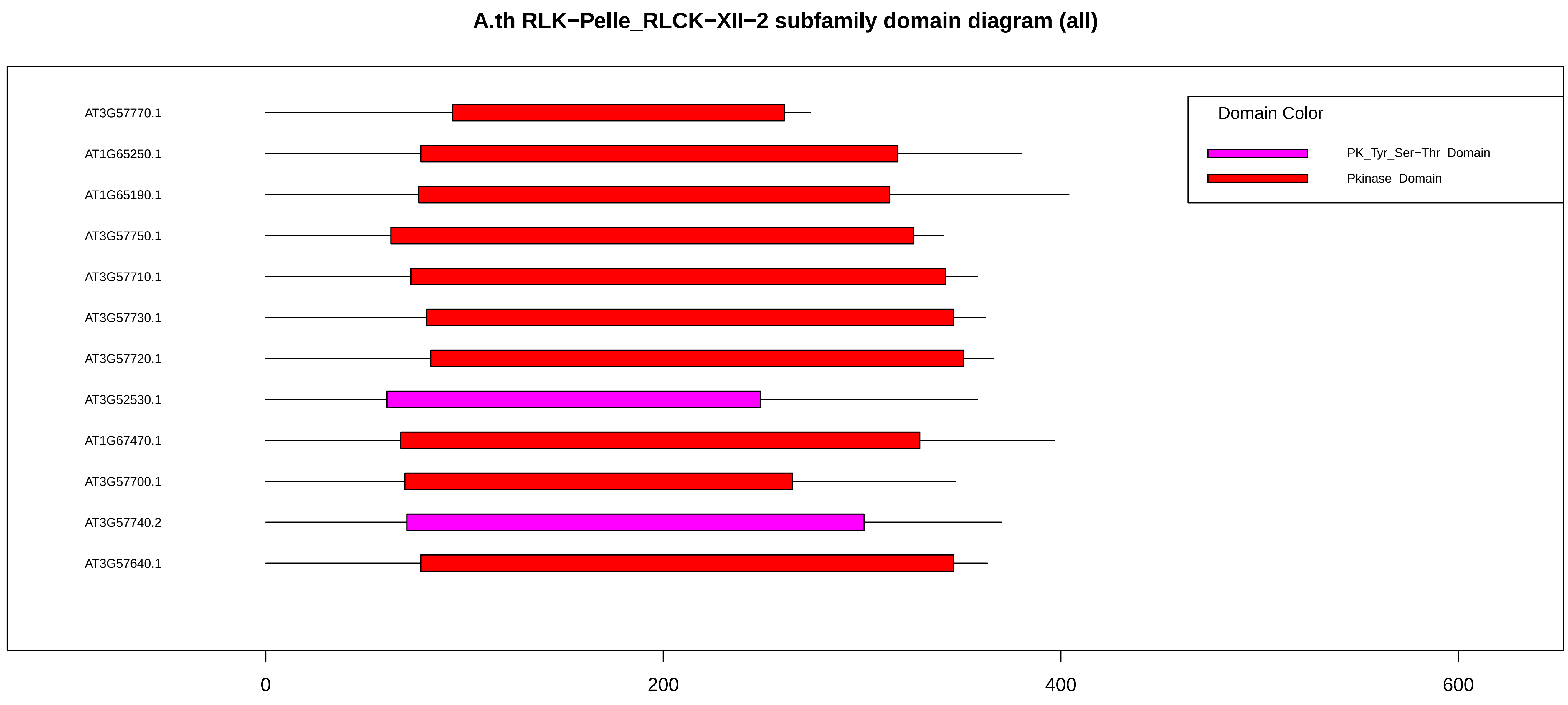
A.th RLK−Pelle_RLCK−XII−2 subfamily domain diagram (all)
AT3G57770.1
AT1G65250.1
AT1G65190.1
AT3G57750.1
AT3G57710.1
AT3G57730.1
AT3G57720.1
AT3G52530.1
AT1G67470.1
AT3G57700.1
AT3G57740.2
AT3G57640.1
Domain Color
PK_Tyr_Ser−Thr Domain
Pkinase Domain
0
200
400
600

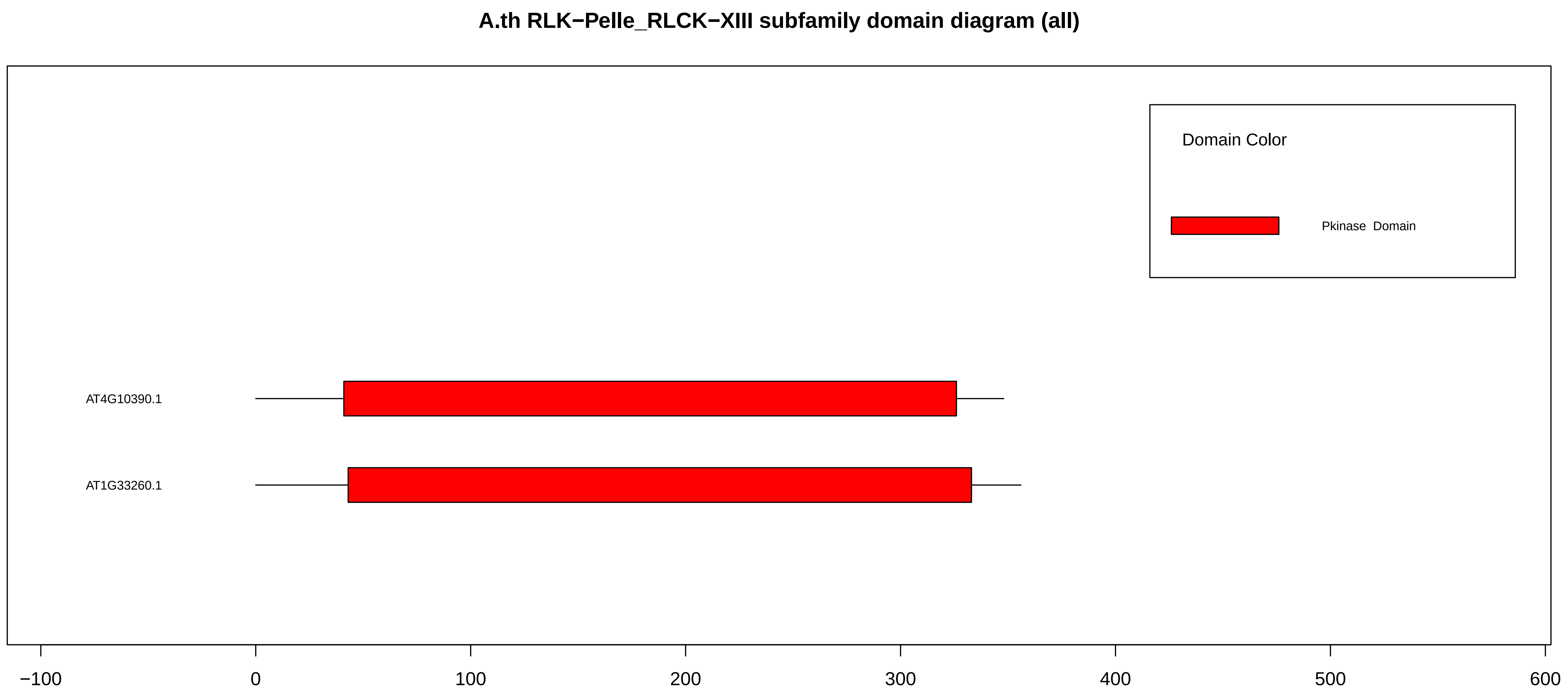
A.th RLK−Pelle_RLCK−XIII subfamily domain diagram (all)
Domain Color
Pkinase Domain
AT4G10390.1
AT1G33260.1
−100
0
100
200
300
400
500
600

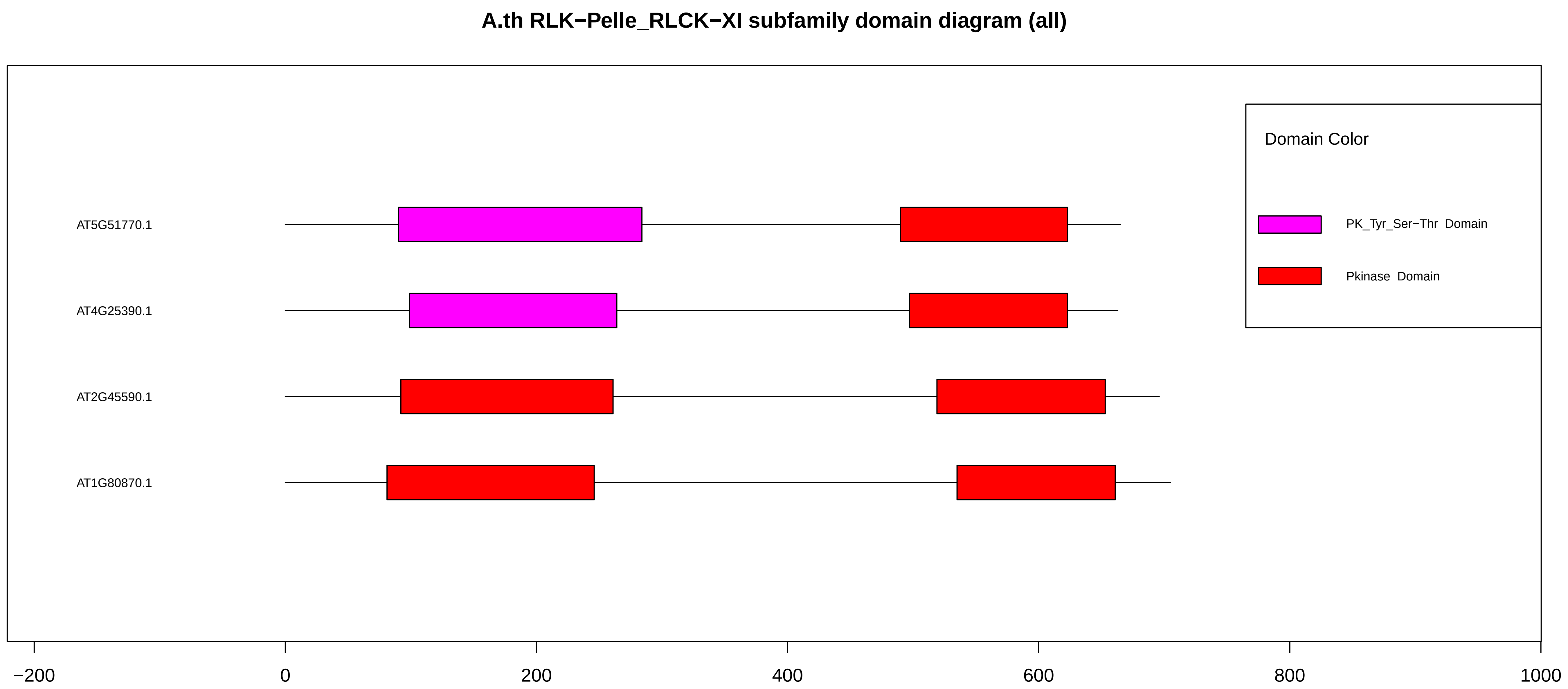
A.th RLK−Pelle_RLCK−XI subfamily domain diagram (all)
AT5G51770.1
AT4G25390.1
AT2G45590.1
AT1G80870.1
Domain Color
PK_Tyr_Ser−Thr Domain
Pkinase Domain
−200
0
200
400
600
800
1000

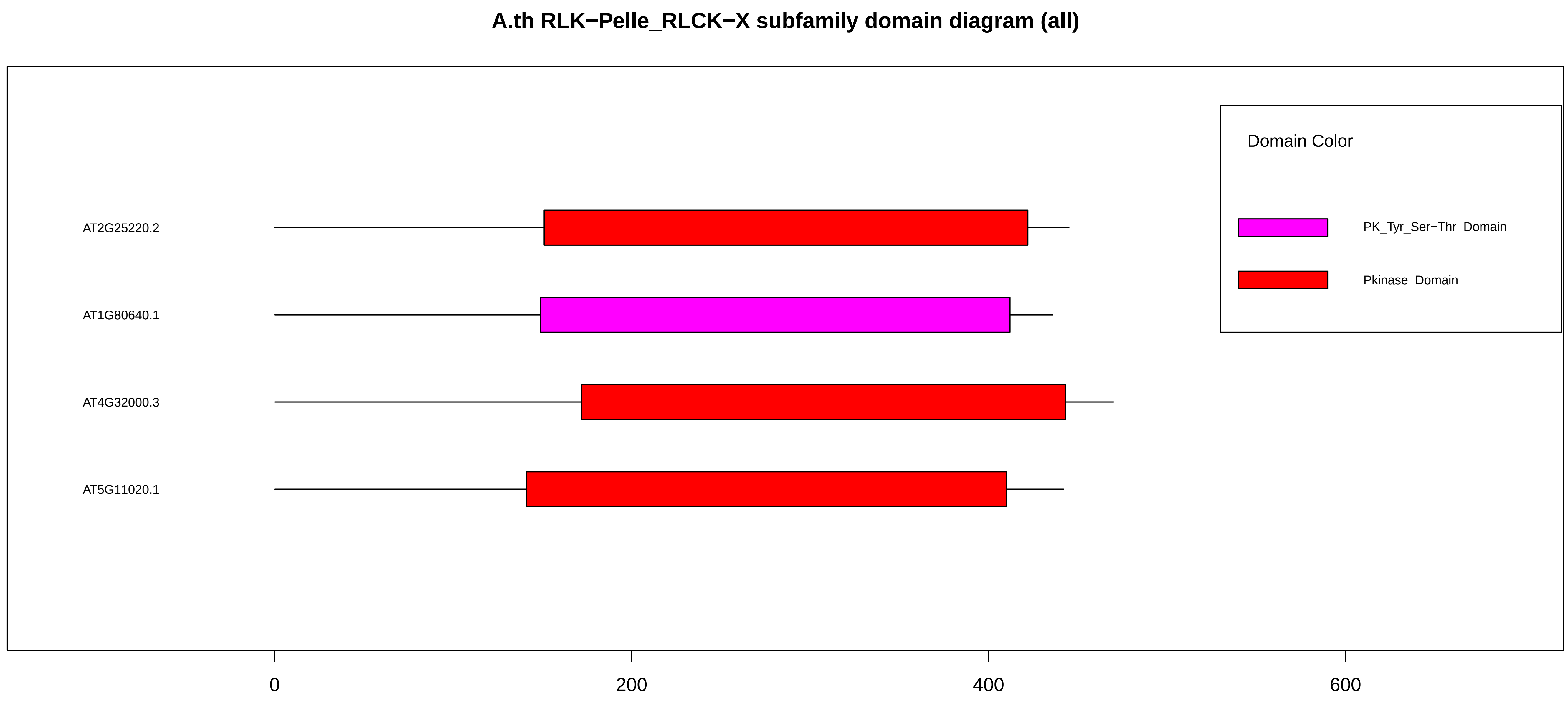
A.th RLK−Pelle_RLCK−X subfamily domain diagram (all)
Domain Color
PK_Tyr_Ser−Thr Domain
Pkinase Domain
AT2G25220.2
AT1G80640.1
AT4G32000.3
AT5G11020.1
0
200
400
600

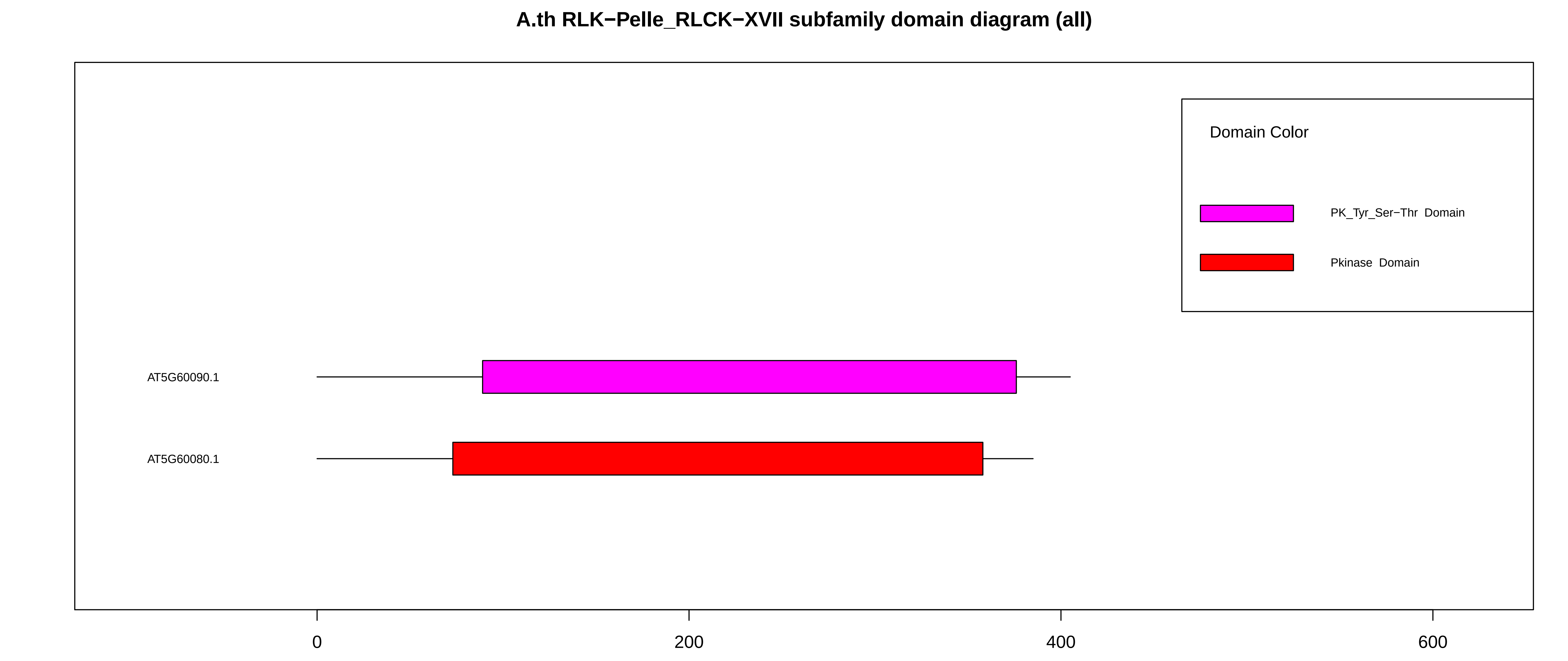
A.th RLK−Pelle_RLCK−XVII subfamily domain diagram (all)
Domain Color
PK_Tyr_Ser−Thr Domain
Pkinase Domain
AT5G60090.1
AT5G60080.1
0
200
400
600

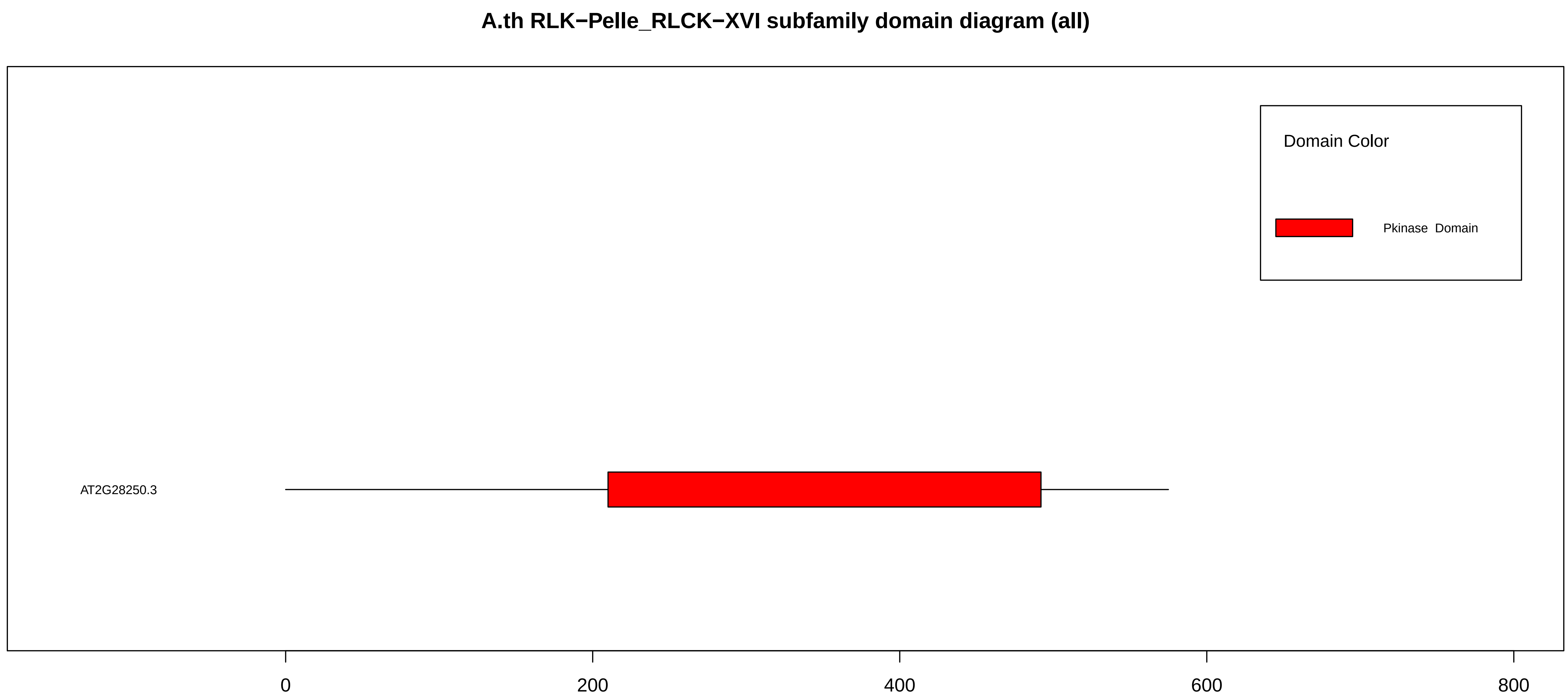

A.th RLK−Pelle_RLCK−XVI subfamily domain diagram (all)
Domain Color
Pkinase Domain
AT2G28250.3
0
200
400
600
800

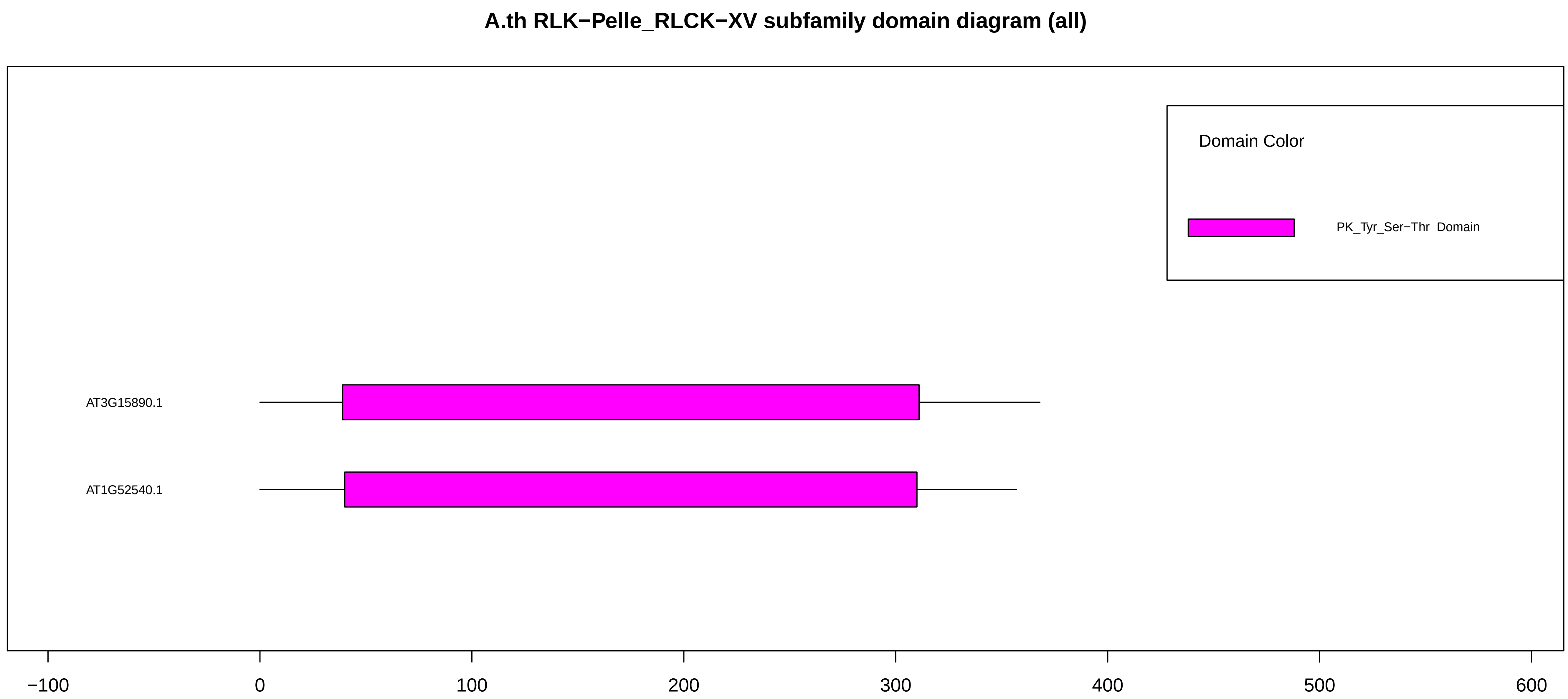
A.th RLK−Pelle_RLCK−XV subfamily domain diagram (all)
Domain Color
PK_Tyr_Ser−Thr Domain
AT3G15890.1
AT1G52540.1
−100
0
100
200
300
400
500
600

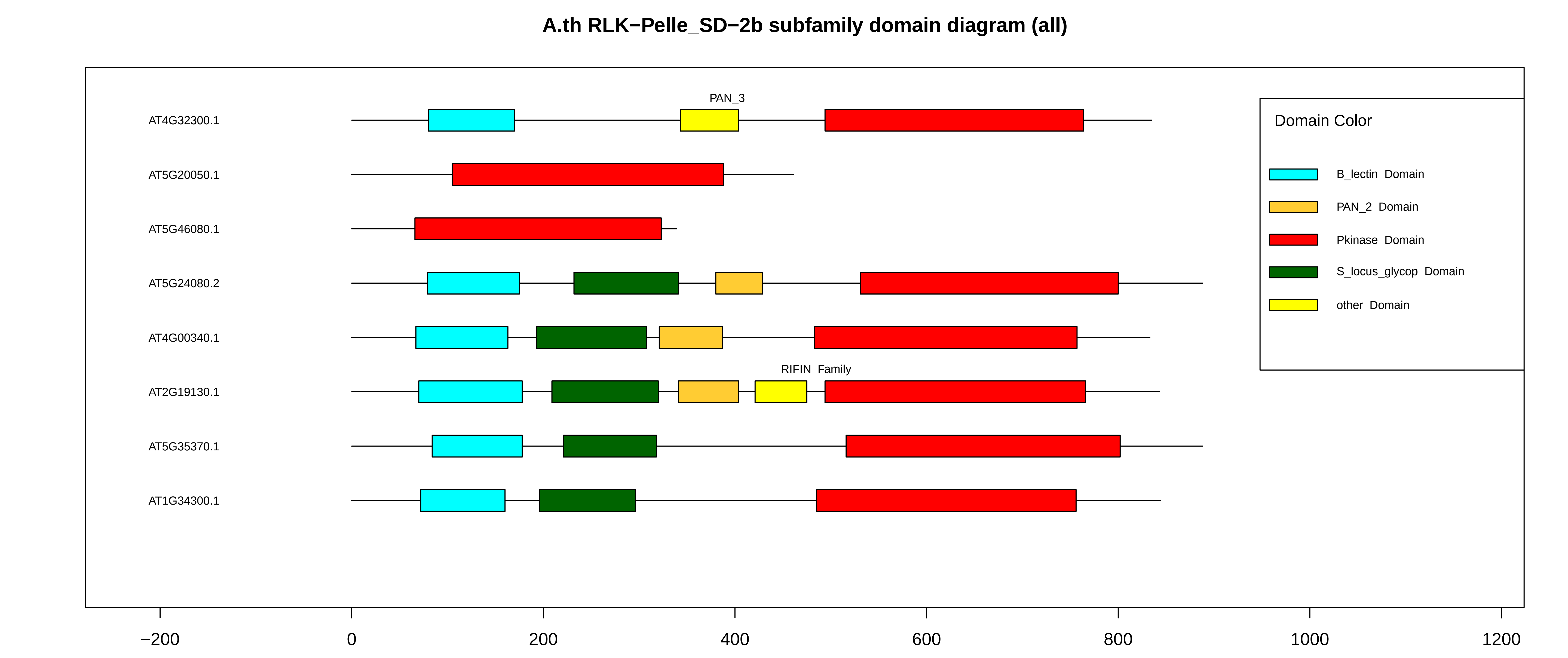
A.th RLK−Pelle_SD−2b subfamily domain diagram (all)
AT4G32300.1
PAN_3
AT5G20050.1
AT5G46080.1
AT5G24080.2
AT4G00340.1
RIFIN Family
AT2G19130.1
AT5G35370.1
AT1G34300.1
Domain Color
B_lectin Domain
PAN_2 Domain
Pkinase Domain
S_locus_glycop Domain
other Domain
−200
0
200
400
600
800
1000
1200

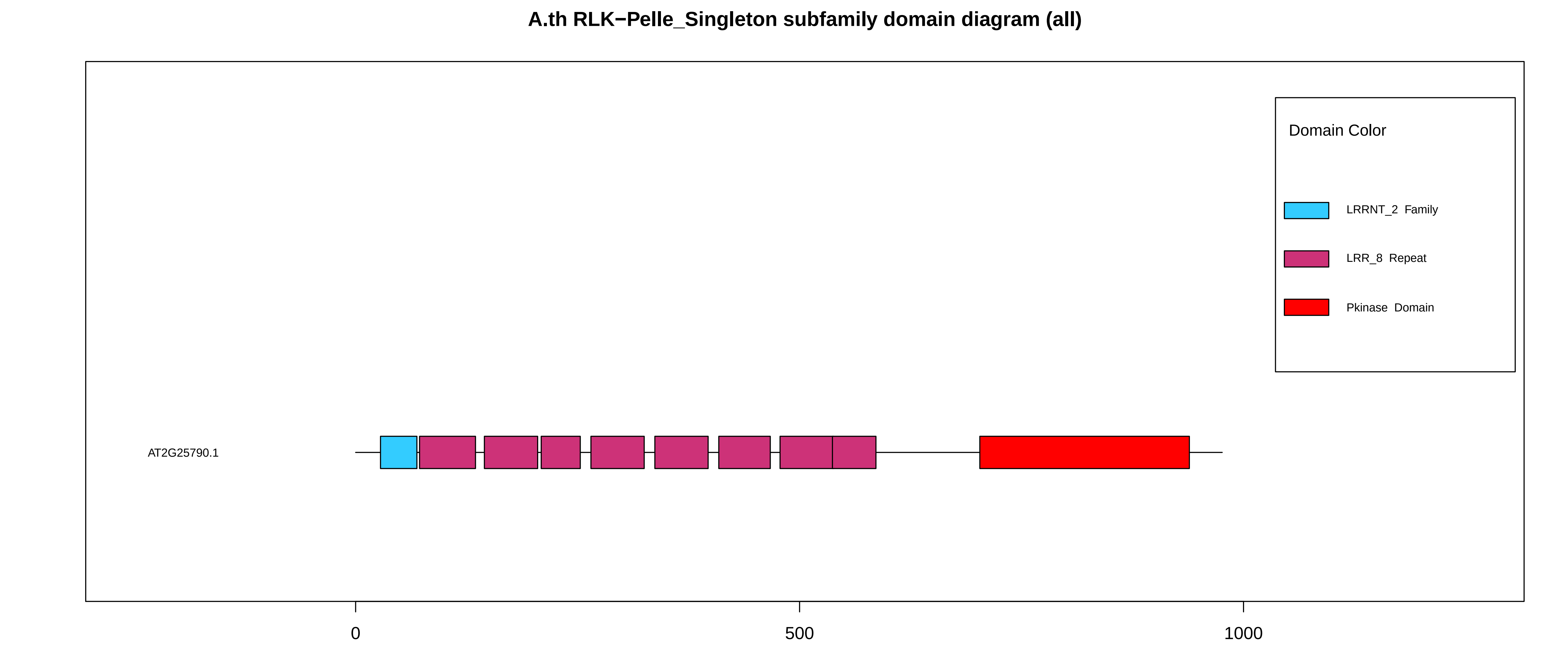
A.th RLK−Pelle_Singleton subfamily domain diagram (all)
Domain Color
LRRNT_2 Family
LRR_8 Repeat
Pkinase Domain
AT2G25790.1
0
500
1000

A.th RLK−Pelle_URK−1 subfamily domain diagram (all)

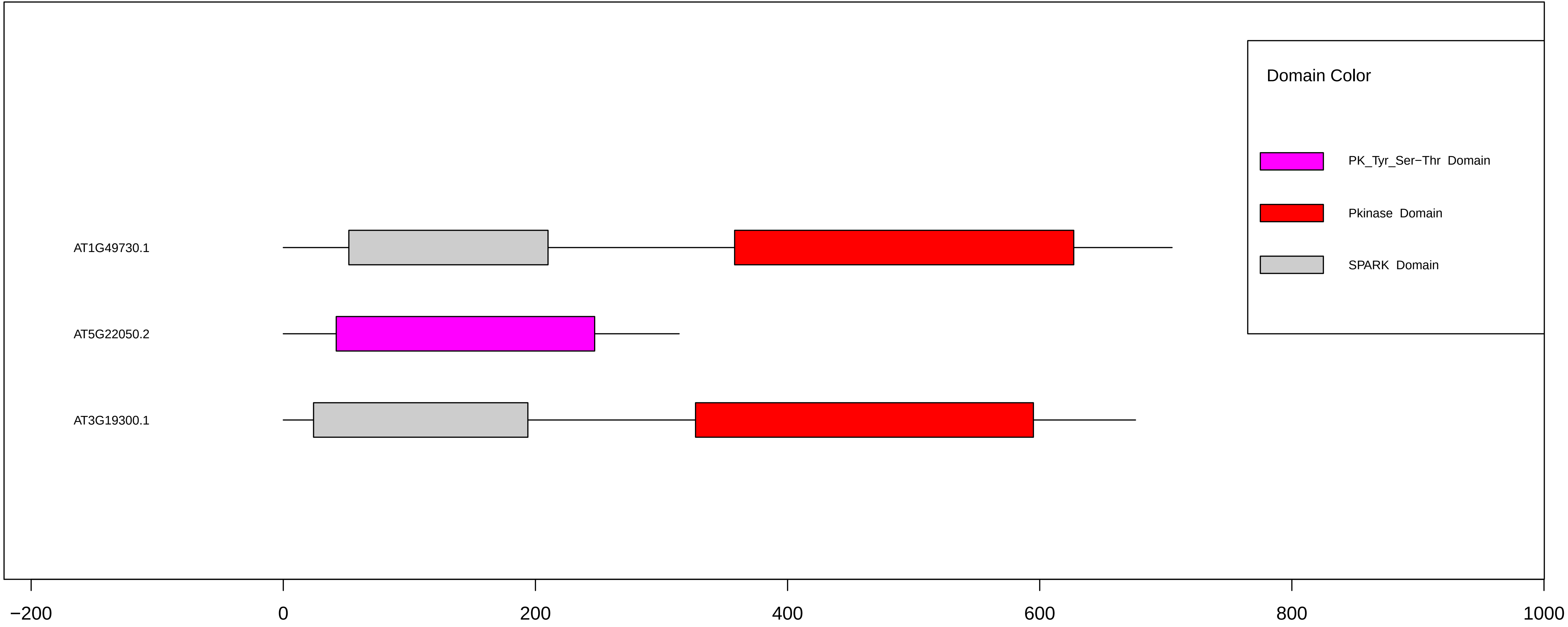

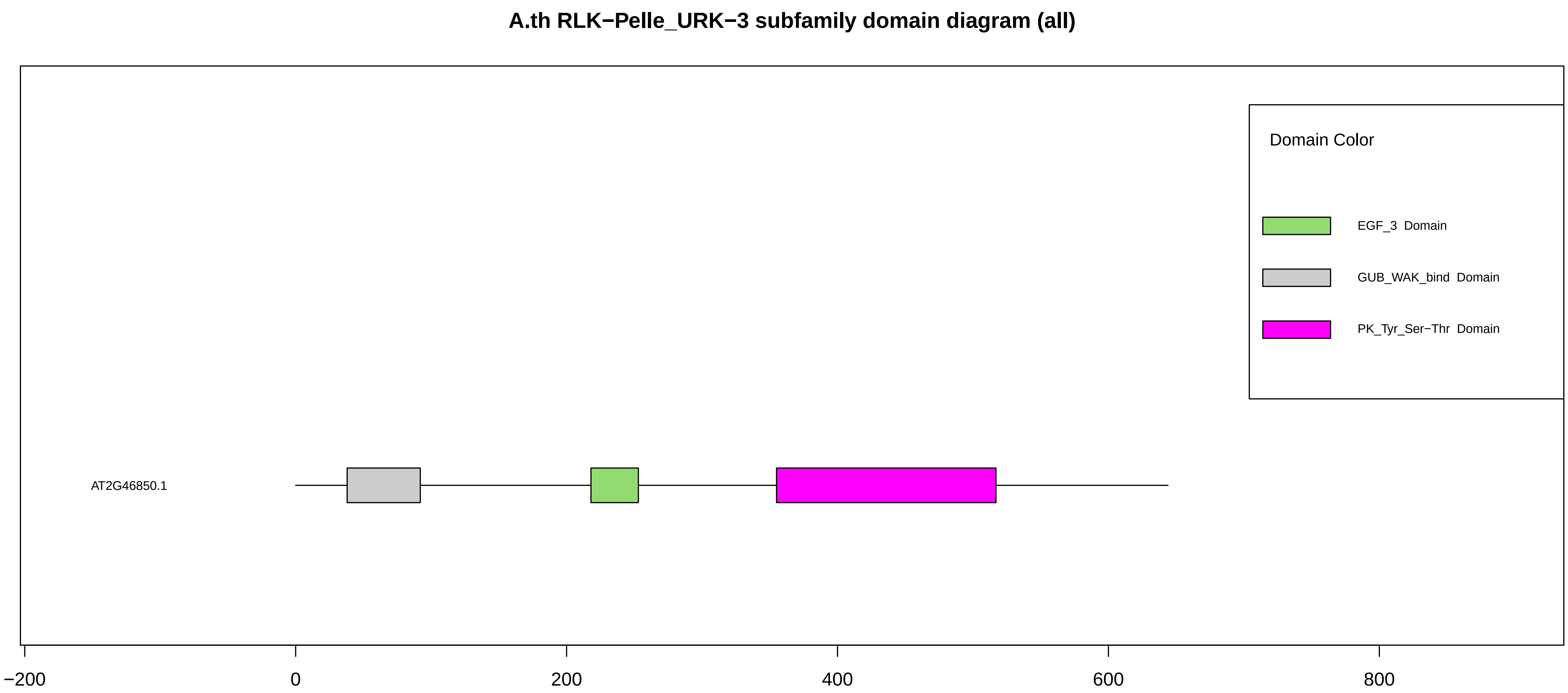

A.th RLK−Pelle_URK−3 subfamily domain diagram (all)
Domain Color
EGF_3 Domain
GUB_WAK_bind Domain
PK_Tyr_Ser−Thr Domain
AT2G46850.1
−200
0
200
400
600
800

A.th RLK−Pelle_WAK_LRK10L−1 subfamily domain diagram (all)

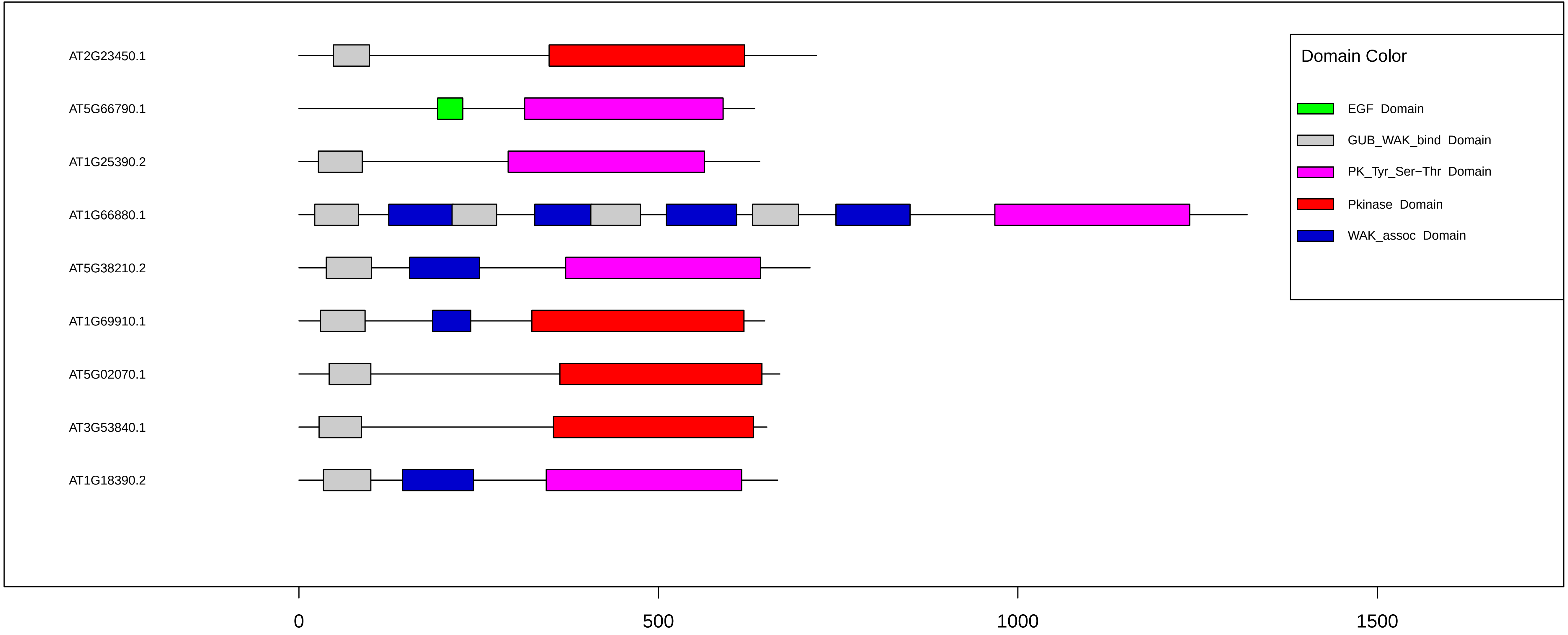

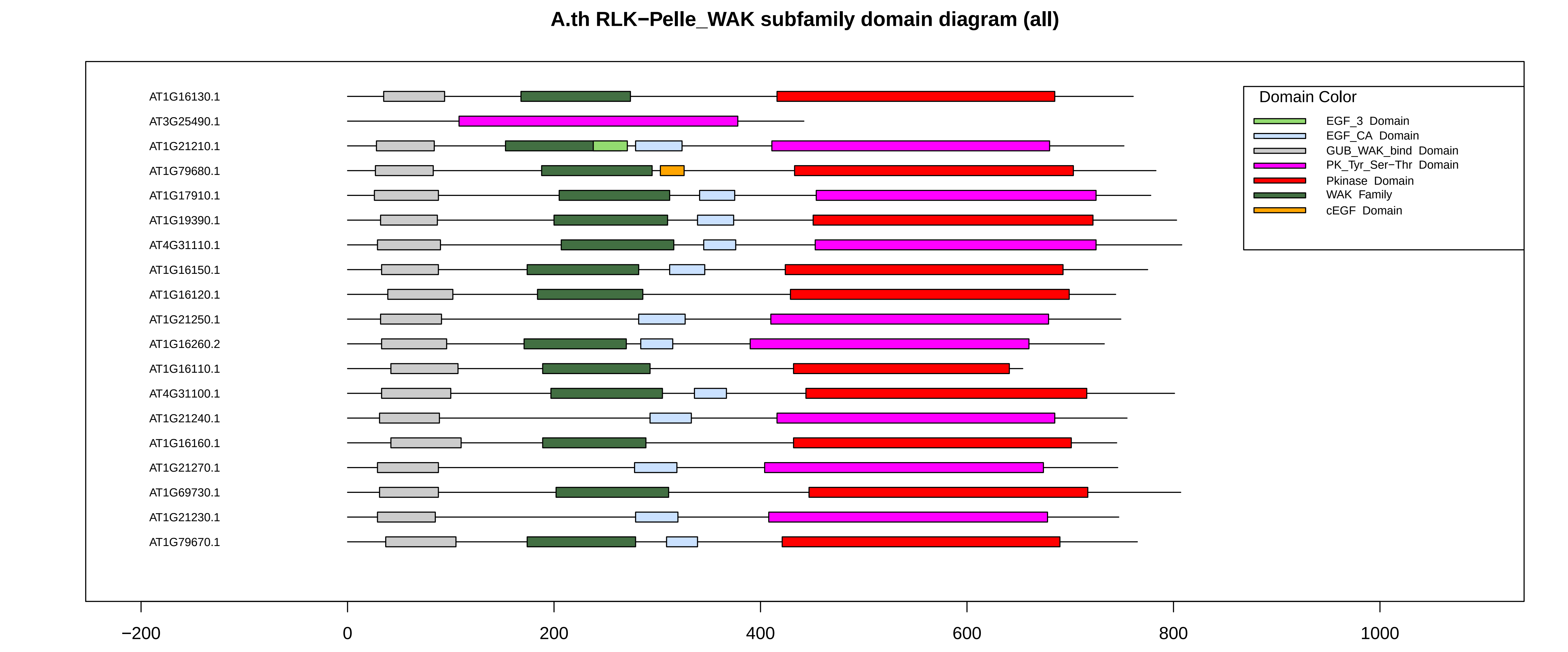

A.th RLK−Pelle_WAK subfamily domain diagram (all)
AT1G16130.1
AT3G25490.1
AT1G21210.1
AT1G79680.1
AT1G17910.1
AT1G19390.1
AT4G31110.1
AT1G16150.1
AT1G16120.1
AT1G21250.1
AT1G16260.2
AT1G16110.1
AT4G31100.1
AT1G21240.1
AT1G16160.1
AT1G21270.1
AT1G69730.1
AT1G21230.1
AT1G79670.1
Domain Color
EGF_3 Domain
EGF_CA Domain
GUB_WAK_bind Domain
PK_Tyr_Ser−Thr Domain
Pkinase Domain
WAK Family
cEGF Domain
−200
0
200
400
600
800
1000

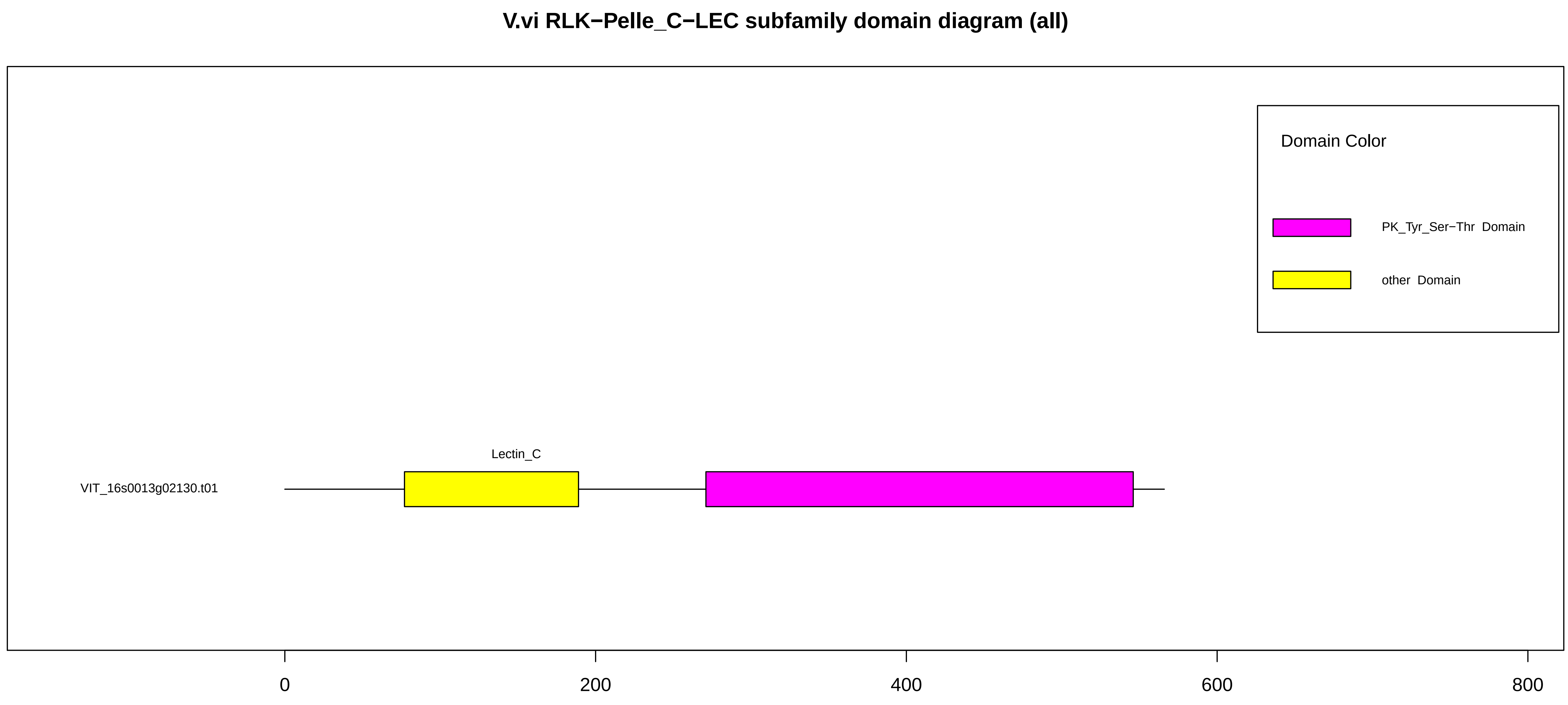

V.vi RLK−Pelle_C−LEC subfamily domain diagram (all)
Domain Color
PK_Tyr_Ser−Thr Domain
other Domain
Lectin_C
VIT_16s0013g02130.t01
0
200
400
600
800

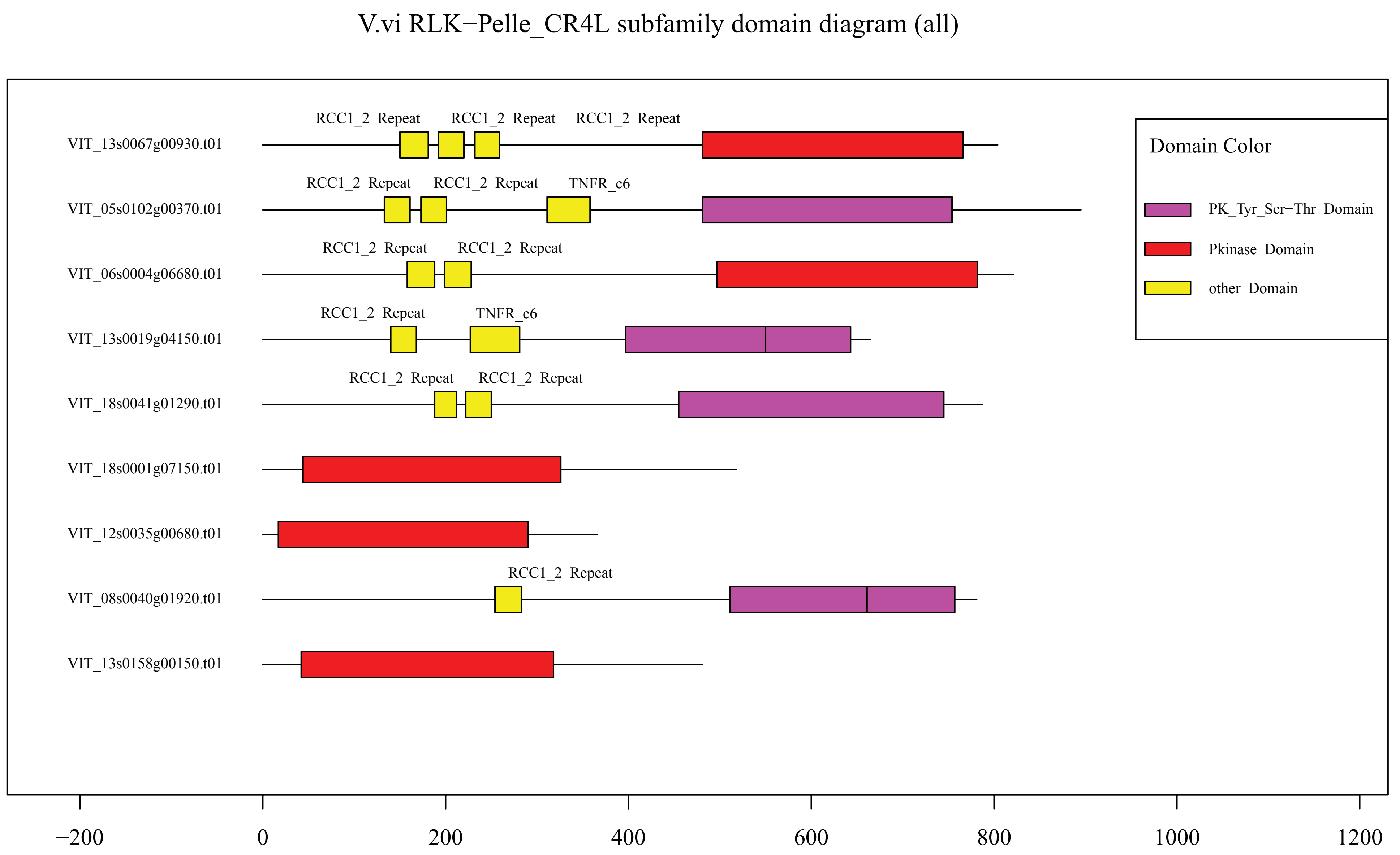
V.vi RLK−Pelle_CR4L subfamily domain diagram (all)
VIT_13s0067g00930.t01
VIT_05s0102g00370.t01
VIT_06s0004g06680.t01
VIT_13s0019g04150.t01
VIT_18s0041g01290.t01
VIT_18s0001g07150.t01
VIT_12s0035g00680.t01
VIT_08s0040g01920.t01
VIT_13s0158g00150.t01
RCC1_2 Repeat
TNFR_c6
Domain Color
PK_Tyr_Ser−Thr Domain
Pkinase Domain
other Domain
−200
0
200
400
600
800
1000
1200

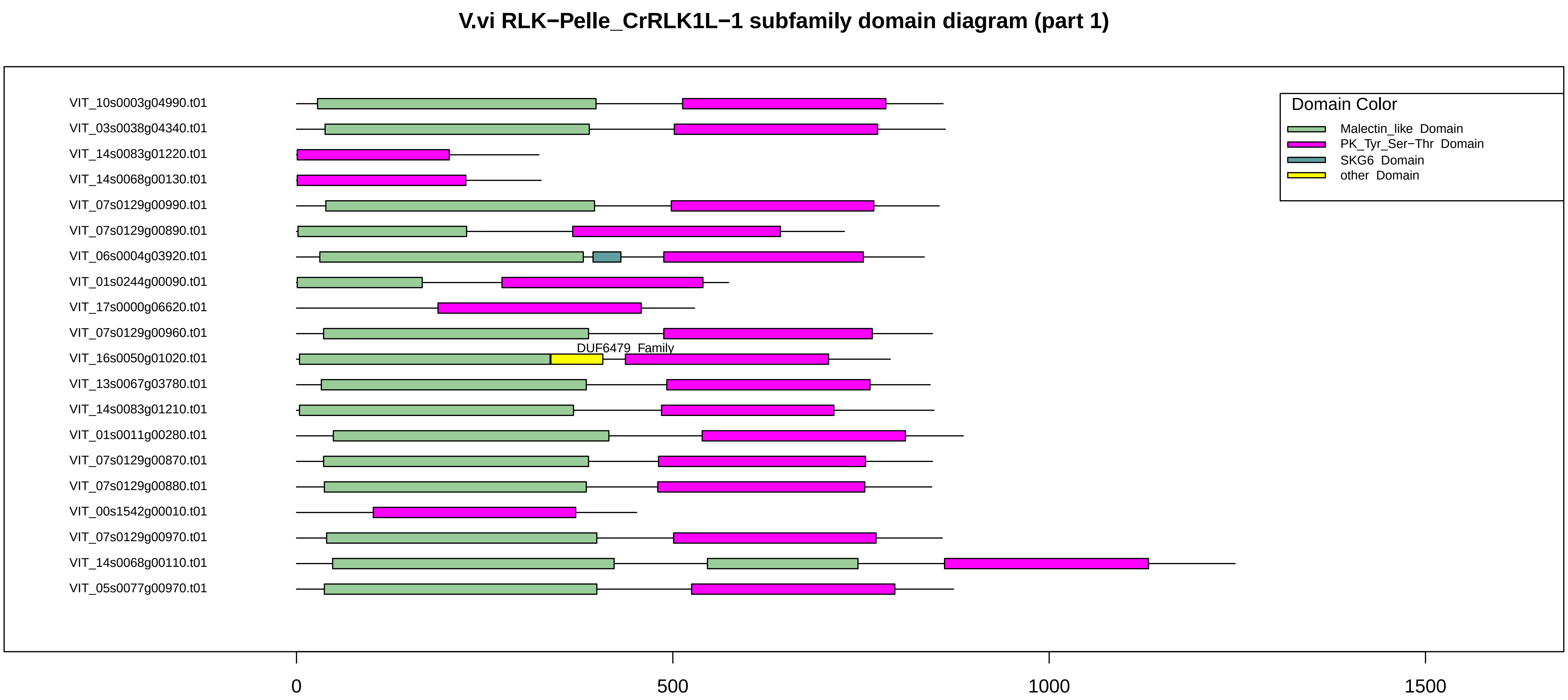

V.vi RLK−Pelle_CrRLK1L−1 subfamily domain diagram (part 1)
VIT_10s0003g04990.t01
VIT_03s0038g04340.t01
VIT_14s0083g01220.t01
VIT_14s0068g00130.t01
VIT_07s0129g00990.t01
VIT_07s0129g00890.t01
VIT_06s0004g03920.t01
VIT_01s0244g00090.t01
VIT_17s0000g06620.t01
VIT_07s0129g00960.t01
VIT_16s0050g01020.t01
DUF6479_Family
VIT_13s0067g03780.t01
VIT_14s0083g01210.t01
VIT_01s0011g00280.t01
VIT_07s0129g00870.t01
VIT_07s0129g00880.t01
VIT_00s1542g00010.t01
VIT_07s0129g00970.t01
VIT_14s0068g00110.t01
VIT_05s0077g00970.t01
Domain Color
Malectin_like Domain
PK_Tyr_Ser−Thr Domain
SKG6 Domain
other Domain
0
500
1000
1500

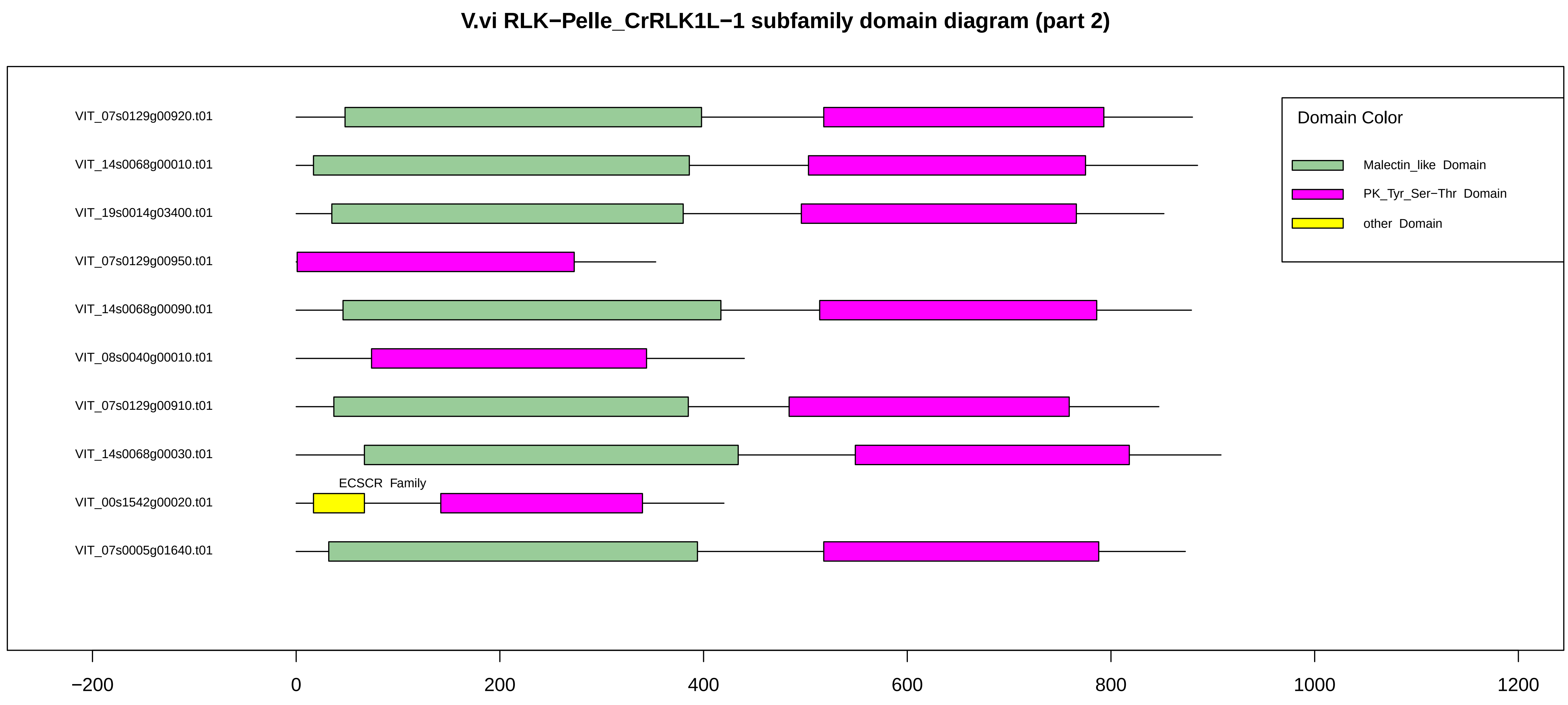
V.vi RLK−Pelle_CrRLK1L−1 subfamily domain diagram (part 2)
VIT_07s0129g00920.t01
VIT_14s0068g00010.t01
VIT_19s0014g03400.t01
VIT_07s0129g00950.t01
VIT_14s0068g00090.t01
VIT_08s0040g00010.t01
VIT_07s0129g00910.t01
VIT_14s0068g00030.t01
ECSCR Family
VIT_00s1542g00020.t01
VIT_07s0005g01640.t01
Domain Color
Malectin_like Domain
PK_Tyr_Ser−Thr Domain
other Domain
−200
0
200
400
600
800
1000
1200

V.vi RLK−Pelle_DLSV subfamily domain diagram (part 1)

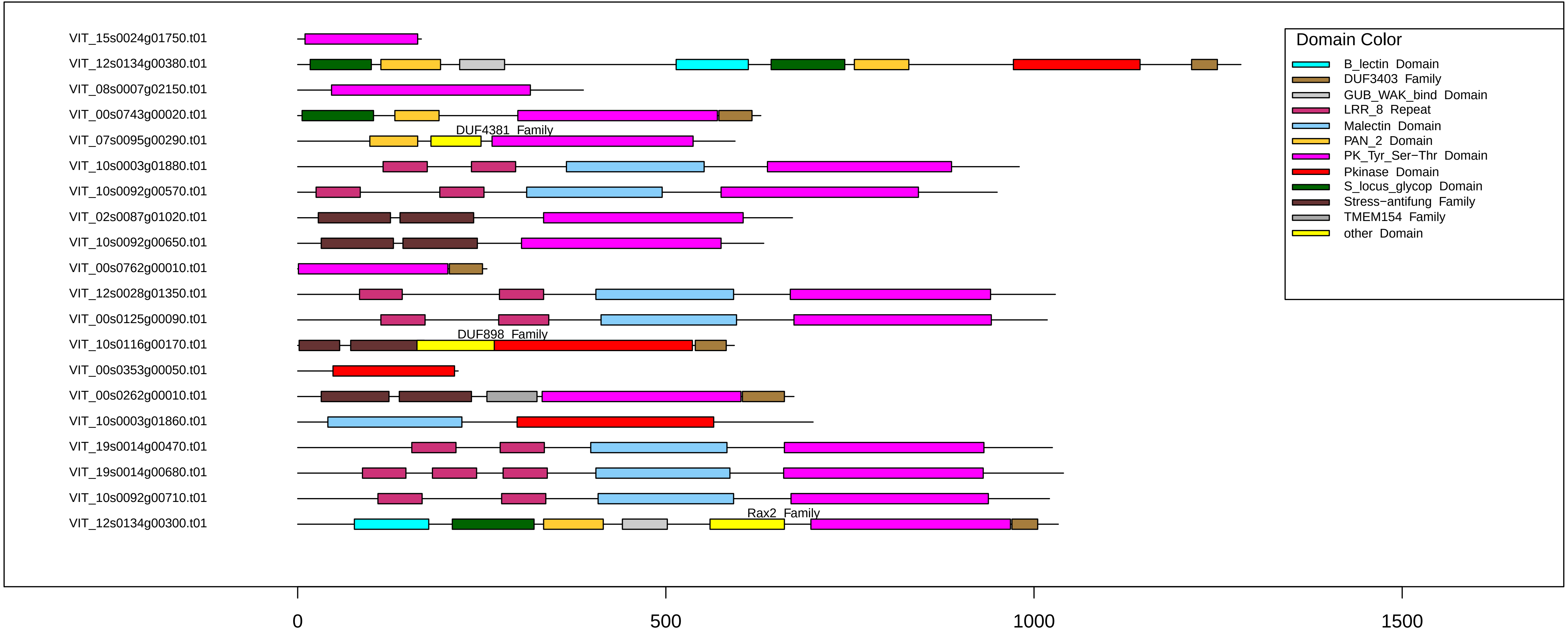

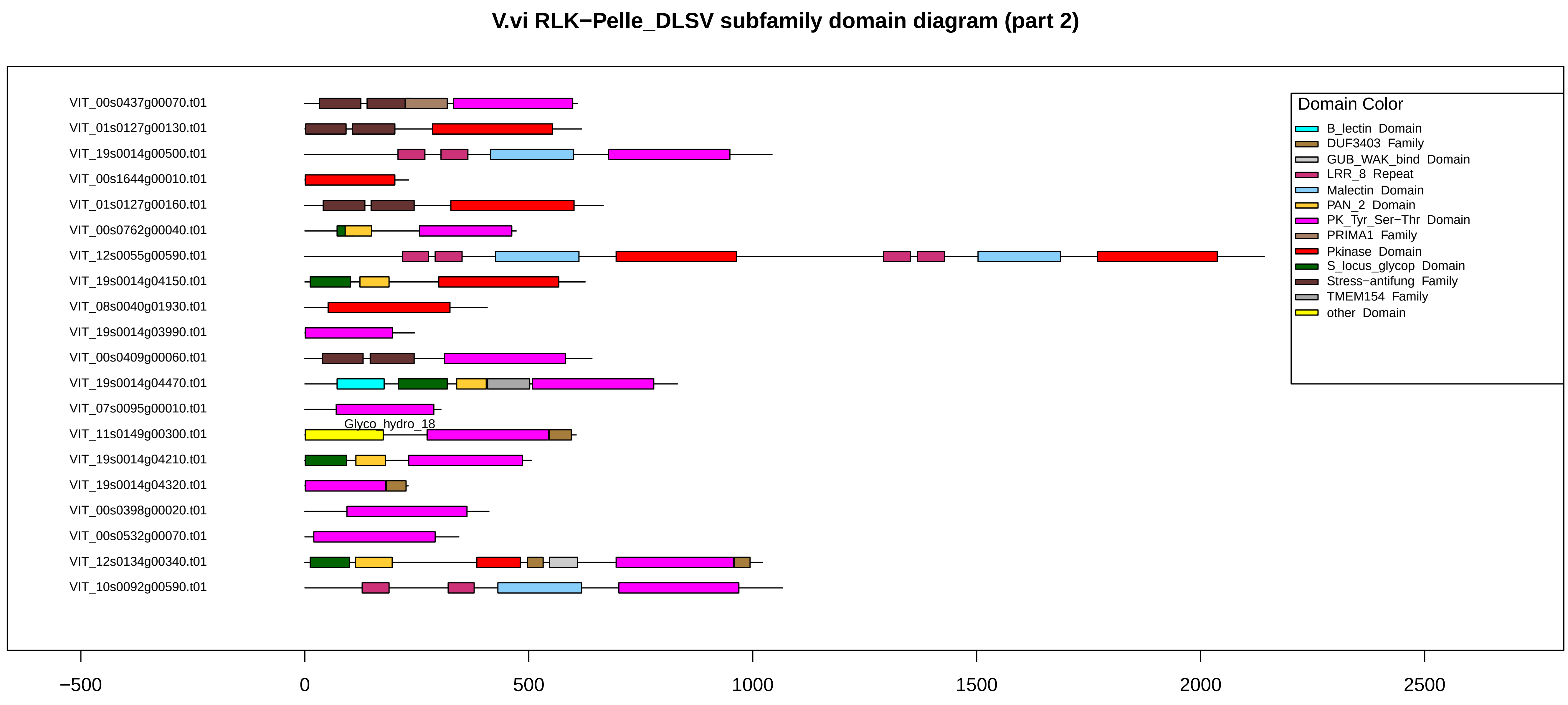
V.vi RLK−Pelle_DLSV subfamily domain diagram (part 2)
VIT_00s0437g00070.t01
VIT_01s0127g00130.t01
VIT_19s0014g00500.t01
VIT_00s1644g00010.t01
VIT_01s0127g00160.t01
VIT_00s0762g00040.t01
VIT_12s0055g00590.t01
VIT_19s0014g04150.t01
VIT_08s0040g01930.t01
VIT_19s0014g03990.t01
VIT_00s0409g00060.t01
VIT_19s0014g04470.t01
VIT_07s0095g00010.t01
VIT_11s0149g00300.t01
Glyco_hydro_18
VIT_19s0014g04210.t01
VIT_19s0014g04320.t01
VIT_00s0398g00020.t01
VIT_00s0532g00070.t01
VIT_12s0134g00340.t01
VIT_10s0092g00590.t01
Domain Color
B_lectin Domain
DUF3403 Family
GUB_WAK_bind Domain
LRR_8 Repeat
Malectin Domain
PAN_2 Domain
PK_Tyr_Ser−Thr Domain
PRIMA1 Family
Pkinase Domain
S_locus_glycop Domain
Stress−antifung Family
TMEM154 Family
other Domain
−500
0
500
1000
1500
2000
2500

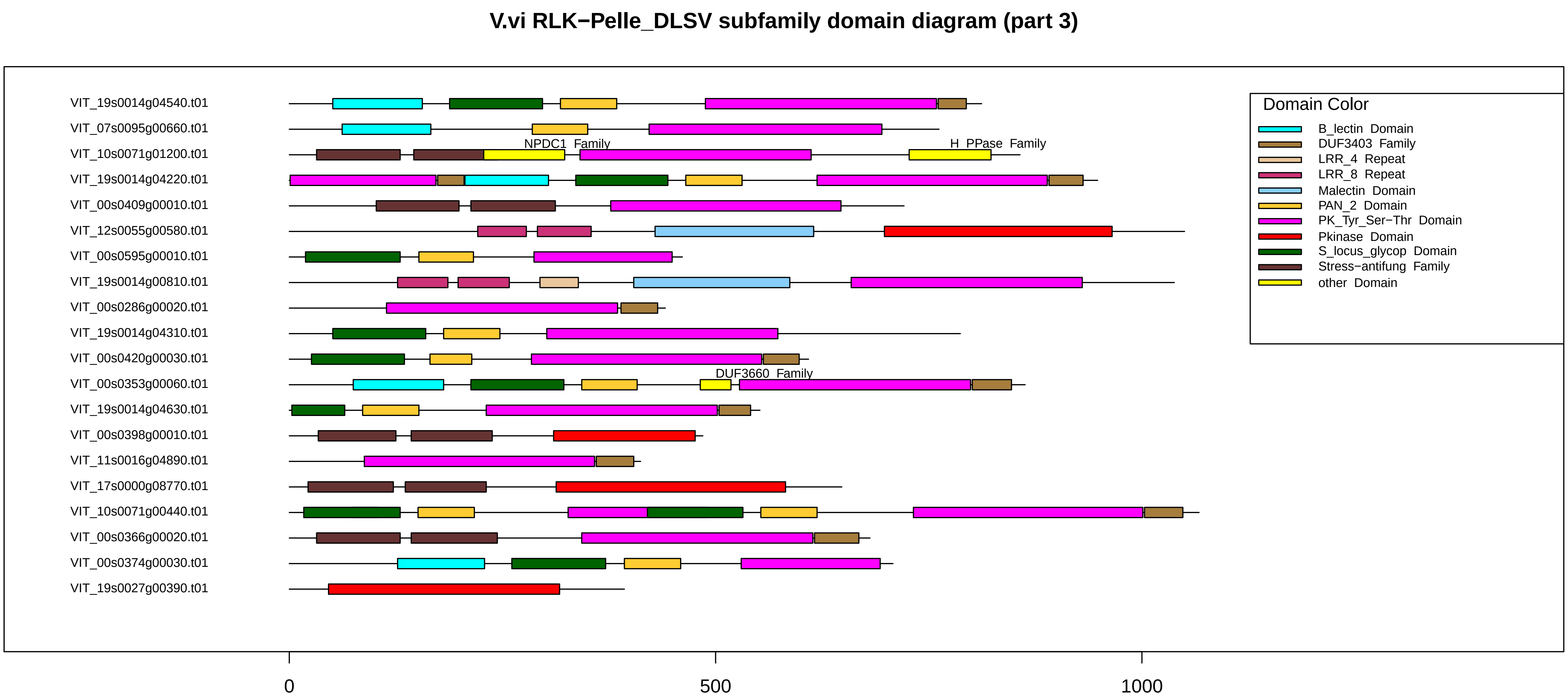
V.vi RLK−Pelle_DLSV subfamily domain diagram (part 3)
VIT_19s0014g04540.t01
VIT_07s0095g00660.t01
VIT_10s0071g01200.t01
VIT_19s0014g04220.t01
VIT_00s0409g00010.t01
VIT_12s0055g00580.t01
VIT_00s0595g00010.t01
VIT_19s0014g00810.t01
VIT_00s0286g00020.t01
VIT_19s0014g04310.t01
VIT_00s0420g00030.t01
VIT_00s0353g00060.t01
VIT_19s0014g04630.t01
VIT_00s0398g00010.t01
VIT_11s0016g04890.t01
VIT_17s0000g08770.t01
VIT_10s0071g00440.t01
VIT_00s0366g00020.t01
VIT_00s0374g00030.t01
VIT_19s0027g00390.t01
NPDC1 Family
H_PPase Family
DUF3660 Family
Domain Color
B_lectin Domain
DUF3403 Family
LRR_4 Repeat
LRR_8 Repeat
Malectin Domain
PAN_2 Domain
PK_Tyr_Ser−Thr Domain
Pkinase Domain
S_locus_glycop Domain
Stress−antifung Family
other Domain
0
500
1000

V.vi RLK−Pelle_DLSV subfamily domain diagram (part 4)

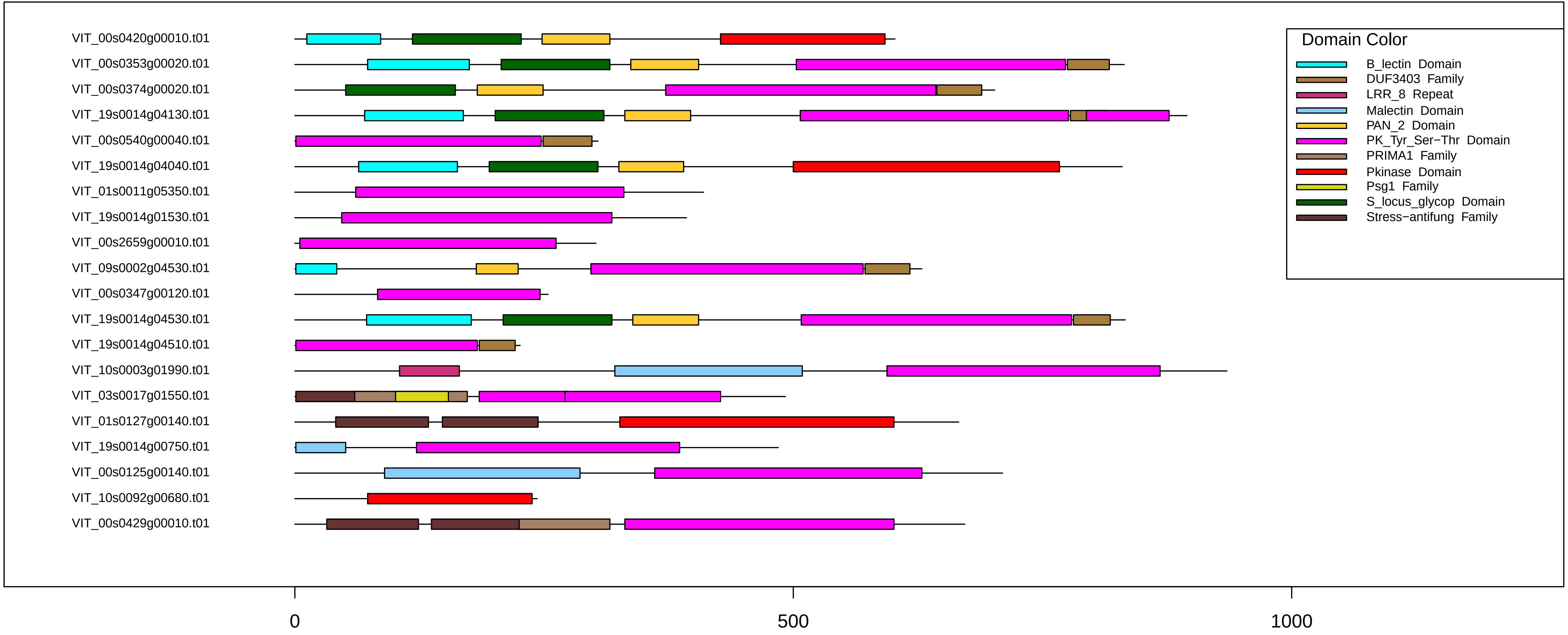

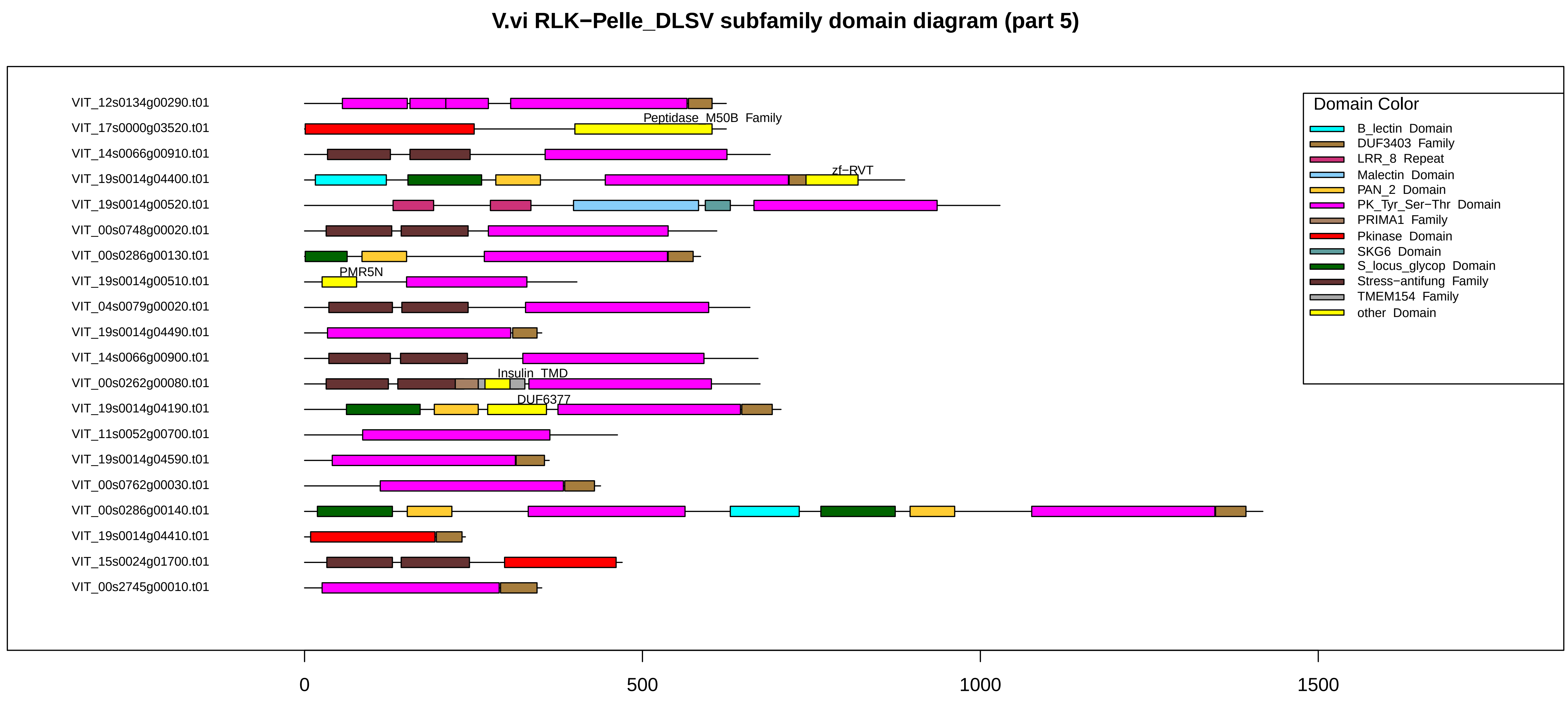

V.vi RLK−Pelle_DLSV subfamily domain diagram (part 5)
VIT_12s0134g00290.t01
VIT_17s0000g03520.t01
VIT_14s0066g00910.t01
VIT_19s0014g04400.t01
VIT_19s0014g00520.t01
VIT_00s0748g00020.t01
VIT_00s0286g00130.t01
VIT_19s0014g00510.t01
VIT_04s0079g00020.t01
VIT_19s0014g04490.t01
VIT_14s0066g00900.t01
VIT_00s0262g00080.t01
VIT_19s0014g04190.t01
VIT_11s0052g00700.t01
VIT_19s0014g04590.t01
VIT_00s0762g00030.t01
VIT_00s0286g00140.t01
VIT_19s0014g04410.t01
VIT_15s0024g01700.t01
VIT_00s2745g00010.t01
Peptidase_M50B Family
zf−RVT
PMR5N
Insulin_TMD
DUF6377
Domain Color
B_lectin Domain
DUF3403 Family
LRR_8 Repeat
Malectin Domain
PAN_2 Domain
PK_Tyr_Ser−Thr Domain
PRIMA1 Family
Pkinase Domain
SKG6 Domain
S_locus_glycop Domain
Stress−antifung Family
TMEM154 Family
other Domain
0
500
1000
1500

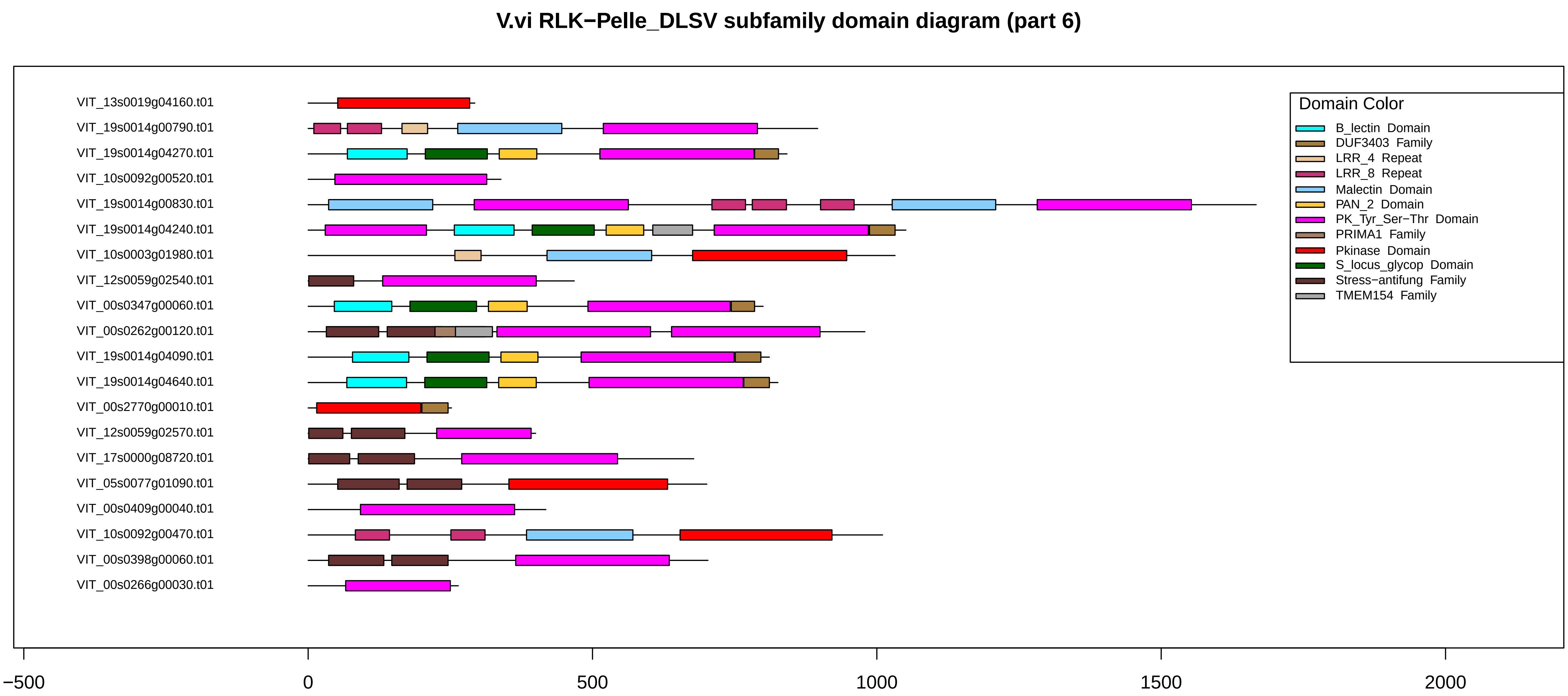
V.vi RLK−Pelle_DLSV subfamily domain diagram (part 6)
VIT_13s0019g04160.t01
VIT_19s0014g00790.t01
VIT_19s0014g04270.t01
VIT_10s0092g00520.t01
VIT_19s0014g00830.t01
VIT_19s0014g04240.t01
VIT_10s0003g01980.t01
VIT_12s0059g02540.t01
VIT_00s0347g00060.t01
VIT_00s0262g00120.t01
VIT_19s0014g04090.t01
VIT_19s0014g04640.t01
VIT_00s2770g00010.t01
VIT_12s0059g02570.t01
VIT_17s0000g08720.t01
VIT_05s0077g01090.t01
VIT_00s0409g00040.t01
VIT_10s0092g00470.t01
VIT_00s0398g00060.t01
VIT_00s0266g00030.t01
Domain Color
B_lectin Domain
DUF3403 Family
LRR_4 Repeat
LRR_8 Repeat
Malectin Domain
PAN_2 Domain
PK_Tyr_Ser−Thr Domain
PRIMA1 Family
Pkinase Domain
S_locus_glycop Domain
Stress−antifung Family
TMEM154 Family
−500
0
500
1000
1500
2000

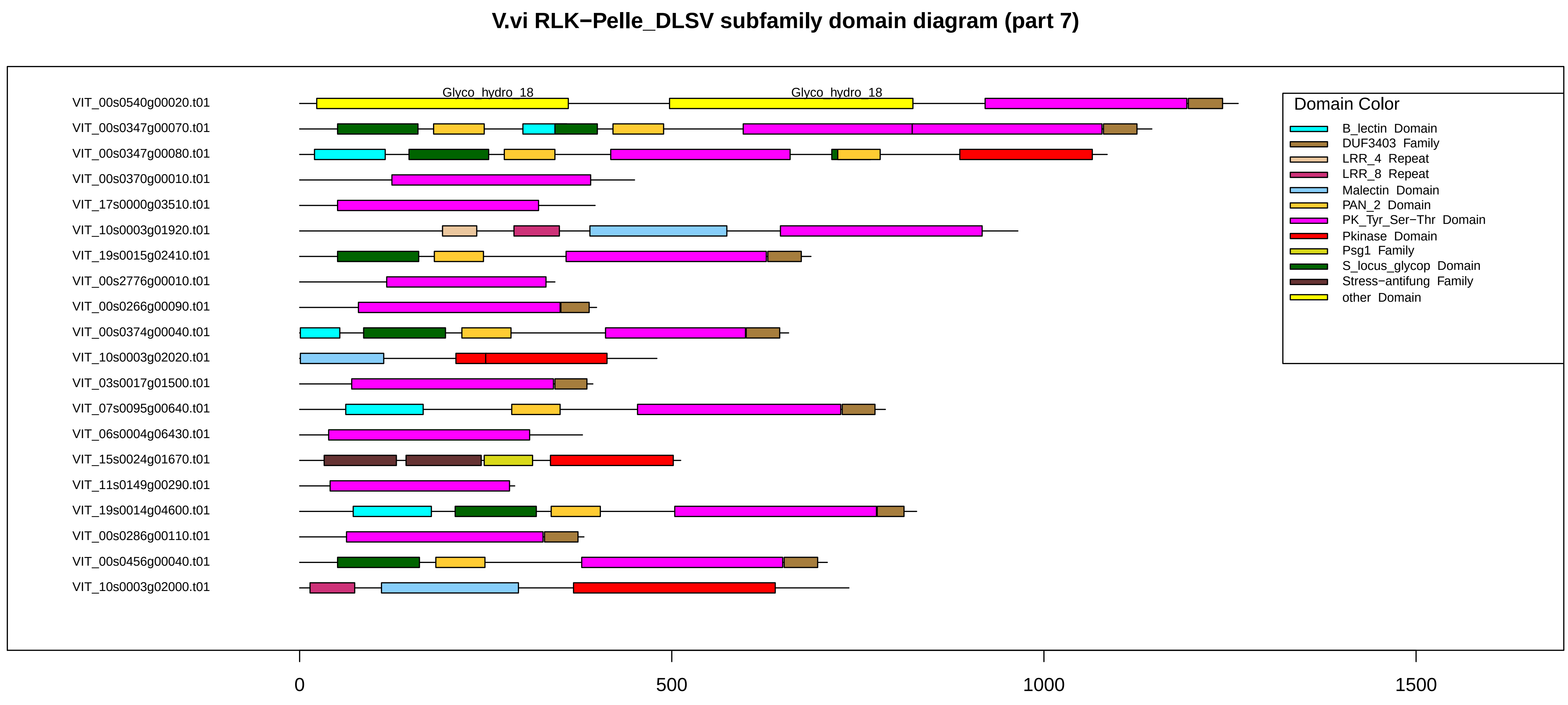
V.vi RLK−Pelle_DLSV subfamily domain diagram (part 7)
Glyco_hydro_18
Glyco_hydro_18
VIT_00s0540g00020.t01
VIT_00s0347g00070.t01
VIT_00s0347g00080.t01
VIT_00s0370g00010.t01
VIT_17s0000g03510.t01
VIT_10s0003g01920.t01
VIT_19s0015g02410.t01
VIT_00s2776g00010.t01
VIT_00s0266g00090.t01
VIT_00s0374g00040.t01
VIT_10s0003g02020.t01
VIT_03s0017g01500.t01
VIT_07s0095g00640.t01
VIT_06s0004g06430.t01
VIT_15s0024g01670.t01
VIT_11s0149g00290.t01
VIT_19s0014g04600.t01
VIT_00s0286g00110.t01
VIT_00s0456g00040.t01
VIT_10s0003g02000.t01
Domain Color
B_lectin Domain
DUF3403 Family
LRR_4 Repeat
LRR_8 Repeat
Malectin Domain
PAN_2 Domain
PK_Tyr_Ser−Thr Domain
Pkinase Domain
Psg1 Family
S_locus_glycop Domain
Stress−antifung Family
other Domain
0
500
1000
1500

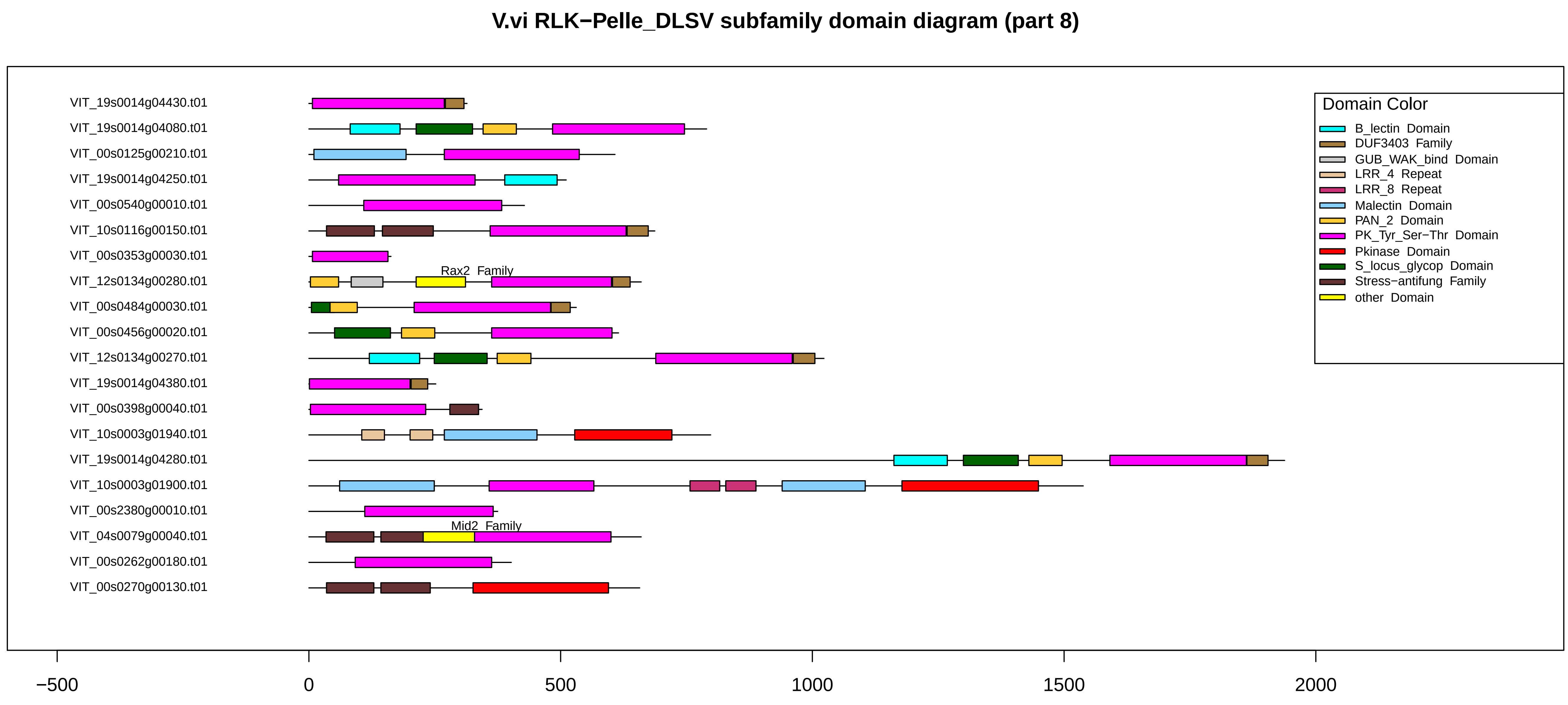

V.vi RLK−Pelle_DLSV subfamily domain diagram (part 8)
VIT_19s0014g04430.t01
VIT_19s0014g04080.t01
VIT_00s0125g00210.t01
VIT_19s0014g04250.t01
VIT_00s0540g00010.t01
VIT_10s0116g00150.t01
VIT_00s0353g00030.t01
VIT_12s0134g00280.t01
Rax2 Family
VIT_00s0484g00030.t01
VIT_00s0456g00020.t01
VIT_12s0134g00270.t01
VIT_19s0014g04380.t01
VIT_00s0398g00040.t01
VIT_10s0003g01940.t01
VIT_19s0014g04280.t01
VIT_10s0003g01900.t01
VIT_00s2380g00010.t01
VIT_04s0079g00040.t01
Mid2 Family
VIT_00s0262g00180.t01
VIT_00s0270g00130.t01
Domain Color
B_lectin Domain
DUF3403 Family
GUB_WAK_bind Domain
LRR_4 Repeat
LRR_8 Repeat
Malectin Domain
PAN_2 Domain
PK_Tyr_Ser−Thr Domain
Pkinase Domain
S_locus_glycop Domain
Stress−antifung Family
other Domain
−500
0
500
1000
1500
2000

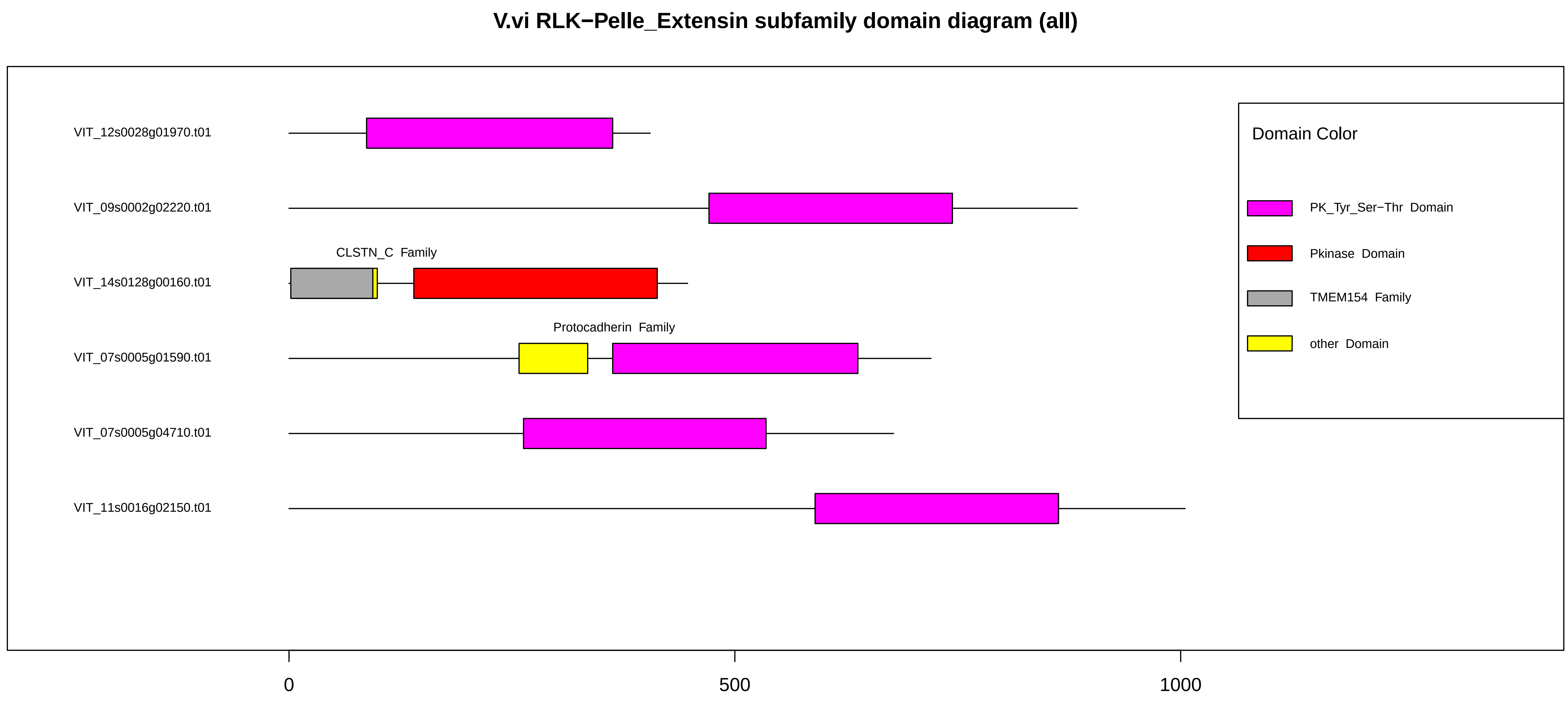
V.vi RLK−Pelle_Extensin subfamily domain diagram (all)
VIT_12s0028g01970.t01
VIT_09s0002g02220.t01
VIT_14s0128g00160.t01
VIT_07s0005g01590.t01
VIT_07s0005g04710.t01
VIT_11s0016g02150.t01
CLSTN_C Family
Protocadherin Family
Domain Color
PK_Tyr_Ser−Thr Domain
Pkinase Domain
TMEM154 Family
other Domain
0
500
1000

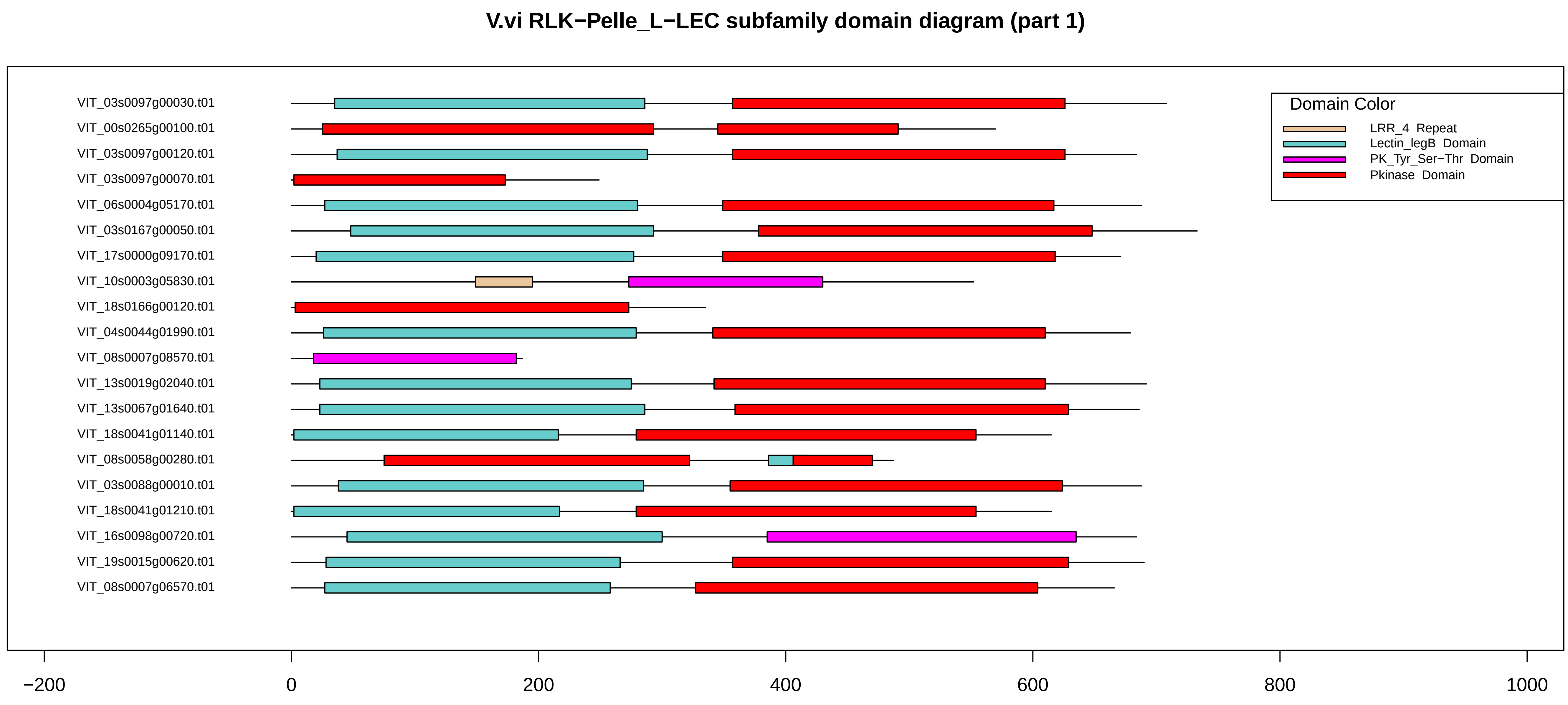
V.vi RLK−Pelle_L−LEC subfamily domain diagram (part 1)
VIT_03s0097g00030.t01
VIT_00s0265g00100.t01
VIT_03s0097g00120.t01
VIT_03s0097g00070.t01
VIT_06s0004g05170.t01
VIT_03s0167g00050.t01
VIT_17s0000g09170.t01
VIT_10s0003g05830.t01
VIT_18s0166g00120.t01
VIT_04s0044g01990.t01
VIT_08s0007g08570.t01
VIT_13s0019g02040.t01
VIT_13s0067g01640.t01
VIT_18s0041g01140.t01
VIT_08s0058g00280.t01
VIT_03s0088g00010.t01
VIT_18s0041g01210.t01
VIT_16s0098g00720.t01
VIT_19s0015g00620.t01
VIT_08s0007g06570.t01
Domain Color
LRR_4 Repeat
Lectin_legB Domain
PK_Tyr_Ser−Thr Domain
Pkinase Domain
−200
0
200
400
600
800
1000

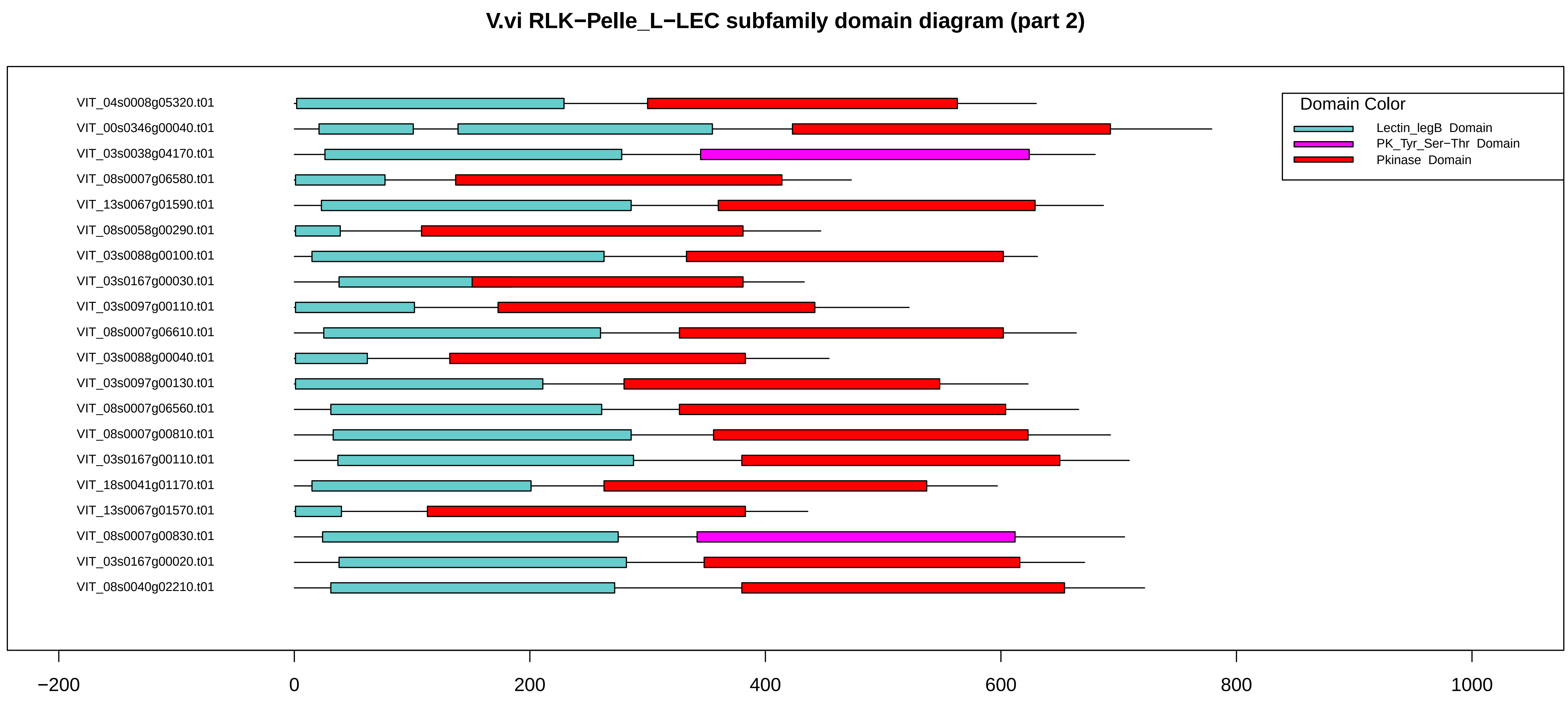

V.vi RLK−Pelle_L−LEC subfamily domain diagram (part 2)
VIT_04s0008g05320.t01
VIT_00s0346g00040.t01
VIT_03s0038g04170.t01
VIT_08s0007g06580.t01
VIT_13s0067g01590.t01
VIT_08s0058g00290.t01
VIT_03s0088g00100.t01
VIT_03s0167g00030.t01
VIT_03s0097g00110.t01
VIT_08s0007g06610.t01
VIT_03s0088g00040.t01
VIT_03s0097g00130.t01
VIT_08s0007g06560.t01
VIT_08s0007g00810.t01
VIT_03s0167g00110.t01
VIT_18s0041g01170.t01
VIT_13s0067g01570.t01
VIT_08s0007g00830.t01
VIT_03s0167g00020.t01
VIT_08s0040g02210.t01
Domain Color
Lectin_legB Domain
PK_Tyr_Ser−Thr Domain
Pkinase Domain
−200
0
200
400
600
800
1000

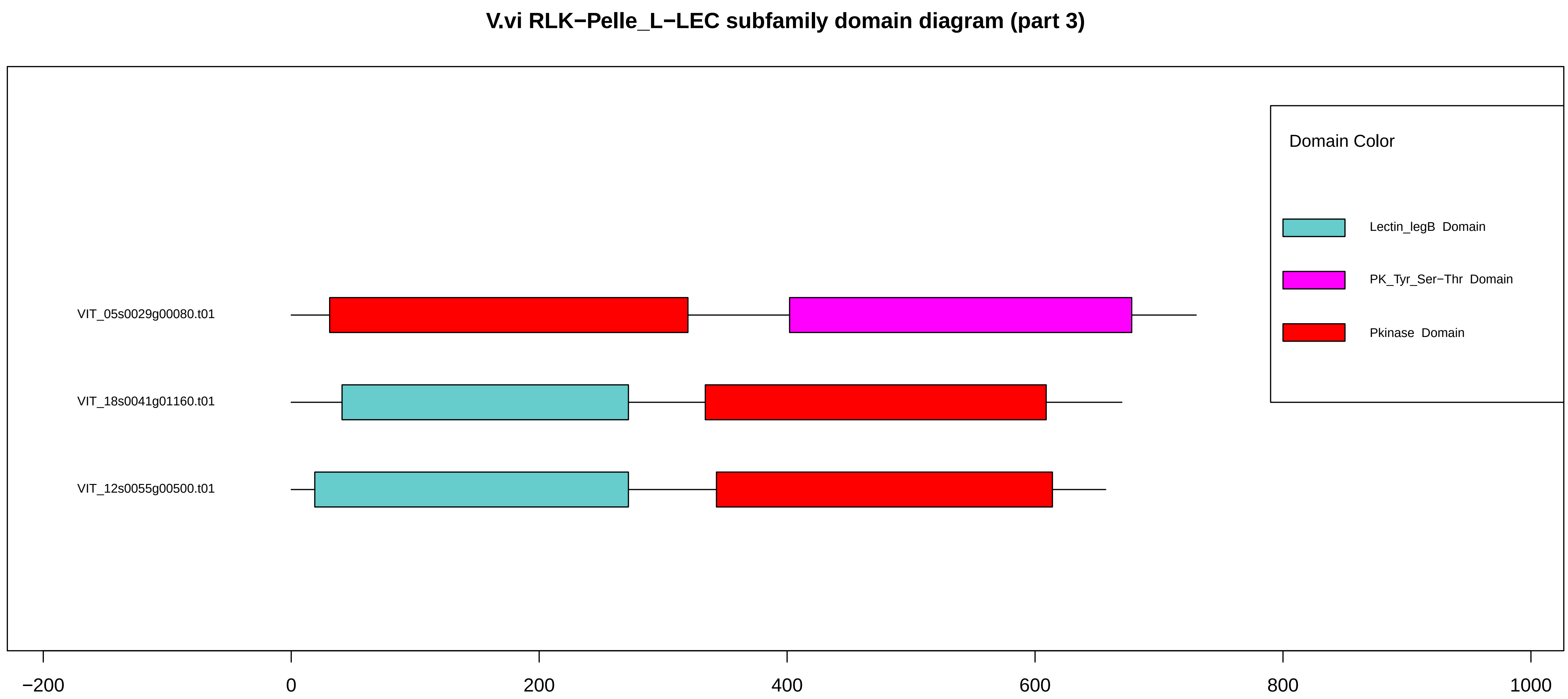
V.vi RLK−Pelle_L−LEC subfamily domain diagram (part 3)
Domain Color
Lectin_legB Domain
PK_Tyr_Ser−Thr Domain
Pkinase Domain
VIT_05s0029g00080.t01
VIT_18s0041g01160.t01
VIT_12s0055g00500.t01
−200
0
200
400
600
800
1000

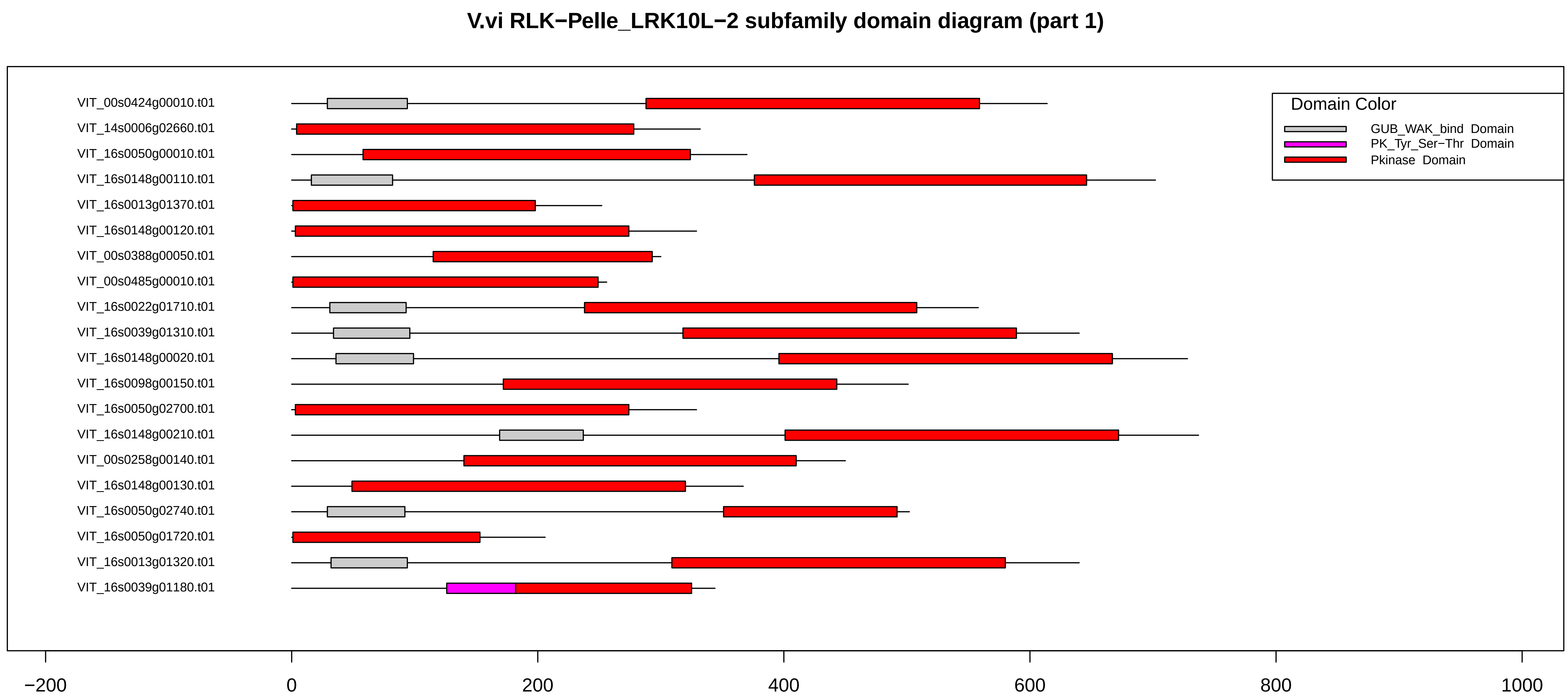

V.vi RLK−Pelle_LRK10L−2 subfamily domain diagram (part 1)
VIT_00s0424g00010.t01
VIT_14s0006g02660.t01
VIT_16s0050g00010.t01
VIT_16s0148g00110.t01
VIT_16s0013g01370.t01
VIT_16s0148g00120.t01
VIT_00s0388g00050.t01
VIT_00s0485g00010.t01
VIT_16s0022g01710.t01
VIT_16s0039g01310.t01
VIT_16s0148g00020.t01
VIT_16s0098g00150.t01
VIT_16s0050g02700.t01
VIT_16s0148g00210.t01
VIT_00s0258g00140.t01
VIT_16s0148g00130.t01
VIT_16s0050g02740.t01
VIT_16s0050g01720.t01
VIT_16s0013g01320.t01
VIT_16s0039g01180.t01
Domain Color
GUB_WAK_bind Domain
PK_Tyr_Ser−Thr Domain
Pkinase Domain
−200
0
200
400
600
800
1000

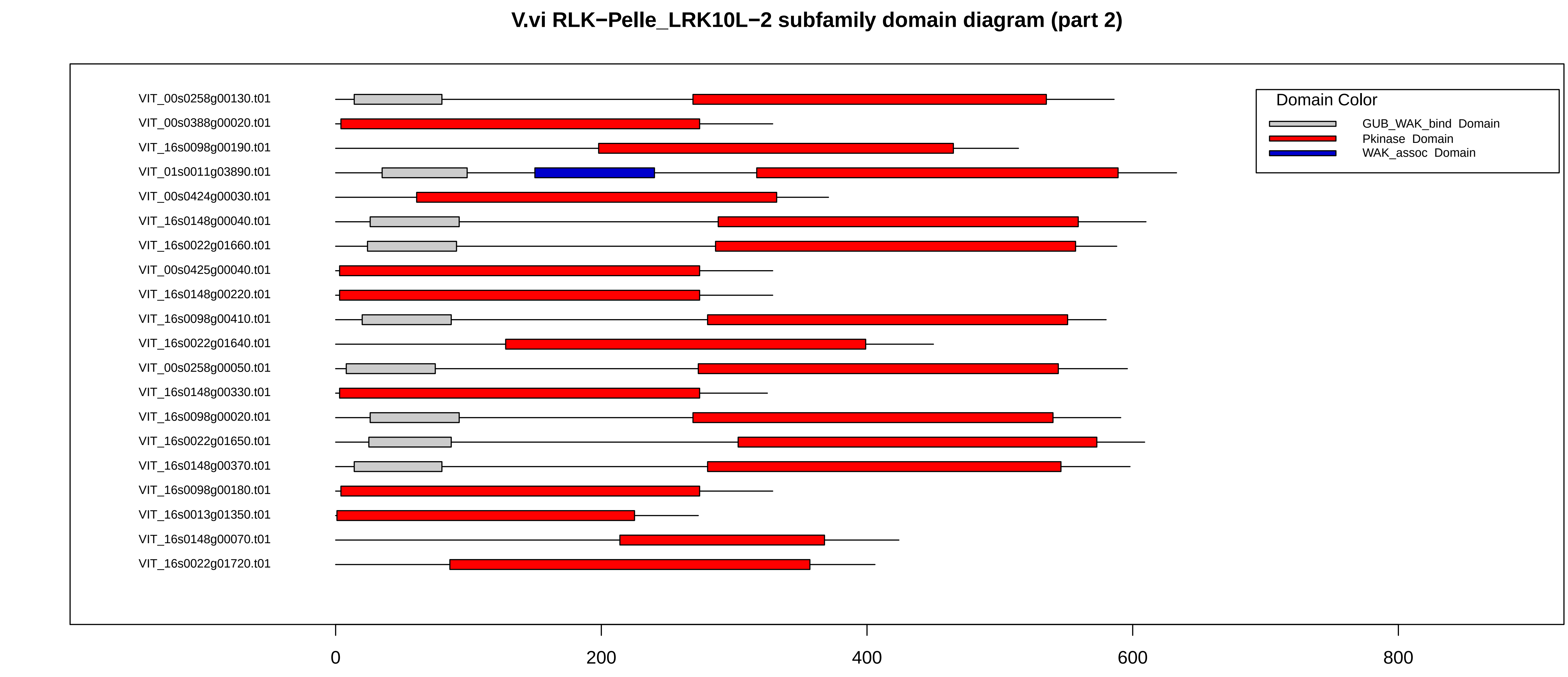
V.vi RLK−Pelle_LRK10L−2 subfamily domain diagram (part 2)
VIT_00s0258g00130.t01
VIT_00s0388g00020.t01
VIT_16s0098g00190.t01
VIT_01s0011g03890.t01
VIT_00s0424g00030.t01
VIT_16s0148g00040.t01
VIT_16s0022g01660.t01
VIT_00s0425g00040.t01
VIT_16s0148g00220.t01
VIT_16s0098g00410.t01
VIT_16s0022g01640.t01
VIT_00s0258g00050.t01
VIT_16s0148g00330.t01
VIT_16s0098g00020.t01
VIT_16s0022g01650.t01
VIT_16s0148g00370.t01
VIT_16s0098g00180.t01
VIT_16s0013g01350.t01
VIT_16s0148g00070.t01
VIT_16s0022g01720.t01
Domain Color
GUB_WAK_bind Domain
Pkinase Domain
WAK_assoc Domain
0
200
400
600
800

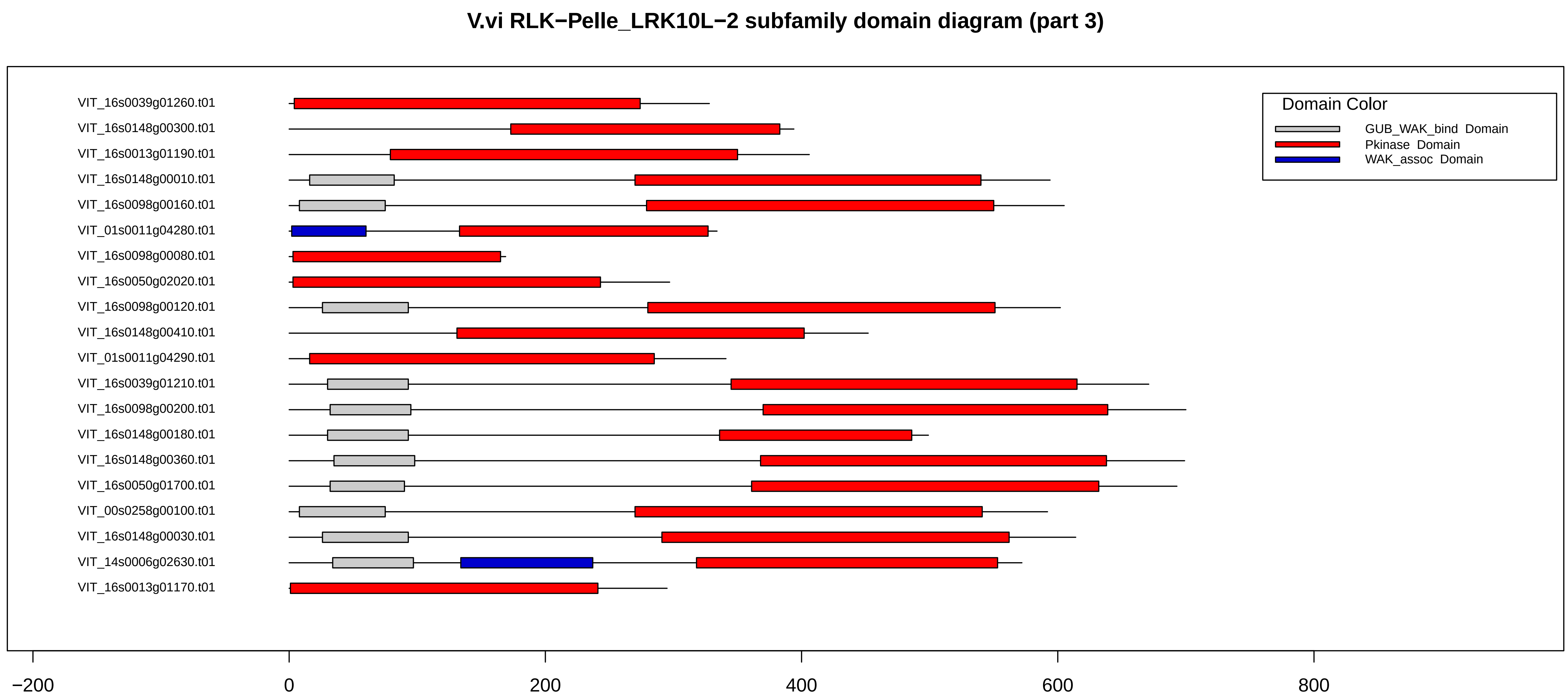
V.vi RLK−Pelle_LRK10L−2 subfamily domain diagram (part 3)
VIT_16s0039g01260.t01
VIT_16s0148g00300.t01
VIT_16s0013g01190.t01
VIT_16s0148g00010.t01
VIT_16s0098g00160.t01
VIT_01s0011g04280.t01
VIT_16s0098g00080.t01
VIT_16s0050g02020.t01
VIT_16s0098g00120.t01
VIT_16s0148g00410.t01
VIT_01s0011g04290.t01
VIT_16s0039g01210.t01
VIT_16s0098g00200.t01
VIT_16s0148g00180.t01
VIT_16s0148g00360.t01
VIT_16s0050g01700.t01
VIT_00s0258g00100.t01
VIT_16s0148g00030.t01
VIT_14s0006g02630.t01
VIT_16s0013g01170.t01
Domain Color
GUB_WAK_bind Domain
Pkinase Domain
WAK_assoc Domain
−200
0
200
400
600
800

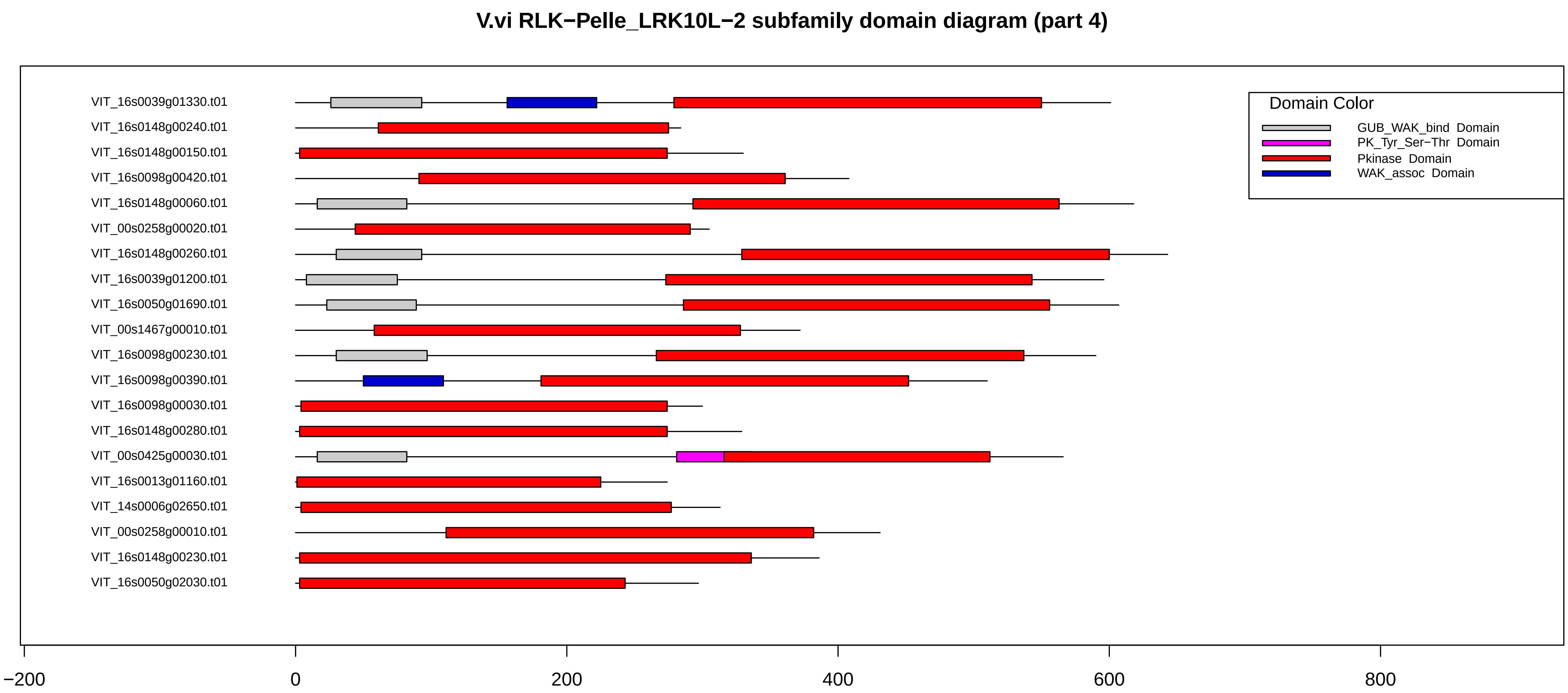
V.vi RLK−Pelle_LRK10L−2 subfamily domain diagram (part 4)
VIT_16s0039g01330.t01
VIT_16s0148g00240.t01
VIT_16s0148g00150.t01
VIT_16s0098g00420.t01
VIT_16s0148g00060.t01
VIT_00s0258g00020.t01
VIT_16s0148g00260.t01
VIT_16s0039g01200.t01
VIT_16s0050g01690.t01
VIT_00s1467g00010.t01
VIT_16s0098g00230.t01
VIT_16s0098g00390.t01
VIT_16s0098g00030.t01
VIT_16s0148g00280.t01
VIT_00s0425g00030.t01
VIT_16s0013g01160.t01
VIT_14s0006g02650.t01
VIT_00s0258g00010.t01
VIT_16s0148g00230.t01
VIT_16s0050g02030.t01
Domain Color
GUB_WAK_bind Domain
PK_Tyr_Ser−Thr Domain
Pkinase Domain
WAK_assoc Domain
−200
0
200
400
600
800

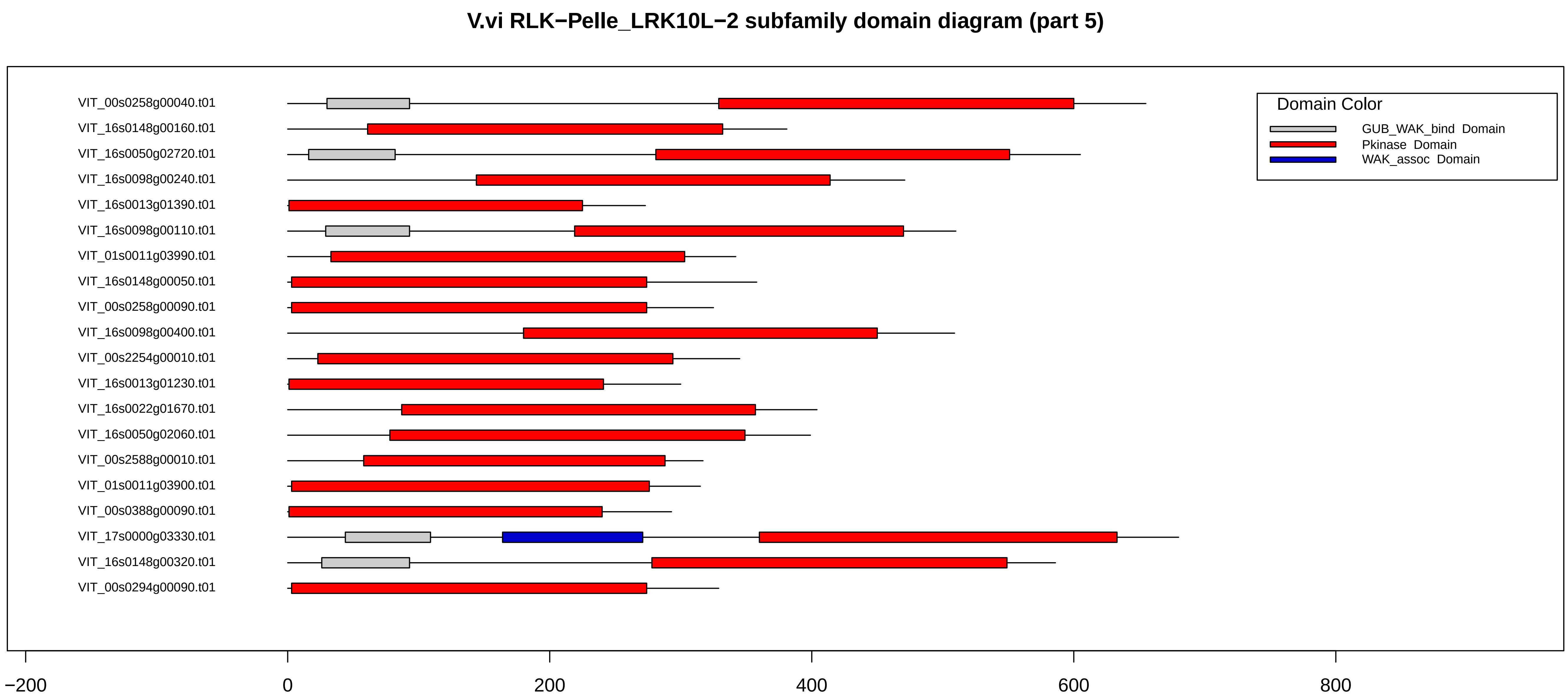

V.vi RLK−Pelle_LRK10L−2 subfamily domain diagram (part 5)
VIT_00s0258g00040.t01
VIT_16s0148g00160.t01
VIT_16s0050g02720.t01
VIT_16s0098g00240.t01
VIT_16s0013g01390.t01
VIT_16s0098g00110.t01
VIT_01s0011g03990.t01
VIT_16s0148g00050.t01
VIT_00s0258g00090.t01
VIT_16s0098g00400.t01
VIT_00s2254g00010.t01
VIT_16s0013g01230.t01
VIT_16s0022g01670.t01
VIT_16s0050g02060.t01
VIT_00s2588g00010.t01
VIT_01s0011g03900.t01
VIT_00s0388g00090.t01
VIT_17s0000g03330.t01
VIT_16s0148g00320.t01
VIT_00s0294g00090.t01
Domain Color
GUB_WAK_bind Domain
Pkinase Domain
WAK_assoc Domain
−200
0
200
400
600
800

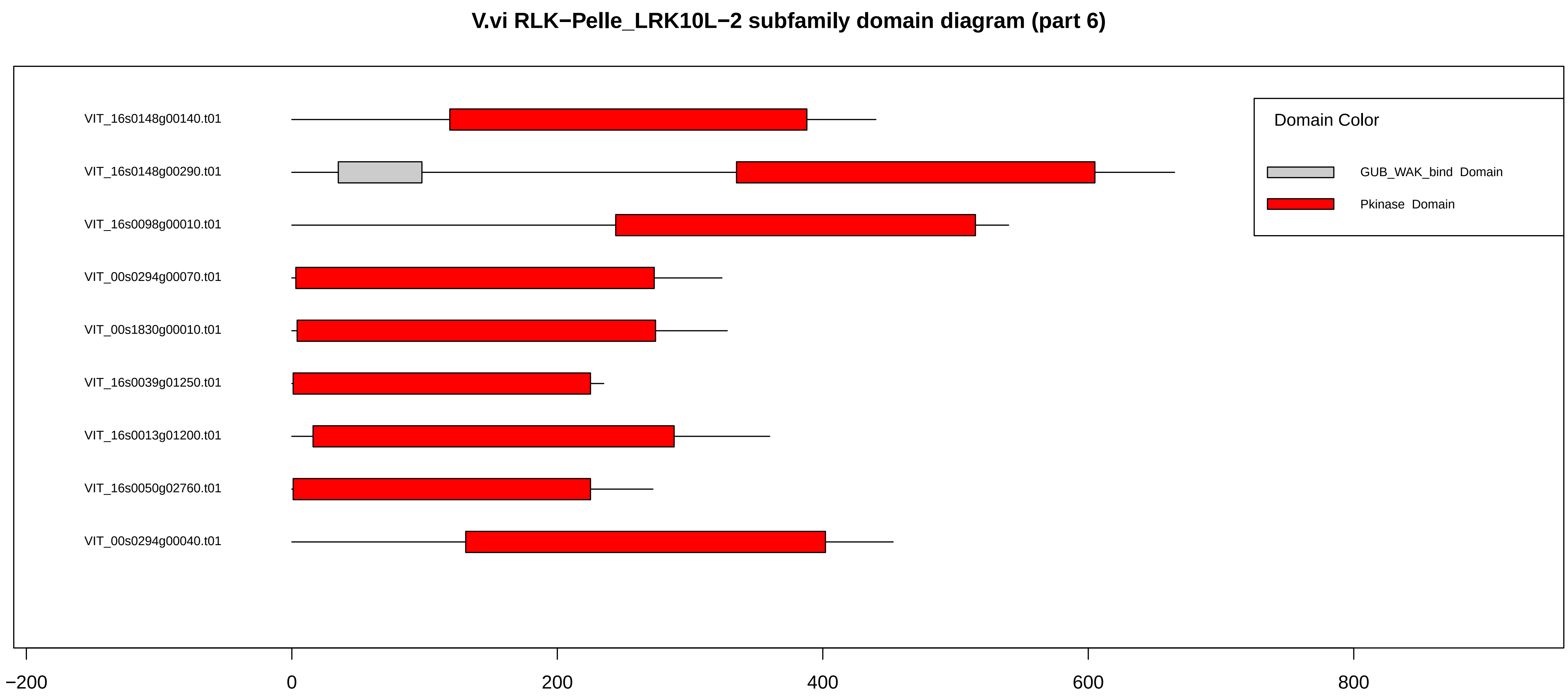
V.vi RLK−Pelle_LRK10L−2 subfamily domain diagram (part 6)
VIT_16s0148g00140.t01
VIT_16s0148g00290.t01
VIT_16s0098g00010.t01
VIT_00s0294g00070.t01
VIT_00s1830g00010.t01
VIT_16s0039g01250.t01
VIT_16s0013g01200.t01
VIT_16s0050g02760.t01
VIT_00s0294g00040.t01
Domain Color
GUB_WAK_bind Domain
Pkinase Domain
−200
0
200
400
600
800

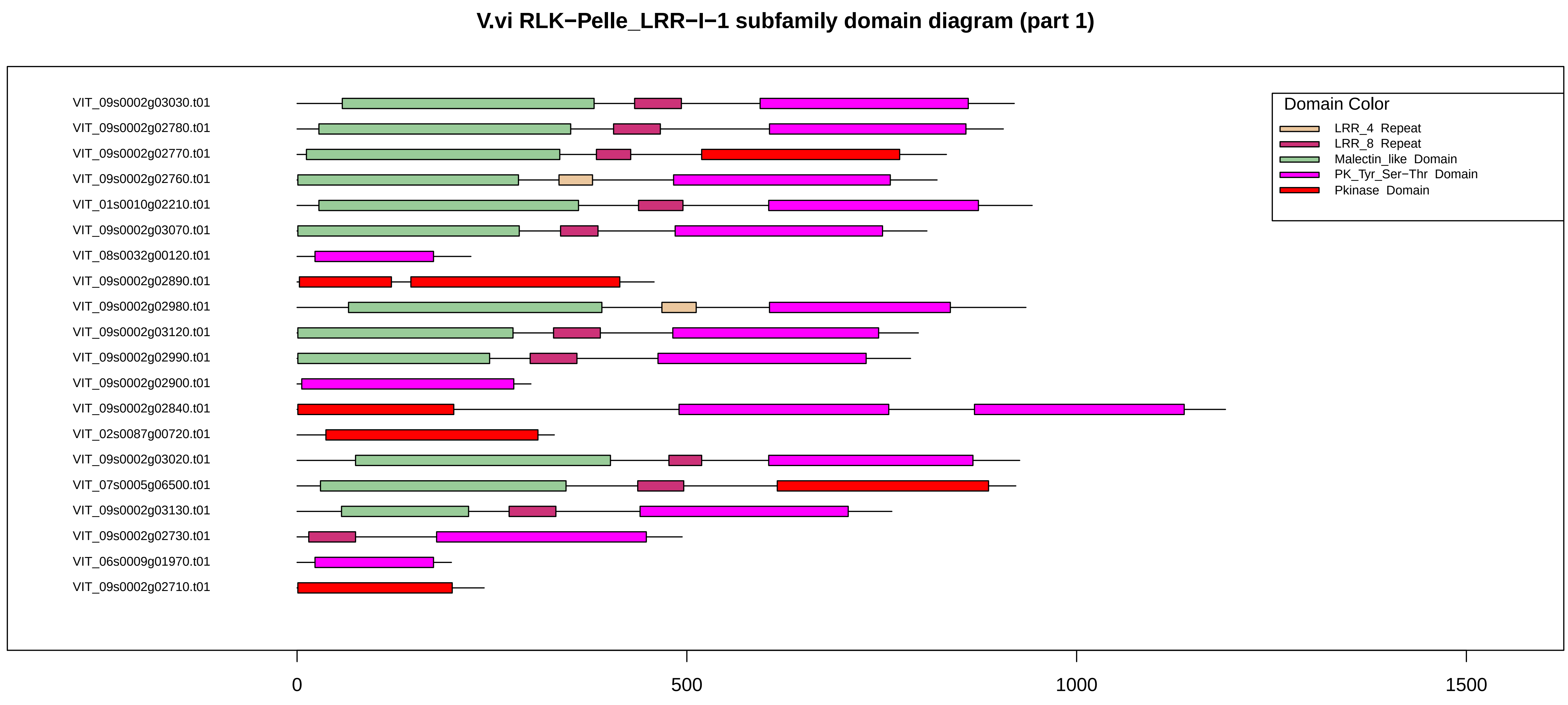
V.vi RLK−Pelle_LRR−I−1 subfamily domain diagram (part 1)
VIT_09s0002g03030.t01
VIT_09s0002g02780.t01
VIT_09s0002g02770.t01
VIT_09s0002g02760.t01
VIT_01s0010g02210.t01
VIT_09s0002g03070.t01
VIT_08s0032g00120.t01
VIT_09s0002g02890.t01
VIT_09s0002g02980.t01
VIT_09s0002g03120.t01
VIT_09s0002g02990.t01
VIT_09s0002g02900.t01
VIT_09s0002g02840.t01
VIT_02s0087g00720.t01
VIT_09s0002g03020.t01
VIT_07s0005g06500.t01
VIT_09s0002g03130.t01
VIT_09s0002g02730.t01
VIT_06s0009g01970.t01
VIT_09s0002g02710.t01
Domain Color
LRR_4 Repeat
LRR_8 Repeat
Malectin_like Domain
PK_Tyr_Ser−Thr Domain
Pkinase Domain
0
500
1000
1500

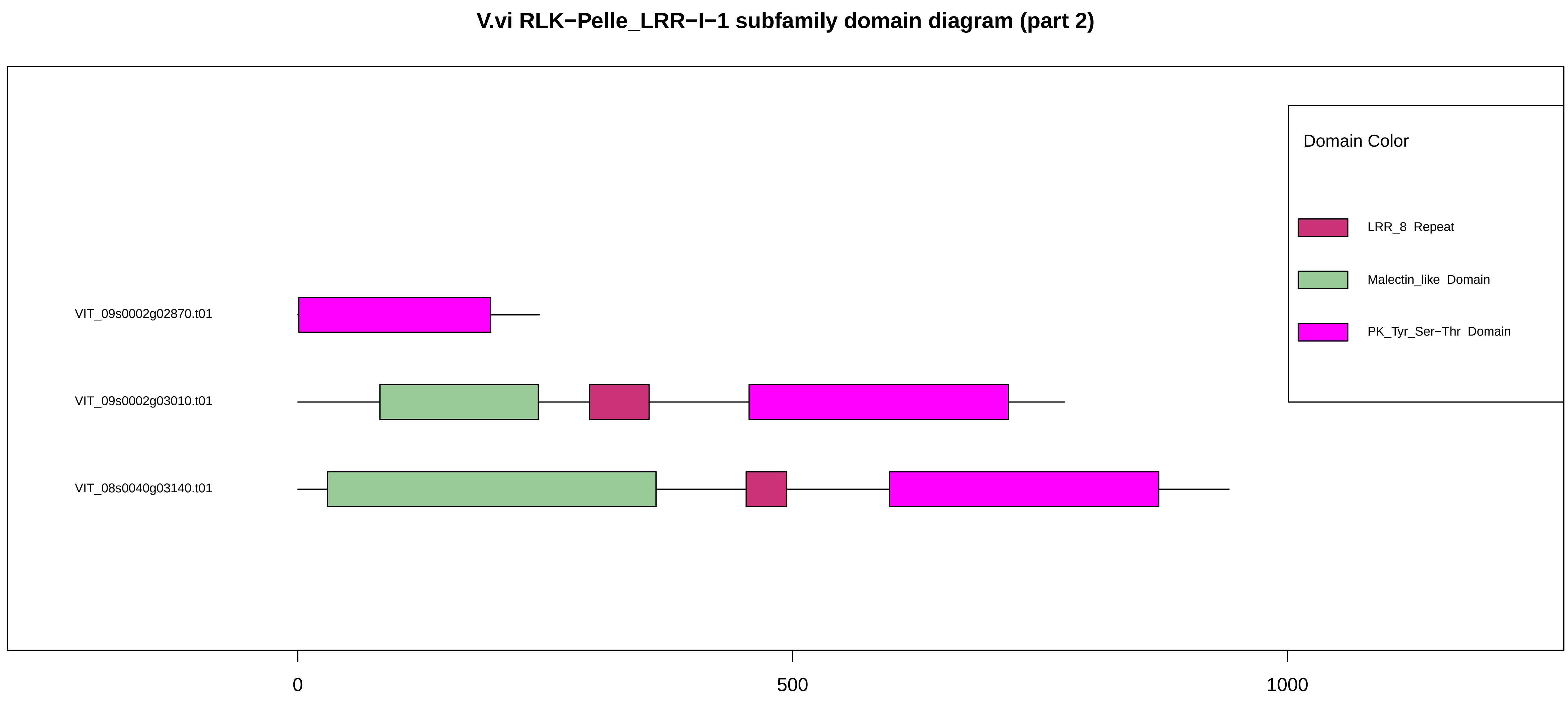

V.vi RLK−Pelle_LRR−I−1 subfamily domain diagram (part 2)
Domain Color
LRR_8 Repeat
Malectin_like Domain
PK_Tyr_Ser−Thr Domain
VIT_09s0002g02870.t01
VIT_09s0002g03010.t01
VIT_08s0040g03140.t01
0
500
1000

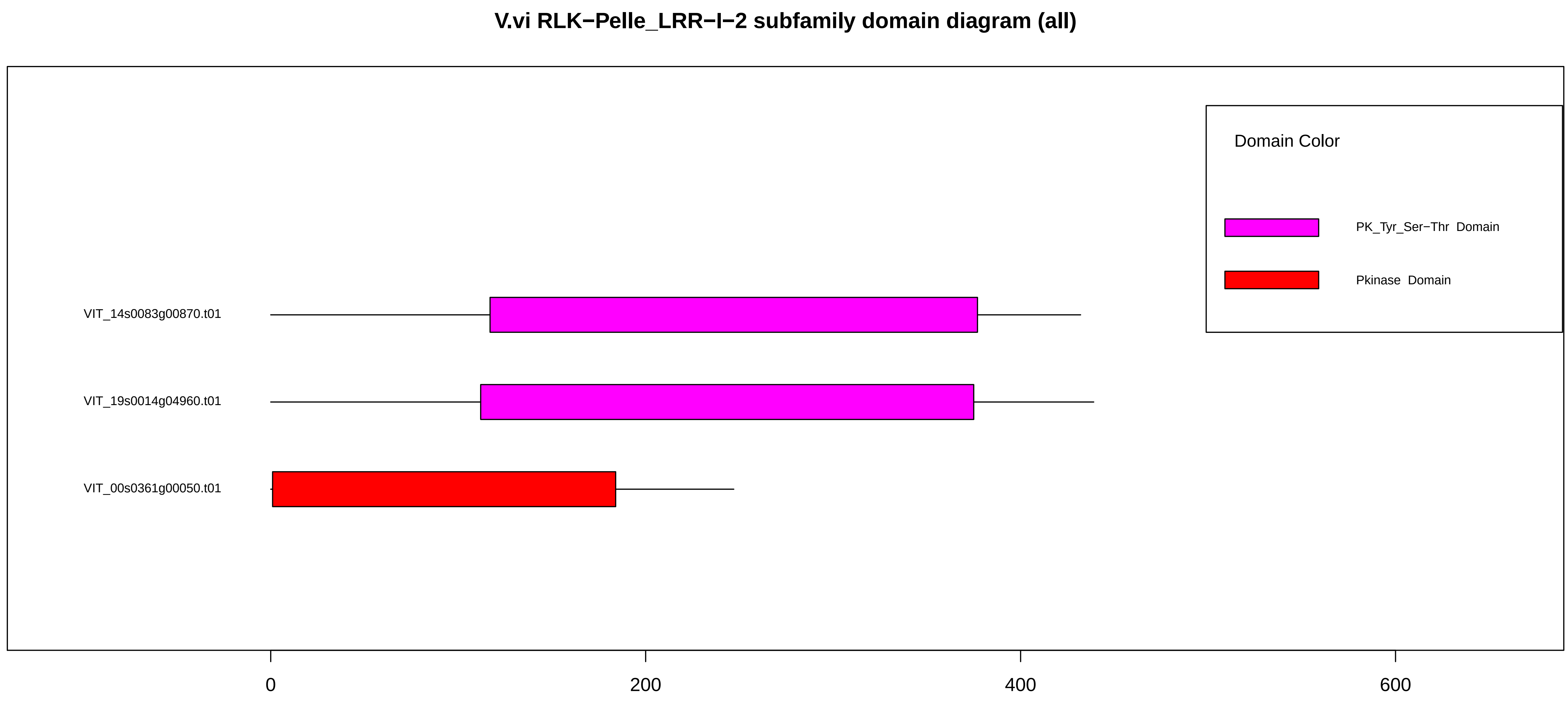
V.vi RLK−Pelle_LRR−I−2 subfamily domain diagram (all)
Domain Color
PK_Tyr_Ser−Thr Domain
Pkinase Domain
VIT_14s0083g00870.t01
VIT_19s0014g04960.t01
VIT_00s0361g00050.t01
0
200
400
600

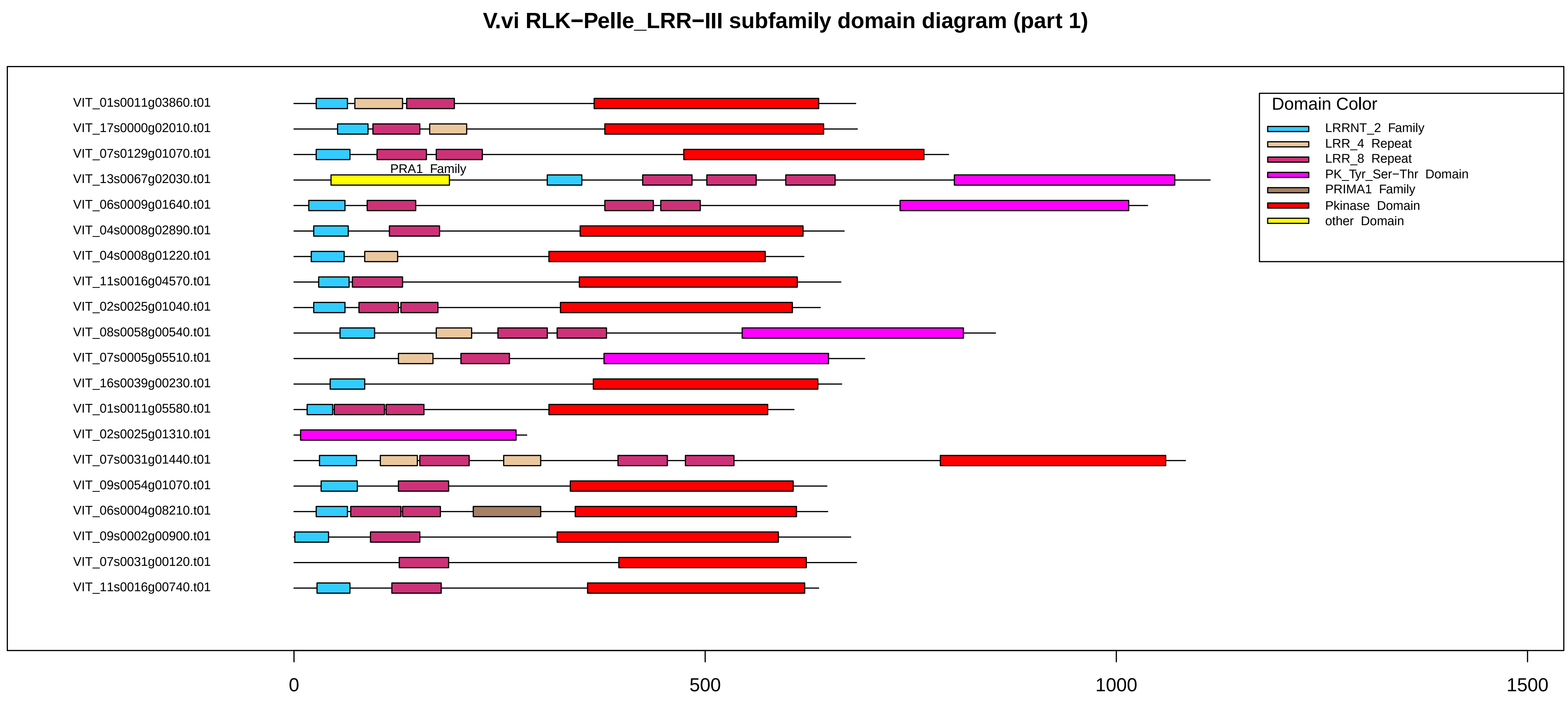

V.vi RLK−Pelle_LRR−III subfamily domain diagram (part 1)
VIT_01s0011g03860.t01
VIT_17s0000g02010.t01
VIT_07s0129g01070.t01
VIT_13s0067g02030.t01
PRA1_Family
VIT_06s0009g01640.t01
VIT_04s0008g02890.t01
VIT_04s0008g01220.t01
VIT_11s0016g04570.t01
VIT_02s0025g01040.t01
VIT_08s0058g00540.t01
VIT_07s0005g05510.t01
VIT_16s0039g00230.t01
VIT_01s0011g05580.t01
VIT_02s0025g01310.t01
VIT_07s0031g01440.t01
VIT_09s0054g01070.t01
VIT_06s0004g08210.t01
VIT_09s0002g00900.t01
VIT_07s0031g00120.t01
VIT_11s0016g00740.t01
Domain Color
LRRNT_2 Family
LRR_4 Repeat
LRR_8 Repeat
PK_Tyr_Ser−Thr Domain
PRIMA1 Family
Pkinase Domain
other Domain
0
500
1000
1500

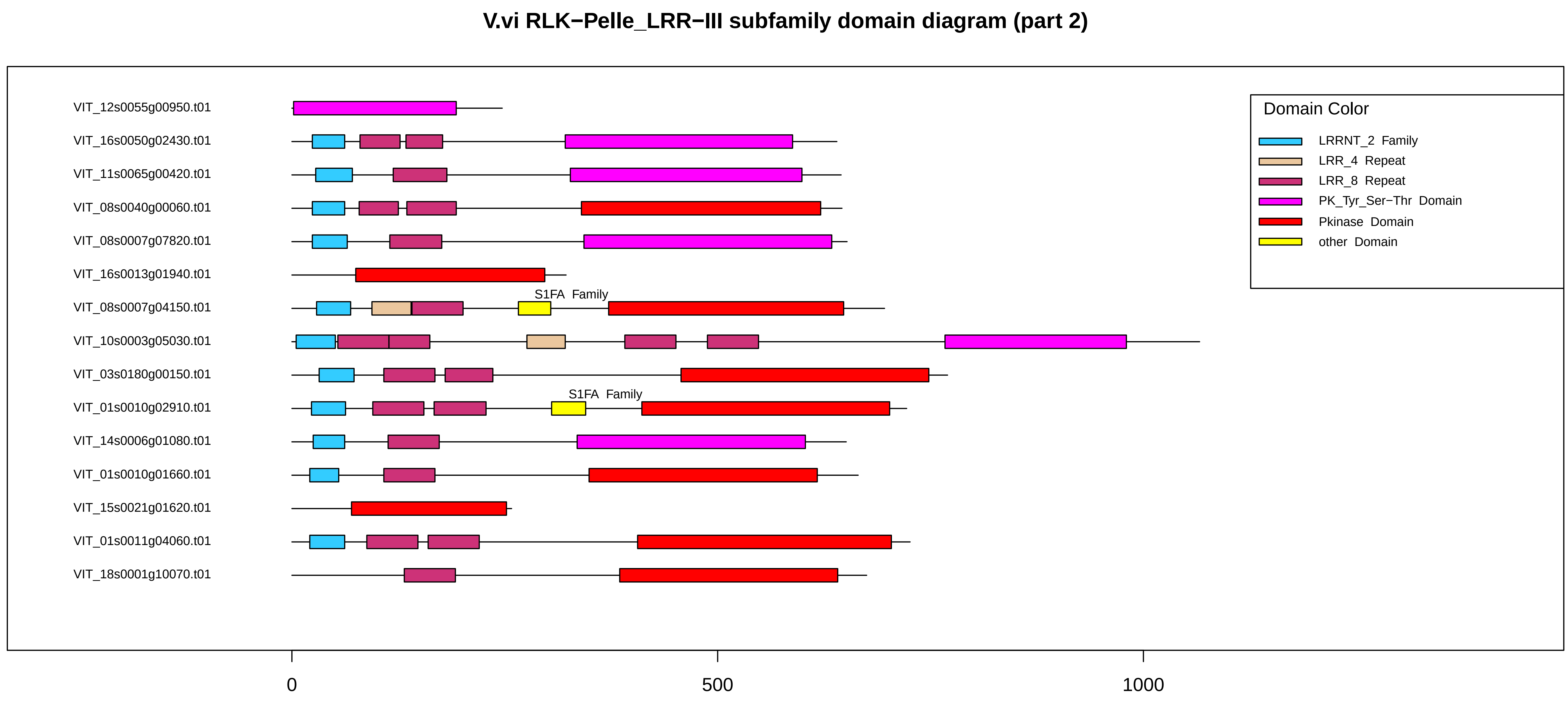
V.vi RLK−Pelle_LRR−III subfamily domain diagram (part 2)
VIT_12s0055g00950.t01
VIT_16s0050g02430.t01
VIT_11s0065g00420.t01
VIT_08s0040g00060.t01
VIT_08s0007g07820.t01
VIT_16s0013g01940.t01
VIT_08s0007g04150.t01
S1FA Family
VIT_10s0003g05030.t01
VIT_03s0180g00150.t01
VIT_01s0010g02910.t01
S1FA Family
VIT_14s0006g01080.t01
VIT_01s0010g01660.t01
VIT_15s0021g01620.t01
VIT_01s0011g04060.t01
VIT_18s0001g10070.t01
Domain Color
LRRNT_2 Family
LRR_4 Repeat
LRR_8 Repeat
PK_Tyr_Ser−Thr Domain
Pkinase Domain
other Domain
0
500
1000

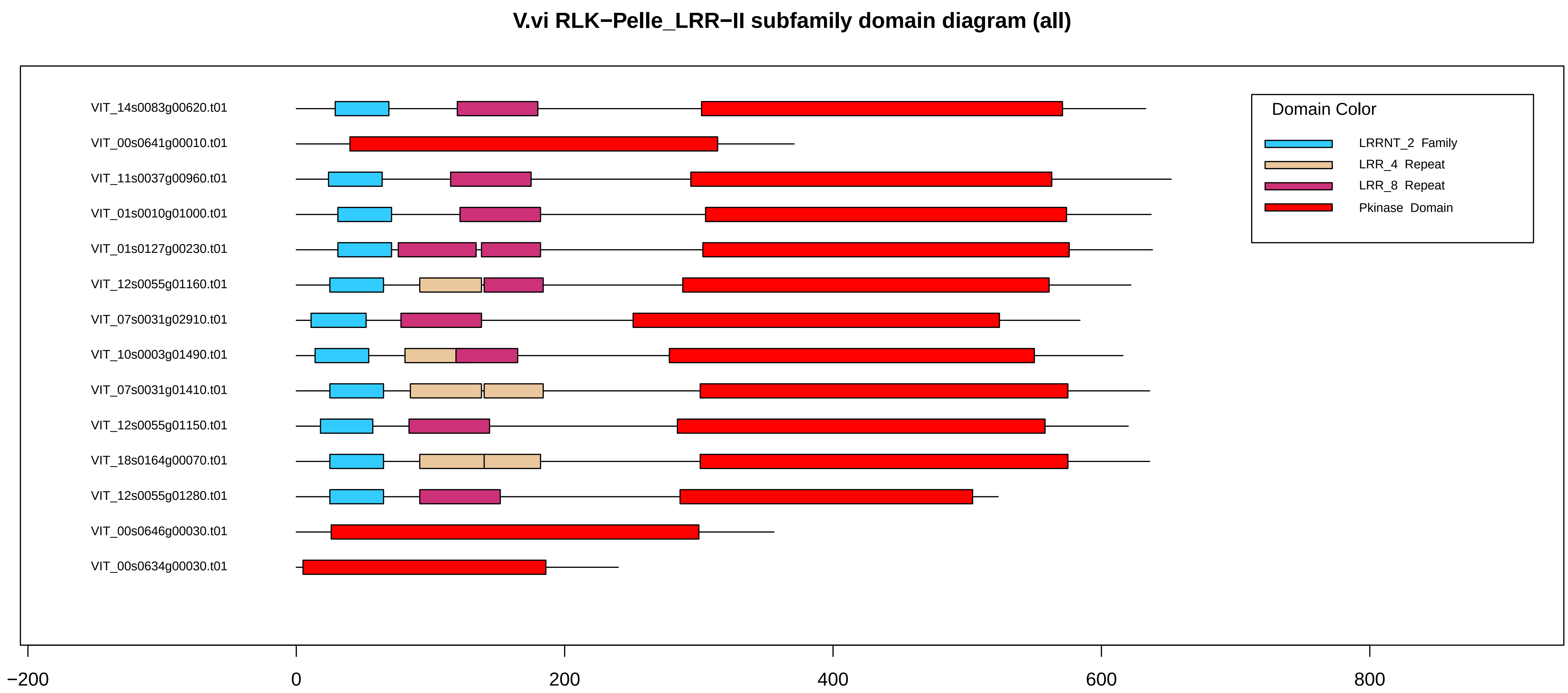
V.vi RLK−Pelle_LRR−II subfamily domain diagram (all)
VIT_14s0083g00620.t01
VIT_00s0641g00010.t01
VIT_11s0037g00960.t01
VIT_01s0010g01000.t01
VIT_01s0127g00230.t01
VIT_12s0055g01160.t01
VIT_07s0031g02910.t01
VIT_10s0003g01490.t01
VIT_07s0031g01410.t01
VIT_12s0055g01150.t01
VIT_18s0164g00070.t01
VIT_12s0055g01280.t01
VIT_00s0646g00030.t01
VIT_00s0634g00030.t01
Domain Color
LRRNT_2 Family
LRR_4 Repeat
LRR_8 Repeat
Pkinase Domain
−200
0
200
400
600
800

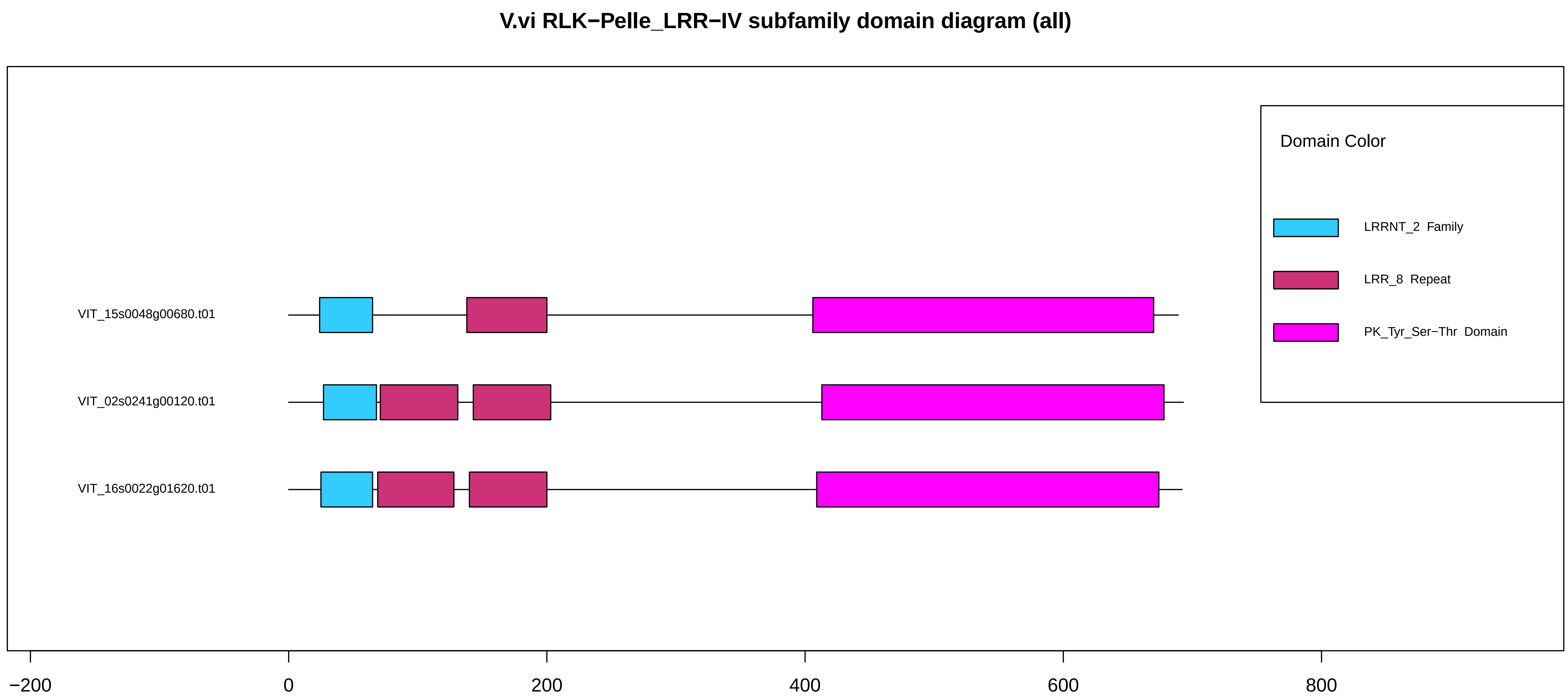

V.vi RLK−Pelle_LRR−IV subfamily domain diagram (all)
VIT_15s0048g00680.t01
VIT_02s0241g00120.t01
VIT_16s0022g01620.t01
Domain Color
LRRNT_2 Family
LRR_8 Repeat
PK_Tyr_Ser−Thr Domain
−200
0
200
400
600
800

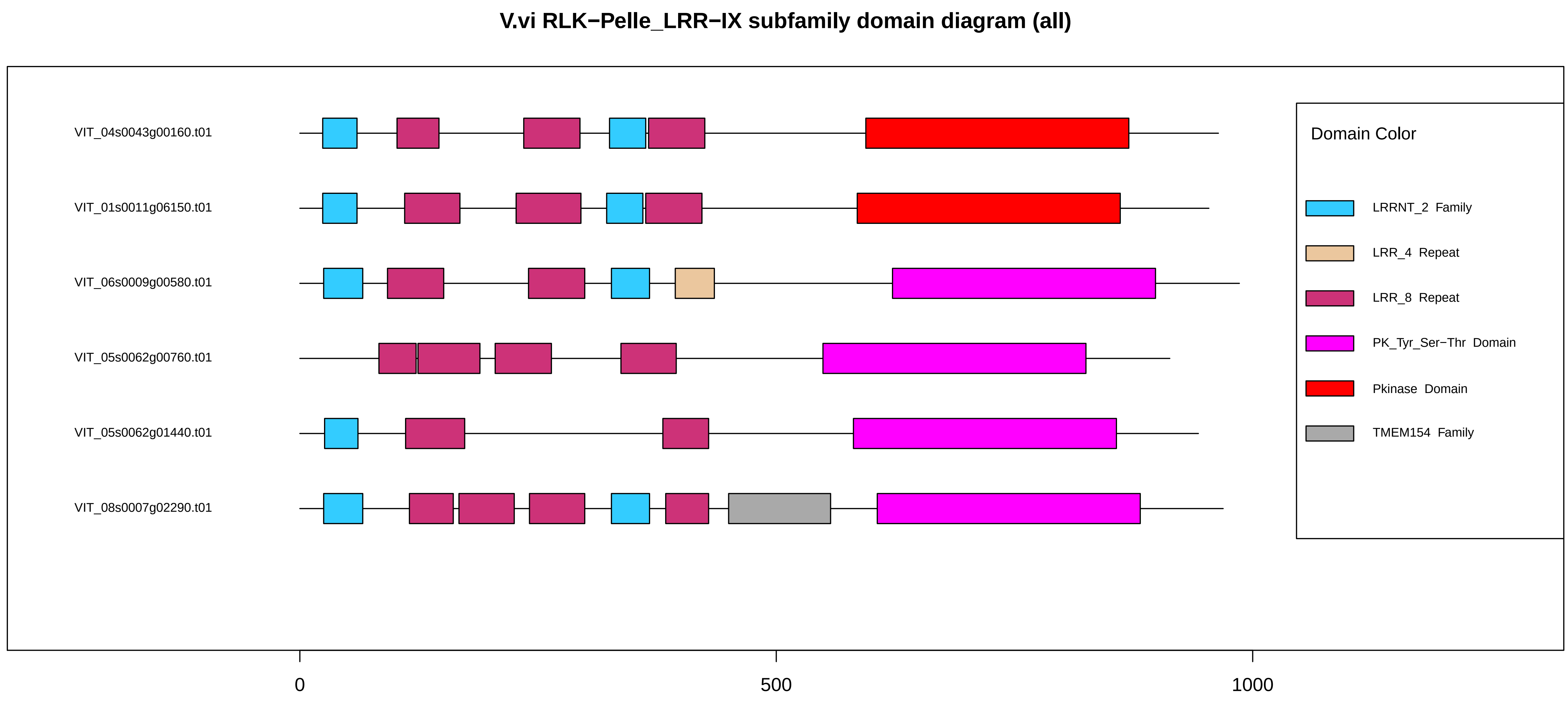
V.vi RLK−Pelle_LRR−IX subfamily domain diagram (all)
VIT_04s0043g00160.t01
VIT_01s0011g06150.t01
VIT_06s0009g00580.t01
VIT_05s0062g00760.t01
VIT_05s0062g01440.t01
VIT_08s0007g02290.t01
Domain Color
LRRNT_2 Family
LRR_4 Repeat
LRR_8 Repeat
PK_Tyr_Ser−Thr Domain
Pkinase Domain
TMEM154 Family
0
500
1000

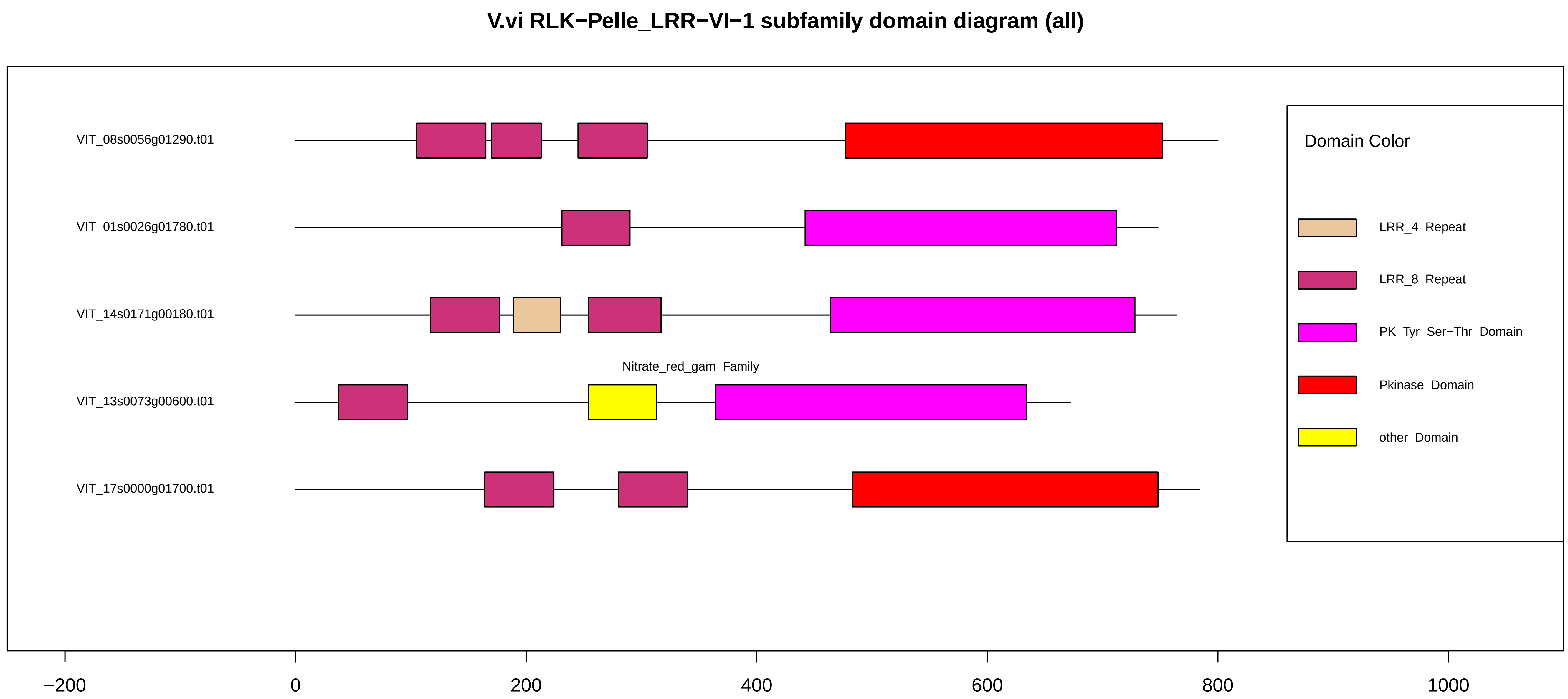
V.vi RLK−Pelle_LRR−VI−1 subfamily domain diagram (all)
VIT_08s0056g01290.t01
VIT_01s0026g01780.t01
VIT_14s0171g00180.t01
Nitrate_red_gam Family
VIT_13s0073g00600.t01
VIT_17s0000g01700.t01
Domain Color
LRR_4 Repeat
LRR_8 Repeat
PK_Tyr_Ser−Thr Domain
Pkinase Domain
other Domain
−200
0
200
400
600
800
1000

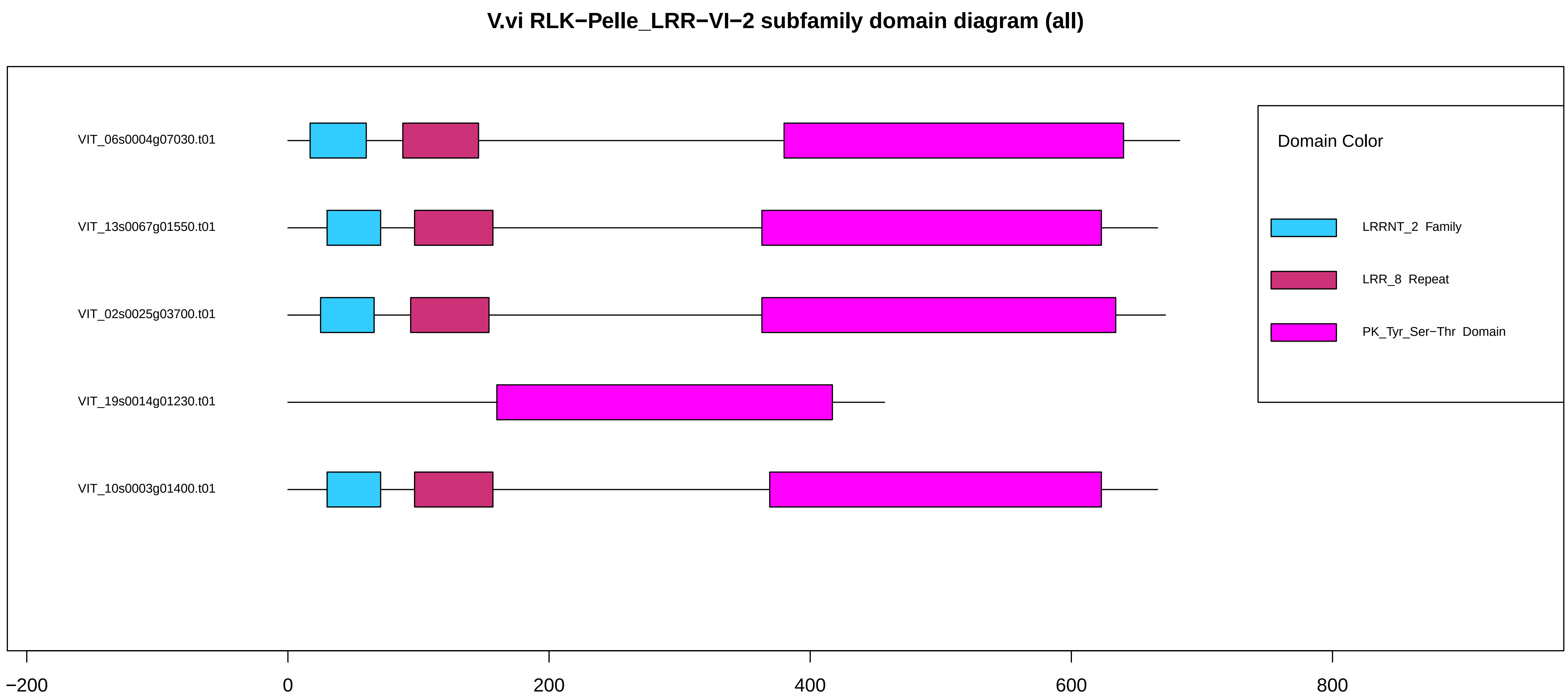
V.vi RLK−Pelle_LRR−VI−2 subfamily domain diagram (all)
VIT_06s0004g07030.t01
VIT_13s0067g01550.t01
VIT_02s0025g03700.t01
VIT_19s0014g01230.t01
VIT_10s0003g01400.t01
Domain Color
LRRNT_2 Family
LRR_8 Repeat
PK_Tyr_Ser−Thr Domain
−200
0
200
400
600
800

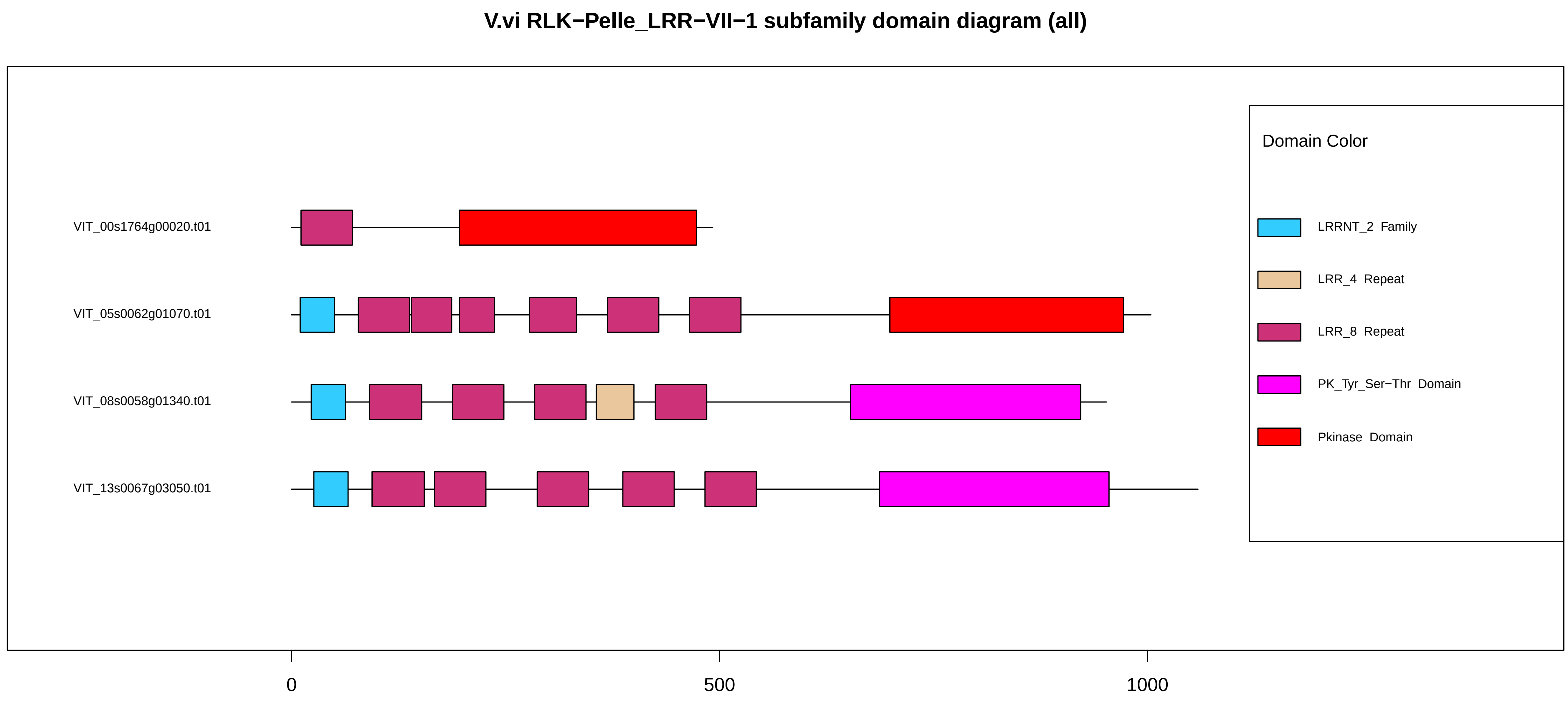
V.vi RLK−Pelle_LRR−VII−1 subfamily domain diagram (all)
VIT_00s1764g00020.t01
VIT_05s0062g01070.t01
VIT_08s0058g01340.t01
VIT_13s0067g03050.t01
Domain Color
LRRNT_2 Family
LRR_4 Repeat
LRR_8 Repeat
PK_Tyr_Ser−Thr Domain
Pkinase Domain
0
500
1000

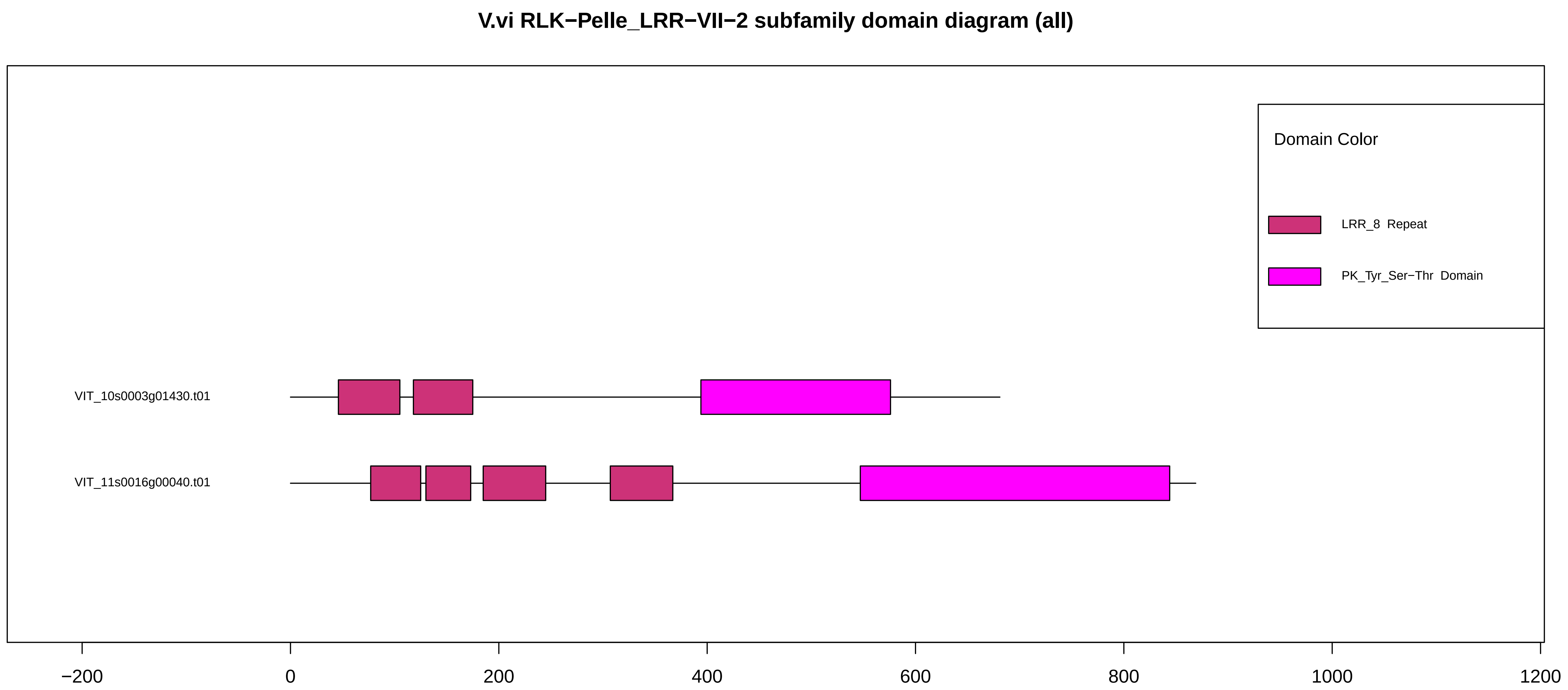
V.vi RLK−Pelle_LRR−VII−2 subfamily domain diagram (all)
Domain Color
LRR_8 Repeat
PK_Tyr_Ser−Thr Domain
VIT_10s0003g01430.t01
VIT_11s0016g00040.t01
−200
0
200
400
600
800
1000
1200

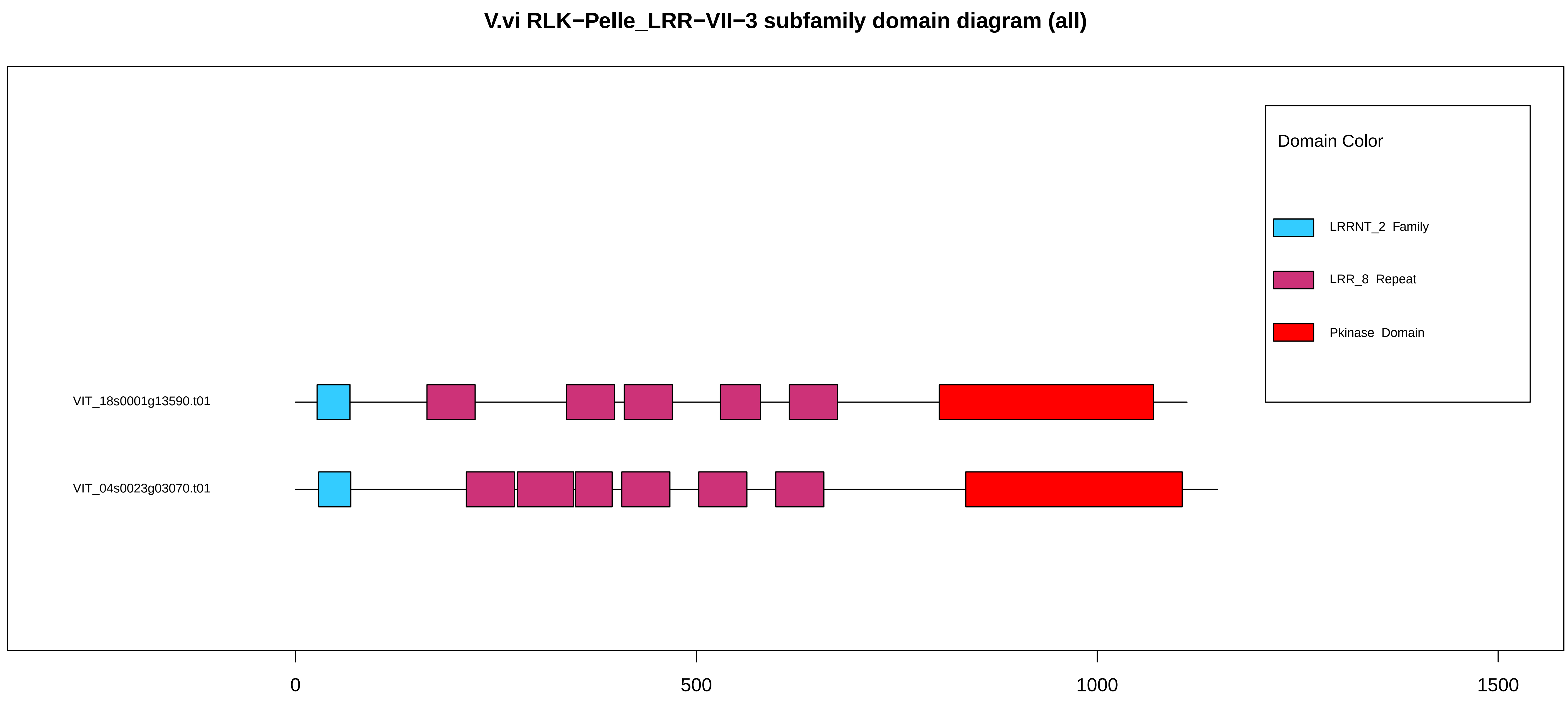
V.vi RLK−Pelle_LRR−VII−3 subfamily domain diagram (all)
Domain Color
LRRNT_2 Family
LRR_8 Repeat
Pkinase Domain
VIT_18s0001g13590.t01
VIT_04s0023g03070.t01
0
500
1000
1500

V.vi RLK−Pelle_LRR−VIII−1 subfamily domain diagram (all)

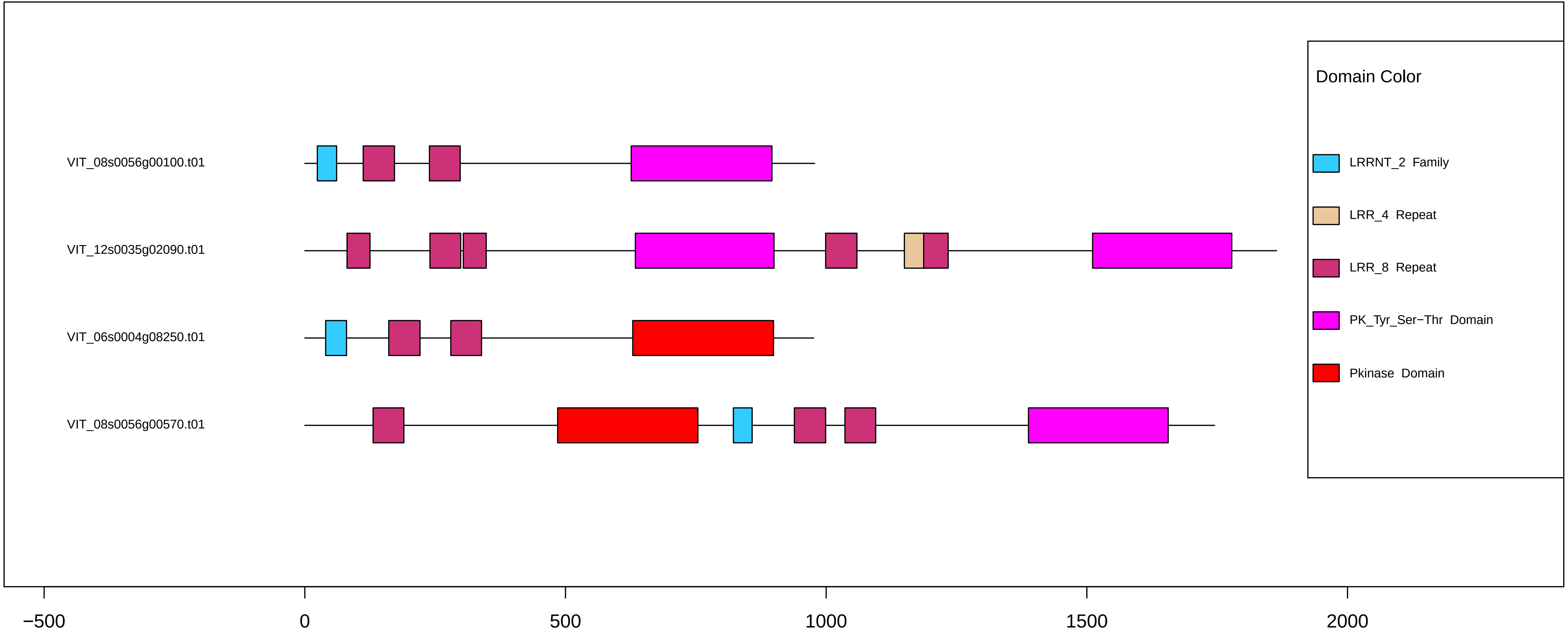

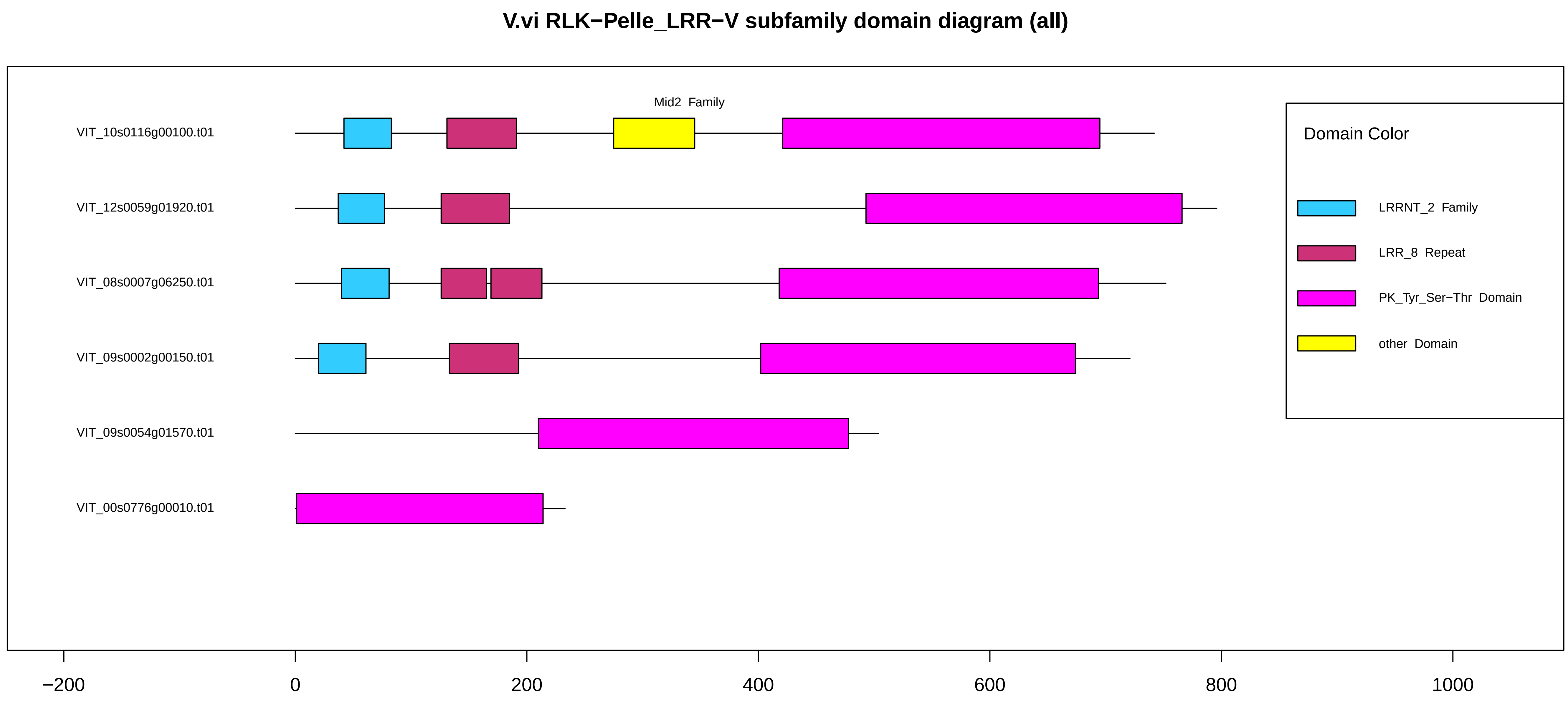
V.vi RLK−Pelle_LRR−V subfamily domain diagram (all)
Mid2 Family
VIT_10s0116g00100.t01
VIT_12s0059g01920.t01
VIT_08s0007g06250.t01
VIT_09s0002g00150.t01
VIT_09s0054g01570.t01
VIT_00s0776g00010.t01
Domain Color
LRRNT_2 Family
LRR_8 Repeat
PK_Tyr_Ser−Thr Domain
other Domain
−200
0
200
400
600
800
1000

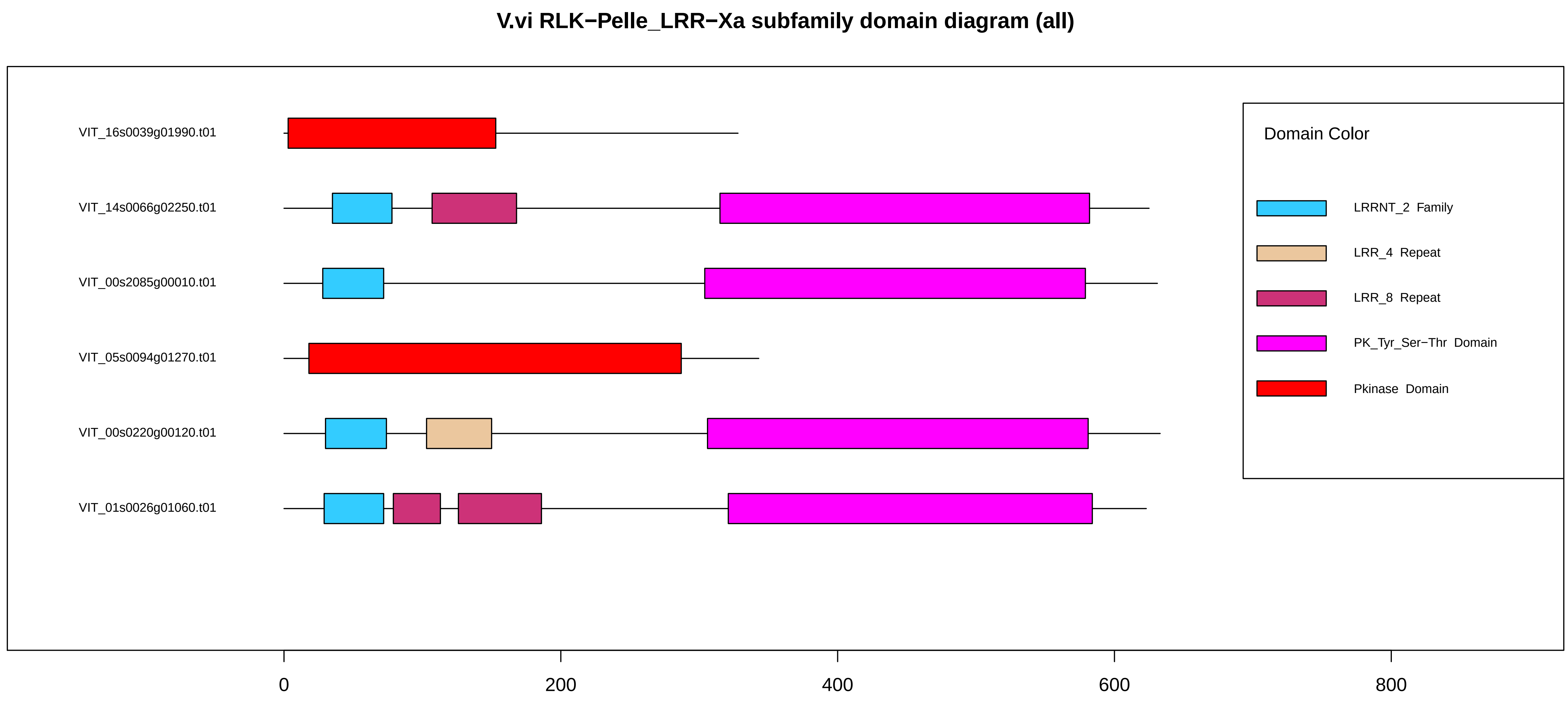

V.vi RLK−Pelle_LRR−Xa subfamily domain diagram (all)
VIT_16s0039g01990.t01
VIT_14s0066g02250.t01
VIT_00s2085g00010.t01
VIT_05s0094g01270.t01
VIT_00s0220g00120.t01
VIT_01s0026g01060.t01
Domain Color
LRRNT_2 Family
LRR_4 Repeat
LRR_8 Repeat
PK_Tyr_Ser−Thr Domain
Pkinase Domain
0
200
400
600
800

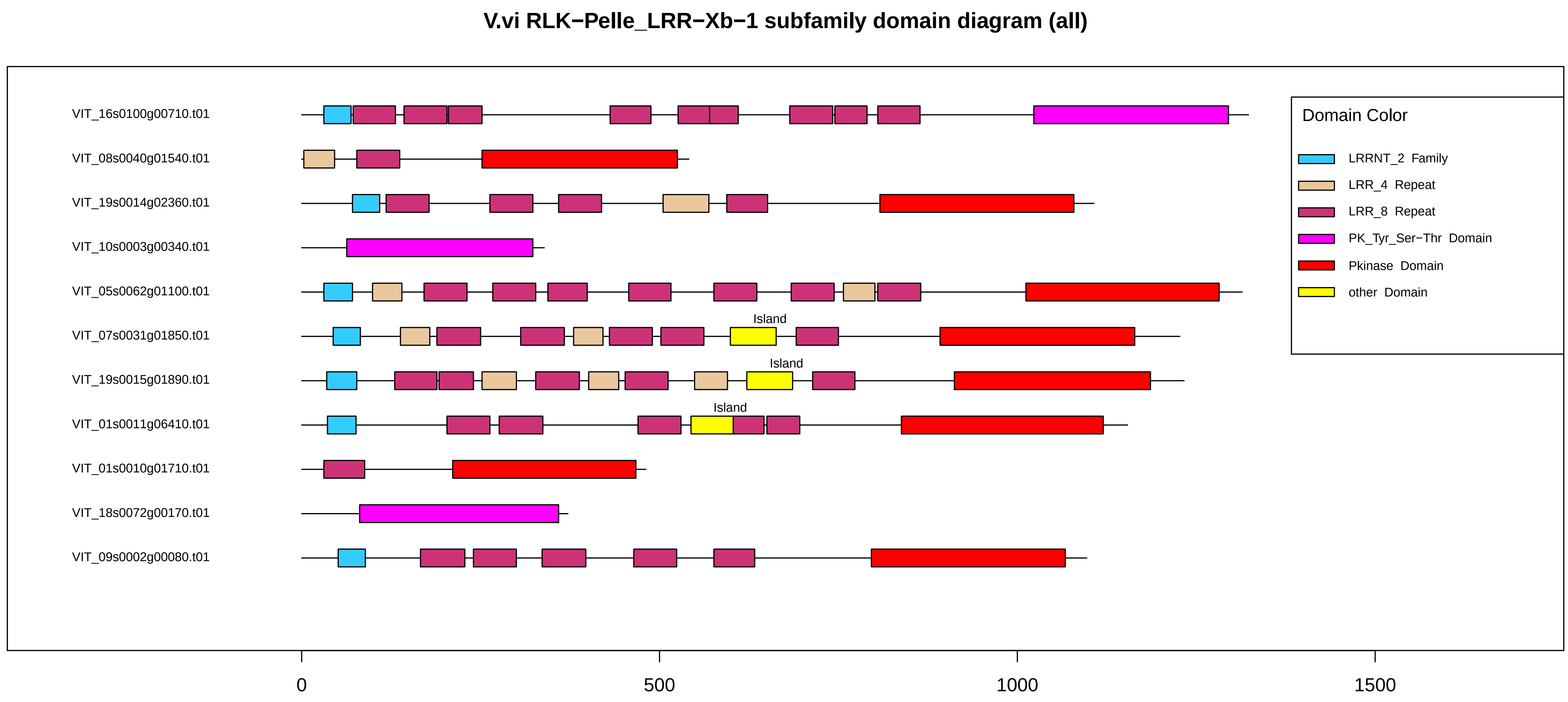
V.vi RLK−Pelle_LRR−Xb−1 subfamily domain diagram (all)
VIT_16s0100g00710.t01
VIT_08s0040g01540.t01
VIT_19s0014g02360.t01
VIT_10s0003g00340.t01
VIT_05s0062g01100.t01
VIT_07s0031g01850.t01
VIT_19s0015g01890.t01
VIT_01s0011g06410.t01
VIT_01s0010g01710.t01
VIT_18s0072g00170.t01
VIT_09s0002g00080.t01
Island
Island
Island
Domain Color
LRRNT_2 Family
LRR_4 Repeat
LRR_8 Repeat
PK_Tyr_Ser−Thr Domain
Pkinase Domain
other Domain
0
500
1000
1500

V.vi RLK−Pelle_LRR−Xb−2 subfamily domain diagram (all)

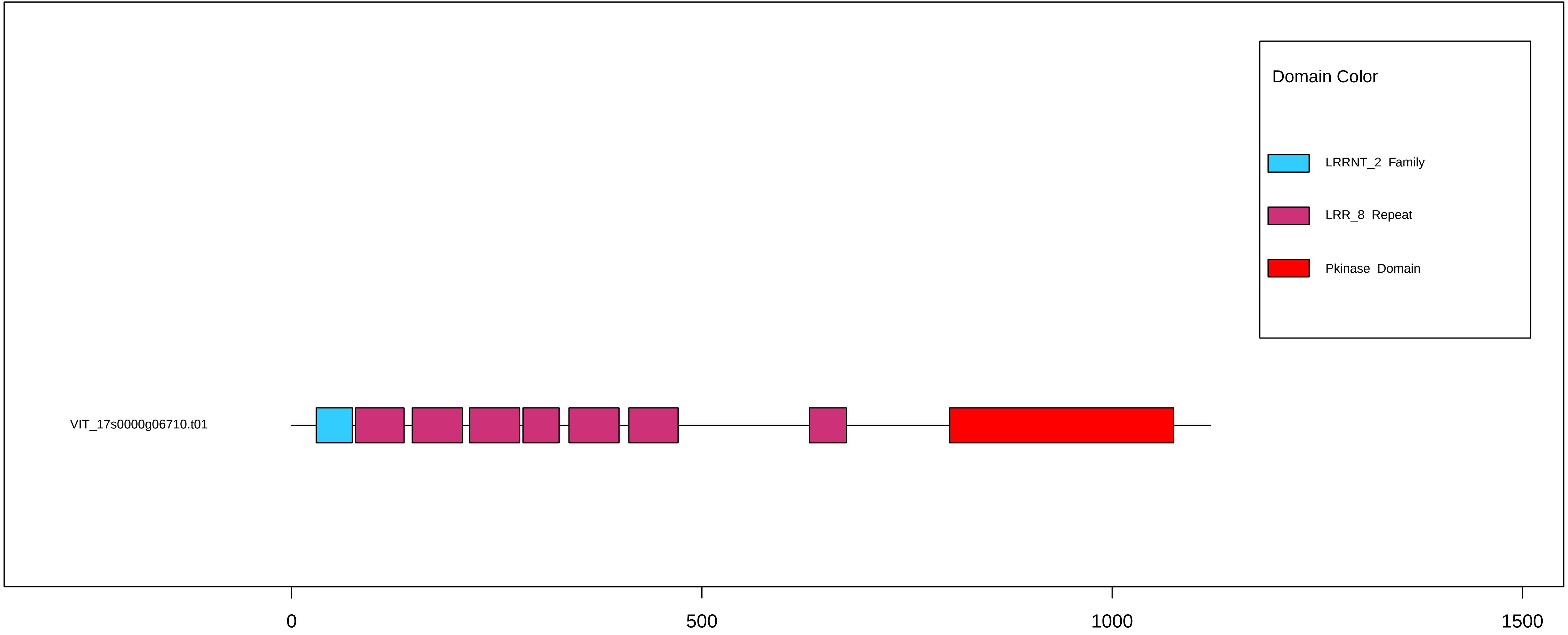

V.vi RLK−Pelle_LRR−XI−1 subfamily domain diagram (part 1)

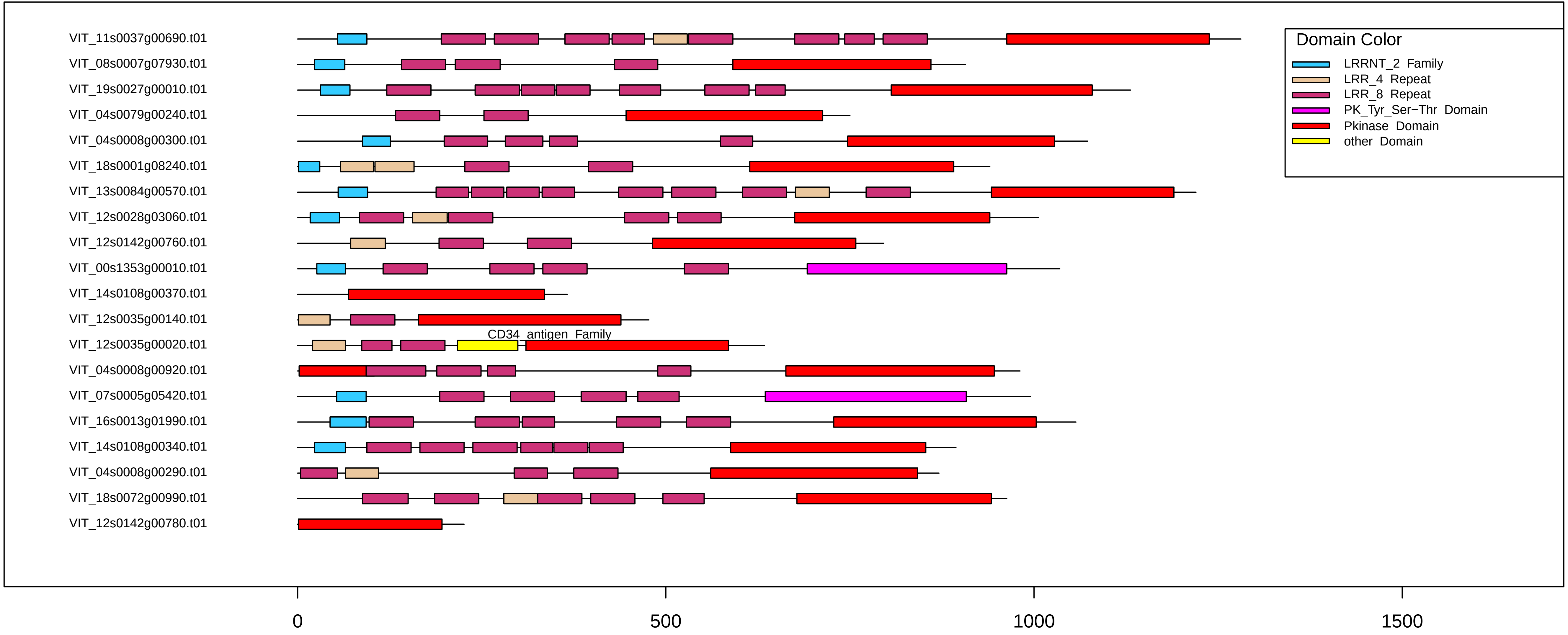

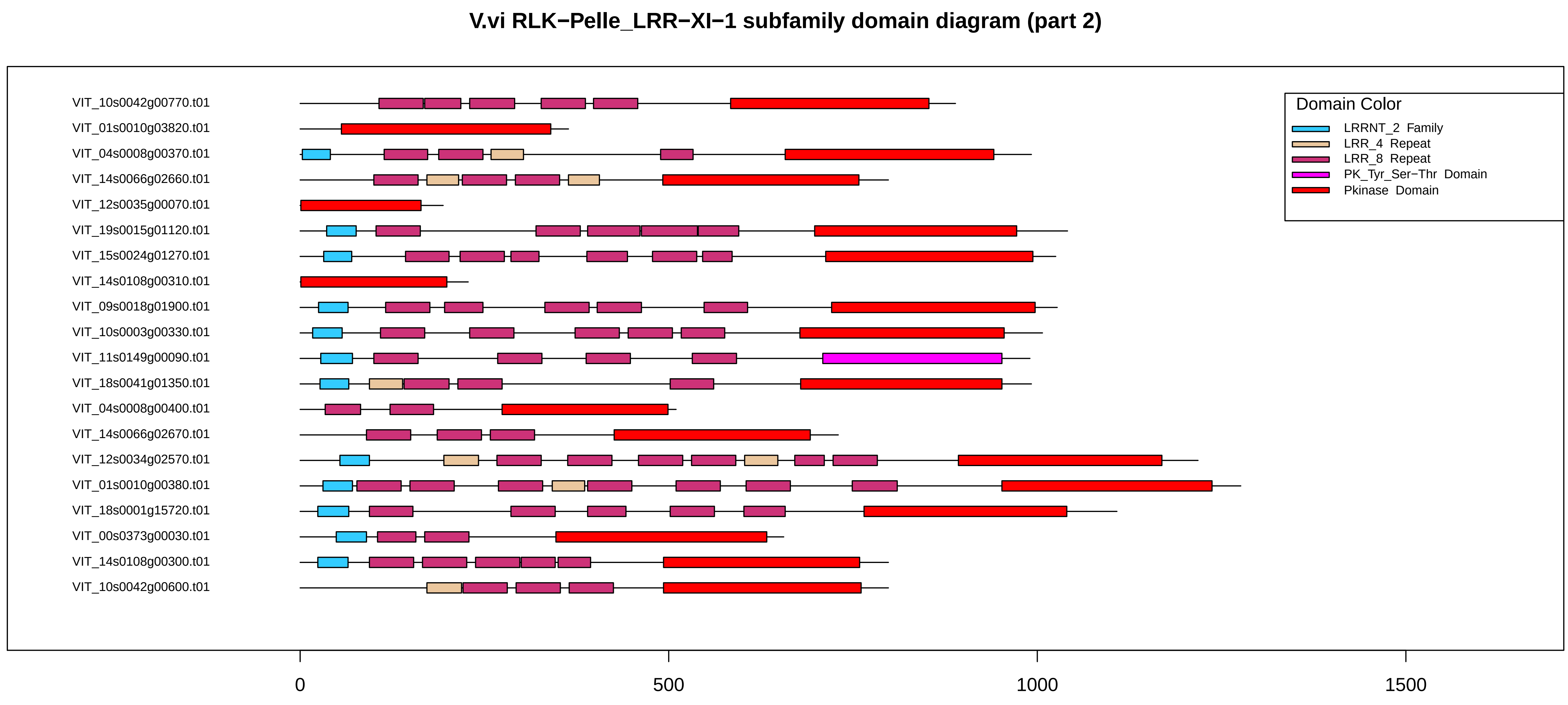
V.vi RLK−Pelle_LRR−XI−1 subfamily domain diagram (part 2)
VIT_10s0042g00770.t01
VIT_01s0010g03820.t01
VIT_04s0008g00370.t01
VIT_14s0066g02660.t01
VIT_12s0035g00070.t01
VIT_19s0015g01120.t01
VIT_15s0024g01270.t01
VIT_14s0108g00310.t01
VIT_09s0018g01900.t01
VIT_10s0003g00330.t01
VIT_11s0149g00090.t01
VIT_18s0041g01350.t01
VIT_04s0008g00400.t01
VIT_14s0066g02670.t01
VIT_12s0034g02570.t01
VIT_01s0010g00380.t01
VIT_18s0001g15720.t01
VIT_00s0373g00030.t01
VIT_14s0108g00300.t01
VIT_10s0042g00600.t01
Domain Color
LRRNT_2 Family
LRR_4 Repeat
LRR_8 Repeat
PK_Tyr_Ser−Thr Domain
Pkinase Domain
0
500
1000
1500

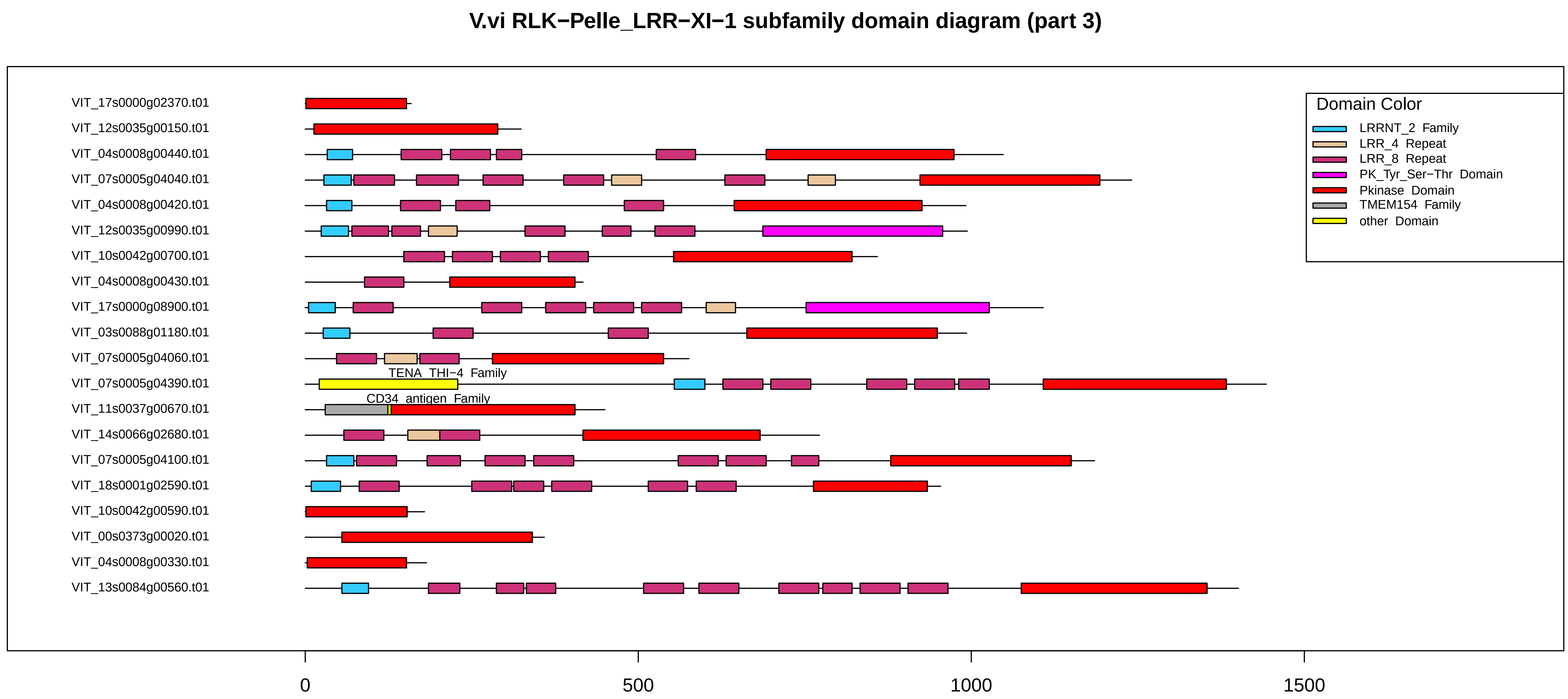

V.vi RLK−Pelle_LRR−XI−1 subfamily domain diagram (part 3)
VIT_17s0000g02370.t01
VIT_12s0035g00150.t01
VIT_04s0008g00440.t01
VIT_07s0005g04040.t01
VIT_04s0008g00420.t01
VIT_12s0035g00990.t01
VIT_10s0042g00700.t01
VIT_04s0008g00430.t01
VIT_17s0000g08900.t01
VIT_03s0088g01180.t01
VIT_07s0005g04060.t01
VIT_07s0005g04390.t01
VIT_11s0037g00670.t01
VIT_14s0066g02680.t01
VIT_07s0005g04100.t01
VIT_18s0001g02590.t01
VIT_10s0042g00590.t01
VIT_00s0373g00020.t01
VIT_04s0008g00330.t01
VIT_13s0084g00560.t01
TENA_THI−4 Family
CD34_antigen Family
Domain Color
LRRNT_2 Family
LRR_4 Repeat
LRR_8 Repeat
PK_Tyr_Ser−Thr Domain
Pkinase Domain
TMEM154 Family
other Domain
0
500
1000
1500

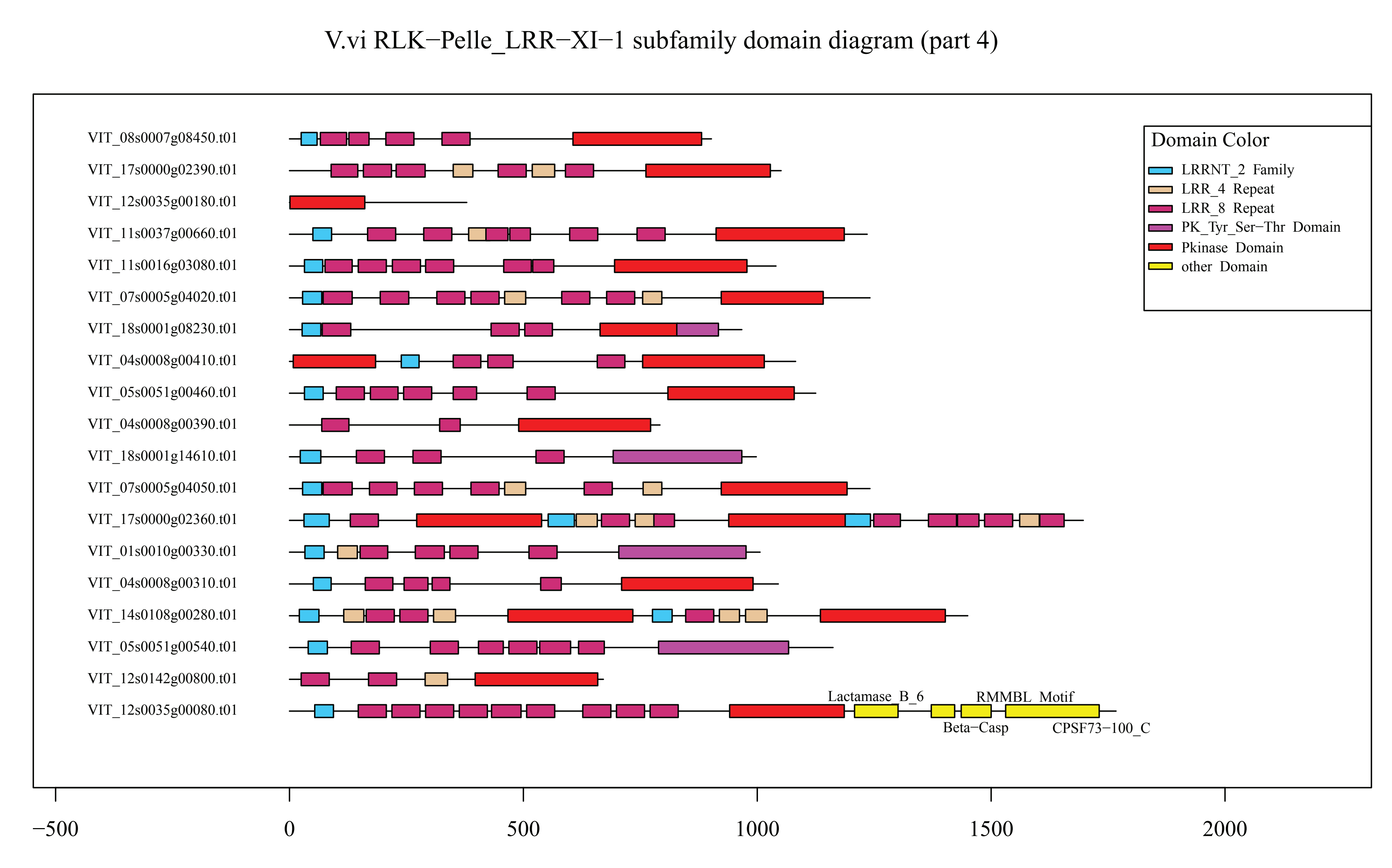

V.vi RLK−Pelle_LRR−XI−1 subfamily domain diagram (part 4)
VIT_08s0007g08450.t01
VIT_17s0000g02390.t01
VIT_12s0035g00180.t01
VIT_11s0037g00660.t01
VIT_11s0016g03080.t01
VIT_07s0005g04020.t01
VIT_18s0001g08230.t01
VIT_04s0008g00410.t01
VIT_05s0051g00460.t01
VIT_04s0008g00390.t01
VIT_18s0001g14610.t01
VIT_07s0005g04050.t01
VIT_17s0000g02360.t01
VIT_01s0010g00330.t01
VIT_04s0008g00310.t01
VIT_14s0108g00280.t01
VIT_05s0051g00540.t01
VIT_12s0142g00800.t01
VIT_12s0035g00080.t01
Lactamase_B_6
RMMBL Motif
Beta−Casp
CPSF73−100_C
Domain Color
LRRNT_2 Family
LRR_4 Repeat
LRR_8 Repeat
PK_Tyr_Ser−Thr Domain
Pkinase Domain
other Domain
−500
0
500
1000
1500
2000

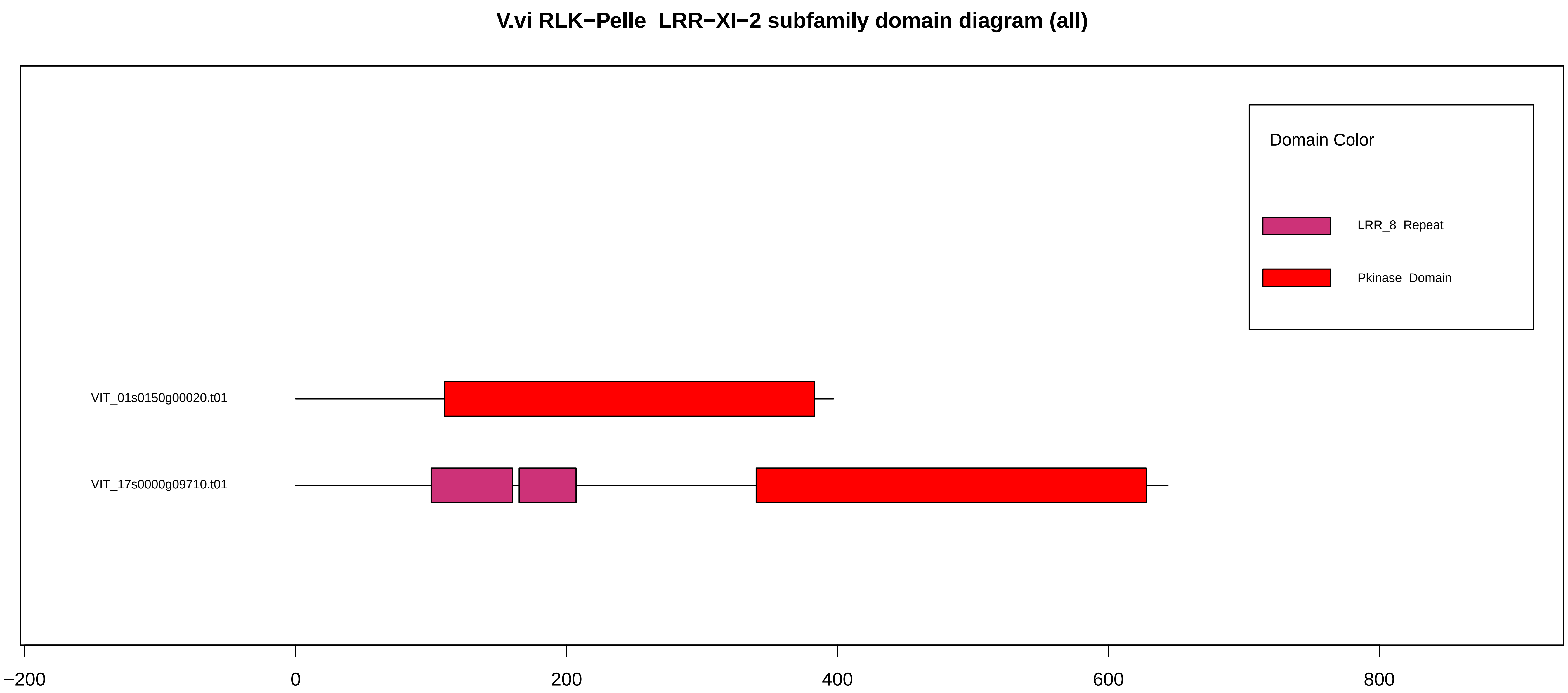
V.vi RLK−Pelle_LRR−XI−2 subfamily domain diagram (all)
Domain Color
LRR_8 Repeat
Pkinase Domain
VIT_01s0150g00020.t01
VIT_17s0000g09710.t01
−200
0
200
400
600
800

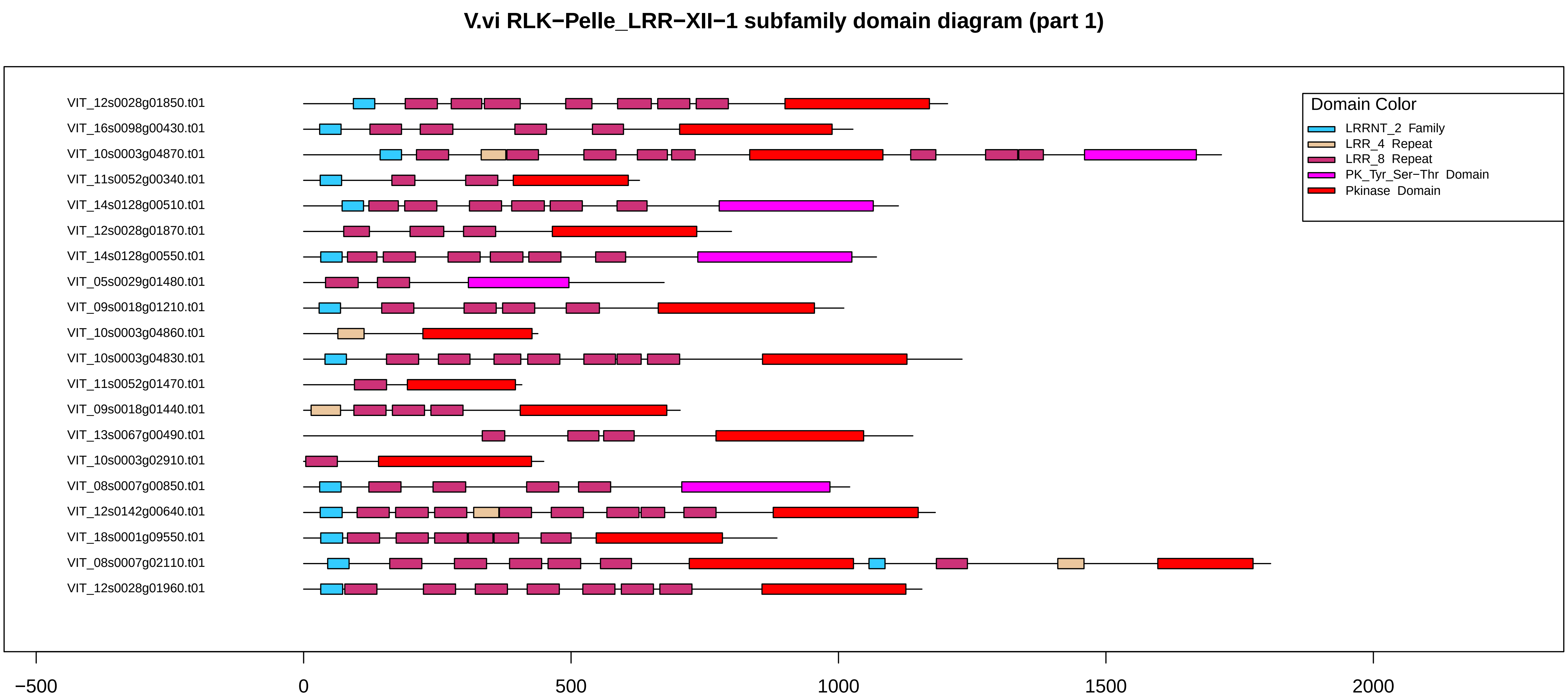
V.vi RLK−Pelle_LRR−XII−1 subfamily domain diagram (part 1)
VIT_12s0028g01850.t01
VIT_16s0098g00430.t01
VIT_10s0003g04870.t01
VIT_11s0052g00340.t01
VIT_14s0128g00510.t01
VIT_12s0028g01870.t01
VIT_14s0128g00550.t01
VIT_05s0029g01480.t01
VIT_09s0018g01210.t01
VIT_10s0003g04860.t01
VIT_10s0003g04830.t01
VIT_11s0052g01470.t01
VIT_09s0018g01440.t01
VIT_13s0067g00490.t01
VIT_10s0003g02910.t01
VIT_08s0007g00850.t01
VIT_12s0142g00640.t01
VIT_18s0001g09550.t01
VIT_08s0007g02110.t01
VIT_12s0028g01960.t01
Domain Color
LRRNT_2 Family
LRR_4 Repeat
LRR_8 Repeat
PK_Tyr_Ser−Thr Domain
Pkinase Domain
−500
0
500
1000
1500
2000

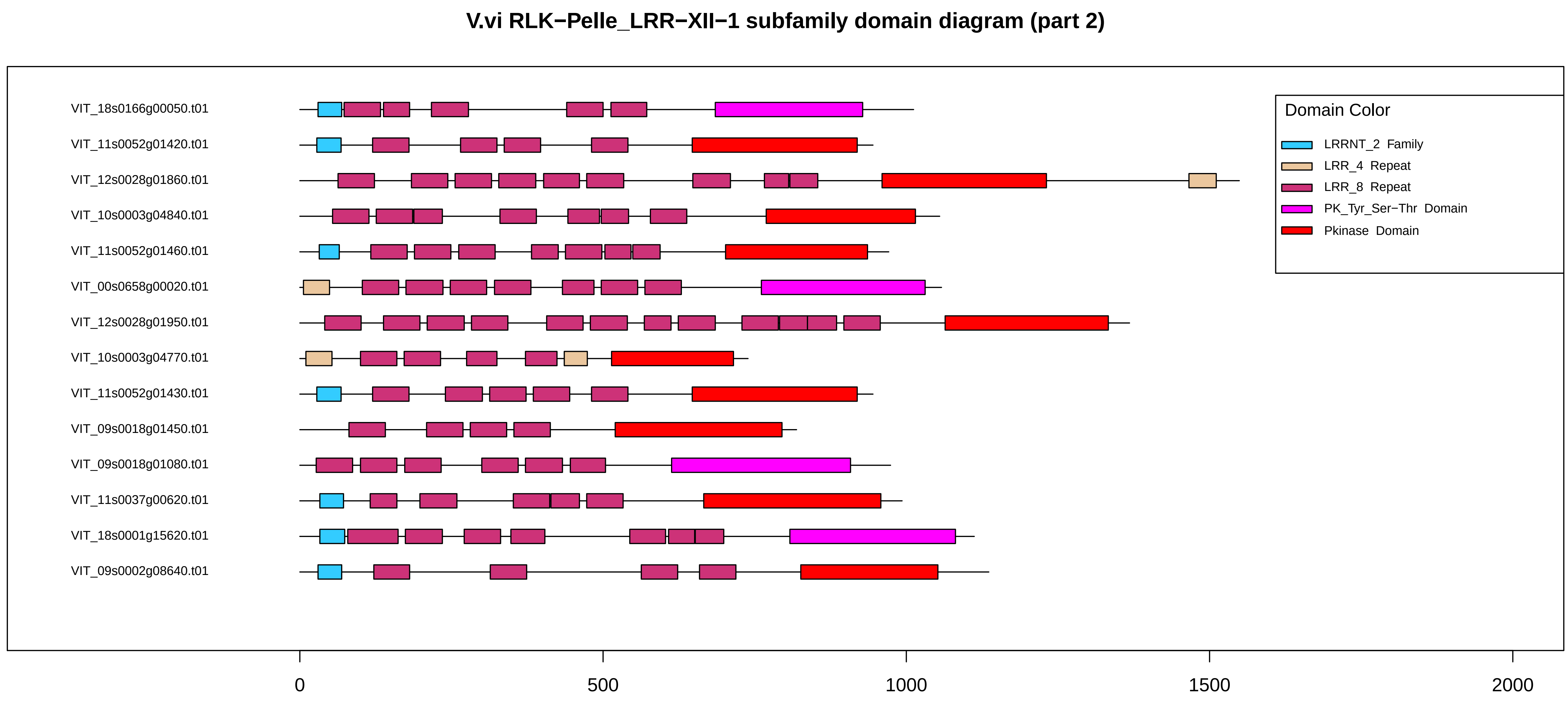
V.vi RLK−Pelle_LRR−XII−1 subfamily domain diagram (part 2)
VIT_18s0166g00050.t01
VIT_11s0052g01420.t01
VIT_12s0028g01860.t01
VIT_10s0003g04840.t01
VIT_11s0052g01460.t01
VIT_00s0658g00020.t01
VIT_12s0028g01950.t01
VIT_10s0003g04770.t01
VIT_11s0052g01430.t01
VIT_09s0018g01450.t01
VIT_09s0018g01080.t01
VIT_11s0037g00620.t01
VIT_18s0001g15620.t01
VIT_09s0002g08640.t01
Domain Color
LRRNT_2 Family
LRR_4 Repeat
LRR_8 Repeat
PK_Tyr_Ser−Thr Domain
Pkinase Domain
0
500
1000
1500
2000

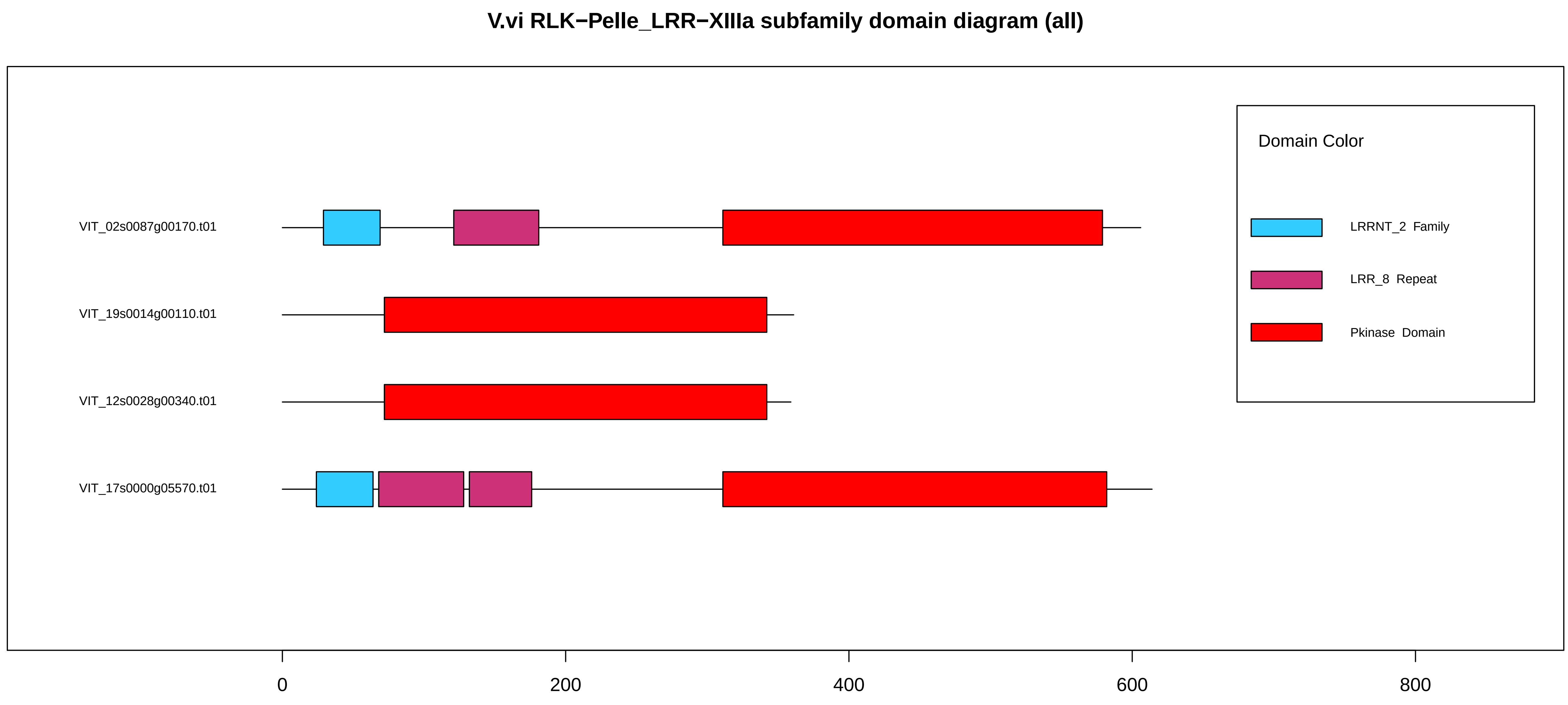
V.vi RLK−Pelle_LRR−XIIIa subfamily domain diagram (all)
VIT_02s0087g00170.t01
VIT_19s0014g00110.t01
VIT_12s0028g00340.t01
VIT_17s0000g05570.t01
Domain Color
LRRNT_2 Family
LRR_8 Repeat
Pkinase Domain
0
200
400
600
800

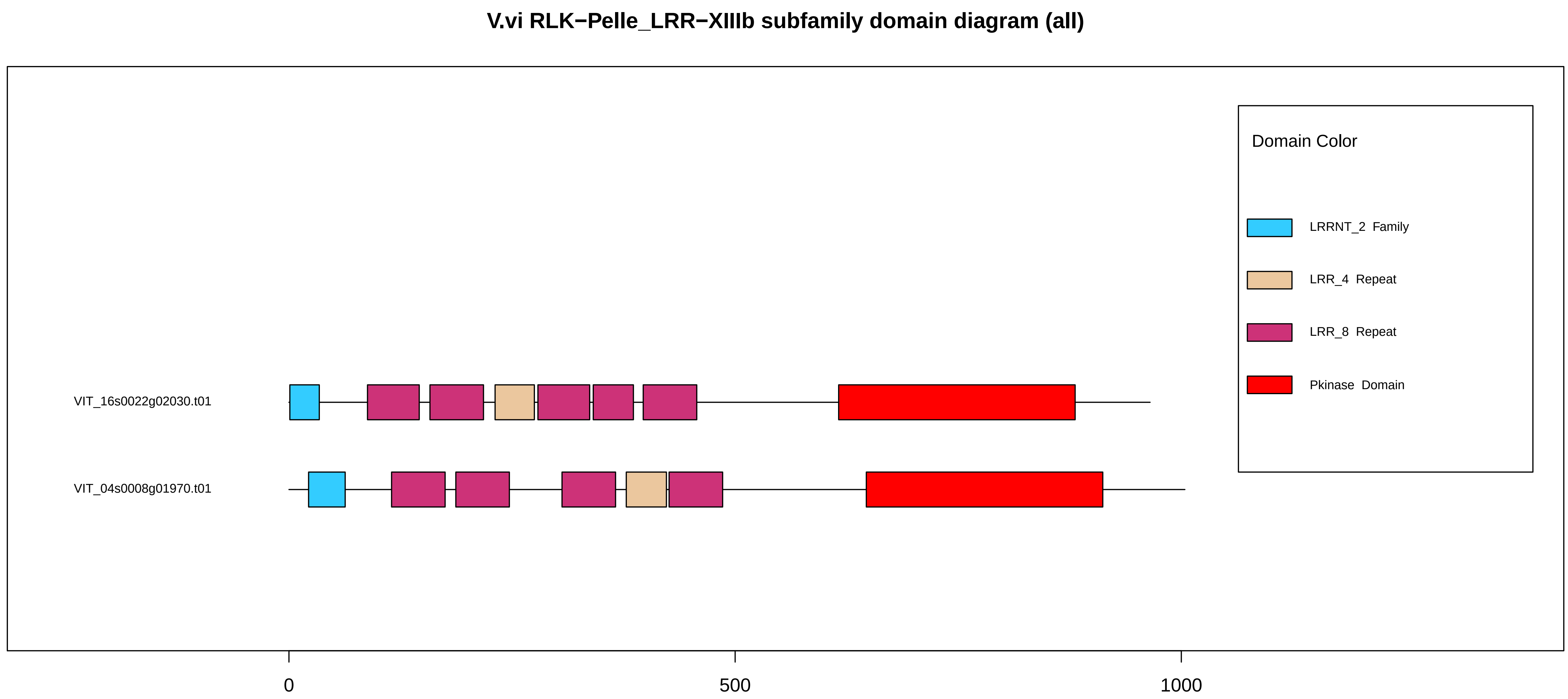
V.vi RLK−Pelle_LRR−XIIIb subfamily domain diagram (all)
Domain Color
LRRNT_2 Family
LRR_4 Repeat
LRR_8 Repeat
Pkinase Domain
VIT_16s0022g02030.t01
VIT_04s0008g01970.t01
0
500
1000

V.vi RLK−Pelle_LRR−XIV subfamily domain diagram (all)

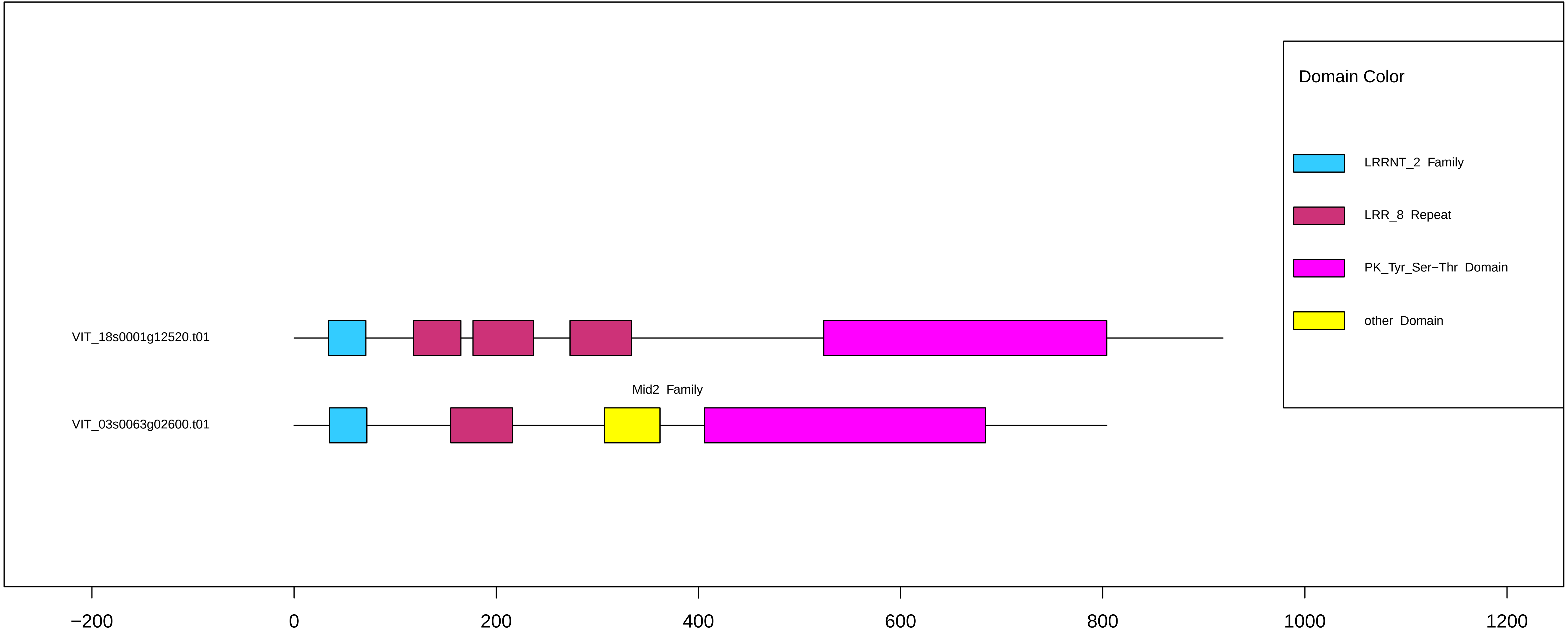

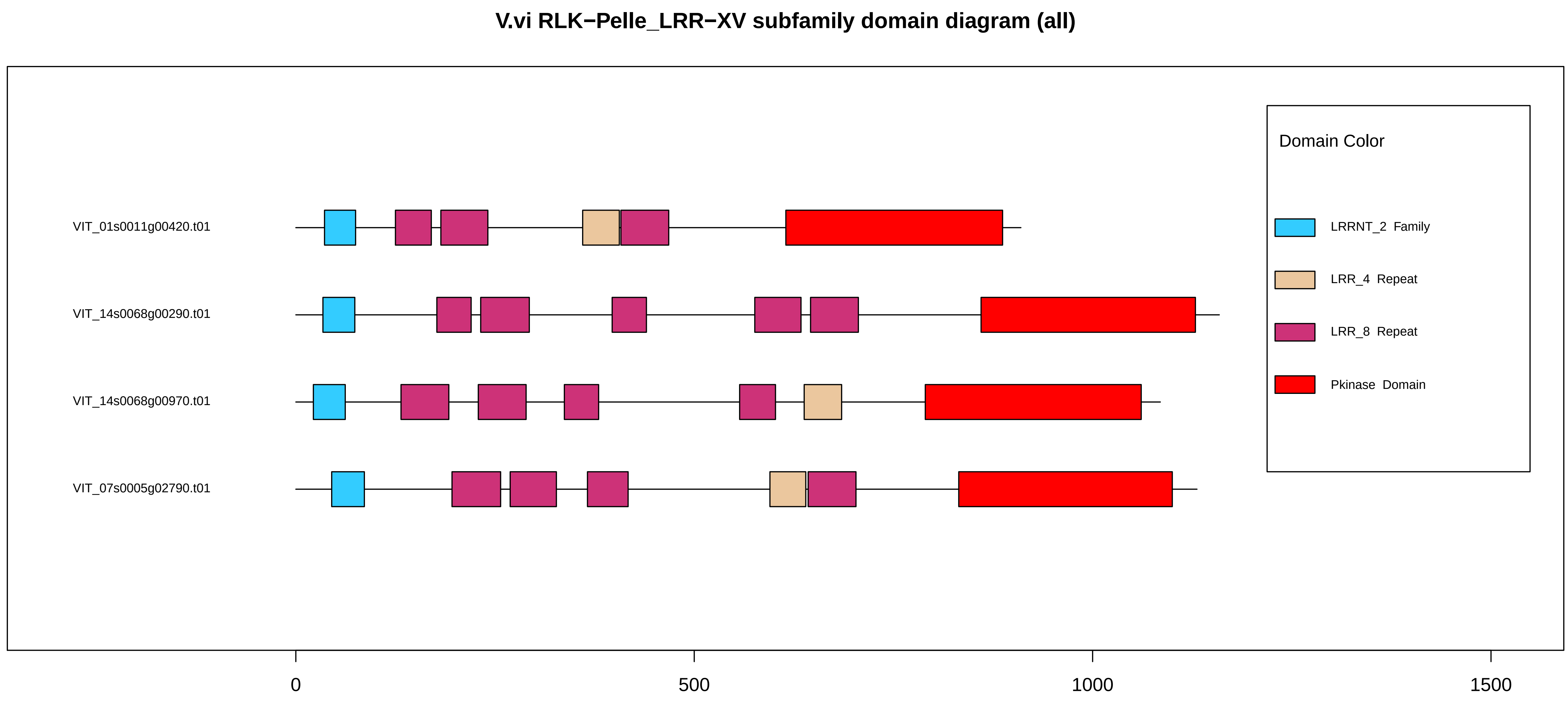

V.vi RLK−Pelle_LRR−XV subfamily domain diagram (all)
VIT_01s0011g00420.t01
VIT_14s0068g00290.t01
VIT_14s0068g00970.t01
VIT_07s0005g02790.t01
Domain Color
LRRNT_2 Family
LRR_4 Repeat
LRR_8 Repeat
Pkinase Domain
0
500
1000
1500

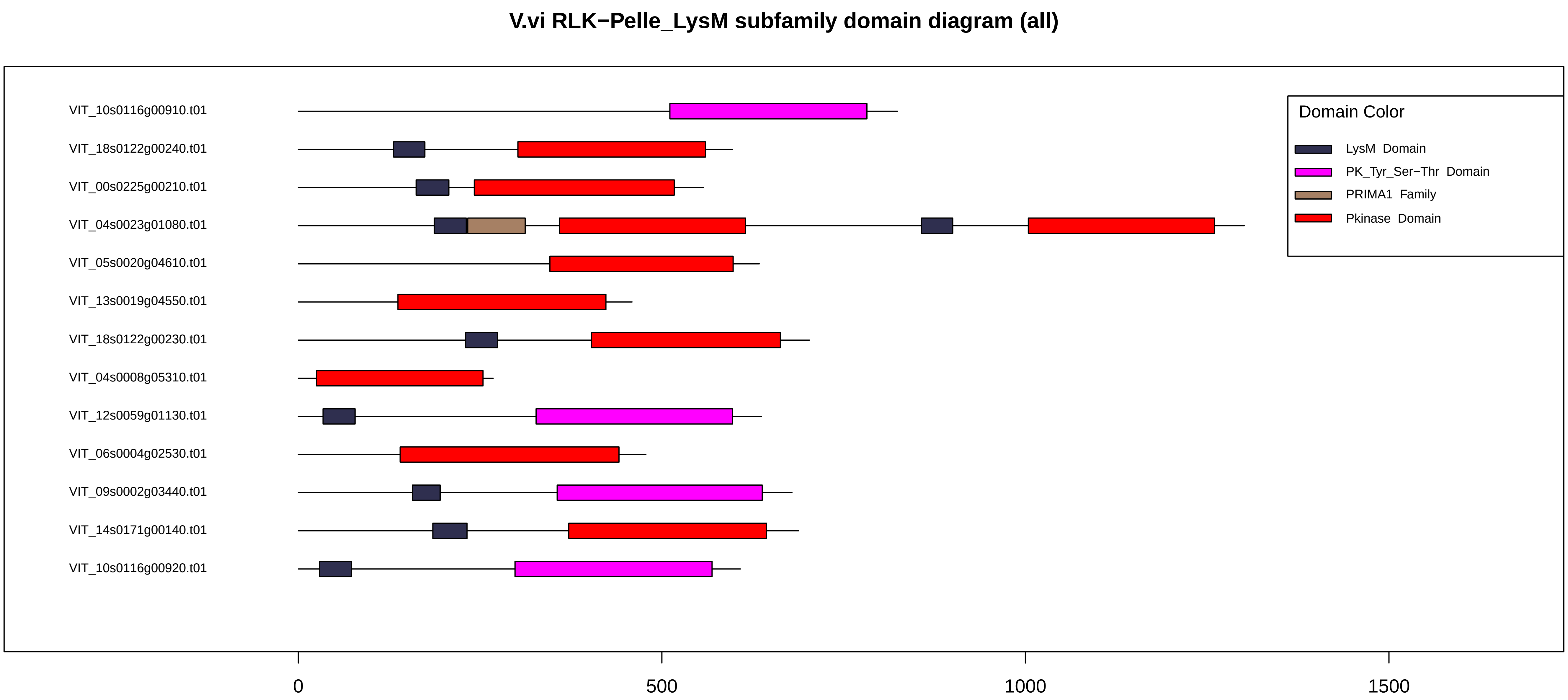
V.vi RLK−Pelle_LysM subfamily domain diagram (all)
VIT_10s0116g00910.t01
VIT_18s0122g00240.t01
VIT_00s0225g00210.t01
VIT_04s0023g01080.t01
VIT_05s0020g04610.t01
VIT_13s0019g04550.t01
VIT_18s0122g00230.t01
VIT_04s0008g05310.t01
VIT_12s0059g01130.t01
VIT_06s0004g02530.t01
VIT_09s0002g03440.t01
VIT_14s0171g00140.t01
VIT_10s0116g00920.t01
Domain Color
LysM Domain
PK_Tyr_Ser−Thr Domain
PRIMA1 Family
Pkinase Domain
0
500
1000
1500

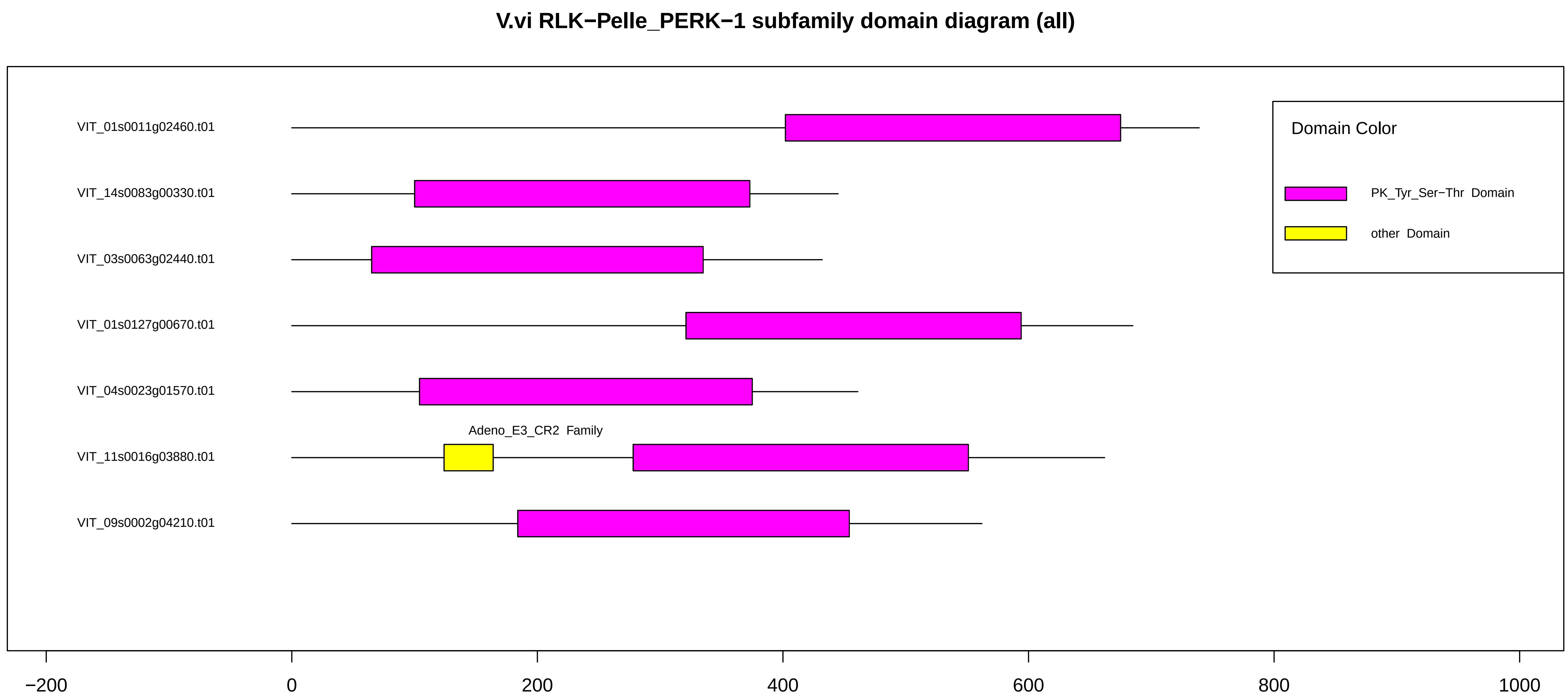

V.vi RLK−Pelle_PERK−1 subfamily domain diagram (all)
VIT_01s0011g02460.t01
VIT_14s0083g00330.t01
VIT_03s0063g02440.t01
VIT_01s0127g00670.t01
VIT_04s0023g01570.t01
Adeno_E3_CR2 Family
VIT_11s0016g03880.t01
VIT_09s0002g04210.t01
Domain Color
PK_Tyr_Ser−Thr Domain
other Domain
−200
0
200
400
600
800
1000

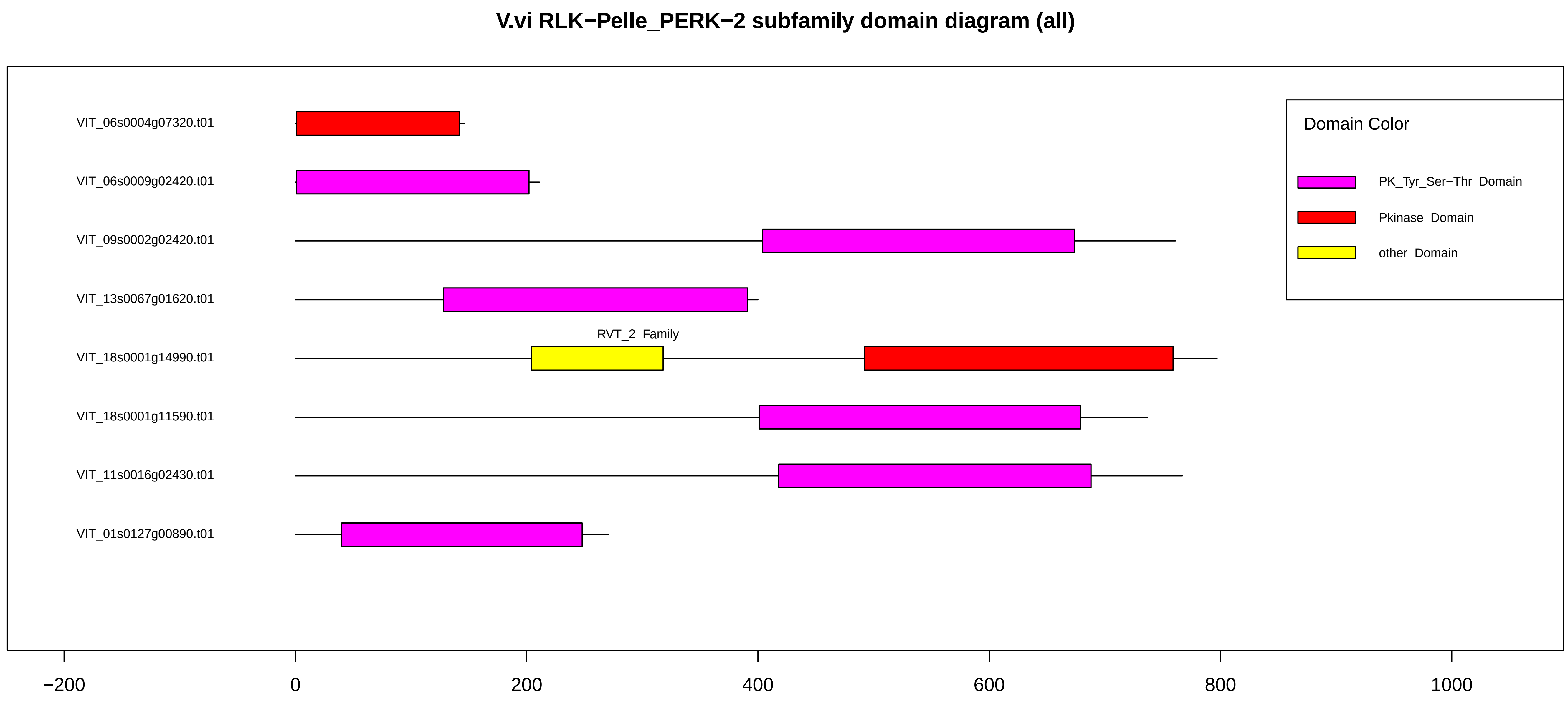
V.vi RLK−Pelle_PERK−2 subfamily domain diagram (all)
VIT_06s0004g07320.t01
VIT_06s0009g02420.t01
VIT_09s0002g02420.t01
VIT_13s0067g01620.t01
RVT_2 Family
VIT_18s0001g14990.t01
VIT_18s0001g11590.t01
VIT_11s0016g02430.t01
VIT_01s0127g00890.t01
Domain Color
PK_Tyr_Ser−Thr Domain
Pkinase Domain
other Domain
−200
0
200
400
600
800
1000

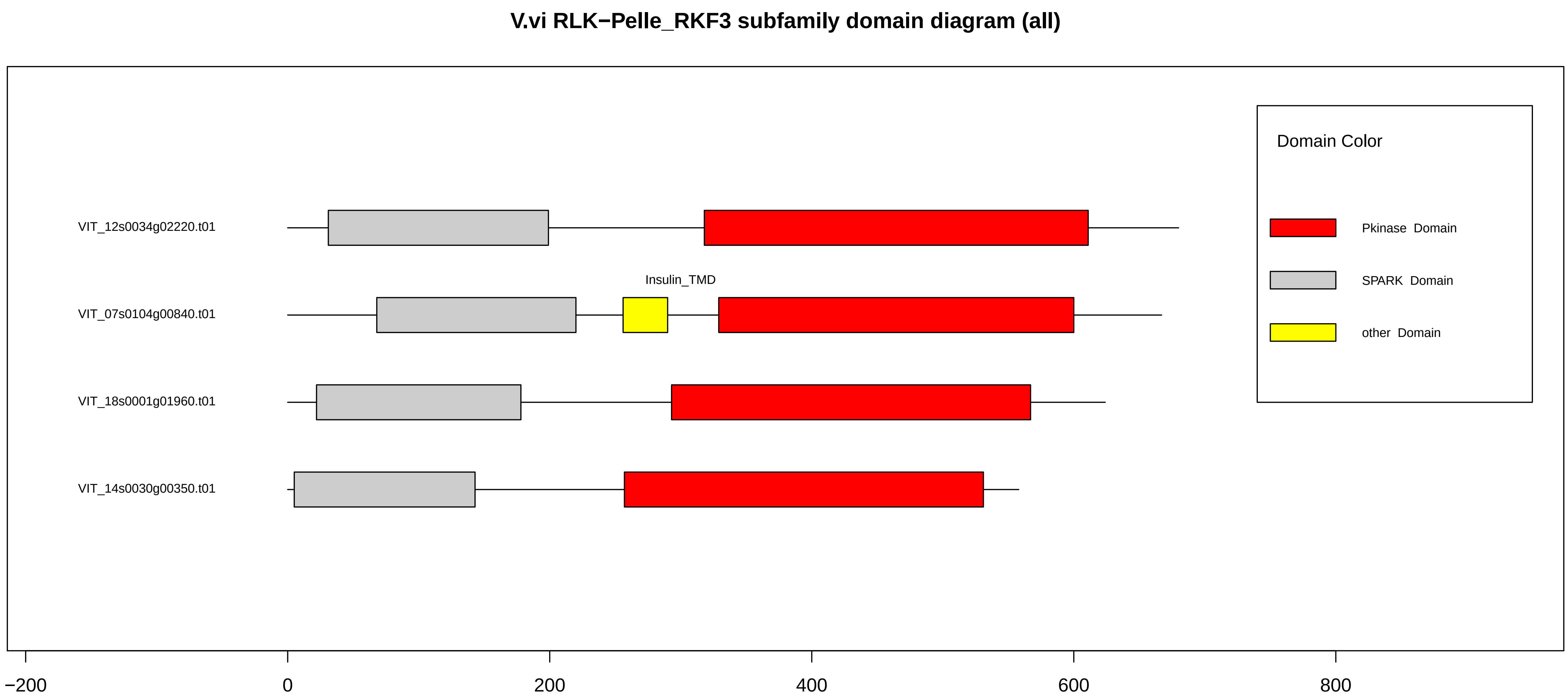

V.vi RLK−Pelle_RKF3 subfamily domain diagram (all)
VIT_12s0034g02220.t01
VIT_07s0104g00840.t01
VIT_18s0001g01960.t01
VIT_14s0030g00350.t01
Insulin_TMD
Domain Color
Pkinase Domain
SPARK Domain
other Domain
−200
0
200
400
600
800

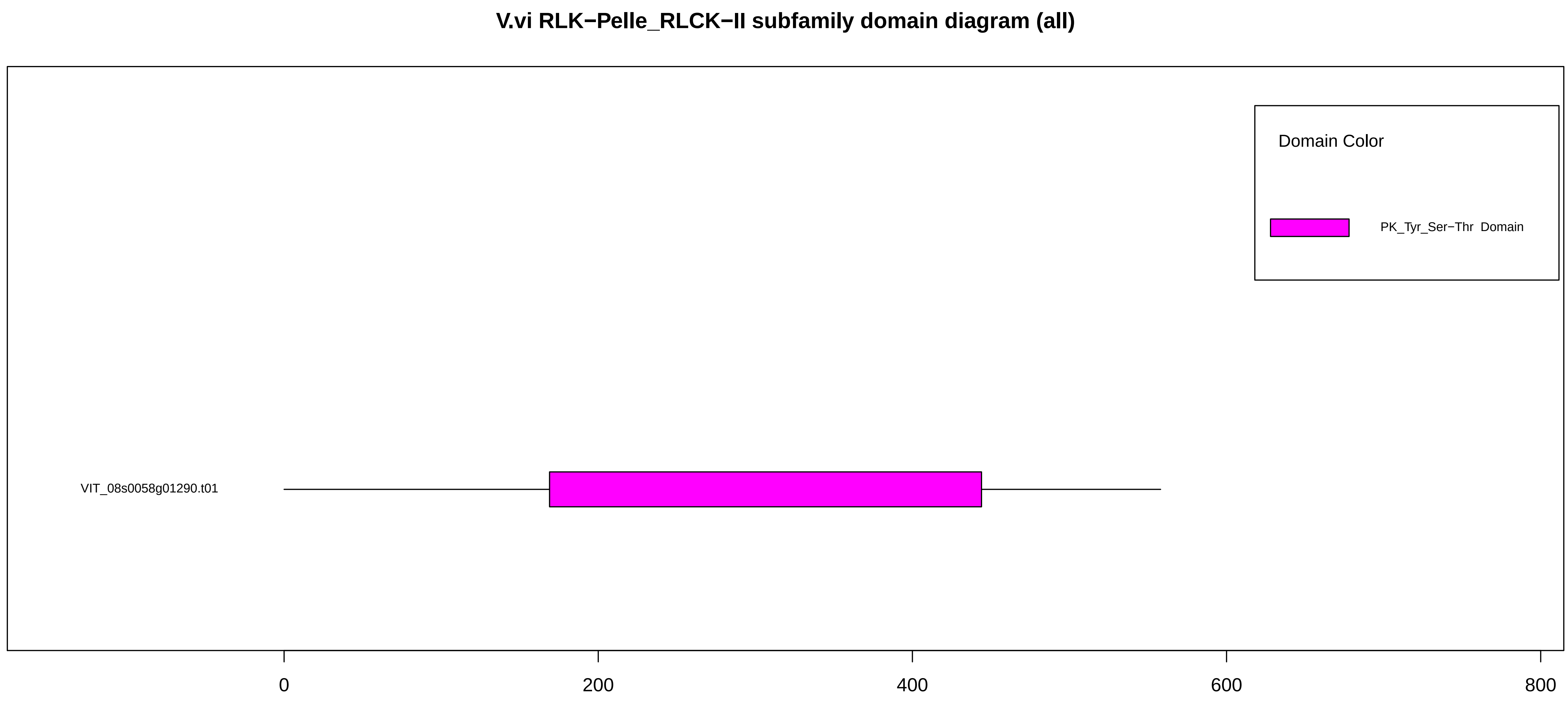
V.vi RLK−Pelle_RLCK−II subfamily domain diagram (all)
Domain Color
PK_Tyr_Ser−Thr Domain
VIT_08s0058g01290.t01
0
200
400
600
800

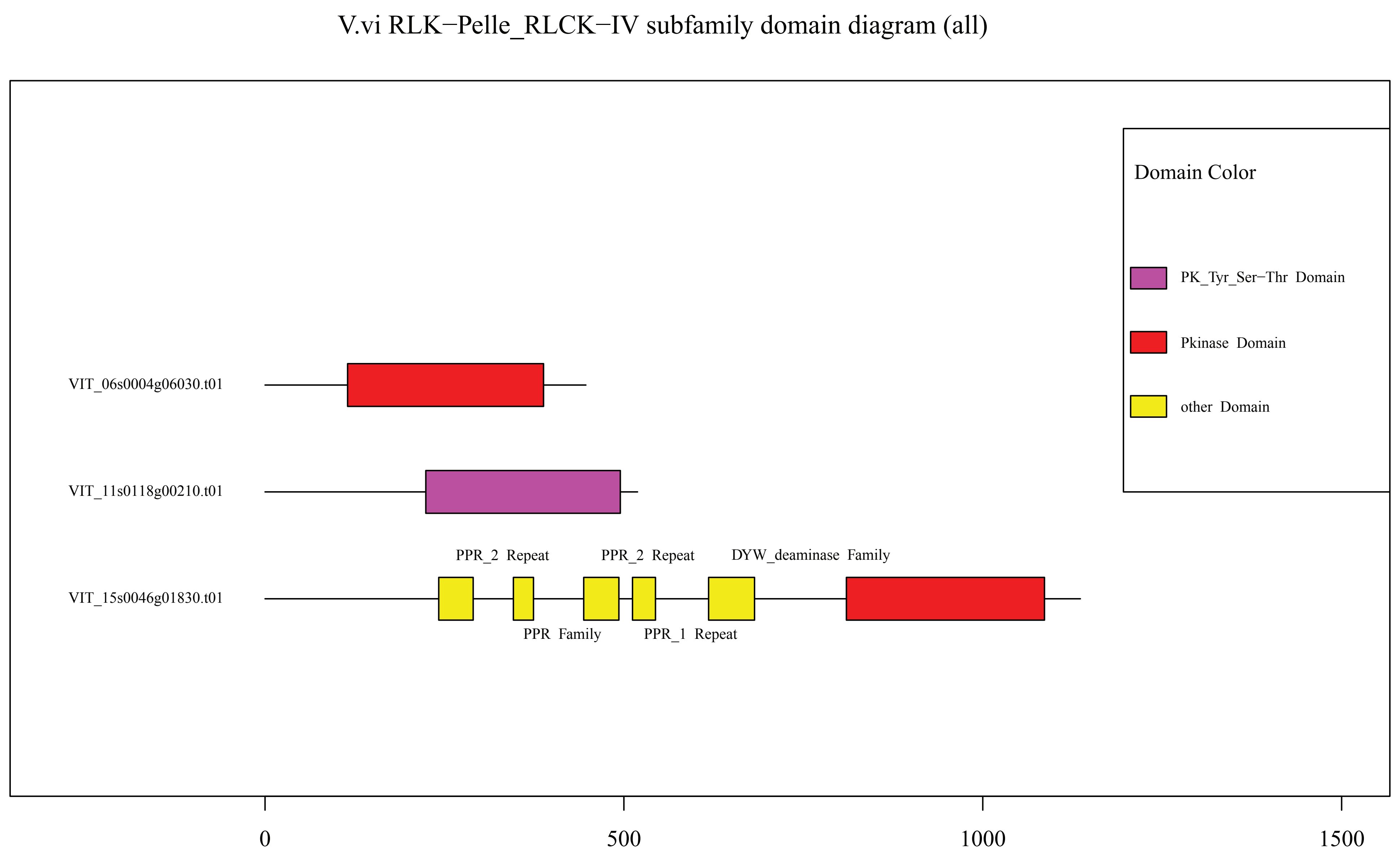
V.vi RLK−Pelle_RLCK−IV subfamily domain diagram (all)
Domain Color
PK_Tyr_Ser−Thr Domain
Pkinase Domain
other Domain
VIT_06s0004g06030.t01
VIT_11s0118g00210.t01
VIT_15s0046g01830.t01
PPR_2 Repeat
PPR_2 Repeat
DYW_deaminase Family
PPR Family
PPR_1 Repeat
0
500
1000
1500

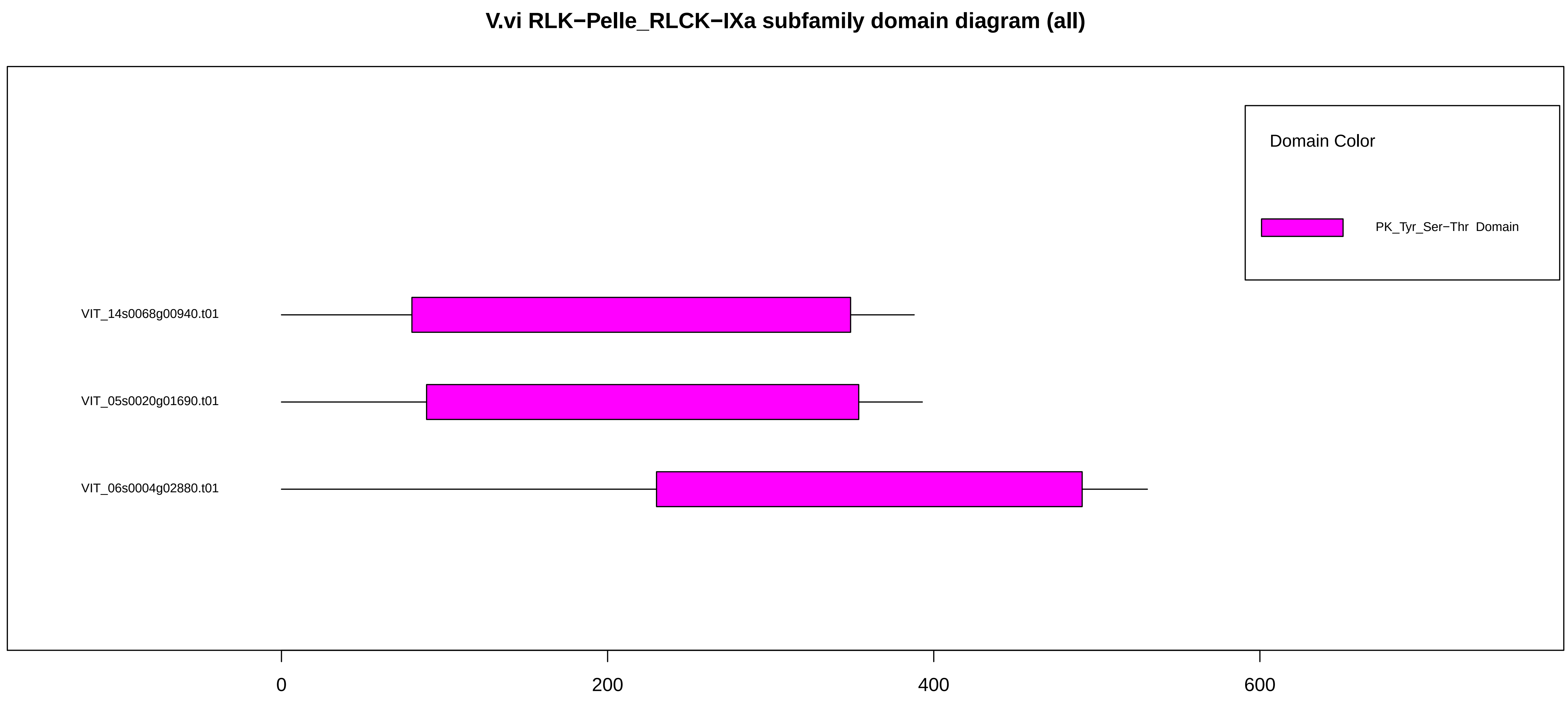

V.vi RLK−Pelle_RLCK−IXa subfamily domain diagram (all)
Domain Color
PK_Tyr_Ser−Thr Domain
VIT_14s0068g00940.t01
VIT_05s0020g01690.t01
VIT_06s0004g02880.t01
0
200
400
600

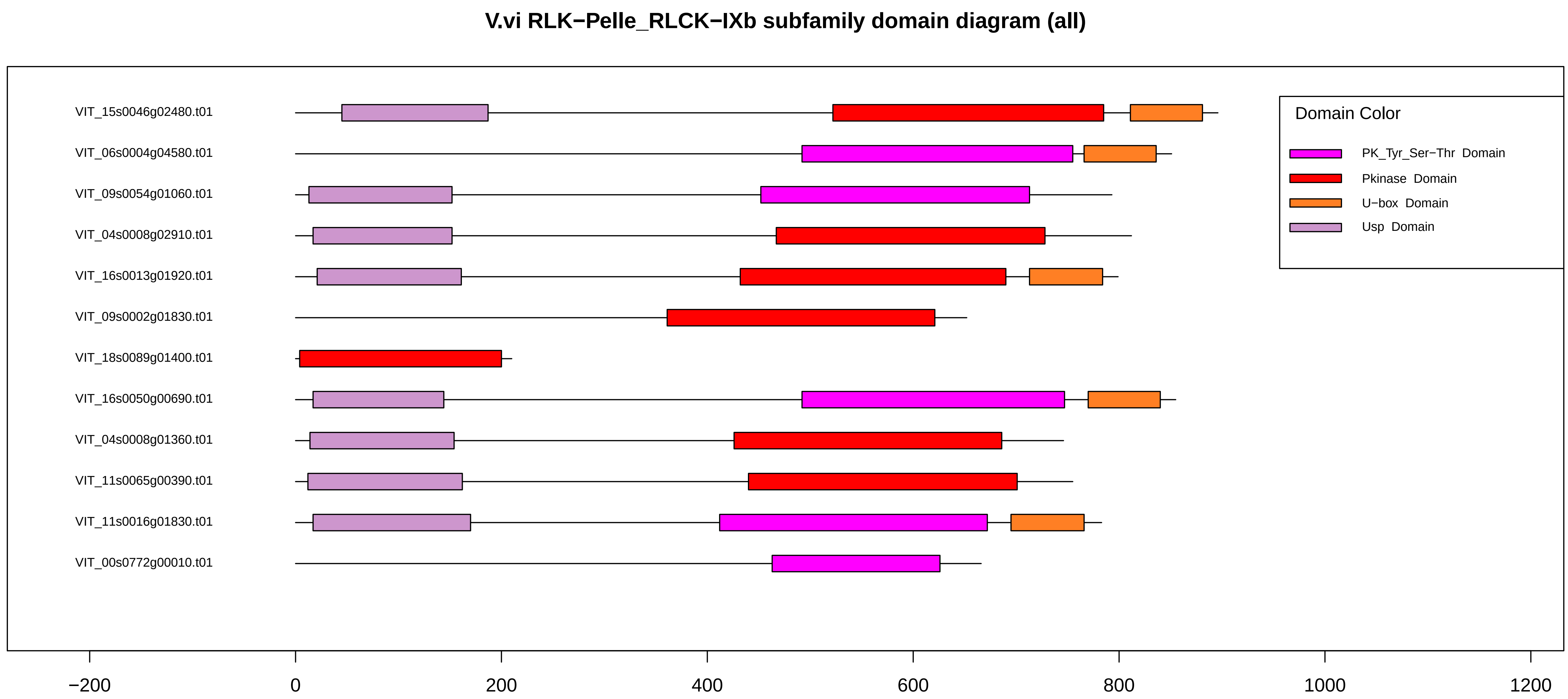
V.vi RLK−Pelle_RLCK−IXb subfamily domain diagram (all)
VIT_15s0046g02480.t01
VIT_06s0004g04580.t01
VIT_09s0054g01060.t01
VIT_04s0008g02910.t01
VIT_16s0013g01920.t01
VIT_09s0002g01830.t01
VIT_18s0089g01400.t01
VIT_16s0050g00690.t01
VIT_04s0008g01360.t01
VIT_11s0065g00390.t01
VIT_11s0016g01830.t01
VIT_00s0772g00010.t01
Domain Color
PK_Tyr_Ser−Thr Domain
Pkinase Domain
U−box Domain
Usp Domain
−200
0
200
400
600
800
1000
1200

V.vi RLK−Pelle_RLCK−Os subfamily domain diagram (all)

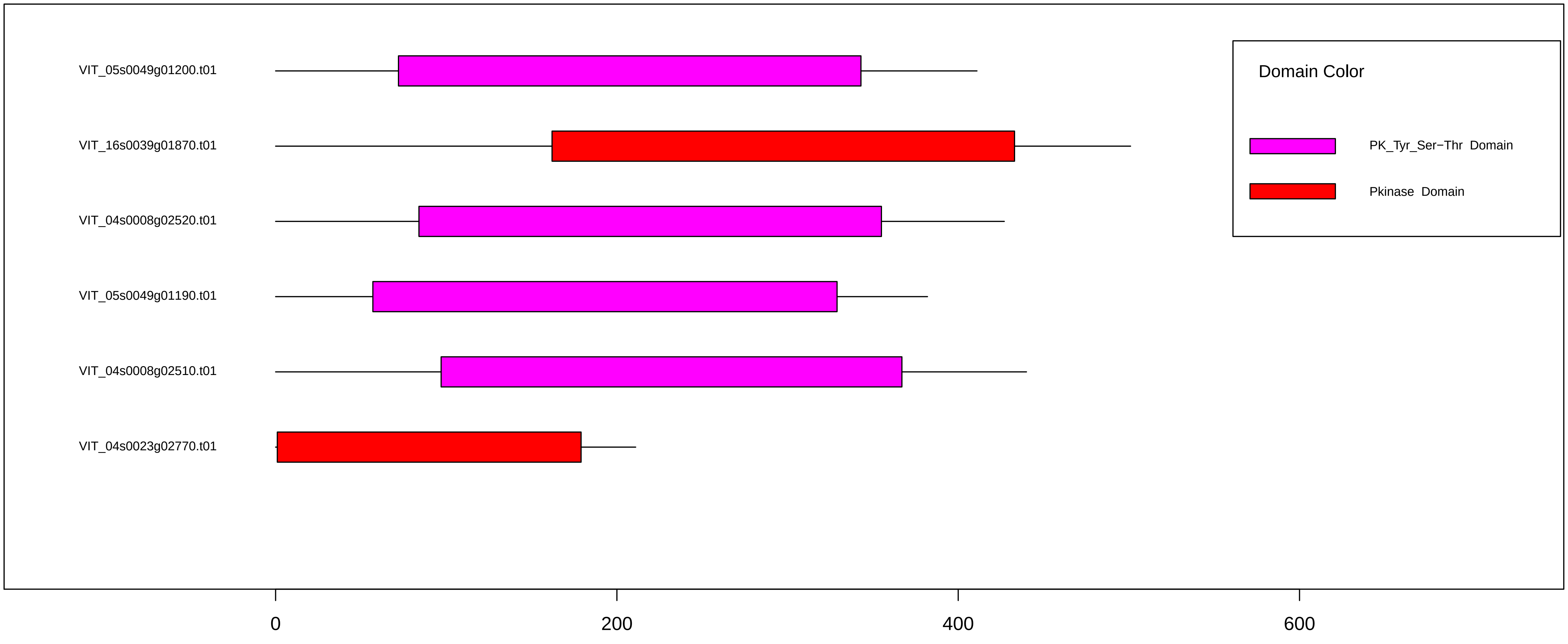

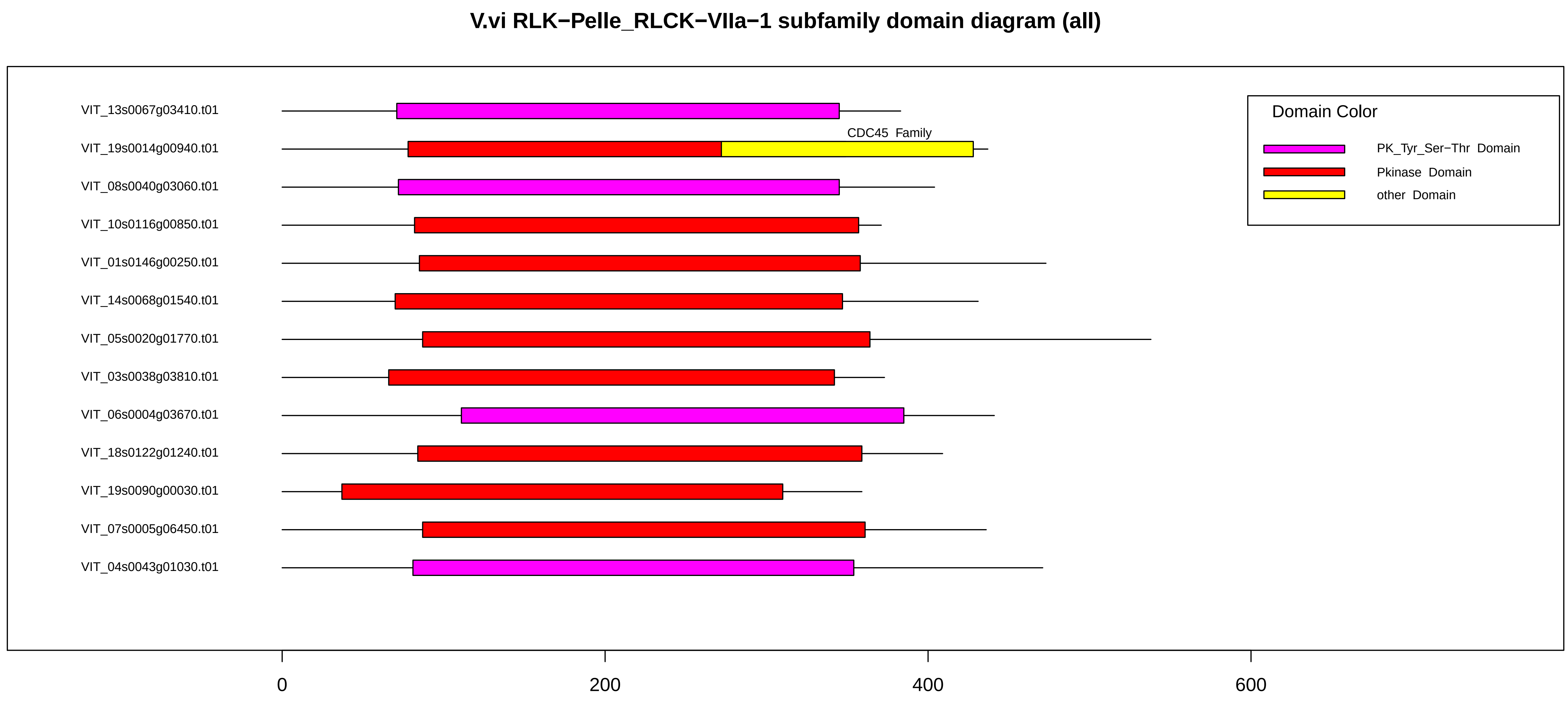
V.vi RLK−Pelle_RLCK−VIIa−1 subfamily domain diagram (all)
VIT_13s0067g03410.t01
VIT_19s0014g00940.t01
VIT_08s0040g03060.t01
VIT_10s0116g00850.t01
VIT_01s0146g00250.t01
VIT_14s0068g01540.t01
VIT_05s0020g01770.t01
VIT_03s0038g03810.t01
VIT_06s0004g03670.t01
VIT_18s0122g01240.t01
VIT_19s0090g00030.t01
VIT_07s0005g06450.t01
VIT_04s0043g01030.t01
CDC45 Family
Domain Color
PK_Tyr_Ser−Thr Domain
Pkinase Domain
other Domain
0
200
400
600

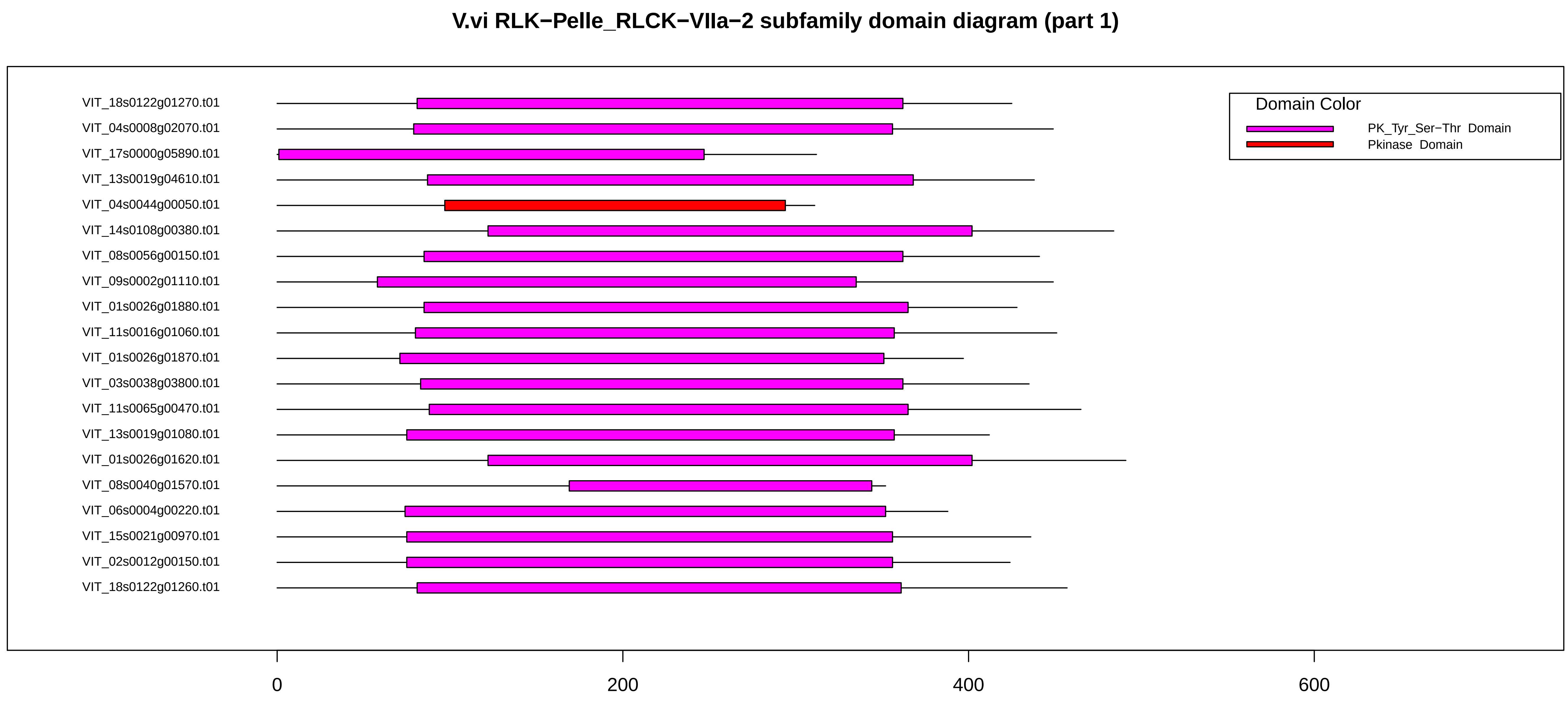
V.vi RLK−Pelle_RLCK−VIIa−2 subfamily domain diagram (part 1)
VIT_18s0122g01270.t01
VIT_04s0008g02070.t01
VIT_17s0000g05890.t01
VIT_13s0019g04610.t01
VIT_04s0044g00050.t01
VIT_14s0108g00380.t01
VIT_08s0056g00150.t01
VIT_09s0002g01110.t01
VIT_01s0026g01880.t01
VIT_11s0016g01060.t01
VIT_01s0026g01870.t01
VIT_03s0038g03800.t01
VIT_11s0065g00470.t01
VIT_13s0019g01080.t01
VIT_01s0026g01620.t01
VIT_08s0040g01570.t01
VIT_06s0004g00220.t01
VIT_15s0021g00970.t01
VIT_02s0012g00150.t01
VIT_18s0122g01260.t01
Domain Color
PK_Tyr_Ser−Thr Domain
Pkinase Domain
0
200
400
600

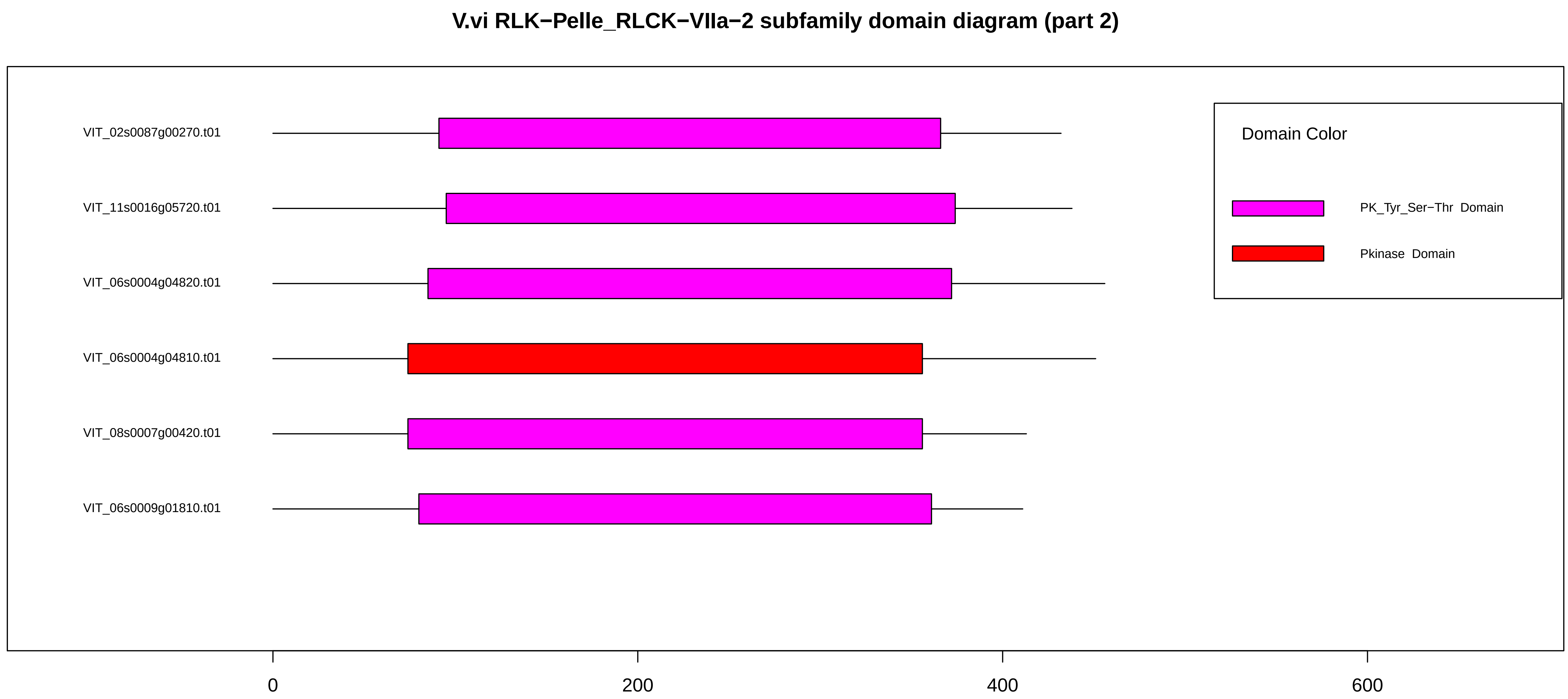

V.vi RLK−Pelle_RLCK−VIIa−2 subfamily domain diagram (part 2)
VIT_02s0087g00270.t01
VIT_11s0016g05720.t01
VIT_06s0004g04820.t01
VIT_06s0004g04810.t01
VIT_08s0007g00420.t01
VIT_06s0009g01810.t01
Domain Color
PK_Tyr_Ser−Thr Domain
Pkinase Domain
0
200
400
600

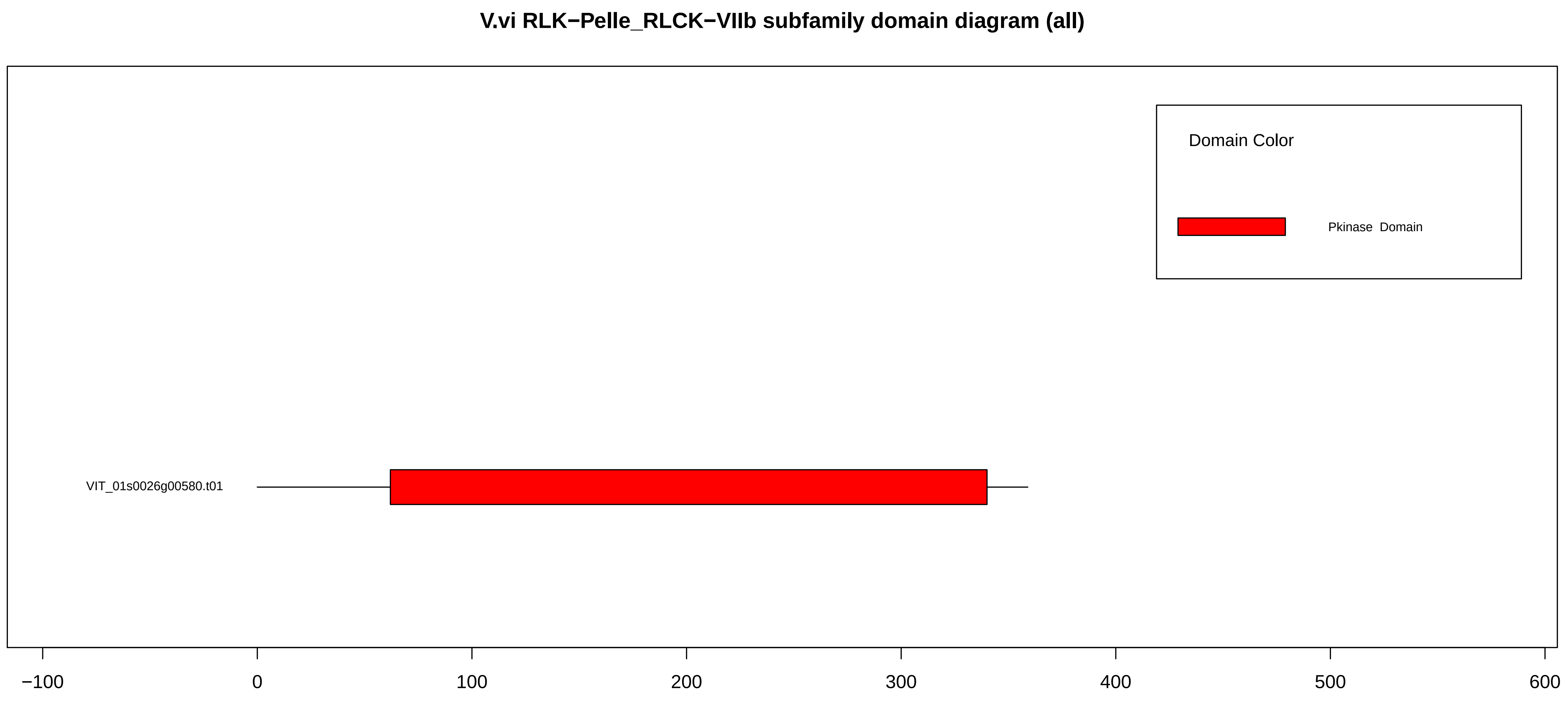
V.vi RLK−Pelle_RLCK−VIIb subfamily domain diagram (all)
Domain Color
Pkinase Domain
VIT_01s0026g00580.t01
−100
0
100
200
300
400
500
600

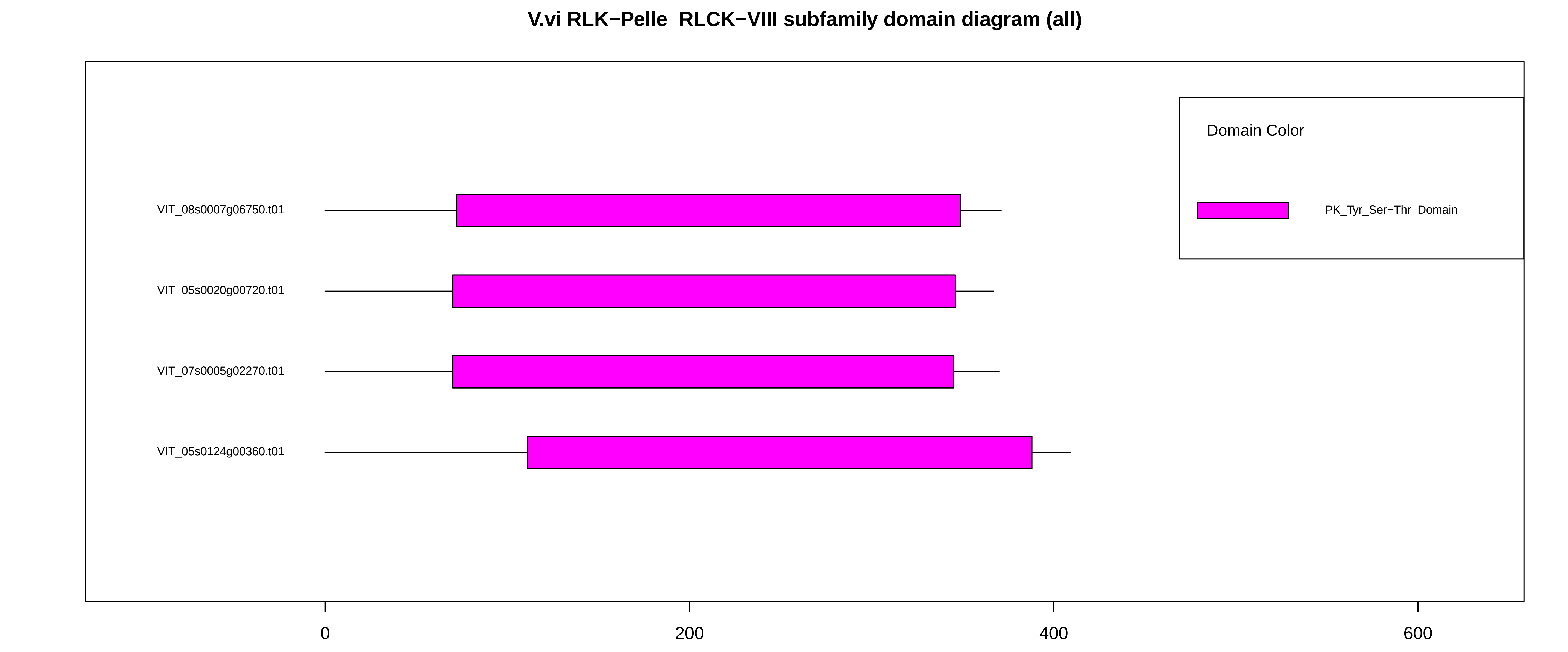
V.vi RLK−Pelle_RLCK−VIII subfamily domain diagram (all)
VIT_08s0007g06750.t01
VIT_05s0020g00720.t01
VIT_07s0005g02270.t01
VIT_05s0124g00360.t01
Domain Color
PK_Tyr_Ser−Thr Domain
0
200
400
600

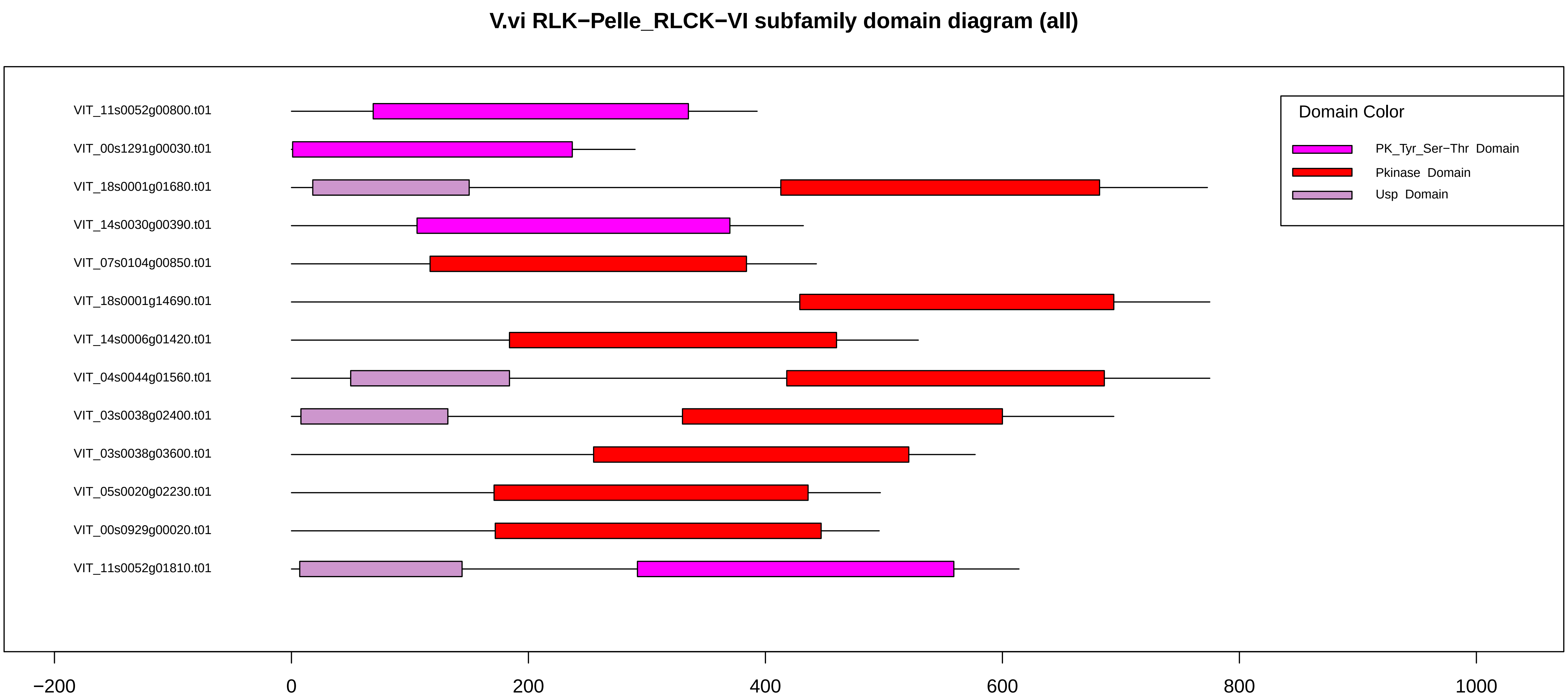
V.vi RLK−Pelle_RLCK−VI subfamily domain diagram (all)
VIT_11s0052g00800.t01
VIT_00s1291g00030.t01
VIT_18s0001g01680.t01
VIT_14s0030g00390.t01
VIT_07s0104g00850.t01
VIT_18s0001g14690.t01
VIT_14s0006g01420.t01
VIT_04s0044g01560.t01
VIT_03s0038g02400.t01
VIT_03s0038g03600.t01
VIT_05s0020g02230.t01
VIT_00s0929g00020.t01
VIT_11s0052g01810.t01
Domain Color
PK_Tyr_Ser−Thr Domain
Pkinase Domain
Usp Domain
−200
0
200
400
600
800
1000

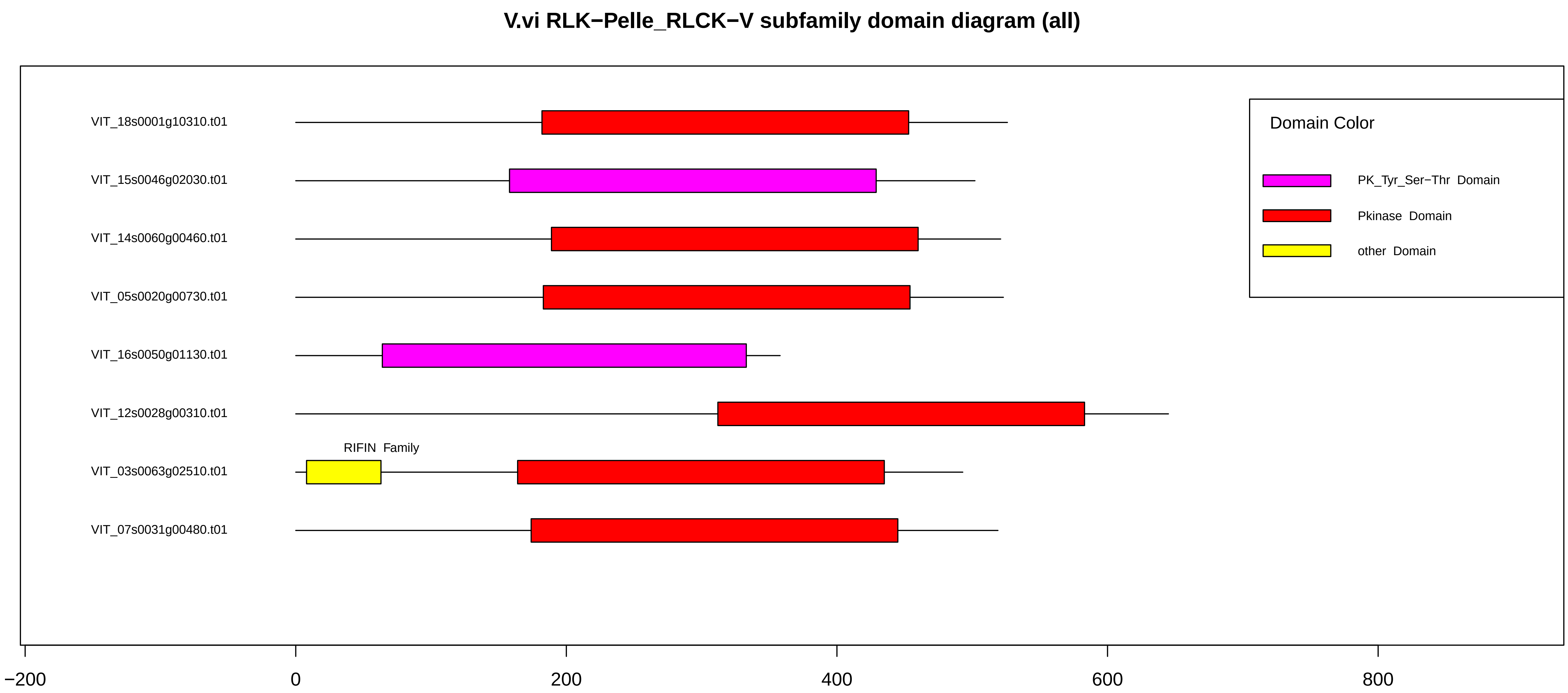
V.vi RLK−Pelle_RLCK−V subfamily domain diagram (all)
VIT_18s0001g10310.t01
VIT_15s0046g02030.t01
VIT_14s0060g00460.t01
VIT_05s0020g00730.t01
VIT_16s0050g01130.t01
VIT_12s0028g00310.t01
RIFIN Family
VIT_03s0063g02510.t01
VIT_07s0031g00480.t01
Domain Color
PK_Tyr_Ser−Thr Domain
Pkinase Domain
other Domain
−200
0
200
400
600
800

V.vi RLK−Pelle_RLCK−XII−1 subfamily domain diagram (all)

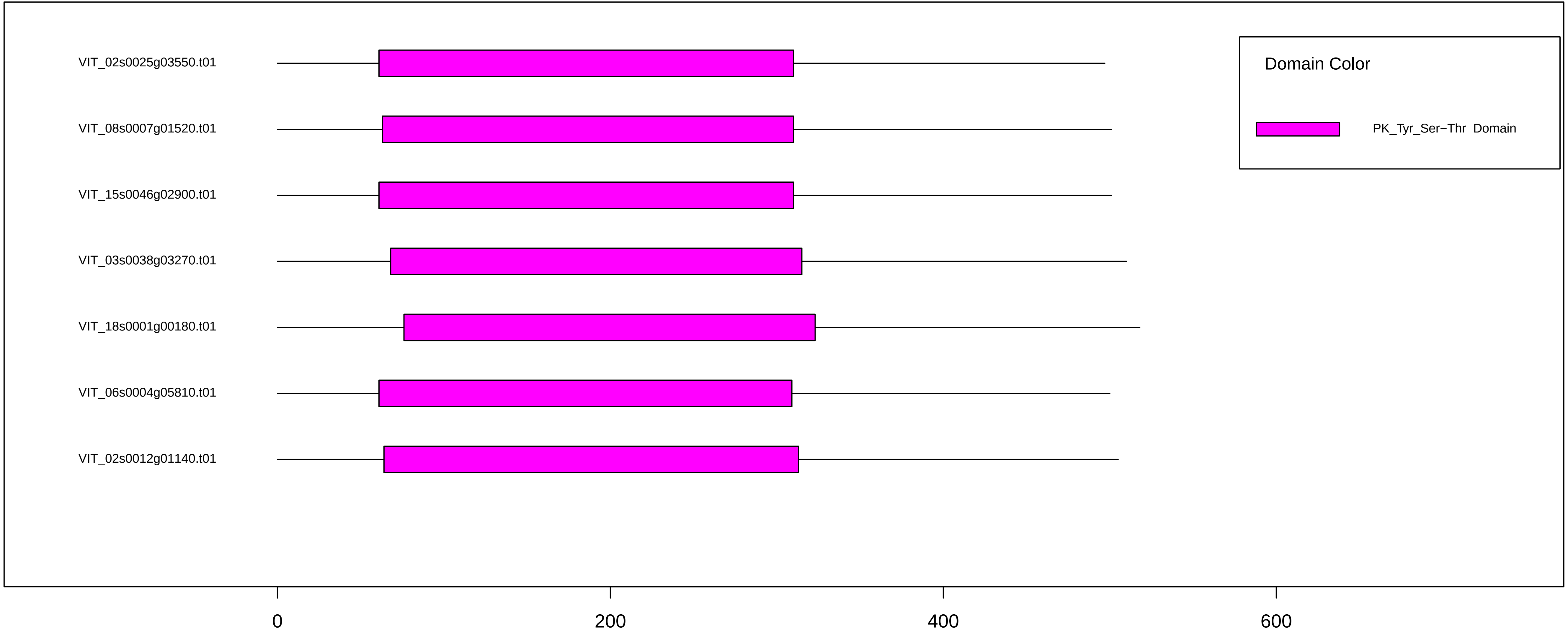

V.vi RLK−Pelle_RLCK−XII−2 subfamily domain diagram (all)

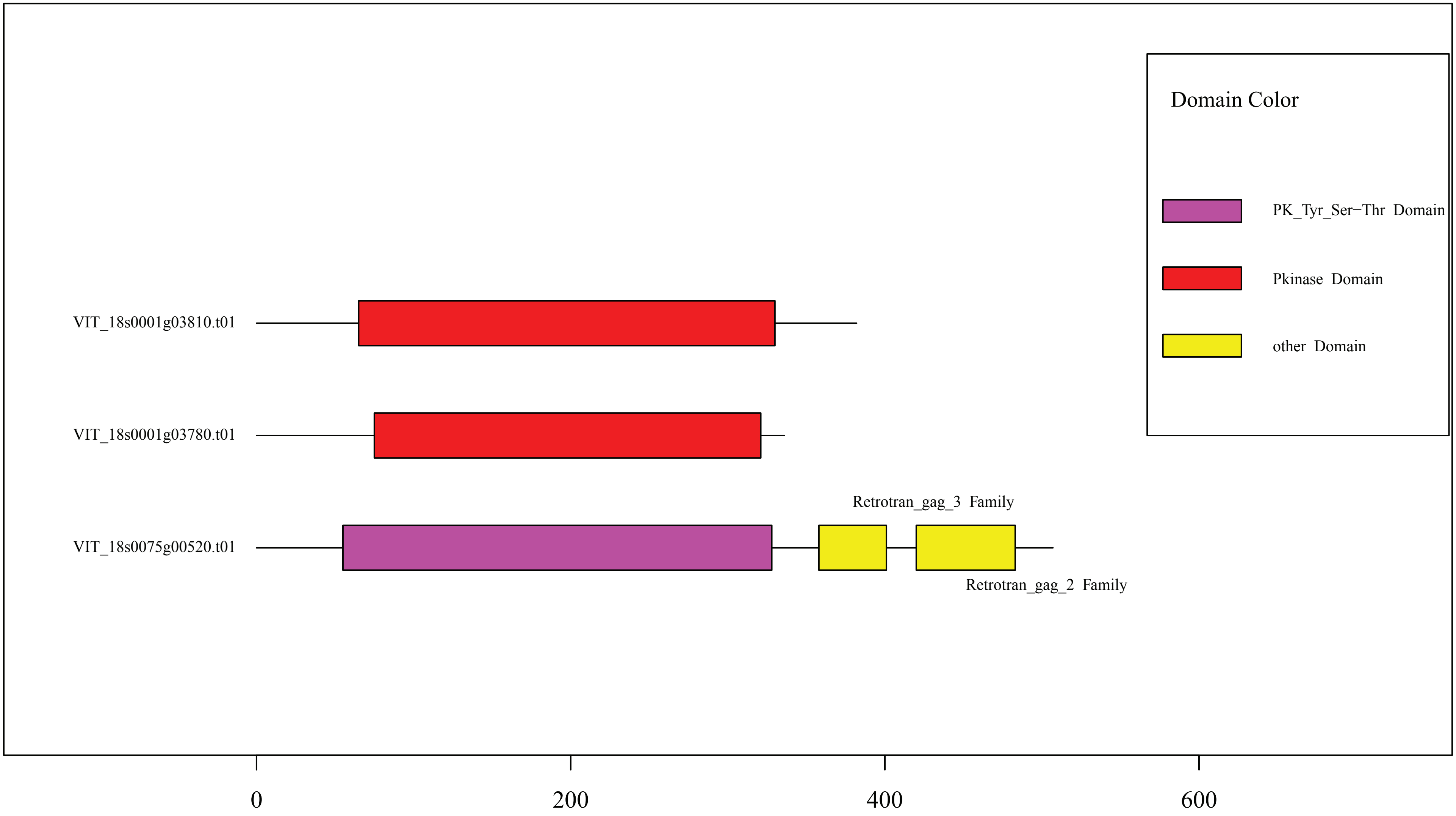

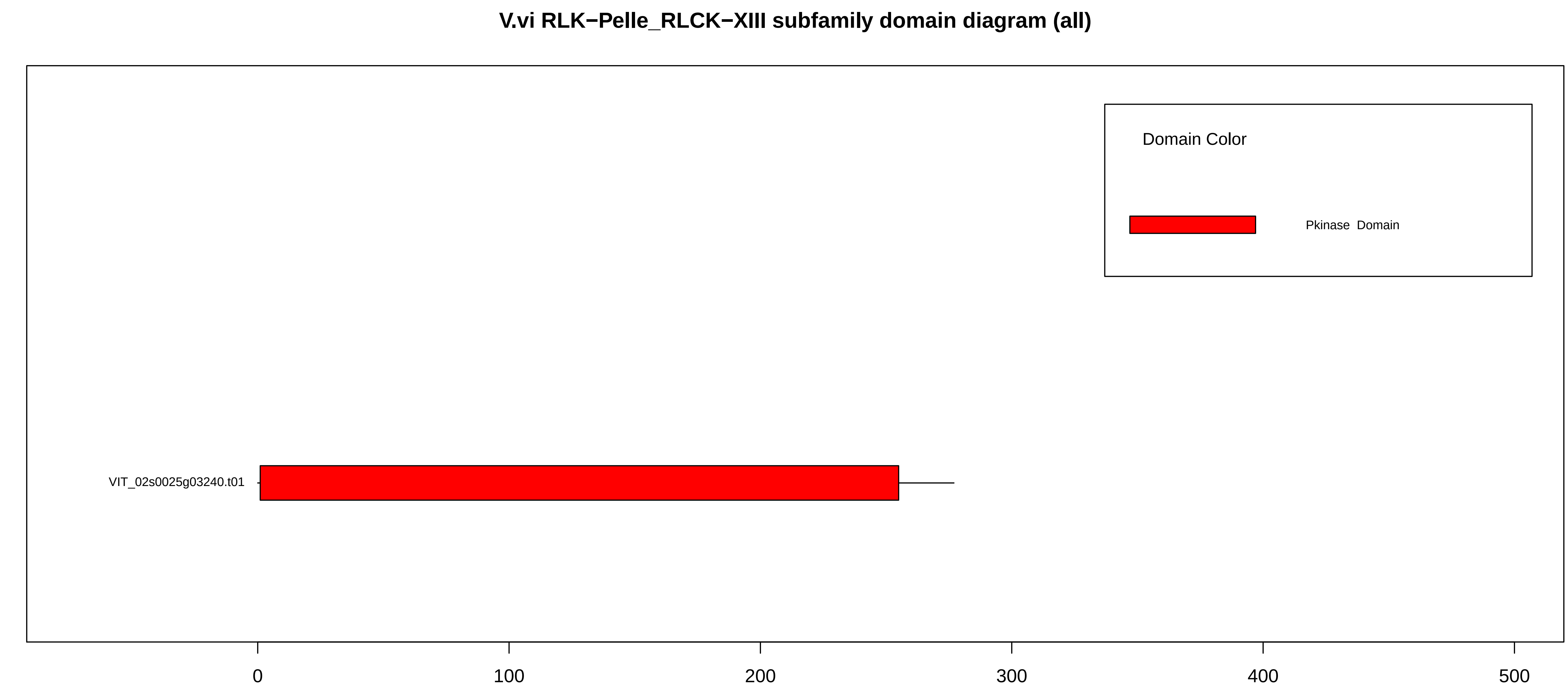
V.vi RLK−Pelle_RLCK−XIII subfamily domain diagram (all)
Domain Color
Pkinase Domain
VIT_02s0025g03240.t01
0
100
200
300
400
500

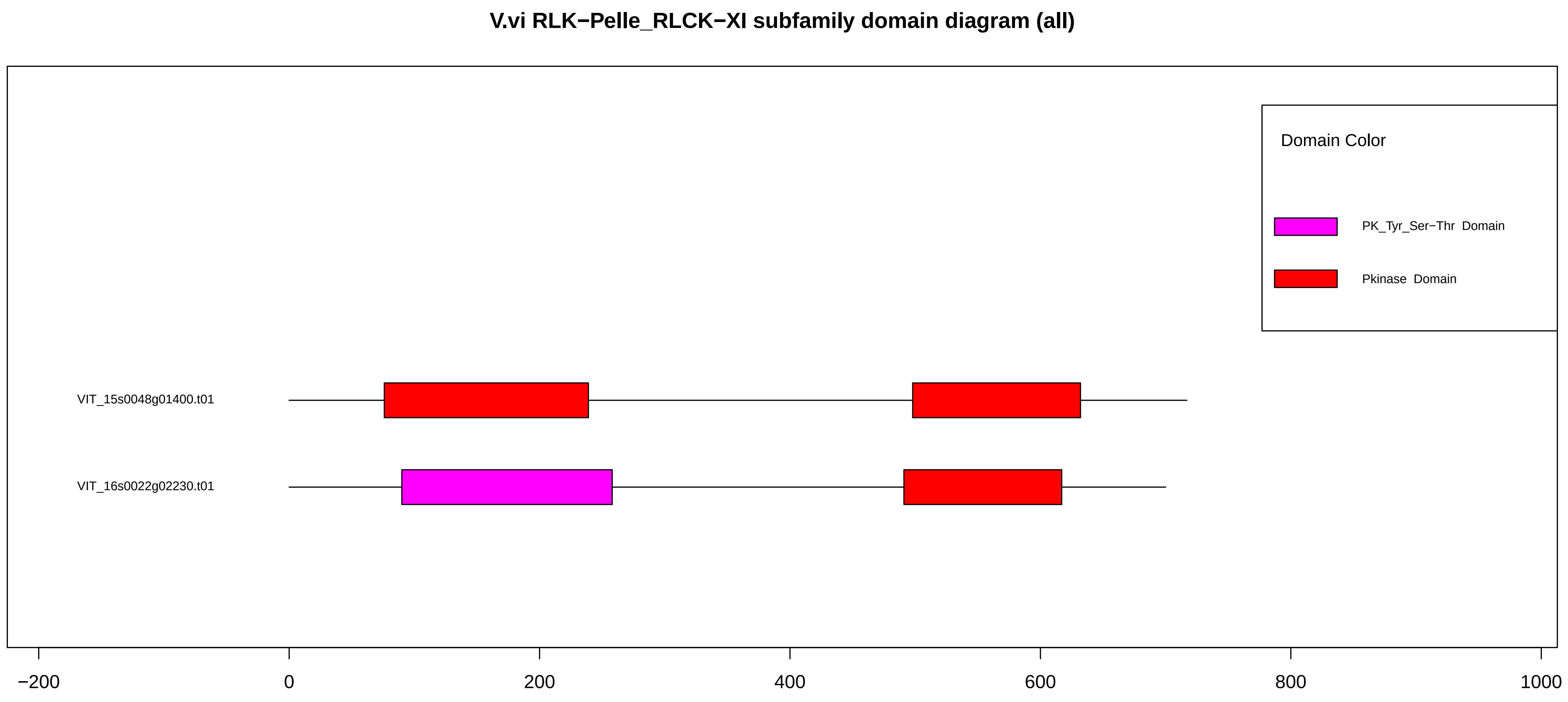
V.vi RLK−Pelle_RLCK−XI subfamily domain diagram (all)
Domain Color
PK_Tyr_Ser−Thr Domain
Pkinase Domain
VIT_15s0048g01400.t01
VIT_16s0022g02230.t01
−200
0
200
400
600
800
1000

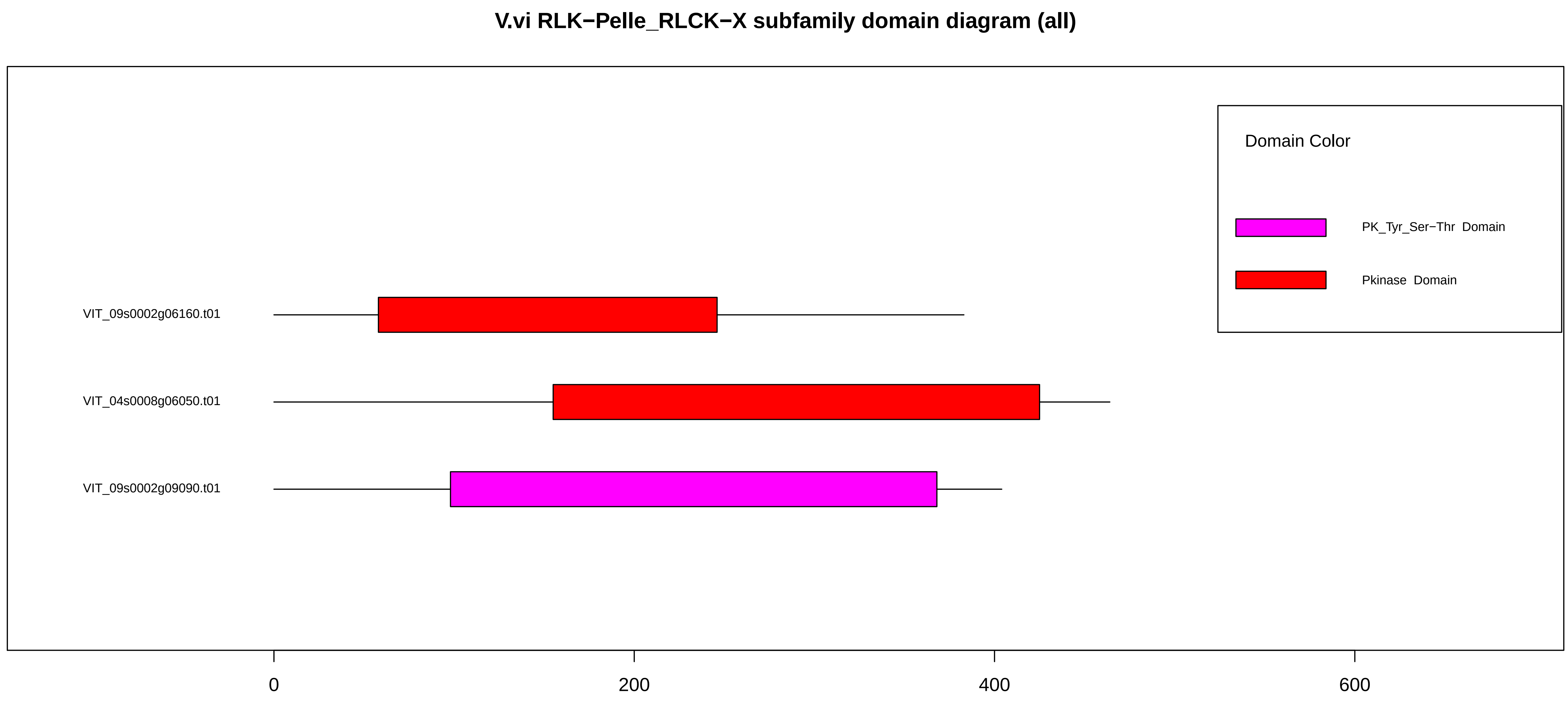
V.vi RLK−Pelle_RLCK−X subfamily domain diagram (all)
Domain Color
PK_Tyr_Ser−Thr Domain
Pkinase Domain
VIT_09s0002g06160.t01
VIT_04s0008g06050.t01
VIT_09s0002g09090.t01
0
200
400
600

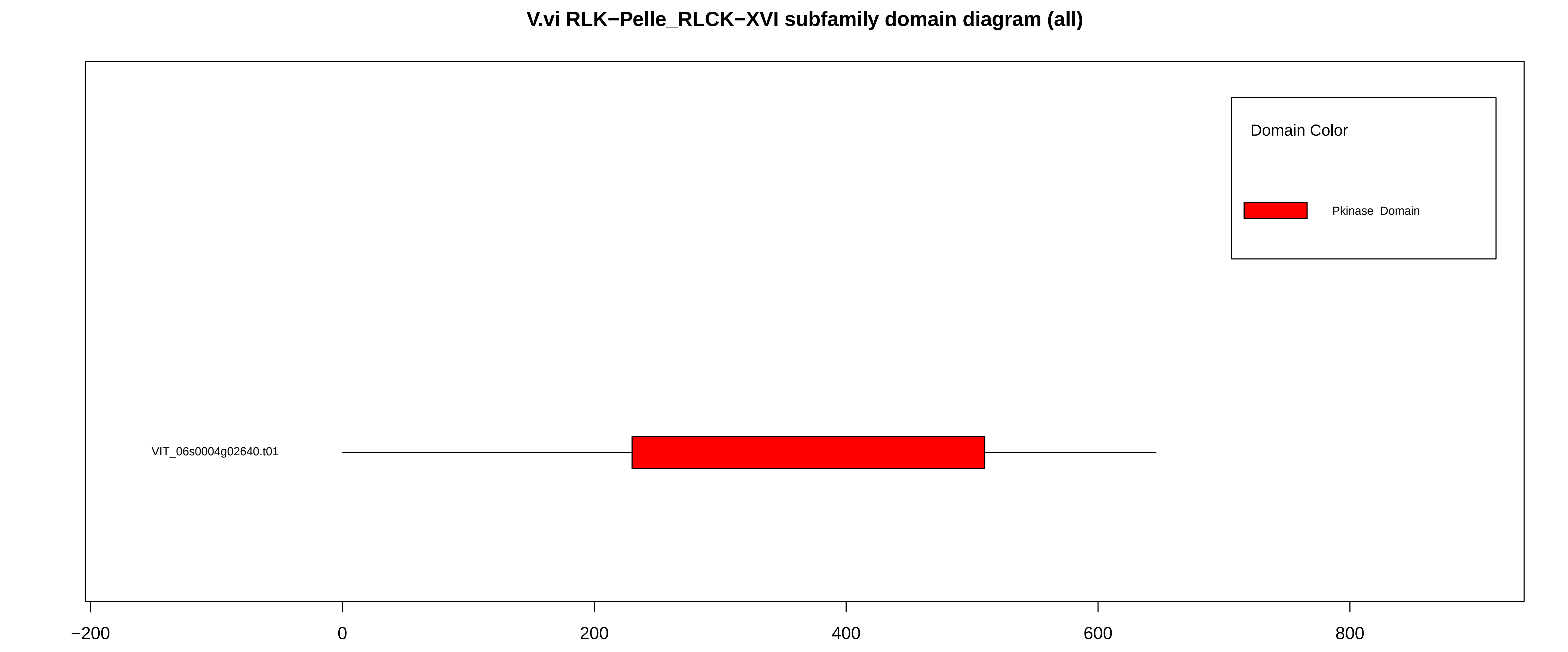
V.vi RLK−Pelle_RLCK−XVI subfamily domain diagram (all)
Domain Color
Pkinase Domain
VIT_06s0004g02640.t01
−200
0
200
400
600
800

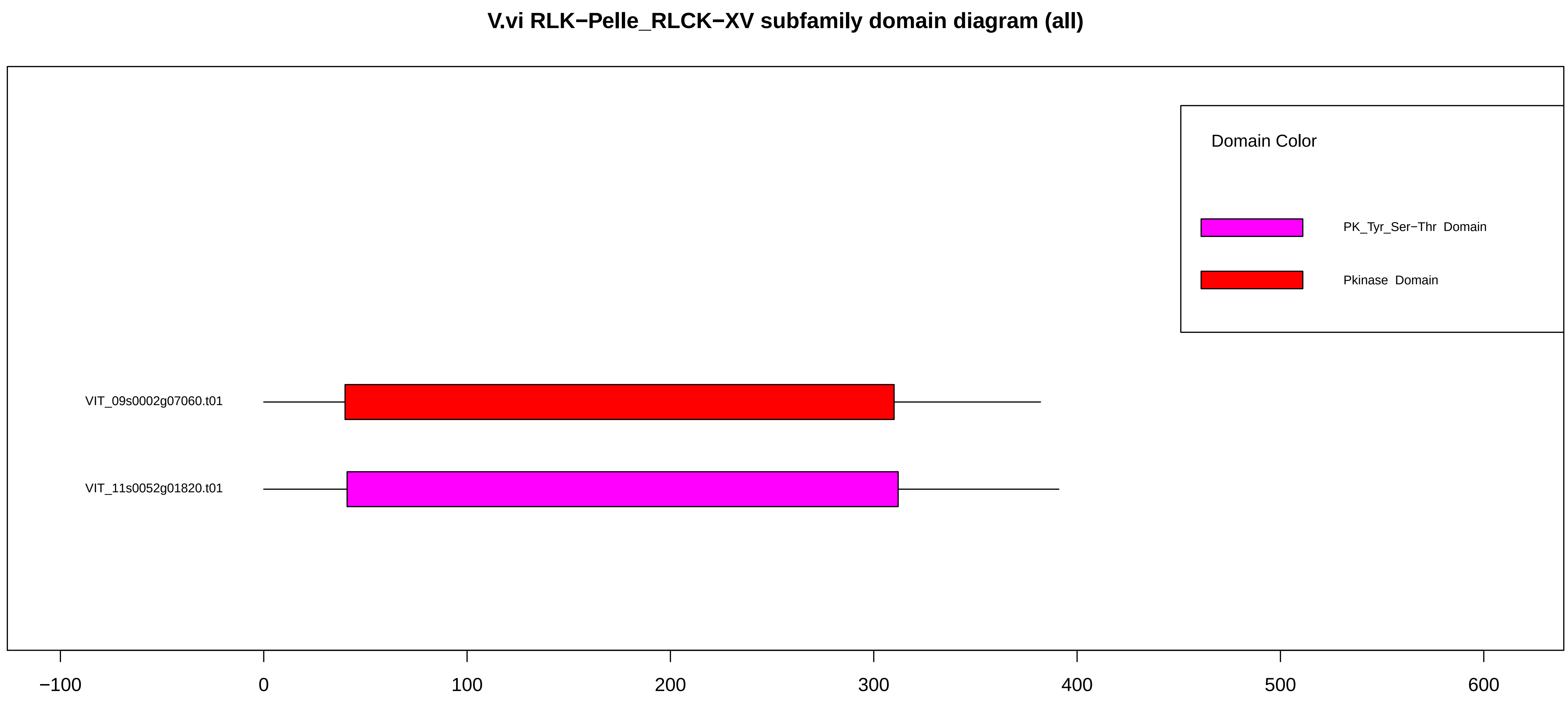
V.vi RLK−Pelle_RLCK−XV subfamily domain diagram (all)
Domain Color
PK_Tyr_Ser−Thr Domain
Pkinase Domain
VIT_09s0002g07060.t01
VIT_11s0052g01820.t01
−100
0
100
200
300
400
500
600

V.vi RLK−Pelle_SD−2b subfamily domain diagram (part 1)

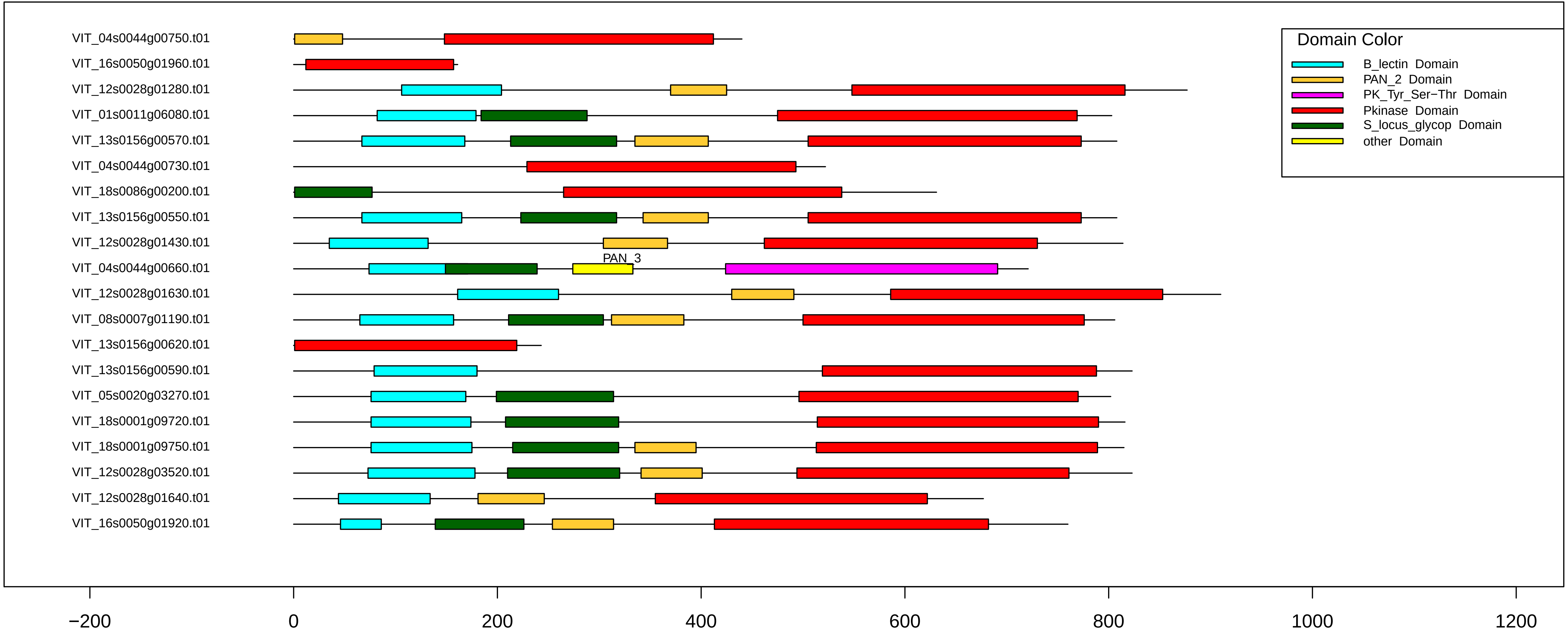

V.vi RLK−Pelle_SD−2b subfamily domain diagram (part 2)

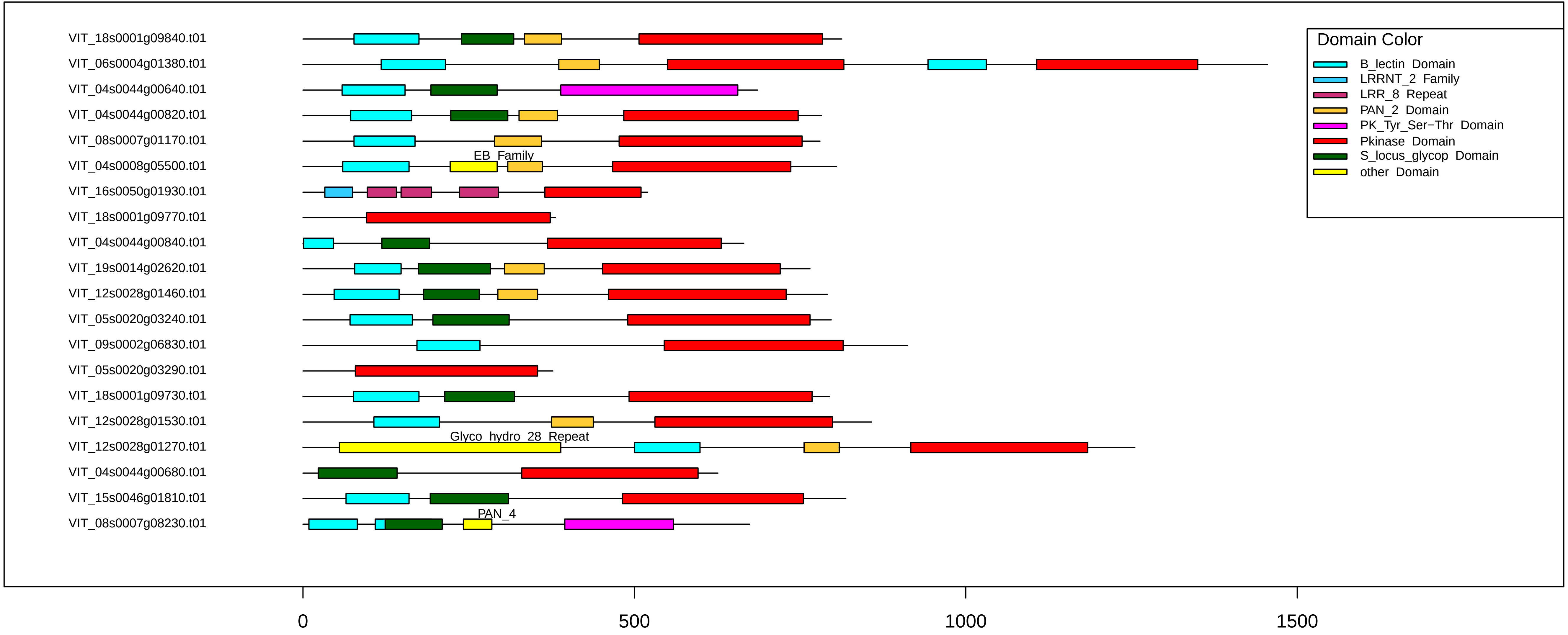

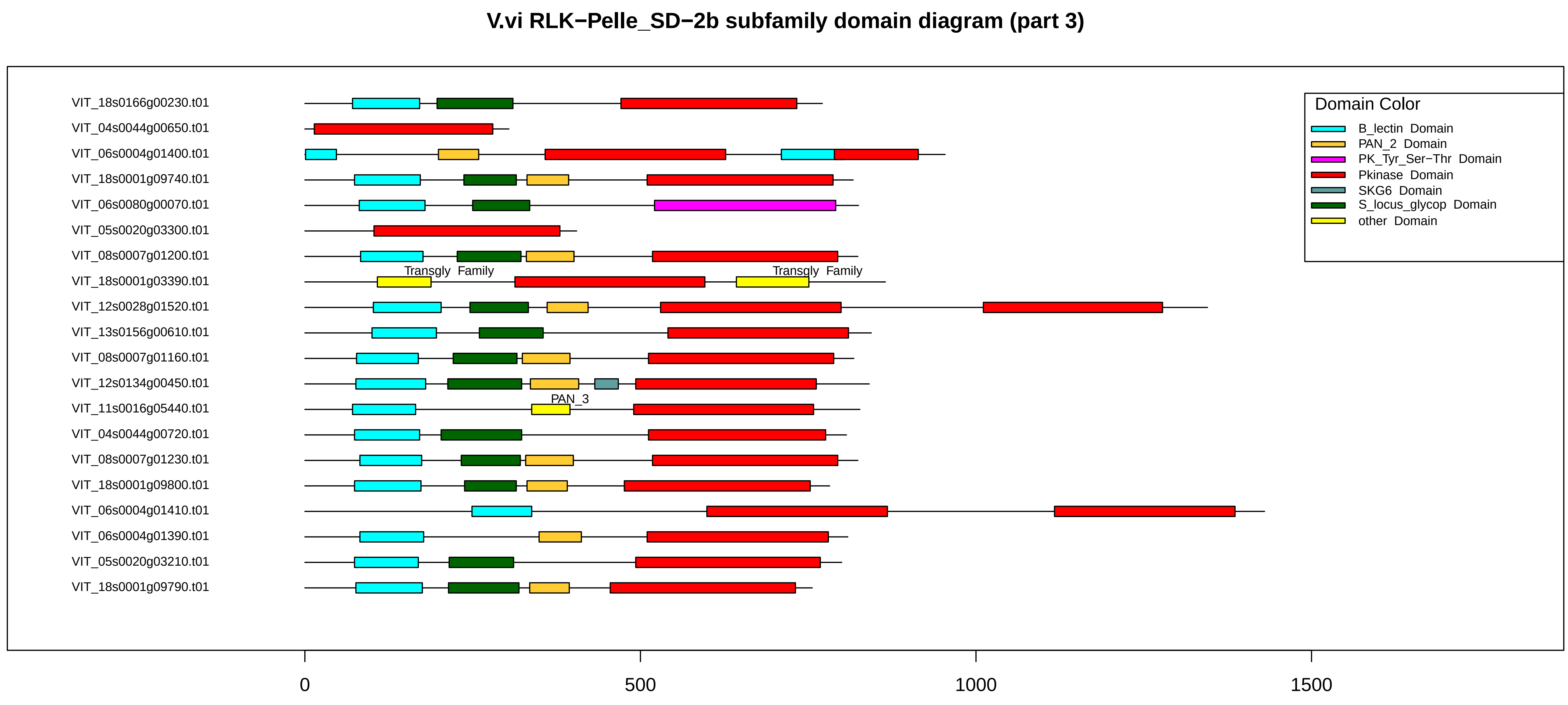
V.vi RLK−Pelle_SD−2b subfamily domain diagram (part 3)
VIT_18s0166g00230.t01
VIT_04s0044g00650.t01
VIT_06s0004g01400.t01
VIT_18s0001g09740.t01
VIT_06s0080g00070.t01
VIT_05s0020g03300.t01
VIT_08s0007g01200.t01
VIT_18s0001g03390.t01
VIT_12s0028g01520.t01
VIT_13s0156g00610.t01
VIT_08s0007g01160.t01
VIT_12s0134g00450.t01
VIT_11s0016g05440.t01
VIT_04s0044g00720.t01
VIT_08s0007g01230.t01
VIT_18s0001g09800.t01
VIT_06s0004g01410.t01
VIT_06s0004g01390.t01
VIT_05s0020g03210.t01
VIT_18s0001g09790.t01
Transgly Family
Transgly Family
PAN_3
Domain Color
B_lectin Domain
PAN_2 Domain
PK_Tyr_Ser−Thr Domain
Pkinase Domain
SKG6 Domain
S_locus_glycop Domain
other Domain
0
500
1000
1500

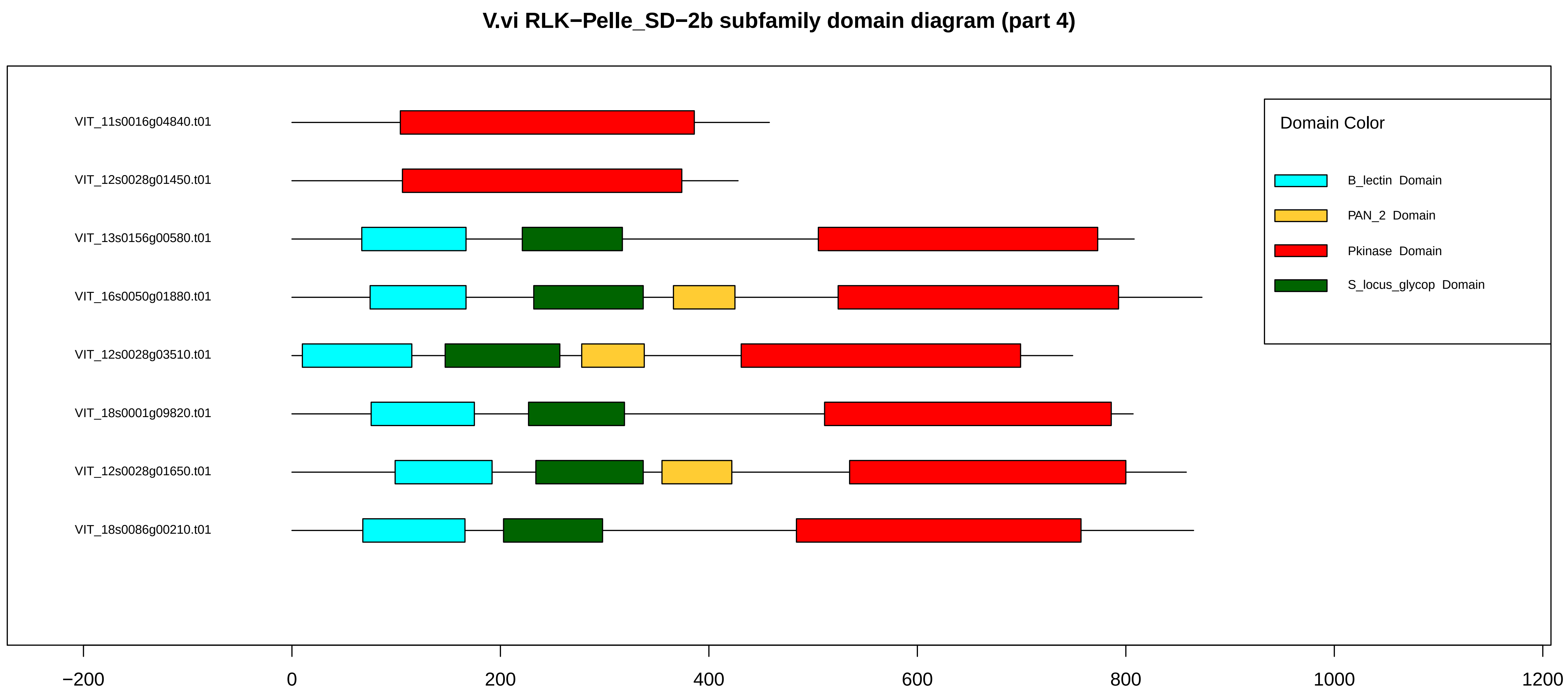
V.vi RLK−Pelle_SD−2b subfamily domain diagram (part 4)
VIT_11s0016g04840.t01
VIT_12s0028g01450.t01
VIT_13s0156g00580.t01
VIT_16s0050g01880.t01
VIT_12s0028g03510.t01
VIT_18s0001g09820.t01
VIT_12s0028g01650.t01
VIT_18s0086g00210.t01
Domain Color
B_lectin Domain
PAN_2 Domain
Pkinase Domain
S_locus_glycop Domain
−200
0
200
400
600
800
1000
1200

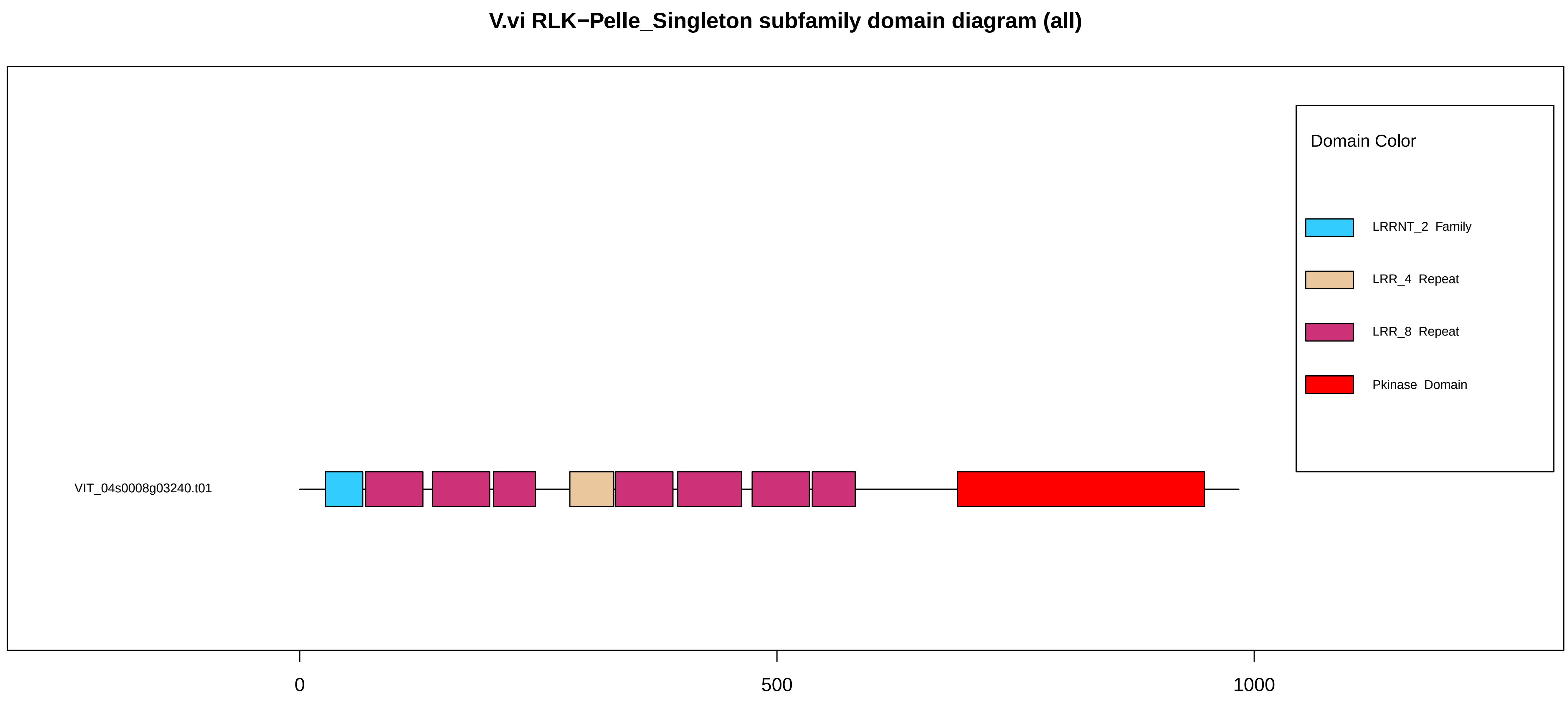

V.vi RLK−Pelle_Singleton subfamily domain diagram (all)
Domain Color
LRRNT_2 Family
LRR_4 Repeat
LRR_8 Repeat
Pkinase Domain
VIT_04s0008g03240.t01
0
500
1000

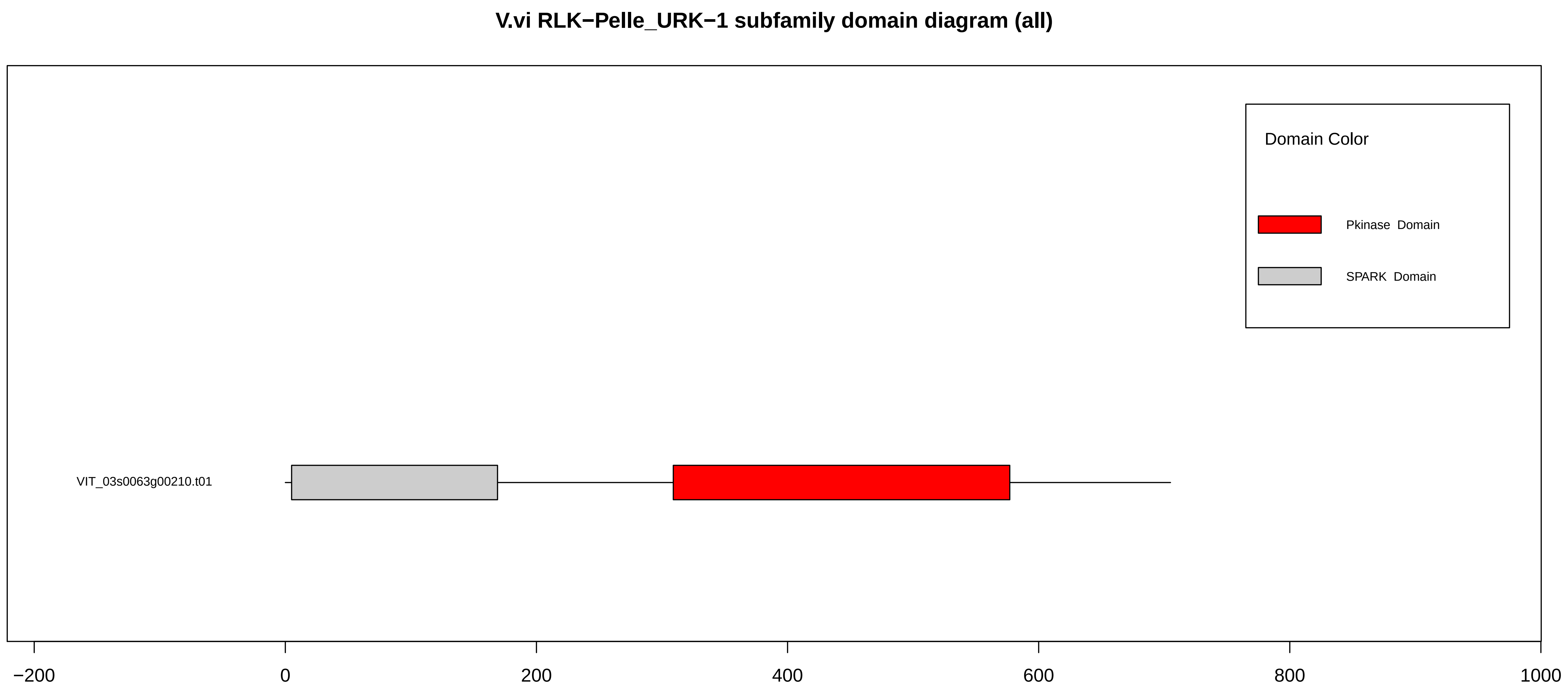
V.vi RLK−Pelle_URK−1 subfamily domain diagram (all)
Domain Color
Pkinase Domain
SPARK Domain
VIT_03s0063g00210.t01
−200
0
200
400
600
800
1000

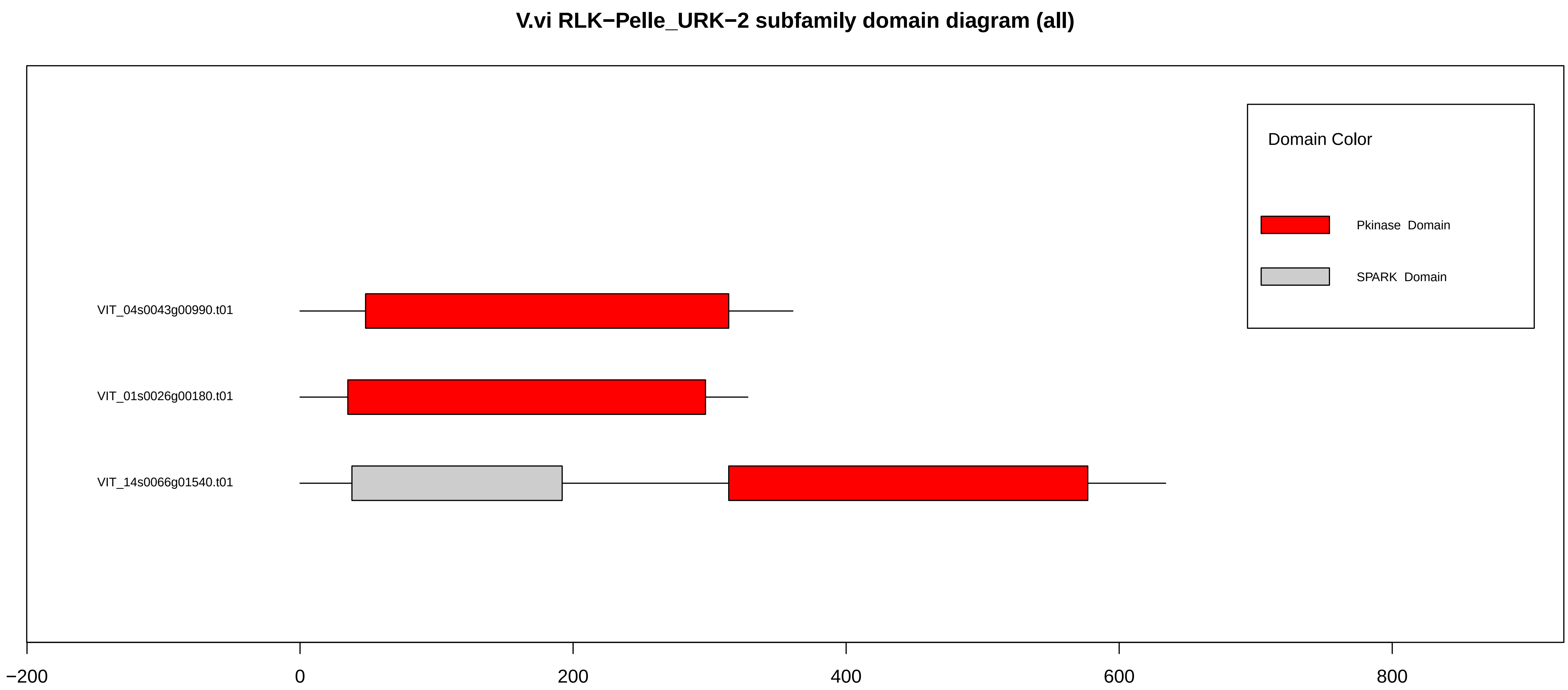
V.vi RLK−Pelle_URK−2 subfamily domain diagram (all)
Domain Color
Pkinase Domain
SPARK Domain
VIT_04s0043g00990.t01
VIT_01s0026g00180.t01
VIT_14s0066g01540.t01
−200
0
200
400
600
800

V.vi RLK−Pelle_URK−3 subfamily domain diagram (all)

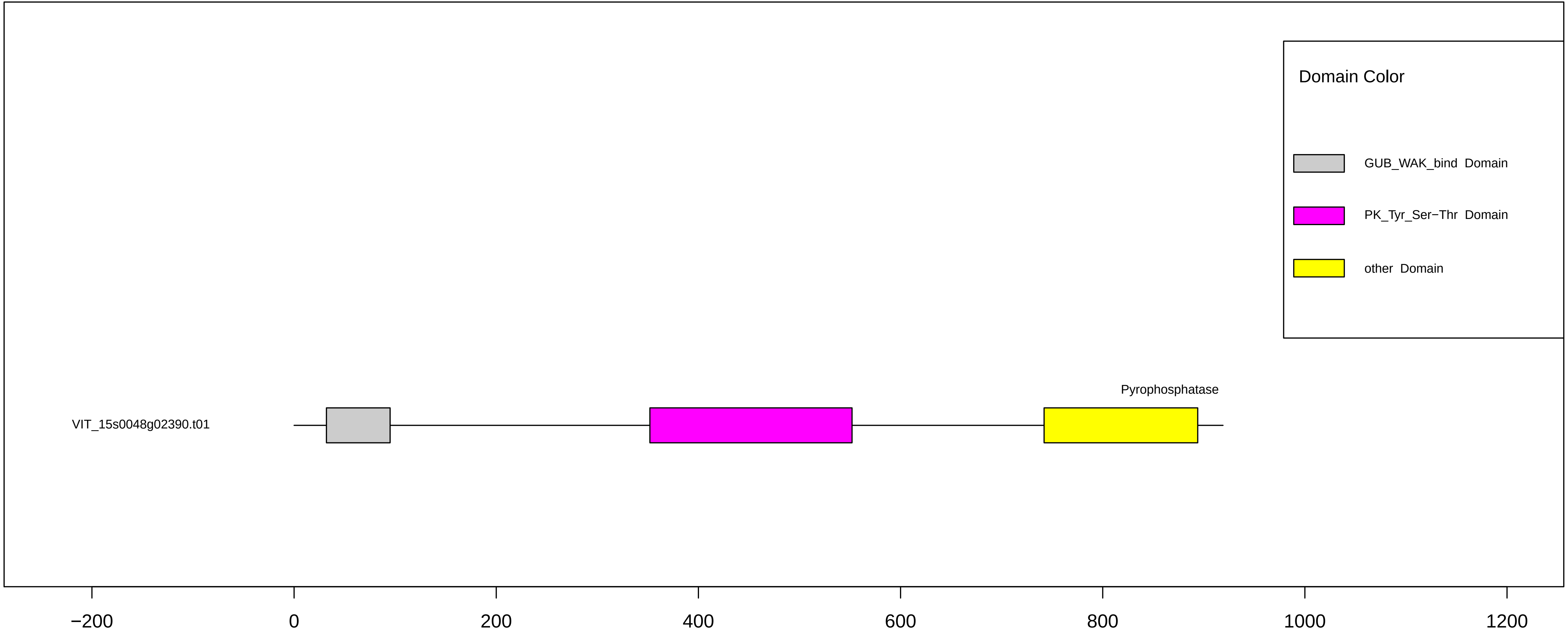

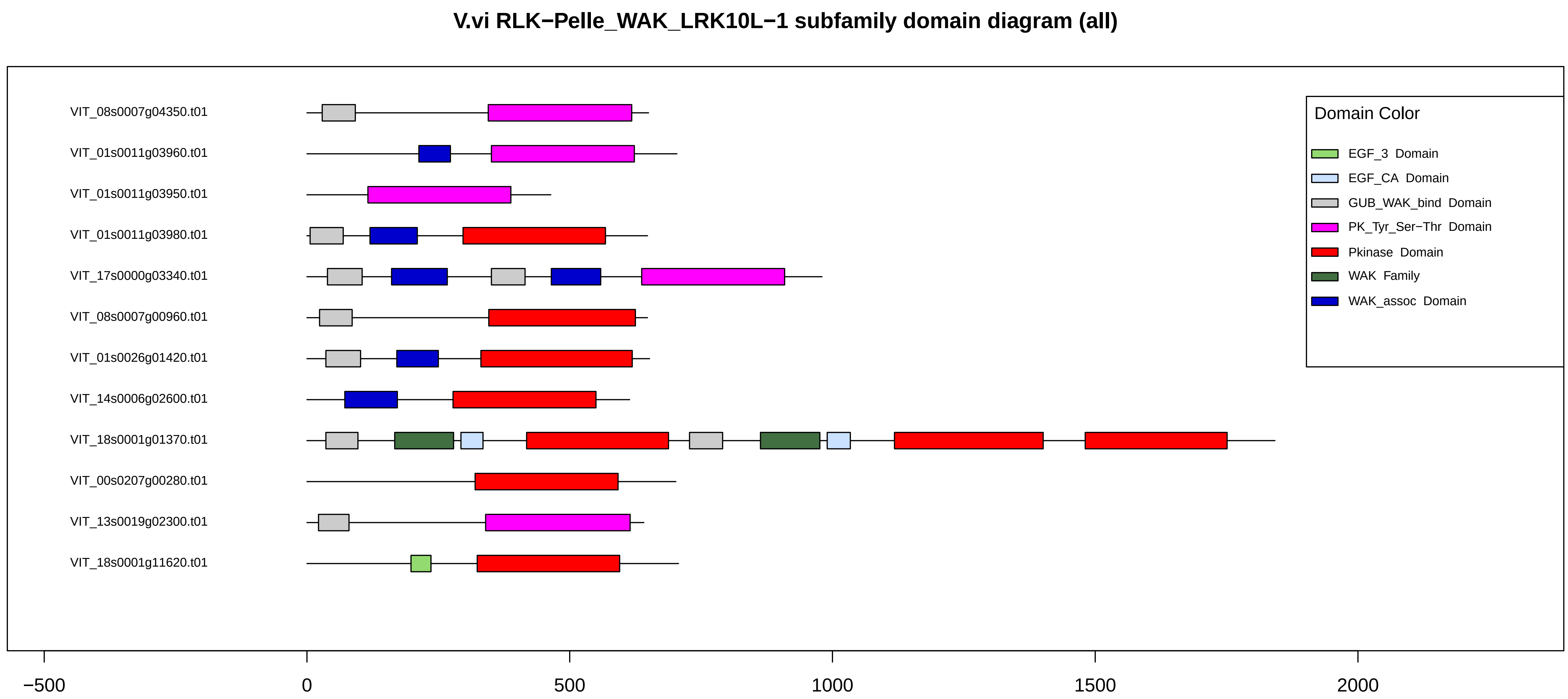

V.vi RLK−Pelle_WAK_LRK10L−1 subfamily domain diagram (all)
VIT_08s0007g04350.t01
VIT_01s0011g03960.t01
VIT_01s0011g03950.t01
VIT_01s0011g03980.t01
VIT_17s0000g03340.t01
VIT_08s0007g00960.t01
VIT_01s0026g01420.t01
VIT_14s0006g02600.t01
VIT_18s0001g01370.t01
VIT_00s0207g00280.t01
VIT_13s0019g02300.t01
VIT_18s0001g11620.t01
Domain Color
EGF_3 Domain
EGF_CA Domain
GUB_WAK_bind Domain
PK_Tyr_Ser−Thr Domain
Pkinase Domain
WAK Family
WAK_assoc Domain
−500
0
500
1000
1500
2000

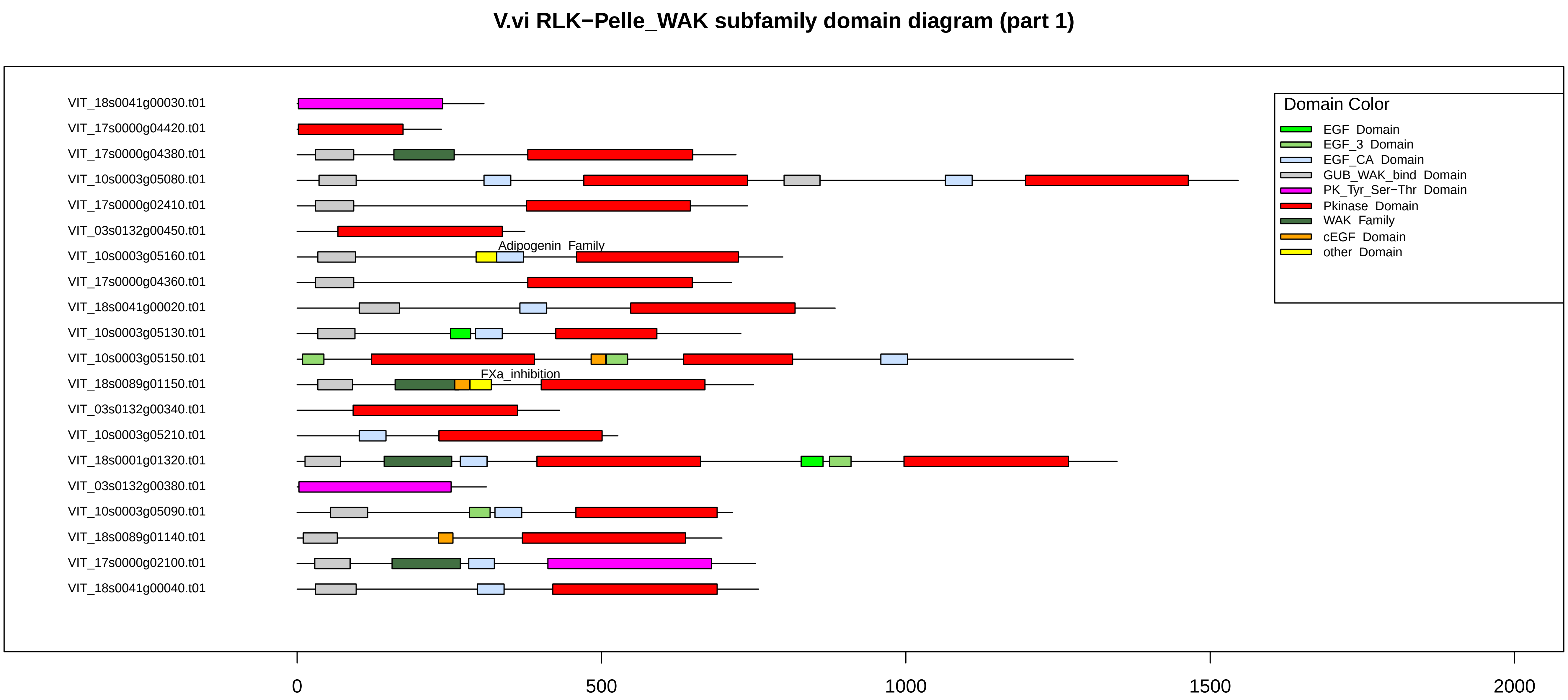

V.vi RLK−Pelle_WAK subfamily domain diagram (part 1)
VIT_18s0041g00030.t01
VIT_17s0000g04420.t01
VIT_17s0000g04380.t01
VIT_10s0003g05080.t01
VIT_17s0000g02410.t01
VIT_03s0132g00450.t01
VIT_10s0003g05160.t01
Adipogenin Family
VIT_17s0000g04360.t01
VIT_18s0041g00020.t01
VIT_10s0003g05130.t01
VIT_10s0003g05150.t01
VIT_18s0089g01150.t01
FXa_inhibition
VIT_03s0132g00340.t01
VIT_10s0003g05210.t01
VIT_18s0001g01320.t01
VIT_03s0132g00380.t01
VIT_10s0003g05090.t01
VIT_18s0089g01140.t01
VIT_17s0000g02100.t01
VIT_18s0041g00040.t01
Domain Color
EGF Domain
EGF_3 Domain
EGF_CA Domain
GUB_WAK_bind Domain
PK_Tyr_Ser−Thr Domain
Pkinase Domain
WAK Family
cEGF Domain
other Domain
0
500
1000
1500
2000

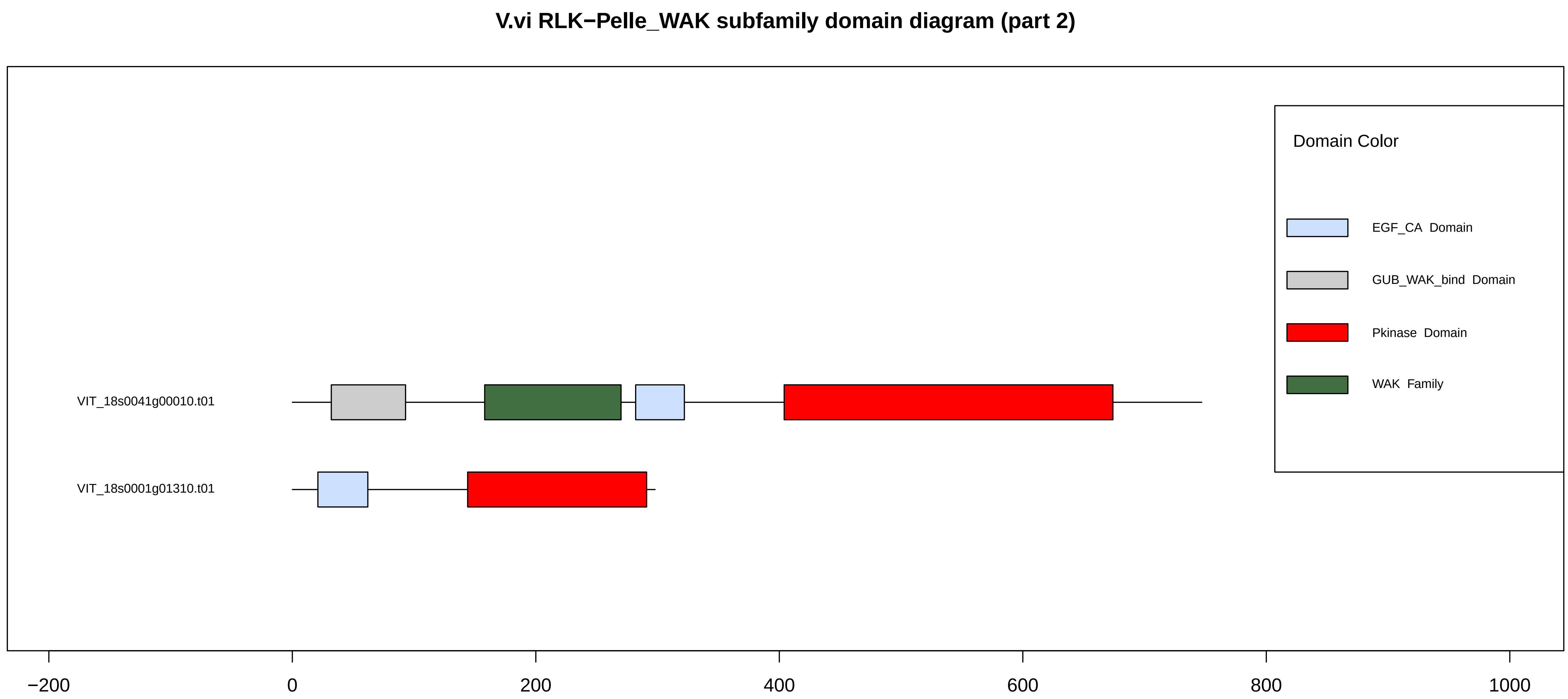

V.vi RLK−Pelle_WAK subfamily domain diagram (part 2)
Domain Color
EGF_CA Domain
GUB_WAK_bind Domain
Pkinase Domain
WAK Family
VIT_18s0041g00010.t01
VIT_18s0001g01310.t01
−200
0
200
400
600
800
1000

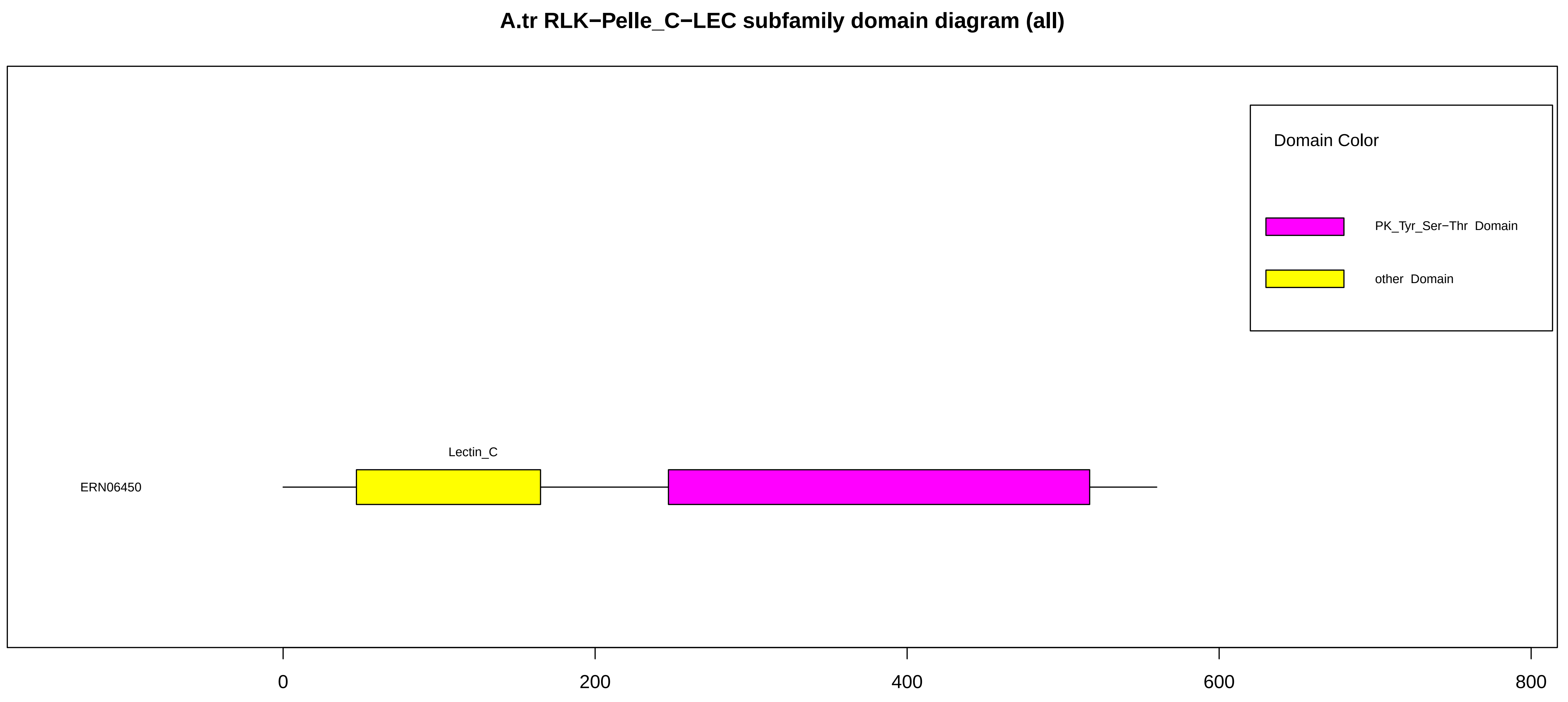

A.tr RLK−Pelle_C−LEC subfamily domain diagram (all)
Domain Color
PK_Tyr_Ser−Thr Domain
other Domain
Lectin_C
ERN06450
0
200
400
600
800

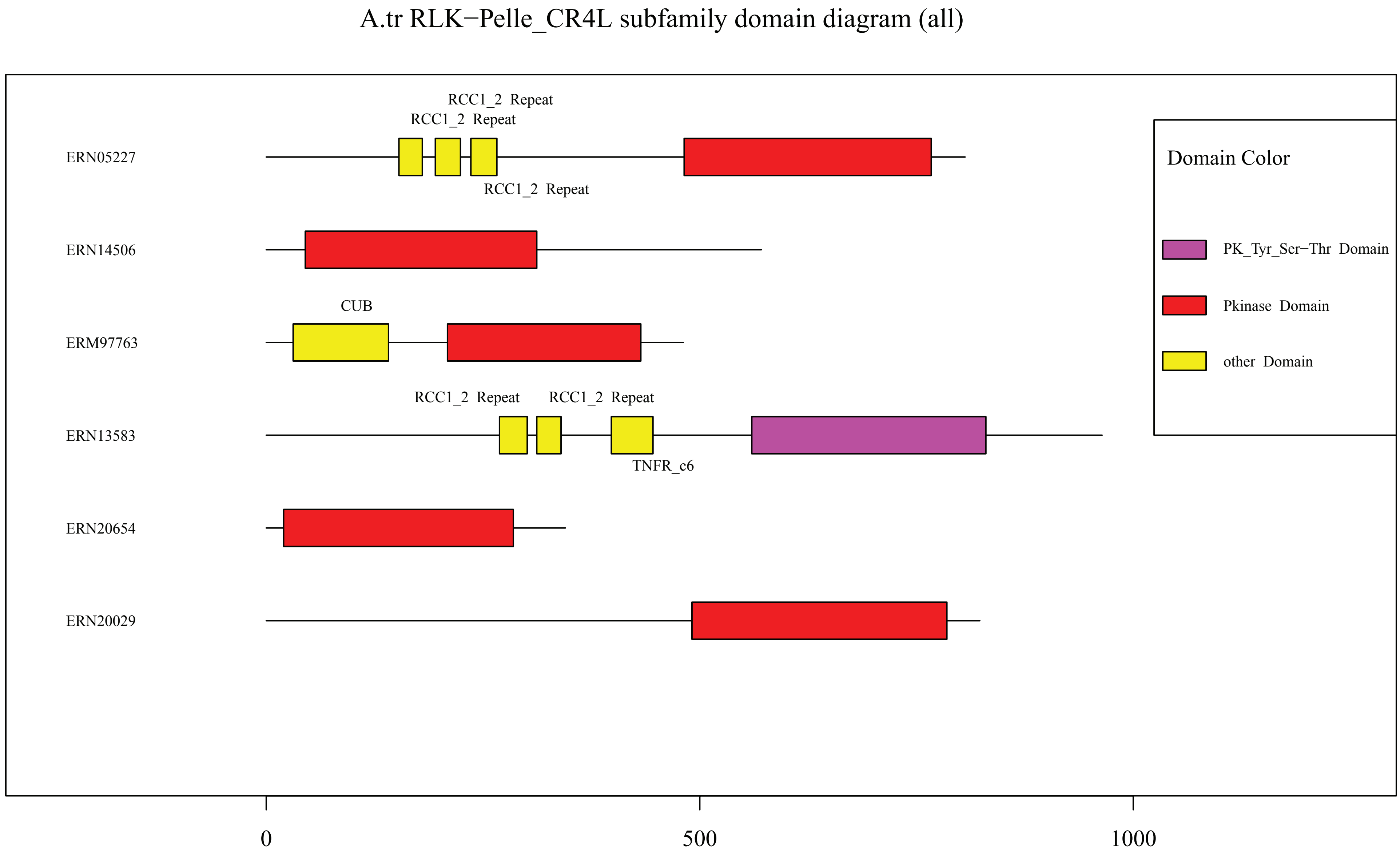

A.tr RLK−Pelle_CR4L subfamily domain diagram (all)
RCC1_2 Repeat
RCC1_2 Repeat
ERN05227
RCC1_2 Repeat
ERN14506
CUB
ERM97763
RCC1_2 Repeat
RCC1_2 Repeat
ERN13583
TNFR_c6
ERN20654
ERN20029
Domain Color
PK_Tyr_Ser−Thr Domain
Pkinase Domain
other Domain
0
500
1000

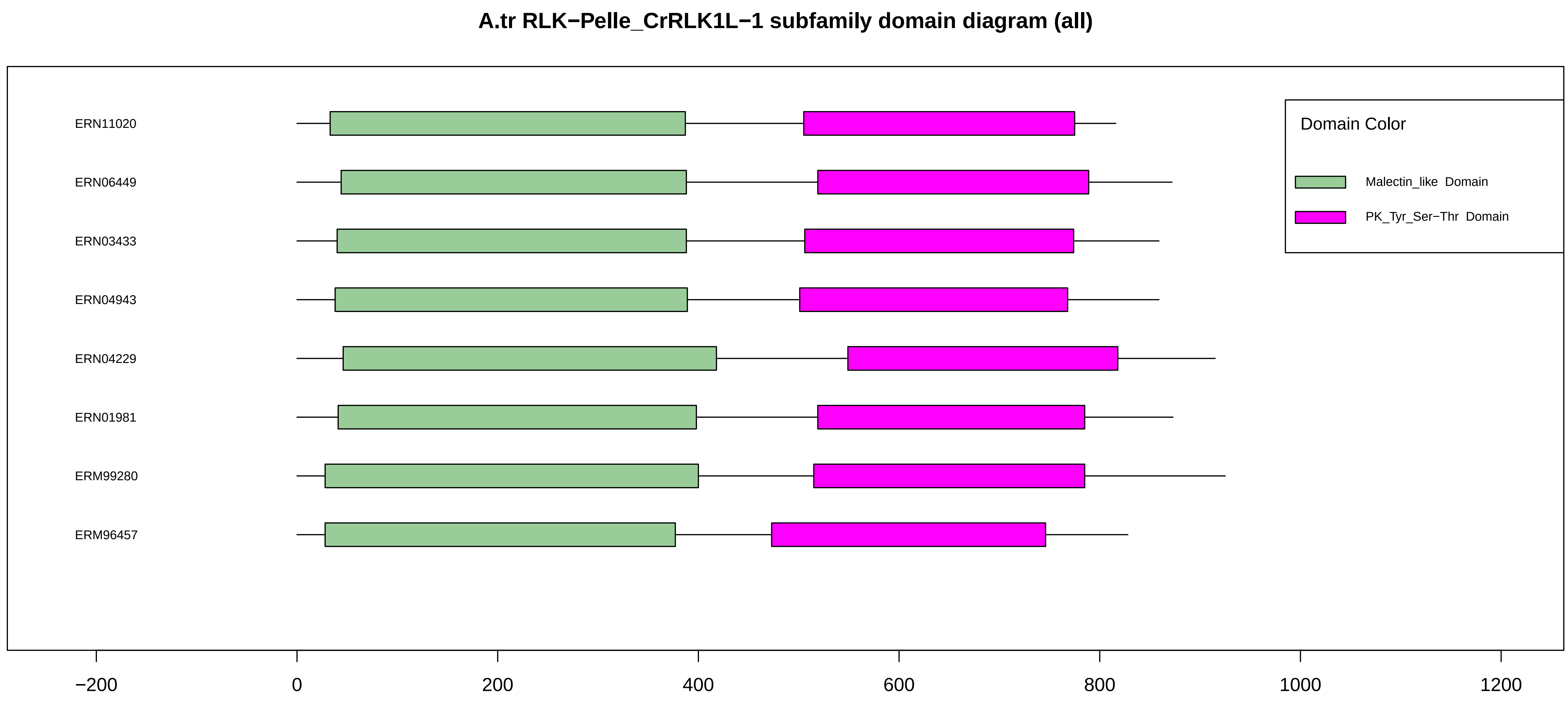

A.tr RLK−Pelle_CrRLK1L−1 subfamily domain diagram (all)
ERN11020
ERN06449
ERN03433
ERN04943
ERN04229
ERN01981
ERM99280
ERM96457
Domain Color
Malectin_like Domain
PK_Tyr_Ser−Thr Domain
−200
0
200
400
600
800
1000
1200

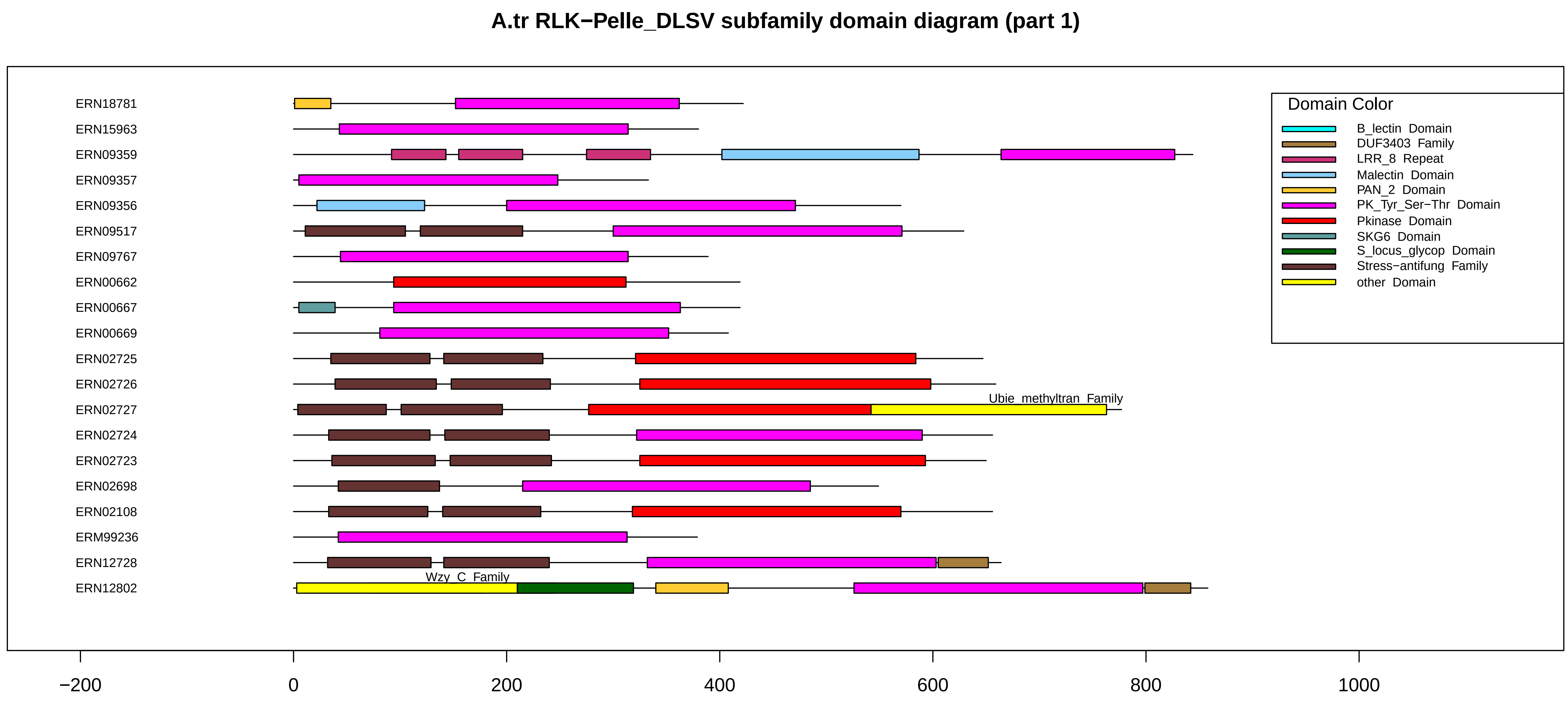

A.tr RLK−Pelle_DLSV subfamily domain diagram (part 1)
ERN18781
ERN15963
ERN09359
ERN09357
ERN09356
ERN09517
ERN09767
ERN00662
ERN00667
ERN00669
ERN02725
ERN02726
ERN02727
ERN02724
ERN02723
ERN02698
ERN02108
ERM99236
ERN12728
ERN12802
Ubie_methyltran_Family
Wzy_C_Family
Domain Color
B_lectin Domain
DUF3403 Family
LRR_8 Repeat
Malectin Domain
PAN_2 Domain
PK_Tyr_Ser−Thr Domain
Pkinase Domain
SKG6 Domain
S_locus_glycop Domain
Stress−antifung Family
other Domain
−200
0
200
400
600
800
1000

A.tr RLK−Pelle_DLSV subfamily domain diagram (part 2)

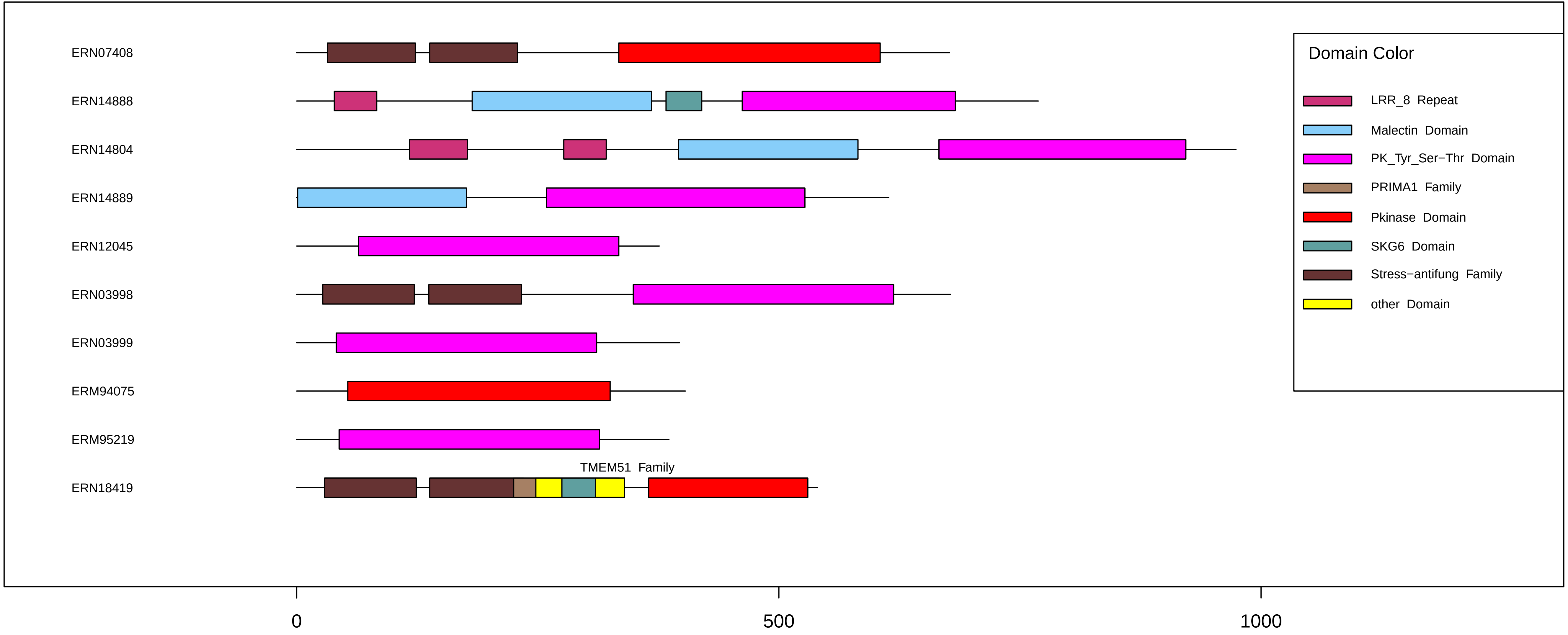

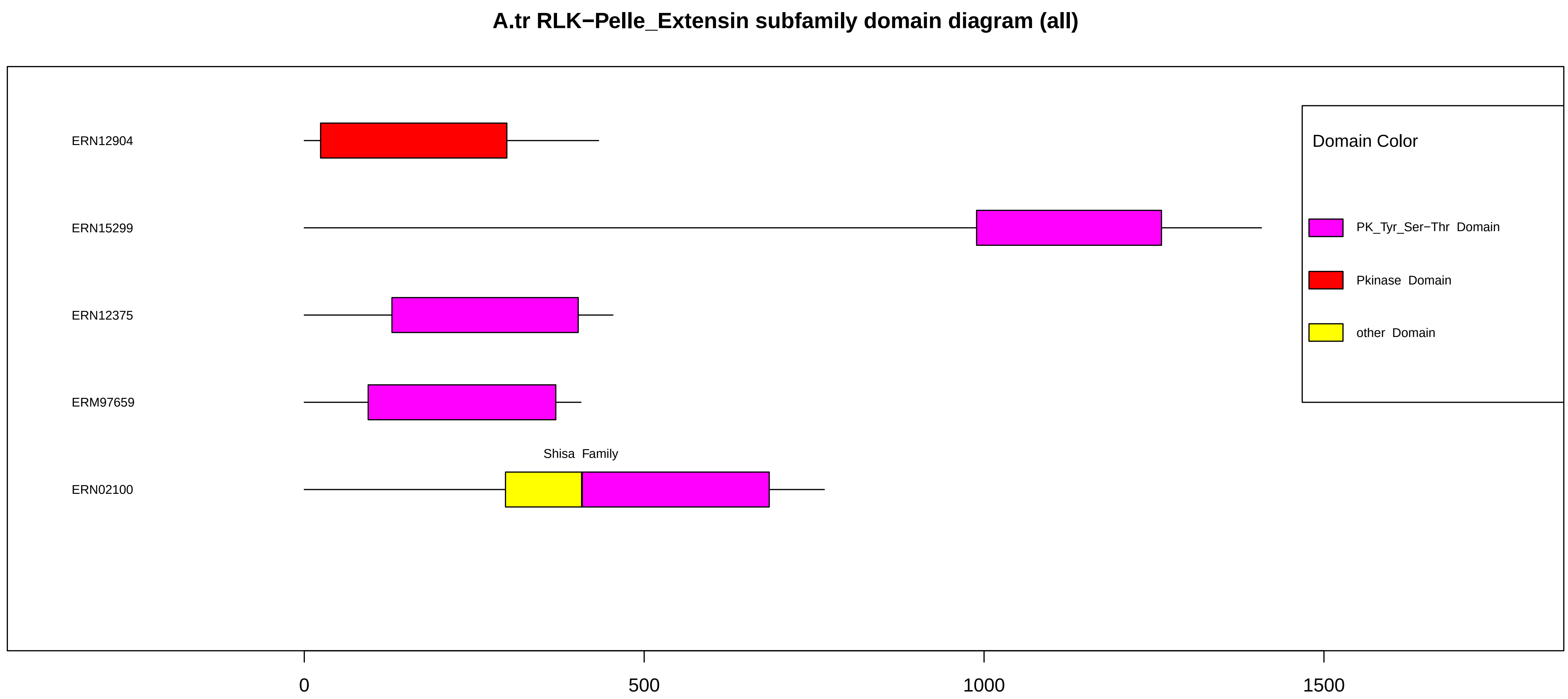
A.tr RLK−Pelle_Extensin subfamily domain diagram (all)
ERN12904
ERN15299
ERN12375
ERM97659
ERN02100
Shisa Family
Domain Color
PK_Tyr_Ser−Thr Domain
Pkinase Domain
other Domain
0
500
1000
1500

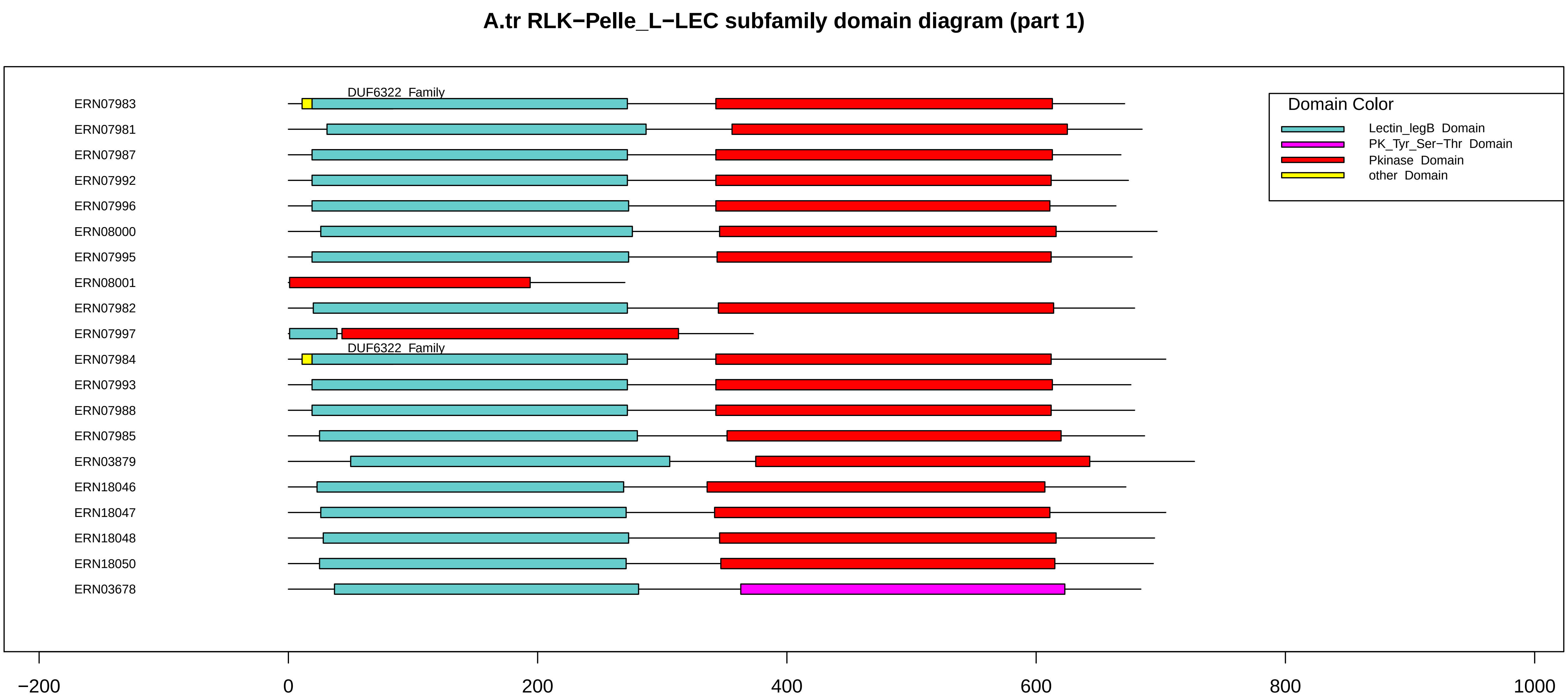
A.tr RLK−Pelle_L−LEC subfamily domain diagram (part 1)
DUF6322_Family
ERN07983
ERN07981
ERN07987
ERN07992
ERN07996
ERN08000
ERN07995
ERN08001
ERN07982
ERN07997
ERN07984
ERN07993
ERN07988
ERN07985
ERN03879
ERN18046
ERN18047
ERN18048
ERN18050
ERN03678
Domain Color
Lectin_legB Domain
PK_Tyr_Ser−Thr Domain
Pkinase Domain
other Domain
−200
0
200
400
600
800
1000

A.tr RLK−Pelle_L−LEC subfamily domain diagram (part 2)

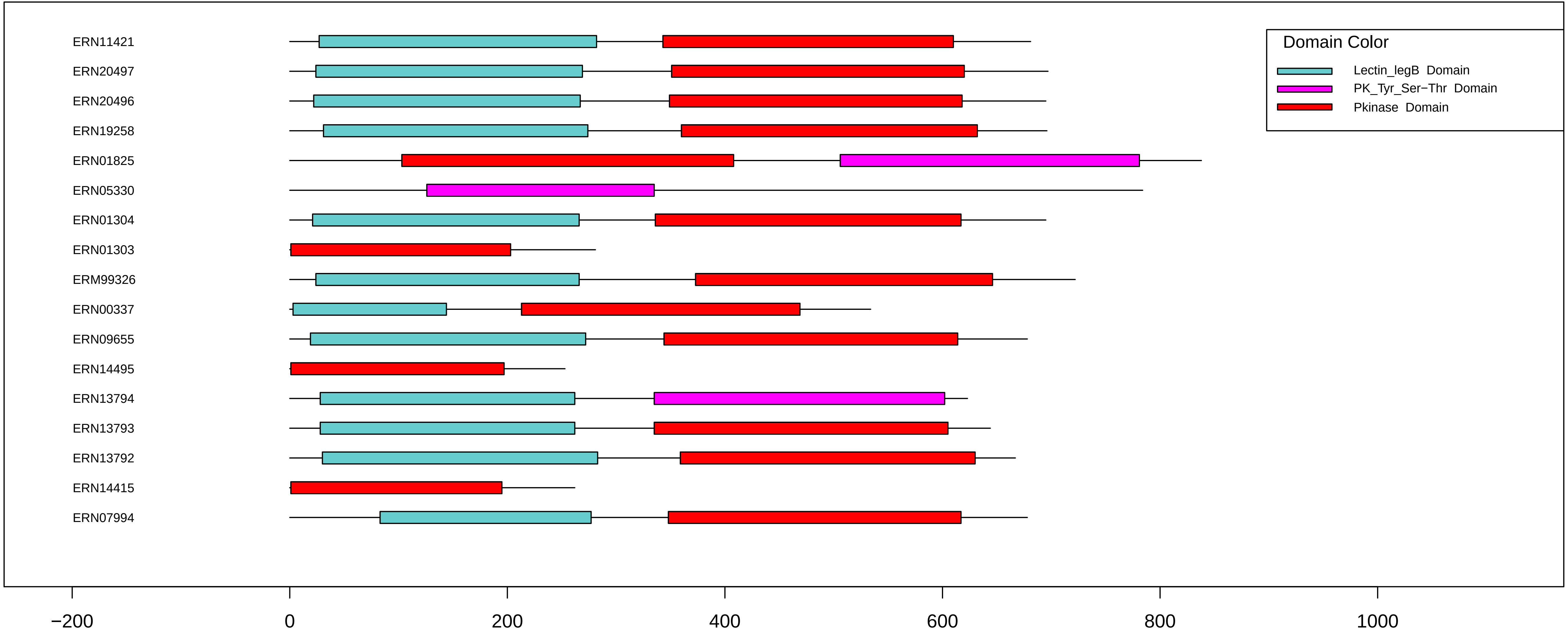

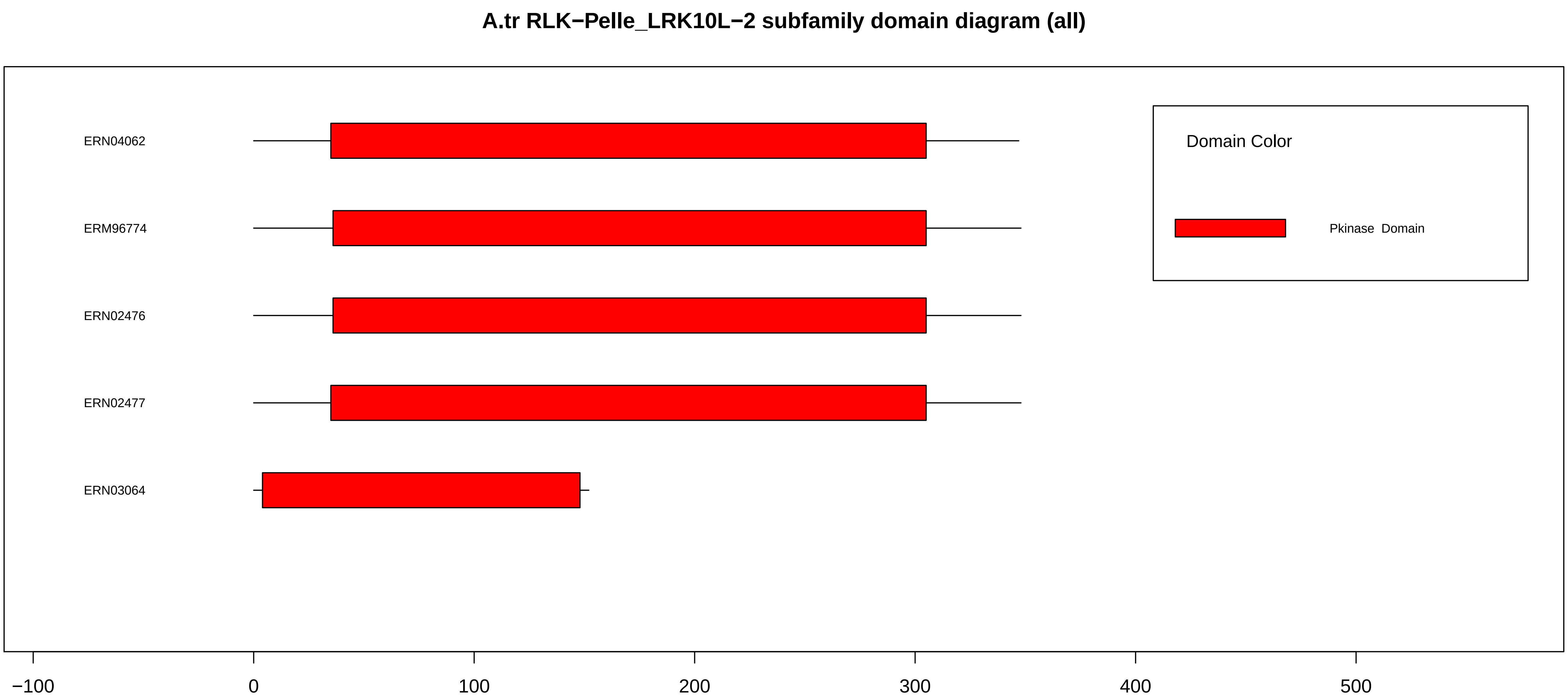
A.tr RLK−Pelle_LRK10L−2 subfamily domain diagram (all)
ERN04062
ERM96774
ERN02476
ERN02477
ERN03064
Domain Color
Pkinase Domain
−100
0
100
200
300
400
500

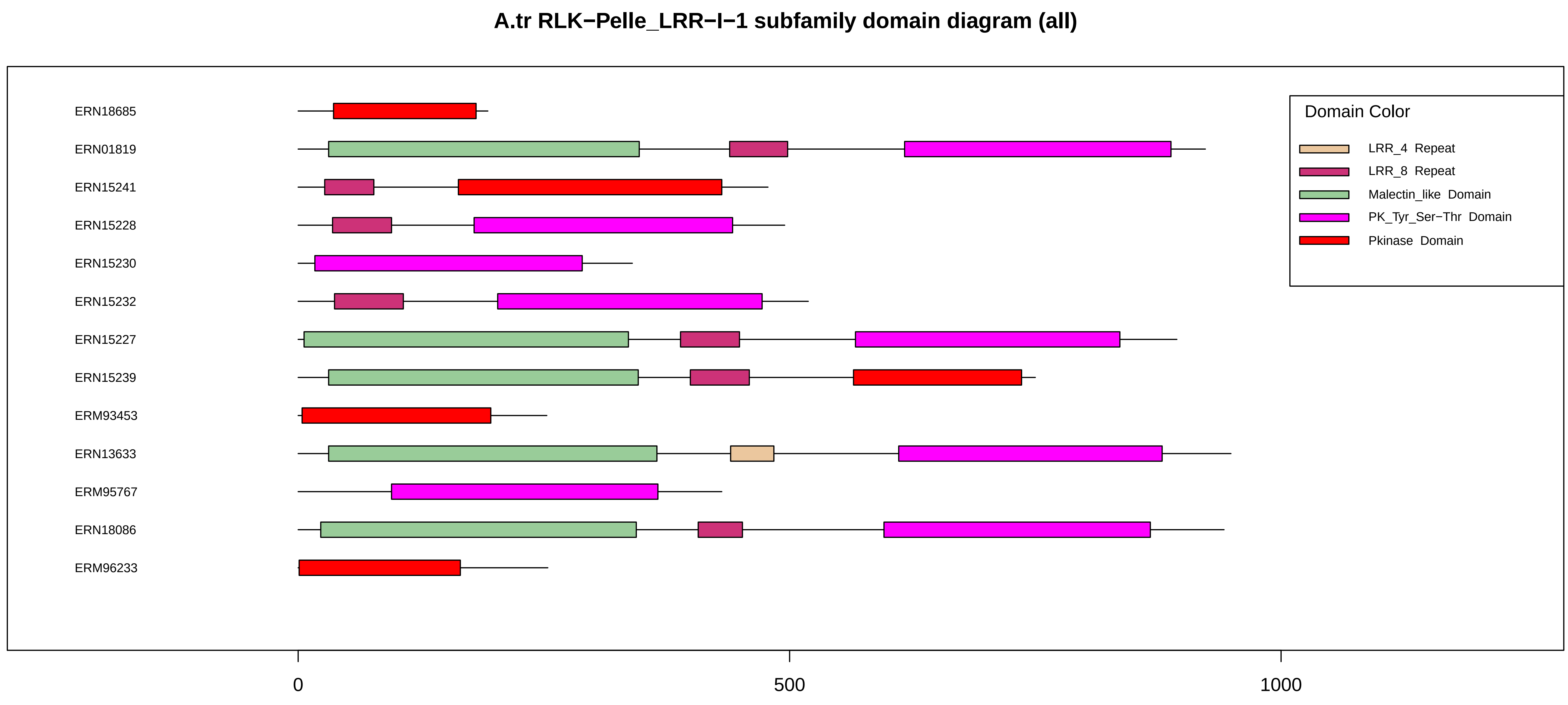

A.tr RLK−Pelle_LRR−I−1 subfamily domain diagram (all)
ERN18685
ERN01819
ERN15241
ERN15228
ERN15230
ERN15232
ERN15227
ERN15239
ERM93453
ERN13633
ERM95767
ERN18086
ERM96233
Domain Color
LRR_4 Repeat
LRR_8 Repeat
Malectin_like Domain
PK_Tyr_Ser−Thr Domain
Pkinase Domain
0
500
1000

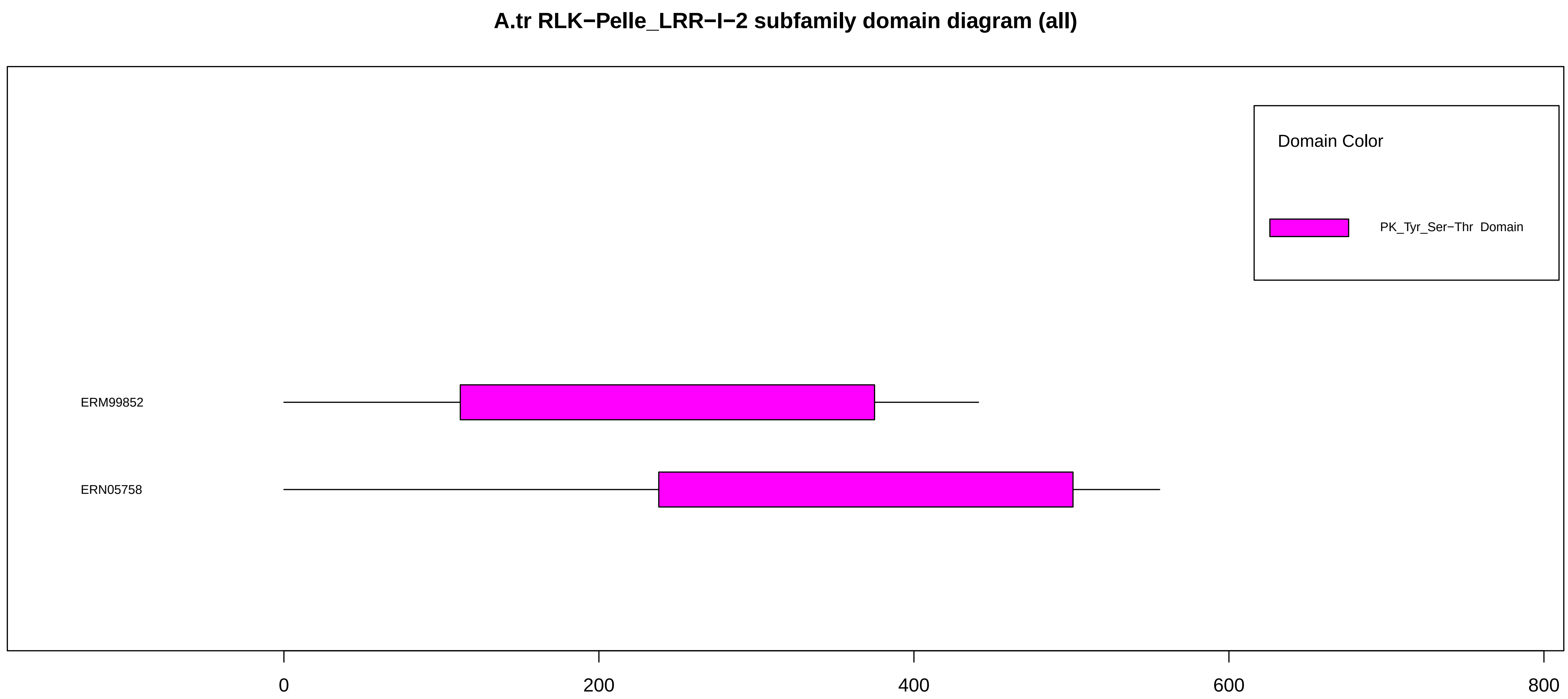
A.tr RLK−Pelle_LRR−I−2 subfamily domain diagram (all)
Domain Color
PK_Tyr_Ser−Thr Domain
ERM99852
ERN05758
0
200
400
600
800

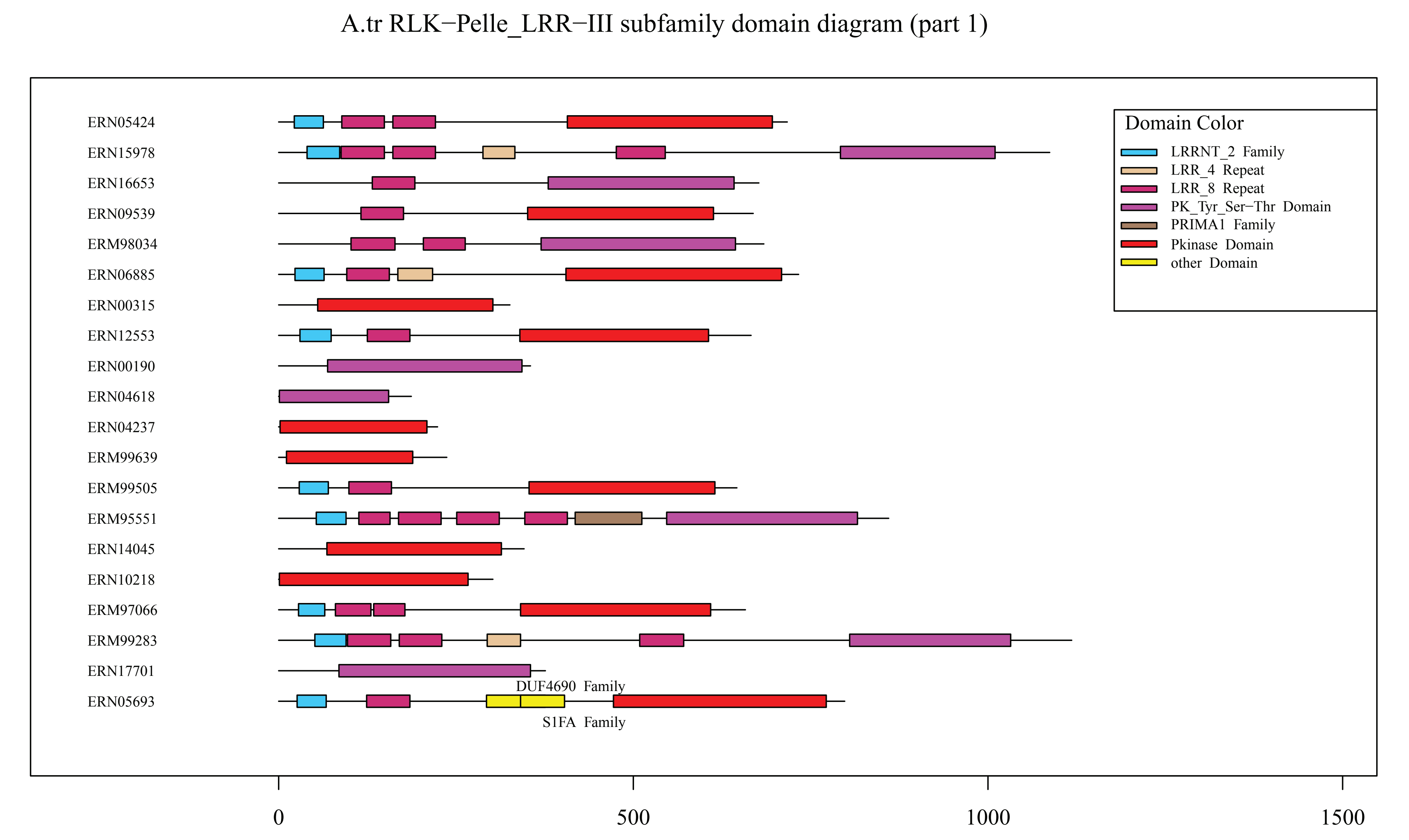
A.tr RLK−Pelle_LRR−III subfamily domain diagram (part 1)
ERN05424
ERN15978
ERN16653
ERN09539
ERM98034
ERN06885
ERN00315
ERN12553
ERN00190
ERN04618
ERN04237
ERM99639
ERM99505
ERM95551
ERN14045
ERN10218
ERM97066
ERM99283
ERN17701
ERN05693
DUF4690 Family
S1FA Family
Domain Color
LRRNT_2 Family
LRR_4 Repeat
LRR_8 Repeat
PK_Tyr_Ser−Thr Domain
PRIMA1 Family
Pkinase Domain
other Domain
0
500
1000
1500

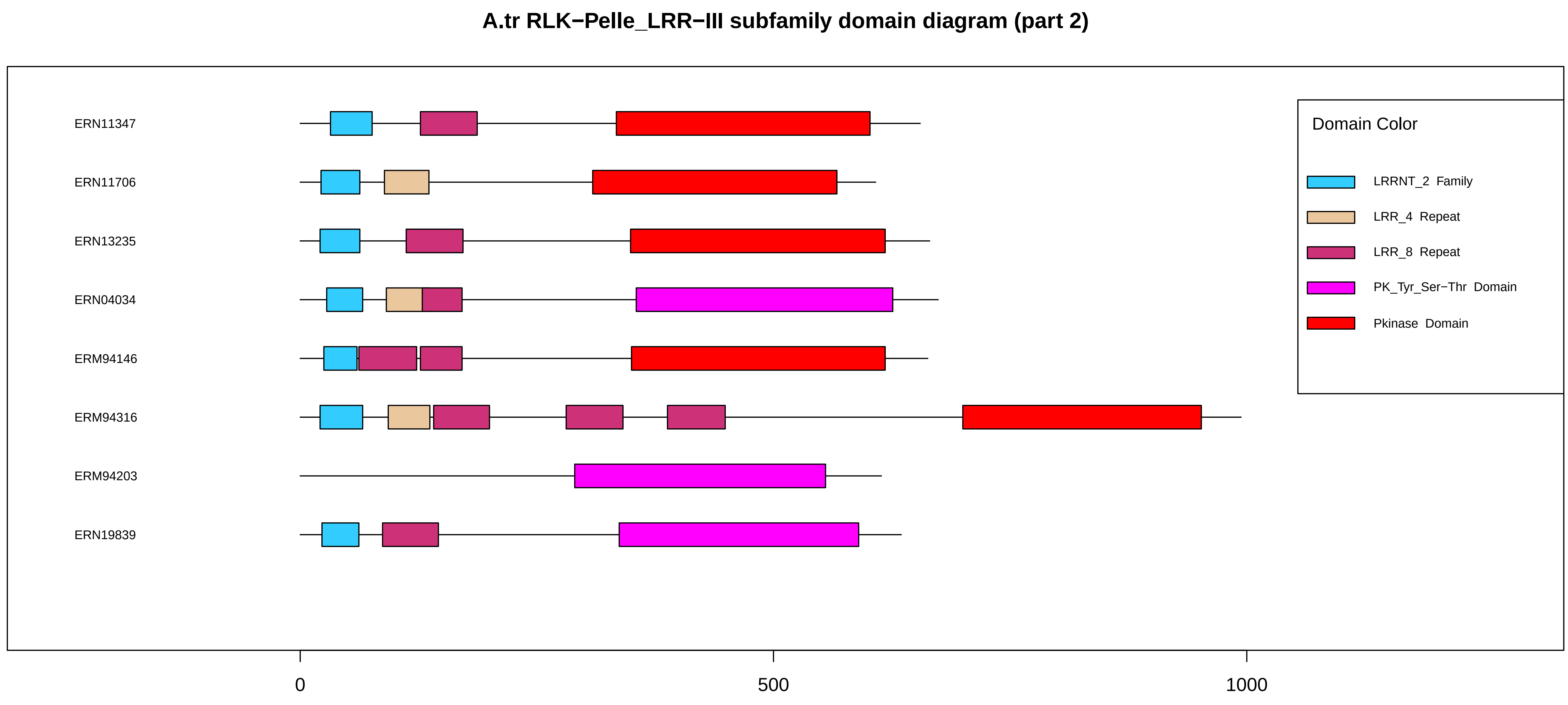
A.tr RLK−Pelle_LRR−III subfamily domain diagram (part 2)
ERN11347
ERN11706
ERN13235
ERN04034
ERM94146
ERM94316
ERM94203
ERN19839
Domain Color
LRRNT_2 Family
LRR_4 Repeat
LRR_8 Repeat
PK_Tyr_Ser−Thr Domain
Pkinase Domain
0
500
1000

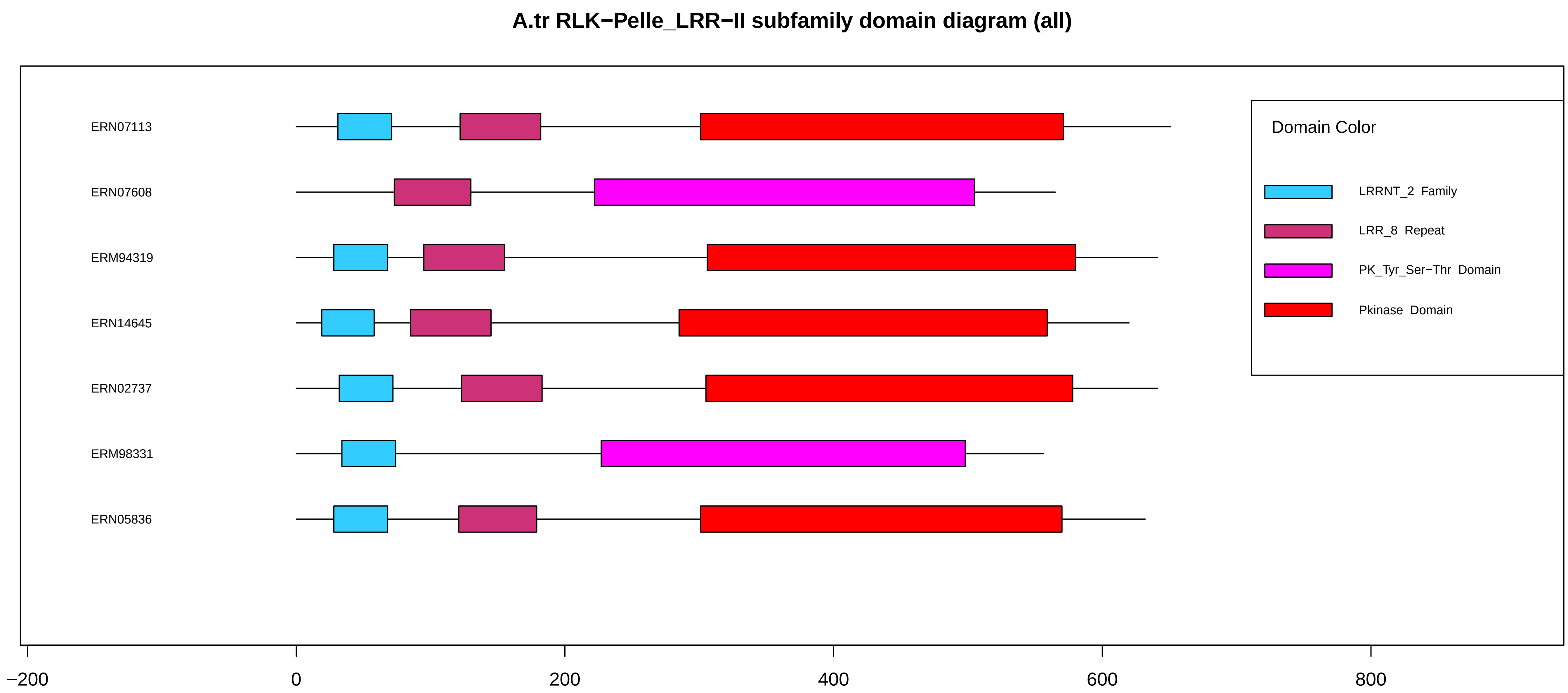

A.tr RLK−Pelle_LRR−II subfamily domain diagram (all)
ERN07113
ERN07608
ERM94319
ERN14645
ERN02737
ERM98331
ERN05836
Domain Color
LRRNT_2 Family
LRR_8 Repeat
PK_Tyr_Ser−Thr Domain
Pkinase Domain
−200
0
200
400
600
800

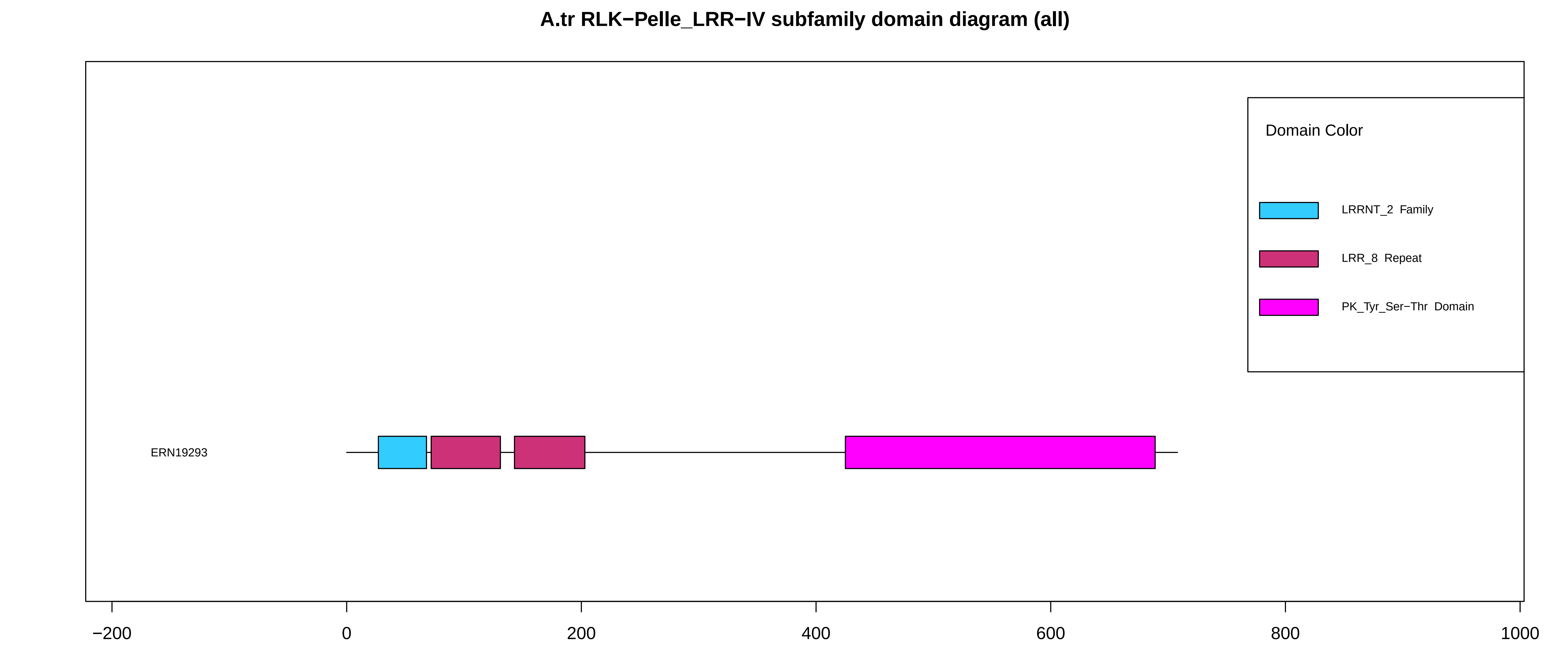

A.tr RLK−Pelle_LRR−IV subfamily domain diagram (all)
Domain Color
LRRNT_2 Family
LRR_8 Repeat
PK_Tyr_Ser−Thr Domain
ERN19293
−200
0
200
400
600
800
1000

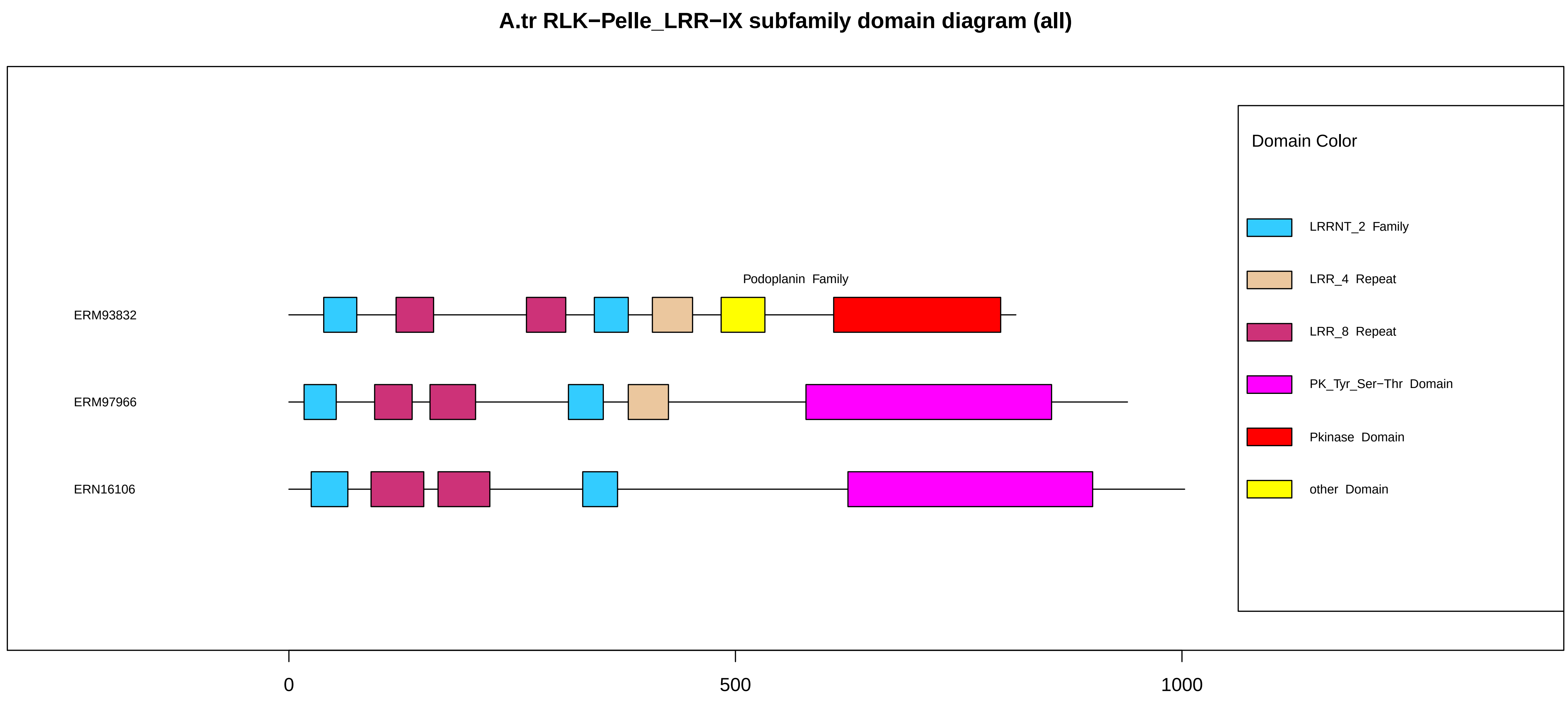
A.tr RLK−Pelle_LRR−IX subfamily domain diagram (all)
Podoplanin Family
ERM93832
ERM97966
ERN16106
Domain Color
LRRNT_2 Family
LRR_4 Repeat
LRR_8 Repeat
PK_Tyr_Ser−Thr Domain
Pkinase Domain
other Domain
0
500
1000

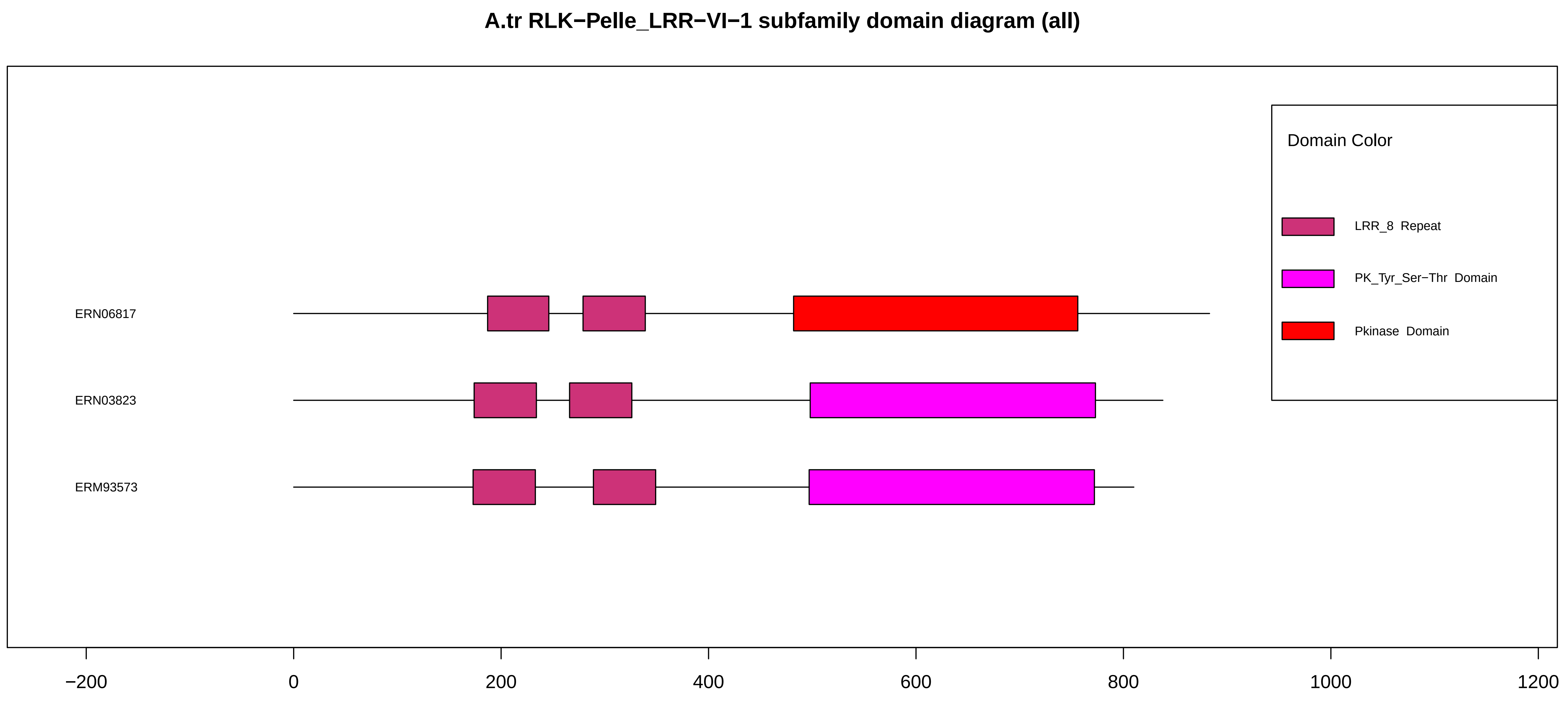
A.tr RLK−Pelle_LRR−VI−1 subfamily domain diagram (all)
ERN06817
ERN03823
ERM93573
Domain Color
LRR_8 Repeat
PK_Tyr_Ser−Thr Domain
Pkinase Domain
−200
0
200
400
600
800
1000
1200

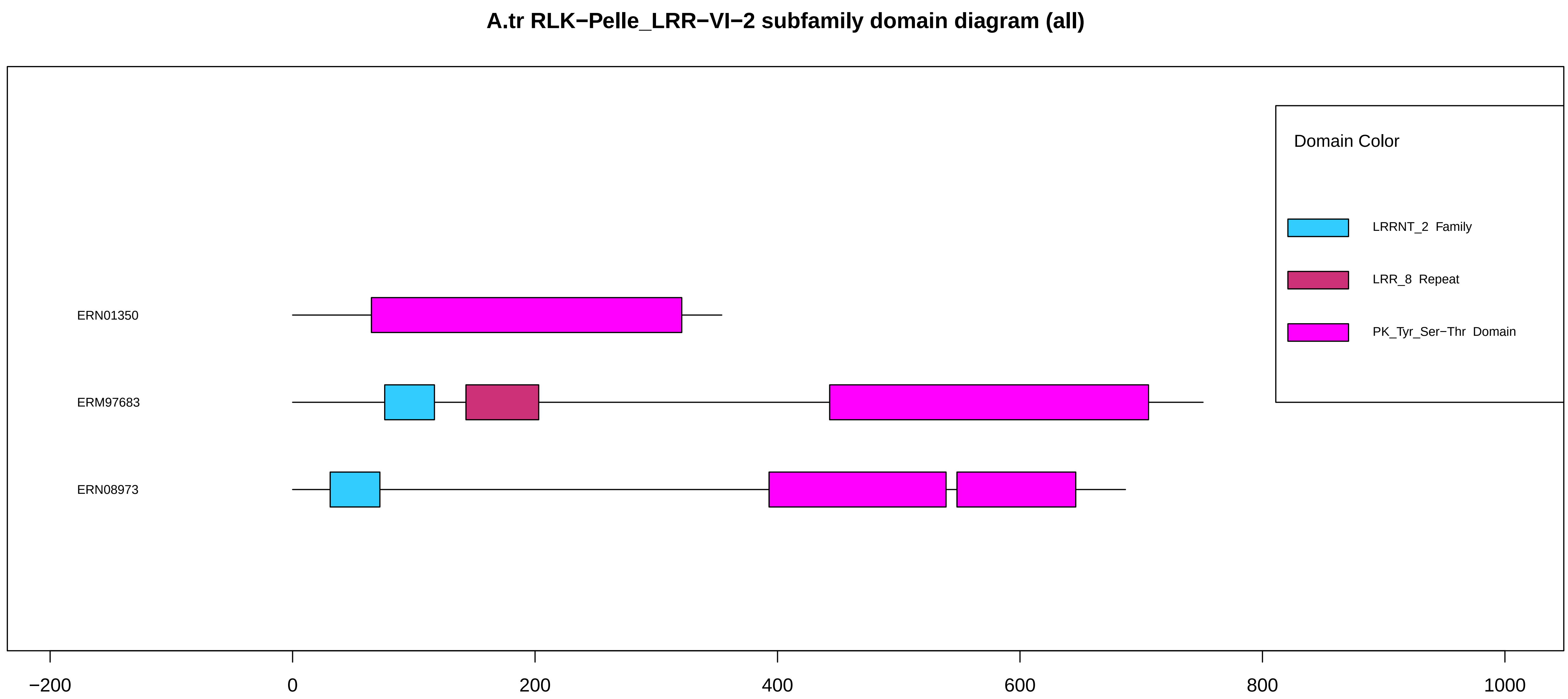
A.tr RLK−Pelle_LRR−VI−2 subfamily domain diagram (all)
Domain Color
LRRNT_2 Family
LRR_8 Repeat
PK_Tyr_Ser−Thr Domain
ERN01350
ERM97683
ERN08973
−200
0
200
400
600
800
1000

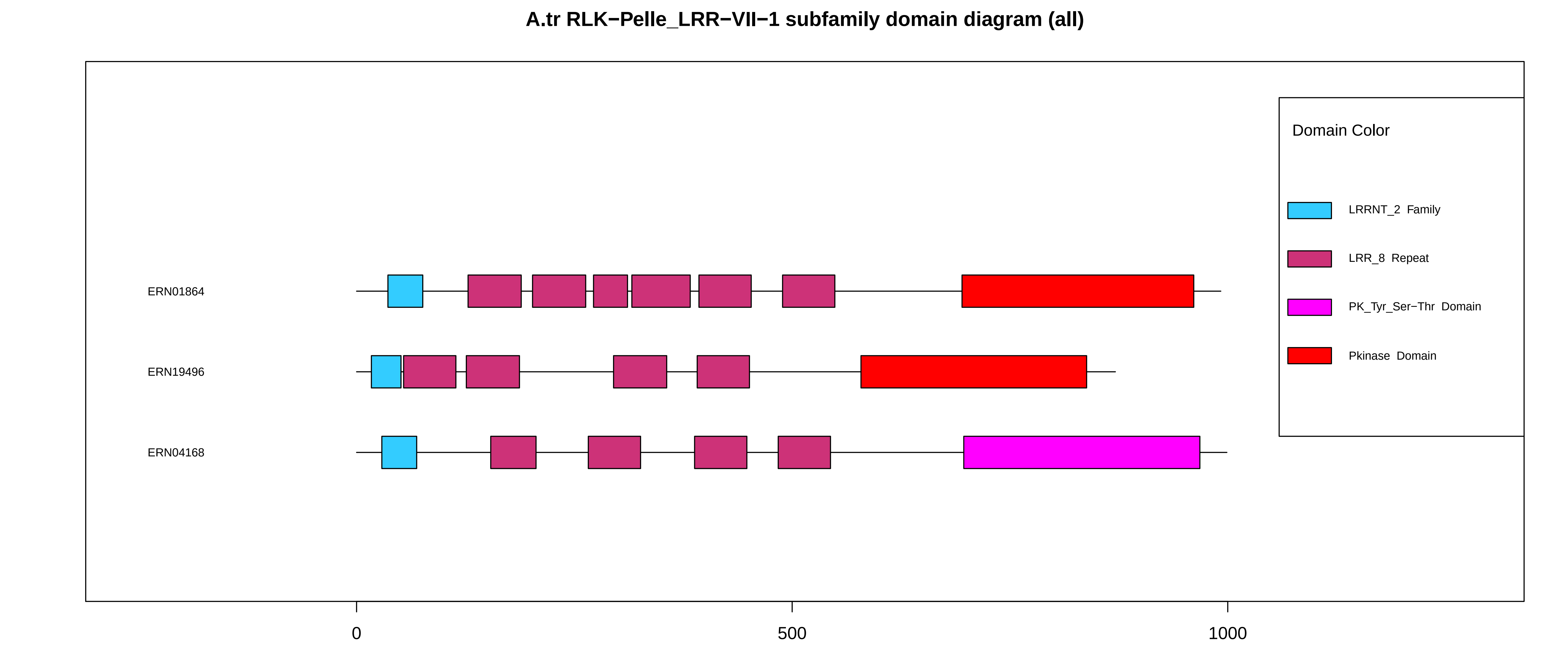
A.tr RLK−Pelle_LRR−VII−1 subfamily domain diagram (all)
ERN01864
ERN19496
ERN04168
Domain Color
LRRNT_2 Family
LRR_8 Repeat
PK_Tyr_Ser−Thr Domain
Pkinase Domain
0
500
1000

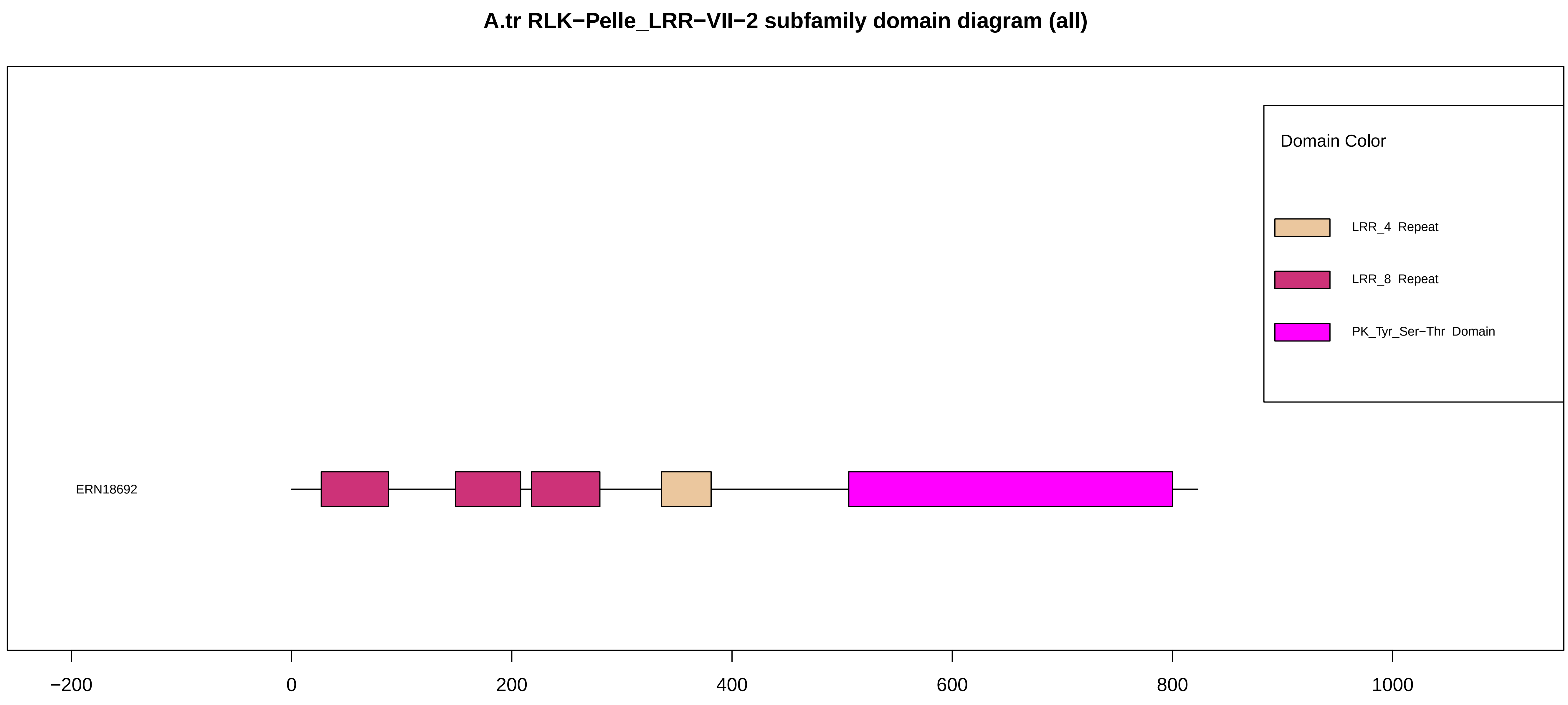

A.tr RLK−Pelle_LRR−VII−2 subfamily domain diagram (all)
Domain Color
LRR_4 Repeat
LRR_8 Repeat
PK_Tyr_Ser−Thr Domain
ERN18692
−200
0
200
400
600
800
1000

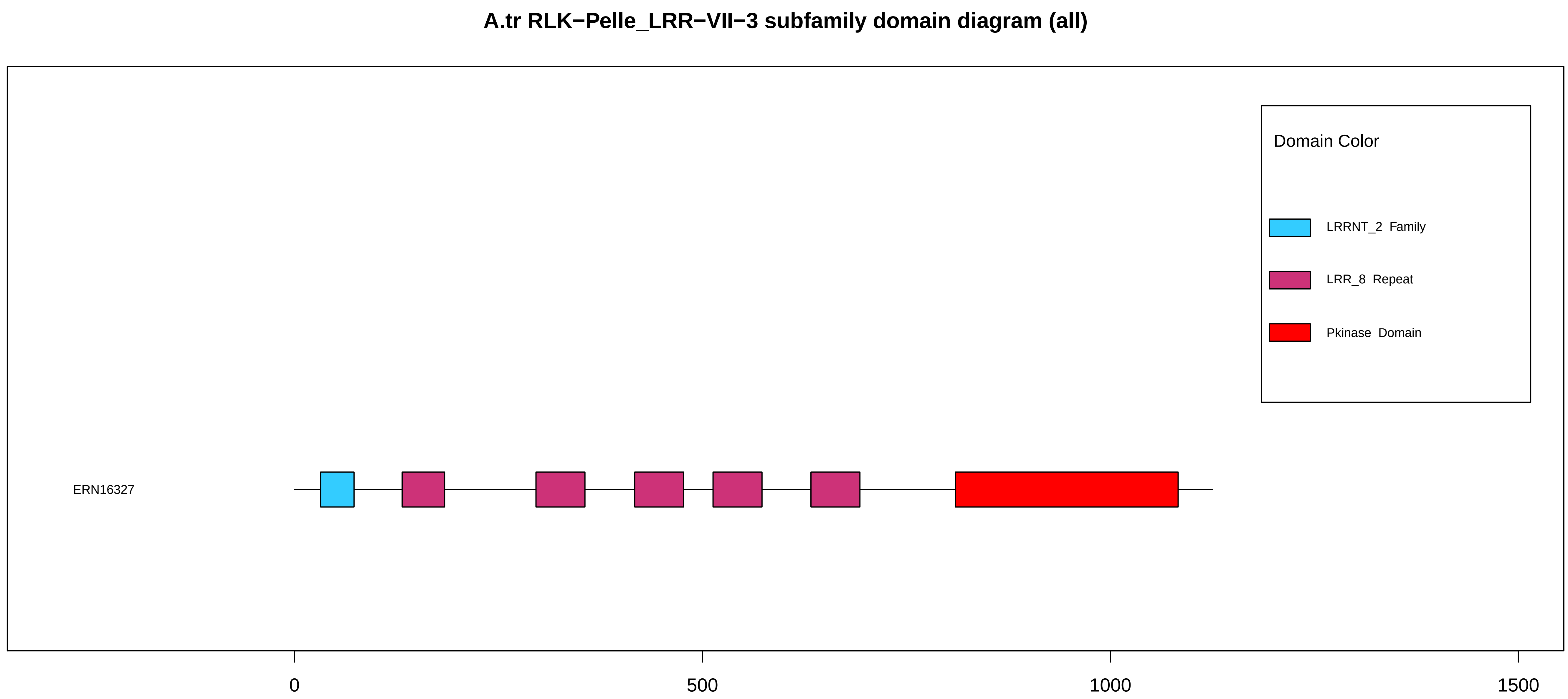

A.tr RLK−Pelle_LRR−VII−3 subfamily domain diagram (all)
Domain Color
LRRNT_2 Family
LRR_8 Repeat
Pkinase Domain
ERN16327
0
500
1000
1500

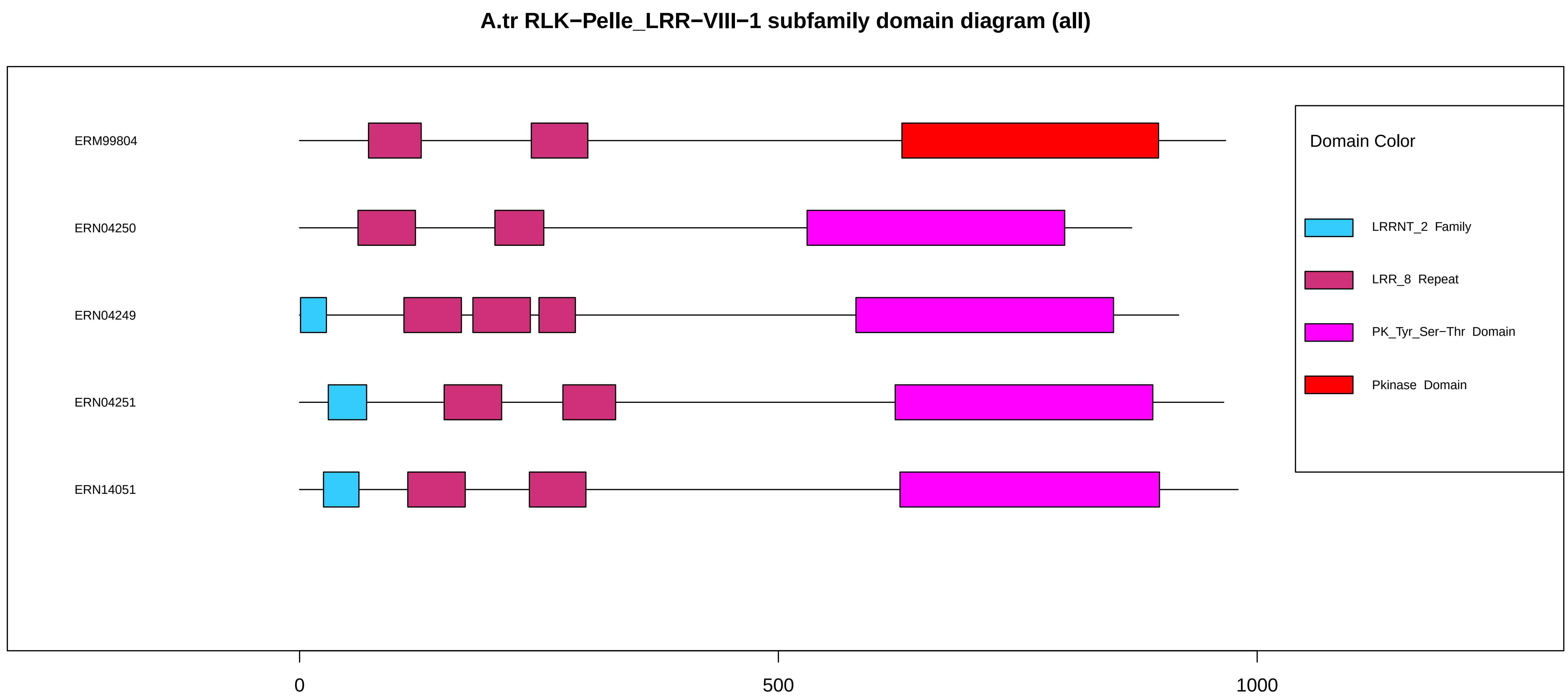
A.tr RLK−Pelle_LRR−VIII−1 subfamily domain diagram (all)
ERM99804
ERN04250
ERN04249
ERN04251
ERN14051
Domain Color
LRRNT_2 Family
LRR_8 Repeat
PK_Tyr_Ser−Thr Domain
Pkinase Domain
0
500
1000

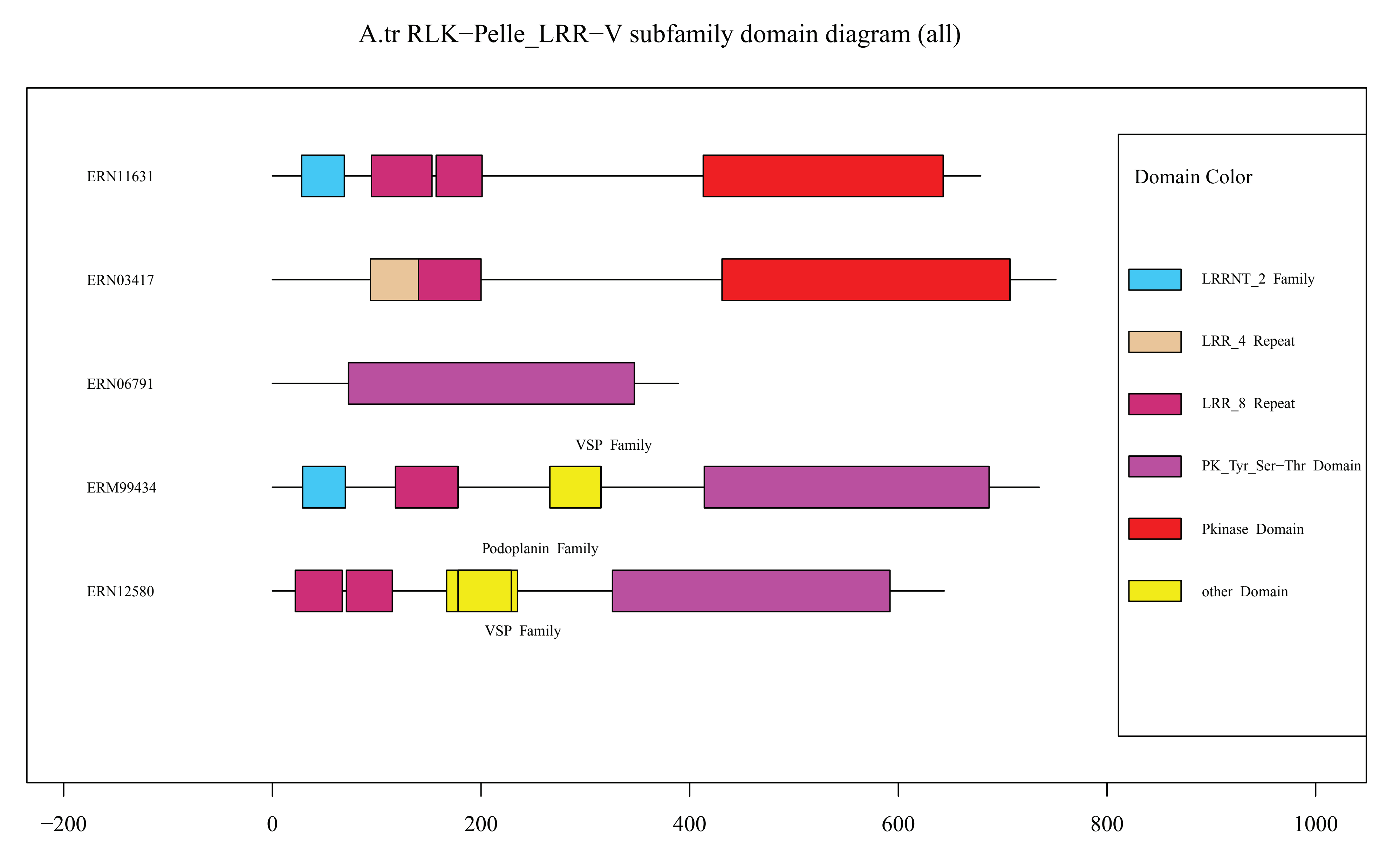
A.tr RLK−Pelle_LRR−V subfamily domain diagram (all)
ERN11631
ERN03417
ERN06791
ERM99434
ERN12580
VSP Family
Podoplanin Family
VSP Family
Domain Color
LRRNT_2 Family
LRR_4 Repeat
LRR_8 Repeat
PK_Tyr_Ser−Thr Domain
Pkinase Domain
other Domain
−200
0
200
400
600
800
1000

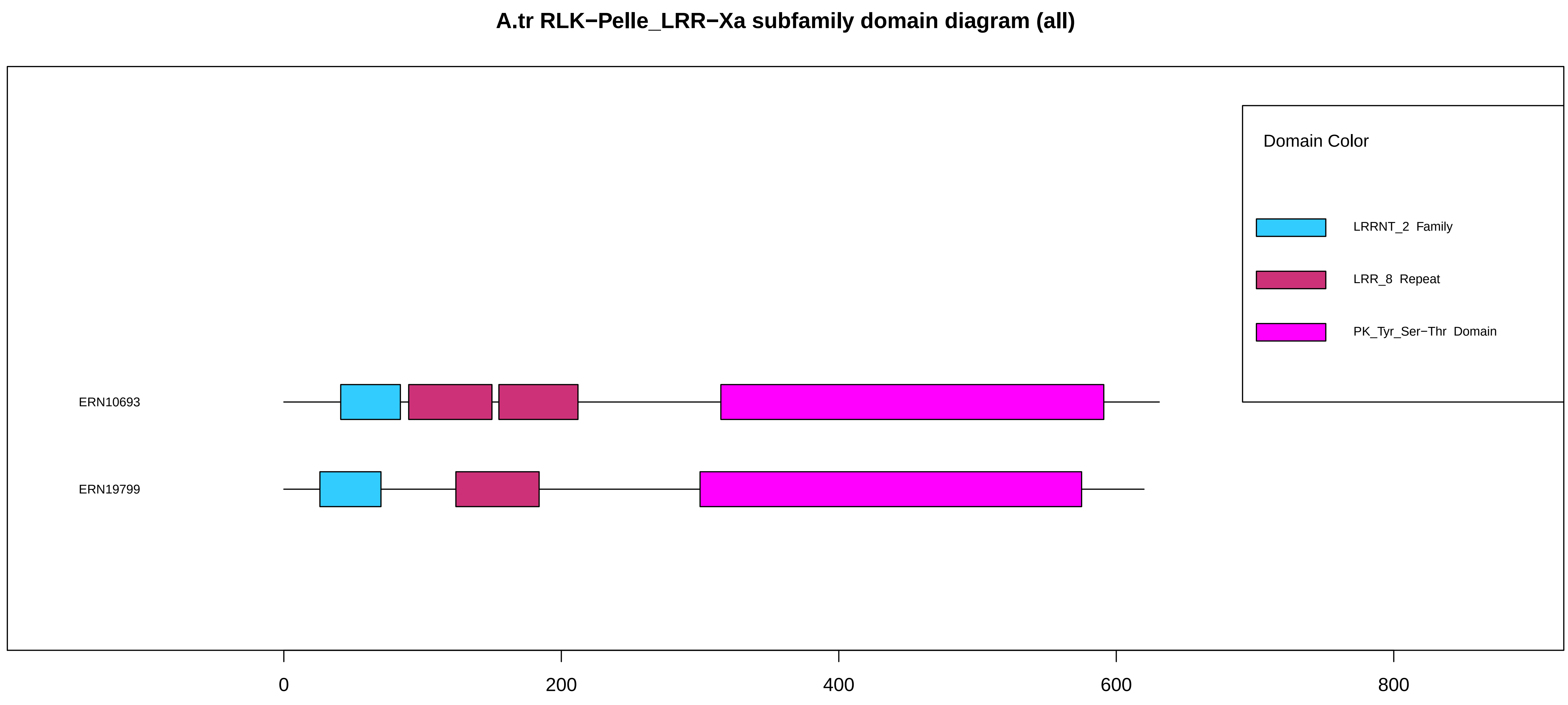
A.tr RLK−Pelle_LRR−Xa subfamily domain diagram (all)
Domain Color
LRRNT_2 Family
LRR_8 Repeat
PK_Tyr_Ser−Thr Domain
ERN10693
ERN19799
0
200
400
600
800

A.tr RLK−Pelle_LRR−Xb−1 subfamily domain diagram (all)

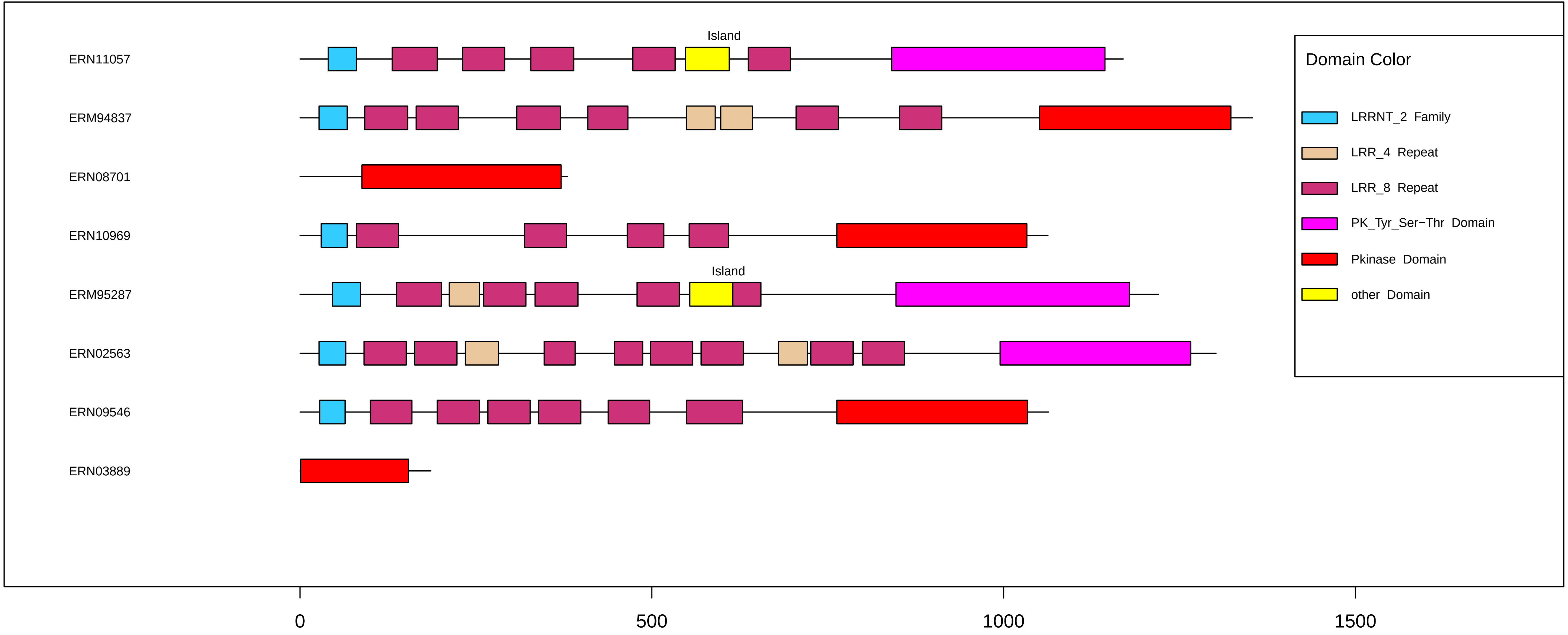

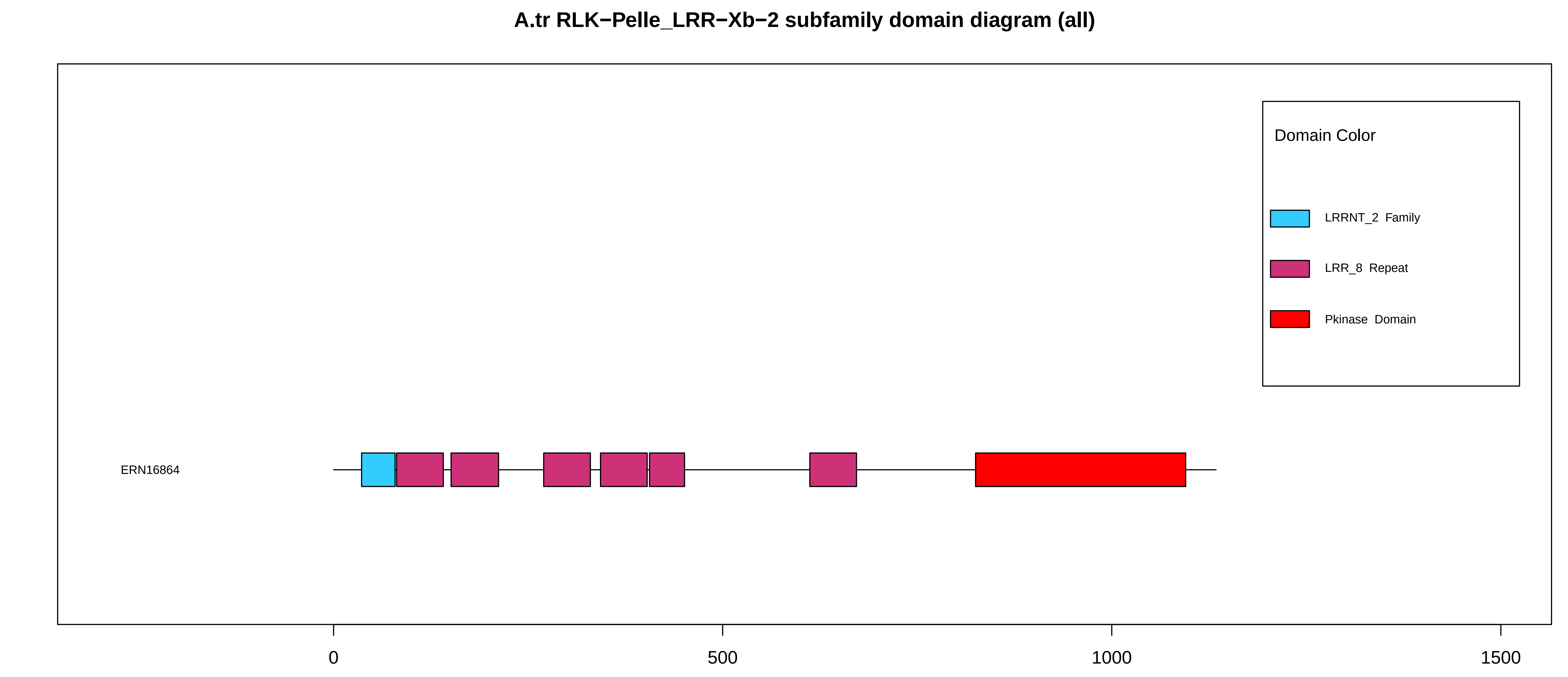

A.tr RLK−Pelle_LRR−Xb−2 subfamily domain diagram (all)
Domain Color
LRRNT_2 Family
LRR_8 Repeat
Pkinase Domain
ERN16864
0
500
1000
1500

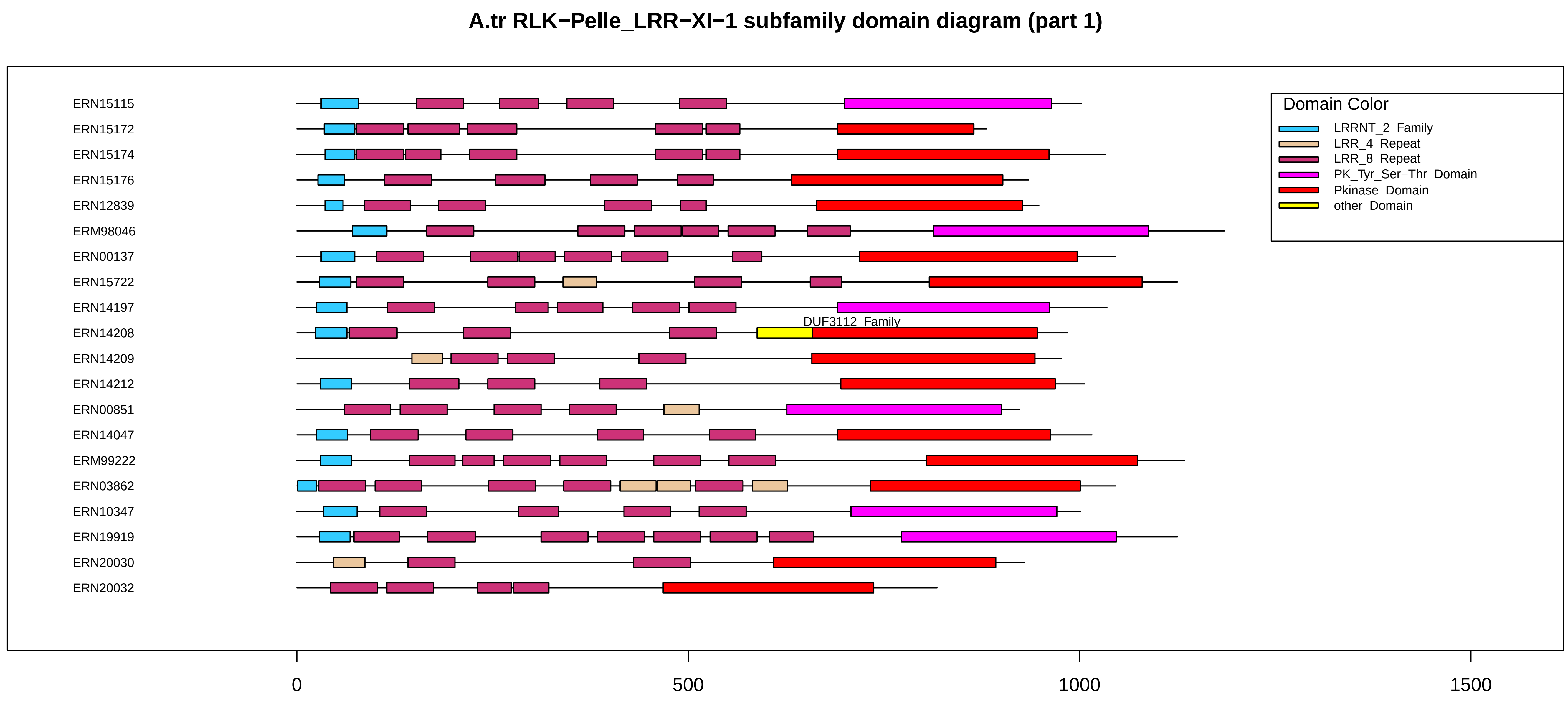
A.tr RLK−Pelle_LRR−XI−1 subfamily domain diagram (part 1)
ERN15115
ERN15172
ERN15174
ERN15176
ERN12839
ERM98046
ERN00137
ERN15722
ERN14197
ERN14208
ERN14209
ERN14212
ERN00851
ERN14047
ERM99222
ERN03862
ERN10347
ERN19919
ERN20030
ERN20032
DUF3112 Family
Domain Color
LRRNT_2 Family
LRR_4 Repeat
LRR_8 Repeat
PK_Tyr_Ser−Thr Domain
Pkinase Domain
other Domain
0
500
1000
1500

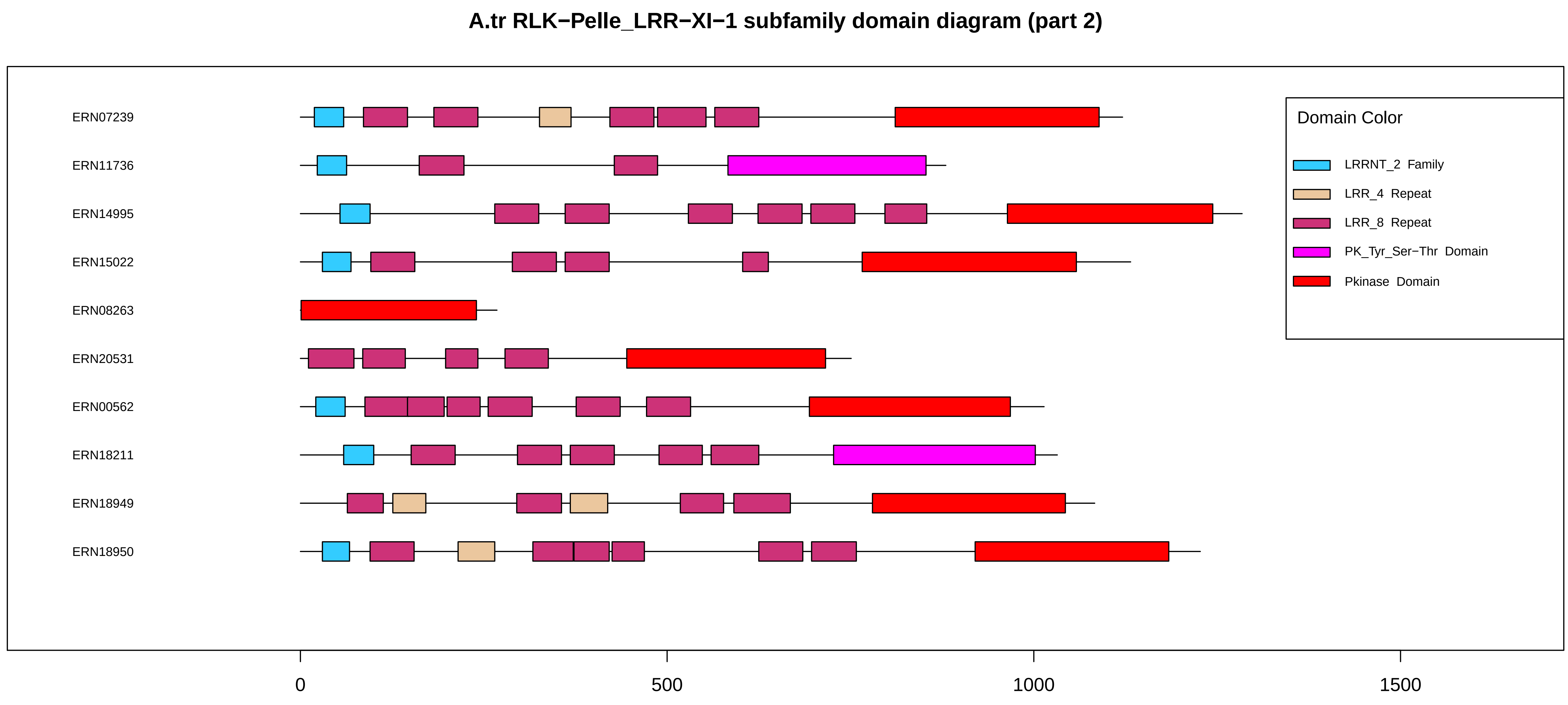
A.tr RLK−Pelle_LRR−XI−1 subfamily domain diagram (part 2)
ERN07239
ERN11736
ERN14995
ERN15022
ERN08263
ERN20531
ERN00562
ERN18211
ERN18949
ERN18950
Domain Color
LRRNT_2 Family
LRR_4 Repeat
LRR_8 Repeat
PK_Tyr_Ser−Thr Domain
Pkinase Domain
0
500
1000
1500

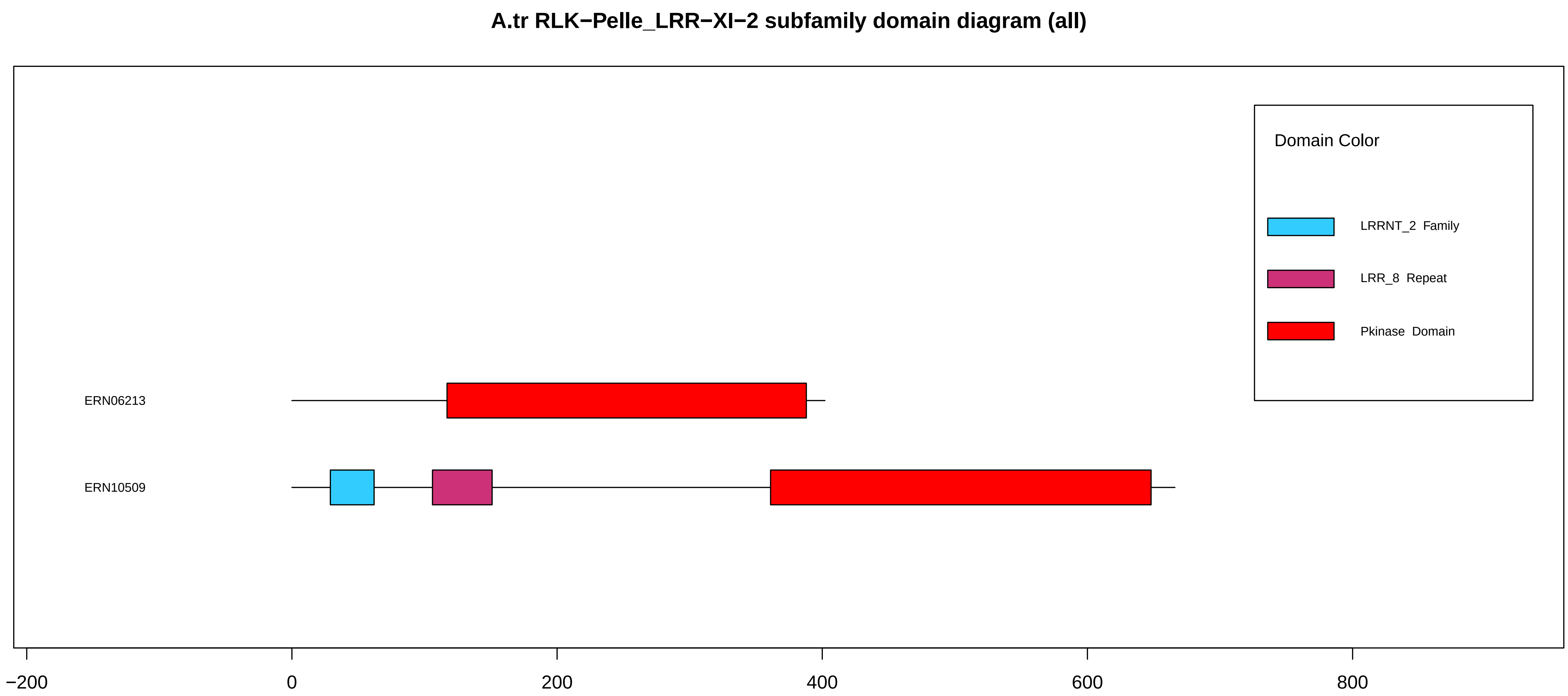

A.tr RLK−Pelle_LRR−XI−2 subfamily domain diagram (all)
Domain Color
LRRNT_2 Family
LRR_8 Repeat
Pkinase Domain
ERN06213
ERN10509
−200
0
200
400
600
800

A.tr RLK−Pelle_LRR−XII−1 subfamily domain diagram (all)

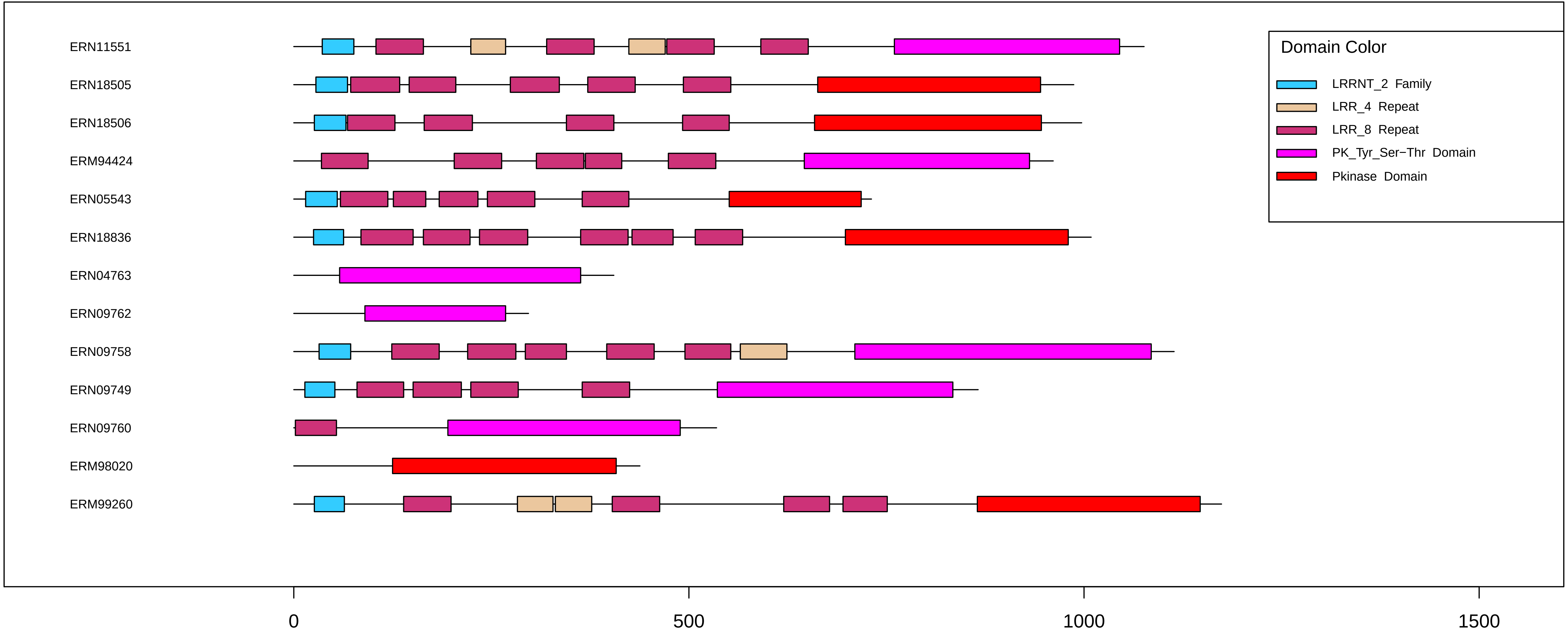

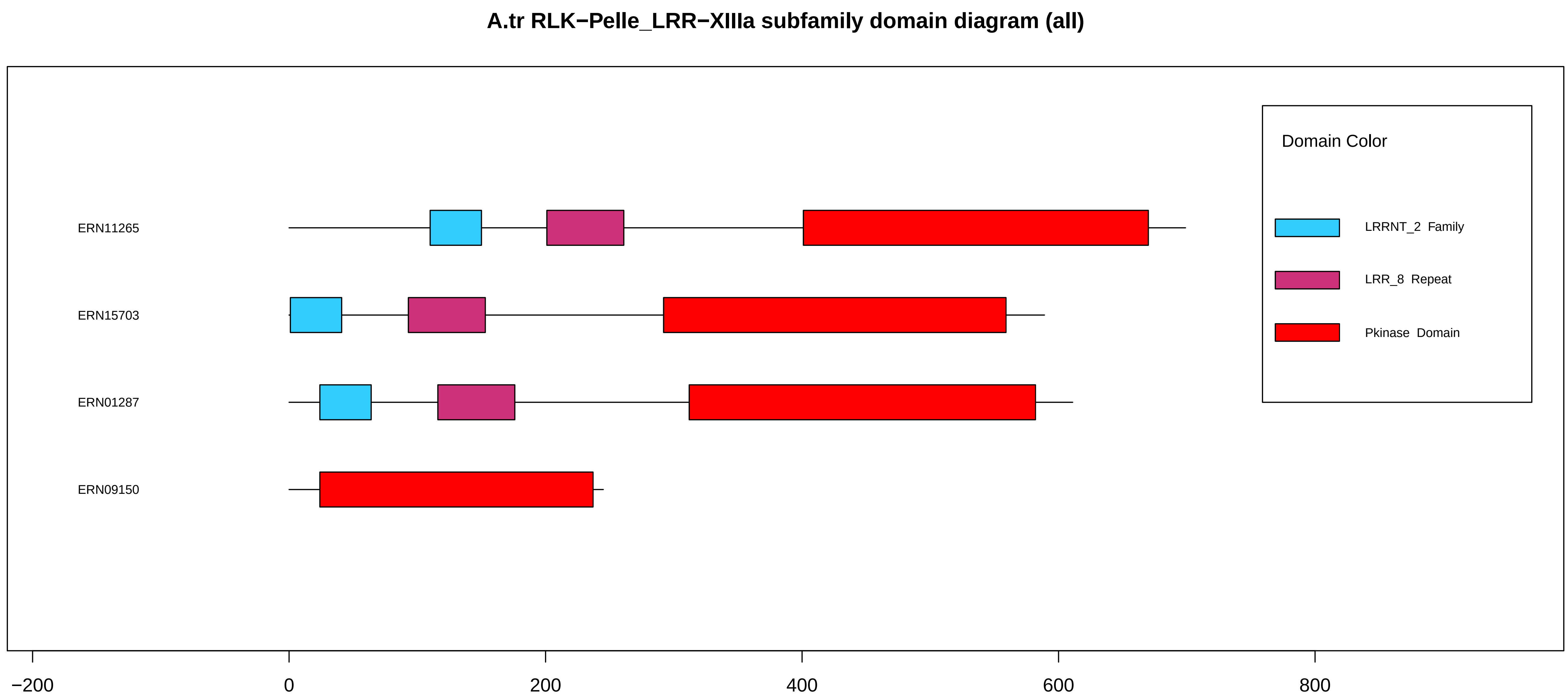
A.tr RLK−Pelle_LRR−XIIIa subfamily domain diagram (all)
ERN11265
ERN15703
ERN01287
ERN09150
Domain Color
LRRNT_2 Family
LRR_8 Repeat
Pkinase Domain
−200
0
200
400
600
800

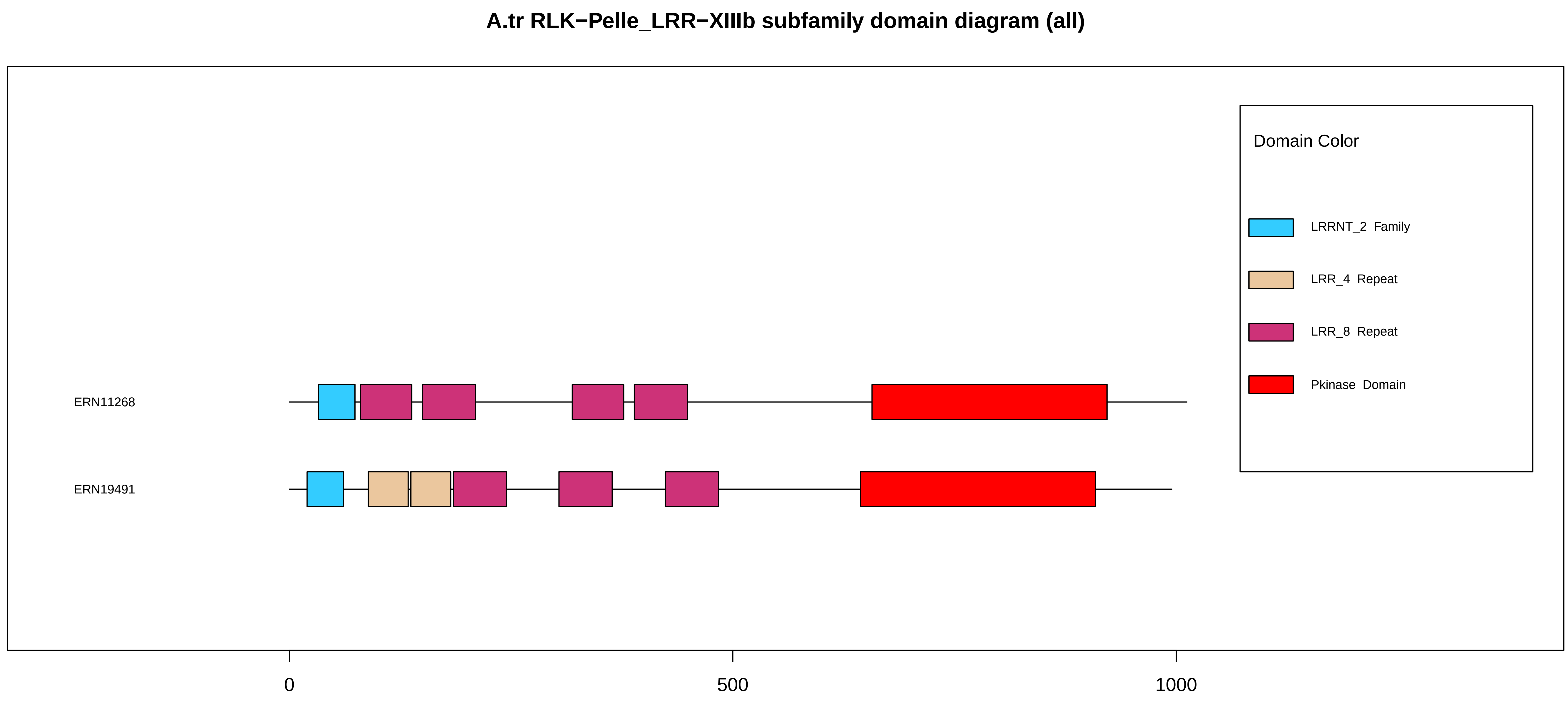
A.tr RLK−Pelle_LRR−XIIIb subfamily domain diagram (all)
ERN11268
ERN19491
0
500
1000
Domain Color
LRRNT_2 Family
LRR_4 Repeat
LRR_8 Repeat
Pkinase Domain

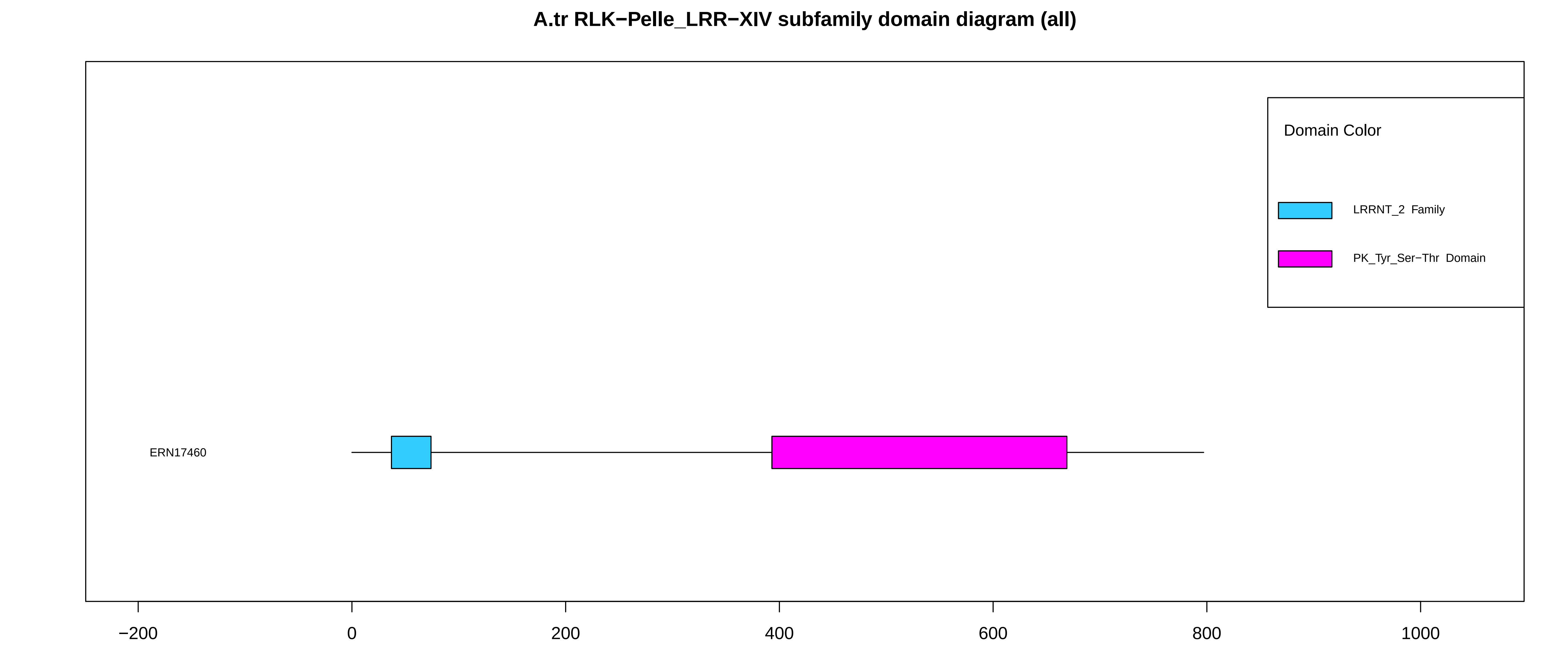
A.tr RLK−Pelle_LRR−XIV subfamily domain diagram (all)
Domain Color
LRRNT_2 Family
PK_Tyr_Ser−Thr Domain
ERN17460
−200
0
200
400
600
800
1000

A.tr RLK−Pelle_LRR−XV subfamily domain diagram (all)

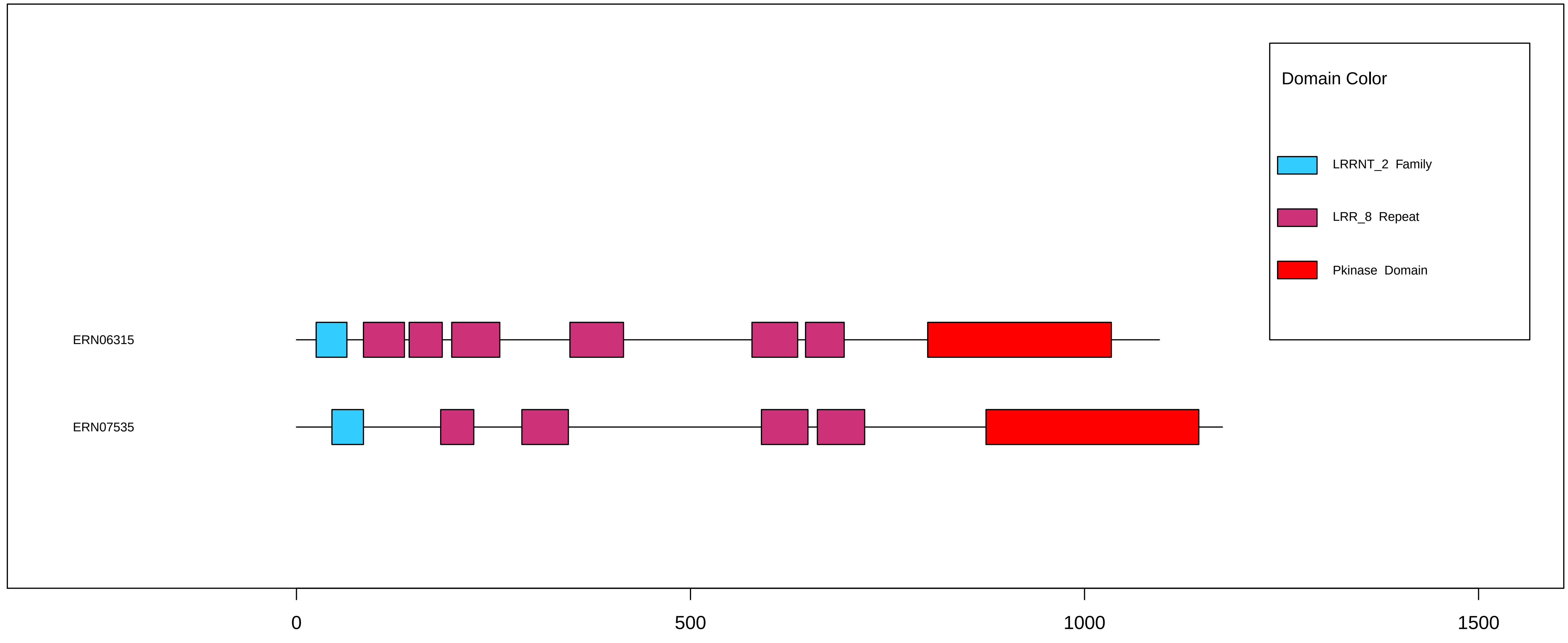

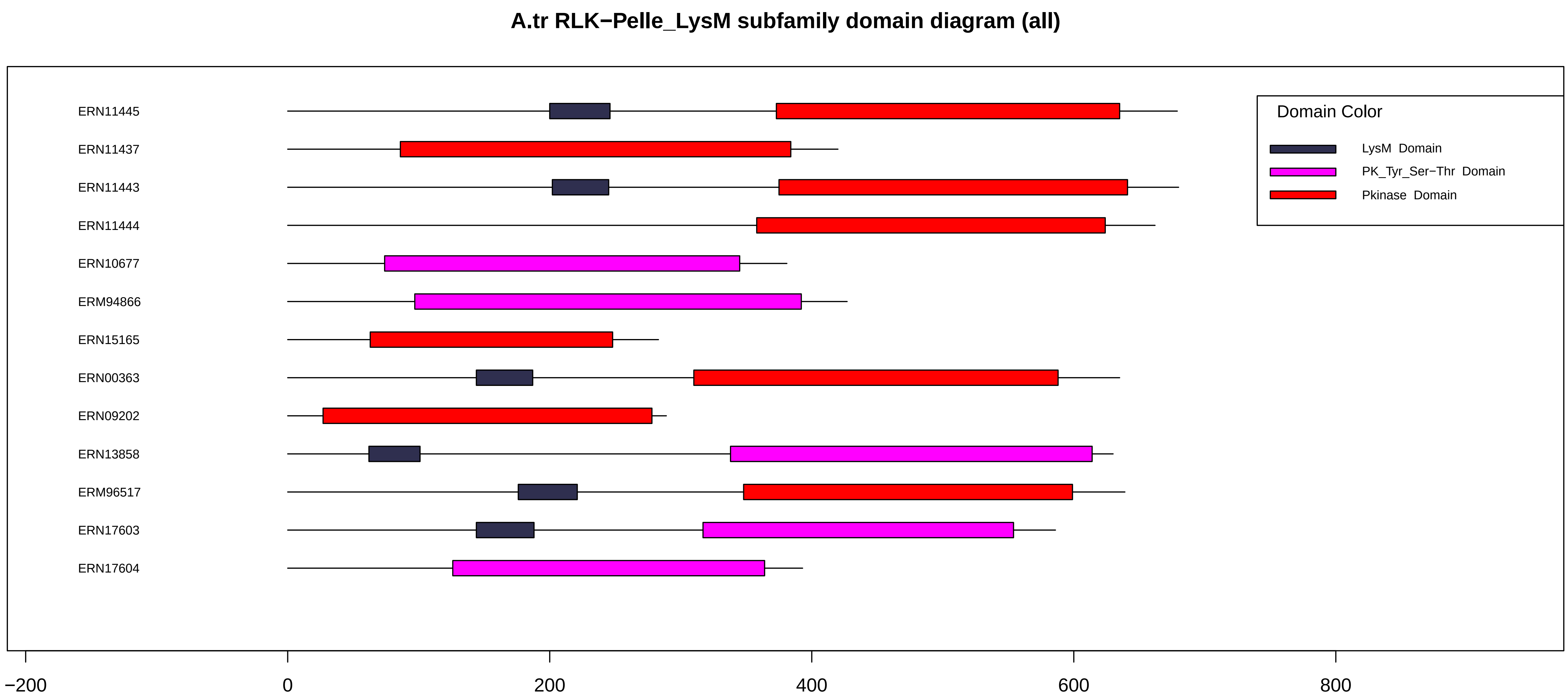
A.tr RLK−Pelle_LysM subfamily domain diagram (all)
ERN11445
ERN11437
ERN11443
ERN11444
ERN10677
ERM94866
ERN15165
ERN00363
ERN09202
ERN13858
ERM96517
ERN17603
ERN17604
Domain Color
LysM Domain
PK_Tyr_Ser−Thr Domain
Pkinase Domain
−200
0
200
400
600
800

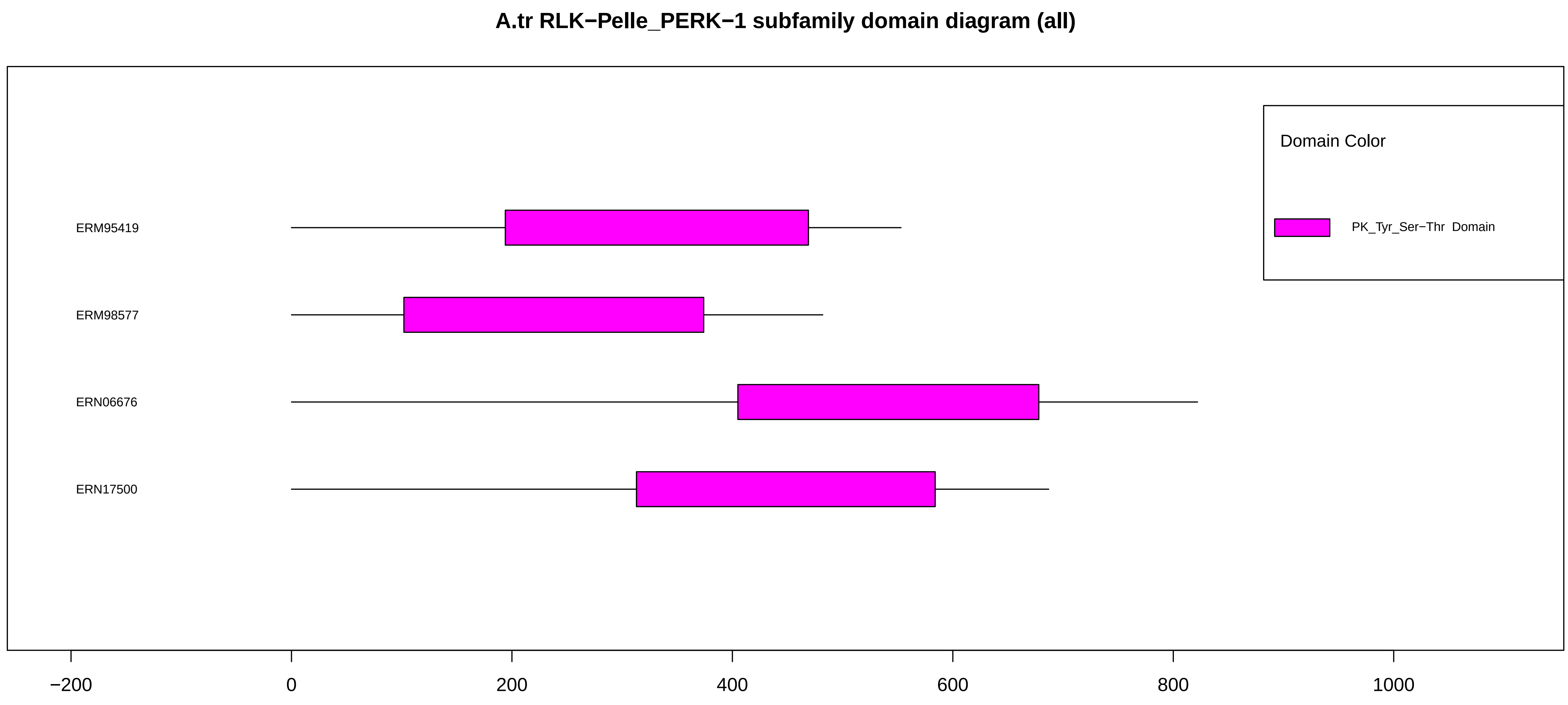

A.tr RLK−Pelle_PERK−1 subfamily domain diagram (all)
ERM95419
ERM98577
ERN06676
ERN17500
Domain Color
PK_Tyr_Ser−Thr Domain
−200
0
200
400
600
800
1000

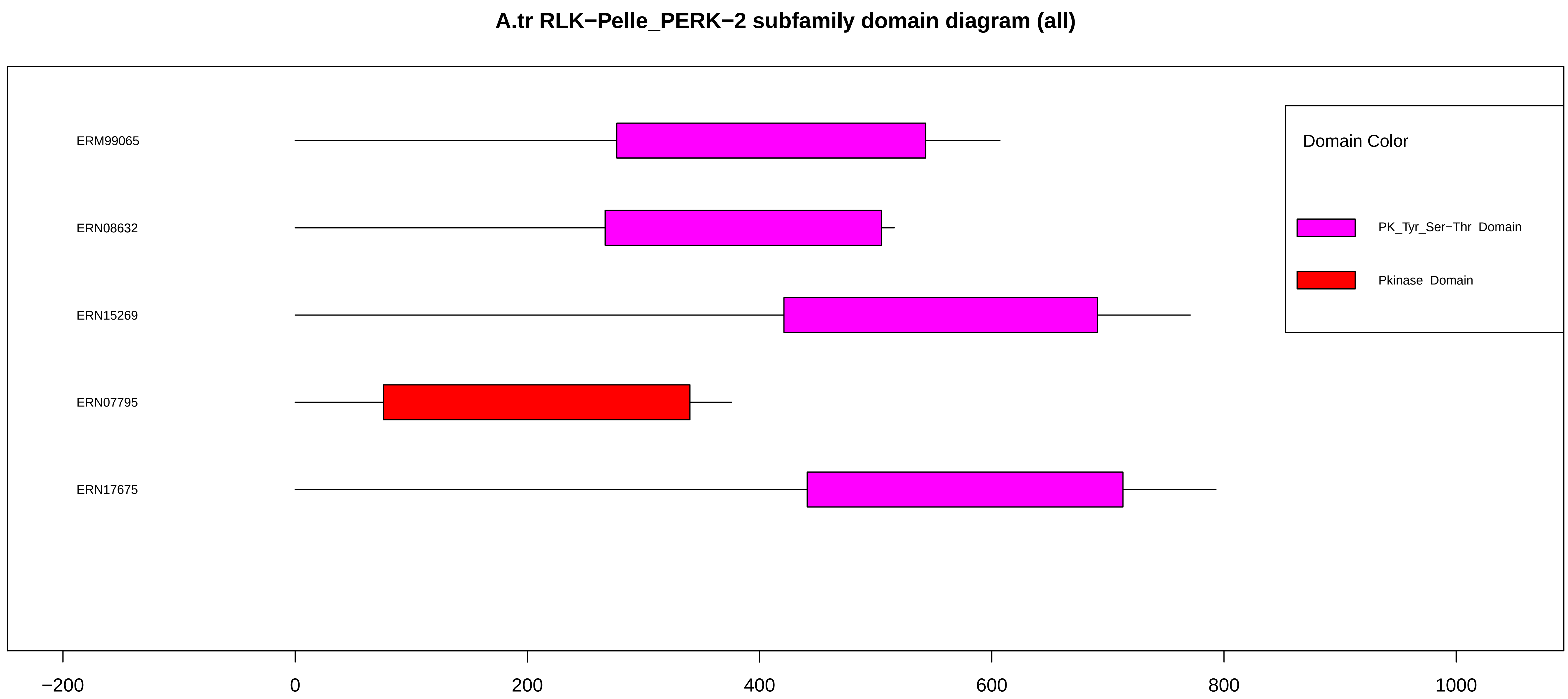
A.tr RLK−Pelle_PERK−2 subfamily domain diagram (all)
ERM99065
ERN08632
ERN15269
ERN07795
ERN17675
Domain Color
PK_Tyr_Ser−Thr Domain
Pkinase Domain
−200
0
200
400
600
800
1000

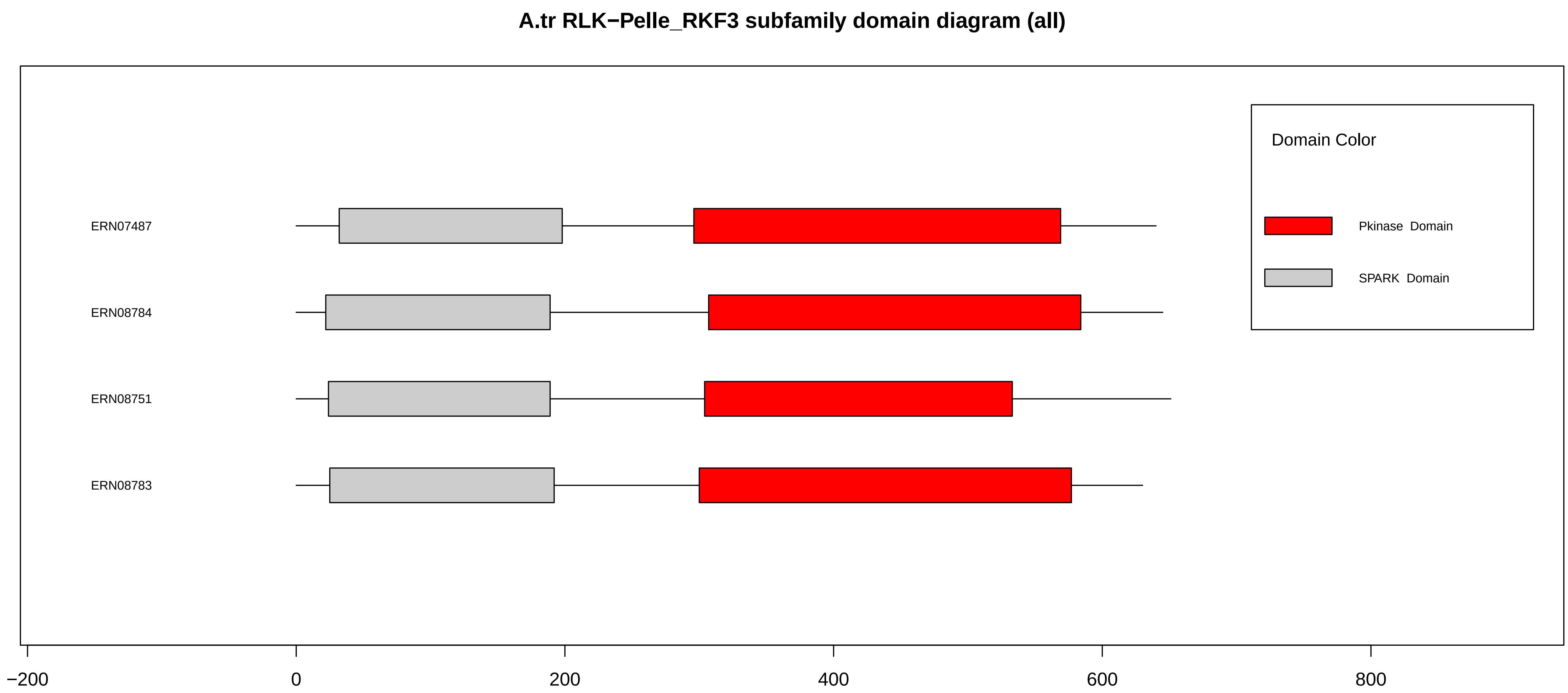
A.tr RLK−Pelle_RKF3 subfamily domain diagram (all)
ERN07487
ERN08784
ERN08751
ERN08783
Domain Color
Pkinase Domain
SPARK Domain
−200
0
200
400
600
800

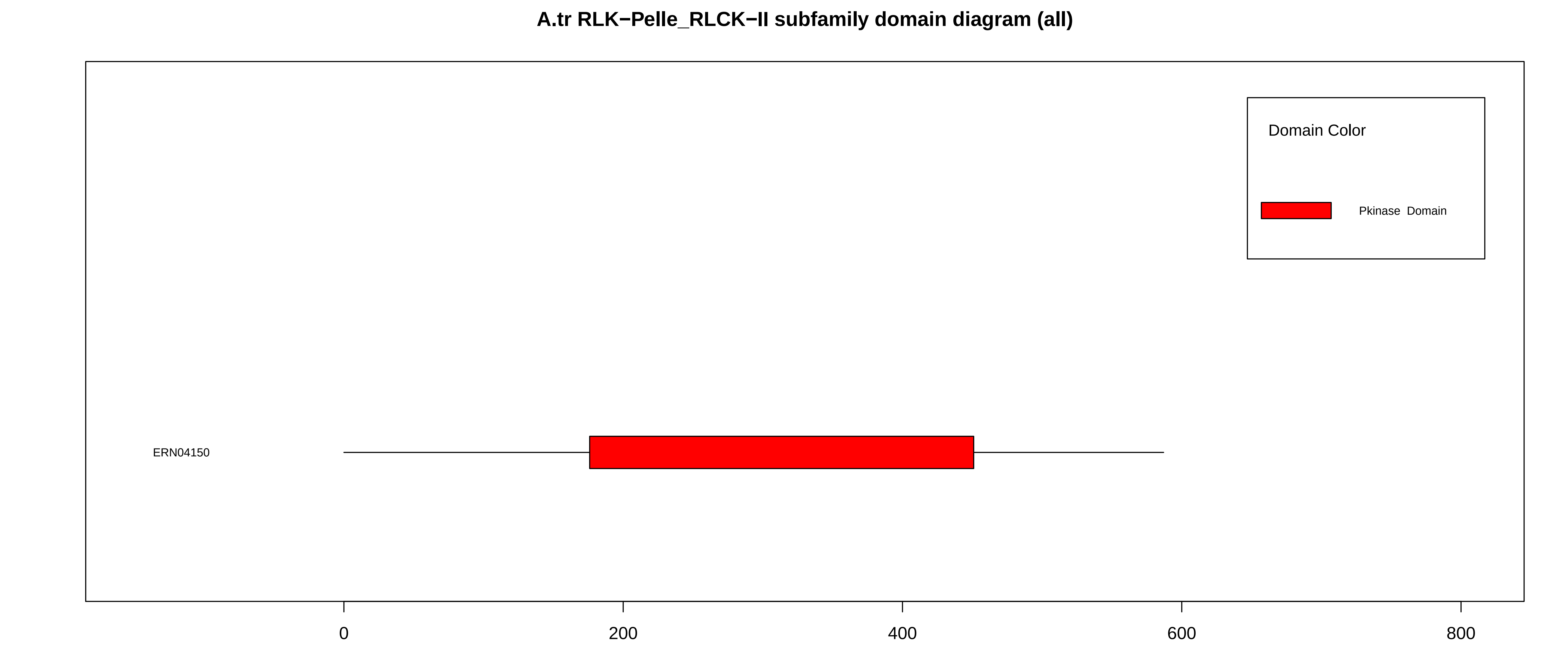
A.tr RLK−Pelle_RLCK−II subfamily domain diagram (all)
Domain Color
Pkinase Domain
ERN04150
0
200
400
600
800

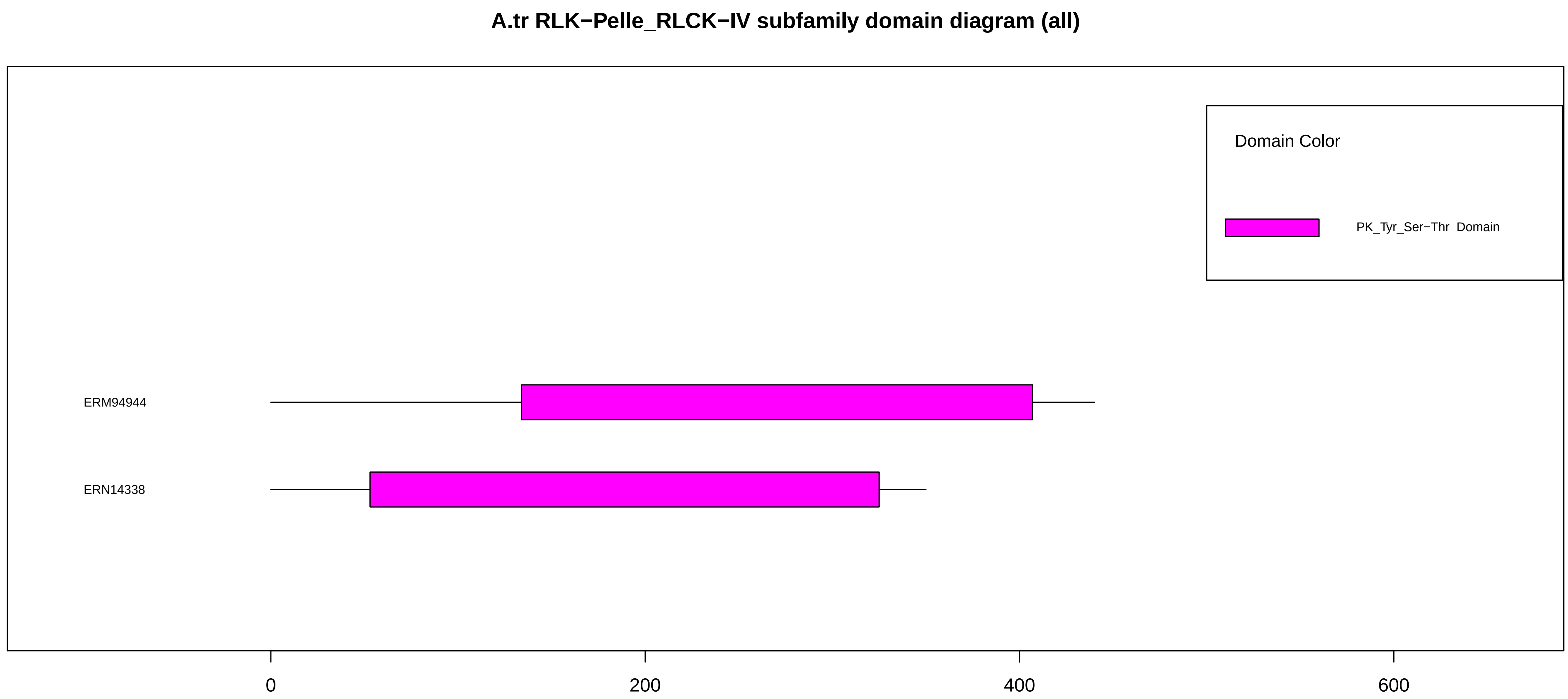
A.tr RLK−Pelle_RLCK−IV subfamily domain diagram (all)
Domain Color
PK_Tyr_Ser−Thr Domain
ERM94944
ERN14338
0
200
400
600

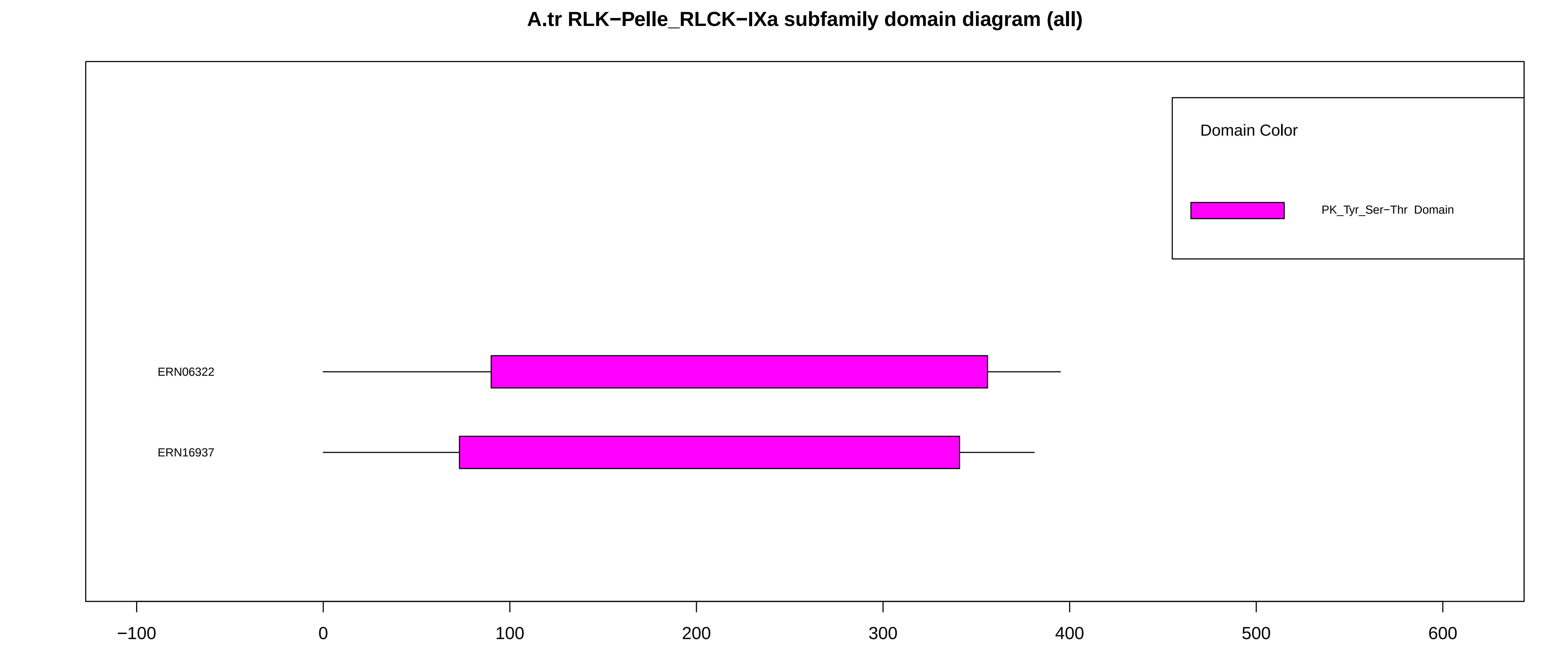
A.tr RLK−Pelle_RLCK−IXa subfamily domain diagram (all)
Domain Color
PK_Tyr_Ser−Thr Domain
ERN06322
ERN16937
−100
0
100
200
300
400
500
600

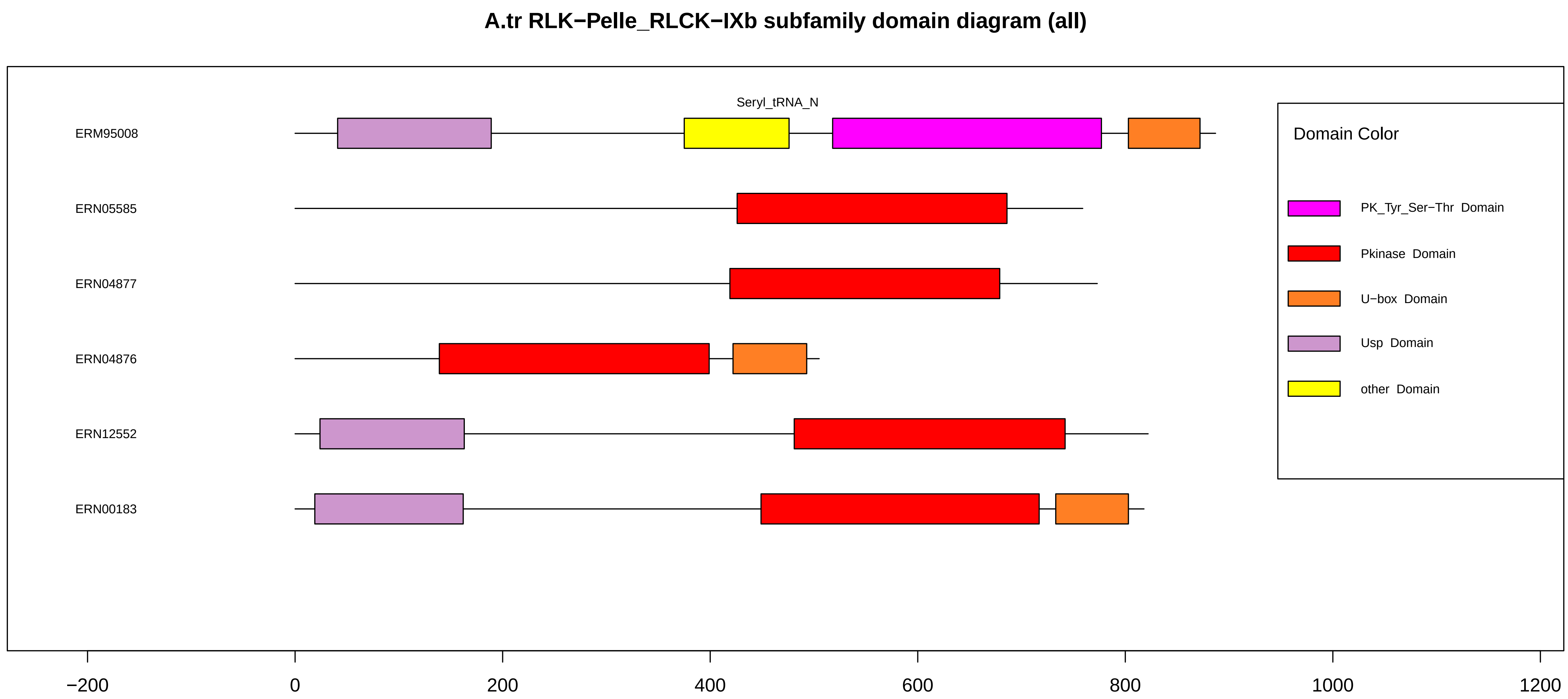
A.tr RLK−Pelle_RLCK−IXb subfamily domain diagram (all)
Seryl_tRNA_N
ERM95008
ERN05585
ERN04877
ERN04876
ERN12552
ERN00183
Domain Color
PK_Tyr_Ser−Thr Domain
Pkinase Domain
U−box Domain
Usp Domain
other Domain
−200
0
200
400
600
800
1000
1200

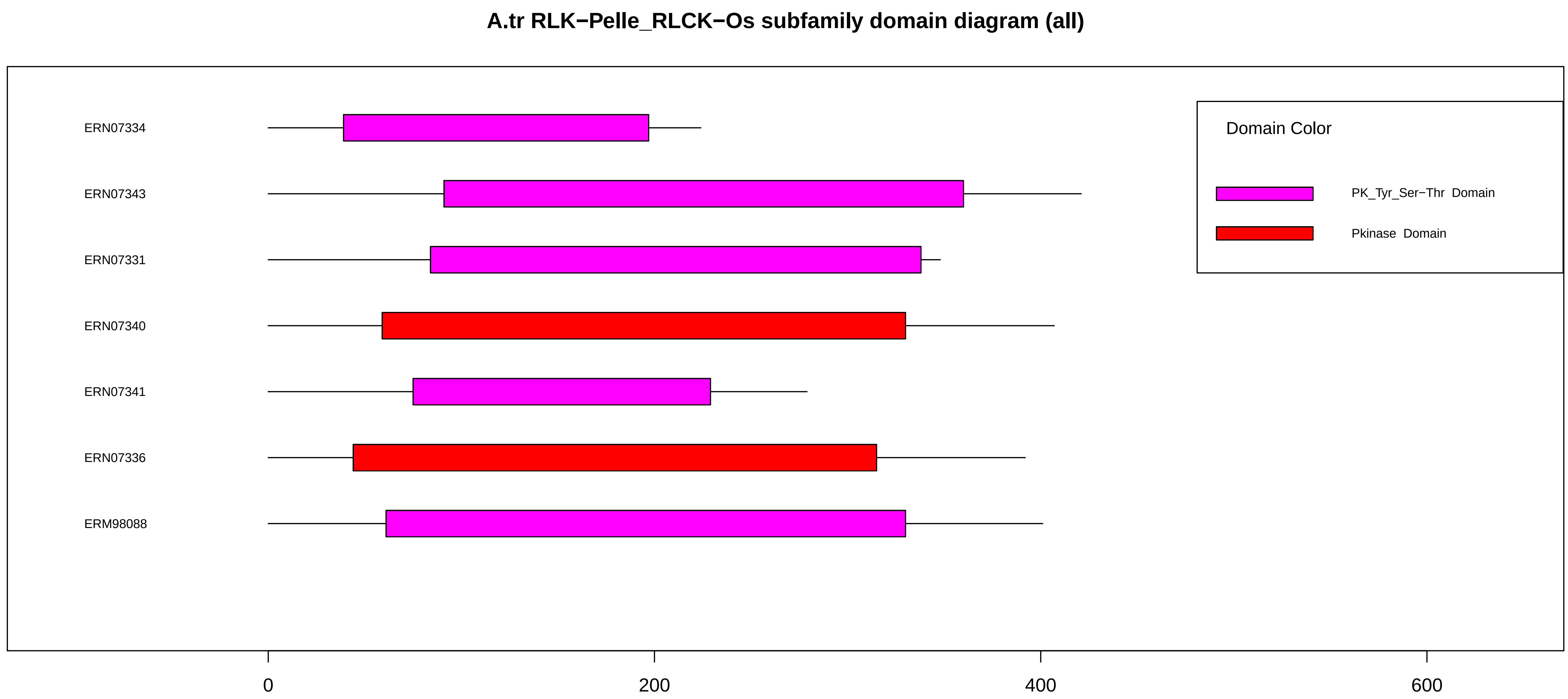
A.tr RLK−Pelle_RLCK−Os subfamily domain diagram (all)
ERN07334
ERN07343
ERN07331
ERN07340
ERN07341
ERN07336
ERM98088
Domain Color
PK_Tyr_Ser−Thr Domain
Pkinase Domain
0
200
400
600

A.tr RLK−Pelle_RLCK−VIIa−1 subfamily domain diagram (all)

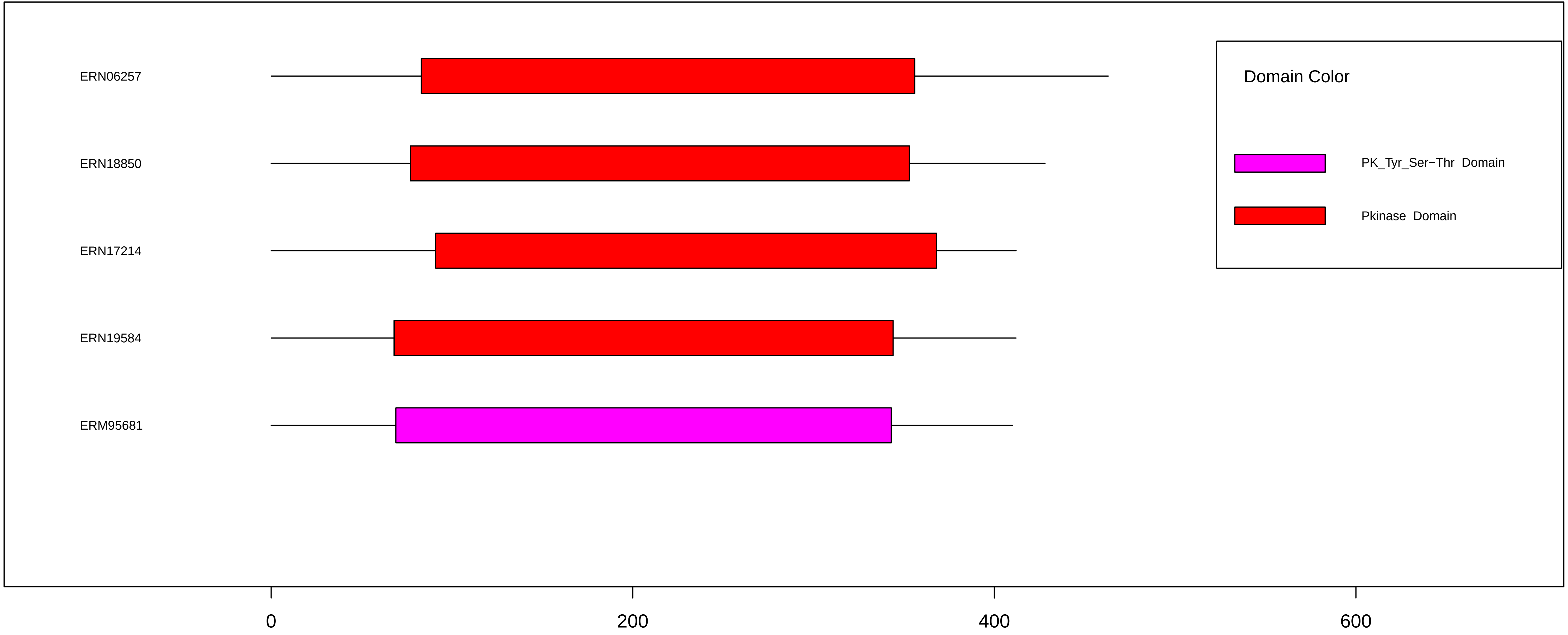

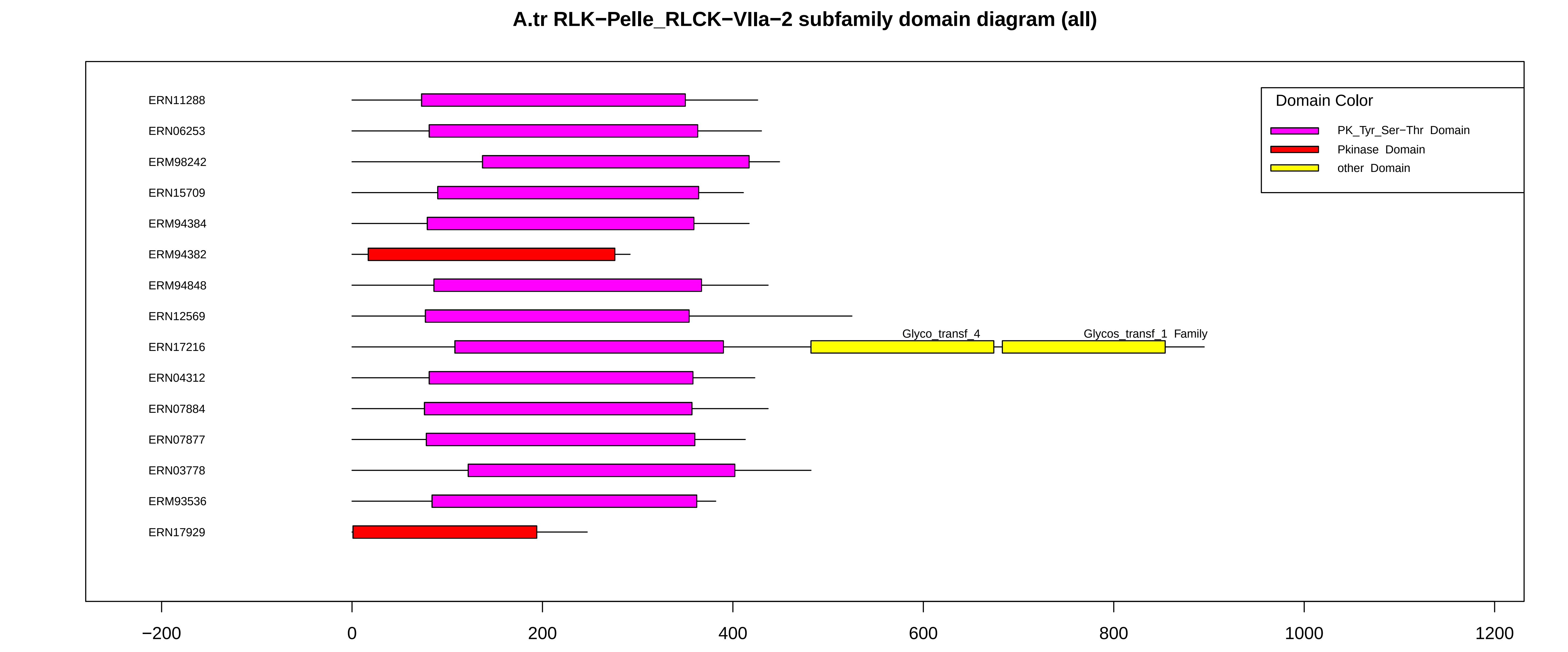

A.tr RLK−Pelle_RLCK−VIIa−2 subfamily domain diagram (all)
ERN11288
ERN06253
ERM98242
ERN15709
ERM94384
ERM94382
ERM94848
ERN12569
ERN17216
ERN04312
ERN07884
ERN07877
ERN03778
ERM93536
ERN17929
Glyco_transf_4
Glycos_transf_1 Family
Domain Color
PK_Tyr_Ser−Thr Domain
Pkinase Domain
other Domain
−200
0
200
400
600
800
1000
1200

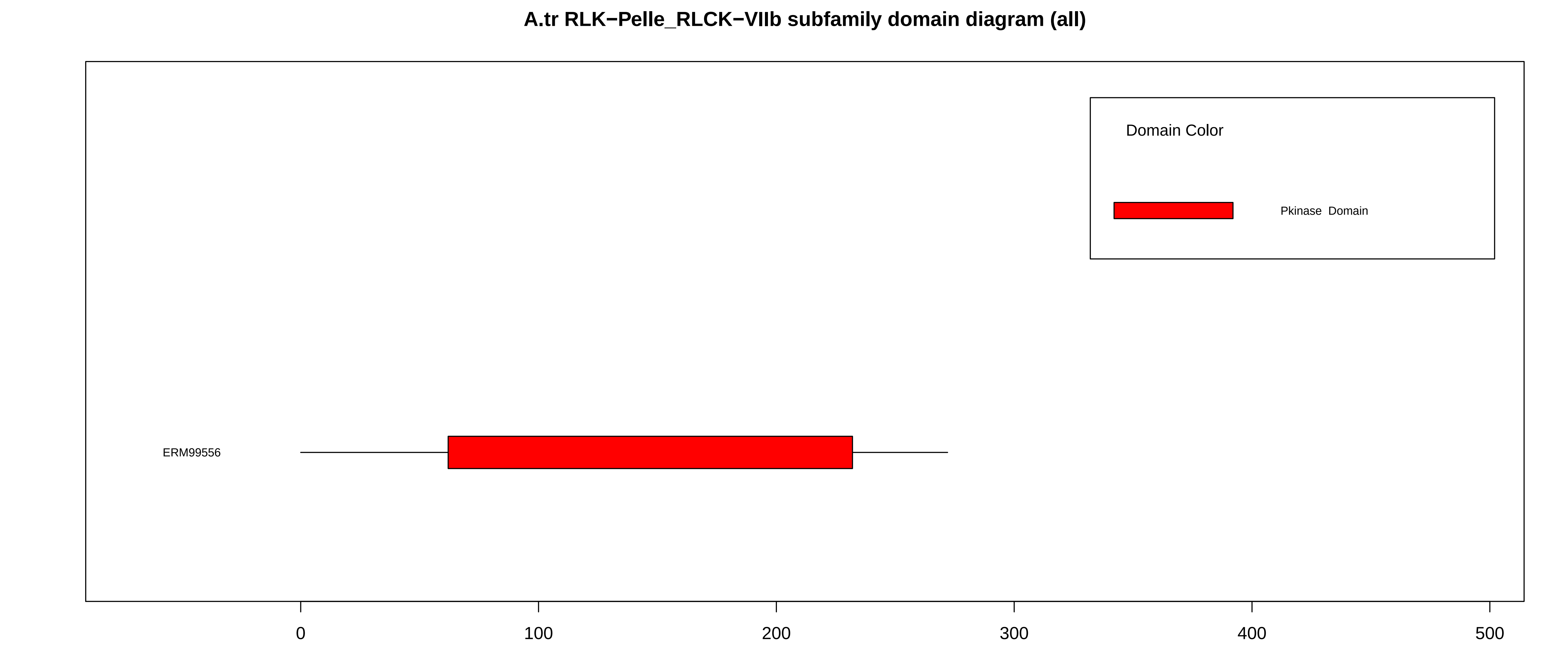
A.tr RLK−Pelle_RLCK−VIIb subfamily domain diagram (all)
Domain Color
Pkinase Domain
ERM99556
0
100
200
300
400
500

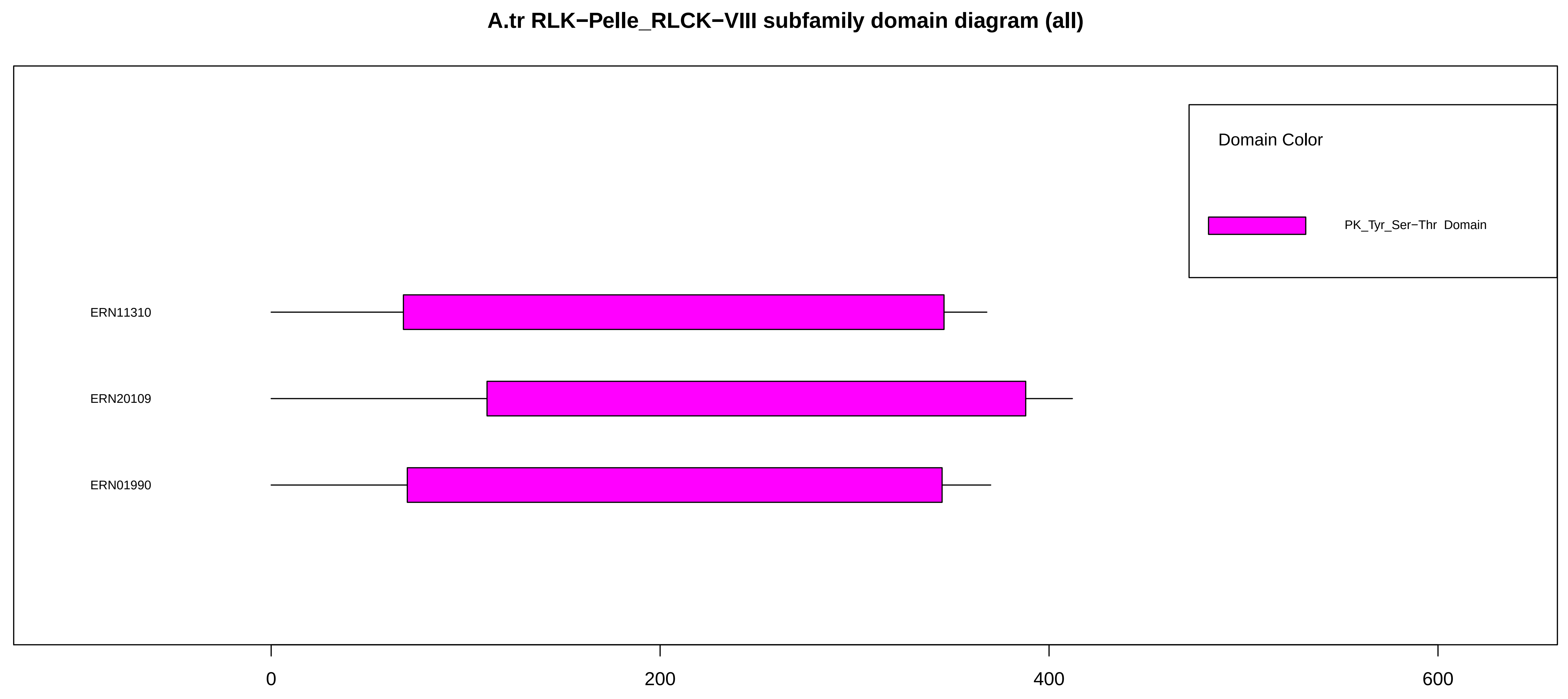
A.tr RLK−Pelle_RLCK−VIII subfamily domain diagram (all)
Domain Color
PK_Tyr_Ser−Thr Domain
ERN11310
ERN20109
ERN01990
0
200
400
600

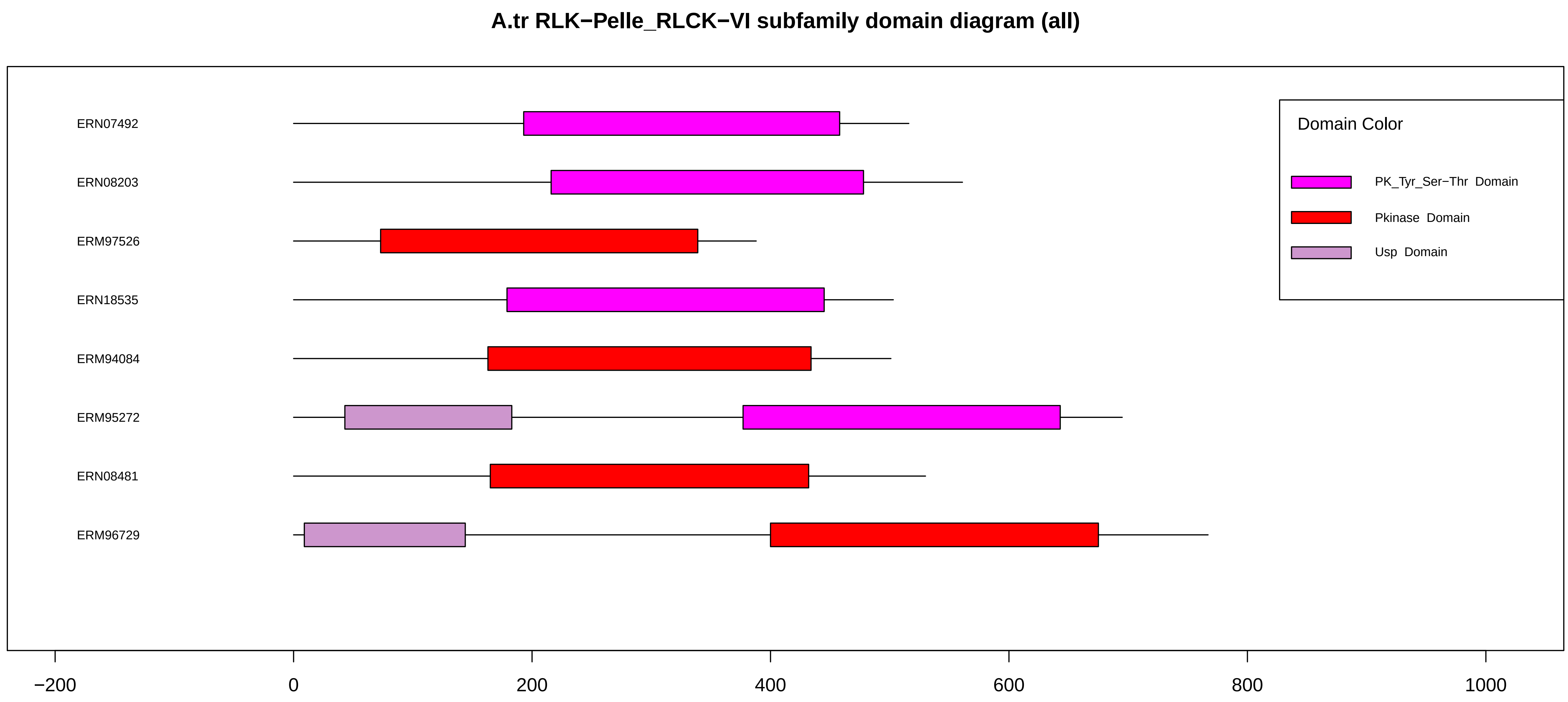
A.tr RLK−Pelle_RLCK−VI subfamily domain diagram (all)
ERN07492
ERN08203
ERM97526
ERN18535
ERM94084
ERM95272
ERN08481
ERM96729
Domain Color
PK_Tyr_Ser−Thr Domain
Pkinase Domain
Usp Domain
−200
0
200
400
600
800
1000

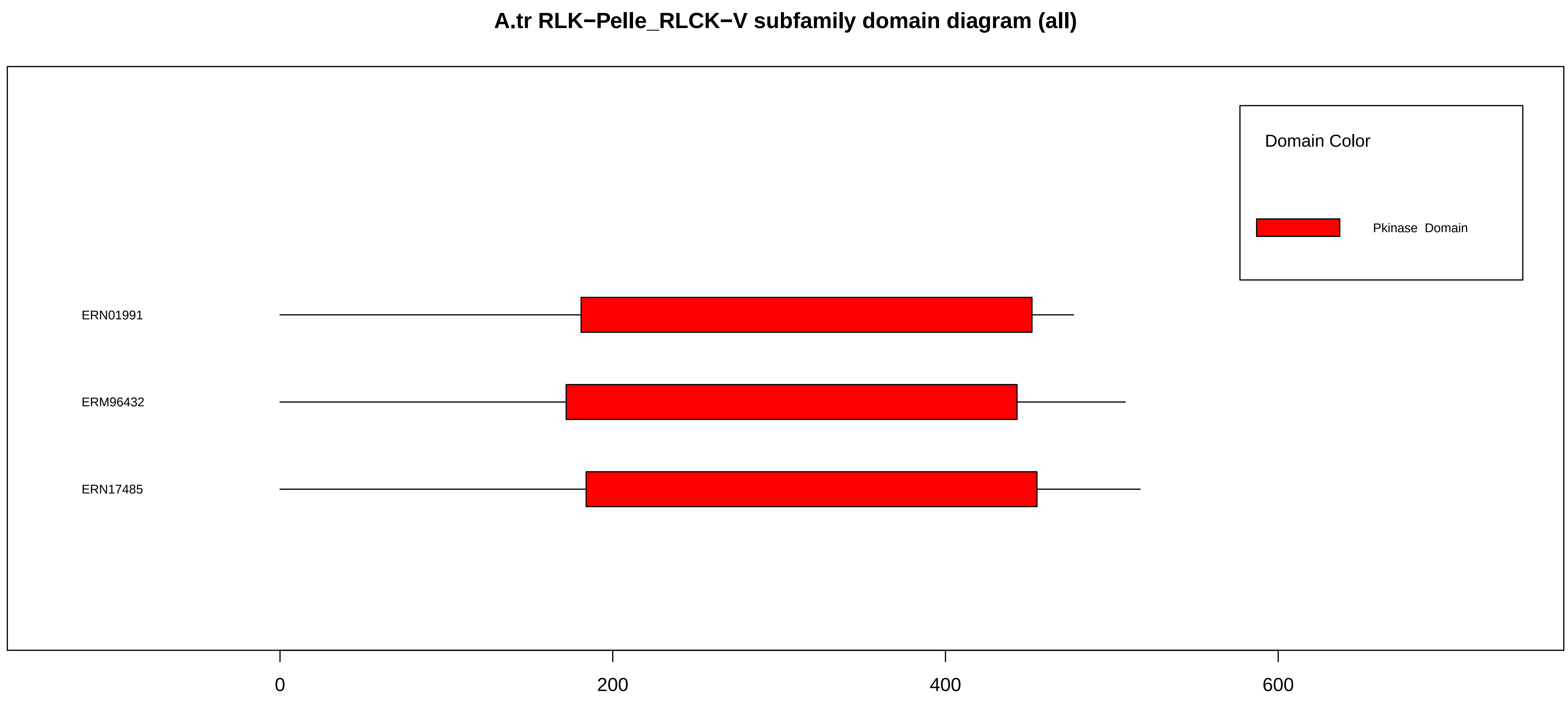

A.tr RLK−Pelle_RLCK−V subfamily domain diagram (all)
Domain Color
Pkinase Domain
ERN01991
ERM96432
ERN17485
0
200
400
600

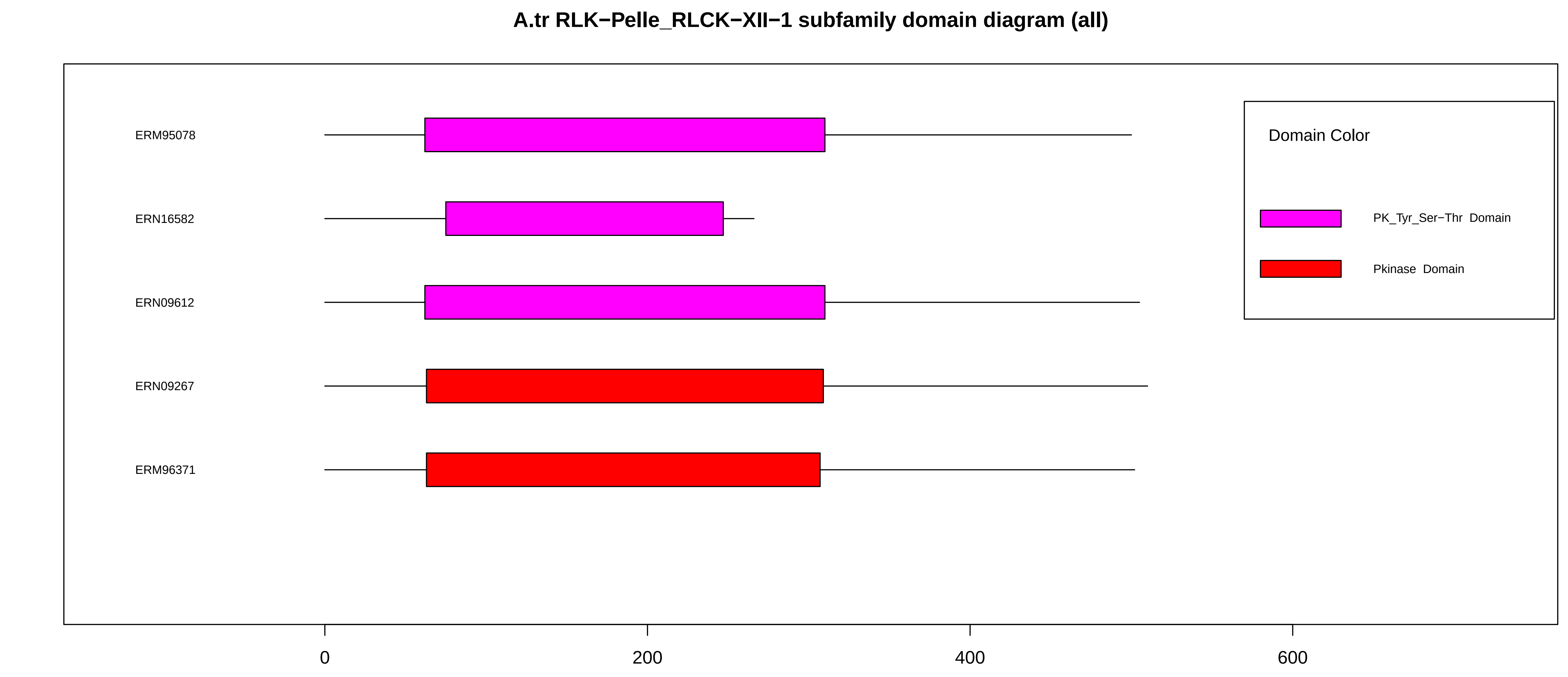
A.tr RLK−Pelle_RLCK−XII−1 subfamily domain diagram (all)
ERM95078
ERN16582
ERN09612
ERN09267
ERM96371
Domain Color
PK_Tyr_Ser−Thr Domain
Pkinase Domain
0
200
400
600

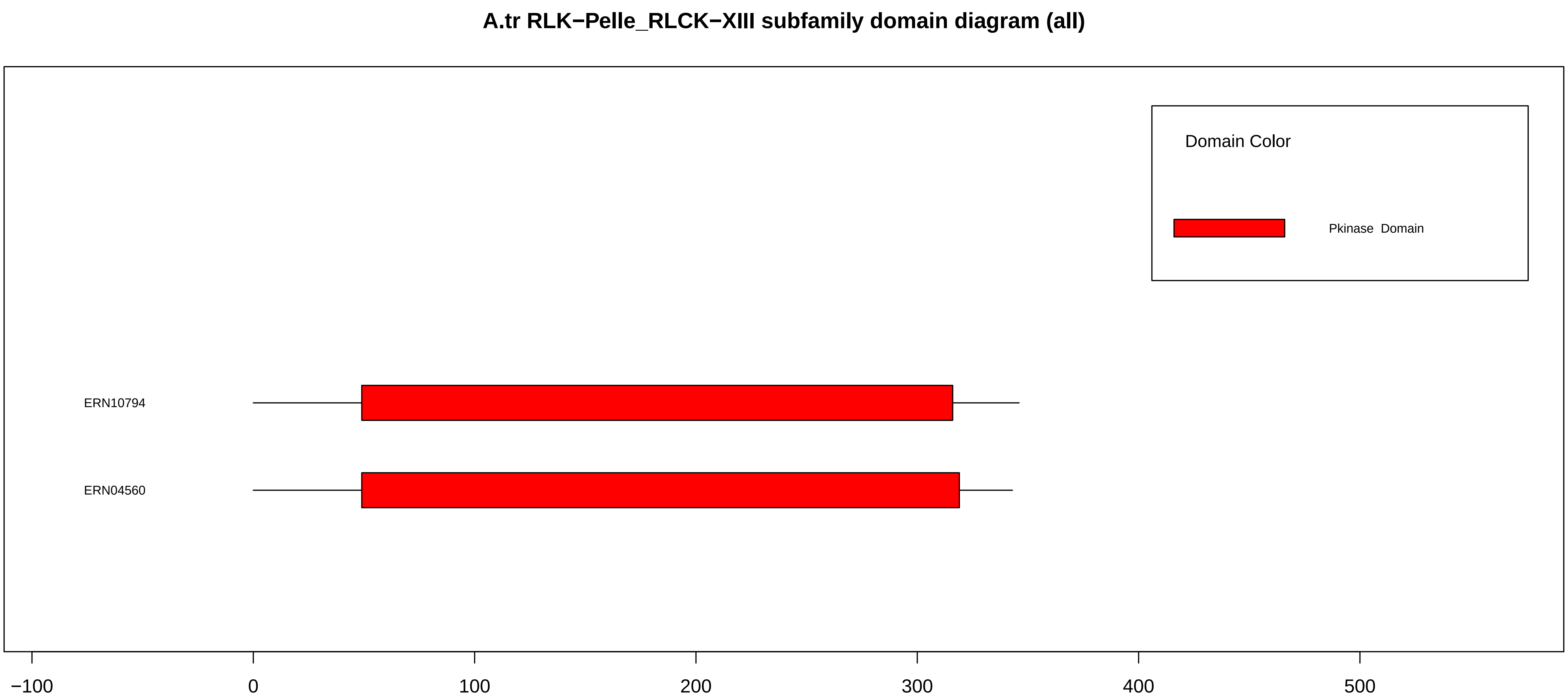

A.tr RLK−Pelle_RLCK−XIII subfamily domain diagram (all)
Domain Color
Pkinase Domain
ERN10794
ERN04560
−100
0
100
200
300
400
500

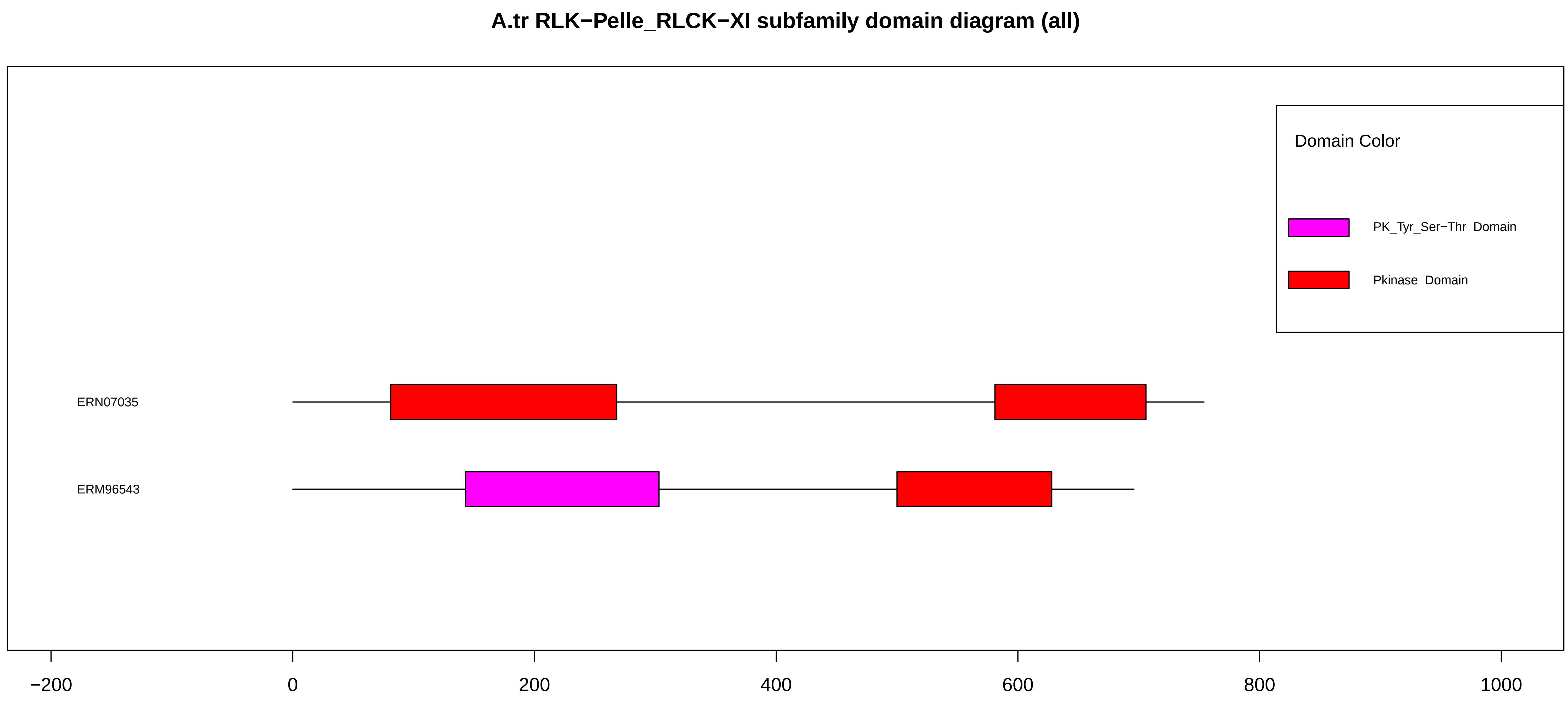
A.tr RLK−Pelle_RLCK−XI subfamily domain diagram (all)
Domain Color
PK_Tyr_Ser−Thr Domain
Pkinase Domain
ERN07035
ERM96543
−200
0
200
400
600
800
1000

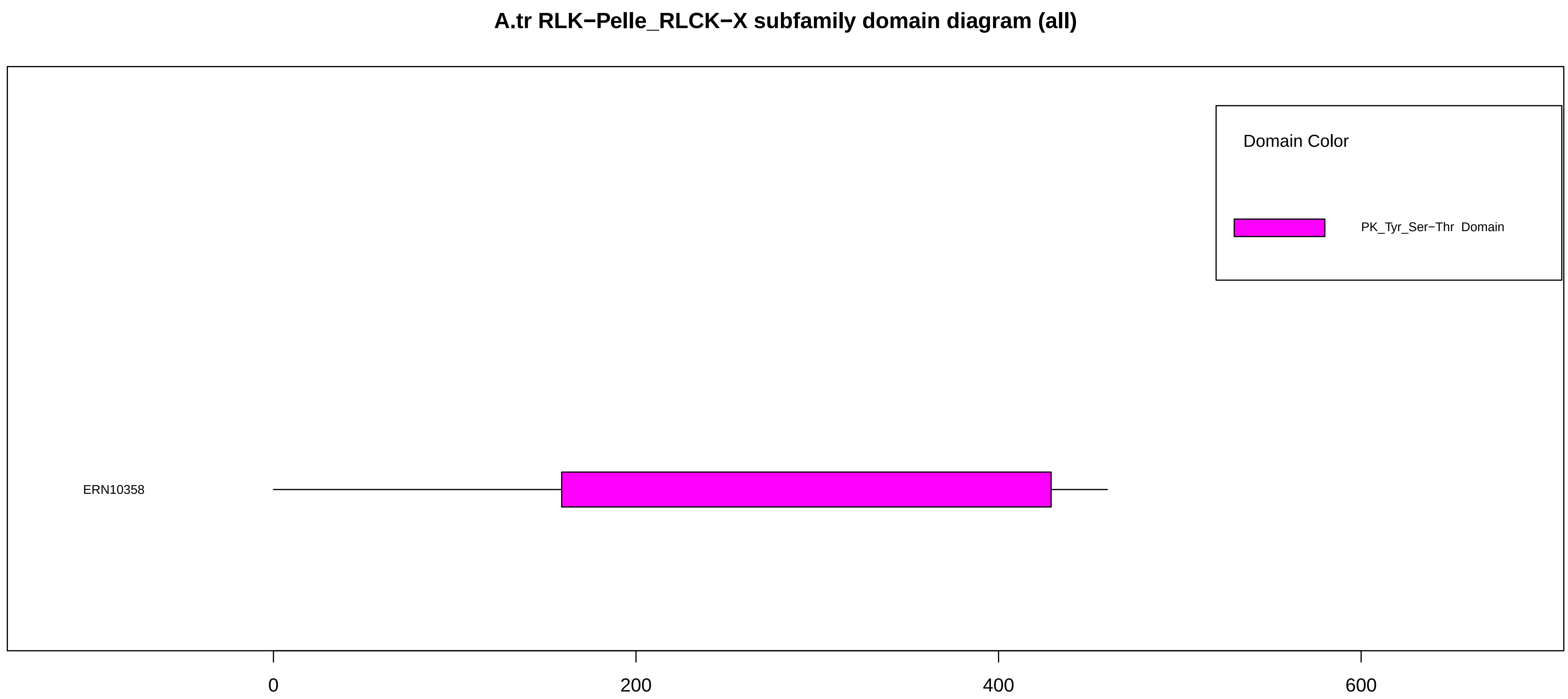
A.tr RLK−Pelle_RLCK−X subfamily domain diagram (all)
Domain Color
PK_Tyr_Ser−Thr Domain
ERN10358
0
200
400
600

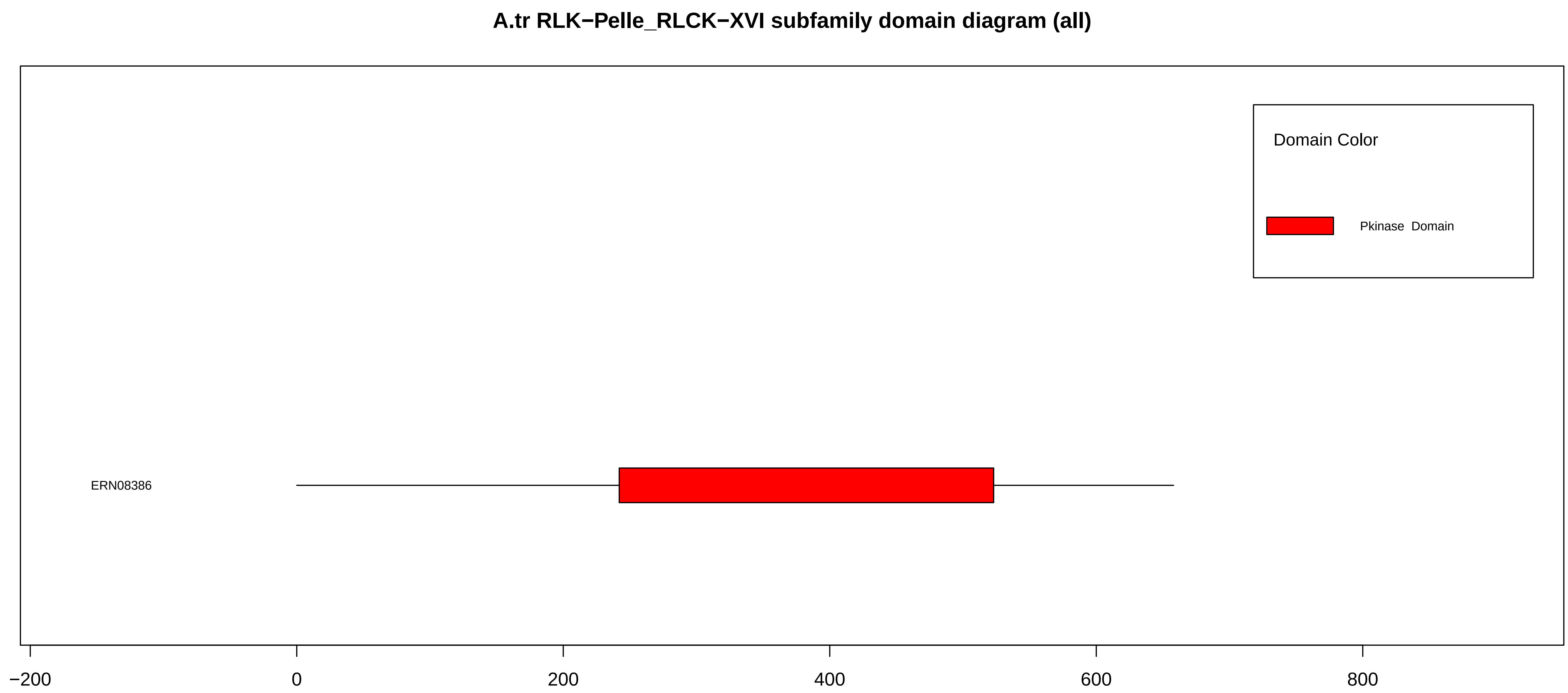
A.tr RLK−Pelle_RLCK−XVI subfamily domain diagram (all)
Domain Color
Pkinase Domain
ERN08386
−200
0
200
400
600
800

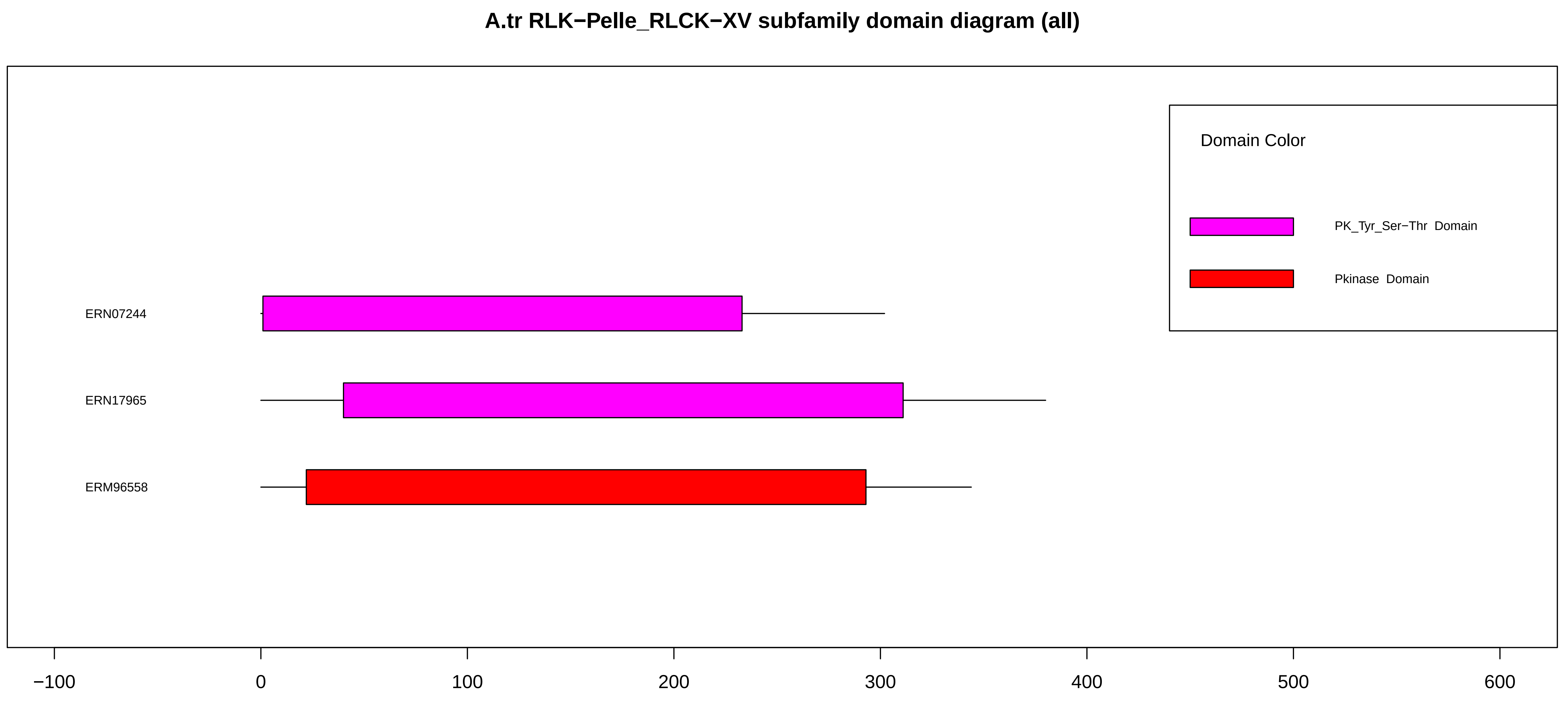
A.tr RLK−Pelle_RLCK−XV subfamily domain diagram (all)
Domain Color
PK_Tyr_Ser−Thr Domain
Pkinase Domain
ERN07244
ERN17965
ERM96558
−100
0
100
200
300
400
500
600

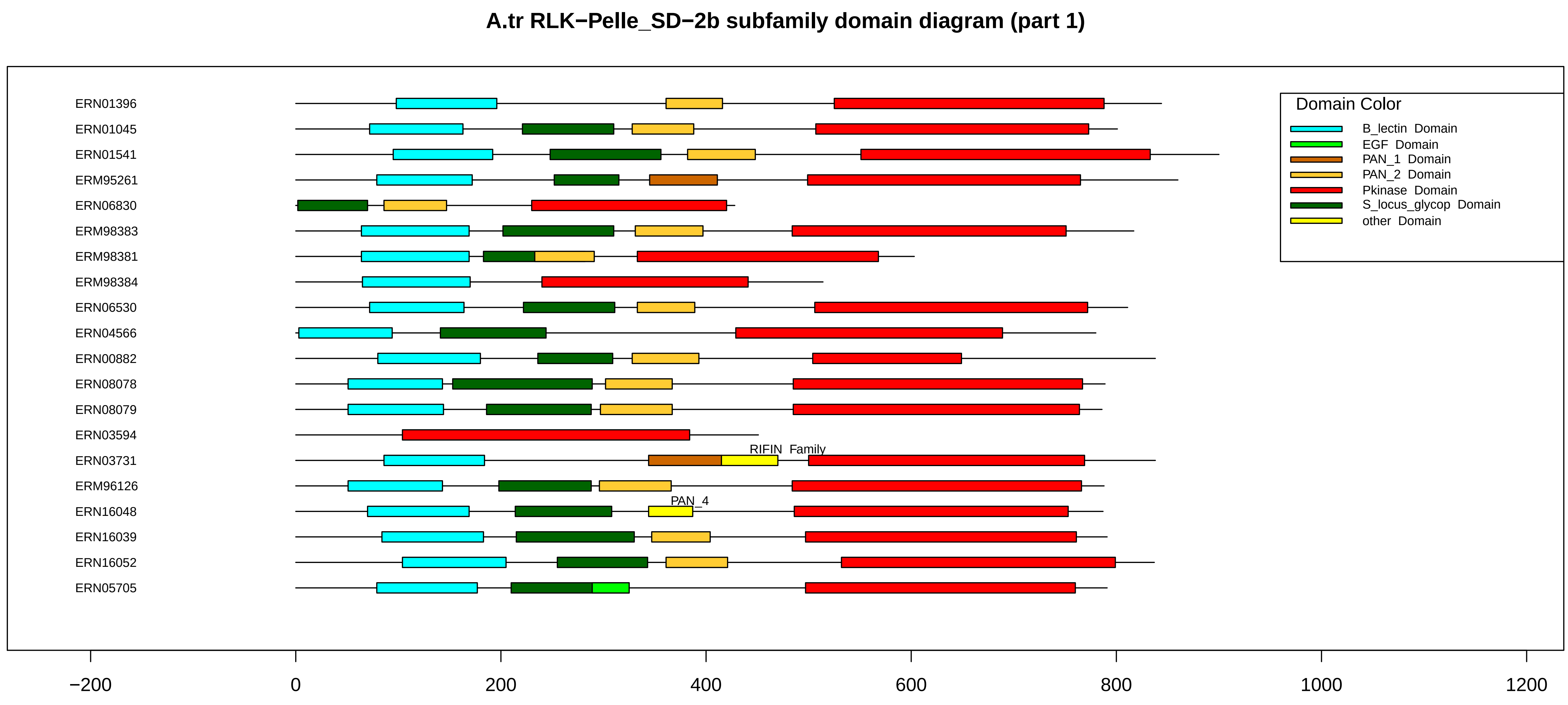

A.tr RLK−Pelle_SD−2b subfamily domain diagram (part 1)
ERN01396
ERN01045
ERN01541
ERM95261
ERN06830
ERM98383
ERM98381
ERM98384
ERN06530
ERN04566
ERN00882
ERN08078
ERN08079
ERN03594
ERN03731
ERM96126
ERN16048
ERN16039
ERN16052
ERN05705
RIFIN Family
PAN_4
Domain Color
B_lectin Domain
EGF Domain
PAN_1 Domain
PAN_2 Domain
Pkinase Domain
S_locus_glycop Domain
other Domain
−200
0
200
400
600
800
1000
1200

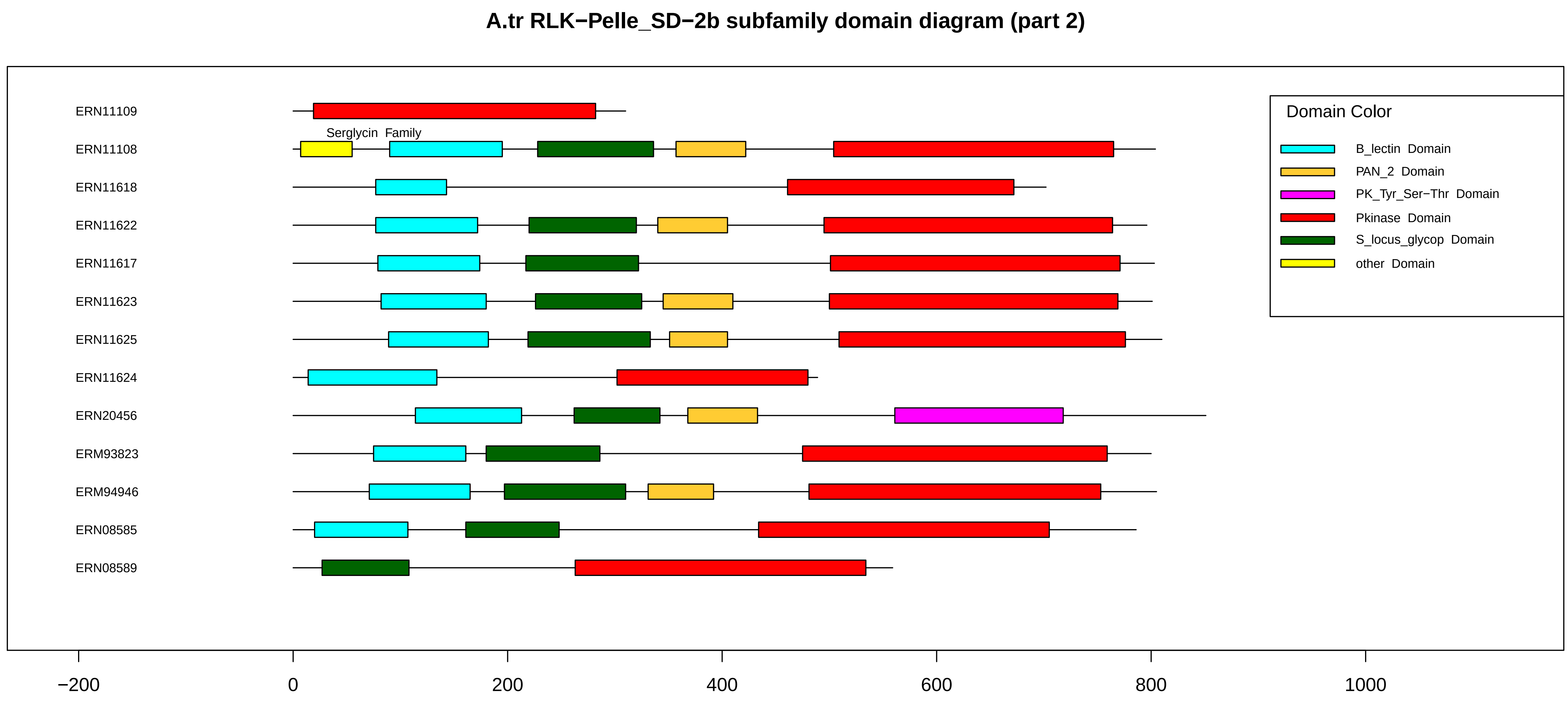
A.tr RLK−Pelle_SD−2b subfamily domain diagram (part 2)
ERN11109
ERN11108
Serglycin Family
ERN11618
ERN11622
ERN11617
ERN11623
ERN11625
ERN11624
ERN20456
ERM93823
ERM94946
ERN08585
ERN08589
Domain Color
B_lectin Domain
PAN_2 Domain
PK_Tyr_Ser−Thr Domain
Pkinase Domain
S_locus_glycop Domain
other Domain
−200
0
200
400
600
800
1000

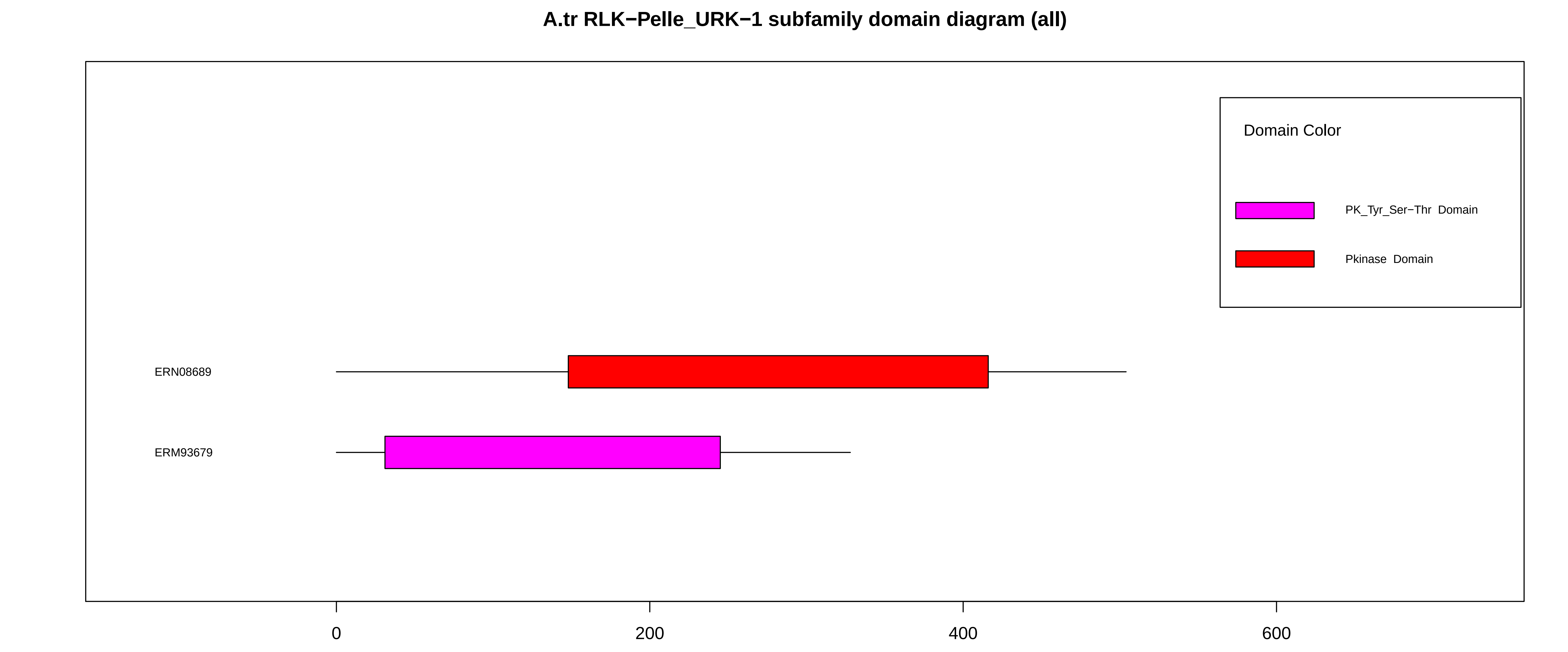

A.tr RLK−Pelle_URK−1 subfamily domain diagram (all)
Domain Color
PK_Tyr_Ser−Thr Domain
Pkinase Domain
ERN08689
ERM93679
0
200
400
600

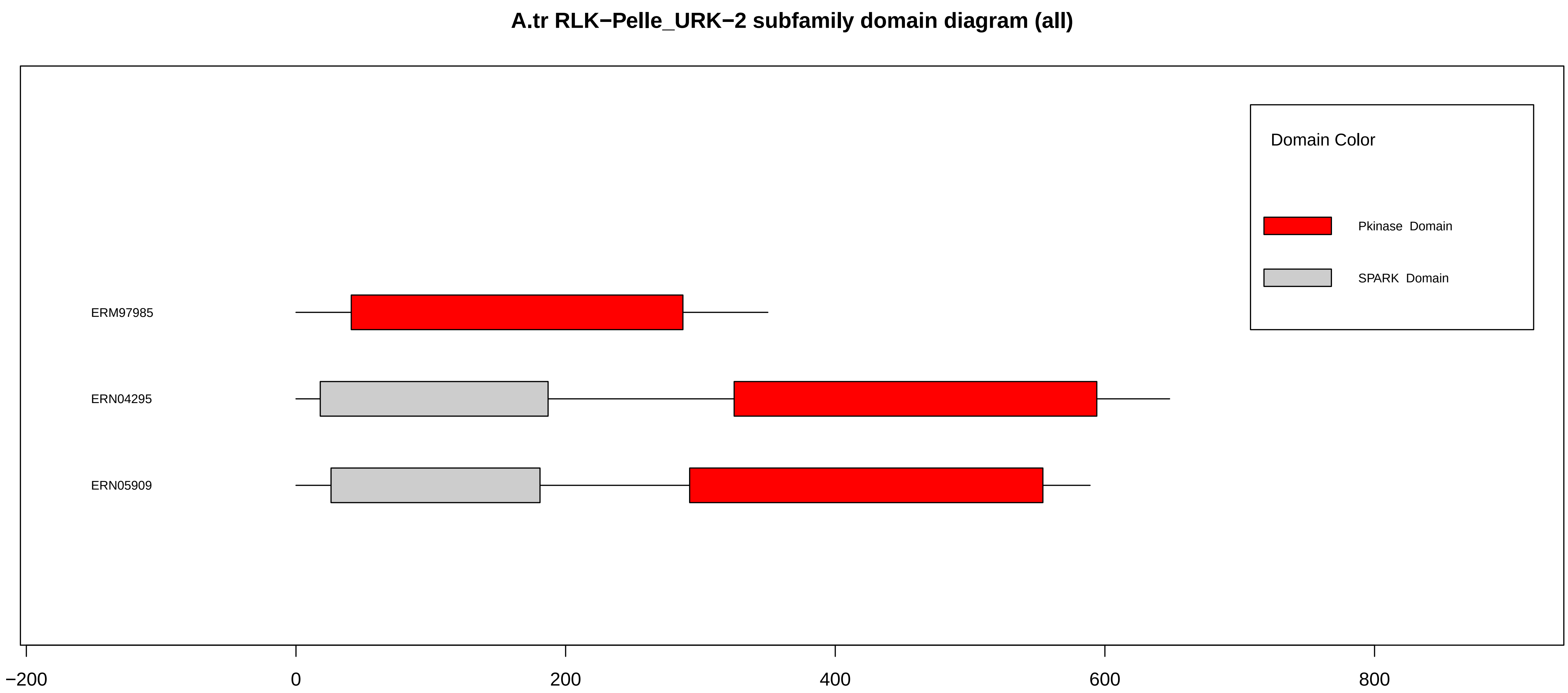

A.tr RLK−Pelle_URK−2 subfamily domain diagram (all)
Domain Color
Pkinase Domain
SPARK Domain
ERM97985
ERN04295
ERN05909
−200
0
200
400
600
800

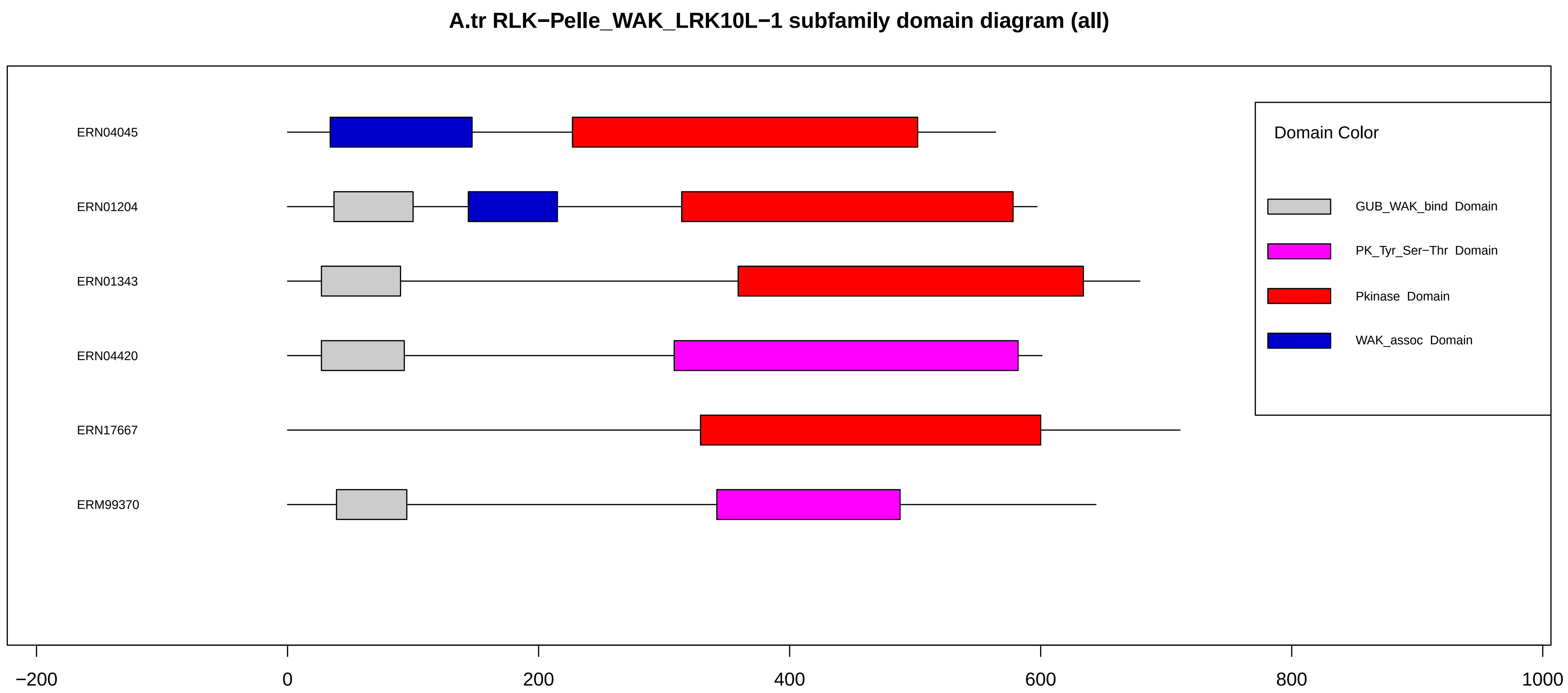
A.tr RLK−Pelle_WAK_LRK10L−1 subfamily domain diagram (all)
ERN04045
ERN01204
ERN01343
ERN04420
ERN17667
ERM99370
Domain Color
GUB_WAK_bind Domain
PK_Tyr_Ser−Thr Domain
Pkinase Domain
WAK_assoc Domain
−200
0
200
400
600
800
1000

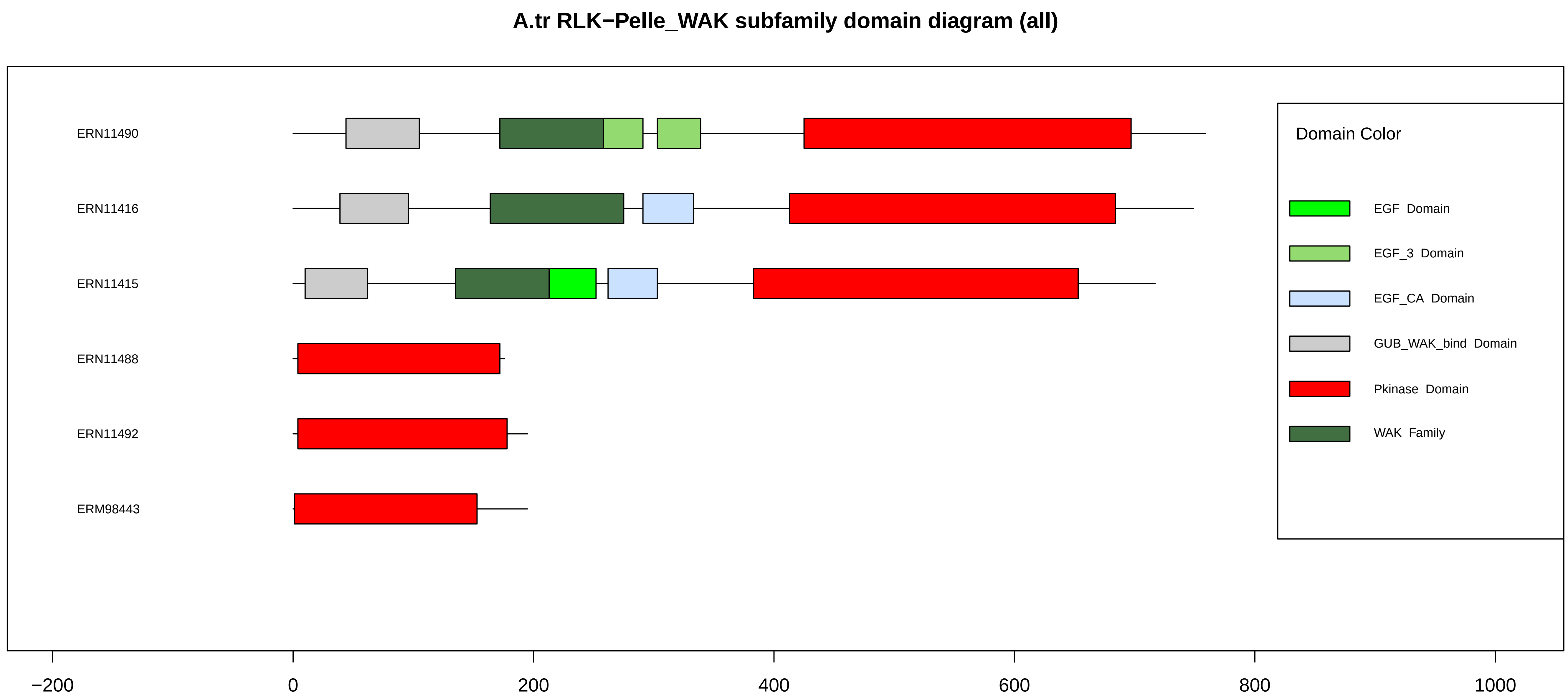

A.tr RLK−Pelle_WAK subfamily domain diagram (all)
ERN11490
ERN11416
ERN11415
ERN11488
ERN11492
ERM98443
Domain Color
EGF Domain
EGF_3 Domain
EGF_CA Domain
GUB_WAK_bind Domain
Pkinase Domain
WAK Family
−200
0
200
400
600
800
1000

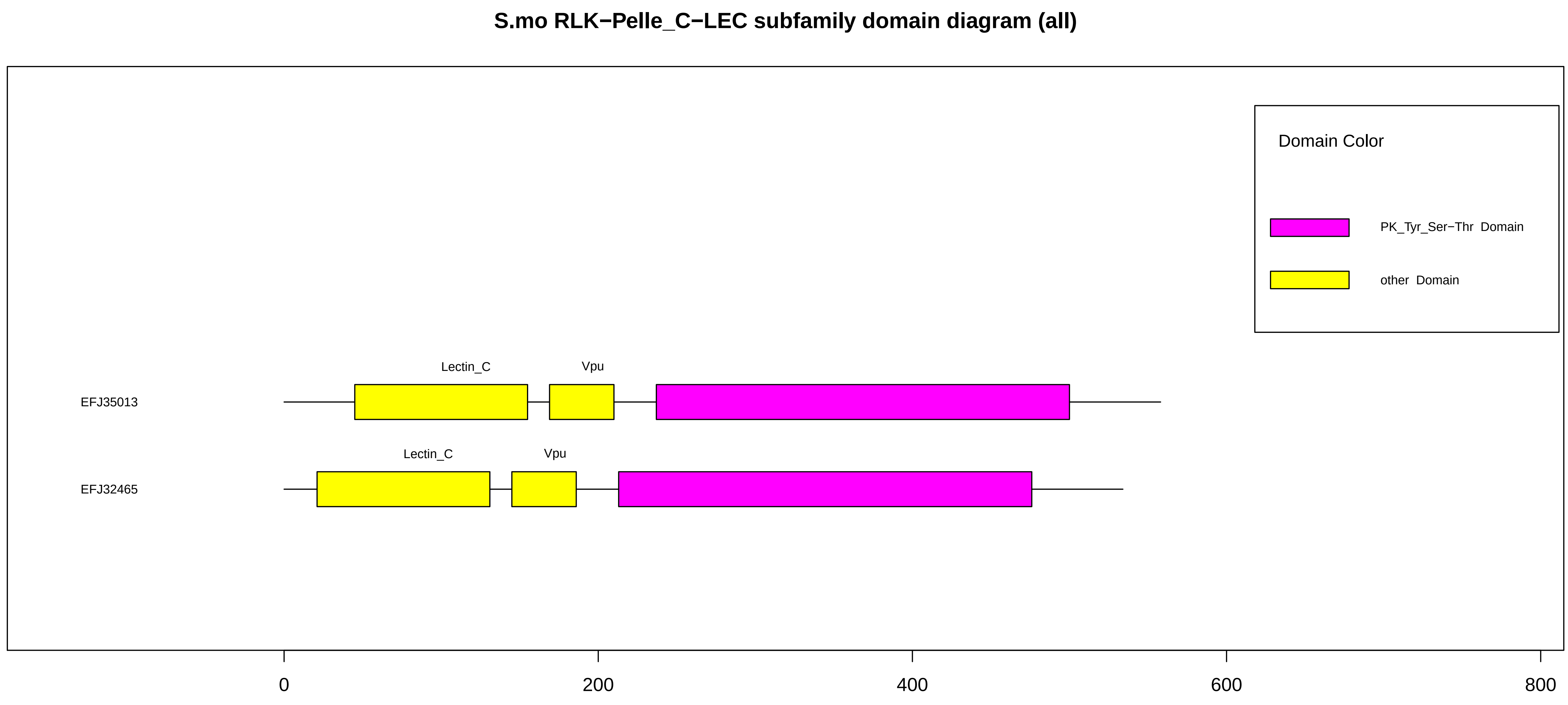
S.mo RLK−Pelle_C−LEC subfamily domain diagram (all)
Domain Color
PK_Tyr_Ser−Thr Domain
other Domain
Lectin_C
Vpu
EFJ35013
Lectin_C
Vpu
EFJ32465
0
200
400
600
800

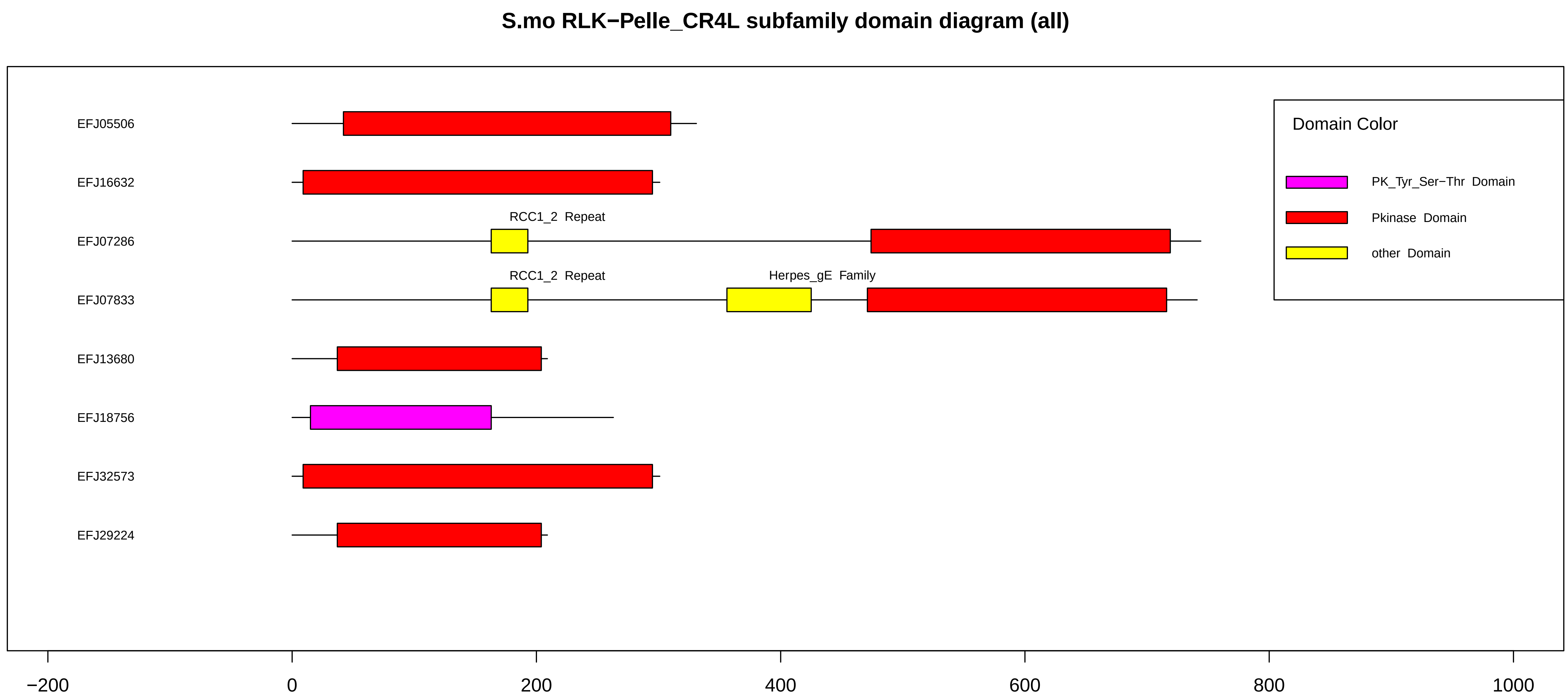
S.mo RLK−Pelle_CR4L subfamily domain diagram (all)
EFJ05506
EFJ16632
EFJ07286
EFJ07833
EFJ13680
EFJ18756
EFJ32573
EFJ29224
RCC1_2 Repeat
RCC1_2 Repeat
Herpes_gE Family
Domain Color
PK_Tyr_Ser−Thr Domain
Pkinase Domain
other Domain
−200
0
200
400
600
800
1000

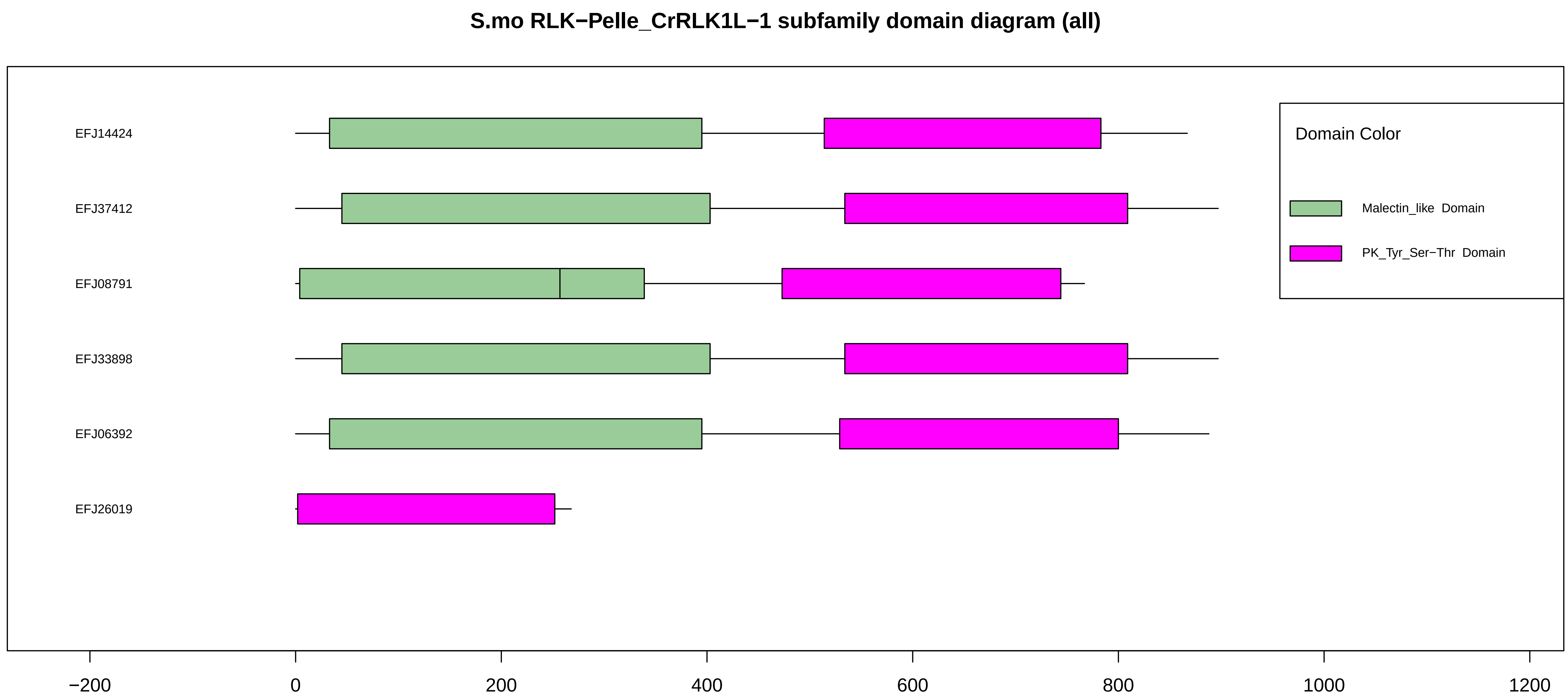
S.mo RLK−Pelle_CrRLK1L−1 subfamily domain diagram (all)
EFJ14424
EFJ37412
EFJ08791
EFJ33898
EFJ06392
EFJ26019
Domain Color
Malectin_like Domain
PK_Tyr_Ser−Thr Domain
−200
0
200
400
600
800
1000
1200

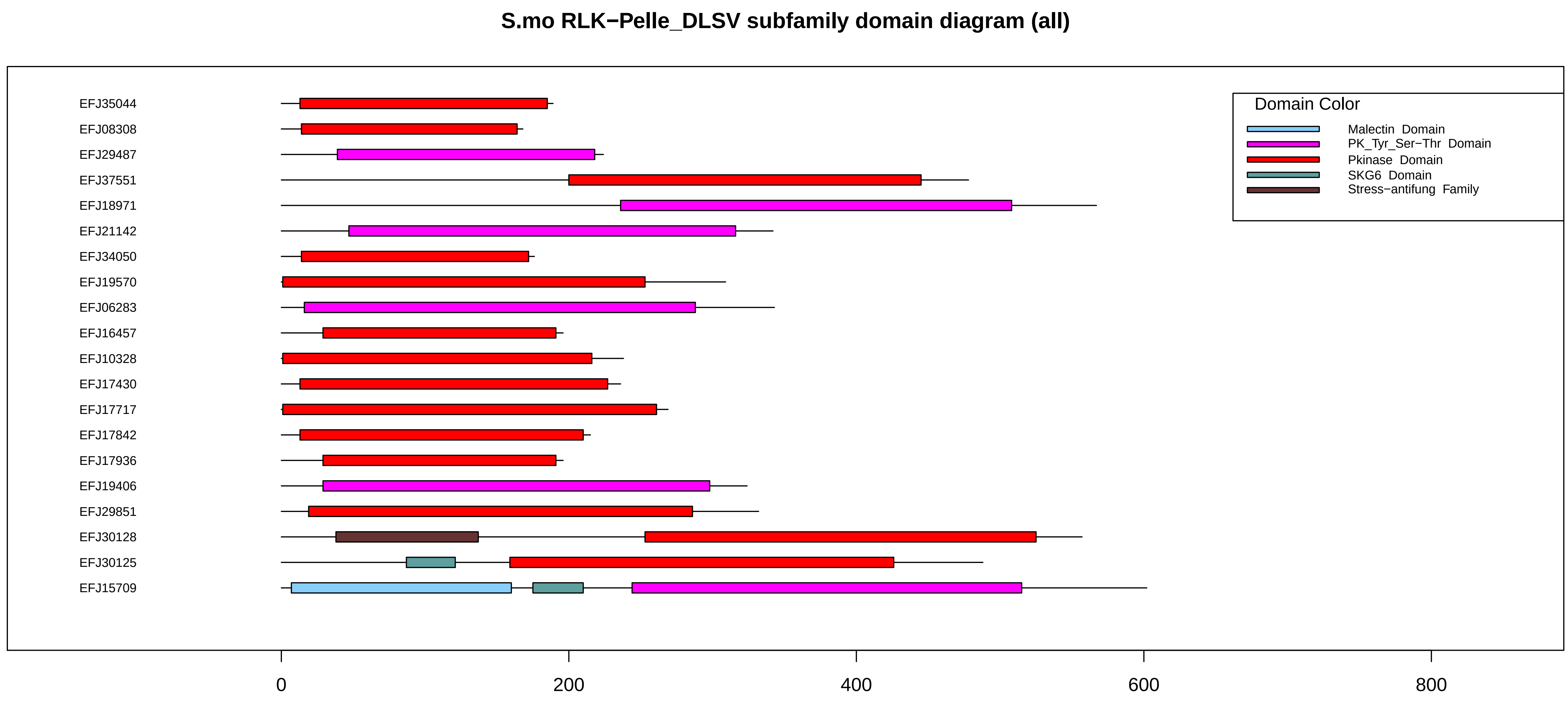
S.mo RLK−Pelle_DLSV subfamily domain diagram (all)
EFJ35044
EFJ08308
EFJ29487
EFJ37551
EFJ18971
EFJ21142
EFJ34050
EFJ19570
EFJ06283
EFJ16457
EFJ10328
EFJ17430
EFJ17717
EFJ17842
EFJ17936
EFJ19406
EFJ29851
EFJ30128
EFJ30125
EFJ15709
Domain Color
Malectin Domain
PK_Tyr_Ser−Thr Domain
Pkinase Domain
SKG6 Domain
Stress−antifung Family
0
200
400
600
800

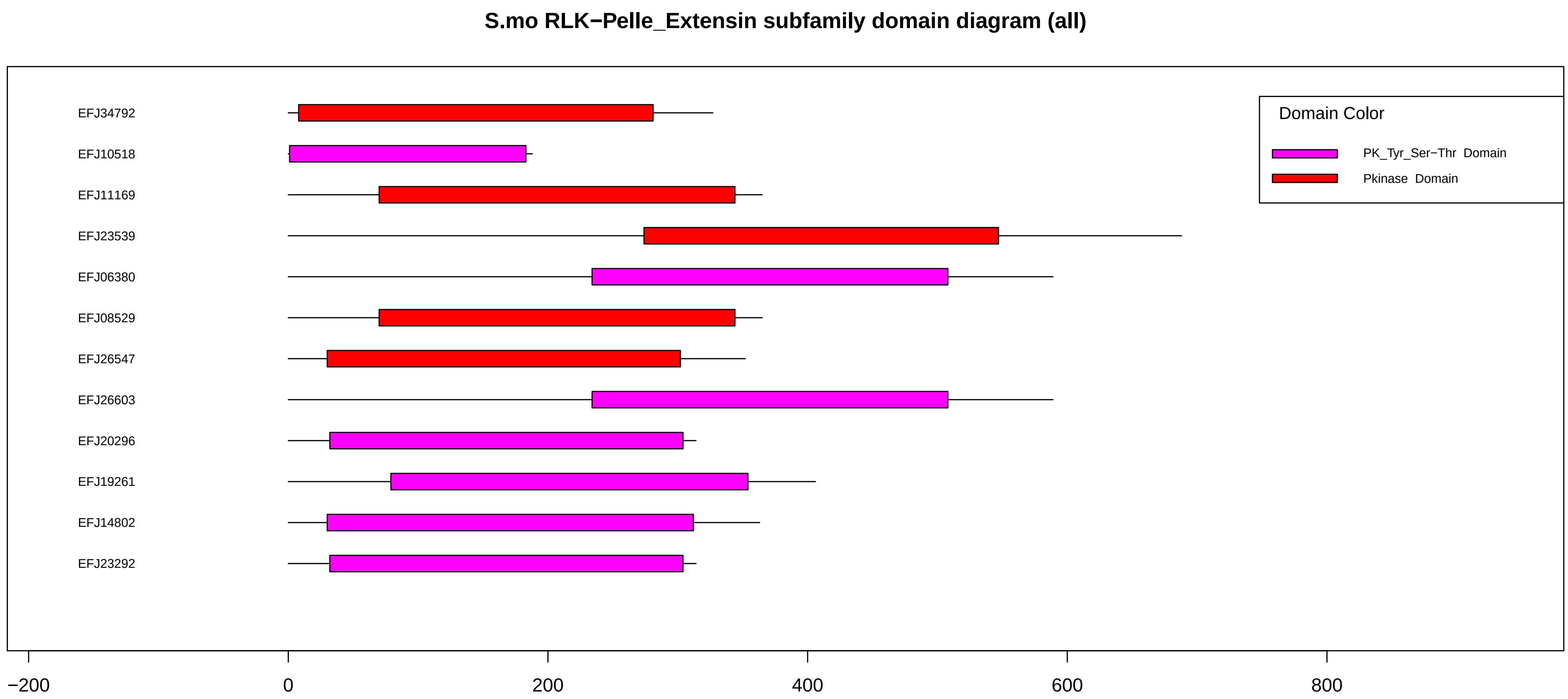

S.mo RLK−Pelle_Extensin subfamily domain diagram (all)
EFJ34792
EFJ10518
EFJ11169
EFJ23539
EFJ06380
EFJ08529
EFJ26547
EFJ26603
EFJ20296
EFJ19261
EFJ14802
EFJ23292
Domain Color
PK_Tyr_Ser−Thr Domain
Pkinase Domain
−200
0
200
400
600
800

S.mo RLK−Pelle_L−LEC subfamily domain diagram (part 1)

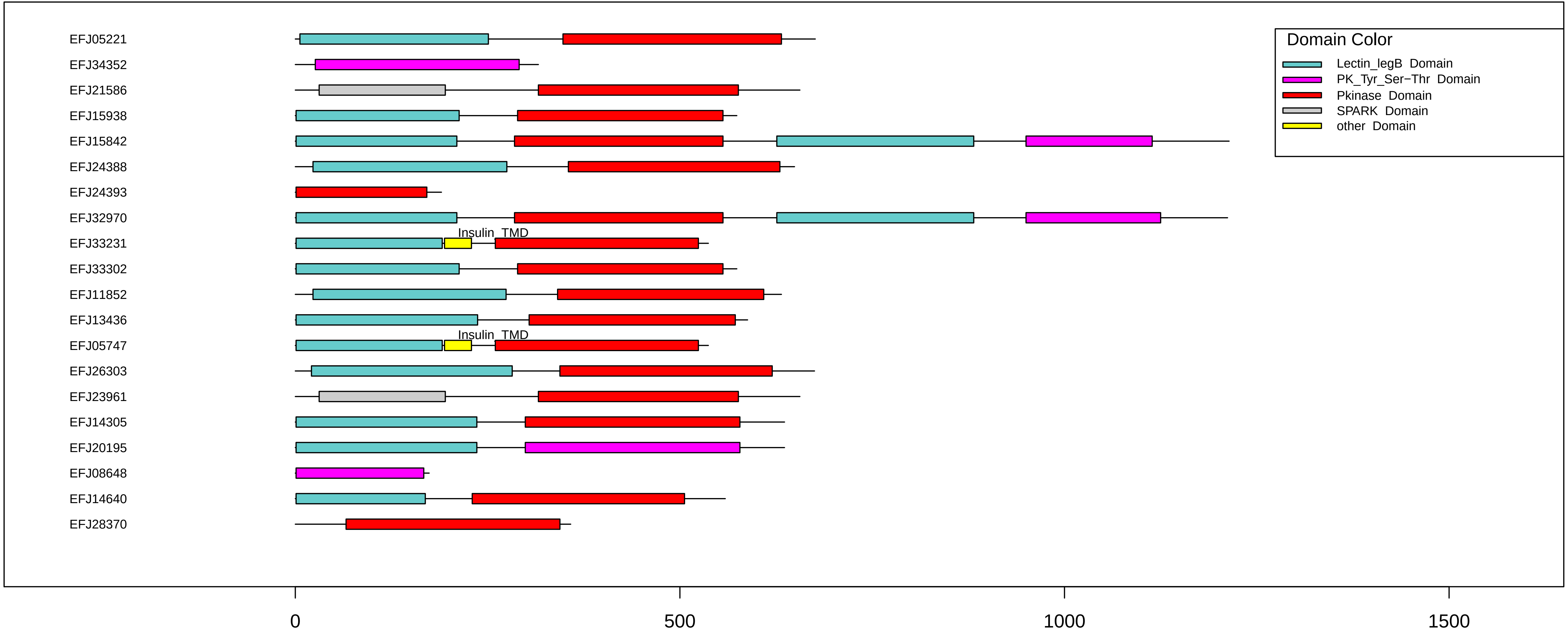

S.mo RLK−Pelle_L−LEC subfamily domain diagram (part 2)

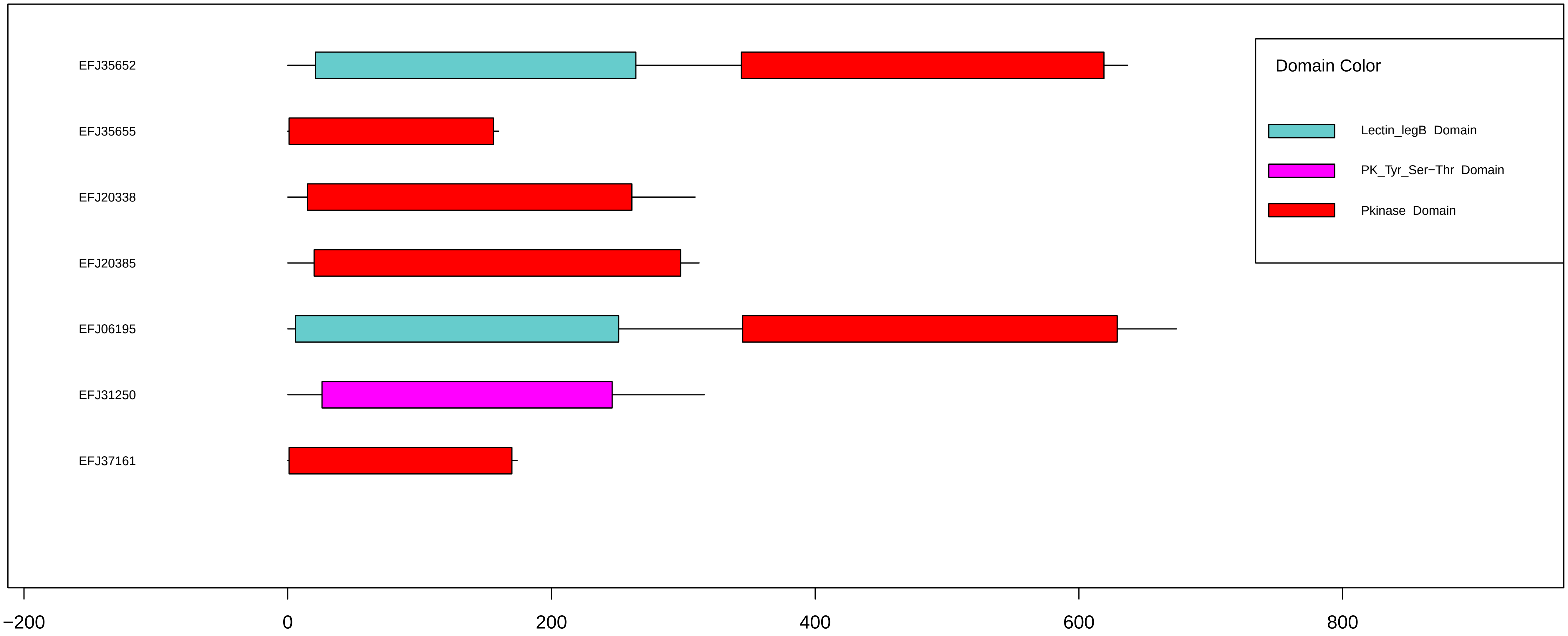

S.mo RLK−Pelle_LRR−I−1 subfamily domain diagram (all)

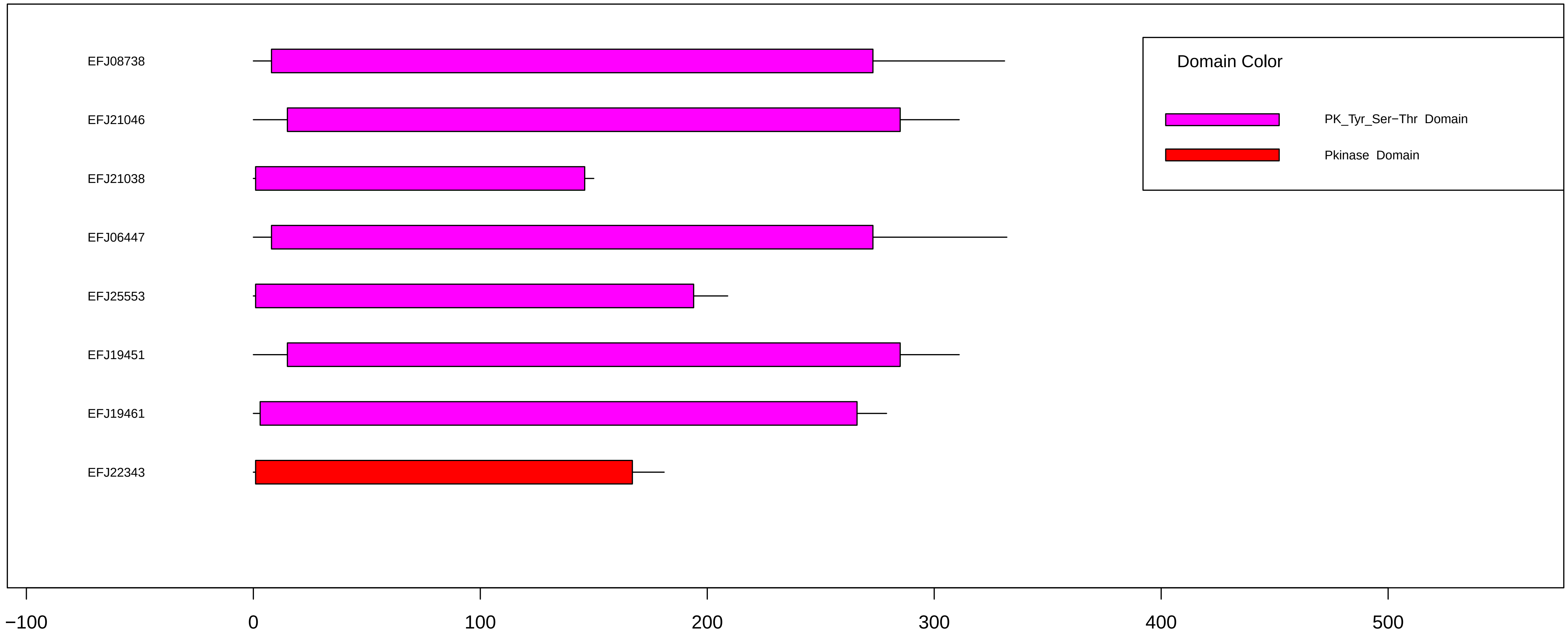

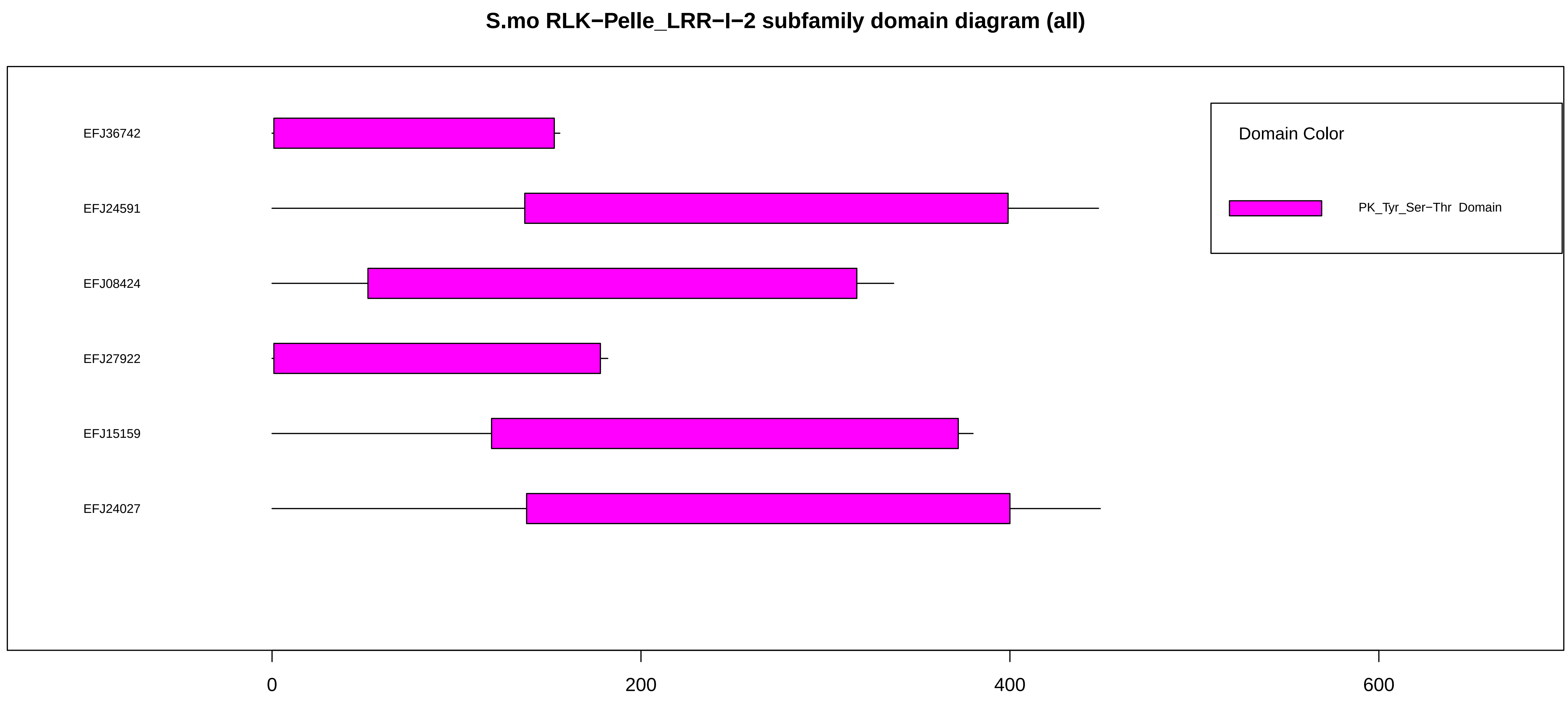
S.mo RLK−Pelle_LRR−I−2 subfamily domain diagram (all)
EFJ36742
EFJ24591
EFJ08424
EFJ27922
EFJ15159
EFJ24027
Domain Color
PK_Tyr_Ser−Thr Domain
0
200
400
600

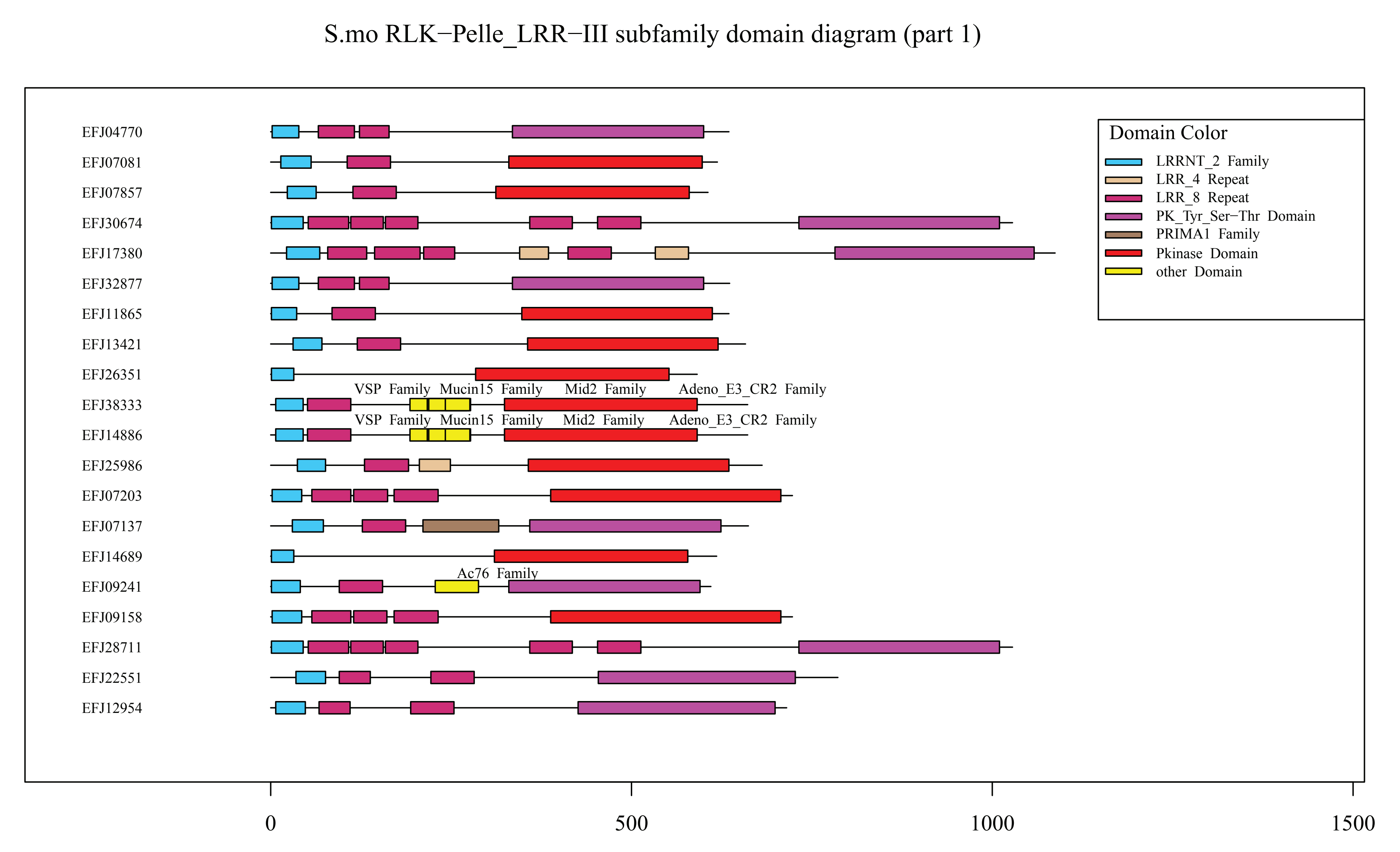

S.mo RLK−Pelle_LRR−III subfamily domain diagram (part 1)
EFJ04770
EFJ07081
EFJ07857
EFJ30674
EFJ17380
EFJ32877
EFJ11865
EFJ13421
EFJ26351
EFJ38333
EFJ14886
EFJ25986
EFJ07203
EFJ07137
EFJ14689
EFJ09241
EFJ09158
EFJ28711
EFJ22551
EFJ12954
VSP Family
Mucin15 Family
Mid2 Family
Adeno_E3_CR2 Family
VSP Family
Mucin15 Family
Mid2 Family
Adeno_E3_CR2 Family
Ac76 Family
Domain Color
LRRNT_2 Family
LRR_4 Repeat
LRR_8 Repeat
PK_Tyr_Ser−Thr Domain
PRIMA1 Family
Pkinase Domain
other Domain
0
500
1000
1500

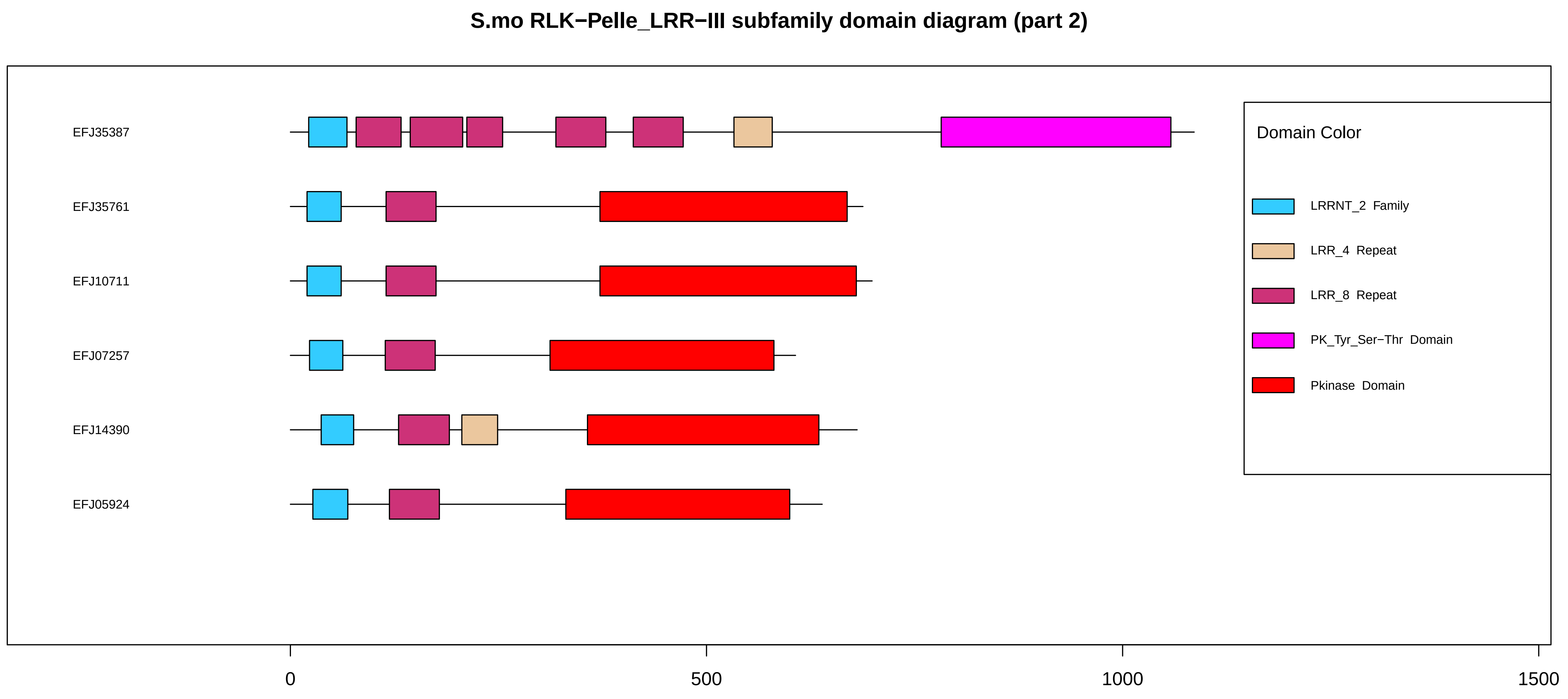
S.mo RLK−Pelle_LRR−III subfamily domain diagram (part 2)
EFJ35387
EFJ35761
EFJ10711
EFJ07257
EFJ14390
EFJ05924
Domain Color
LRRNT_2 Family
LRR_4 Repeat
LRR_8 Repeat
PK_Tyr_Ser−Thr Domain
Pkinase Domain
0
500
1000
1500

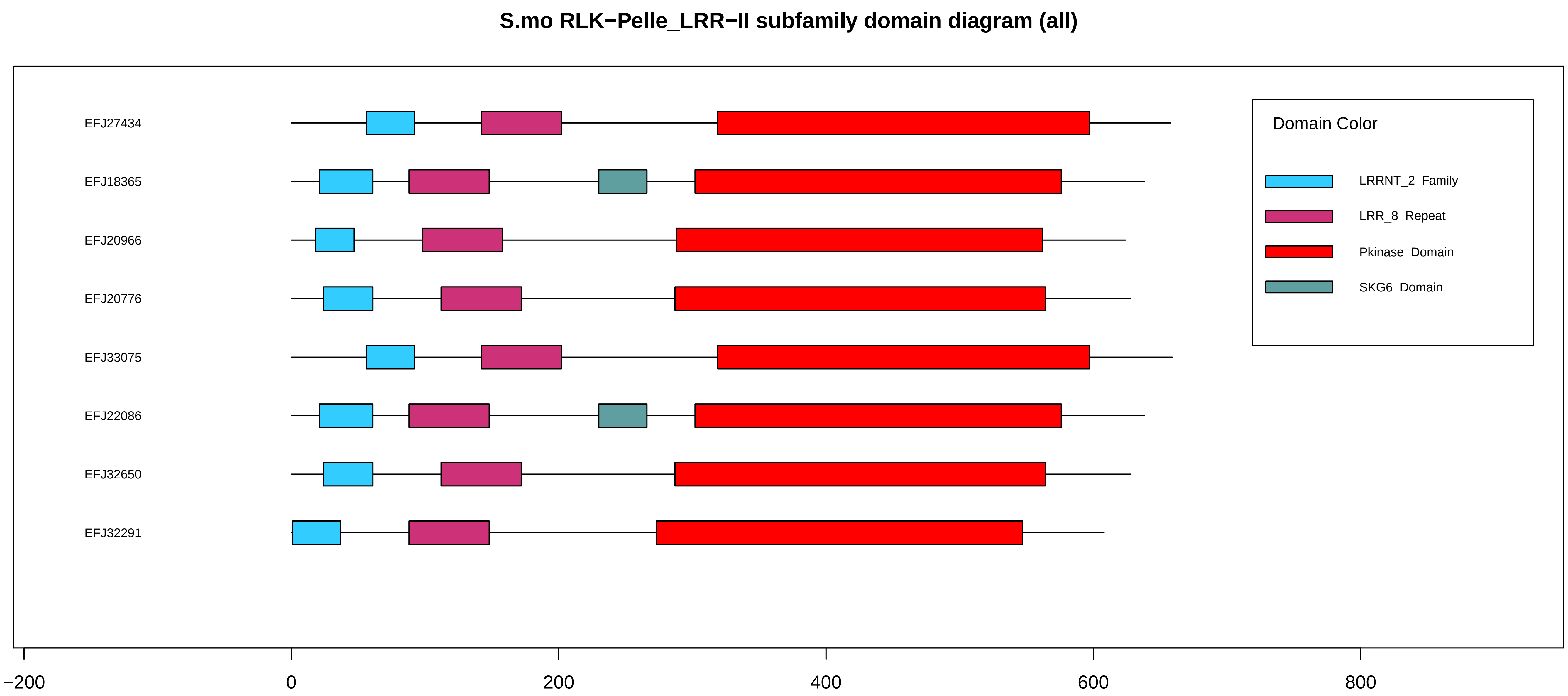
S.mo RLK−Pelle_LRR−II subfamily domain diagram (all)
EFJ27434
EFJ18365
EFJ20966
EFJ20776
EFJ33075
EFJ22086
EFJ32650
EFJ32291
Domain Color
LRRNT_2 Family
LRR_8 Repeat
Pkinase Domain
SKG6 Domain
−200
0
200
400
600
800

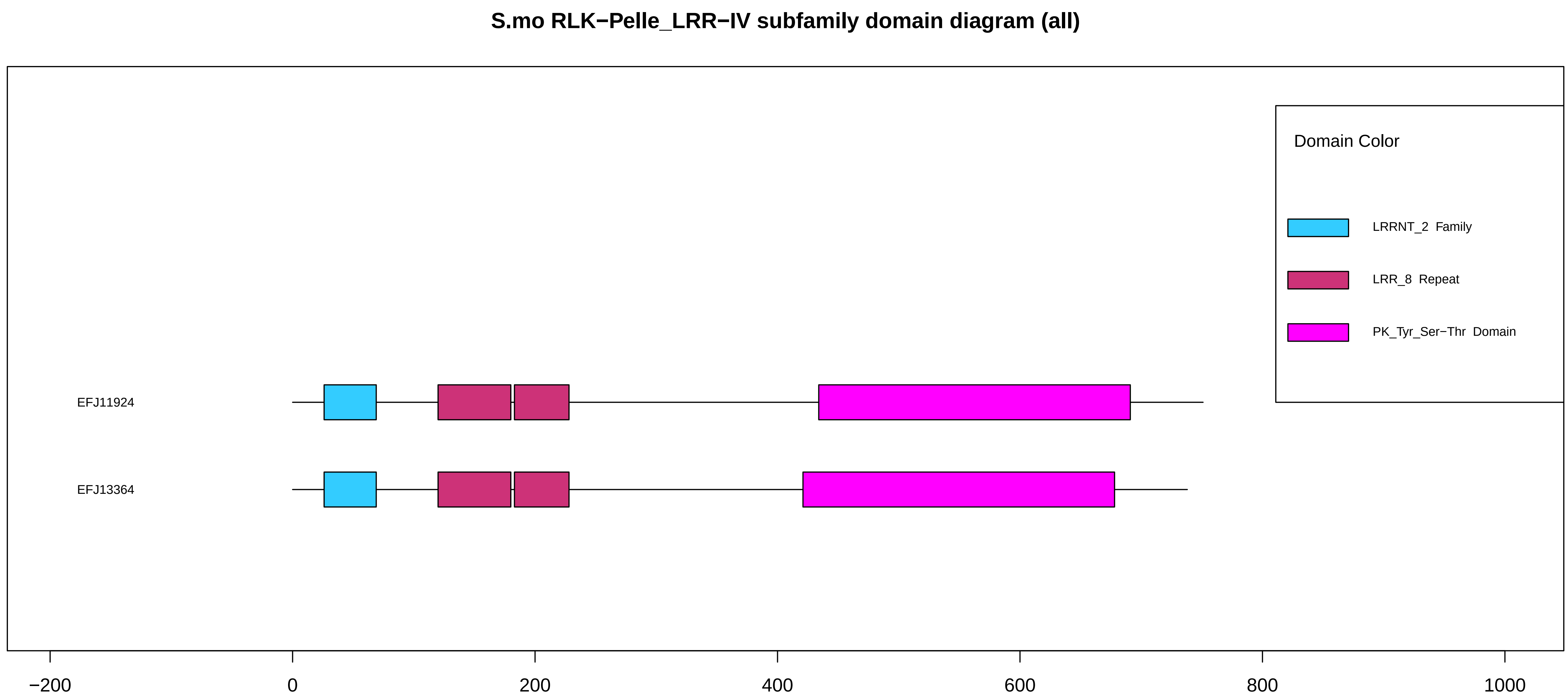
S.mo RLK−Pelle_LRR−IV subfamily domain diagram (all)
Domain Color
LRRNT_2 Family
LRR_8 Repeat
PK_Tyr_Ser−Thr Domain
EFJ11924
EFJ13364
−200
0
200
400
600
800
1000

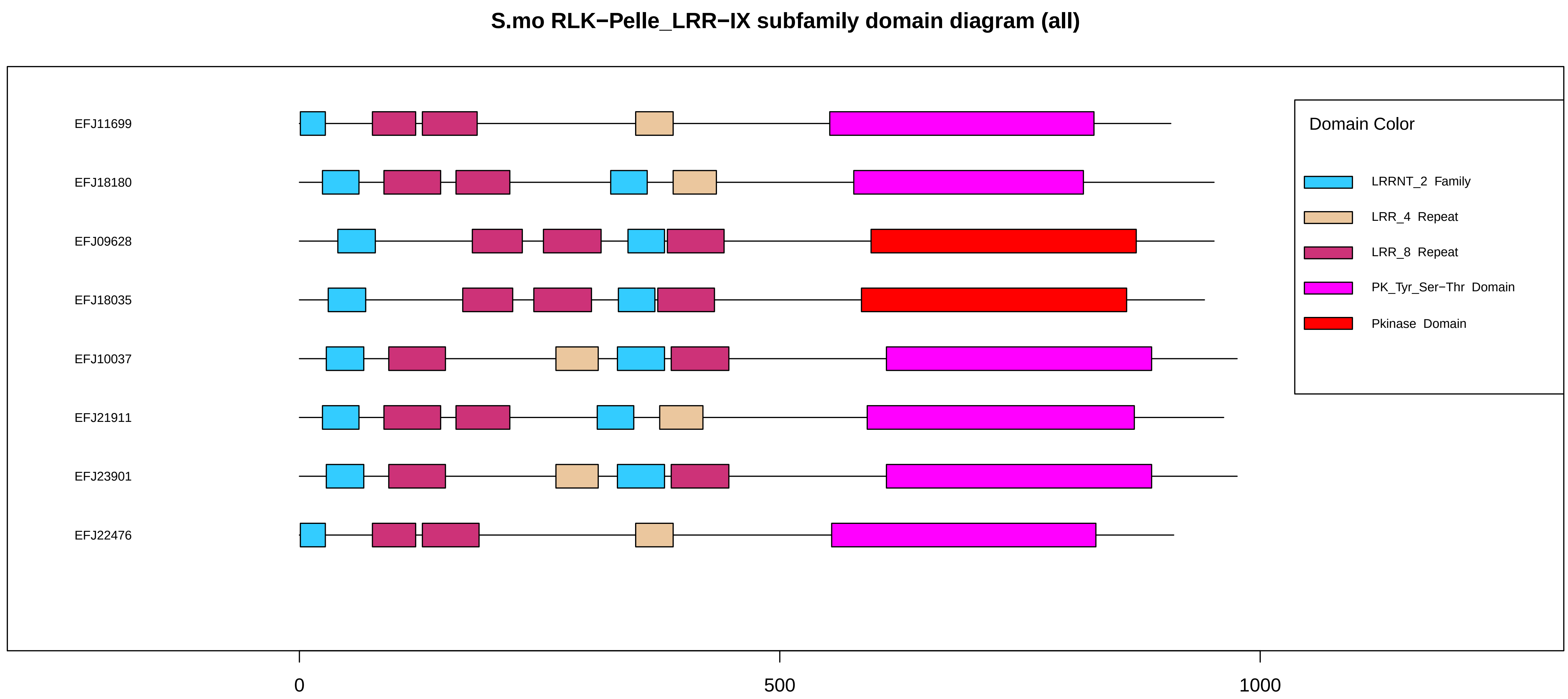
S.mo RLK−Pelle_LRR−IX subfamily domain diagram (all)
EFJ11699
EFJ18180
EFJ09628
EFJ18035
EFJ10037
EFJ21911
EFJ23901
EFJ22476
Domain Color
LRRNT_2 Family
LRR_4 Repeat
LRR_8 Repeat
PK_Tyr_Ser−Thr Domain
Pkinase Domain
0
500
1000

S.mo RLK−Pelle_LRR−VI−1 subfamily domain diagram (all)

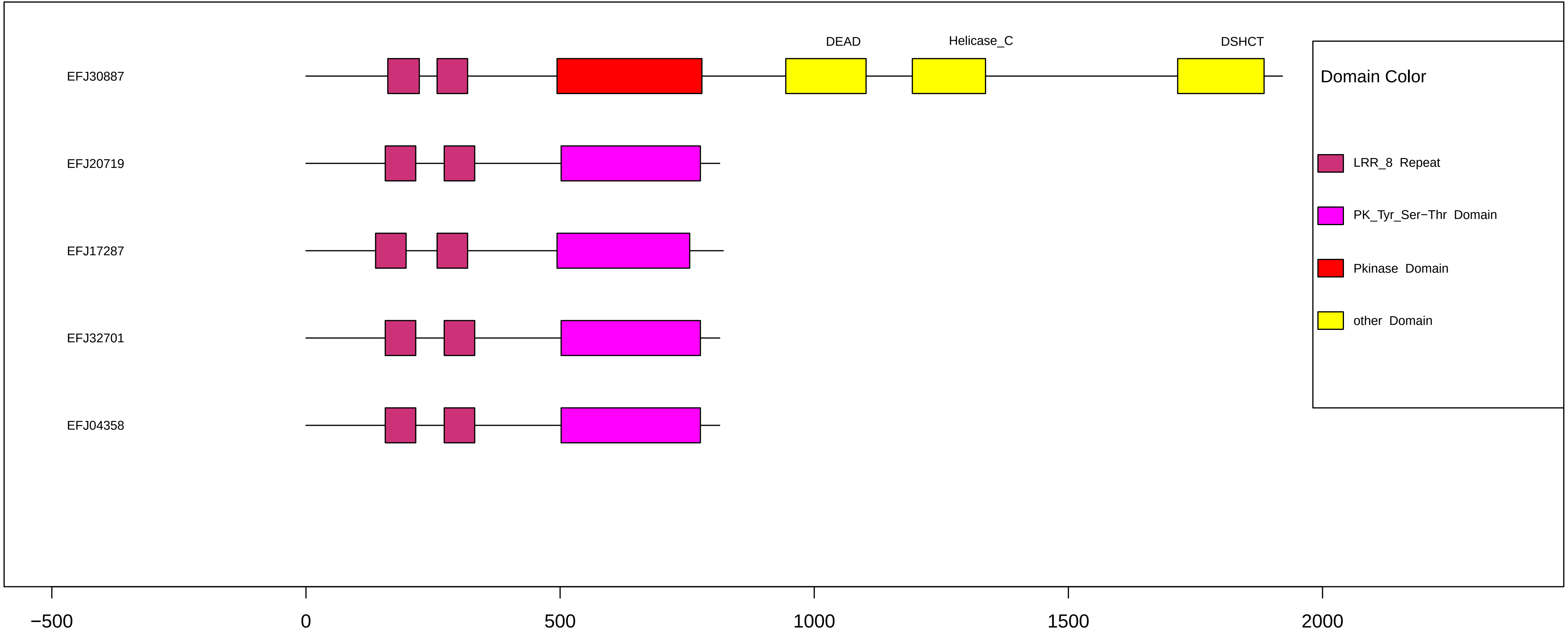

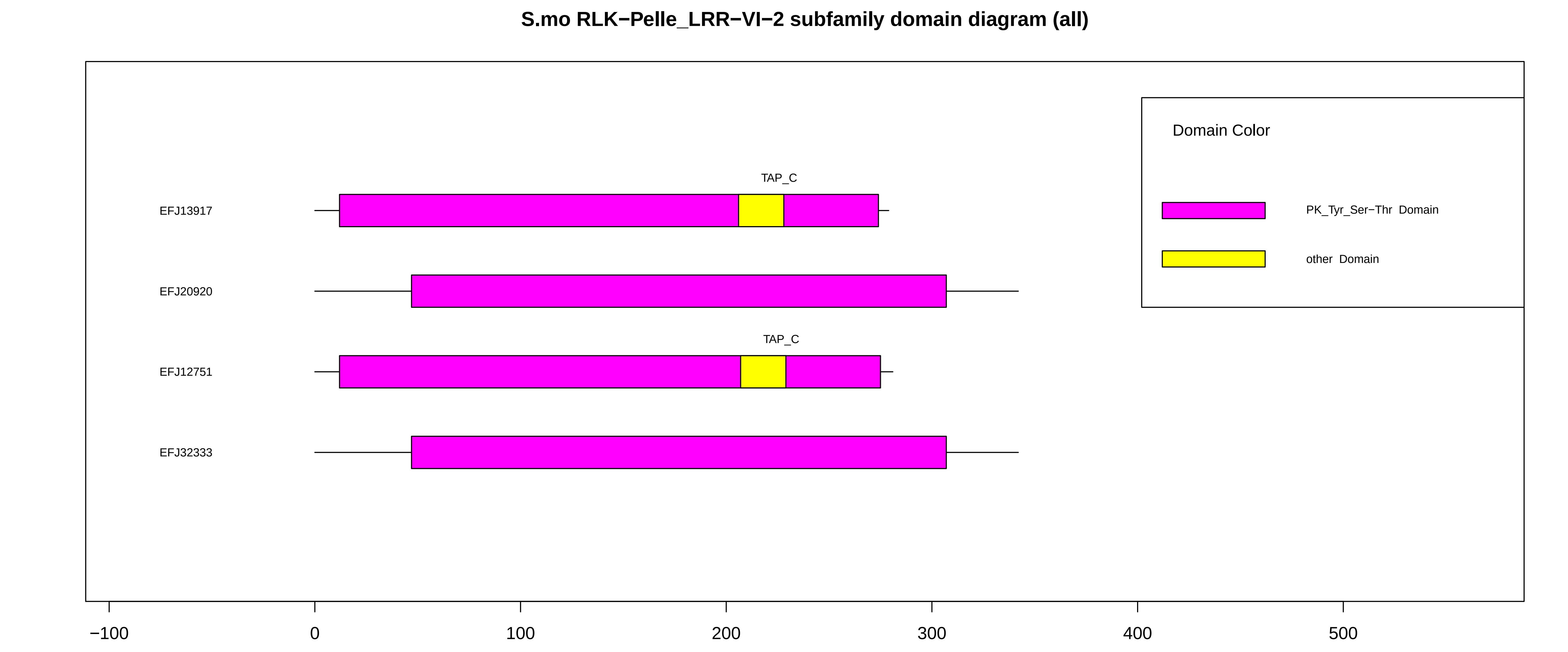

S.mo RLK−Pelle_LRR−VI−2 subfamily domain diagram (all)
Domain Color
PK_Tyr_Ser−Thr Domain
other Domain
TAP_C
EFJ13917
EFJ20920
TAP_C
EFJ12751
EFJ32333
−100
0
100
200
300
400
500

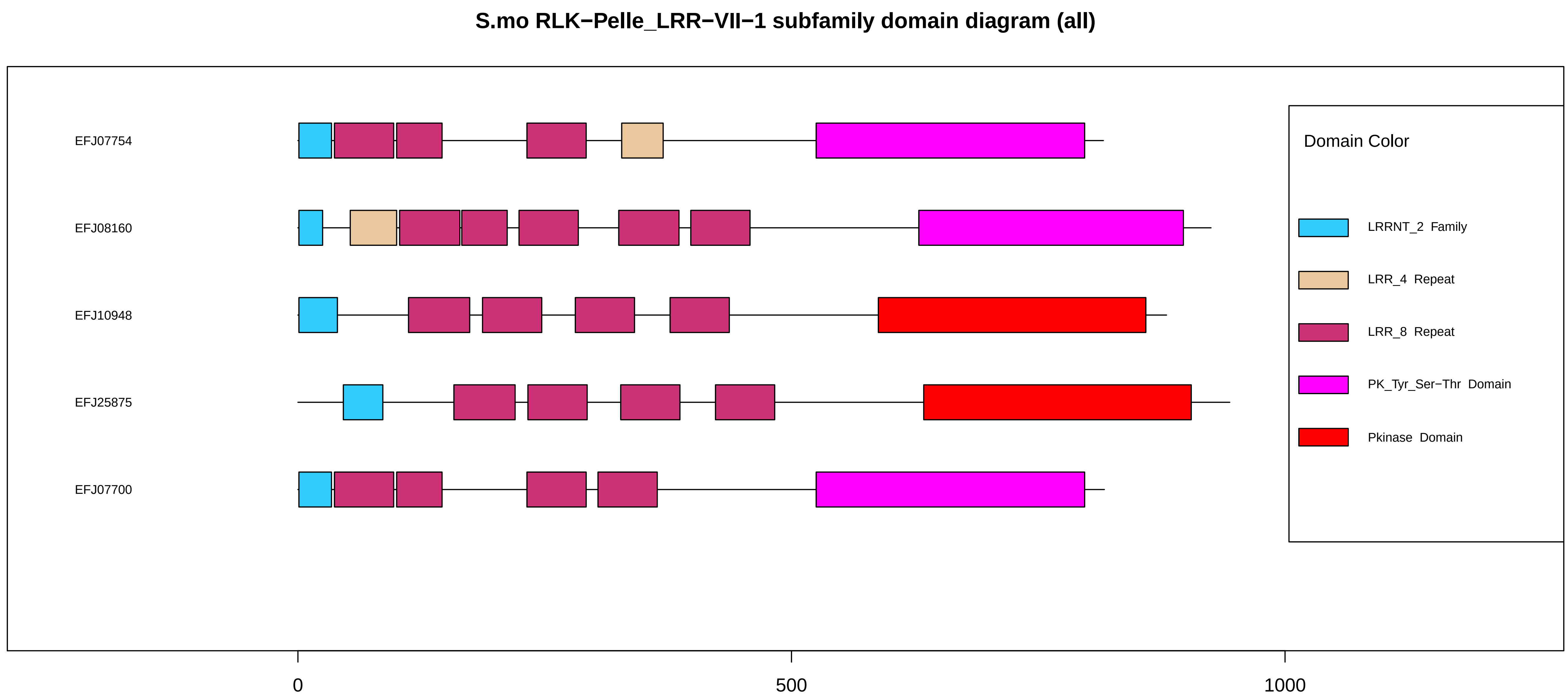
S.mo RLK−Pelle_LRR−VII−1 subfamily domain diagram (all)
EFJ07754
EFJ08160
EFJ10948
EFJ25875
EFJ07700
Domain Color
LRRNT_2 Family
LRR_4 Repeat
LRR_8 Repeat
PK_Tyr_Ser−Thr Domain
Pkinase Domain
0
500
1000

S.mo RLK−Pelle_LRR−VII−2 subfamily domain diagram (all)

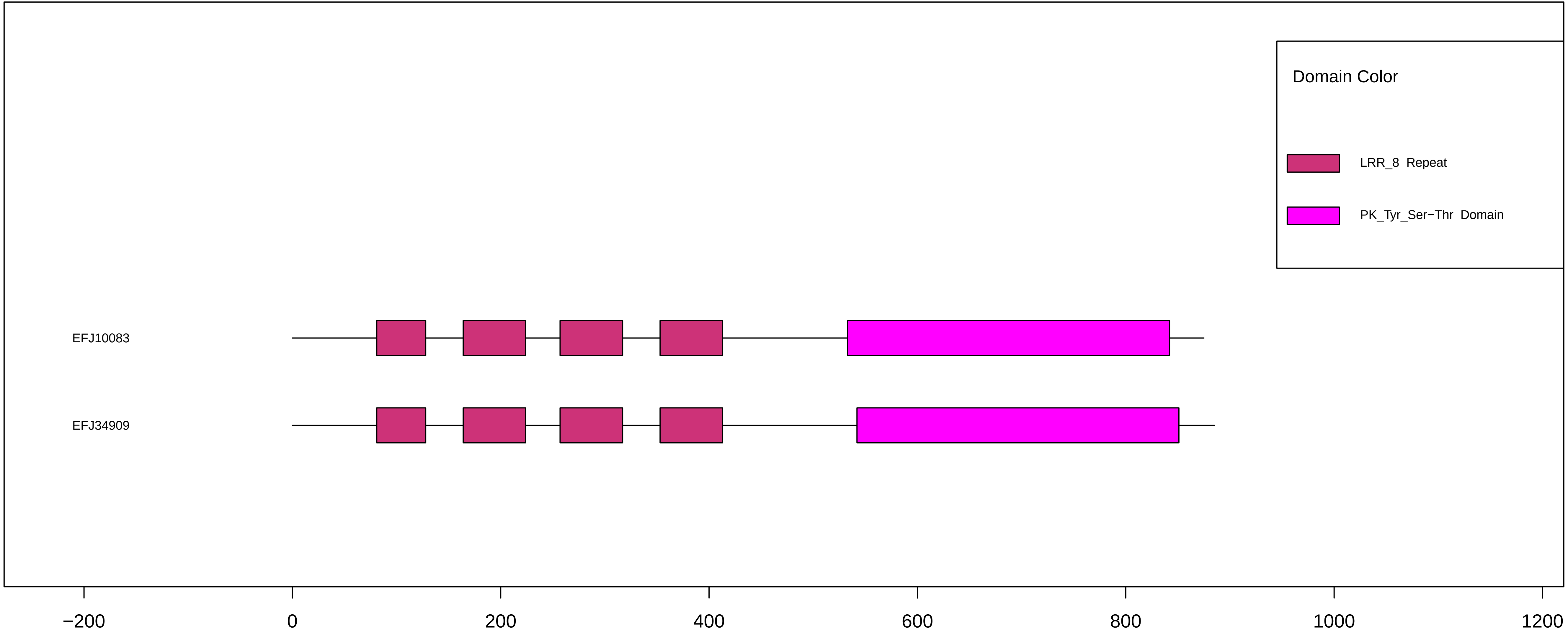

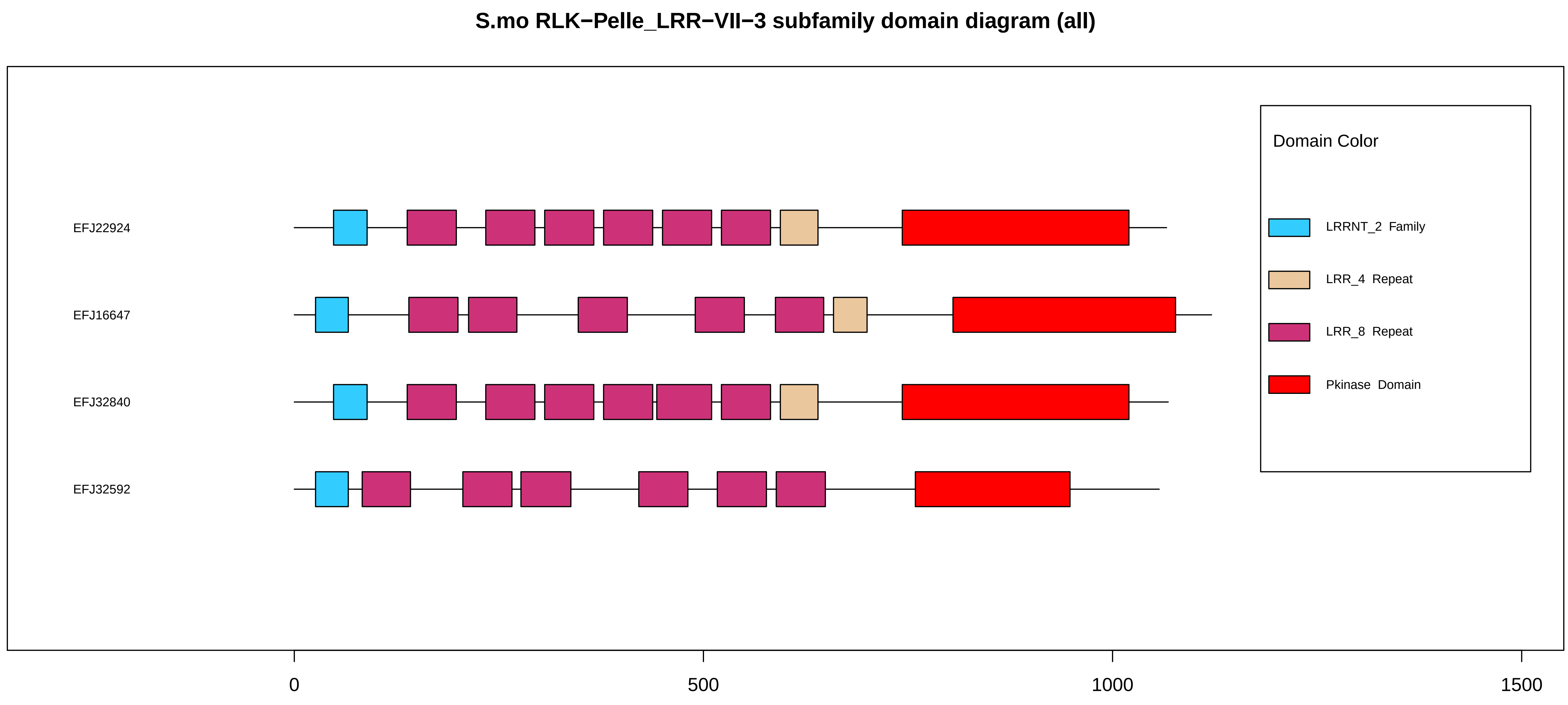
S.mo RLK−Pelle_LRR−VII−3 subfamily domain diagram (all)
EFJ22924
EFJ16647
EFJ32840
EFJ32592
Domain Color
LRRNT_2 Family
LRR_4 Repeat
LRR_8 Repeat
Pkinase Domain
0
500
1000
1500

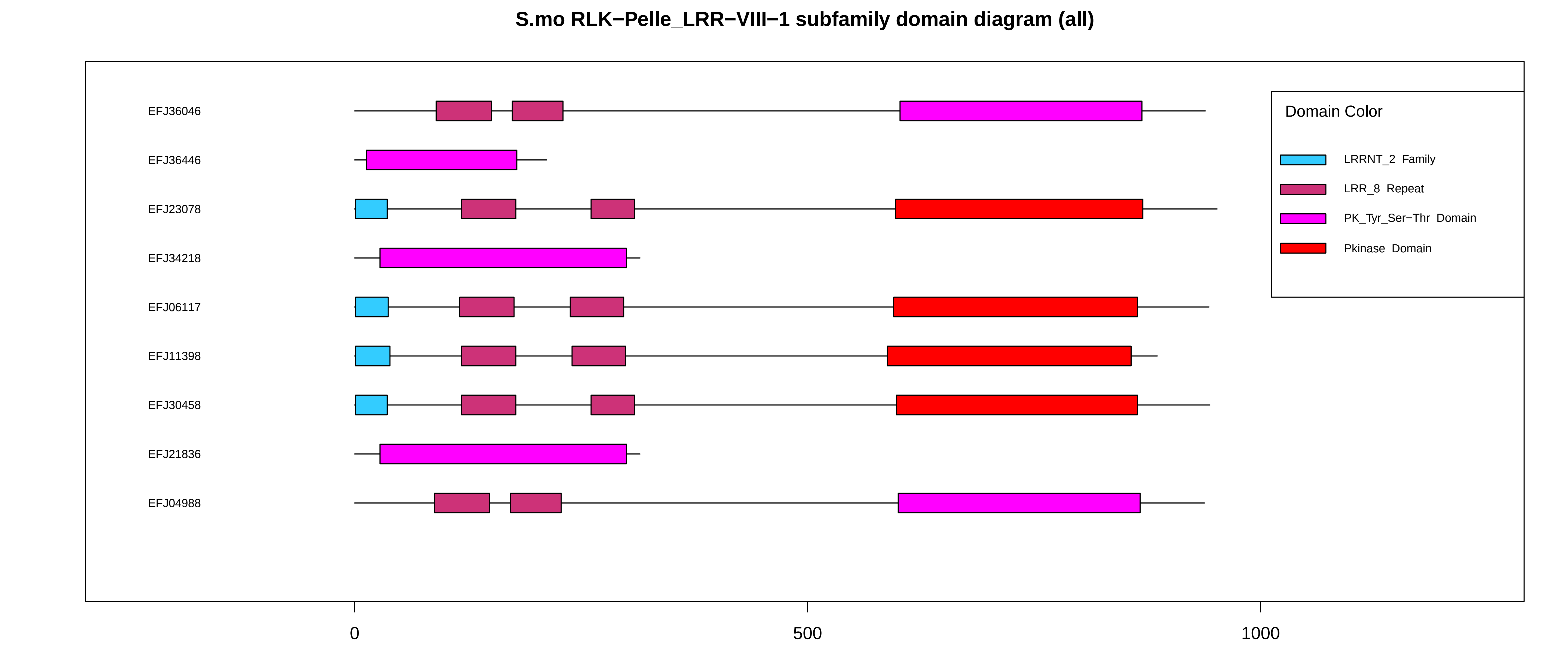

S.mo RLK−Pelle_LRR−VIII−1 subfamily domain diagram (all)
EFJ36046
EFJ36446
EFJ23078
EFJ34218
EFJ06117
EFJ11398
EFJ30458
EFJ21836
EFJ04988
Domain Color
LRRNT_2 Family
LRR_8 Repeat
PK_Tyr_Ser−Thr Domain
Pkinase Domain
0
500
1000

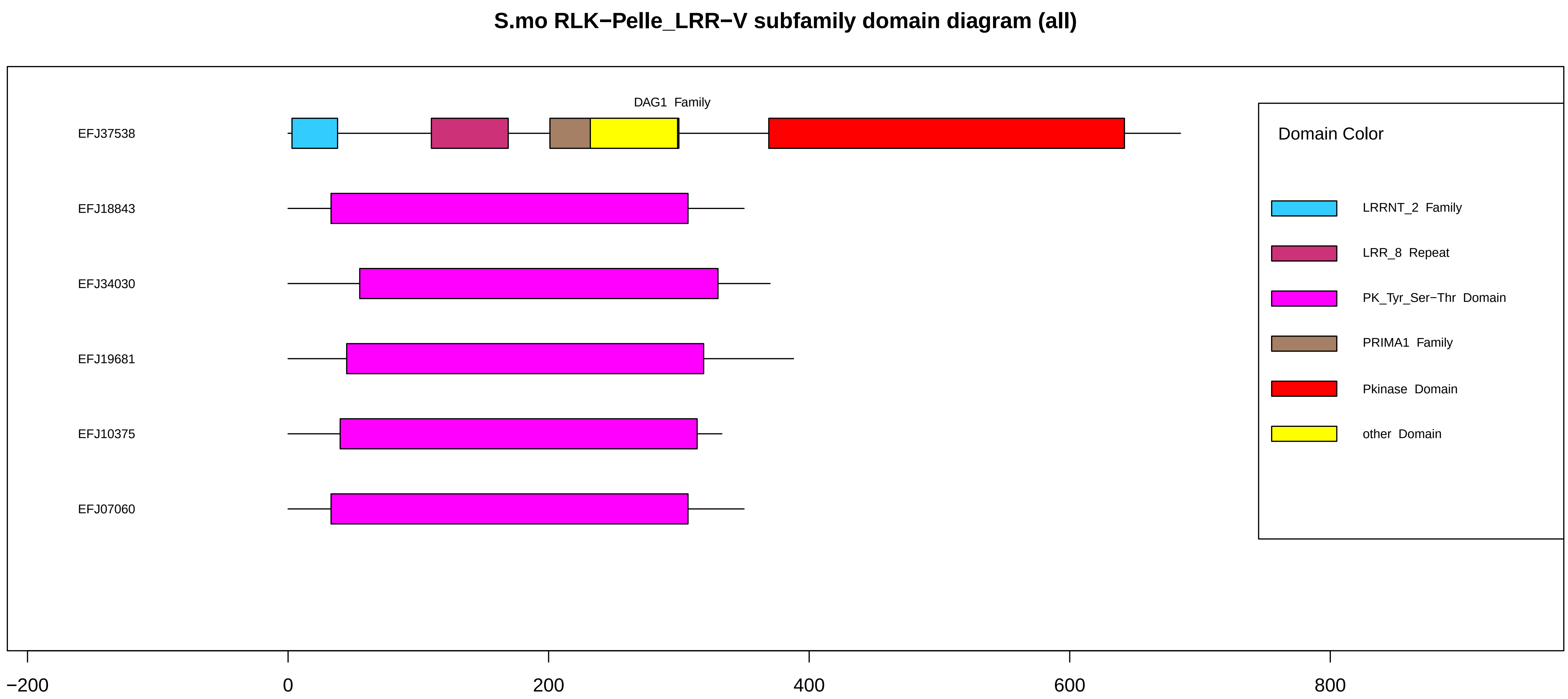
S.mo RLK−Pelle_LRR−V subfamily domain diagram (all)
DAG1 Family
EFJ37538
EFJ18843
EFJ34030
EFJ19681
EFJ10375
EFJ07060
Domain Color
LRRNT_2 Family
LRR_8 Repeat
PK_Tyr_Ser−Thr Domain
PRIMA1 Family
Pkinase Domain
other Domain
−200
0
200
400
600
800

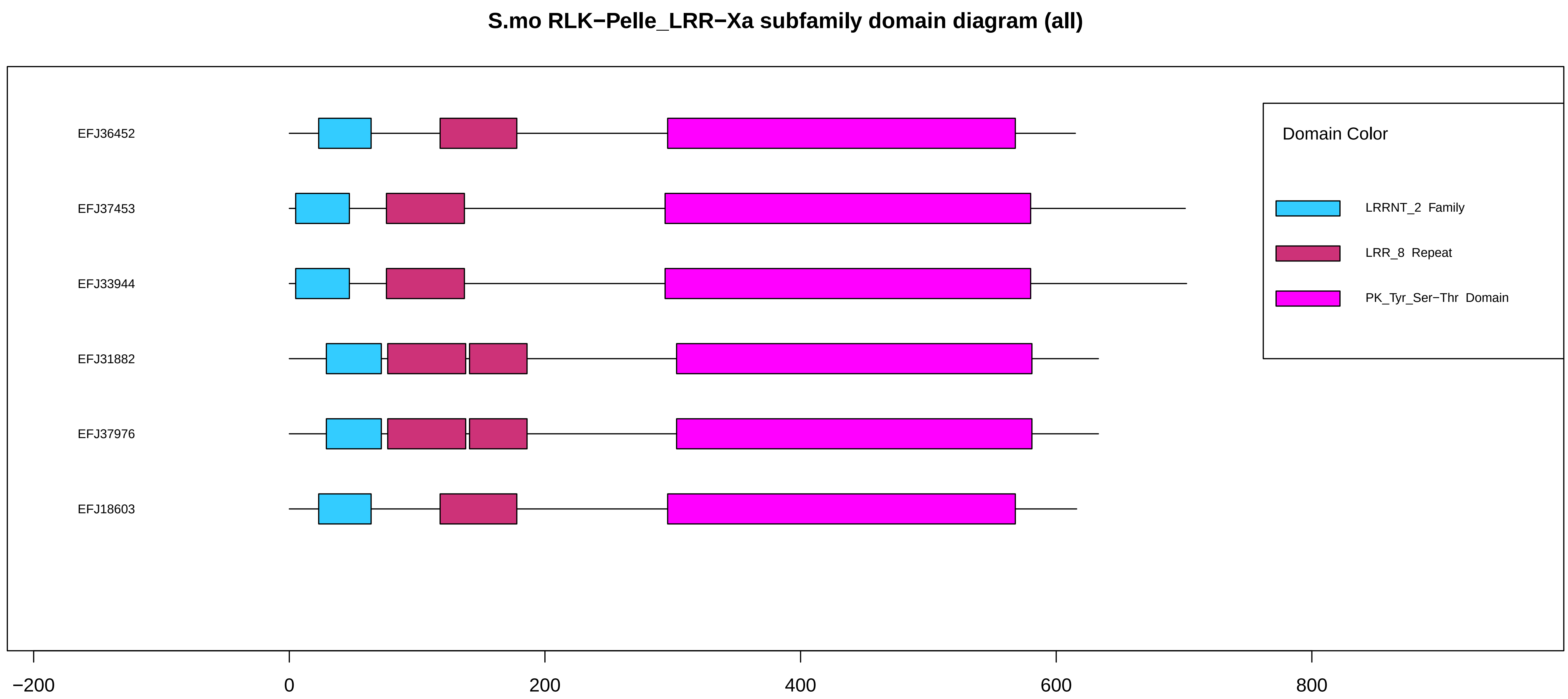

S.mo RLK−Pelle_LRR−Xa subfamily domain diagram (all)
EFJ36452
EFJ37453
EFJ33944
EFJ31882
EFJ37976
EFJ18603
Domain Color
LRRNT_2 Family
LRR_8 Repeat
PK_Tyr_Ser−Thr Domain
−200
0
200
400
600
800

S.mo RLK−Pelle_LRR−Xb−1 subfamily domain diagram (all)

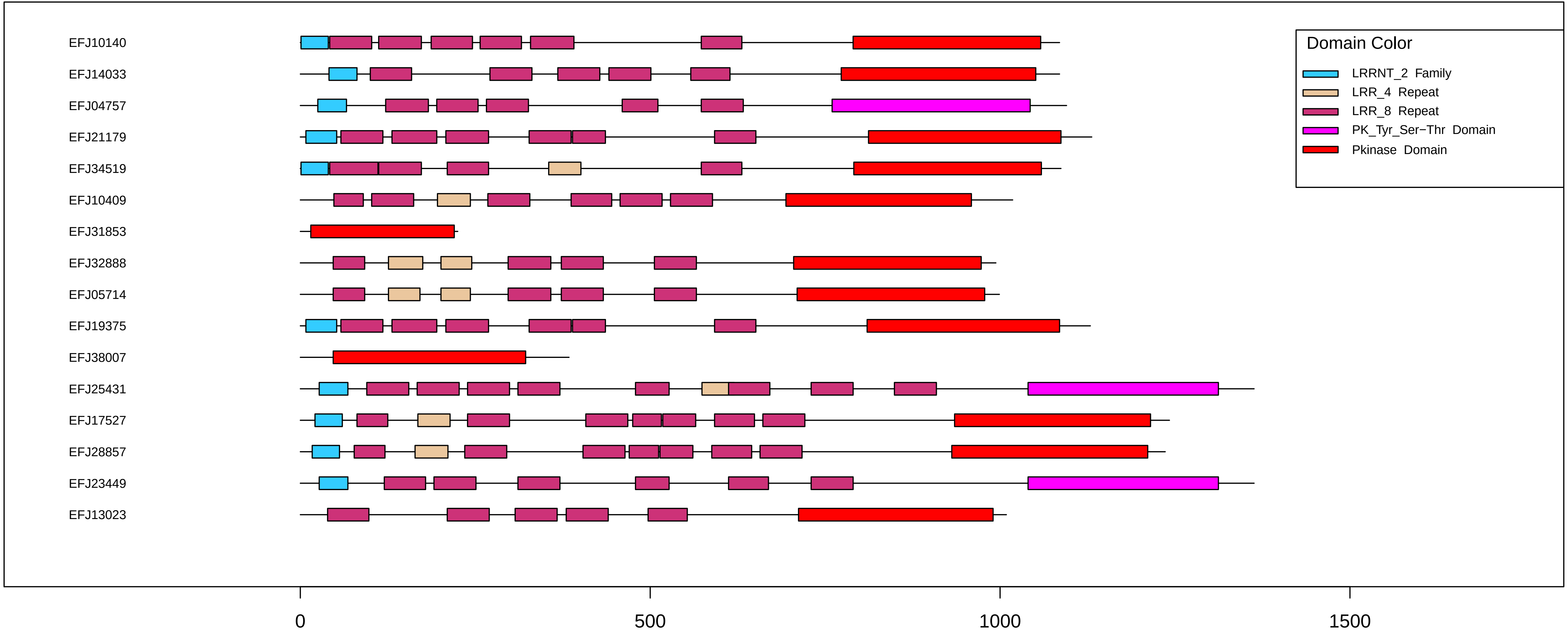

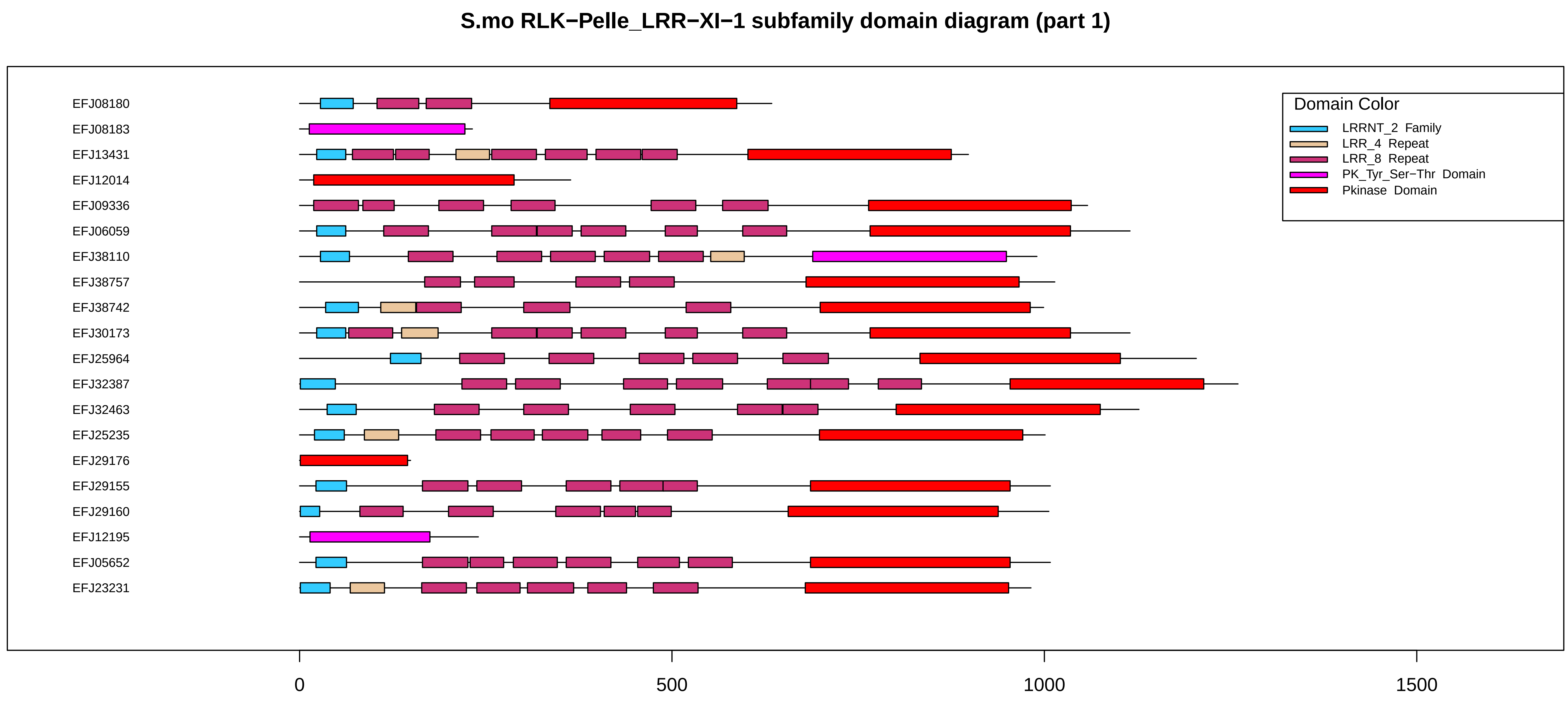
S.mo RLK−Pelle_LRR−XI−1 subfamily domain diagram (part 1)
Domain Color
LRRNT_2 Family
LRR_4 Repeat
LRR_8 Repeat
PK_Tyr_Ser−Thr Domain
Pkinase Domain
EFJ08180
EFJ08183
EFJ13431
EFJ12014
EFJ09336
EFJ06059
EFJ38110
EFJ38757
EFJ38742
EFJ30173
EFJ25964
EFJ32387
EFJ32463
EFJ25235
EFJ29176
EFJ29155
EFJ29160
EFJ12195
EFJ05652
EFJ23231
0
500
1000
1500

S.mo RLK−Pelle_LRR−XI−1 subfamily domain diagram (part 2)

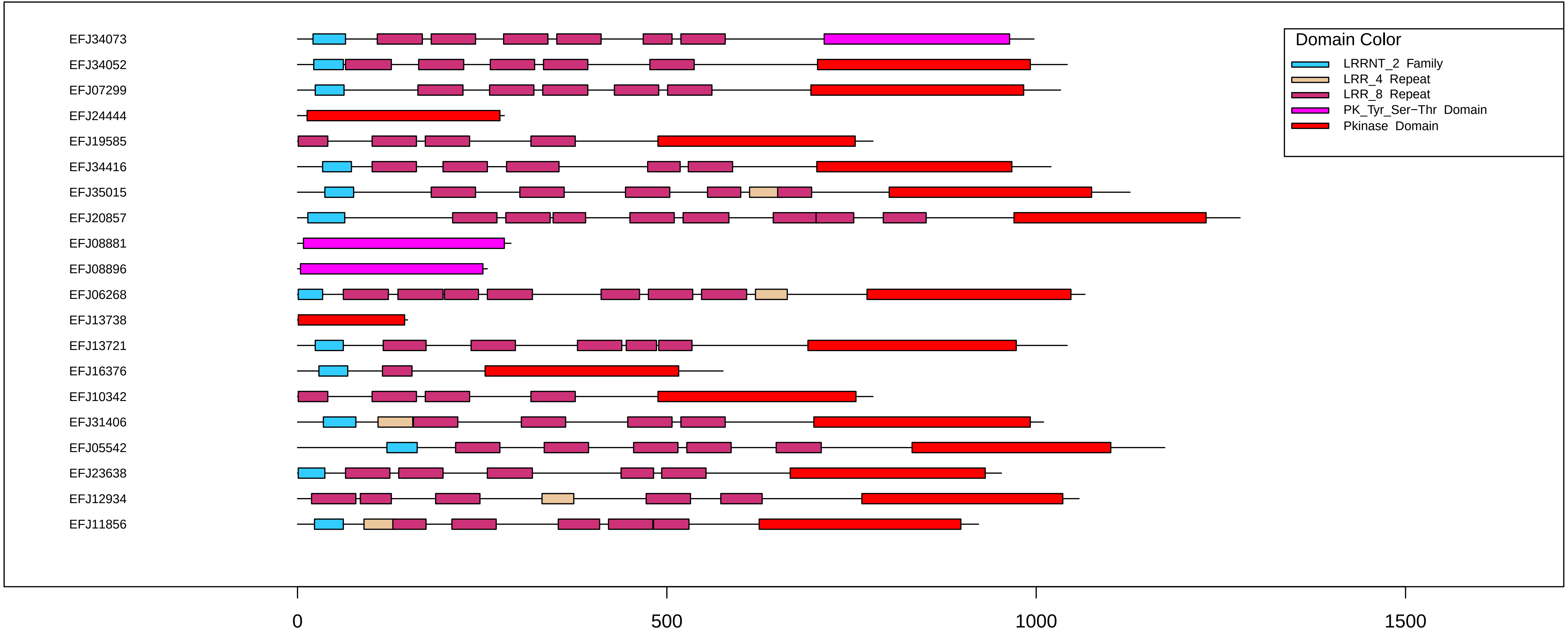

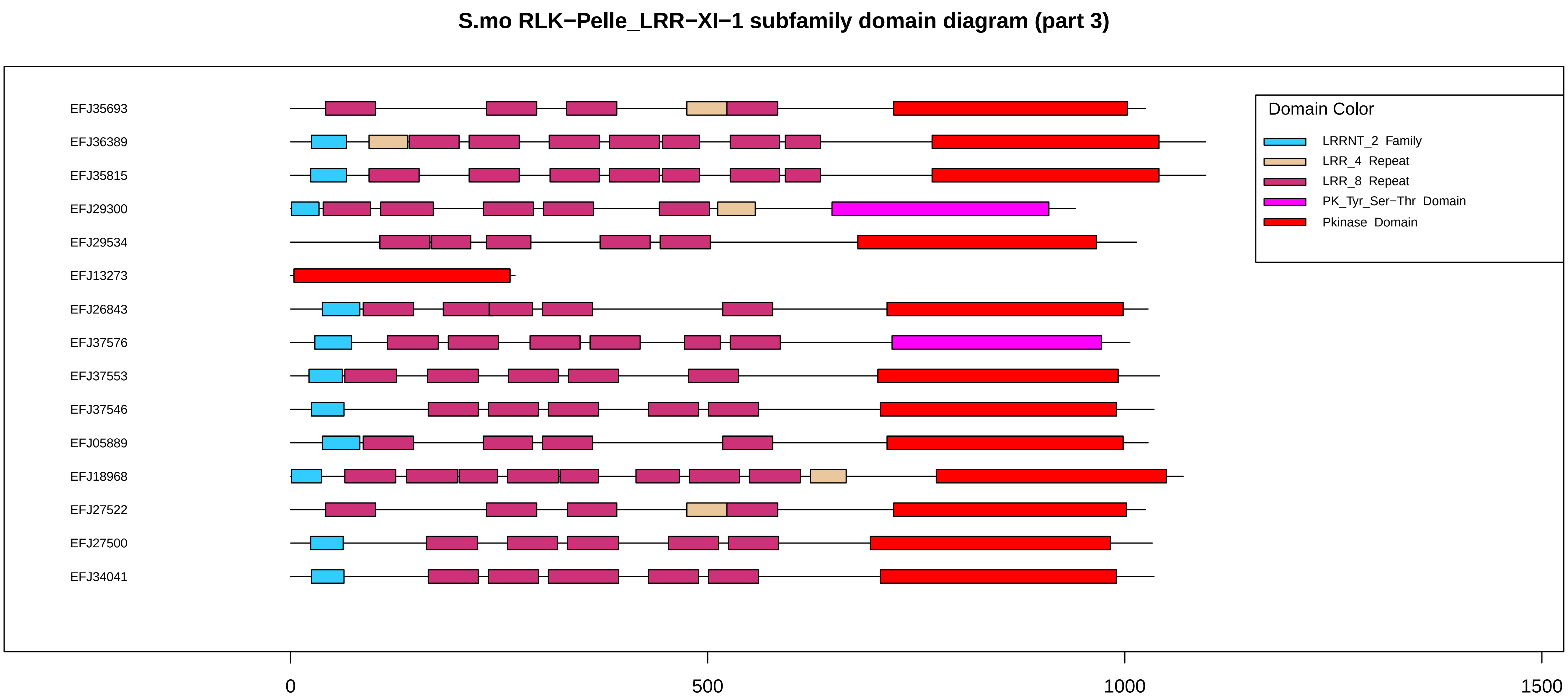

S.mo RLK−Pelle_LRR−XI−1 subfamily domain diagram (part 3)
EFJ35693
EFJ36389
EFJ35815
EFJ29300
EFJ29534
EFJ13273
EFJ26843
EFJ37576
EFJ37553
EFJ37546
EFJ05889
EFJ18968
EFJ27522
EFJ27500
EFJ34041
Domain Color
LRRNT_2 Family
LRR_4 Repeat
LRR_8 Repeat
PK_Tyr_Ser−Thr Domain
Pkinase Domain
0
500
1000
1500

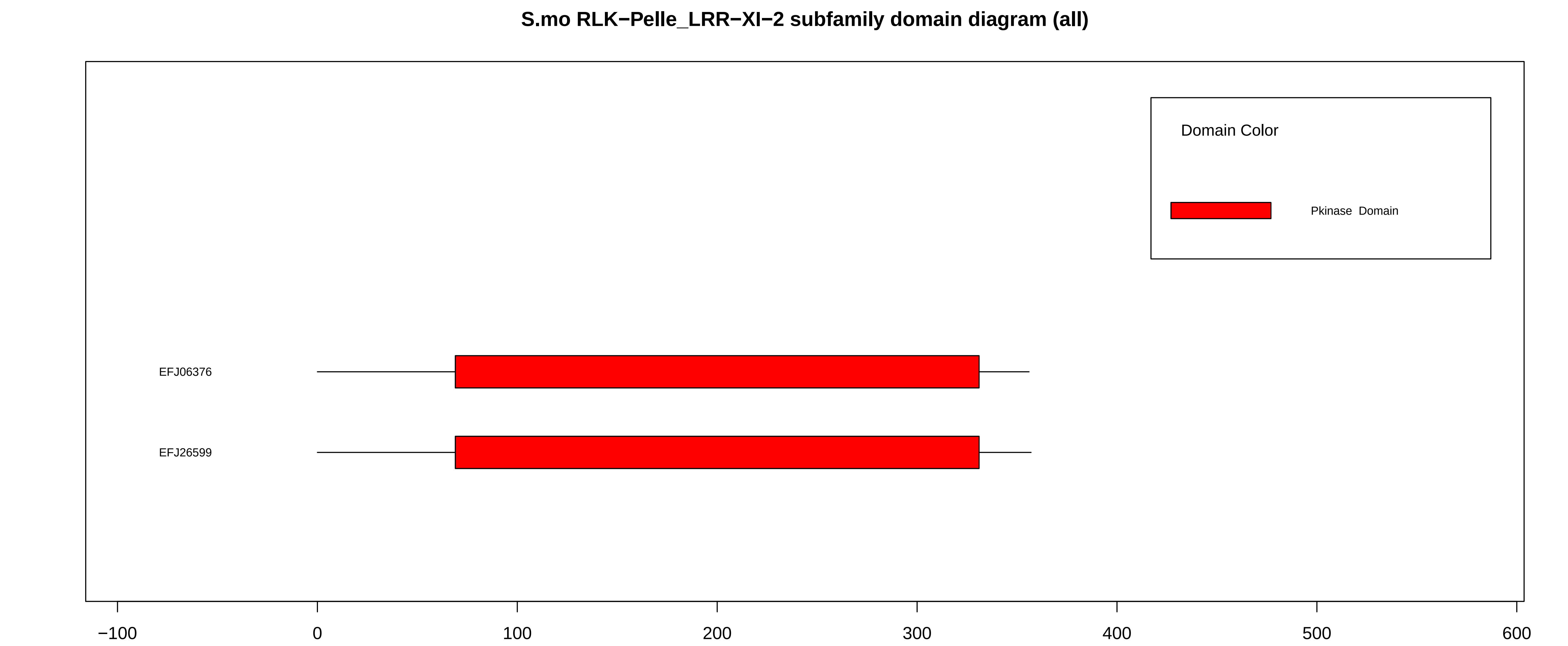
S.mo RLK−Pelle_LRR−XI−2 subfamily domain diagram (all)
Domain Color
Pkinase Domain
EFJ06376
EFJ26599
−100
0
100
200
300
400
500
600

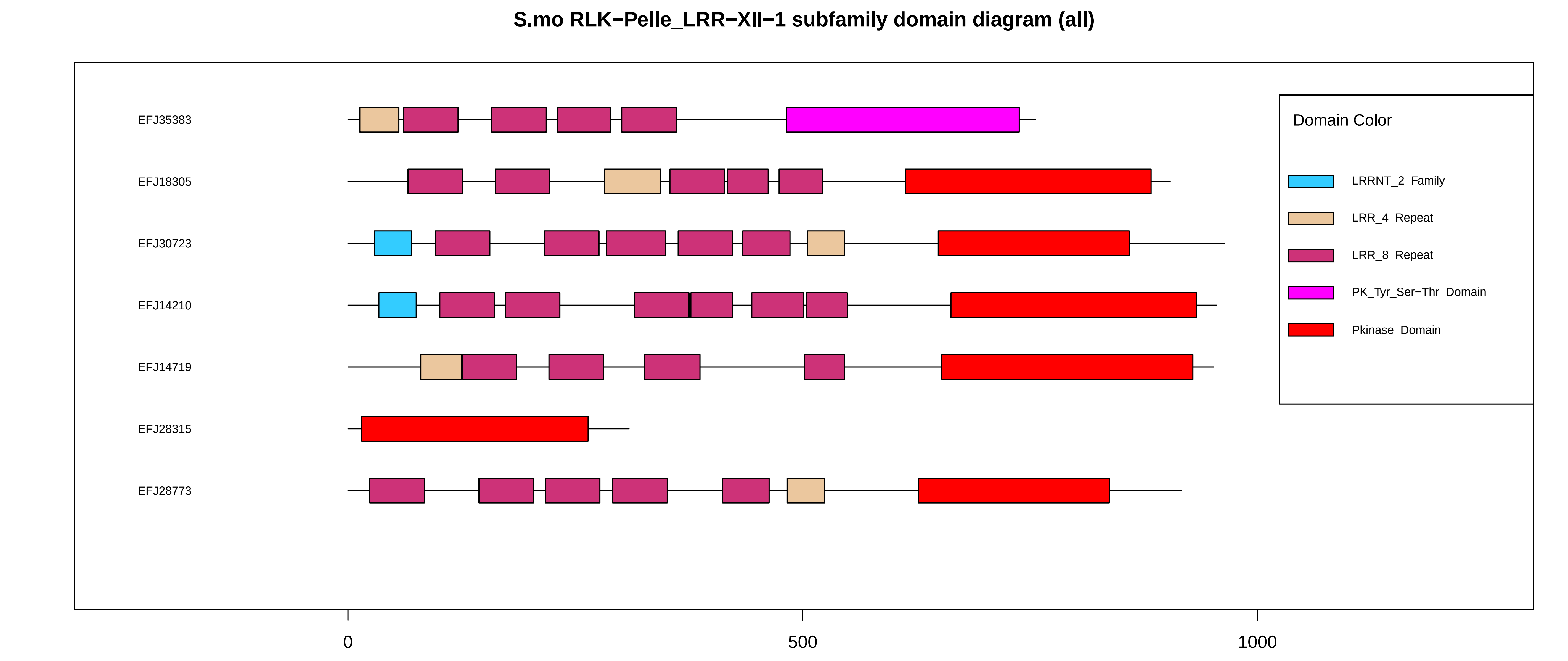
S.mo RLK−Pelle_LRR−XII−1 subfamily domain diagram (all)
EFJ35383
EFJ18305
EFJ30723
EFJ14210
EFJ14719
EFJ28315
EFJ28773
Domain Color
LRRNT_2 Family
LRR_4 Repeat
LRR_8 Repeat
PK_Tyr_Ser−Thr Domain
Pkinase Domain
0
500
1000

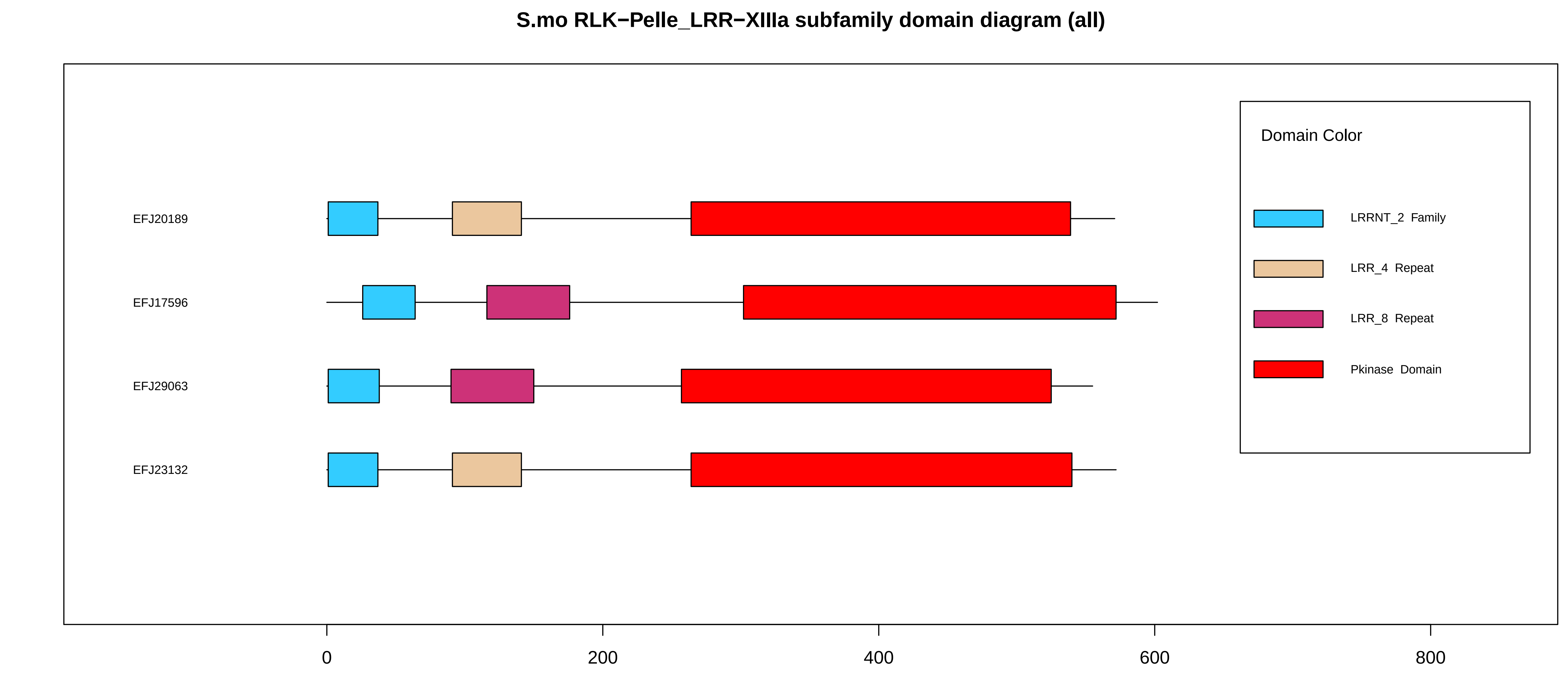
S.mo RLK−Pelle_LRR−XIIIa subfamily domain diagram (all)
EFJ20189
EFJ17596
EFJ29063
EFJ23132
Domain Color
LRRNT_2 Family
LRR_4 Repeat
LRR_8 Repeat
Pkinase Domain
0
200
400
600
800

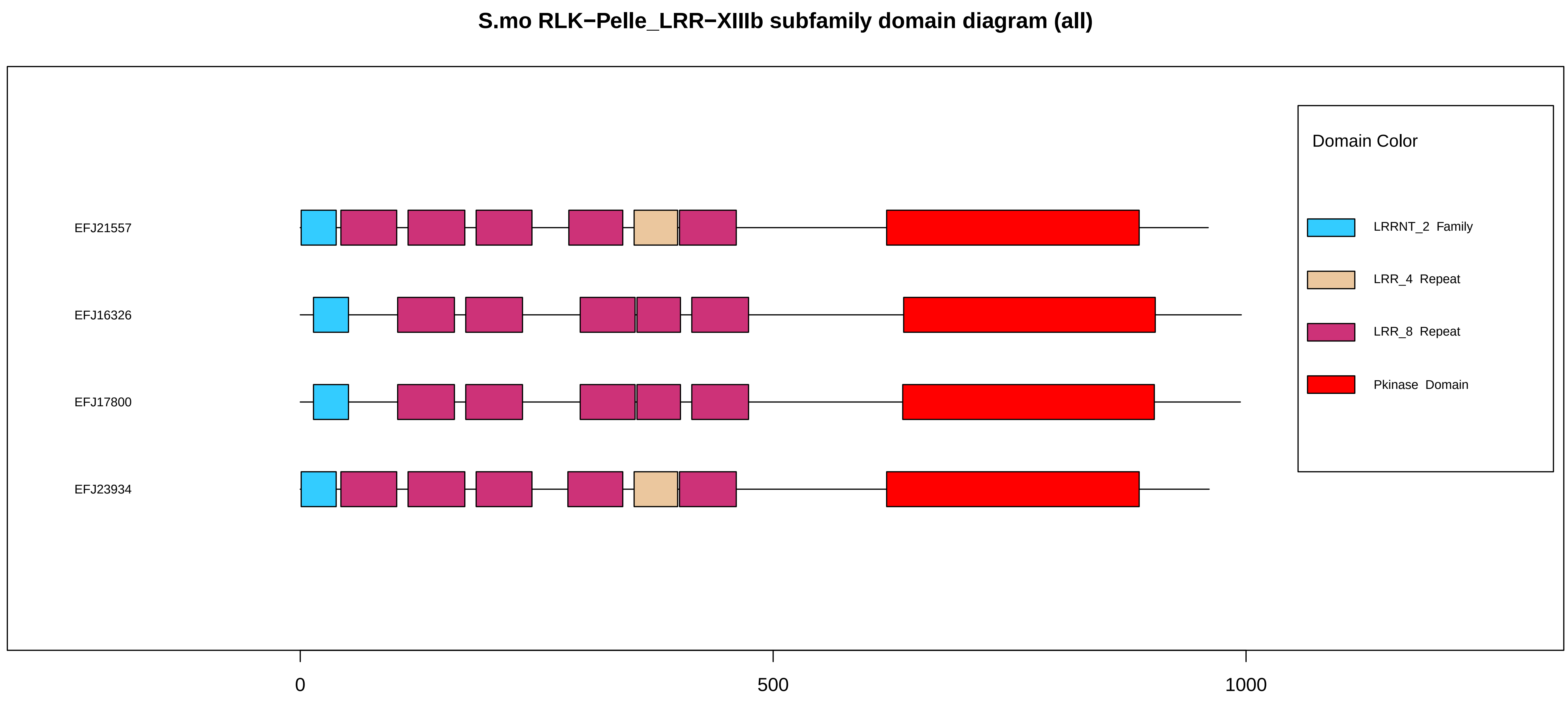
S.mo RLK−Pelle_LRR−XIIIb subfamily domain diagram (all)
EFJ21557
EFJ16326
EFJ17800
EFJ23934
Domain Color
LRRNT_2 Family
LRR_4 Repeat
LRR_8 Repeat
Pkinase Domain
0
500
1000

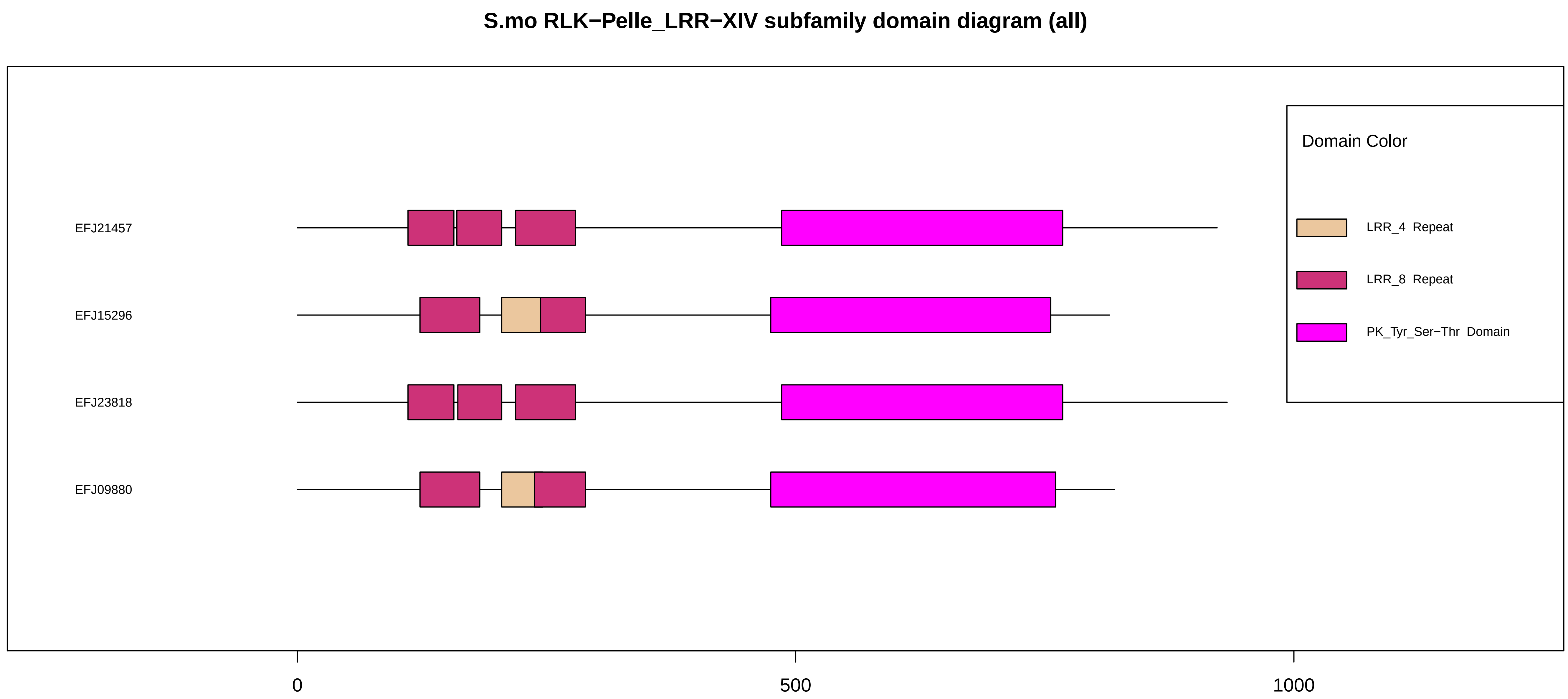
S.mo RLK−Pelle_LRR−XIV subfamily domain diagram (all)
EFJ21457
EFJ15296
EFJ23818
EFJ09880
Domain Color
LRR_4 Repeat
LRR_8 Repeat
PK_Tyr_Ser−Thr Domain
0
500
1000

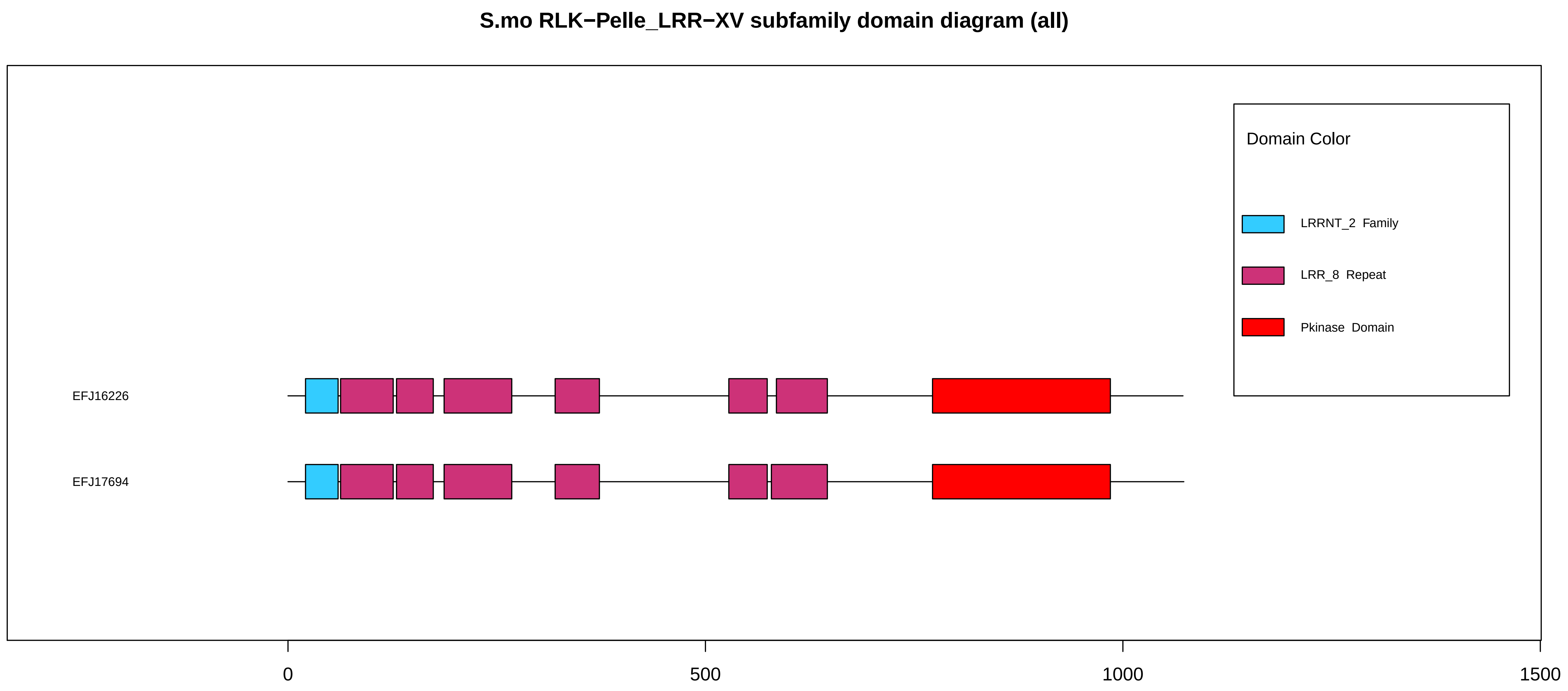
S.mo RLK−Pelle_LRR−XV subfamily domain diagram (all)
Domain Color
LRRNT_2 Family
LRR_8 Repeat
Pkinase Domain
EFJ16226
EFJ17694
0
500
1000
1500

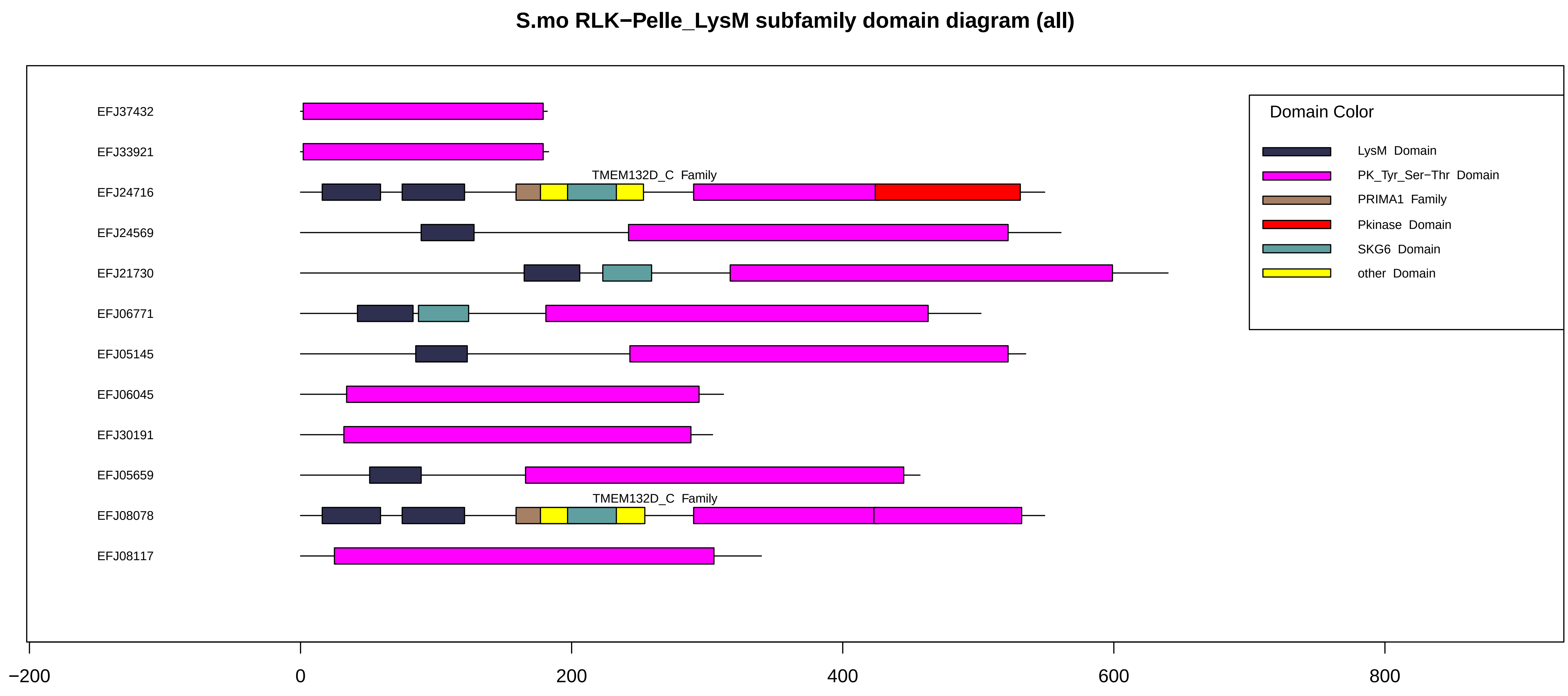

S.mo RLK−Pelle_LysM subfamily domain diagram (all)
EFJ37432
EFJ33921
EFJ24716
TMEM132D_C Family
EFJ24569
EFJ21730
EFJ06771
EFJ05145
EFJ06045
EFJ30191
EFJ05659
EFJ08078
TMEM132D_C Family
EFJ08117
Domain Color
LysM Domain
PK_Tyr_Ser−Thr Domain
PRIMA1 Family
Pkinase Domain
SKG6 Domain
other Domain
−200
0
200
400
600
800

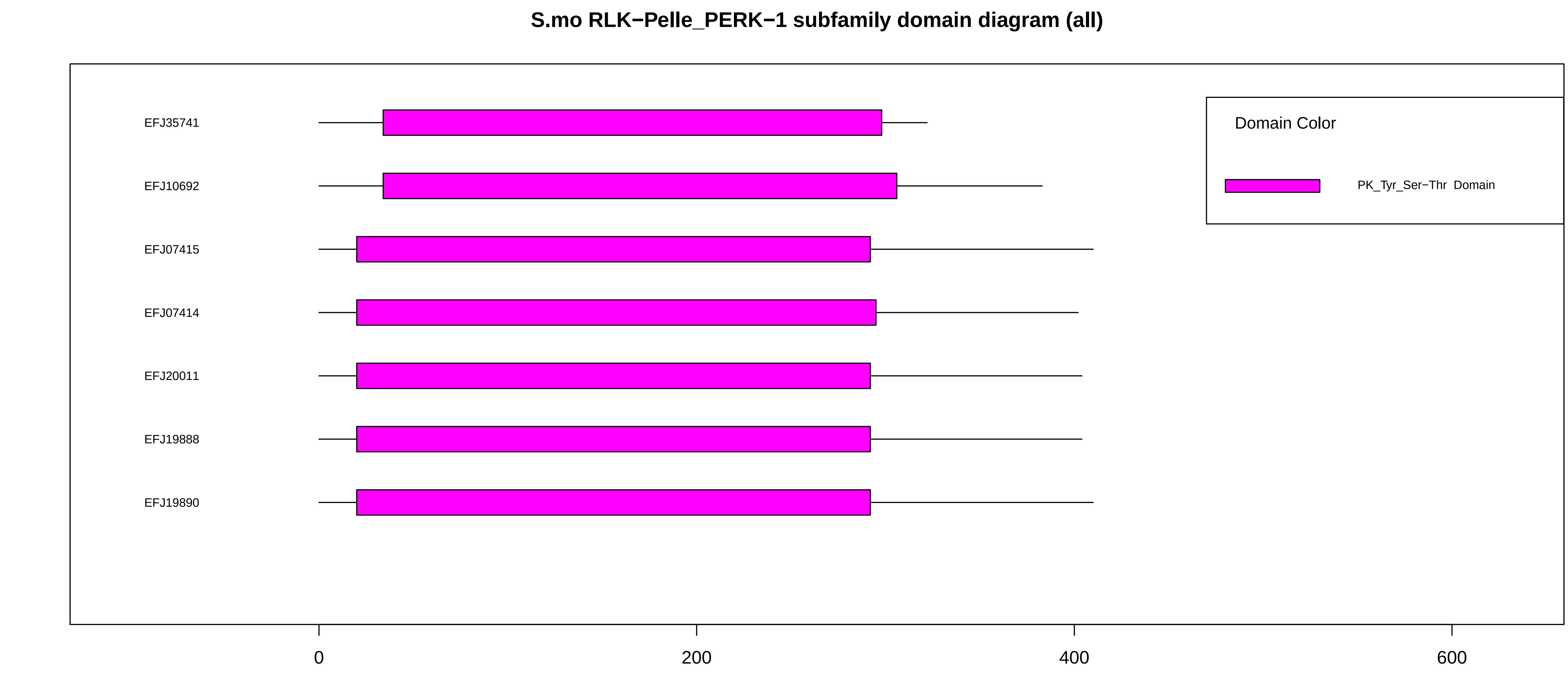

S.mo RLK−Pelle_PERK−1 subfamily domain diagram (all)
EFJ35741
EFJ10692
EFJ07415
EFJ07414
EFJ20011
EFJ19888
EFJ19890
Domain Color
PK_Tyr_Ser−Thr Domain
0
200
400
600

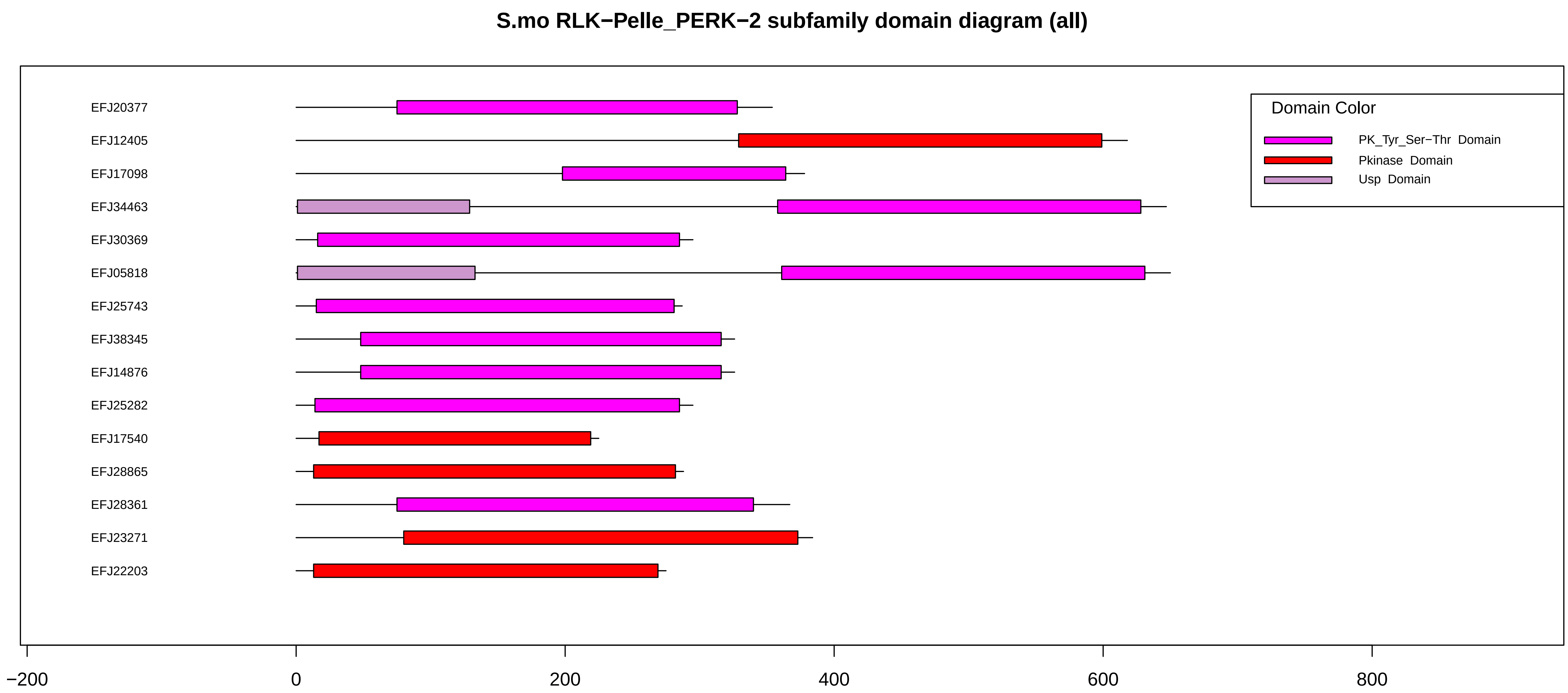
S.mo RLK−Pelle_PERK−2 subfamily domain diagram (all)
EFJ20377
EFJ12405
EFJ17098
EFJ34463
EFJ30369
EFJ05818
EFJ25743
EFJ38345
EFJ14876
EFJ25282
EFJ17540
EFJ28865
EFJ28361
EFJ23271
EFJ22203
Domain Color
PK_Tyr_Ser−Thr Domain
Pkinase Domain
Usp Domain
−200
0
200
400
600
800

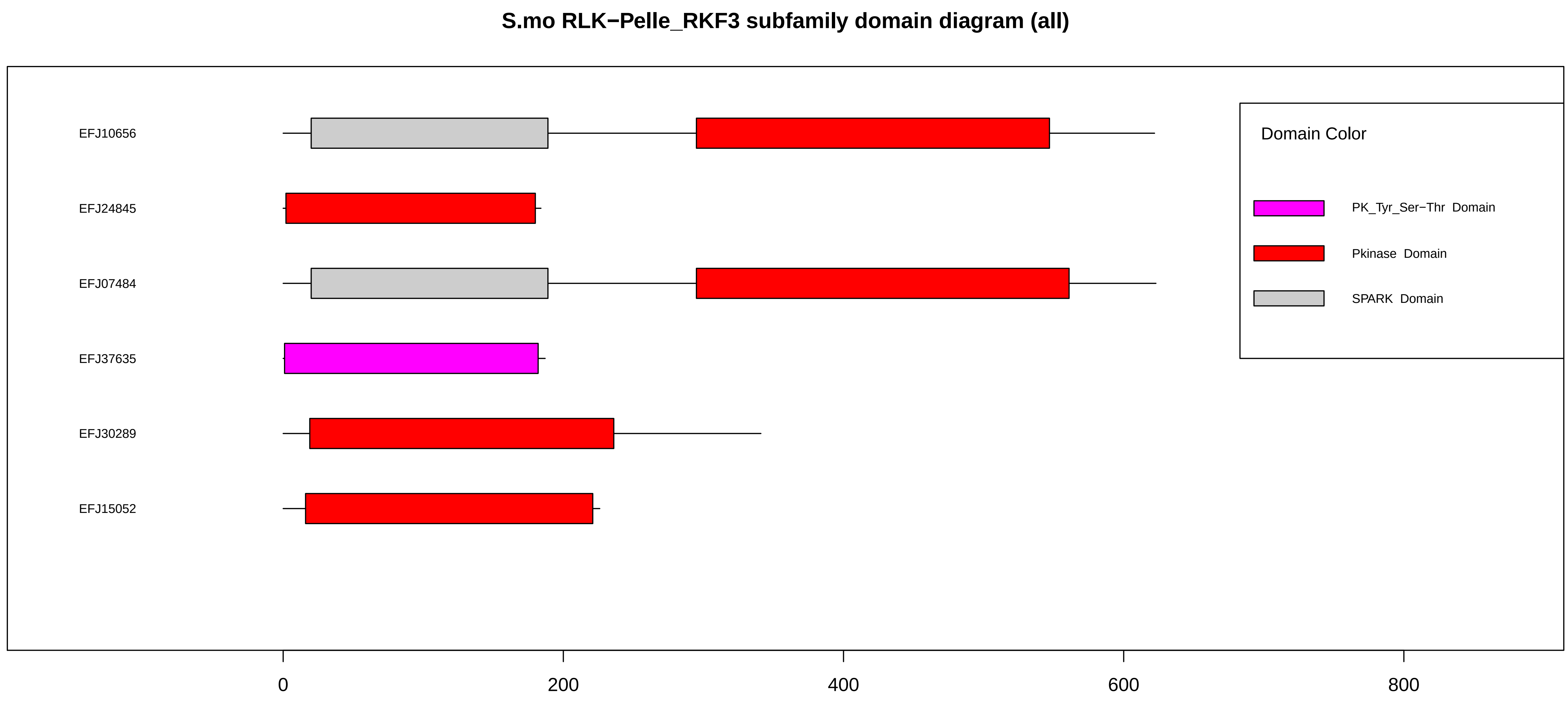
S.mo RLK−Pelle_RKF3 subfamily domain diagram (all)
EFJ10656
EFJ24845
EFJ07484
EFJ37635
EFJ30289
EFJ15052
Domain Color
PK_Tyr_Ser−Thr Domain
Pkinase Domain
SPARK Domain
0
200
400
600
800

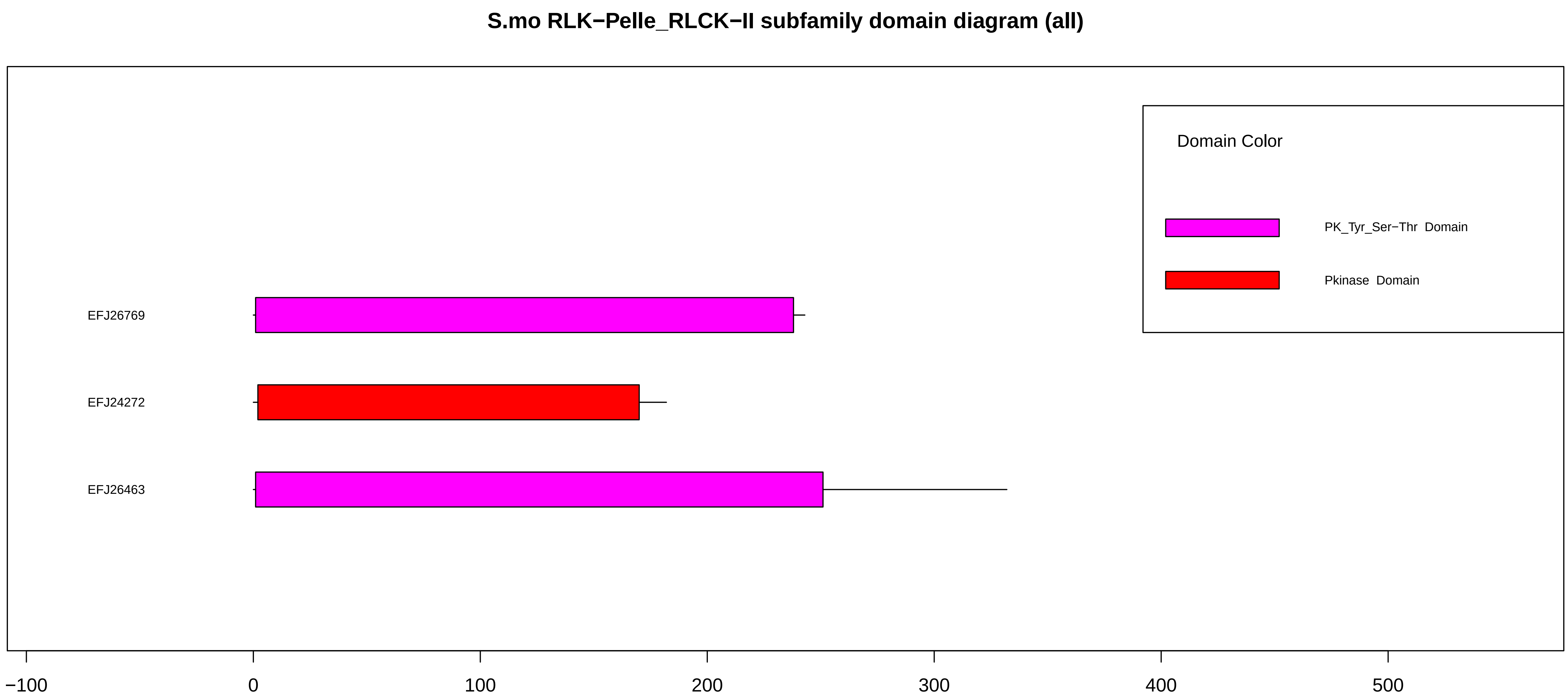

S.mo RLK−Pelle_RLCK−II subfamily domain diagram (all)
Domain Color
PK_Tyr_Ser−Thr Domain
Pkinase Domain
EFJ26769
EFJ24272
EFJ26463
−100
0
100
200
300
400
500

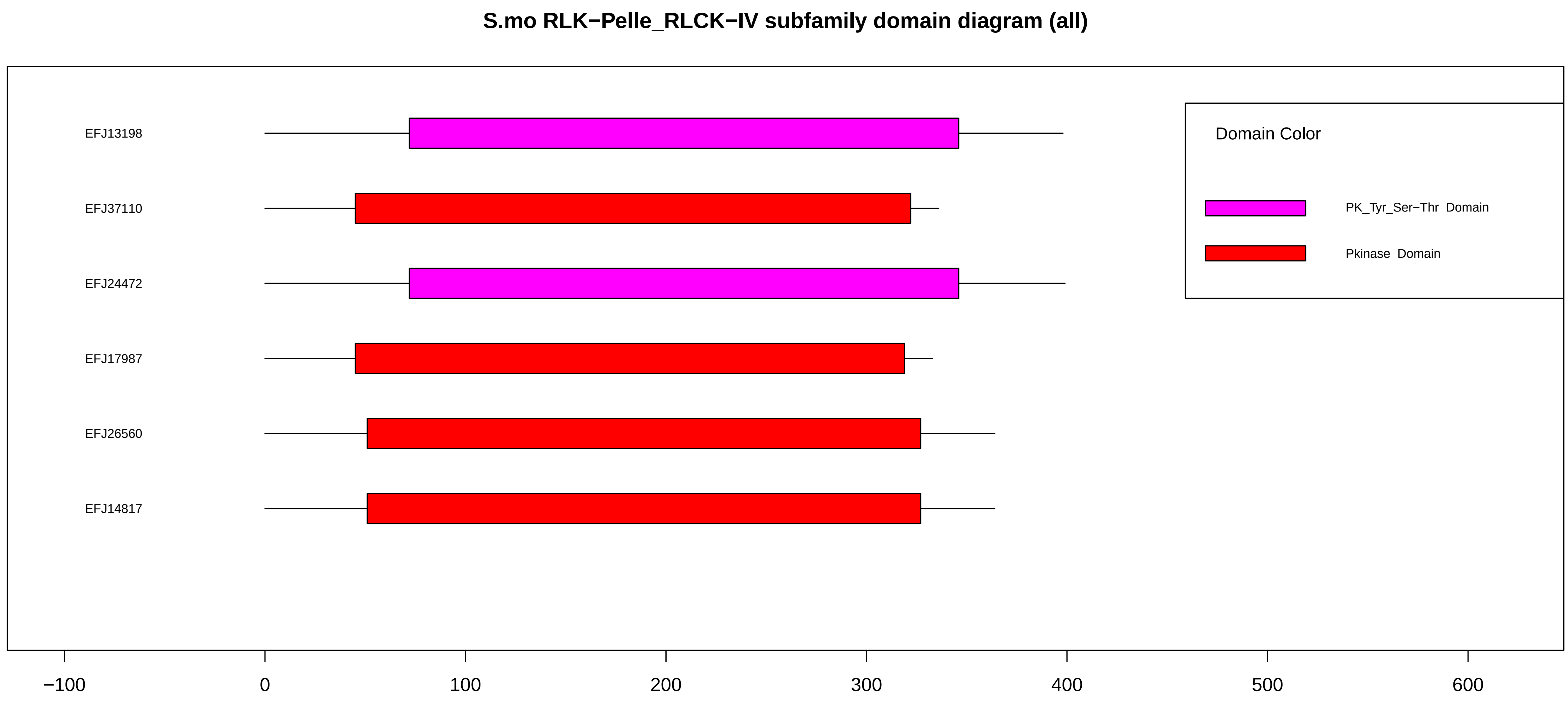
S.mo RLK−Pelle_RLCK−IV subfamily domain diagram (all)
EFJ13198
EFJ37110
EFJ24472
EFJ17987
EFJ26560
EFJ14817
Domain Color
PK_Tyr_Ser−Thr Domain
Pkinase Domain
−100
0
100
200
300
400
500
600

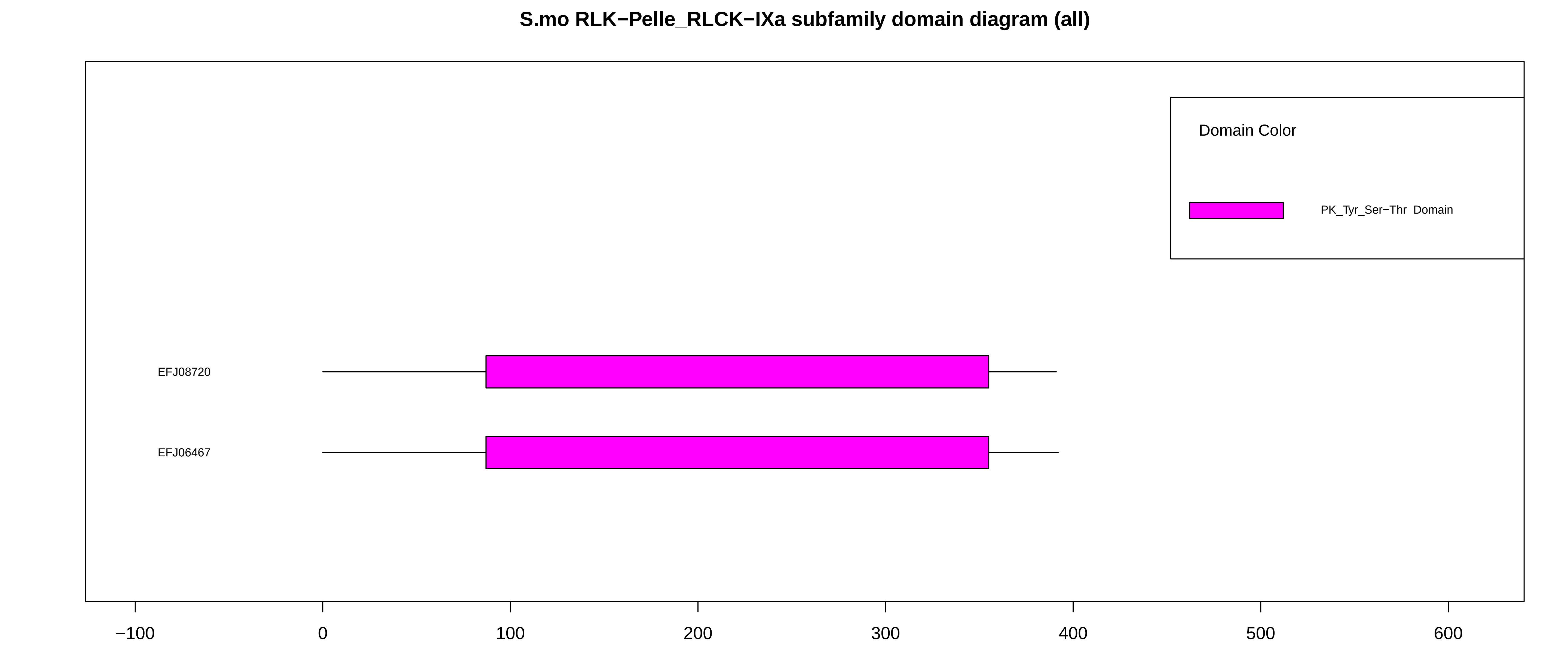

S.mo RLK−Pelle_RLCK−IXa subfamily domain diagram (all)
Domain Color
PK_Tyr_Ser−Thr Domain
EFJ08720
EFJ06467
−100
0
100
200
300
400
500
600

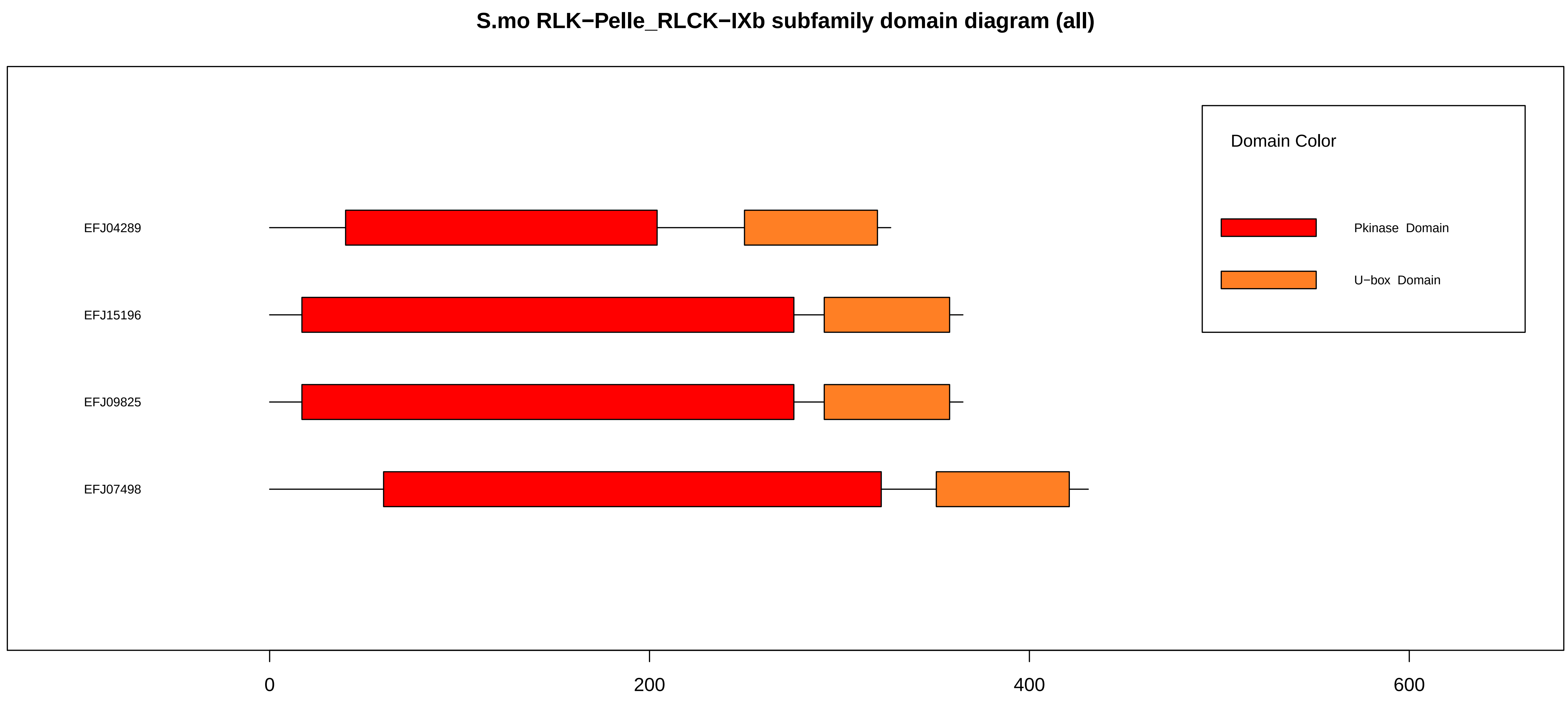
S.mo RLK−Pelle_RLCK−IXb subfamily domain diagram (all)
Domain Color
Pkinase Domain
U−box Domain
EFJ04289
EFJ15196
EFJ09825
EFJ07498
0
200
400
600

S.mo RLK−Pelle_RLCK−VIIa−1 subfamily domain diagram (all)

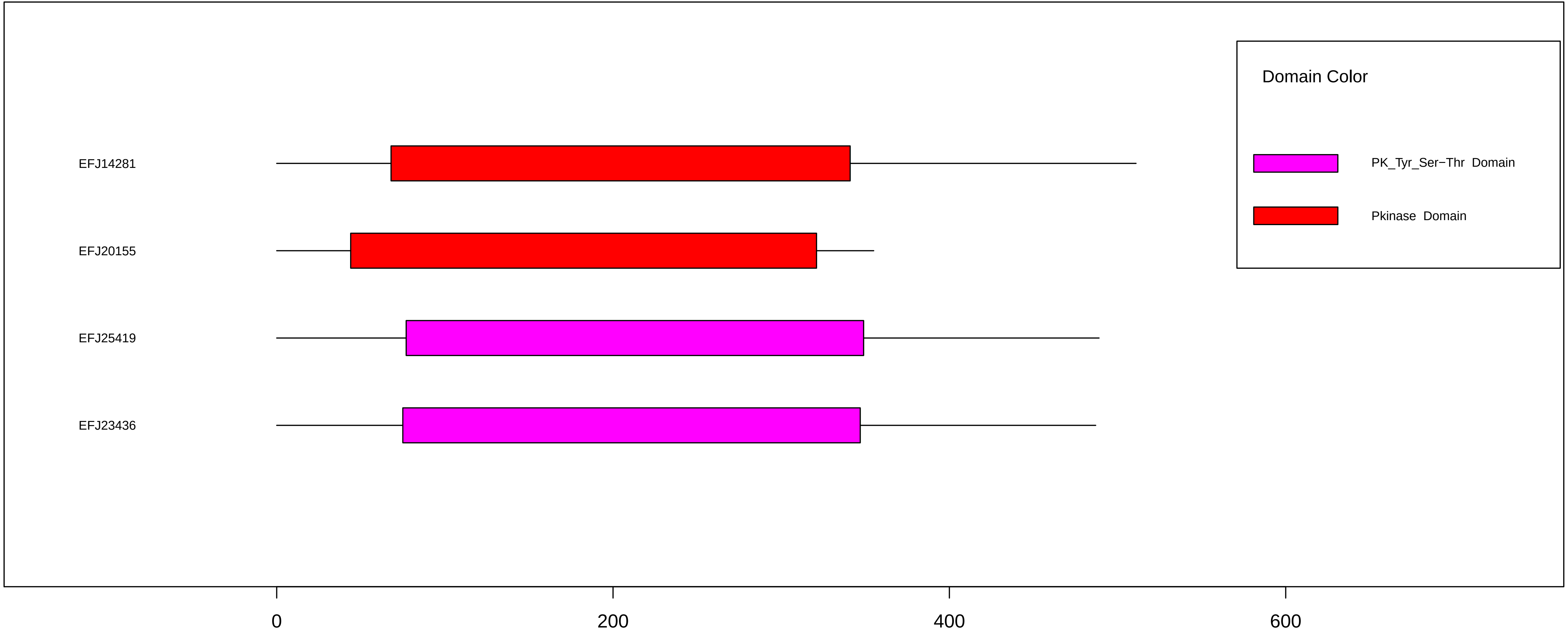

S.mo RLK−Pelle_RLCK−VIIa−2 subfamily domain diagram (all)

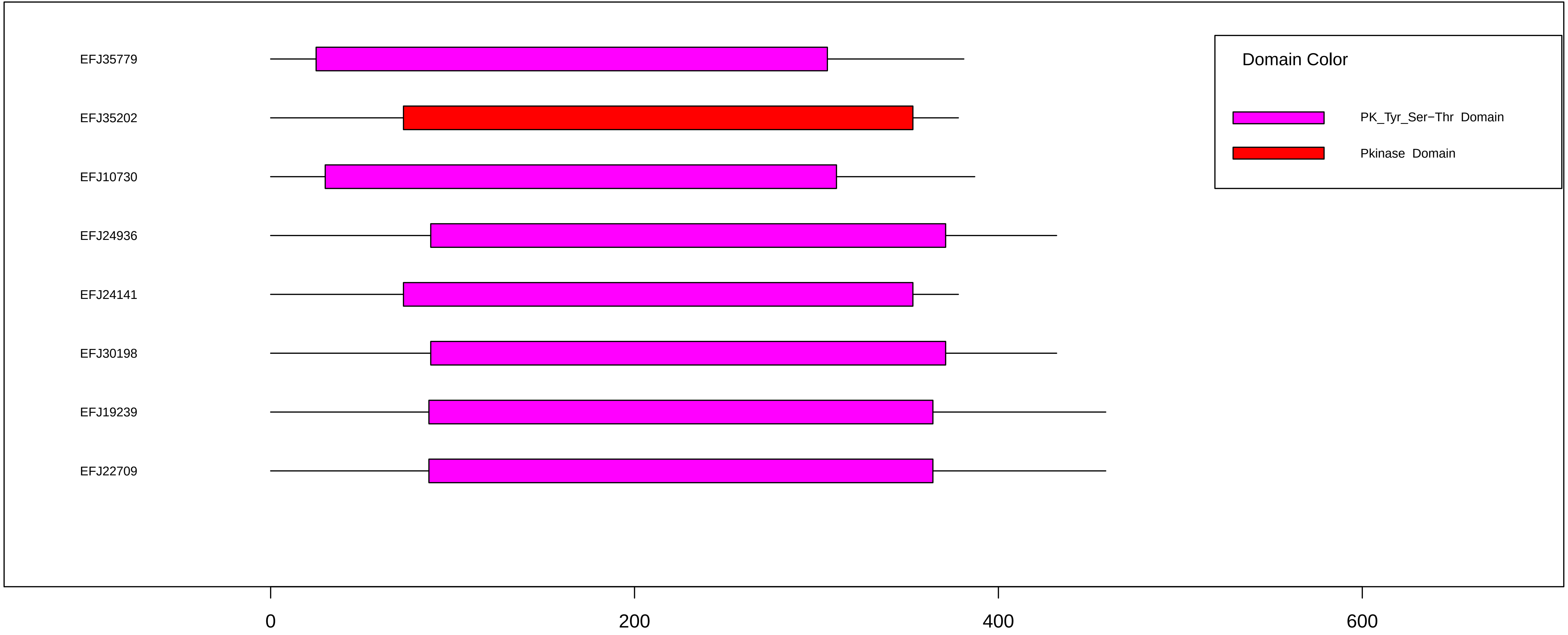

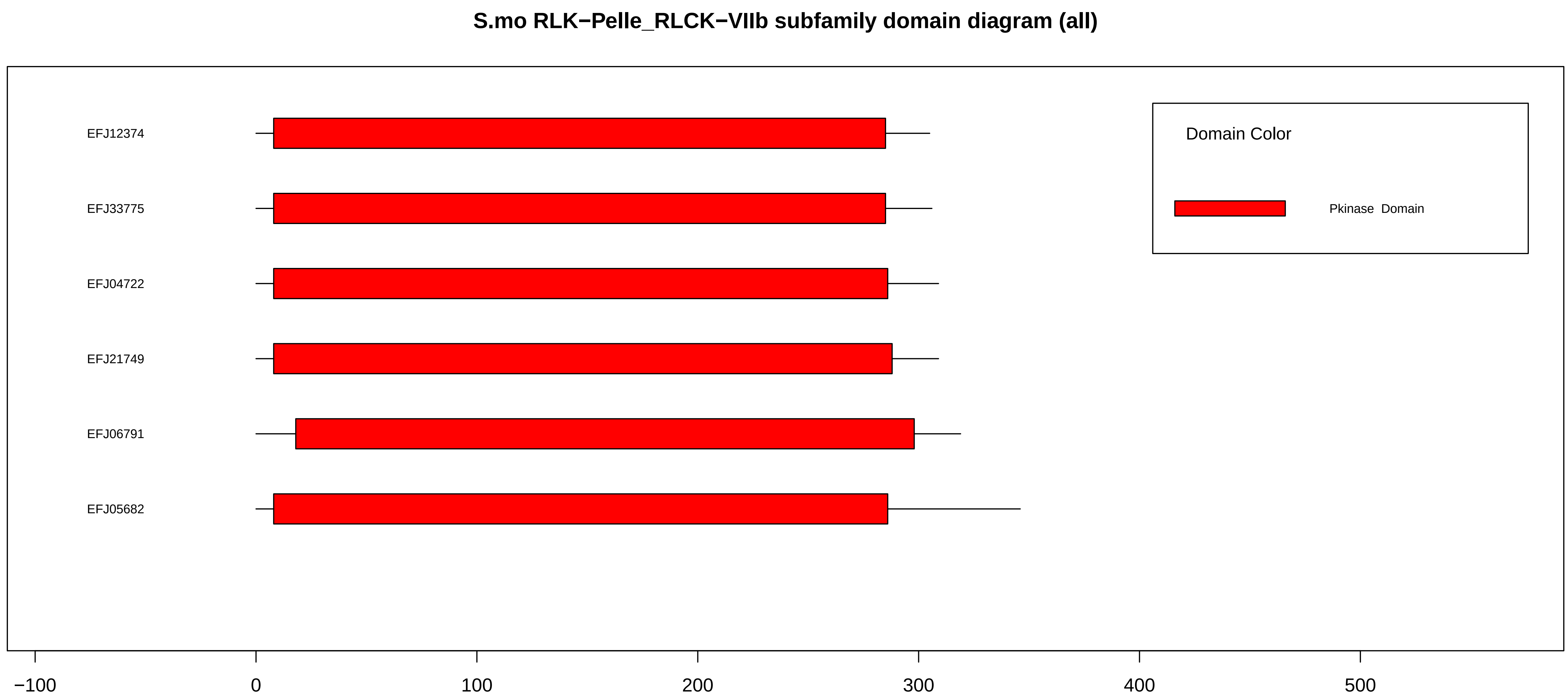
S.mo RLK−Pelle_RLCK−VIIb subfamily domain diagram (all)
EFJ12374
EFJ33775
EFJ04722
EFJ21749
EFJ06791
EFJ05682
Domain Color
Pkinase Domain
−100
0
100
200
300
400
500

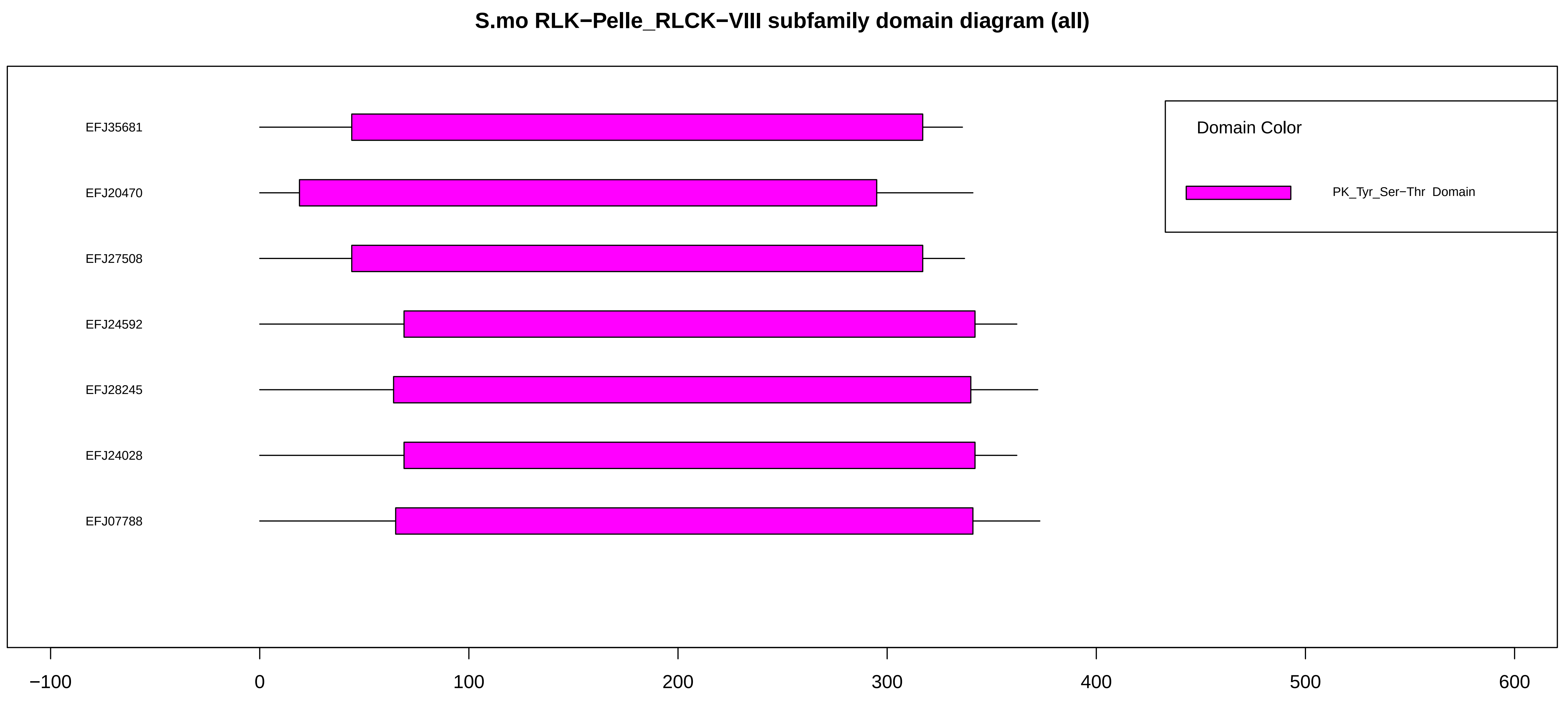
S.mo RLK−Pelle_RLCK−VIII subfamily domain diagram (all)
EFJ35681
EFJ20470
EFJ27508
EFJ24592
EFJ28245
EFJ24028
EFJ07788
Domain Color
PK_Tyr_Ser−Thr Domain
−100
0
100
200
300
400
500
600

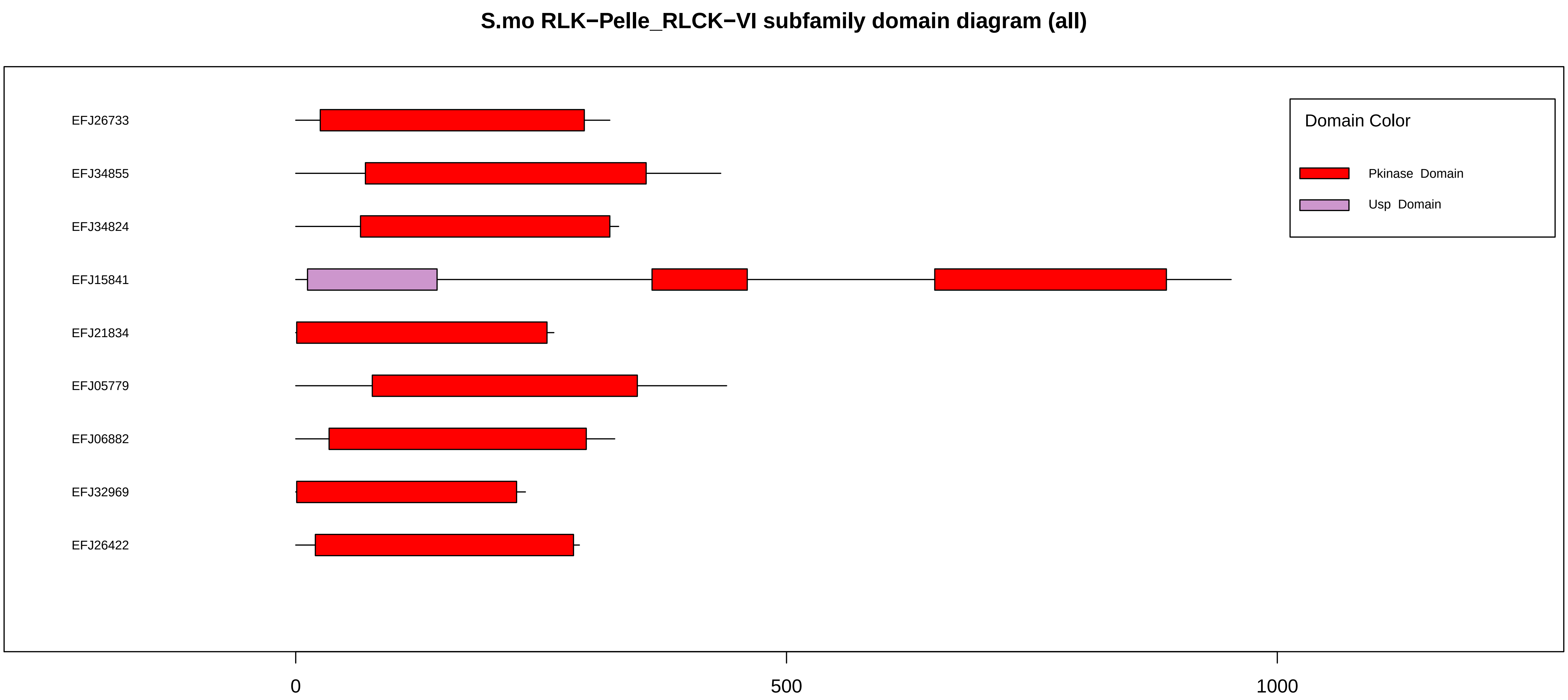

S.mo RLK−Pelle_RLCK−VI subfamily domain diagram (all)
EFJ26733
EFJ34855
EFJ34824
EFJ15841
EFJ21834
EFJ05779
EFJ06882
EFJ32969
EFJ26422
Domain Color
Pkinase Domain
Usp Domain
0
500
1000

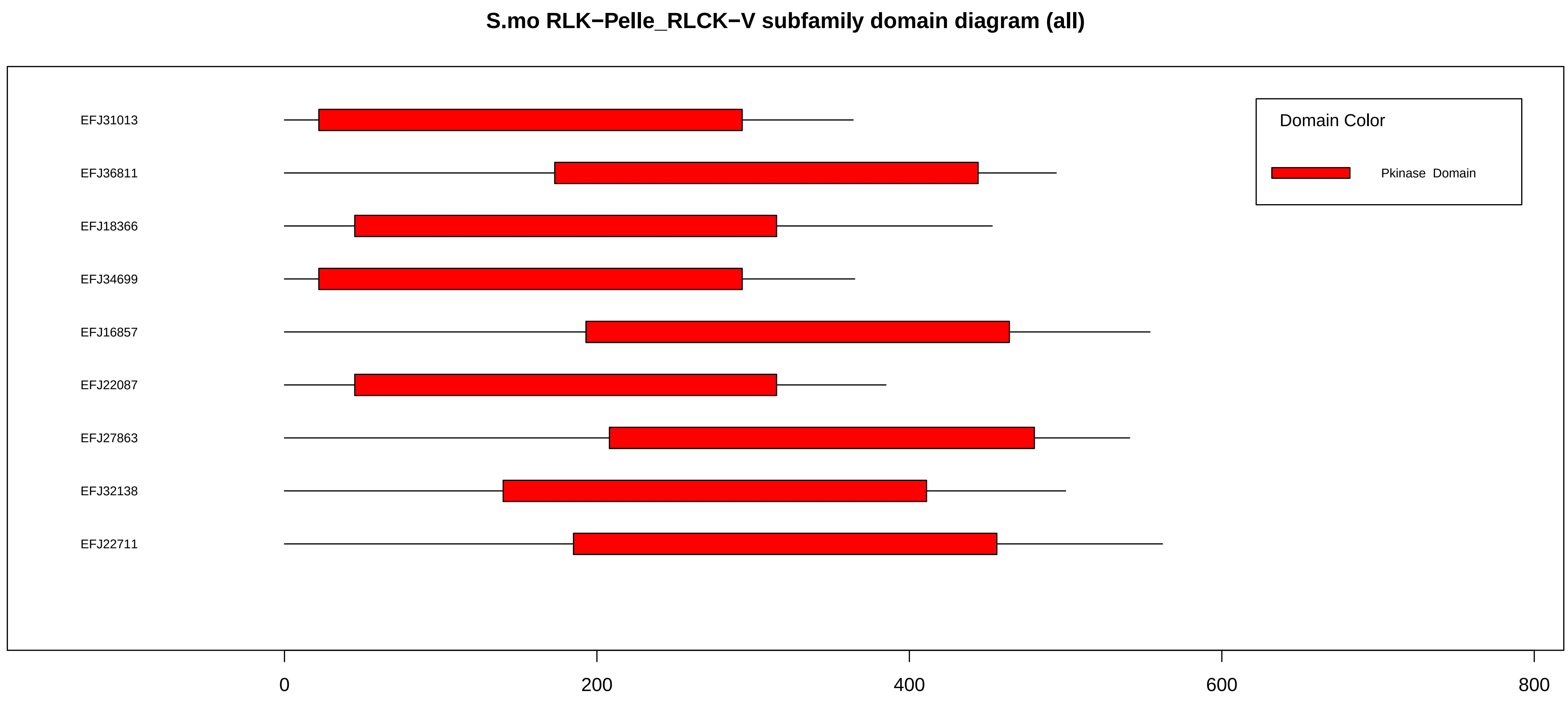
S.mo RLK−Pelle_RLCK−V subfamily domain diagram (all)
EFJ31013
EFJ36811
EFJ18366
EFJ34699
EFJ16857
EFJ22087
EFJ27863
EFJ32138
EFJ22711
Domain Color
Pkinase Domain
0
200
400
600
800

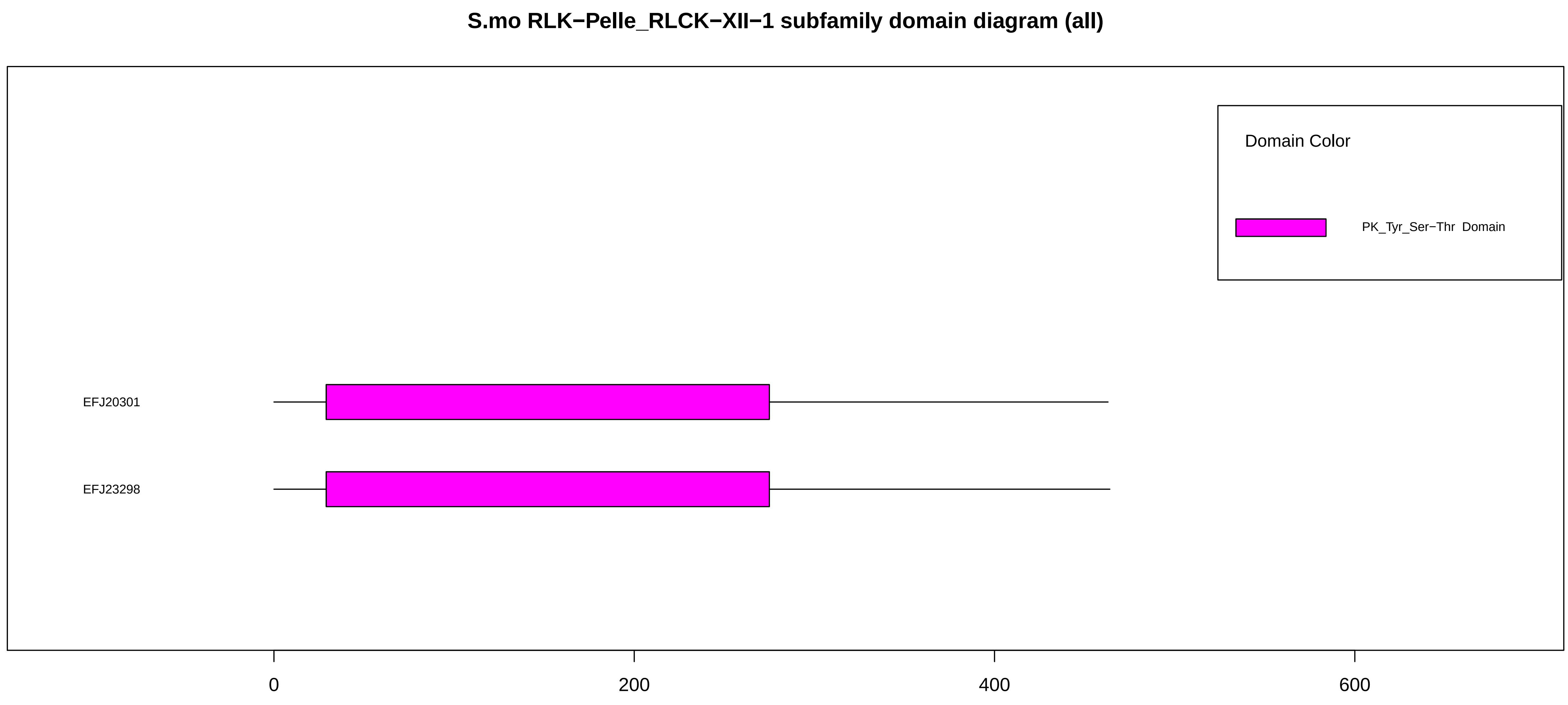
S.mo RLK−Pelle_RLCK−XII−1 subfamily domain diagram (all)
Domain Color
PK_Tyr_Ser−Thr Domain
EFJ20301
EFJ23298
0
200
400
600

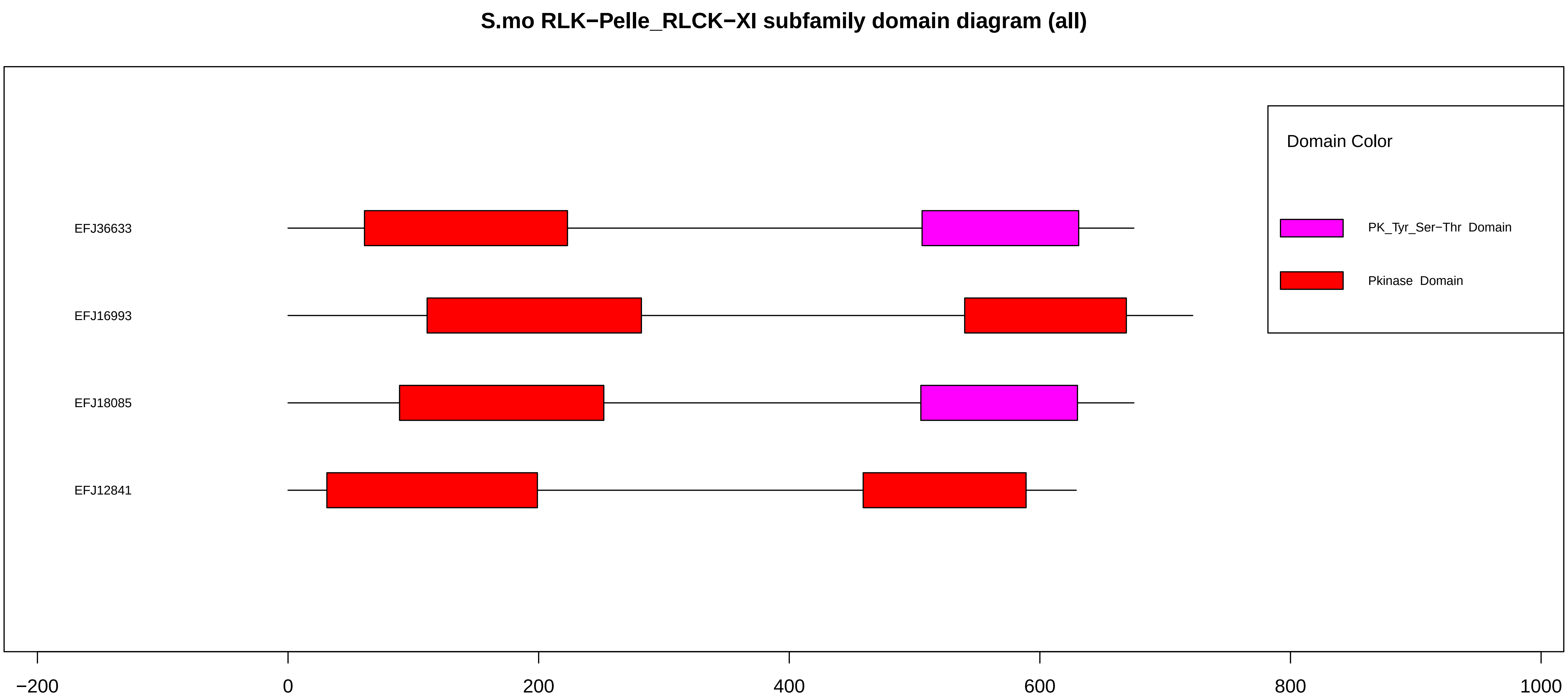
S.mo RLK−Pelle_RLCK−XI subfamily domain diagram (all)
EFJ36633
EFJ16993
EFJ18085
EFJ12841
Domain Color
PK_Tyr_Ser−Thr Domain
Pkinase Domain
−200
0
200
400
600
800
1000

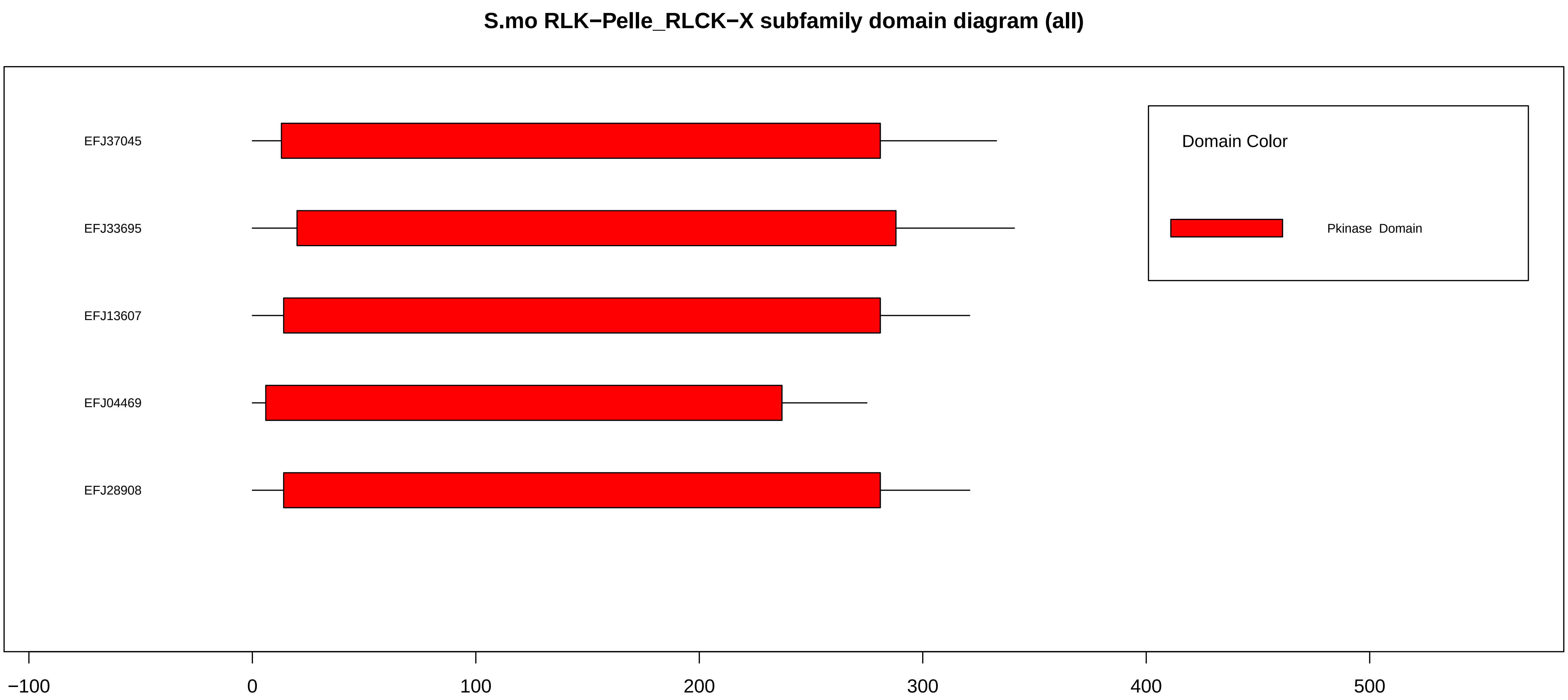
S.mo RLK−Pelle_RLCK−X subfamily domain diagram (all)
EFJ37045
EFJ33695
EFJ13607
EFJ04469
EFJ28908
Domain Color
Pkinase Domain
−100
0
100
200
300
400
500

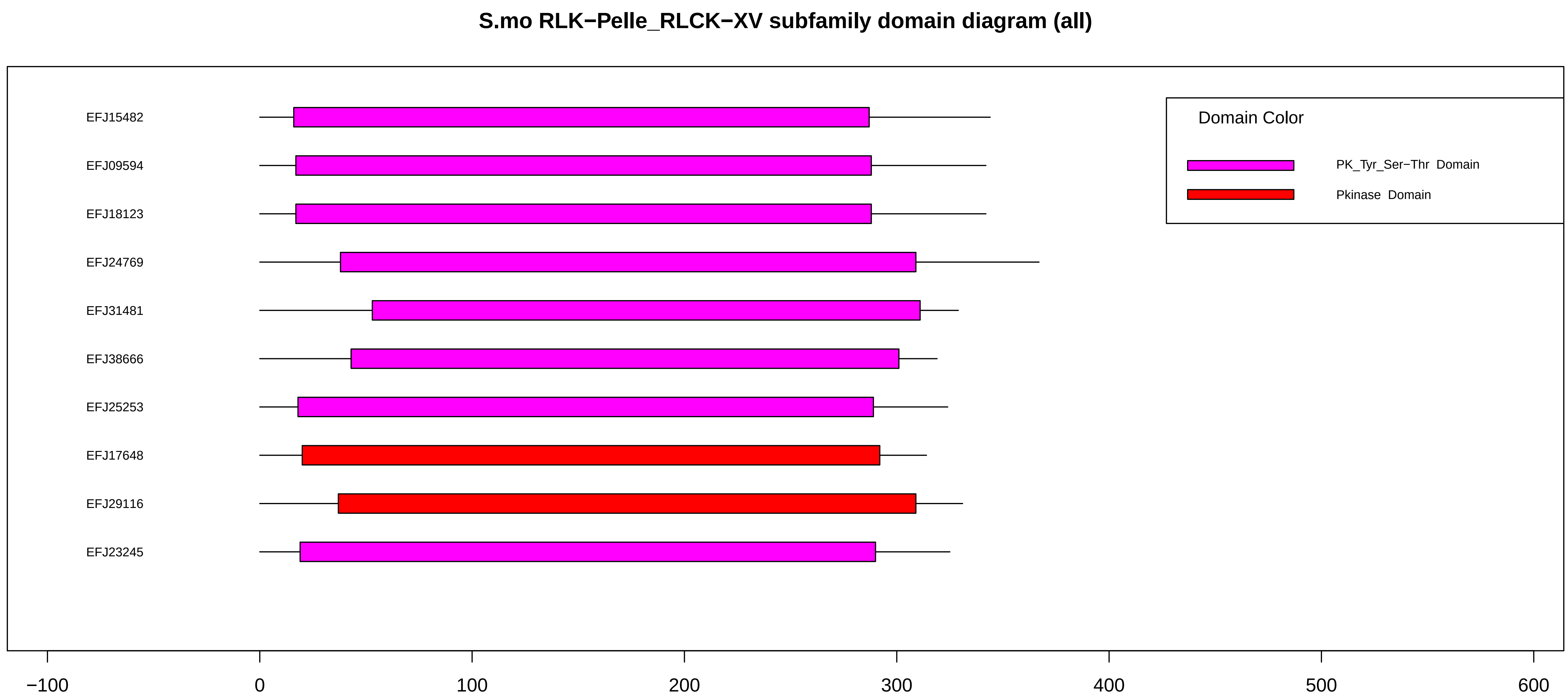

S.mo RLK−Pelle_RLCK−XV subfamily domain diagram (all)
EFJ15482
EFJ09594
EFJ18123
EFJ24769
EFJ31481
EFJ38666
EFJ25253
EFJ17648
EFJ29116
EFJ23245
Domain Color
PK_Tyr_Ser−Thr Domain
Pkinase Domain
−100
0
100
200
300
400
500
600

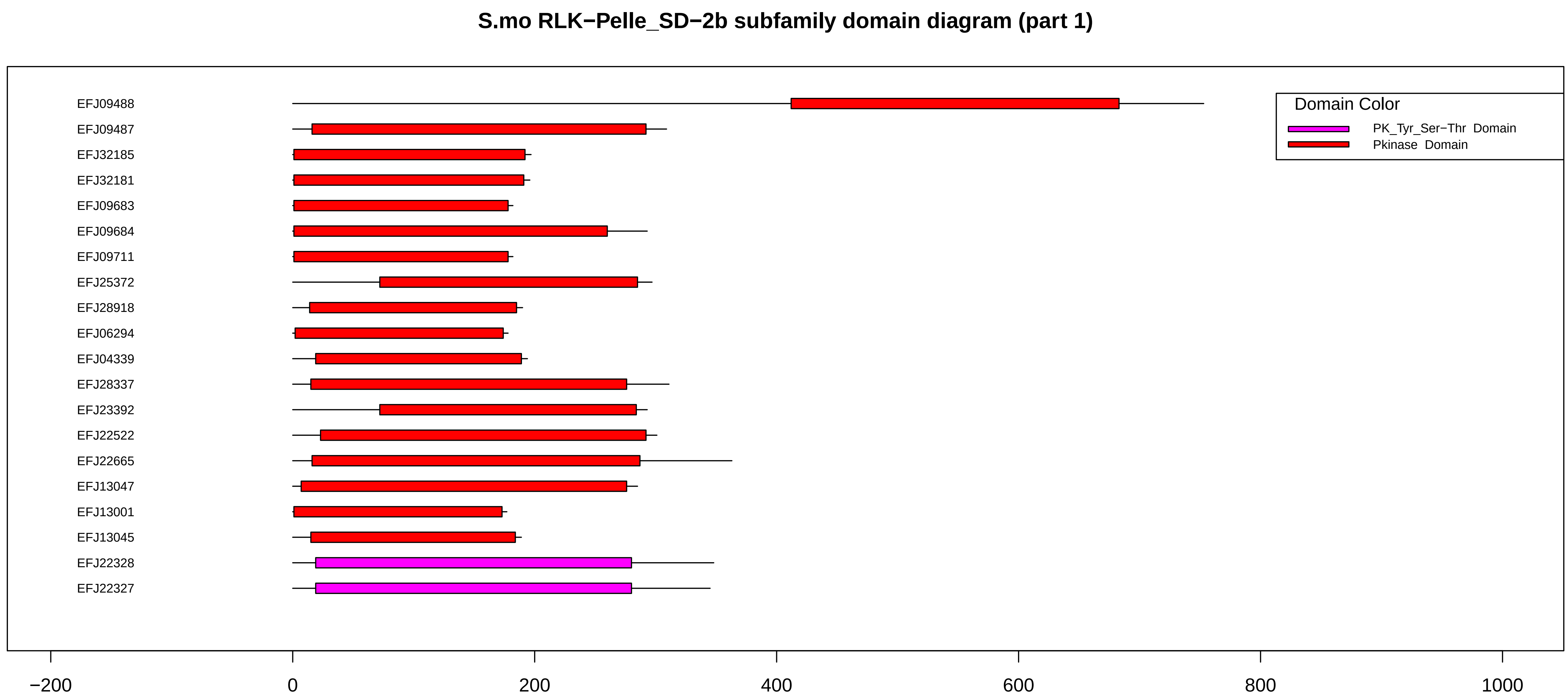
S.mo RLK−Pelle_SD−2b subfamily domain diagram (part 1)
EFJ09488
EFJ09487
EFJ32185
EFJ32181
EFJ09683
EFJ09684
EFJ09711
EFJ25372
EFJ28918
EFJ06294
EFJ04339
EFJ28337
EFJ23392
EFJ22522
EFJ22665
EFJ13047
EFJ13001
EFJ13045
EFJ22328
EFJ22327
Domain Color
PK_Tyr_Ser−Thr Domain
Pkinase Domain
−200
0
200
400
600
800
1000

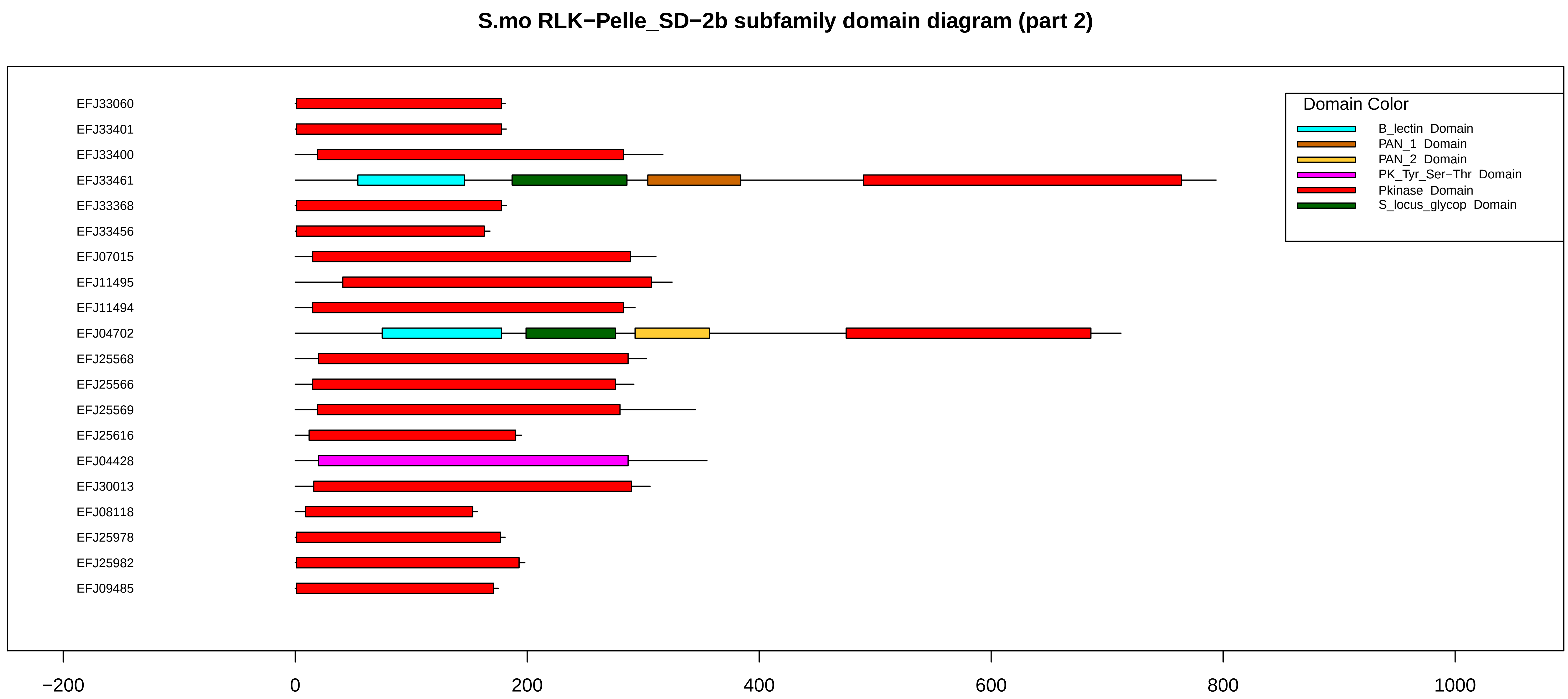
S.mo RLK−Pelle_SD−2b subfamily domain diagram (part 2)
EFJ33060
EFJ33401
EFJ33400
EFJ33461
EFJ33368
EFJ33456
EFJ07015
EFJ11495
EFJ11494
EFJ04702
EFJ25568
EFJ25566
EFJ25569
EFJ25616
EFJ04428
EFJ30013
EFJ08118
EFJ25978
EFJ25982
EFJ09485
Domain Color
B_lectin Domain
PAN_1 Domain
PAN_2 Domain
PK_Tyr_Ser−Thr Domain
Pkinase Domain
S_locus_glycop Domain
−200
0
200
400
600
800
1000

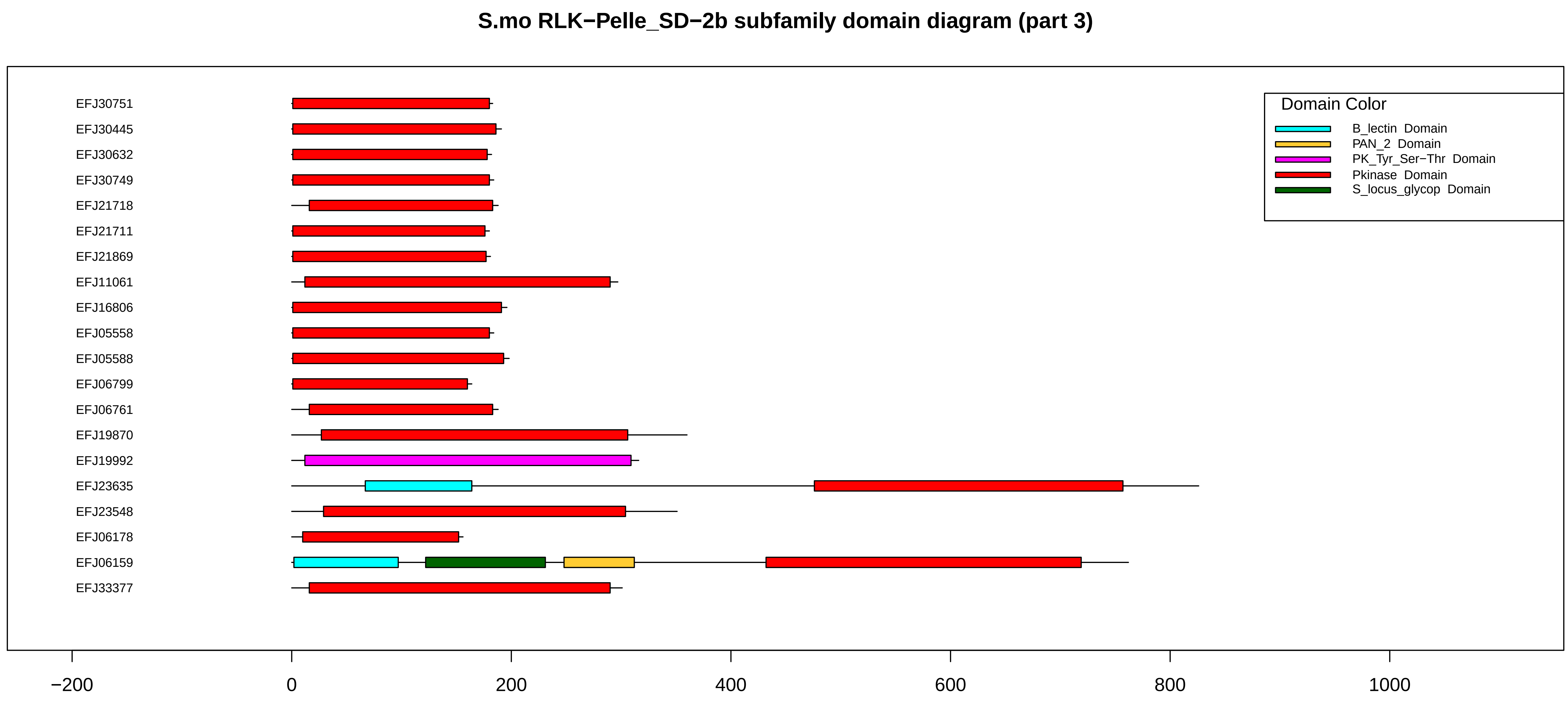
S.mo RLK−Pelle_SD−2b subfamily domain diagram (part 3)
Domain Color
B_lectin Domain
PAN_2 Domain
PK_Tyr_Ser−Thr Domain
Pkinase Domain
S_locus_glycop Domain
EFJ30751
EFJ30445
EFJ30632
EFJ30749
EFJ21718
EFJ21711
EFJ21869
EFJ11061
EFJ16806
EFJ05558
EFJ05588
EFJ06799
EFJ06761
EFJ19870
EFJ19992
EFJ23635
EFJ23548
EFJ06178
EFJ06159
EFJ33377
−200
0
200
400
600
800
1000

S.mo RLK−Pelle_SD−2b subfamily domain diagram (part 4)

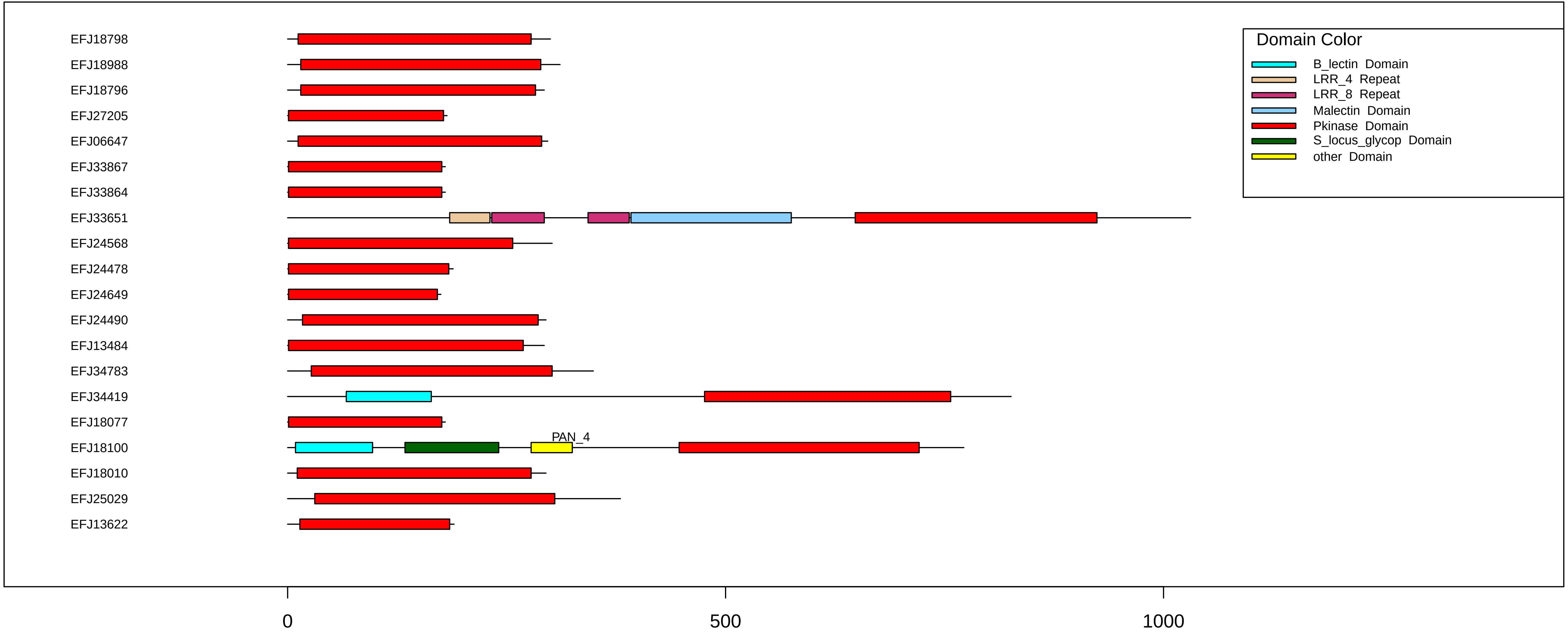

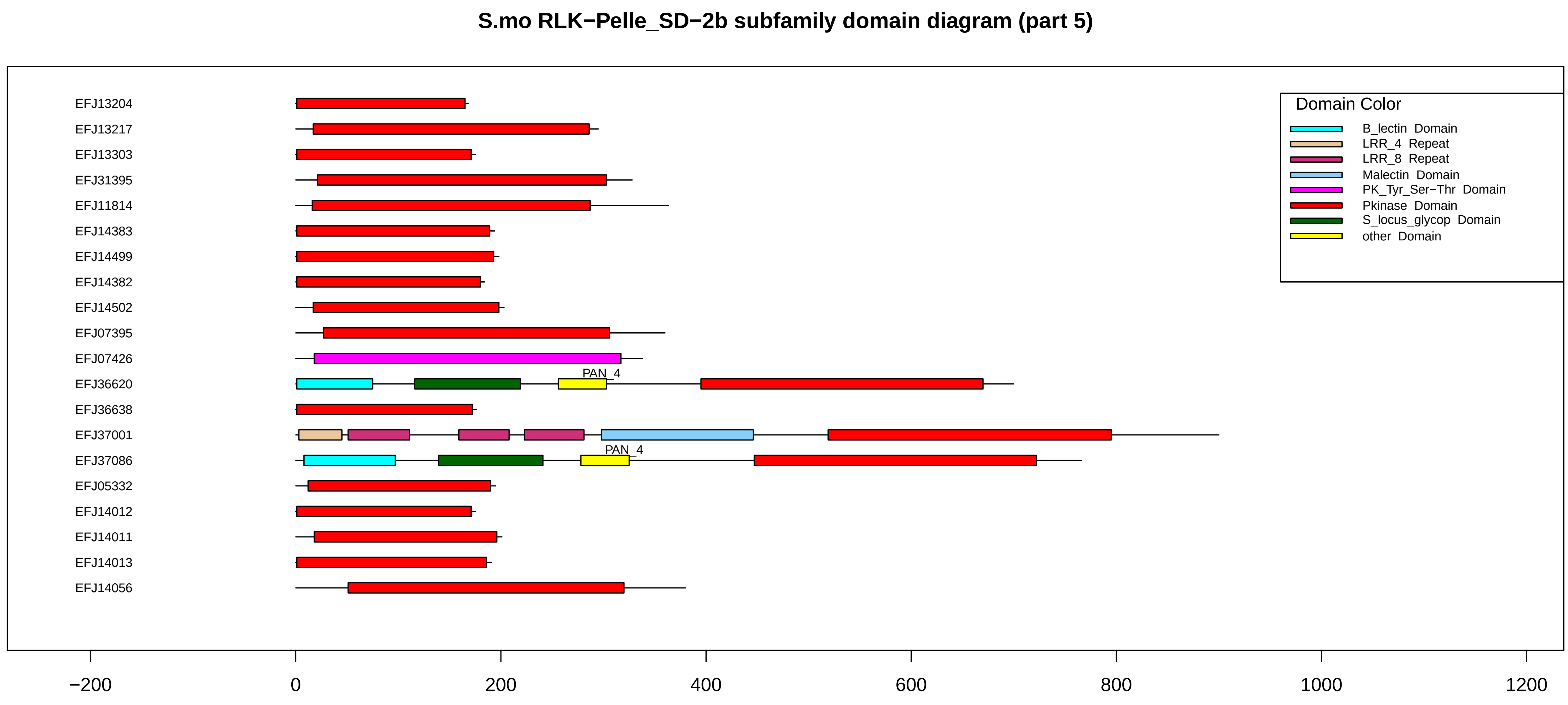
S.mo RLK−Pelle_SD−2b subfamily domain diagram (part 5)
EFJ13204
EFJ13217
EFJ13303
EFJ31395
EFJ11814
EFJ14383
EFJ14499
EFJ14382
EFJ14502
EFJ07395
EFJ07426
EFJ36620
EFJ36638
EFJ37001
EFJ37086
EFJ05332
EFJ14012
EFJ14011
EFJ14013
EFJ14056
PAN_4
PAN_4
Domain Color
B_lectin Domain
LRR_4 Repeat
LRR_8 Repeat
Malectin Domain
PK_Tyr_Ser−Thr Domain
Pkinase Domain
S_locus_glycop Domain
other Domain
−200
0
200
400
600
800
1000
1200

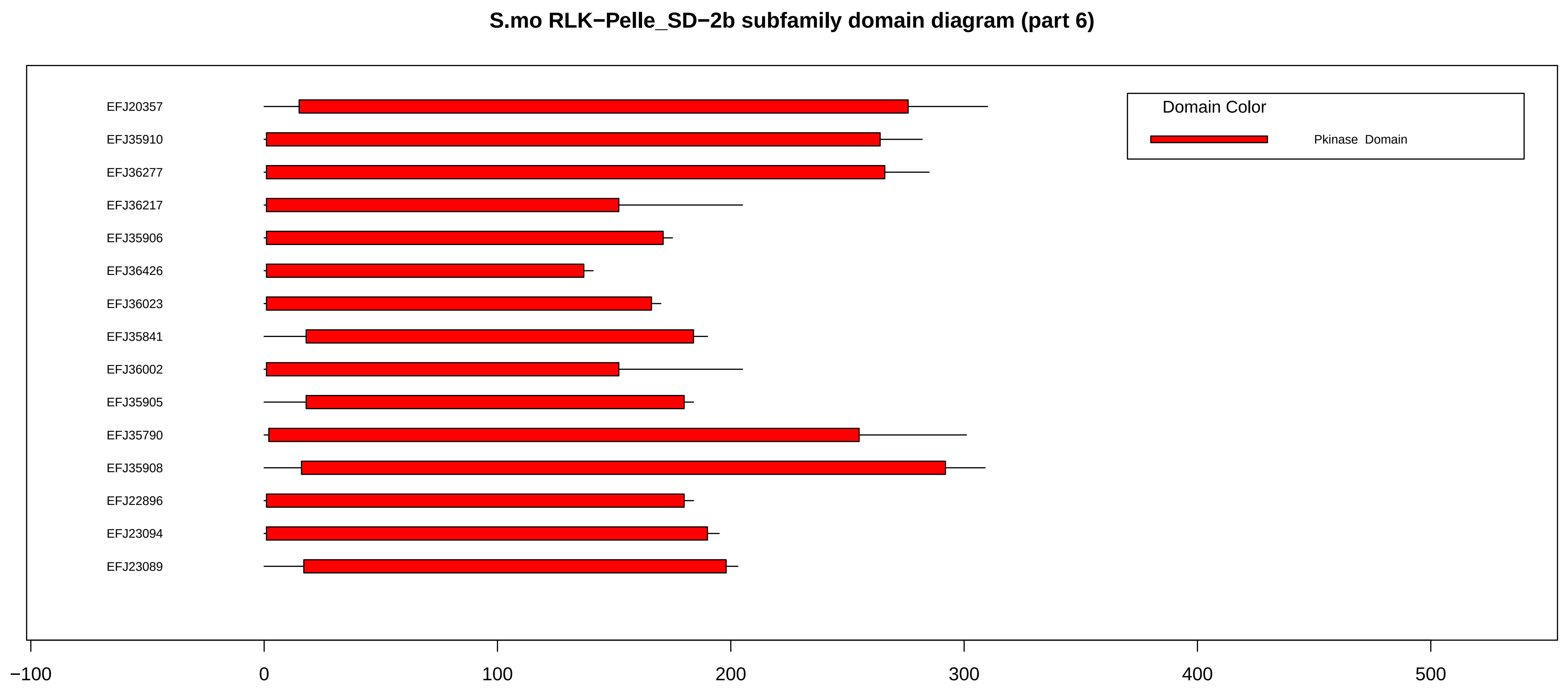
S.mo RLK−Pelle_SD−2b subfamily domain diagram (part 6)
Domain Color
Pkinase Domain
EFJ20357
EFJ35910
EFJ36277
EFJ36217
EFJ35906
EFJ36426
EFJ36023
EFJ35841
EFJ36002
EFJ35905
EFJ35790
EFJ35908
EFJ22896
EFJ23094
EFJ23089
−100
0
100
200
300
400
500

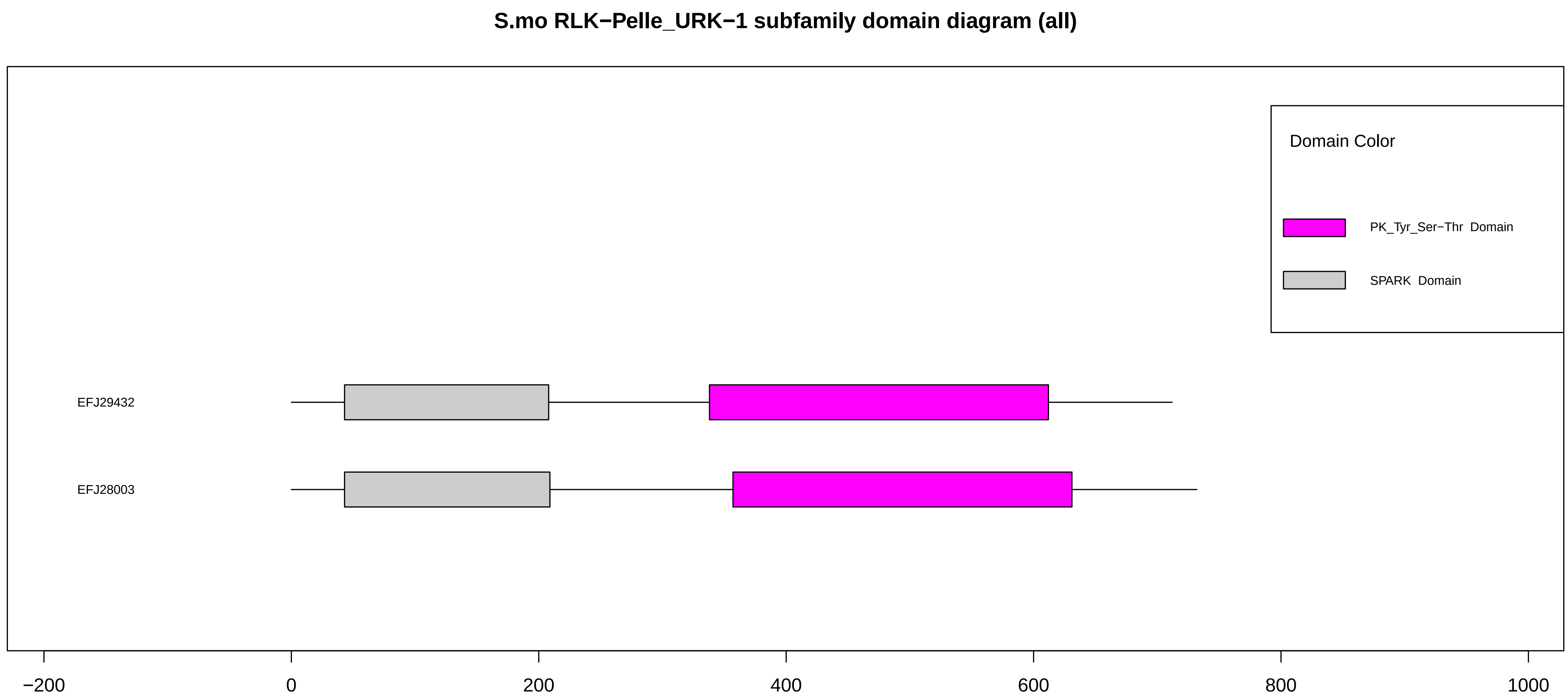

S.mo RLK−Pelle_URK−1 subfamily domain diagram (all)
Domain Color
PK_Tyr_Ser−Thr Domain
SPARK Domain
EFJ29432
EFJ28003
−200
0
200
400
600
800
1000

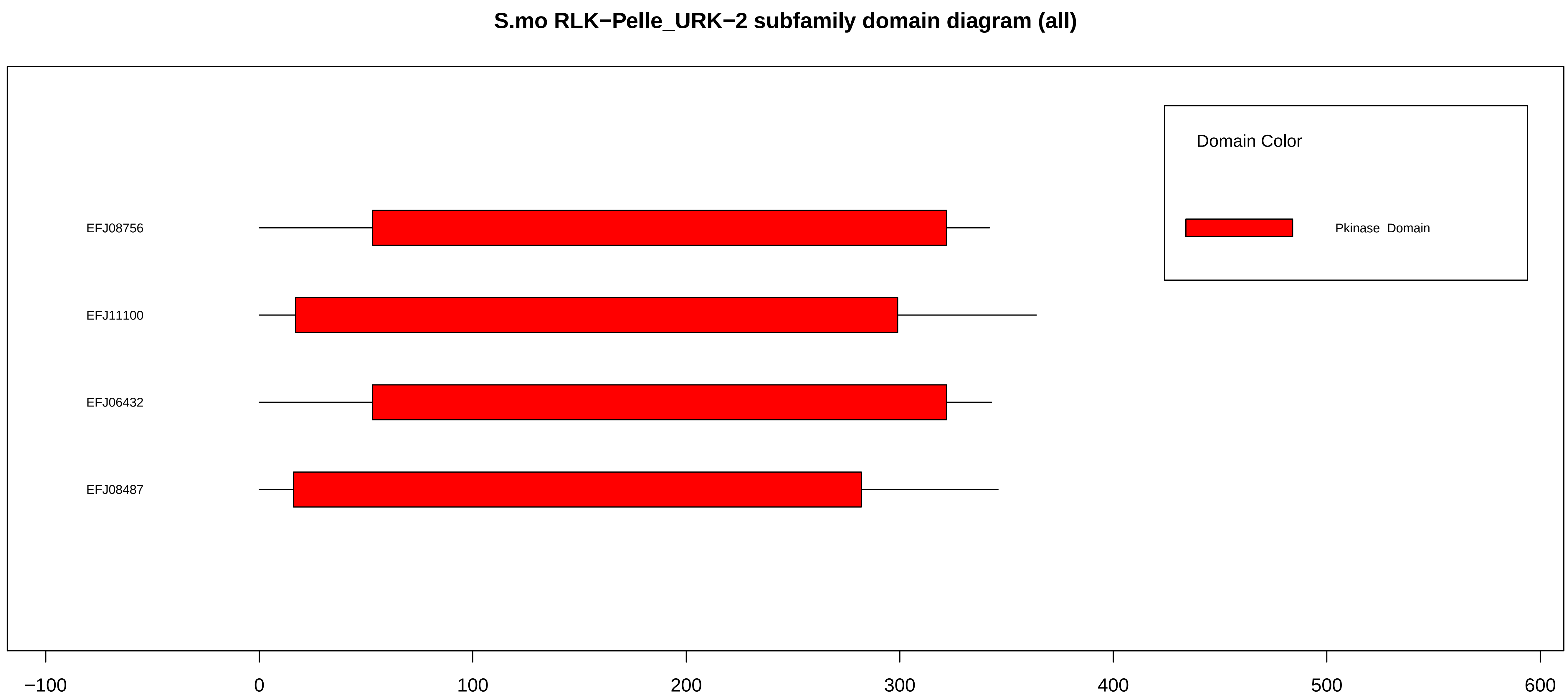

S.mo RLK−Pelle_URK−2 subfamily domain diagram (all)
Domain Color
Pkinase Domain
EFJ08756
EFJ11100
EFJ06432
EFJ08487
−100
0
100
200
300
400
500
600

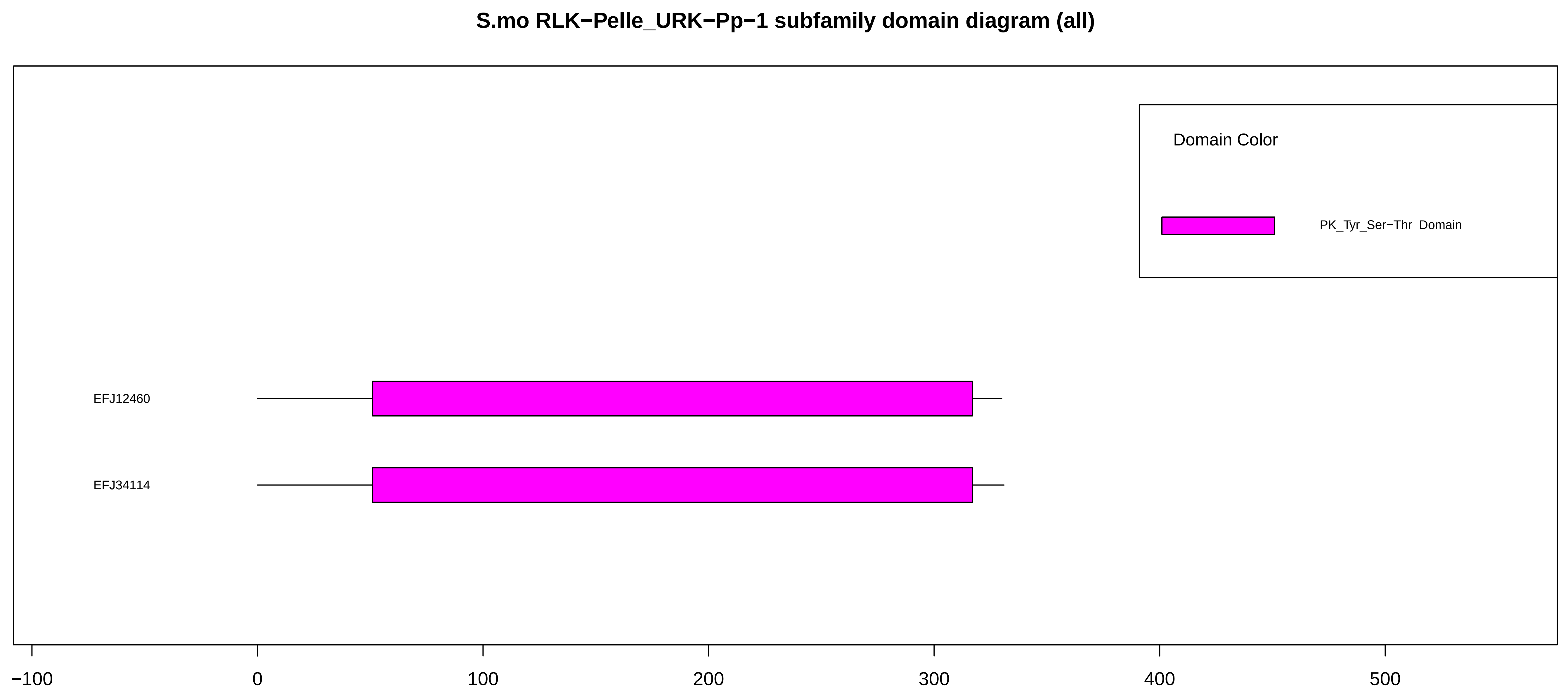
S.mo RLK−Pelle_URK−Pp−1 subfamily domain diagram (all)
Domain Color
PK_Tyr_Ser−Thr Domain
EFJ12460
EFJ34114
−100
0
100
200
300
400
500

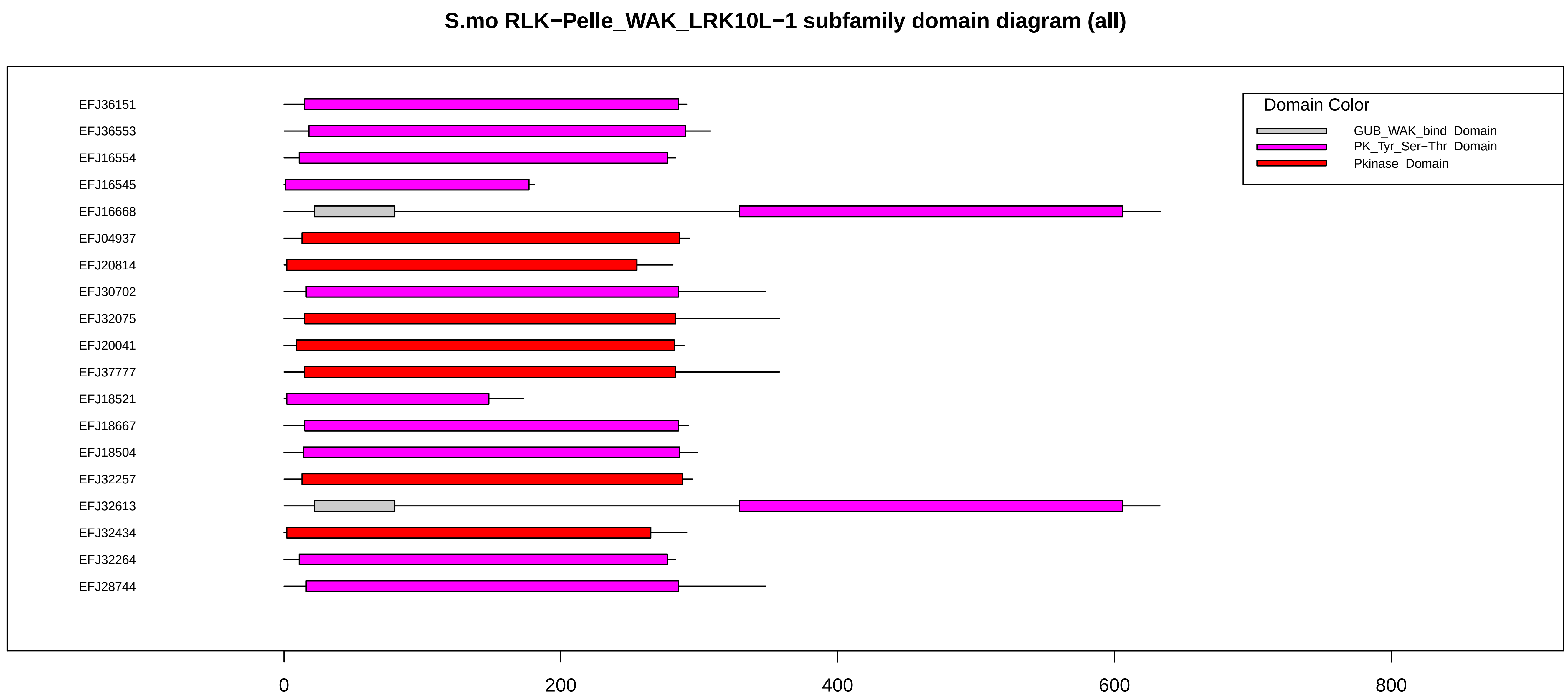
S.mo RLK−Pelle_WAK_LRK10L−1 subfamily domain diagram (all)
Domain Color
GUB_WAK_bind Domain
PK_Tyr_Ser−Thr Domain
Pkinase Domain
EFJ36151
EFJ36553
EFJ16554
EFJ16545
EFJ16668
EFJ04937
EFJ20814
EFJ30702
EFJ32075
EFJ20041
EFJ37777
EFJ18521
EFJ18667
EFJ18504
EFJ32257
EFJ32613
EFJ32434
EFJ32264
EFJ28744
0
200
400
600
800

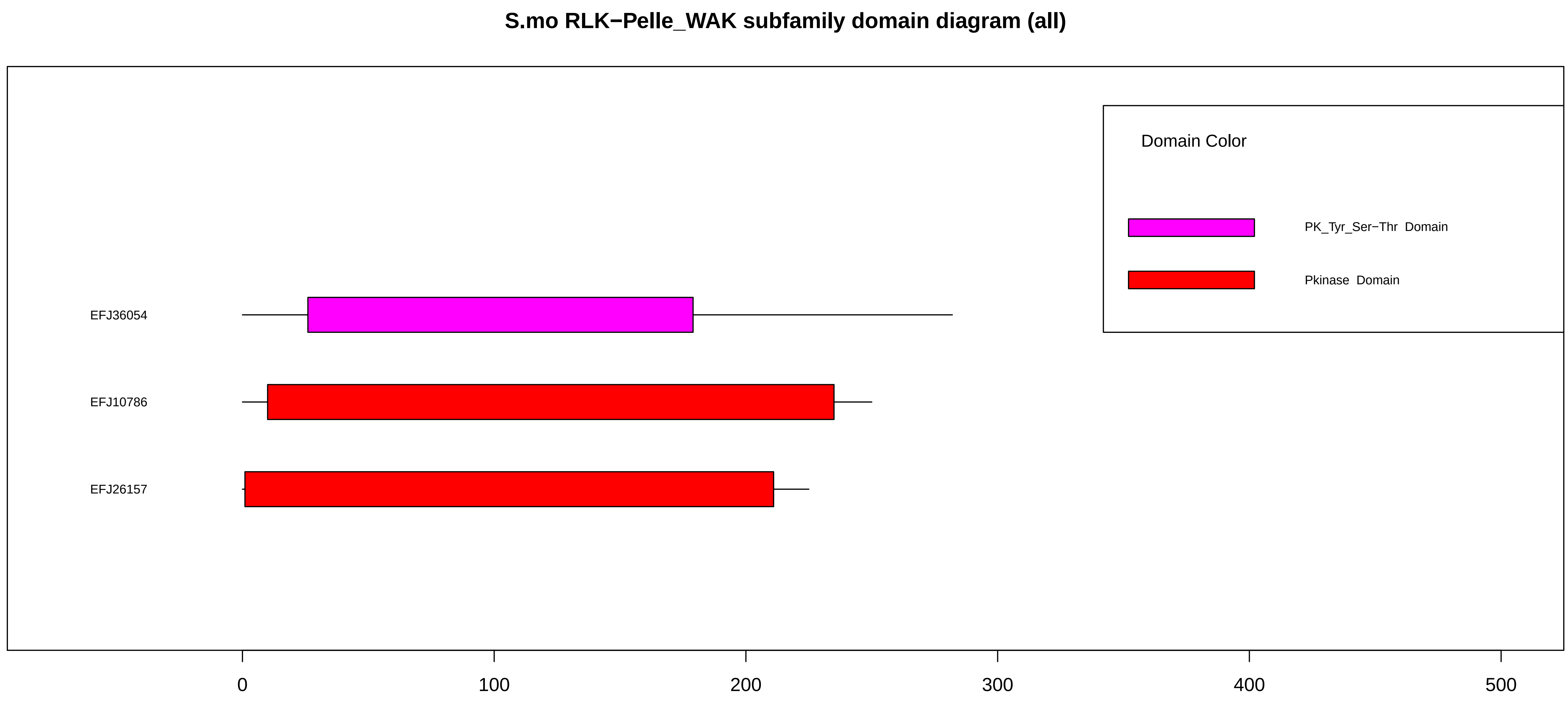
S.mo RLK−Pelle_WAK subfamily domain diagram (all)
Domain Color
PK_Tyr_Ser−Thr Domain
Pkinase Domain
EFJ36054
EFJ10786
EFJ26157
0
100
200
300
400
500

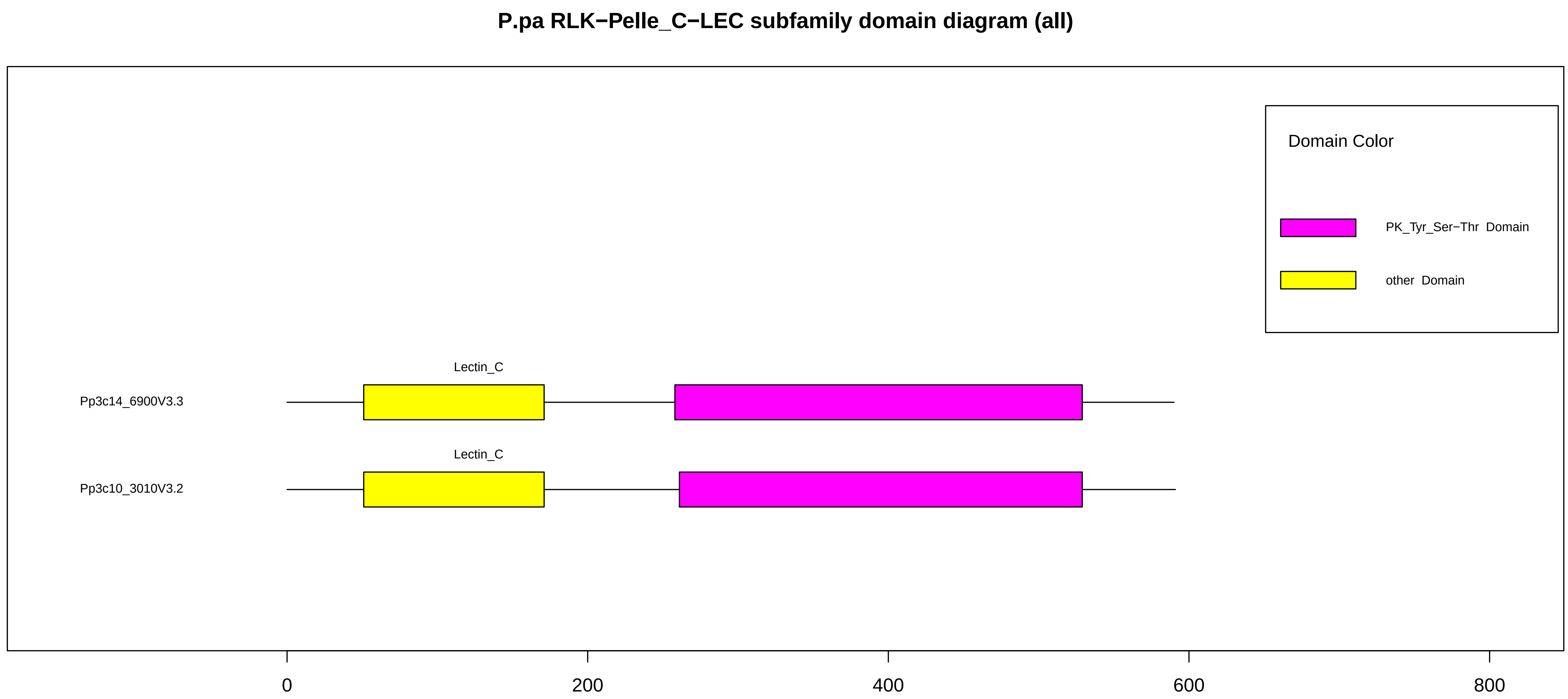
P.pa RLK−Pelle_C−LEC subfamily domain diagram (all)
Domain Color
PK_Tyr_Ser−Thr Domain
other Domain
Lectin_C
Pp3c14_6900V3.3
Lectin_C
Pp3c10_3010V3.2
0
200
400
600
800

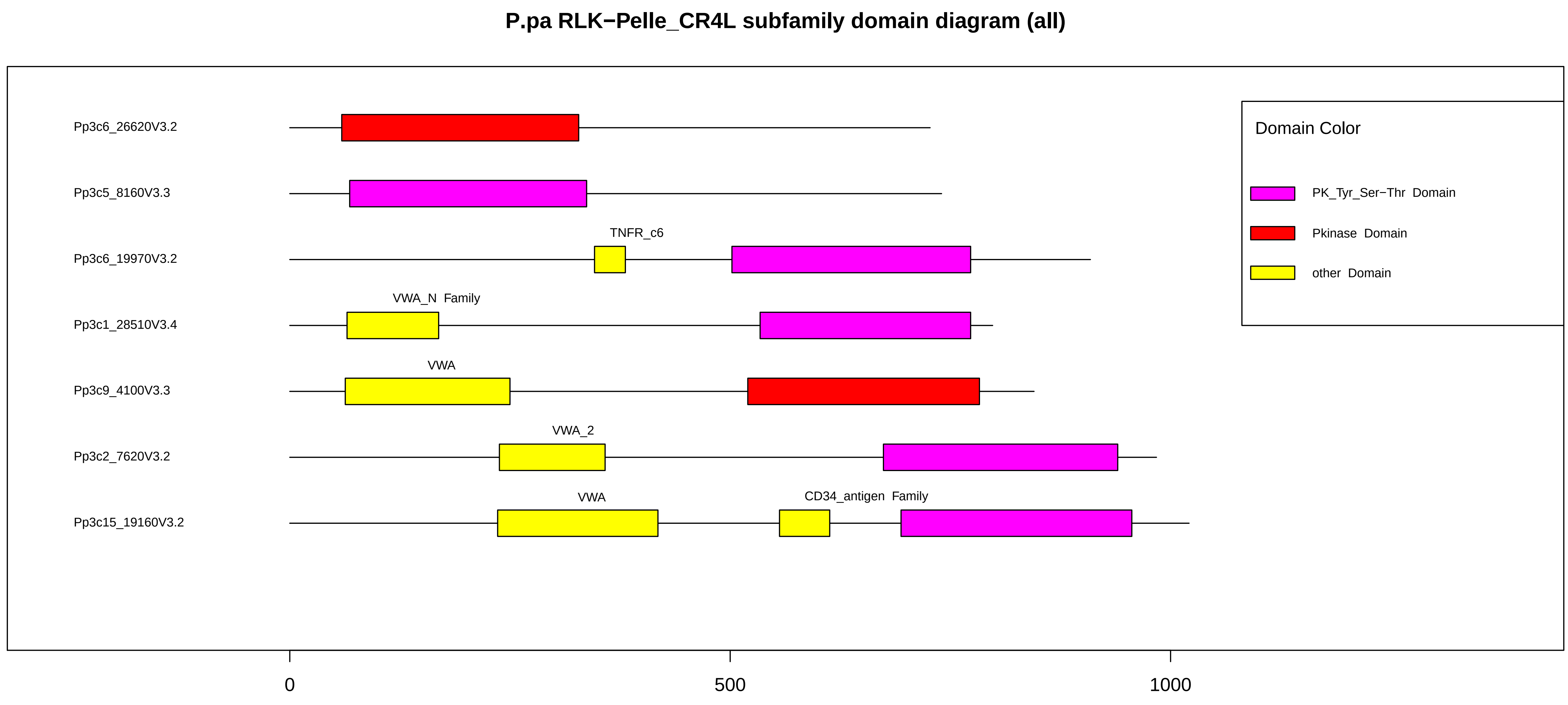
P.pa RLK−Pelle_CR4L subfamily domain diagram (all)
Pp3c6_26620V3.2
Pp3c5_8160V3.3
Pp3c6_19970V3.2
Pp3c1_28510V3.4
Pp3c9_4100V3.3
Pp3c2_7620V3.2
Pp3c15_19160V3.2
TNFR_c6
VWA_N Family
VWA
VWA_2
VWA
CD34_antigen Family
Domain Color
PK_Tyr_Ser−Thr Domain
Pkinase Domain
other Domain
0
500
1000

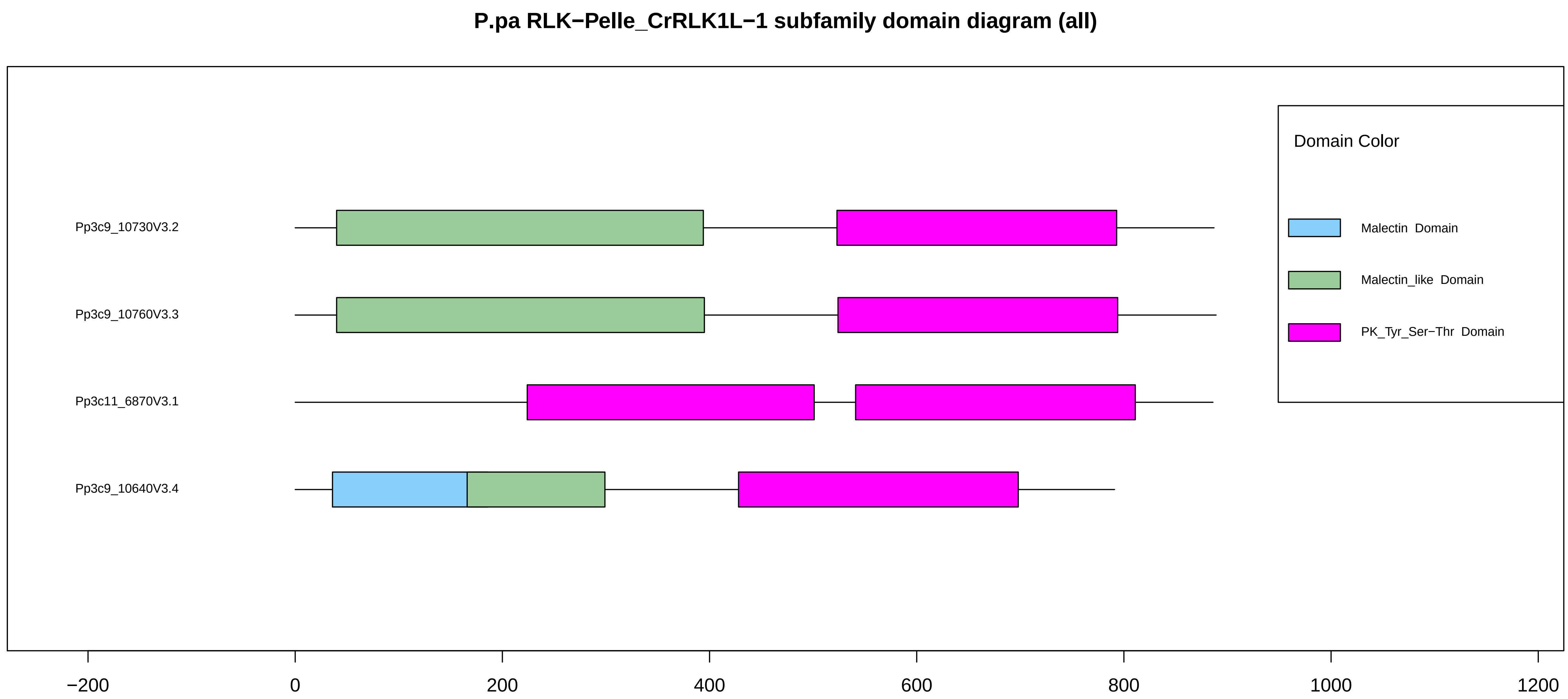
P.pa RLK−Pelle_CrRLK1L−1 subfamily domain diagram (all)
Pp3c9_10730V3.2
Pp3c9_10760V3.3
Pp3c11_6870V3.1
Pp3c9_10640V3.4
Domain Color
Malectin Domain
Malectin_like Domain
PK_Tyr_Ser−Thr Domain
−200
0
200
400
600
800
1000
1200

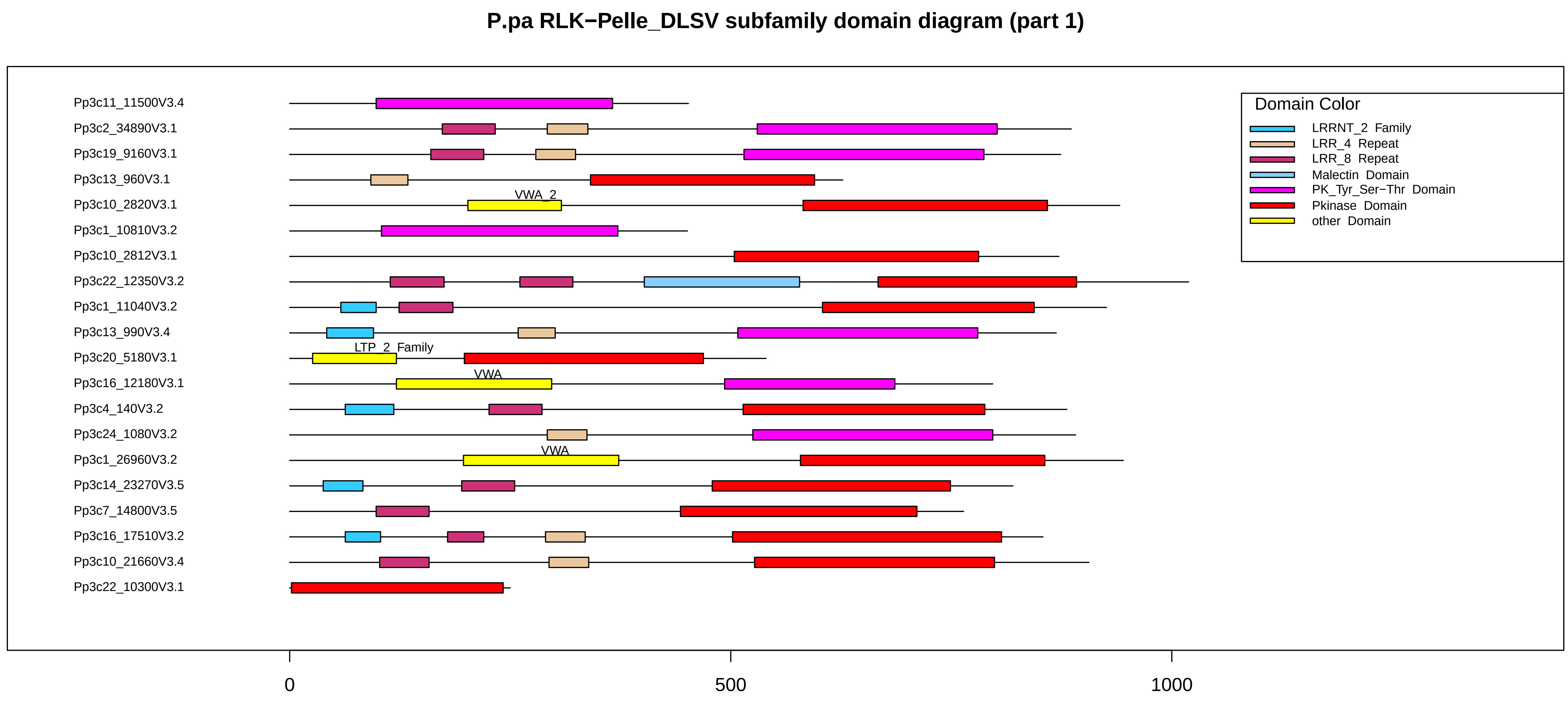
P.pa RLK−Pelle_DLSV subfamily domain diagram (part 1)
Pp3c11_11500V3.4
Pp3c2_34890V3.1
Pp3c19_9160V3.1
Pp3c13_960V3.1
Pp3c10_2820V3.1
VWA_2
Pp3c1_10810V3.2
Pp3c10_2812V3.1
Pp3c22_12350V3.2
Pp3c1_11040V3.2
Pp3c13_990V3.4
Pp3c20_5180V3.1
LTP_2_Family
Pp3c16_12180V3.1
VWA
Pp3c4_140V3.2
Pp3c24_1080V3.2
Pp3c1_26960V3.2
VWA
Pp3c14_23270V3.5
Pp3c7_14800V3.5
Pp3c16_17510V3.2
Pp3c10_21660V3.4
Pp3c22_10300V3.1
Domain Color
LRRNT_2 Family
LRR_4 Repeat
LRR_8 Repeat
Malectin Domain
PK_Tyr_Ser−Thr Domain
Pkinase Domain
other Domain
0
500
1000

P.pa RLK−Pelle_DLSV subfamily domain diagram (part 2)

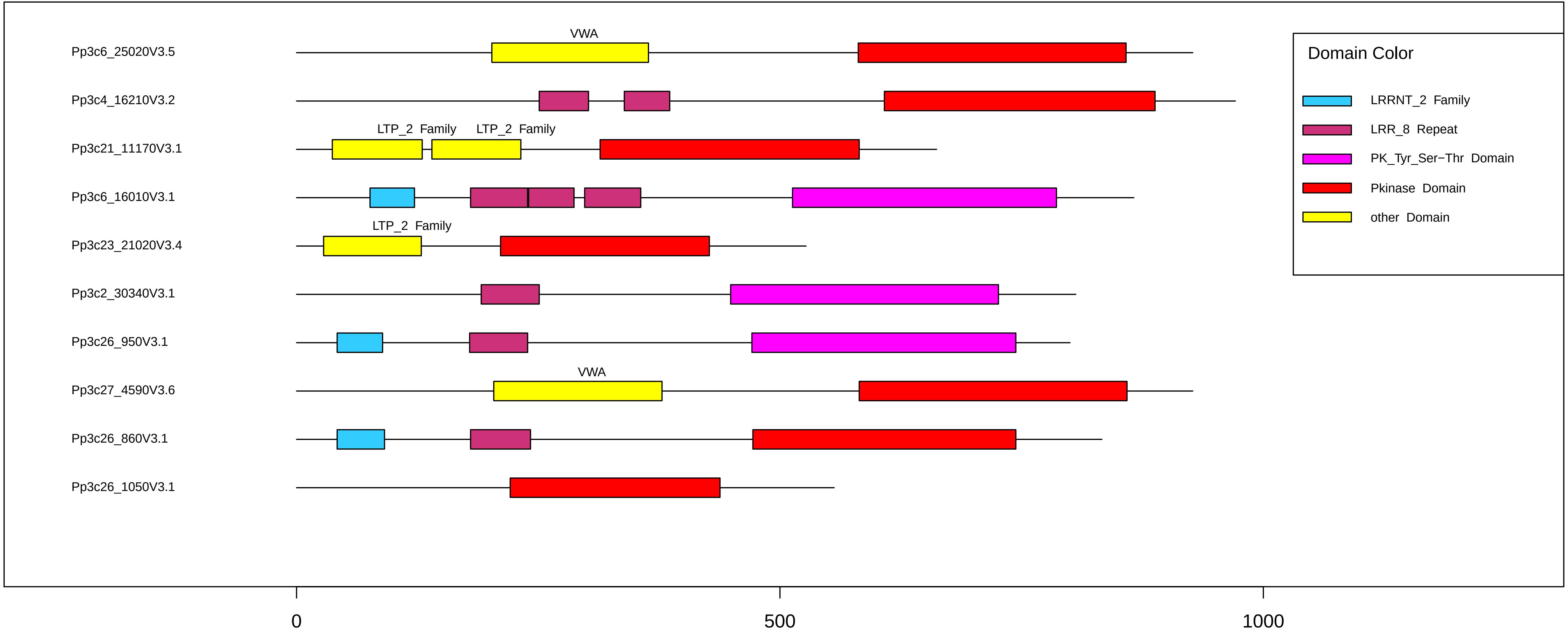

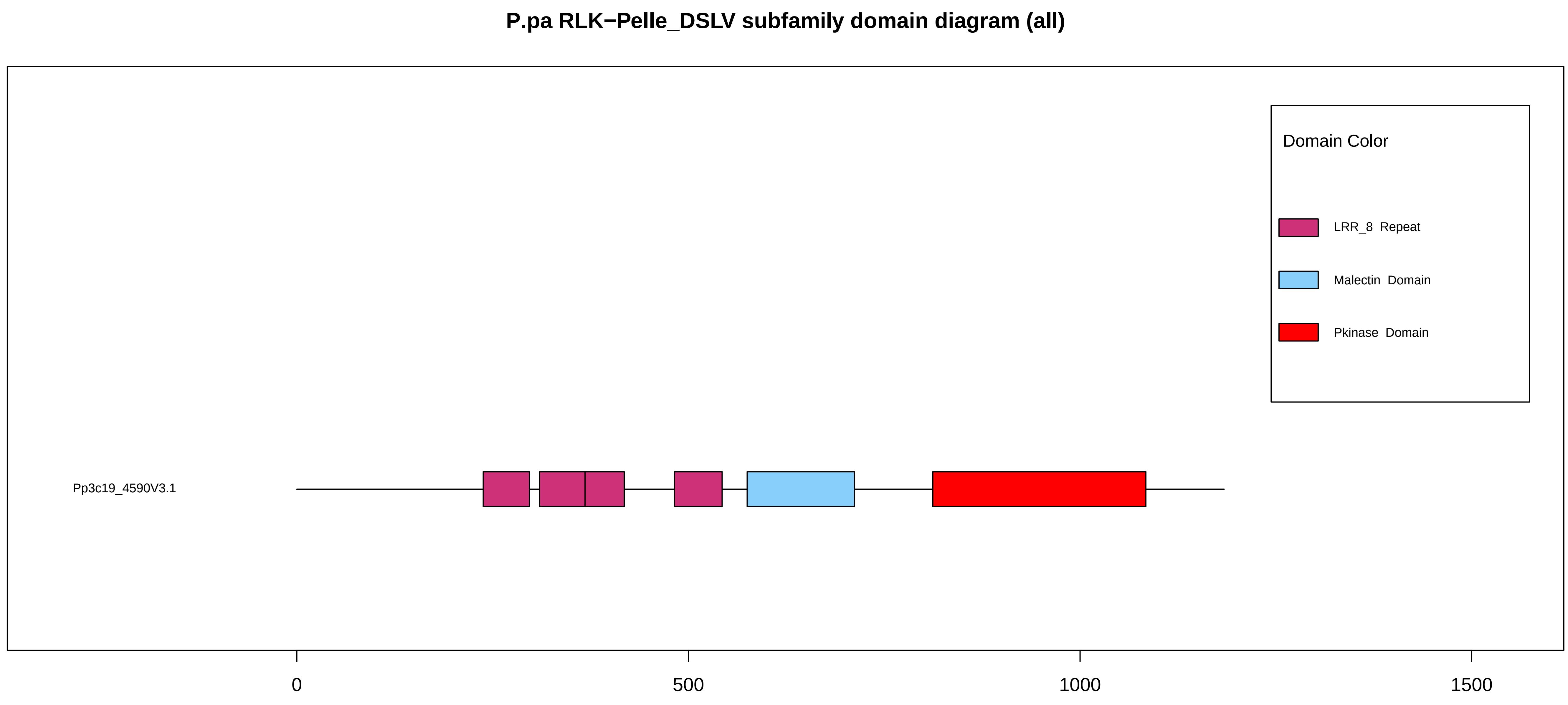

P.pa RLK−Pelle_DSLV subfamily domain diagram (all)
Domain Color
LRR_8 Repeat
Malectin Domain
Pkinase Domain
Pp3c19_4590V3.1
0
500
1000
1500

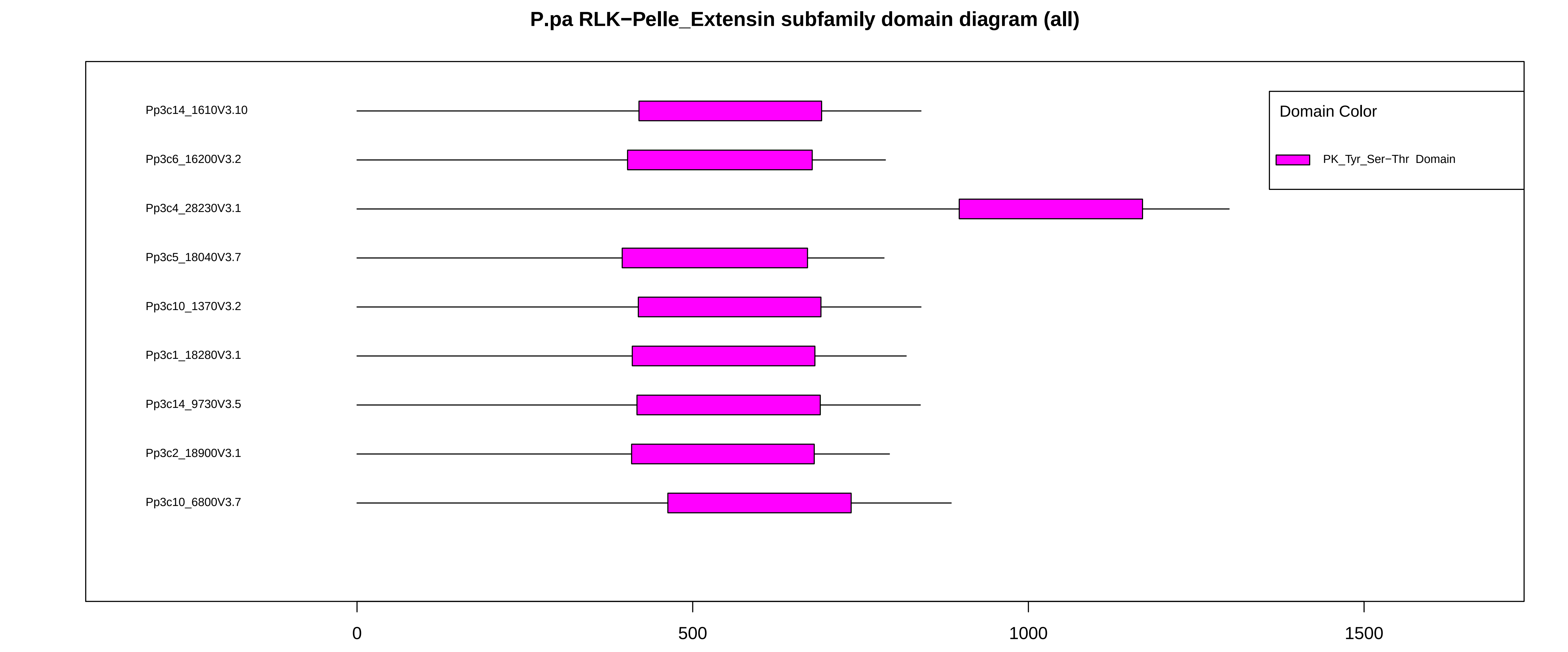

P.pa RLK−Pelle_Extensin subfamily domain diagram (all)
Pp3c14_1610V3.10
Pp3c6_16200V3.2
Pp3c4_28230V3.1
Pp3c5_18040V3.7
Pp3c10_1370V3.2
Pp3c1_18280V3.1
Pp3c14_9730V3.5
Pp3c2_18900V3.1
Pp3c10_6800V3.7
Domain Color
PK_Tyr_Ser−Thr Domain
0
500
1000
1500

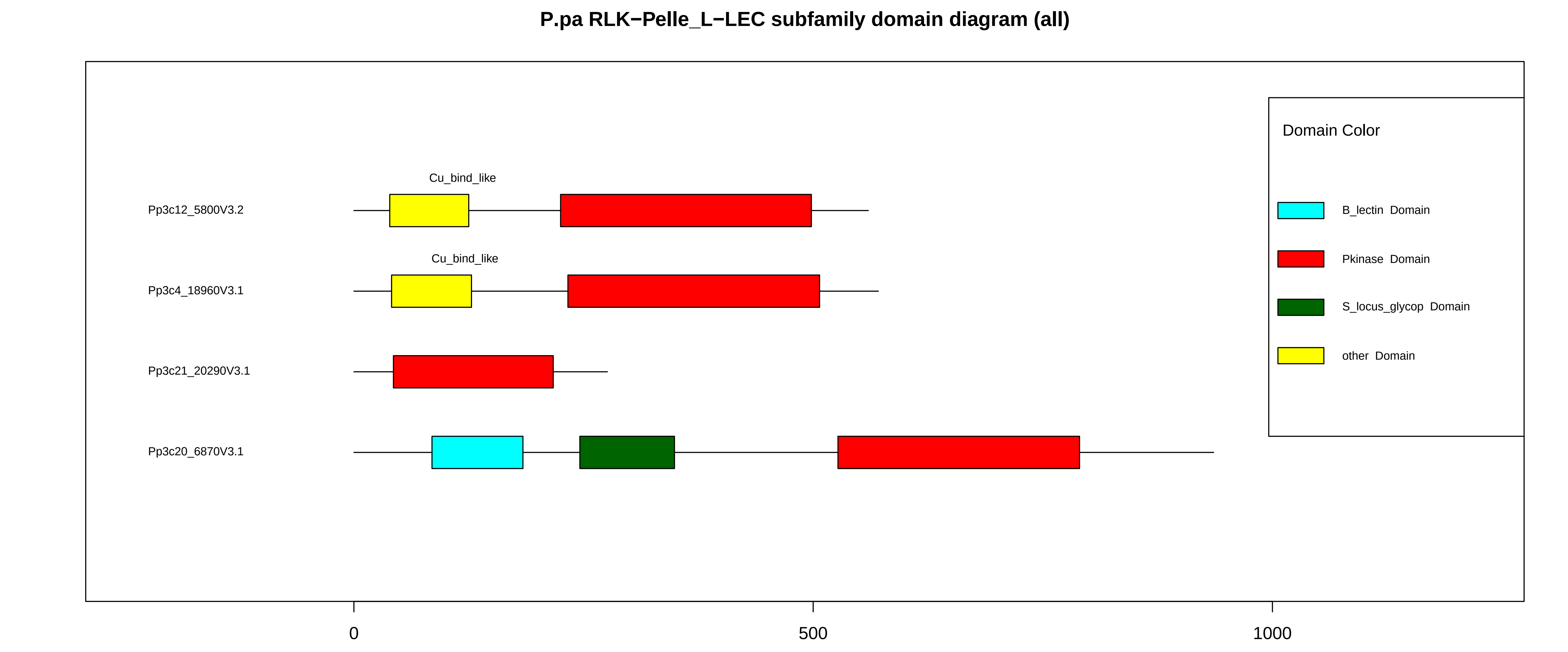
P.pa RLK−Pelle_L−LEC subfamily domain diagram (all)
Cu_bind_like
Pp3c12_5800V3.2
Cu_bind_like
Pp3c4_18960V3.1
Pp3c21_20290V3.1
Pp3c20_6870V3.1
Domain Color
B_lectin Domain
Pkinase Domain
S_locus_glycop Domain
other Domain
0
500
1000

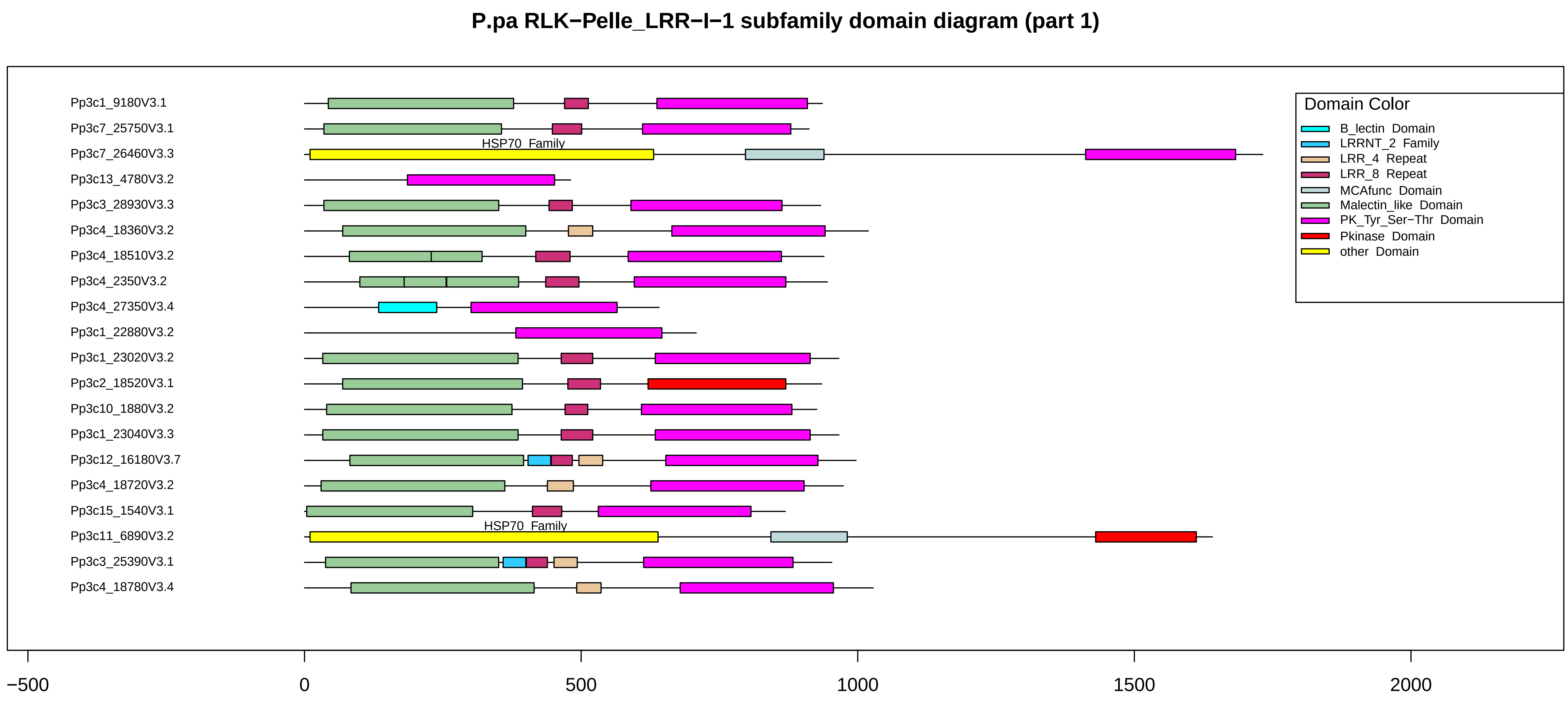
P.pa RLK−Pelle_LRR−I−1 subfamily domain diagram (part 1)
Pp3c1_9180V3.1
Pp3c7_25750V3.1
Pp3c7_26460V3.3
HSP70_Family
Pp3c13_4780V3.2
Pp3c3_28930V3.3
Pp3c4_18360V3.2
Pp3c4_18510V3.2
Pp3c4_2350V3.2
Pp3c4_27350V3.4
Pp3c1_22880V3.2
Pp3c1_23020V3.2
Pp3c2_18520V3.1
Pp3c10_1880V3.2
Pp3c1_23040V3.3
Pp3c12_16180V3.7
Pp3c4_18720V3.2
Pp3c15_1540V3.1
Pp3c11_6890V3.2
HSP70_Family
Pp3c3_25390V3.1
Pp3c4_18780V3.4
Domain Color
B_lectin_Domain
LRRNT_2_Family
LRR_4_Repeat
LRR_8_Repeat
MCAfunc_Domain
Malectin_like_Domain
PK_Tyr_Ser−Thr_Domain
Pkinase_Domain
other_Domain
−500
0
500
1000
1500
2000

P.pa RLK−Pelle_LRR−I−1 subfamily domain diagram (part 2)

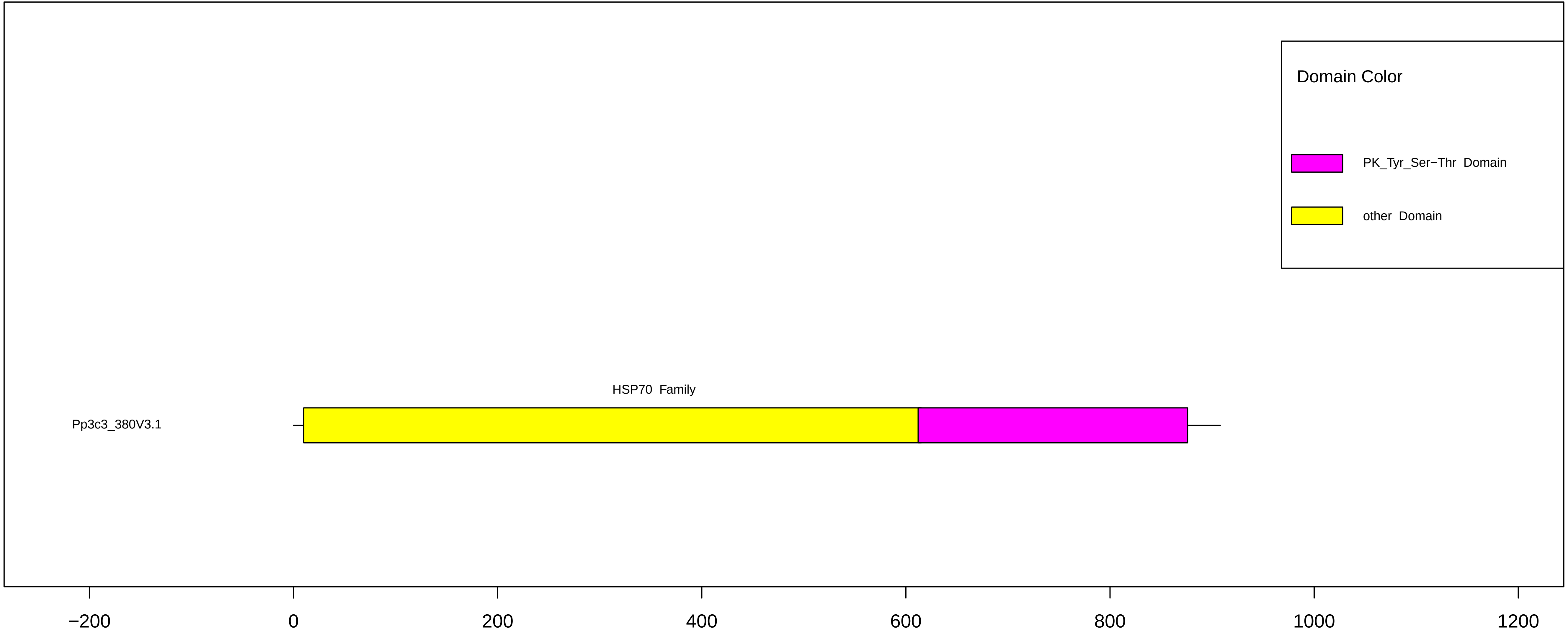

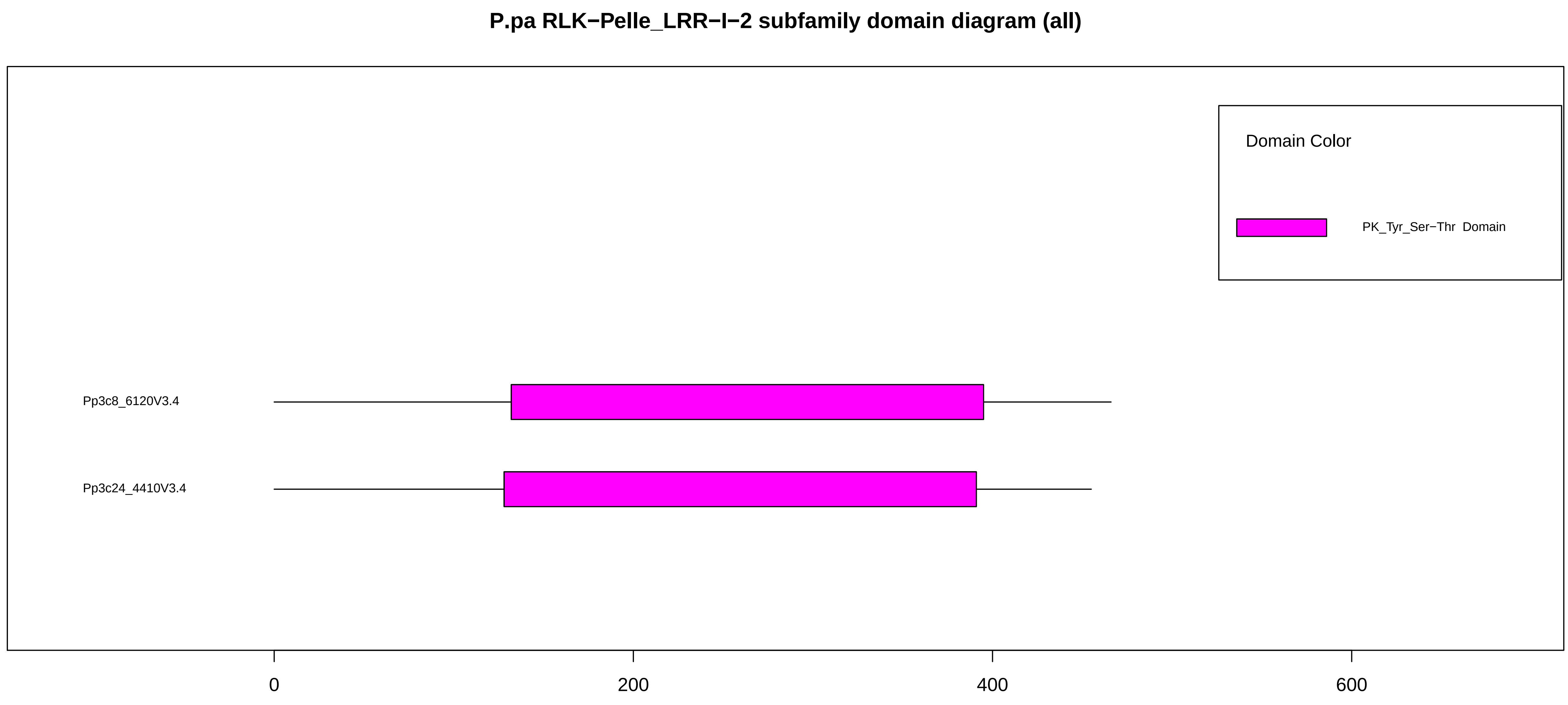
P.pa RLK−Pelle_LRR−I−2 subfamily domain diagram (all)
Domain Color
PK_Tyr_Ser−Thr Domain
Pp3c8_6120V3.4
Pp3c24_4410V3.4
0
200
400
600

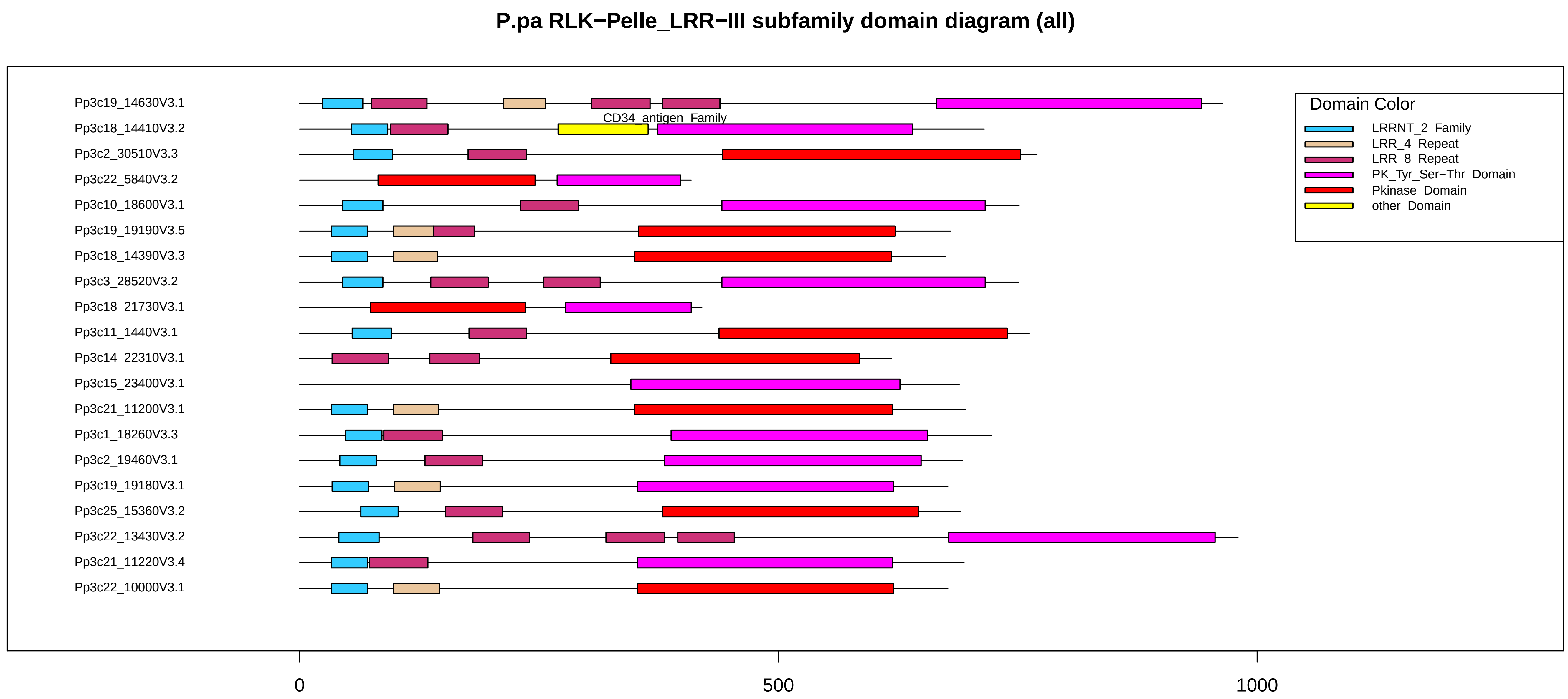
P.pa RLK−Pelle_LRR−III subfamily domain diagram (all)
Pp3c19_14630V3.1
Pp3c18_14410V3.2
Pp3c2_30510V3.3
Pp3c22_5840V3.2
Pp3c10_18600V3.1
Pp3c19_19190V3.5
Pp3c18_14390V3.3
Pp3c3_28520V3.2
Pp3c18_21730V3.1
Pp3c11_1440V3.1
Pp3c14_22310V3.1
Pp3c15_23400V3.1
Pp3c21_11200V3.1
Pp3c1_18260V3.3
Pp3c2_19460V3.1
Pp3c19_19180V3.1
Pp3c25_15360V3.2
Pp3c22_13430V3.2
Pp3c21_11220V3.4
Pp3c22_10000V3.1
CD34_antigen_Family
Domain Color
LRRNT_2 Family
LRR_4 Repeat
LRR_8 Repeat
PK_Tyr_Ser−Thr Domain
Pkinase Domain
other Domain
0
500
1000

P.pa RLK−Pelle_LRR−II subfamily domain diagram (all)

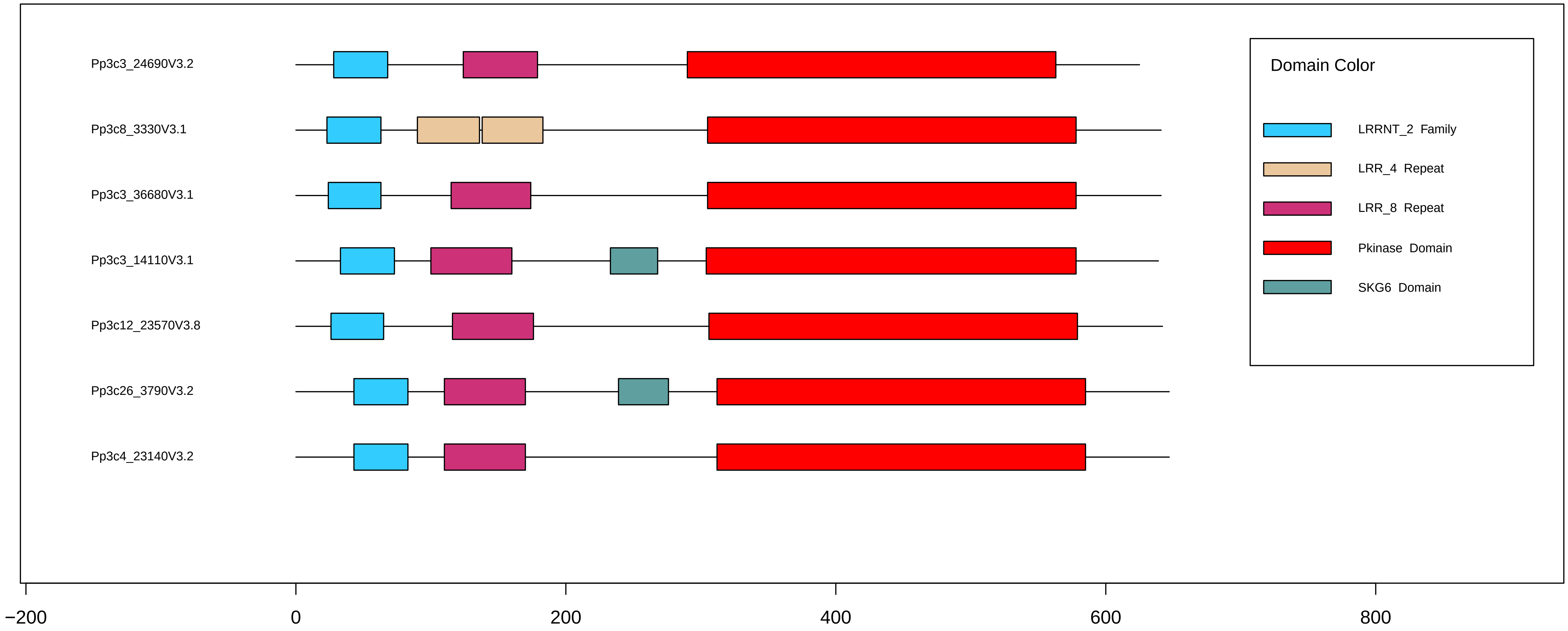

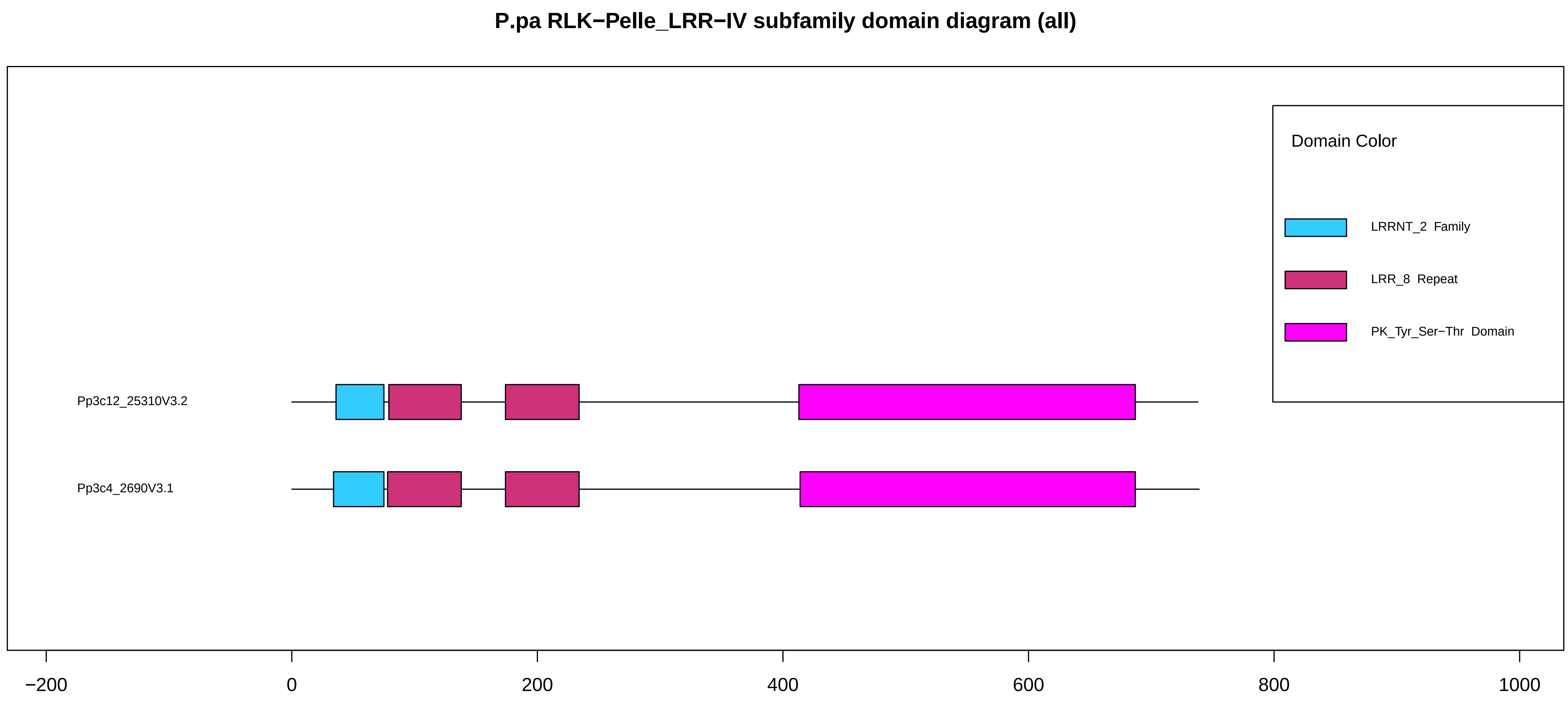
P.pa RLK−Pelle_LRR−IV subfamily domain diagram (all)
Domain Color
LRRNT_2 Family
LRR_8 Repeat
PK_Tyr_Ser−Thr Domain
Pp3c12_25310V3.2
Pp3c4_2690V3.1
−200
0
200
400
600
800
1000

P.pa RLK−Pelle_LRR−IX subfamily domain diagram (all)

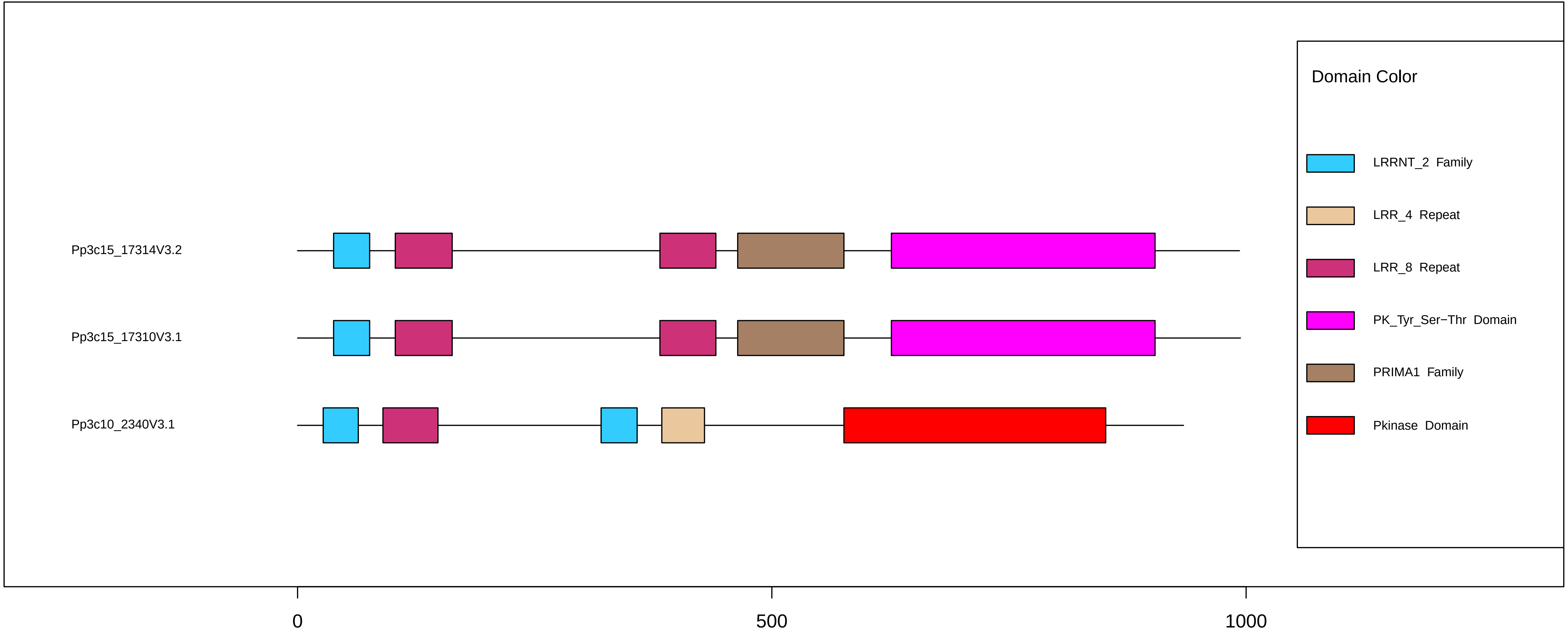

P.pa RLK−Pelle_LRR−VI−1 subfamily domain diagram (all)

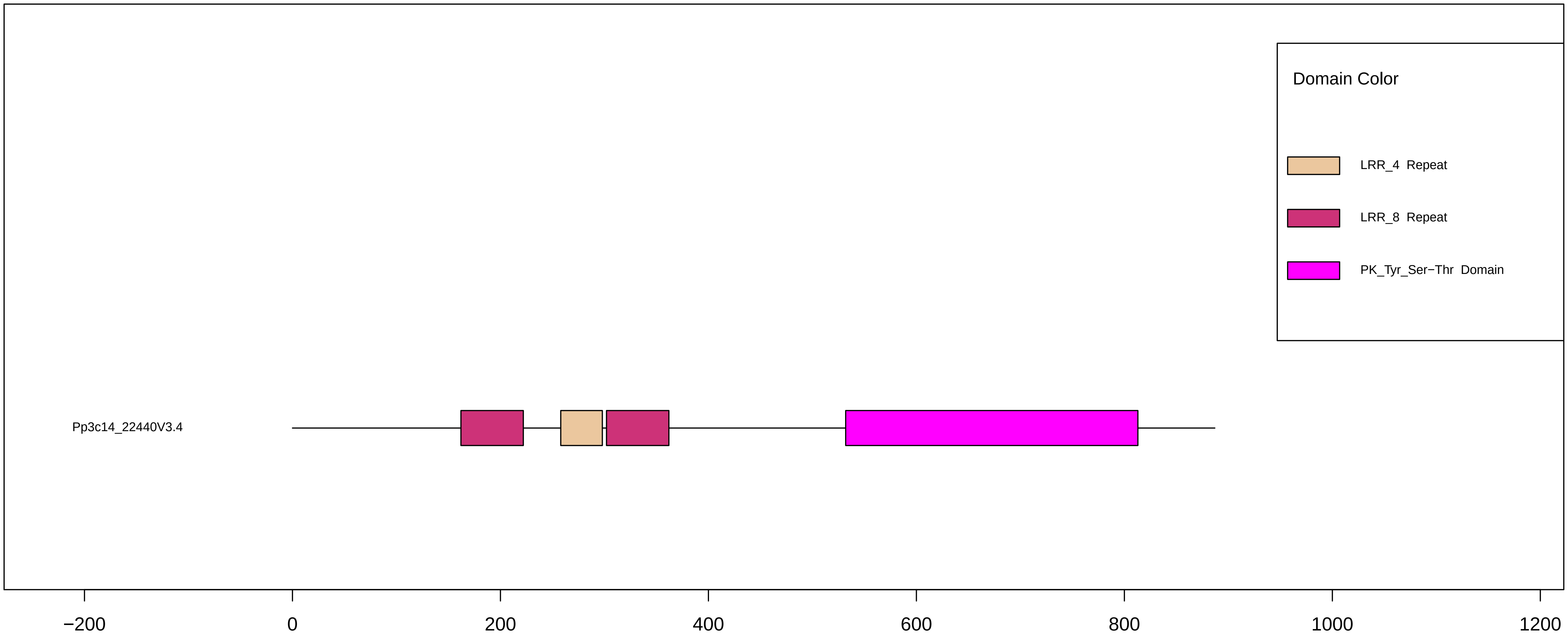

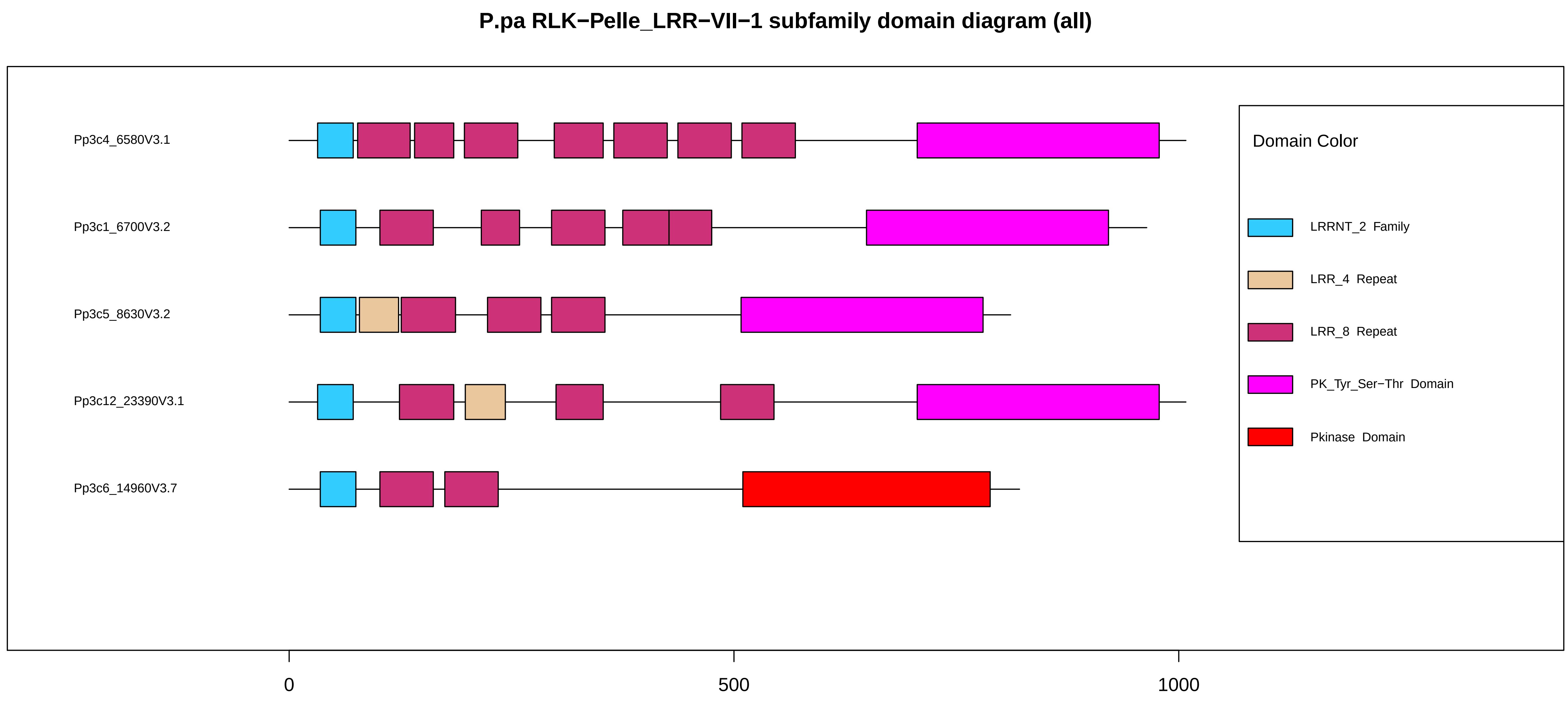
P.pa RLK−Pelle_LRR−VII−1 subfamily domain diagram (all)
Pp3c4_6580V3.1
Pp3c1_6700V3.2
Pp3c5_8630V3.2
Pp3c12_23390V3.1
Pp3c6_14960V3.7
Domain Color
LRRNT_2 Family
LRR_4 Repeat
LRR_8 Repeat
PK_Tyr_Ser−Thr Domain
Pkinase Domain
0
500
1000

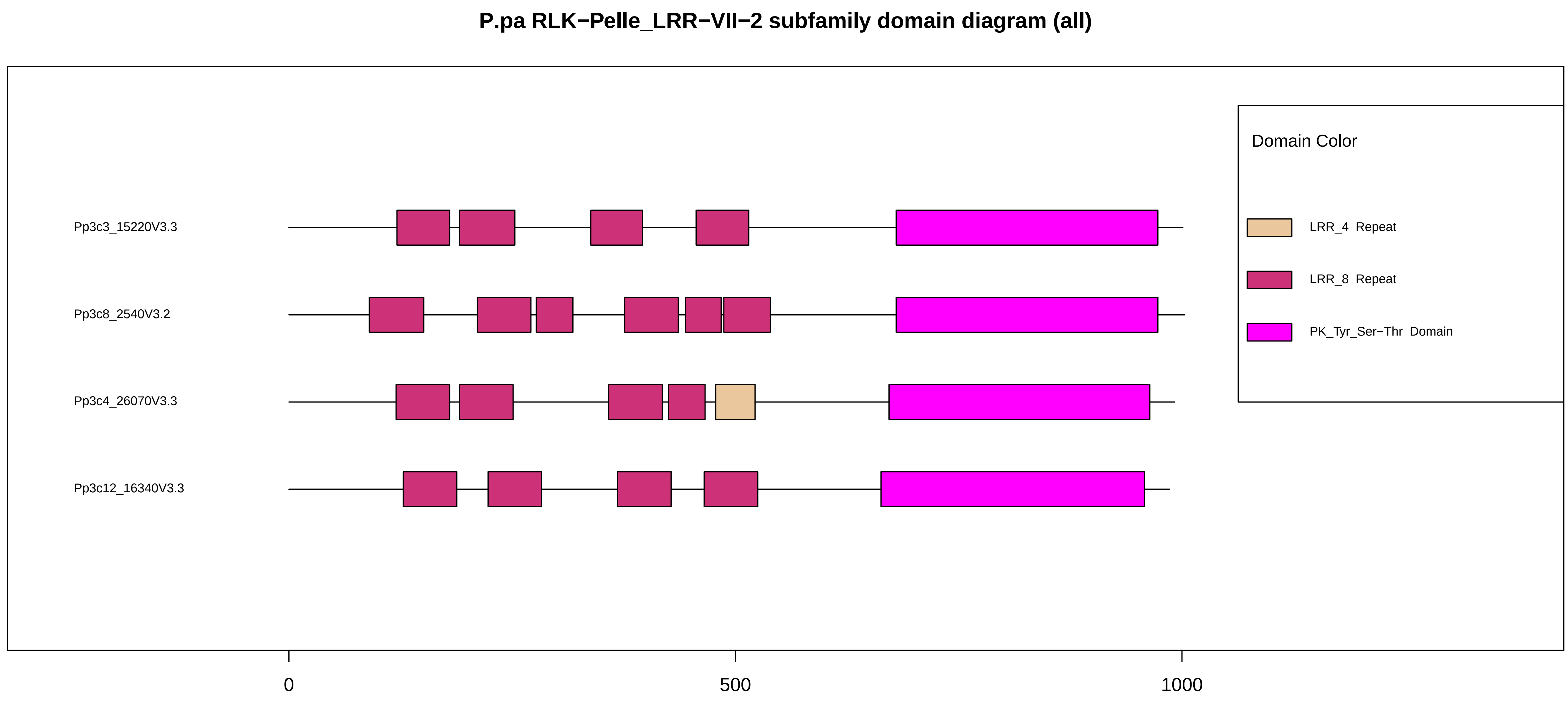

P.pa RLK−Pelle_LRR−VII−2 subfamily domain diagram (all)
Pp3c3_15220V3.3
Pp3c8_2540V3.2
Pp3c4_26070V3.3
Pp3c12_16340V3.3
Domain Color
LRR_4 Repeat
LRR_8 Repeat
PK_Tyr_Ser−Thr Domain
0
500
1000

P.pa RLK−Pelle_LRR−VII−3 subfamily domain diagram (all)

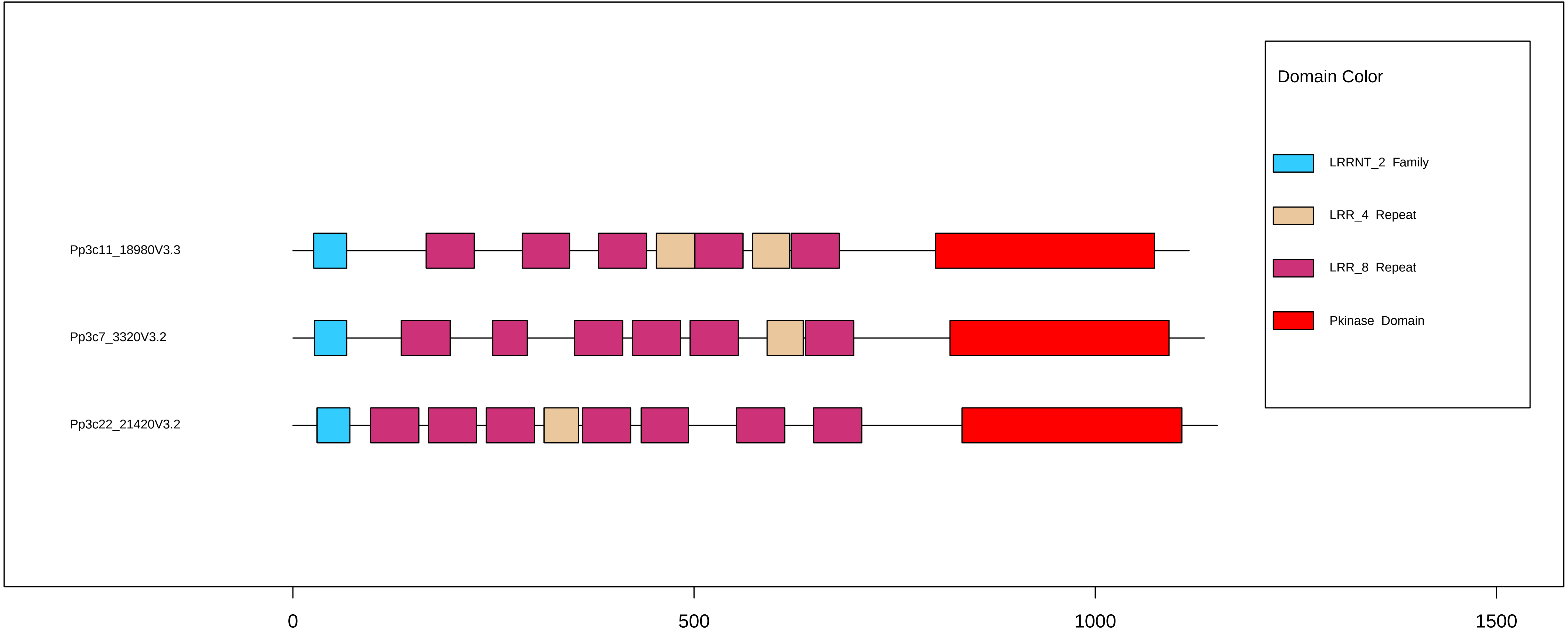

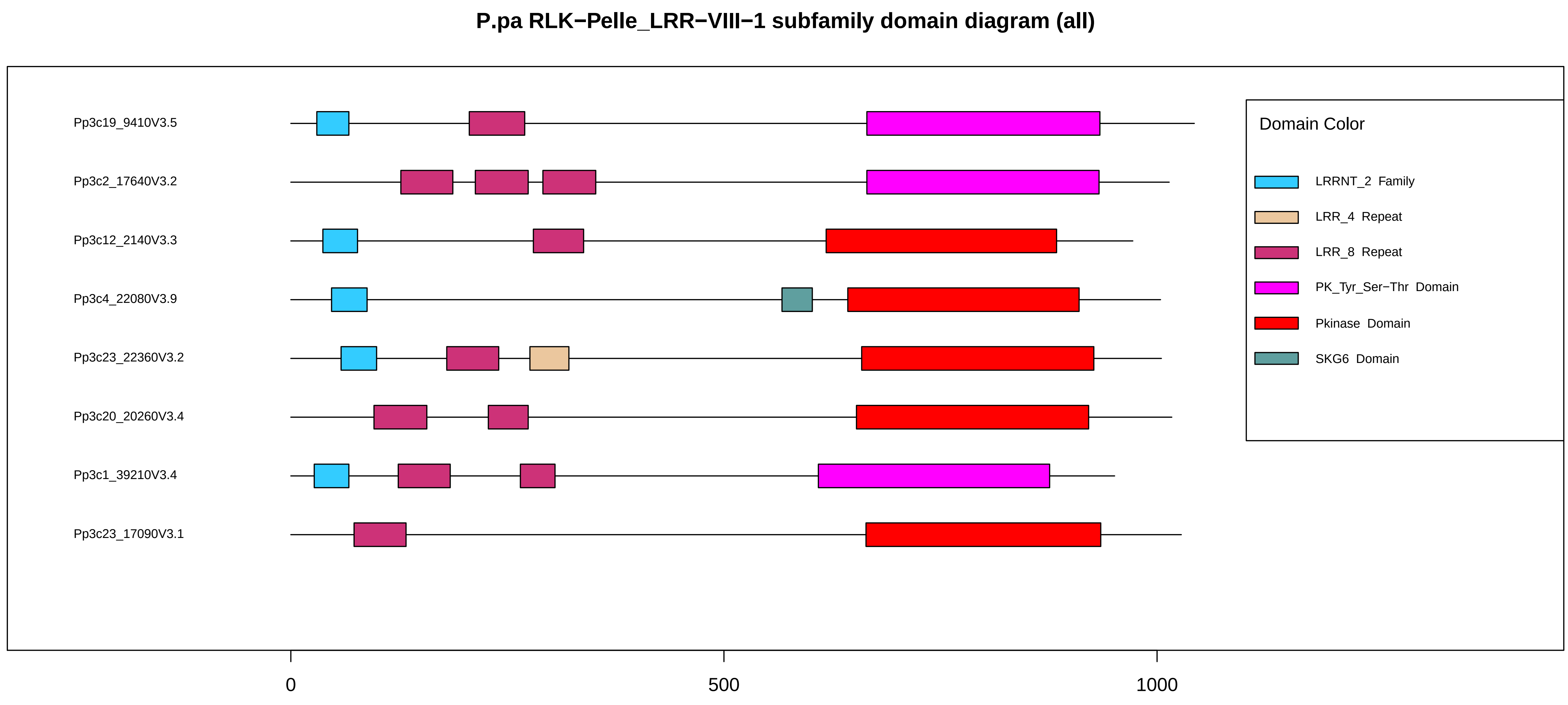

P.pa RLK−Pelle_LRR−VIII−1 subfamily domain diagram (all)
Pp3c19_9410V3.5
Pp3c2_17640V3.2
Pp3c12_2140V3.3
Pp3c4_22080V3.9
Pp3c23_22360V3.2
Pp3c20_20260V3.4
Pp3c1_39210V3.4
Pp3c23_17090V3.1
Domain Color
LRRNT_2 Family
LRR_4 Repeat
LRR_8 Repeat
PK_Tyr_Ser−Thr Domain
Pkinase Domain
SKG6 Domain
0
500
1000

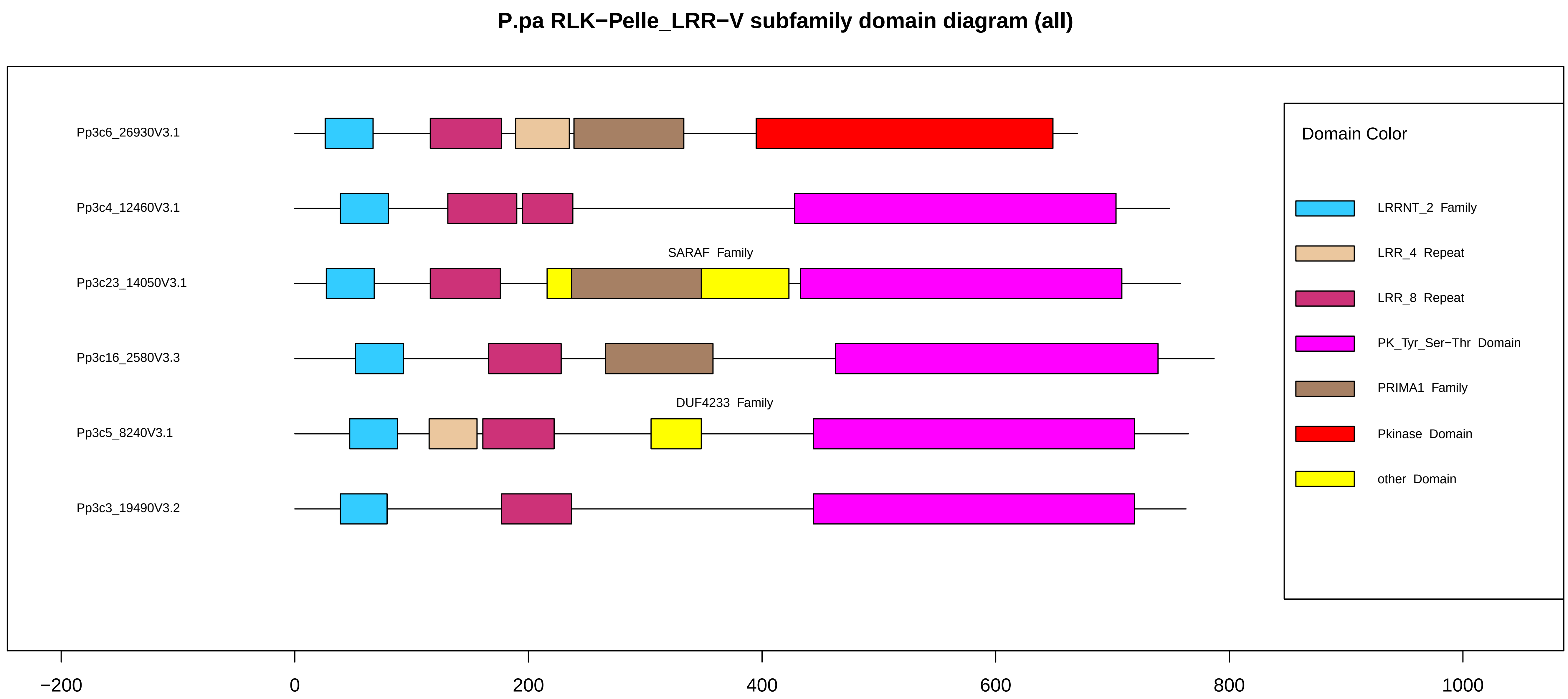

P.pa RLK−Pelle_LRR−V subfamily domain diagram (all)
Pp3c6_26930V3.1
Pp3c4_12460V3.1
Pp3c23_14050V3.1
Pp3c16_2580V3.3
Pp3c5_8240V3.1
Pp3c3_19490V3.2
SARAF Family
DUF4233 Family
Domain Color
LRRNT_2 Family
LRR_4 Repeat
LRR_8 Repeat
PK_Tyr_Ser−Thr Domain
PRIMA1 Family
Pkinase Domain
other Domain
−200
0
200
400
600
800
1000

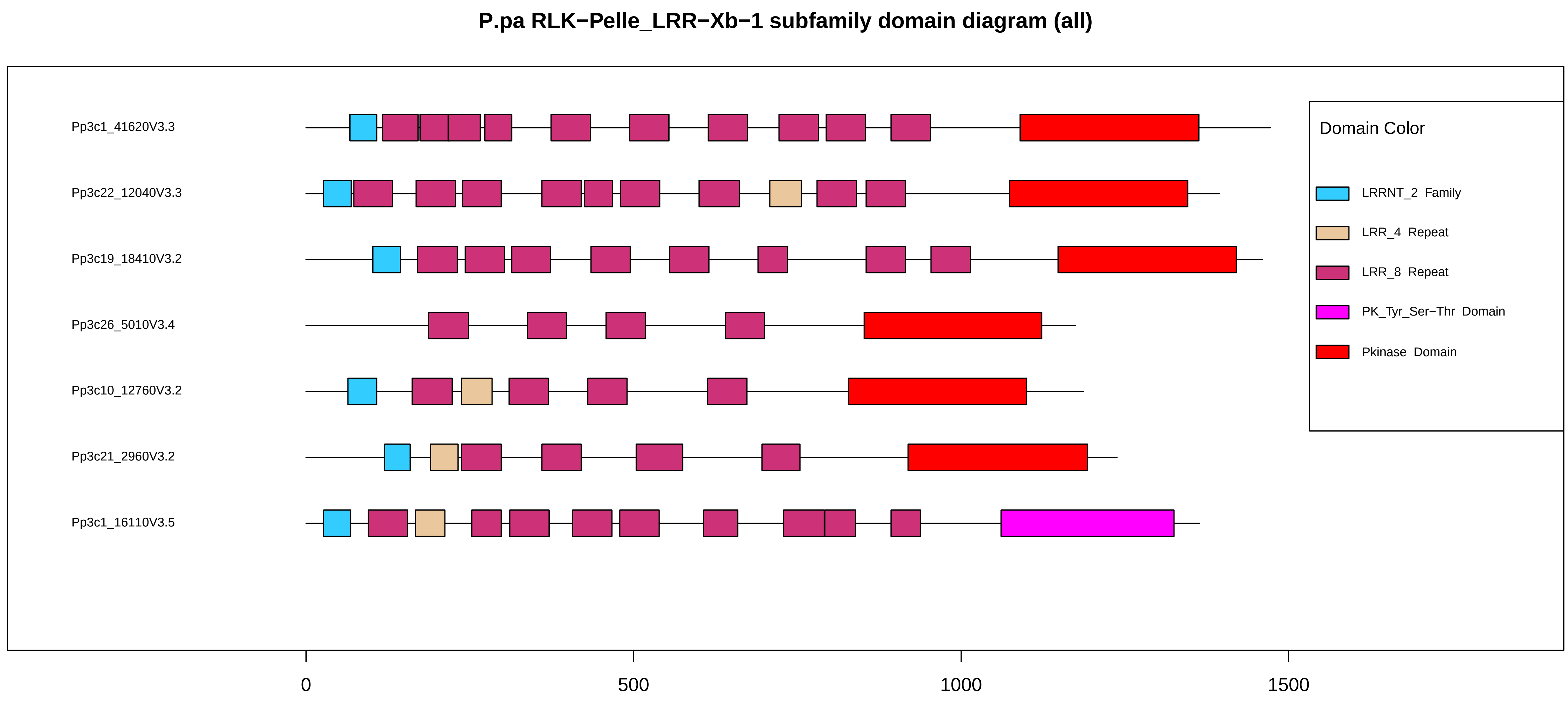
P.pa RLK−Pelle_LRR−Xb−1 subfamily domain diagram (all)
Pp3c1_41620V3.3
Pp3c22_12040V3.3
Pp3c19_18410V3.2
Pp3c26_5010V3.4
Pp3c10_12760V3.2
Pp3c21_2960V3.2
Pp3c1_16110V3.5
Domain Color
LRRNT_2 Family
LRR_4 Repeat
LRR_8 Repeat
PK_Tyr_Ser−Thr Domain
Pkinase Domain
0
500
1000
1500

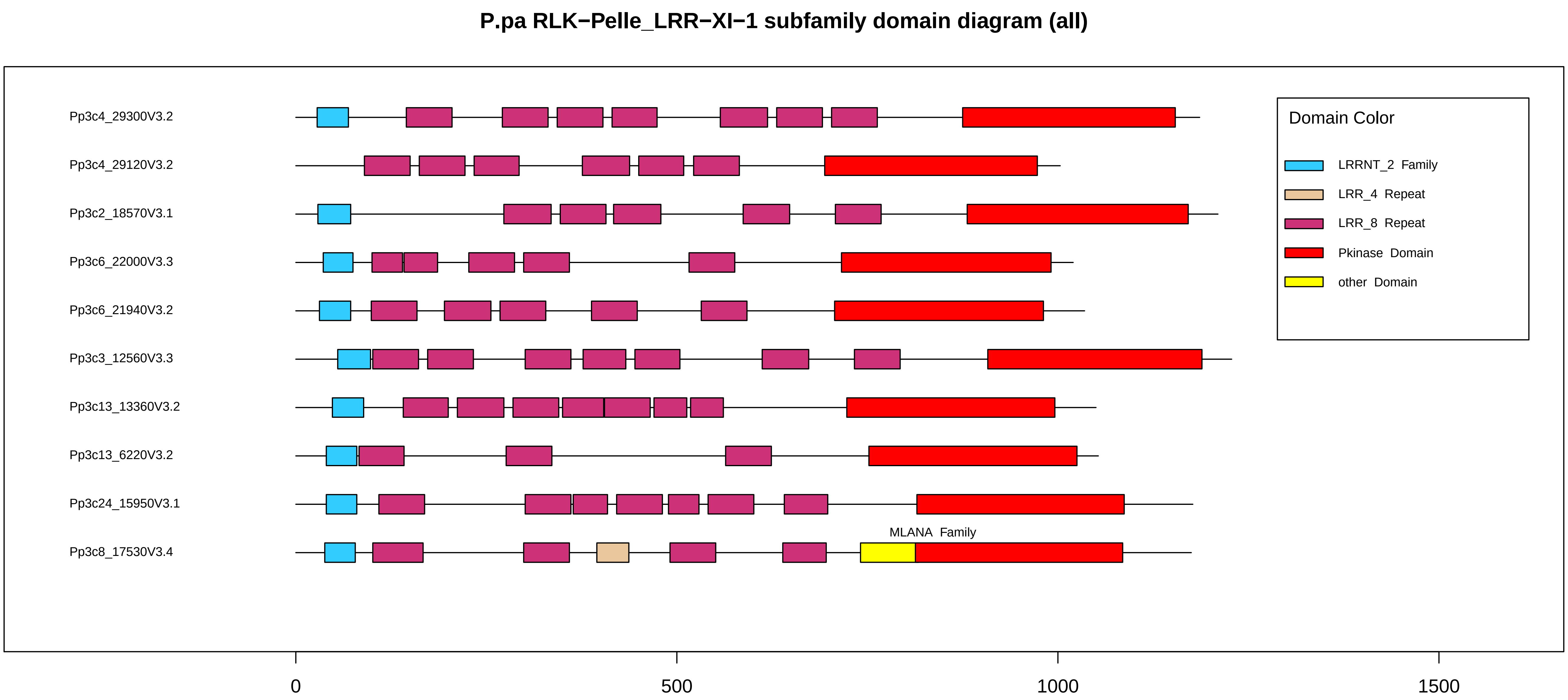

P.pa RLK−Pelle_LRR−XI−1 subfamily domain diagram (all)
Pp3c4_29300V3.2
Pp3c4_29120V3.2
Pp3c2_18570V3.1
Pp3c6_22000V3.3
Pp3c6_21940V3.2
Pp3c3_12560V3.3
Pp3c13_13360V3.2
Pp3c13_6220V3.2
Pp3c24_15950V3.1
Pp3c8_17530V3.4
MLANA Family
Domain Color
LRRNT_2 Family
LRR_4 Repeat
LRR_8 Repeat
Pkinase Domain
other Domain
0
500
1000
1500

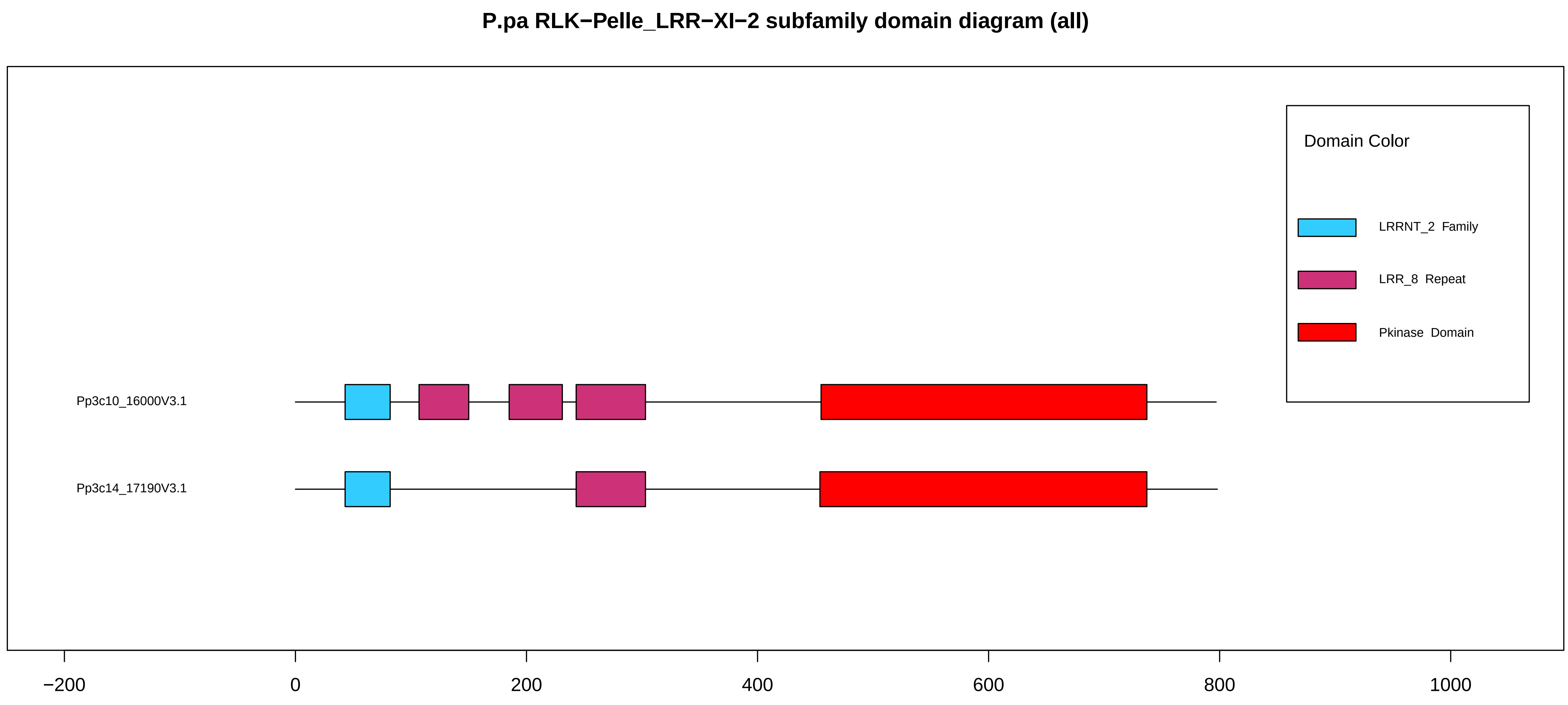
P.pa RLK−Pelle_LRR−XI−2 subfamily domain diagram (all)
Domain Color
LRRNT_2 Family
LRR_8 Repeat
Pkinase Domain
Pp3c10_16000V3.1
Pp3c14_17190V3.1
−200
0
200
400
600
800
1000

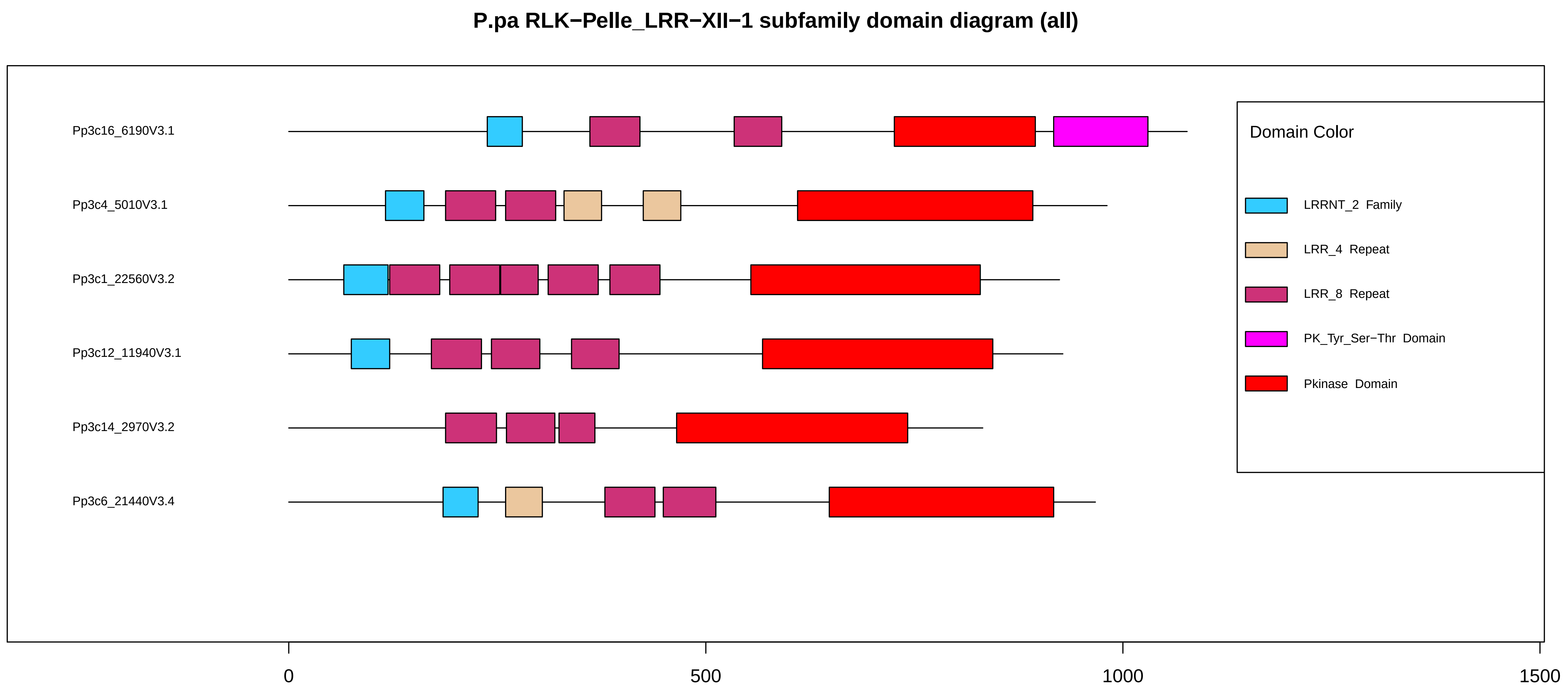
P.pa RLK−Pelle_LRR−XII−1 subfamily domain diagram (all)
Pp3c16_6190V3.1
Pp3c4_5010V3.1
Pp3c1_22560V3.2
Pp3c12_11940V3.1
Pp3c14_2970V3.2
Pp3c6_21440V3.4
Domain Color
LRRNT_2 Family
LRR_4 Repeat
LRR_8 Repeat
PK_Tyr_Ser−Thr Domain
Pkinase Domain
0
500
1000
1500

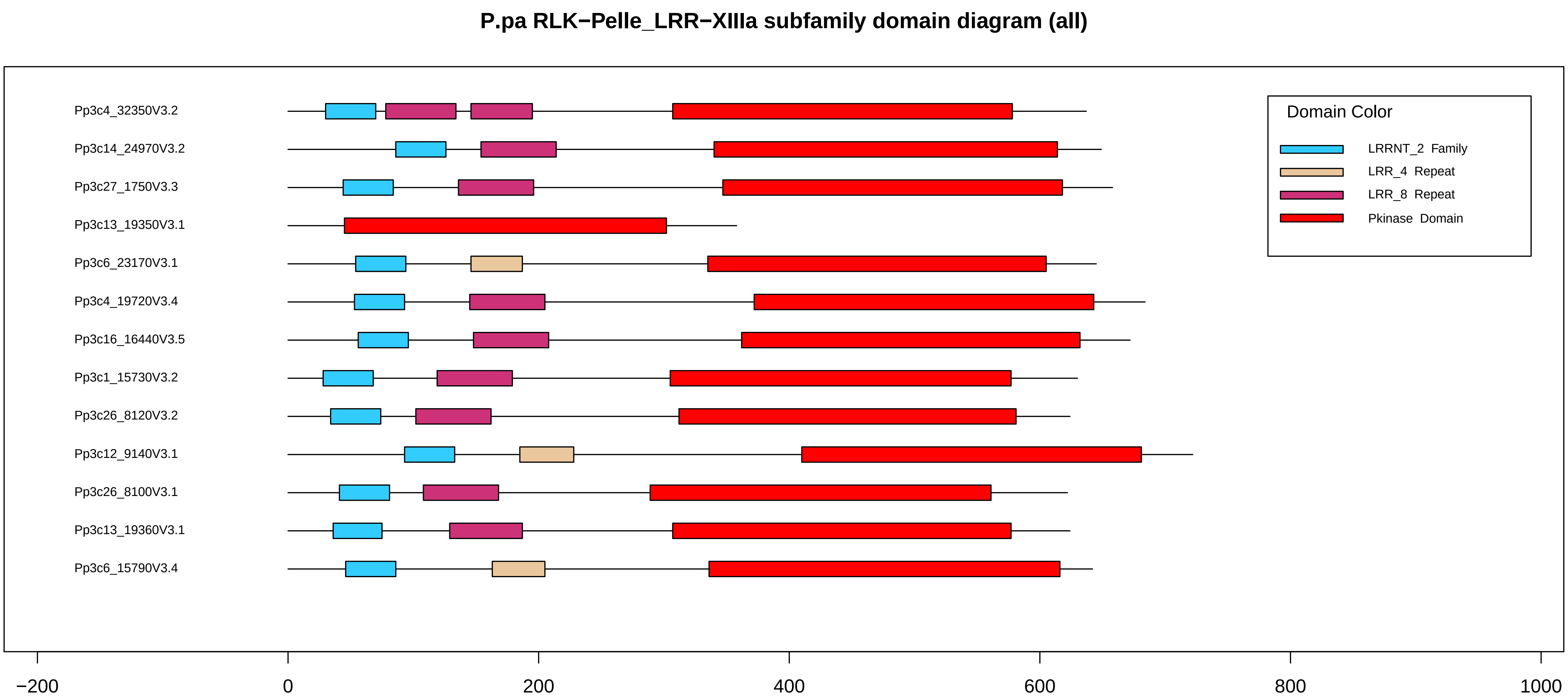

P.pa RLK−Pelle_LRR−XIIIa subfamily domain diagram (all)
Pp3c4_32350V3.2
Pp3c14_24970V3.2
Pp3c27_1750V3.3
Pp3c13_19350V3.1
Pp3c6_23170V3.1
Pp3c4_19720V3.4
Pp3c16_16440V3.5
Pp3c1_15730V3.2
Pp3c26_8120V3.2
Pp3c12_9140V3.1
Pp3c26_8100V3.1
Pp3c13_19360V3.1
Pp3c6_15790V3.4
Domain Color
LRRNT_2 Family
LRR_4 Repeat
LRR_8 Repeat
Pkinase Domain
−200
0
200
400
600
800
1000

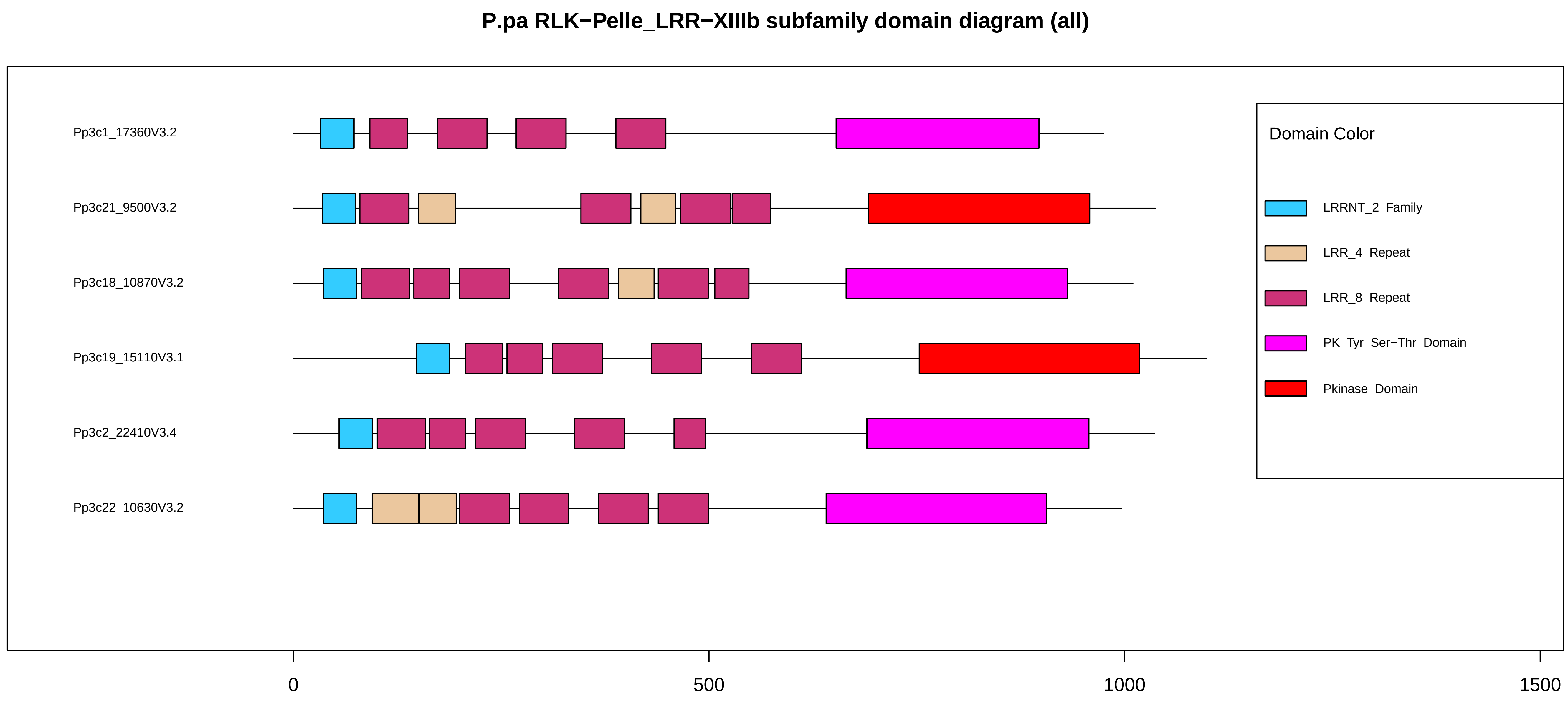

P.pa RLK−Pelle_LRR−XIIIb subfamily domain diagram (all)
Pp3c1_17360V3.2
Pp3c21_9500V3.2
Pp3c18_10870V3.2
Pp3c19_15110V3.1
Pp3c2_22410V3.4
Pp3c22_10630V3.2
Domain Color
LRRNT_2 Family
LRR_4 Repeat
LRR_8 Repeat
PK_Tyr_Ser−Thr Domain
Pkinase Domain
0
500
1000
1500

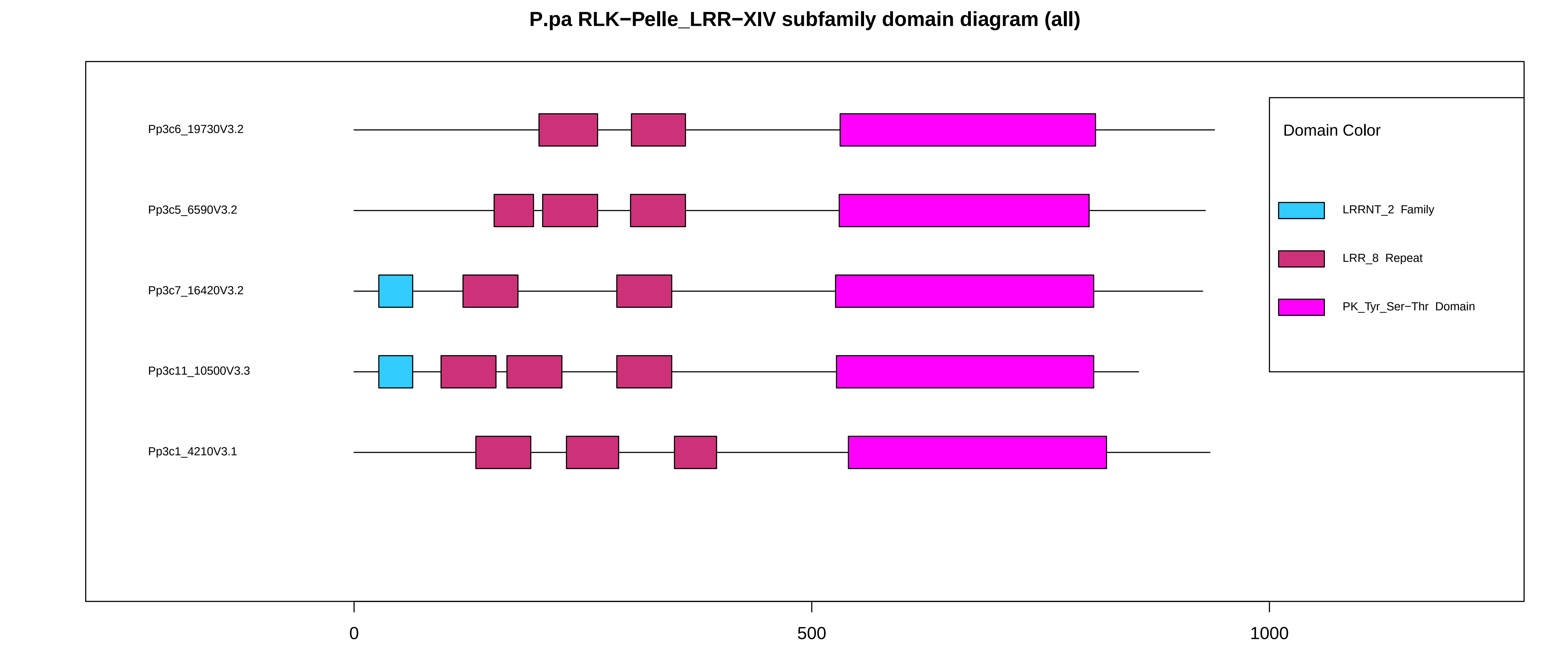
P.pa RLK−Pelle_LRR−XIV subfamily domain diagram (all)
Pp3c6_19730V3.2
Pp3c5_6590V3.2
Pp3c7_16420V3.2
Pp3c11_10500V3.3
Pp3c1_4210V3.1
Domain Color
LRRNT_2 Family
LRR_8 Repeat
PK_Tyr_Ser−Thr Domain
0
500
1000

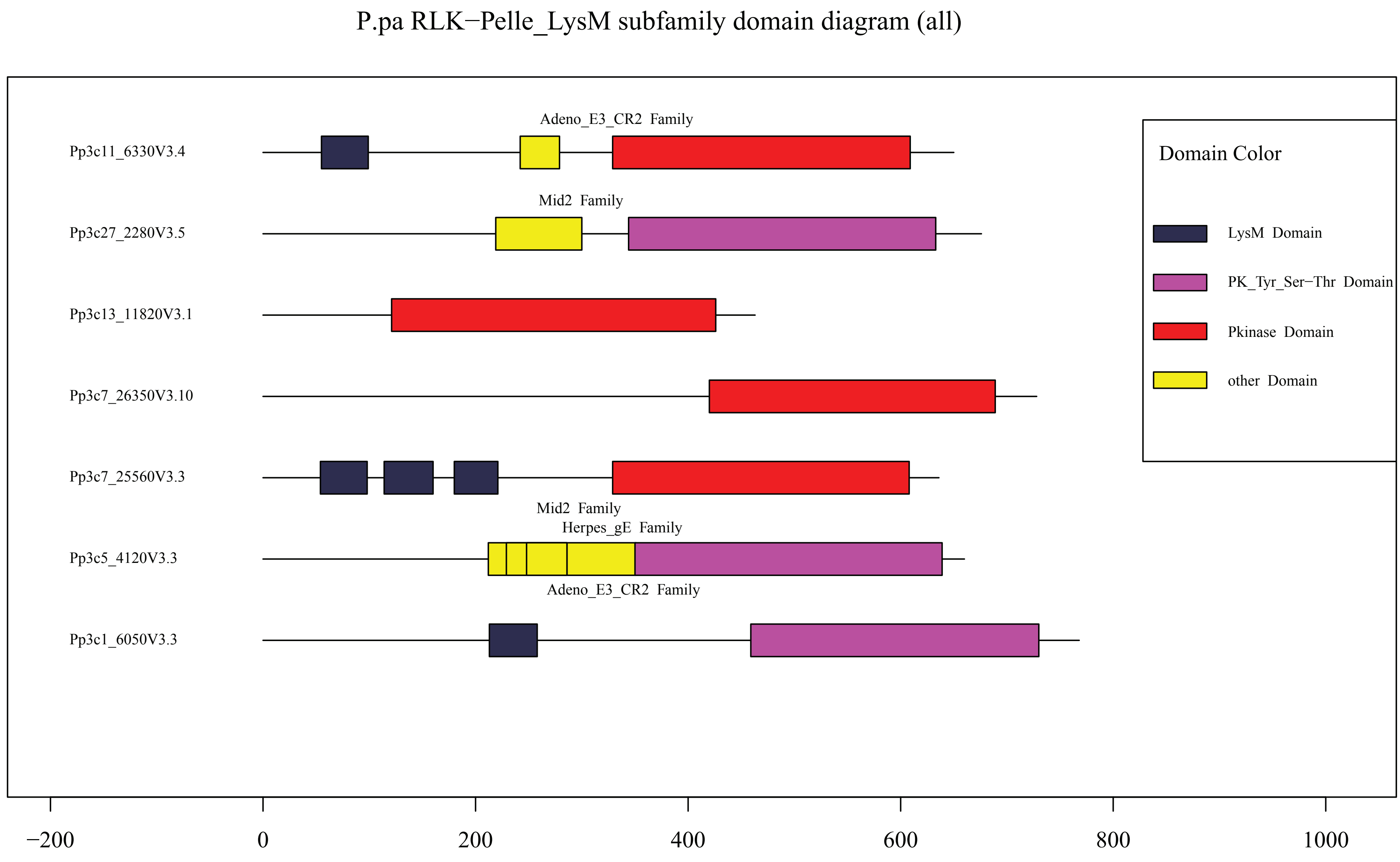
P.pa RLK−Pelle_LysM subfamily domain diagram (all)
Adeno_E3_CR2 Family
Pp3c11_6330V3.4
Mid2 Family
Pp3c27_2280V3.5
Pp3c13_11820V3.1
Pp3c7_26350V3.10
Pp3c7_25560V3.3
Mid2 Family
Herpes_gE Family
Pp3c5_4120V3.3
Adeno_E3_CR2 Family
Pp3c1_6050V3.3
Domain Color
LysM Domain
PK_Tyr_Ser−Thr Domain
Pkinase Domain
other Domain
−200
0
200
400
600
800
1000

P.pa RLK−Pelle_PERK−1 subfamily domain diagram (all)

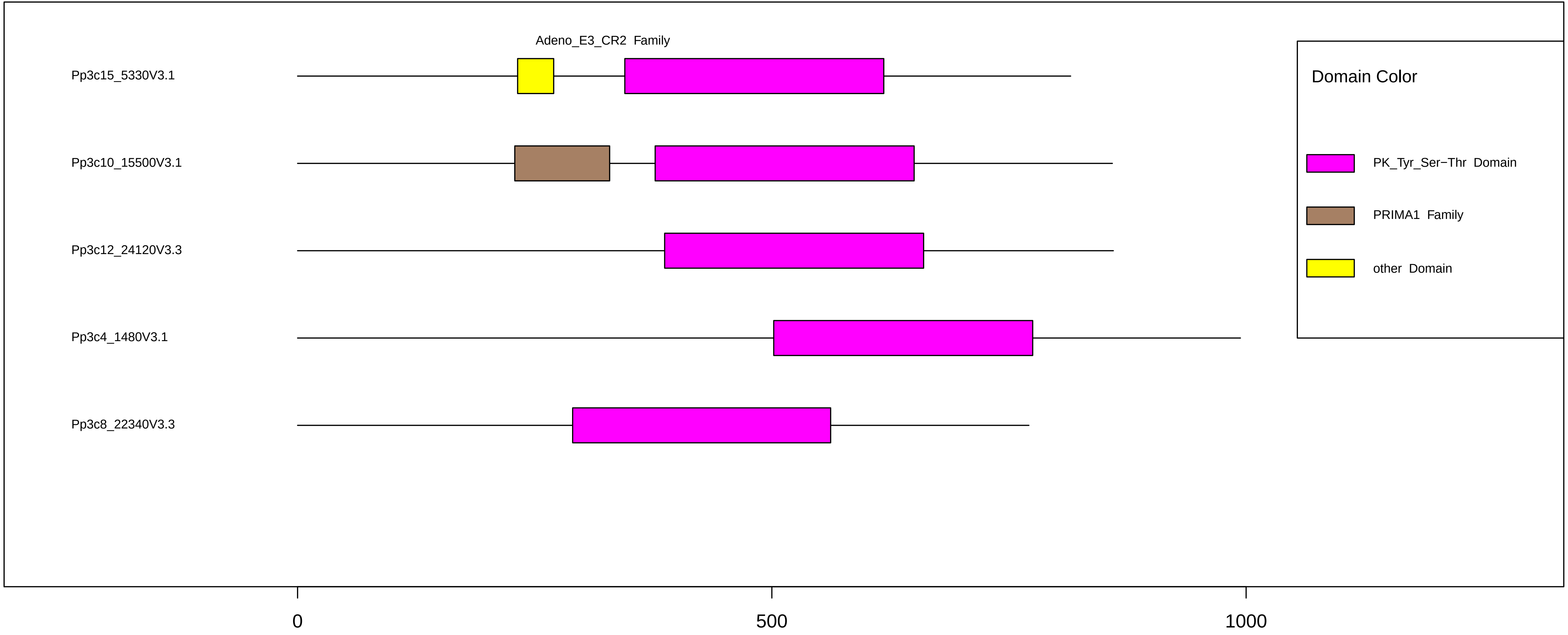

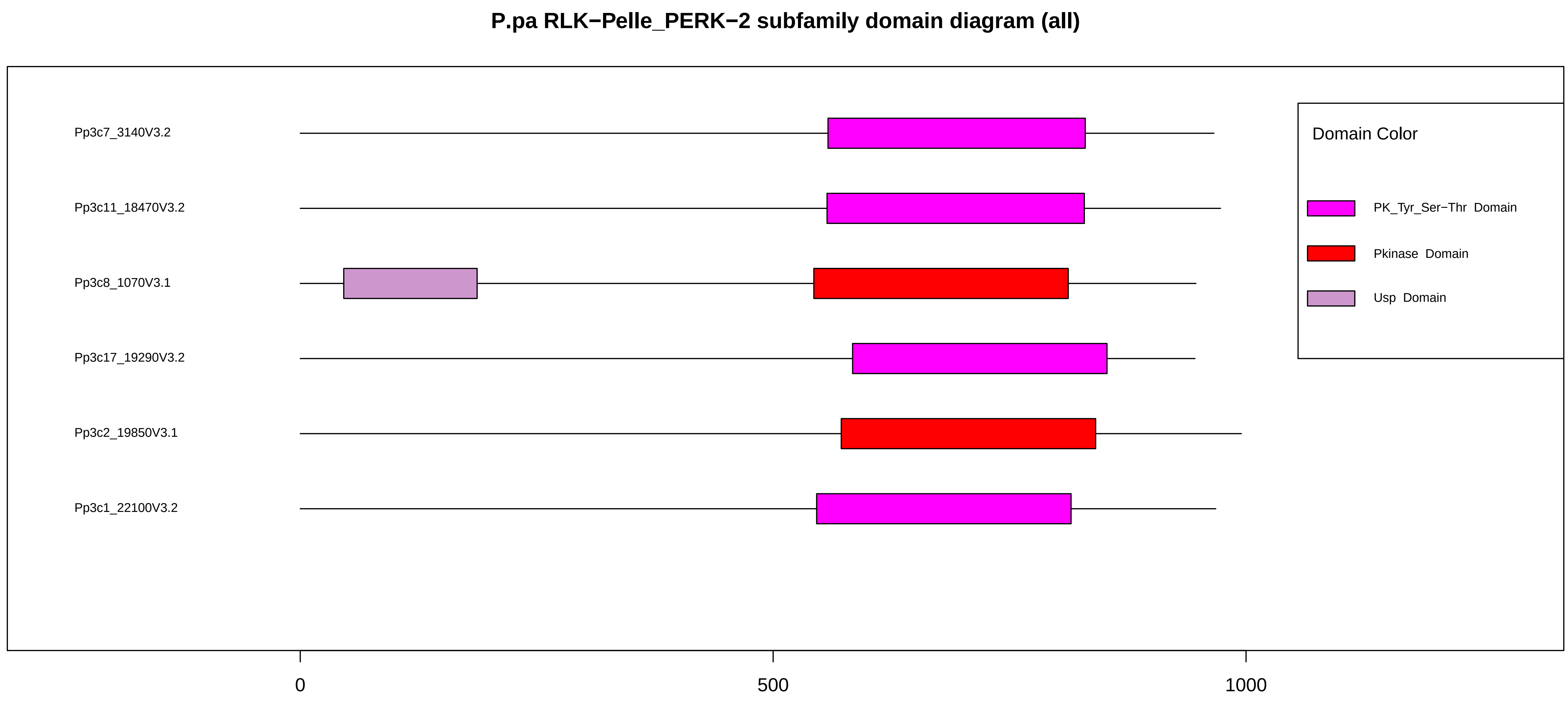

P.pa RLK−Pelle_PERK−2 subfamily domain diagram (all)
Pp3c7_3140V3.2
Pp3c11_18470V3.2
Pp3c8_1070V3.1
Pp3c17_19290V3.2
Pp3c2_19850V3.1
Pp3c1_22100V3.2
Domain Color
PK_Tyr_Ser−Thr Domain
Pkinase Domain
Usp Domain
0
500
1000

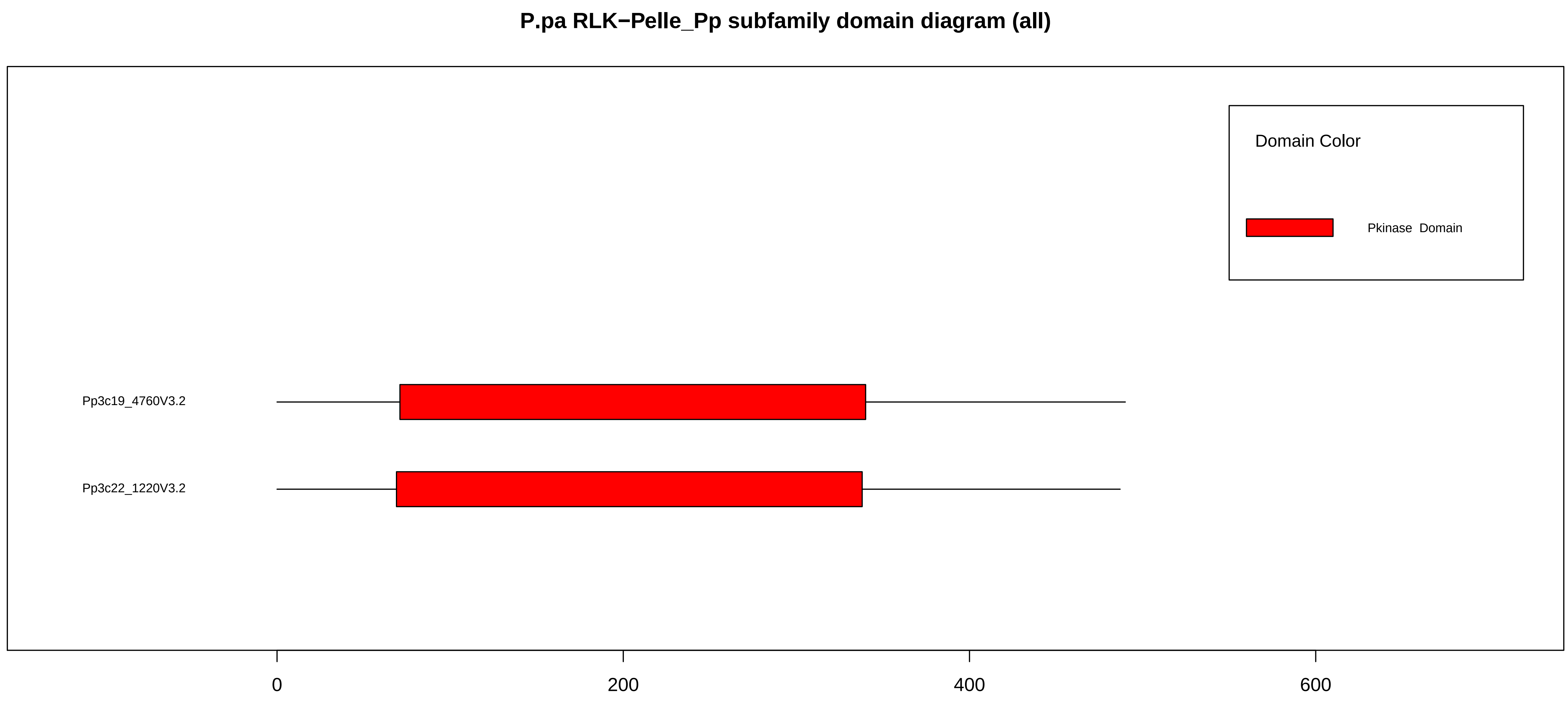
P.pa RLK−Pelle_Pp subfamily domain diagram (all)
Domain Color
Pkinase Domain
Pp3c19_4760V3.2
Pp3c22_1220V3.2
0
200
400
600

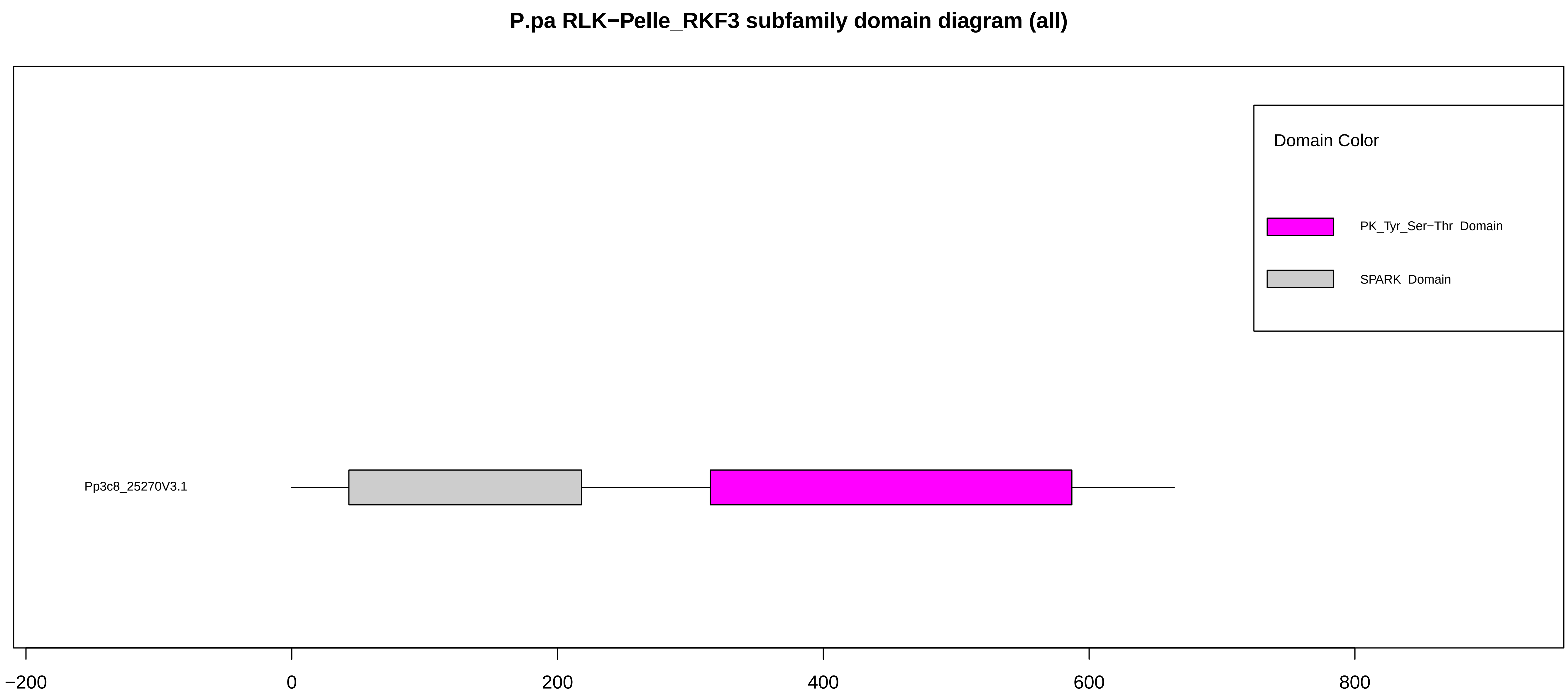
P.pa RLK−Pelle_RKF3 subfamily domain diagram (all)
Domain Color
PK_Tyr_Ser−Thr Domain
SPARK Domain
Pp3c8_25270V3.1
−200
0
200
400
600
800

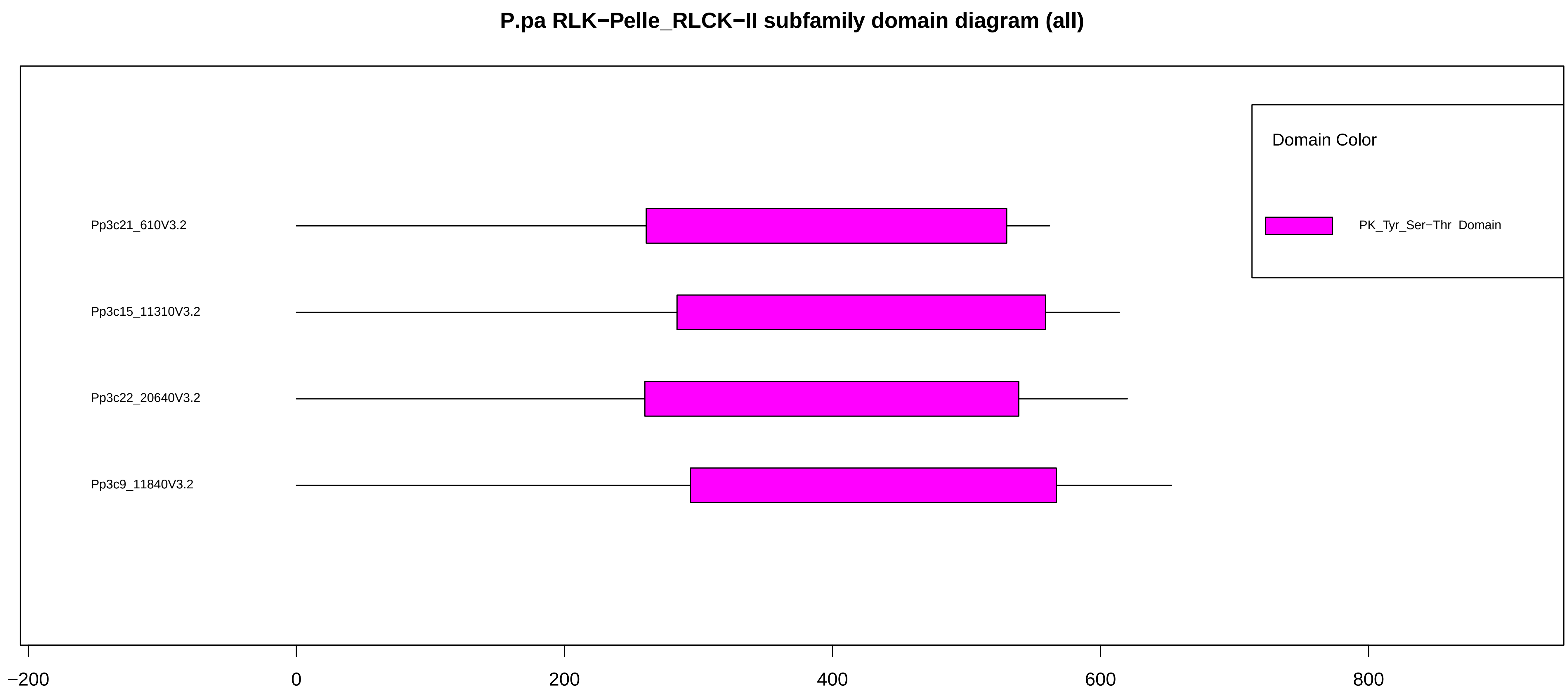

P.pa RLK−Pelle_RLCK−II subfamily domain diagram (all)
Domain Color
PK_Tyr_Ser−Thr Domain
Pp3c21_610V3.2
Pp3c15_11310V3.2
Pp3c22_20640V3.2
Pp3c9_11840V3.2
−200
0
200
400
600
800

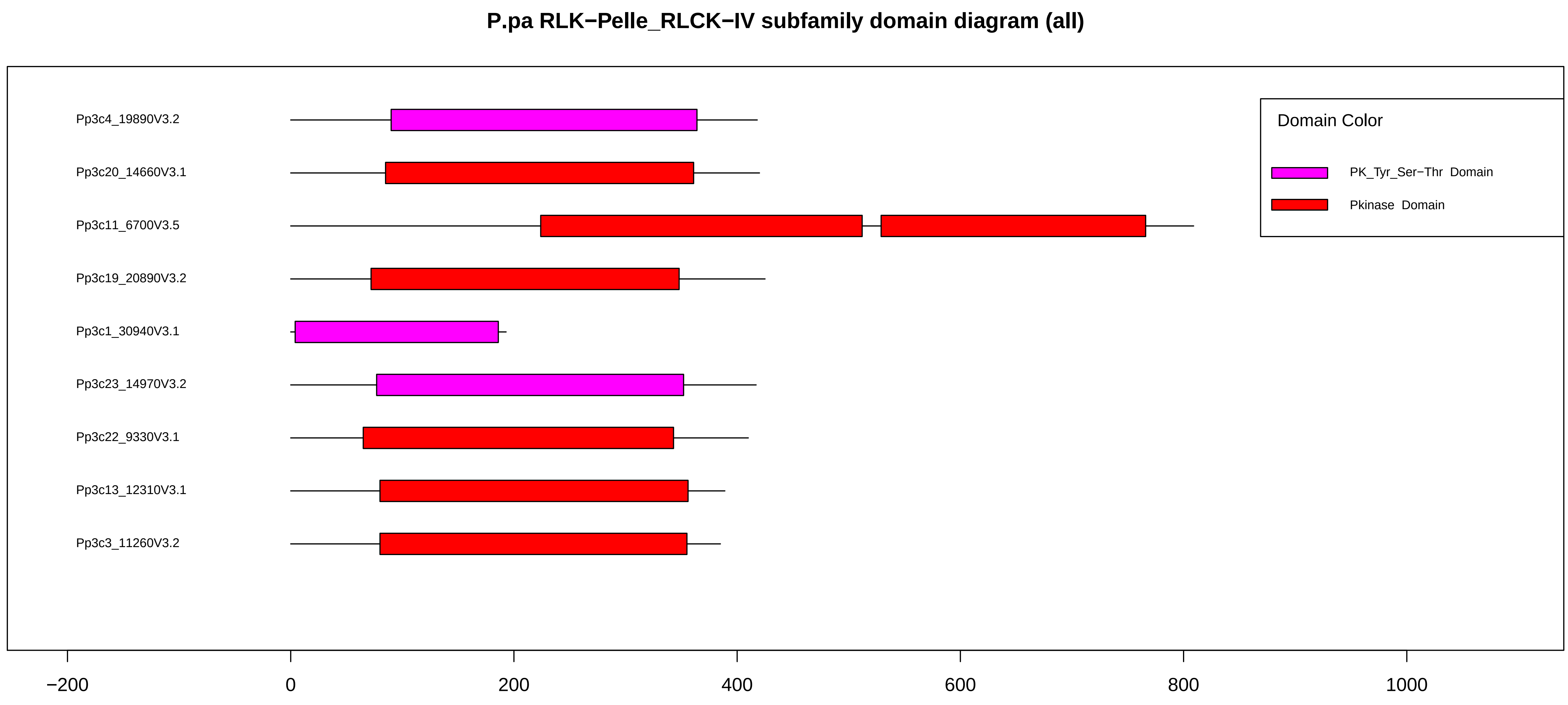
P.pa RLK−Pelle_RLCK−IV subfamily domain diagram (all)
Pp3c4_19890V3.2
Pp3c20_14660V3.1
Pp3c11_6700V3.5
Pp3c19_20890V3.2
Pp3c1_30940V3.1
Pp3c23_14970V3.2
Pp3c22_9330V3.1
Pp3c13_12310V3.1
Pp3c3_11260V3.2
Domain Color
PK_Tyr_Ser−Thr Domain
Pkinase Domain
−200
0
200
400
600
800
1000

P.pa RLK−Pelle_RLCK−Pp subfamily domain diagram (all)

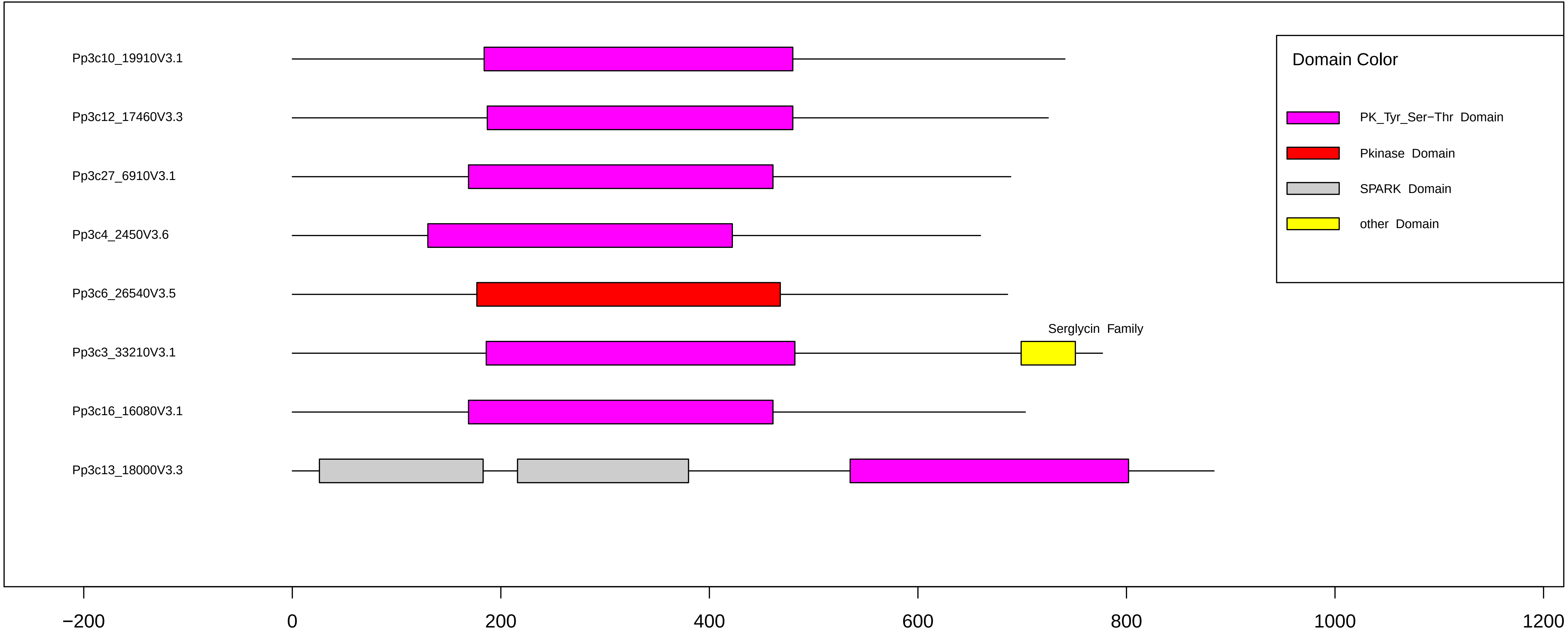

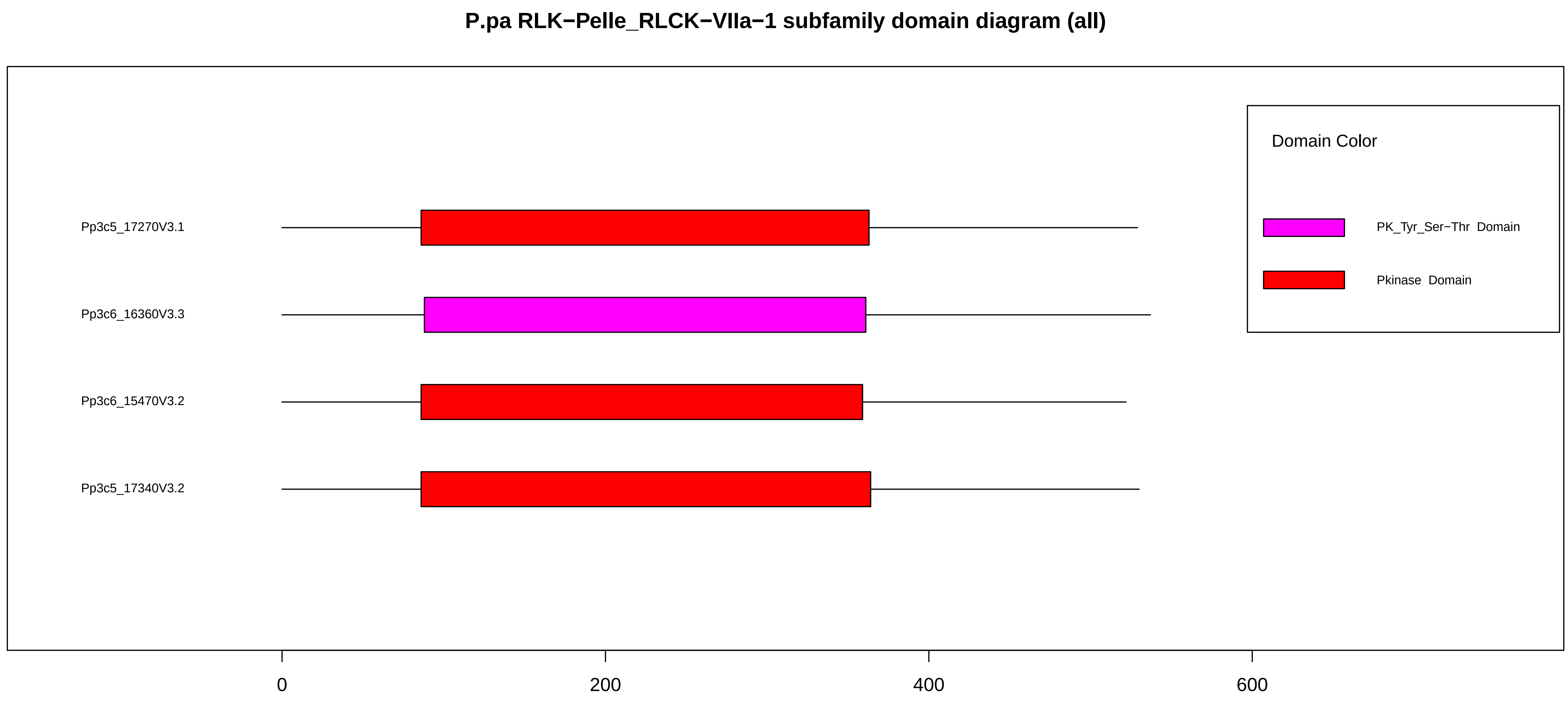
P.pa RLK−Pelle_RLCK−VIIa−1 subfamily domain diagram (all)
Domain Color
PK_Tyr_Ser−Thr Domain
Pkinase Domain
Pp3c5_17270V3.1
Pp3c6_16360V3.3
Pp3c6_15470V3.2
Pp3c5_17340V3.2
0
200
400
600

P.pa RLK−Pelle_RLCK−VIIa−2 subfamily domain diagram (all)

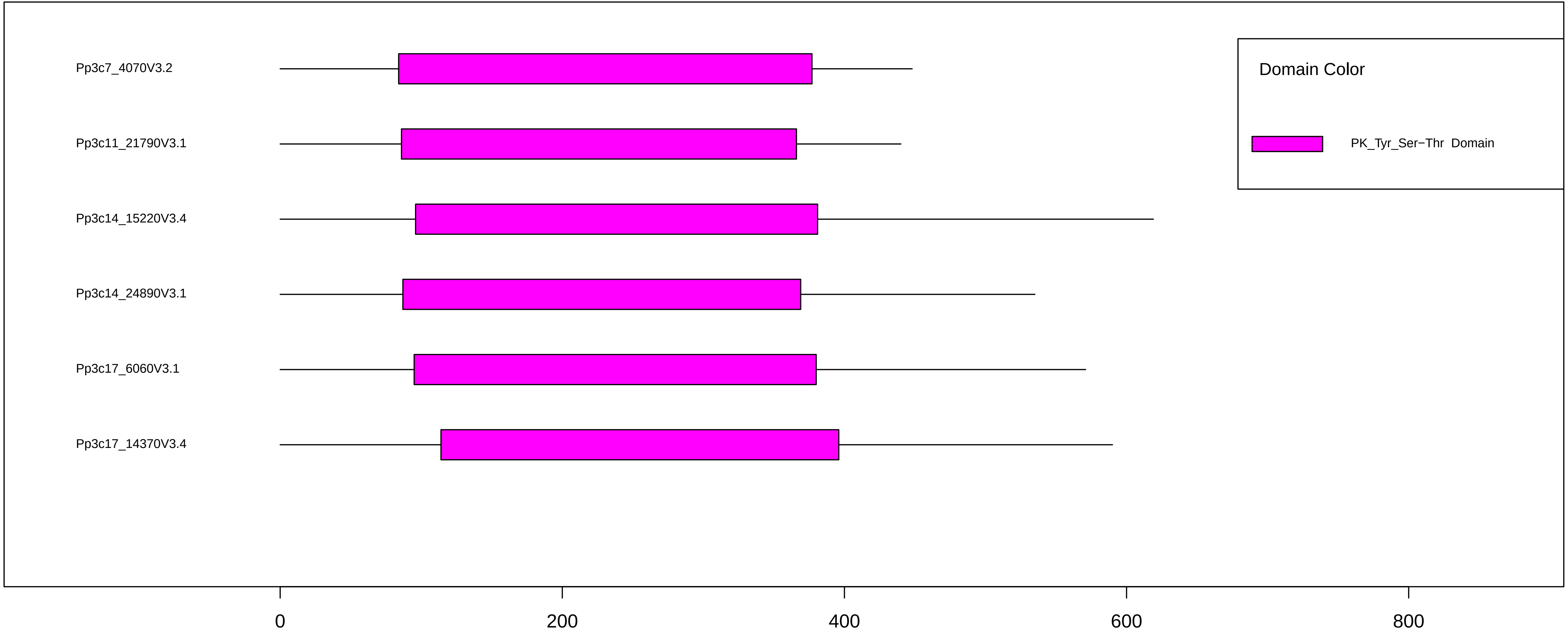

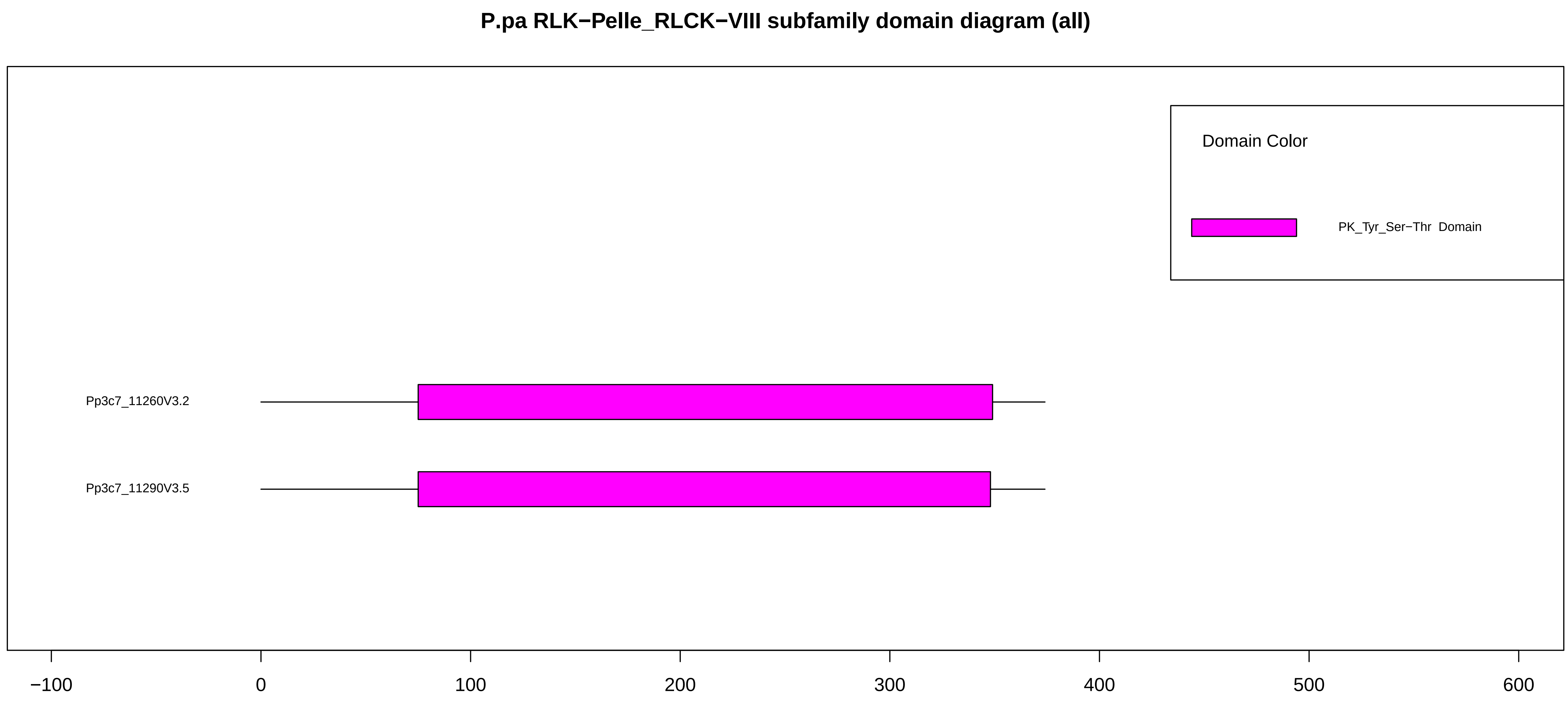

P.pa RLK−Pelle_RLCK−VIII subfamily domain diagram (all)
Domain Color
PK_Tyr_Ser−Thr Domain
Pp3c7_11260V3.2
Pp3c7_11290V3.5
−100
0
100
200
300
400
500
600

P.pa RLK−Pelle_RLCK−VI subfamily domain diagram (all)

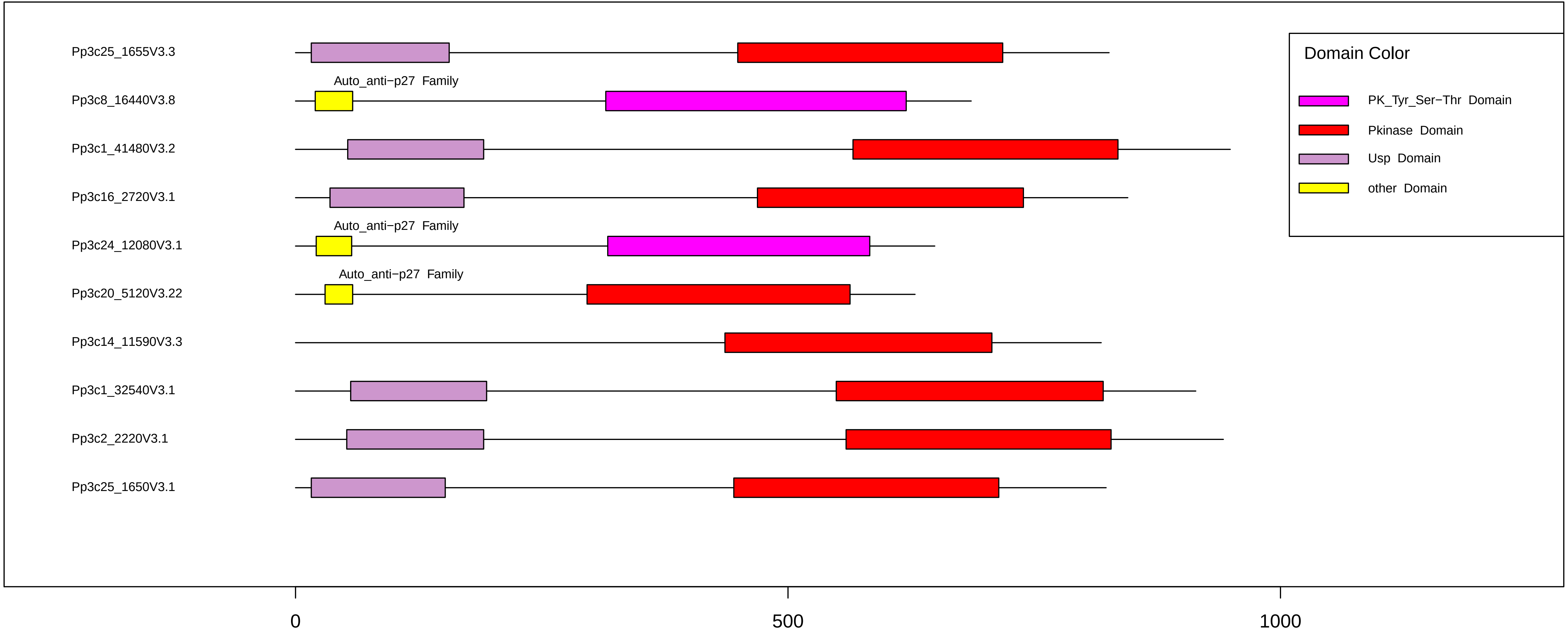

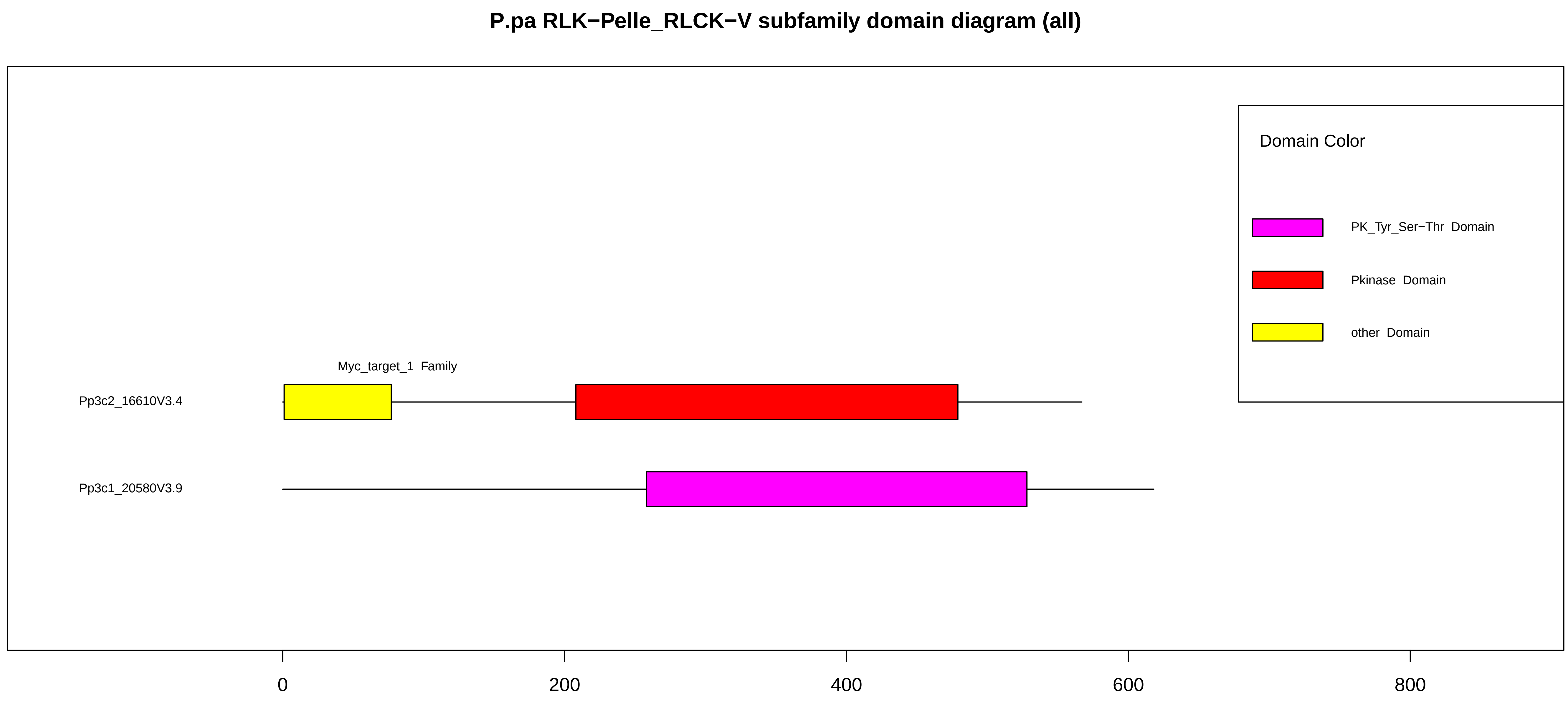

P.pa RLK−Pelle_RLCK−V subfamily domain diagram (all)
Domain Color
PK_Tyr_Ser−Thr Domain
Pkinase Domain
other Domain
Myc_target_1 Family
Pp3c2_16610V3.4
Pp3c1_20580V3.9
0
200
400
600
800

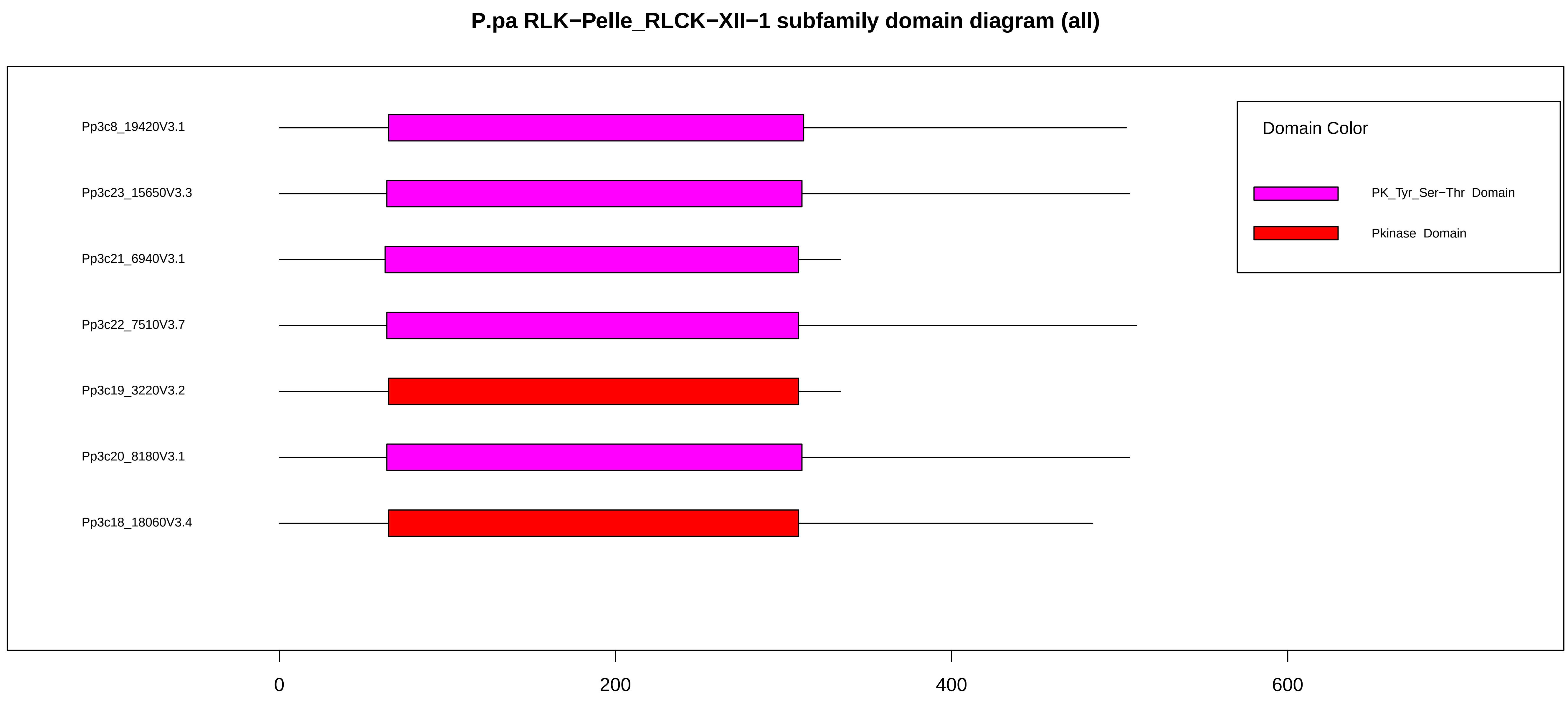
P.pa RLK−Pelle_RLCK−XII−1 subfamily domain diagram (all)
Pp3c8_19420V3.1
Pp3c23_15650V3.3
Pp3c21_6940V3.1
Pp3c22_7510V3.7
Pp3c19_3220V3.2
Pp3c20_8180V3.1
Pp3c18_18060V3.4
Domain Color
PK_Tyr_Ser−Thr Domain
Pkinase Domain
0
200
400
600

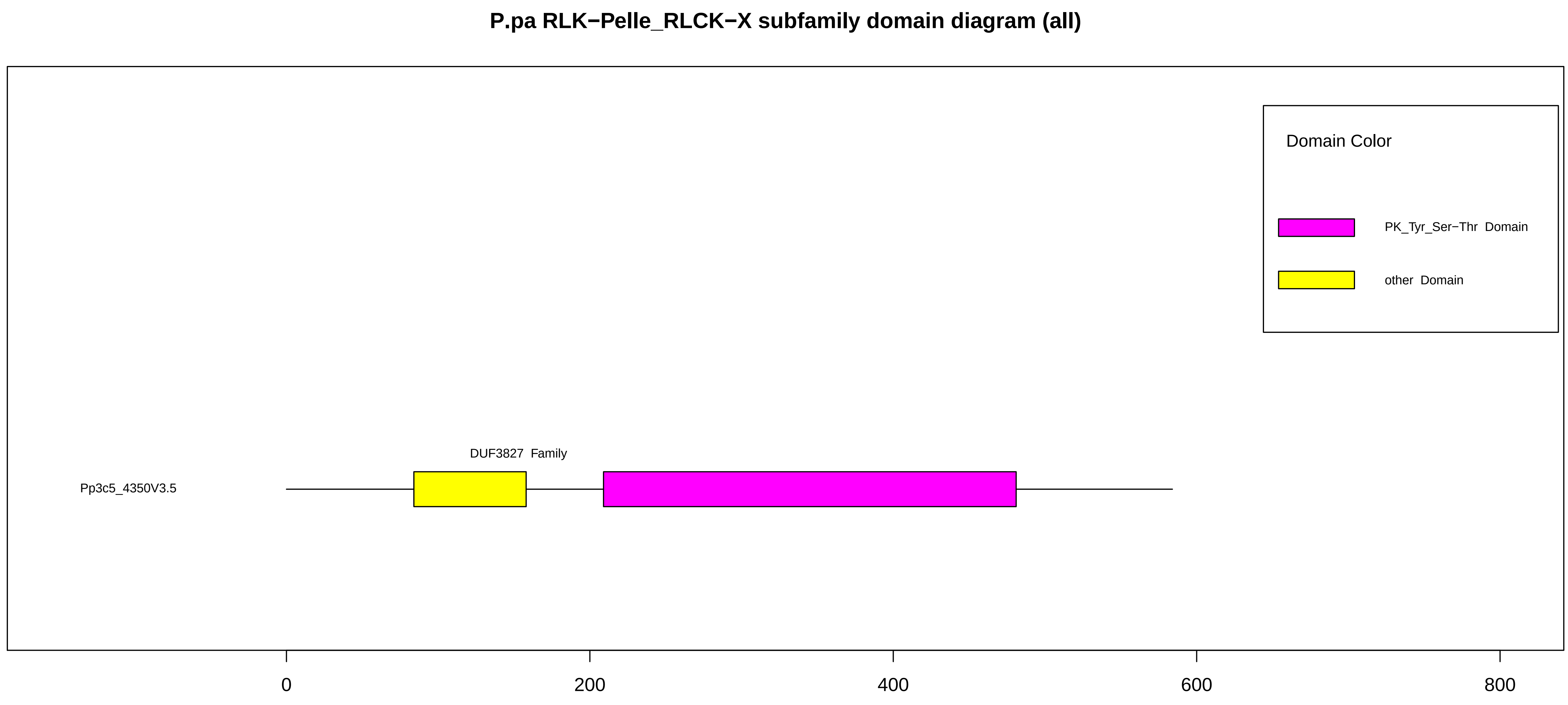
P.pa RLK−Pelle_RLCK−X subfamily domain diagram (all)
Domain Color
PK_Tyr_Ser−Thr Domain
other Domain
DUF3827 Family
Pp3c5_4350V3.5
0
200
400
600
800

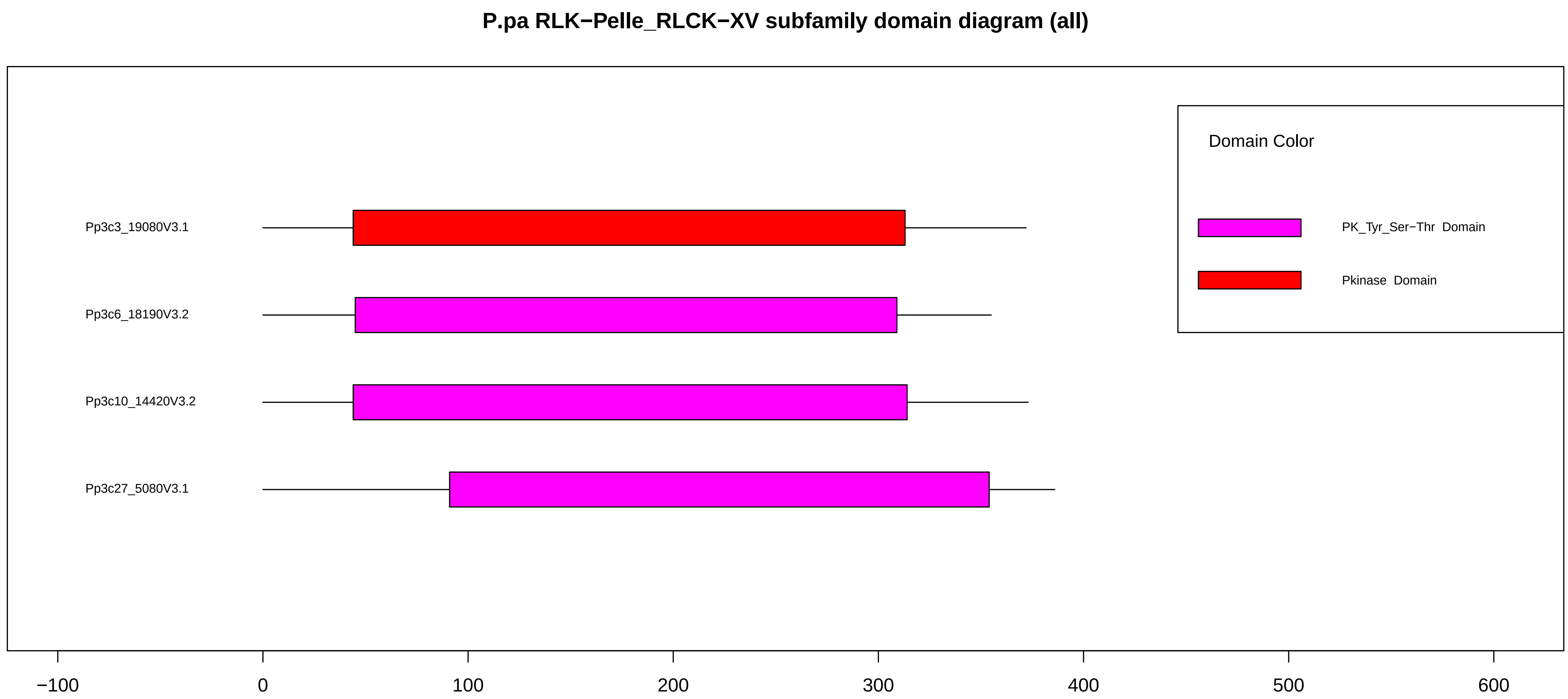

P.pa RLK−Pelle_RLCK−XV subfamily domain diagram (all)
Pp3c3_19080V3.1
Pp3c6_18190V3.2
Pp3c10_14420V3.2
Pp3c27_5080V3.1
Domain Color
PK_Tyr_Ser−Thr Domain
Pkinase Domain
−100
0
100
200
300
400
500
600

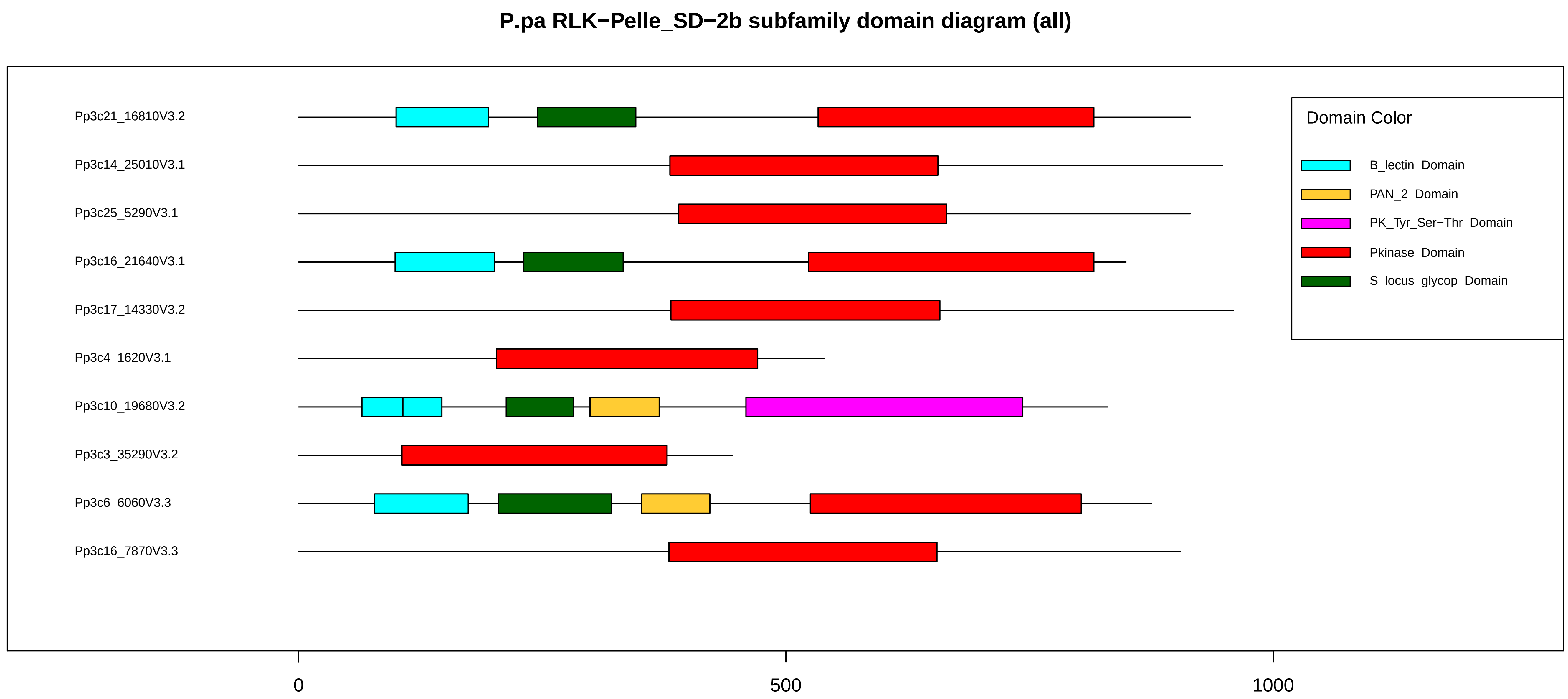
P.pa RLK−Pelle_SD−2b subfamily domain diagram (all)
Pp3c21_16810V3.2
Pp3c14_25010V3.1
Pp3c25_5290V3.1
Pp3c16_21640V3.1
Pp3c17_14330V3.2
Pp3c4_1620V3.1
Pp3c10_19680V3.2
Pp3c3_35290V3.2
Pp3c6_6060V3.3
Pp3c16_7870V3.3
Domain Color
B_lectin Domain
PAN_2 Domain
PK_Tyr_Ser−Thr Domain
Pkinase Domain
S_locus_glycop Domain
0
500
1000

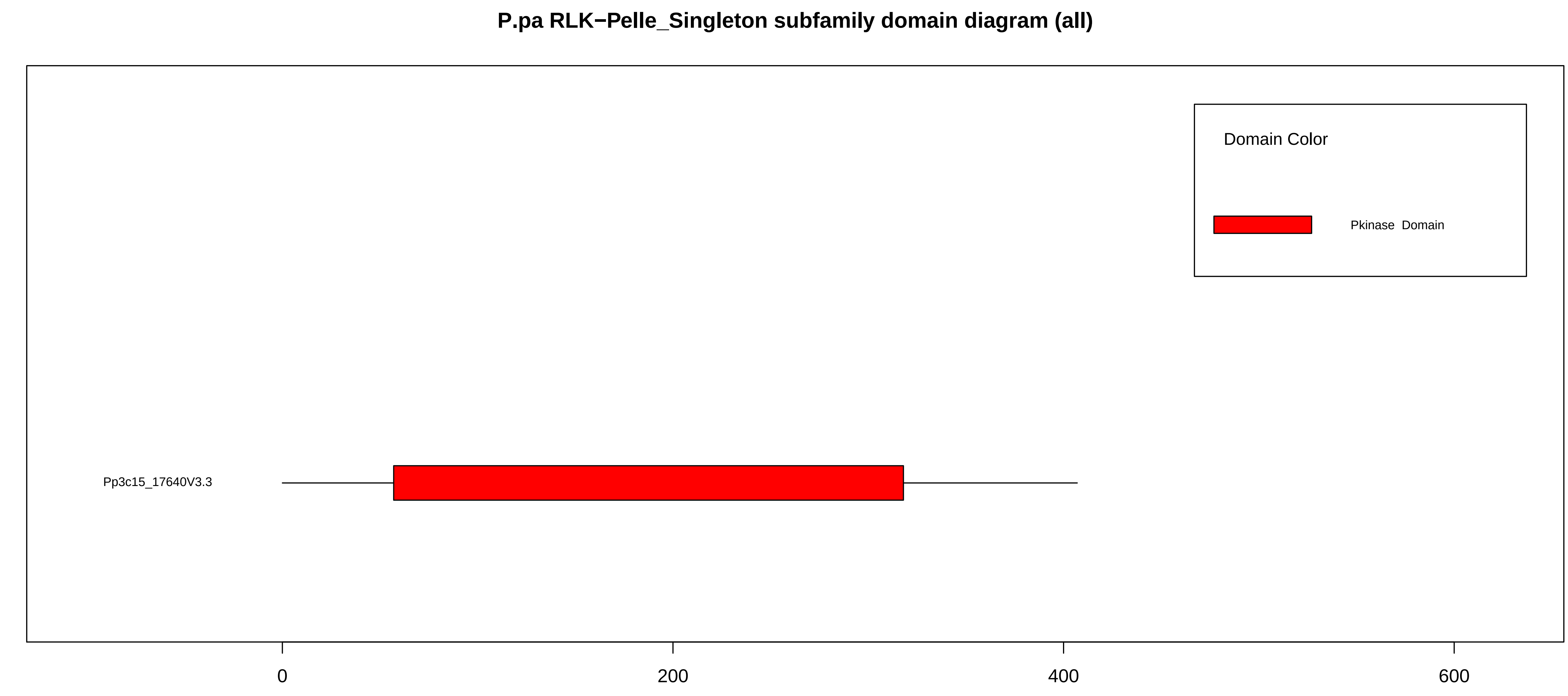
P.pa RLK−Pelle_Singleton subfamily domain diagram (all)
Domain Color
Pkinase Domain
Pp3c15_17640V3.3
0
200
400
600

P.pa RLK−Pelle_URK−Pp−1 subfamily domain diagram (all)

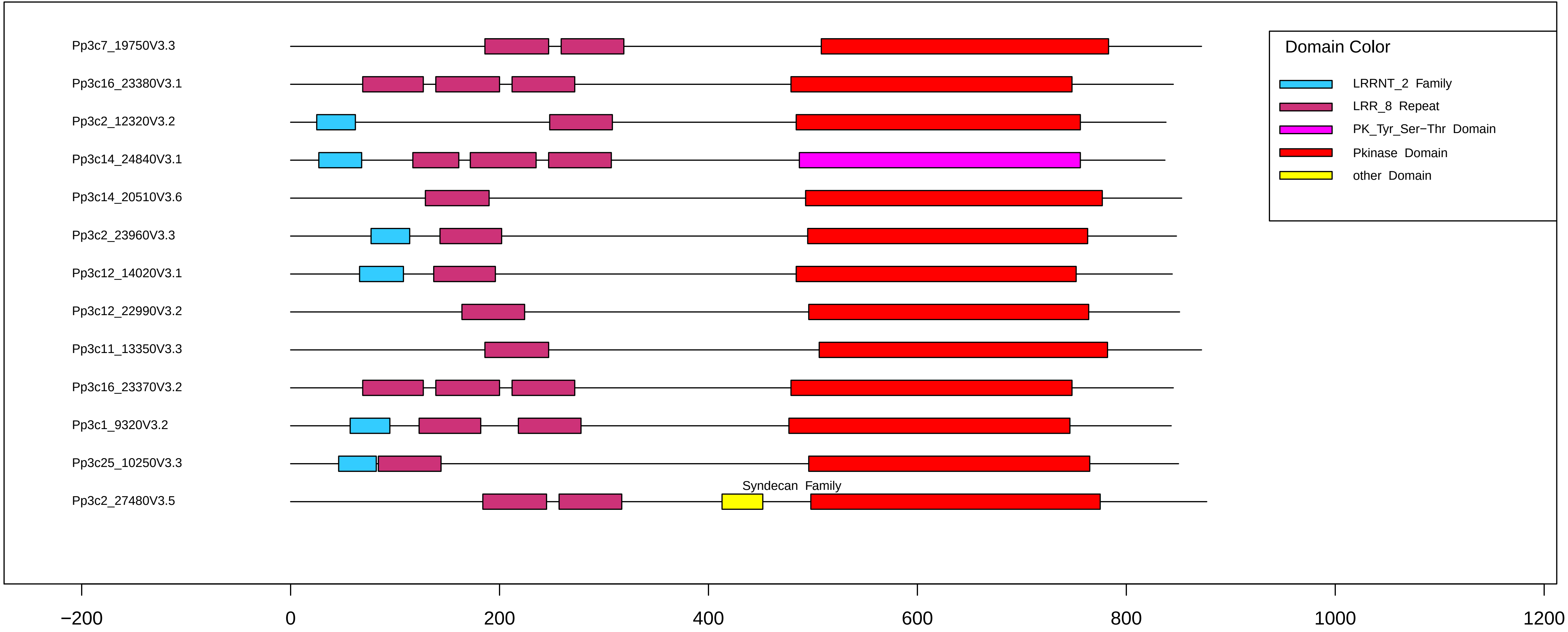

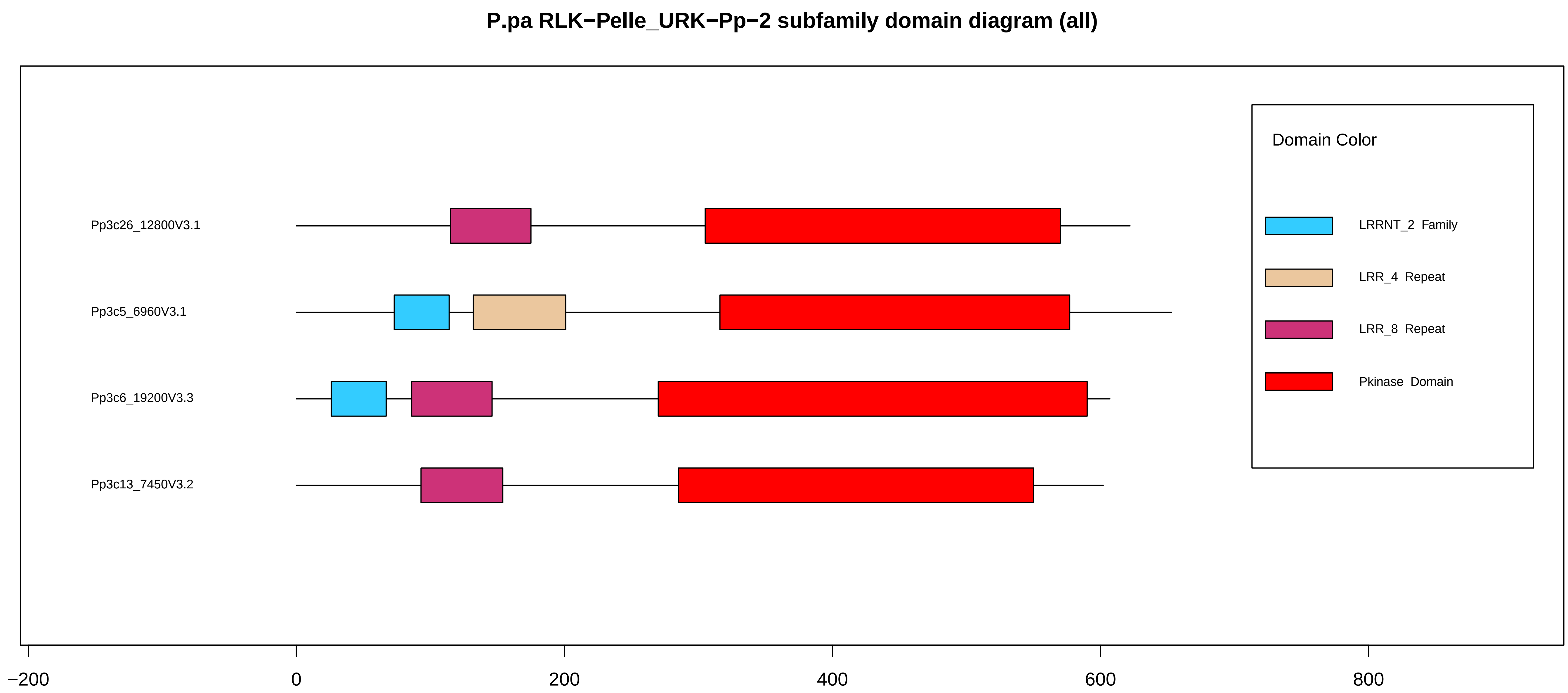
P.pa RLK−Pelle_URK−Pp−2 subfamily domain diagram (all)
Pp3c26_12800V3.1
Pp3c5_6960V3.1
Pp3c6_19200V3.3
Pp3c13_7450V3.2
Domain Color
LRRNT_2 Family
LRR_4 Repeat
LRR_8 Repeat
Pkinase Domain
−200
0
200
400
600
800

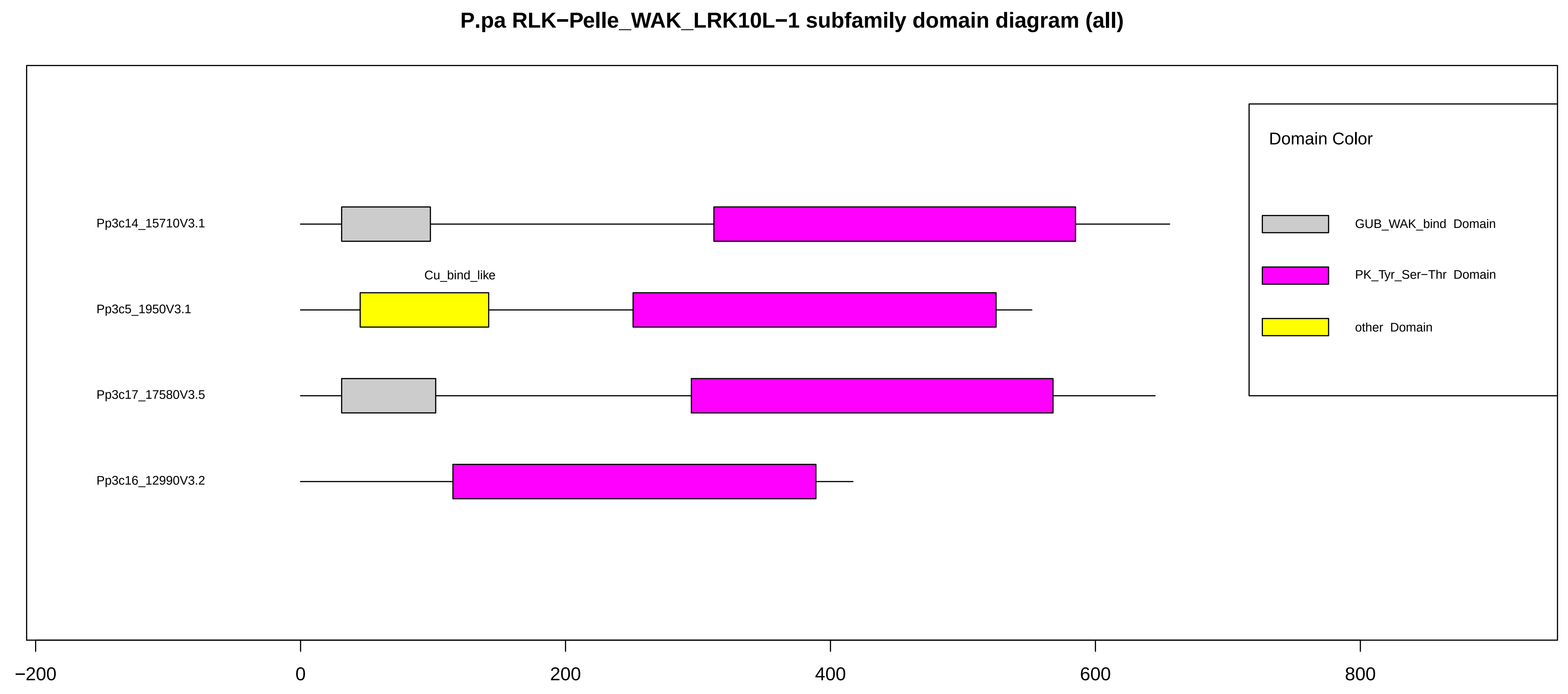
P.pa RLK−Pelle_WAK_LRK10L−1 subfamily domain diagram (all)
Pp3c14_15710V3.1
Cu_bind_like
Pp3c5_1950V3.1
Pp3c17_17580V3.5
Pp3c16_12990V3.2
Domain Color
GUB_WAK_bind Domain
PK_Tyr_Ser−Thr Domain
other Domain
−200
0
200
400
600
800

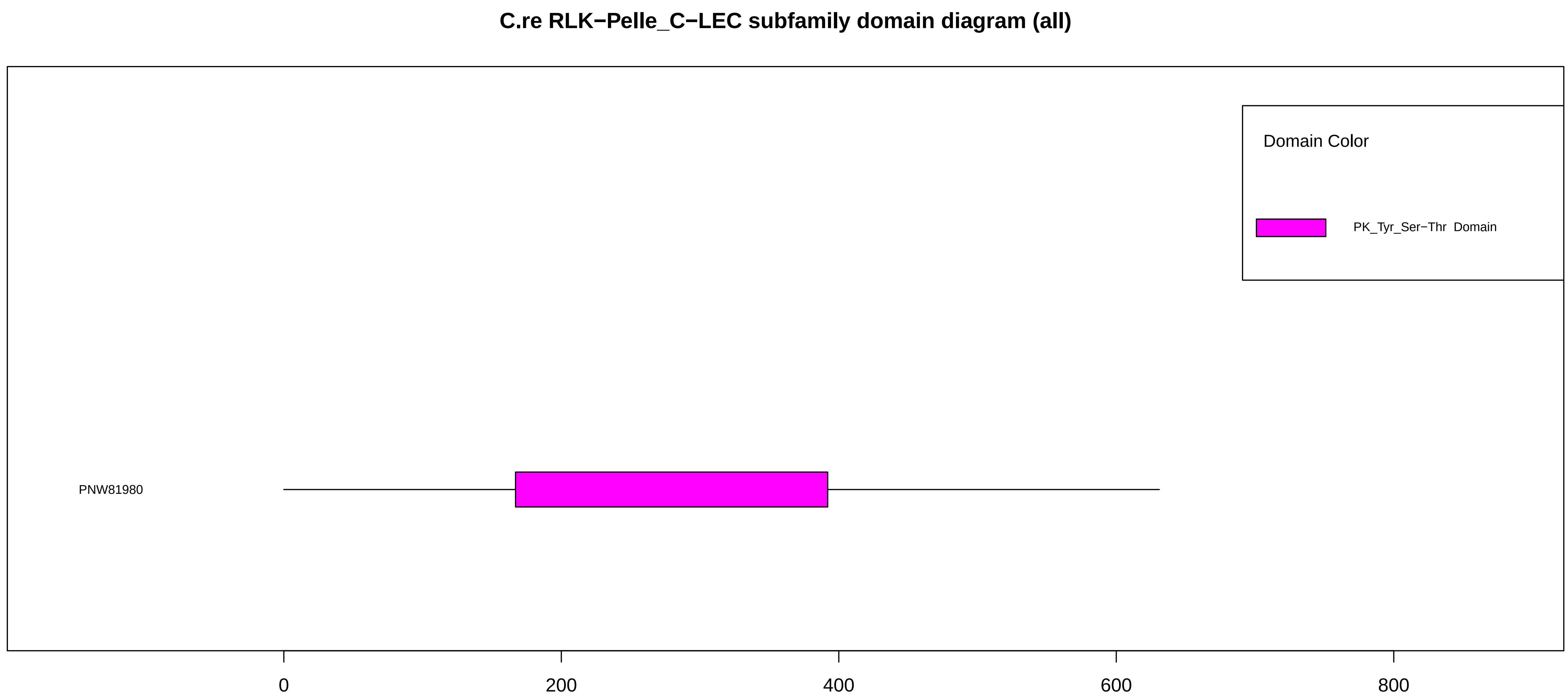
C.re RLK−Pelle_C−LEC subfamily domain diagram (all)
Domain Color
PK_Tyr_Ser−Thr Domain
PNW81980
0
200
400
600
800

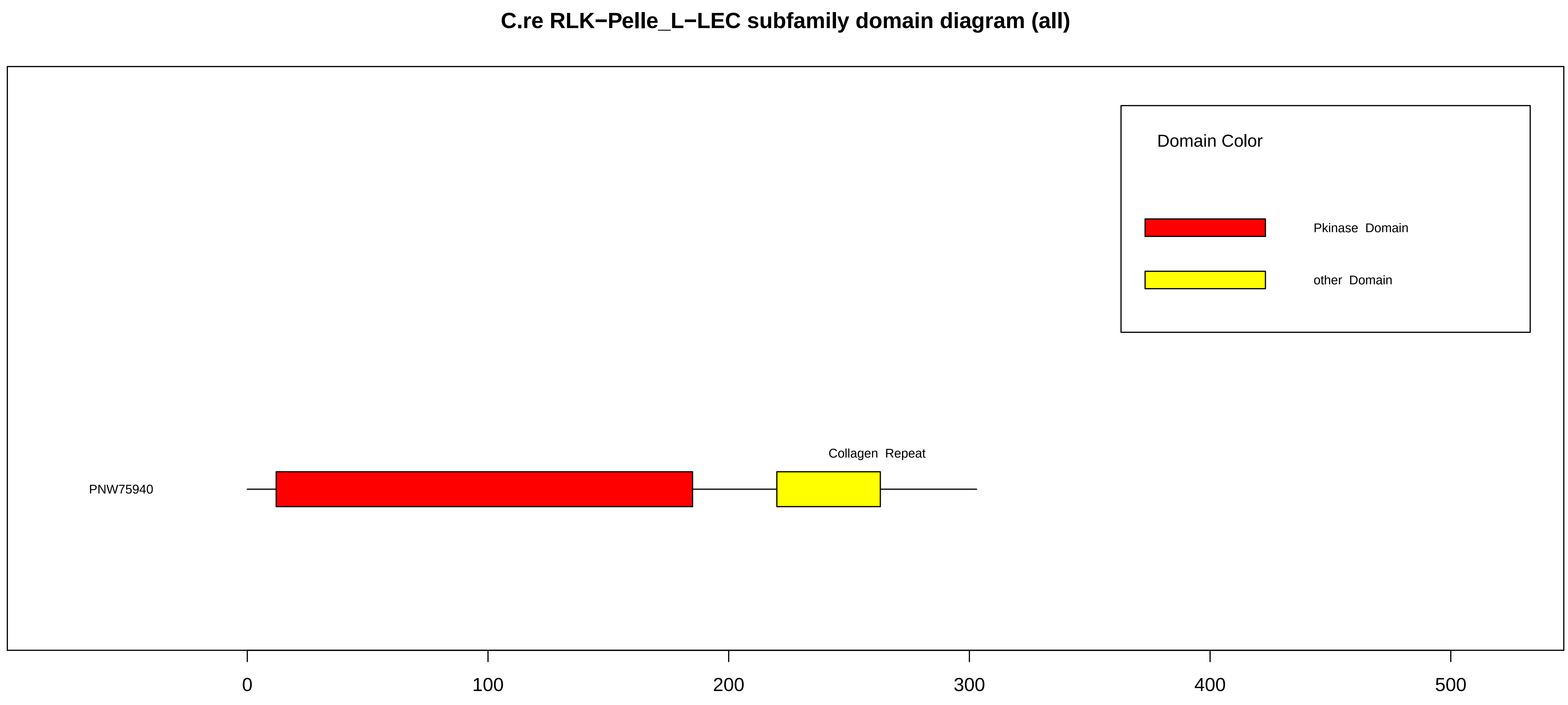
C.re RLK−Pelle_L−LEC subfamily domain diagram (all)
Domain Color
Pkinase Domain
other Domain
Collagen Repeat
PNW75940
0
100
200
300
400
500

C.re RLK−Pelle_RLCK−IXb subfamily domain diagram (all)

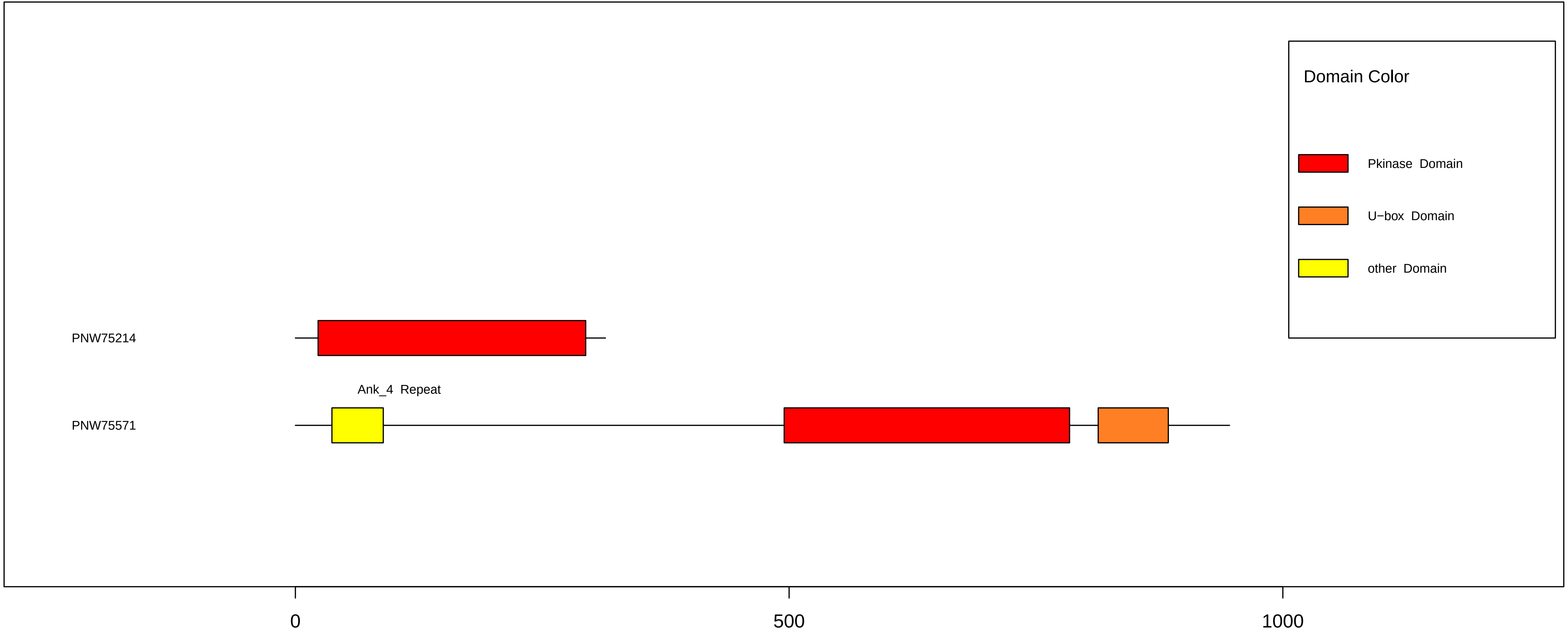

Supplement: Supplementary file 4 — Additional file 4: Figure S4. Domain diagrams of RLKs in 15 plants. Different domains are represented by boxes with different colours. [file 12864_2023_9303_MOESM4_ESM.pdf]
