# Supplementary material for: Phylogeny of the plant receptor-like kinase (RLK) gene family and expression analysis of wheat RLK genes in response to biotic and abiotic stresses
Source: BMC Genomics. 2023 May 1;24:224. doi: 10.1186/s12864-023-09303-7 (PMC10152718; doi:10.1186/s12864-023-09303-7)

A

Drought tolerance mechanisms in two broadly adapted wheat cultivars "TAM 111" and "TAM 112" (Bioproject: 659916)

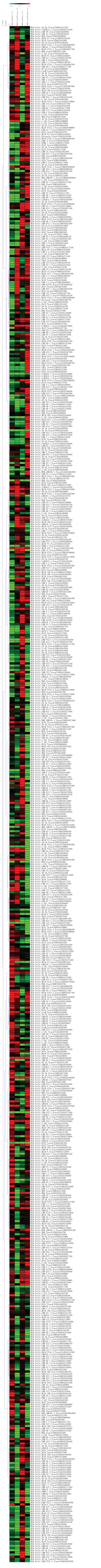

B

Drought tolerance mechanisms in two wheat genotypes "Svevo" and "IL20-2" (Bioproject: 686121)

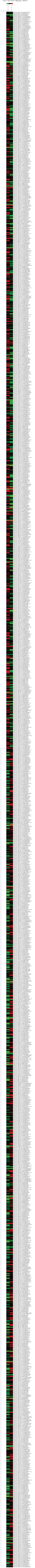

C

Drought tolerance mechanisms in two wheat genotypes "L-82" and "Marvdasht" (Bioproject: 450487)

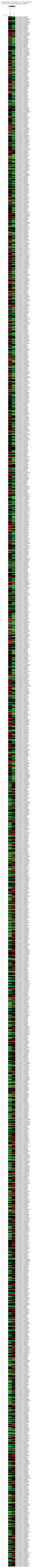

Supplement: Supplementary file 9 — Additional file 9: Figure S9. Heatmap of the expression patterns of individual T. aestivum RLK genes under drought stress treatments. (A) In wheat cultivars “TAM 111” and “TAM 112”; (B) in two wheat genotypes “Svevo” and “IL20-2”; and (C) in two wheat genotypes “L-82” and “Marvdasht”. [file 12864_2023_9303_MOESM9_ESM.pdf]
