# Supplementary material for: Phylogeny of the plant receptor-like kinase (RLK) gene family and expression analysis of wheat RLK genes in response to biotic and abiotic stresses
Source: BMC Genomics. 2023 May 1;24:224. doi: 10.1186/s12864-023-09303-7 (PMC10152718; doi:10.1186/s12864-023-09303-7)

A

Drought tolerance mechanisms in two *Aegilops tauschii* cultivars "XJ98" and "XJ2" (Bioproject: 482066)

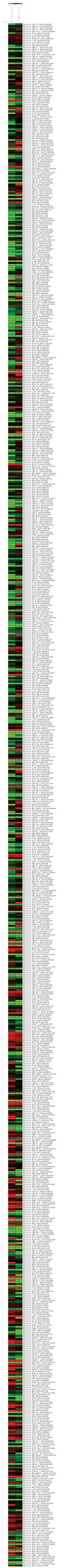

B

Drought tolerance mechanisms in three *Brachypodium distachyon* cultivars “ABR4”, “ABR8” and “KOZ1” (Bioproject: 524106)

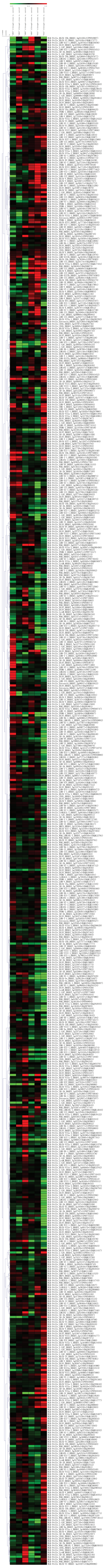

Supplement: Supplementary file 10 — Additional file 10: Figure S10. Heatmap of the expression patterns of individual Ae. tauschii and B. distachyon RLK genes under drought stress treatments. (A) In two Ae. tauschii cultivars “XJ98” and “XJ2” and (B) in three B. distachyon cultivars “ABR4”, “ABR8” and “KOZ1”. [file 12864_2023_9303_MOESM10_ESM.pdf]
