# Supplementary material for: Phylogeny of the plant receptor-like kinase (RLK) gene family and expression analysis of wheat RLK genes in response to biotic and abiotic stresses
Source: BMC Genomics. 2023 May 1;24:224. doi: 10.1186/s12864-023-09303-7 (PMC10152718; doi:10.1186/s12864-023-09303-7)

A

Heat stress on transcriptome of developing wheat grain (Bioproject: 598150)

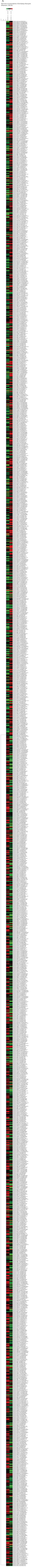

B

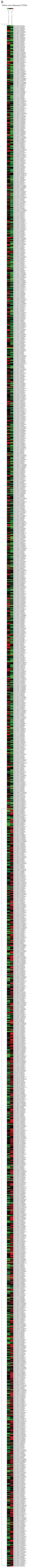
Salinity stress (Bioproject:573996)
-2.0
0.0
2.0
root_(salinity stress vs. control)
leaf_(salinity stress vs. control)
-1.0
0.0
1.0

C

Different wheat varieties in response to waterlogging stress (Bioproject:604012)

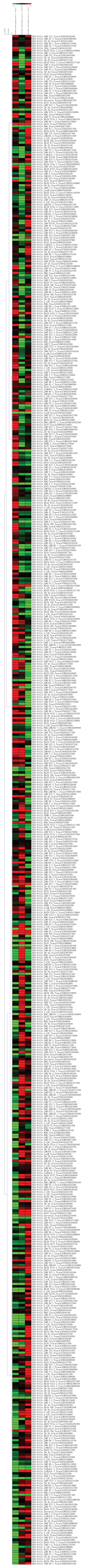

Supplement: Supplementary file 11 — Additional file 11: Figure S11. Heatmap of the expression patterns of individual T. aestivum RLK genes under other abiotic stress treatments. (A) Heat stress; (B) salinity stress; and (C) waterlogging stress. [file 12864_2023_9303_MOESM11_ESM.pdf]
