# Supplementary material for: Phylogeny of the plant receptor-like kinase (RLK) gene family and expression analysis of wheat RLK genes in response to biotic and abiotic stresses
Source: BMC Genomics. 2023 May 1;24:224. doi: 10.1186/s12864-023-09303-7 (PMC10152718; doi:10.1186/s12864-023-09303-7)

A

Fusarium graminearum (EBI study accession:PRJEB12358)

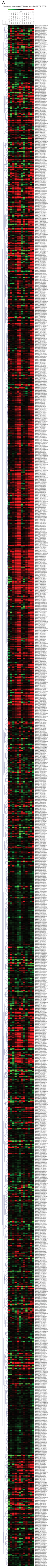

B

Stripe rust (Bioproject:613349)

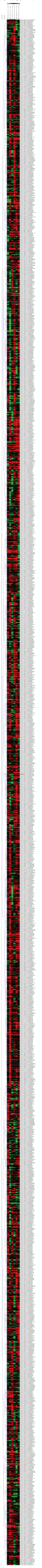

C

Interactions with mycorhizal fungi (*Funneliformis mossae*) with and without a pathogen attack by *Xanthomonas translucens* (Bioproject:474303)

Control：Wheat+*Mycorhizal* Fungi

Treatment：Wheat+*Mycorhizal* Fungi+*Xanthomonas*

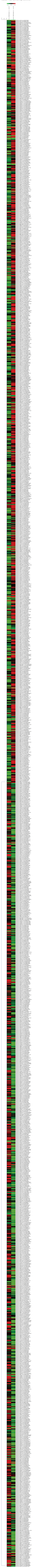

D

Xanthomonas translucens infection (Bioproject:401247)

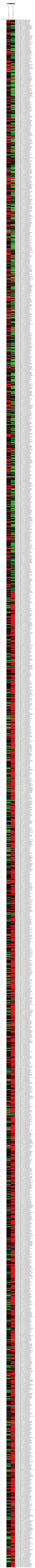

Supplement: Supplementary file 12 — Additional file 12: Figure S12. Heatmap of the expression patterns of individual T. aestivum RLK genes under biotic stress treatments. (A) F. graminearum infection; (B) stripe rust; (C) interactions with mycorhizal fungi (F. mossae) with and without pathogen attack by X. translucens; and (D) X. translucens infection. [file 12864_2023_9303_MOESM12_ESM.pdf]
